# Supplementary material for: Investigating the role of gut microbiota in diabetic nephropathy through plasma proteome mediated analysis
Source: Sci Rep. 2025 Feb 14;15:5457. doi: 10.1038/s41598-025-90306-7 (PMC11828962; doi:10.1038/s41598-025-90306-7)
Supplement: Supplementary file 3 — Supplementary Material 3 [file 41598_2025_90306_MOESM3_ESM.pdf]

Supplementary Figure 3: Forest plots of all MR analyses in this study

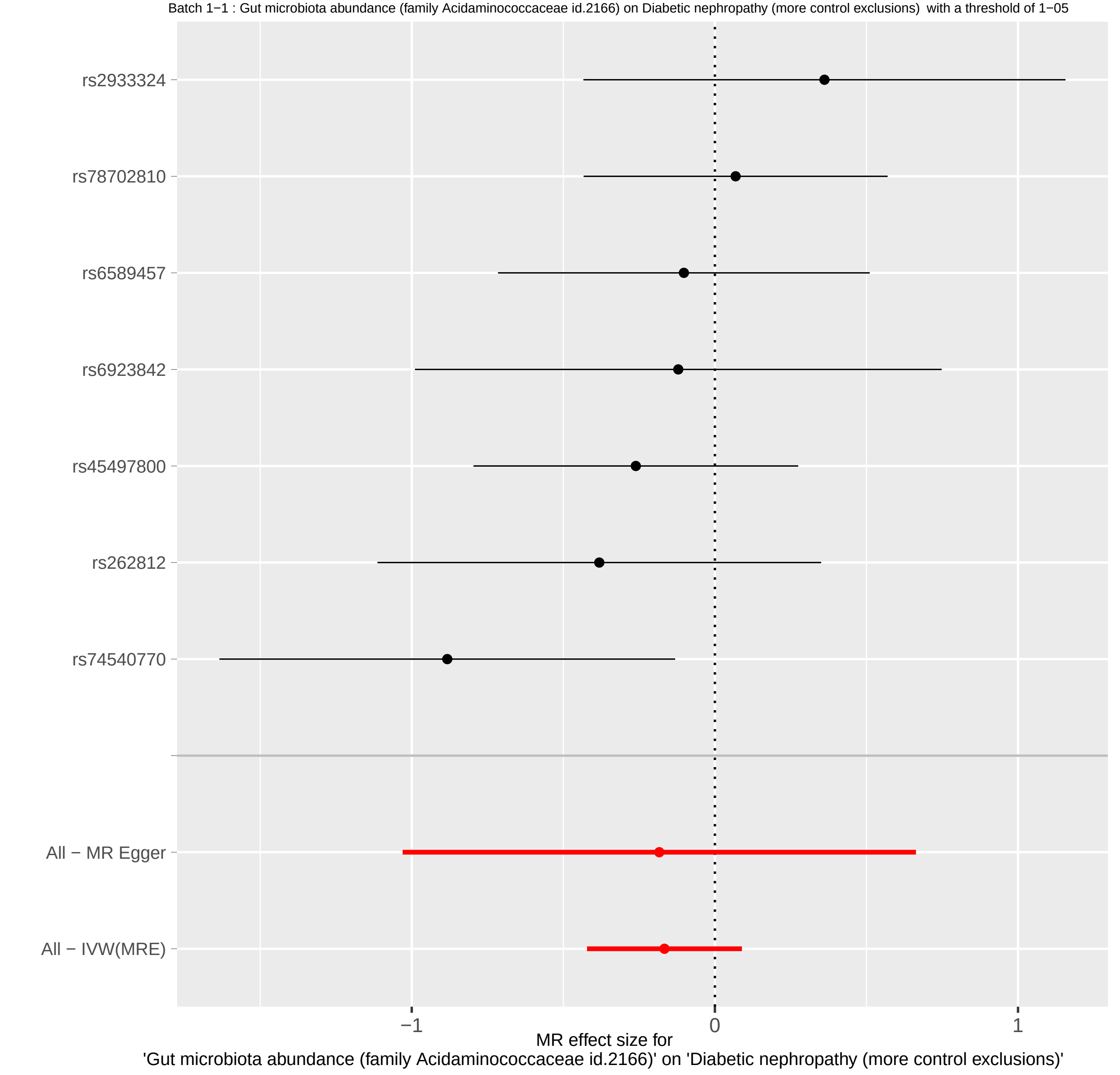

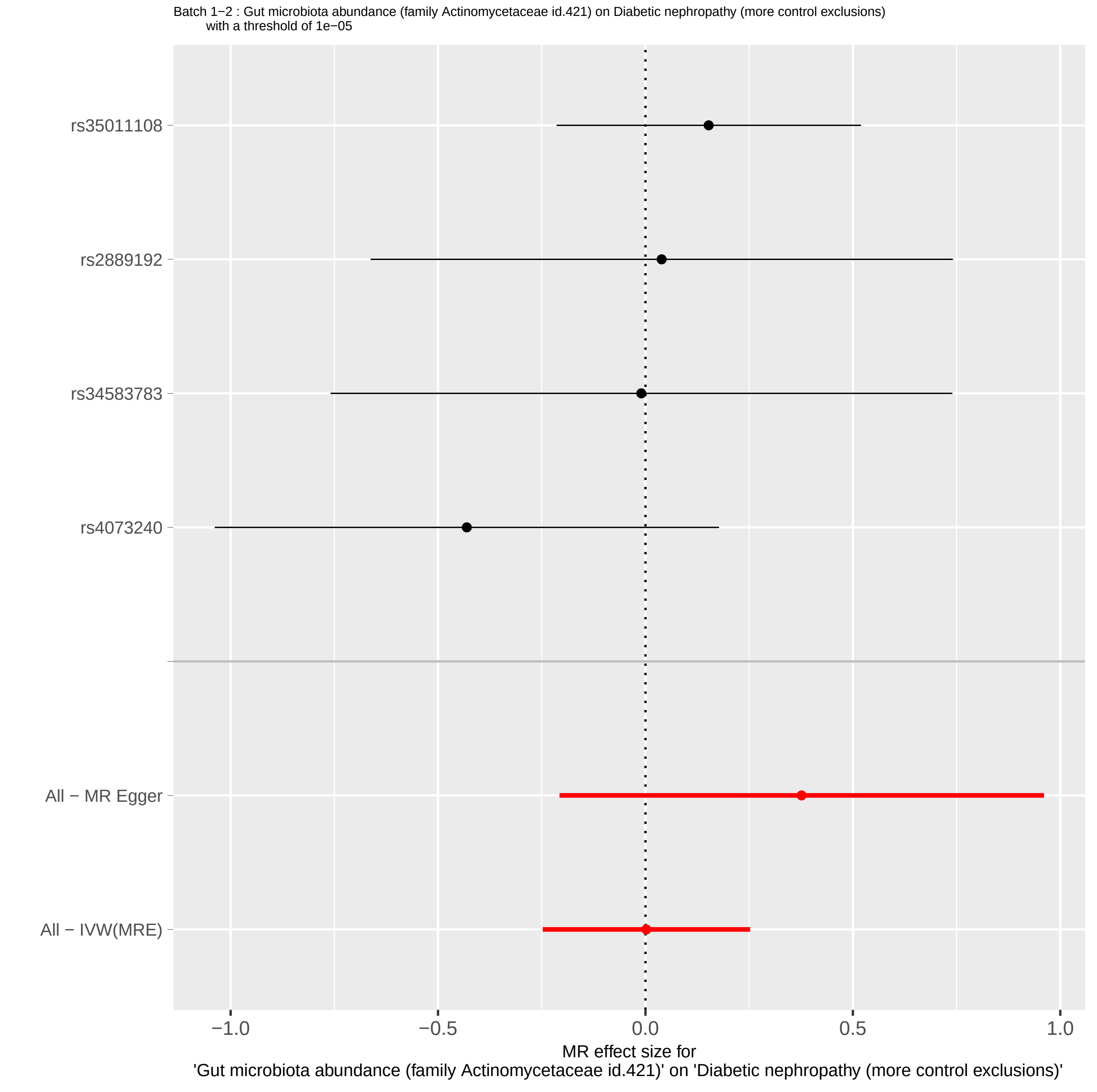

Batch 1−2 : Gut microbiota abundance (family Actinomycetaceae id.421) on Diabetic nephropathy (more control exclusions)
with a threshold of 1e−05
rs35011108
rs2889192
rs34583783
rs4073240
All − MR Egger
All − IVW(MRE)
−1.0
−0.5
0.0
0.5
1.0
MR effect size for
'Gut microbiota abundance (family Actinomycetaceae id.421)' on 'Diabetic nephropathy (more control exclusions)'

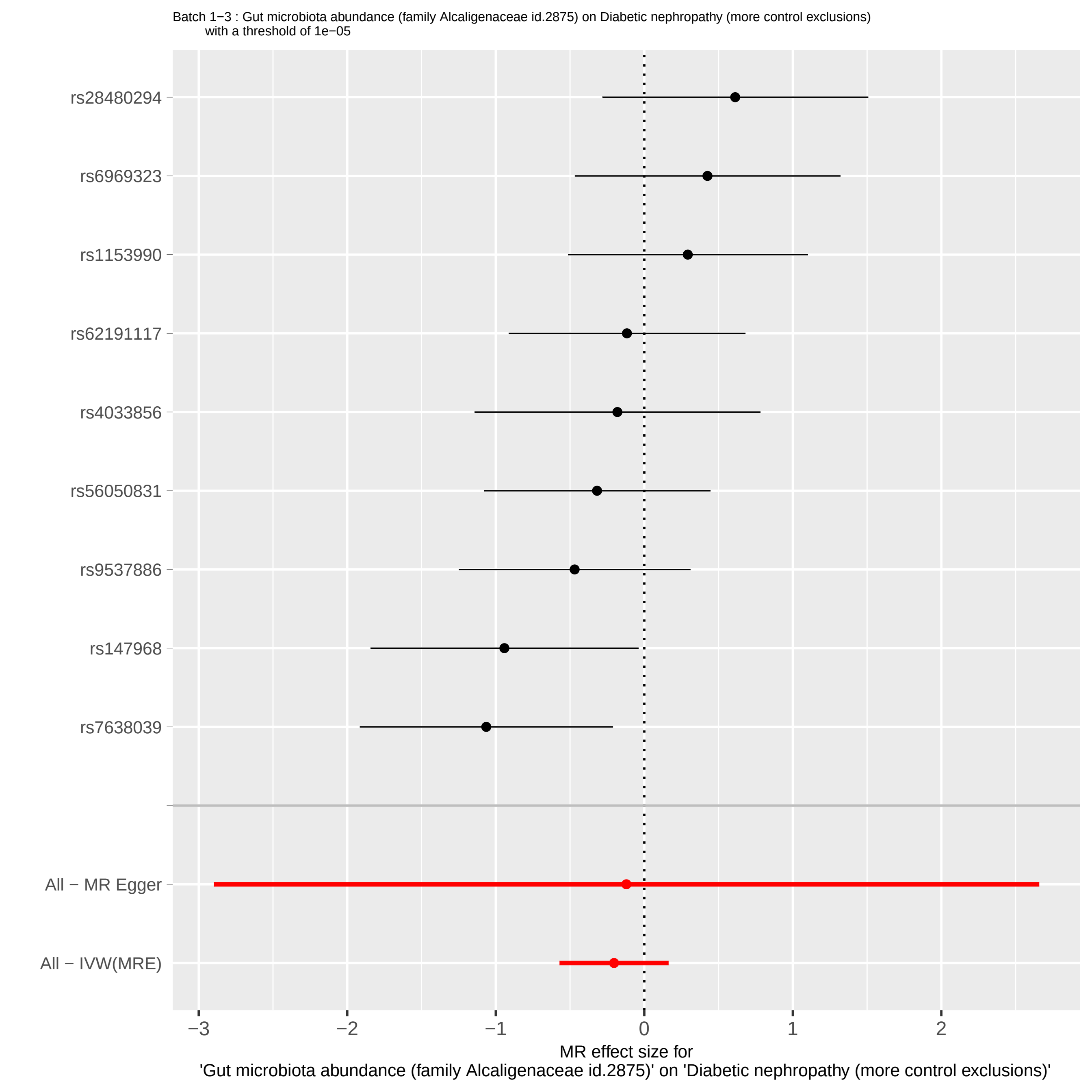
Batch 1−3 : Gut microbiota abundance (family Alcaligenaceae id.2875) on Diabetic nephropathy (more control exclusions) with a threshold of 1e−05
rs28480294
rs6969323
rs1153990
rs62191117
rs4033856
rs56050831
rs9537886
rs147968
rs7638039
All − MR Egger
All − IVW(MRE)
−3
−2
−1
0
1
2
MR effect size for
'Gut microbiota abundance (family Alcaligenaceae id.2875)' on 'Diabetic nephropathy (more control exclusions)'

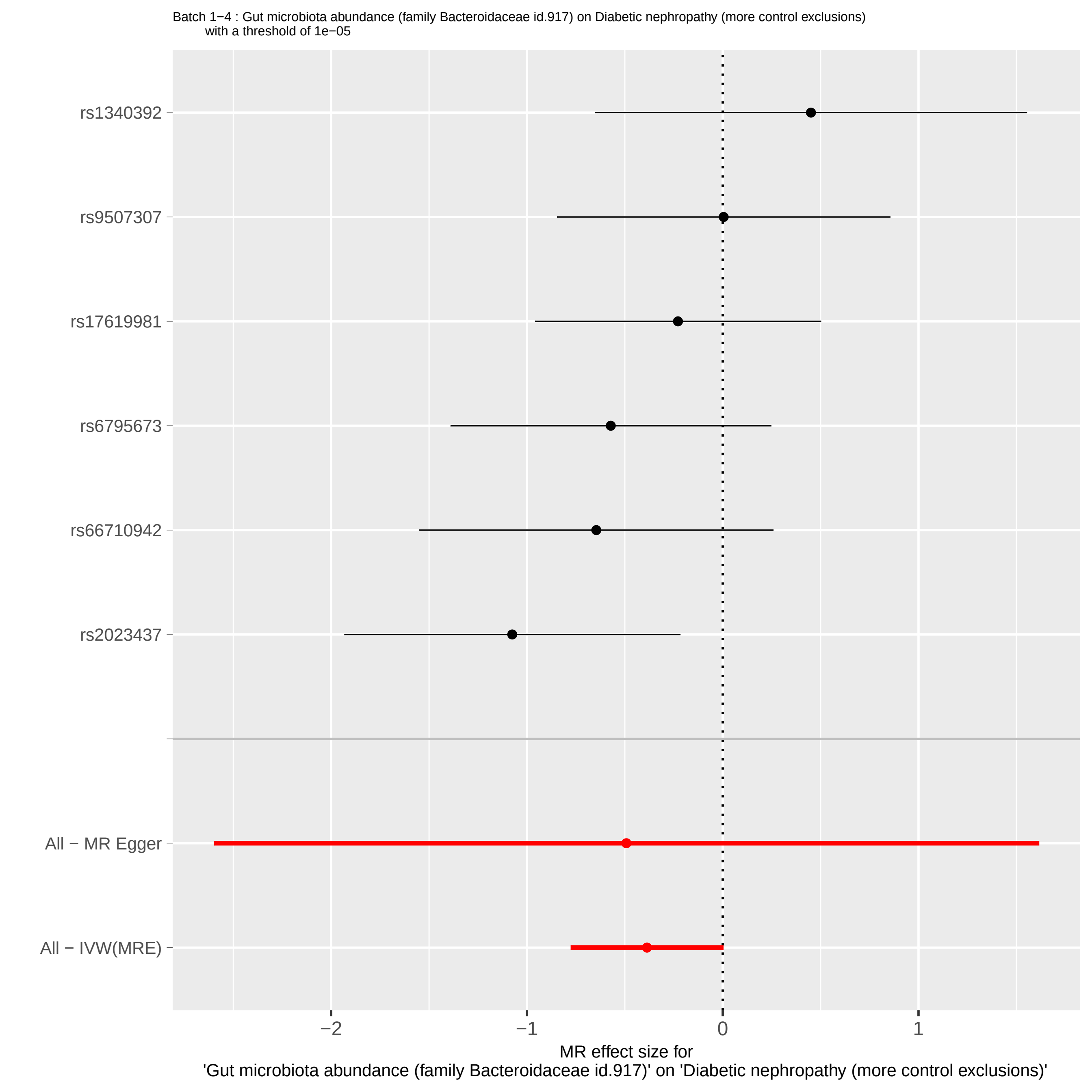
Batch 1−4 : Gut microbiota abundance (family Bacteroidaceae id.917) on Diabetic nephropathy (more control exclusions)
with a threshold of 1e−05
rs1340392
rs9507307
rs17619981
rs6795673
rs66710942
rs2023437
All − MR Egger
All − IVW(MRE)
−2
−1
0
1
MR effect size for
'Gut microbiota abundance (family Bacteroidaceae id.917)' on 'Diabetic nephropathy (more control exclusions)'

Batch 1−5 : Gut microbiota abundance (family Bacteroidales S24 7group id.11173) on Diabetic nephropathy (more control exclusions) with a threshold of 1e−05

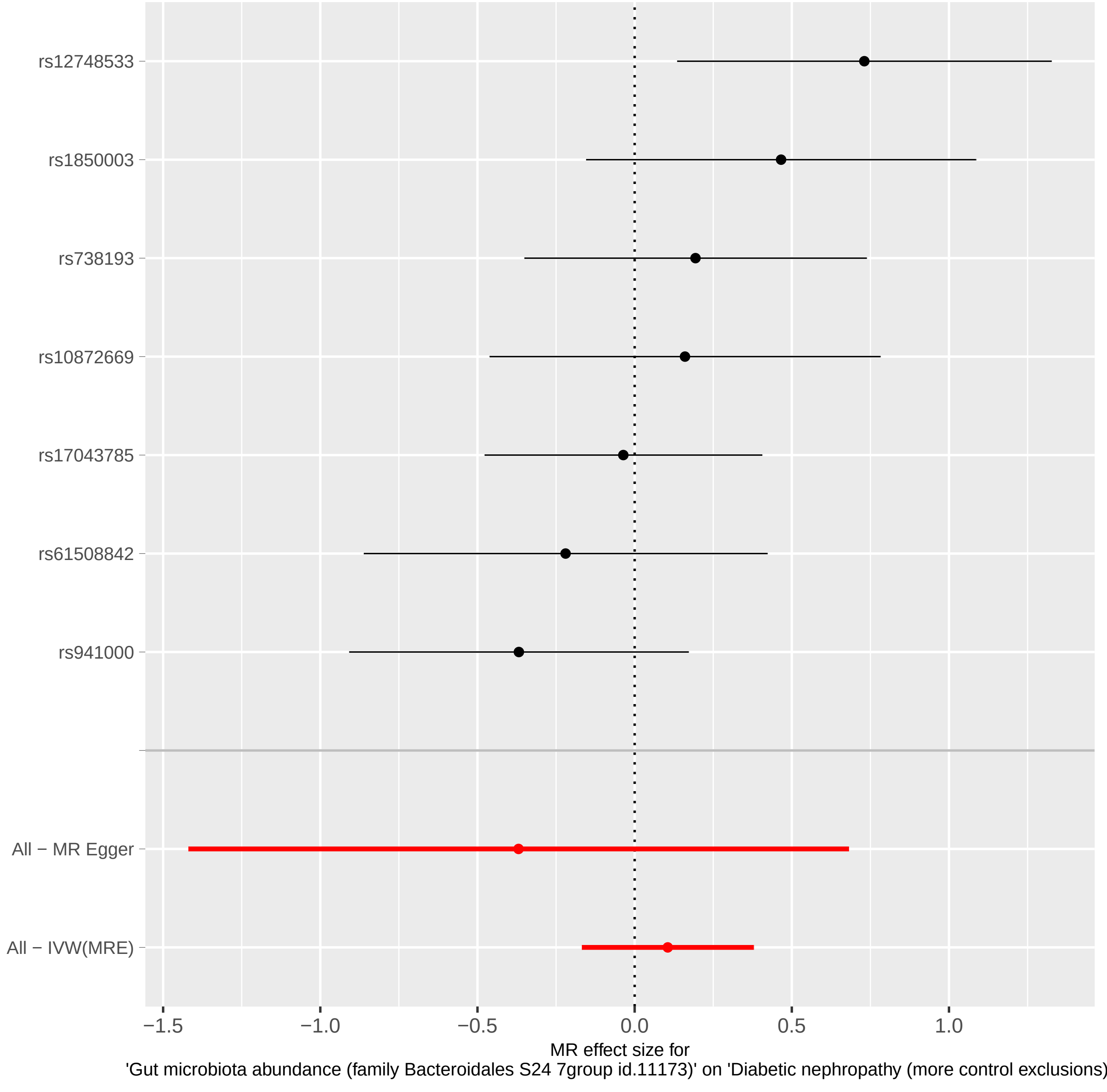

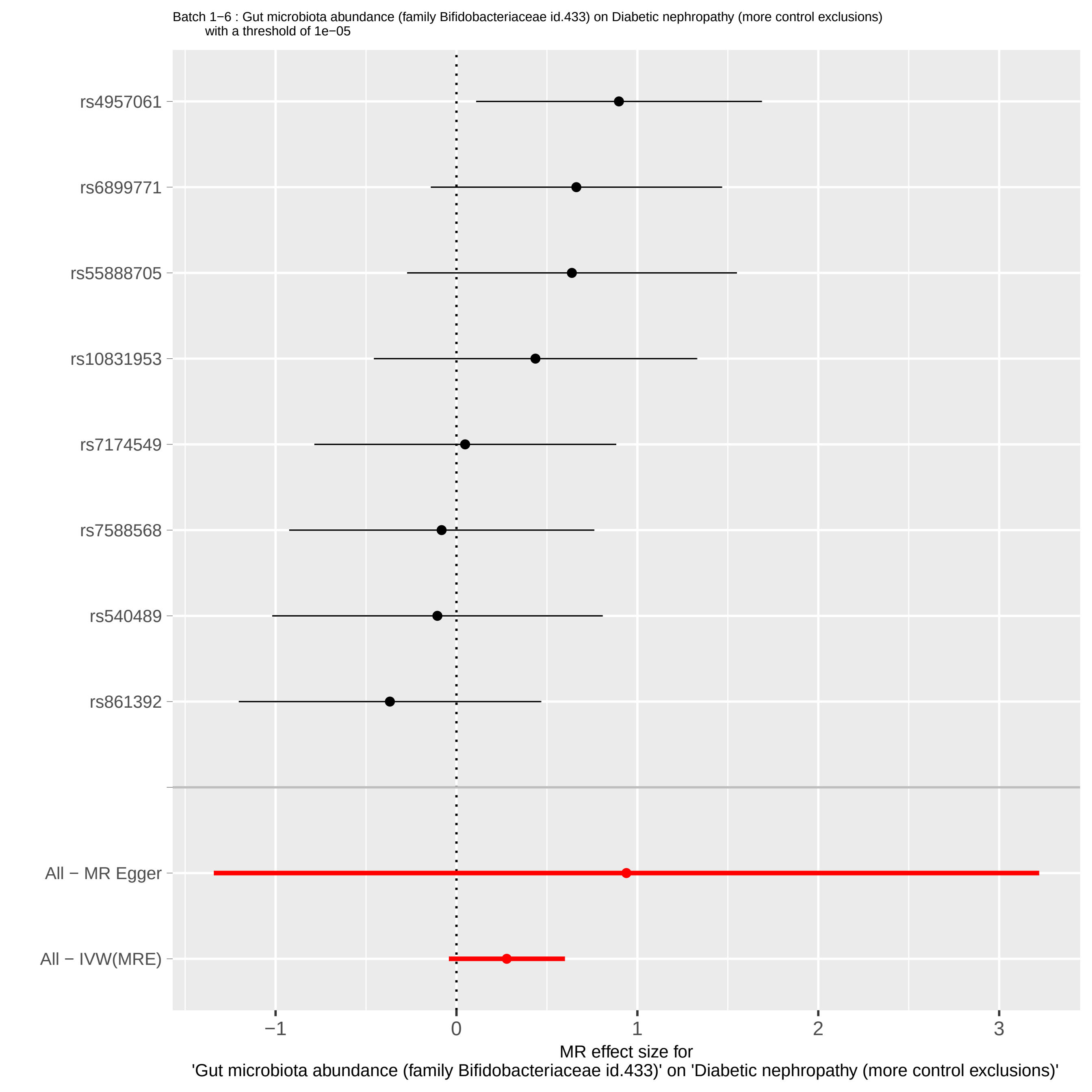

Batch 1−6 : Gut microbiota abundance (family Bifidobacteriaceae id.433) on Diabetic nephropathy (more control exclusions)
with a threshold of 1e−05
rs4957061
rs6899771
rs55888705
rs10831953
rs7174549
rs7588568
rs540489
rs861392
All − MR Egger
All − IVW(MRE)
−1
0
1
2
3
MR effect size for
'Gut microbiota abundance (family Bifidobacteriaceae id.433)' on 'Diabetic nephropathy (more control exclusions)'

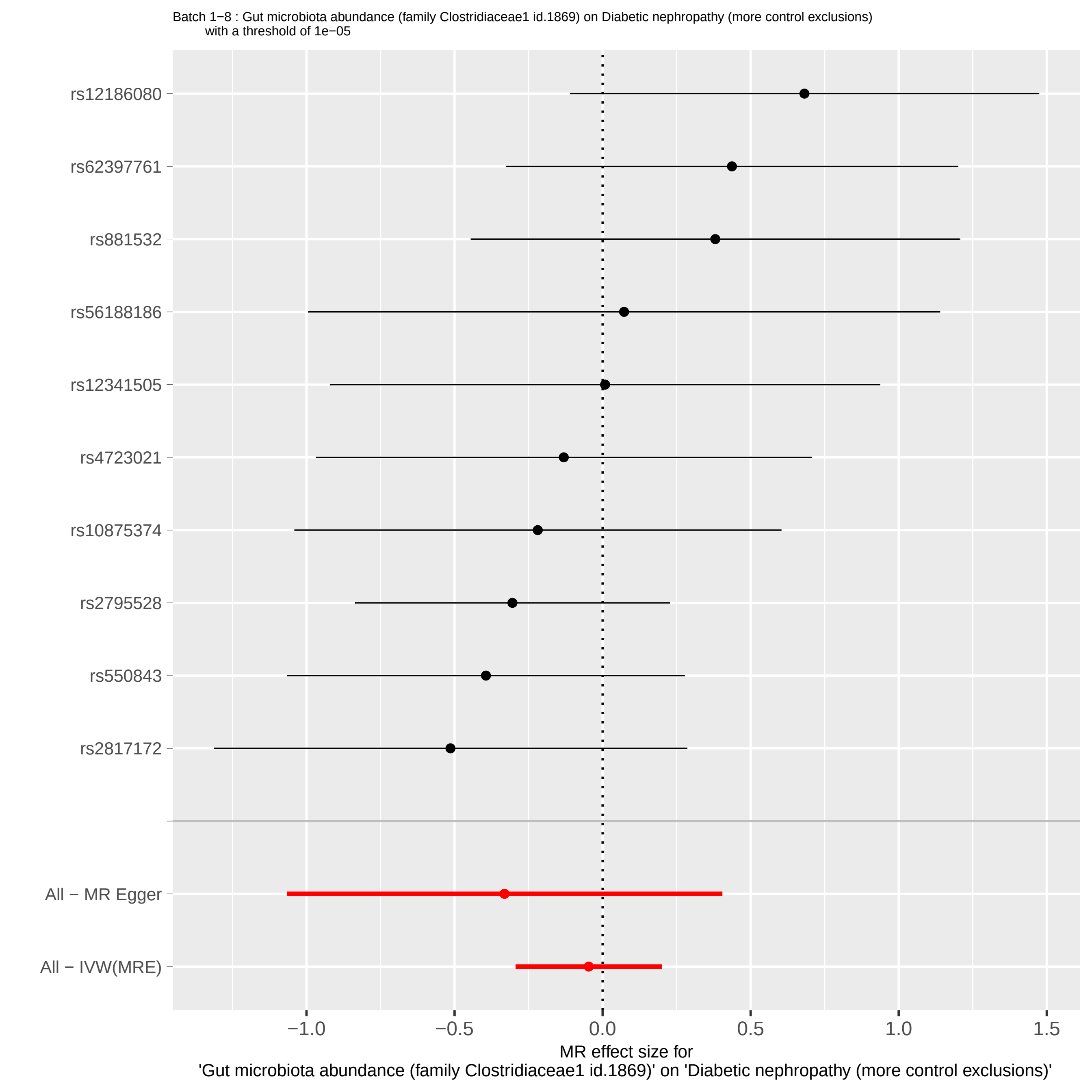
Batch 1−8 : Gut microbiota abundance (family Clostridiaceae1 id.1869) on Diabetic nephropathy (more control exclusions)
with a threshold of 1e−05
rs12186080
rs62397761
rs881532
rs56188186
rs12341505
rs4723021
rs10875374
rs2795528
rs550843
rs2817172
All − MR Egger
All − IVW(MRE)
−1.0
−0.5
0.0
0.5
1.0
1.5
MR effect size for
'Gut microbiota abundance (family Clostridiaceae1 id.1869)' on 'Diabetic nephropathy (more control exclusions)'

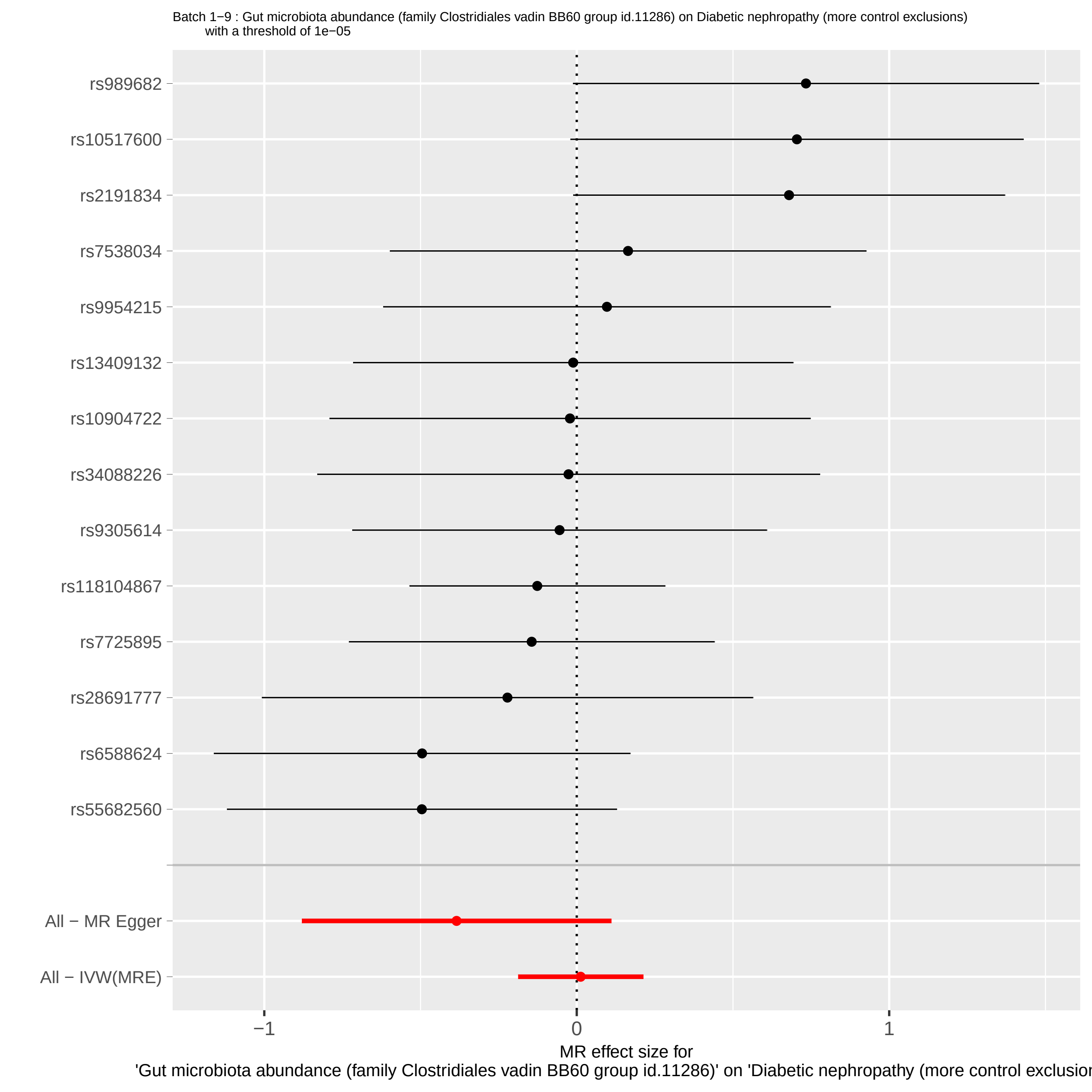
Batch 1−9 : Gut microbiota abundance (family Clostridiales vadin BB60 group id.11286) on Diabetic nephropathy (more control exclusions) with a threshold of 1e−05
rs989682
rs10517600
rs2191834
rs7538034
rs9954215
rs13409132
rs10904722
rs34088226
rs9305614
rs118104867
rs7725895
rs28691777
rs6588624
rs55682560
All − MR Egger
All − IVW(MRE)
−1
0
1
MR effect size for
'Gut microbiota abundance (family Clostridiales vadin BB60 group id.11286)' on 'Diabetic nephropathy (more control exclusion

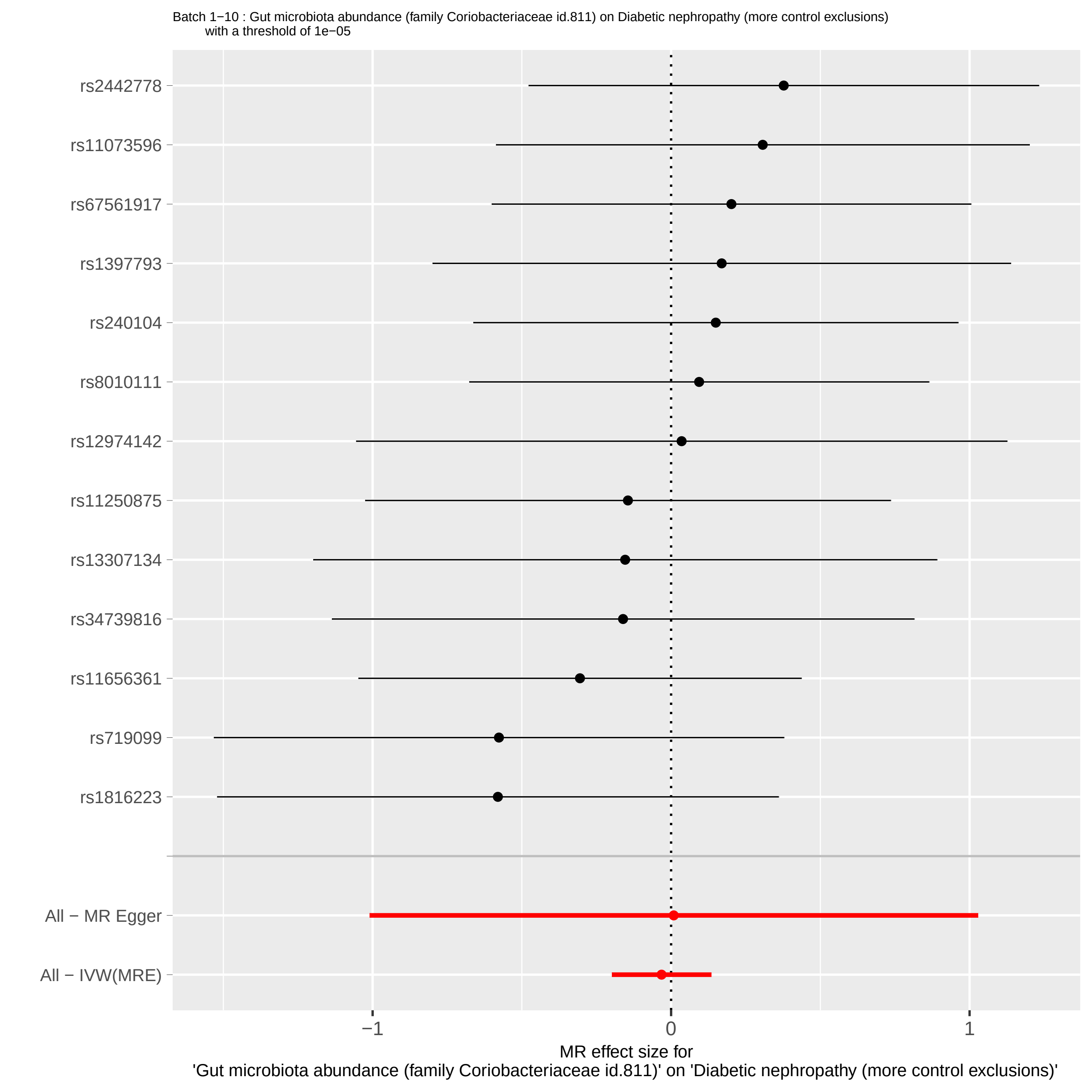
Batch 1−10 : Gut microbiota abundance (family Coriobacteriaceae id.811) on Diabetic nephropathy (more control exclusions) with a threshold of 1e−05
rs2442778
rs11073596
rs67561917
rs1397793
rs240104
rs8010111
rs12974142
rs11250875
rs13307134
rs34739816
rs11656361
rs719099
rs1816223
All − MR Egger
All − IVW(MRE)
−1
0
1
MR effect size for
'Gut microbiota abundance (family Coriobacteriaceae id.811)' on 'Diabetic nephropathy (more control exclusions)'

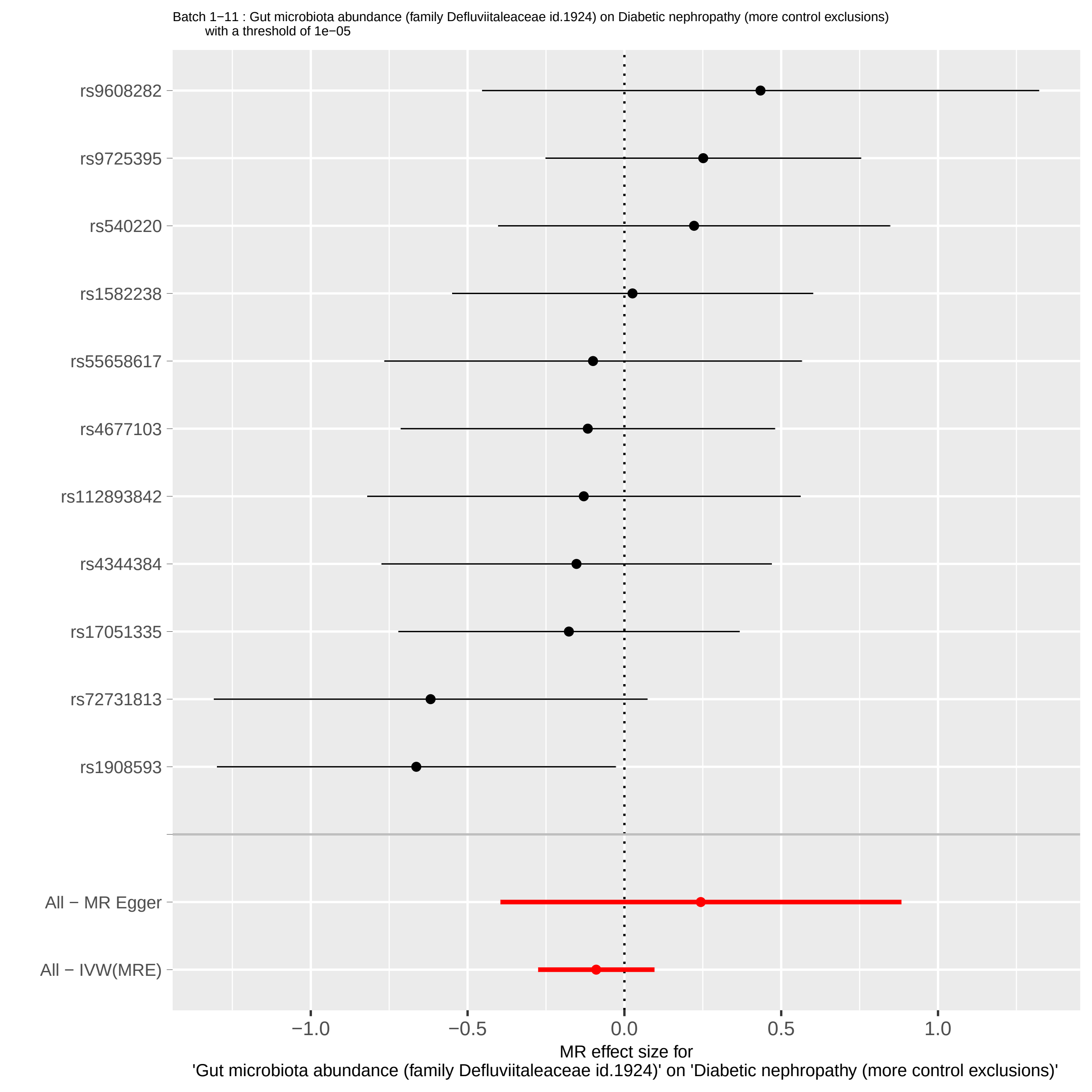

Batch 1−11 : Gut microbiota abundance (family Defluviitaleaceae id.1924) on Diabetic nephropathy (more control exclusions) with a threshold of 1e−05
rs9608282
rs9725395
rs540220
rs1582238
rs55658617
rs4677103
rs112893842
rs4344384
rs17051335
rs72731813
rs1908593
All − MR Egger
All − IVW(MRE)
−1.0
−0.5
0.0
0.5
1.0
MR effect size for
'Gut microbiota abundance (family Defluviitaleaceae id.1924)' on 'Diabetic nephropathy (more control exclusions)'

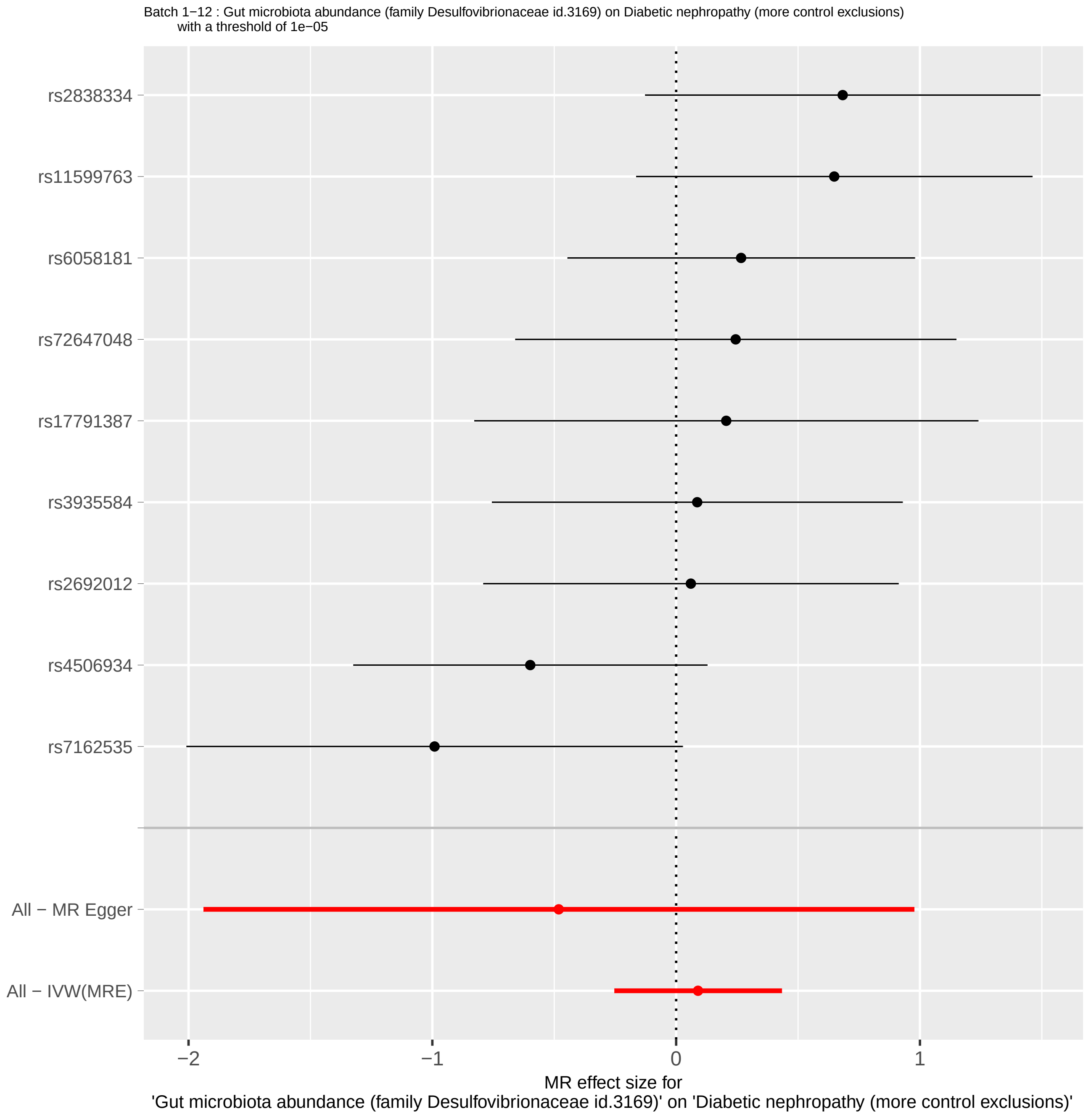
Batch 1−12 : Gut microbiota abundance (family Desulfovibrionaceae id.3169) on Diabetic nephropathy (more control exclusions)
with a threshold of 1e−05
rs2838334
rs11599763
rs6058181
rs72647048
rs17791387
rs3935584
rs2692012
rs4506934
rs7162535
All − MR Egger
All − IVW(MRE)
−2
−1
0
1
MR effect size for
'Gut microbiota abundance (family Desulfovibrionaceae id.3169)' on 'Diabetic nephropathy (more control exclusions)'

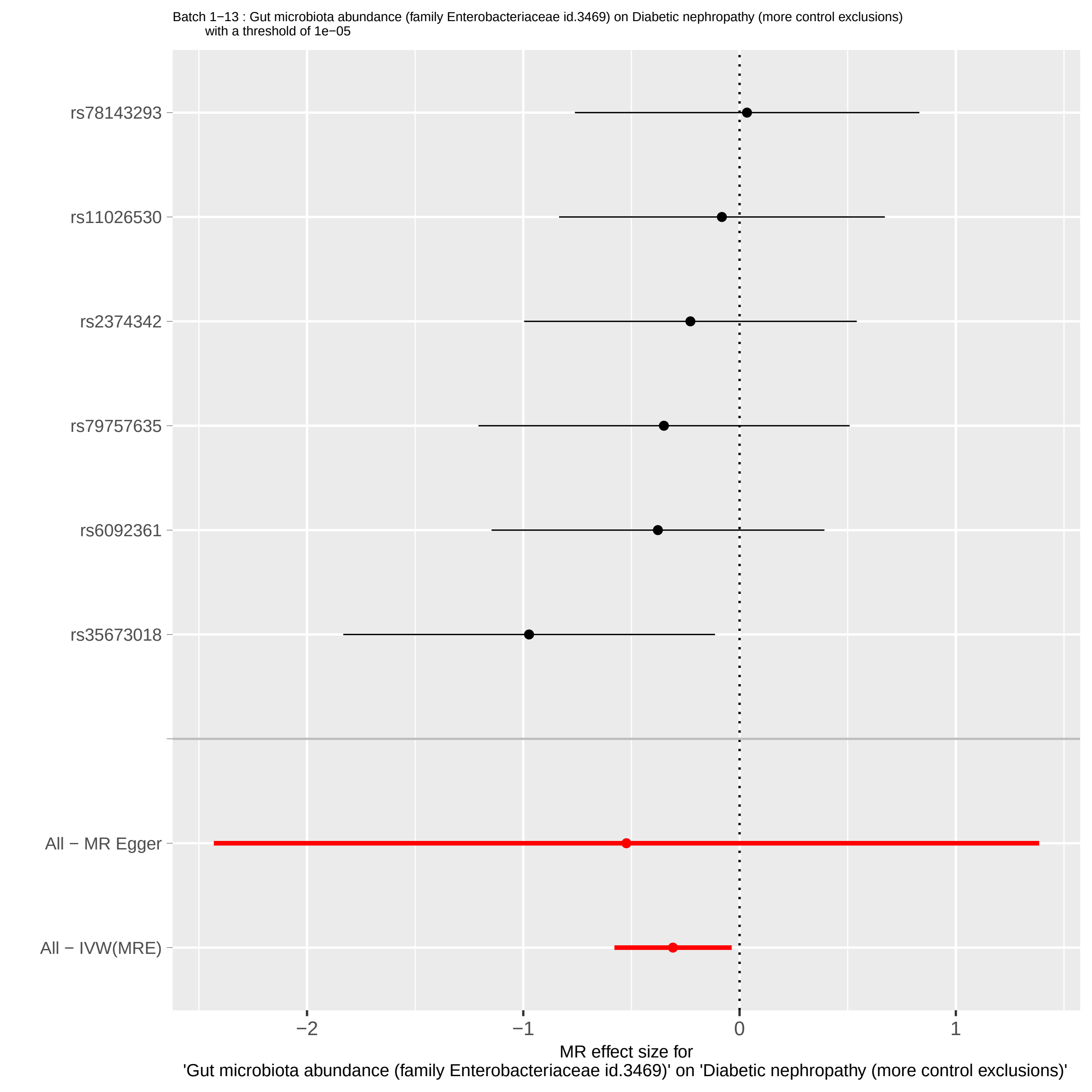

Batch 1−13 : Gut microbiota abundance (family Enterobacteriaceae id.3469) on Diabetic nephropathy (more control exclusions)
with a threshold of 1e−05
rs78143293
rs11026530
rs2374342
rs79757635
rs6092361
rs35673018
All − MR Egger
All − IVW(MRE)
−2
−1
0
1
MR effect size for
'Gut microbiota abundance (family Enterobacteriaceae id.3469)' on 'Diabetic nephropathy (more control exclusions)'

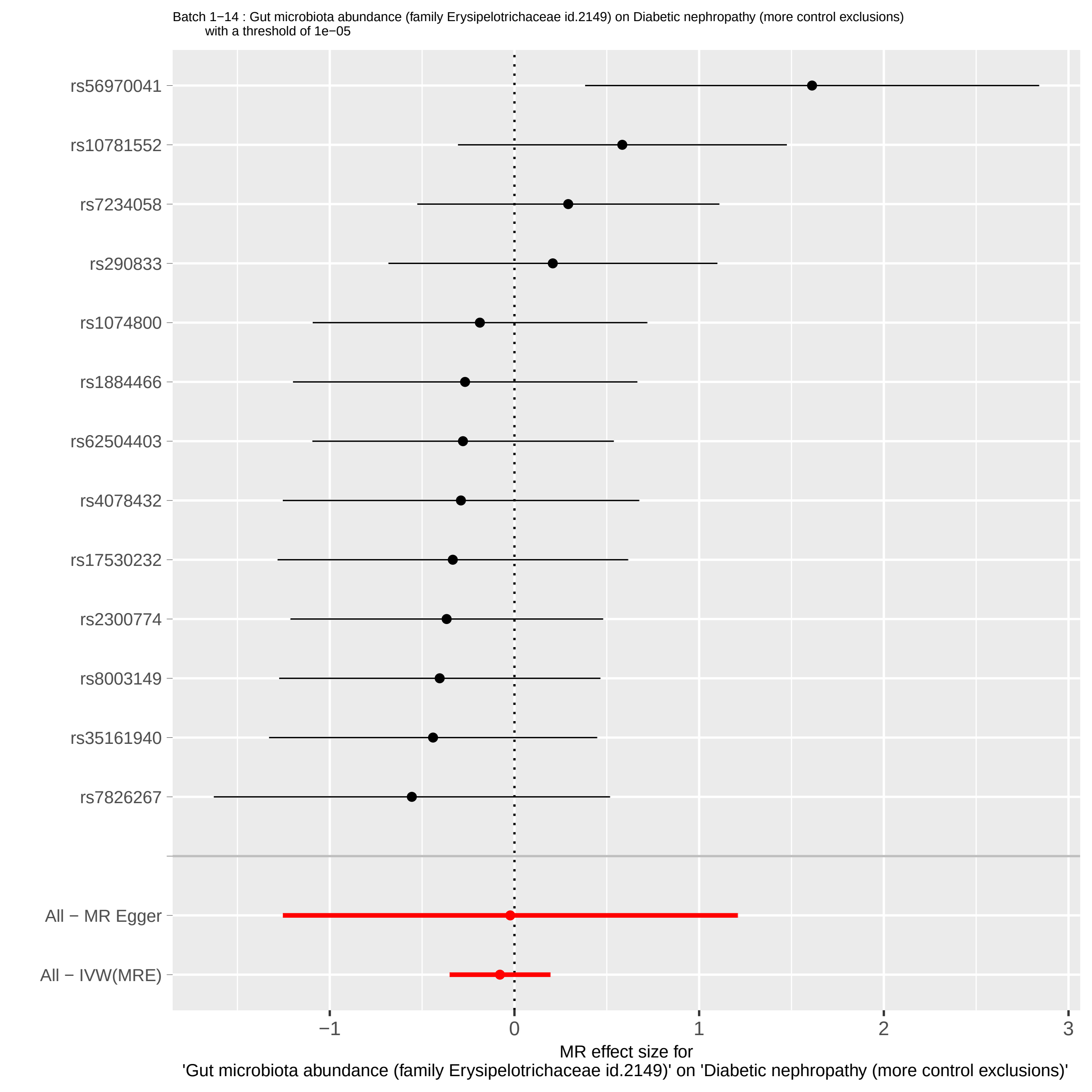
Batch 1−14 : Gut microbiota abundance (family Erysipelotrichaceae id.2149) on Diabetic nephropathy (more control exclusions)
with a threshold of 1e−05
rs56970041
rs10781552
rs7234058
rs290833
rs1074800
rs1884466
rs62504403
rs4078432
rs17530232
rs2300774
rs8003149
rs35161940
rs7826267
All − MR Egger
All − IVW(MRE)
−1
0
1
2
3
MR effect size for
'Gut microbiota abundance (family Erysipelotrichaceae id.2149)' on 'Diabetic nephropathy (more control exclusions)'

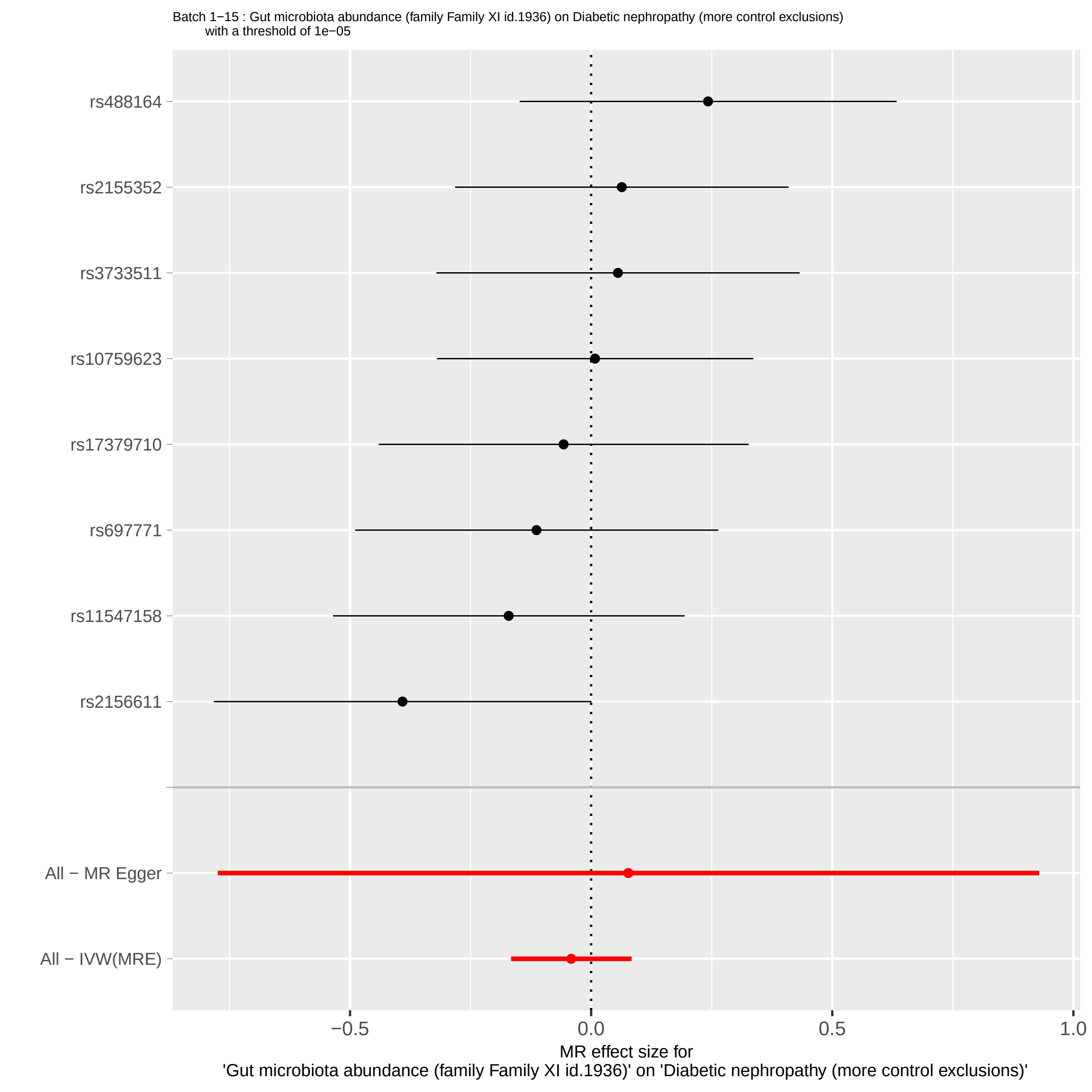

Batch 1−15 : Gut microbiota abundance (family Family XI id.1936) on Diabetic nephropathy (more control exclusions) with a threshold of 1e−05
rs488164
rs2155352
rs3733511
rs10759623
rs17379710
rs697771
rs11547158
rs2156611
All − MR Egger
All − IVW(MRE)
−0.5
0.0
0.5
1.0
MR effect size for
'Gut microbiota abundance (family Family XI id.1936)' on 'Diabetic nephropathy (more control exclusions)'

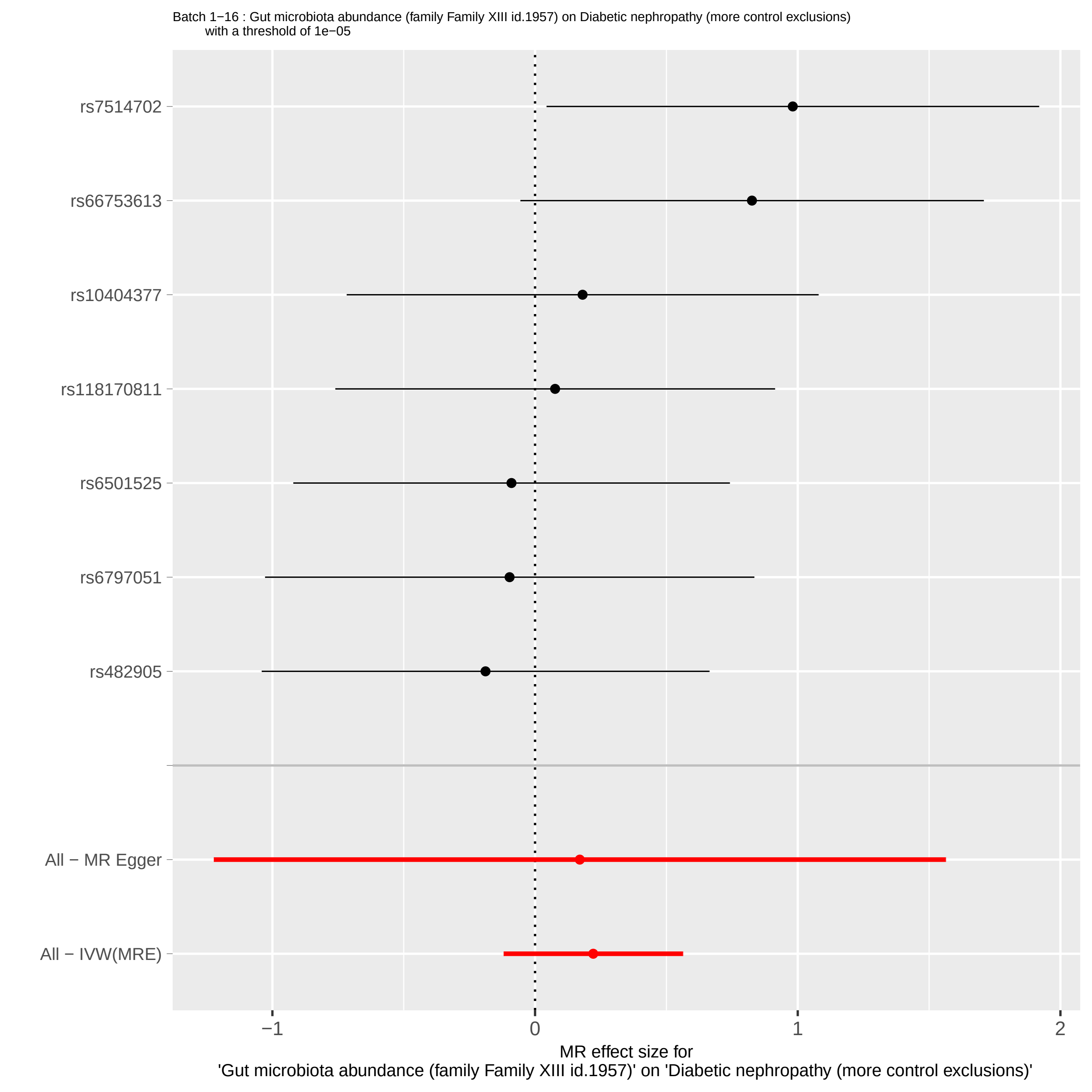

Batch 1−16 : Gut microbiota abundance (family Family XIII id.1957) on Diabetic nephropathy (more control exclusions)
with a threshold of 1e−05
rs7514702
rs66753613
rs10404377
rs118170811
rs6501525
rs6797051
rs482905
All − MR Egger
All − IVW(MRE)
−1
0
1
2
MR effect size for
'Gut microbiota abundance (family Family XIII id.1957)' on 'Diabetic nephropathy (more control exclusions)'

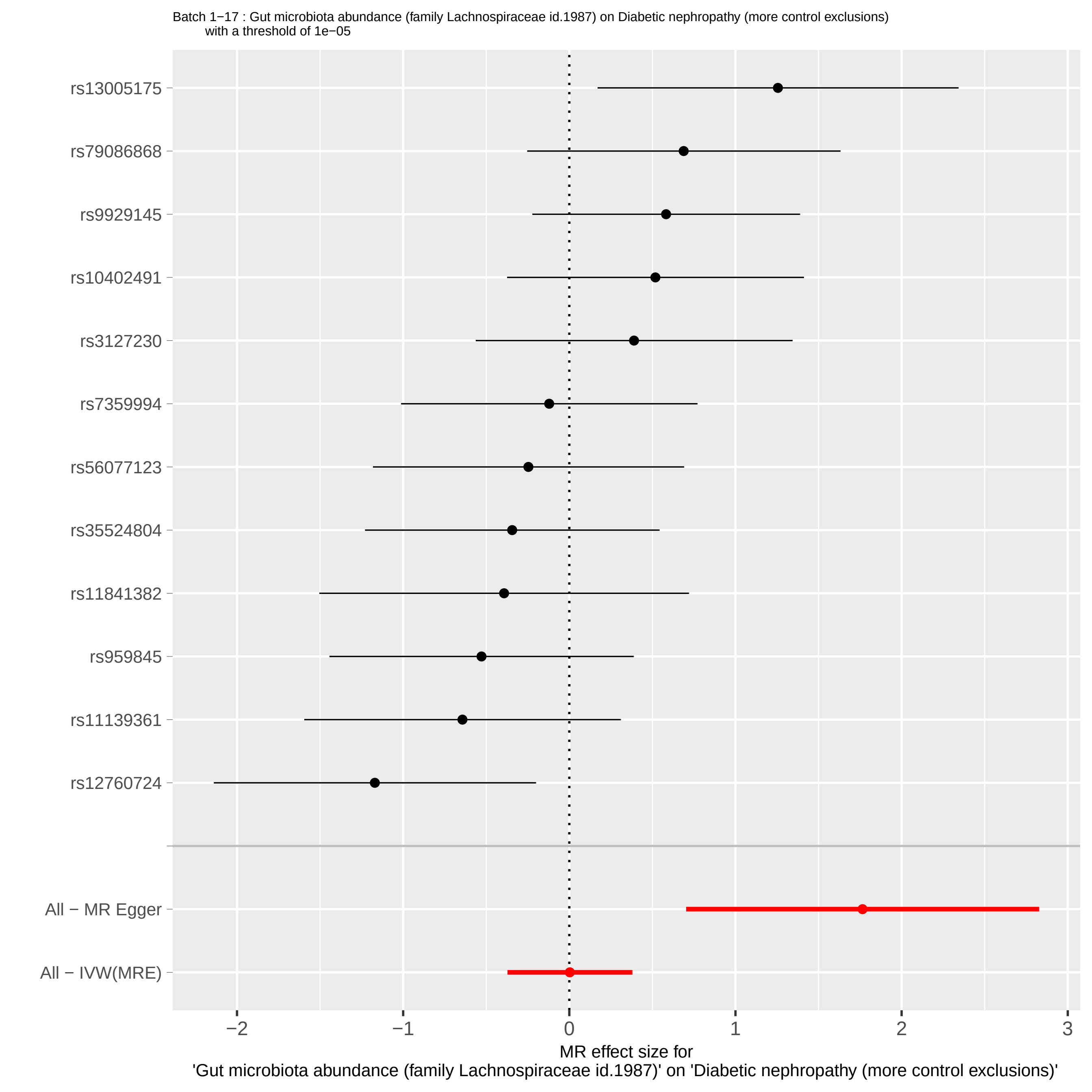
Batch 1−17 : Gut microbiota abundance (family Lachnospiraceae id.1987) on Diabetic nephropathy (more control exclusions) with a threshold of 1e−05
rs13005175
rs79086868
rs9929145
rs10402491
rs3127230
rs7359994
rs56077123
rs35524804
rs11841382
rs959845
rs11139361
rs12760724
All − MR Egger
All − IVW(MRE)
−2
−1
0
1
2
3
MR effect size for
'Gut microbiota abundance (family Lachnospiraceae id.1987)' on 'Diabetic nephropathy (more control exclusions)'

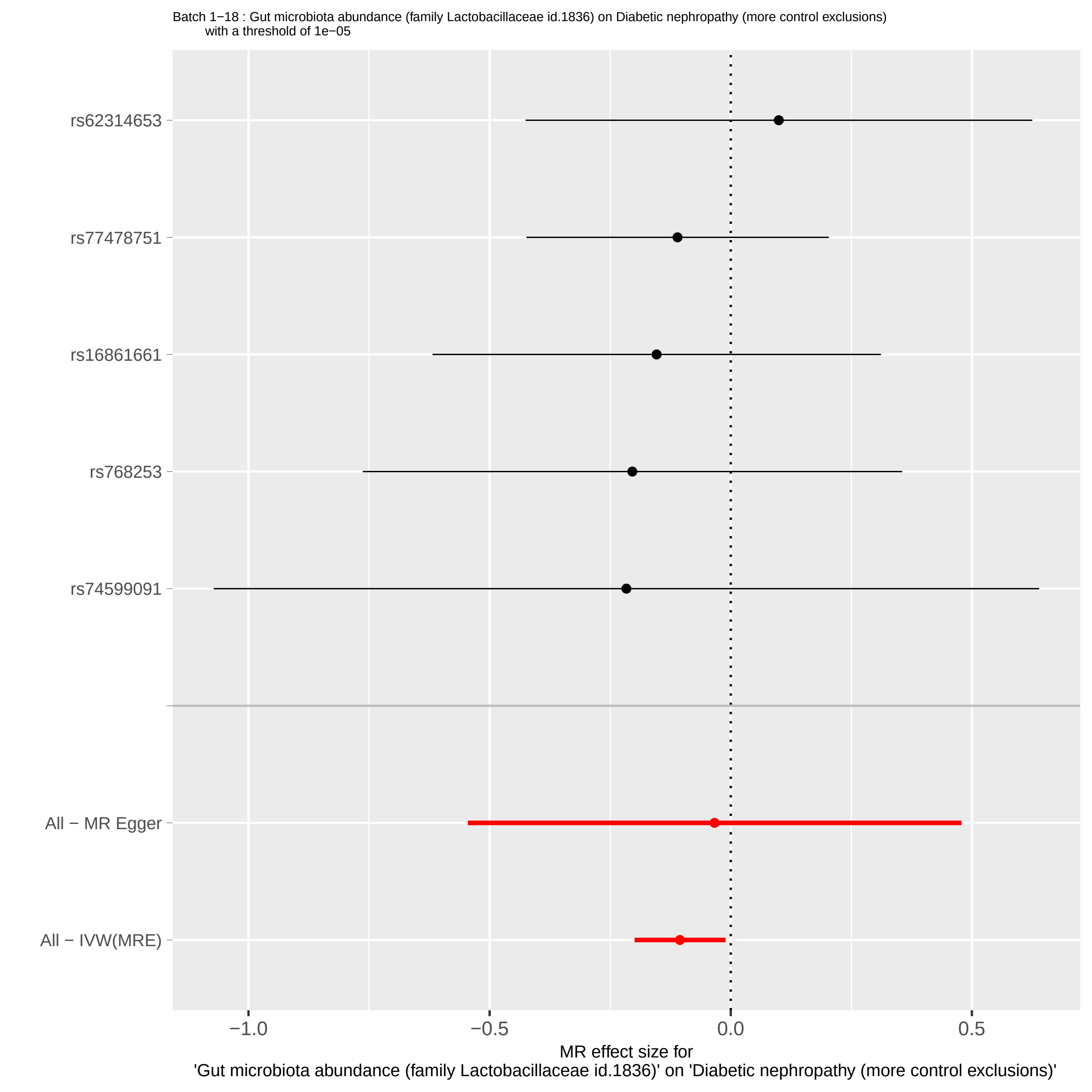

Batch 1−18 : Gut microbiota abundance (family Lactobacillaceae id.1836) on Diabetic nephropathy (more control exclusions)
with a threshold of 1e−05
rs62314653
rs77478751
rs16861661
rs768253
rs74599091
All − MR Egger
All − IVW(MRE)
−1.0
−0.5
0.0
0.5
MR effect size for
'Gut microbiota abundance (family Lactobacillaceae id.1836)' on 'Diabetic nephropathy (more control exclusions)'

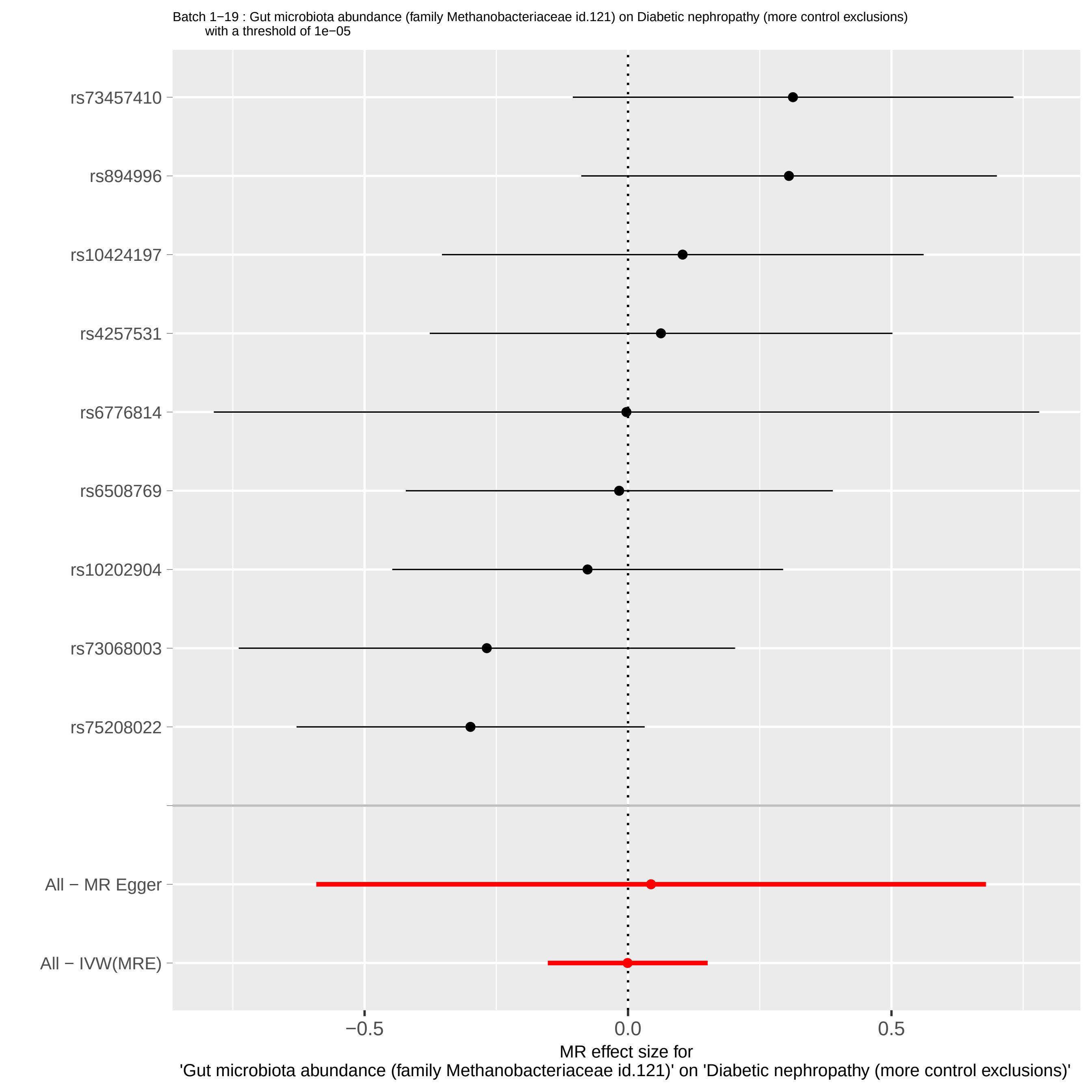
Batch 1−19 : Gut microbiota abundance (family Methanobacteriaceae id.121) on Diabetic nephropathy (more control exclusions) with a threshold of 1e−05
rs73457410
rs894996
rs10424197
rs4257531
rs6776814
rs6508769
rs10202904
rs73068003
rs75208022
All − MR Egger
All − IVW(MRE)
−0.5
0.0
0.5
MR effect size for
'Gut microbiota abundance (family Methanobacteriaceae id.121)' on 'Diabetic nephropathy (more control exclusions)'

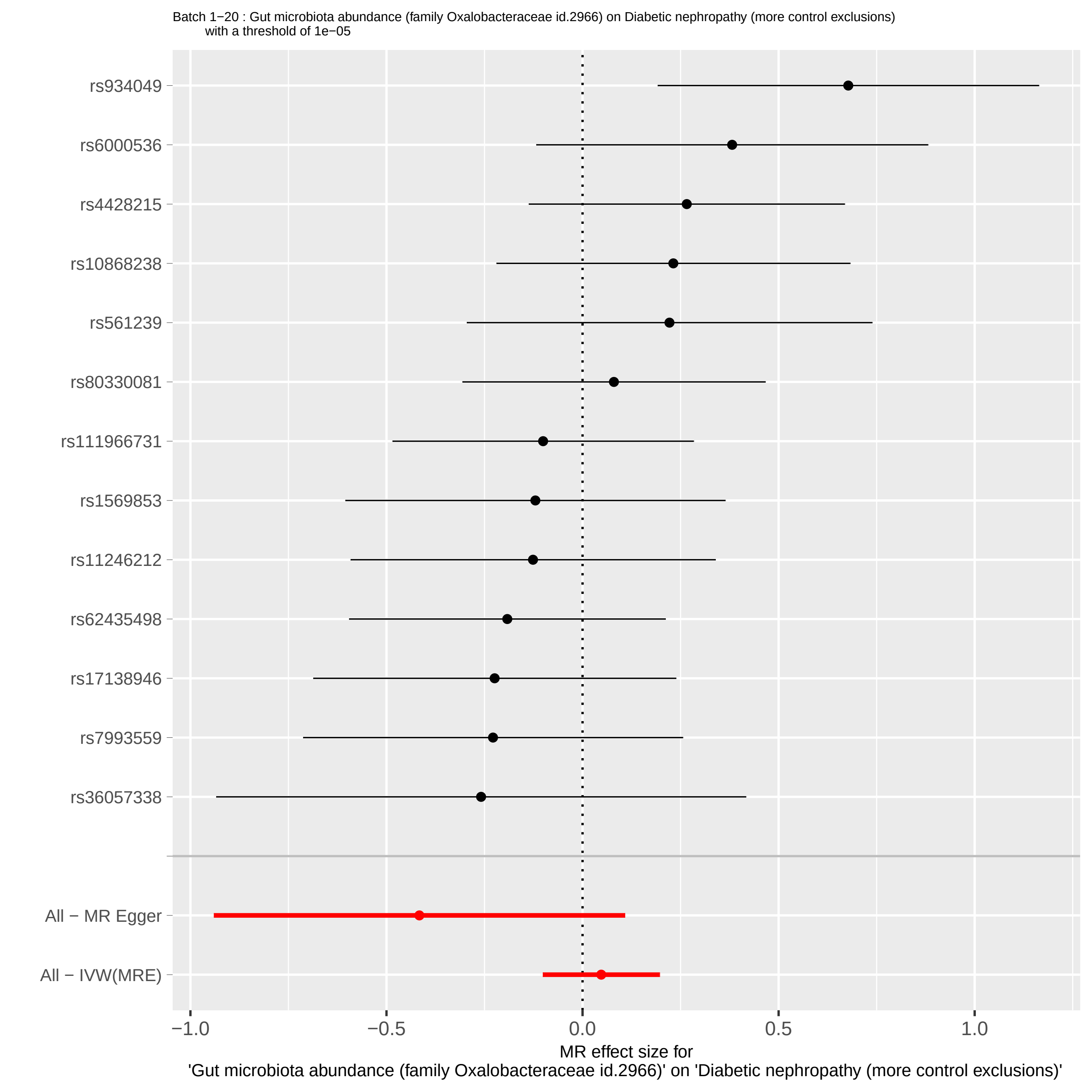

Batch 1−20 : Gut microbiota abundance (family Oxalobacteraceae id.2966) on Diabetic nephropathy (more control exclusions)
with a threshold of 1e−05
rs934049
rs6000536
rs4428215
rs10868238
rs561239
rs80330081
rs111966731
rs1569853
rs11246212
rs62435498
rs17138946
rs7993559
rs36057338
All − MR Egger
All − IVW(MRE)
−1.0
−0.5
0.0
0.5
1.0
MR effect size for
'Gut microbiota abundance (family Oxalobacteraceae id.2966)' on 'Diabetic nephropathy (more control exclusions)'

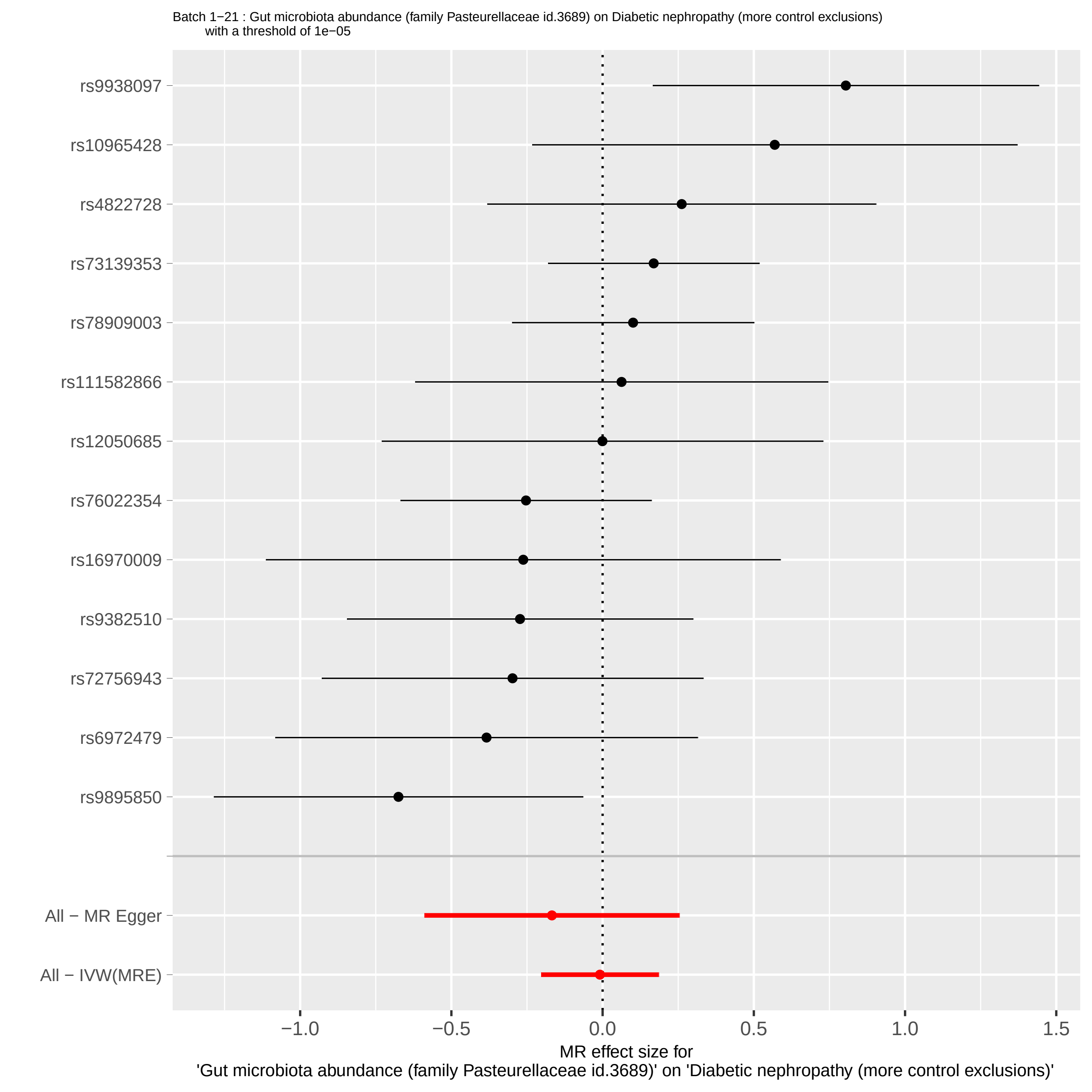
Batch 1−21 : Gut microbiota abundance (family Pasteurellaceae id.3689) on Diabetic nephropathy (more control exclusions) with a threshold of 1e−05
rs9938097
rs10965428
rs4822728
rs73139353
rs78909003
rs111582866
rs12050685
rs76022354
rs16970009
rs9382510
rs72756943
rs6972479
rs9895850
All − MR Egger
All − IVW(MRE)
−1.0
−0.5
0.0
0.5
1.0
1.5
MR effect size for
'Gut microbiota abundance (family Pasteurellaceae id.3689)' on 'Diabetic nephropathy (more control exclusions)'

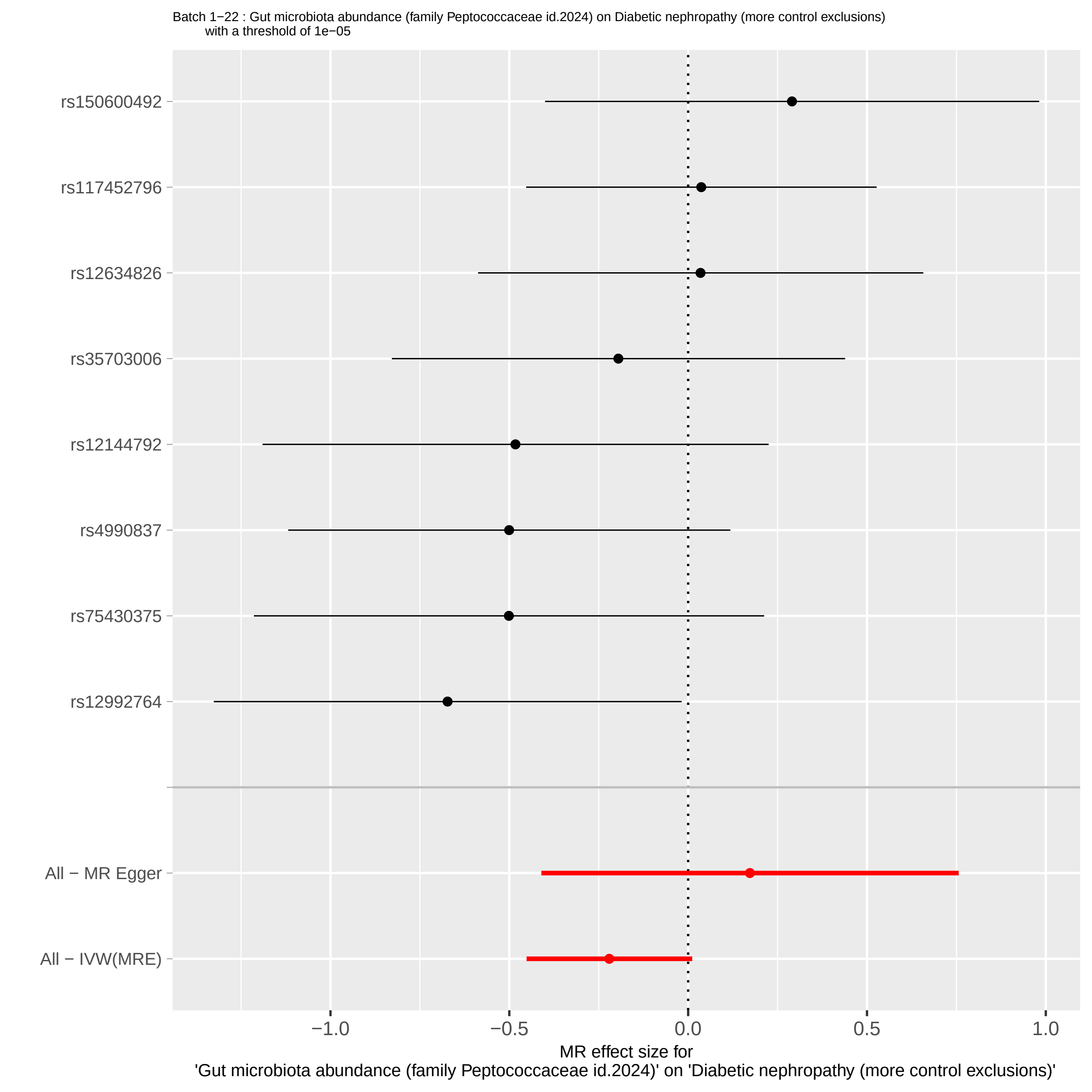

Batch 1−22 : Gut microbiota abundance (family Peptococcaceae id.2024) on Diabetic nephropathy (more control exclusions) with a threshold of 1e−05
rs150600492
rs117452796
rs12634826
rs35703006
rs12144792
rs4990837
rs75430375
rs12992764
All − MR Egger
All − IVW(MRE)
−1.0
−0.5
0.0
0.5
1.0
MR effect size for
'Gut microbiota abundance (family Peptococcaceae id.2024)' on 'Diabetic nephropathy (more control exclusions)'

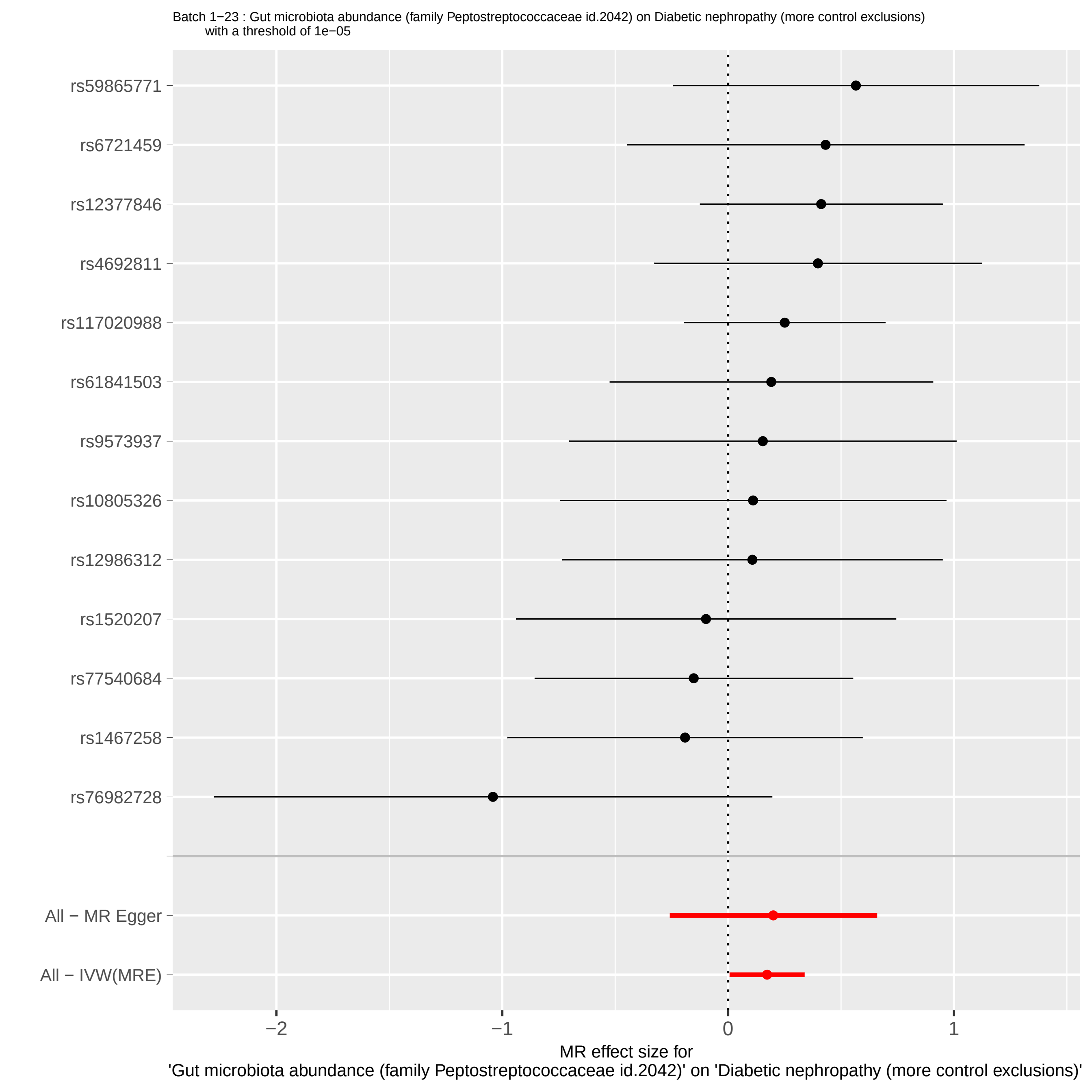

Batch 1−23 : Gut microbiota abundance (family Peptostreptococcaceae id.2042) on Diabetic nephropathy (more control exclusions)
with a threshold of 1e−05
rs59865771
rs6721459
rs12377846
rs4692811
rs117020988
rs61841503
rs9573937
rs10805326
rs12986312
rs1520207
rs77540684
rs1467258
rs76982728
All − MR Egger
All − IVW(MRE)
−2
−1
0
1
MR effect size for
'Gut microbiota abundance (family Peptostreptococcaceae id.2042)' on 'Diabetic nephropathy (more control exclusions)'

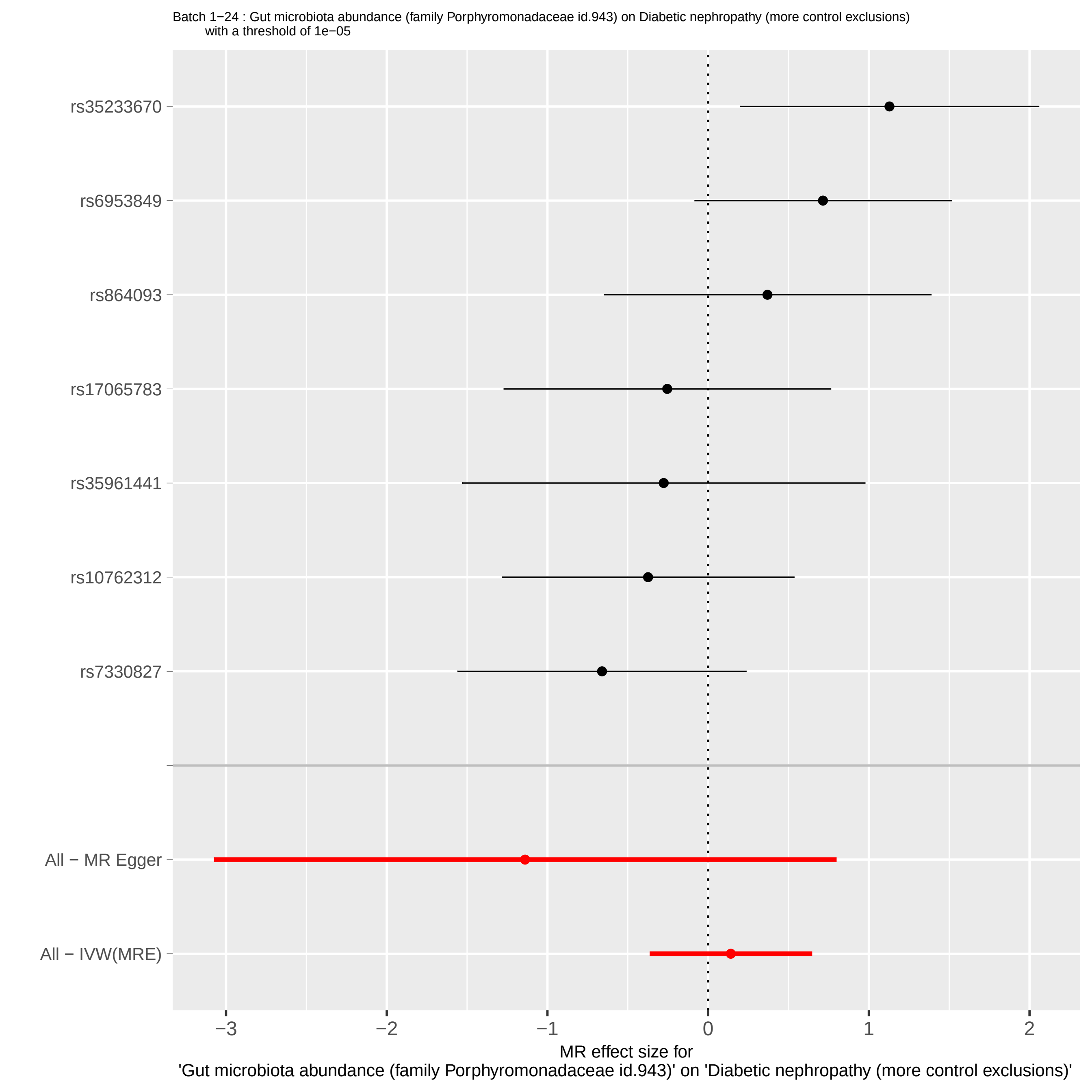

Batch 1−24 : Gut microbiota abundance (family Porphyromonadaceae id.943) on Diabetic nephropathy (more control exclusions) with a threshold of 1e−05
rs35233670
rs6953849
rs864093
rs17065783
rs35961441
rs10762312
rs7330827
All − MR Egger
All − IVW(MRE)
−3
−2
−1
0
1
2
MR effect size for
'Gut microbiota abundance (family Porphyromonadaceae id.943)' on 'Diabetic nephropathy (more control exclusions)'

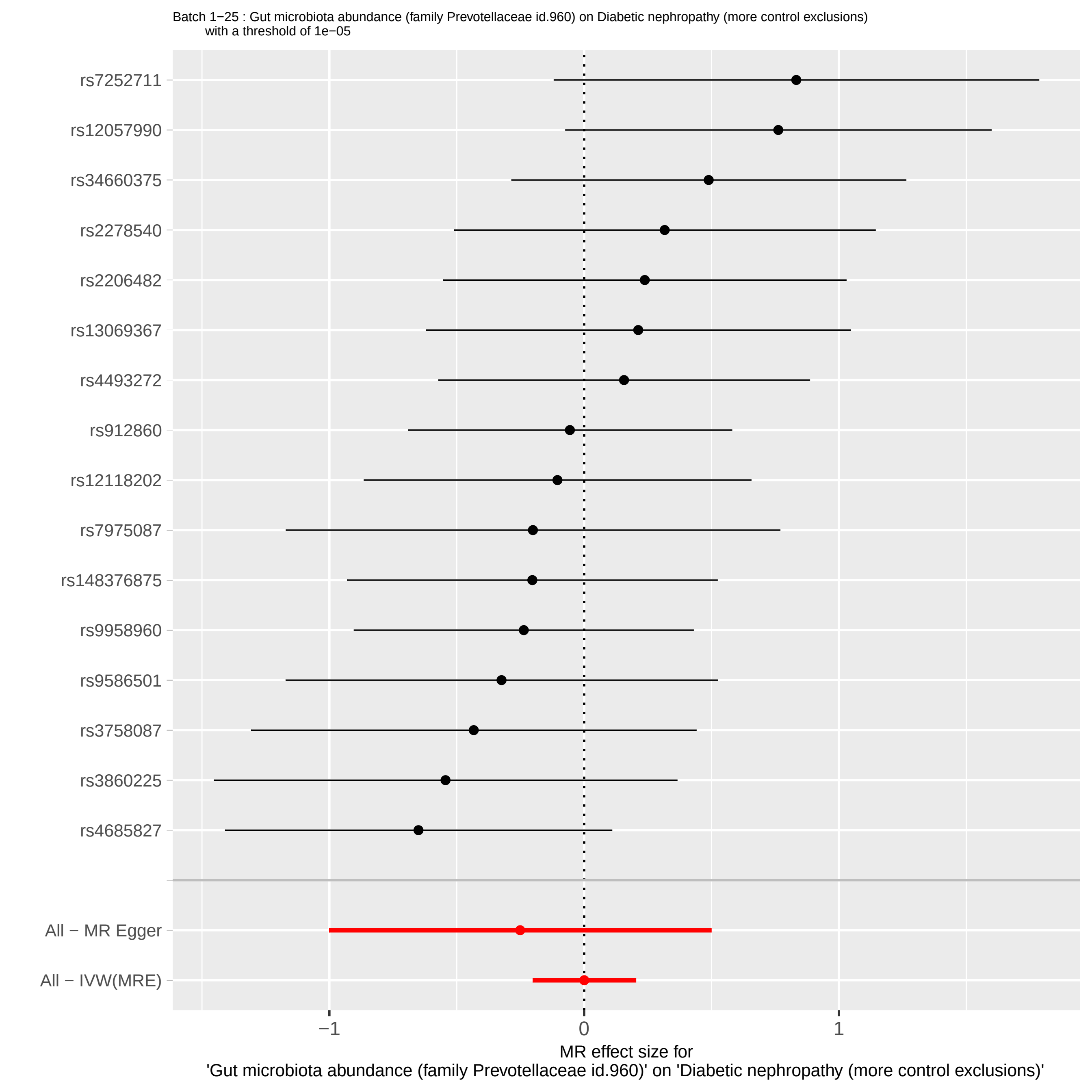
Batch 1−25 : Gut microbiota abundance (family Prevotellaceae id.960) on Diabetic nephropathy (more control exclusions)
with a threshold of 1e−05
rs7252711
rs12057990
rs34660375
rs2278540
rs2206482
rs13069367
rs4493272
rs912860
rs12118202
rs7975087
rs148376875
rs9958960
rs9586501
rs3758087
rs3860225
rs4685827
All − MR Egger
All − IVW(MRE)
−1
0
1
MR effect size for
'Gut microbiota abundance (family Prevotellaceae id.960)' on 'Diabetic nephropathy (more control exclusions)'

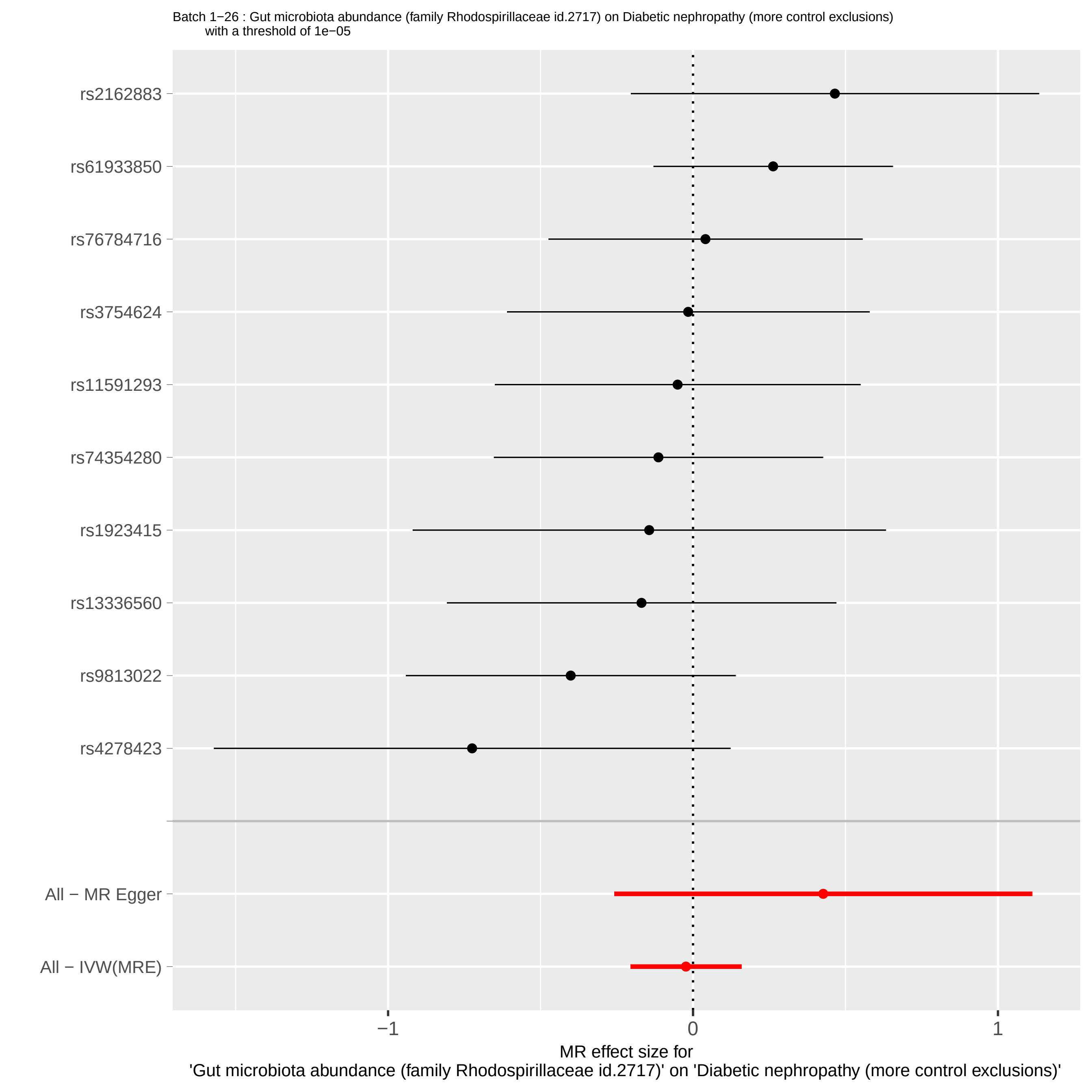
Batch 1−26 : Gut microbiota abundance (family Rhodospirillaceae id.2717) on Diabetic nephropathy (more control exclusions)
with a threshold of 1e−05
rs2162883
rs61933850
rs76784716
rs3754624
rs11591293
rs74354280
rs1923415
rs13336560
rs9813022
rs4278423
All − MR Egger
All − IVW(MRE)
−1
0
1
MR effect size for
'Gut microbiota abundance (family Rhodospirillaceae id.2717)' on 'Diabetic nephropathy (more control exclusions)'

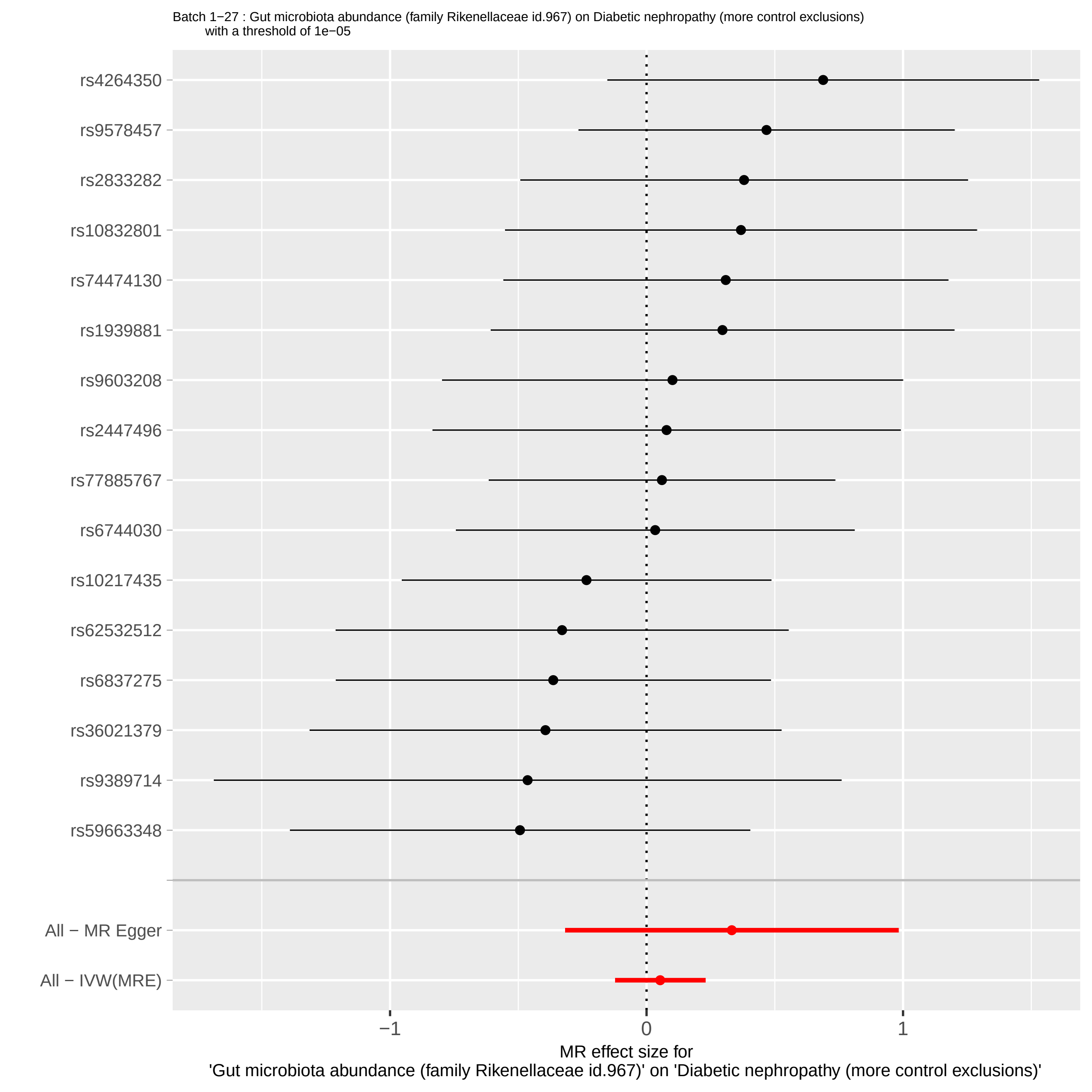

Batch 1−27 : Gut microbiota abundance (family Rikenellaceae id.967) on Diabetic nephropathy (more control exclusions)
with a threshold of 1e−05
rs4264350
rs9578457
rs2833282
rs10832801
rs74474130
rs1939881
rs9603208
rs2447496
rs77885767
rs6744030
rs10217435
rs62532512
rs6837275
rs36021379
rs9389714
rs59663348
All − MR Egger
All − IVW(MRE)
−1
0
1
MR effect size for
'Gut microbiota abundance (family Rikenellaceae id.967)' on 'Diabetic nephropathy (more control exclusions)'

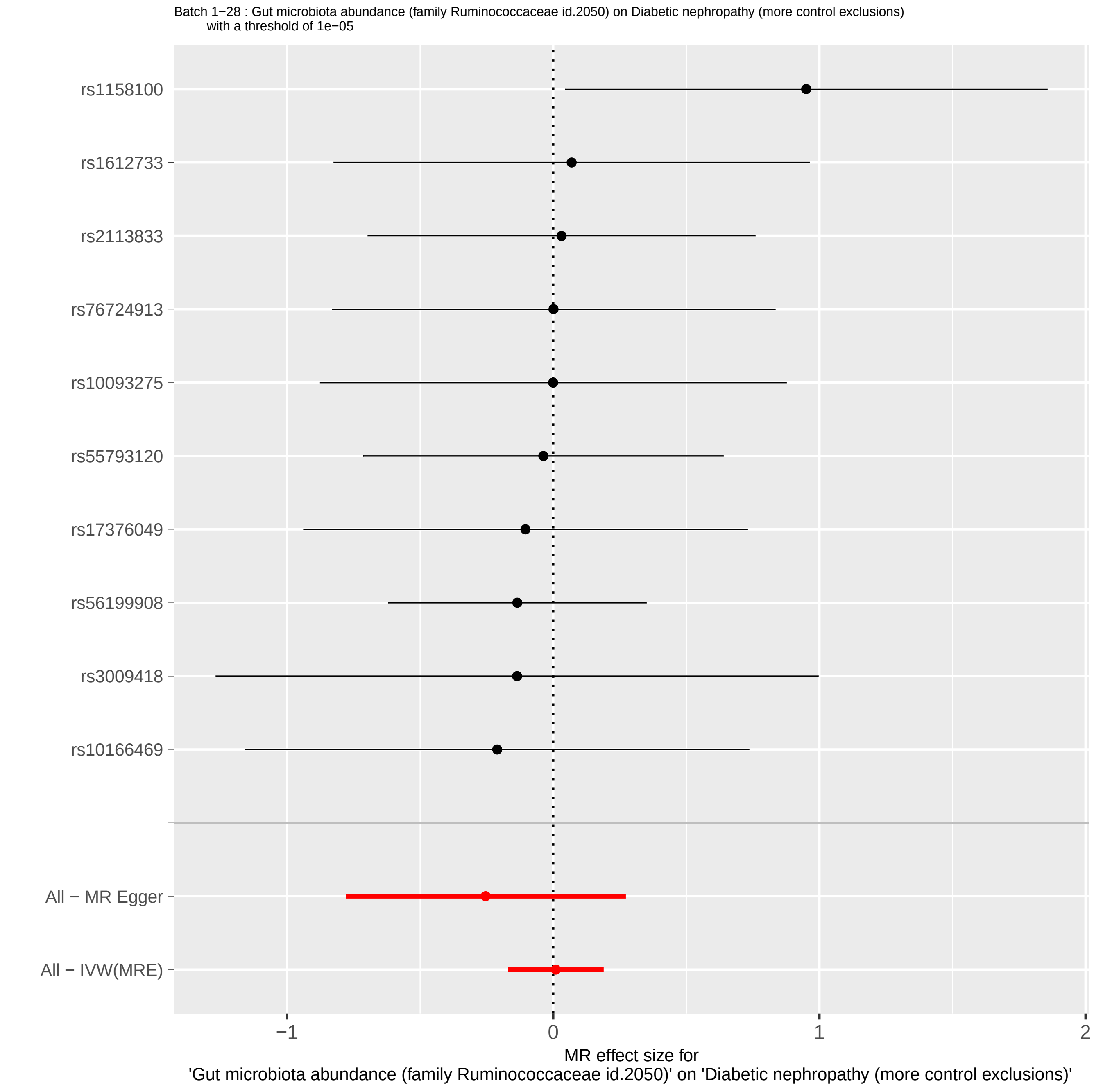

Batch 1−28 : Gut microbiota abundance (family Ruminococcaceae id.2050) on Diabetic nephropathy (more control exclusions)
with a threshold of 1e−05
rs1158100
rs1612733
rs2113833
rs76724913
rs10093275
rs55793120
rs17376049
rs56199908
rs3009418
rs10166469
All − MR Egger
All − IVW(MRE)
−1
0
1
2
MR effect size for
'Gut microbiota abundance (family Ruminococcaceae id.2050)' on 'Diabetic nephropathy (more control exclusions)'

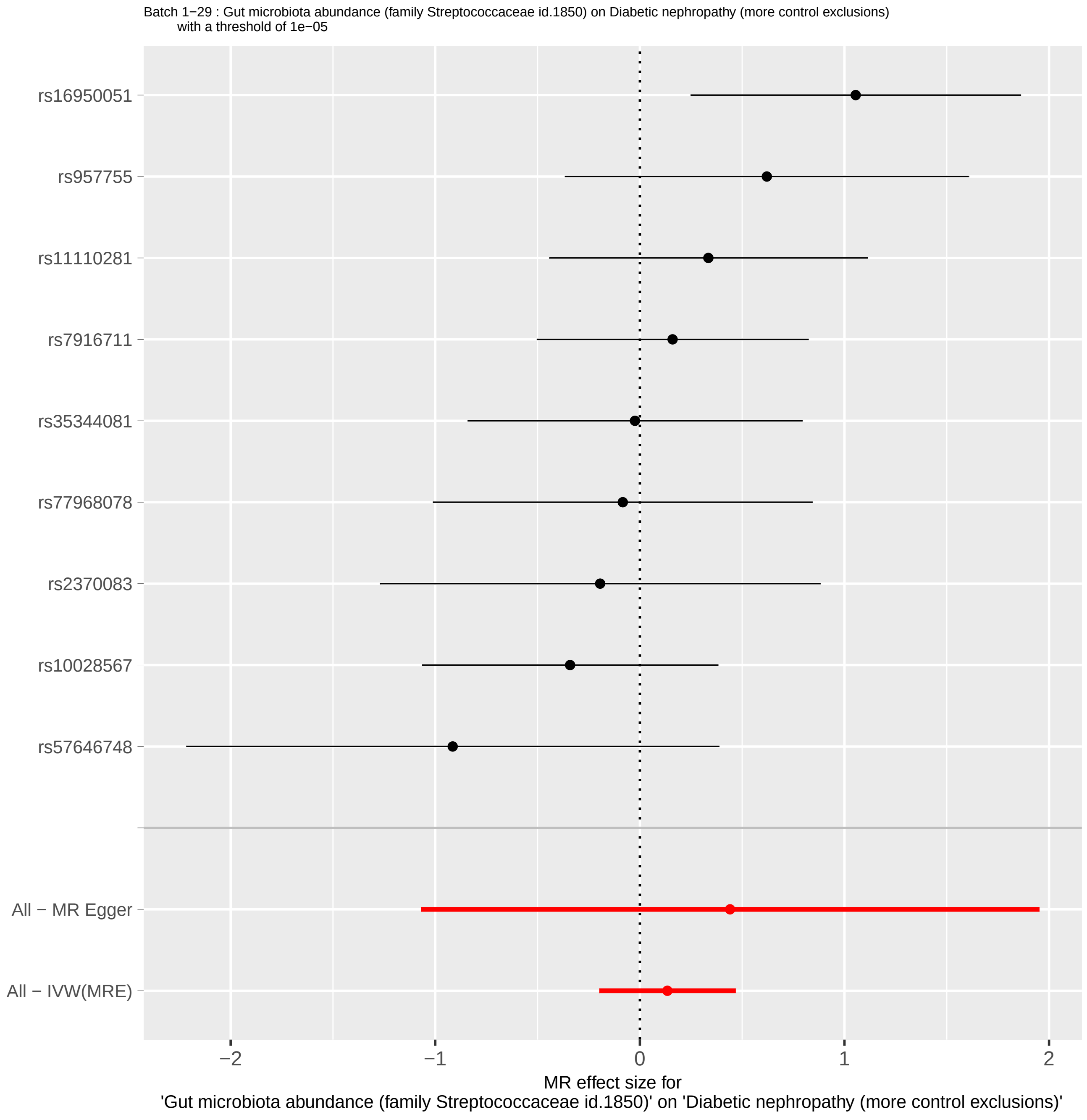

Batch 1−29 : Gut microbiota abundance (family Streptococcaceae id.1850) on Diabetic nephropathy (more control exclusions)
with a threshold of 1e−05
rs16950051
rs957755
rs11110281
rs7916711
rs35344081
rs77968078
rs2370083
rs10028567
rs57646748
All − MR Egger
All − IVW(MRE)
−2
−1
0
1
2
MR effect size for
'Gut microbiota abundance (family Streptococcaceae id.1850)' on 'Diabetic nephropathy (more control exclusions)'

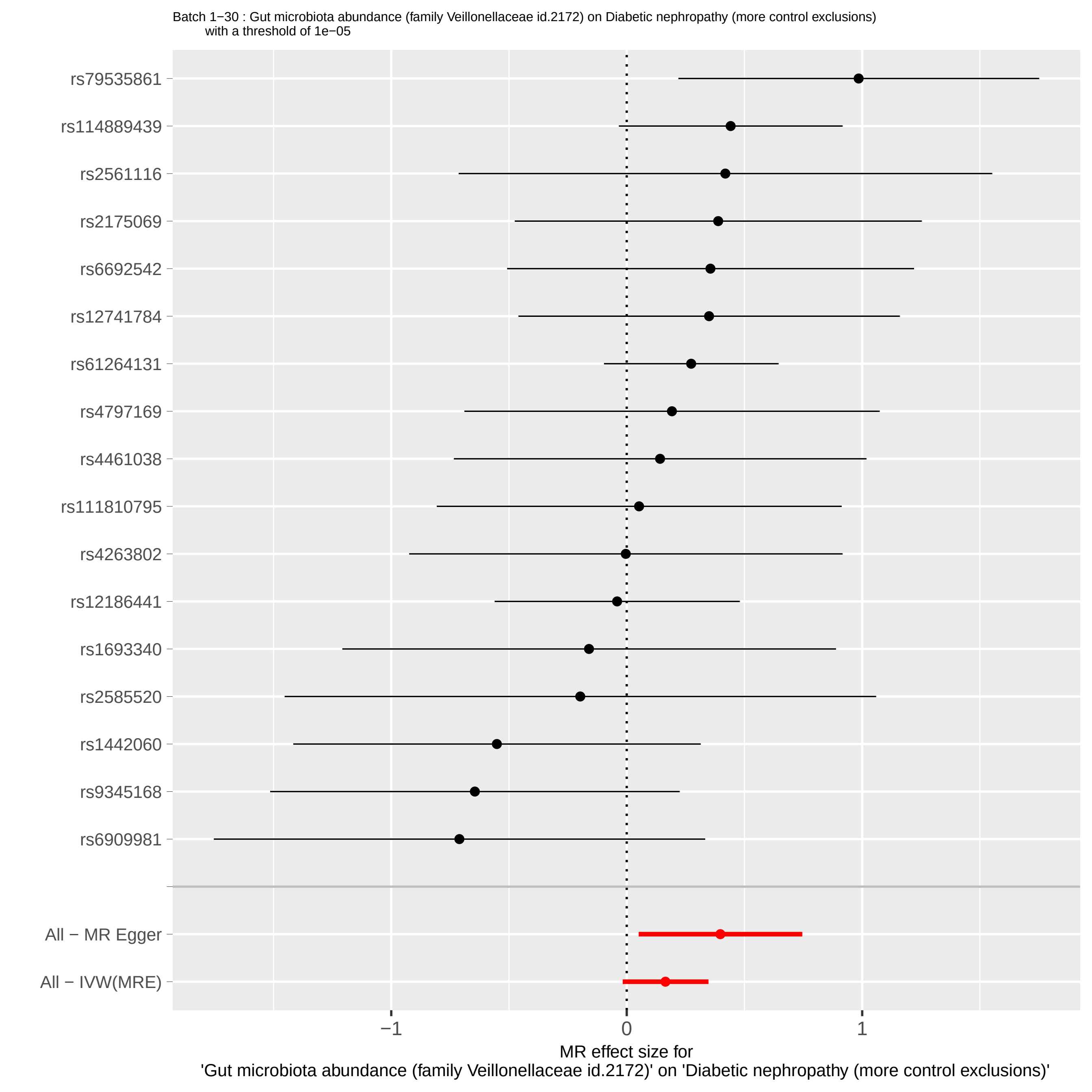

Batch 1−30 : Gut microbiota abundance (family Veillonellaceae id.2172) on Diabetic nephropathy (more control exclusions)
with a threshold of 1e−05
rs79535861
rs114889439
rs2561116
rs2175069
rs6692542
rs12741784
rs61264131
rs4797169
rs4461038
rs111810795
rs4263802
rs12186441
rs1693340
rs2585520
rs1442060
rs9345168
rs6909981
All − MR Egger
All − IVW(MRE)
−1
0
1
MR effect size for
'Gut microbiota abundance (family Veillonellaceae id.2172)' on 'Diabetic nephropathy (more control exclusions)'

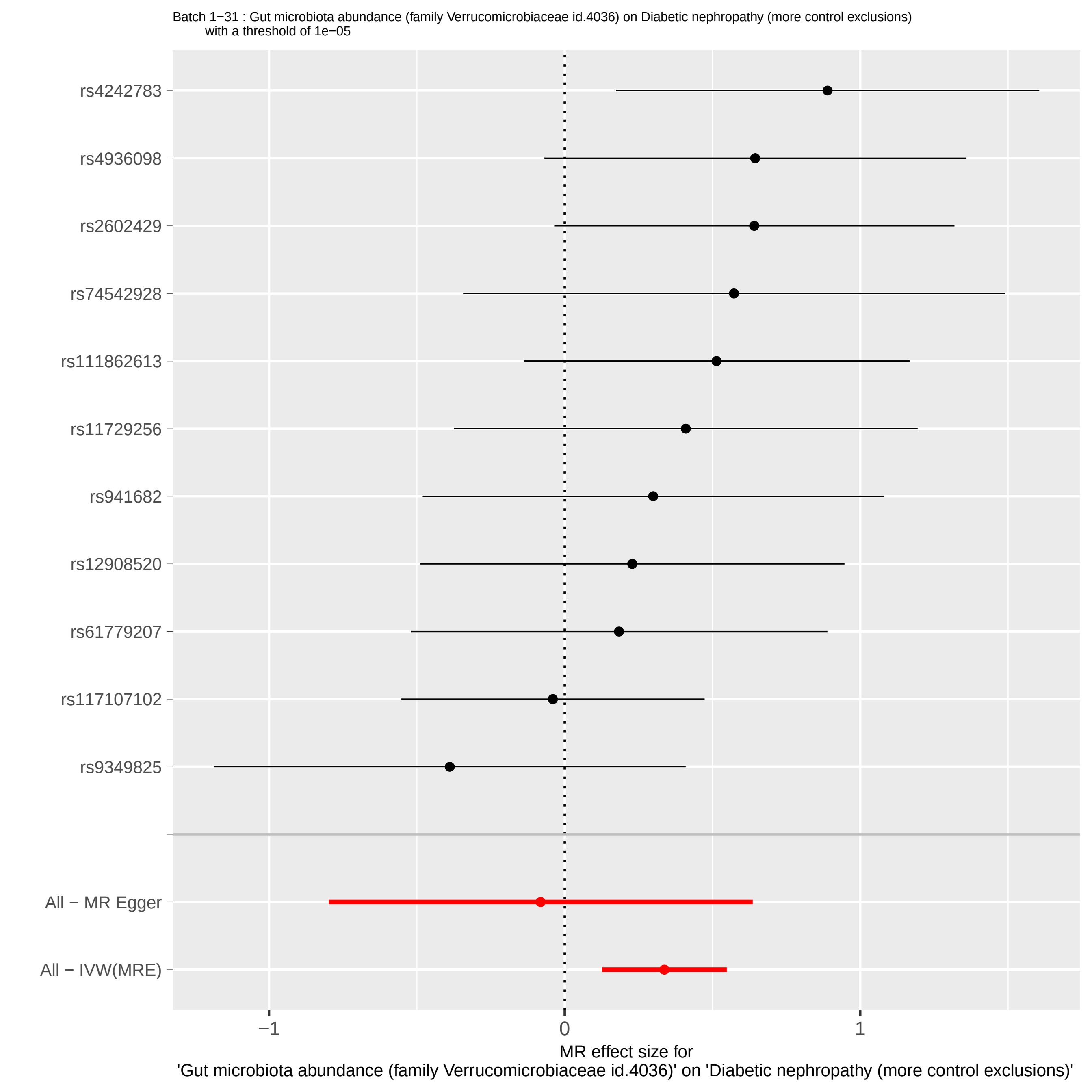
Batch 1−31 : Gut microbiota abundance (family Verrucomicrobiaceae id.4036) on Diabetic nephropathy (more control exclusions) with a threshold of 1e−05
rs4242783
rs4936098
rs2602429
rs74542928
rs111862613
rs11729256
rs941682
rs12908520
rs61779207
rs117107102
rs9349825
All − MR Egger
All − IVW(MRE)
−1
0
1
MR effect size for
'Gut microbiota abundance (family Verrucomicrobiaceae id.4036)' on 'Diabetic nephropathy (more control exclusions)'

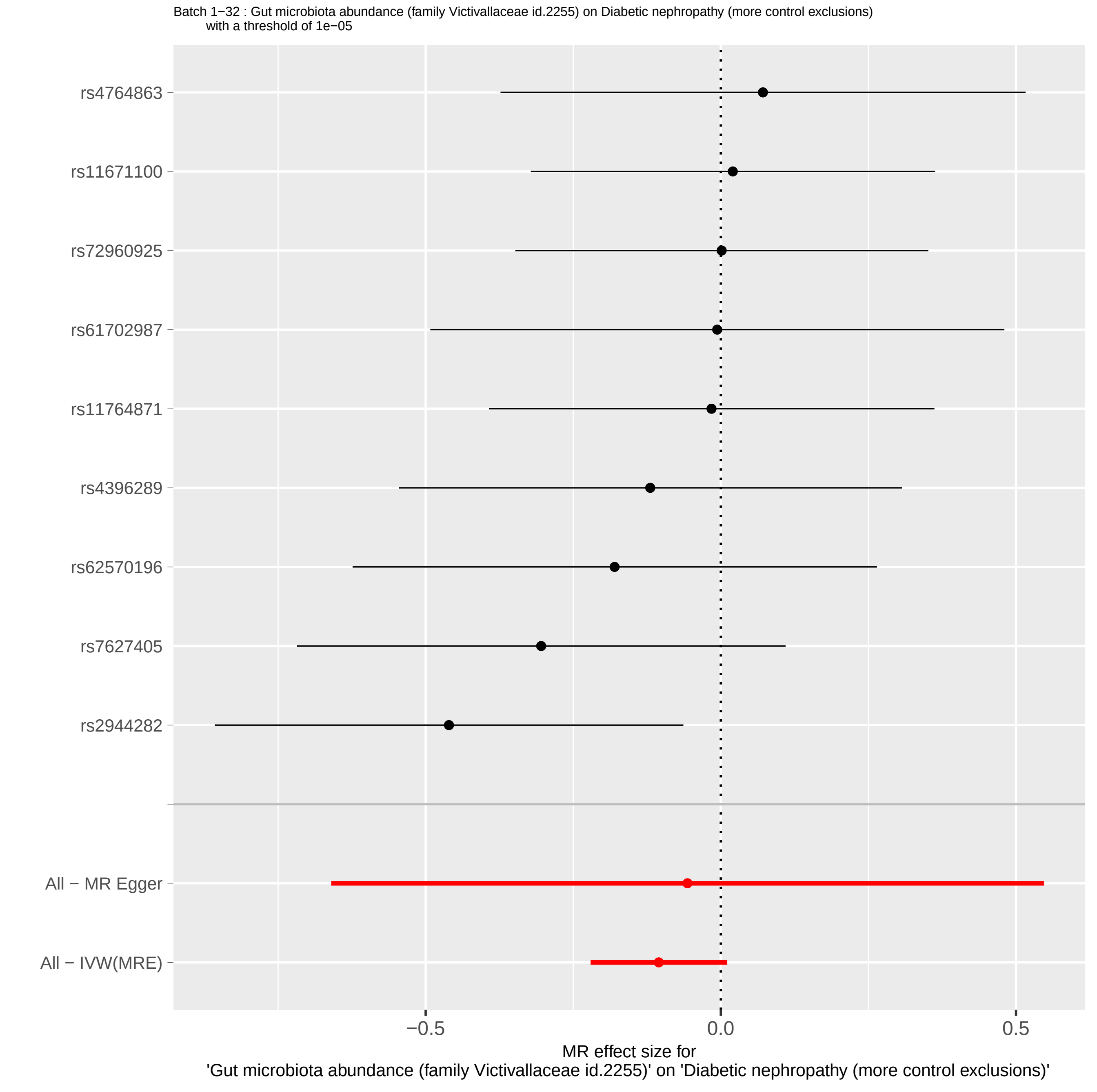

Batch 1−32 : Gut microbiota abundance (family Victivallaceae id.2255) on Diabetic nephropathy (more control exclusions) with a threshold of 1e−05
rs4764863
rs11671100
rs72960925
rs61702987
rs11764871
rs4396289
rs62570196
rs7627405
rs2944282
All − MR Egger
All − IVW(MRE)
−0.5
0.0
0.5
MR effect size for 'Gut microbiota abundance (family Victivallaceae id.2255)' on 'Diabetic nephropathy (more control exclusions)'

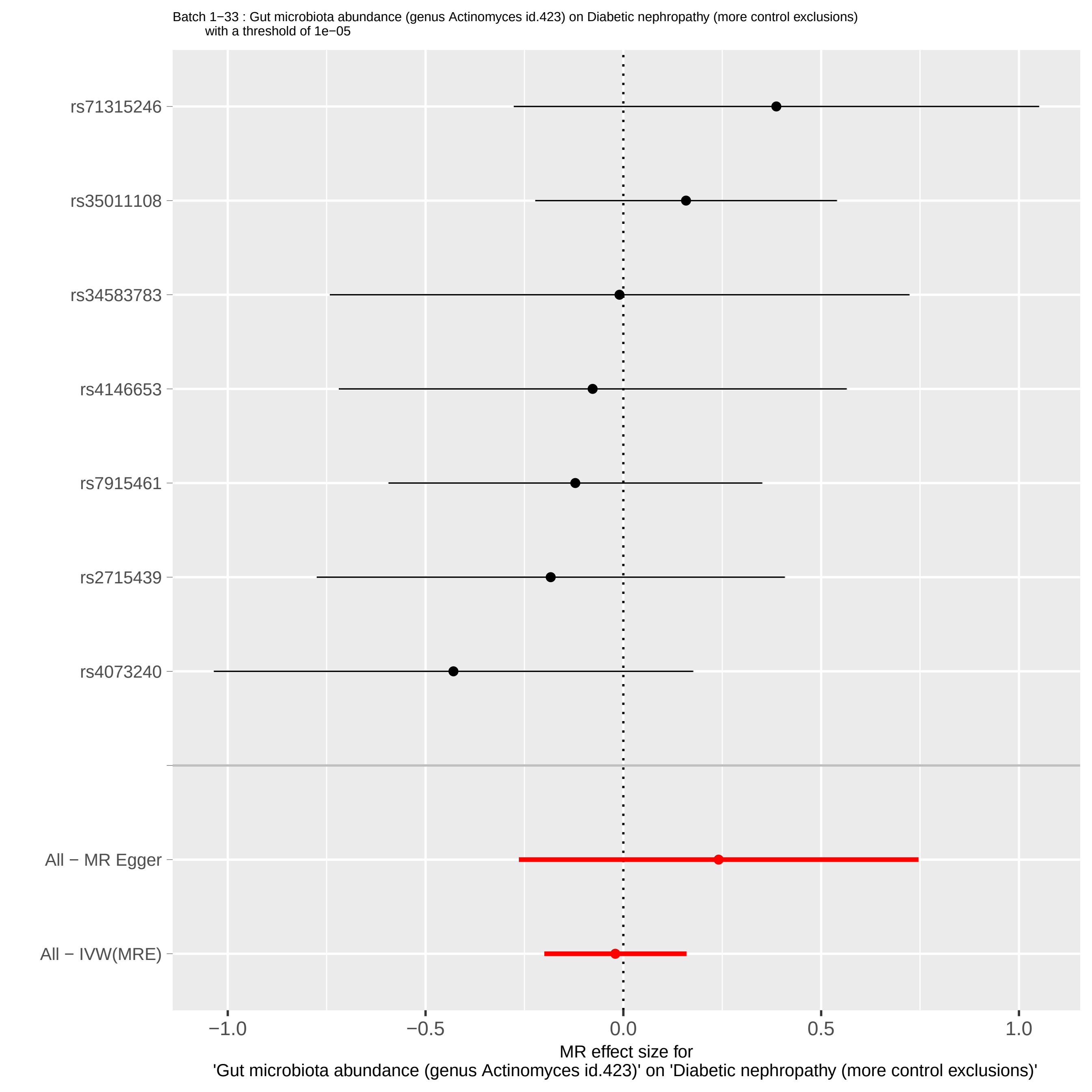

Batch 1−33 : Gut microbiota abundance (genus Actinomyces id.423) on Diabetic nephropathy (more control exclusions)
with a threshold of 1e−05
rs71315246
rs35011108
rs34583783
rs4146653
rs7915461
rs2715439
rs4073240
All − MR Egger
All − IVW(MRE)
−1.0
−0.5
0.0
0.5
1.0
MR effect size for
'Gut microbiota abundance (genus Actinomyces id.423)' on 'Diabetic nephropathy (more control exclusions)'

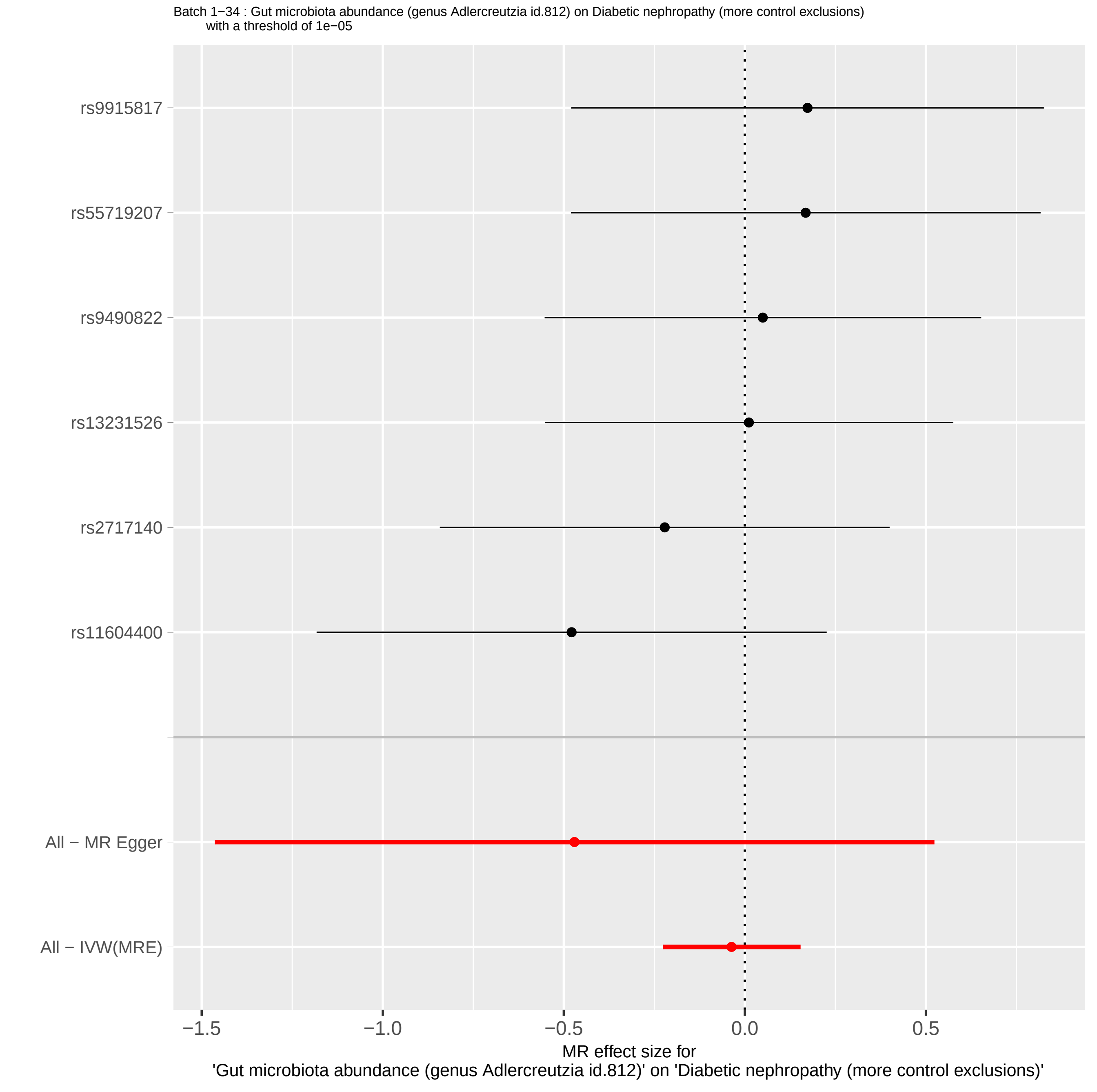

Batch 1−34 : Gut microbiota abundance (genus Adlercreutzia id.812) on Diabetic nephropathy (more control exclusions)
with a threshold of 1e−05
rs9915817
rs55719207
rs9490822
rs13231526
rs2717140
rs11604400
All − MR Egger
All − IVW(MRE)
−1.5
−1.0
−0.5
0.0
0.5
MR effect size for
'Gut microbiota abundance (genus Adlercreutzia id.812)' on 'Diabetic nephropathy (more control exclusions)'

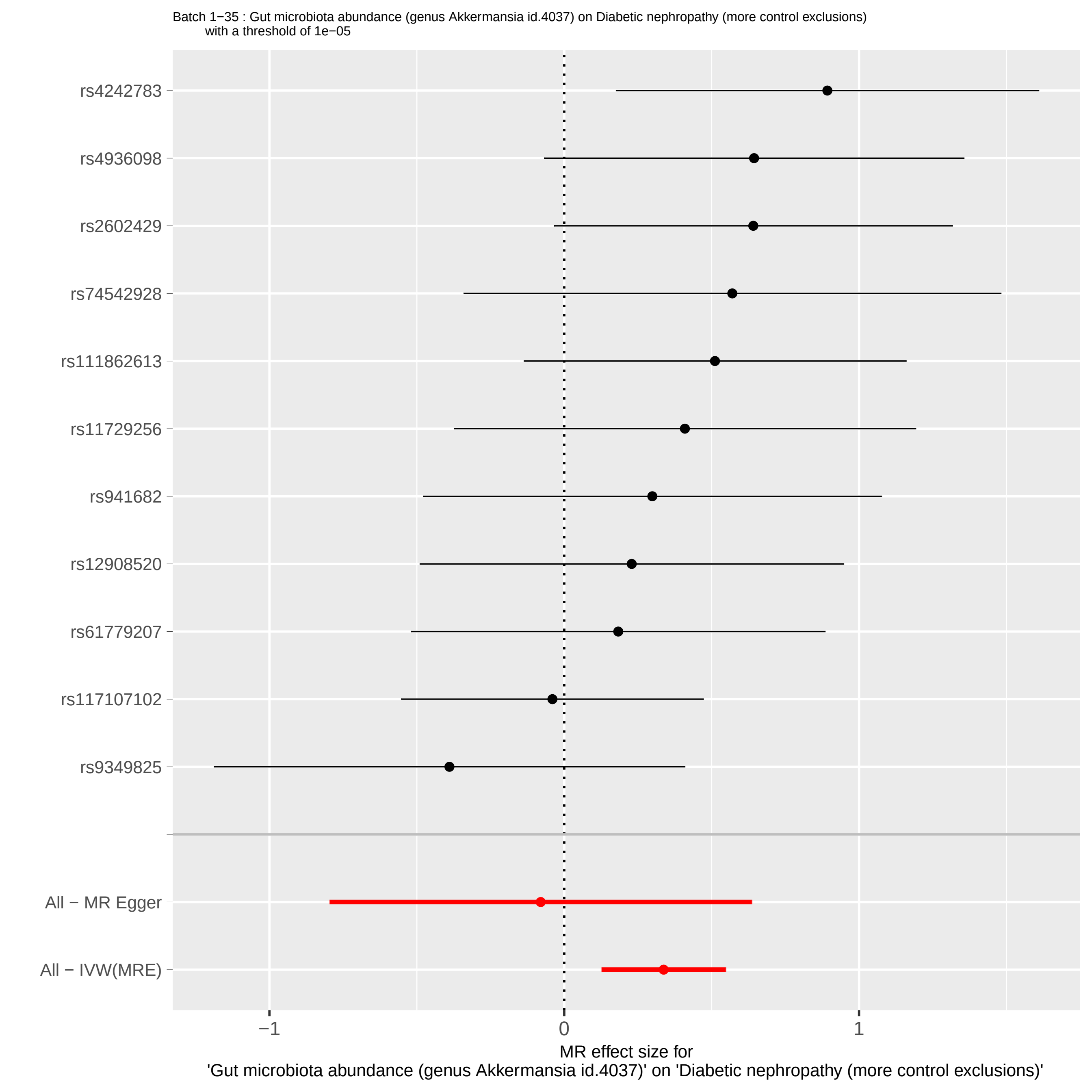

Batch 1−35 : Gut microbiota abundance (genus Akkermansia id.4037) on Diabetic nephropathy (more control exclusions)
with a threshold of 1e−05
rs4242783
rs4936098
rs2602429
rs74542928
rs111862613
rs11729256
rs941682
rs12908520
rs61779207
rs117107102
rs9349825
All − MR Egger
All − IVW(MRE)
−1
0
1
MR effect size for
'Gut microbiota abundance (genus Akkermansia id.4037)' on 'Diabetic nephropathy (more control exclusions)'

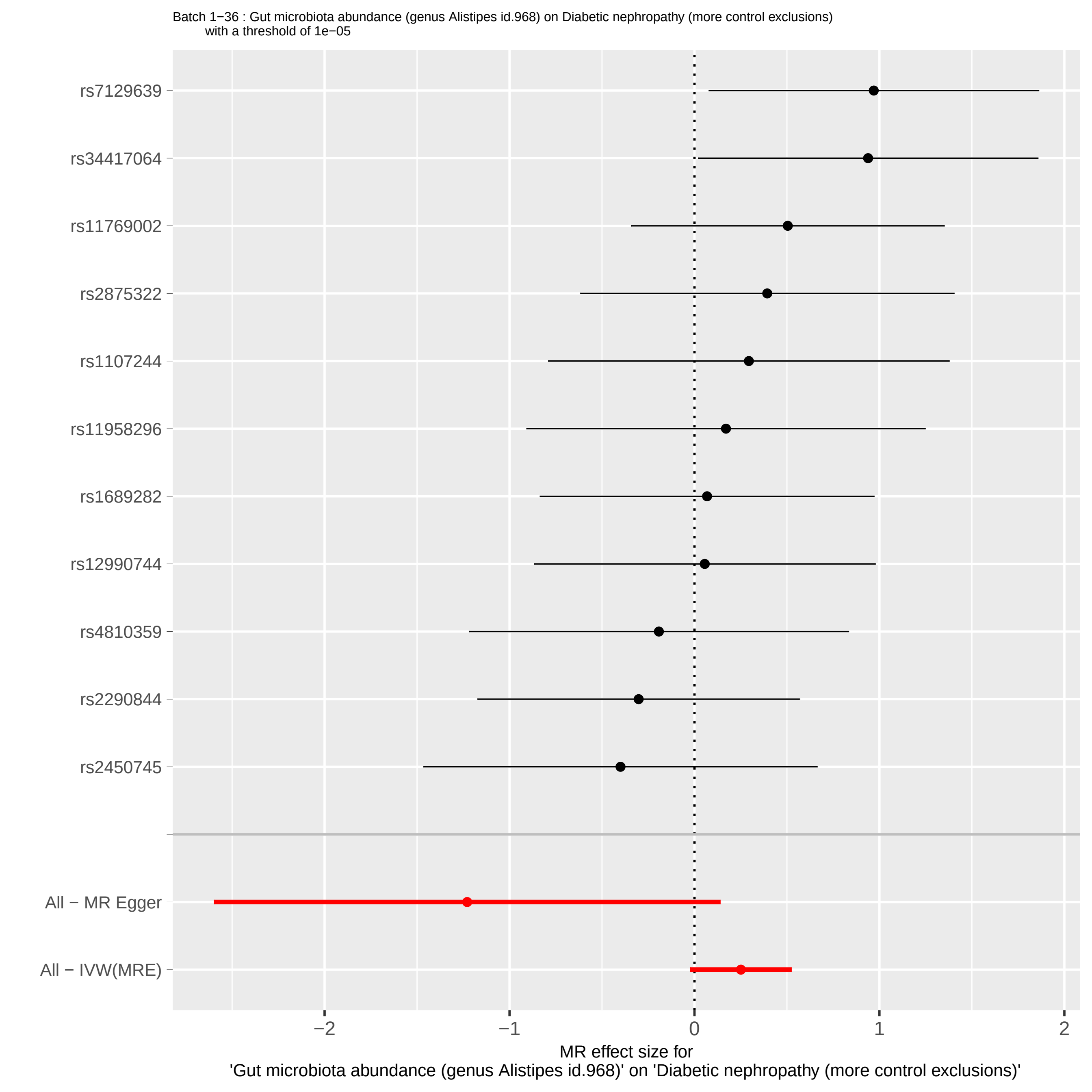

Batch 1−36 : Gut microbiota abundance (genus Alistipes id.968) on Diabetic nephropathy (more control exclusions)
with a threshold of 1e−05
rs7129639
rs34417064
rs11769002
rs2875322
rs1107244
rs11958296
rs1689282
rs12990744
rs4810359
rs2290844
rs2450745
All − MR Egger
All − IVW(MRE)
−2
−1
0
1
2
MR effect size for
'Gut microbiota abundance (genus Alistipes id.968)' on 'Diabetic nephropathy (more control exclusions)'

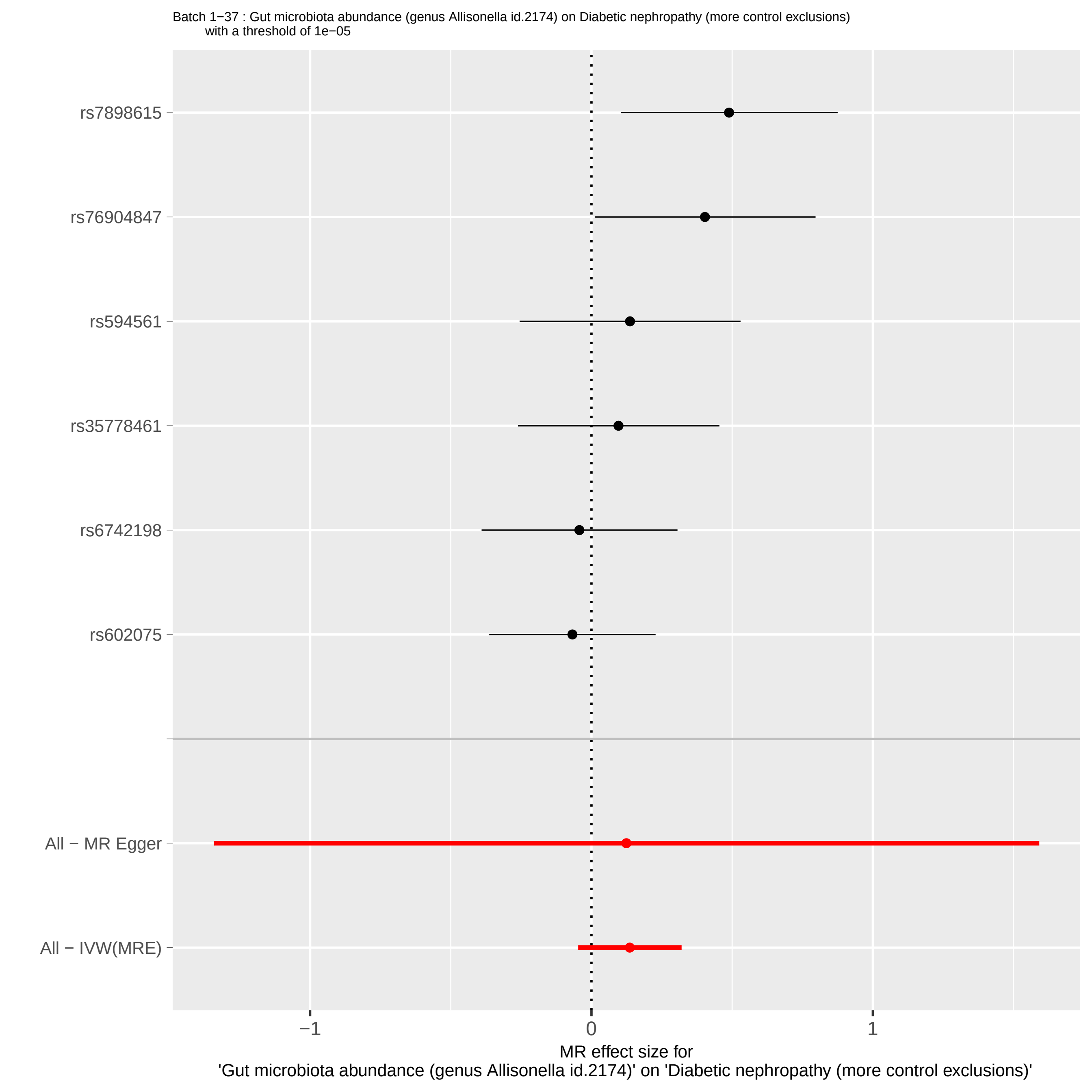

Batch 1−37 : Gut microbiota abundance (genus Allisonella id.2174) on Diabetic nephropathy (more control exclusions)
with a threshold of 1e−05
rs7898615
rs76904847
rs594561
rs35778461
rs6742198
rs602075
All − MR Egger
All − IVW(MRE)
−1
0
1
MR effect size for
'Gut microbiota abundance (genus Allisonella id.2174)' on 'Diabetic nephropathy (more control exclusions)'

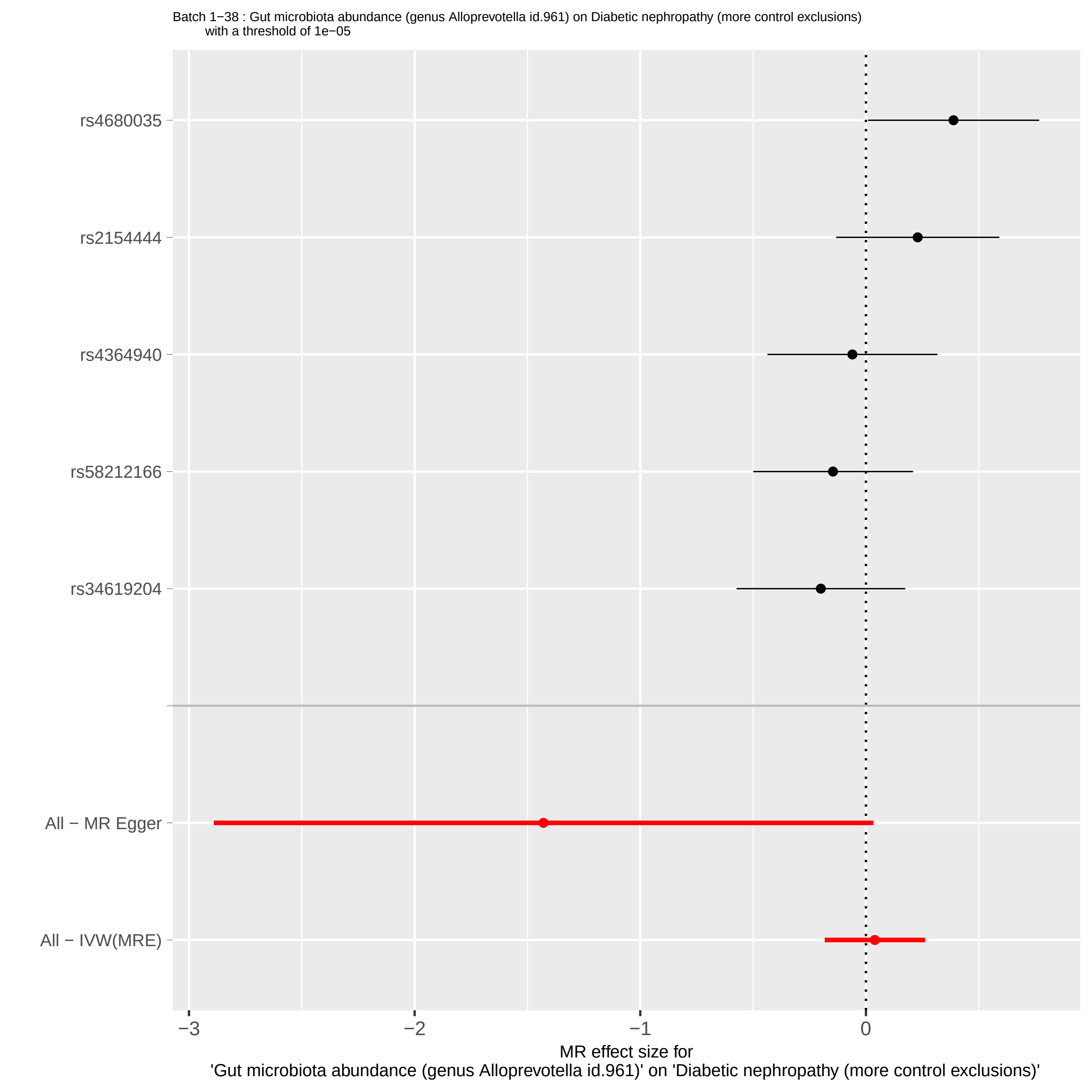
Batch 1−38 : Gut microbiota abundance (genus Alloprevotella id.961) on Diabetic nephropathy (more control exclusions) with a threshold of 1e−05
rs4680035
rs2154444
rs4364940
rs58212166
rs34619204
All − MR Egger
All − IVW(MRE)
−3
−2
−1
0
MR effect size for
'Gut microbiota abundance (genus Alloprevotella id.961)' on 'Diabetic nephropathy (more control exclusions)'

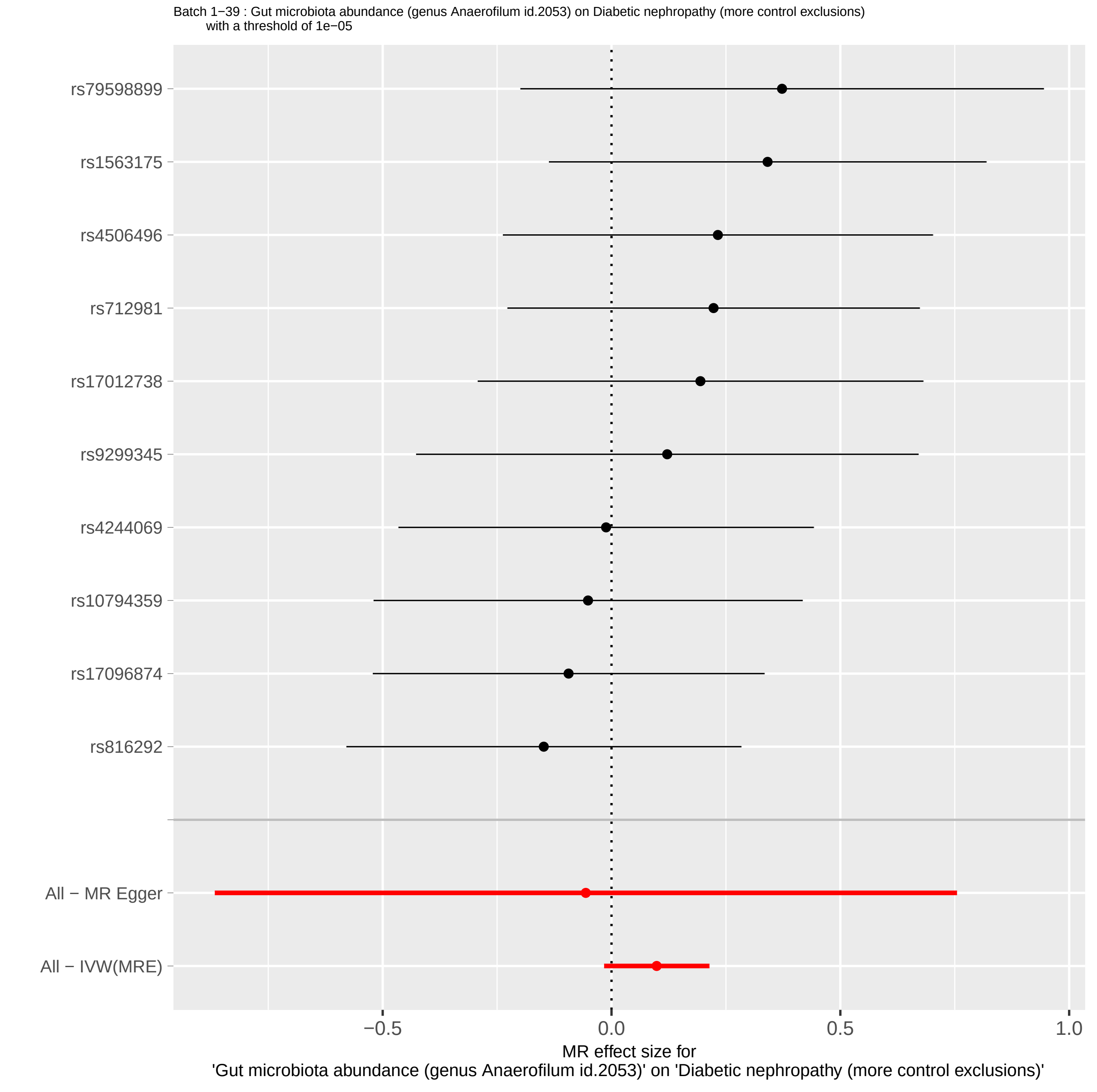
Batch 1−39 : Gut microbiota abundance (genus Anaerofilum id.2053) on Diabetic nephropathy (more control exclusions)
with a threshold of 1e−05
rs79598899
rs1563175
rs4506496
rs712981
rs17012738
rs9299345
rs4244069
rs10794359
rs17096874
rs816292
All − MR Egger
All − IVW(MRE)
−0.5
0.0
0.5
1.0
MR effect size for
'Gut microbiota abundance (genus Anaerofilum id.2053)' on 'Diabetic nephropathy (more control exclusions)'

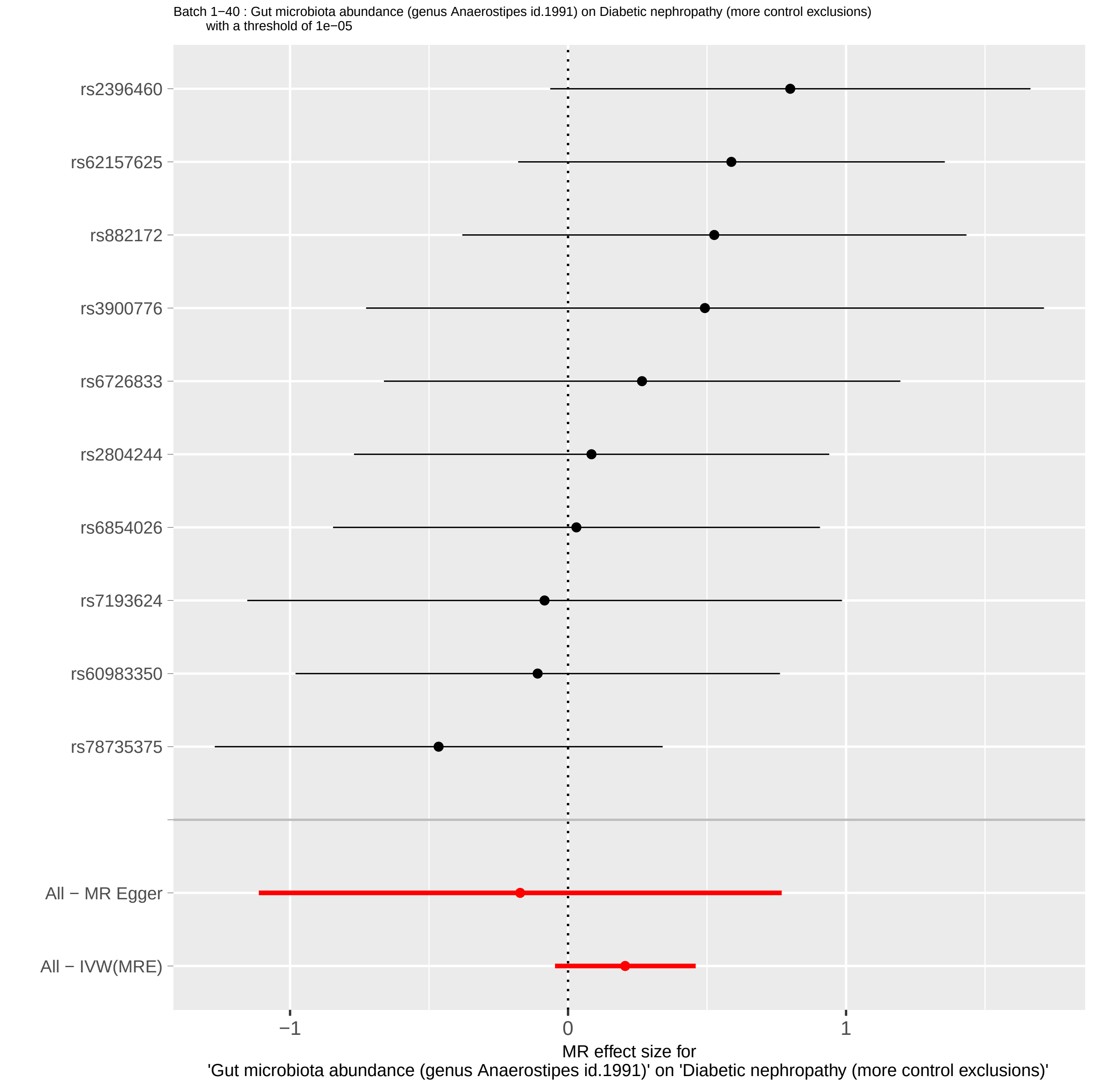

Batch 1−40 : Gut microbiota abundance (genus Anaerostipes id.1991) on Diabetic nephropathy (more control exclusions)
with a threshold of 1e−05
rs2396460
rs62157625
rs882172
rs3900776
rs6726833
rs2804244
rs6854026
rs7193624
rs60983350
rs78735375
All − MR Egger
All − IVW(MRE)
−1
0
1
MR effect size for
'Gut microbiota abundance (genus Anaerostipes id.1991)' on 'Diabetic nephropathy (more control exclusions)'

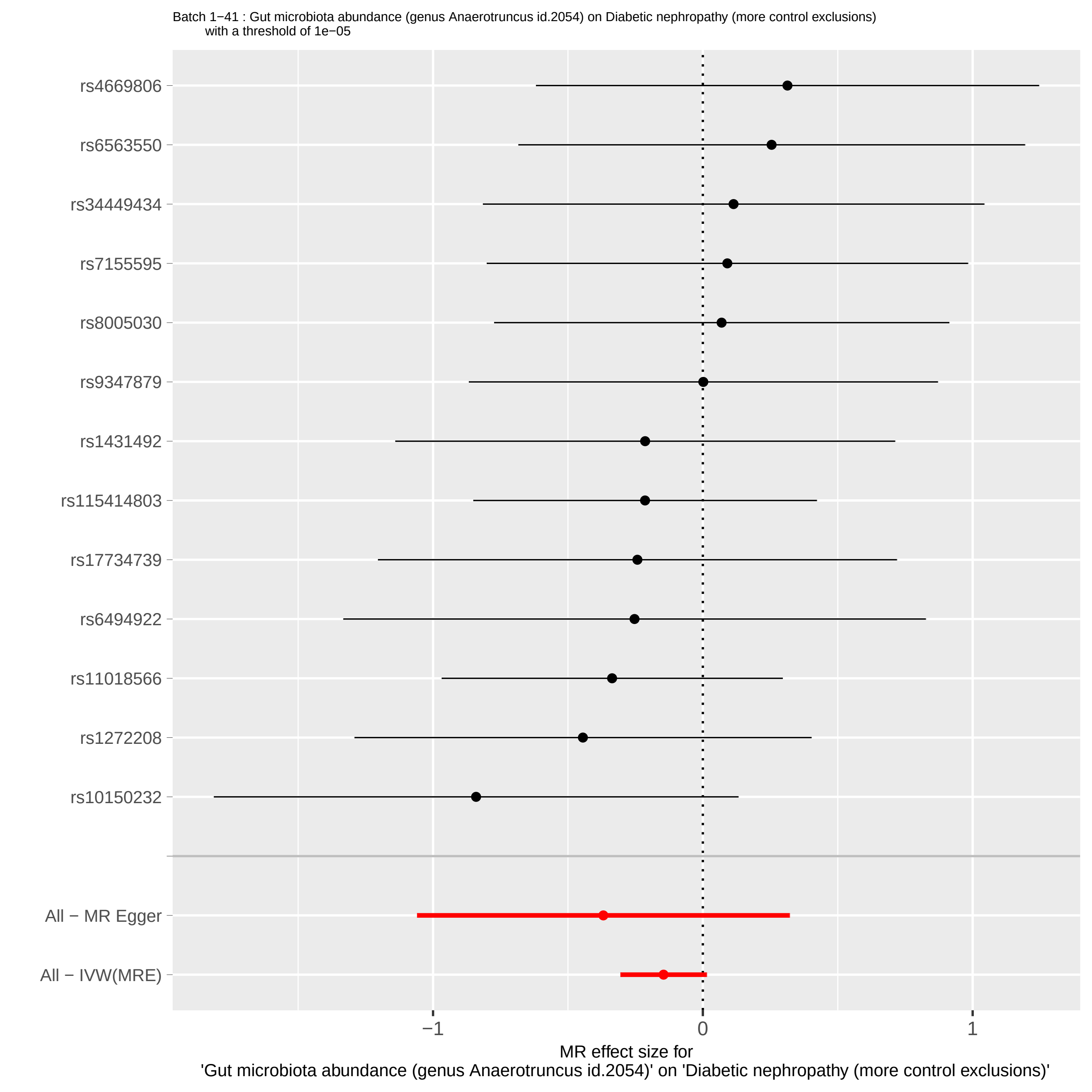
Batch 1−41 : Gut microbiota abundance (genus Anaerotruncus id.2054) on Diabetic nephropathy (more control exclusions) with a threshold of 1e−05
rs4669806
rs6563550
rs34449434
rs7155595
rs8005030
rs9347879
rs1431492
rs115414803
rs17734739
rs6494922
rs11018566
rs1272208
rs10150232
All − MR Egger
All − IVW(MRE)
−1
0
1
MR effect size for
'Gut microbiota abundance (genus Anaerotruncus id.2054)' on 'Diabetic nephropathy (more control exclusions)'

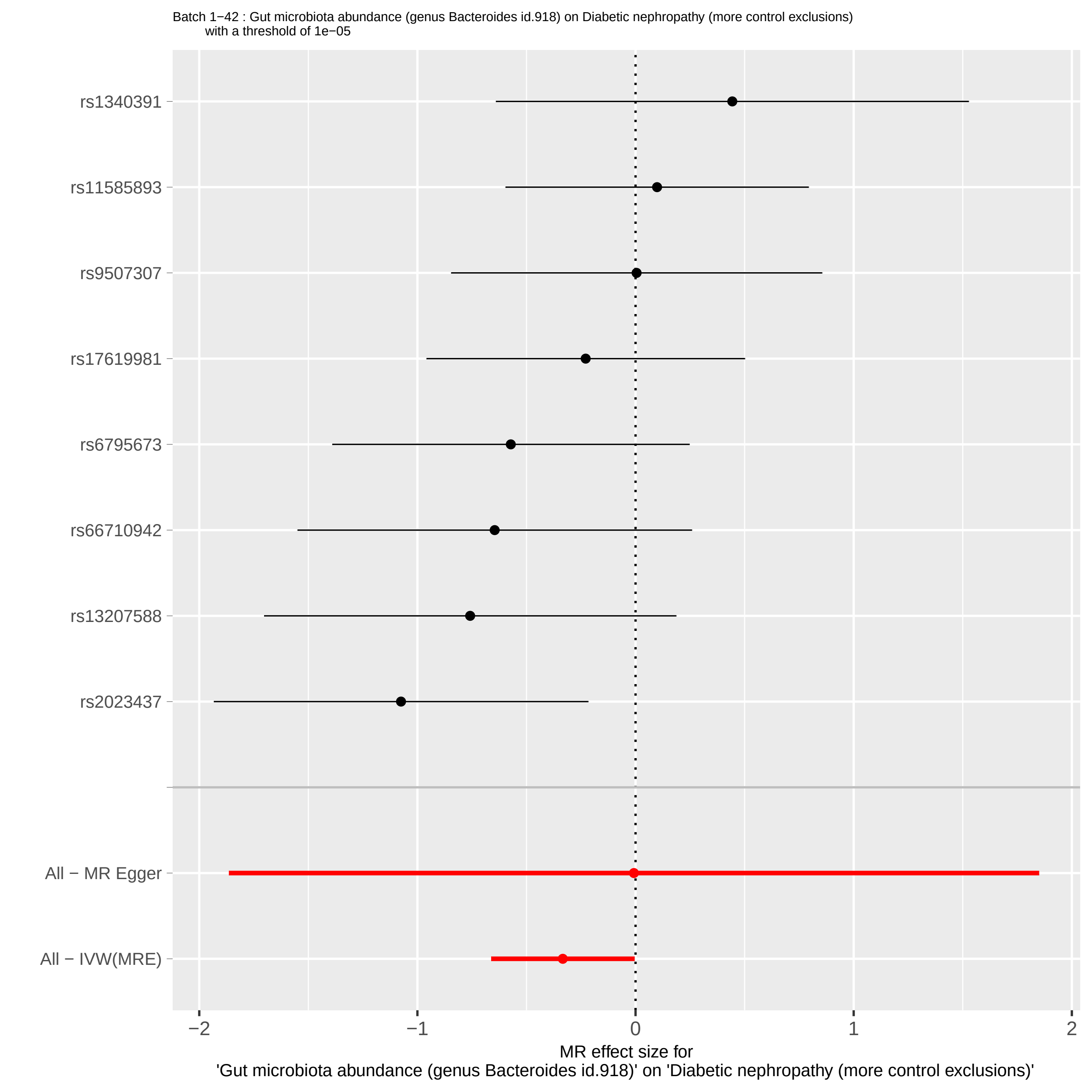
Batch 1−42 : Gut microbiota abundance (genus Bacteroides id.918) on Diabetic nephropathy (more control exclusions)
with a threshold of 1e−05
rs1340391
rs11585893
rs9507307
rs17619981
rs6795673
rs66710942
rs13207588
rs2023437
All − MR Egger
All − IVW(MRE)
−2
−1
0
1
2
MR effect size for
'Gut microbiota abundance (genus Bacteroides id.918)' on 'Diabetic nephropathy (more control exclusions)'

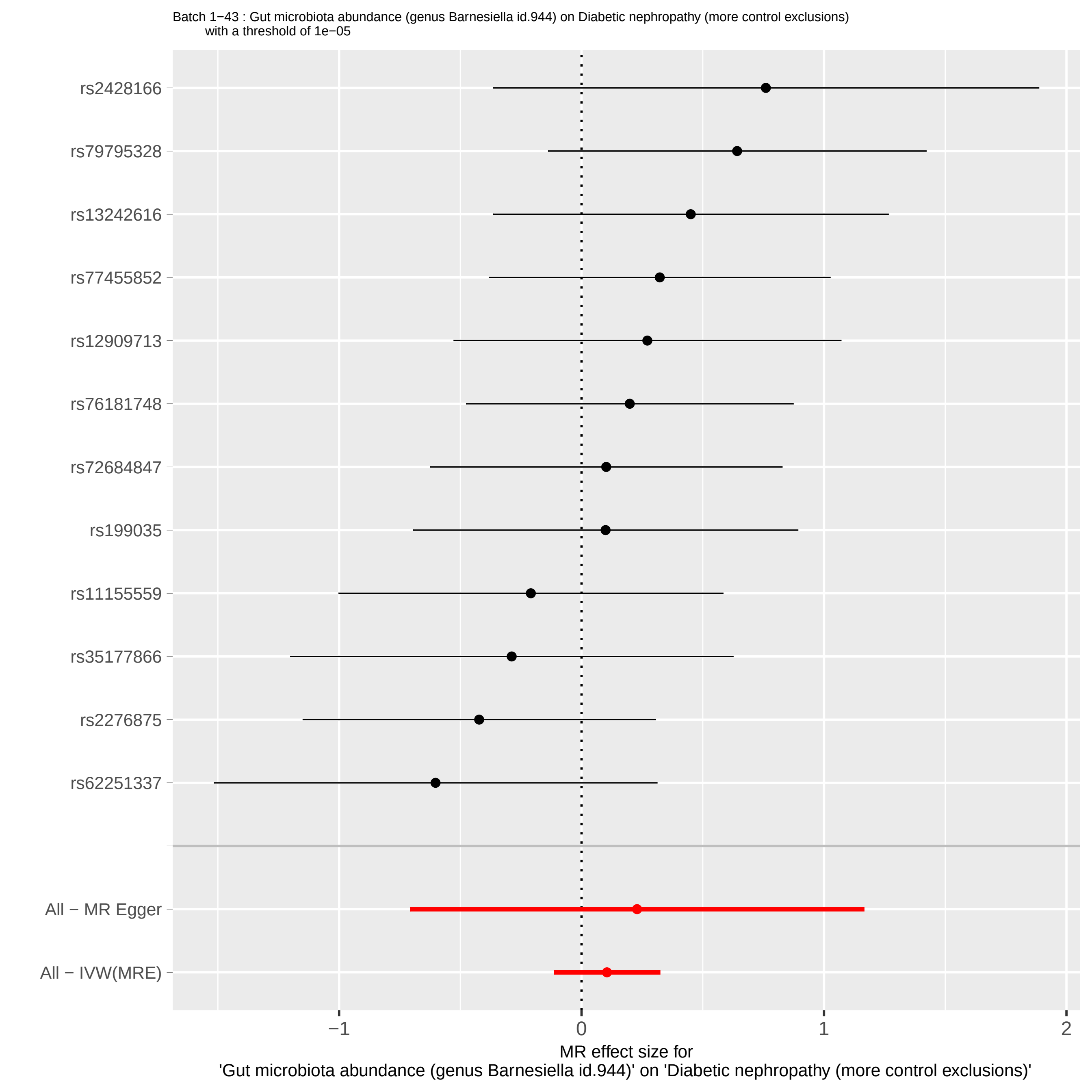
Batch 1−43 : Gut microbiota abundance (genus Barnesiella id.944) on Diabetic nephropathy (more control exclusions)
with a threshold of 1e−05
rs2428166
rs79795328
rs13242616
rs77455852
rs12909713
rs76181748
rs72684847
rs199035
rs11155559
rs35177866
rs2276875
rs62251337
All − MR Egger
All − IVW(MRE)
−1
0
1
2
MR effect size for
'Gut microbiota abundance (genus Barnesiella id.944)' on 'Diabetic nephropathy (more control exclusions)'

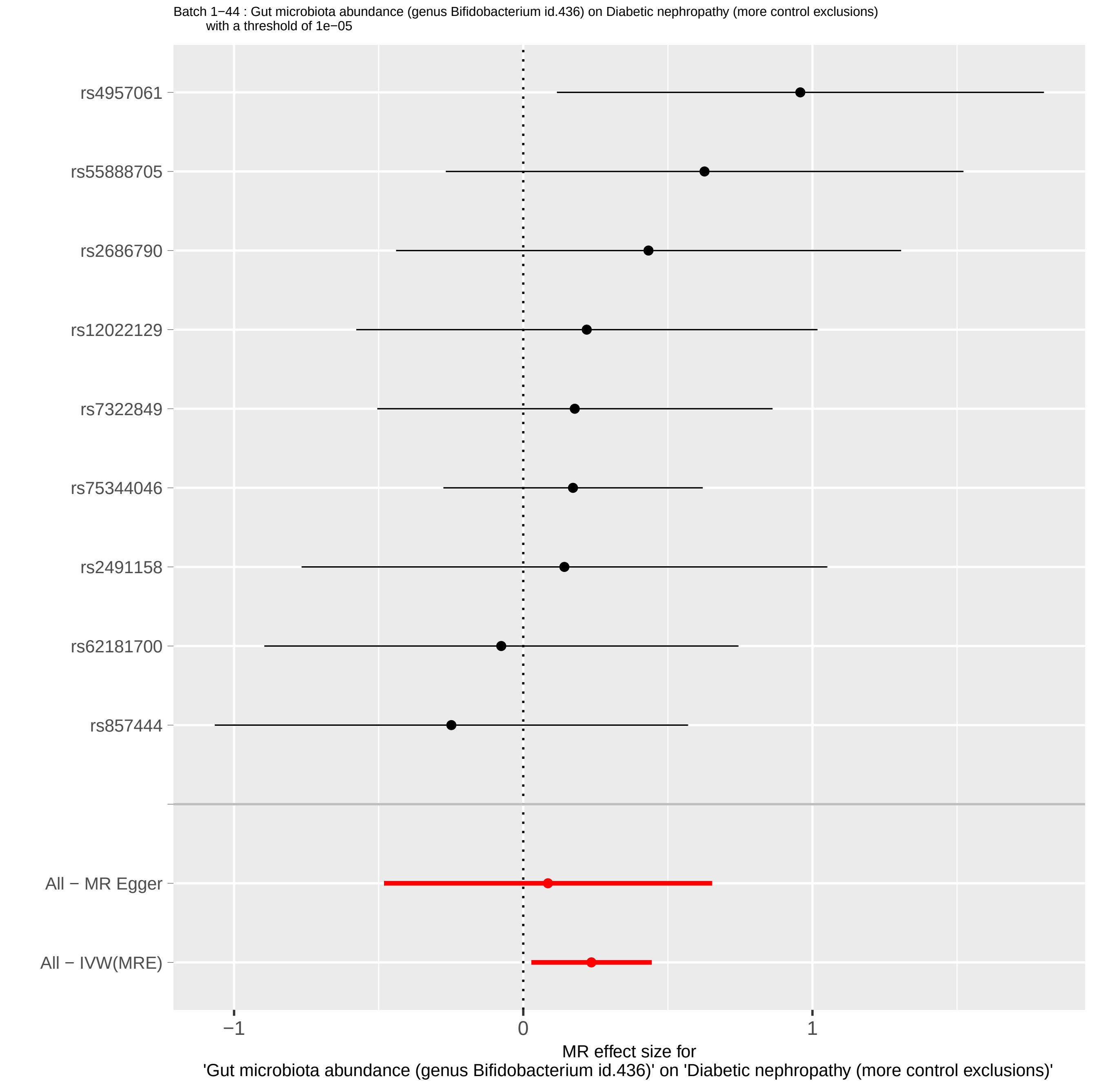

Batch 1−44 : Gut microbiota abundance (genus Bifidobacterium id.436) on Diabetic nephropathy (more control exclusions) with a threshold of 1e−05
rs4957061
rs55888705
rs2686790
rs12022129
rs7322849
rs75344046
rs2491158
rs62181700
rs857444
All − MR Egger
All − IVW(MRE)
−1
0
1
MR effect size for
'Gut microbiota abundance (genus Bifidobacterium id.436)' on 'Diabetic nephropathy (more control exclusions)'

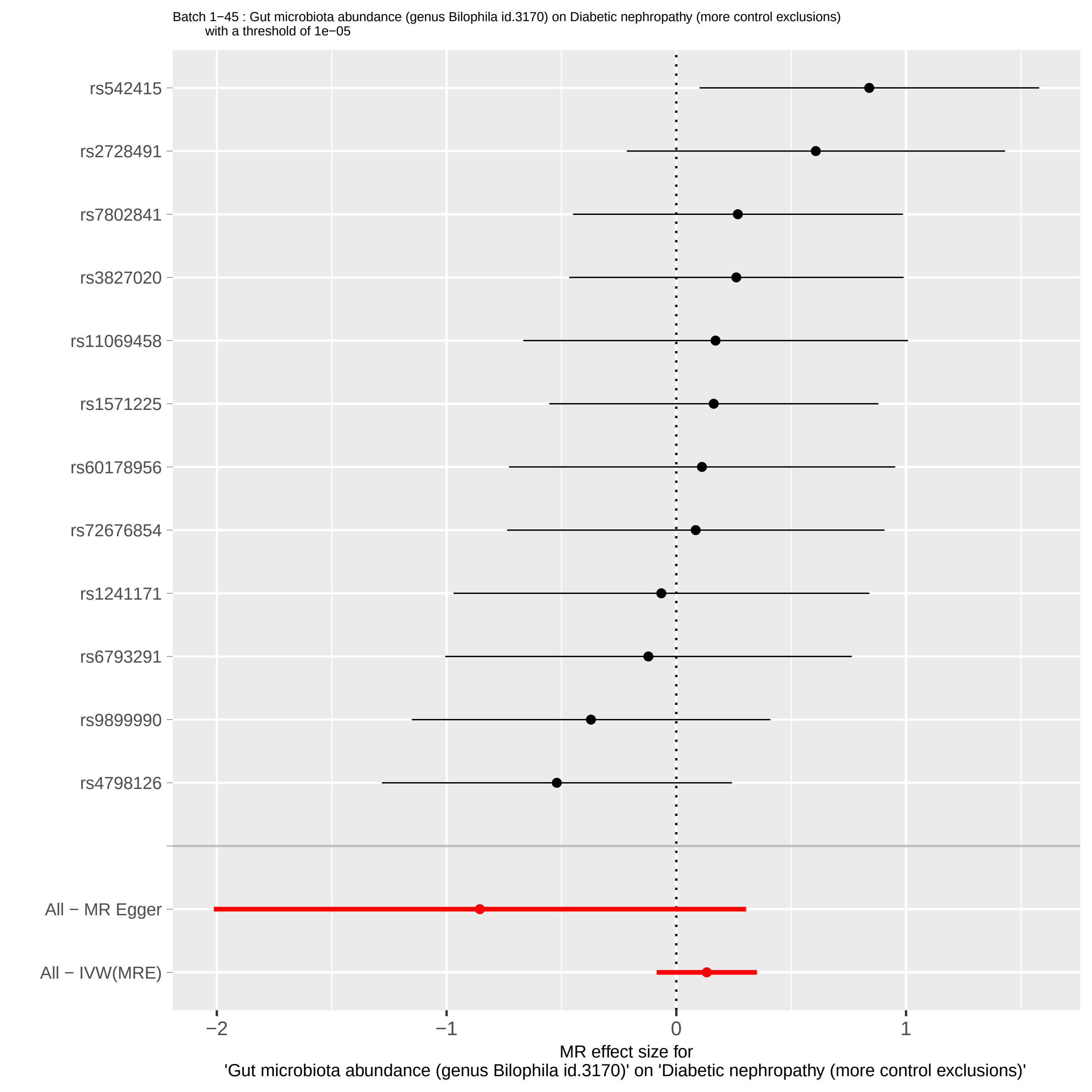

Batch 1−45 : Gut microbiota abundance (genus Bilophila id.3170) on Diabetic nephropathy (more control exclusions) with a threshold of 1e−05
rs542415
rs2728491
rs7802841
rs3827020
rs11069458
rs1571225
rs60178956
rs72676854
rs1241171
rs6793291
rs9899990
rs4798126
All − MR Egger
All − IVW(MRE)
−2
−1
0
1
MR effect size for
'Gut microbiota abundance (genus Bilophila id.3170)' on 'Diabetic nephropathy (more control exclusions)'

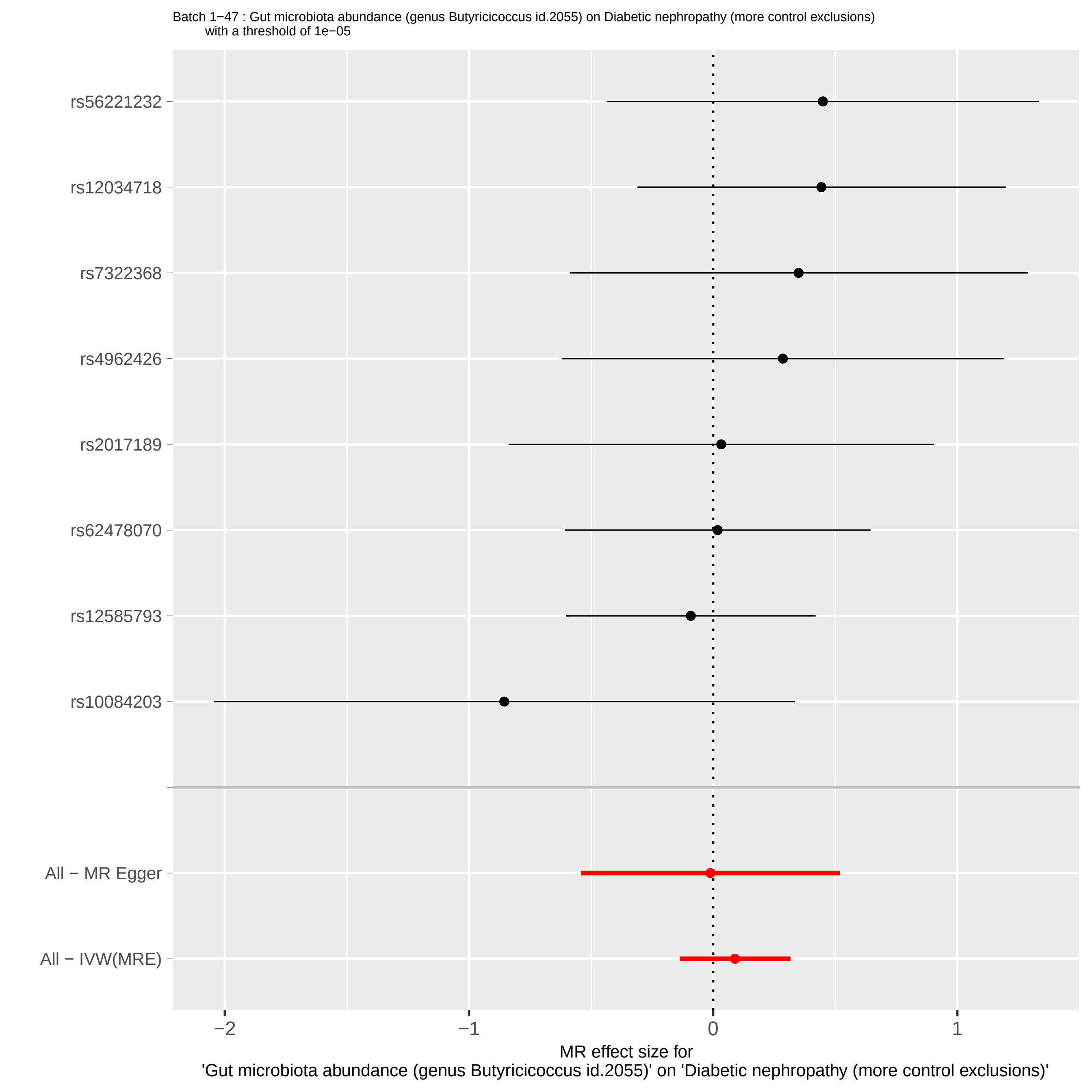

Batch 1−47 : Gut microbiota abundance (genus Butyricicoccus id.2055) on Diabetic nephropathy (more control exclusions) with a threshold of 1e−05
rs56221232
rs12034718
rs7322368
rs4962426
rs2017189
rs62478070
rs12585793
rs10084203
All − MR Egger
All − IVW(MRE)
−2
−1
0
1
MR effect size for
'Gut microbiota abundance (genus Butyricicoccus id.2055)' on 'Diabetic nephropathy (more control exclusions)'

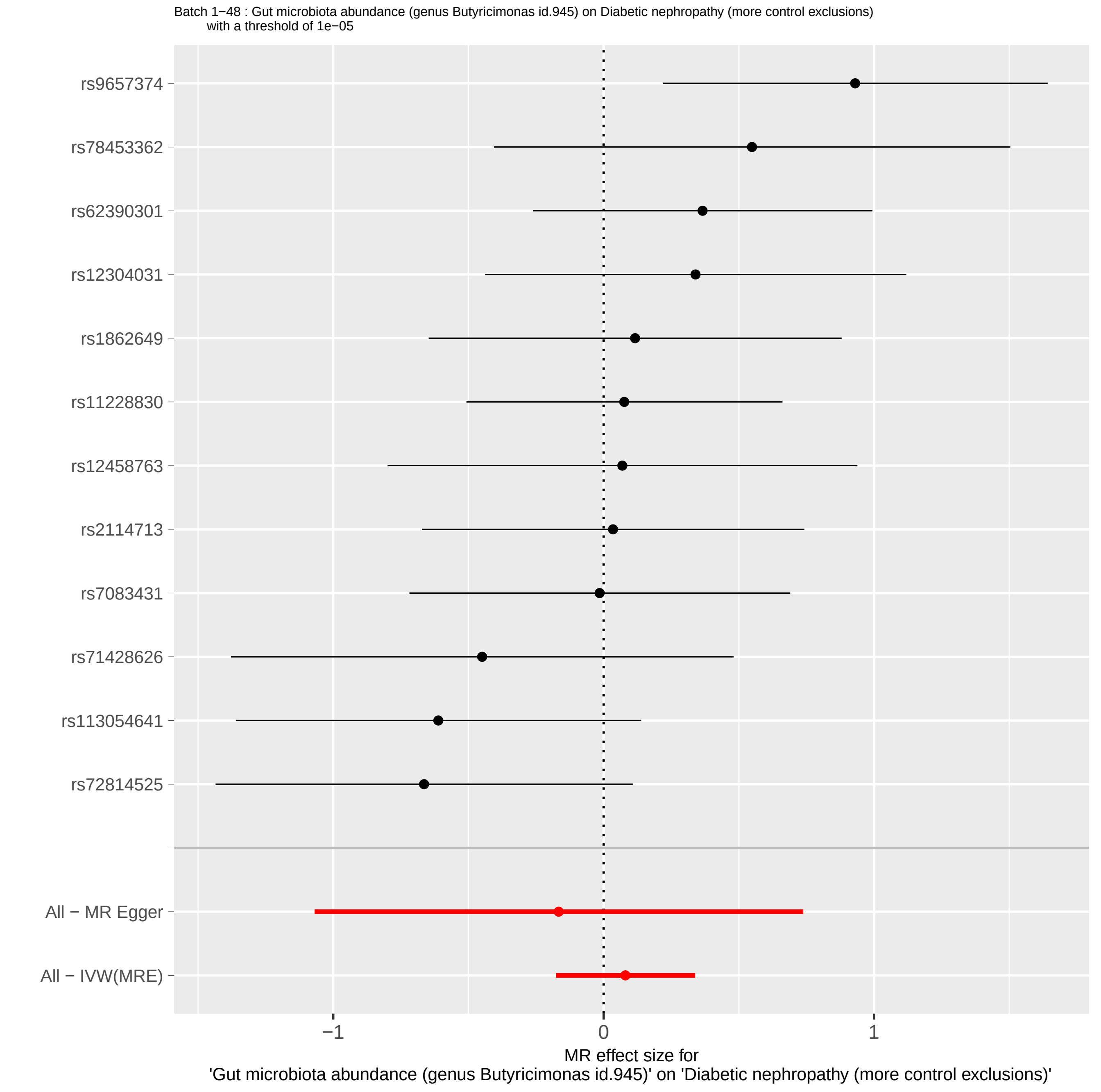

Batch 1−48 : Gut microbiota abundance (genus Butyricimonas id.945) on Diabetic nephropathy (more control exclusions) with a threshold of 1e−05
rs9657374
rs78453362
rs62390301
rs12304031
rs1862649
rs11228830
rs12458763
rs2114713
rs7083431
rs71428626
rs113054641
rs72814525
All − MR Egger
All − IVW(MRE)
−1
0
1
MR effect size for
'Gut microbiota abundance (genus Butyricimonas id.945)' on 'Diabetic nephropathy (more control exclusions)'

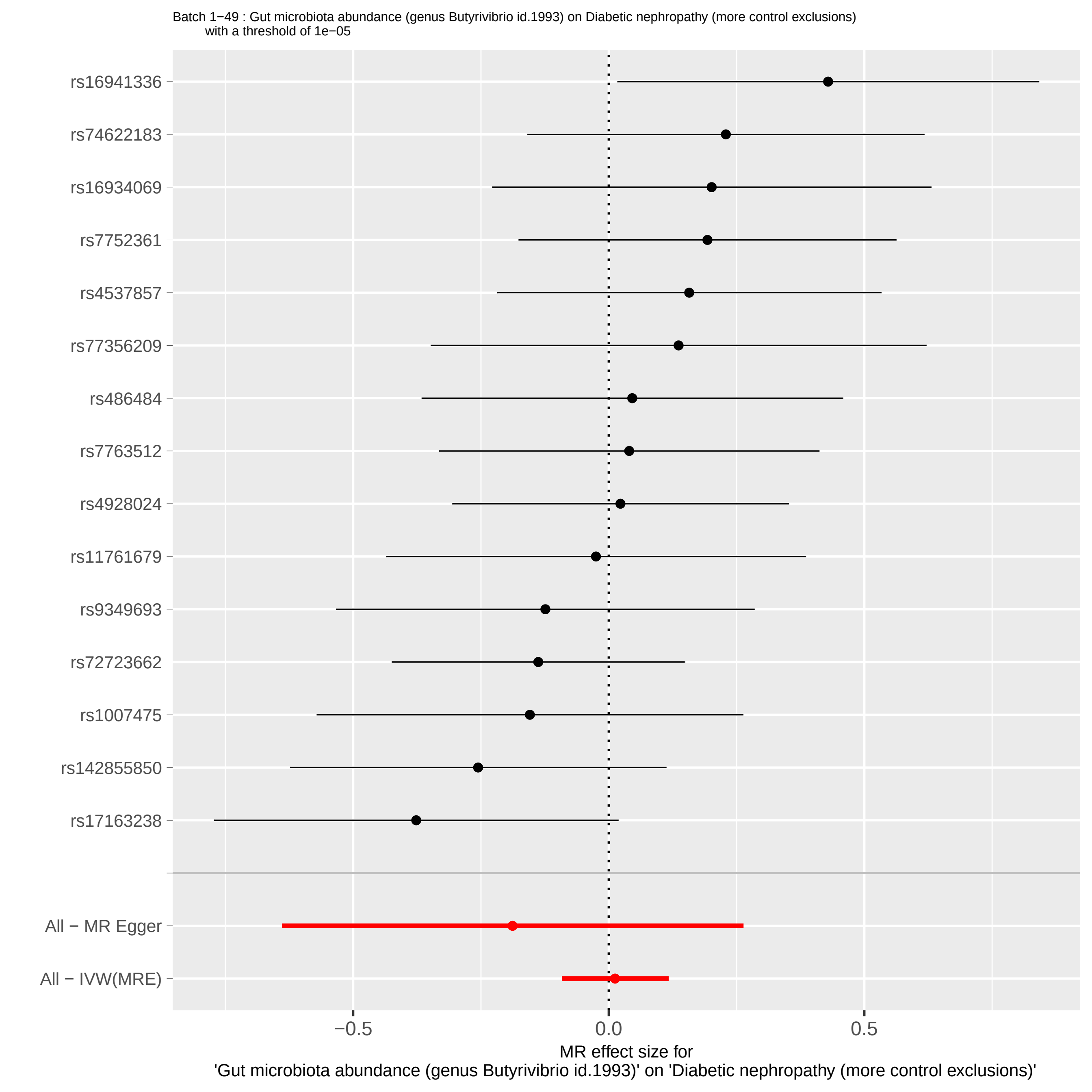
Batch 1−49 : Gut microbiota abundance (genus Butyrivibrio id.1993) on Diabetic nephropathy (more control exclusions)
with a threshold of 1e−05
rs16941336
rs74622183
rs16934069
rs7752361
rs4537857
rs77356209
rs486484
rs7763512
rs4928024
rs11761679
rs9349693
rs72723662
rs1007475
rs142855850
rs17163238
All − MR Egger
All − IVW(MRE)
−0.5
0.0
0.5
MR effect size for
'Gut microbiota abundance (genus Butyrivibrio id.1993)' on 'Diabetic nephropathy (more control exclusions)'

Batch 1−50 : Gut microbiota abundance (genus Candidatus Soleaferrea id.11350) on Diabetic nephropathy (more control exclusions) with a threshold of 1e−05

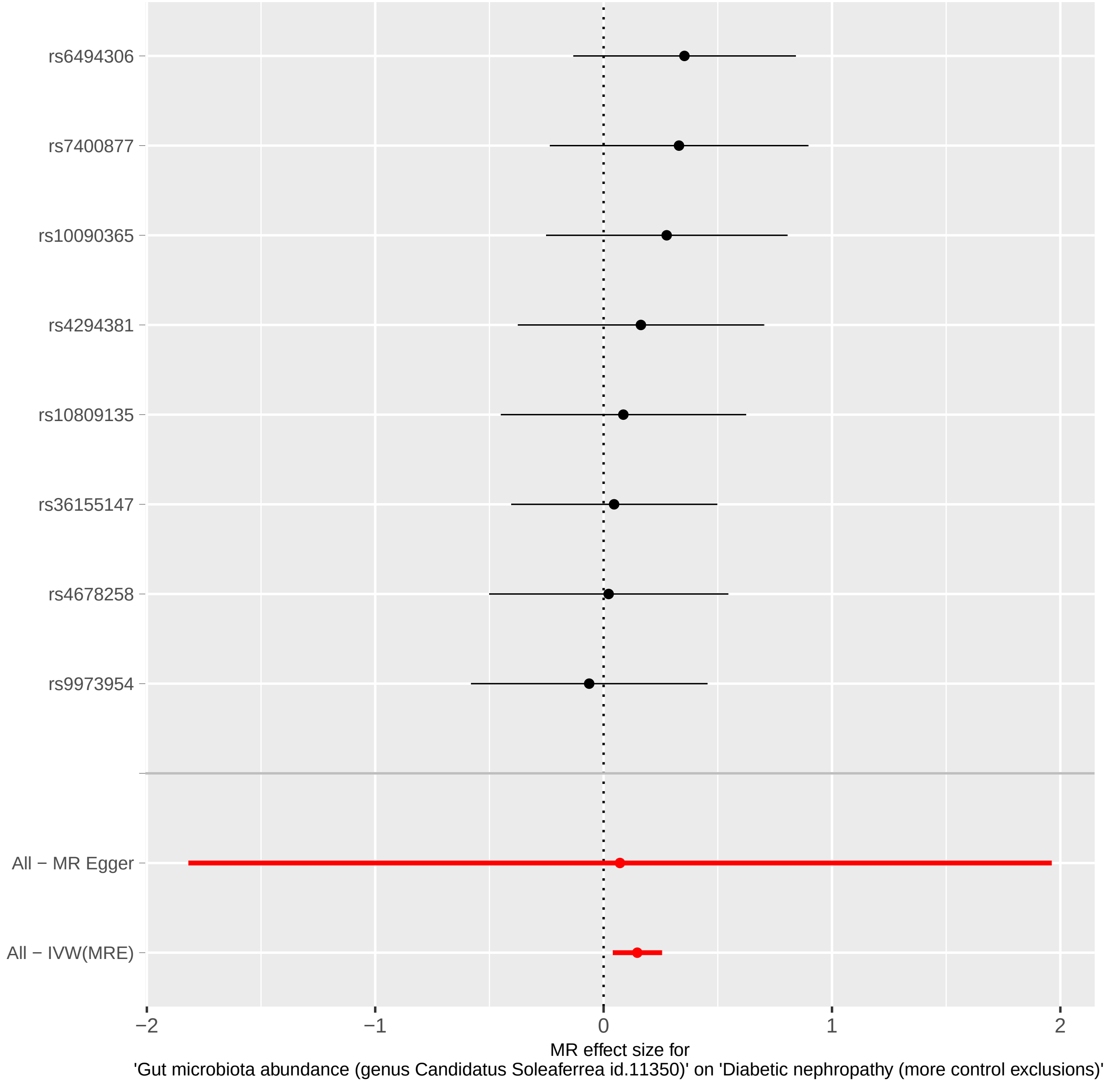

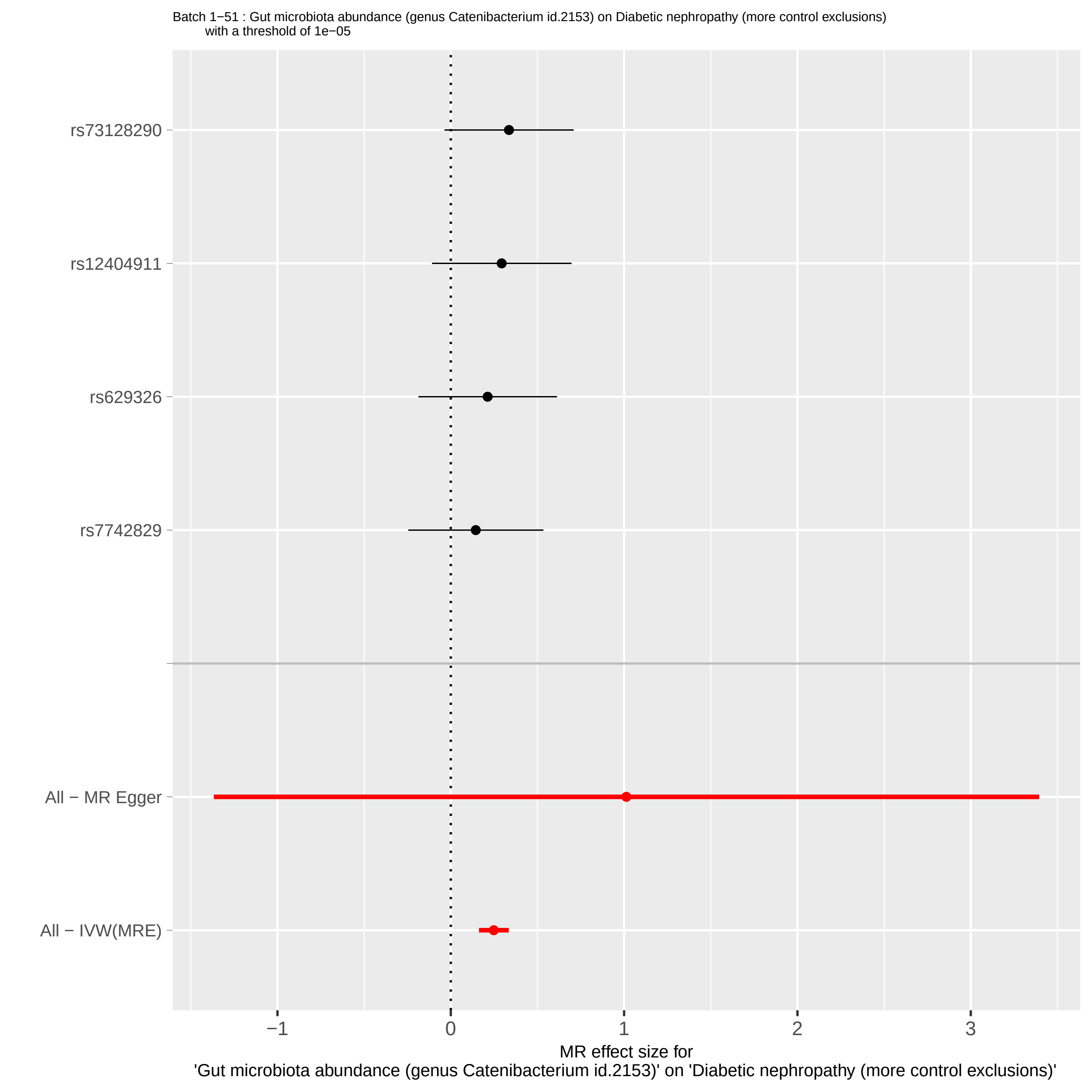
Batch 1−51 : Gut microbiota abundance (genus Catenibacterium id.2153) on Diabetic nephropathy (more control exclusions) with a threshold of 1e−05
rs73128290
rs12404911
rs629326
rs7742829
All − MR Egger
All − IVW(MRE)
−1
0
1
2
3
MR effect size for
'Gut microbiota abundance (genus Catenibacterium id.2153)' on 'Diabetic nephropathy (more control exclusions)'

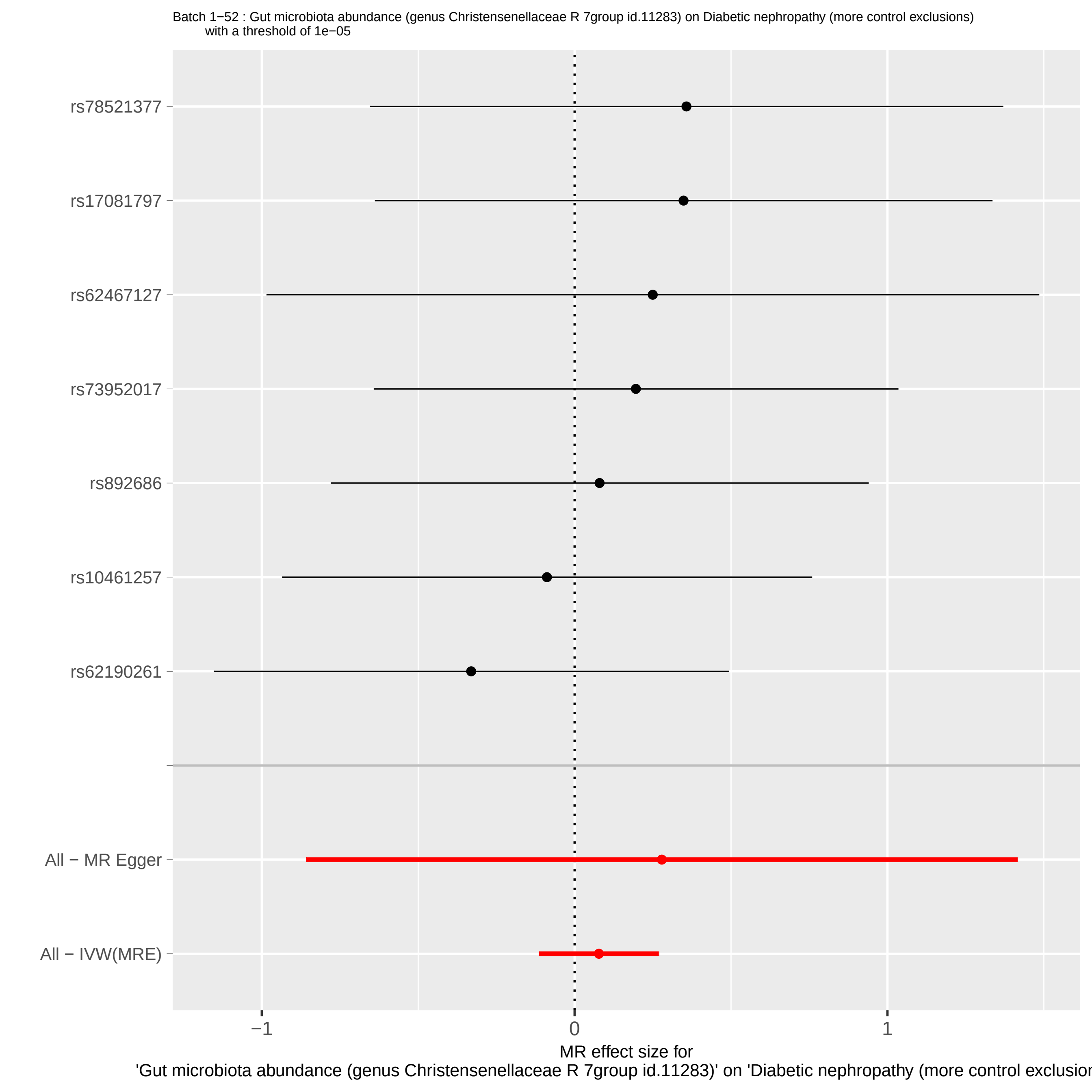
Batch 1−52 : Gut microbiota abundance (genus Christensenellaceae R 7group id.11283) on Diabetic nephropathy (more control exclusions) with a threshold of 1e−05
rs78521377
rs17081797
rs62467127
rs73952017
rs892686
rs10461257
rs62190261
All − MR Egger
All − IVW(MRE)
−1
0
1
MR effect size for
'Gut microbiota abundance (genus Christensenellaceae R 7group id.11283)' on 'Diabetic nephropathy (more control exclusion

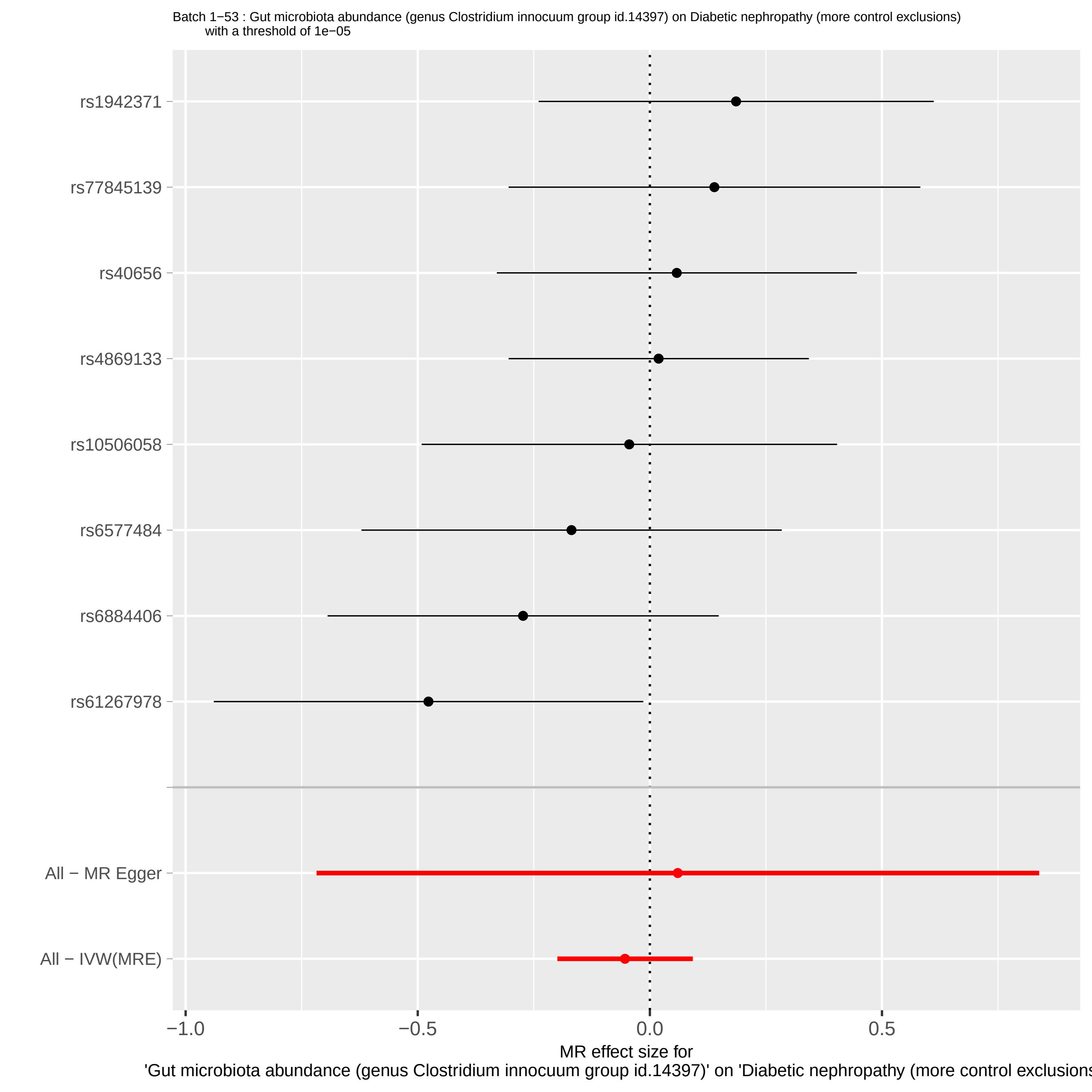

Batch 1−53 : Gut microbiota abundance (genus Clostridium innocuum group id.14397) on Diabetic nephropathy (more control exclusions)
with a threshold of 1e−05
rs1942371
rs77845139
rs40656
rs4869133
rs10506058
rs6577484
rs6884406
rs61267978
All − MR Egger
All − IVW(MRE)
−1.0
−0.5
0.0
0.5
MR effect size for
'Gut microbiota abundance (genus Clostridium innocuum group id.14397)' on 'Diabetic nephropathy (more control exclusions

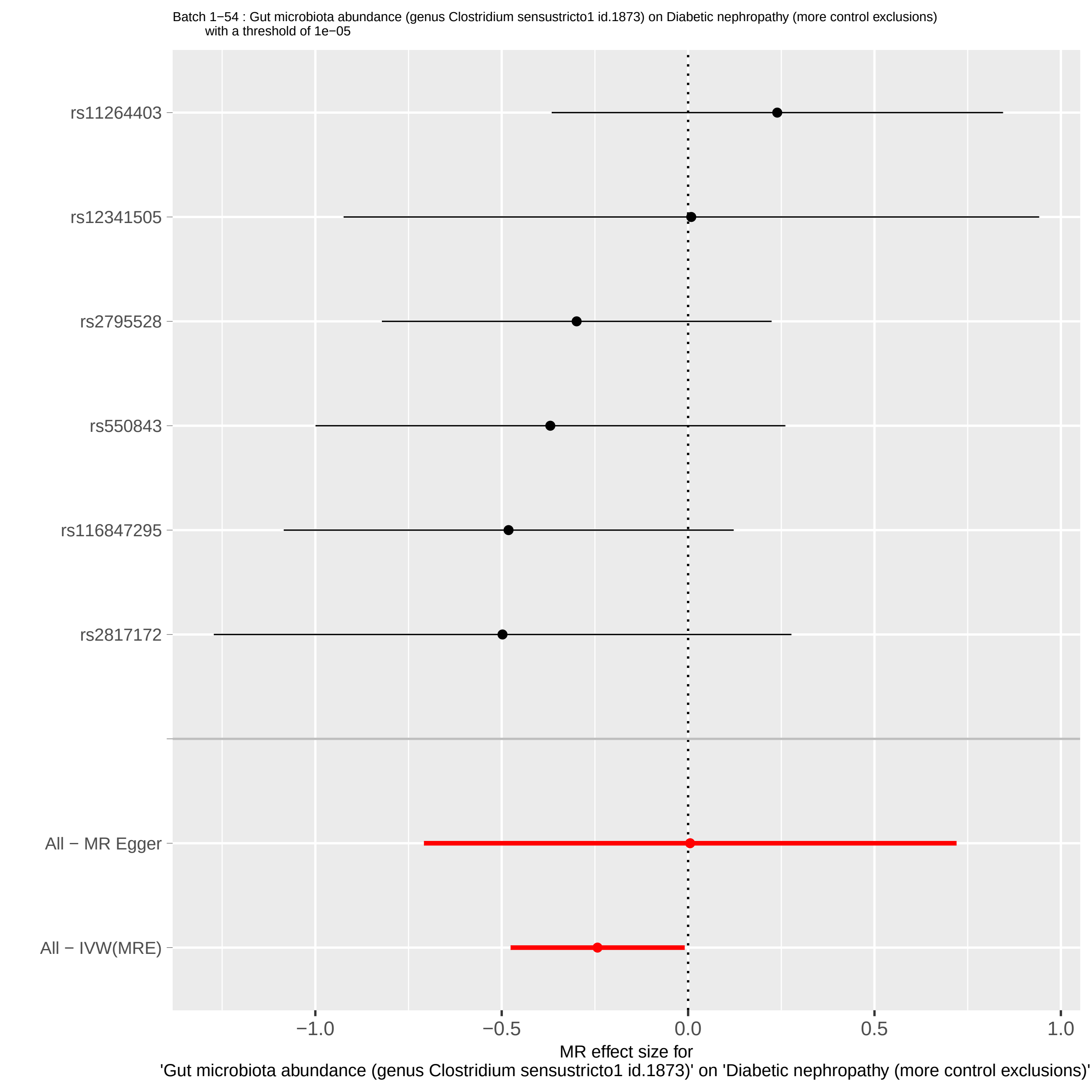

Batch 1−54 : Gut microbiota abundance (genus Clostridium sensustricto1 id.1873) on Diabetic nephropathy (more control exclusions)
with a threshold of 1e−05
rs11264403
rs12341505
rs2795528
rs550843
rs116847295
rs2817172
All − MR Egger
All − IVW(MRE)
−1.0
−0.5
0.0
0.5
1.0
MR effect size for
'Gut microbiota abundance (genus Clostridium sensustricto1 id.1873)' on 'Diabetic nephropathy (more control exclusions)'

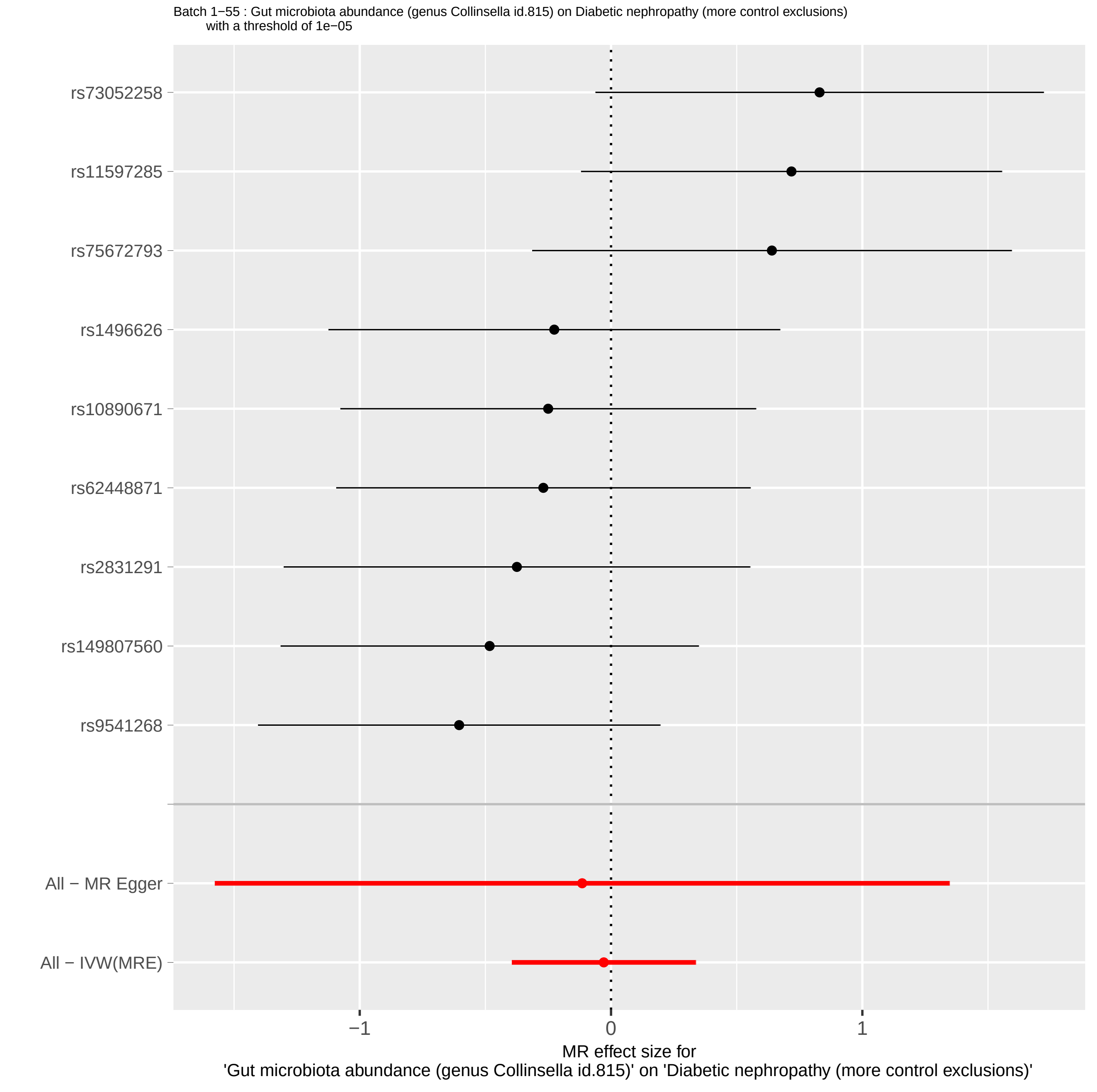
Batch 1−55 : Gut microbiota abundance (genus Collinsella id.815) on Diabetic nephropathy (more control exclusions)
with a threshold of 1e−05
rs73052258
rs11597285
rs75672793
rs1496626
rs10890671
rs62448871
rs2831291
rs149807560
rs9541268
All − MR Egger
All − IVW(MRE)
−1
0
1
MR effect size for
'Gut microbiota abundance (genus Collinsella id.815)' on 'Diabetic nephropathy (more control exclusions)'

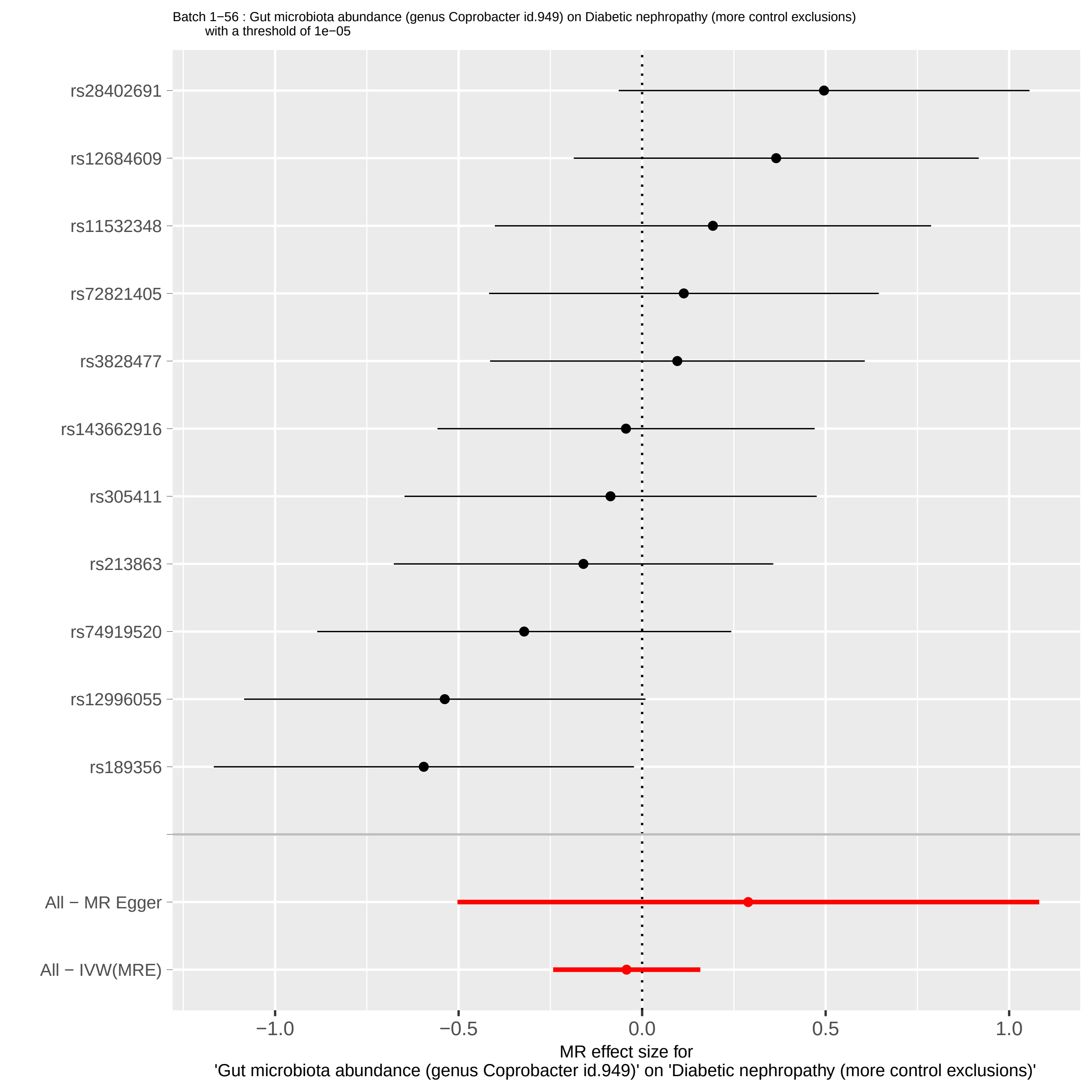

Batch 1−56 : Gut microbiota abundance (genus Coprobacter id.949) on Diabetic nephropathy (more control exclusions) with a threshold of 1e−05
rs28402691
rs12684609
rs11532348
rs72821405
rs3828477
rs143662916
rs305411
rs213863
rs74919520
rs12996055
rs189356
All − MR Egger
All − IVW(MRE)
−1.0
−0.5
0.0
0.5
1.0
MR effect size for
'Gut microbiota abundance (genus Coprobacter id.949)' on 'Diabetic nephropathy (more control exclusions)'

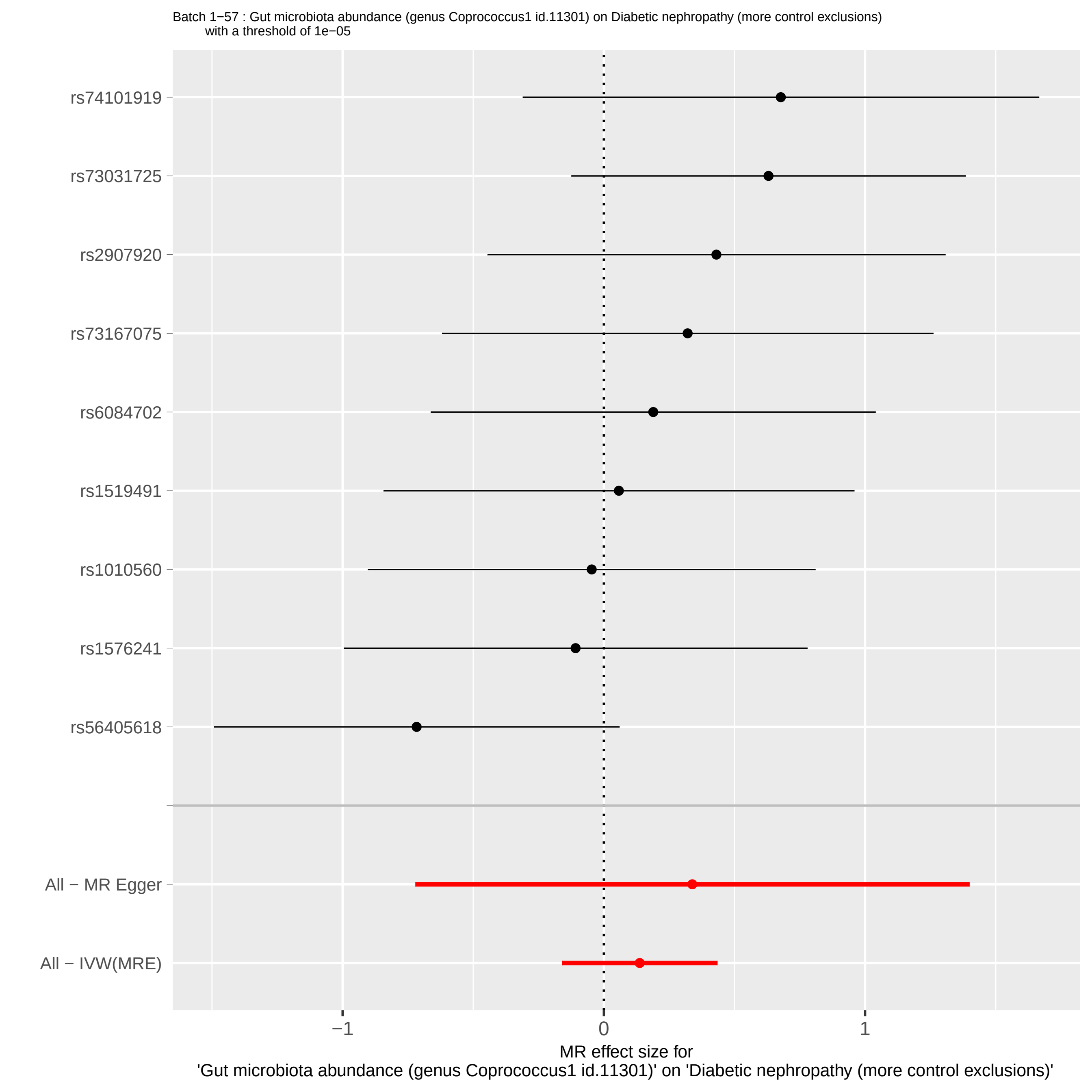

Batch 1−57 : Gut microbiota abundance (genus Coprococcus1 id.11301) on Diabetic nephropathy (more control exclusions)
with a threshold of 1e−05
rs74101919
rs73031725
rs2907920
rs73167075
rs6084702
rs1519491
rs1010560
rs1576241
rs56405618
All − MR Egger
All − IVW(MRE)
−1
0
1
MR effect size for
'Gut microbiota abundance (genus Coprococcus1 id.11301)' on 'Diabetic nephropathy (more control exclusions)'

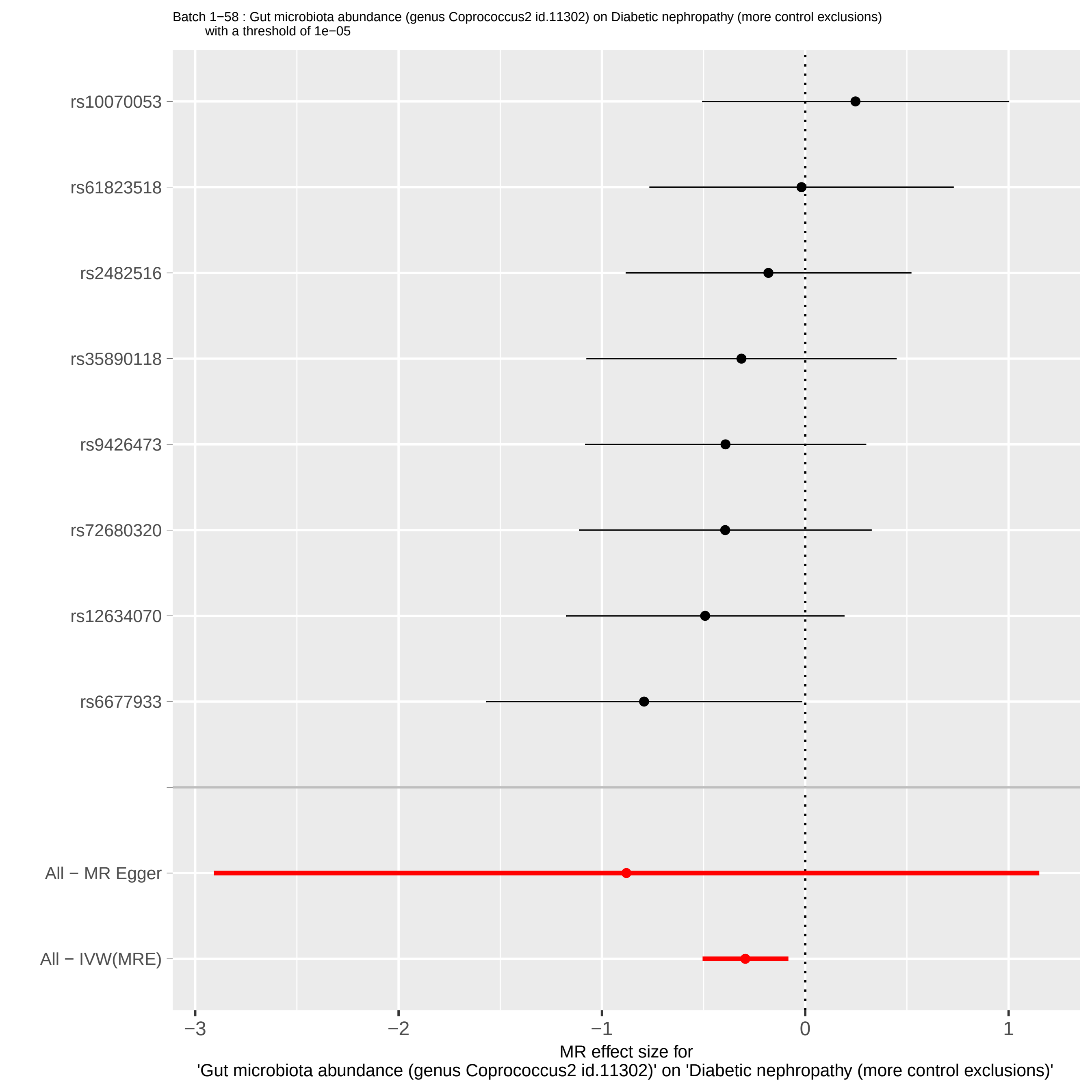

Batch 1−58 : Gut microbiota abundance (genus Coprococcus2 id.11302) on Diabetic nephropathy (more control exclusions) with a threshold of 1e−05
rs10070053
rs61823518
rs2482516
rs35890118
rs9426473
rs72680320
rs12634070
rs6677933
All − MR Egger
All − IVW(MRE)
−3
−2
−1
0
1
MR effect size for
'Gut microbiota abundance (genus Coprococcus2 id.11302)' on 'Diabetic nephropathy (more control exclusions)'

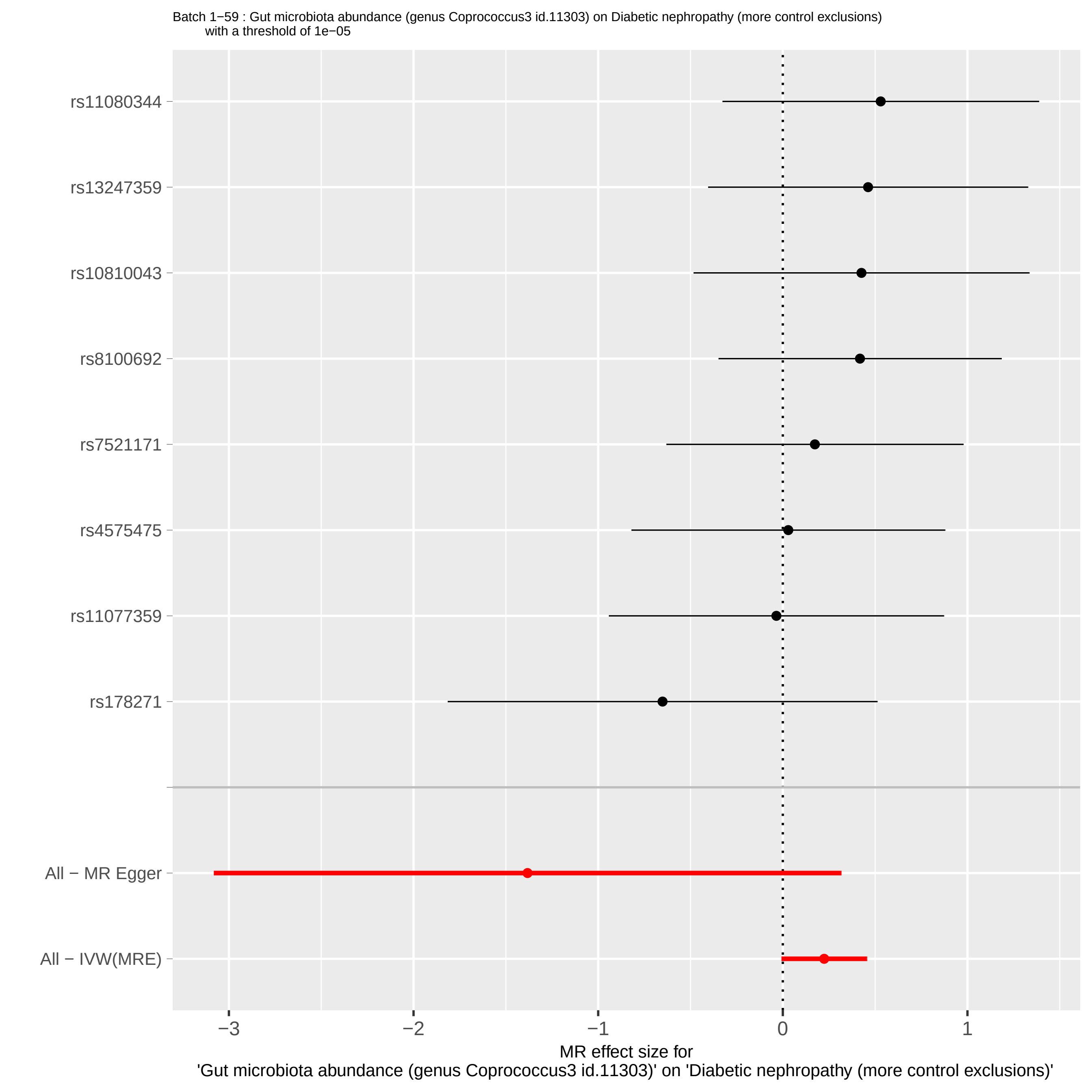

Batch 1−59 : Gut microbiota abundance (genus Coprococcus3 id.11303) on Diabetic nephropathy (more control exclusions)
with a threshold of 1e−05
rs11080344
rs13247359
rs10810043
rs8100692
rs7521171
rs4575475
rs11077359
rs178271
All − MR Egger
All − IVW(MRE)
−3
−2
−1
0
1
MR effect size for
'Gut microbiota abundance (genus Coprococcus3 id.11303)' on 'Diabetic nephropathy (more control exclusions)'

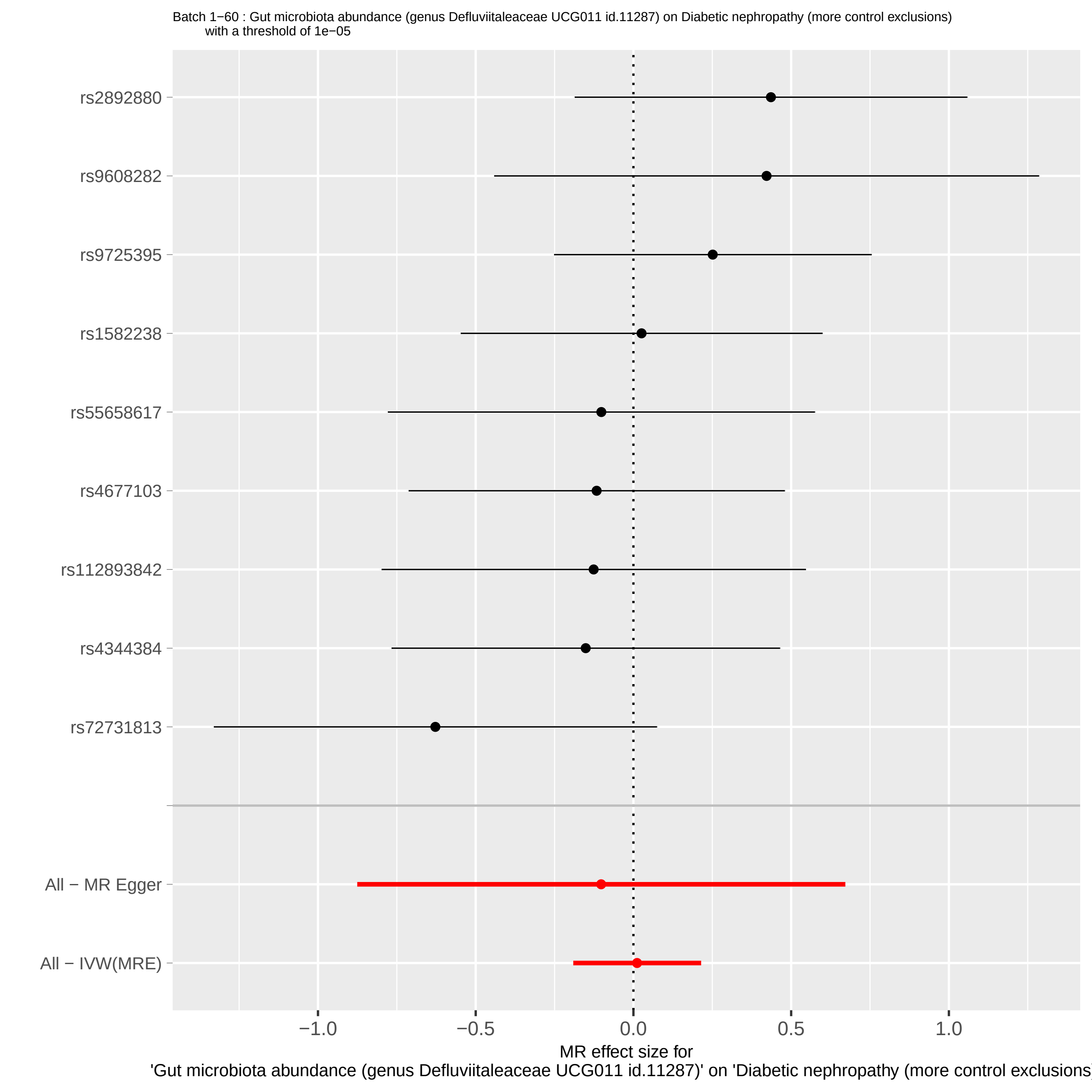

Batch 1−60 : Gut microbiota abundance (genus Defluviitaleaceae UCG011 id.11287) on Diabetic nephropathy (more control exclusions)
with a threshold of 1e−05
rs2892880
rs9608282
rs9725395
rs1582238
rs55658617
rs4677103
rs112893842
rs4344384
rs72731813
All − MR Egger
All − IVW(MRE)
−1.0
−0.5
0.0
0.5
1.0
MR effect size for
'Gut microbiota abundance (genus Defluviitaleaceae UCG011 id.11287)' on 'Diabetic nephropathy (more control exclusions)'

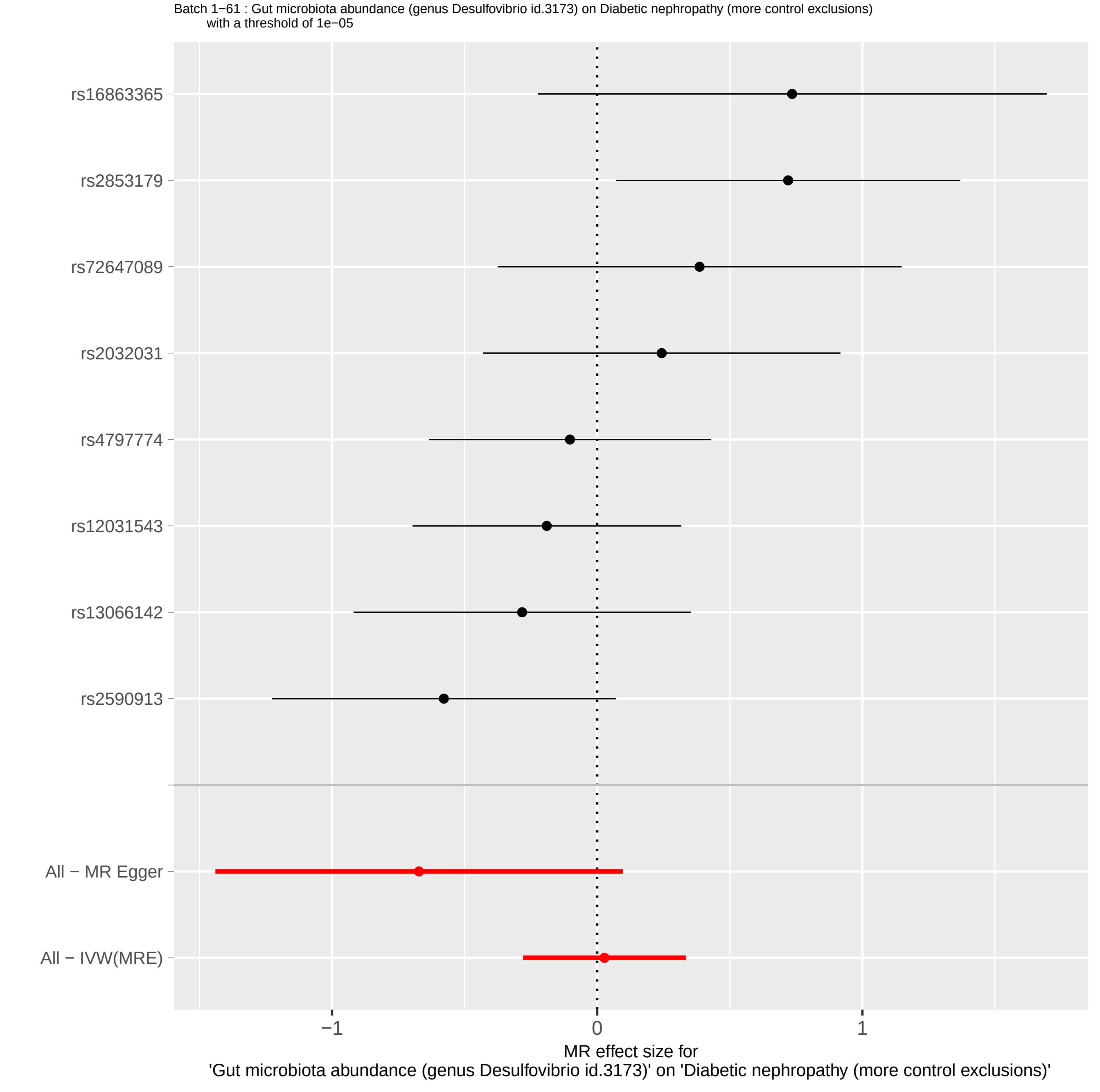

Batch 1−61 : Gut microbiota abundance (genus Desulfovibrio id.3173) on Diabetic nephropathy (more control exclusions) with a threshold of 1e−05
rs16863365
rs2853179
rs72647089
rs2032031
rs4797774
rs12031543
rs13066142
rs2590913
All − MR Egger
All − IVW(MRE)
−1
0
1
MR effect size for 'Gut microbiota abundance (genus Desulfovibrio id.3173)' on 'Diabetic nephropathy (more control exclusions)'

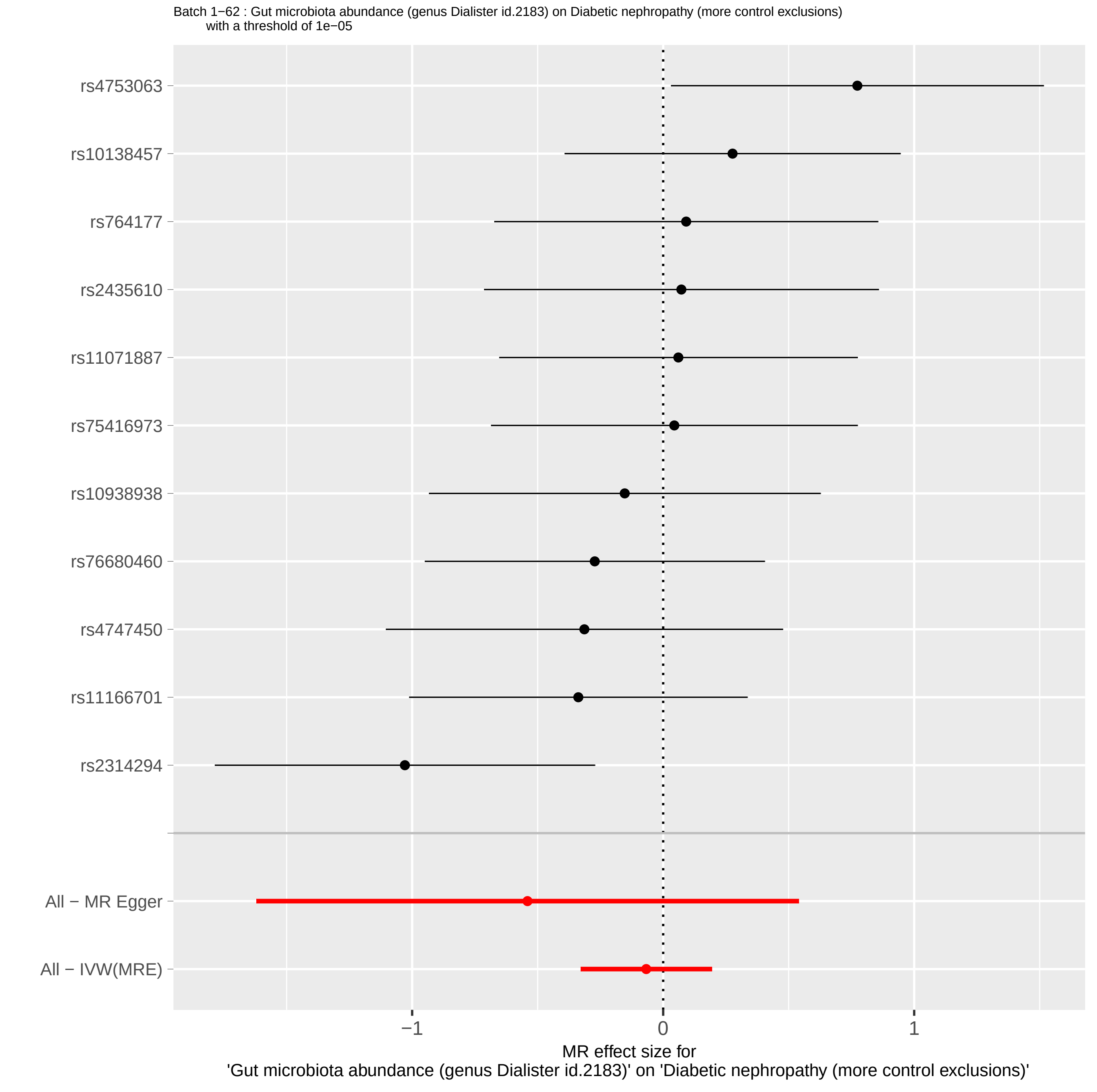

Batch 1−62 : Gut microbiota abundance (genus Dialister id.2183) on Diabetic nephropathy (more control exclusions)
with a threshold of 1e−05
rs4753063
rs10138457
rs764177
rs2435610
rs11071887
rs75416973
rs10938938
rs76680460
rs4747450
rs11166701
rs2314294
All − MR Egger
All − IVW(MRE)
−1
0
1
MR effect size for
'Gut microbiota abundance (genus Dialister id.2183)' on 'Diabetic nephropathy (more control exclusions)'

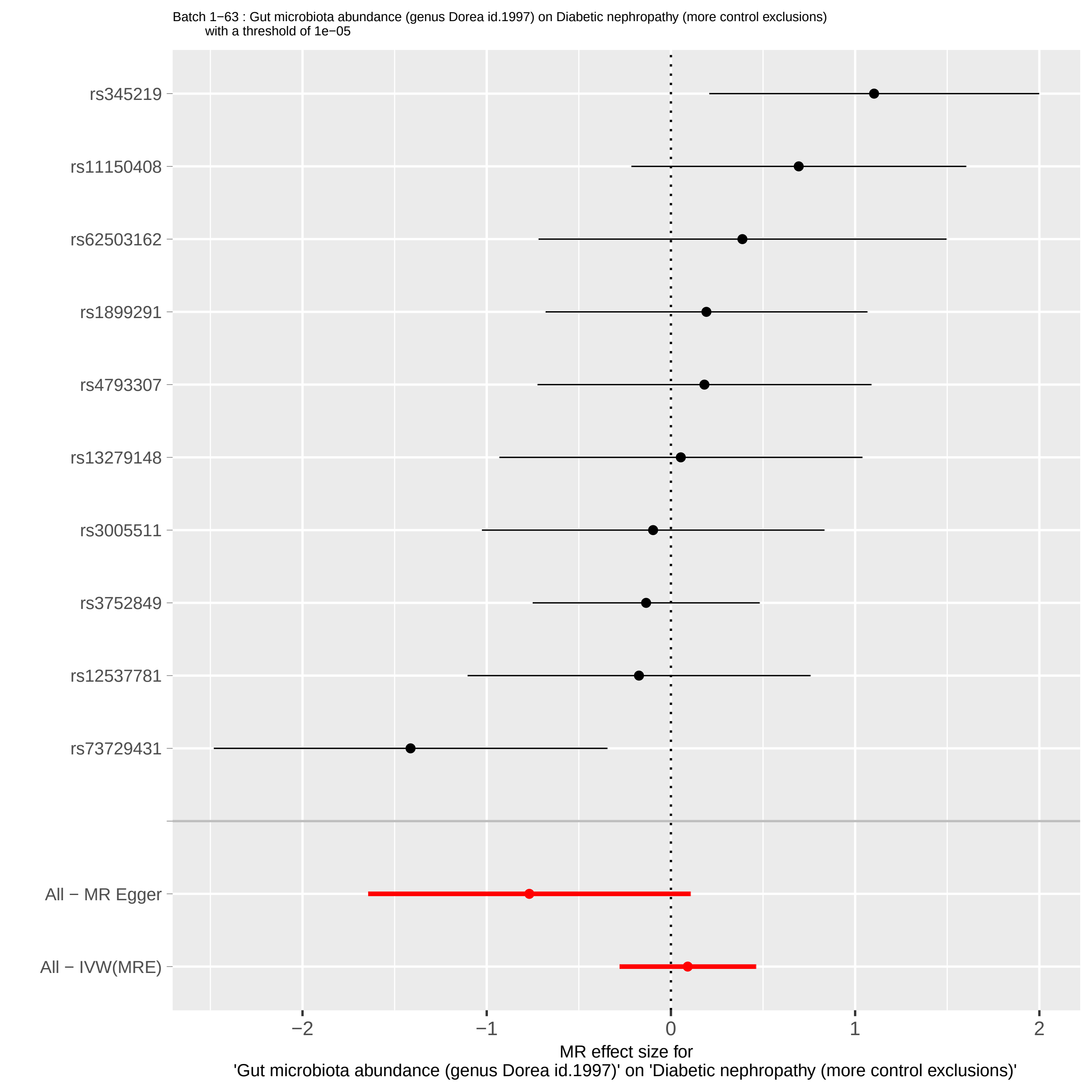

Batch 1−63 : Gut microbiota abundance (genus Dorea id.1997) on Diabetic nephropathy (more control exclusions)
with a threshold of 1e−05
rs345219
rs11150408
rs62503162
rs1899291
rs4793307
rs13279148
rs3005511
rs3752849
rs12537781
rs73729431
All − MR Egger
All − IVW(MRE)
−2
−1
0
1
2
MR effect size for
'Gut microbiota abundance (genus Dorea id.1997)' on 'Diabetic nephropathy (more control exclusions)'

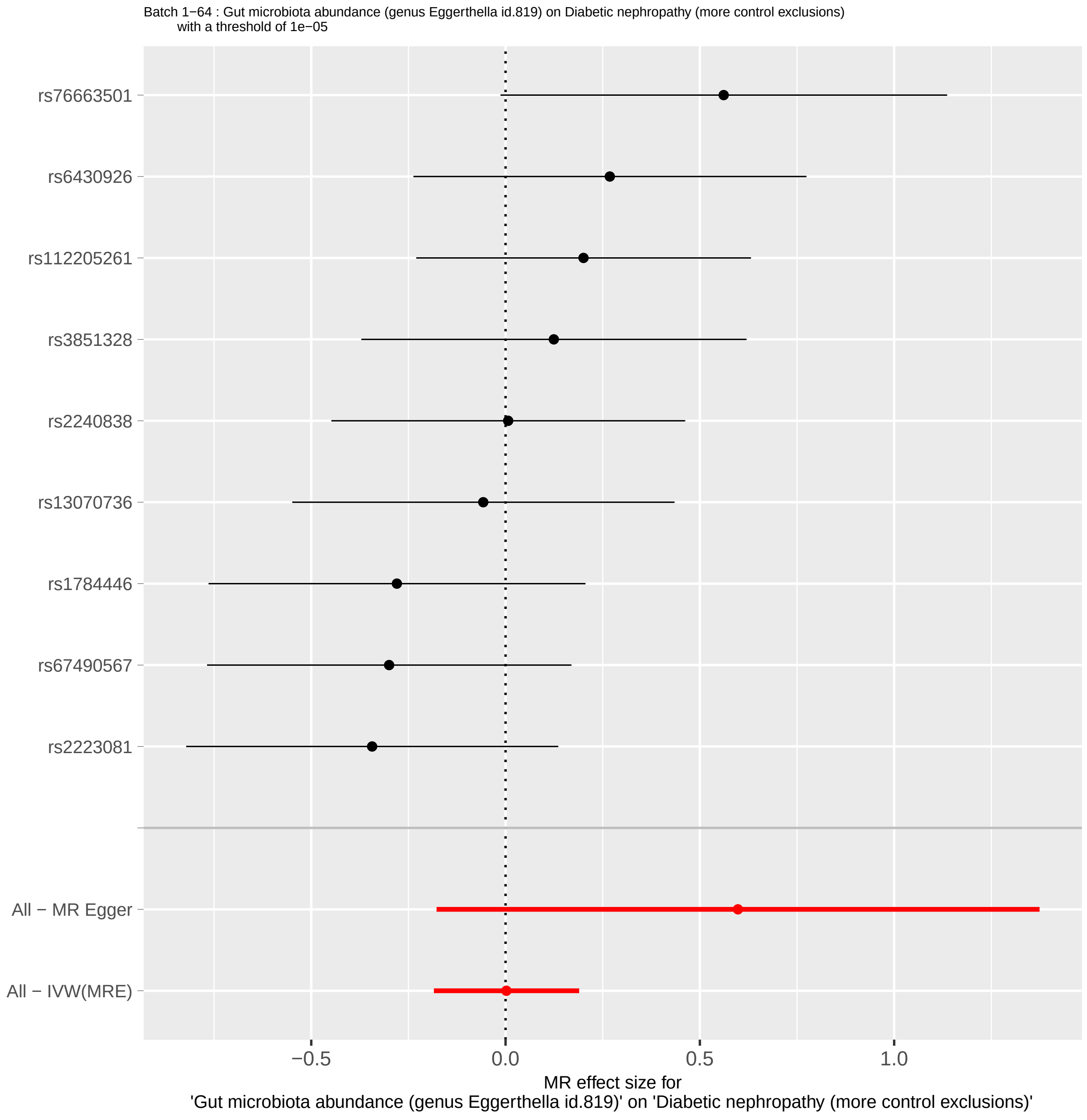
Batch 1−64 : Gut microbiota abundance (genus Eggerthella id.819) on Diabetic nephropathy (more control exclusions) with a threshold of 1e−05
rs76663501
rs6430926
rs112205261
rs3851328
rs2240838
rs13070736
rs1784446
rs67490567
rs2223081
All − MR Egger
All − IVW(MRE)
−0.5
0.0
0.5
1.0
MR effect size for
'Gut microbiota abundance (genus Eggerthella id.819)' on 'Diabetic nephropathy (more control exclusions)'

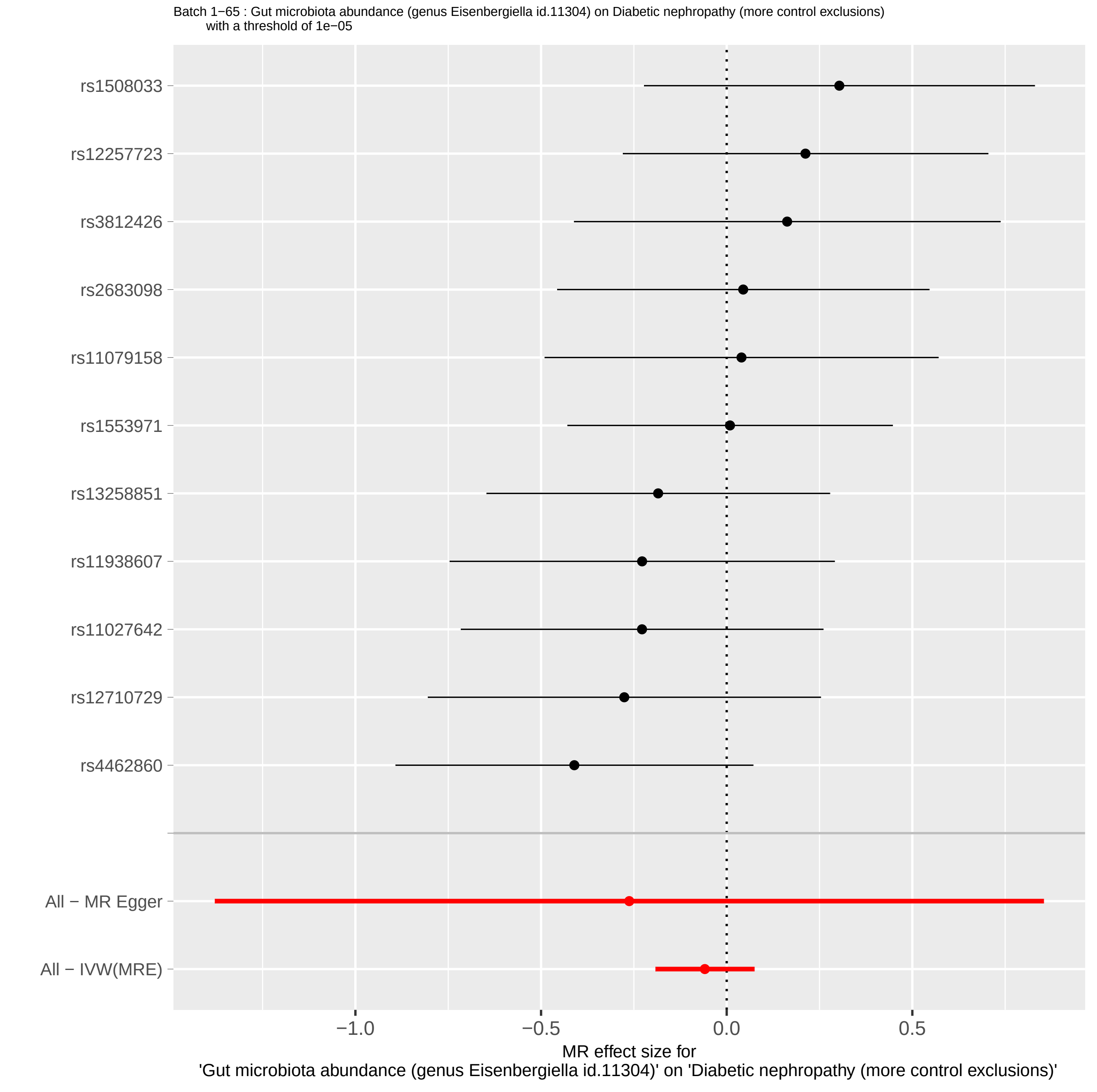

Batch 1−65 : Gut microbiota abundance (genus Eisenbergiella id.11304) on Diabetic nephropathy (more control exclusions) with a threshold of 1e−05
rs1508033
rs12257723
rs3812426
rs2683098
rs11079158
rs1553971
rs13258851
rs11938607
rs11027642
rs12710729
rs4462860
All − MR Egger
All − IVW(MRE)
−1.0
−0.5
0.0
0.5
MR effect size for
'Gut microbiota abundance (genus Eisenbergiella id.11304)' on 'Diabetic nephropathy (more control exclusions)'

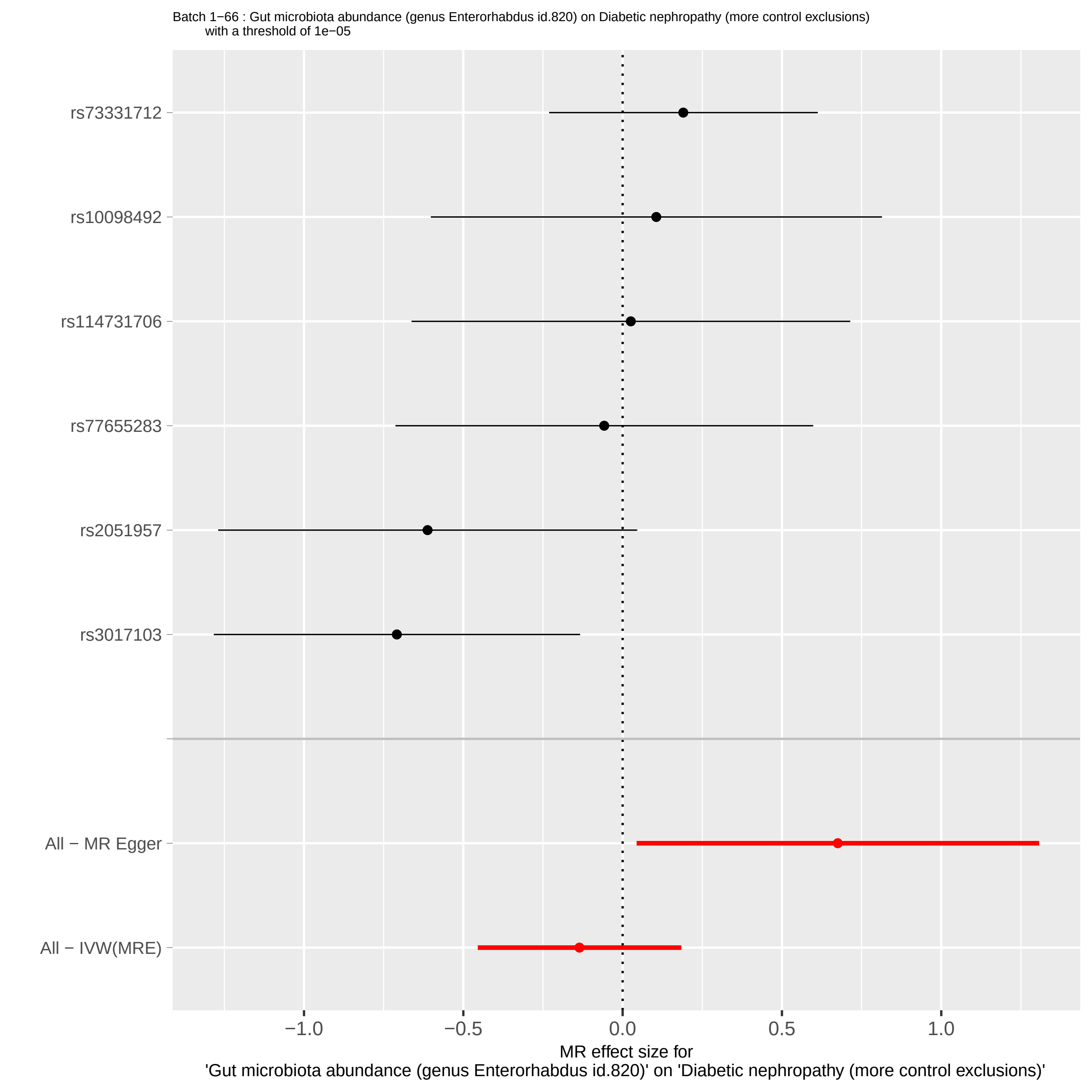

Batch 1−66 : Gut microbiota abundance (genus Enterorhabdus id.820) on Diabetic nephropathy (more control exclusions)
with a threshold of 1e−05
rs73331712
rs10098492
rs114731706
rs77655283
rs2051957
rs3017103
All − MR Egger
All − IVW(MRE)
−1.0
−0.5
0.0
0.5
1.0
MR effect size for
'Gut microbiota abundance (genus Enterorhabdus id.820)' on 'Diabetic nephropathy (more control exclusions)'

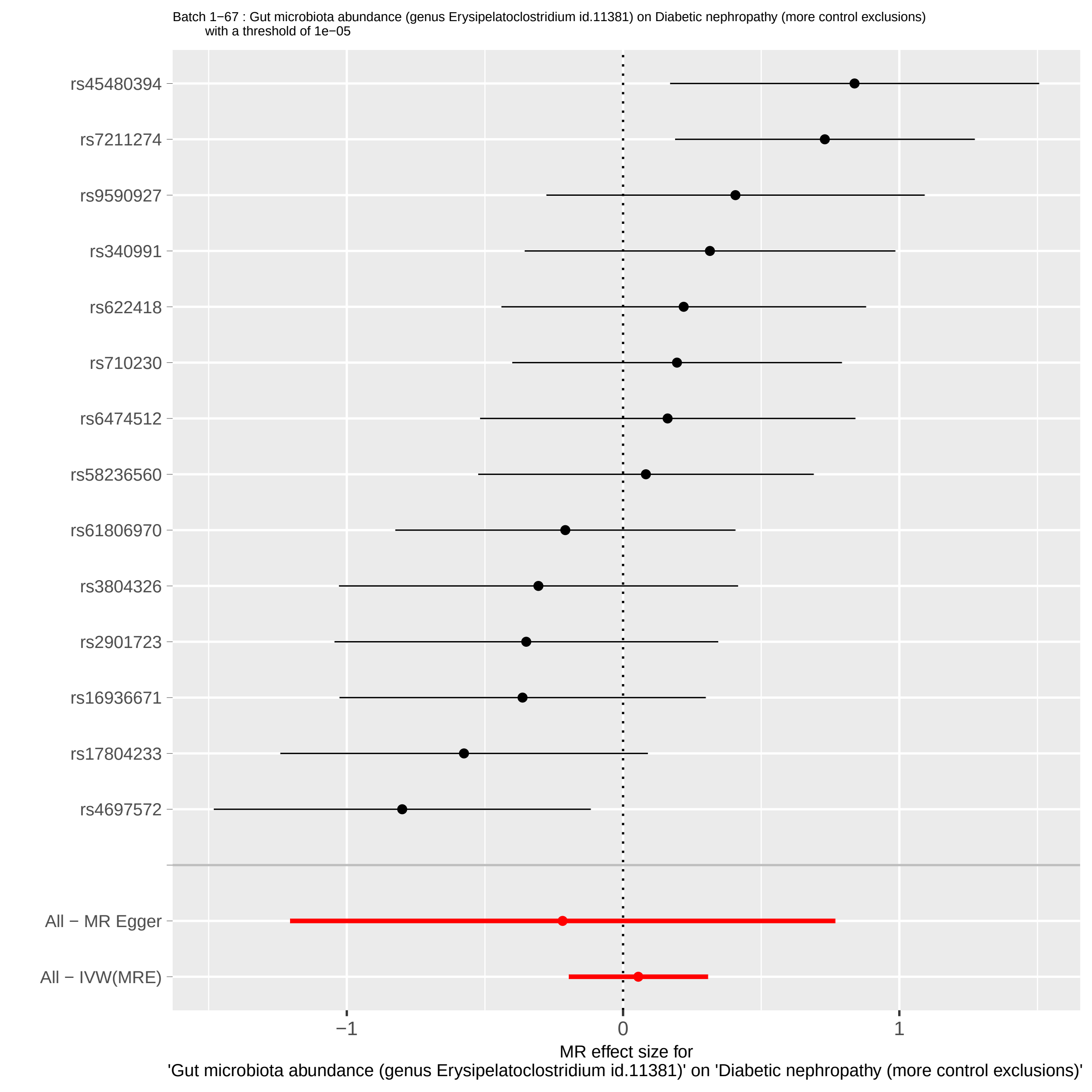
Batch 1−67 : Gut microbiota abundance (genus Erysipelatoclostridium id.11381) on Diabetic nephropathy (more control exclusions)
with a threshold of 1e−05
rs45480394
rs7211274
rs9590927
rs340991
rs622418
rs710230
rs6474512
rs58236560
rs61806970
rs3804326
rs2901723
rs16936671
rs17804233
rs4697572
All − MR Egger
All − IVW(MRE)
−1
0
1
MR effect size for
'Gut microbiota abundance (genus Erysipelatoclostridium id.11381)' on 'Diabetic nephropathy (more control exclusions)'

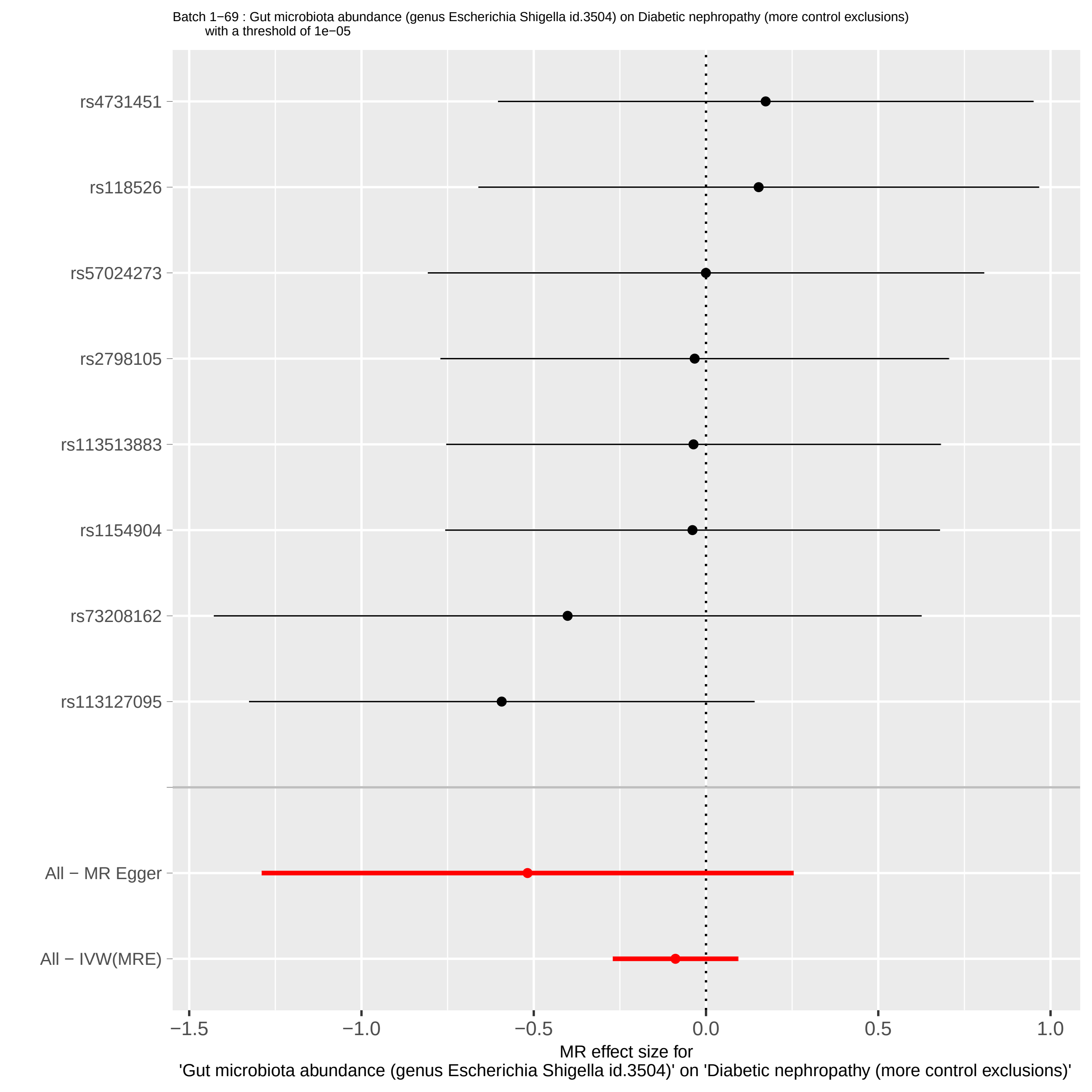

Batch 1−69 : Gut microbiota abundance (genus Escherichia Shigella id.3504) on Diabetic nephropathy (more control exclusions) with a threshold of 1e−05
rs4731451
rs118526
rs57024273
rs2798105
rs113513883
rs1154904
rs73208162
rs113127095
All − MR Egger
All − IVW(MRE)
−1.5
−1.0
−0.5
0.0
0.5
1.0
MR effect size for
'Gut microbiota abundance (genus Escherichia Shigella id.3504)' on 'Diabetic nephropathy (more control exclusions)'

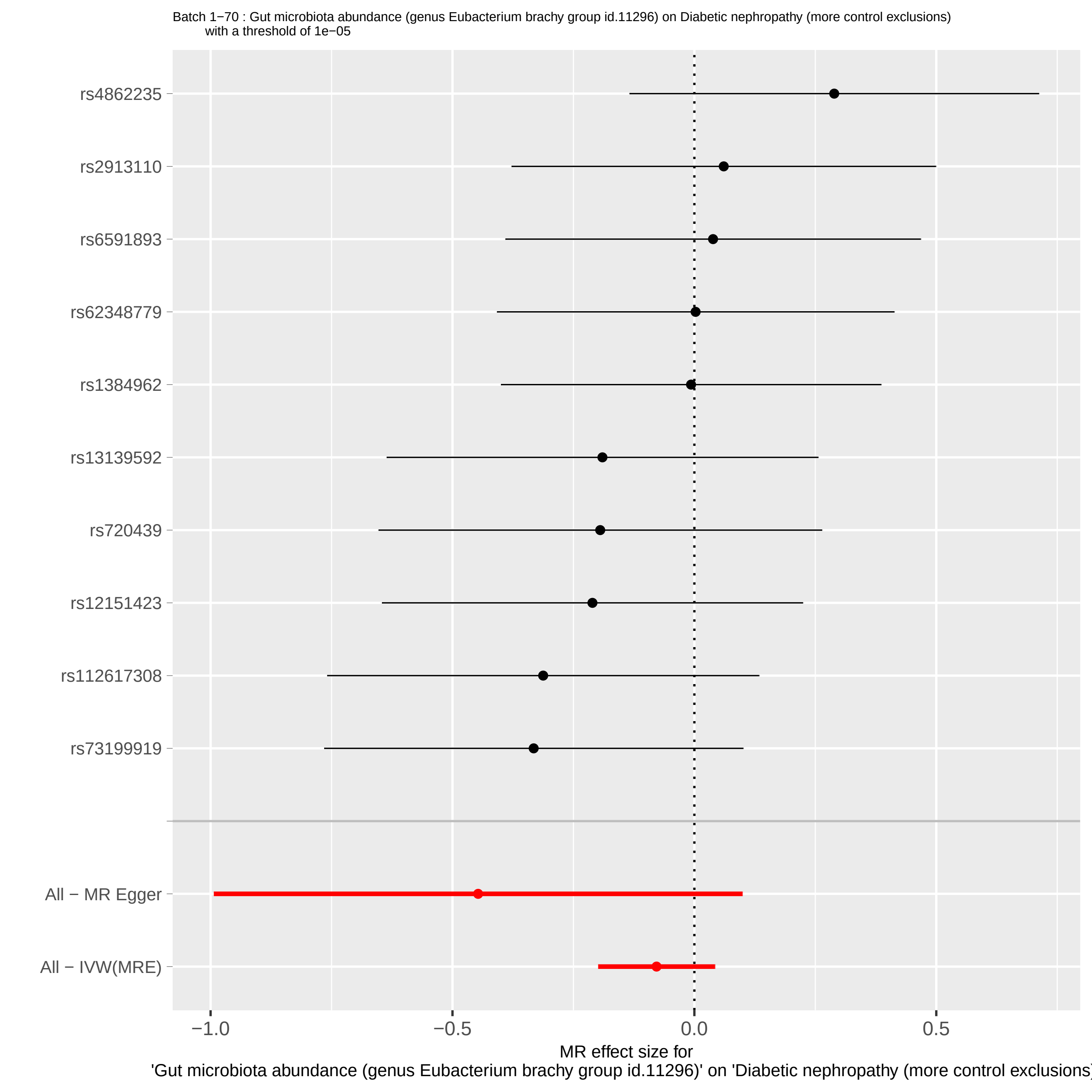

Batch 1−70 : Gut microbiota abundance (genus Eubacterium brachy group id.11296) on Diabetic nephropathy (more control exclusions)
with a threshold of 1e−05
rs4862235
rs2913110
rs6591893
rs62348779
rs1384962
rs13139592
rs720439
rs12151423
rs112617308
rs73199919
All − MR Egger
All − IVW(MRE)
−1.0
−0.5
0.0
0.5
MR effect size for
'Gut microbiota abundance (genus Eubacterium brachy group id.11296)' on 'Diabetic nephropathy (more control exclusions)

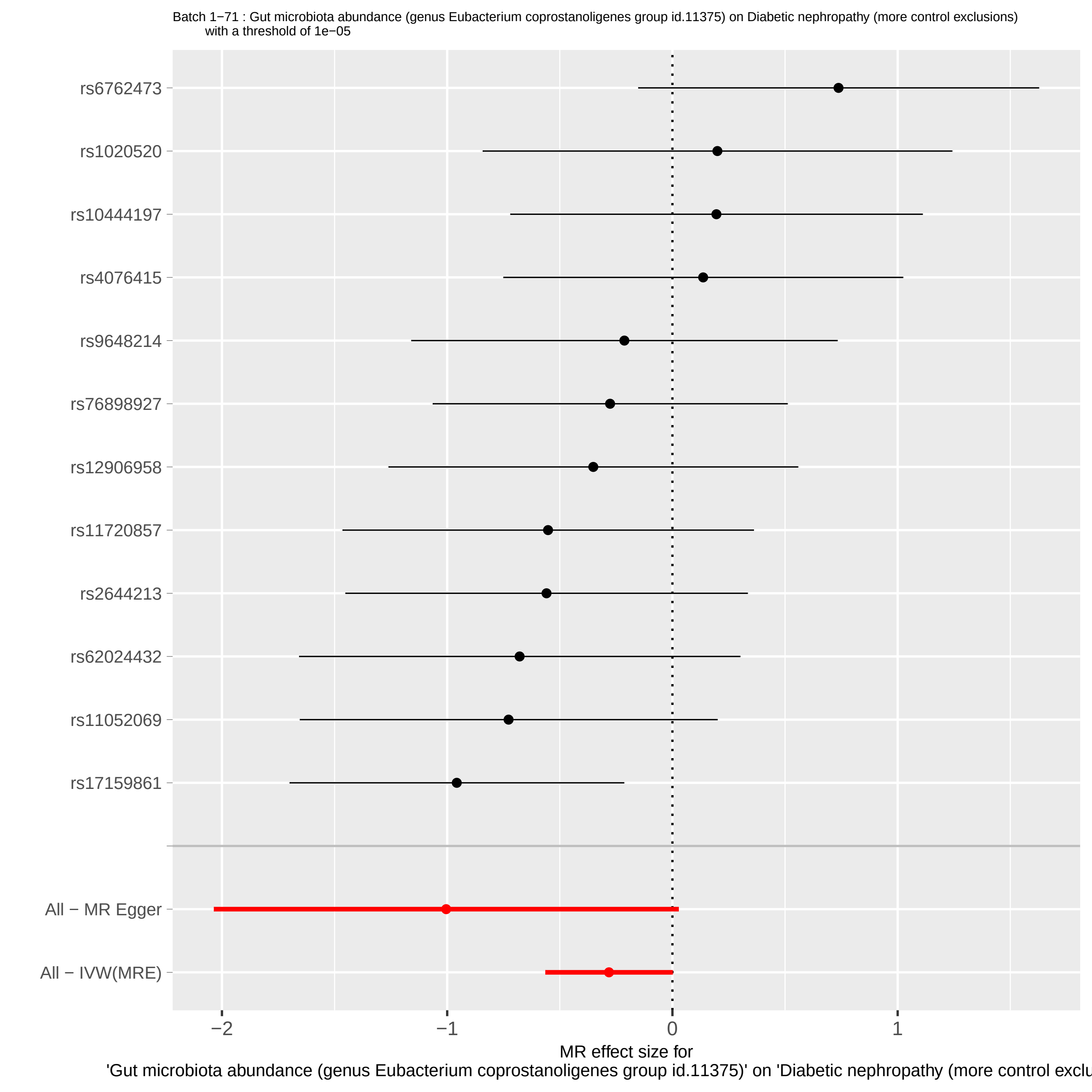

Batch 1−71 : Gut microbiota abundance (genus Eubacterium coprostanoligenes group id.11375) on Diabetic nephropathy (more control exclusions) with a threshold of 1e−05
rs6762473
rs1020520
rs10444197
rs4076415
rs9648214
rs76898927
rs12906958
rs11720857
rs2644213
rs62024432
rs11052069
rs17159861
All − MR Egger
All − IVW(MRE)
−2
−1
0
1
MR effect size for
'Gut microbiota abundance (genus Eubacterium coprostanoligenes group id.11375)' on 'Diabetic nephropathy (more control exclu

Batch 1−72 : Gut microbiota abundance (genus Eubacterium eligens group id.14372) on Diabetic nephropathy (more control exclusions) with a threshold of 1e−05

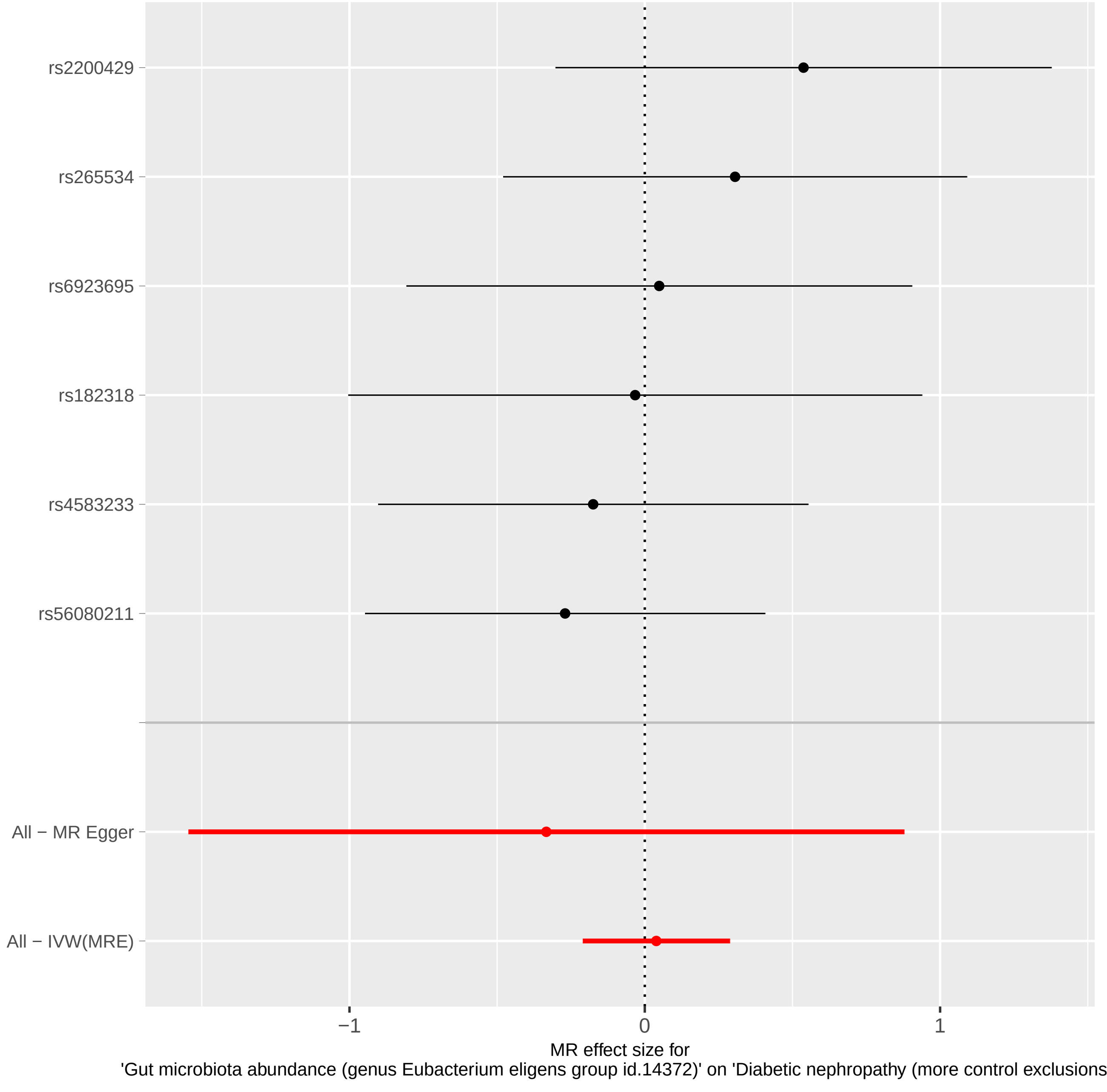

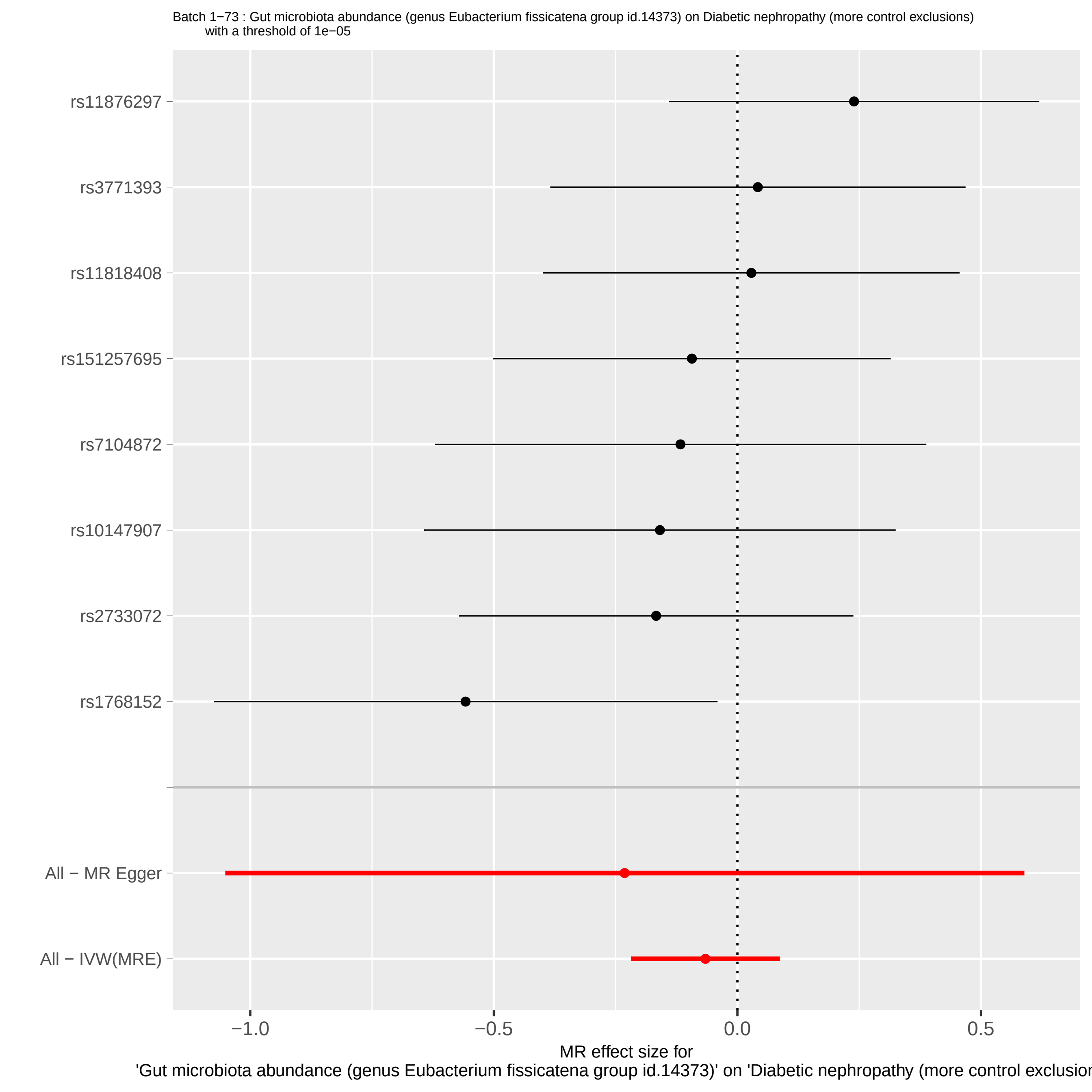

Batch 1−73 : Gut microbiota abundance (genus Eubacterium fissicatena group id.14373) on Diabetic nephropathy (more control exclusions)
with a threshold of 1e−05
rs11876297
rs3771393
rs11818408
rs151257695
rs7104872
rs10147907
rs2733072
rs1768152
All − MR Egger
All − IVW(MRE)
−1.0
−0.5
0.0
0.5
MR effect size for
'Gut microbiota abundance (genus Eubacterium fissicatena group id.14373)' on 'Diabetic nephropathy (more control exclusio

Batch 1−74 : Gut microbiota abundance (genus Eubacterium hallii group id.11338) on Diabetic nephropathy (more control exclusions) with a threshold of 1e−05

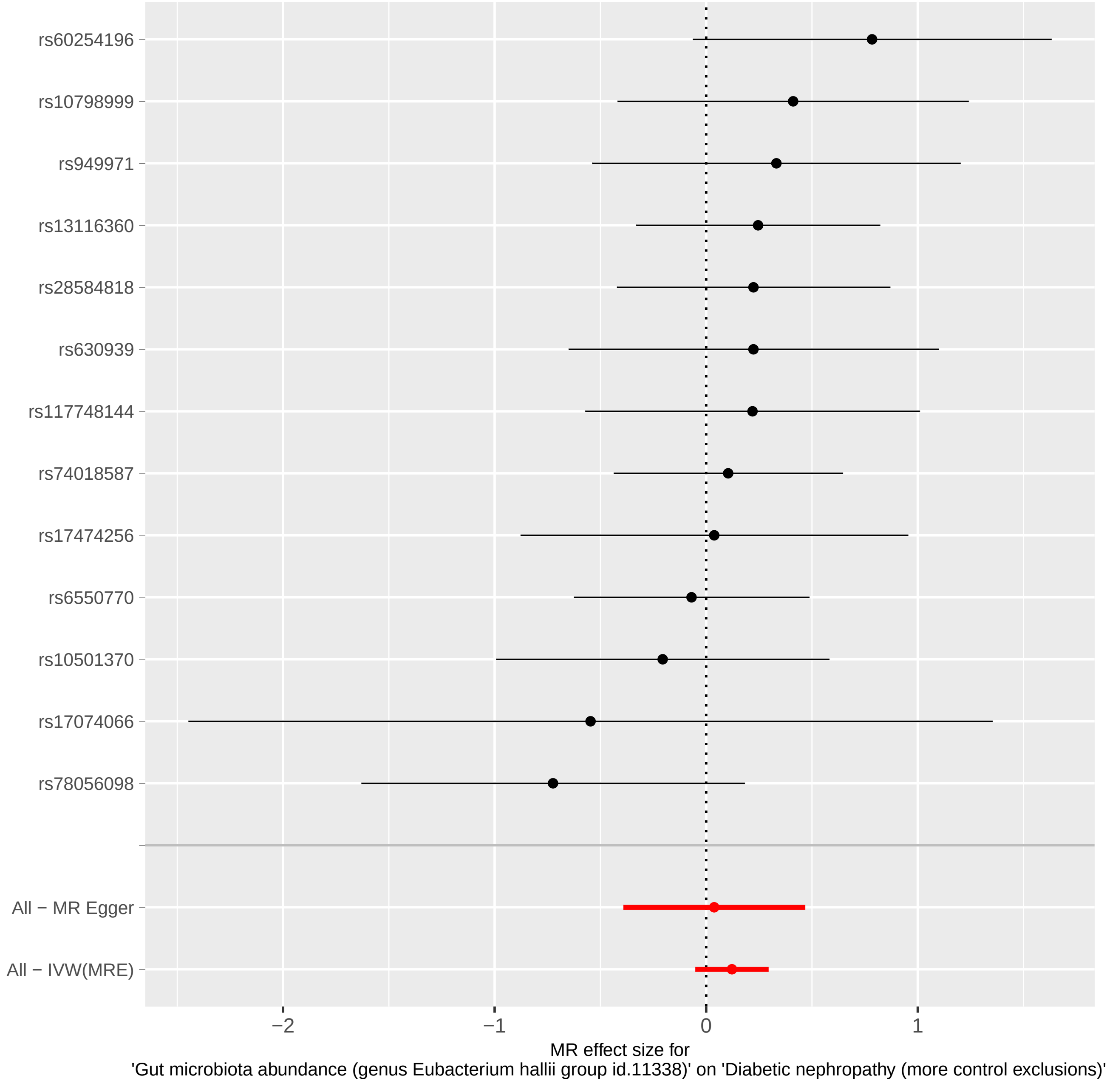

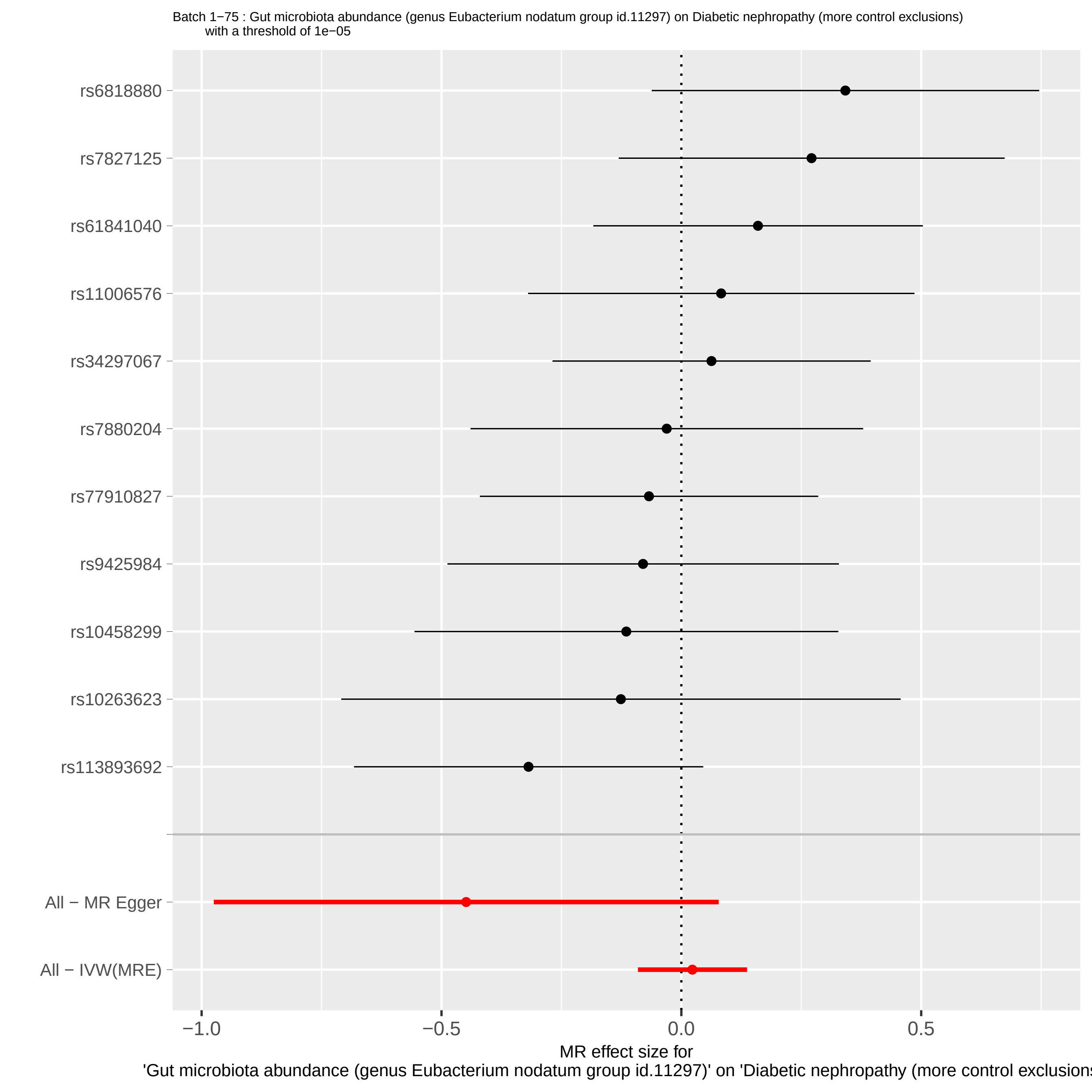

Batch 1−75 : Gut microbiota abundance (genus Eubacterium nodatum group id.11297) on Diabetic nephropathy (more control exclusions) with a threshold of 1e−05
rs6818880
rs7827125
rs61841040
rs11006576
rs34297067
rs7880204
rs77910827
rs9425984
rs10458299
rs10263623
rs113893692
All − MR Egger
All − IVW(MRE)
−1.0
−0.5
0.0
0.5
MR effect size for
'Gut microbiota abundance (genus Eubacterium nodatum group id.11297)' on 'Diabetic nephropathy (more control exclusions

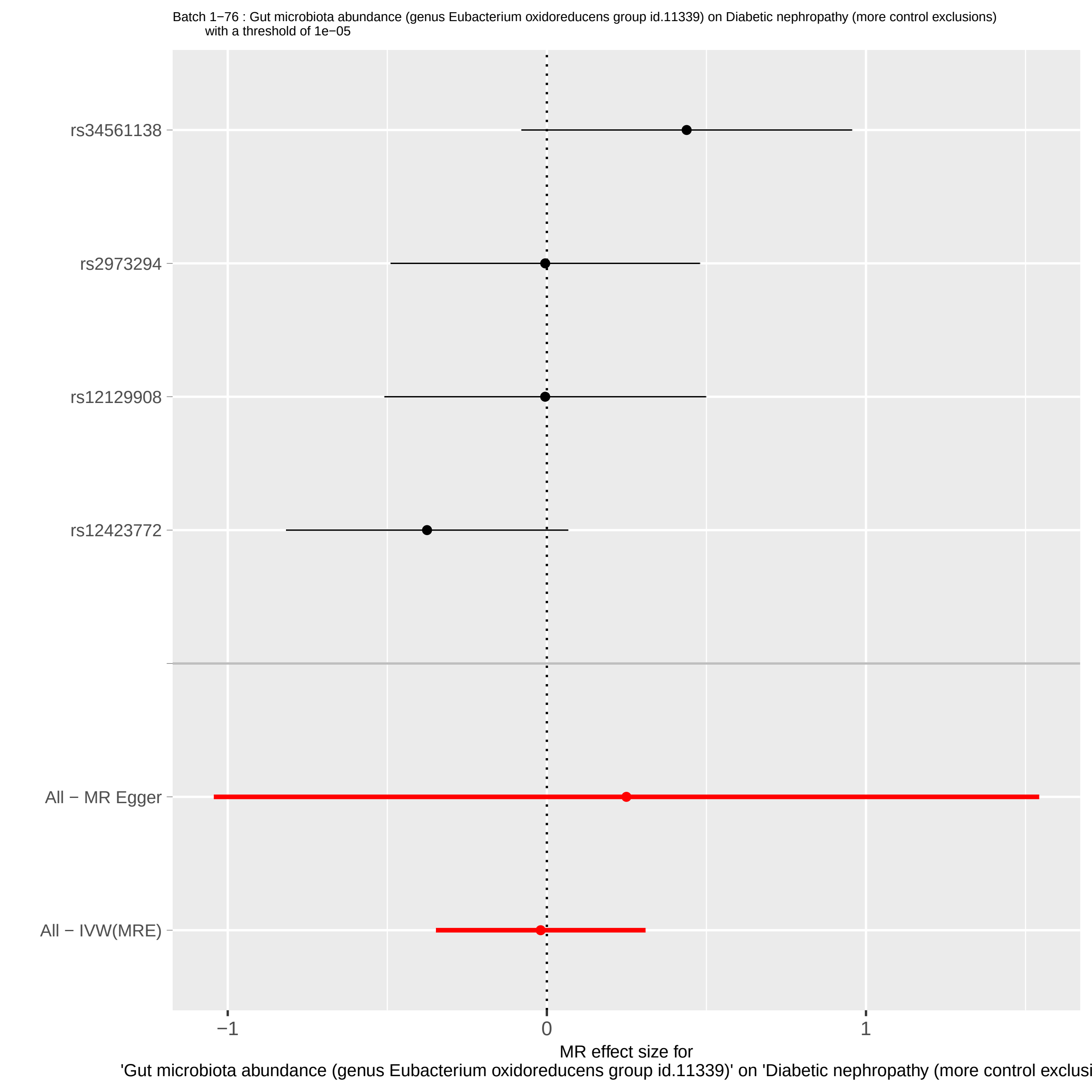

Batch 1−76 : Gut microbiota abundance (genus Eubacterium oxidoreducens group id.11339) on Diabetic nephropathy (more control exclusions)
with a threshold of 1e−05
rs34561138
rs2973294
rs12129908
rs12423772
All − MR Egger
All − IVW(MRE)
−1
0
1
MR effect size for
'Gut microbiota abundance (genus Eubacterium oxidoreducens group id.11339)' on 'Diabetic nephropathy (more control exclusi

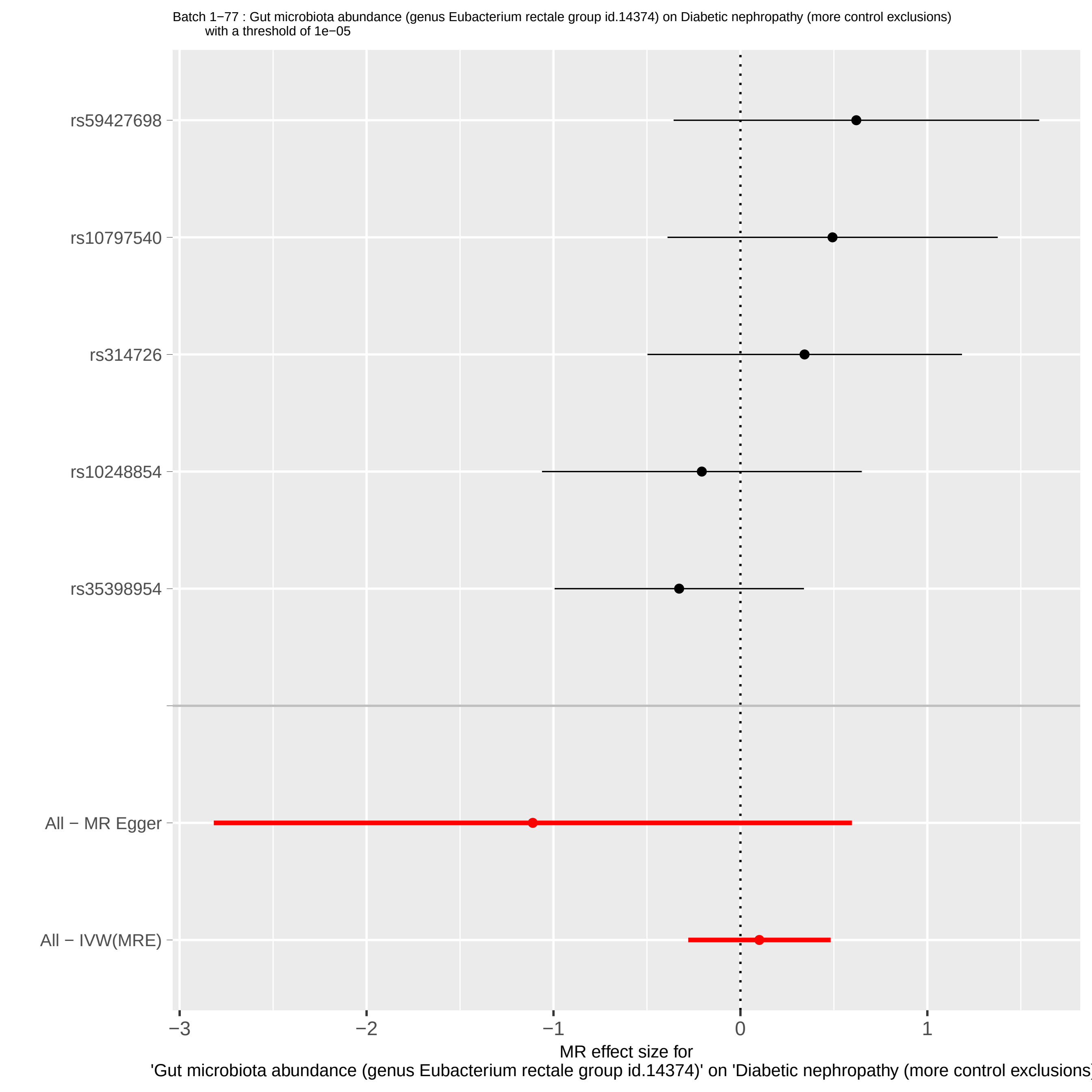
Batch 1−77 : Gut microbiota abundance (genus Eubacterium rectale group id.14374) on Diabetic nephropathy (more control exclusions)
with a threshold of 1e−05
rs59427698
rs10797540
rs314726
rs10248854
rs35398954
All − MR Egger
All − IVW(MRE)
−3
−2
−1
0
1
MR effect size for
'Gut microbiota abundance (genus Eubacterium rectale group id.14374)' on 'Diabetic nephropathy (more control exclusions)'

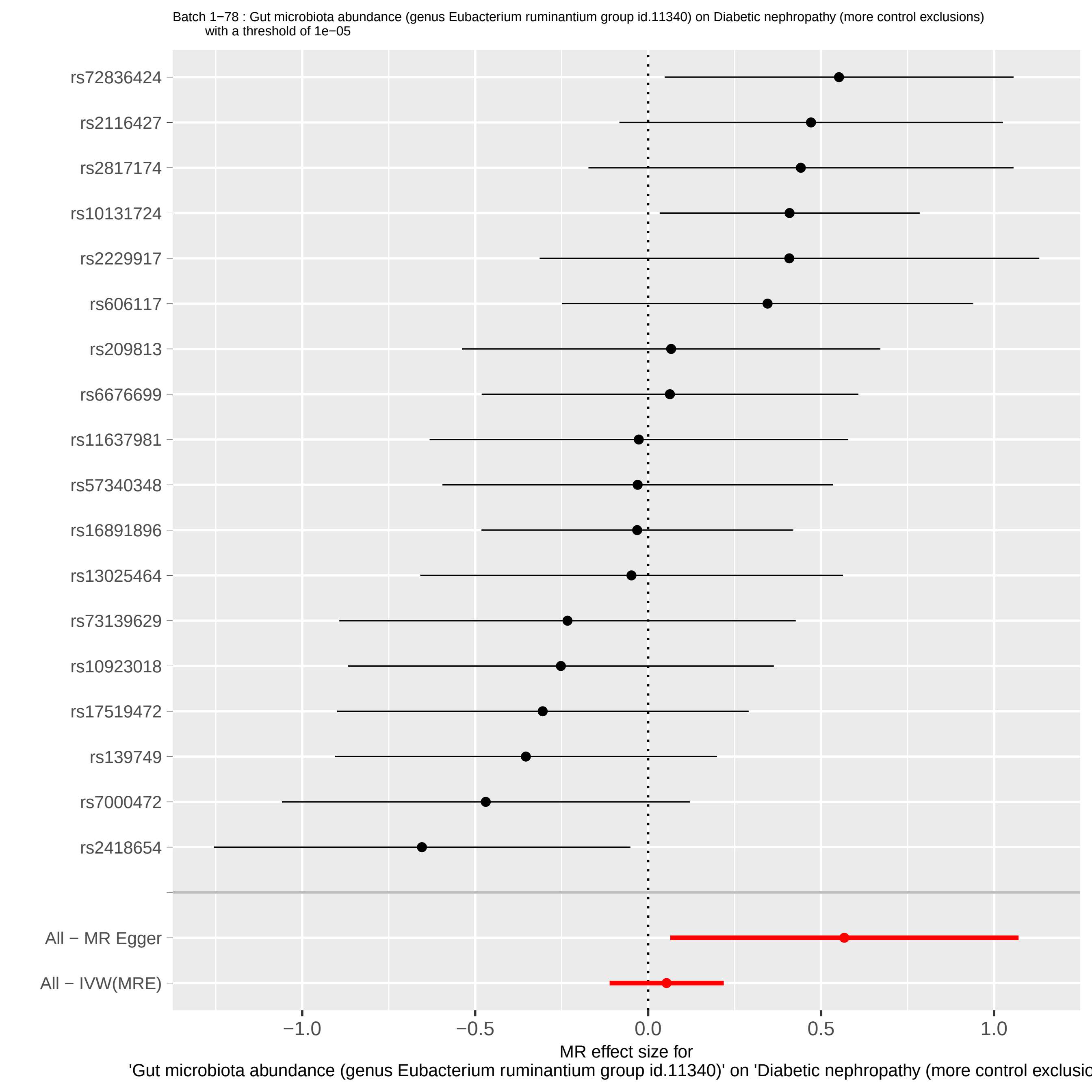

Batch 1−78 : Gut microbiota abundance (genus Eubacterium ruminantium group id.11340) on Diabetic nephropathy (more control exclusions)
with a threshold of 1e−05
rs72836424
rs2116427
rs2817174
rs10131724
rs2229917
rs606117
rs209813
rs6676699
rs11637981
rs57340348
rs16891896
rs13025464
rs73139629
rs10923018
rs17519472
rs139749
rs7000472
rs2418654
All − MR Egger
All − IVW(MRE)
−1.0
−0.5
0.0
0.5
1.0
MR effect size for
'Gut microbiota abundance (genus Eubacterium ruminantium group id.11340)' on 'Diabetic nephropathy (more control exclusio

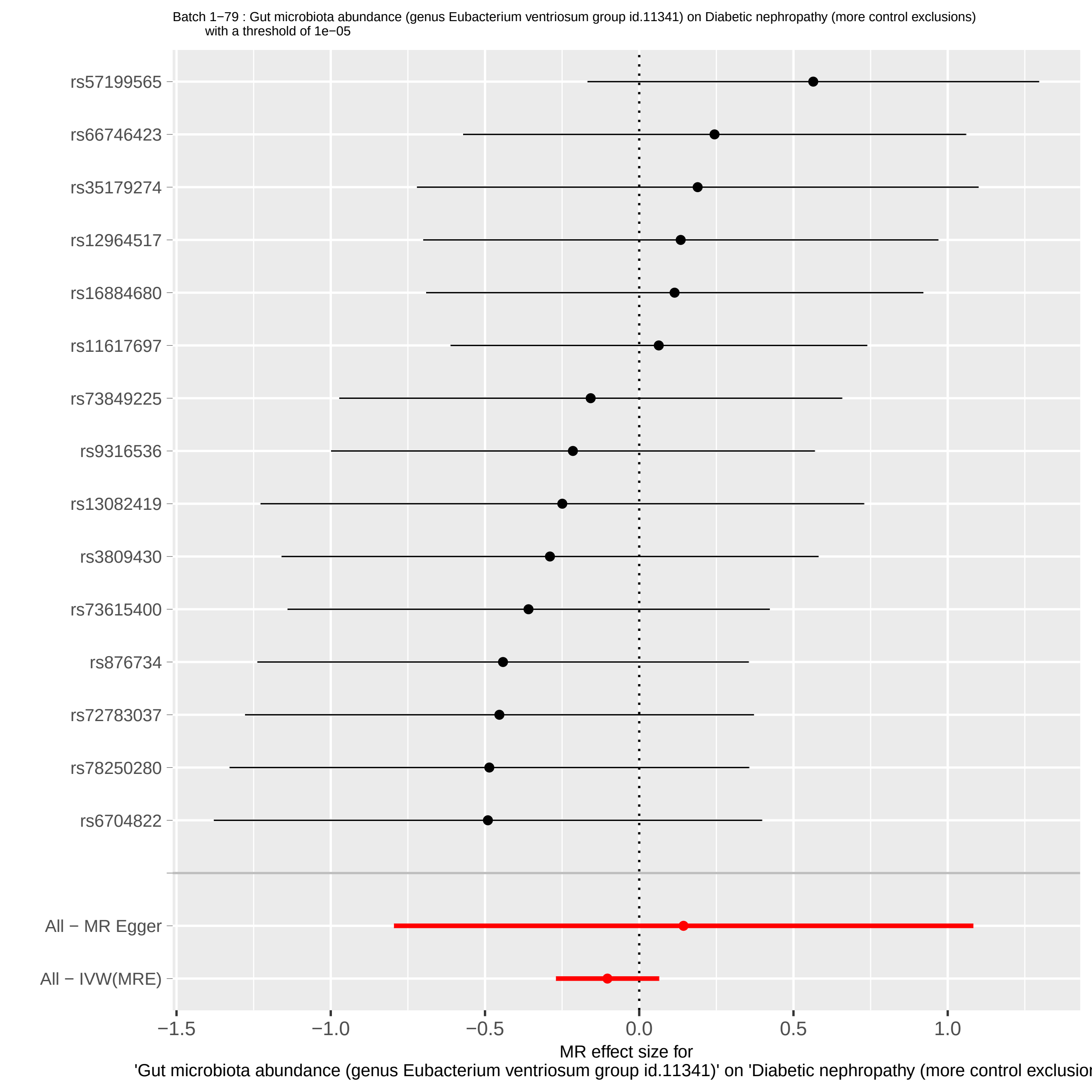
Batch 1−79 : Gut microbiota abundance (genus Eubacterium ventriosum group id.11341) on Diabetic nephropathy (more control exclusions)
with a threshold of 1e−05
rs57199565
rs66746423
rs35179274
rs12964517
rs16884680
rs11617697
rs73849225
rs9316536
rs13082419
rs3809430
rs73615400
rs876734
rs72783037
rs78250280
rs6704822
All − MR Egger
All − IVW(MRE)
−1.5
−1.0
−0.5
0.0
0.5
1.0
MR effect size for
'Gut microbiota abundance (genus Eubacterium ventriosum group id.11341)' on 'Diabetic nephropathy (more control exclusio

Batch 1−80 : Gut microbiota abundance (genus Eubacterium xylanophilum group id.14375) on Diabetic nephropathy (more control exclusions) with a threshold of 1e−05

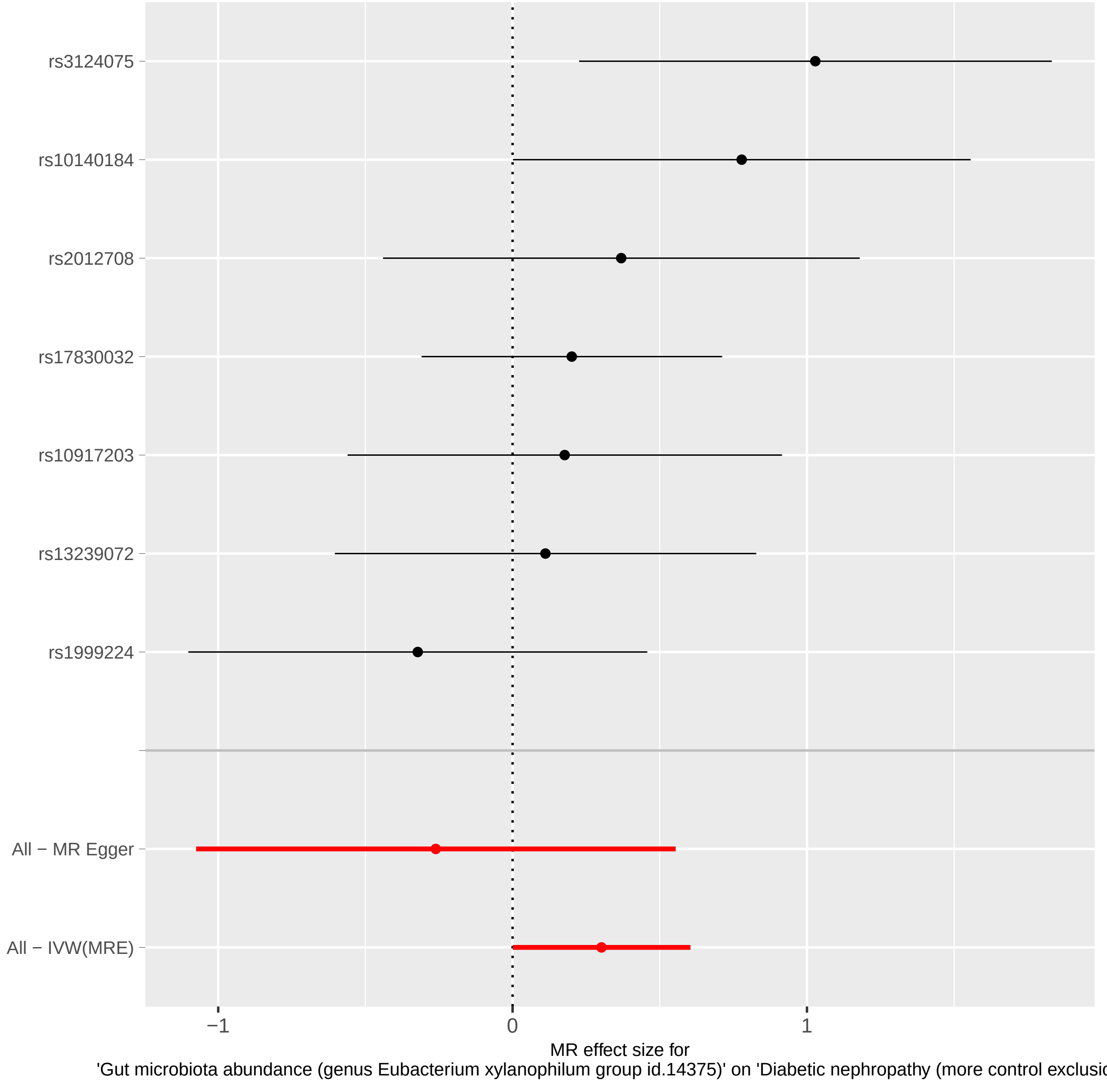

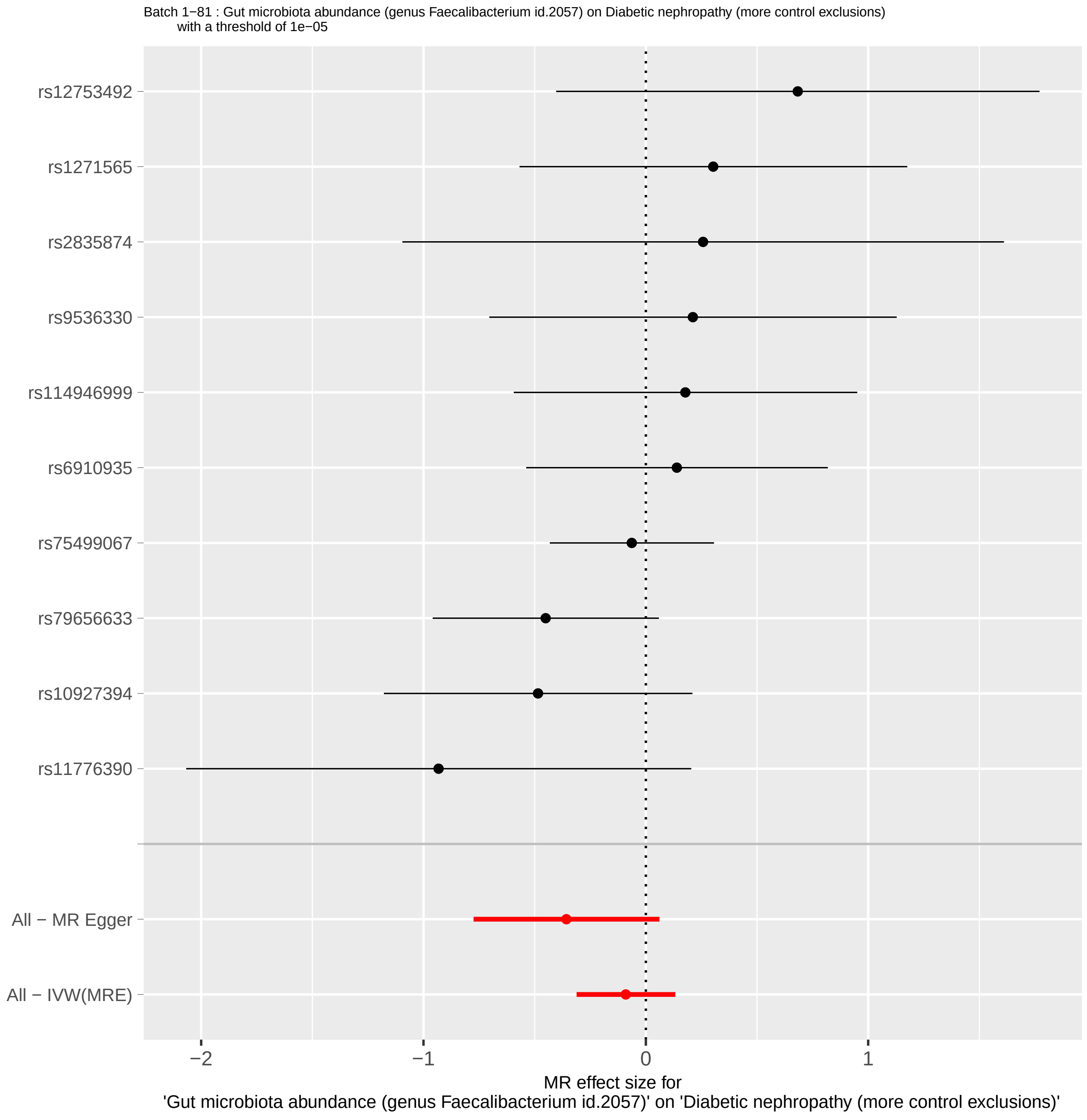
Batch 1−81 : Gut microbiota abundance (genus Faecalibacterium id.2057) on Diabetic nephropathy (more control exclusions) with a threshold of 1e−05
rs12753492
rs1271565
rs2835874
rs9536330
rs114946999
rs6910935
rs75499067
rs79656633
rs10927394
rs11776390
All − MR Egger
All − IVW(MRE)
−2
−1
0
1
MR effect size for
'Gut microbiota abundance (genus Faecalibacterium id.2057)' on 'Diabetic nephropathy (more control exclusions)'

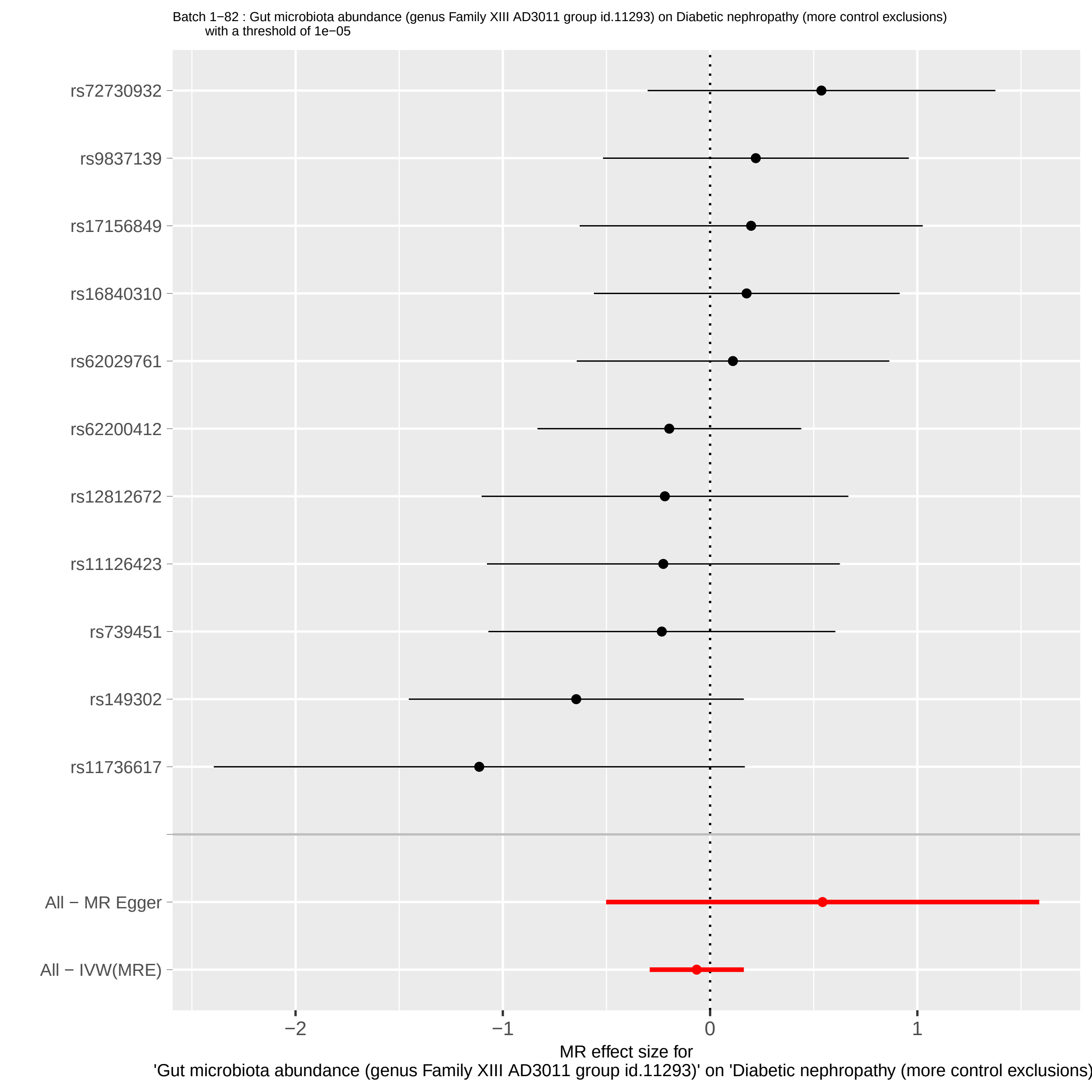
Batch 1−82 : Gut microbiota abundance (genus Family XIII AD3011 group id.11293) on Diabetic nephropathy (more control exclusions) with a threshold of 1e−05
rs72730932
rs9837139
rs17156849
rs16840310
rs62029761
rs62200412
rs12812672
rs11126423
rs739451
rs149302
rs11736617
All − MR Egger
All − IVW(MRE)
−2
−1
0
1
MR effect size for
'Gut microbiota abundance (genus Family XIII AD3011 group id.11293)' on 'Diabetic nephropathy (more control exclusions)'

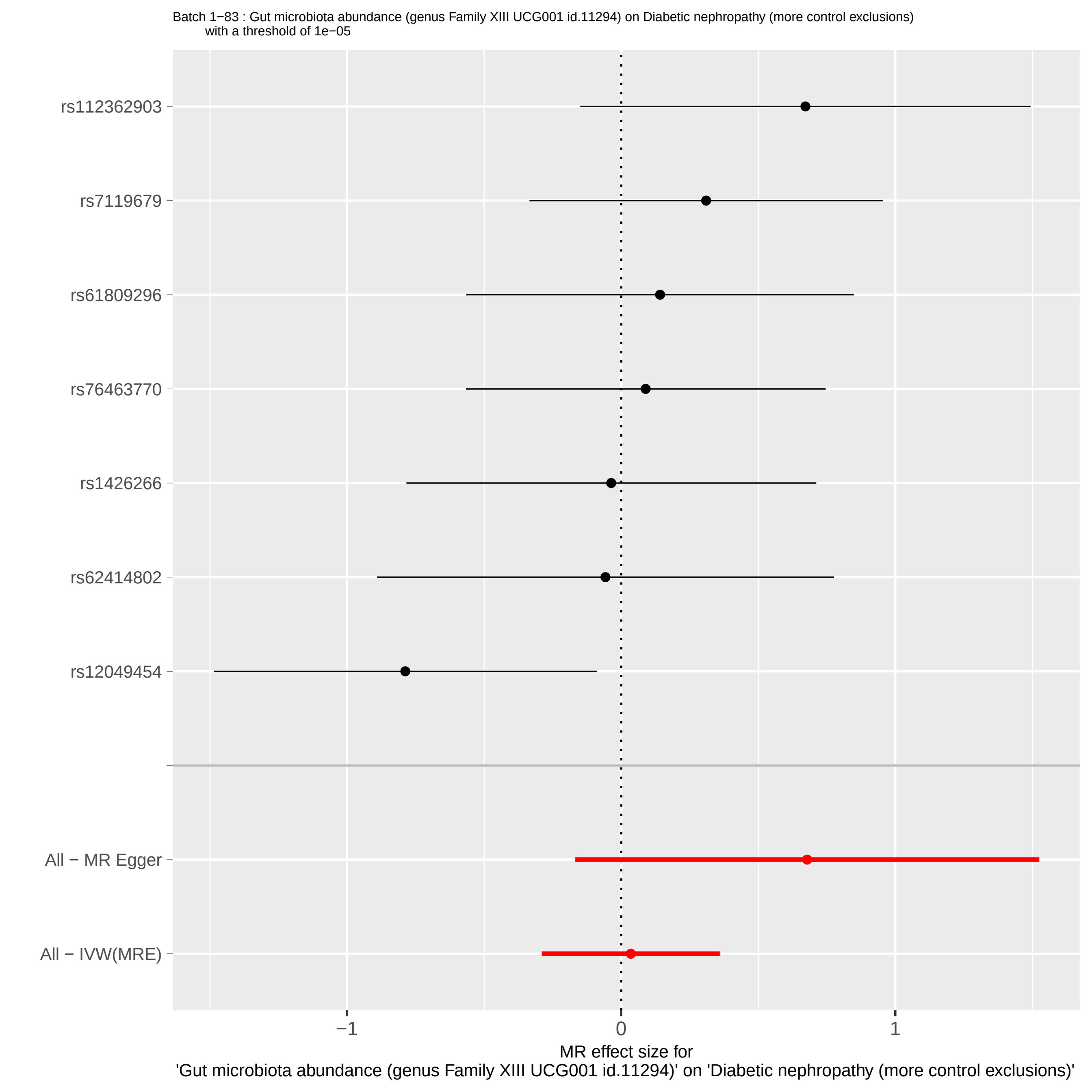
Batch 1−83 : Gut microbiota abundance (genus Family XIII UCG001 id.11294) on Diabetic nephropathy (more control exclusions)
with a threshold of 1e−05
rs112362903
rs7119679
rs61809296
rs76463770
rs1426266
rs62414802
rs12049454
All − MR Egger
All − IVW(MRE)
−1
0
1
MR effect size for
'Gut microbiota abundance (genus Family XIII UCG001 id.11294)' on 'Diabetic nephropathy (more control exclusions)'

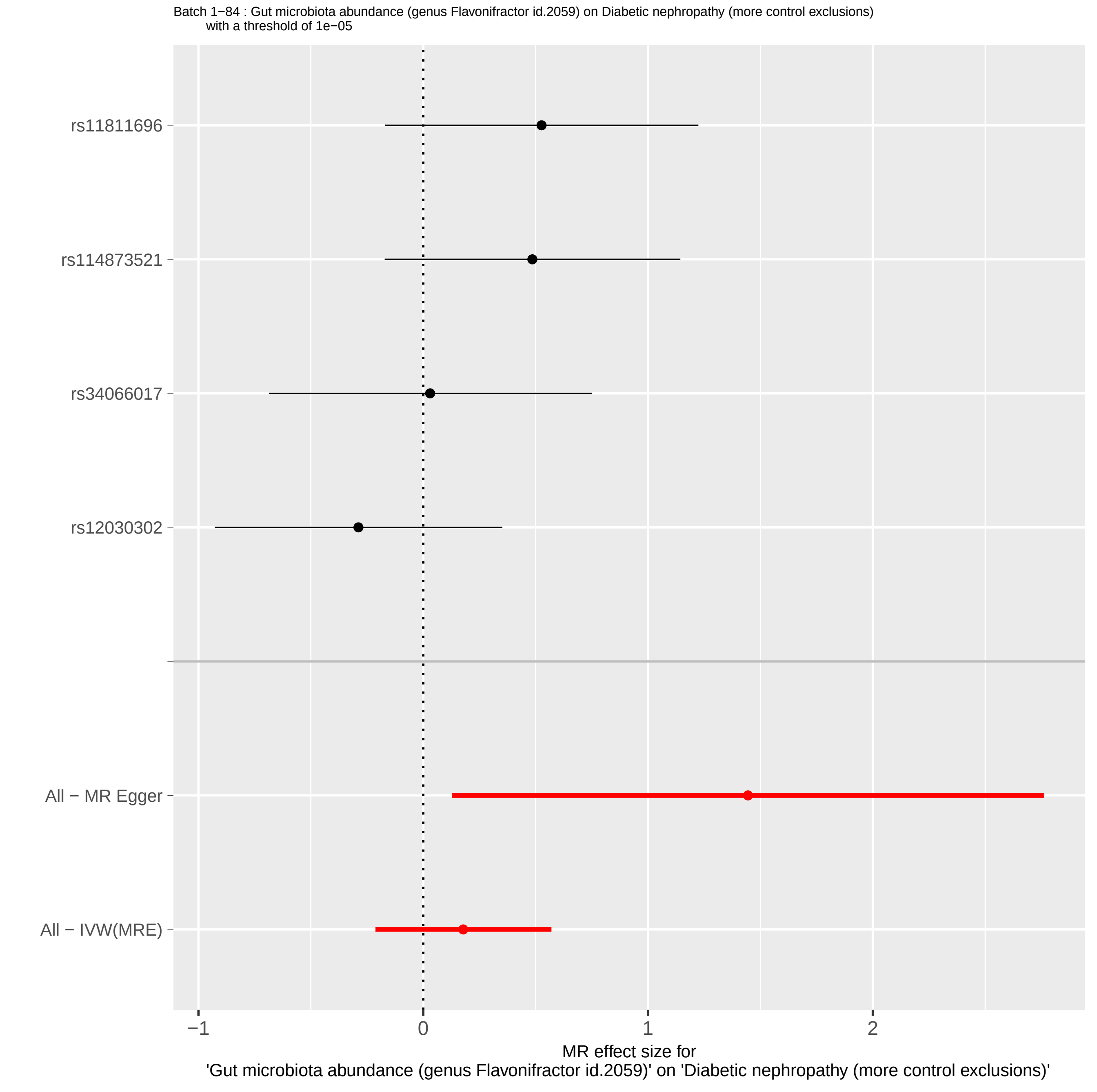
Batch 1−84 : Gut microbiota abundance (genus Flavonifractor id.2059) on Diabetic nephropathy (more control exclusions) with a threshold of 1e−05
rs11811696
rs114873521
rs34066017
rs12030302
All − MR Egger
All − IVW(MRE)
−1
0
1
2
MR effect size for
'Gut microbiota abundance (genus Flavonifractor id.2059)' on 'Diabetic nephropathy (more control exclusions)'

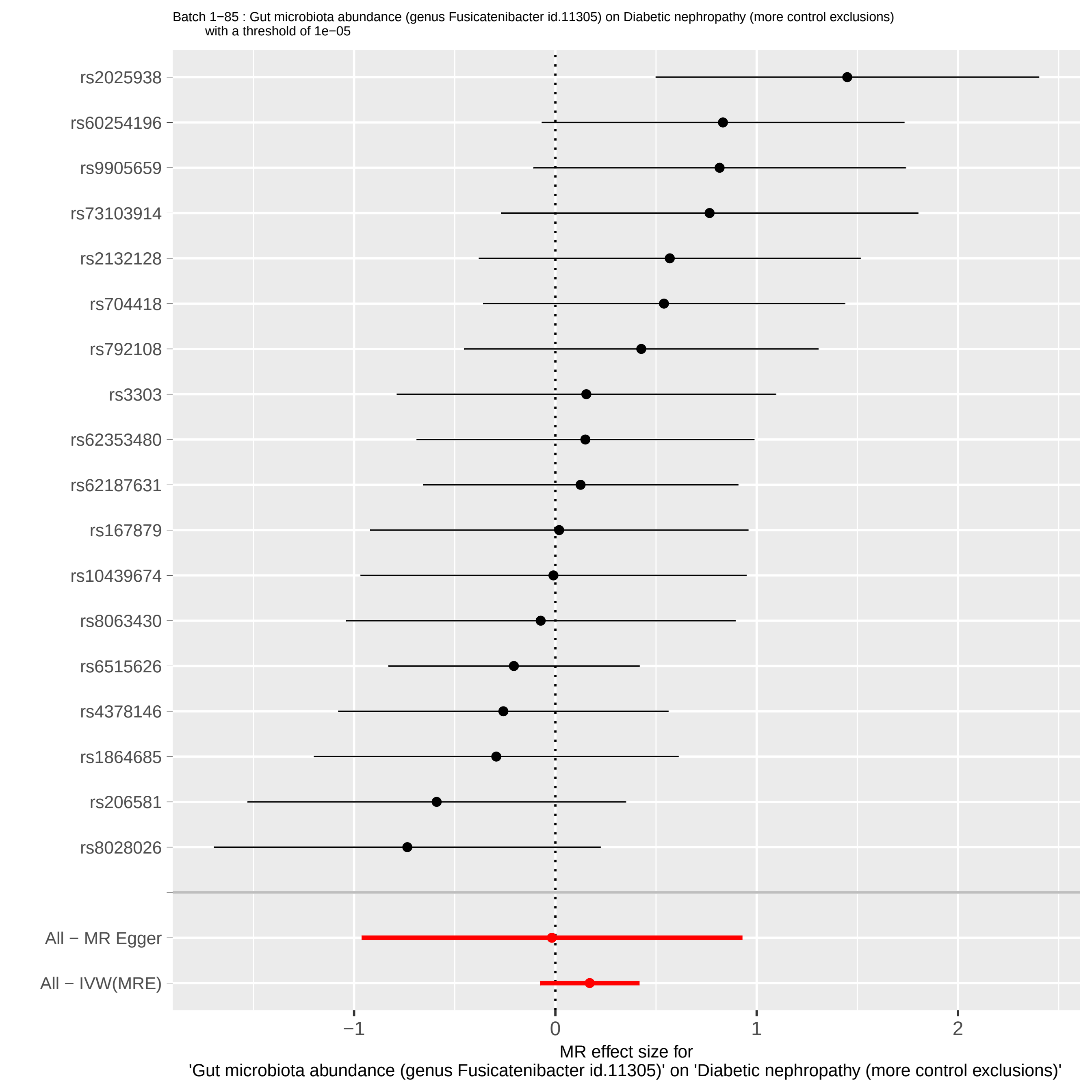
Batch 1−85 : Gut microbiota abundance (genus Fusicatenibacter id.11305) on Diabetic nephropathy (more control exclusions)
with a threshold of 1e−05
rs2025938
rs60254196
rs9905659
rs73103914
rs2132128
rs704418
rs792108
rs3303
rs62353480
rs62187631
rs167879
rs10439674
rs8063430
rs6515626
rs4378146
rs1864685
rs206581
rs8028026
All − MR Egger
All − IVW(MRE)
−1
0
1
2
MR effect size for
'Gut microbiota abundance (genus Fusicatenibacter id.11305)' on 'Diabetic nephropathy (more control exclusions)'

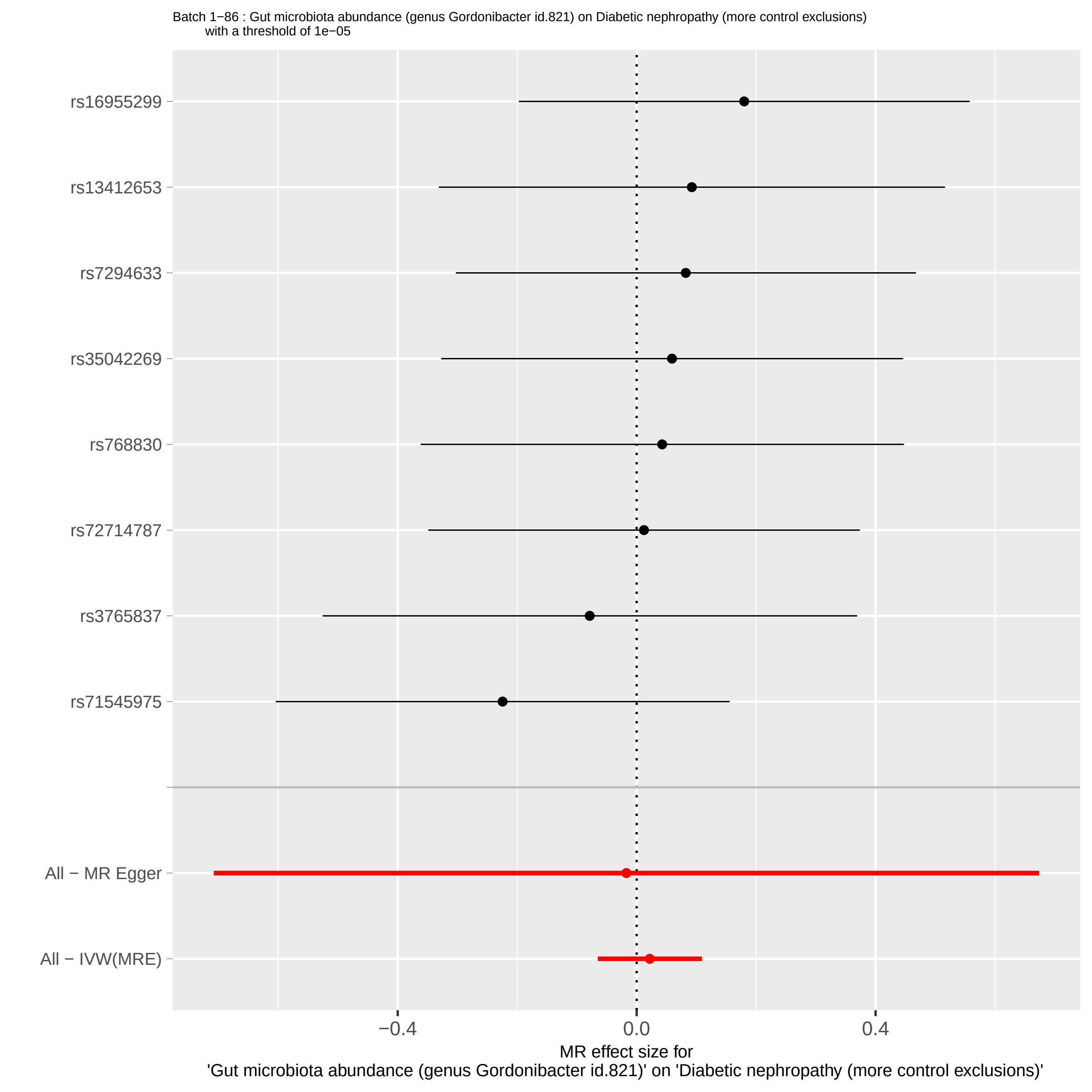
Batch 1−86 : Gut microbiota abundance (genus Gordonibacter id.821) on Diabetic nephropathy (more control exclusions) with a threshold of 1e−05
rs16955299
rs13412653
rs7294633
rs35042269
rs768830
rs72714787
rs3765837
rs71545975
All − MR Egger
All − IVW(MRE)
−0.4
0.0
0.4
MR effect size for
'Gut microbiota abundance (genus Gordonibacter id.821)' on 'Diabetic nephropathy (more control exclusions)'

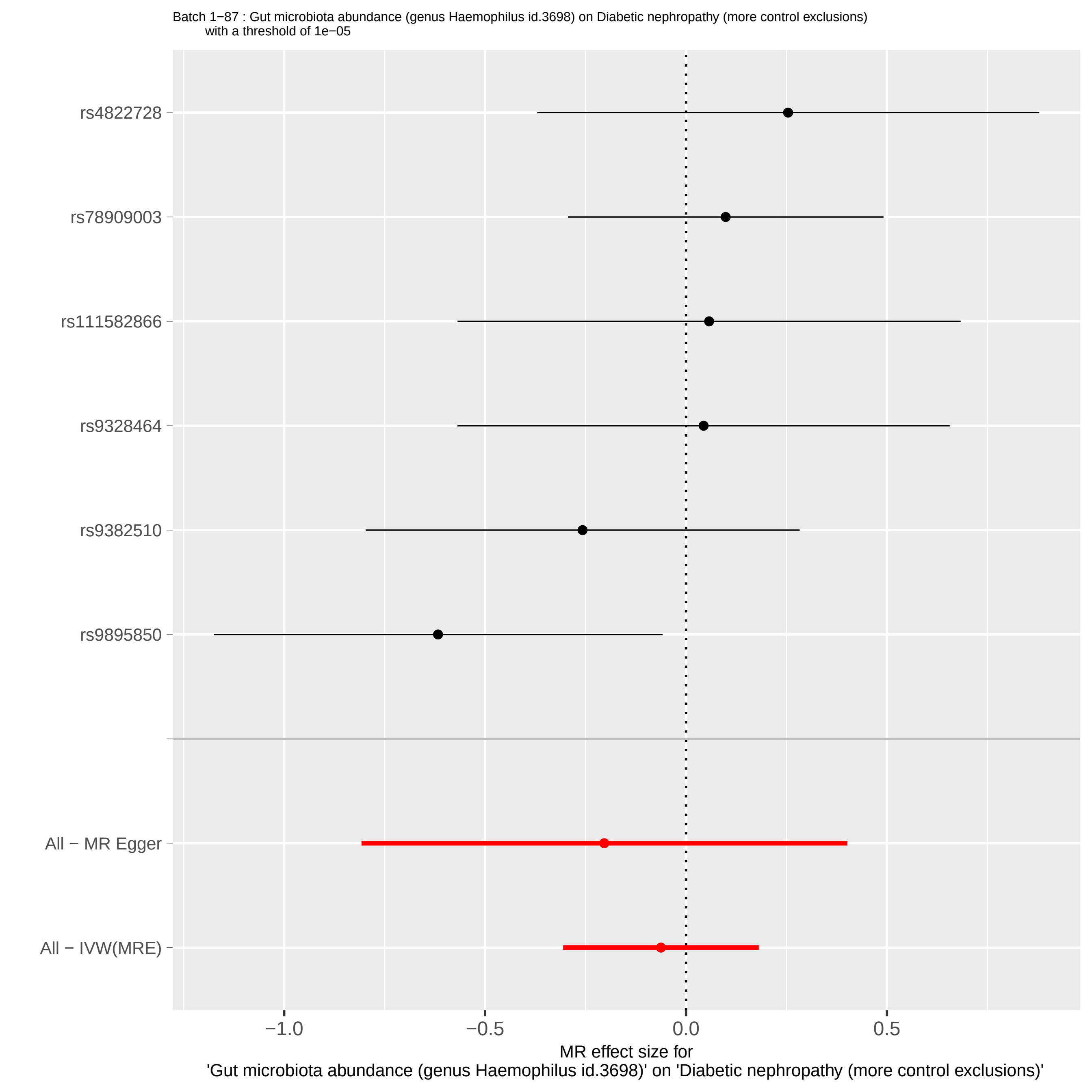
Batch 1−87 : Gut microbiota abundance (genus Haemophilus id.3698) on Diabetic nephropathy (more control exclusions)
with a threshold of 1e−05
rs4822728
rs78909003
rs111582866
rs9328464
rs9382510
rs9895850
All − MR Egger
All − IVW(MRE)
−1.0
−0.5
0.0
0.5
MR effect size for
'Gut microbiota abundance (genus Haemophilus id.3698)' on 'Diabetic nephropathy (more control exclusions)'

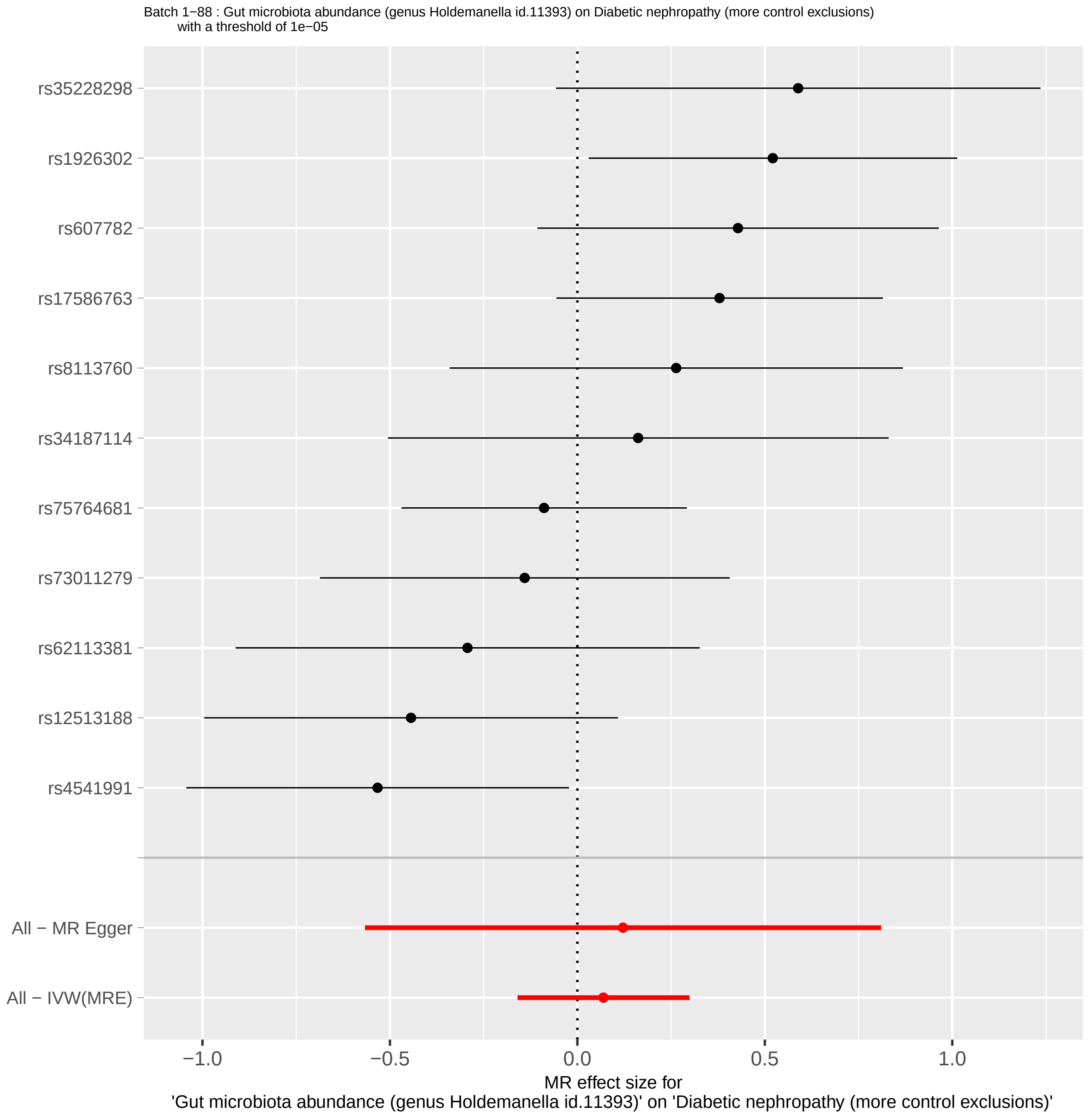

Batch 1−88 : Gut microbiota abundance (genus Holdemanella id.11393) on Diabetic nephropathy (more control exclusions)
with a threshold of 1e−05
rs35228298
rs1926302
rs607782
rs17586763
rs8113760
rs34187114
rs75764681
rs73011279
rs62113381
rs12513188
rs4541991
All − MR Egger
All − IVW(MRE)
−1.0
−0.5
0.0
0.5
1.0
MR effect size for
'Gut microbiota abundance (genus Holdemanella id.11393)' on 'Diabetic nephropathy (more control exclusions)'

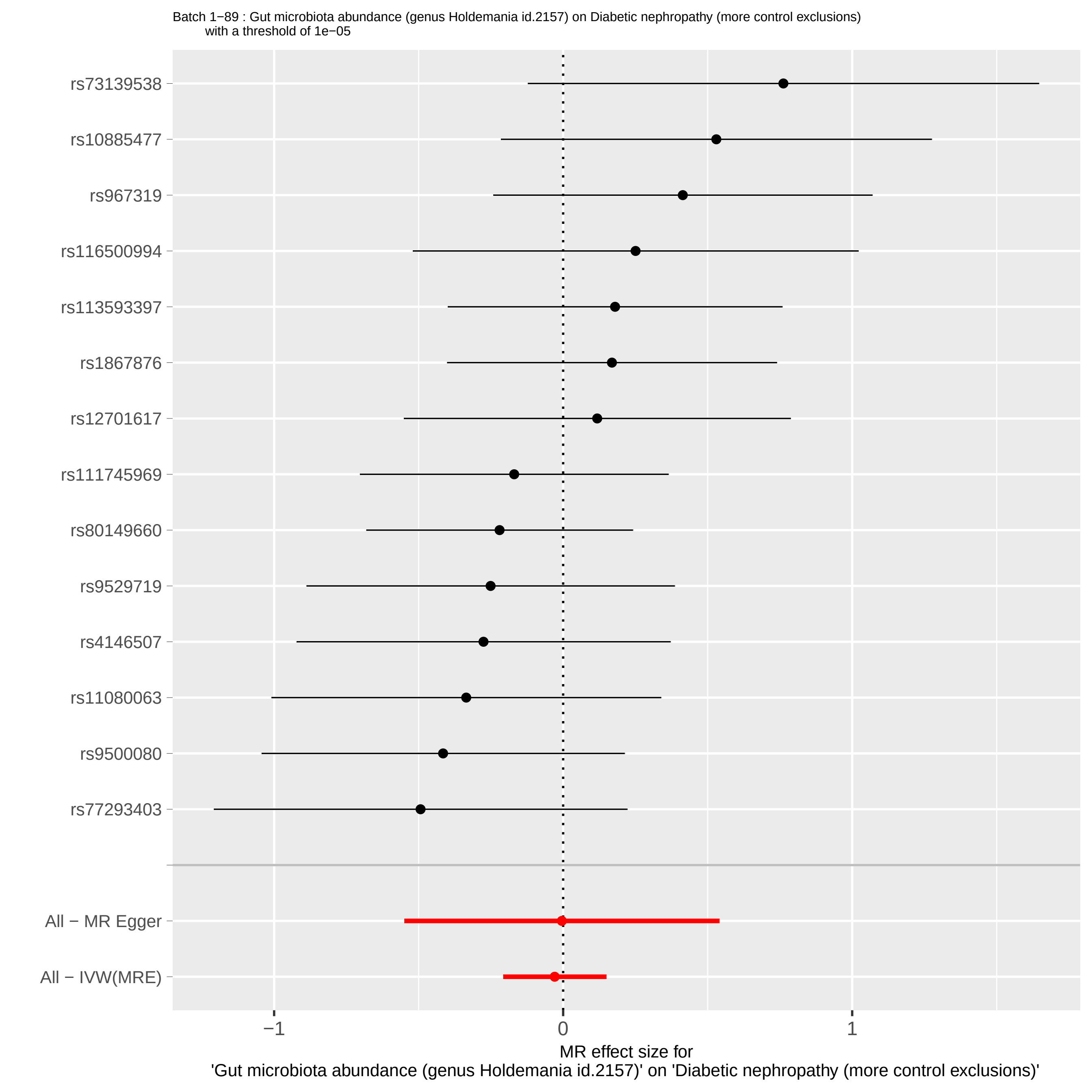

Batch 1−89 : Gut microbiota abundance (genus Holdemania id.2157) on Diabetic nephropathy (more control exclusions)
with a threshold of 1e−05
rs73139538
rs10885477
rs967319
rs116500994
rs113593397
rs1867876
rs12701617
rs111745969
rs80149660
rs9529719
rs4146507
rs11080063
rs9500080
rs77293403
All − MR Egger
All − IVW(MRE)
−1
0
1
MR effect size for
'Gut microbiota abundance (genus Holdemania id.2157)' on 'Diabetic nephropathy (more control exclusions)'

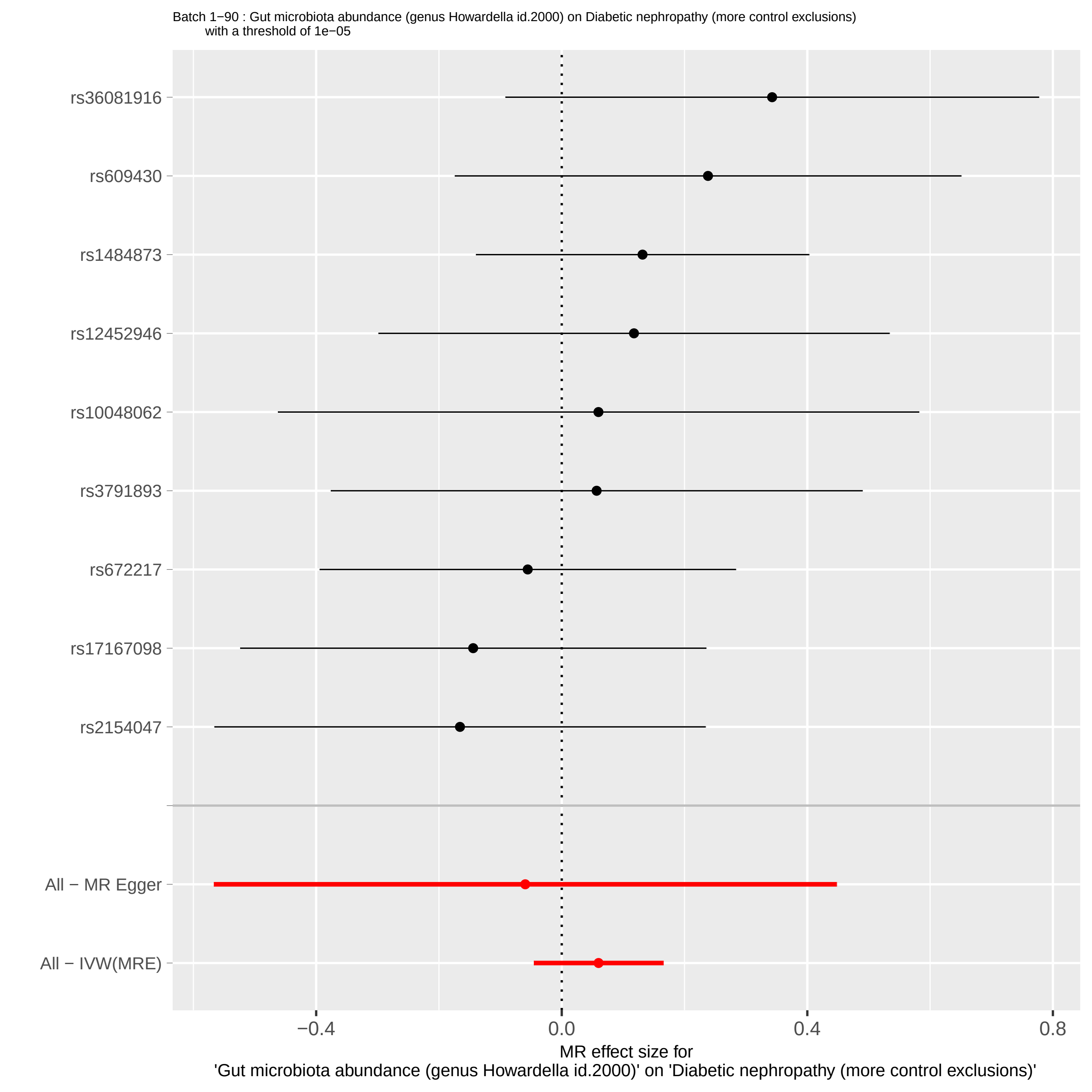

Batch 1−90 : Gut microbiota abundance (genus Howardella id.2000) on Diabetic nephropathy (more control exclusions)
with a threshold of 1e−05
rs36081916
rs609430
rs1484873
rs12452946
rs10048062
rs3791893
rs672217
rs17167098
rs2154047
All − MR Egger
All − IVW(MRE)
−0.4
0.0
0.4
0.8
MR effect size for
'Gut microbiota abundance (genus Howardella id.2000)' on 'Diabetic nephropathy (more control exclusions)'

Batch 1−91 : Gut microbiota abundance (genus Hungatella id.11306) on Diabetic nephropathy (more control exclusions)
with a threshold of 1e−05

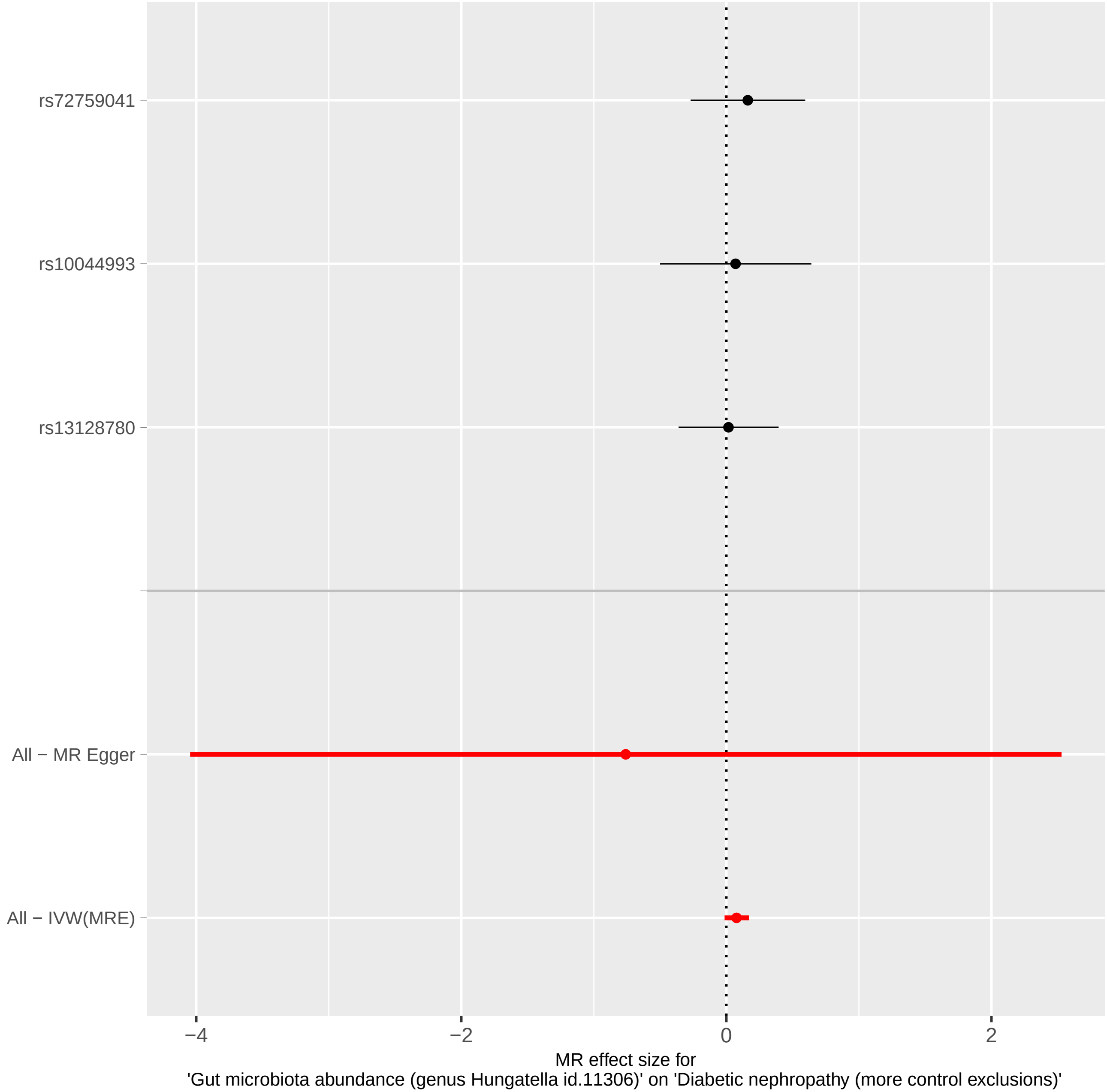

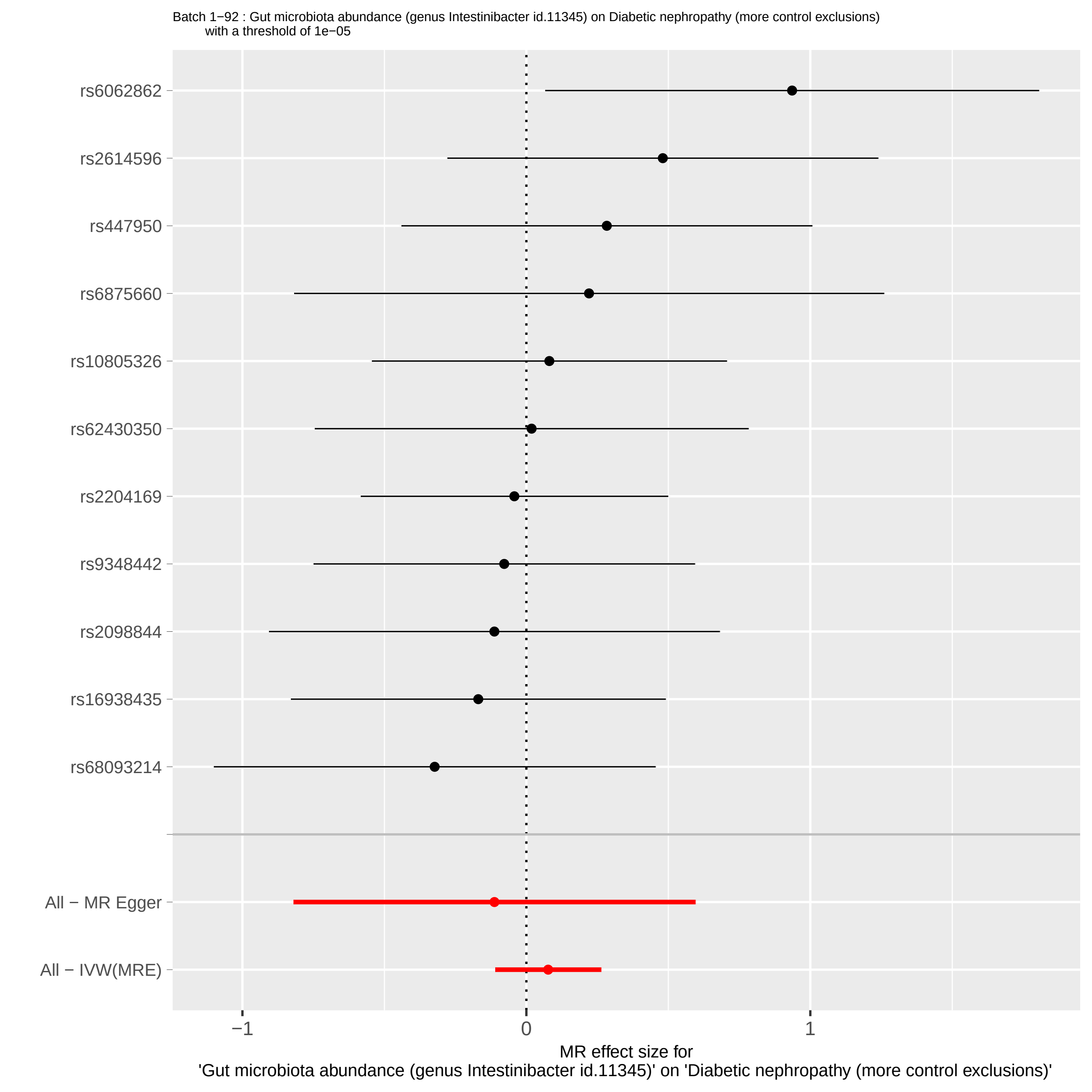

Batch 1−92 : Gut microbiota abundance (genus Intestinibacter id.11345) on Diabetic nephropathy (more control exclusions) with a threshold of 1e−05
rs6062862
rs2614596
rs447950
rs6875660
rs10805326
rs62430350
rs2204169
rs9348442
rs2098844
rs16938435
rs68093214
All − MR Egger
All − IVW(MRE)
−1
0
1
MR effect size for
'Gut microbiota abundance (genus Intestinibacter id.11345)' on 'Diabetic nephropathy (more control exclusions)'

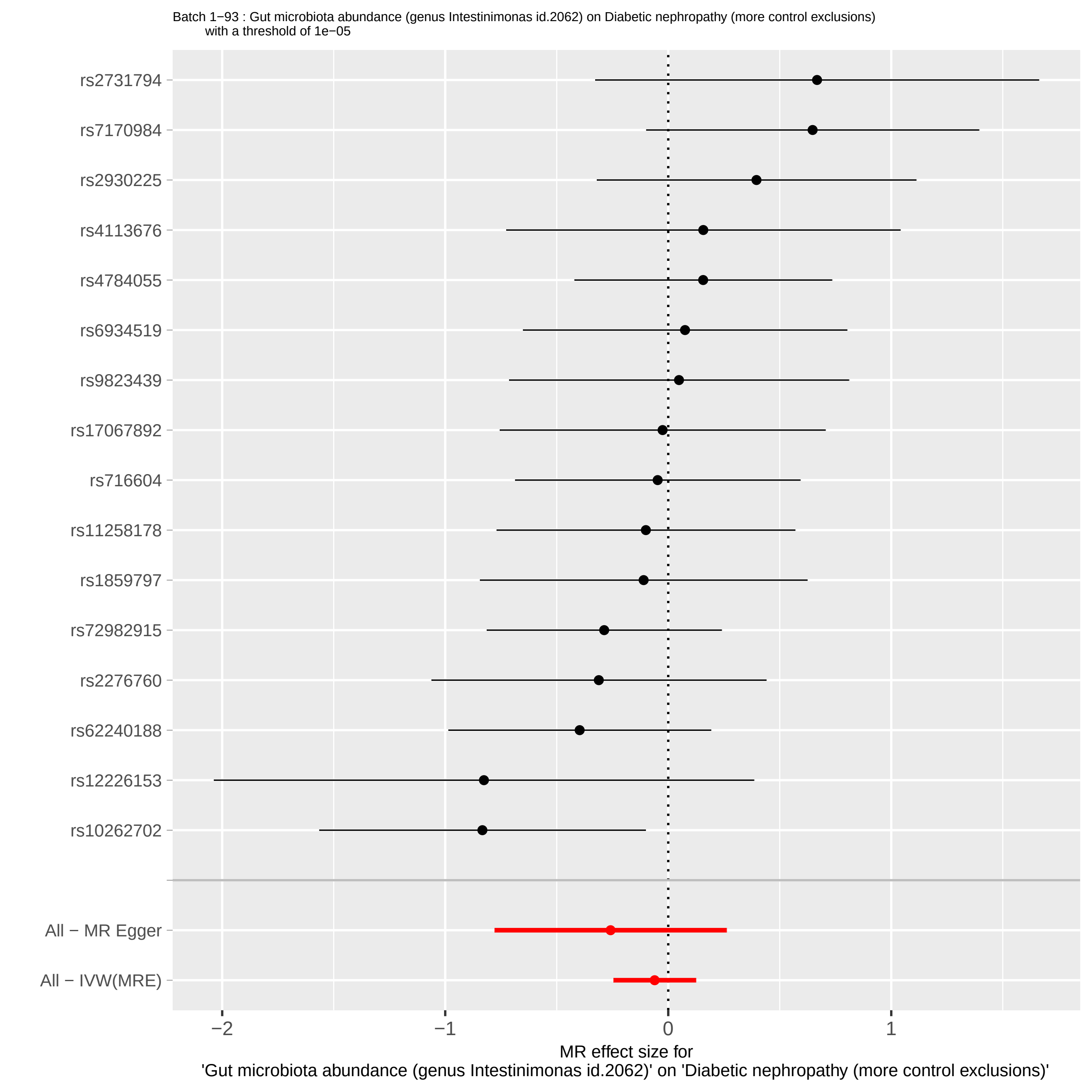

Batch 1−93 : Gut microbiota abundance (genus Intestinimonas id.2062) on Diabetic nephropathy (more control exclusions)
with a threshold of 1e−05
rs2731794
rs7170984
rs2930225
rs4113676
rs4784055
rs6934519
rs9823439
rs17067892
rs716604
rs11258178
rs1859797
rs72982915
rs2276760
rs62240188
rs12226153
rs10262702
All − MR Egger
All − IVW(MRE)
−2
−1
0
1
MR effect size for
'Gut microbiota abundance (genus Intestinimonas id.2062)' on 'Diabetic nephropathy (more control exclusions)'

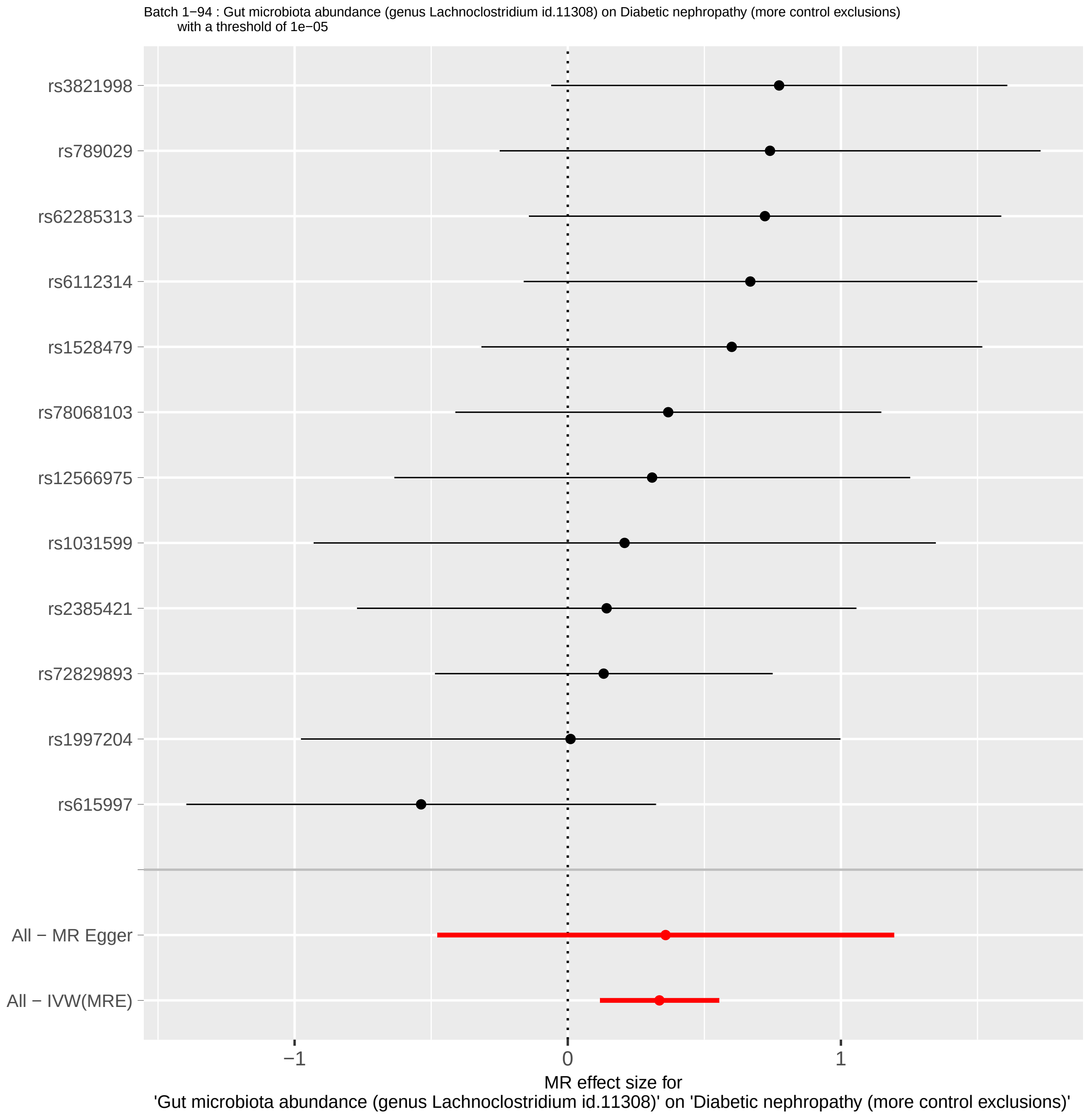

Batch 1−94 : Gut microbiota abundance (genus Lachnoclostridium id.11308) on Diabetic nephropathy (more control exclusions)
with a threshold of 1e−05
rs3821998
rs789029
rs62285313
rs6112314
rs1528479
rs78068103
rs12566975
rs1031599
rs2385421
rs72829893
rs1997204
rs615997
All − MR Egger
All − IVW(MRE)
−1
0
1
MR effect size for
'Gut microbiota abundance (genus Lachnoclostridium id.11308)' on 'Diabetic nephropathy (more control exclusions)'

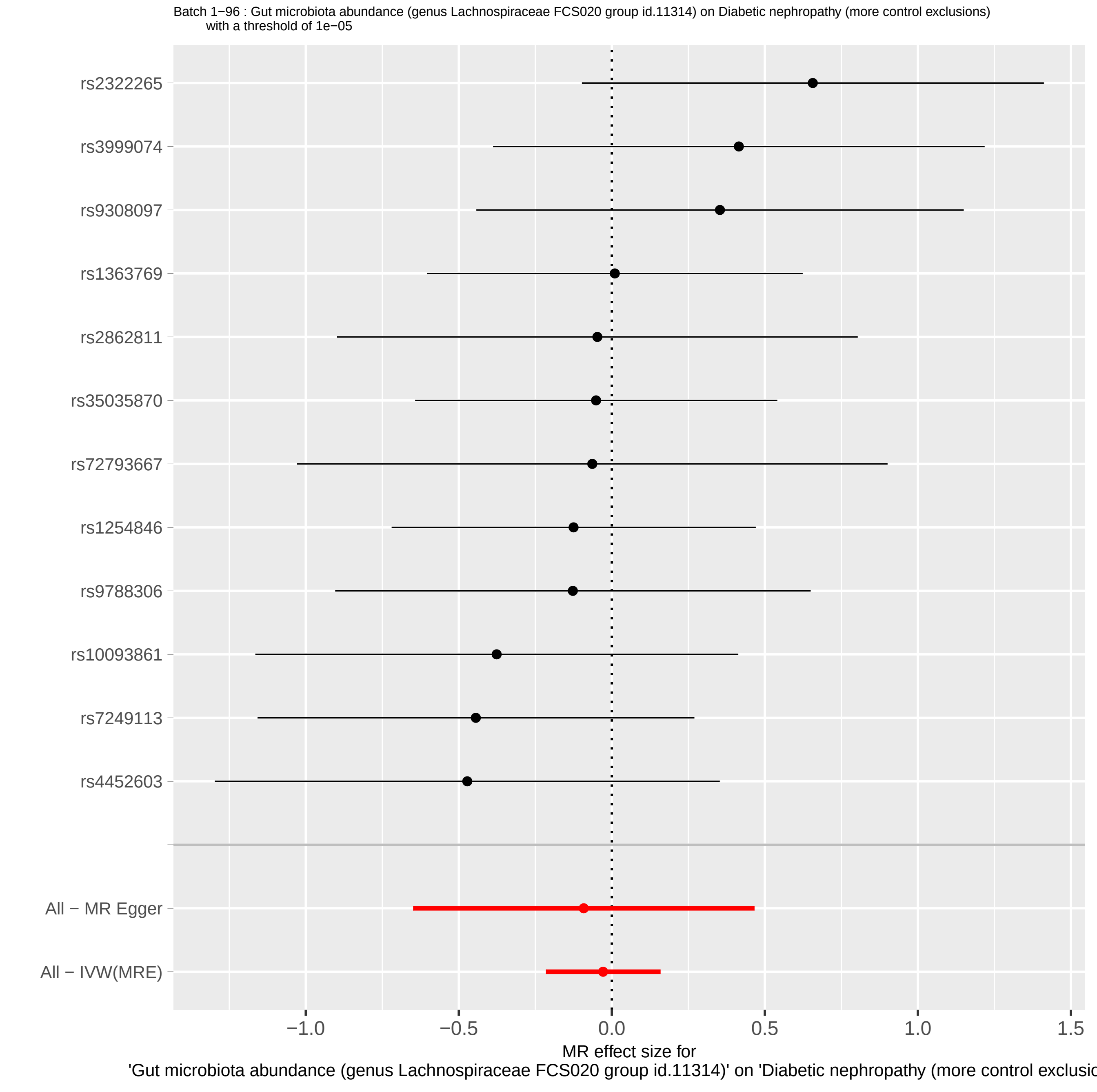

Batch 1−96 : Gut microbiota abundance (genus Lachnospiraceae FCS020 group id.11314) on Diabetic nephropathy (more control exclusions)
with a threshold of 1e−05
rs2322265
rs3999074
rs9308097
rs1363769
rs2862811
rs35035870
rs72793667
rs1254846
rs9788306
rs10093861
rs7249113
rs4452603
All − MR Egger
All − IVW(MRE)
−1.0
−0.5
0.0
0.5
1.0
1.5
MR effect size for
'Gut microbiota abundance (genus Lachnospiraceae FCS020 group id.11314)' on 'Diabetic nephropathy (more control exclusio

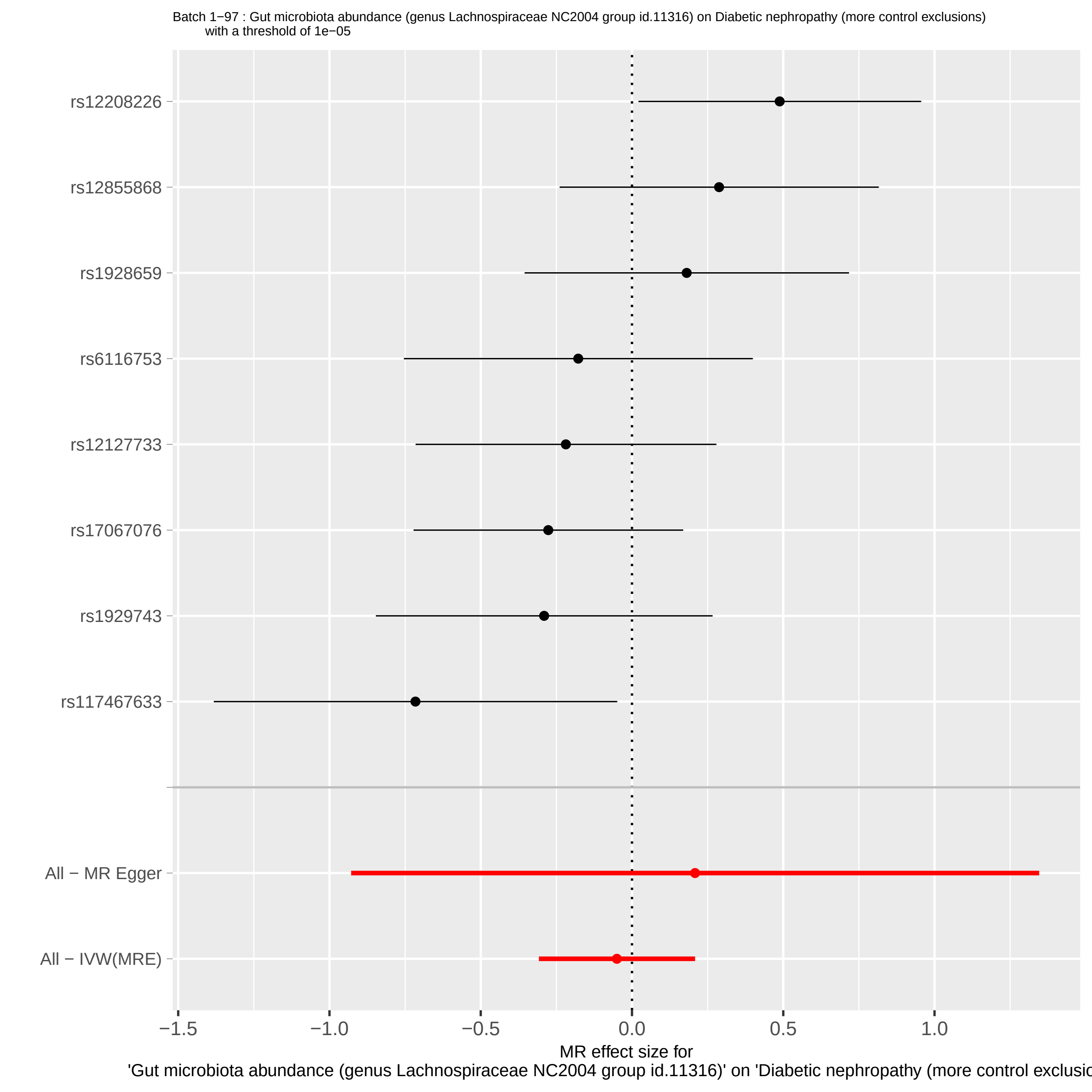

Batch 1−97 : Gut microbiota abundance (genus Lachnospiraceae NC2004 group id.11316) on Diabetic nephropathy (more control exclusions)
with a threshold of 1e−05
rs12208226
rs12855868
rs1928659
rs6116753
rs12127733
rs17067076
rs1929743
rs117467633
All − MR Egger
All − IVW(MRE)
−1.5
−1.0
−0.5
0.0
0.5
1.0
MR effect size for
'Gut microbiota abundance (genus Lachnospiraceae NC2004 group id.11316)' on 'Diabetic nephropathy (more control exclusio

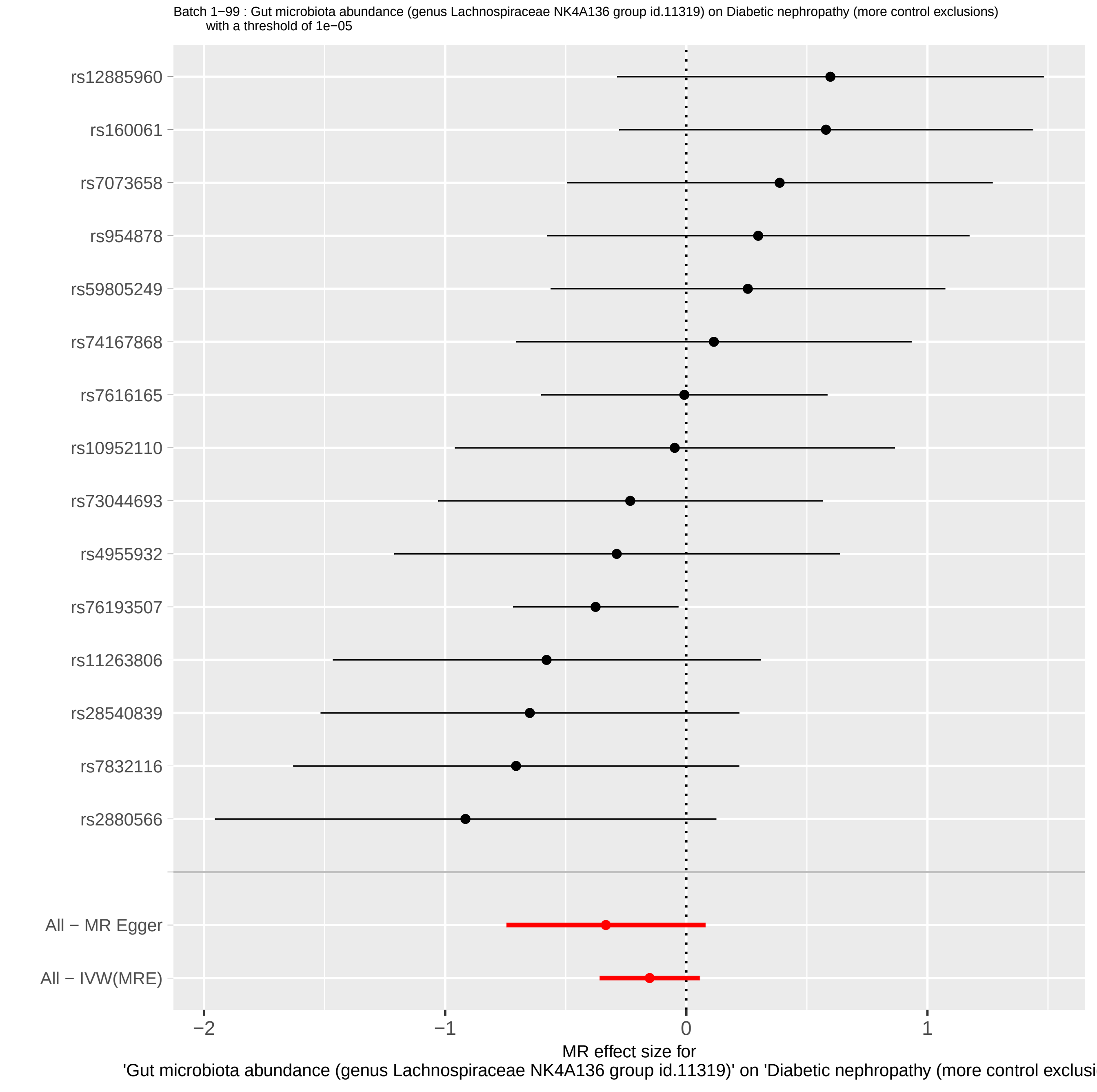

Batch 1−99 : Gut microbiota abundance (genus Lachnospiraceae NK4A136 group id.11319) on Diabetic nephropathy (more control exclusions) with a threshold of 1e−05
rs12885960
rs160061
rs7073658
rs954878
rs59805249
rs74167868
rs7616165
rs10952110
rs73044693
rs4955932
rs76193507
rs11263806
rs28540839
rs7832116
rs2880566
All − MR Egger
All − IVW(MRE)
−2
−1
0
1
MR effect size for
'Gut microbiota abundance (genus Lachnospiraceae NK4A136 group id.11319)' on 'Diabetic nephropathy (more control exclusi

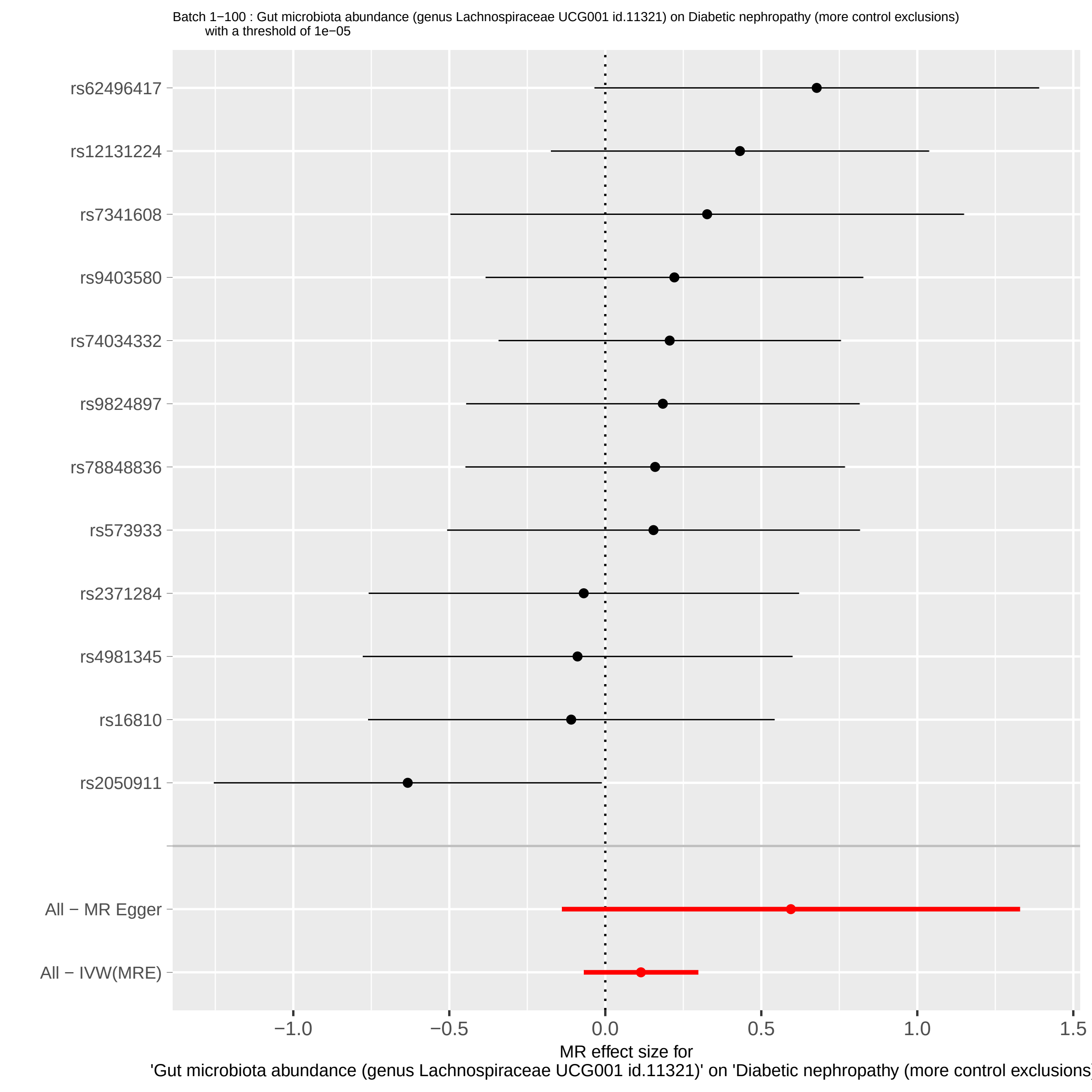

Batch 1−100 : Gut microbiota abundance (genus Lachnospiraceae UCG001 id.11321) on Diabetic nephropathy (more control exclusions)
with a threshold of 1e−05
rs62496417
rs12131224
rs7341608
rs9403580
rs74034332
rs9824897
rs78848836
rs573933
rs2371284
rs4981345
rs16810
rs2050911
All − MR Egger
All − IVW(MRE)
−1.0
−0.5
0.0
0.5
1.0
1.5
MR effect size for
'Gut microbiota abundance (genus Lachnospiraceae UCG001 id.11321)' on 'Diabetic nephropathy (more control exclusions)'

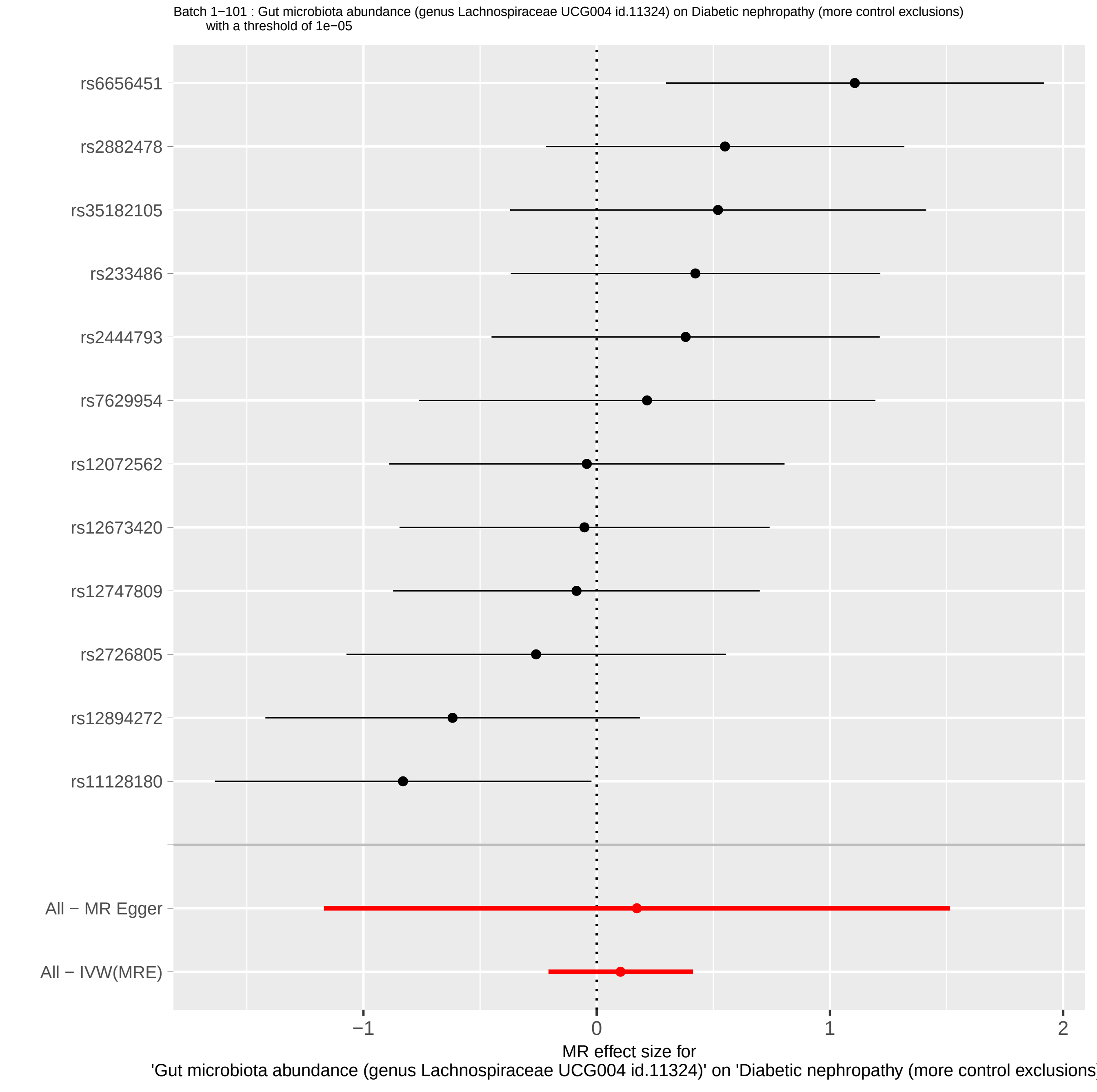
Batch 1−101 : Gut microbiota abundance (genus Lachnospiraceae UCG004 id.11324) on Diabetic nephropathy (more control exclusions) with a threshold of 1e−05
rs6656451
rs2882478
rs35182105
rs233486
rs2444793
rs7629954
rs12072562
rs12673420
rs12747809
rs2726805
rs12894272
rs11128180
All − MR Egger
All − IVW(MRE)
−1
0
1
2
MR effect size for
'Gut microbiota abundance (genus Lachnospiraceae UCG004 id.11324)' on 'Diabetic nephropathy (more control exclusions)

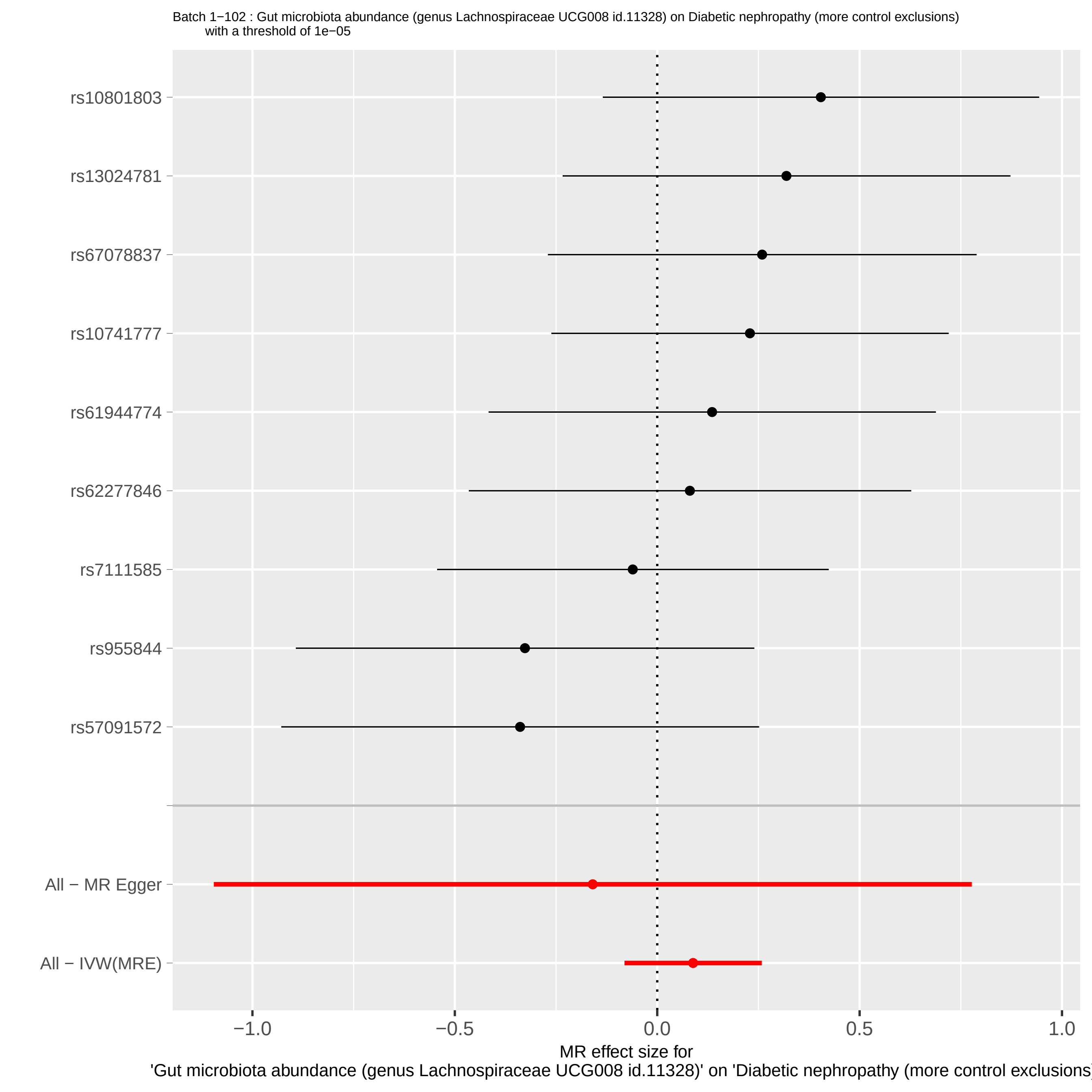
Batch 1−102 : Gut microbiota abundance (genus Lachnospiraceae UCG008 id.11328) on Diabetic nephropathy (more control exclusions)
with a threshold of 1e−05
rs10801803
rs13024781
rs67078837
rs10741777
rs61944774
rs62277846
rs7111585
rs955844
rs57091572
All − MR Egger
All − IVW(MRE)
−1.0
−0.5
0.0
0.5
1.0
MR effect size for
'Gut microbiota abundance (genus Lachnospiraceae UCG008 id.11328)' on 'Diabetic nephropathy (more control exclusions)

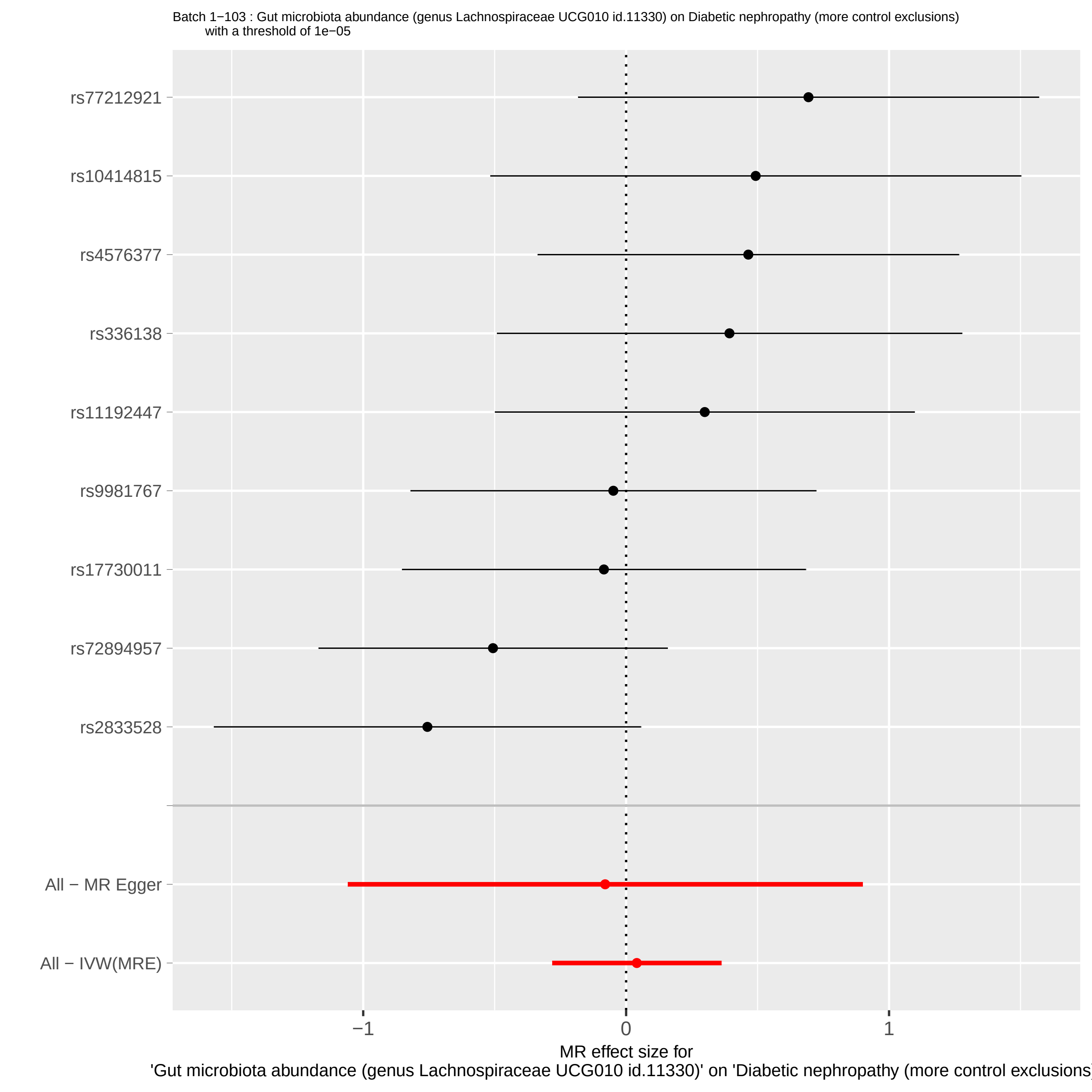
Batch 1−103 : Gut microbiota abundance (genus Lachnospiraceae UCG010 id.11330) on Diabetic nephropathy (more control exclusions)
with a threshold of 1e−05
rs77212921
rs10414815
rs4576377
rs336138
rs11192447
rs9981767
rs17730011
rs72894957
rs2833528
All − MR Egger
All − IVW(MRE)
−1
0
1
MR effect size for
'Gut microbiota abundance (genus Lachnospiraceae UCG010 id.11330)' on 'Diabetic nephropathy (more control exclusions)

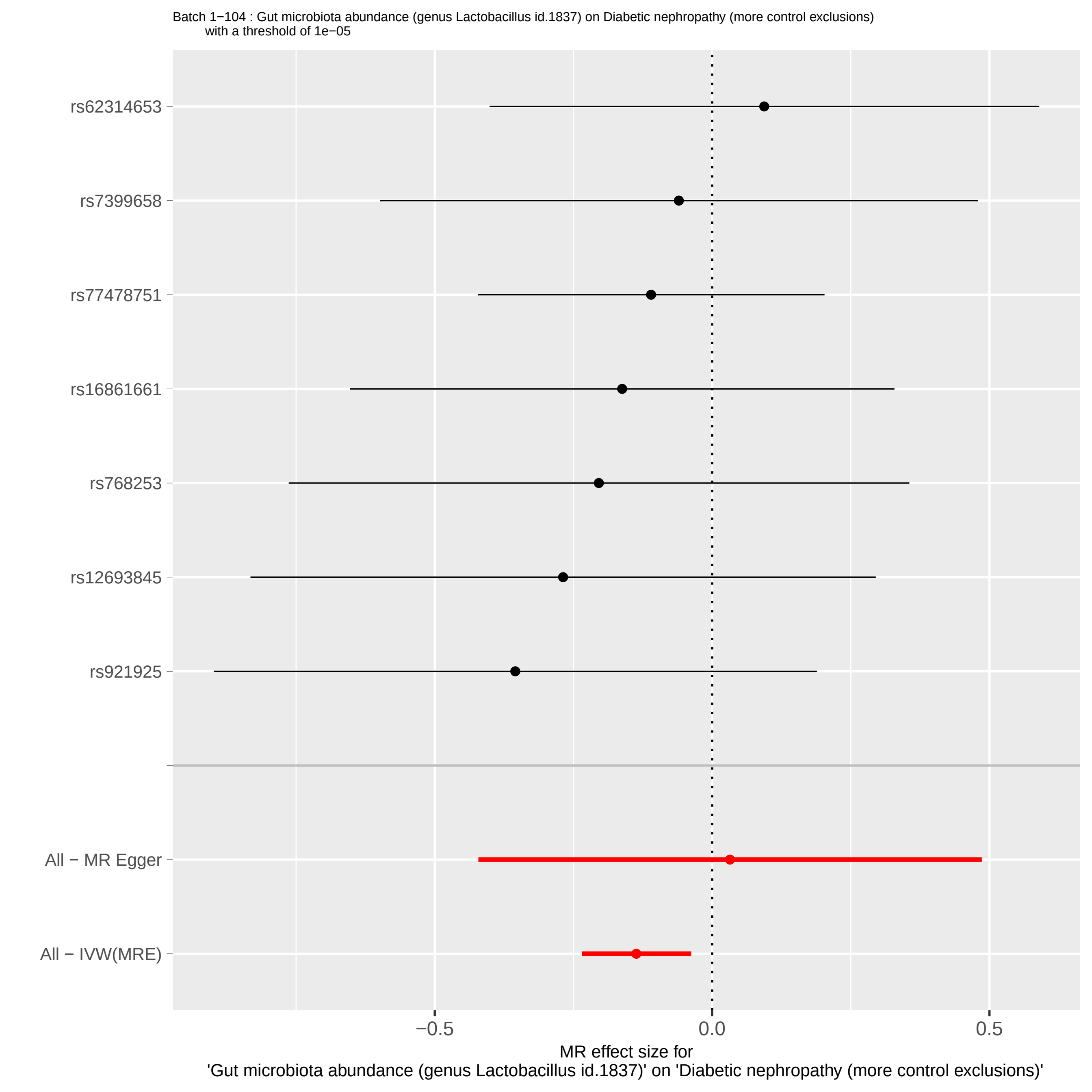

Batch 1−104 : Gut microbiota abundance (genus Lactobacillus id.1837) on Diabetic nephropathy (more control exclusions)
with a threshold of 1e−05
rs62314653
rs7399658
rs77478751
rs16861661
rs768253
rs12693845
rs921925
All − MR Egger
All − IVW(MRE)
−0.5
0.0
0.5
MR effect size for
'Gut microbiota abundance (genus Lactobacillus id.1837)' on 'Diabetic nephropathy (more control exclusions)'

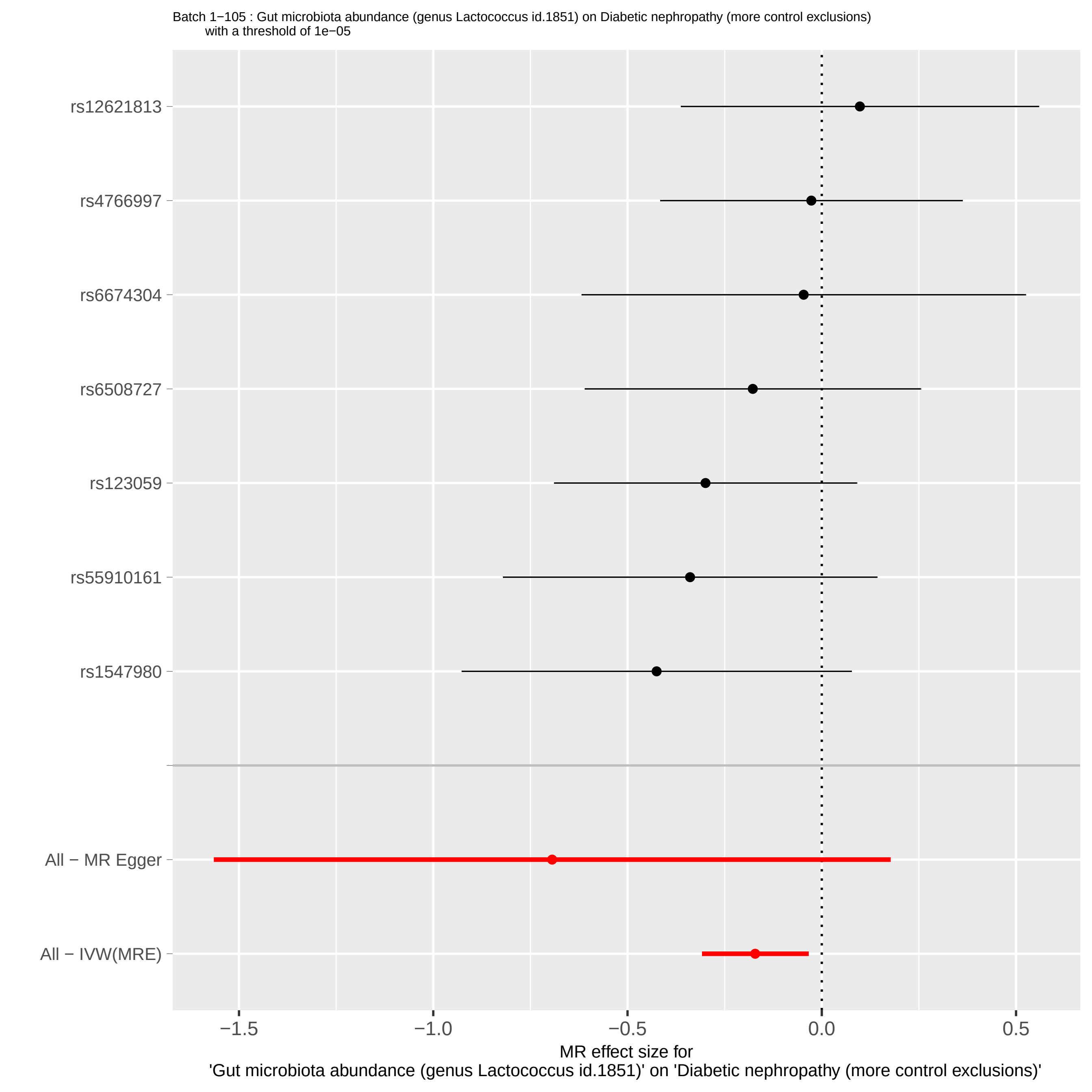

Batch 1−105 : Gut microbiota abundance (genus Lactococcus id.1851) on Diabetic nephropathy (more control exclusions) with a threshold of 1e−05
rs12621813
rs4766997
rs6674304
rs6508727
rs123059
rs55910161
rs1547980
All − MR Egger
All − IVW(MRE)
−1.5
−1.0
−0.5
0.0
0.5
MR effect size for
'Gut microbiota abundance (genus Lactococcus id.1851)' on 'Diabetic nephropathy (more control exclusions)'

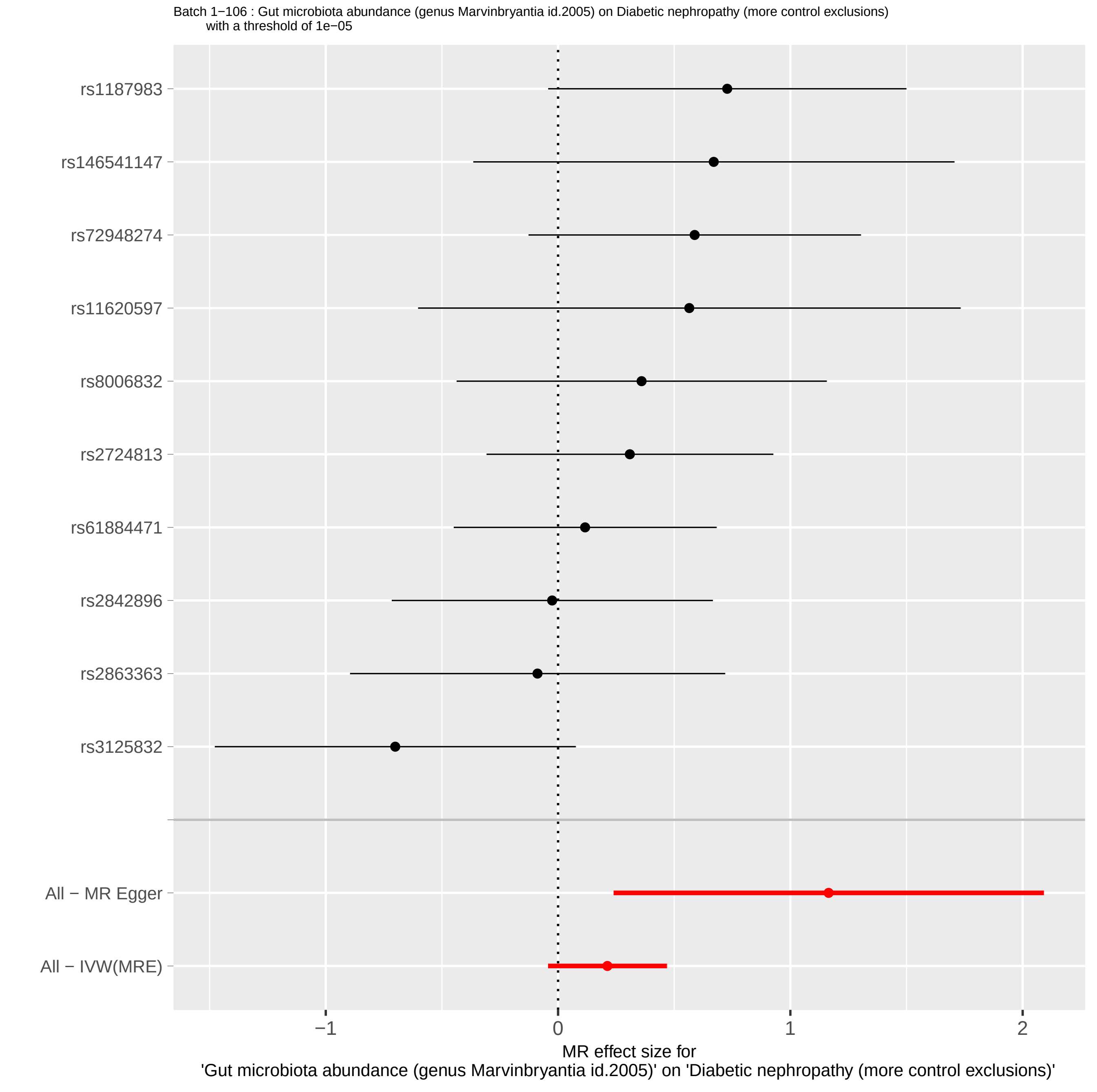
Batch 1−106 : Gut microbiota abundance (genus Marvinbryantia id.2005) on Diabetic nephropathy (more control exclusions)
with a threshold of 1e−05
rs1187983
rs146541147
rs72948274
rs11620597
rs8006832
rs2724813
rs61884471
rs2842896
rs2863363
rs3125832
All − MR Egger
All − IVW(MRE)
−1
0
1
2
MR effect size for
'Gut microbiota abundance (genus Marvinbryantia id.2005)' on 'Diabetic nephropathy (more control exclusions)'

Batch 1−107 : Gut microbiota abundance (genus Methanobrevibacter id.123) on Diabetic nephropathy (more control exclusions) with a threshold of 1e−05

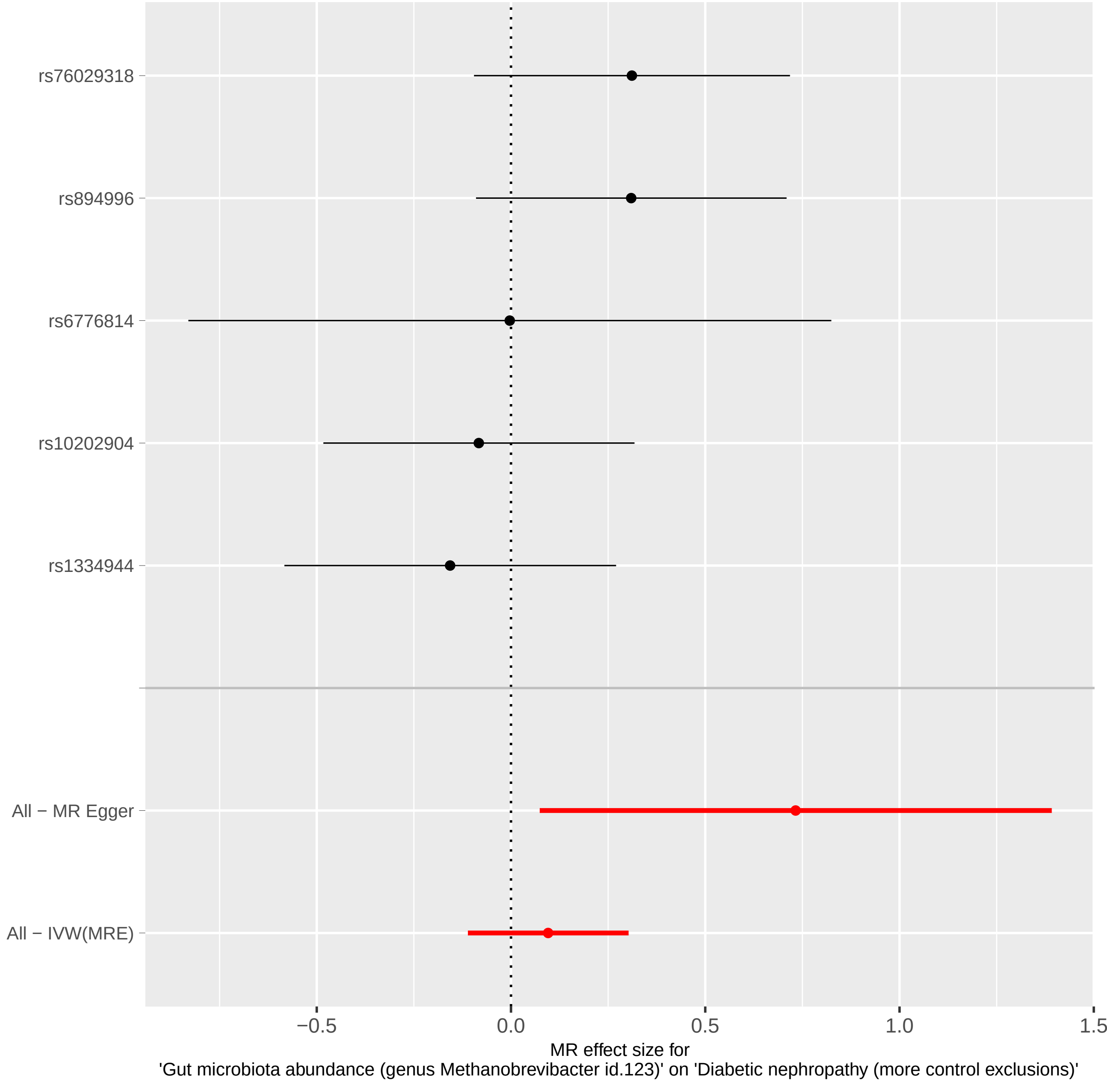

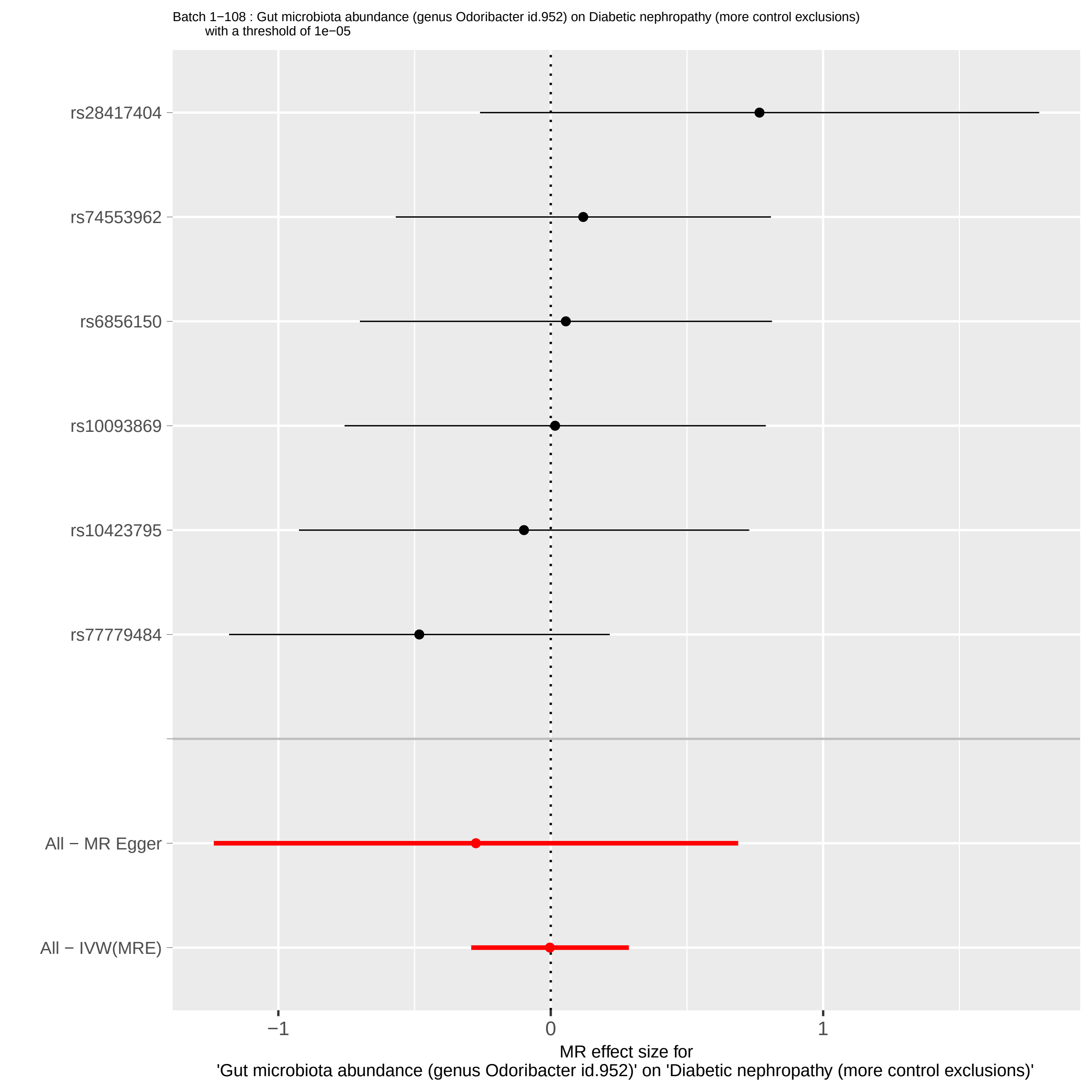

Batch 1−108 : Gut microbiota abundance (genus Odoribacter id.952) on Diabetic nephropathy (more control exclusions) with a threshold of 1e−05
rs28417404
rs74553962
rs6856150
rs10093869
rs10423795
rs77779484
All − MR Egger
All − IVW(MRE)
−1
0
1
MR effect size for
'Gut microbiota abundance (genus Odoribacter id.952)' on 'Diabetic nephropathy (more control exclusions)'

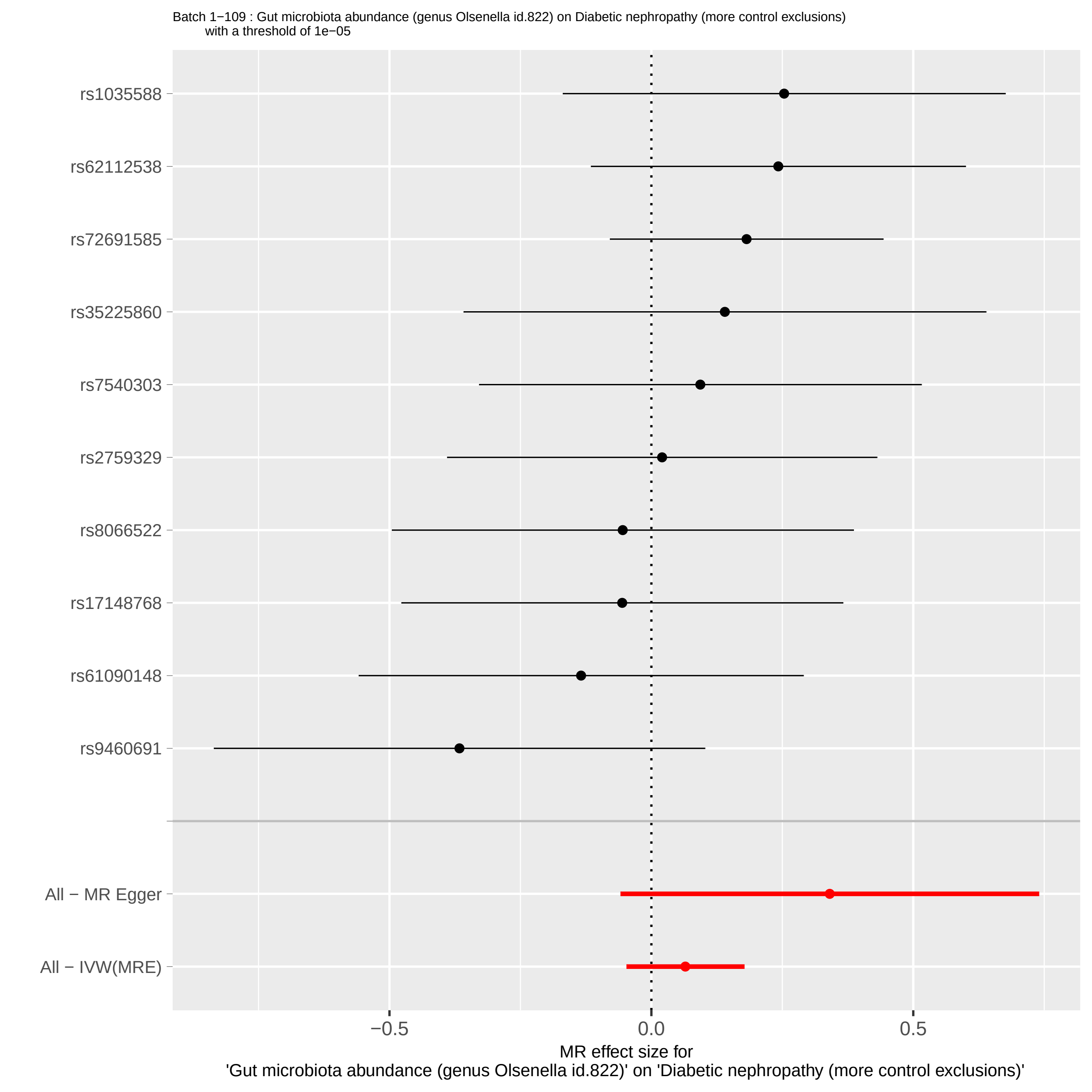
Batch 1−109 : Gut microbiota abundance (genus Olsenella id.822) on Diabetic nephropathy (more control exclusions)
with a threshold of 1e−05
rs1035588
rs62112538
rs72691585
rs35225860
rs7540303
rs2759329
rs8066522
rs17148768
rs61090148
rs9460691
All − MR Egger
All − IVW(MRE)
−0.5
0.0
0.5
MR effect size for
'Gut microbiota abundance (genus Olsenella id.822)' on 'Diabetic nephropathy (more control exclusions)'

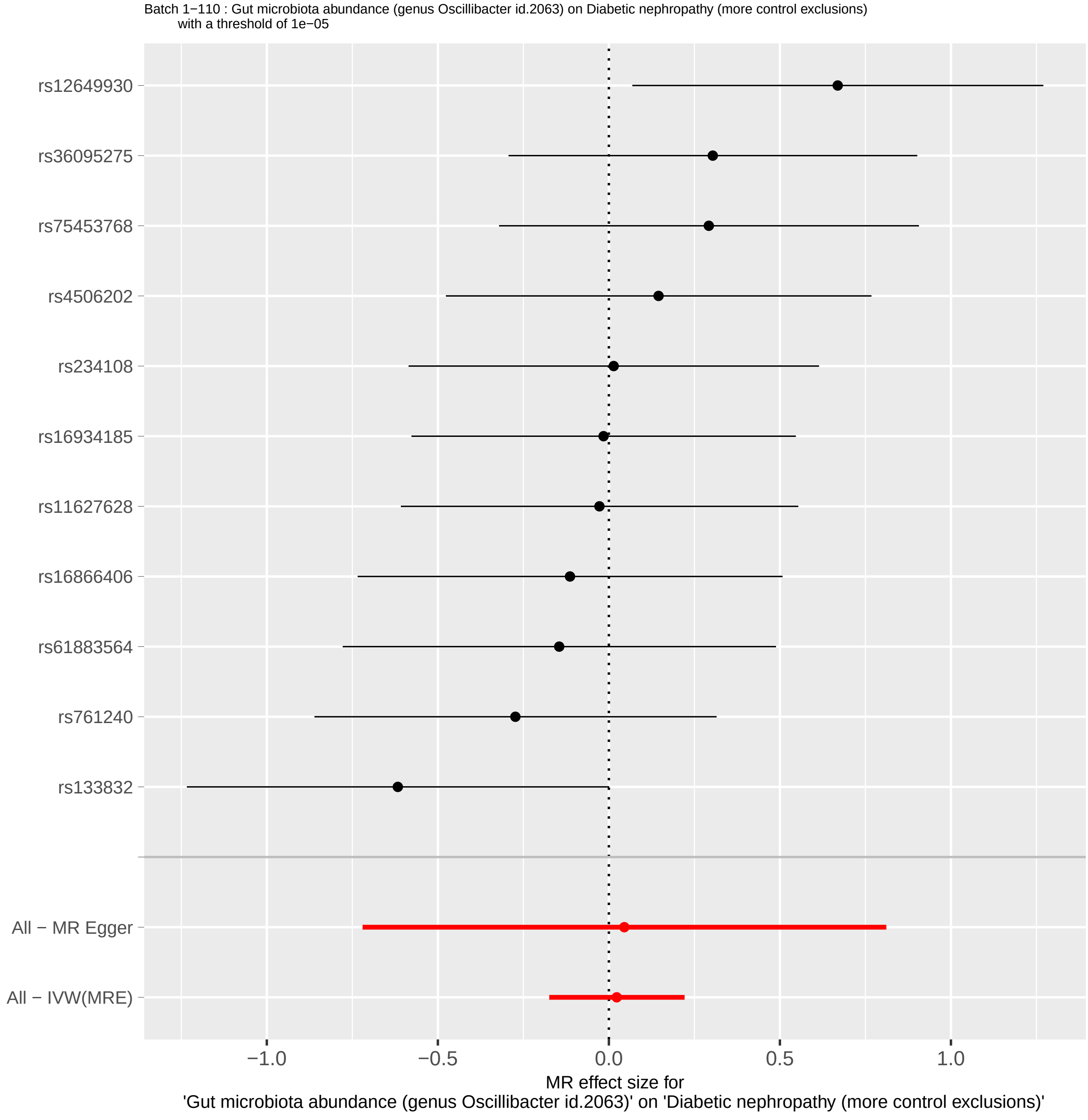
Batch 1−110 : Gut microbiota abundance (genus Oscillibacter id.2063) on Diabetic nephropathy (more control exclusions)
with a threshold of 1e−05
rs12649930
rs36095275
rs75453768
rs4506202
rs234108
rs16934185
rs11627628
rs16866406
rs61883564
rs761240
rs133832
All − MR Egger
All − IVW(MRE)
−1.0
−0.5
0.0
0.5
1.0
MR effect size for
'Gut microbiota abundance (genus Oscillibacter id.2063)' on 'Diabetic nephropathy (more control exclusions)'

Batch 1−111 : Gut microbiota abundance (genus Oscillospira id.2064) on Diabetic nephropathy (more control exclusions)
with a threshold of 1e−05

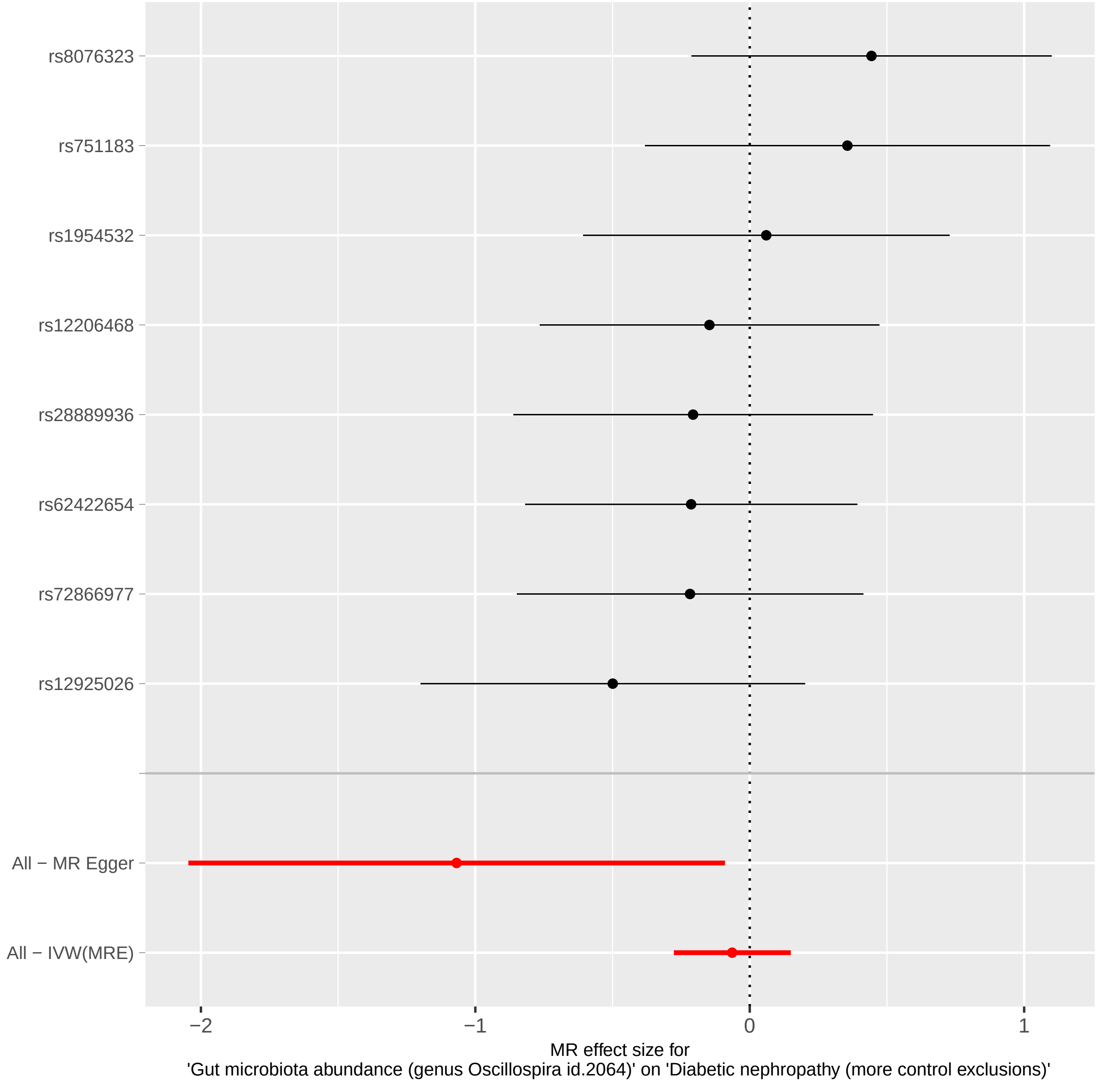

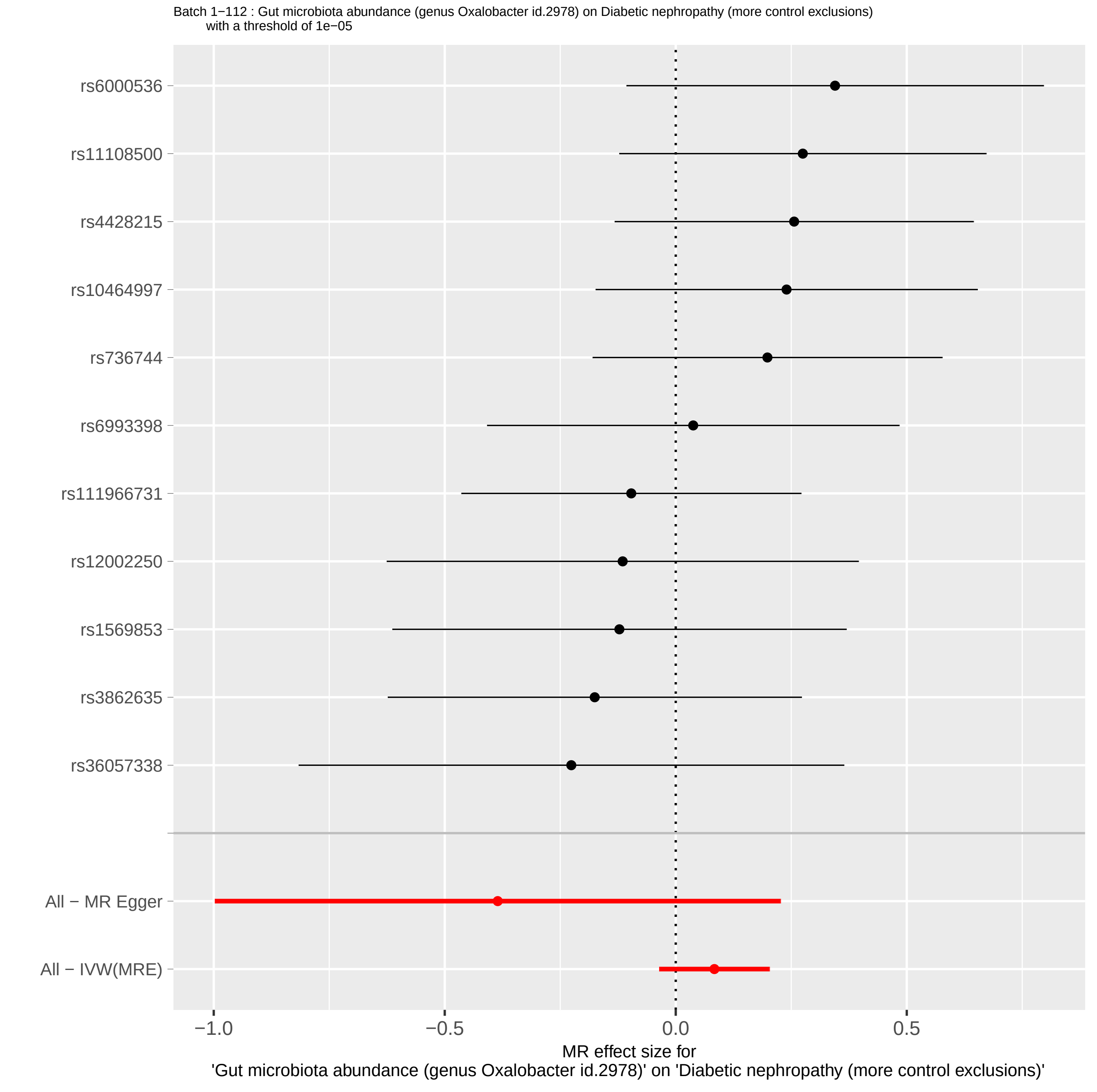

Batch 1−112 : Gut microbiota abundance (genus Oxalobacter id.2978) on Diabetic nephropathy (more control exclusions)
with a threshold of 1e−05
rs6000536
rs11108500
rs4428215
rs10464997
rs736744
rs6993398
rs111966731
rs12002250
rs1569853
rs3862635
rs36057338
All − MR Egger
All − IVW(MRE)
−1.0
−0.5
0.0
0.5
MR effect size for
'Gut microbiota abundance (genus Oxalobacter id.2978)' on 'Diabetic nephropathy (more control exclusions)'

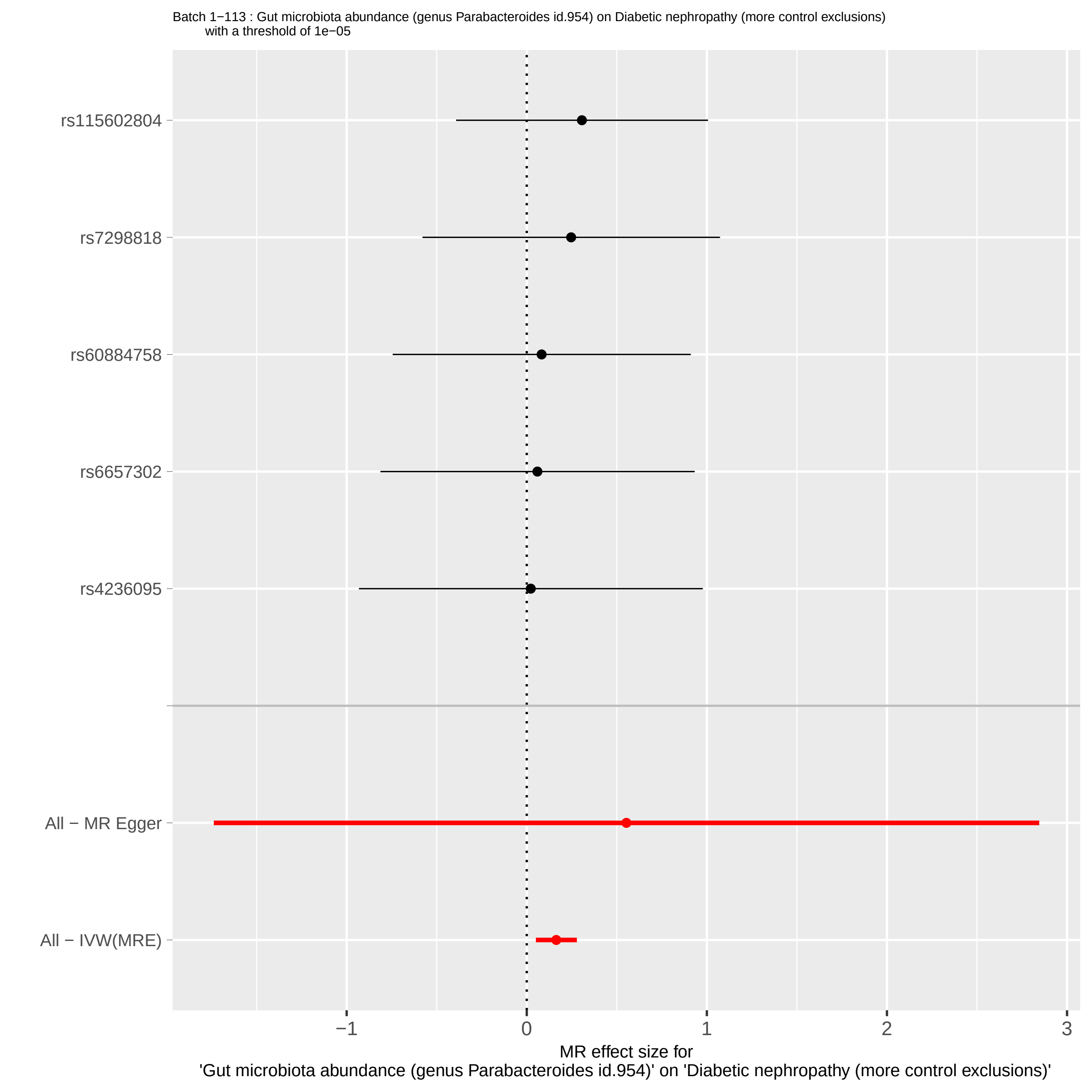
Batch 1−113 : Gut microbiota abundance (genus Parabacteroides id.954) on Diabetic nephropathy (more control exclusions)
with a threshold of 1e−05
rs115602804
rs7298818
rs60884758
rs6657302
rs4236095
All − MR Egger
All − IVW(MRE)
−1
0
1
2
3
MR effect size for
'Gut microbiota abundance (genus Parabacteroides id.954)' on 'Diabetic nephropathy (more control exclusions)'

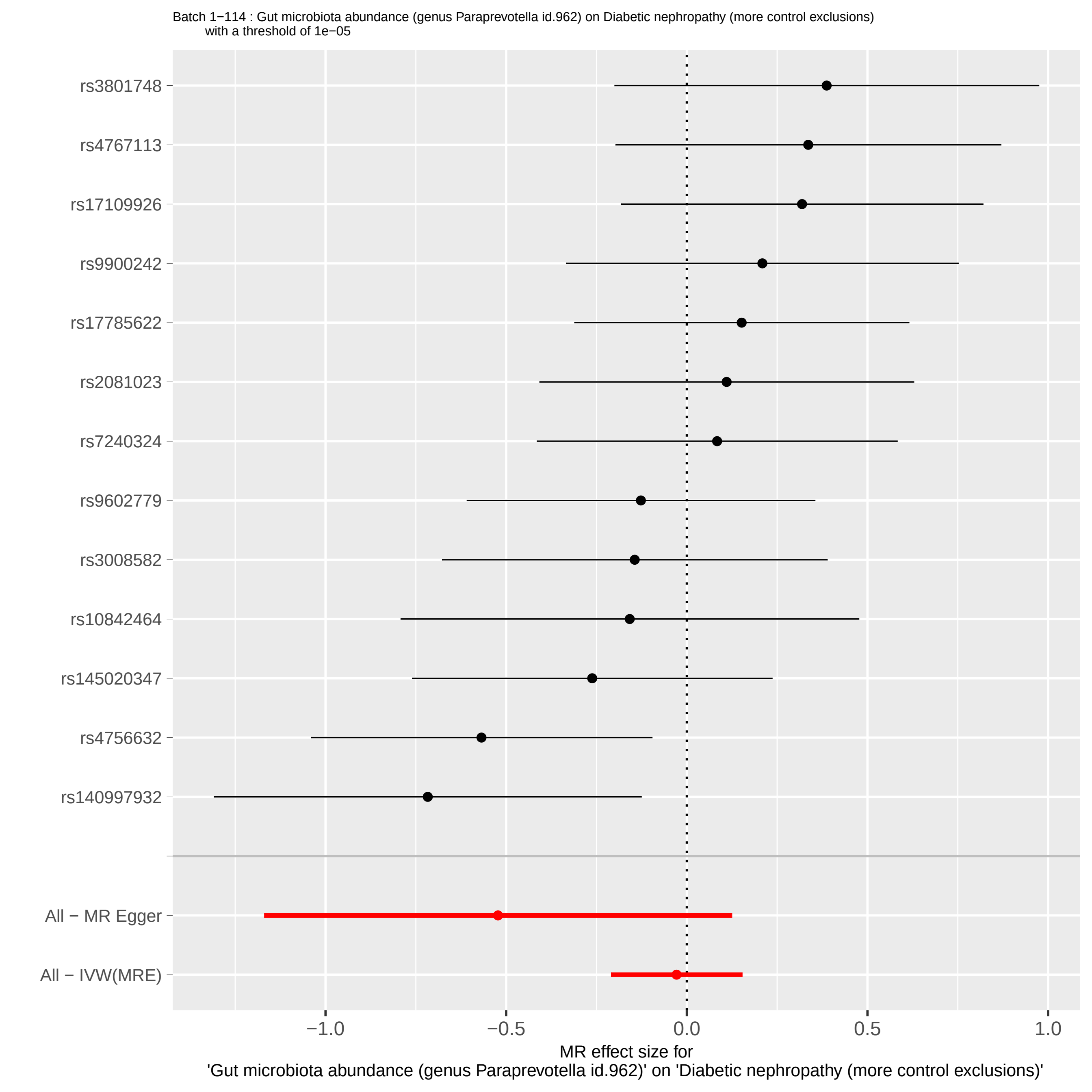

Batch 1−114 : Gut microbiota abundance (genus Paraprevotella id.962) on Diabetic nephropathy (more control exclusions) with a threshold of 1e−05
rs3801748
rs4767113
rs17109926
rs9900242
rs17785622
rs2081023
rs7240324
rs9602779
rs3008582
rs10842464
rs145020347
rs4756632
rs140997932
All − MR Egger
All − IVW(MRE)
−1.0
−0.5
0.0
0.5
1.0
MR effect size for
'Gut microbiota abundance (genus Paraprevotella id.962)' on 'Diabetic nephropathy (more control exclusions)'

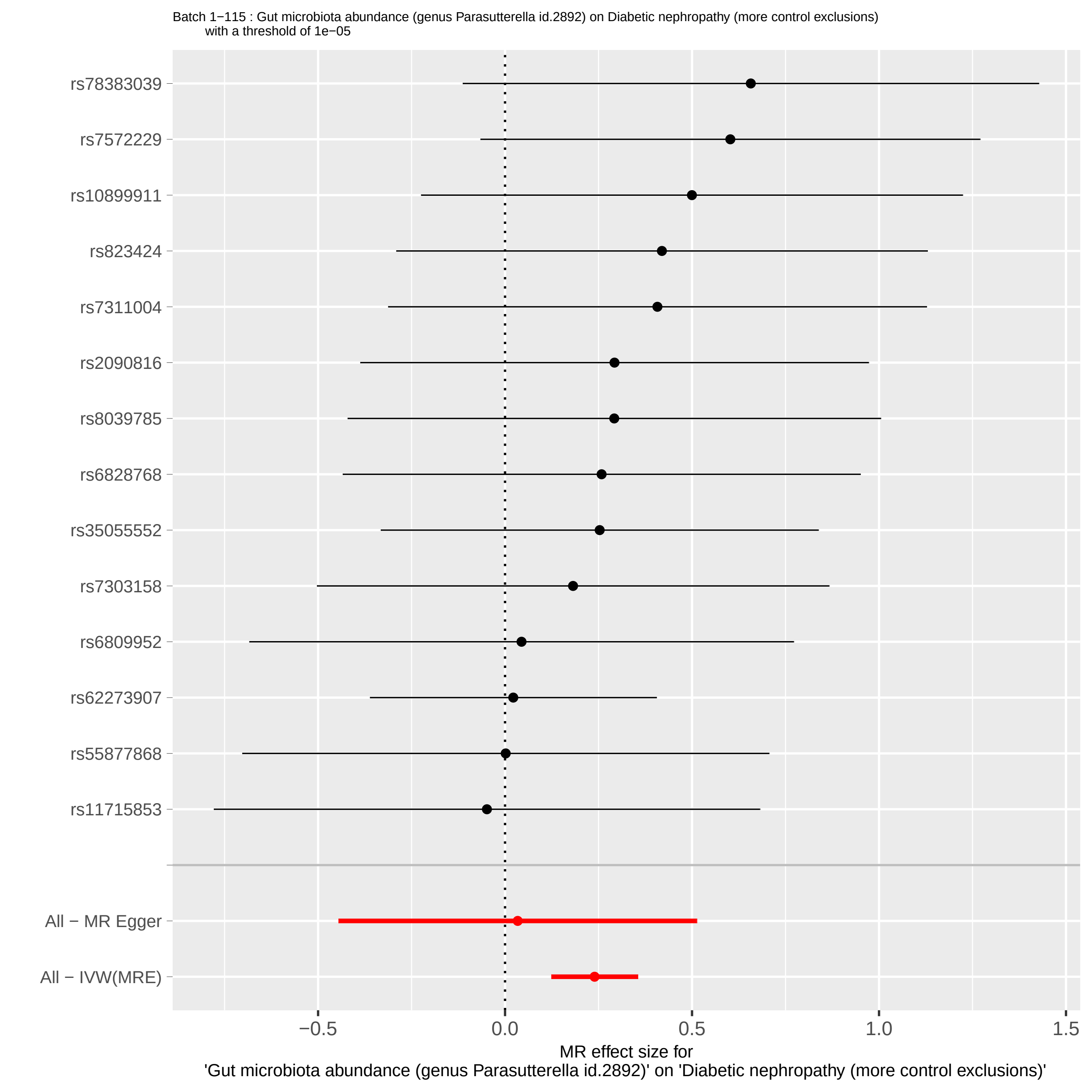

Batch 1−115 : Gut microbiota abundance (genus Parasutterella id.2892) on Diabetic nephropathy (more control exclusions)
with a threshold of 1e−05
rs78383039
rs7572229
rs10899911
rs823424
rs7311004
rs2090816
rs8039785
rs6828768
rs35055552
rs7303158
rs6809952
rs62273907
rs55877868
rs11715853
All − MR Egger
All − IVW(MRE)
−0.5
0.0
0.5
1.0
1.5
MR effect size for
'Gut microbiota abundance (genus Parasutterella id.2892)' on 'Diabetic nephropathy (more control exclusions)'

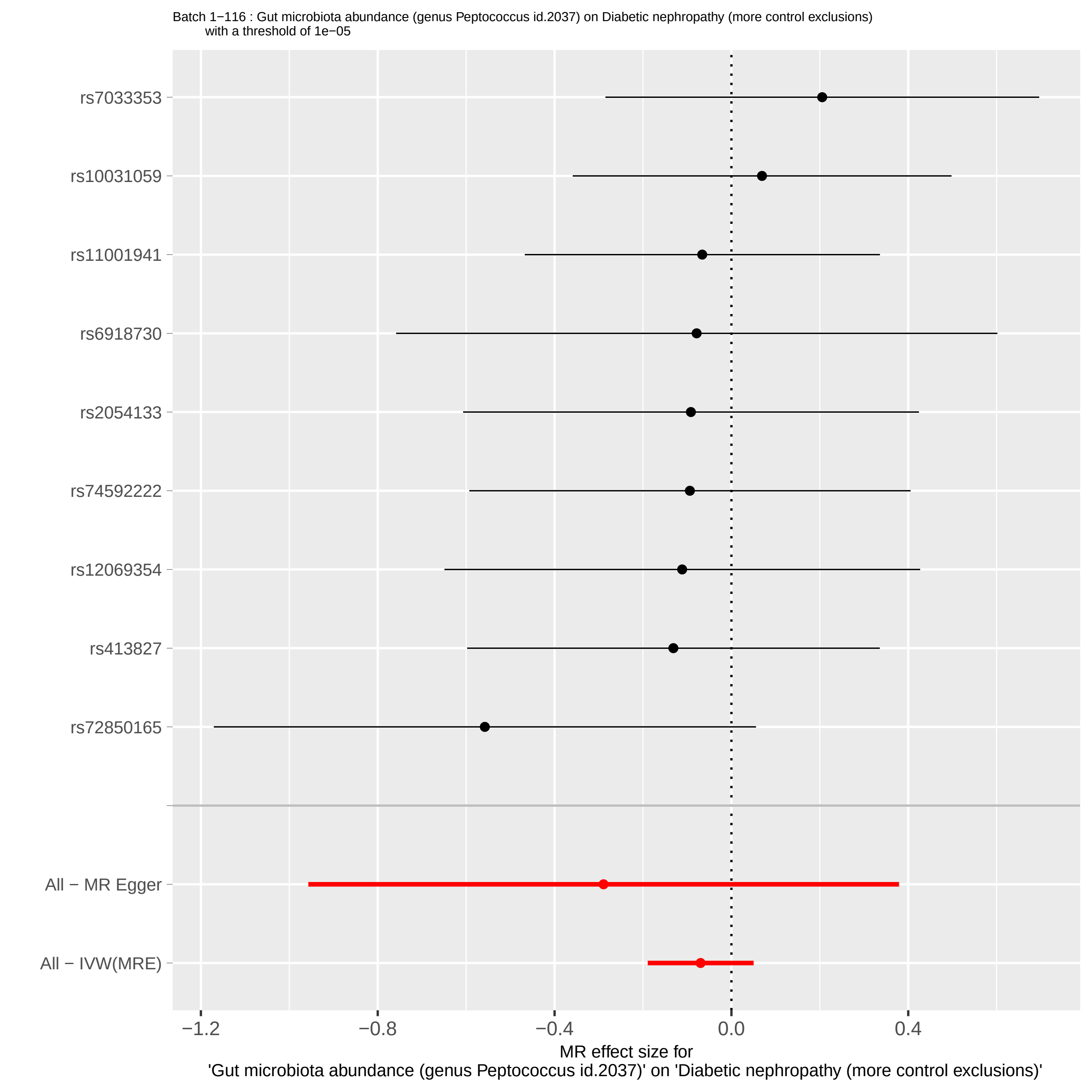

Batch 1−116 : Gut microbiota abundance (genus Peptococcus id.2037) on Diabetic nephropathy (more control exclusions)
with a threshold of 1e−05
rs7033353
rs10031059
rs11001941
rs6918730
rs2054133
rs74592222
rs12069354
rs413827
rs72850165
All − MR Egger
All − IVW(MRE)
−1.2
−0.8
−0.4
0.0
0.4
MR effect size for
'Gut microbiota abundance (genus Peptococcus id.2037)' on 'Diabetic nephropathy (more control exclusions)'

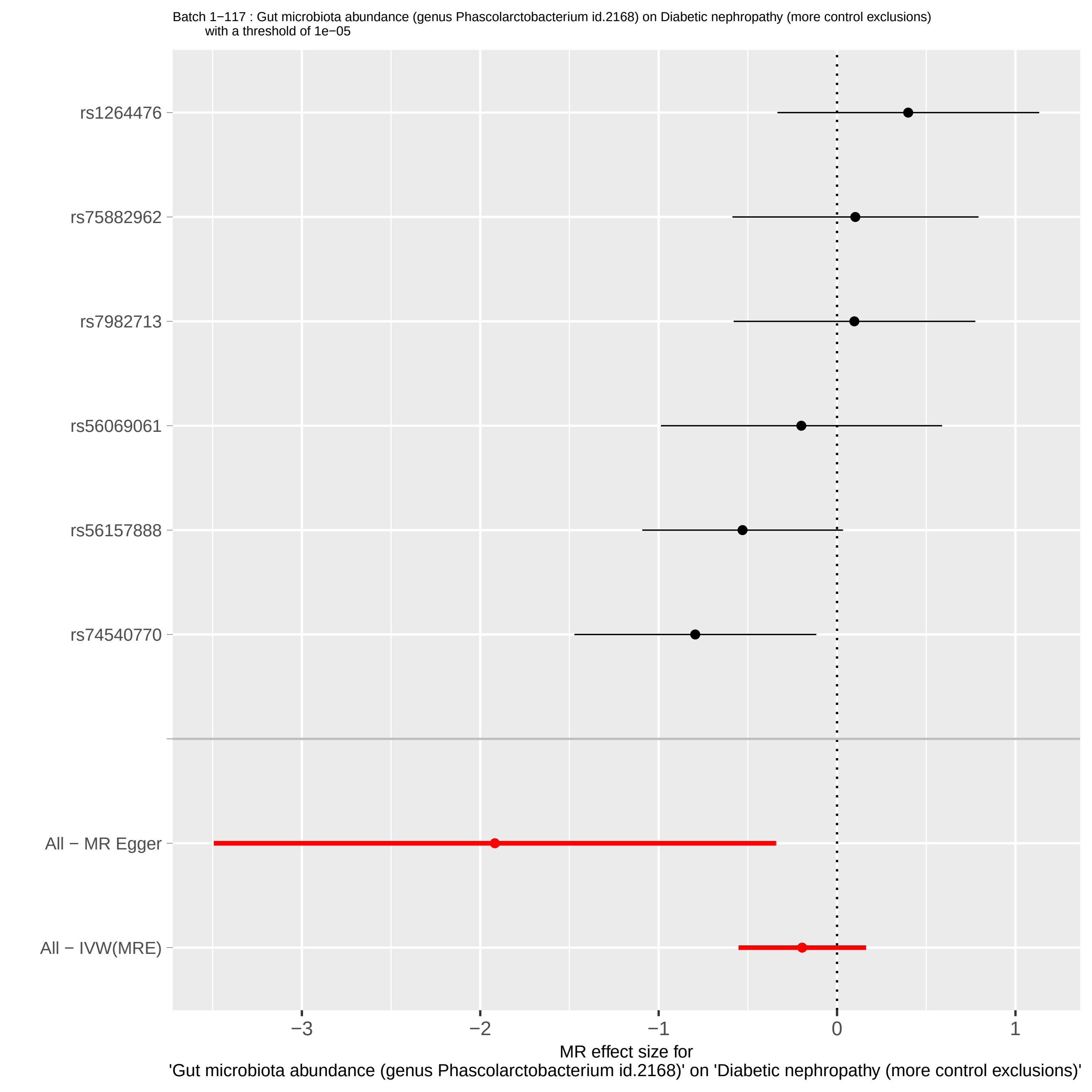

Batch 1−117 : Gut microbiota abundance (genus Phascolarctobacterium id.2168) on Diabetic nephropathy (more control exclusions)
with a threshold of 1e−05
rs1264476
rs75882962
rs7982713
rs56069061
rs56157888
rs74540770
All − MR Egger
All − IVW(MRE)
−3
−2
−1
0
1
MR effect size for
'Gut microbiota abundance (genus Phascolarctobacterium id.2168)' on 'Diabetic nephropathy (more control exclusions)'

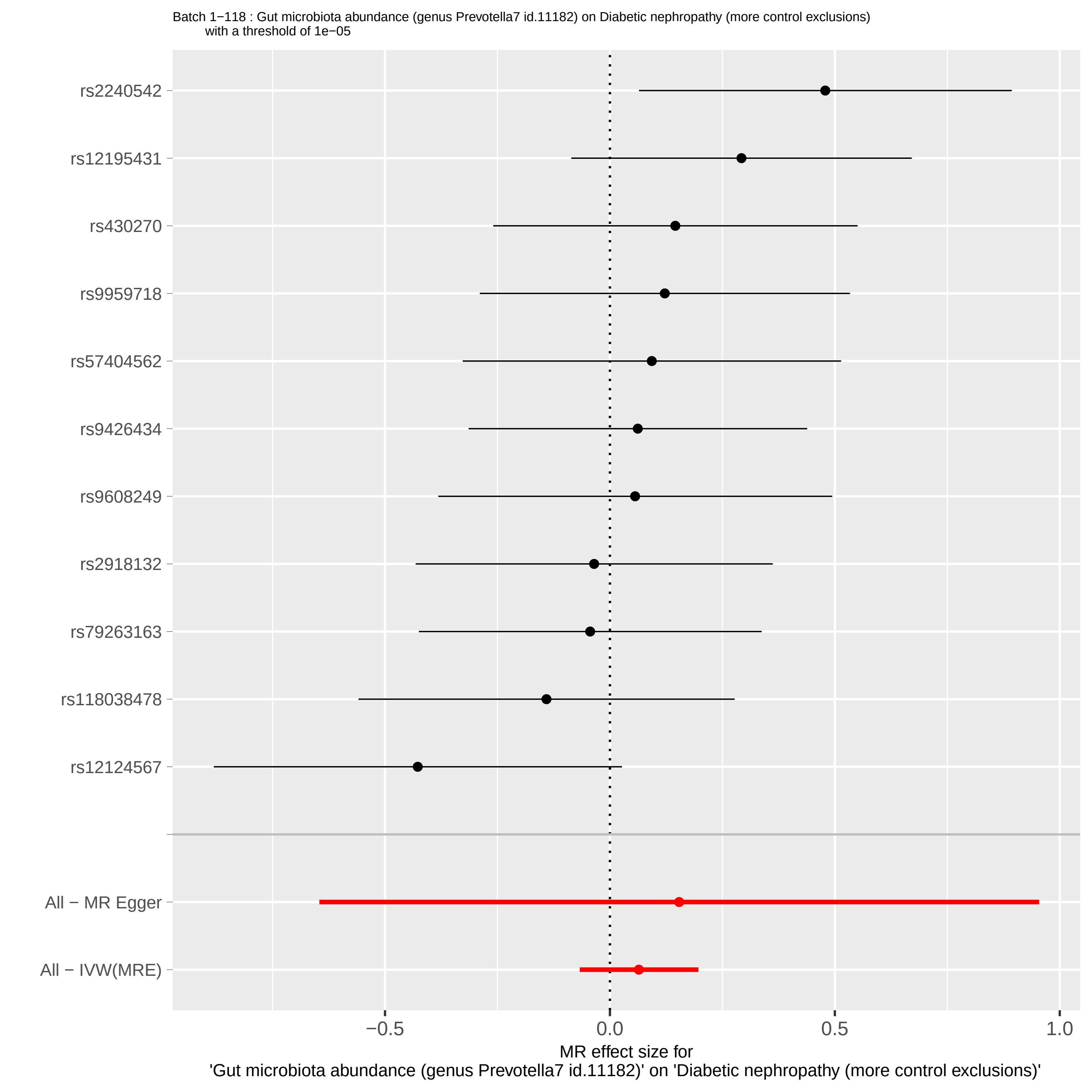

Batch 1−118 : Gut microbiota abundance (genus Prevotella7 id.11182) on Diabetic nephropathy (more control exclusions)
with a threshold of 1e−05
rs2240542
rs12195431
rs430270
rs9959718
rs57404562
rs9426434
rs9608249
rs2918132
rs79263163
rs118038478
rs12124567
All − MR Egger
All − IVW(MRE)
−0.5
0.0
0.5
1.0
MR effect size for
'Gut microbiota abundance (genus Prevotella7 id.11182)' on 'Diabetic nephropathy (more control exclusions)'

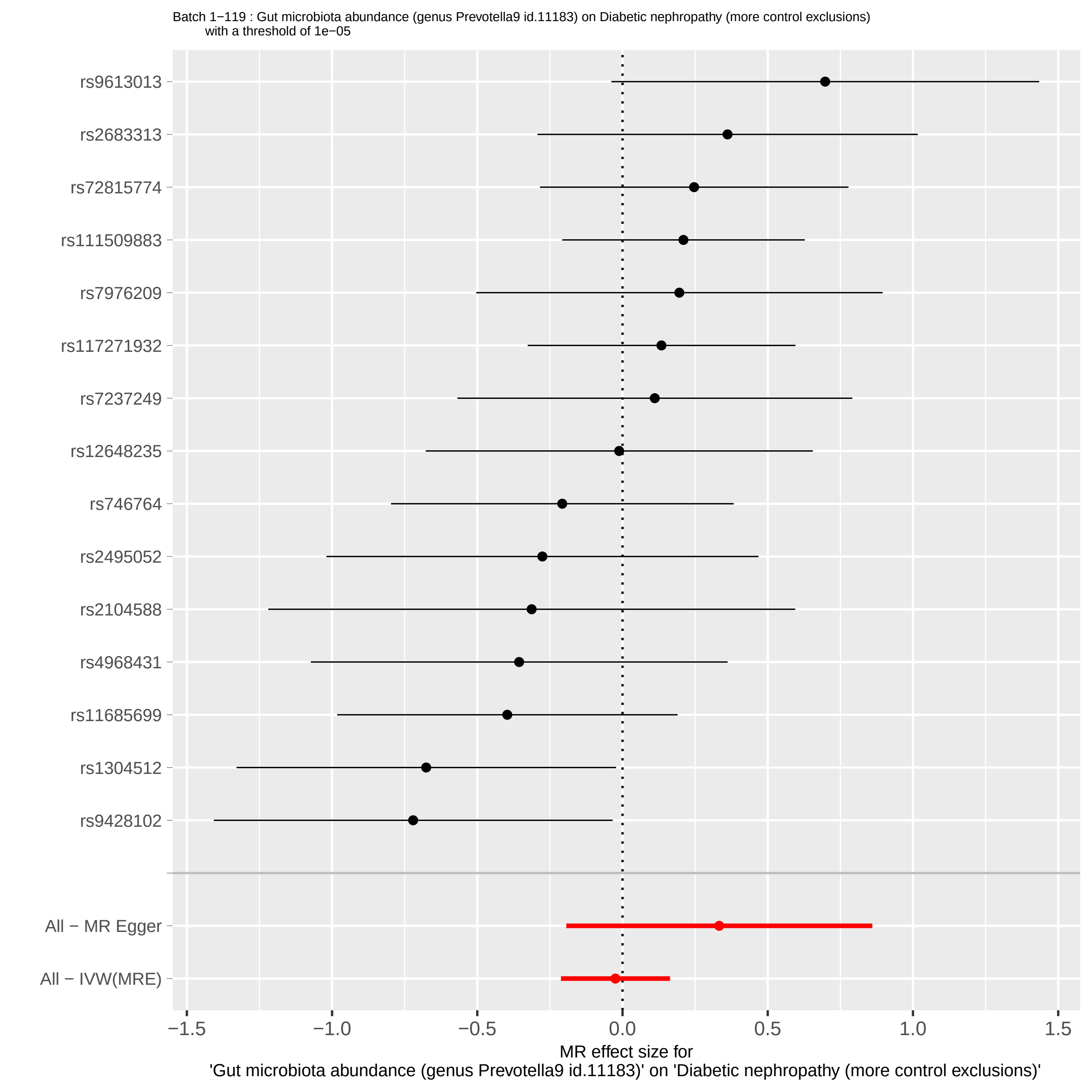

Batch 1−119 : Gut microbiota abundance (genus Prevotella9 id.11183) on Diabetic nephropathy (more control exclusions) with a threshold of 1e−05
rs9613013
rs2683313
rs72815774
rs111509883
rs7976209
rs117271932
rs7237249
rs12648235
rs746764
rs2495052
rs2104588
rs4968431
rs11685699
rs1304512
rs9428102
All − MR Egger
All − IVW(MRE)
−1.5
−1.0
−0.5
0.0
0.5
1.0
1.5
MR effect size for
'Gut microbiota abundance (genus Prevotella9 id.11183)' on 'Diabetic nephropathy (more control exclusions)'

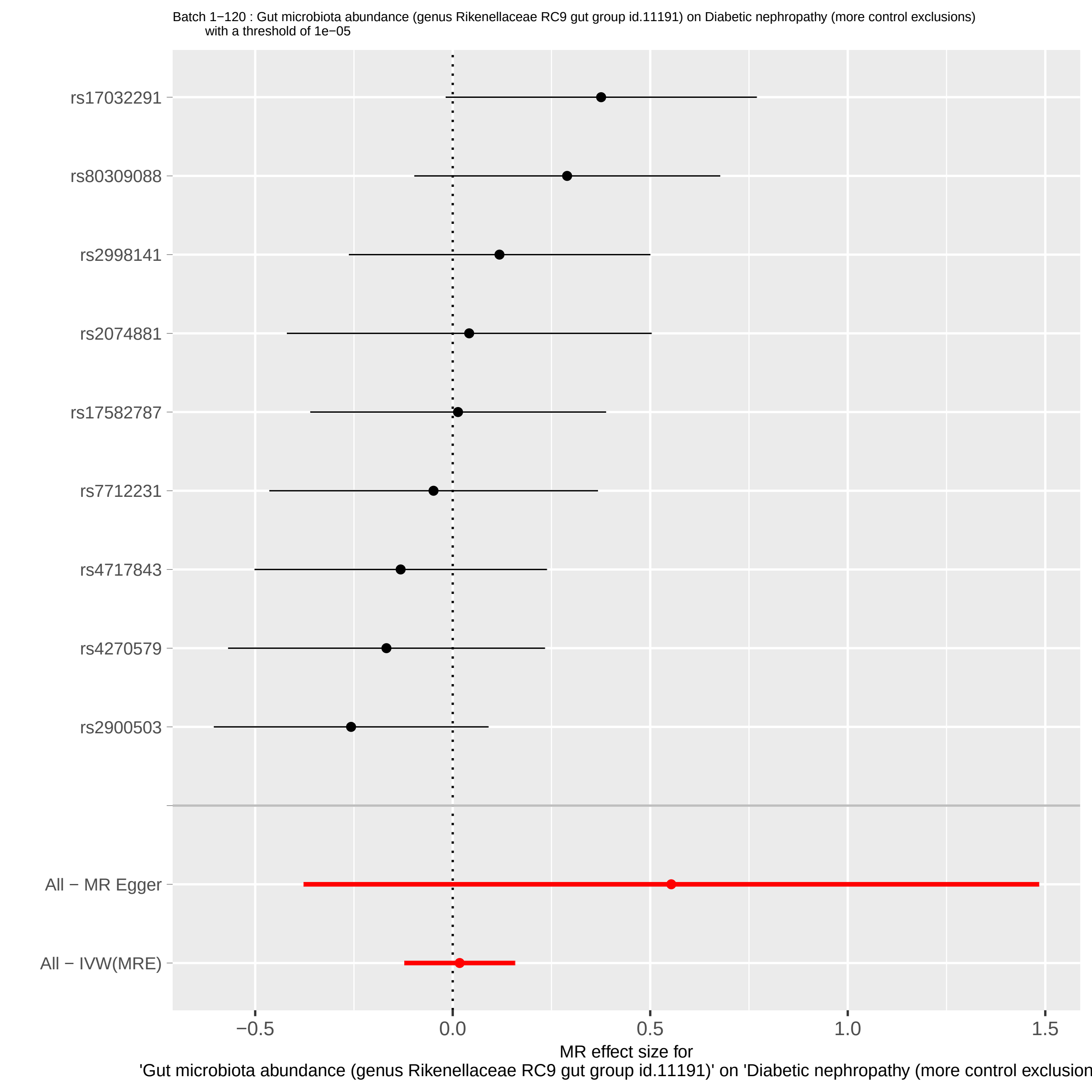

Batch 1−120 : Gut microbiota abundance (genus Rikenellaceae RC9 gut group id.11191) on Diabetic nephropathy (more control exclusions) with a threshold of 1e−05
rs17032291
rs80309088
rs2998141
rs2074881
rs17582787
rs7712231
rs4717843
rs4270579
rs2900503
All − MR Egger
All − IVW(MRE)
−0.5
0.0
0.5
1.0
1.5
MR effect size for
'Gut microbiota abundance (genus Rikenellaceae RC9 gut group id.11191)' on 'Diabetic nephropathy (more control exclusion

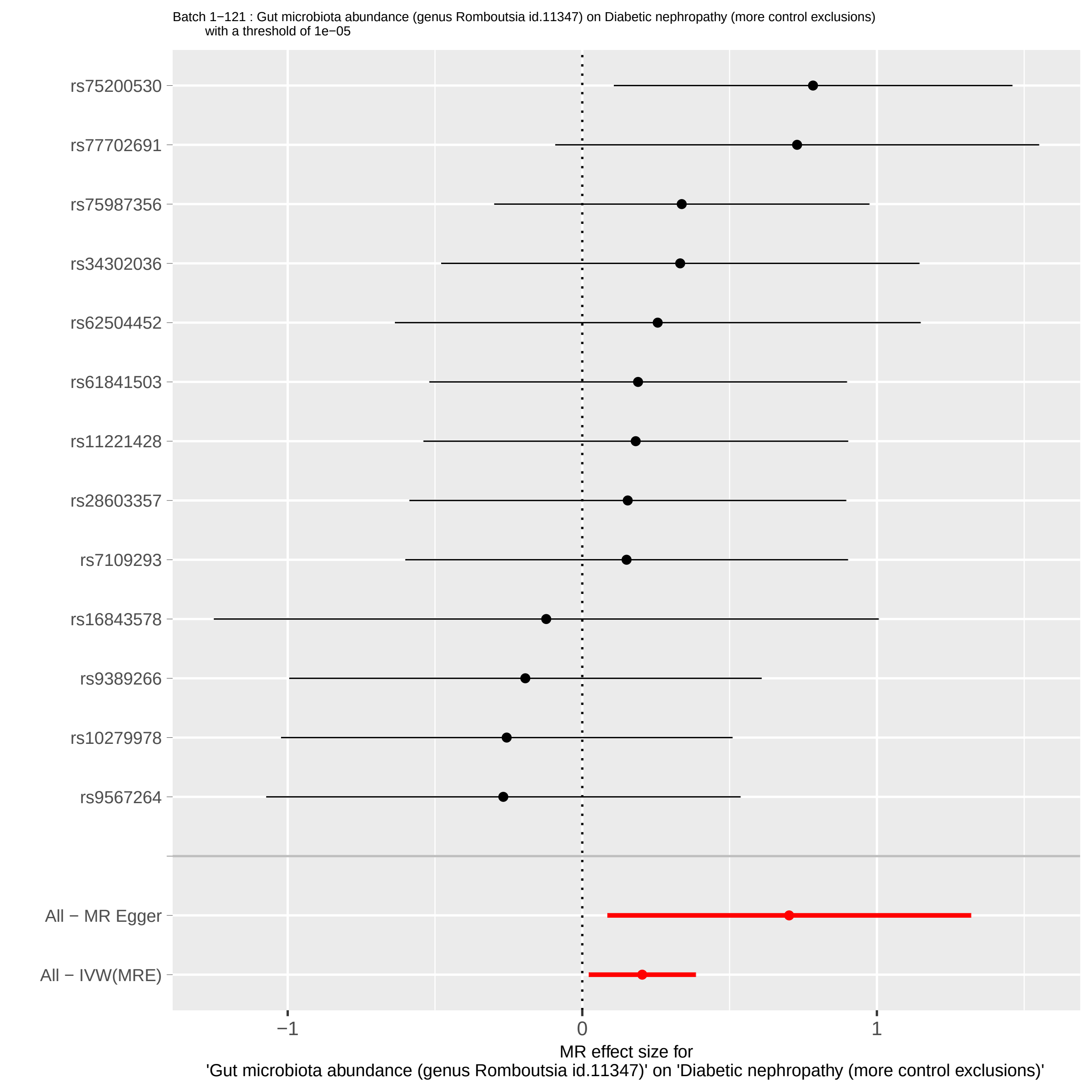
Batch 1−121 : Gut microbiota abundance (genus Romboutsia id.11347) on Diabetic nephropathy (more control exclusions)
with a threshold of 1e−05
rs75200530
rs77702691
rs75987356
rs34302036
rs62504452
rs61841503
rs11221428
rs28603357
rs7109293
rs16843578
rs9389266
rs10279978
rs9567264
All − MR Egger
All − IVW(MRE)
−1
0
1
MR effect size for
'Gut microbiota abundance (genus Romboutsia id.11347)' on 'Diabetic nephropathy (more control exclusions)'

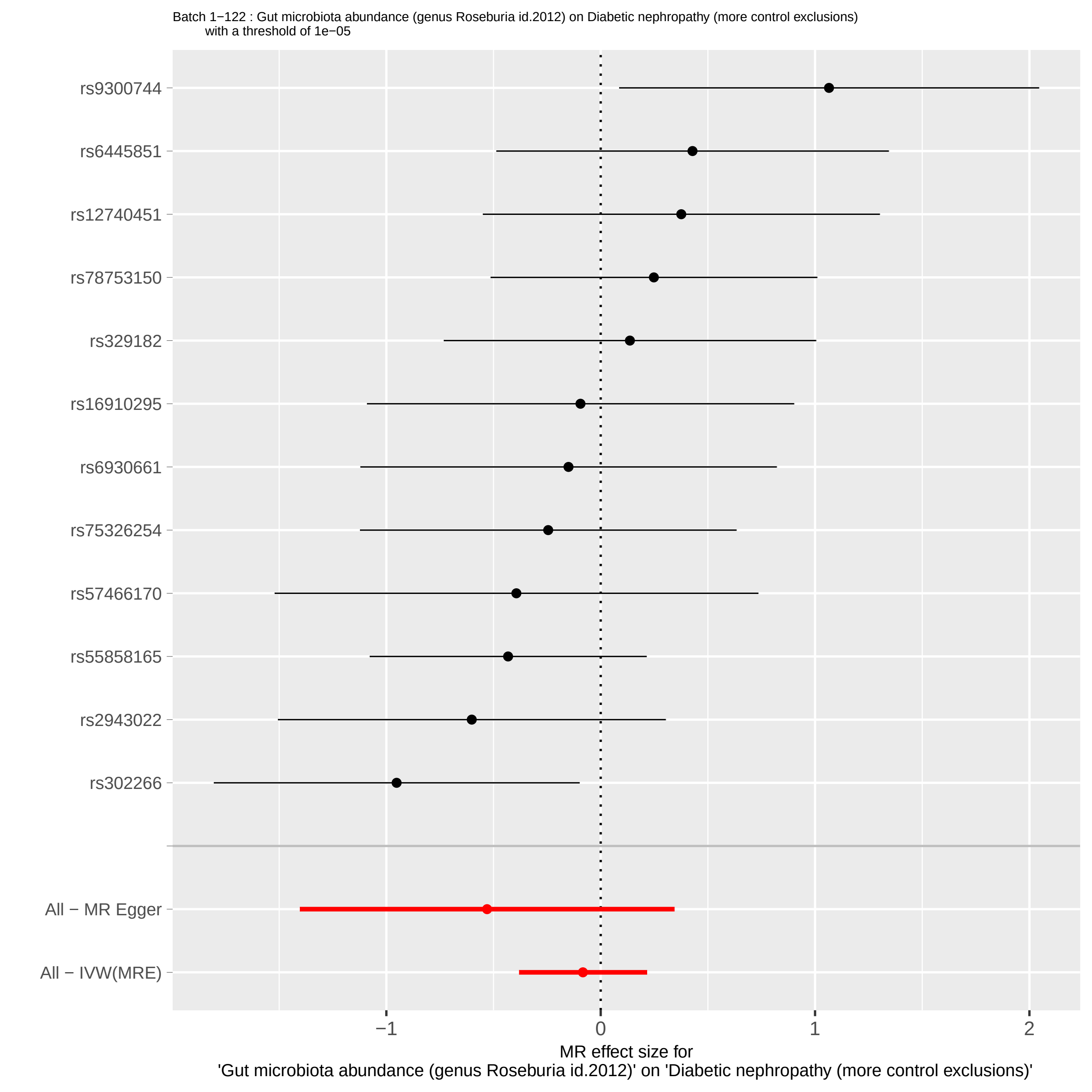

Batch 1−122 : Gut microbiota abundance (genus Roseburia id.2012) on Diabetic nephropathy (more control exclusions)
with a threshold of 1e−05
rs9300744
rs6445851
rs12740451
rs78753150
rs329182
rs16910295
rs6930661
rs75326254
rs57466170
rs55858165
rs2943022
rs302266
All − MR Egger
All − IVW(MRE)
−1
0
1
2
MR effect size for
'Gut microbiota abundance (genus Roseburia id.2012)' on 'Diabetic nephropathy (more control exclusions)'

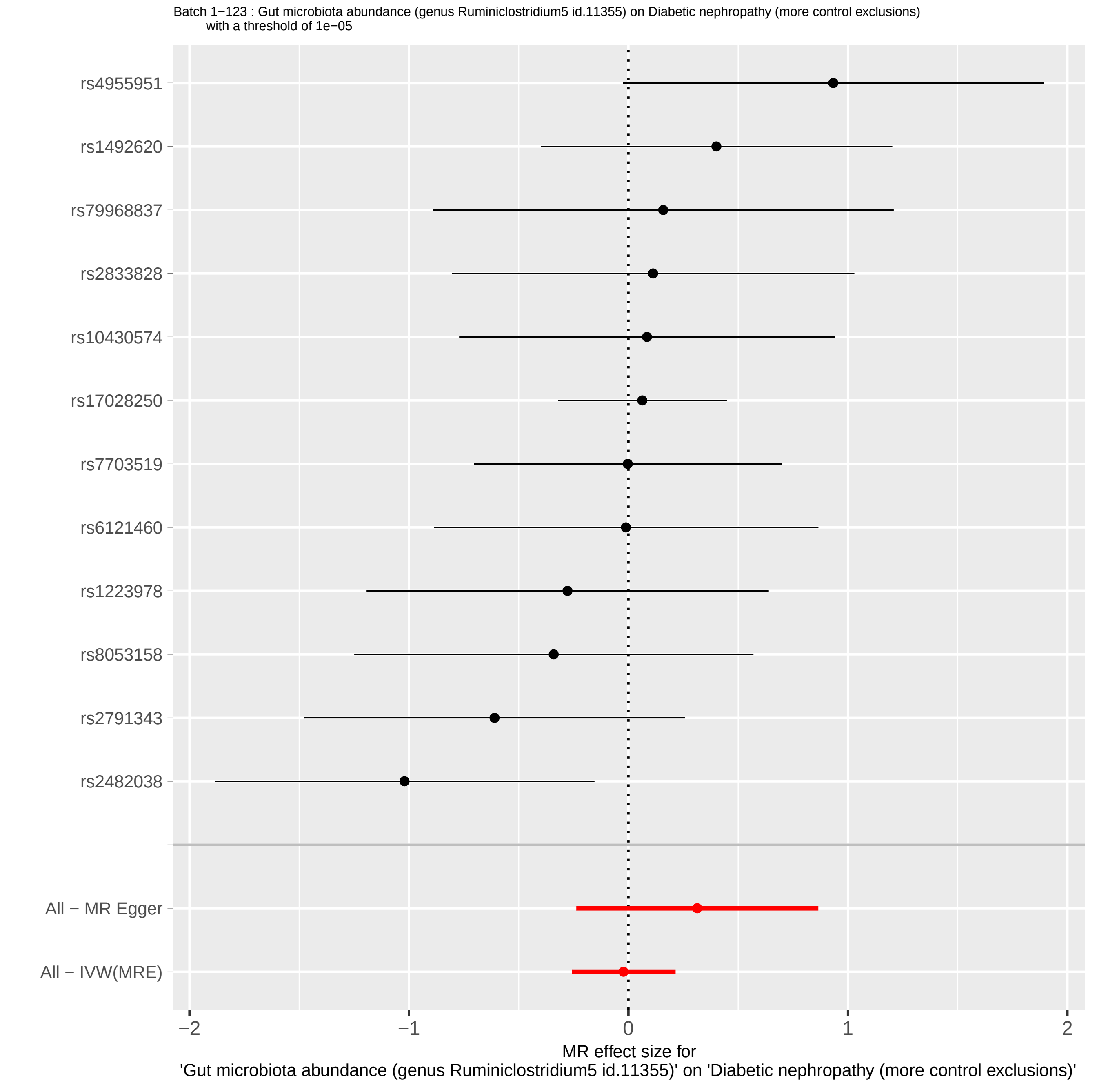

Batch 1−123 : Gut microbiota abundance (genus Ruminiclostridium5 id.11355) on Diabetic nephropathy (more control exclusions) with a threshold of 1e−05
rs4955951
rs1492620
rs79968837
rs2833828
rs10430574
rs17028250
rs7703519
rs6121460
rs1223978
rs8053158
rs2791343
rs2482038
All − MR Egger
All − IVW(MRE)
−2
−1
0
1
2
MR effect size for
'Gut microbiota abundance (genus Ruminiclostridium5 id.11355)' on 'Diabetic nephropathy (more control exclusions)'

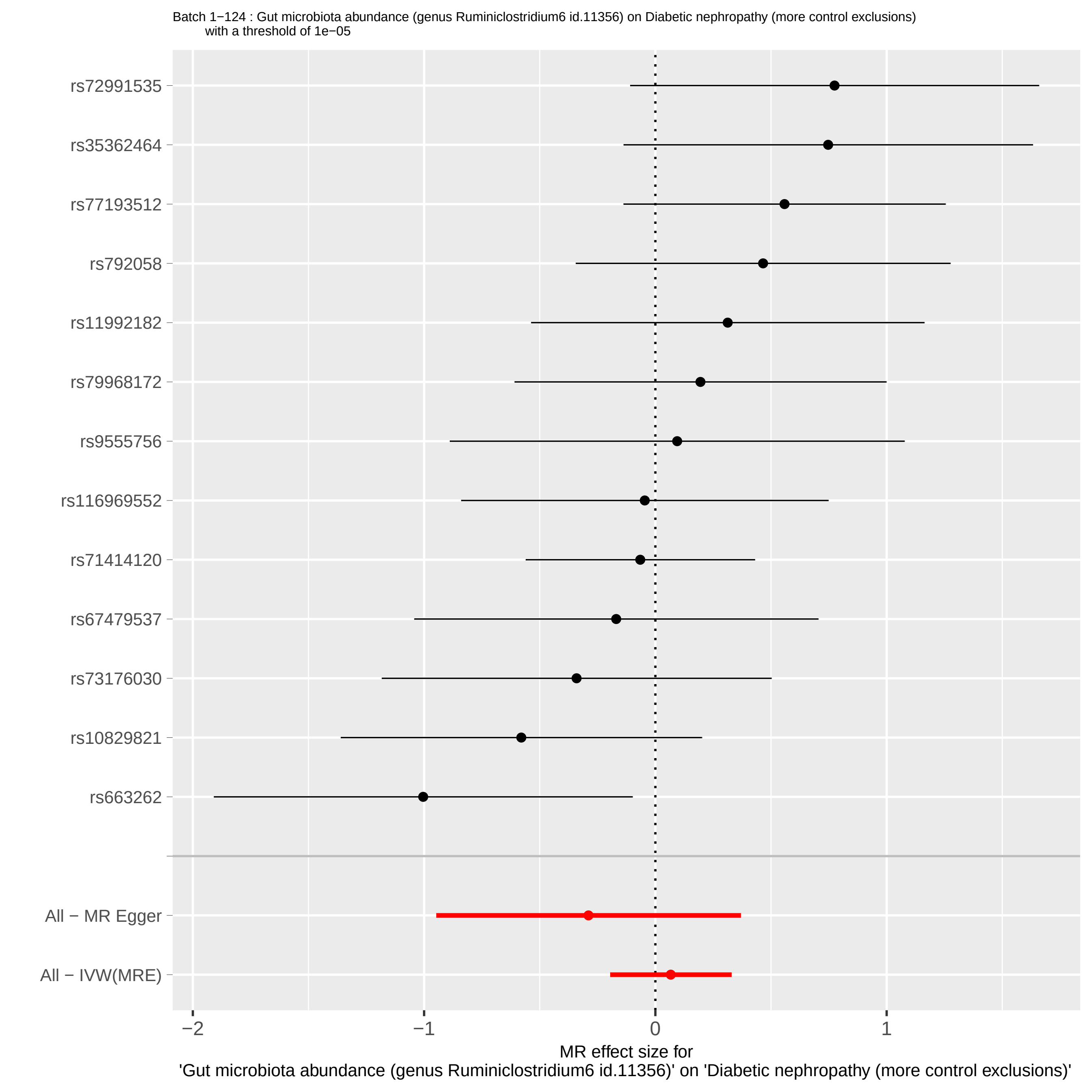

Batch 1−124 : Gut microbiota abundance (genus Ruminiclostridium6 id.11356) on Diabetic nephropathy (more control exclusions) with a threshold of 1e−05
rs72991535
rs35362464
rs77193512
rs792058
rs11992182
rs79968172
rs9555756
rs116969552
rs71414120
rs67479537
rs73176030
rs10829821
rs663262
All − MR Egger
All − IVW(MRE)
−2
−1
0
1
MR effect size for
'Gut microbiota abundance (genus Ruminiclostridium6 id.11356)' on 'Diabetic nephropathy (more control exclusions)'

Batch 1−125 : Gut microbiota abundance (genus Ruminiclostridium9 id.11357) on Diabetic nephropathy (more control exclusions) with a threshold of 1e−05

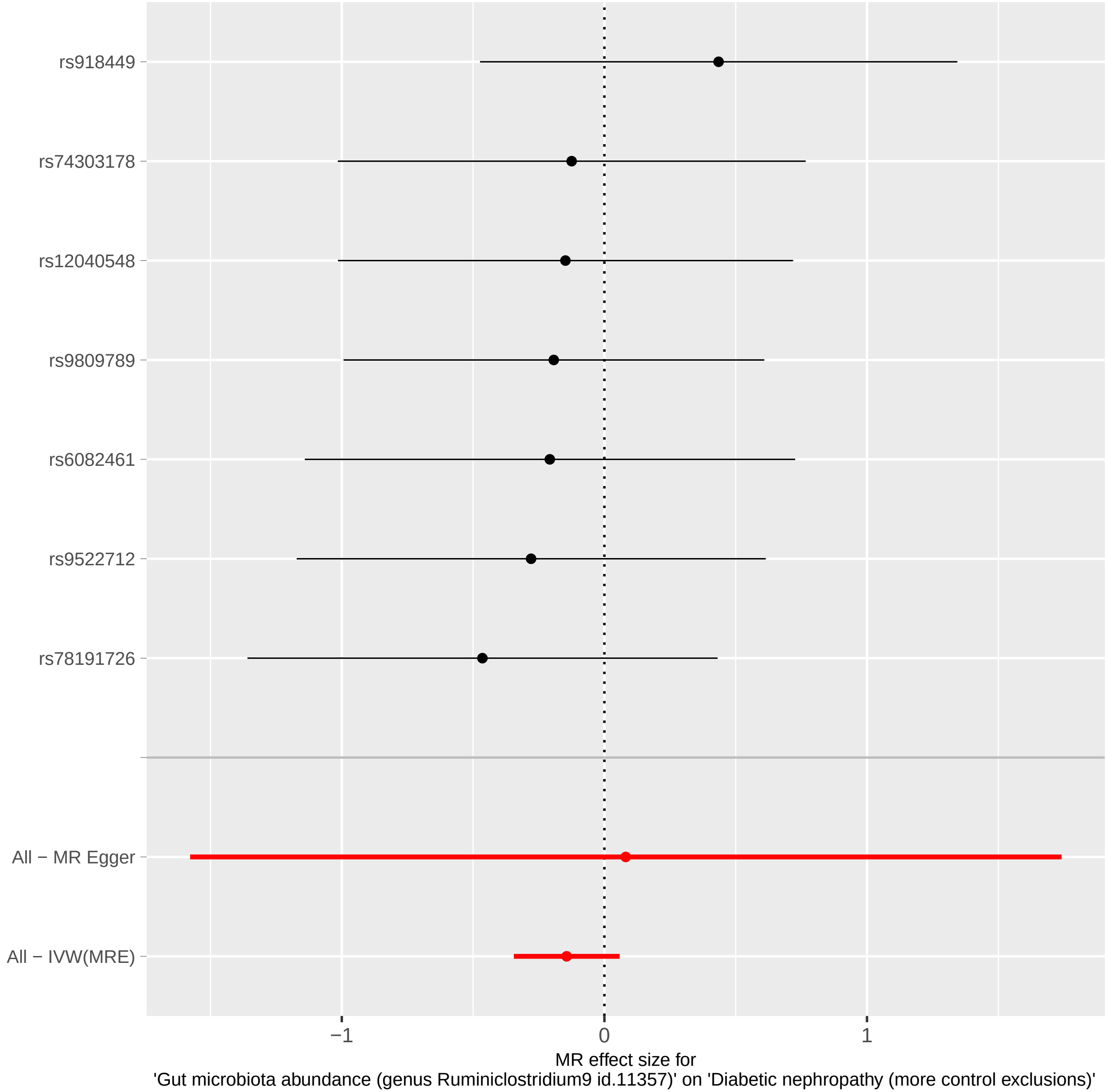

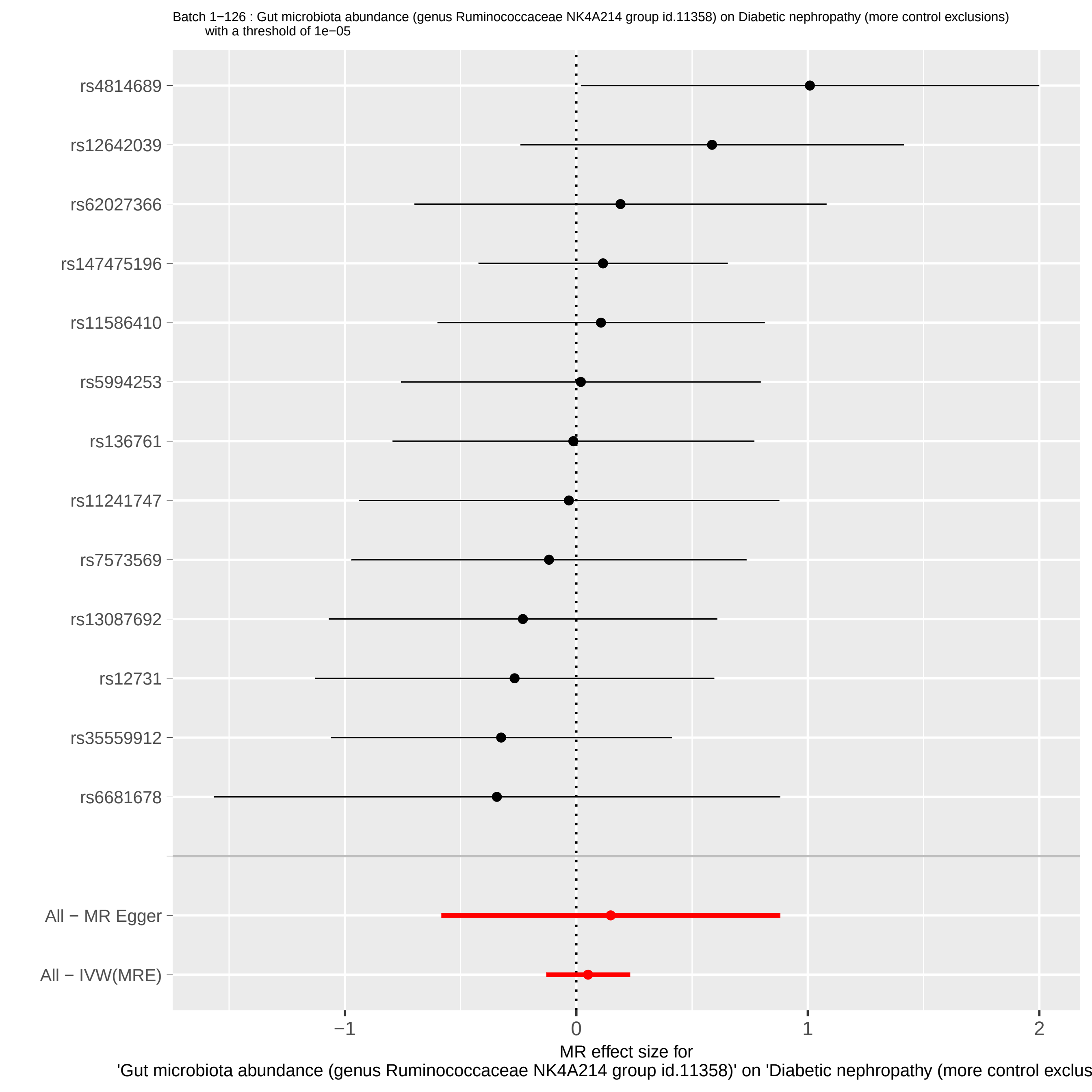
Batch 1−126 : Gut microbiota abundance (genus Ruminococcaceae NK4A214 group id.11358) on Diabetic nephropathy (more control exclusions) with a threshold of 1e−05
rs4814689
rs12642039
rs62027366
rs147475196
rs11586410
rs5994253
rs136761
rs11241747
rs7573569
rs13087692
rs12731
rs35559912
rs6681678
All − MR Egger
All − IVW(MRE)
−1
0
1
2
MR effect size for
'Gut microbiota abundance (genus Ruminococcaceae NK4A214 group id.11358)' on 'Diabetic nephropathy (more control exclus

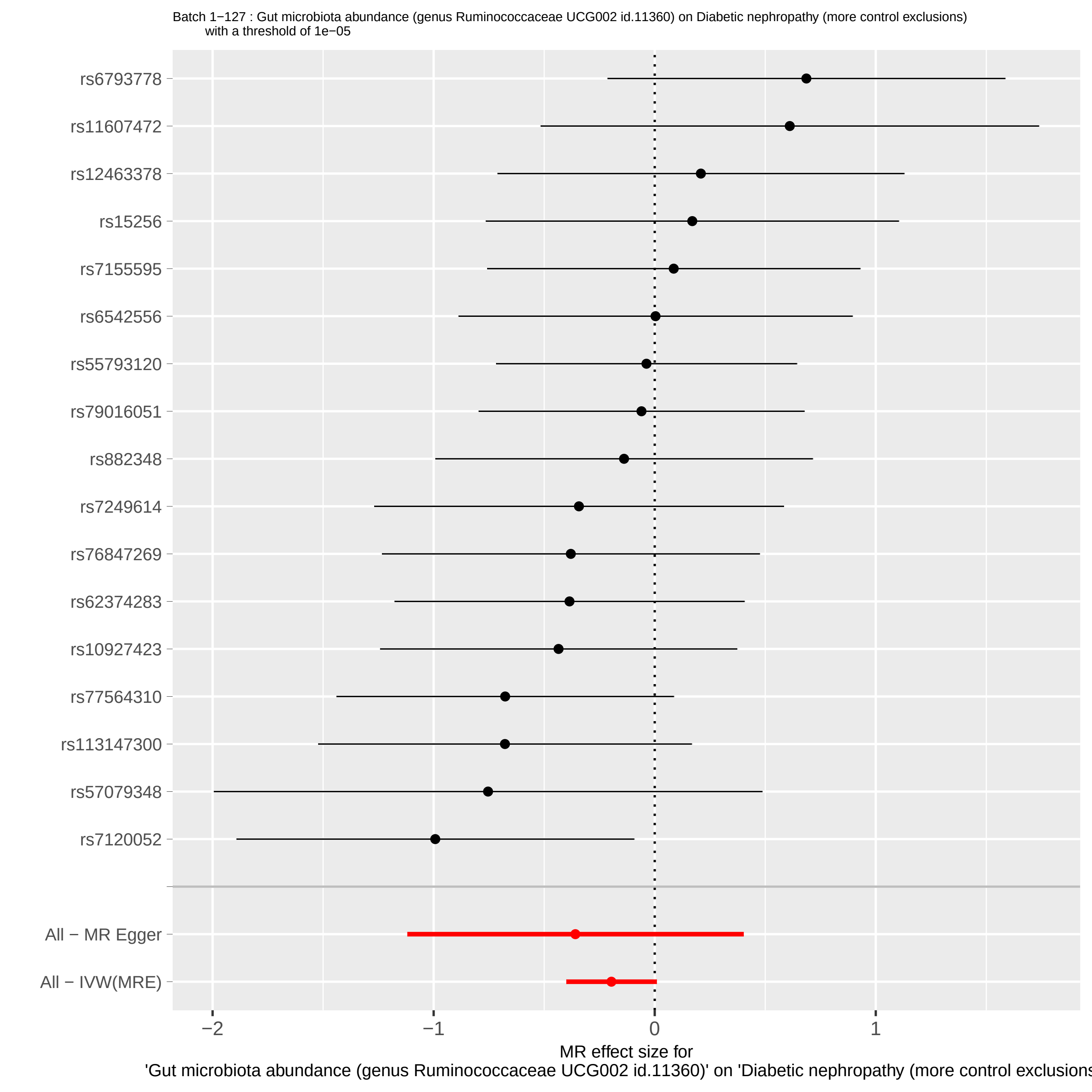

Batch 1−127 : Gut microbiota abundance (genus Ruminococcaceae UCG002 id.11360) on Diabetic nephropathy (more control exclusions)
with a threshold of 1e−05
rs6793778
rs11607472
rs12463378
rs15256
rs7155595
rs6542556
rs55793120
rs79016051
rs882348
rs7249614
rs76847269
rs62374283
rs10927423
rs77564310
rs113147300
rs57079348
rs7120052
All − MR Egger
All − IVW(MRE)
−2
−1
0
1
MR effect size for
'Gut microbiota abundance (genus Ruminococcaceae UCG002 id.11360)' on 'Diabetic nephropathy (more control exclusions

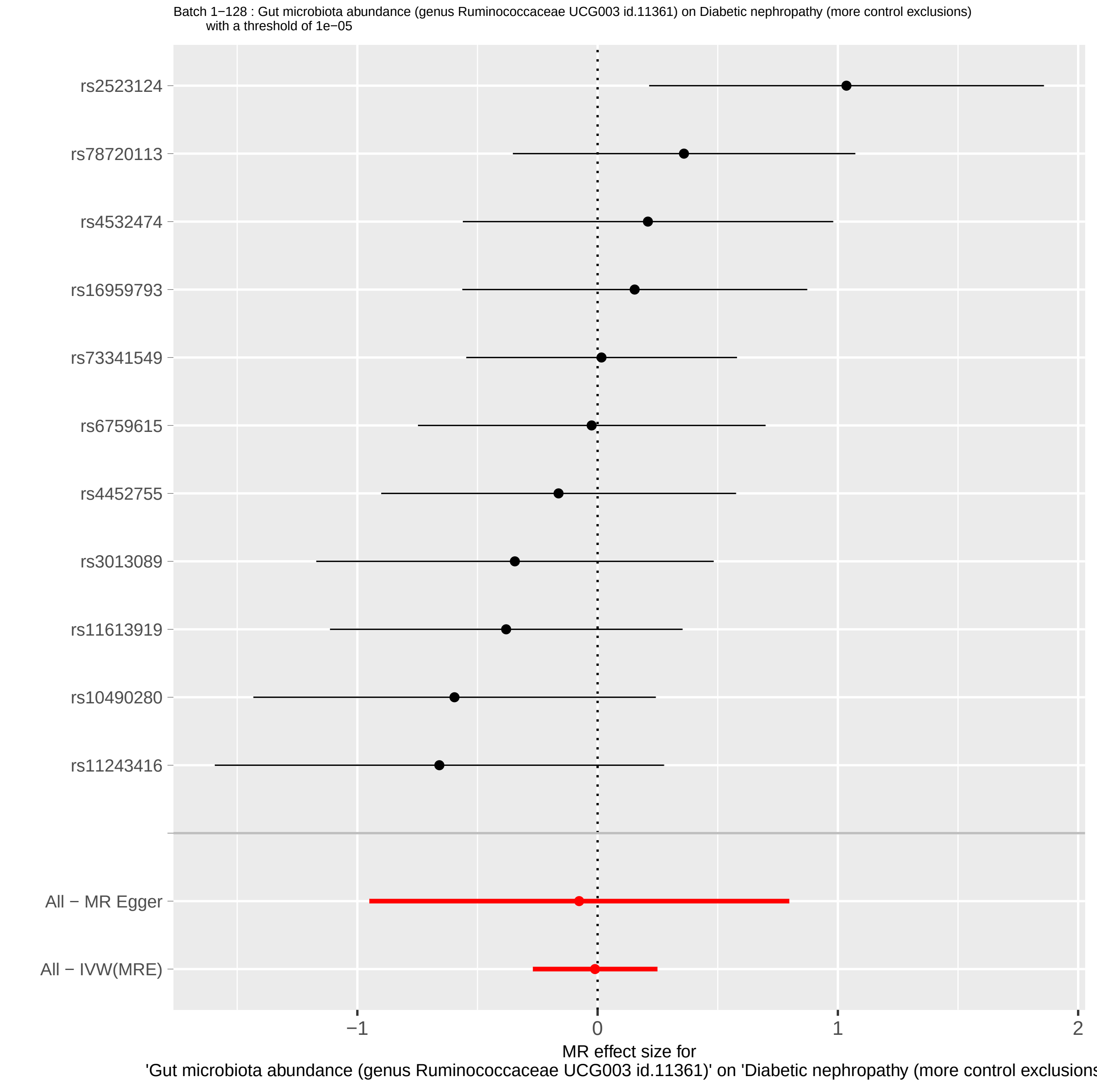
Batch 1−128 : Gut microbiota abundance (genus Ruminococcaceae UCG003 id.11361) on Diabetic nephropathy (more control exclusions) with a threshold of 1e−05
rs2523124
rs78720113
rs4532474
rs16959793
rs73341549
rs6759615
rs4452755
rs3013089
rs11613919
rs10490280
rs11243416
All − MR Egger
All − IVW(MRE)
−1
0
1
2
MR effect size for
'Gut microbiota abundance (genus Ruminococcaceae UCG003 id.11361)' on 'Diabetic nephropathy (more control exclusions

Batch 1−129 : Gut microbiota abundance (genus Ruminococcaceae UCG004 id.11362) on Diabetic nephropathy (more control exclusions) with a threshold of 1e−05

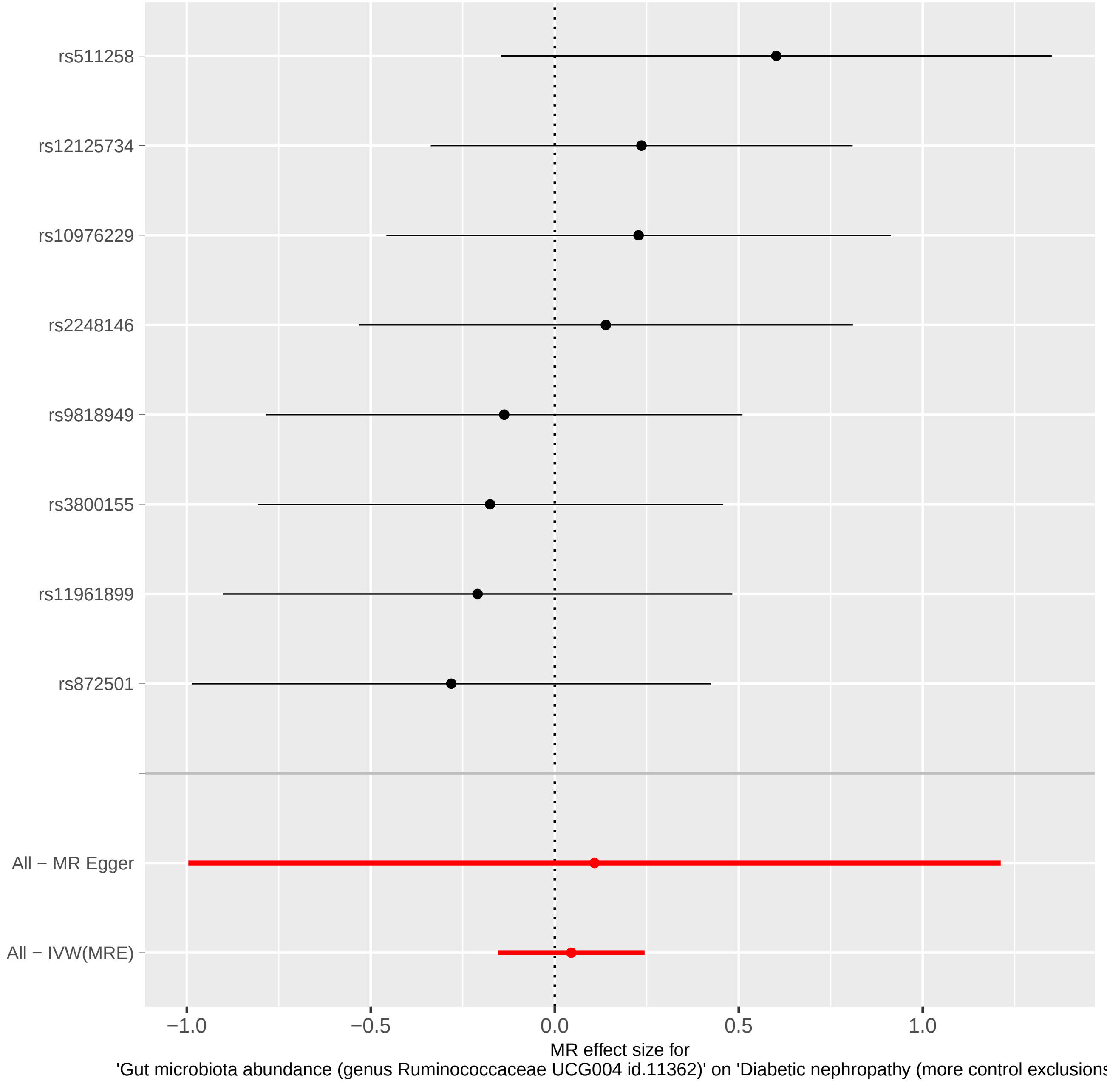

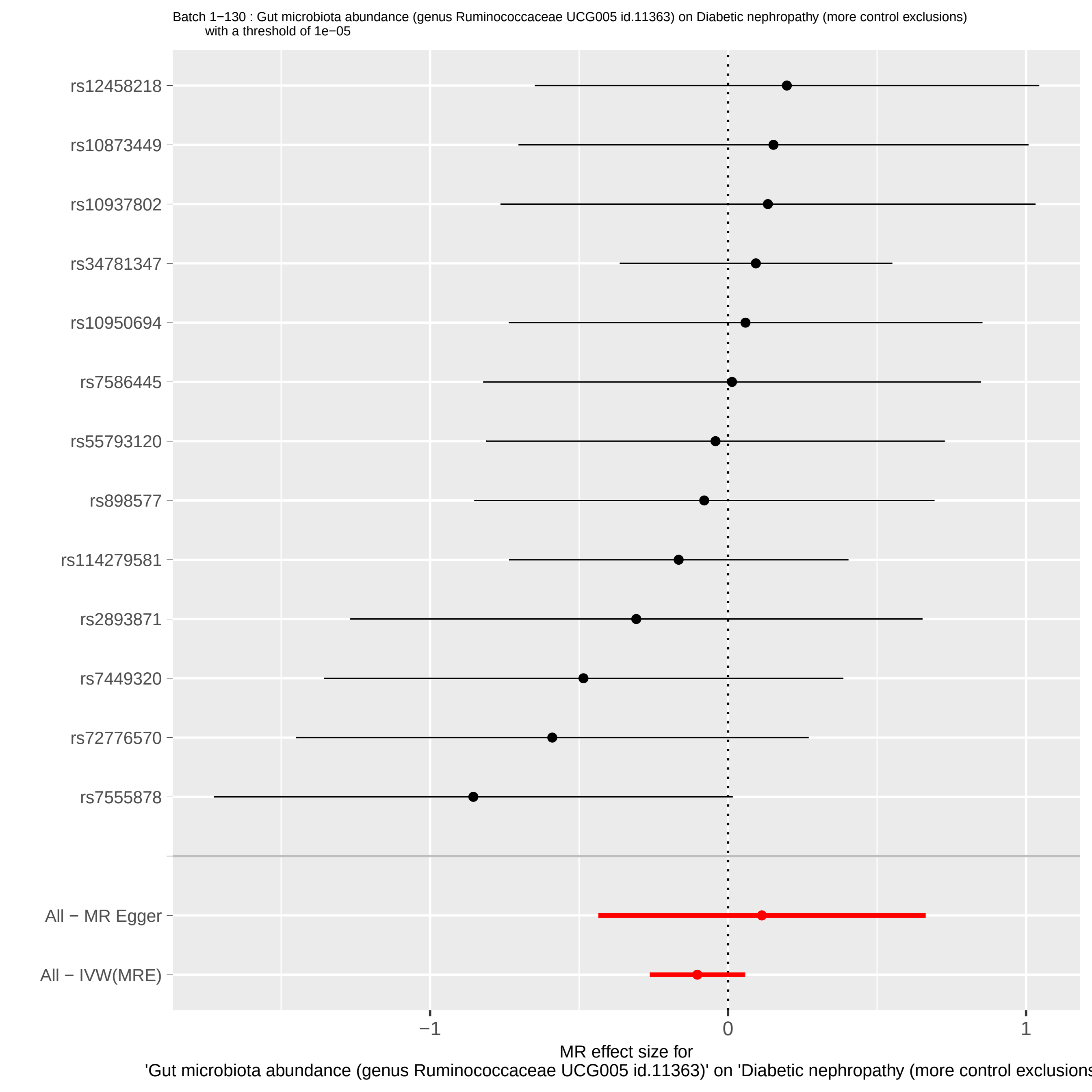

Batch 1−130 : Gut microbiota abundance (genus Ruminococcaceae UCG005 id.11363) on Diabetic nephropathy (more control exclusions)
with a threshold of 1e−05
rs12458218
rs10873449
rs10937802
rs34781347
rs10950694
rs7586445
rs55793120
rs898577
rs114279581
rs2893871
rs7449320
rs72776570
rs7555878
All − MR Egger
All − IVW(MRE)
−1
0
1
MR effect size for
'Gut microbiota abundance (genus Ruminococcaceae UCG005 id.11363)' on 'Diabetic nephropathy (more control exclusions

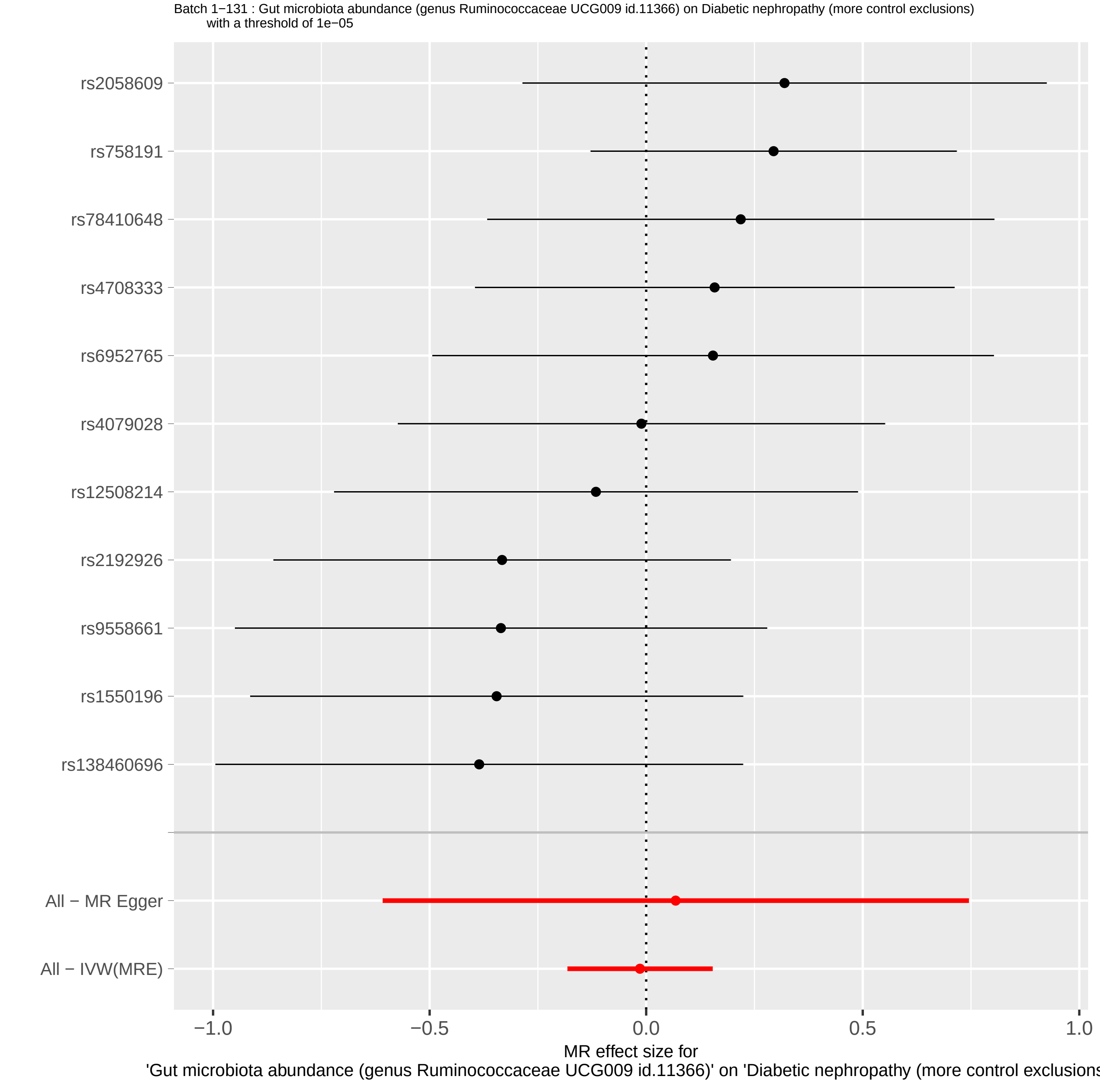

Batch 1−131 : Gut microbiota abundance (genus Ruminococcaceae UCG009 id.11366) on Diabetic nephropathy (more control exclusions) with a threshold of 1e−05
rs2058609
rs758191
rs78410648
rs4708333
rs6952765
rs4079028
rs12508214
rs2192926
rs9558661
rs1550196
rs138460696
All − MR Egger
All − IVW(MRE)
−1.0
−0.5
0.0
0.5
1.0
MR effect size for
'Gut microbiota abundance (genus Ruminococcaceae UCG009 id.11366)' on 'Diabetic nephropathy (more control exclusions

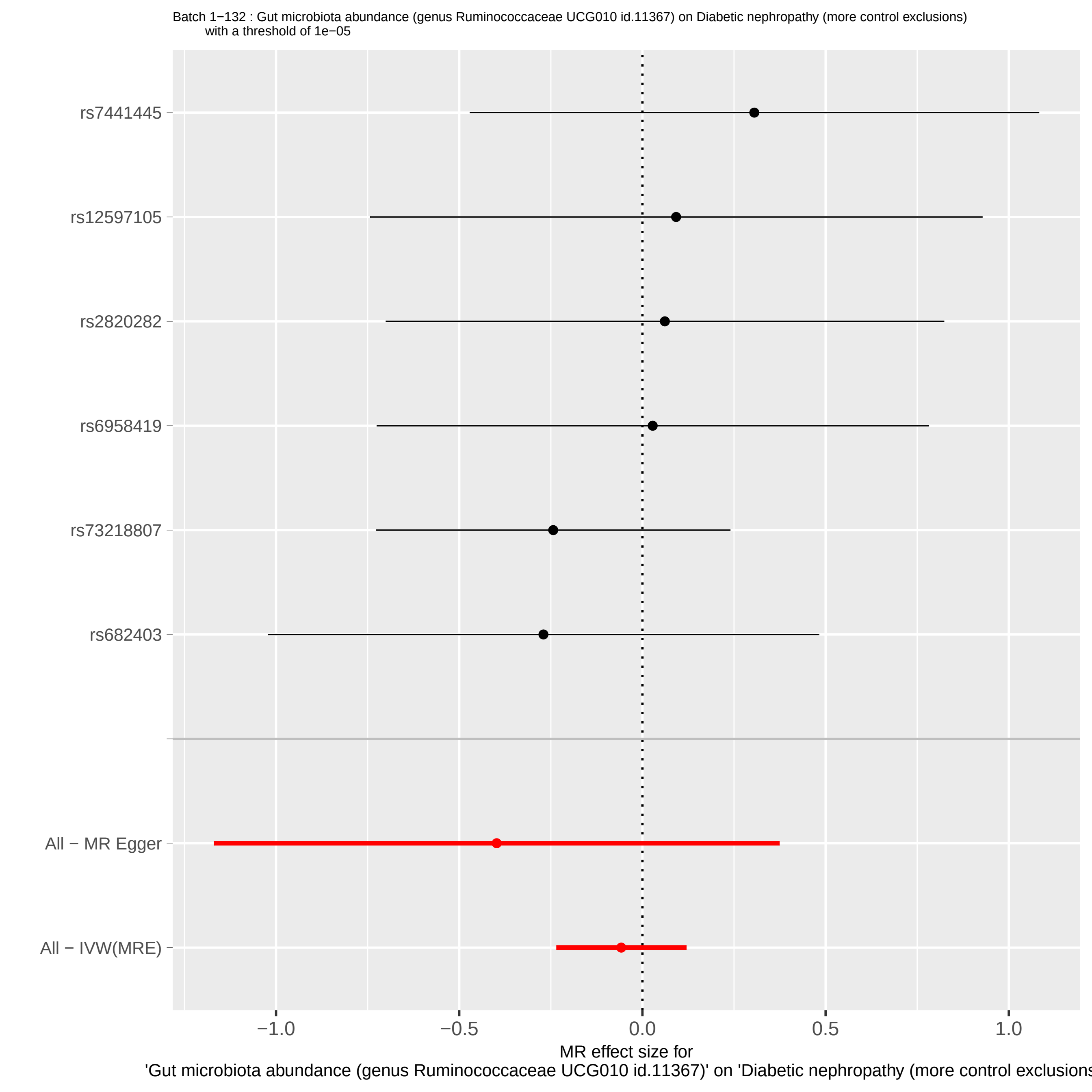

Batch 1−132 : Gut microbiota abundance (genus Ruminococcaceae UCG010 id.11367) on Diabetic nephropathy (more control exclusions) with a threshold of 1e−05
rs7441445
rs12597105
rs2820282
rs6958419
rs73218807
rs682403
All − MR Egger
All − IVW(MRE)
−1.0
−0.5
0.0
0.5
1.0
MR effect size for
'Gut microbiota abundance (genus Ruminococcaceae UCG010 id.11367)' on 'Diabetic nephropathy (more control exclusions

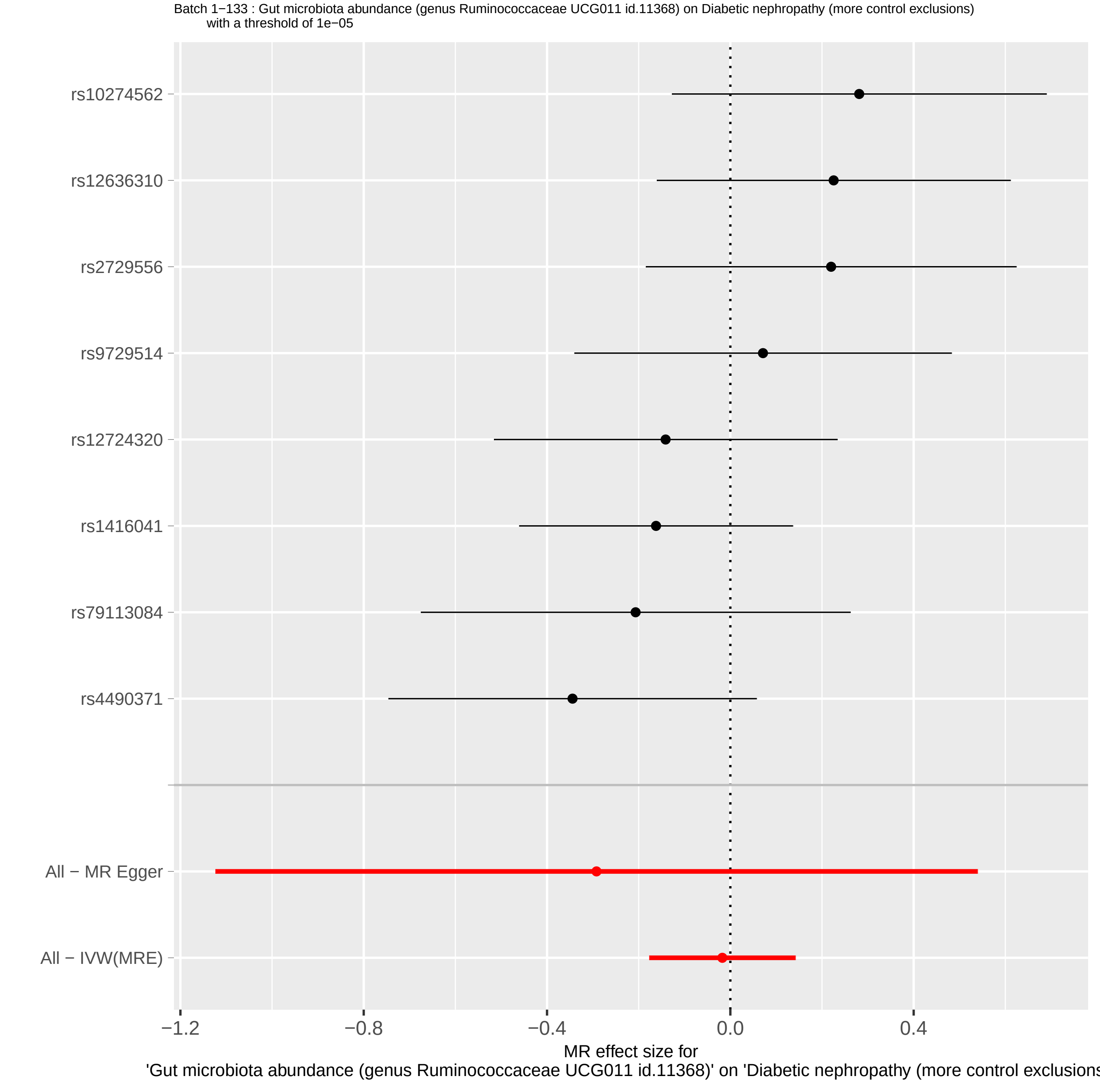

Batch 1−133 : Gut microbiota abundance (genus Ruminococcaceae UCG011 id.11368) on Diabetic nephropathy (more control exclusions) with a threshold of 1e−05
rs10274562
rs12636310
rs2729556
rs9729514
rs12724320
rs1416041
rs79113084
rs4490371
All − MR Egger
All − IVW(MRE)
−1.2
−0.8
−0.4
0.0
0.4
MR effect size for
'Gut microbiota abundance (genus Ruminococcaceae UCG011 id.11368)' on 'Diabetic nephropathy (more control exclusions

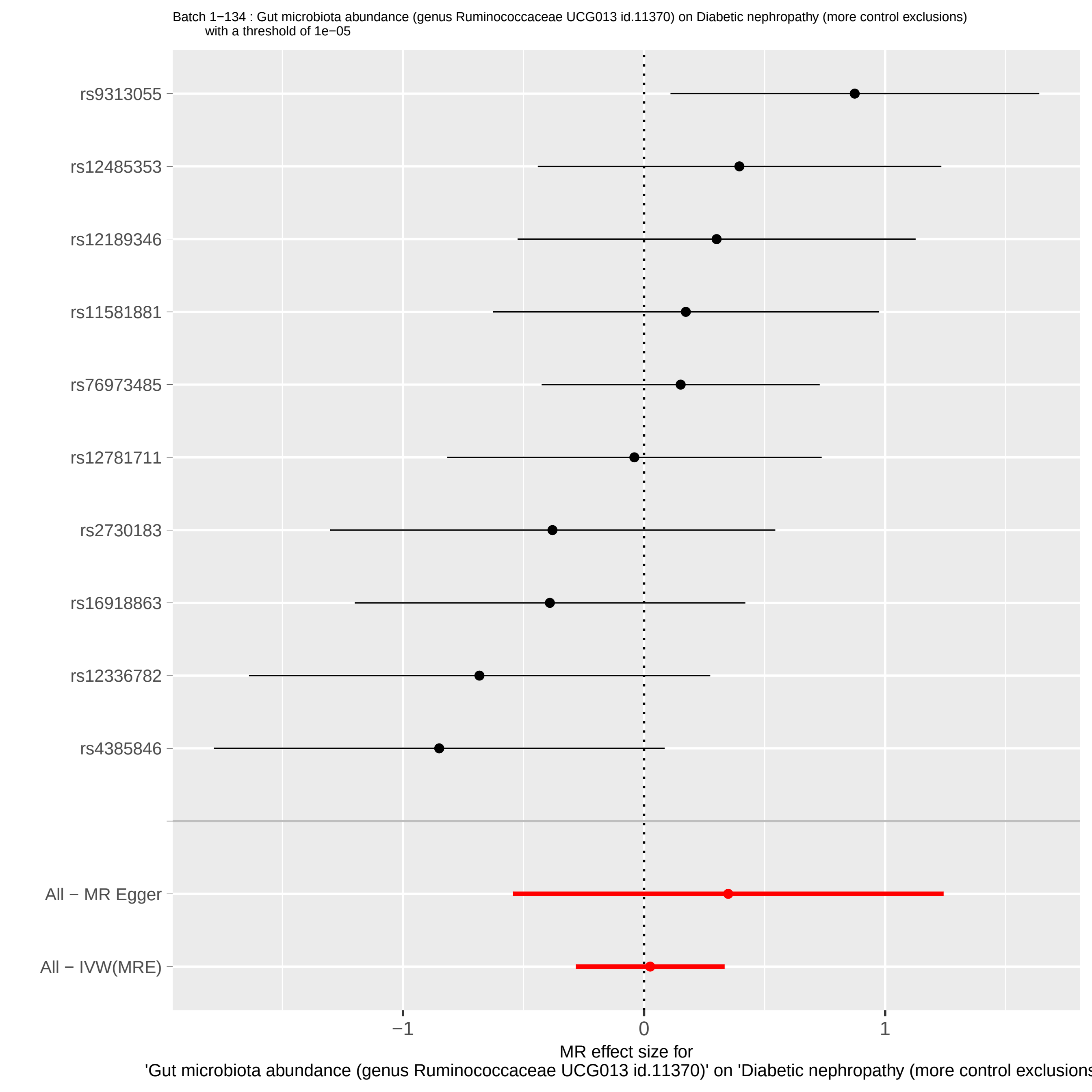

Batch 1−134 : Gut microbiota abundance (genus Ruminococcaceae UCG013 id.11370) on Diabetic nephropathy (more control exclusions)
with a threshold of 1e−05
rs9313055
rs12485353
rs12189346
rs11581881
rs76973485
rs12781711
rs2730183
rs16918863
rs12336782
rs4385846
All − MR Egger
All − IVW(MRE)
−1
0
1
MR effect size for
'Gut microbiota abundance (genus Ruminococcaceae UCG013 id.11370)' on 'Diabetic nephropathy (more control exclusions

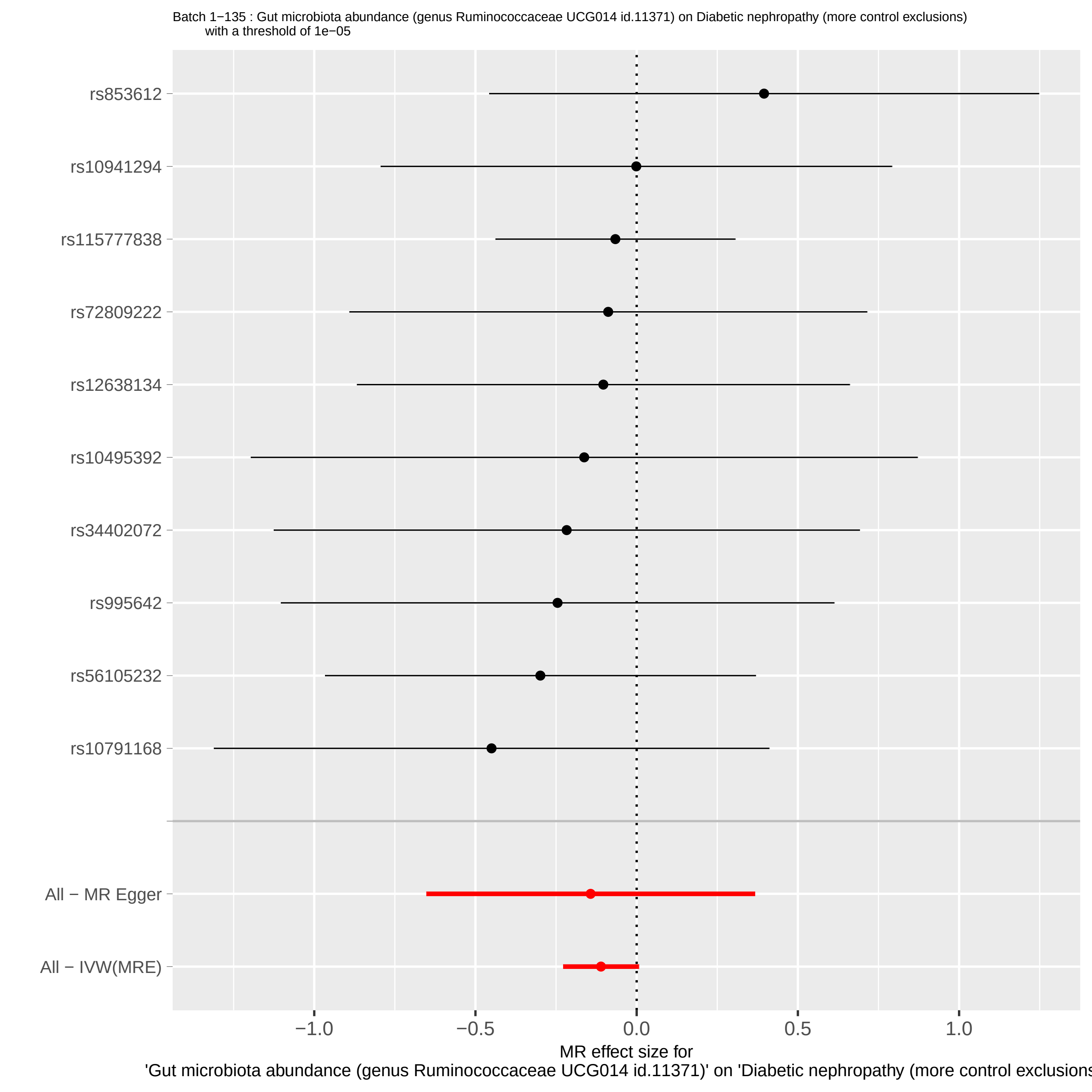

Batch 1−135 : Gut microbiota abundance (genus Ruminococcaceae UCG014 id.11371) on Diabetic nephropathy (more control exclusions) with a threshold of 1e−05
rs853612
rs10941294
rs115777838
rs72809222
rs12638134
rs10495392
rs34402072
rs995642
rs56105232
rs10791168
All − MR Egger
All − IVW(MRE)
−1.0
−0.5
0.0
0.5
1.0
MR effect size for
'Gut microbiota abundance (genus Ruminococcaceae UCG014 id.11371)' on 'Diabetic nephropathy (more control exclusions

Batch 1−136 : Gut microbiota abundance (genus Ruminococcus gauvreauii group id.11342) on Diabetic nephropathy (more control exclusions) with a threshold of 1e−05

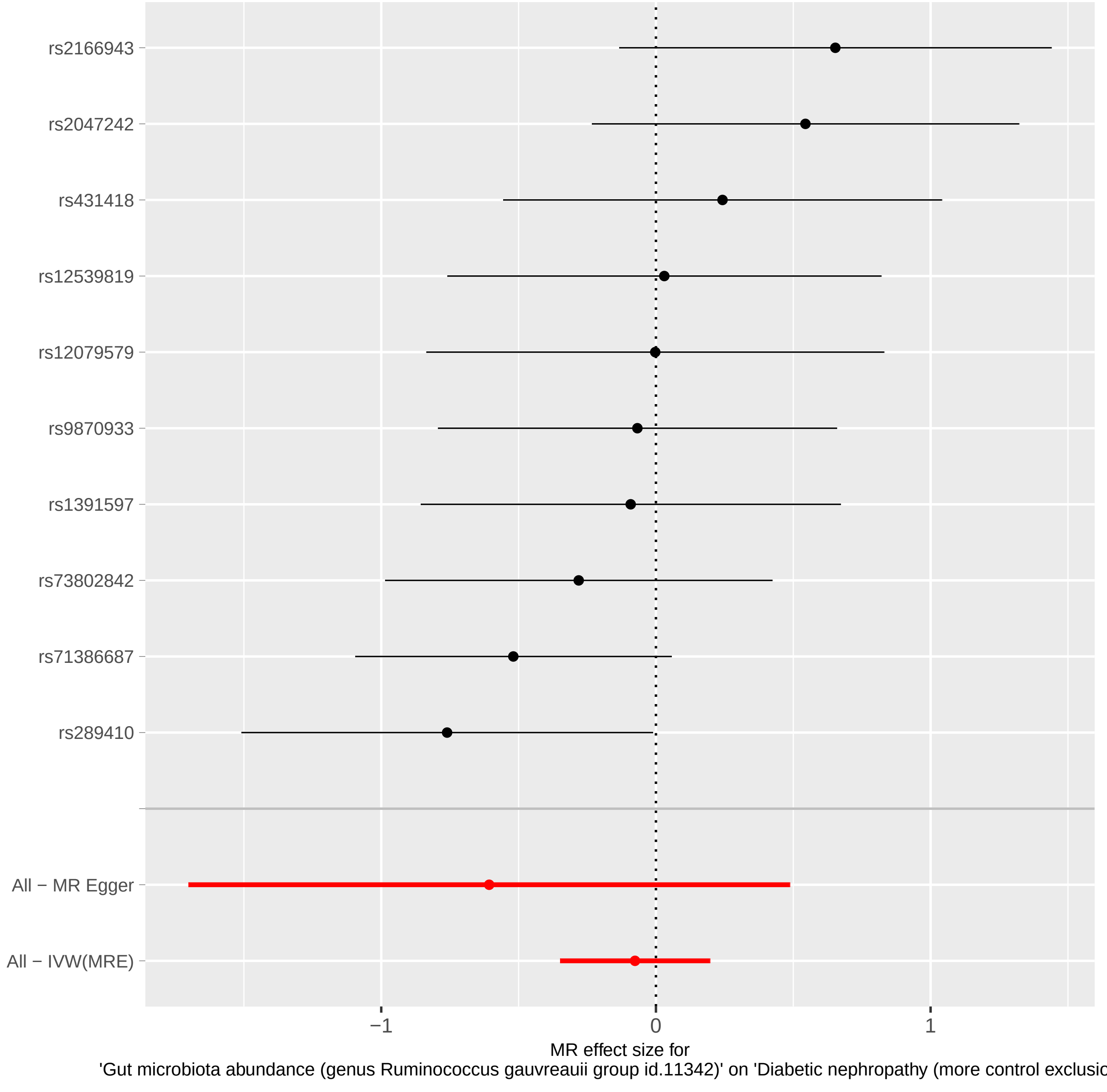

Batch 1−137 : Gut microbiota abundance (genus Ruminococcus gnavus group id.14376) on Diabetic nephropathy (more control exclusions)
with a threshold of 1e−05

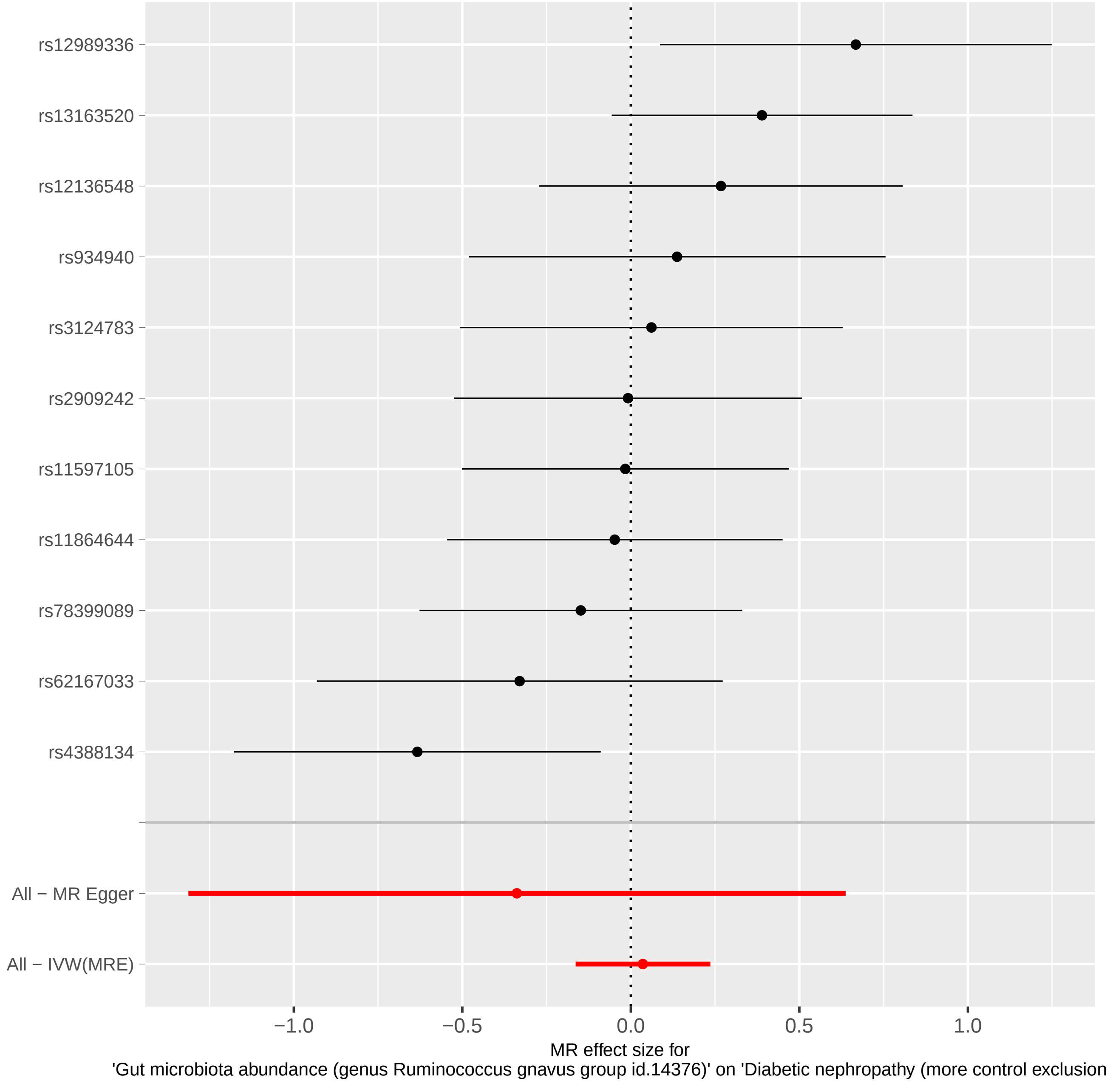

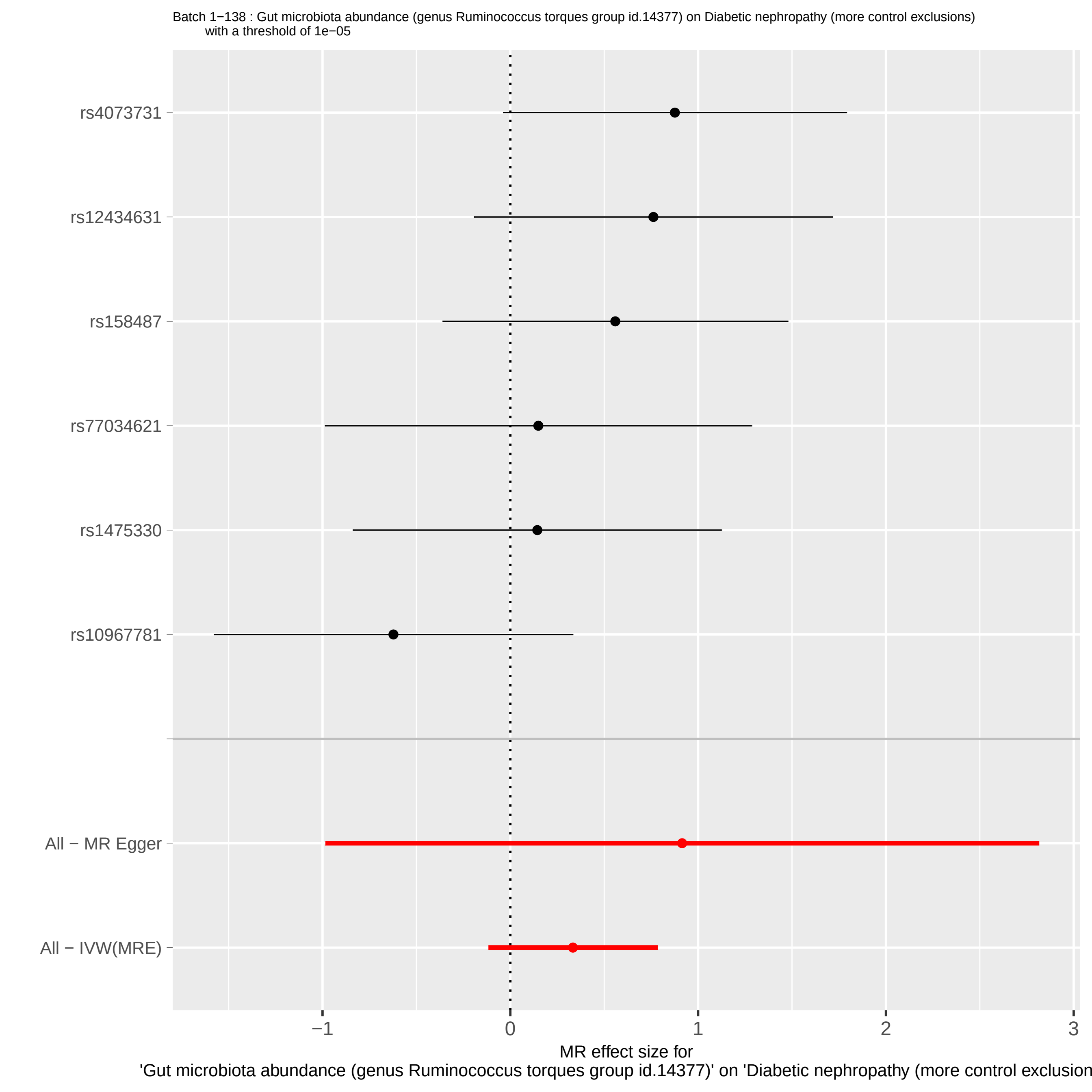

Batch 1−138 : Gut microbiota abundance (genus Ruminococcus torques group id.14377) on Diabetic nephropathy (more control exclusions)
with a threshold of 1e−05
rs4073731
rs12434631
rs158487
rs77034621
rs1475330
rs10967781
All − MR Egger
All − IVW(MRE)
−1
0
1
2
3
MR effect size for
'Gut microbiota abundance (genus Ruminococcus torques group id.14377)' on 'Diabetic nephropathy (more control exclusion

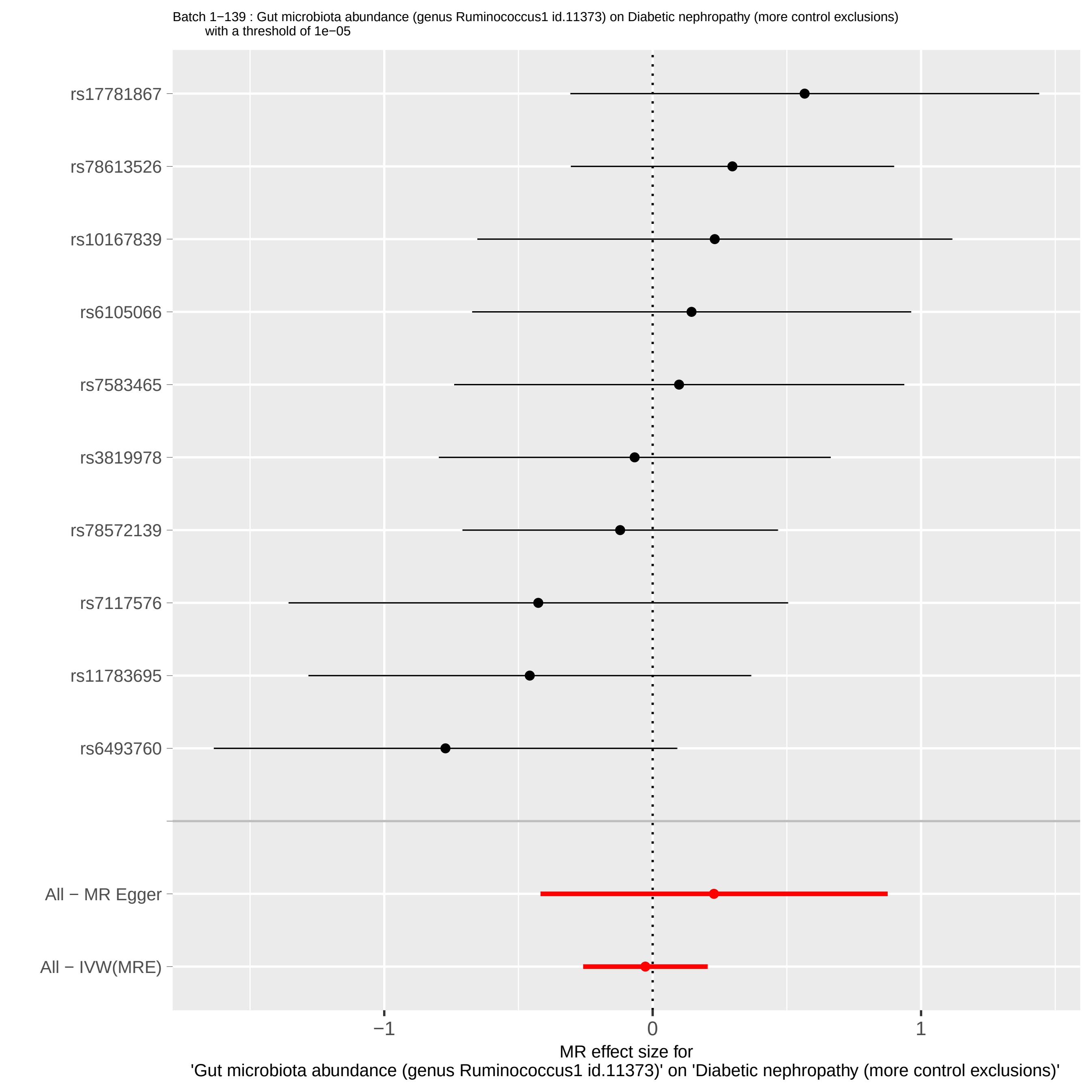

Batch 1−139 : Gut microbiota abundance (genus Ruminococcus1 id.11373) on Diabetic nephropathy (more control exclusions) with a threshold of 1e−05
rs17781867
rs78613526
rs10167839
rs6105066
rs7583465
rs3819978
rs78572139
rs7117576
rs11783695
rs6493760
All − MR Egger
All − IVW(MRE)
−1
0
1
MR effect size for
'Gut microbiota abundance (genus Ruminococcus1 id.11373)' on 'Diabetic nephropathy (more control exclusions)'

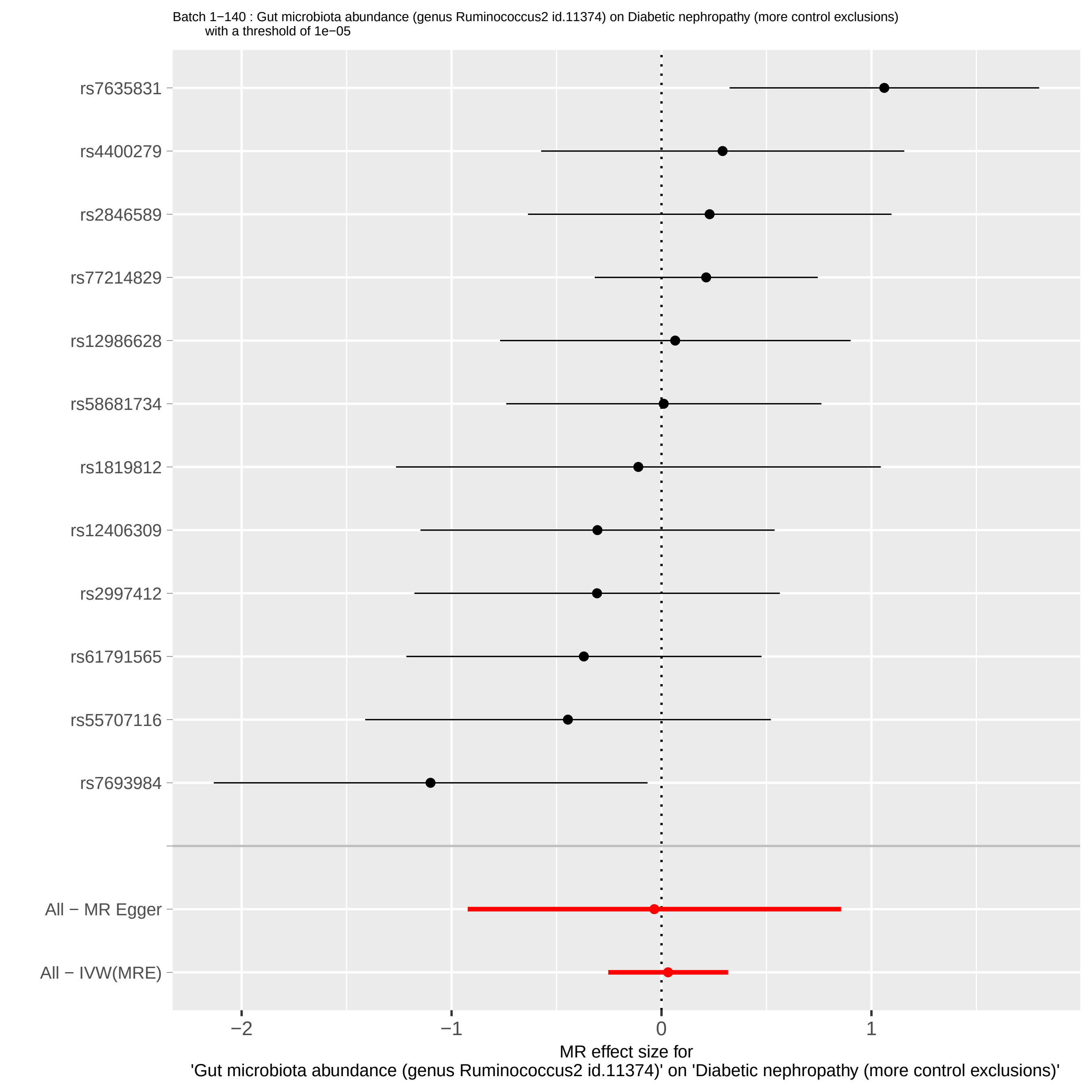

Batch 1−140 : Gut microbiota abundance (genus Ruminococcus2 id.11374) on Diabetic nephropathy (more control exclusions)
with a threshold of 1e−05
rs7635831
rs4400279
rs2846589
rs77214829
rs12986628
rs58681734
rs1819812
rs12406309
rs2997412
rs61791565
rs55707116
rs7693984
All − MR Egger
All − IVW(MRE)
−2
−1
0
1
MR effect size for
'Gut microbiota abundance (genus Ruminococcus2 id.11374)' on 'Diabetic nephropathy (more control exclusions)'

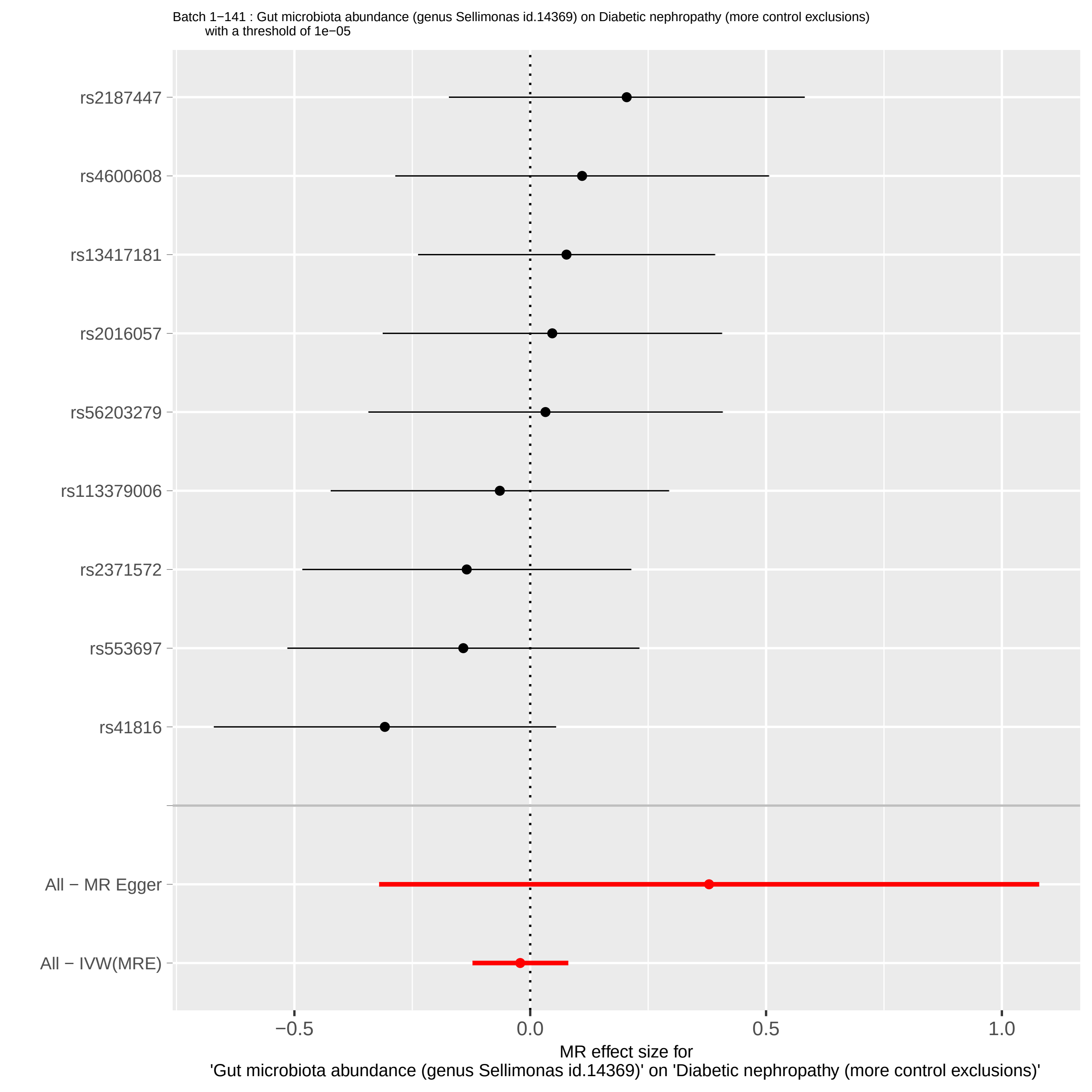

Batch 1−141 : Gut microbiota abundance (genus Sellimonas id.14369) on Diabetic nephropathy (more control exclusions) with a threshold of 1e−05
rs2187447
rs4600608
rs13417181
rs2016057
rs56203279
rs113379006
rs2371572
rs553697
rs41816
All − MR Egger
All − IVW(MRE)
−0.5
0.0
0.5
1.0
MR effect size for
'Gut microbiota abundance (genus Sellimonas id.14369)' on 'Diabetic nephropathy (more control exclusions)'

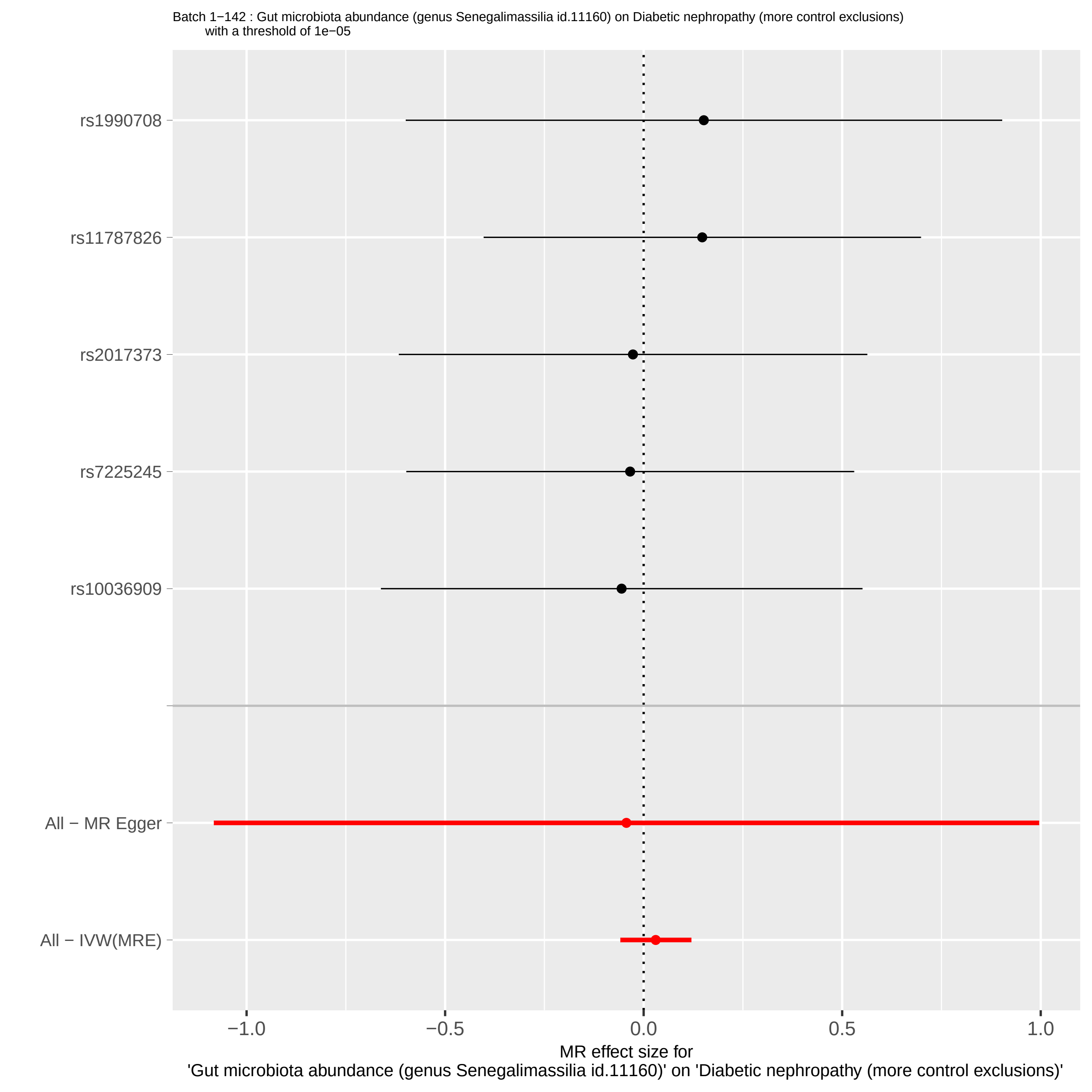

Batch 1−142 : Gut microbiota abundance (genus Senegalimassilia id.11160) on Diabetic nephropathy (more control exclusions)
with a threshold of 1e−05
rs1990708
rs11787826
rs2017373
rs7225245
rs10036909
All − MR Egger
All − IVW(MRE)
−1.0
−0.5
0.0
0.5
1.0
MR effect size for
'Gut microbiota abundance (genus Senegalimassilia id.11160)' on 'Diabetic nephropathy (more control exclusions)'

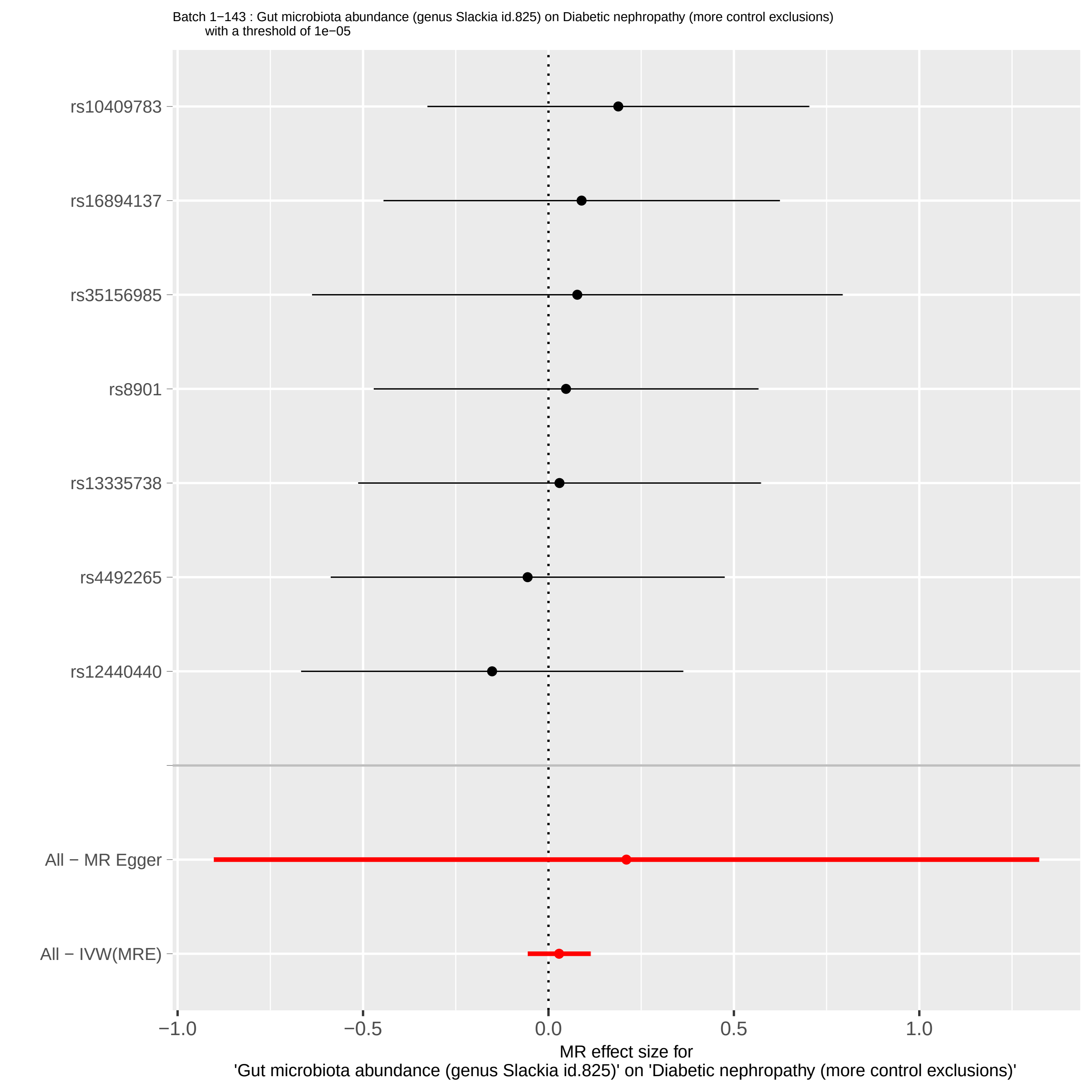

Batch 1−143 : Gut microbiota abundance (genus Slackia id.825) on Diabetic nephropathy (more control exclusions)
with a threshold of 1e−05
rs10409783
rs16894137
rs35156985
rs8901
rs13335738
rs4492265
rs12440440
All − MR Egger
All − IVW(MRE)
−1.0
−0.5
0.0
0.5
1.0
MR effect size for
'Gut microbiota abundance (genus Slackia id.825)' on 'Diabetic nephropathy (more control exclusions)'

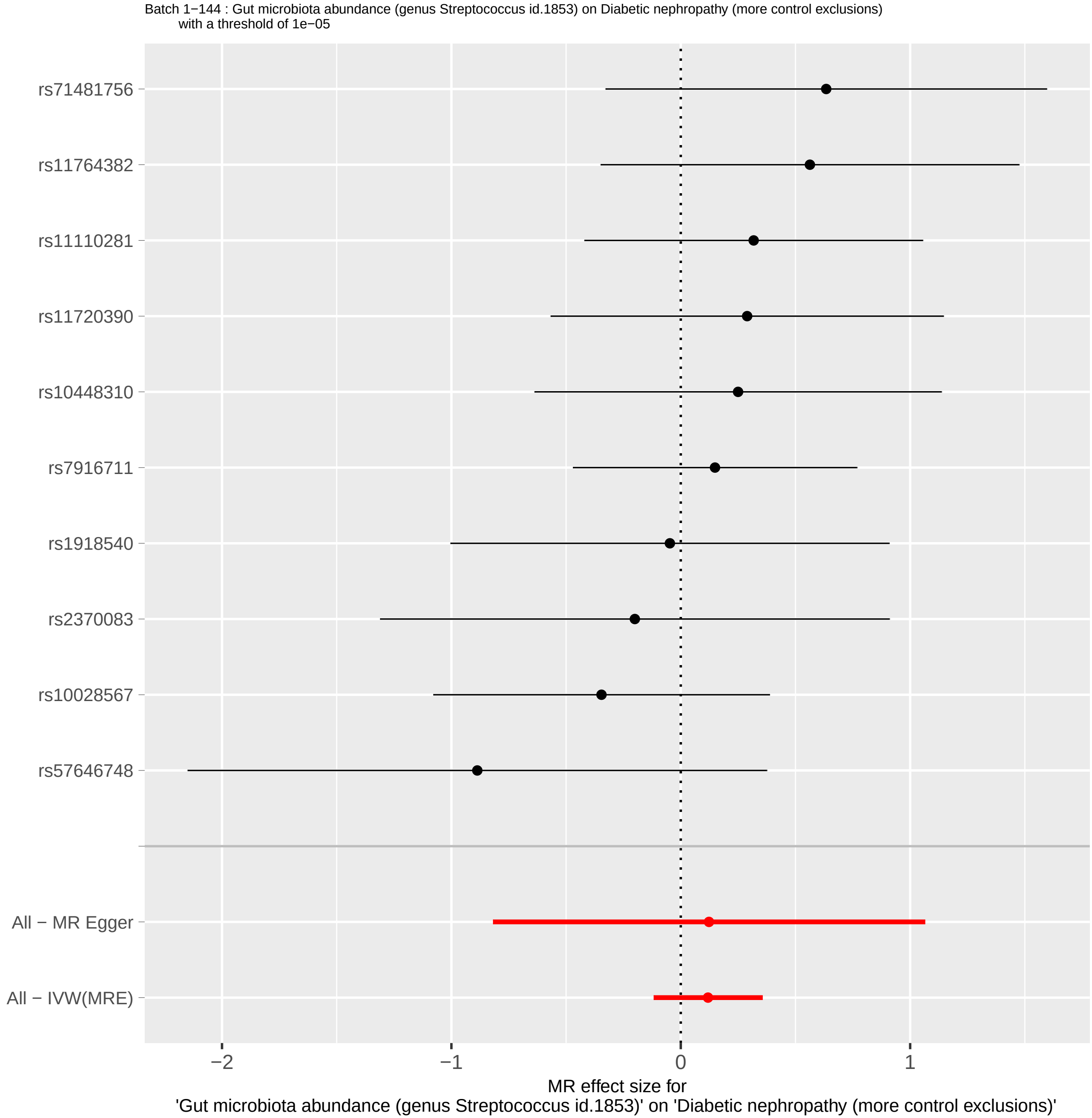
Batch 1−144 : Gut microbiota abundance (genus Streptococcus id.1853) on Diabetic nephropathy (more control exclusions)
with a threshold of 1e−05
rs71481756
rs11764382
rs11110281
rs11720390
rs10448310
rs7916711
rs1918540
rs2370083
rs10028567
rs57646748
All − MR Egger
All − IVW(MRE)
−2
−1
0
1
MR effect size for
'Gut microbiota abundance (genus Streptococcus id.1853)' on 'Diabetic nephropathy (more control exclusions)'

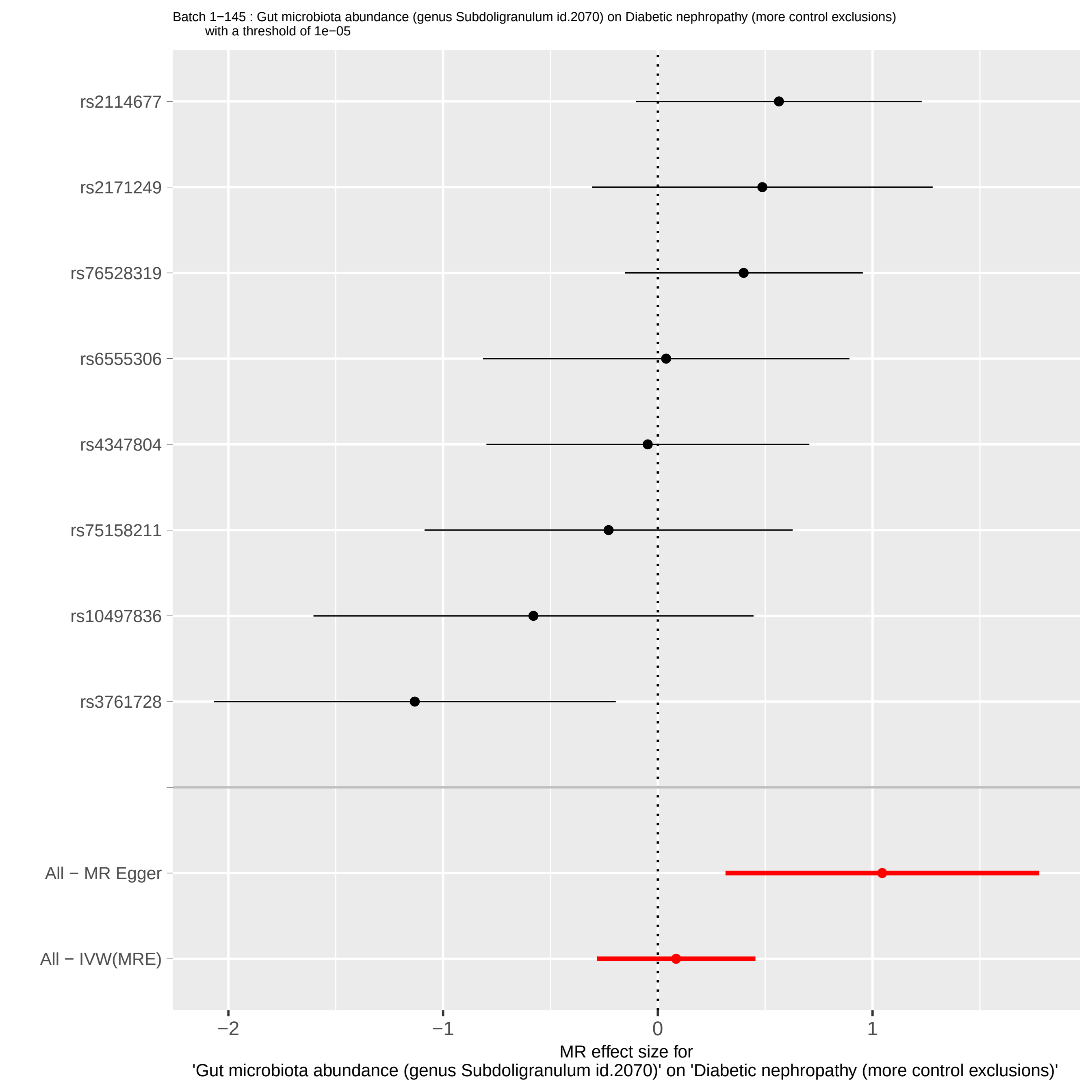
Batch 1−145 : Gut microbiota abundance (genus Subdoligranulum id.2070) on Diabetic nephropathy (more control exclusions)
with a threshold of 1e−05
rs2114677
rs2171249
rs76528319
rs6555306
rs4347804
rs75158211
rs10497836
rs3761728
All − MR Egger
All − IVW(MRE)
−2
−1
0
1
MR effect size for
'Gut microbiota abundance (genus Subdoligranulum id.2070)' on 'Diabetic nephropathy (more control exclusions)'

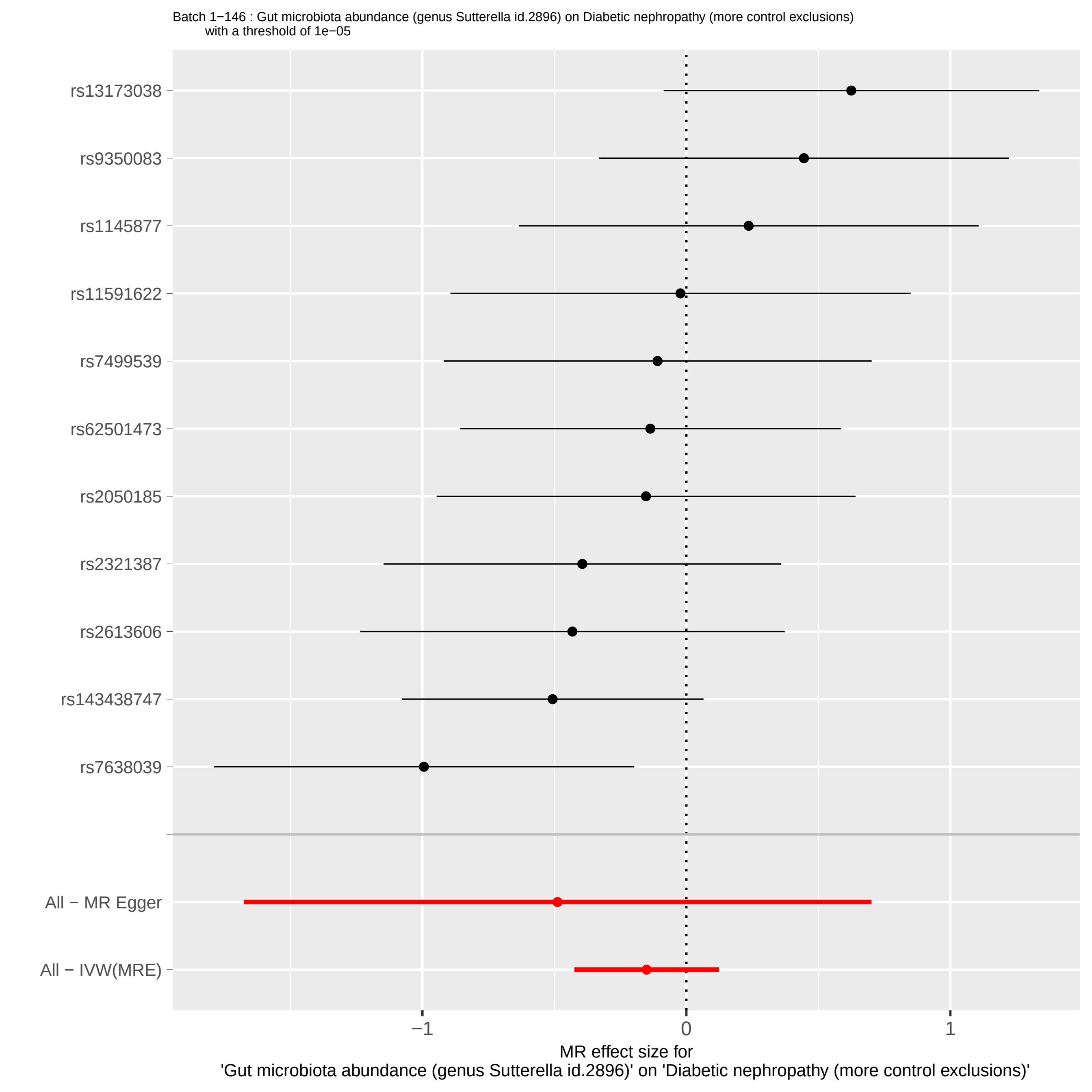

Batch 1−146 : Gut microbiota abundance (genus Sutterella id.2896) on Diabetic nephropathy (more control exclusions)
with a threshold of 1e−05
rs13173038
rs9350083
rs1145877
rs11591622
rs7499539
rs62501473
rs2050185
rs2321387
rs2613606
rs143438747
rs7638039
All − MR Egger
All − IVW(MRE)
−1
0
1
MR effect size for
'Gut microbiota abundance (genus Sutterella id.2896)' on 'Diabetic nephropathy (more control exclusions)'

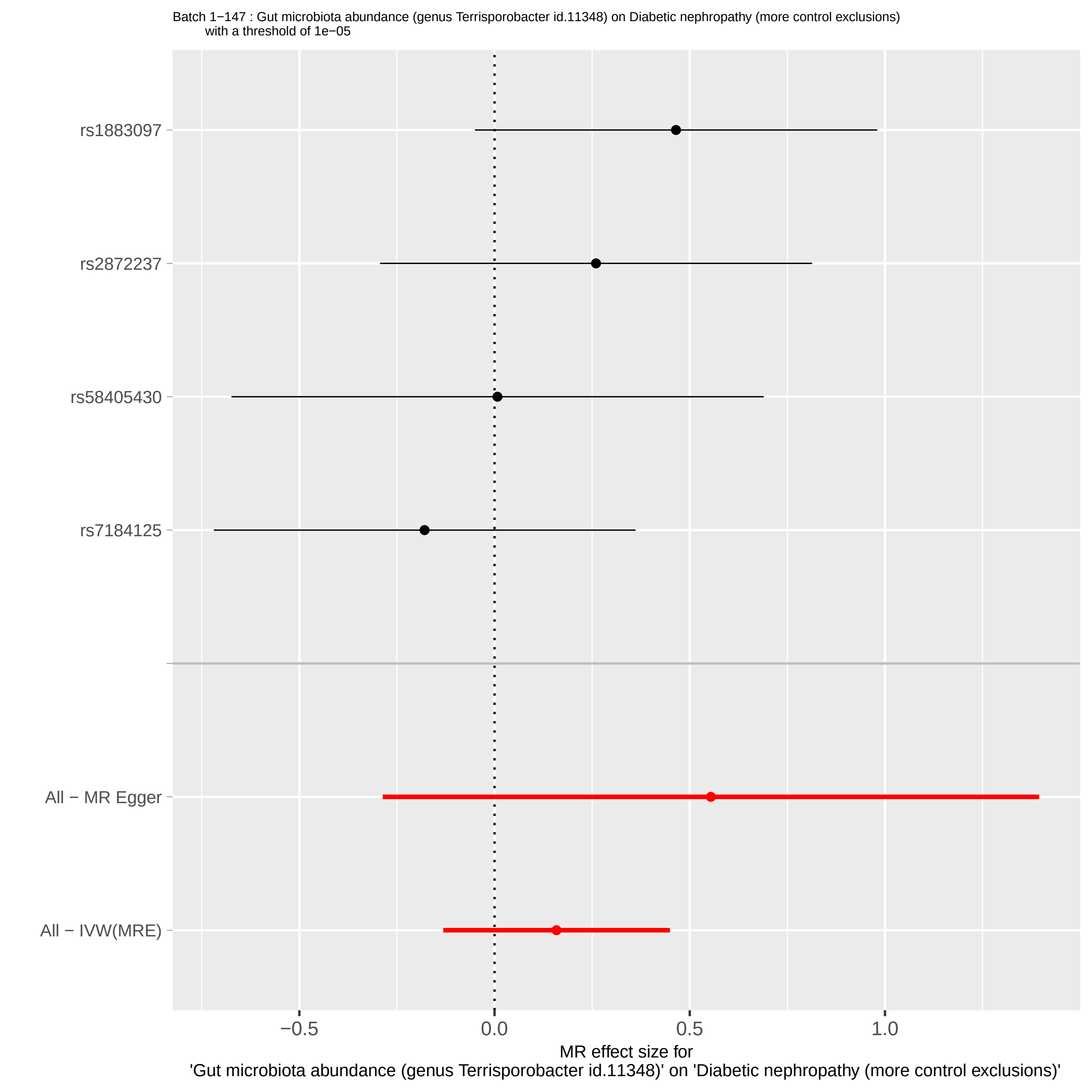

Batch 1−147 : Gut microbiota abundance (genus Terrisporobacter id.11348) on Diabetic nephropathy (more control exclusions) with a threshold of 1e−05
rs1883097
rs2872237
rs58405430
rs7184125
All − MR Egger
All − IVW(MRE)
−0.5
0.0
0.5
1.0
MR effect size for
'Gut microbiota abundance (genus Terrisporobacter id.11348)' on 'Diabetic nephropathy (more control exclusions)'

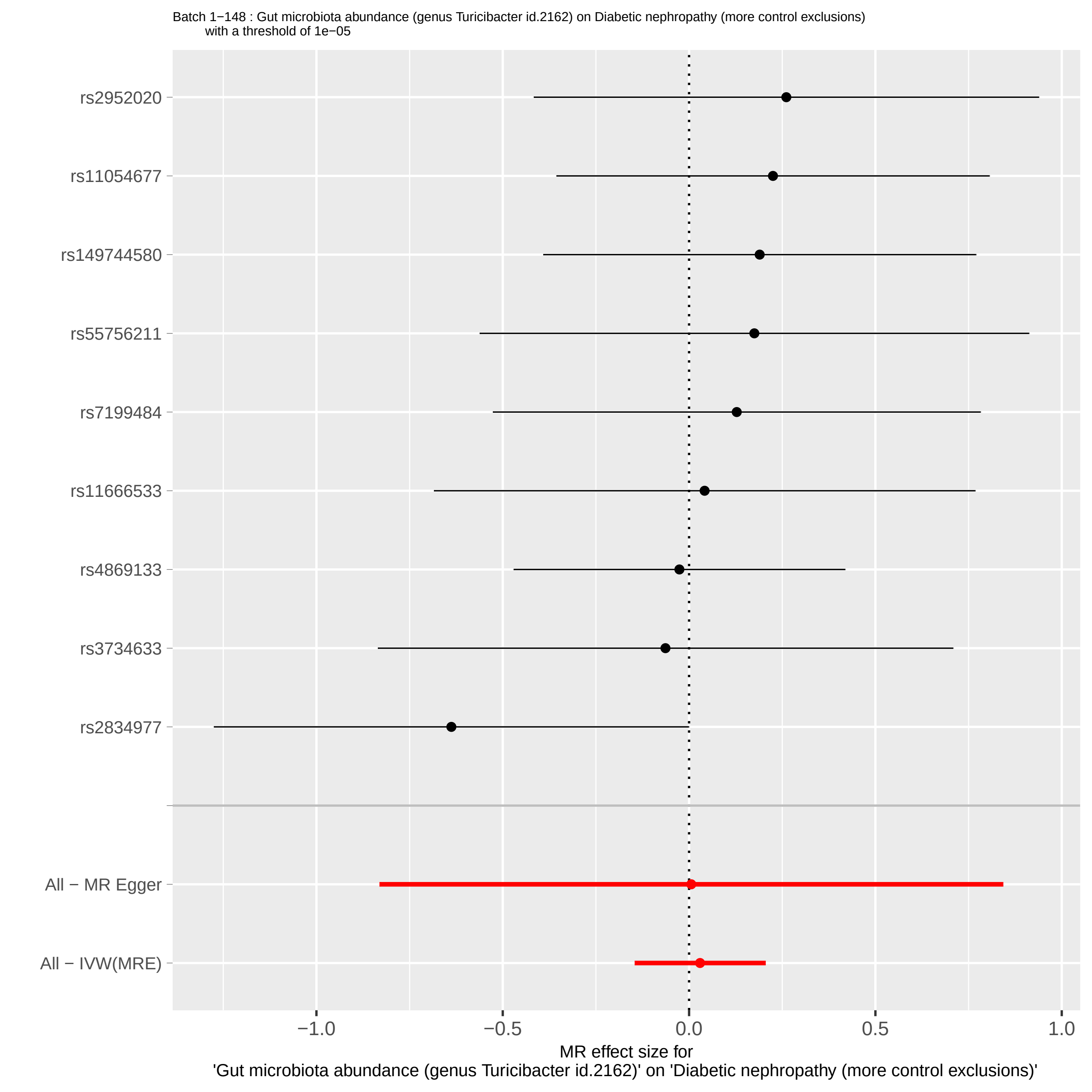

Batch 1−148 : Gut microbiota abundance (genus Turicibacter id.2162) on Diabetic nephropathy (more control exclusions)
with a threshold of 1e−05
rs2952020
rs11054677
rs149744580
rs55756211
rs7199484
rs11666533
rs4869133
rs3734633
rs2834977
All − MR Egger
All − IVW(MRE)
−1.0
−0.5
0.0
0.5
1.0
MR effect size for
'Gut microbiota abundance (genus Turicibacter id.2162)' on 'Diabetic nephropathy (more control exclusions)'

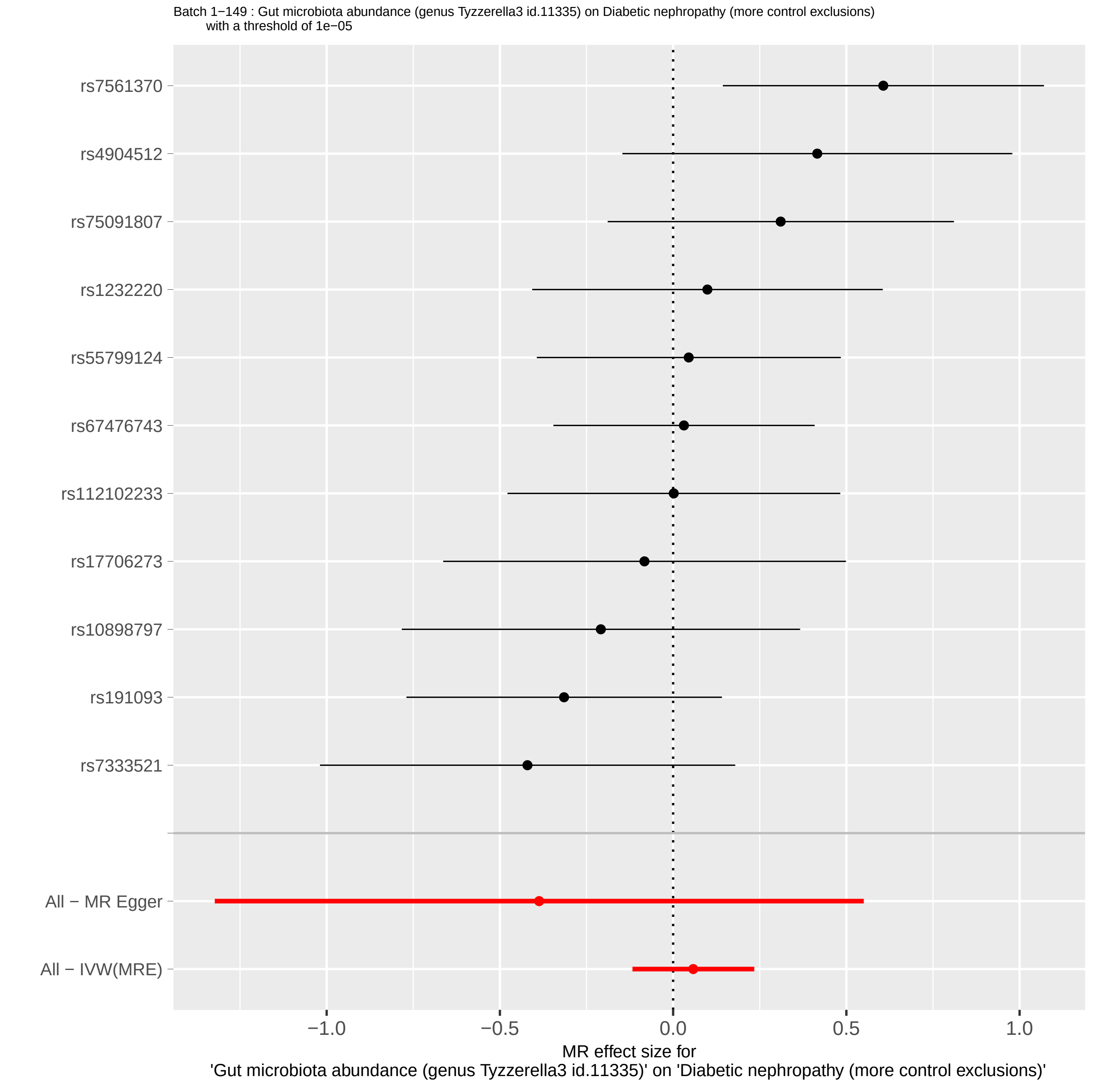

Batch 1−149 : Gut microbiota abundance (genus Tyzzerella3 id.11335) on Diabetic nephropathy (more control exclusions) with a threshold of 1e−05
rs7561370
rs4904512
rs75091807
rs1232220
rs55799124
rs67476743
rs112102233
rs17706273
rs10898797
rs191093
rs7333521
All − MR Egger
All − IVW(MRE)
−1.0
−0.5
0.0
0.5
1.0
MR effect size for
'Gut microbiota abundance (genus Tyzzerella3 id.11335)' on 'Diabetic nephropathy (more control exclusions)'

Batch 1−150 : Gut microbiota abundance (genus Veillonella id.2198) on Diabetic nephropathy (more control exclusions)
with a threshold of 1e−05

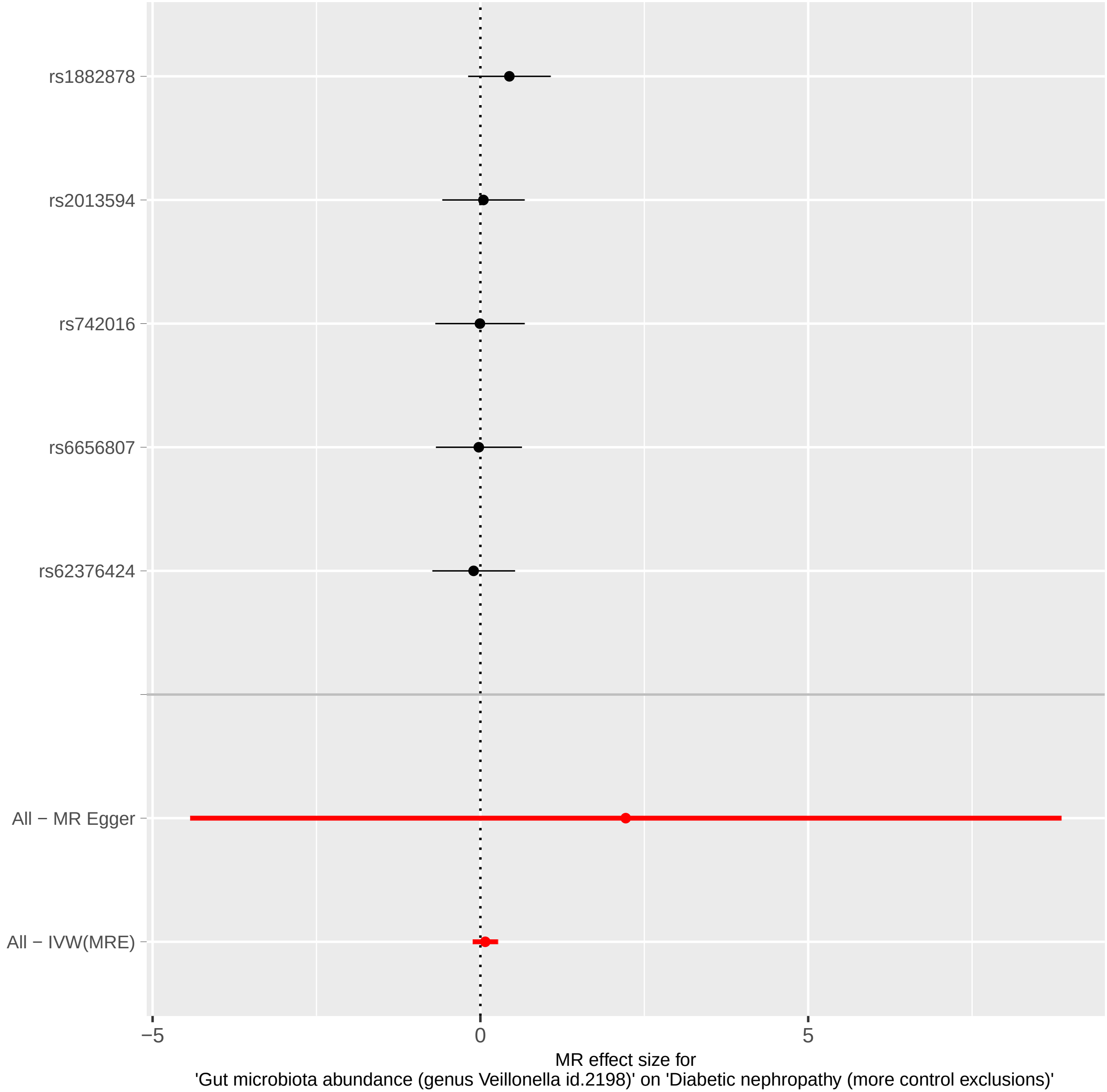

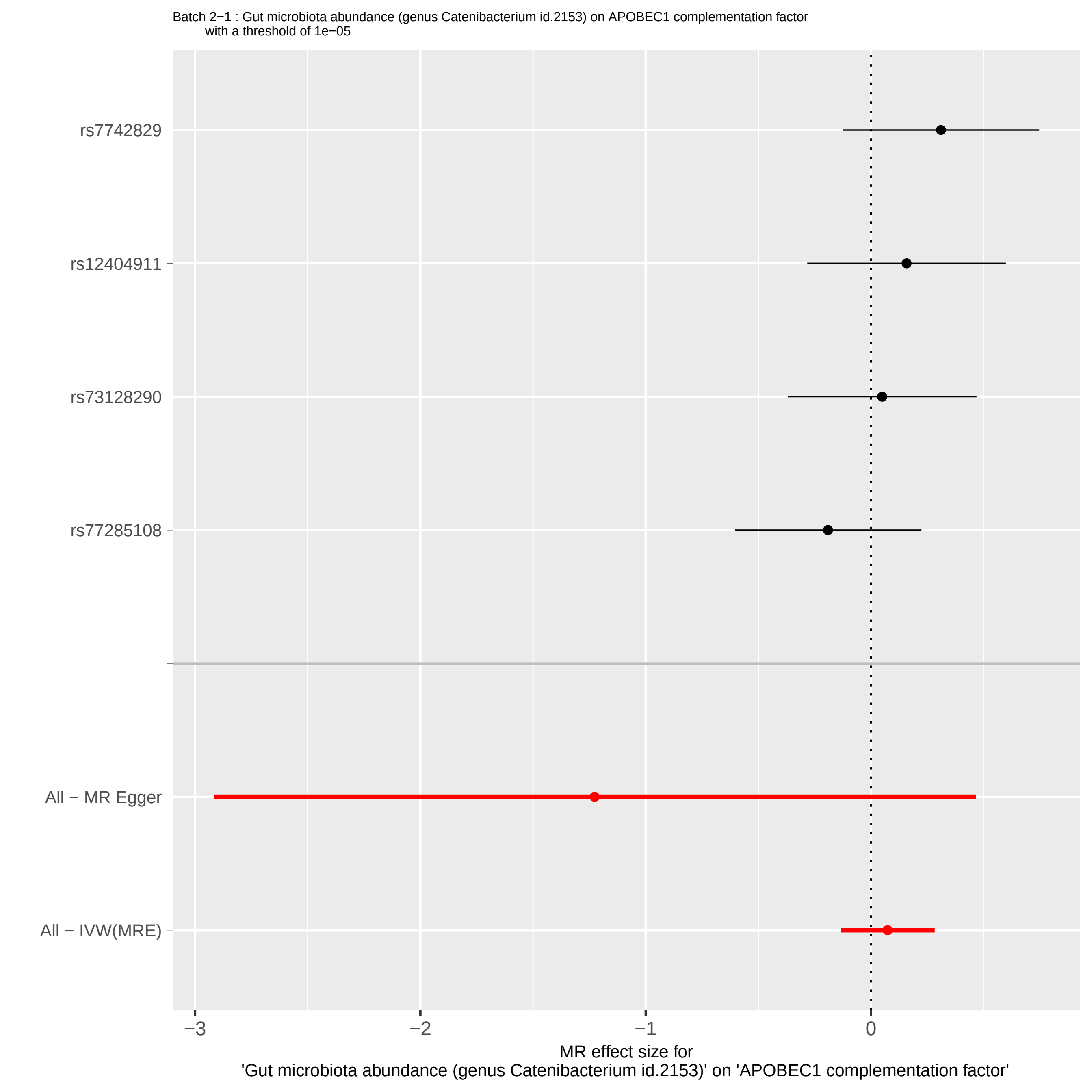
Batch 2−1 : Gut microbiota abundance (genus Catenibacterium id.2153) on APOBEC1 complementation factor
with a threshold of 1e−05
rs7742829
rs12404911
rs73128290
rs77285108
All − MR Egger
All − IVW(MRE)
−3
−2
−1
0
MR effect size for
'Gut microbiota abundance (genus Catenibacterium id.2153)' on 'APOBEC1 complementation factor'

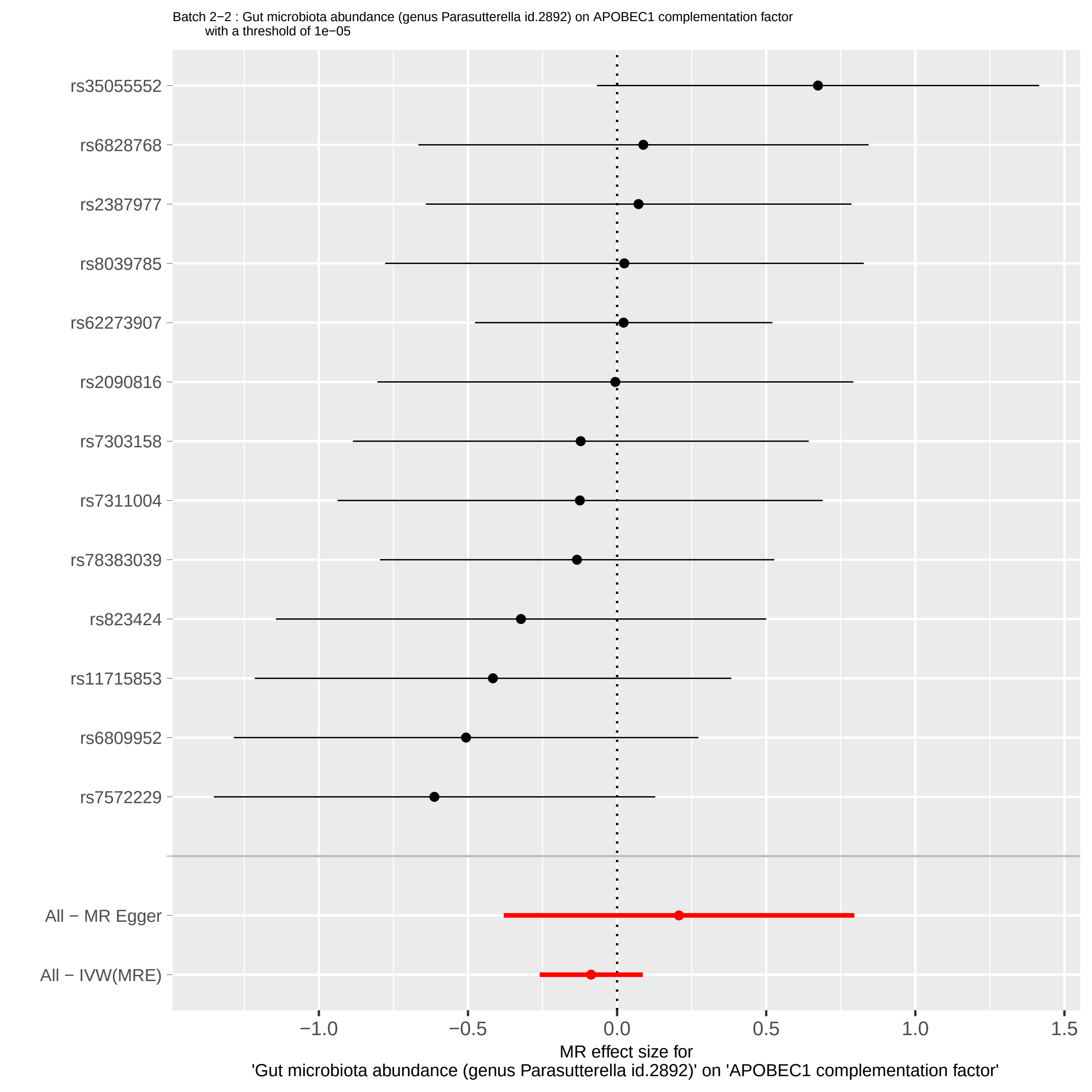

Batch 2−2 : Gut microbiota abundance (genus Parasutterella id.2892) on APOBEC1 complementation factor
with a threshold of 1e−05
rs35055552
rs6828768
rs2387977
rs8039785
rs62273907
rs2090816
rs7303158
rs7311004
rs78383039
rs823424
rs11715853
rs6809952
rs7572229
All − MR Egger
All − IVW(MRE)
−1.0
−0.5
0.0
0.5
1.0
1.5
MR effect size for
'Gut microbiota abundance (genus Parasutterella id.2892)' on 'APOBEC1 complementation factor'

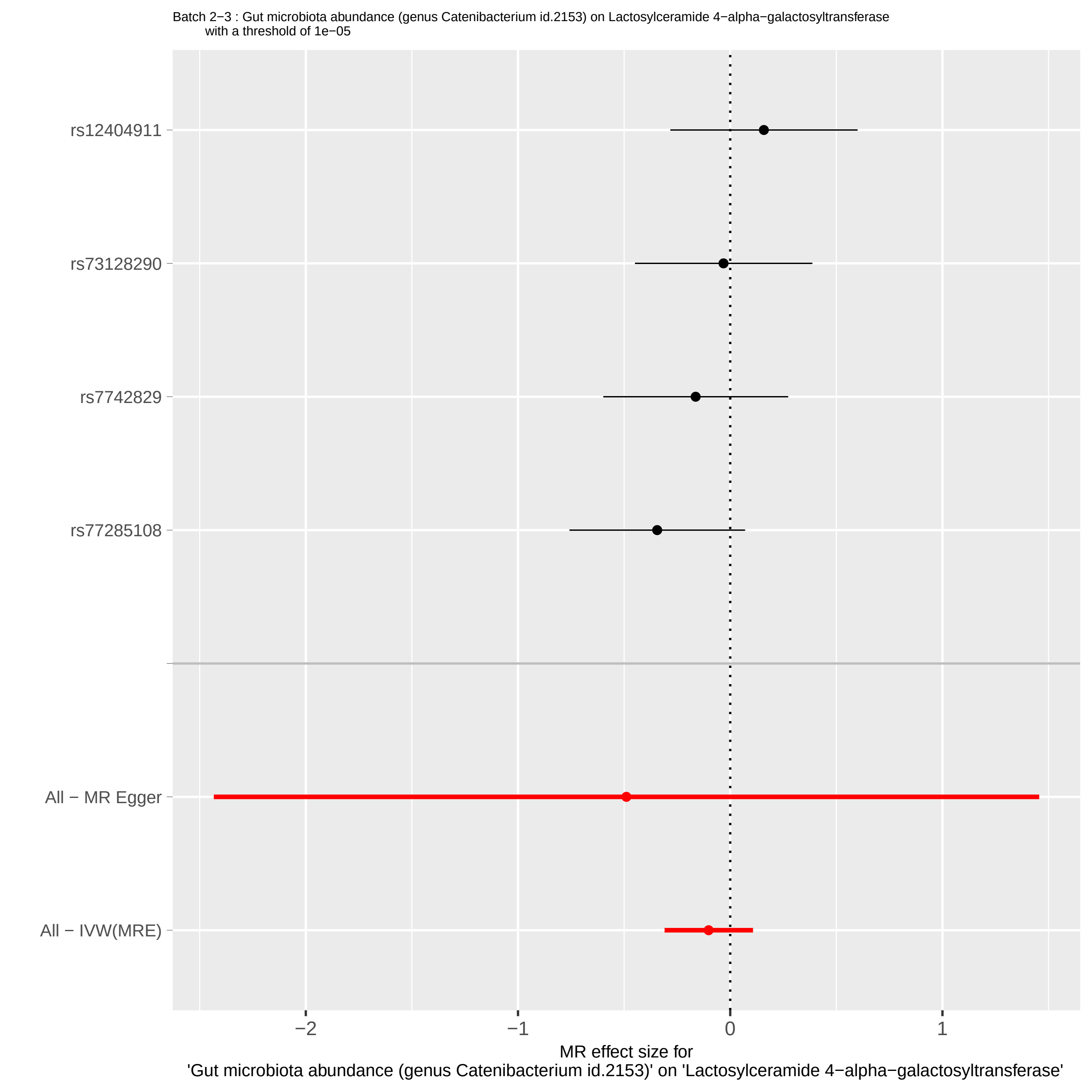

Batch 2−3 : Gut microbiota abundance (genus Catenibacterium id.2153) on Lactosylceramide 4−alpha−galactosyltransferase
with a threshold of 1e−05
rs12404911
rs73128290
rs7742829
rs77285108
All − MR Egger
All − IVW(MRE)
−2
−1
0
1
MR effect size for
'Gut microbiota abundance (genus Catenibacterium id.2153)' on 'Lactosylceramide 4−alpha−galactosyltransferase'

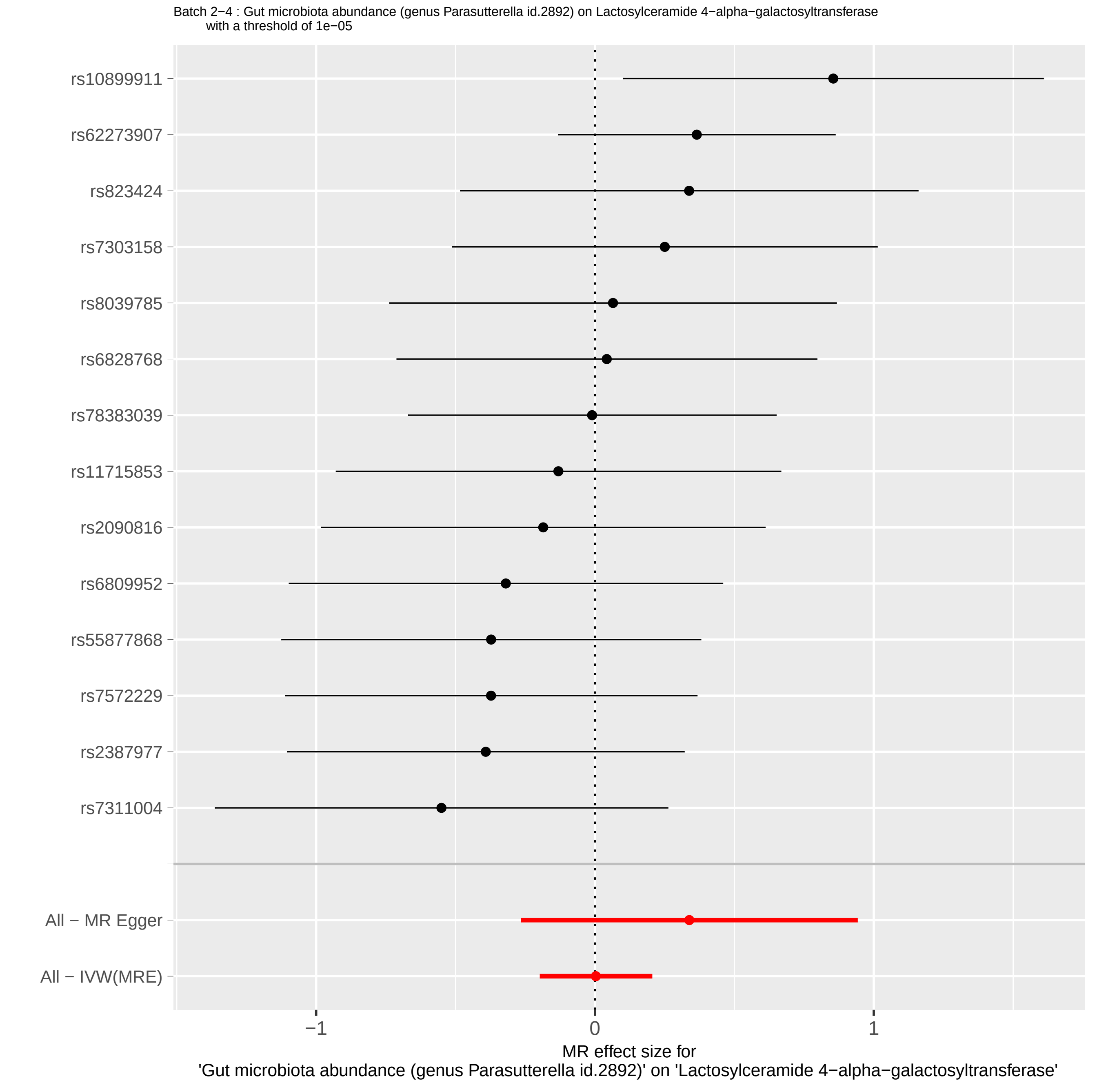
Batch 2−4 : Gut microbiota abundance (genus Parasutterella id.2892) on Lactosylceramide 4−alpha−galactosyltransferase with a threshold of 1e−05
rs10899911
rs62273907
rs823424
rs7303158
rs8039785
rs6828768
rs78383039
rs11715853
rs2090816
rs6809952
rs55877868
rs7572229
rs2387977
rs7311004
All − MR Egger
All − IVW(MRE)
−1
0
1
MR effect size for
'Gut microbiota abundance (genus Parasutterella id.2892)' on 'Lactosylceramide 4−alpha−galactosyltransferase'

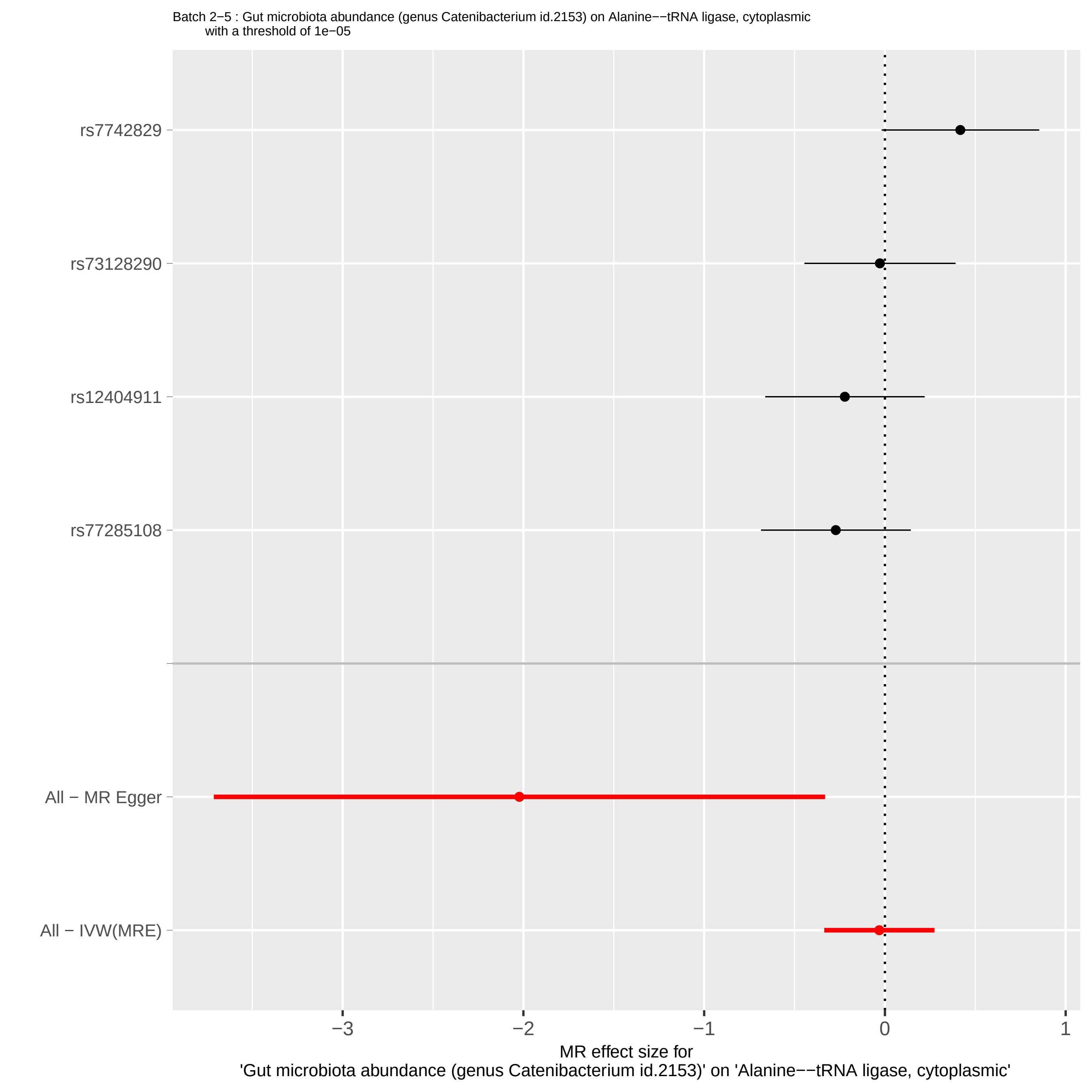

Batch 2−5 : Gut microbiota abundance (genus Catenibacterium id.2153) on Alanine−−tRNA ligase, cytoplasmic
with a threshold of 1e−05
rs7742829
rs73128290
rs12404911
rs77285108
All − MR Egger
All − IVW(MRE)
−3
−2
−1
0
1
MR effect size for
'Gut microbiota abundance (genus Catenibacterium id.2153)' on 'Alanine−−tRNA ligase, cytoplasmic'

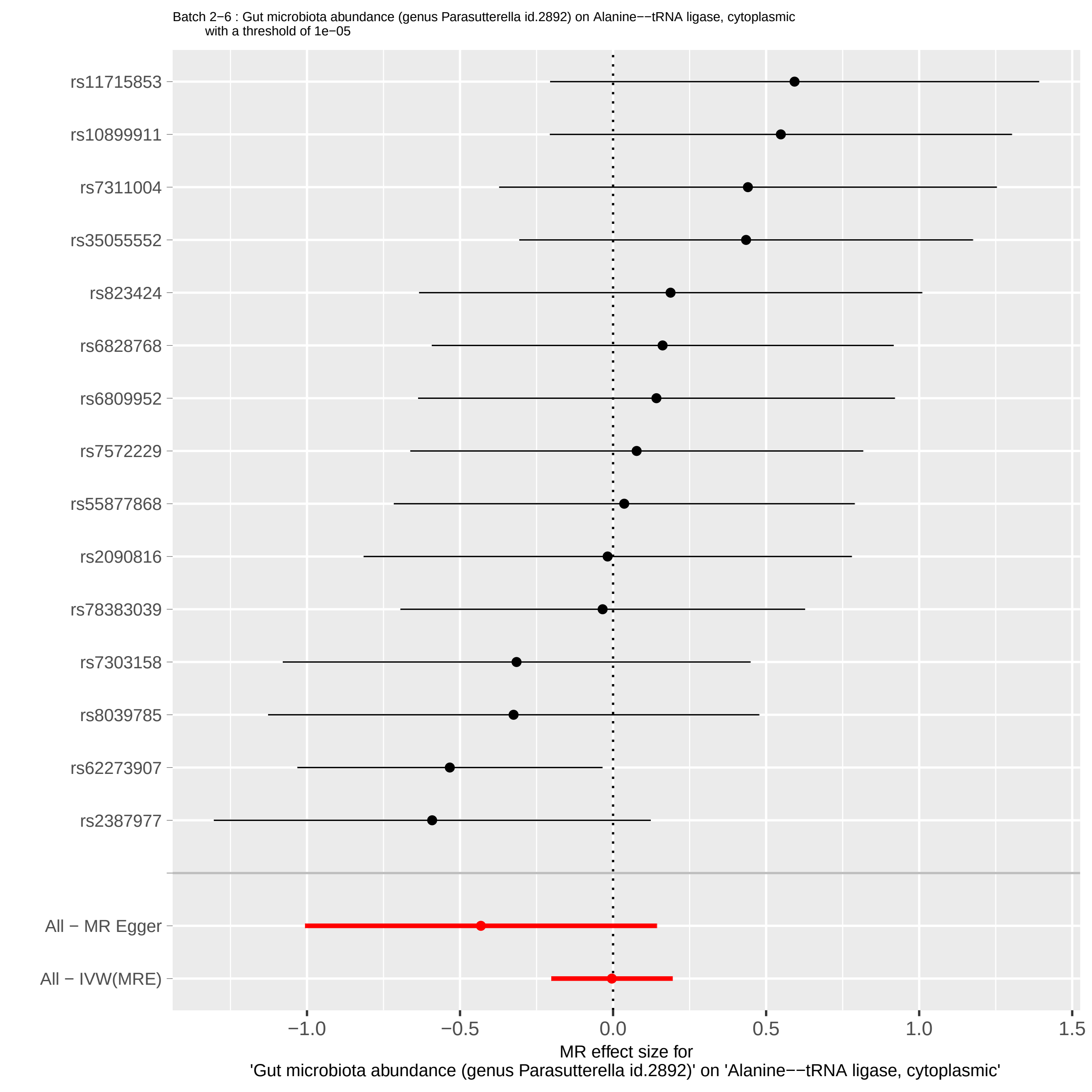

Batch 2−6 : Gut microbiota abundance (genus Parasutterella id.2892) on Alanine−−tRNA ligase, cytoplasmic
with a threshold of 1e−05
rs11715853
rs10899911
rs7311004
rs35055552
rs823424
rs6828768
rs6809952
rs7572229
rs55877868
rs2090816
rs78383039
rs7303158
rs8039785
rs62273907
rs2387977
All − MR Egger
All − IVW(MRE)
−1.0
−0.5
0.0
0.5
1.0
1.5
MR effect size for
'Gut microbiota abundance (genus Parasutterella id.2892)' on 'Alanine−−tRNA ligase, cytoplasmic'

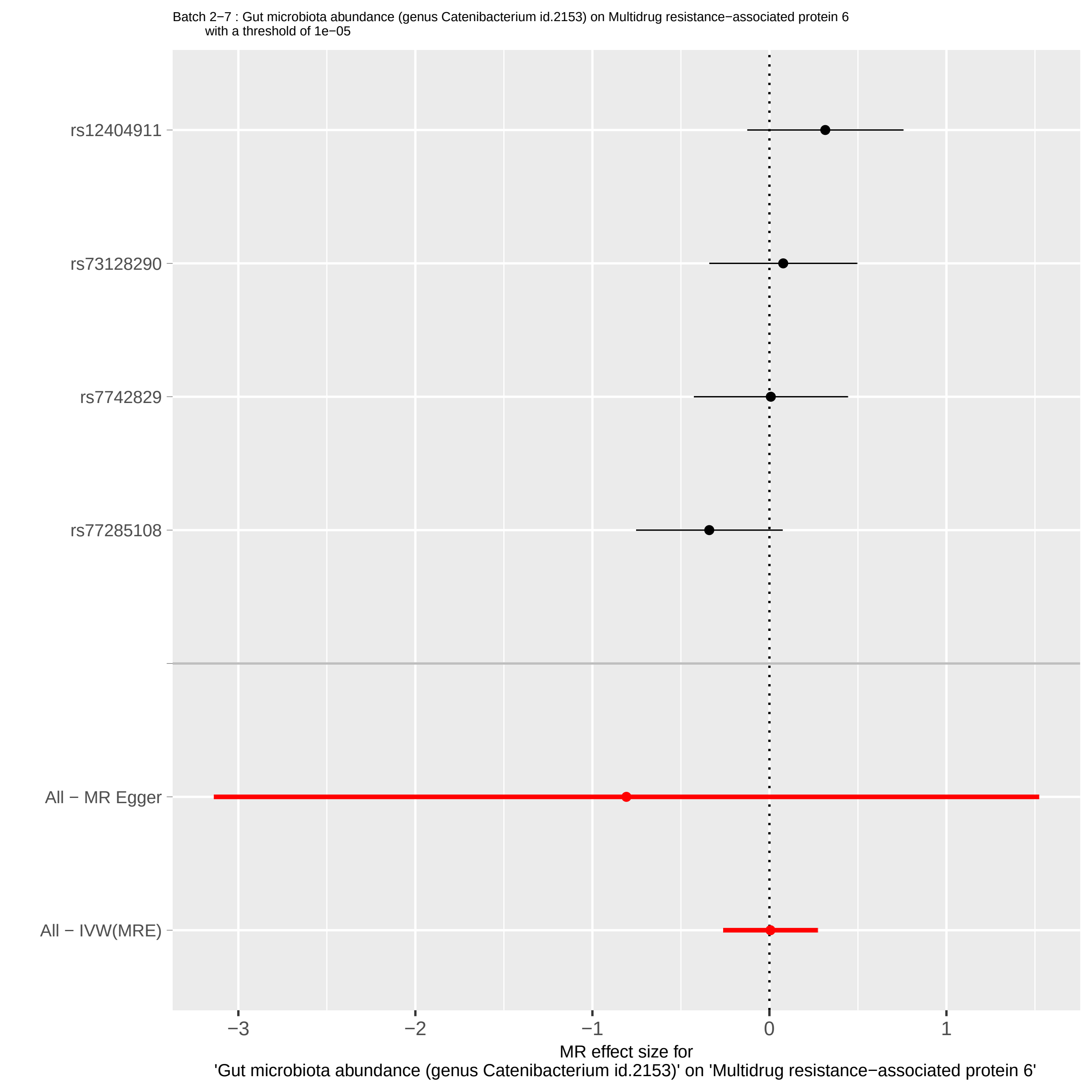

Batch 2−7 : Gut microbiota abundance (genus Catenibacterium id.2153) on Multidrug resistance−associated protein 6
with a threshold of 1e−05
rs12404911
rs73128290
rs7742829
rs77285108
All − MR Egger
All − IVW(MRE)
−3
−2
−1
0
1
MR effect size for
'Gut microbiota abundance (genus Catenibacterium id.2153)' on 'Multidrug resistance−associated protein 6'

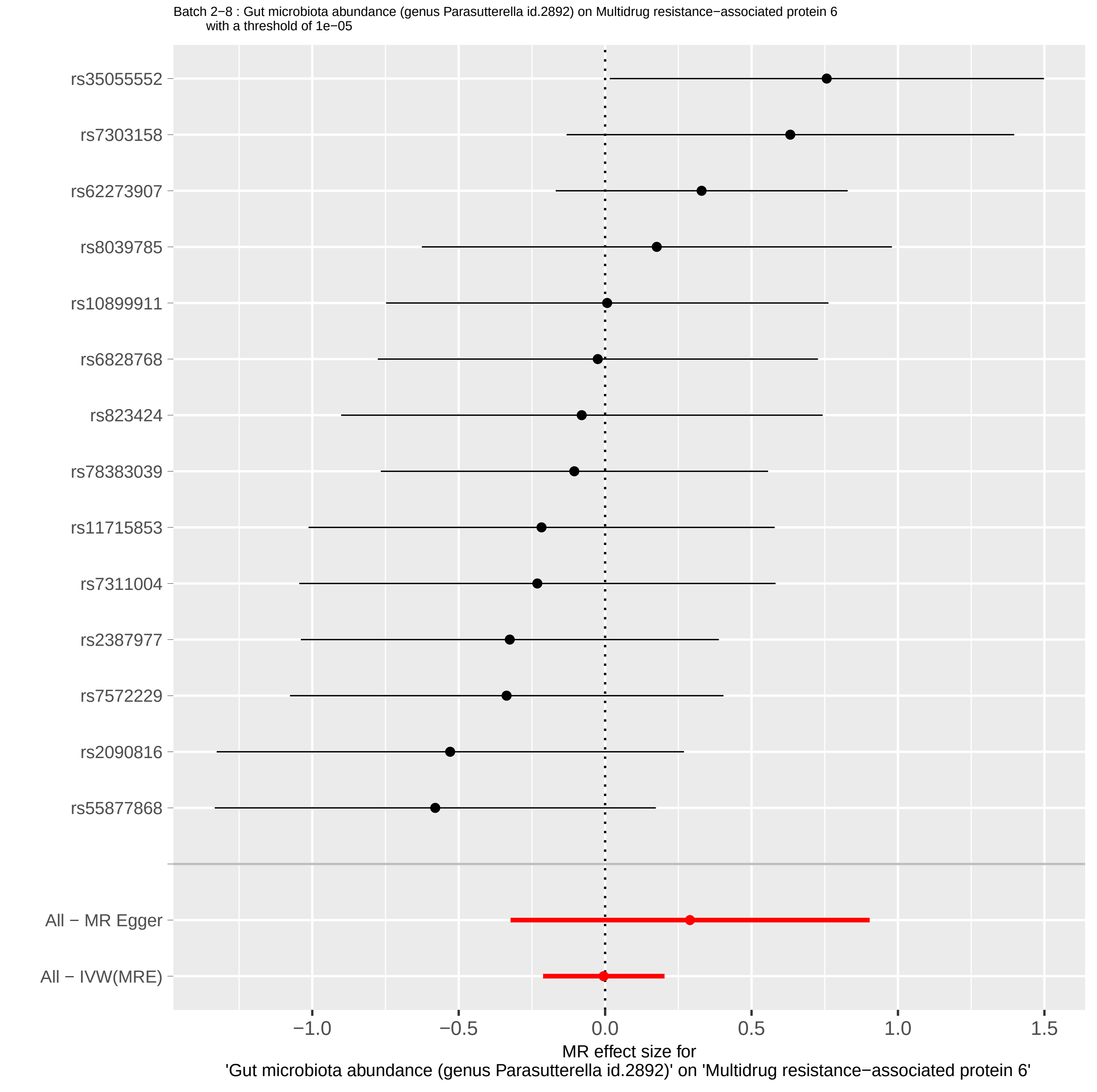
Batch 2−8 : Gut microbiota abundance (genus Parasutterella id.2892) on Multidrug resistance−associated protein 6
with a threshold of 1e−05
rs35055552
rs7303158
rs62273907
rs8039785
rs10899911
rs6828768
rs823424
rs78383039
rs11715853
rs7311004
rs2387977
rs7572229
rs2090816
rs55877868
All − MR Egger
All − IVW(MRE)
−1.0
−0.5
0.0
0.5
1.0
1.5
MR effect size for
'Gut microbiota abundance (genus Parasutterella id.2892)' on 'Multidrug resistance−associated protein 6'

Batch 2−9 : Gut microbiota abundance (genus Catenibacterium id.2153) on Monoacylglycerol lipase ABHD12
with a threshold of 1e−05

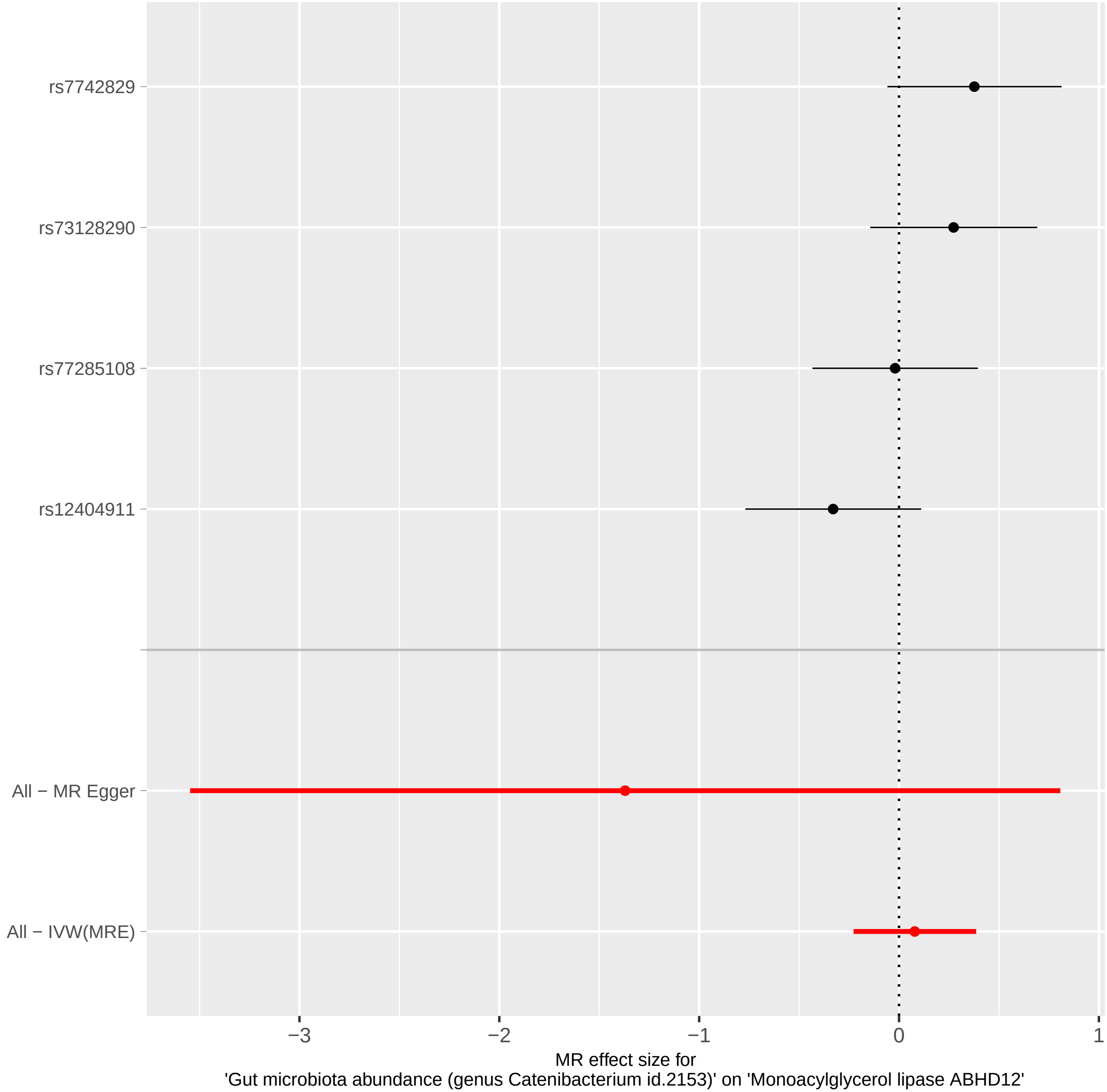

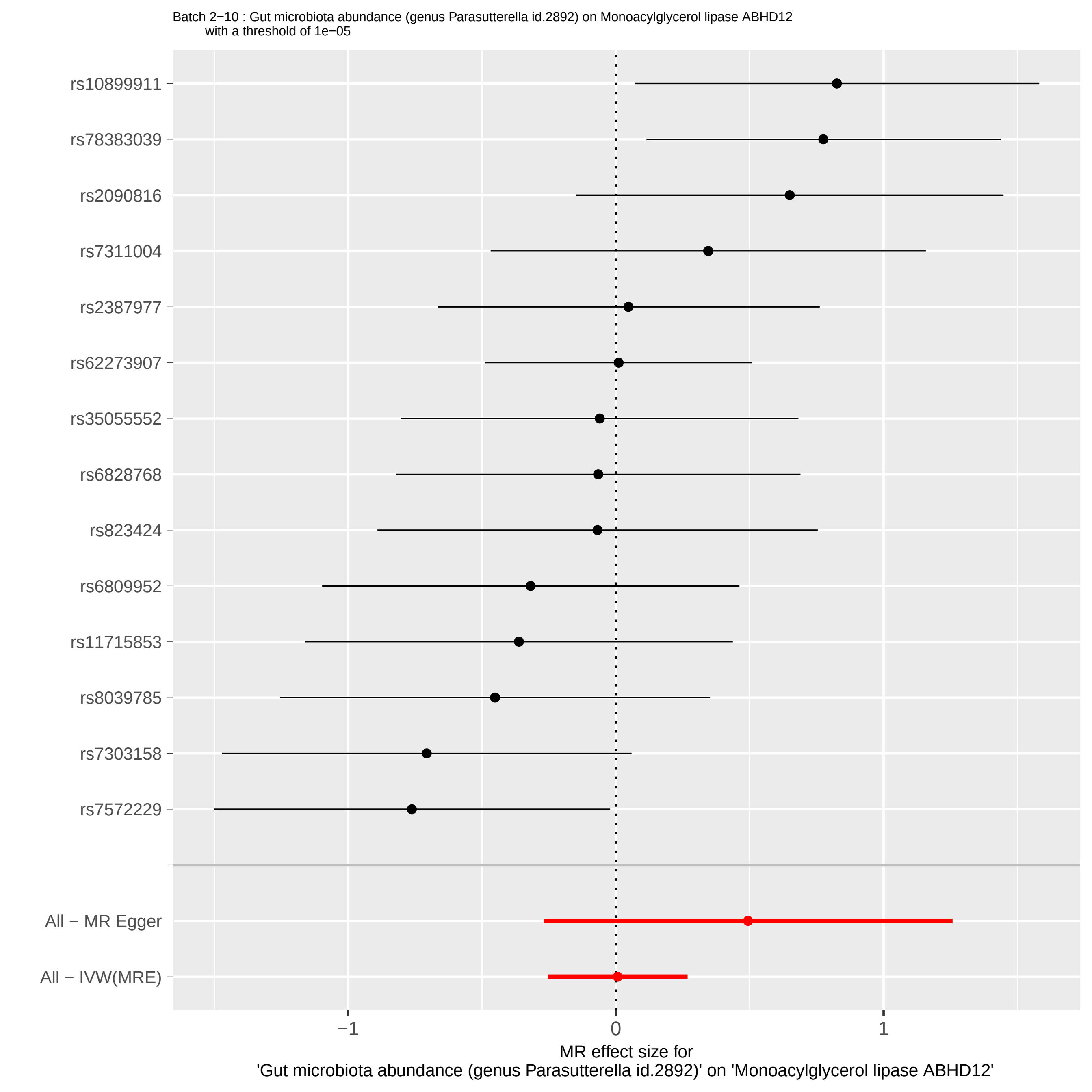

Batch 2−10 : Gut microbiota abundance (genus Parasutterella id.2892) on Monoacylglycerol lipase ABHD12
with a threshold of 1e−05
rs10899911
rs78383039
rs2090816
rs7311004
rs2387977
rs62273907
rs35055552
rs6828768
rs823424
rs6809952
rs11715853
rs8039785
rs7303158
rs7572229
All − MR Egger
All − IVW(MRE)
−1
0
1
MR effect size for
'Gut microbiota abundance (genus Parasutterella id.2892)' on 'Monoacylglycerol lipase ABHD12'

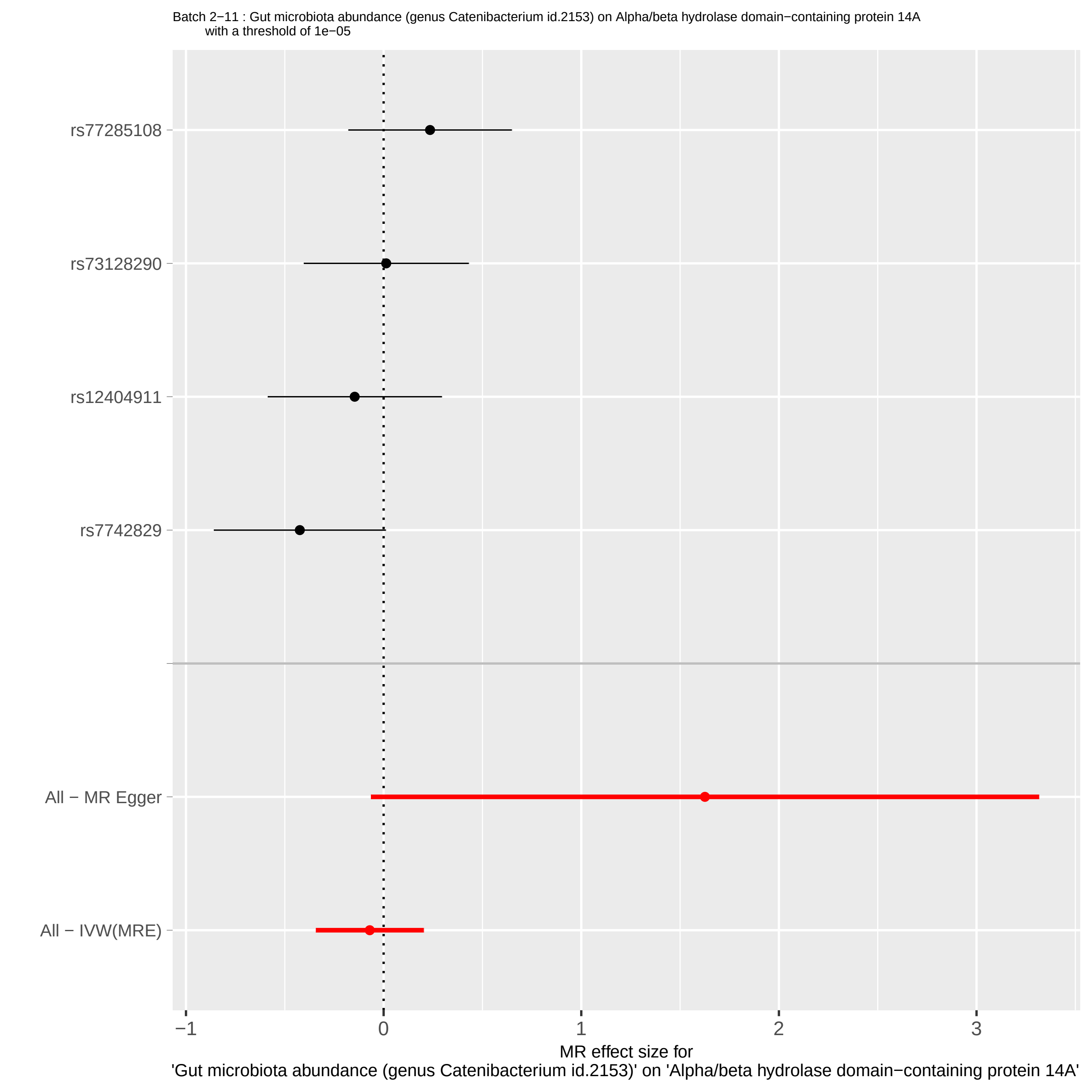

Batch 2−11 : Gut microbiota abundance (genus Catenibacterium id.2153) on Alpha/beta hydrolase domain−containing protein 14A
with a threshold of 1e−05
rs77285108
rs73128290
rs12404911
rs7742829
All − MR Egger
All − IVW(MRE)
−1
0
1
2
3
MR effect size for
'Gut microbiota abundance (genus Catenibacterium id.2153)' on 'Alpha/beta hydrolase domain−containing protein 14A'

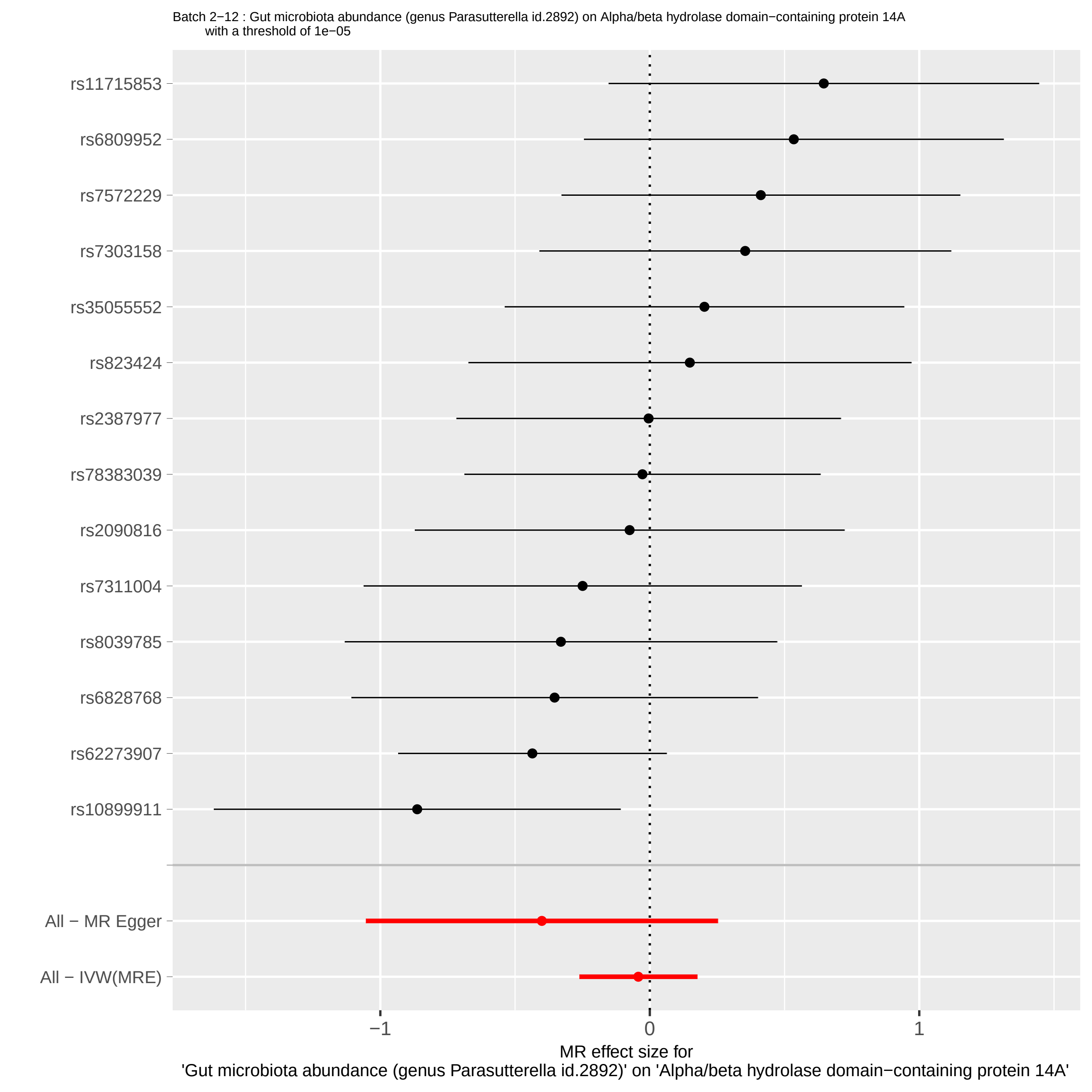

Batch 2−12 : Gut microbiota abundance (genus Parasutterella id.2892) on Alpha/beta hydrolase domain−containing protein 14A
with a threshold of 1e−05
rs11715853
rs6809952
rs7572229
rs7303158
rs35055552
rs823424
rs2387977
rs78383039
rs2090816
rs7311004
rs8039785
rs6828768
rs62273907
rs10899911
All − MR Egger
All − IVW(MRE)
−1
0
1
MR effect size for
'Gut microbiota abundance (genus Parasutterella id.2892)' on 'Alpha/beta hydrolase domain−containing protein 14A'

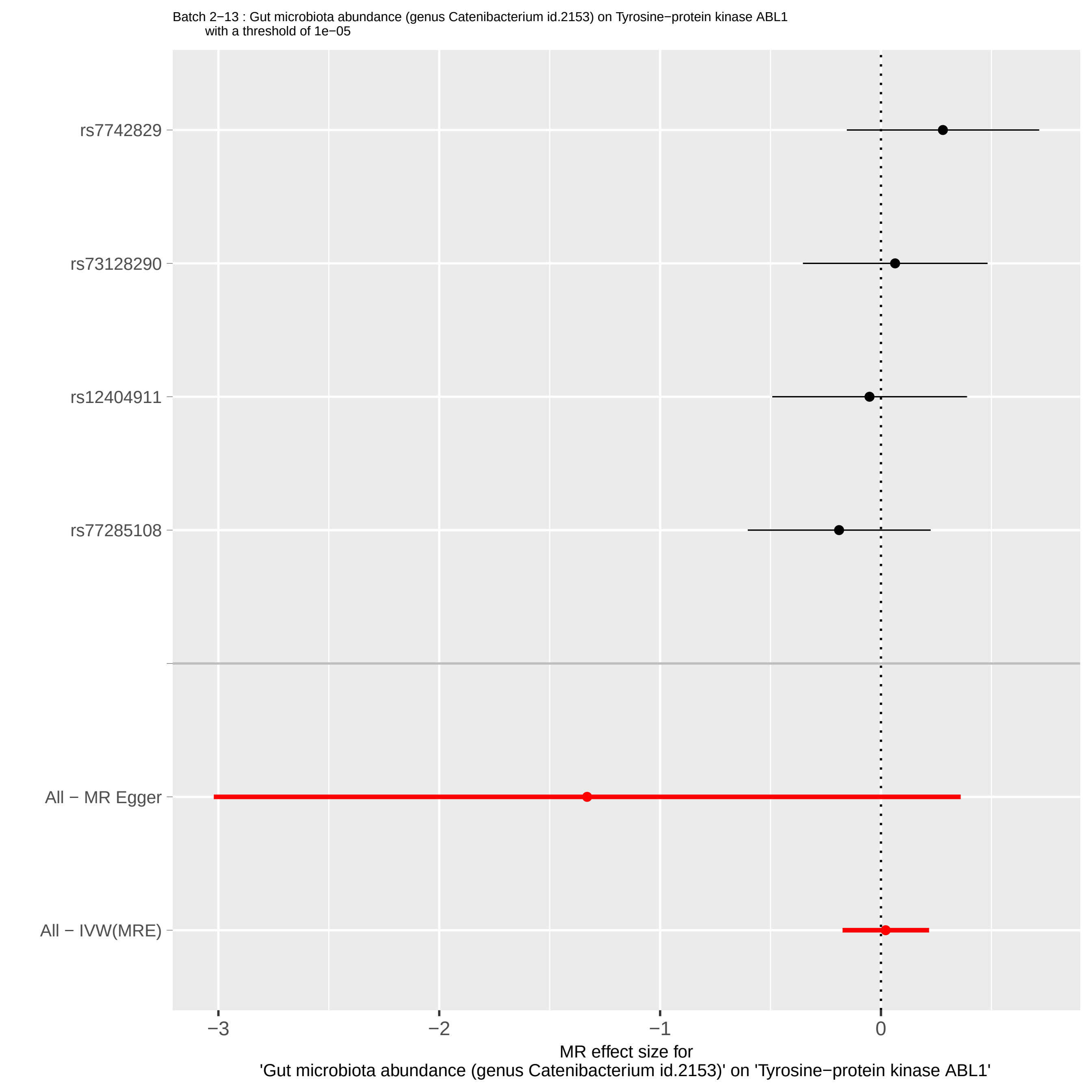

Batch 2−13 : Gut microbiota abundance (genus Catenibacterium id.2153) on Tyrosine−protein kinase ABL1
with a threshold of 1e−05
rs7742829
rs73128290
rs12404911
rs77285108
All − MR Egger
All − IVW(MRE)
−3
−2
−1
0
MR effect size for
'Gut microbiota abundance (genus Catenibacterium id.2153)' on 'Tyrosine−protein kinase ABL1'

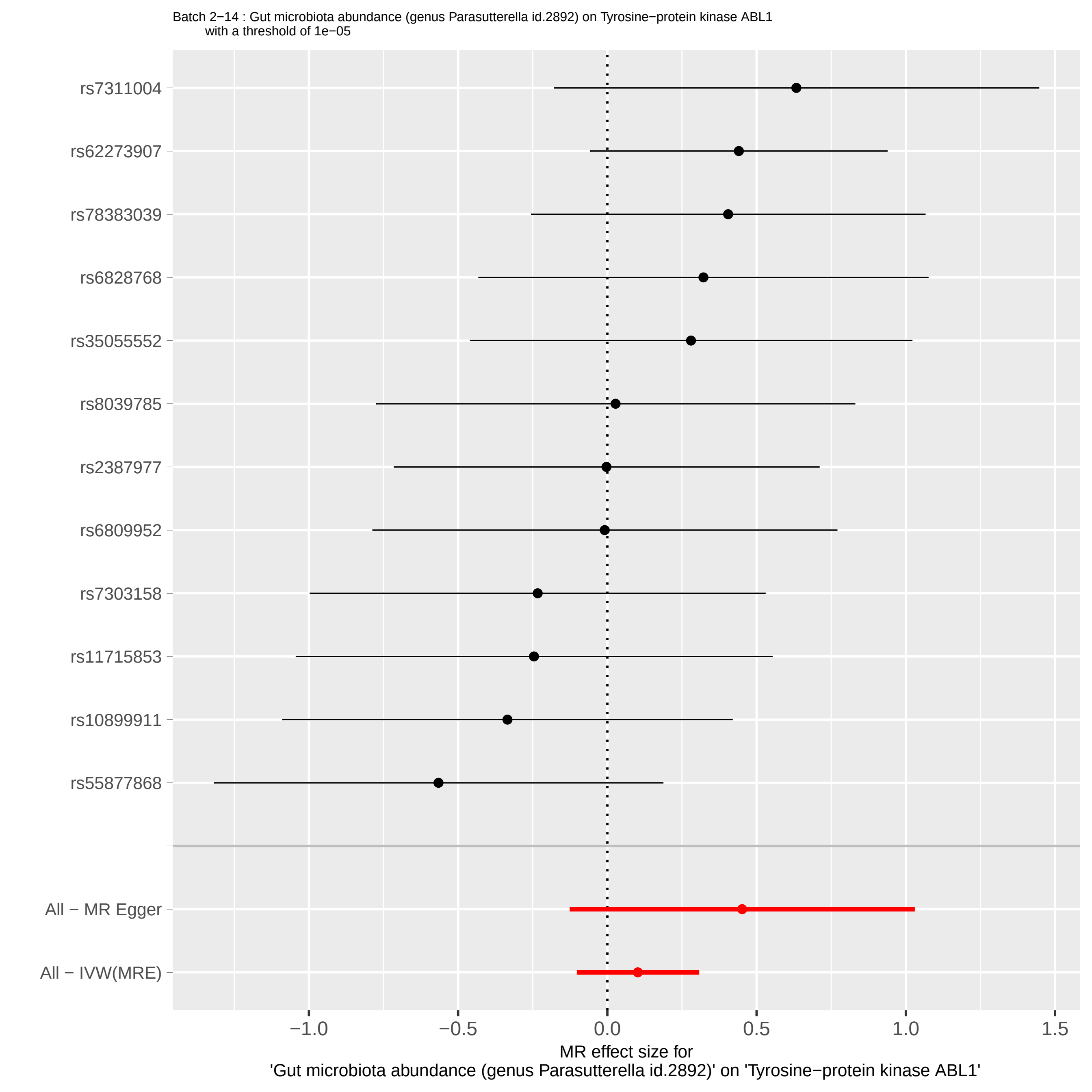
Batch 2−14 : Gut microbiota abundance (genus Parasutterella id.2892) on Tyrosine−protein kinase ABL1
with a threshold of 1e−05
rs7311004
rs62273907
rs78383039
rs6828768
rs35055552
rs8039785
rs2387977
rs6809952
rs7303158
rs11715853
rs10899911
rs55877868
All − MR Egger
All − IVW(MRE)
−1.0
−0.5
0.0
0.5
1.0
1.5
MR effect size for
'Gut microbiota abundance (genus Parasutterella id.2892)' on 'Tyrosine−protein kinase ABL1'

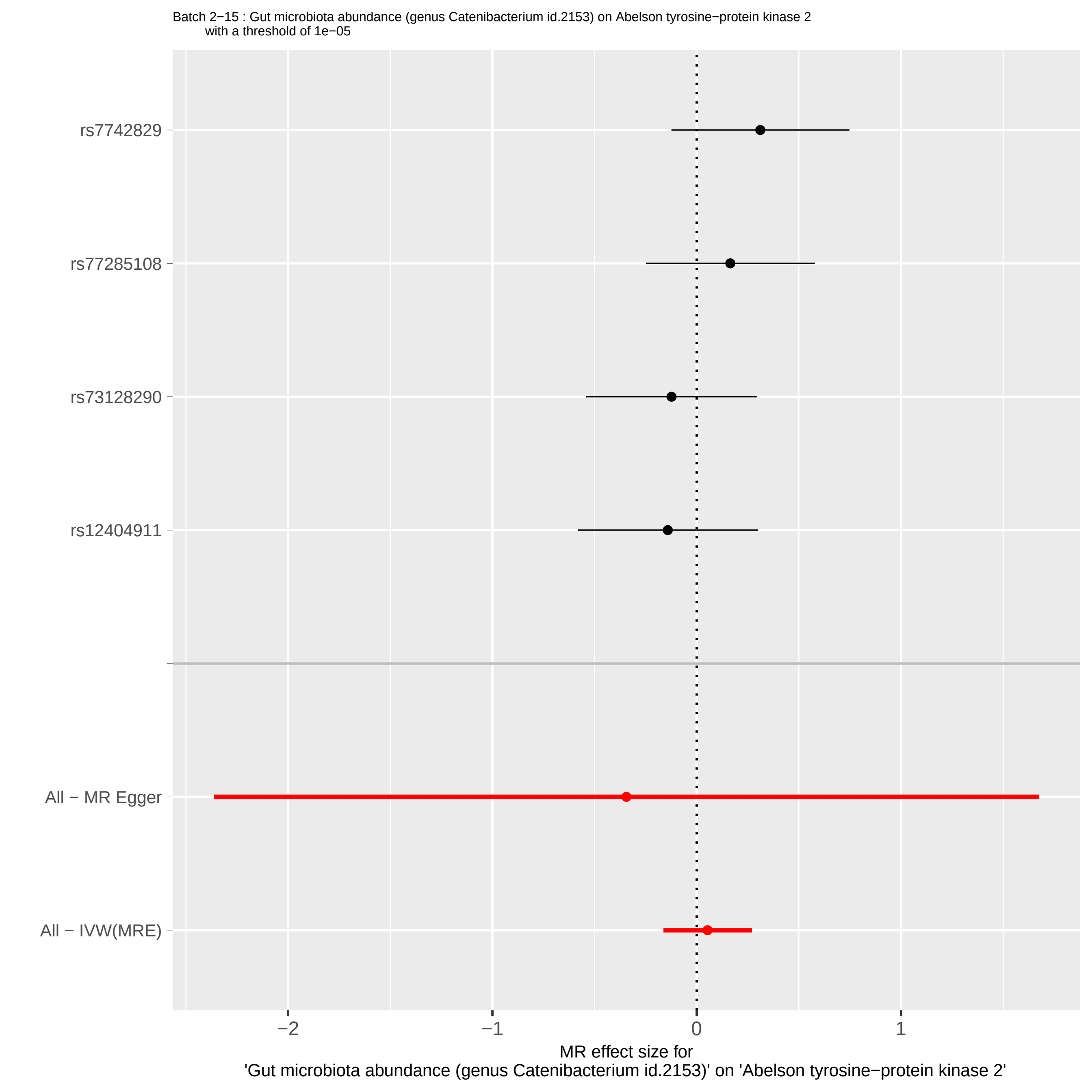

Batch 2−15 : Gut microbiota abundance (genus Catenibacterium id.2153) on Abelson tyrosine−protein kinase 2
with a threshold of 1e−05
rs7742829
rs77285108
rs73128290
rs12404911
All − MR Egger
All − IVW(MRE)
−2
−1
0
1
MR effect size for
'Gut microbiota abundance (genus Catenibacterium id.2153)' on 'Abelson tyrosine−protein kinase 2'

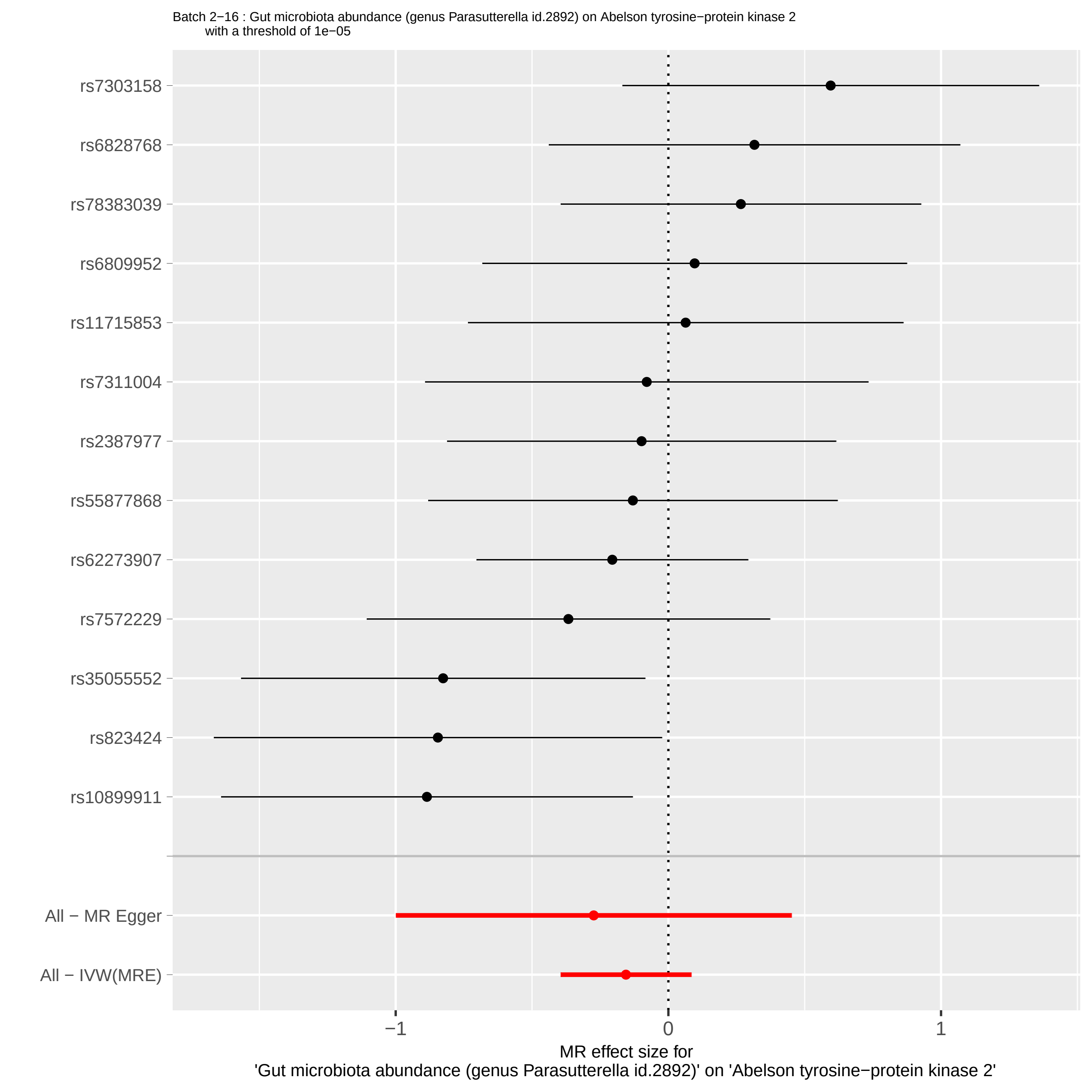
Batch 2−16 : Gut microbiota abundance (genus Parasutterella id.2892) on Abelson tyrosine−protein kinase 2
with a threshold of 1e−05
rs7303158
rs6828768
rs78383039
rs6809952
rs11715853
rs7311004
rs2387977
rs55877868
rs62273907
rs7572229
rs35055552
rs823424
rs10899911
All − MR Egger
All − IVW(MRE)
−1
0
1
MR effect size for
'Gut microbiota abundance (genus Parasutterella id.2892)' on 'Abelson tyrosine−protein kinase 2'

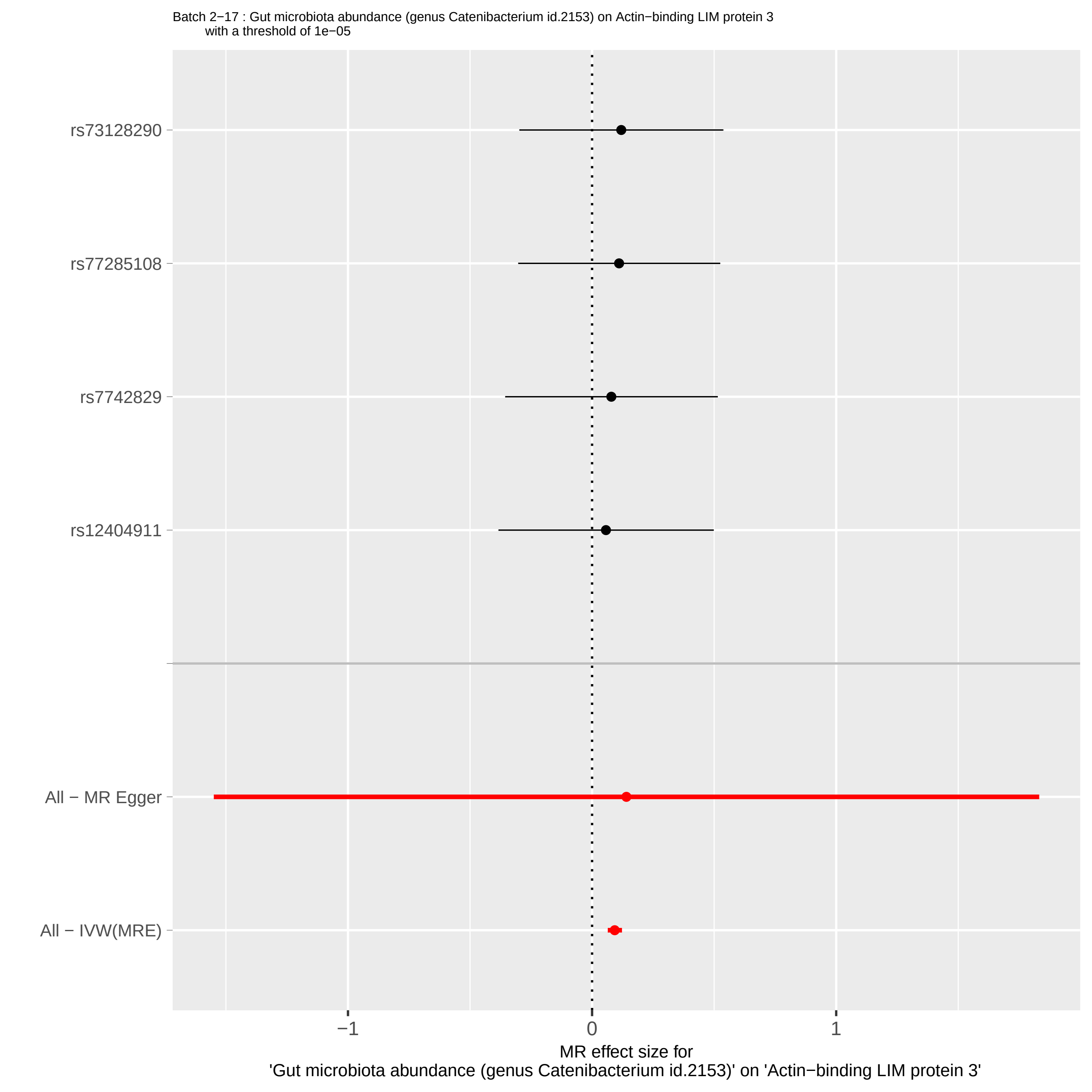

Batch 2−17 : Gut microbiota abundance (genus Catenibacterium id.2153) on Actin−binding LIM protein 3
with a threshold of 1e−05
rs73128290
rs77285108
rs7742829
rs12404911
All − MR Egger
All − IVW(MRE)
−1
0
1
MR effect size for
'Gut microbiota abundance (genus Catenibacterium id.2153)' on 'Actin−binding LIM protein 3'

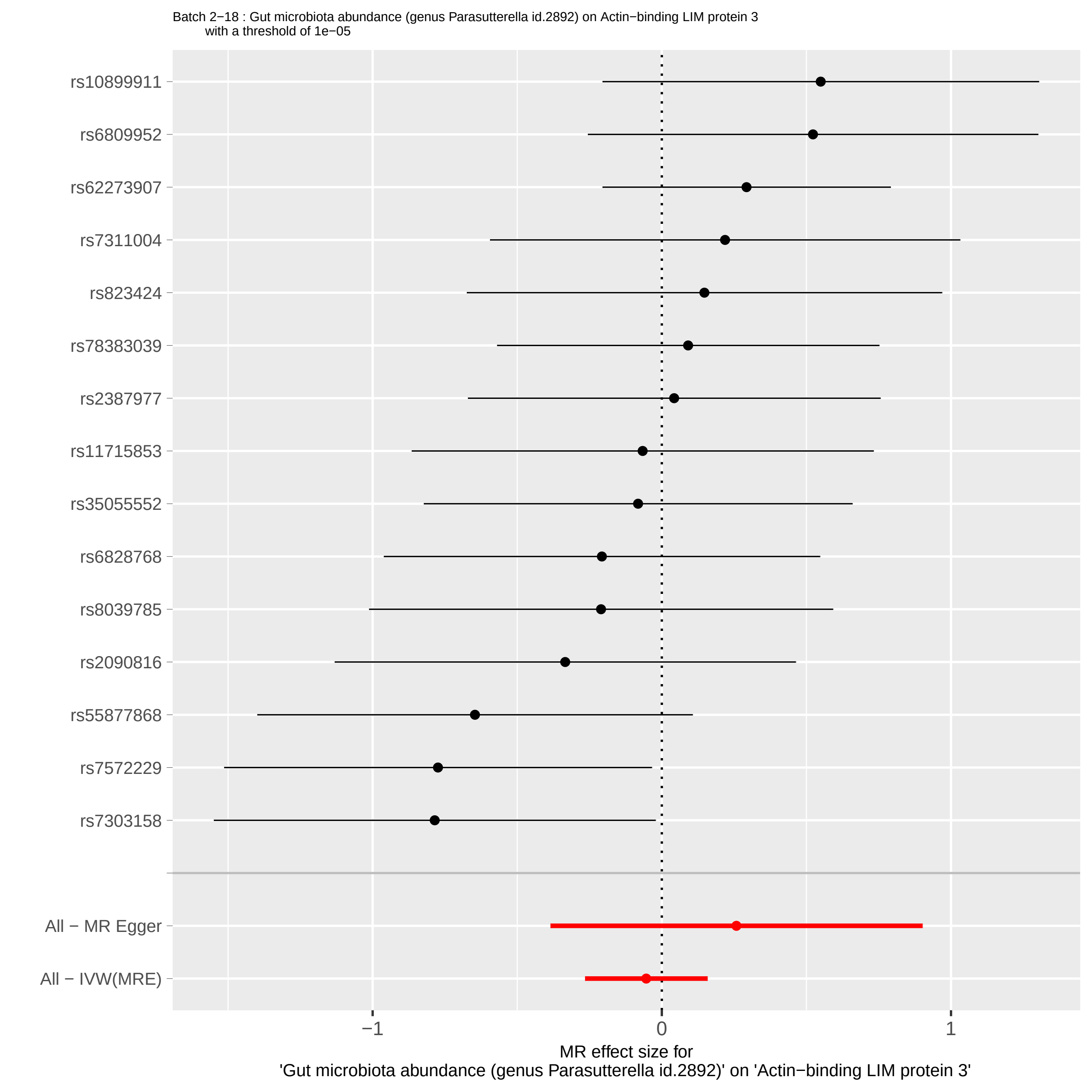
Batch 2−18 : Gut microbiota abundance (genus Parasutterella id.2892) on Actin−binding LIM protein 3
with a threshold of 1e−05
rs10899911
rs6809952
rs62273907
rs7311004
rs823424
rs78383039
rs2387977
rs11715853
rs35055552
rs6828768
rs8039785
rs2090816
rs55877868
rs7572229
rs7303158
All − MR Egger
All − IVW(MRE)
−1
0
1
MR effect size for
'Gut microbiota abundance (genus Parasutterella id.2892)' on 'Actin−binding LIM protein 3'

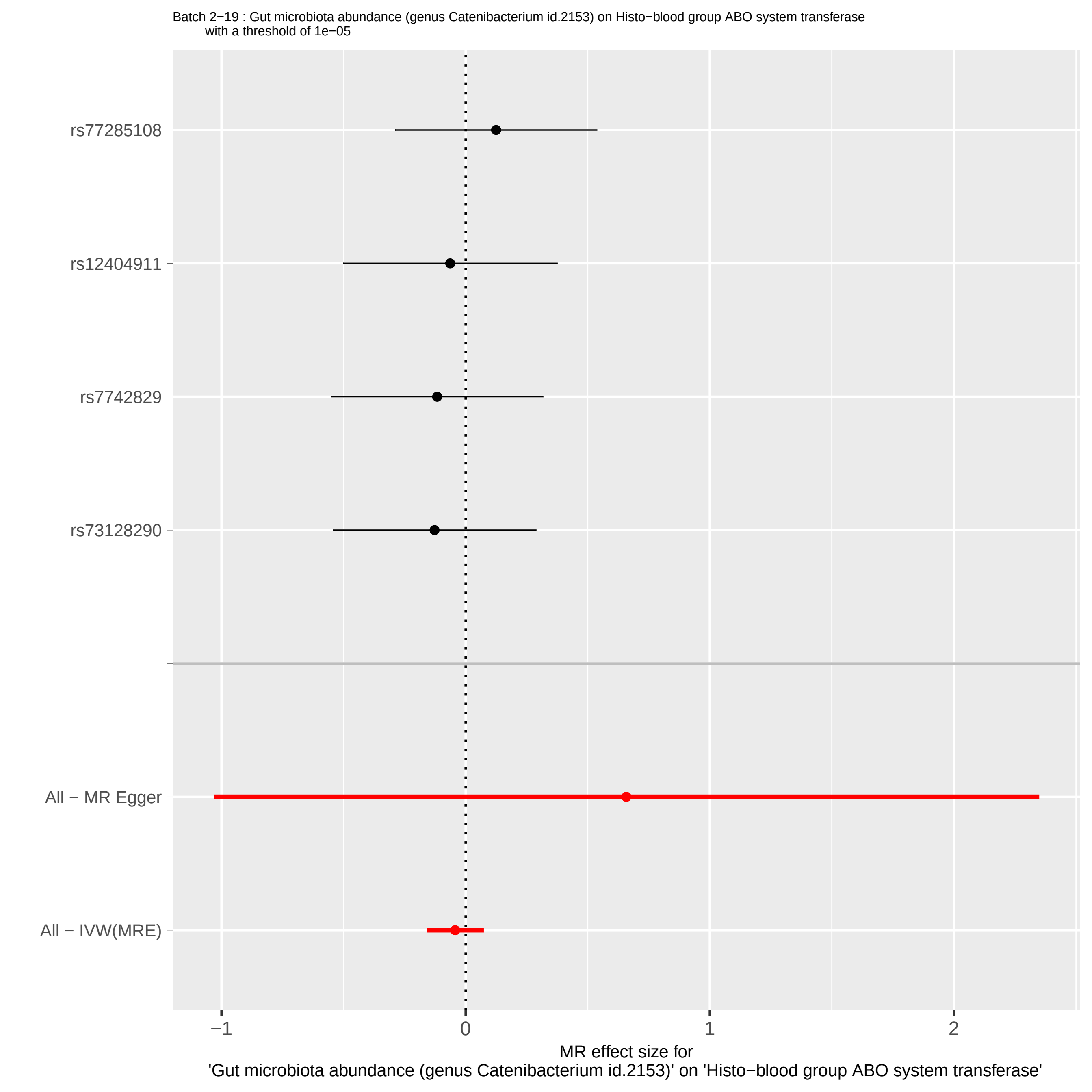

Batch 2−19 : Gut microbiota abundance (genus Catenibacterium id.2153) on Histo−blood group ABO system transferase
with a threshold of 1e−05
rs77285108
rs12404911
rs7742829
rs73128290
All − MR Egger
All − IVW(MRE)
−1
0
1
2
MR effect size for
'Gut microbiota abundance (genus Catenibacterium id.2153)' on 'Histo−blood group ABO system transferase'

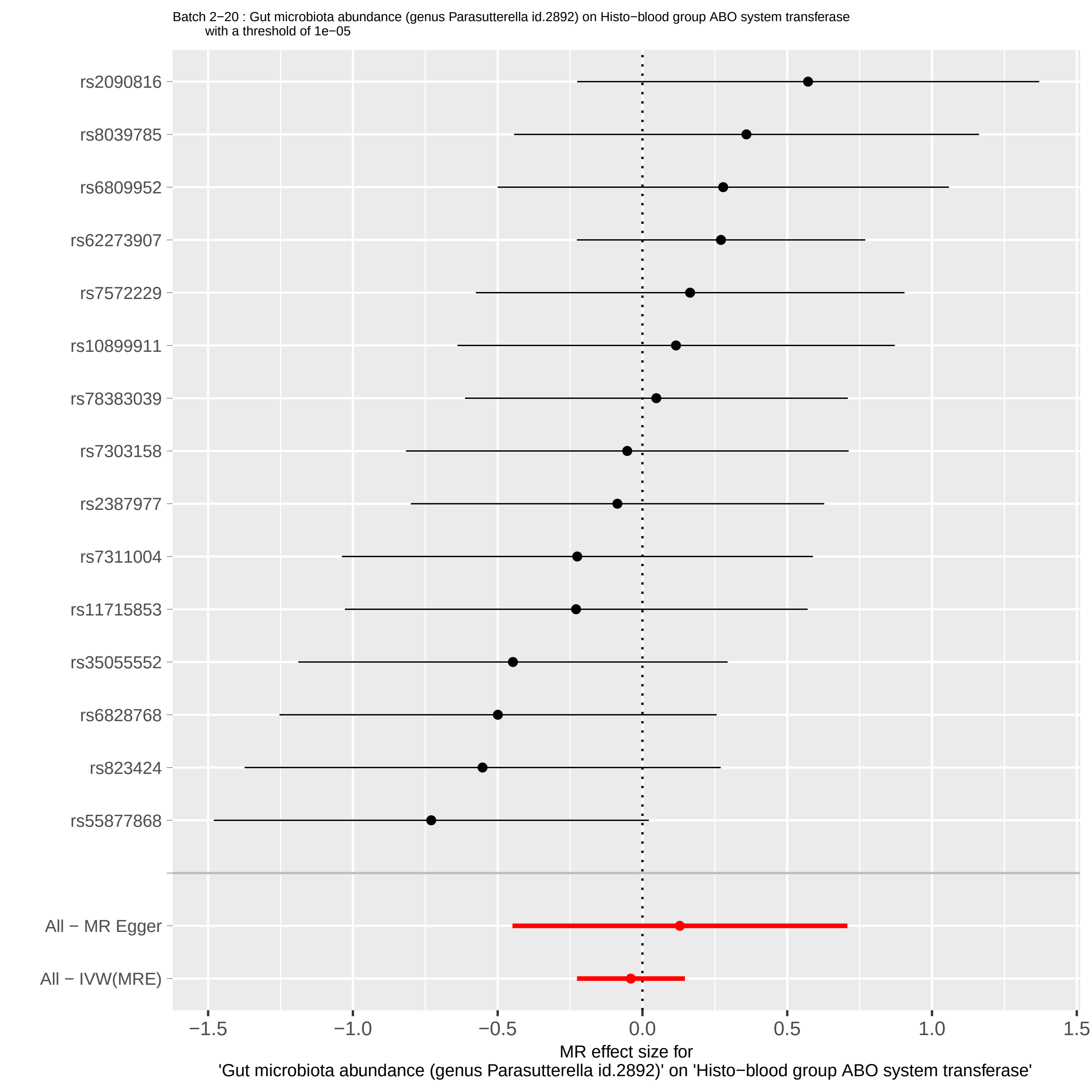
Batch 2−20 : Gut microbiota abundance (genus Parasutterella id.2892) on Histo−blood group ABO system transferase with a threshold of 1e−05
rs2090816
rs8039785
rs6809952
rs62273907
rs7572229
rs10899911
rs78383039
rs7303158
rs2387977
rs7311004
rs11715853
rs35055552
rs6828768
rs823424
rs55877868
All − MR Egger
All − IVW(MRE)
−1.5
−1.0
−0.5
0.0
0.5
1.0
1.5
MR effect size for
'Gut microbiota abundance (genus Parasutterella id.2892)' on 'Histo−blood group ABO system transferase'

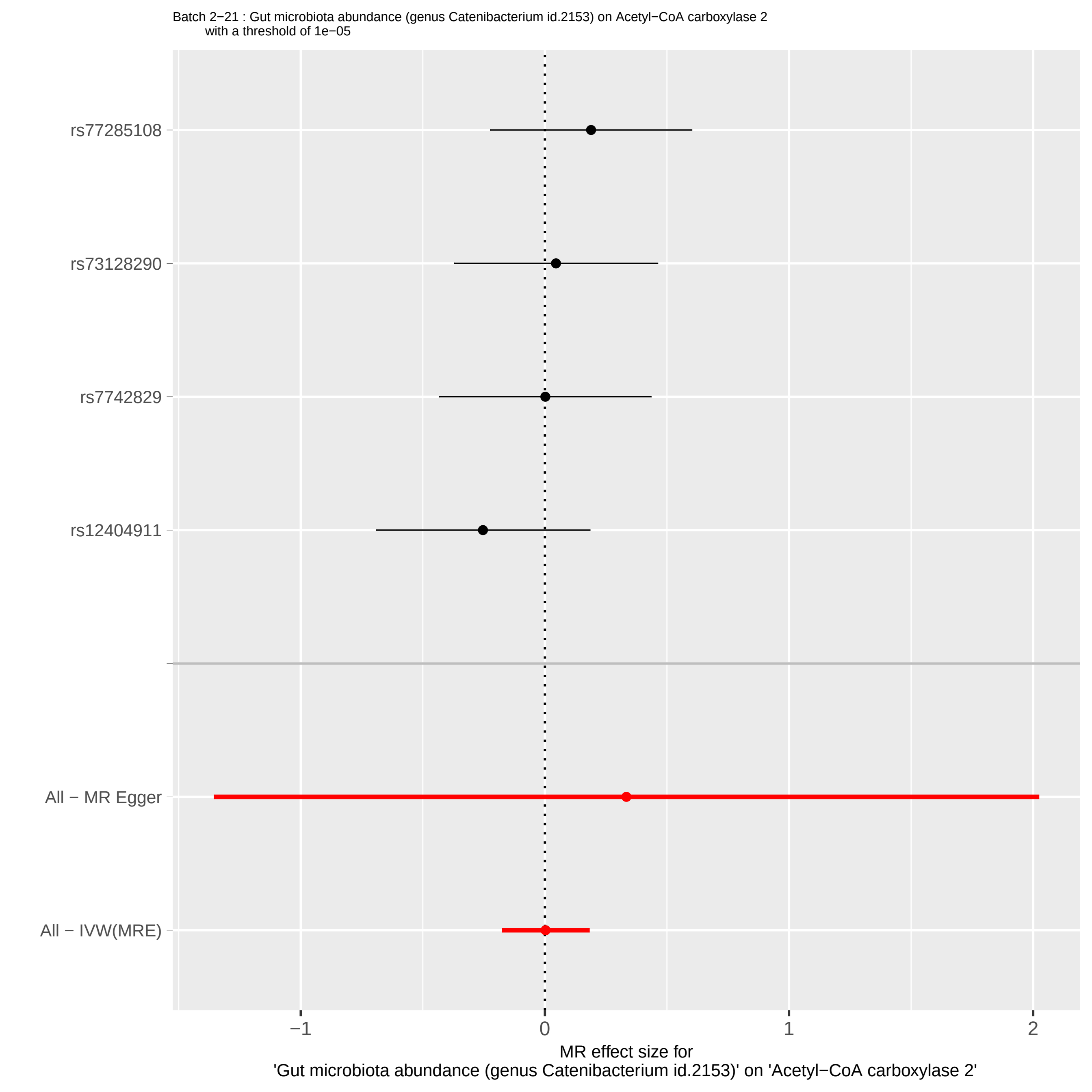
Batch 2−21 : Gut microbiota abundance (genus Catenibacterium id.2153) on Acetyl−CoA carboxylase 2
with a threshold of 1e−05
rs77285108
rs73128290
rs7742829
rs12404911
All − MR Egger
All − IVW(MRE)
−1
0
1
2
MR effect size for
'Gut microbiota abundance (genus Catenibacterium id.2153)' on 'Acetyl−CoA carboxylase 2'

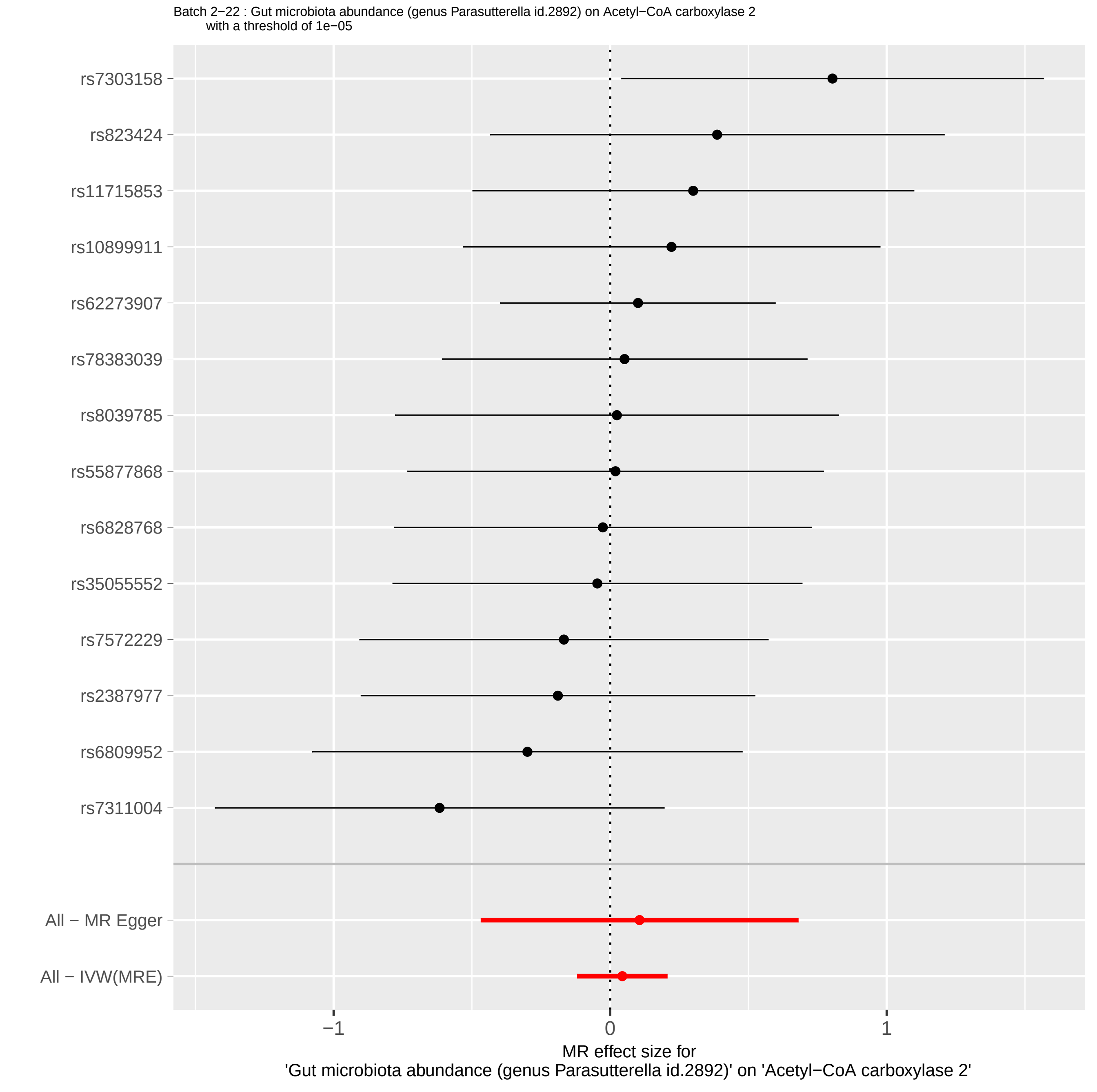
Batch 2−22 : Gut microbiota abundance (genus Parasutterella id.2892) on Acetyl−CoA carboxylase 2
with a threshold of 1e−05
rs7303158
rs823424
rs11715853
rs10899911
rs62273907
rs78383039
rs8039785
rs55877868
rs6828768
rs35055552
rs7572229
rs2387977
rs6809952
rs7311004
All − MR Egger
All − IVW(MRE)
−1
0
1
MR effect size for
'Gut microbiota abundance (genus Parasutterella id.2892)' on 'Acetyl−CoA carboxylase 2'

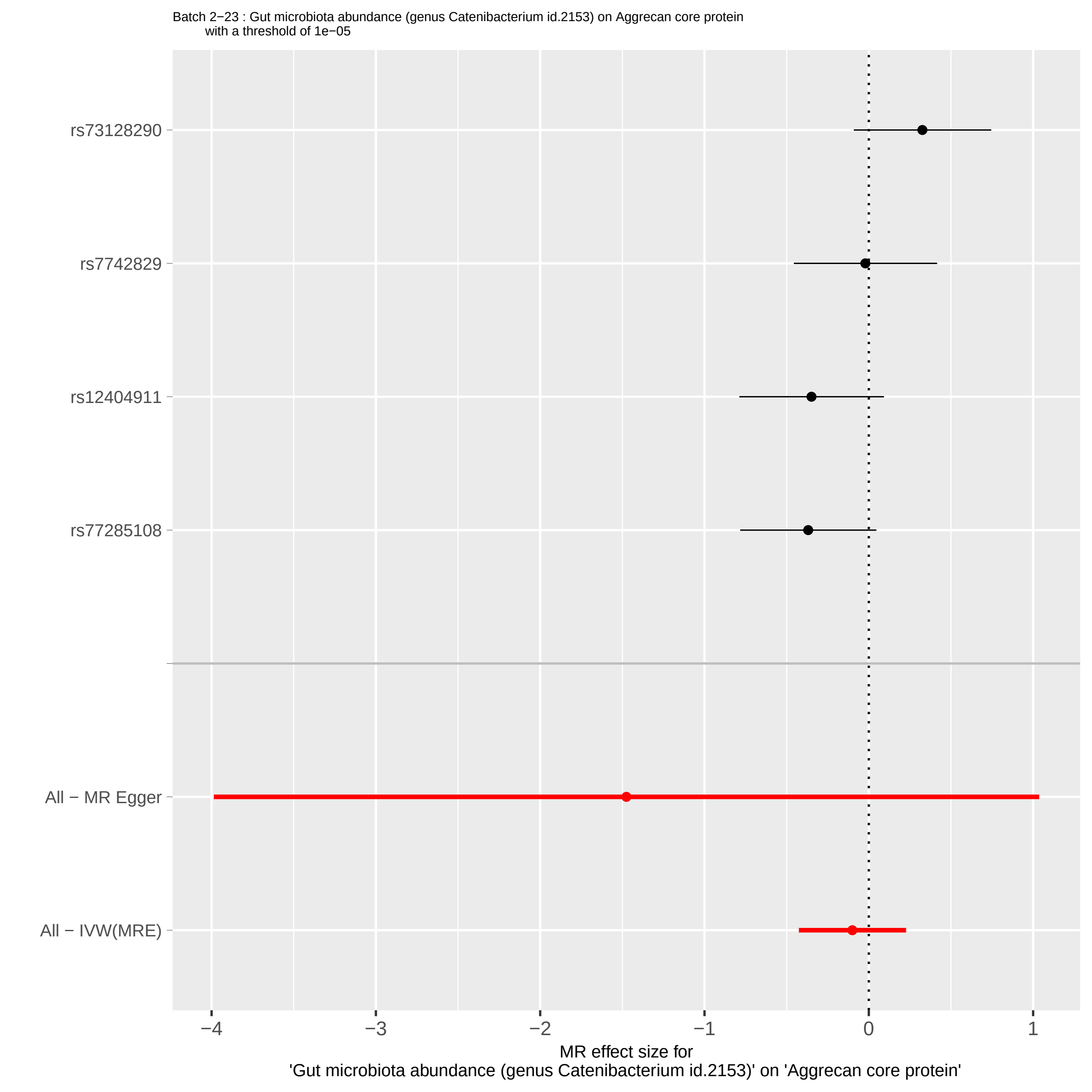
Batch 2−23 : Gut microbiota abundance (genus Catenibacterium id.2153) on Aggrecan core protein
with a threshold of 1e−05
rs73128290
rs7742829
rs12404911
rs77285108
All − MR Egger
All − IVW(MRE)
−4
−3
−2
−1
0
1
MR effect size for
'Gut microbiota abundance (genus Catenibacterium id.2153)' on 'Aggrecan core protein'

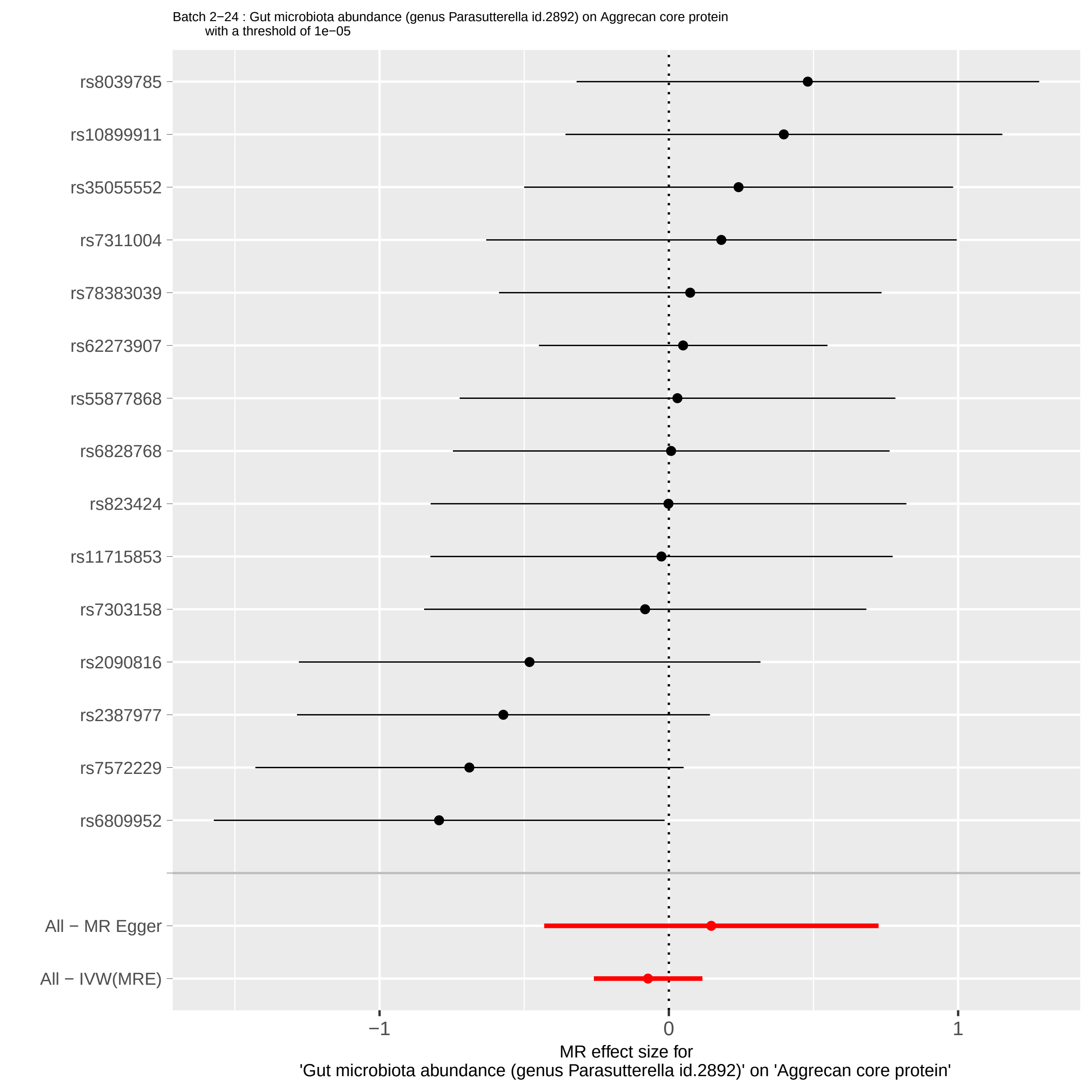
Batch 2−24 : Gut microbiota abundance (genus Parasutterella id.2892) on Aggrecan core protein
with a threshold of 1e−05
rs8039785
rs10899911
rs35055552
rs7311004
rs78383039
rs62273907
rs55877868
rs6828768
rs823424
rs11715853
rs7303158
rs2090816
rs2387977
rs7572229
rs6809952
All − MR Egger
All − IVW(MRE)
−1
0
1
MR effect size for
'Gut microbiota abundance (genus Parasutterella id.2892)' on 'Aggrecan core protein'

Batch 2−25 : Gut microbiota abundance (genus Catenibacterium id.2153) on Arf−GAP with coiled−coil, ANK repeat and PH domain−containing protein 2 with a threshold of 1e−05

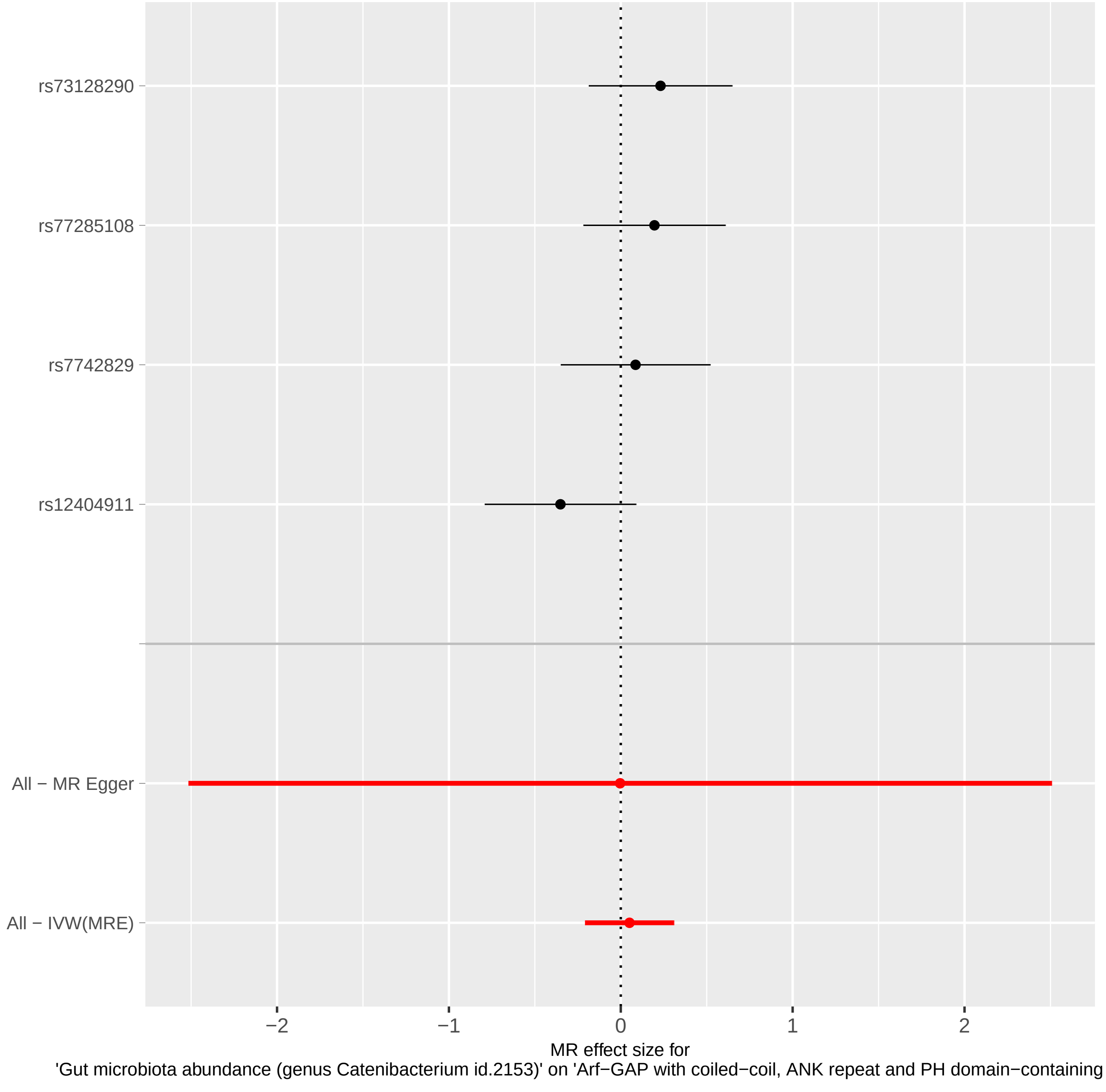

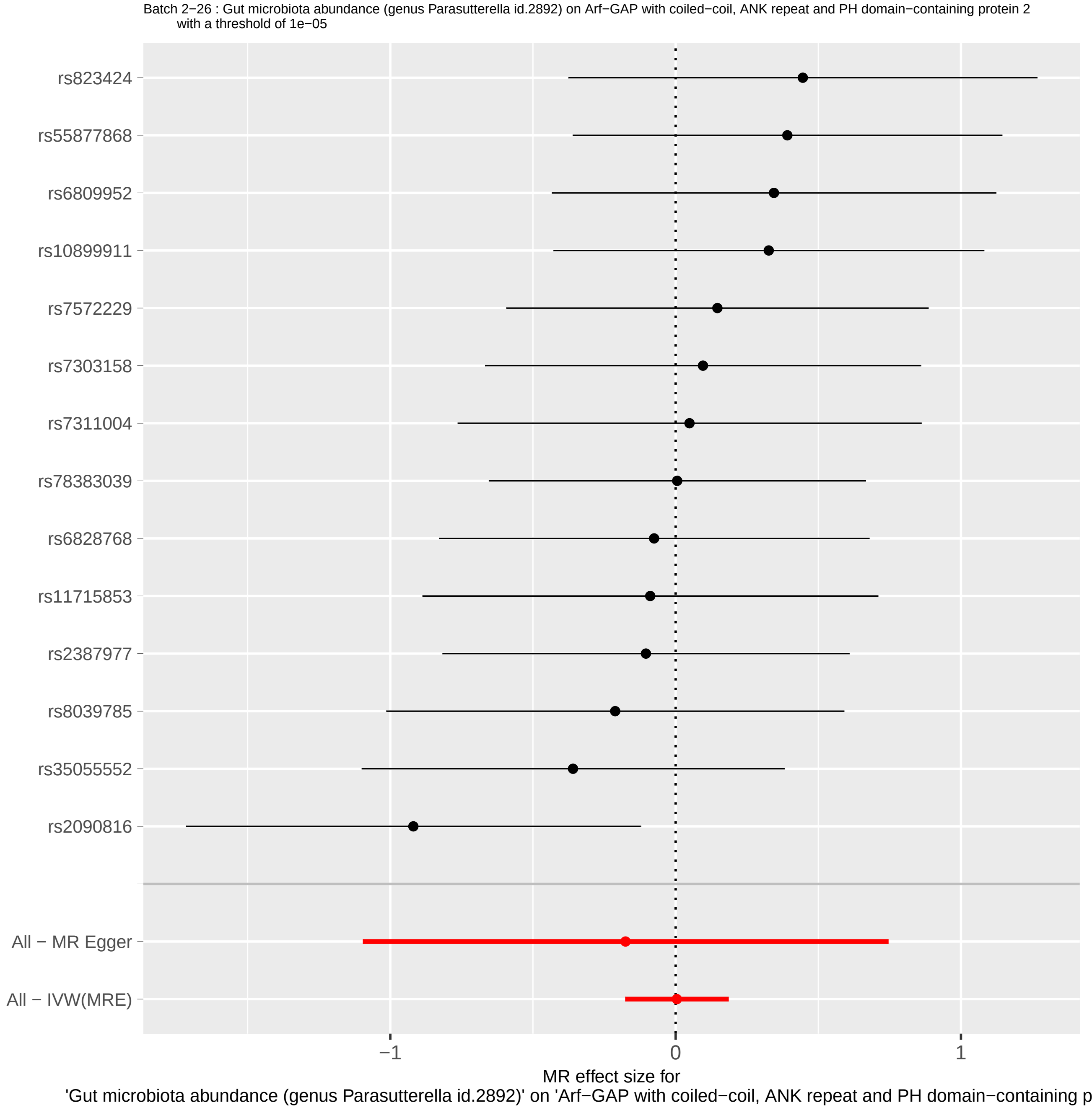

Batch 2−26 : Gut microbiota abundance (genus Parasutterella id.2892) on Arf−GAP with coiled−coil, ANK repeat and PH domain−containing protein 2 with a threshold of 1e−05
rs823424
rs55877868
rs6809952
rs10899911
rs7572229
rs7303158
rs7311004
rs78383039
rs6828768
rs11715853
rs2387977
rs8039785
rs35055552
rs2090816
All − MR Egger
All − IVW(MRE)
−1
0
1
MR effect size for
'Gut microbiota abundance (genus Parasutterella id.2892)' on 'Arf−GAP with coiled−coil, ANK repeat and PH domain−containing p

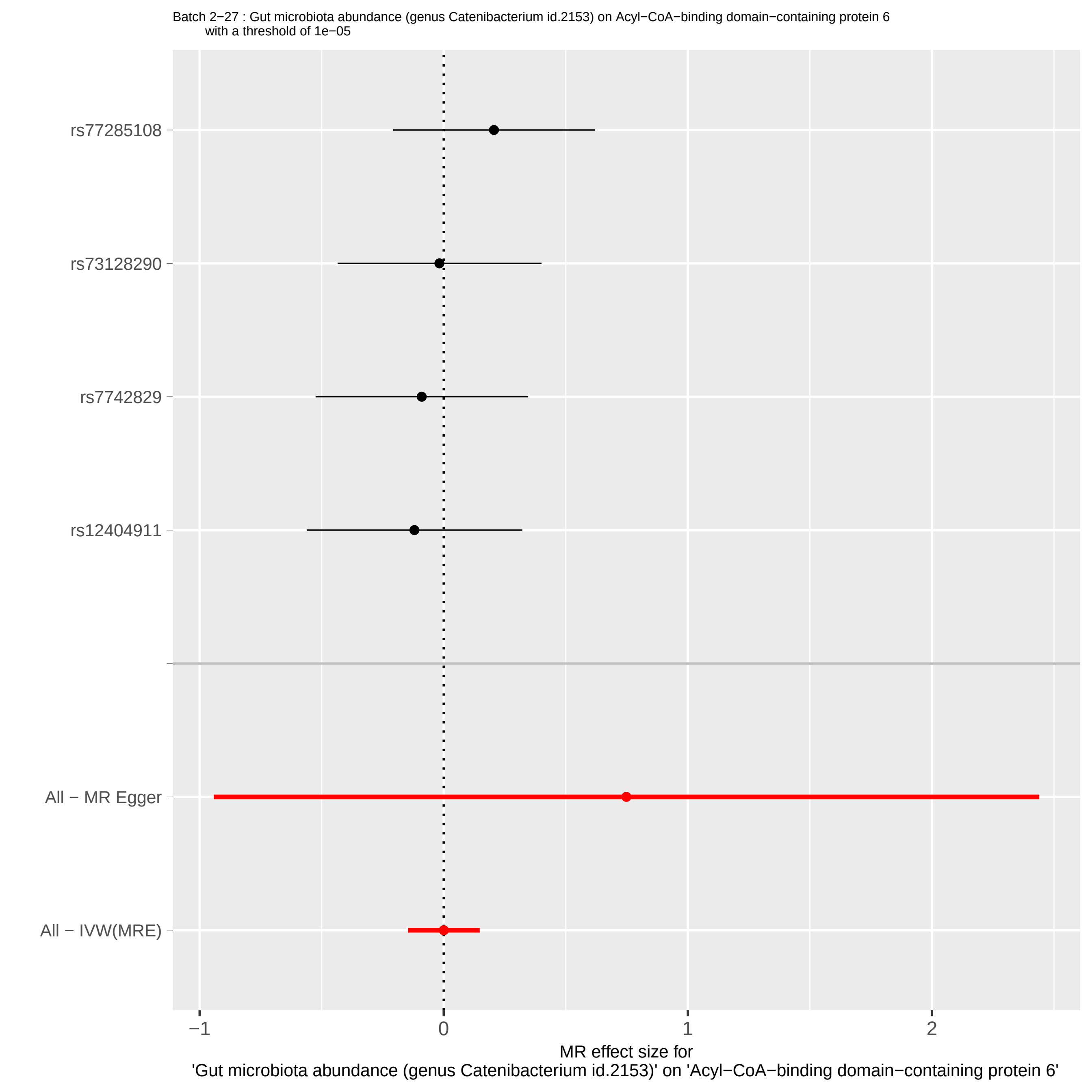

Batch 2−27 : Gut microbiota abundance (genus Catenibacterium id.2153) on Acyl−CoA−binding domain−containing protein 6
with a threshold of 1e−05
rs77285108
rs73128290
rs7742829
rs12404911
All − MR Egger
All − IVW(MRE)
−1
0
1
2
MR effect size for
'Gut microbiota abundance (genus Catenibacterium id.2153)' on 'Acyl−CoA−binding domain−containing protein 6'

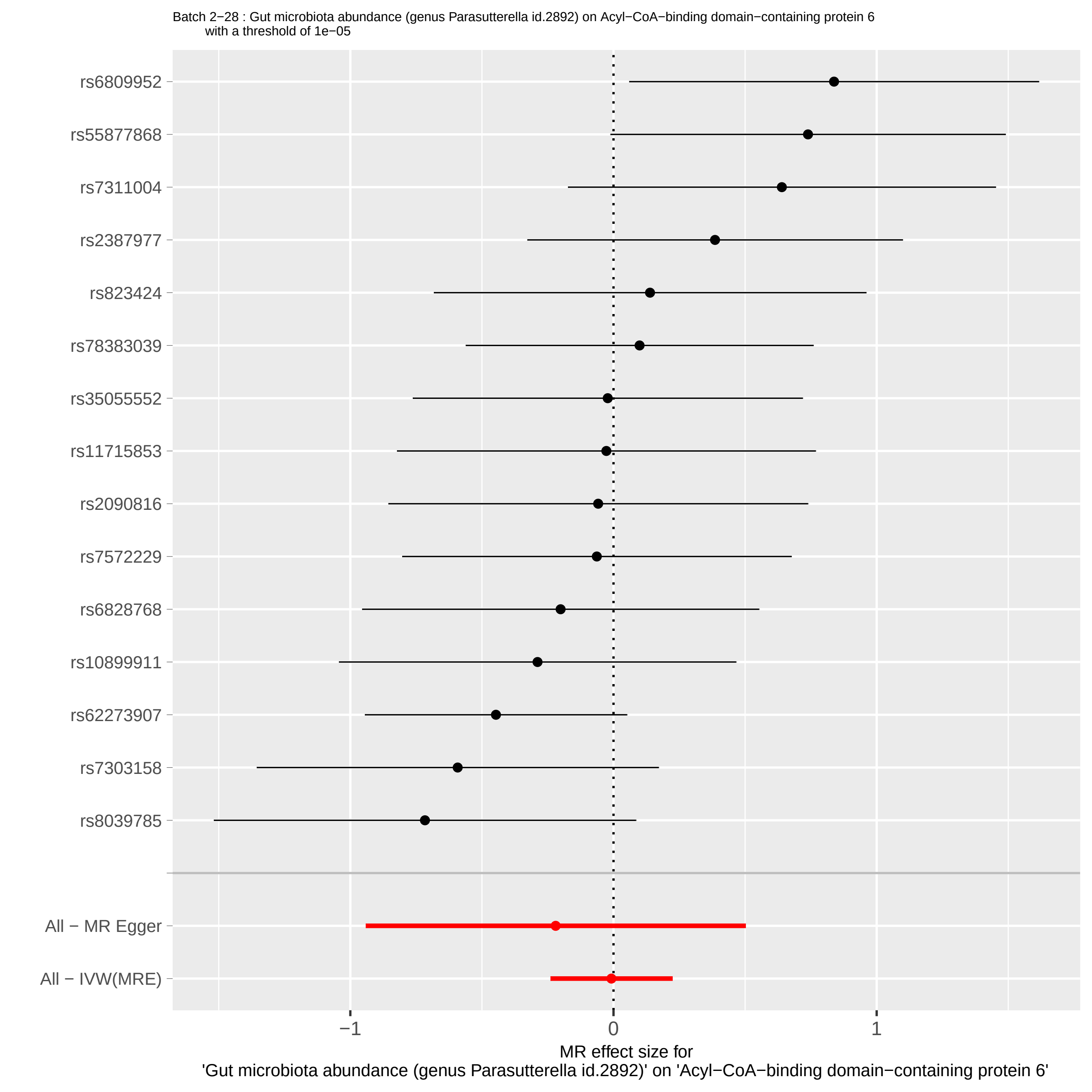

Batch 2−28 : Gut microbiota abundance (genus Parasutterella id.2892) on Acyl−CoA−binding domain−containing protein 6
with a threshold of 1e−05
rs6809952
rs55877868
rs7311004
rs2387977
rs823424
rs78383039
rs35055552
rs11715853
rs2090816
rs7572229
rs6828768
rs10899911
rs62273907
rs7303158
rs8039785
All − MR Egger
All − IVW(MRE)
−1
0
1
MR effect size for
'Gut microbiota abundance (genus Parasutterella id.2892)' on 'Acyl−CoA−binding domain−containing protein 6'

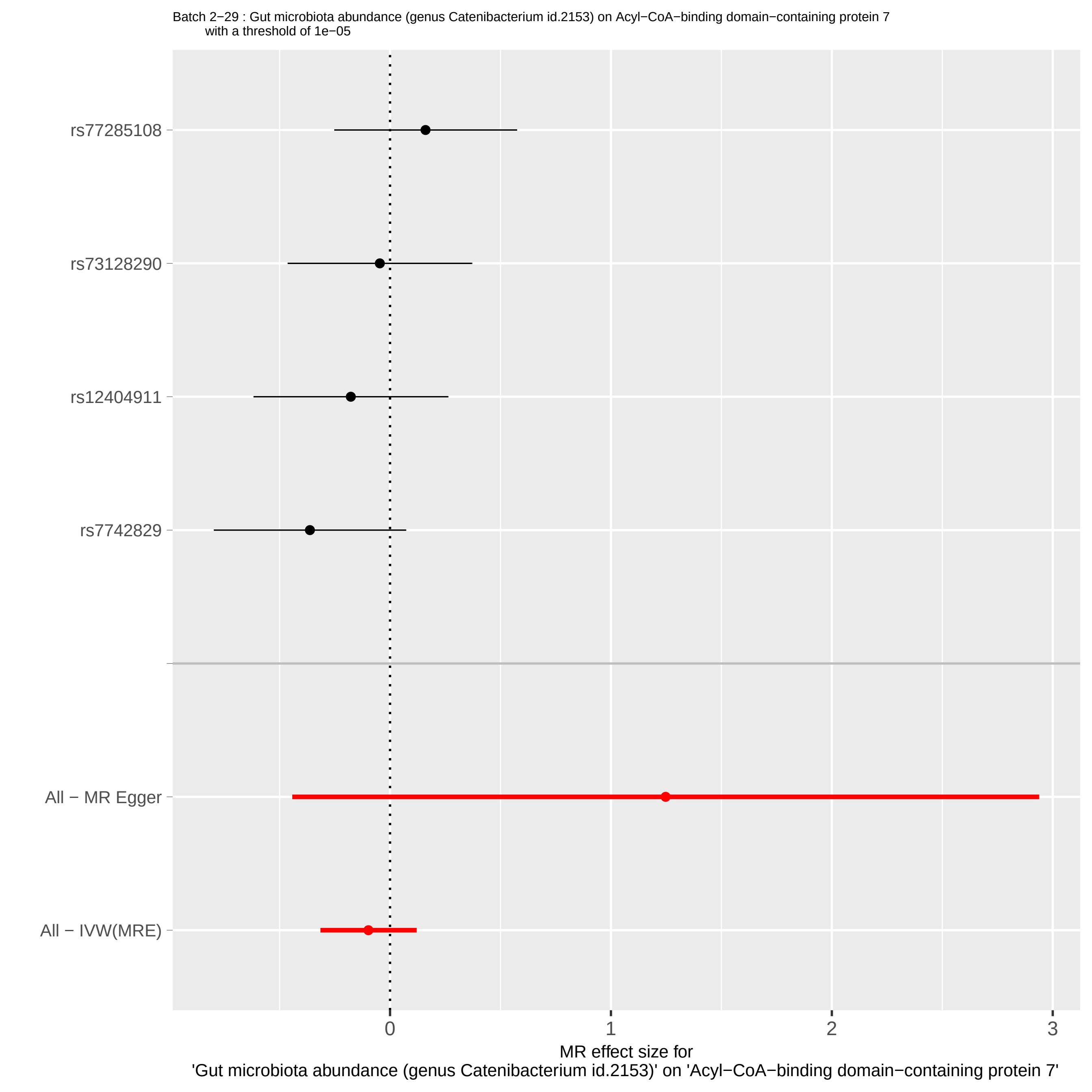

Batch 2−29 : Gut microbiota abundance (genus Catenibacterium id.2153) on Acyl−CoA−binding domain−containing protein 7
with a threshold of 1e−05
rs77285108
rs73128290
rs12404911
rs7742829
All − MR Egger
All − IVW(MRE)
0
1
2
3
MR effect size for
'Gut microbiota abundance (genus Catenibacterium id.2153)' on 'Acyl−CoA−binding domain−containing protein 7'

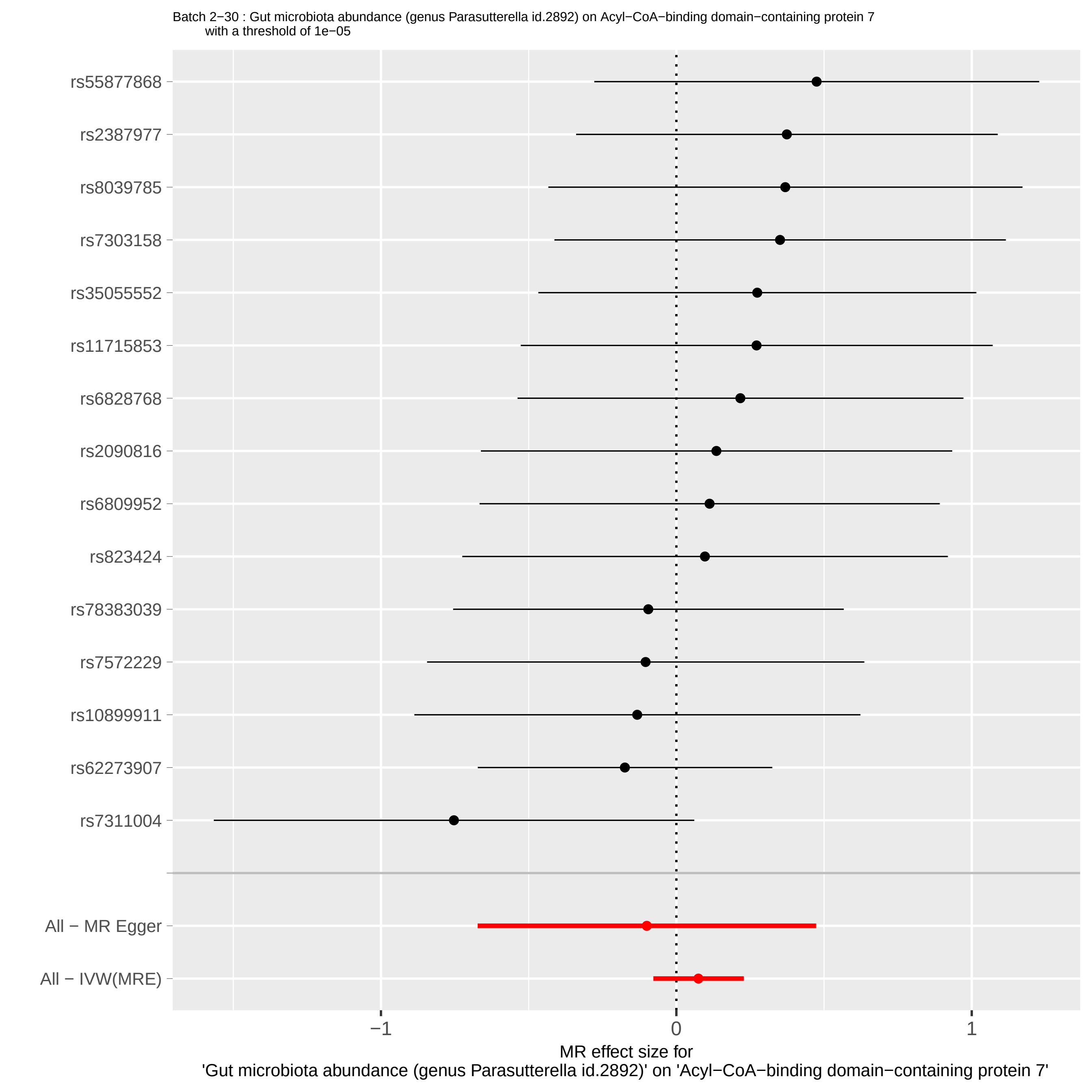
Batch 2−30 : Gut microbiota abundance (genus Parasutterella id.2892) on Acyl−CoA−binding domain−containing protein 7
with a threshold of 1e−05
rs55877868
rs2387977
rs8039785
rs7303158
rs35055552
rs11715853
rs6828768
rs2090816
rs6809952
rs823424
rs78383039
rs7572229
rs10899911
rs62273907
rs7311004
All − MR Egger
All − IVW(MRE)
−1
0
1
MR effect size for
'Gut microbiota abundance (genus Parasutterella id.2892)' on 'Acyl−CoA−binding domain−containing protein 7'

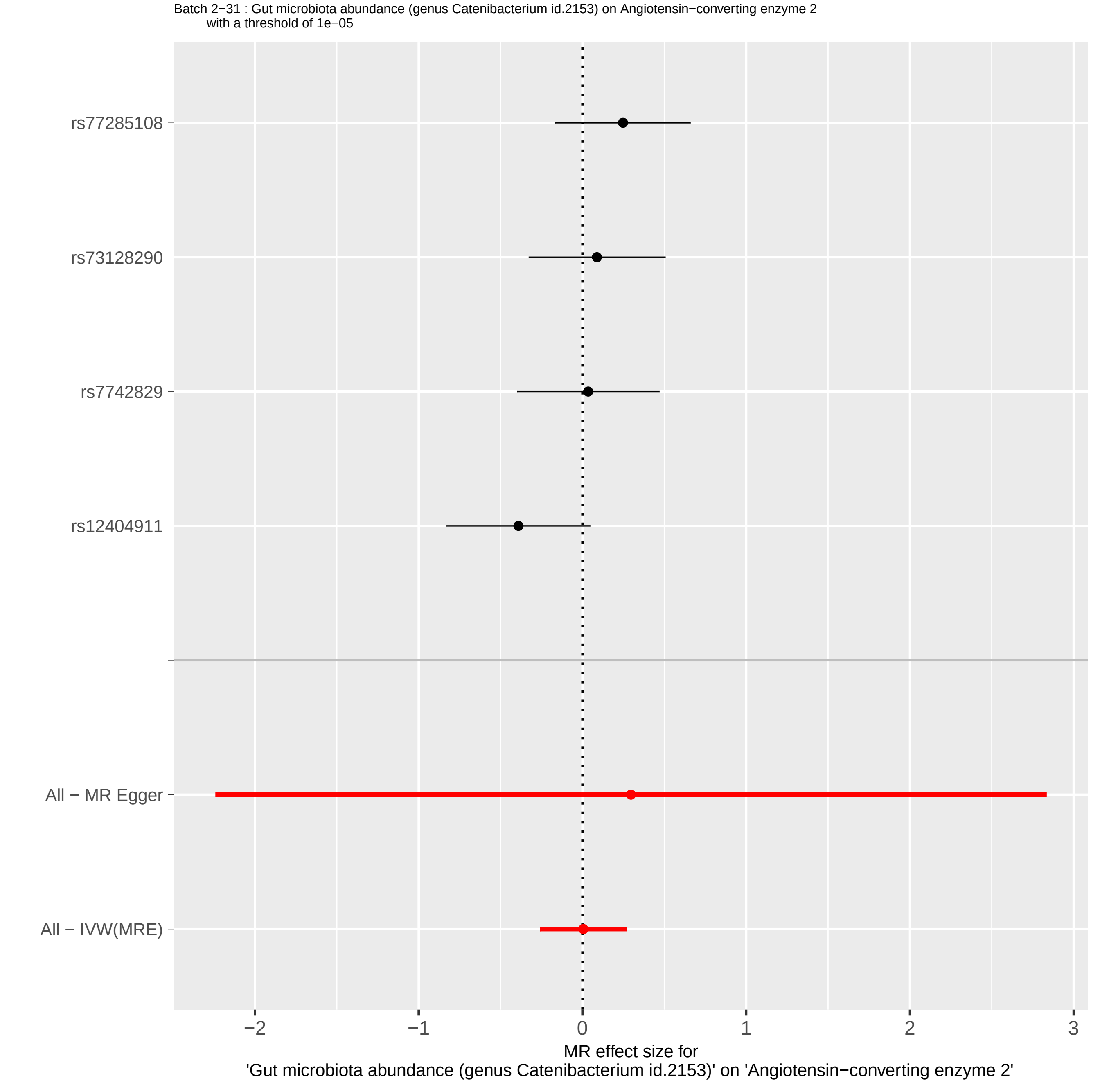

Batch 2−31 : Gut microbiota abundance (genus Catenibacterium id.2153) on Angiotensin−converting enzyme 2
with a threshold of 1e−05
rs77285108
rs73128290
rs7742829
rs12404911
All − MR Egger
All − IVW(MRE)
−2
−1
0
1
2
3
MR effect size for
'Gut microbiota abundance (genus Catenibacterium id.2153)' on 'Angiotensin−converting enzyme 2'

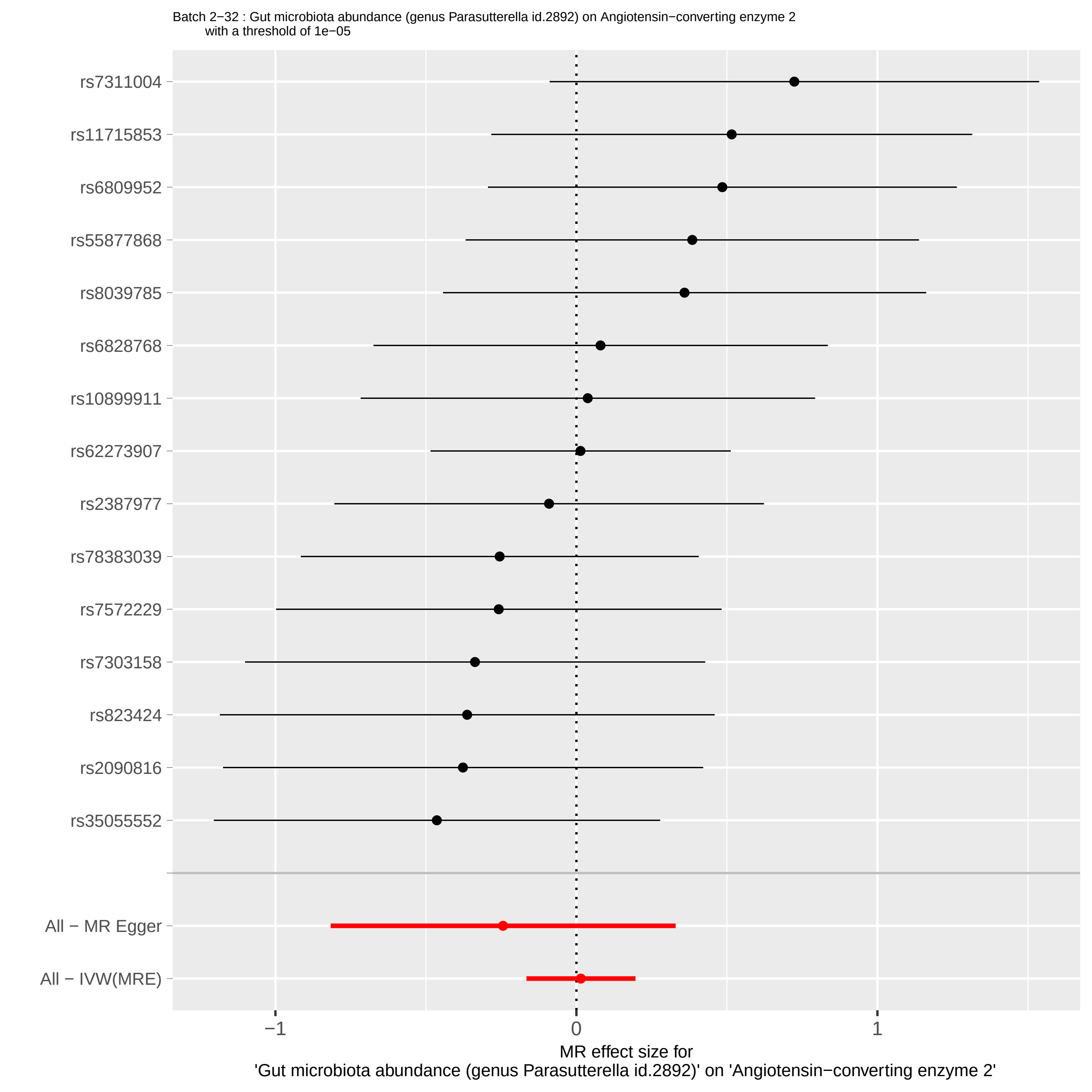
Batch 2−32 : Gut microbiota abundance (genus Parasutterella id.2892) on Angiotensin−converting enzyme 2
with a threshold of 1e−05
rs7311004
rs11715853
rs6809952
rs55877868
rs8039785
rs6828768
rs10899911
rs62273907
rs2387977
rs78383039
rs7572229
rs7303158
rs823424
rs2090816
rs35055552
All − MR Egger
All − IVW(MRE)
−1
0
1
MR effect size for
'Gut microbiota abundance (genus Parasutterella id.2892)' on 'Angiotensin−converting enzyme 2'

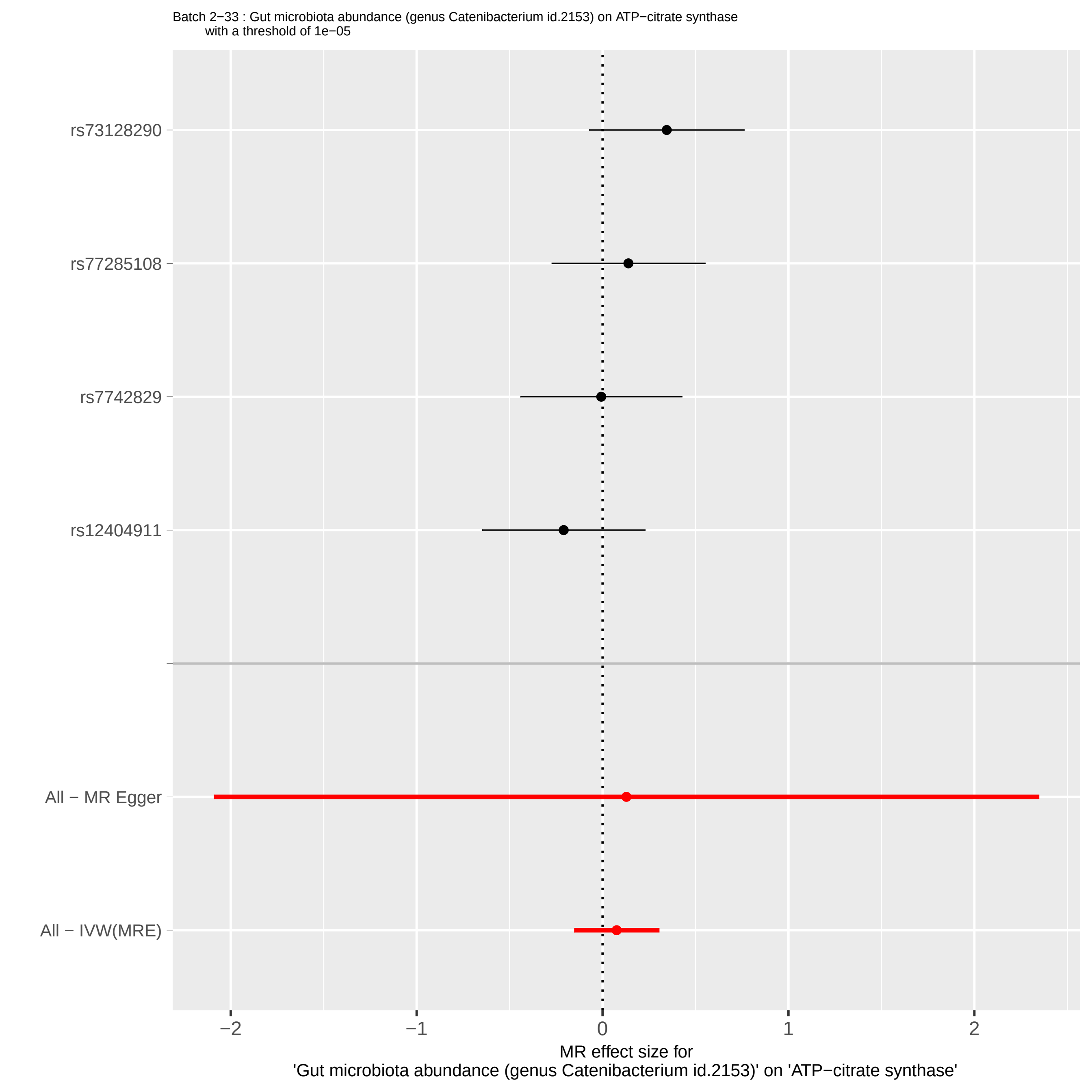
Batch 2−33 : Gut microbiota abundance (genus Catenibacterium id.2153) on ATP−citrate synthase
with a threshold of 1e−05
rs73128290
rs77285108
rs7742829
rs12404911
All − MR Egger
All − IVW(MRE)
−2
−1
0
1
2
MR effect size for
'Gut microbiota abundance (genus Catenibacterium id.2153)' on 'ATP−citrate synthase'

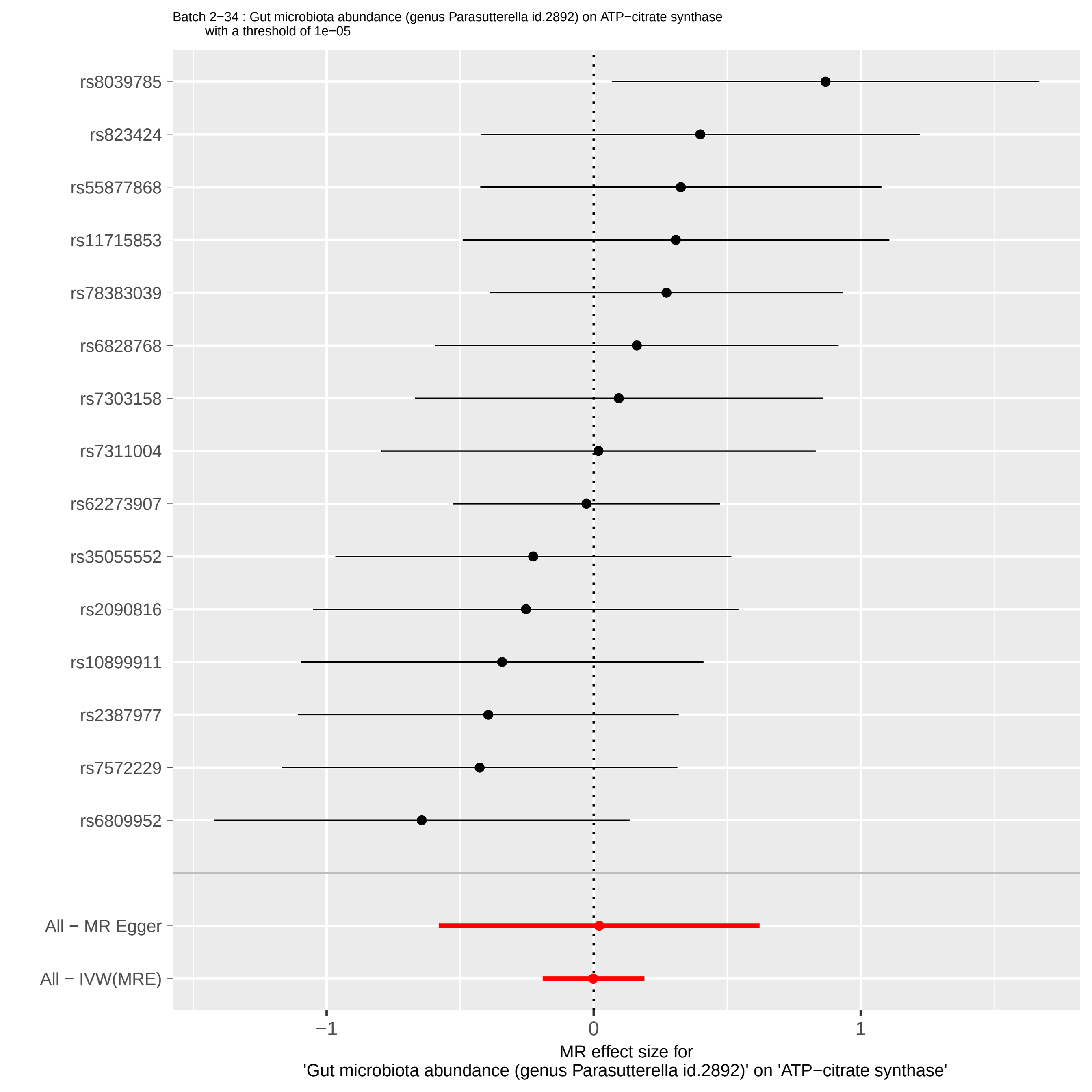
Batch 2−34 : Gut microbiota abundance (genus Parasutterella id.2892) on ATP−citrate synthase
with a threshold of 1e−05
rs8039785
rs823424
rs55877868
rs11715853
rs78383039
rs6828768
rs7303158
rs7311004
rs62273907
rs35055552
rs2090816
rs10899911
rs2387977
rs7572229
rs6809952
All − MR Egger
All − IVW(MRE)
−1
0
1
MR effect size for
'Gut microbiota abundance (genus Parasutterella id.2892)' on 'ATP−citrate synthase'

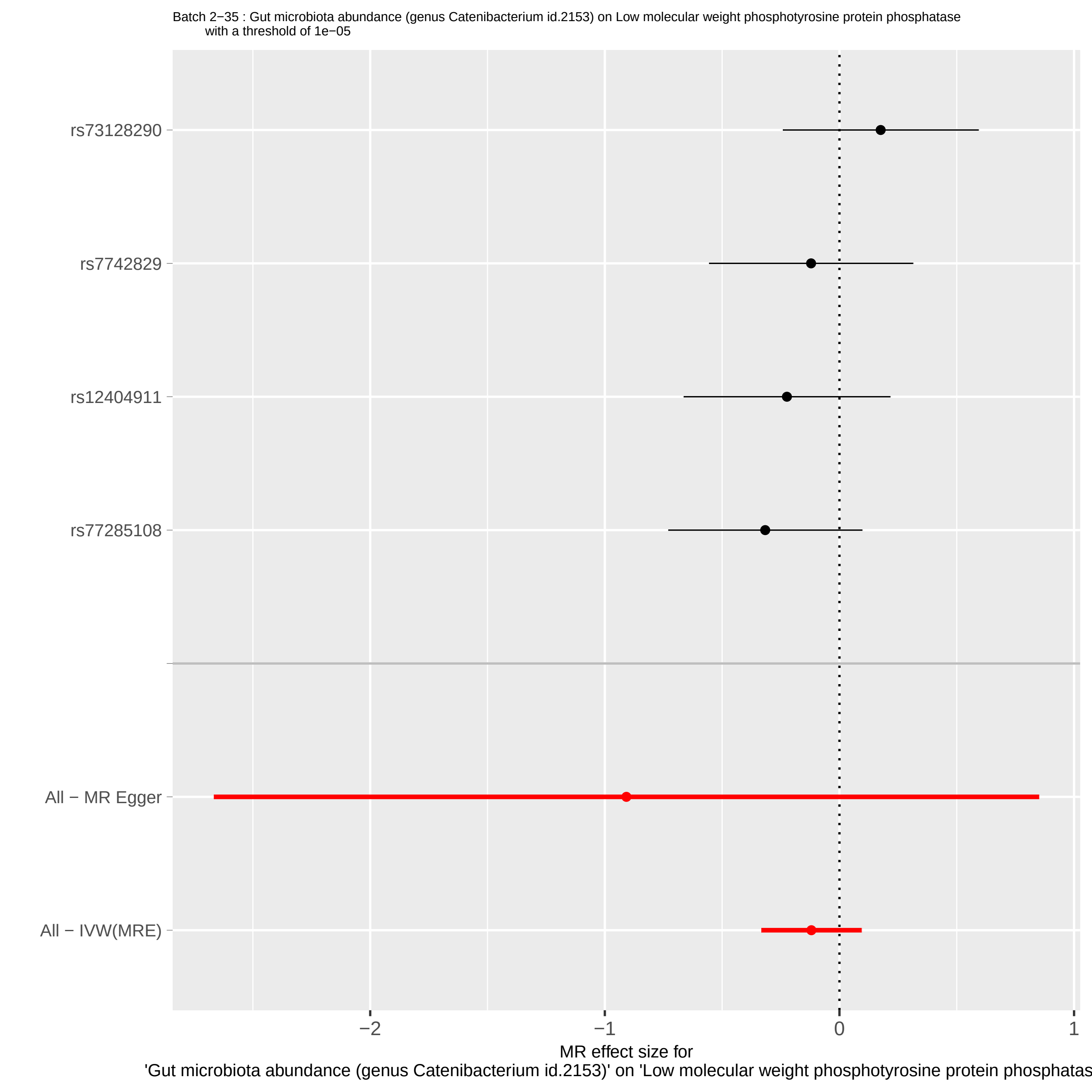

Batch 2−35 : Gut microbiota abundance (genus Catenibacterium id.2153) on Low molecular weight phosphotyrosine protein phosphatase
with a threshold of 1e−05
rs73128290
rs7742829
rs12404911
rs77285108
All − MR Egger
All − IVW(MRE)
−2
−1
0
1
MR effect size for
'Gut microbiota abundance (genus Catenibacterium id.2153)' on 'Low molecular weight phosphotyrosine protein phosphatas

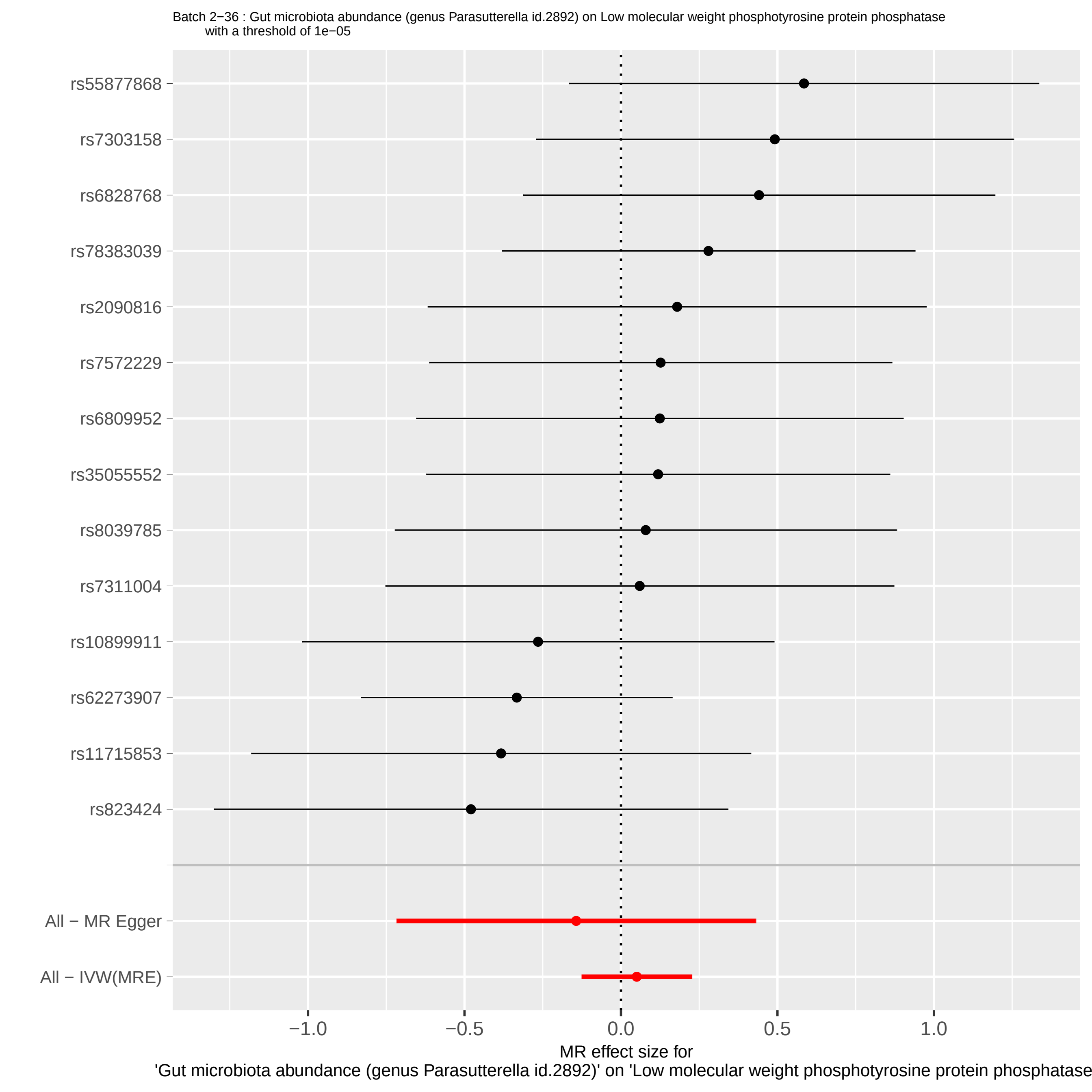
Batch 2−36 : Gut microbiota abundance (genus Parasutterella id.2892) on Low molecular weight phosphotyrosine protein phosphatase with a threshold of 1e−05
rs55877868
rs7303158
rs6828768
rs78383039
rs2090816
rs7572229
rs6809952
rs35055552
rs8039785
rs7311004
rs10899911
rs62273907
rs11715853
rs823424
All − MR Egger
All − IVW(MRE)
−1.0
−0.5
0.0
0.5
1.0
MR effect size for
'Gut microbiota abundance (genus Parasutterella id.2892)' on 'Low molecular weight phosphotyrosine protein phosphatase'

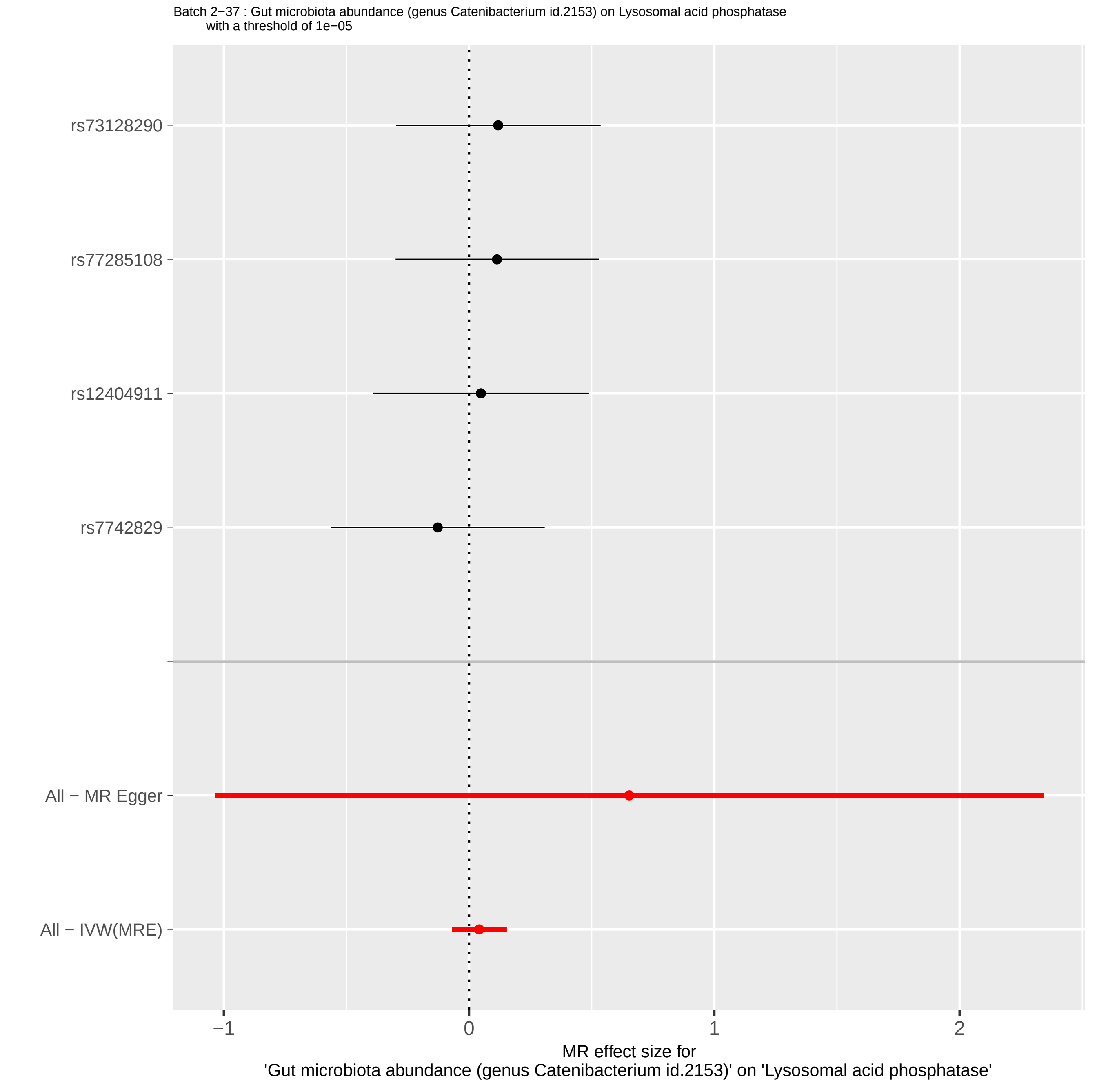

Batch 2−37 : Gut microbiota abundance (genus Catenibacterium id.2153) on Lysosomal acid phosphatase
with a threshold of 1e−05
rs73128290
rs77285108
rs12404911
rs7742829
All − MR Egger
All − IVW(MRE)
−1
0
1
2
MR effect size for
'Gut microbiota abundance (genus Catenibacterium id.2153)' on 'Lysosomal acid phosphatase'

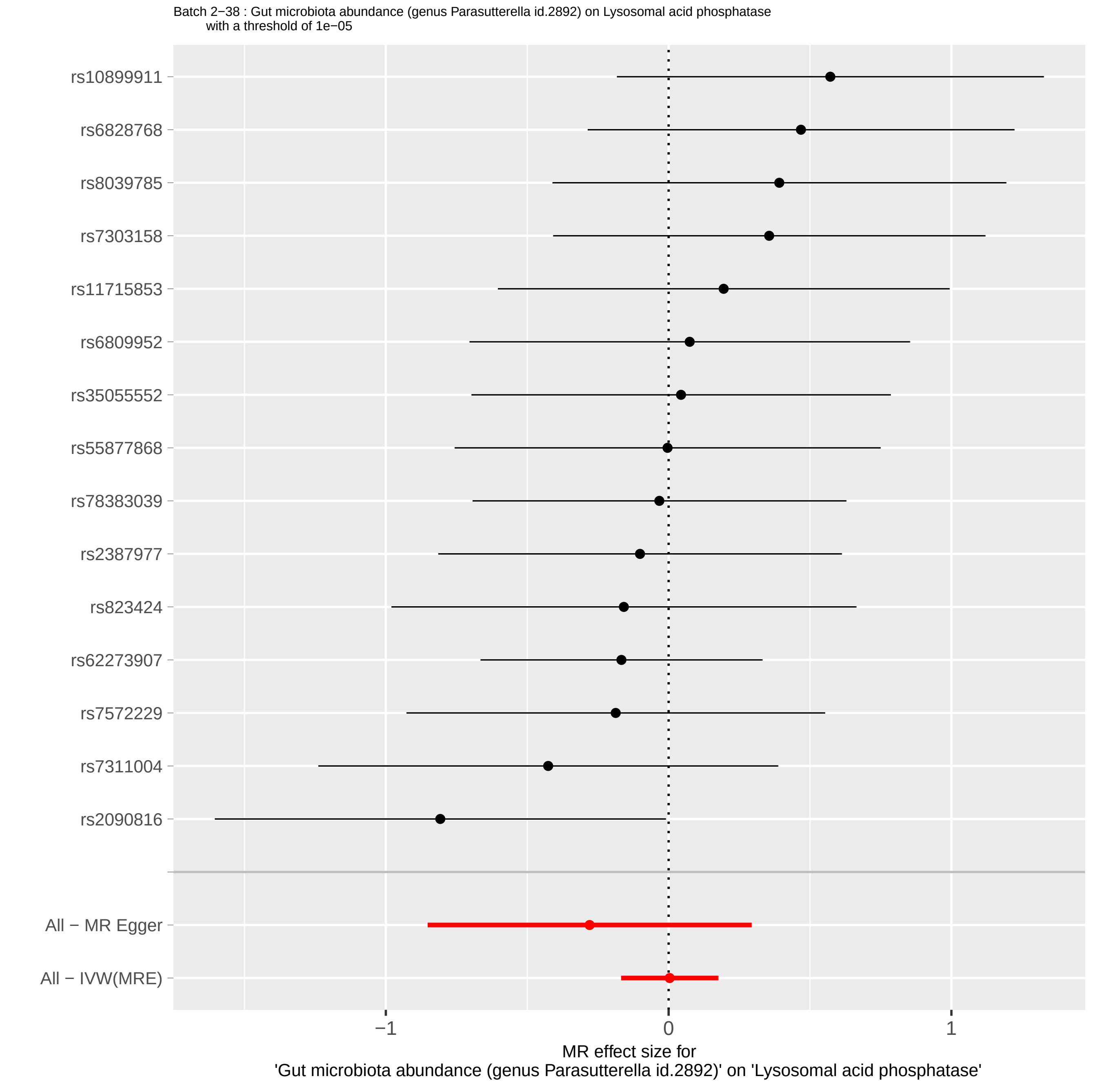

Batch 2−38 : Gut microbiota abundance (genus Parasutterella id.2892) on Lysosomal acid phosphatase
with a threshold of 1e−05
rs10899911
rs6828768
rs8039785
rs7303158
rs11715853
rs6809952
rs35055552
rs55877868
rs78383039
rs2387977
rs823424
rs62273907
rs7572229
rs7311004
rs2090816
All − MR Egger
All − IVW(MRE)
−1
0
1
MR effect size for
'Gut microbiota abundance (genus Parasutterella id.2892)' on 'Lysosomal acid phosphatase'

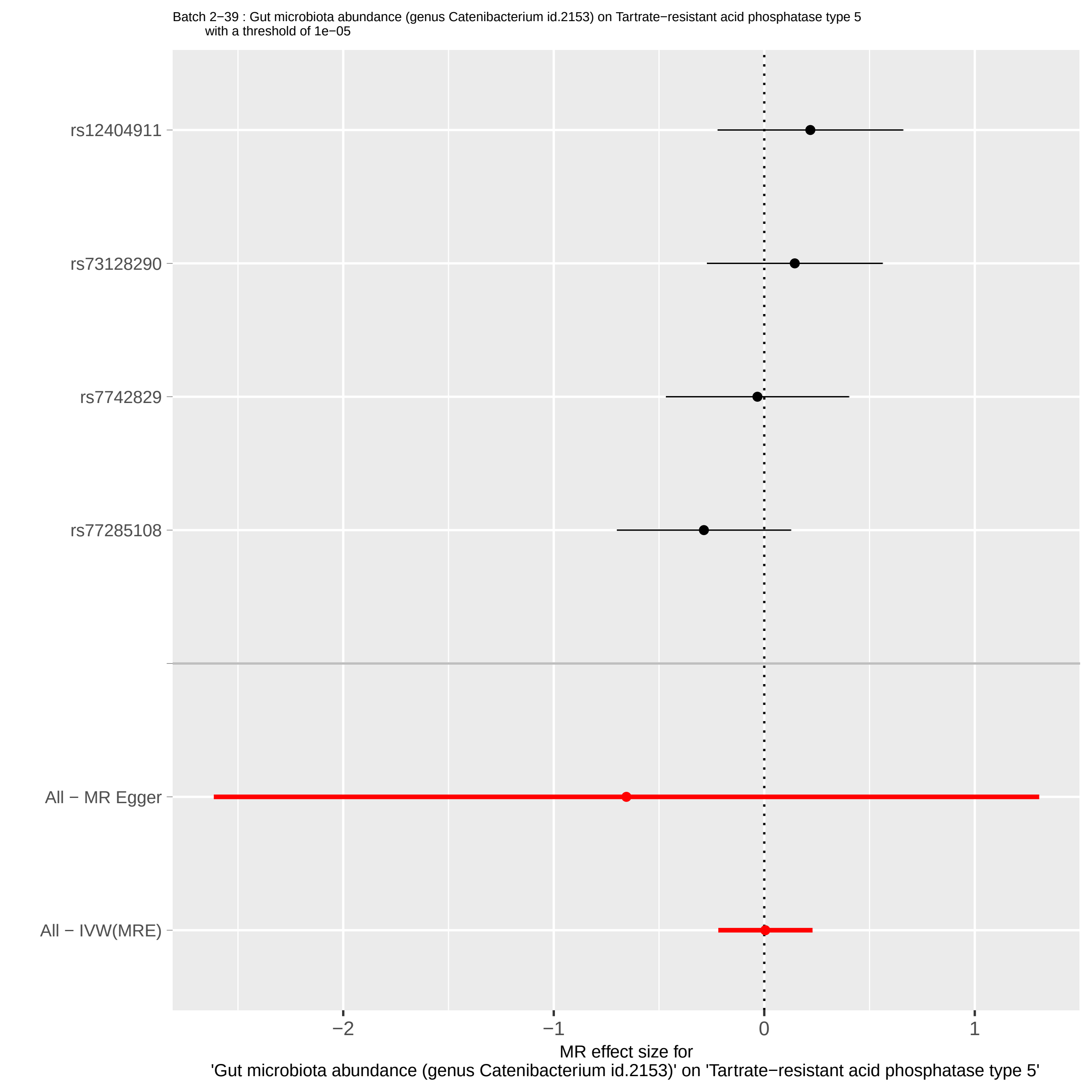

Batch 2−39 : Gut microbiota abundance (genus Catenibacterium id.2153) on Tartrate−resistant acid phosphatase type 5
with a threshold of 1e−05
rs12404911
rs73128290
rs7742829
rs77285108
All − MR Egger
All − IVW(MRE)
−2
−1
0
1
MR effect size for
'Gut microbiota abundance (genus Catenibacterium id.2153)' on 'Tartrate−resistant acid phosphatase type 5'

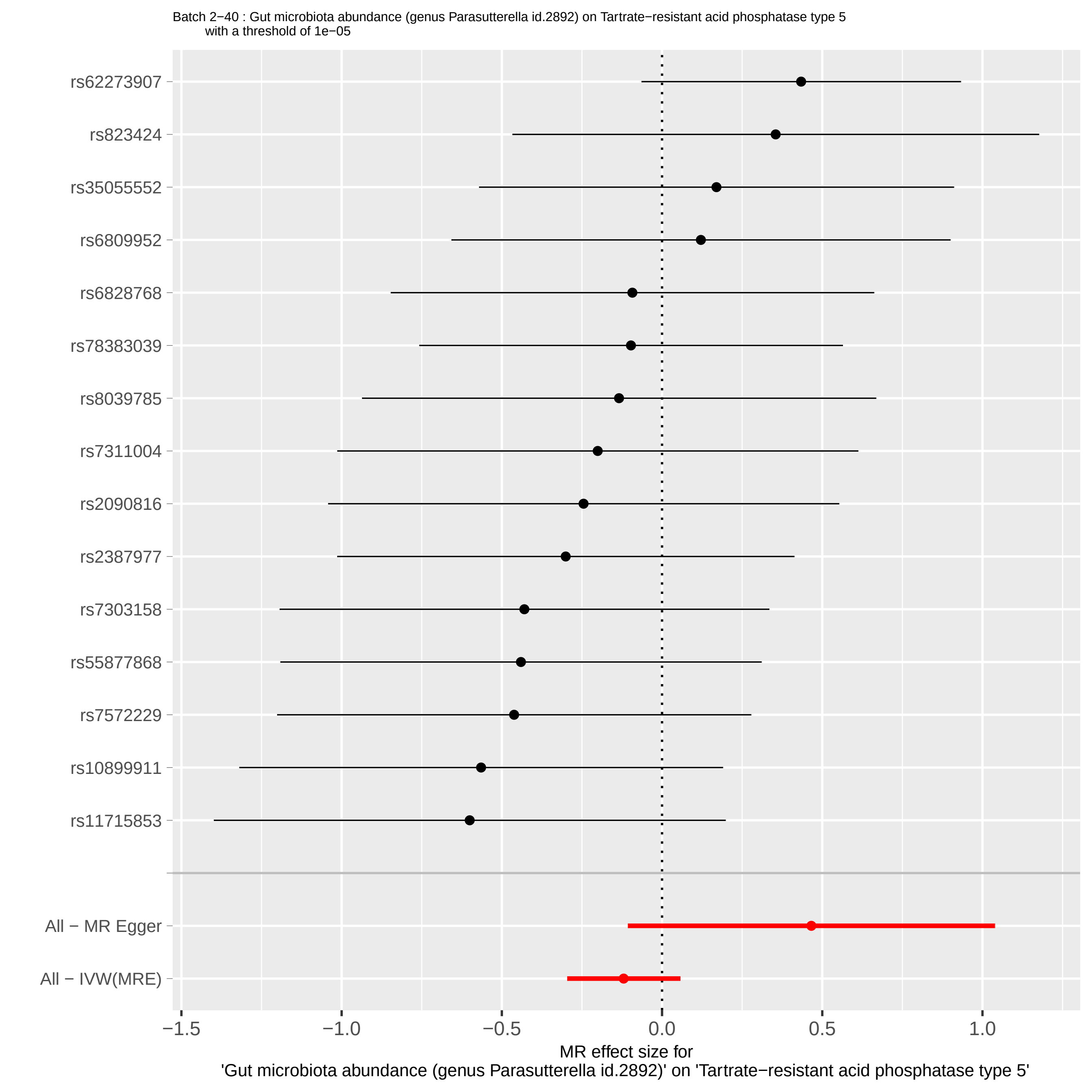
Batch 2−40 : Gut microbiota abundance (genus Parasutterella id.2892) on Tartrate−resistant acid phosphatase type 5
with a threshold of 1e−05
rs62273907
rs823424
rs35055552
rs6809952
rs6828768
rs78383039
rs8039785
rs7311004
rs2090816
rs2387977
rs7303158
rs55877868
rs7572229
rs10899911
rs11715853
All − MR Egger
All − IVW(MRE)
−1.5
−1.0
−0.5
0.0
0.5
1.0
MR effect size for
'Gut microbiota abundance (genus Parasutterella id.2892)' on 'Tartrate−resistant acid phosphatase type 5'

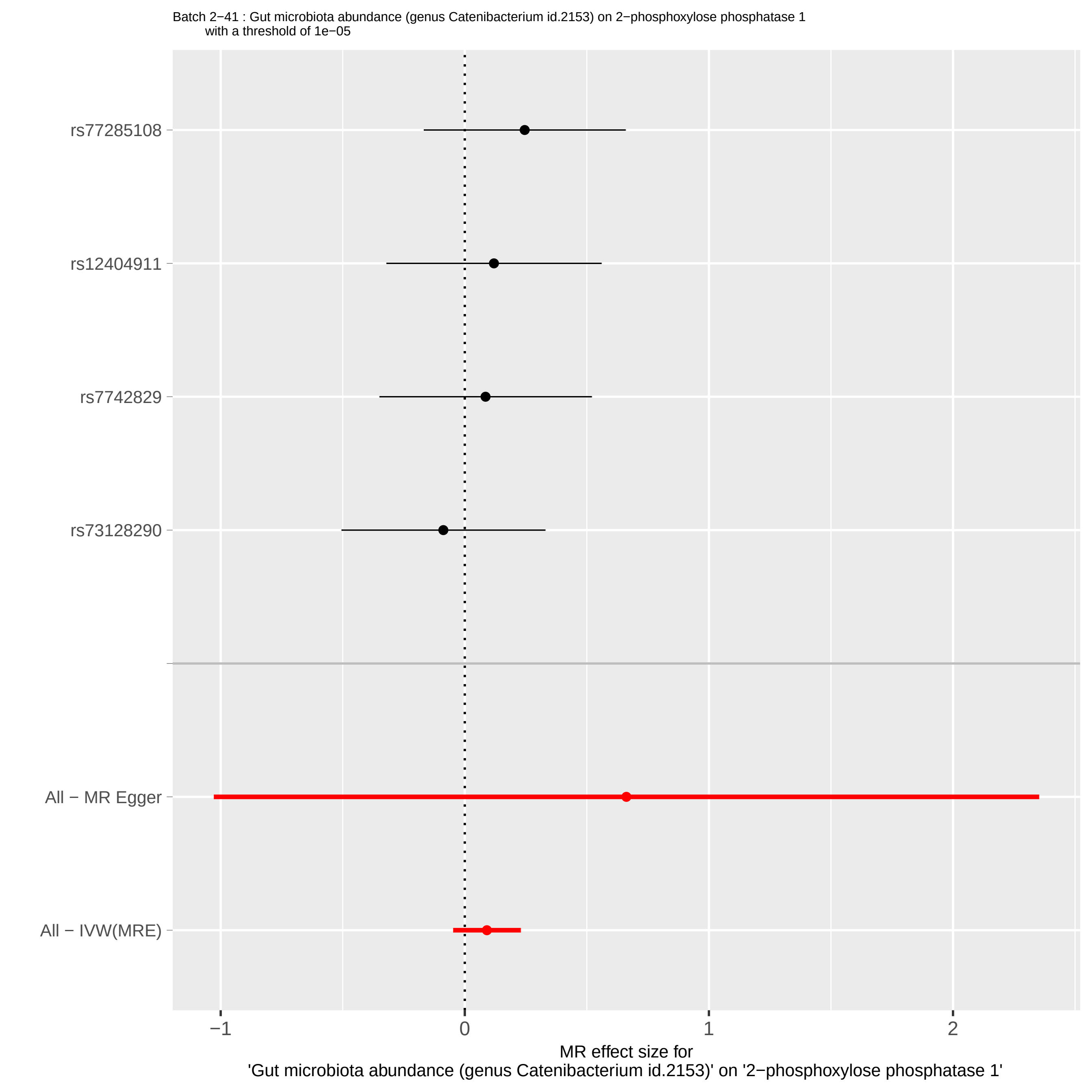
Batch 2−41 : Gut microbiota abundance (genus Catenibacterium id.2153) on 2−phosphoxylose phosphatase 1
with a threshold of 1e−05
rs77285108
rs12404911
rs7742829
rs73128290
All − MR Egger
All − IVW(MRE)
−1
0
1
2
MR effect size for
'Gut microbiota abundance (genus Catenibacterium id.2153)' on '2−phosphoxylose phosphatase 1'

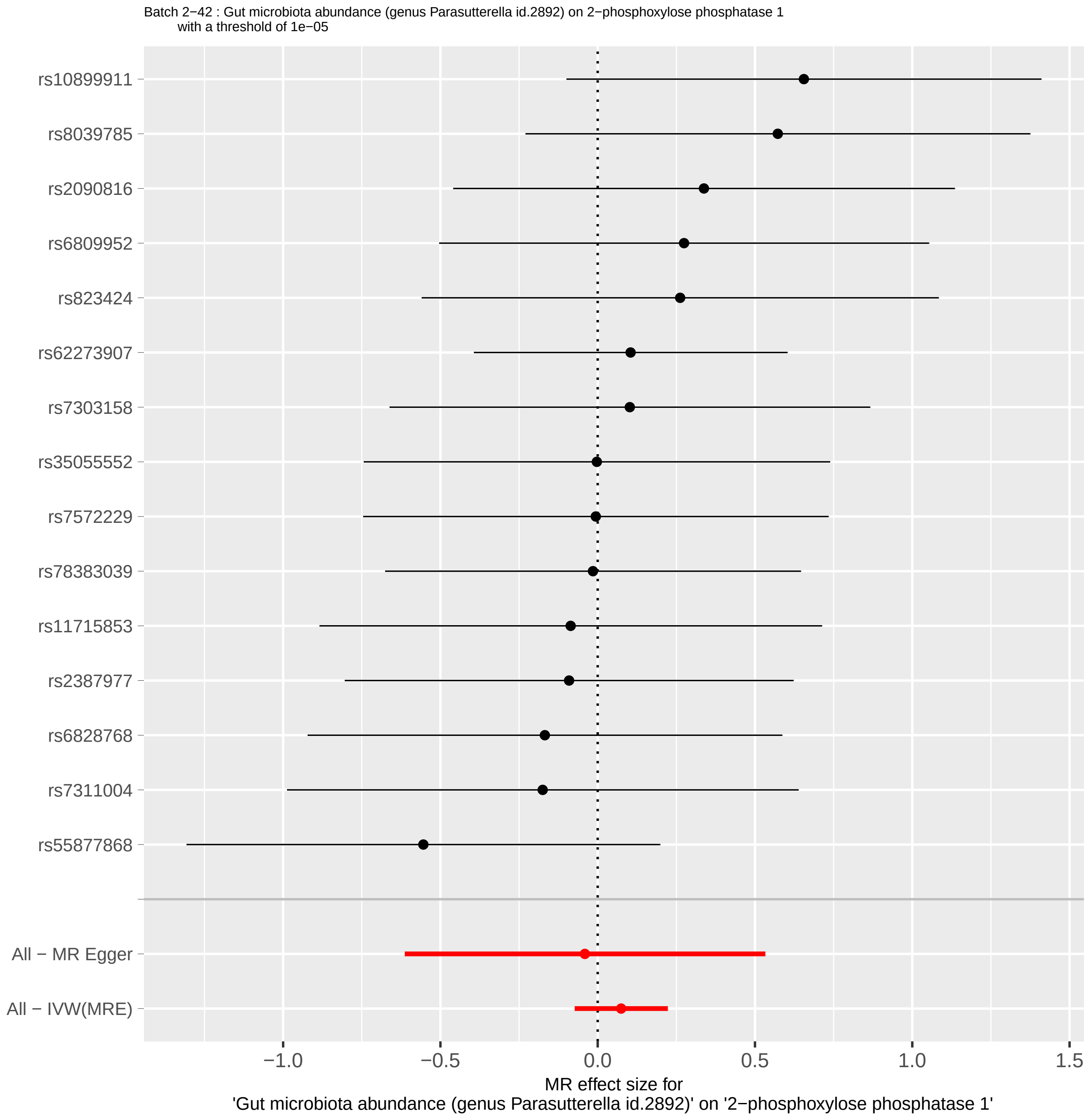

Batch 2−42 : Gut microbiota abundance (genus Parasutterella id.2892) on 2−phosphoxylose phosphatase 1
with a threshold of 1e−05
rs10899911
rs8039785
rs2090816
rs6809952
rs823424
rs62273907
rs7303158
rs35055552
rs7572229
rs78383039
rs11715853
rs2387977
rs6828768
rs7311004
rs55877868
All − MR Egger
All − IVW(MRE)
−1.0
−0.5
0.0
0.5
1.0
1.5
MR effect size for
'Gut microbiota abundance (genus Parasutterella id.2892)' on '2−phosphoxylose phosphatase 1'

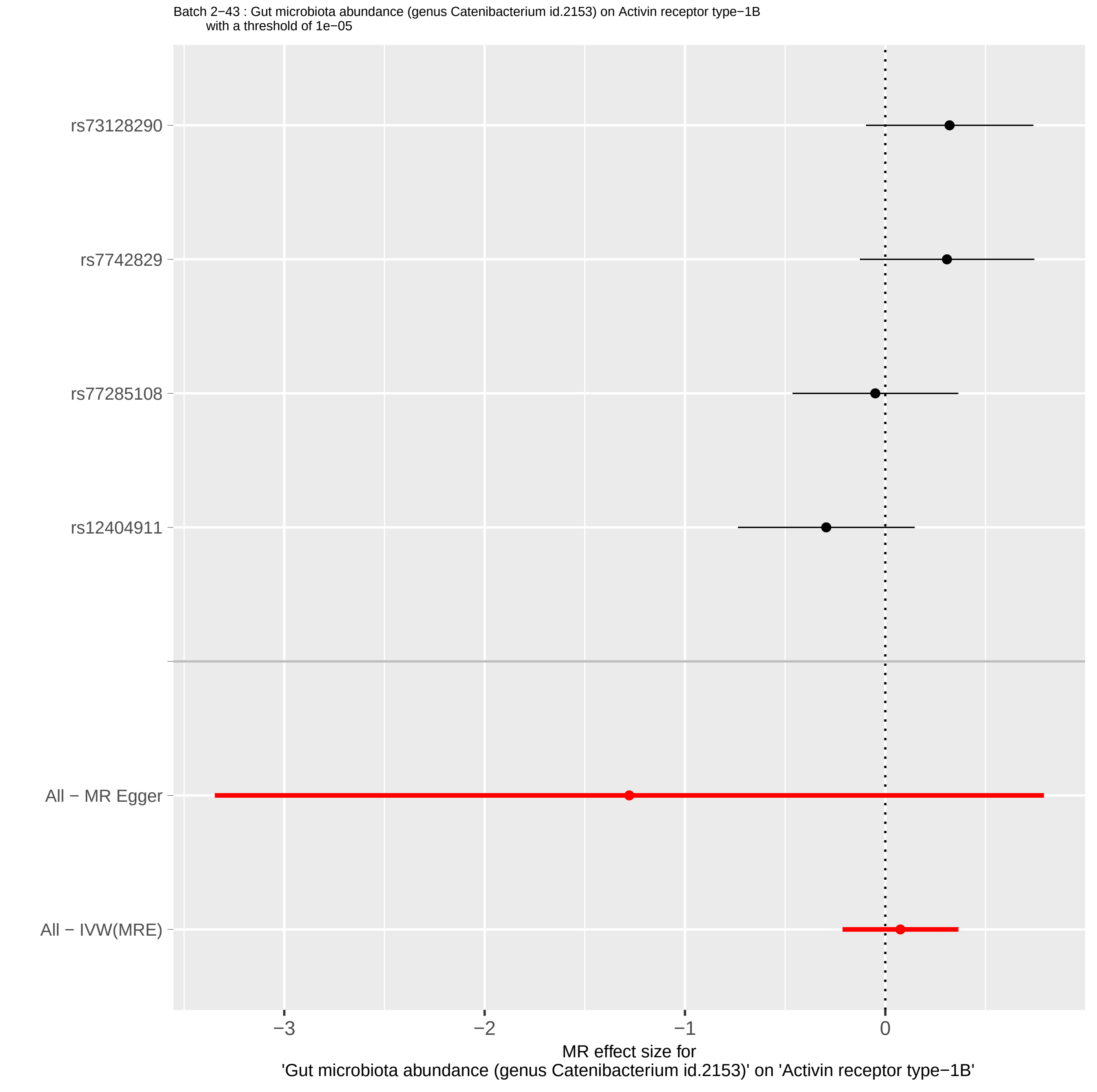
Batch 2−43 : Gut microbiota abundance (genus Catenibacterium id.2153) on Activin receptor type−1B
with a threshold of 1e−05
rs73128290
rs7742829
rs77285108
rs12404911
All − MR Egger
All − IVW(MRE)
−3
−2
−1
0
MR effect size for
'Gut microbiota abundance (genus Catenibacterium id.2153)' on 'Activin receptor type−1B'

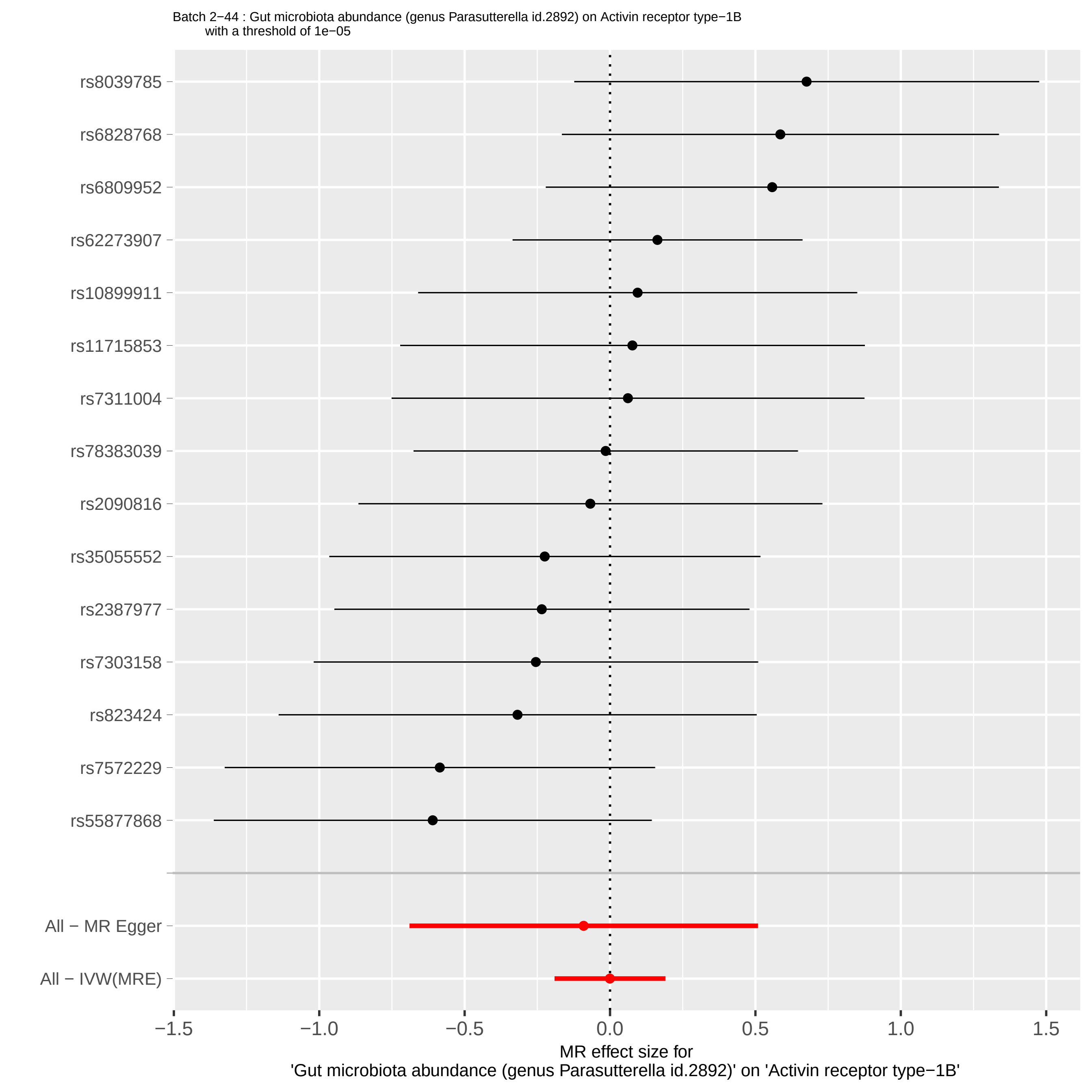

Batch 2−44 : Gut microbiota abundance (genus Parasutterella id.2892) on Activin receptor type−1B
with a threshold of 1e−05
rs8039785
rs6828768
rs6809952
rs62273907
rs10899911
rs11715853
rs7311004
rs78383039
rs2090816
rs35055552
rs2387977
rs7303158
rs823424
rs7572229
rs55877868
All − MR Egger
All − IVW(MRE)
−1.5
−1.0
−0.5
0.0
0.5
1.0
1.5
MR effect size for
'Gut microbiota abundance (genus Parasutterella id.2892)' on 'Activin receptor type−1B'

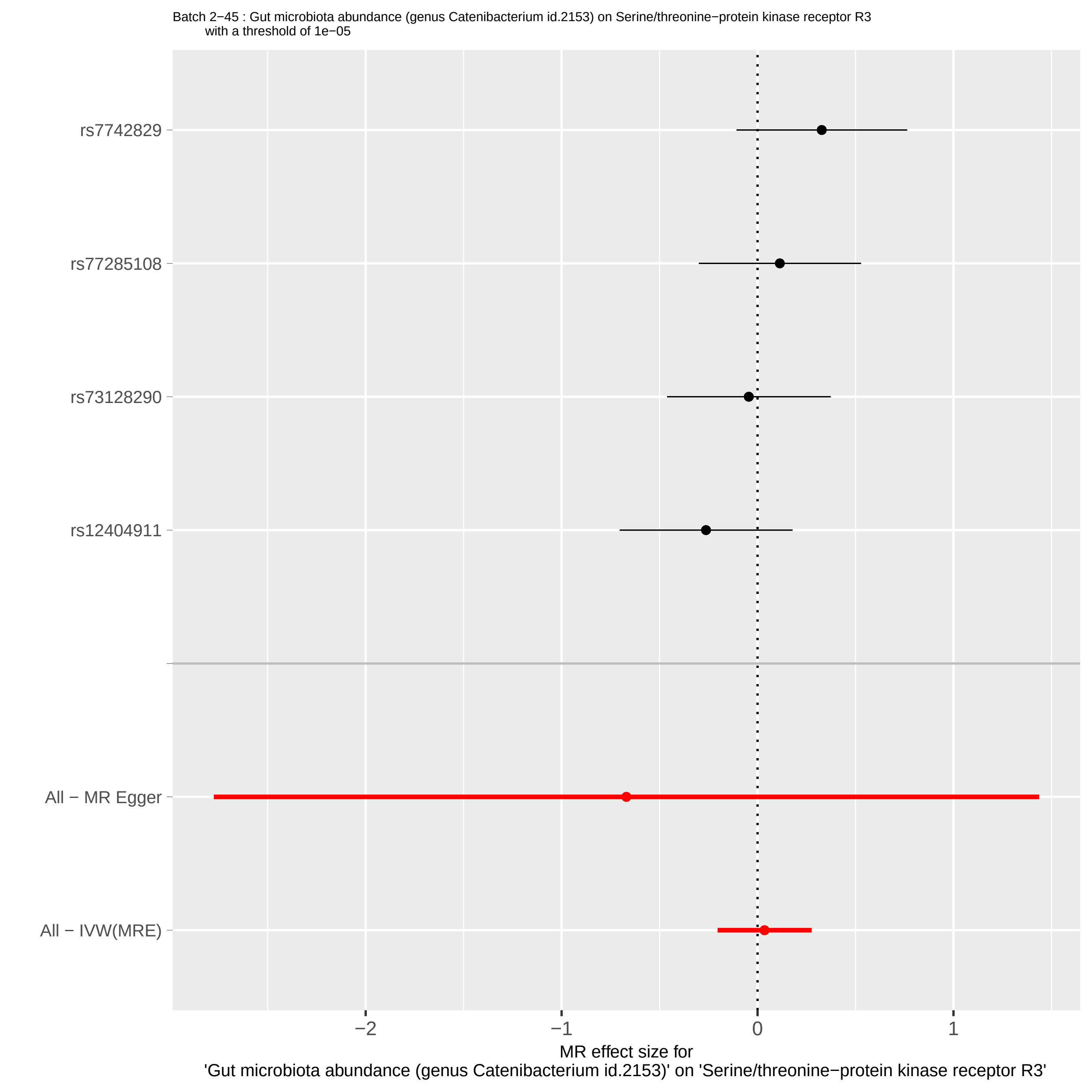
Batch 2−45 : Gut microbiota abundance (genus Catenibacterium id.2153) on Serine/threonine−protein kinase receptor R3
with a threshold of 1e−05
rs7742829
rs77285108
rs73128290
rs12404911
All − MR Egger
All − IVW(MRE)
−2
−1
0
1
MR effect size for
'Gut microbiota abundance (genus Catenibacterium id.2153)' on 'Serine/threonine−protein kinase receptor R3'

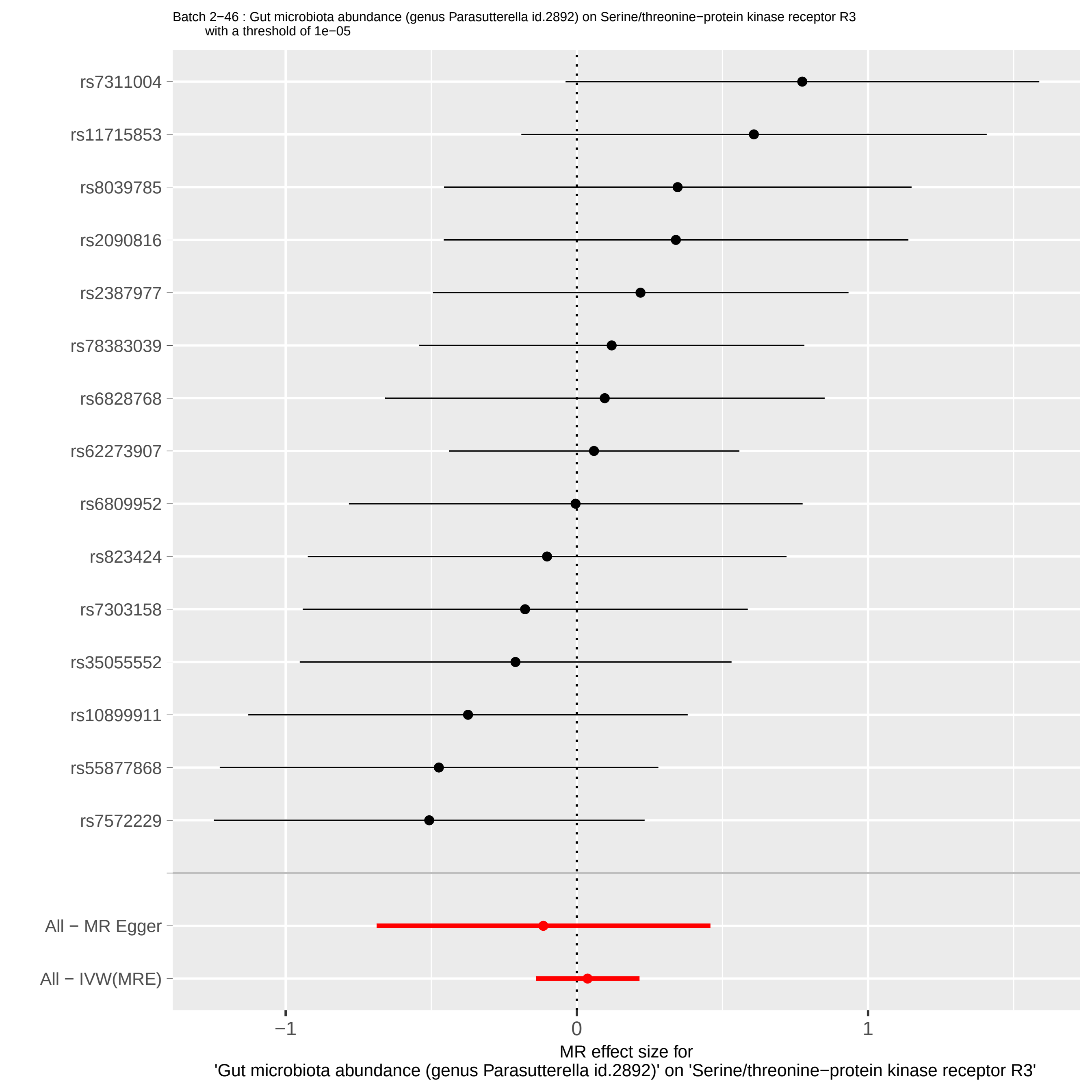

Batch 2−46 : Gut microbiota abundance (genus Parasutterella id.2892) on Serine/threonine−protein kinase receptor R3
with a threshold of 1e−05
rs7311004
rs11715853
rs8039785
rs2090816
rs2387977
rs78383039
rs6828768
rs62273907
rs6809952
rs823424
rs7303158
rs35055552
rs10899911
rs55877868
rs7572229
All − MR Egger
All − IVW(MRE)
−1
0
1
MR effect size for
'Gut microbiota abundance (genus Parasutterella id.2892)' on 'Serine/threonine−protein kinase receptor R3'

Batch 2−47 : Gut microbiota abundance (genus Catenibacterium id.2153) on Acylphosphatase−2
with a threshold of 1e−05

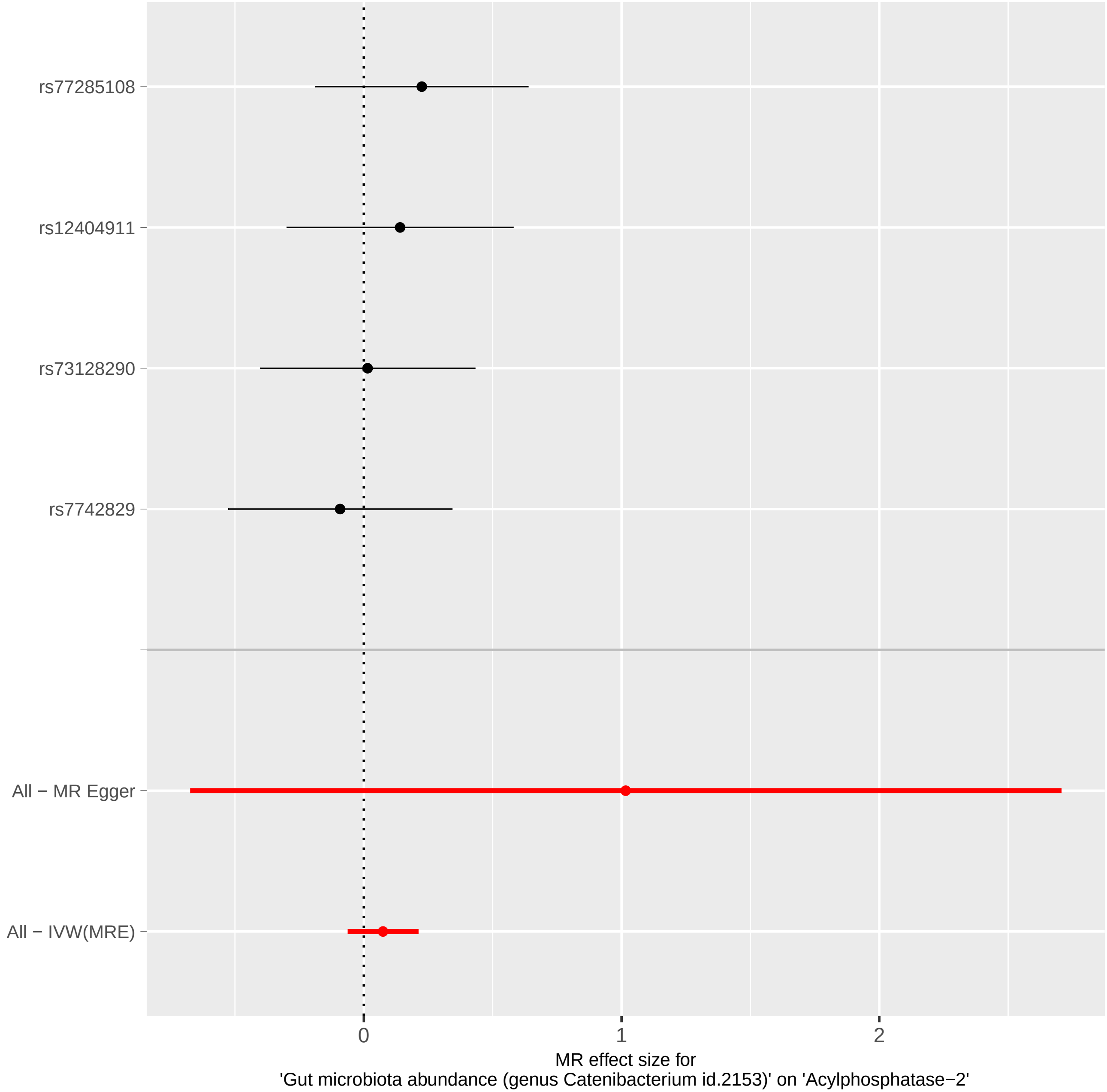

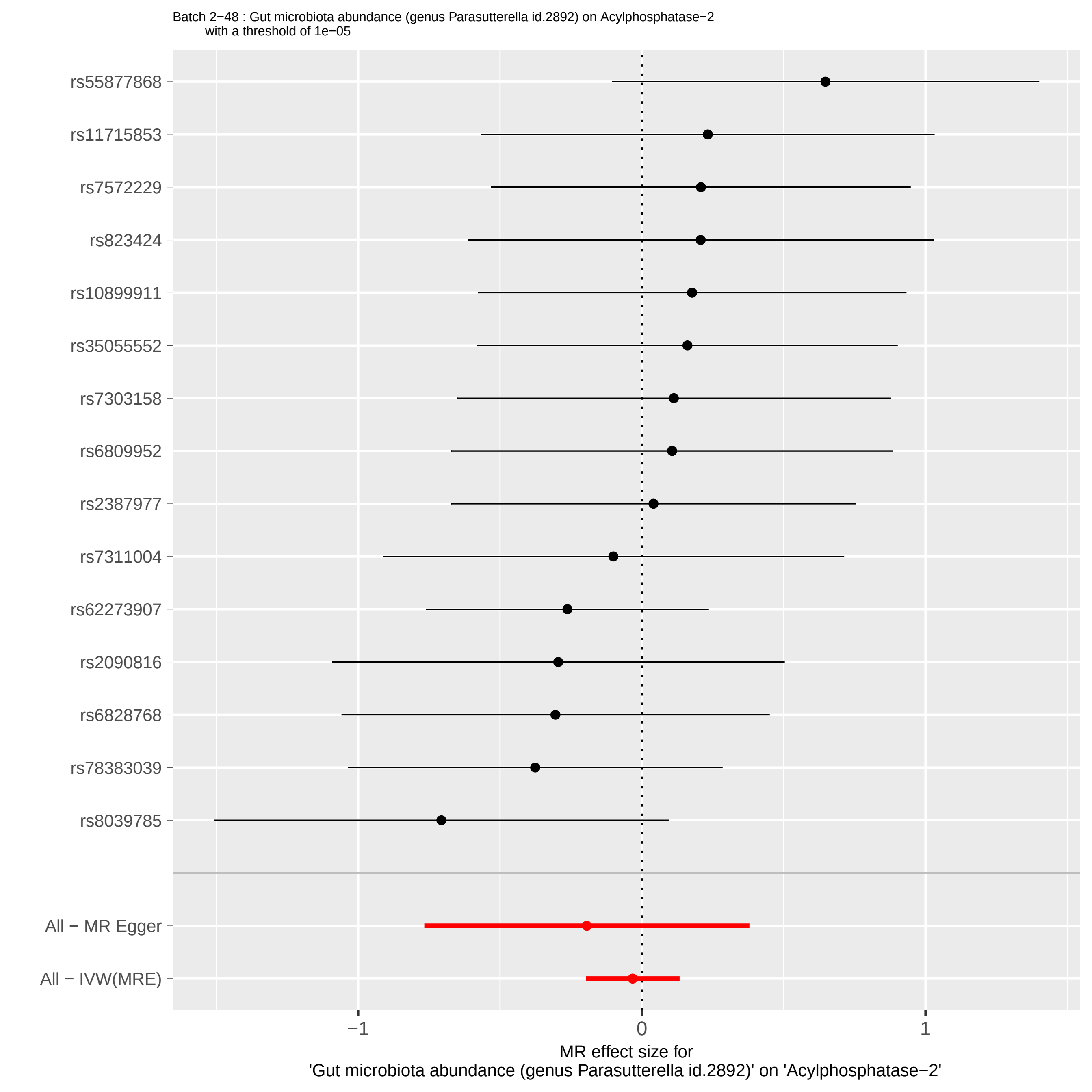
Batch 2−48 : Gut microbiota abundance (genus Parasutterella id.2892) on Acylphosphatase−2
with a threshold of 1e−05
rs55877868
rs11715853
rs7572229
rs823424
rs10899911
rs35055552
rs7303158
rs6809952
rs2387977
rs7311004
rs62273907
rs2090816
rs6828768
rs78383039
rs8039785
All − MR Egger
All − IVW(MRE)
−1
0
1
MR effect size for
'Gut microbiota abundance (genus Parasutterella id.2892)' on 'Acylphosphatase−2'

Batch 2−49 : Gut microbiota abundance (genus Catenibacterium id.2153) on Disintegrin and metalloproteinase domain−containing protein 11
with a threshold of 1e−05

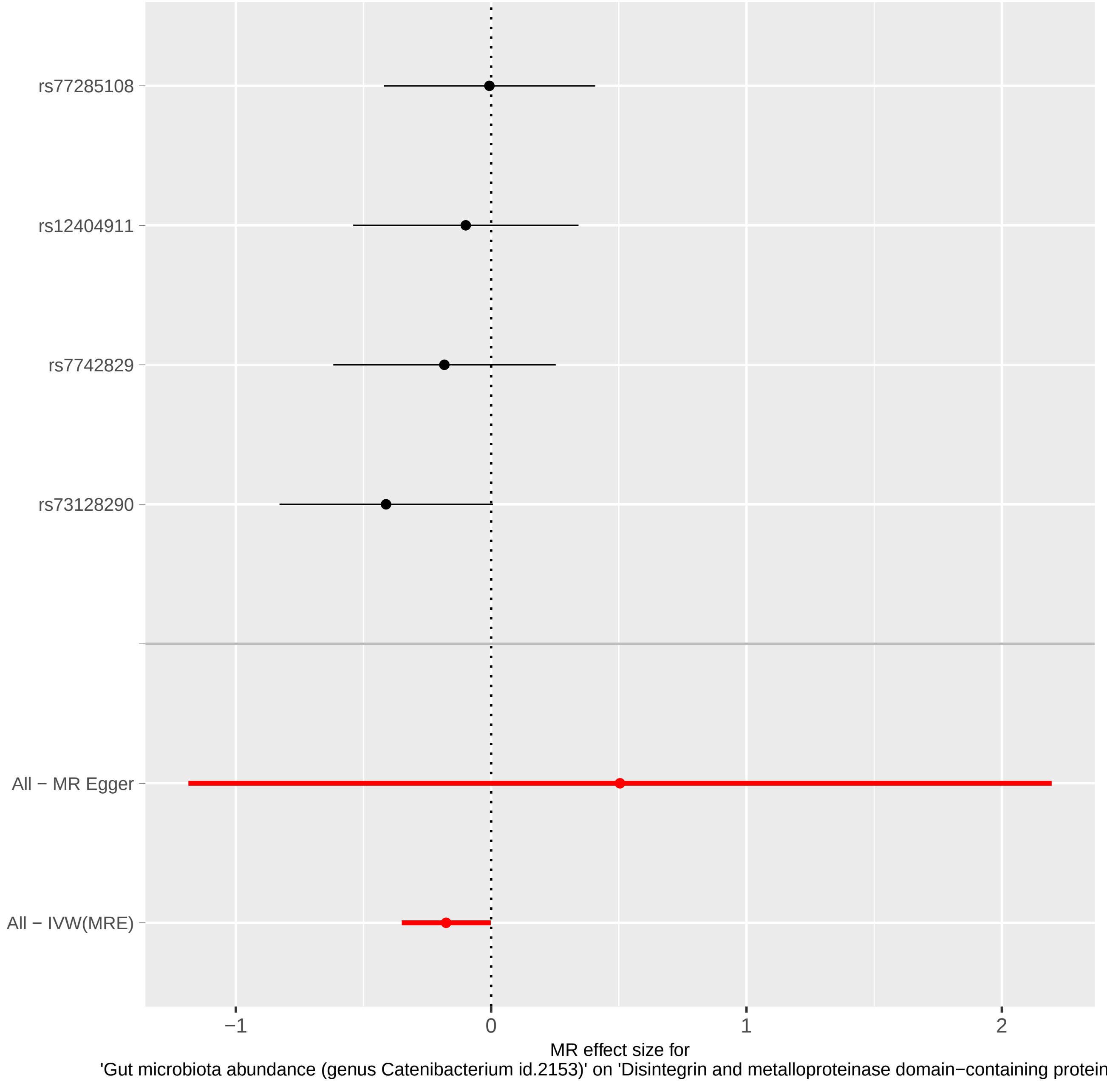

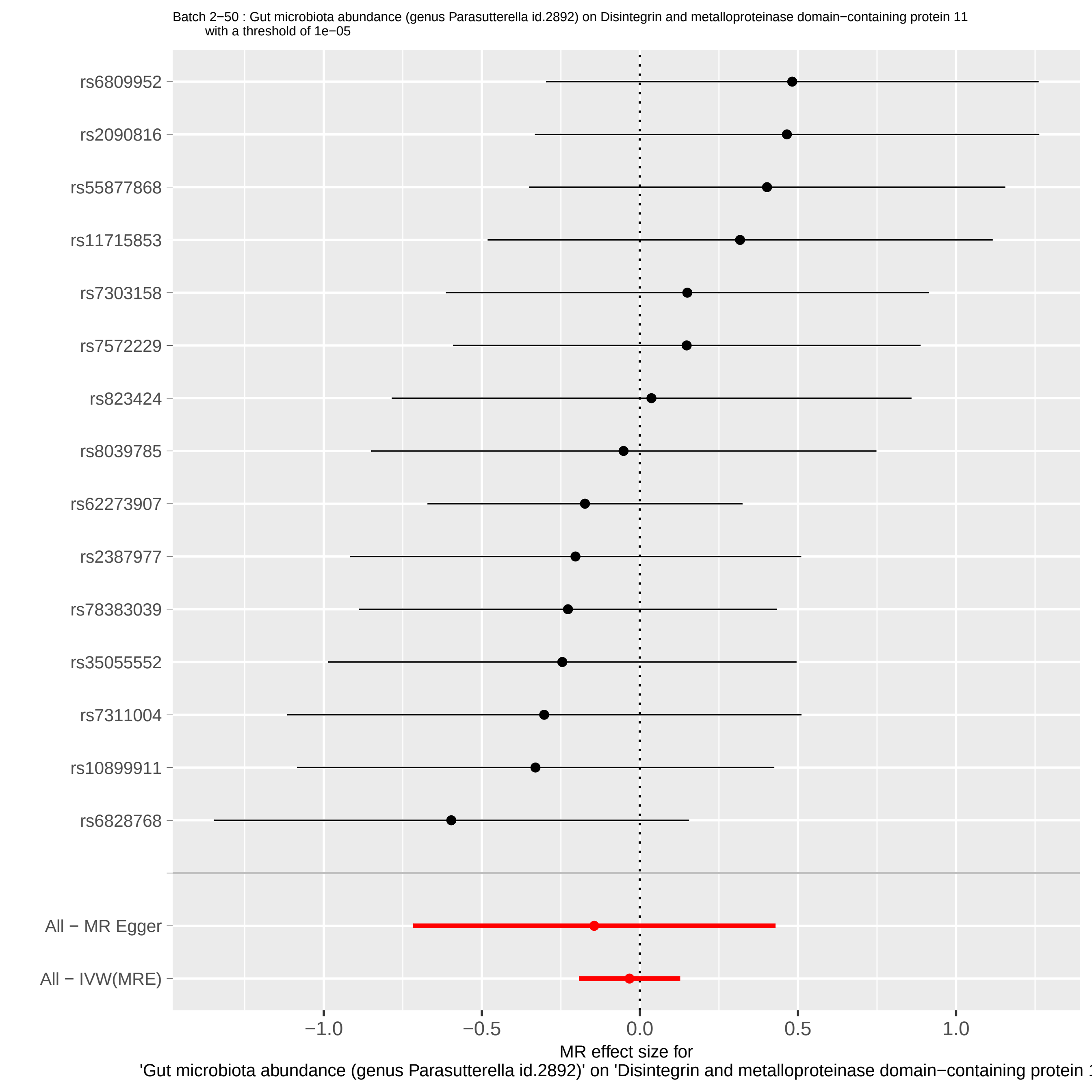

Batch 2−50 : Gut microbiota abundance (genus Parasutterella id.2892) on Disintegrin and metalloproteinase domain−containing protein 11
with a threshold of 1e−05
rs6809952
rs2090816
rs55877868
rs11715853
rs7303158
rs7572229
rs823424
rs8039785
rs62273907
rs2387977
rs78383039
rs35055552
rs7311004
rs10899911
rs6828768
All − MR Egger
All − IVW(MRE)
−1.0
−0.5
0.0
0.5
1.0
MR effect size for
'Gut microbiota abundance (genus Parasutterella id.2892)' on 'Disintegrin and metalloproteinase domain−containing protein 1

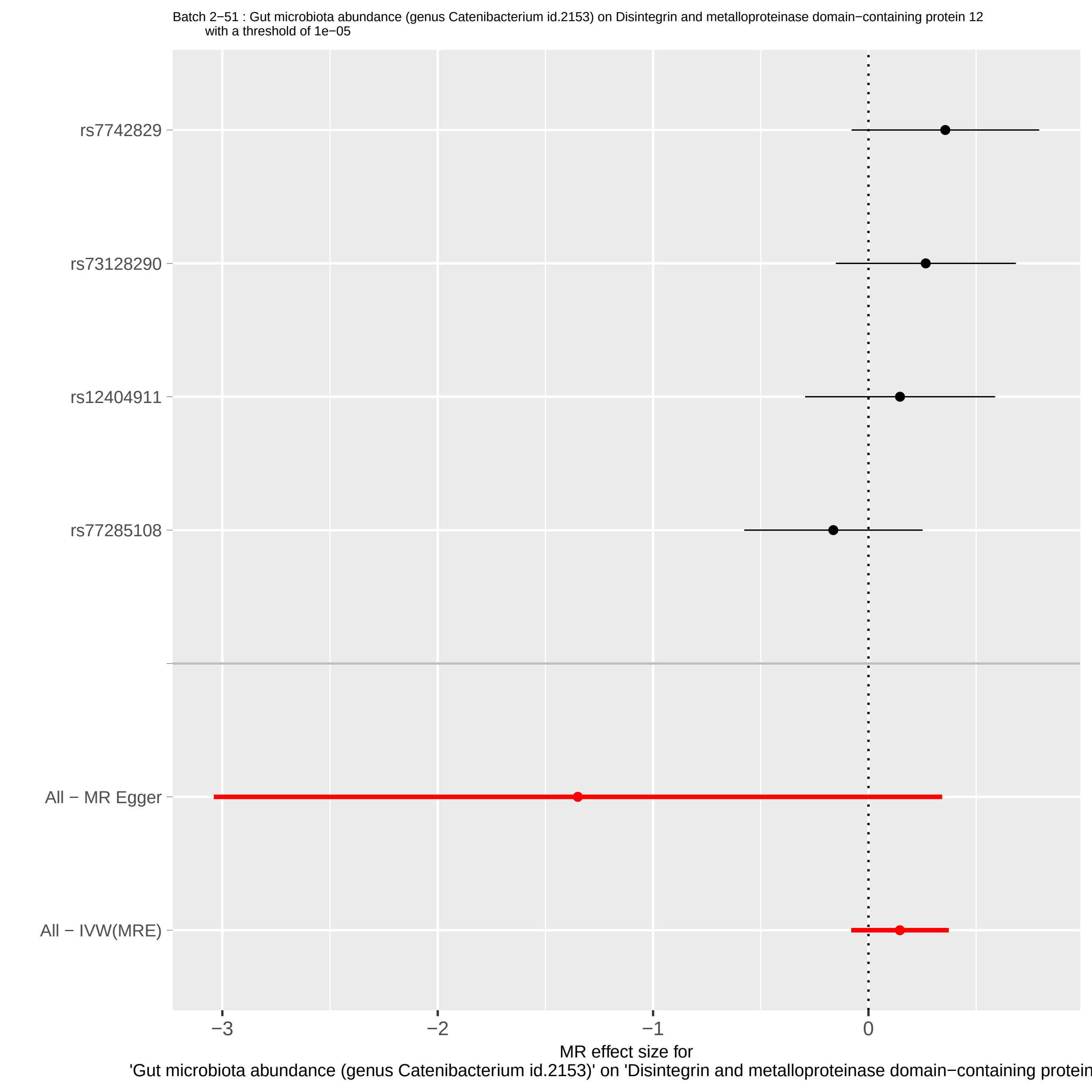
Batch 2−51 : Gut microbiota abundance (genus Catenibacterium id.2153) on Disintegrin and metalloproteinase domain−containing protein 12
with a threshold of 1e−05
rs7742829
rs73128290
rs12404911
rs77285108
All − MR Egger
All − IVW(MRE)
−3
−2
−1
0
MR effect size for
'Gut microbiota abundance (genus Catenibacterium id.2153)' on 'Disintegrin and metalloproteinase domain−containing protein

Batch 2−52 : Gut microbiota abundance (genus Parasutterella id.2892) on Disintegrin and metalloproteinase domain−containing protein 12 with a threshold of 1e−05

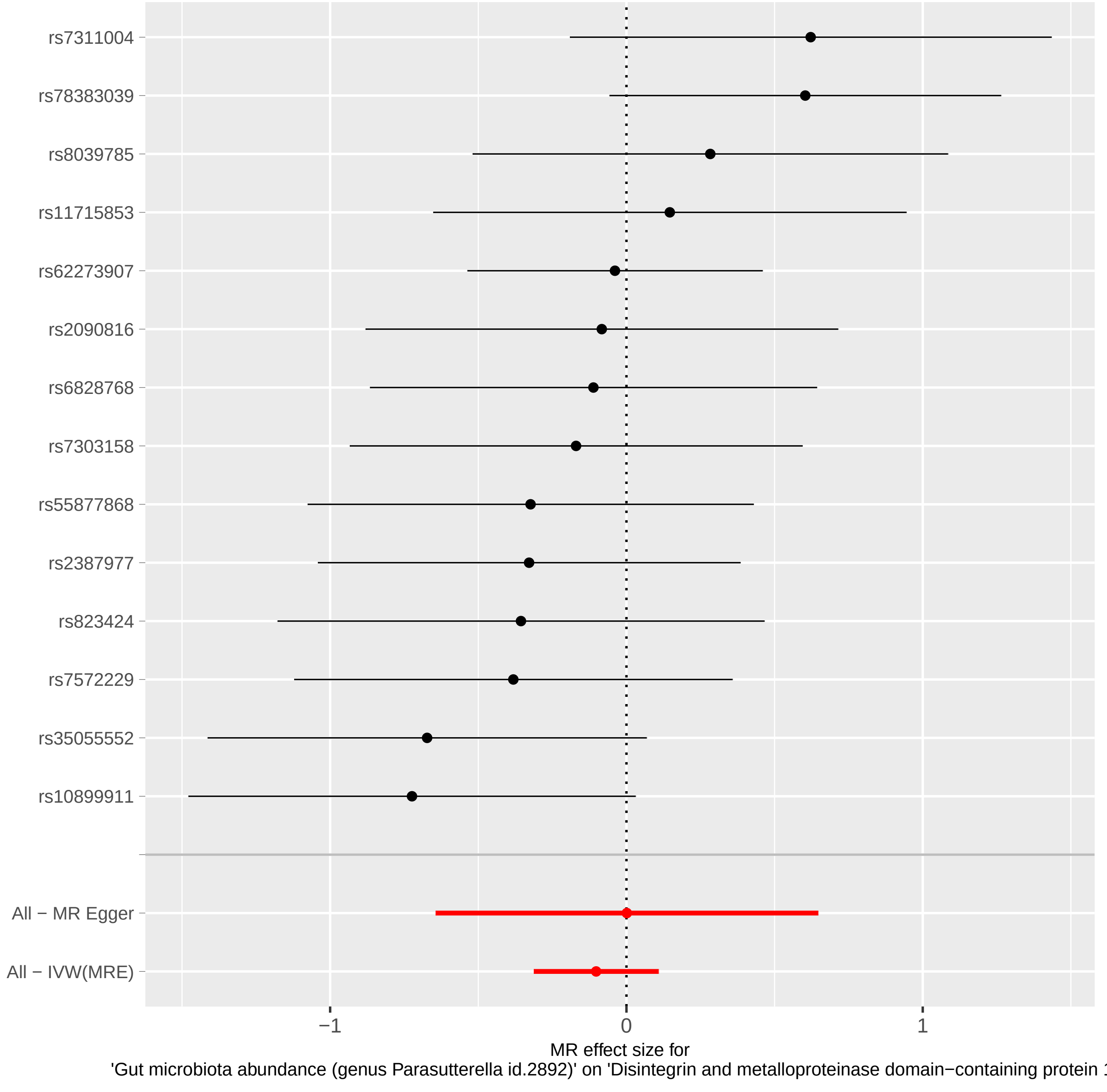

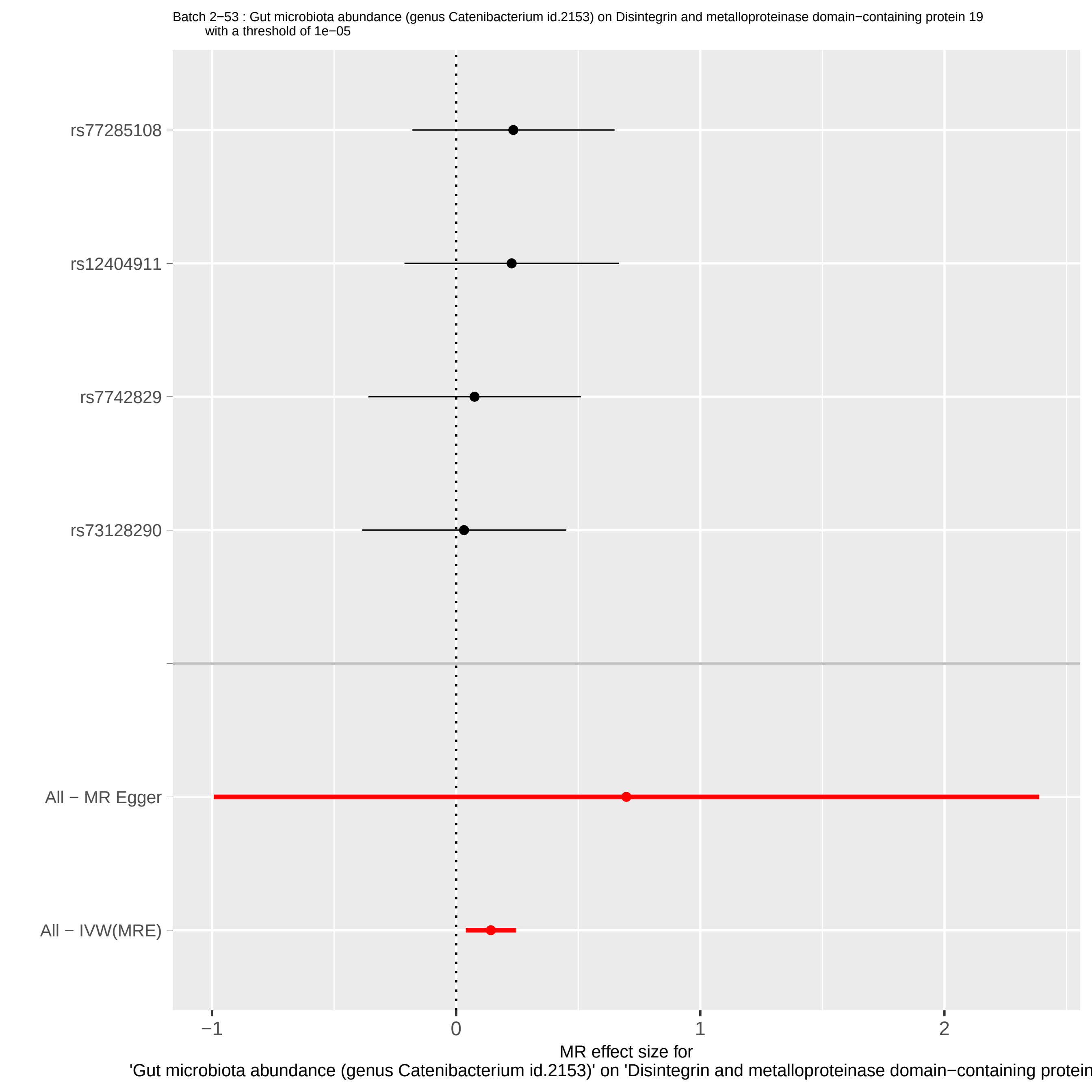

Batch 2−53 : Gut microbiota abundance (genus Catenibacterium id.2153) on Disintegrin and metalloproteinase domain−containing protein 19
with a threshold of 1e−05
rs77285108
rs12404911
rs7742829
rs73128290
All − MR Egger
All − IVW(MRE)
−1
0
1
2
MR effect size for
'Gut microbiota abundance (genus Catenibacterium id.2153)' on 'Disintegrin and metalloproteinase domain−containing protein

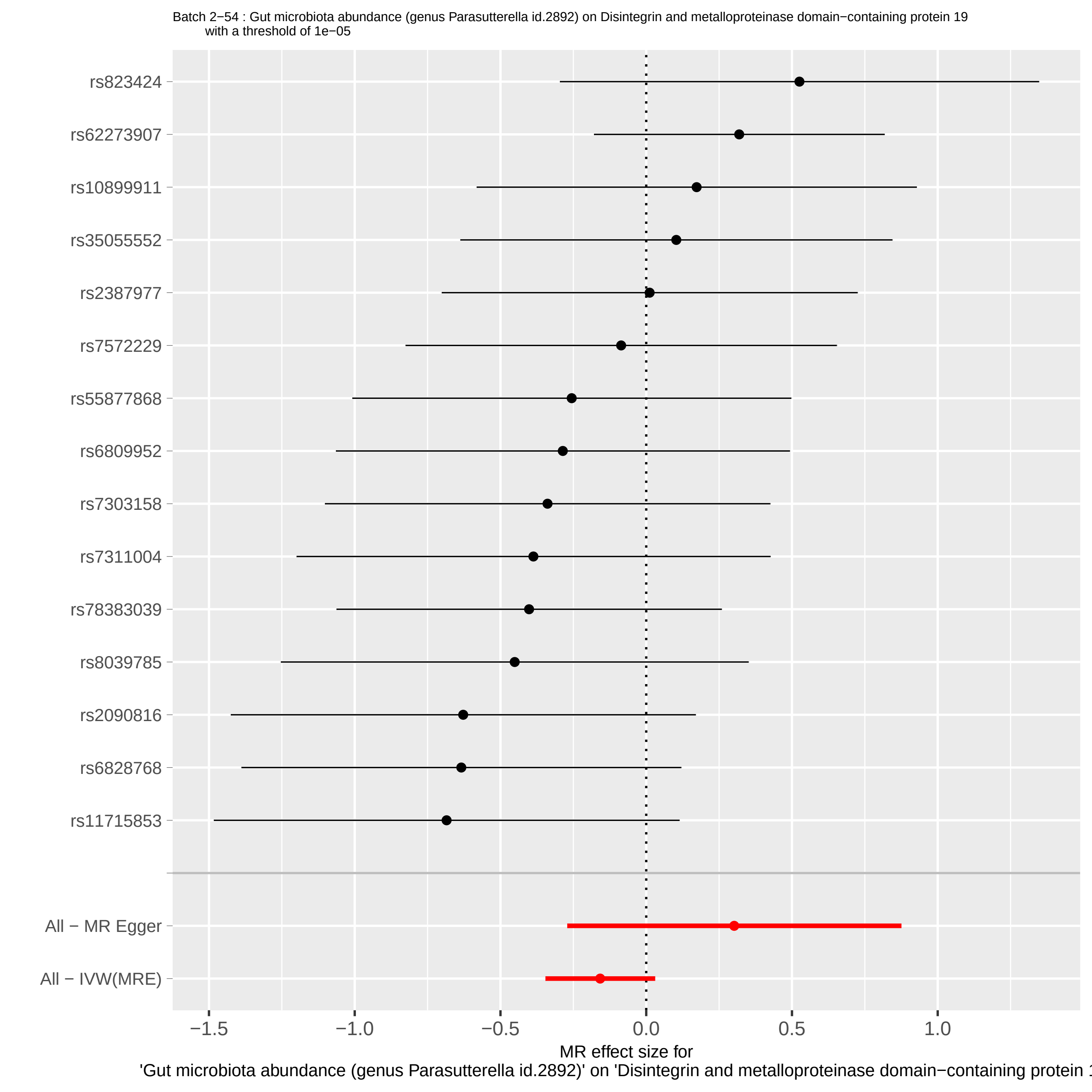
Batch 2−54 : Gut microbiota abundance (genus Parasutterella id.2892) on Disintegrin and metalloproteinase domain−containing protein 19
with a threshold of 1e−05
rs823424
rs62273907
rs10899911
rs35055552
rs2387977
rs7572229
rs55877868
rs6809952
rs7303158
rs7311004
rs78383039
rs8039785
rs2090816
rs6828768
rs11715853
All − MR Egger
All − IVW(MRE)
−1.5
−1.0
−0.5
0.0
0.5
1.0
MR effect size for
'Gut microbiota abundance (genus Parasutterella id.2892)' on 'Disintegrin and metalloproteinase domain−containing protein 1

Batch 2−55 : Gut microbiota abundance (genus Catenibacterium id.2153) on Disintegrin and metalloproteinase domain−containing protein 23 with a threshold of 1e−05

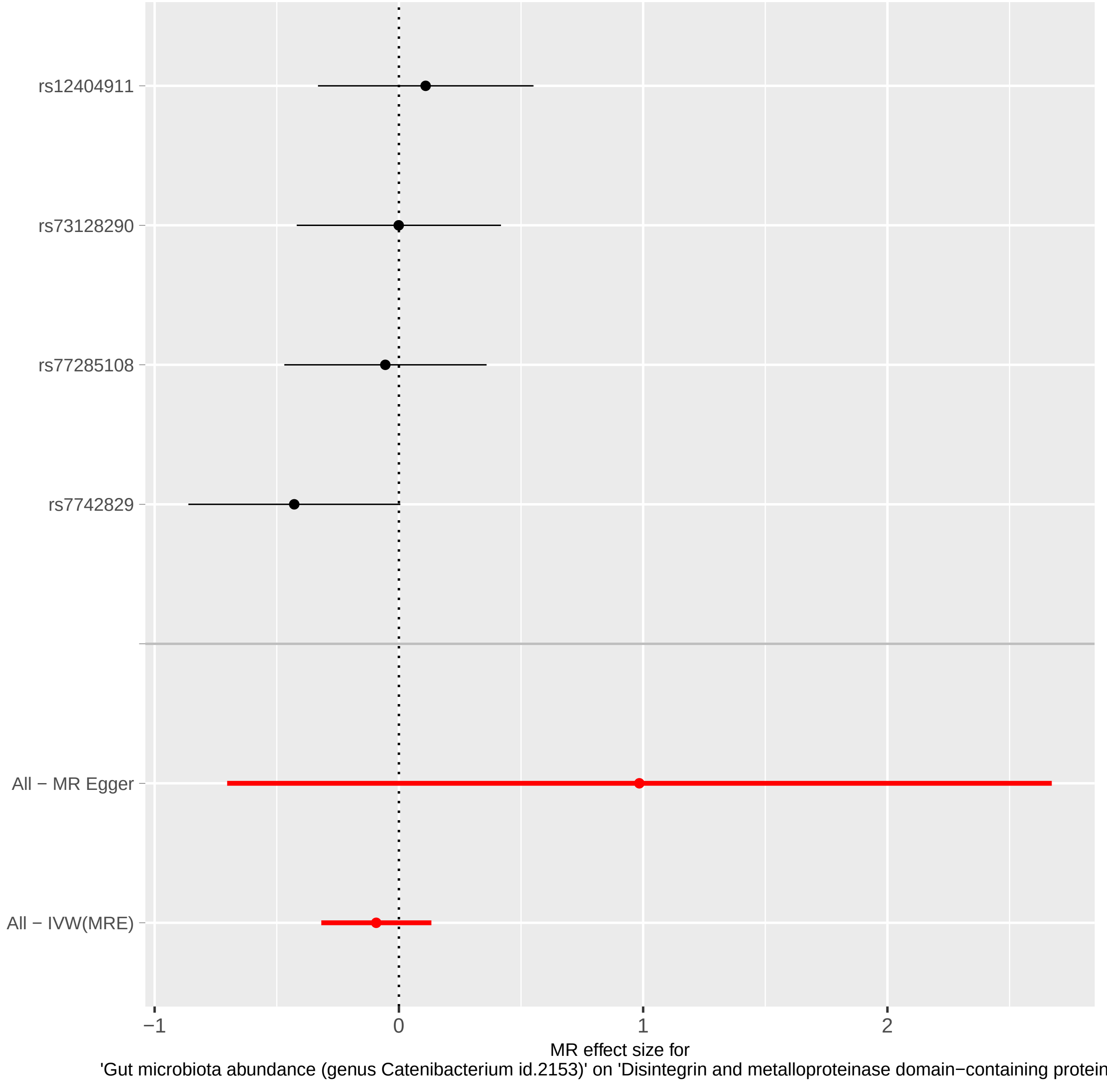

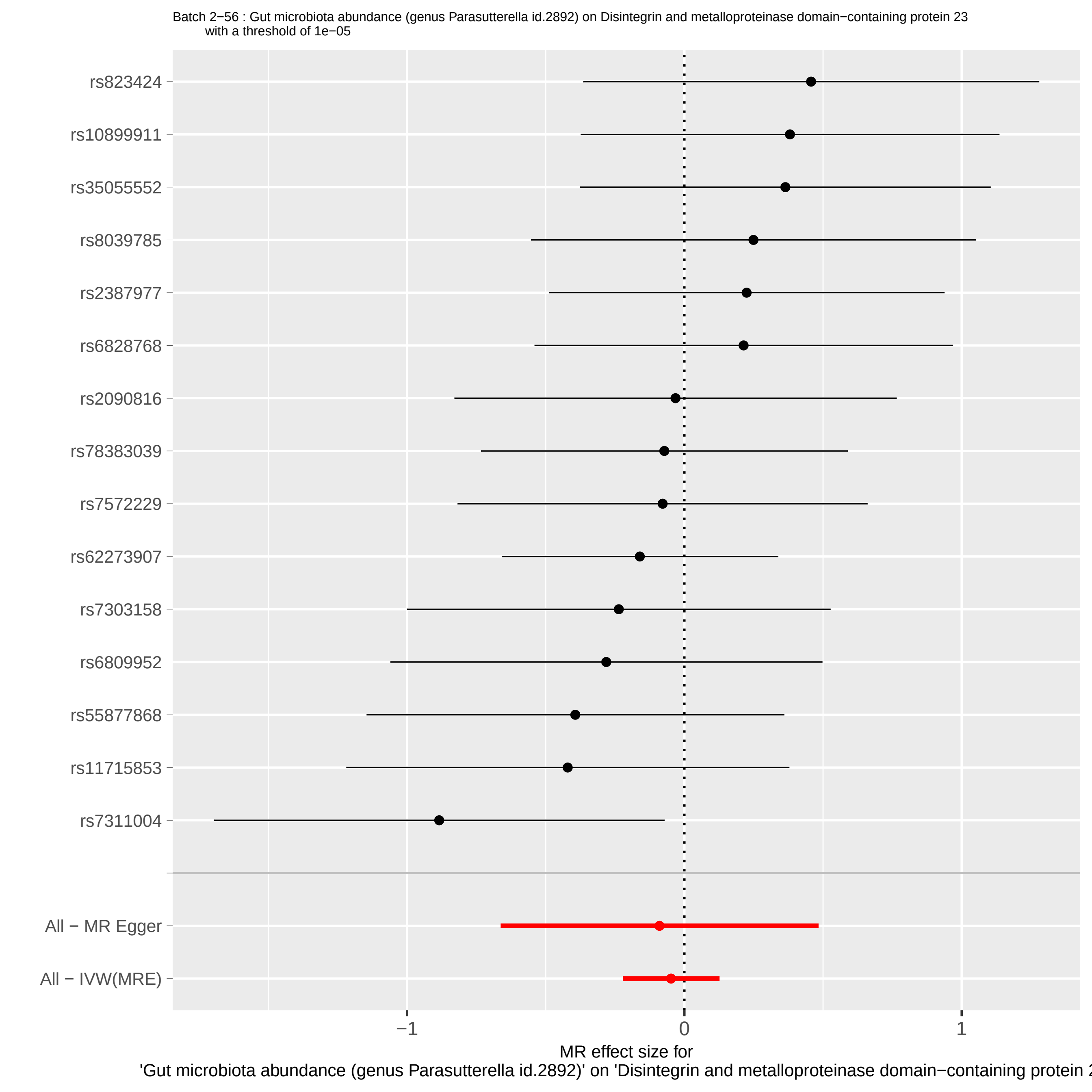

Batch 2−56 : Gut microbiota abundance (genus Parasutterella id.2892) on Disintegrin and metalloproteinase domain−containing protein 23
with a threshold of 1e−05
rs823424
rs10899911
rs35055552
rs8039785
rs2387977
rs6828768
rs2090816
rs78383039
rs7572229
rs62273907
rs7303158
rs6809952
rs55877868
rs11715853
rs7311004
All − MR Egger
All − IVW(MRE)
−1
0
1
MR effect size for
'Gut microbiota abundance (genus Parasutterella id.2892)' on 'Disintegrin and metalloproteinase domain−containing protein 2

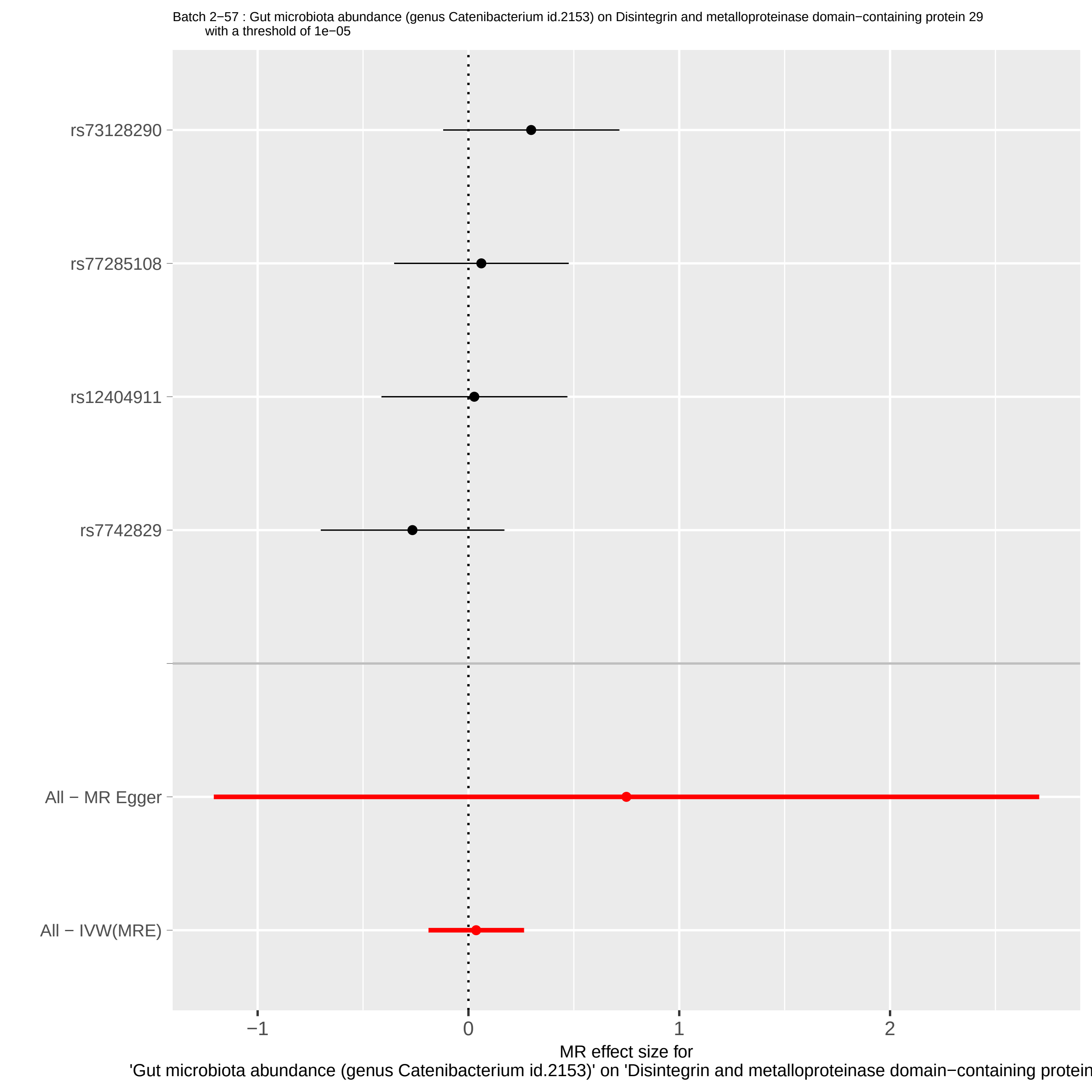
Batch 2−57 : Gut microbiota abundance (genus Catenibacterium id.2153) on Disintegrin and metalloproteinase domain−containing protein 29
with a threshold of 1e−05
rs73128290
rs77285108
rs12404911
rs7742829
All − MR Egger
All − IVW(MRE)
−1
0
1
2
MR effect size for
'Gut microbiota abundance (genus Catenibacterium id.2153)' on 'Disintegrin and metalloproteinase domain−containing protein

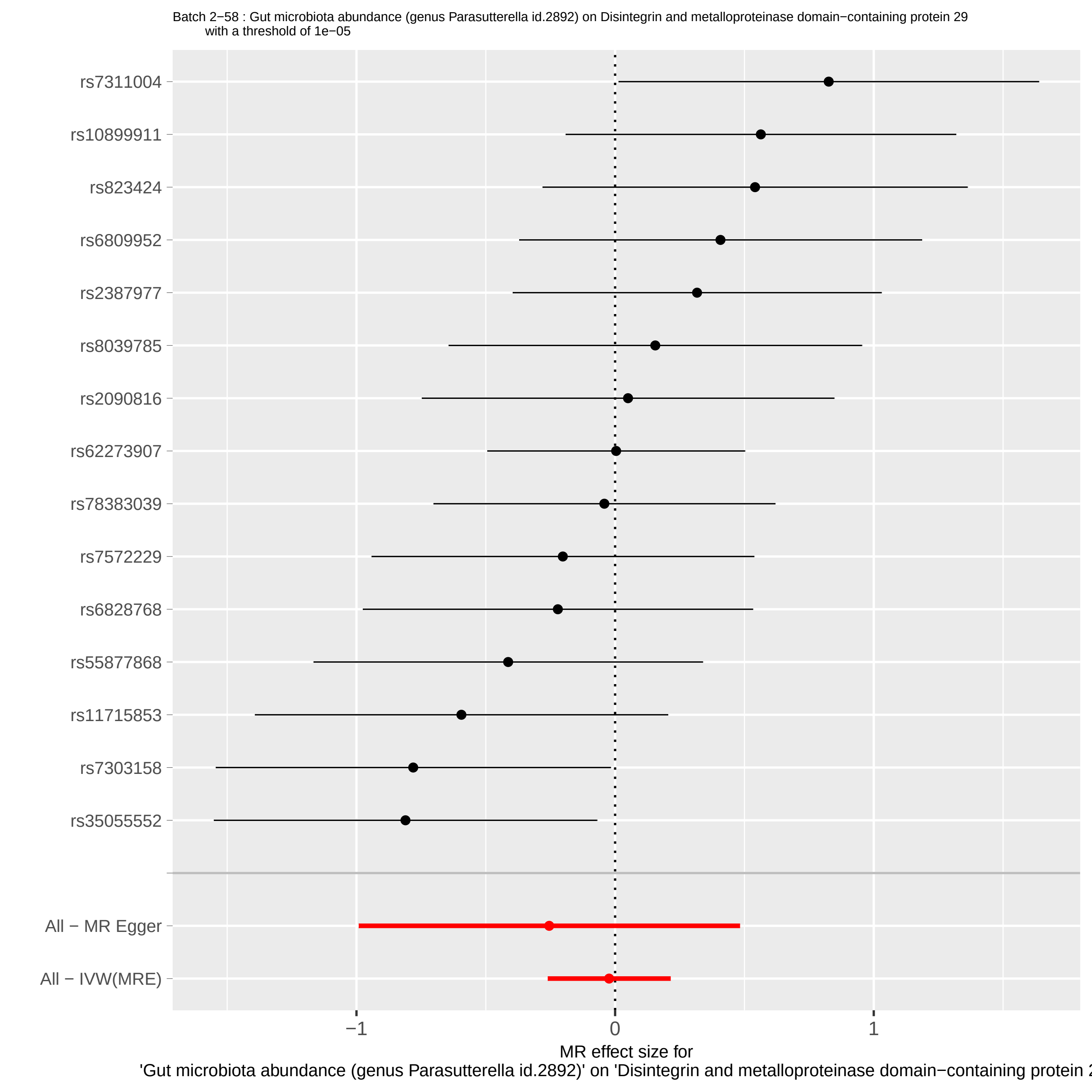

Batch 2−58 : Gut microbiota abundance (genus Parasutterella id.2892) on Disintegrin and metalloproteinase domain−containing protein 29 with a threshold of 1e−05
rs7311004
rs10899911
rs823424
rs6809952
rs2387977
rs8039785
rs2090816
rs62273907
rs78383039
rs7572229
rs6828768
rs55877868
rs11715853
rs7303158
rs35055552
All − MR Egger
All − IVW(MRE)
−1
0
1
MR effect size for
'Gut microbiota abundance (genus Parasutterella id.2892)' on 'Disintegrin and metalloproteinase domain−containing protein 2

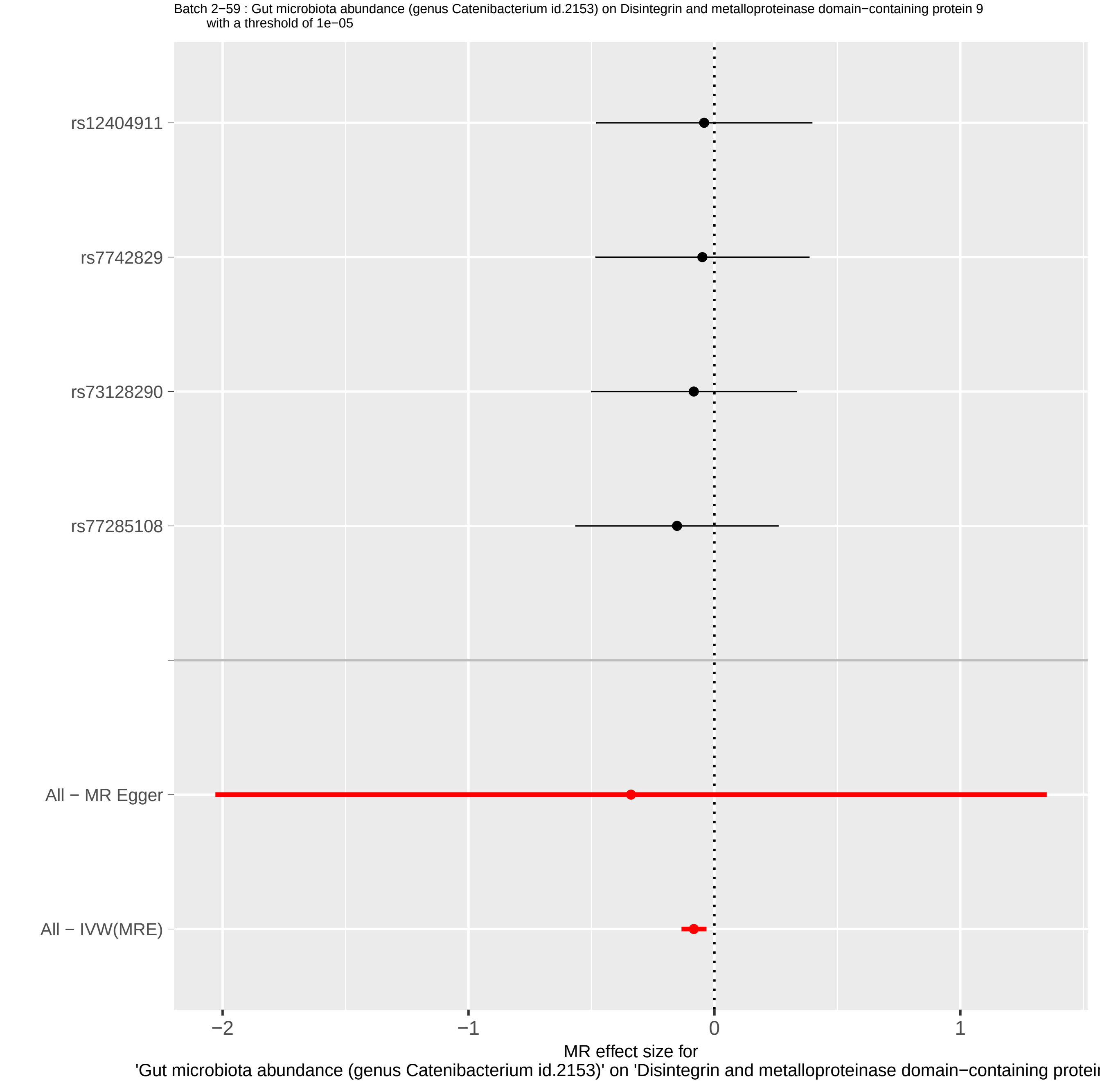
Batch 2−59 : Gut microbiota abundance (genus Catenibacterium id.2153) on Disintegrin and metalloproteinase domain−containing protein 9
with a threshold of 1e−05
rs12404911
rs7742829
rs73128290
rs77285108
All − MR Egger
All − IVW(MRE)
−2
−1
0
1
MR effect size for
'Gut microbiota abundance (genus Catenibacterium id.2153)' on 'Disintegrin and metalloproteinase domain−containing protei

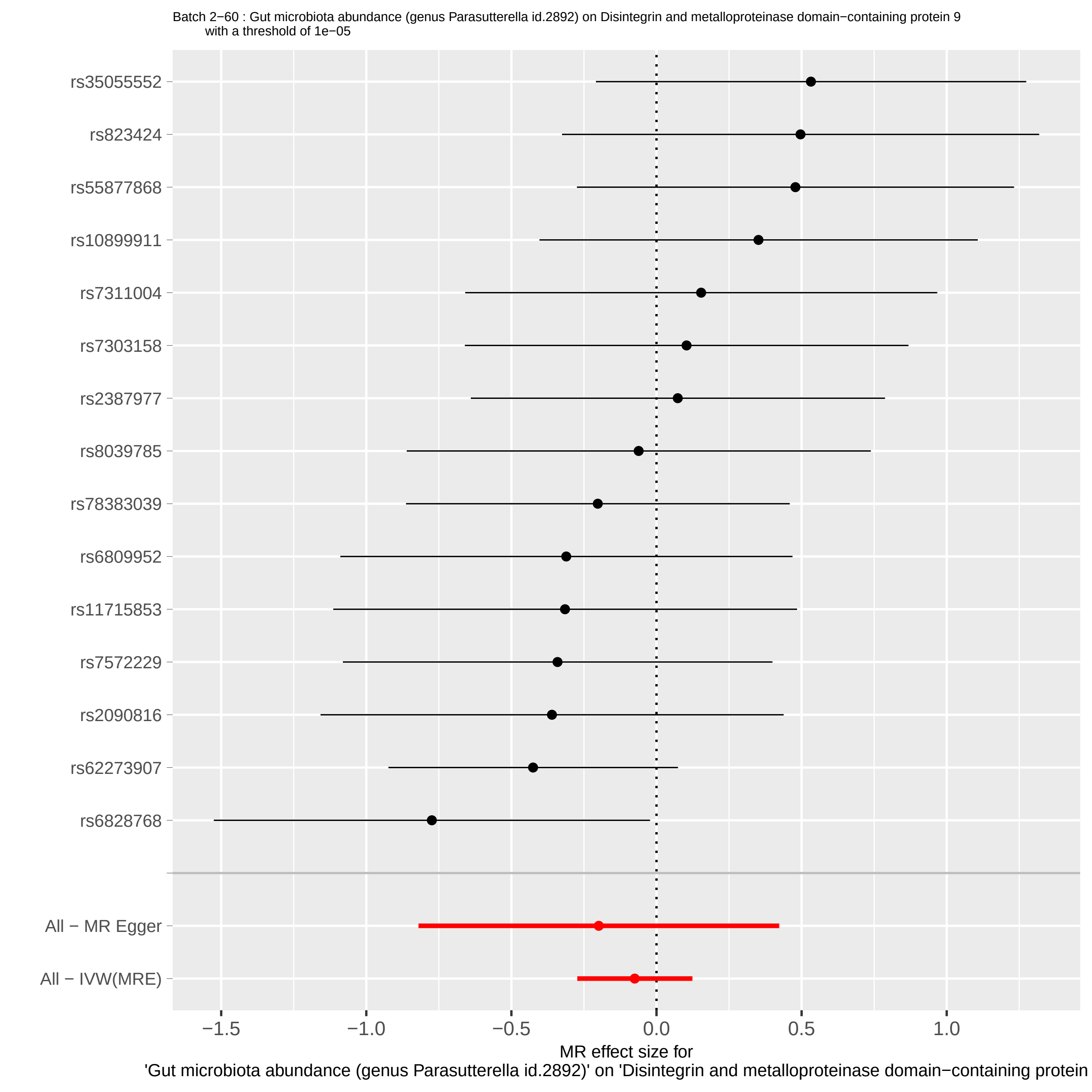

Batch 2−60 : Gut microbiota abundance (genus Parasutterella id.2892) on Disintegrin and metalloproteinase domain−containing protein 9
with a threshold of 1e−05
rs35055552
rs823424
rs55877868
rs10899911
rs7311004
rs7303158
rs2387977
rs8039785
rs78383039
rs6809952
rs11715853
rs7572229
rs2090816
rs62273907
rs6828768
All − MR Egger
All − IVW(MRE)
−1.5
−1.0
−0.5
0.0
0.5
1.0
MR effect size for
'Gut microbiota abundance (genus Parasutterella id.2892)' on 'Disintegrin and metalloproteinase domain−containing protein

Batch 2−61 : Gut microbiota abundance (genus Catenibacterium id.2153) on A disintegrin and metalloproteinase with thrombospondin motifs 1 with a threshold of 1e−05

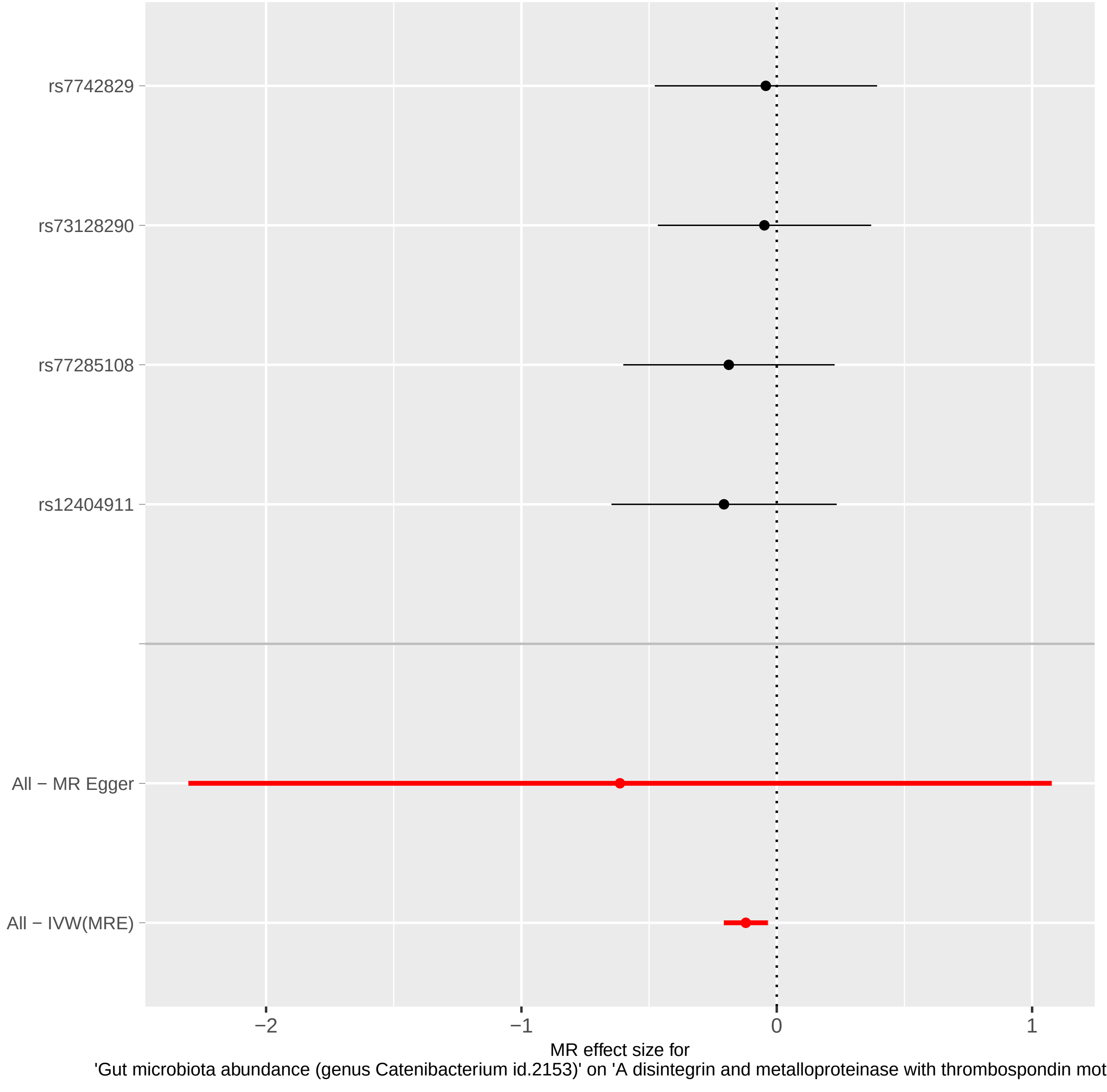

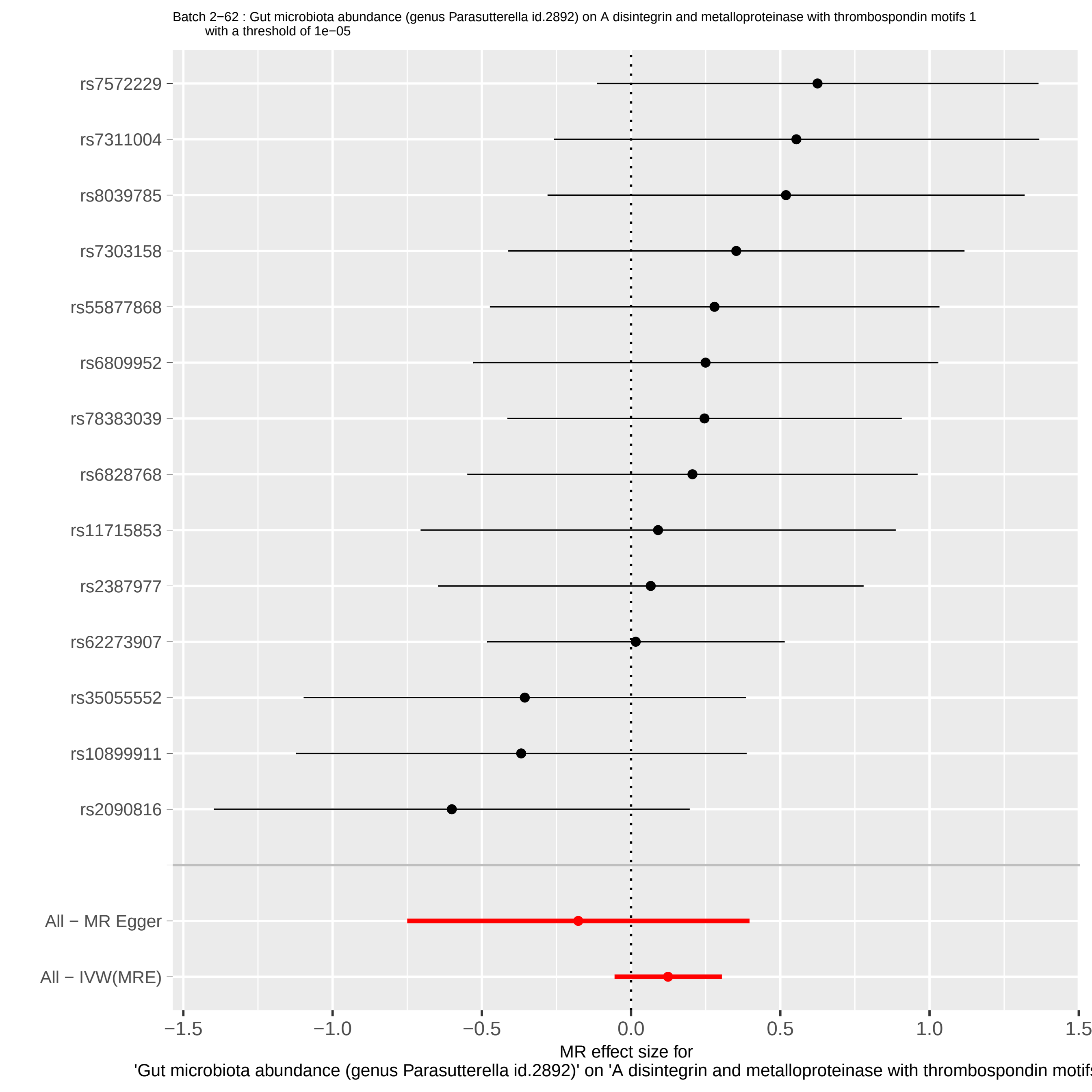

Batch 2−62 : Gut microbiota abundance (genus Parasutterella id.2892) on A disintegrin and metalloproteinase with thrombospondin motifs 1 with a threshold of 1e−05
rs7572229
rs7311004
rs8039785
rs7303158
rs55877868
rs6809952
rs78383039
rs6828768
rs11715853
rs2387977
rs62273907
rs35055552
rs10899911
rs2090816
All − MR Egger
All − IVW(MRE)
−1.5
−1.0
−0.5
0.0
0.5
1.0
1.5
MR effect size for
'Gut microbiota abundance (genus Parasutterella id.2892)' on 'A disintegrin and metalloproteinase with thrombospondin motifs

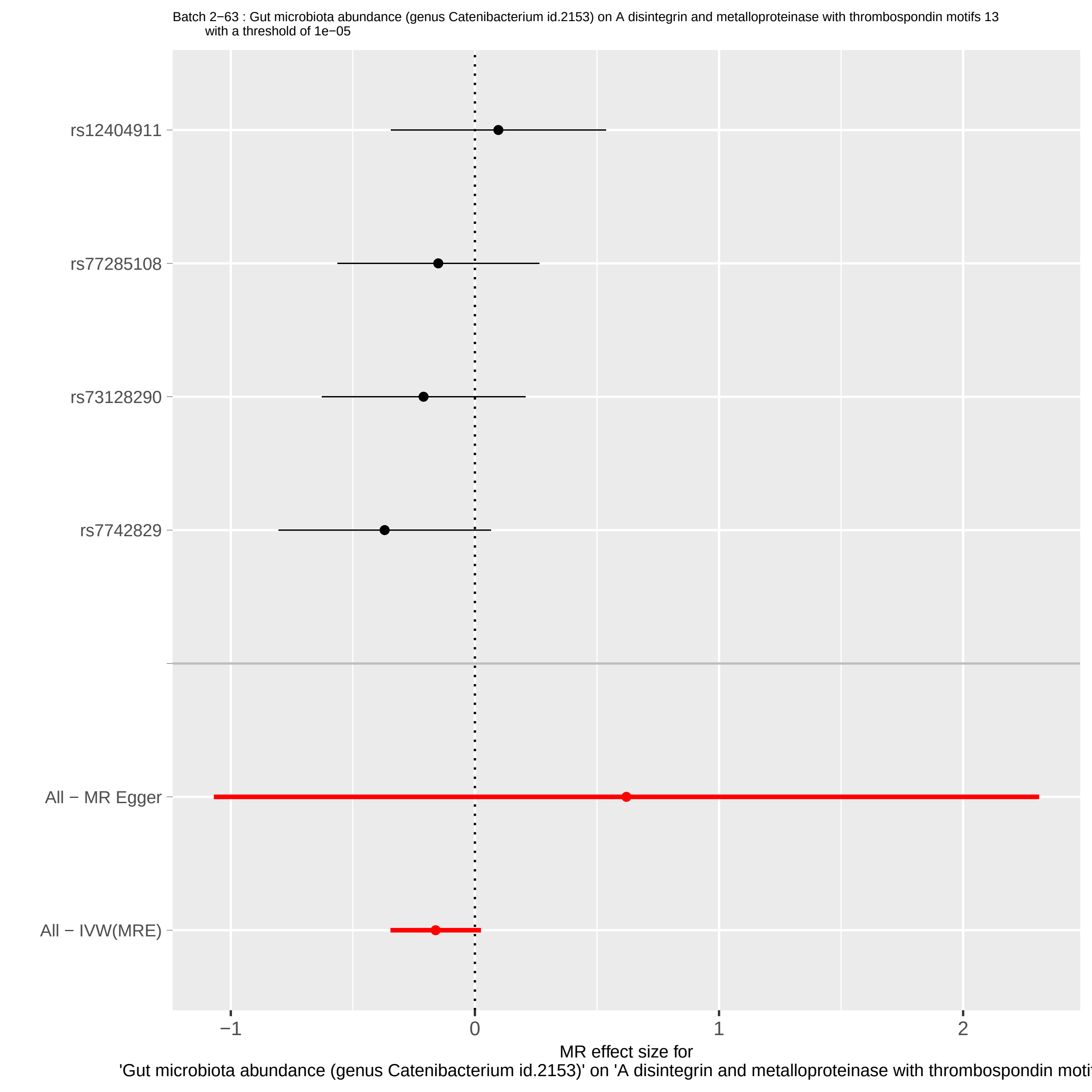

Batch 2−63 : Gut microbiota abundance (genus Catenibacterium id.2153) on A disintegrin and metalloproteinase with thrombospondin motifs 13
with a threshold of 1e−05
rs12404911
rs77285108
rs73128290
rs7742829
All − MR Egger
All − IVW(MRE)
−1
0
1
2
MR effect size for
'Gut microbiota abundance (genus Catenibacterium id.2153)' on 'A disintegrin and metalloproteinase with thrombospondin moti

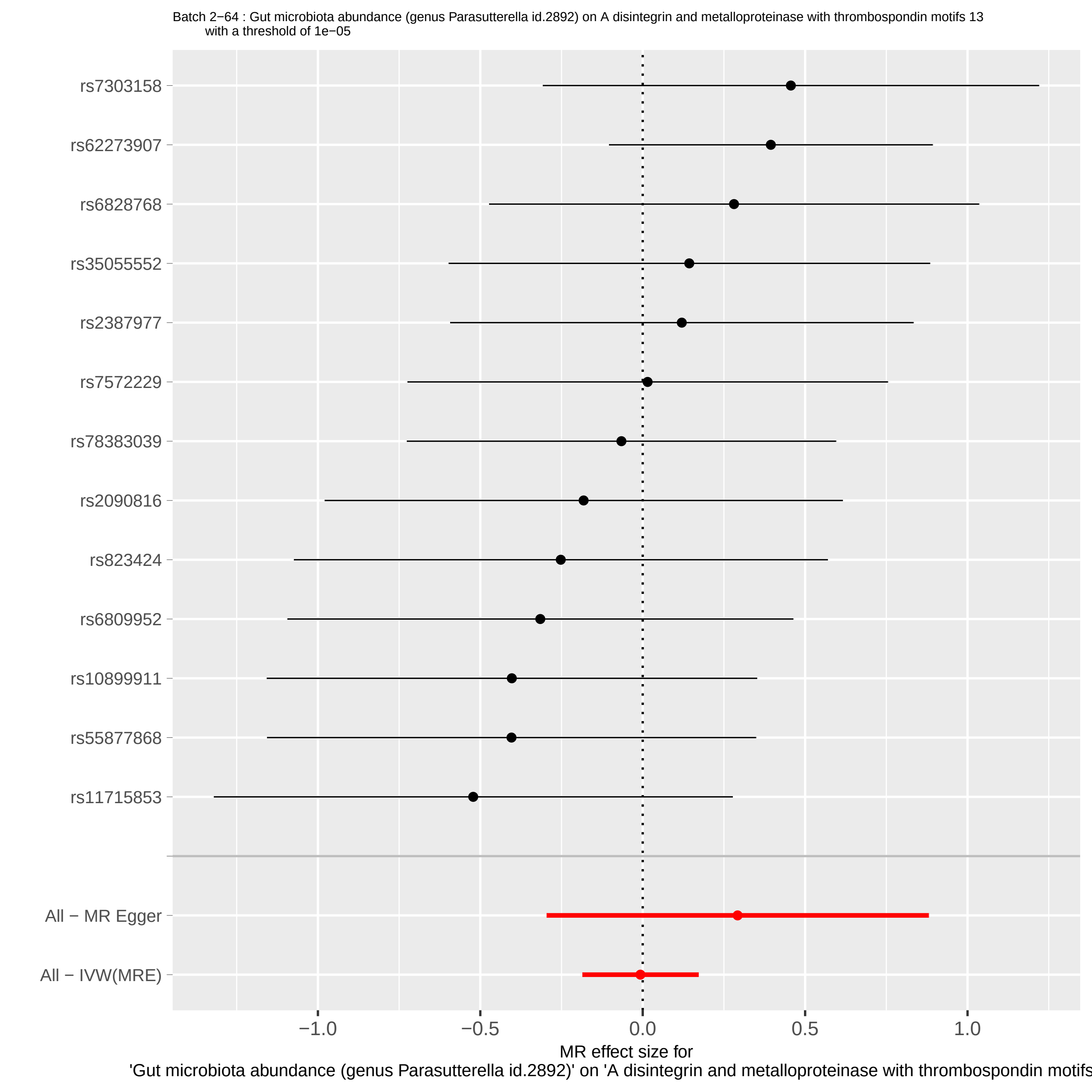

Batch 2−64 : Gut microbiota abundance (genus Parasutterella id.2892) on A disintegrin and metalloproteinase with thrombospondin motifs 13
with a threshold of 1e−05
rs7303158
rs62273907
rs6828768
rs35055552
rs2387977
rs7572229
rs78383039
rs2090816
rs823424
rs6809952
rs10899911
rs55877868
rs11715853
All − MR Egger
All − IVW(MRE)
−1.0
−0.5
0.0
0.5
1.0
MR effect size for
'Gut microbiota abundance (genus Parasutterella id.2892)' on 'A disintegrin and metalloproteinase with thrombospondin motifs

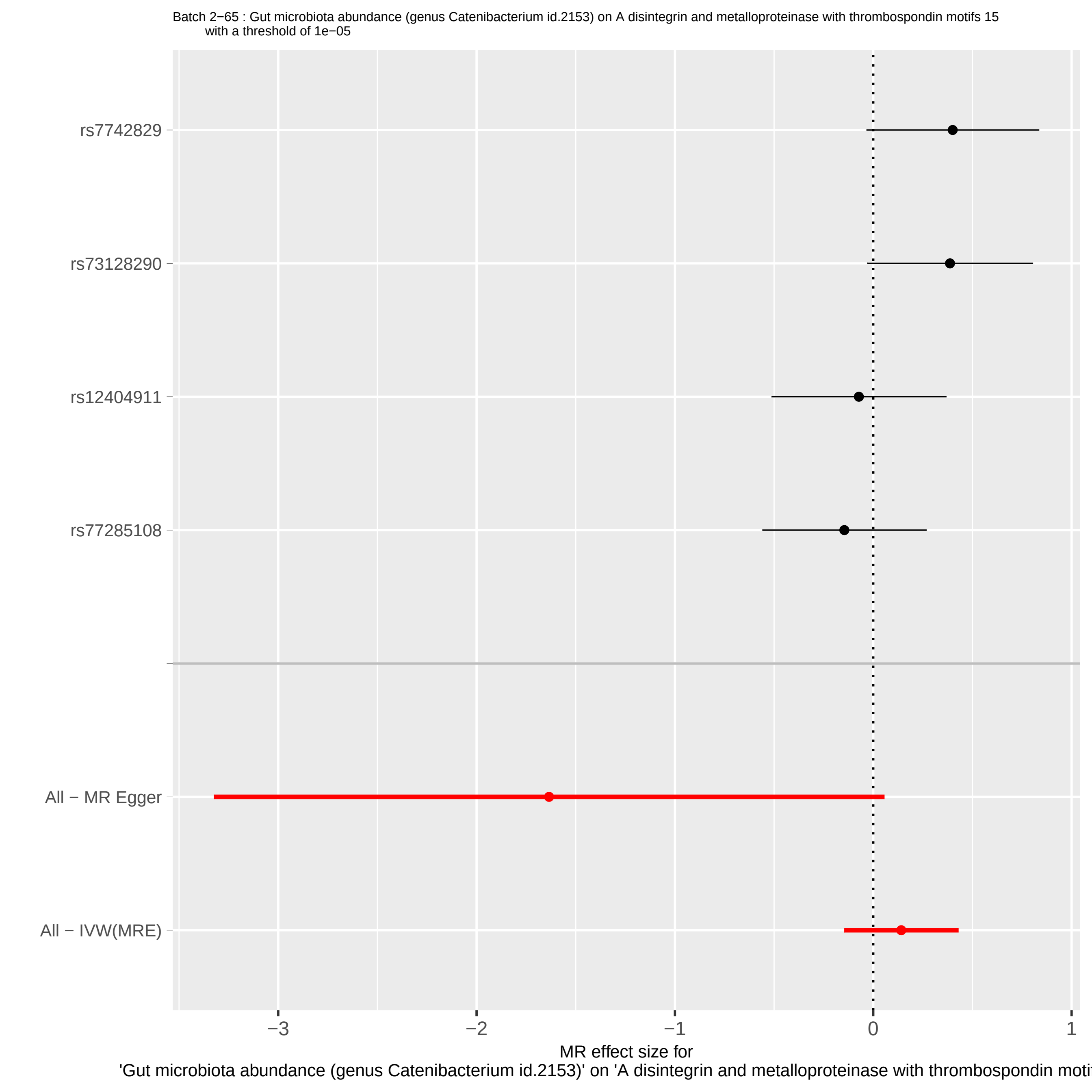
Batch 2−65 : Gut microbiota abundance (genus Catenibacterium id.2153) on A disintegrin and metalloproteinase with thrombospondin motifs 15
with a threshold of 1e−05
rs7742829
rs73128290
rs12404911
rs77285108
All − MR Egger
All − IVW(MRE)
−3
−2
−1
0
1
MR effect size for
'Gut microbiota abundance (genus Catenibacterium id.2153)' on 'A disintegrin and metalloproteinase with thrombospondin moti

Batch 2−66 : Gut microbiota abundance (genus Parasutterella id.2892) on A disintegrin and metalloproteinase with thrombospondin motifs 15 with a threshold of 1e−05

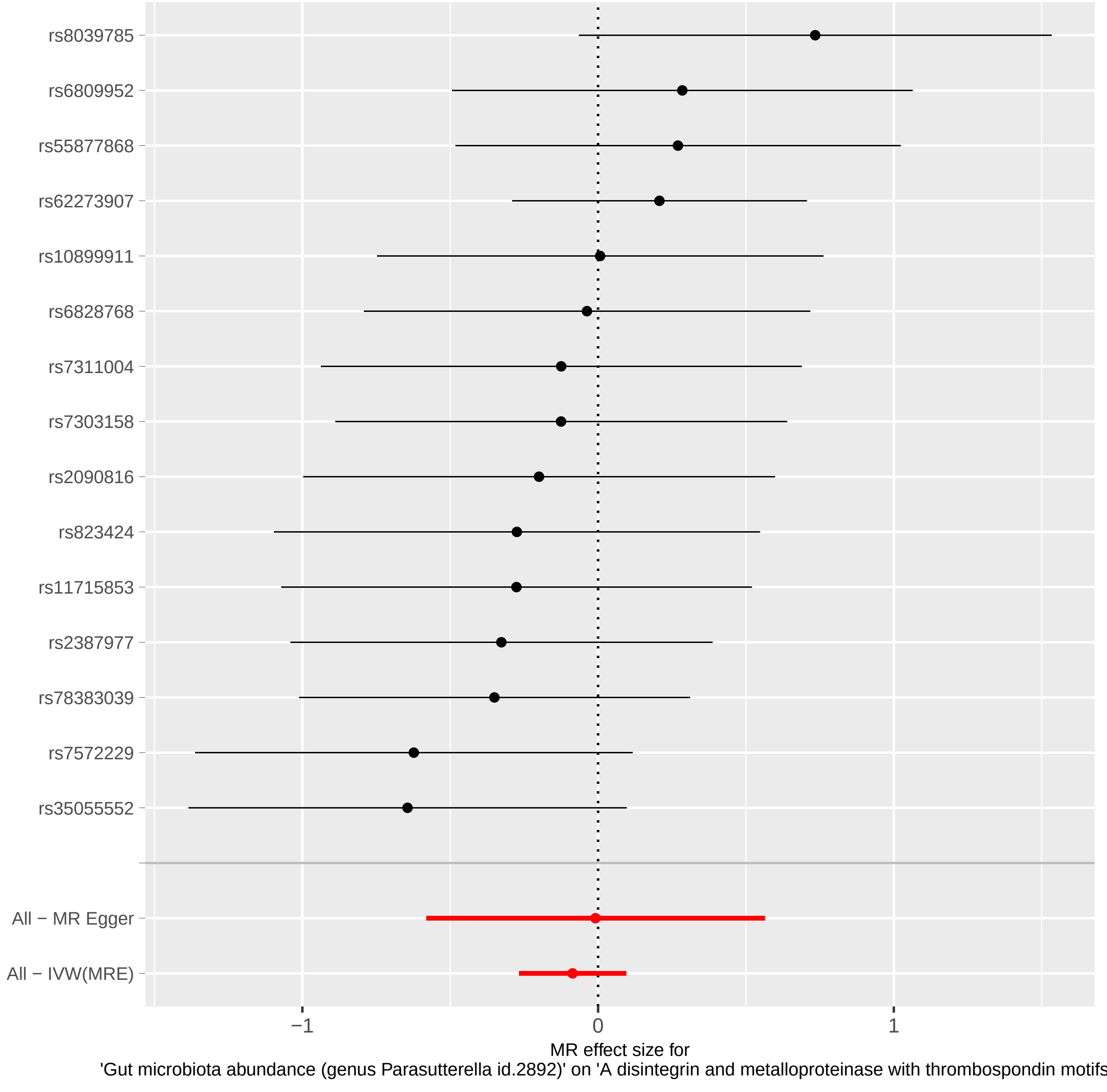

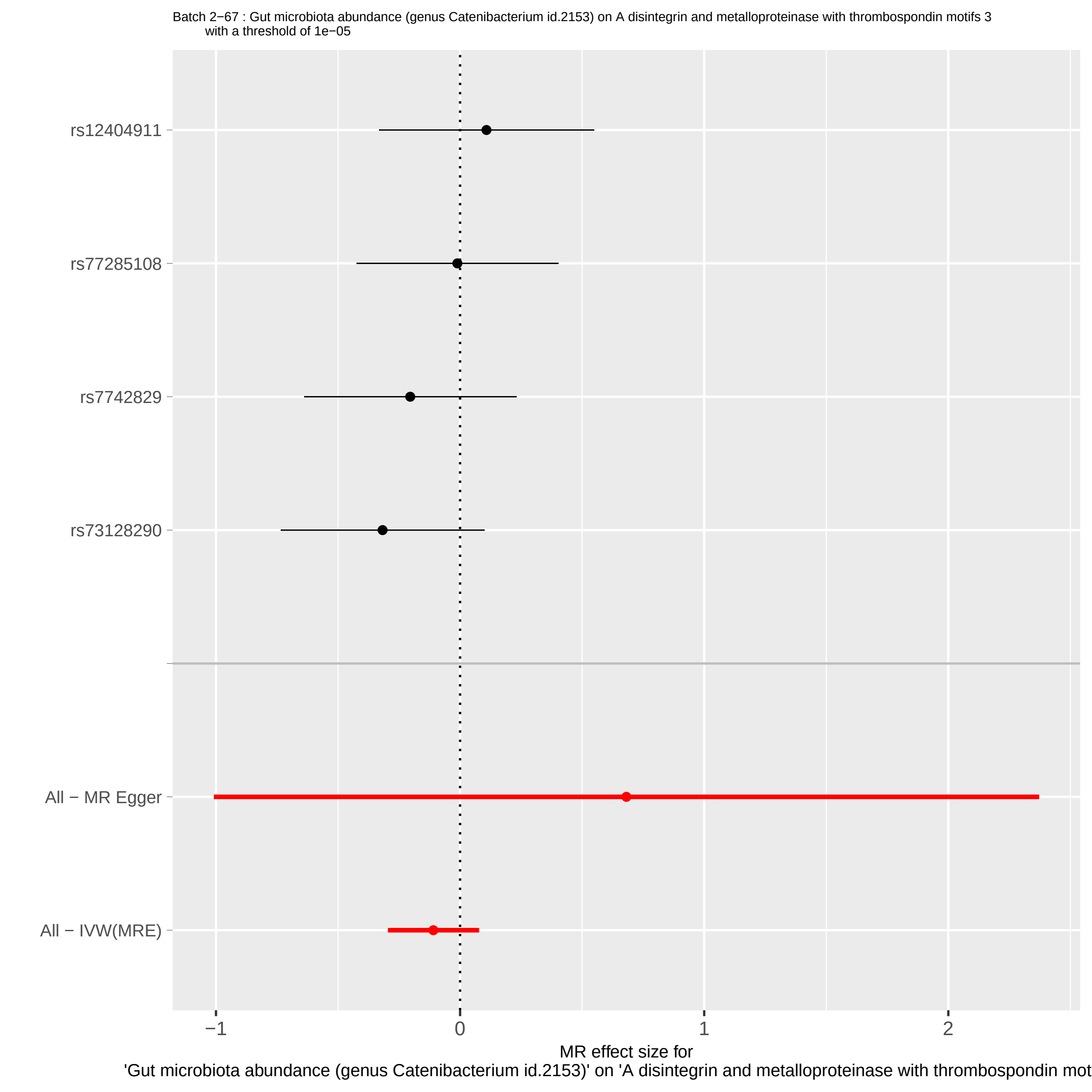
Batch 2−67 : Gut microbiota abundance (genus Catenibacterium id.2153) on A disintegrin and metalloproteinase with thrombospondin motifs 3
with a threshold of 1e−05
rs12404911
rs77285108
rs7742829
rs73128290
All − MR Egger
All − IVW(MRE)
−1
0
1
2
MR effect size for
'Gut microbiota abundance (genus Catenibacterium id.2153)' on 'A disintegrin and metalloproteinase with thrombospondin mot

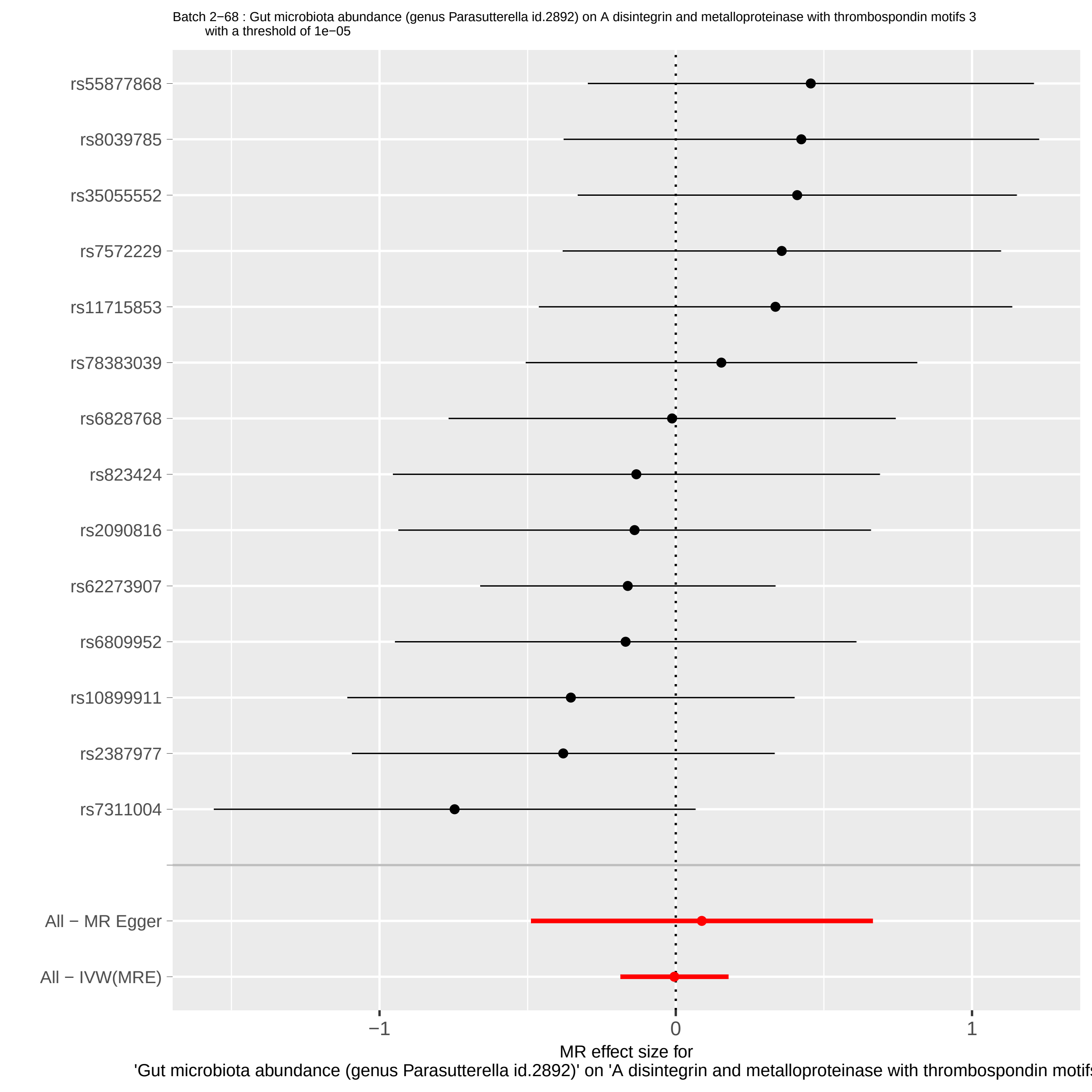
Batch 2−68 : Gut microbiota abundance (genus Parasutterella id.2892) on A disintegrin and metalloproteinase with thrombospondin motifs 3 with a threshold of 1e−05
rs55877868
rs8039785
rs35055552
rs7572229
rs11715853
rs78383039
rs6828768
rs823424
rs2090816
rs62273907
rs6809952
rs10899911
rs2387977
rs7311004
All − MR Egger
All − IVW(MRE)
−1
0
1
MR effect size for
'Gut microbiota abundance (genus Parasutterella id.2892)' on 'A disintegrin and metalloproteinase with thrombospondin motifs

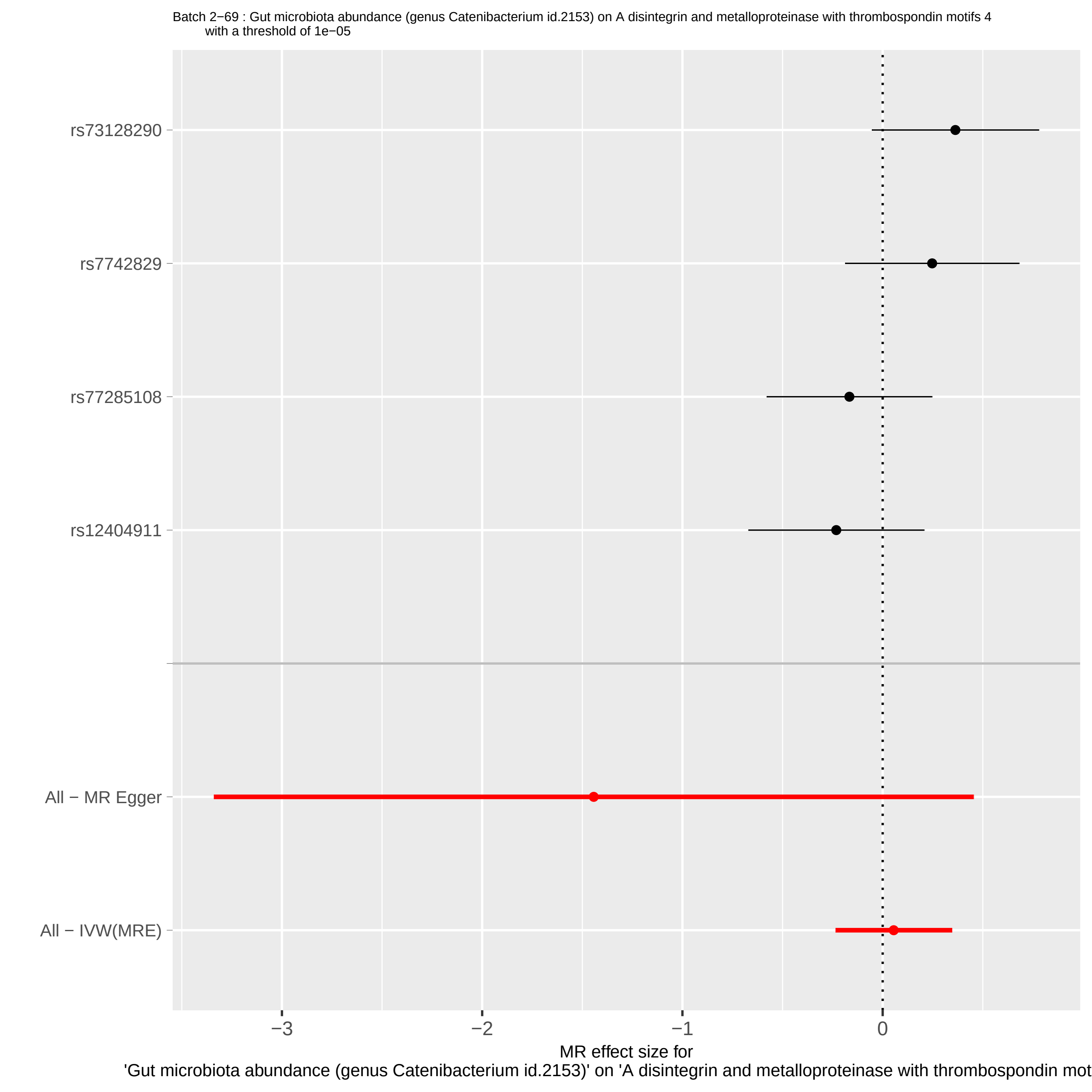

Batch 2−69 : Gut microbiota abundance (genus Catenibacterium id.2153) on A disintegrin and metalloproteinase with thrombospondin motifs 4
with a threshold of 1e−05
rs73128290
rs7742829
rs77285108
rs12404911
All − MR Egger
All − IVW(MRE)
−3
−2
−1
0
MR effect size for
'Gut microbiota abundance (genus Catenibacterium id.2153)' on 'A disintegrin and metalloproteinase with thrombospondin mot

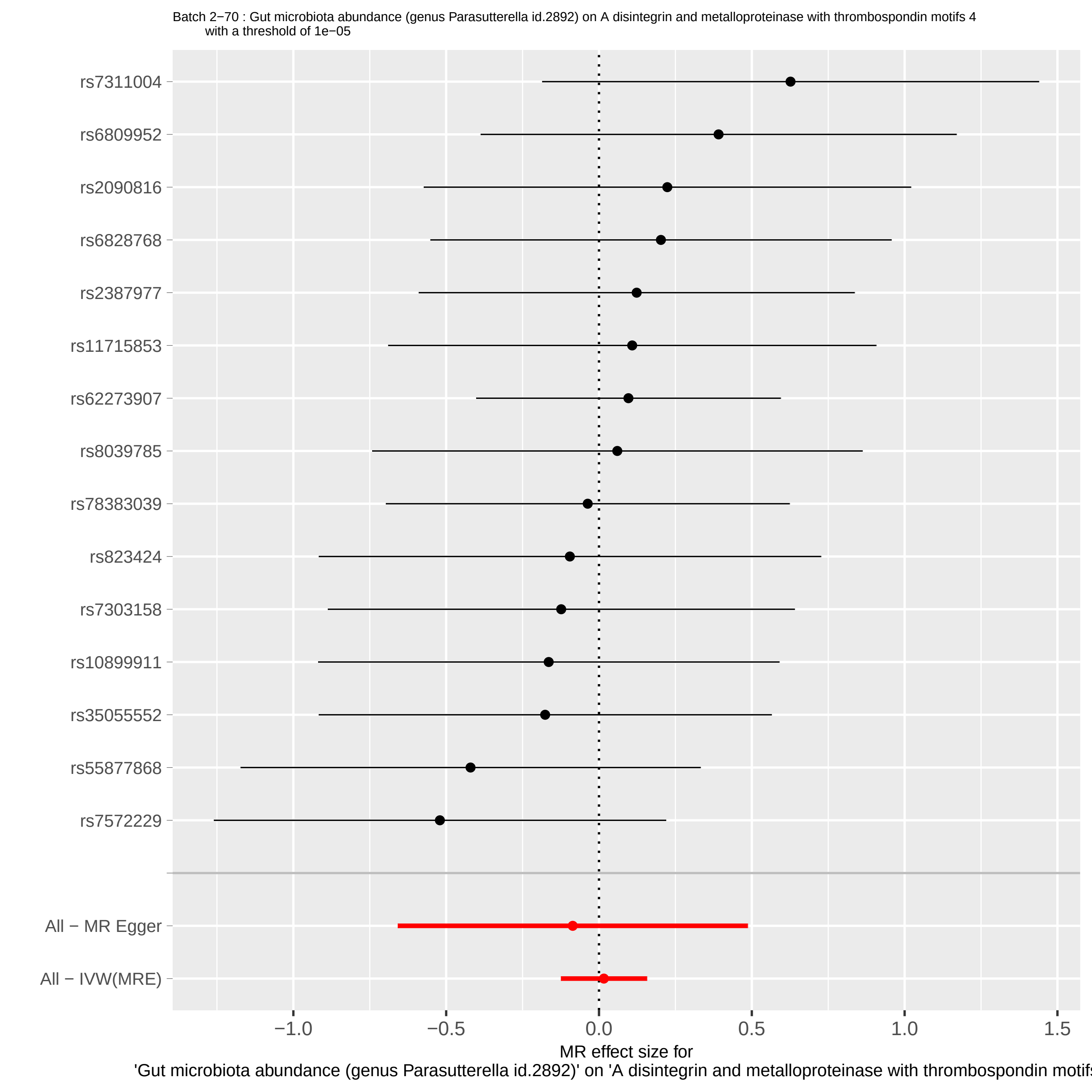

Batch 2−70 : Gut microbiota abundance (genus Parasutterella id.2892) on A disintegrin and metalloproteinase with thrombospondin motifs 4 with a threshold of 1e−05
rs7311004
rs6809952
rs2090816
rs6828768
rs2387977
rs11715853
rs62273907
rs8039785
rs78383039
rs823424
rs7303158
rs10899911
rs35055552
rs55877868
rs7572229
All − MR Egger
All − IVW(MRE)
−1.0
−0.5
0.0
0.5
1.0
1.5
MR effect size for
'Gut microbiota abundance (genus Parasutterella id.2892)' on 'A disintegrin and metalloproteinase with thrombospondin motifs

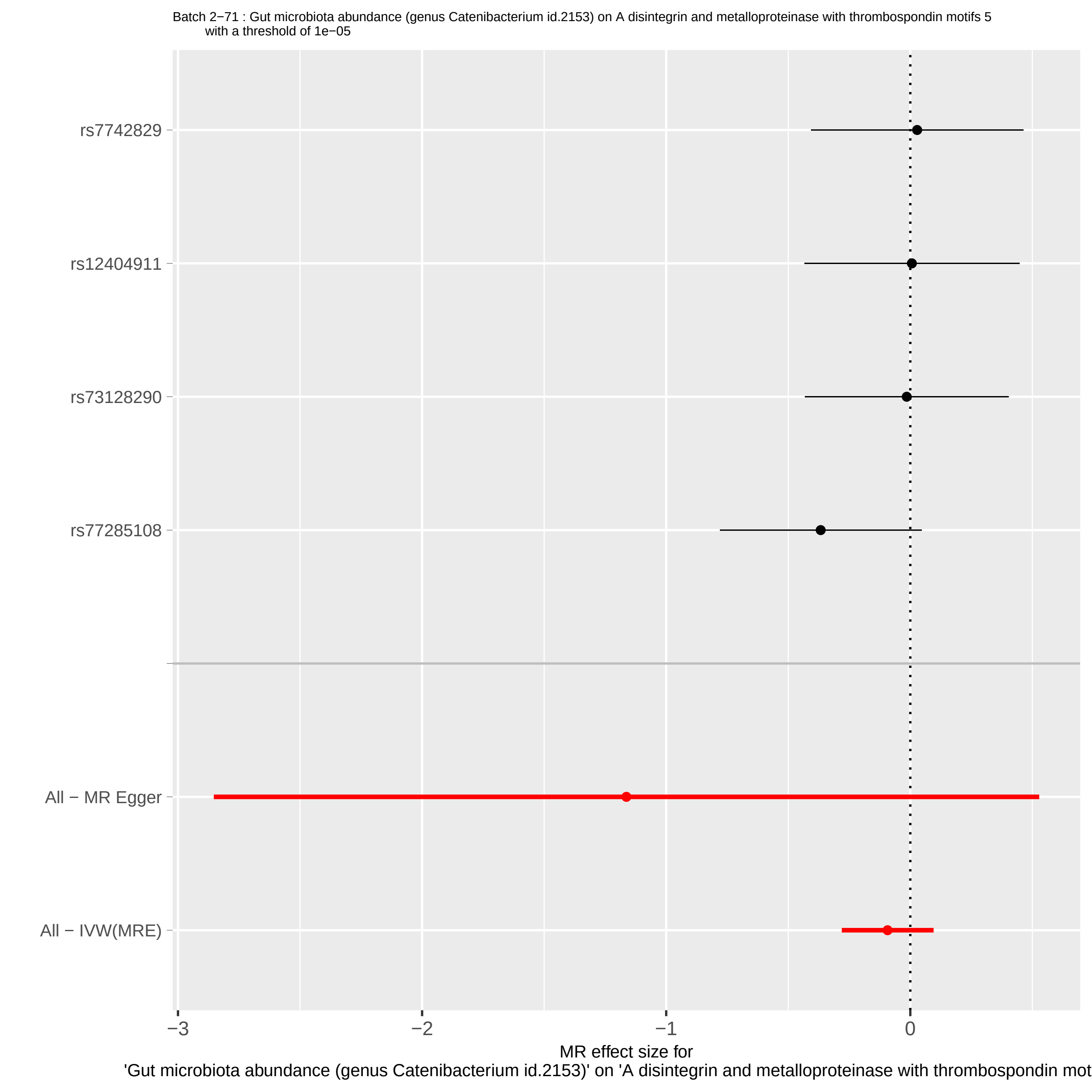
Batch 2−71 : Gut microbiota abundance (genus Catenibacterium id.2153) on A disintegrin and metalloproteinase with thrombospondin motifs 5
with a threshold of 1e−05
rs7742829
rs12404911
rs73128290
rs77285108
All − MR Egger
All − IVW(MRE)
−3
−2
−1
0
MR effect size for
'Gut microbiota abundance (genus Catenibacterium id.2153)' on 'A disintegrin and metalloproteinase with thrombospondin mot

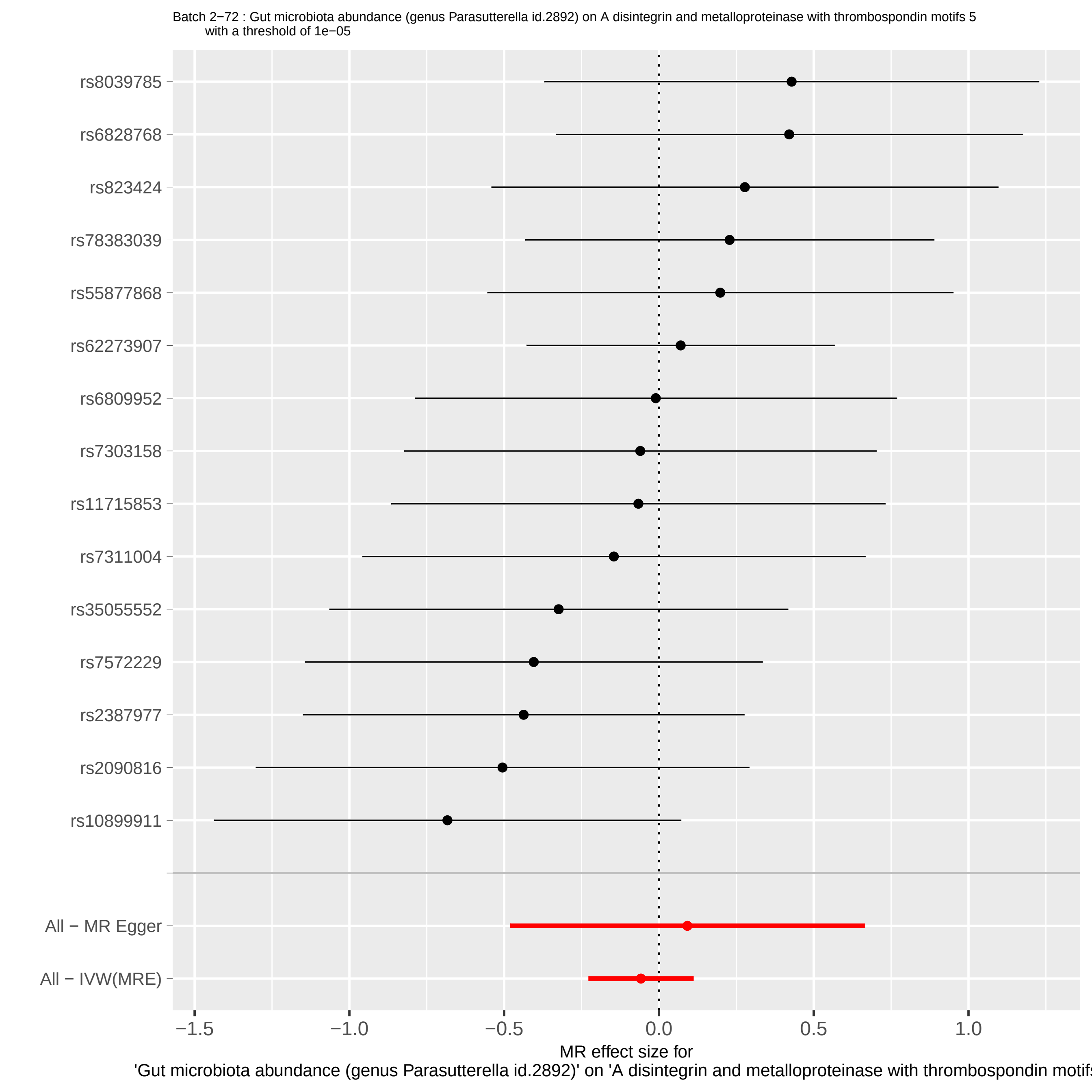
Batch 2−72 : Gut microbiota abundance (genus Parasutterella id.2892) on A disintegrin and metalloproteinase with thrombospondin motifs 5 with a threshold of 1e−05
rs8039785
rs6828768
rs823424
rs78383039
rs55877868
rs62273907
rs6809952
rs7303158
rs11715853
rs7311004
rs35055552
rs7572229
rs2387977
rs2090816
rs10899911
All − MR Egger
All − IVW(MRE)
−1.5
−1.0
−0.5
0.0
0.5
1.0
MR effect size for
'Gut microbiota abundance (genus Parasutterella id.2892)' on 'A disintegrin and metalloproteinase with thrombospondin motifs

Batch 2−73 : Gut microbiota abundance (genus Catenibacterium id.2153) on A disintegrin and metalloproteinase with thrombospondin motifs 6 with a threshold of 1e−05

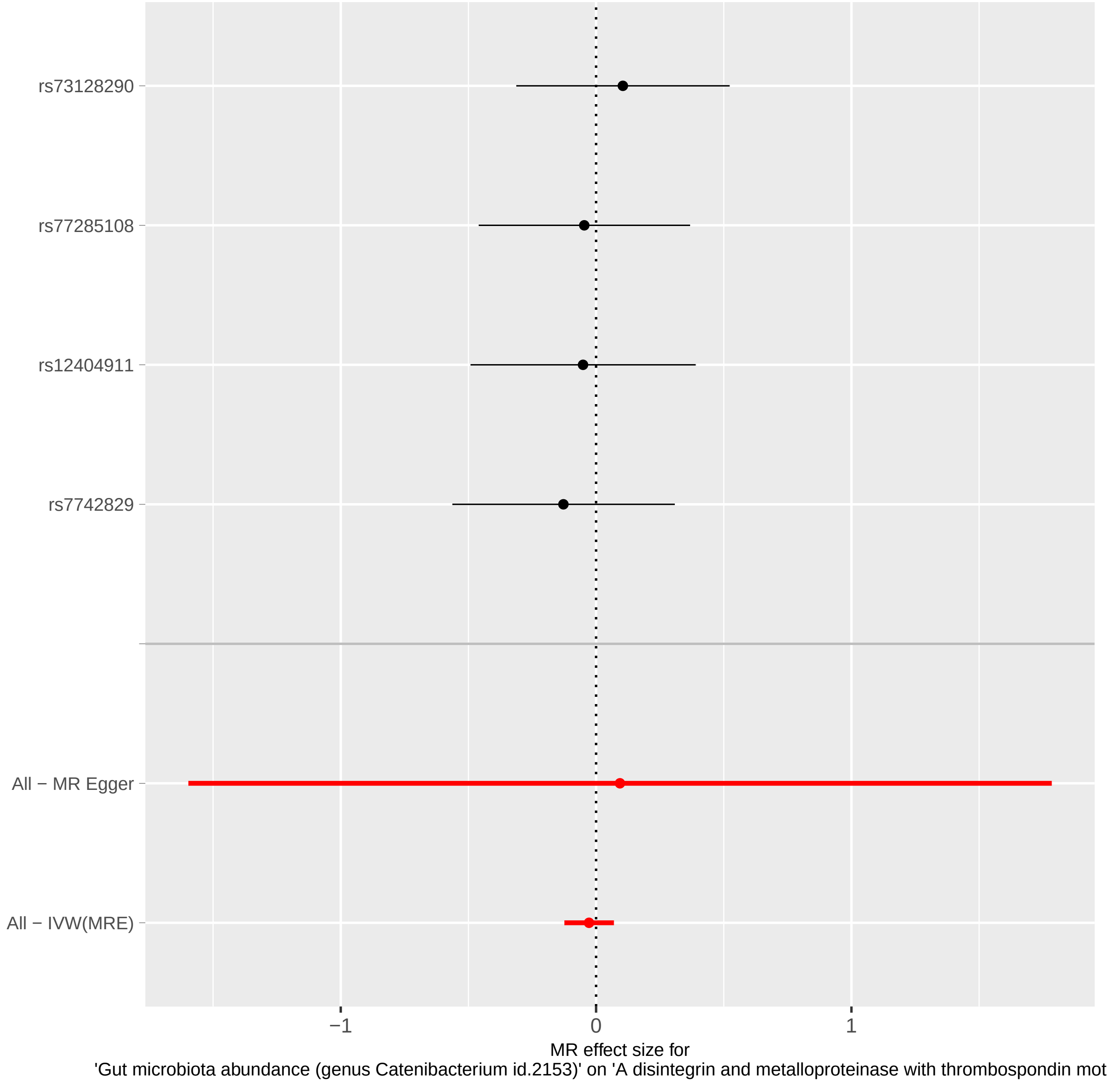

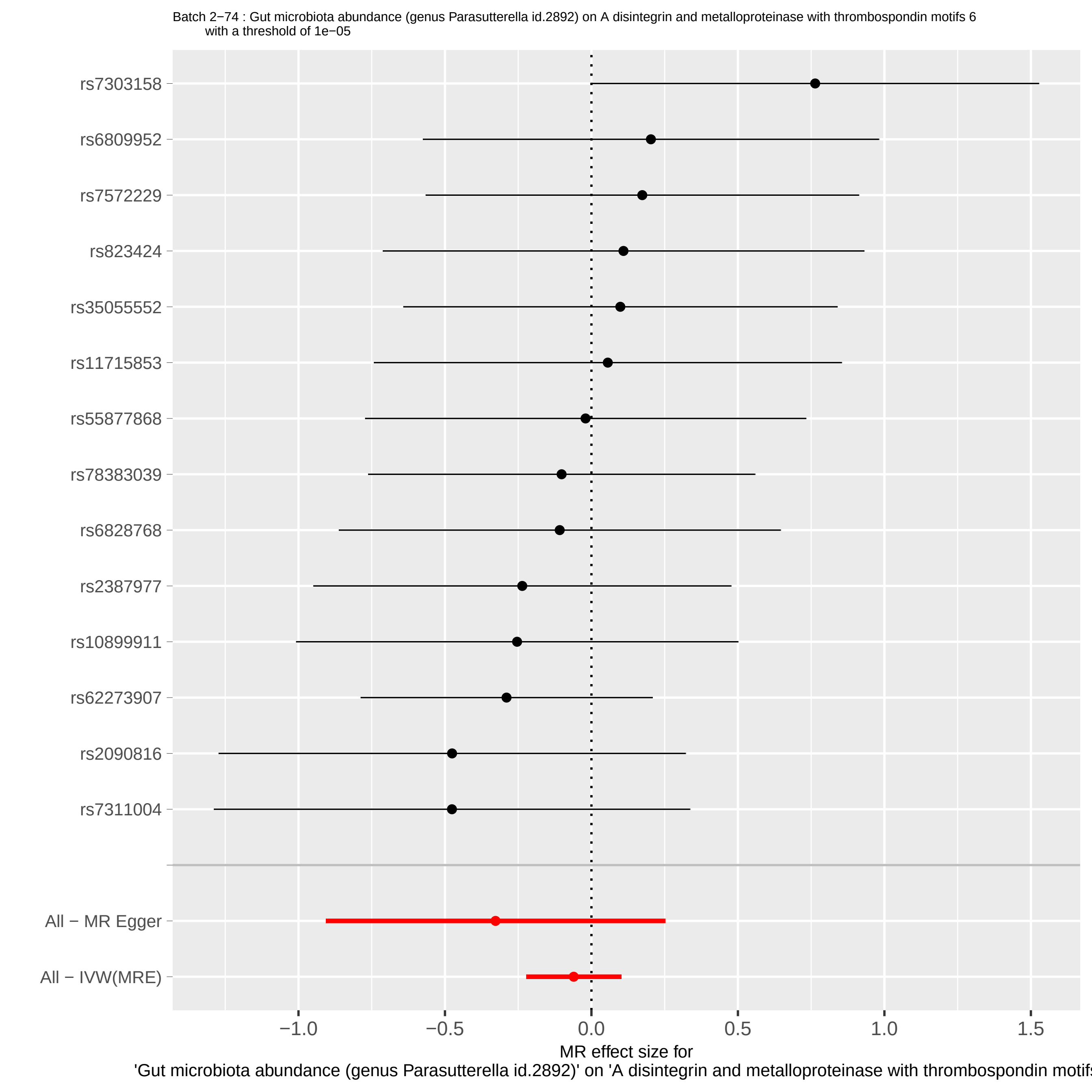

Batch 2−74 : Gut microbiota abundance (genus Parasutterella id.2892) on A disintegrin and metalloproteinase with thrombospondin motifs 6 with a threshold of 1e−05
rs7303158
rs6809952
rs7572229
rs823424
rs35055552
rs11715853
rs55877868
rs78383039
rs6828768
rs2387977
rs10899911
rs62273907
rs2090816
rs7311004
All − MR Egger
All − IVW(MRE)
−1.0
−0.5
0.0
0.5
1.0
1.5
MR effect size for
'Gut microbiota abundance (genus Parasutterella id.2892)' on 'A disintegrin and metalloproteinase with thrombospondin motifs

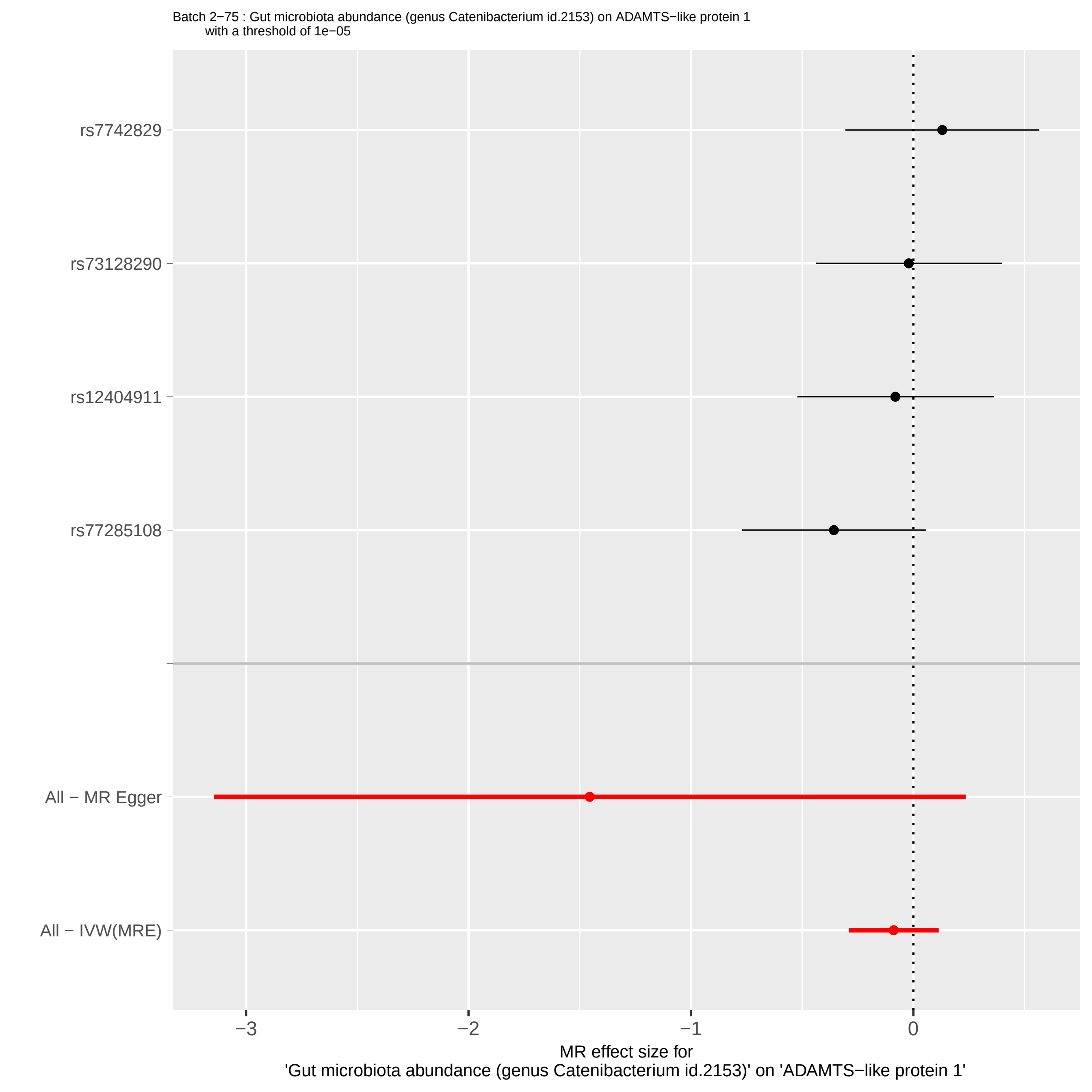

Batch 2−75 : Gut microbiota abundance (genus Catenibacterium id.2153) on ADAMTS−like protein 1
with a threshold of 1e−05
rs7742829
rs73128290
rs12404911
rs77285108
All − MR Egger
All − IVW(MRE)
−3
−2
−1
0
MR effect size for
'Gut microbiota abundance (genus Catenibacterium id.2153)' on 'ADAMTS−like protein 1'

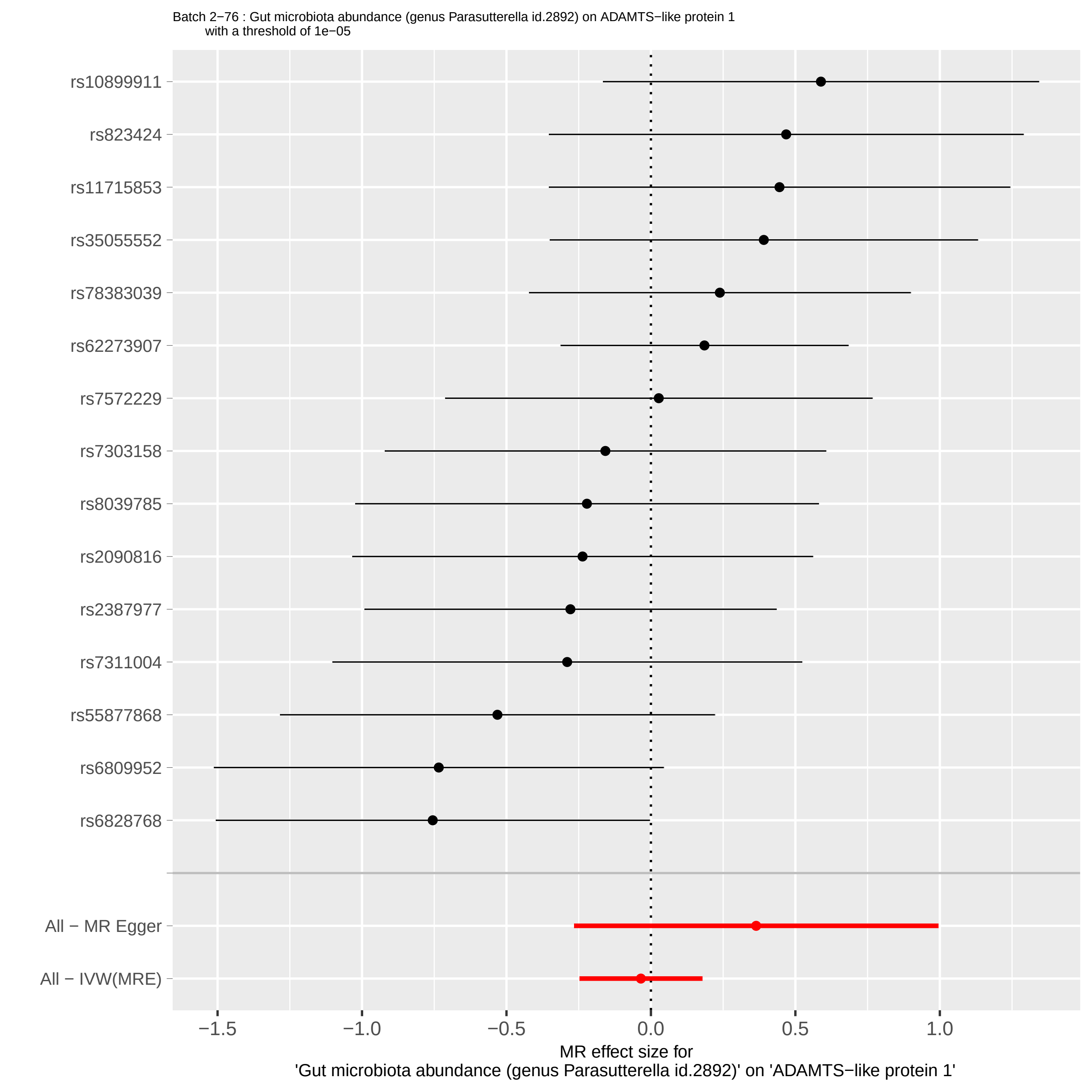
Batch 2−76 : Gut microbiota abundance (genus Parasutterella id.2892) on ADAMTS−like protein 1
with a threshold of 1e−05
rs10899911
rs823424
rs11715853
rs35055552
rs78383039
rs62273907
rs7572229
rs7303158
rs8039785
rs2090816
rs2387977
rs7311004
rs55877868
rs6809952
rs6828768
All − MR Egger
All − IVW(MRE)
−1.5
−1.0
−0.5
0.0
0.5
1.0
MR effect size for
'Gut microbiota abundance (genus Parasutterella id.2892)' on 'ADAMTS−like protein 1'

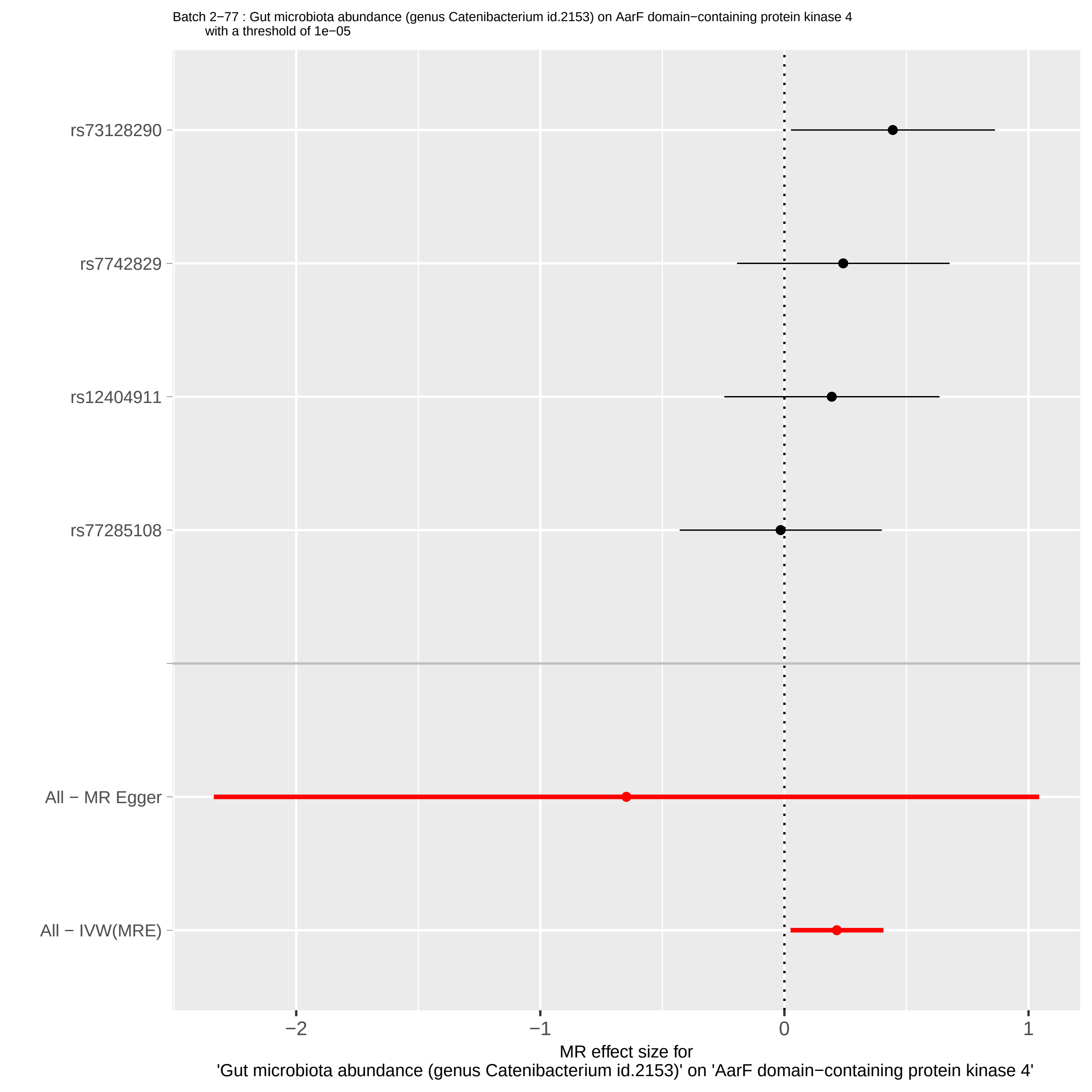

Batch 2−77 : Gut microbiota abundance (genus Catenibacterium id.2153) on AarF domain−containing protein kinase 4
with a threshold of 1e−05
rs73128290
rs7742829
rs12404911
rs77285108
All − MR Egger
All − IVW(MRE)
−2
−1
0
1
MR effect size for
'Gut microbiota abundance (genus Catenibacterium id.2153)' on 'AarF domain−containing protein kinase 4'

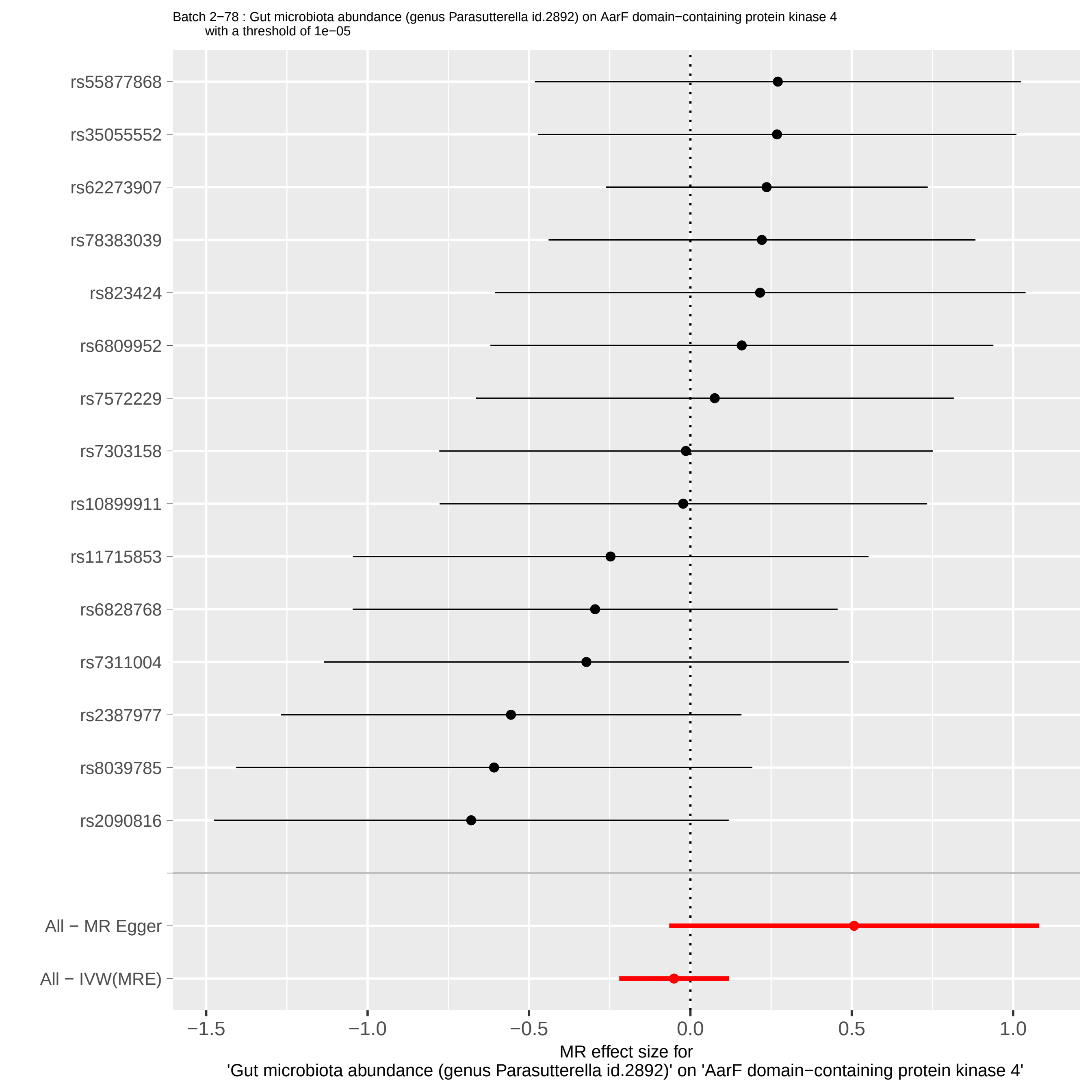

Batch 2−78 : Gut microbiota abundance (genus Parasutterella id.2892) on AarF domain−containing protein kinase 4
with a threshold of 1e−05
rs55877868
rs35055552
rs62273907
rs78383039
rs823424
rs6809952
rs7572229
rs7303158
rs10899911
rs11715853
rs6828768
rs7311004
rs2387977
rs8039785
rs2090816
All − MR Egger
All − IVW(MRE)
−1.5
−1.0
−0.5
0.0
0.5
1.0
MR effect size for
'Gut microbiota abundance (genus Parasutterella id.2892)' on 'AarF domain−containing protein kinase 4'

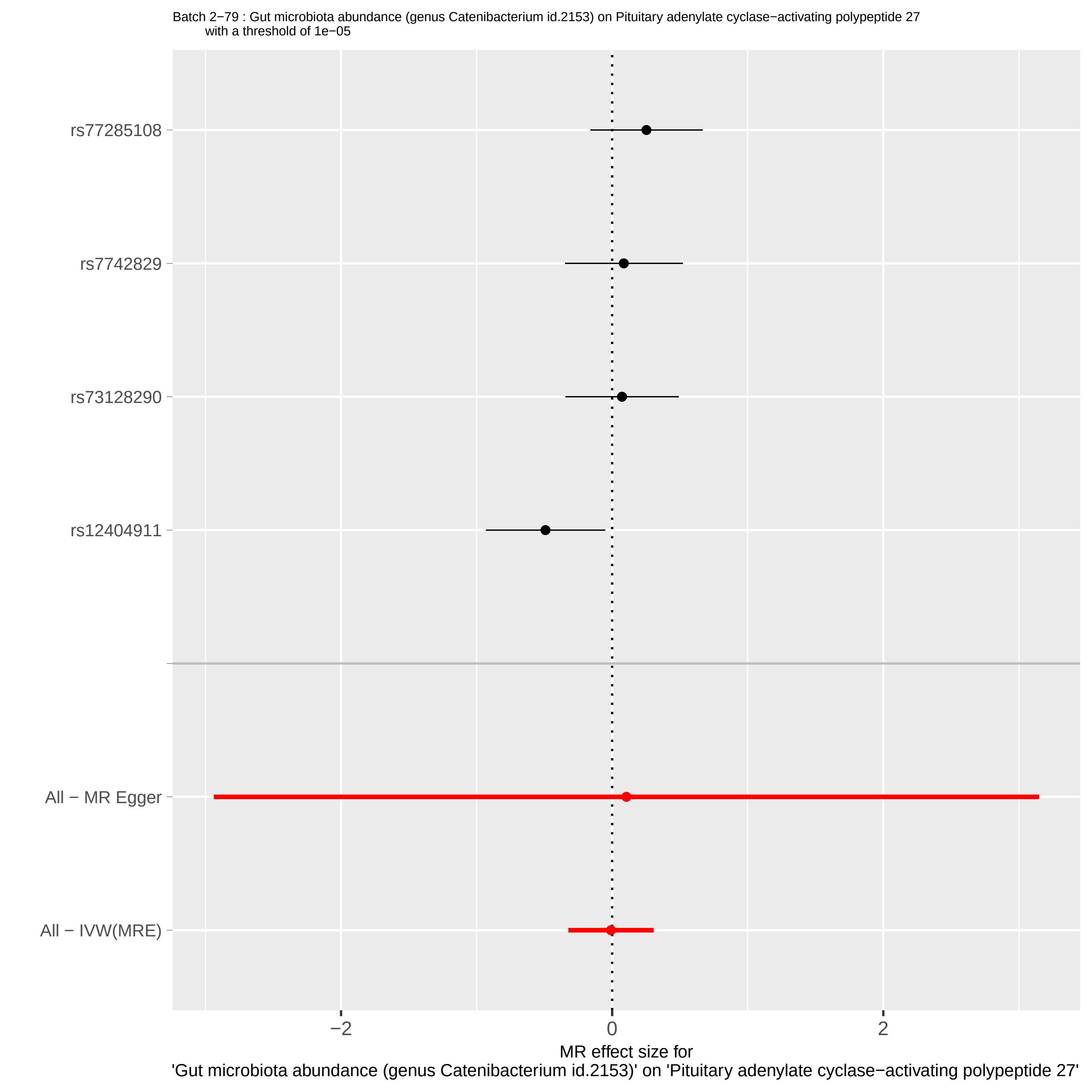

Batch 2−79 : Gut microbiota abundance (genus Catenibacterium id.2153) on Pituitary adenylate cyclase−activating polypeptide 27
with a threshold of 1e−05
rs77285108
rs7742829
rs73128290
rs12404911
All − MR Egger
All − IVW(MRE)
−2
0
2
MR effect size for
'Gut microbiota abundance (genus Catenibacterium id.2153)' on 'Pituitary adenylate cyclase−activating polypeptide 27'

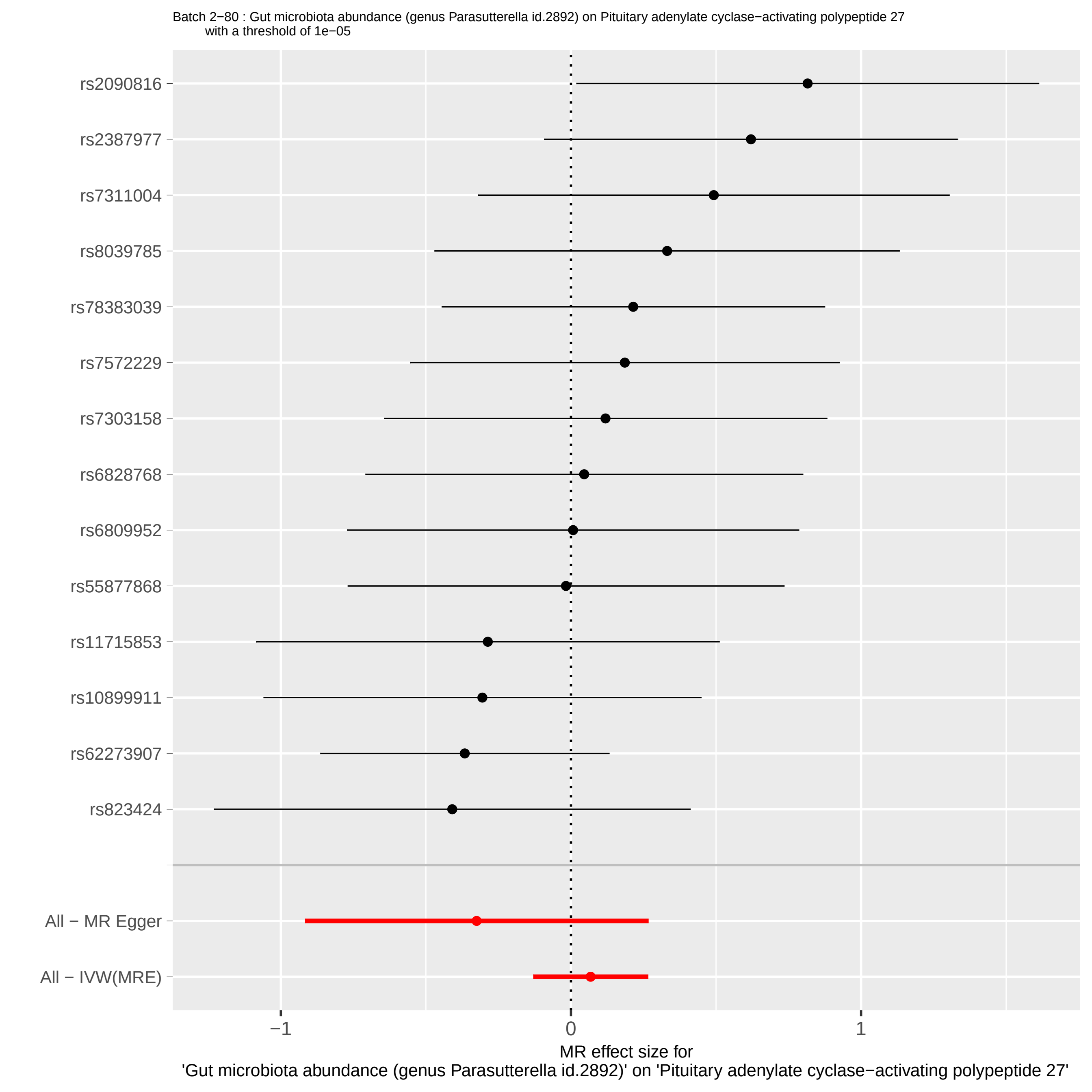

Batch 2−80 : Gut microbiota abundance (genus Parasutterella id.2892) on Pituitary adenylate cyclase−activating polypeptide 27
with a threshold of 1e−05
rs2090816
rs2387977
rs7311004
rs8039785
rs78383039
rs7572229
rs7303158
rs6828768
rs6809952
rs55877868
rs11715853
rs10899911
rs62273907
rs823424
All − MR Egger
All − IVW(MRE)
−1
0
1
MR effect size for
'Gut microbiota abundance (genus Parasutterella id.2892)' on 'Pituitary adenylate cyclase−activating polypeptide 27'

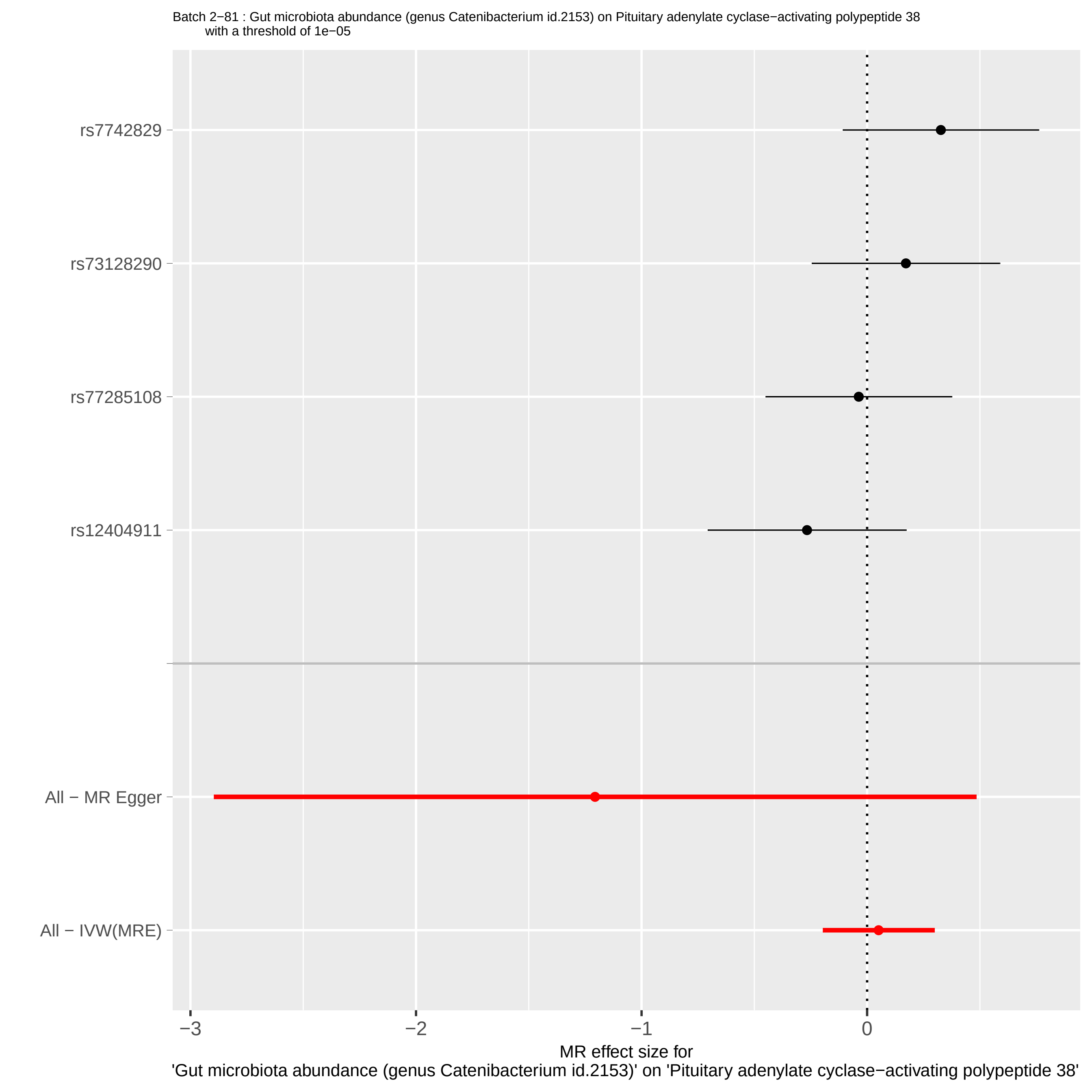

Batch 2−81 : Gut microbiota abundance (genus Catenibacterium id.2153) on Pituitary adenylate cyclase−activating polypeptide 38
with a threshold of 1e−05
rs7742829
rs73128290
rs77285108
rs12404911
All − MR Egger
All − IVW(MRE)
−3
−2
−1
0
MR effect size for
'Gut microbiota abundance (genus Catenibacterium id.2153)' on 'Pituitary adenylate cyclase−activating polypeptide 38'

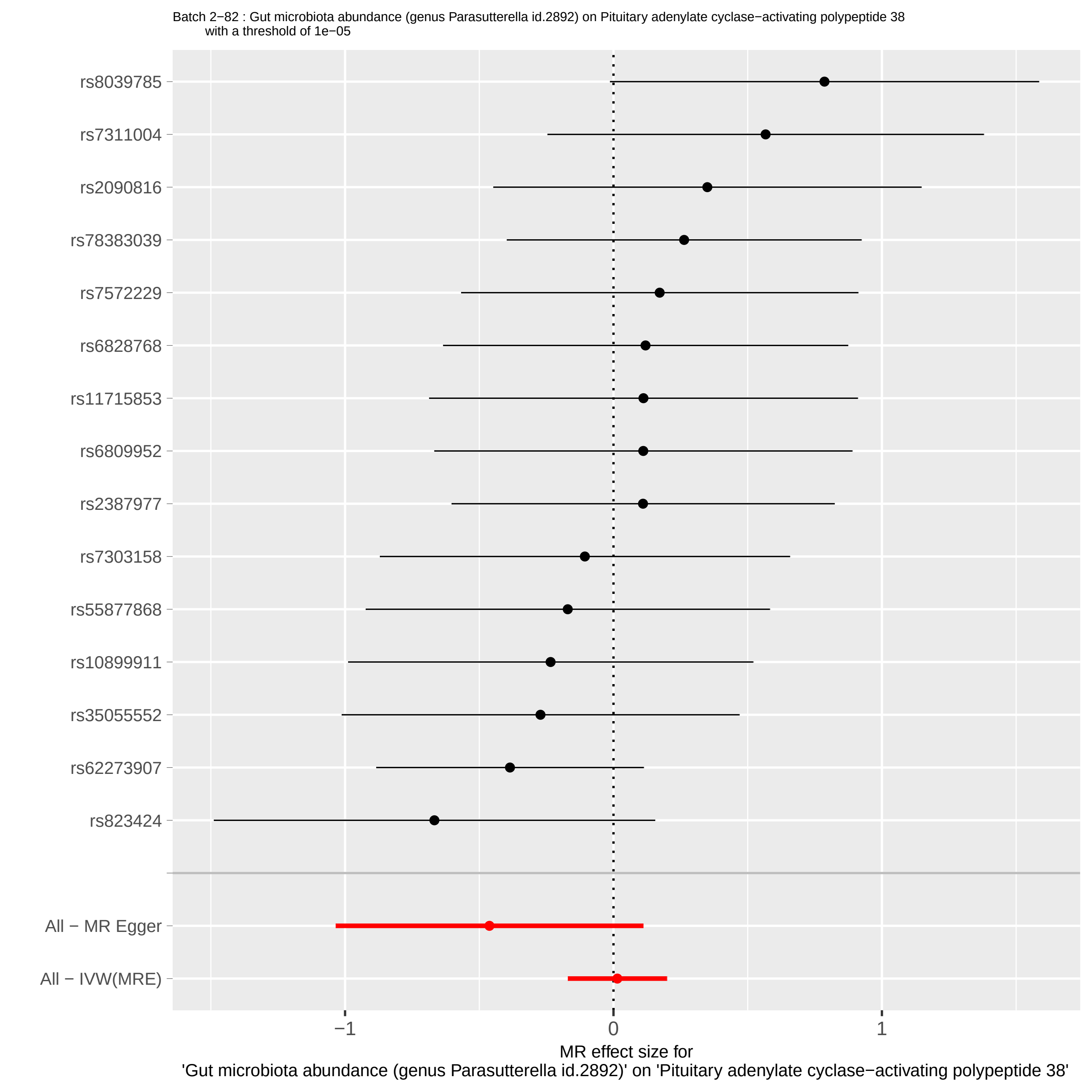
Batch 2−82 : Gut microbiota abundance (genus Parasutterella id.2892) on Pituitary adenylate cyclase−activating polypeptide 38
with a threshold of 1e−05
rs8039785
rs7311004
rs2090816
rs78383039
rs7572229
rs6828768
rs11715853
rs6809952
rs2387977
rs7303158
rs55877868
rs10899911
rs35055552
rs62273907
rs823424
All − MR Egger
All − IVW(MRE)
−1
0
1
MR effect size for
'Gut microbiota abundance (genus Parasutterella id.2892)' on 'Pituitary adenylate cyclase−activating polypeptide 38'

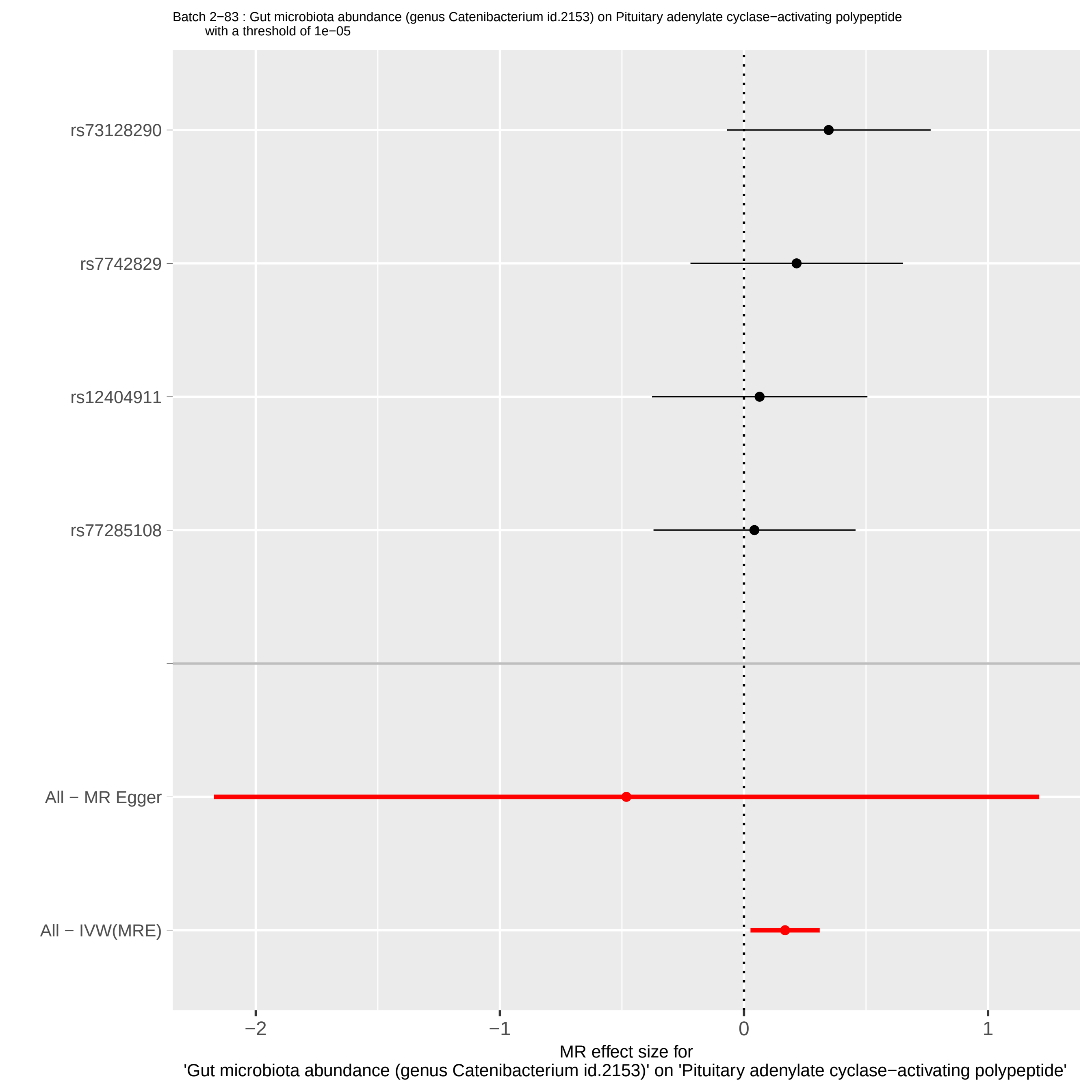

Batch 2−83 : Gut microbiota abundance (genus Catenibacterium id.2153) on Pituitary adenylate cyclase−activating polypeptide
with a threshold of 1e−05
rs73128290
rs7742829
rs12404911
rs77285108
All − MR Egger
All − IVW(MRE)
−2
−1
0
1
MR effect size for
'Gut microbiota abundance (genus Catenibacterium id.2153)' on 'Pituitary adenylate cyclase−activating polypeptide'

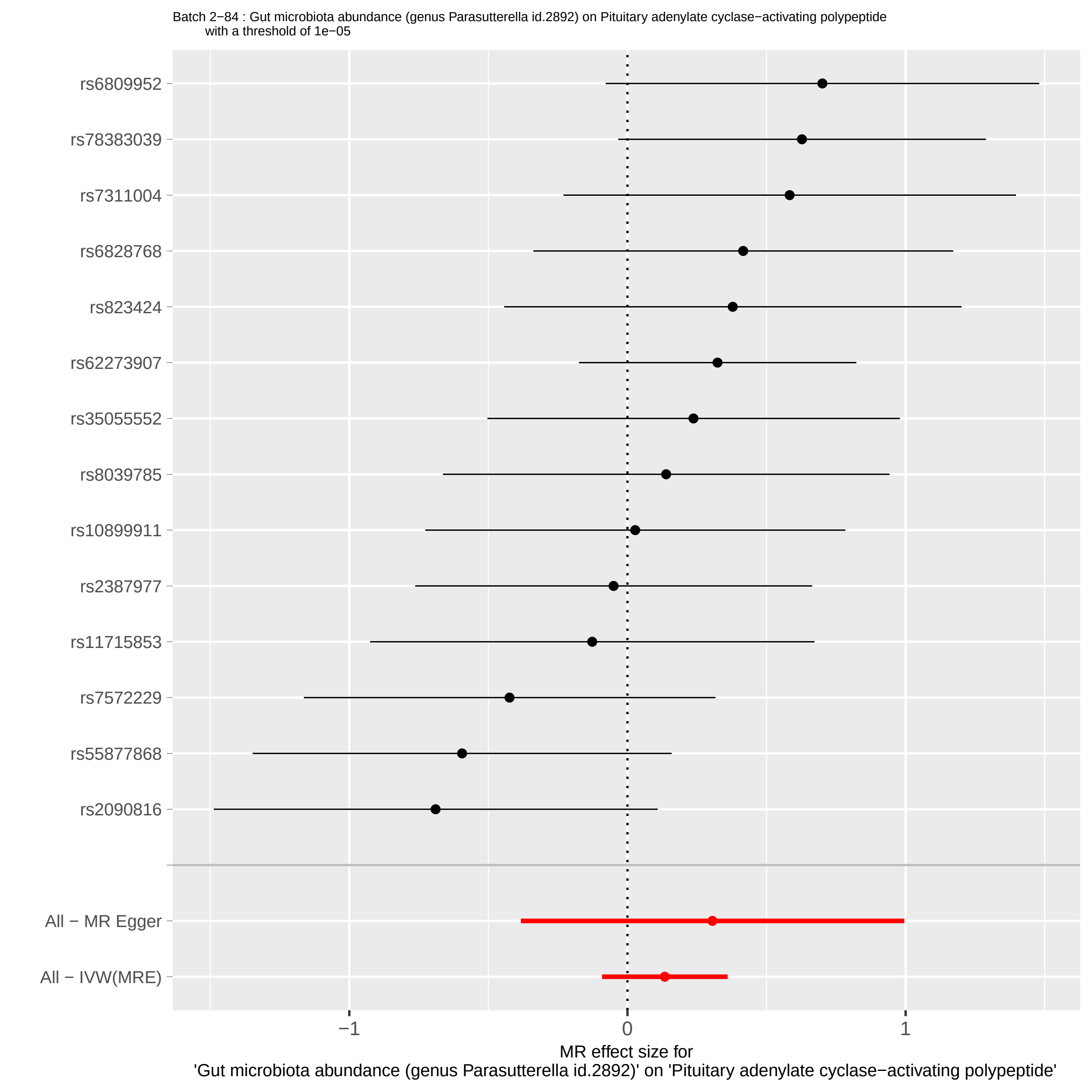
Batch 2−84 : Gut microbiota abundance (genus Parasutterella id.2892) on Pituitary adenylate cyclase−activating polypeptide with a threshold of 1e−05
rs6809952
rs78383039
rs7311004
rs6828768
rs823424
rs62273907
rs35055552
rs8039785
rs10899911
rs2387977
rs11715853
rs7572229
rs55877868
rs2090816
All − MR Egger
All − IVW(MRE)
−1
0
1
MR effect size for
'Gut microbiota abundance (genus Parasutterella id.2892)' on 'Pituitary adenylate cyclase−activating polypeptide'

Batch 2−85 : Gut microbiota abundance (genus Catenibacterium id.2153) on Adhesion G−protein coupled receptor F1
with a threshold of 1e−05

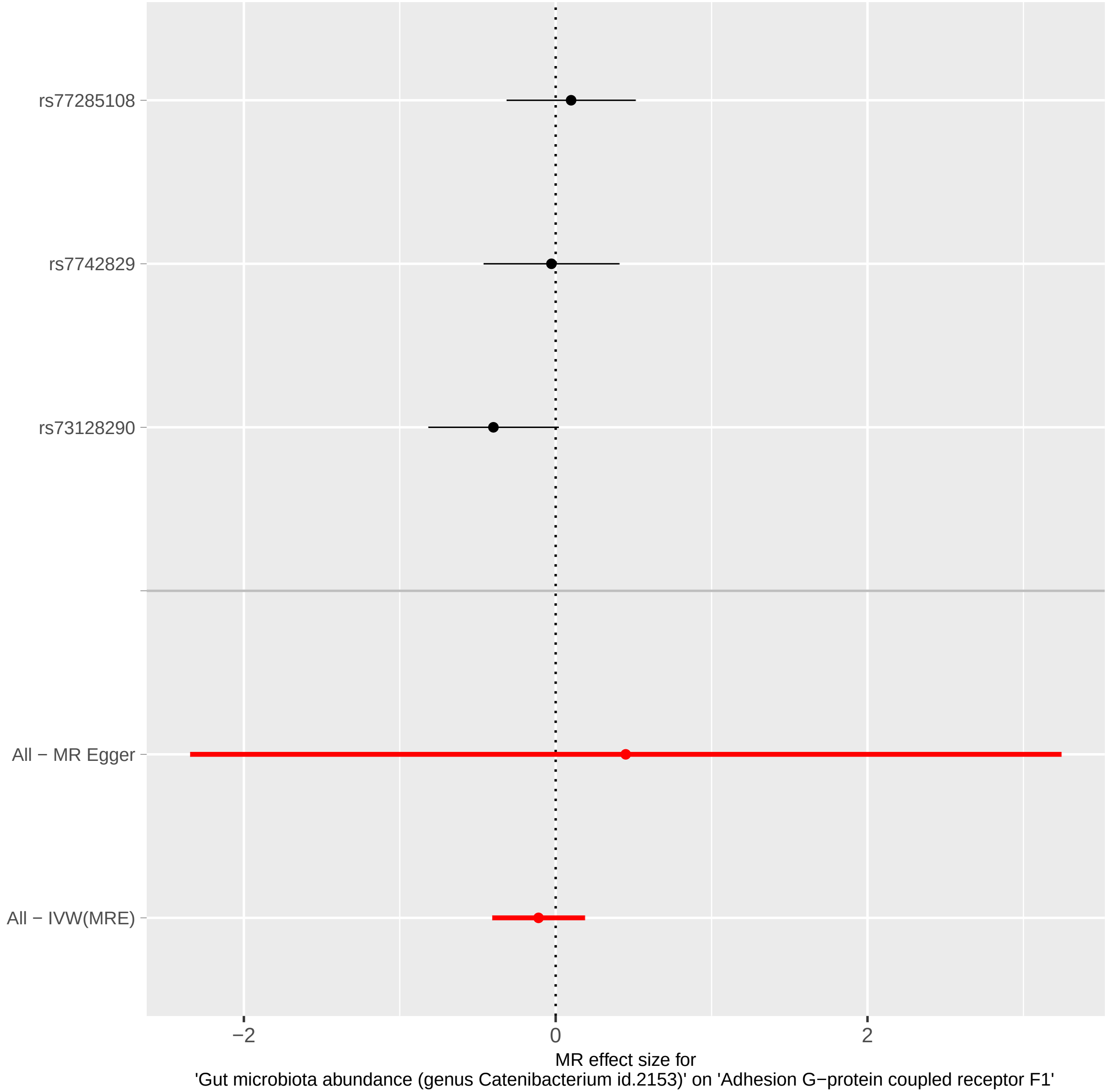

Batch 2−86 : Gut microbiota abundance (genus Parasutterella id.2892) on Adhesion G−protein coupled receptor F1
with a threshold of 1e−05

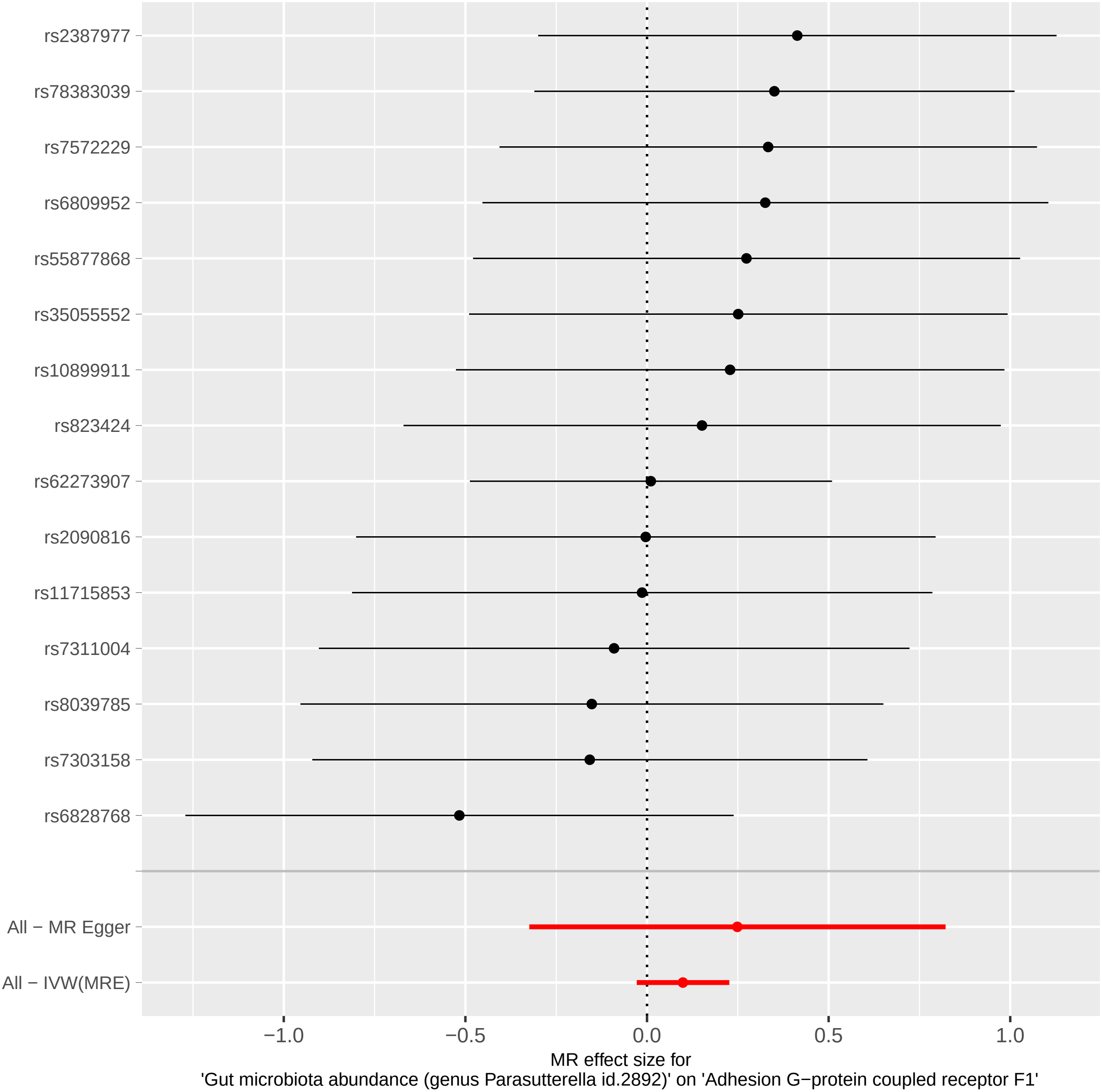

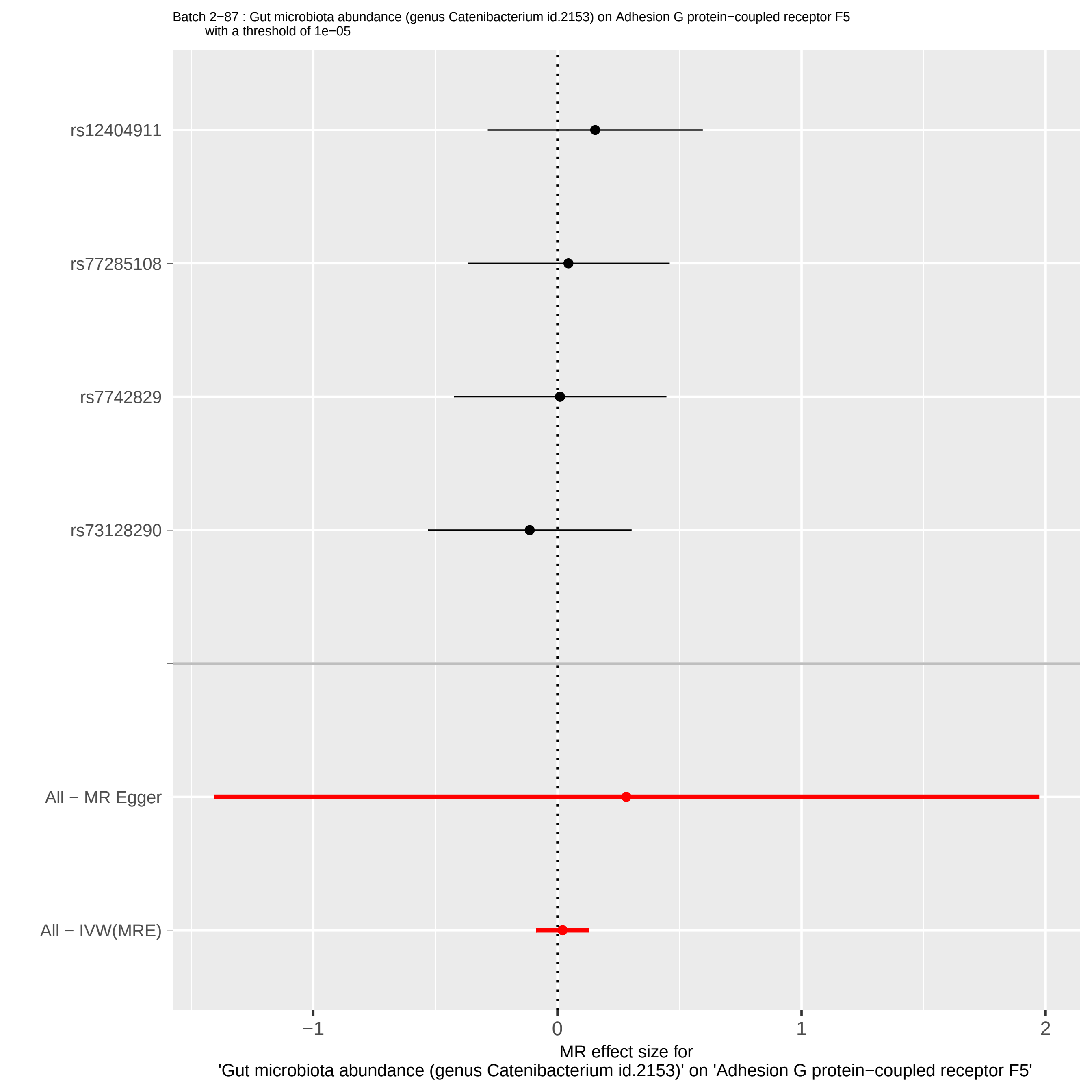
Batch 2−87 : Gut microbiota abundance (genus Catenibacterium id.2153) on Adhesion G protein−coupled receptor F5
with a threshold of 1e−05
rs12404911
rs77285108
rs7742829
rs73128290
All − MR Egger
All − IVW(MRE)
−1
0
1
2
MR effect size for
'Gut microbiota abundance (genus Catenibacterium id.2153)' on 'Adhesion G protein−coupled receptor F5'

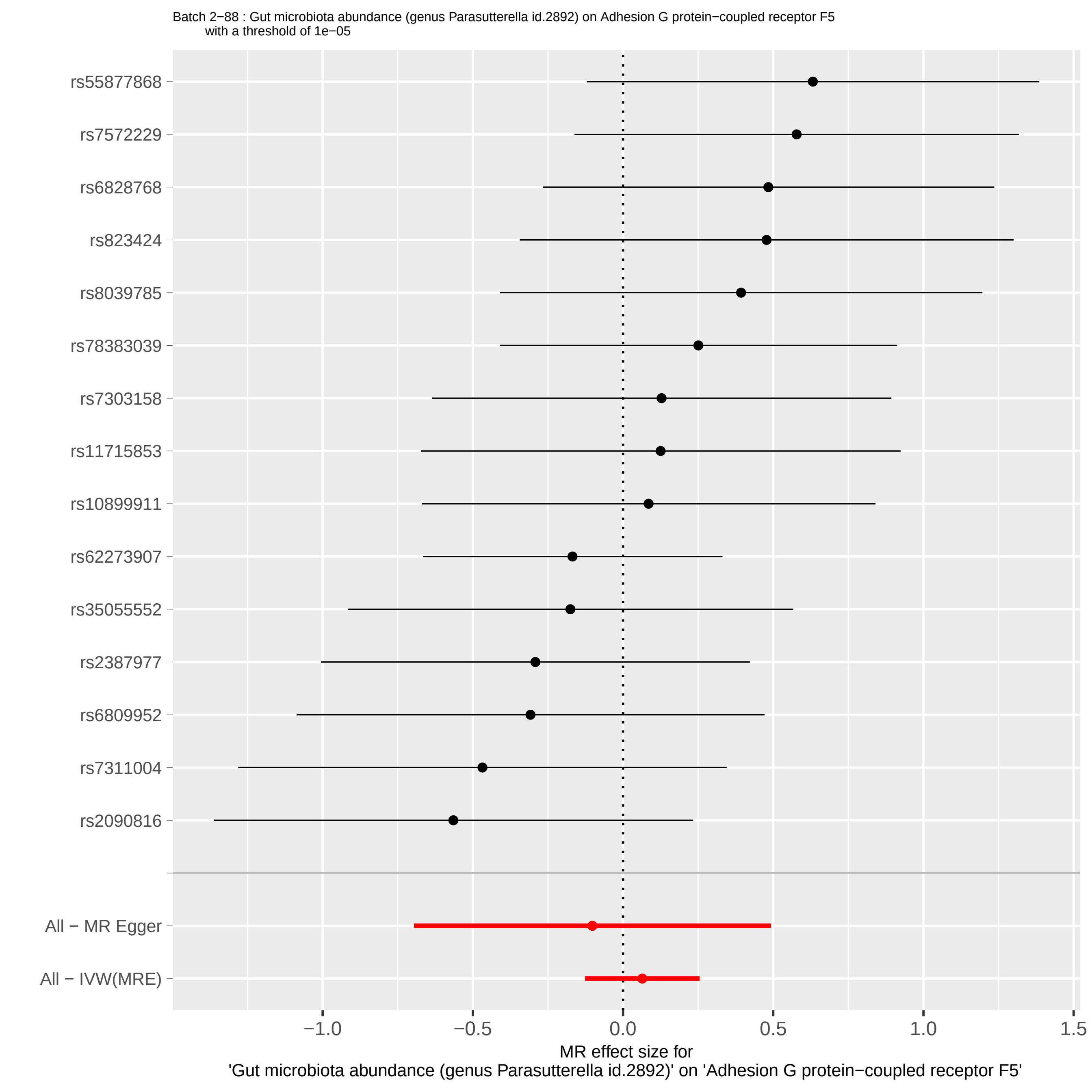
Batch 2−88 : Gut microbiota abundance (genus Parasutterella id.2892) on Adhesion G protein−coupled receptor F5
with a threshold of 1e−05
rs55877868
rs7572229
rs6828768
rs823424
rs8039785
rs78383039
rs7303158
rs11715853
rs10899911
rs62273907
rs35055552
rs2387977
rs6809952
rs7311004
rs2090816
All − MR Egger
All − IVW(MRE)
−1.0
−0.5
0.0
0.5
1.0
1.5
MR effect size for
'Gut microbiota abundance (genus Parasutterella id.2892)' on 'Adhesion G protein−coupled receptor F5'

Batch 2−89 : Gut microbiota abundance (genus Catenibacterium id.2153) on Adhesion G−protein coupled receptor G5
with a threshold of 1e−05

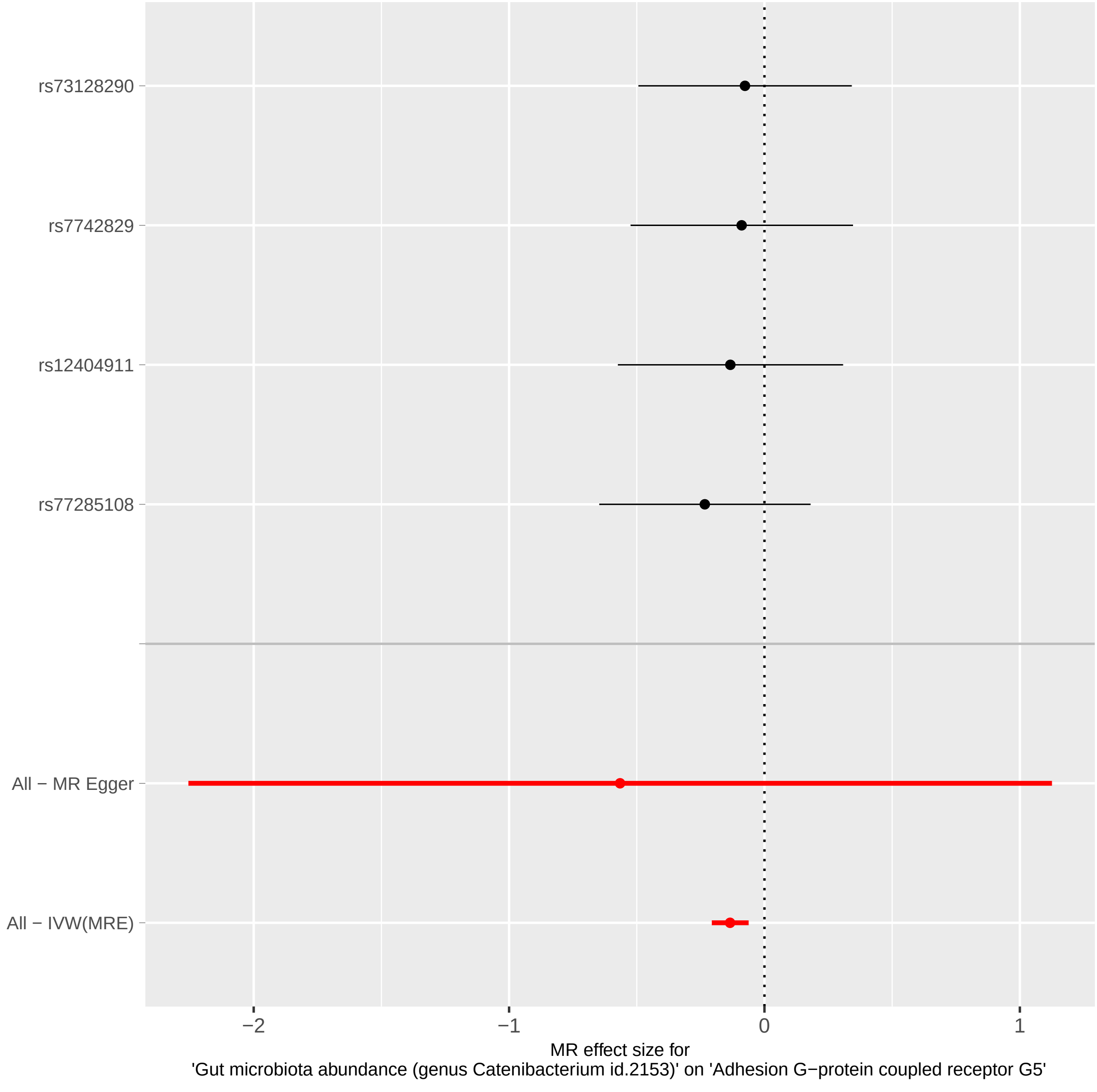

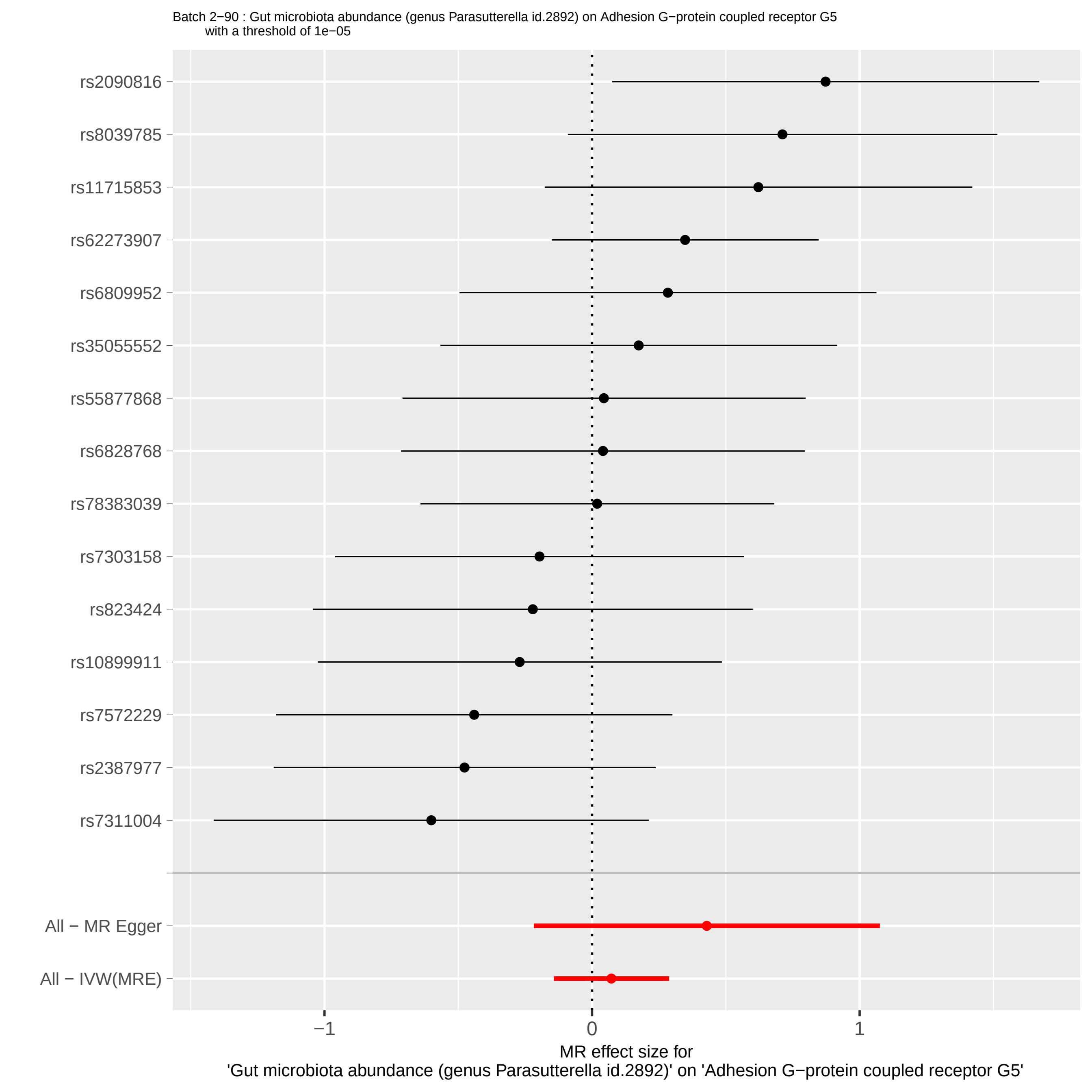
Batch 2−90 : Gut microbiota abundance (genus Parasutterella id.2892) on Adhesion G−protein coupled receptor G5
with a threshold of 1e−05
rs2090816
rs8039785
rs11715853
rs62273907
rs6809952
rs35055552
rs55877868
rs6828768
rs78383039
rs7303158
rs823424
rs10899911
rs7572229
rs2387977
rs7311004
All − MR Egger
All − IVW(MRE)
−1
0
1
MR effect size for
'Gut microbiota abundance (genus Parasutterella id.2892)' on 'Adhesion G−protein coupled receptor G5'

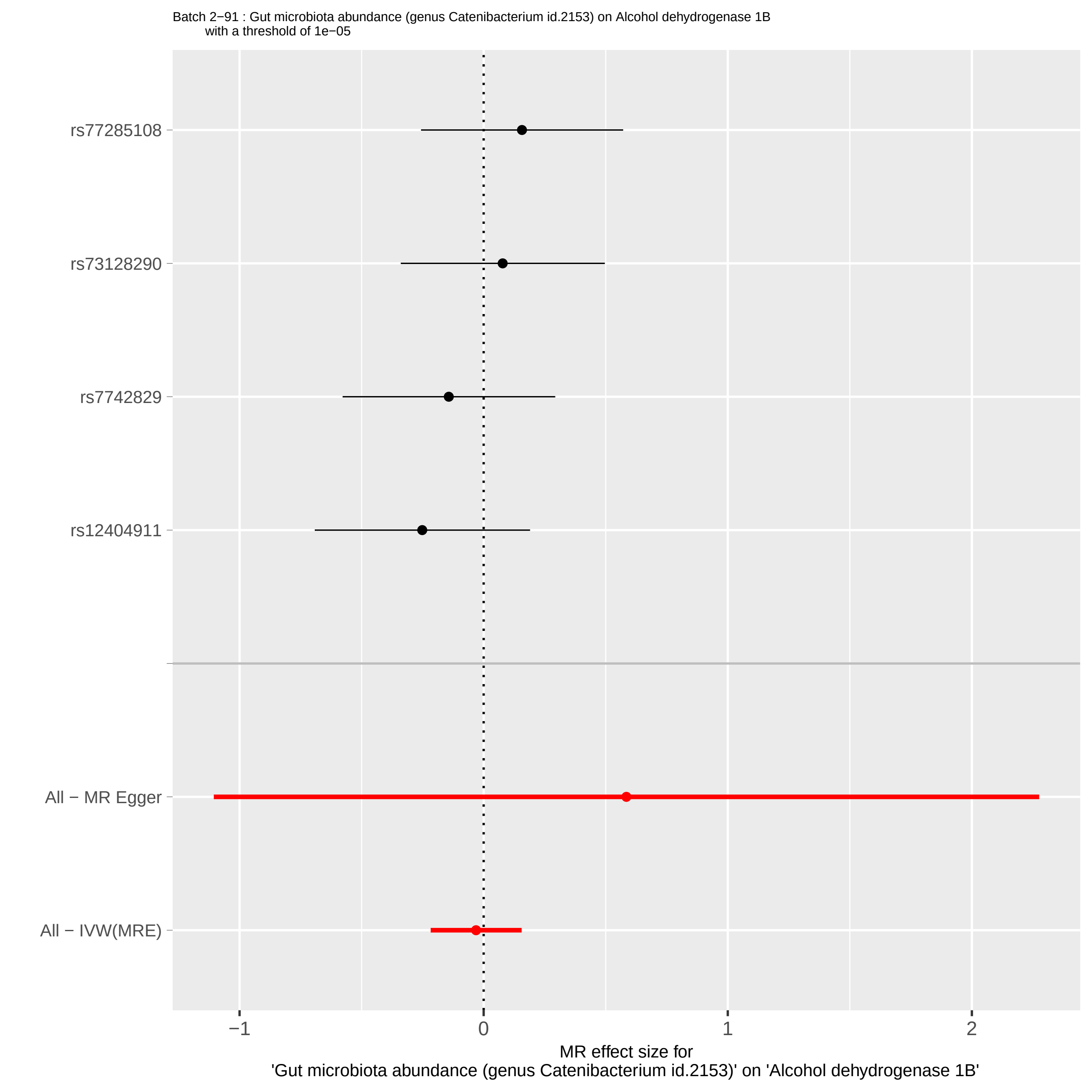
Batch 2−91 : Gut microbiota abundance (genus Catenibacterium id.2153) on Alcohol dehydrogenase 1B
with a threshold of 1e−05
rs77285108
rs73128290
rs7742829
rs12404911
All − MR Egger
All − IVW(MRE)
−1
0
1
2
MR effect size for
'Gut microbiota abundance (genus Catenibacterium id.2153)' on 'Alcohol dehydrogenase 1B'

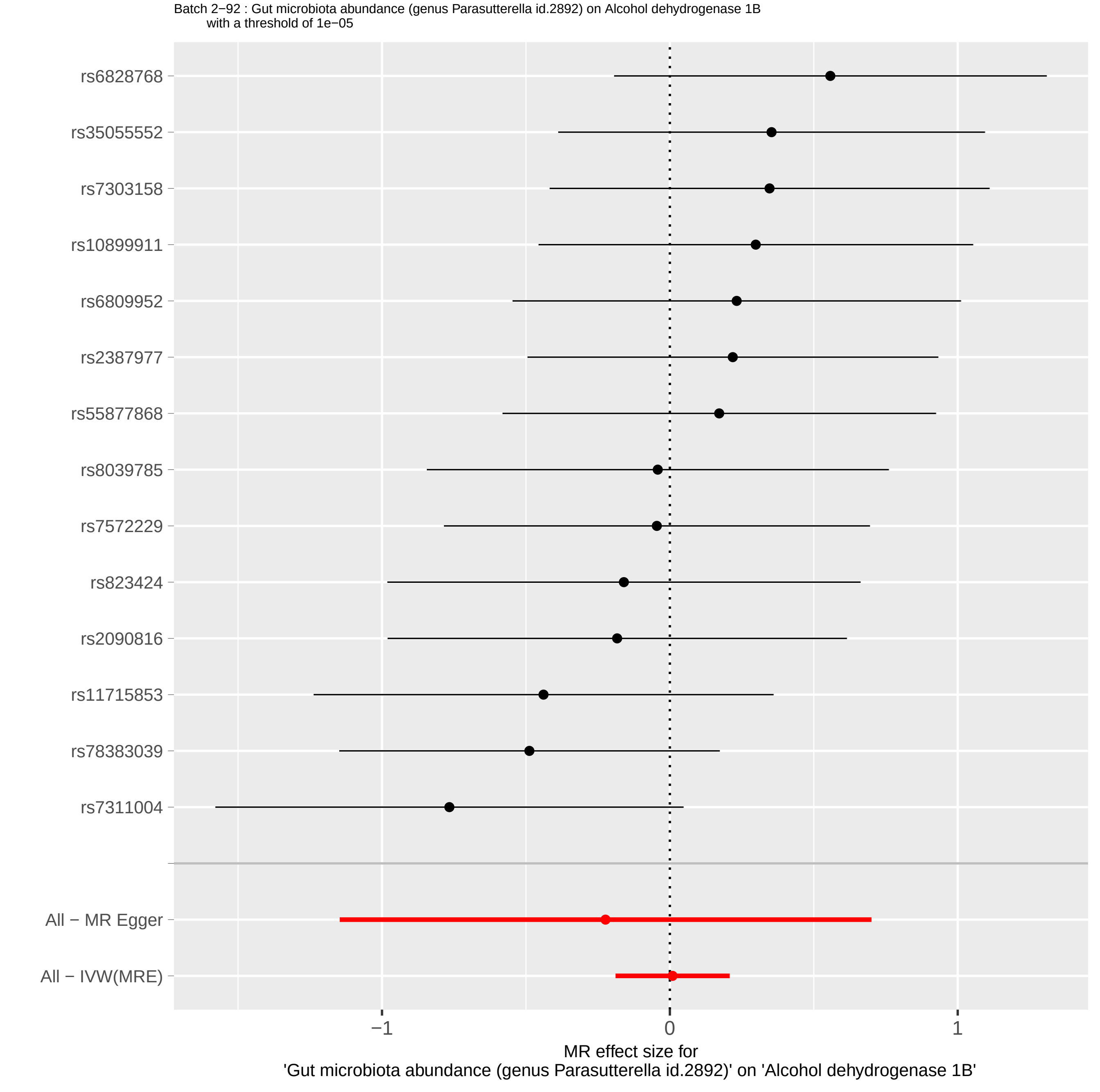

Batch 2−92 : Gut microbiota abundance (genus Parasutterella id.2892) on Alcohol dehydrogenase 1B
with a threshold of 1e−05
rs6828768
rs35055552
rs7303158
rs10899911
rs6809952
rs2387977
rs55877868
rs8039785
rs7572229
rs823424
rs2090816
rs11715853
rs78383039
rs7311004
All − MR Egger
All − IVW(MRE)
−1
0
1
MR effect size for
'Gut microbiota abundance (genus Parasutterella id.2892)' on 'Alcohol dehydrogenase 1B'

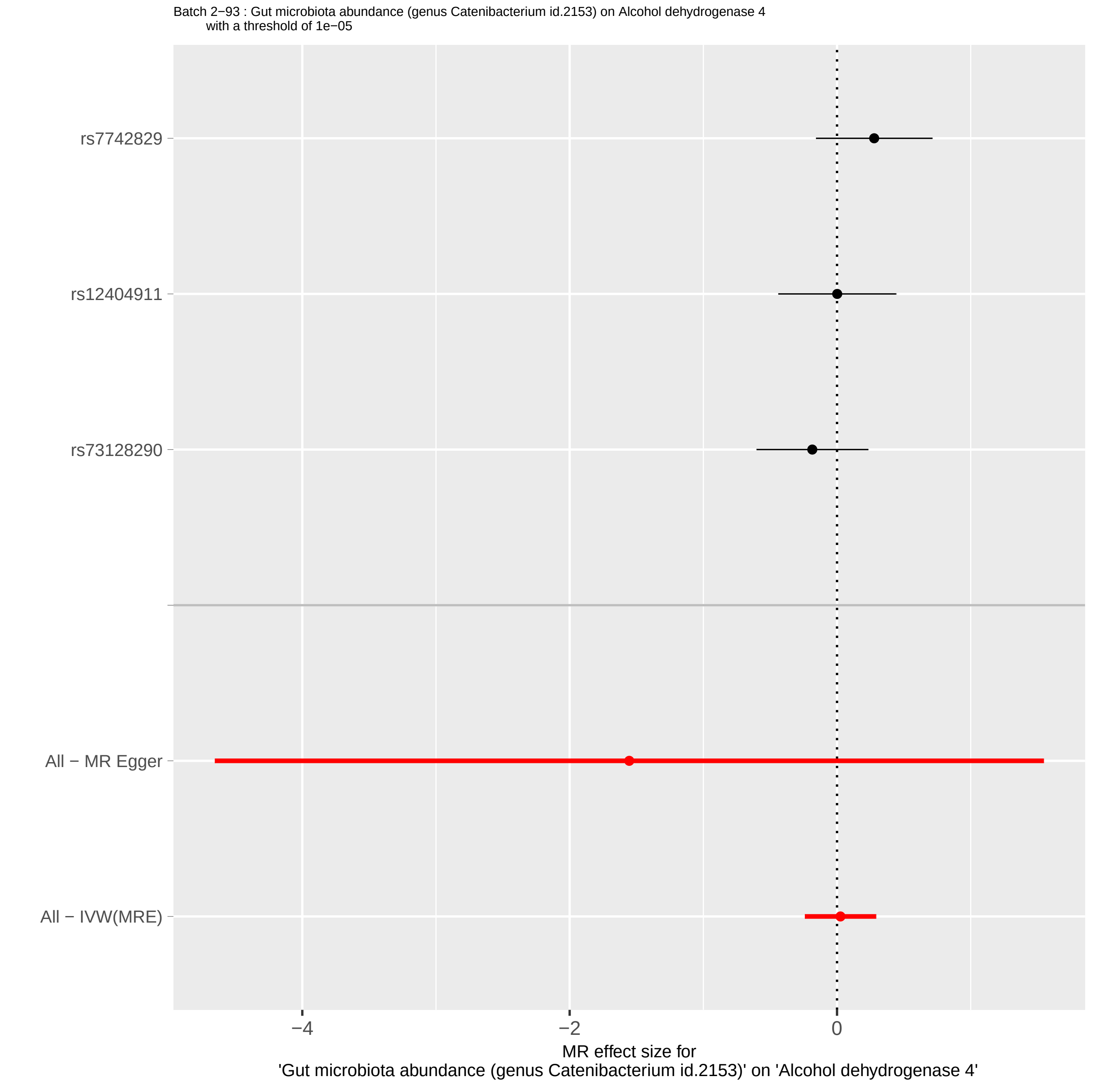
Batch 2−93 : Gut microbiota abundance (genus Catenibacterium id.2153) on Alcohol dehydrogenase 4
with a threshold of 1e−05
rs7742829
rs12404911
rs73128290
All − MR Egger
All − IVW(MRE)
−4
−2
0
MR effect size for
'Gut microbiota abundance (genus Catenibacterium id.2153)' on 'Alcohol dehydrogenase 4'

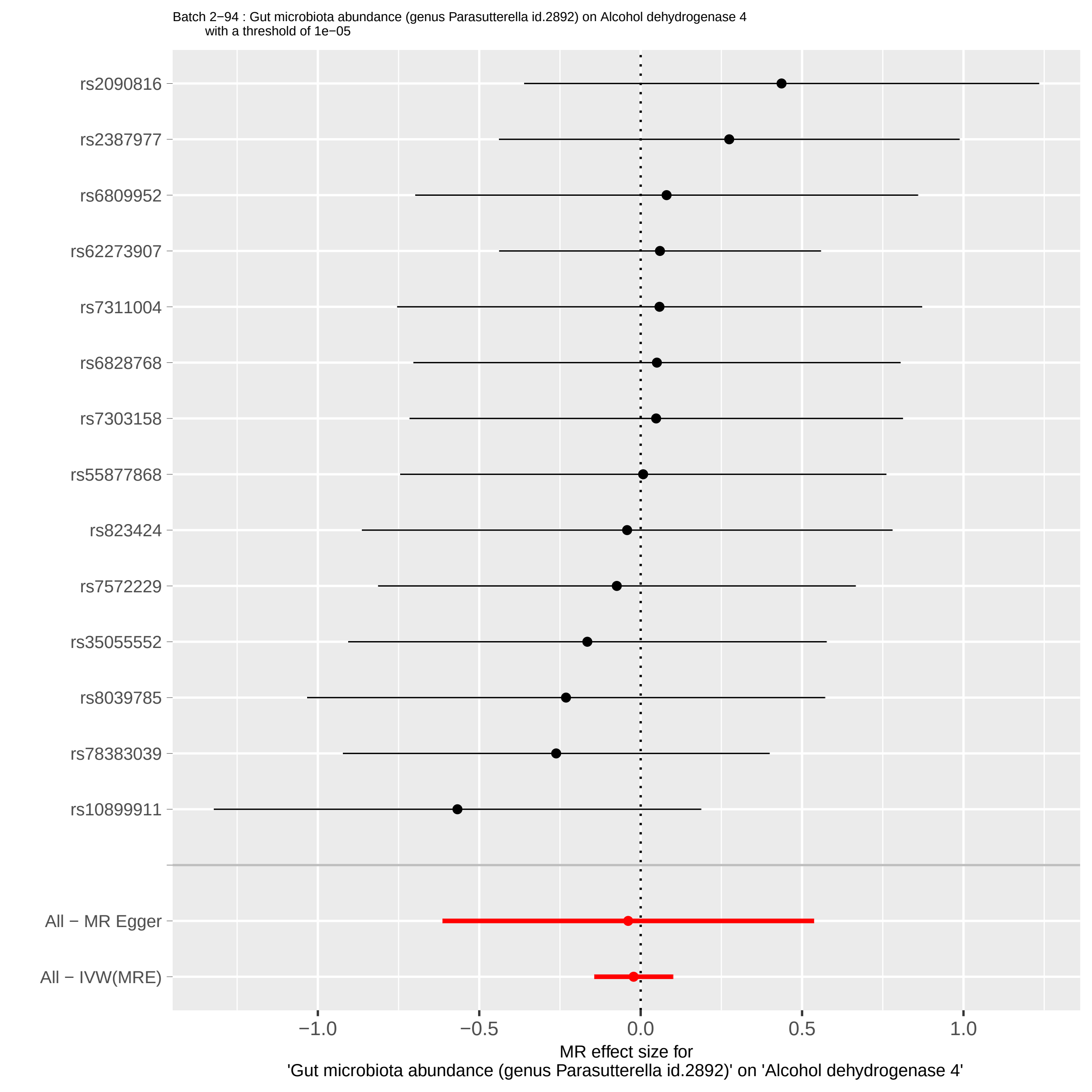

Batch 2−94 : Gut microbiota abundance (genus Parasutterella id.2892) on Alcohol dehydrogenase 4
with a threshold of 1e−05
rs2090816
rs2387977
rs6809952
rs62273907
rs7311004
rs6828768
rs7303158
rs55877868
rs823424
rs7572229
rs35055552
rs8039785
rs78383039
rs10899911
All − MR Egger
All − IVW(MRE)
−1.0
−0.5
0.0
0.5
1.0
MR effect size for
'Gut microbiota abundance (genus Parasutterella id.2892)' on 'Alcohol dehydrogenase 4'

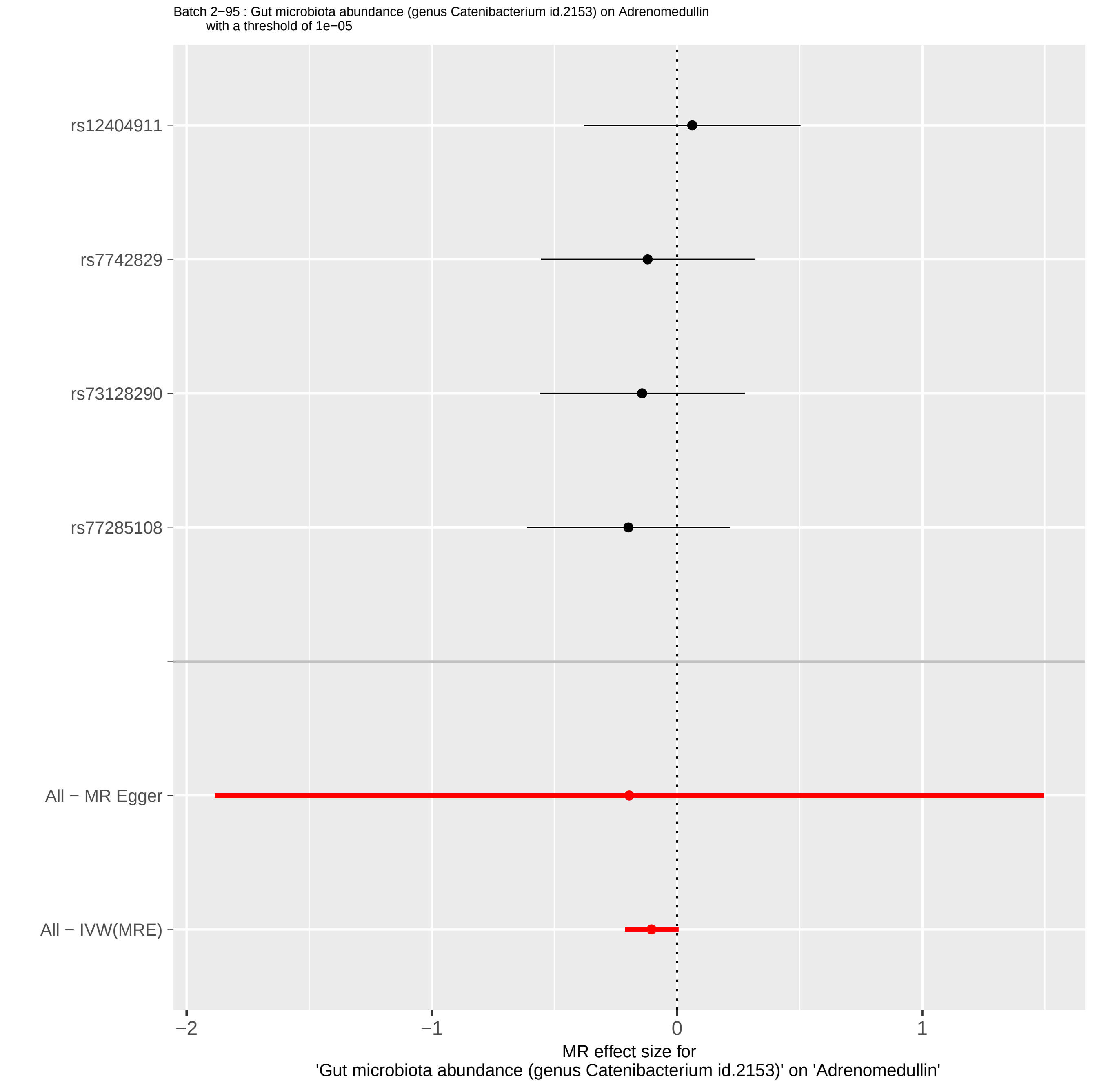

Batch 2−95 : Gut microbiota abundance (genus Catenibacterium id.2153) on Adrenomedullin
with a threshold of 1e−05
rs12404911
rs7742829
rs73128290
rs77285108
All − MR Egger
All − IVW(MRE)
−2
−1
0
1
MR effect size for
'Gut microbiota abundance (genus Catenibacterium id.2153)' on 'Adrenomedullin'

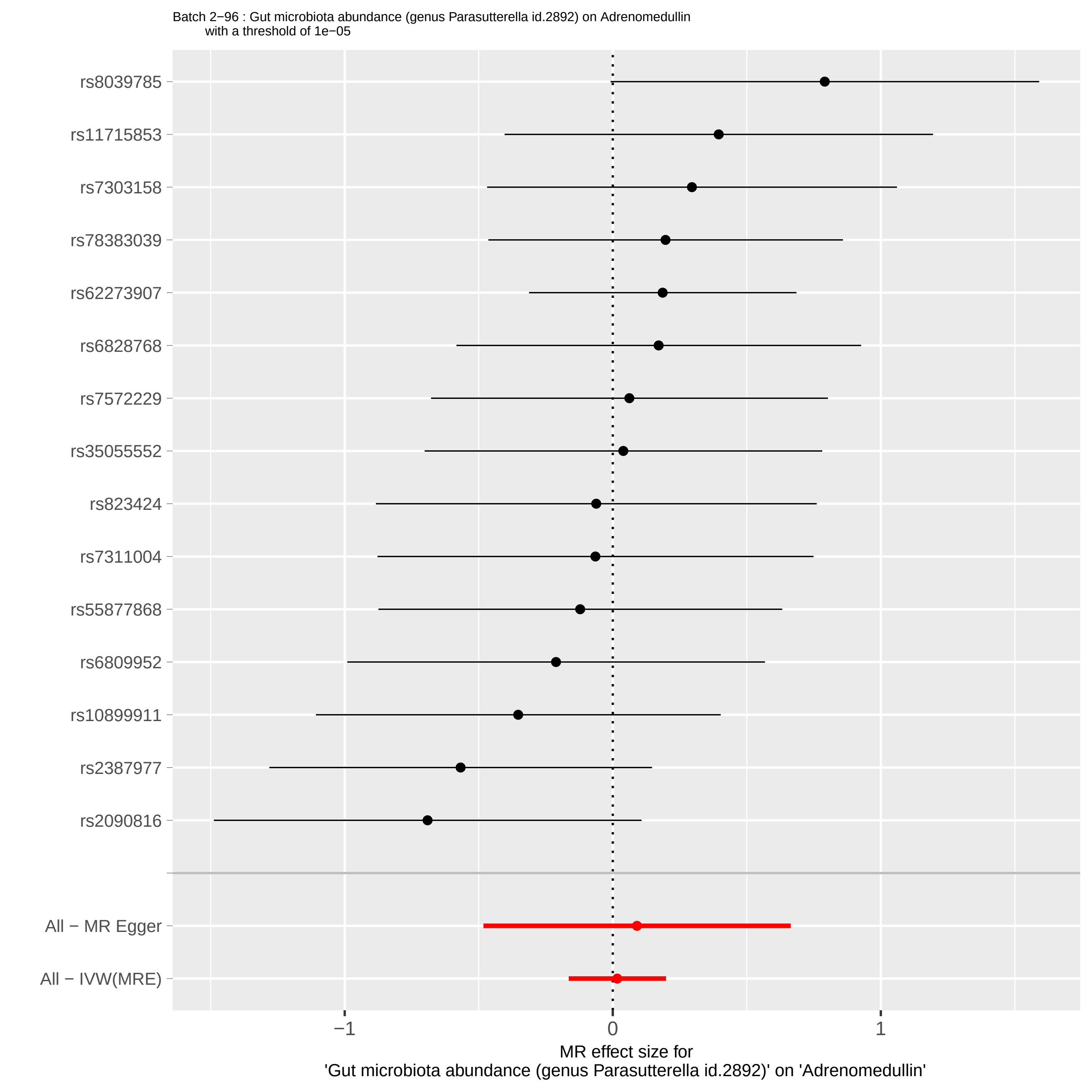

Batch 2−96 : Gut microbiota abundance (genus Parasutterella id.2892) on Adrenomedullin
with a threshold of 1e−05
rs8039785
rs11715853
rs7303158
rs78383039
rs62273907
rs6828768
rs7572229
rs35055552
rs823424
rs7311004
rs55877868
rs6809952
rs10899911
rs2387977
rs2090816
All − MR Egger
All − IVW(MRE)
−1
0
1
MR effect size for
'Gut microbiota abundance (genus Parasutterella id.2892)' on 'Adrenomedullin'

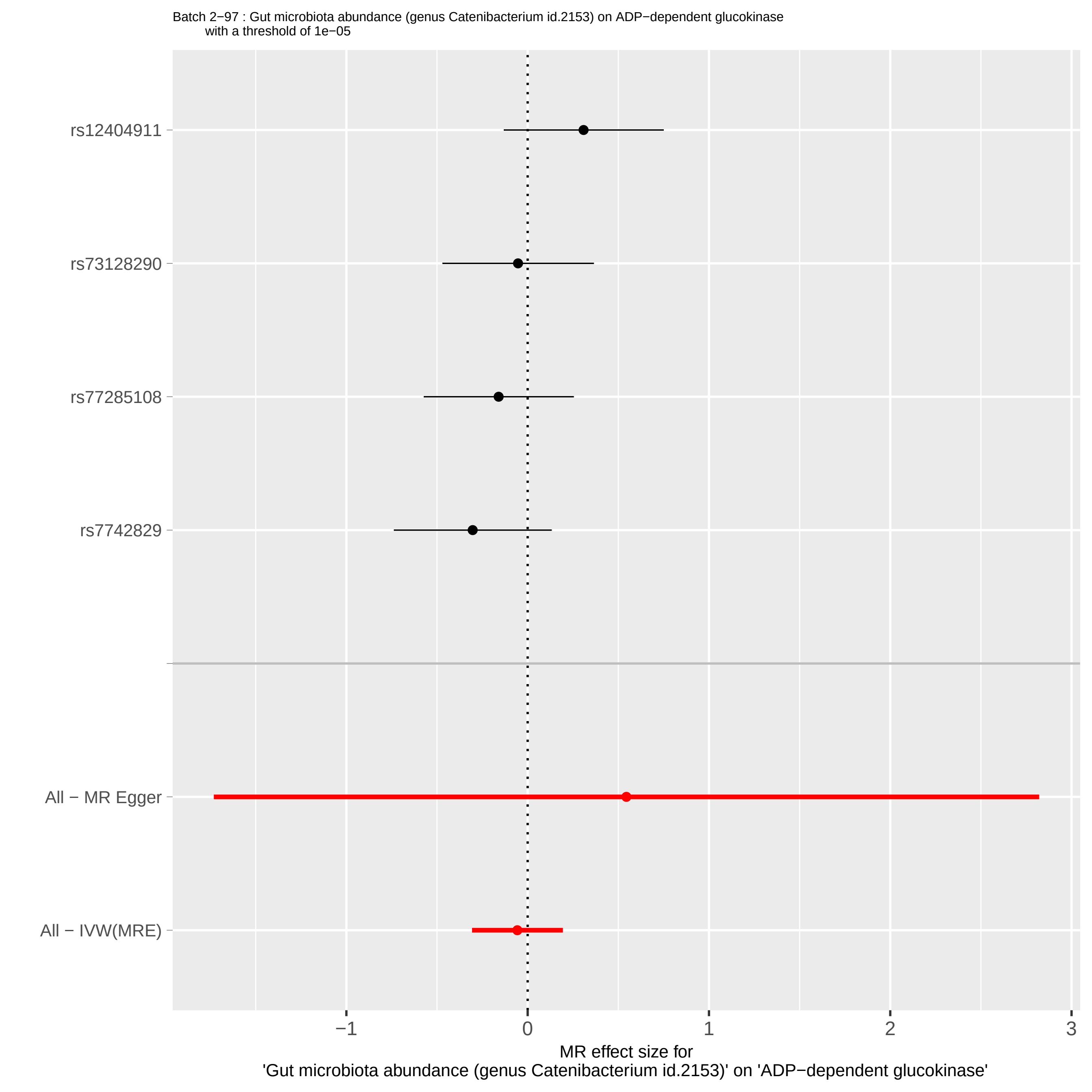

Batch 2−97 : Gut microbiota abundance (genus Catenibacterium id.2153) on ADP−dependent glucokinase
with a threshold of 1e−05
rs12404911
rs73128290
rs77285108
rs7742829
All − MR Egger
All − IVW(MRE)
−1
0
1
2
3
MR effect size for
'Gut microbiota abundance (genus Catenibacterium id.2153)' on 'ADP−dependent glucokinase'

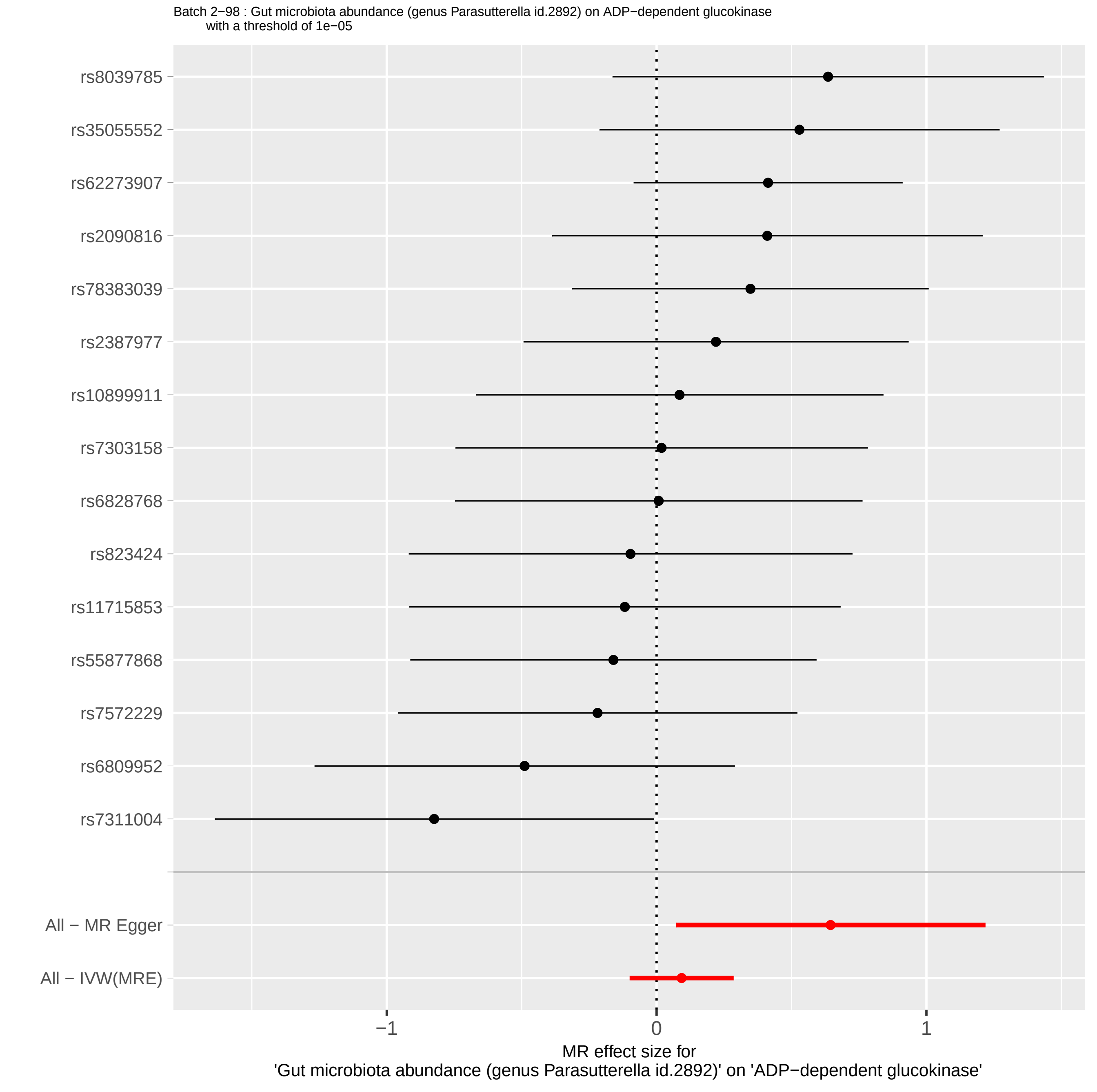
Batch 2−98 : Gut microbiota abundance (genus Parasutterella id.2892) on ADP−dependent glucokinase
with a threshold of 1e−05
rs8039785
rs35055552
rs62273907
rs2090816
rs78383039
rs2387977
rs10899911
rs7303158
rs6828768
rs823424
rs11715853
rs55877868
rs7572229
rs6809952
rs7311004
All − MR Egger
All − IVW(MRE)
−1
0
1
MR effect size for
'Gut microbiota abundance (genus Parasutterella id.2892)' on 'ADP−dependent glucokinase'

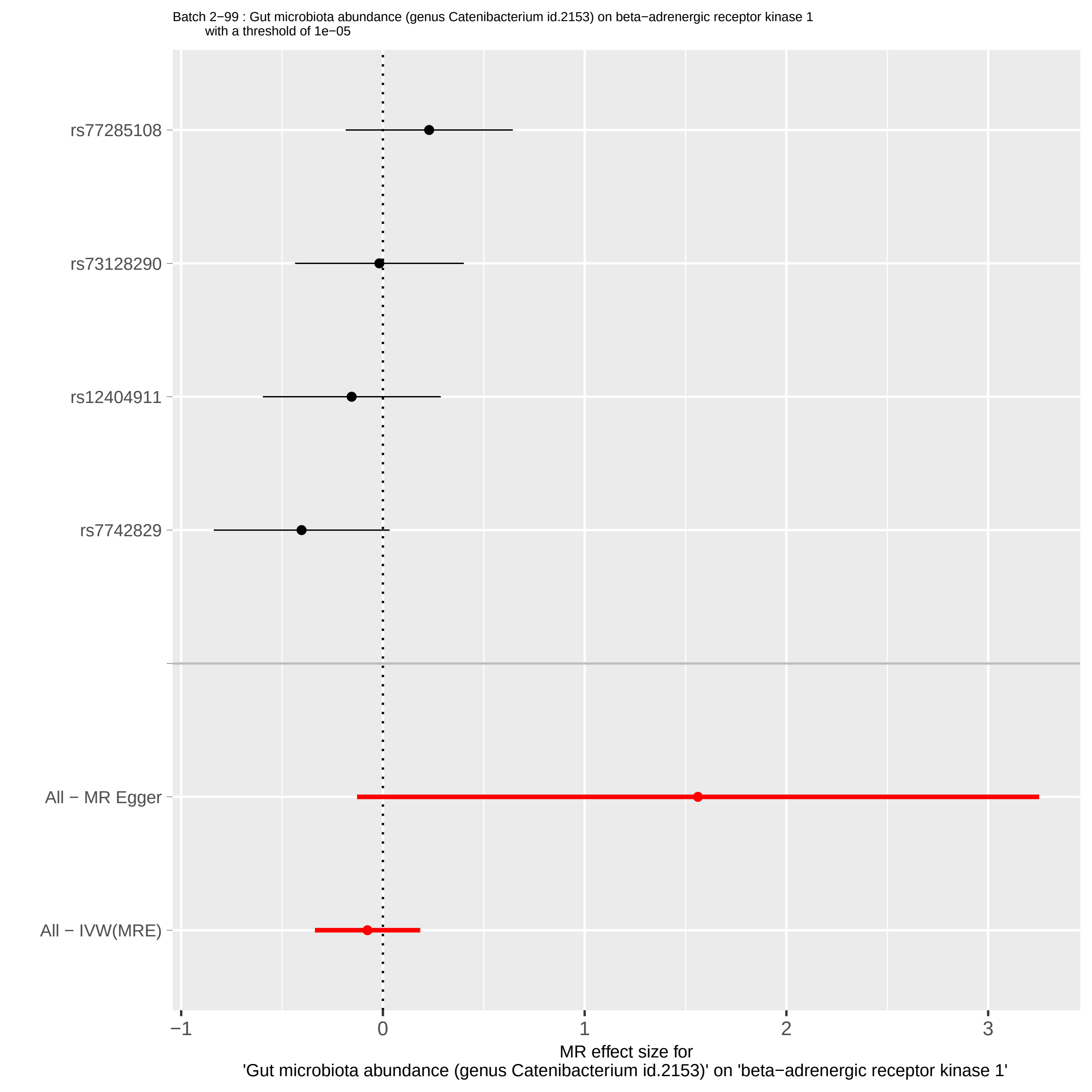

Batch 2−99 : Gut microbiota abundance (genus Catenibacterium id.2153) on beta−adrenergic receptor kinase 1
with a threshold of 1e−05
rs77285108
rs73128290
rs12404911
rs7742829
All − MR Egger
All − IVW(MRE)
−1
0
1
2
3
MR effect size for
'Gut microbiota abundance (genus Catenibacterium id.2153)' on 'beta−adrenergic receptor kinase 1'

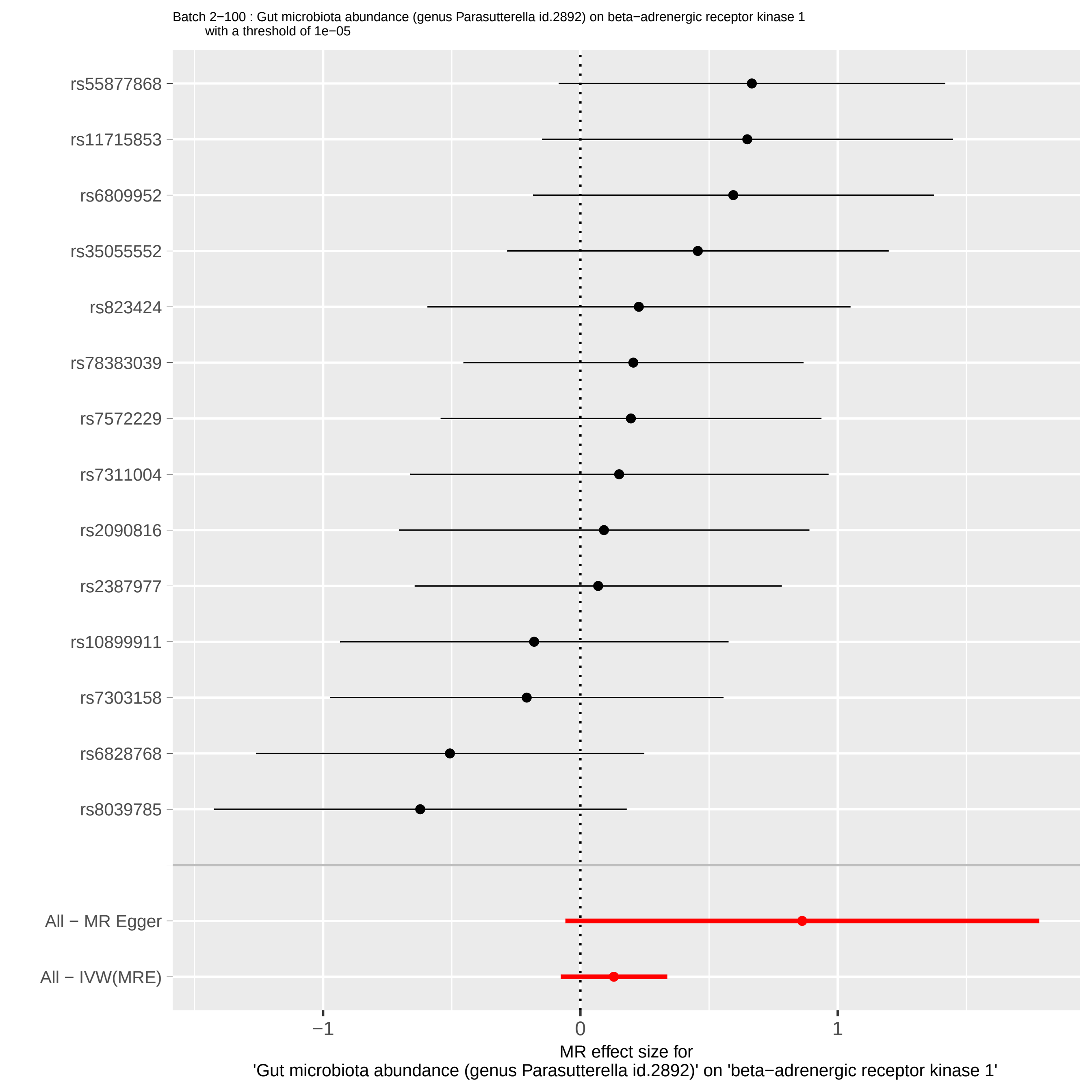

Batch 2−100 : Gut microbiota abundance (genus Parasutterella id.2892) on beta−adrenergic receptor kinase 1
with a threshold of 1e−05
rs55877868
rs11715853
rs6809952
rs35055552
rs823424
rs78383039
rs7572229
rs7311004
rs2090816
rs2387977
rs10899911
rs7303158
rs6828768
rs8039785
All − MR Egger
All − IVW(MRE)
−1
0
1
MR effect size for
'Gut microbiota abundance (genus Parasutterella id.2892)' on 'beta−adrenergic receptor kinase 1'

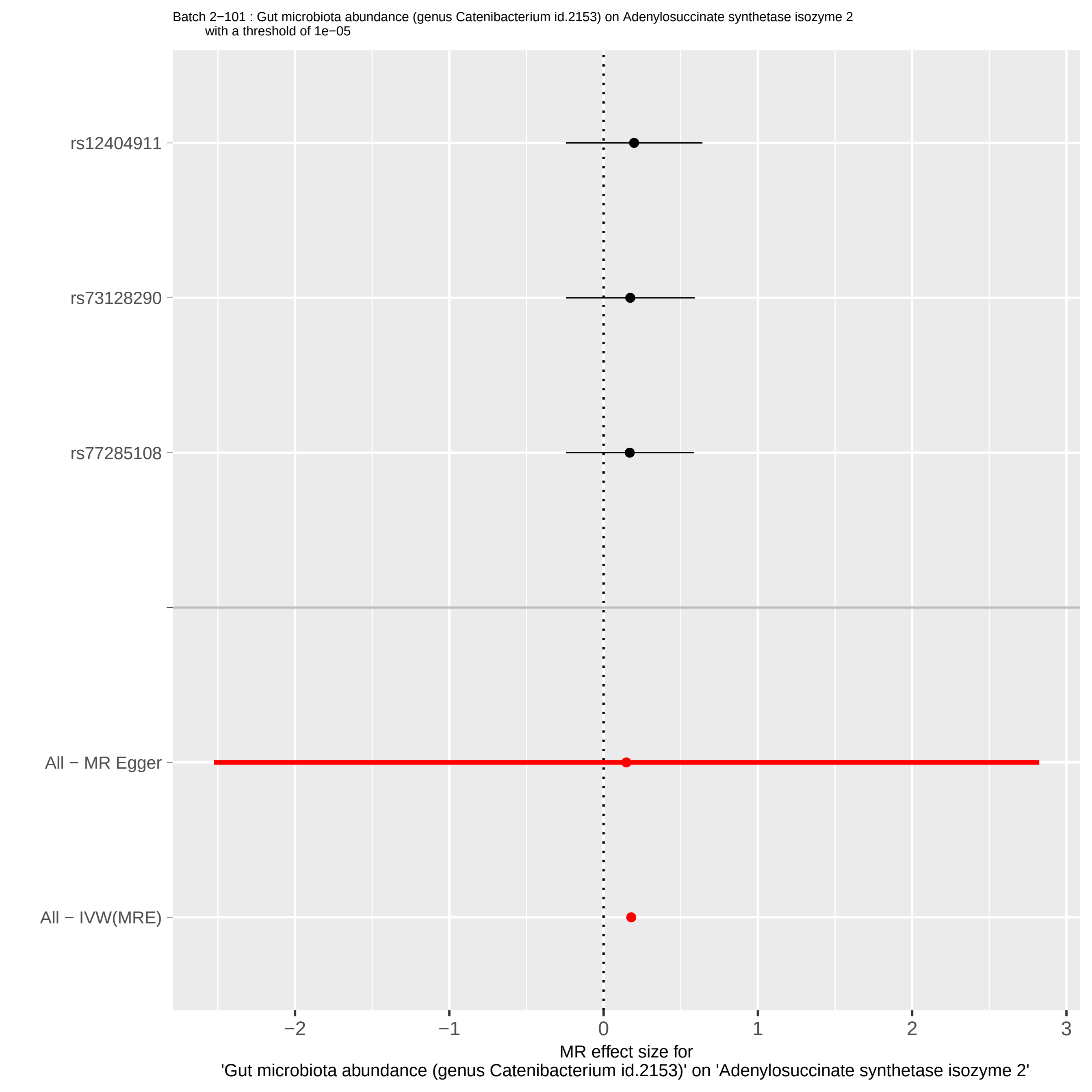

Batch 2−101 : Gut microbiota abundance (genus Catenibacterium id.2153) on Adenylosuccinate synthetase isozyme 2
with a threshold of 1e−05
rs12404911
rs73128290
rs77285108
All − MR Egger
All − IVW(MRE)
−2
−1
0
1
2
3
MR effect size for
'Gut microbiota abundance (genus Catenibacterium id.2153)' on 'Adenylosuccinate synthetase isozyme 2'

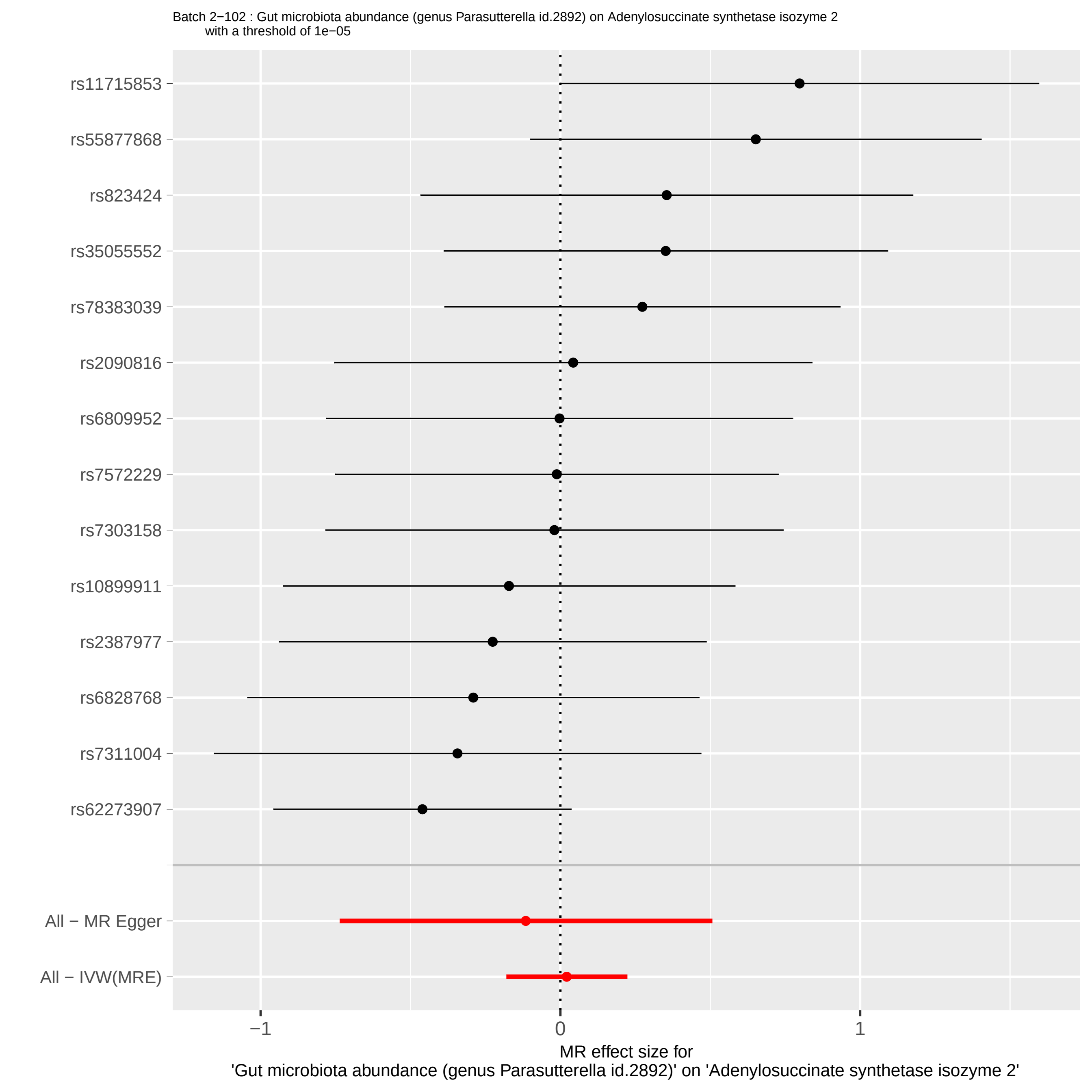

Batch 2−102 : Gut microbiota abundance (genus Parasutterella id.2892) on Adenylosuccinate synthetase isozyme 2
with a threshold of 1e−05
rs11715853
rs55877868
rs823424
rs35055552
rs78383039
rs2090816
rs6809952
rs7572229
rs7303158
rs10899911
rs2387977
rs6828768
rs7311004
rs62273907
All − MR Egger
All − IVW(MRE)
−1
0
1
MR effect size for
'Gut microbiota abundance (genus Parasutterella id.2892)' on 'Adenylosuccinate synthetase isozyme 2'

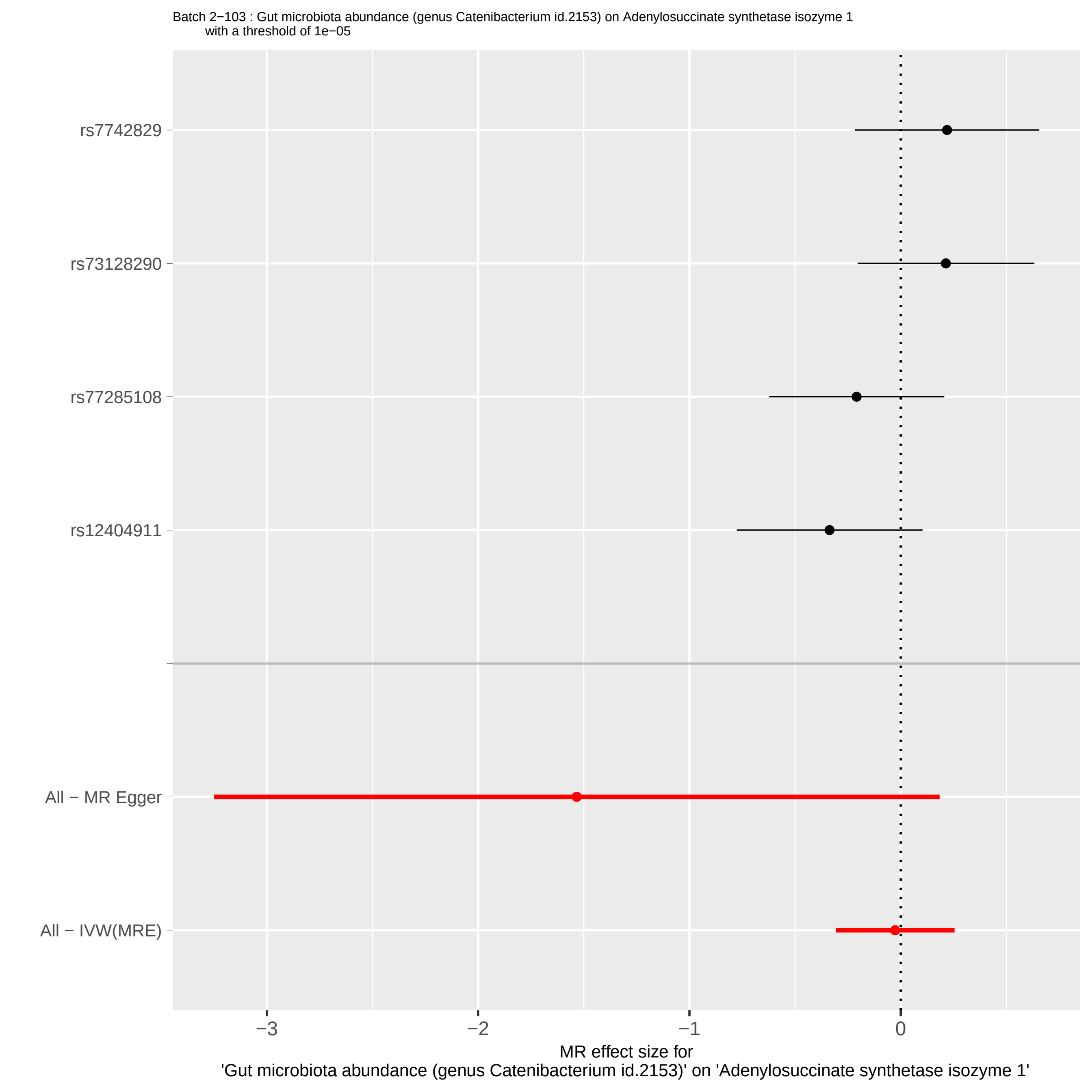

Batch 2−103 : Gut microbiota abundance (genus Catenibacterium id.2153) on Adenylosuccinate synthetase isozyme 1
with a threshold of 1e−05
rs7742829
rs73128290
rs77285108
rs12404911
All − MR Egger
All − IVW(MRE)
−3
−2
−1
0
MR effect size for
'Gut microbiota abundance (genus Catenibacterium id.2153)' on 'Adenylosuccinate synthetase isozyme 1'

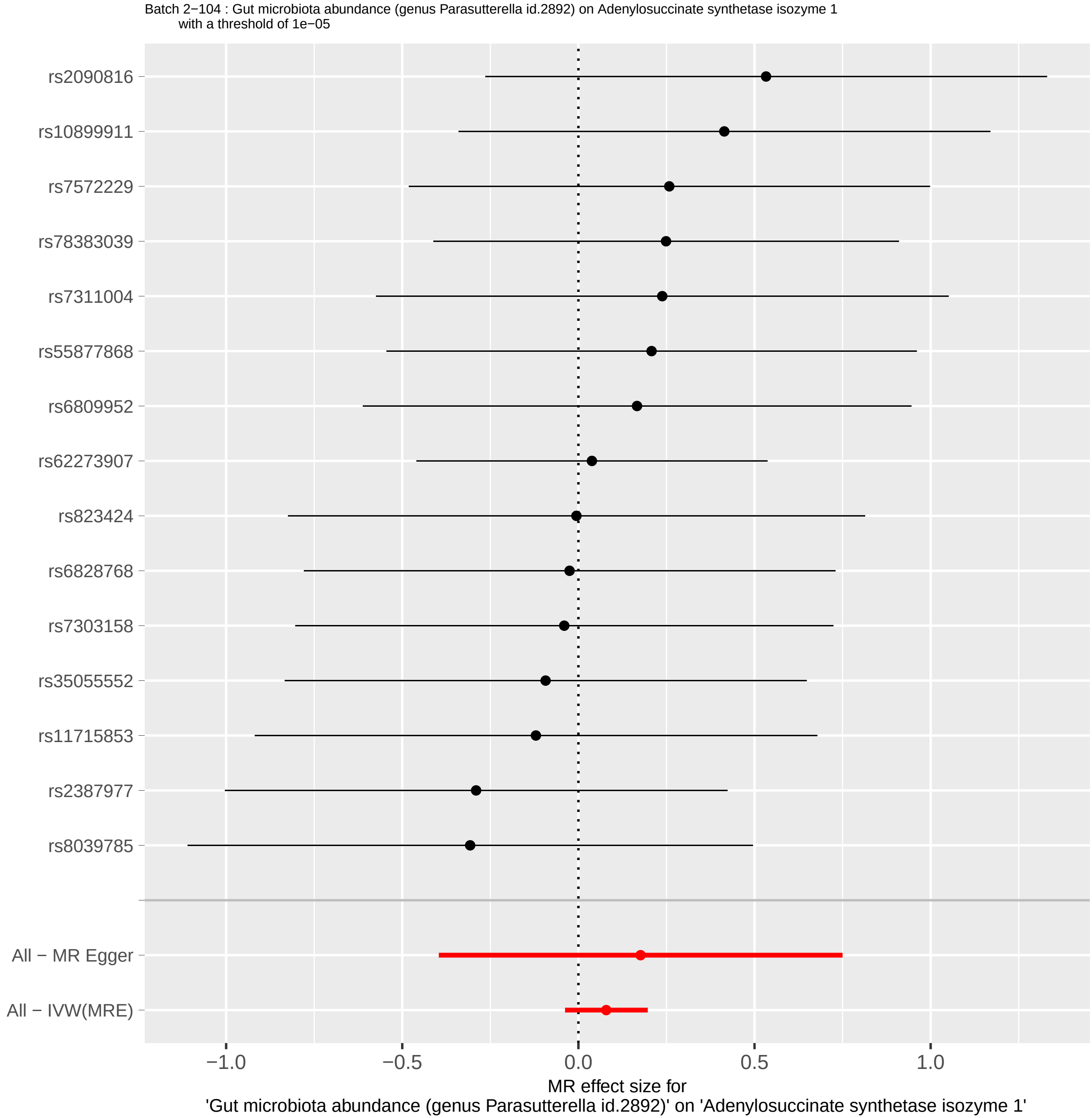

Batch 2−104 : Gut microbiota abundance (genus Parasutterella id.2892) on Adenylosuccinate synthetase isozyme 1
with a threshold of 1e−05
rs2090816
rs10899911
rs7572229
rs78383039
rs7311004
rs55877868
rs6809952
rs62273907
rs823424
rs6828768
rs7303158
rs35055552
rs11715853
rs2387977
rs8039785
All − MR Egger
All − IVW(MRE)
−1.0
−0.5
0.0
0.5
1.0
MR effect size for
'Gut microbiota abundance (genus Parasutterella id.2892)' on 'Adenylosuccinate synthetase isozyme 1'

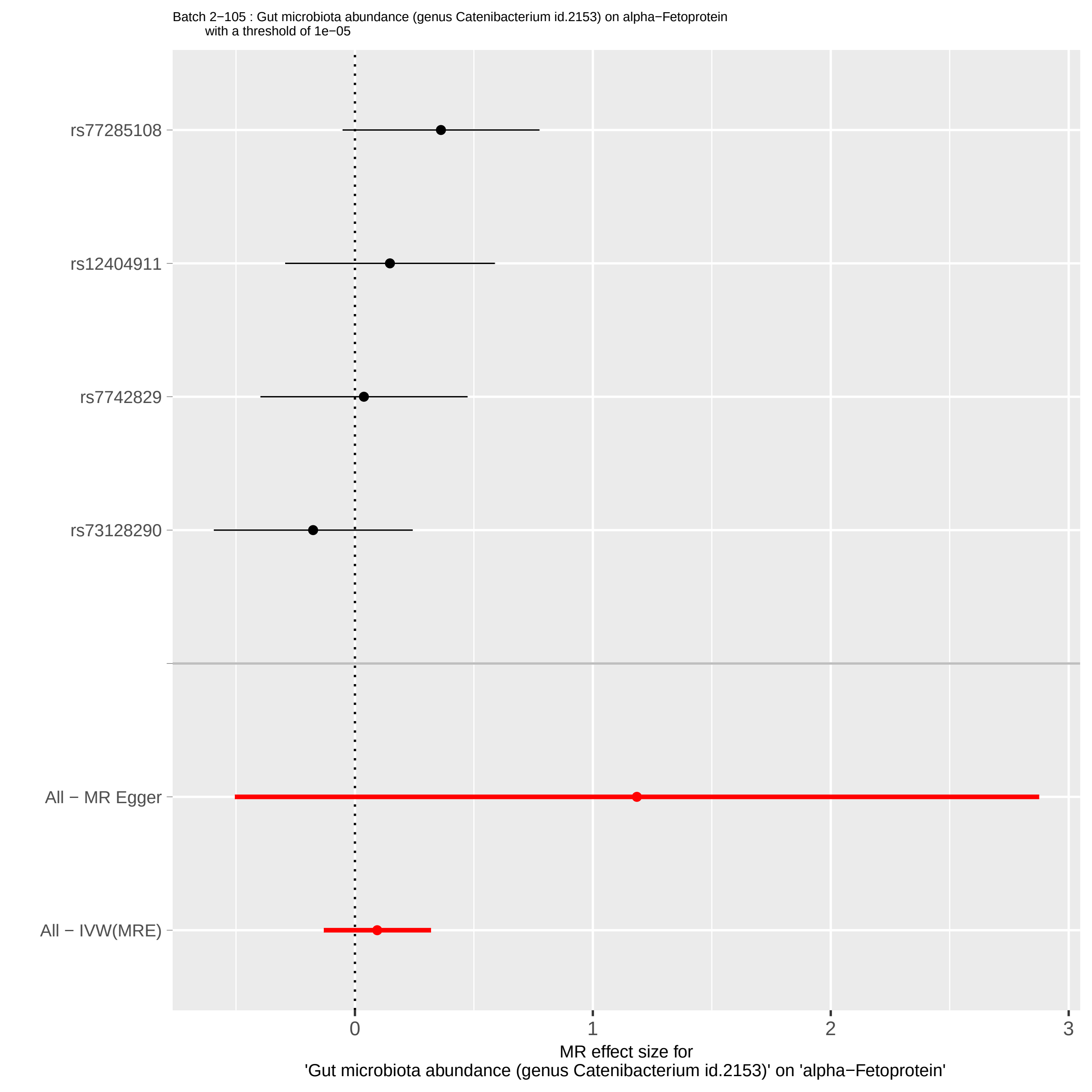

Batch 2−105 : Gut microbiota abundance (genus Catenibacterium id.2153) on alpha−Fetoprotein
with a threshold of 1e−05
rs77285108
rs12404911
rs7742829
rs73128290
All − MR Egger
All − IVW(MRE)
0
1
2
3
MR effect size for
'Gut microbiota abundance (genus Catenibacterium id.2153)' on 'alpha−Fetoprotein'

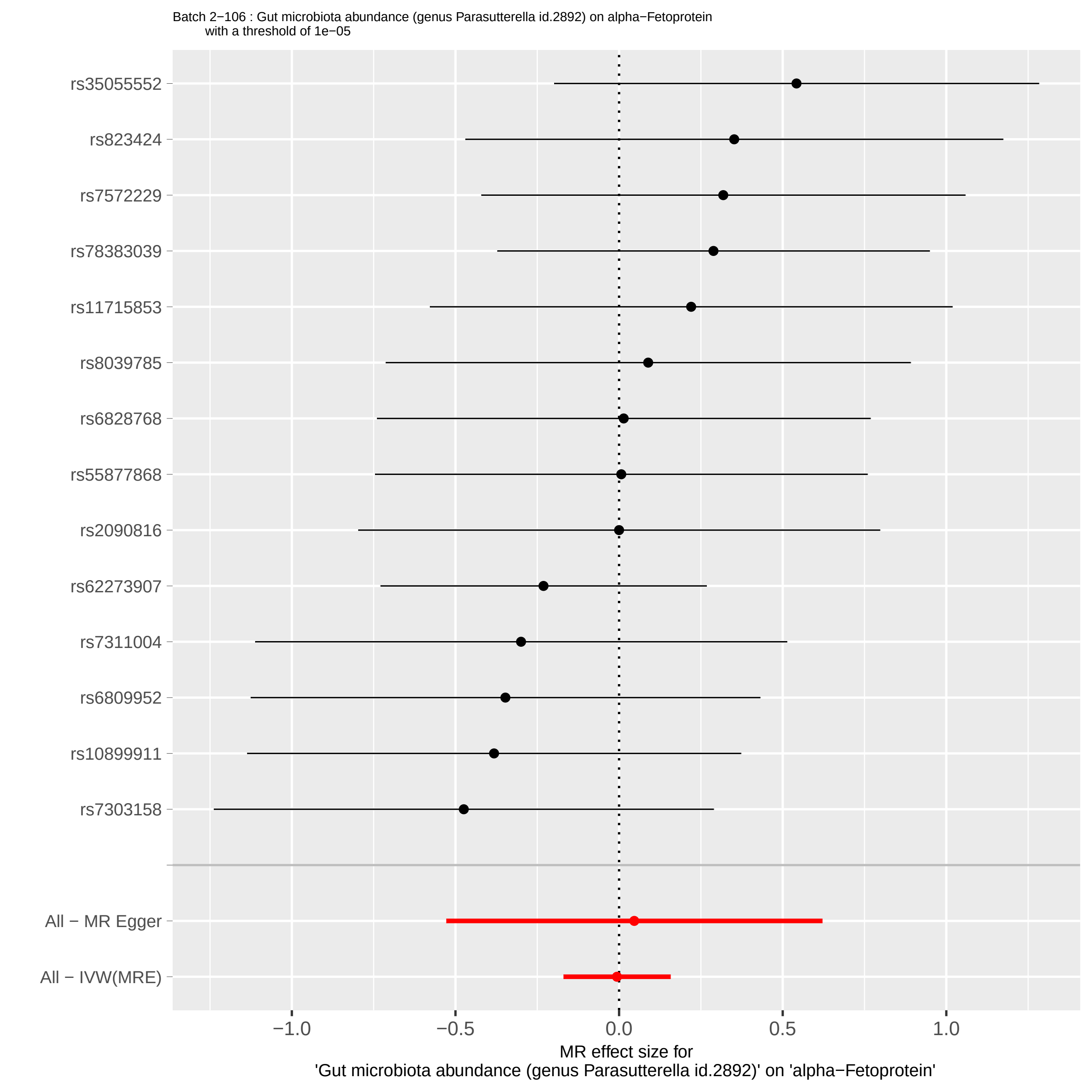

Batch 2−106 : Gut microbiota abundance (genus Parasutterella id.2892) on alpha−Fetoprotein
with a threshold of 1e−05
rs35055552
rs823424
rs7572229
rs78383039
rs11715853
rs8039785
rs6828768
rs55877868
rs2090816
rs62273907
rs7311004
rs6809952
rs10899911
rs7303158
All − MR Egger
All − IVW(MRE)
−1.0
−0.5
0.0
0.5
1.0
MR effect size for
'Gut microbiota abundance (genus Parasutterella id.2892)' on 'alpha−Fetoprotein'

Batch 2−107 : Gut microbiota abundance (genus Catenibacterium id.2153) on Advanced glycosylation end product−specific receptor, soluble
with a threshold of 1e−05

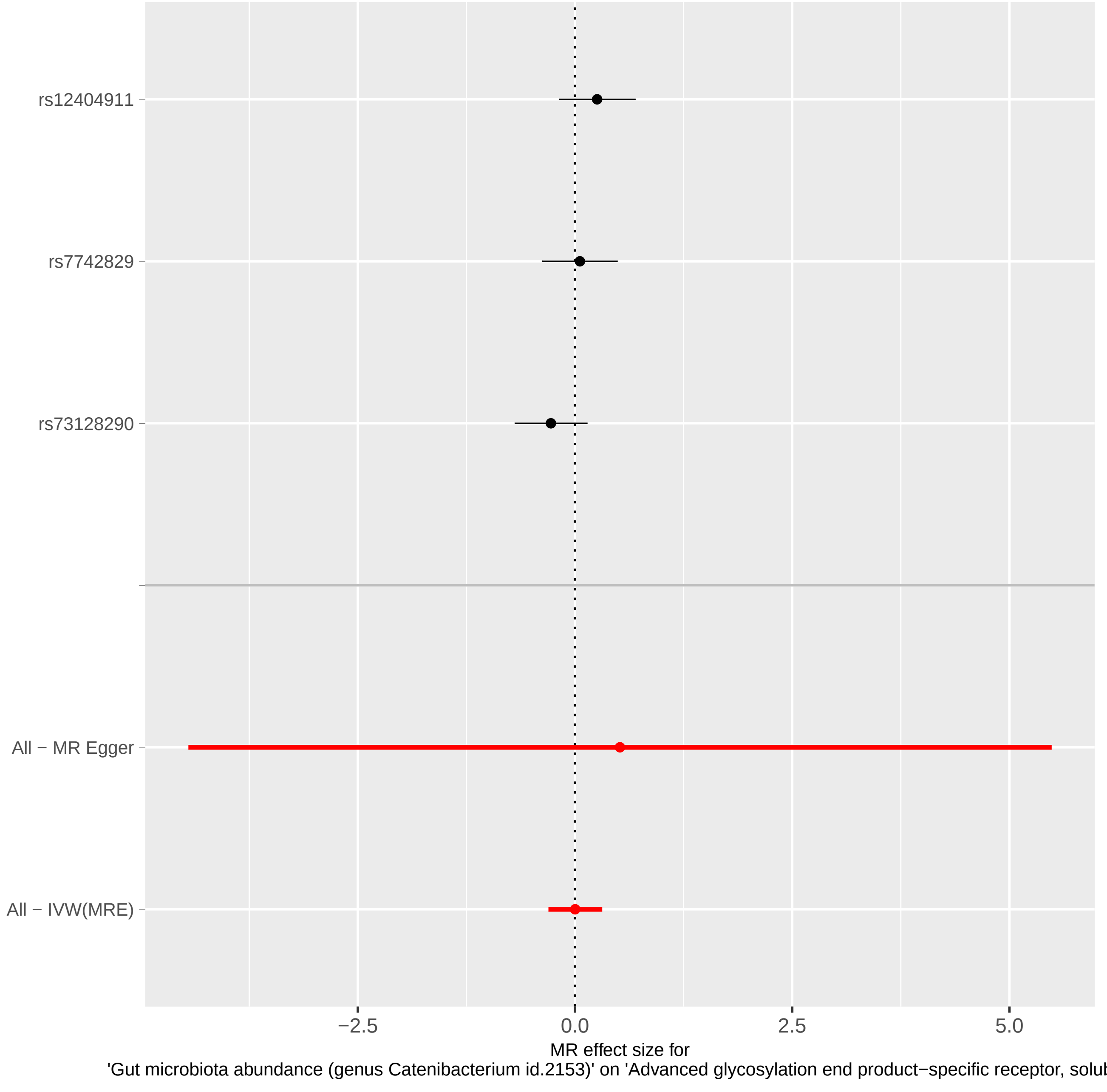

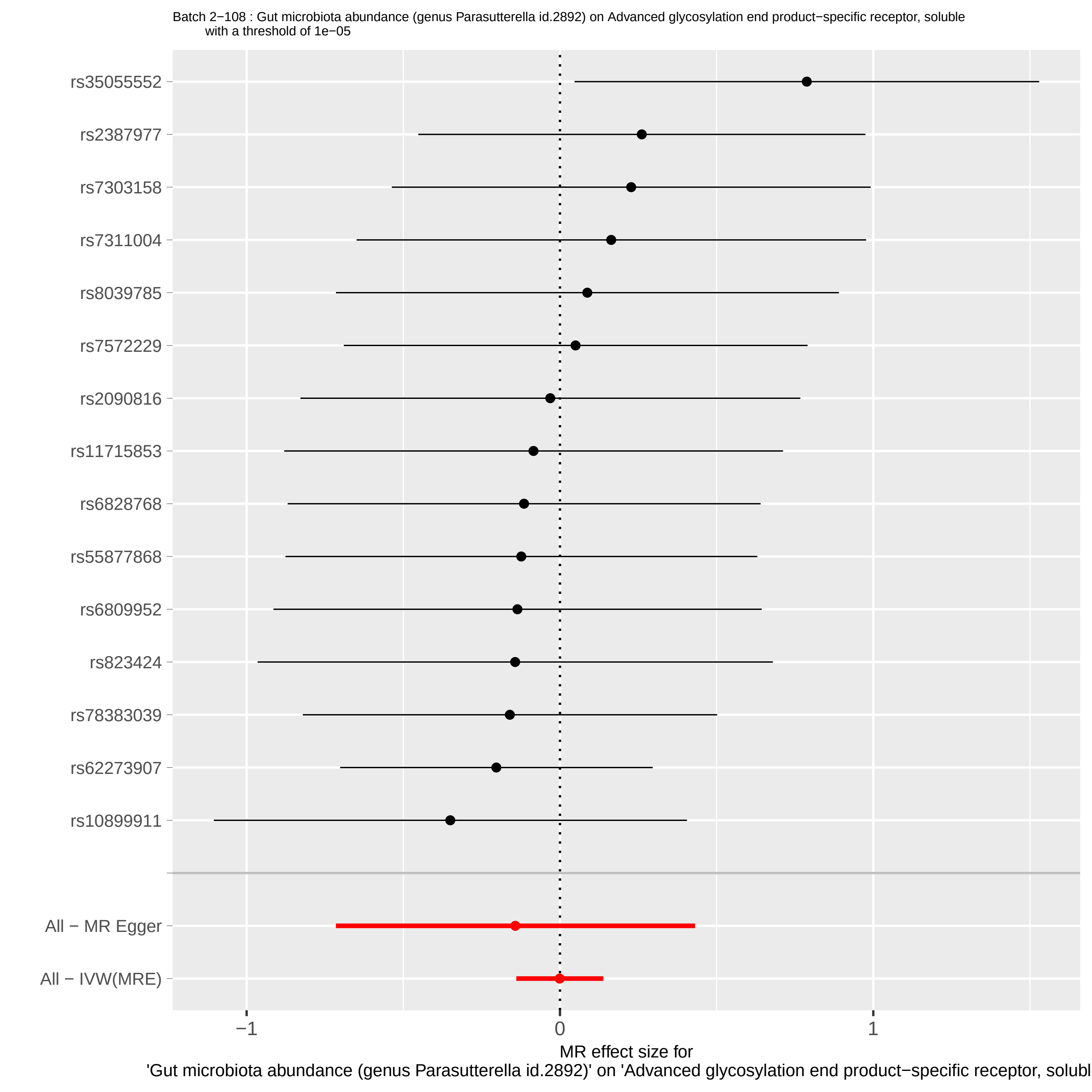

Batch 2−108 : Gut microbiota abundance (genus Parasutterella id.2892) on Advanced glycosylation end product−specific receptor, soluble
with a threshold of 1e−05
rs35055552
rs2387977
rs7303158
rs7311004
rs8039785
rs7572229
rs2090816
rs11715853
rs6828768
rs55877868
rs6809952
rs823424
rs78383039
rs62273907
rs10899911
All − MR Egger
All − IVW(MRE)
−1
0
1
MR effect size for
'Gut microbiota abundance (genus Parasutterella id.2892)' on 'Advanced glycosylation end product−specific receptor, solubl

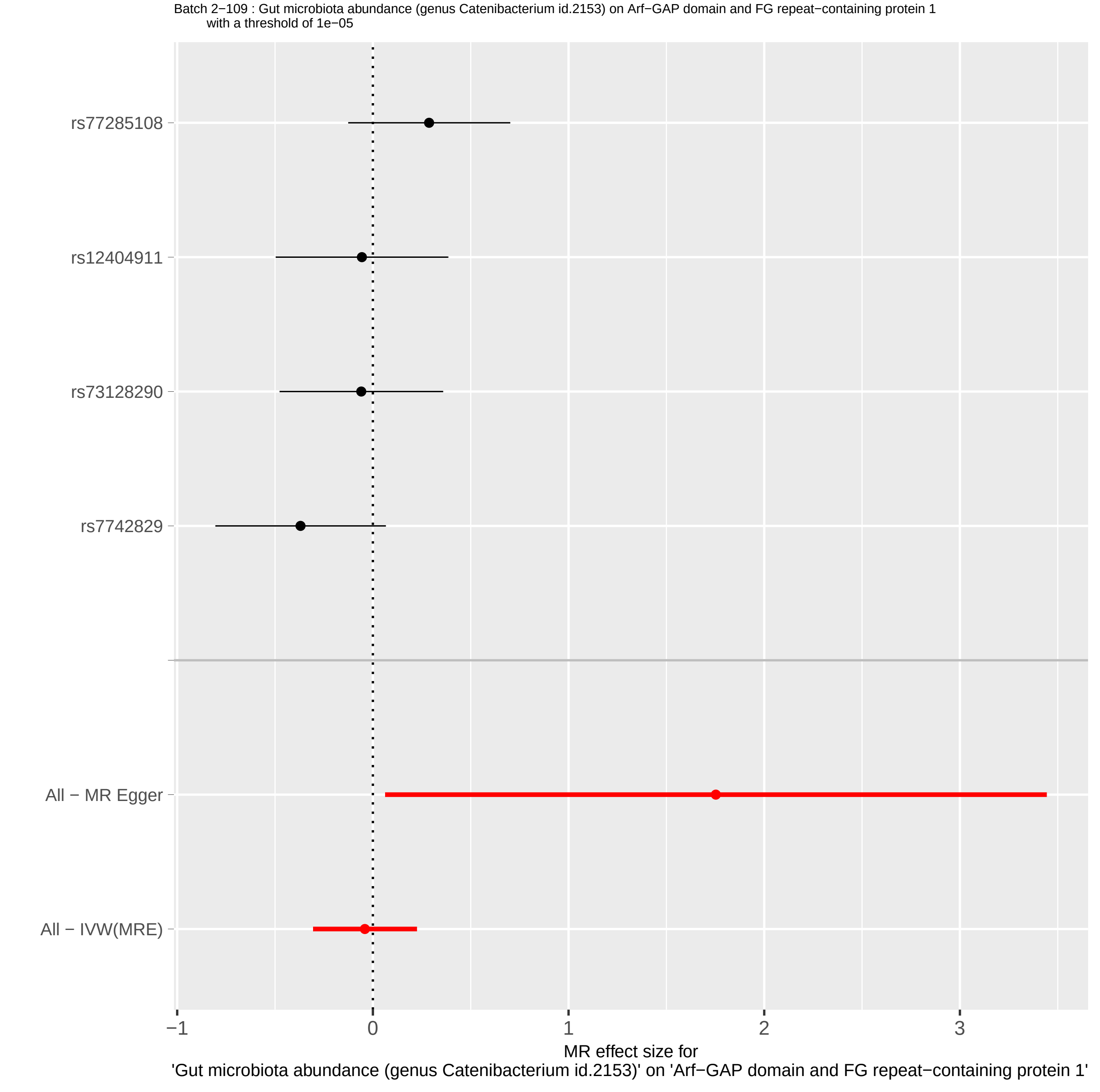

Batch 2−109 : Gut microbiota abundance (genus Catenibacterium id.2153) on Arf−GAP domain and FG repeat−containing protein 1 with a threshold of 1e−05
rs77285108
rs12404911
rs73128290
rs7742829
All − MR Egger
All − IVW(MRE)
−1
0
1
2
3
MR effect size for
'Gut microbiota abundance (genus Catenibacterium id.2153)' on 'Arf−GAP domain and FG repeat−containing protein 1'

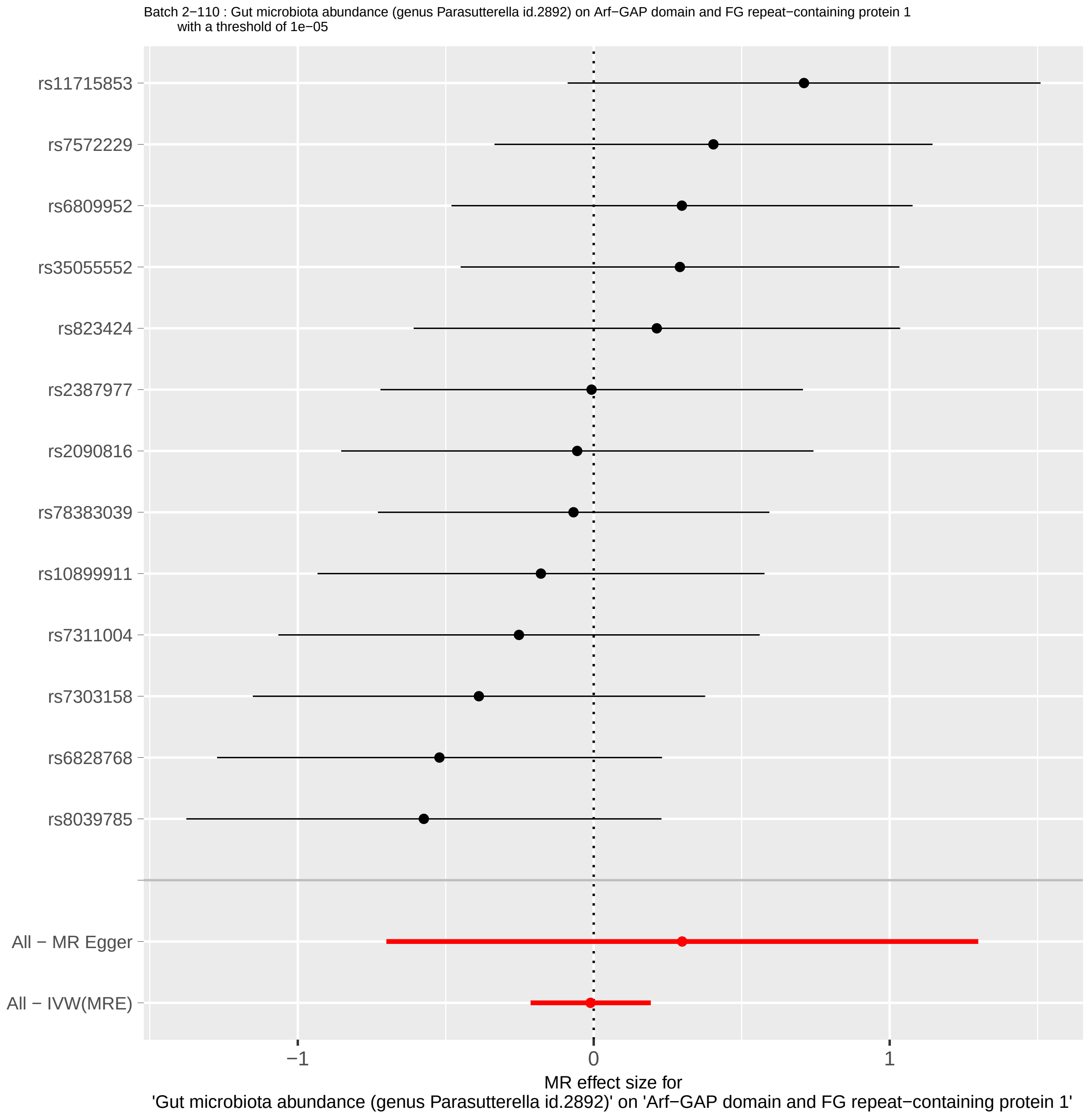

Batch 2−110 : Gut microbiota abundance (genus Parasutterella id.2892) on Arf−GAP domain and FG repeat−containing protein 1
with a threshold of 1e−05
rs11715853
rs7572229
rs6809952
rs35055552
rs823424
rs2387977
rs2090816
rs78383039
rs10899911
rs7311004
rs7303158
rs6828768
rs8039785
All − MR Egger
All − IVW(MRE)
−1
0
1
MR effect size for
'Gut microbiota abundance (genus Parasutterella id.2892)' on 'Arf−GAP domain and FG repeat−containing protein 1'

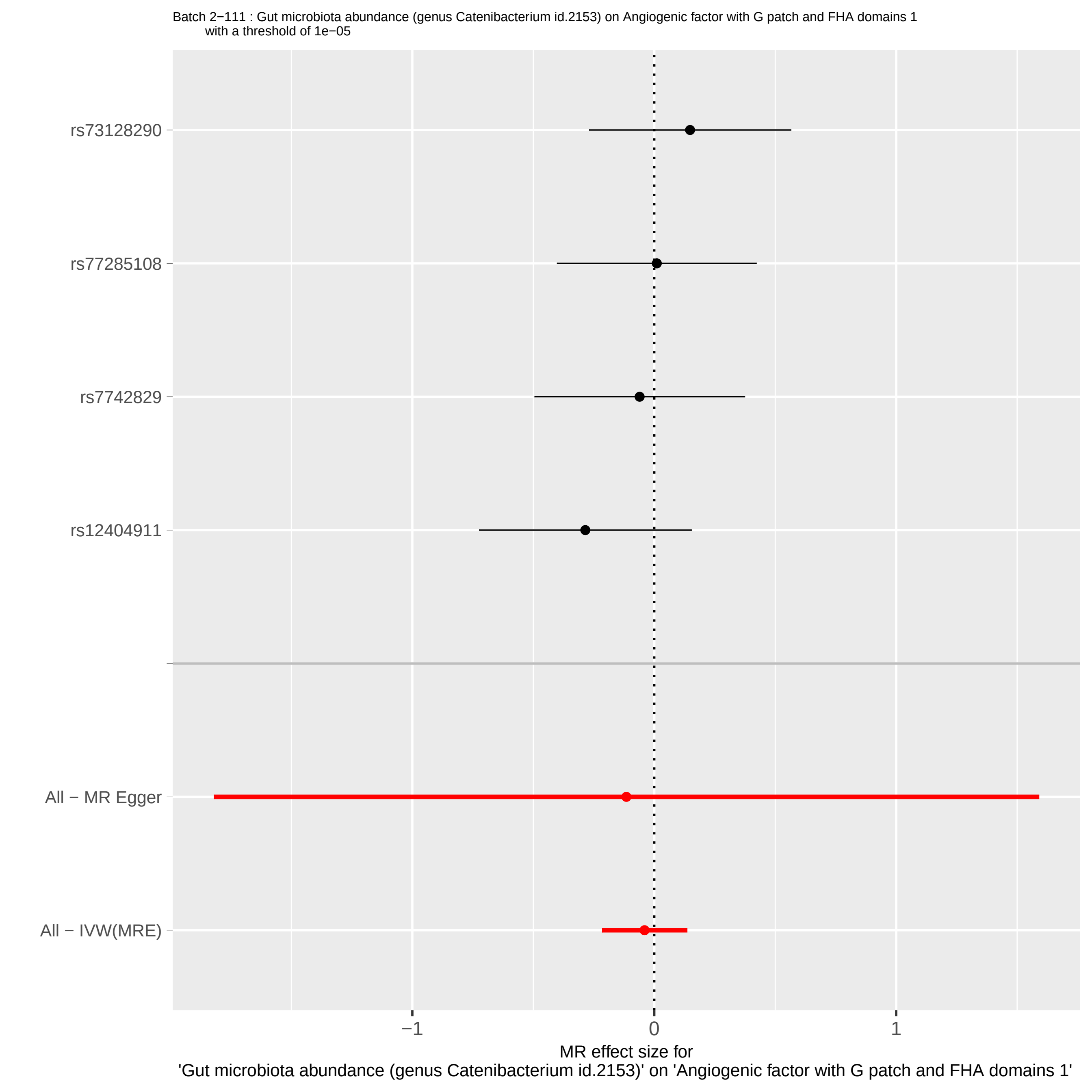
Batch 2−111 : Gut microbiota abundance (genus Catenibacterium id.2153) on Angiogenic factor with G patch and FHA domains 1
with a threshold of 1e−05
rs73128290
rs77285108
rs7742829
rs12404911
All − MR Egger
All − IVW(MRE)
−1
0
1
MR effect size for
'Gut microbiota abundance (genus Catenibacterium id.2153)' on 'Angiogenic factor with G patch and FHA domains 1'

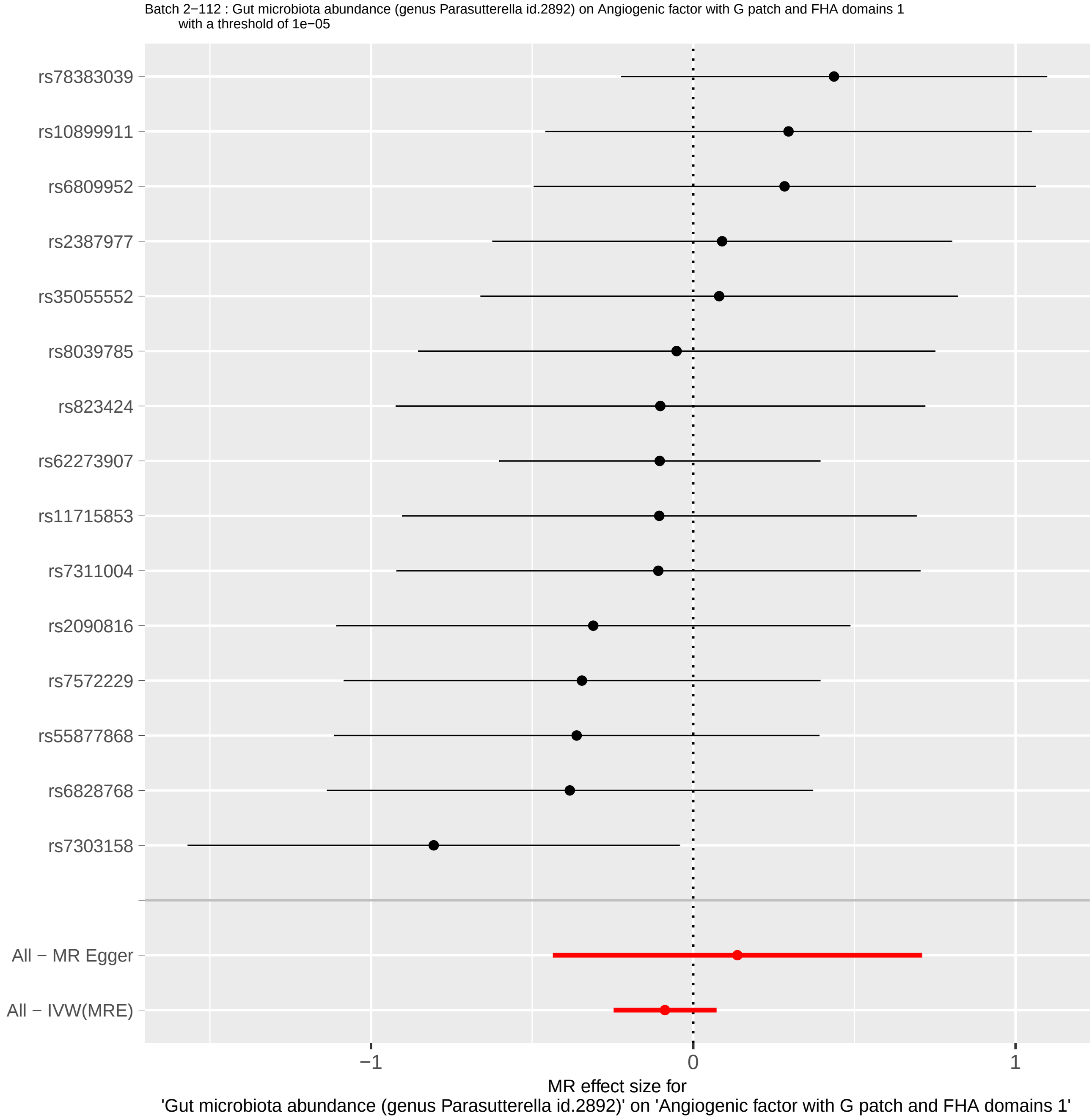

Batch 2−112 : Gut microbiota abundance (genus Parasutterella id.2892) on Angiogenic factor with G patch and FHA domains 1
with a threshold of 1e−05
rs78383039
rs10899911
rs6809952
rs2387977
rs35055552
rs8039785
rs823424
rs62273907
rs11715853
rs7311004
rs2090816
rs7572229
rs55877868
rs6828768
rs7303158
All − MR Egger
All − IVW(MRE)
−1
0
1
MR effect size for
'Gut microbiota abundance (genus Parasutterella id.2892)' on 'Angiogenic factor with G patch and FHA domains 1'

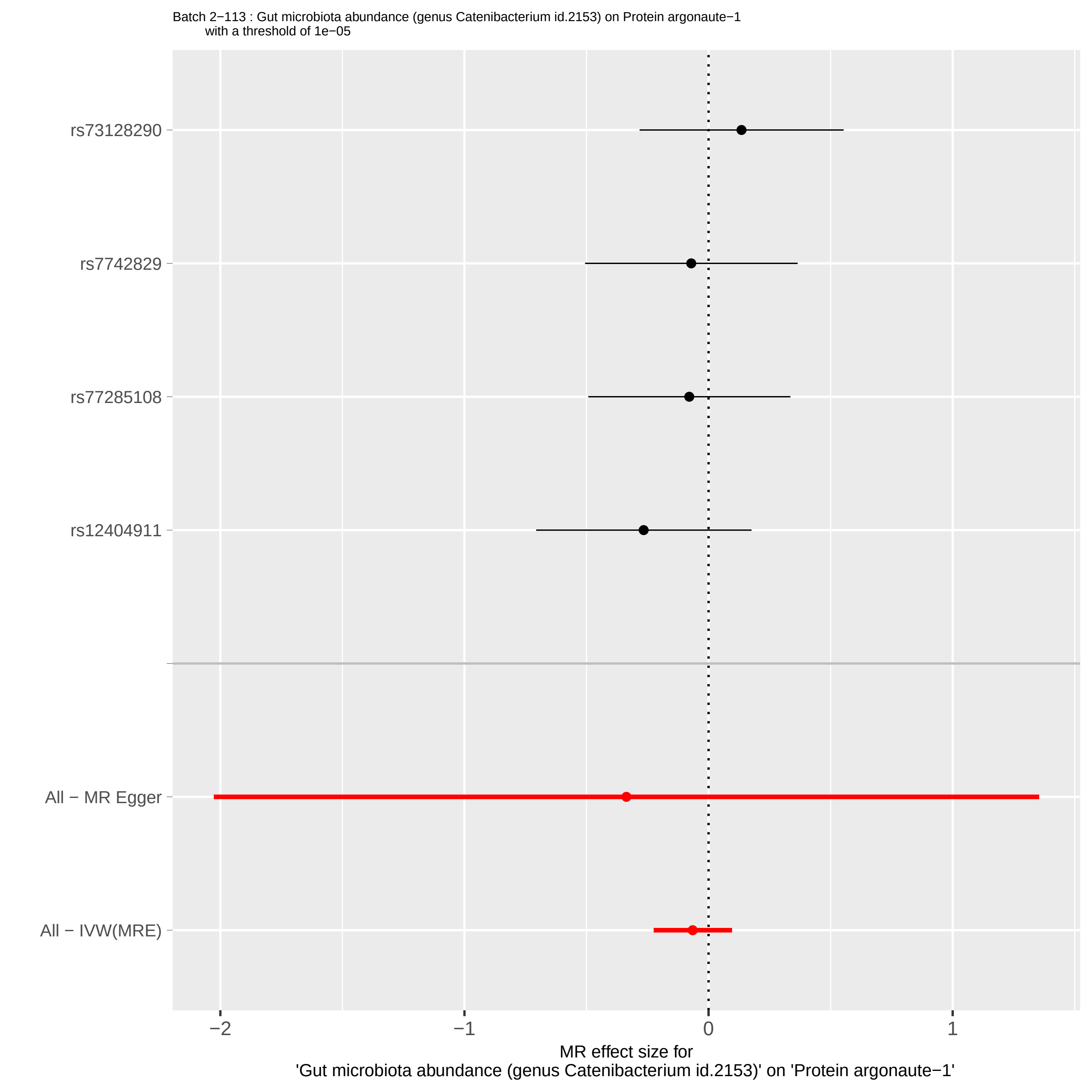
Batch 2−113 : Gut microbiota abundance (genus Catenibacterium id.2153) on Protein argonaute−1
with a threshold of 1e−05
rs73128290
rs7742829
rs77285108
rs12404911
All − MR Egger
All − IVW(MRE)
−2
−1
0
1
MR effect size for
'Gut microbiota abundance (genus Catenibacterium id.2153)' on 'Protein argonaute−1'

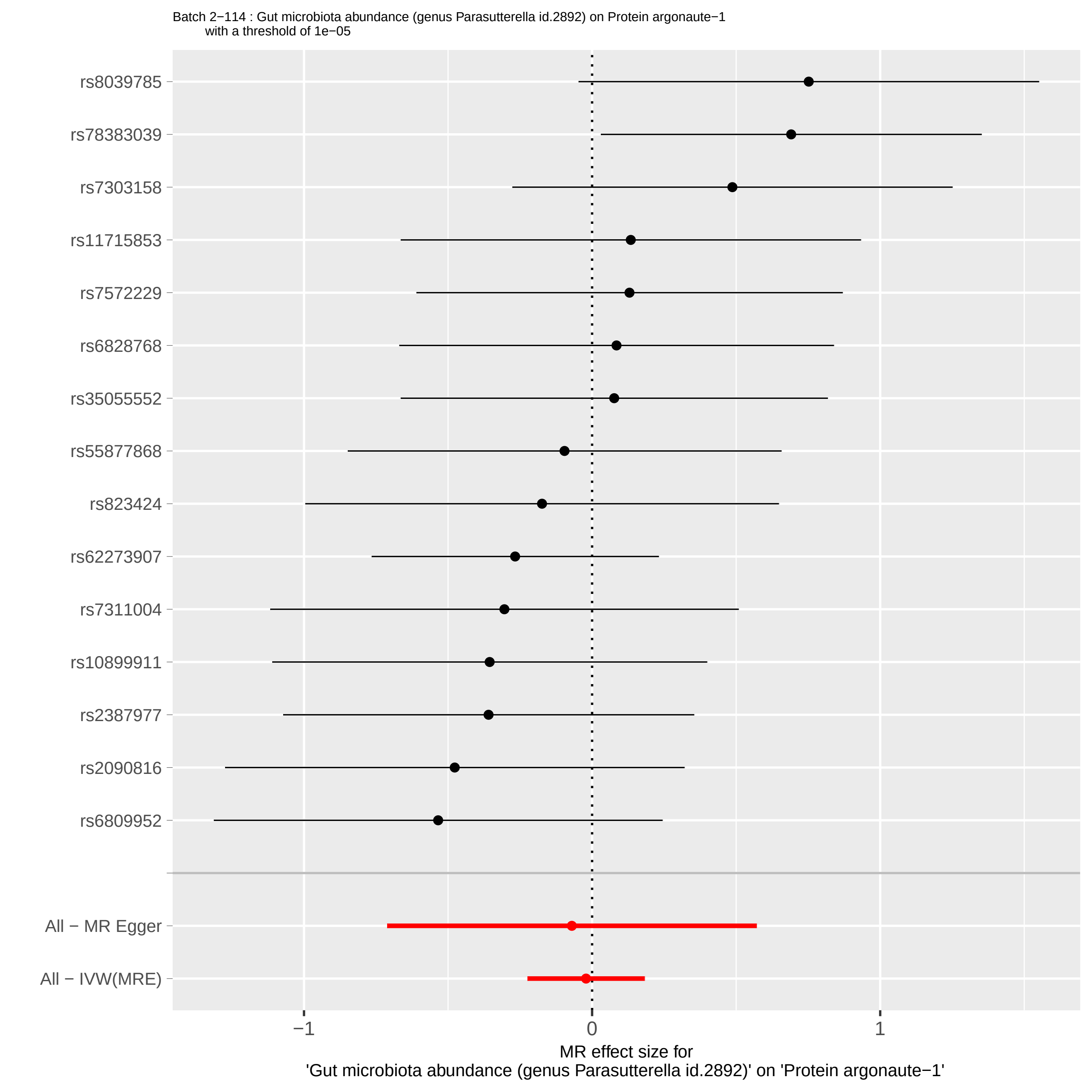

Batch 2−114 : Gut microbiota abundance (genus Parasutterella id.2892) on Protein argonaute−1
with a threshold of 1e−05
rs8039785
rs78383039
rs7303158
rs11715853
rs7572229
rs6828768
rs35055552
rs55877868
rs823424
rs62273907
rs7311004
rs10899911
rs2387977
rs2090816
rs6809952
All − MR Egger
All − IVW(MRE)
−1
0
1
MR effect size for
'Gut microbiota abundance (genus Parasutterella id.2892)' on 'Protein argonaute−1'

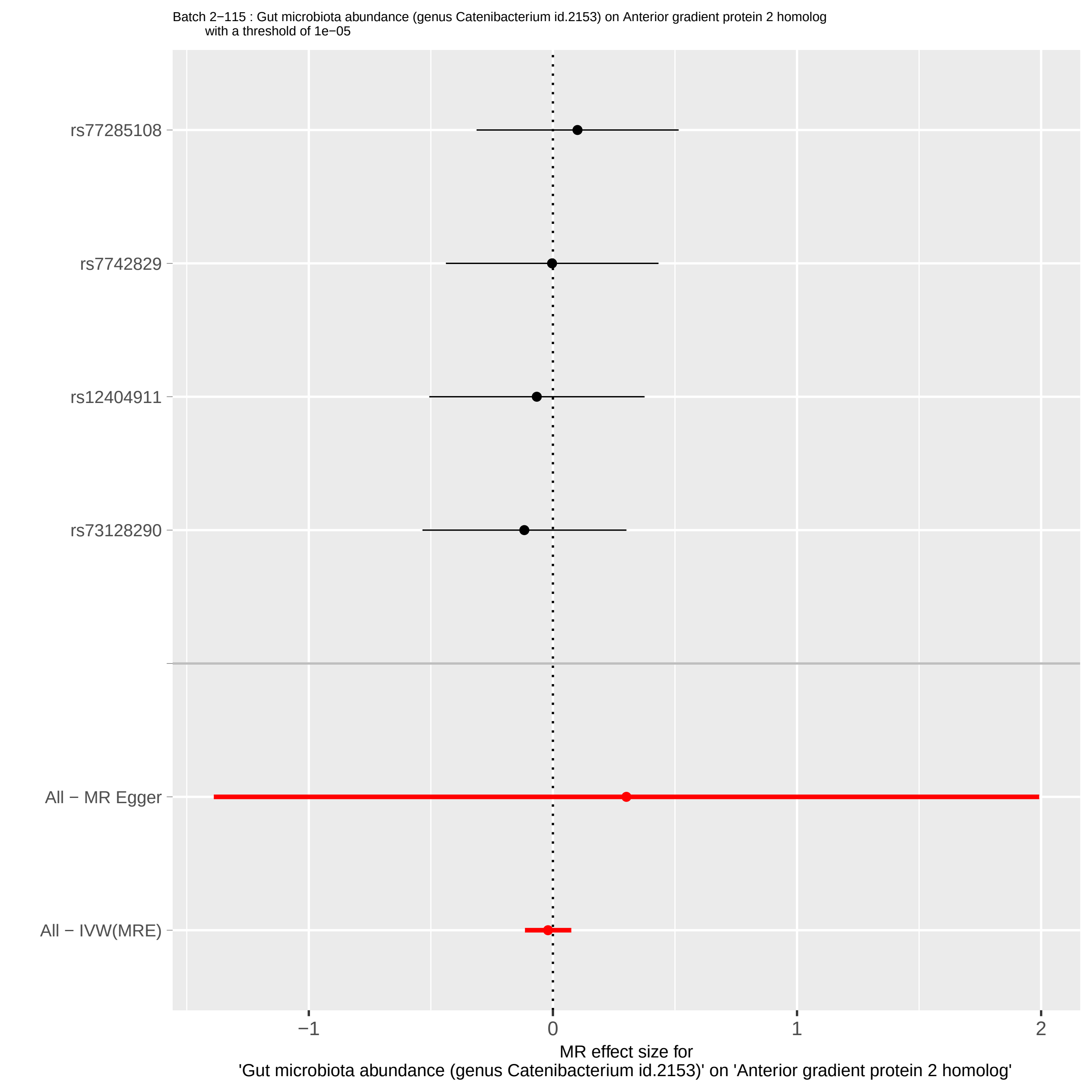
Batch 2−115 : Gut microbiota abundance (genus Catenibacterium id.2153) on Anterior gradient protein 2 homolog
with a threshold of 1e−05
rs77285108
rs7742829
rs12404911
rs73128290
All − MR Egger
All − IVW(MRE)
−1
0
1
2
MR effect size for
'Gut microbiota abundance (genus Catenibacterium id.2153)' on 'Anterior gradient protein 2 homolog'

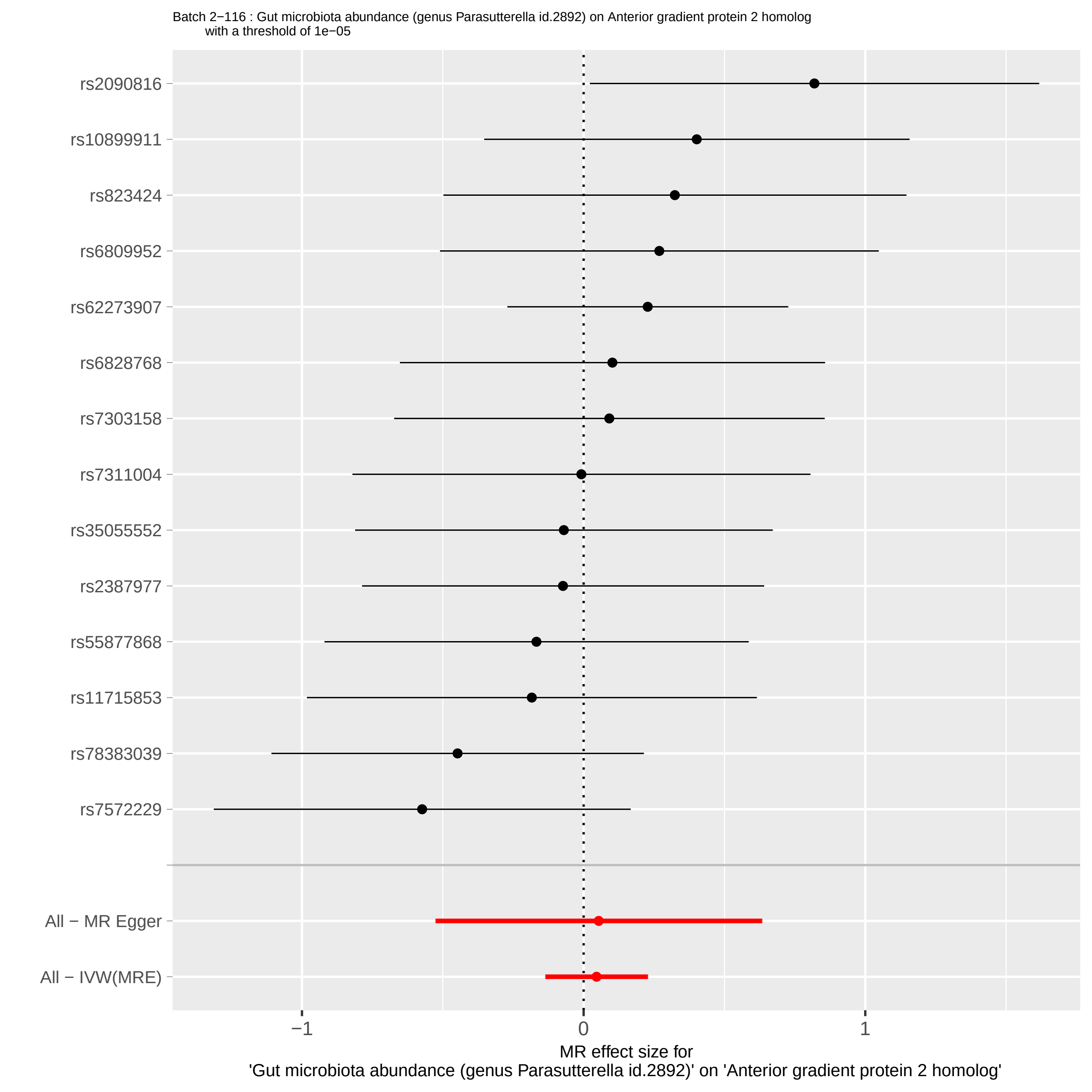

Batch 2−116 : Gut microbiota abundance (genus Parasutterella id.2892) on Anterior gradient protein 2 homolog
with a threshold of 1e−05
rs2090816
rs10899911
rs823424
rs6809952
rs62273907
rs6828768
rs7303158
rs7311004
rs35055552
rs2387977
rs55877868
rs11715853
rs78383039
rs7572229
All − MR Egger
All − IVW(MRE)
−1
0
1
MR effect size for
'Gut microbiota abundance (genus Parasutterella id.2892)' on 'Anterior gradient protein 2 homolog'

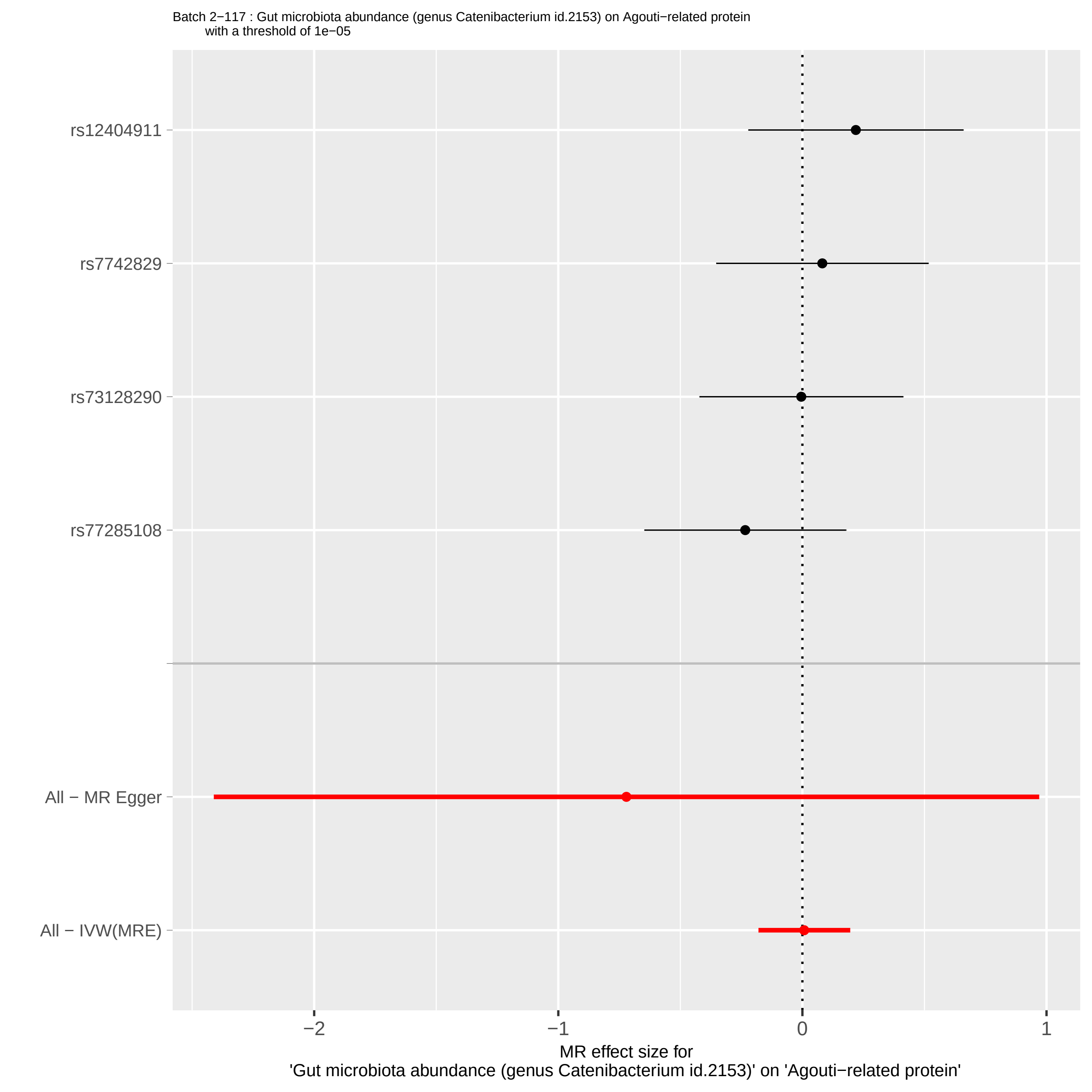

Batch 2−117 : Gut microbiota abundance (genus Catenibacterium id.2153) on Agouti−related protein
with a threshold of 1e−05
rs12404911
rs7742829
rs73128290
rs77285108
All − MR Egger
All − IVW(MRE)
−2
−1
0
1
MR effect size for
'Gut microbiota abundance (genus Catenibacterium id.2153)' on 'Agouti−related protein'

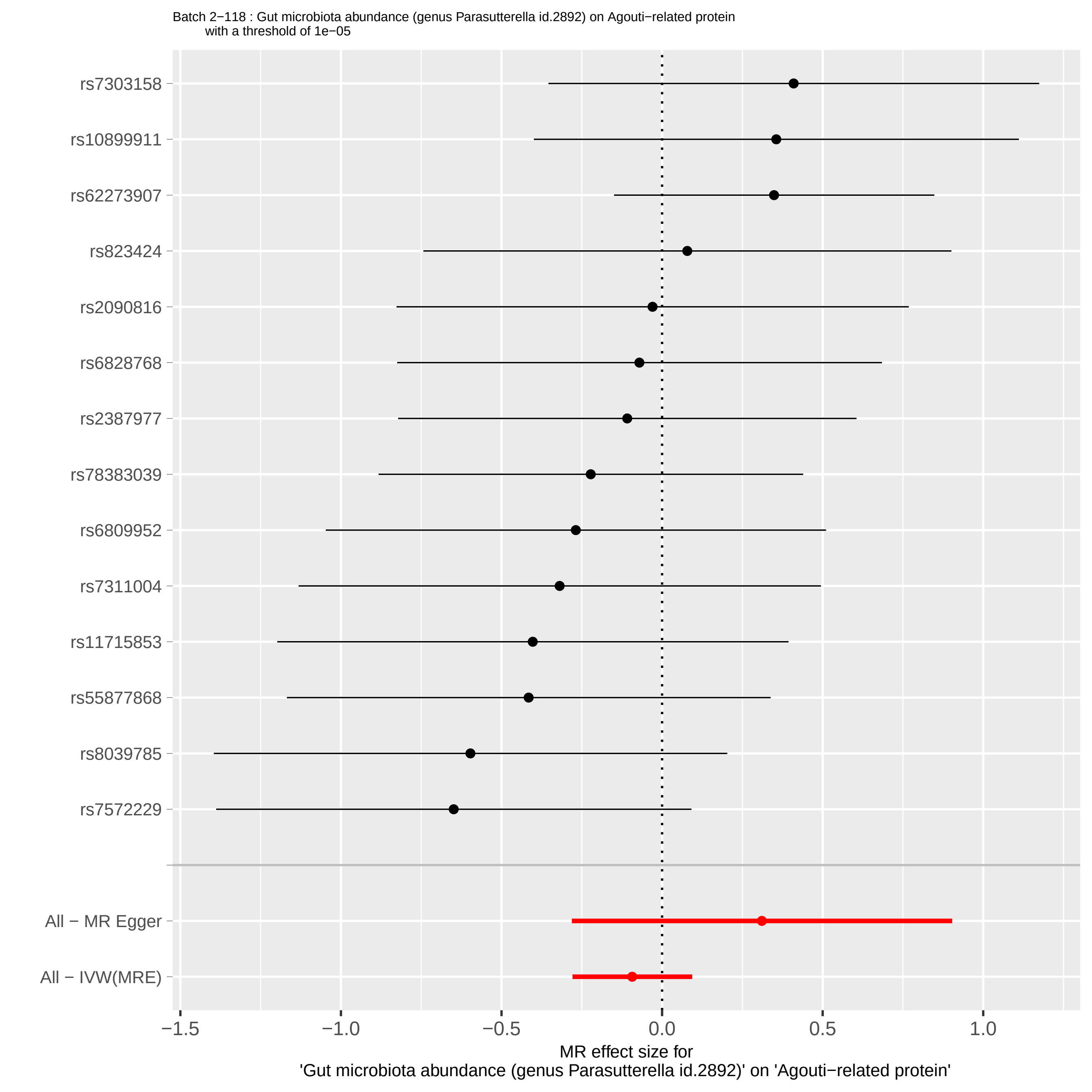
Batch 2−118 : Gut microbiota abundance (genus Parasutterella id.2892) on Agouti−related protein
with a threshold of 1e−05
rs7303158
rs10899911
rs62273907
rs823424
rs2090816
rs6828768
rs2387977
rs78383039
rs6809952
rs7311004
rs11715853
rs55877868
rs8039785
rs7572229
All − MR Egger
All − IVW(MRE)
−1.5
−1.0
−0.5
0.0
0.5
1.0
MR effect size for
'Gut microbiota abundance (genus Parasutterella id.2892)' on 'Agouti−related protein'

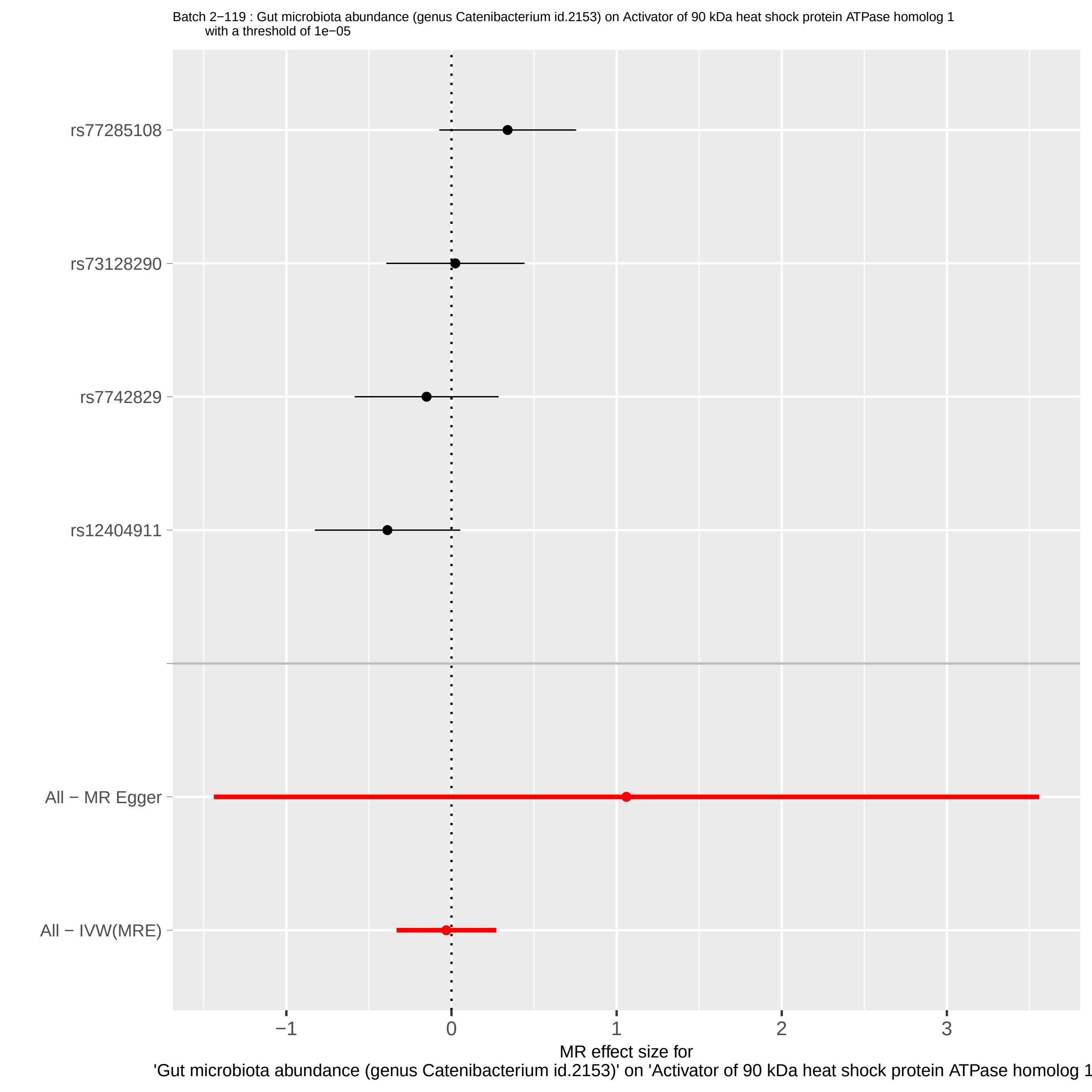

Batch 2−119 : Gut microbiota abundance (genus Catenibacterium id.2153) on Activator of 90 kDa heat shock protein ATPase homolog 1
with a threshold of 1e−05
rs77285108
rs73128290
rs7742829
rs12404911
All − MR Egger
All − IVW(MRE)
−1
0
1
2
3
MR effect size for
'Gut microbiota abundance (genus Catenibacterium id.2153)' on 'Activator of 90 kDa heat shock protein ATPase homolog 1

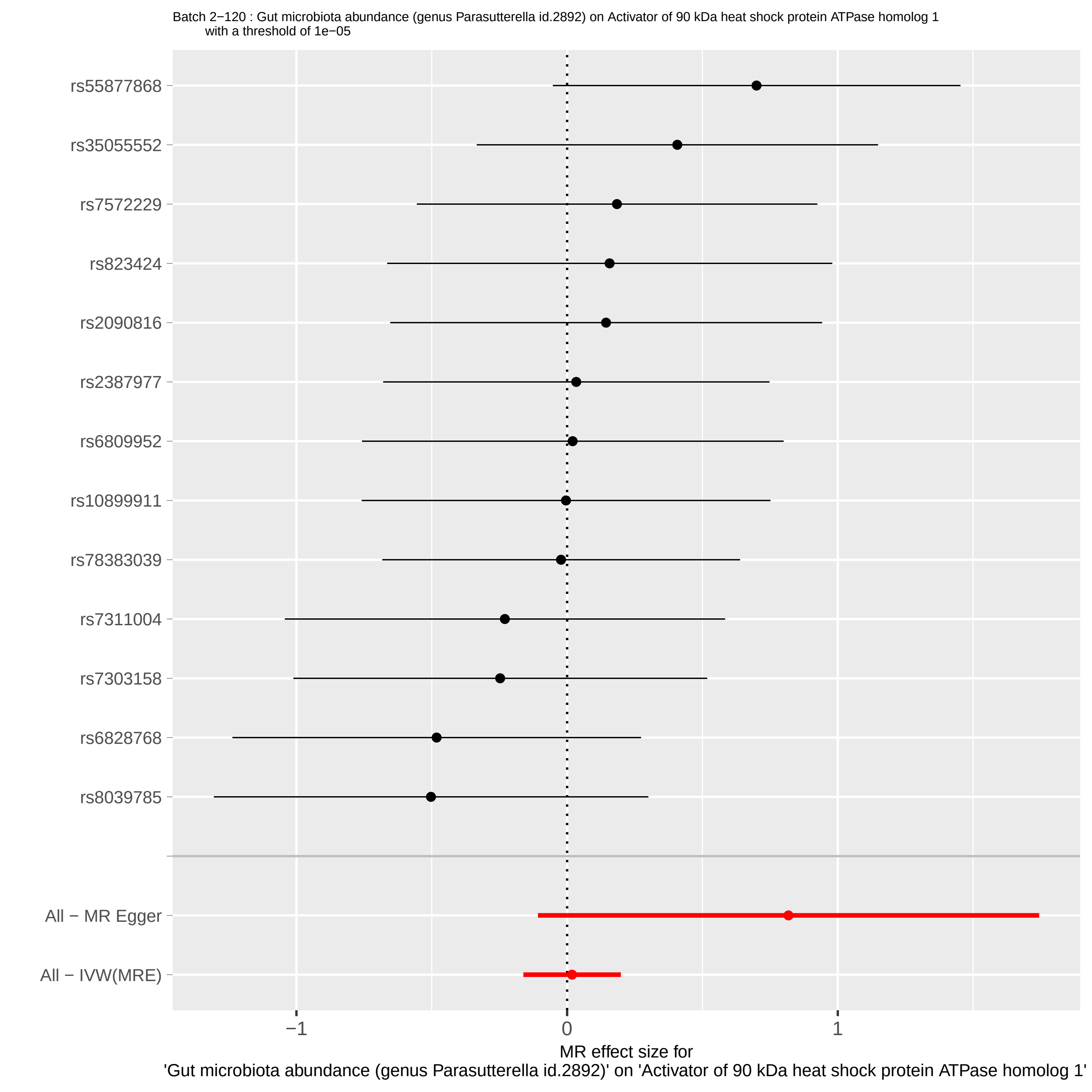
Batch 2−120 : Gut microbiota abundance (genus Parasutterella id.2892) on Activator of 90 kDa heat shock protein ATPase homolog 1
with a threshold of 1e−05
rs55877868
rs35055552
rs7572229
rs823424
rs2090816
rs2387977
rs6809952
rs10899911
rs78383039
rs7311004
rs7303158
rs6828768
rs8039785
All − MR Egger
All − IVW(MRE)
−1
0
1
MR effect size for
'Gut microbiota abundance (genus Parasutterella id.2892)' on 'Activator of 90 kDa heat shock protein ATPase homolog 1'

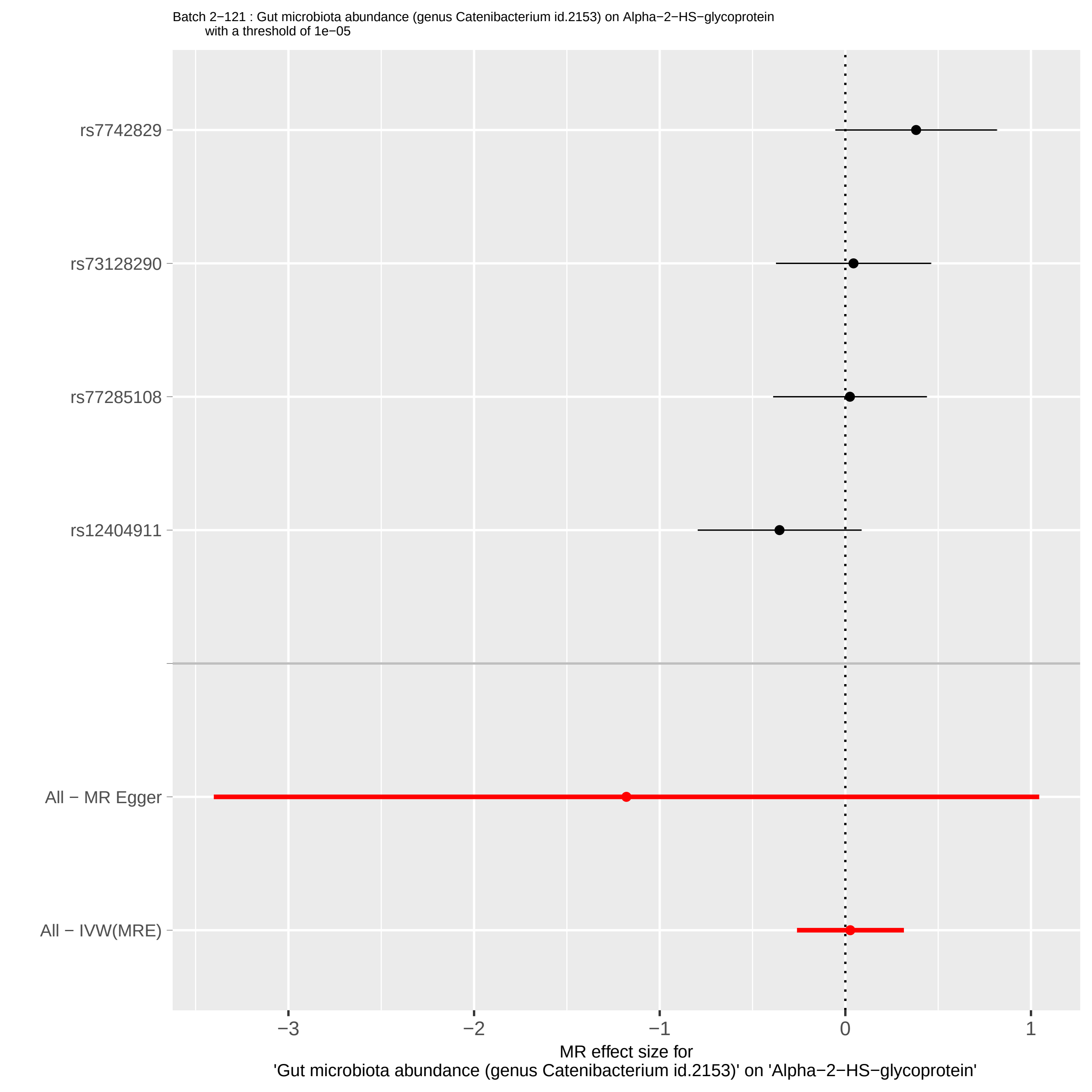
Batch 2−121 : Gut microbiota abundance (genus Catenibacterium id.2153) on Alpha−2−HS−glycoprotein
with a threshold of 1e−05
rs7742829
rs73128290
rs77285108
rs12404911
All − MR Egger
All − IVW(MRE)
−3
−2
−1
0
1
MR effect size for
'Gut microbiota abundance (genus Catenibacterium id.2153)' on 'Alpha−2−HS−glycoprotein'

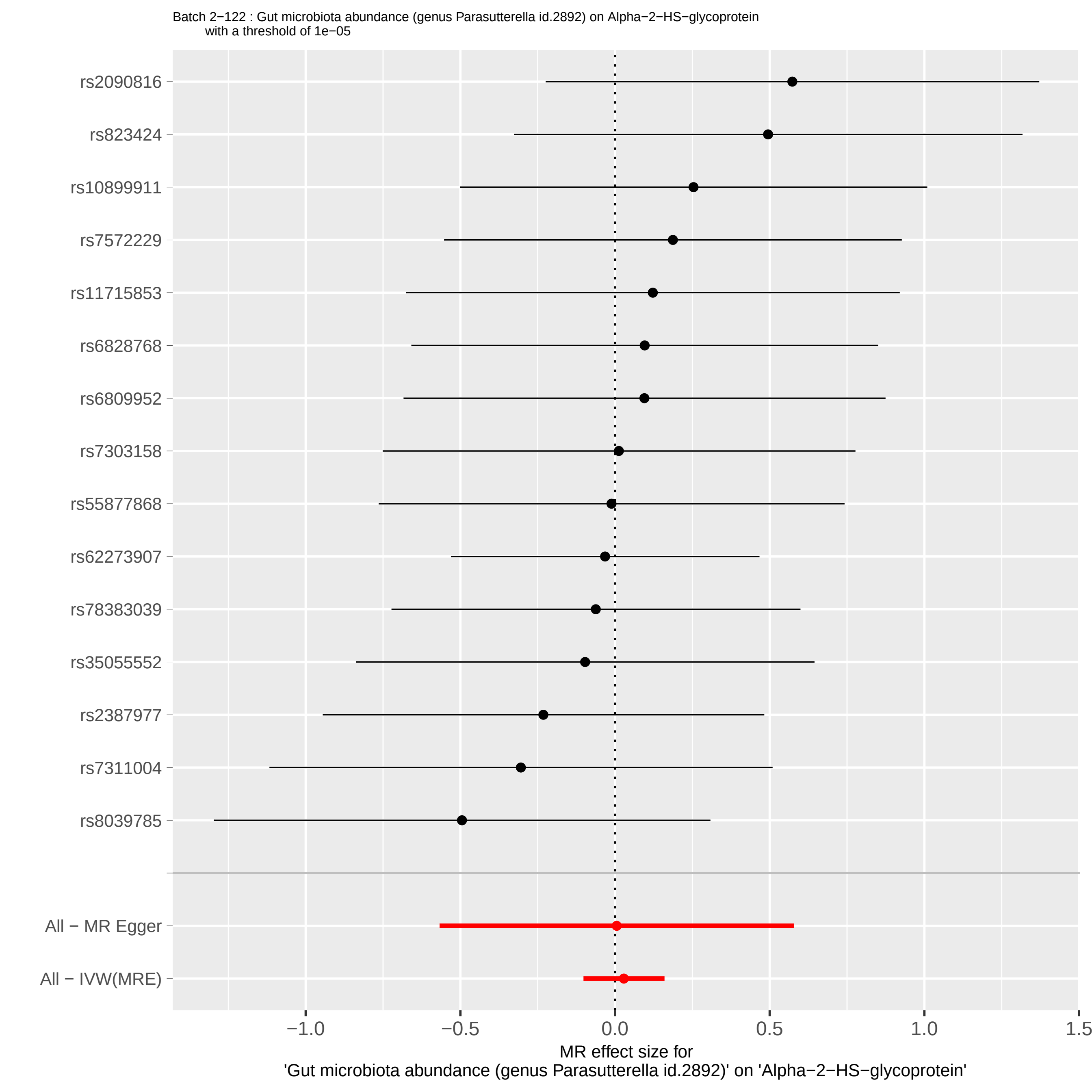

Batch 2−122 : Gut microbiota abundance (genus Parasutterella id.2892) on Alpha−2−HS−glycoprotein
with a threshold of 1e−05
rs2090816
rs823424
rs10899911
rs7572229
rs11715853
rs6828768
rs6809952
rs7303158
rs55877868
rs62273907
rs78383039
rs35055552
rs2387977
rs7311004
rs8039785
All − MR Egger
All − IVW(MRE)
−1.0
−0.5
0.0
0.5
1.0
1.5
MR effect size for
'Gut microbiota abundance (genus Parasutterella id.2892)' on 'Alpha−2−HS−glycoprotein'

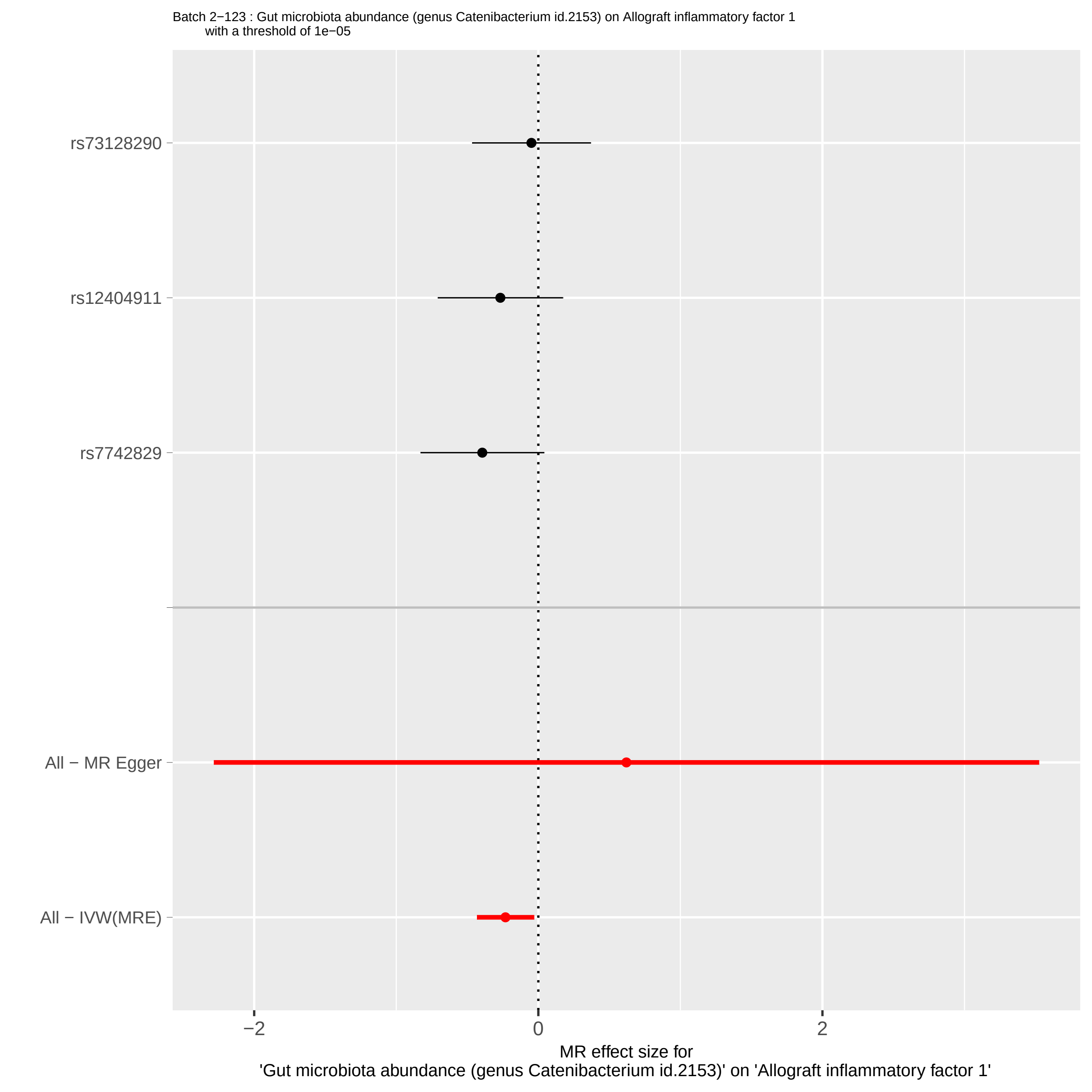

Batch 2−123 : Gut microbiota abundance (genus Catenibacterium id.2153) on Allograft inflammatory factor 1
with a threshold of 1e−05
rs73128290
rs12404911
rs7742829
All − MR Egger
All − IVW(MRE)
−2
0
2
MR effect size for
'Gut microbiota abundance (genus Catenibacterium id.2153)' on 'Allograft inflammatory factor 1'

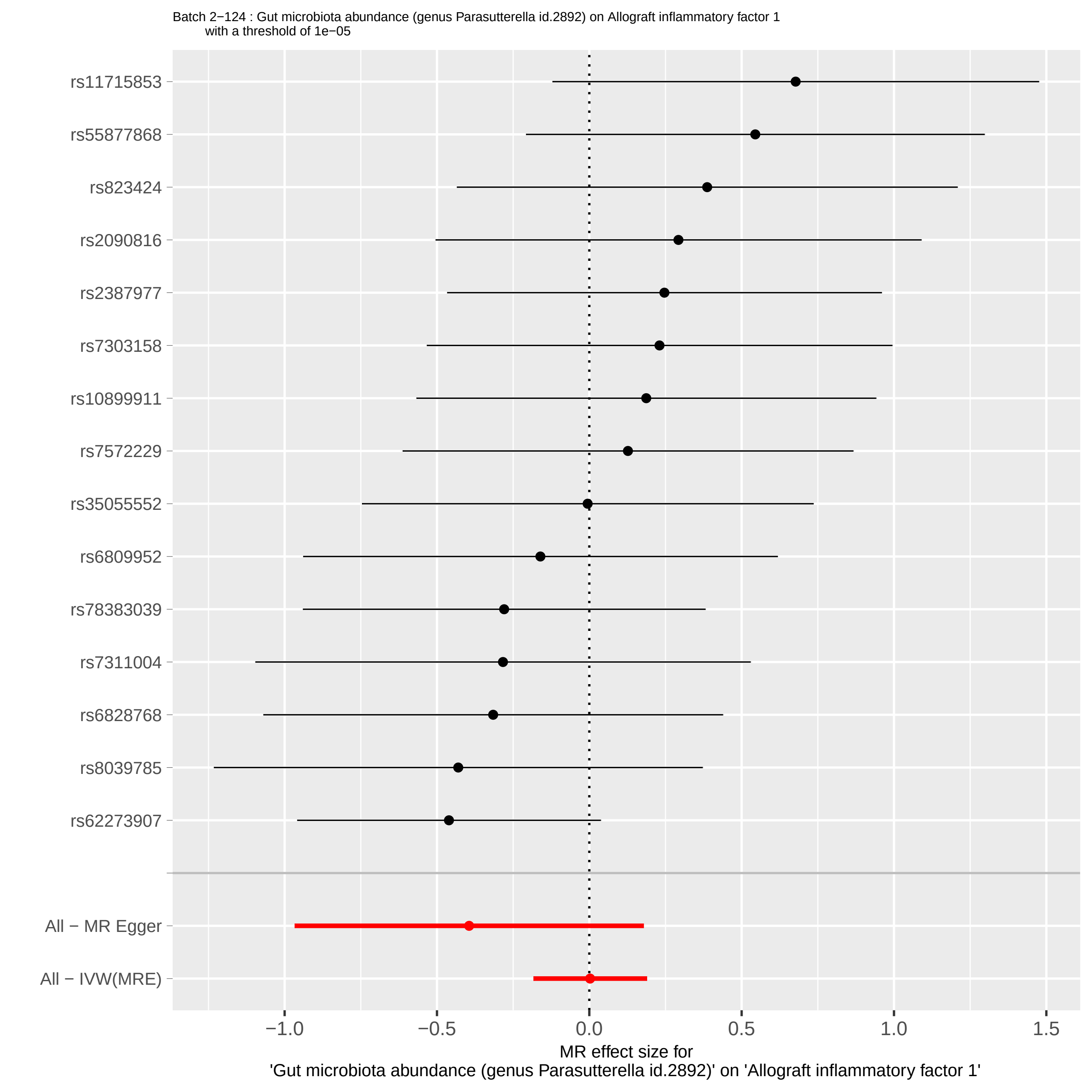

Batch 2−124 : Gut microbiota abundance (genus Parasutterella id.2892) on Allograft inflammatory factor 1
with a threshold of 1e−05
rs11715853
rs55877868
rs823424
rs2090816
rs2387977
rs7303158
rs10899911
rs7572229
rs35055552
rs6809952
rs78383039
rs7311004
rs6828768
rs8039785
rs62273907
All − MR Egger
All − IVW(MRE)
−1.0
−0.5
0.0
0.5
1.0
1.5
MR effect size for
'Gut microbiota abundance (genus Parasutterella id.2892)' on 'Allograft inflammatory factor 1'

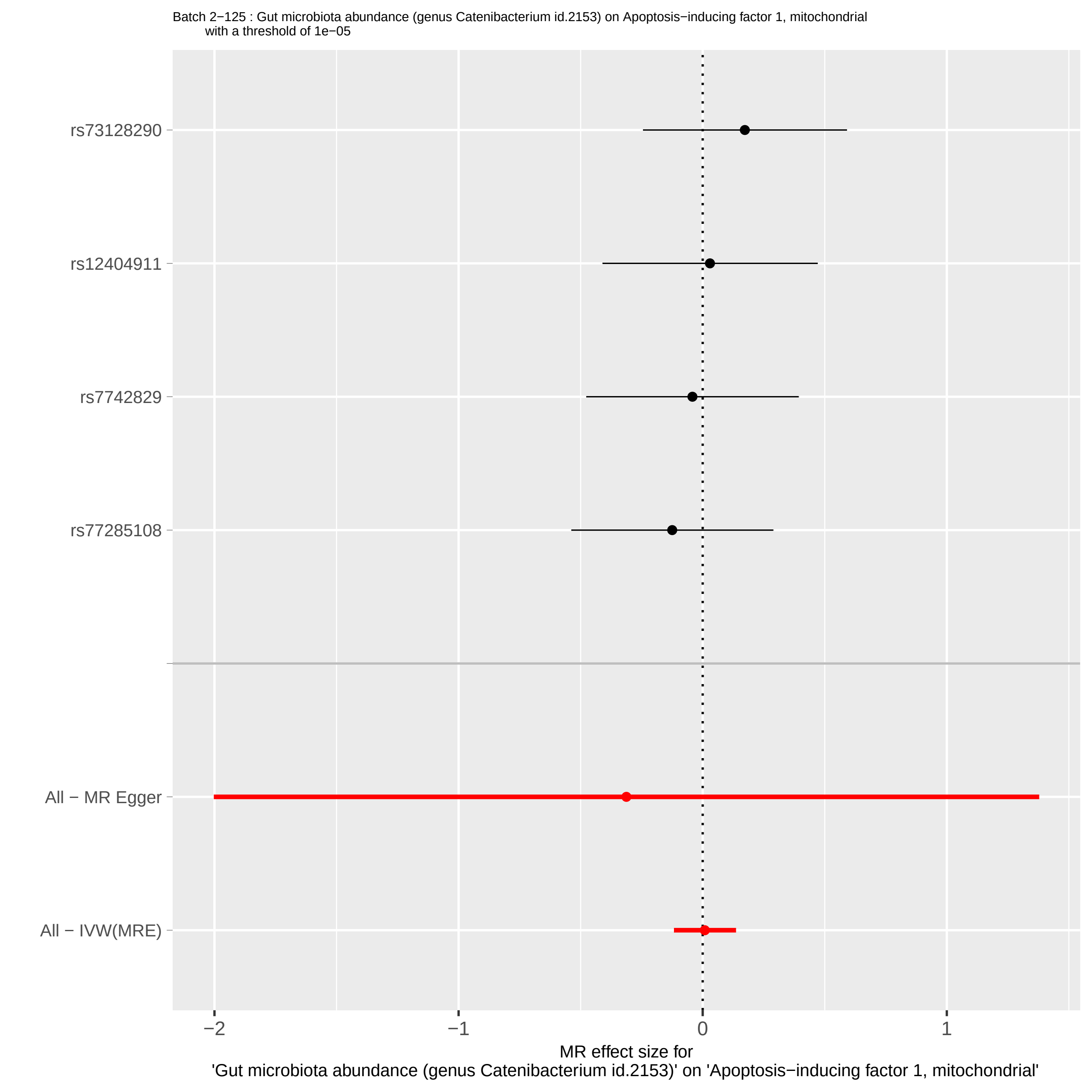

Batch 2−125 : Gut microbiota abundance (genus Catenibacterium id.2153) on Apoptosis−inducing factor 1, mitochondrial
with a threshold of 1e−05
rs73128290
rs12404911
rs7742829
rs77285108
All − MR Egger
All − IVW(MRE)
−2
−1
0
1
MR effect size for
'Gut microbiota abundance (genus Catenibacterium id.2153)' on 'Apoptosis−inducing factor 1, mitochondrial'

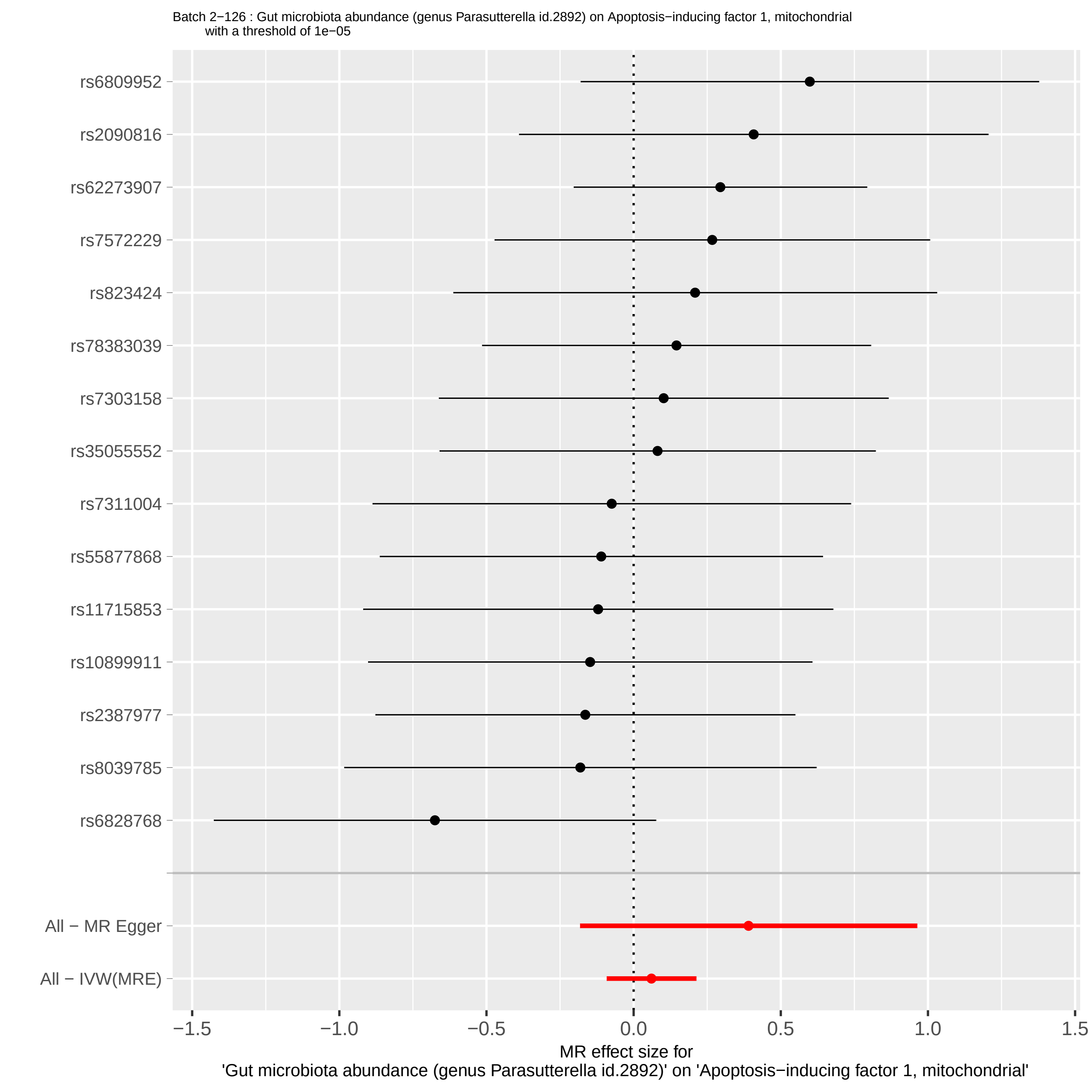
Batch 2−126 : Gut microbiota abundance (genus Parasutterella id.2892) on Apoptosis−inducing factor 1, mitochondrial
with a threshold of 1e−05
rs6809952
rs2090816
rs62273907
rs7572229
rs823424
rs78383039
rs7303158
rs35055552
rs7311004
rs55877868
rs11715853
rs10899911
rs2387977
rs8039785
rs6828768
All − MR Egger
All − IVW(MRE)
−1.5
−1.0
−0.5
0.0
0.5
1.0
1.5
MR effect size for
'Gut microbiota abundance (genus Parasutterella id.2892)' on 'Apoptosis−inducing factor 1, mitochondrial'

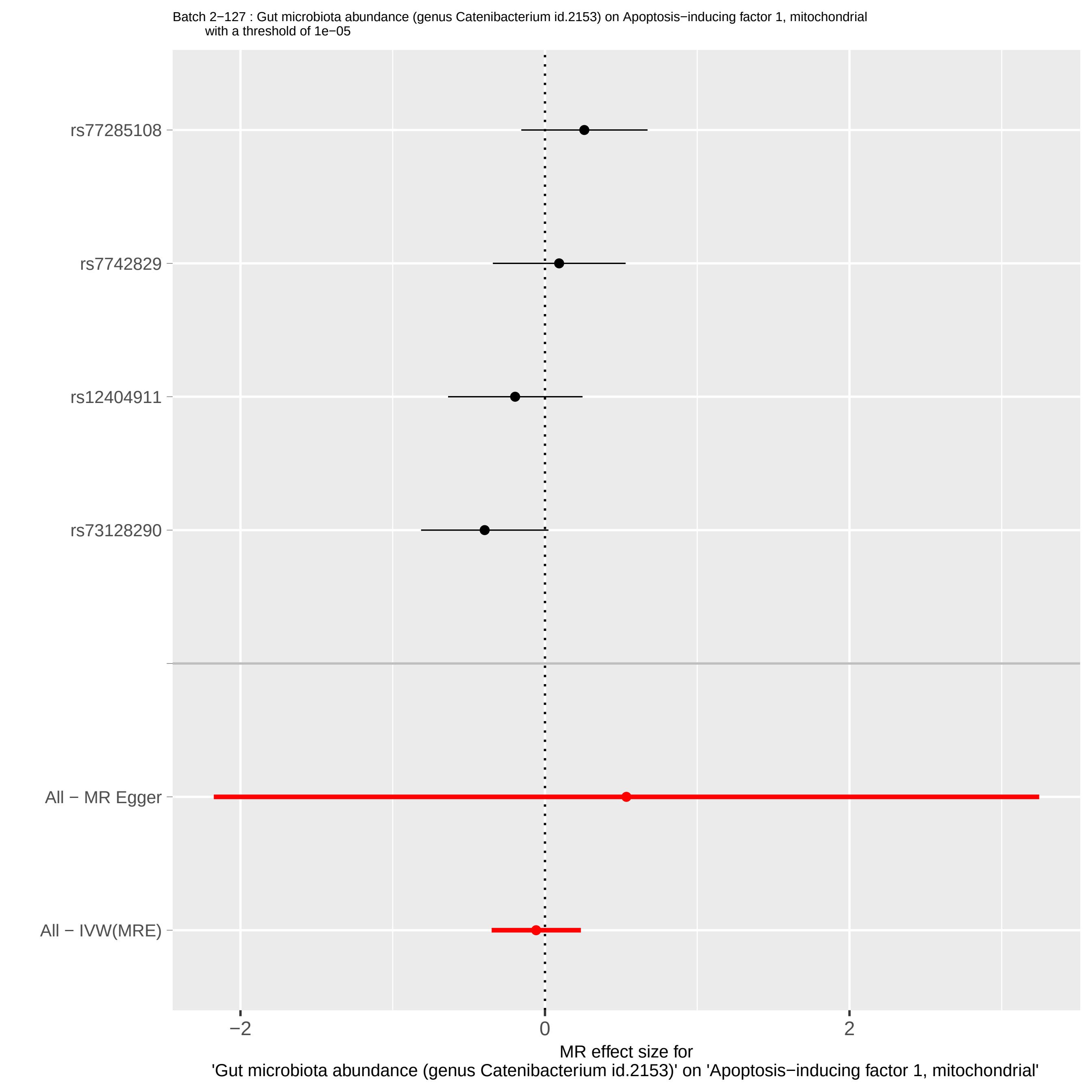
Batch 2−127 : Gut microbiota abundance (genus Catenibacterium id.2153) on Apoptosis−inducing factor 1, mitochondrial
with a threshold of 1e−05
rs77285108
rs7742829
rs12404911
rs73128290
All − MR Egger
All − IVW(MRE)
−2
0
2
MR effect size for
'Gut microbiota abundance (genus Catenibacterium id.2153)' on 'Apoptosis−inducing factor 1, mitochondrial'

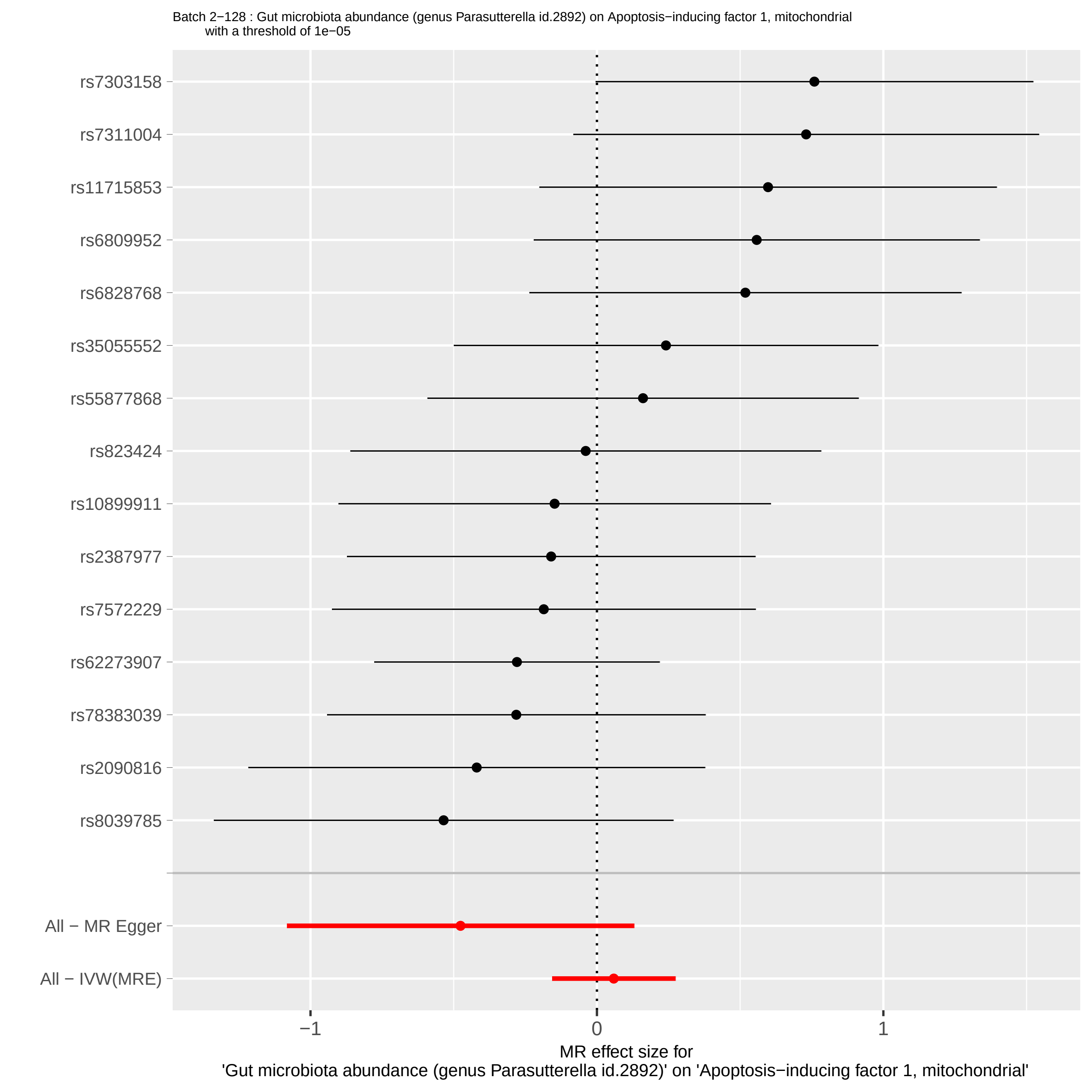
Batch 2−128 : Gut microbiota abundance (genus Parasutterella id.2892) on Apoptosis−inducing factor 1, mitochondrial
with a threshold of 1e−05
rs7303158
rs7311004
rs11715853
rs6809952
rs6828768
rs35055552
rs55877868
rs823424
rs10899911
rs2387977
rs7572229
rs62273907
rs78383039
rs2090816
rs8039785
All − MR Egger
All − IVW(MRE)
−1
0
1
MR effect size for
'Gut microbiota abundance (genus Parasutterella id.2892)' on 'Apoptosis−inducing factor 1, mitochondrial'

Batch 2−129 : Gut microbiota abundance (genus Catenibacterium id.2153) on Endothelial monocyte−activating polypeptide 2
with a threshold of 1e−05

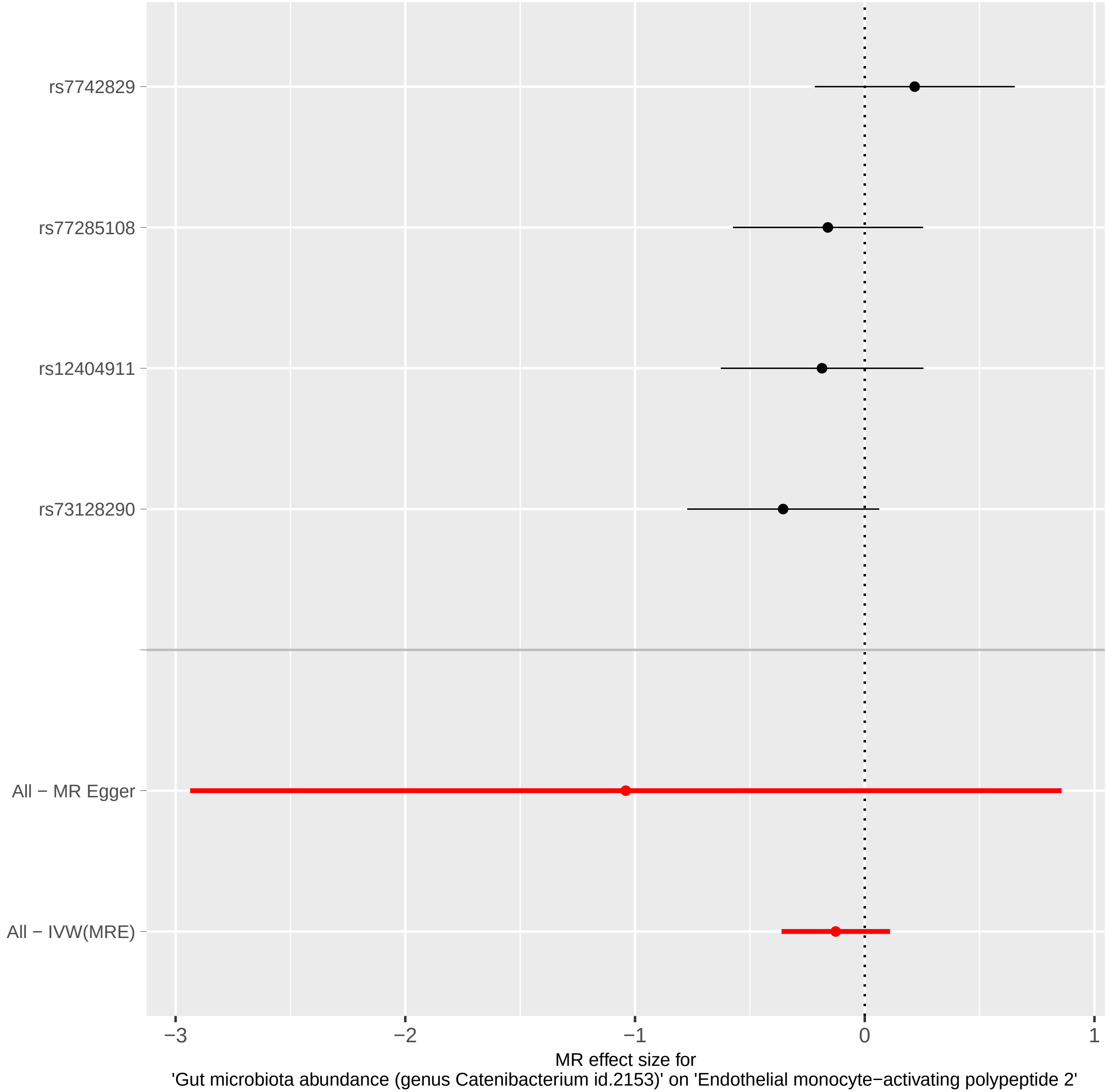

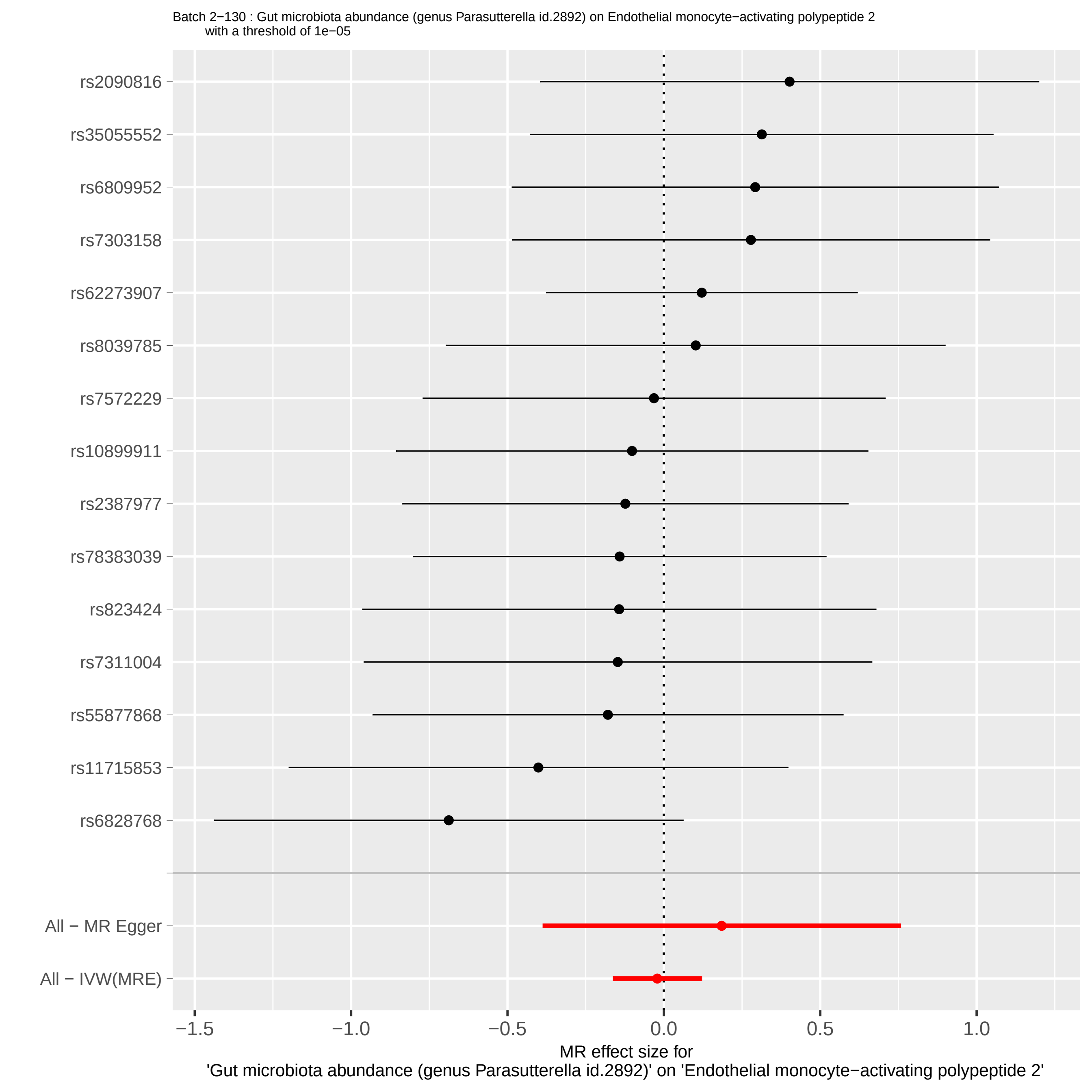
Batch 2−130 : Gut microbiota abundance (genus Parasutterella id.2892) on Endothelial monocyte−activating polypeptide 2
with a threshold of 1e−05
rs2090816
rs35055552
rs6809952
rs7303158
rs62273907
rs8039785
rs7572229
rs10899911
rs2387977
rs78383039
rs823424
rs7311004
rs55877868
rs11715853
rs6828768
All − MR Egger
All − IVW(MRE)
−1.5
−1.0
−0.5
0.0
0.5
1.0
MR effect size for
'Gut microbiota abundance (genus Parasutterella id.2892)' on 'Endothelial monocyte−activating polypeptide 2'

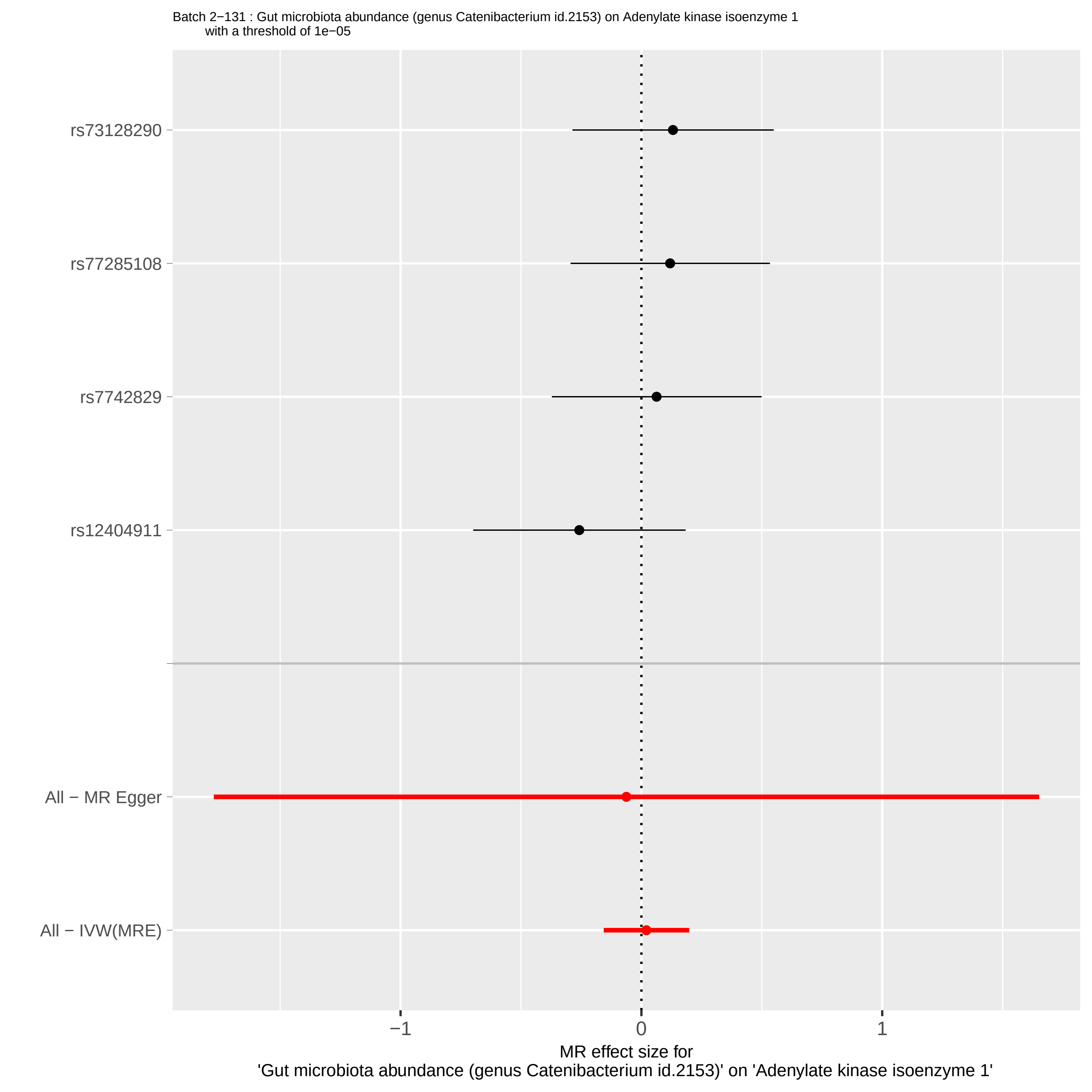

Batch 2−131 : Gut microbiota abundance (genus Catenibacterium id.2153) on Adenylate kinase isoenzyme 1
with a threshold of 1e−05
rs73128290
rs77285108
rs7742829
rs12404911
All − MR Egger
All − IVW(MRE)
−1
0
1
MR effect size for
'Gut microbiota abundance (genus Catenibacterium id.2153)' on 'Adenylate kinase isoenzyme 1'

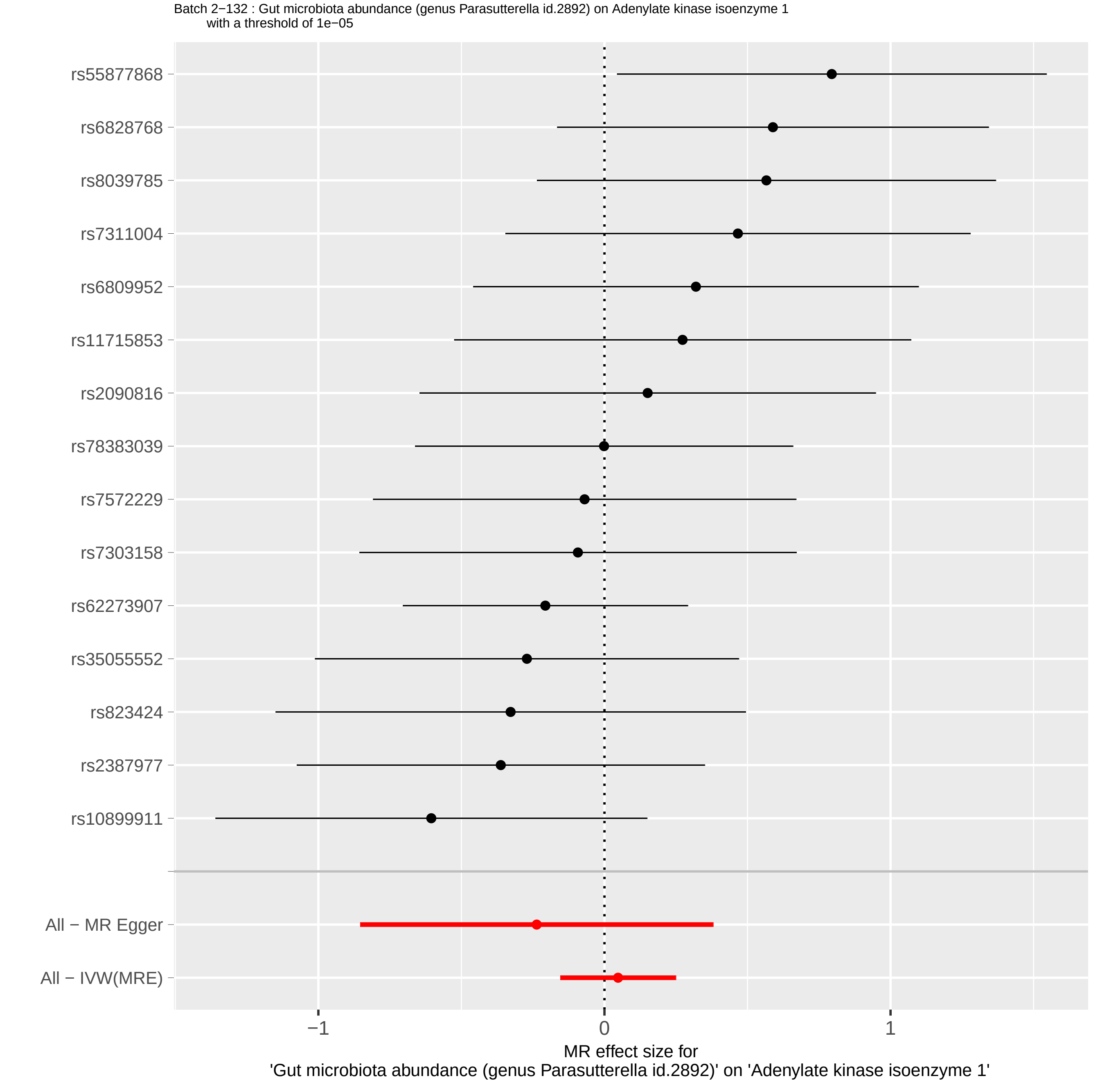

Batch 2−132 : Gut microbiota abundance (genus Parasutterella id.2892) on Adenylate kinase isoenzyme 1
with a threshold of 1e−05
rs55877868
rs6828768
rs8039785
rs7311004
rs6809952
rs11715853
rs2090816
rs78383039
rs7572229
rs7303158
rs62273907
rs35055552
rs823424
rs2387977
rs10899911
All − MR Egger
All − IVW(MRE)
−1
0
1
MR effect size for
'Gut microbiota abundance (genus Parasutterella id.2892)' on 'Adenylate kinase isoenzyme 1'

Batch 2−133 : Gut microbiota abundance (genus Catenibacterium id.2153) on Alcohol dehydrogenase [NADP(+)]
with a threshold of 1e−05

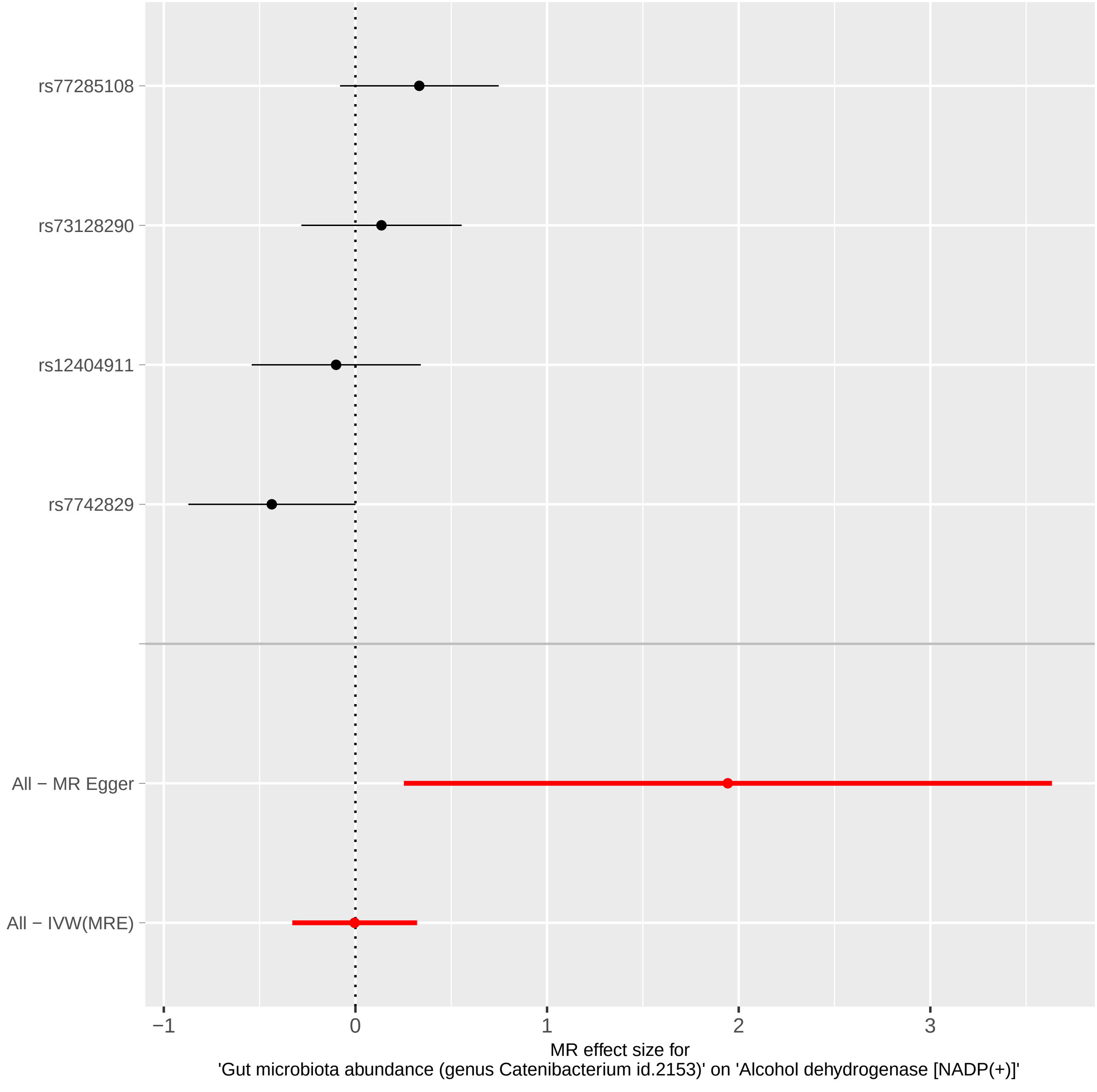

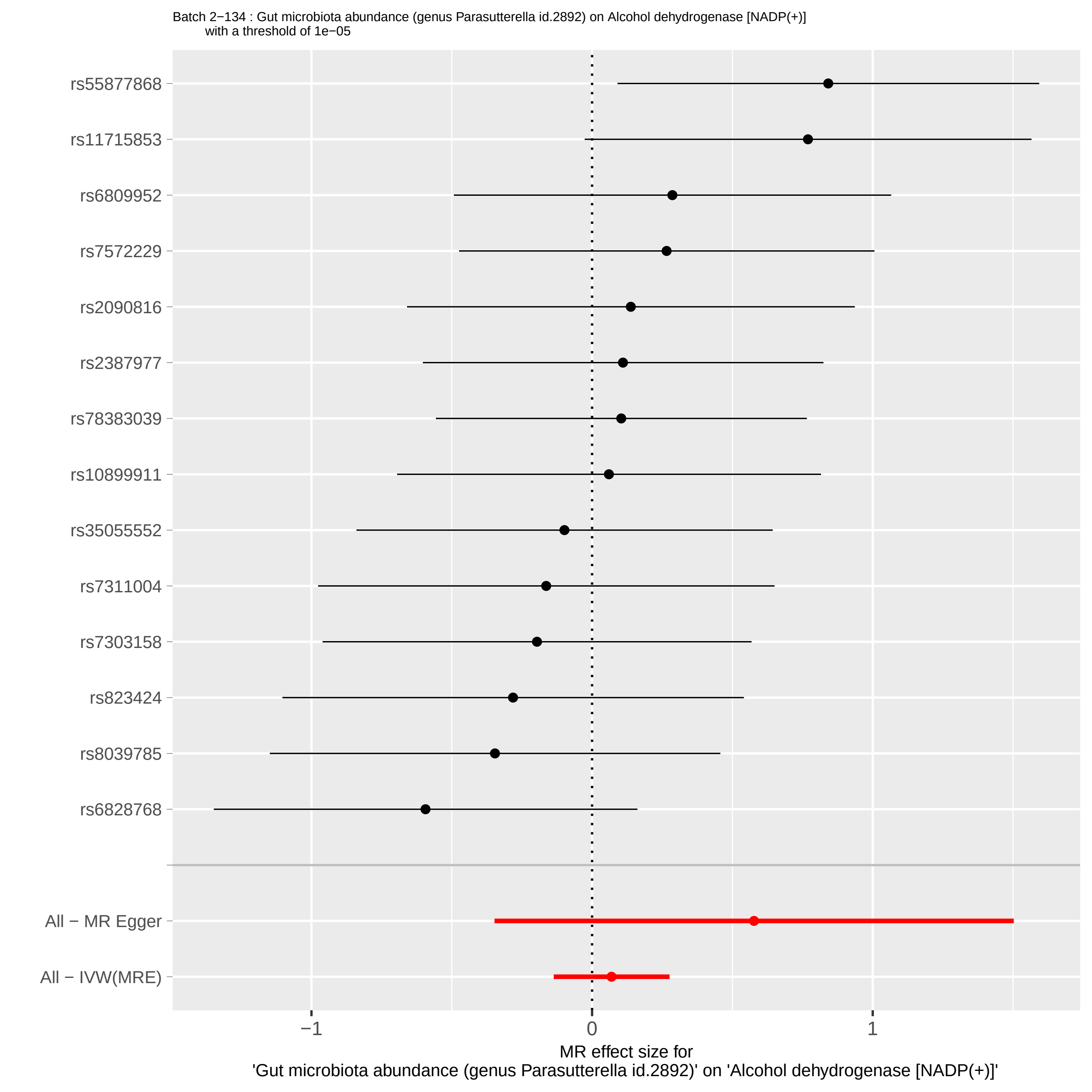
Batch 2−134 : Gut microbiota abundance (genus Parasutterella id.2892) on Alcohol dehydrogenase [NADP(+)]
with a threshold of 1e−05
rs55877868
rs11715853
rs6809952
rs7572229
rs2090816
rs2387977
rs78383039
rs10899911
rs35055552
rs7311004
rs7303158
rs823424
rs8039785
rs6828768
All − MR Egger
All − IVW(MRE)
−1
0
1
MR effect size for
'Gut microbiota abundance (genus Parasutterella id.2892)' on 'Alcohol dehydrogenase [NADP(+)]'

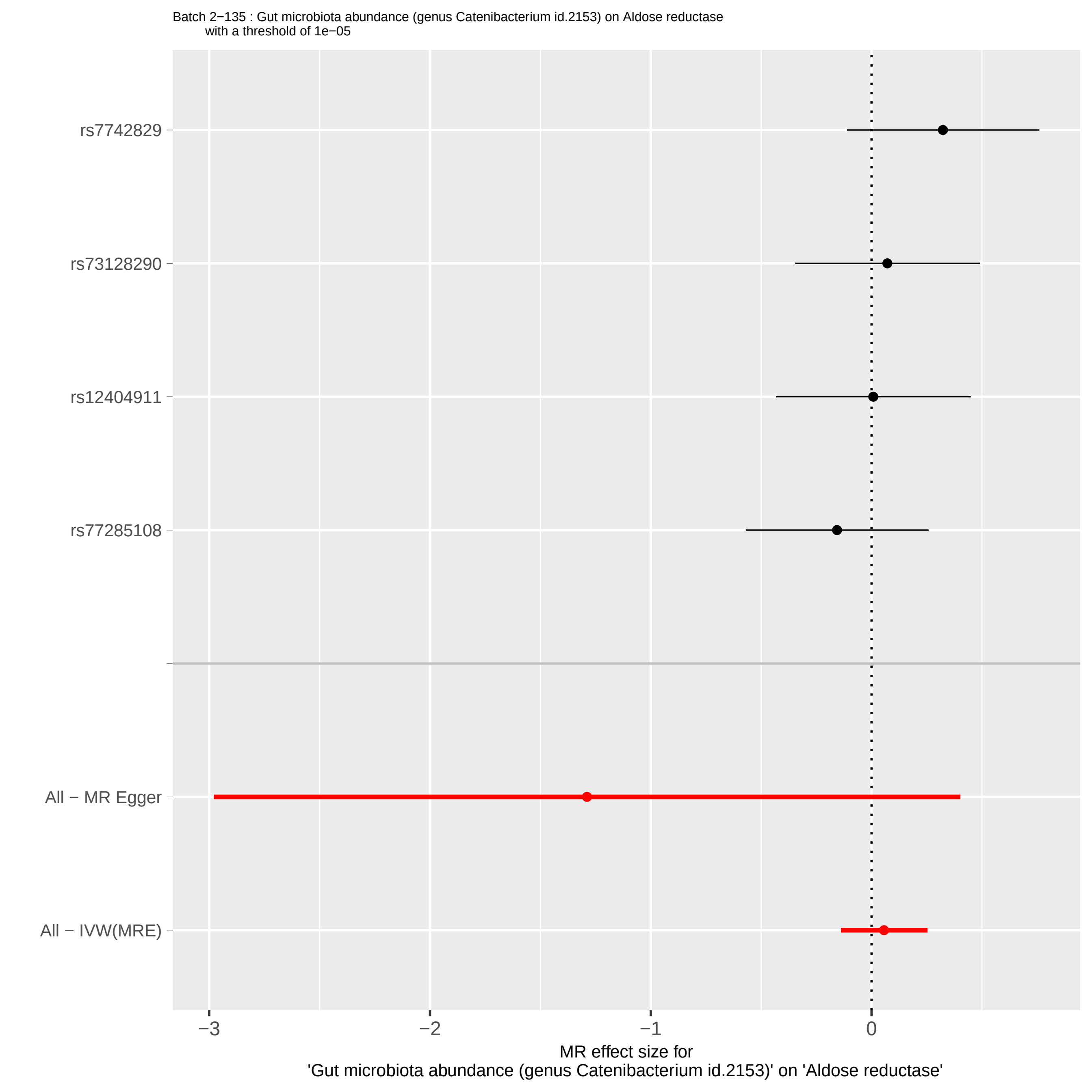

Batch 2−135 : Gut microbiota abundance (genus Catenibacterium id.2153) on Aldose reductase
with a threshold of 1e−05
rs7742829
rs73128290
rs12404911
rs77285108
All − MR Egger
All − IVW(MRE)
−3
−2
−1
0
MR effect size for
'Gut microbiota abundance (genus Catenibacterium id.2153)' on 'Aldose reductase'

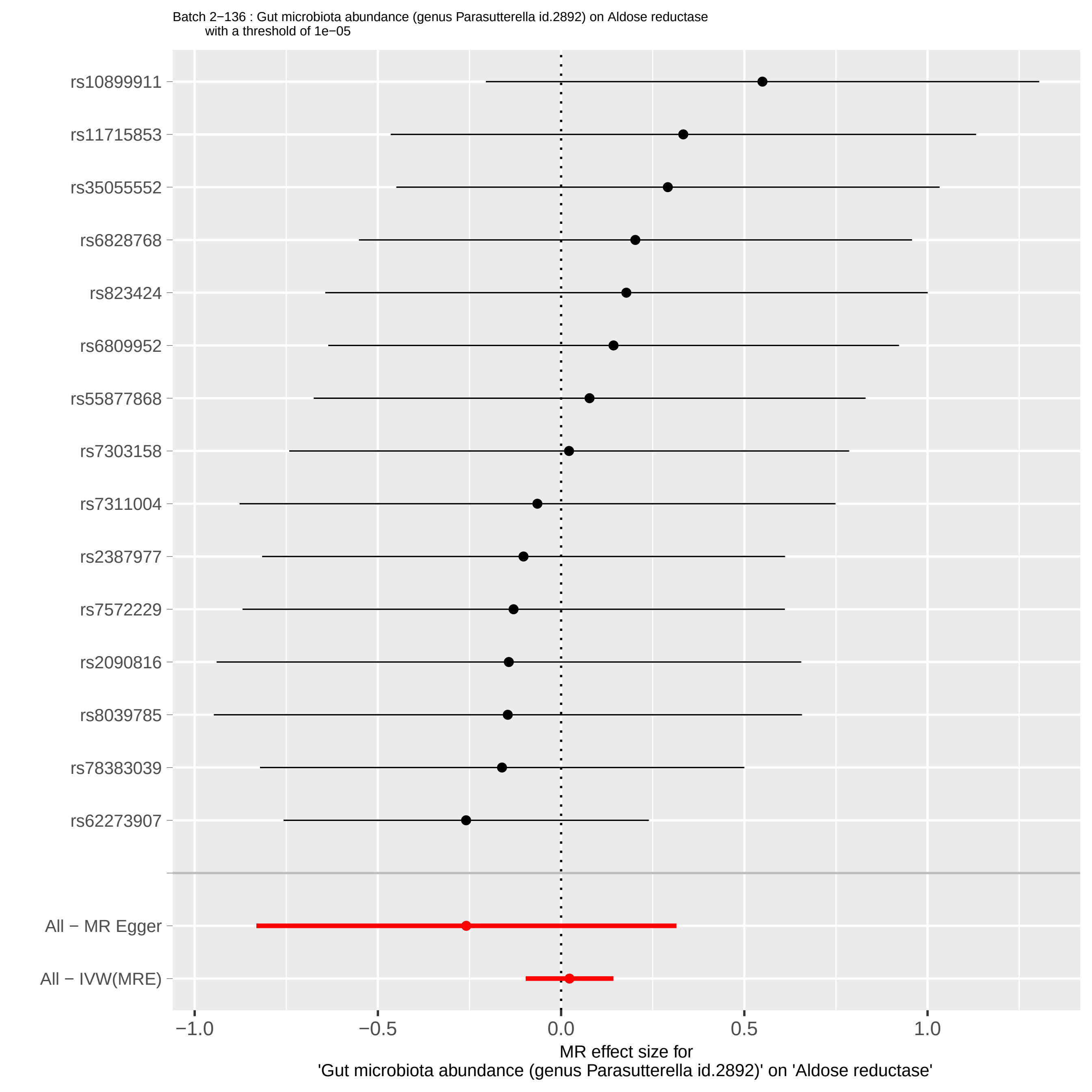

Batch 2−136 : Gut microbiota abundance (genus Parasutterella id.2892) on Aldose reductase
with a threshold of 1e−05
rs10899911
rs11715853
rs35055552
rs6828768
rs823424
rs6809952
rs55877868
rs7303158
rs7311004
rs2387977
rs7572229
rs2090816
rs8039785
rs78383039
rs62273907
All − MR Egger
All − IVW(MRE)
−1.0
−0.5
0.0
0.5
1.0
MR effect size for
'Gut microbiota abundance (genus Parasutterella id.2892)' on 'Aldose reductase'

Batch 2−137 : Gut microbiota abundance (genus Catenibacterium id.2153) on Aldo−keto reductase family 1 member C1 with a threshold of 1e−05

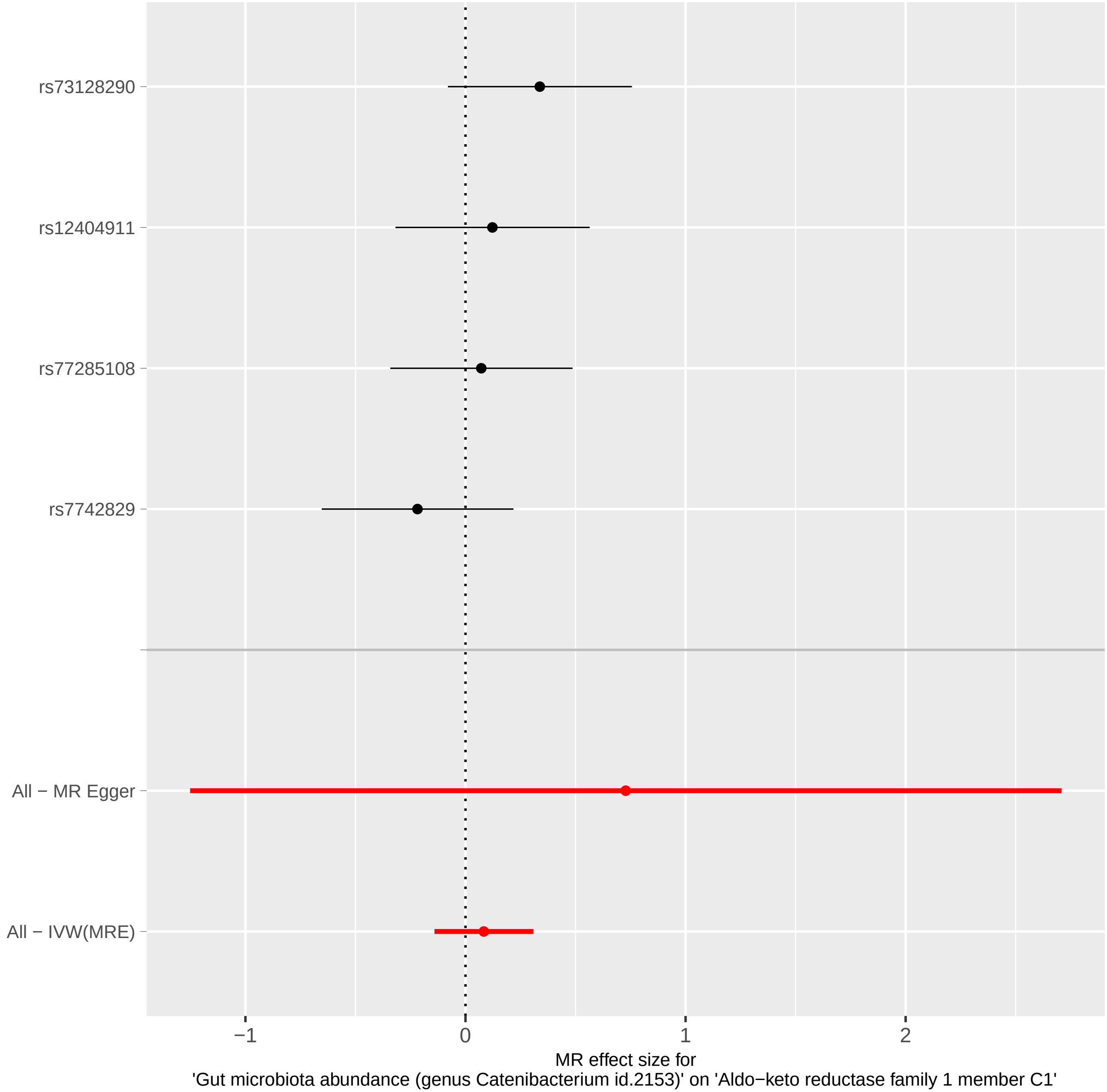

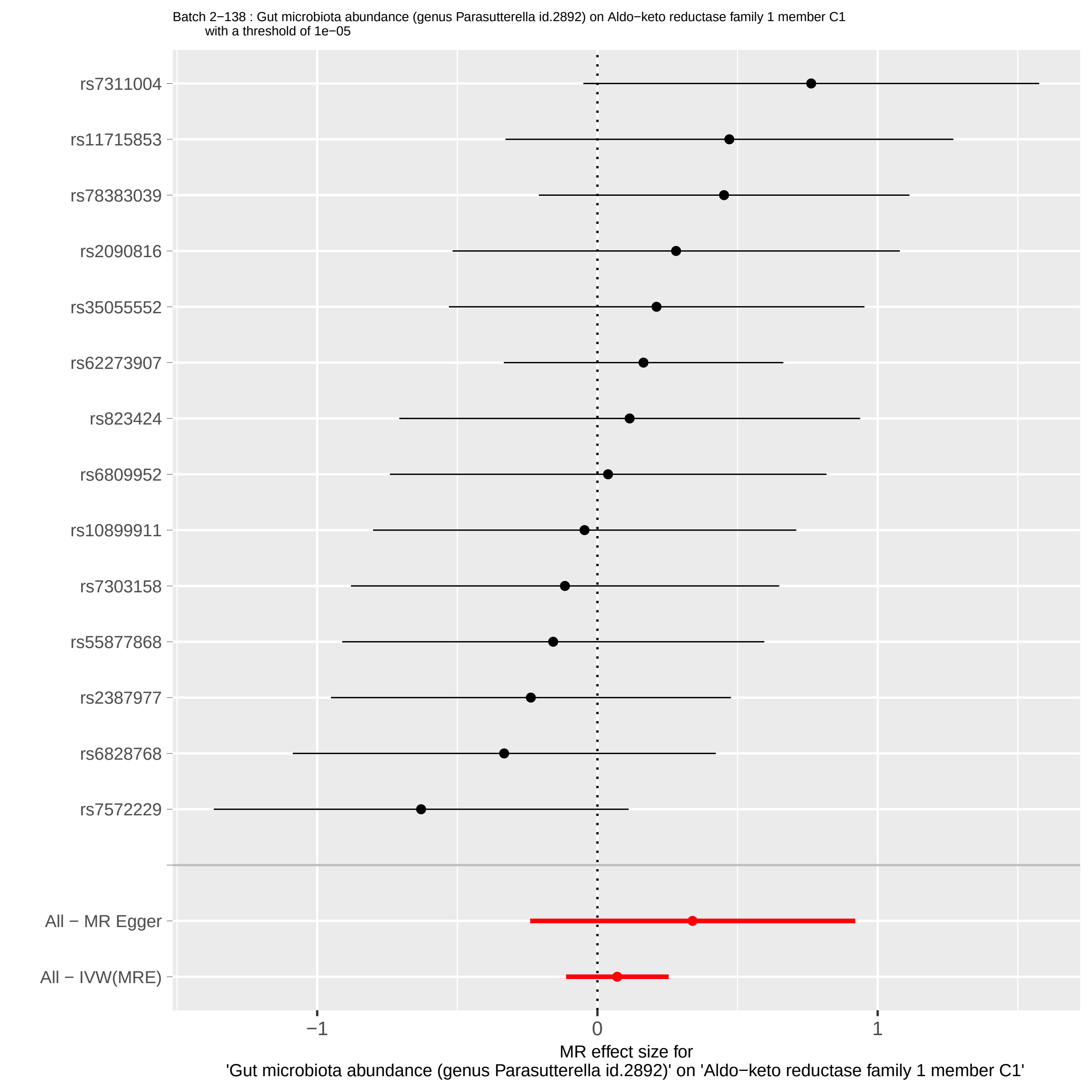
Batch 2−138 : Gut microbiota abundance (genus Parasutterella id.2892) on Aldo−keto reductase family 1 member C1
with a threshold of 1e−05
rs7311004
rs11715853
rs78383039
rs2090816
rs35055552
rs62273907
rs823424
rs6809952
rs10899911
rs7303158
rs55877868
rs2387977
rs6828768
rs7572229
All − MR Egger
All − IVW(MRE)
−1
0
1
MR effect size for
'Gut microbiota abundance (genus Parasutterella id.2892)' on 'Aldo−keto reductase family 1 member C1'

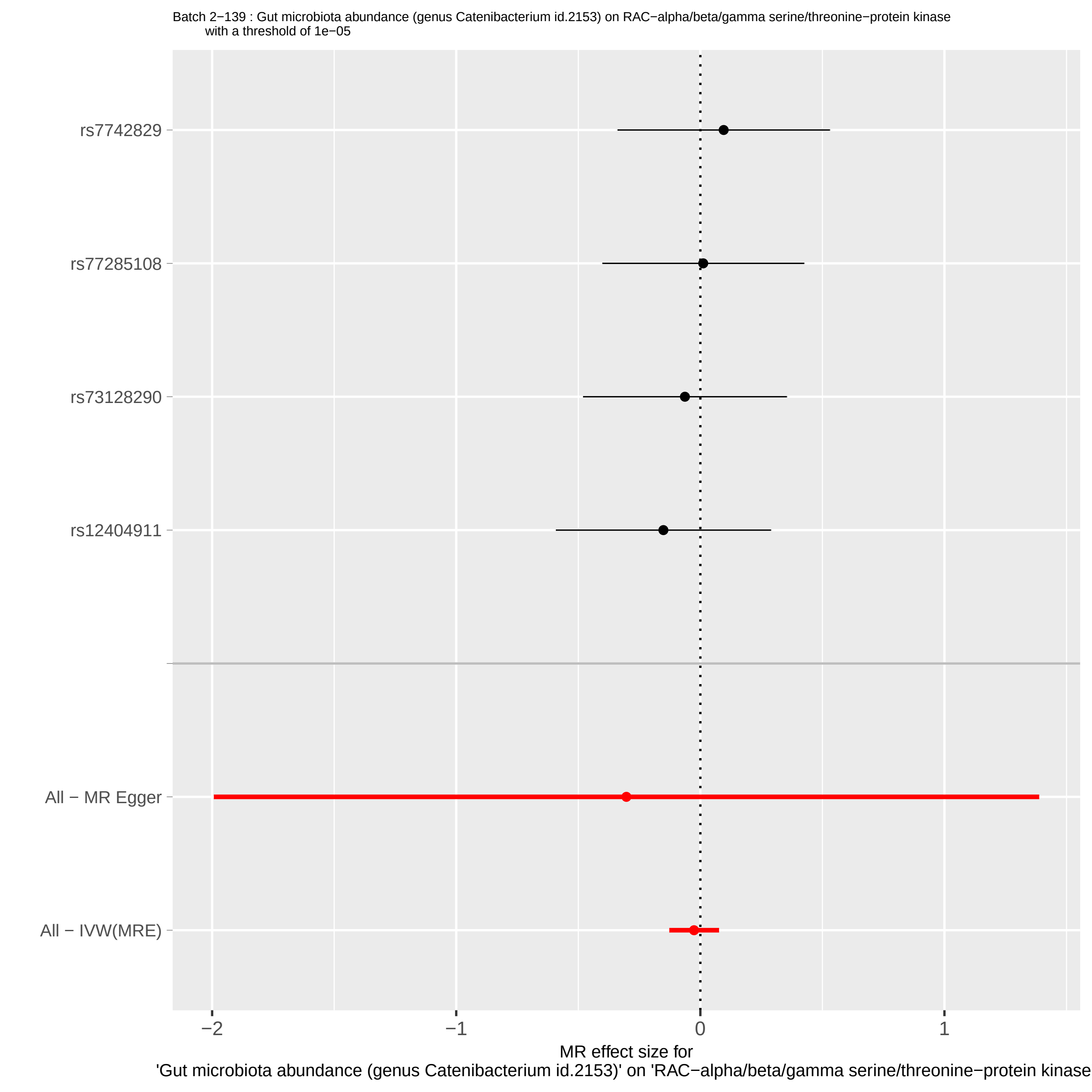

Batch 2−139 : Gut microbiota abundance (genus Catenibacterium id.2153) on RAC−alpha/beta/gamma serine/threonine−protein kinase with a threshold of 1e−05
rs7742829
rs77285108
rs73128290
rs12404911
All − MR Egger
All − IVW(MRE)
−2
−1
0
1
MR effect size for
'Gut microbiota abundance (genus Catenibacterium id.2153)' on 'RAC−alpha/beta/gamma serine/threonine−protein kinase'

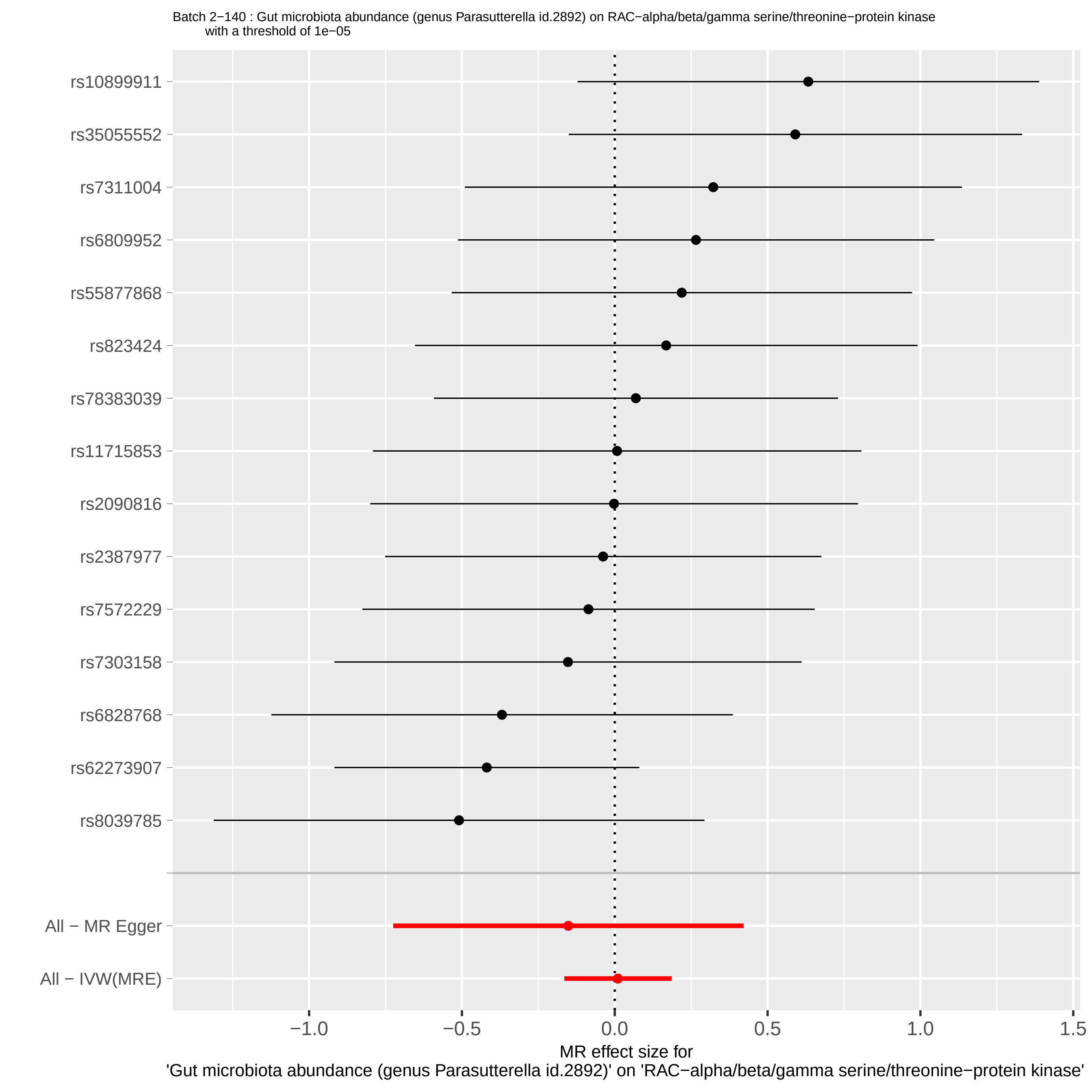

Batch 2−140 : Gut microbiota abundance (genus Parasutterella id.2892) on RAC−alpha/beta/gamma serine/threonine−protein kinase with a threshold of 1e−05
rs10899911
rs35055552
rs7311004
rs6809952
rs55877868
rs823424
rs78383039
rs11715853
rs2090816
rs2387977
rs7572229
rs7303158
rs6828768
rs62273907
rs8039785
All − MR Egger
All − IVW(MRE)
−1.0
−0.5
0.0
0.5
1.0
1.5
MR effect size for
'Gut microbiota abundance (genus Parasutterella id.2892)' on 'RAC−alpha/beta/gamma serine/threonine−protein kinase'

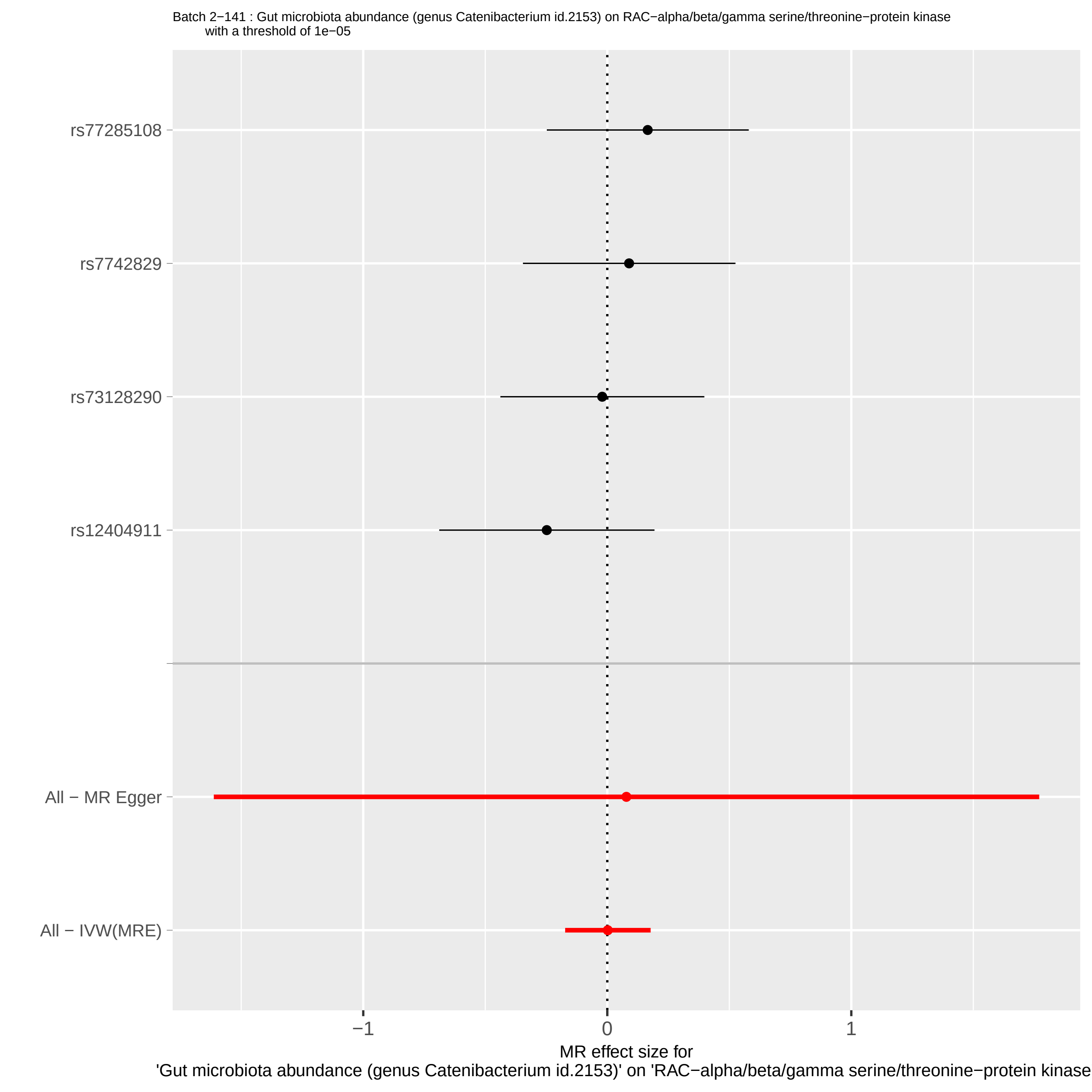

Batch 2−141 : Gut microbiota abundance (genus Catenibacterium id.2153) on RAC−alpha/beta/gamma serine/threonine−protein kinase with a threshold of 1e−05
rs77285108
rs7742829
rs73128290
rs12404911
All − MR Egger
All − IVW(MRE)
−1
0
1
MR effect size for
'Gut microbiota abundance (genus Catenibacterium id.2153)' on 'RAC−alpha/beta/gamma serine/threonine−protein kinase

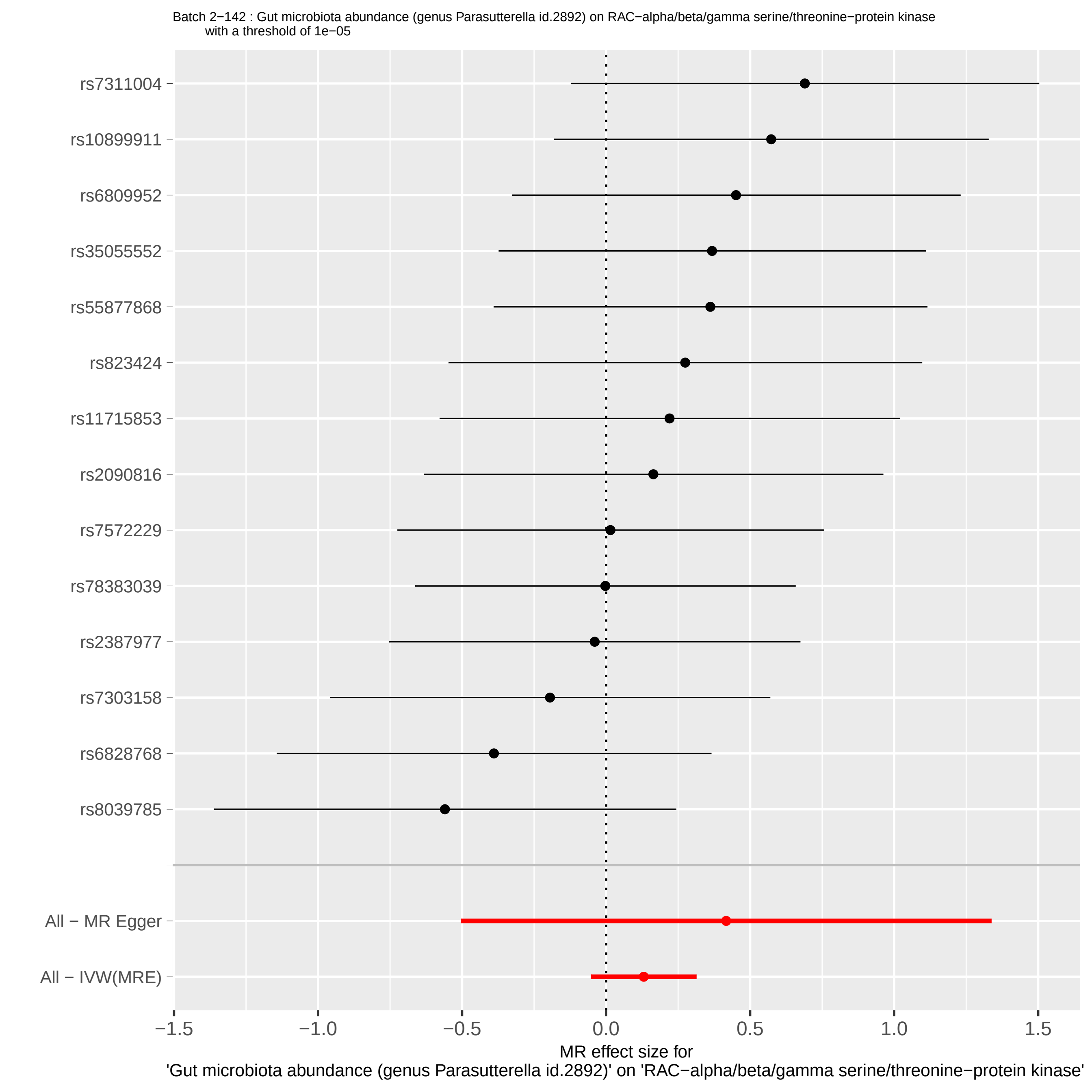

Batch 2−142 : Gut microbiota abundance (genus Parasutterella id.2892) on RAC−alpha/beta/gamma serine/threonine−protein kinase
with a threshold of 1e−05
rs7311004
rs10899911
rs6809952
rs35055552
rs55877868
rs823424
rs11715853
rs2090816
rs7572229
rs78383039
rs2387977
rs7303158
rs6828768
rs8039785
All − MR Egger
All − IVW(MRE)
−1.5
−1.0
−0.5
0.0
0.5
1.0
1.5
MR effect size for
'Gut microbiota abundance (genus Parasutterella id.2892)' on 'RAC−alpha/beta/gamma serine/threonine−protein kinase'

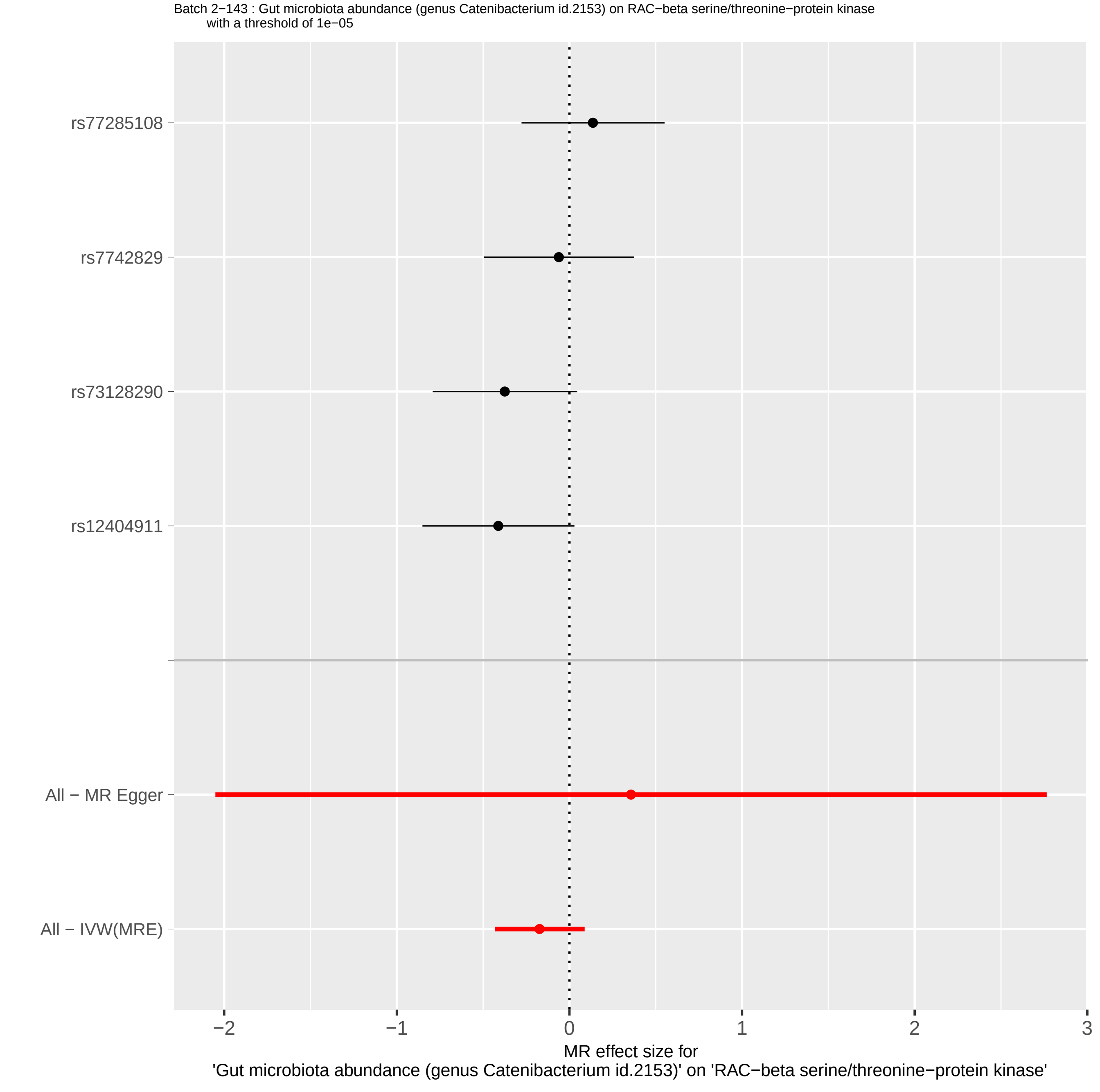

Batch 2−143 : Gut microbiota abundance (genus Catenibacterium id.2153) on RAC−beta serine/threonine−protein kinase with a threshold of 1e−05
rs77285108
rs7742829
rs73128290
rs12404911
All − MR Egger
All − IVW(MRE)
−2
−1
0
1
2
3
MR effect size for
'Gut microbiota abundance (genus Catenibacterium id.2153)' on 'RAC−beta serine/threonine−protein kinase'

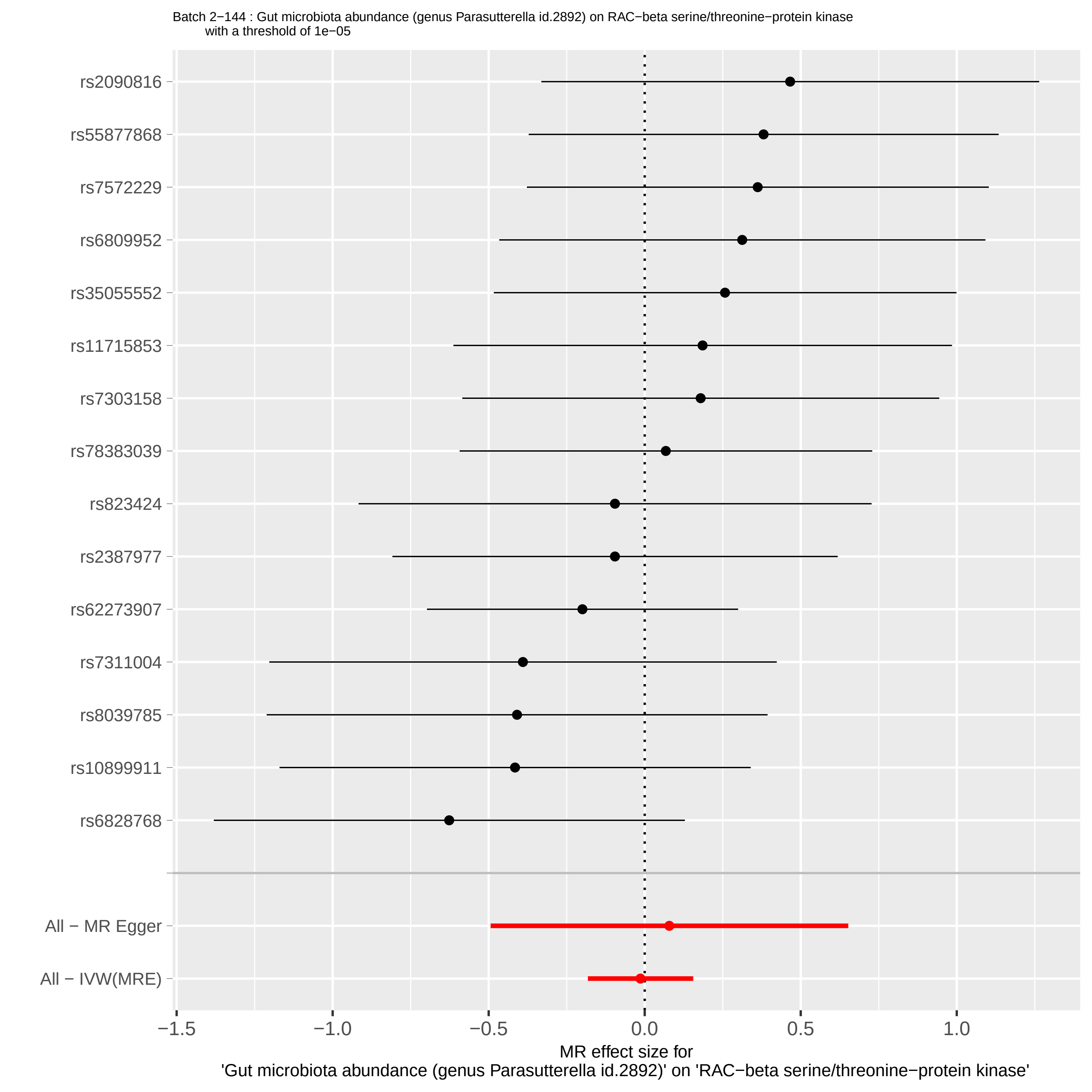

Batch 2−144 : Gut microbiota abundance (genus Parasutterella id.2892) on RAC−beta serine/threonine−protein kinase
with a threshold of 1e−05
rs2090816
rs55877868
rs7572229
rs6809952
rs35055552
rs11715853
rs7303158
rs78383039
rs823424
rs2387977
rs62273907
rs7311004
rs8039785
rs10899911
rs6828768
All − MR Egger
All − IVW(MRE)
−1.5
−1.0
−0.5
0.0
0.5
1.0
MR effect size for
'Gut microbiota abundance (genus Parasutterella id.2892)' on 'RAC−beta serine/threonine−protein kinase'

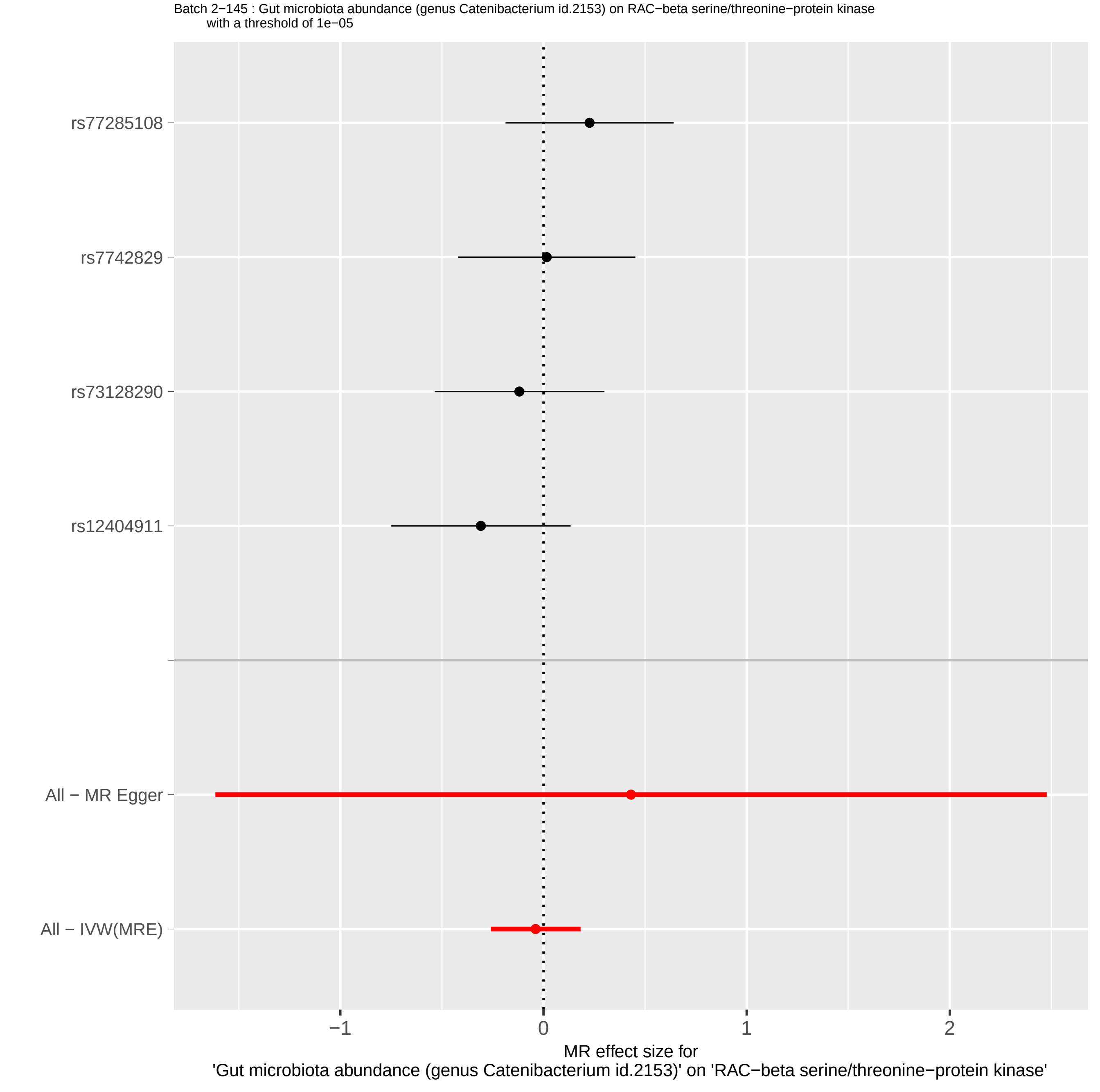

Batch 2−145 : Gut microbiota abundance (genus Catenibacterium id.2153) on RAC−beta serine/threonine−protein kinase
with a threshold of 1e−05
rs77285108
rs7742829
rs73128290
rs12404911
All − MR Egger
All − IVW(MRE)
−1
0
1
2
MR effect size for
'Gut microbiota abundance (genus Catenibacterium id.2153)' on 'RAC−beta serine/threonine−protein kinase'

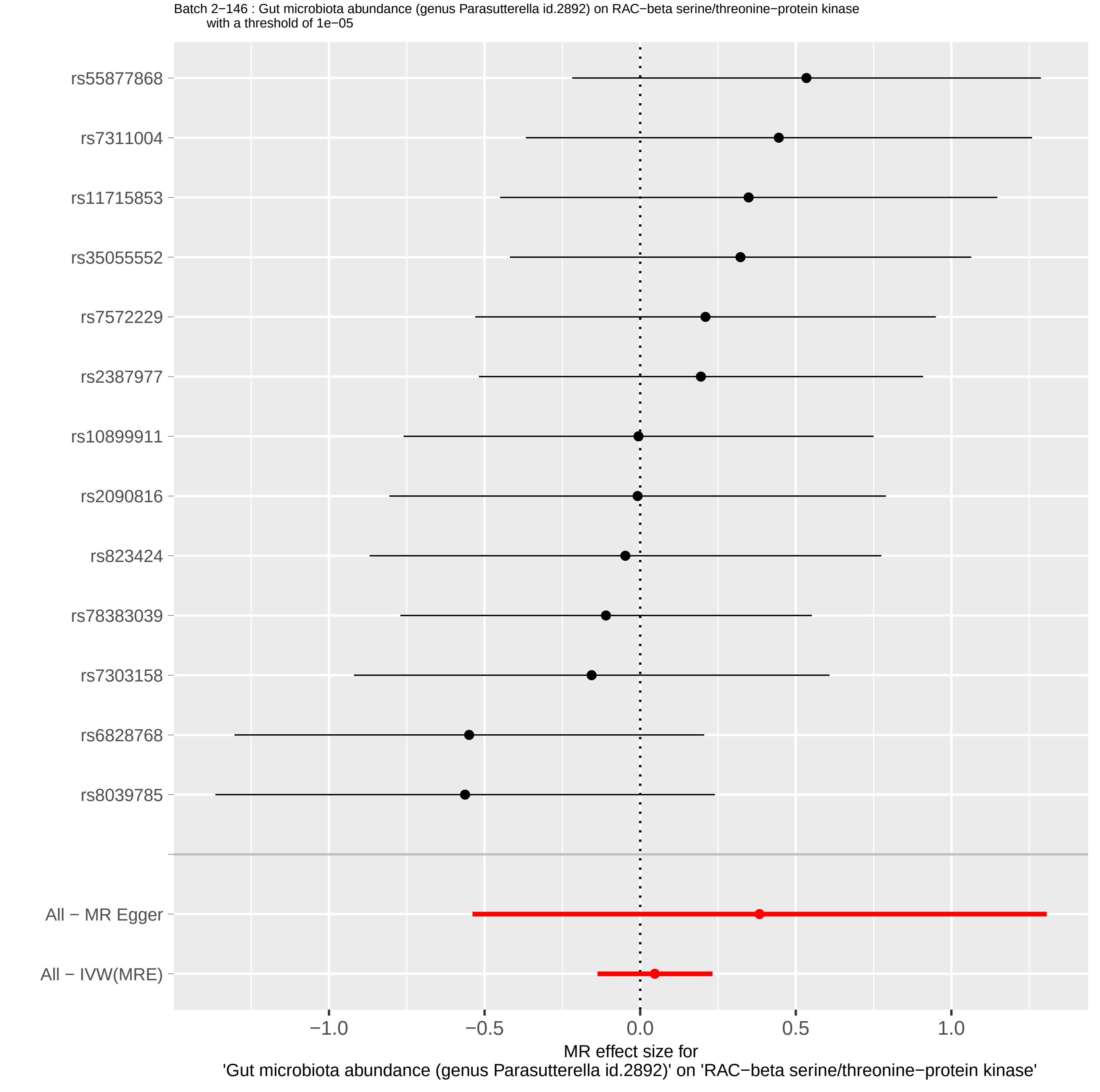

Batch 2−146 : Gut microbiota abundance (genus Parasutterella id.2892) on RAC−beta serine/threonine−protein kinase with a threshold of 1e−05
rs55877868
rs7311004
rs11715853
rs35055552
rs7572229
rs2387977
rs10899911
rs2090816
rs823424
rs78383039
rs7303158
rs6828768
rs8039785
All − MR Egger
All − IVW(MRE)
−1.0
−0.5
0.0
0.5
1.0
MR effect size for
'Gut microbiota abundance (genus Parasutterella id.2892)' on 'RAC−beta serine/threonine−protein kinase'

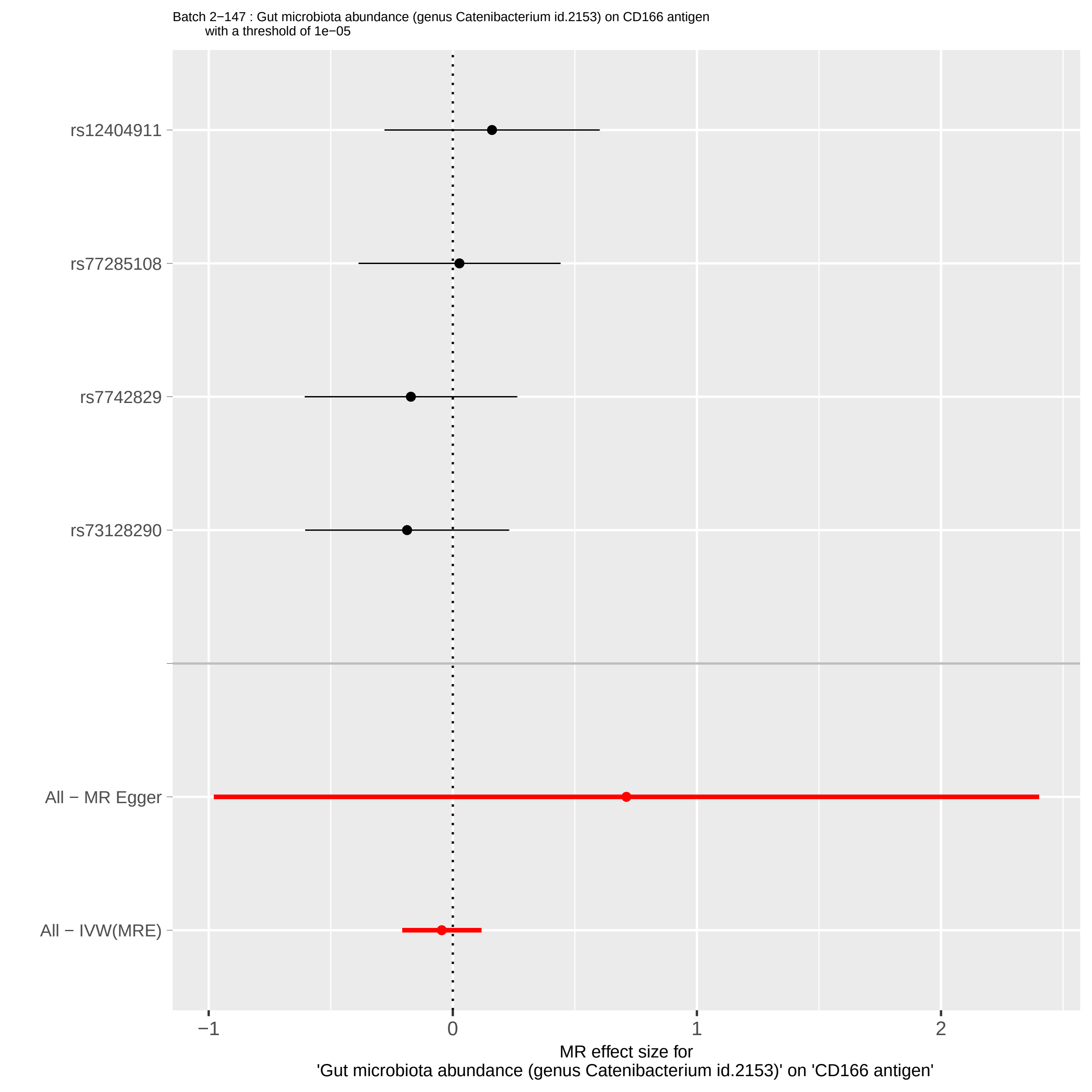
Batch 2−147 : Gut microbiota abundance (genus Catenibacterium id.2153) on CD166 antigen
with a threshold of 1e−05
rs12404911
rs77285108
rs7742829
rs73128290
All − MR Egger
All − IVW(MRE)
−1
0
1
2
MR effect size for
'Gut microbiota abundance (genus Catenibacterium id.2153)' on 'CD166 antigen'

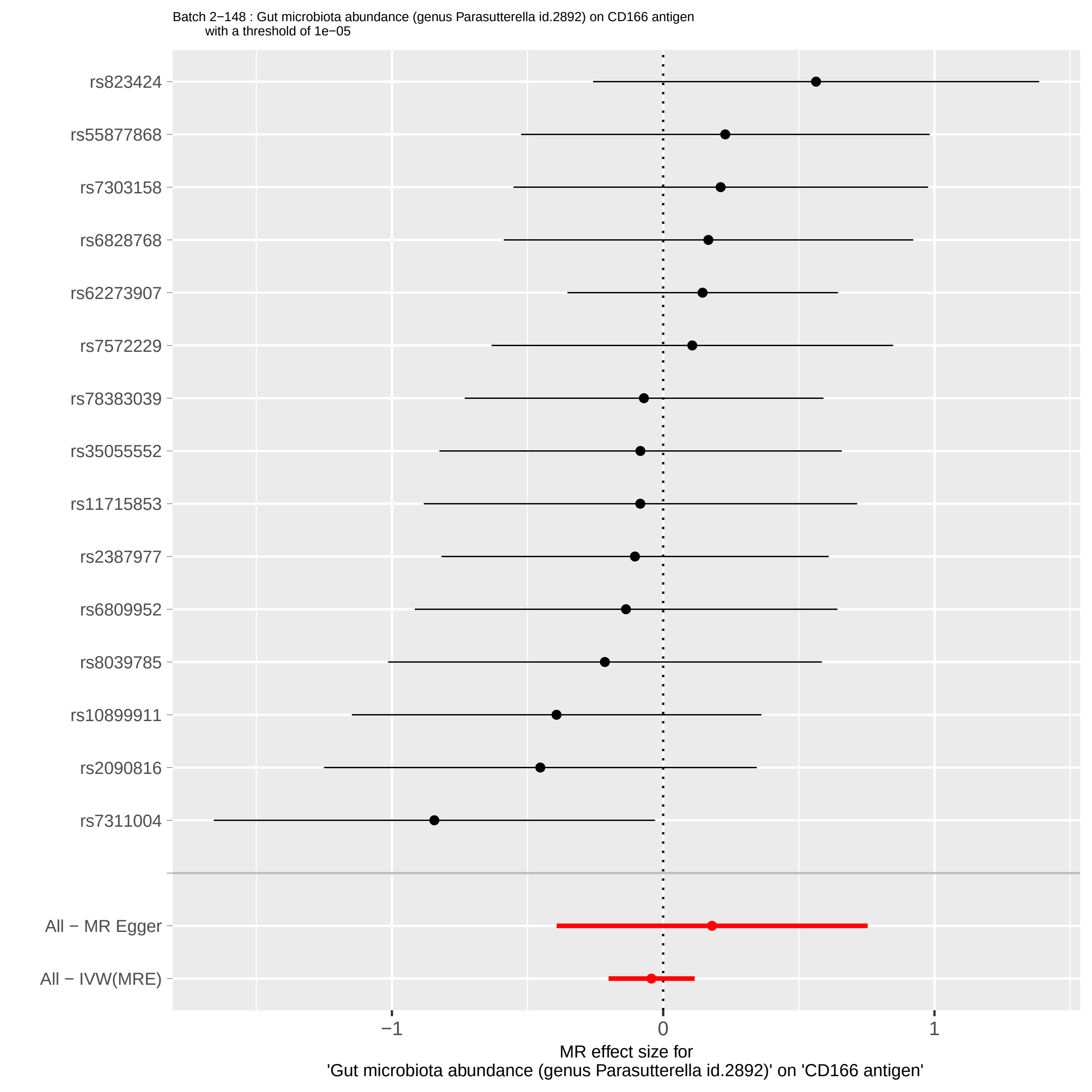

Batch 2−148 : Gut microbiota abundance (genus Parasutterella id.2892) on CD166 antigen
with a threshold of 1e−05
rs823424
rs55877868
rs7303158
rs6828768
rs62273907
rs7572229
rs78383039
rs35055552
rs11715853
rs2387977
rs6809952
rs8039785
rs10899911
rs2090816
rs7311004
All − MR Egger
All − IVW(MRE)
−1
0
1
MR effect size for
'Gut microbiota abundance (genus Parasutterella id.2892)' on 'CD166 antigen'

Batch 2−149 : Gut microbiota abundance (genus Catenibacterium id.2153) on Aldehyde dehydrogenase, dimeric NADP−preferring
with a threshold of 1e−05

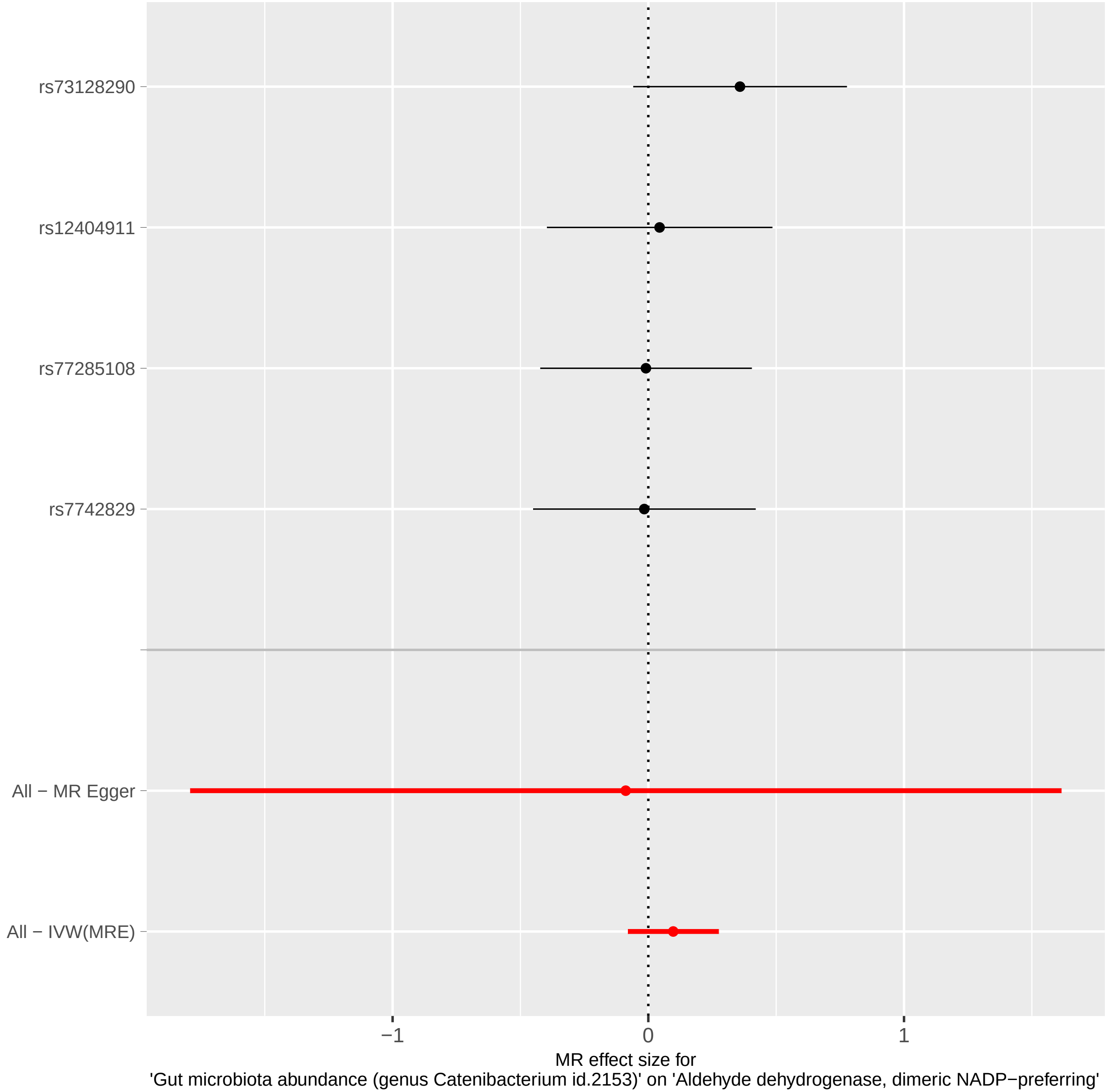

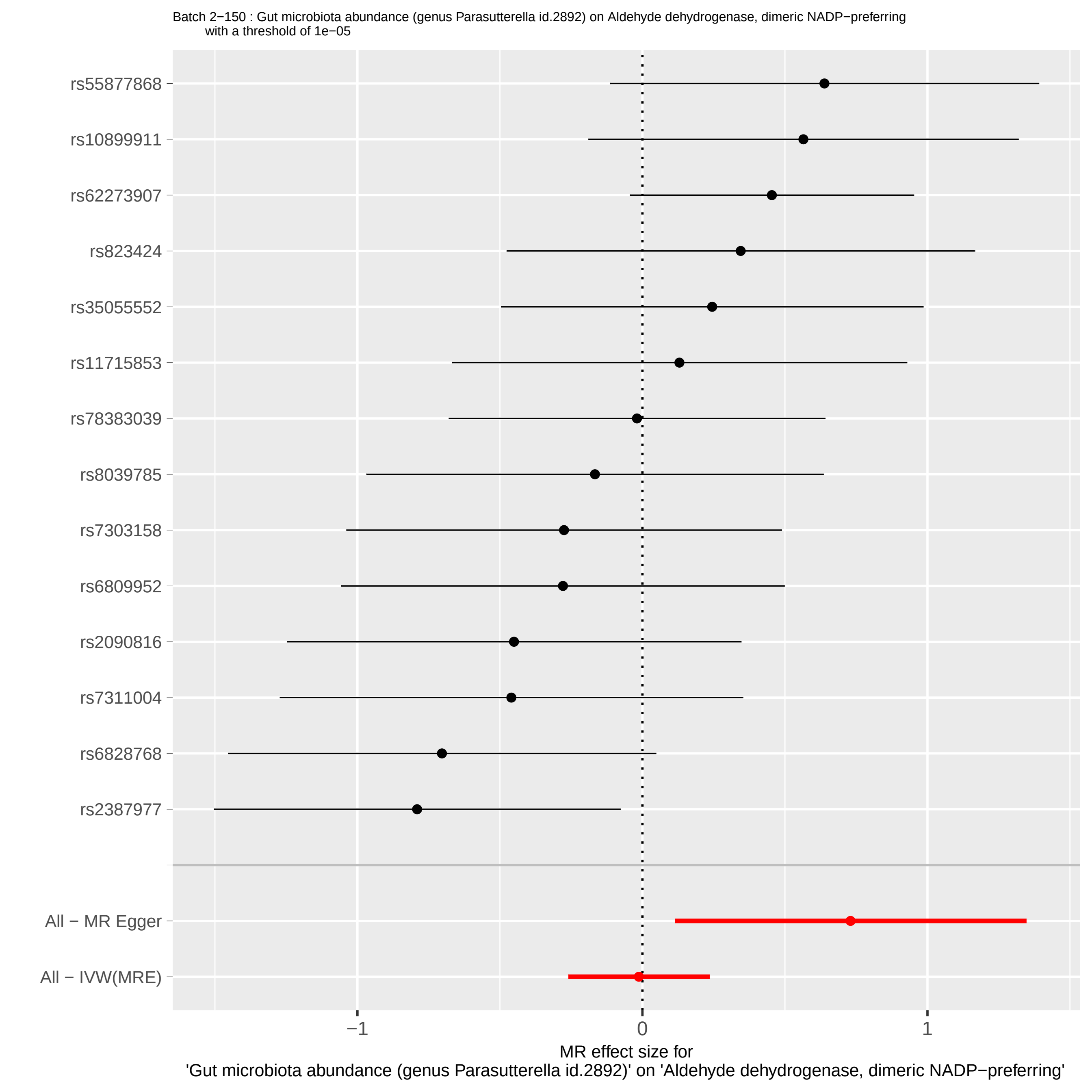

Batch 2−150 : Gut microbiota abundance (genus Parasutterella id.2892) on Aldehyde dehydrogenase, dimeric NADP−preferring
with a threshold of 1e−05
rs55877868
rs10899911
rs62273907
rs823424
rs35055552
rs11715853
rs78383039
rs8039785
rs7303158
rs6809952
rs2090816
rs7311004
rs6828768
rs2387977
All − MR Egger
All − IVW(MRE)
−1
0
1
MR effect size for
'Gut microbiota abundance (genus Parasutterella id.2892)' on 'Aldehyde dehydrogenase, dimeric NADP−preferring'

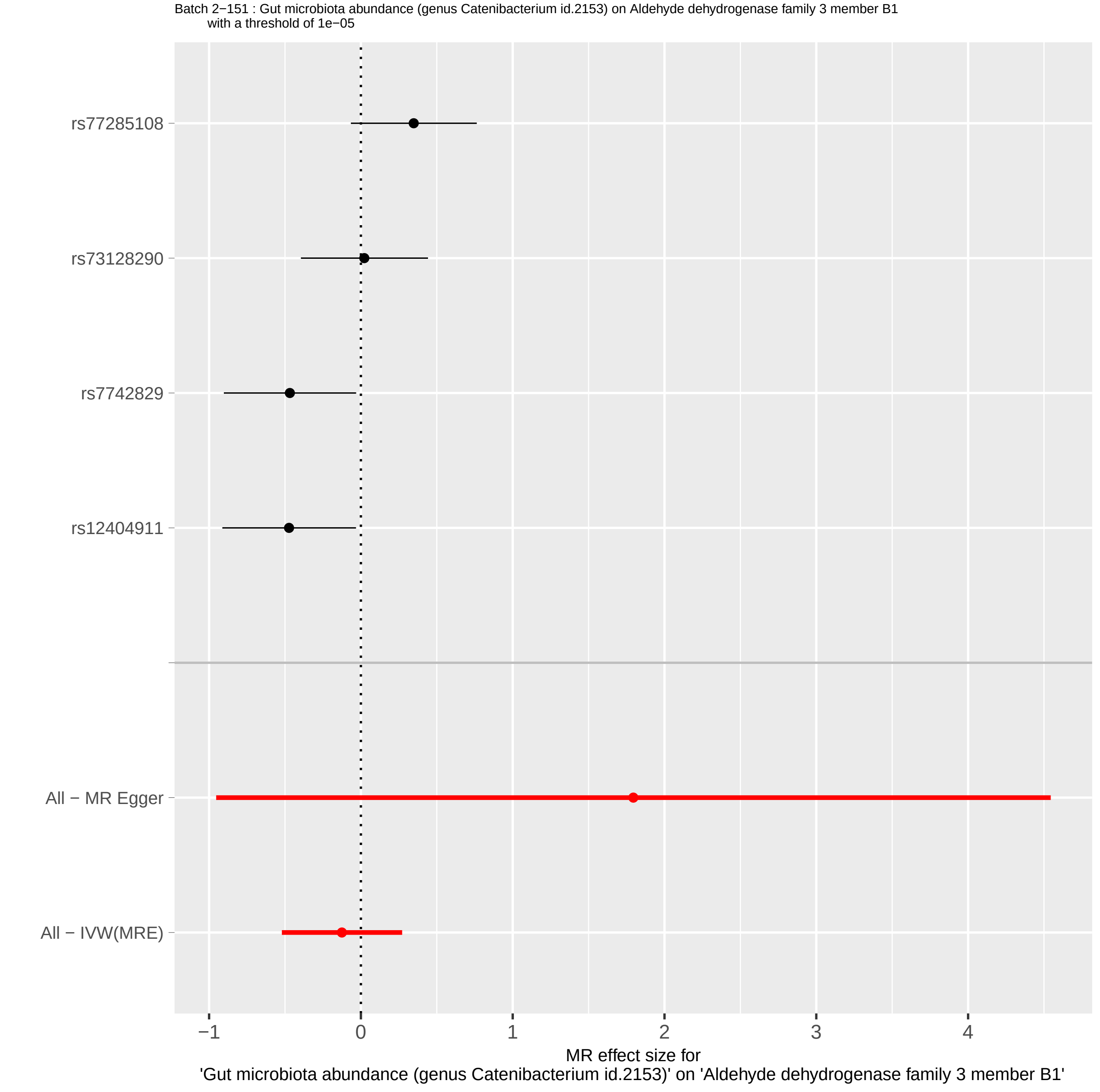

Batch 2−151 : Gut microbiota abundance (genus Catenibacterium id.2153) on Aldehyde dehydrogenase family 3 member B1
with a threshold of 1e−05
rs77285108
rs73128290
rs7742829
rs12404911
All − MR Egger
All − IVW(MRE)
−1
0
1
2
3
4
MR effect size for
'Gut microbiota abundance (genus Catenibacterium id.2153)' on 'Aldehyde dehydrogenase family 3 member B1'

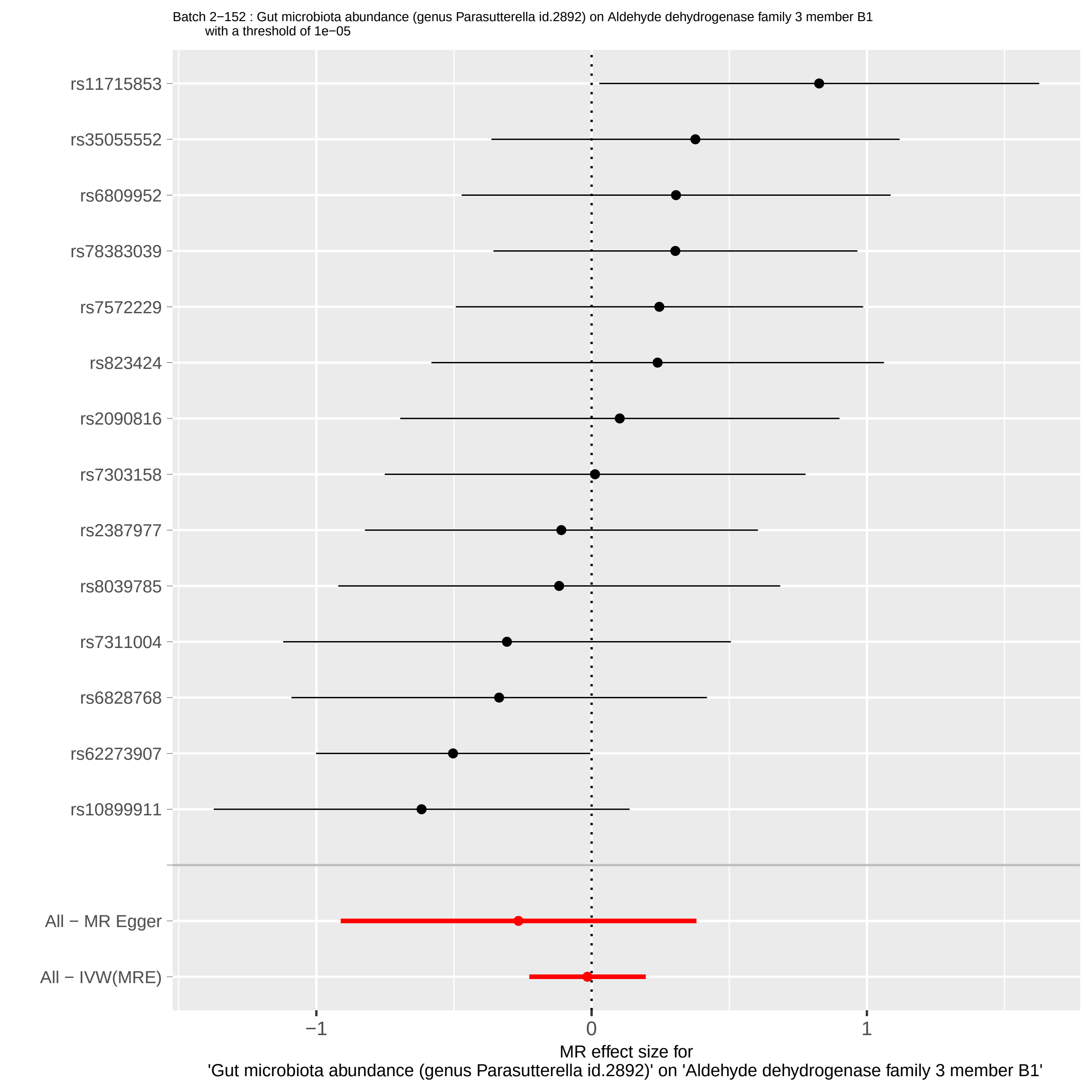
Batch 2−152 : Gut microbiota abundance (genus Parasutterella id.2892) on Aldehyde dehydrogenase family 3 member B1
with a threshold of 1e−05
rs11715853
rs35055552
rs6809952
rs78383039
rs7572229
rs823424
rs2090816
rs7303158
rs2387977
rs8039785
rs7311004
rs6828768
rs62273907
rs10899911
All − MR Egger
All − IVW(MRE)
−1
0
1
MR effect size for
'Gut microbiota abundance (genus Parasutterella id.2892)' on 'Aldehyde dehydrogenase family 3 member B1'

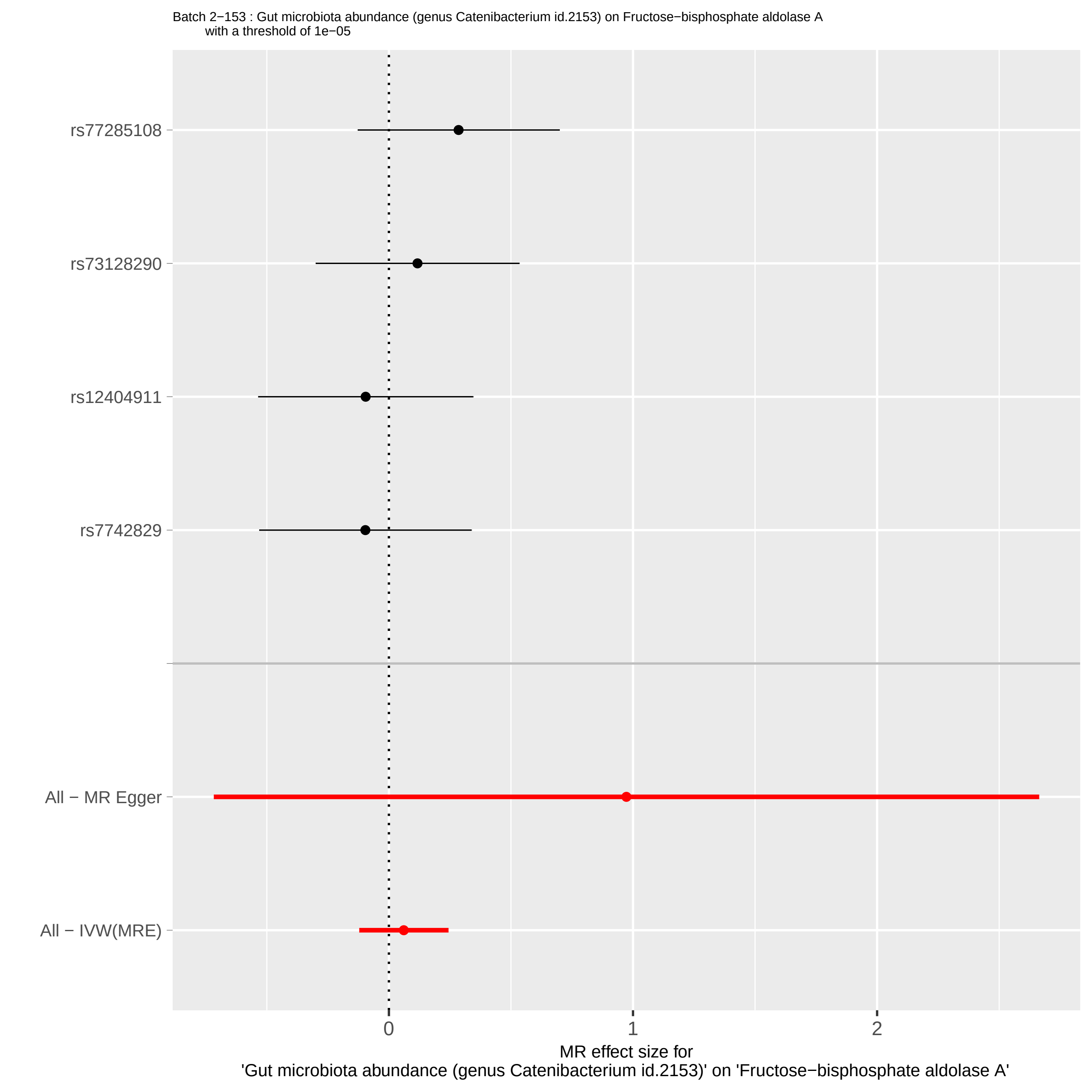

Batch 2−153 : Gut microbiota abundance (genus Catenibacterium id.2153) on Fructose−bisphosphate aldolase A
with a threshold of 1e−05
rs77285108
rs73128290
rs12404911
rs7742829
All − MR Egger
All − IVW(MRE)
0
1
2
MR effect size for
'Gut microbiota abundance (genus Catenibacterium id.2153)' on 'Fructose−bisphosphate aldolase A'

Batch 2−154 : Gut microbiota abundance (genus Parasutterella id.2892) on Fructose−bisphosphate aldolase A
with a threshold of 1e−05

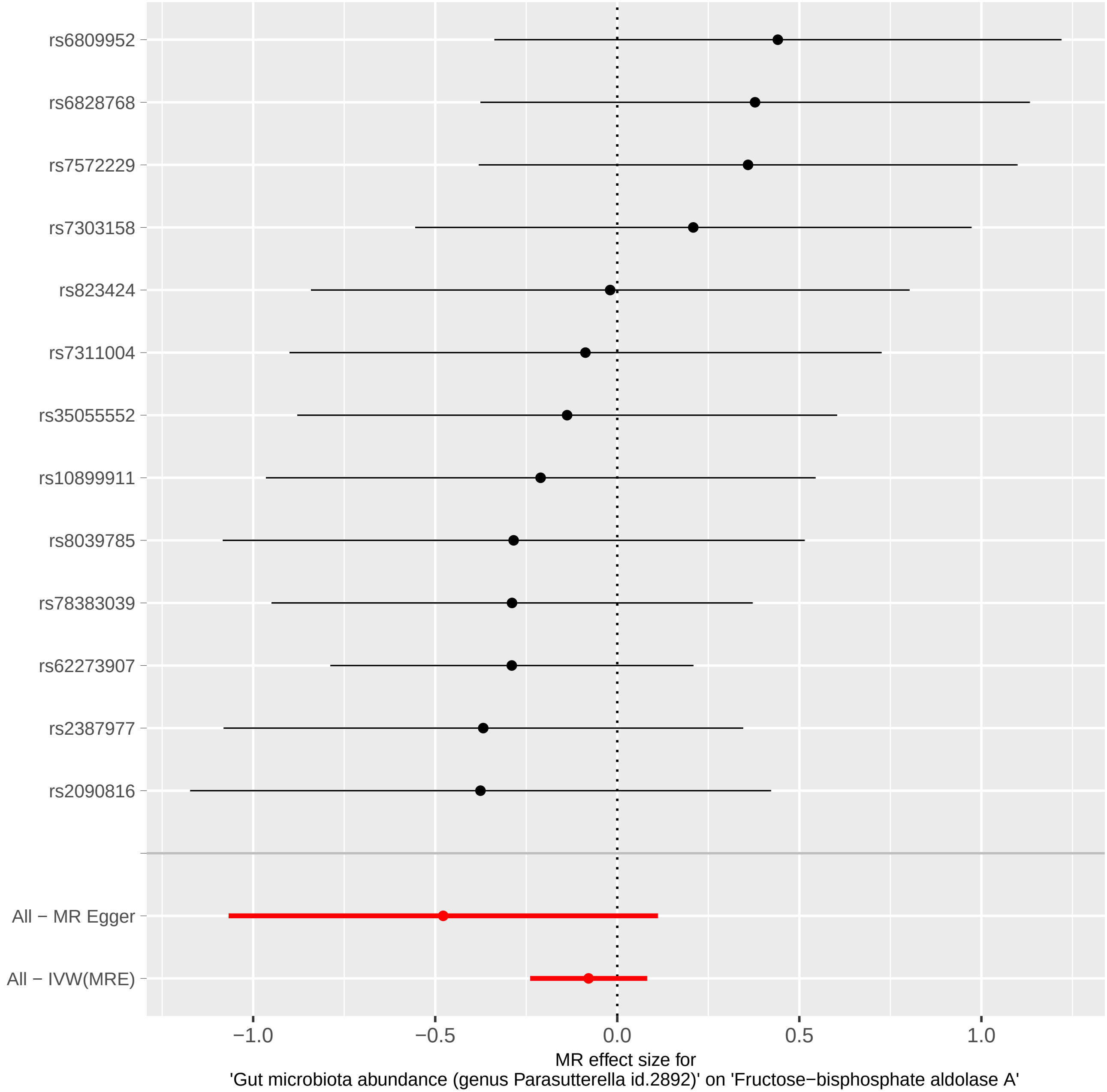

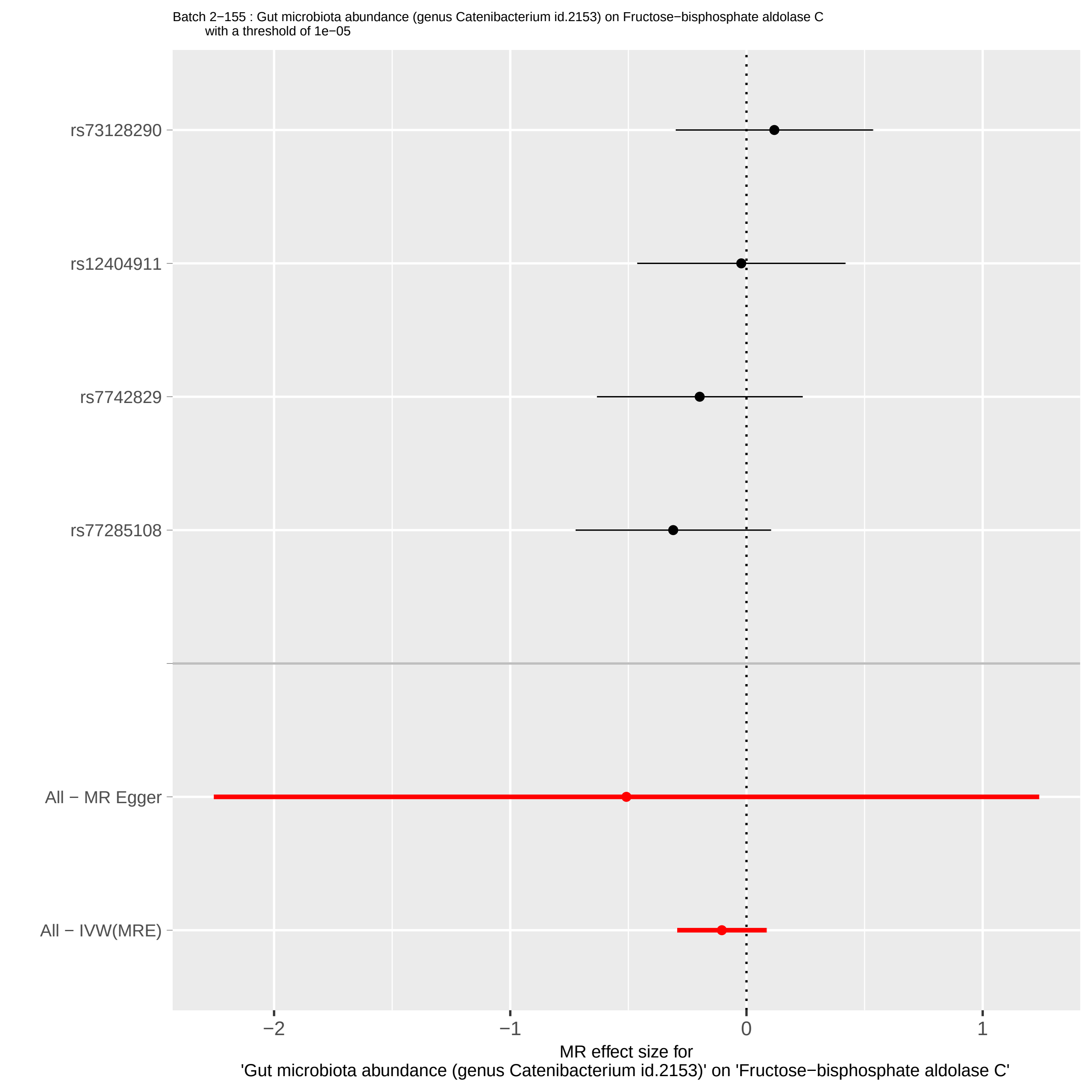

Batch 2−155 : Gut microbiota abundance (genus Catenibacterium id.2153) on Fructose−bisphosphate aldolase C
with a threshold of 1e−05
rs73128290
rs12404911
rs7742829
rs77285108
All − MR Egger
All − IVW(MRE)
−2
−1
0
1
MR effect size for
'Gut microbiota abundance (genus Catenibacterium id.2153)' on 'Fructose−bisphosphate aldolase C'

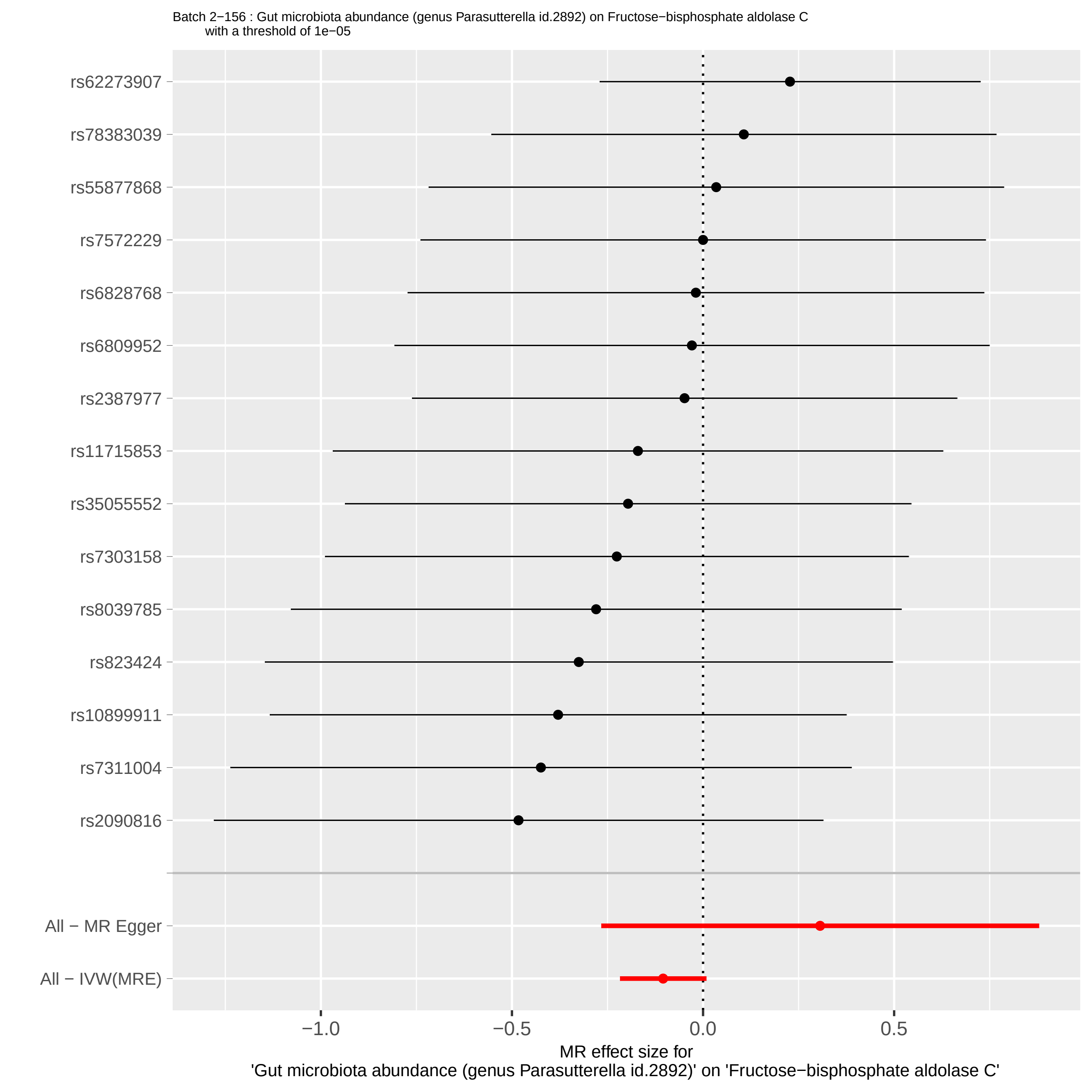

Batch 2−156 : Gut microbiota abundance (genus Parasutterella id.2892) on Fructose−bisphosphate aldolase C
with a threshold of 1e−05
rs62273907
rs78383039
rs55877868
rs7572229
rs6828768
rs6809952
rs2387977
rs11715853
rs35055552
rs7303158
rs8039785
rs823424
rs10899911
rs7311004
rs2090816
All − MR Egger
All − IVW(MRE)
−1.0
−0.5
0.0
0.5
MR effect size for
'Gut microbiota abundance (genus Parasutterella id.2892)' on 'Fructose−bisphosphate aldolase C'

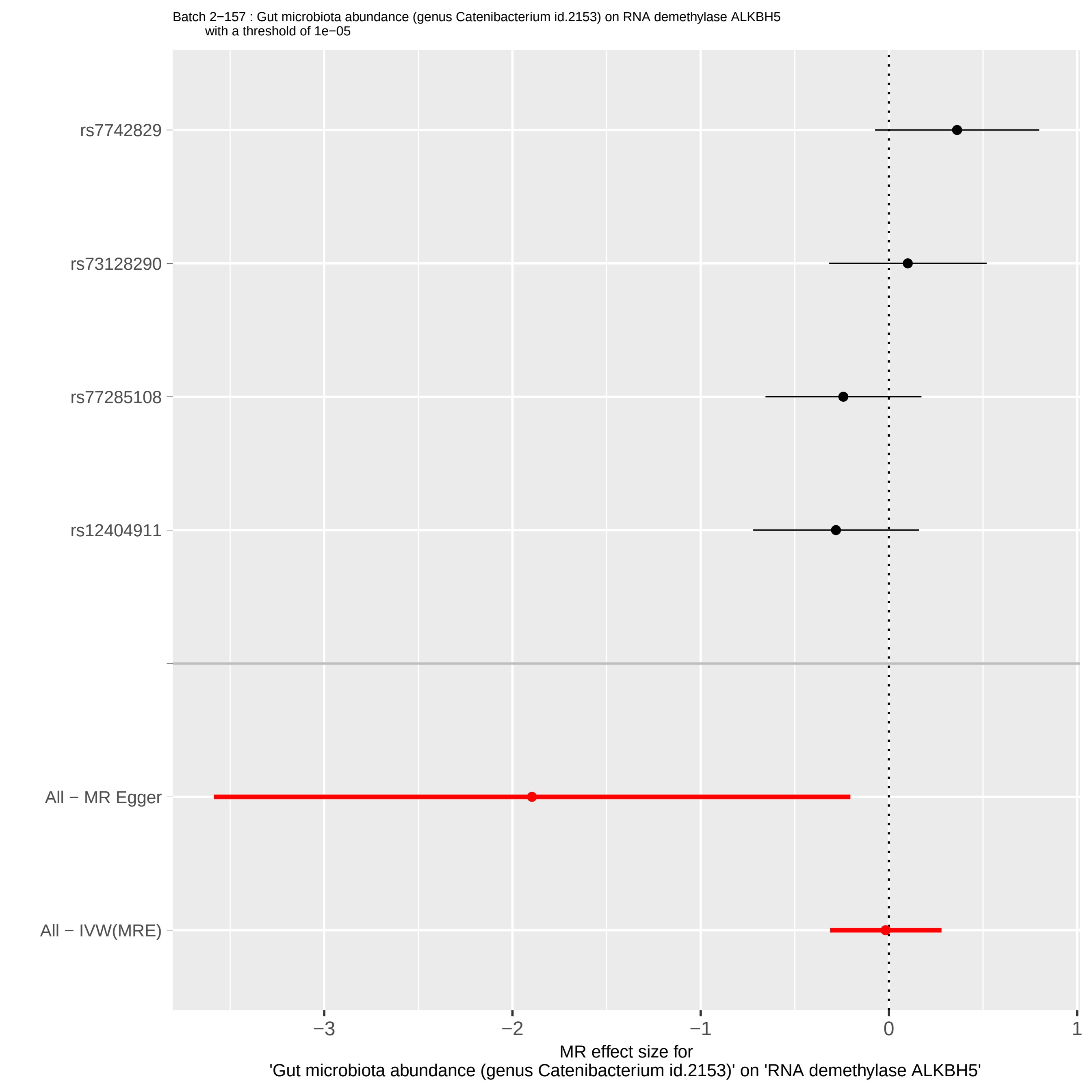
Batch 2−157 : Gut microbiota abundance (genus Catenibacterium id.2153) on RNA demethylase ALKBH5
with a threshold of 1e−05
rs7742829
rs73128290
rs77285108
rs12404911
All − MR Egger
All − IVW(MRE)
−3
−2
−1
0
1
MR effect size for
'Gut microbiota abundance (genus Catenibacterium id.2153)' on 'RNA demethylase ALKBH5'

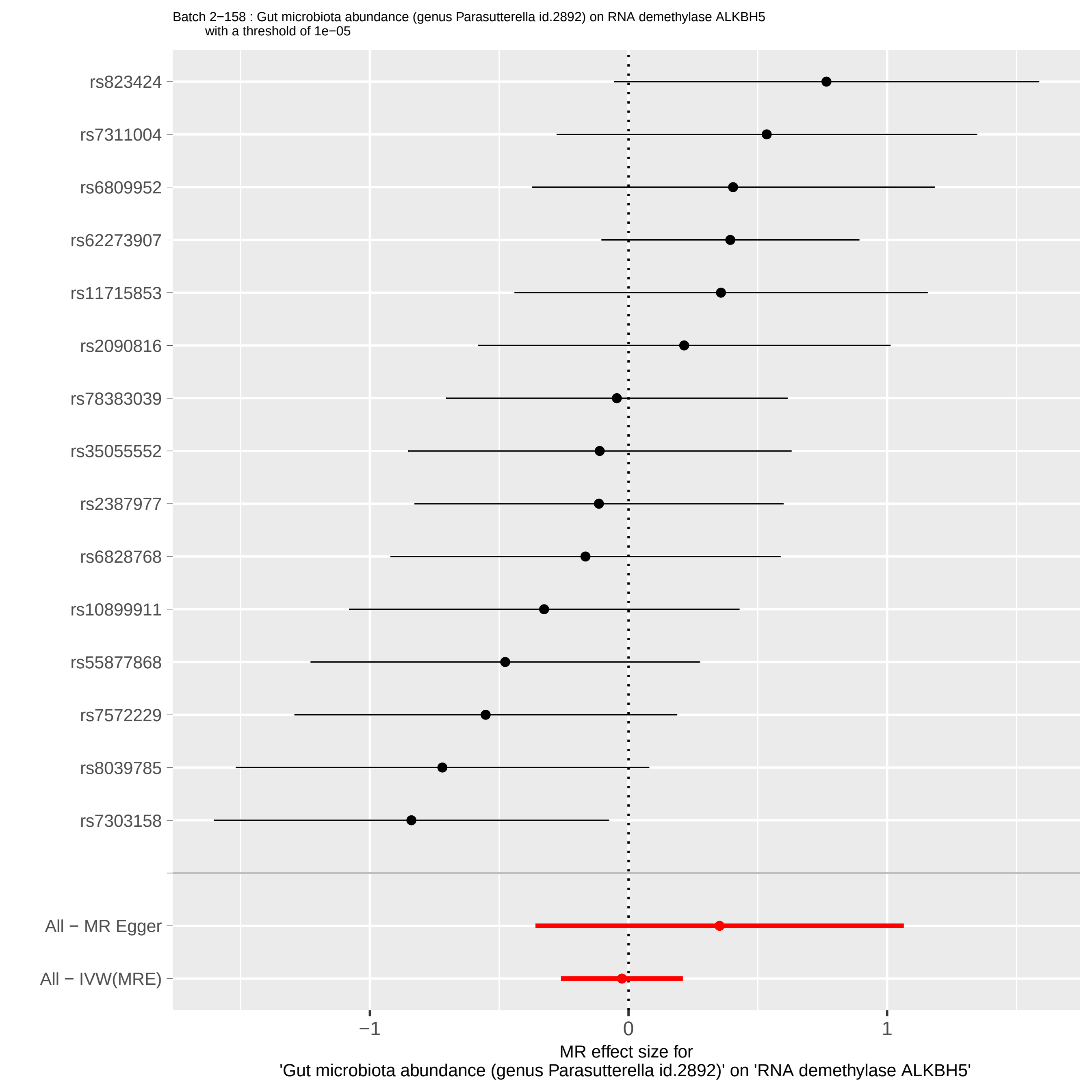

Batch 2−158 : Gut microbiota abundance (genus Parasutterella id.2892) on RNA demethylase ALKBH5
with a threshold of 1e−05
rs823424
rs7311004
rs6809952
rs62273907
rs11715853
rs2090816
rs78383039
rs35055552
rs2387977
rs6828768
rs10899911
rs55877868
rs7572229
rs8039785
rs7303158
All − MR Egger
All − IVW(MRE)
−1
0
1
MR effect size for
'Gut microbiota abundance (genus Parasutterella id.2892)' on 'RNA demethylase ALKBH5'

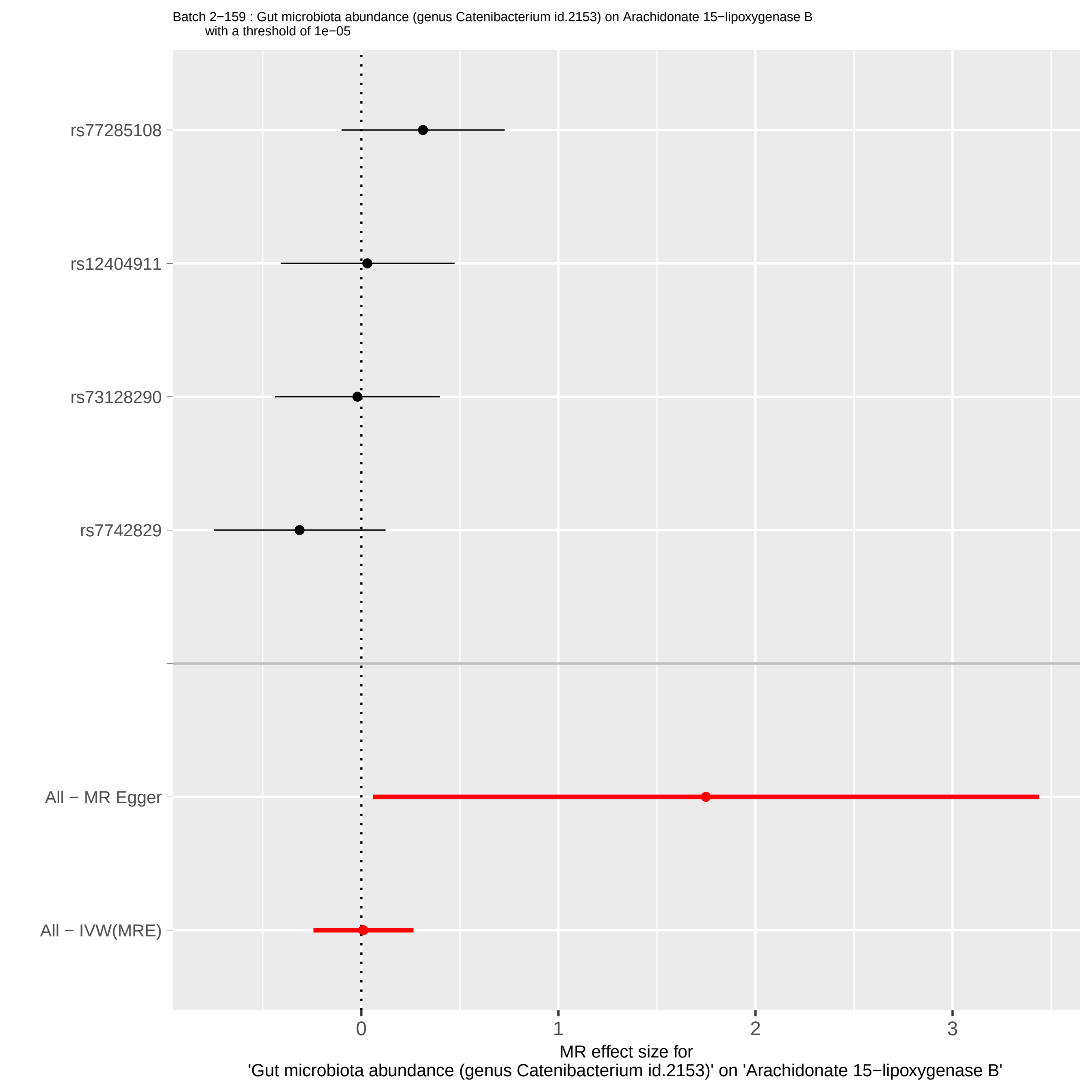

Batch 2−159 : Gut microbiota abundance (genus Catenibacterium id.2153) on Arachidonate 15−lipoxygenase B
with a threshold of 1e−05
rs77285108
rs12404911
rs73128290
rs7742829
All − MR Egger
All − IVW(MRE)
0
1
2
3
MR effect size for
'Gut microbiota abundance (genus Catenibacterium id.2153)' on 'Arachidonate 15−lipoxygenase B'

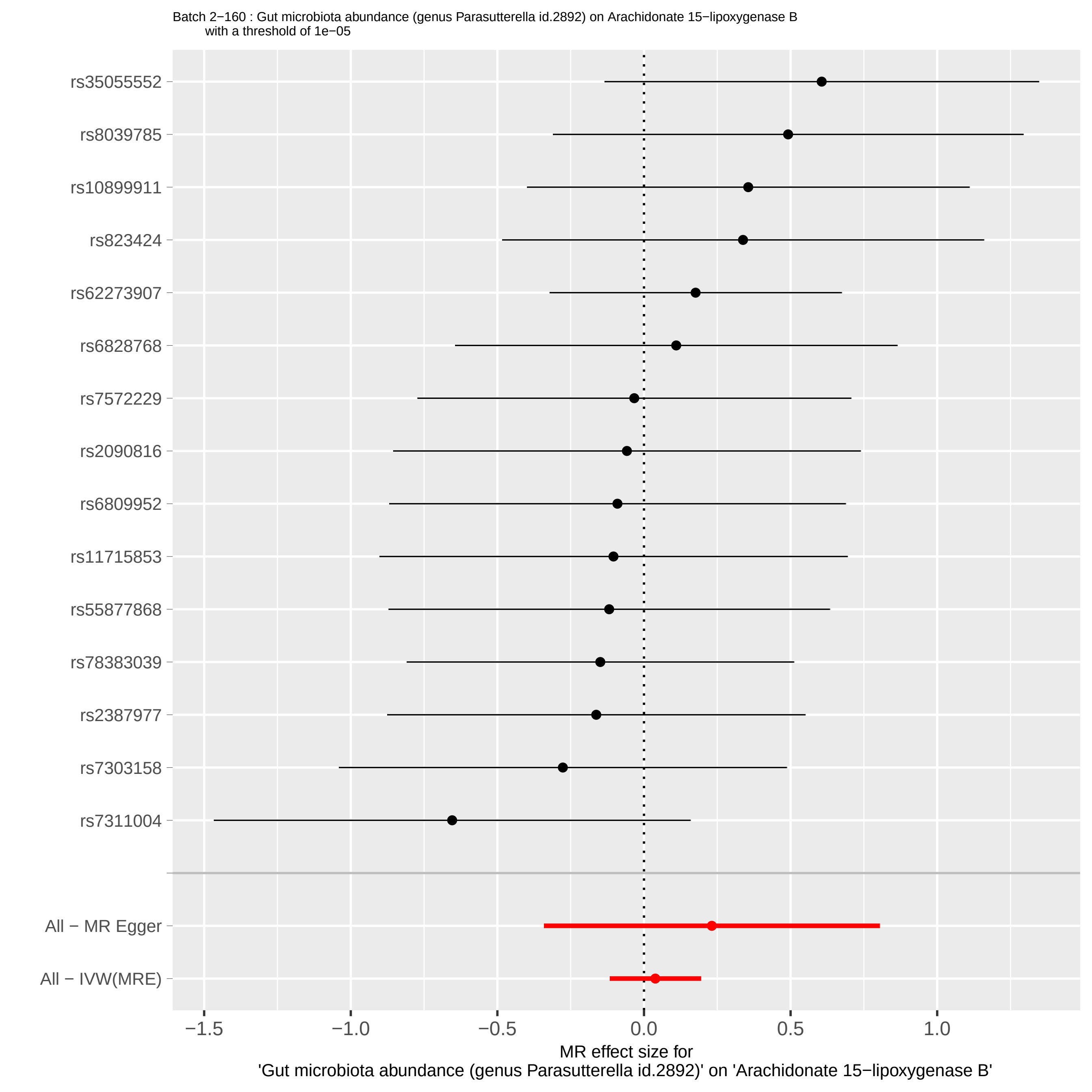

Batch 2−160 : Gut microbiota abundance (genus Parasutterella id.2892) on Arachidonate 15−lipoxygenase B with a threshold of 1e−05
rs35055552
rs8039785
rs10899911
rs823424
rs62273907
rs6828768
rs7572229
rs2090816
rs6809952
rs11715853
rs55877868
rs78383039
rs2387977
rs7303158
rs7311004
All − MR Egger
All − IVW(MRE)
−1.5
−1.0
−0.5
0.0
0.5
1.0
MR effect size for
'Gut microbiota abundance (genus Parasutterella id.2892)' on 'Arachidonate 15−lipoxygenase B'

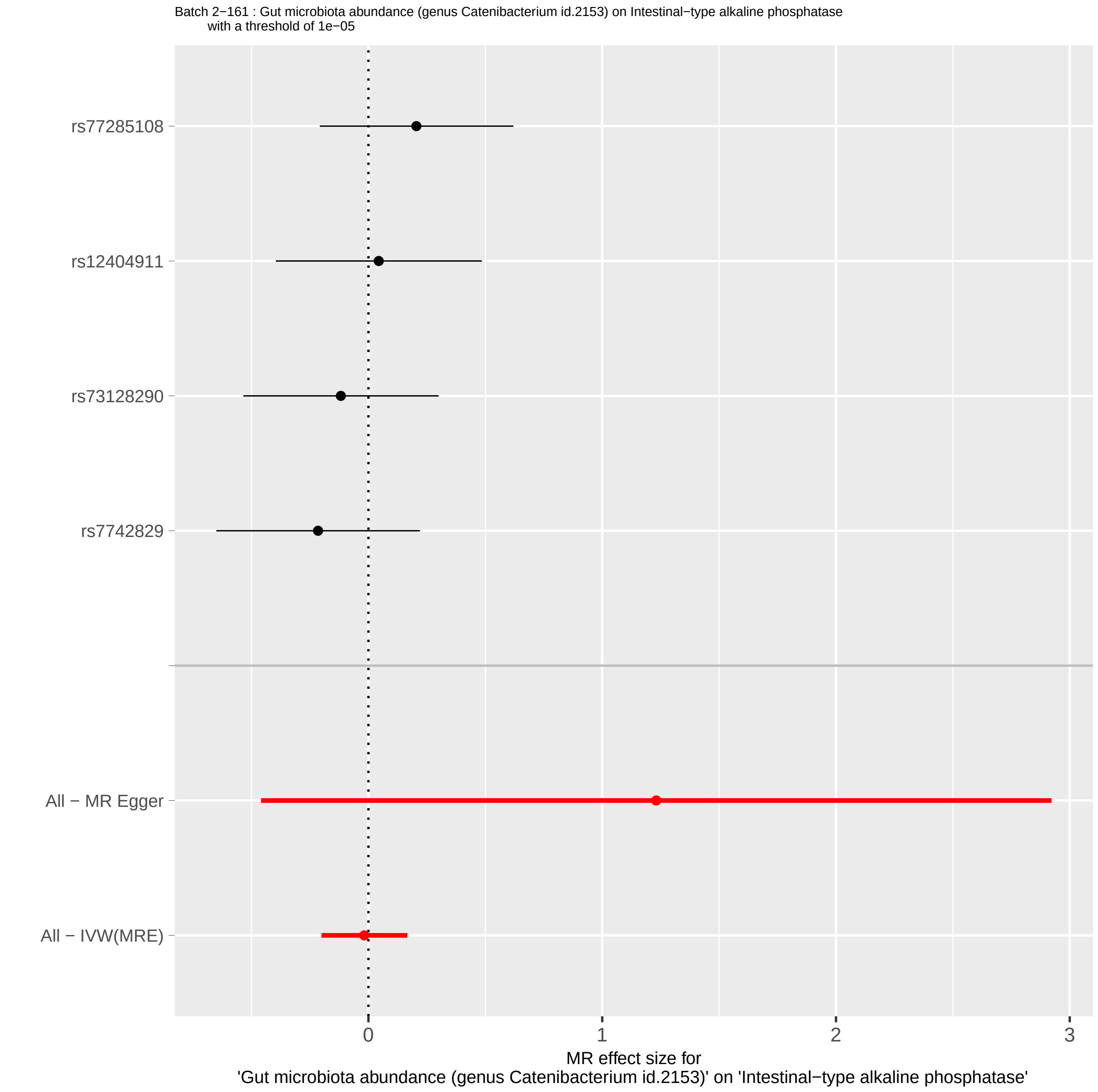

Batch 2−161 : Gut microbiota abundance (genus Catenibacterium id.2153) on Intestinal−type alkaline phosphatase
with a threshold of 1e−05
rs77285108
rs12404911
rs73128290
rs7742829
All − MR Egger
All − IVW(MRE)
0
1
2
3
MR effect size for
'Gut microbiota abundance (genus Catenibacterium id.2153)' on 'Intestinal−type alkaline phosphatase'

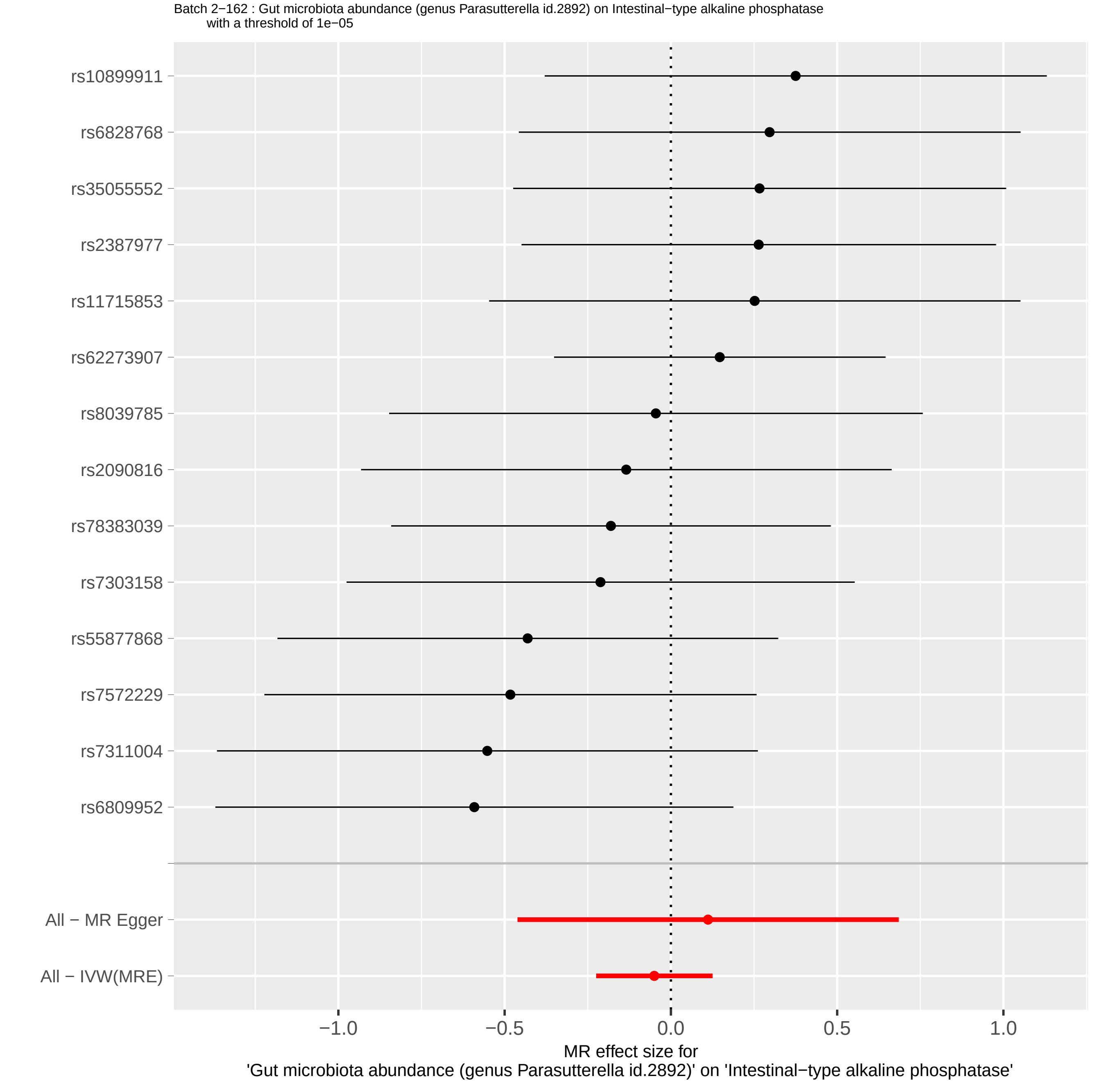

Batch 2−162 : Gut microbiota abundance (genus Parasutterella id.2892) on Intestinal−type alkaline phosphatase
with a threshold of 1e−05
rs10899911
rs6828768
rs35055552
rs2387977
rs11715853
rs62273907
rs8039785
rs2090816
rs78383039
rs7303158
rs55877868
rs7572229
rs7311004
rs6809952
All − MR Egger
All − IVW(MRE)
−1.0
−0.5
0.0
0.5
1.0
MR effect size for
'Gut microbiota abundance (genus Parasutterella id.2892)' on 'Intestinal−type alkaline phosphatase'

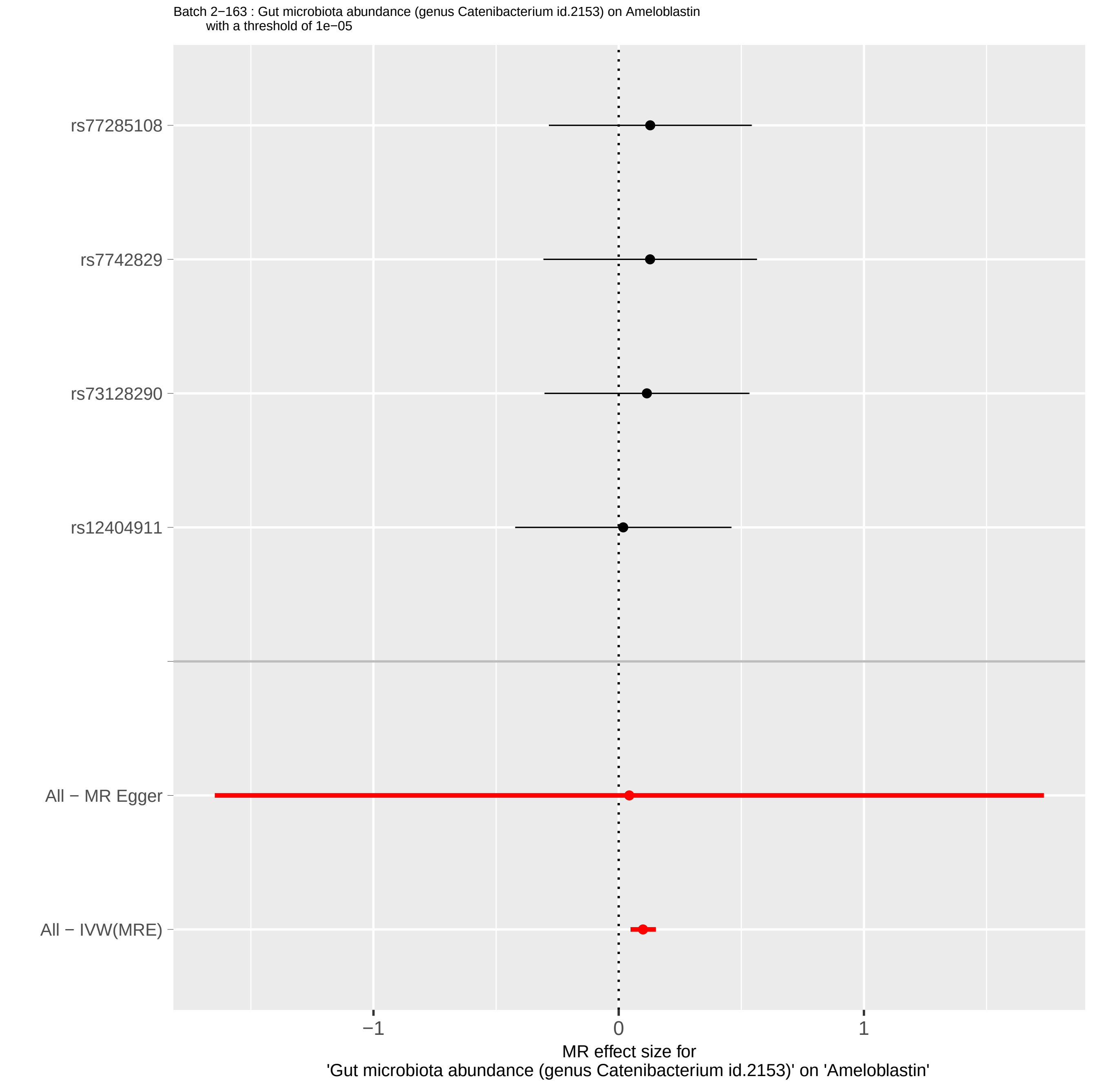

Batch 2−163 : Gut microbiota abundance (genus Catenibacterium id.2153) on Ameloblastin
with a threshold of 1e−05
rs77285108
rs7742829
rs73128290
rs12404911
All − MR Egger
All − IVW(MRE)
−1
0
1
MR effect size for
'Gut microbiota abundance (genus Catenibacterium id.2153)' on 'Ameloblastin'

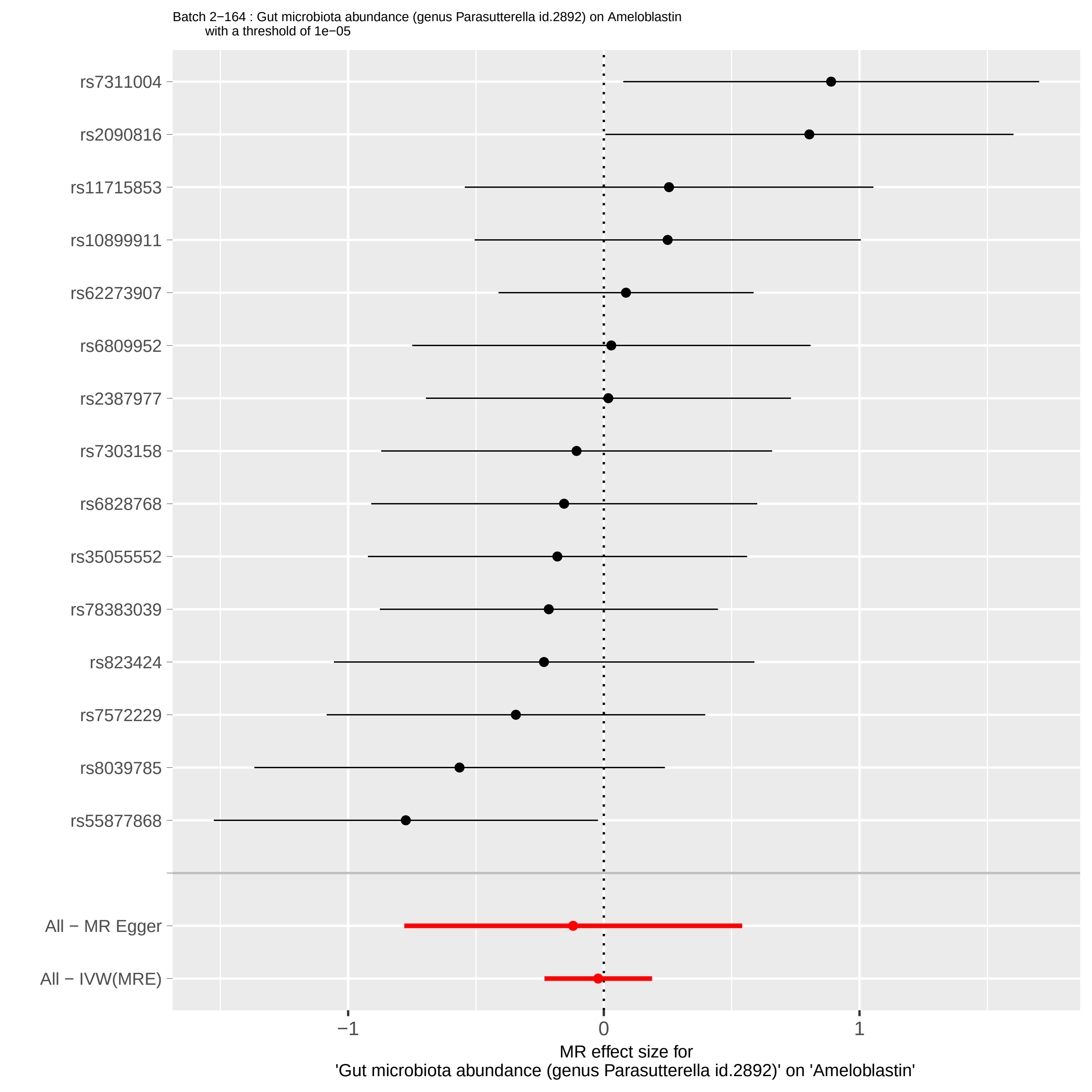
Batch 2−164 : Gut microbiota abundance (genus Parasutterella id.2892) on Ameloblastin
with a threshold of 1e−05
rs7311004
rs2090816
rs11715853
rs10899911
rs62273907
rs6809952
rs2387977
rs7303158
rs6828768
rs35055552
rs78383039
rs823424
rs7572229
rs8039785
rs55877868
All − MR Egger
All − IVW(MRE)
−1
0
1
MR effect size for
'Gut microbiota abundance (genus Parasutterella id.2892)' on 'Ameloblastin'

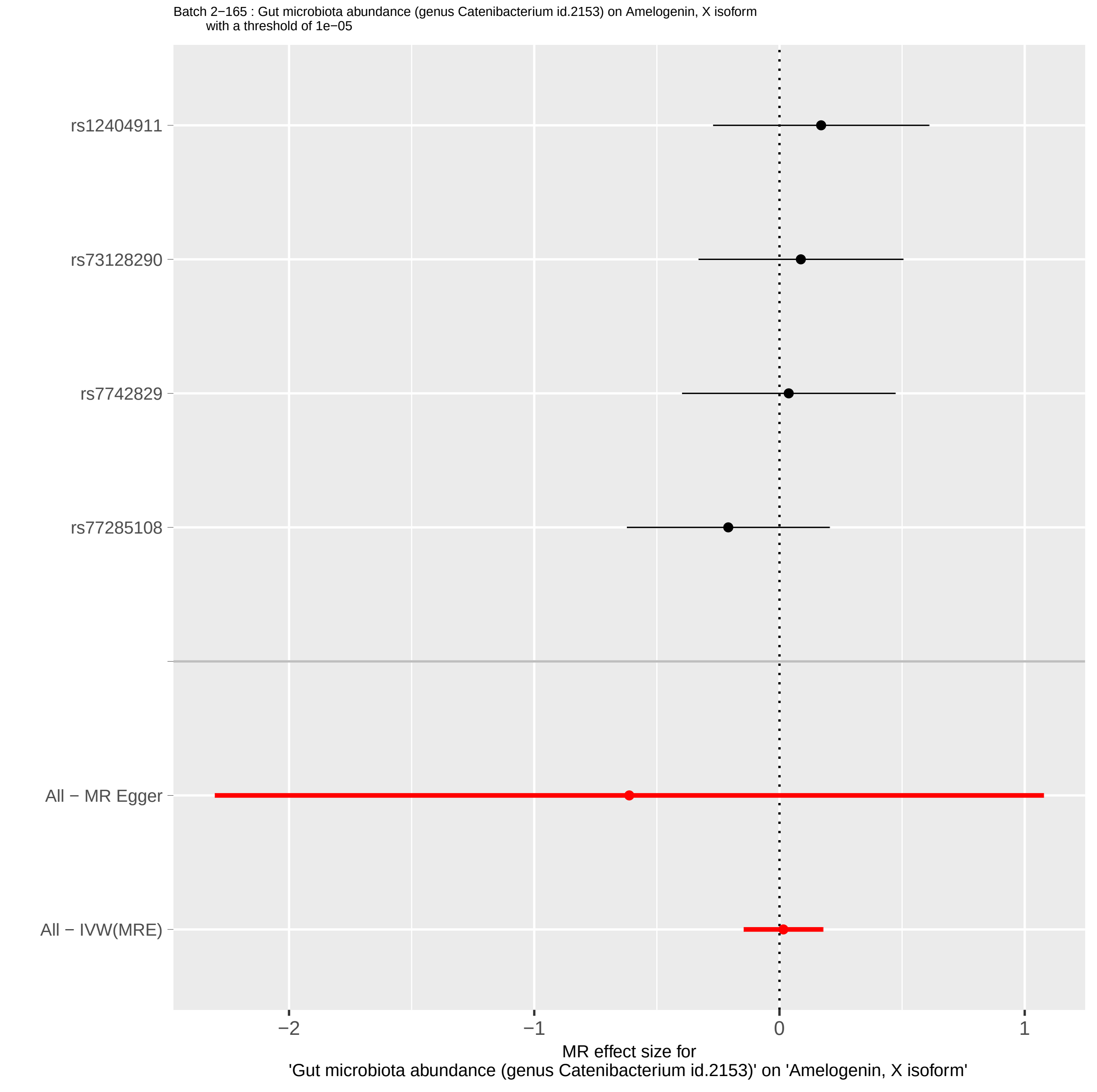
Batch 2−165 : Gut microbiota abundance (genus Catenibacterium id.2153) on Amelogenin, X isoform
with a threshold of 1e−05
rs12404911
rs73128290
rs7742829
rs77285108
All − MR Egger
All − IVW(MRE)
−2
−1
0
1
MR effect size for
'Gut microbiota abundance (genus Catenibacterium id.2153)' on 'Amelogenin, X isoform'

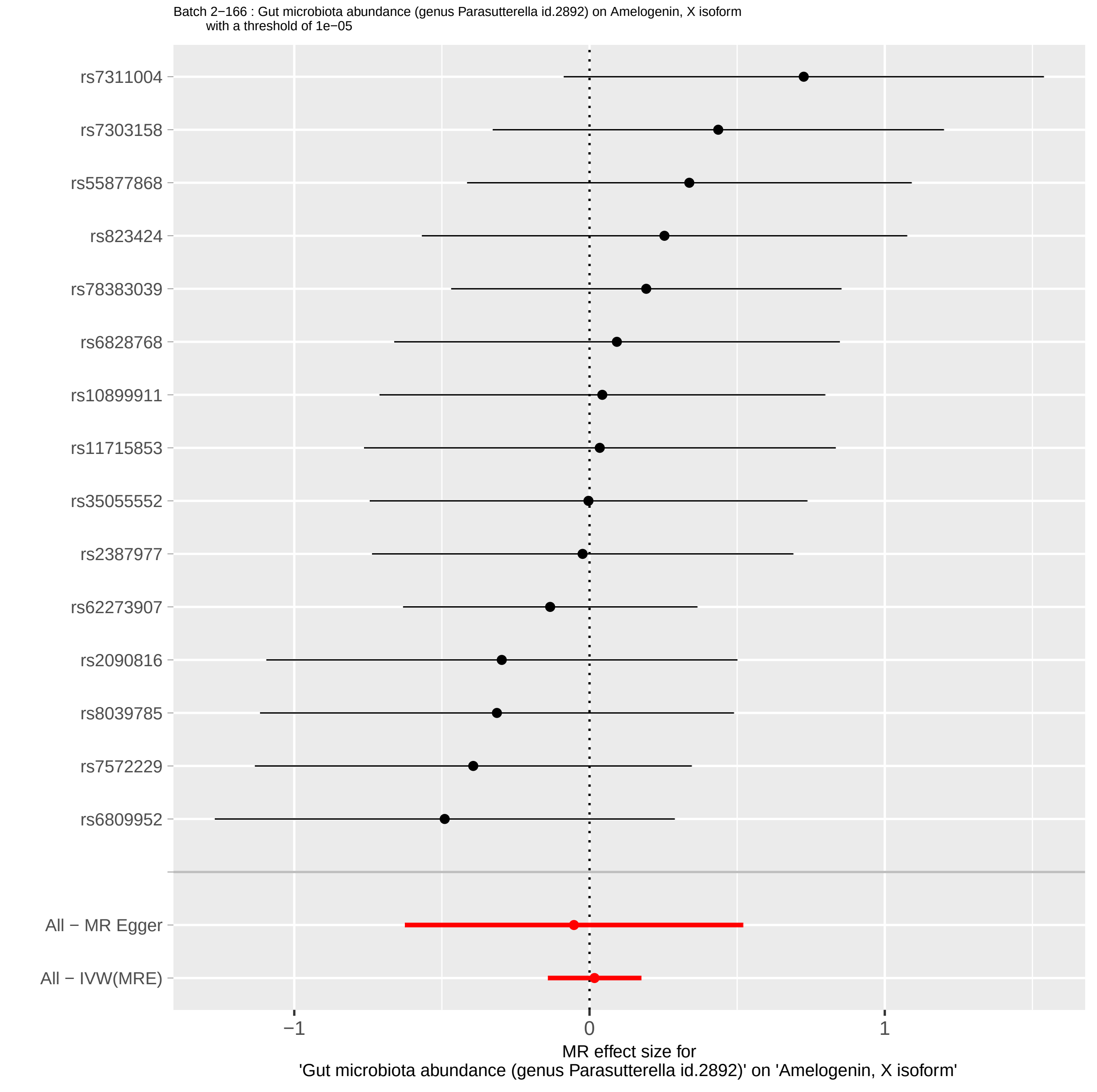
Batch 2−166 : Gut microbiota abundance (genus Parasutterella id.2892) on Amelogenin, X isoform
with a threshold of 1e−05
rs7311004
rs7303158
rs55877868
rs823424
rs78383039
rs6828768
rs10899911
rs11715853
rs35055552
rs2387977
rs62273907
rs2090816
rs8039785
rs7572229
rs6809952
All − MR Egger
All − IVW(MRE)
−1
0
1
MR effect size for
'Gut microbiota abundance (genus Parasutterella id.2892)' on 'Amelogenin, X isoform'

Batch 2−167 : Gut microbiota abundance (genus Catenibacterium id.2153) on Muellerian−inhibiting factor
with a threshold of 1e−05

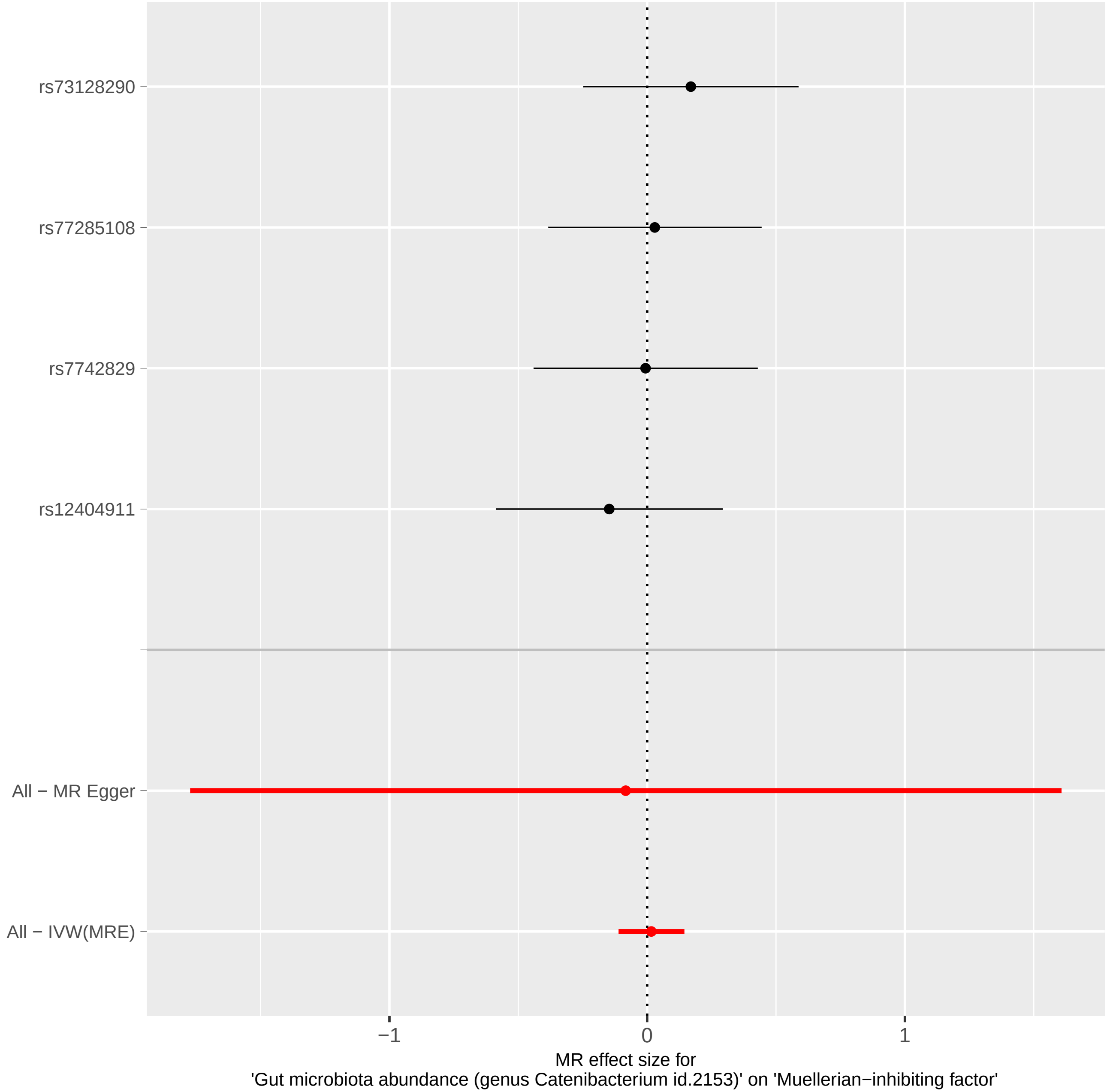

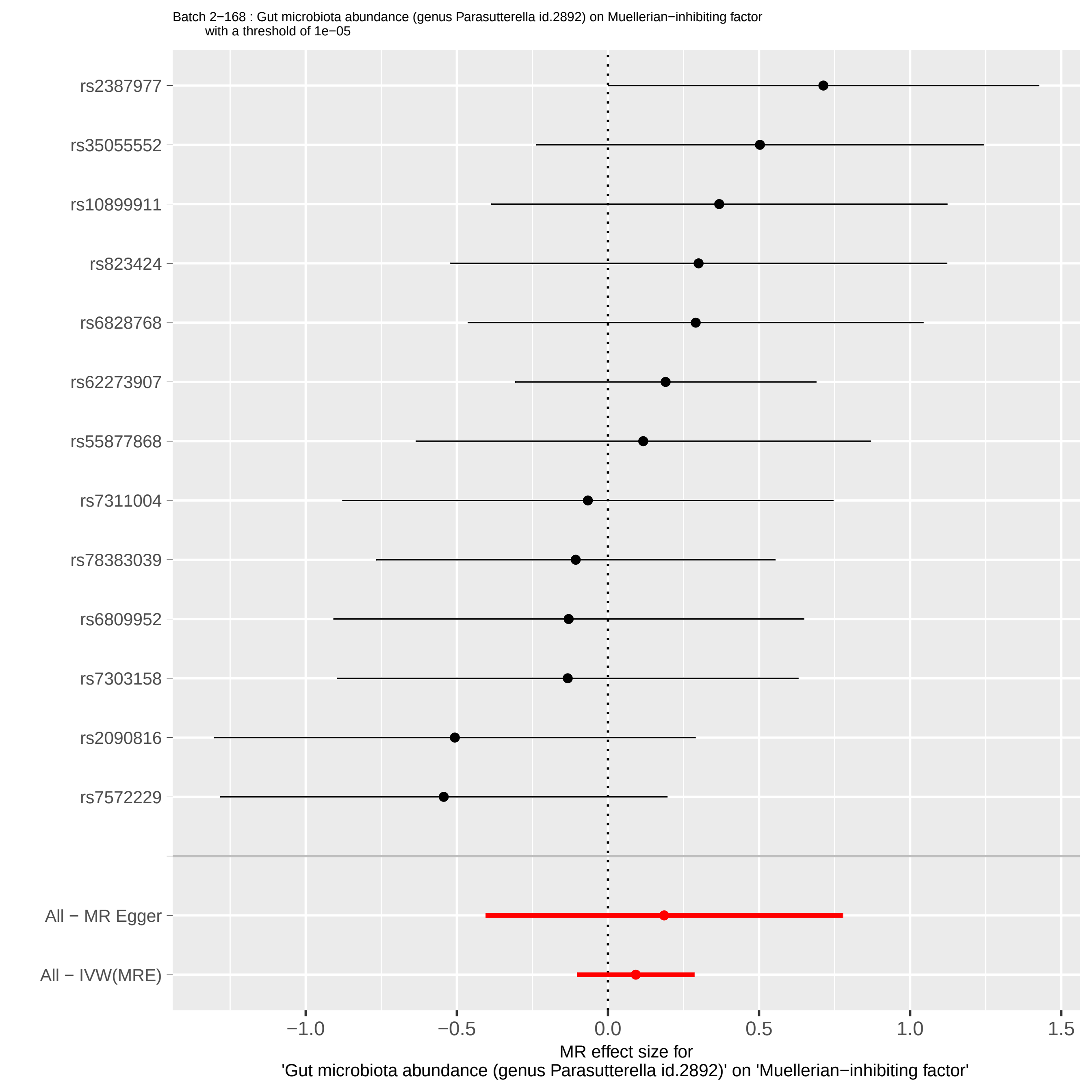
Batch 2−168 : Gut microbiota abundance (genus Parasutterella id.2892) on Muellerian−inhibiting factor
with a threshold of 1e−05
rs2387977
rs35055552
rs10899911
rs823424
rs6828768
rs62273907
rs55877868
rs7311004
rs78383039
rs6809952
rs7303158
rs2090816
rs7572229
All − MR Egger
All − IVW(MRE)
−1.0
−0.5
0.0
0.5
1.0
1.5
MR effect size for
'Gut microbiota abundance (genus Parasutterella id.2892)' on 'Muellerian−inhibiting factor

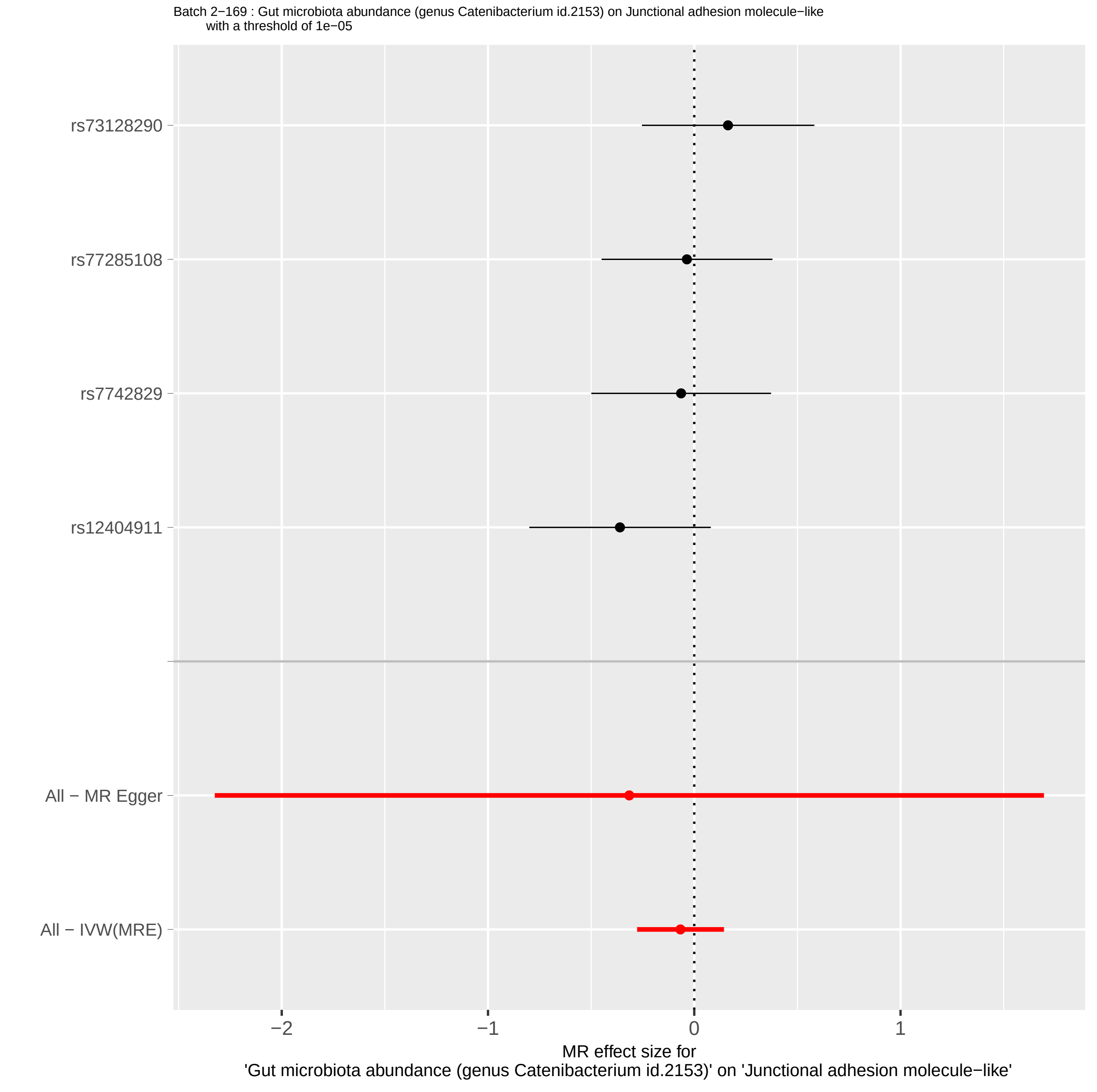
Batch 2−169 : Gut microbiota abundance (genus Catenibacterium id.2153) on Junctional adhesion molecule−like
with a threshold of 1e−05
rs73128290
rs77285108
rs7742829
rs12404911
All − MR Egger
All − IVW(MRE)
−2
−1
0
1
MR effect size for
'Gut microbiota abundance (genus Catenibacterium id.2153)' on 'Junctional adhesion molecule−like'

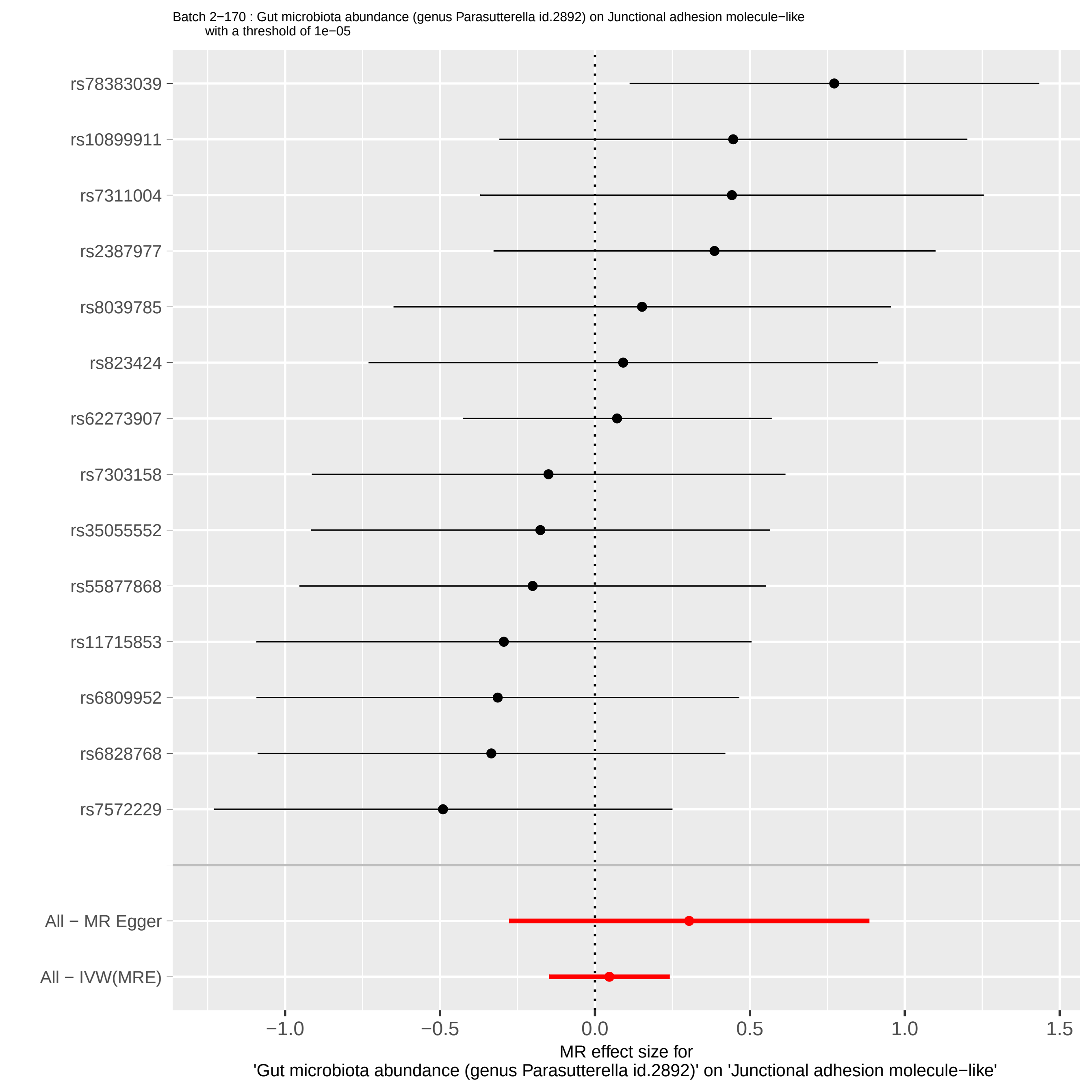

Batch 2−170 : Gut microbiota abundance (genus Parasutterella id.2892) on Junctional adhesion molecule−like
with a threshold of 1e−05
rs78383039
rs10899911
rs7311004
rs2387977
rs8039785
rs823424
rs62273907
rs7303158
rs35055552
rs55877868
rs11715853
rs6809952
rs6828768
rs7572229
All − MR Egger
All − IVW(MRE)
−1.0
−0.5
0.0
0.5
1.0
1.5
MR effect size for
'Gut microbiota abundance (genus Parasutterella id.2892)' on 'Junctional adhesion molecule−like'

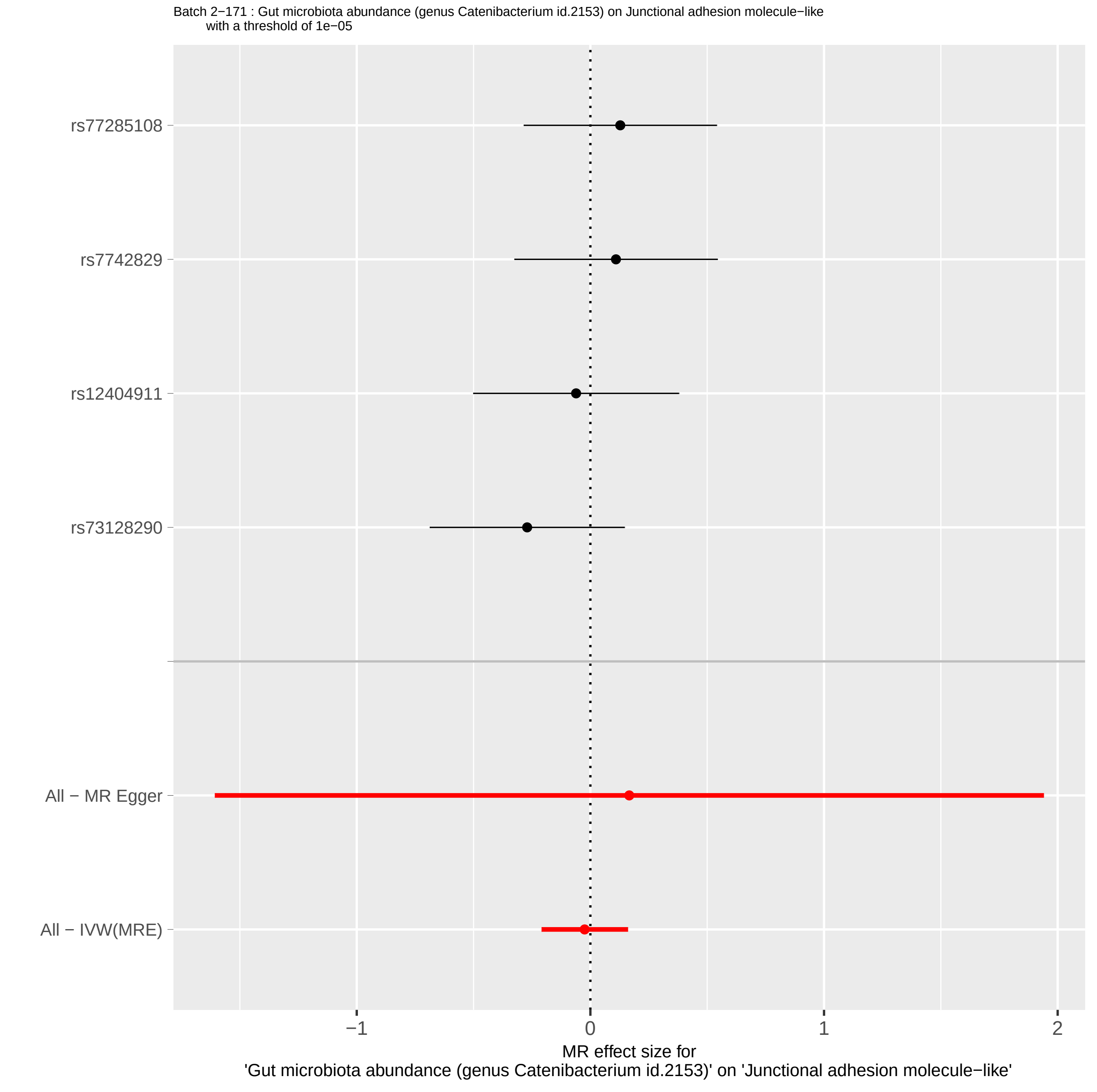

Batch 2−171 : Gut microbiota abundance (genus Catenibacterium id.2153) on Junctional adhesion molecule−like
with a threshold of 1e−05
rs77285108
rs7742829
rs12404911
rs73128290
All − MR Egger
All − IVW(MRE)
−1
0
1
2
MR effect size for
'Gut microbiota abundance (genus Catenibacterium id.2153)' on 'Junctional adhesion molecule−like'

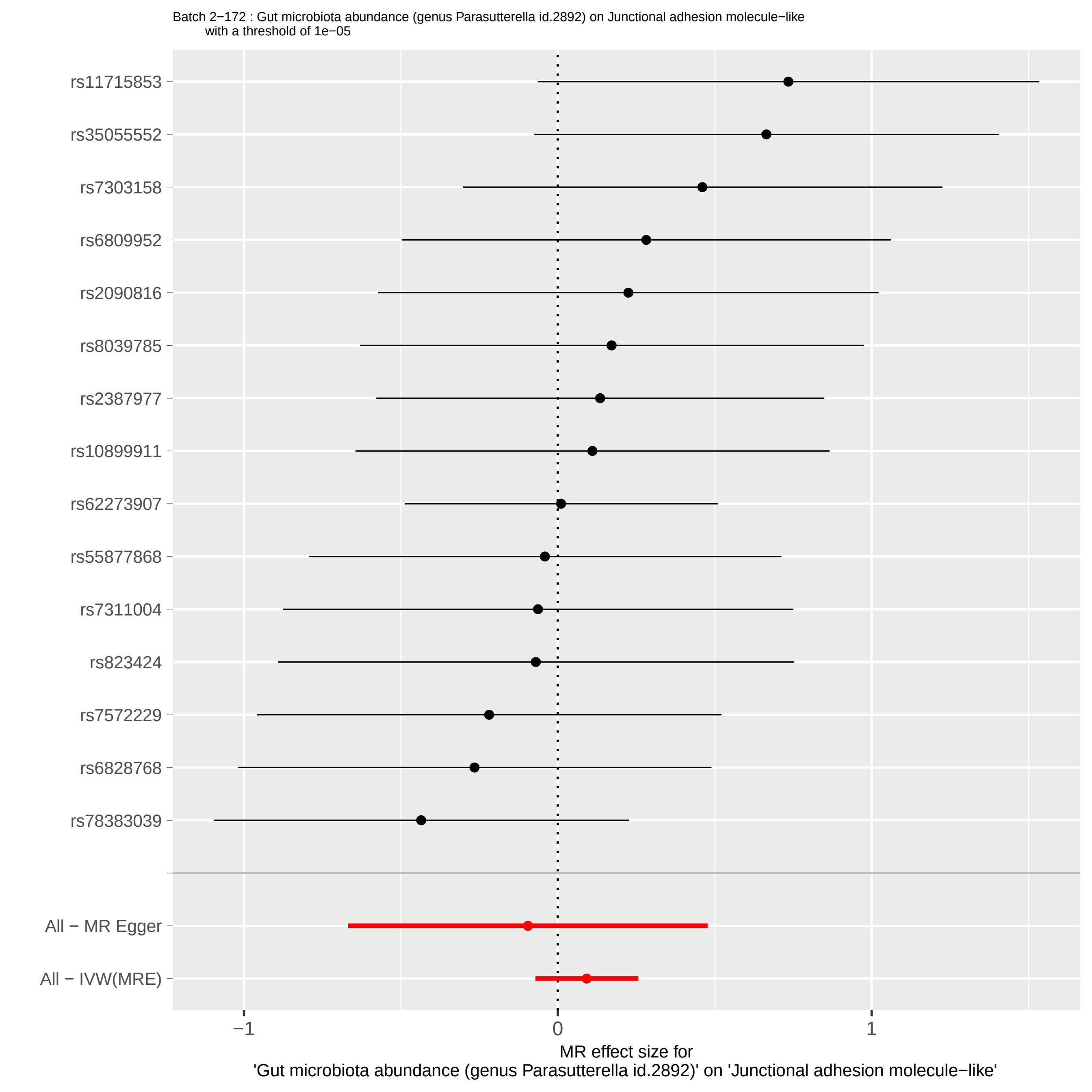

Batch 2−172 : Gut microbiota abundance (genus Parasutterella id.2892) on Junctional adhesion molecule−like
with a threshold of 1e−05
rs11715853
rs35055552
rs7303158
rs6809952
rs2090816
rs8039785
rs2387977
rs10899911
rs62273907
rs55877868
rs7311004
rs823424
rs7572229
rs6828768
rs78383039
All − MR Egger
All − IVW(MRE)
−1
0
1
MR effect size for
'Gut microbiota abundance (genus Parasutterella id.2892)' on 'Junctional adhesion molecule−like'

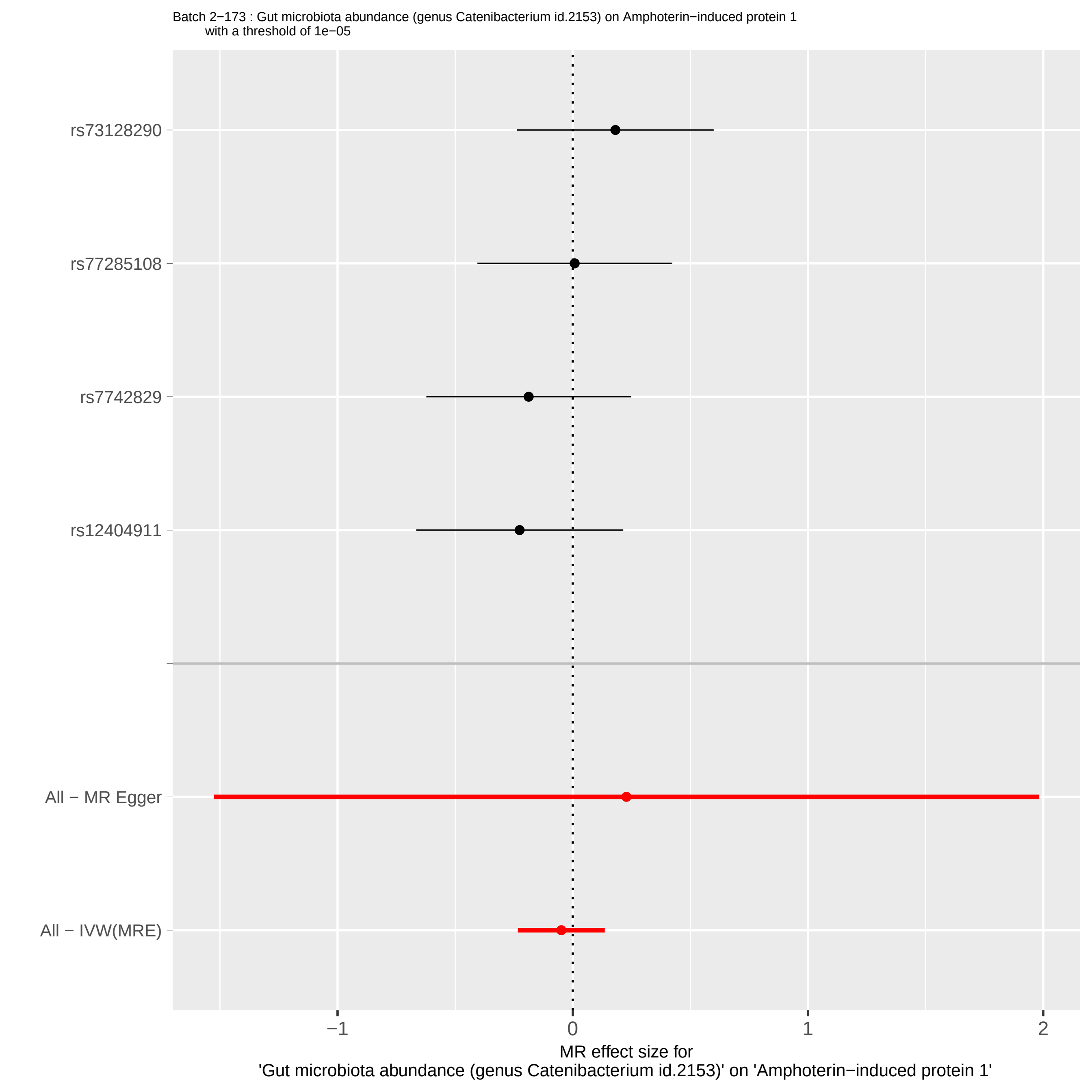

Batch 2−173 : Gut microbiota abundance (genus Catenibacterium id.2153) on Amphoterin−induced protein 1
with a threshold of 1e−05
rs73128290
rs77285108
rs7742829
rs12404911
All − MR Egger
All − IVW(MRE)
−1
0
1
2
MR effect size for
'Gut microbiota abundance (genus Catenibacterium id.2153)' on 'Amphoterin−induced protein 1'

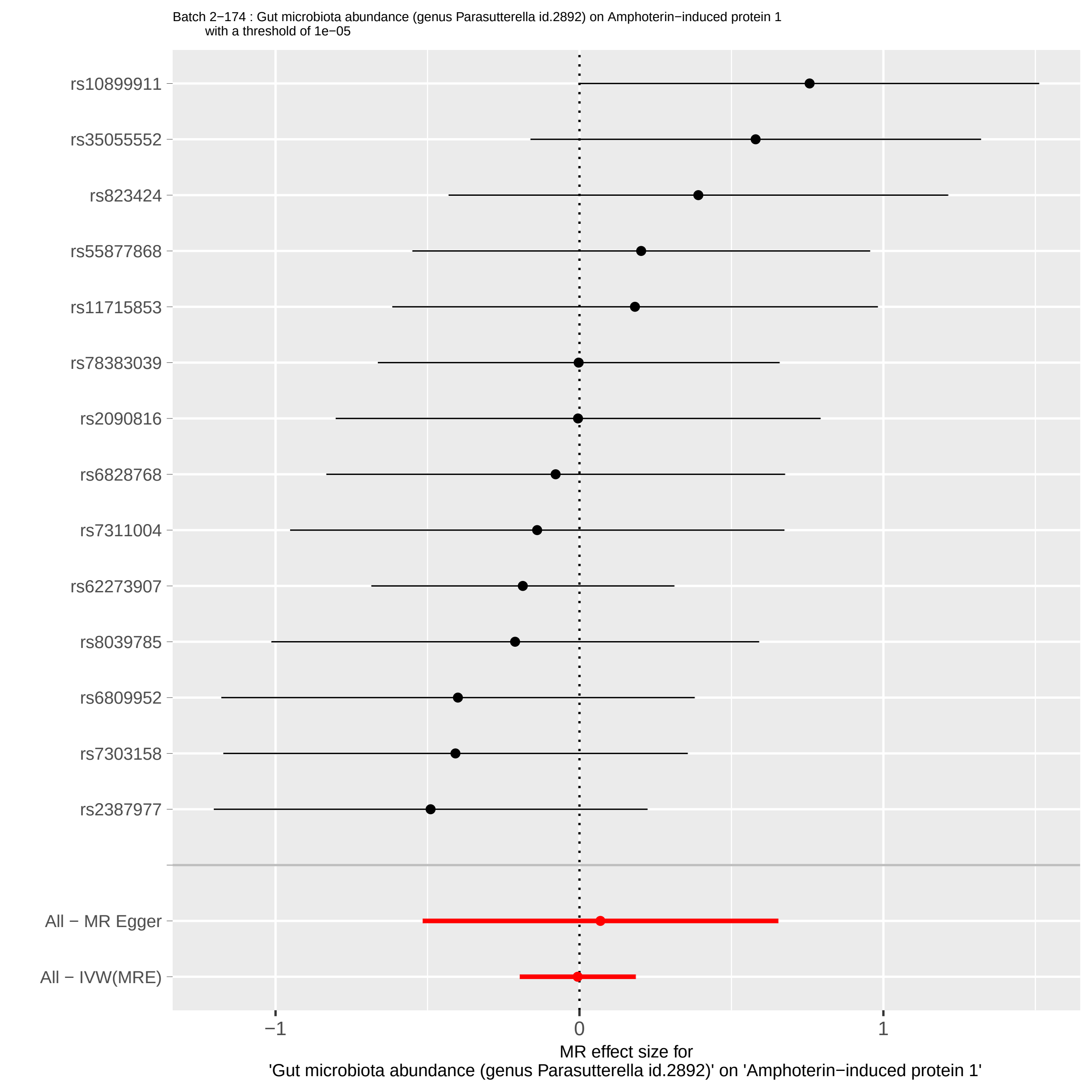
Batch 2−174 : Gut microbiota abundance (genus Parasutterella id.2892) on Amphoterin−induced protein 1
with a threshold of 1e−05
rs10899911
rs35055552
rs823424
rs55877868
rs11715853
rs78383039
rs2090816
rs6828768
rs7311004
rs62273907
rs8039785
rs6809952
rs7303158
rs2387977
All − MR Egger
All − IVW(MRE)
−1
0
1
MR effect size for
'Gut microbiota abundance (genus Parasutterella id.2892)' on 'Amphoterin−induced protein 1'

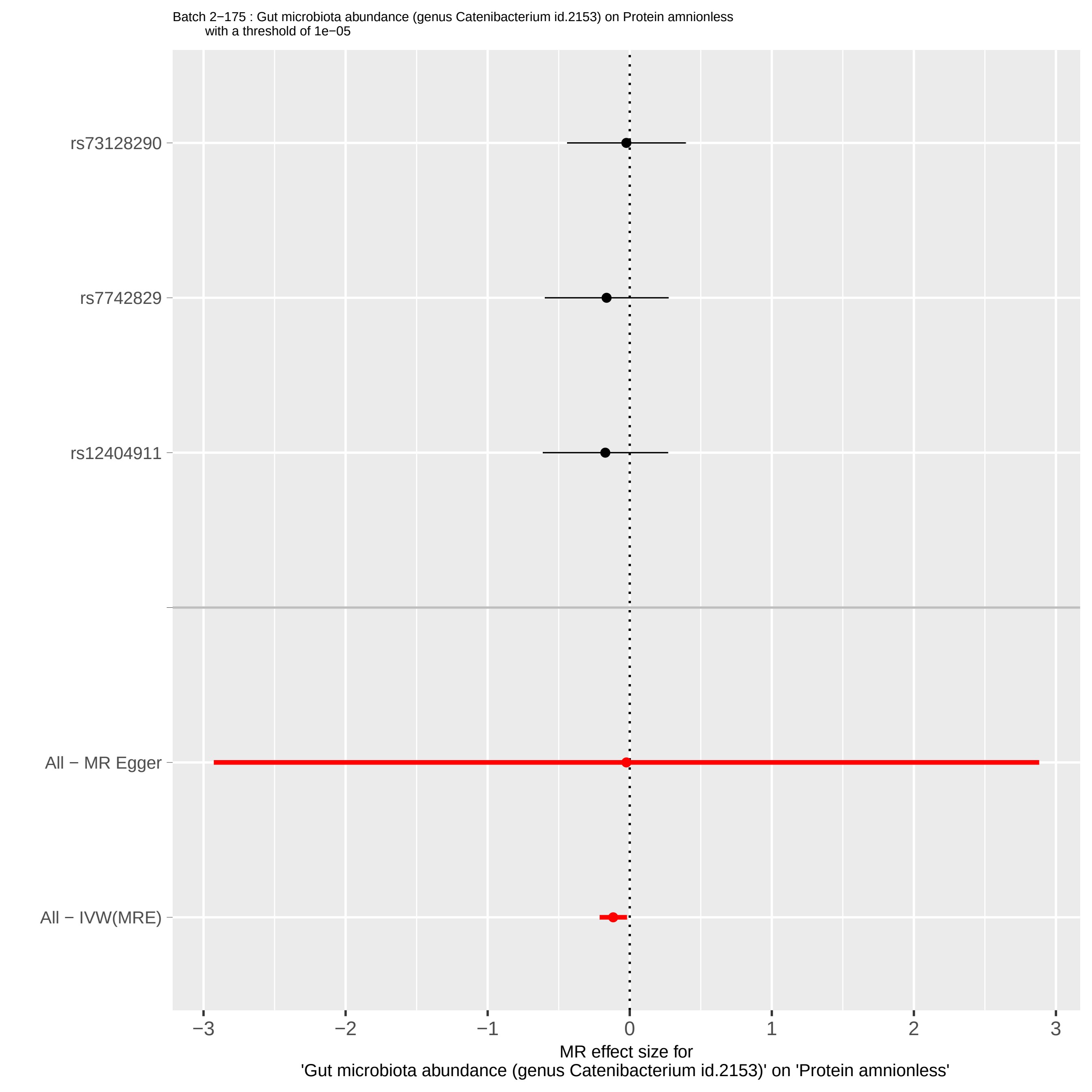

Batch 2−175 : Gut microbiota abundance (genus Catenibacterium id.2153) on Protein amnionless
with a threshold of 1e−05
rs73128290
rs7742829
rs12404911
All − MR Egger
All − IVW(MRE)
−3
−2
−1
0
1
2
3
MR effect size for
'Gut microbiota abundance (genus Catenibacterium id.2153)' on 'Protein amnionless'

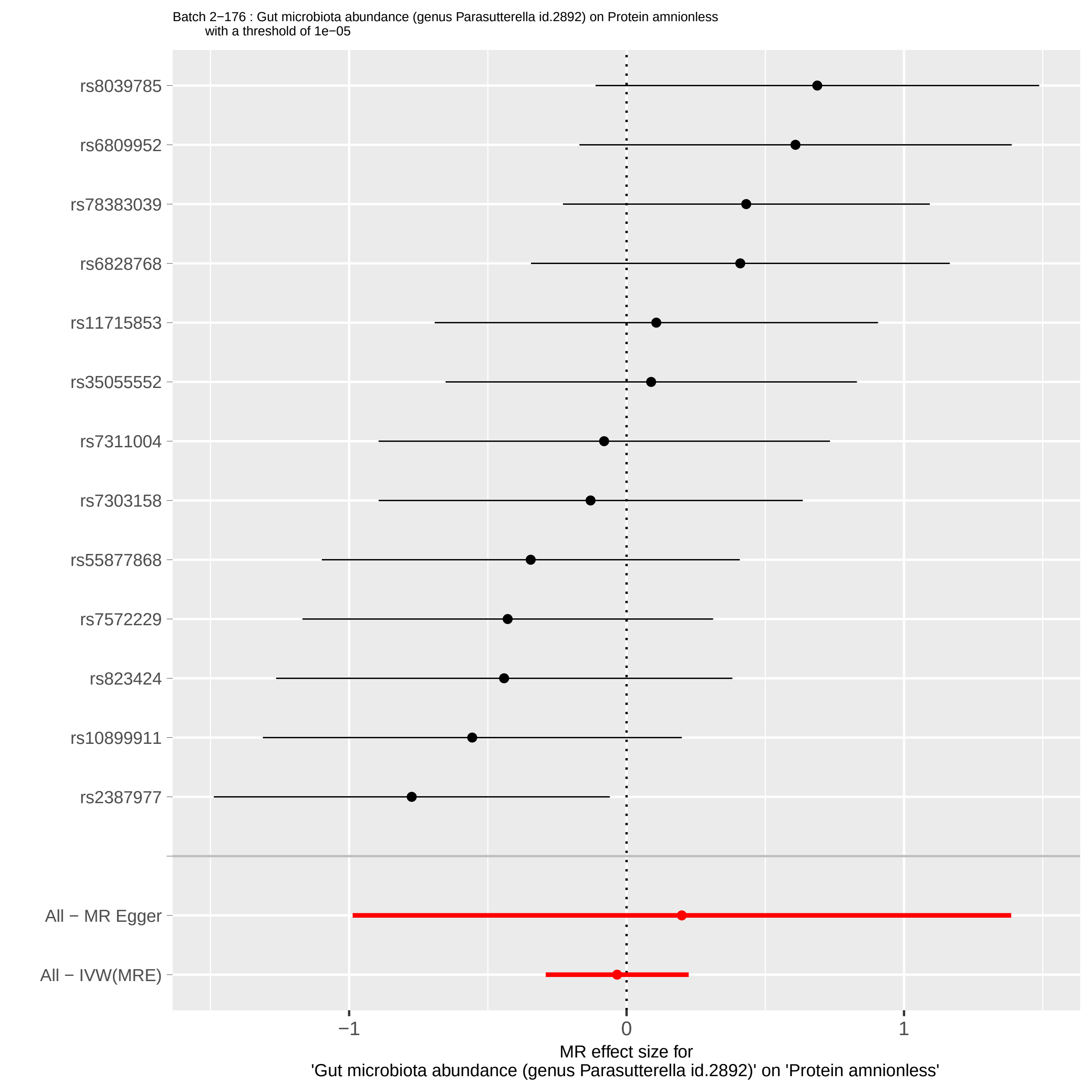

Batch 2−176 : Gut microbiota abundance (genus Parasutterella id.2892) on Protein amnionless
with a threshold of 1e−05
rs8039785
rs6809952
rs78383039
rs6828768
rs11715853
rs35055552
rs7311004
rs7303158
rs55877868
rs7572229
rs823424
rs10899911
rs2387977
All − MR Egger
All − IVW(MRE)
−1
0
1
MR effect size for
'Gut microbiota abundance (genus Parasutterella id.2892)' on 'Protein amnionless'

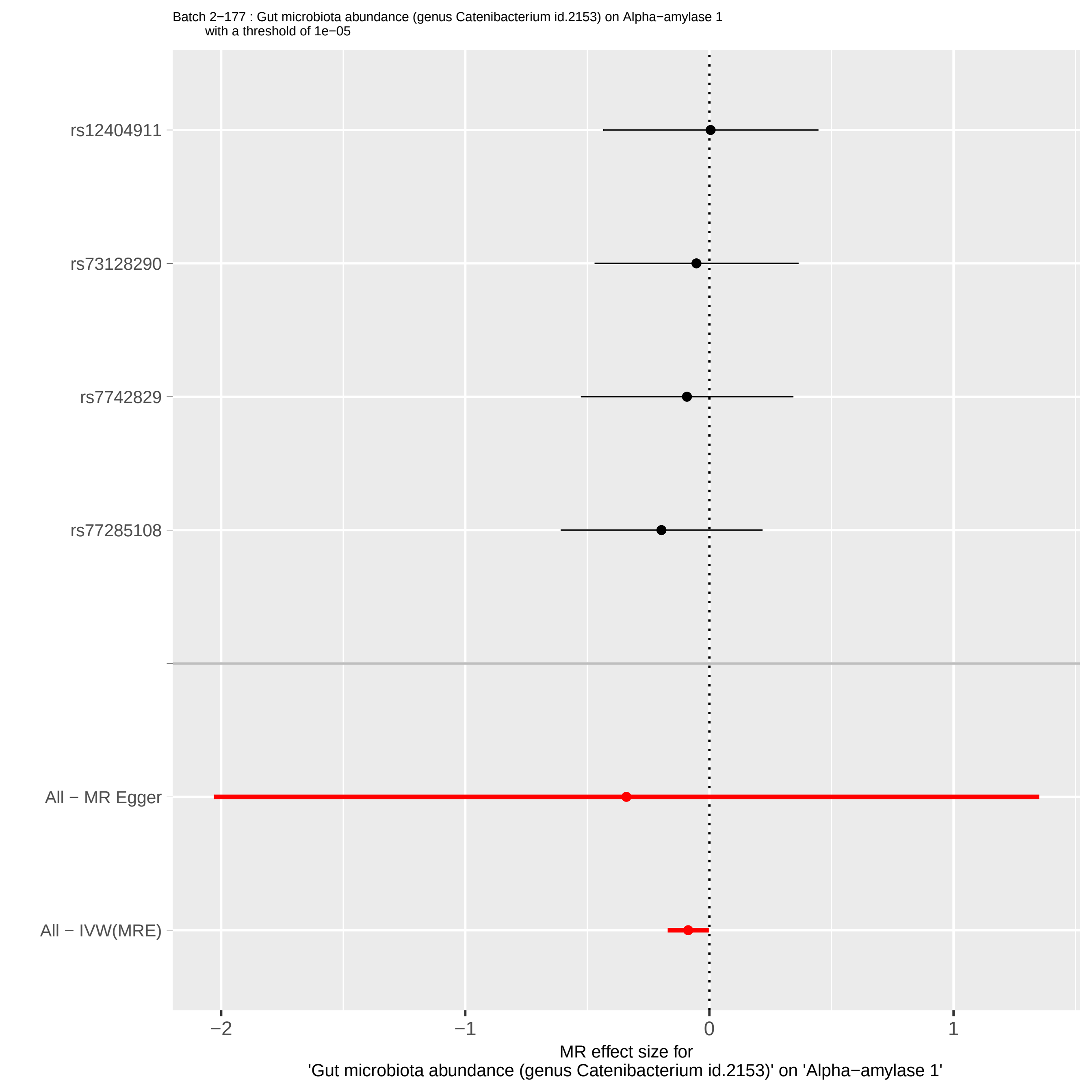
Batch 2−177 : Gut microbiota abundance (genus Catenibacterium id.2153) on Alpha−amylase 1
with a threshold of 1e−05
rs12404911
rs73128290
rs7742829
rs77285108
All − MR Egger
All − IVW(MRE)
−2
−1
0
1
MR effect size for
'Gut microbiota abundance (genus Catenibacterium id.2153)' on 'Alpha−amylase 1'

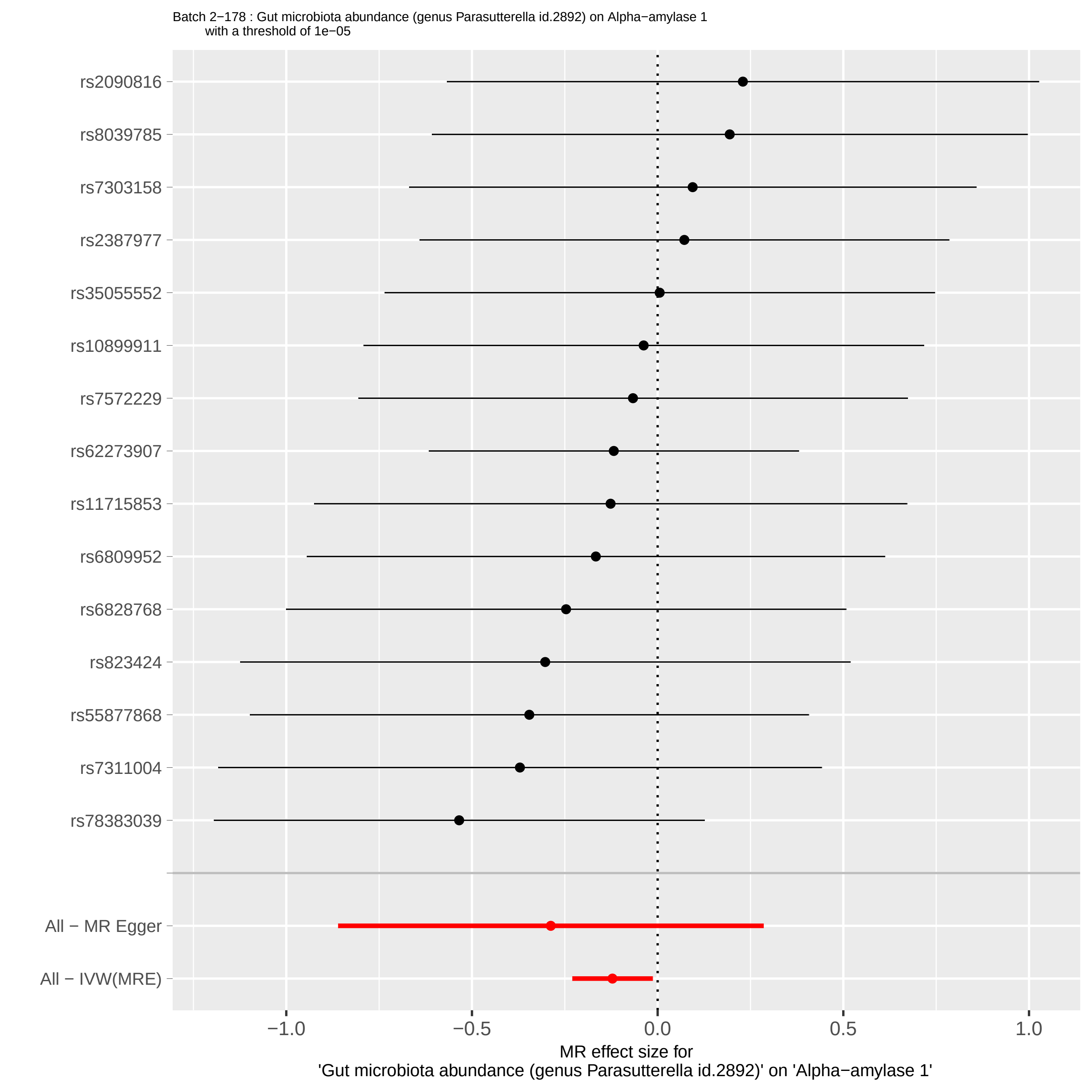
Batch 2−178 : Gut microbiota abundance (genus Parasutterella id.2892) on Alpha−amylase 1
with a threshold of 1e−05
rs2090816
rs8039785
rs7303158
rs2387977
rs35055552
rs10899911
rs7572229
rs62273907
rs11715853
rs6809952
rs6828768
rs823424
rs55877868
rs7311004
rs78383039
All − MR Egger
All − IVW(MRE)
−1.0
−0.5
0.0
0.5
1.0
MR effect size for
'Gut microbiota abundance (genus Parasutterella id.2892)' on 'Alpha−amylase 1'

Batch 2−179 : Gut microbiota abundance (genus Catenibacterium id.2153) on Anaphase−promoting complex subunit 10
with a threshold of 1e−05

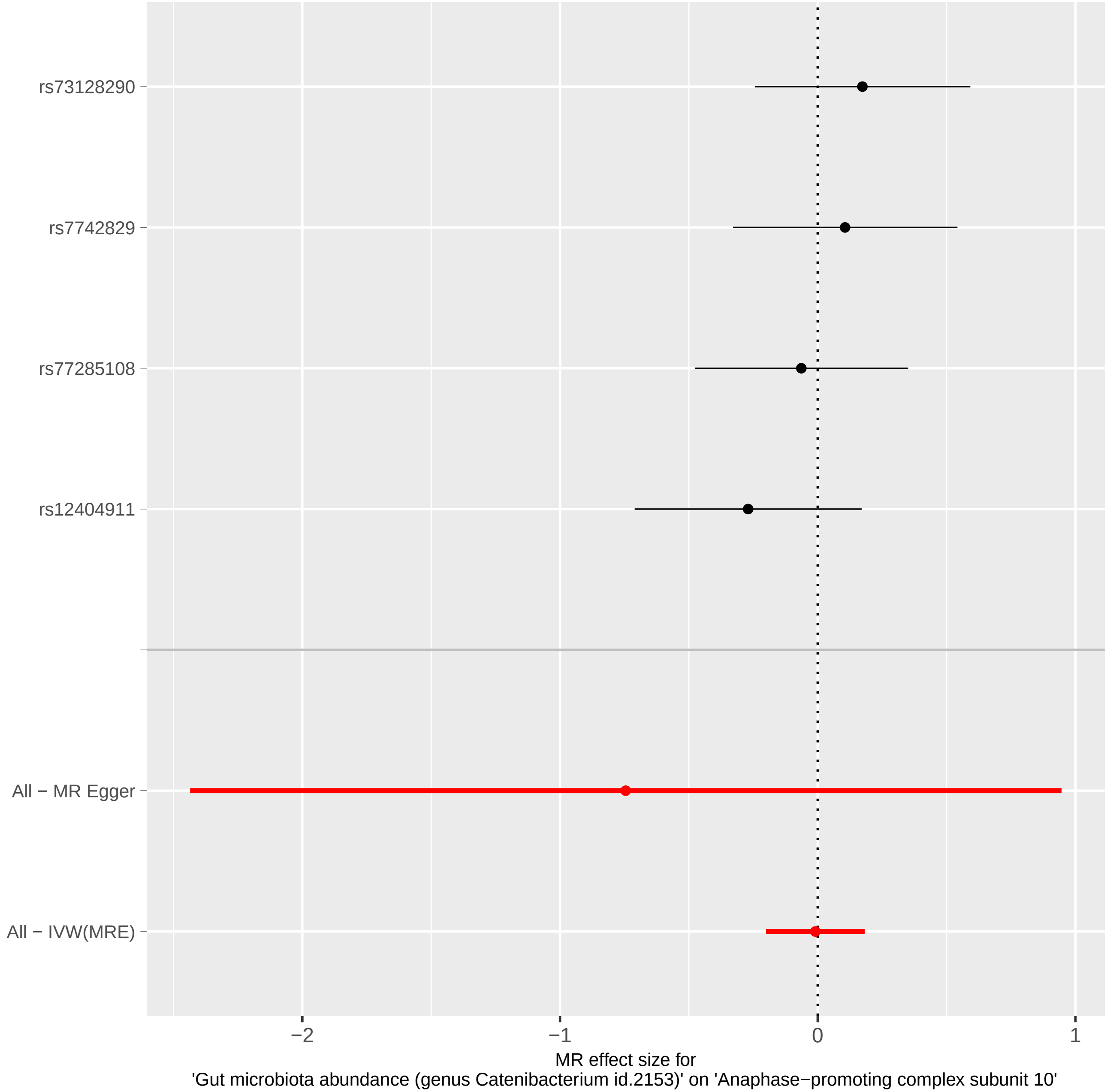

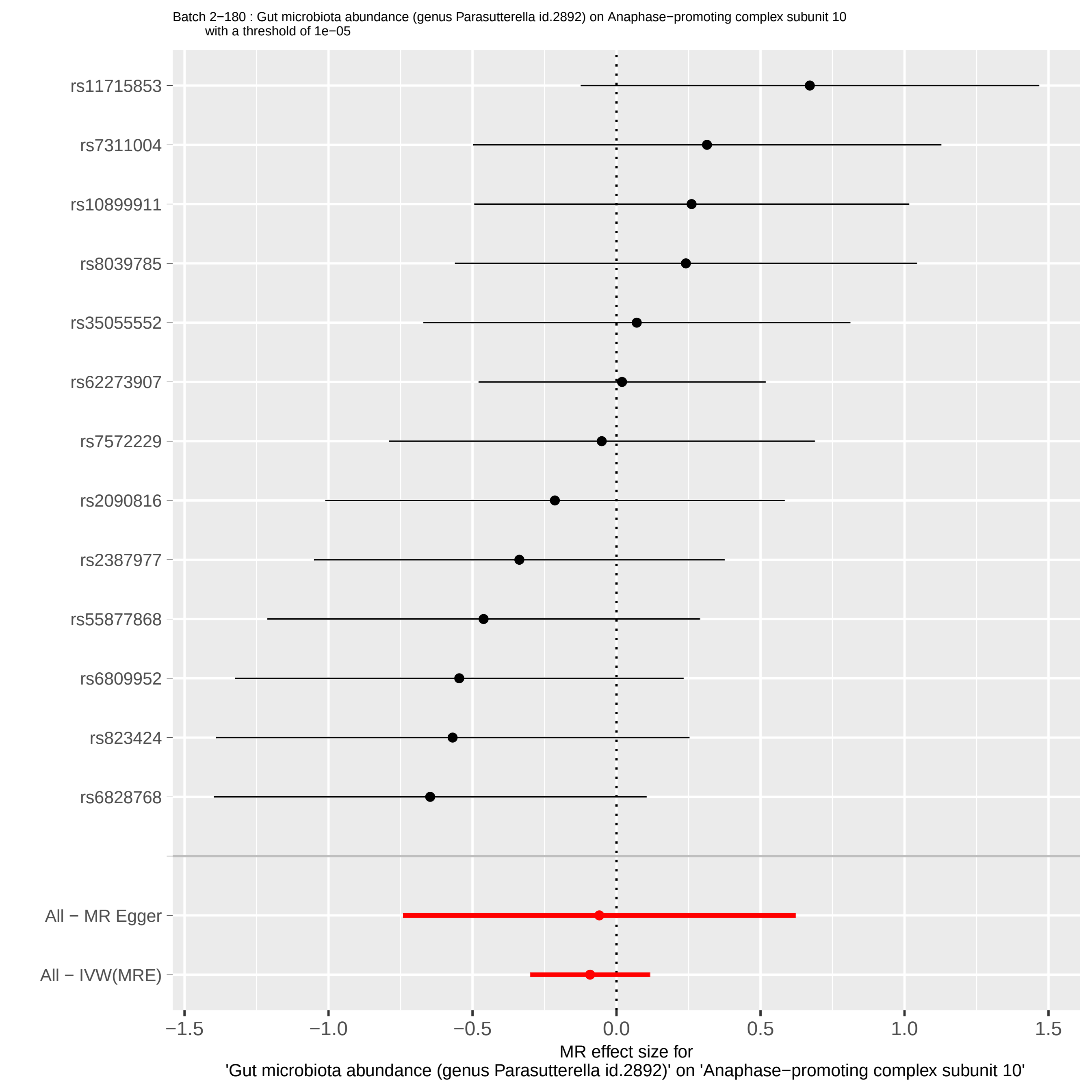

Batch 2−180 : Gut microbiota abundance (genus Parasutterella id.2892) on Anaphase−promoting complex subunit 10
with a threshold of 1e−05
rs11715853
rs7311004
rs10899911
rs8039785
rs35055552
rs62273907
rs7572229
rs2090816
rs2387977
rs55877868
rs6809952
rs823424
rs6828768
All − MR Egger
All − IVW(MRE)
−1.5
−1.0
−0.5
0.0
0.5
1.0
1.5
MR effect size for
'Gut microbiota abundance (genus Parasutterella id.2892)' on 'Anaphase−promoting complex subunit 10'

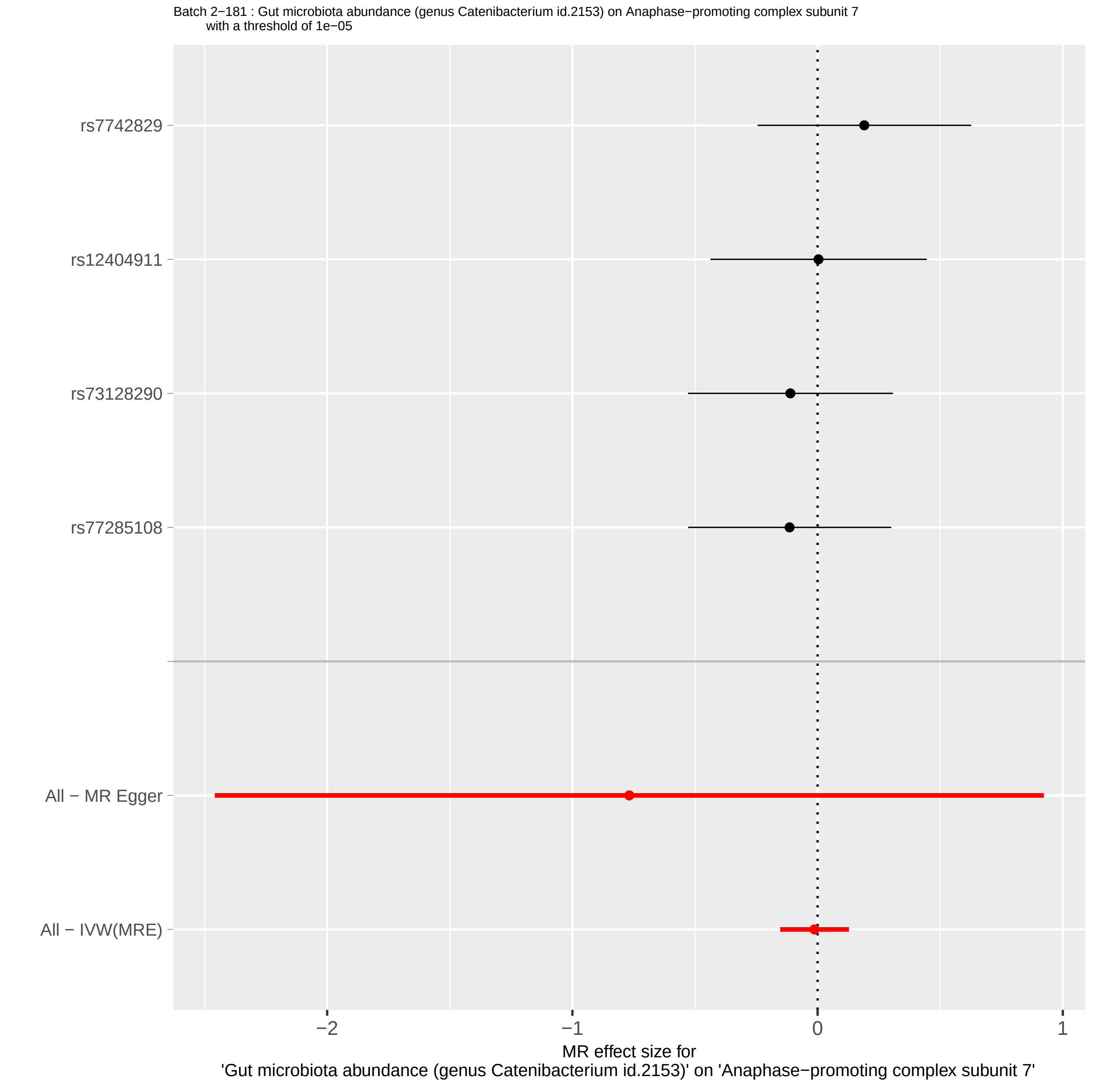

Batch 2−181 : Gut microbiota abundance (genus Catenibacterium id.2153) on Anaphase−promoting complex subunit 7
with a threshold of 1e−05
rs7742829
rs12404911
rs73128290
rs77285108
All − MR Egger
All − IVW(MRE)
−2
−1
0
1
MR effect size for
'Gut microbiota abundance (genus Catenibacterium id.2153)' on 'Anaphase−promoting complex subunit 7'

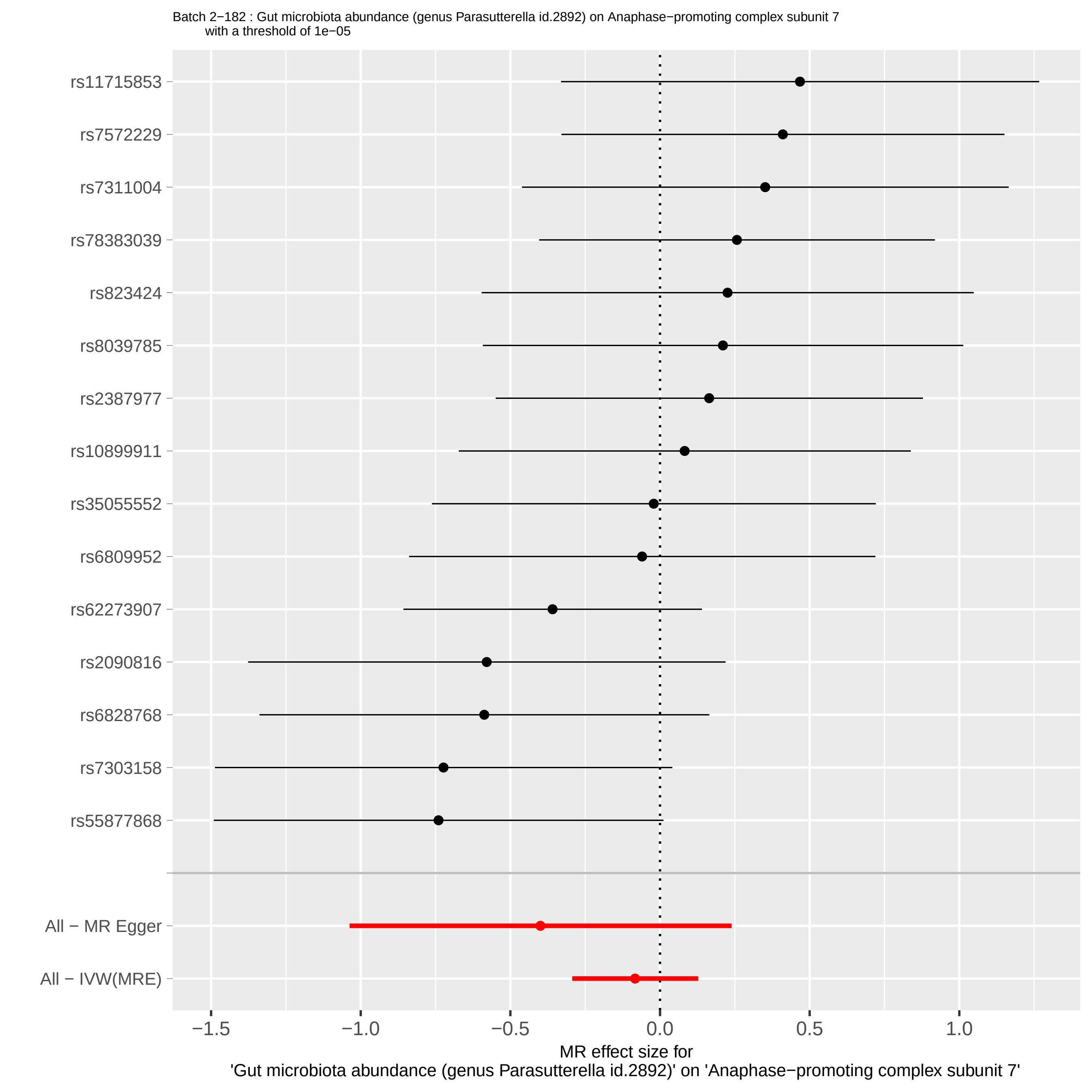

Batch 2−182 : Gut microbiota abundance (genus Parasutterella id.2892) on Anaphase−promoting complex subunit 7
with a threshold of 1e−05
rs11715853
rs7572229
rs7311004
rs78383039
rs823424
rs8039785
rs2387977
rs10899911
rs35055552
rs6809952
rs62273907
rs2090816
rs6828768
rs7303158
rs55877868
All − MR Egger
All − IVW(MRE)
−1.5
−1.0
−0.5
0.0
0.5
1.0
MR effect size for
'Gut microbiota abundance (genus Parasutterella id.2892)' on 'Anaphase−promoting complex subunit 7'

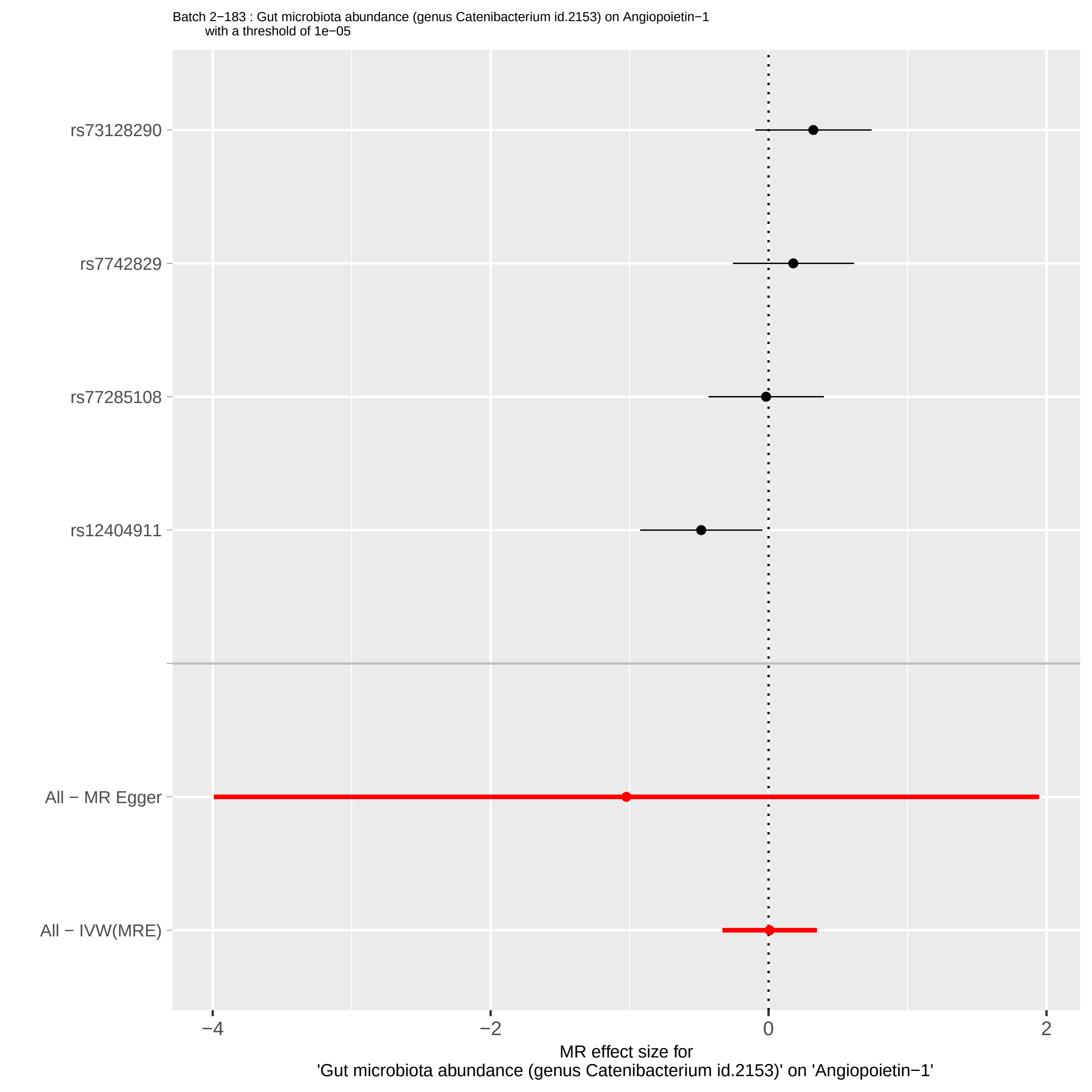
Batch 2−183 : Gut microbiota abundance (genus Catenibacterium id.2153) on Angiopoietin−1
with a threshold of 1e−05
rs73128290
rs7742829
rs77285108
rs12404911
All − MR Egger
All − IVW(MRE)
−4
−2
0
2
MR effect size for
'Gut microbiota abundance (genus Catenibacterium id.2153)' on 'Angiopoietin−1'

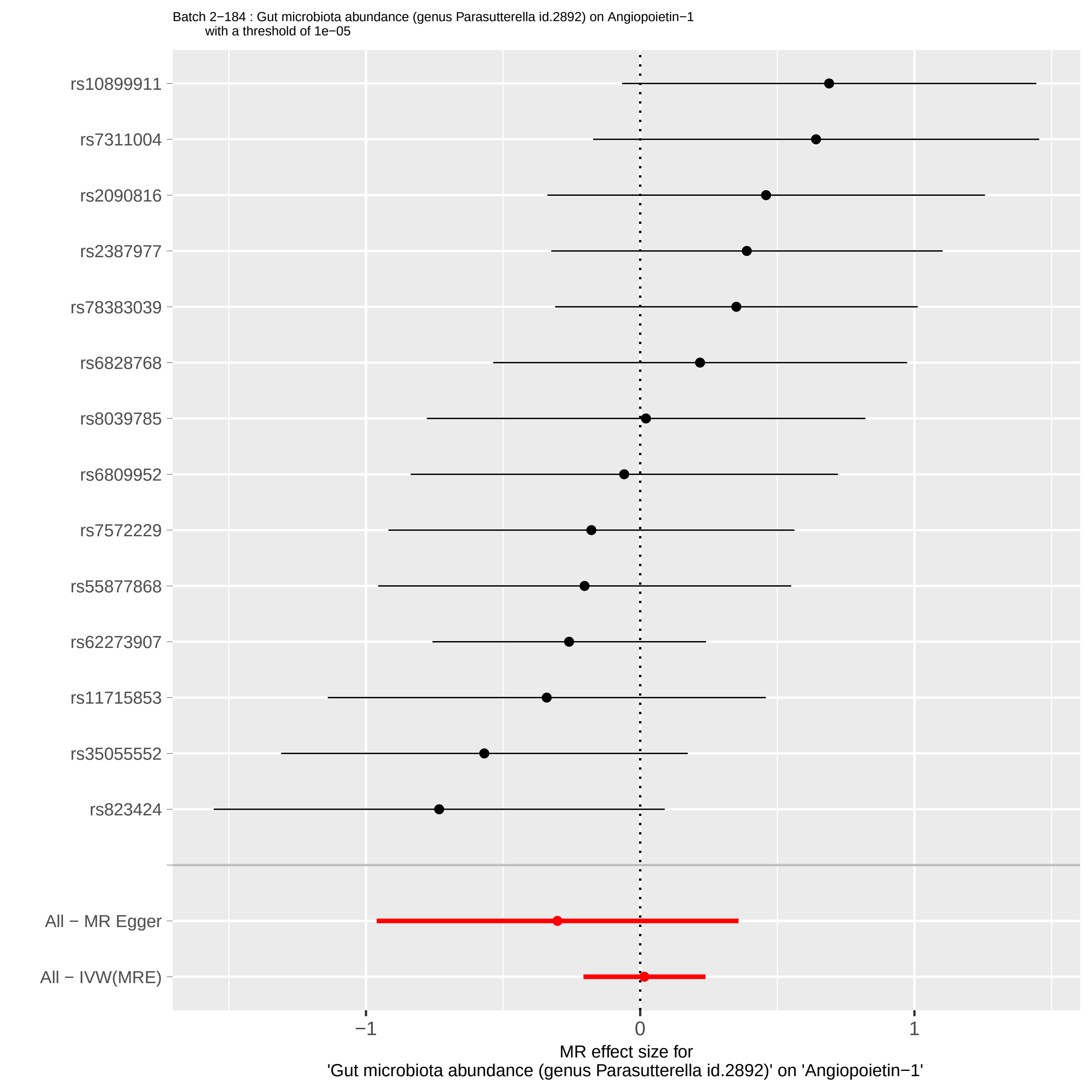
Batch 2−184 : Gut microbiota abundance (genus Parasutterella id.2892) on Angiopoietin−1
with a threshold of 1e−05
rs10899911
rs7311004
rs2090816
rs2387977
rs78383039
rs6828768
rs8039785
rs6809952
rs7572229
rs55877868
rs62273907
rs11715853
rs35055552
rs823424
All − MR Egger
All − IVW(MRE)
−1
0
1
MR effect size for
'Gut microbiota abundance (genus Parasutterella id.2892)' on 'Angiopoietin−1'

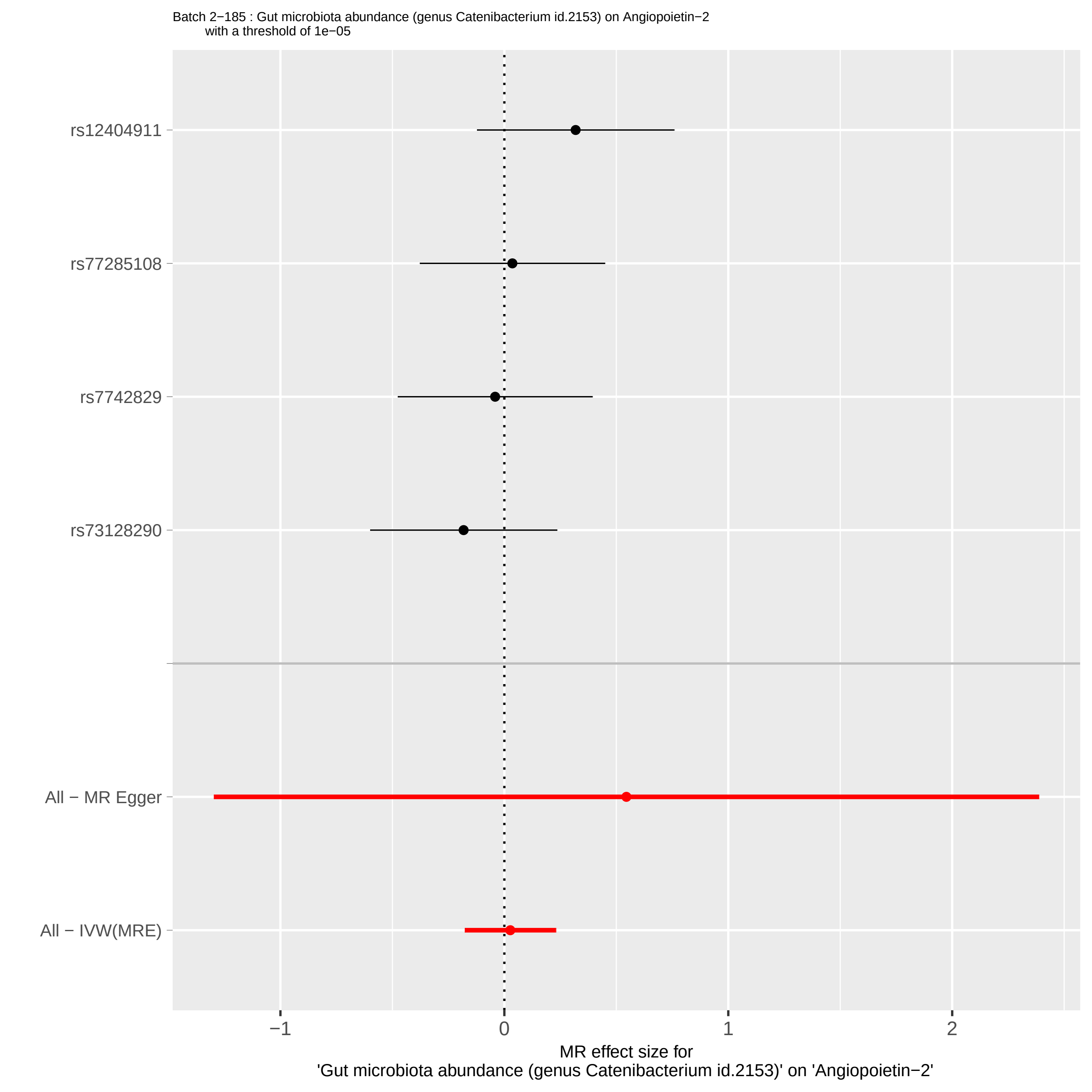

Batch 2−185 : Gut microbiota abundance (genus Catenibacterium id.2153) on Angiopoietin−2
with a threshold of 1e−05
rs12404911
rs77285108
rs7742829
rs73128290
All − MR Egger
All − IVW(MRE)
−1
0
1
2
MR effect size for
'Gut microbiota abundance (genus Catenibacterium id.2153)' on 'Angiopoietin−2'

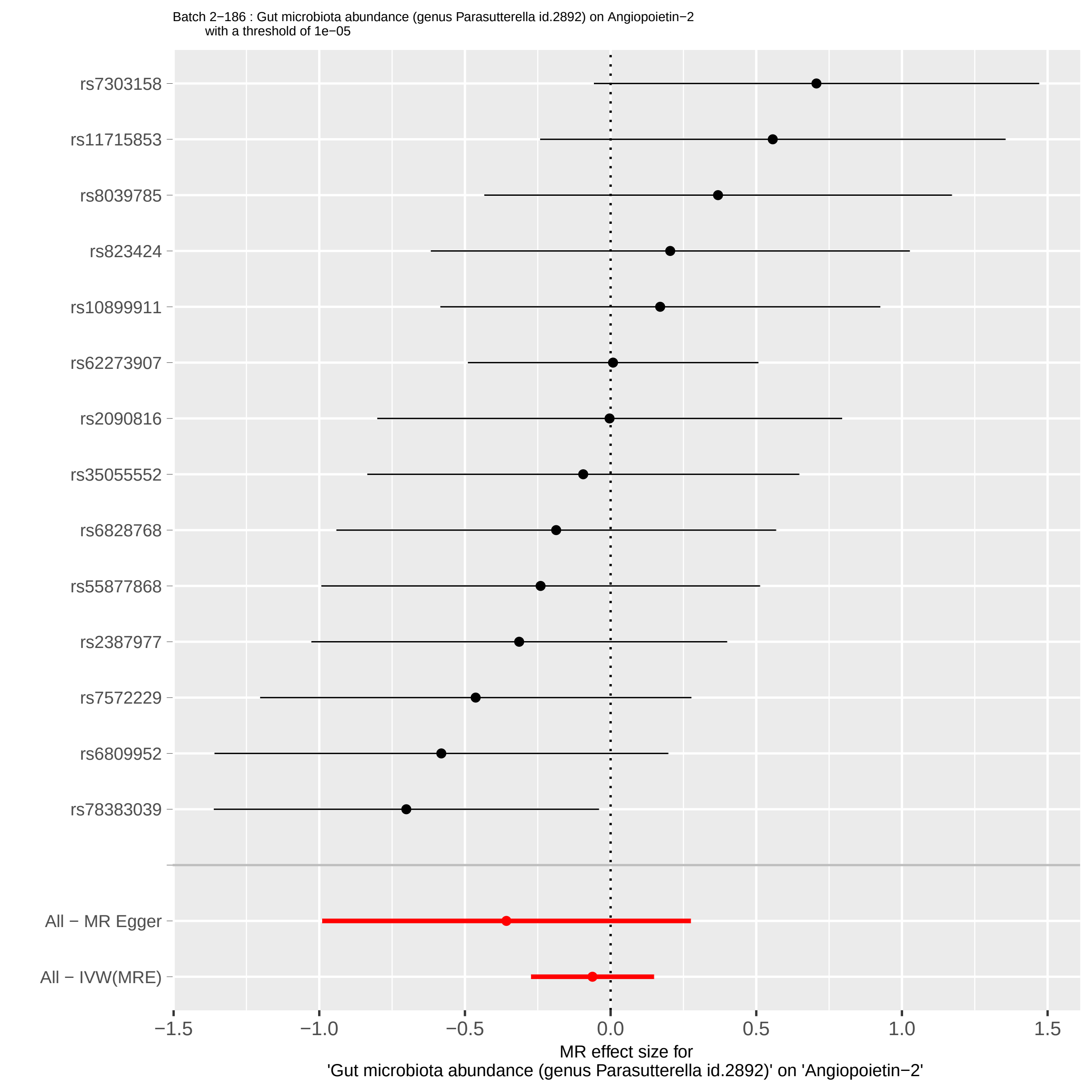

Batch 2−186 : Gut microbiota abundance (genus Parasutterella id.2892) on Angiopoietin−2
with a threshold of 1e−05
rs7303158
rs11715853
rs8039785
rs823424
rs10899911
rs62273907
rs2090816
rs35055552
rs6828768
rs55877868
rs2387977
rs7572229
rs6809952
rs78383039
All − MR Egger
All − IVW(MRE)
−1.5
−1.0
−0.5
0.0
0.5
1.0
1.5
MR effect size for
'Gut microbiota abundance (genus Parasutterella id.2892)' on 'Angiopoietin−2'

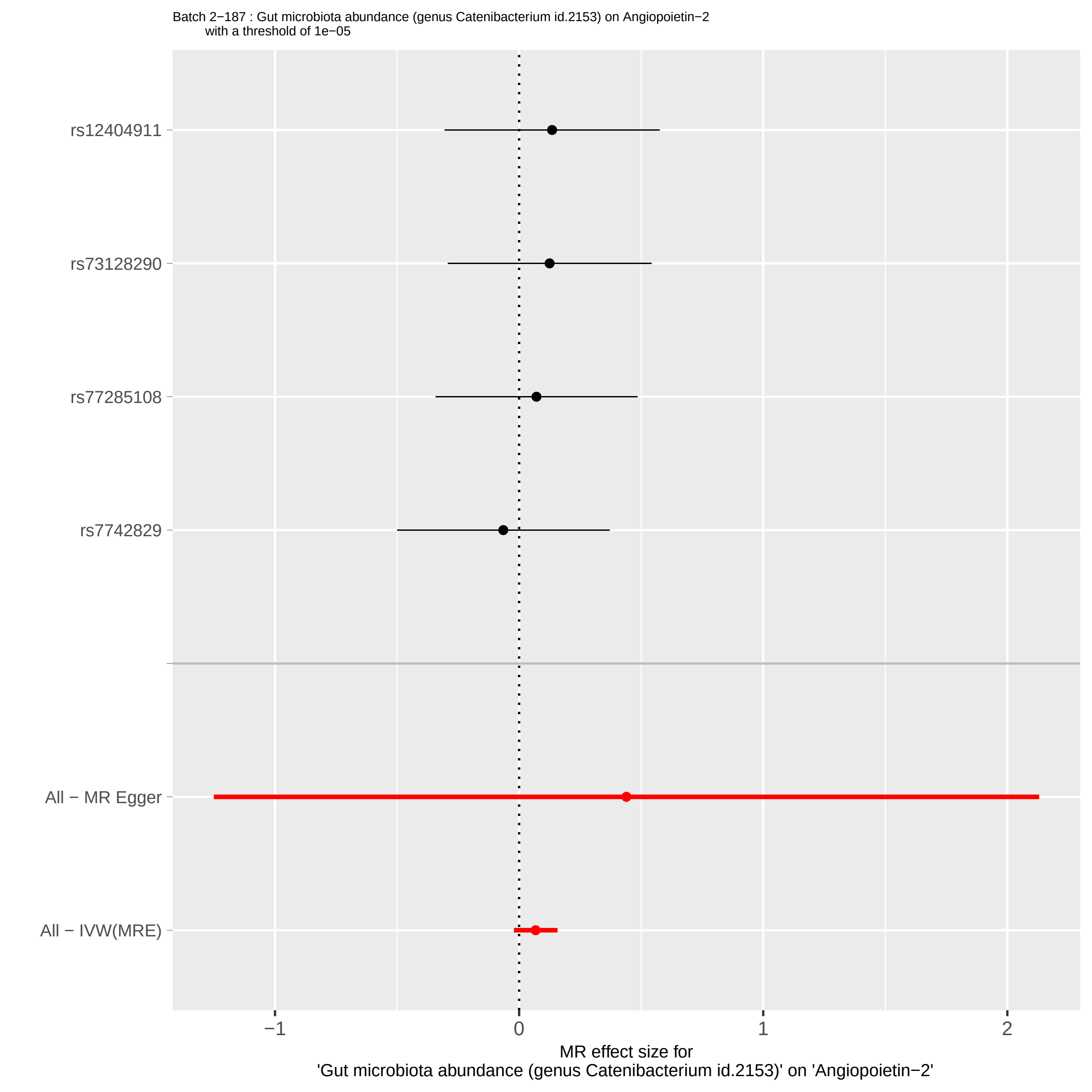

Batch 2−187 : Gut microbiota abundance (genus Catenibacterium id.2153) on Angiopoietin−2
with a threshold of 1e−05
rs12404911
rs73128290
rs77285108
rs7742829
All − MR Egger
All − IVW(MRE)
−1
0
1
2
MR effect size for
'Gut microbiota abundance (genus Catenibacterium id.2153)' on 'Angiopoietin−2'

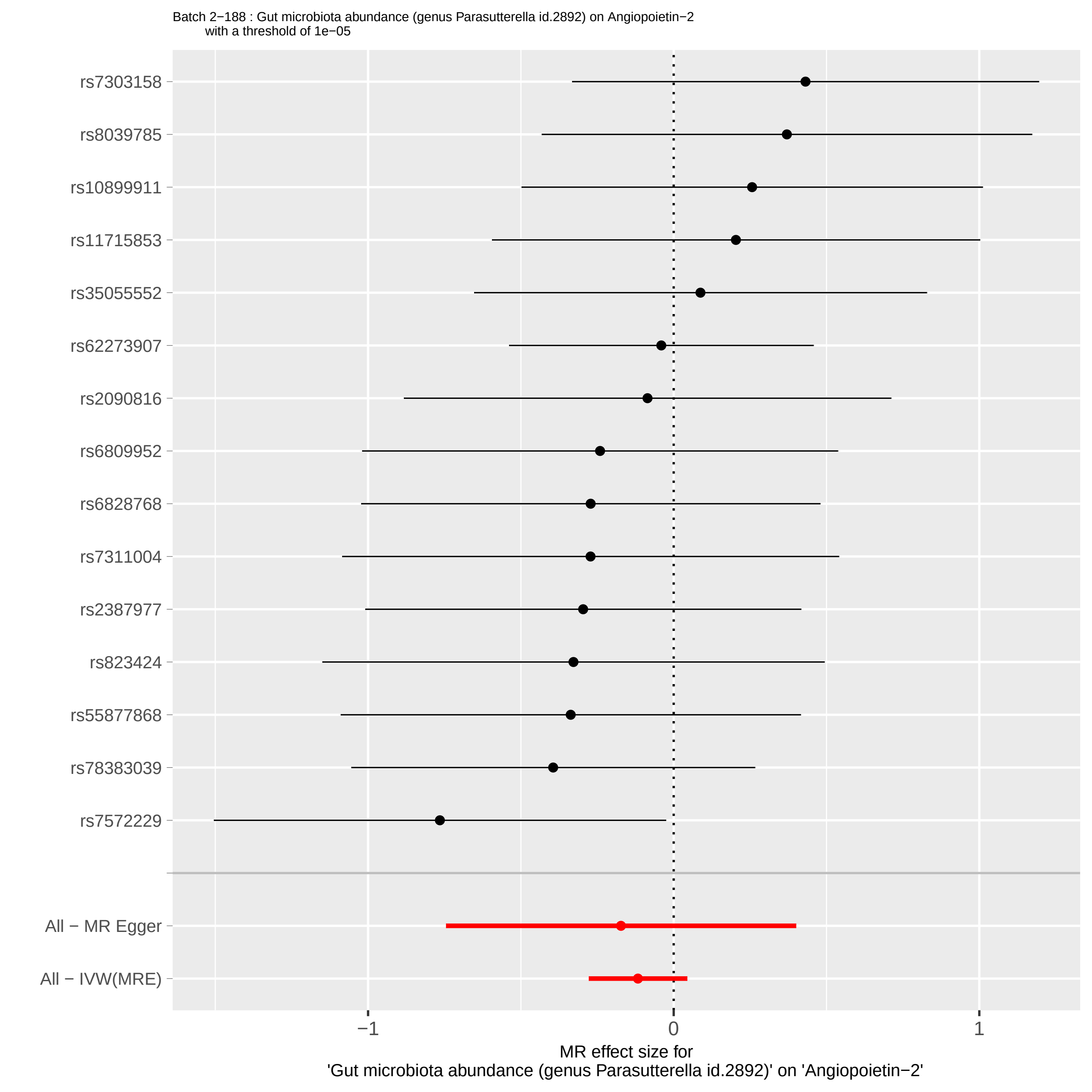

Batch 2−188 : Gut microbiota abundance (genus Parasutterella id.2892) on Angiopoietin−2
with a threshold of 1e−05
rs7303158
rs8039785
rs10899911
rs11715853
rs35055552
rs62273907
rs2090816
rs6809952
rs6828768
rs7311004
rs2387977
rs823424
rs55877868
rs78383039
rs7572229
All − MR Egger
All − IVW(MRE)
−1
0
1
MR effect size for
'Gut microbiota abundance (genus Parasutterella id.2892)' on 'Angiopoietin−2'

Batch 2−189 : Gut microbiota abundance (genus Catenibacterium id.2153) on Angiopoietin−4
with a threshold of 1e−05

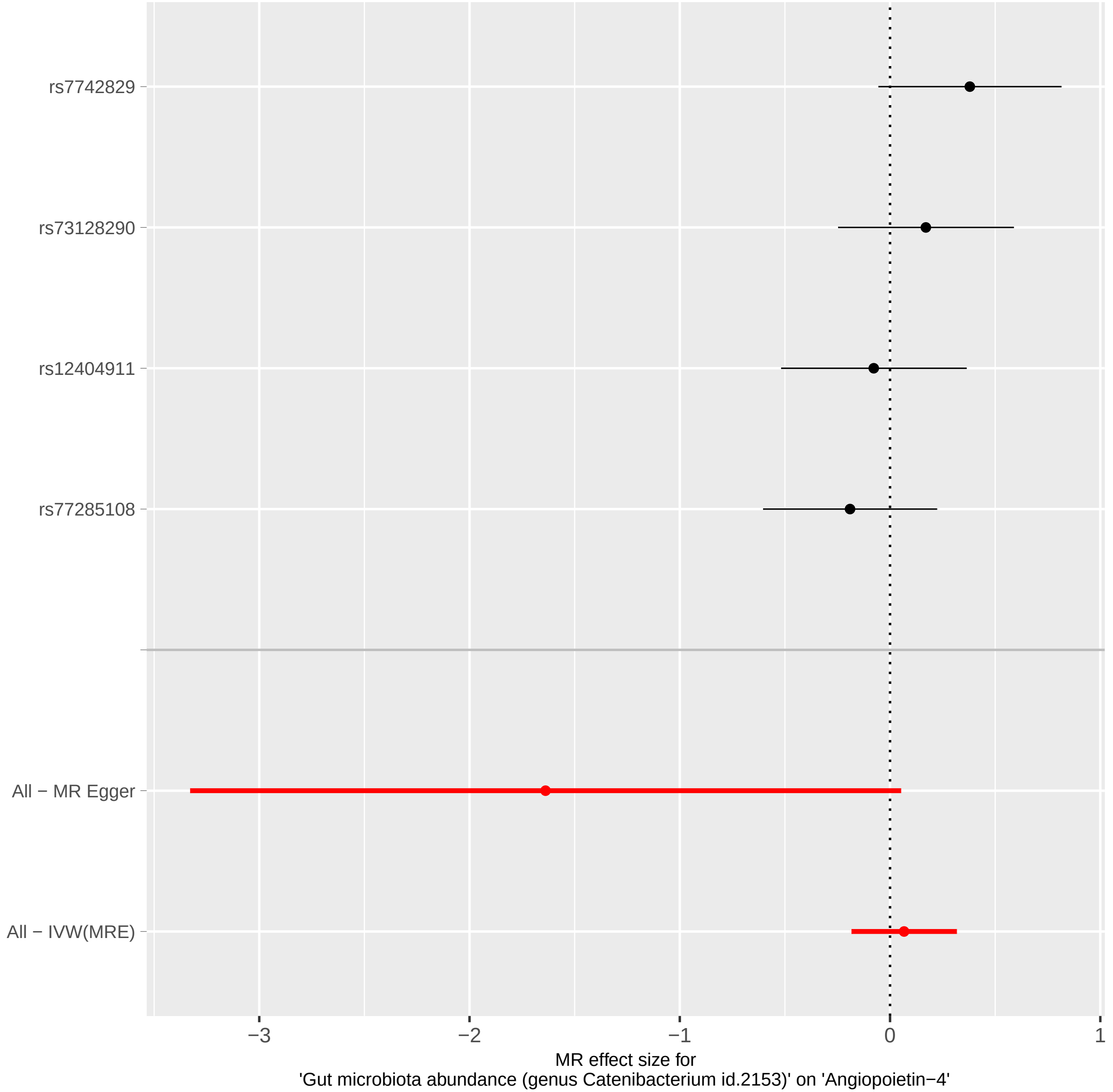

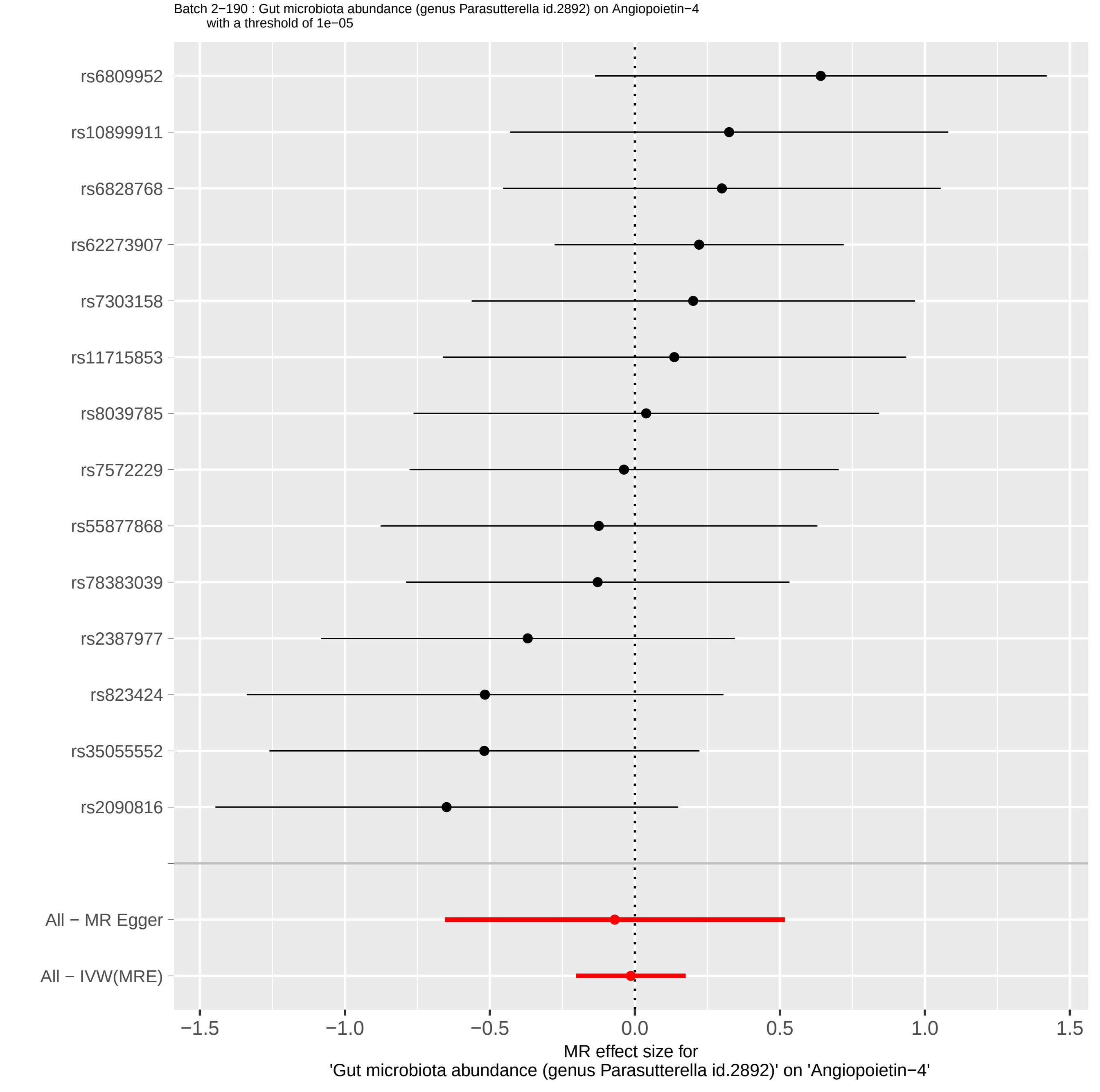

Batch 2−190 : Gut microbiota abundance (genus Parasutterella id.2892) on Angiopoietin−4
with a threshold of 1e−05
rs6809952
rs10899911
rs6828768
rs62273907
rs7303158
rs11715853
rs8039785
rs7572229
rs55877868
rs78383039
rs2387977
rs823424
rs35055552
rs2090816
All − MR Egger
All − IVW(MRE)
−1.5
−1.0
−0.5
0.0
0.5
1.0
1.5
MR effect size for
'Gut microbiota abundance (genus Parasutterella id.2892)' on 'Angiopoietin−4'

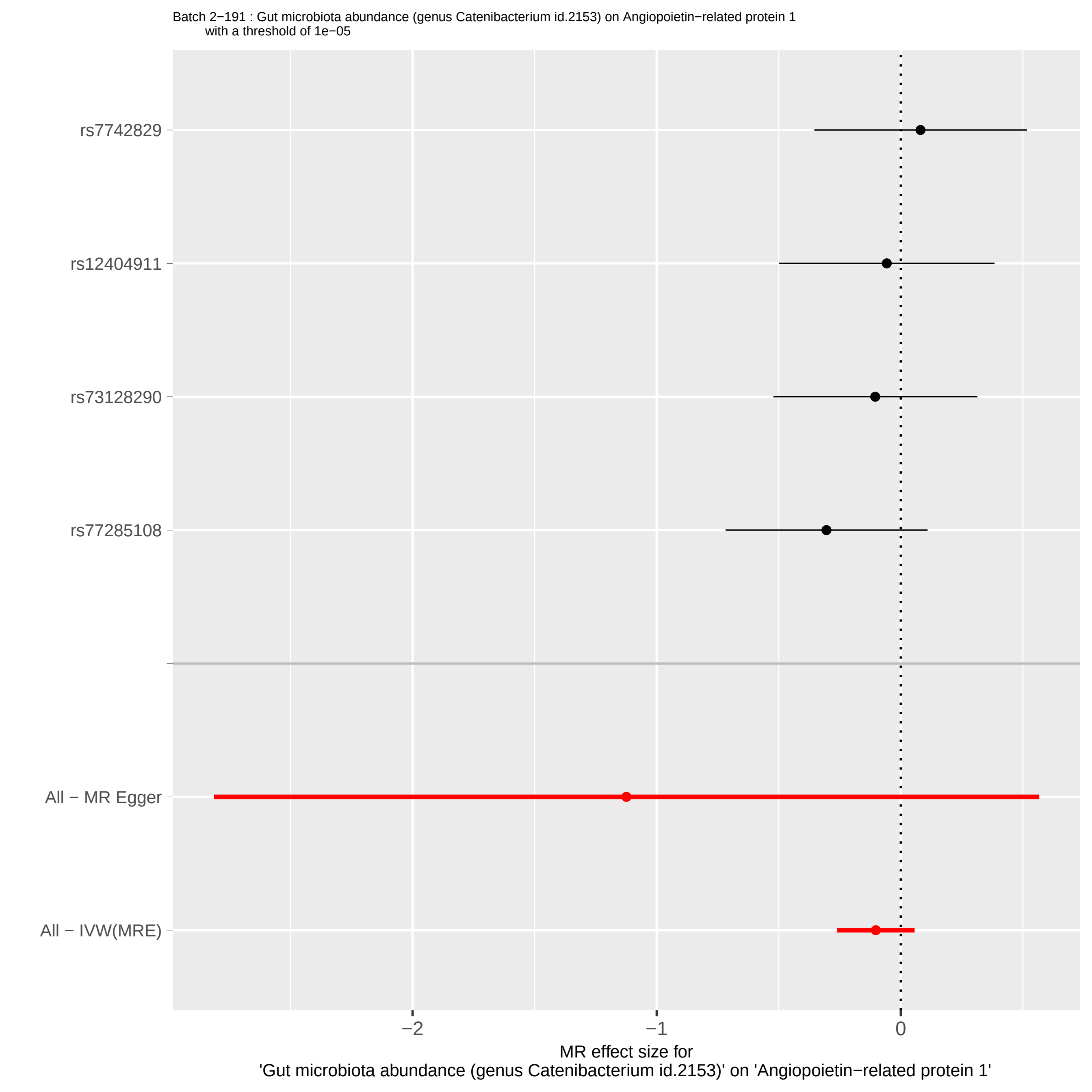

Batch 2−191 : Gut microbiota abundance (genus Catenibacterium id.2153) on Angiopoietin−related protein 1
with a threshold of 1e−05
rs7742829
rs12404911
rs73128290
rs77285108
All − MR Egger
All − IVW(MRE)
−2
−1
0
MR effect size for
'Gut microbiota abundance (genus Catenibacterium id.2153)' on 'Angiopoietin−related protein 1'

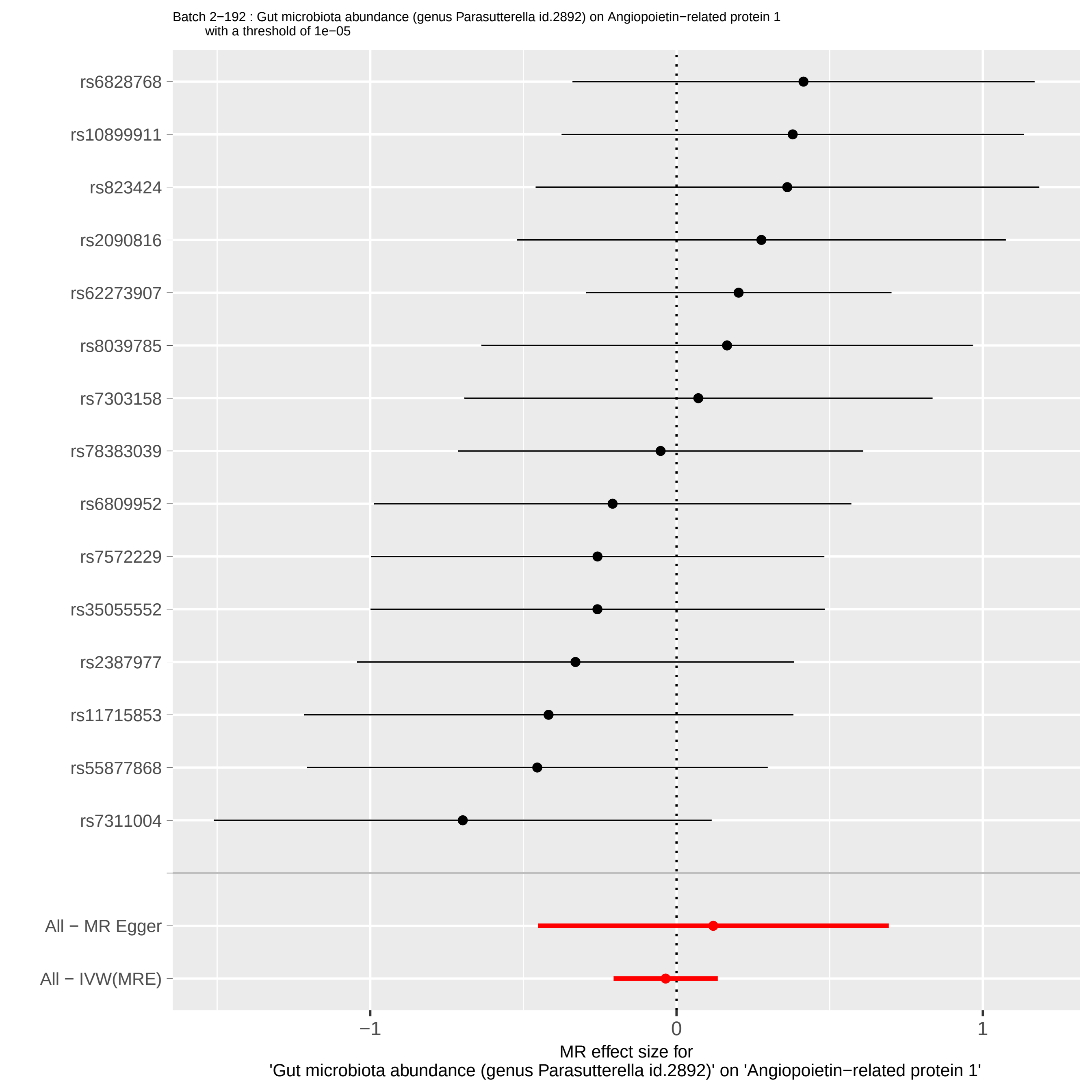

Batch 2−192 : Gut microbiota abundance (genus Parasutterella id.2892) on Angiopoietin−related protein 1
with a threshold of 1e−05
rs6828768
rs10899911
rs823424
rs2090816
rs62273907
rs8039785
rs7303158
rs78383039
rs6809952
rs7572229
rs35055552
rs2387977
rs11715853
rs55877868
rs7311004
All − MR Egger
All − IVW(MRE)
−1
0
1
MR effect size for
'Gut microbiota abundance (genus Parasutterella id.2892)' on 'Angiopoietin−related protein 1'

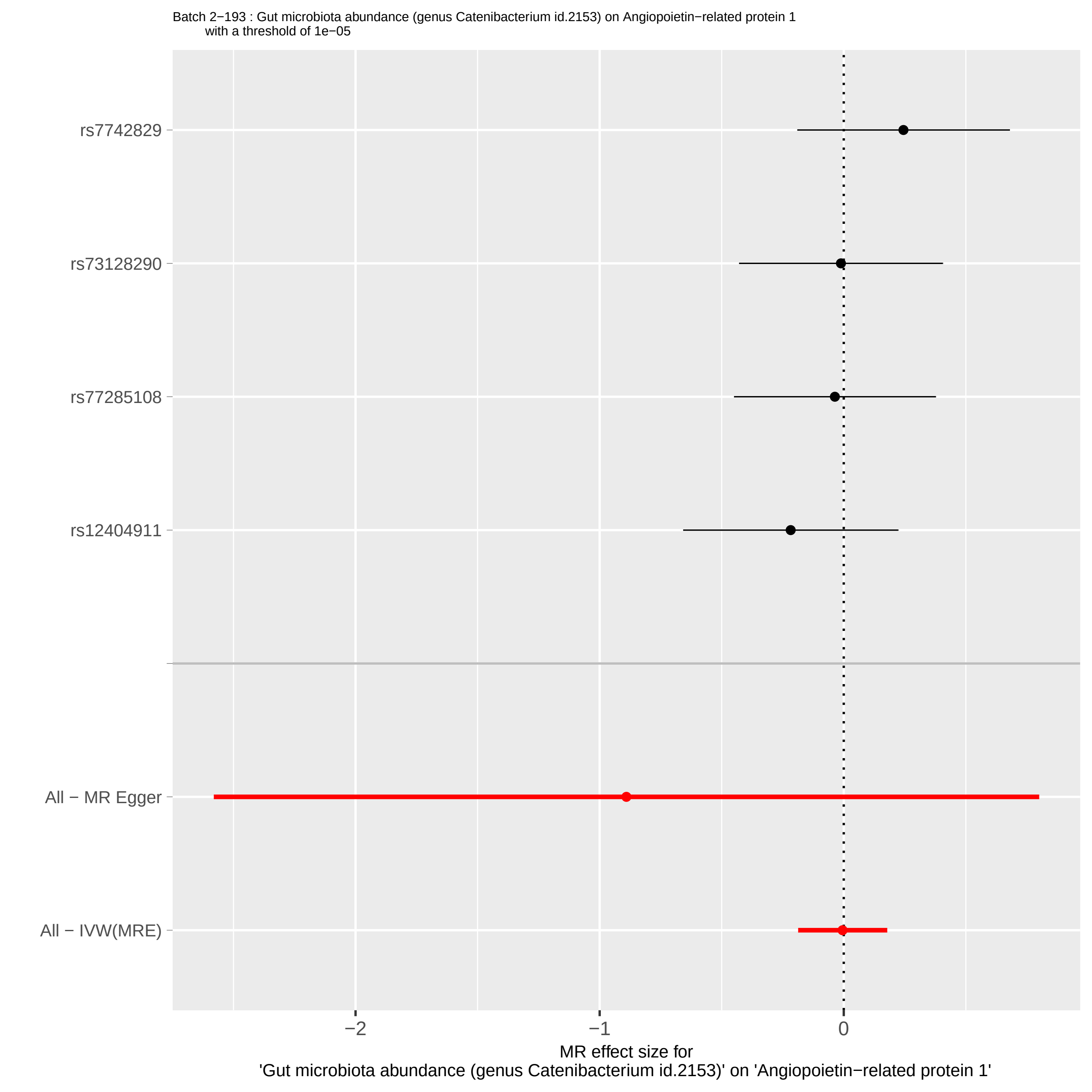

Batch 2−193 : Gut microbiota abundance (genus Catenibacterium id.2153) on Angiopoietin−related protein 1
with a threshold of 1e−05
rs7742829
rs73128290
rs77285108
rs12404911
All − MR Egger
All − IVW(MRE)
−2
−1
0
MR effect size for
'Gut microbiota abundance (genus Catenibacterium id.2153)' on 'Angiopoietin−related protein 1'

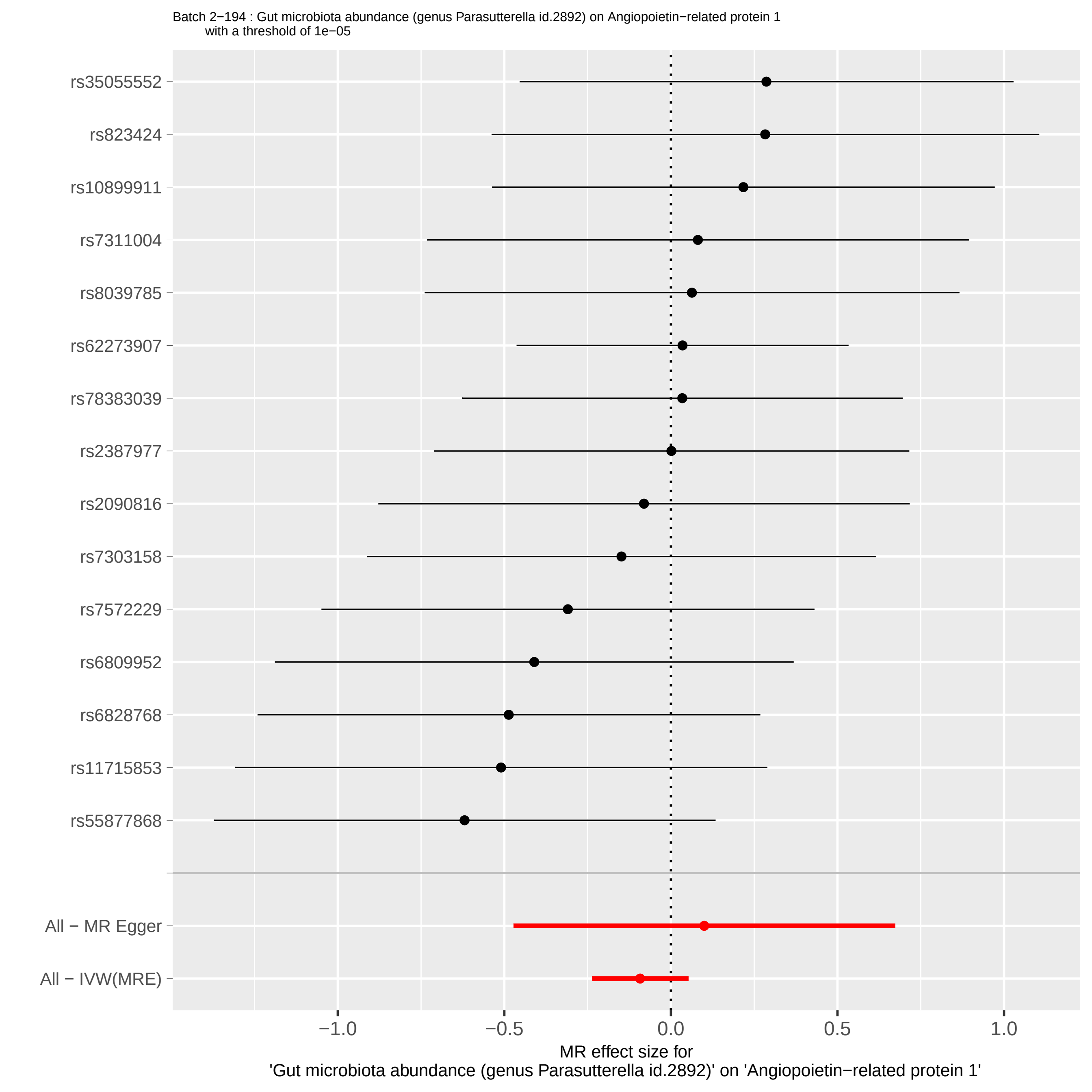

Batch 2−194 : Gut microbiota abundance (genus Parasutterella id.2892) on Angiopoietin−related protein 1
with a threshold of 1e−05
rs35055552
rs823424
rs10899911
rs7311004
rs8039785
rs62273907
rs78383039
rs2387977
rs2090816
rs7303158
rs7572229
rs6809952
rs6828768
rs11715853
rs55877868
All − MR Egger
All − IVW(MRE)
−1.0
−0.5
0.0
0.5
1.0
MR effect size for
'Gut microbiota abundance (genus Parasutterella id.2892)' on 'Angiopoietin−related protein 1'

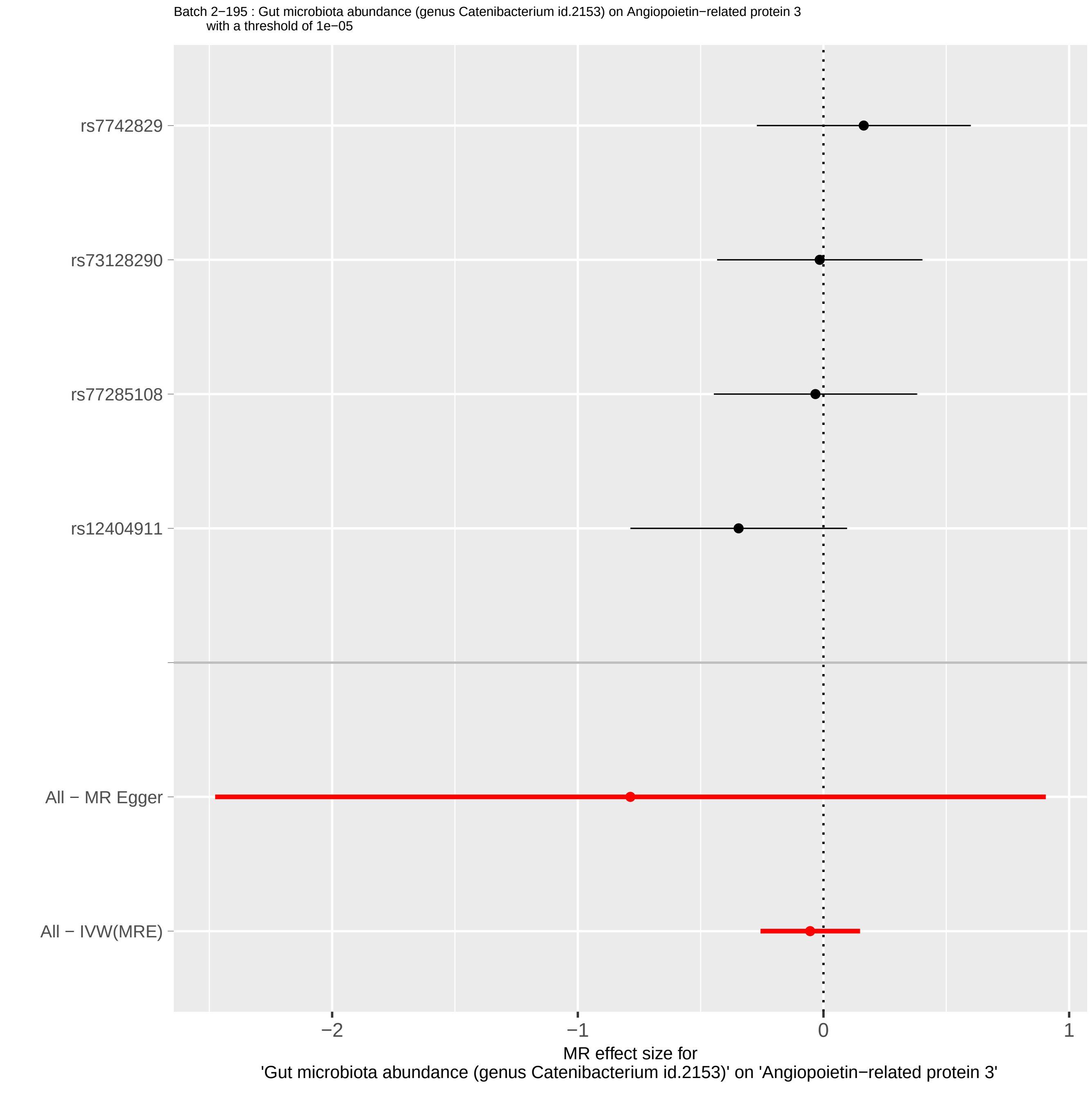
Batch 2−195 : Gut microbiota abundance (genus Catenibacterium id.2153) on Angiopoietin−related protein 3
with a threshold of 1e−05
rs7742829
rs73128290
rs77285108
rs12404911
All − MR Egger
All − IVW(MRE)
−2
−1
0
1
MR effect size for
'Gut microbiota abundance (genus Catenibacterium id.2153)' on 'Angiopoietin−related protein 3'

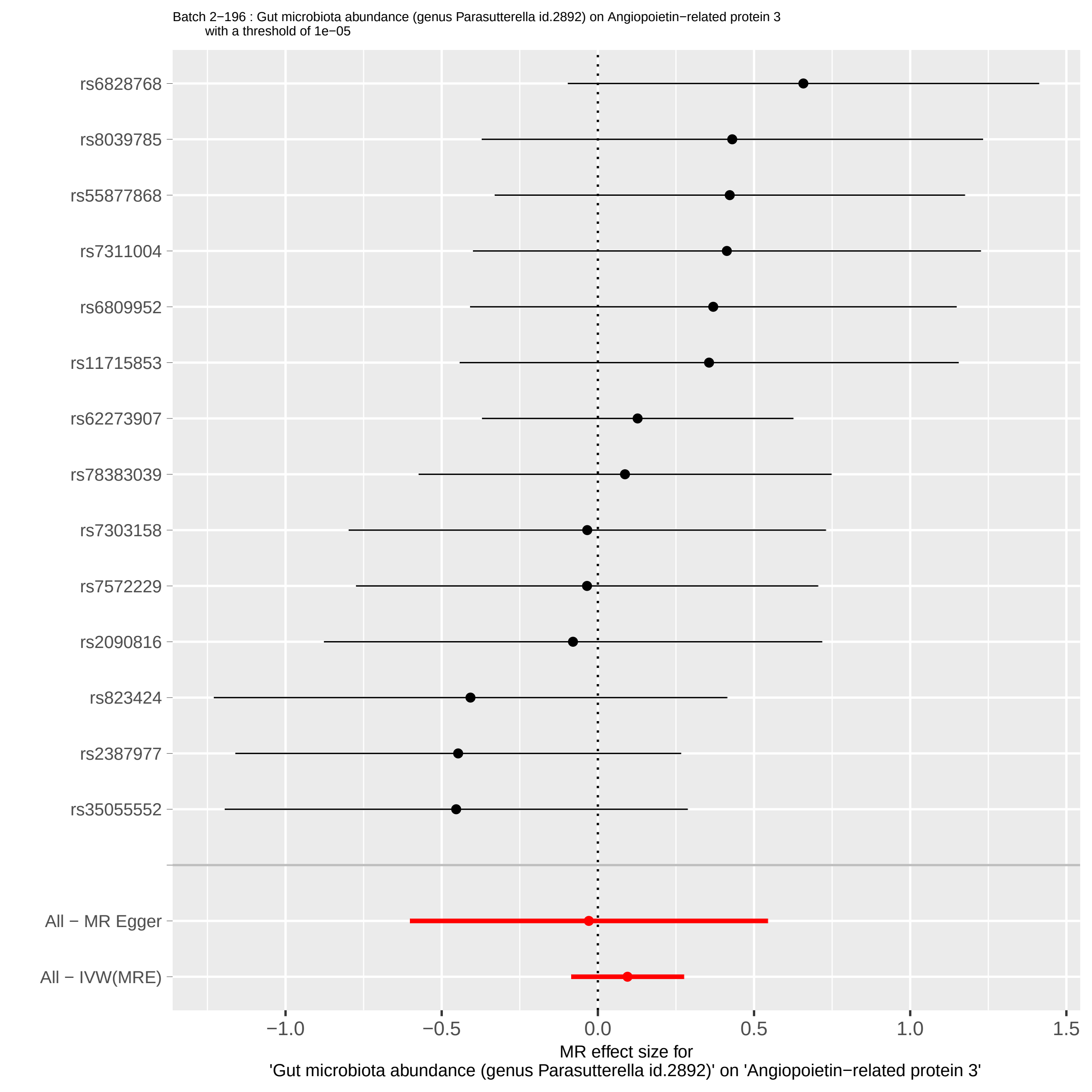

Batch 2−196 : Gut microbiota abundance (genus Parasutterella id.2892) on Angiopoietin−related protein 3
with a threshold of 1e−05
rs6828768
rs8039785
rs55877868
rs7311004
rs6809952
rs11715853
rs62273907
rs78383039
rs7303158
rs7572229
rs2090816
rs823424
rs2387977
rs35055552
All − MR Egger
All − IVW(MRE)
−1.0
−0.5
0.0
0.5
1.0
1.5
MR effect size for
'Gut microbiota abundance (genus Parasutterella id.2892)' on 'Angiopoietin−related protein 3'

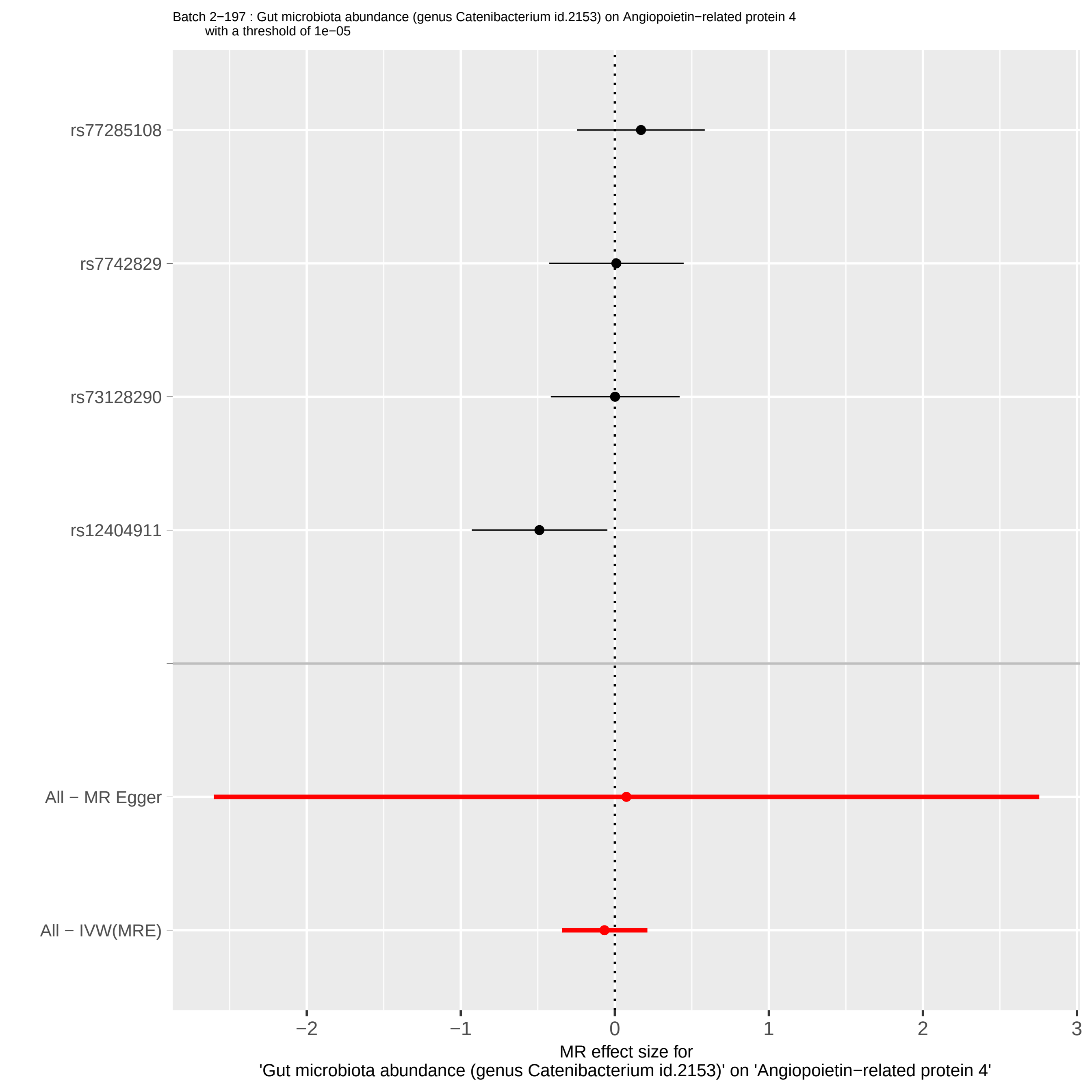

Batch 2−197 : Gut microbiota abundance (genus Catenibacterium id.2153) on Angiopoietin−related protein 4
with a threshold of 1e−05
rs77285108
rs7742829
rs73128290
rs12404911
All − MR Egger
All − IVW(MRE)
−2
−1
0
1
2
3
MR effect size for
'Gut microbiota abundance (genus Catenibacterium id.2153)' on 'Angiopoietin−related protein 4'

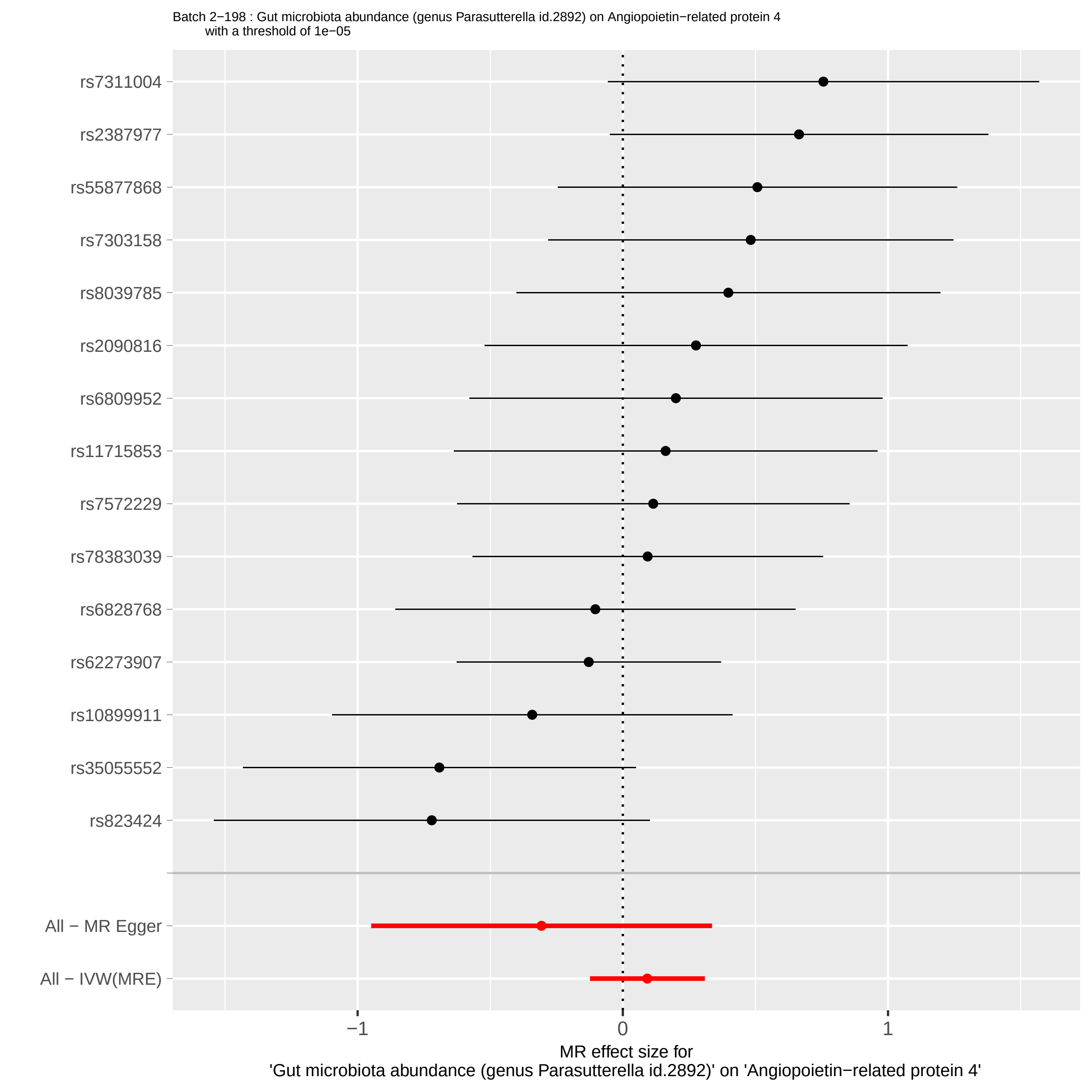

Batch 2−198 : Gut microbiota abundance (genus Parasutterella id.2892) on Angiopoietin−related protein 4
with a threshold of 1e−05
rs7311004
rs2387977
rs55877868
rs7303158
rs8039785
rs2090816
rs6809952
rs11715853
rs7572229
rs78383039
rs6828768
rs62273907
rs10899911
rs35055552
rs823424
All − MR Egger
All − IVW(MRE)
−1
0
1
MR effect size for
'Gut microbiota abundance (genus Parasutterella id.2892)' on 'Angiopoietin−related protein 4'

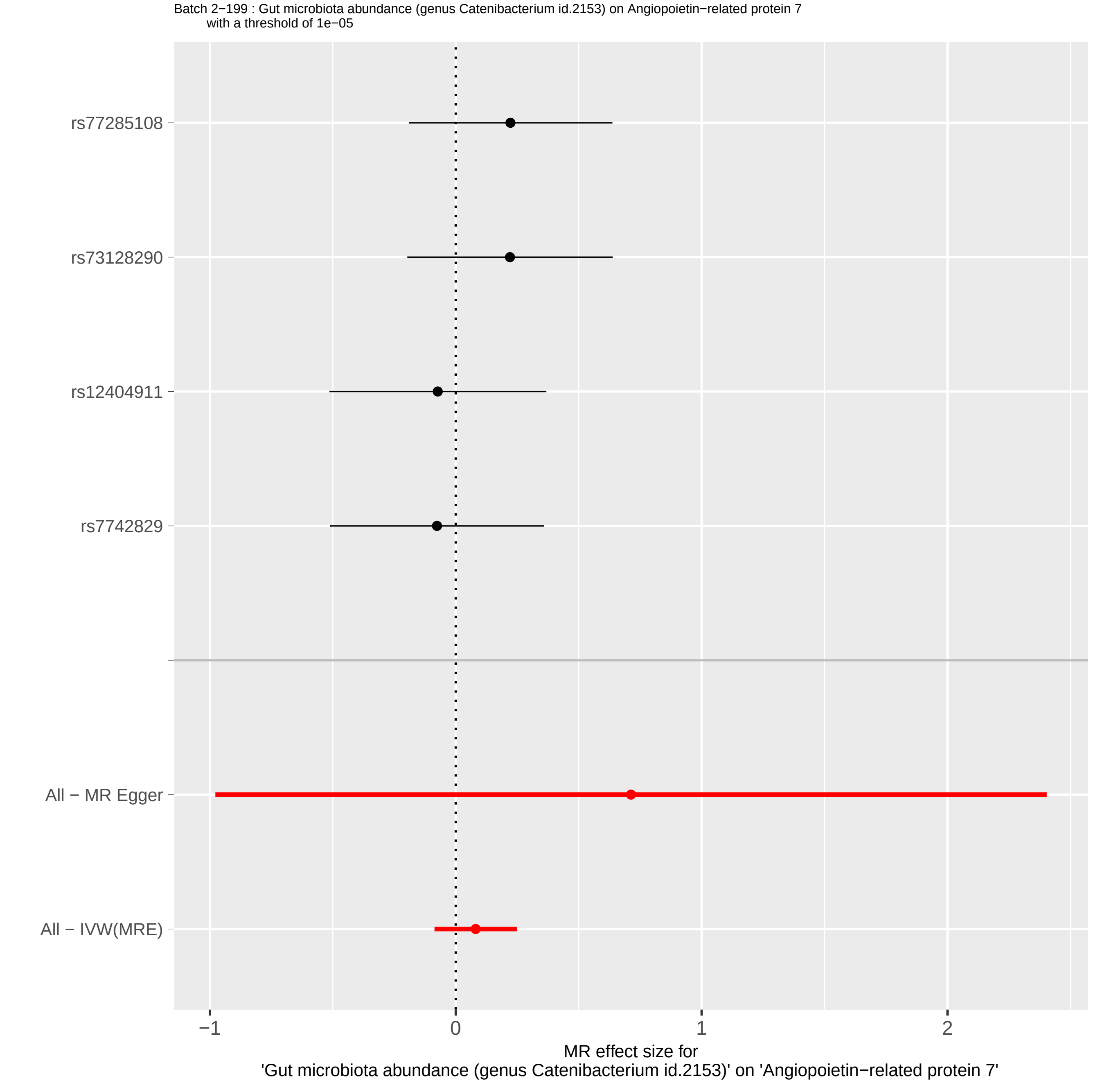

Batch 2−199 : Gut microbiota abundance (genus Catenibacterium id.2153) on Angiopoietin−related protein 7
with a threshold of 1e−05
rs77285108
rs73128290
rs12404911
rs7742829
All − MR Egger
All − IVW(MRE)
−1
0
1
2
MR effect size for
'Gut microbiota abundance (genus Catenibacterium id.2153)' on 'Angiopoietin−related protein 7'

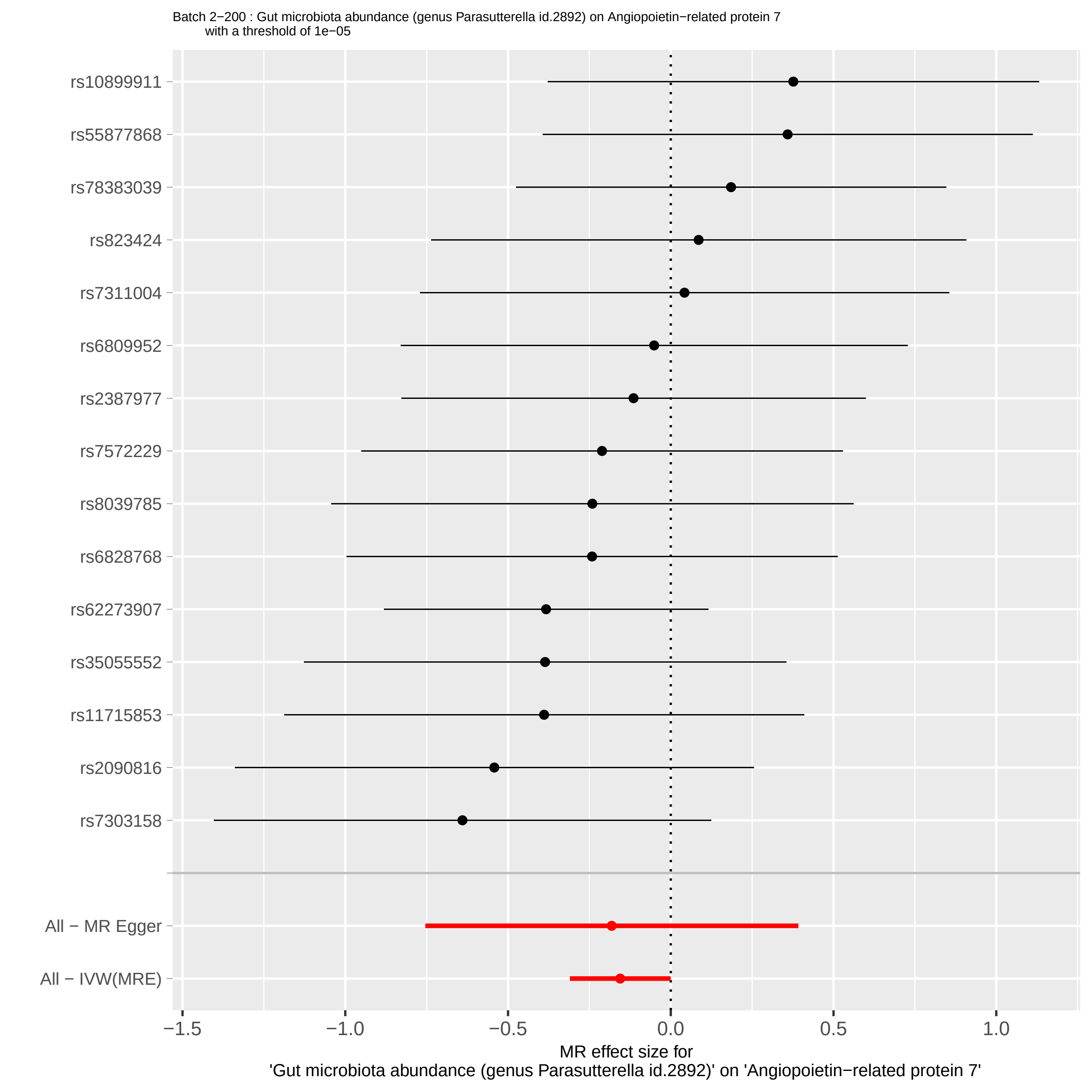
Batch 2−200 : Gut microbiota abundance (genus Parasutterella id.2892) on Angiopoietin−related protein 7
with a threshold of 1e−05
rs10899911
rs55877868
rs78383039
rs823424
rs7311004
rs6809952
rs2387977
rs7572229
rs8039785
rs6828768
rs62273907
rs35055552
rs11715853
rs2090816
rs7303158
All − MR Egger
All − IVW(MRE)
−1.5
−1.0
−0.5
0.0
0.5
1.0
MR effect size for
'Gut microbiota abundance (genus Parasutterella id.2892)' on 'Angiopoietin−related protein 7'

Batch 2−201 : Gut microbiota abundance (genus Catenibacterium id.2153) on Ankyrin−2
with a threshold of 1e−05

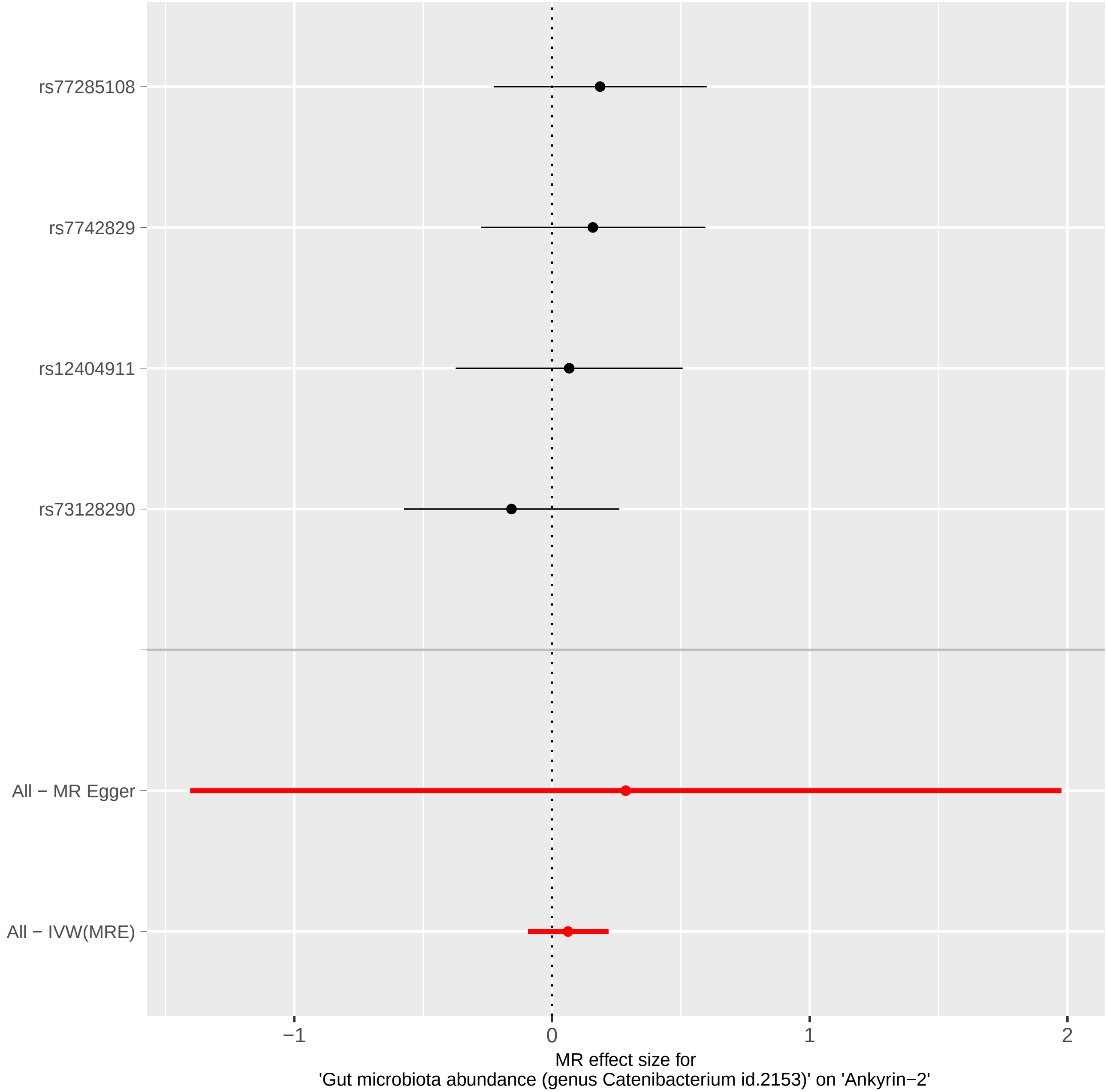

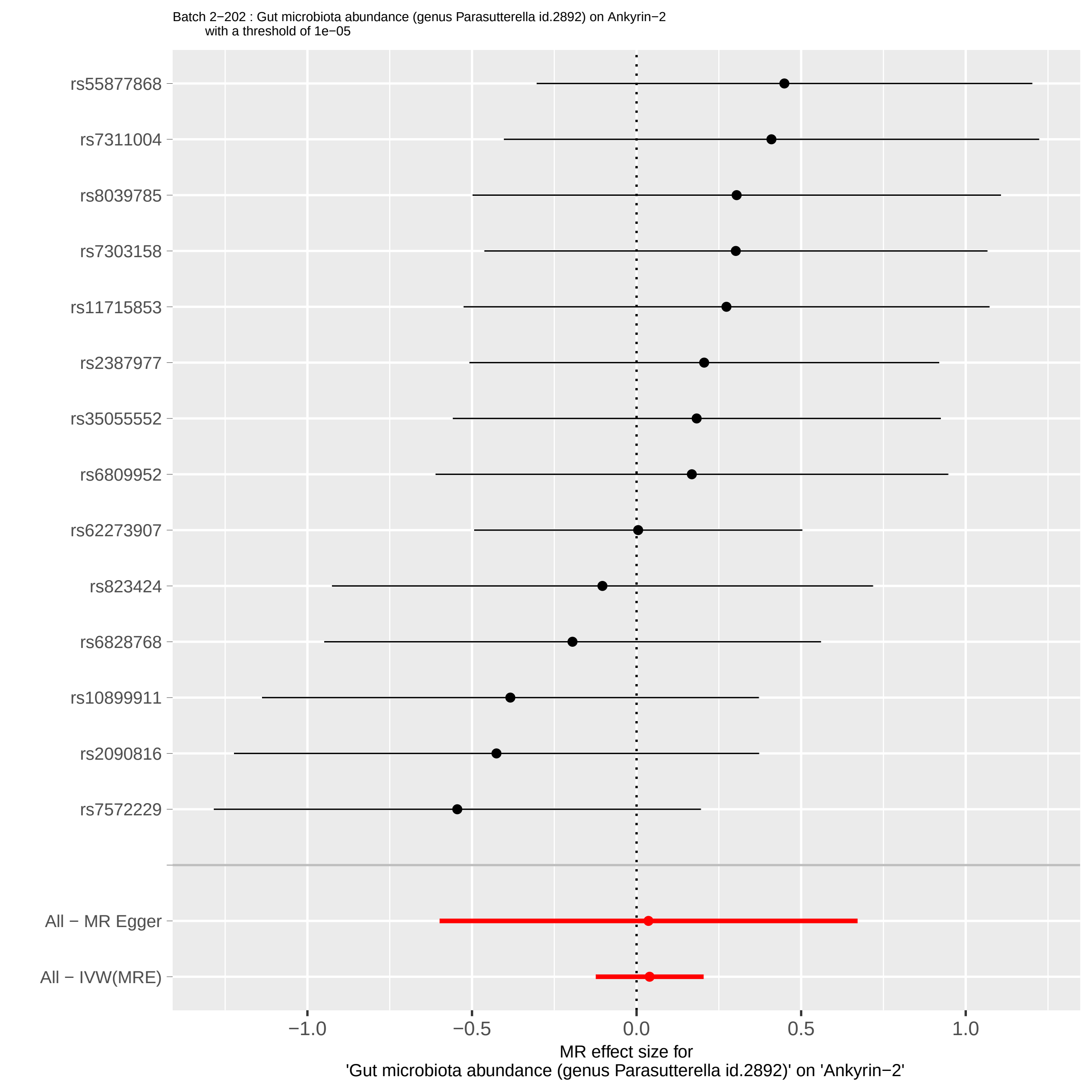

Batch 2−202 : Gut microbiota abundance (genus Parasutterella id.2892) on Ankyrin−2
with a threshold of 1e−05
rs55877868
rs7311004
rs8039785
rs7303158
rs11715853
rs2387977
rs35055552
rs6809952
rs62273907
rs823424
rs6828768
rs10899911
rs2090816
rs7572229
All − MR Egger
All − IVW(MRE)
−1.0
−0.5
0.0
0.5
1.0
MR effect size for
'Gut microbiota abundance (genus Parasutterella id.2892)' on 'Ankyrin−2'

Batch 2−203 : Gut microbiota abundance (genus Catenibacterium id.2153) on Ankyrin repeat domain−containing protein 27
with a threshold of 1e−05

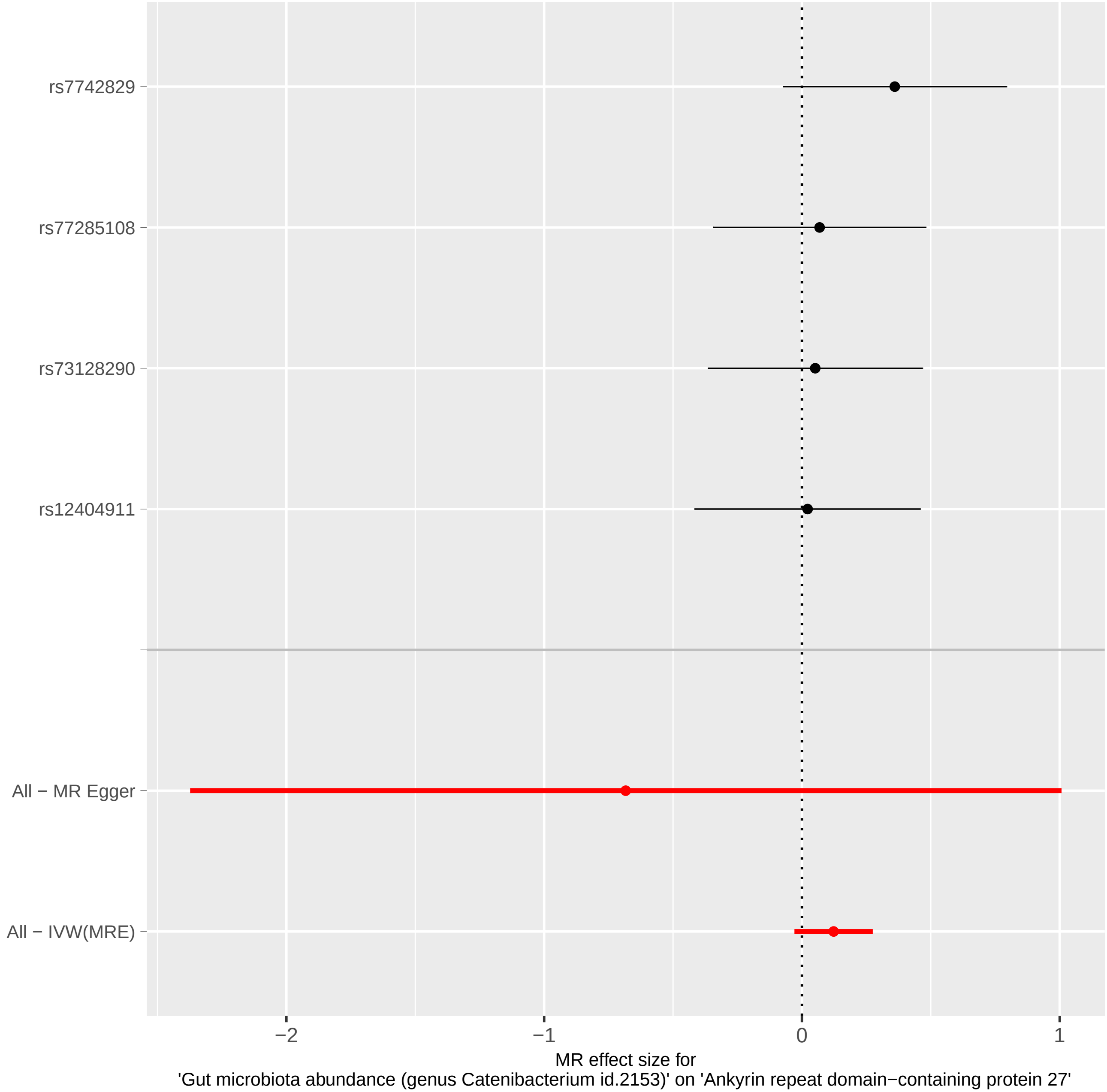

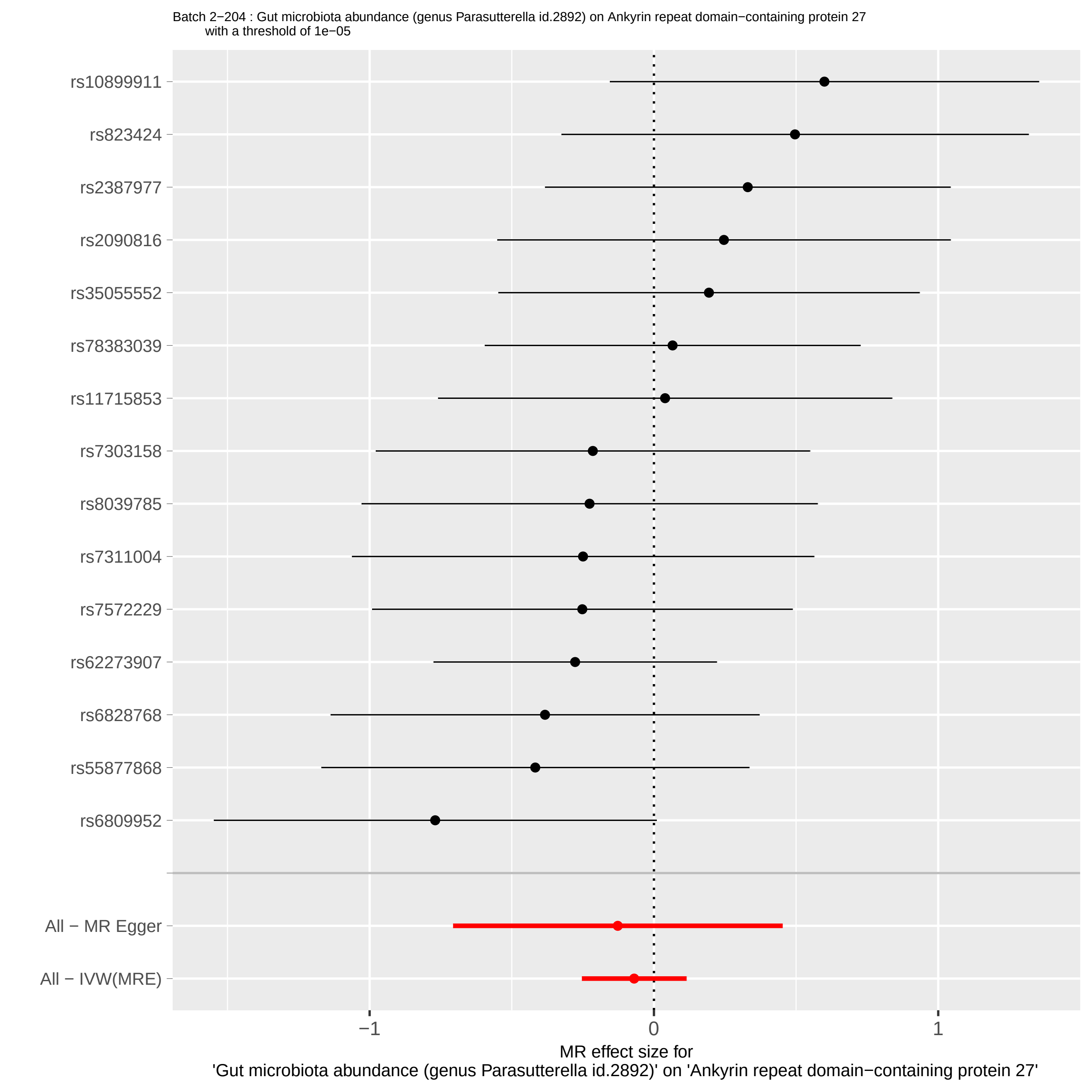

Batch 2−204 : Gut microbiota abundance (genus Parasutterella id.2892) on Ankyrin repeat domain−containing protein 27
with a threshold of 1e−05
rs10899911
rs823424
rs2387977
rs2090816
rs35055552
rs78383039
rs11715853
rs7303158
rs8039785
rs7311004
rs7572229
rs62273907
rs6828768
rs55877868
rs6809952
All − MR Egger
All − IVW(MRE)
−1
0
1
MR effect size for
'Gut microbiota abundance (genus Parasutterella id.2892)' on 'Ankyrin repeat domain−containing protein 27'

Batch 2−205 : Gut microbiota abundance (genus Catenibacterium id.2153) on Ankyrin repeat domain−containing protein 46
with a threshold of 1e−05

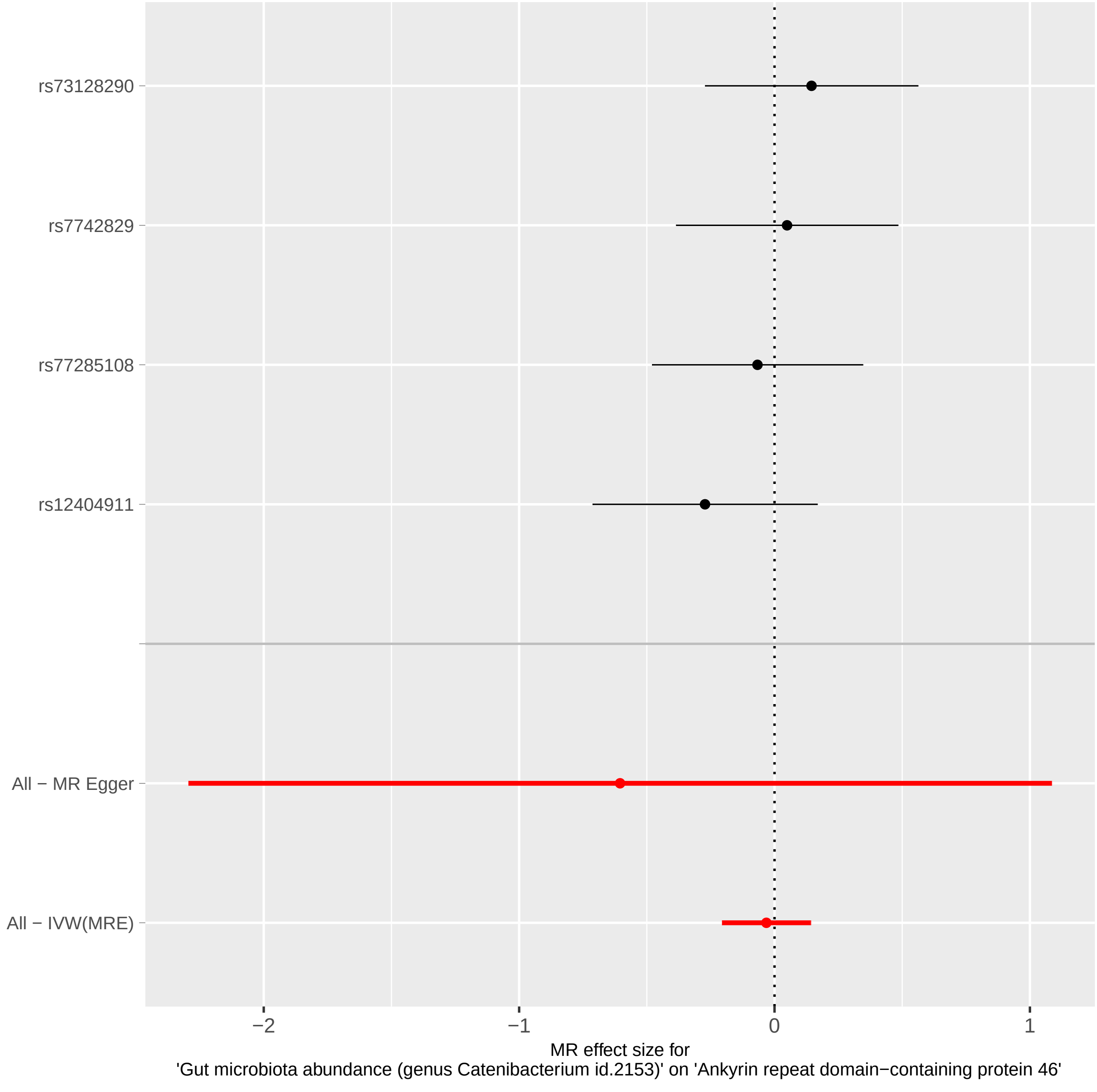

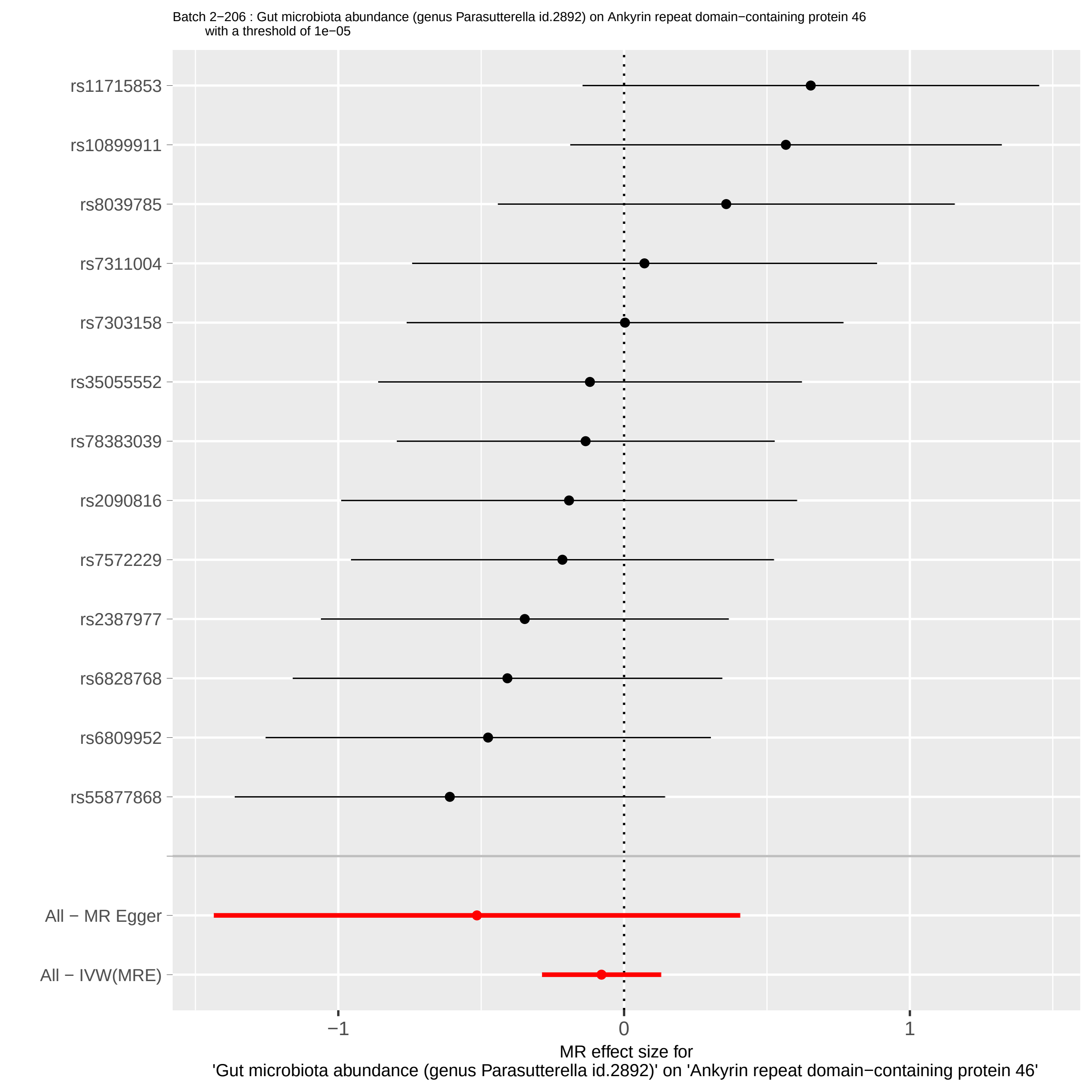

Batch 2−206 : Gut microbiota abundance (genus Parasutterella id.2892) on Ankyrin repeat domain−containing protein 46
with a threshold of 1e−05
rs11715853
rs10899911
rs8039785
rs7311004
rs7303158
rs35055552
rs78383039
rs2090816
rs7572229
rs2387977
rs6828768
rs6809952
rs55877868
All − MR Egger
All − IVW(MRE)
−1
0
1
MR effect size for
'Gut microbiota abundance (genus Parasutterella id.2892)' on 'Ankyrin repeat domain−containing protein 46'

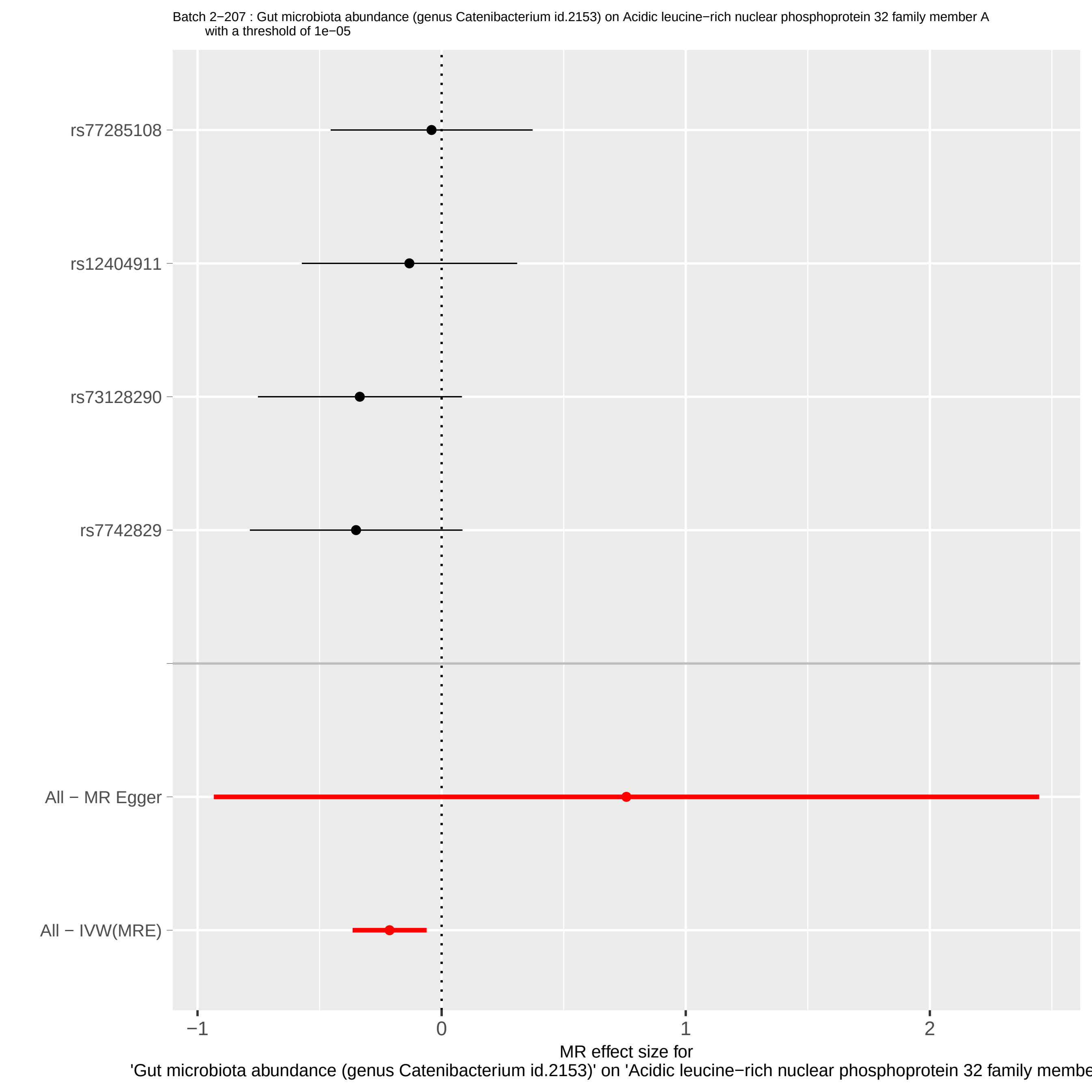
Batch 2−207 : Gut microbiota abundance (genus Catenibacterium id.2153) on Acidic leucine−rich nuclear phosphoprotein 32 family member A
with a threshold of 1e−05
rs77285108
rs12404911
rs73128290
rs7742829
All − MR Egger
All − IVW(MRE)
−1
0
1
2
MR effect size for
'Gut microbiota abundance (genus Catenibacterium id.2153)' on 'Acidic leucine−rich nuclear phosphoprotein 32 family membe

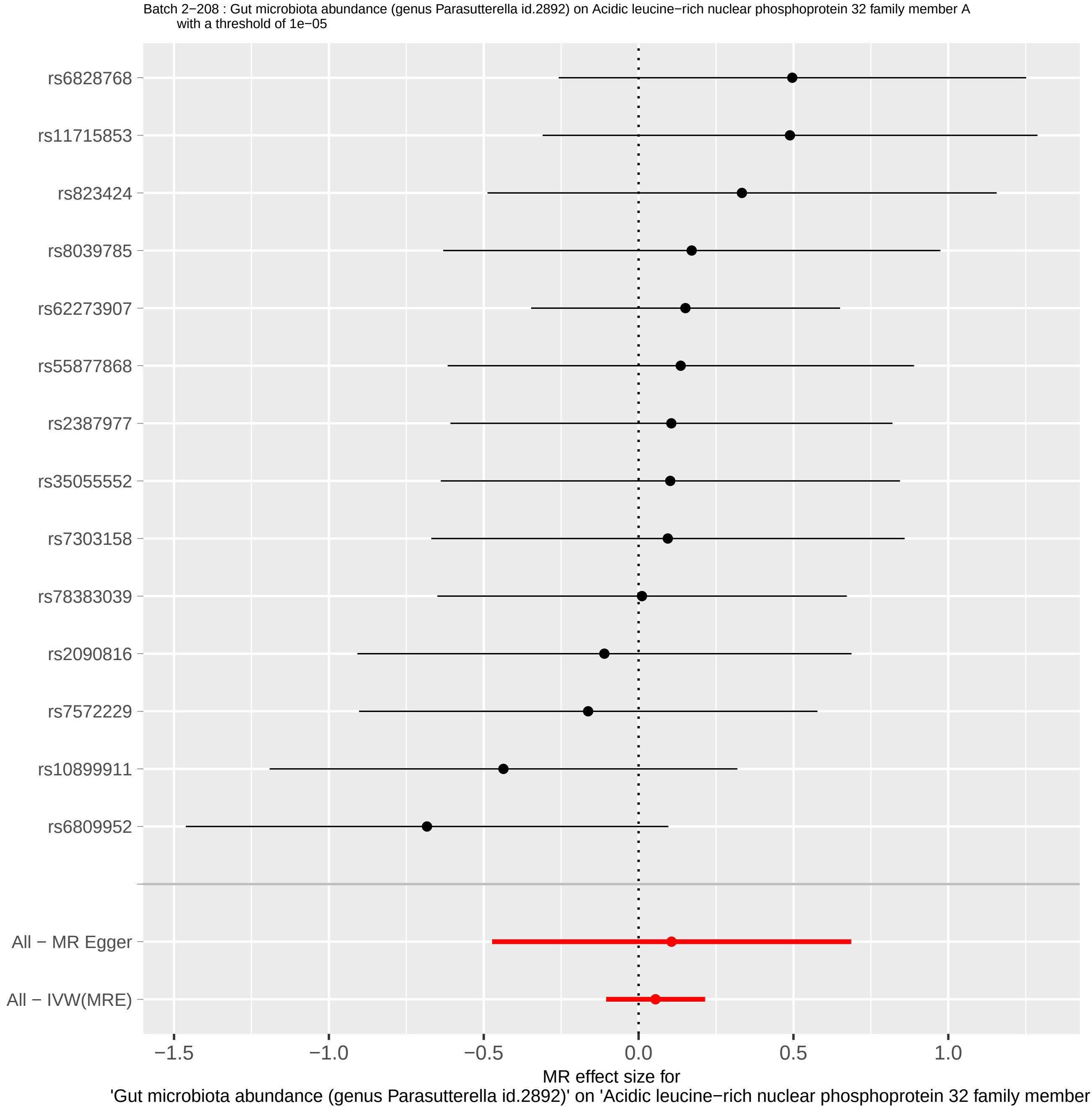

Batch 2−208 : Gut microbiota abundance (genus Parasutterella id.2892) on Acidic leucine−rich nuclear phosphoprotein 32 family member A with a threshold of 1e−05
rs6828768
rs11715853
rs823424
rs8039785
rs62273907
rs55877868
rs2387977
rs35055552
rs7303158
rs78383039
rs2090816
rs7572229
rs10899911
rs6809952
All − MR Egger
All − IVW(MRE)
−1.5
−1.0
−0.5
0.0
0.5
1.0
MR effect size for
'Gut microbiota abundance (genus Parasutterella id.2892)' on 'Acidic leucine−rich nuclear phosphoprotein 32 family member

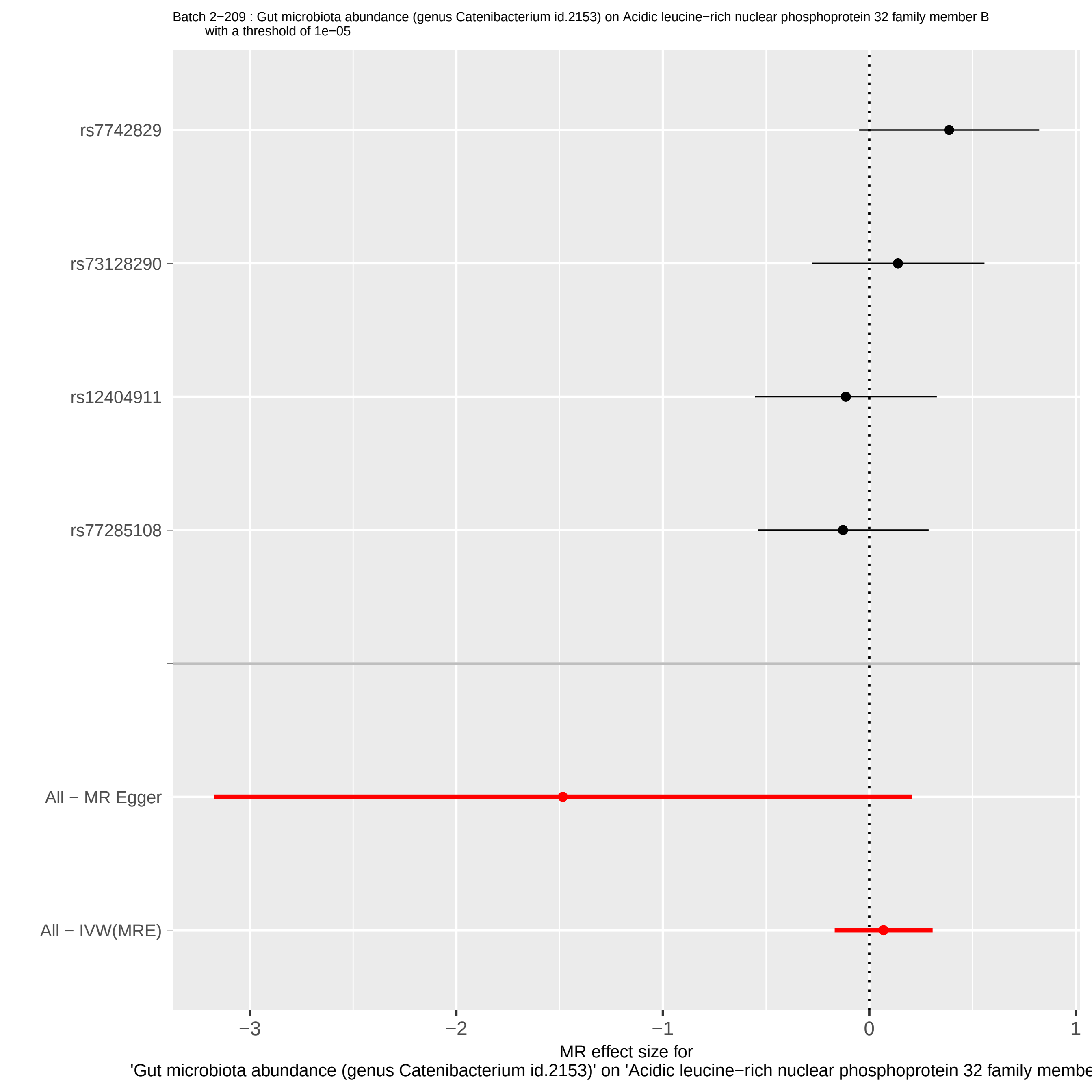
Batch 2−209 : Gut microbiota abundance (genus Catenibacterium id.2153) on Acidic leucine−rich nuclear phosphoprotein 32 family member B
with a threshold of 1e−05
rs7742829
rs73128290
rs12404911
rs77285108
All − MR Egger
All − IVW(MRE)
−3
−2
−1
0
1
MR effect size for
'Gut microbiota abundance (genus Catenibacterium id.2153)' on 'Acidic leucine−rich nuclear phosphoprotein 32 family membe

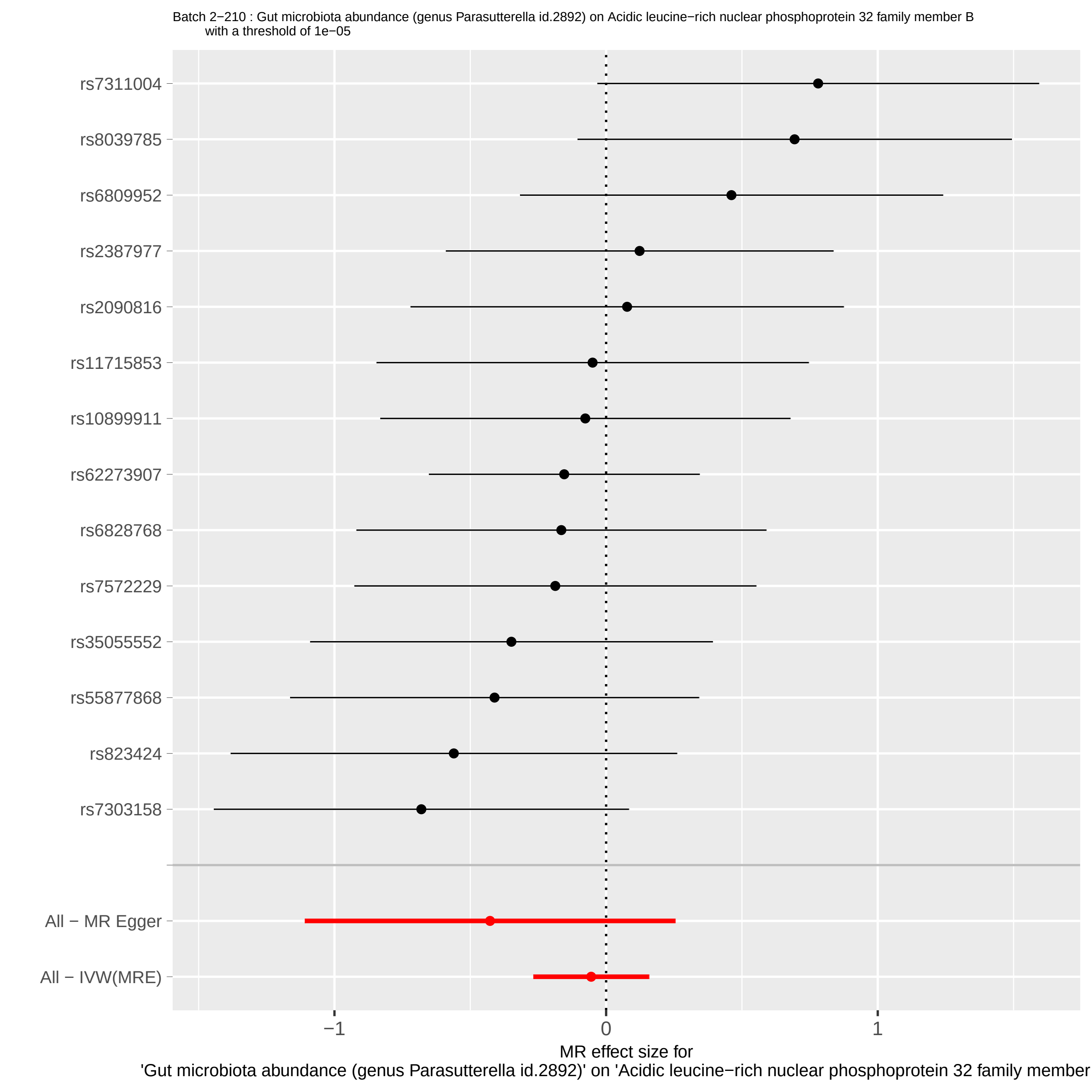

Batch 2−210 : Gut microbiota abundance (genus Parasutterella id.2892) on Acidic leucine−rich nuclear phosphoprotein 32 family member B
with a threshold of 1e−05
rs7311004
rs8039785
rs6809952
rs2387977
rs2090816
rs11715853
rs10899911
rs62273907
rs6828768
rs7572229
rs35055552
rs55877868
rs823424
rs7303158
All − MR Egger
All − IVW(MRE)
−1
0
1
MR effect size for
'Gut microbiota abundance (genus Parasutterella id.2892)' on 'Acidic leucine−rich nuclear phosphoprotein 32 family member

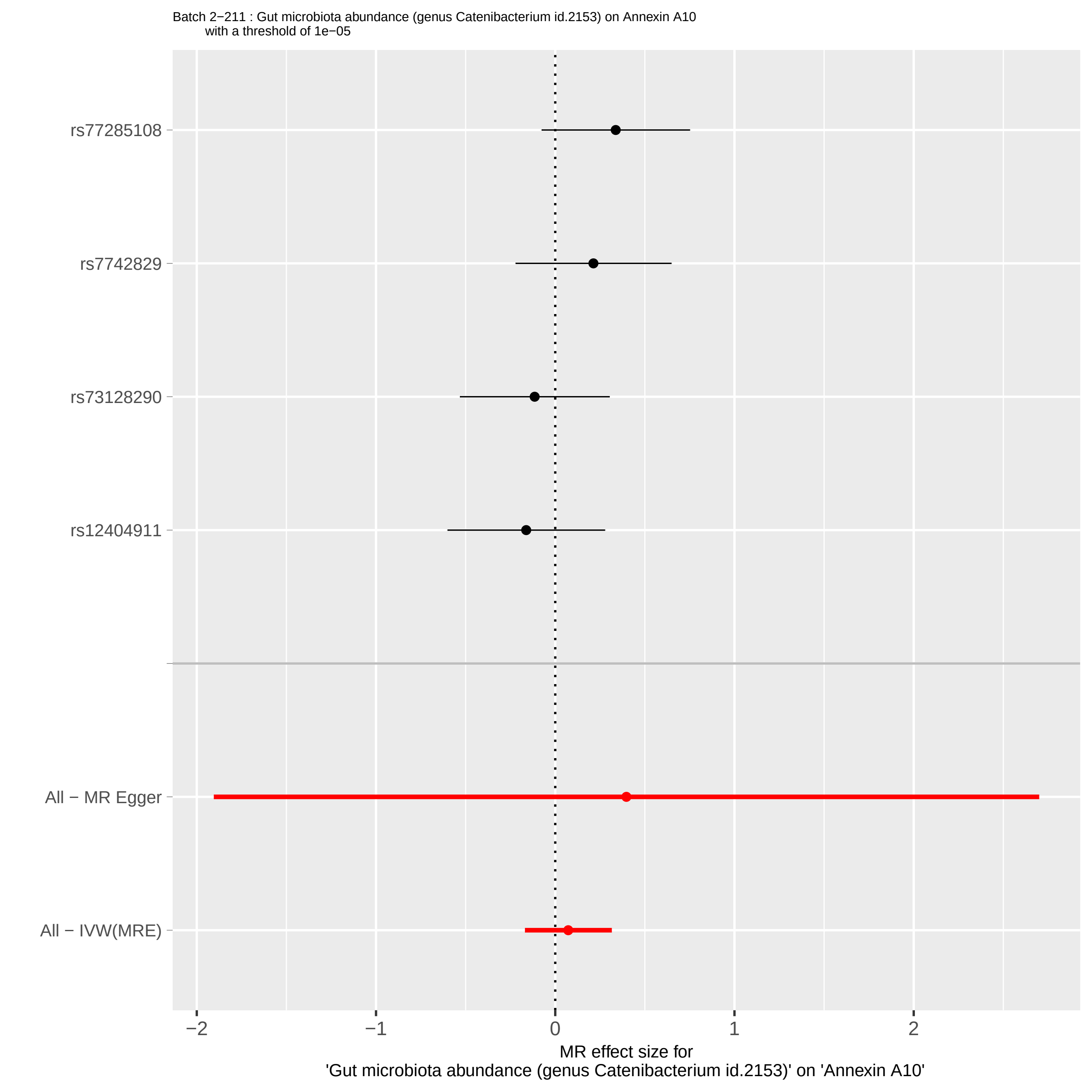
Batch 2−211 : Gut microbiota abundance (genus Catenibacterium id.2153) on Annexin A10
with a threshold of 1e−05
rs77285108
rs7742829
rs73128290
rs12404911
All − MR Egger
All − IVW(MRE)
−2
−1
0
1
2
MR effect size for
'Gut microbiota abundance (genus Catenibacterium id.2153)' on 'Annexin A10'

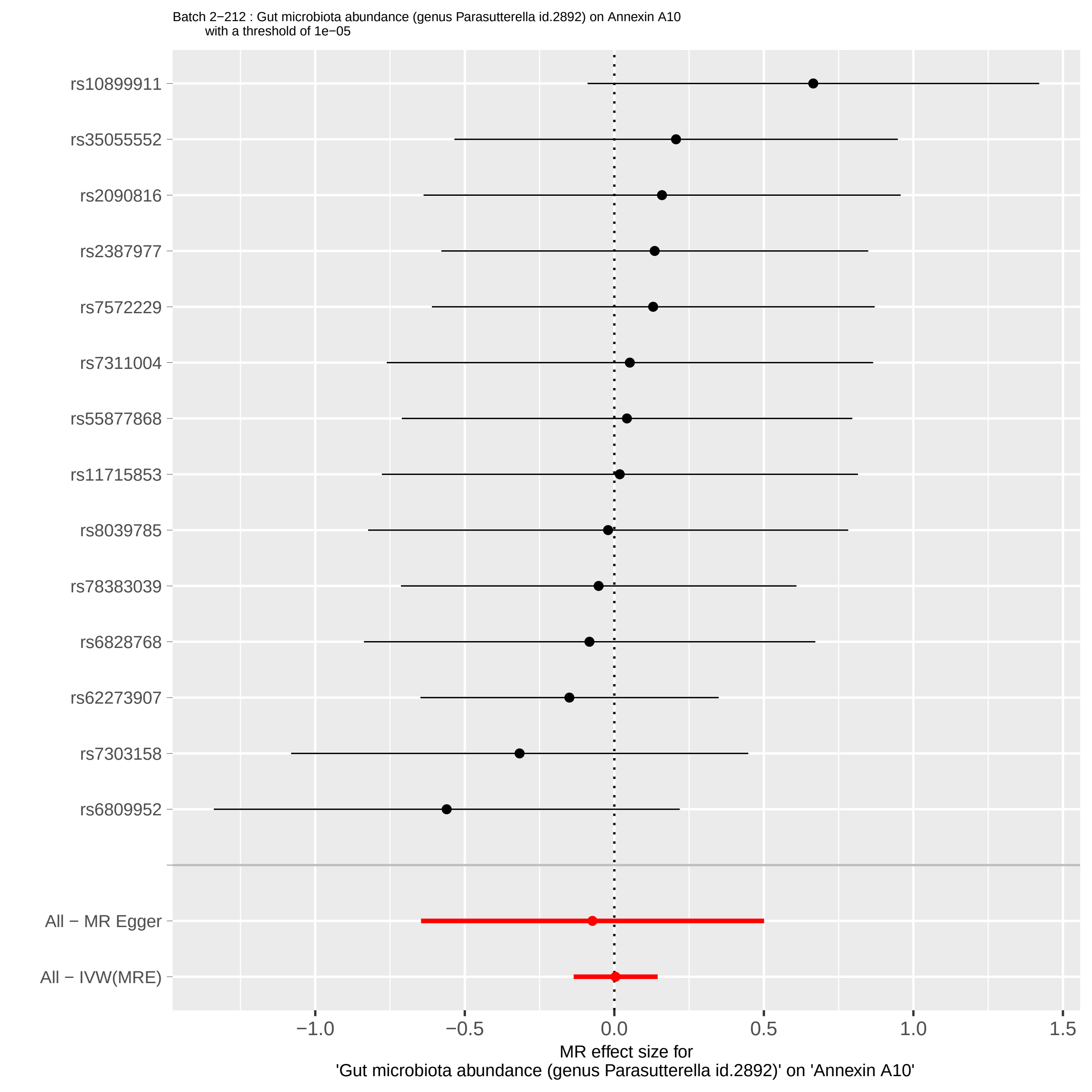
Batch 2−212 : Gut microbiota abundance (genus Parasutterella id.2892) on Annexin A10
with a threshold of 1e−05
rs10899911
rs35055552
rs2090816
rs2387977
rs7572229
rs7311004
rs55877868
rs11715853
rs8039785
rs78383039
rs6828768
rs62273907
rs7303158
rs6809952
All − MR Egger
All − IVW(MRE)
−1.0
−0.5
0.0
0.5
1.0
1.5
MR effect size for
'Gut microbiota abundance (genus Parasutterella id.2892)' on 'Annexin A10'

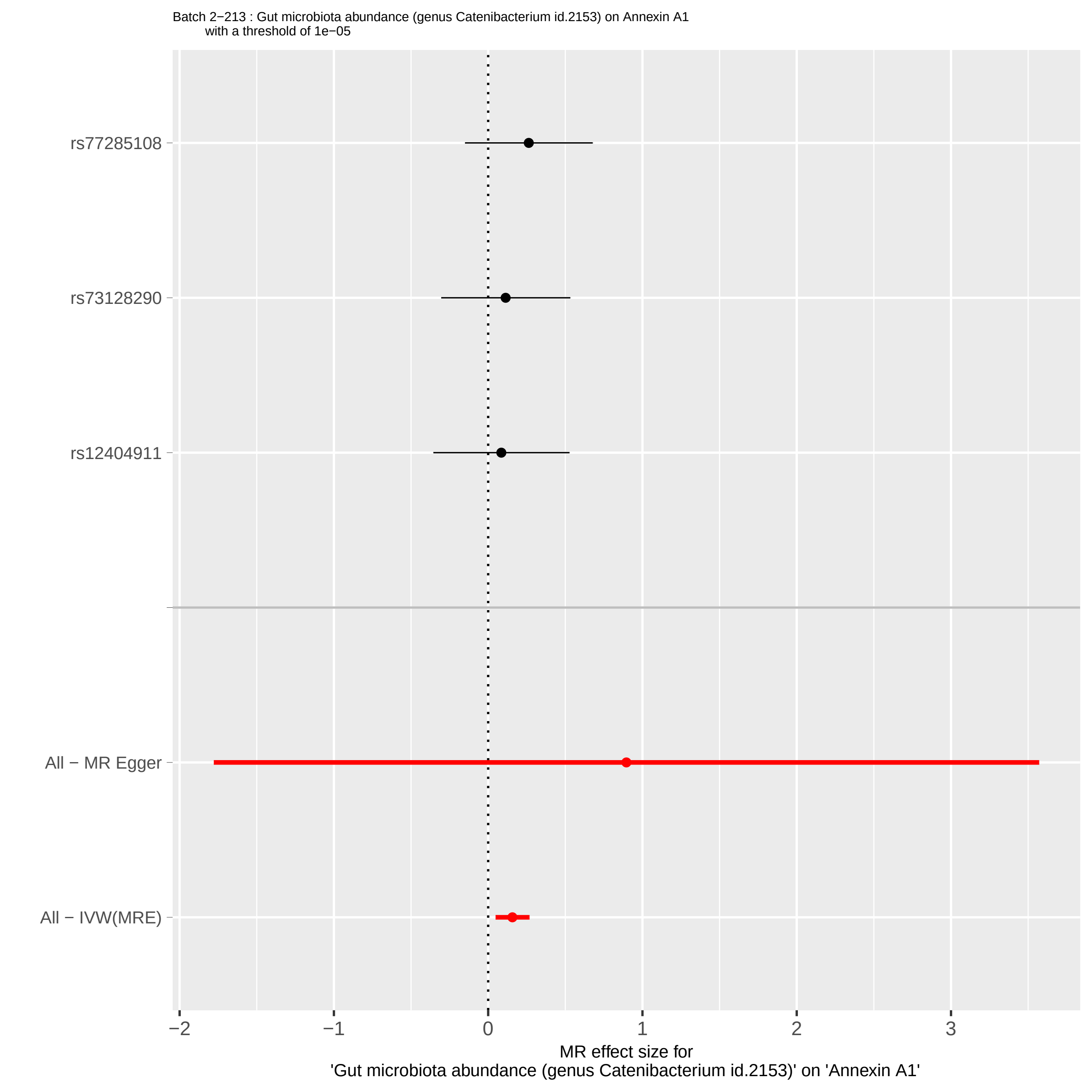

Batch 2−213 : Gut microbiota abundance (genus Catenibacterium id.2153) on Annexin A1
with a threshold of 1e−05
rs77285108
rs73128290
rs12404911
All − MR Egger
All − IVW(MRE)
−2
−1
0
1
2
3
MR effect size for
'Gut microbiota abundance (genus Catenibacterium id.2153)' on 'Annexin A1'

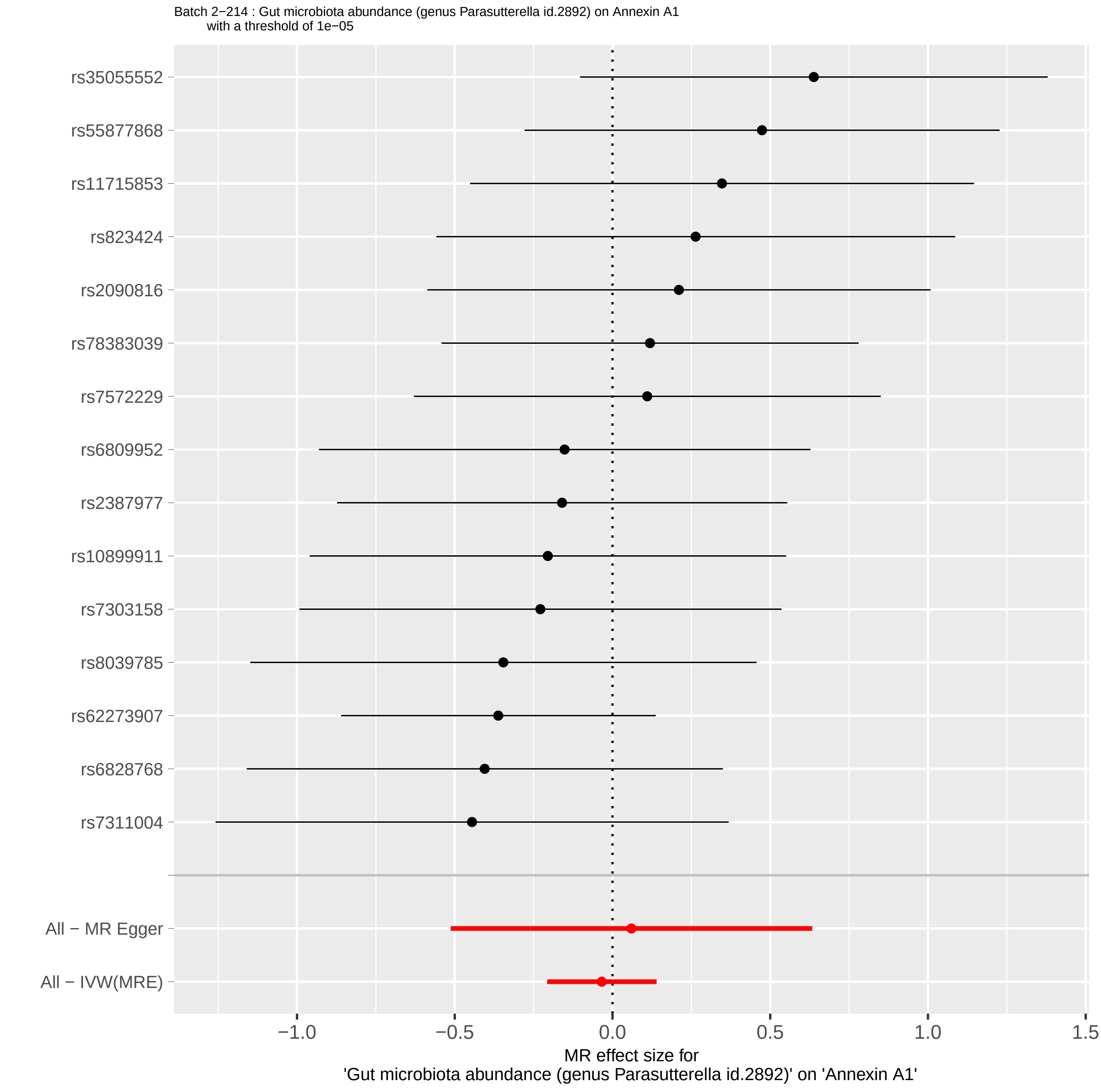
Batch 2−214 : Gut microbiota abundance (genus Parasutterella id.2892) on Annexin A1
with a threshold of 1e−05
rs35055552
rs55877868
rs11715853
rs823424
rs2090816
rs78383039
rs7572229
rs6809952
rs2387977
rs10899911
rs7303158
rs8039785
rs62273907
rs6828768
rs7311004
All − MR Egger
All − IVW(MRE)
−1.0
−0.5
0.0
0.5
1.0
1.5
MR effect size for
'Gut microbiota abundance (genus Parasutterella id.2892)' on 'Annexin A1'

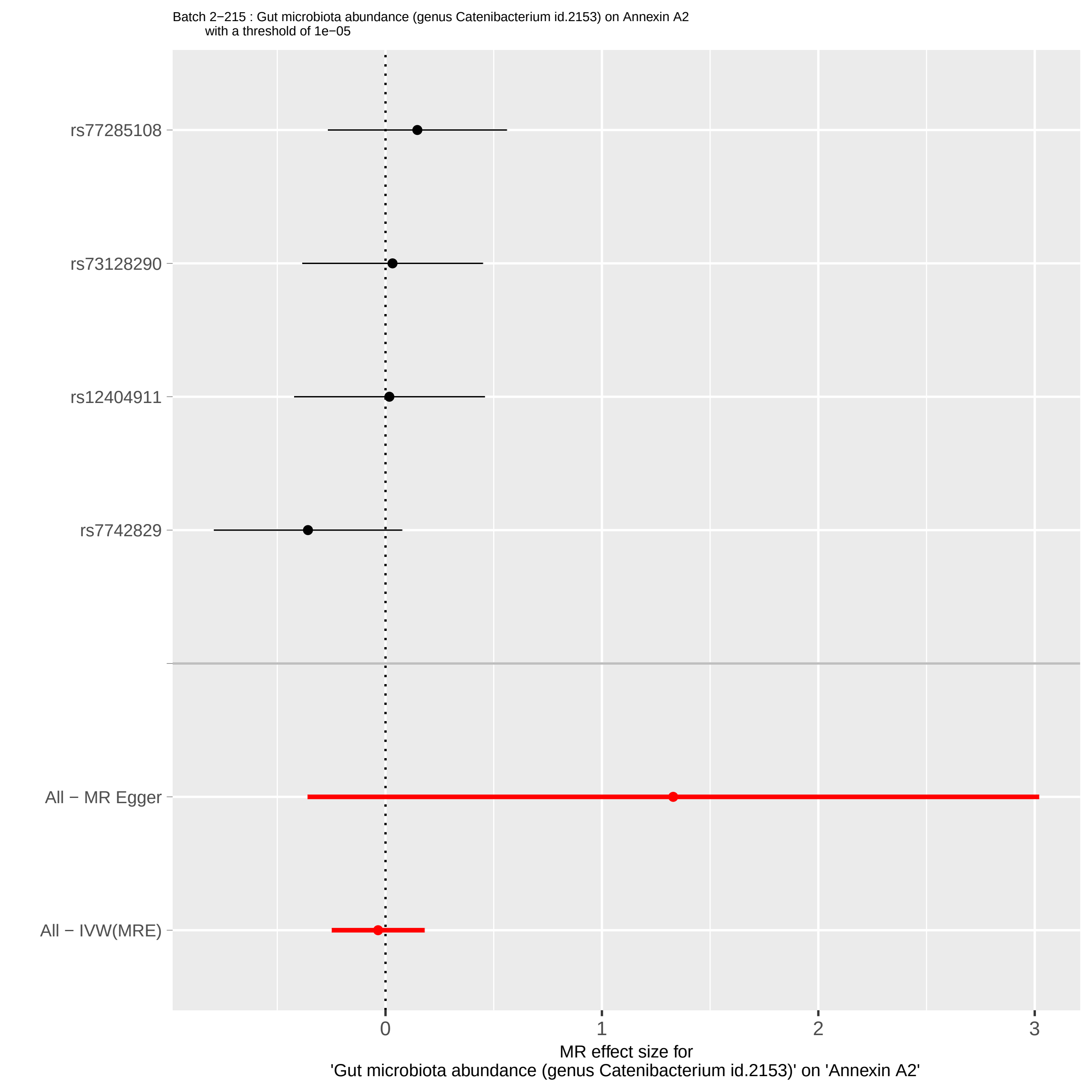

Batch 2−215 : Gut microbiota abundance (genus Catenibacterium id.2153) on Annexin A2
with a threshold of 1e−05
rs77285108
rs73128290
rs12404911
rs7742829
All − MR Egger
All − IVW(MRE)
0
1
2
3
MR effect size for
'Gut microbiota abundance (genus Catenibacterium id.2153)' on 'Annexin A2'

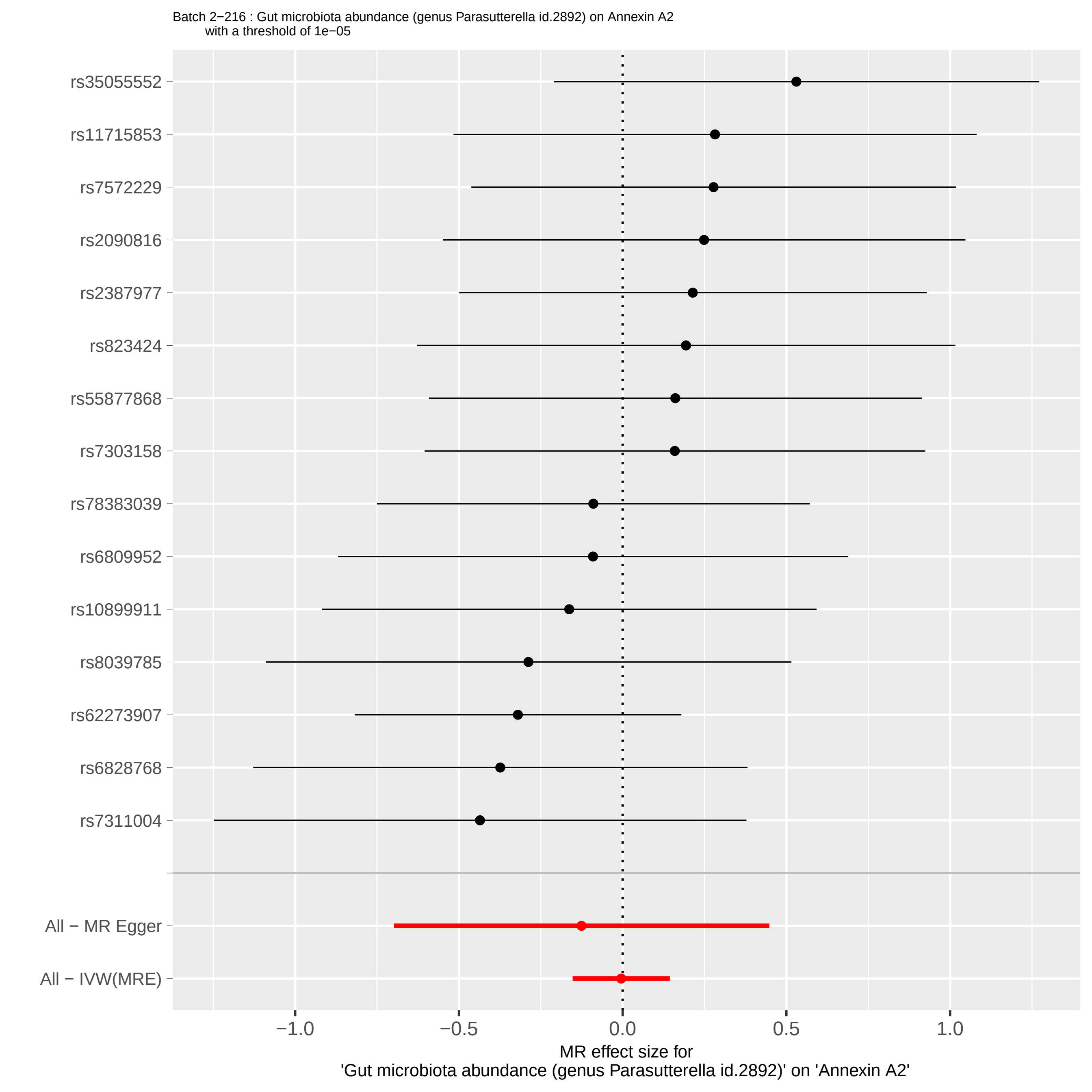
Batch 2−216 : Gut microbiota abundance (genus Parasutterella id.2892) on Annexin A2
with a threshold of 1e−05
rs35055552
rs11715853
rs7572229
rs2090816
rs2387977
rs823424
rs55877868
rs7303158
rs78383039
rs6809952
rs10899911
rs8039785
rs62273907
rs6828768
rs7311004
All − MR Egger
All − IVW(MRE)
−1.0
−0.5
0.0
0.5
1.0
MR effect size for
'Gut microbiota abundance (genus Parasutterella id.2892)' on 'Annexin A2'

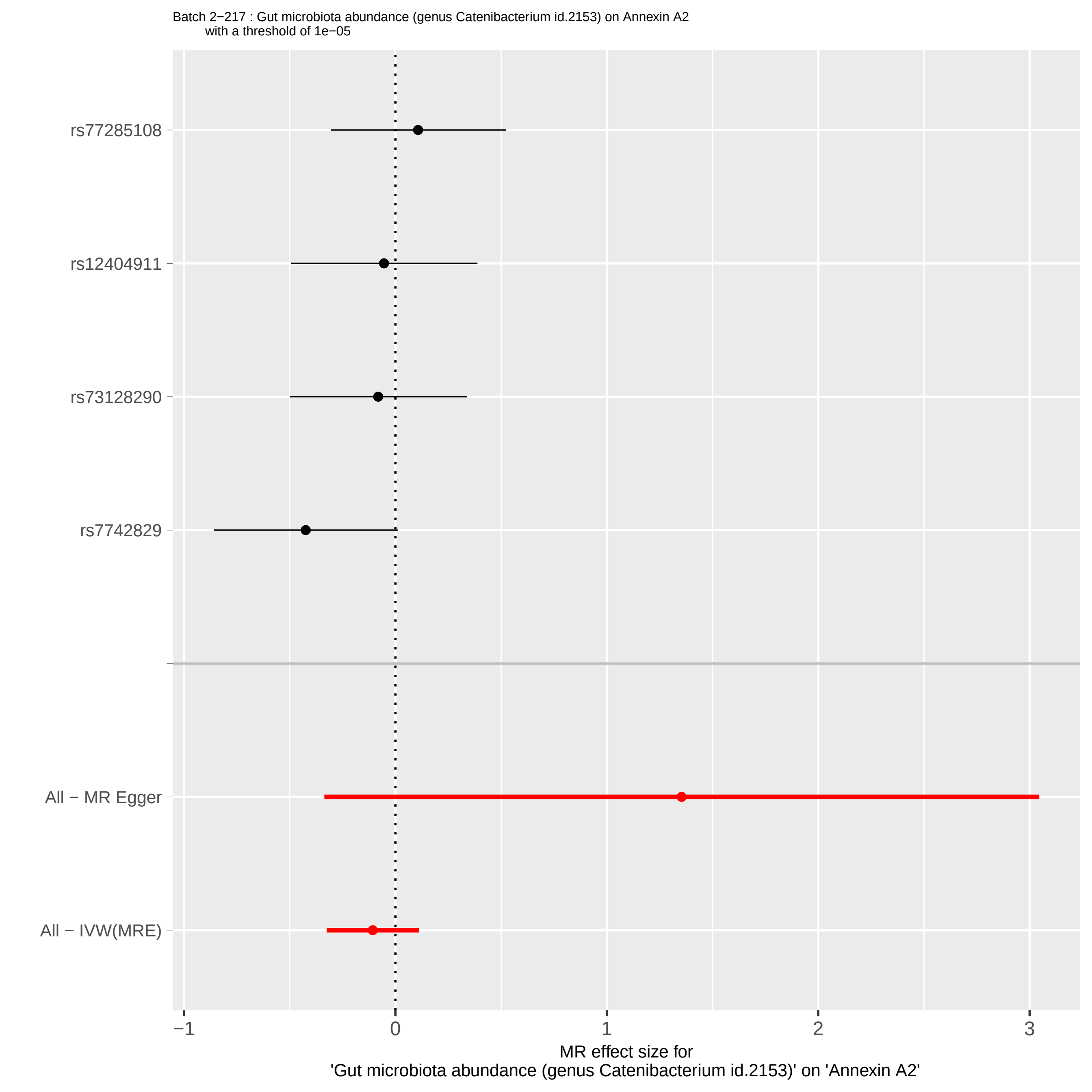

Batch 2−217 : Gut microbiota abundance (genus Catenibacterium id.2153) on Annexin A2
with a threshold of 1e−05
rs77285108
rs12404911
rs73128290
rs7742829
All − MR Egger
All − IVW(MRE)
−1
0
1
2
3
MR effect size for
'Gut microbiota abundance (genus Catenibacterium id.2153)' on 'Annexin A2'

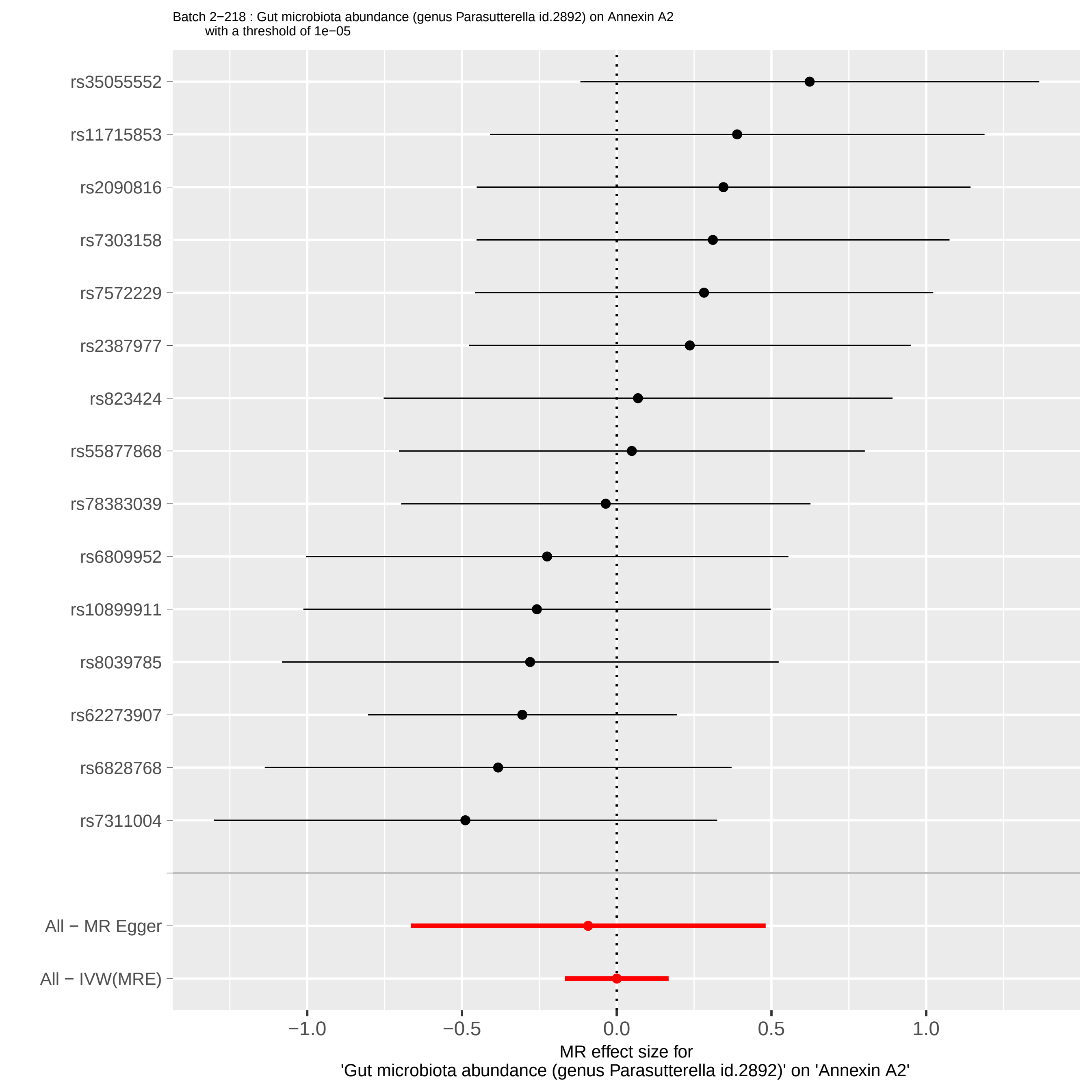
Batch 2−218 : Gut microbiota abundance (genus Parasutterella id.2892) on Annexin A2
with a threshold of 1e−05
rs35055552
rs11715853
rs2090816
rs7303158
rs7572229
rs2387977
rs823424
rs55877868
rs78383039
rs6809952
rs10899911
rs8039785
rs62273907
rs6828768
rs7311004
All − MR Egger
All − IVW(MRE)
−1.0
−0.5
0.0
0.5
1.0
MR effect size for
'Gut microbiota abundance (genus Parasutterella id.2892)' on 'Annexin A2'

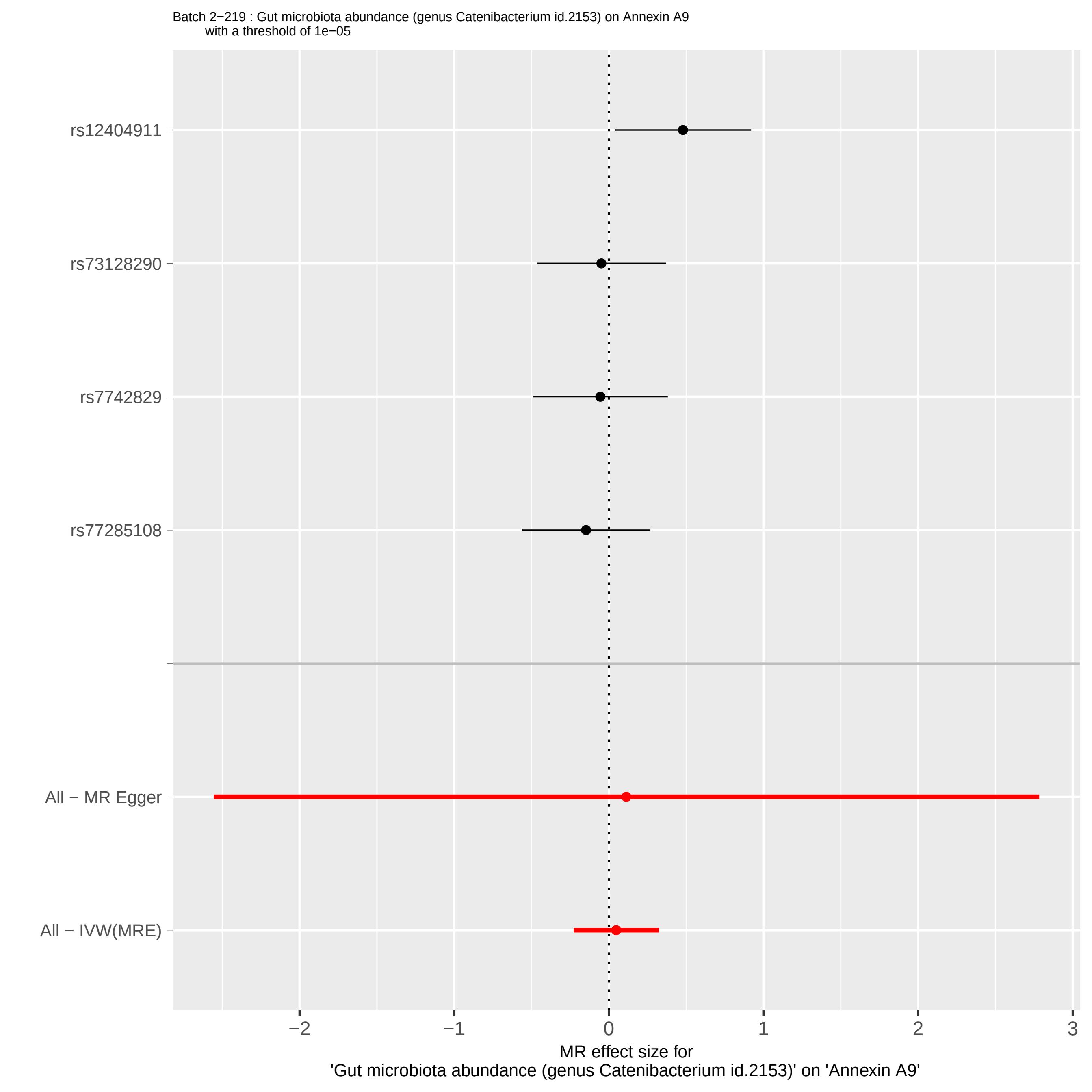

Batch 2−219 : Gut microbiota abundance (genus Catenibacterium id.2153) on Annexin A9
with a threshold of 1e−05
rs12404911
rs73128290
rs7742829
rs77285108
All − MR Egger
All − IVW(MRE)
−2
−1
0
1
2
3
MR effect size for
'Gut microbiota abundance (genus Catenibacterium id.2153)' on 'Annexin A9'

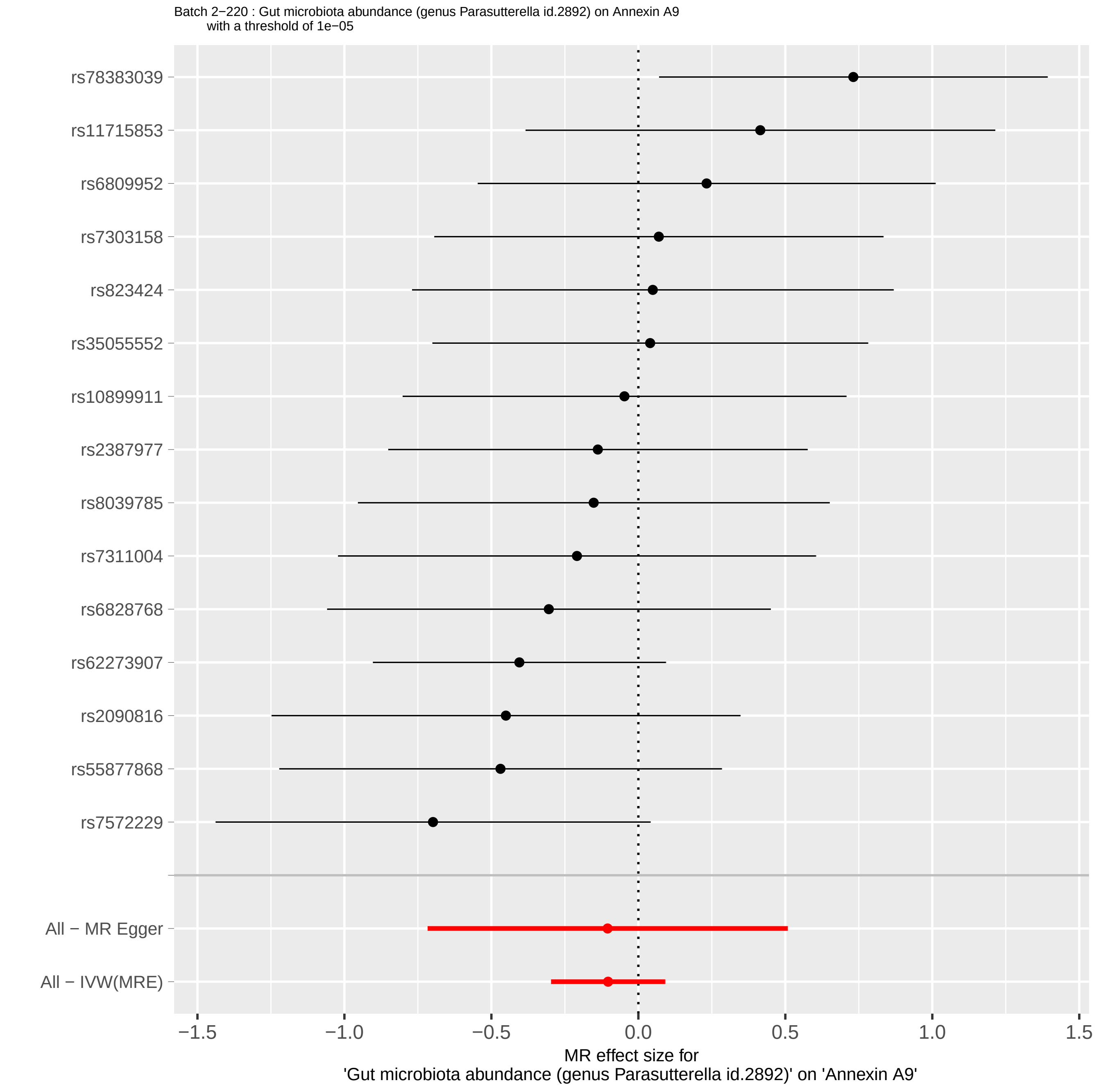

Batch 2−220 : Gut microbiota abundance (genus Parasutterella id.2892) on Annexin A9
with a threshold of 1e−05
rs78383039
rs11715853
rs6809952
rs7303158
rs823424
rs35055552
rs10899911
rs2387977
rs8039785
rs7311004
rs6828768
rs62273907
rs2090816
rs55877868
rs7572229
All − MR Egger
All − IVW(MRE)
−1.5
−1.0
−0.5
0.0
0.5
1.0
1.5
MR effect size for
'Gut microbiota abundance (genus Parasutterella id.2892)' on 'Annexin A9'

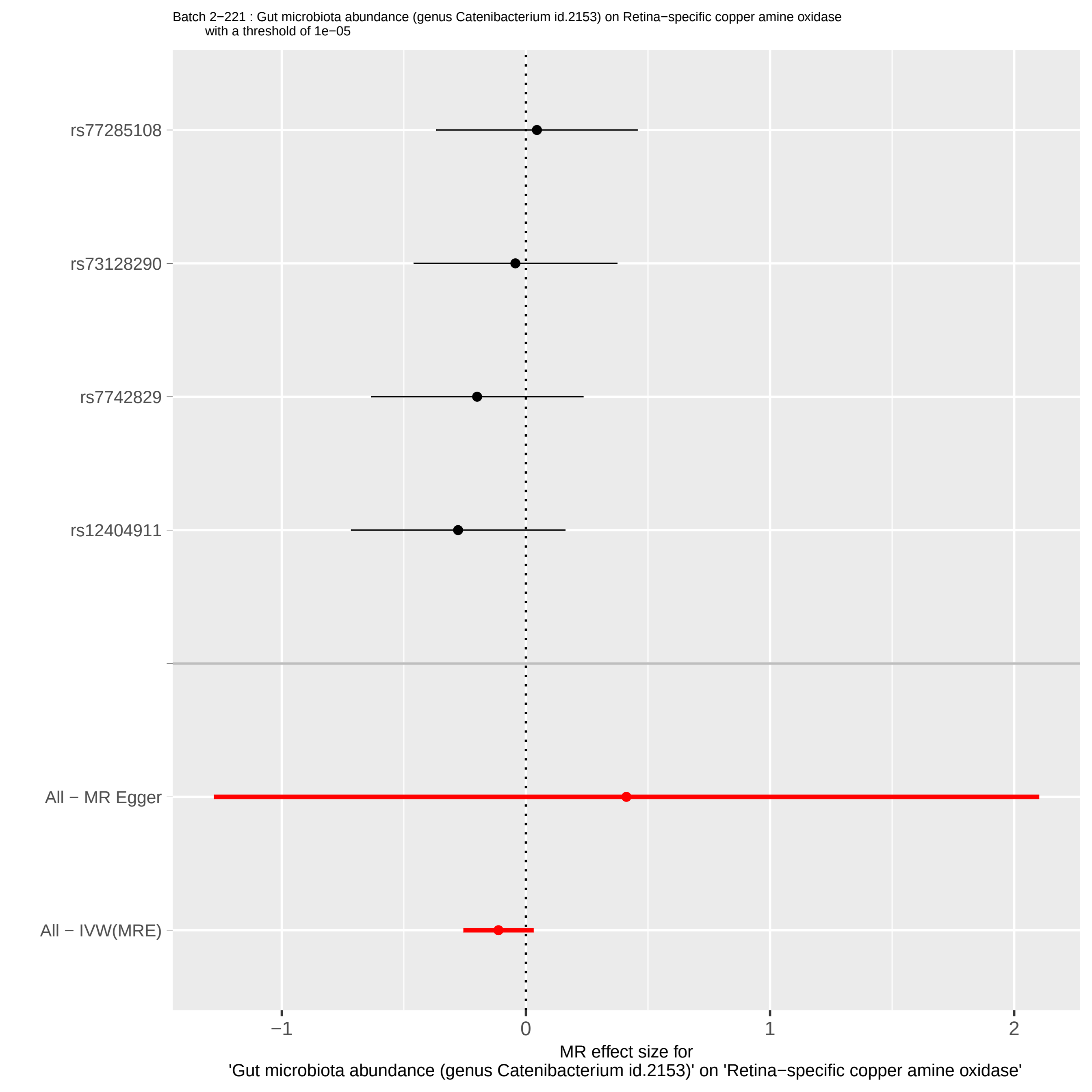

Batch 2−221 : Gut microbiota abundance (genus Catenibacterium id.2153) on Retina−specific copper amine oxidase
with a threshold of 1e−05
rs77285108
rs73128290
rs7742829
rs12404911
All − MR Egger
All − IVW(MRE)
−1
0
1
2
MR effect size for
'Gut microbiota abundance (genus Catenibacterium id.2153)' on 'Retina−specific copper amine oxidase'

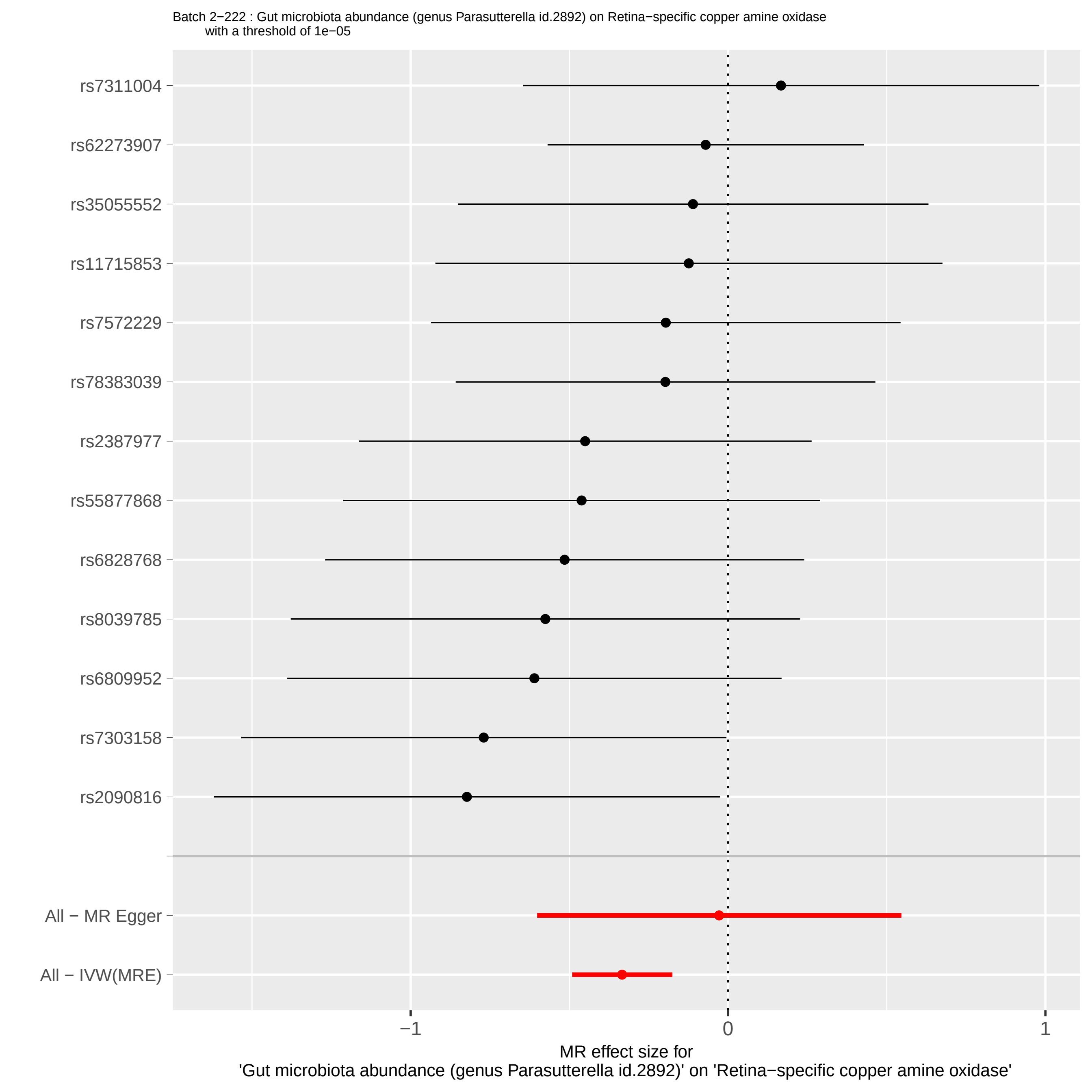

Batch 2−222 : Gut microbiota abundance (genus Parasutterella id.2892) on Retina−specific copper amine oxidase
with a threshold of 1e−05
rs7311004
rs62273907
rs35055552
rs11715853
rs7572229
rs78383039
rs2387977
rs55877868
rs6828768
rs8039785
rs6809952
rs7303158
rs2090816
All − MR Egger
All − IVW(MRE)
−1
0
1
MR effect size for
'Gut microbiota abundance (genus Parasutterella id.2892)' on 'Retina−specific copper amine oxidase'

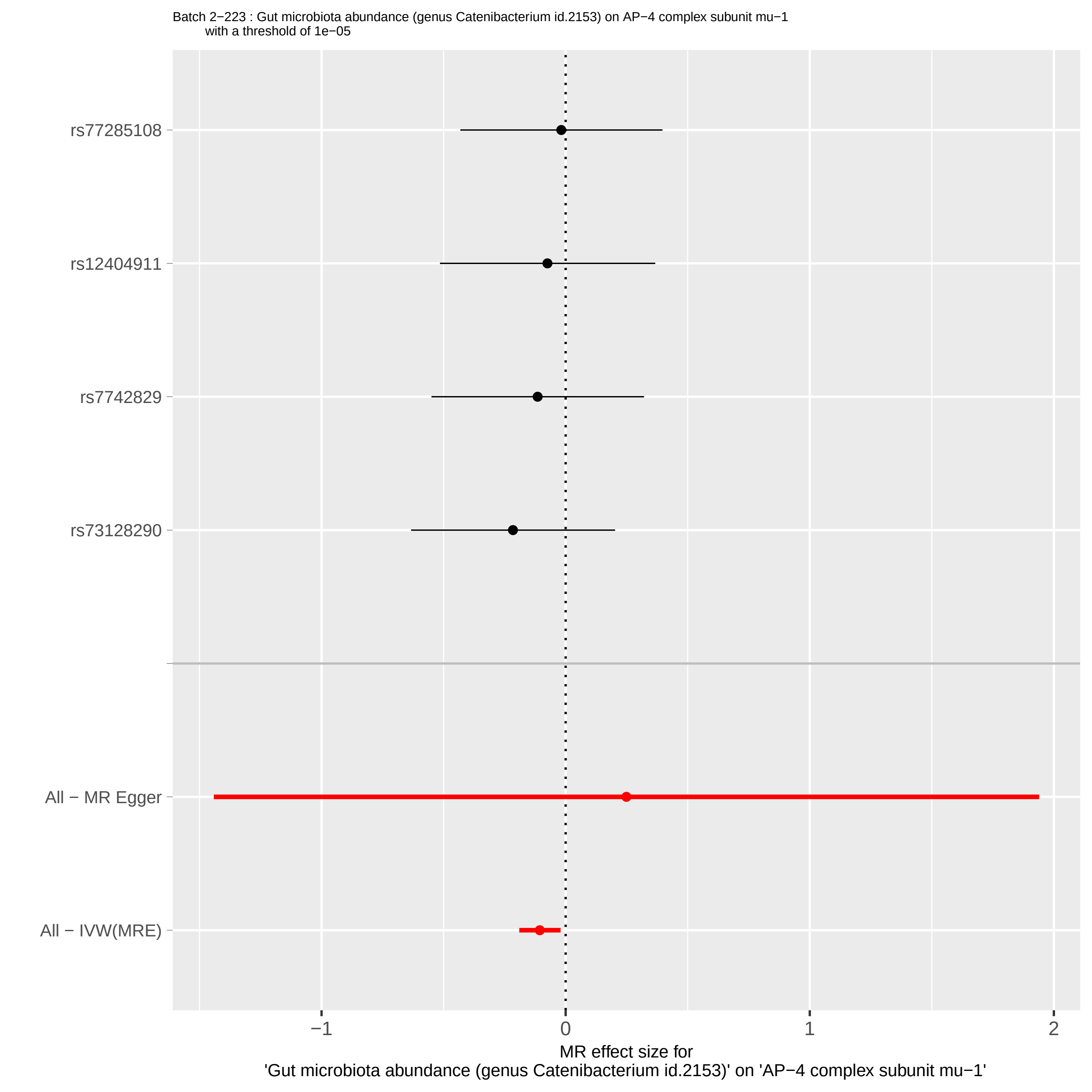
Batch 2−223 : Gut microbiota abundance (genus Catenibacterium id.2153) on AP−4 complex subunit mu−1
with a threshold of 1e−05
rs77285108
rs12404911
rs7742829
rs73128290
All − MR Egger
All − IVW(MRE)
−1
0
1
2
MR effect size for
'Gut microbiota abundance (genus Catenibacterium id.2153)' on 'AP−4 complex subunit mu−1'

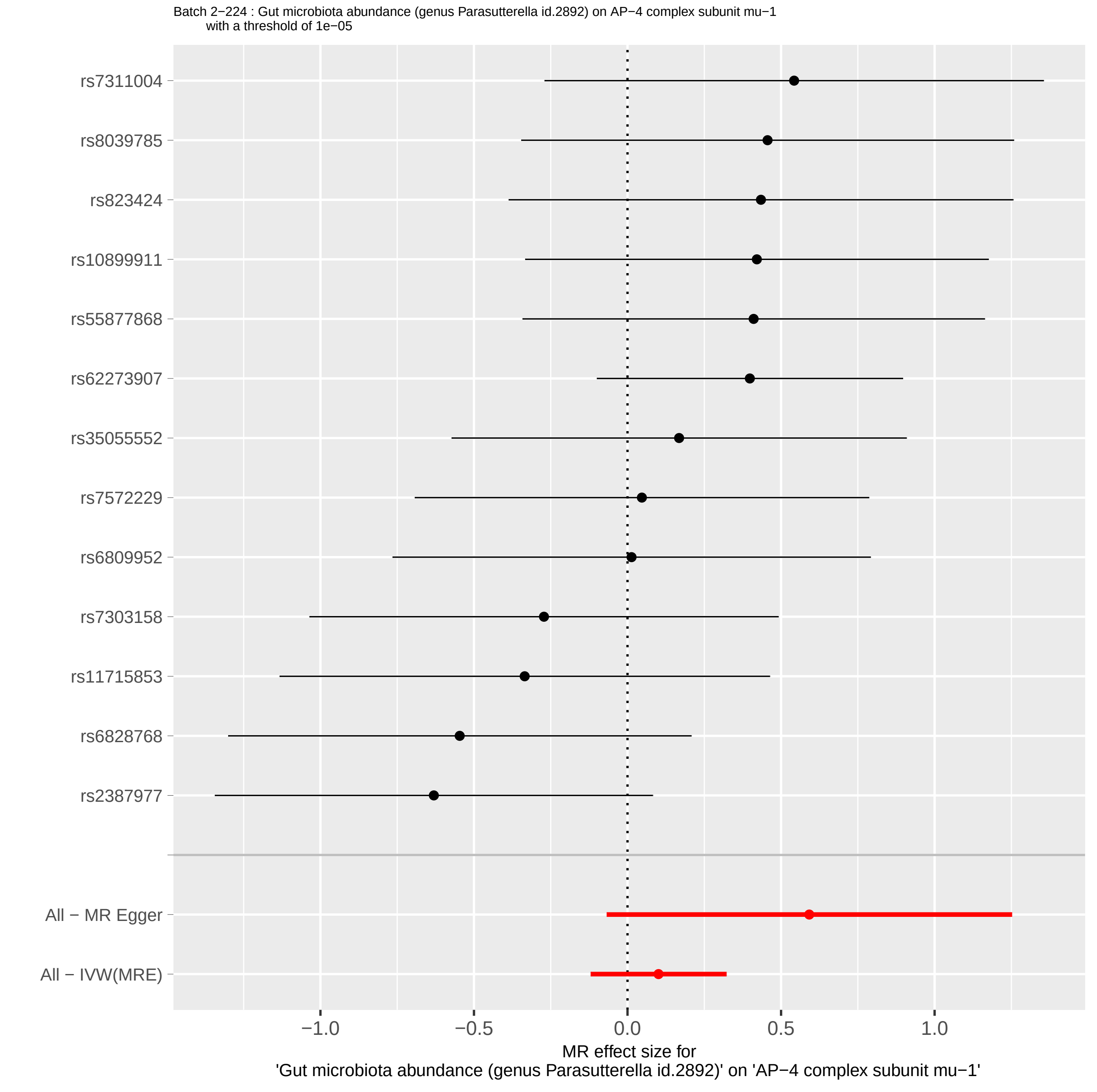

Batch 2−224 : Gut microbiota abundance (genus Parasutterella id.2892) on AP−4 complex subunit mu−1
with a threshold of 1e−05
rs7311004
rs8039785
rs823424
rs10899911
rs55877868
rs62273907
rs35055552
rs7572229
rs6809952
rs7303158
rs11715853
rs6828768
rs2387977
All − MR Egger
All − IVW(MRE)
−1.0
−0.5
0.0
0.5
1.0
MR effect size for
'Gut microbiota abundance (genus Parasutterella id.2892)' on 'AP−4 complex subunit mu−1'

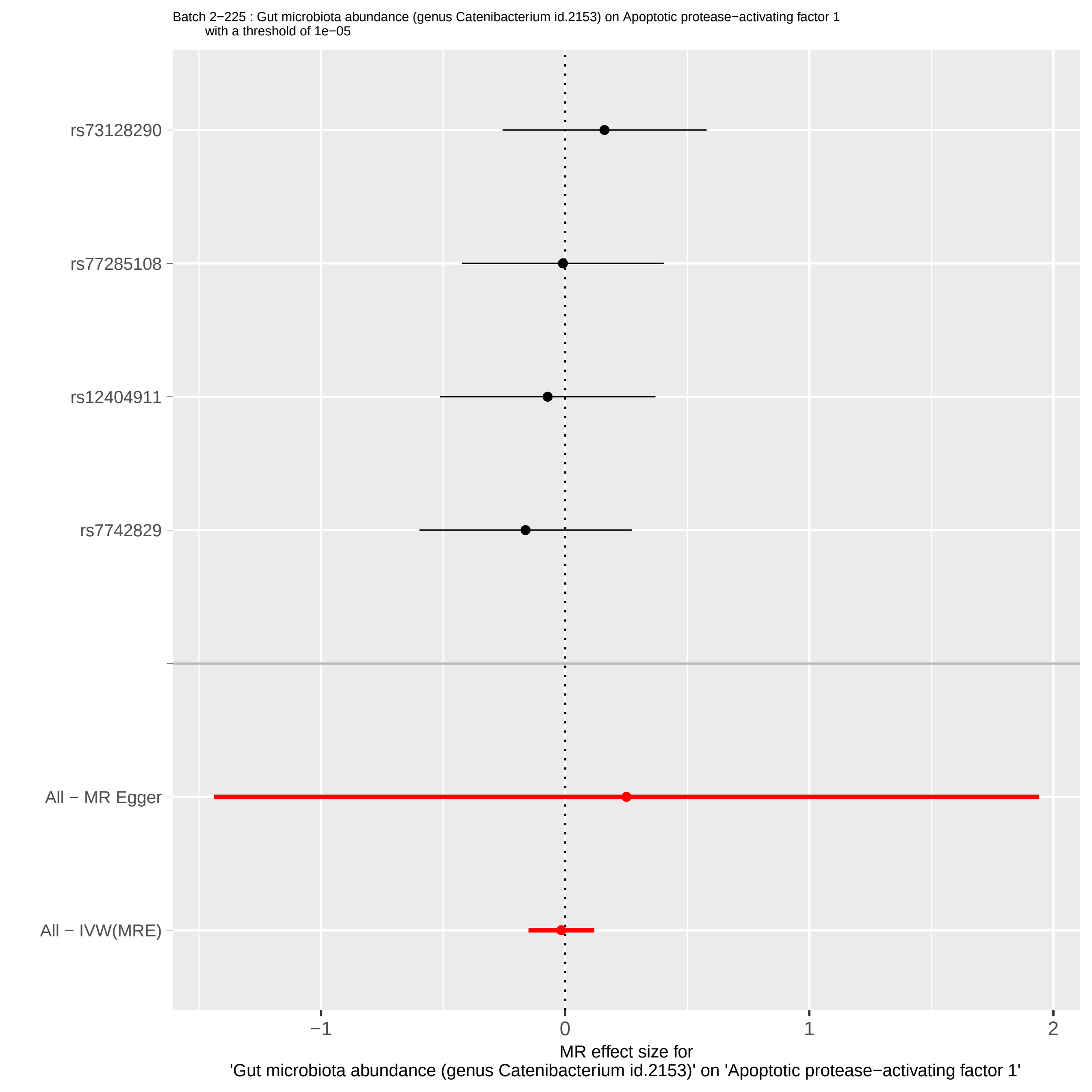

Batch 2−225 : Gut microbiota abundance (genus Catenibacterium id.2153) on Apoptotic protease−activating factor 1
with a threshold of 1e−05
rs73128290
rs77285108
rs12404911
rs7742829
All − MR Egger
All − IVW(MRE)
−1
0
1
2
MR effect size for
'Gut microbiota abundance (genus Catenibacterium id.2153)' on 'Apoptotic protease−activating factor 1'

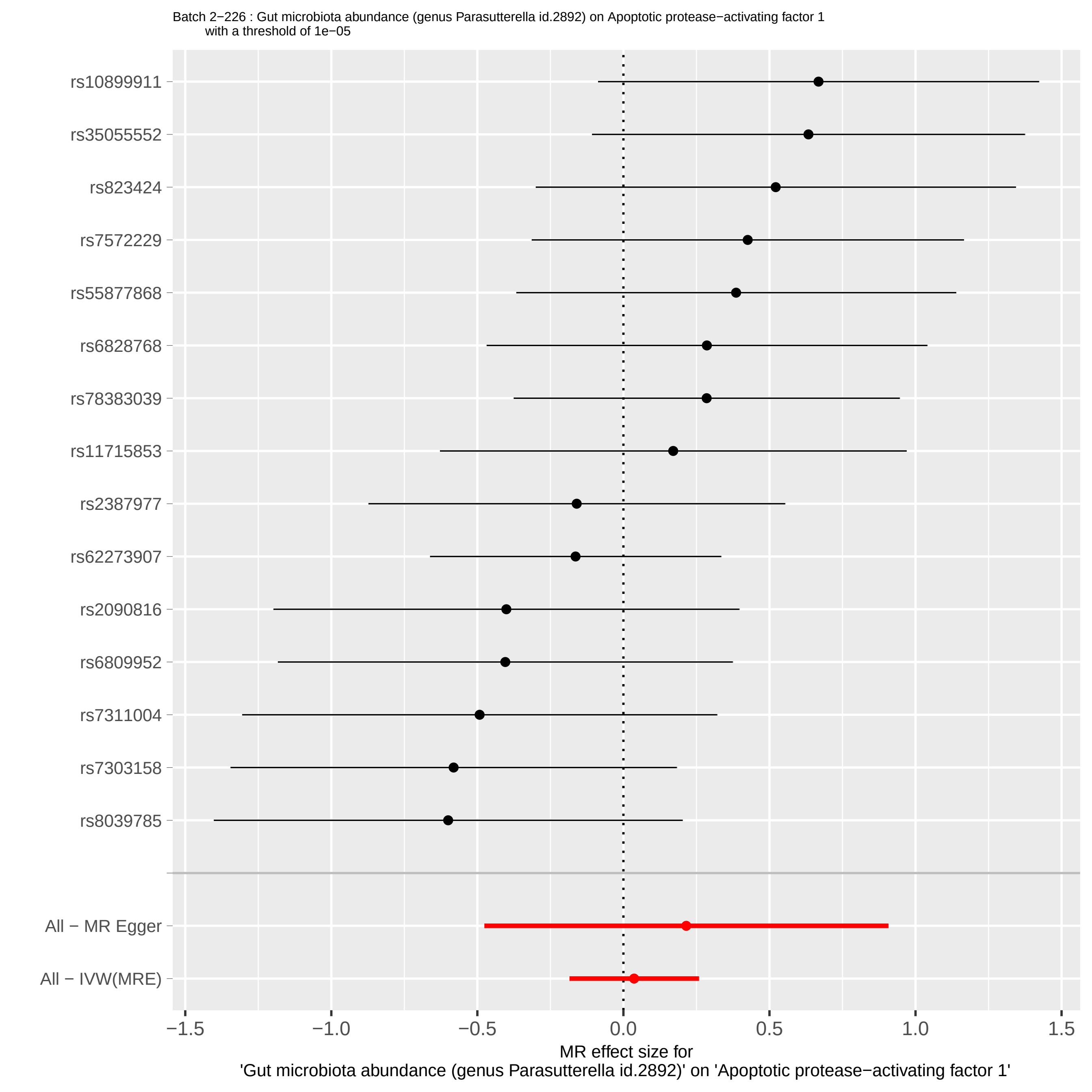
Batch 2−226 : Gut microbiota abundance (genus Parasutterella id.2892) on Apoptotic protease−activating factor 1
with a threshold of 1e−05
rs10899911
rs35055552
rs823424
rs7572229
rs55877868
rs6828768
rs78383039
rs11715853
rs2387977
rs62273907
rs2090816
rs6809952
rs7311004
rs7303158
rs8039785
All − MR Egger
All − IVW(MRE)
−1.5
−1.0
−0.5
0.0
0.5
1.0
1.5
MR effect size for
'Gut microbiota abundance (genus Parasutterella id.2892)' on 'Apoptotic protease−activating factor 1'

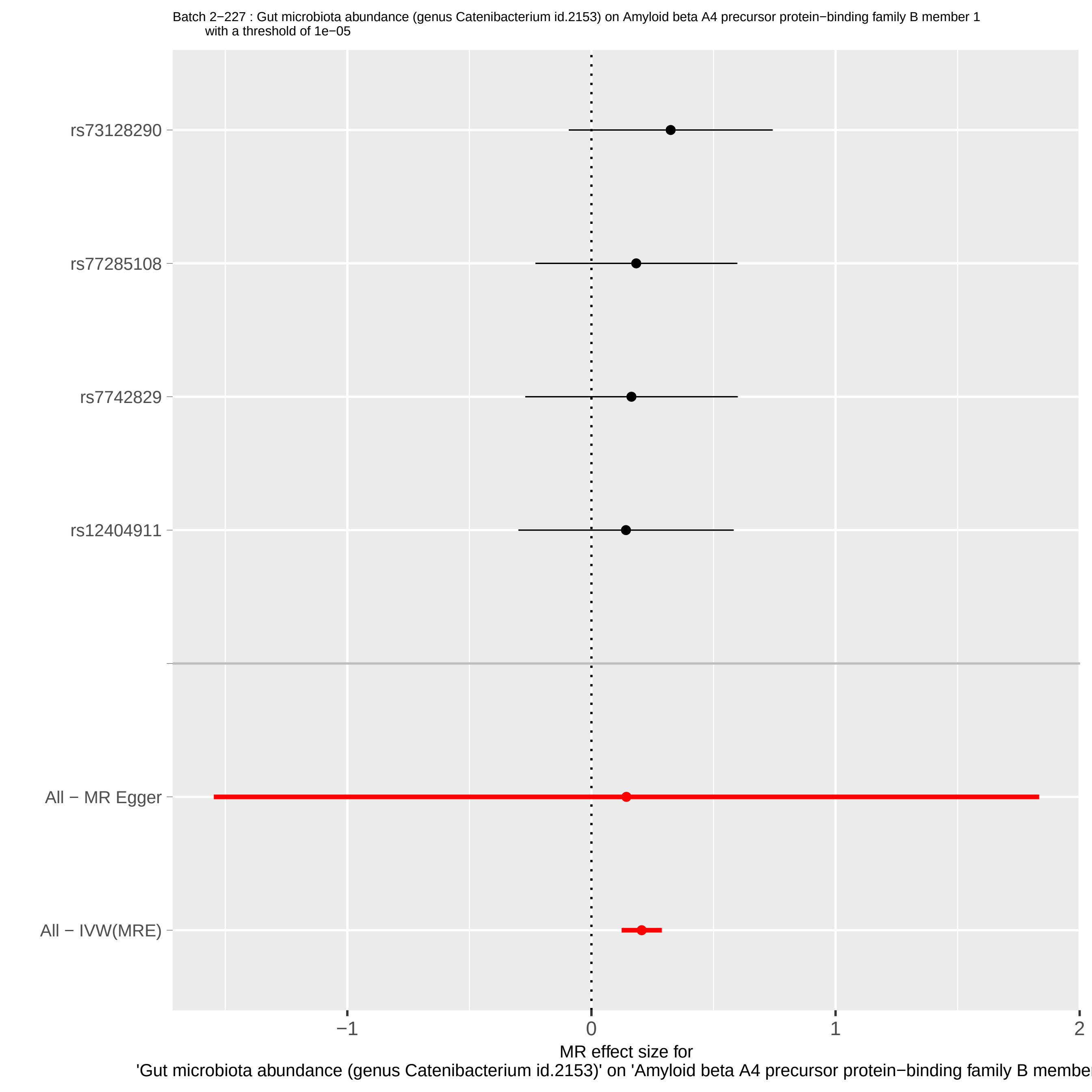

Batch 2−227 : Gut microbiota abundance (genus Catenibacterium id.2153) on Amyloid beta A4 precursor protein−binding family B member 1
with a threshold of 1e−05
rs73128290
rs77285108
rs7742829
rs12404911
All − MR Egger
All − IVW(MRE)
−1
0
1
2
MR effect size for
'Gut microbiota abundance (genus Catenibacterium id.2153)' on 'Amyloid beta A4 precursor protein−binding family B membe

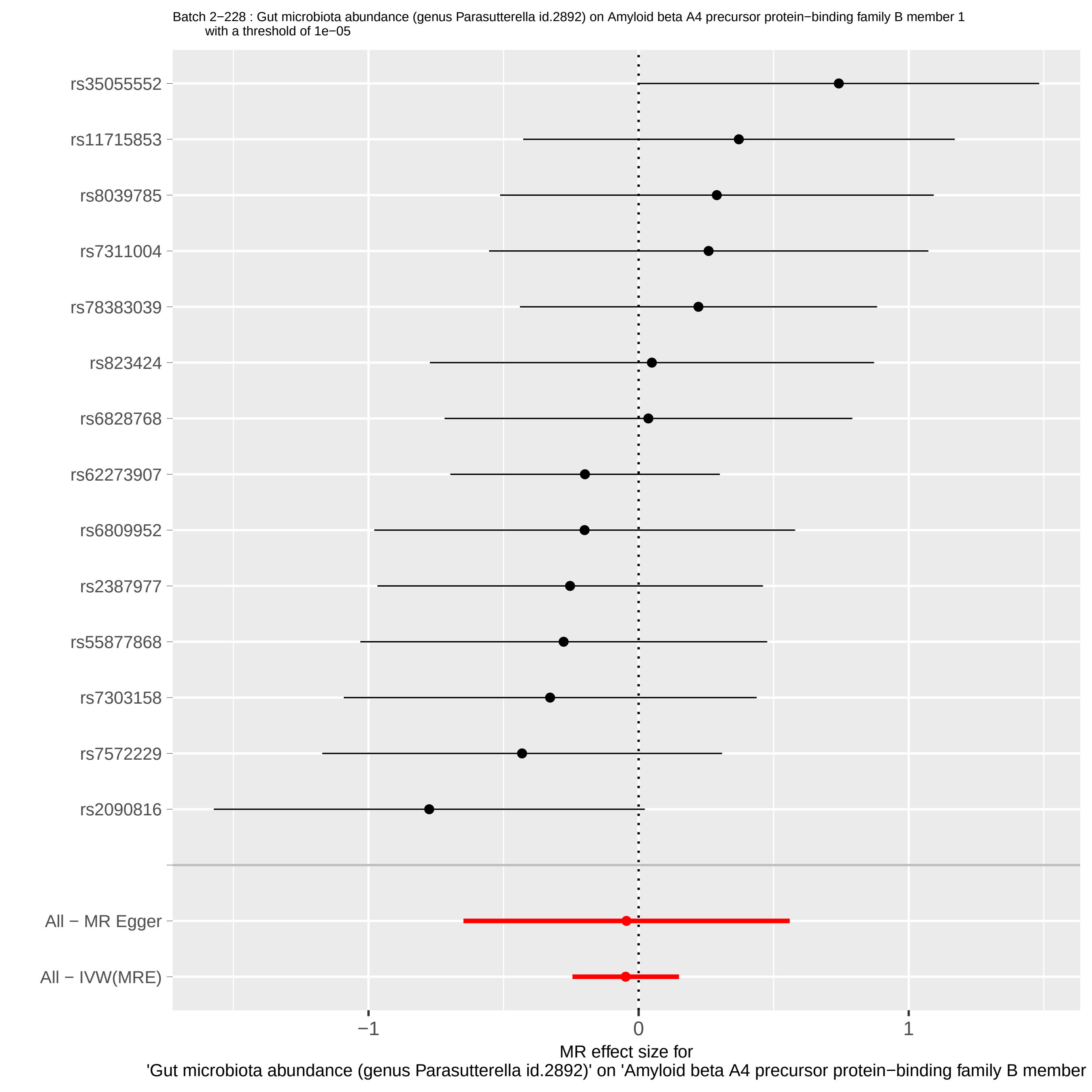

Batch 2−228 : Gut microbiota abundance (genus Parasutterella id.2892) on Amyloid beta A4 precursor protein−binding family B member 1 with a threshold of 1e−05
rs35055552
rs11715853
rs8039785
rs7311004
rs78383039
rs823424
rs6828768
rs62273907
rs6809952
rs2387977
rs55877868
rs7303158
rs7572229
rs2090816
All − MR Egger
All − IVW(MRE)
−1
0
1
MR effect size for
'Gut microbiota abundance (genus Parasutterella id.2892)' on 'Amyloid beta A4 precursor protein−binding family B member

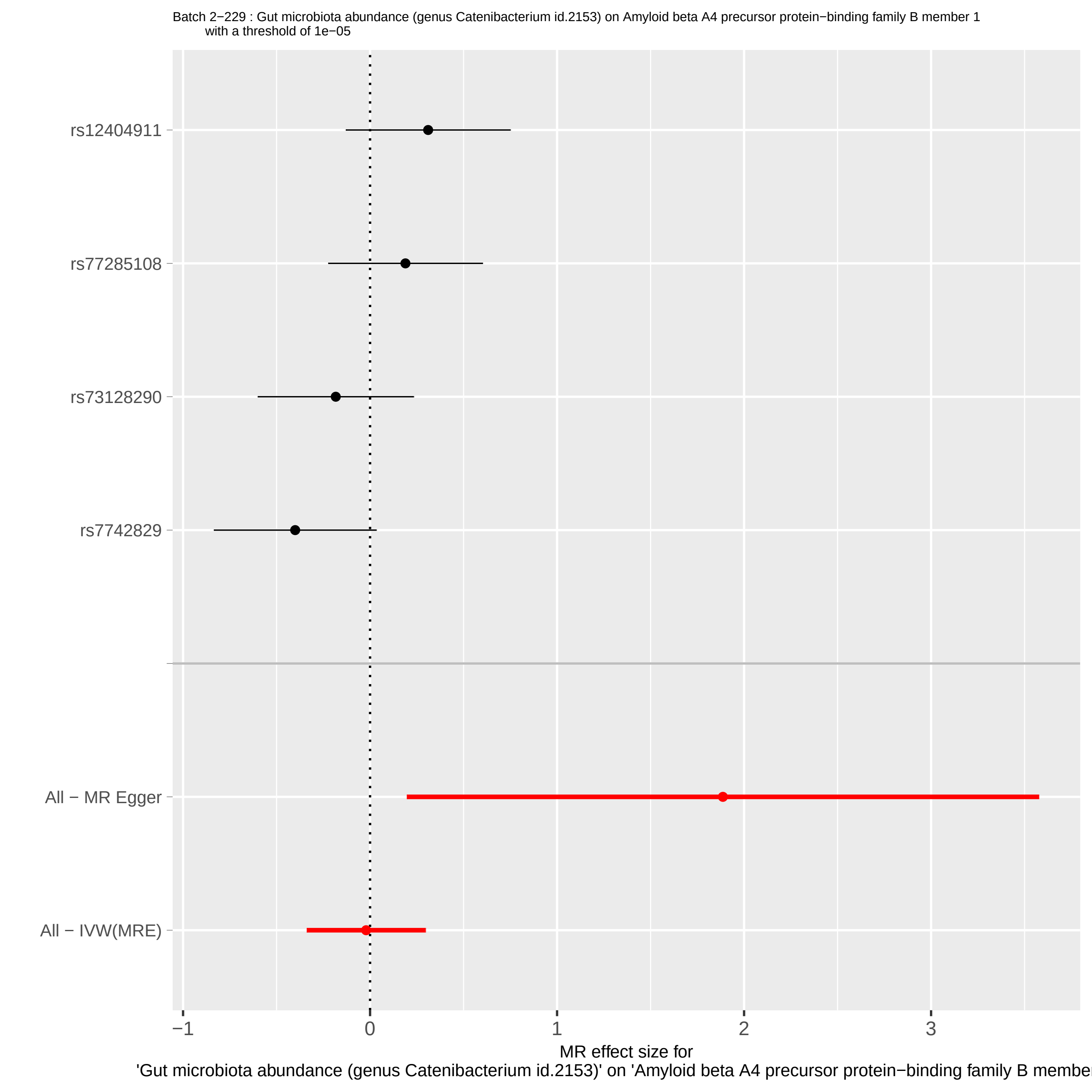

Batch 2−229 : Gut microbiota abundance (genus Catenibacterium id.2153) on Amyloid beta A4 precursor protein−binding family B member 1
with a threshold of 1e−05
rs12404911
rs77285108
rs73128290
rs7742829
All − MR Egger
All − IVW(MRE)
−1
0
1
2
3
MR effect size for
'Gut microbiota abundance (genus Catenibacterium id.2153)' on 'Amyloid beta A4 precursor protein−binding family B membe

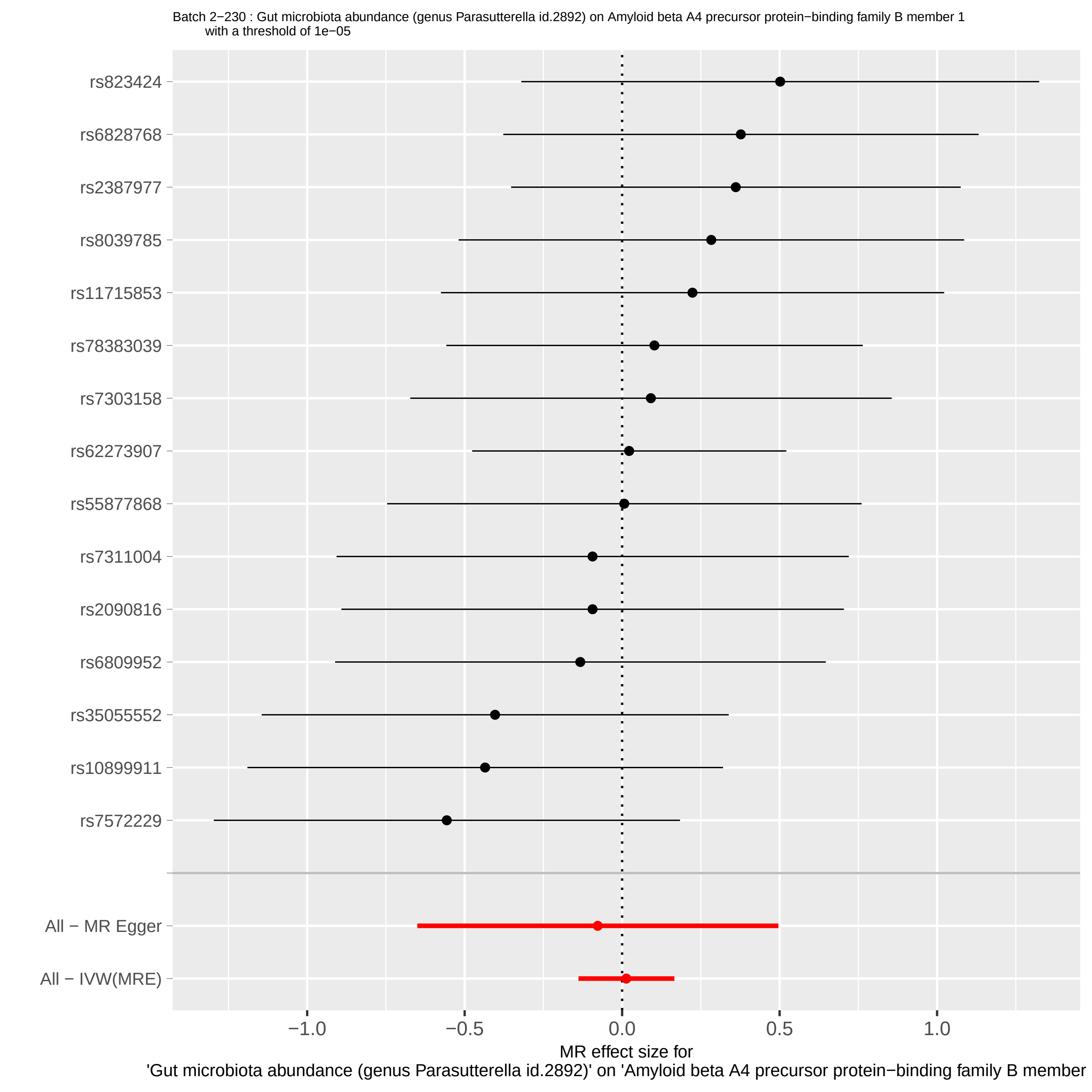
Batch 2−230 : Gut microbiota abundance (genus Parasutterella id.2892) on Amyloid beta A4 precursor protein−binding family B member 1
with a threshold of 1e−05
rs823424
rs6828768
rs2387977
rs8039785
rs11715853
rs78383039
rs7303158
rs62273907
rs55877868
rs7311004
rs2090816
rs6809952
rs35055552
rs10899911
rs7572229
All − MR Egger
All − IVW(MRE)
−1.0
−0.5
0.0
0.5
1.0
MR effect size for
'Gut microbiota abundance (genus Parasutterella id.2892)' on 'Amyloid beta A4 precursor protein−binding family B member

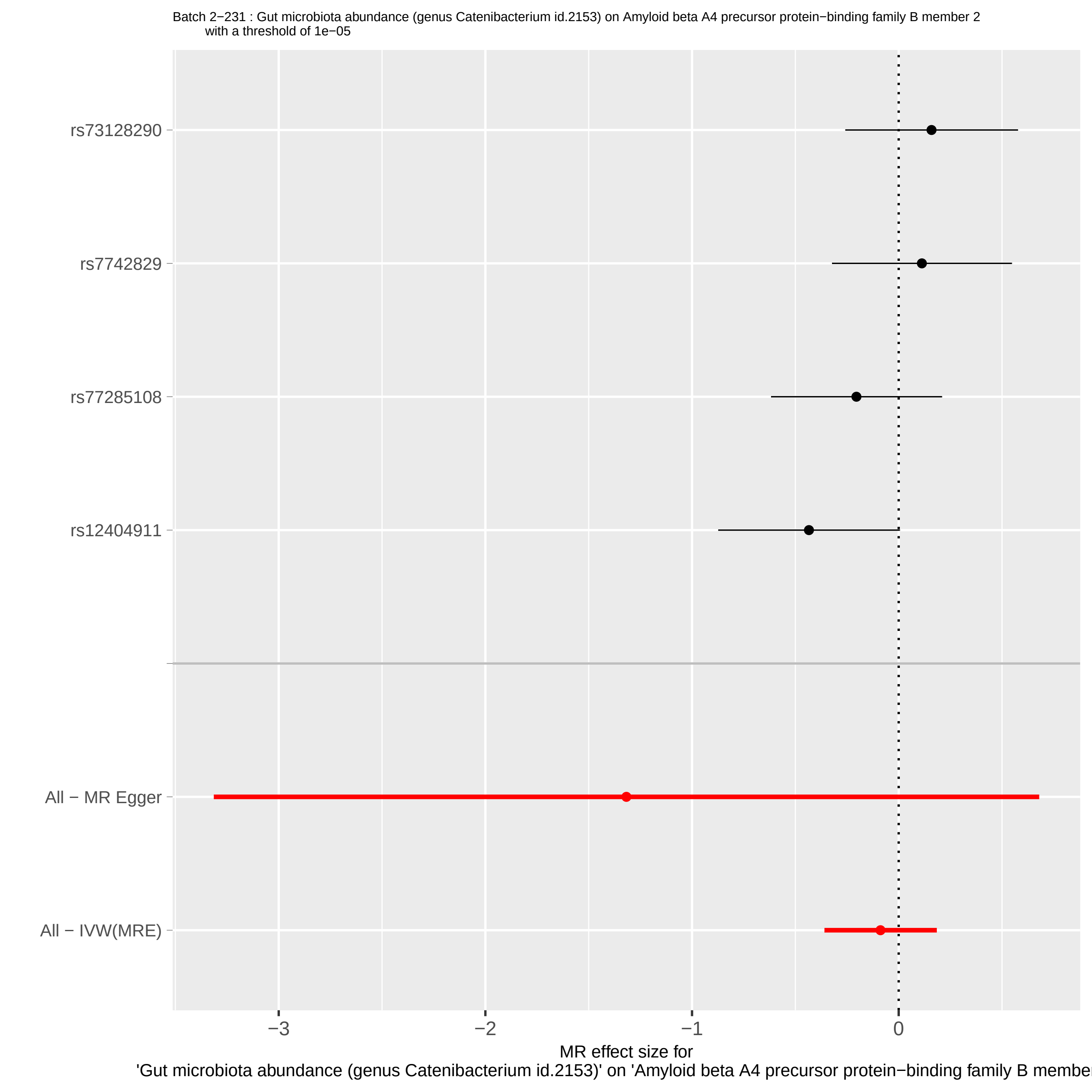
Batch 2−231 : Gut microbiota abundance (genus Catenibacterium id.2153) on Amyloid beta A4 precursor protein−binding family B member 2
with a threshold of 1e−05
rs73128290
rs7742829
rs77285108
rs12404911
All − MR Egger
All − IVW(MRE)
−3
−2
−1
0
MR effect size for
'Gut microbiota abundance (genus Catenibacterium id.2153)' on 'Amyloid beta A4 precursor protein−binding family B membe

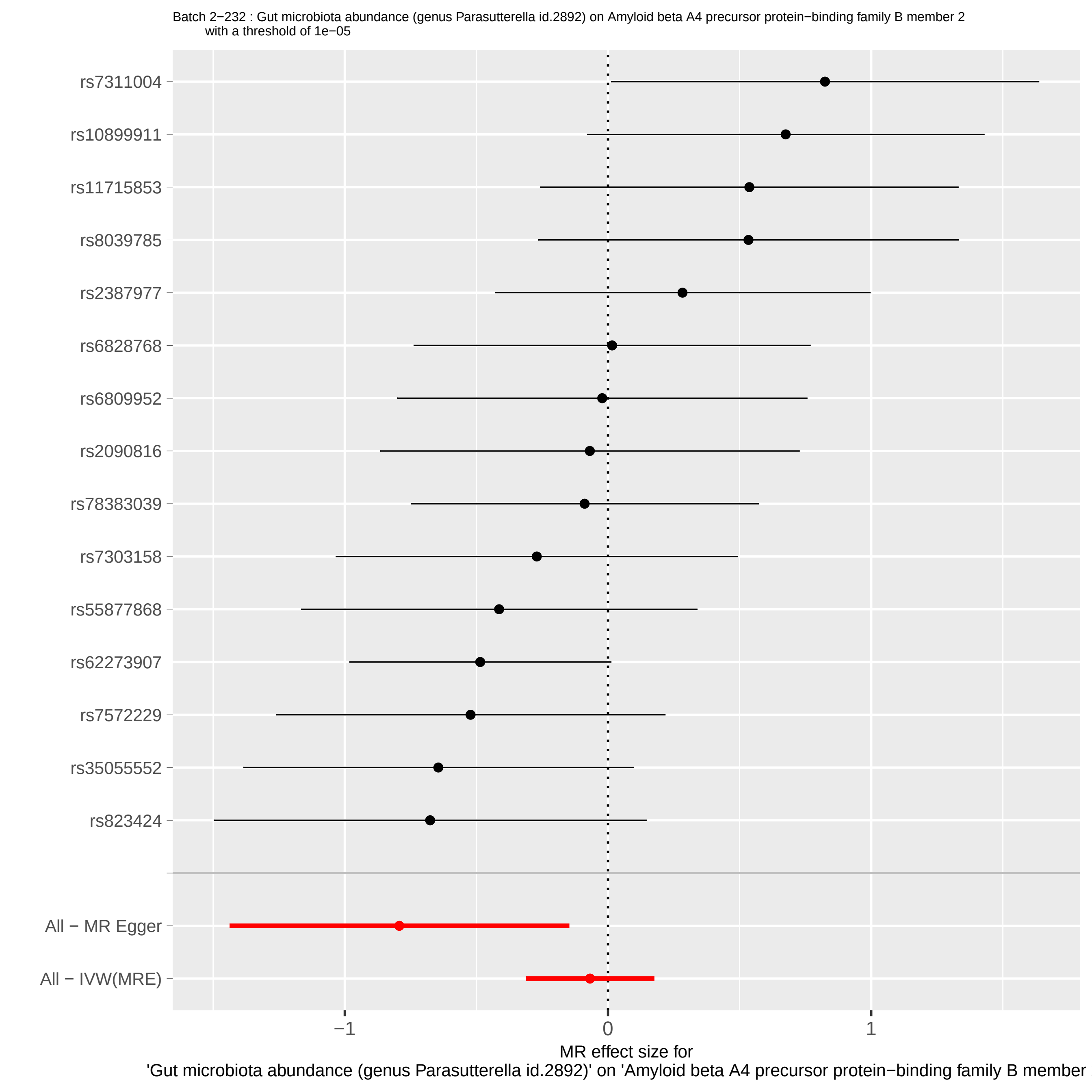

Batch 2−232 : Gut microbiota abundance (genus Parasutterella id.2892) on Amyloid beta A4 precursor protein−binding family B member 2
with a threshold of 1e−05
rs7311004
rs10899911
rs11715853
rs8039785
rs2387977
rs6828768
rs6809952
rs2090816
rs78383039
rs7303158
rs55877868
rs62273907
rs7572229
rs35055552
rs823424
All − MR Egger
All − IVW(MRE)
−1
0
1
MR effect size for
'Gut microbiota abundance (genus Parasutterella id.2892)' on 'Amyloid beta A4 precursor protein−binding family B member

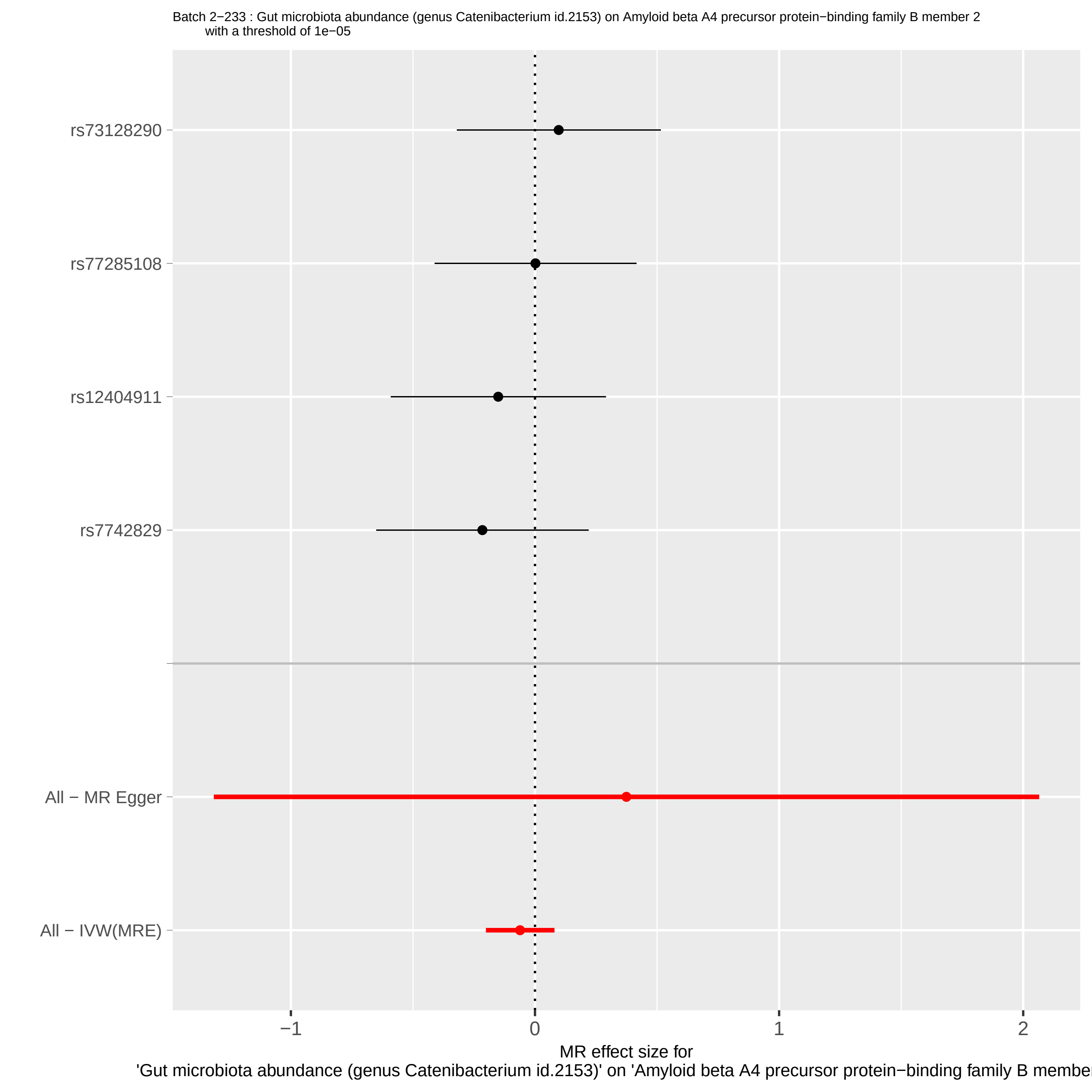

Batch 2−233 : Gut microbiota abundance (genus Catenibacterium id.2153) on Amyloid beta A4 precursor protein−binding family B member 2
with a threshold of 1e−05
rs73128290
rs77285108
rs12404911
rs7742829
All − MR Egger
All − IVW(MRE)
−1
0
1
2
MR effect size for
'Gut microbiota abundance (genus Catenibacterium id.2153)' on 'Amyloid beta A4 precursor protein−binding family B membe

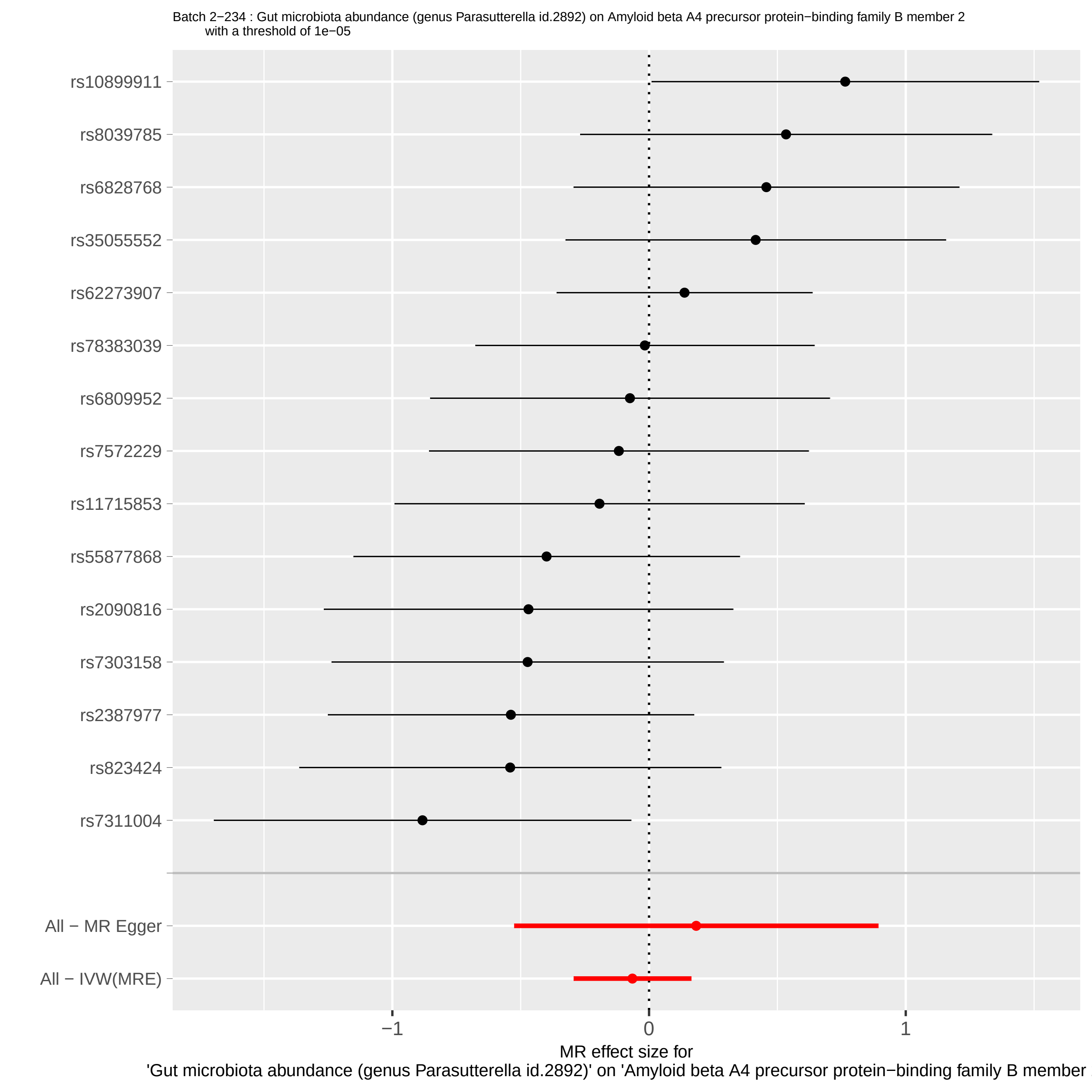

Batch 2−234 : Gut microbiota abundance (genus Parasutterella id.2892) on Amyloid beta A4 precursor protein−binding family B member 2
with a threshold of 1e−05
rs10899911
rs8039785
rs6828768
rs35055552
rs62273907
rs78383039
rs6809952
rs7572229
rs11715853
rs55877868
rs2090816
rs7303158
rs2387977
rs823424
rs7311004
All − MR Egger
All − IVW(MRE)
−1
0
1
MR effect size for
'Gut microbiota abundance (genus Parasutterella id.2892)' on 'Amyloid beta A4 precursor protein−binding family B member

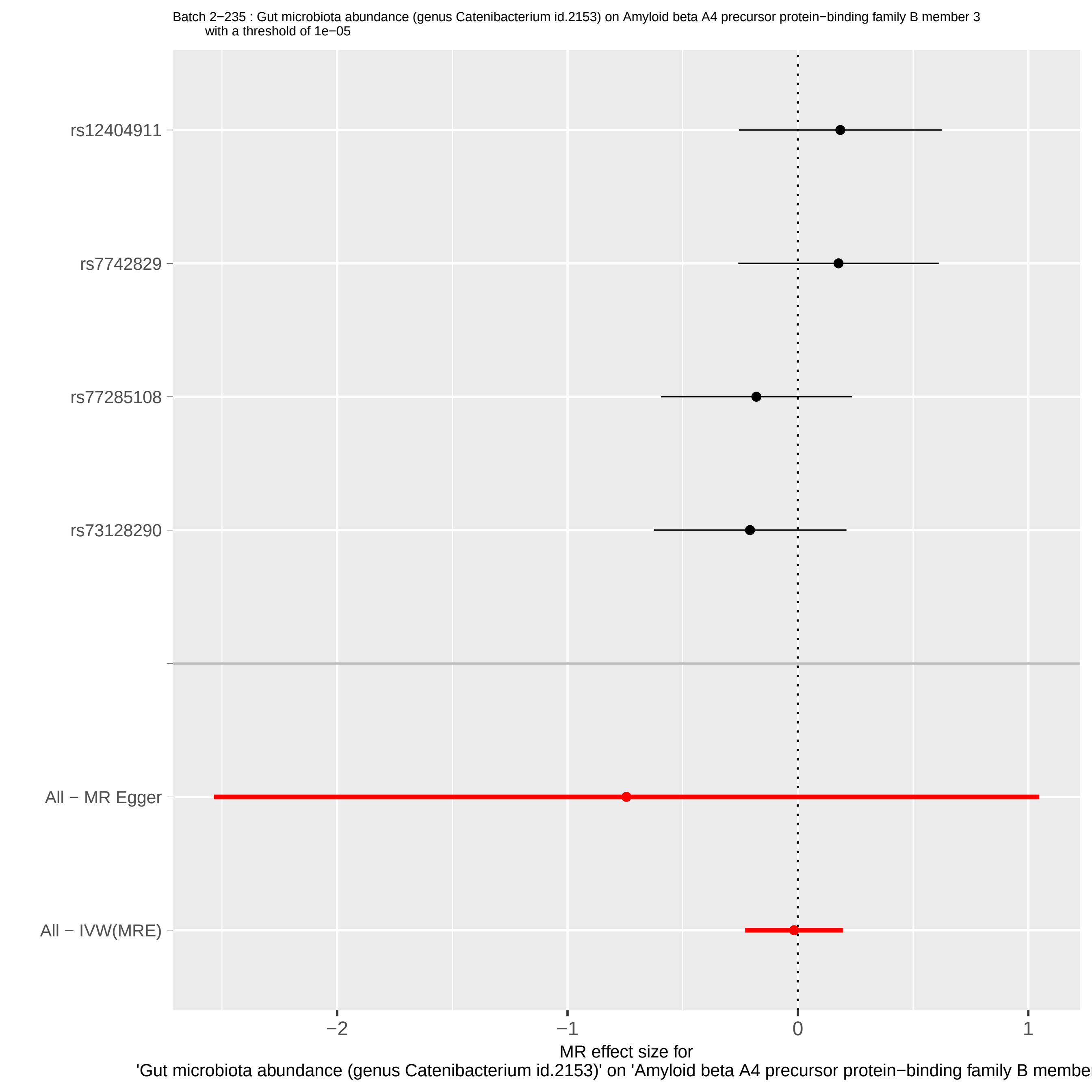

Batch 2−235 : Gut microbiota abundance (genus Catenibacterium id.2153) on Amyloid beta A4 precursor protein−binding family B member 3
with a threshold of 1e−05
rs12404911
rs7742829
rs77285108
rs73128290
All − MR Egger
All − IVW(MRE)
−2
−1
0
1
MR effect size for
'Gut microbiota abundance (genus Catenibacterium id.2153)' on 'Amyloid beta A4 precursor protein−binding family B membe

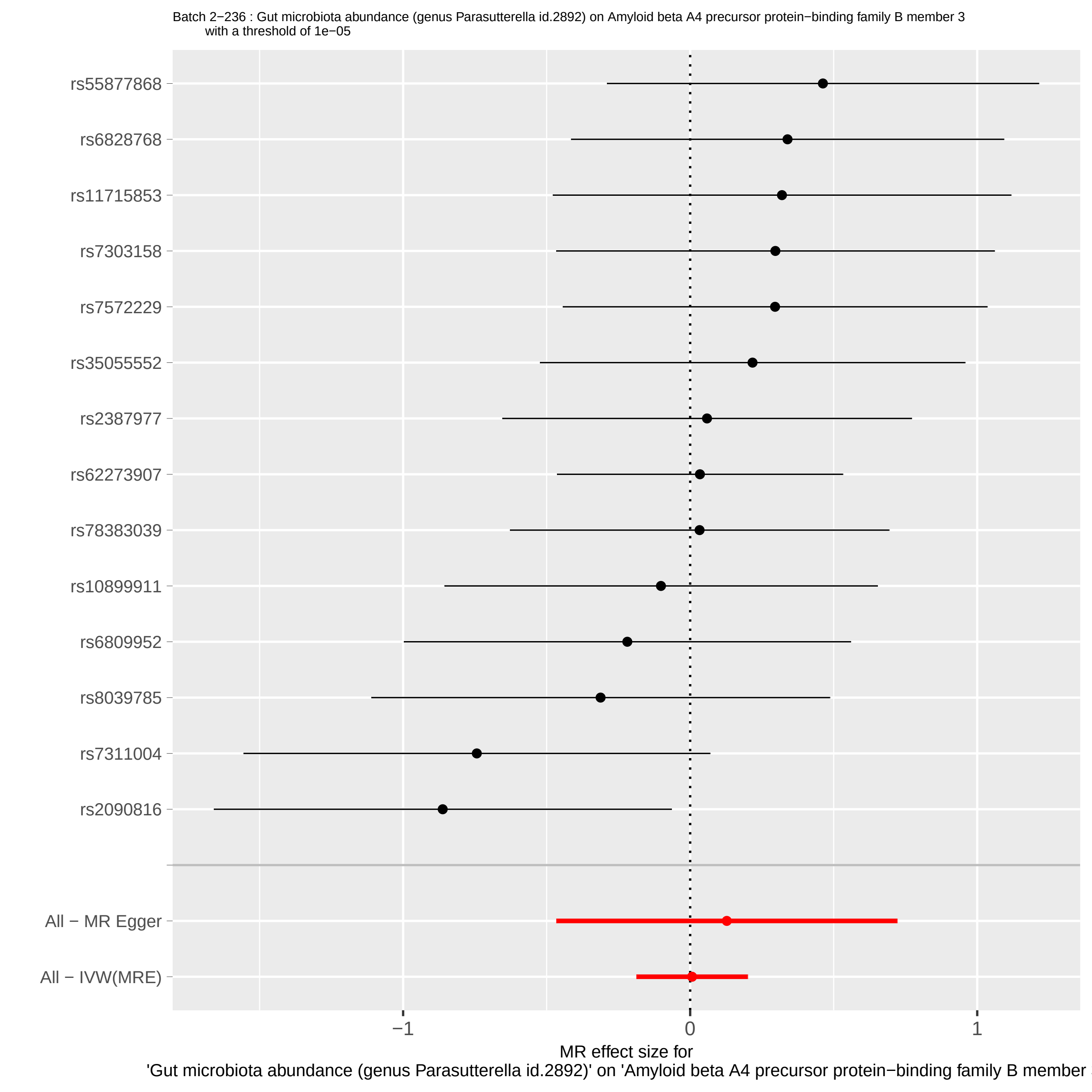

Batch 2−236 : Gut microbiota abundance (genus Parasutterella id.2892) on Amyloid beta A4 precursor protein−binding family B member 3
with a threshold of 1e−05
rs55877868
rs6828768
rs11715853
rs7303158
rs7572229
rs35055552
rs2387977
rs62273907
rs78383039
rs10899911
rs6809952
rs8039785
rs7311004
rs2090816
All − MR Egger
All − IVW(MRE)
−1
0
1
MR effect size for
'Gut microbiota abundance (genus Parasutterella id.2892)' on 'Amyloid beta A4 precursor protein−binding family B member

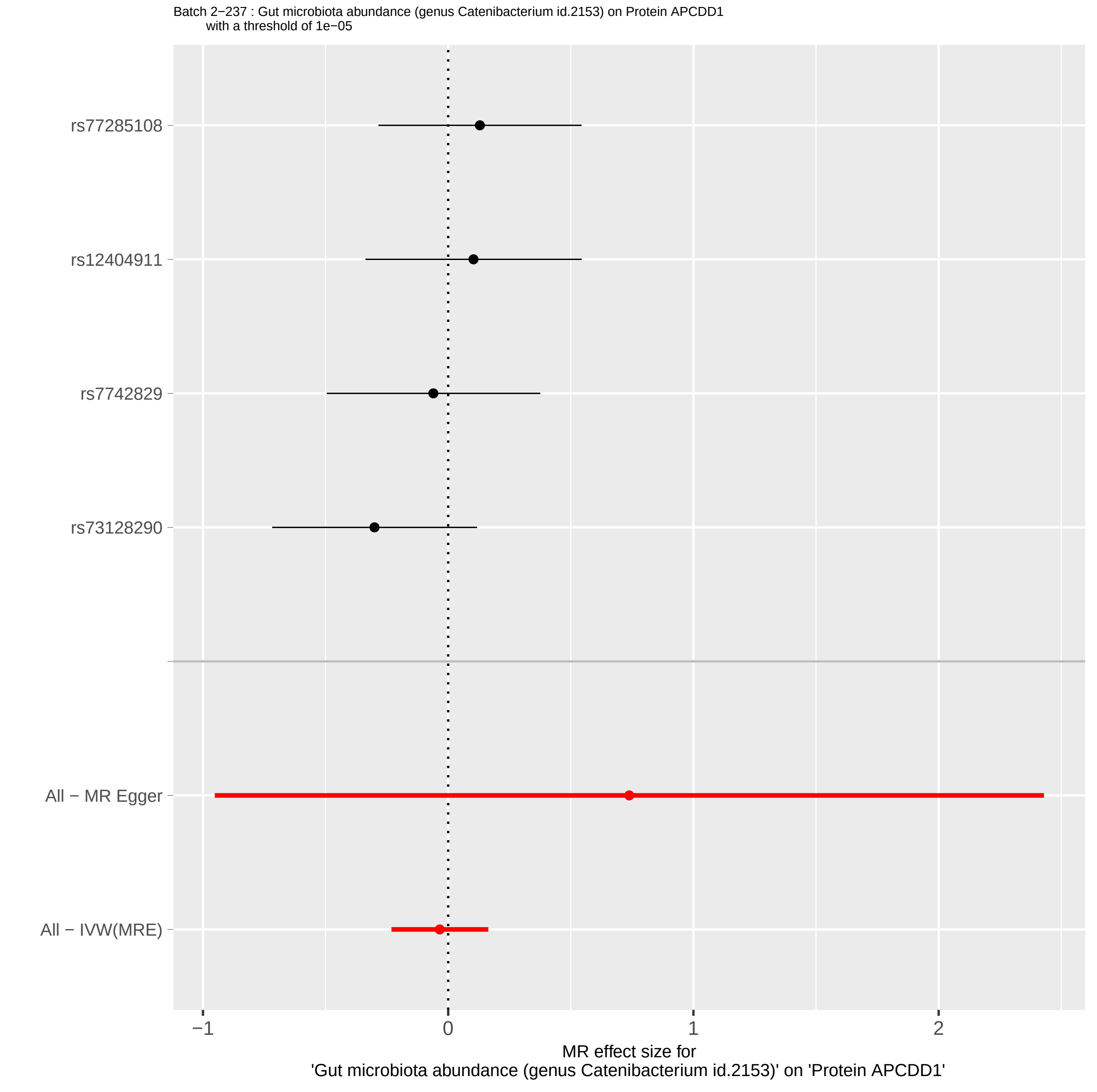

Batch 2−237 : Gut microbiota abundance (genus Catenibacterium id.2153) on Protein APCDD1
with a threshold of 1e−05
rs77285108
rs12404911
rs7742829
rs73128290
All − MR Egger
All − IVW(MRE)
−1
0
1
2
MR effect size for
'Gut microbiota abundance (genus Catenibacterium id.2153)' on 'Protein APCDD1'

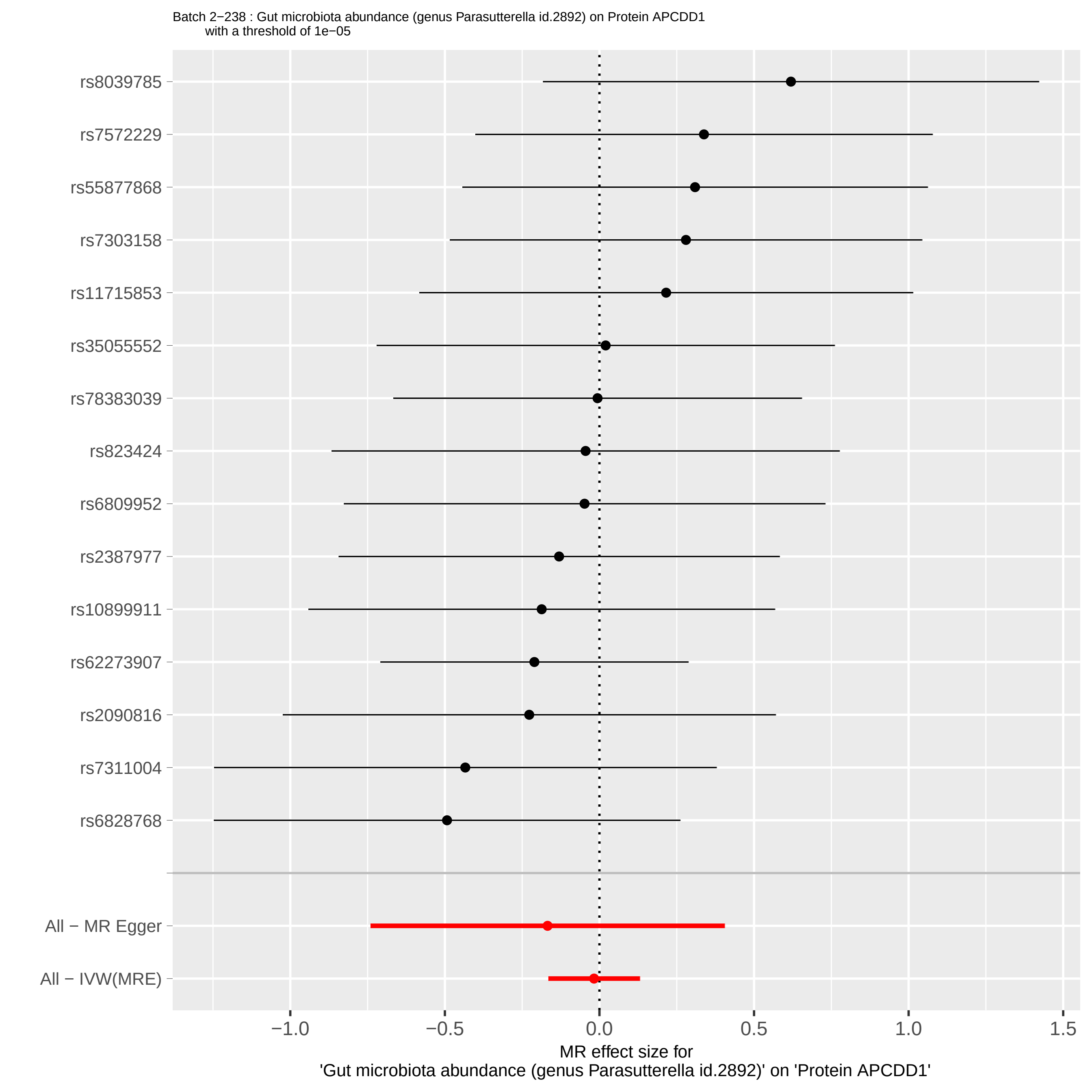

Batch 2−238 : Gut microbiota abundance (genus Parasutterella id.2892) on Protein APCDD1
with a threshold of 1e−05
rs8039785
rs7572229
rs55877868
rs7303158
rs11715853
rs35055552
rs78383039
rs823424
rs6809952
rs2387977
rs10899911
rs62273907
rs2090816
rs7311004
rs6828768
All − MR Egger
All − IVW(MRE)
−1.0
−0.5
0.0
0.5
1.0
1.5
MR effect size for
'Gut microbiota abundance (genus Parasutterella id.2892)' on 'Protein APCDD1'

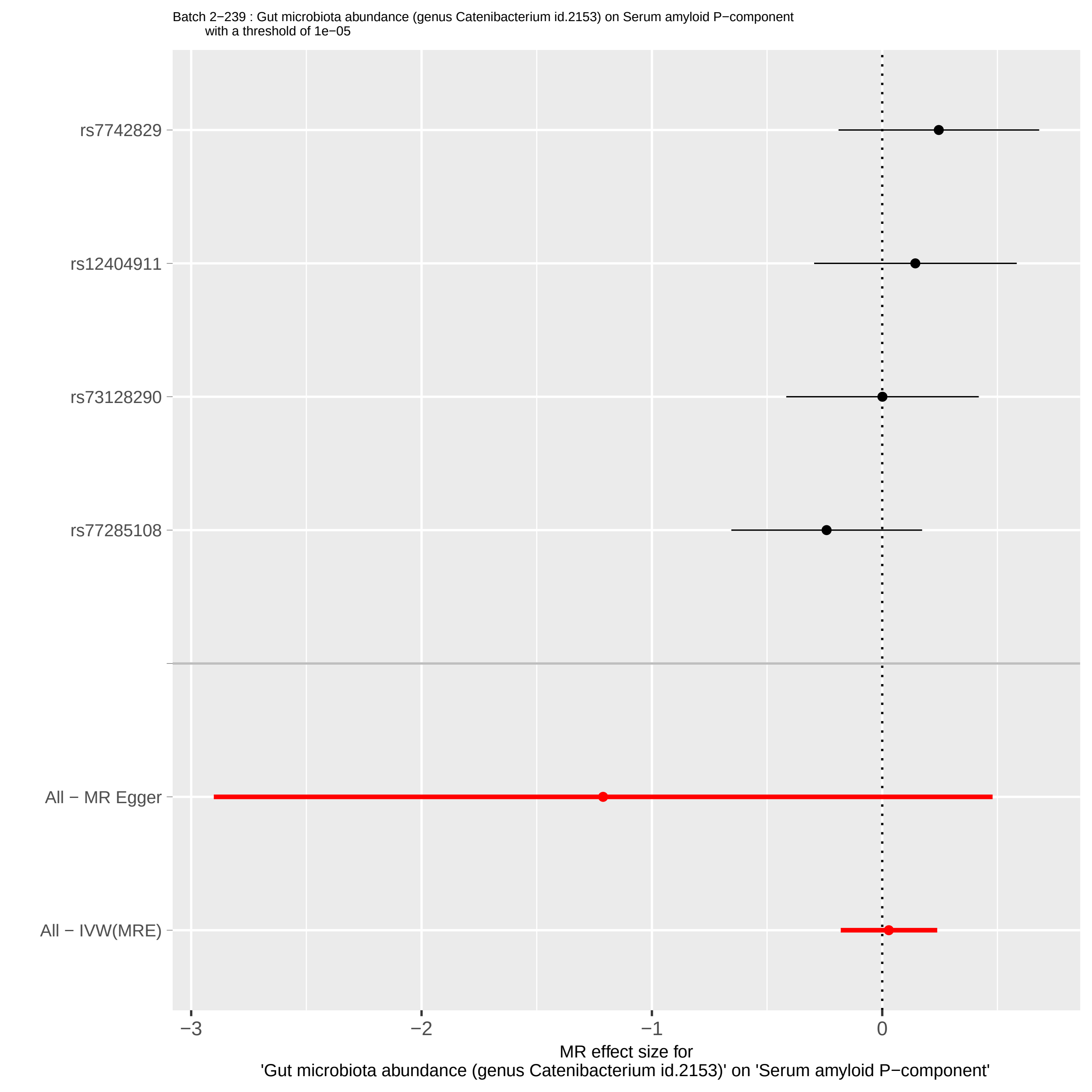

Batch 2−239 : Gut microbiota abundance (genus Catenibacterium id.2153) on Serum amyloid P−component
with a threshold of 1e−05
rs7742829
rs12404911
rs73128290
rs77285108
All − MR Egger
All − IVW(MRE)
−3
−2
−1
0
MR effect size for
'Gut microbiota abundance (genus Catenibacterium id.2153)' on 'Serum amyloid P−component'

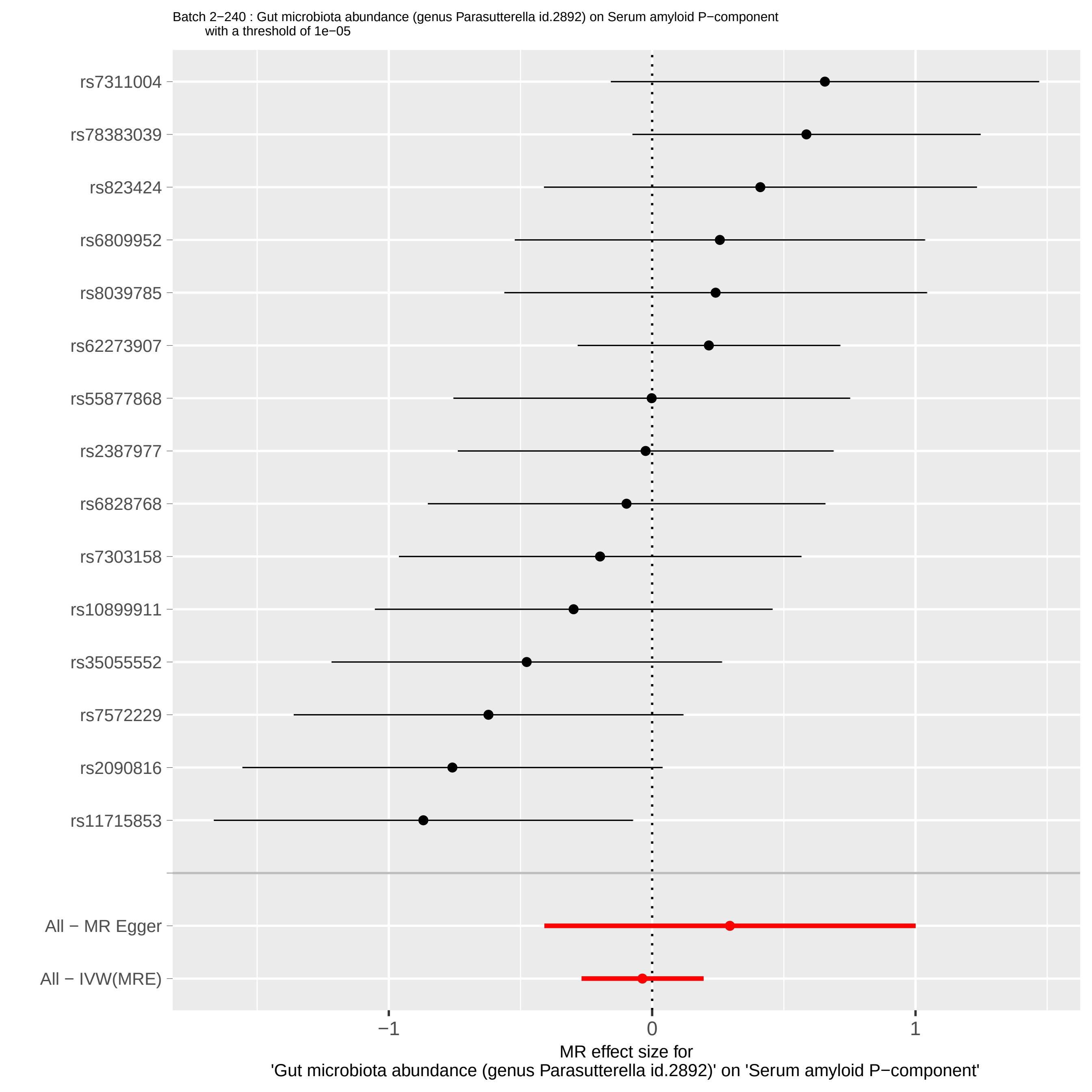

Batch 2−240 : Gut microbiota abundance (genus Parasutterella id.2892) on Serum amyloid P−component
with a threshold of 1e−05
rs7311004
rs78383039
rs823424
rs6809952
rs8039785
rs62273907
rs55877868
rs2387977
rs6828768
rs7303158
rs10899911
rs35055552
rs7572229
rs2090816
rs11715853
All − MR Egger
All − IVW(MRE)
−1
0
1
MR effect size for
'Gut microbiota abundance (genus Parasutterella id.2892)' on 'Serum amyloid P−component'

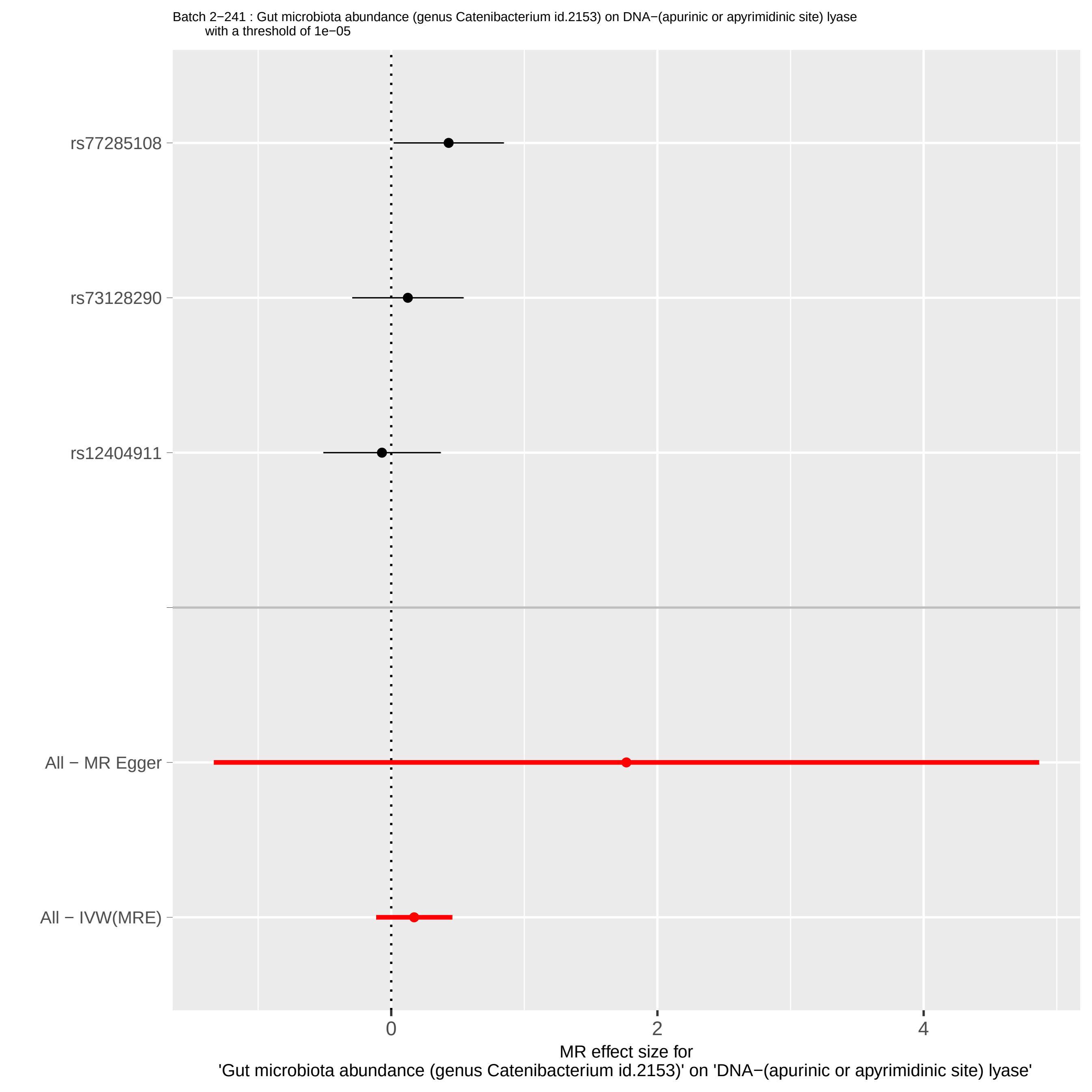

Batch 2−241 : Gut microbiota abundance (genus Catenibacterium id.2153) on DNA−(apurinic or apyrimidinic site) lyase with a threshold of 1e−05
rs77285108
rs73128290
rs12404911
All − MR Egger
All − IVW(MRE)
0
2
4
MR effect size for
'Gut microbiota abundance (genus Catenibacterium id.2153)' on 'DNA−(apurinic or apyrimidinic site) lyase'

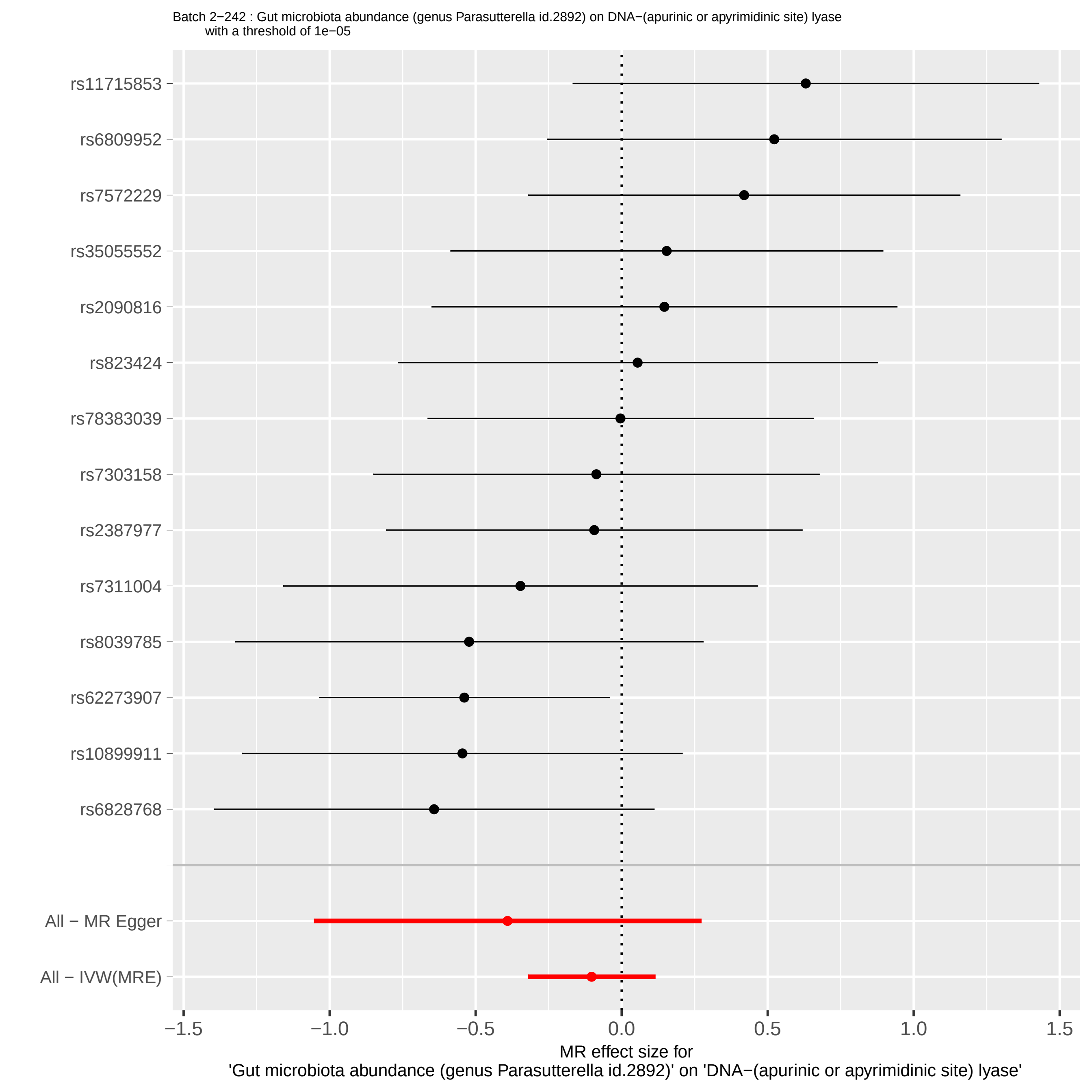

Batch 2−242 : Gut microbiota abundance (genus Parasutterella id.2892) on DNA−(apurinic or apyrimidinic site) lyase with a threshold of 1e−05
rs11715853
rs6809952
rs7572229
rs35055552
rs2090816
rs823424
rs78383039
rs7303158
rs2387977
rs7311004
rs8039785
rs62273907
rs10899911
rs6828768
All − MR Egger
All − IVW(MRE)
−1.5
−1.0
−0.5
0.0
0.5
1.0
1.5
MR effect size for
'Gut microbiota abundance (genus Parasutterella id.2892)' on 'DNA−(apurinic or apyrimidinic site) lyase'

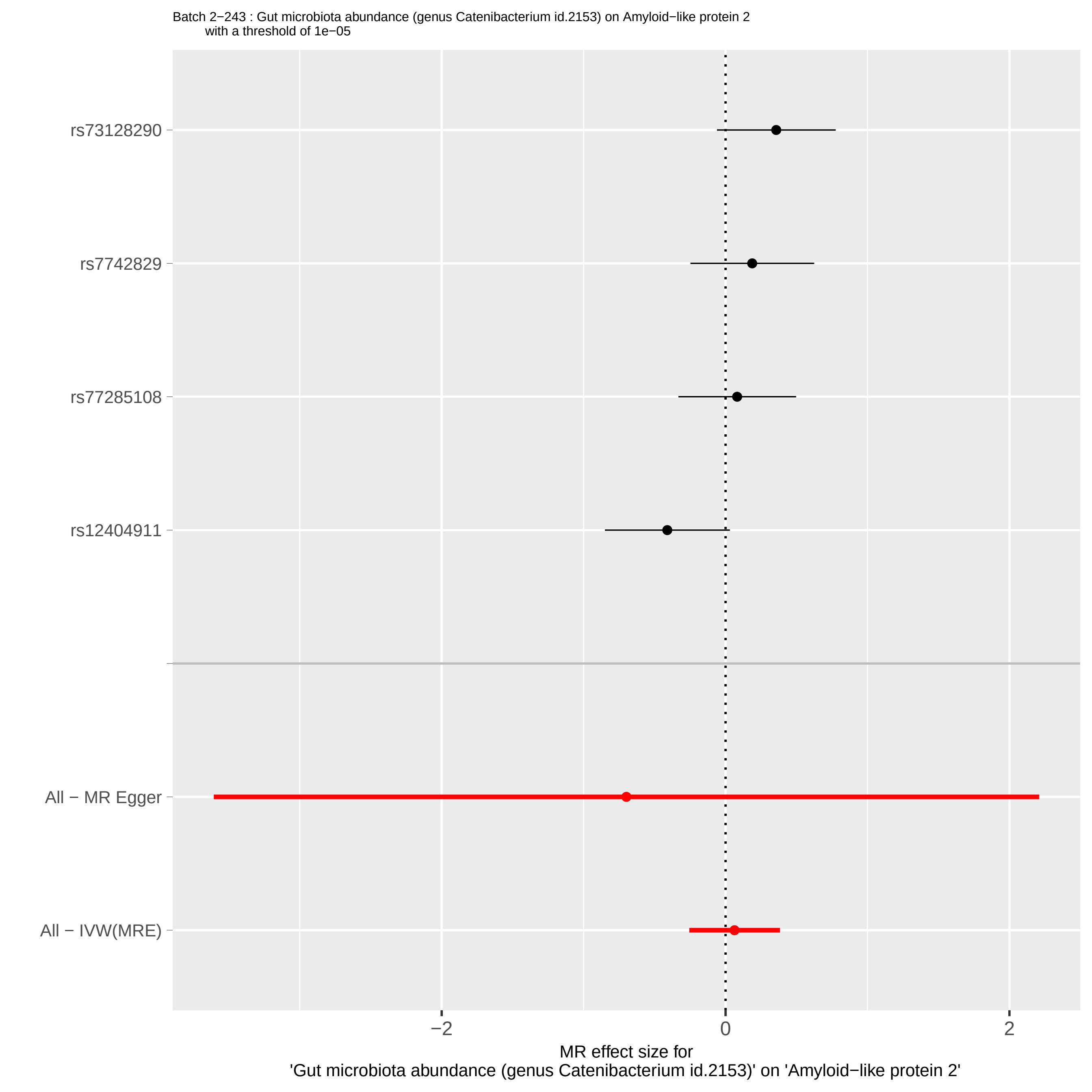
Batch 2−243 : Gut microbiota abundance (genus Catenibacterium id.2153) on Amyloid−like protein 2
with a threshold of 1e−05
rs73128290
rs7742829
rs77285108
rs12404911
All − MR Egger
All − IVW(MRE)
−2
0
2
MR effect size for
'Gut microbiota abundance (genus Catenibacterium id.2153)' on 'Amyloid−like protein 2'

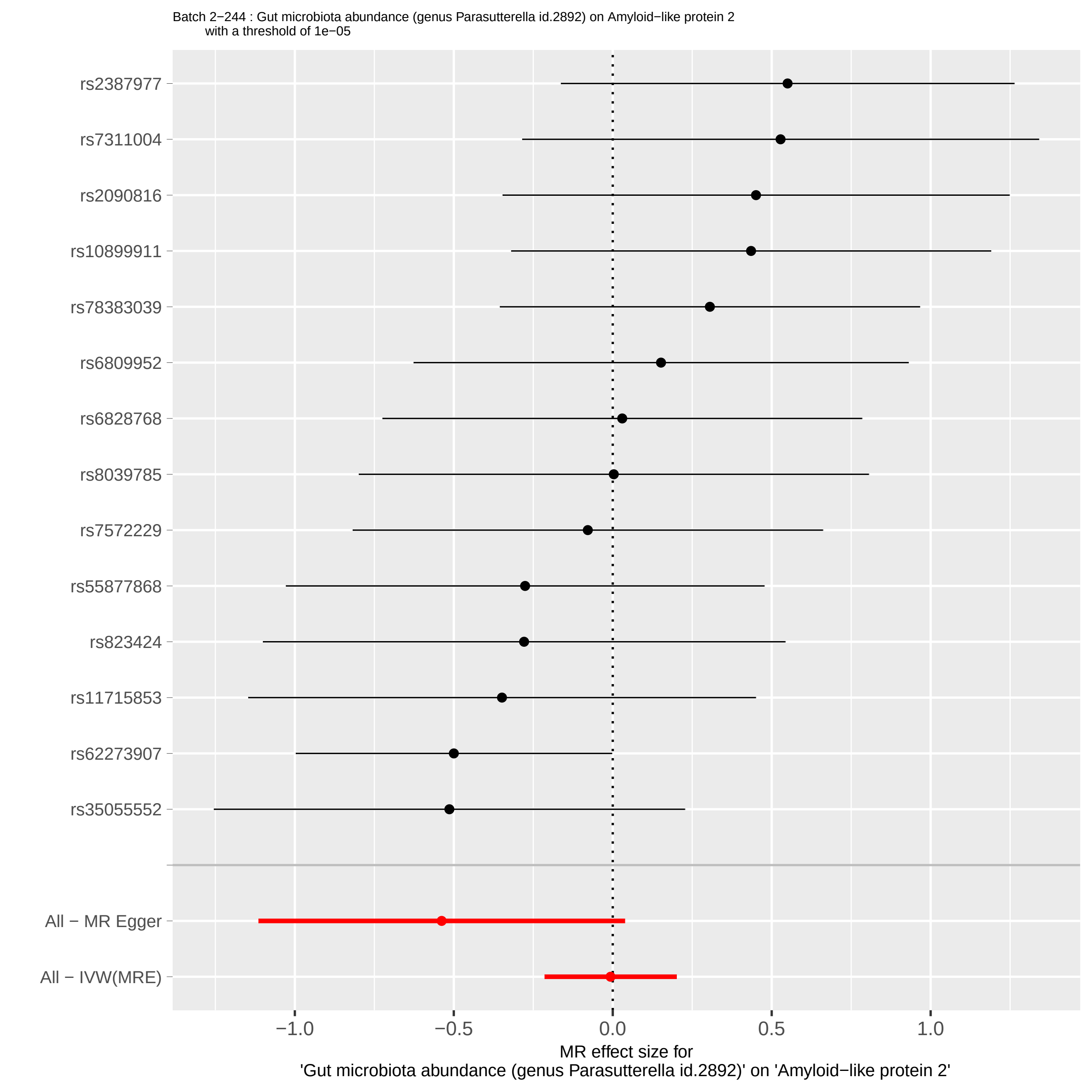

Batch 2−244 : Gut microbiota abundance (genus Parasutterella id.2892) on Amyloid−like protein 2
with a threshold of 1e−05
rs2387977
rs7311004
rs2090816
rs10899911
rs78383039
rs6809952
rs6828768
rs8039785
rs7572229
rs55877868
rs823424
rs11715853
rs62273907
rs35055552
All − MR Egger
All − IVW(MRE)
−1.0
−0.5
0.0
0.5
1.0
MR effect size for
'Gut microbiota abundance (genus Parasutterella id.2892)' on 'Amyloid−like protein 2'

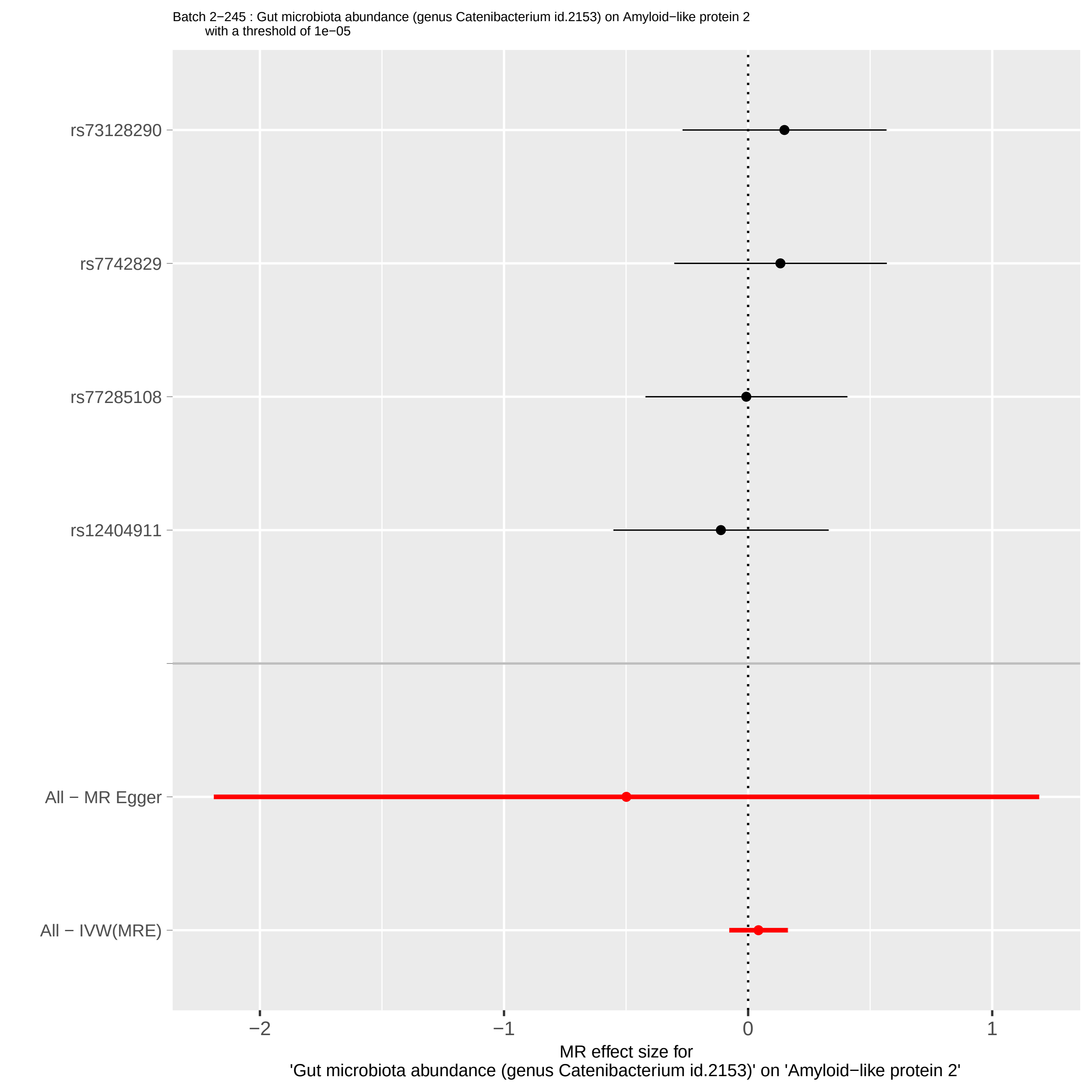

Batch 2−245 : Gut microbiota abundance (genus Catenibacterium id.2153) on Amyloid−like protein 2
with a threshold of 1e−05
rs73128290
rs7742829
rs77285108
rs12404911
All − MR Egger
All − IVW(MRE)
−2
−1
0
1
MR effect size for
'Gut microbiota abundance (genus Catenibacterium id.2153)' on 'Amyloid−like protein 2'

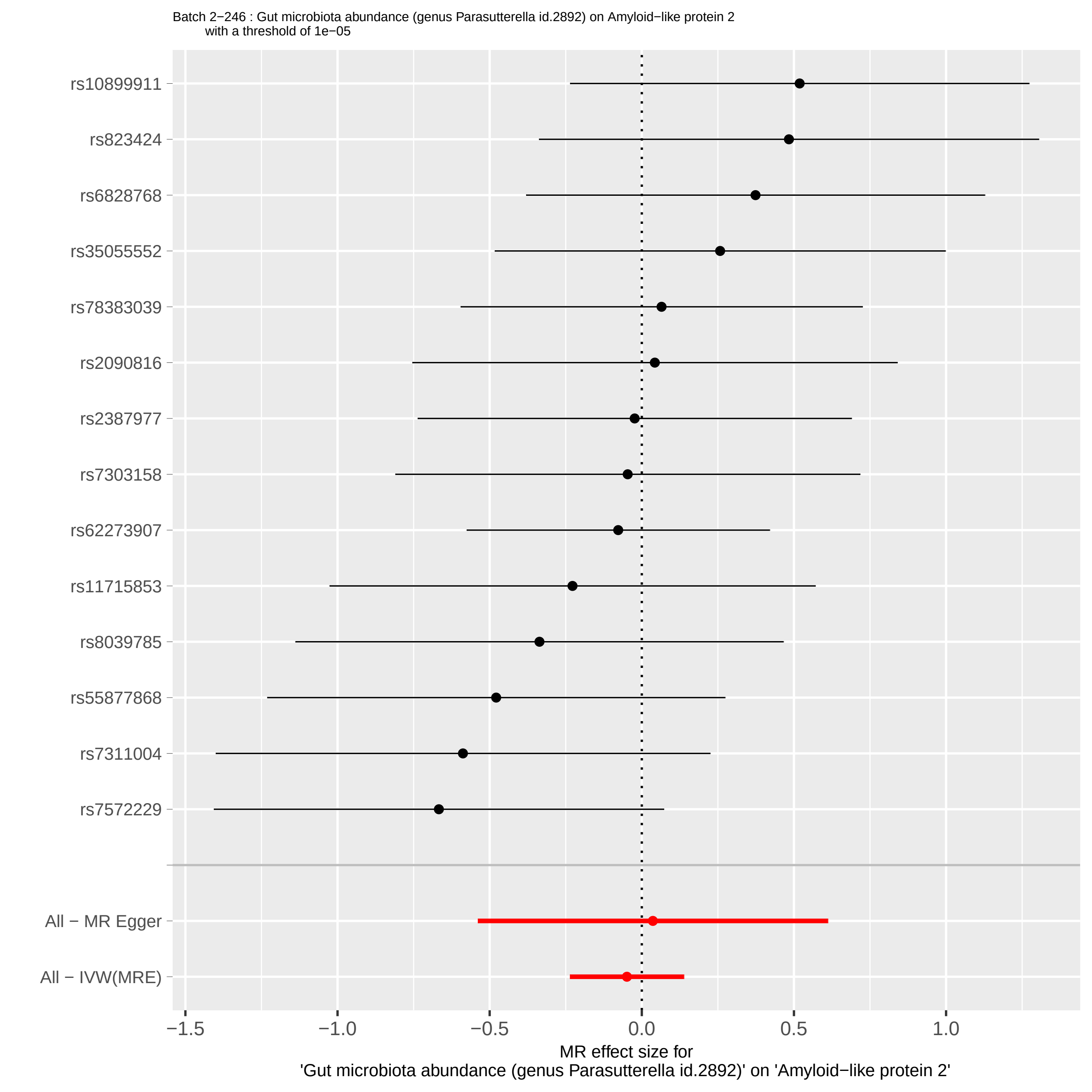

Batch 2−246 : Gut microbiota abundance (genus Parasutterella id.2892) on Amyloid−like protein 2
with a threshold of 1e−05
rs10899911
rs823424
rs6828768
rs35055552
rs78383039
rs2090816
rs2387977
rs7303158
rs62273907
rs11715853
rs8039785
rs55877868
rs7311004
rs7572229
All − MR Egger
All − IVW(MRE)
−1.5
−1.0
−0.5
0.0
0.5
1.0
MR effect size for
'Gut microbiota abundance (genus Parasutterella id.2892)' on 'Amyloid−like protein 2'

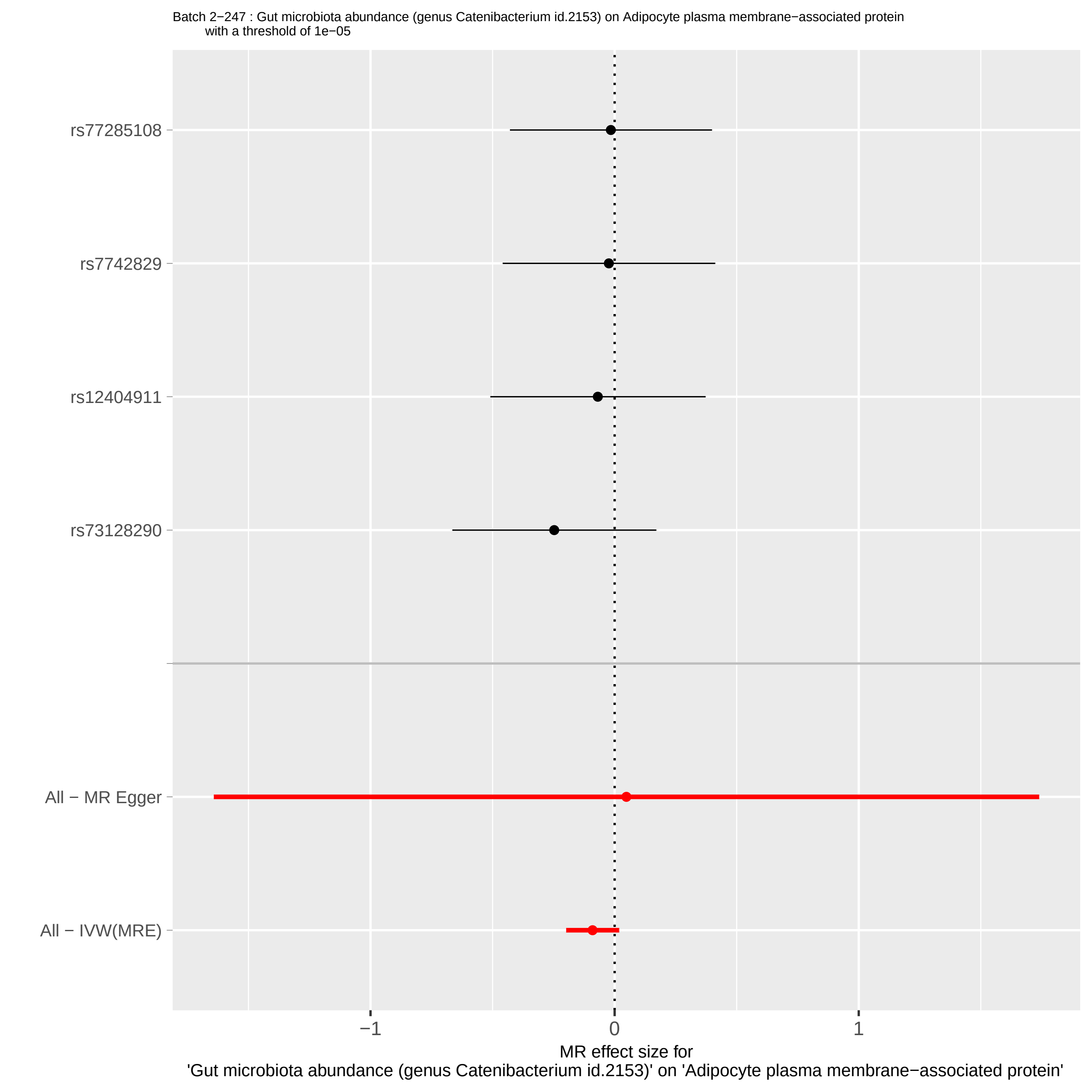
Batch 2−247 : Gut microbiota abundance (genus Catenibacterium id.2153) on Adipocyte plasma membrane−associated protein
with a threshold of 1e−05
rs77285108
rs7742829
rs12404911
rs73128290
All − MR Egger
All − IVW(MRE)
−1
0
1
MR effect size for
'Gut microbiota abundance (genus Catenibacterium id.2153)' on 'Adipocyte plasma membrane−associated protein'

Batch 2−248 : Gut microbiota abundance (genus Parasutterella id.2892) on Adipocyte plasma membrane−associated protein
with a threshold of 1e−05

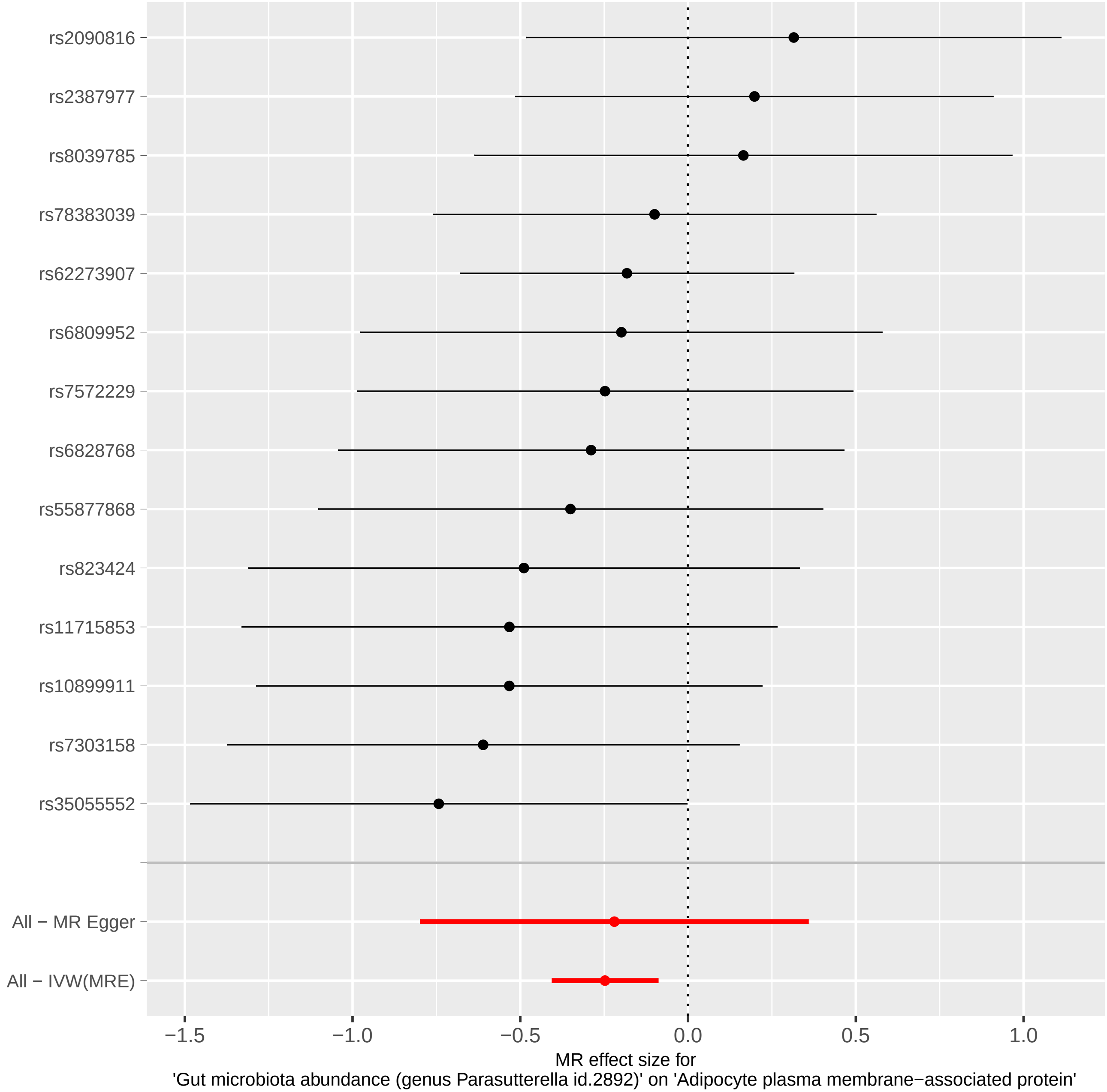

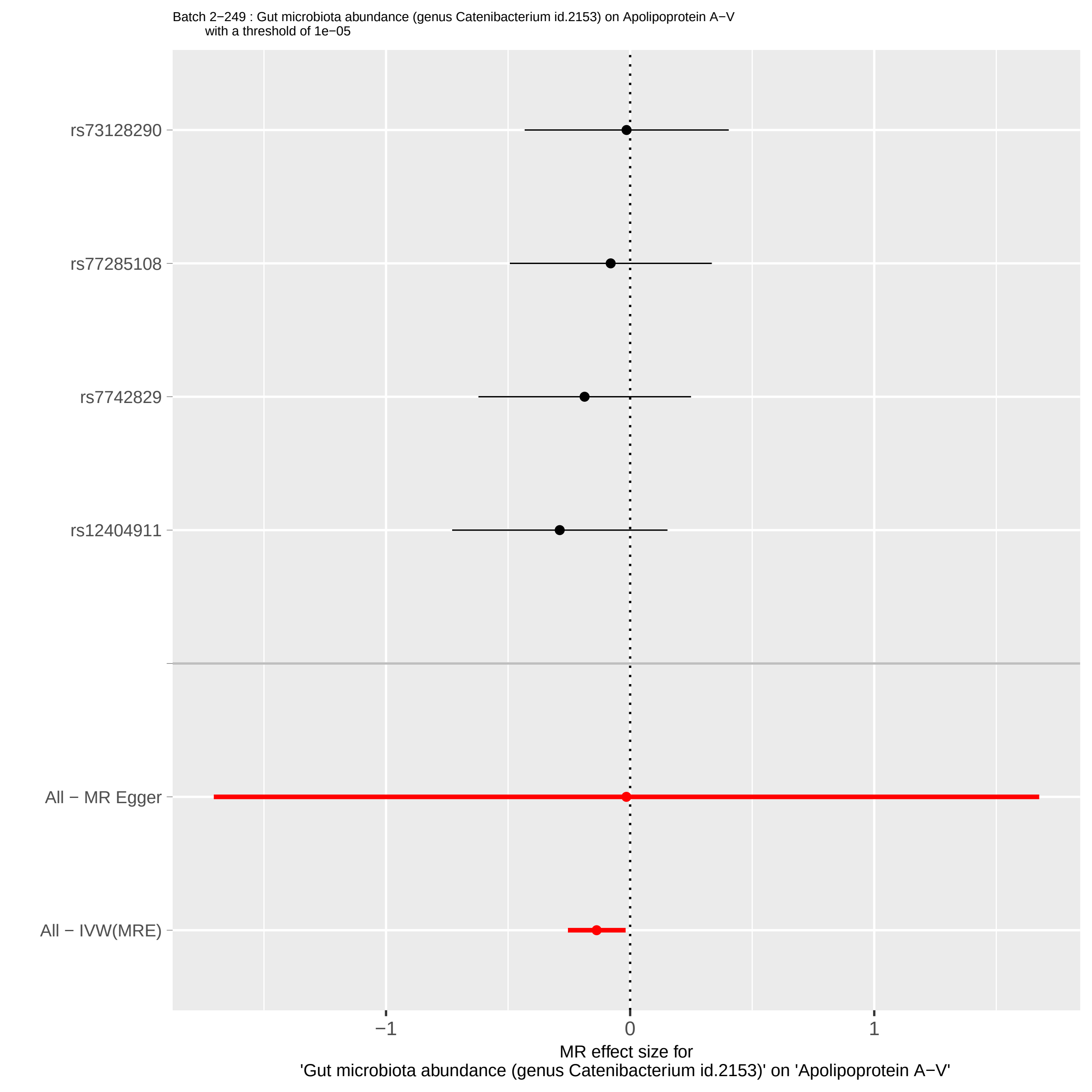

Batch 2−249 : Gut microbiota abundance (genus Catenibacterium id.2153) on Apolipoprotein A−V
with a threshold of 1e−05
rs73128290
rs77285108
rs7742829
rs12404911
All − MR Egger
All − IVW(MRE)
−1
0
1
MR effect size for
'Gut microbiota abundance (genus Catenibacterium id.2153)' on 'Apolipoprotein A−V'

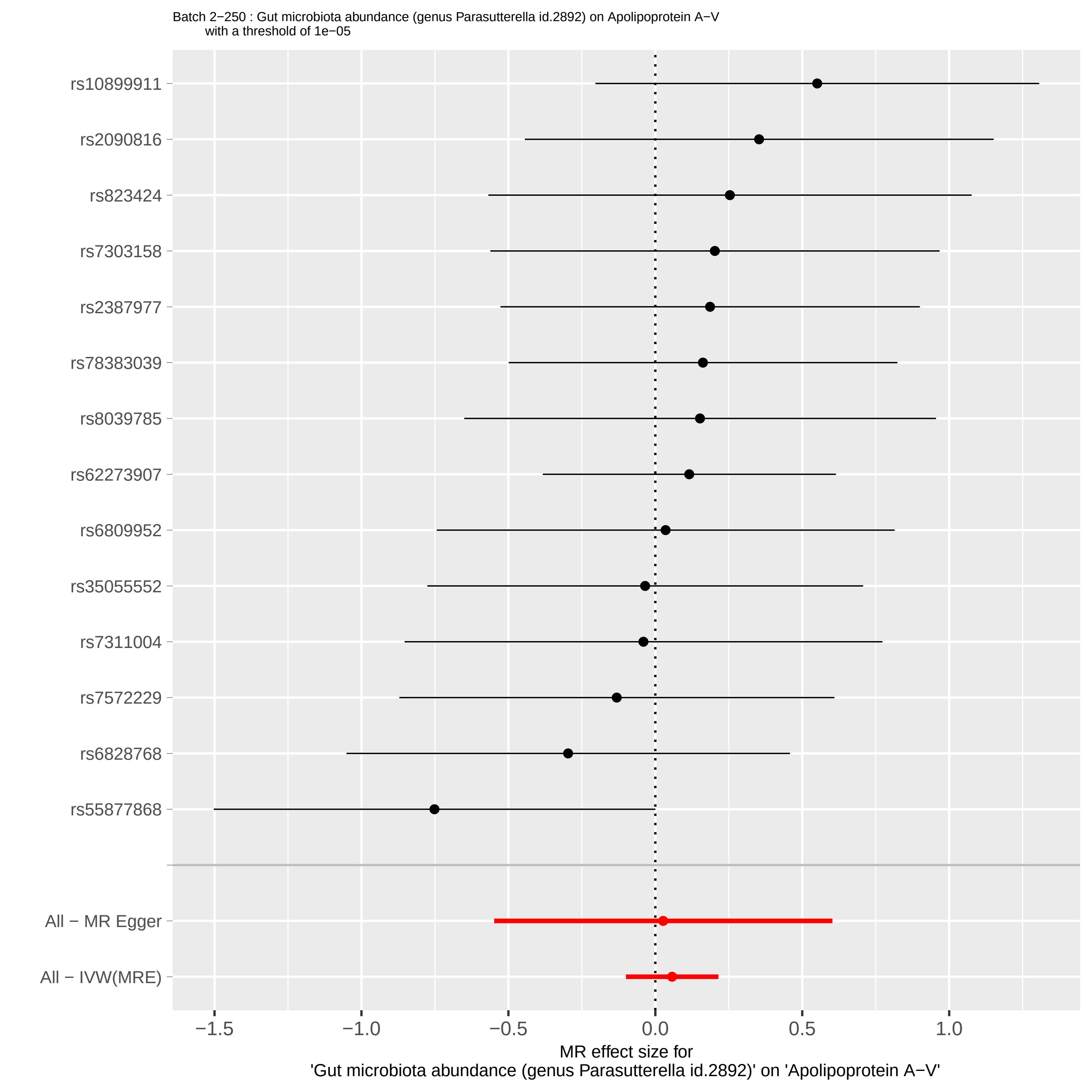

Batch 2−250 : Gut microbiota abundance (genus Parasutterella id.2892) on Apolipoprotein A−V
with a threshold of 1e−05
rs10899911
rs2090816
rs823424
rs7303158
rs2387977
rs78383039
rs8039785
rs62273907
rs6809952
rs35055552
rs7311004
rs7572229
rs6828768
rs55877868
All − MR Egger
All − IVW(MRE)
−1.5
−1.0
−0.5
0.0
0.5
1.0
MR effect size for
'Gut microbiota abundance (genus Parasutterella id.2892)' on 'Apolipoprotein A−V'

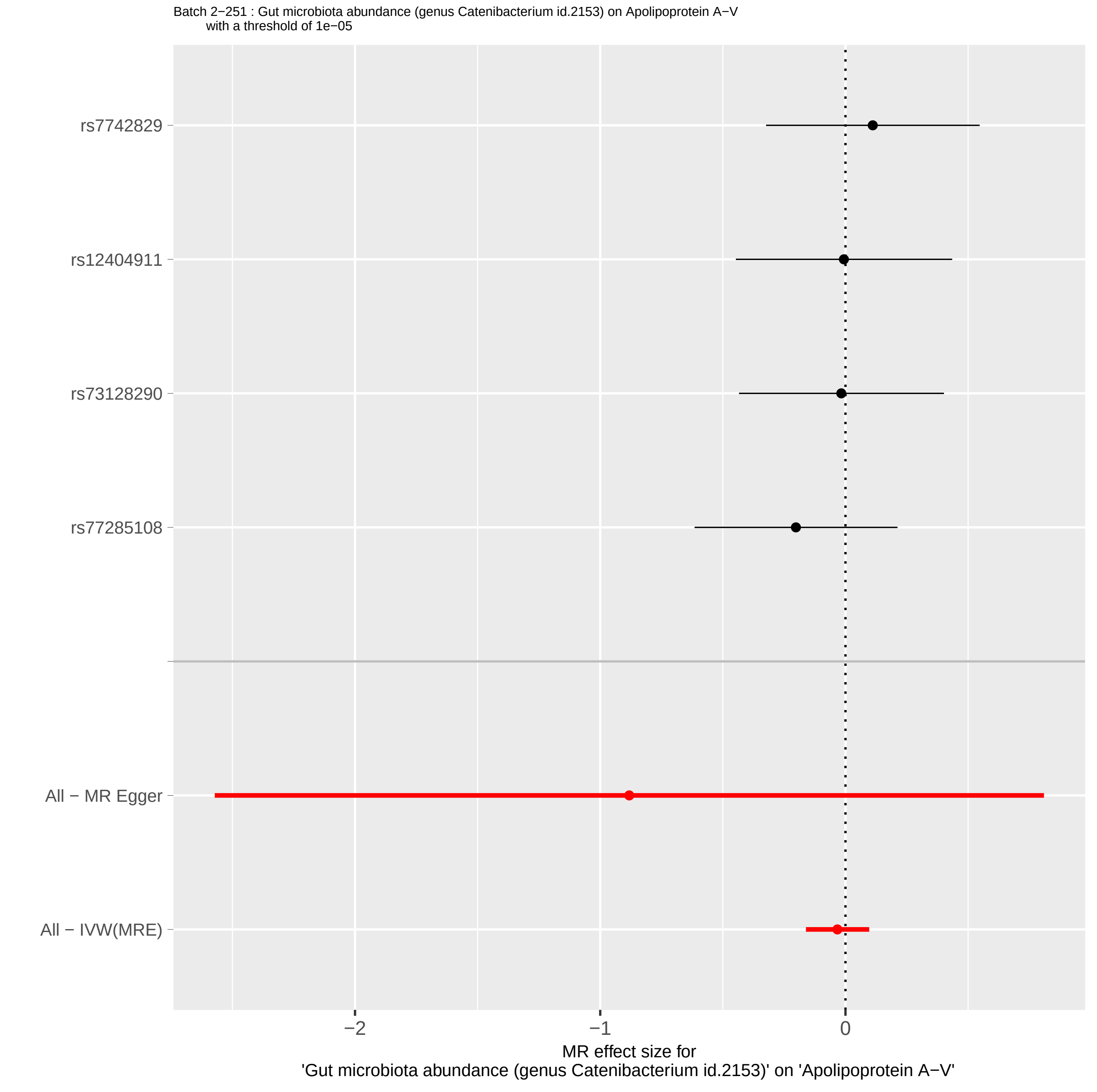
Batch 2−251 : Gut microbiota abundance (genus Catenibacterium id.2153) on Apolipoprotein A−V
with a threshold of 1e−05
rs7742829
rs12404911
rs73128290
rs77285108
All − MR Egger
All − IVW(MRE)
−2
−1
0
MR effect size for
'Gut microbiota abundance (genus Catenibacterium id.2153)' on 'Apolipoprotein A−V'

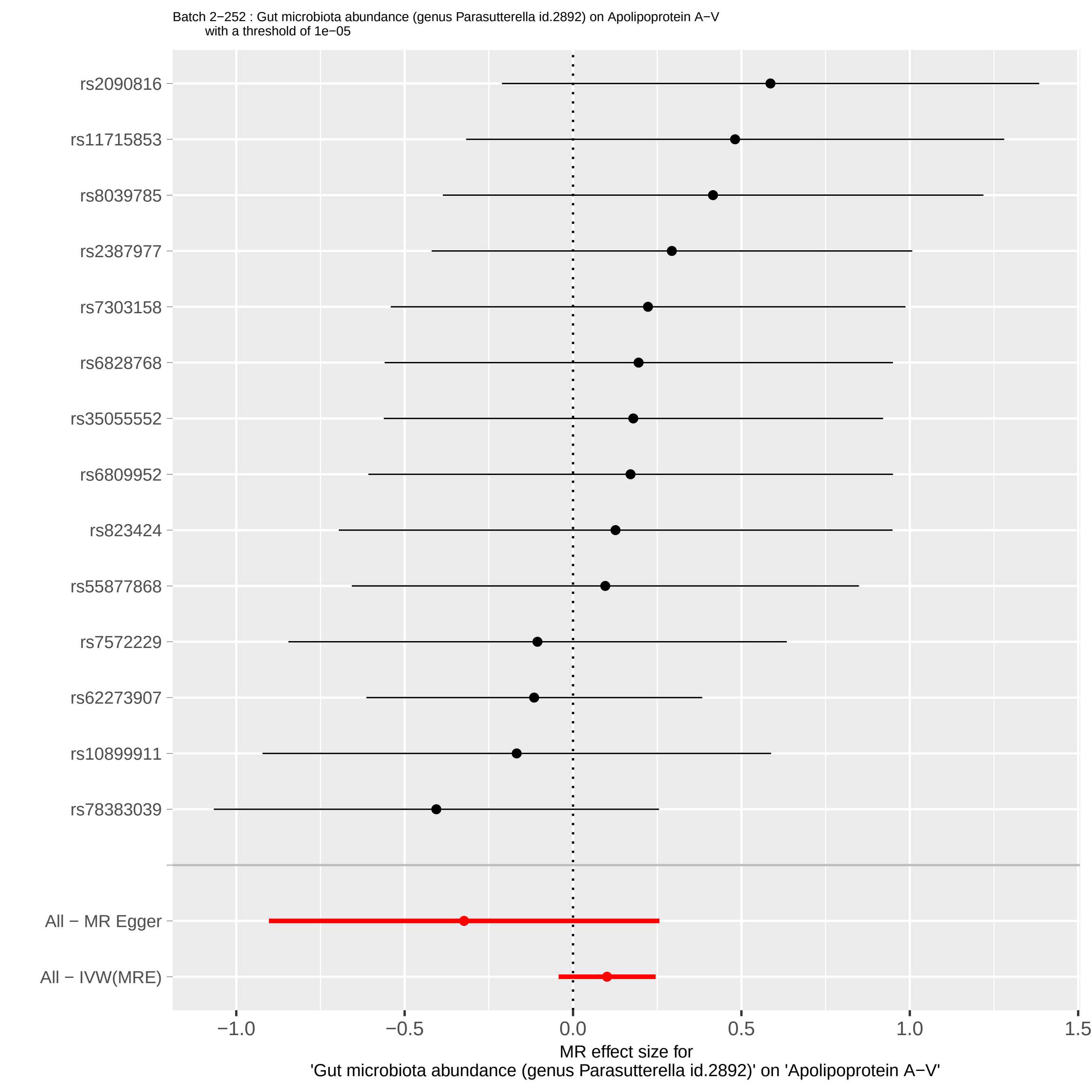
Batch 2−252 : Gut microbiota abundance (genus Parasutterella id.2892) on Apolipoprotein A−V
with a threshold of 1e−05
rs2090816
rs11715853
rs8039785
rs2387977
rs7303158
rs6828768
rs35055552
rs6809952
rs823424
rs55877868
rs7572229
rs62273907
rs10899911
rs78383039
All − MR Egger
All − IVW(MRE)
−1.0
−0.5
0.0
0.5
1.0
1.5
MR effect size for
'Gut microbiota abundance (genus Parasutterella id.2892)' on 'Apolipoprotein A−V'

Batch 2−253 : Gut microbiota abundance (genus Catenibacterium id.2153) on Apolipoprotein B
with a threshold of 1e−05

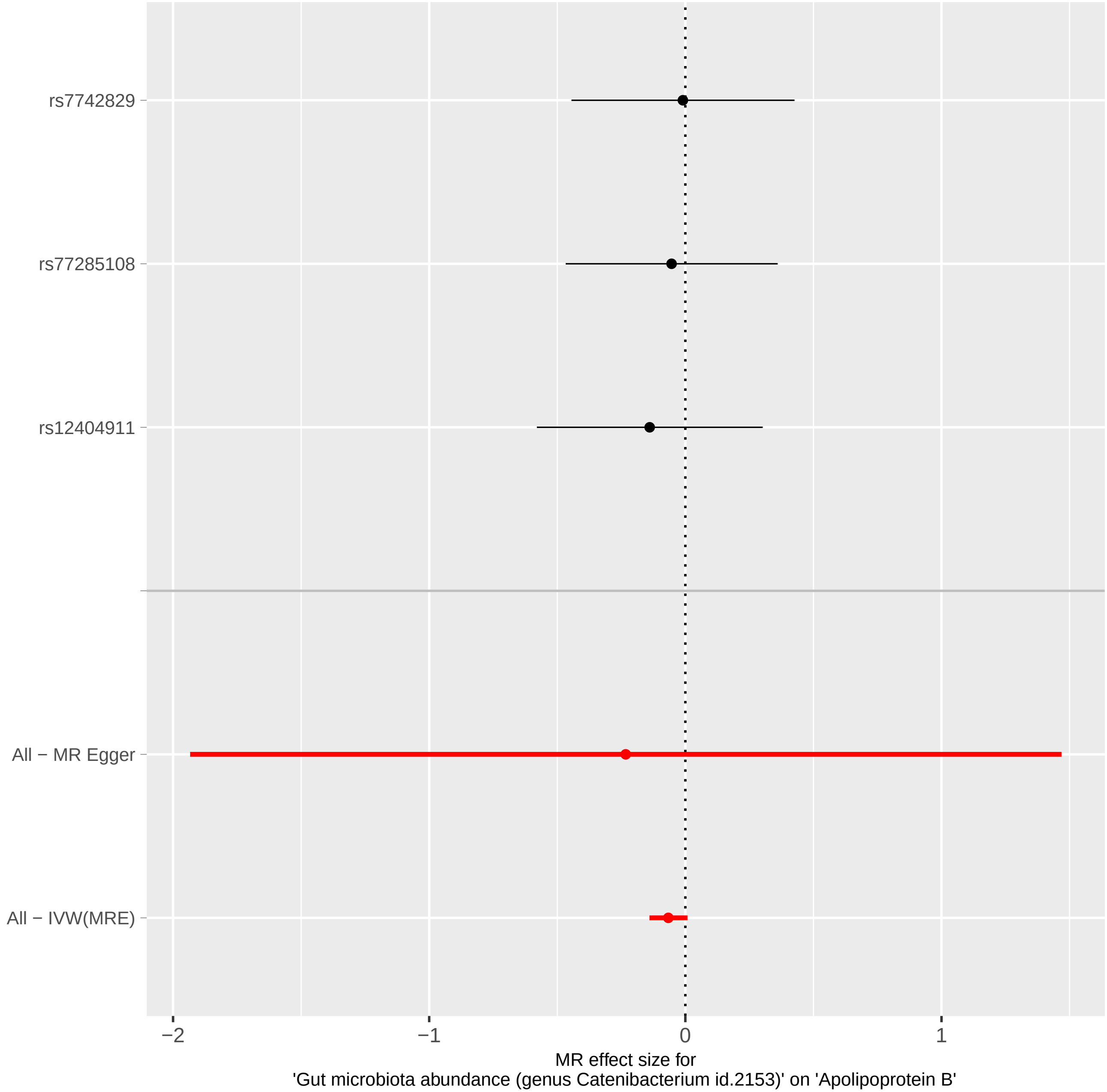

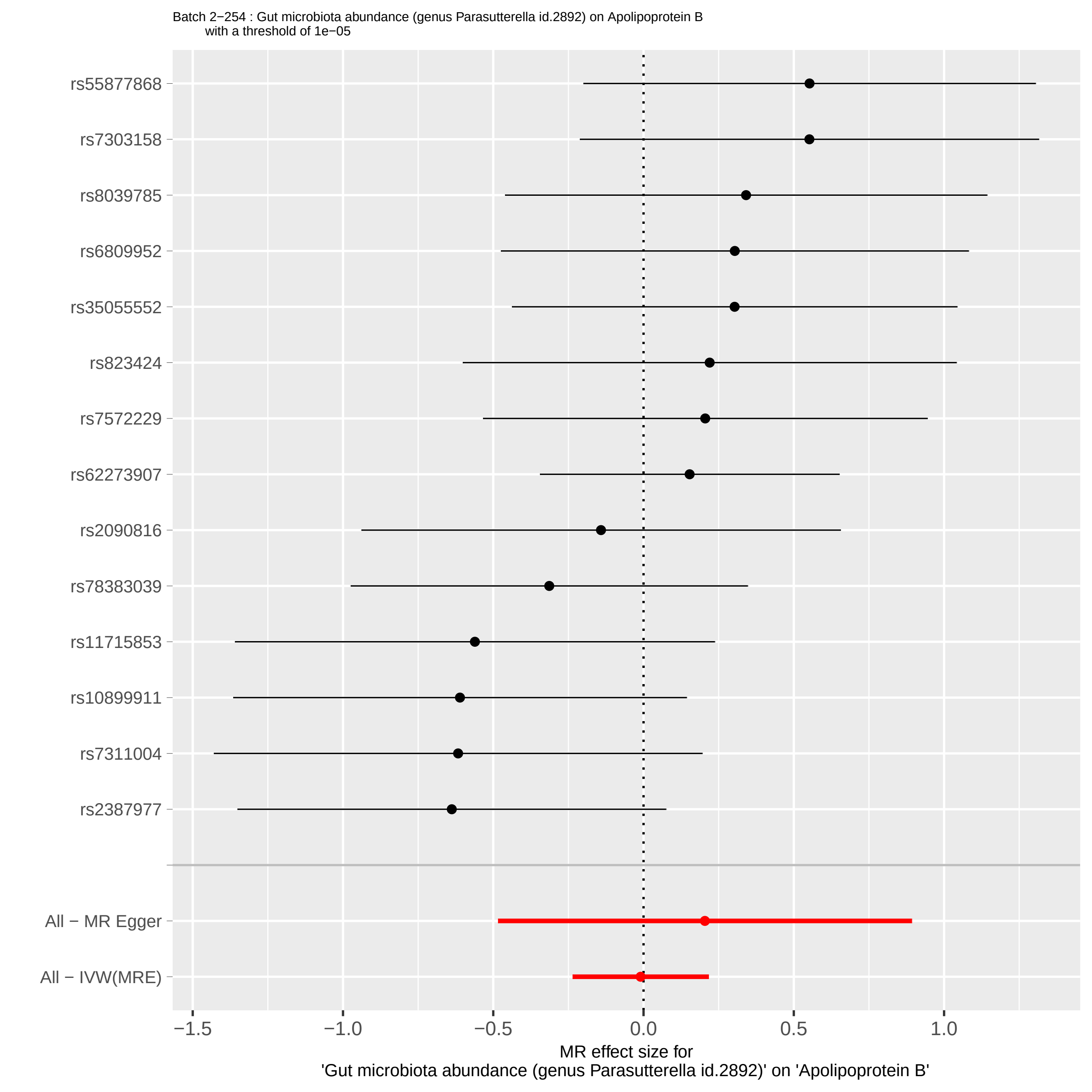

Batch 2−254 : Gut microbiota abundance (genus Parasutterella id.2892) on Apolipoprotein B
with a threshold of 1e−05
rs55877868
rs7303158
rs8039785
rs6809952
rs35055552
rs823424
rs7572229
rs62273907
rs2090816
rs78383039
rs11715853
rs10899911
rs7311004
rs2387977
All − MR Egger
All − IVW(MRE)
−1.5
−1.0
−0.5
0.0
0.5
1.0
MR effect size for
'Gut microbiota abundance (genus Parasutterella id.2892)' on 'Apolipoprotein B'

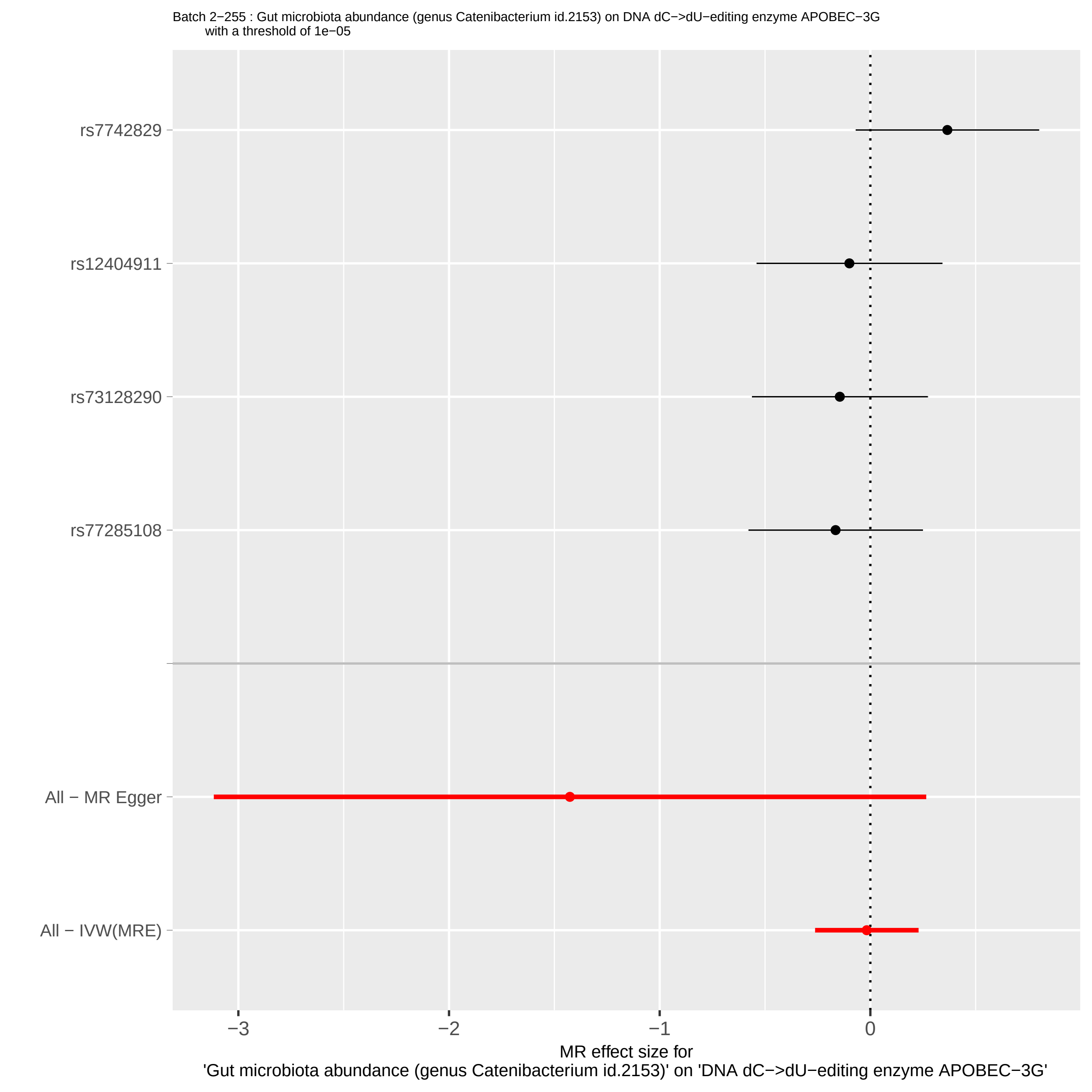
Batch 2−255 : Gut microbiota abundance (genus Catenibacterium id.2153) on DNA dC−>dU−editing enzyme APOBEC−3G
with a threshold of 1e−05
rs7742829
rs12404911
rs73128290
rs77285108
All − MR Egger
All − IVW(MRE)
−3
−2
−1
0
MR effect size for
'Gut microbiota abundance (genus Catenibacterium id.2153)' on 'DNA dC−>dU−editing enzyme APOBEC−3G'

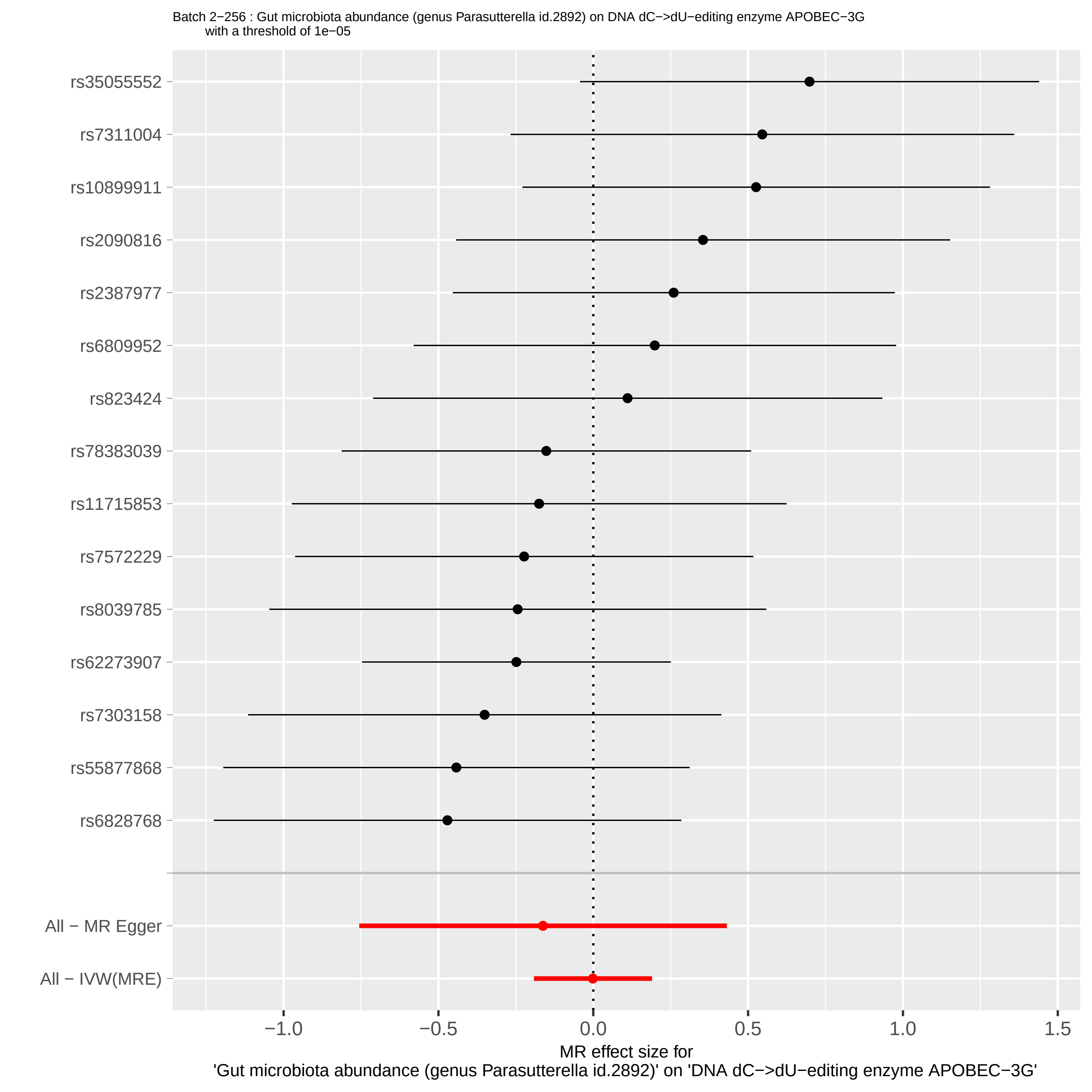
Batch 2−256 : Gut microbiota abundance (genus Parasutterella id.2892) on DNA dC−>dU−editing enzyme APOBEC−3G
with a threshold of 1e−05
rs35055552
rs7311004
rs10899911
rs2090816
rs2387977
rs6809952
rs823424
rs78383039
rs11715853
rs7572229
rs8039785
rs62273907
rs7303158
rs55877868
rs6828768
All − MR Egger
All − IVW(MRE)
−1.0
−0.5
0.0
0.5
1.0
1.5
MR effect size for
'Gut microbiota abundance (genus Parasutterella id.2892)' on 'DNA dC−>dU−editing enzyme APOBEC−3G'

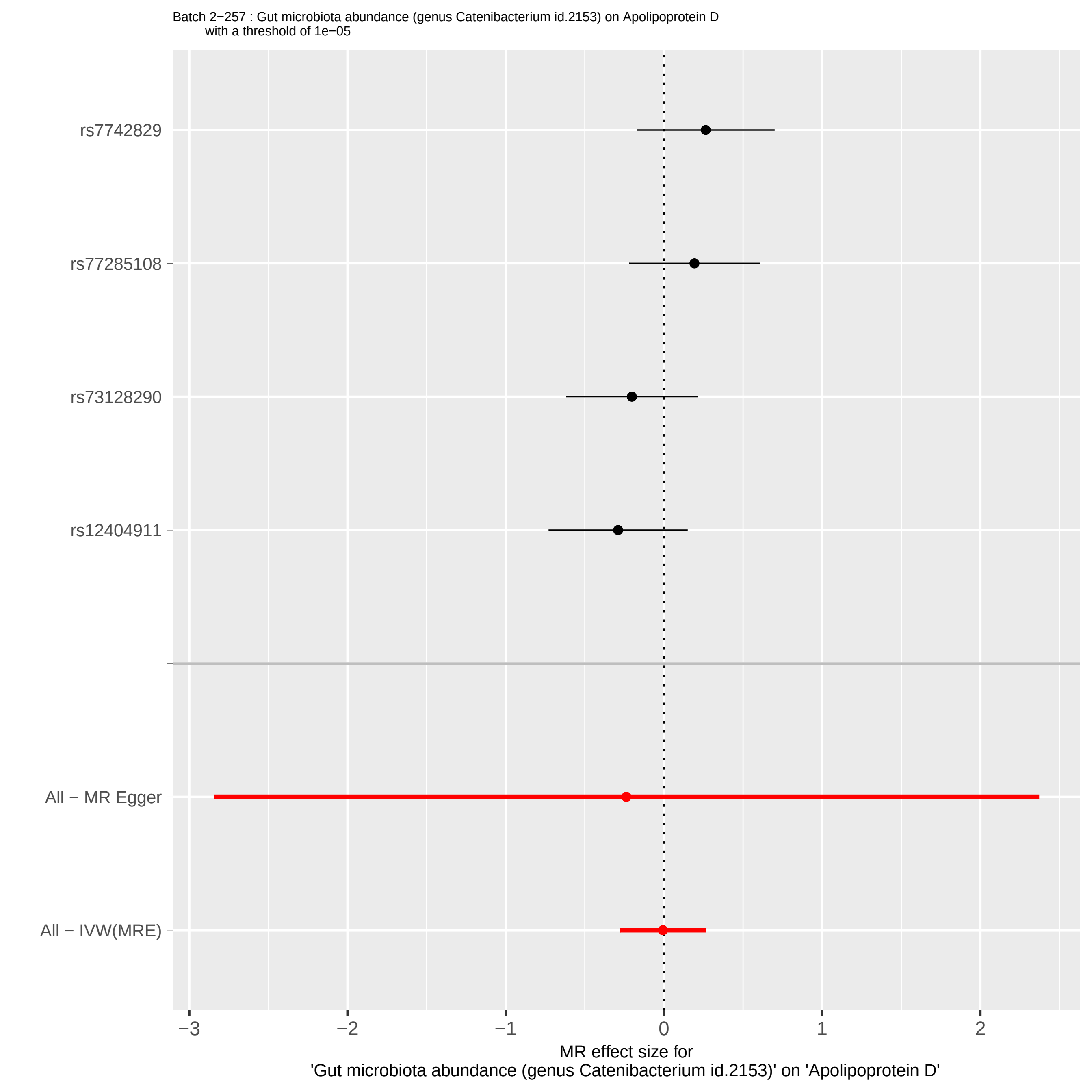

Batch 2−257 : Gut microbiota abundance (genus Catenibacterium id.2153) on Apolipoprotein D
with a threshold of 1e−05
rs7742829
rs77285108
rs73128290
rs12404911
All − MR Egger
All − IVW(MRE)
−3
−2
−1
0
1
2
MR effect size for
'Gut microbiota abundance (genus Catenibacterium id.2153)' on 'Apolipoprotein D'

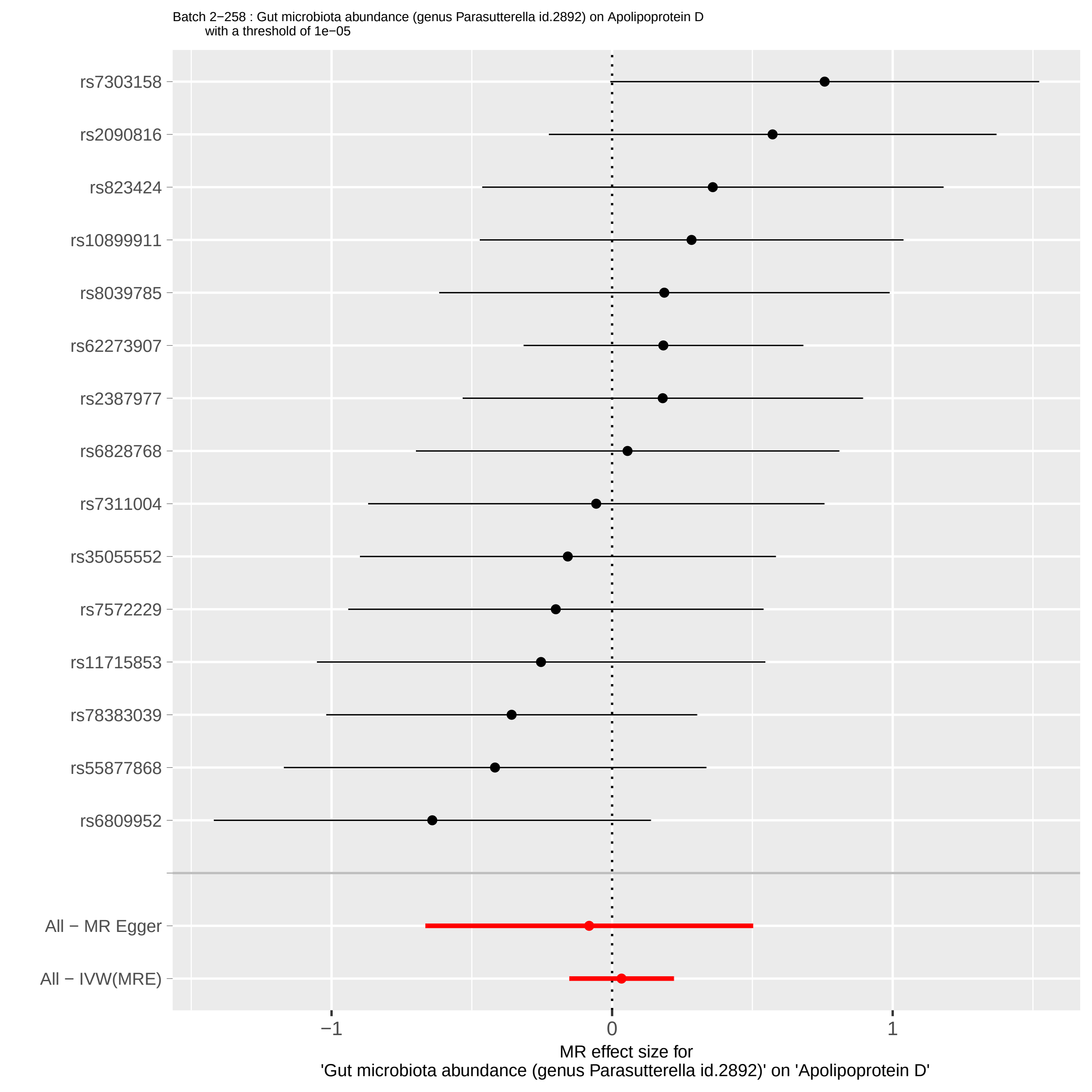
Batch 2−258 : Gut microbiota abundance (genus Parasutterella id.2892) on Apolipoprotein D
with a threshold of 1e−05
rs7303158
rs2090816
rs823424
rs10899911
rs8039785
rs62273907
rs2387977
rs6828768
rs7311004
rs35055552
rs7572229
rs11715853
rs78383039
rs55877868
rs6809952
All − MR Egger
All − IVW(MRE)
−1
0
1
MR effect size for
'Gut microbiota abundance (genus Parasutterella id.2892)' on 'Apolipoprotein D'

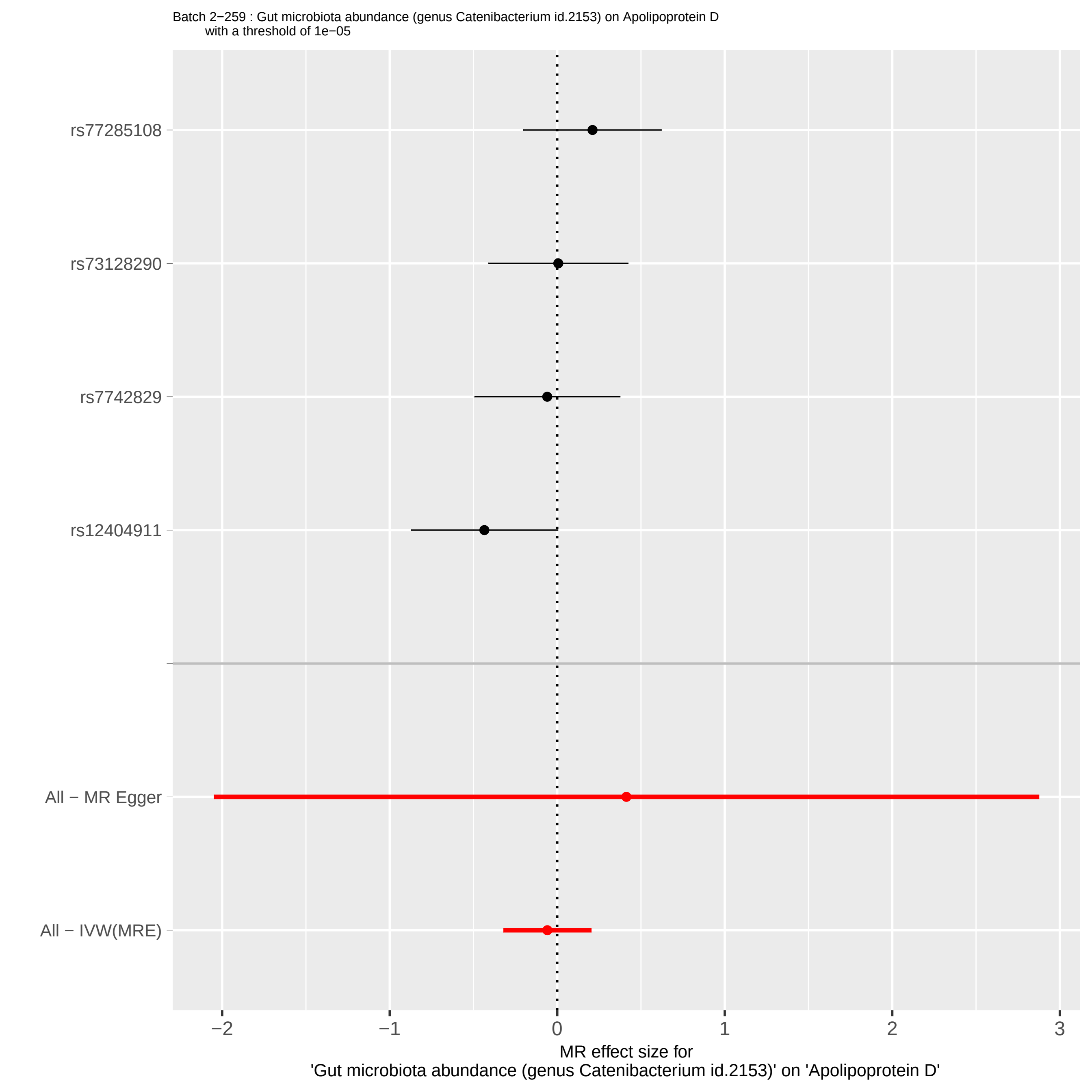

Batch 2−259 : Gut microbiota abundance (genus Catenibacterium id.2153) on Apolipoprotein D
with a threshold of 1e−05
rs77285108
rs73128290
rs7742829
rs12404911
All − MR Egger
All − IVW(MRE)
−2
−1
0
1
2
3
MR effect size for
'Gut microbiota abundance (genus Catenibacterium id.2153)' on 'Apolipoprotein D'

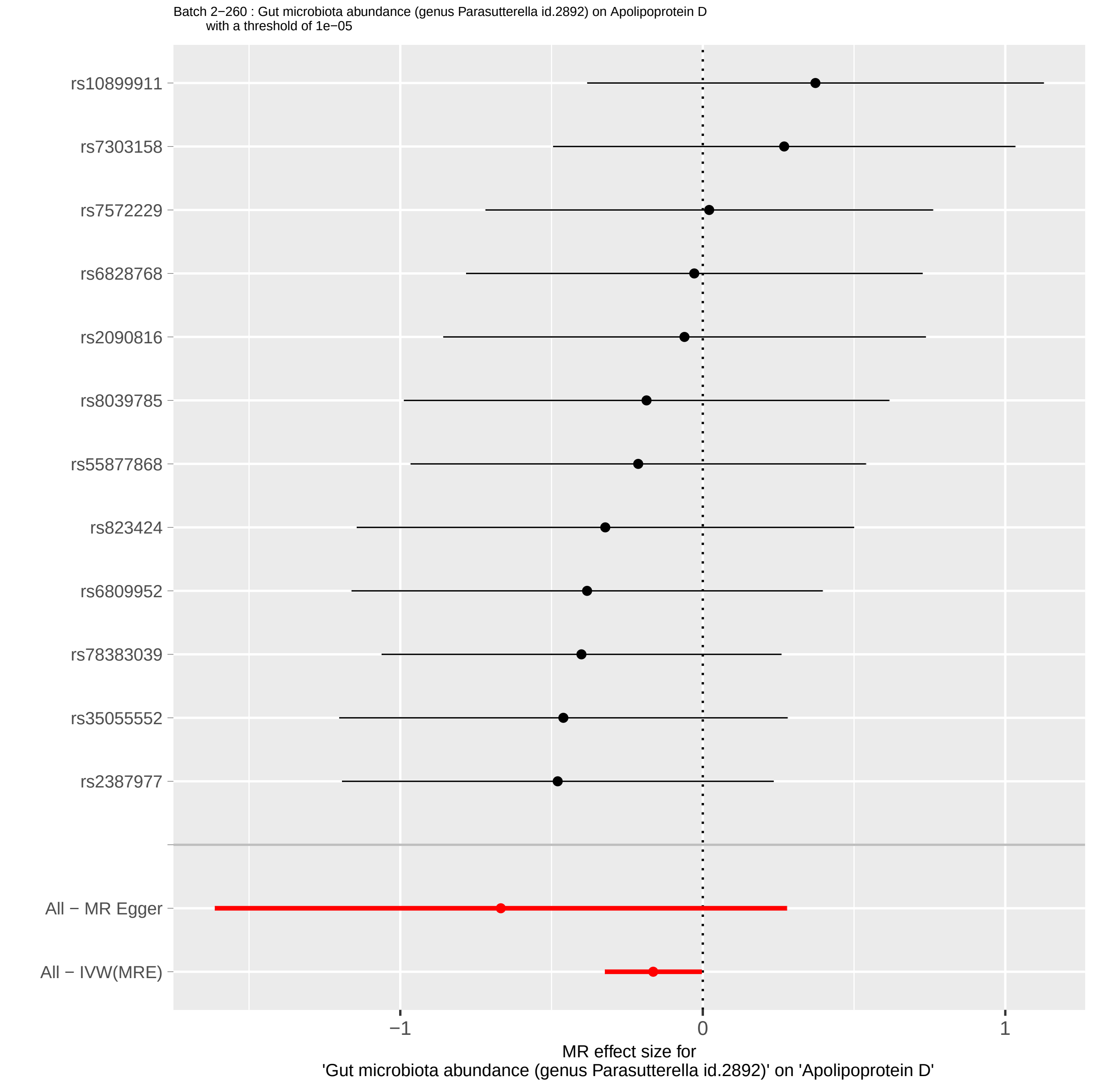

Batch 2−260 : Gut microbiota abundance (genus Parasutterella id.2892) on Apolipoprotein D
with a threshold of 1e−05
rs10899911
rs7303158
rs7572229
rs6828768
rs2090816
rs8039785
rs55877868
rs823424
rs6809952
rs78383039
rs35055552
rs2387977
All − MR Egger
All − IVW(MRE)
−1
0
1
MR effect size for
'Gut microbiota abundance (genus Parasutterella id.2892)' on 'Apolipoprotein D'

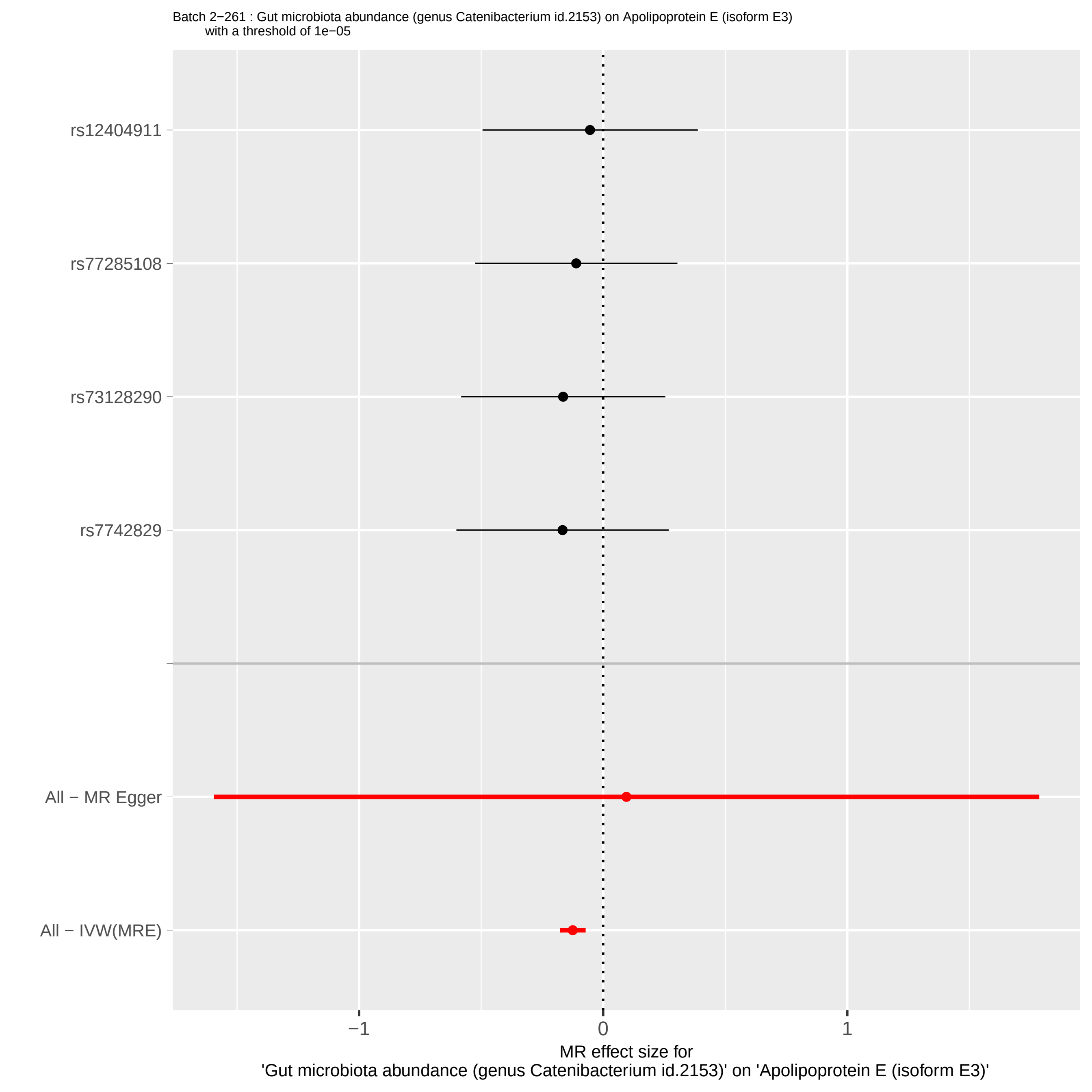
Batch 2−261 : Gut microbiota abundance (genus Catenibacterium id.2153) on Apolipoprotein E (isoform E3)
with a threshold of 1e−05
rs12404911
rs77285108
rs73128290
rs7742829
All − MR Egger
All − IVW(MRE)
−1
0
1
MR effect size for
'Gut microbiota abundance (genus Catenibacterium id.2153)' on 'Apolipoprotein E (isoform E3)'

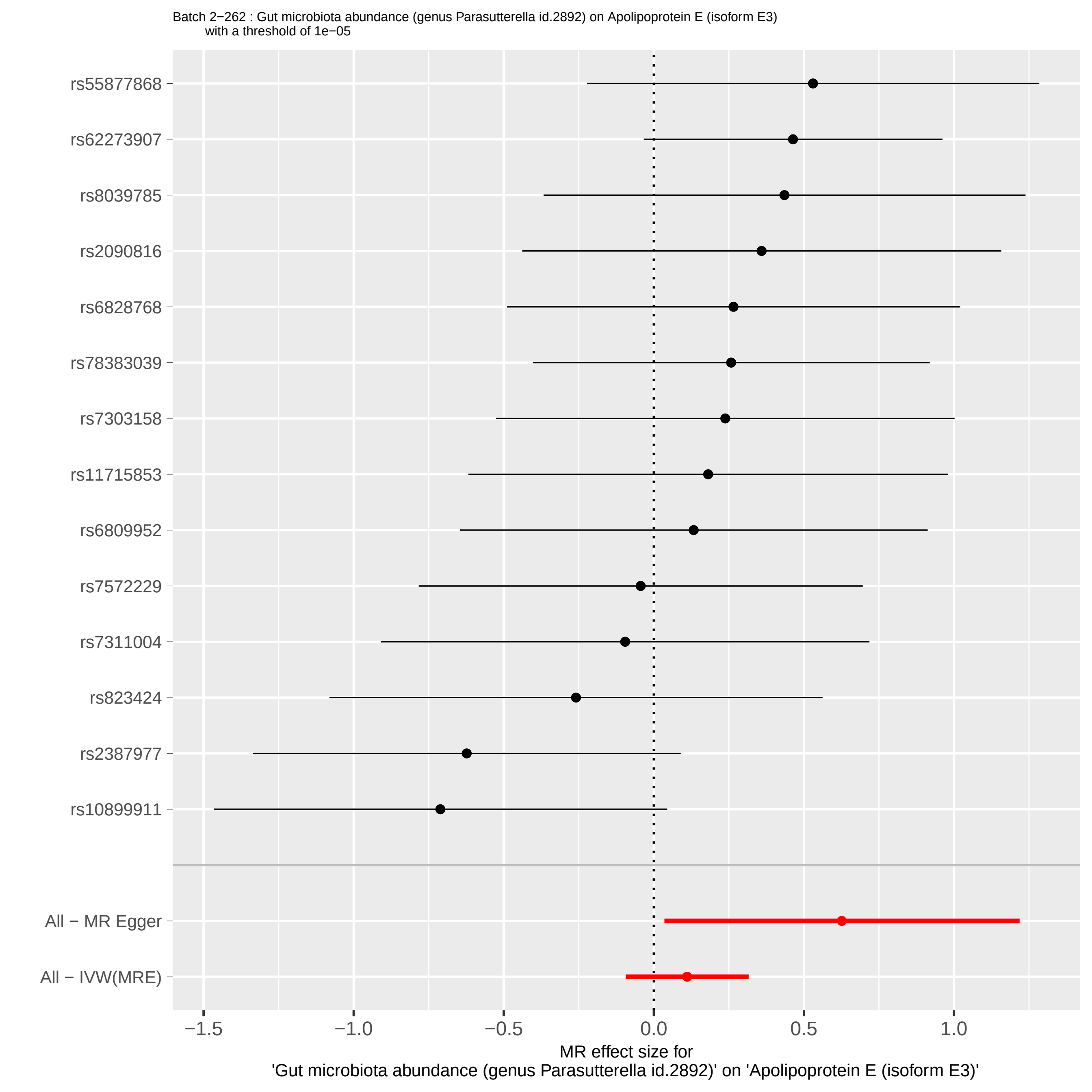
Batch 2−262 : Gut microbiota abundance (genus Parasutterella id.2892) on Apolipoprotein E (isoform E3)
with a threshold of 1e−05
rs55877868
rs62273907
rs8039785
rs2090816
rs6828768
rs78383039
rs7303158
rs11715853
rs6809952
rs7572229
rs7311004
rs823424
rs2387977
rs10899911
All − MR Egger
All − IVW(MRE)
−1.5
−1.0
−0.5
0.0
0.5
1.0
MR effect size for
'Gut microbiota abundance (genus Parasutterella id.2892)' on 'Apolipoprotein E (isoform E3)'

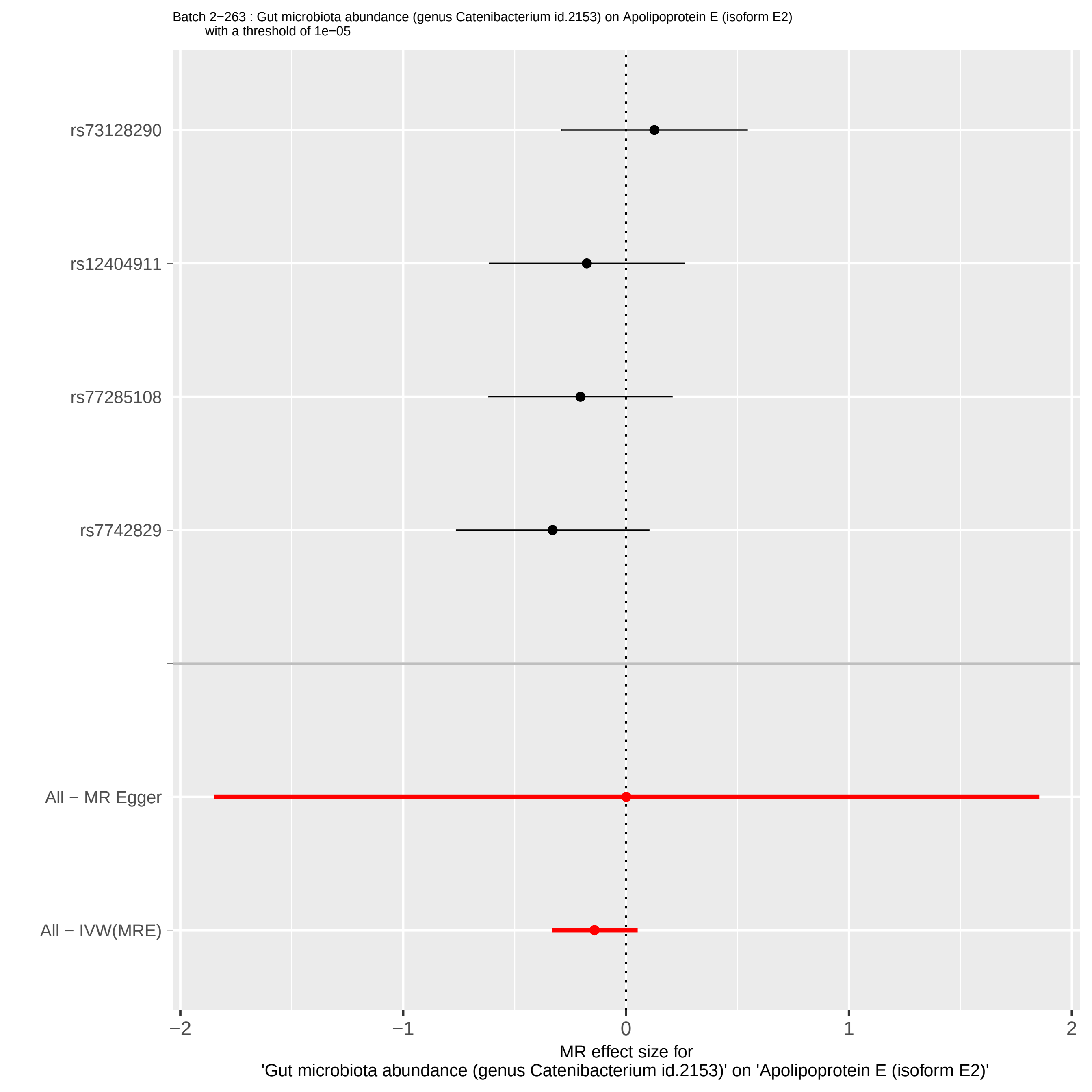

Batch 2−263 : Gut microbiota abundance (genus Catenibacterium id.2153) on Apolipoprotein E (isoform E2)
with a threshold of 1e−05
rs73128290
rs12404911
rs77285108
rs7742829
All − MR Egger
All − IVW(MRE)
−2
−1
0
1
2
MR effect size for
'Gut microbiota abundance (genus Catenibacterium id.2153)' on 'Apolipoprotein E (isoform E2)'

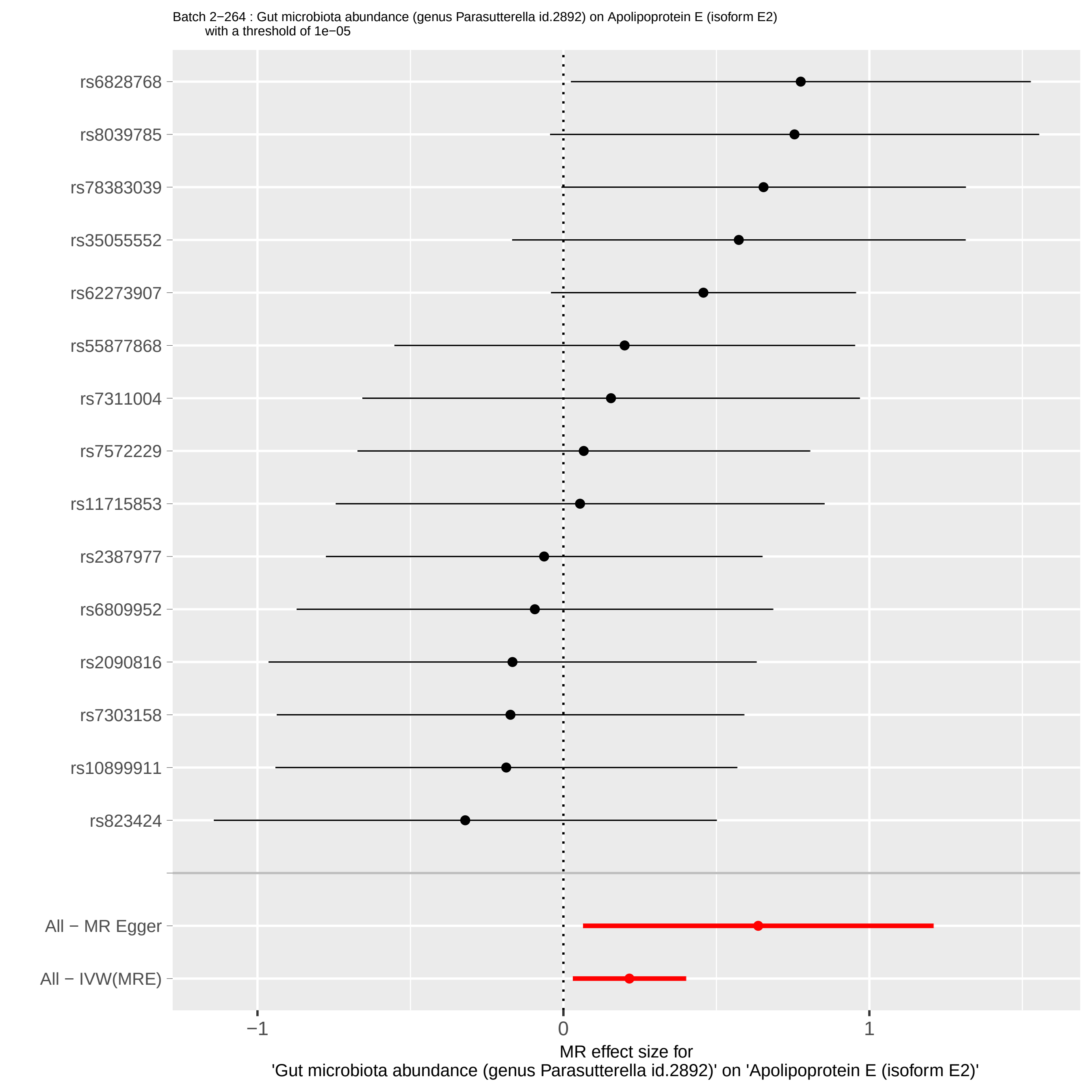

Batch 2−264 : Gut microbiota abundance (genus Parasutterella id.2892) on Apolipoprotein E (isoform E2)
with a threshold of 1e−05
rs6828768
rs8039785
rs78383039
rs35055552
rs62273907
rs55877868
rs7311004
rs7572229
rs11715853
rs2387977
rs6809952
rs2090816
rs7303158
rs10899911
rs823424
All − MR Egger
All − IVW(MRE)
−1
0
1
MR effect size for
'Gut microbiota abundance (genus Parasutterella id.2892)' on 'Apolipoprotein E (isoform E2)'

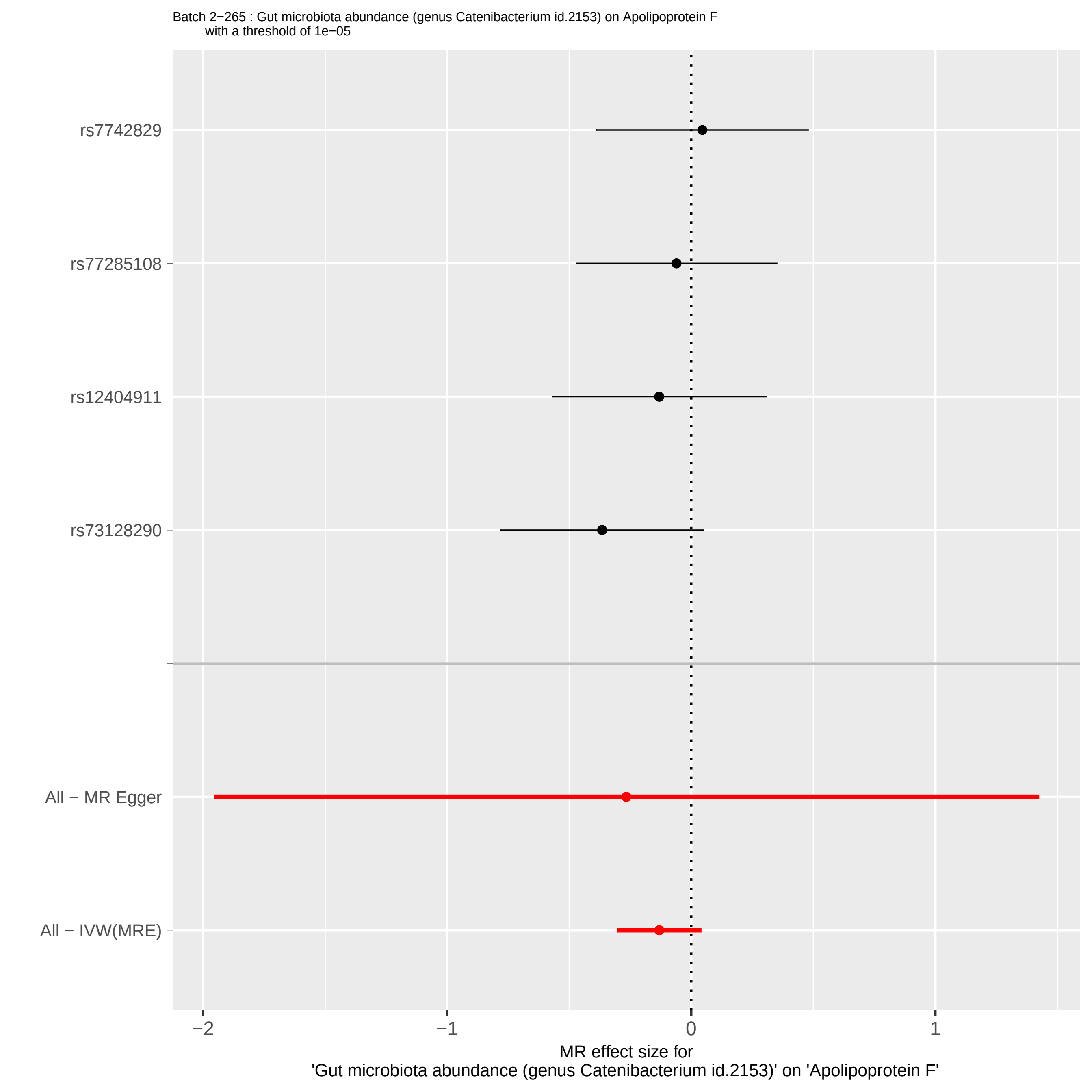
Batch 2−265 : Gut microbiota abundance (genus Catenibacterium id.2153) on Apolipoprotein F
with a threshold of 1e−05
rs7742829
rs77285108
rs12404911
rs73128290
All − MR Egger
All − IVW(MRE)
−2
−1
0
1
MR effect size for
'Gut microbiota abundance (genus Catenibacterium id.2153)' on 'Apolipoprotein F'

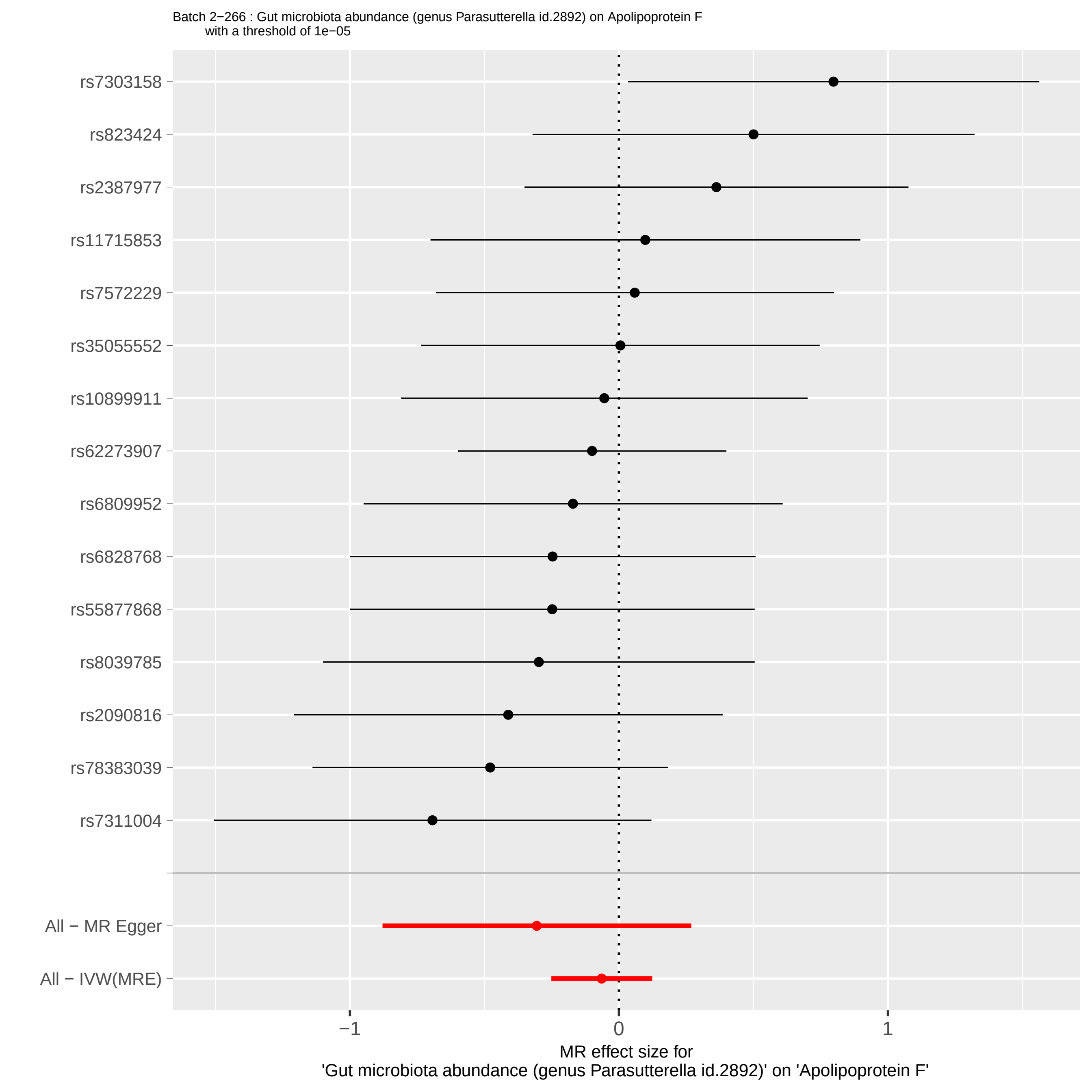
Batch 2−266 : Gut microbiota abundance (genus Parasutterella id.2892) on Apolipoprotein F
with a threshold of 1e−05
rs7303158
rs823424
rs2387977
rs11715853
rs7572229
rs35055552
rs10899911
rs62273907
rs6809952
rs6828768
rs55877868
rs8039785
rs2090816
rs78383039
rs7311004
All − MR Egger
All − IVW(MRE)
−1
0
1
MR effect size for
'Gut microbiota abundance (genus Parasutterella id.2892)' on 'Apolipoprotein F'

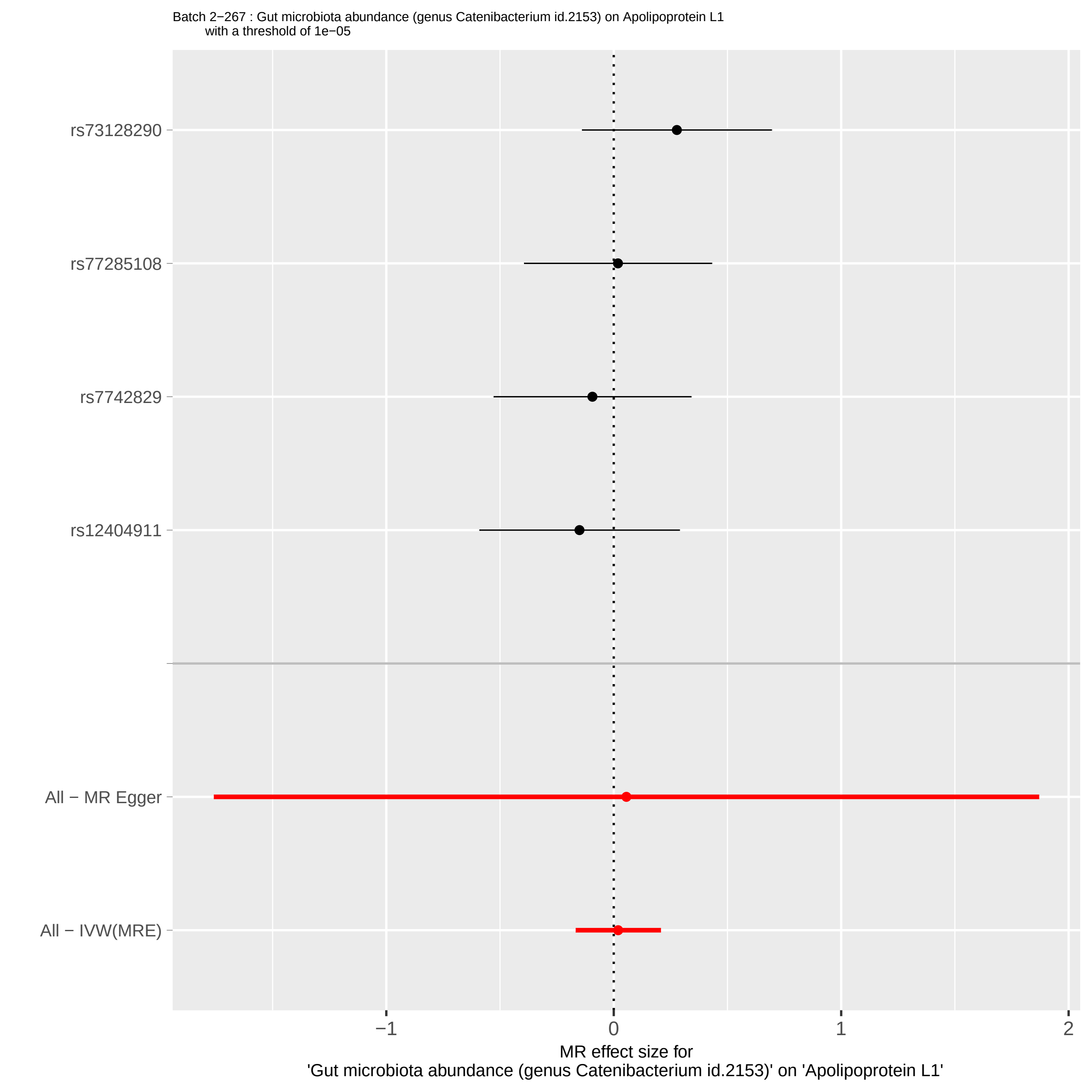

Batch 2−267 : Gut microbiota abundance (genus Catenibacterium id.2153) on Apolipoprotein L1
with a threshold of 1e−05
rs73128290
rs77285108
rs7742829
rs12404911
All − MR Egger
All − IVW(MRE)
−1
0
1
2
MR effect size for
'Gut microbiota abundance (genus Catenibacterium id.2153)' on 'Apolipoprotein L1'

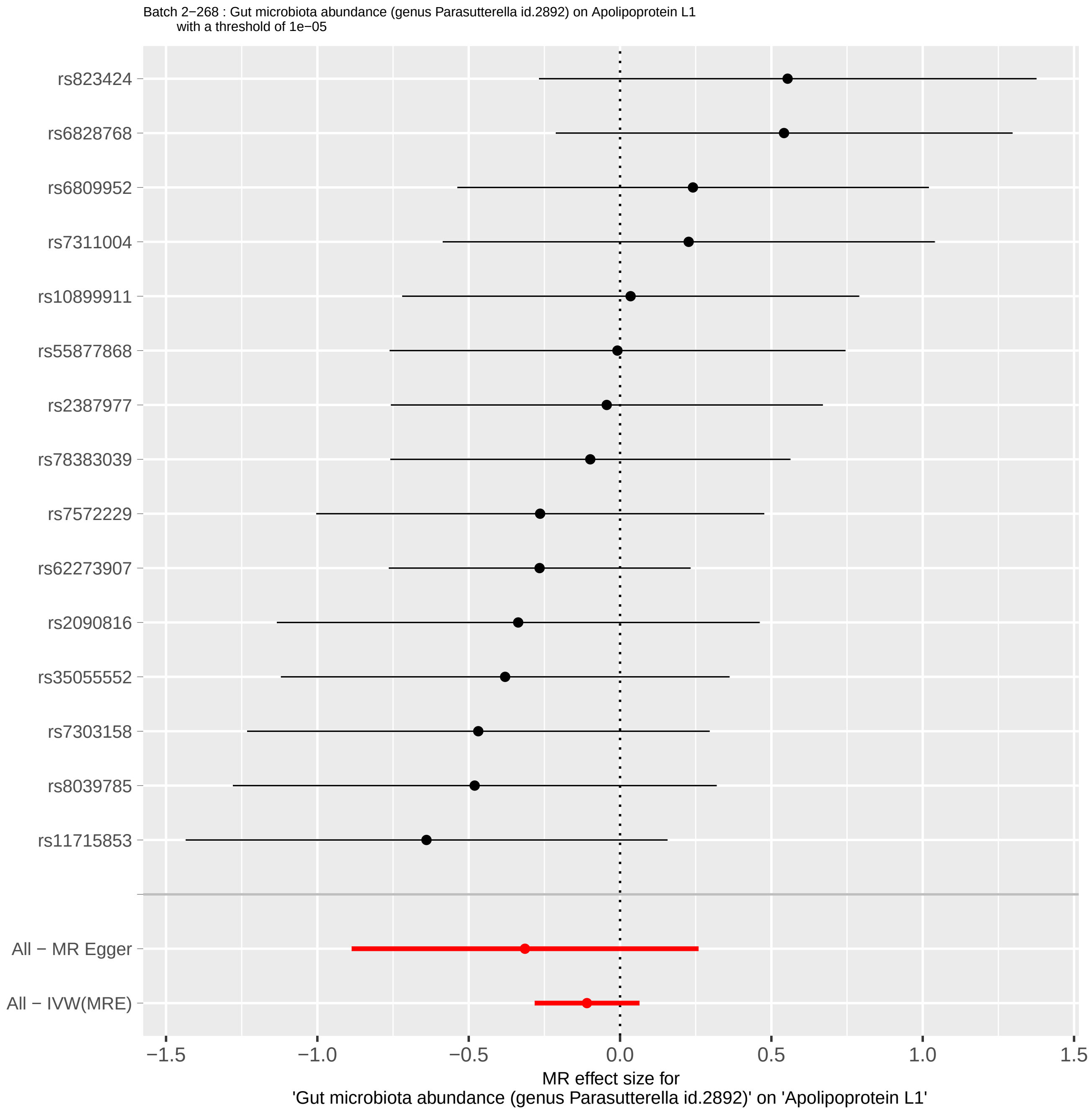

Batch 2−268 : Gut microbiota abundance (genus Parasutterella id.2892) on Apolipoprotein L1
with a threshold of 1e−05
rs823424
rs6828768
rs6809952
rs7311004
rs10899911
rs55877868
rs2387977
rs78383039
rs7572229
rs62273907
rs2090816
rs35055552
rs7303158
rs8039785
rs11715853
All − MR Egger
All − IVW(MRE)
−1.5
−1.0
−0.5
0.0
0.5
1.0
1.5
MR effect size for
'Gut microbiota abundance (genus Parasutterella id.2892)' on 'Apolipoprotein L1'

Batch 2−269 : Gut microbiota abundance (genus Catenibacterium id.2153) on Apolipoprotein L1
with a threshold of 1e−05

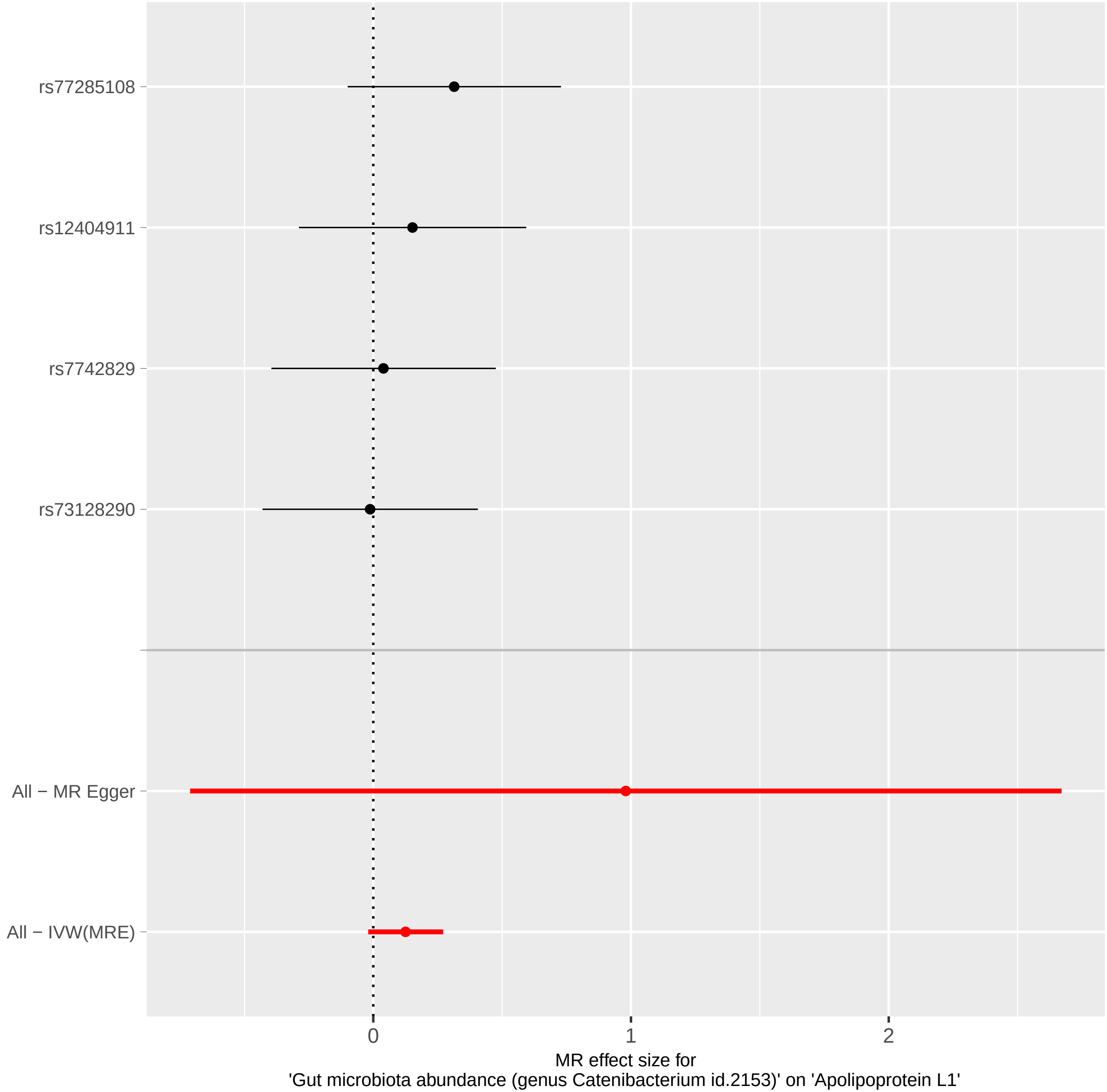

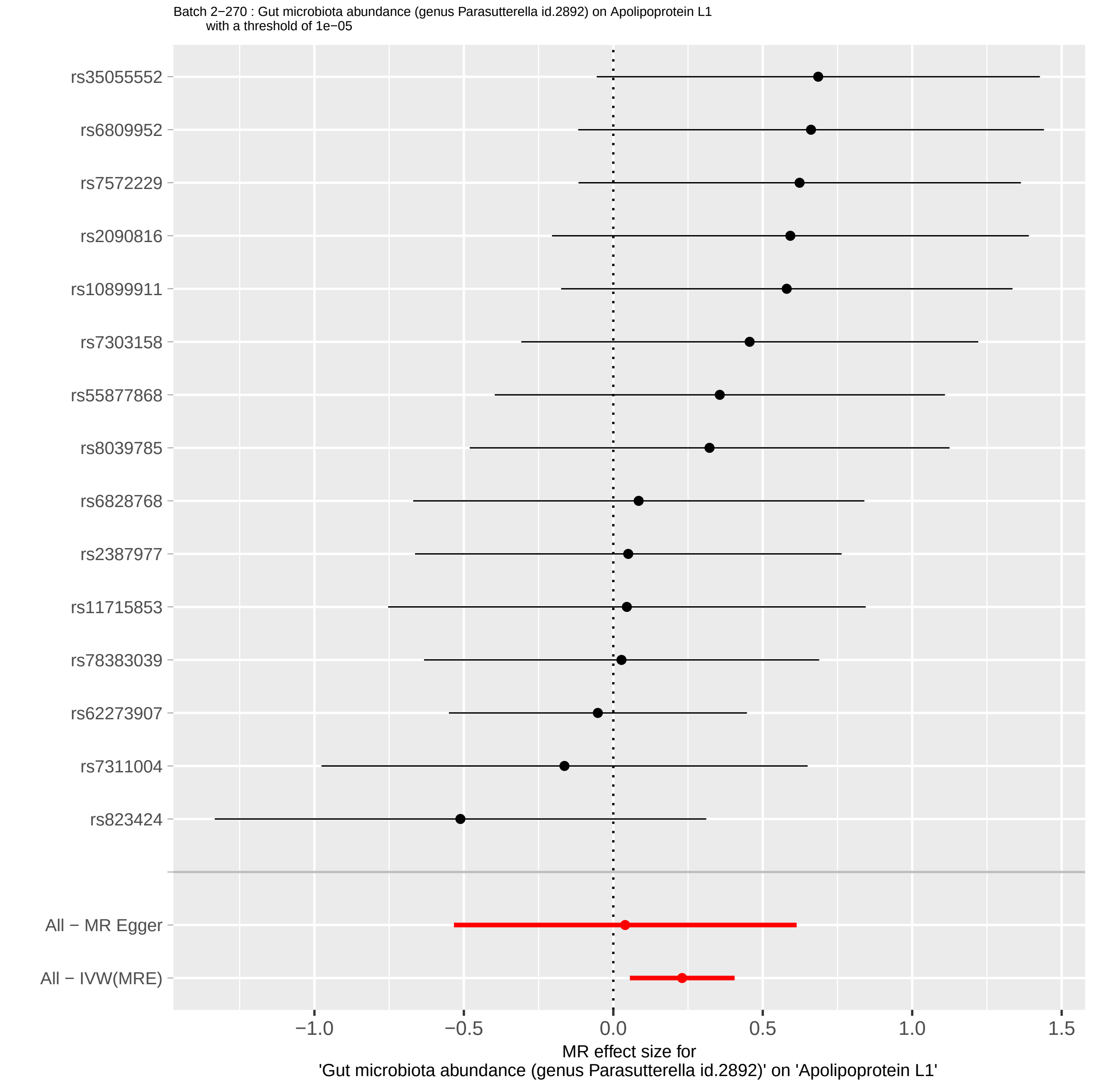

Batch 2−270 : Gut microbiota abundance (genus Parasutterella id.2892) on Apolipoprotein L1
with a threshold of 1e−05
rs35055552
rs6809952
rs7572229
rs2090816
rs10899911
rs7303158
rs55877868
rs8039785
rs6828768
rs2387977
rs11715853
rs78383039
rs62273907
rs7311004
rs823424
All − MR Egger
All − IVW(MRE)
−1.0
−0.5
0.0
0.5
1.0
1.5
MR effect size for
'Gut microbiota abundance (genus Parasutterella id.2892)' on 'Apolipoprotein L1'

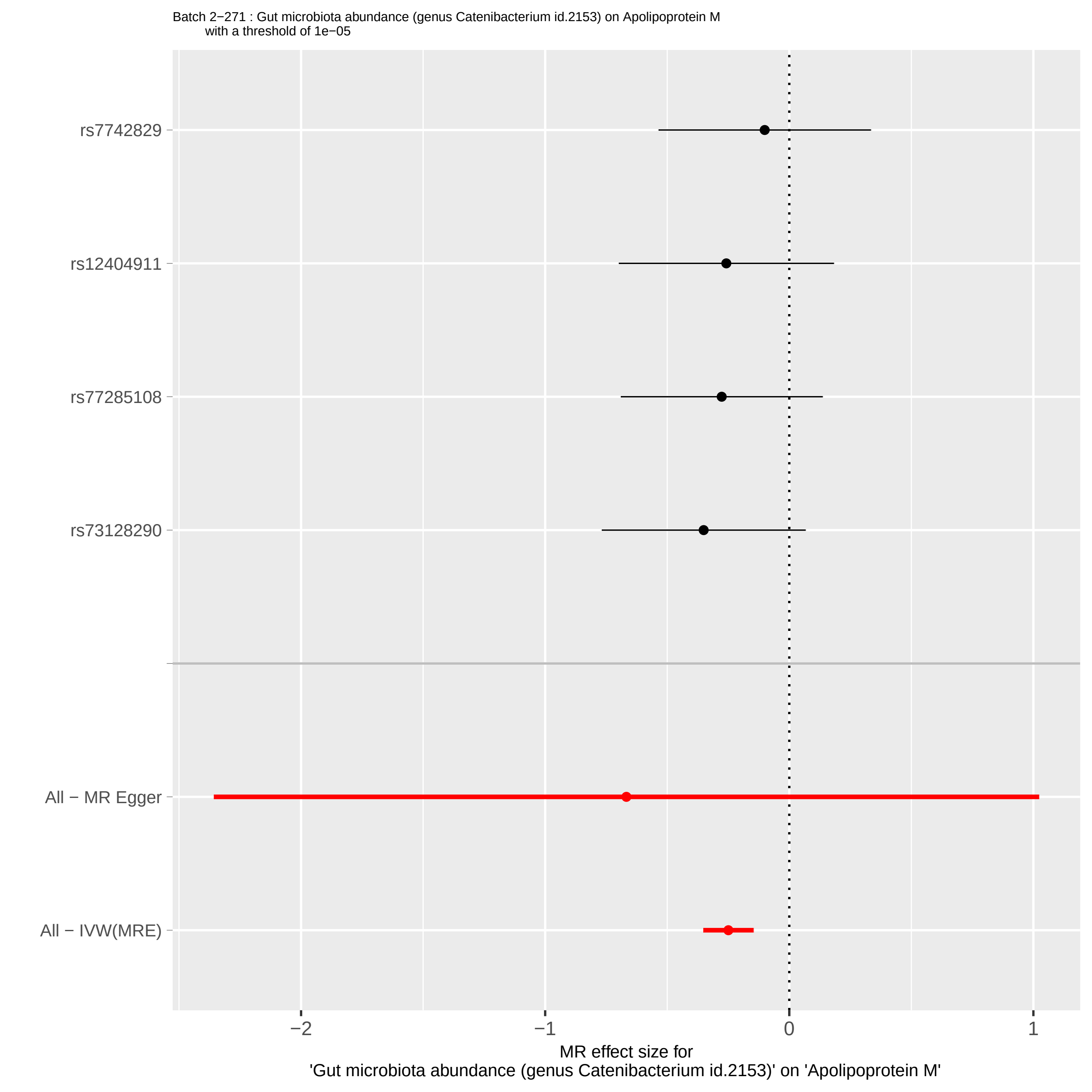

Batch 2−271 : Gut microbiota abundance (genus Catenibacterium id.2153) on Apolipoprotein M
with a threshold of 1e−05
rs7742829
rs12404911
rs77285108
rs73128290
All − MR Egger
All − IVW(MRE)
−2
−1
0
1
MR effect size for
'Gut microbiota abundance (genus Catenibacterium id.2153)' on 'Apolipoprotein M'

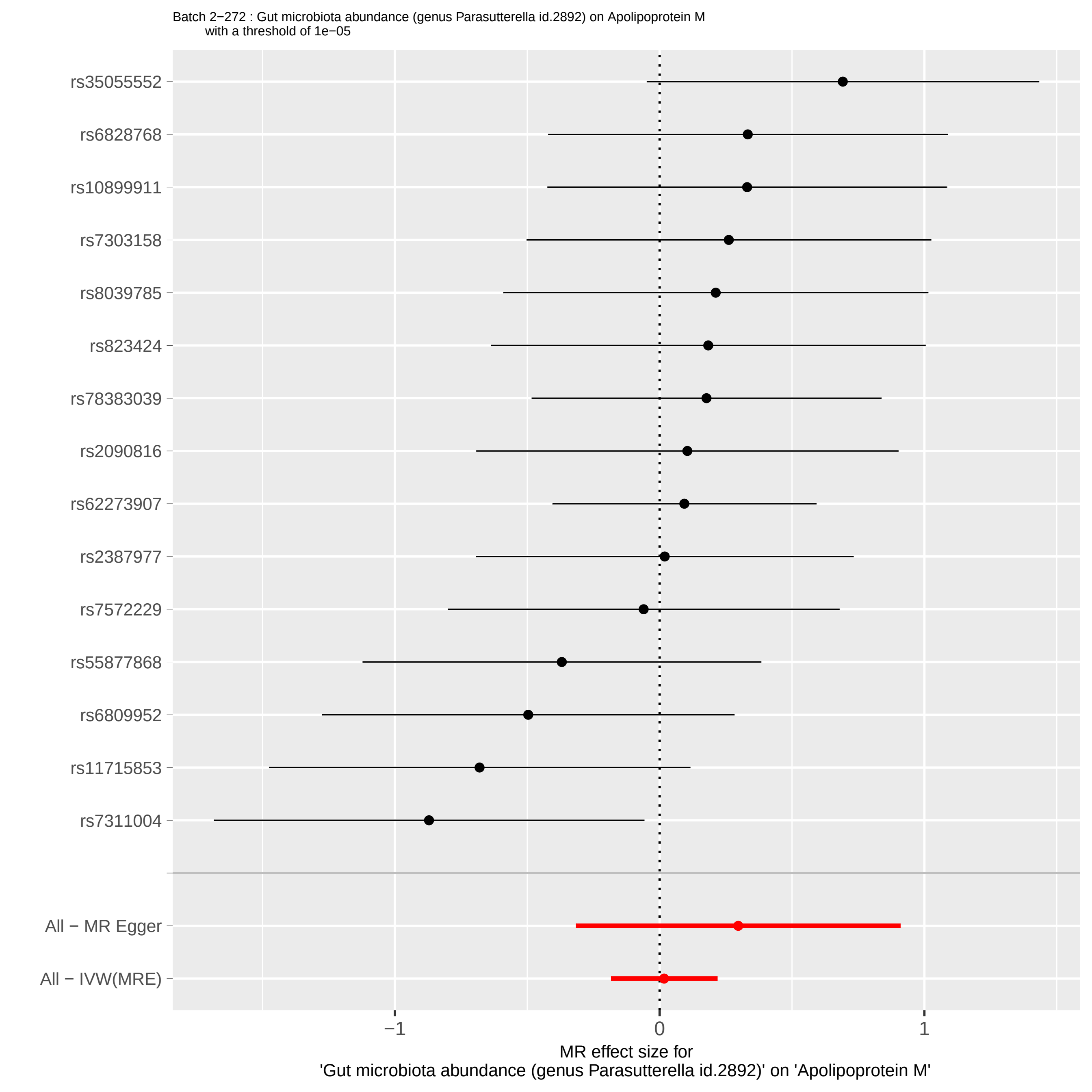

Batch 2−272 : Gut microbiota abundance (genus Parasutterella id.2892) on Apolipoprotein M
with a threshold of 1e−05
rs35055552
rs6828768
rs10899911
rs7303158
rs8039785
rs823424
rs78383039
rs2090816
rs62273907
rs2387977
rs7572229
rs55877868
rs6809952
rs11715853
rs7311004
All − MR Egger
All − IVW(MRE)
−1
0
1
MR effect size for
'Gut microbiota abundance (genus Parasutterella id.2892)' on 'Apolipoprotein M'

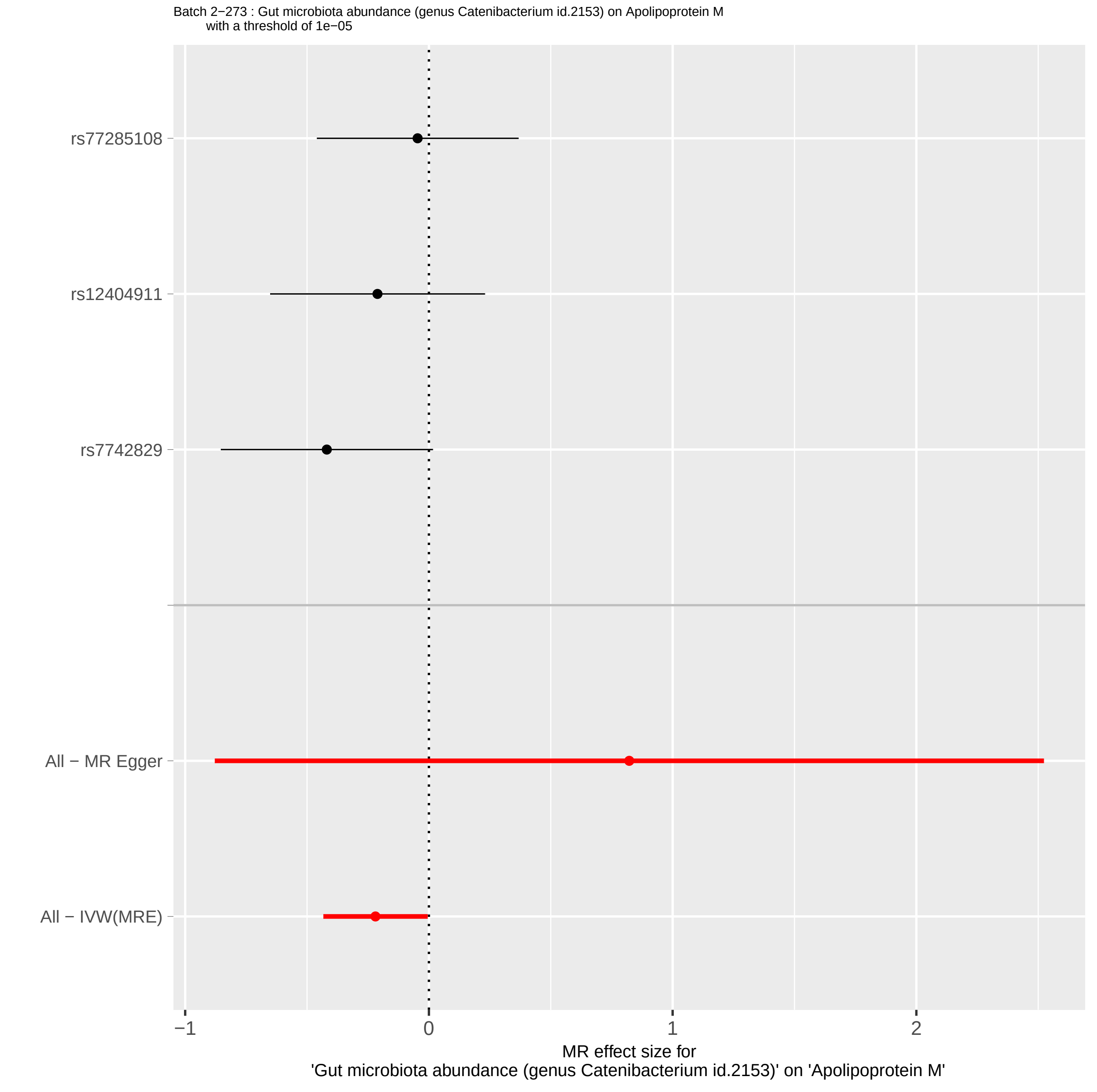
Batch 2−273 : Gut microbiota abundance (genus Catenibacterium id.2153) on Apolipoprotein M
with a threshold of 1e−05
rs77285108
rs12404911
rs7742829
All − MR Egger
All − IVW(MRE)
−1
0
1
2
MR effect size for
'Gut microbiota abundance (genus Catenibacterium id.2153)' on 'Apolipoprotein M'

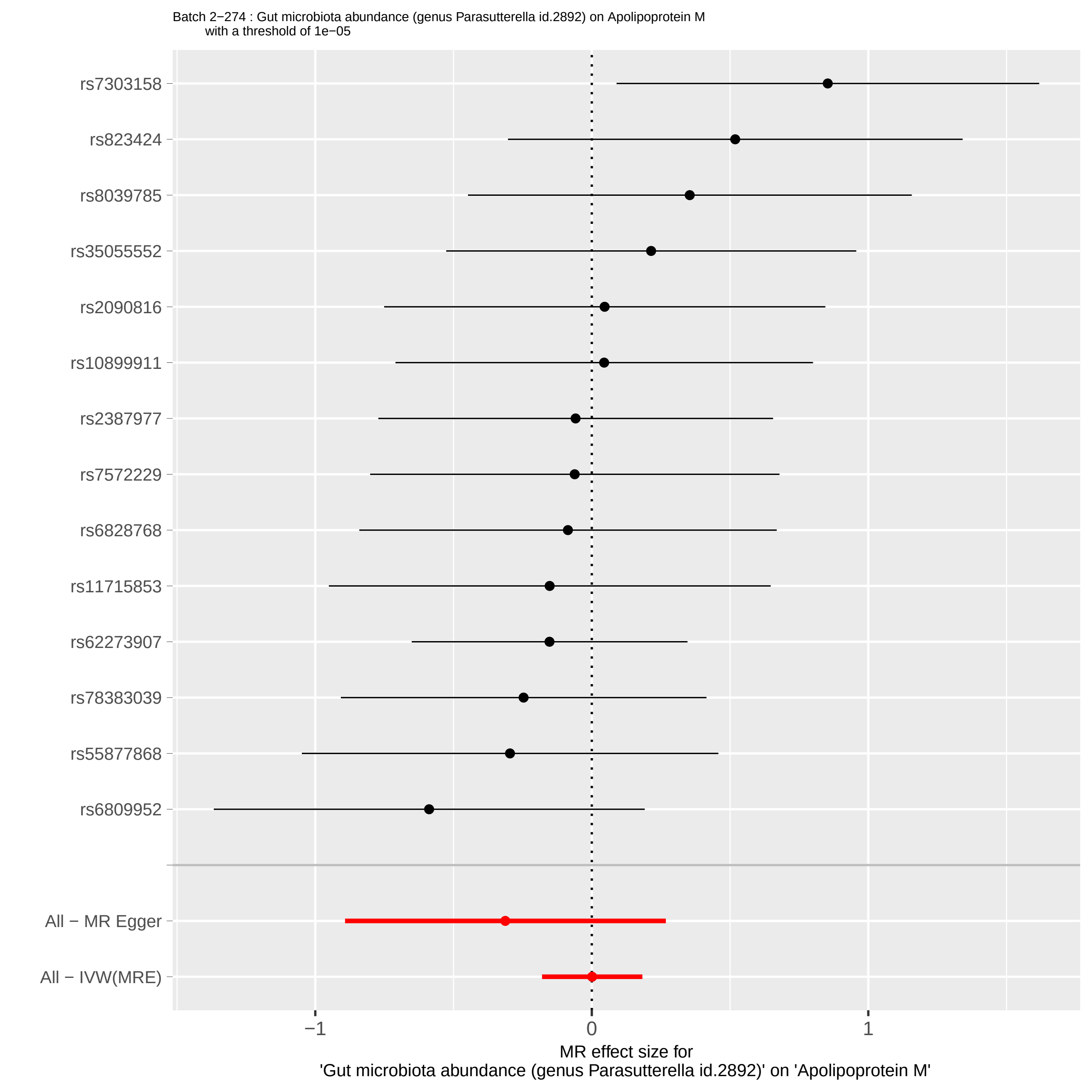

Batch 2−274 : Gut microbiota abundance (genus Parasutterella id.2892) on Apolipoprotein M
with a threshold of 1e−05
rs7303158
rs823424
rs8039785
rs35055552
rs2090816
rs10899911
rs2387977
rs7572229
rs6828768
rs11715853
rs62273907
rs78383039
rs55877868
rs6809952
All − MR Egger
All − IVW(MRE)
−1
0
1
MR effect size for
'Gut microbiota abundance (genus Parasutterella id.2892)' on 'Apolipoprotein M'

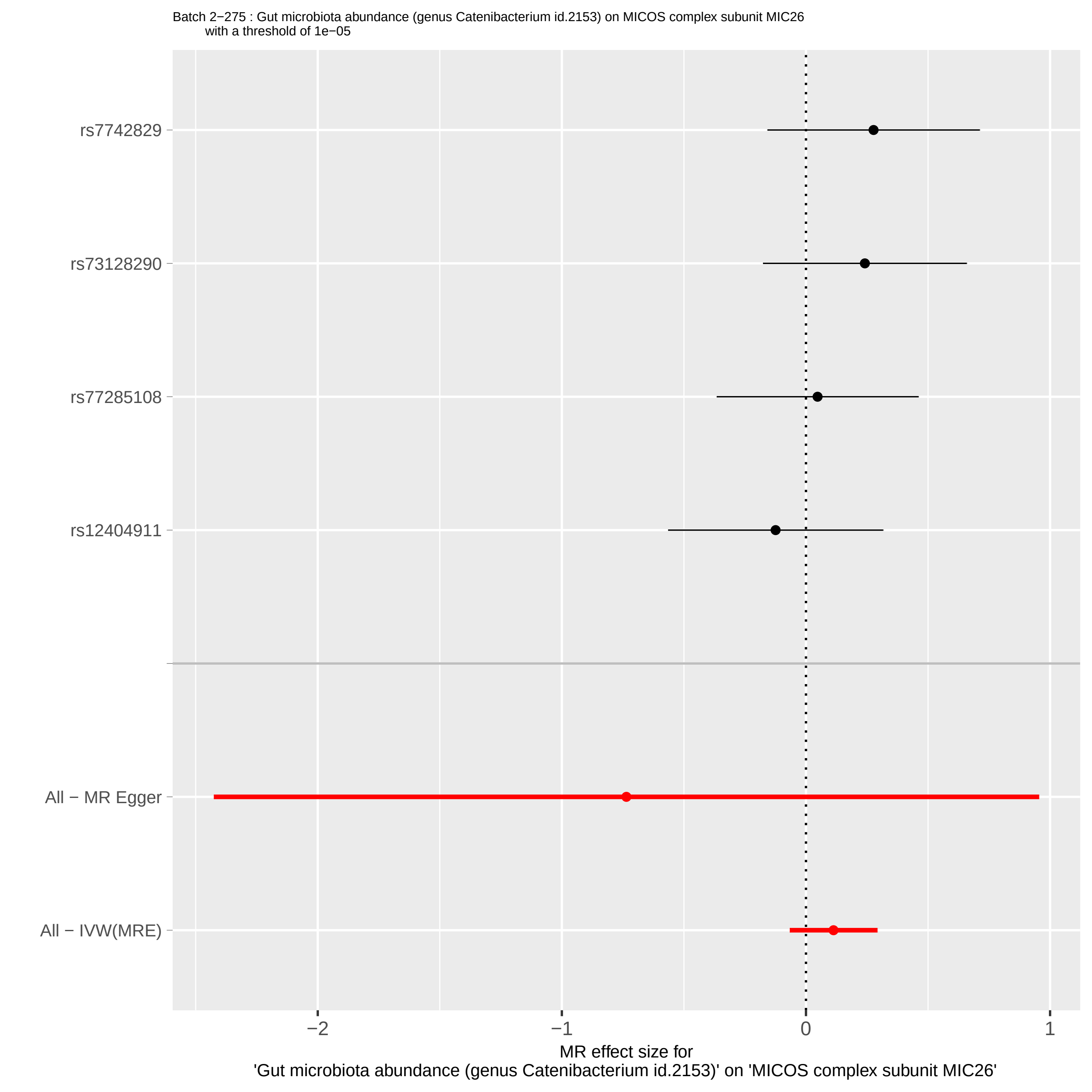

Batch 2−275 : Gut microbiota abundance (genus Catenibacterium id.2153) on MICOS complex subunit MIC26
with a threshold of 1e−05
rs7742829
rs73128290
rs77285108
rs12404911
All − MR Egger
All − IVW(MRE)
−2
−1
0
1
MR effect size for
'Gut microbiota abundance (genus Catenibacterium id.2153)' on 'MICOS complex subunit MIC26'

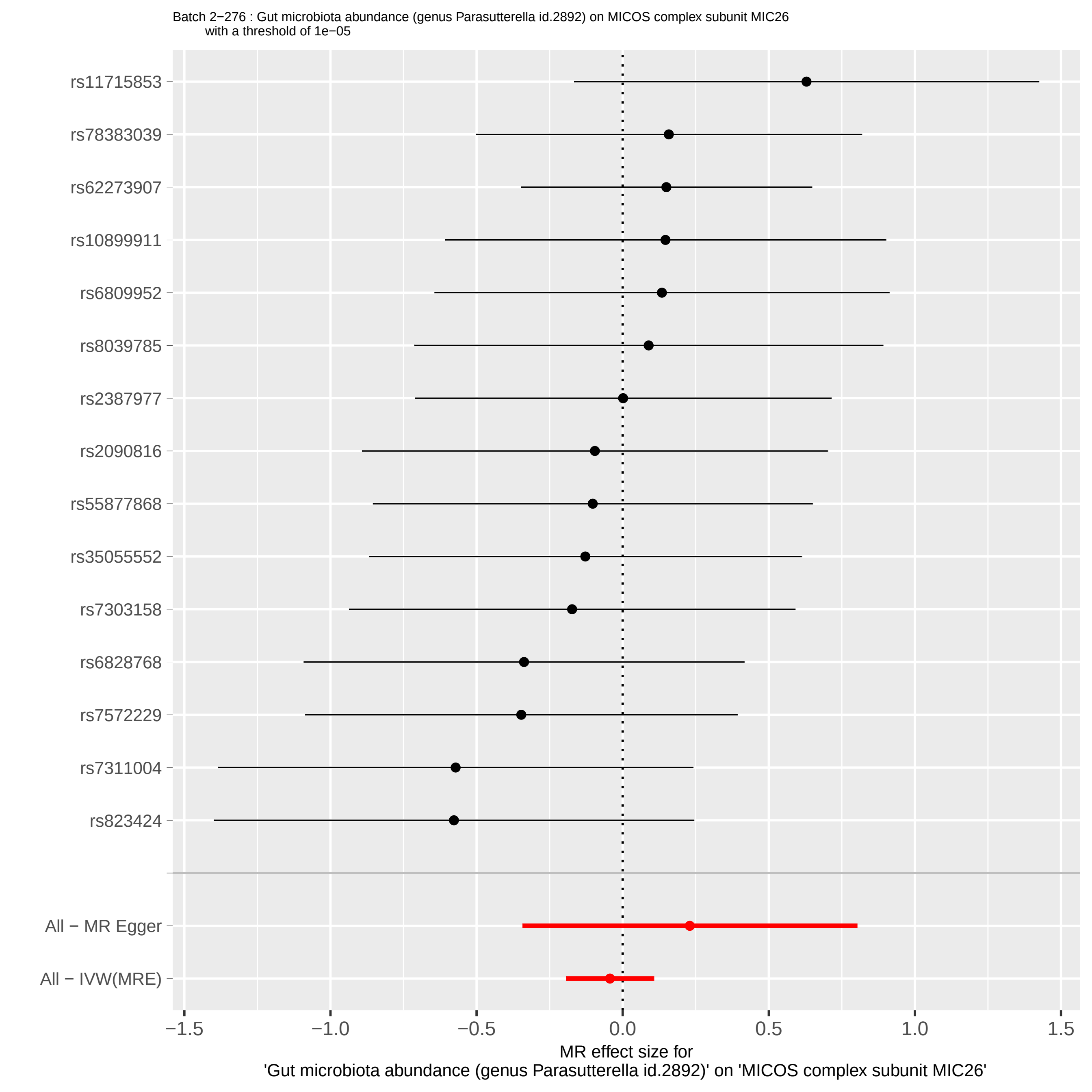

Batch 2−276 : Gut microbiota abundance (genus Parasutterella id.2892) on MICOS complex subunit MIC26
with a threshold of 1e−05
rs11715853
rs78383039
rs62273907
rs10899911
rs6809952
rs8039785
rs2387977
rs2090816
rs55877868
rs35055552
rs7303158
rs6828768
rs7572229
rs7311004
rs823424
All − MR Egger
All − IVW(MRE)
−1.5
−1.0
−0.5
0.0
0.5
1.0
1.5
MR effect size for
'Gut microbiota abundance (genus Parasutterella id.2892)' on 'MICOS complex subunit MIC26'

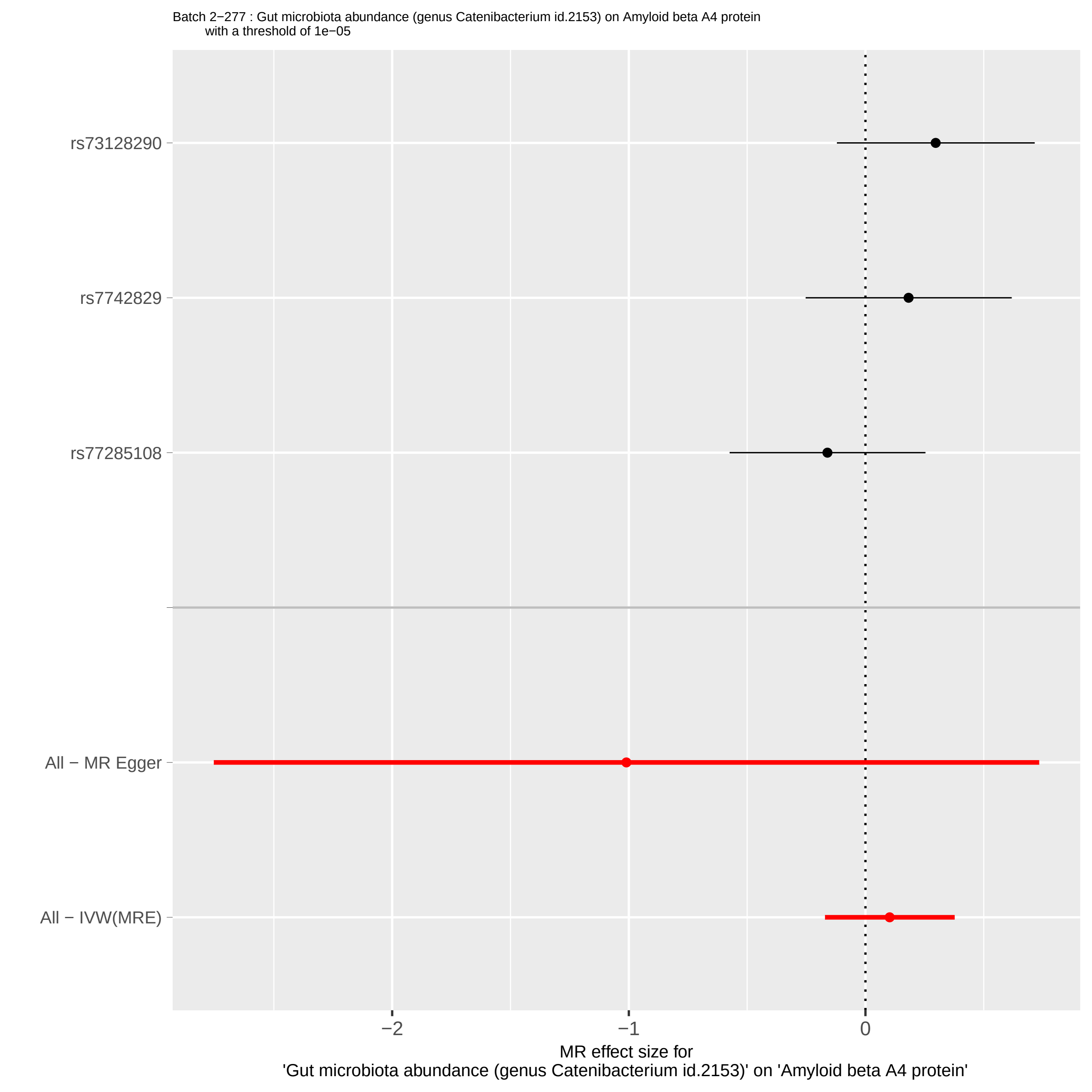

Batch 2−277 : Gut microbiota abundance (genus Catenibacterium id.2153) on Amyloid beta A4 protein
with a threshold of 1e−05
rs73128290
rs7742829
rs77285108
All − MR Egger
All − IVW(MRE)
−2
−1
0
MR effect size for
'Gut microbiota abundance (genus Catenibacterium id.2153)' on 'Amyloid beta A4 protein'

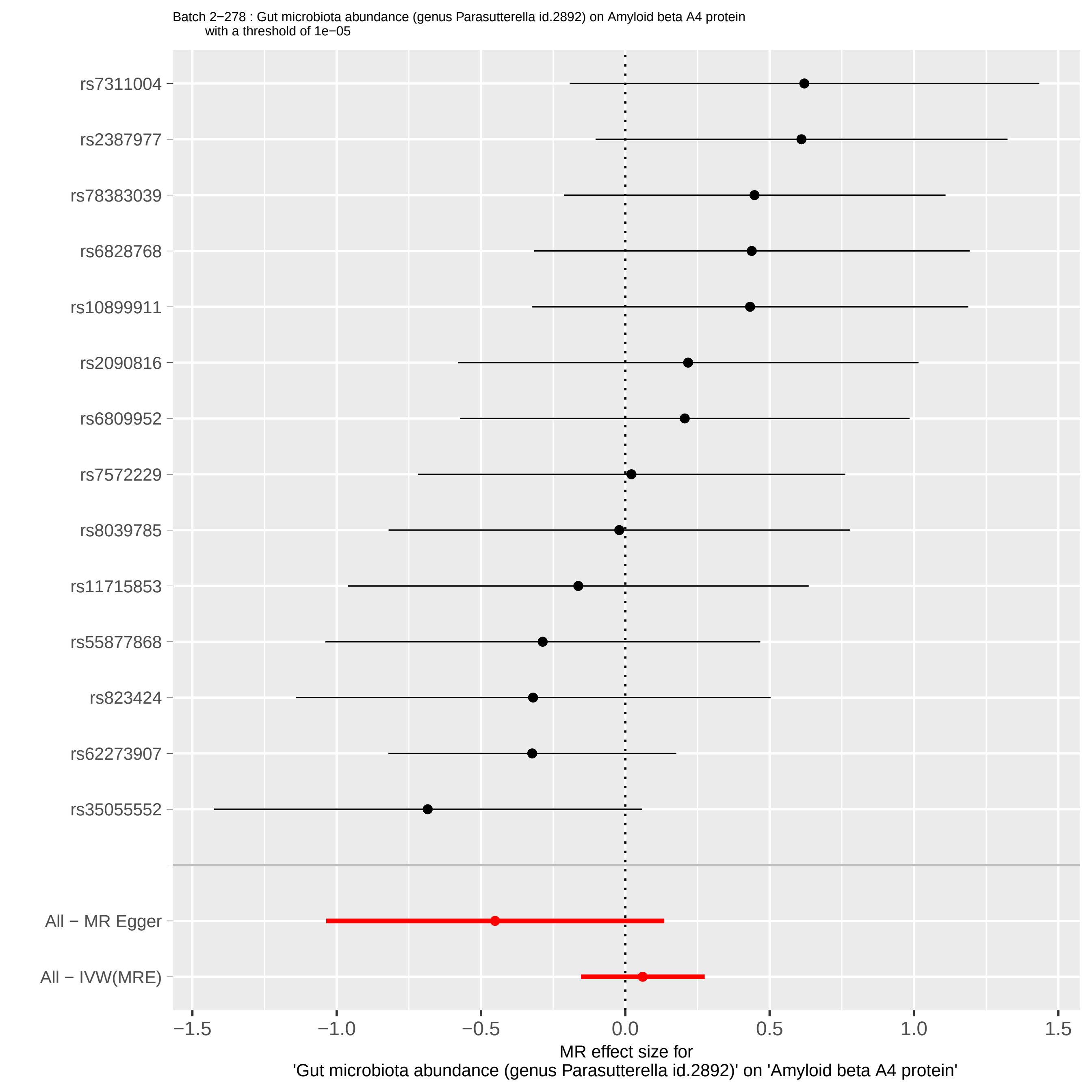

Batch 2−278 : Gut microbiota abundance (genus Parasutterella id.2892) on Amyloid beta A4 protein
with a threshold of 1e−05
rs7311004
rs2387977
rs78383039
rs6828768
rs10899911
rs2090816
rs6809952
rs7572229
rs8039785
rs11715853
rs55877868
rs823424
rs62273907
rs35055552
All − MR Egger
All − IVW(MRE)
−1.5
−1.0
−0.5
0.0
0.5
1.0
1.5
MR effect size for
'Gut microbiota abundance (genus Parasutterella id.2892)' on 'Amyloid beta A4 protein'

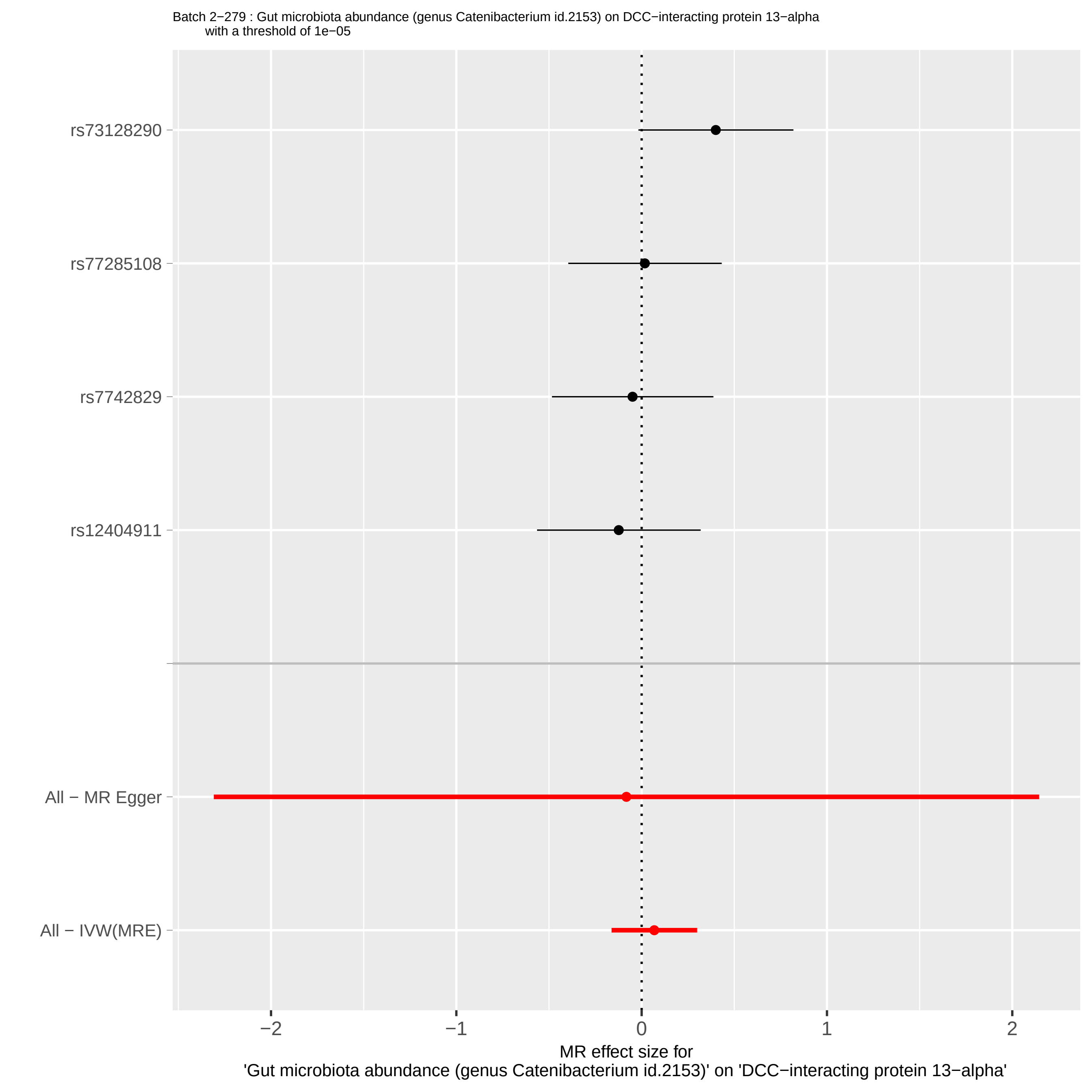

Batch 2−279 : Gut microbiota abundance (genus Catenibacterium id.2153) on DCC−interacting protein 13−alpha
with a threshold of 1e−05
rs73128290
rs77285108
rs7742829
rs12404911
All − MR Egger
All − IVW(MRE)
−2
−1
0
1
2
MR effect size for
'Gut microbiota abundance (genus Catenibacterium id.2153)' on 'DCC−interacting protein 13−alpha'

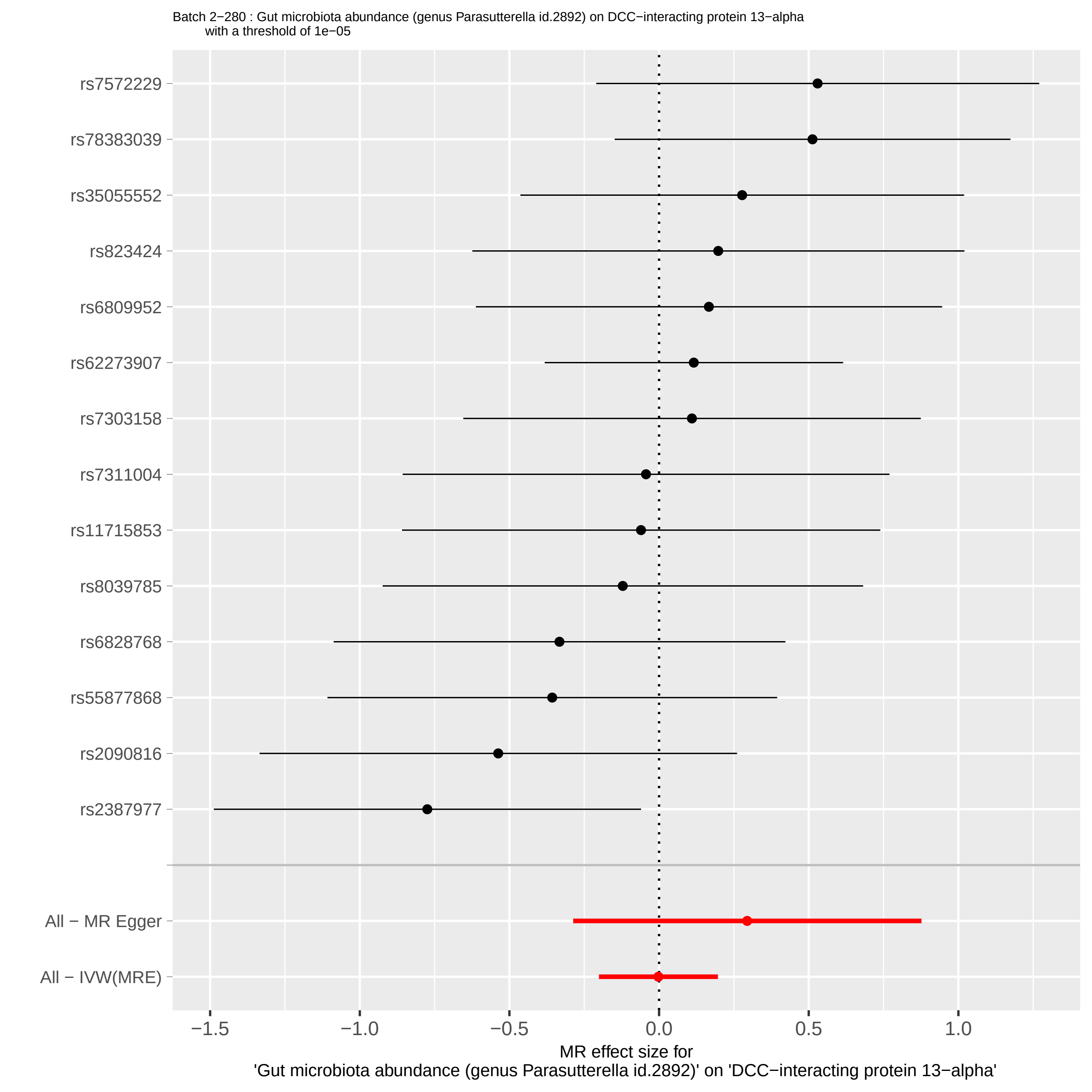

Batch 2−280 : Gut microbiota abundance (genus Parasutterella id.2892) on DCC−interacting protein 13−alpha
with a threshold of 1e−05
rs7572229
rs78383039
rs35055552
rs823424
rs6809952
rs62273907
rs7303158
rs7311004
rs11715853
rs8039785
rs6828768
rs55877868
rs2090816
rs2387977
All − MR Egger
All − IVW(MRE)
−1.5
−1.0
−0.5
0.0
0.5
1.0
MR effect size for
'Gut microbiota abundance (genus Parasutterella id.2892)' on 'DCC−interacting protein 13−alpha'

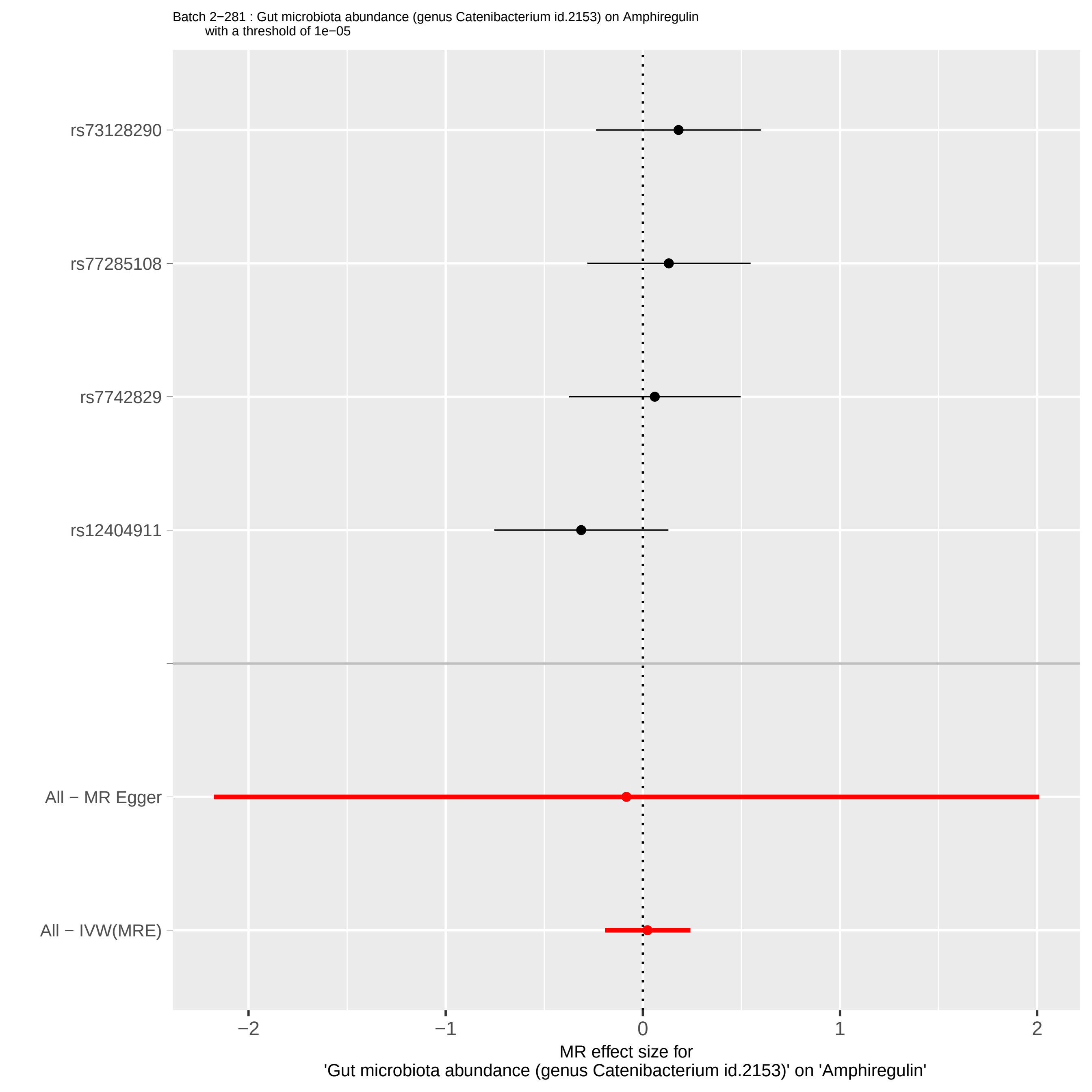

Batch 2−281 : Gut microbiota abundance (genus Catenibacterium id.2153) on Amphiregulin
with a threshold of 1e−05
rs73128290
rs77285108
rs7742829
rs12404911
All − MR Egger
All − IVW(MRE)
−2
−1
0
1
2
MR effect size for
'Gut microbiota abundance (genus Catenibacterium id.2153)' on 'Amphiregulin'

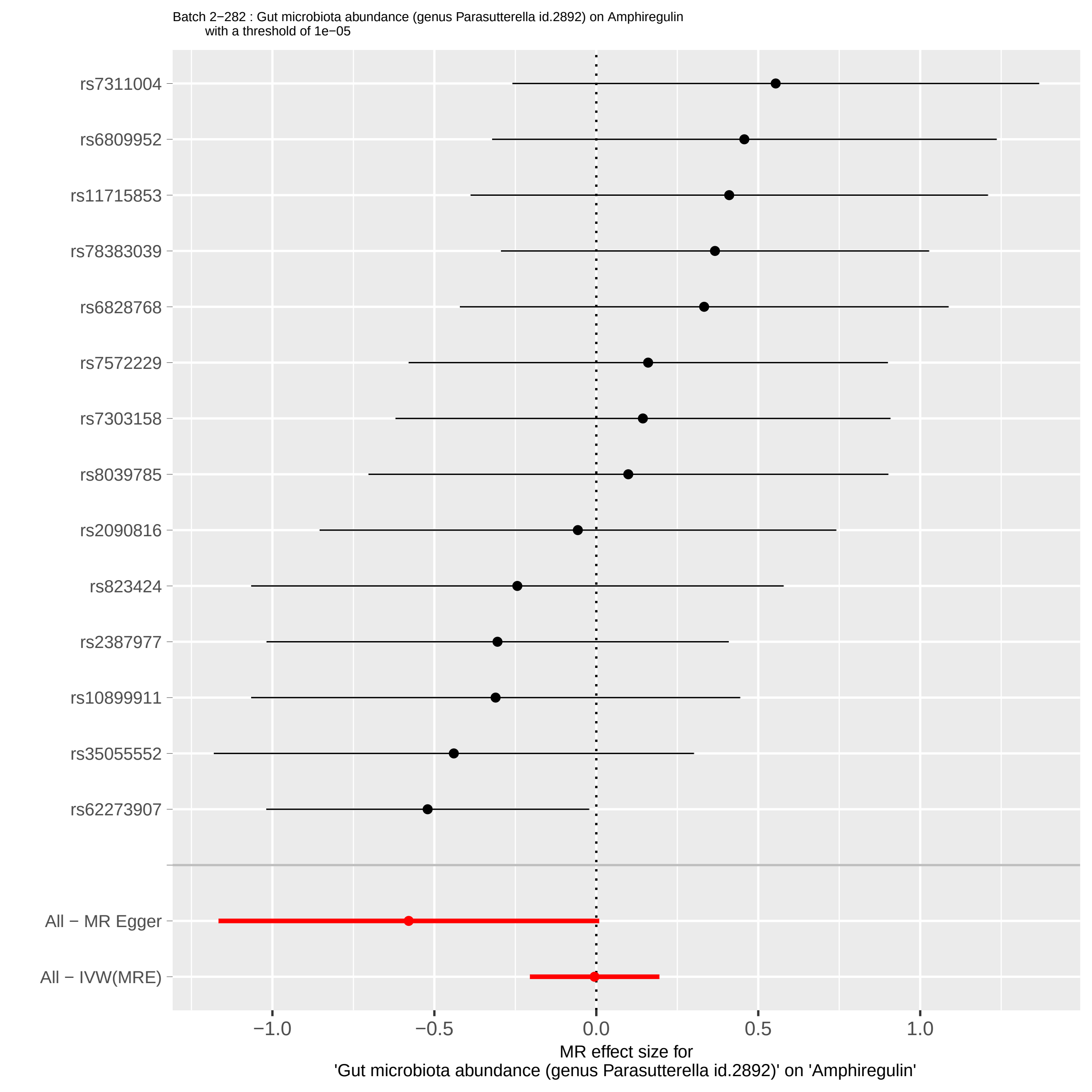

Batch 2−282 : Gut microbiota abundance (genus Parasutterella id.2892) on Amphiregulin
with a threshold of 1e−05
rs7311004
rs6809952
rs11715853
rs78383039
rs6828768
rs7572229
rs7303158
rs8039785
rs2090816
rs823424
rs2387977
rs10899911
rs35055552
rs62273907
All − MR Egger
All − IVW(MRE)
−1.0
−0.5
0.0
0.5
1.0
MR effect size for
'Gut microbiota abundance (genus Parasutterella id.2892)' on 'Amphiregulin'

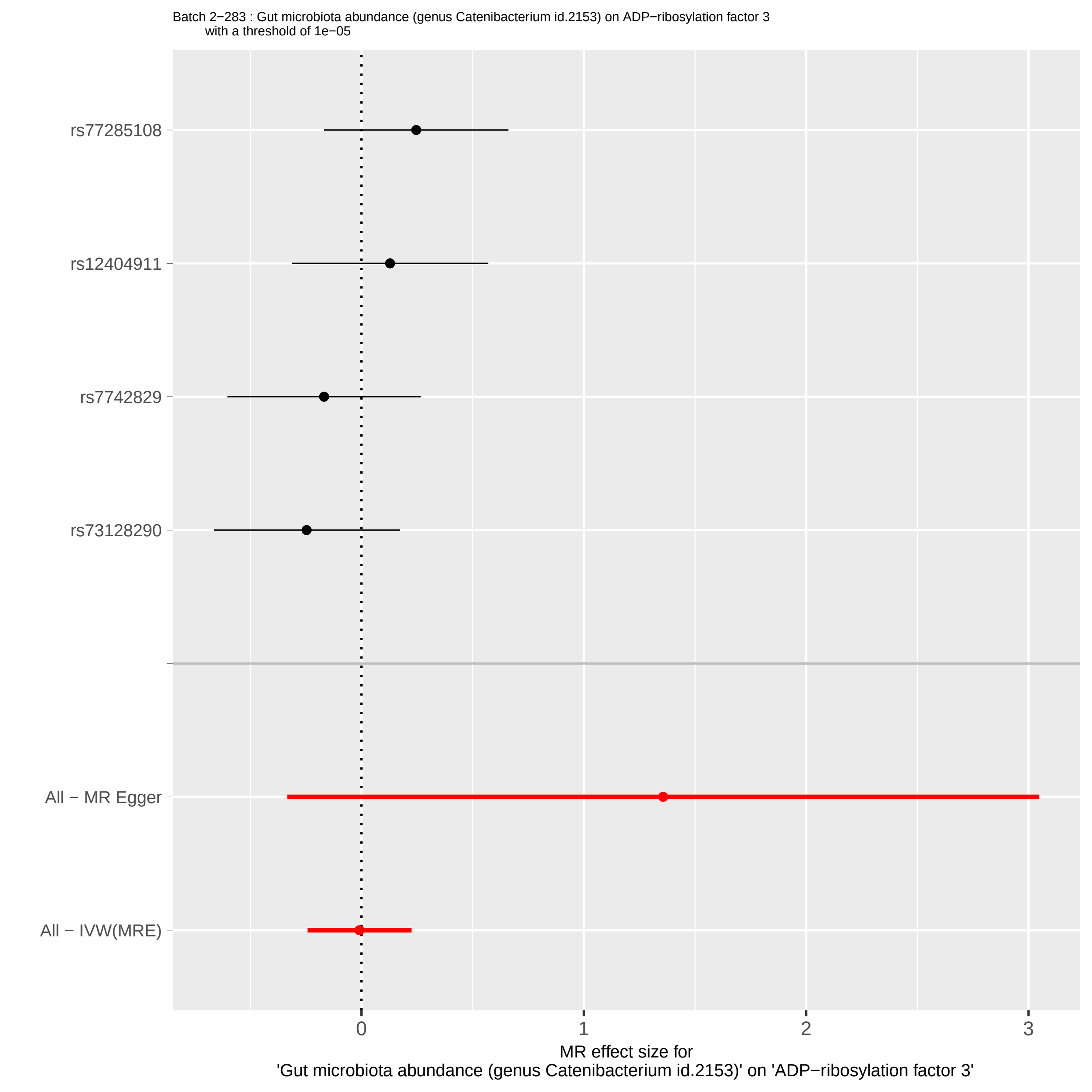

Batch 2−283 : Gut microbiota abundance (genus Catenibacterium id.2153) on ADP−ribosylation factor 3
with a threshold of 1e−05
rs77285108
rs12404911
rs7742829
rs73128290
All − MR Egger
All − IVW(MRE)
0
1
2
3
MR effect size for
'Gut microbiota abundance (genus Catenibacterium id.2153)' on 'ADP−ribosylation factor 3'

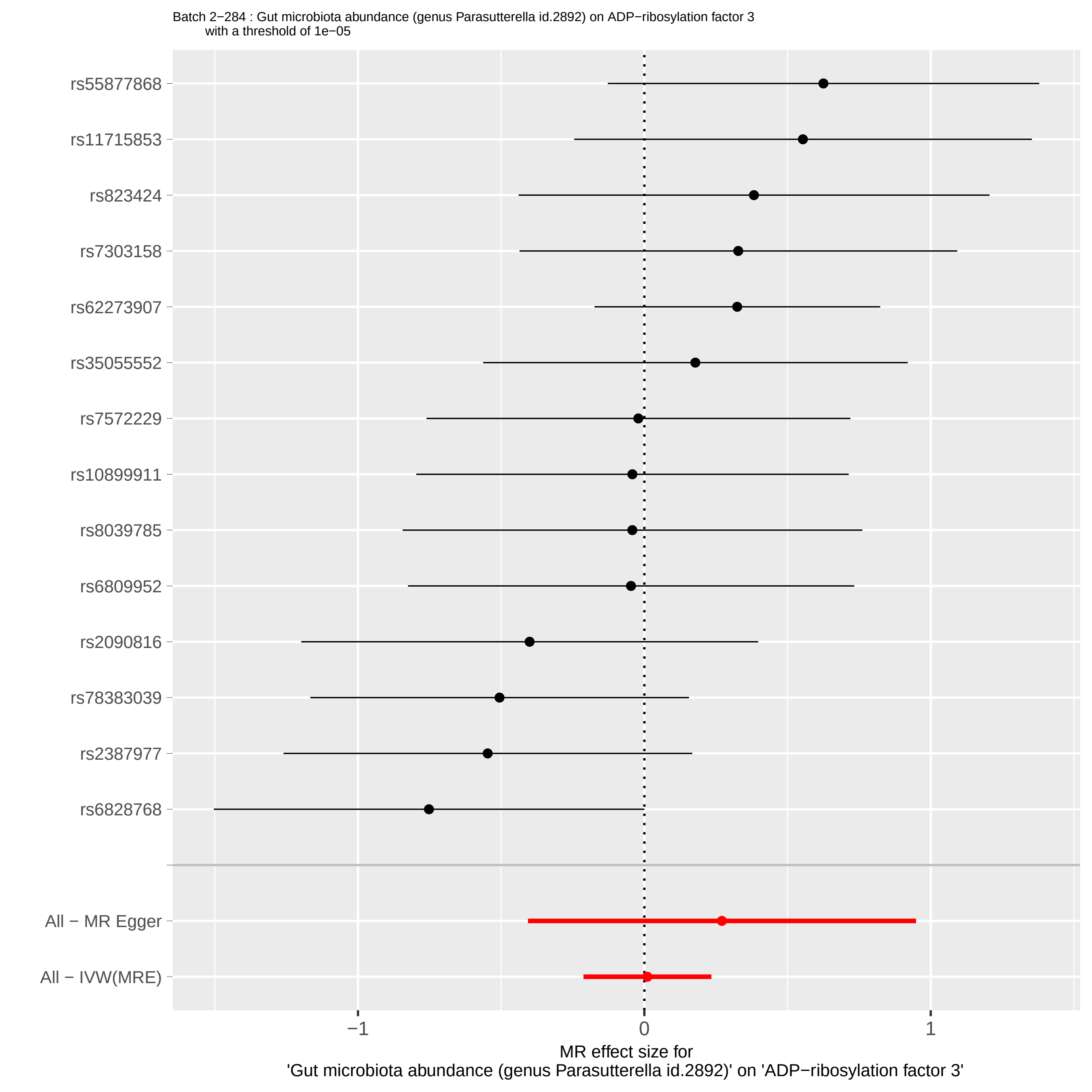

Batch 2−284 : Gut microbiota abundance (genus Parasutterella id.2892) on ADP−ribosylation factor 3
with a threshold of 1e−05
rs55877868
rs11715853
rs823424
rs7303158
rs62273907
rs35055552
rs7572229
rs10899911
rs8039785
rs6809952
rs2090816
rs78383039
rs2387977
rs6828768
All − MR Egger
All − IVW(MRE)
−1
0
1
MR effect size for
'Gut microbiota abundance (genus Parasutterella id.2892)' on 'ADP−ribosylation factor 3'

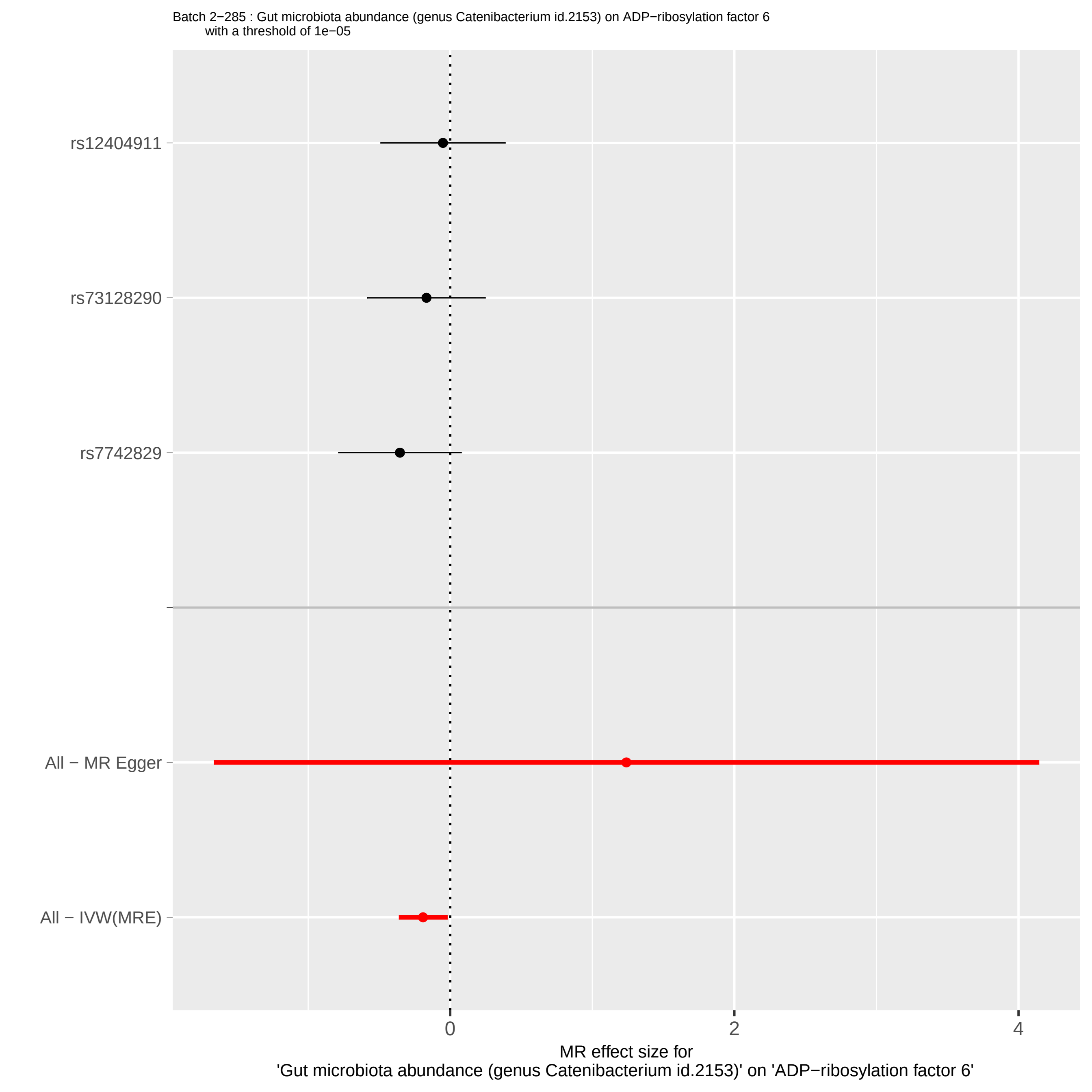

Batch 2−285 : Gut microbiota abundance (genus Catenibacterium id.2153) on ADP−ribosylation factor 6
with a threshold of 1e−05
rs12404911
rs73128290
rs7742829
All − MR Egger
All − IVW(MRE)
0
2
4
MR effect size for
'Gut microbiota abundance (genus Catenibacterium id.2153)' on 'ADP−ribosylation factor 6'

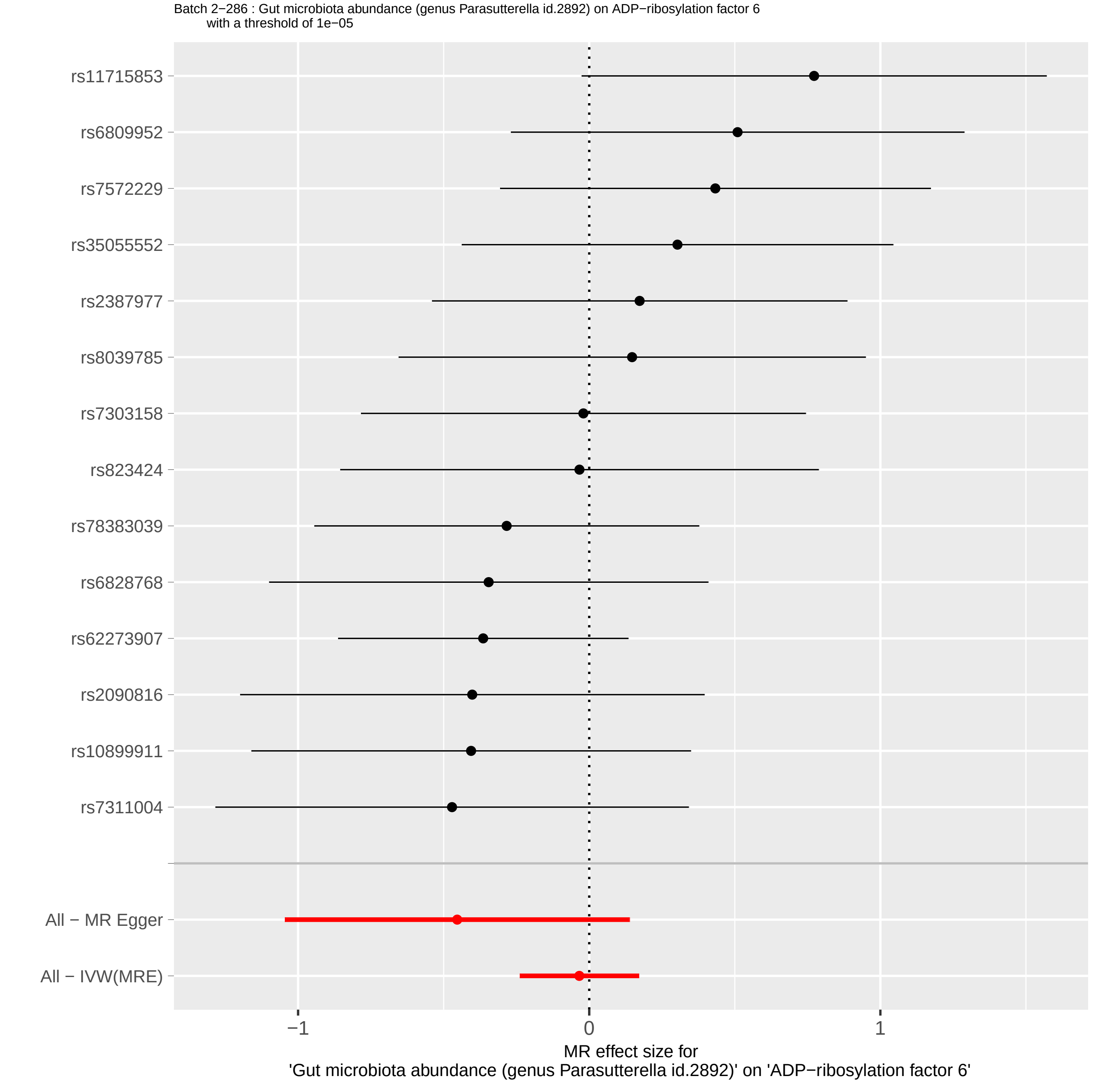

Batch 2−286 : Gut microbiota abundance (genus Parasutterella id.2892) on ADP−ribosylation factor 6
with a threshold of 1e−05
rs11715853
rs6809952
rs7572229
rs35055552
rs2387977
rs8039785
rs7303158
rs823424
rs78383039
rs6828768
rs62273907
rs2090816
rs10899911
rs7311004
All − MR Egger
All − IVW(MRE)
−1
0
1
MR effect size for
'Gut microbiota abundance (genus Parasutterella id.2892)' on 'ADP−ribosylation factor 6'

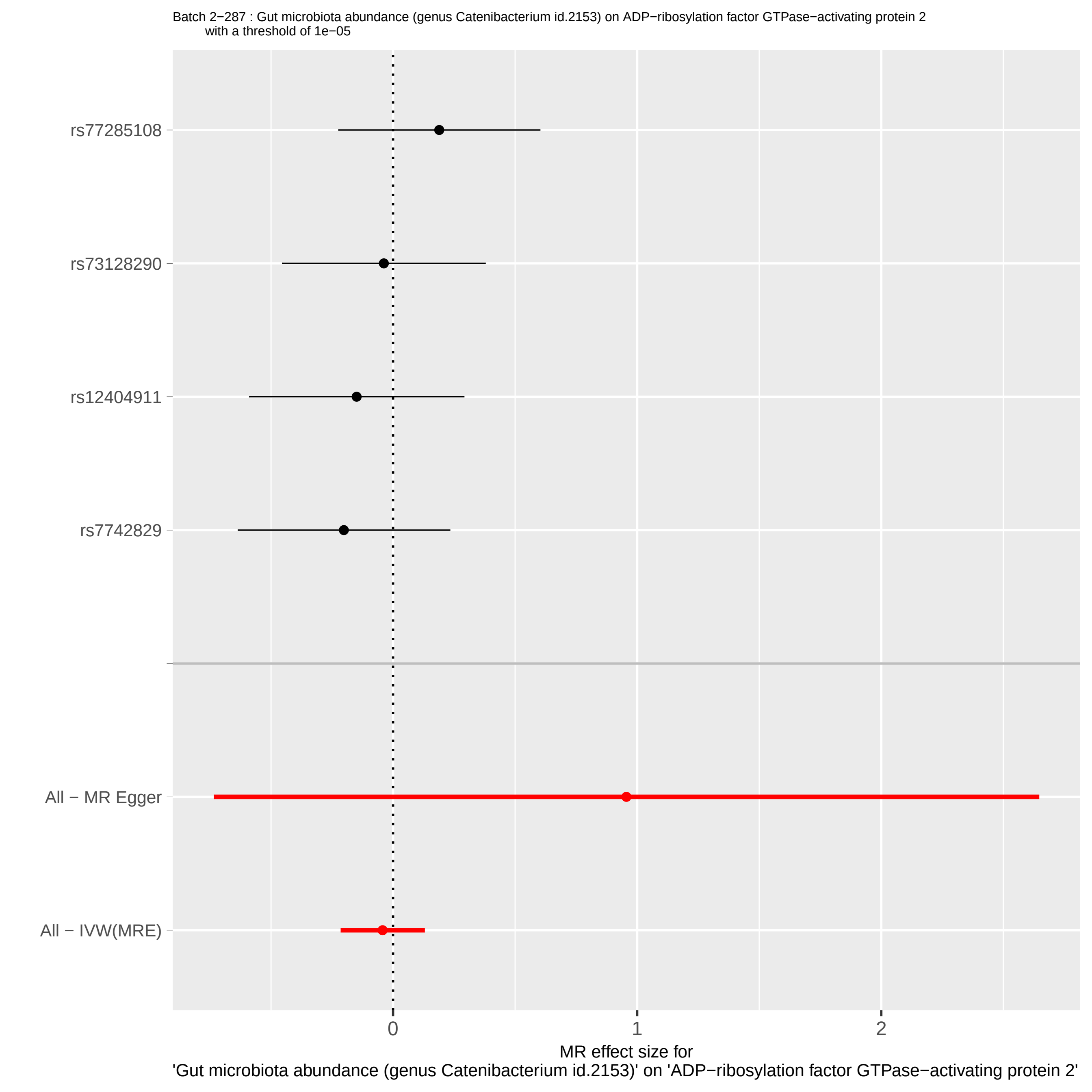

Batch 2−287 : Gut microbiota abundance (genus Catenibacterium id.2153) on ADP−ribosylation factor GTPase−activating protein 2
with a threshold of 1e−05
rs77285108
rs73128290
rs12404911
rs7742829
All − MR Egger
All − IVW(MRE)
0
1
2
MR effect size for
'Gut microbiota abundance (genus Catenibacterium id.2153)' on 'ADP−ribosylation factor GTPase−activating protein 2'

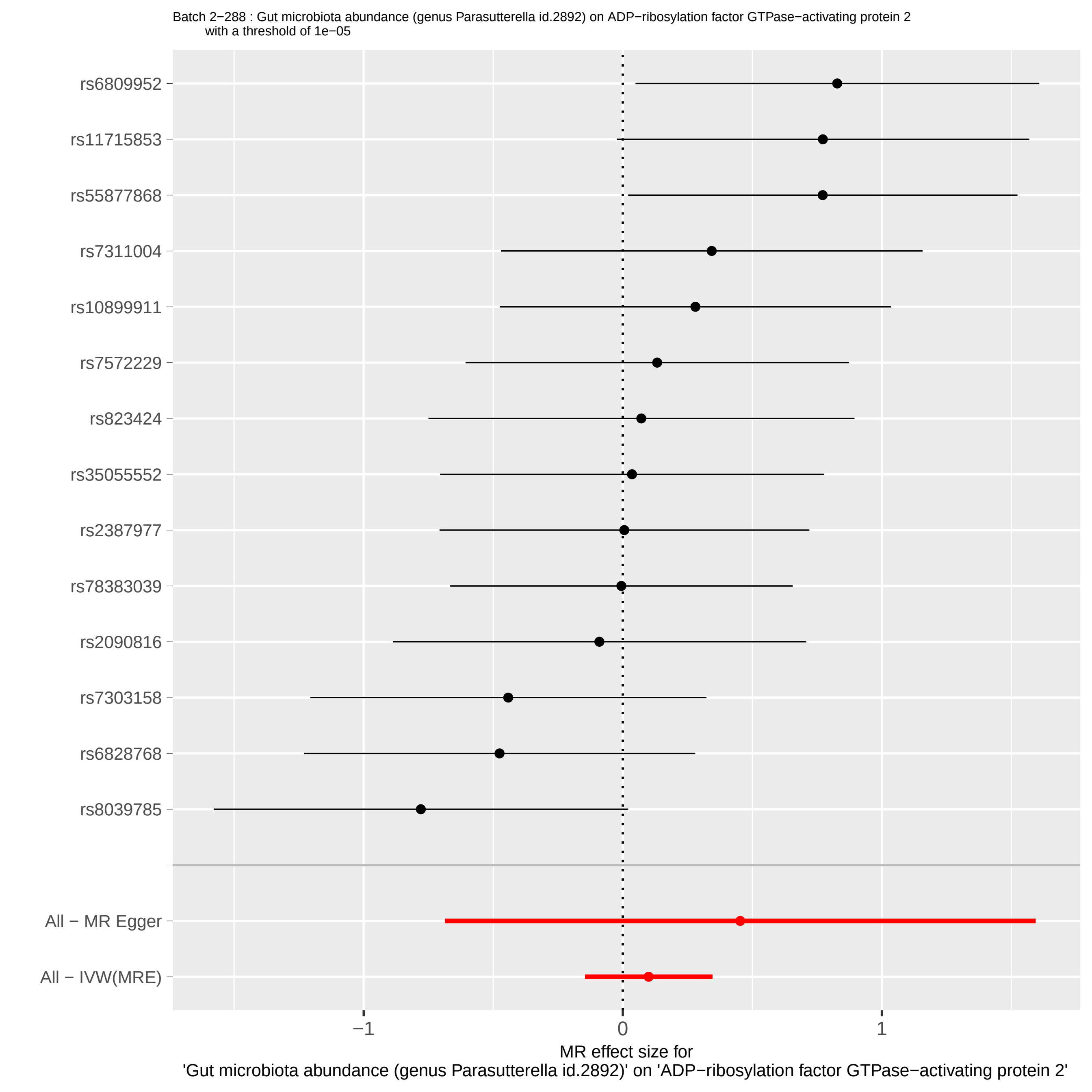
Batch 2−288 : Gut microbiota abundance (genus Parasutterella id.2892) on ADP−ribosylation factor GTPase−activating protein 2
with a threshold of 1e−05
rs6809952
rs11715853
rs55877868
rs7311004
rs10899911
rs7572229
rs823424
rs35055552
rs2387977
rs78383039
rs2090816
rs7303158
rs6828768
rs8039785
All − MR Egger
All − IVW(MRE)
−1
0
1
MR effect size for
'Gut microbiota abundance (genus Parasutterella id.2892)' on 'ADP−ribosylation factor GTPase−activating protein 2'

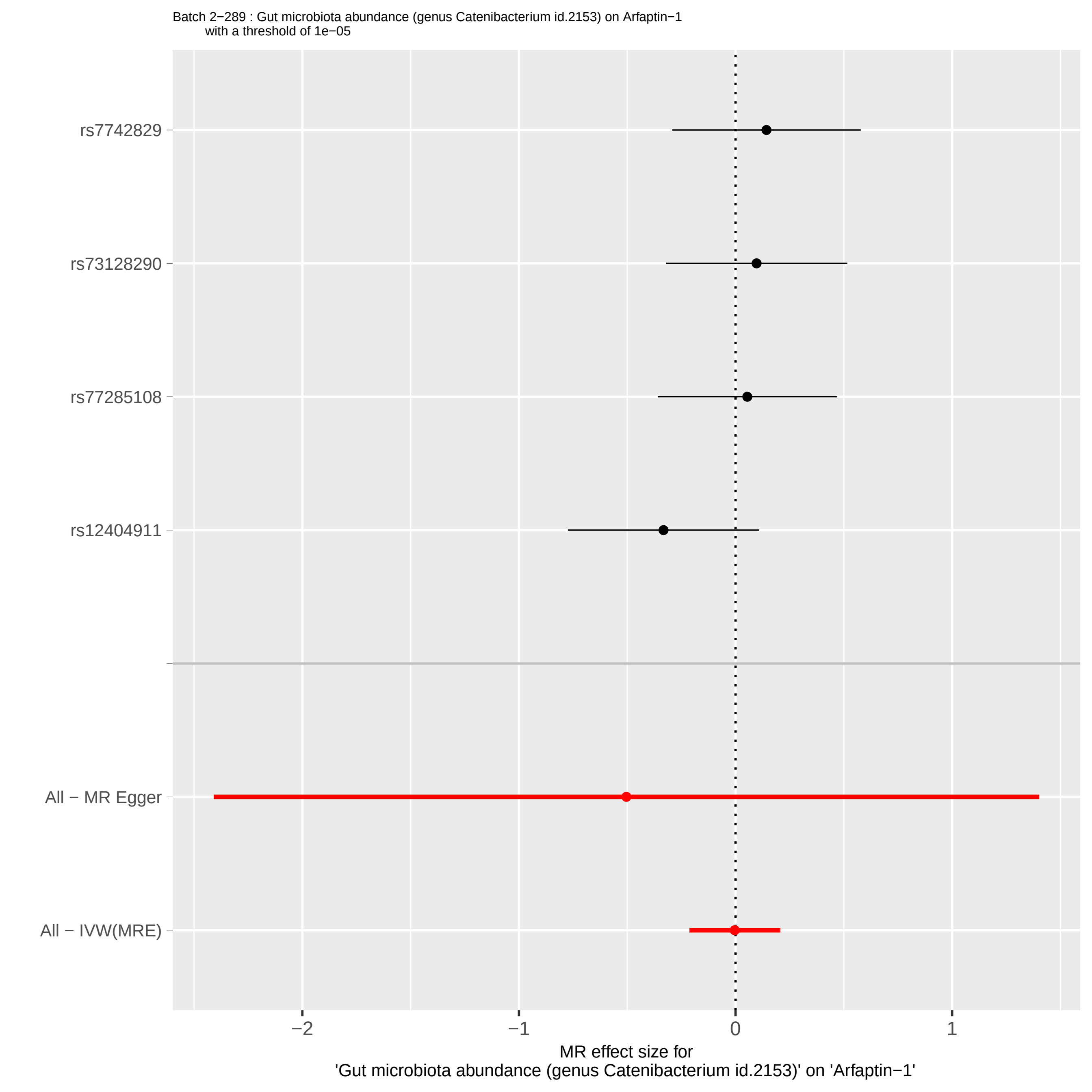
Batch 2−289 : Gut microbiota abundance (genus Catenibacterium id.2153) on Arfaptin−1
with a threshold of 1e−05
rs7742829
rs73128290
rs77285108
rs12404911
All − MR Egger
All − IVW(MRE)
−2
−1
0
1
MR effect size for
'Gut microbiota abundance (genus Catenibacterium id.2153)' on 'Arfaptin−1'

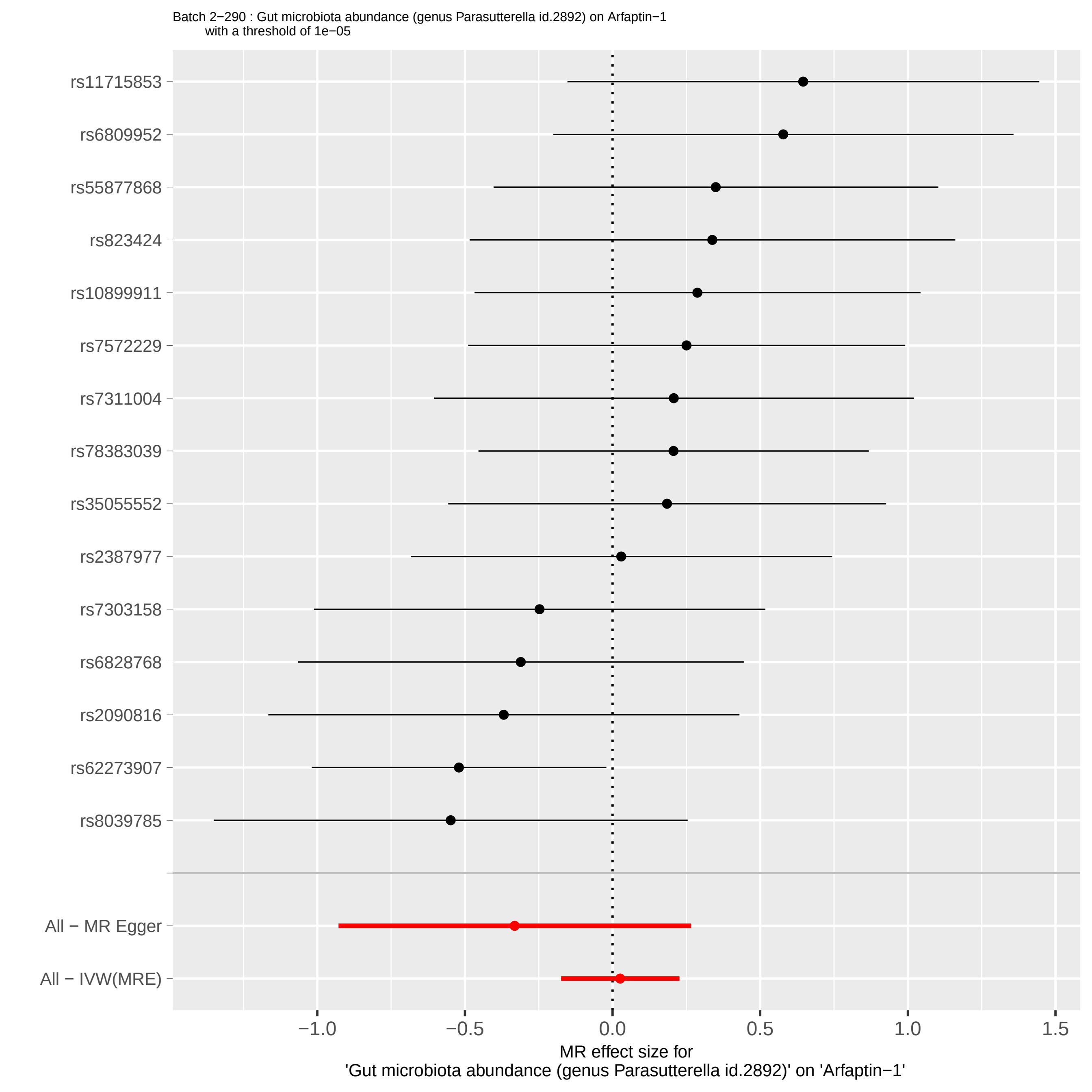

Batch 2−290 : Gut microbiota abundance (genus Parasutterella id.2892) on Arfaptin−1
with a threshold of 1e−05
rs11715853
rs6809952
rs55877868
rs823424
rs10899911
rs7572229
rs7311004
rs78383039
rs35055552
rs2387977
rs7303158
rs6828768
rs2090816
rs62273907
rs8039785
All − MR Egger
All − IVW(MRE)
−1.0
−0.5
0.0
0.5
1.0
1.5
MR effect size for
'Gut microbiota abundance (genus Parasutterella id.2892)' on 'Arfaptin−1'

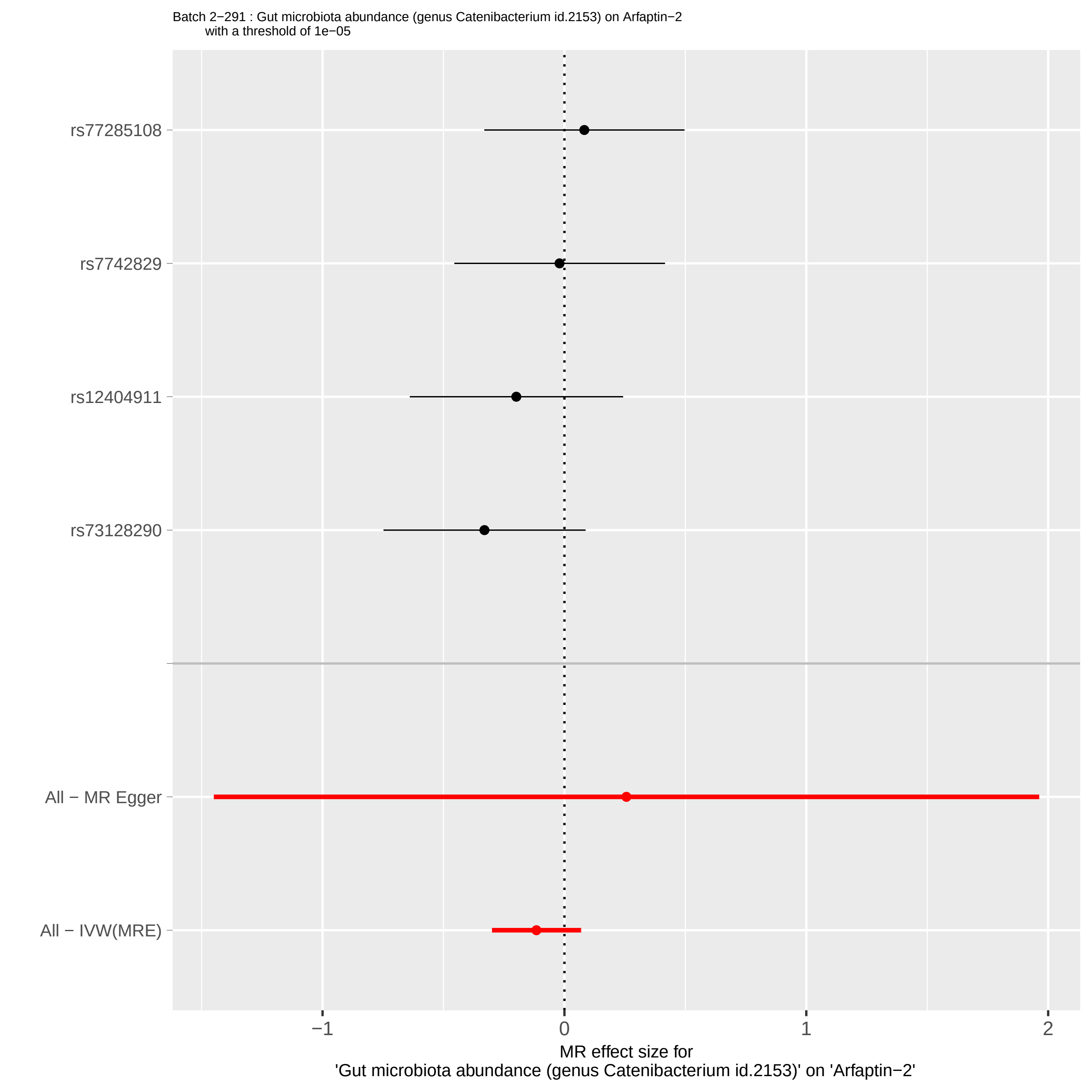

Batch 2−291 : Gut microbiota abundance (genus Catenibacterium id.2153) on Arfaptin−2
with a threshold of 1e−05
rs77285108
rs7742829
rs12404911
rs73128290
All − MR Egger
All − IVW(MRE)
−1
0
1
2
MR effect size for
'Gut microbiota abundance (genus Catenibacterium id.2153)' on 'Arfaptin−2'

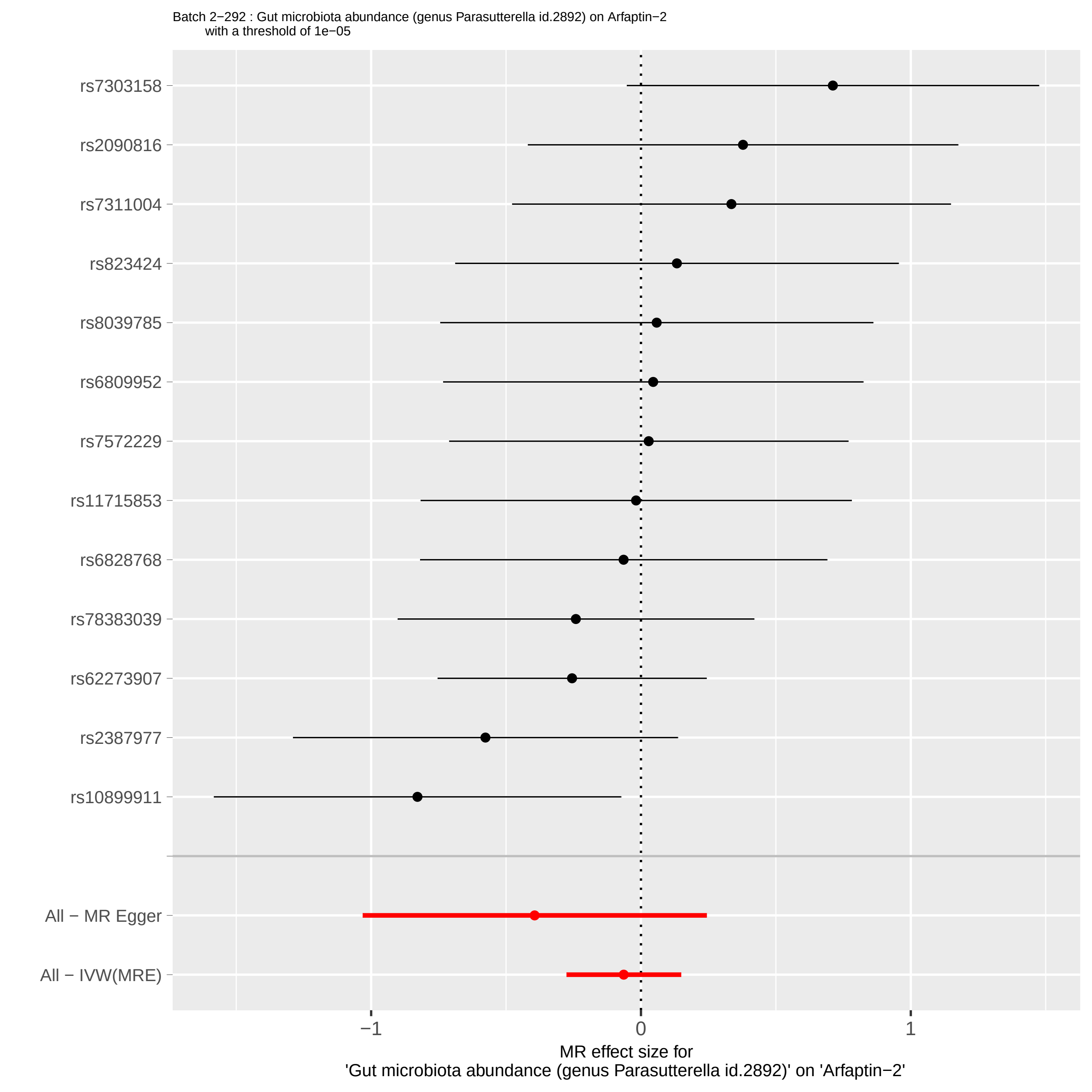
Batch 2−292 : Gut microbiota abundance (genus Parasutterella id.2892) on Arfaptin−2
with a threshold of 1e−05
rs7303158
rs2090816
rs7311004
rs823424
rs8039785
rs6809952
rs7572229
rs11715853
rs6828768
rs78383039
rs62273907
rs2387977
rs10899911
All − MR Egger
All − IVW(MRE)
−1
0
1
MR effect size for
'Gut microbiota abundance (genus Parasutterella id.2892)' on 'Arfaptin−2'

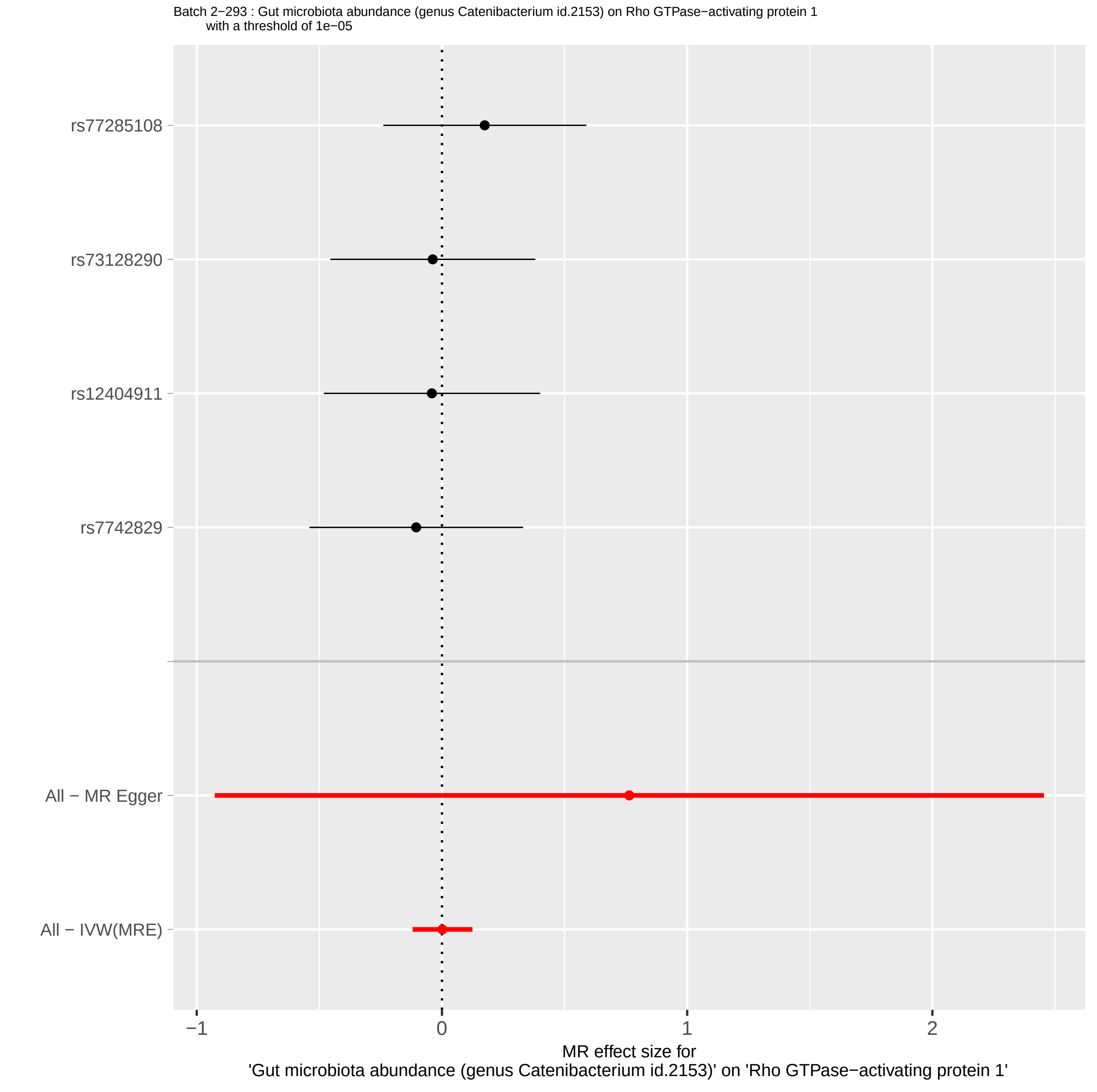

Batch 2−293 : Gut microbiota abundance (genus Catenibacterium id.2153) on Rho GTPase−activating protein 1
with a threshold of 1e−05
rs77285108
rs73128290
rs12404911
rs7742829
All − MR Egger
All − IVW(MRE)
−1
0
1
2
MR effect size for
'Gut microbiota abundance (genus Catenibacterium id.2153)' on 'Rho GTPase−activating protein 1'

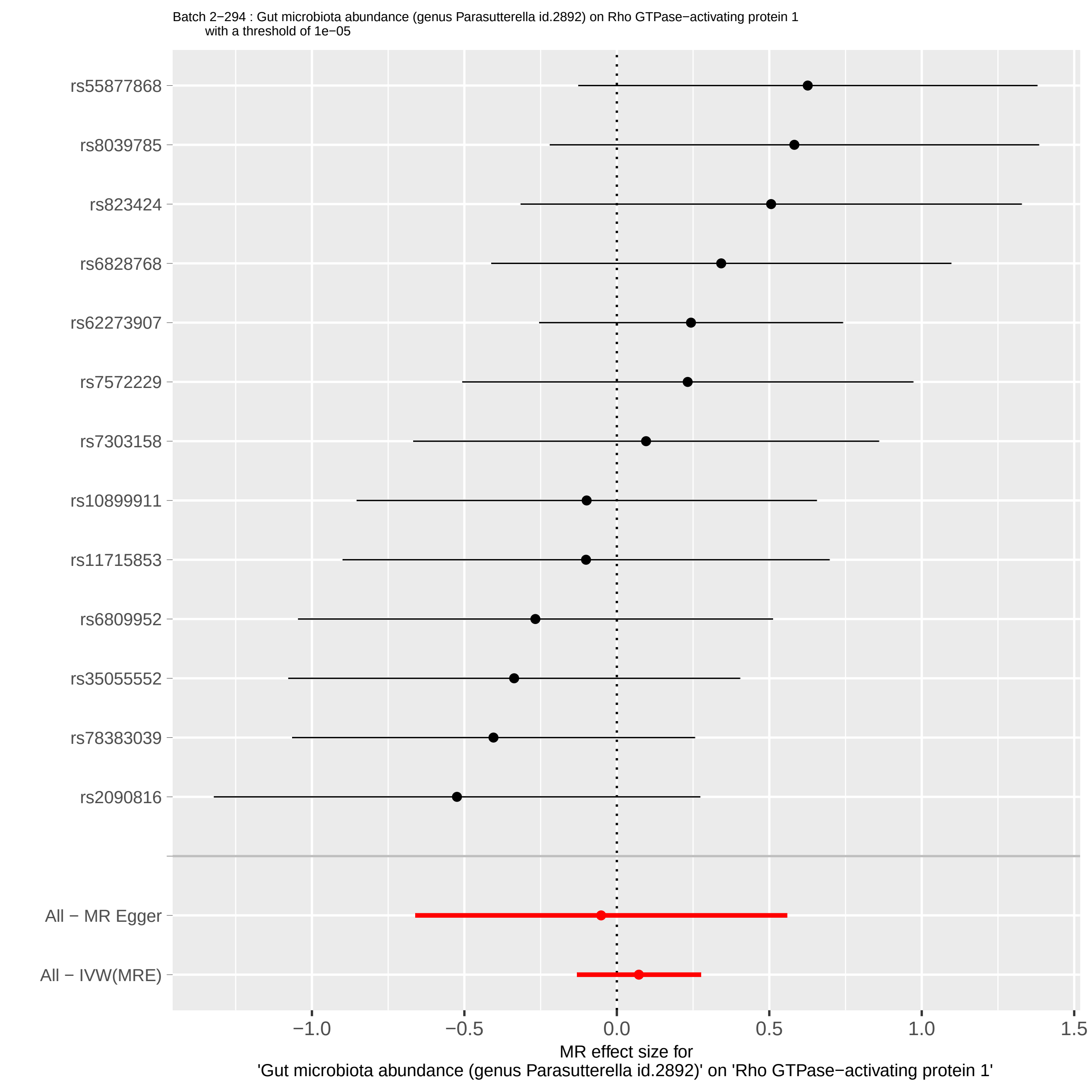

Batch 2−294 : Gut microbiota abundance (genus Parasutterella id.2892) on Rho GTPase−activating protein 1
with a threshold of 1e−05
rs55877868
rs8039785
rs823424
rs6828768
rs62273907
rs7572229
rs7303158
rs10899911
rs11715853
rs6809952
rs35055552
rs78383039
rs2090816
All − MR Egger
All − IVW(MRE)
−1.0
−0.5
0.0
0.5
1.0
1.5
MR effect size for
'Gut microbiota abundance (genus Parasutterella id.2892)' on 'Rho GTPase−activating protein 1'

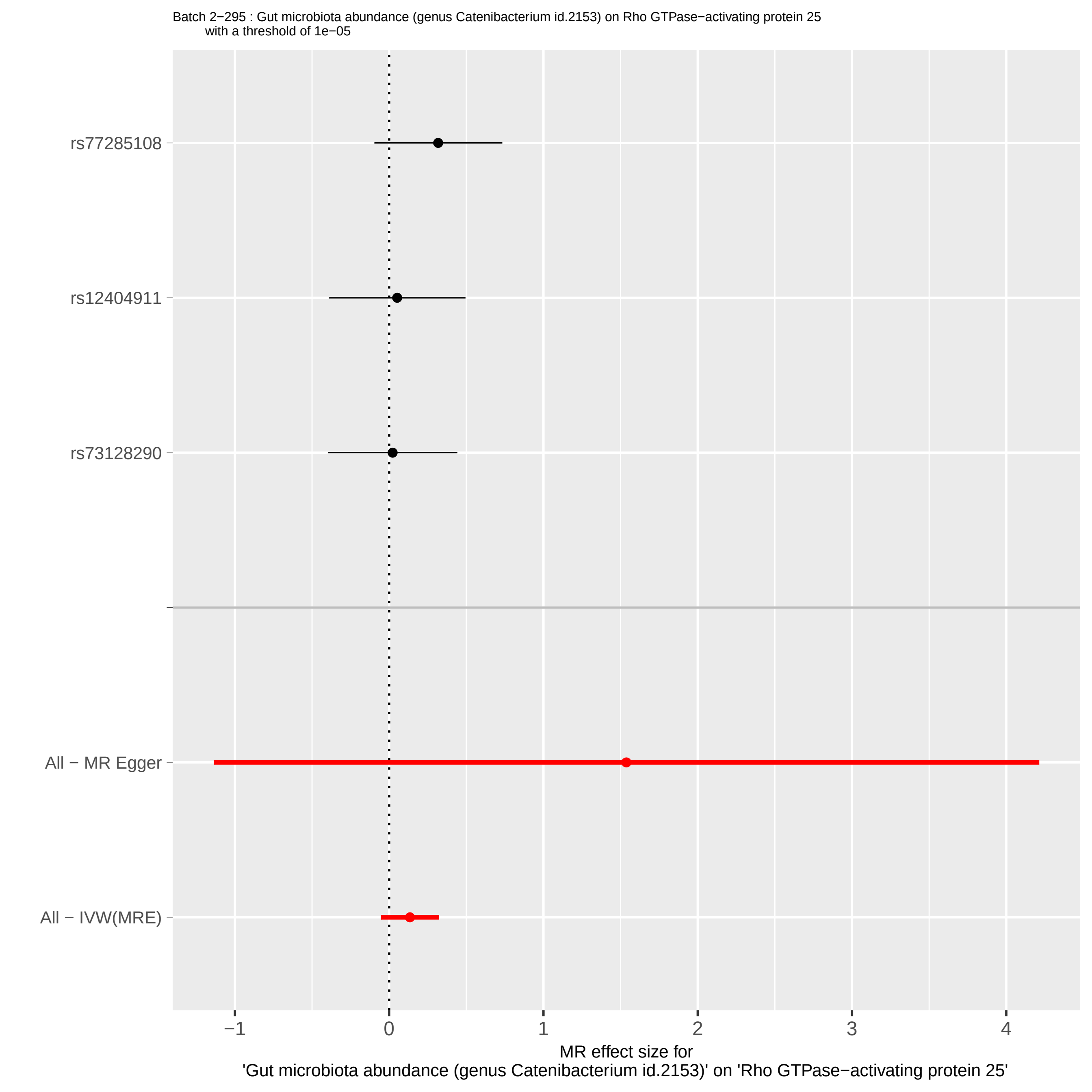

Batch 2−295 : Gut microbiota abundance (genus Catenibacterium id.2153) on Rho GTPase−activating protein 25
with a threshold of 1e−05
rs77285108
rs12404911
rs73128290
All − MR Egger
All − IVW(MRE)
−1
0
1
2
3
4
MR effect size for
'Gut microbiota abundance (genus Catenibacterium id.2153)' on 'Rho GTPase−activating protein 25'

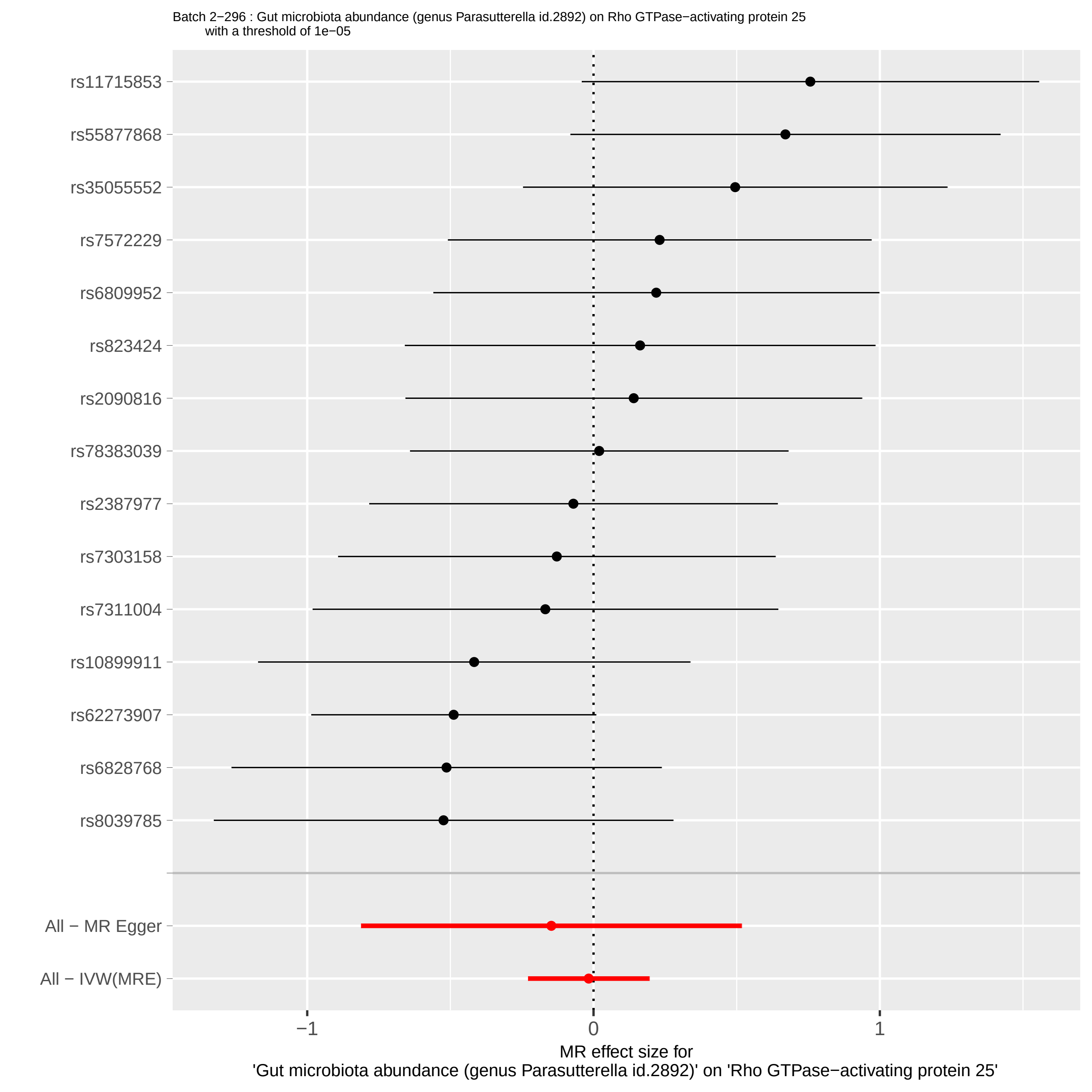

Batch 2−296 : Gut microbiota abundance (genus Parasutterella id.2892) on Rho GTPase−activating protein 25
with a threshold of 1e−05
rs11715853
rs55877868
rs35055552
rs7572229
rs6809952
rs823424
rs2090816
rs78383039
rs2387977
rs7303158
rs7311004
rs10899911
rs62273907
rs6828768
rs8039785
All − MR Egger
All − IVW(MRE)
−1
0
1
MR effect size for
'Gut microbiota abundance (genus Parasutterella id.2892)' on 'Rho GTPase−activating protein 25'

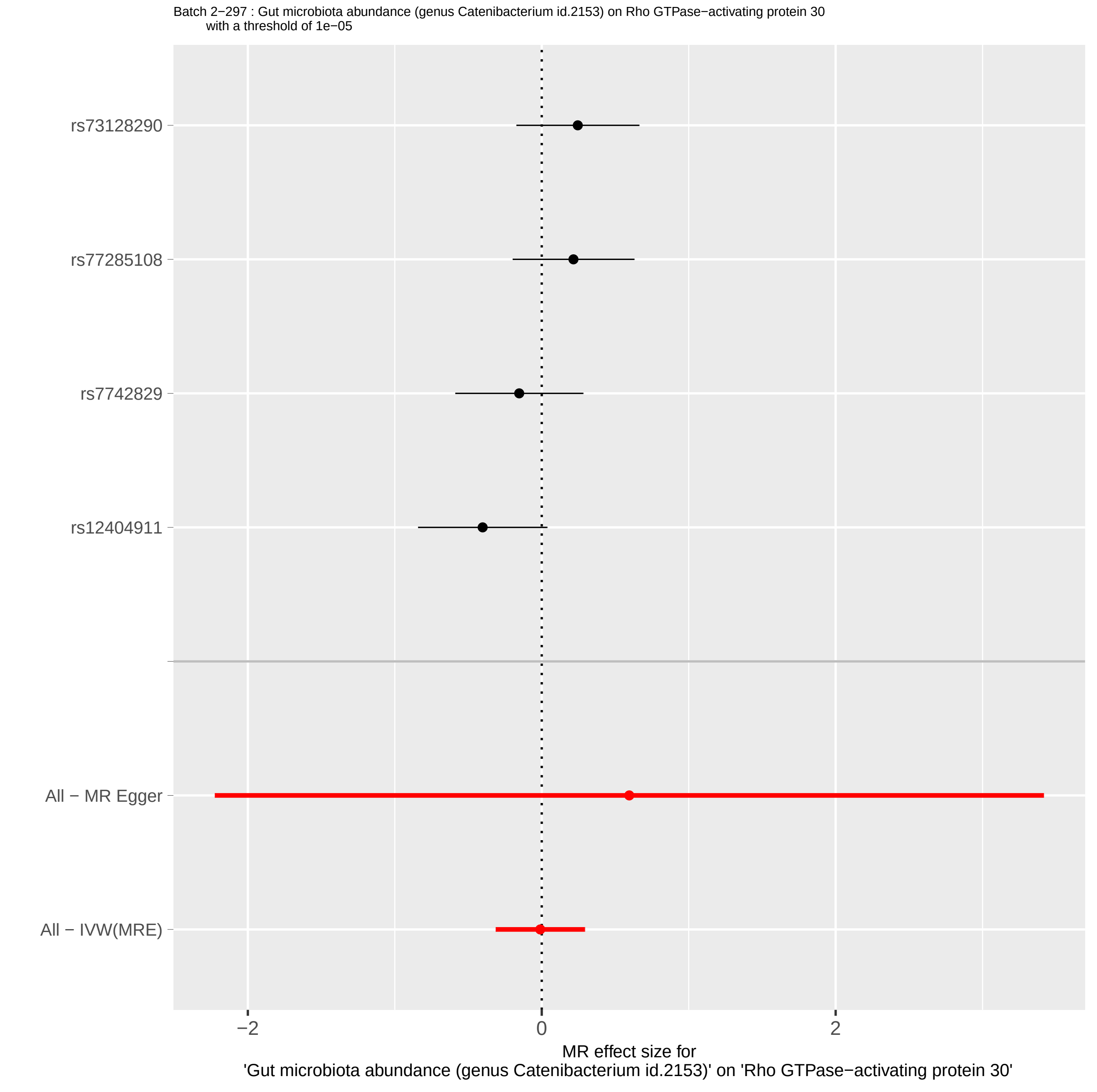
Batch 2−297 : Gut microbiota abundance (genus Catenibacterium id.2153) on Rho GTPase−activating protein 30
with a threshold of 1e−05
rs73128290
rs77285108
rs7742829
rs12404911
All − MR Egger
All − IVW(MRE)
−2
0
2
MR effect size for
'Gut microbiota abundance (genus Catenibacterium id.2153)' on 'Rho GTPase−activating protein 30'

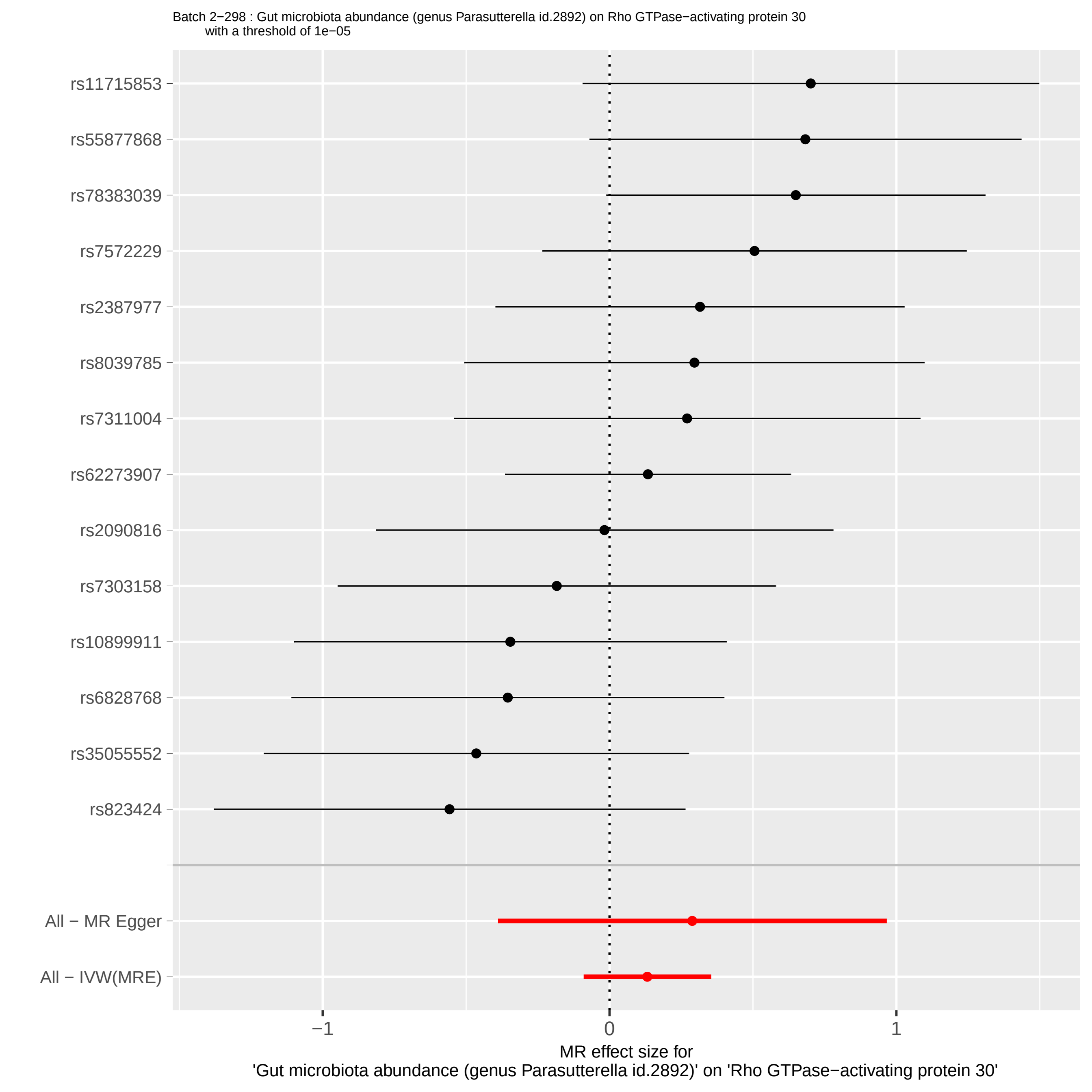

Batch 2−298 : Gut microbiota abundance (genus Parasutterella id.2892) on Rho GTPase−activating protein 30
with a threshold of 1e−05
rs11715853
rs55877868
rs78383039
rs7572229
rs2387977
rs8039785
rs7311004
rs62273907
rs2090816
rs7303158
rs10899911
rs6828768
rs35055552
rs823424
All − MR Egger
All − IVW(MRE)
−1
0
1
MR effect size for
'Gut microbiota abundance (genus Parasutterella id.2892)' on 'Rho GTPase−activating protein 30'

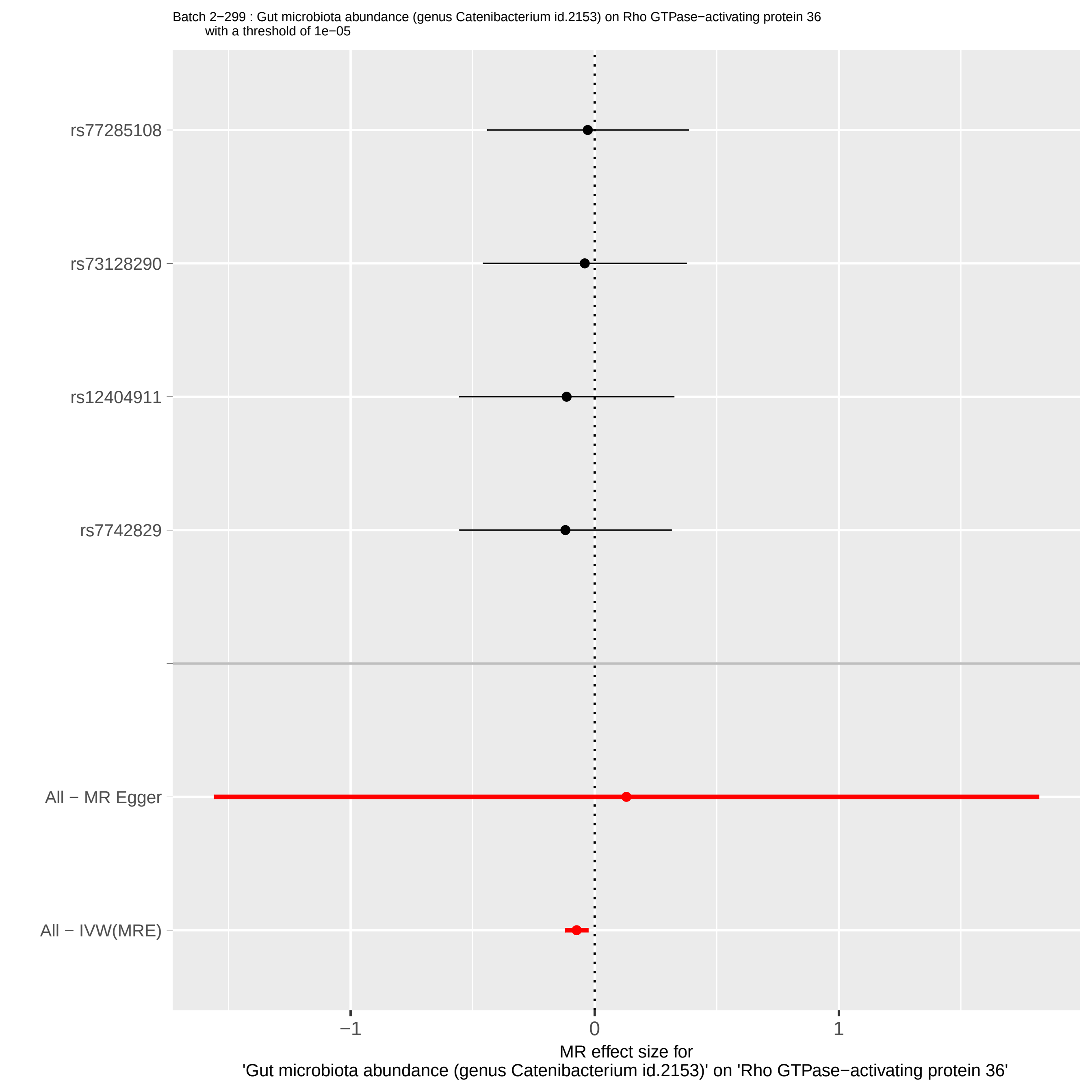

Batch 2−299 : Gut microbiota abundance (genus Catenibacterium id.2153) on Rho GTPase−activating protein 36
with a threshold of 1e−05
rs77285108
rs73128290
rs12404911
rs7742829
All − MR Egger
All − IVW(MRE)
−1
0
1
MR effect size for
'Gut microbiota abundance (genus Catenibacterium id.2153)' on 'Rho GTPase−activating protein 36'

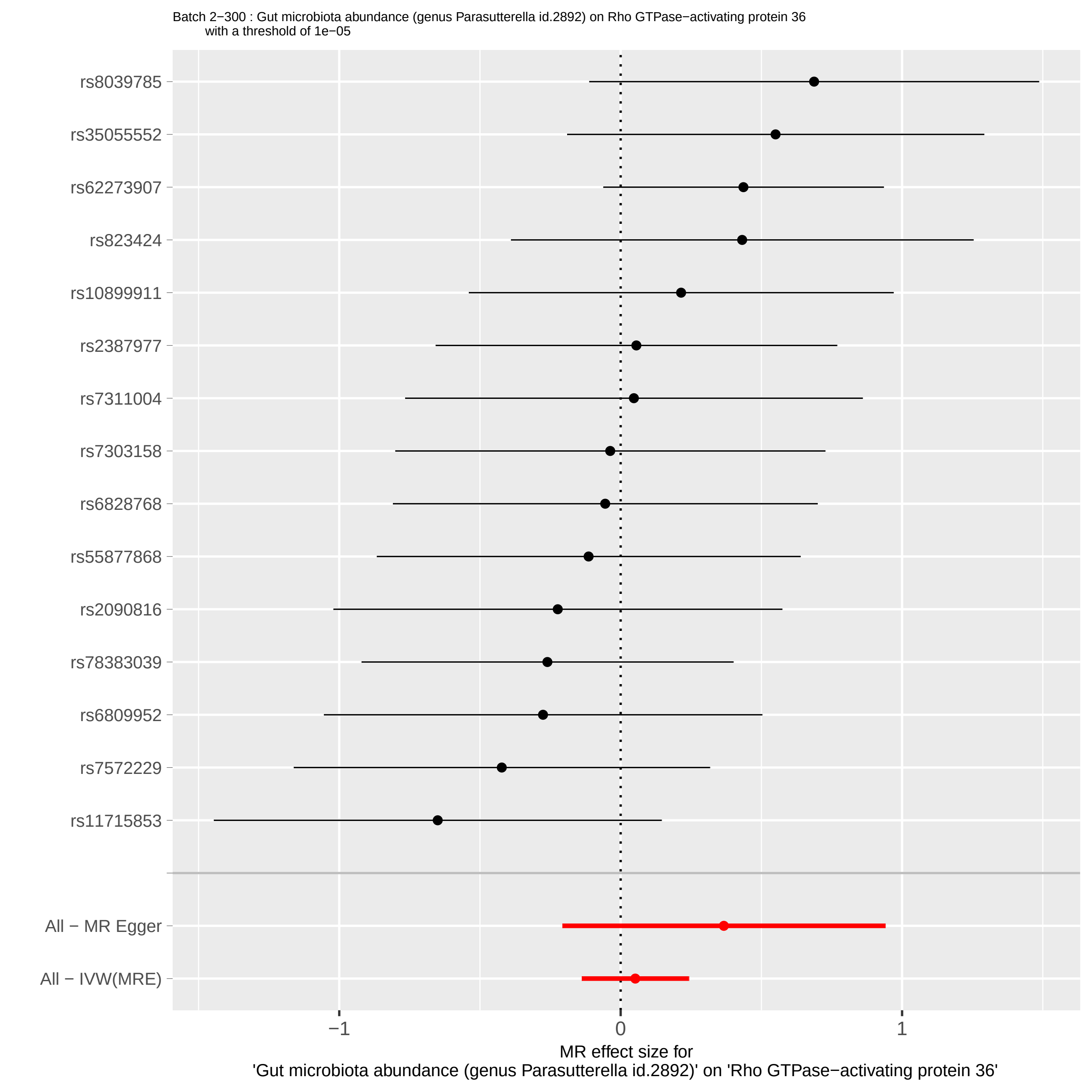
Batch 2−300 : Gut microbiota abundance (genus Parasutterella id.2892) on Rho GTPase−activating protein 36
with a threshold of 1e−05
rs8039785
rs35055552
rs62273907
rs823424
rs10899911
rs2387977
rs7311004
rs7303158
rs6828768
rs55877868
rs2090816
rs78383039
rs6809952
rs7572229
rs11715853
All − MR Egger
All − IVW(MRE)
−1
0
1
MR effect size for
'Gut microbiota abundance (genus Parasutterella id.2892)' on 'Rho GTPase−activating protein 36'

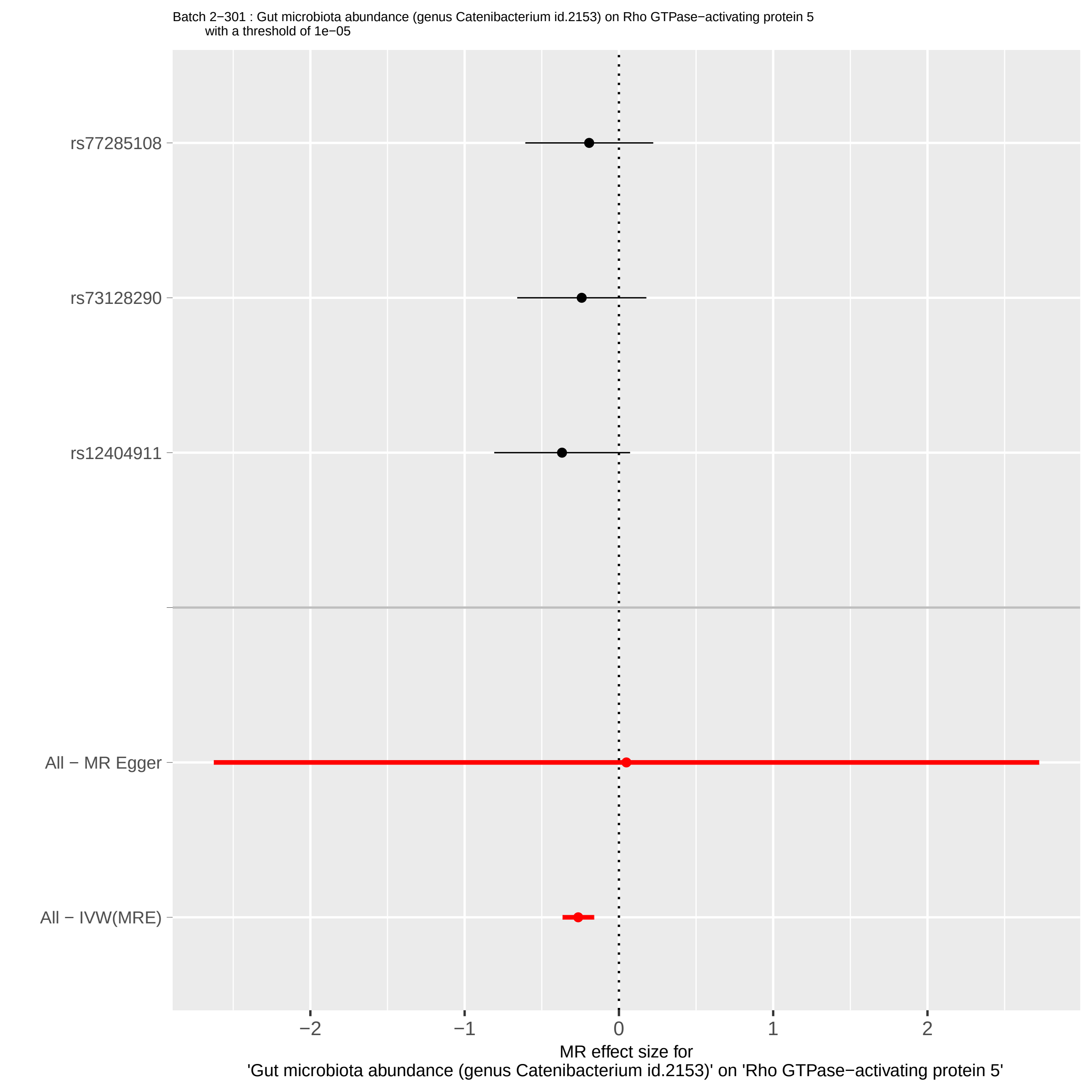

Batch 2−301 : Gut microbiota abundance (genus Catenibacterium id.2153) on Rho GTPase−activating protein 5
with a threshold of 1e−05
rs77285108
rs73128290
rs12404911
All − MR Egger
All − IVW(MRE)
−2
−1
0
1
2
MR effect size for
'Gut microbiota abundance (genus Catenibacterium id.2153)' on 'Rho GTPase−activating protein 5'

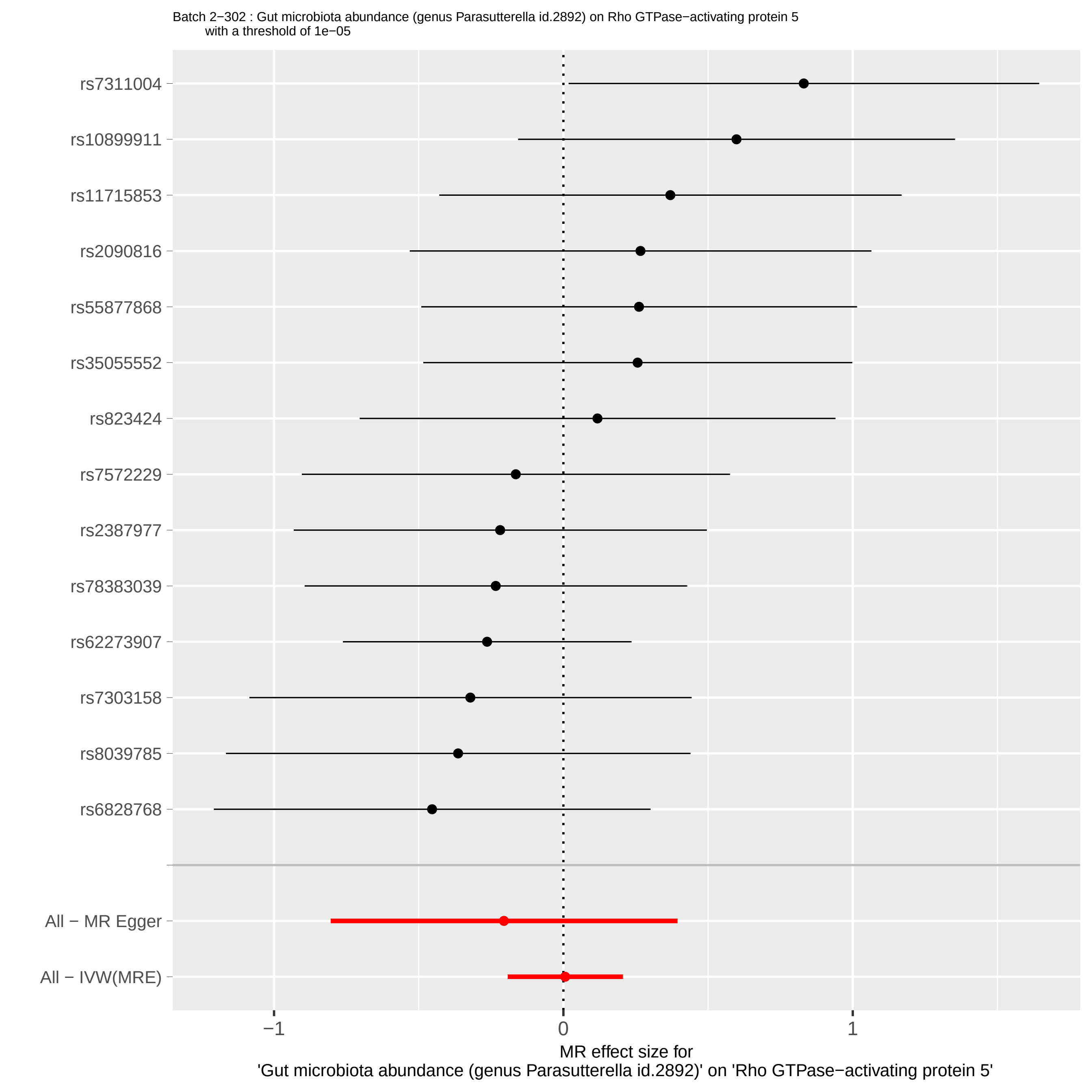

Batch 2−302 : Gut microbiota abundance (genus Parasutterella id.2892) on Rho GTPase−activating protein 5
with a threshold of 1e−05
rs7311004
rs10899911
rs11715853
rs2090816
rs55877868
rs35055552
rs823424
rs7572229
rs2387977
rs78383039
rs62273907
rs7303158
rs8039785
rs6828768
All − MR Egger
All − IVW(MRE)
−1
0
1
MR effect size for
'Gut microbiota abundance (genus Parasutterella id.2892)' on 'Rho GTPase−activating protein 5'

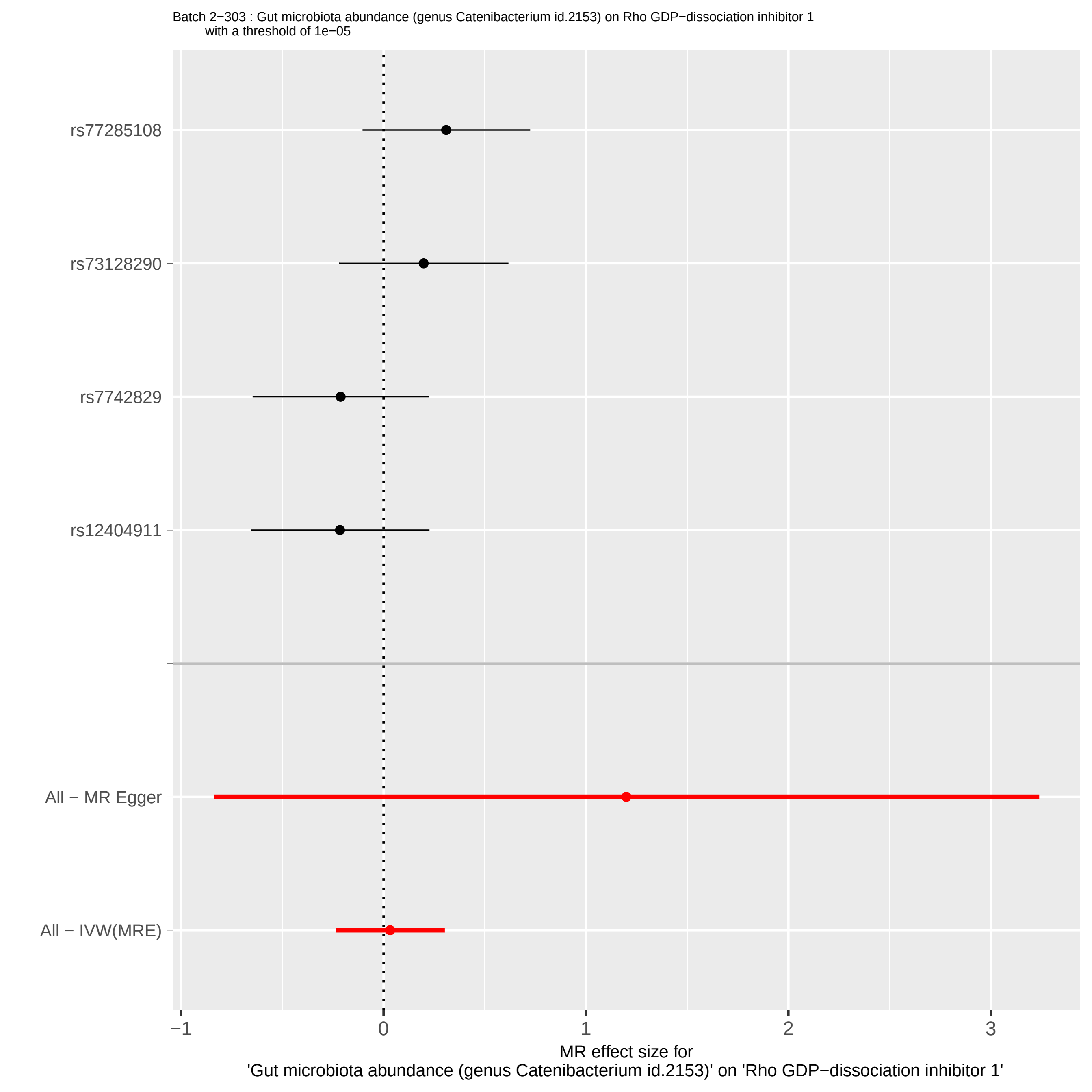
Batch 2−303 : Gut microbiota abundance (genus Catenibacterium id.2153) on Rho GDP−dissociation inhibitor 1
with a threshold of 1e−05
rs77285108
rs73128290
rs7742829
rs12404911
All − MR Egger
All − IVW(MRE)
−1
0
1
2
3
MR effect size for
'Gut microbiota abundance (genus Catenibacterium id.2153)' on 'Rho GDP−dissociation inhibitor 1'

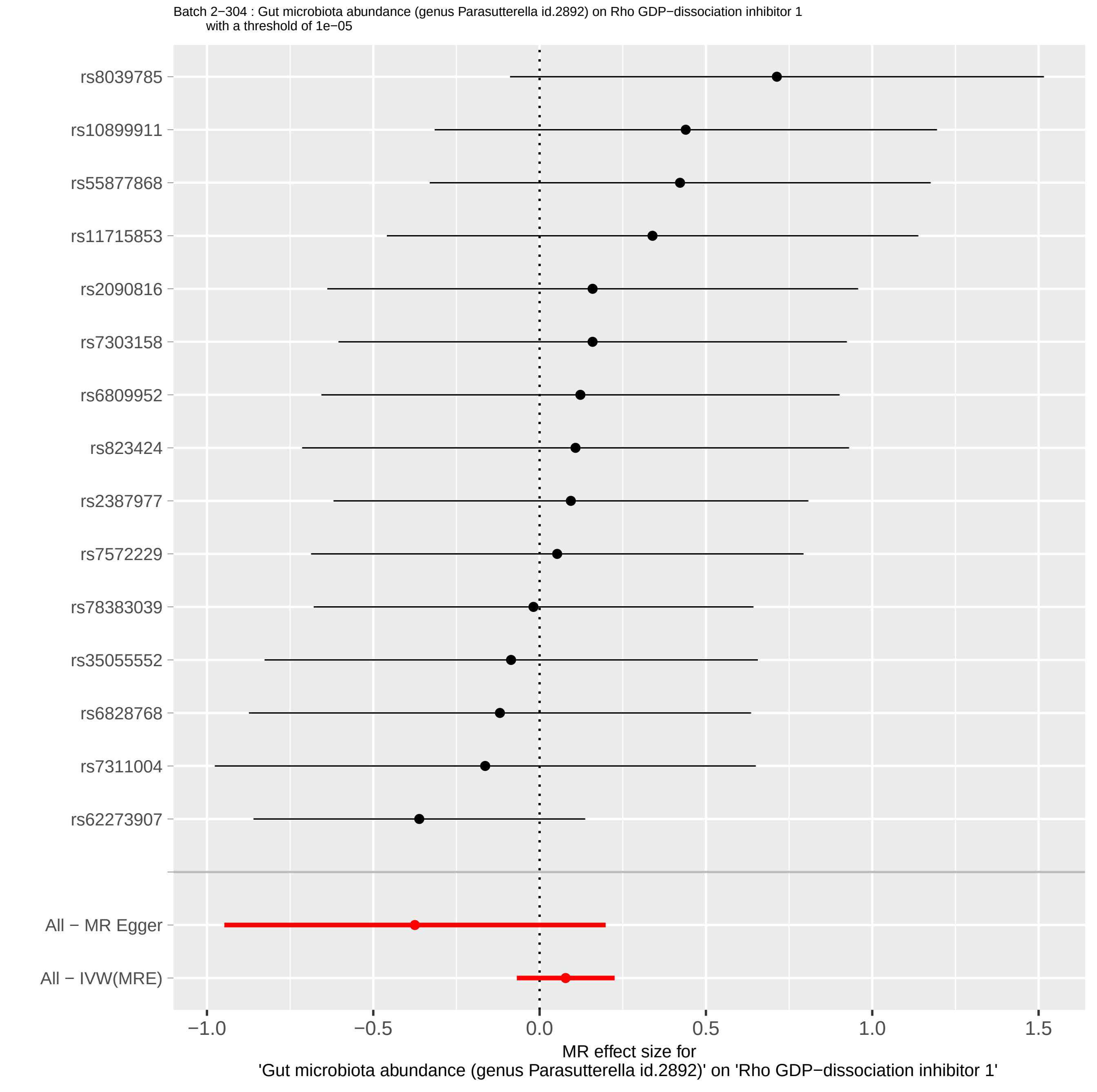

Batch 2−304 : Gut microbiota abundance (genus Parasutterella id.2892) on Rho GDP−dissociation inhibitor 1 with a threshold of 1e−05
rs8039785
rs10899911
rs55877868
rs11715853
rs2090816
rs7303158
rs6809952
rs823424
rs2387977
rs7572229
rs78383039
rs35055552
rs6828768
rs7311004
rs62273907
All − MR Egger
All − IVW(MRE)
−1.0
−0.5
0.0
0.5
1.0
1.5
MR effect size for
'Gut microbiota abundance (genus Parasutterella id.2892)' on 'Rho GDP−dissociation inhibitor 1'

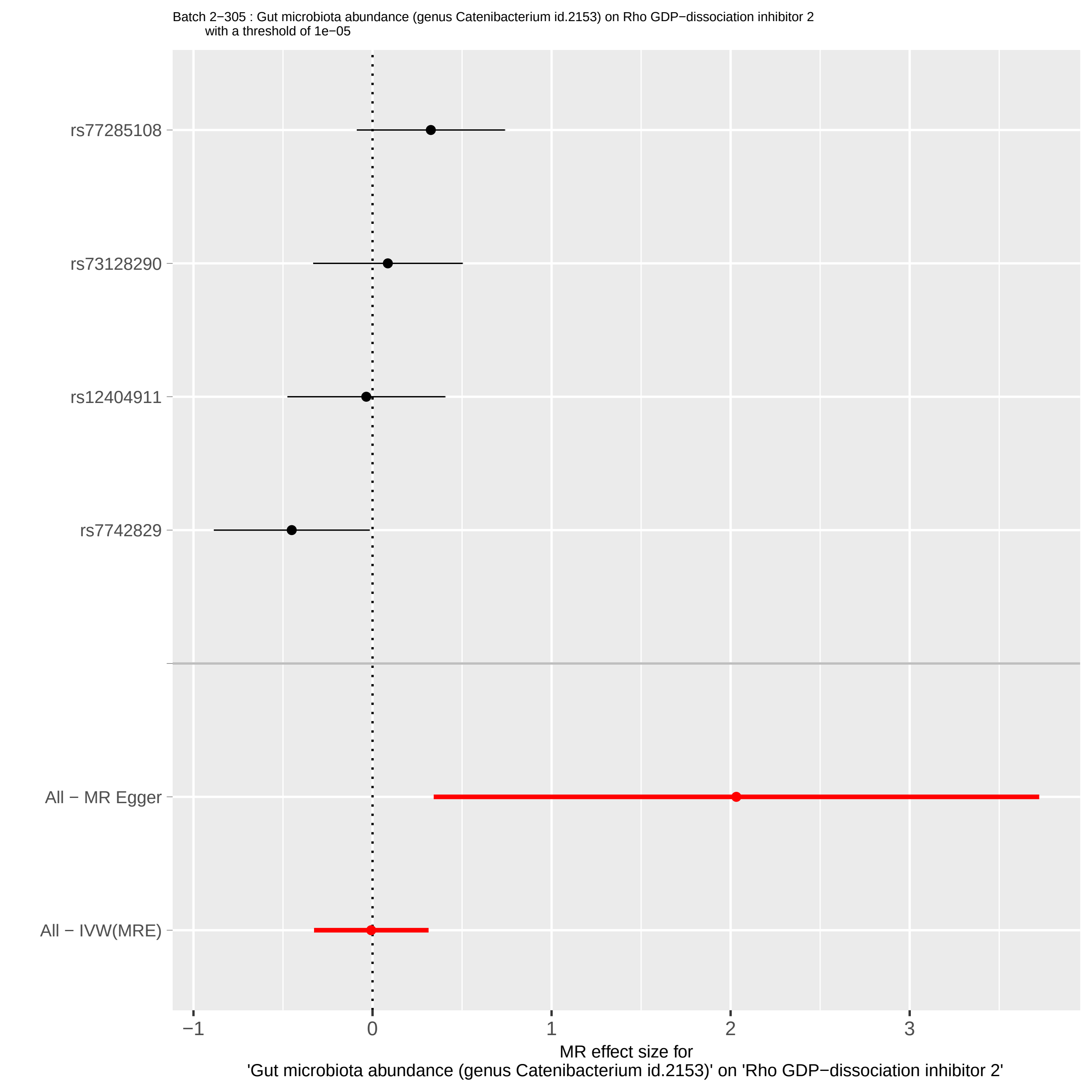

Batch 2−305 : Gut microbiota abundance (genus Catenibacterium id.2153) on Rho GDP−dissociation inhibitor 2
with a threshold of 1e−05
rs77285108
rs73128290
rs12404911
rs7742829
All − MR Egger
All − IVW(MRE)
−1
0
1
2
3
MR effect size for
'Gut microbiota abundance (genus Catenibacterium id.2153)' on 'Rho GDP−dissociation inhibitor 2'

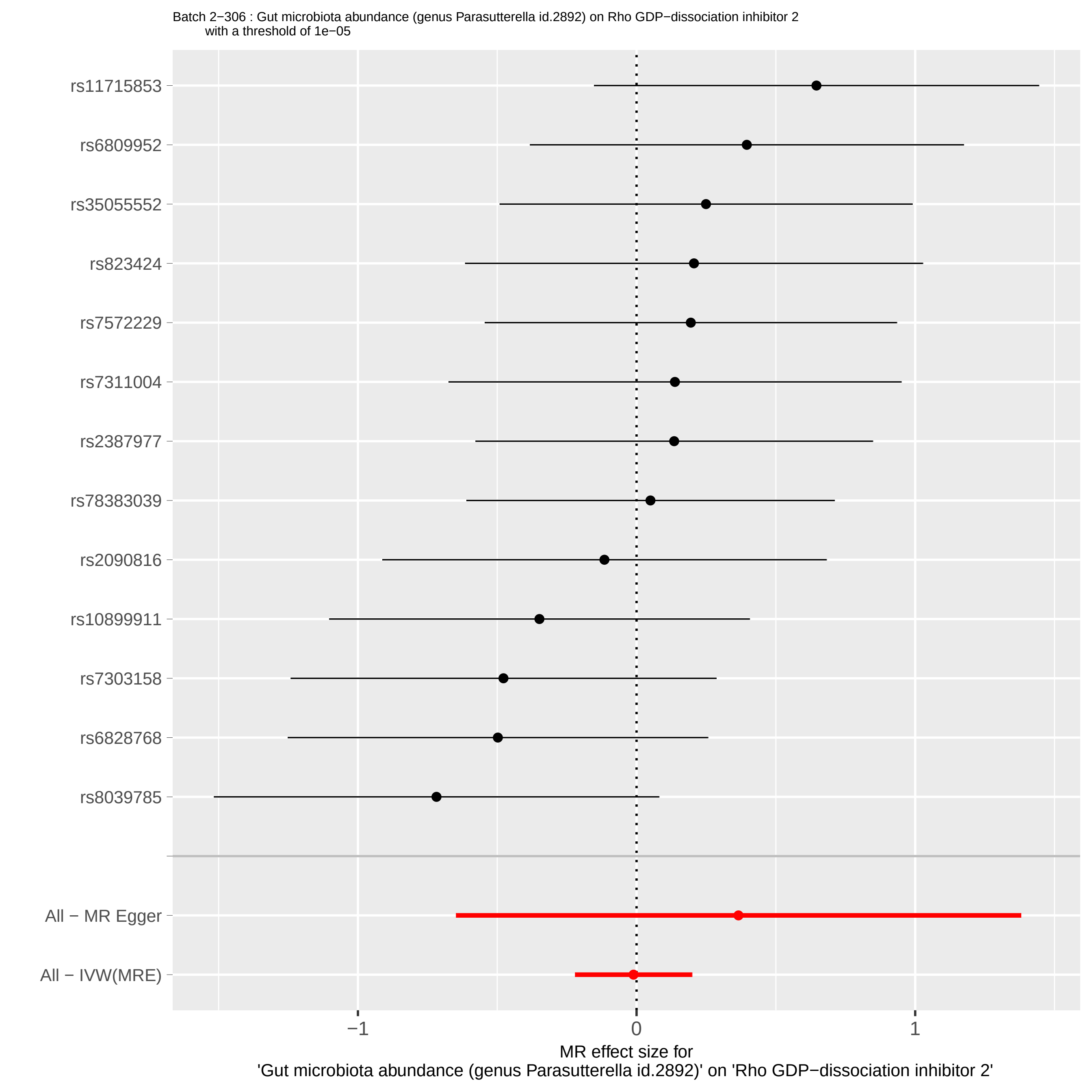

Batch 2−306 : Gut microbiota abundance (genus Parasutterella id.2892) on Rho GDP−dissociation inhibitor 2
with a threshold of 1e−05
rs11715853
rs6809952
rs35055552
rs823424
rs7572229
rs7311004
rs2387977
rs78383039
rs2090816
rs10899911
rs7303158
rs6828768
rs8039785
All − MR Egger
All − IVW(MRE)
−1
0
1
MR effect size for
'Gut microbiota abundance (genus Parasutterella id.2892)' on 'Rho GDP−dissociation inhibitor 2'

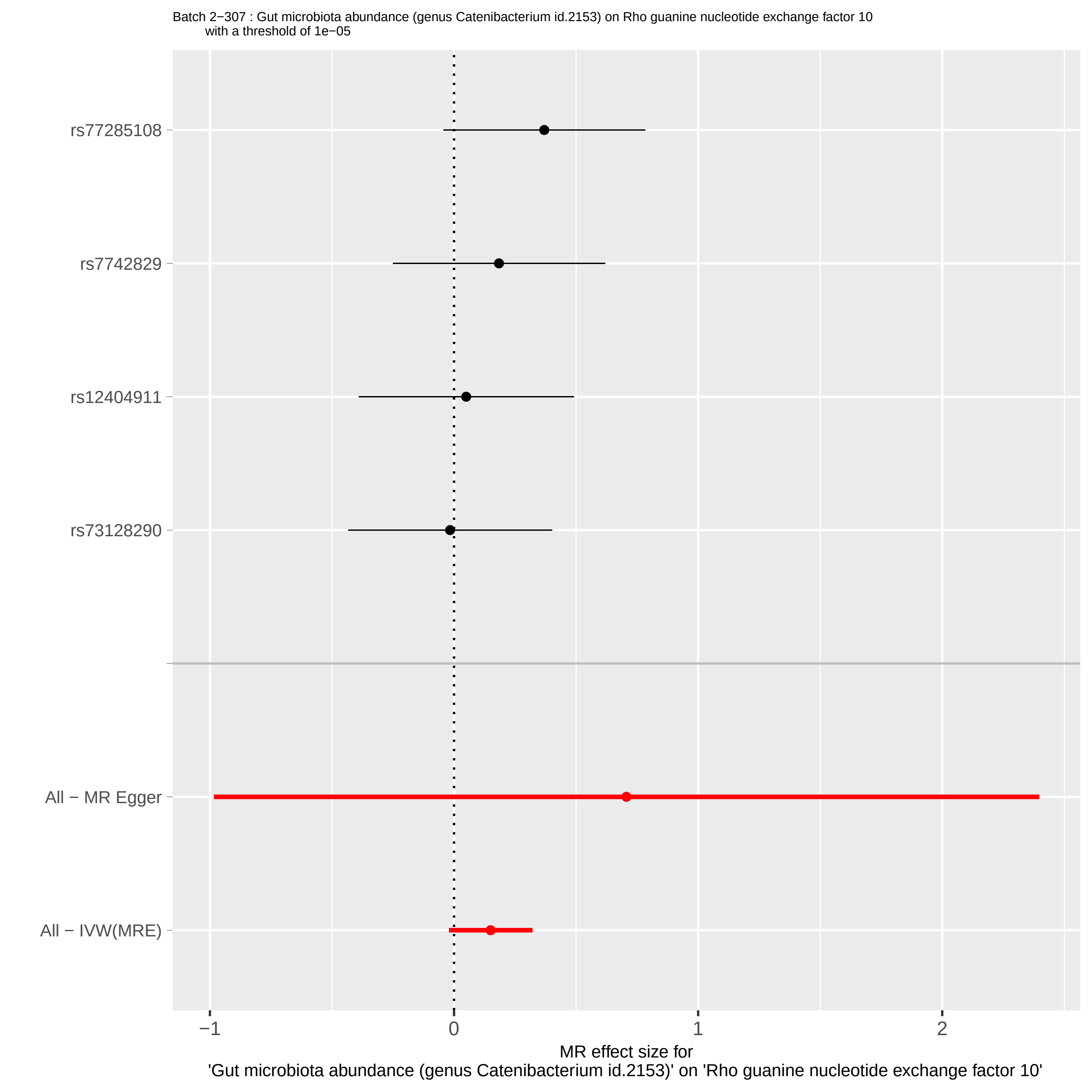
Batch 2−307 : Gut microbiota abundance (genus Catenibacterium id.2153) on Rho guanine nucleotide exchange factor 10
with a threshold of 1e−05
rs77285108
rs7742829
rs12404911
rs73128290
All − MR Egger
All − IVW(MRE)
−1
0
1
2
MR effect size for
'Gut microbiota abundance (genus Catenibacterium id.2153)' on 'Rho guanine nucleotide exchange factor 10'

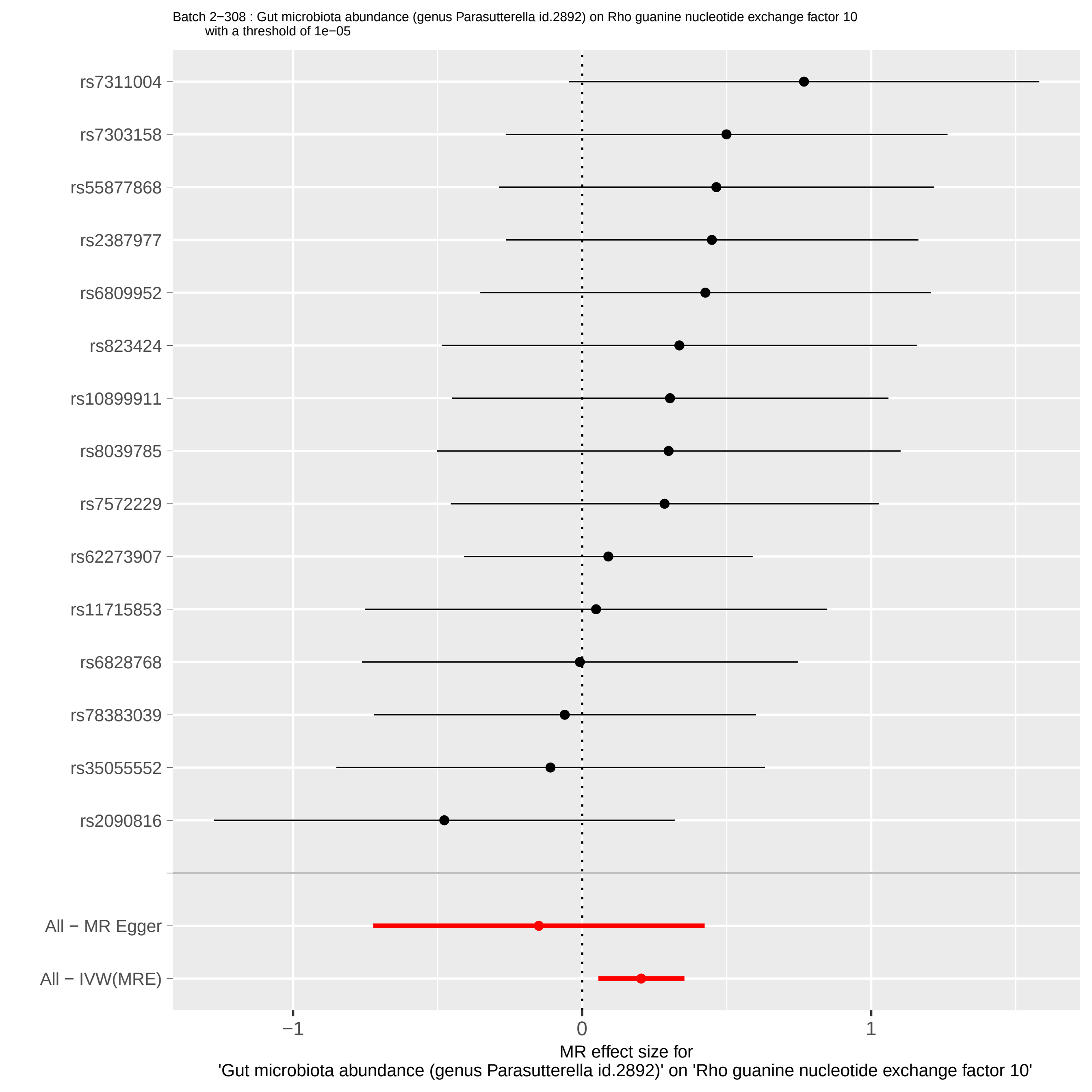

Batch 2−308 : Gut microbiota abundance (genus Parasutterella id.2892) on Rho guanine nucleotide exchange factor 10
with a threshold of 1e−05
rs7311004
rs7303158
rs55877868
rs2387977
rs6809952
rs823424
rs10899911
rs8039785
rs7572229
rs62273907
rs11715853
rs6828768
rs78383039
rs35055552
rs2090816
All − MR Egger
All − IVW(MRE)
−1
0
1
MR effect size for
'Gut microbiota abundance (genus Parasutterella id.2892)' on 'Rho guanine nucleotide exchange factor 10'

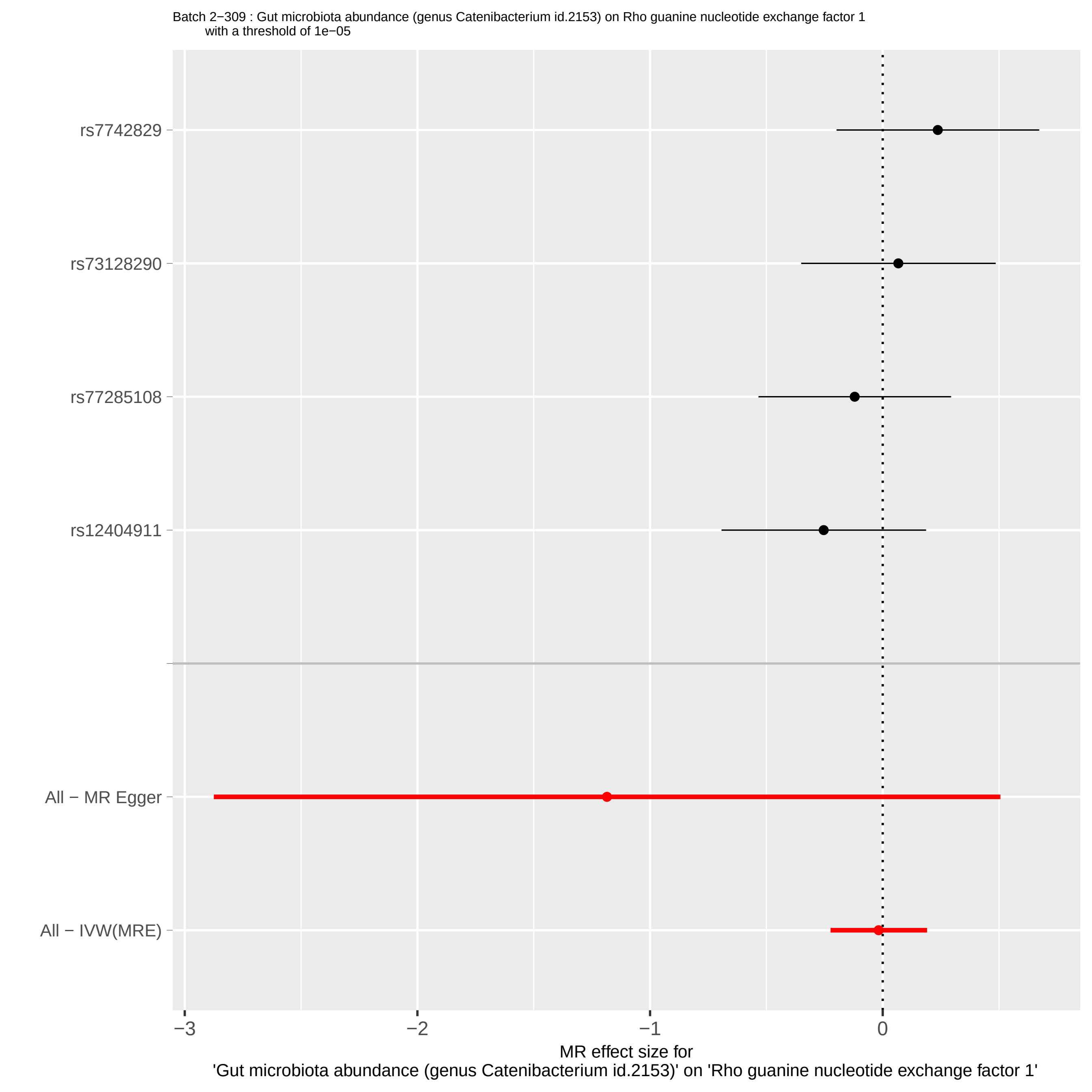

Batch 2−309 : Gut microbiota abundance (genus Catenibacterium id.2153) on Rho guanine nucleotide exchange factor 1
with a threshold of 1e−05
rs7742829
rs73128290
rs77285108
rs12404911
All − MR Egger
All − IVW(MRE)
−3
−2
−1
0
MR effect size for
'Gut microbiota abundance (genus Catenibacterium id.2153)' on 'Rho guanine nucleotide exchange factor 1'

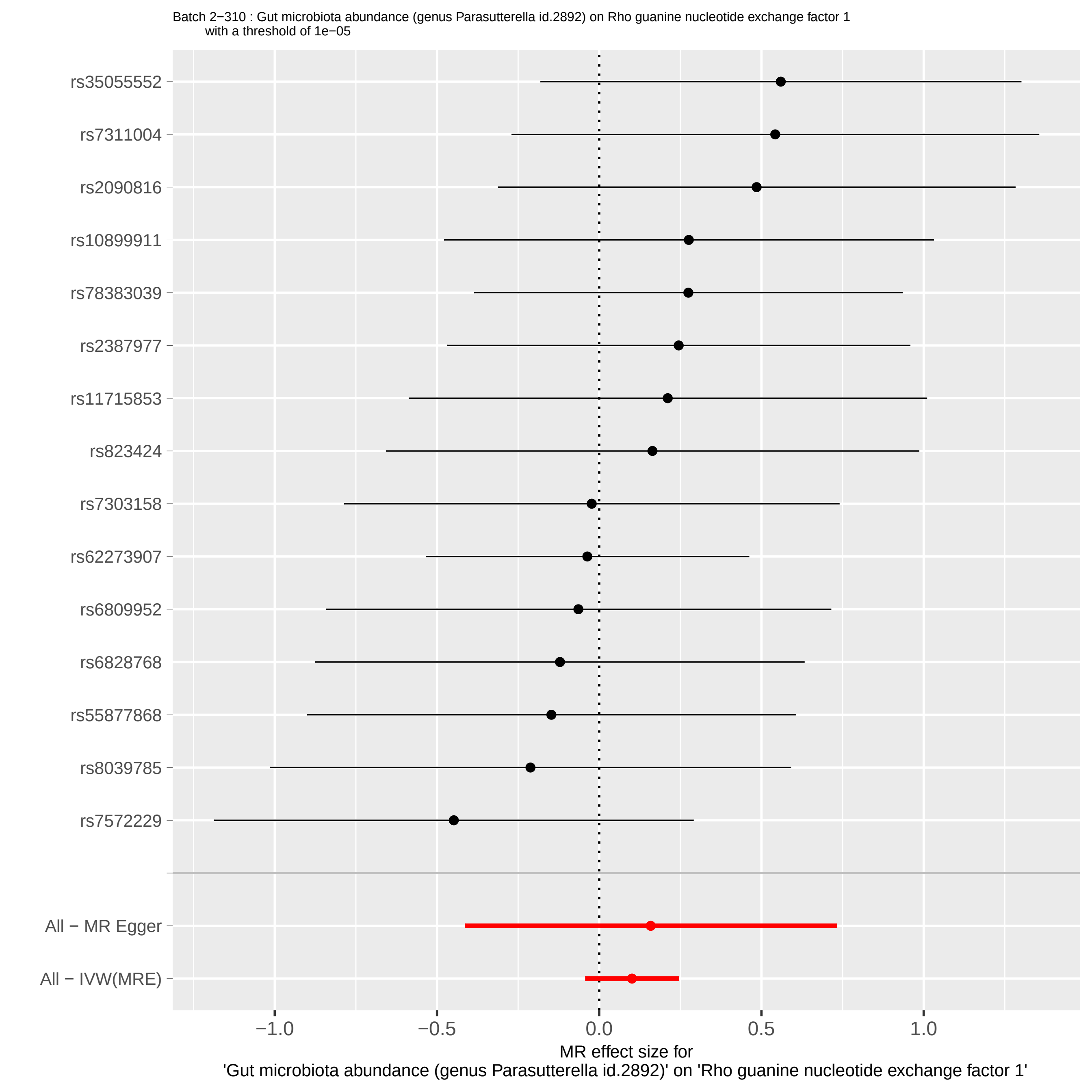

Batch 2−310 : Gut microbiota abundance (genus Parasutterella id.2892) on Rho guanine nucleotide exchange factor 1 with a threshold of 1e−05
rs35055552
rs7311004
rs2090816
rs10899911
rs78383039
rs2387977
rs11715853
rs823424
rs7303158
rs62273907
rs6809952
rs6828768
rs55877868
rs8039785
rs7572229
All − MR Egger
All − IVW(MRE)
−1.0
−0.5
0.0
0.5
1.0
MR effect size for
'Gut microbiota abundance (genus Parasutterella id.2892)' on 'Rho guanine nucleotide exchange factor 1'

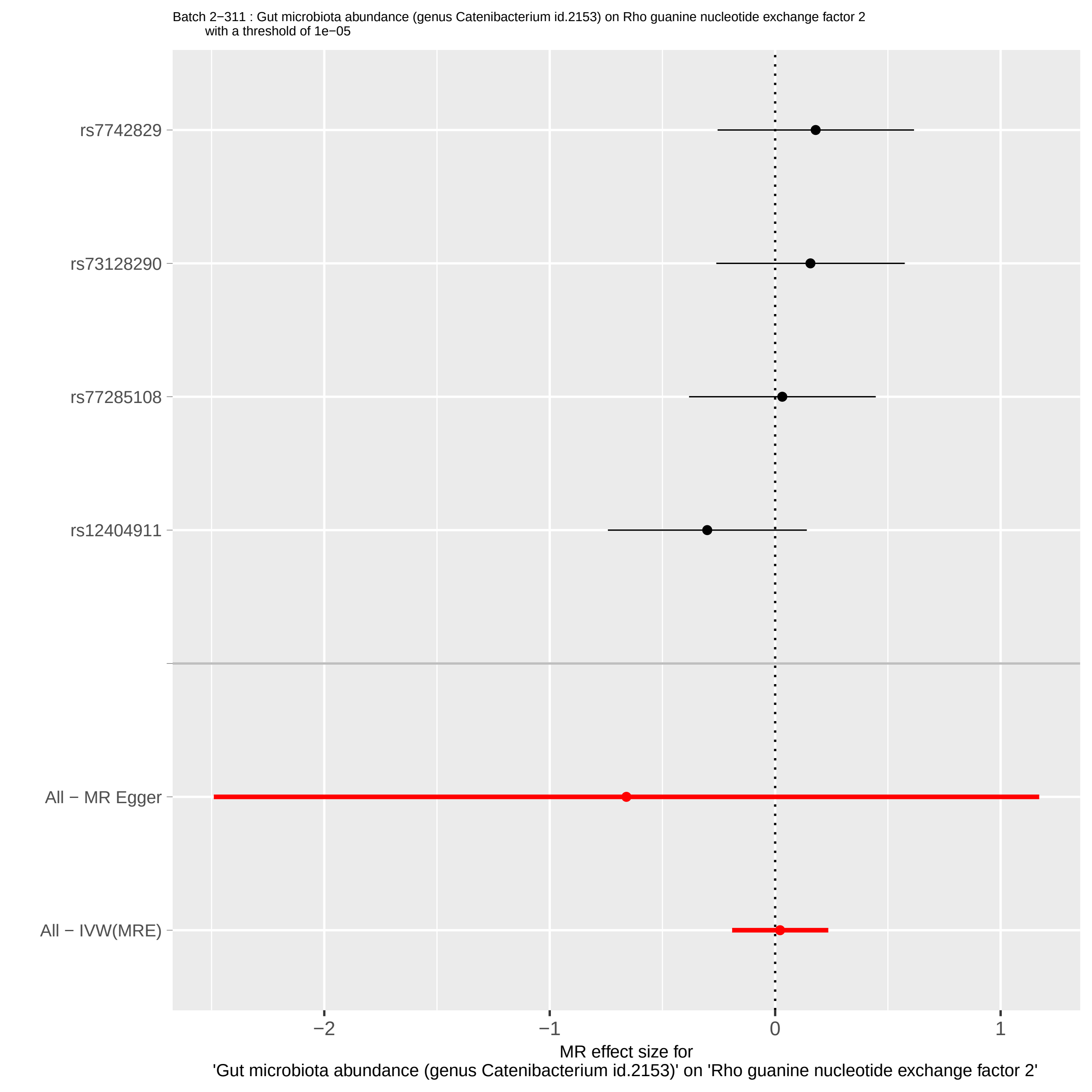

Batch 2−311 : Gut microbiota abundance (genus Catenibacterium id.2153) on Rho guanine nucleotide exchange factor 2
with a threshold of 1e−05
rs7742829
rs73128290
rs77285108
rs12404911
All − MR Egger
All − IVW(MRE)
−2
−1
0
1
MR effect size for
'Gut microbiota abundance (genus Catenibacterium id.2153)' on 'Rho guanine nucleotide exchange factor 2'

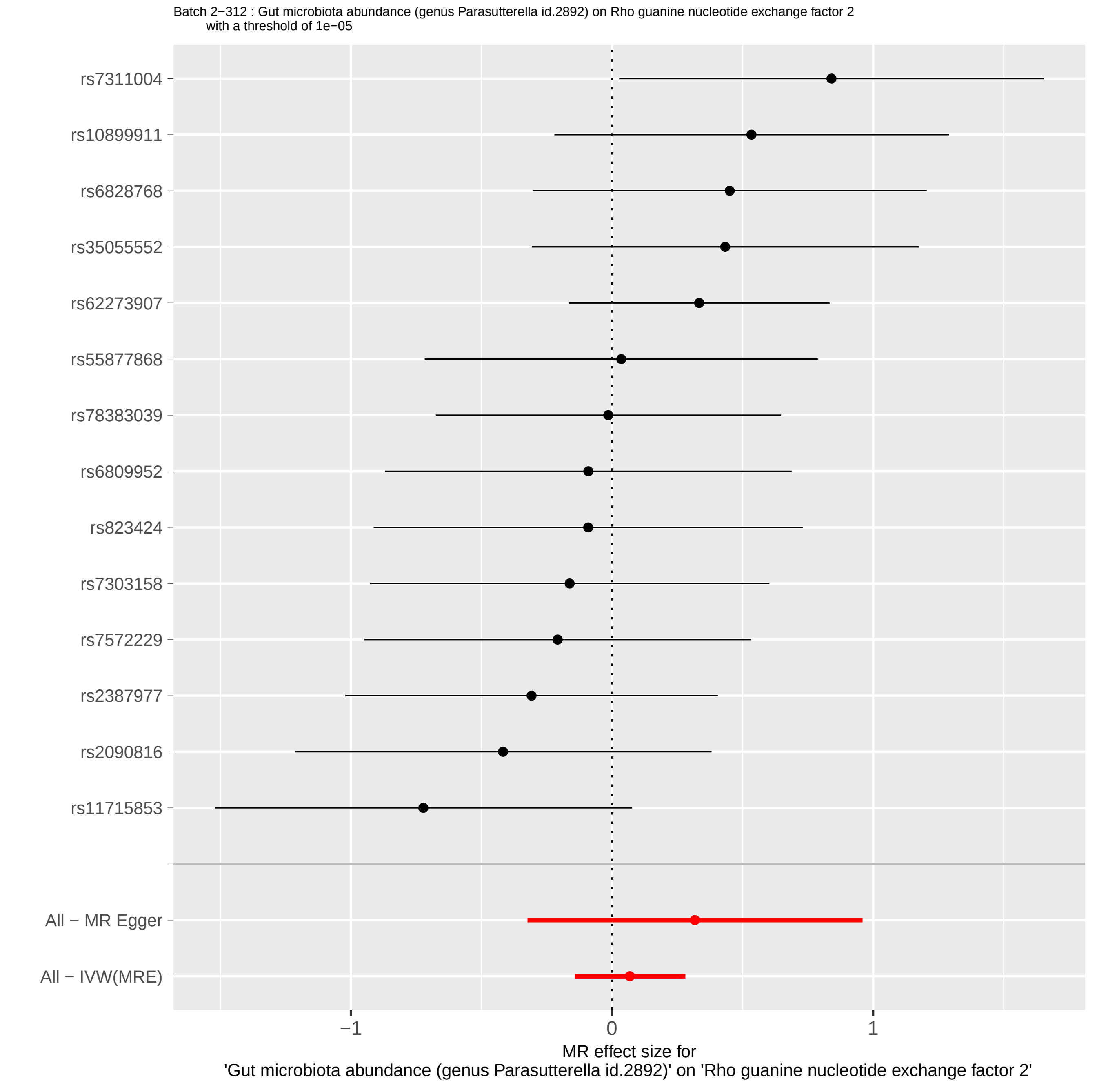

Batch 2−312 : Gut microbiota abundance (genus Parasutterella id.2892) on Rho guanine nucleotide exchange factor 2
with a threshold of 1e−05
rs7311004
rs10899911
rs6828768
rs35055552
rs62273907
rs55877868
rs78383039
rs6809952
rs823424
rs7303158
rs7572229
rs2387977
rs2090816
rs11715853
All − MR Egger
All − IVW(MRE)
−1
0
1
MR effect size for
'Gut microbiota abundance (genus Parasutterella id.2892)' on 'Rho guanine nucleotide exchange factor 2'

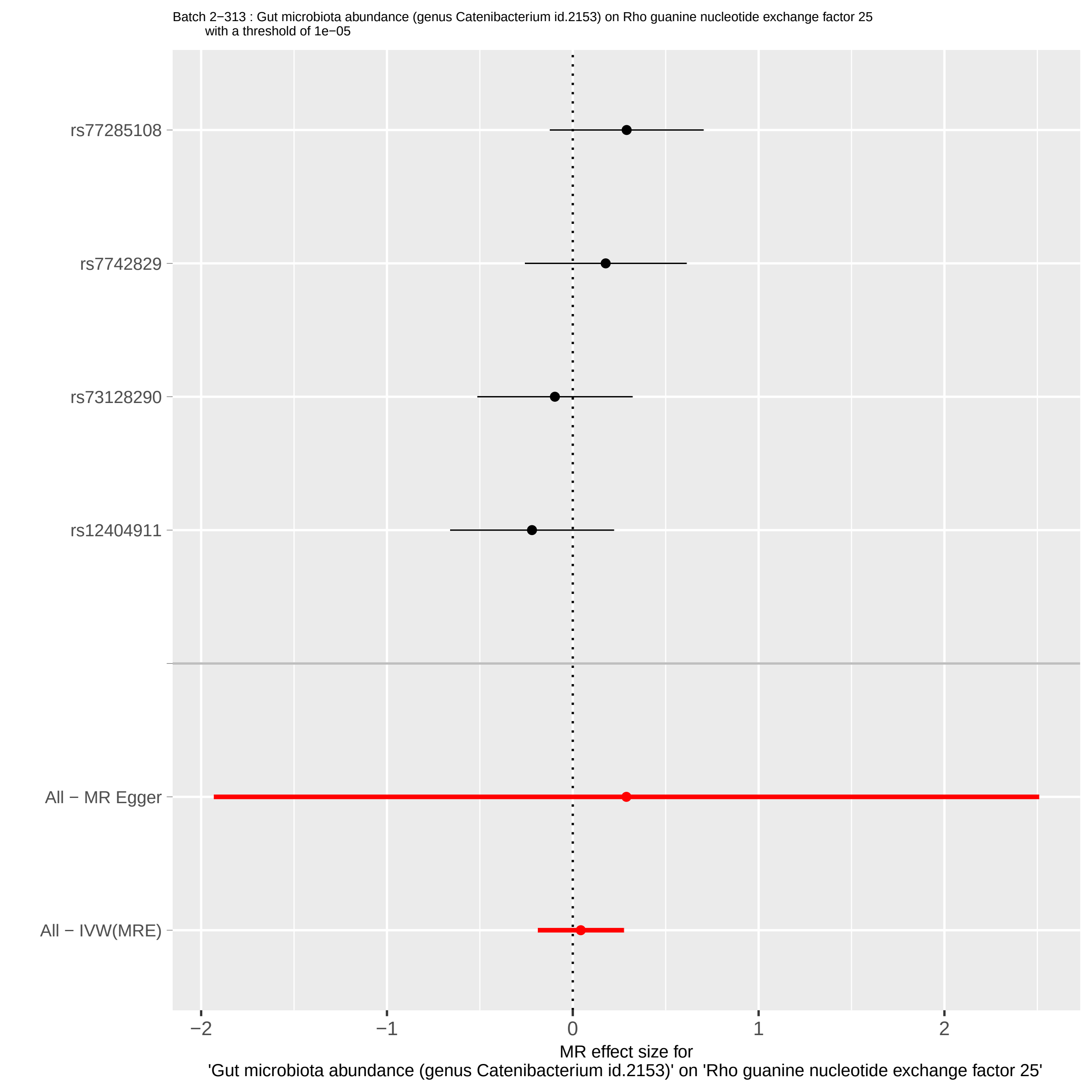
Batch 2−313 : Gut microbiota abundance (genus Catenibacterium id.2153) on Rho guanine nucleotide exchange factor 25
with a threshold of 1e−05
rs77285108
rs7742829
rs73128290
rs12404911
All − MR Egger
All − IVW(MRE)
−2
−1
0
1
2
MR effect size for
'Gut microbiota abundance (genus Catenibacterium id.2153)' on 'Rho guanine nucleotide exchange factor 25'

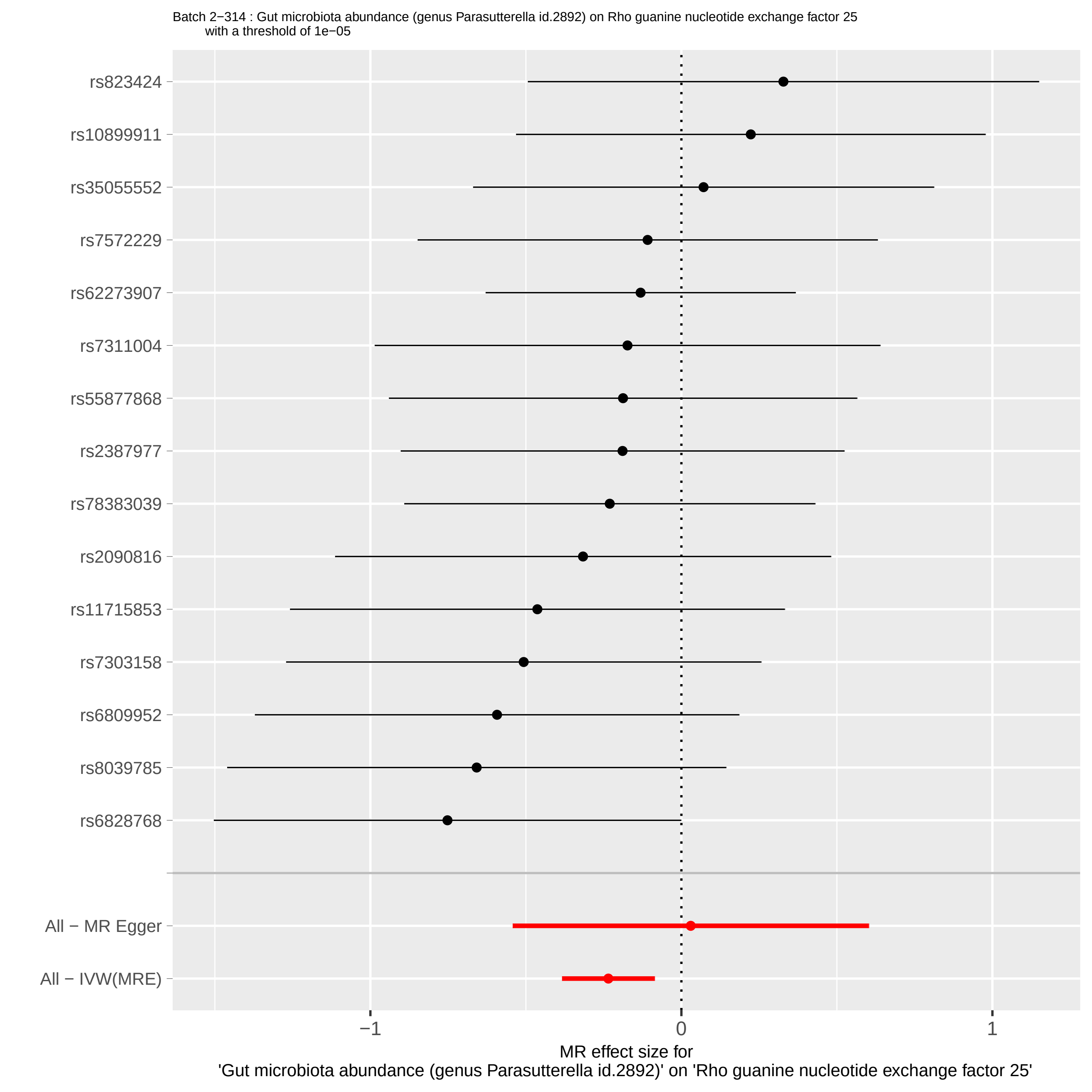
Batch 2−314 : Gut microbiota abundance (genus Parasutterella id.2892) on Rho guanine nucleotide exchange factor 25
with a threshold of 1e−05
rs823424
rs10899911
rs35055552
rs7572229
rs62273907
rs7311004
rs55877868
rs2387977
rs78383039
rs2090816
rs11715853
rs7303158
rs6809952
rs8039785
rs6828768
All − MR Egger
All − IVW(MRE)
−1
0
1
MR effect size for
'Gut microbiota abundance (genus Parasutterella id.2892)' on 'Rho guanine nucleotide exchange factor 25'

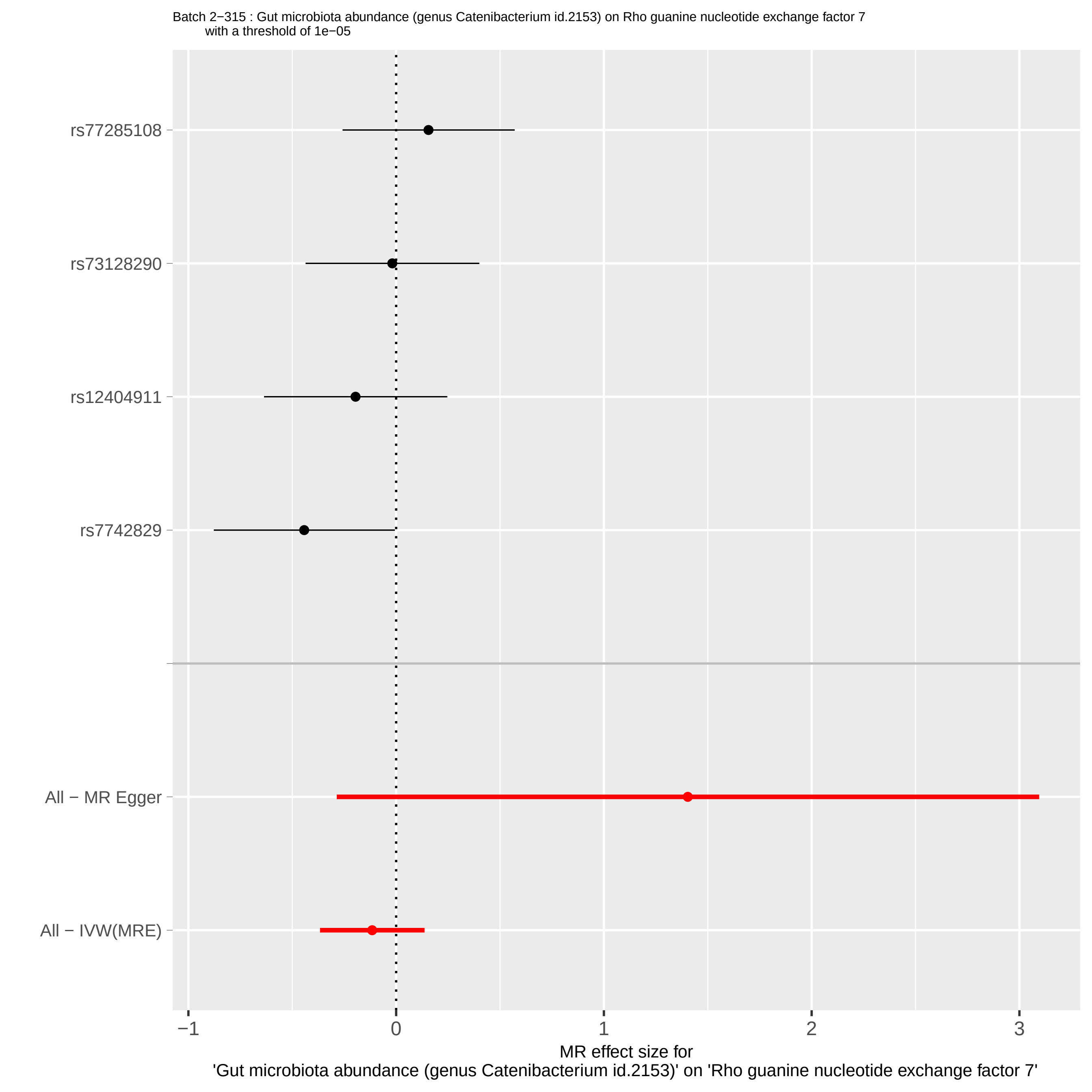

Batch 2−315 : Gut microbiota abundance (genus Catenibacterium id.2153) on Rho guanine nucleotide exchange factor 7
with a threshold of 1e−05
rs77285108
rs73128290
rs12404911
rs7742829
All − MR Egger
All − IVW(MRE)
−1
0
1
2
3
MR effect size for
'Gut microbiota abundance (genus Catenibacterium id.2153)' on 'Rho guanine nucleotide exchange factor 7'

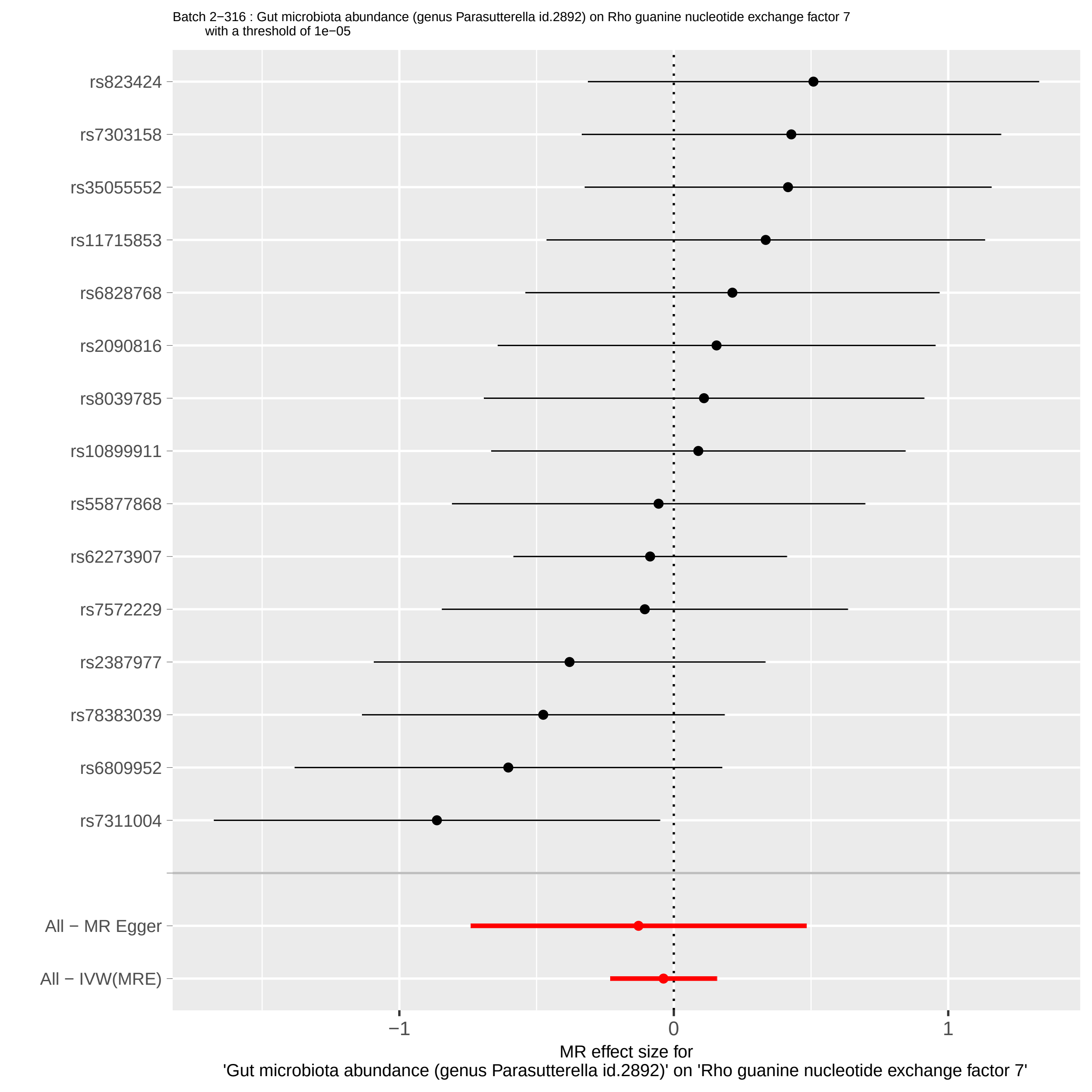
Batch 2−316 : Gut microbiota abundance (genus Parasutterella id.2892) on Rho guanine nucleotide exchange factor 7
with a threshold of 1e−05
rs823424
rs7303158
rs35055552
rs11715853
rs6828768
rs2090816
rs8039785
rs10899911
rs55877868
rs62273907
rs7572229
rs2387977
rs78383039
rs6809952
rs7311004
All − MR Egger
All − IVW(MRE)
−1
0
1
MR effect size for
'Gut microbiota abundance (genus Parasutterella id.2892)' on 'Rho guanine nucleotide exchange factor 7'

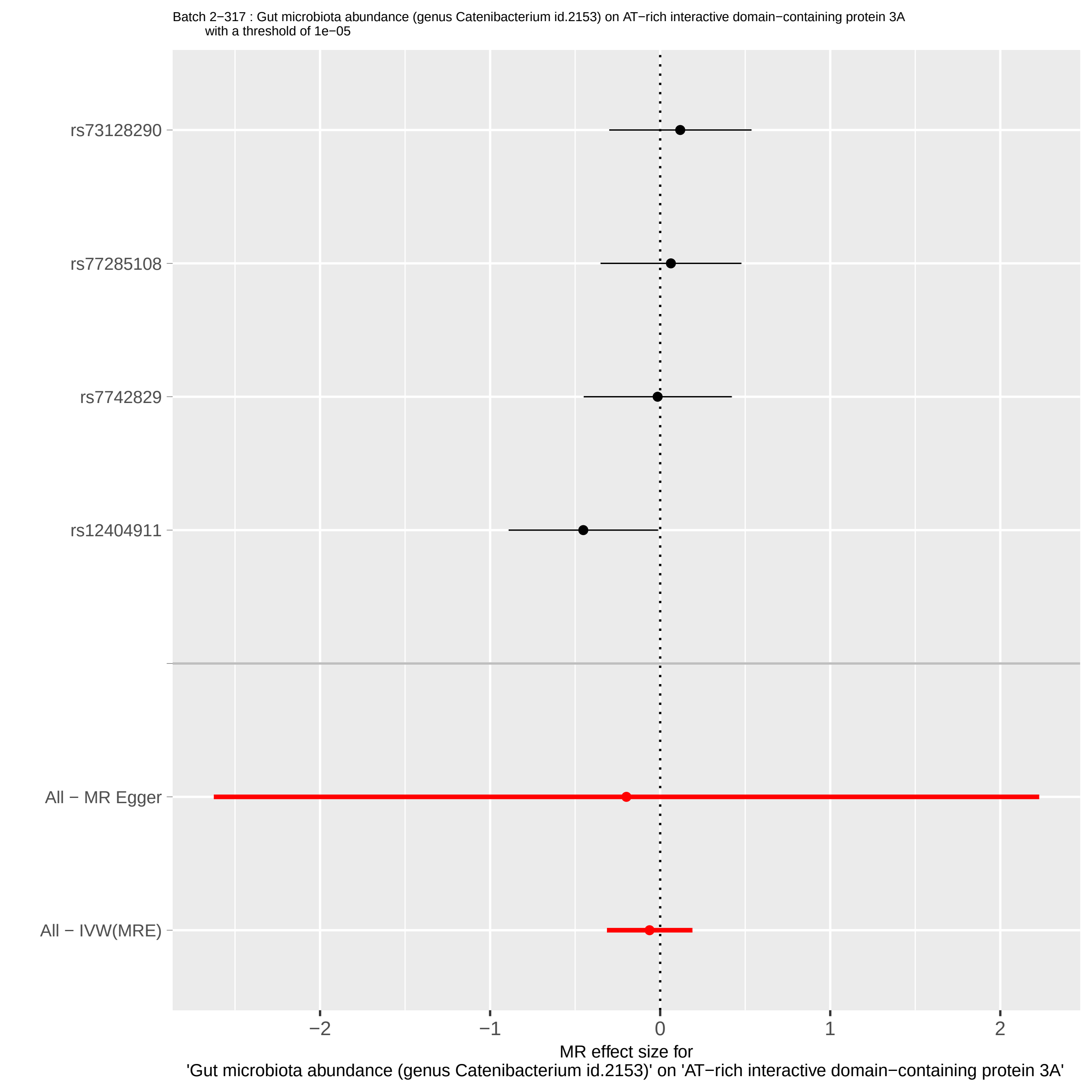
Batch 2−317 : Gut microbiota abundance (genus Catenibacterium id.2153) on AT−rich interactive domain−containing protein 3A
with a threshold of 1e−05
rs73128290
rs77285108
rs7742829
rs12404911
All − MR Egger
All − IVW(MRE)
−2
−1
0
1
2
MR effect size for
'Gut microbiota abundance (genus Catenibacterium id.2153)' on 'AT−rich interactive domain−containing protein 3A'

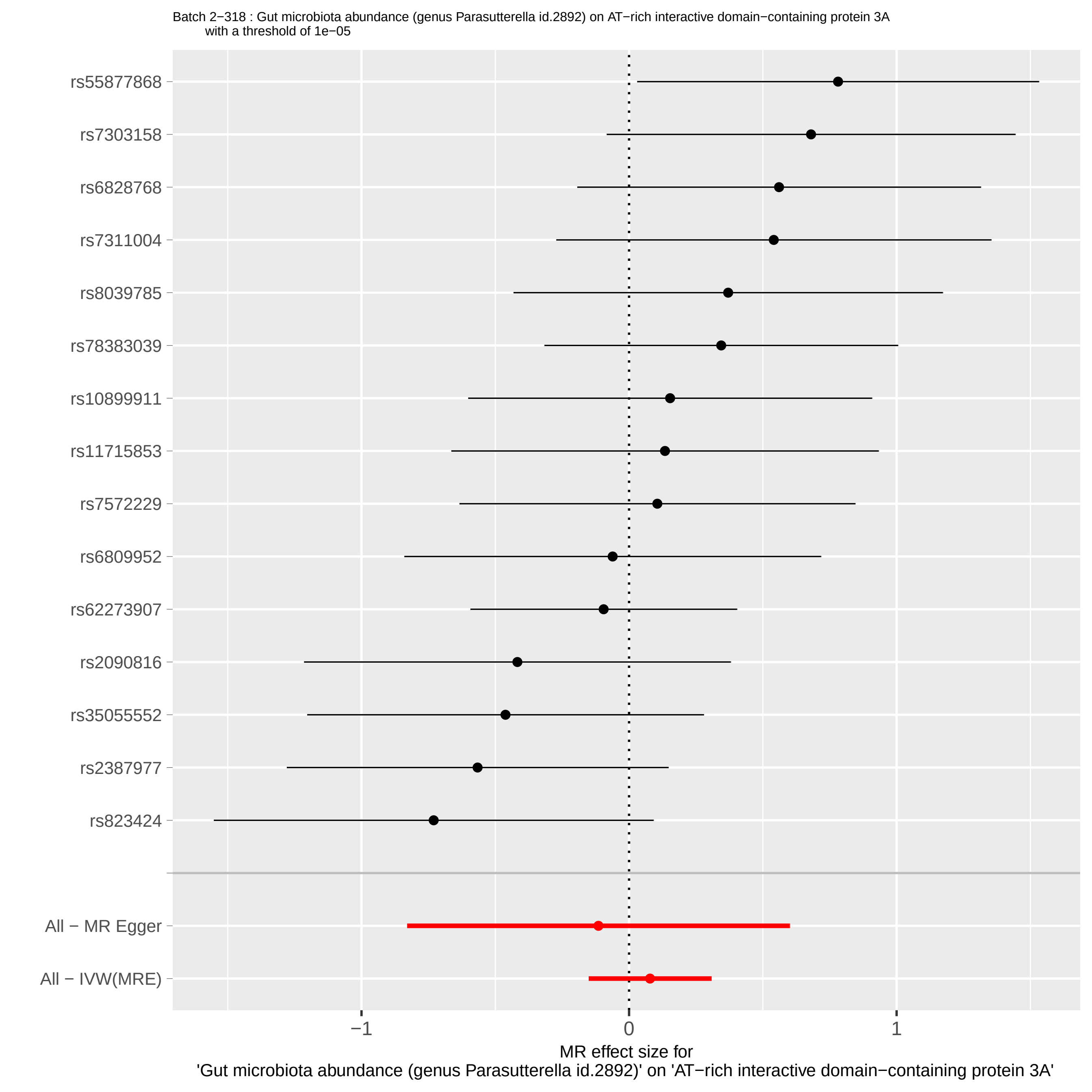

Batch 2−318 : Gut microbiota abundance (genus Parasutterella id.2892) on AT−rich interactive domain−containing protein 3A
with a threshold of 1e−05
rs55877868
rs7303158
rs6828768
rs7311004
rs8039785
rs78383039
rs10899911
rs11715853
rs7572229
rs6809952
rs62273907
rs2090816
rs35055552
rs2387977
rs823424
All − MR Egger
All − IVW(MRE)
−1
0
1
MR effect size for
'Gut microbiota abundance (genus Parasutterella id.2892)' on 'AT−rich interactive domain−containing protein 3A'

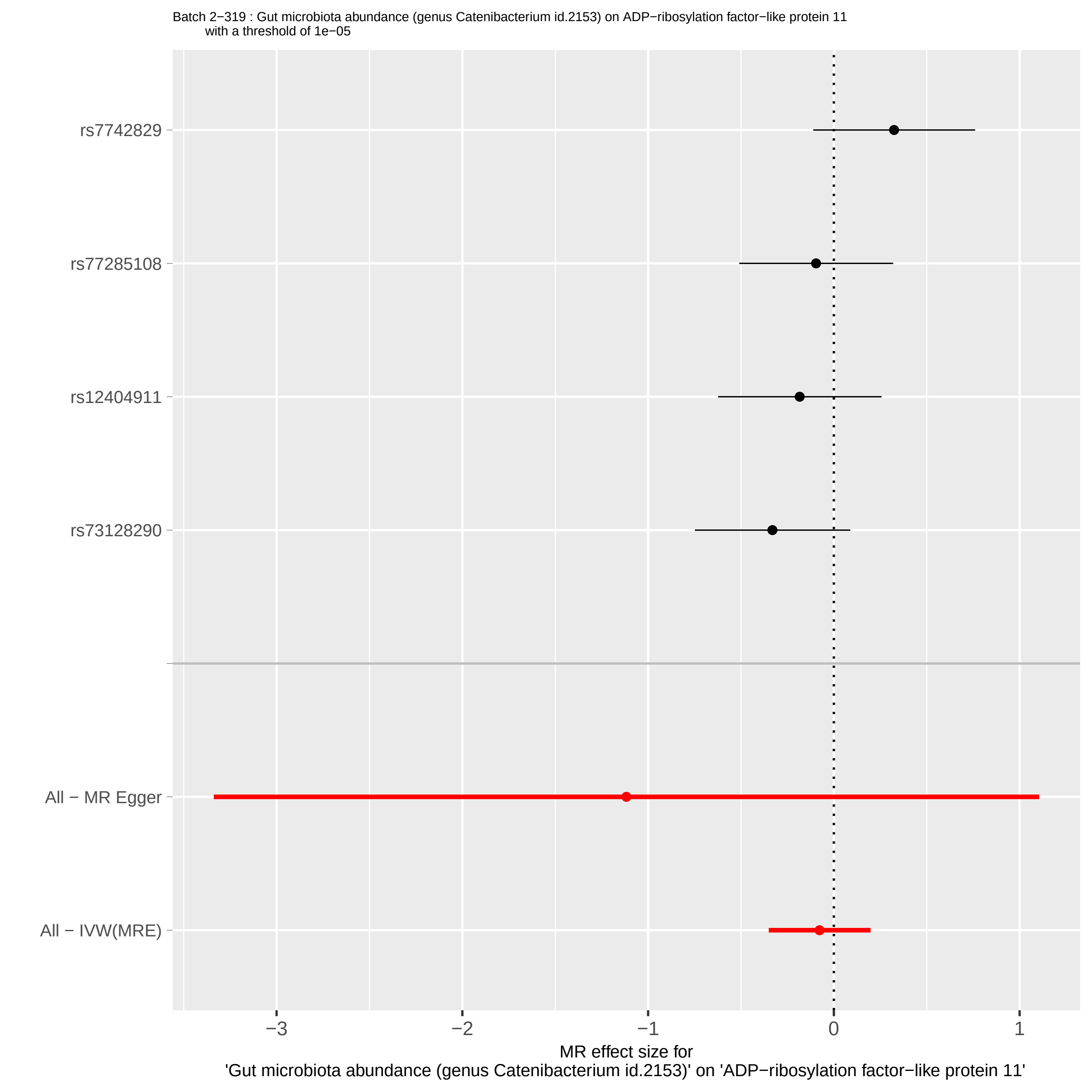

Batch 2−319 : Gut microbiota abundance (genus Catenibacterium id.2153) on ADP−ribosylation factor−like protein 11
with a threshold of 1e−05
rs7742829
rs77285108
rs12404911
rs73128290
All − MR Egger
All − IVW(MRE)
−3
−2
−1
0
1
MR effect size for
'Gut microbiota abundance (genus Catenibacterium id.2153)' on 'ADP−ribosylation factor−like protein 11'

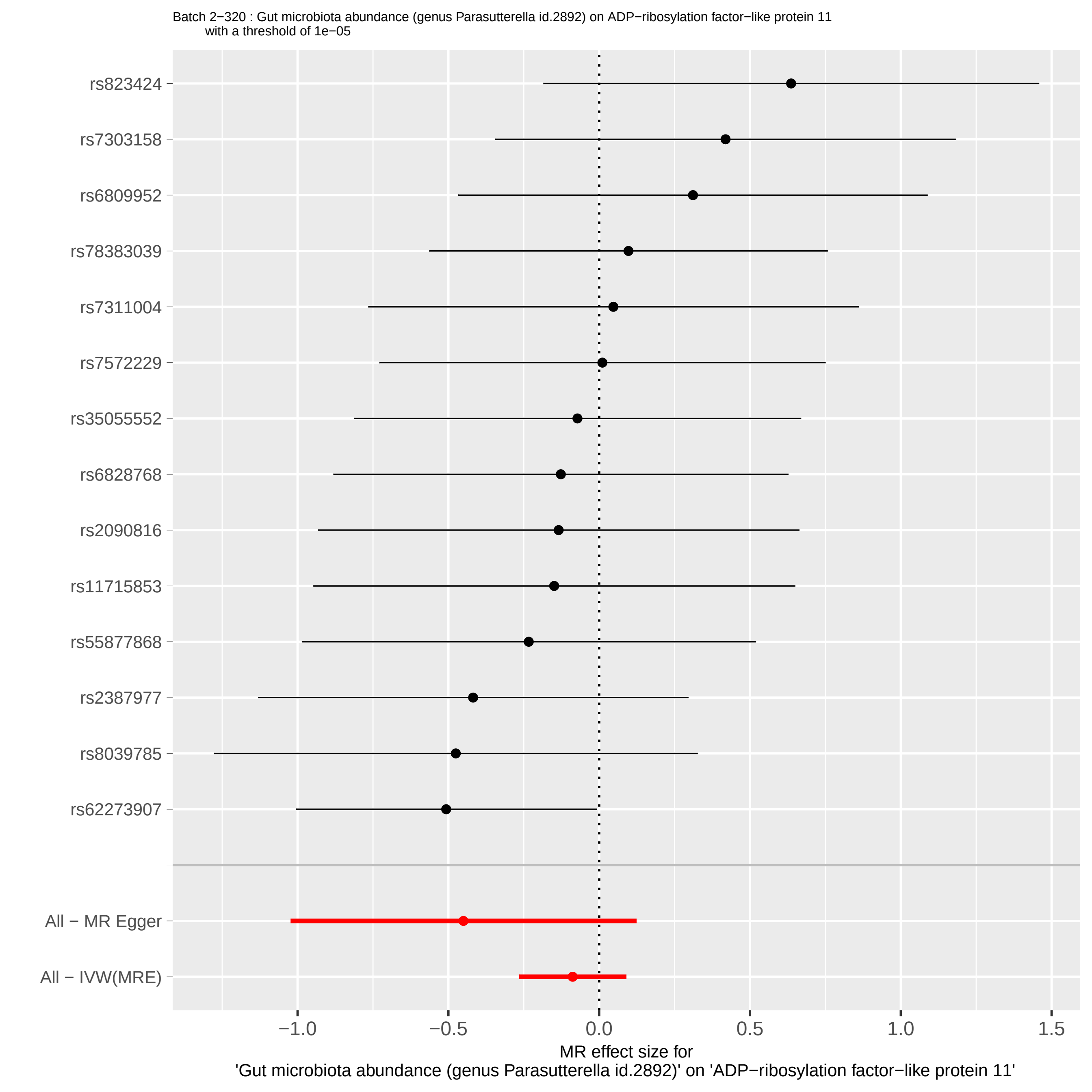

Batch 2−320 : Gut microbiota abundance (genus Parasutterella id.2892) on ADP−ribosylation factor−like protein 11
with a threshold of 1e−05
rs823424
rs7303158
rs6809952
rs78383039
rs7311004
rs7572229
rs35055552
rs6828768
rs2090816
rs11715853
rs55877868
rs2387977
rs8039785
rs62273907
All − MR Egger
All − IVW(MRE)
−1.0
−0.5
0.0
0.5
1.0
1.5
MR effect size for
'Gut microbiota abundance (genus Parasutterella id.2892)' on 'ADP−ribosylation factor−like protein 11'

Batch 2−321 : Gut microbiota abundance (genus Catenibacterium id.2153) on ADP−ribosylation factor−like protein 1
with a threshold of 1e−05

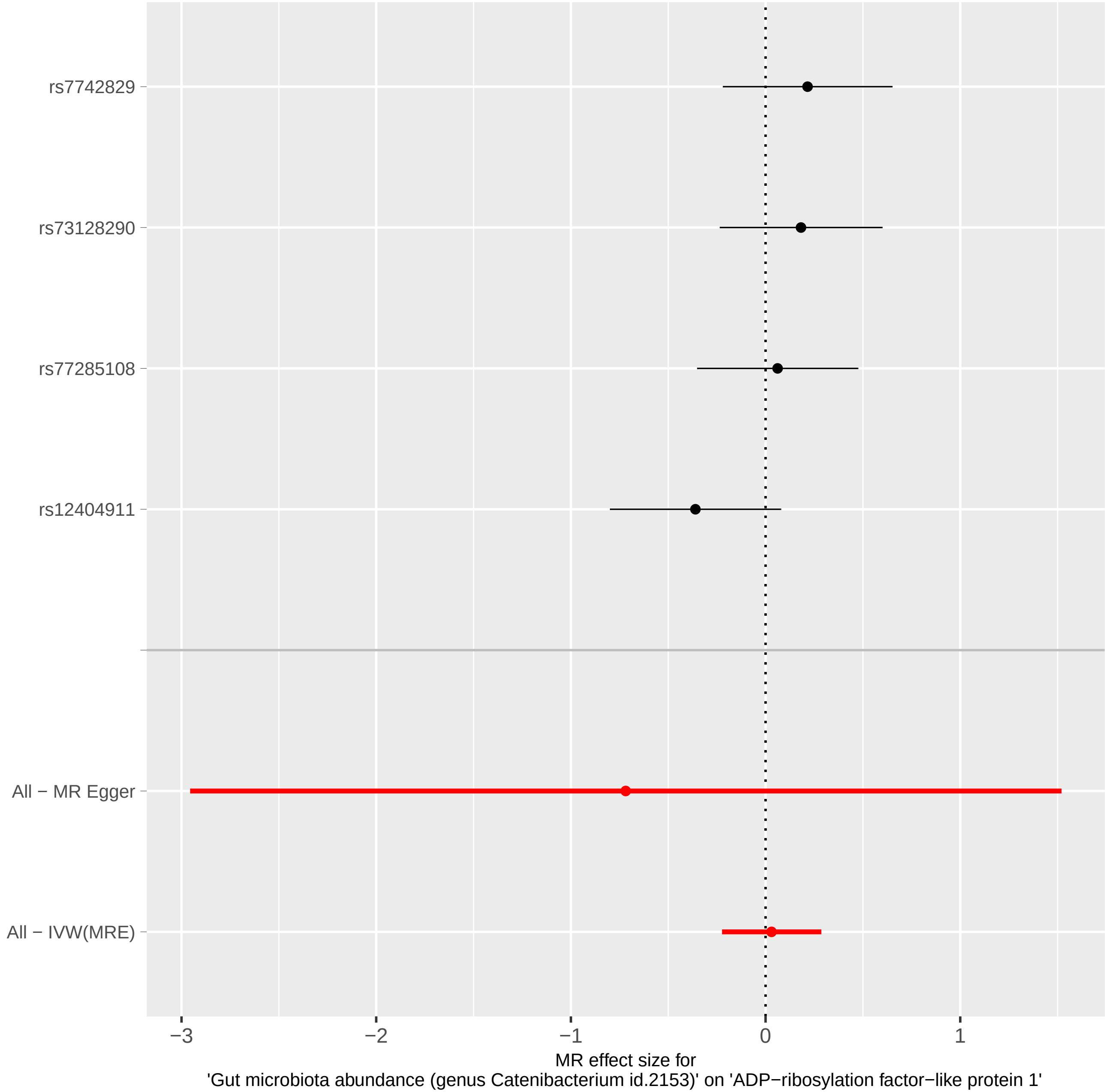

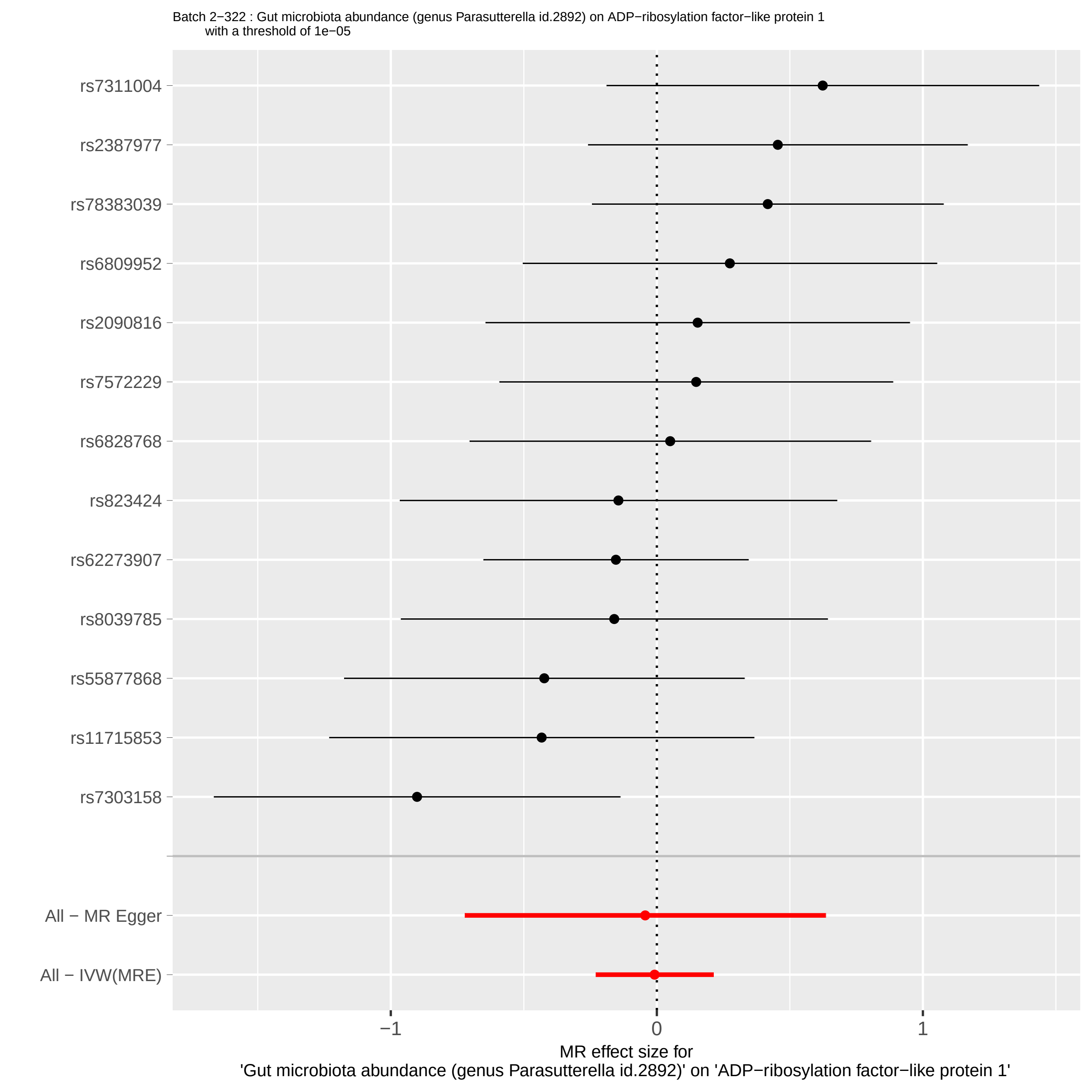

Batch 2−322 : Gut microbiota abundance (genus Parasutterella id.2892) on ADP−ribosylation factor−like protein 1 with a threshold of 1e−05
rs7311004
rs2387977
rs78383039
rs6809952
rs2090816
rs7572229
rs6828768
rs823424
rs62273907
rs8039785
rs55877868
rs11715853
rs7303158
All − MR Egger
All − IVW(MRE)
−1
0
1
MR effect size for
'Gut microbiota abundance (genus Parasutterella id.2892)' on 'ADP−ribosylation factor−like protein 1'

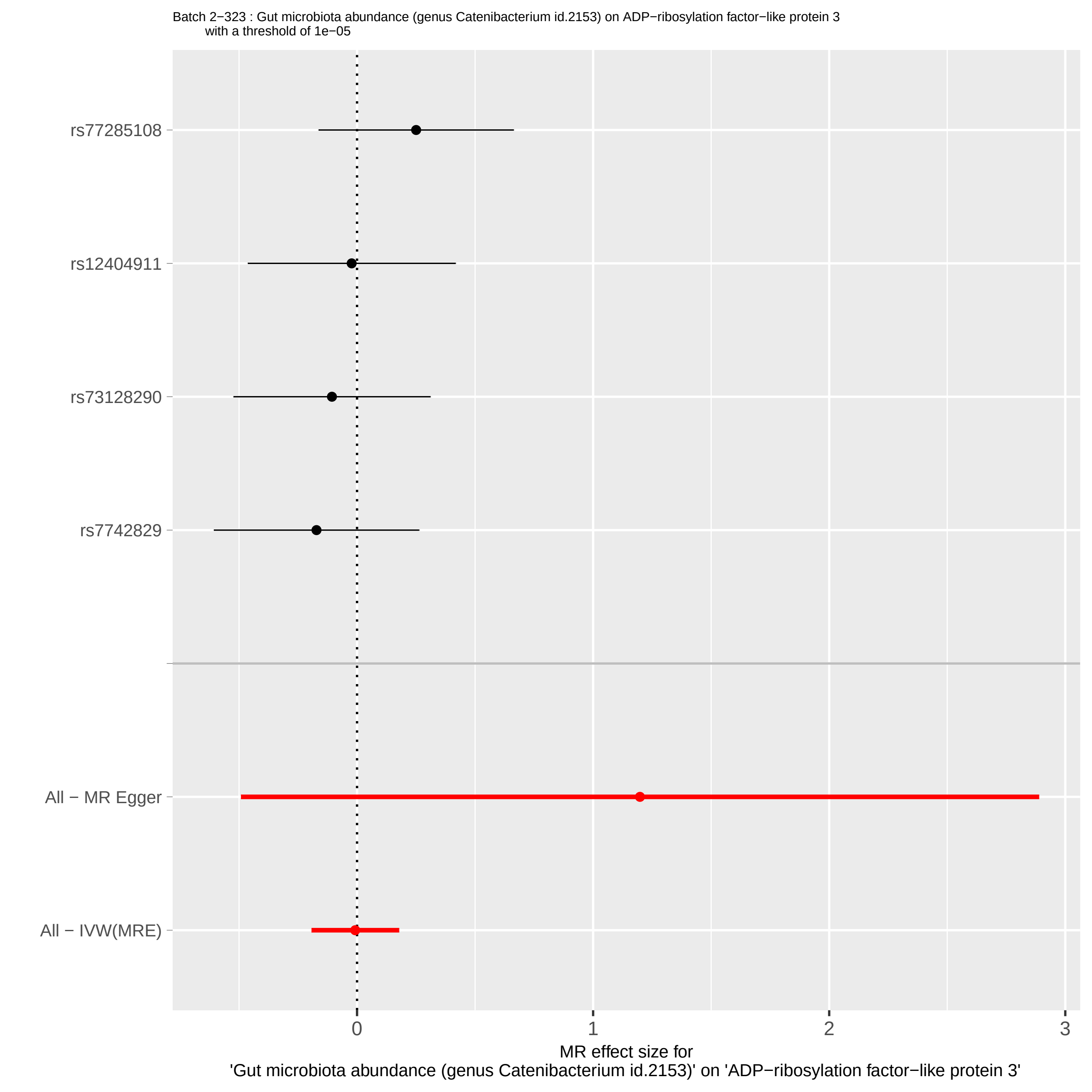

Batch 2−323 : Gut microbiota abundance (genus Catenibacterium id.2153) on ADP−ribosylation factor−like protein 3
with a threshold of 1e−05
rs77285108
rs12404911
rs73128290
rs7742829
All − MR Egger
All − IVW(MRE)
0
1
2
3
MR effect size for
'Gut microbiota abundance (genus Catenibacterium id.2153)' on 'ADP−ribosylation factor−like protein 3'

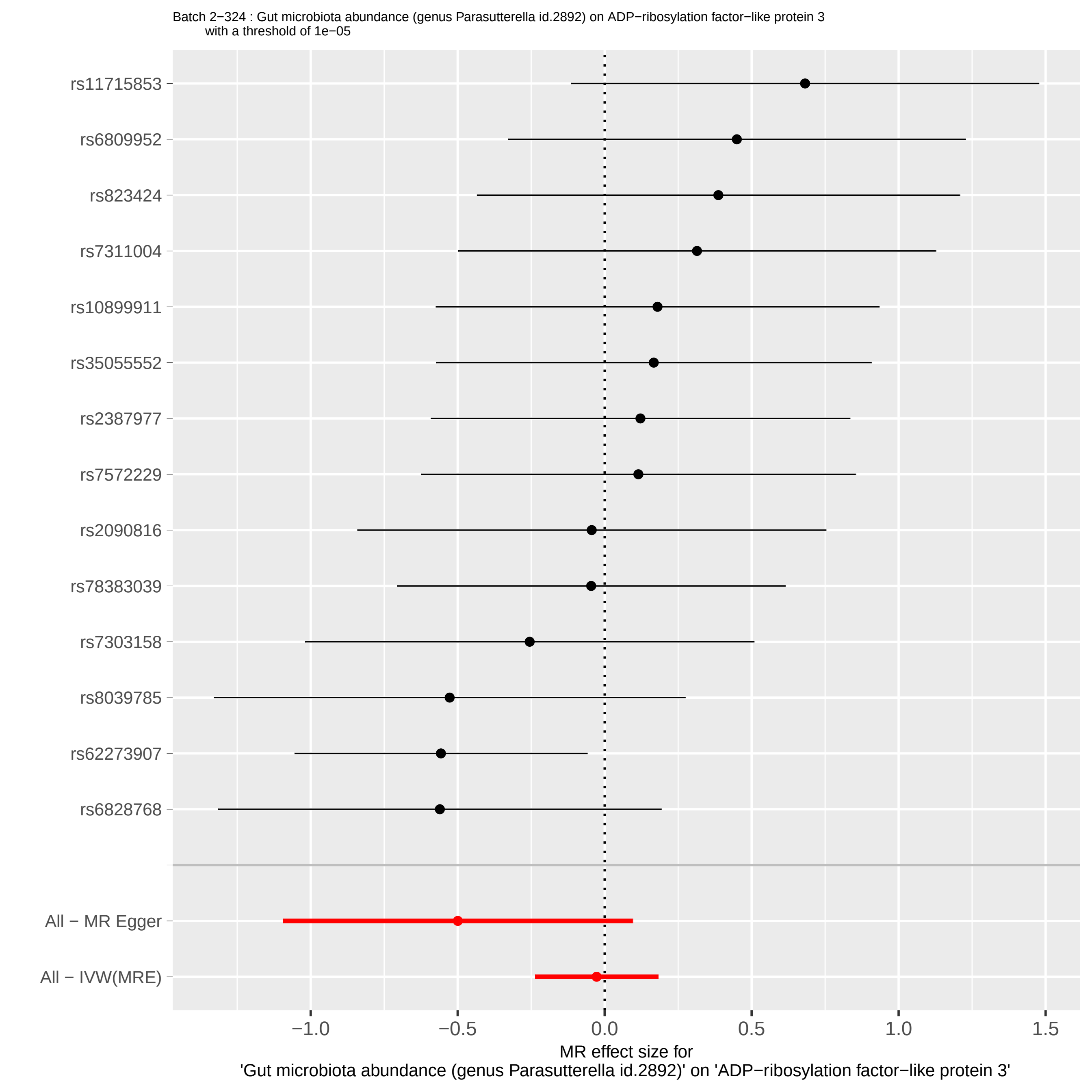

Batch 2−324 : Gut microbiota abundance (genus Parasutterella id.2892) on ADP−ribosylation factor−like protein 3
with a threshold of 1e−05
rs11715853
rs6809952
rs823424
rs7311004
rs10899911
rs35055552
rs2387977
rs7572229
rs2090816
rs78383039
rs7303158
rs8039785
rs62273907
rs6828768
All − MR Egger
All − IVW(MRE)
−1.0
−0.5
0.0
0.5
1.0
1.5
MR effect size for
'Gut microbiota abundance (genus Parasutterella id.2892)' on 'ADP−ribosylation factor−like protein 3'

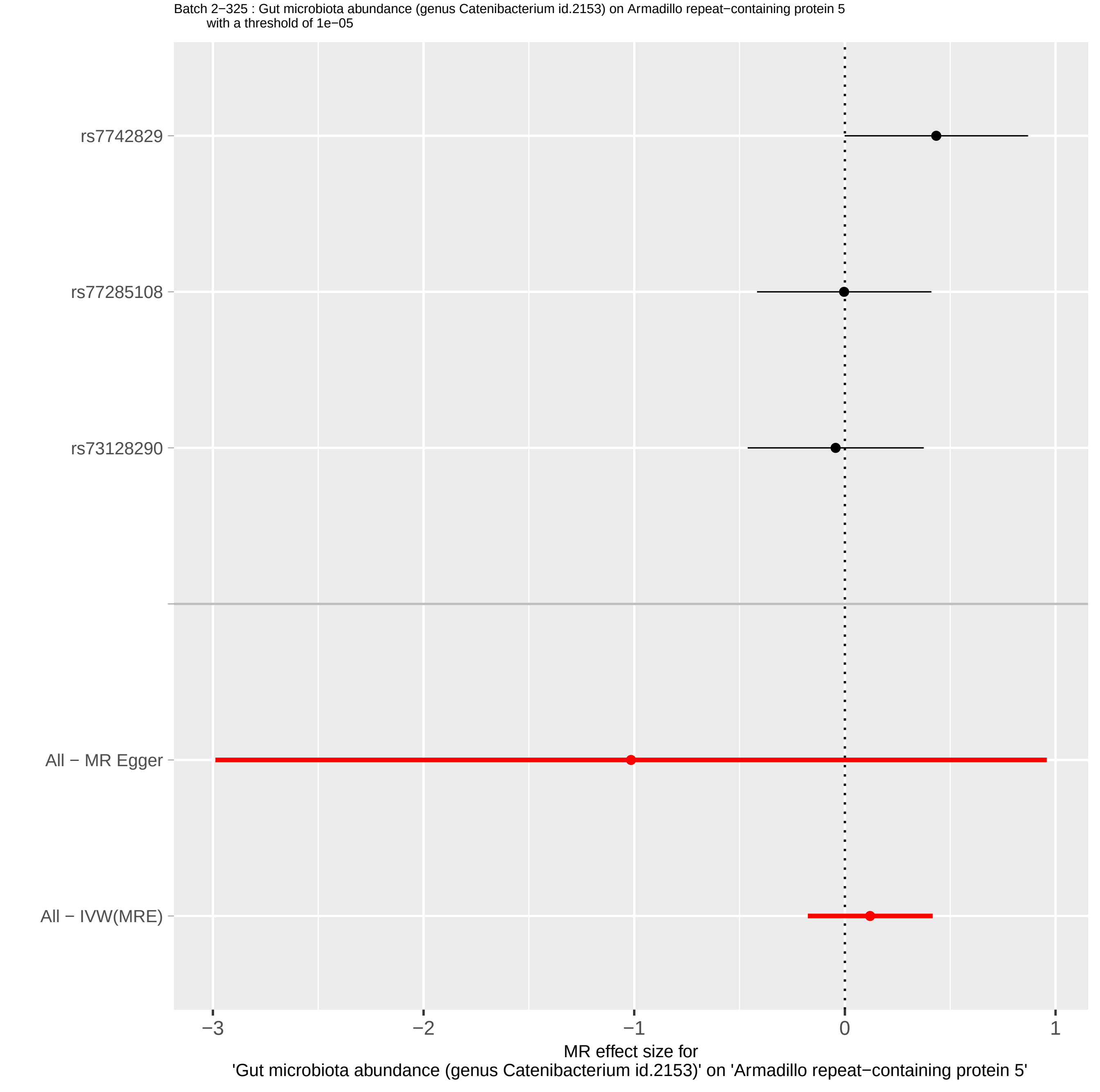
Batch 2−325 : Gut microbiota abundance (genus Catenibacterium id.2153) on Armadillo repeat−containing protein 5
with a threshold of 1e−05
rs7742829
rs77285108
rs73128290
All − MR Egger
All − IVW(MRE)
−3
−2
−1
0
1
MR effect size for
'Gut microbiota abundance (genus Catenibacterium id.2153)' on 'Armadillo repeat−containing protein 5'

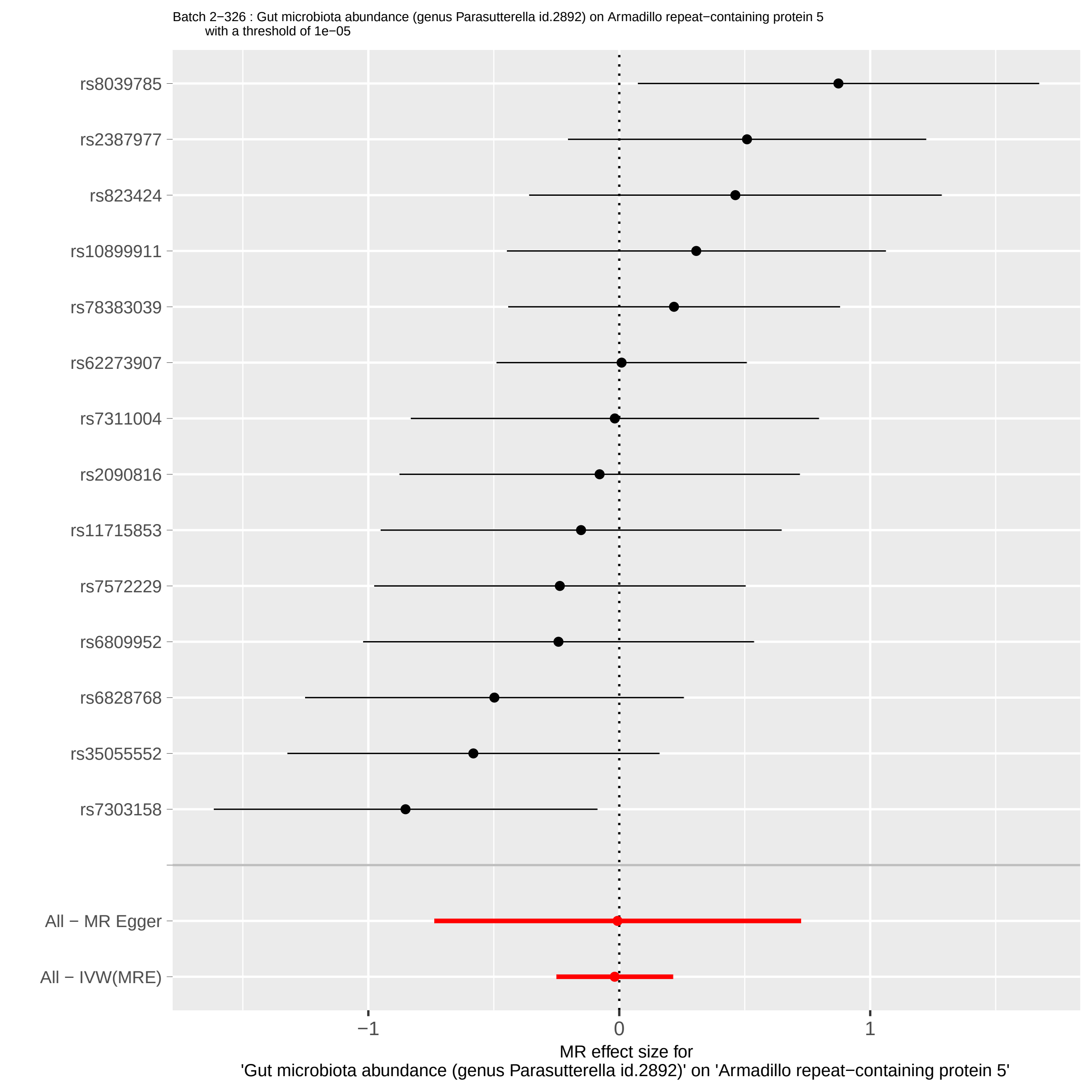

Batch 2−326 : Gut microbiota abundance (genus Parasutterella id.2892) on Armadillo repeat−containing protein 5
with a threshold of 1e−05
rs8039785
rs2387977
rs823424
rs10899911
rs78383039
rs62273907
rs7311004
rs2090816
rs11715853
rs7572229
rs6809952
rs6828768
rs35055552
rs7303158
All − MR Egger
All − IVW(MRE)
−1
0
1
MR effect size for
'Gut microbiota abundance (genus Parasutterella id.2892)' on 'Armadillo repeat−containing protein 5'

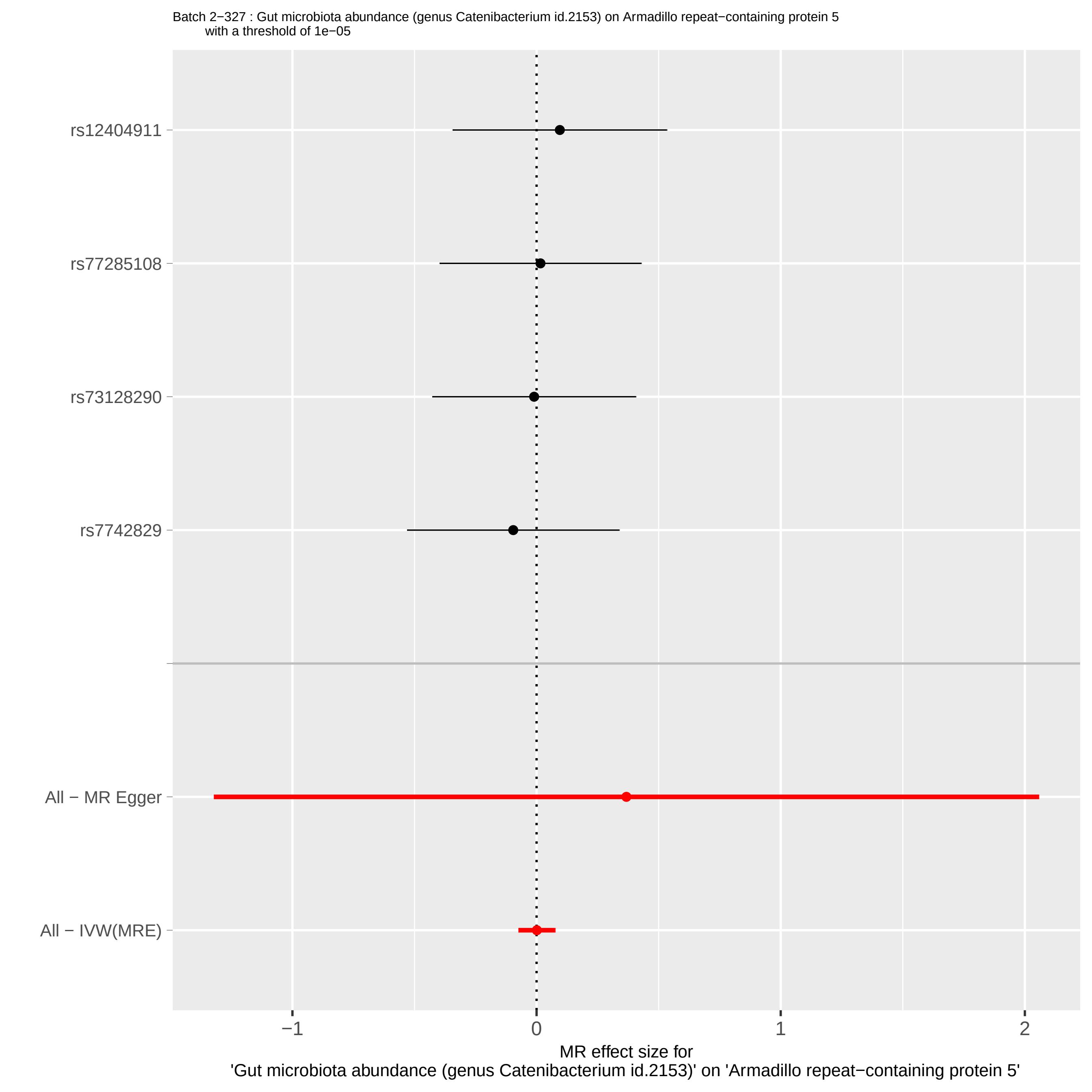

Batch 2−327 : Gut microbiota abundance (genus Catenibacterium id.2153) on Armadillo repeat−containing protein 5
with a threshold of 1e−05
rs12404911
rs77285108
rs73128290
rs7742829
All − MR Egger
All − IVW(MRE)
−1
0
1
2
MR effect size for
'Gut microbiota abundance (genus Catenibacterium id.2153)' on 'Armadillo repeat−containing protein 5'

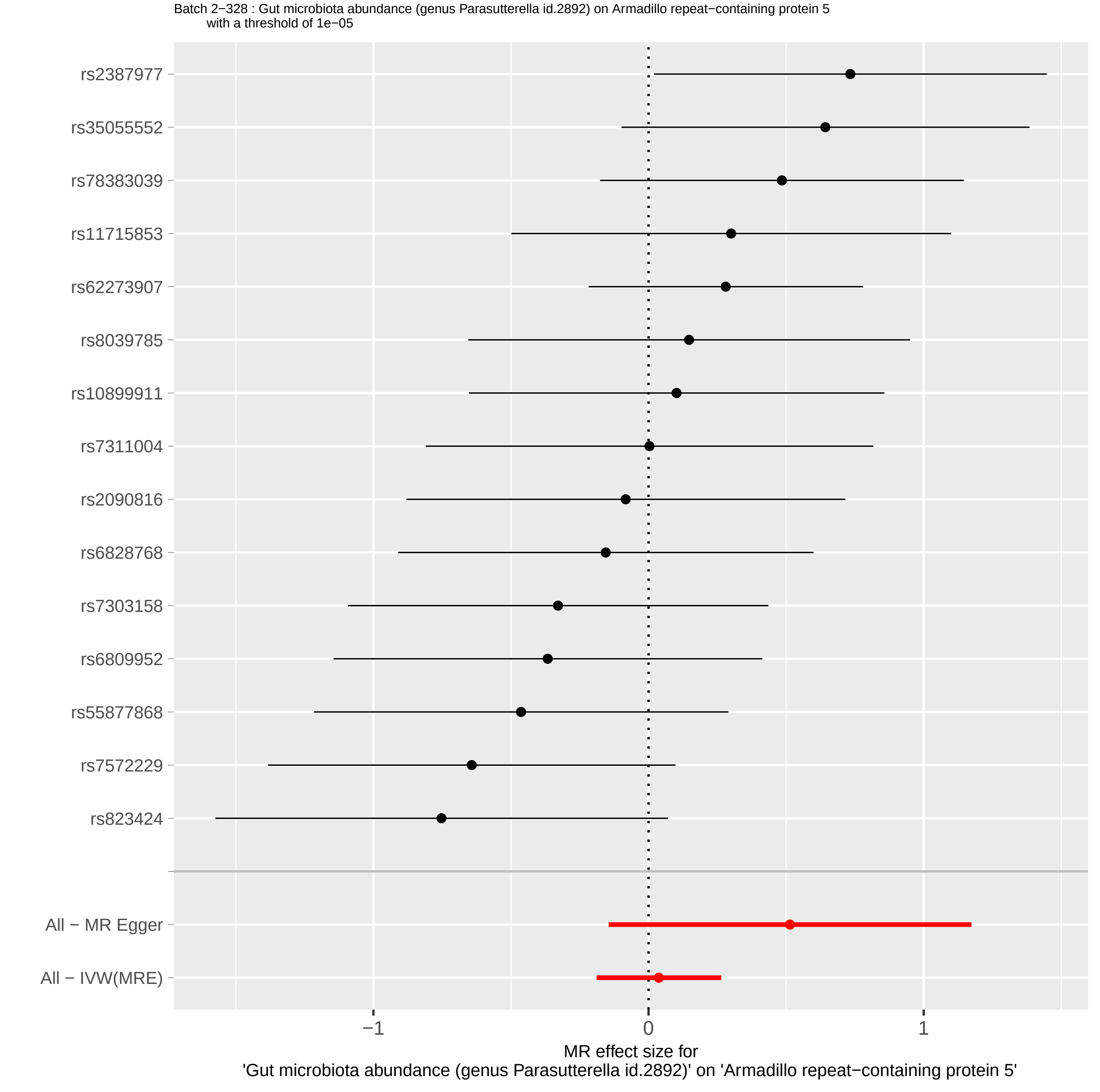

Batch 2−328 : Gut microbiota abundance (genus Parasutterella id.2892) on Armadillo repeat−containing protein 5
with a threshold of 1e−05
rs2387977
rs35055552
rs78383039
rs11715853
rs62273907
rs8039785
rs10899911
rs7311004
rs2090816
rs6828768
rs7303158
rs6809952
rs55877868
rs7572229
rs823424
All − MR Egger
All − IVW(MRE)
−1
0
1
MR effect size for
'Gut microbiota abundance (genus Parasutterella id.2892)' on 'Armadillo repeat−containing protein 5'

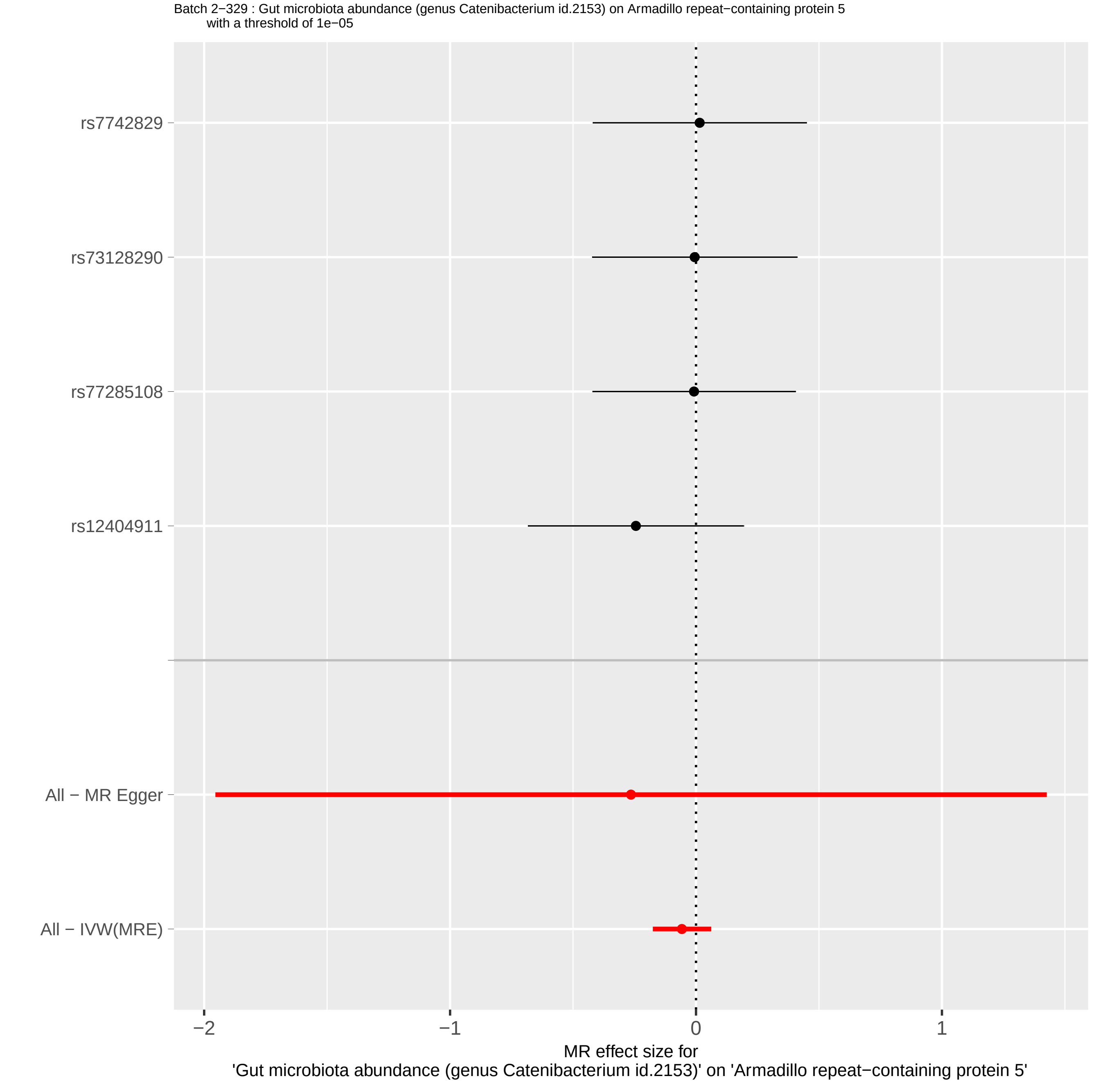

Batch 2−329 : Gut microbiota abundance (genus Catenibacterium id.2153) on Armadillo repeat−containing protein 5
with a threshold of 1e−05
rs7742829
rs73128290
rs77285108
rs12404911
All − MR Egger
All − IVW(MRE)
−2
−1
0
1
MR effect size for
'Gut microbiota abundance (genus Catenibacterium id.2153)' on 'Armadillo repeat−containing protein 5'

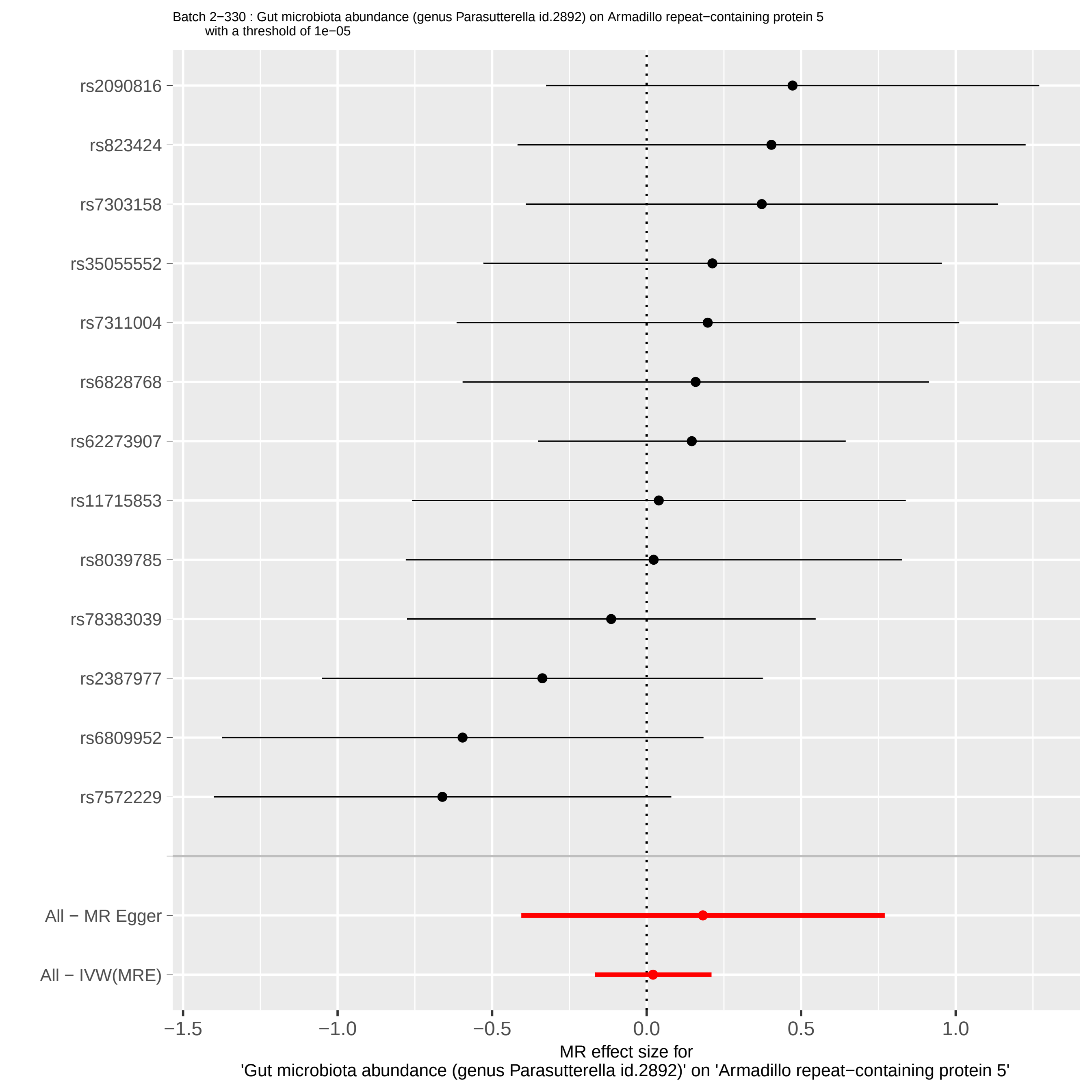

Batch 2−330 : Gut microbiota abundance (genus Parasutterella id.2892) on Armadillo repeat−containing protein 5
with a threshold of 1e−05
rs2090816
rs823424
rs7303158
rs35055552
rs7311004
rs6828768
rs62273907
rs11715853
rs8039785
rs78383039
rs2387977
rs6809952
rs7572229
All − MR Egger
All − IVW(MRE)
−1.5
−1.0
−0.5
0.0
0.5
1.0
MR effect size for
'Gut microbiota abundance (genus Parasutterella id.2892)' on 'Armadillo repeat−containing protein 5'

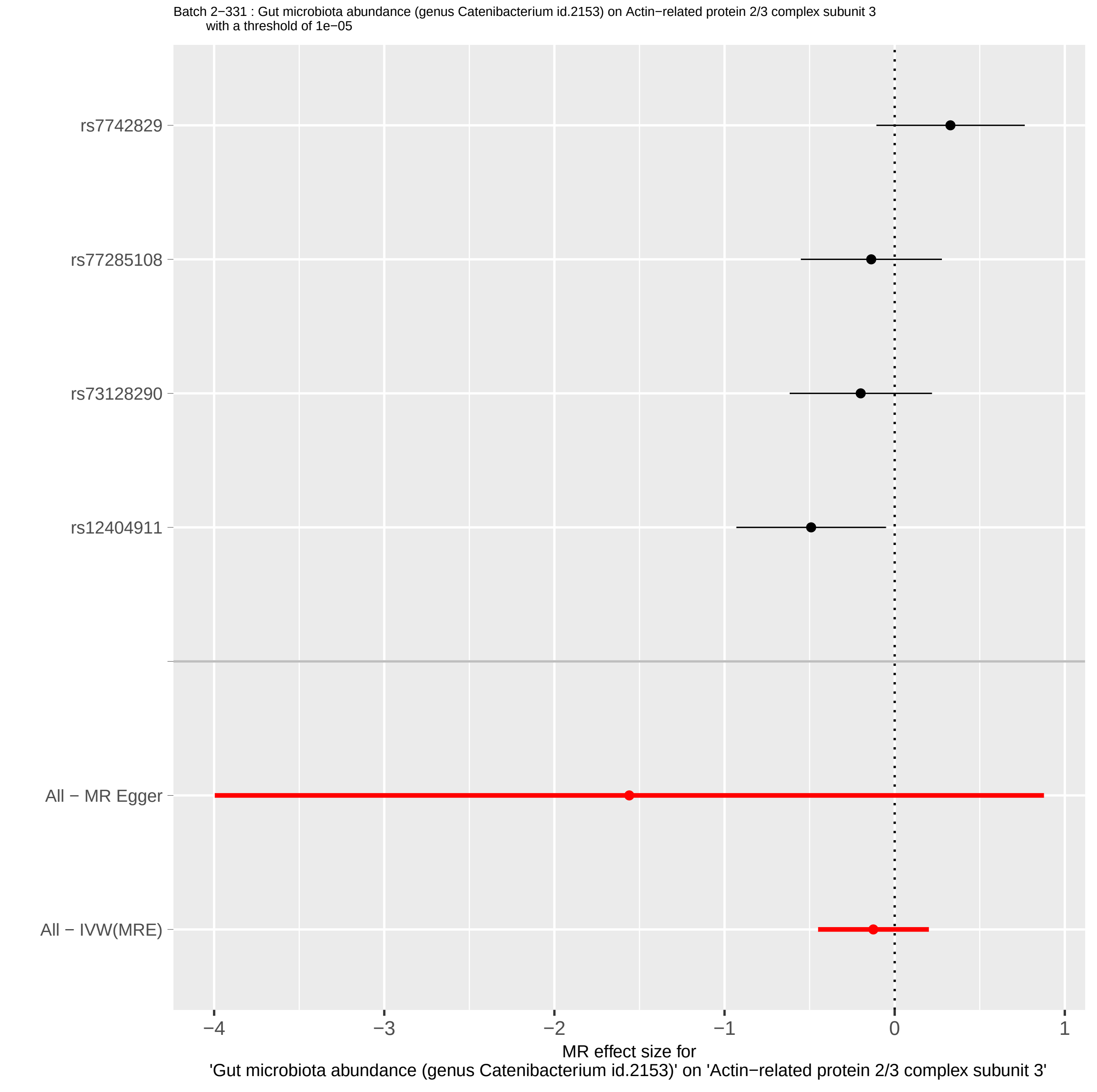

Batch 2−331 : Gut microbiota abundance (genus Catenibacterium id.2153) on Actin−related protein 2/3 complex subunit 3
with a threshold of 1e−05
rs7742829
rs77285108
rs73128290
rs12404911
All − MR Egger
All − IVW(MRE)
−4
−3
−2
−1
0
1
MR effect size for
'Gut microbiota abundance (genus Catenibacterium id.2153)' on 'Actin−related protein 2/3 complex subunit 3'

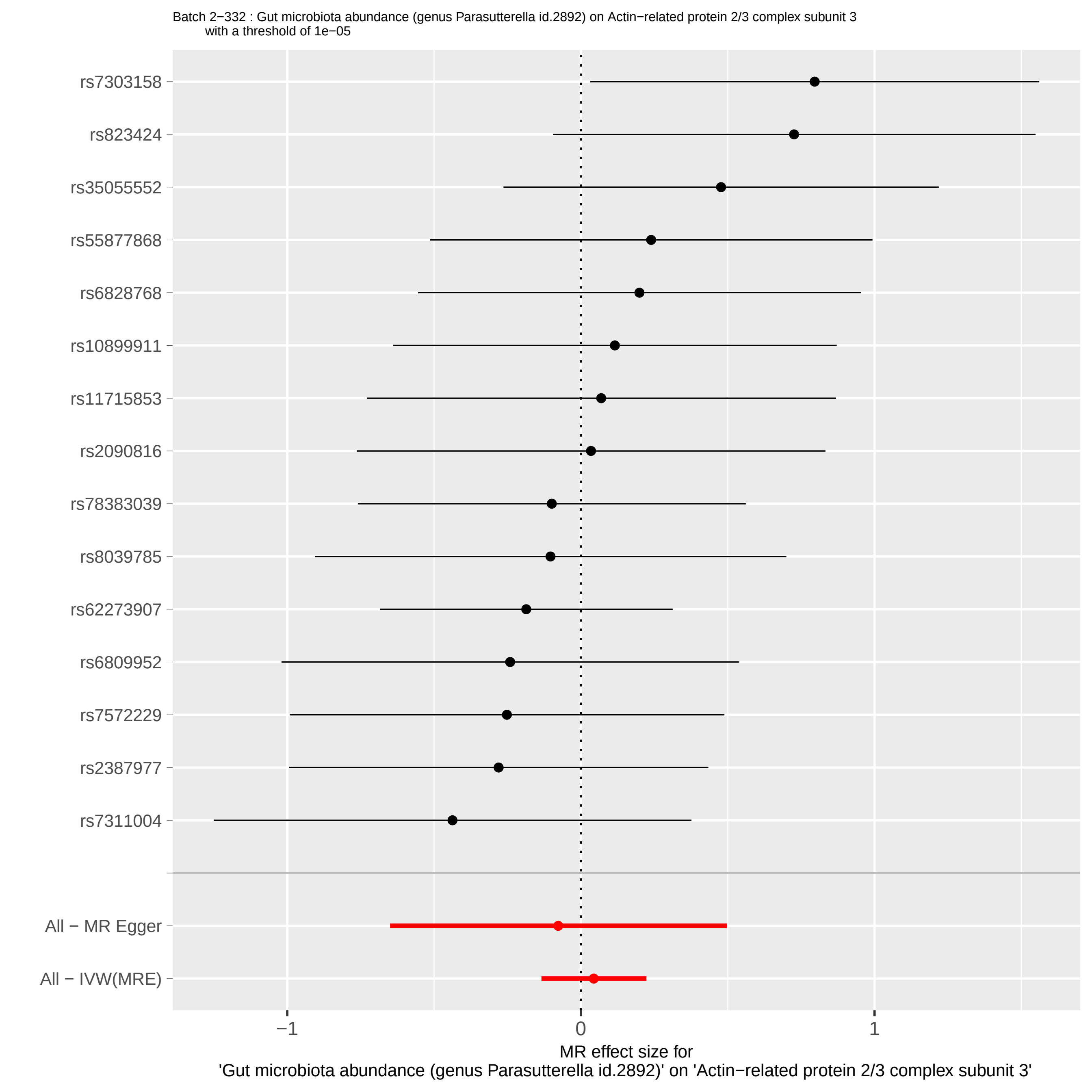

Batch 2−332 : Gut microbiota abundance (genus Parasutterella id.2892) on Actin−related protein 2/3 complex subunit 3
with a threshold of 1e−05
rs7303158
rs823424
rs35055552
rs55877868
rs6828768
rs10899911
rs11715853
rs2090816
rs78383039
rs8039785
rs62273907
rs6809952
rs7572229
rs2387977
rs7311004
All − MR Egger
All − IVW(MRE)
−1
0
1
MR effect size for
'Gut microbiota abundance (genus Parasutterella id.2892)' on 'Actin−related protein 2/3 complex subunit 3'

Batch 2−333 : Gut microbiota abundance (genus Catenibacterium id.2153) on cAMP−regulated phosphoprotein 19
with a threshold of 1e−05

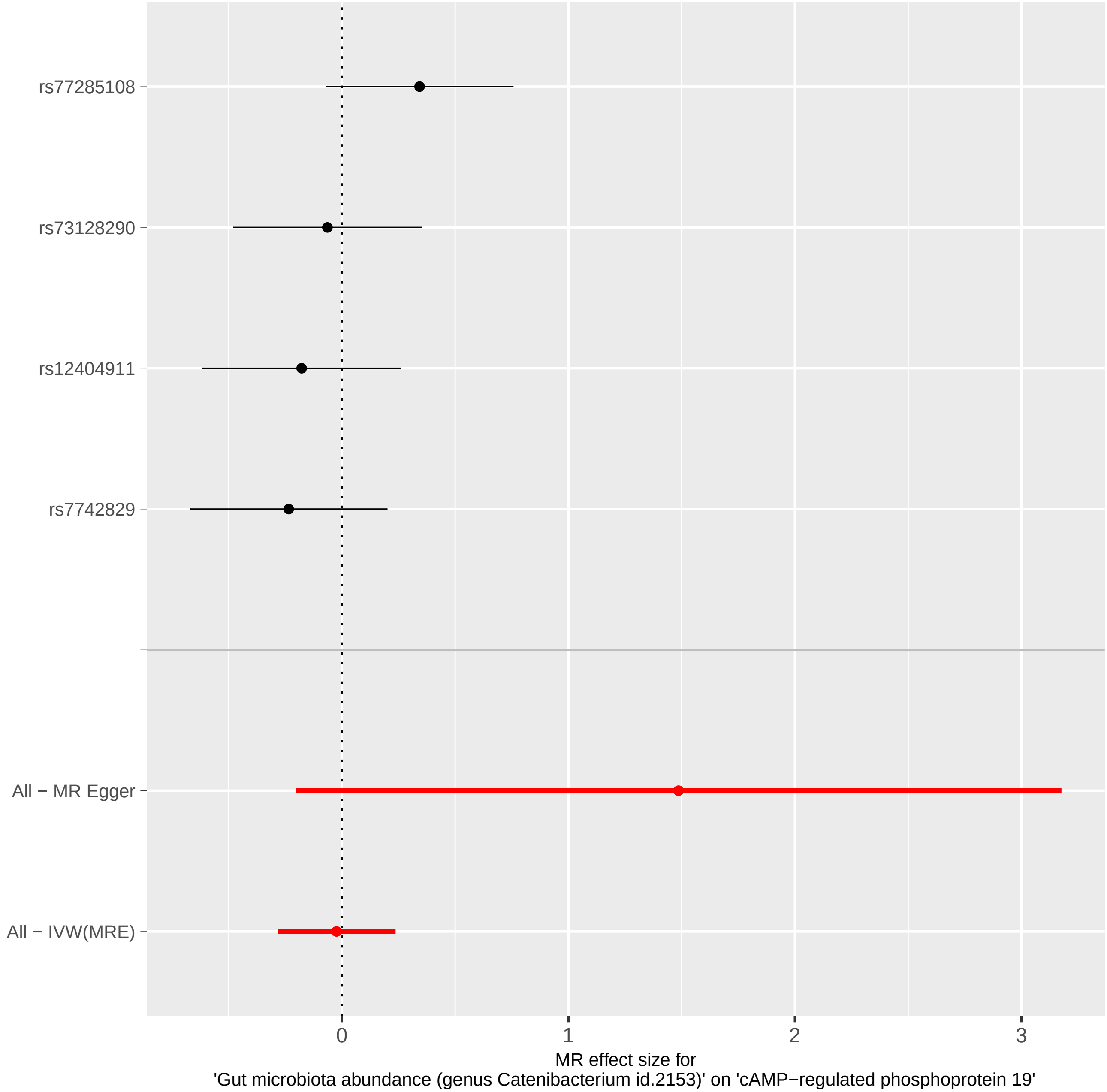

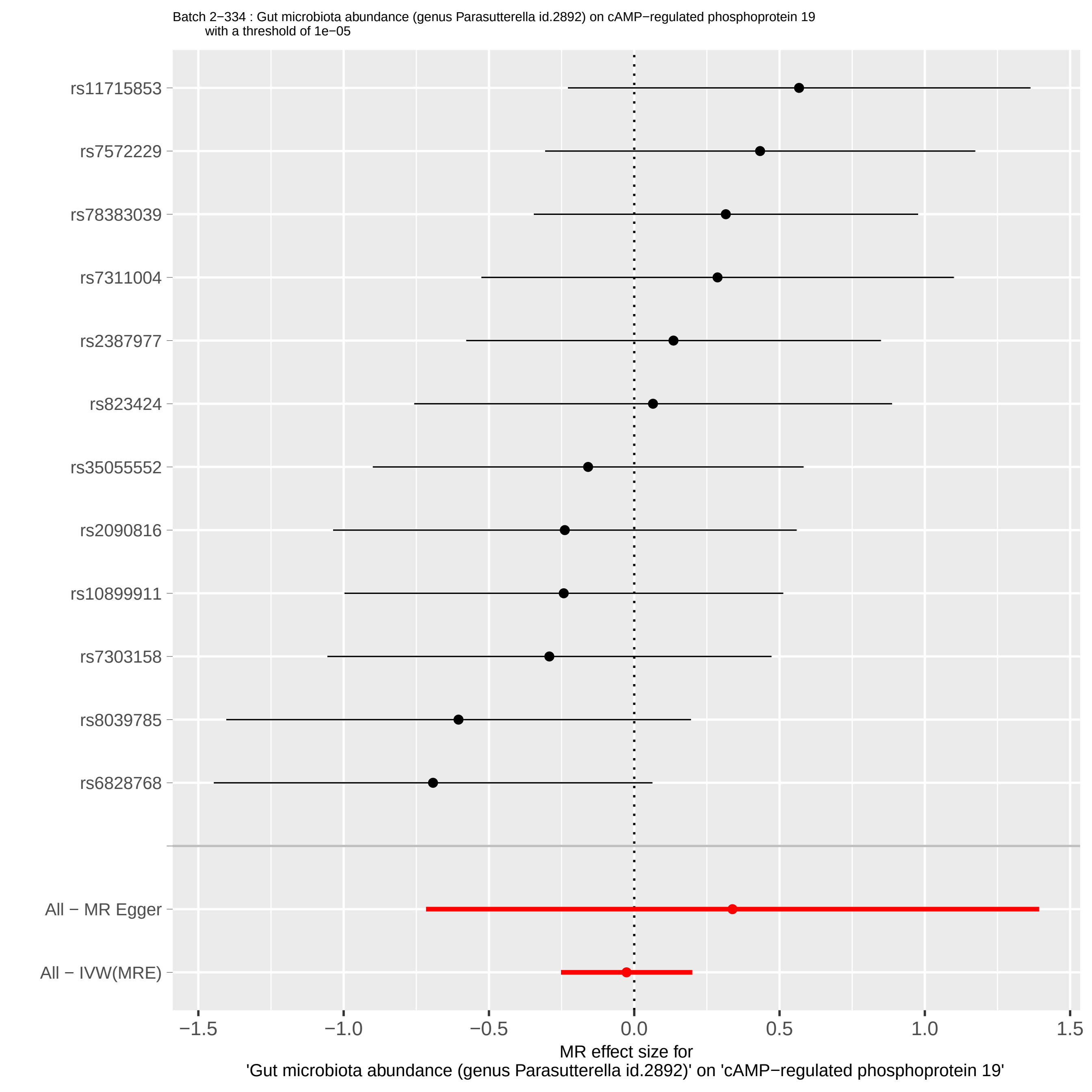

Batch 2−334 : Gut microbiota abundance (genus Parasutterella id.2892) on cAMP−regulated phosphoprotein 19
with a threshold of 1e−05
rs11715853
rs7572229
rs78383039
rs7311004
rs2387977
rs823424
rs35055552
rs2090816
rs10899911
rs7303158
rs8039785
rs6828768
All − MR Egger
All − IVW(MRE)
−1.5
−1.0
−0.5
0.0
0.5
1.0
1.5
MR effect size for
'Gut microbiota abundance (genus Parasutterella id.2892)' on 'cAMP−regulated phosphoprotein 19'

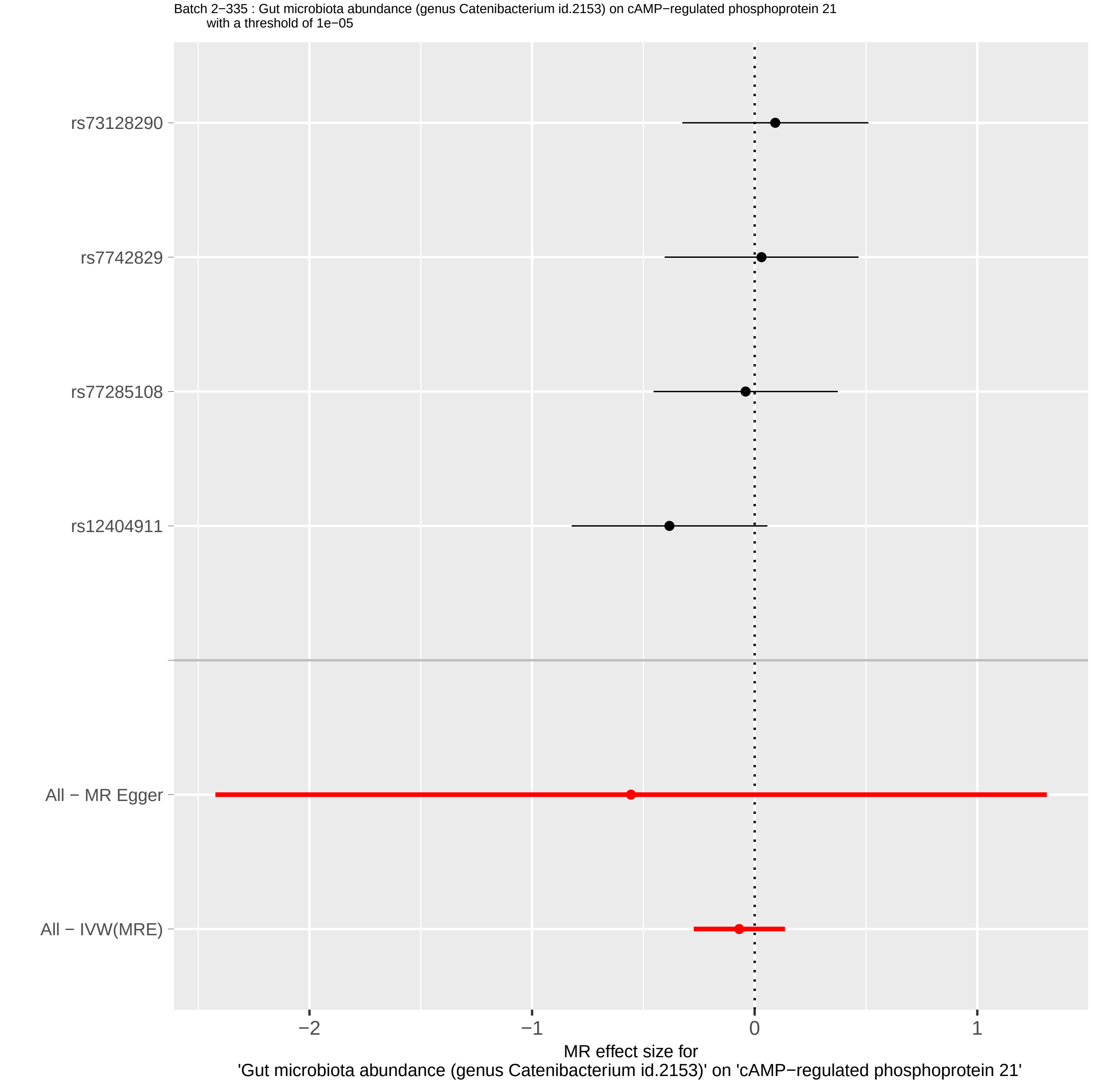
Batch 2−335 : Gut microbiota abundance (genus Catenibacterium id.2153) on cAMP−regulated phosphoprotein 21
with a threshold of 1e−05
rs73128290
rs7742829
rs77285108
rs12404911
All − MR Egger
All − IVW(MRE)
−2
−1
0
1
MR effect size for
'Gut microbiota abundance (genus Catenibacterium id.2153)' on 'cAMP−regulated phosphoprotein 21'

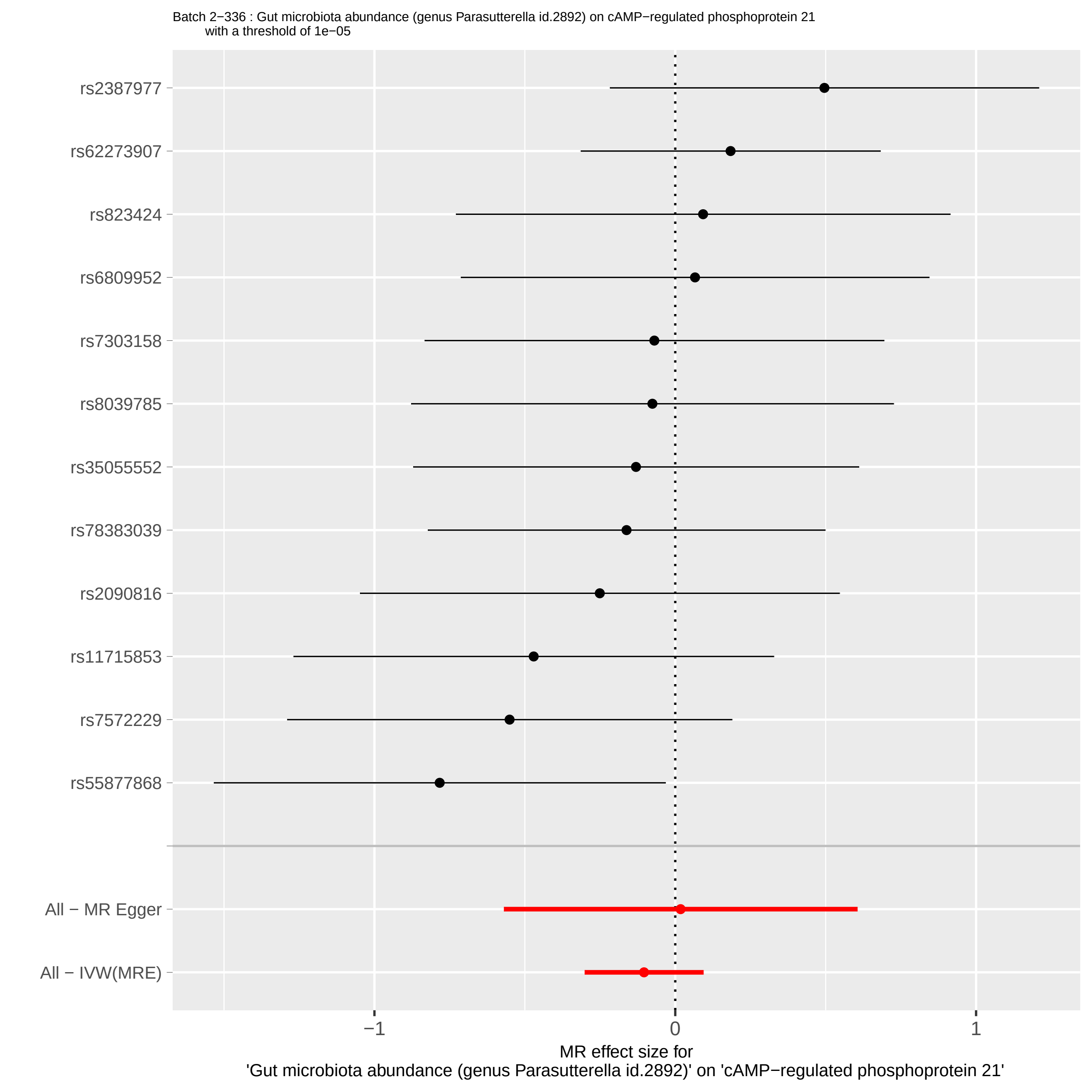

Batch 2−336 : Gut microbiota abundance (genus Parasutterella id.2892) on cAMP−regulated phosphoprotein 21
with a threshold of 1e−05
rs2387977
rs62273907
rs823424
rs6809952
rs7303158
rs8039785
rs35055552
rs78383039
rs2090816
rs11715853
rs7572229
rs55877868
All − MR Egger
All − IVW(MRE)
−1
0
1
MR effect size for
'Gut microbiota abundance (genus Parasutterella id.2892)' on 'cAMP−regulated phosphoprotein 21'

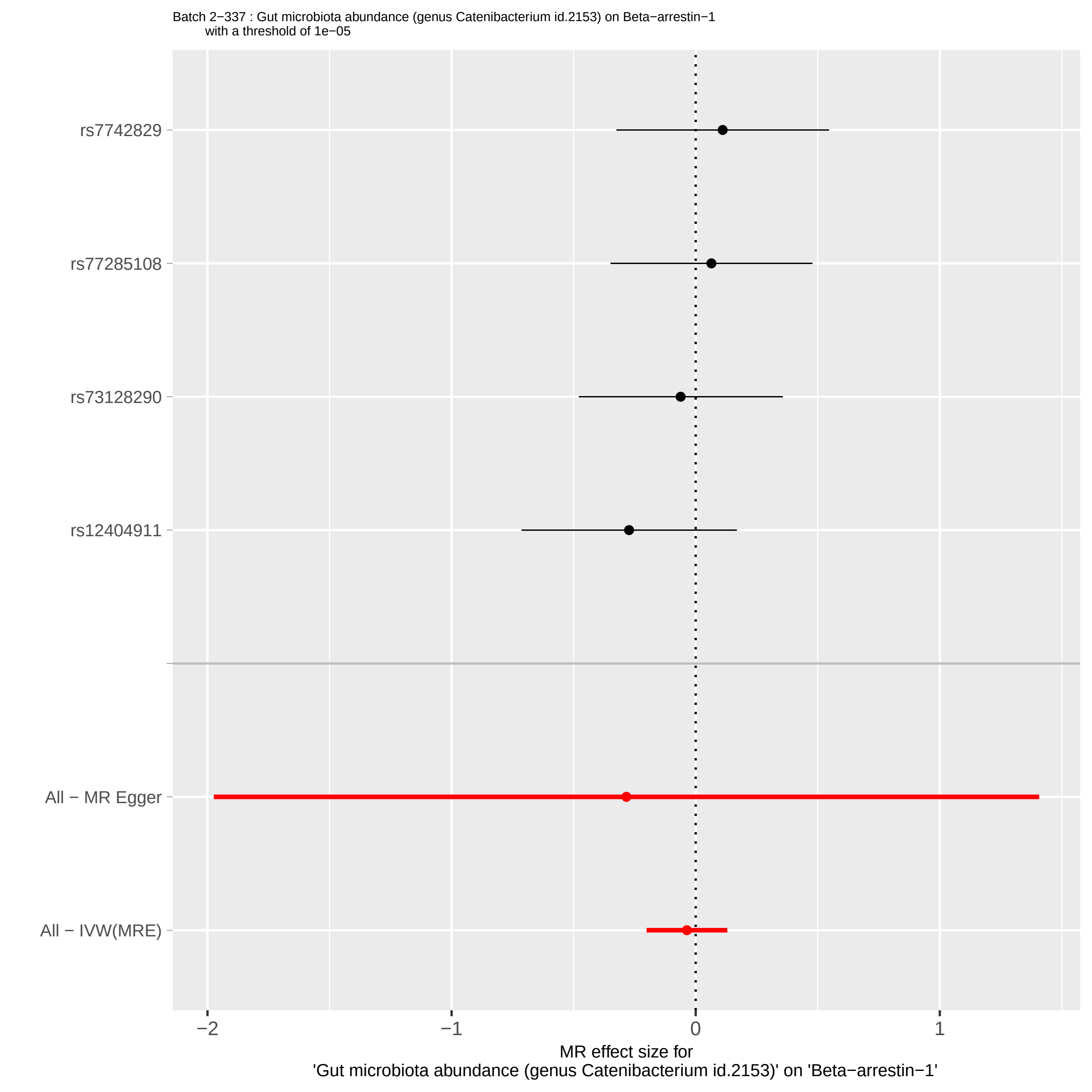

Batch 2−337 : Gut microbiota abundance (genus Catenibacterium id.2153) on Beta−arrestin−1
with a threshold of 1e−05
rs7742829
rs77285108
rs73128290
rs12404911
All − MR Egger
All − IVW(MRE)
−2
−1
0
1
MR effect size for
'Gut microbiota abundance (genus Catenibacterium id.2153)' on 'Beta−arrestin−1'

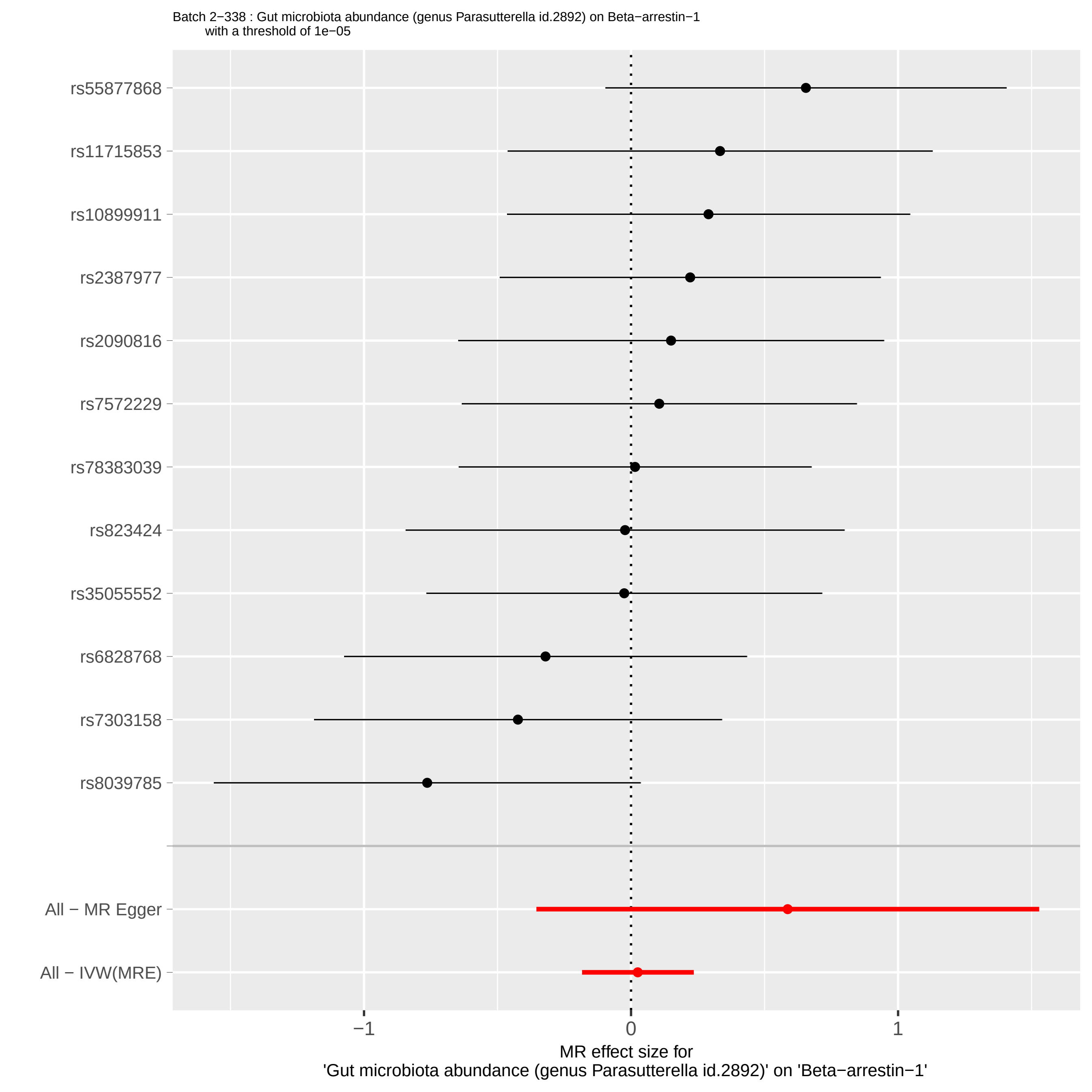

Batch 2−338 : Gut microbiota abundance (genus Parasutterella id.2892) on Beta−arrestin−1
with a threshold of 1e−05
rs55877868
rs11715853
rs10899911
rs2387977
rs2090816
rs7572229
rs78383039
rs823424
rs35055552
rs6828768
rs7303158
rs8039785
All − MR Egger
All − IVW(MRE)
−1
0
1
MR effect size for
'Gut microbiota abundance (genus Parasutterella id.2892)' on 'Beta−arrestin−1'

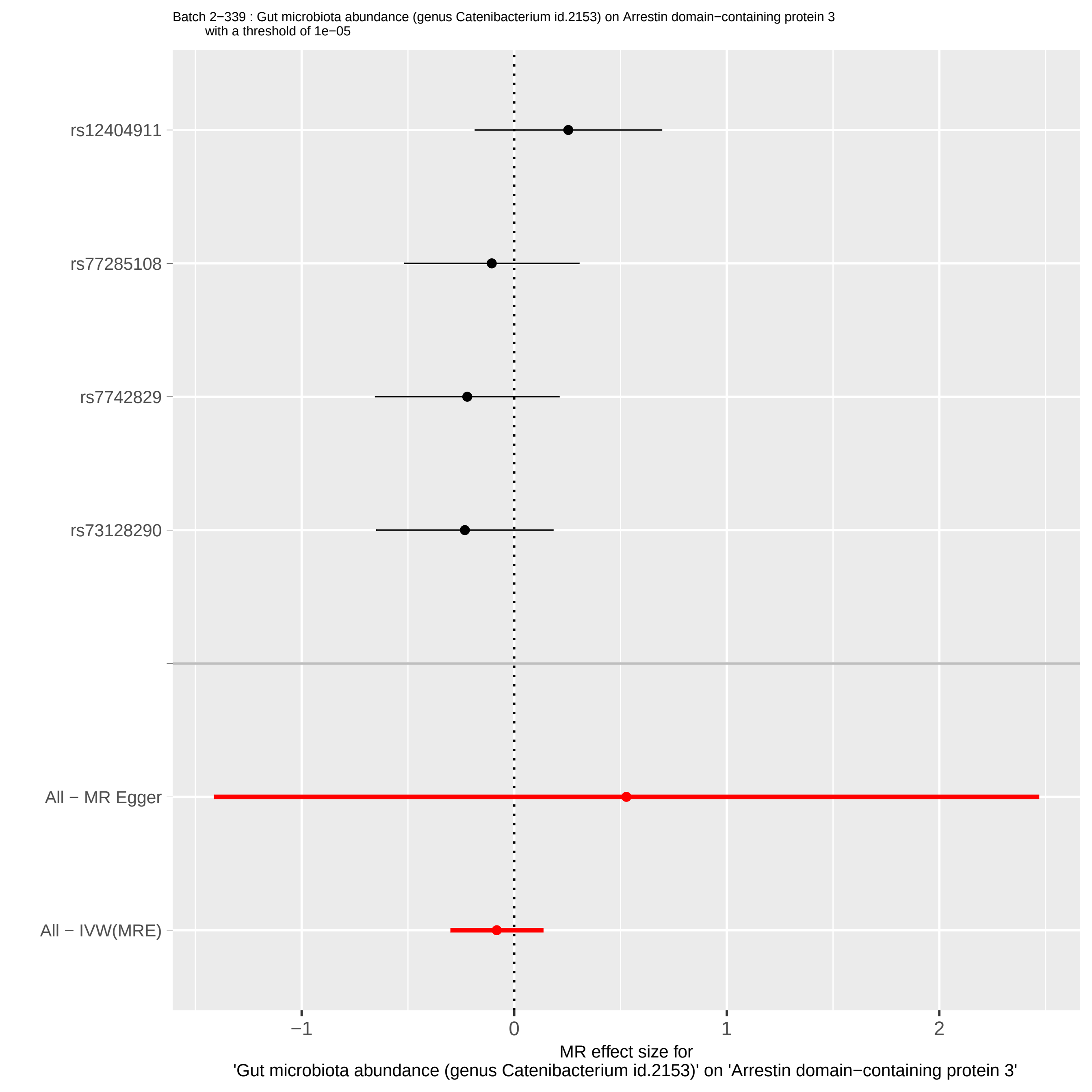

Batch 2−339 : Gut microbiota abundance (genus Catenibacterium id.2153) on Arrestin domain−containing protein 3
with a threshold of 1e−05
rs12404911
rs77285108
rs7742829
rs73128290
All − MR Egger
All − IVW(MRE)
−1
0
1
2
MR effect size for
'Gut microbiota abundance (genus Catenibacterium id.2153)' on 'Arrestin domain−containing protein 3'

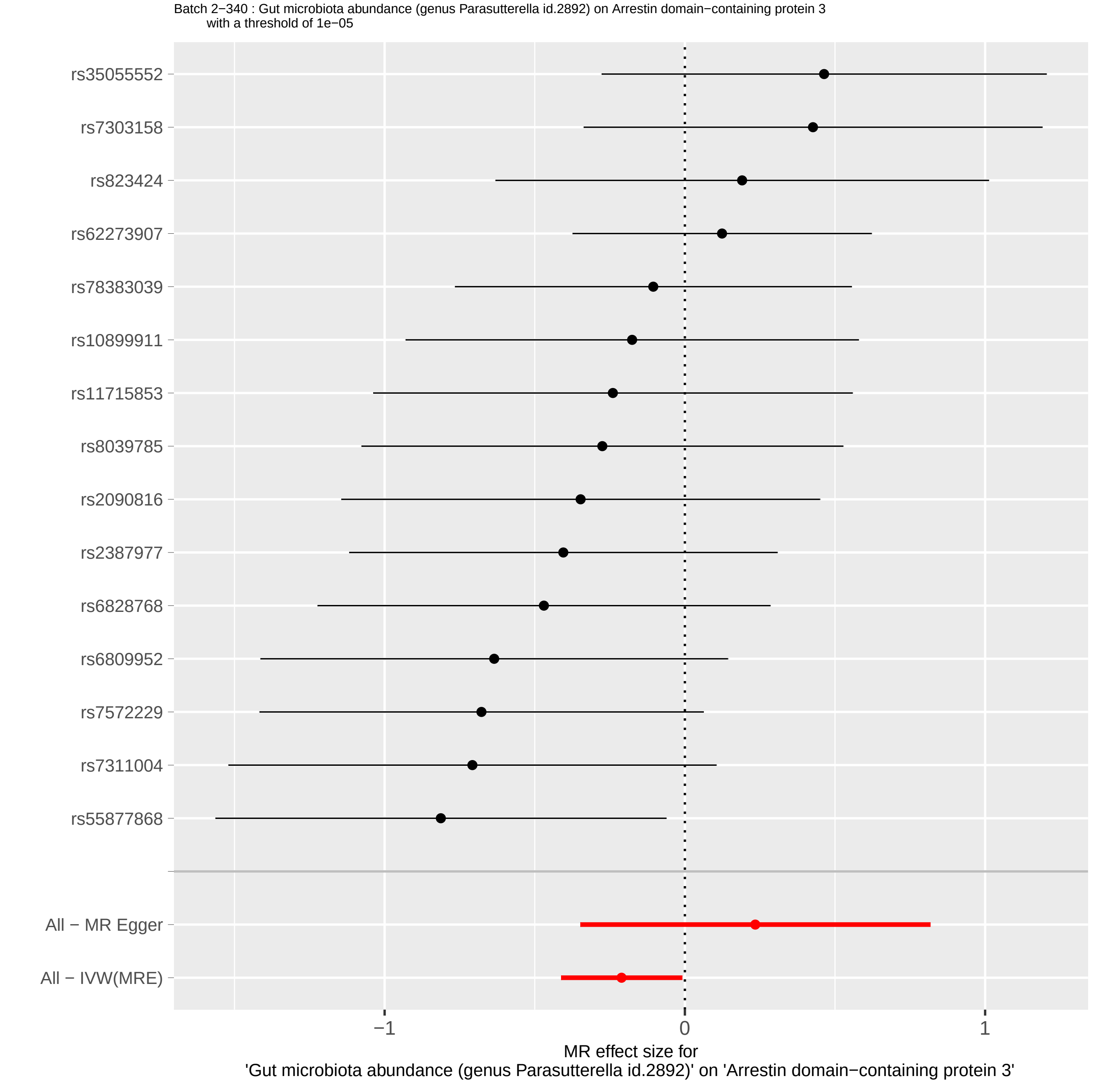

Batch 2−340 : Gut microbiota abundance (genus Parasutterella id.2892) on Arrestin domain−containing protein 3 with a threshold of 1e−05
rs35055552
rs7303158
rs823424
rs62273907
rs78383039
rs10899911
rs11715853
rs8039785
rs2090816
rs2387977
rs6828768
rs6809952
rs7572229
rs7311004
rs55877868
All − MR Egger
All − IVW(MRE)
−1
0
1
MR effect size for
'Gut microbiota abundance (genus Parasutterella id.2892)' on 'Arrestin domain−containing protein 3'

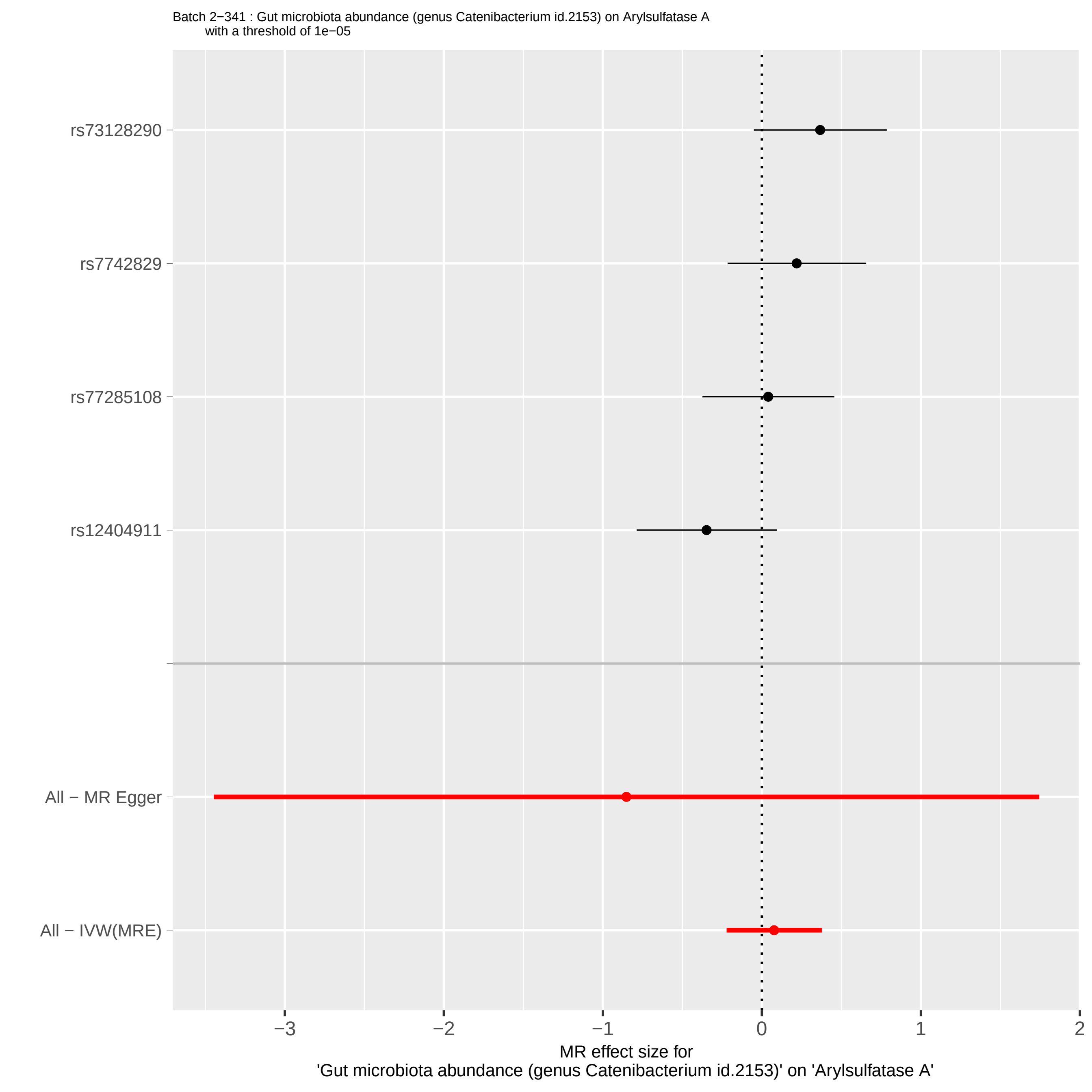

Batch 2−341 : Gut microbiota abundance (genus Catenibacterium id.2153) on Arylsulfatase A
with a threshold of 1e−05
rs73128290
rs7742829
rs77285108
rs12404911
All − MR Egger
All − IVW(MRE)
−3
−2
−1
0
1
2
MR effect size for
'Gut microbiota abundance (genus Catenibacterium id.2153)' on 'Arylsulfatase A'

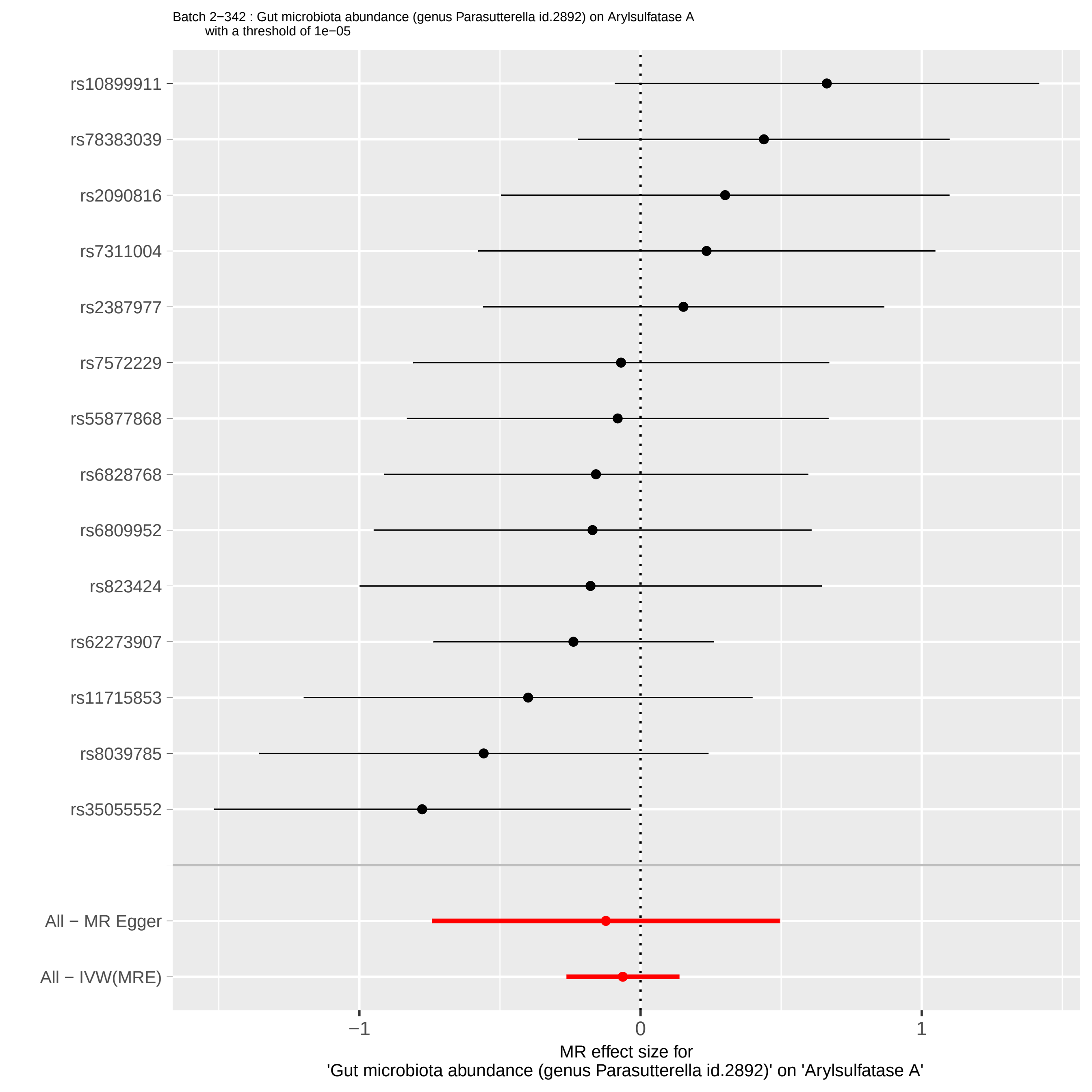
Batch 2−342 : Gut microbiota abundance (genus Parasutterella id.2892) on Arylsulfatase A
with a threshold of 1e−05
rs10899911
rs78383039
rs2090816
rs7311004
rs2387977
rs7572229
rs55877868
rs6828768
rs6809952
rs823424
rs62273907
rs11715853
rs8039785
rs35055552
All − MR Egger
All − IVW(MRE)
−1
0
1
MR effect size for
'Gut microbiota abundance (genus Parasutterella id.2892)' on 'Arylsulfatase A'

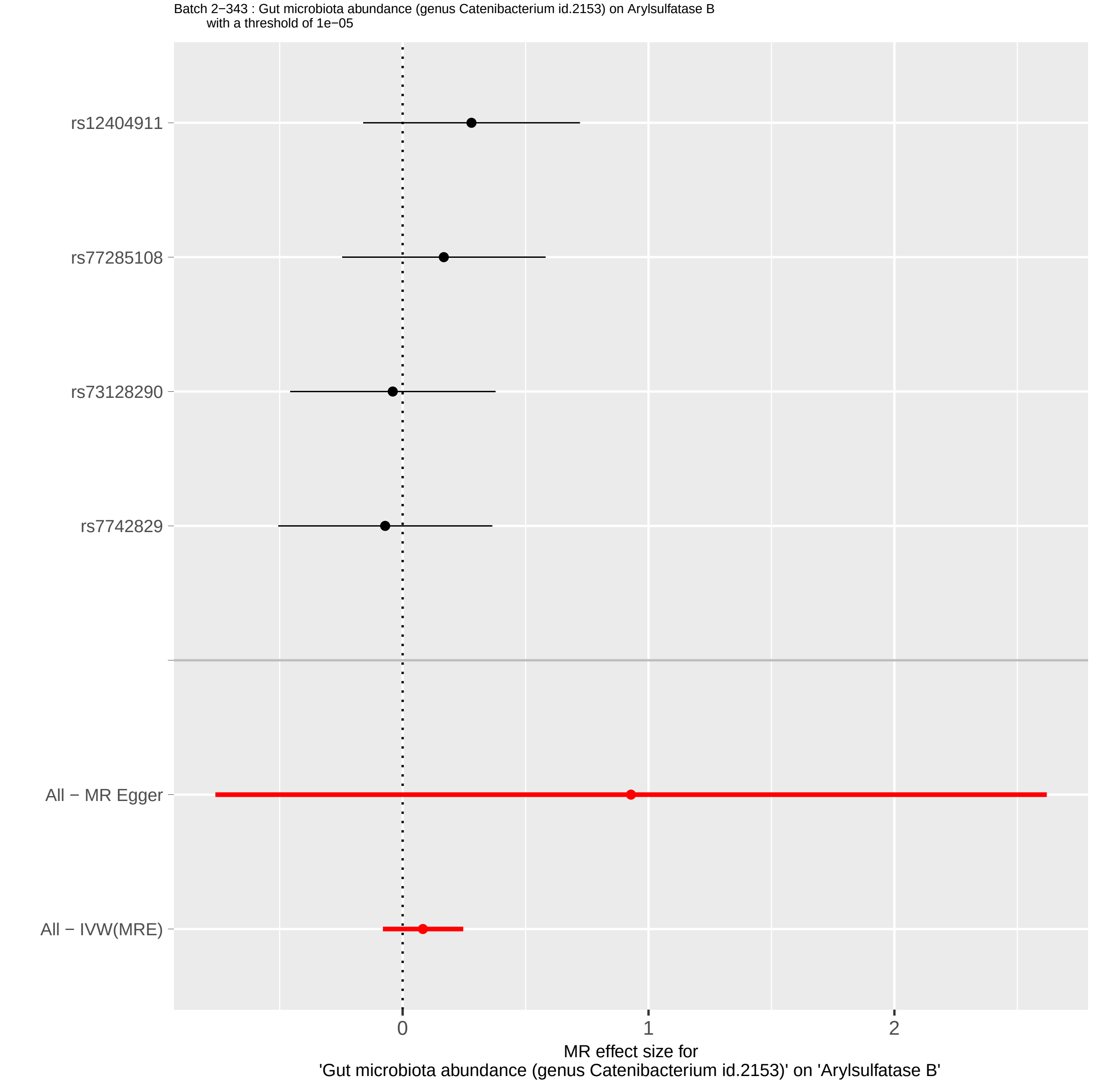

Batch 2−343 : Gut microbiota abundance (genus Catenibacterium id.2153) on Arylsulfatase B
with a threshold of 1e−05
rs12404911
rs77285108
rs73128290
rs7742829
All − MR Egger
All − IVW(MRE)
0
1
2
MR effect size for
'Gut microbiota abundance (genus Catenibacterium id.2153)' on 'Arylsulfatase B'

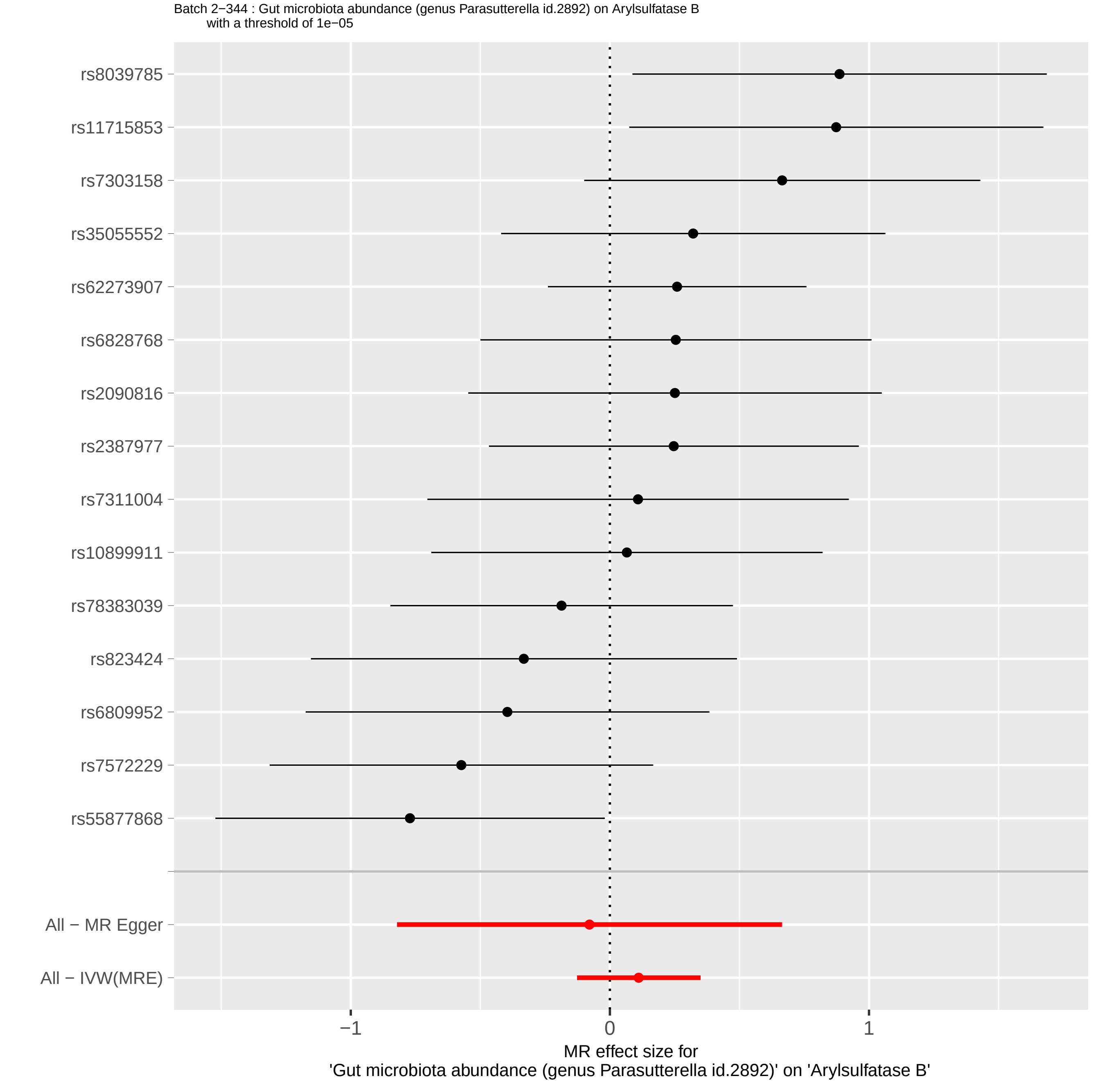

Batch 2−344 : Gut microbiota abundance (genus Parasutterella id.2892) on Arylsulfatase B
with a threshold of 1e−05
rs8039785
rs11715853
rs7303158
rs35055552
rs62273907
rs6828768
rs2090816
rs2387977
rs7311004
rs10899911
rs78383039
rs823424
rs6809952
rs7572229
rs55877868
All − MR Egger
All − IVW(MRE)
−1
0
1
MR effect size for
'Gut microbiota abundance (genus Parasutterella id.2892)' on 'Arylsulfatase B'

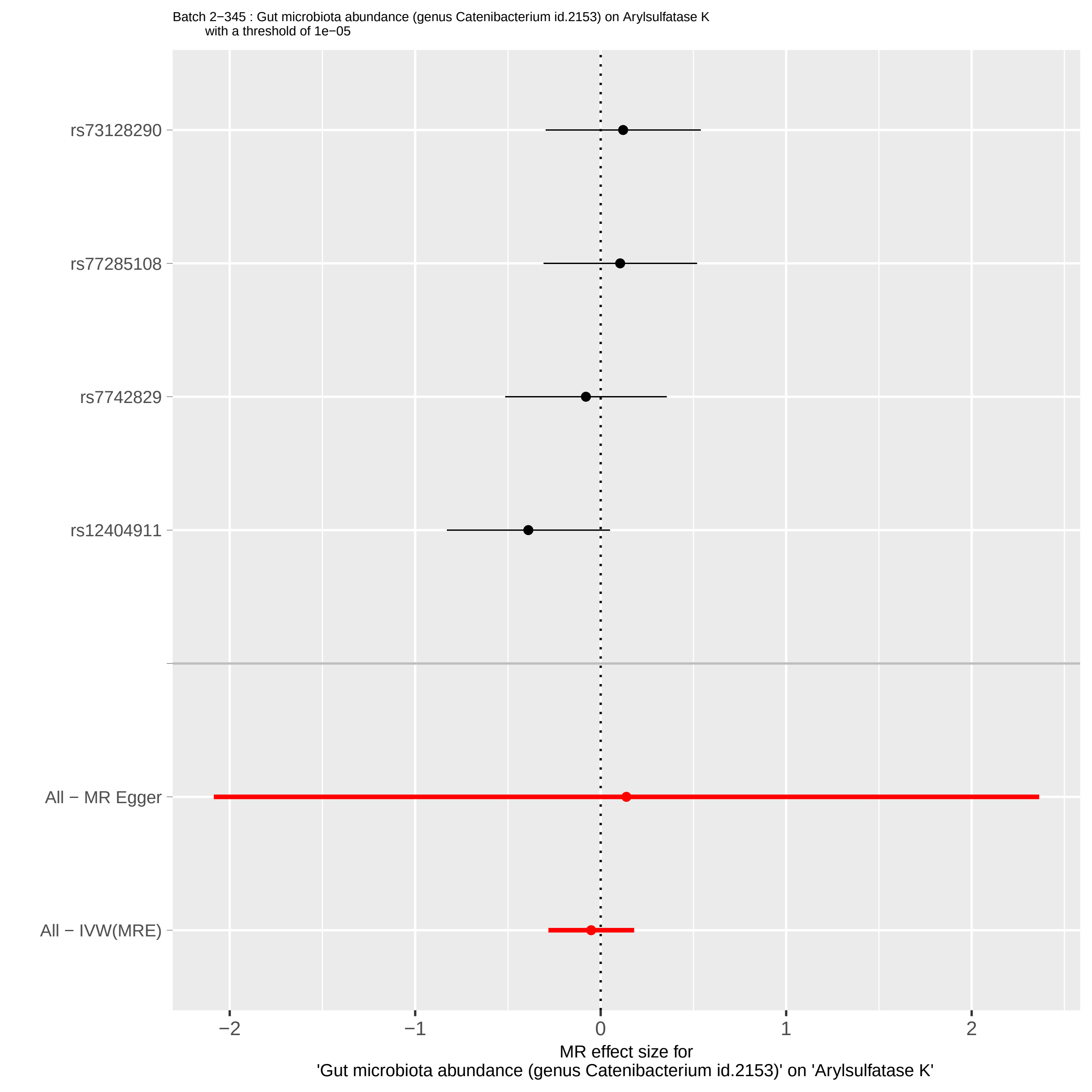

Batch 2−345 : Gut microbiota abundance (genus Catenibacterium id.2153) on Arylsulfatase K
with a threshold of 1e−05
rs73128290
rs77285108
rs7742829
rs12404911
All − MR Egger
All − IVW(MRE)
−2
−1
0
1
2
MR effect size for
'Gut microbiota abundance (genus Catenibacterium id.2153)' on 'Arylsulfatase K'

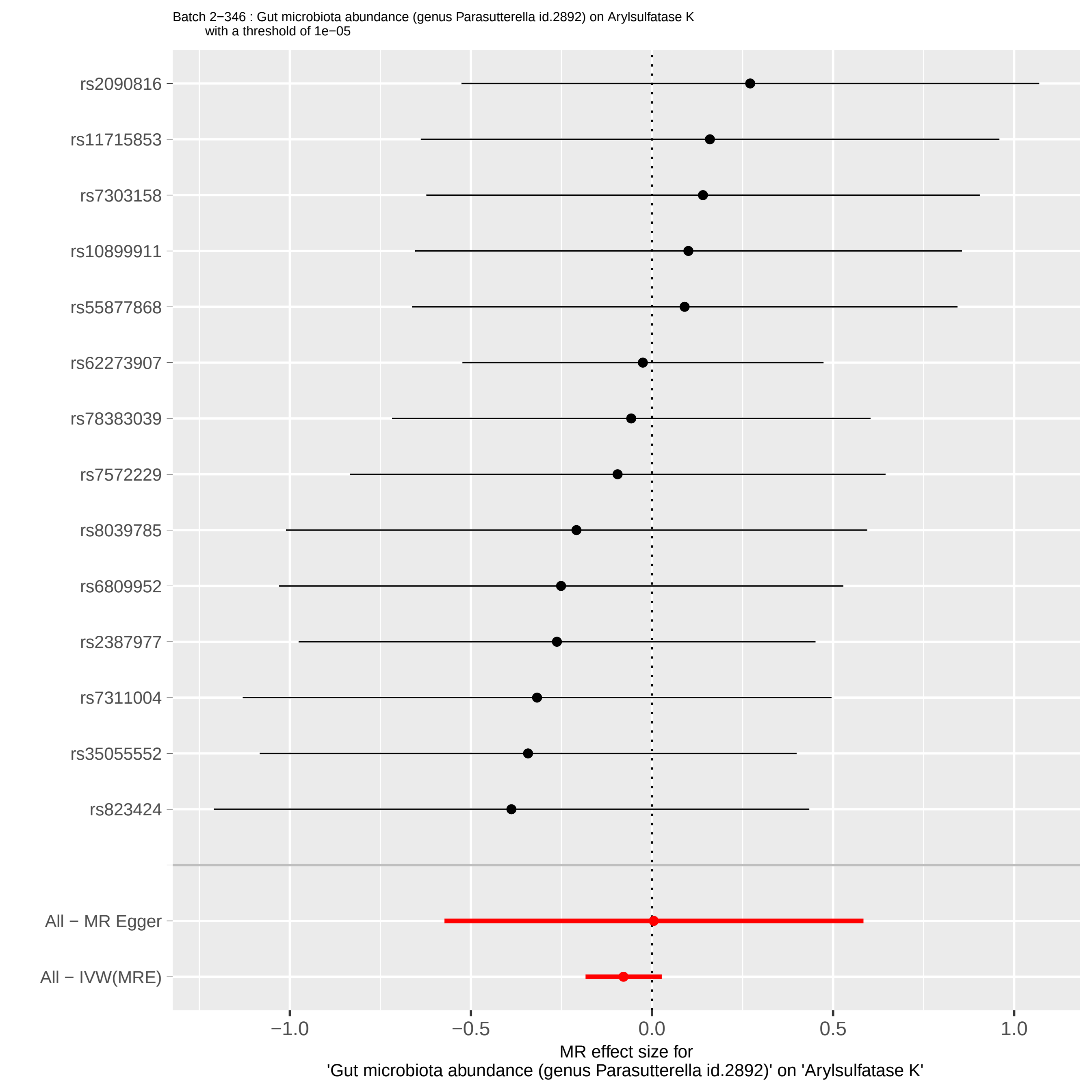

Batch 2−346 : Gut microbiota abundance (genus Parasutterella id.2892) on Arylsulfatase K
with a threshold of 1e−05
rs2090816
rs11715853
rs7303158
rs10899911
rs55877868
rs62273907
rs78383039
rs7572229
rs8039785
rs6809952
rs2387977
rs7311004
rs35055552
rs823424
All − MR Egger
All − IVW(MRE)
−1.0
−0.5
0.0
0.5
1.0
MR effect size for
'Gut microbiota abundance (genus Parasutterella id.2892)' on 'Arylsulfatase K'

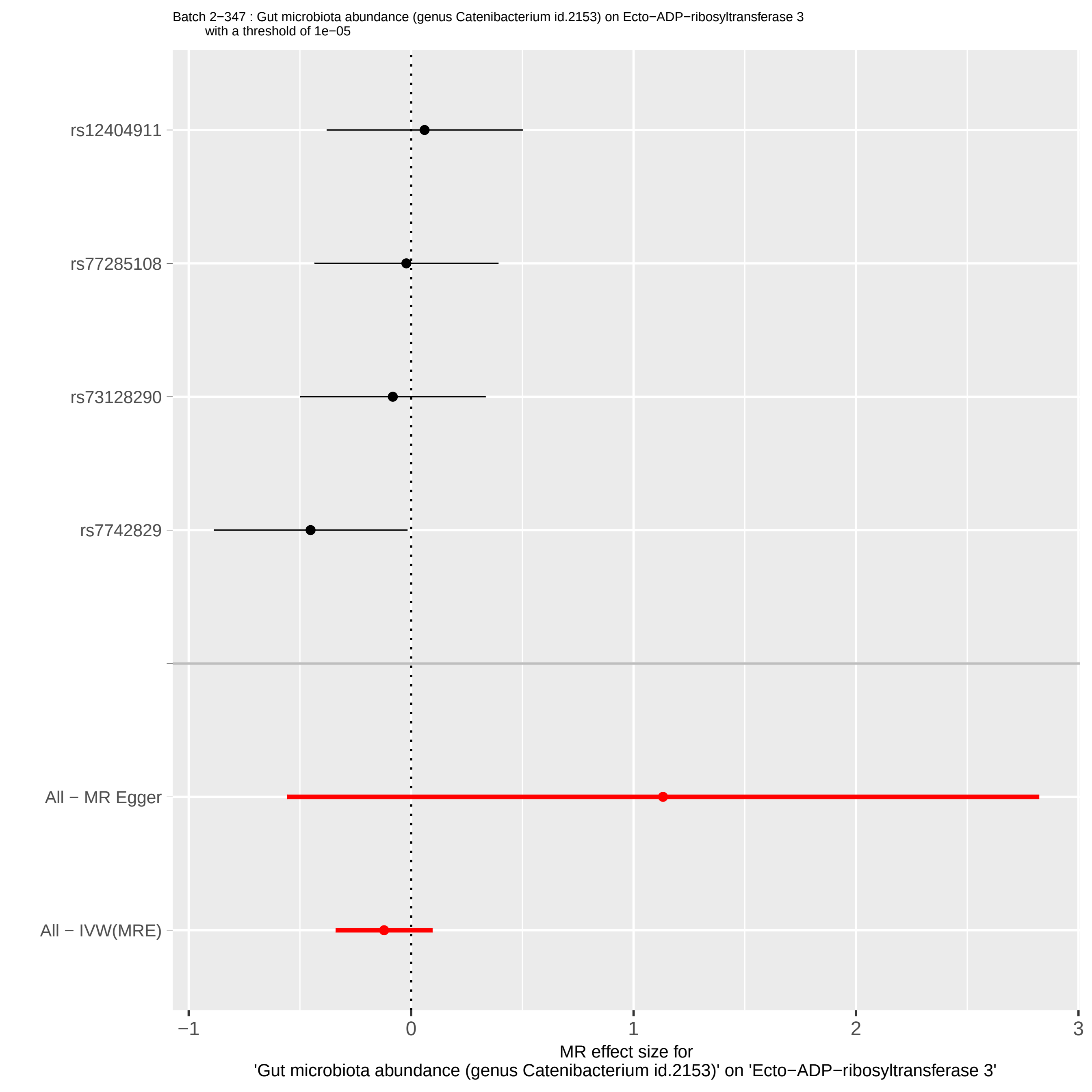

Batch 2−347 : Gut microbiota abundance (genus Catenibacterium id.2153) on Ecto−ADP−ribosyltransferase 3
with a threshold of 1e−05
rs12404911
rs77285108
rs73128290
rs7742829
All − MR Egger
All − IVW(MRE)
−1
0
1
2
3
MR effect size for
'Gut microbiota abundance (genus Catenibacterium id.2153)' on 'Ecto−ADP−ribosyltransferase 3'

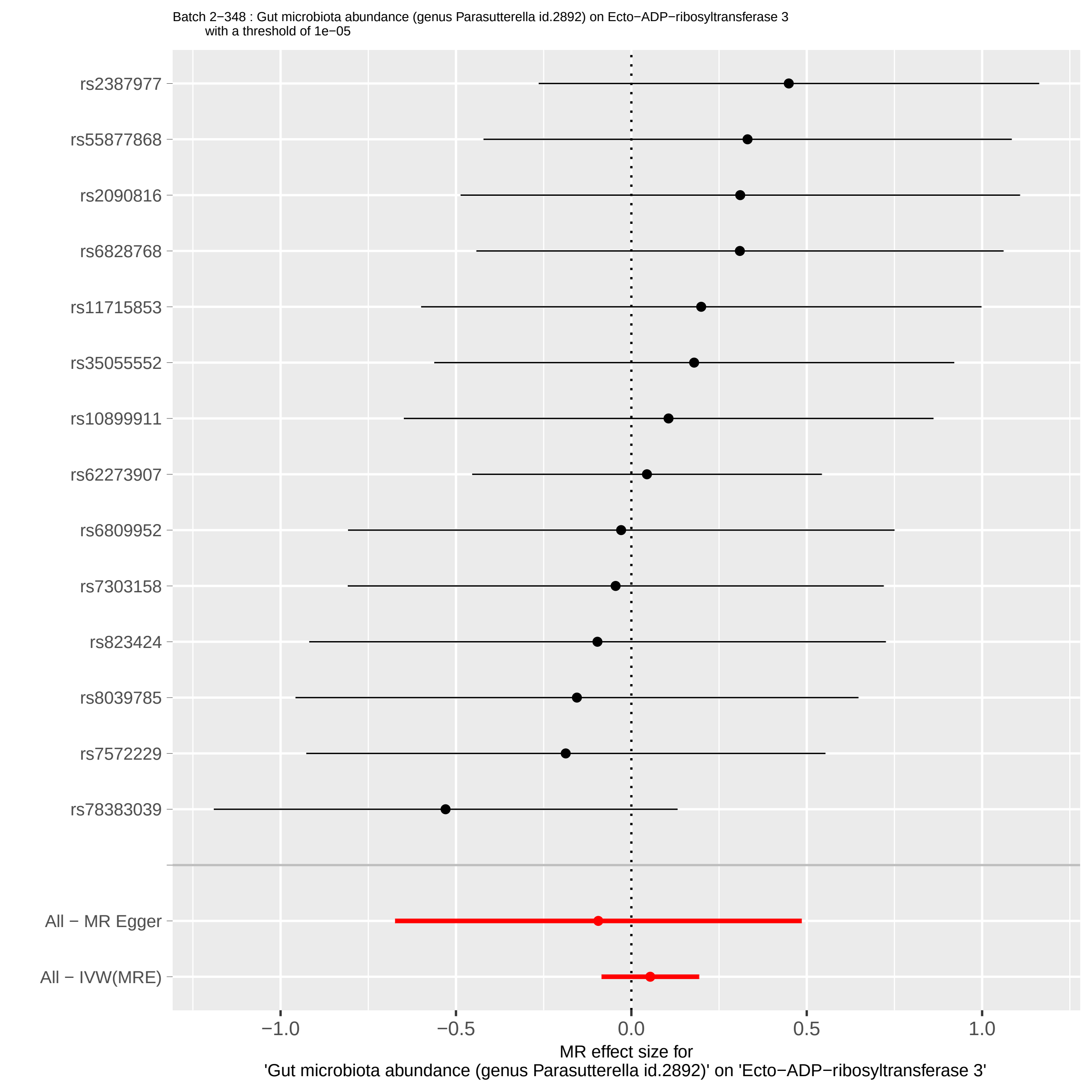

Batch 2−348 : Gut microbiota abundance (genus Parasutterella id.2892) on Ecto−ADP−ribosyltransferase 3
with a threshold of 1e−05
rs2387977
rs55877868
rs2090816
rs6828768
rs11715853
rs35055552
rs10899911
rs62273907
rs6809952
rs7303158
rs823424
rs8039785
rs7572229
rs78383039
All − MR Egger
All − IVW(MRE)
−1.0
−0.5
0.0
0.5
1.0
MR effect size for
'Gut microbiota abundance (genus Parasutterella id.2892)' on 'Ecto−ADP−ribosyltransferase 3'

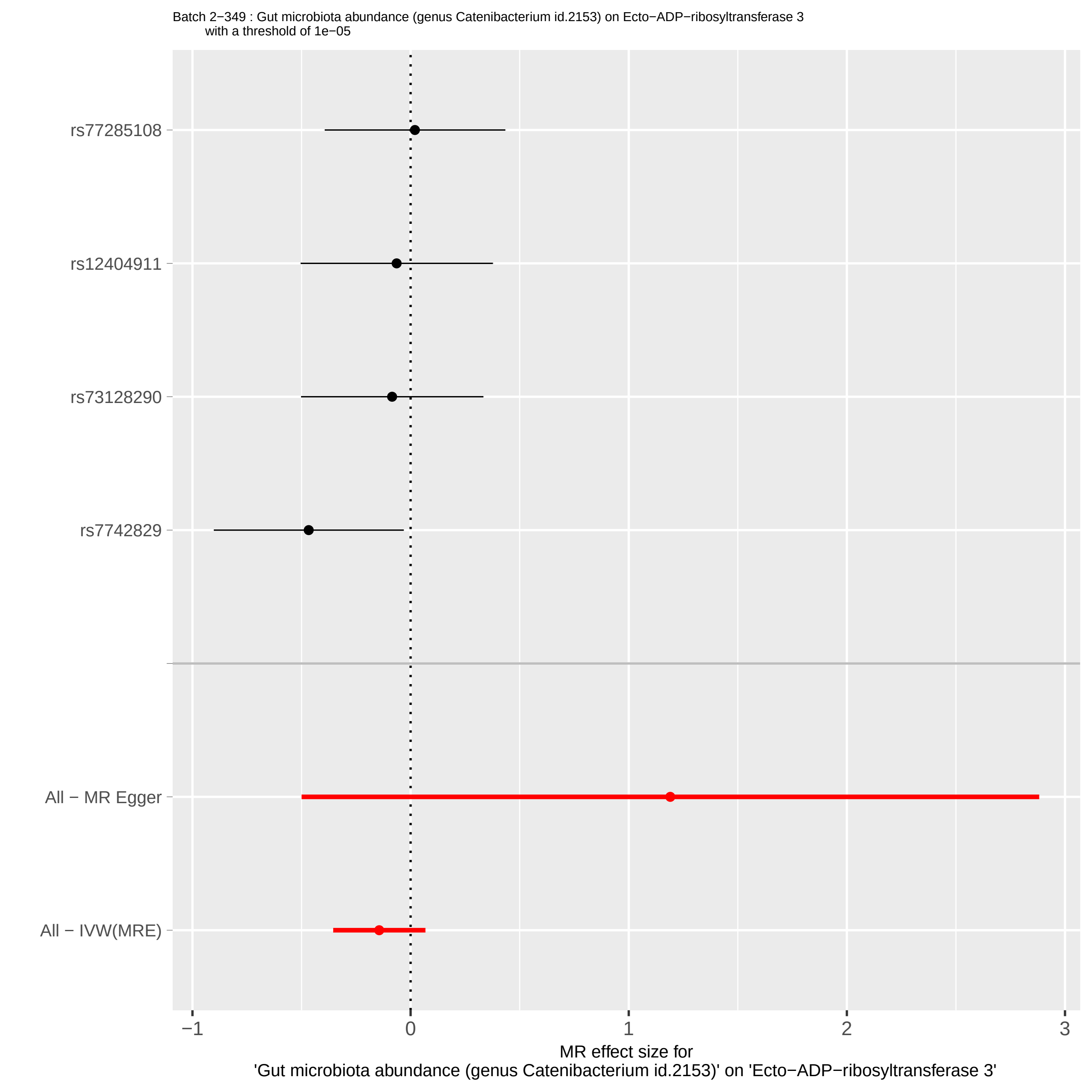

Batch 2−349 : Gut microbiota abundance (genus Catenibacterium id.2153) on Ecto−ADP−ribosyltransferase 3
with a threshold of 1e−05
rs77285108
rs12404911
rs73128290
rs7742829
All − MR Egger
All − IVW(MRE)
−1
0
1
2
3
MR effect size for
'Gut microbiota abundance (genus Catenibacterium id.2153)' on 'Ecto−ADP−ribosyltransferase 3'

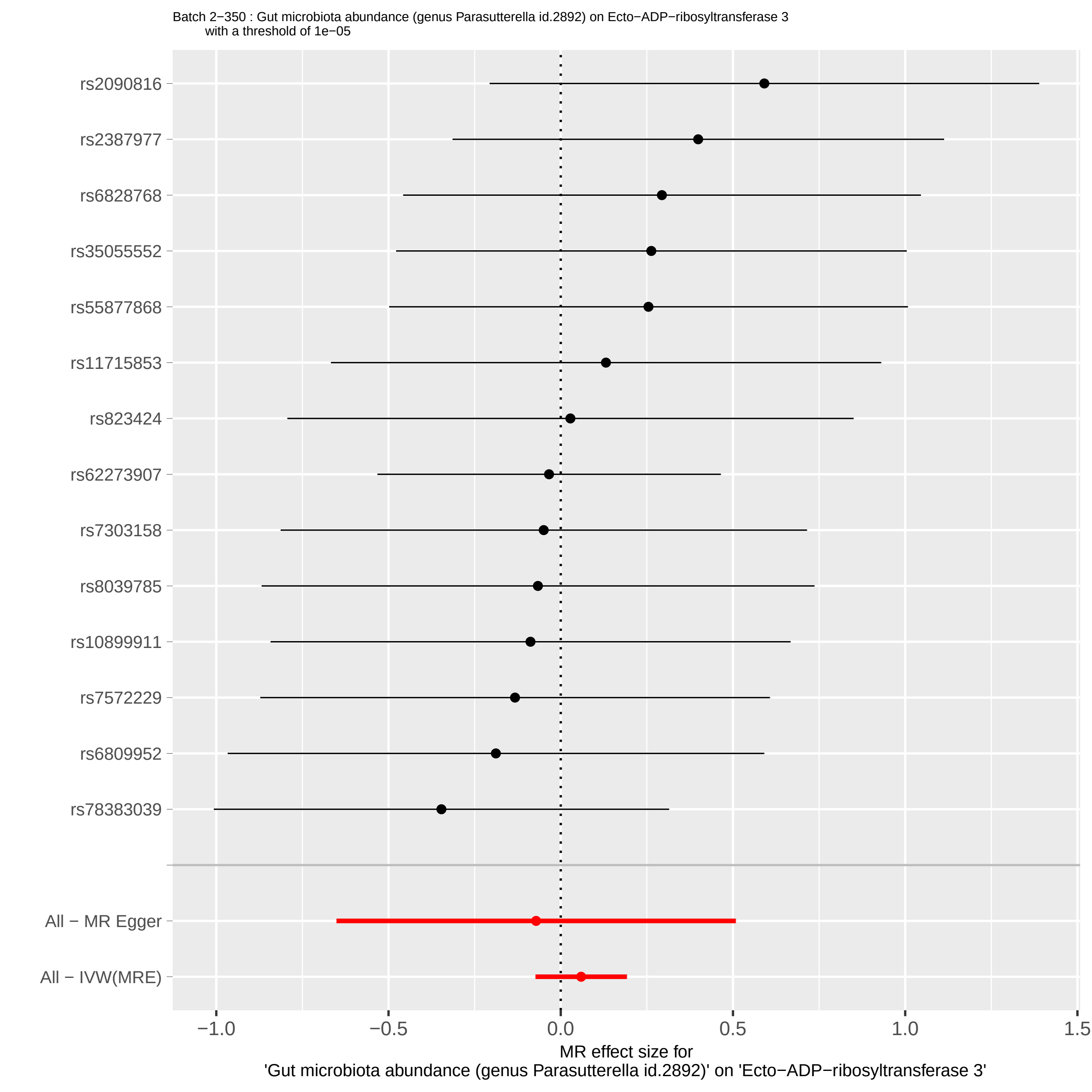

Batch 2−350 : Gut microbiota abundance (genus Parasutterella id.2892) on Ecto−ADP−ribosyltransferase 3
with a threshold of 1e−05
rs2090816
rs2387977
rs6828768
rs35055552
rs55877868
rs11715853
rs823424
rs62273907
rs7303158
rs8039785
rs10899911
rs7572229
rs6809952
rs78383039
All − MR Egger
All − IVW(MRE)
−1.0
−0.5
0.0
0.5
1.0
1.5
MR effect size for
'Gut microbiota abundance (genus Parasutterella id.2892)' on 'Ecto−ADP−ribosyltransferase 3'

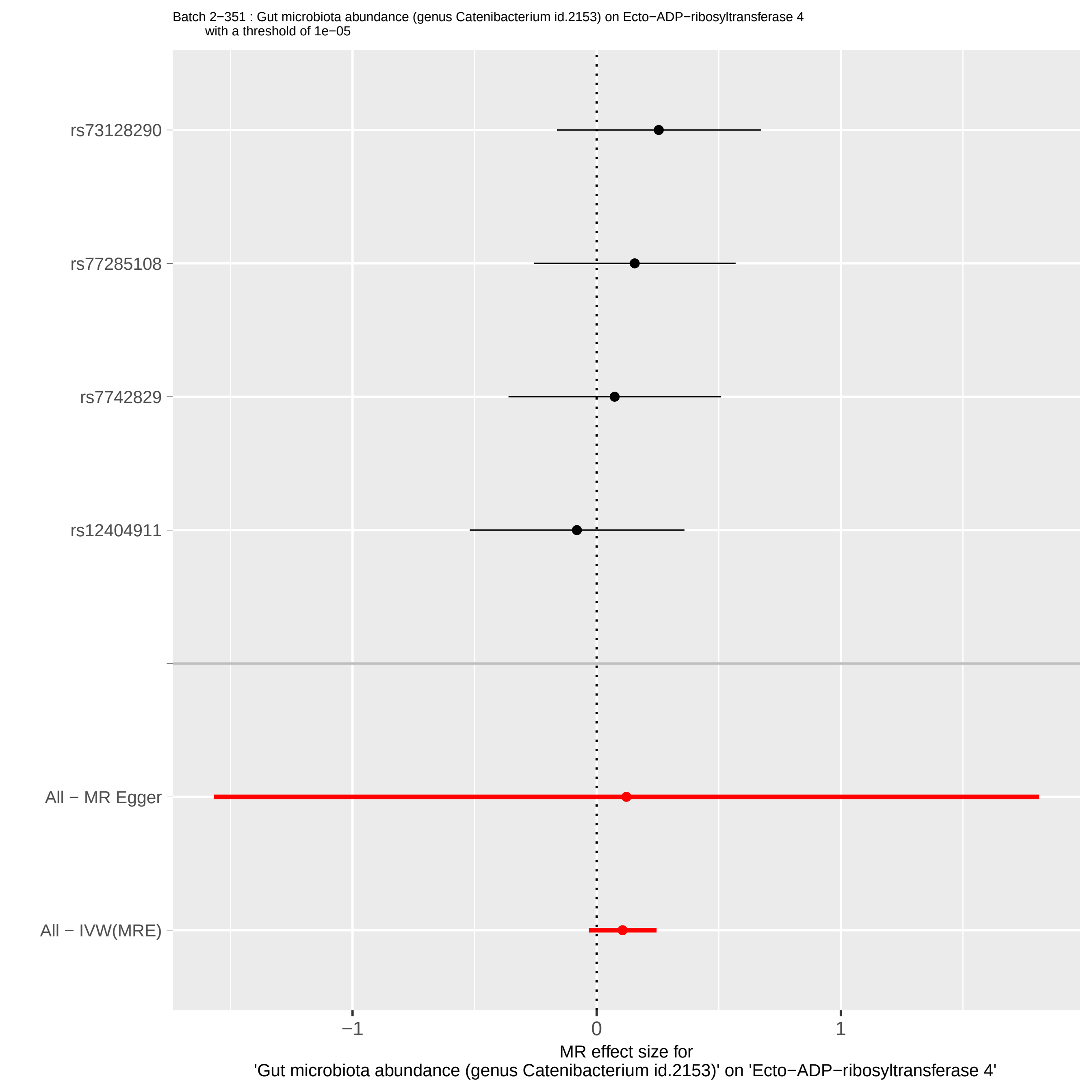
Batch 2−351 : Gut microbiota abundance (genus Catenibacterium id.2153) on Ecto−ADP−ribosyltransferase 4
with a threshold of 1e−05
rs73128290
rs77285108
rs7742829
rs12404911
All − MR Egger
All − IVW(MRE)
−1
0
1
MR effect size for
'Gut microbiota abundance (genus Catenibacterium id.2153)' on 'Ecto−ADP−ribosyltransferase 4'

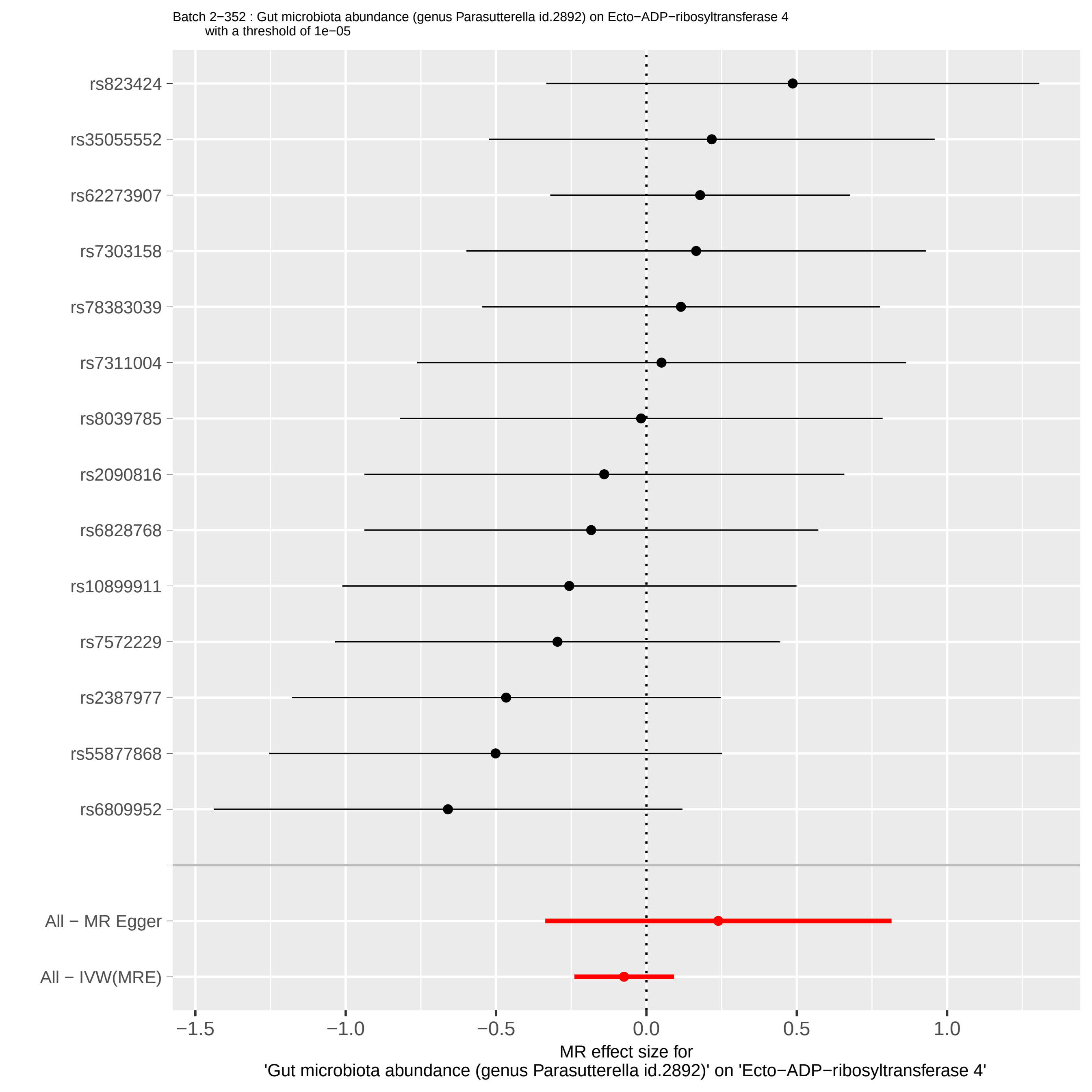
Batch 2−352 : Gut microbiota abundance (genus Parasutterella id.2892) on Ecto−ADP−ribosyltransferase 4
with a threshold of 1e−05
rs823424
rs35055552
rs62273907
rs7303158
rs78383039
rs7311004
rs8039785
rs2090816
rs6828768
rs10899911
rs7572229
rs2387977
rs55877868
rs6809952
All − MR Egger
All − IVW(MRE)
−1.5
−1.0
−0.5
0.0
0.5
1.0
MR effect size for
'Gut microbiota abundance (genus Parasutterella id.2892)' on 'Ecto−ADP−ribosyltransferase 4'

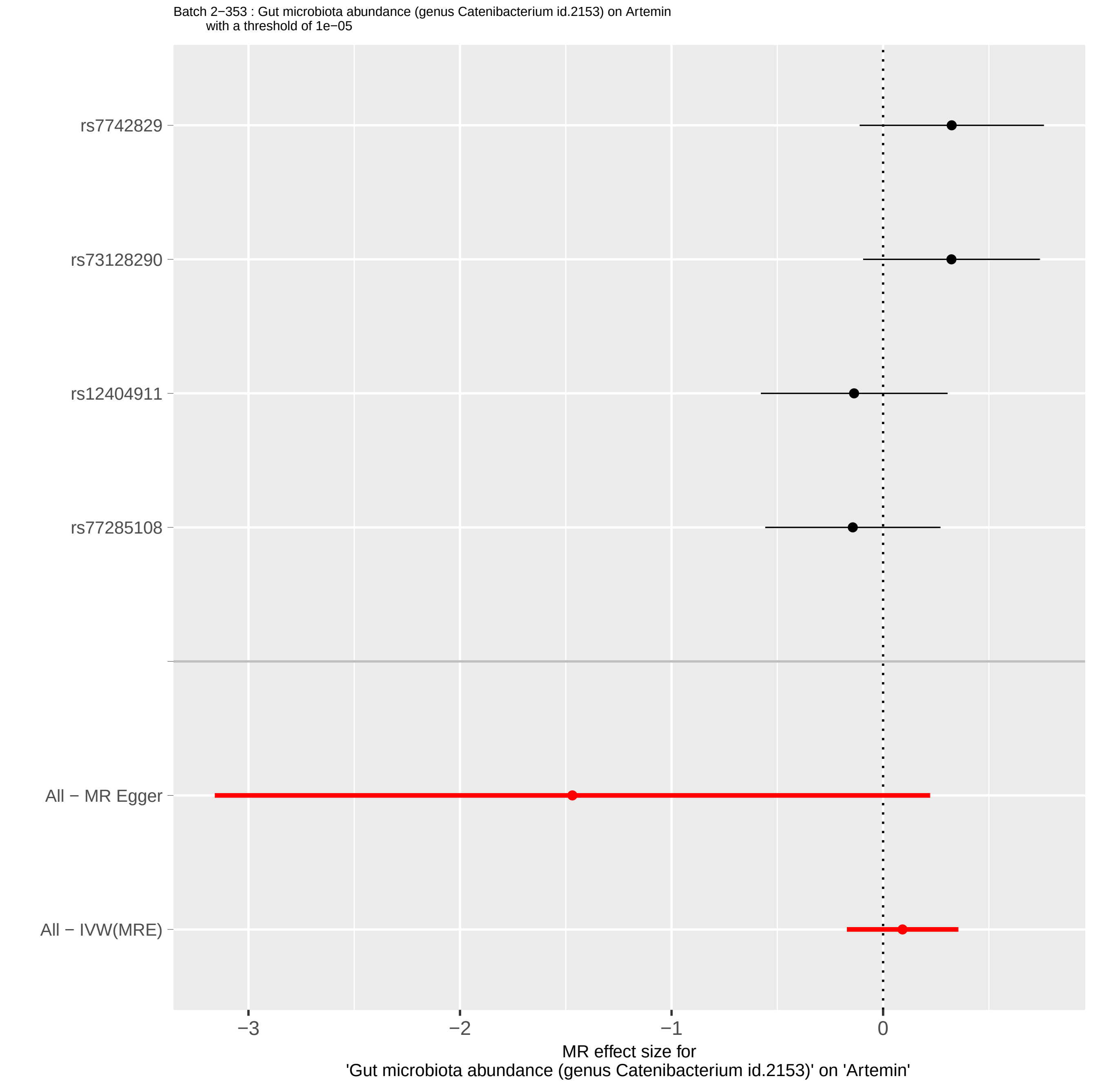

Batch 2−353 : Gut microbiota abundance (genus Catenibacterium id.2153) on Artemin
with a threshold of 1e−05
rs7742829
rs73128290
rs12404911
rs77285108
All − MR Egger
All − IVW(MRE)
−3
−2
−1
0
MR effect size for
'Gut microbiota abundance (genus Catenibacterium id.2153)' on 'Artemin'

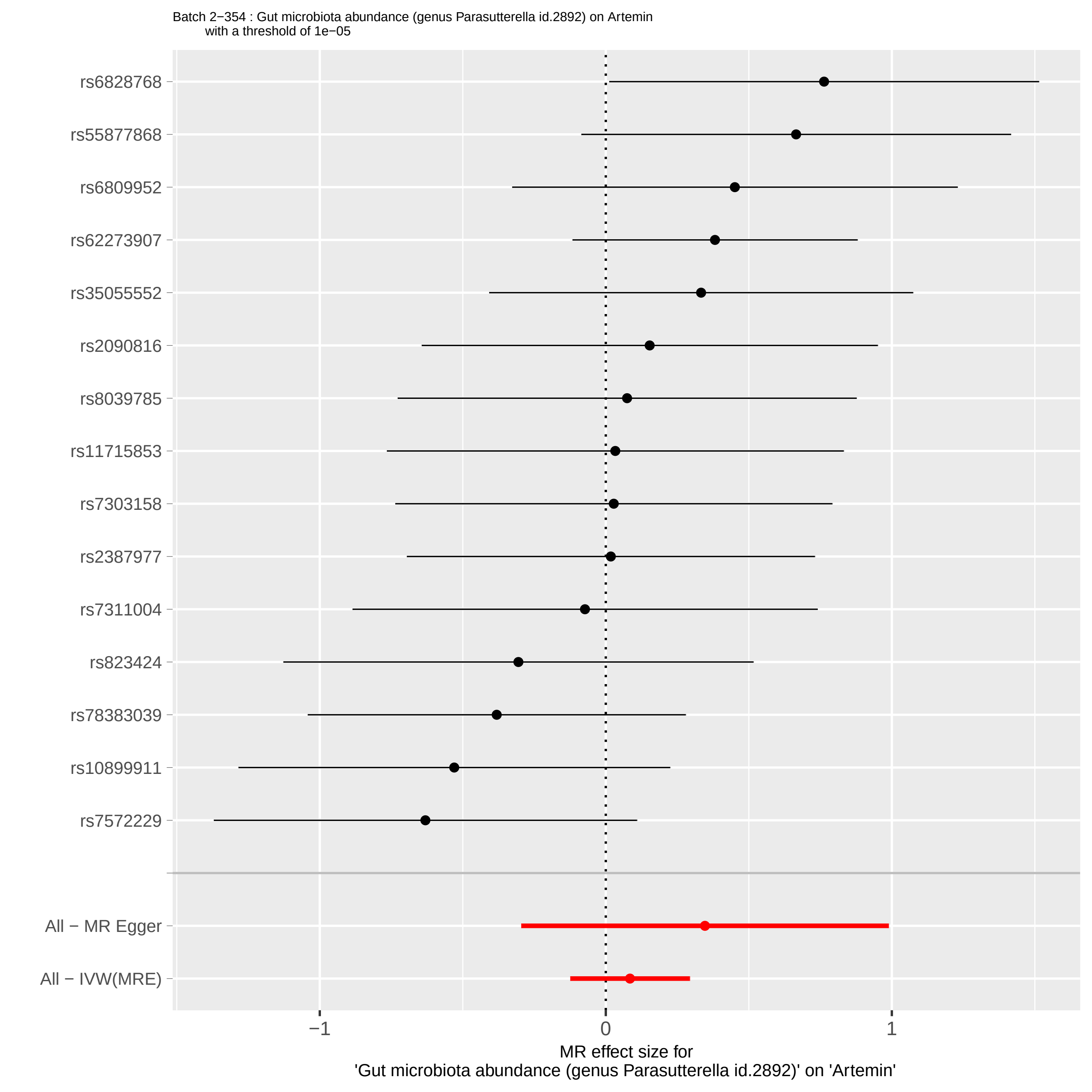
Batch 2−354 : Gut microbiota abundance (genus Parasutterella id.2892) on Artemin
with a threshold of 1e−05
rs6828768
rs55877868
rs6809952
rs62273907
rs35055552
rs2090816
rs8039785
rs11715853
rs7303158
rs2387977
rs7311004
rs823424
rs78383039
rs10899911
rs7572229
All − MR Egger
All − IVW(MRE)
−1
0
1
MR effect size for
'Gut microbiota abundance (genus Parasutterella id.2892)' on 'Artemin'

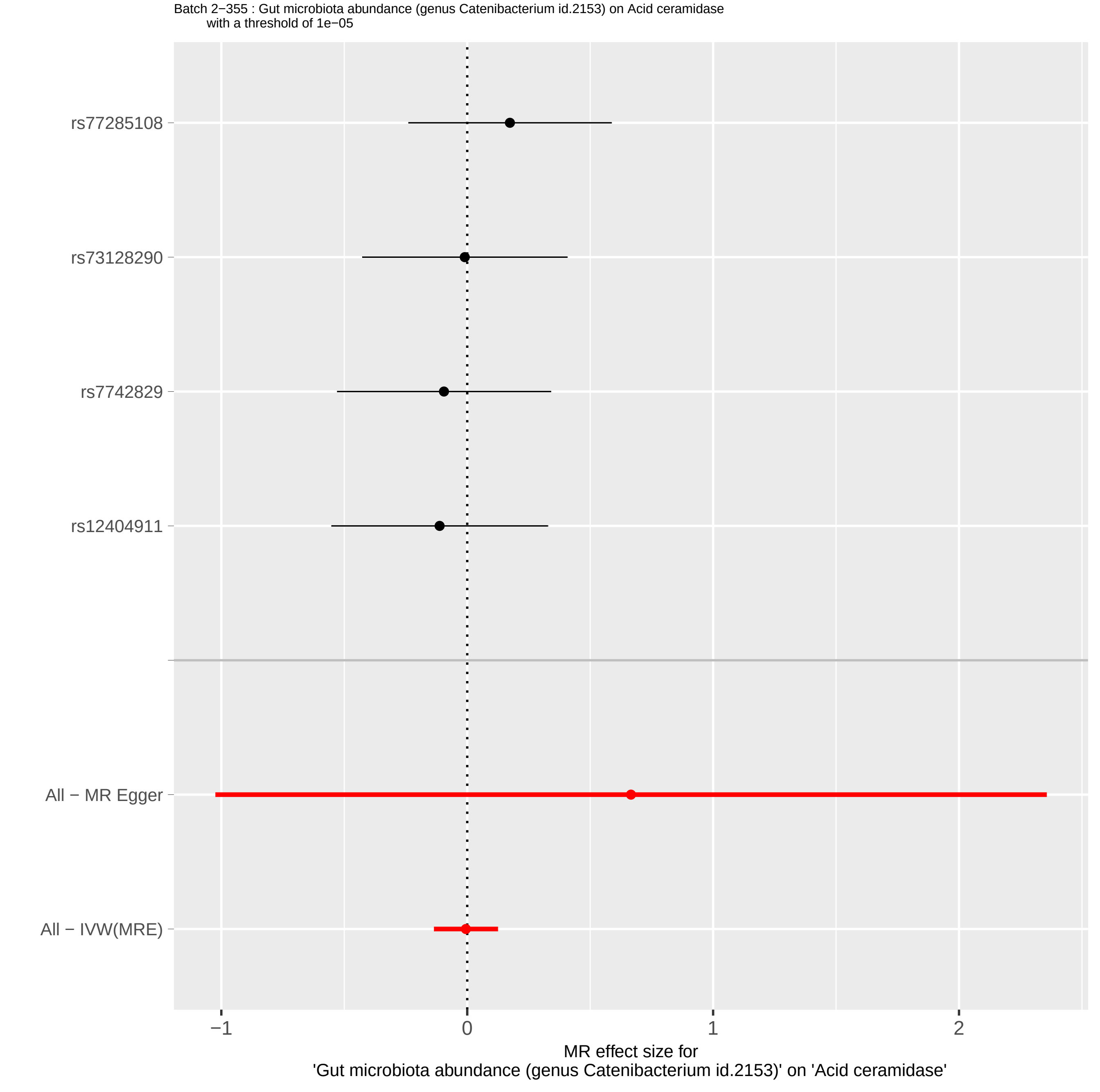

Batch 2−355 : Gut microbiota abundance (genus Catenibacterium id.2153) on Acid ceramidase
with a threshold of 1e−05
rs77285108
rs73128290
rs7742829
rs12404911
All − MR Egger
All − IVW(MRE)
−1
0
1
2
MR effect size for
'Gut microbiota abundance (genus Catenibacterium id.2153)' on 'Acid ceramidase'

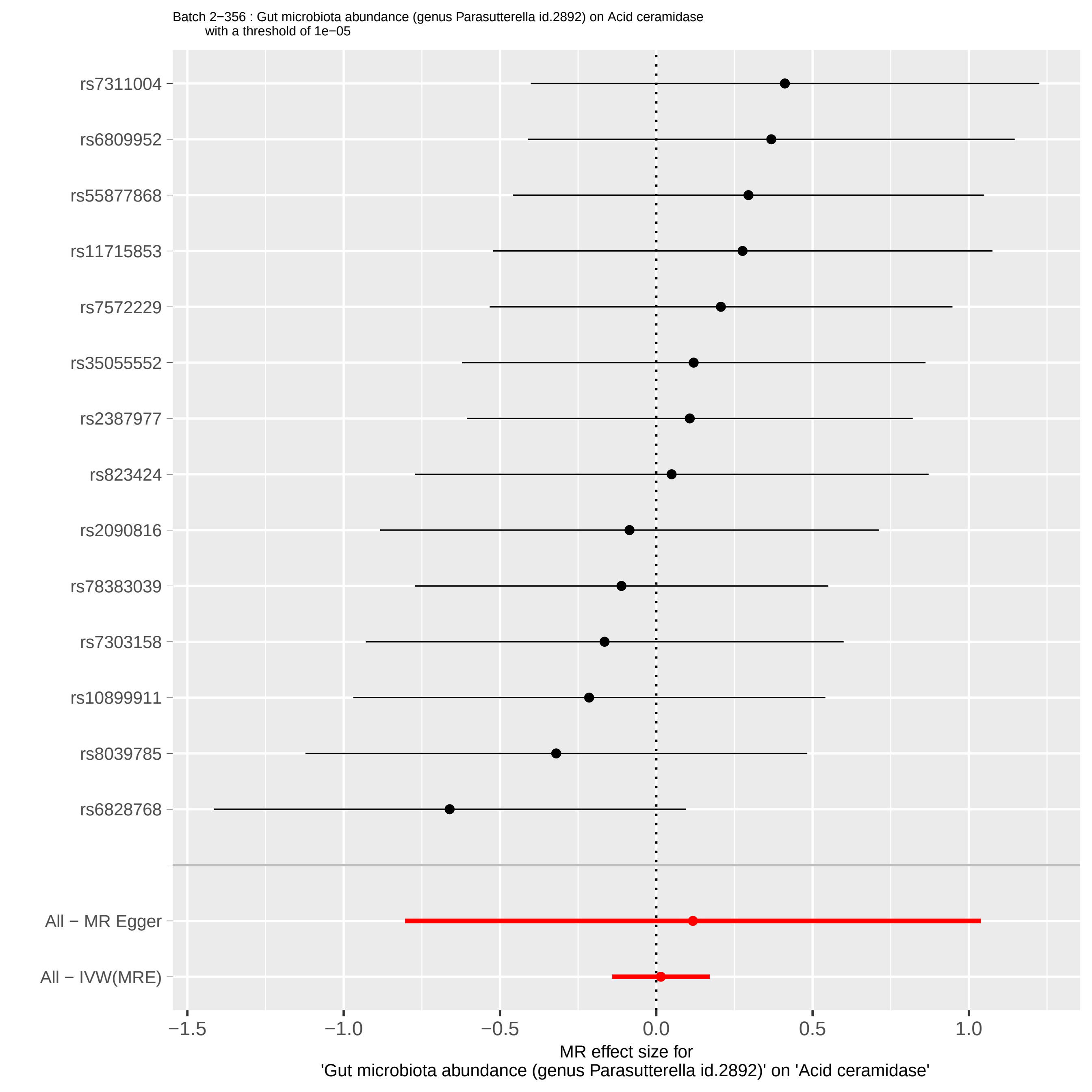

Batch 2−356 : Gut microbiota abundance (genus Parasutterella id.2892) on Acid ceramidase
with a threshold of 1e−05
rs7311004
rs6809952
rs55877868
rs11715853
rs7572229
rs35055552
rs2387977
rs823424
rs2090816
rs78383039
rs7303158
rs10899911
rs8039785
rs6828768
All − MR Egger
All − IVW(MRE)
−1.5
−1.0
−0.5
0.0
0.5
1.0
MR effect size for
'Gut microbiota abundance (genus Parasutterella id.2892)' on 'Acid ceramidase'

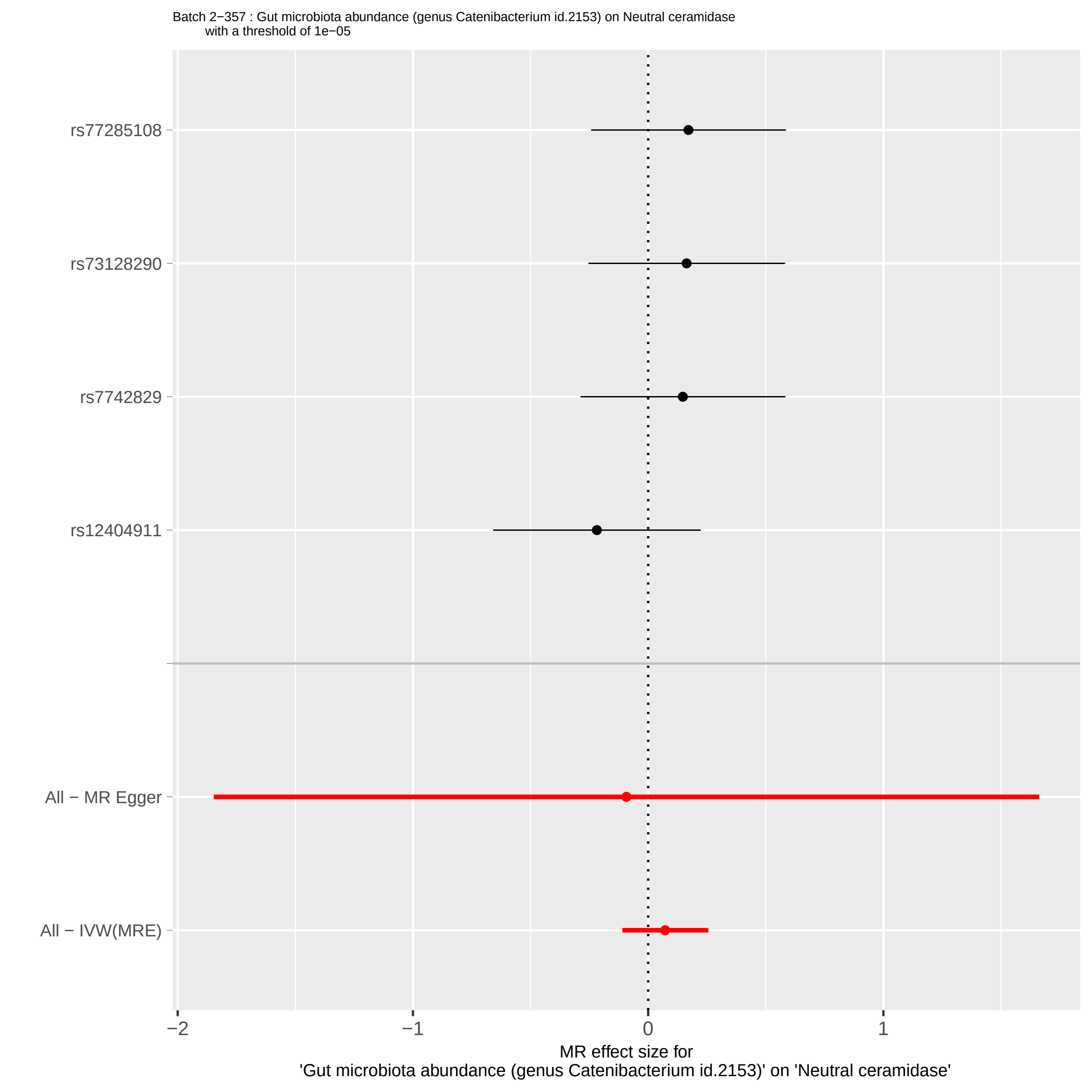
Batch 2−357 : Gut microbiota abundance (genus Catenibacterium id.2153) on Neutral ceramidase
with a threshold of 1e−05
rs77285108
rs73128290
rs7742829
rs12404911
All − MR Egger
All − IVW(MRE)
−2
−1
0
1
MR effect size for
'Gut microbiota abundance (genus Catenibacterium id.2153)' on 'Neutral ceramidase'

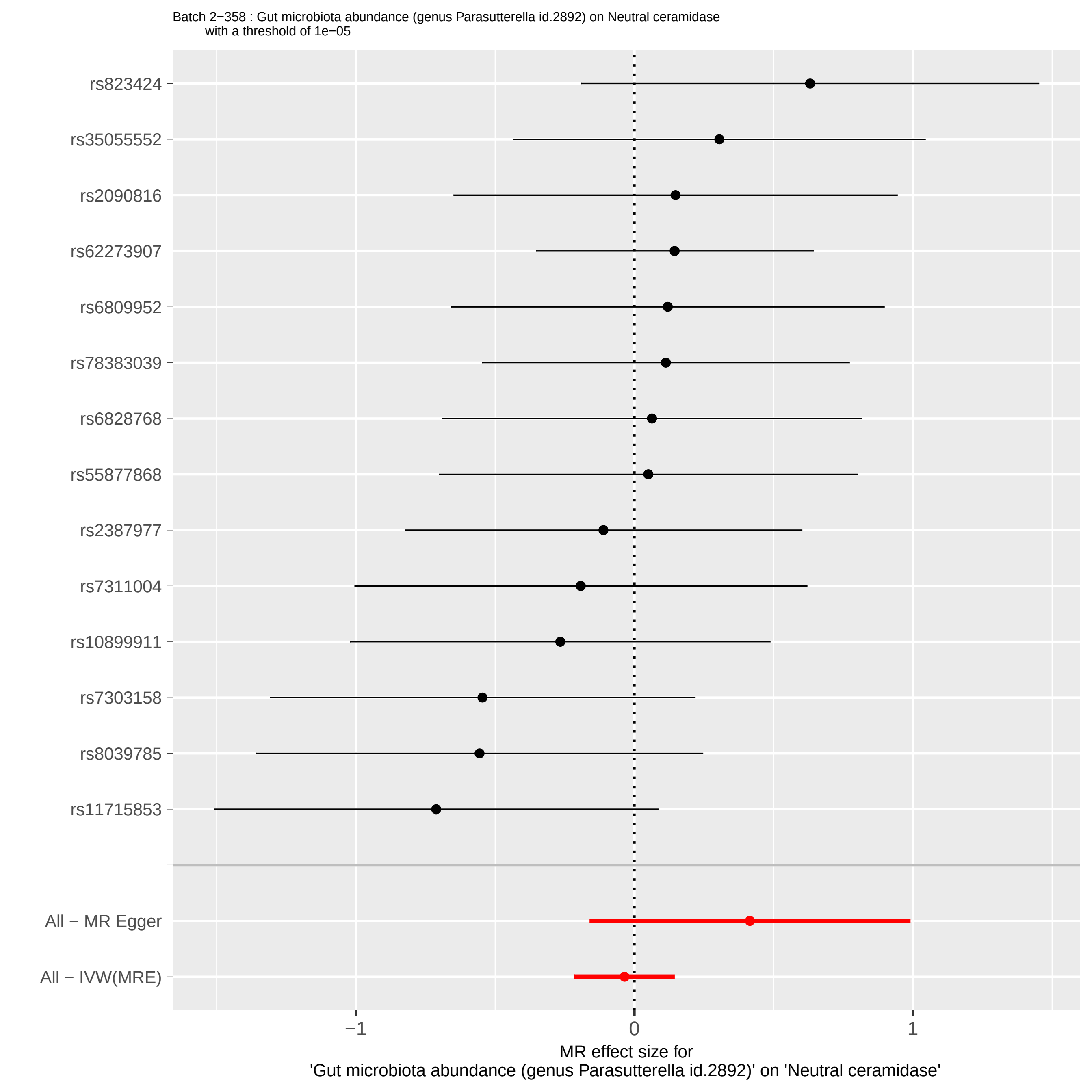

Batch 2−358 : Gut microbiota abundance (genus Parasutterella id.2892) on Neutral ceramidase
with a threshold of 1e−05
rs823424
rs35055552
rs2090816
rs62273907
rs6809952
rs78383039
rs6828768
rs55877868
rs2387977
rs7311004
rs10899911
rs7303158
rs8039785
rs11715853
All − MR Egger
All − IVW(MRE)
−1
0
1
MR effect size for
'Gut microbiota abundance (genus Parasutterella id.2892)' on 'Neutral ceramidase'

Batch 2−359 : Gut microbiota abundance (genus Catenibacterium id.2153) on Arf−GAP with SH3 domain, ANK repeat and PH domain−containing protein 2 with a threshold of 1e−05

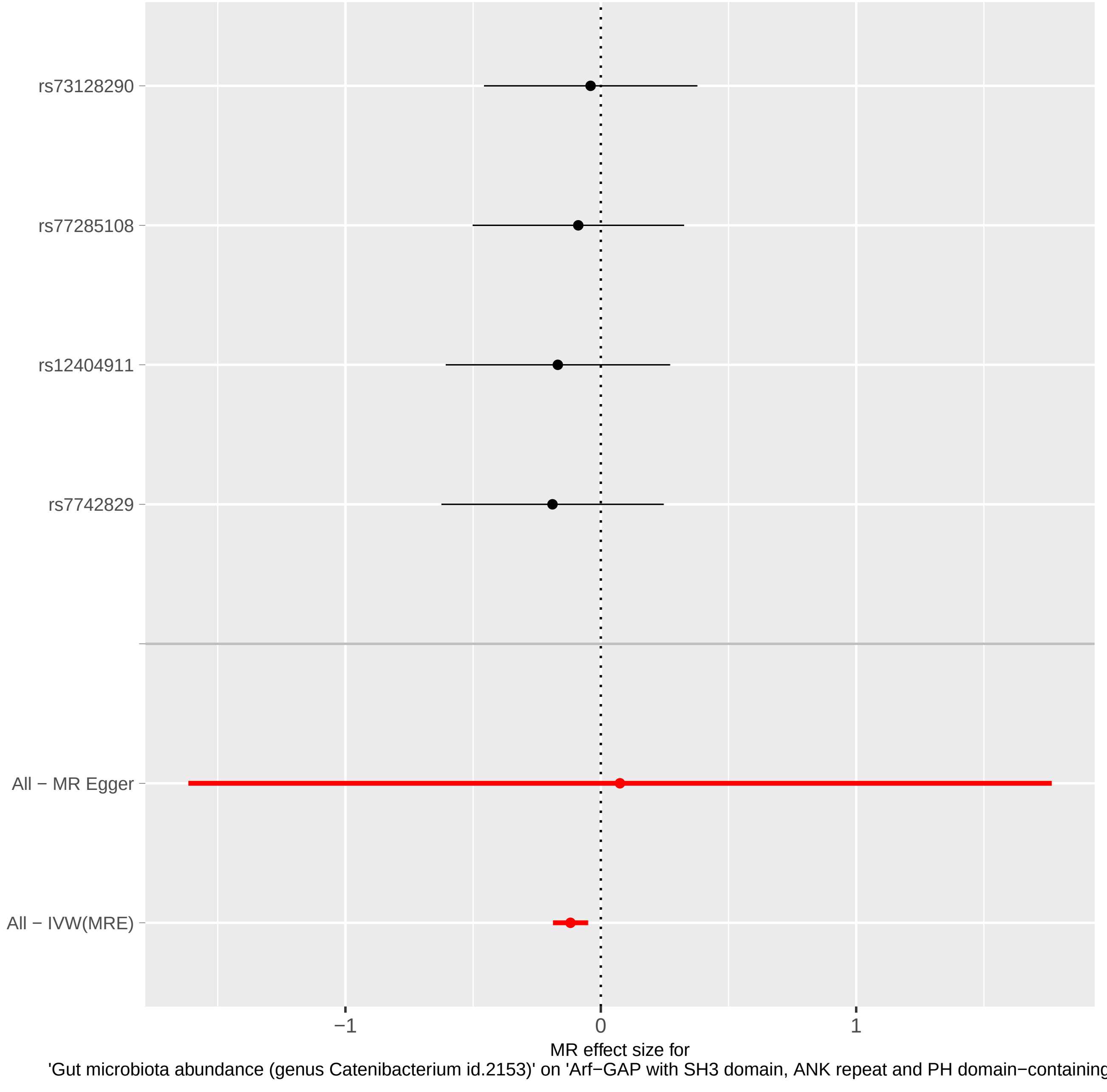

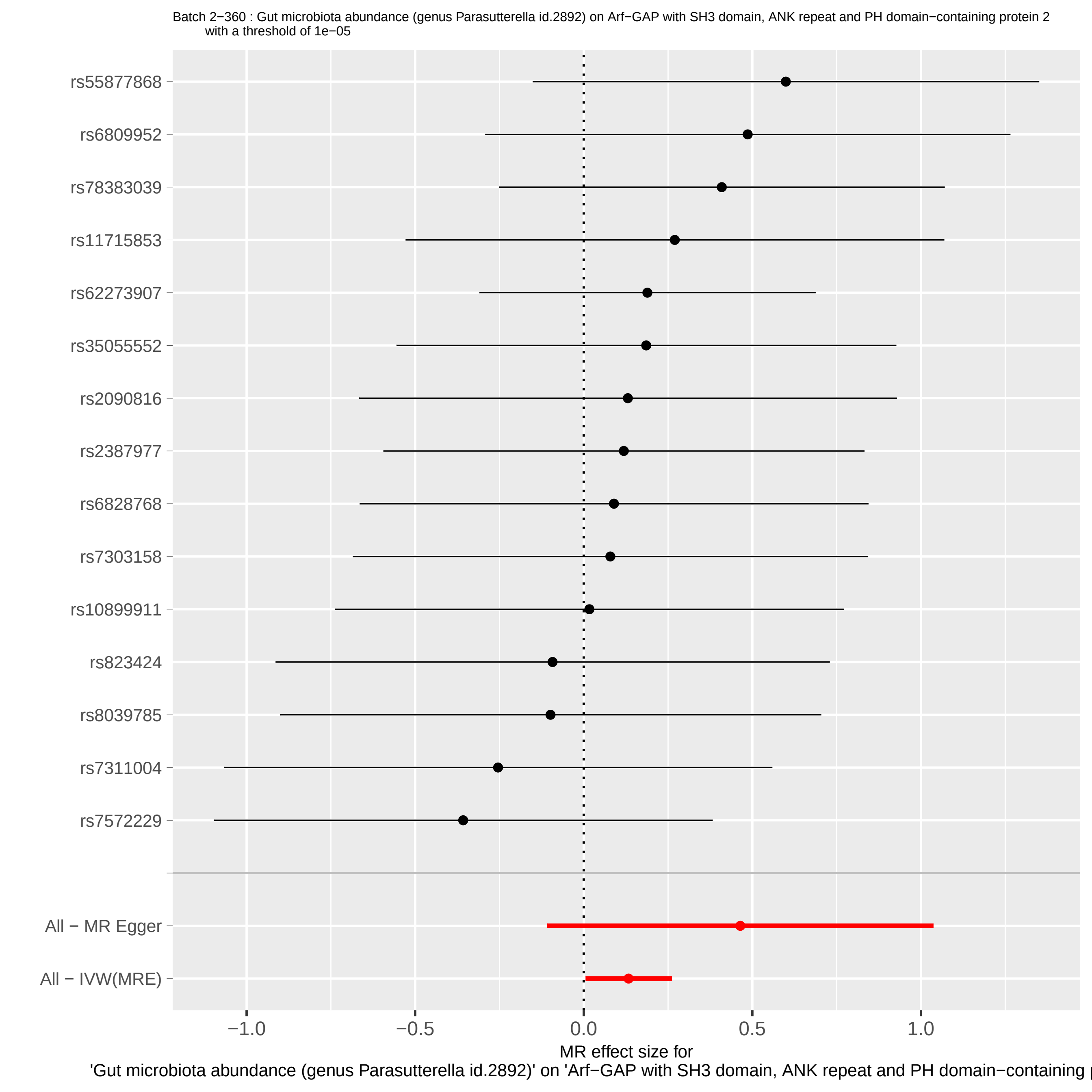

Batch 2−360 : Gut microbiota abundance (genus Parasutterella id.2892) on Arf−GAP with SH3 domain, ANK repeat and PH domain−containing protein 2 with a threshold of 1e−05
rs55877868
rs6809952
rs78383039
rs11715853
rs62273907
rs35055552
rs2090816
rs2387977
rs6828768
rs7303158
rs10899911
rs823424
rs8039785
rs7311004
rs7572229
All − MR Egger
All − IVW(MRE)
−1.0
−0.5
0.0
0.5
1.0
MR effect size for
'Gut microbiota abundance (genus Parasutterella id.2892)' on 'Arf−GAP with SH3 domain, ANK repeat and PH domain−containing p

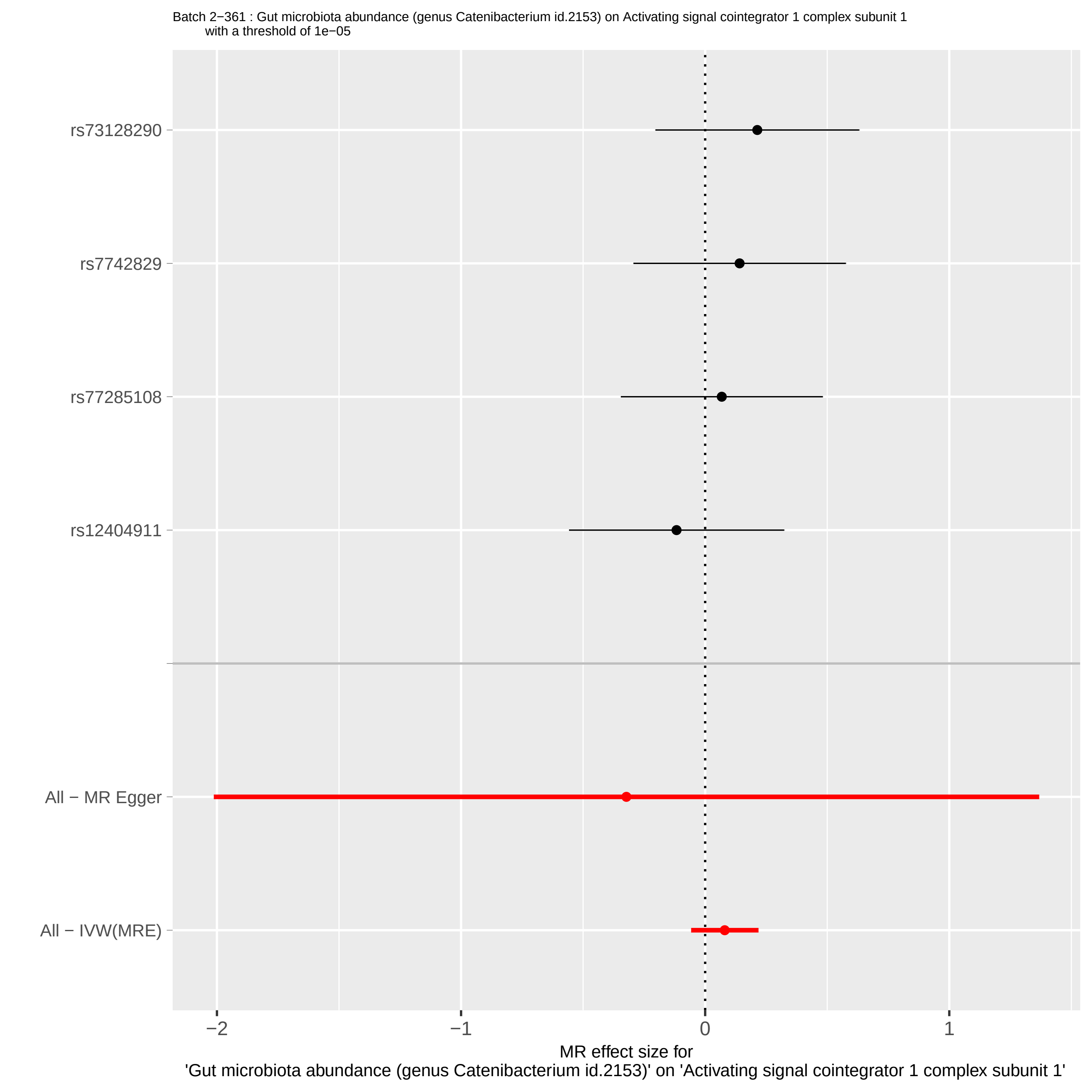
Batch 2−361 : Gut microbiota abundance (genus Catenibacterium id.2153) on Activating signal cointegrator 1 complex subunit 1
with a threshold of 1e−05
rs73128290
rs7742829
rs77285108
rs12404911
All − MR Egger
All − IVW(MRE)
−2
−1
0
1
MR effect size for
'Gut microbiota abundance (genus Catenibacterium id.2153)' on 'Activating signal cointegrator 1 complex subunit 1'

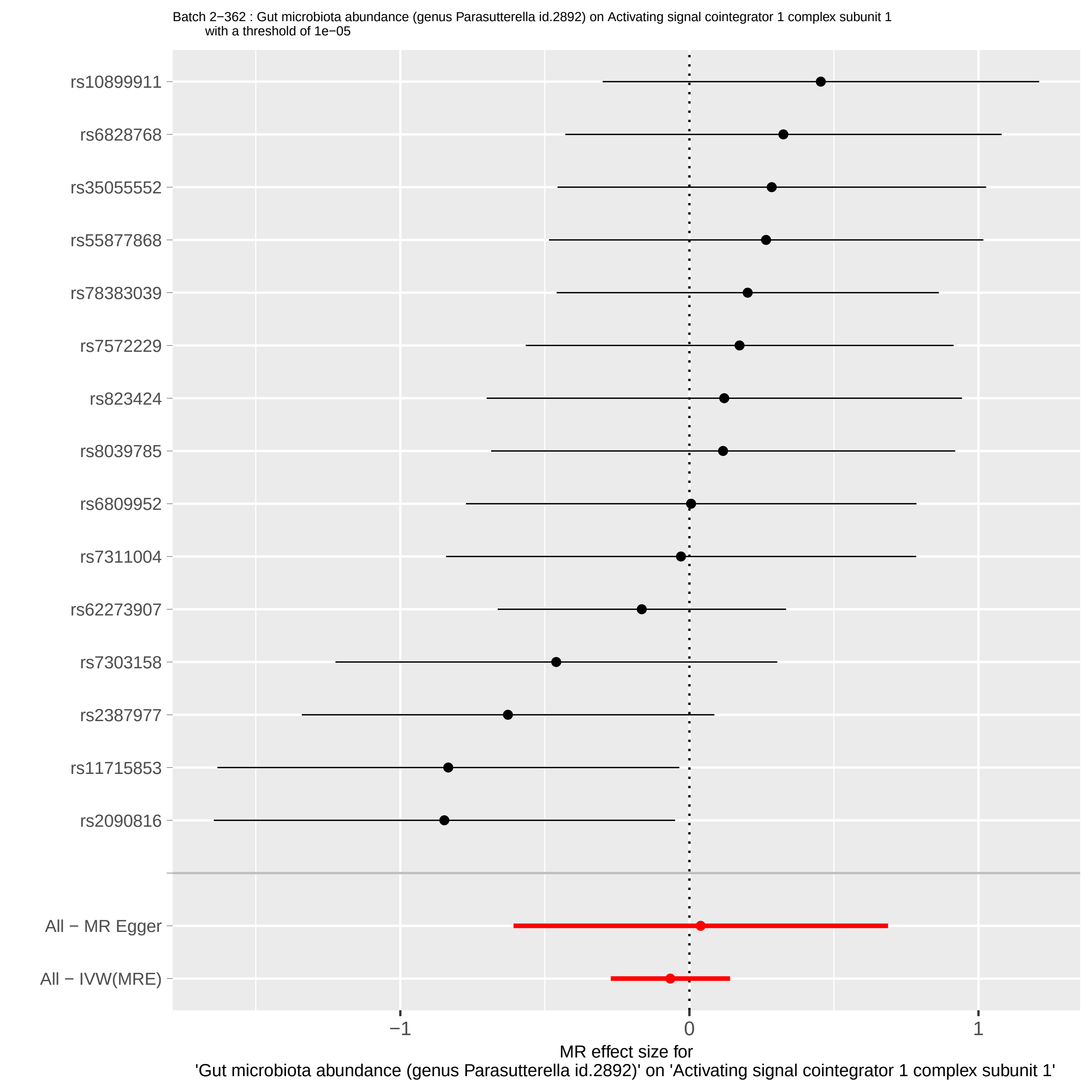
Batch 2−362 : Gut microbiota abundance (genus Parasutterella id.2892) on Activating signal cointegrator 1 complex subunit 1
with a threshold of 1e−05
rs10899911
rs6828768
rs35055552
rs55877868
rs78383039
rs7572229
rs823424
rs8039785
rs6809952
rs7311004
rs62273907
rs7303158
rs2387977
rs11715853
rs2090816
All − MR Egger
All − IVW(MRE)
−1
0
1
MR effect size for
'Gut microbiota abundance (genus Parasutterella id.2892)' on 'Activating signal cointegrator 1 complex subunit 1'

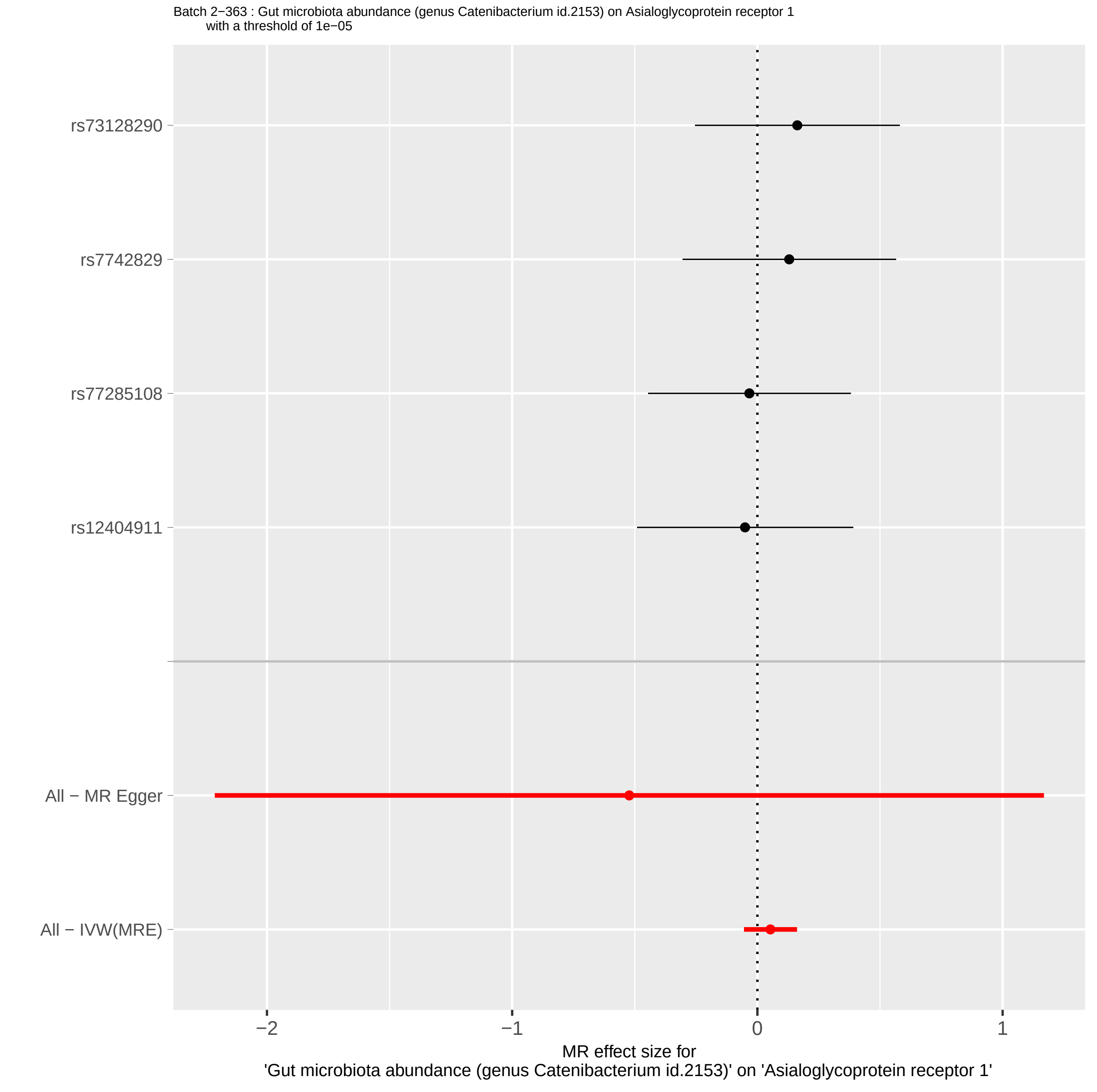

Batch 2−363 : Gut microbiota abundance (genus Catenibacterium id.2153) on Asialoglycoprotein receptor 1
with a threshold of 1e−05
rs73128290
rs7742829
rs77285108
rs12404911
All − MR Egger
All − IVW(MRE)
−2
−1
0
1
MR effect size for
'Gut microbiota abundance (genus Catenibacterium id.2153)' on 'Asialoglycoprotein receptor 1'

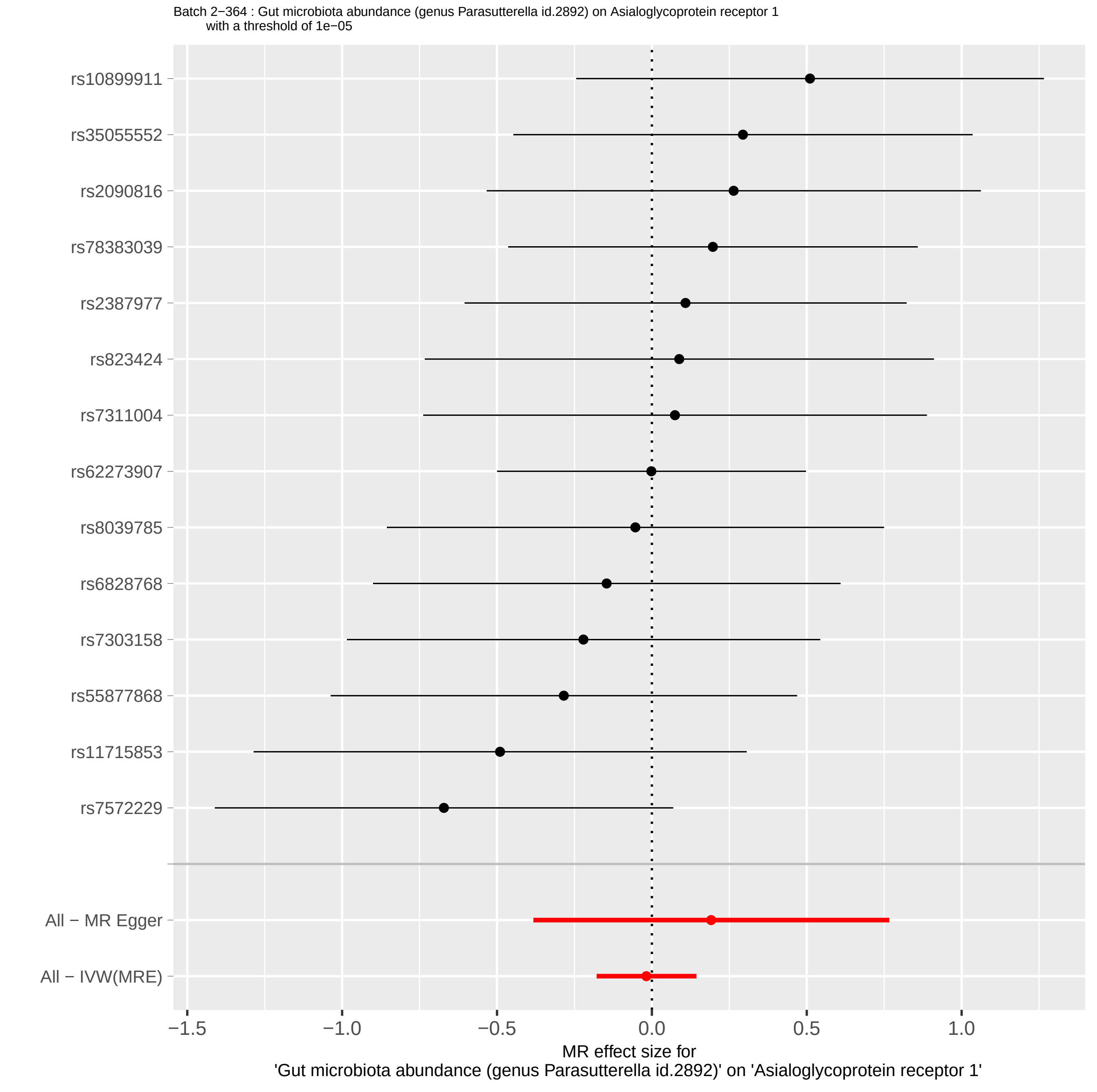
Batch 2−364 : Gut microbiota abundance (genus Parasutterella id.2892) on Asialoglycoprotein receptor 1
with a threshold of 1e−05
rs10899911
rs35055552
rs2090816
rs78383039
rs2387977
rs823424
rs7311004
rs62273907
rs8039785
rs6828768
rs7303158
rs55877868
rs11715853
rs7572229
All − MR Egger
All − IVW(MRE)
−1.5
−1.0
−0.5
0.0
0.5
1.0
MR effect size for
'Gut microbiota abundance (genus Parasutterella id.2892)' on 'Asialoglycoprotein receptor 1'

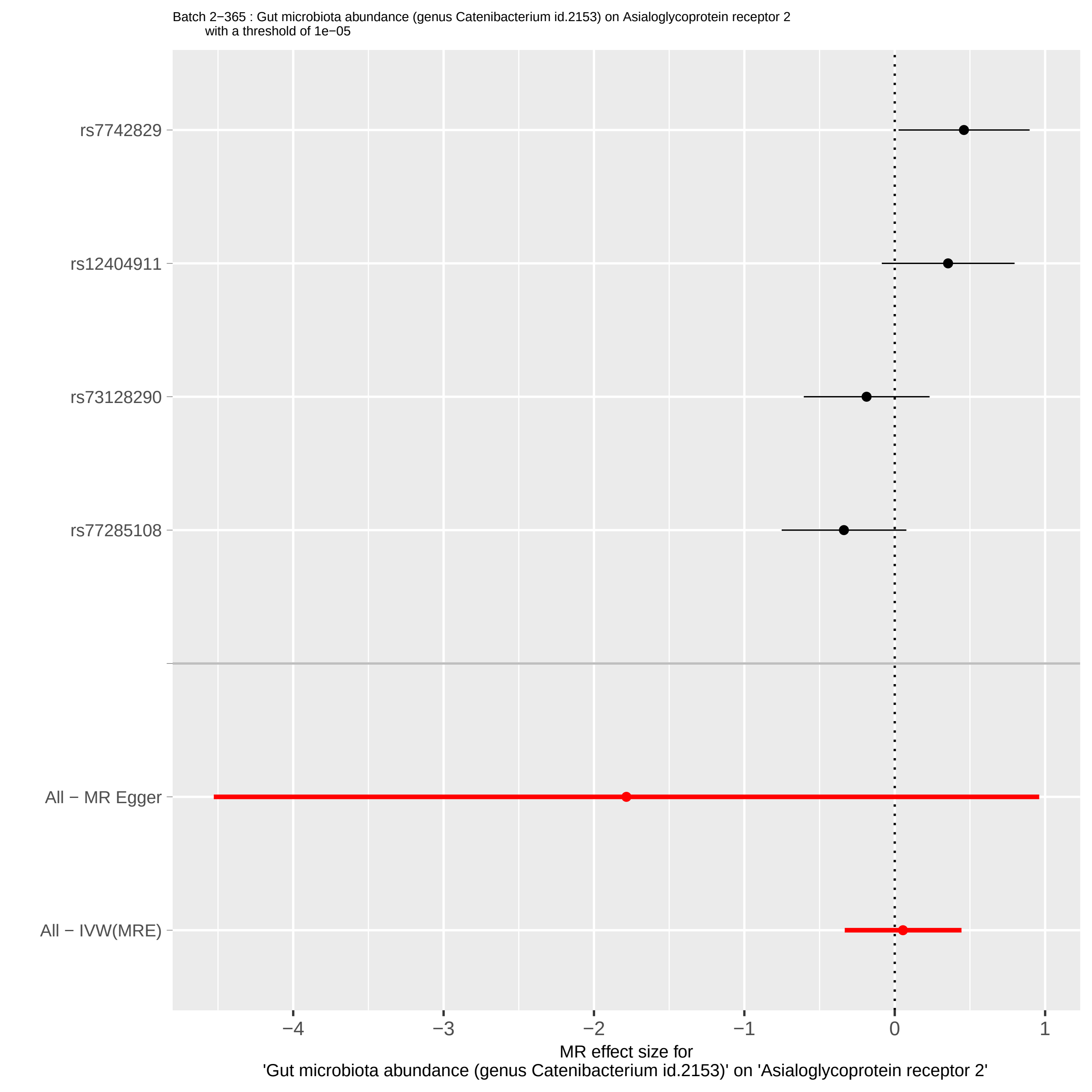

Batch 2−365 : Gut microbiota abundance (genus Catenibacterium id.2153) on Asialoglycoprotein receptor 2
with a threshold of 1e−05
rs7742829
rs12404911
rs73128290
rs77285108
All − MR Egger
All − IVW(MRE)
−4
−3
−2
−1
0
1
MR effect size for
'Gut microbiota abundance (genus Catenibacterium id.2153)' on 'Asialoglycoprotein receptor 2'

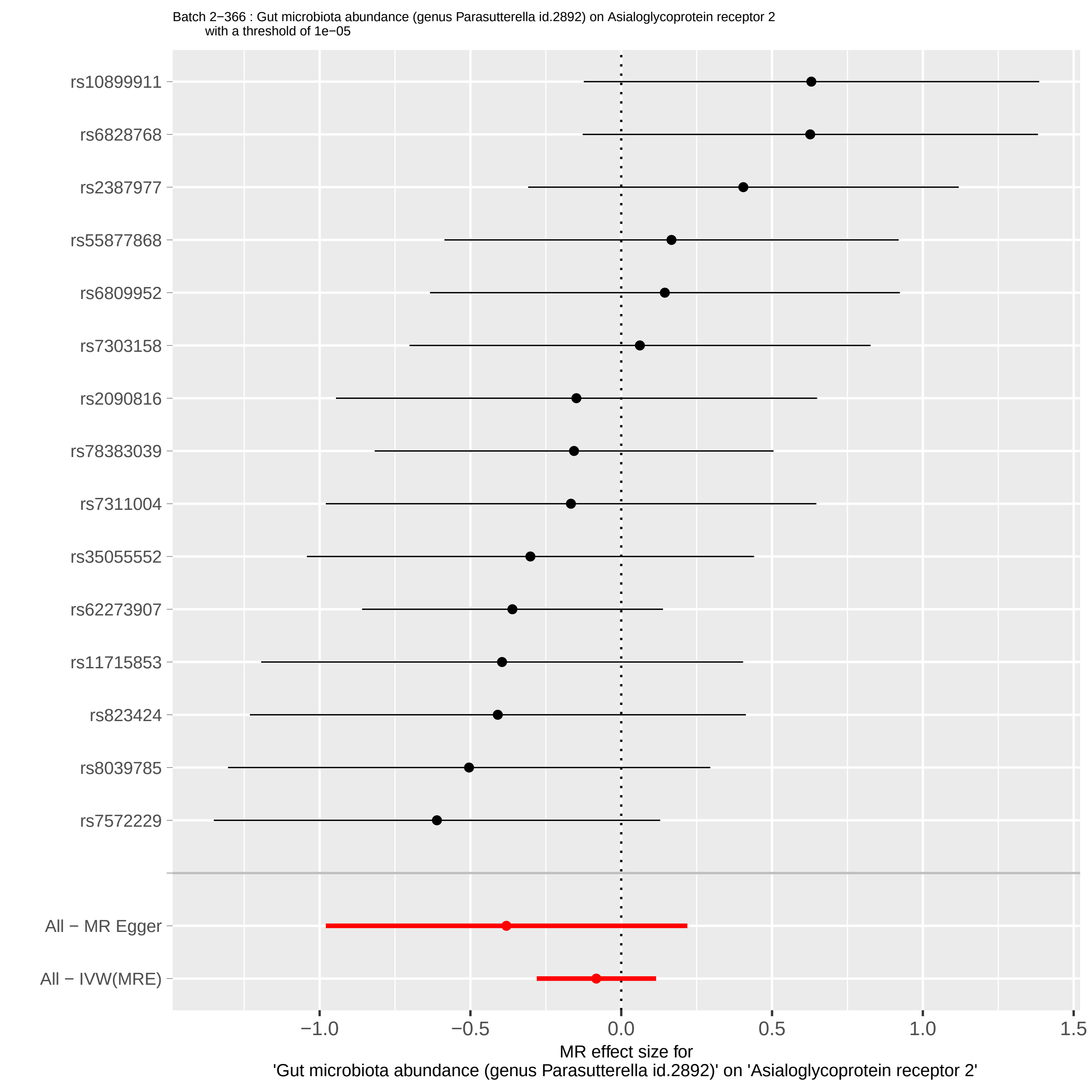

Batch 2−366 : Gut microbiota abundance (genus Parasutterella id.2892) on Asialoglycoprotein receptor 2
with a threshold of 1e−05
rs10899911
rs6828768
rs2387977
rs55877868
rs6809952
rs7303158
rs2090816
rs78383039
rs7311004
rs35055552
rs62273907
rs11715853
rs823424
rs8039785
rs7572229
All − MR Egger
All − IVW(MRE)
−1.0
−0.5
0.0
0.5
1.0
1.5
MR effect size for
'Gut microbiota abundance (genus Parasutterella id.2892)' on 'Asialoglycoprotein receptor 2'

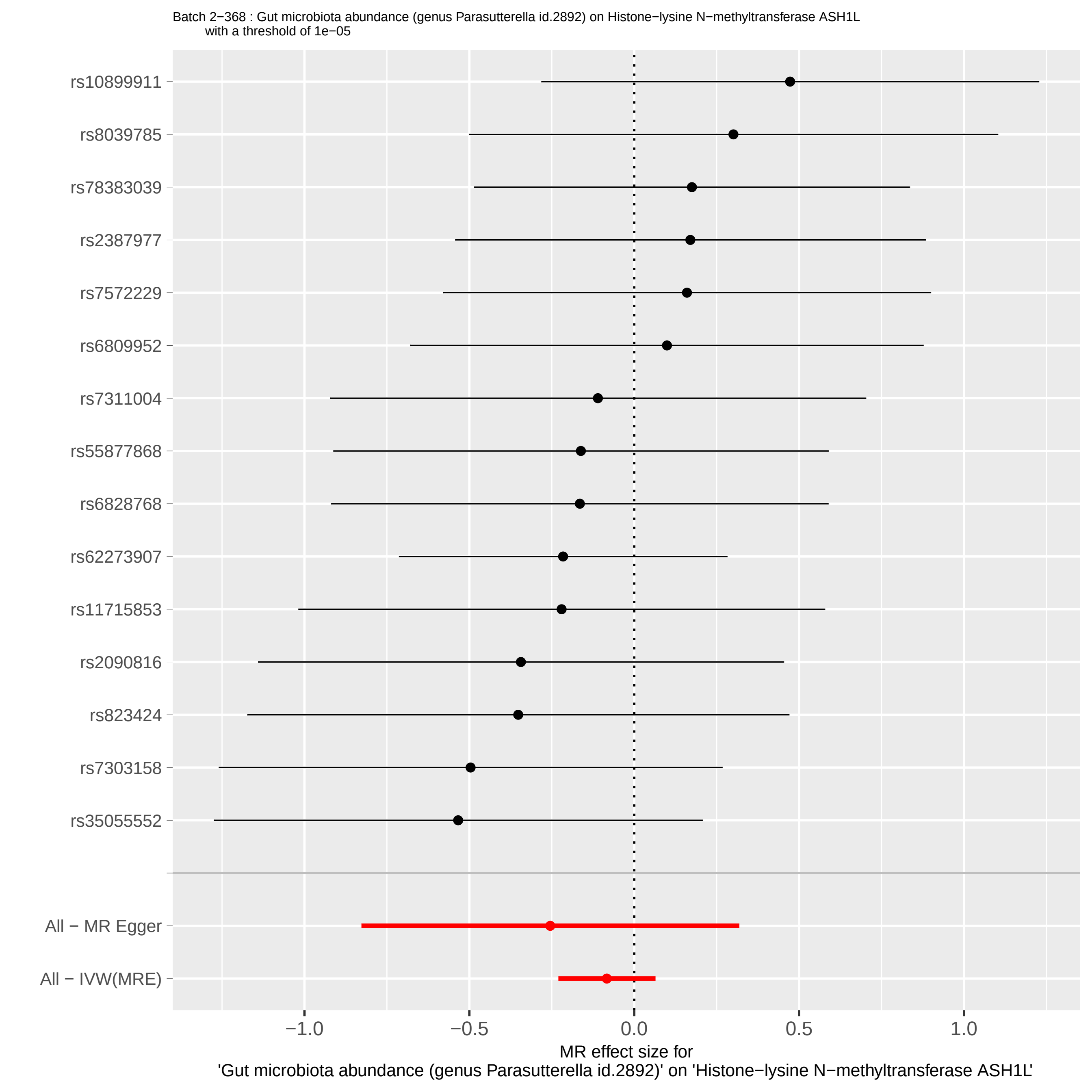

Batch 2−368 : Gut microbiota abundance (genus Parasutterella id.2892) on Histone−lysine N−methyltransferase ASH1L with a threshold of 1e−05
rs10899911
rs8039785
rs78383039
rs2387977
rs7572229
rs6809952
rs7311004
rs55877868
rs6828768
rs62273907
rs11715853
rs2090816
rs823424
rs7303158
rs35055552
All − MR Egger
All − IVW(MRE)
−1.0
−0.5
0.0
0.5
1.0
MR effect size for 'Gut microbiota abundance (genus Parasutterella id.2892)' on 'Histone−lysine N−methyltransferase ASH1L'

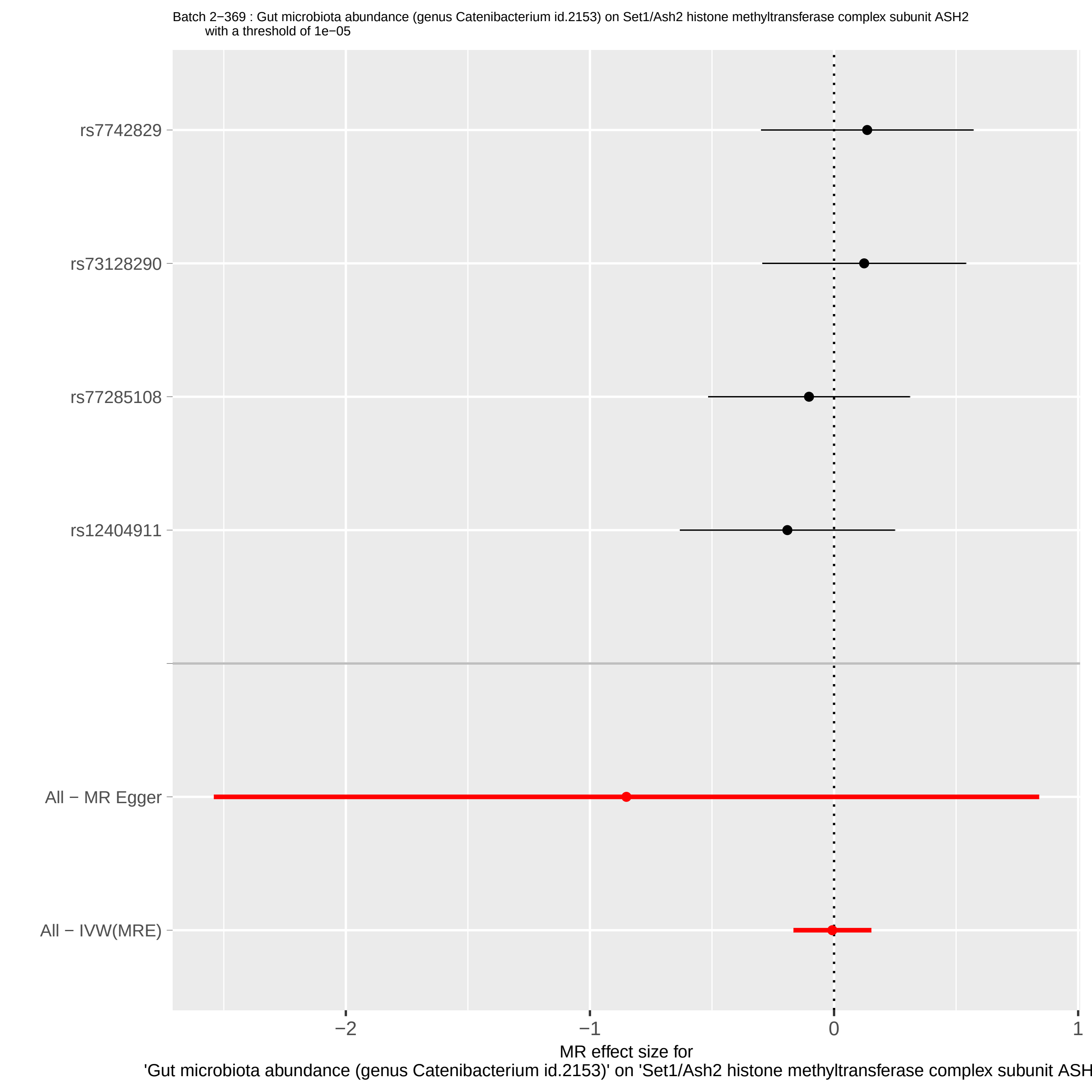

Batch 2−369 : Gut microbiota abundance (genus Catenibacterium id.2153) on Set1/Ash2 histone methyltransferase complex subunit ASH2
with a threshold of 1e−05
rs7742829
rs73128290
rs77285108
rs12404911
All − MR Egger
All − IVW(MRE)
−2
−1
0
1
MR effect size for
'Gut microbiota abundance (genus Catenibacterium id.2153)' on 'Set1/Ash2 histone methyltransferase complex subunit ASH

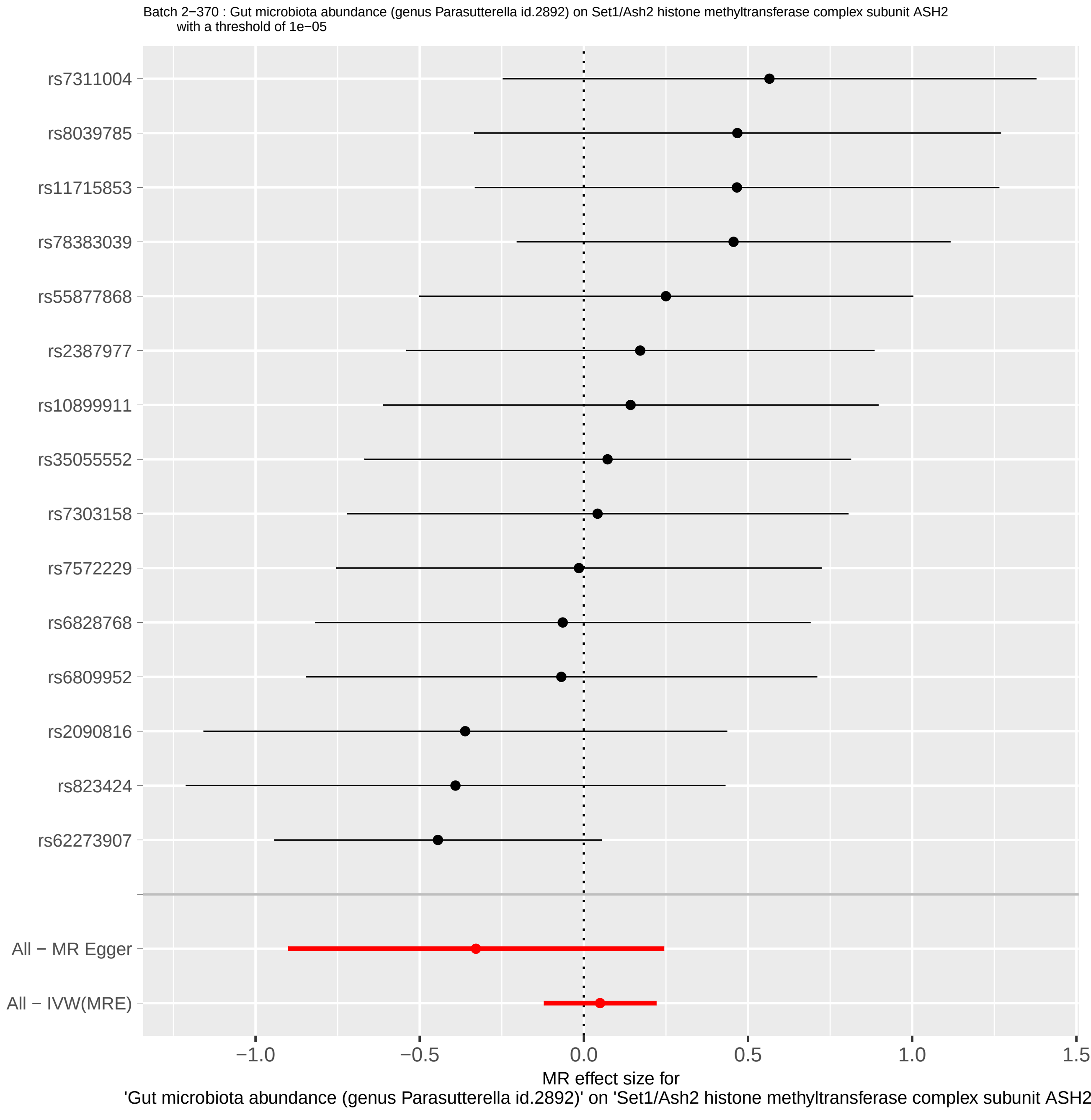
Batch 2−370 : Gut microbiota abundance (genus Parasutterella id.2892) on Set1/Ash2 histone methyltransferase complex subunit ASH2
with a threshold of 1e−05
rs7311004
rs8039785
rs11715853
rs78383039
rs55877868
rs2387977
rs10899911
rs35055552
rs7303158
rs7572229
rs6828768
rs6809952
rs2090816
rs823424
rs62273907
All − MR Egger
All − IVW(MRE)
−1.0
−0.5
0.0
0.5
1.0
1.5
MR effect size for
'Gut microbiota abundance (genus Parasutterella id.2892)' on 'Set1/Ash2 histone methyltransferase complex subunit ASH2

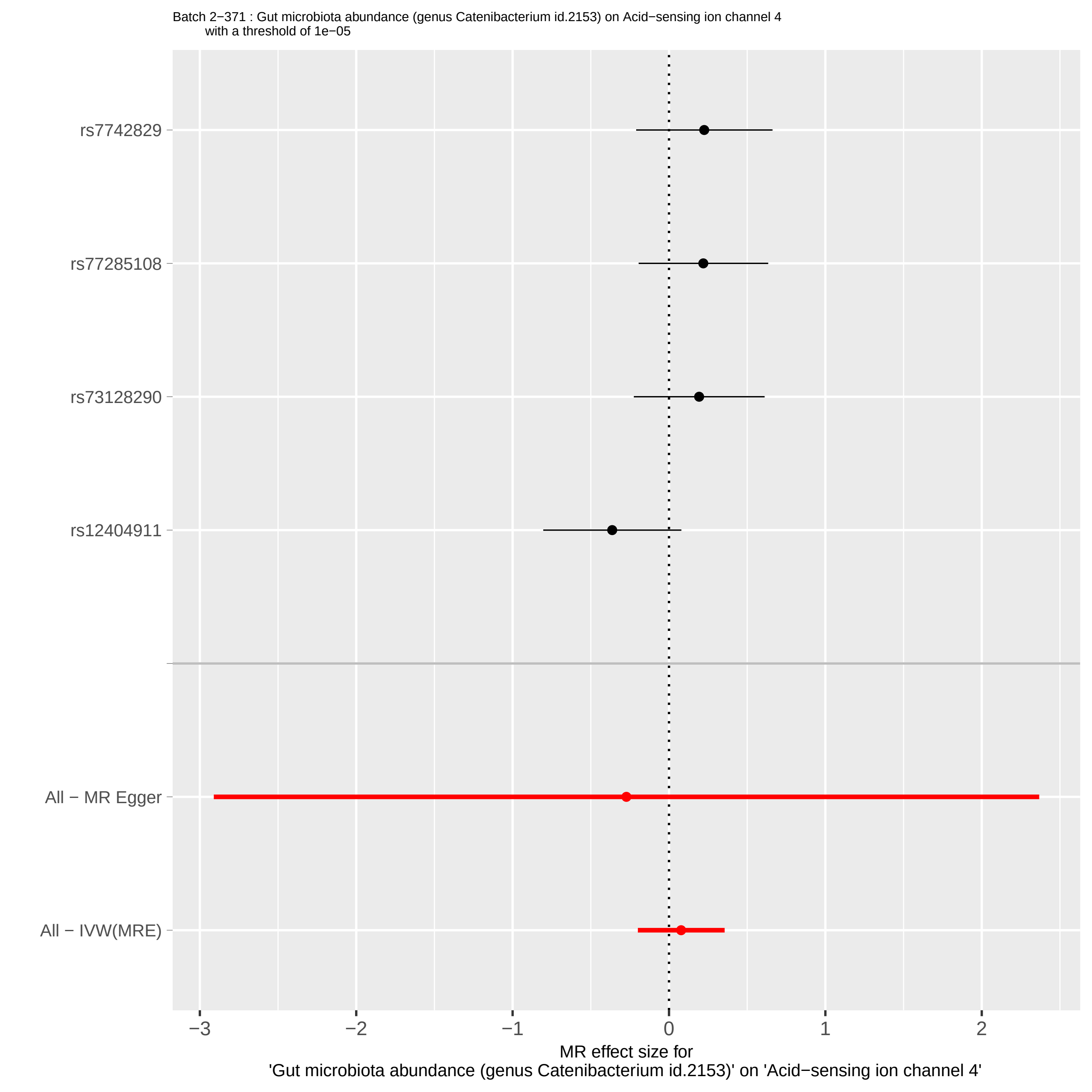

Batch 2−371 : Gut microbiota abundance (genus Catenibacterium id.2153) on Acid−sensing ion channel 4
with a threshold of 1e−05
rs7742829
rs77285108
rs73128290
rs12404911
All − MR Egger
All − IVW(MRE)
−3
−2
−1
0
1
2
MR effect size for
'Gut microbiota abundance (genus Catenibacterium id.2153)' on 'Acid−sensing ion channel 4'

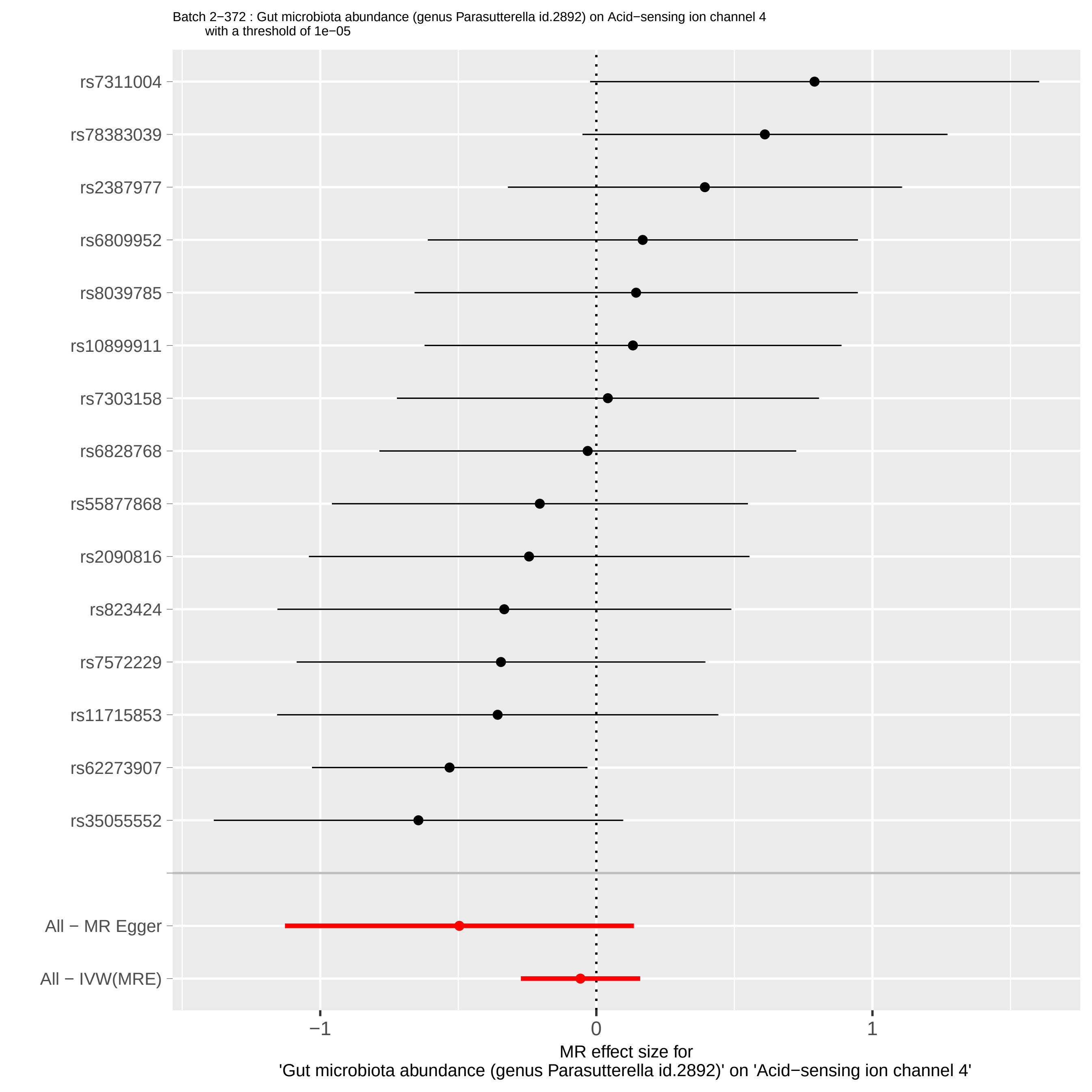

Batch 2−372 : Gut microbiota abundance (genus Parasutterella id.2892) on Acid−sensing ion channel 4
with a threshold of 1e−05
rs7311004
rs78383039
rs2387977
rs6809952
rs8039785
rs10899911
rs7303158
rs6828768
rs55877868
rs2090816
rs823424
rs7572229
rs11715853
rs62273907
rs35055552
All − MR Egger
All − IVW(MRE)
−1
0
1
MR effect size for
'Gut microbiota abundance (genus Parasutterella id.2892)' on 'Acid−sensing ion channel 4'

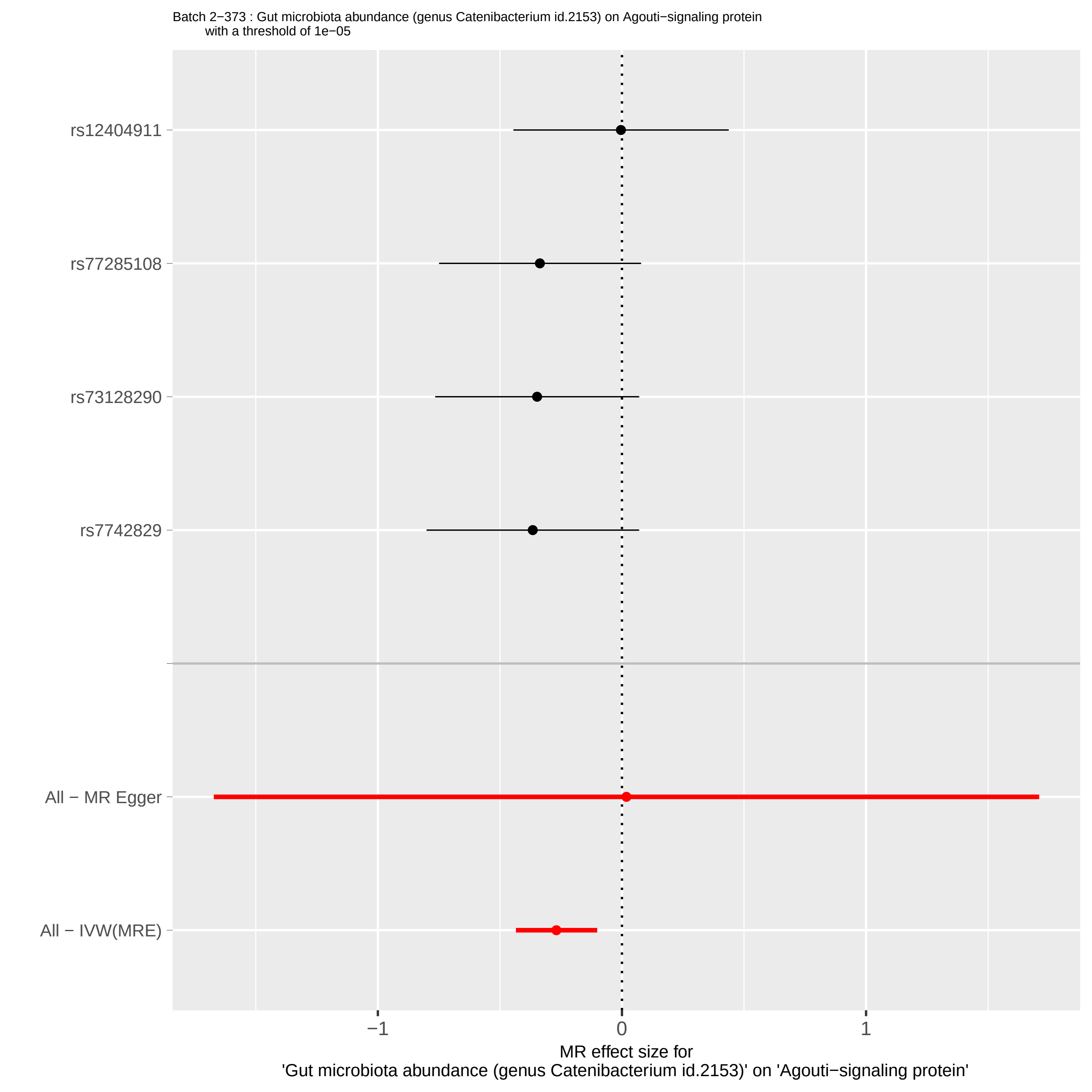

Batch 2−373 : Gut microbiota abundance (genus Catenibacterium id.2153) on Agouti−signaling protein
with a threshold of 1e−05
rs12404911
rs77285108
rs73128290
rs7742829
All − MR Egger
All − IVW(MRE)
−1
0
1
MR effect size for
'Gut microbiota abundance (genus Catenibacterium id.2153)' on 'Agouti−signaling protein'

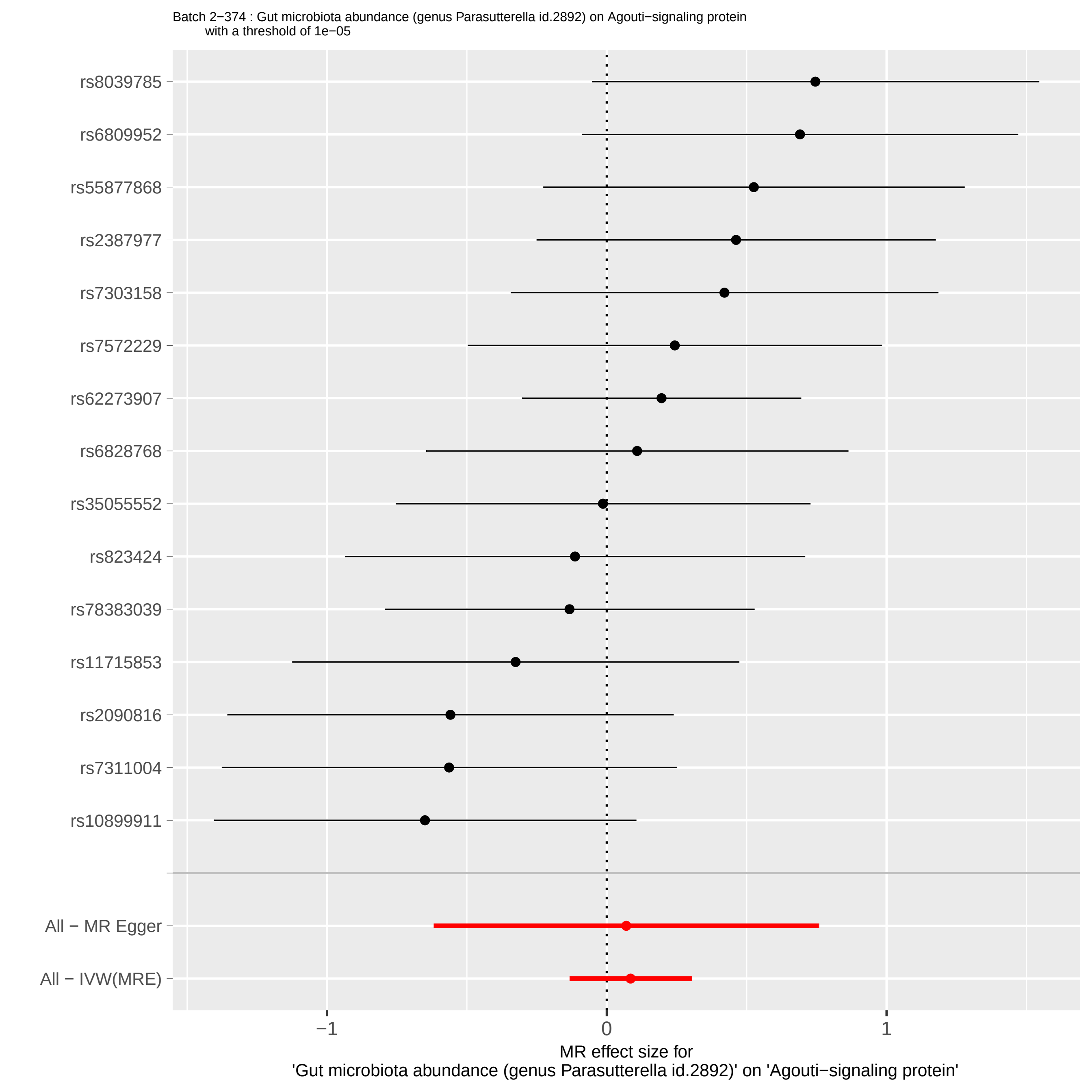

Batch 2−374 : Gut microbiota abundance (genus Parasutterella id.2892) on Agouti−signaling protein
with a threshold of 1e−05
rs8039785
rs6809952
rs55877868
rs2387977
rs7303158
rs7572229
rs62273907
rs6828768
rs35055552
rs823424
rs78383039
rs11715853
rs2090816
rs7311004
rs10899911
All − MR Egger
All − IVW(MRE)
−1
0
1
MR effect size for
'Gut microbiota abundance (genus Parasutterella id.2892)' on 'Agouti−signaling protein'

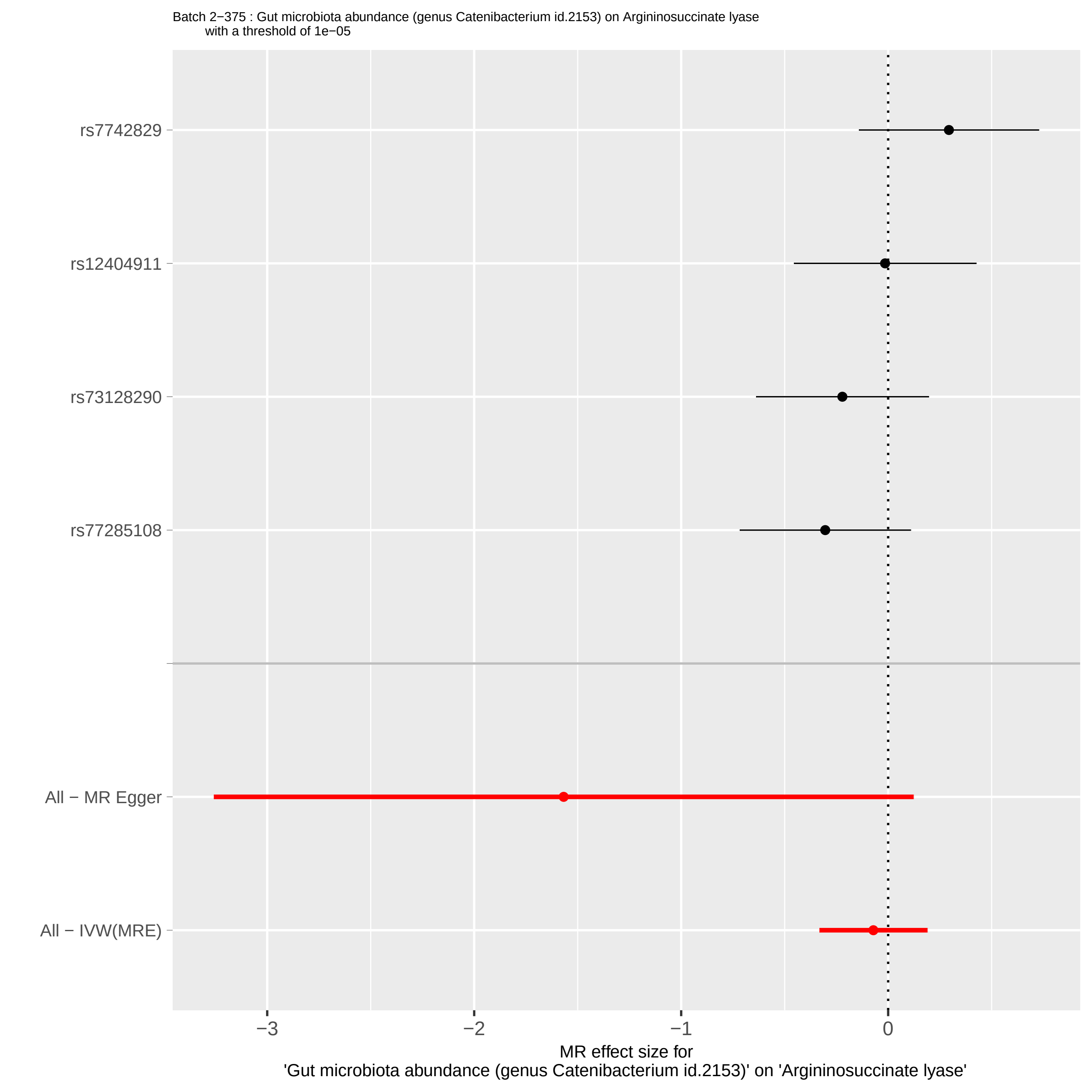
Batch 2−375 : Gut microbiota abundance (genus Catenibacterium id.2153) on Argininosuccinate lyase
with a threshold of 1e−05
rs7742829
rs12404911
rs73128290
rs77285108
All − MR Egger
All − IVW(MRE)
−3
−2
−1
0
MR effect size for
'Gut microbiota abundance (genus Catenibacterium id.2153)' on 'Argininosuccinate lyase'

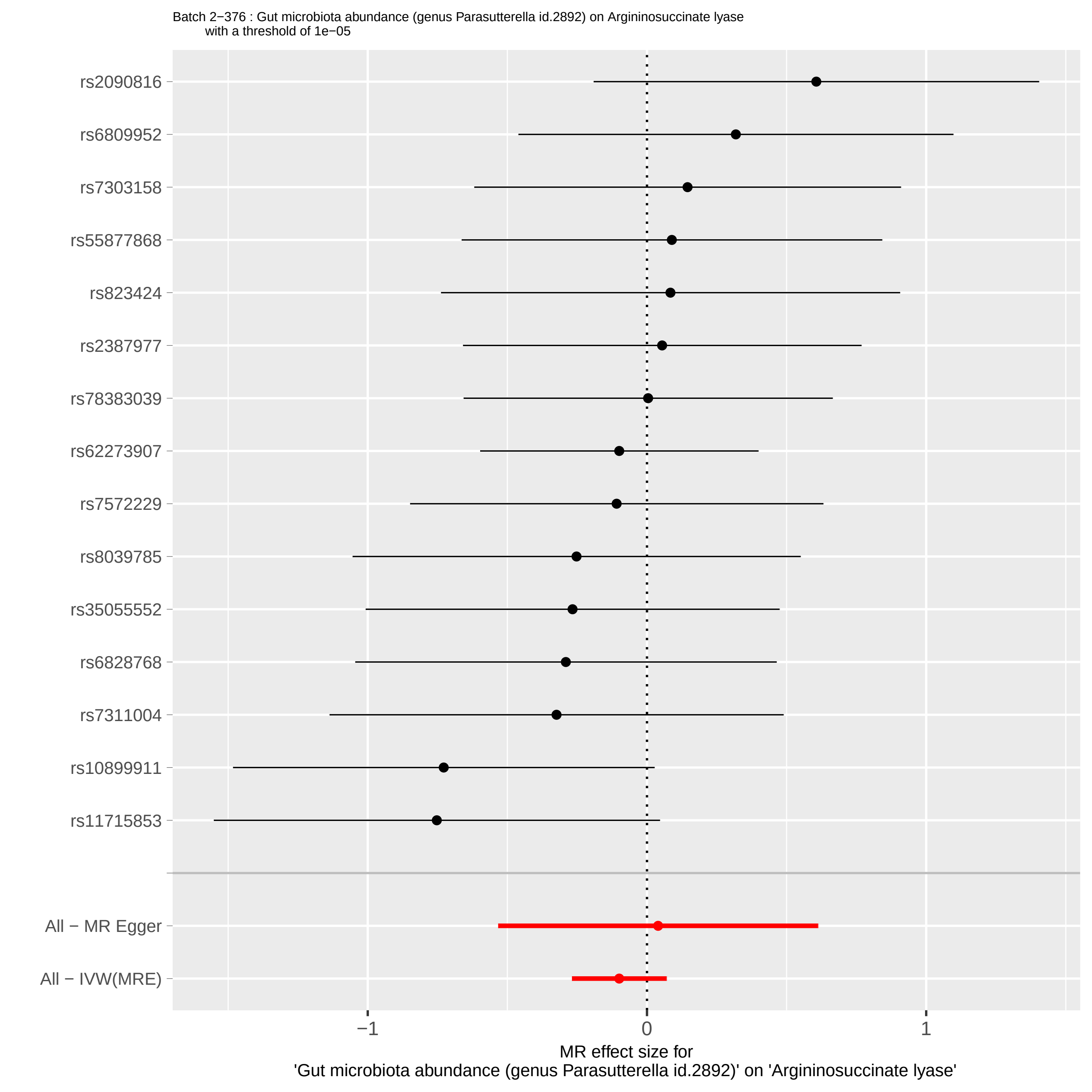

Batch 2−376 : Gut microbiota abundance (genus Parasutterella id.2892) on Argininosuccinate lyase
with a threshold of 1e−05
rs2090816
rs6809952
rs7303158
rs55877868
rs823424
rs2387977
rs78383039
rs62273907
rs7572229
rs8039785
rs35055552
rs6828768
rs7311004
rs10899911
rs11715853
All − MR Egger
All − IVW(MRE)
−1
0
1
MR effect size for
'Gut microbiota abundance (genus Parasutterella id.2892)' on 'Argininosuccinate lyase'

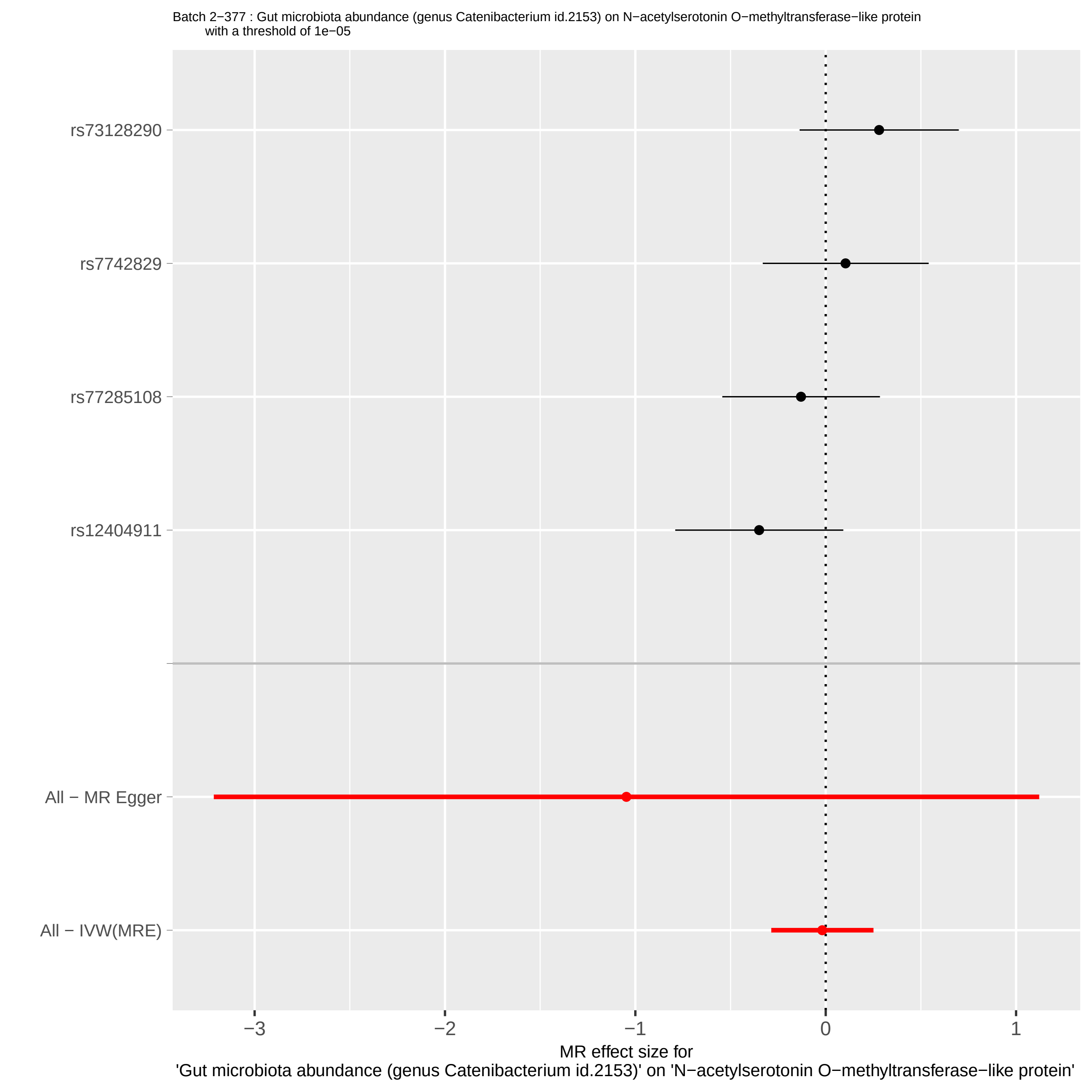

Batch 2−377 : Gut microbiota abundance (genus Catenibacterium id.2153) on N−acetylserotonin O−methyltransferase−like protein
with a threshold of 1e−05
rs73128290
rs7742829
rs77285108
rs12404911
All − MR Egger
All − IVW(MRE)
−3
−2
−1
0
1
MR effect size for
'Gut microbiota abundance (genus Catenibacterium id.2153)' on 'N−acetylserotonin O−methyltransferase−like protein'

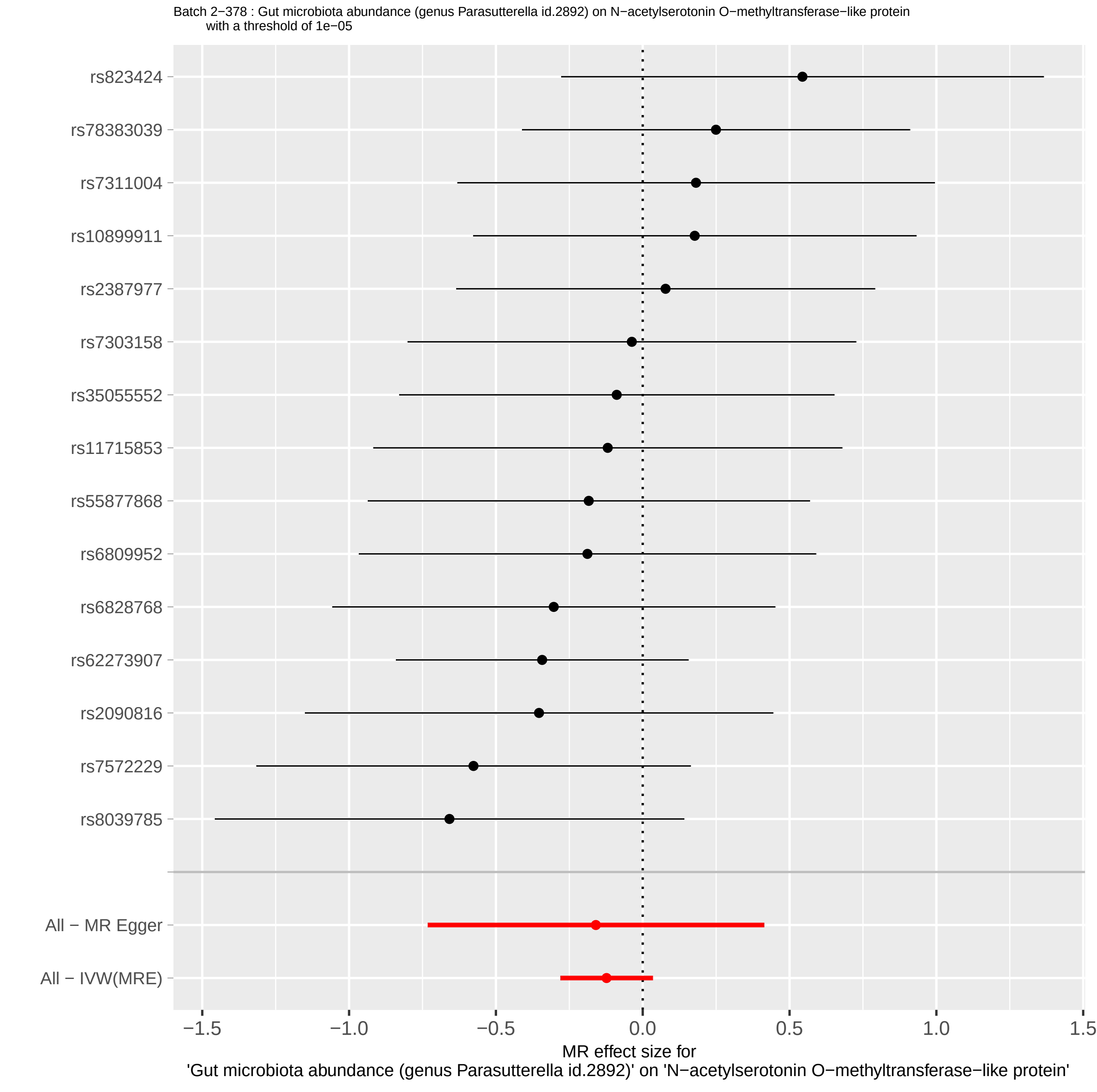

Batch 2−378 : Gut microbiota abundance (genus Parasutterella id.2892) on N−acetylserotonin O−methyltransferase−like protein
with a threshold of 1e−05
rs823424
rs78383039
rs7311004
rs10899911
rs2387977
rs7303158
rs35055552
rs11715853
rs55877868
rs6809952
rs6828768
rs62273907
rs2090816
rs7572229
rs8039785
All − MR Egger
All − IVW(MRE)
−1.5
−1.0
−0.5
0.0
0.5
1.0
1.5
MR effect size for
'Gut microbiota abundance (genus Parasutterella id.2892)' on 'N−acetylserotonin O−methyltransferase−like protein'

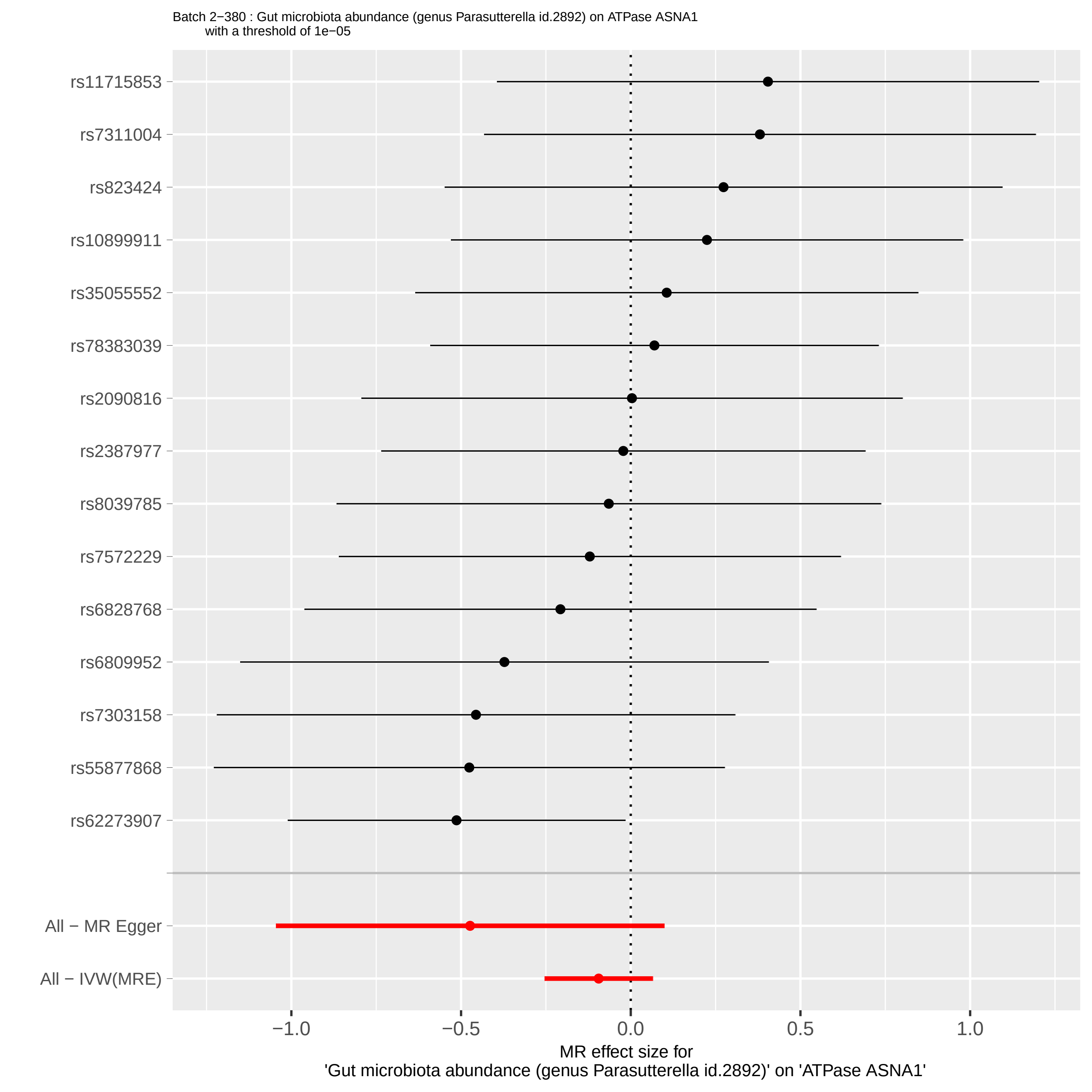

Batch 2−380 : Gut microbiota abundance (genus Parasutterella id.2892) on ATPase ASNA1
with a threshold of 1e−05
rs11715853
rs7311004
rs823424
rs10899911
rs35055552
rs78383039
rs2090816
rs2387977
rs8039785
rs7572229
rs6828768
rs6809952
rs7303158
rs55877868
rs62273907
All − MR Egger
All − IVW(MRE)
−1.0
−0.5
0.0
0.5
1.0
MR effect size for
'Gut microbiota abundance (genus Parasutterella id.2892)' on 'ATPase ASNA1'

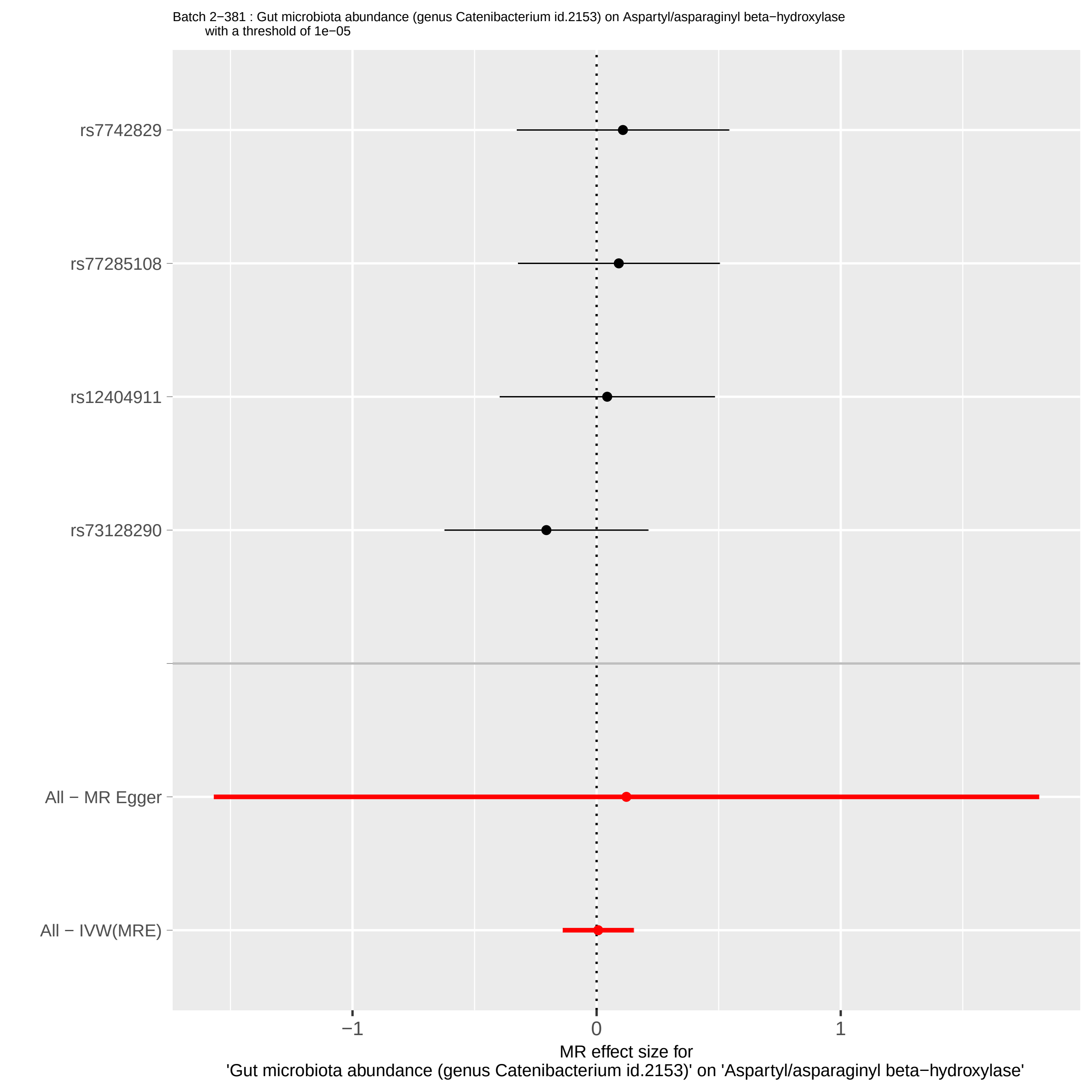

Batch 2−381 : Gut microbiota abundance (genus Catenibacterium id.2153) on Aspartyl/asparaginyl beta−hydroxylase
with a threshold of 1e−05
rs7742829
rs77285108
rs12404911
rs73128290
All − MR Egger
All − IVW(MRE)
−1
0
1
MR effect size for
'Gut microbiota abundance (genus Catenibacterium id.2153)' on 'Aspartyl/asparaginyl beta−hydroxylase'

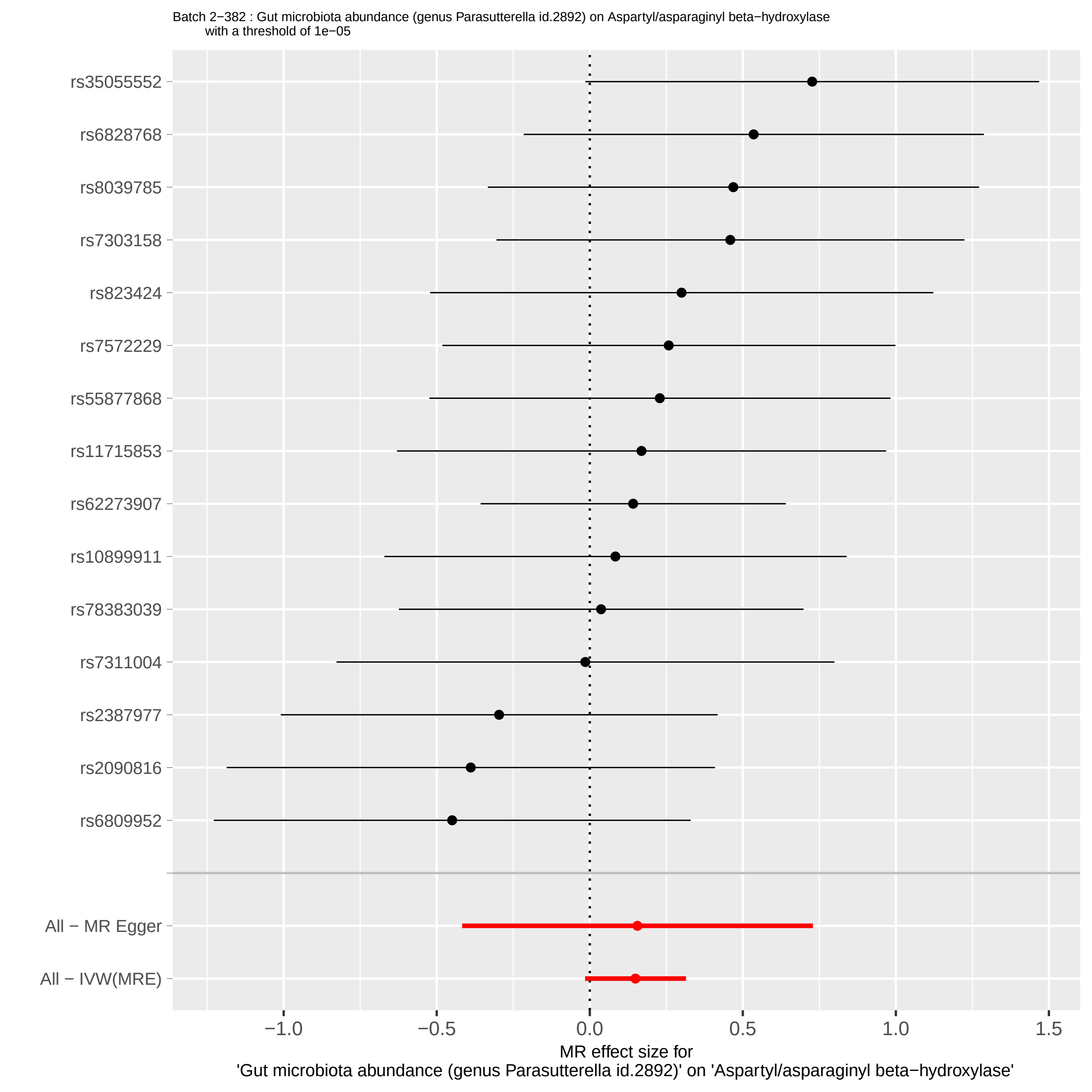

Batch 2−382 : Gut microbiota abundance (genus Parasutterella id.2892) on Aspartyl/asparaginyl beta−hydroxylase
with a threshold of 1e−05
rs35055552
rs6828768
rs8039785
rs7303158
rs823424
rs7572229
rs55877868
rs11715853
rs62273907
rs10899911
rs78383039
rs7311004
rs2387977
rs2090816
rs6809952
All − MR Egger
All − IVW(MRE)
−1.0
−0.5
0.0
0.5
1.0
1.5
MR effect size for
'Gut microbiota abundance (genus Parasutterella id.2892)' on 'Aspartyl/asparaginyl beta−hydroxylase'

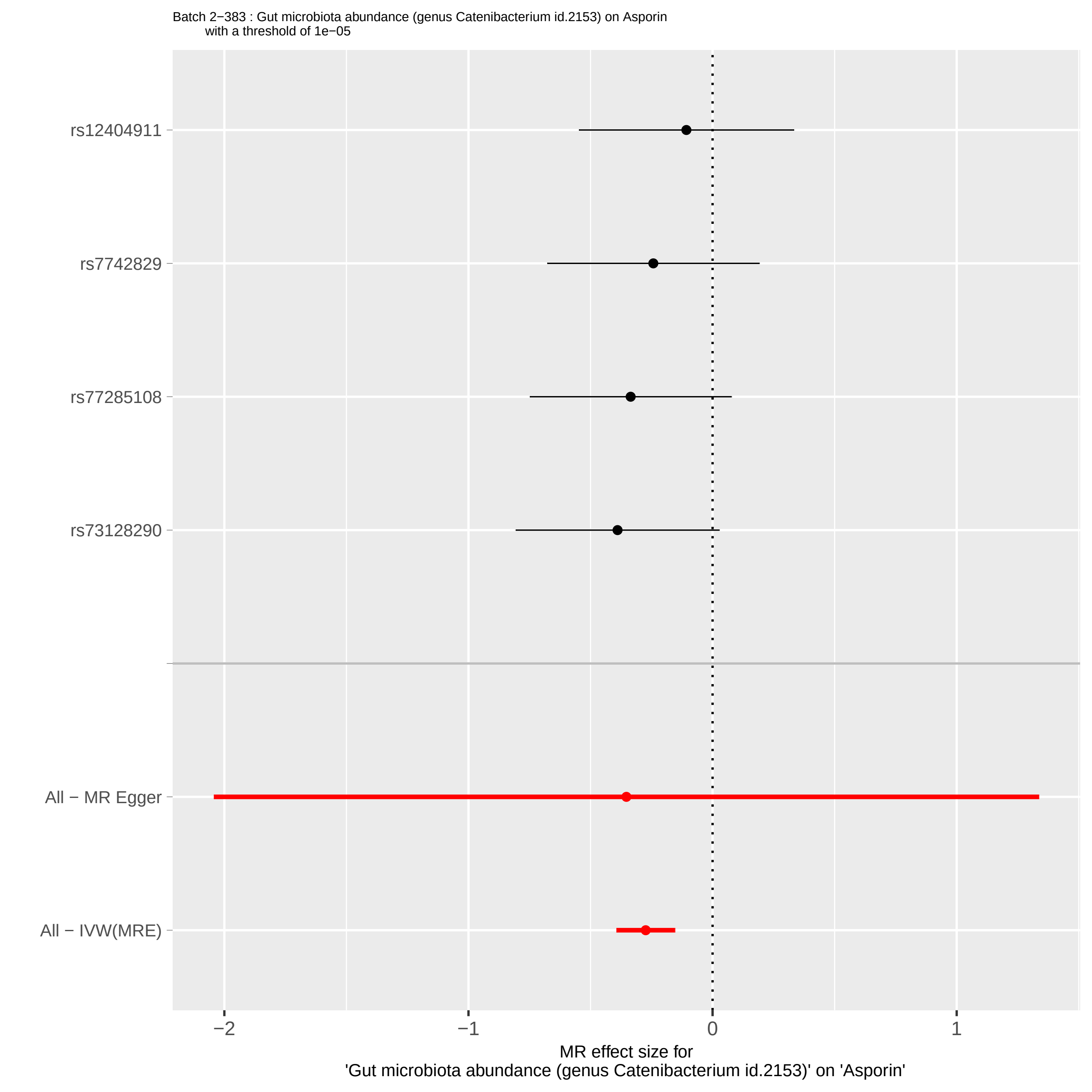

Batch 2−383 : Gut microbiota abundance (genus Catenibacterium id.2153) on Asporin
with a threshold of 1e−05
rs12404911
rs7742829
rs77285108
rs73128290
All − MR Egger
All − IVW(MRE)
−2
−1
0
1
MR effect size for
'Gut microbiota abundance (genus Catenibacterium id.2153)' on 'Asporin'

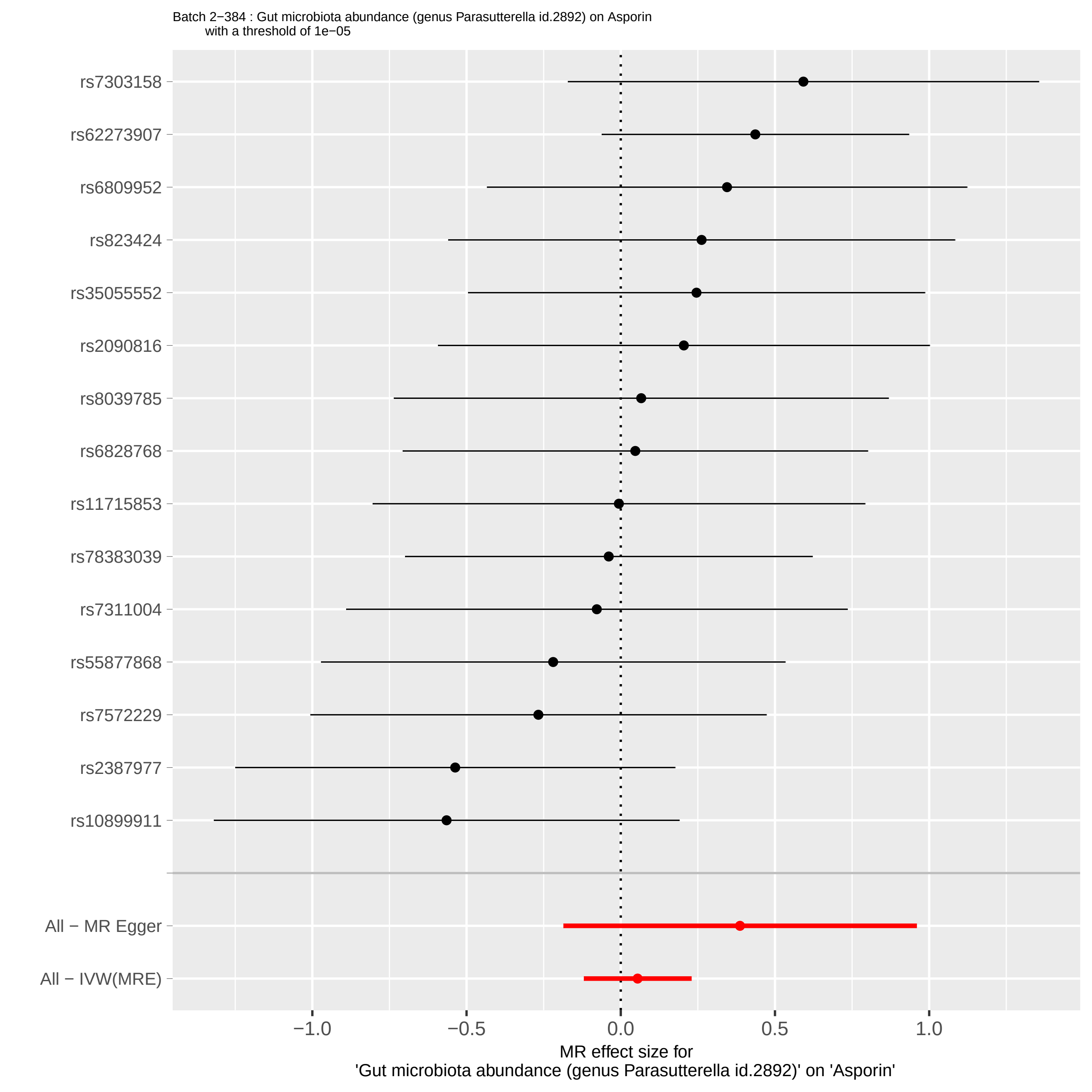

Batch 2−384 : Gut microbiota abundance (genus Parasutterella id.2892) on Asporin
with a threshold of 1e−05
rs7303158
rs62273907
rs6809952
rs823424
rs35055552
rs2090816
rs8039785
rs6828768
rs11715853
rs78383039
rs7311004
rs55877868
rs7572229
rs2387977
rs10899911
All − MR Egger
All − IVW(MRE)
−1.0
−0.5
0.0
0.5
1.0
MR effect size for
'Gut microbiota abundance (genus Parasutterella id.2892)' on 'Asporin'

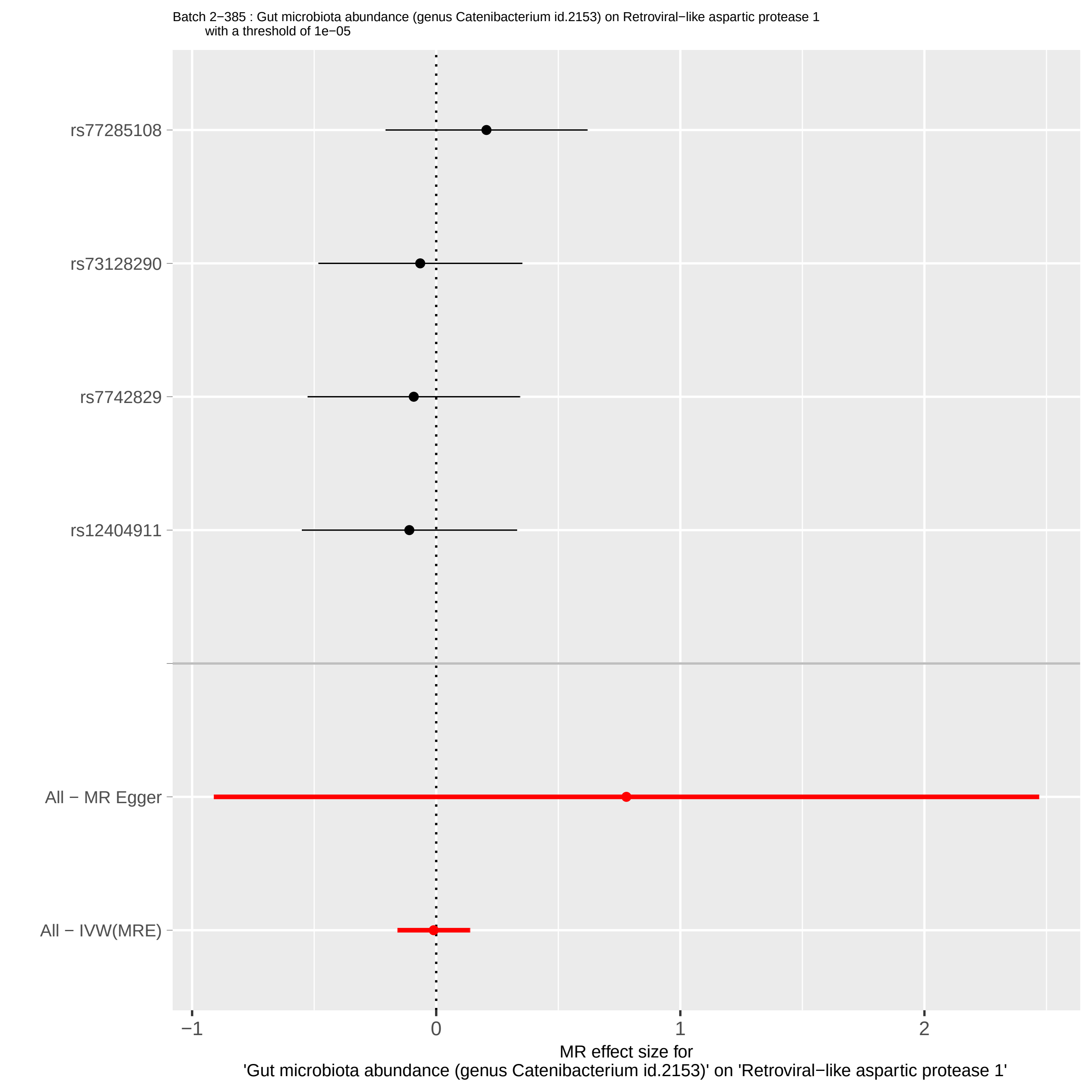
Batch 2−385 : Gut microbiota abundance (genus Catenibacterium id.2153) on Retroviral−like aspartic protease 1
with a threshold of 1e−05
rs77285108
rs73128290
rs7742829
rs12404911
All − MR Egger
All − IVW(MRE)
−1
0
1
2
MR effect size for
'Gut microbiota abundance (genus Catenibacterium id.2153)' on 'Retroviral−like aspartic protease 1'

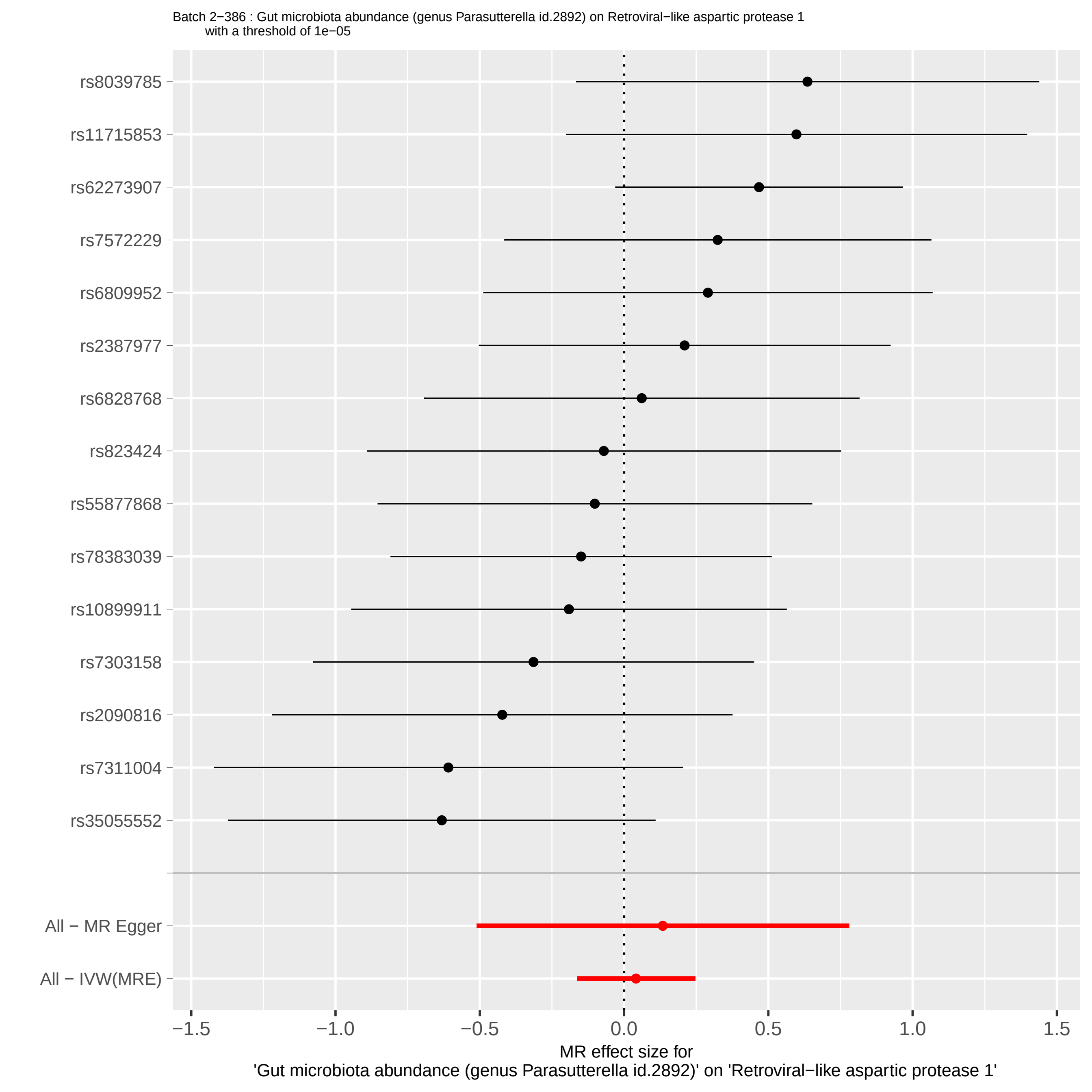

Batch 2−386 : Gut microbiota abundance (genus Parasutterella id.2892) on Retroviral−like aspartic protease 1
with a threshold of 1e−05
rs8039785
rs11715853
rs62273907
rs7572229
rs6809952
rs2387977
rs6828768
rs823424
rs55877868
rs78383039
rs10899911
rs7303158
rs2090816
rs7311004
rs35055552
All − MR Egger
All − IVW(MRE)
−1.5
−1.0
−0.5
0.0
0.5
1.0
1.5
MR effect size for
'Gut microbiota abundance (genus Parasutterella id.2892)' on 'Retroviral−like aspartic protease 1'

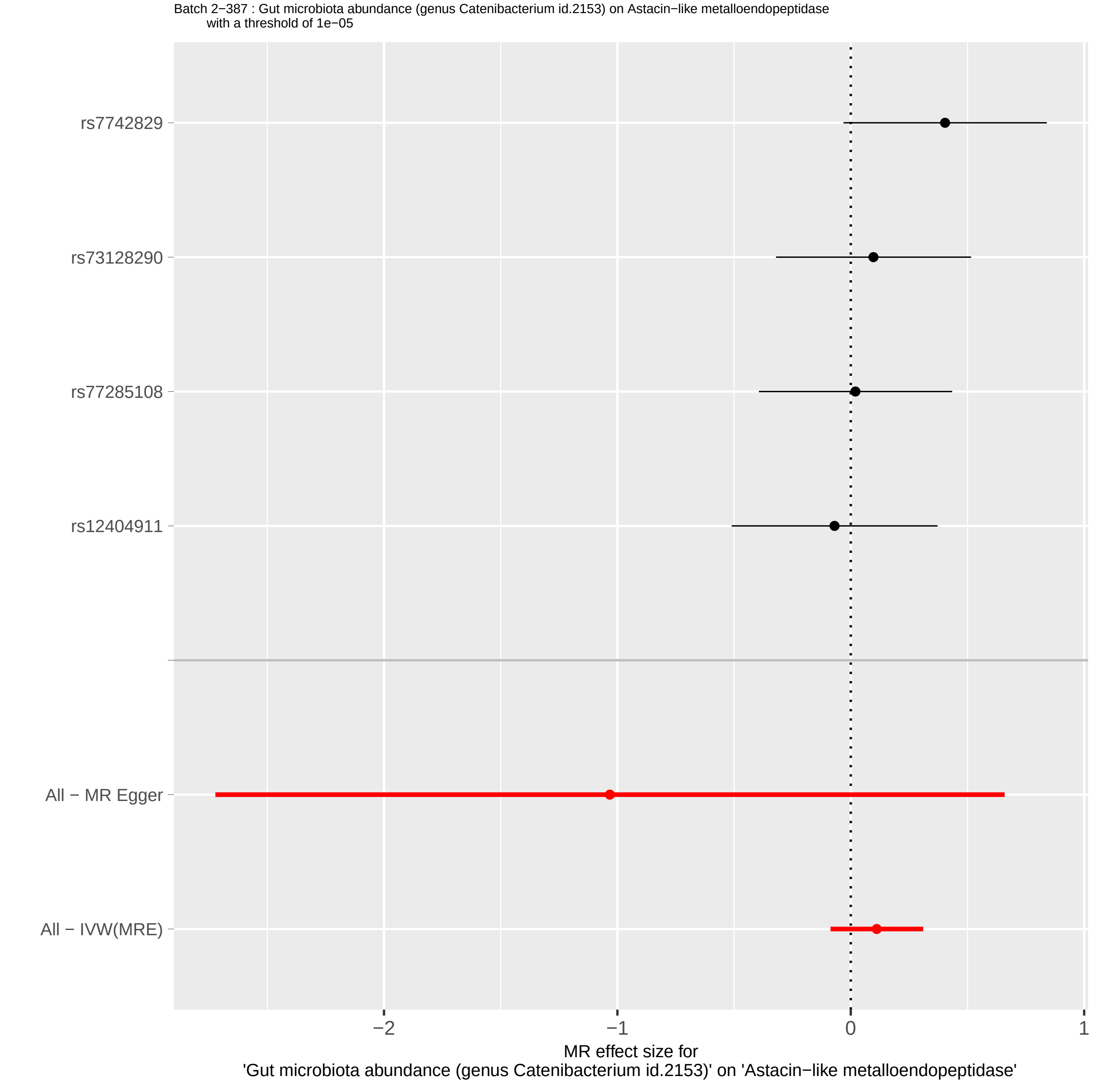

Batch 2−387 : Gut microbiota abundance (genus Catenibacterium id.2153) on Astacin−like metalloendopeptidase
with a threshold of 1e−05
rs7742829
rs73128290
rs77285108
rs12404911
All − MR Egger
All − IVW(MRE)
−2
−1
0
1
MR effect size for
'Gut microbiota abundance (genus Catenibacterium id.2153)' on 'Astacin−like metalloendopeptidase'

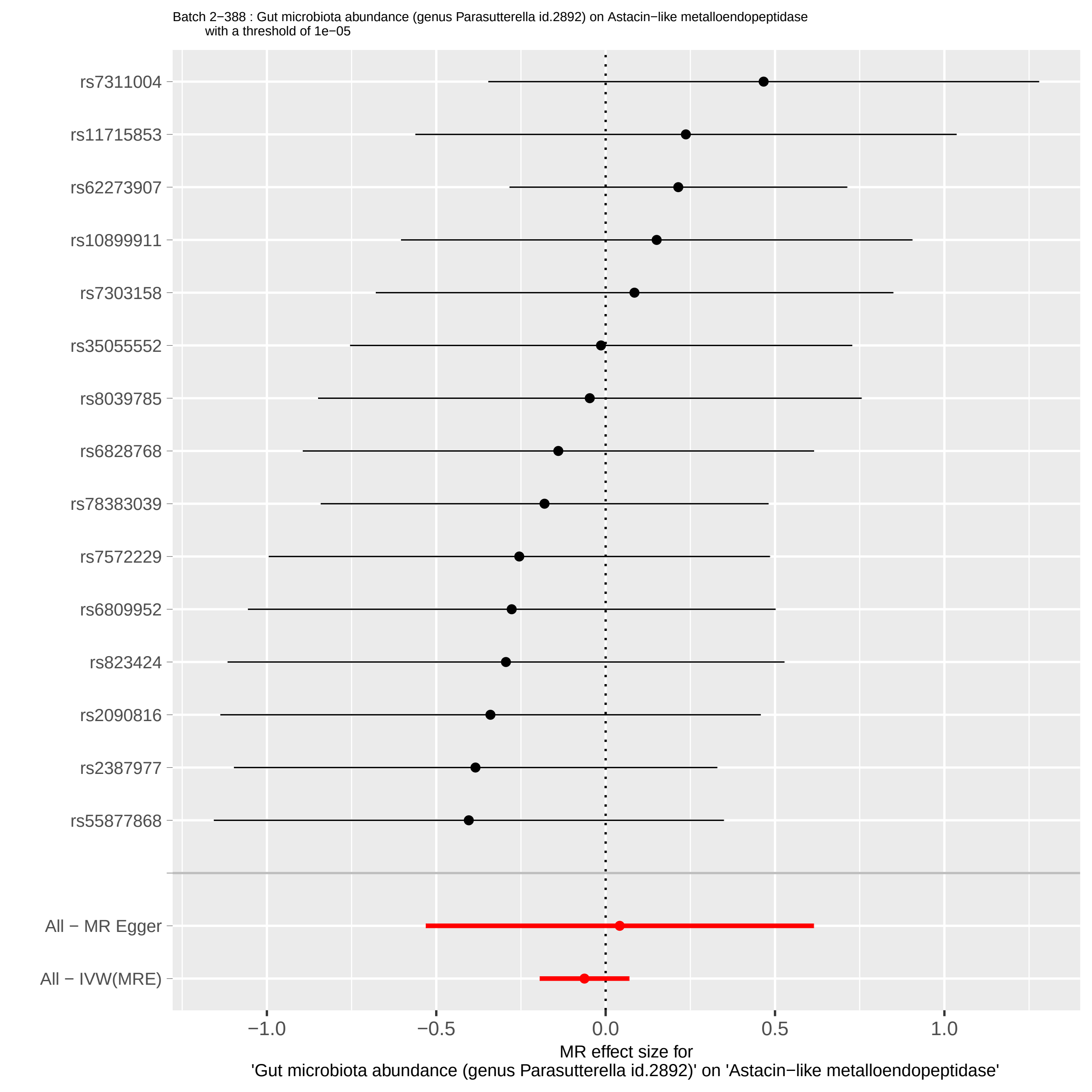

Batch 2−388 : Gut microbiota abundance (genus Parasutterella id.2892) on Astacin−like metalloendopeptidase
with a threshold of 1e−05
rs7311004
rs11715853
rs62273907
rs10899911
rs7303158
rs35055552
rs8039785
rs6828768
rs78383039
rs7572229
rs6809952
rs823424
rs2090816
rs2387977
rs55877868
All − MR Egger
All − IVW(MRE)
−1.0
−0.5
0.0
0.5
1.0
MR effect size for
'Gut microbiota abundance (genus Parasutterella id.2892)' on 'Astacin−like metalloendopeptidase'

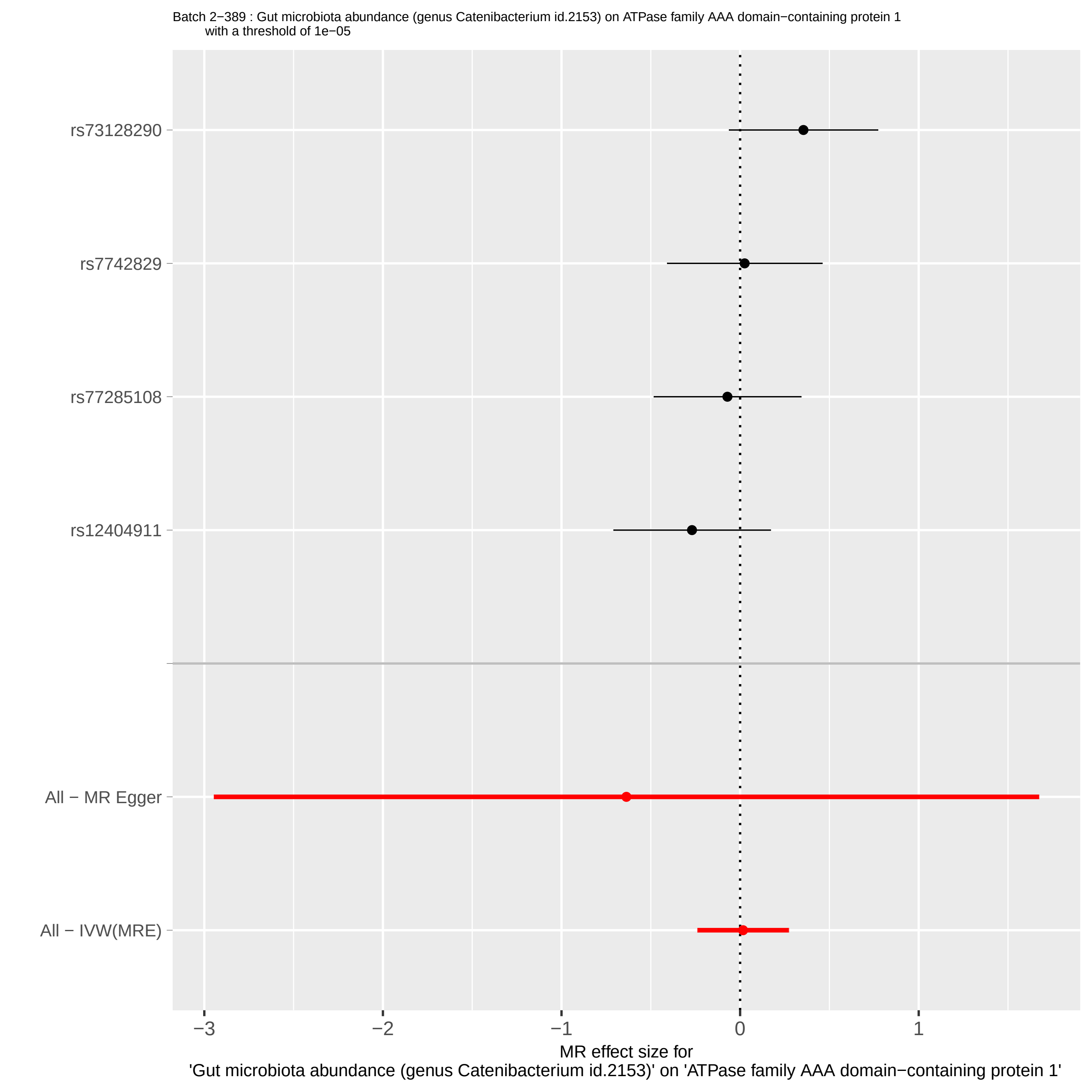

Batch 2−389 : Gut microbiota abundance (genus Catenibacterium id.2153) on ATPase family AAA domain−containing protein 1
with a threshold of 1e−05
rs73128290
rs7742829
rs77285108
rs12404911
All − MR Egger
All − IVW(MRE)
−3
−2
−1
0
1
MR effect size for
'Gut microbiota abundance (genus Catenibacterium id.2153)' on 'ATPase family AAA domain−containing protein 1'

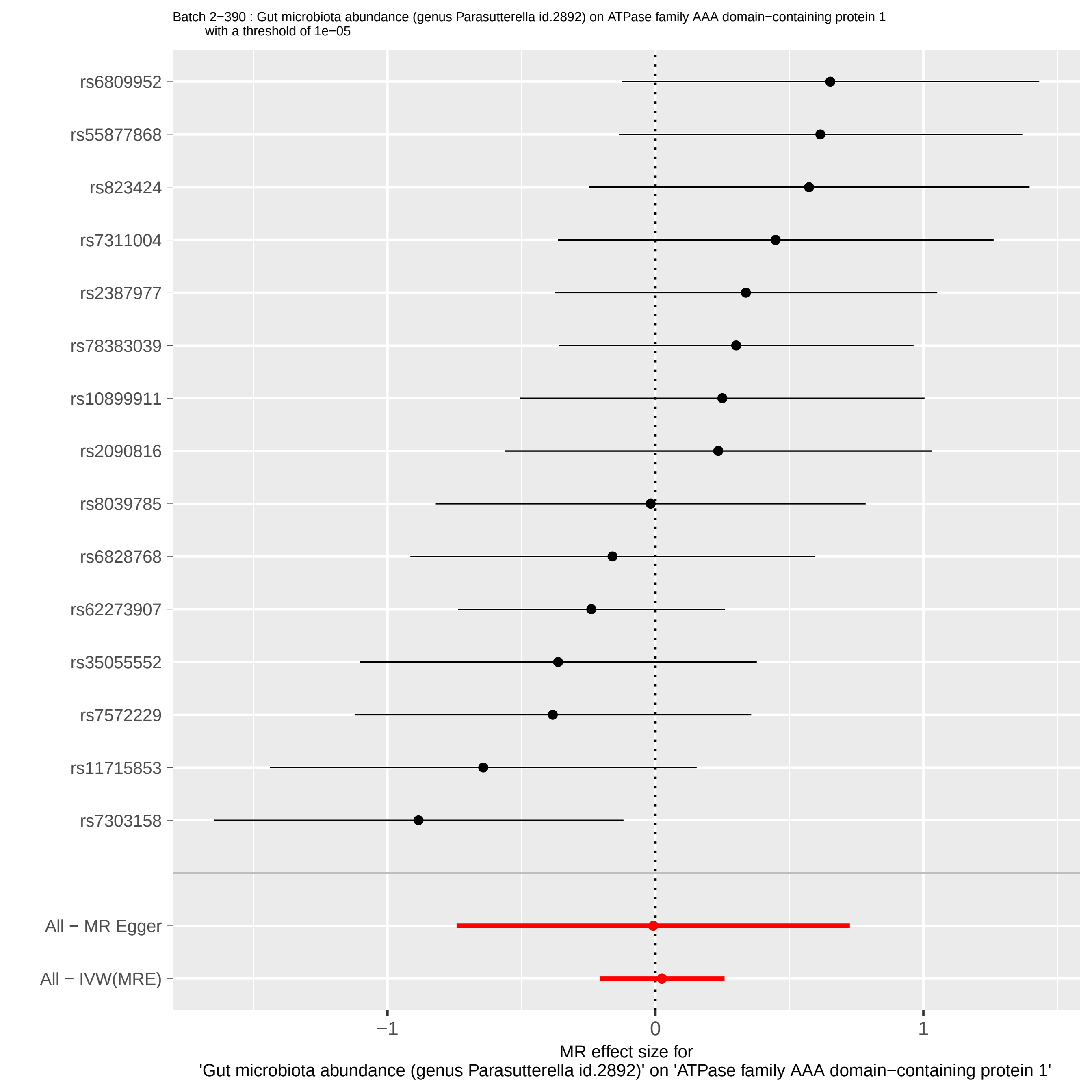

Batch 2−390 : Gut microbiota abundance (genus Parasutterella id.2892) on ATPase family AAA domain−containing protein 1
with a threshold of 1e−05
rs6809952
rs55877868
rs823424
rs7311004
rs2387977
rs78383039
rs10899911
rs2090816
rs8039785
rs6828768
rs62273907
rs35055552
rs7572229
rs11715853
rs7303158
All − MR Egger
All − IVW(MRE)
−1
0
1
MR effect size for
'Gut microbiota abundance (genus Parasutterella id.2892)' on 'ATPase family AAA domain−containing protein 1'

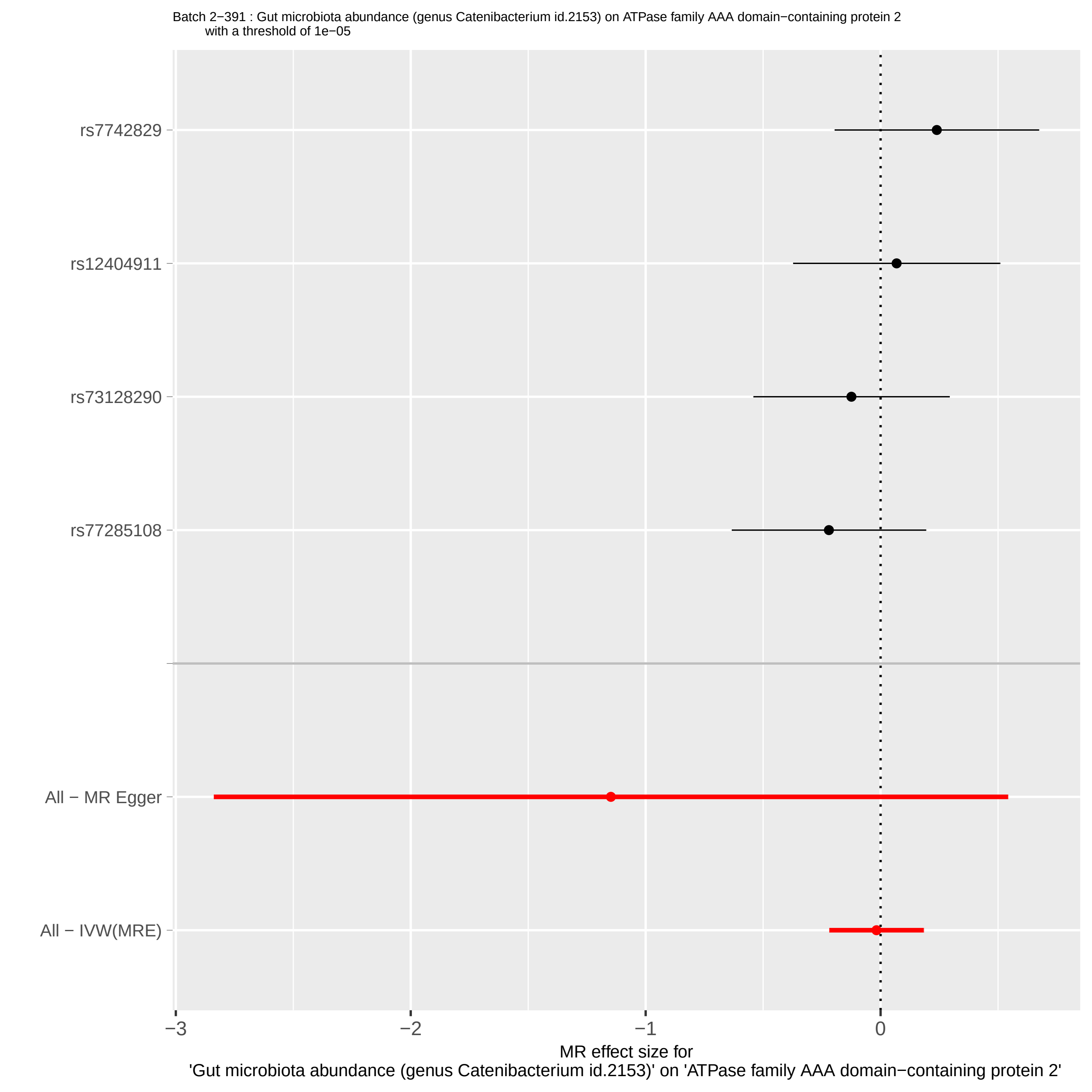

Batch 2−391 : Gut microbiota abundance (genus Catenibacterium id.2153) on ATPase family AAA domain−containing protein 2
with a threshold of 1e−05
rs7742829
rs12404911
rs73128290
rs77285108
All − MR Egger
All − IVW(MRE)
−3
−2
−1
0
MR effect size for
'Gut microbiota abundance (genus Catenibacterium id.2153)' on 'ATPase family AAA domain−containing protein 2'

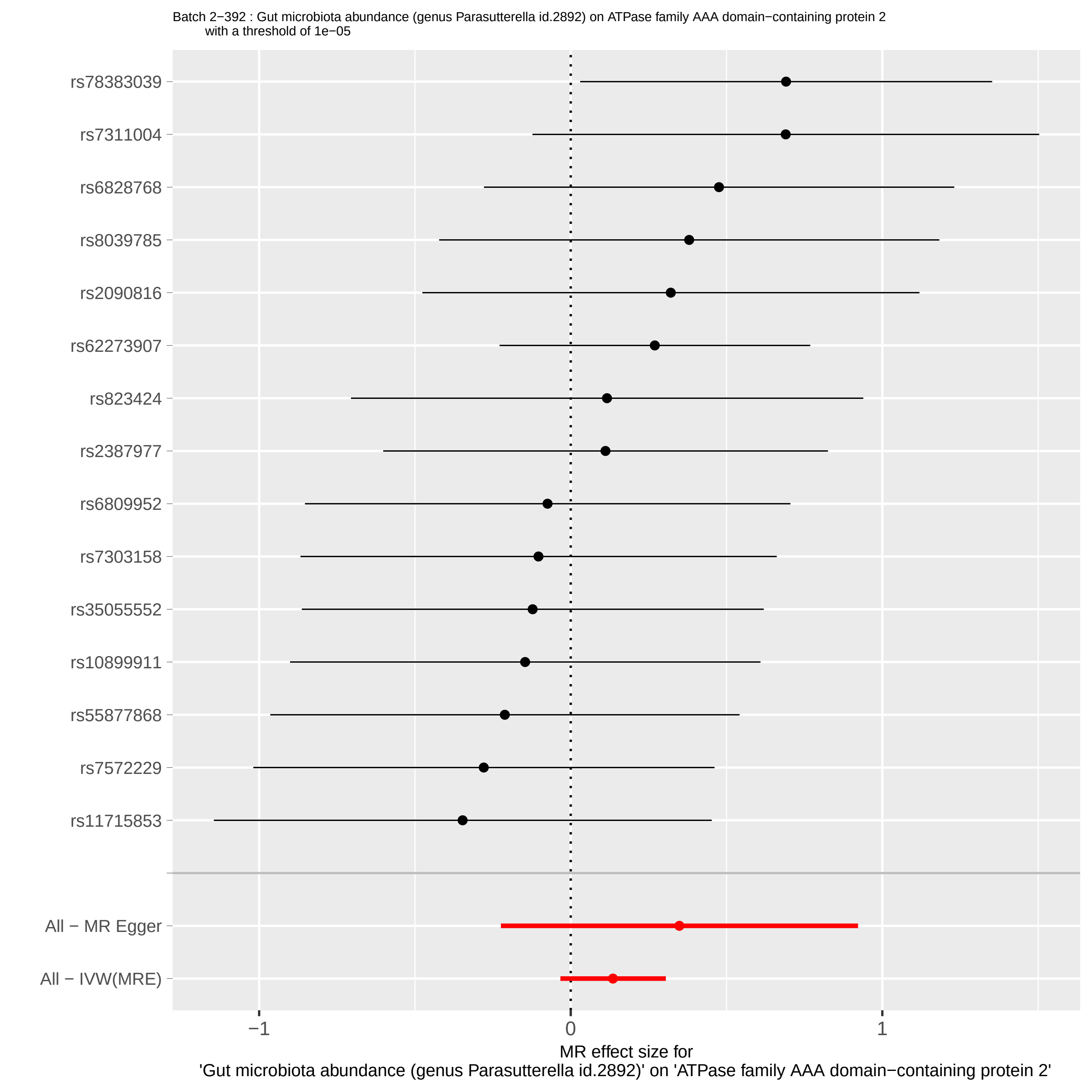

Batch 2−392 : Gut microbiota abundance (genus Parasutterella id.2892) on ATPase family AAA domain−containing protein 2
with a threshold of 1e−05
rs78383039
rs7311004
rs6828768
rs8039785
rs2090816
rs62273907
rs823424
rs2387977
rs6809952
rs7303158
rs35055552
rs10899911
rs55877868
rs7572229
rs11715853
All − MR Egger
All − IVW(MRE)
−1
0
1
MR effect size for
'Gut microbiota abundance (genus Parasutterella id.2892)' on 'ATPase family AAA domain−containing protein 2'

Batch 2−393 : Gut microbiota abundance (genus Catenibacterium id.2153) on Cyclic AMP−dependent transcription factor ATF−6 alpha
with a threshold of 1e−05

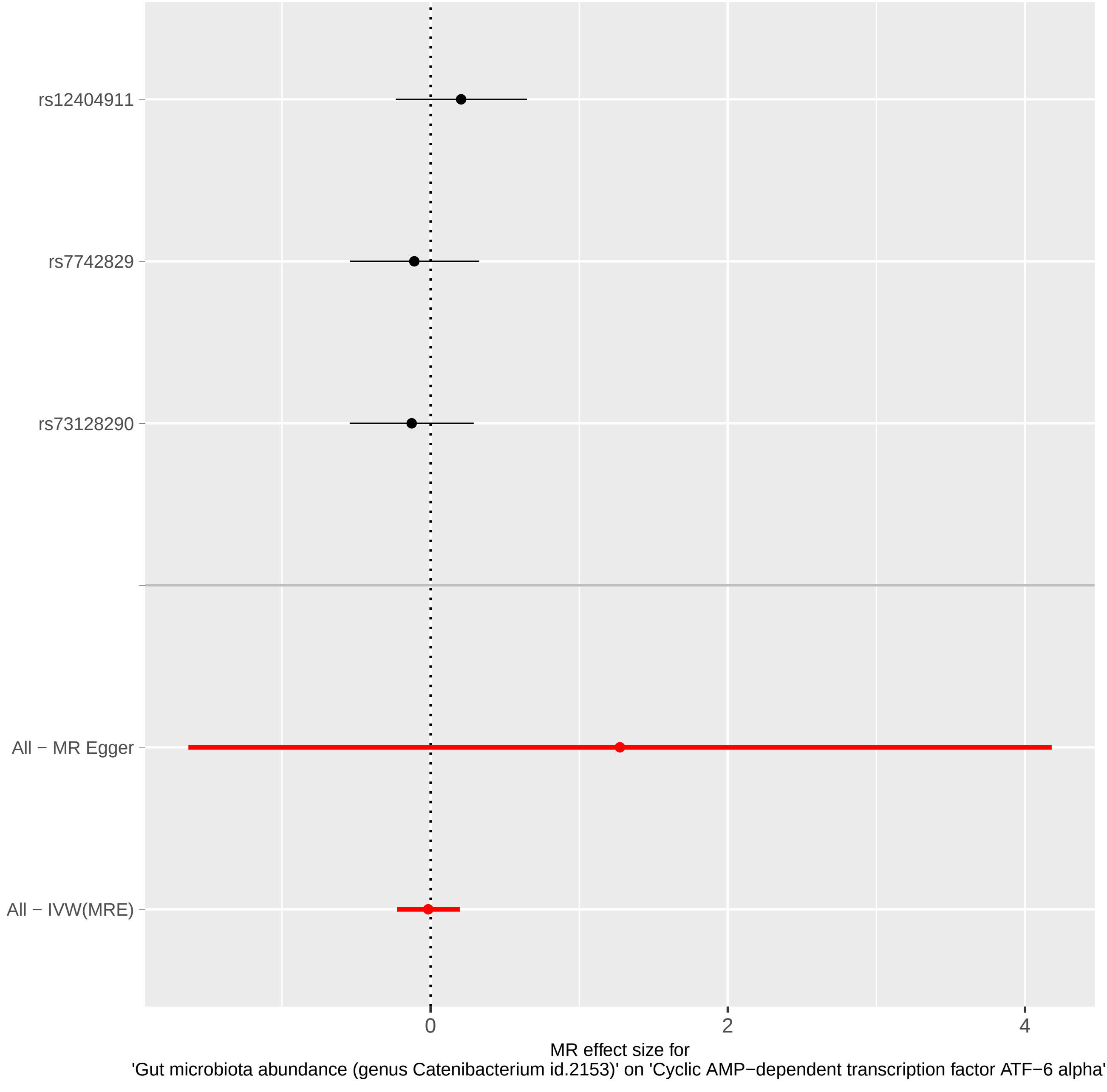

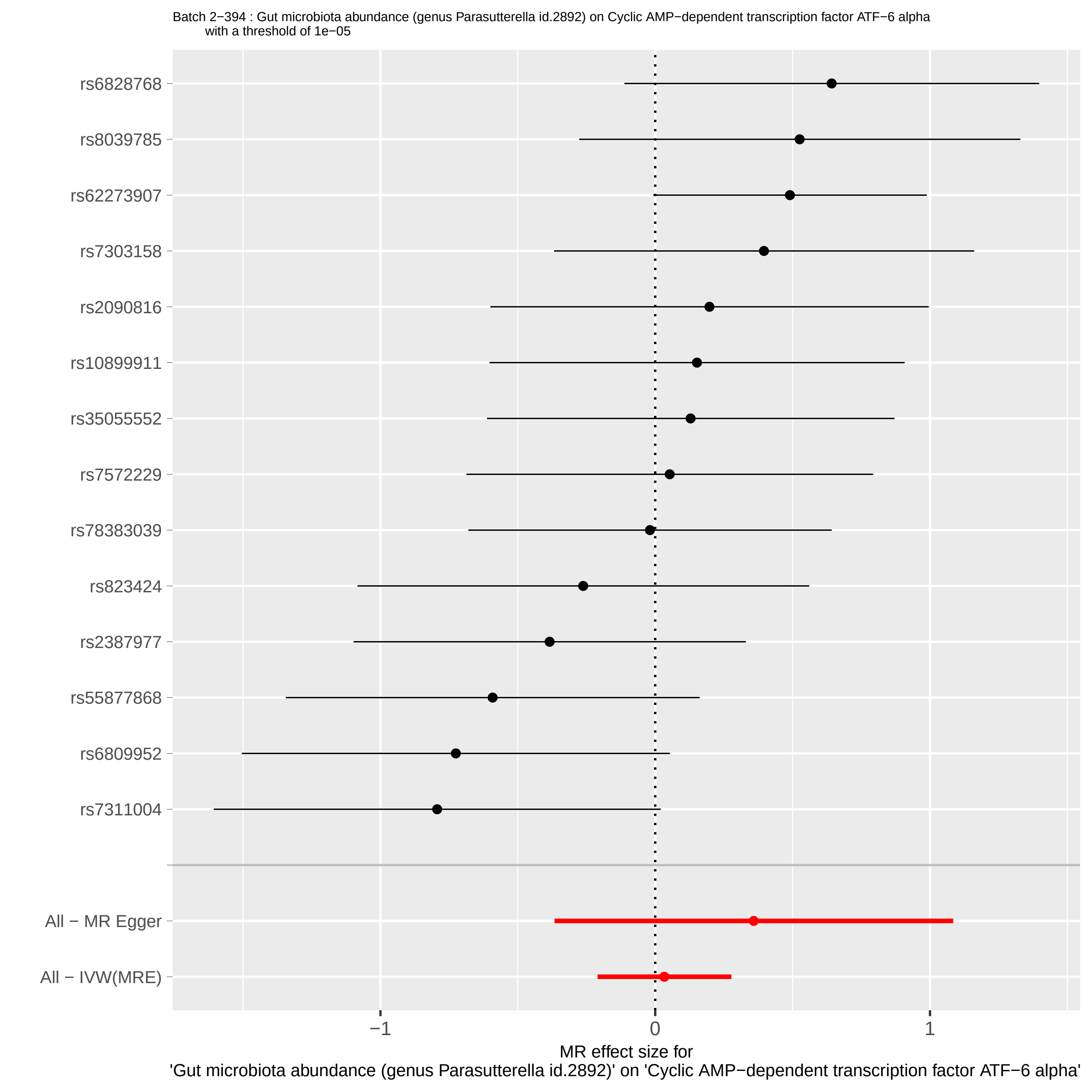

Batch 2−394 : Gut microbiota abundance (genus Parasutterella id.2892) on Cyclic AMP−dependent transcription factor ATF−6 alpha with a threshold of 1e−05
rs6828768
rs8039785
rs62273907
rs7303158
rs2090816
rs10899911
rs35055552
rs7572229
rs78383039
rs823424
rs2387977
rs55877868
rs6809952
rs7311004
All − MR Egger
All − IVW(MRE)
−1
0
1
MR effect size for
'Gut microbiota abundance (genus Parasutterella id.2892)' on 'Cyclic AMP−dependent transcription factor ATF−6 alpha'

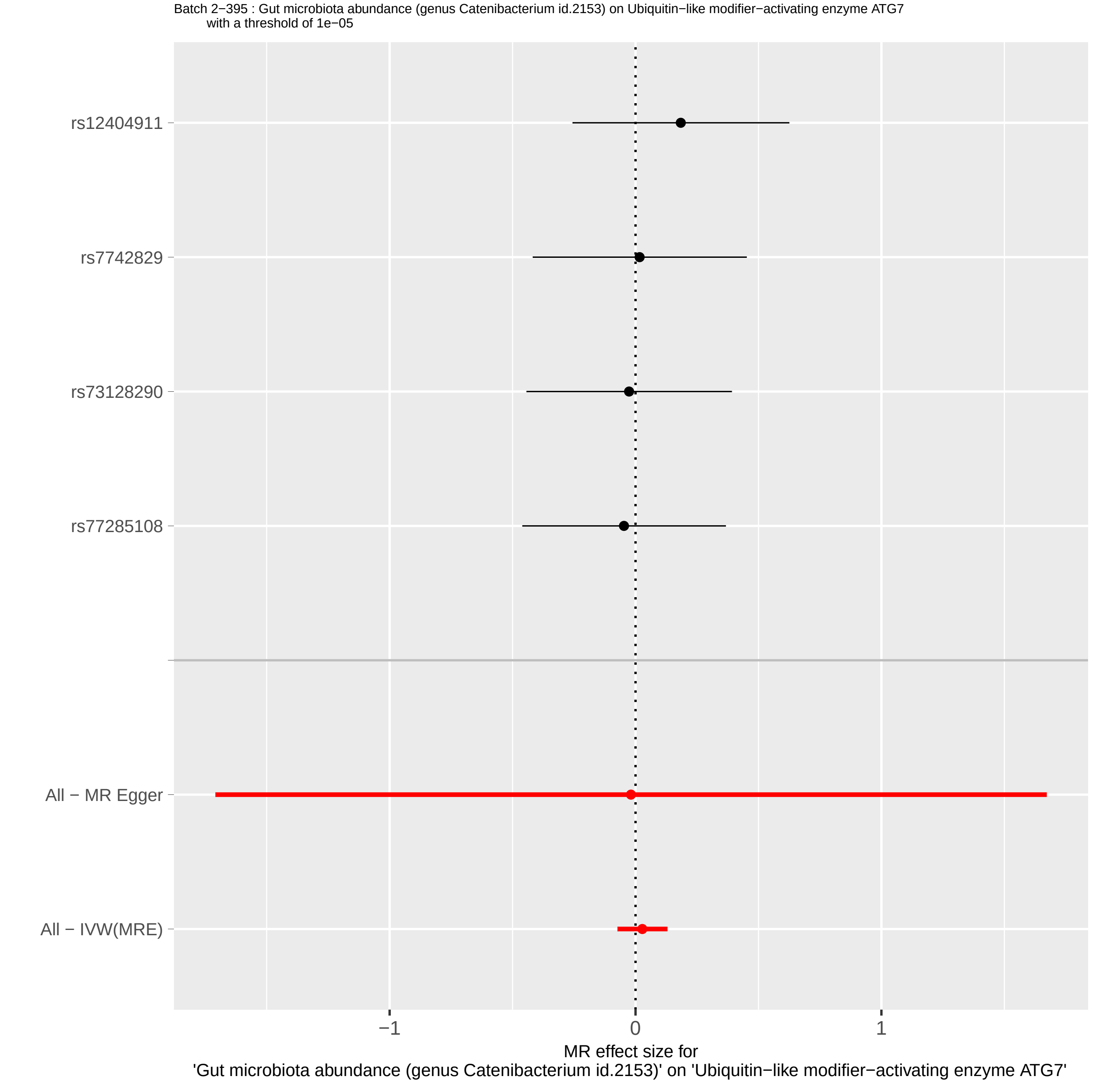

Batch 2−395 : Gut microbiota abundance (genus Catenibacterium id.2153) on Ubiquitin−like modifier−activating enzyme ATG7
with a threshold of 1e−05
rs12404911
rs7742829
rs73128290
rs77285108
All − MR Egger
All − IVW(MRE)
−1
0
1
MR effect size for
'Gut microbiota abundance (genus Catenibacterium id.2153)' on 'Ubiquitin−like modifier−activating enzyme ATG7'

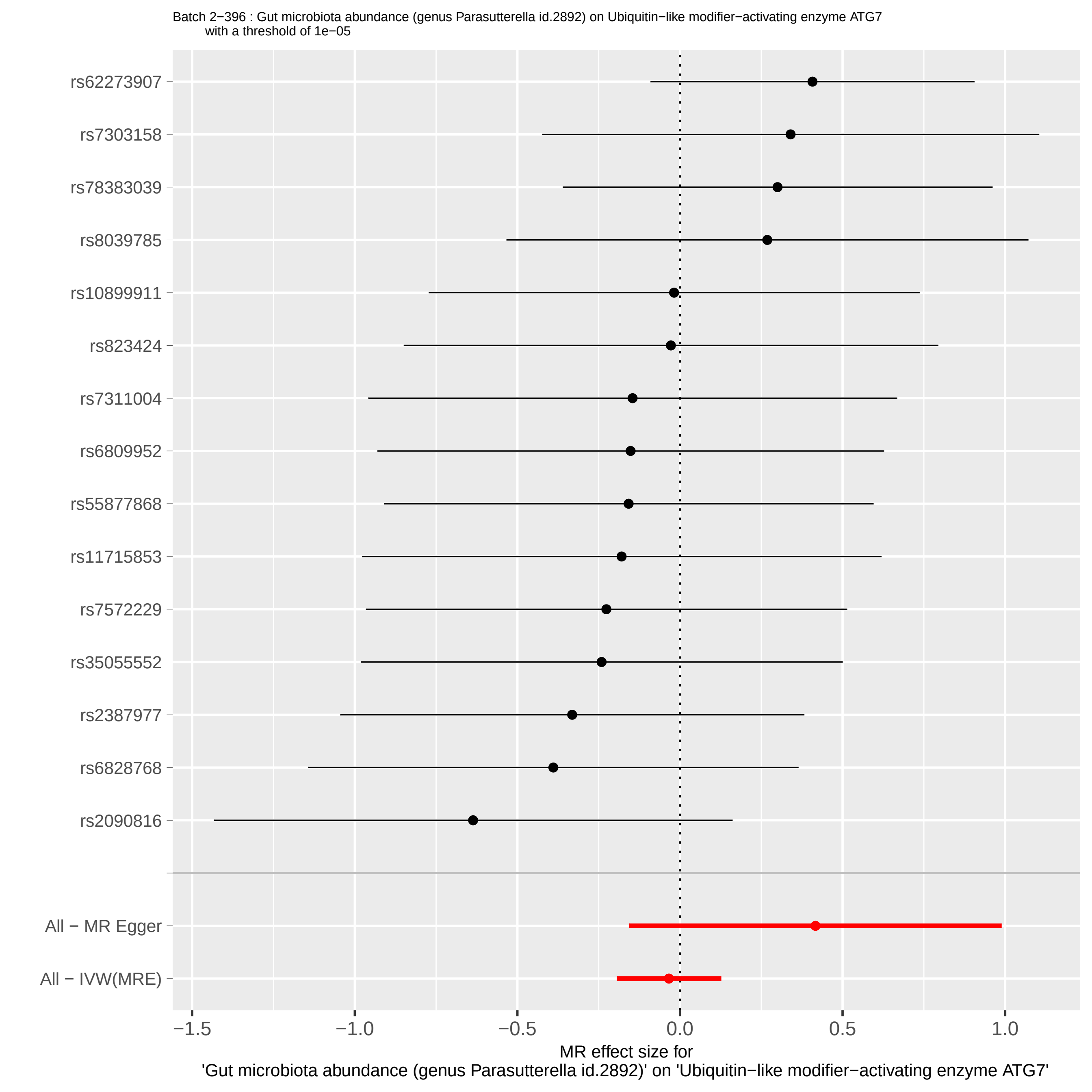

Batch 2−396 : Gut microbiota abundance (genus Parasutterella id.2892) on Ubiquitin−like modifier−activating enzyme ATG7
with a threshold of 1e−05
rs62273907
rs7303158
rs78383039
rs8039785
rs10899911
rs823424
rs7311004
rs6809952
rs55877868
rs11715853
rs7572229
rs35055552
rs2387977
rs6828768
rs2090816
All − MR Egger
All − IVW(MRE)
−1.5
−1.0
−0.5
0.0
0.5
1.0
MR effect size for
'Gut microbiota abundance (genus Parasutterella id.2892)' on 'Ubiquitin−like modifier−activating enzyme ATG7'

Batch 2−397 : Gut microbiota abundance (genus Catenibacterium id.2153) on Sodium/potassium−transporting ATPase subunit alpha−1 with a threshold of 1e−05

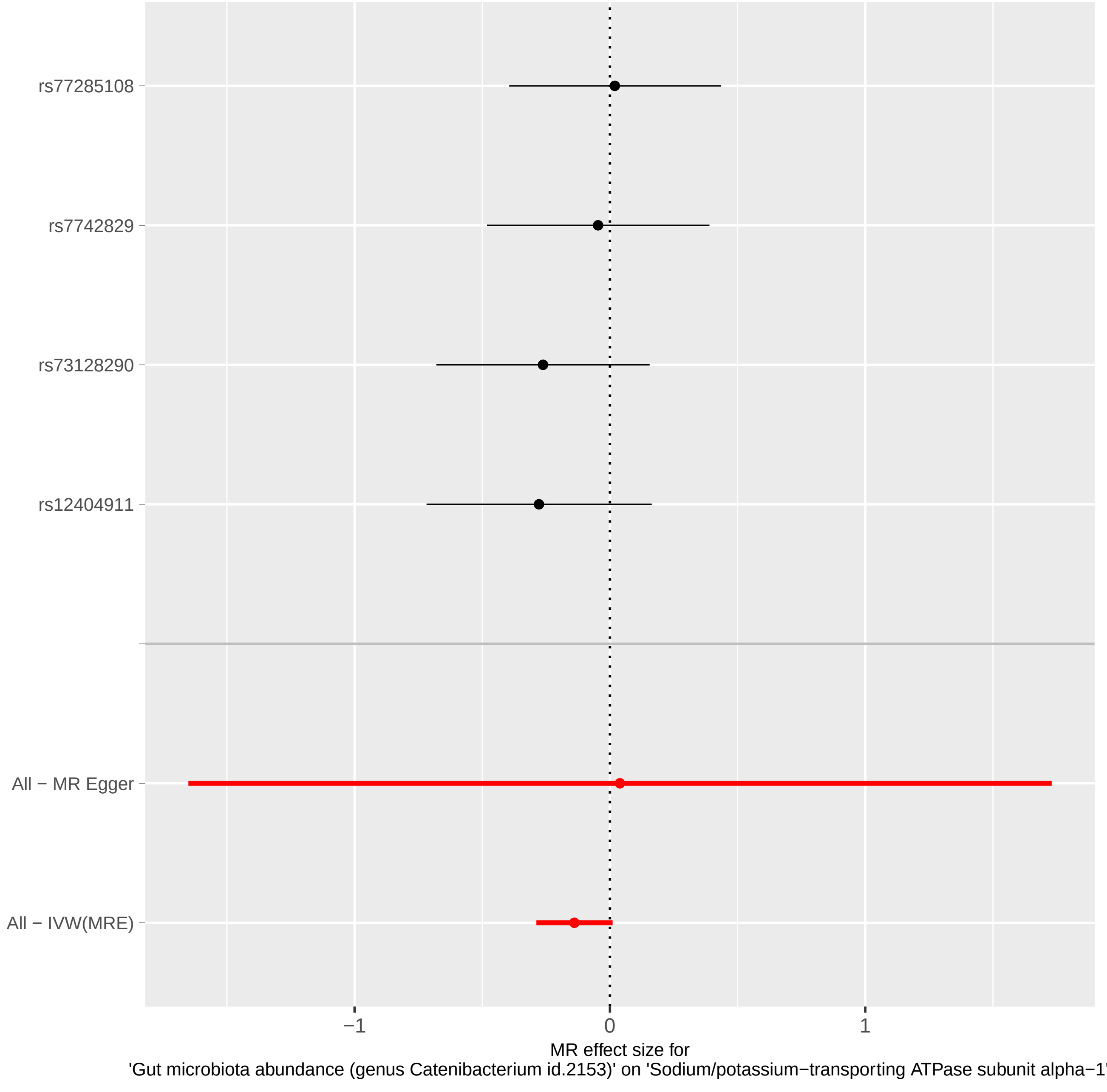

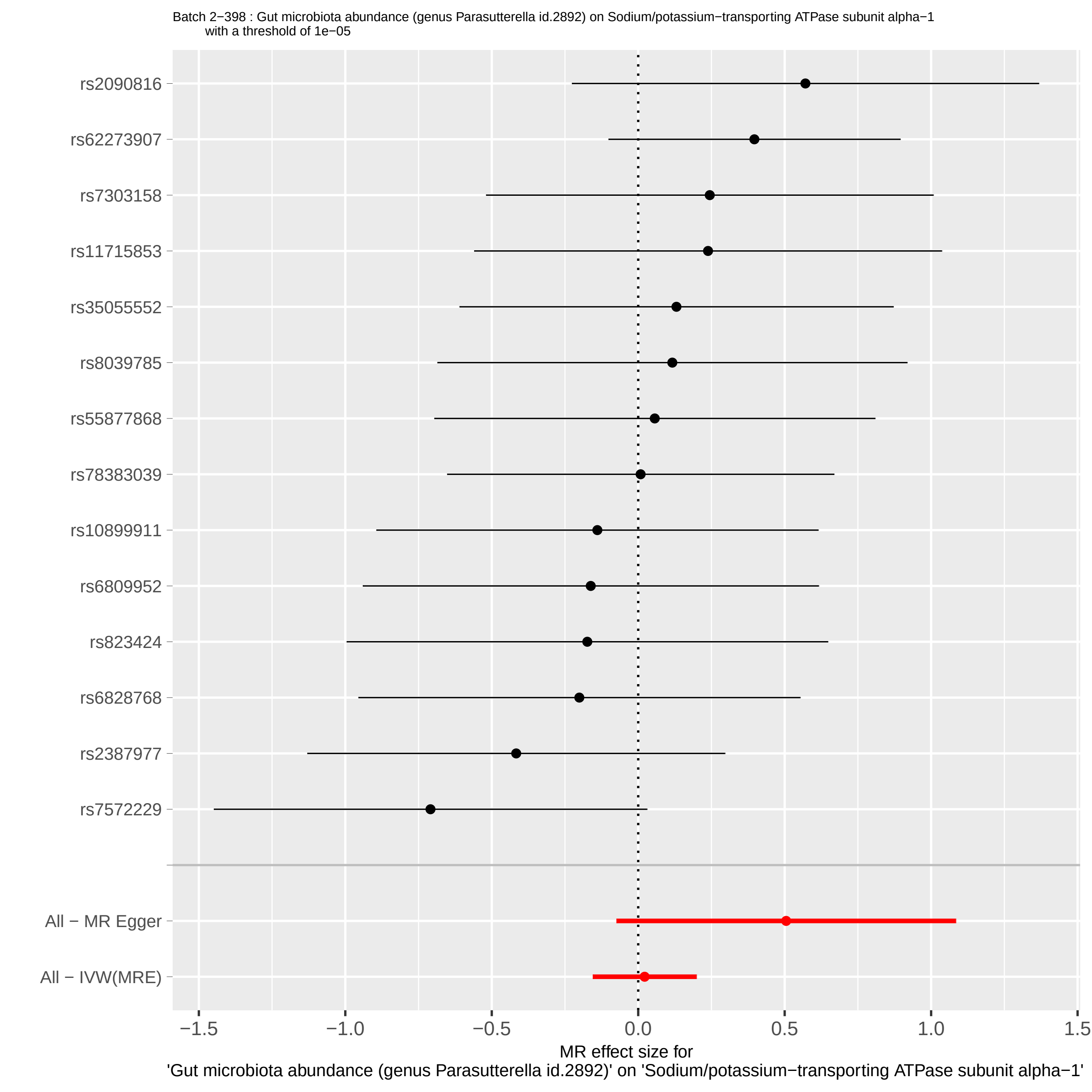
Batch 2−398 : Gut microbiota abundance (genus Parasutterella id.2892) on Sodium/potassium−transporting ATPase subunit alpha−1
with a threshold of 1e−05
rs2090816
rs62273907
rs7303158
rs11715853
rs35055552
rs8039785
rs55877868
rs78383039
rs10899911
rs6809952
rs823424
rs6828768
rs2387977
rs7572229
All − MR Egger
All − IVW(MRE)
−1.5
−1.0
−0.5
0.0
0.5
1.0
1.5
MR effect size for
'Gut microbiota abundance (genus Parasutterella id.2892)' on 'Sodium/potassium−transporting ATPase subunit alpha−1'

Batch 2−399 : Gut microbiota abundance (genus Catenibacterium id.2153) on Sodium/potassium−transporting ATPase subunit beta−2 with a threshold of 1e−05

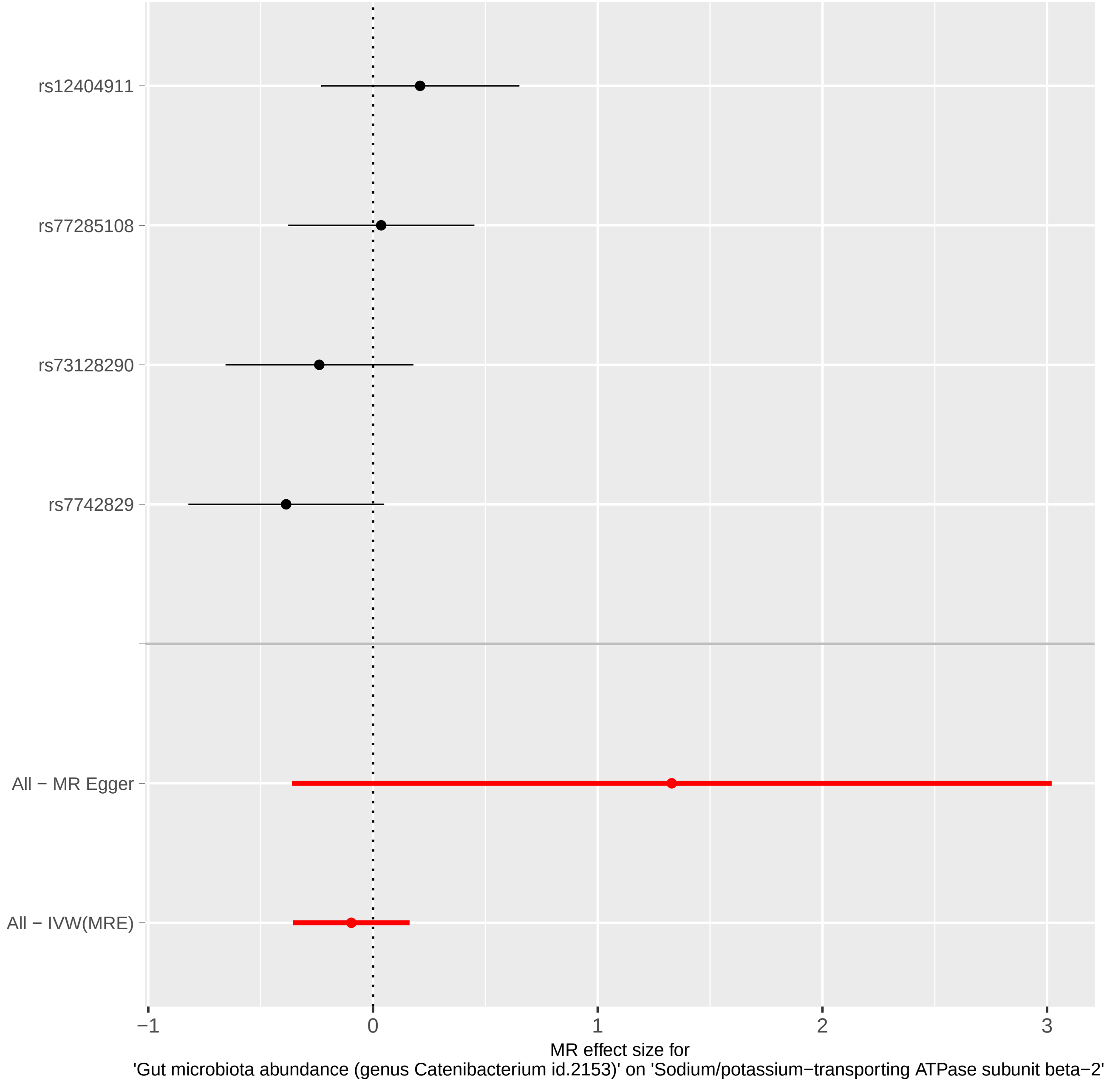

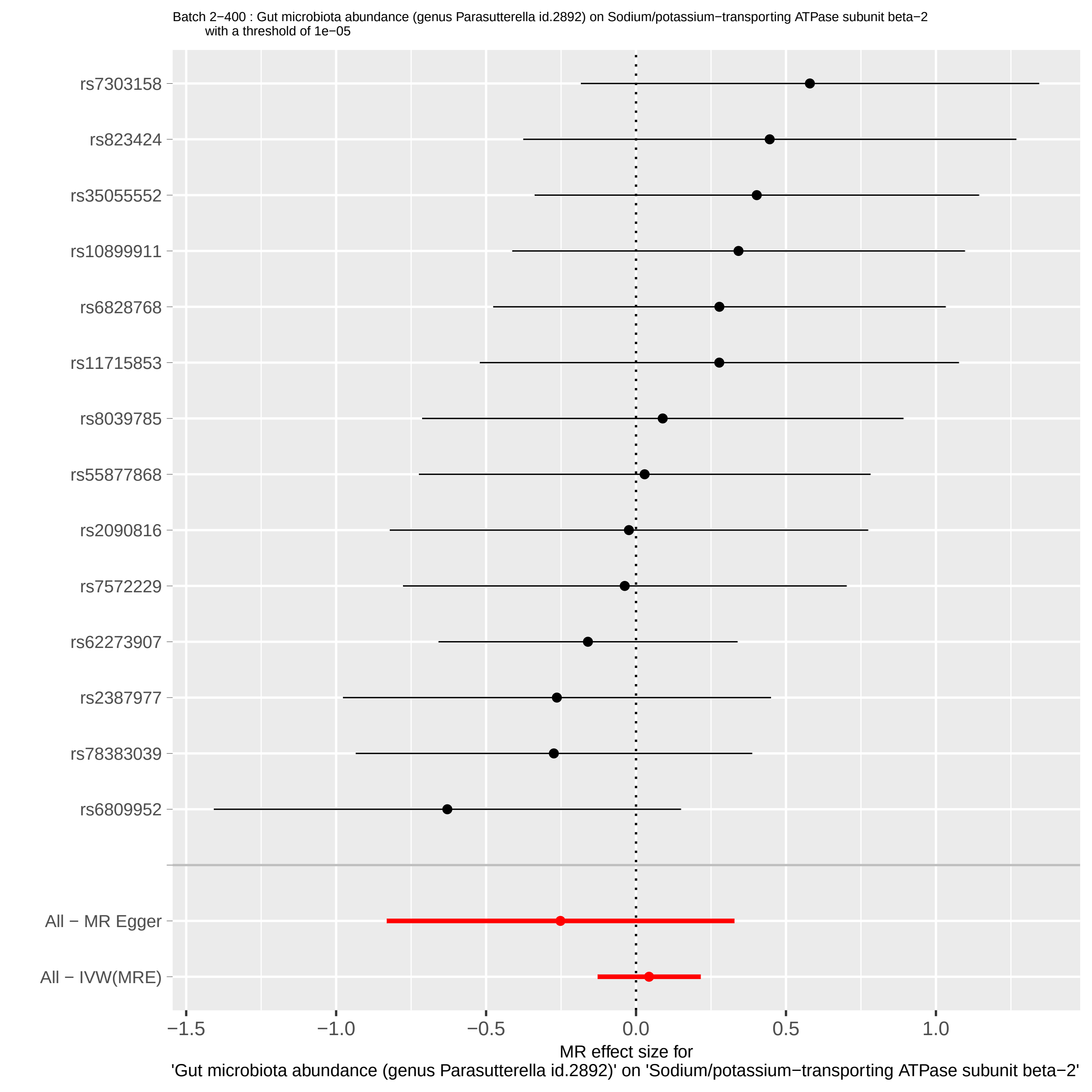

Batch 2−400 : Gut microbiota abundance (genus Parasutterella id.2892) on Sodium/potassium−transporting ATPase subunit beta−2
with a threshold of 1e−05
rs7303158
rs823424
rs35055552
rs10899911
rs6828768
rs11715853
rs8039785
rs55877868
rs2090816
rs7572229
rs62273907
rs2387977
rs78383039
rs6809952
All − MR Egger
All − IVW(MRE)
−1.5
−1.0
−0.5
0.0
0.5
1.0
MR effect size for
'Gut microbiota abundance (genus Parasutterella id.2892)' on 'Sodium/potassium−transporting ATPase subunit beta−2'

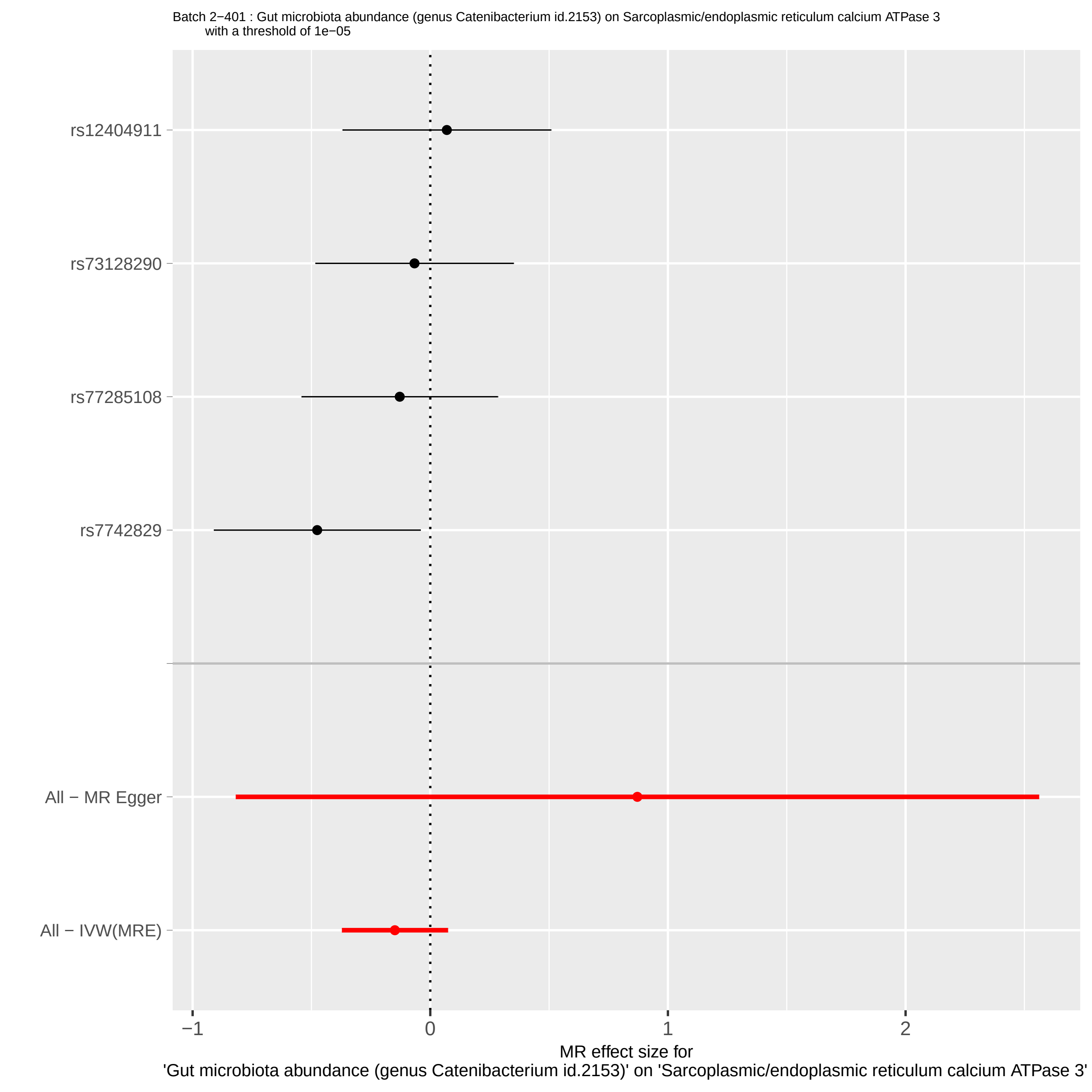

Batch 2−401 : Gut microbiota abundance (genus Catenibacterium id.2153) on Sarcoplasmic/endoplasmic reticulum calcium ATPase 3
with a threshold of 1e−05
rs12404911
rs73128290
rs77285108
rs7742829
All − MR Egger
All − IVW(MRE)
−1
0
1
2
MR effect size for
'Gut microbiota abundance (genus Catenibacterium id.2153)' on 'Sarcoplasmic/endoplasmic reticulum calcium ATPase 3'

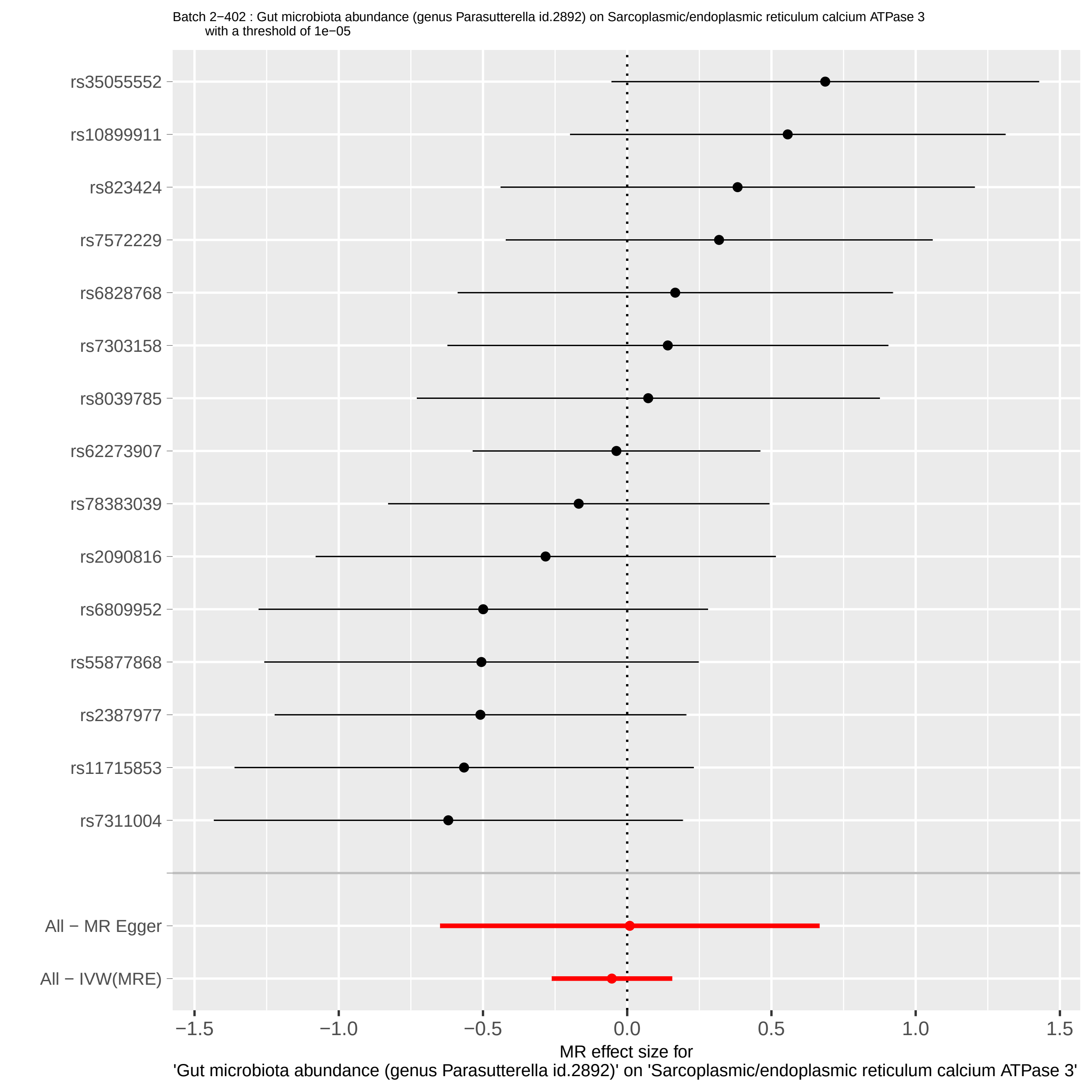

Batch 2−402 : Gut microbiota abundance (genus Parasutterella id.2892) on Sarcoplasmic/endoplasmic reticulum calcium ATPase 3
with a threshold of 1e−05
rs35055552
rs10899911
rs823424
rs7572229
rs6828768
rs7303158
rs8039785
rs62273907
rs78383039
rs2090816
rs6809952
rs55877868
rs2387977
rs11715853
rs7311004
All − MR Egger
All − IVW(MRE)
−1.5
−1.0
−0.5
0.0
0.5
1.0
1.5
MR effect size for
'Gut microbiota abundance (genus Parasutterella id.2892)' on 'Sarcoplasmic/endoplasmic reticulum calcium ATPase 3'

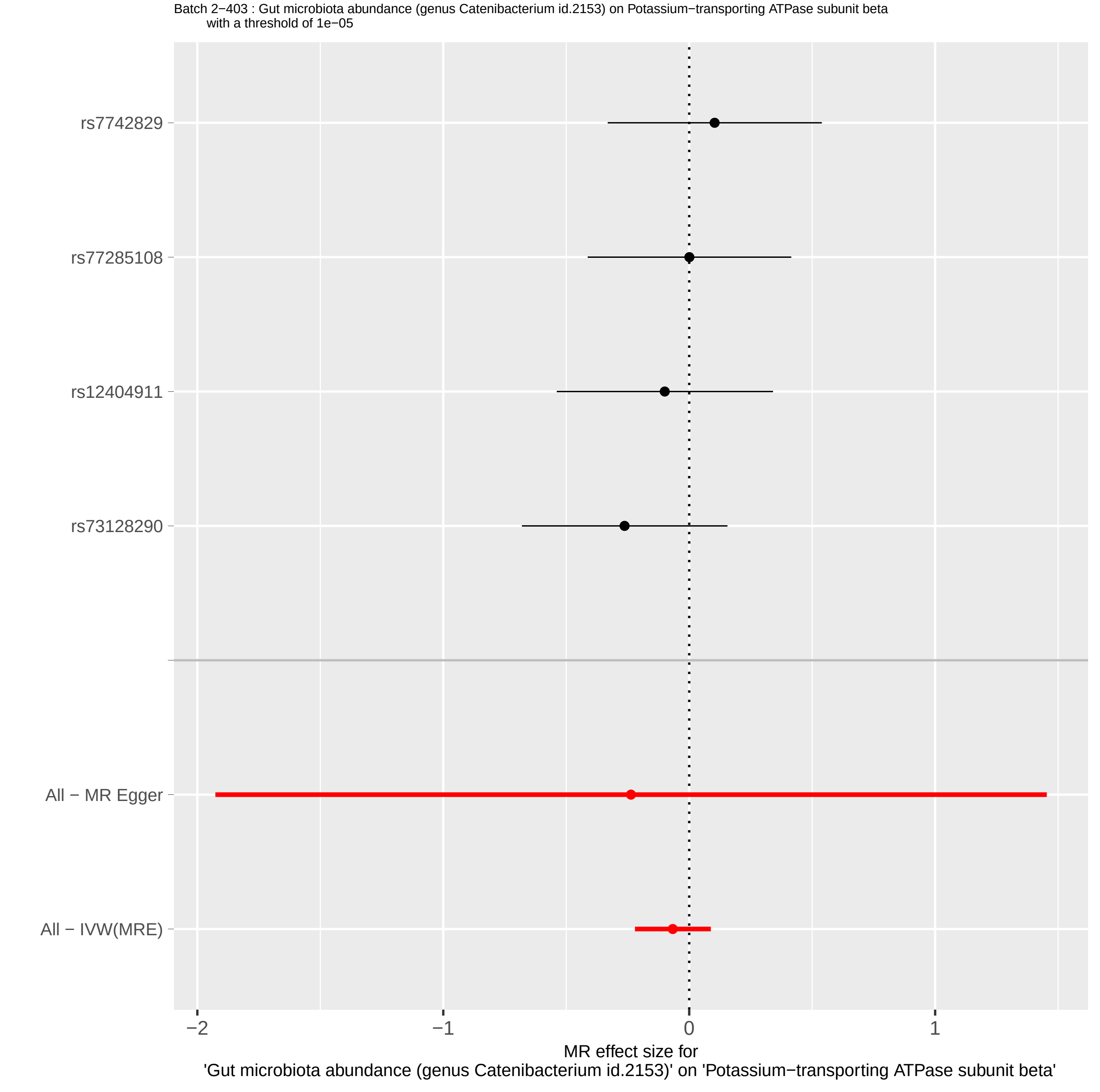
Batch 2−403 : Gut microbiota abundance (genus Catenibacterium id.2153) on Potassium−transporting ATPase subunit beta
with a threshold of 1e−05
rs7742829
rs77285108
rs12404911
rs73128290
All − MR Egger
All − IVW(MRE)
−2
−1
0
1
MR effect size for
'Gut microbiota abundance (genus Catenibacterium id.2153)' on 'Potassium−transporting ATPase subunit beta'

Batch 2−404 : Gut microbiota abundance (genus Parasutterella id.2892) on Potassium−transporting ATPase subunit beta
with a threshold of 1e−05

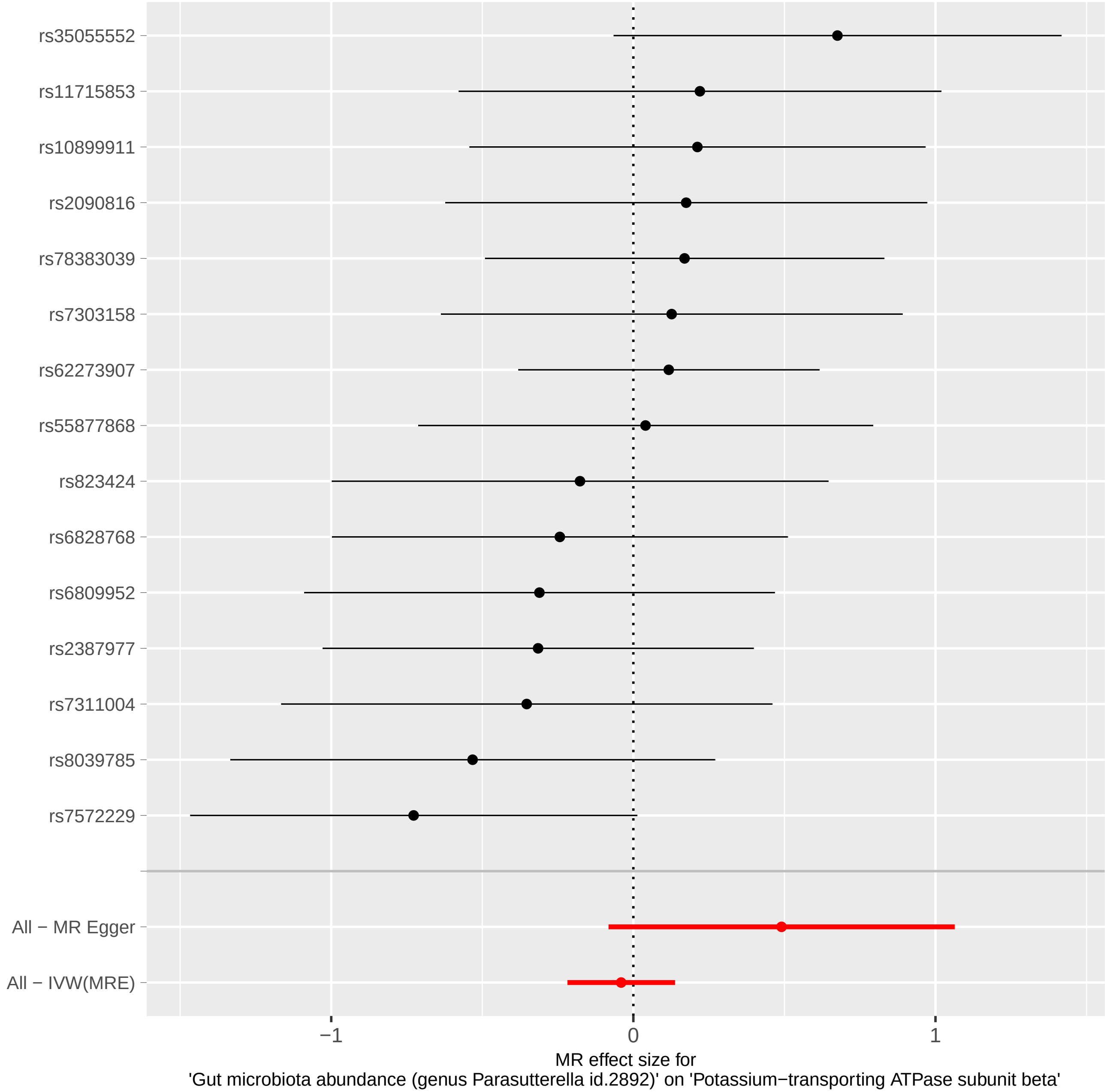

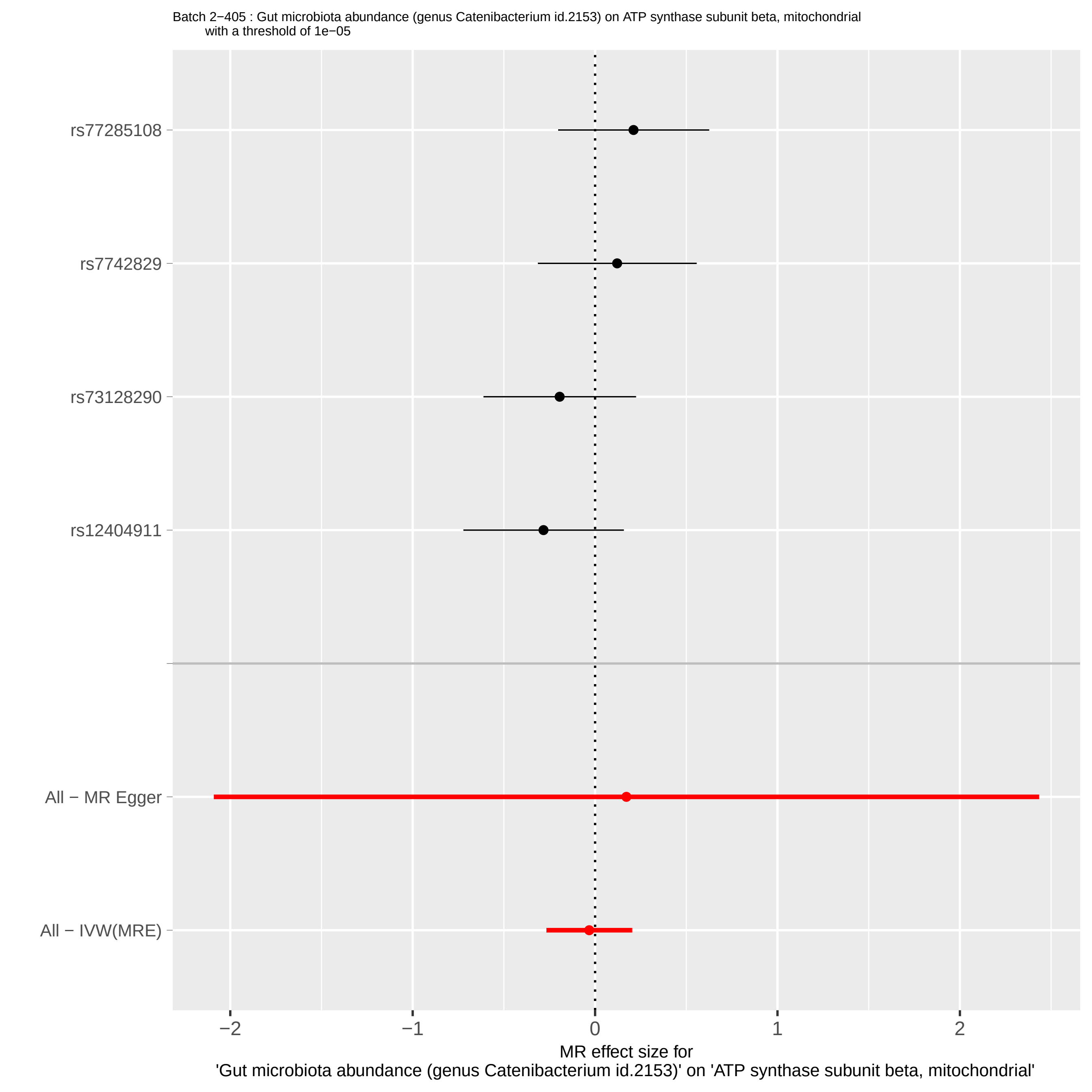

Batch 2−405 : Gut microbiota abundance (genus Catenibacterium id.2153) on ATP synthase subunit beta, mitochondrial
with a threshold of 1e−05
rs77285108
rs7742829
rs73128290
rs12404911
All − MR Egger
All − IVW(MRE)
−2
−1
0
1
2
MR effect size for
'Gut microbiota abundance (genus Catenibacterium id.2153)' on 'ATP synthase subunit beta, mitochondrial'

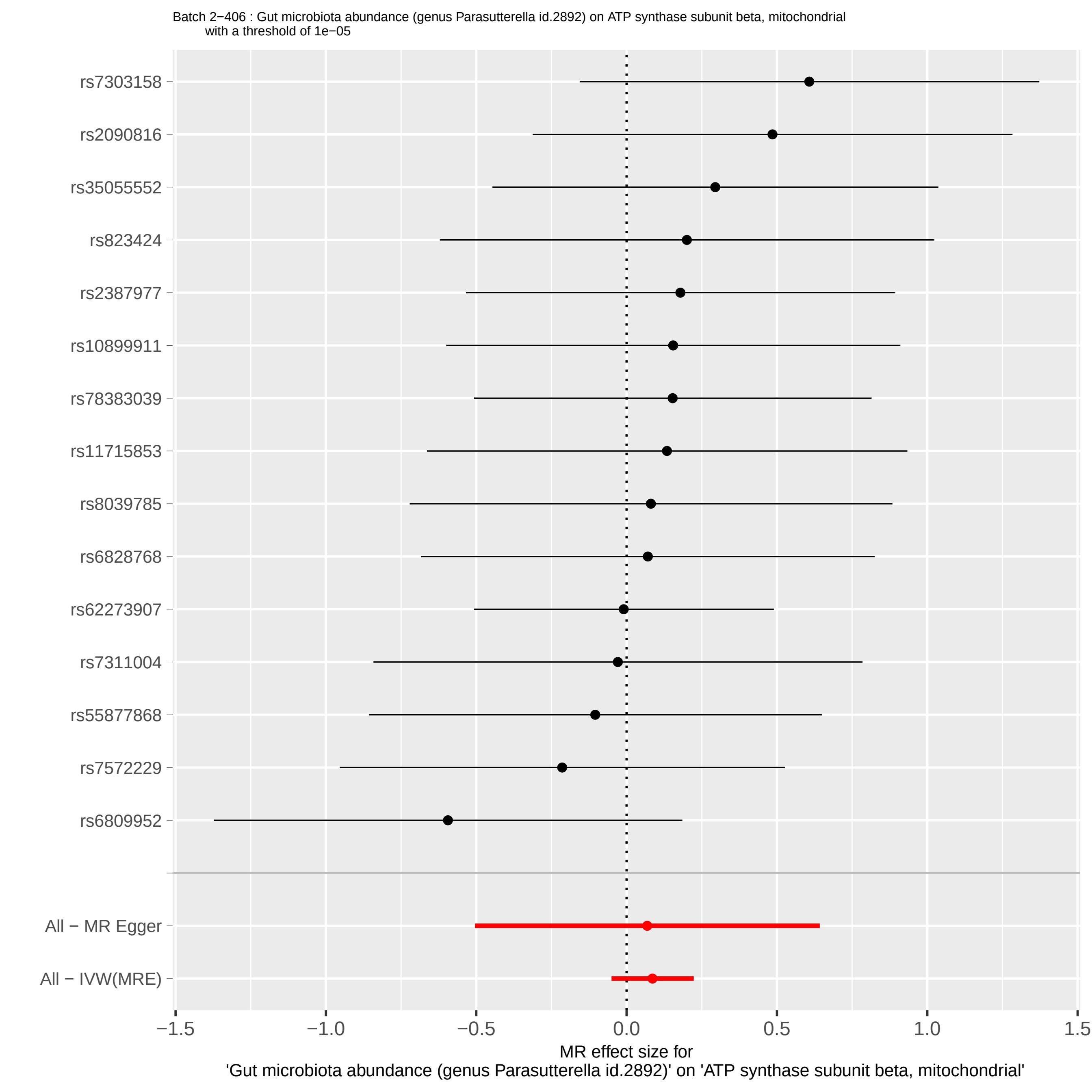

Batch 2−406 : Gut microbiota abundance (genus Parasutterella id.2892) on ATP synthase subunit beta, mitochondrial
with a threshold of 1e−05
rs7303158
rs2090816
rs35055552
rs823424
rs2387977
rs10899911
rs78383039
rs11715853
rs8039785
rs6828768
rs62273907
rs7311004
rs55877868
rs7572229
rs6809952
All − MR Egger
All − IVW(MRE)
−1.5
−1.0
−0.5
0.0
0.5
1.0
1.5
MR effect size for
'Gut microbiota abundance (genus Parasutterella id.2892)' on 'ATP synthase subunit beta, mitochondrial'

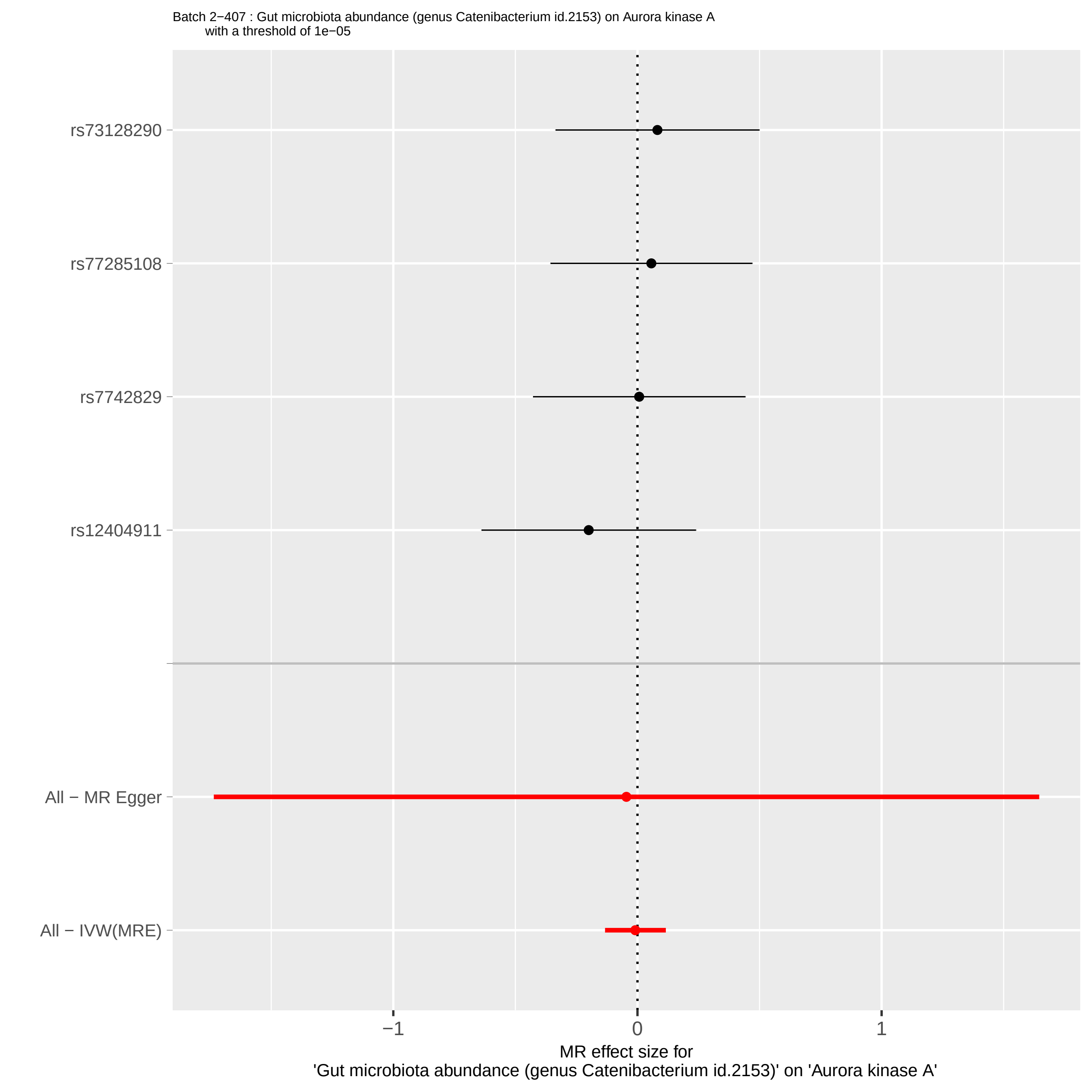
Batch 2−407 : Gut microbiota abundance (genus Catenibacterium id.2153) on Aurora kinase A
with a threshold of 1e−05
rs73128290
rs77285108
rs7742829
rs12404911
All − MR Egger
All − IVW(MRE)
−1
0
1
MR effect size for
'Gut microbiota abundance (genus Catenibacterium id.2153)' on 'Aurora kinase A'

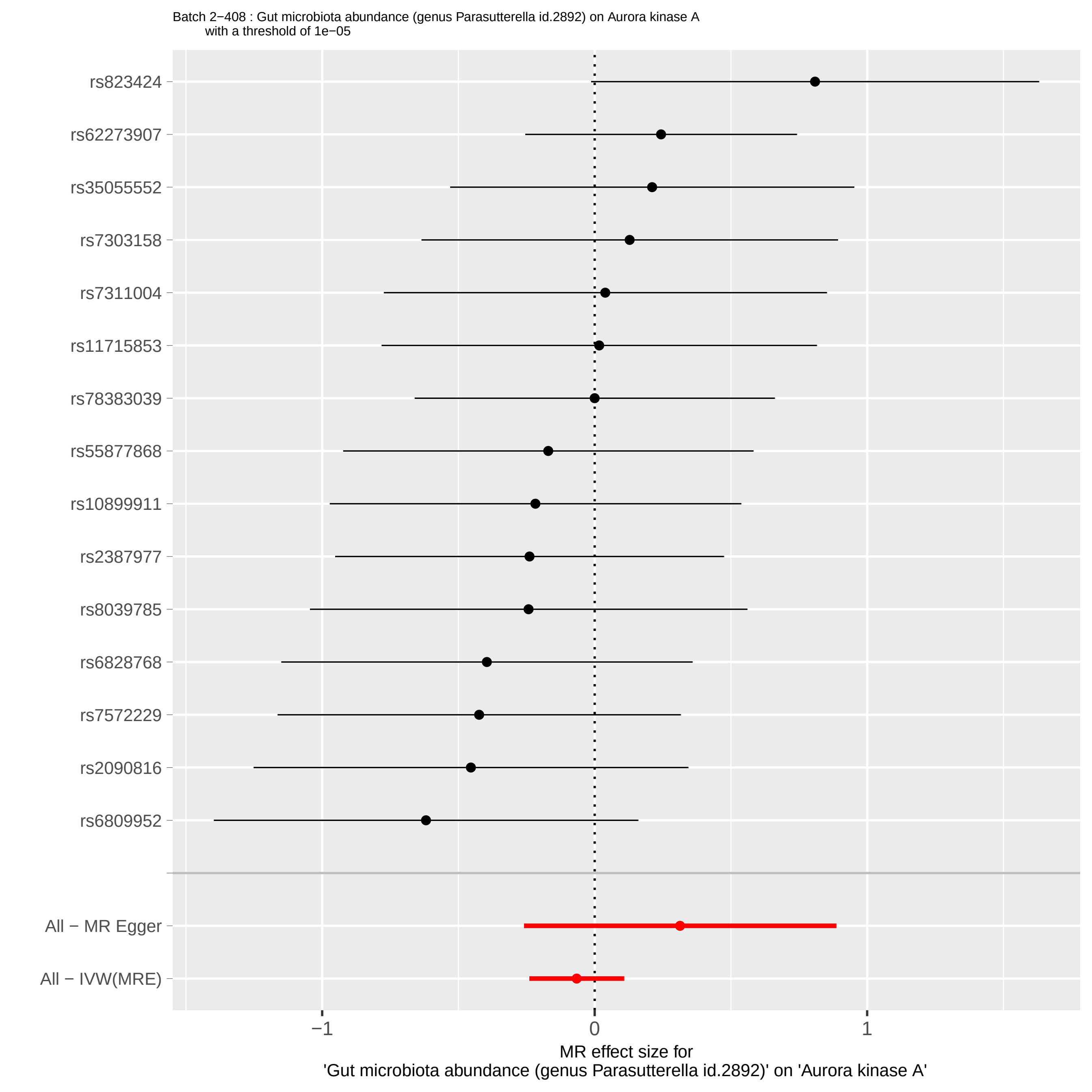

Batch 2−408 : Gut microbiota abundance (genus Parasutterella id.2892) on Aurora kinase A
with a threshold of 1e−05
rs823424
rs62273907
rs35055552
rs7303158
rs7311004
rs11715853
rs78383039
rs55877868
rs10899911
rs2387977
rs8039785
rs6828768
rs7572229
rs2090816
rs6809952
All − MR Egger
All − IVW(MRE)
−1
0
1
MR effect size for
'Gut microbiota abundance (genus Parasutterella id.2892)' on 'Aurora kinase A'

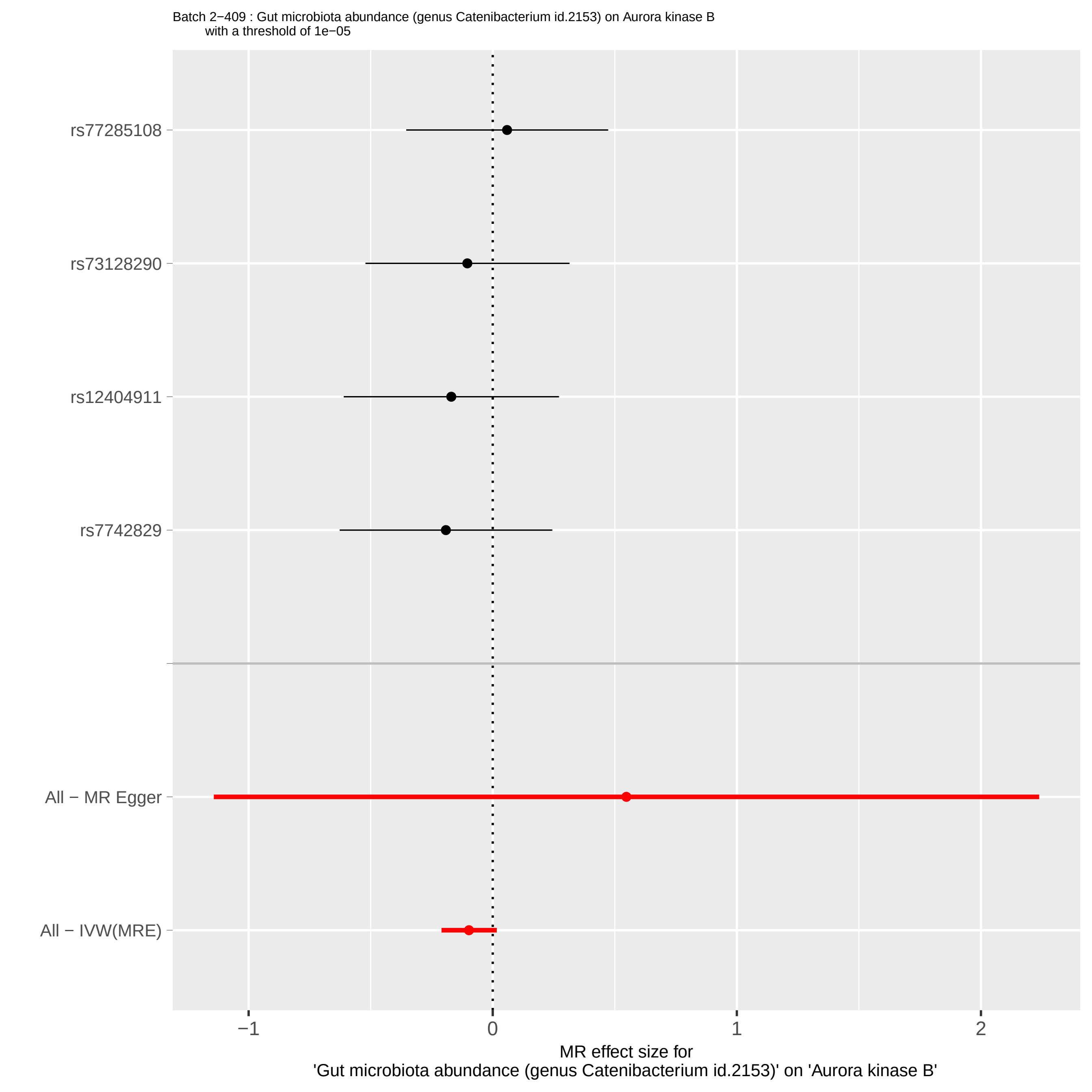

Batch 2−409 : Gut microbiota abundance (genus Catenibacterium id.2153) on Aurora kinase B
with a threshold of 1e−05
rs77285108
rs73128290
rs12404911
rs7742829
All − MR Egger
All − IVW(MRE)
−1
0
1
2
MR effect size for
'Gut microbiota abundance (genus Catenibacterium id.2153)' on 'Aurora kinase B'

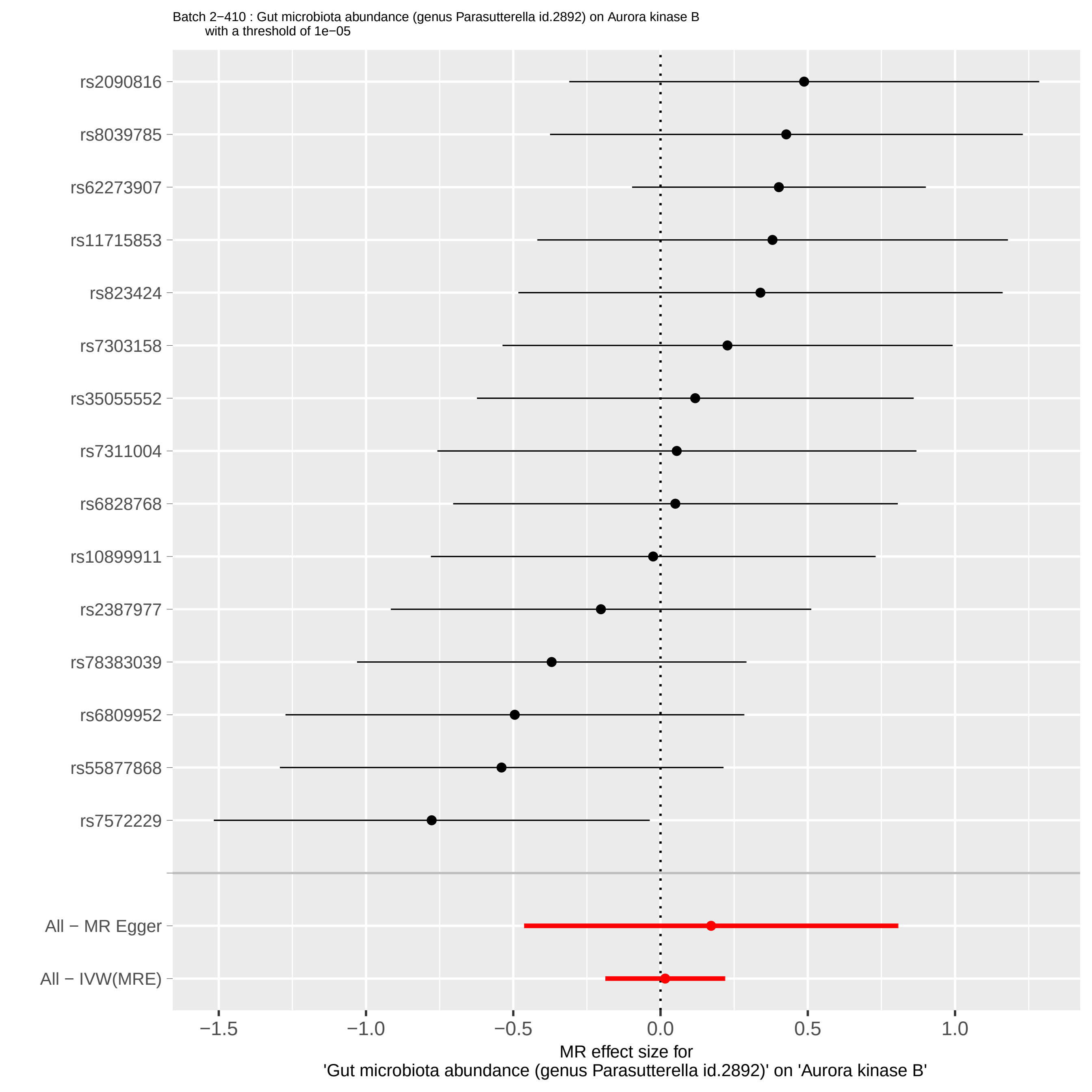

Batch 2−410 : Gut microbiota abundance (genus Parasutterella id.2892) on Aurora kinase B
with a threshold of 1e−05
rs2090816
rs8039785
rs62273907
rs11715853
rs823424
rs7303158
rs35055552
rs7311004
rs6828768
rs10899911
rs2387977
rs78383039
rs6809952
rs55877868
rs7572229
All − MR Egger
All − IVW(MRE)
−1.5
−1.0
−0.5
0.0
0.5
1.0
MR effect size for
'Gut microbiota abundance (genus Parasutterella id.2892)' on 'Aurora kinase B'

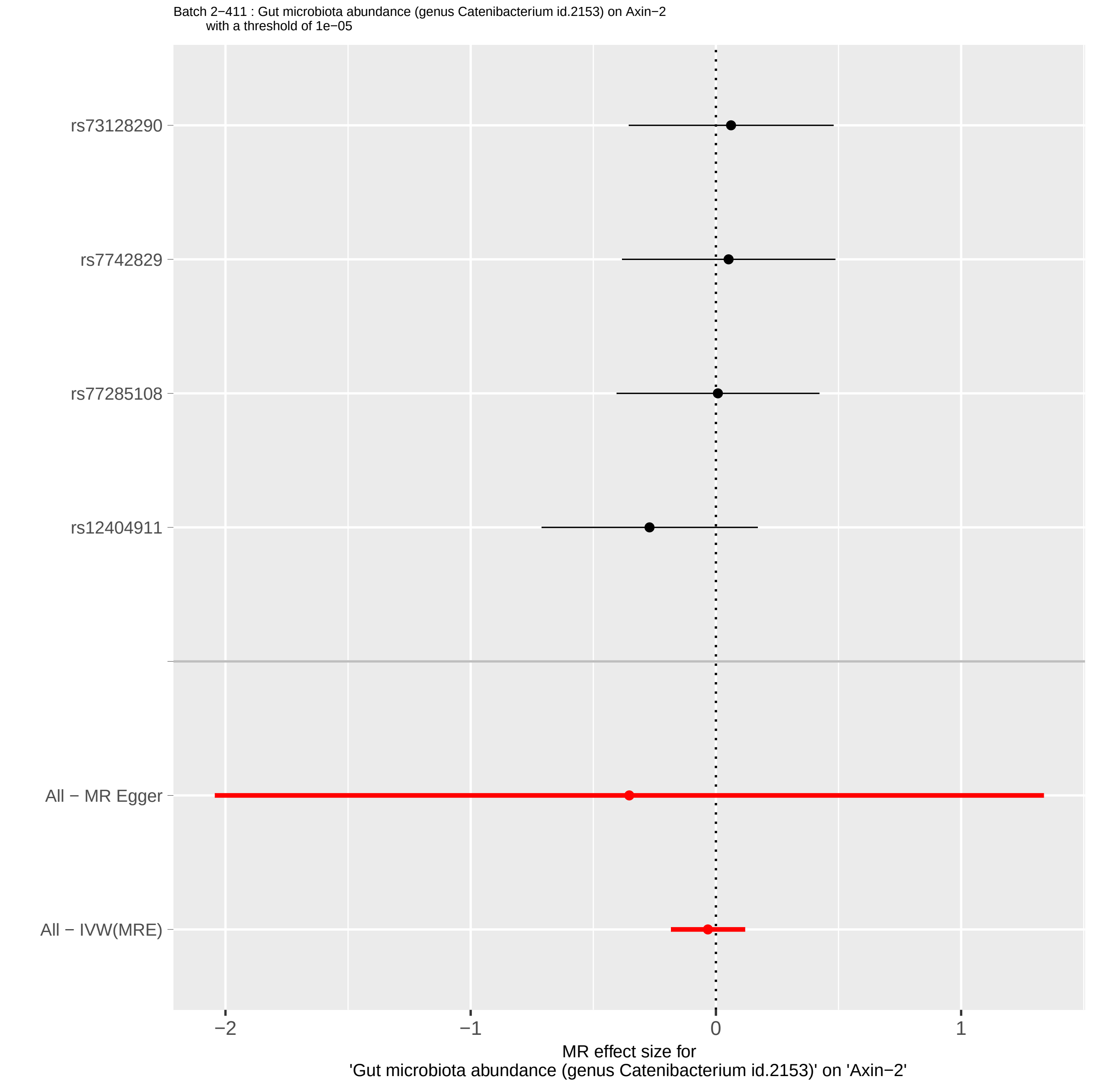

Batch 2−411 : Gut microbiota abundance (genus Catenibacterium id.2153) on Axin−2
with a threshold of 1e−05
rs73128290
rs7742829
rs77285108
rs12404911
All − MR Egger
All − IVW(MRE)
−2
−1
0
1
MR effect size for
'Gut microbiota abundance (genus Catenibacterium id.2153)' on 'Axin−2'

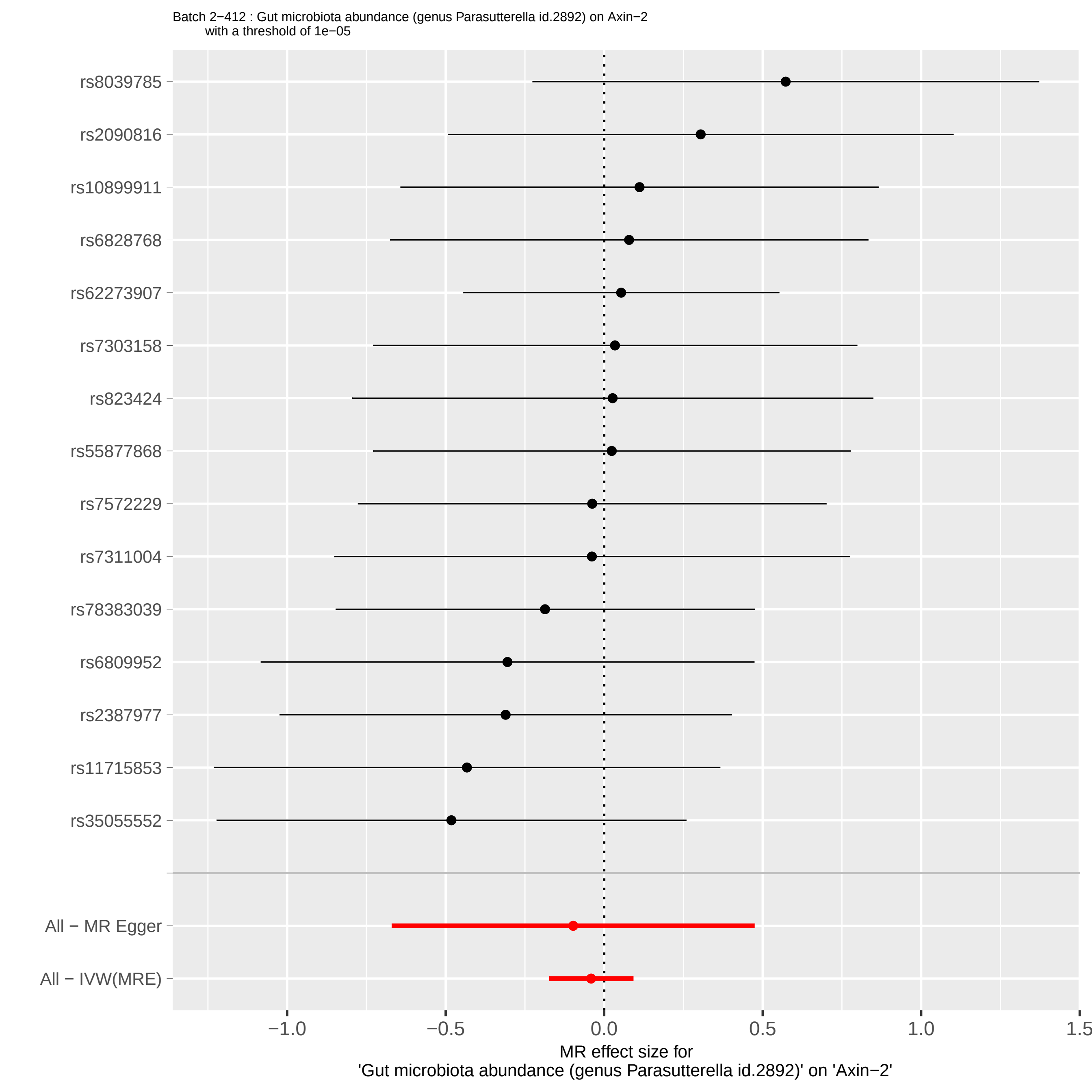
Batch 2−412 : Gut microbiota abundance (genus Parasutterella id.2892) on Axin−2
with a threshold of 1e−05
rs8039785
rs2090816
rs10899911
rs6828768
rs62273907
rs7303158
rs823424
rs55877868
rs7572229
rs7311004
rs78383039
rs6809952
rs2387977
rs11715853
rs35055552
All − MR Egger
All − IVW(MRE)
−1.0
−0.5
0.0
0.5
1.0
1.5
MR effect size for
'Gut microbiota abundance (genus Parasutterella id.2892)' on 'Axin−2'

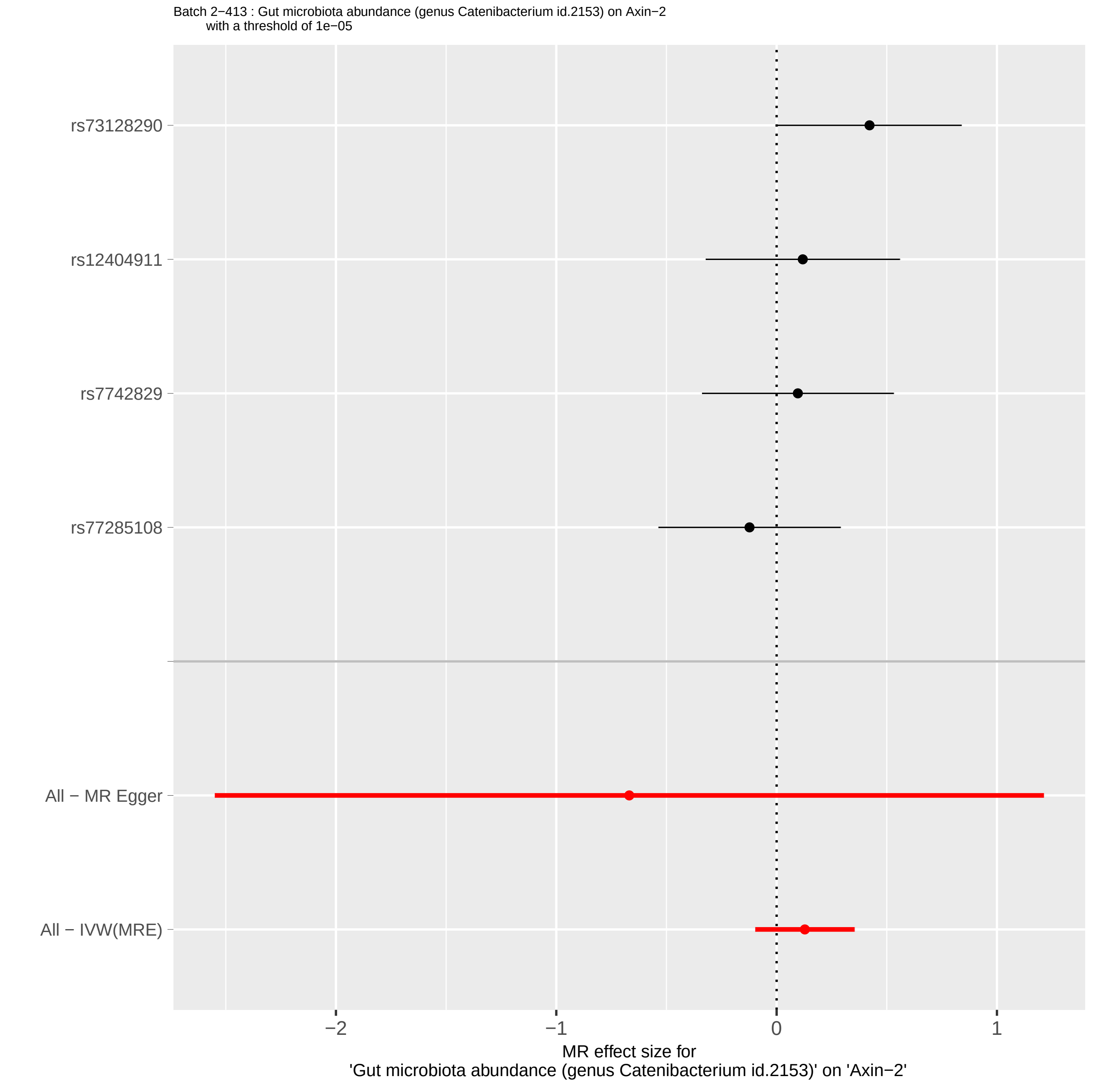
Batch 2−413 : Gut microbiota abundance (genus Catenibacterium id.2153) on Axin−2
with a threshold of 1e−05
rs73128290
rs12404911
rs7742829
rs77285108
All − MR Egger
All − IVW(MRE)
−2
−1
0
1
MR effect size for
'Gut microbiota abundance (genus Catenibacterium id.2153)' on 'Axin−2'

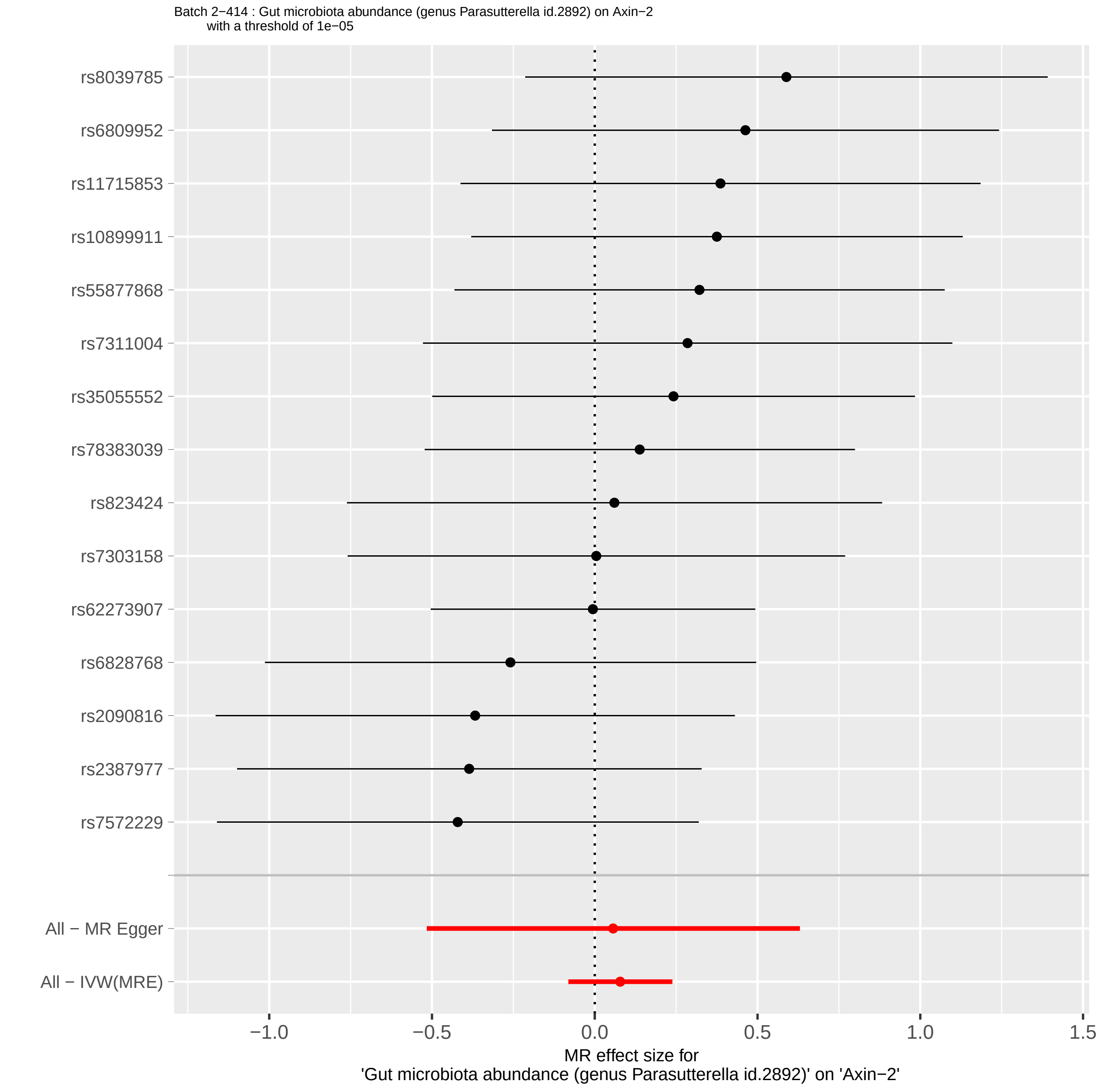

Batch 2−414 : Gut microbiota abundance (genus Parasutterella id.2892) on Axin−2
with a threshold of 1e−05
rs8039785
rs6809952
rs11715853
rs10899911
rs55877868
rs7311004
rs35055552
rs78383039
rs823424
rs7303158
rs62273907
rs6828768
rs2090816
rs2387977
rs7572229
All − MR Egger
All − IVW(MRE)
−1.0
−0.5
0.0
0.5
1.0
1.5
MR effect size for
'Gut microbiota abundance (genus Parasutterella id.2892)' on 'Axin−2'

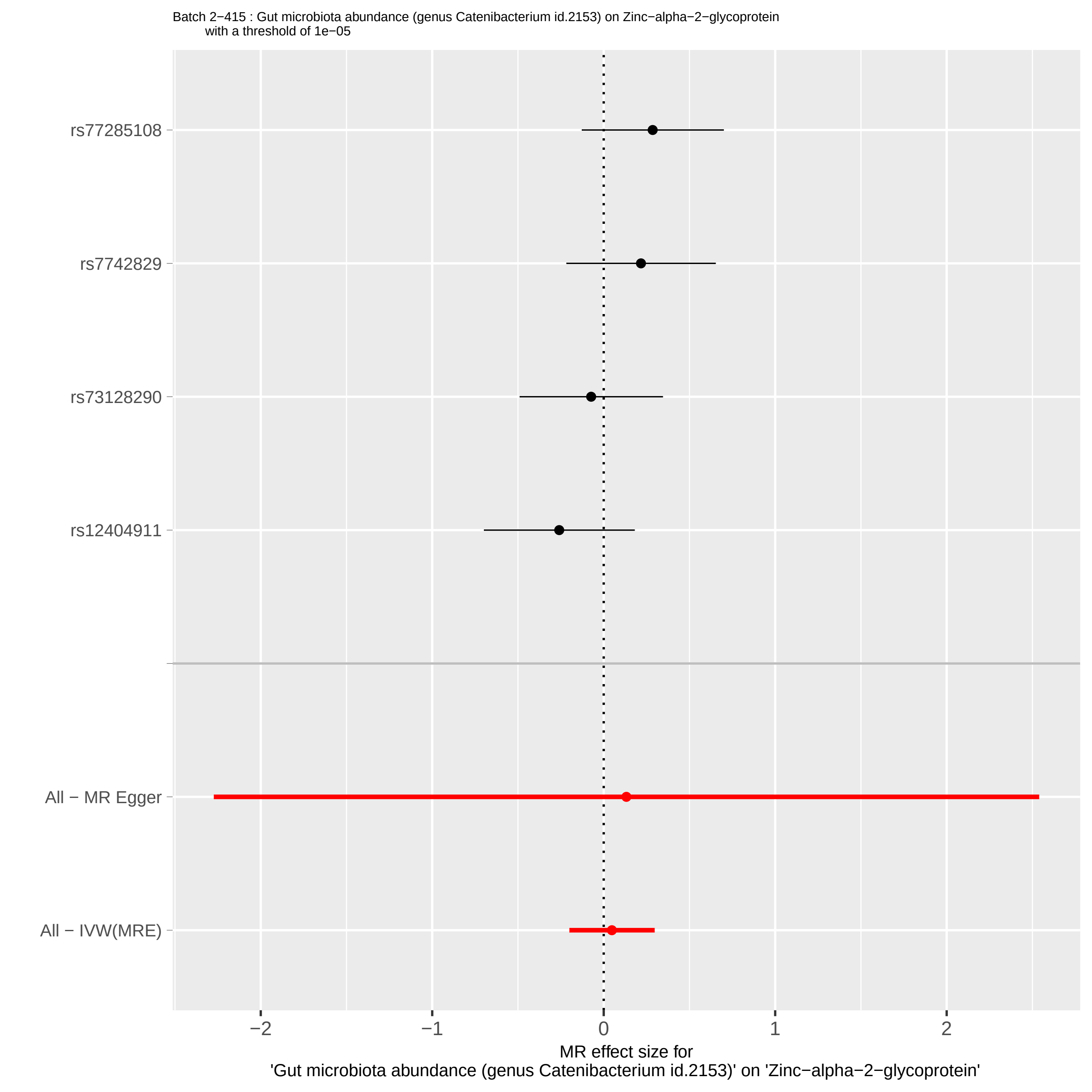

Batch 2−415 : Gut microbiota abundance (genus Catenibacterium id.2153) on Zinc−alpha−2−glycoprotein
with a threshold of 1e−05
rs77285108
rs7742829
rs73128290
rs12404911
All − MR Egger
All − IVW(MRE)
−2
−1
0
1
2
MR effect size for
'Gut microbiota abundance (genus Catenibacterium id.2153)' on 'Zinc−alpha−2−glycoprotein'

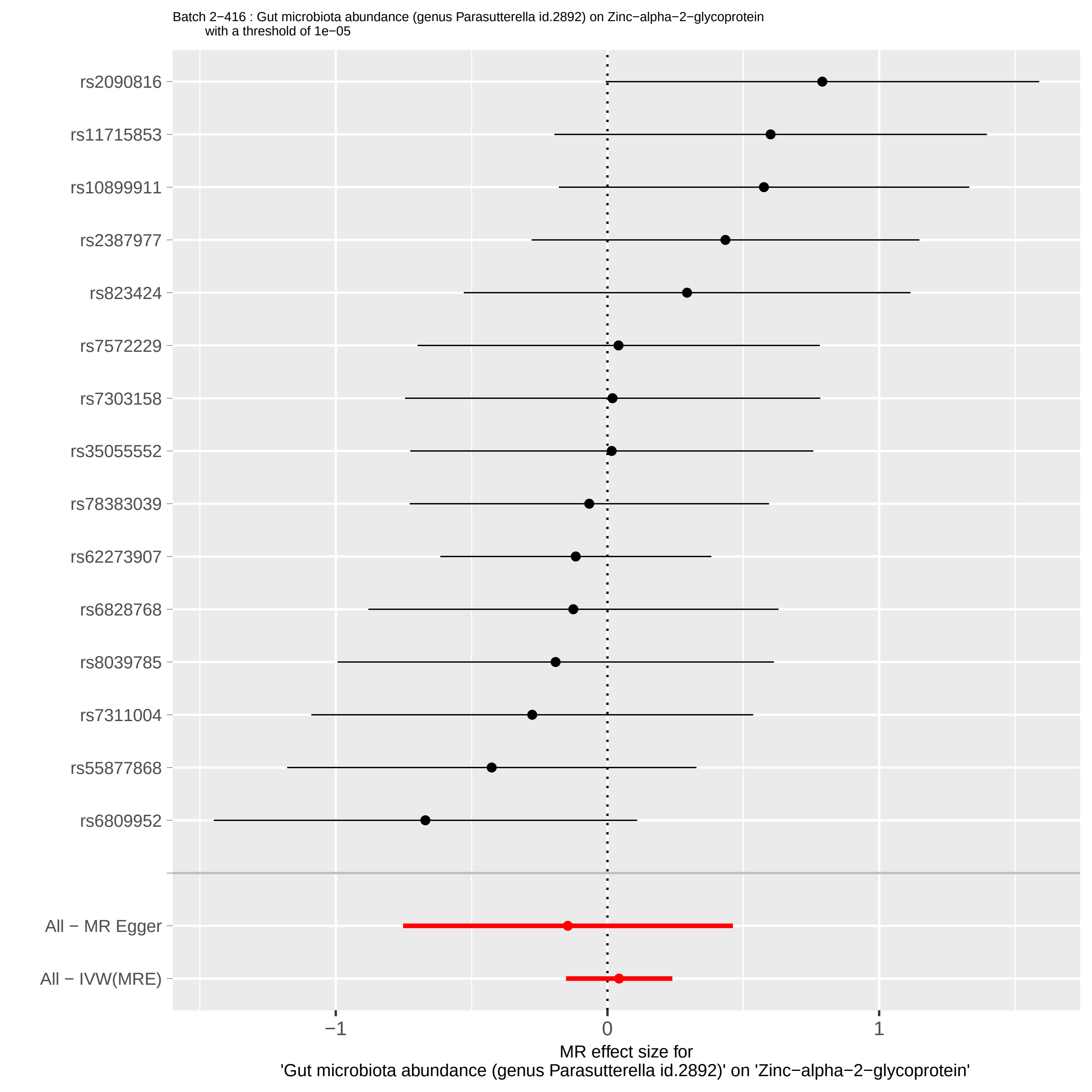
Batch 2−416 : Gut microbiota abundance (genus Parasutterella id.2892) on Zinc−alpha−2−glycoprotein
with a threshold of 1e−05
rs2090816
rs11715853
rs10899911
rs2387977
rs823424
rs7572229
rs7303158
rs35055552
rs78383039
rs62273907
rs6828768
rs8039785
rs7311004
rs55877868
rs6809952
All − MR Egger
All − IVW(MRE)
−1
0
1
MR effect size for
'Gut microbiota abundance (genus Parasutterella id.2892)' on 'Zinc−alpha−2−glycoprotein'

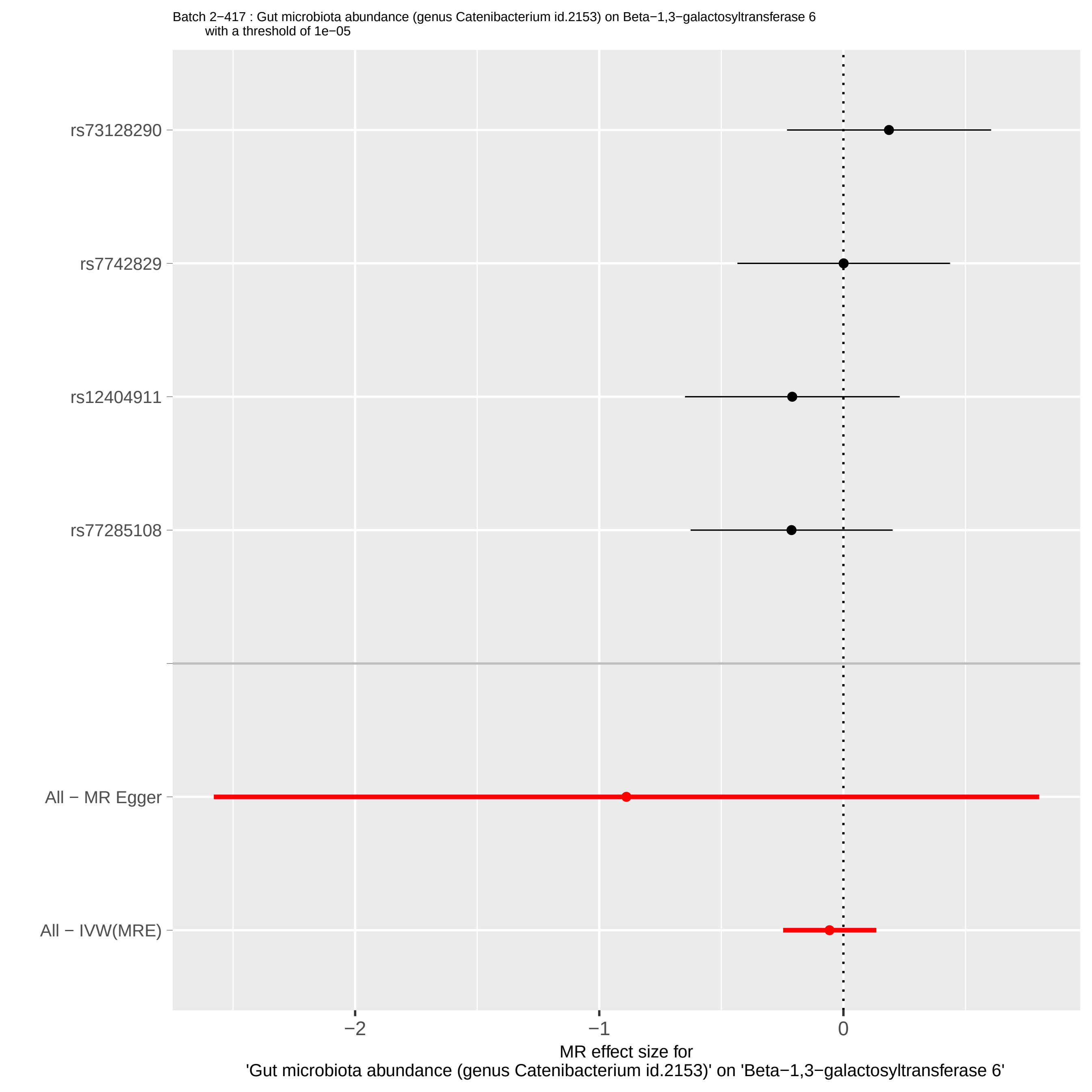

Batch 2−417 : Gut microbiota abundance (genus Catenibacterium id.2153) on Beta−1,3−galactosyltransferase 6
with a threshold of 1e−05
rs73128290
rs7742829
rs12404911
rs77285108
All − MR Egger
All − IVW(MRE)
−2
−1
0
MR effect size for
'Gut microbiota abundance (genus Catenibacterium id.2153)' on 'Beta−1,3−galactosyltransferase 6'

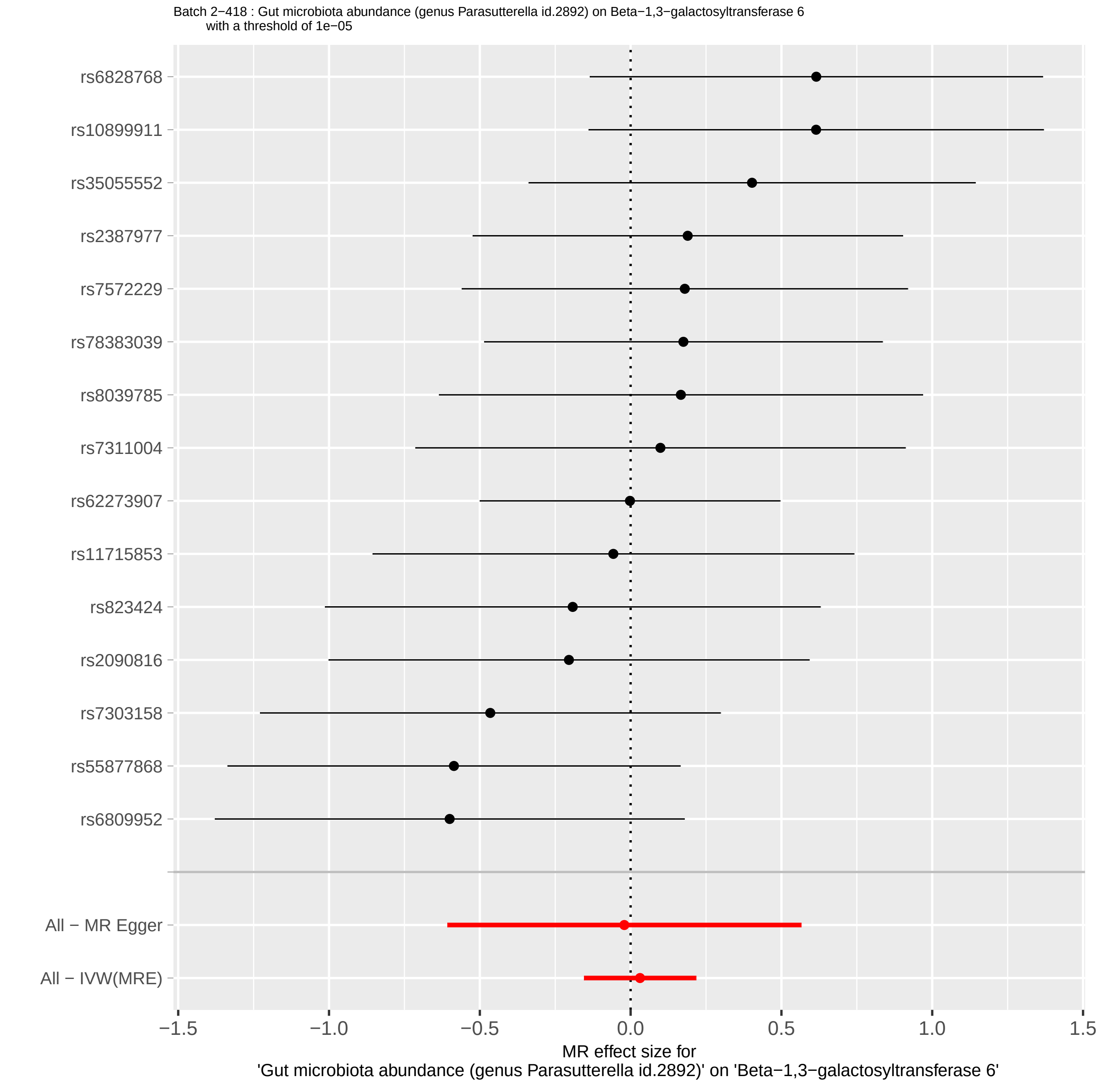
Batch 2−418 : Gut microbiota abundance (genus Parasutterella id.2892) on Beta−1,3−galactosyltransferase 6
with a threshold of 1e−05
rs6828768
rs10899911
rs35055552
rs2387977
rs7572229
rs78383039
rs8039785
rs7311004
rs62273907
rs11715853
rs823424
rs2090816
rs7303158
rs55877868
rs6809952
All − MR Egger
All − IVW(MRE)
−1.5
−1.0
−0.5
0.0
0.5
1.0
1.5
MR effect size for
'Gut microbiota abundance (genus Parasutterella id.2892)' on 'Beta−1,3−galactosyltransferase 6'

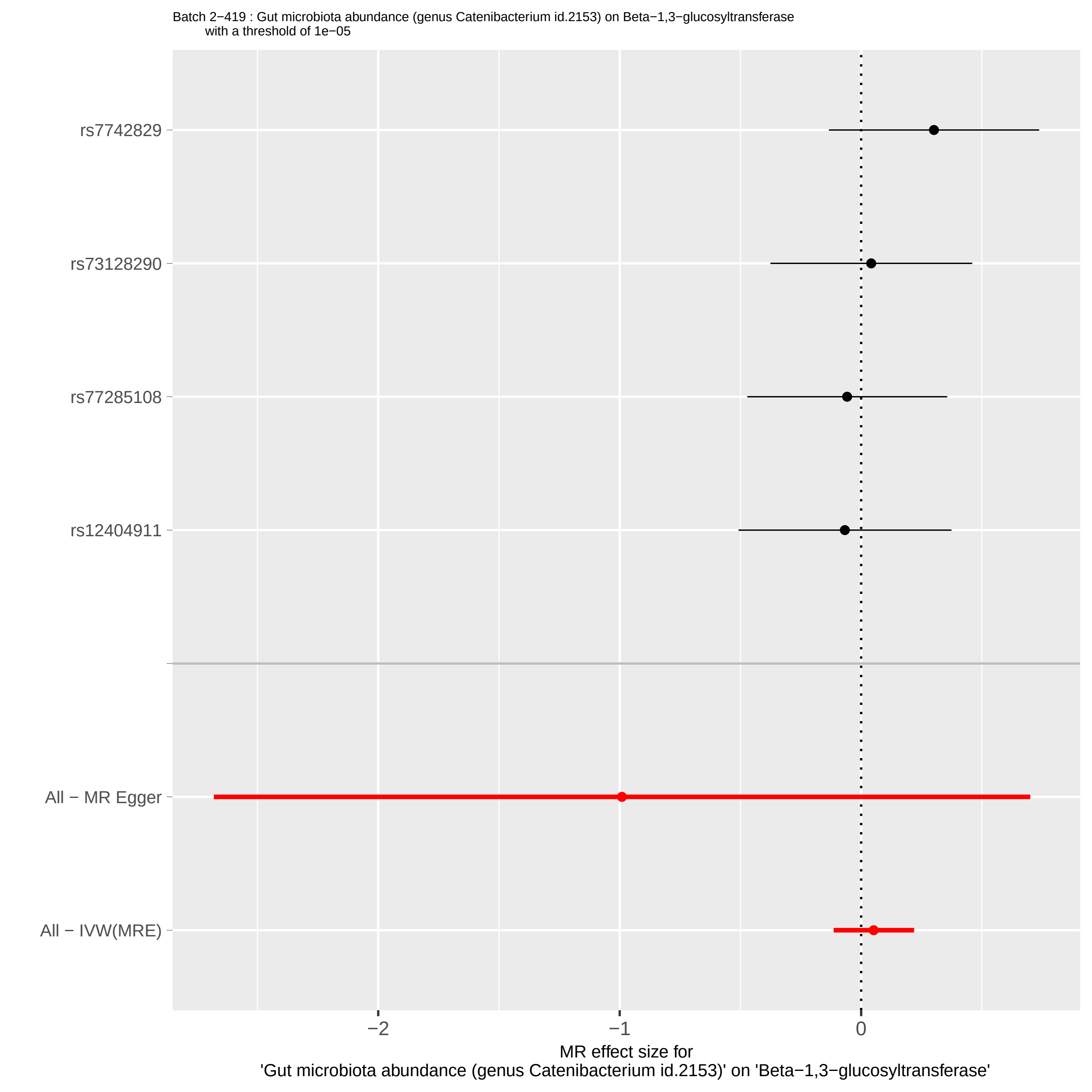

Batch 2−419 : Gut microbiota abundance (genus Catenibacterium id.2153) on Beta−1,3−glucosyltransferase
with a threshold of 1e−05
rs7742829
rs73128290
rs77285108
rs12404911
All − MR Egger
All − IVW(MRE)
−2
−1
0
MR effect size for
'Gut microbiota abundance (genus Catenibacterium id.2153)' on 'Beta−1,3−glucosyltransferase'

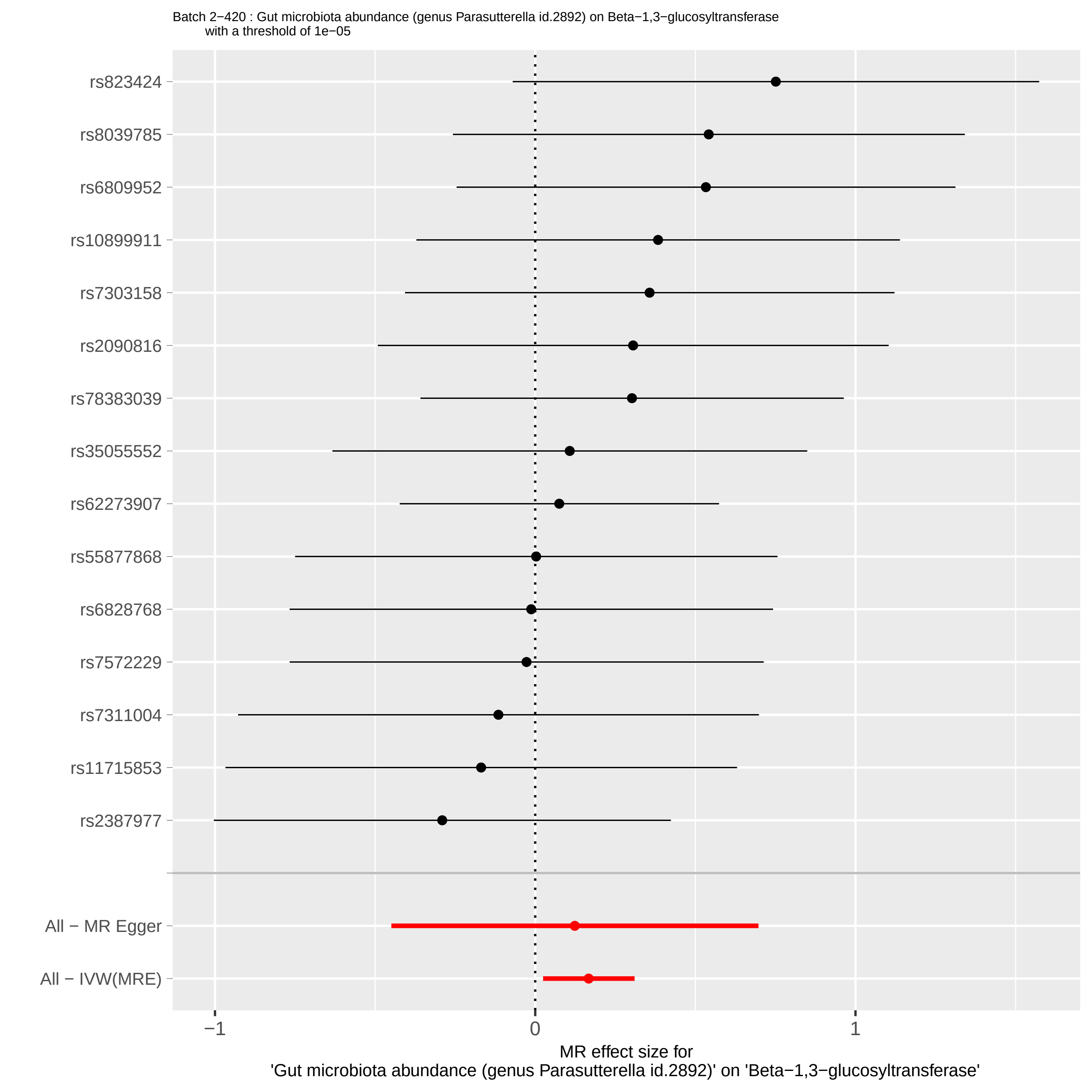

Batch 2−420 : Gut microbiota abundance (genus Parasutterella id.2892) on Beta−1,3−glucosyltransferase
with a threshold of 1e−05
rs823424
rs8039785
rs6809952
rs10899911
rs7303158
rs2090816
rs78383039
rs35055552
rs62273907
rs55877868
rs6828768
rs7572229
rs7311004
rs11715853
rs2387977
All − MR Egger
All − IVW(MRE)
−1
0
1
MR effect size for
'Gut microbiota abundance (genus Parasutterella id.2892)' on 'Beta−1,3−glucosyltransferase'

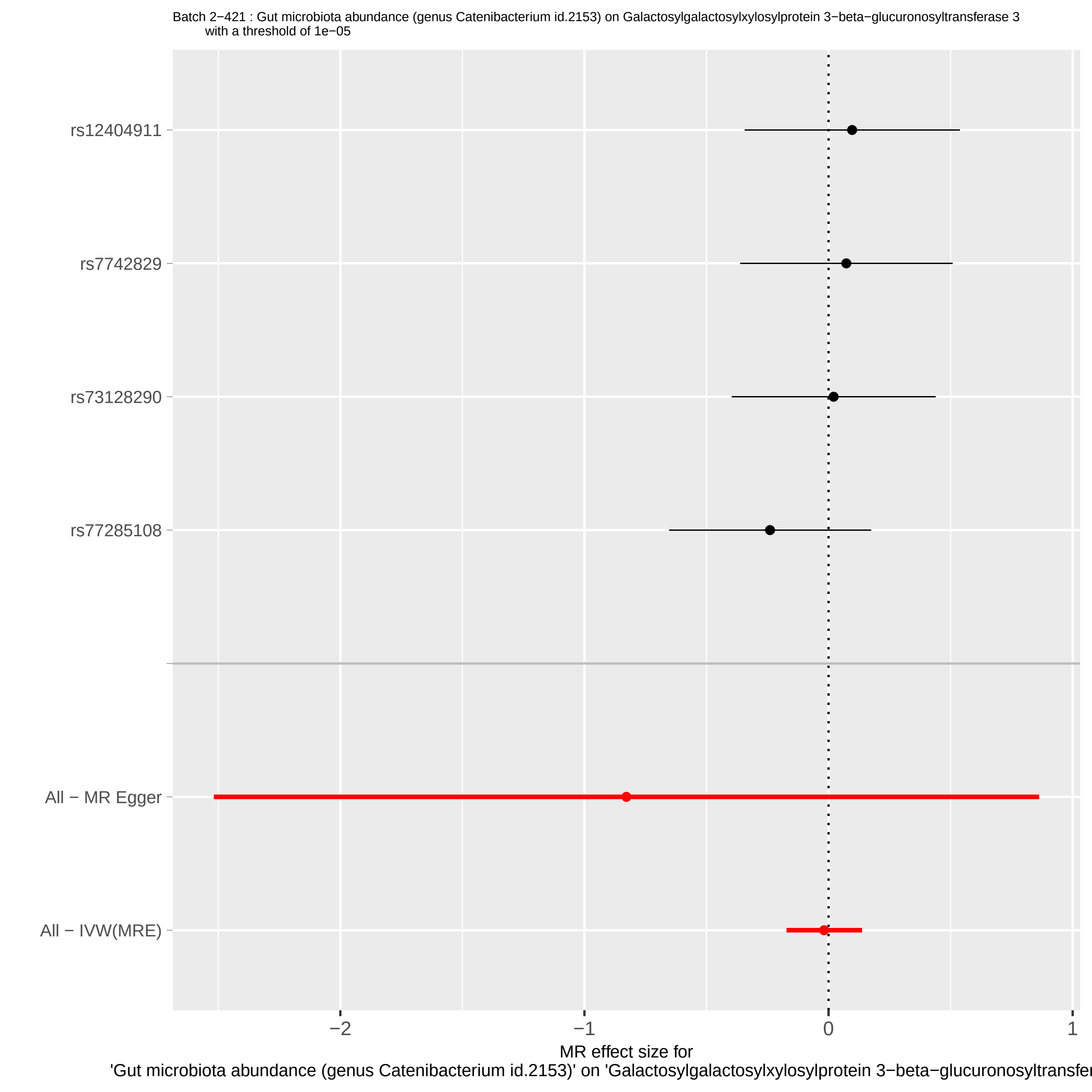
Batch 2−421 : Gut microbiota abundance (genus Catenibacterium id.2153) on Galactosylgalactosylxylosylprotein 3−beta−glucuronosyltransferase 3
with a threshold of 1e−05
rs12404911
rs7742829
rs73128290
rs77285108
All − MR Egger
All − IVW(MRE)
−2
−1
0
1
MR effect size for
'Gut microbiota abundance (genus Catenibacterium id.2153)' on 'Galactosylgalactosylxylosylprotein 3−beta−glucuronosyltransfer

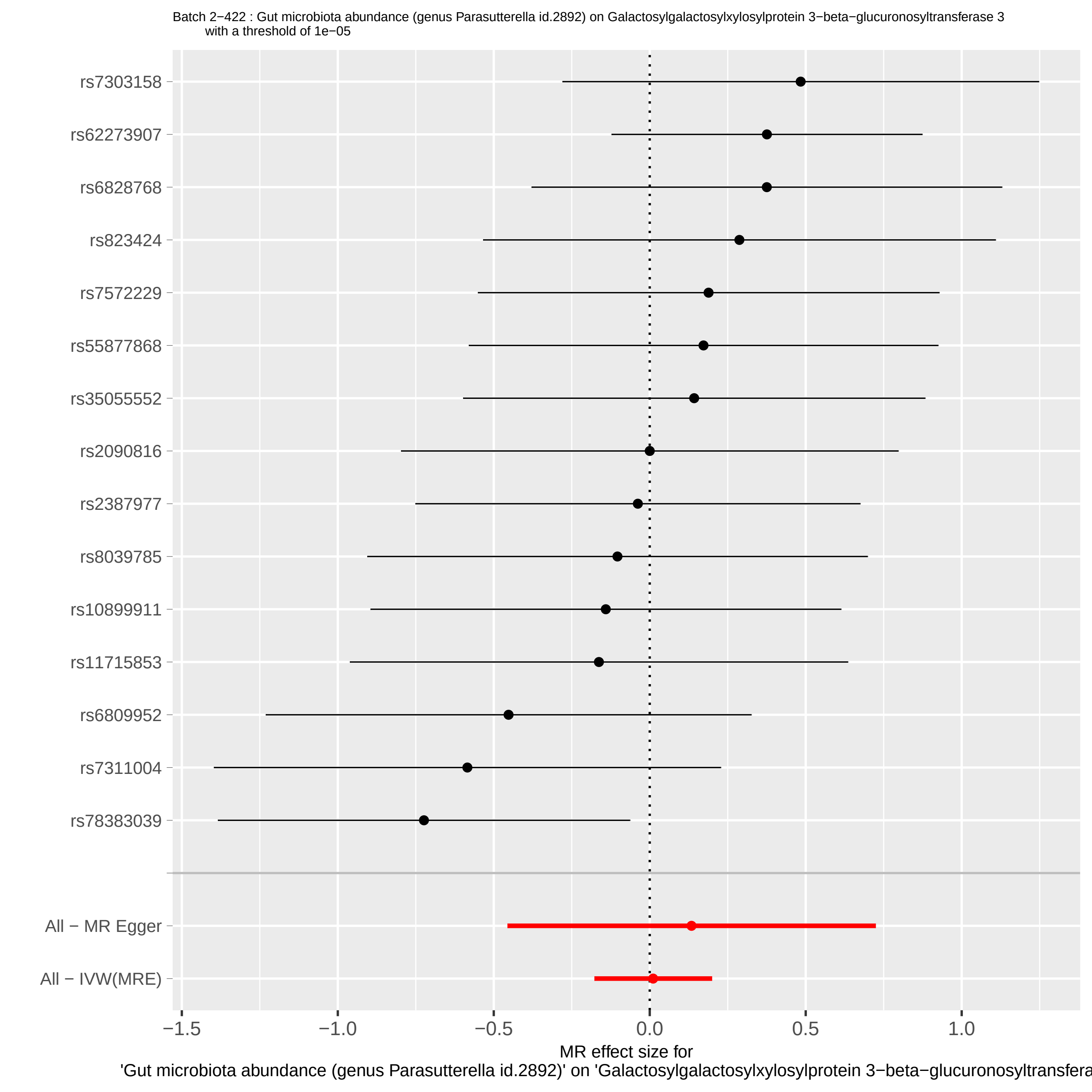
Batch 2−422 : Gut microbiota abundance (genus Parasutterella id.2892) on Galactosylgalactosylxylosylprotein 3−beta−glucuronosyltransferase 3
with a threshold of 1e−05
rs7303158
rs62273907
rs6828768
rs823424
rs7572229
rs55877868
rs35055552
rs2090816
rs2387977
rs8039785
rs10899911
rs11715853
rs6809952
rs7311004
rs78383039
All − MR Egger
All − IVW(MRE)
−1.5
−1.0
−0.5
0.0
0.5
1.0
MR effect size for
'Gut microbiota abundance (genus Parasutterella id.2892)' on 'Galactosylgalactosylxylosylprotein 3−beta−glucuronosyltransfera

Batch 2−423 : Gut microbiota abundance (genus Catenibacterium id.2153) on Beta−1,4−glucuronyltransferase 1
with a threshold of 1e−05

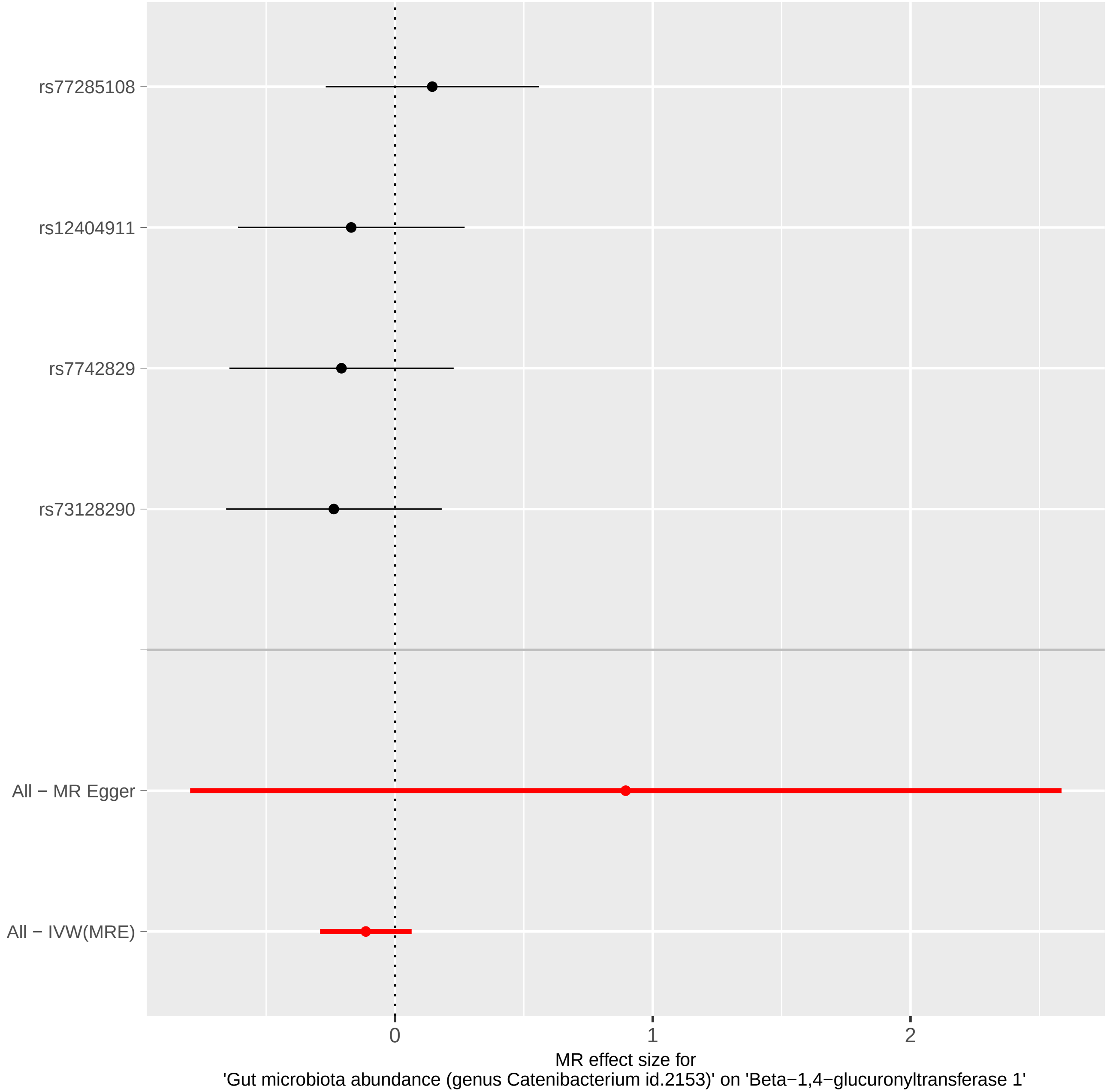

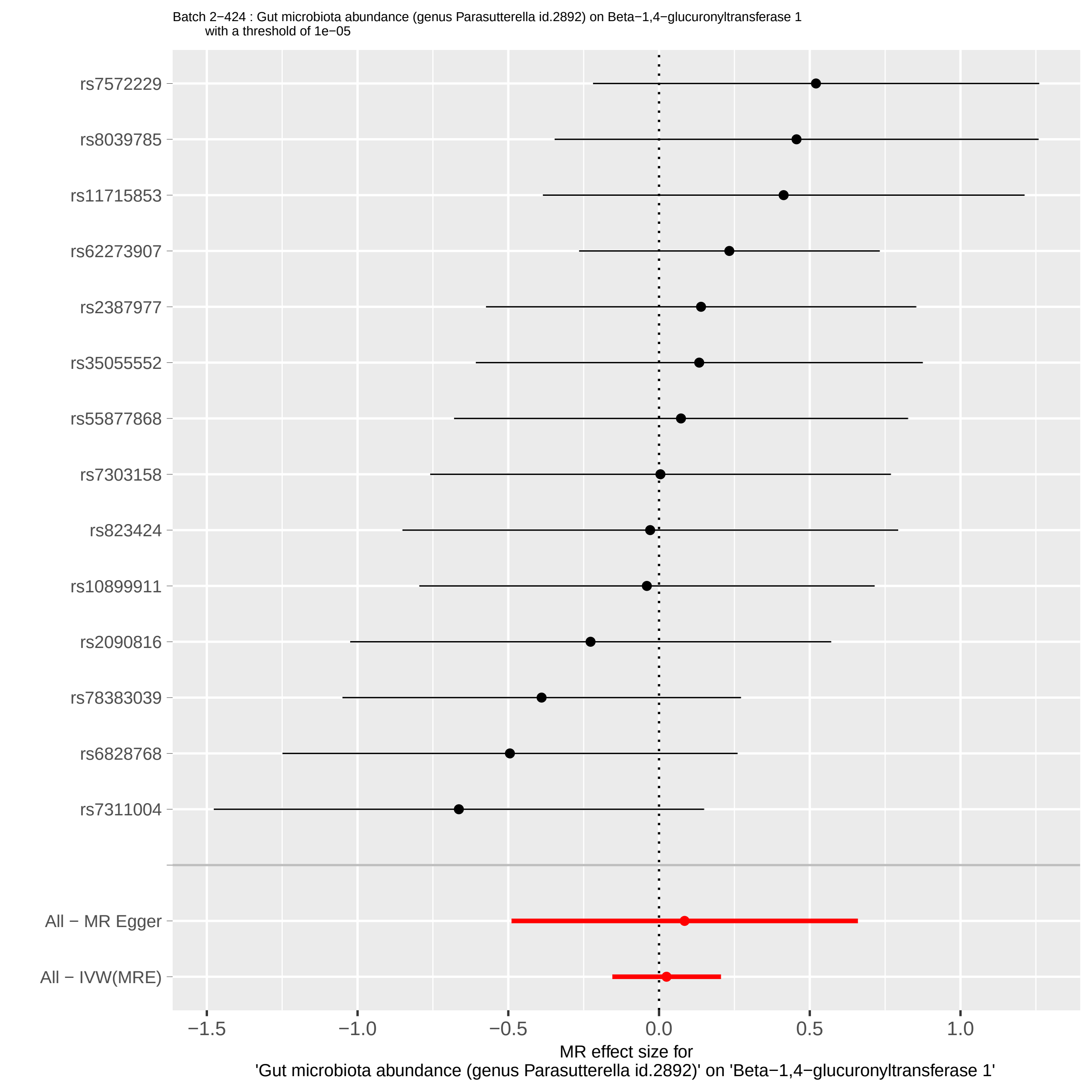
Batch 2−424 : Gut microbiota abundance (genus Parasutterella id.2892) on Beta−1,4−glucuronyltransferase 1
with a threshold of 1e−05
rs7572229
rs8039785
rs11715853
rs62273907
rs2387977
rs35055552
rs55877868
rs7303158
rs823424
rs10899911
rs2090816
rs78383039
rs6828768
rs7311004
All − MR Egger
All − IVW(MRE)
−1.5
−1.0
−0.5
0.0
0.5
1.0
MR effect size for
'Gut microbiota abundance (genus Parasutterella id.2892)' on 'Beta−1,4−glucuronyltransferase 1'

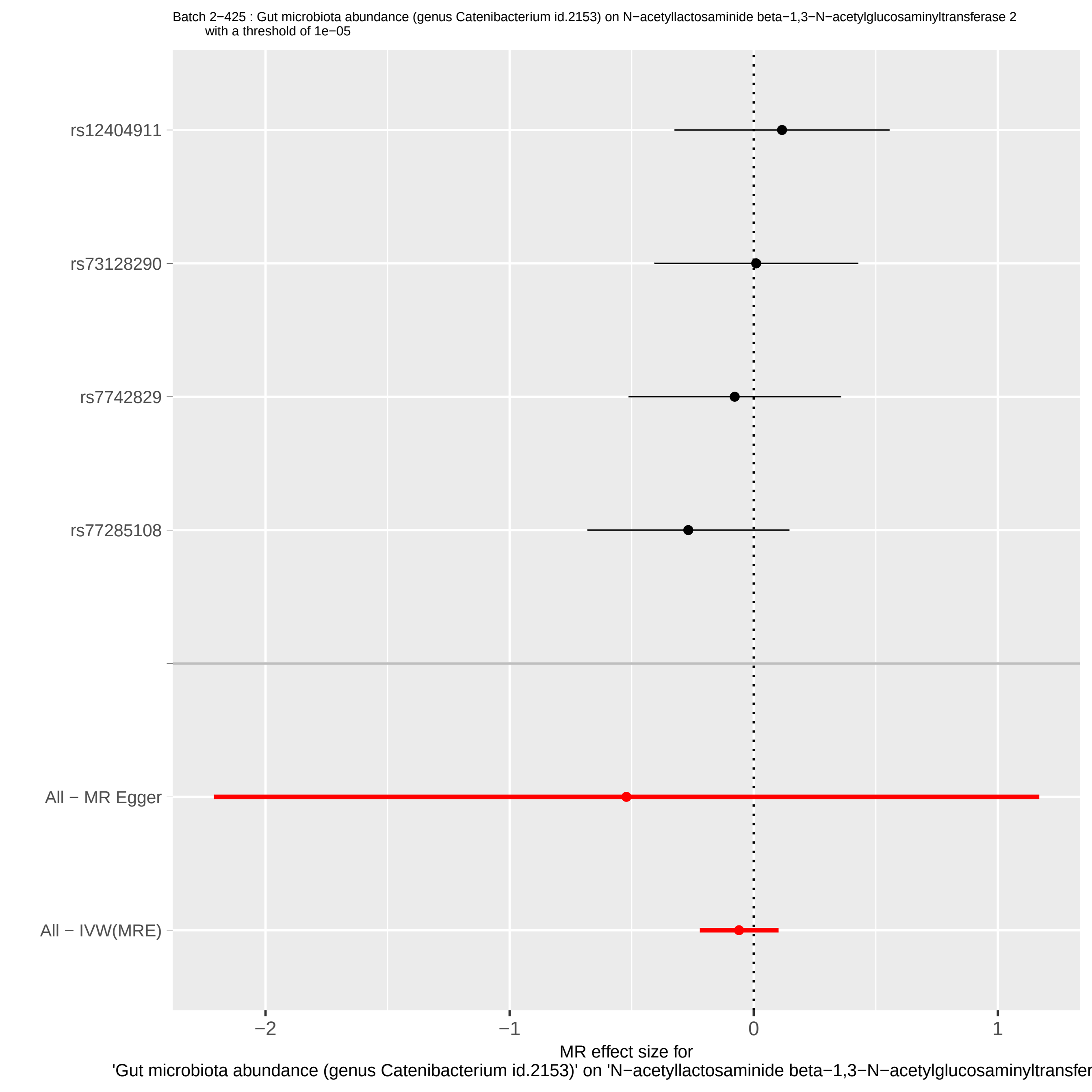

Batch 2−425 : Gut microbiota abundance (genus Catenibacterium id.2153) on N−acetyllactosaminide beta−1,3−N−acetylglucosaminyltransferase 2
with a threshold of 1e−05
rs12404911
rs73128290
rs7742829
rs77285108
All − MR Egger
All − IVW(MRE)
−2
−1
0
1
MR effect size for
'Gut microbiota abundance (genus Catenibacterium id.2153)' on 'N−acetyllactosaminide beta−1,3−N−acetylglucosaminyltransfer

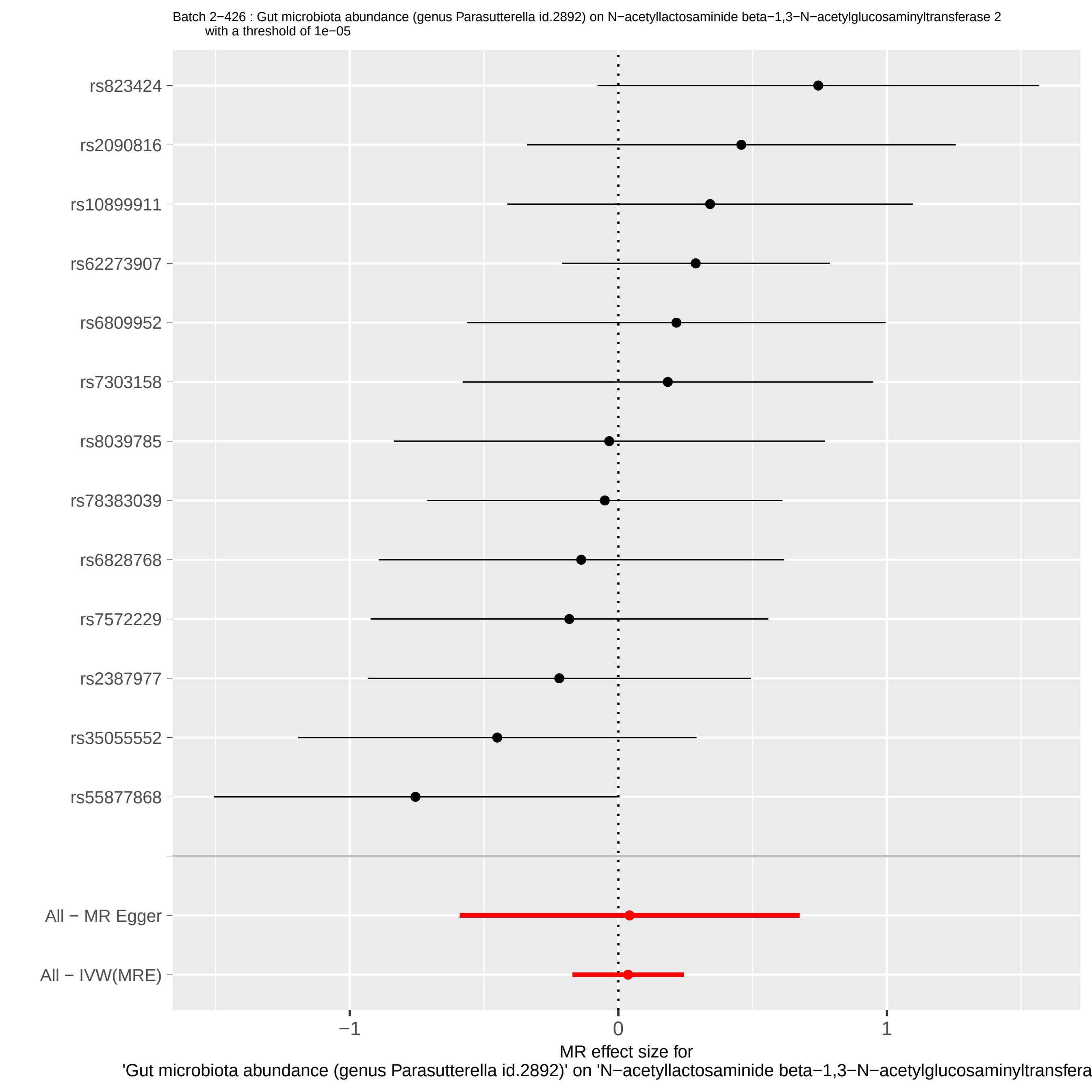

Batch 2−426 : Gut microbiota abundance (genus Parasutterella id.2892) on N−acetyllactosaminide beta−1,3−N−acetylglucosaminyltransferase 2
with a threshold of 1e−05
rs823424
rs2090816
rs10899911
rs62273907
rs6809952
rs7303158
rs8039785
rs78383039
rs6828768
rs7572229
rs2387977
rs35055552
rs55877868
All − MR Egger
All − IVW(MRE)
−1
0
1
MR effect size for
'Gut microbiota abundance (genus Parasutterella id.2892)' on 'N−acetyllactosaminide beta−1,3−N−acetylglucosaminyltransfera

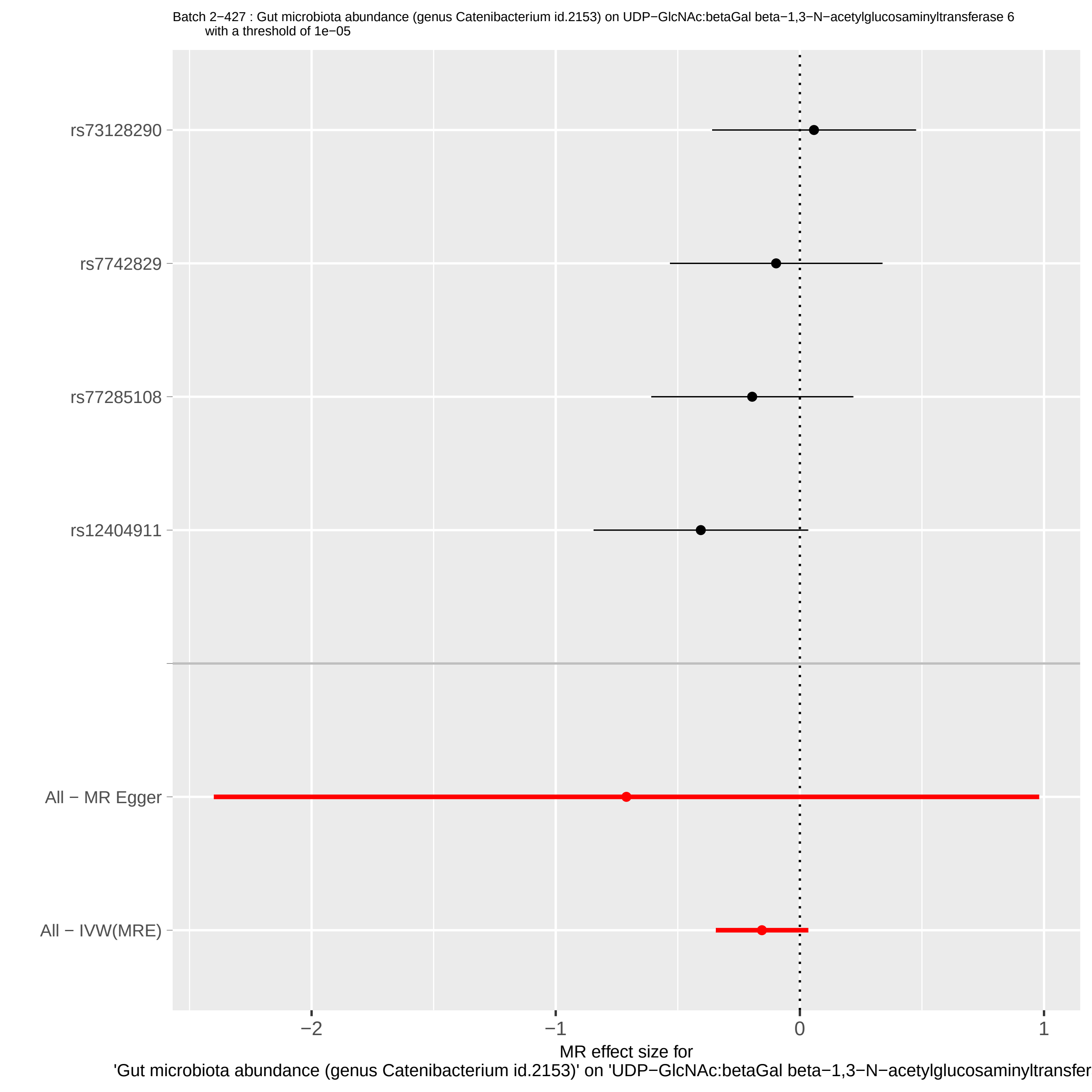

Batch 2−427 : Gut microbiota abundance (genus Catenibacterium id.2153) on UDP−GlcNAc:betaGal beta−1,3−N−acetylglucosaminyltransferase 6
with a threshold of 1e−05
rs73128290
rs7742829
rs77285108
rs12404911
All − MR Egger
All − IVW(MRE)
−2
−1
0
1
MR effect size for
'Gut microbiota abundance (genus Catenibacterium id.2153)' on 'UDP−GlcNAc:betaGal beta−1,3−N−acetylglucosaminyltransfer

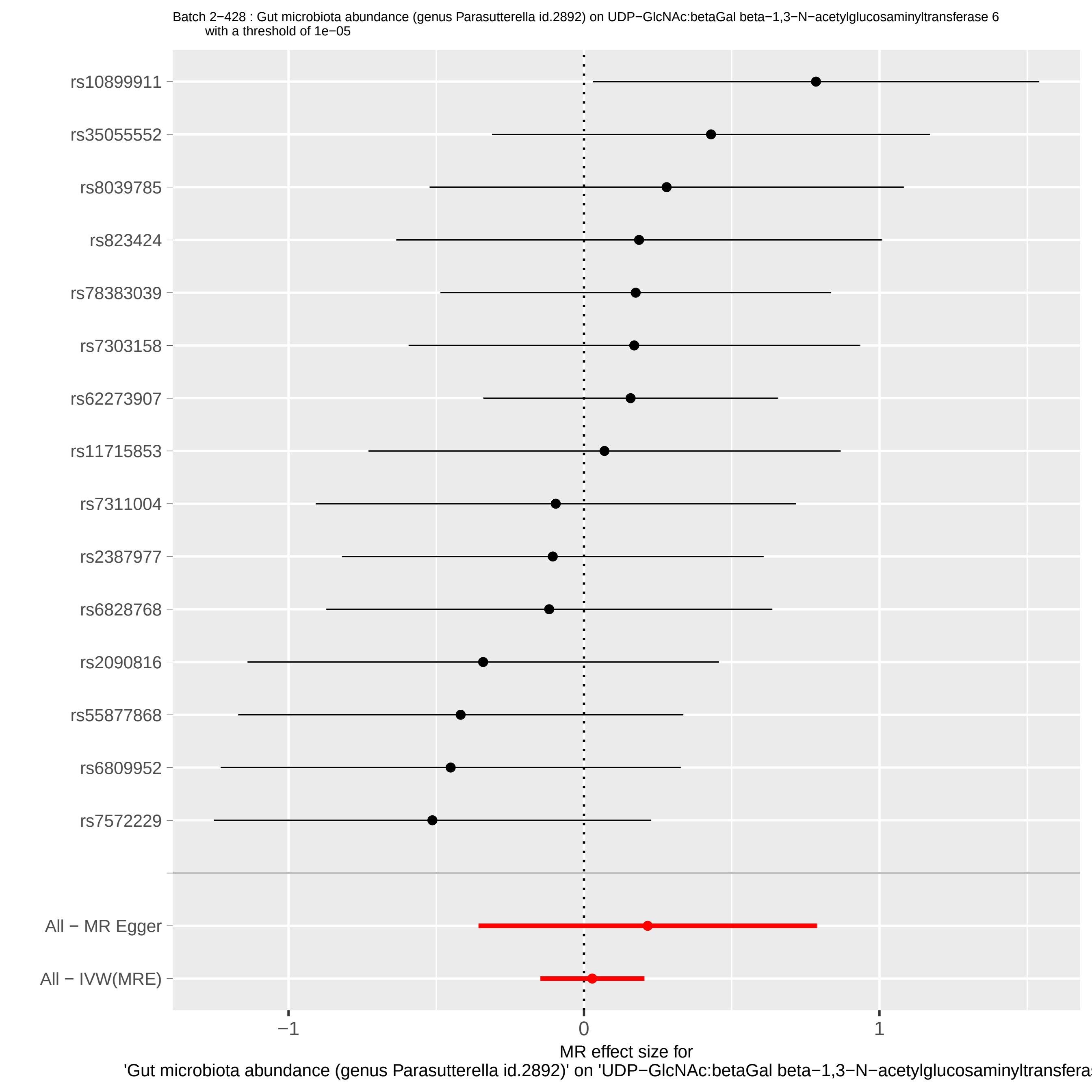

Batch 2−428 : Gut microbiota abundance (genus Parasutterella id.2892) on UDP−GlcNAc:betaGal beta−1,3−N−acetylglucosaminyltransferase 6
with a threshold of 1e−05
rs10899911
rs35055552
rs8039785
rs823424
rs78383039
rs7303158
rs62273907
rs11715853
rs7311004
rs2387977
rs6828768
rs2090816
rs55877868
rs6809952
rs7572229
All − MR Egger
All − IVW(MRE)
−1
0
1
MR effect size for
'Gut microbiota abundance (genus Parasutterella id.2892)' on 'UDP−GlcNAc:betaGal beta−1,3−N−acetylglucosaminyltransfera

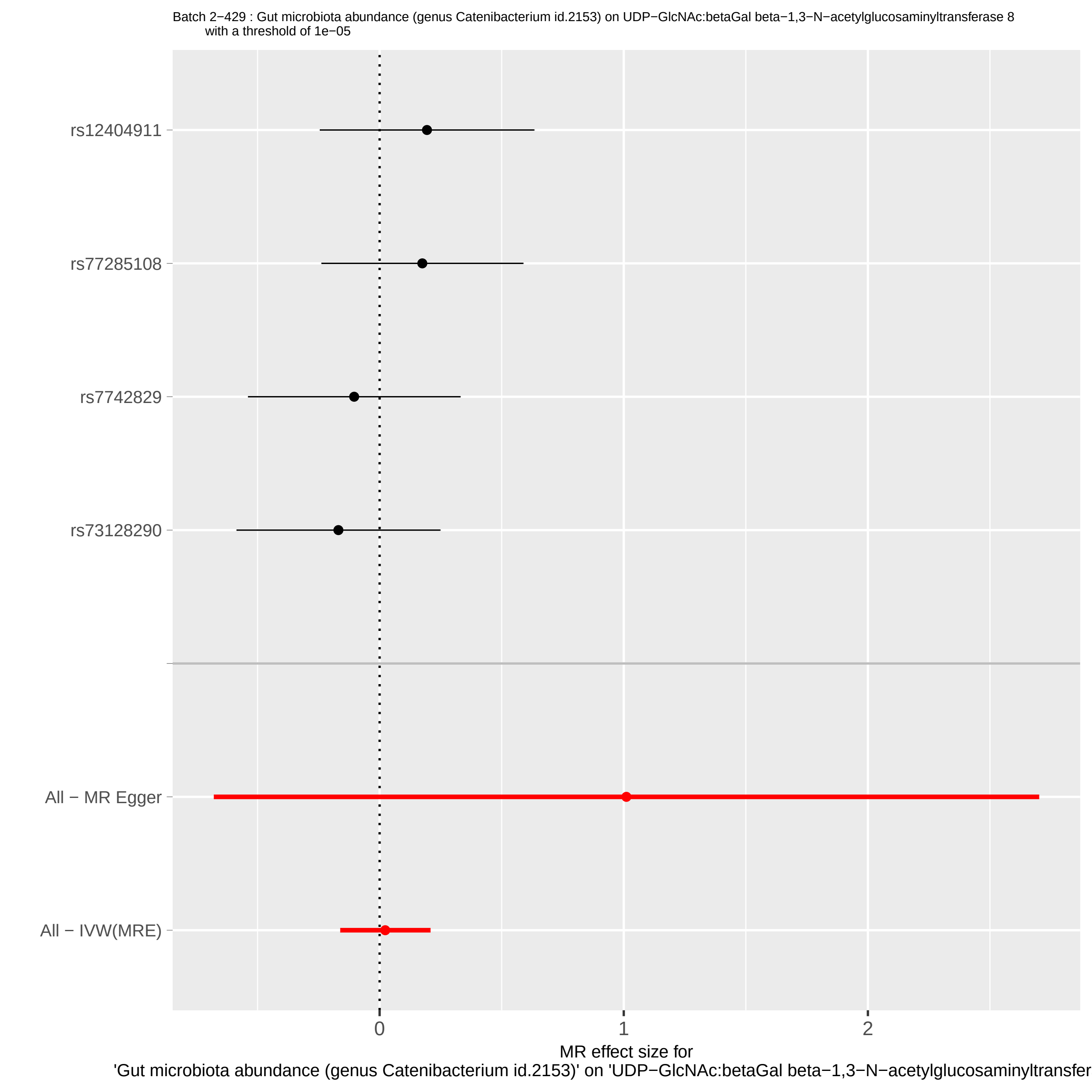
Batch 2−429 : Gut microbiota abundance (genus Catenibacterium id.2153) on UDP−GlcNAc:betaGal beta−1,3−N−acetylglucosaminyltransferase 8
with a threshold of 1e−05
rs12404911
rs77285108
rs7742829
rs73128290
All − MR Egger
All − IVW(MRE)
0
1
2
MR effect size for
'Gut microbiota abundance (genus Catenibacterium id.2153)' on 'UDP−GlcNAc:betaGal beta−1,3−N−acetylglucosaminyltransfer

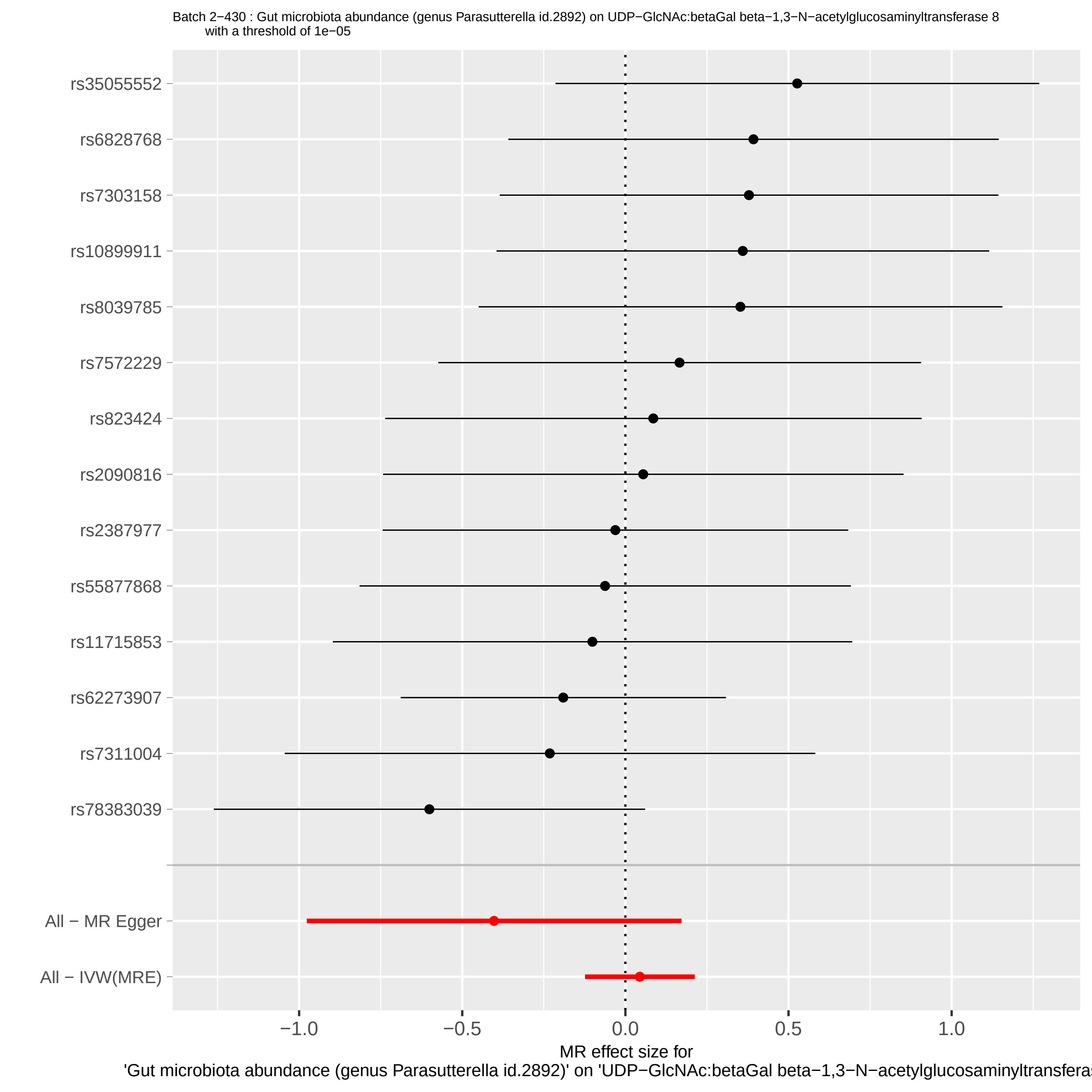

Batch 2−430 : Gut microbiota abundance (genus Parasutterella id.2892) on UDP−GlcNAc:betaGal beta−1,3−N−acetylglucosaminyltransferase 8
with a threshold of 1e−05
rs35055552
rs6828768
rs7303158
rs10899911
rs8039785
rs7572229
rs823424
rs2090816
rs2387977
rs55877868
rs11715853
rs62273907
rs7311004
rs78383039
All − MR Egger
All − IVW(MRE)
−1.0
−0.5
0.0
0.5
1.0
MR effect size for
'Gut microbiota abundance (genus Parasutterella id.2892)' on 'UDP−GlcNAc:betaGal beta−1,3−N−acetylglucosaminyltransfera

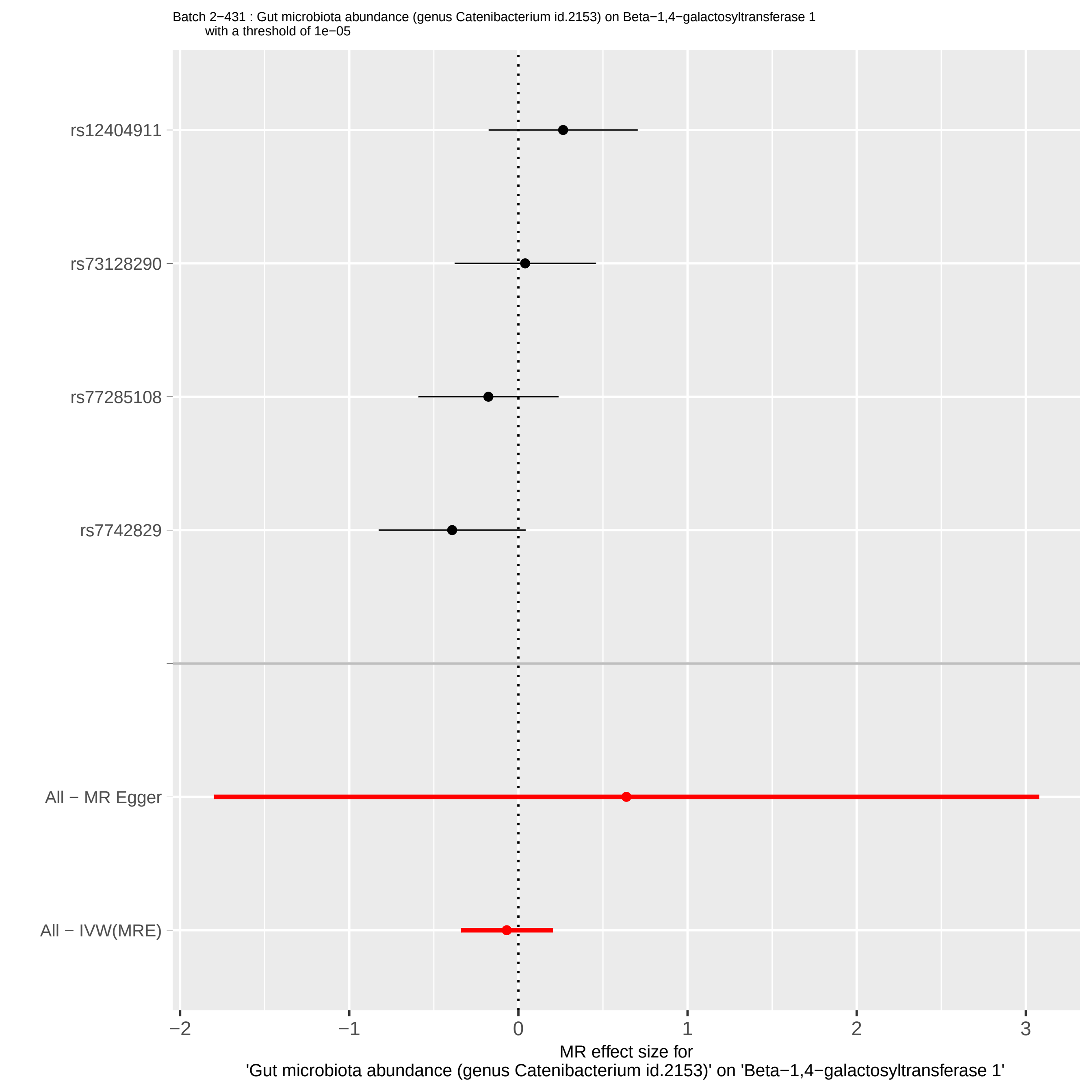

Batch 2−431 : Gut microbiota abundance (genus Catenibacterium id.2153) on Beta−1,4−galactosyltransferase 1
with a threshold of 1e−05
rs12404911
rs73128290
rs77285108
rs7742829
All − MR Egger
All − IVW(MRE)
−2
−1
0
1
2
3
MR effect size for
'Gut microbiota abundance (genus Catenibacterium id.2153)' on 'Beta−1,4−galactosyltransferase 1'

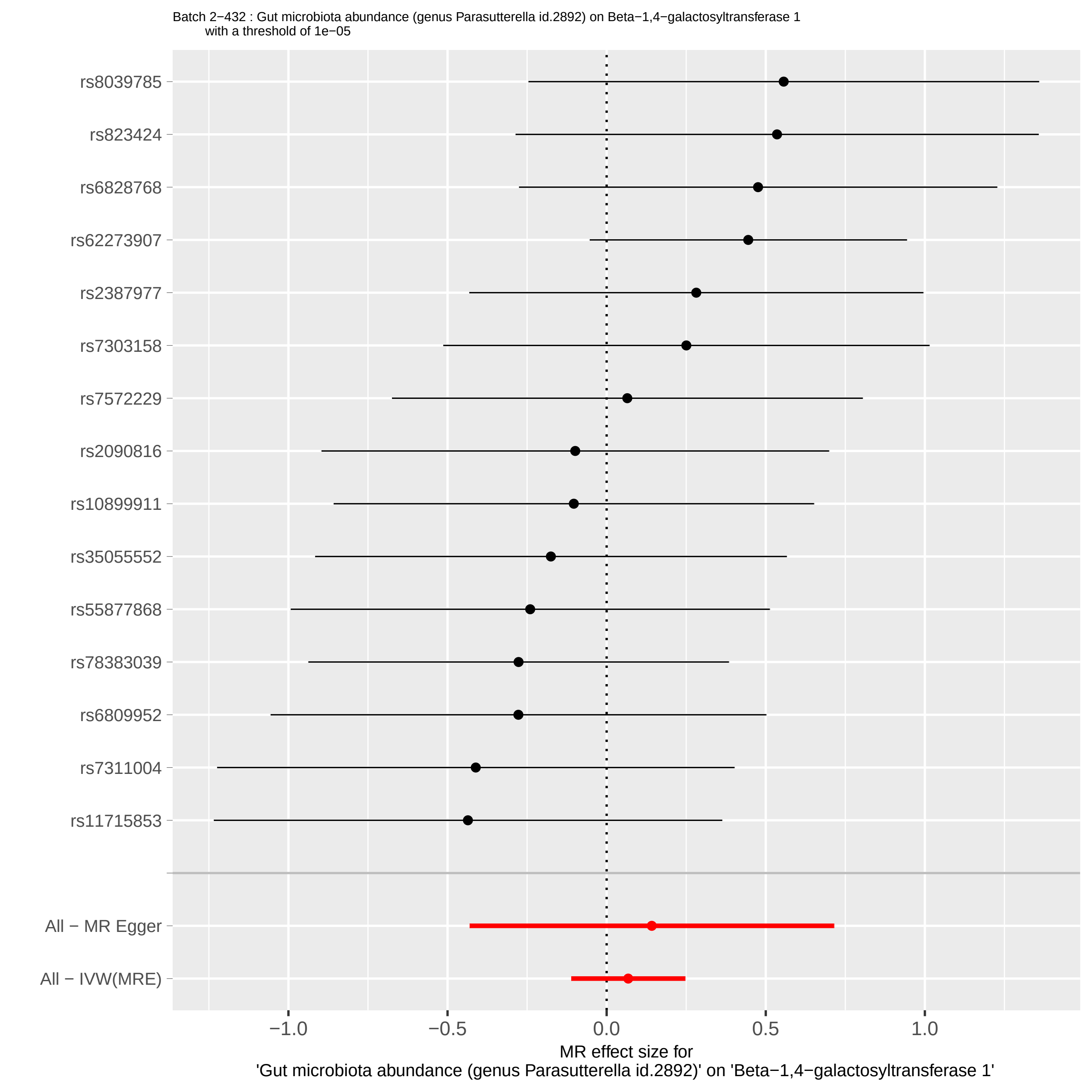

Batch 2−432 : Gut microbiota abundance (genus Parasutterella id.2892) on Beta−1,4−galactosyltransferase 1
with a threshold of 1e−05
rs8039785
rs823424
rs6828768
rs62273907
rs2387977
rs7303158
rs7572229
rs2090816
rs10899911
rs35055552
rs55877868
rs78383039
rs6809952
rs7311004
rs11715853
All − MR Egger
All − IVW(MRE)
−1.0
−0.5
0.0
0.5
1.0
MR effect size for
'Gut microbiota abundance (genus Parasutterella id.2892)' on 'Beta−1,4−galactosyltransferase 1'

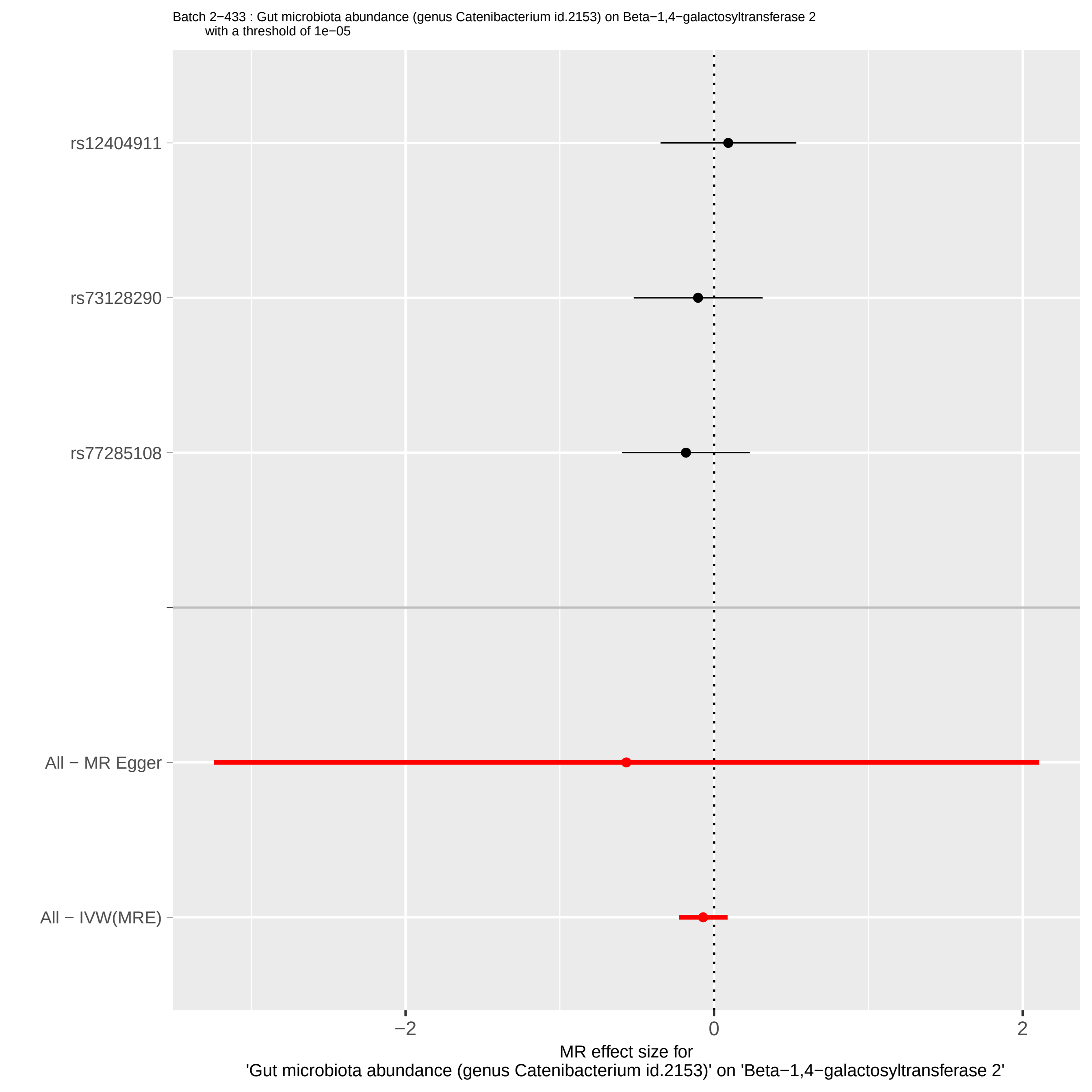
Batch 2−433 : Gut microbiota abundance (genus Catenibacterium id.2153) on Beta−1,4−galactosyltransferase 2
with a threshold of 1e−05
rs12404911
rs73128290
rs77285108
All − MR Egger
All − IVW(MRE)
−2
0
2
MR effect size for
'Gut microbiota abundance (genus Catenibacterium id.2153)' on 'Beta−1,4−galactosyltransferase 2'

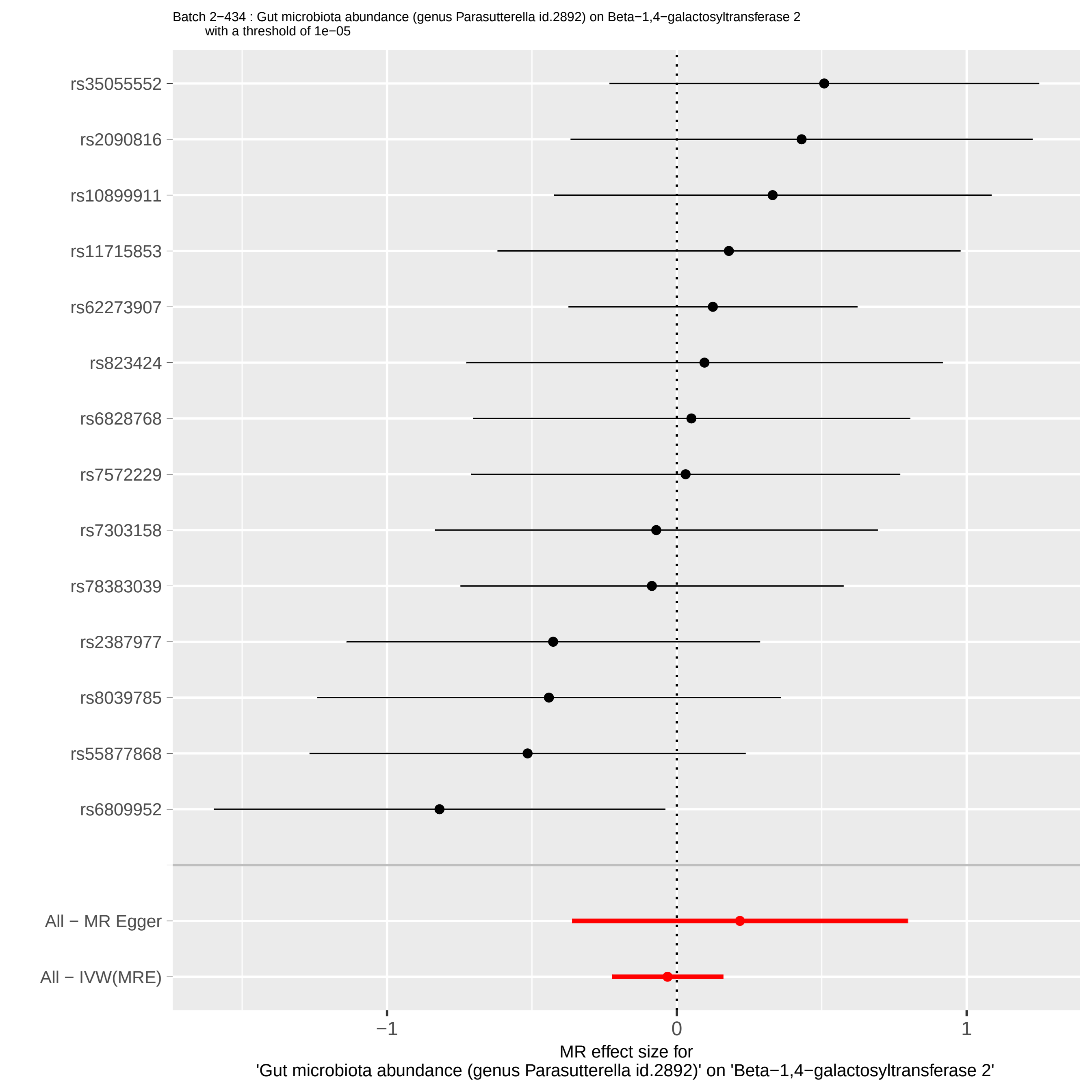

Batch 2−434 : Gut microbiota abundance (genus Parasutterella id.2892) on Beta−1,4−galactosyltransferase 2
with a threshold of 1e−05
rs35055552
rs2090816
rs10899911
rs11715853
rs62273907
rs823424
rs6828768
rs7572229
rs7303158
rs78383039
rs2387977
rs8039785
rs55877868
rs6809952
All − MR Egger
All − IVW(MRE)
−1
0
1
MR effect size for
'Gut microbiota abundance (genus Parasutterella id.2892)' on 'Beta−1,4−galactosyltransferase 2'

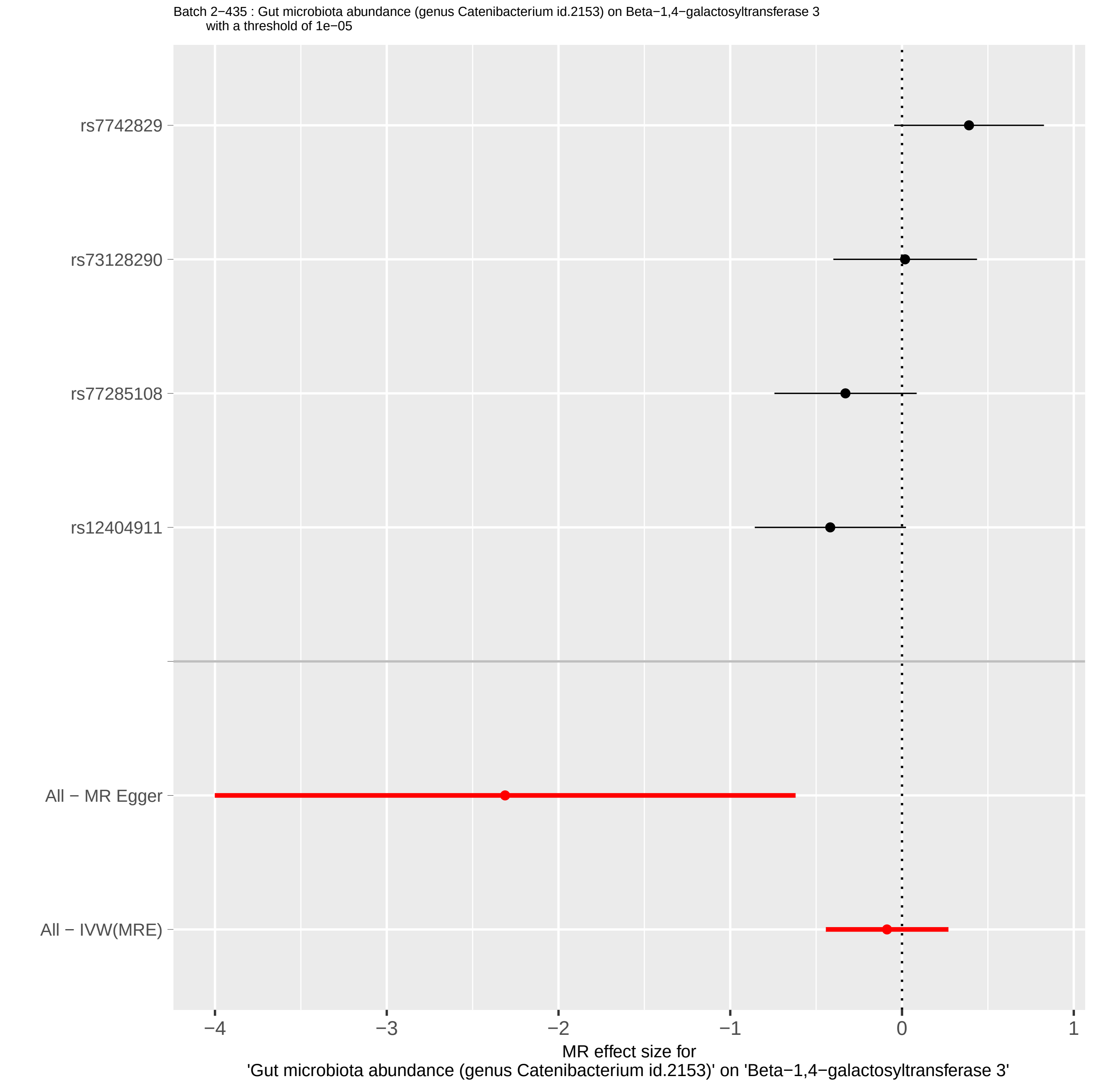

Batch 2−435 : Gut microbiota abundance (genus Catenibacterium id.2153) on Beta−1,4−galactosyltransferase 3
with a threshold of 1e−05
rs7742829
rs73128290
rs77285108
rs12404911
All − MR Egger
All − IVW(MRE)
−4
−3
−2
−1
0
1
MR effect size for
'Gut microbiota abundance (genus Catenibacterium id.2153)' on 'Beta−1,4−galactosyltransferase 3'

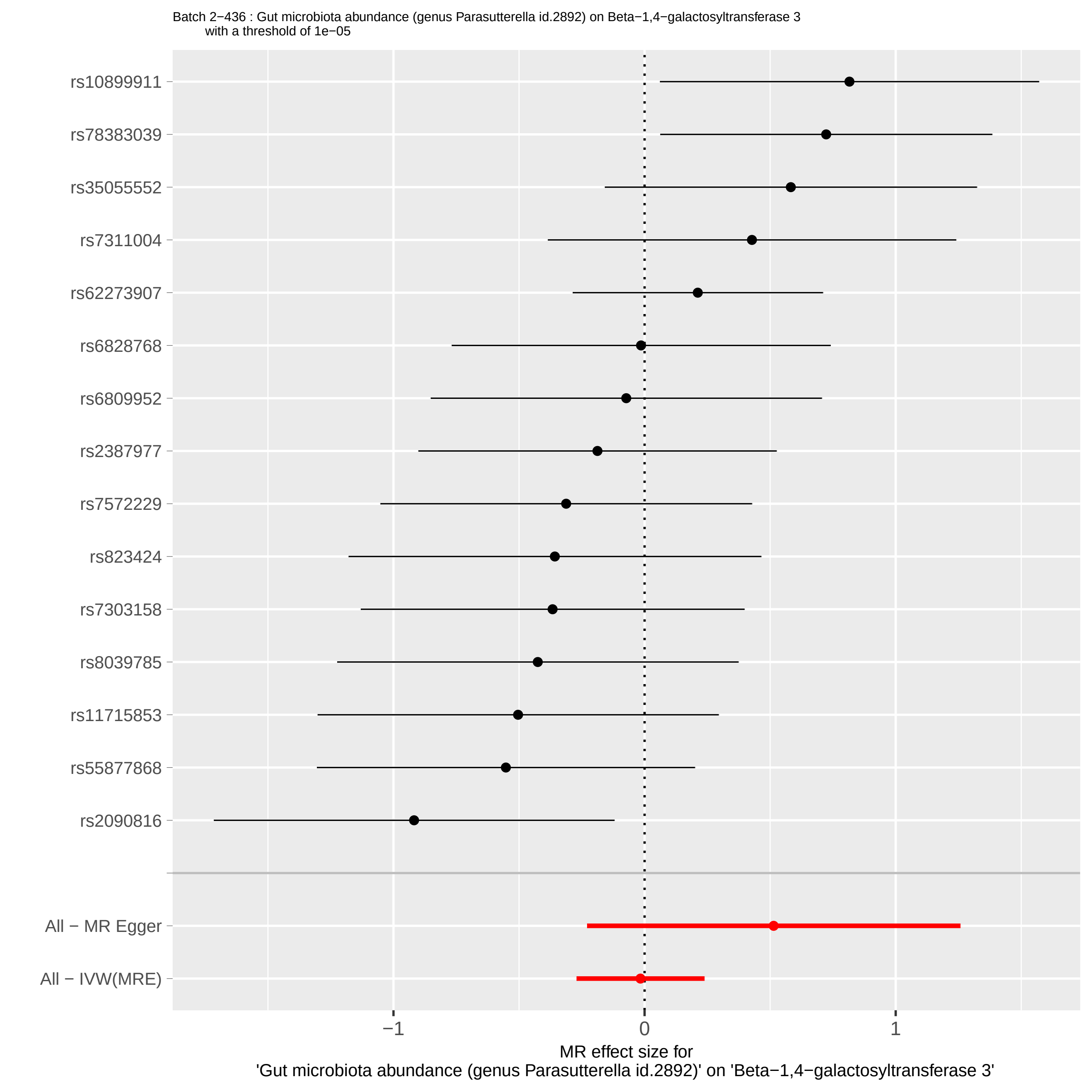
Batch 2−436 : Gut microbiota abundance (genus Parasutterella id.2892) on Beta−1,4−galactosyltransferase 3
with a threshold of 1e−05
rs10899911
rs78383039
rs35055552
rs7311004
rs62273907
rs6828768
rs6809952
rs2387977
rs7572229
rs823424
rs7303158
rs8039785
rs11715853
rs55877868
rs2090816
All − MR Egger
All − IVW(MRE)
−1
0
1
MR effect size for
'Gut microbiota abundance (genus Parasutterella id.2892)' on 'Beta−1,4−galactosyltransferase 3'

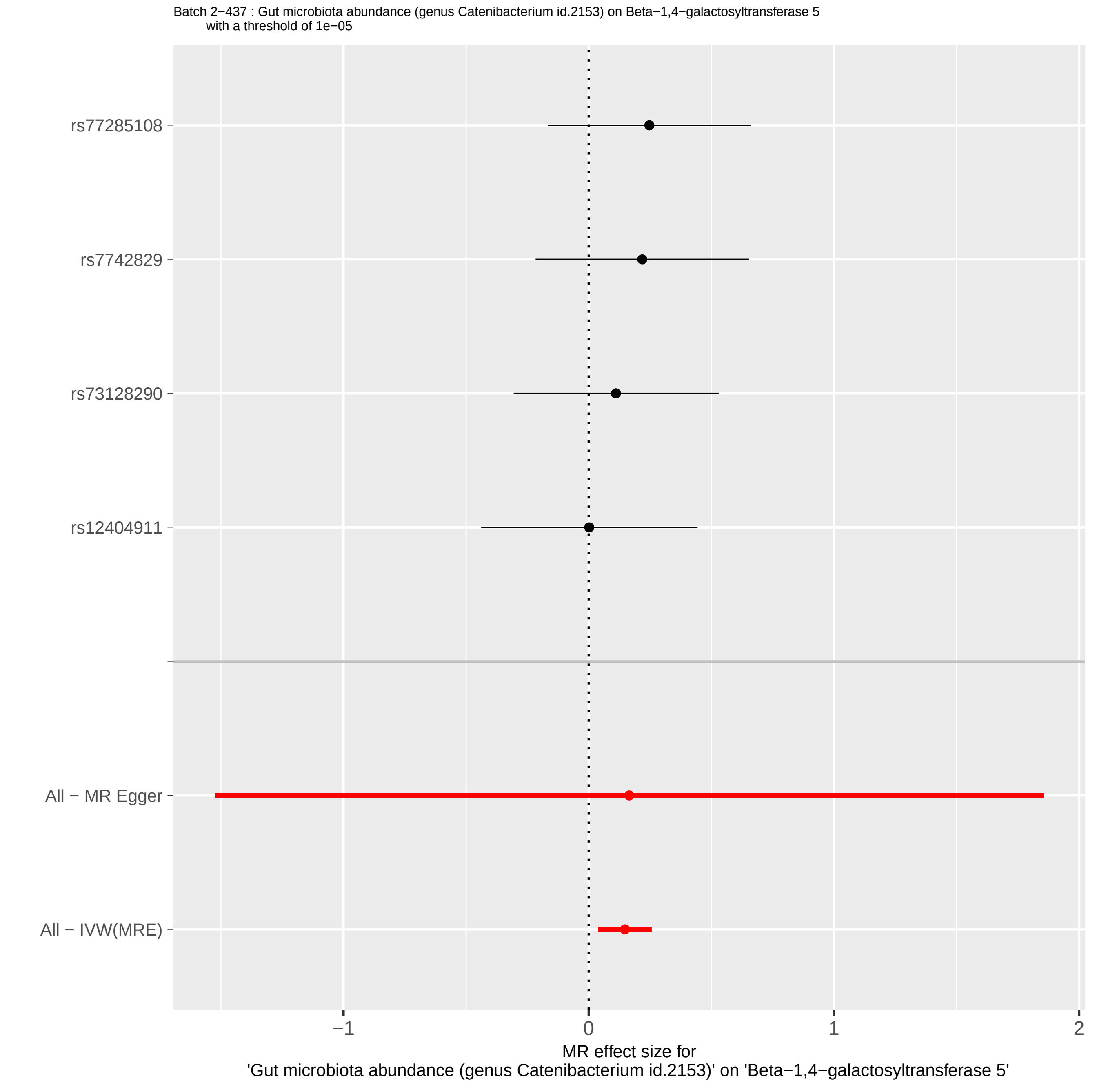

Batch 2−437 : Gut microbiota abundance (genus Catenibacterium id.2153) on Beta−1,4−galactosyltransferase 5
with a threshold of 1e−05
rs77285108
rs7742829
rs73128290
rs12404911
All − MR Egger
All − IVW(MRE)
−1
0
1
2
MR effect size for
'Gut microbiota abundance (genus Catenibacterium id.2153)' on 'Beta−1,4−galactosyltransferase 5'

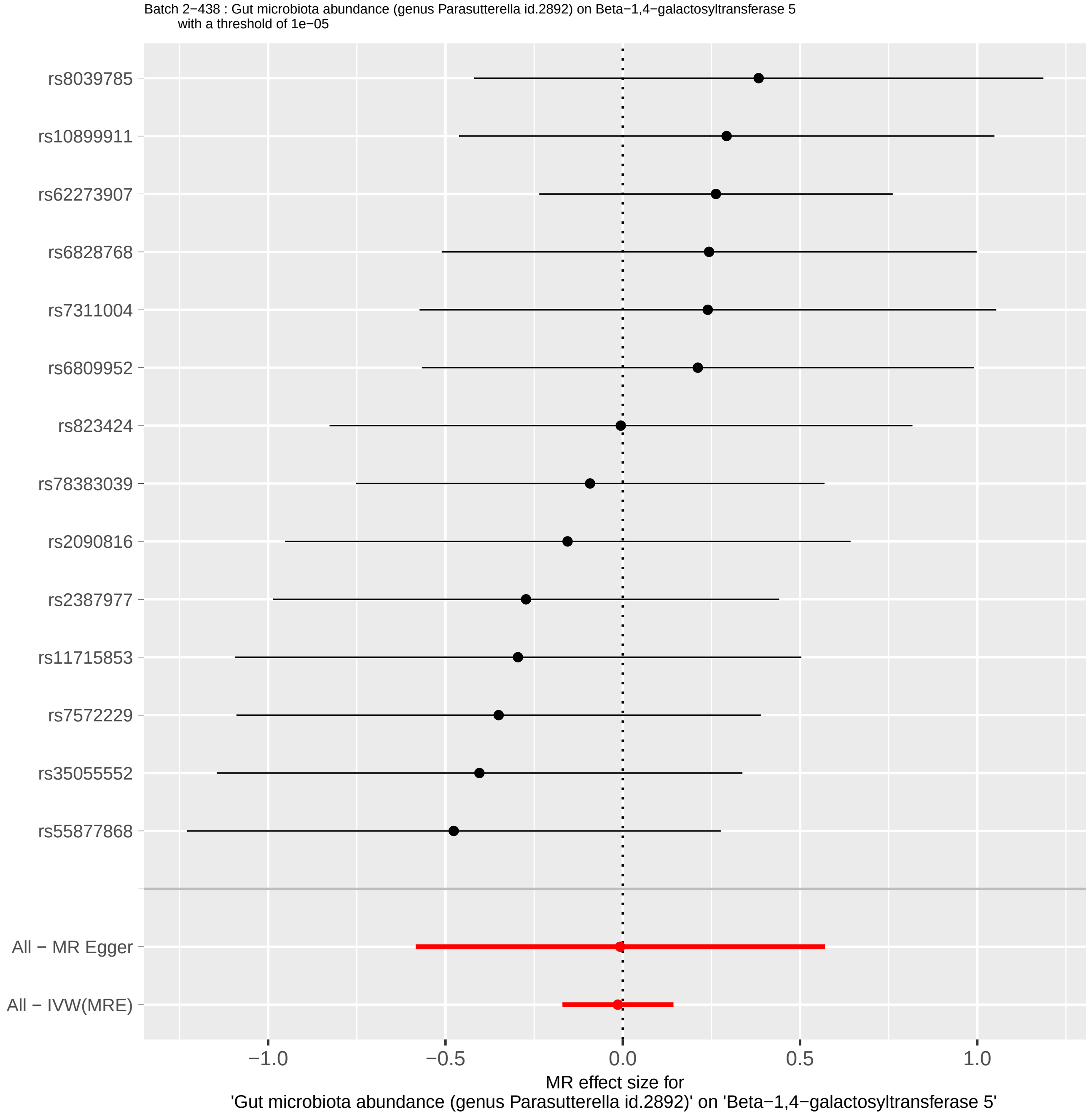

Batch 2−438 : Gut microbiota abundance (genus Parasutterella id.2892) on Beta−1,4−galactosyltransferase 5
with a threshold of 1e−05
rs8039785
rs10899911
rs62273907
rs6828768
rs7311004
rs6809952
rs823424
rs78383039
rs2090816
rs2387977
rs11715853
rs7572229
rs35055552
rs55877868
All − MR Egger
All − IVW(MRE)
−1.0
−0.5
0.0
0.5
1.0
MR effect size for
'Gut microbiota abundance (genus Parasutterella id.2892)' on 'Beta−1,4−galactosyltransferase 5'

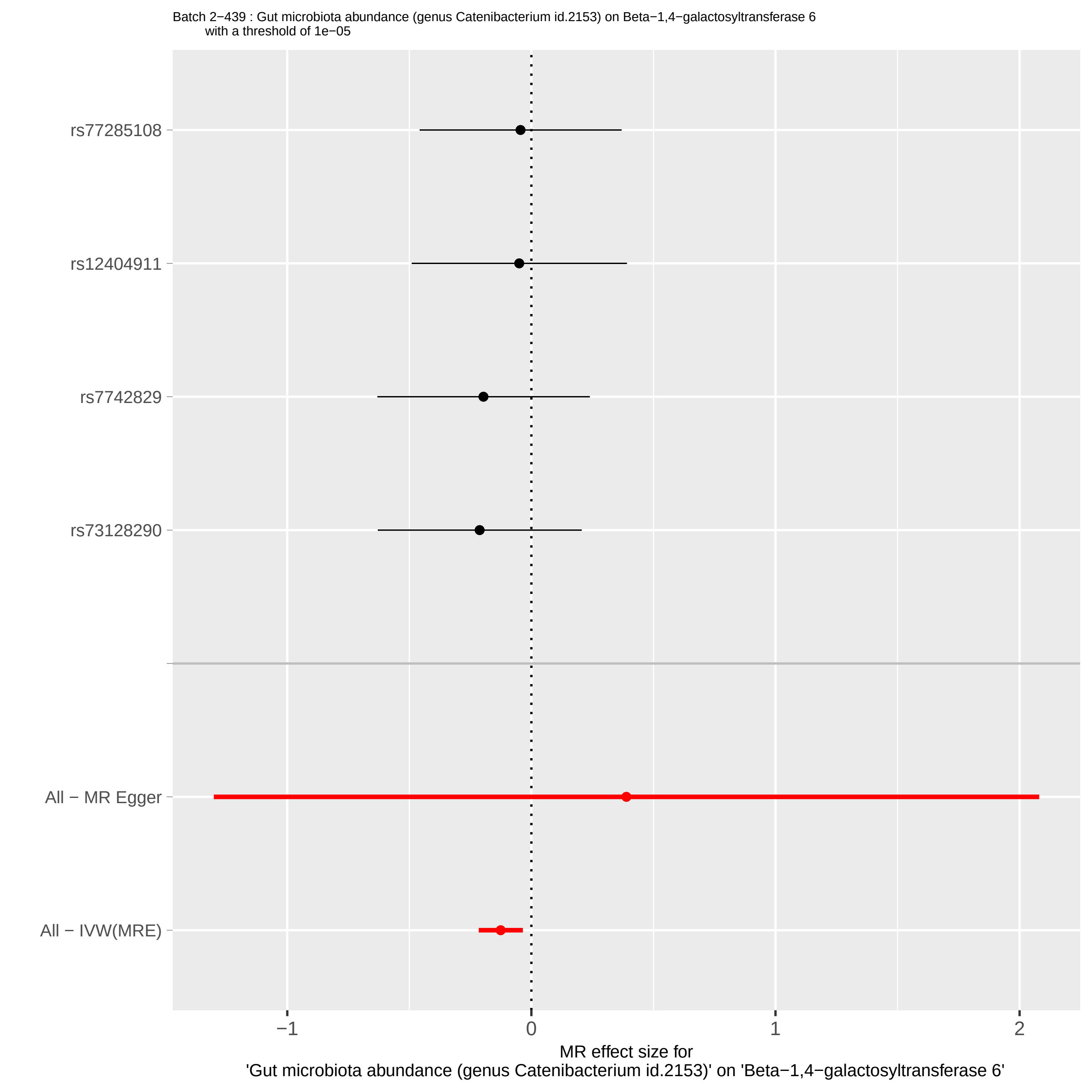

Batch 2−439 : Gut microbiota abundance (genus Catenibacterium id.2153) on Beta−1,4−galactosyltransferase 6
with a threshold of 1e−05
rs77285108
rs12404911
rs7742829
rs73128290
All − MR Egger
All − IVW(MRE)
−1
0
1
2
MR effect size for
'Gut microbiota abundance (genus Catenibacterium id.2153)' on 'Beta−1,4−galactosyltransferase 6'

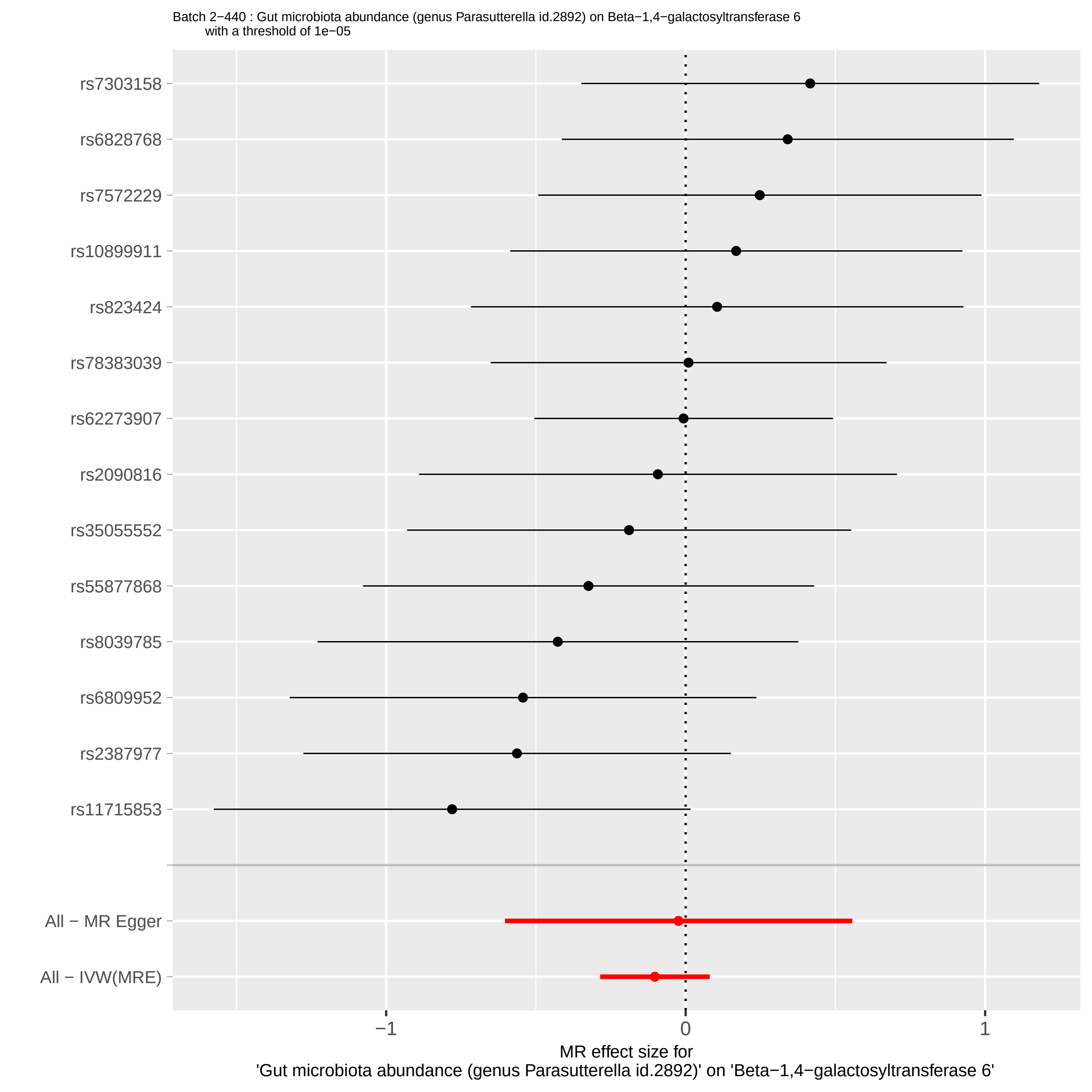

Batch 2−440 : Gut microbiota abundance (genus Parasutterella id.2892) on Beta−1,4−galactosyltransferase 6
with a threshold of 1e−05
rs7303158
rs6828768
rs7572229
rs10899911
rs823424
rs78383039
rs62273907
rs2090816
rs35055552
rs55877868
rs8039785
rs6809952
rs2387977
rs11715853
All − MR Egger
All − IVW(MRE)
−1
0
1
MR effect size for
'Gut microbiota abundance (genus Parasutterella id.2892)' on 'Beta−1,4−galactosyltransferase 6'

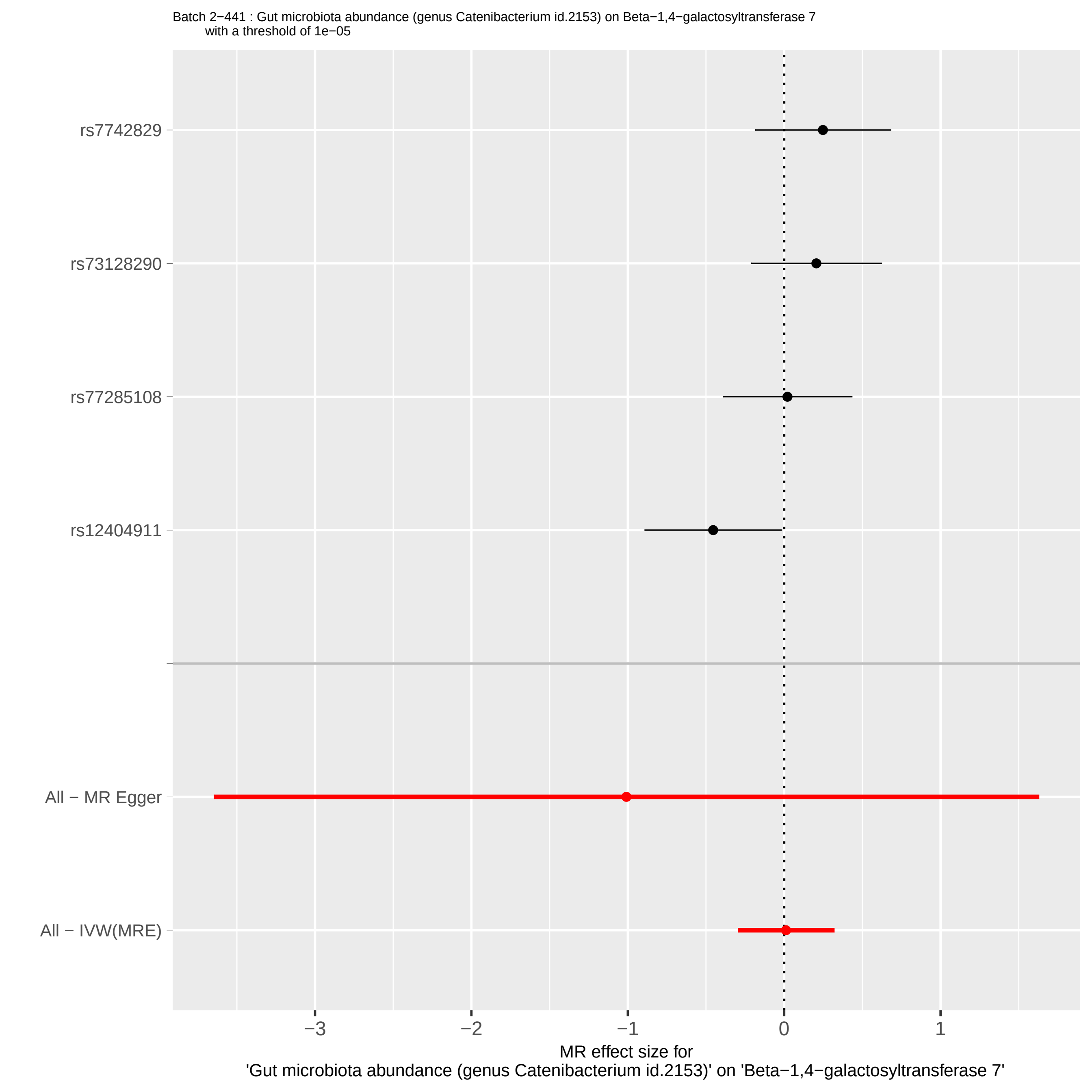

Batch 2−441 : Gut microbiota abundance (genus Catenibacterium id.2153) on Beta−1,4−galactosyltransferase 7
with a threshold of 1e−05
rs7742829
rs73128290
rs77285108
rs12404911
All − MR Egger
All − IVW(MRE)
−3
−2
−1
0
1
MR effect size for
'Gut microbiota abundance (genus Catenibacterium id.2153)' on 'Beta−1,4−galactosyltransferase 7'

Batch 2−442 : Gut microbiota abundance (genus Parasutterella id.2892) on Beta−1,4−galactosyltransferase 7
with a threshold of 1e−05

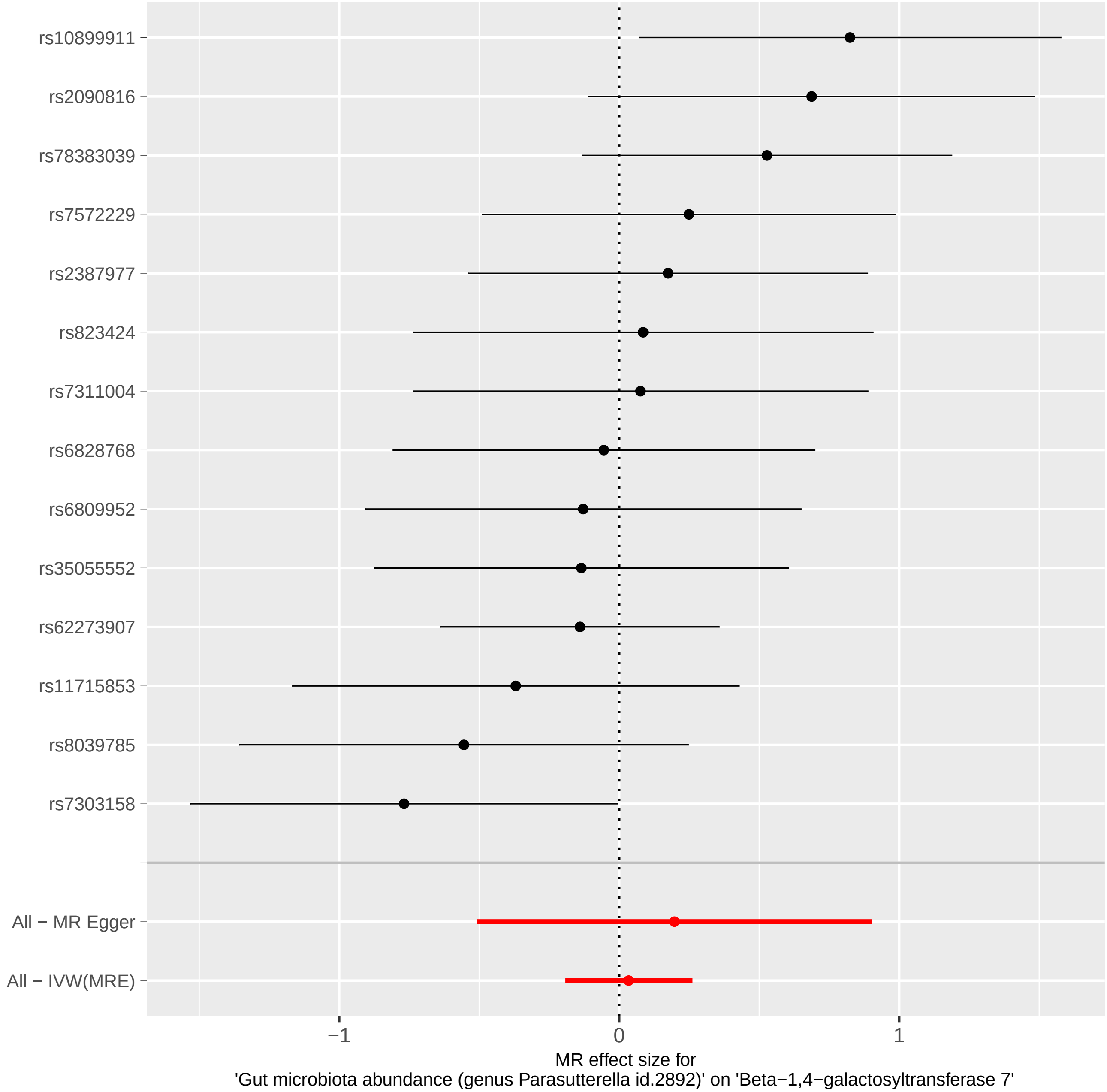

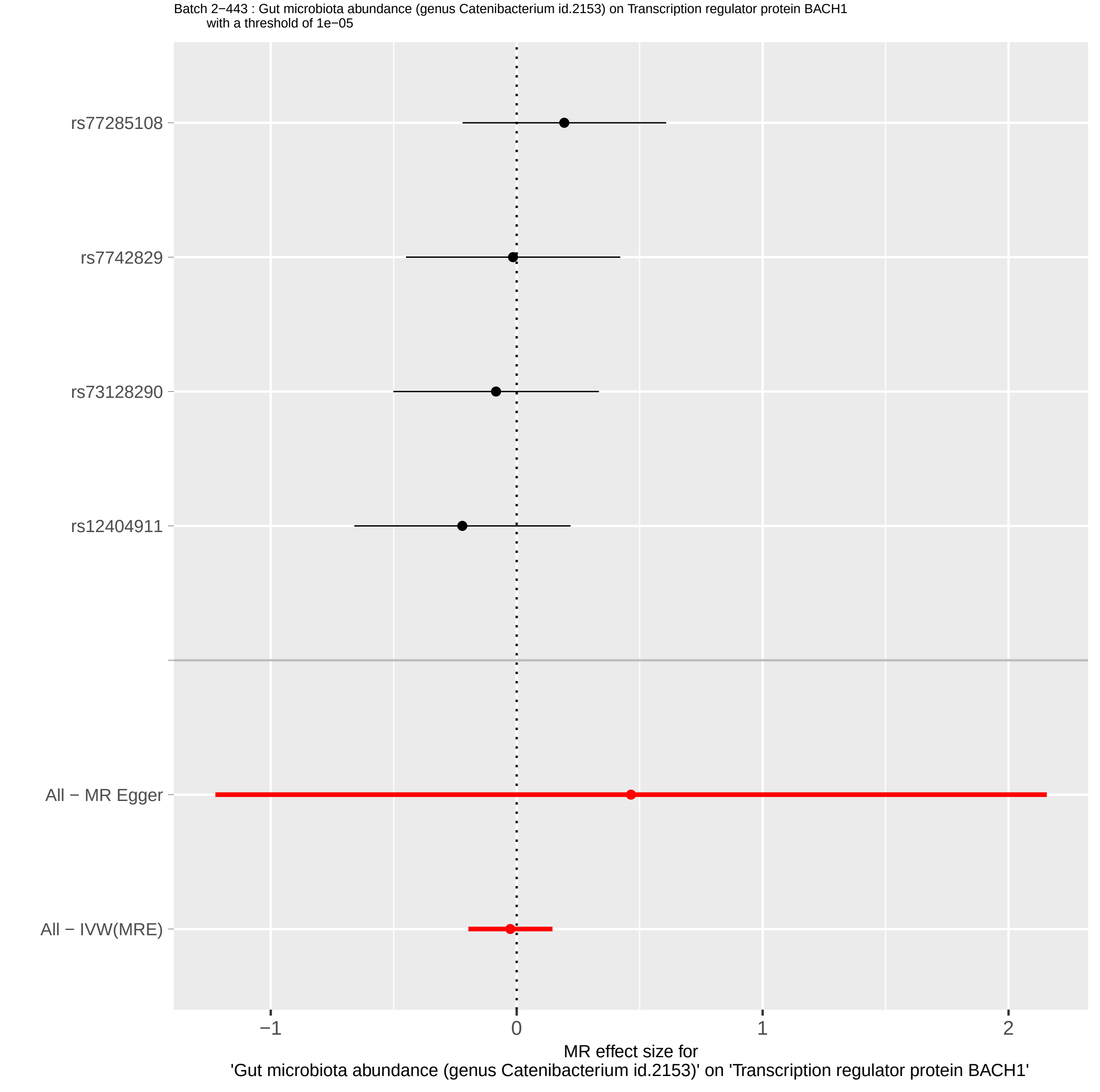

Batch 2−443 : Gut microbiota abundance (genus Catenibacterium id.2153) on Transcription regulator protein BACH1
with a threshold of 1e−05
rs77285108
rs7742829
rs73128290
rs12404911
All − MR Egger
All − IVW(MRE)
−1
0
1
2
MR effect size for
'Gut microbiota abundance (genus Catenibacterium id.2153)' on 'Transcription regulator protein BACH1'

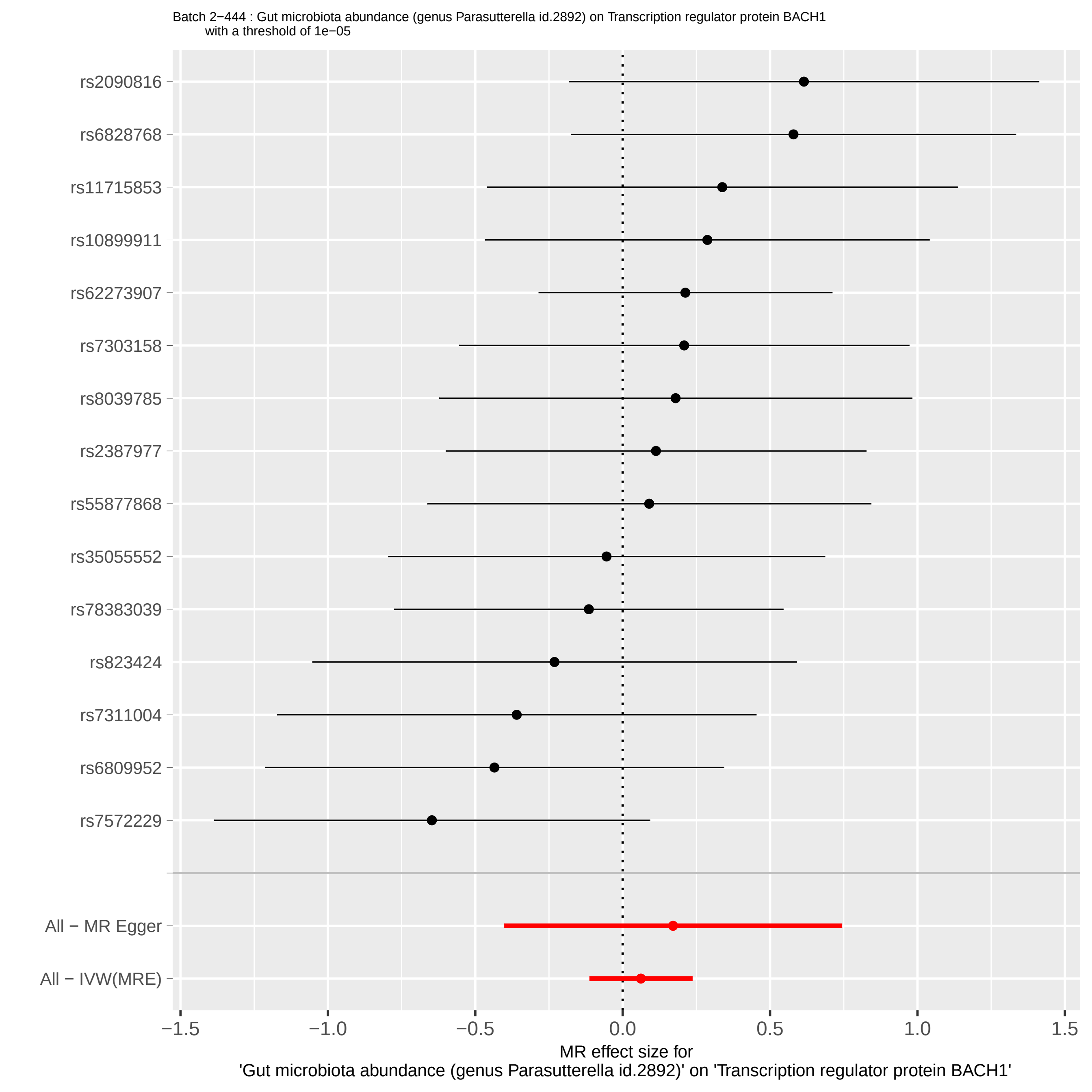

Batch 2−444 : Gut microbiota abundance (genus Parasutterella id.2892) on Transcription regulator protein BACH1
with a threshold of 1e−05
rs2090816
rs6828768
rs11715853
rs10899911
rs62273907
rs7303158
rs8039785
rs2387977
rs55877868
rs35055552
rs78383039
rs823424
rs7311004
rs6809952
rs7572229
All − MR Egger
All − IVW(MRE)
−1.5
−1.0
−0.5
0.0
0.5
1.0
1.5
MR effect size for
'Gut microbiota abundance (genus Parasutterella id.2892)' on 'Transcription regulator protein BACH1'

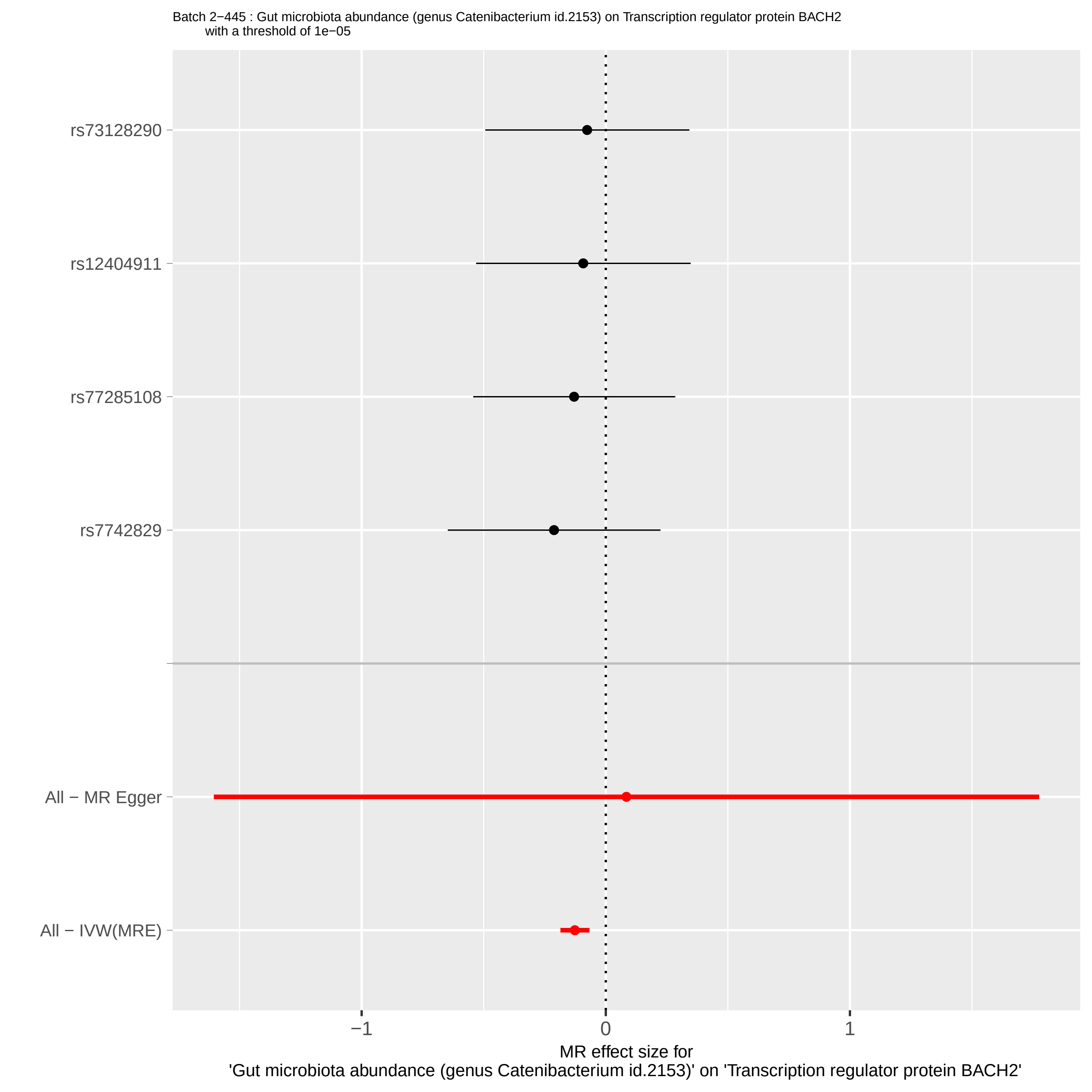
Batch 2−445 : Gut microbiota abundance (genus Catenibacterium id.2153) on Transcription regulator protein BACH2
with a threshold of 1e−05
rs73128290
rs12404911
rs77285108
rs7742829
All − MR Egger
All − IVW(MRE)
−1
0
1
MR effect size for
'Gut microbiota abundance (genus Catenibacterium id.2153)' on 'Transcription regulator protein BACH2'

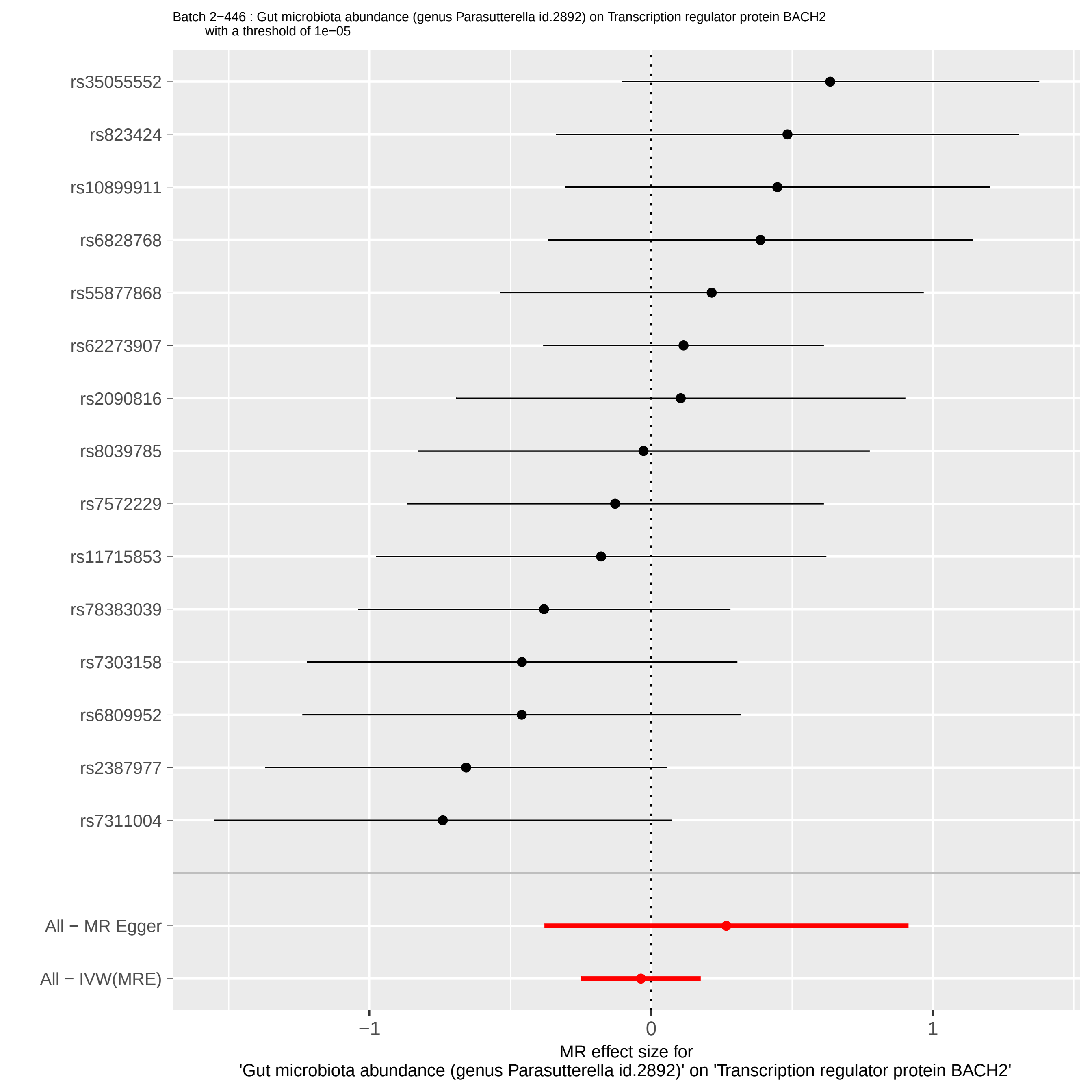

Batch 2−446 : Gut microbiota abundance (genus Parasutterella id.2892) on Transcription regulator protein BACH2 with a threshold of 1e−05
rs35055552
rs823424
rs10899911
rs6828768
rs55877868
rs62273907
rs2090816
rs8039785
rs7572229
rs11715853
rs78383039
rs7303158
rs6809952
rs2387977
rs7311004
All − MR Egger
All − IVW(MRE)
−1
0
1
MR effect size for
'Gut microbiota abundance (genus Parasutterella id.2892)' on 'Transcription regulator protein BACH2'

Batch 2−447 : Gut microbiota abundance (genus Catenibacterium id.2153) on Bcl2−associated agonist of cell death
with a threshold of 1e−05

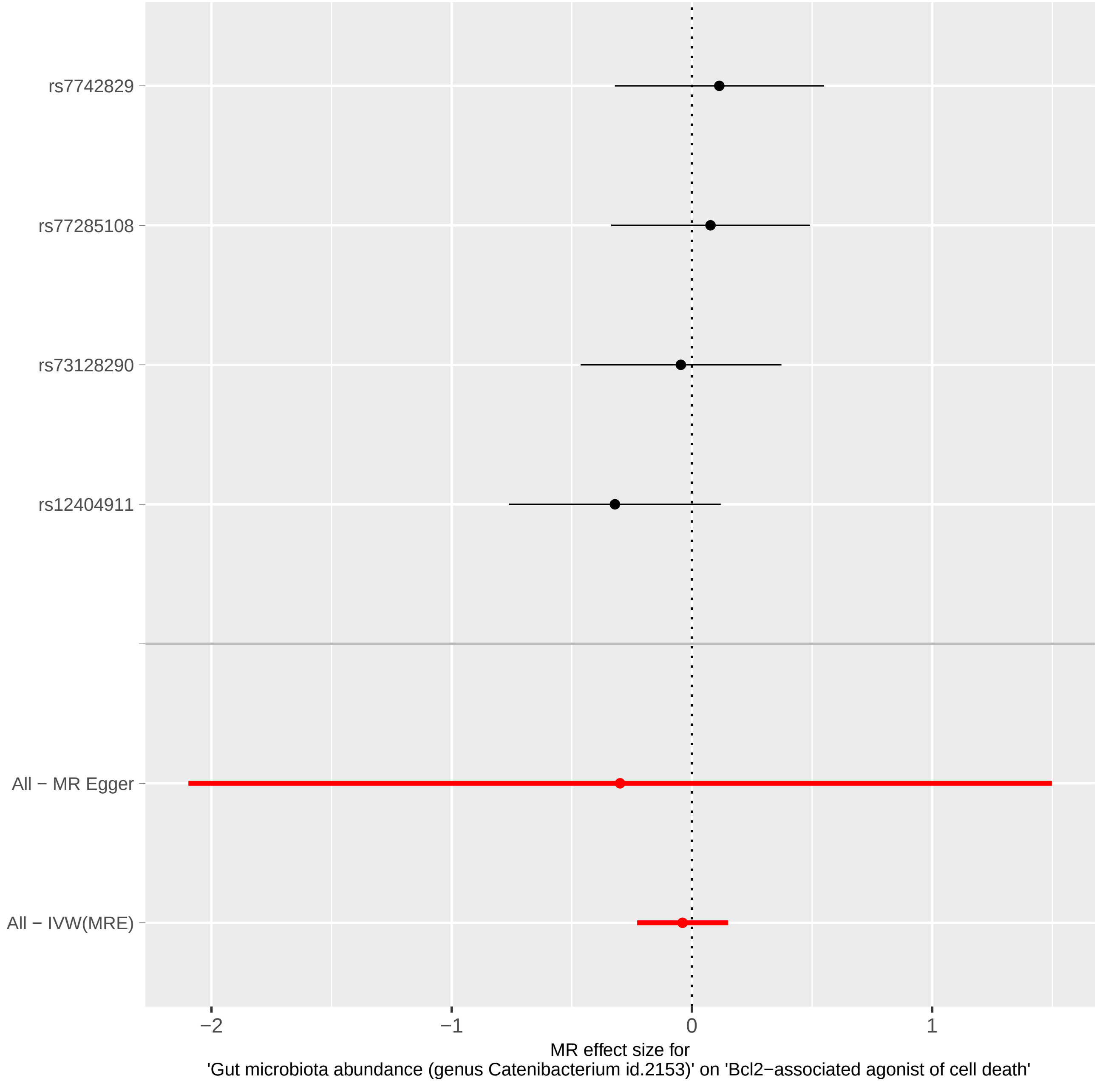

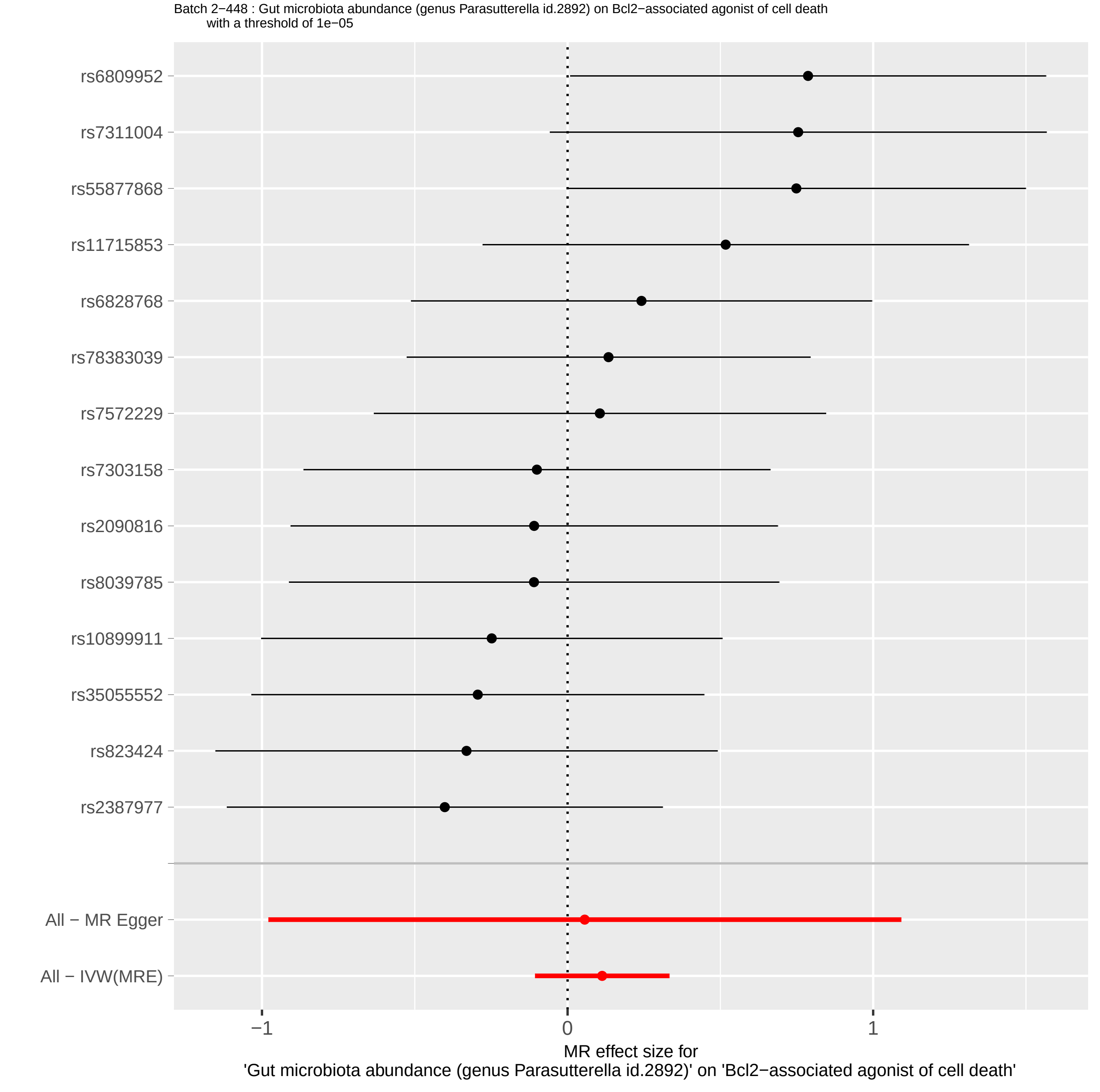
Batch 2−448 : Gut microbiota abundance (genus Parasutterella id.2892) on Bcl2−associated agonist of cell death
with a threshold of 1e−05
rs6809952
rs7311004
rs55877868
rs11715853
rs6828768
rs78383039
rs7572229
rs7303158
rs2090816
rs8039785
rs10899911
rs35055552
rs823424
rs2387977
All − MR Egger
All − IVW(MRE)
−1
0
1
MR effect size for
'Gut microbiota abundance (genus Parasutterella id.2892)' on 'Bcl2−associated agonist of cell death'

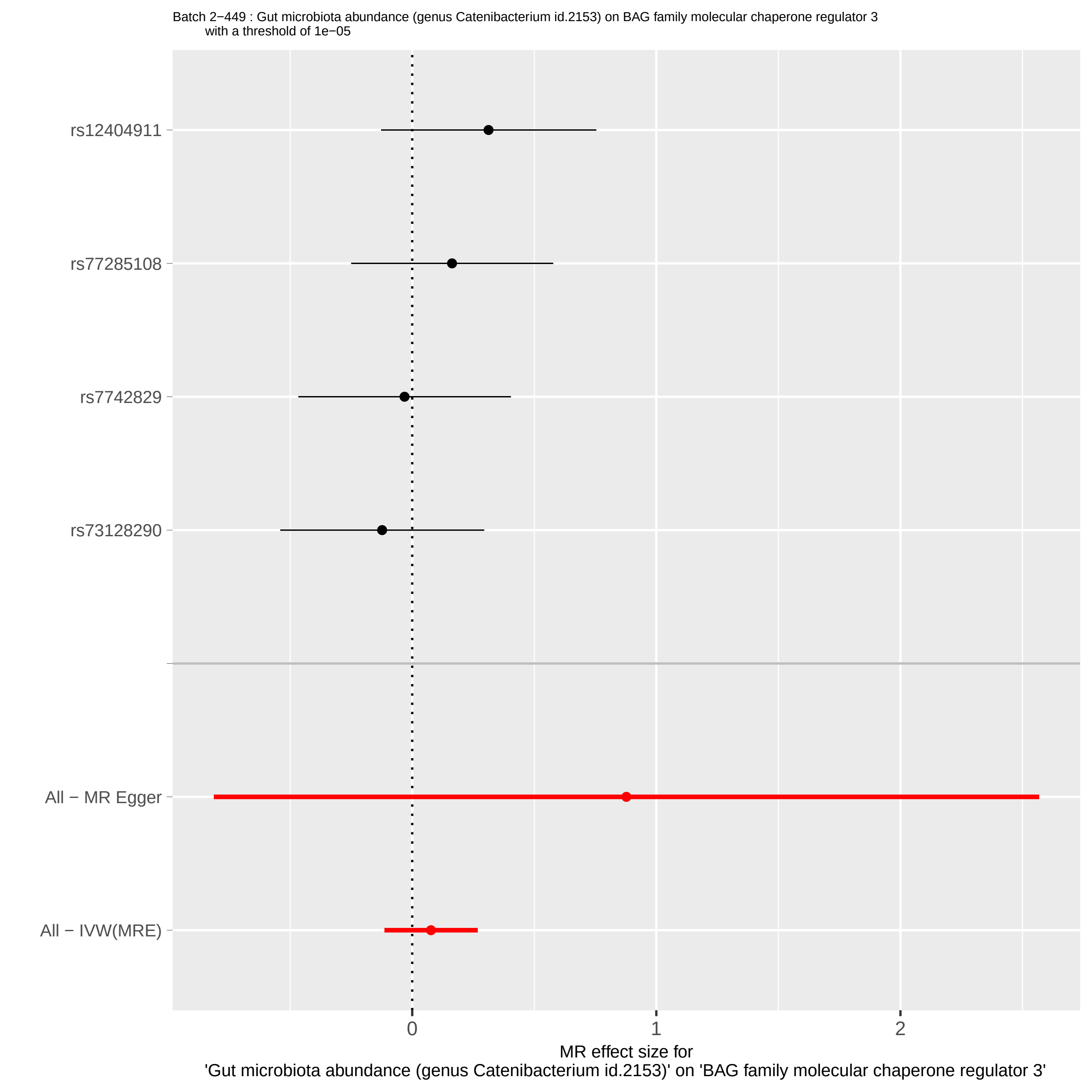
Batch 2−449 : Gut microbiota abundance (genus Catenibacterium id.2153) on BAG family molecular chaperone regulator 3
with a threshold of 1e−05
rs12404911
rs77285108
rs7742829
rs73128290
All − MR Egger
All − IVW(MRE)
0
1
2
MR effect size for
'Gut microbiota abundance (genus Catenibacterium id.2153)' on 'BAG family molecular chaperone regulator 3'

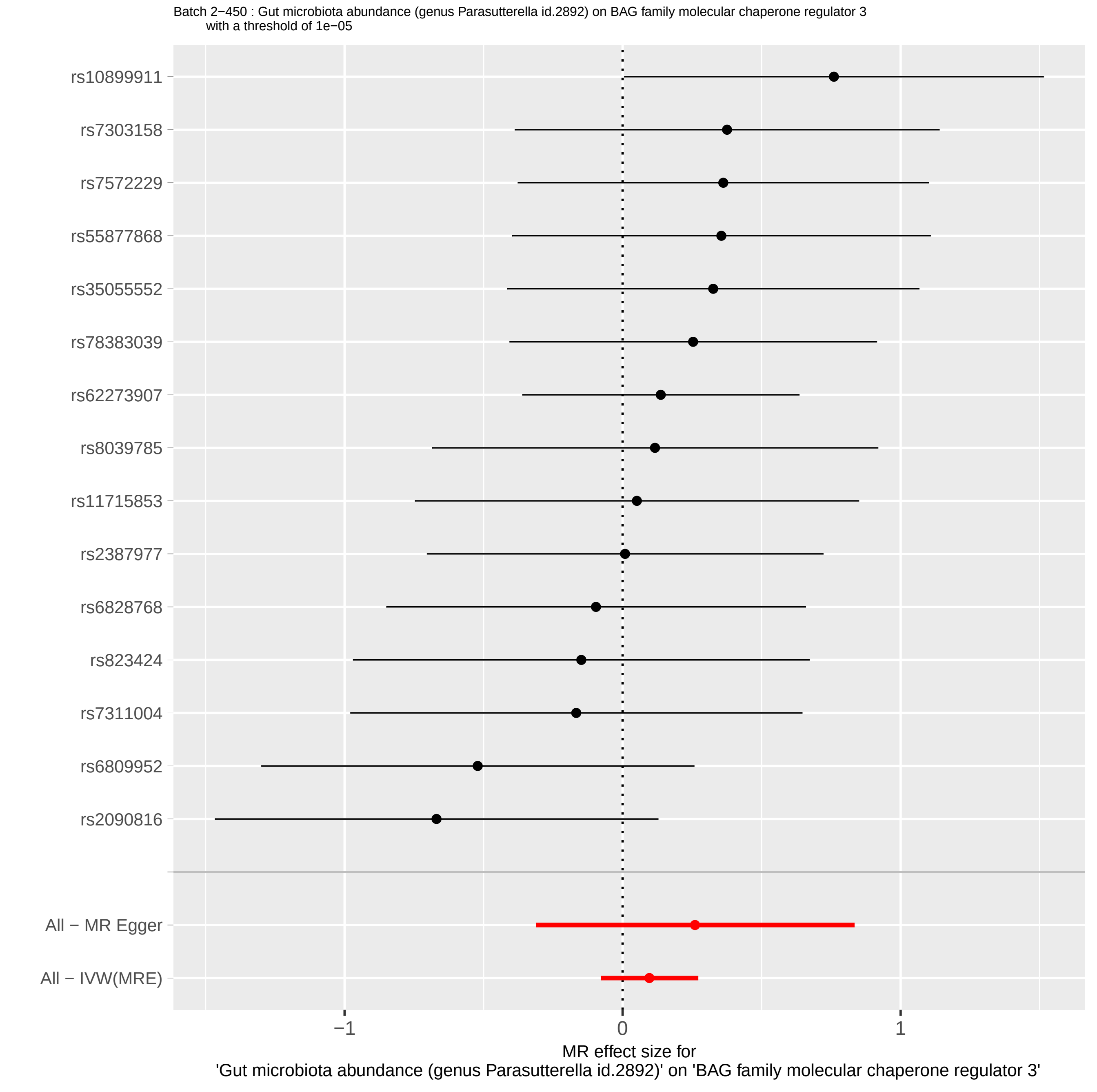

Batch 2−450 : Gut microbiota abundance (genus Parasutterella id.2892) on BAG family molecular chaperone regulator 3
with a threshold of 1e−05
rs10899911
rs7303158
rs7572229
rs55877868
rs35055552
rs78383039
rs62273907
rs8039785
rs11715853
rs2387977
rs6828768
rs823424
rs7311004
rs6809952
rs2090816
All − MR Egger
All − IVW(MRE)
−1
0
1
MR effect size for
'Gut microbiota abundance (genus Parasutterella id.2892)' on 'BAG family molecular chaperone regulator 3'

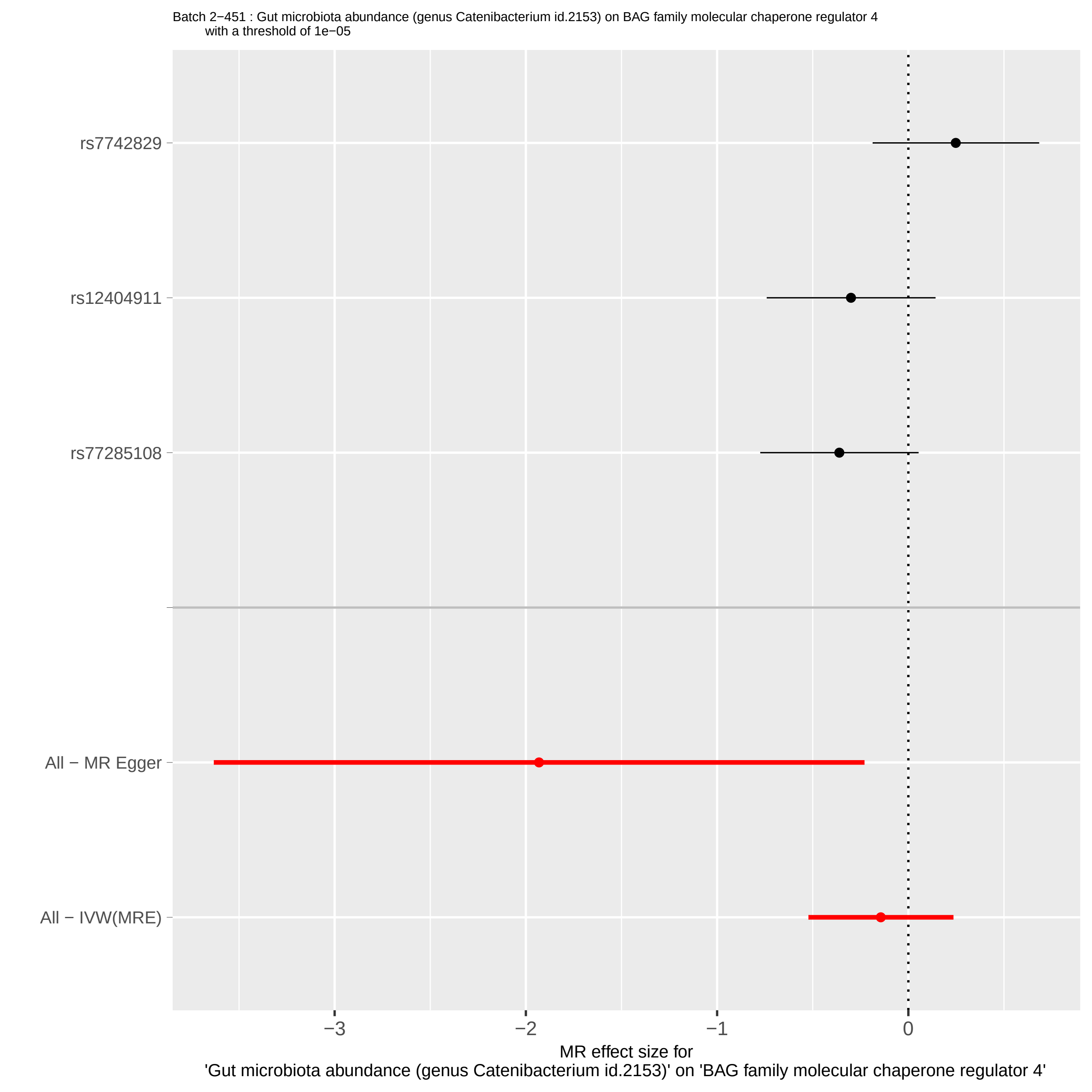
Batch 2−451 : Gut microbiota abundance (genus Catenibacterium id.2153) on BAG family molecular chaperone regulator 4
with a threshold of 1e−05
rs7742829
rs12404911
rs77285108
All − MR Egger
All − IVW(MRE)
−3
−2
−1
0
MR effect size for
'Gut microbiota abundance (genus Catenibacterium id.2153)' on 'BAG family molecular chaperone regulator 4'

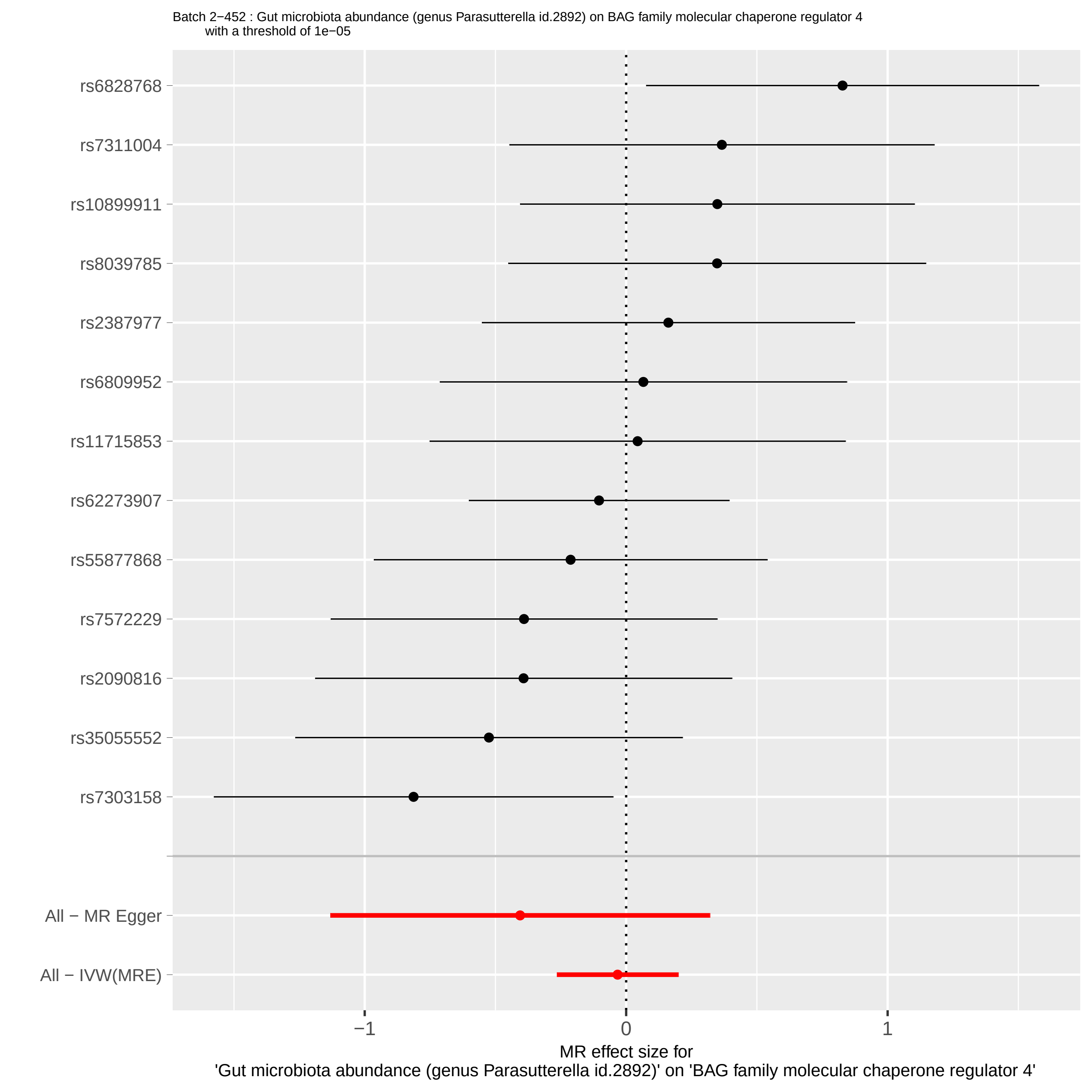

Batch 2−452 : Gut microbiota abundance (genus Parasutterella id.2892) on BAG family molecular chaperone regulator 4
with a threshold of 1e−05
rs6828768
rs7311004
rs10899911
rs8039785
rs2387977
rs6809952
rs11715853
rs62273907
rs55877868
rs7572229
rs2090816
rs35055552
rs7303158
All − MR Egger
All − IVW(MRE)
−1
0
1
MR effect size for
'Gut microbiota abundance (genus Parasutterella id.2892)' on 'BAG family molecular chaperone regulator 4'

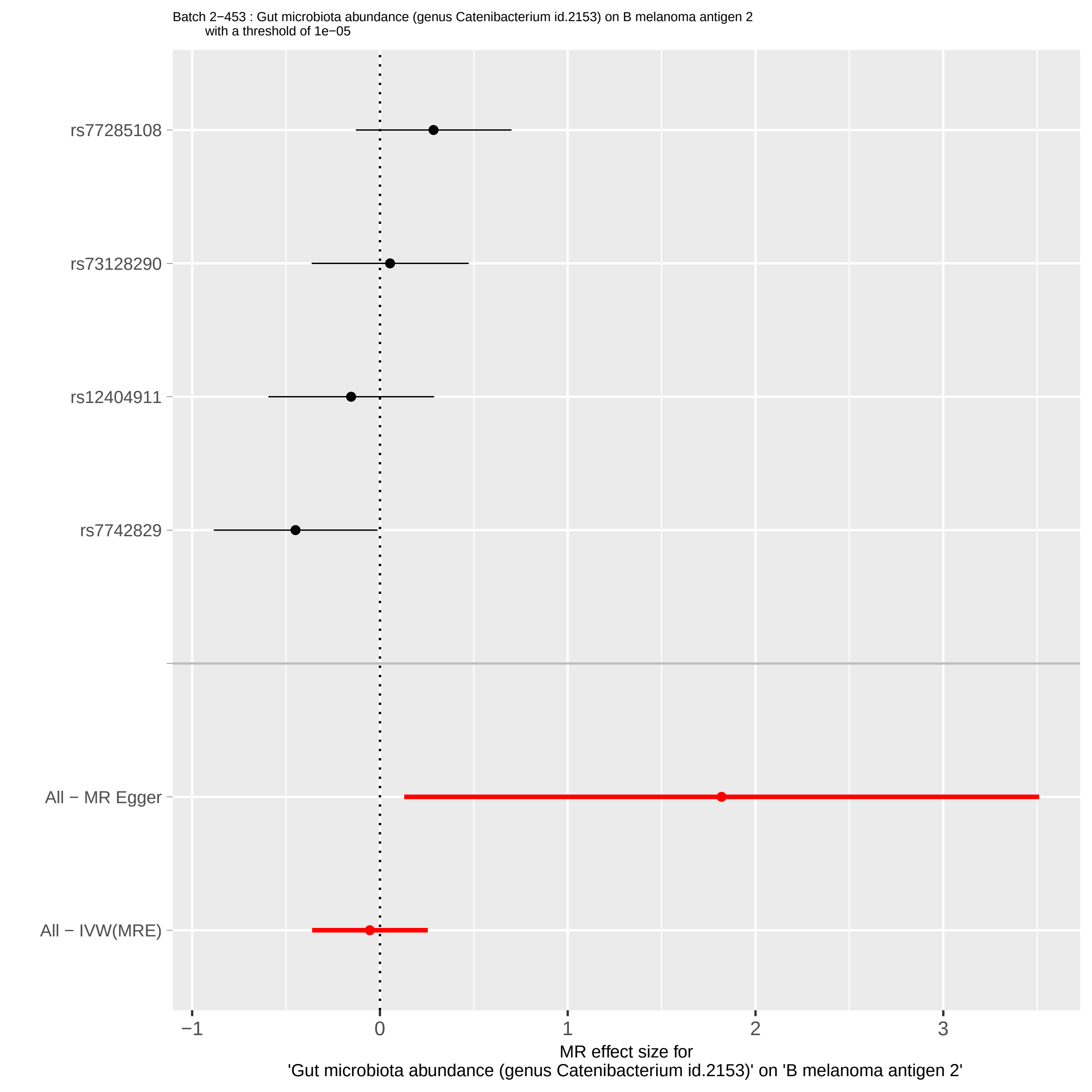

Batch 2−453 : Gut microbiota abundance (genus Catenibacterium id.2153) on B melanoma antigen 2
with a threshold of 1e−05
rs77285108
rs73128290
rs12404911
rs7742829
All − MR Egger
All − IVW(MRE)
−1
0
1
2
3
MR effect size for
'Gut microbiota abundance (genus Catenibacterium id.2153)' on 'B melanoma antigen 2'

Batch 2−454 : Gut microbiota abundance (genus Parasutterella id.2892) on B melanoma antigen 2
with a threshold of 1e−05

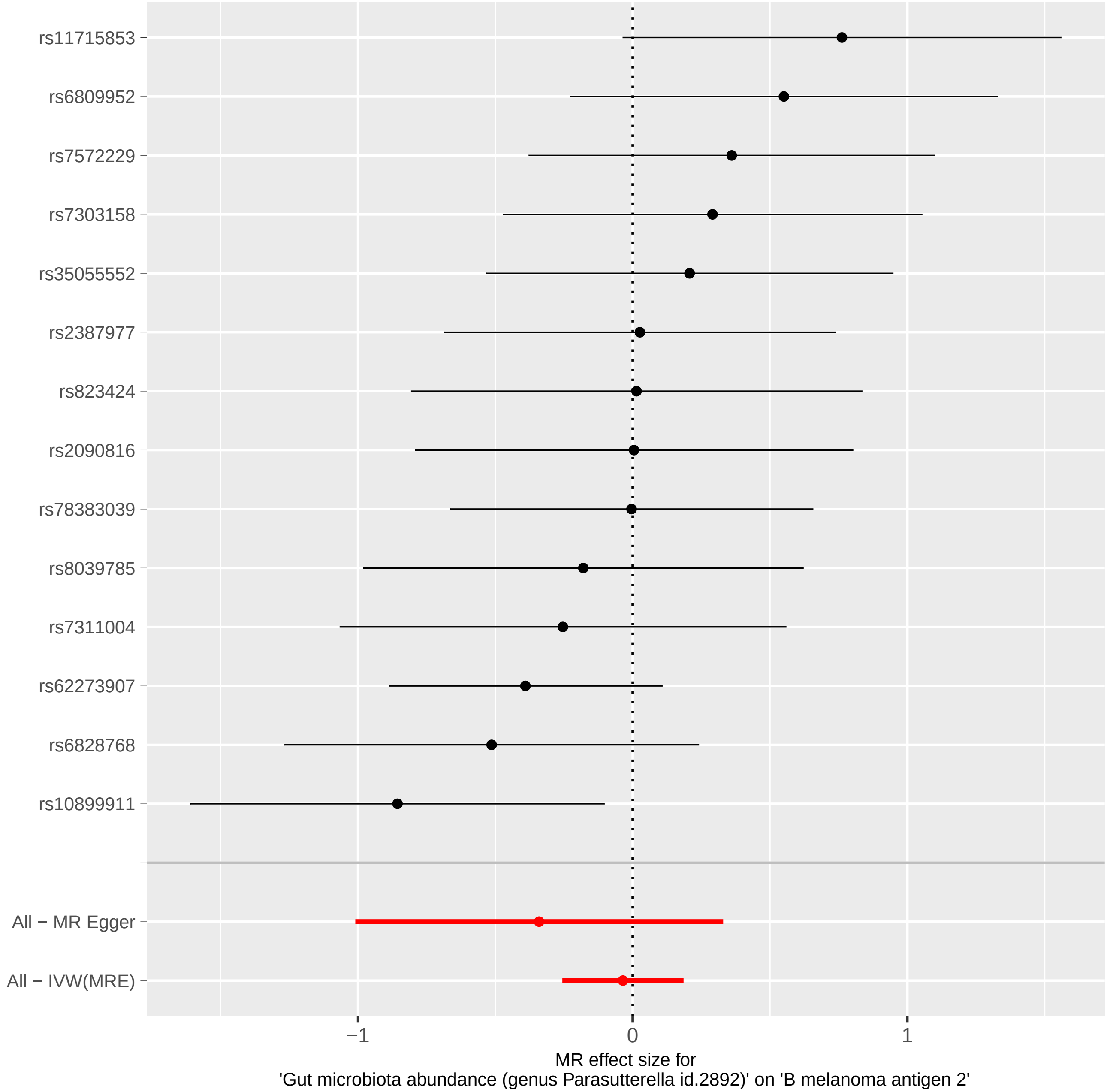

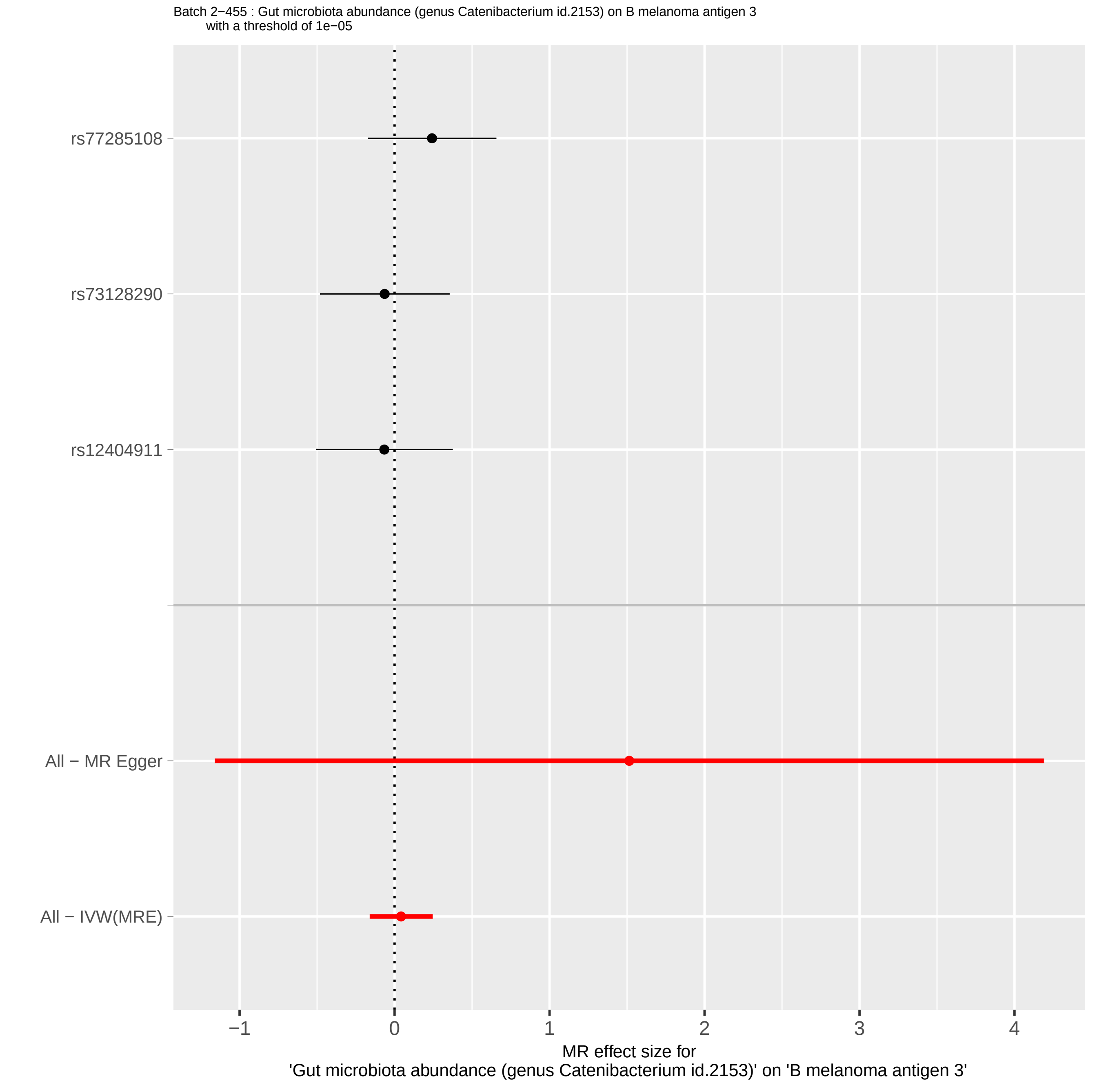

Batch 2−455 : Gut microbiota abundance (genus Catenibacterium id.2153) on B melanoma antigen 3
with a threshold of 1e−05
rs77285108
rs73128290
rs12404911
All − MR Egger
All − IVW(MRE)
−1
0
1
2
3
4
MR effect size for
'Gut microbiota abundance (genus Catenibacterium id.2153)' on 'B melanoma antigen 3'

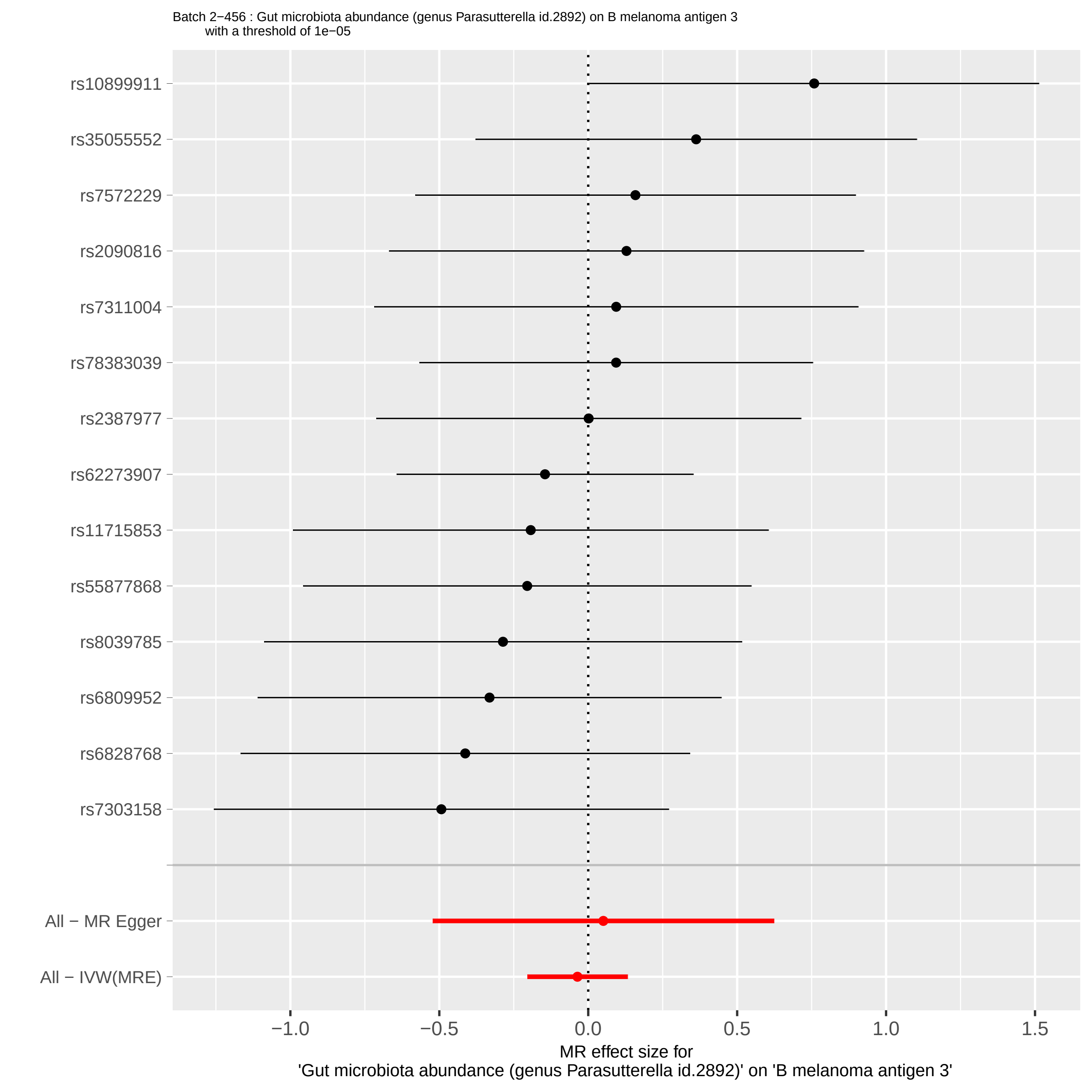

Batch 2−456 : Gut microbiota abundance (genus Parasutterella id.2892) on B melanoma antigen 3
with a threshold of 1e−05
rs10899911
rs35055552
rs7572229
rs2090816
rs7311004
rs78383039
rs2387977
rs62273907
rs11715853
rs55877868
rs8039785
rs6809952
rs6828768
rs7303158
All − MR Egger
All − IVW(MRE)
−1.0
−0.5
0.0
0.5
1.0
1.5
MR effect size for
'Gut microbiota abundance (genus Parasutterella id.2892)' on 'B melanoma antigen 3'

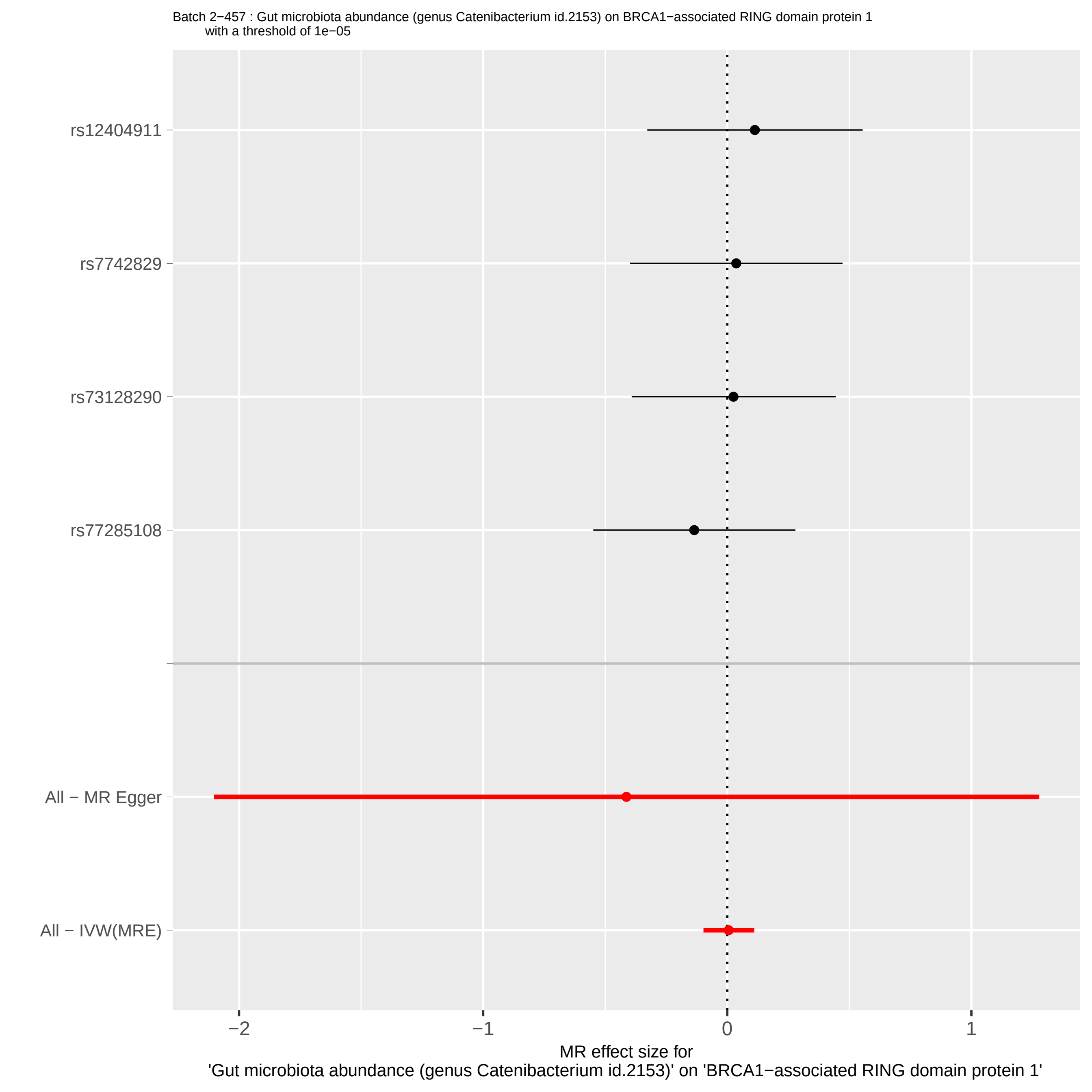
Batch 2−457 : Gut microbiota abundance (genus Catenibacterium id.2153) on BRCA1−associated RING domain protein 1
with a threshold of 1e−05
rs12404911
rs7742829
rs73128290
rs77285108
All − MR Egger
All − IVW(MRE)
−2
−1
0
1
MR effect size for
'Gut microbiota abundance (genus Catenibacterium id.2153)' on 'BRCA1−associated RING domain protein 1'

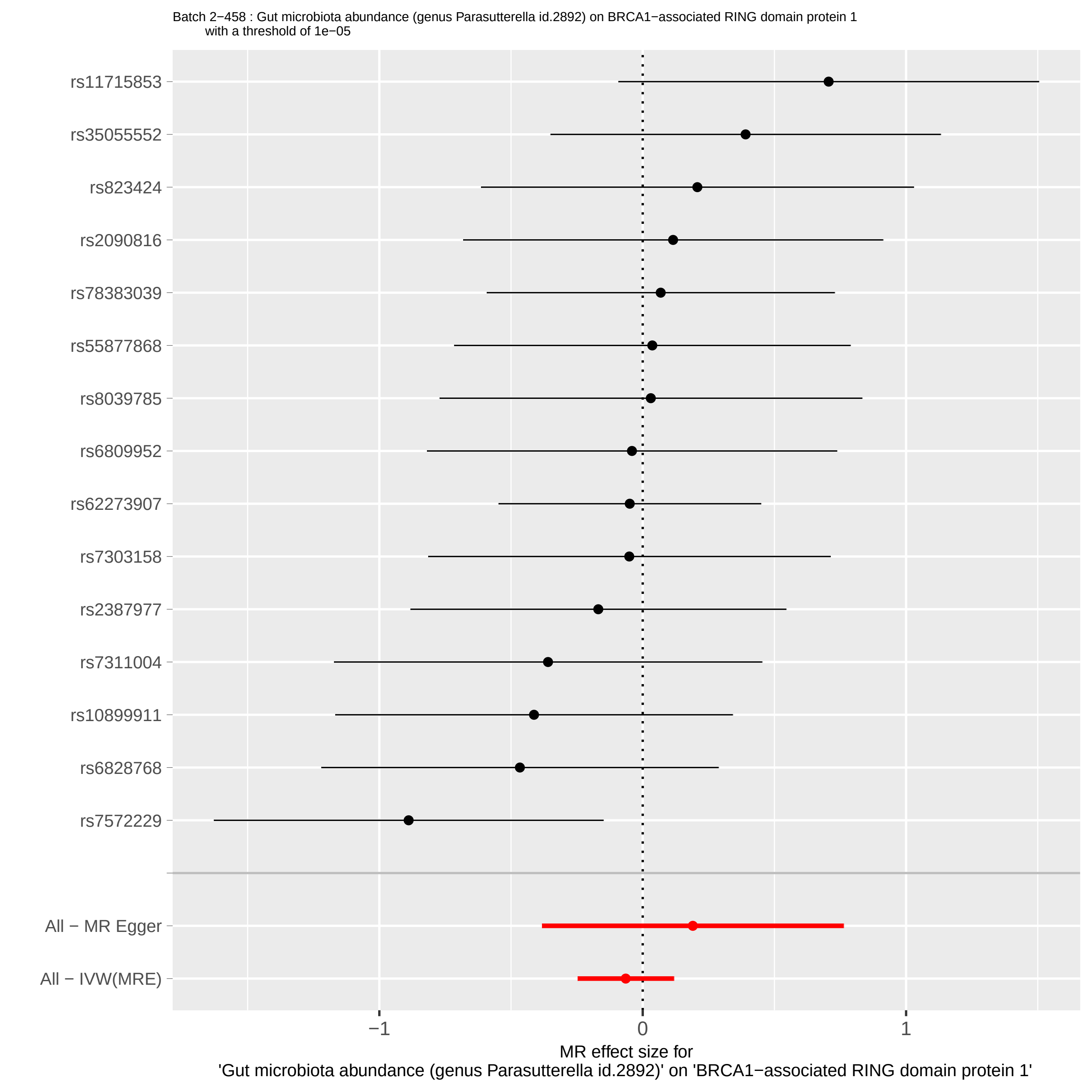
Batch 2−458 : Gut microbiota abundance (genus Parasutterella id.2892) on BRCA1−associated RING domain protein 1
with a threshold of 1e−05
rs11715853
rs35055552
rs823424
rs2090816
rs78383039
rs55877868
rs8039785
rs6809952
rs62273907
rs7303158
rs2387977
rs7311004
rs10899911
rs6828768
rs7572229
All − MR Egger
All − IVW(MRE)
−1
0
1
MR effect size for
'Gut microbiota abundance (genus Parasutterella id.2892)' on 'BRCA1−associated RING domain protein 1'

Batch 2−459 : Gut microbiota abundance (genus Catenibacterium id.2153) on Basic leucine zipper transcriptional factor ATF−like 3
with a threshold of 1e−05

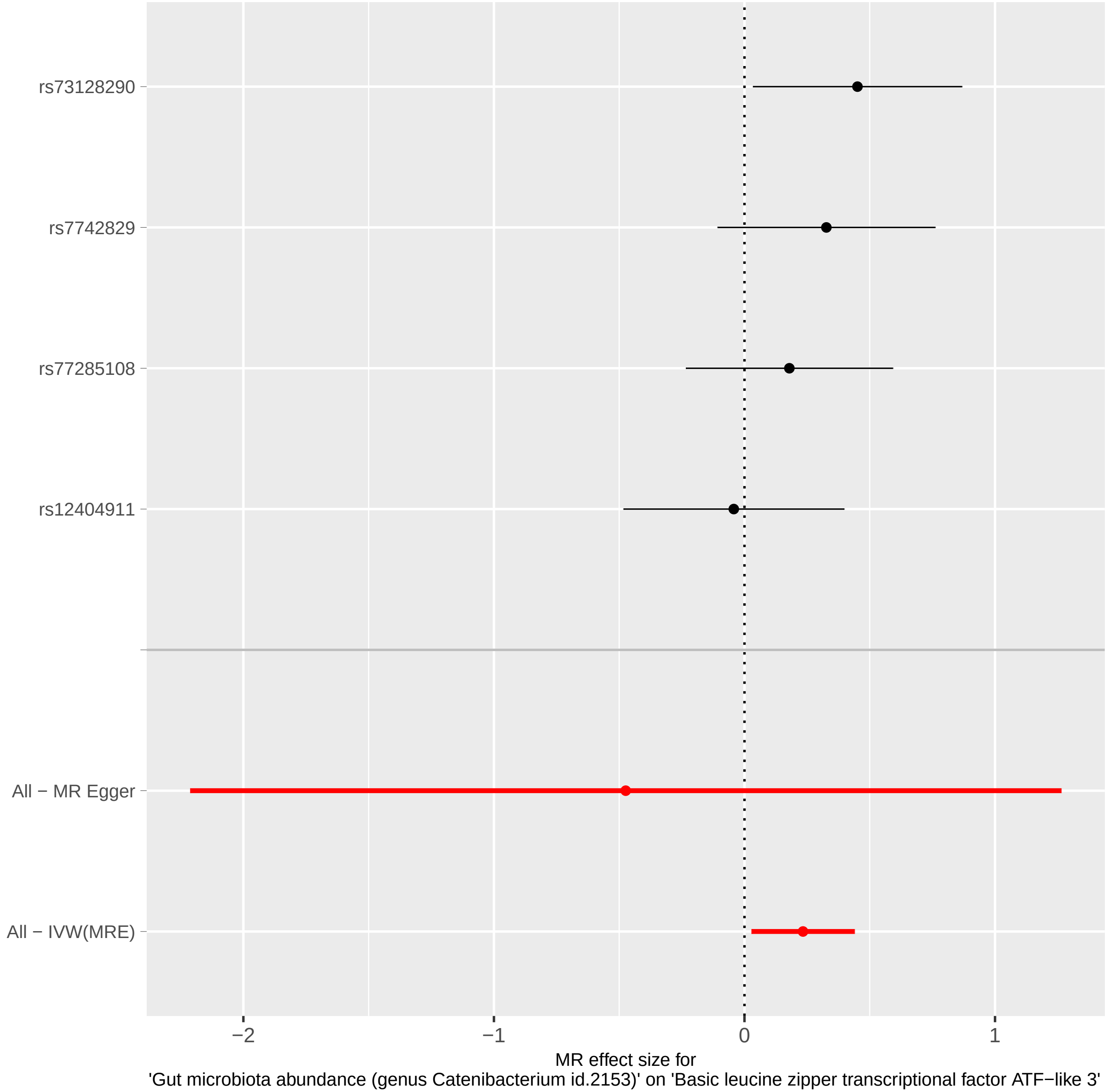

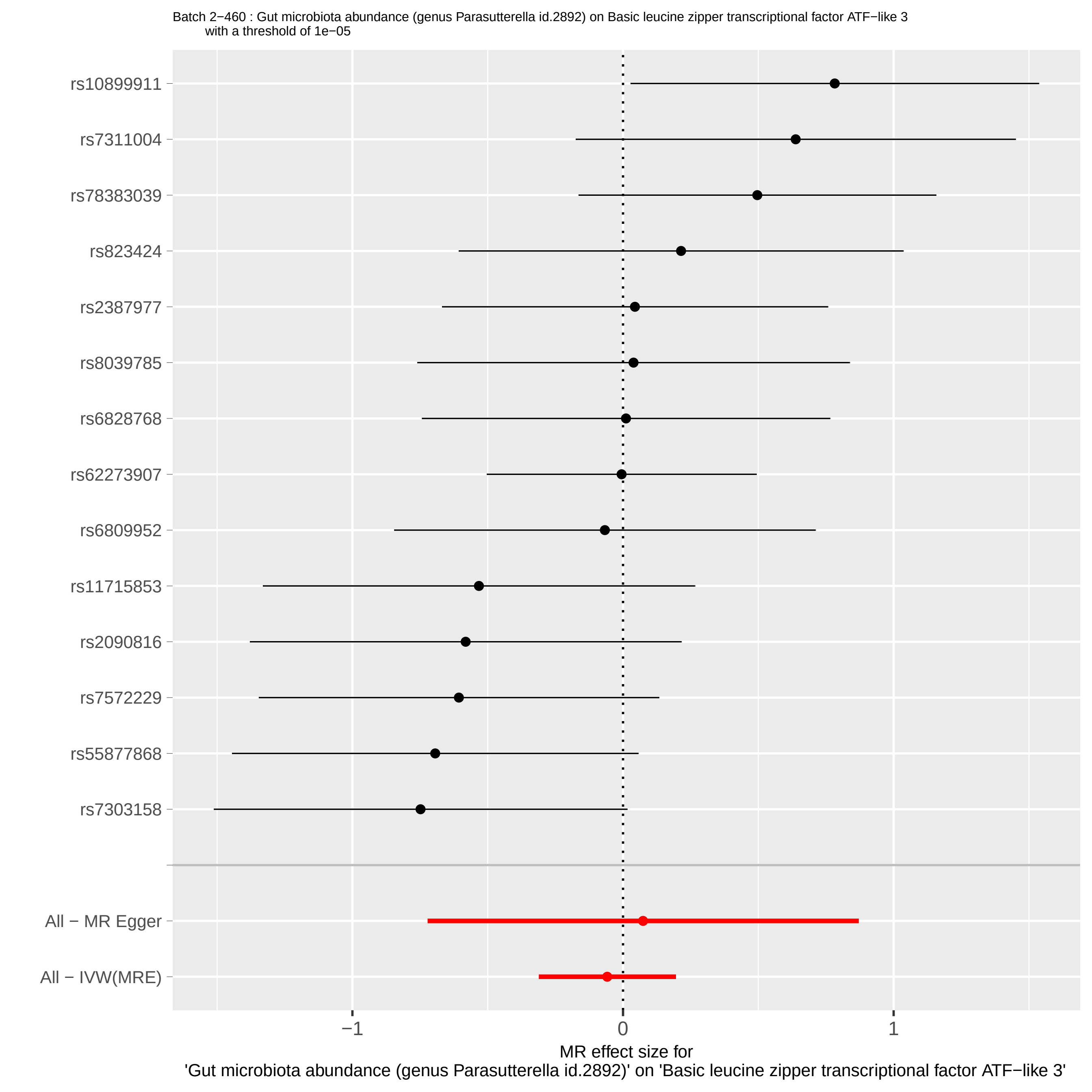

Batch 2−460 : Gut microbiota abundance (genus Parasutterella id.2892) on Basic leucine zipper transcriptional factor ATF−like 3
with a threshold of 1e−05
rs10899911
rs7311004
rs78383039
rs823424
rs2387977
rs8039785
rs6828768
rs62273907
rs6809952
rs11715853
rs2090816
rs7572229
rs55877868
rs7303158
All − MR Egger
All − IVW(MRE)
−1
0
1
MR effect size for
'Gut microbiota abundance (genus Parasutterella id.2892)' on 'Basic leucine zipper transcriptional factor ATF−like 3'

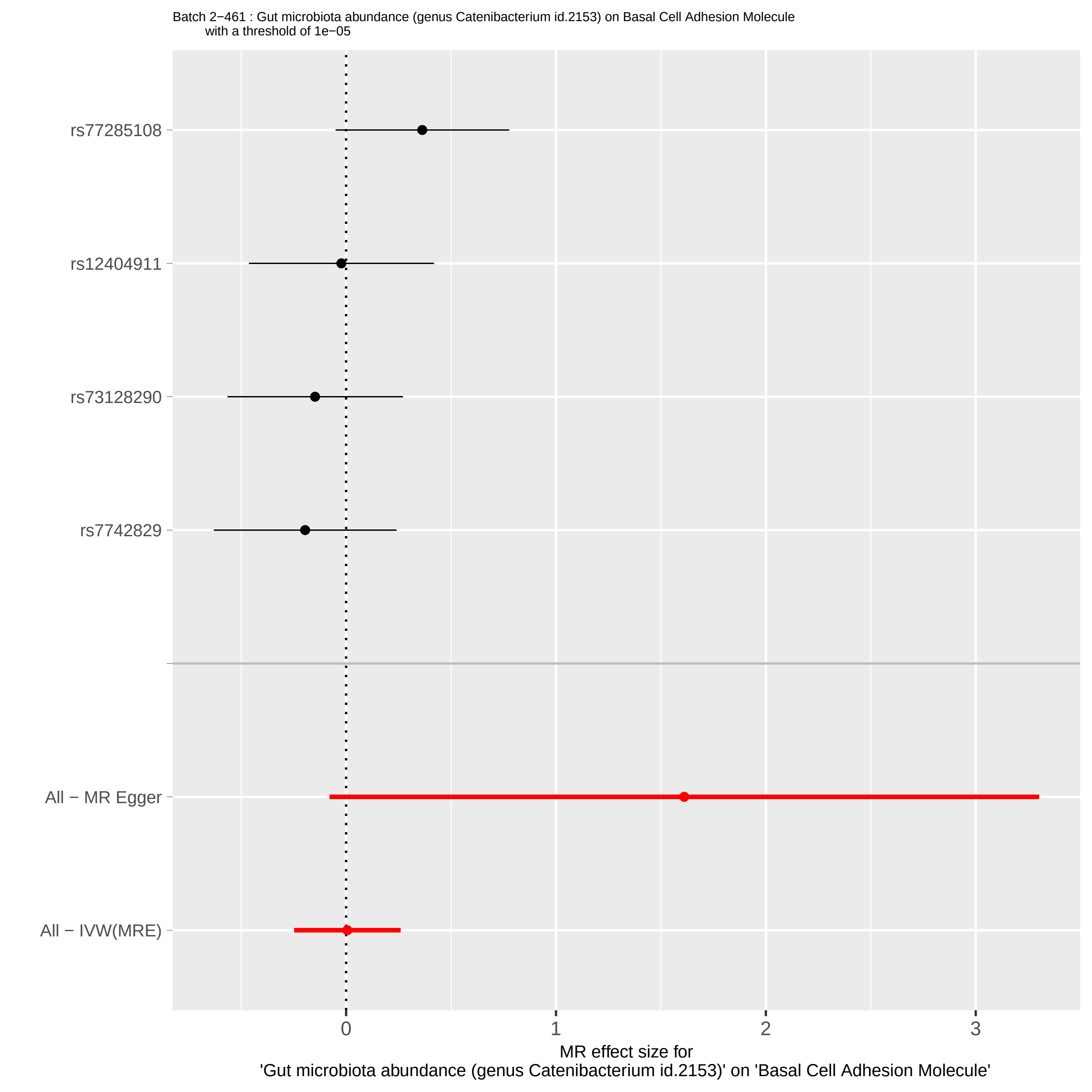

Batch 2−461 : Gut microbiota abundance (genus Catenibacterium id.2153) on Basal Cell Adhesion Molecule
with a threshold of 1e−05
rs77285108
rs12404911
rs73128290
rs7742829
All − MR Egger
All − IVW(MRE)
0
1
2
3
MR effect size for
'Gut microbiota abundance (genus Catenibacterium id.2153)' on 'Basal Cell Adhesion Molecule'

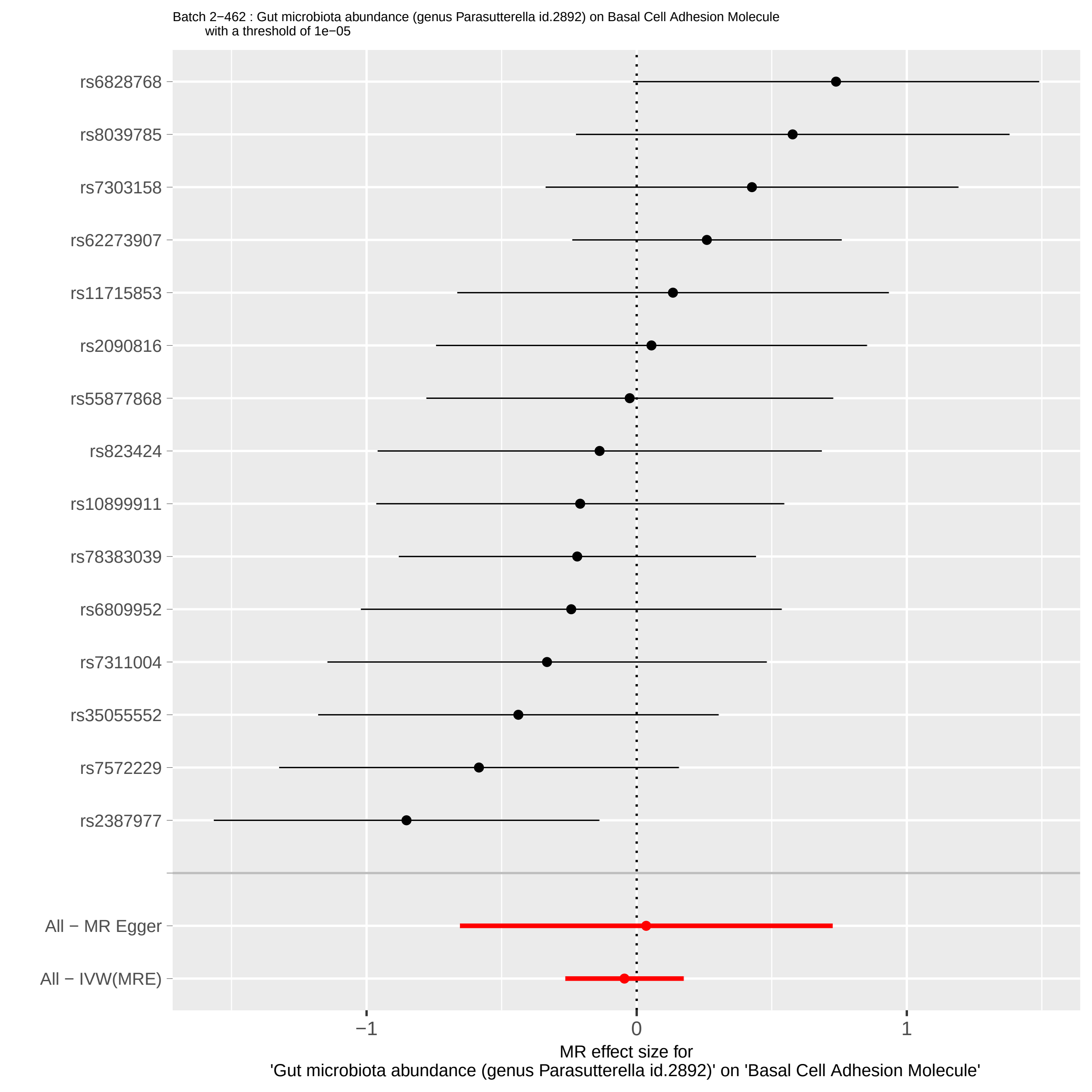

Batch 2−462 : Gut microbiota abundance (genus Parasutterella id.2892) on Basal Cell Adhesion Molecule
with a threshold of 1e−05
rs6828768
rs8039785
rs7303158
rs62273907
rs11715853
rs2090816
rs55877868
rs823424
rs10899911
rs78383039
rs6809952
rs7311004
rs35055552
rs7572229
rs2387977
All − MR Egger
All − IVW(MRE)
−1
0
1
MR effect size for
'Gut microbiota abundance (genus Parasutterella id.2892)' on 'Basal Cell Adhesion Molecule'

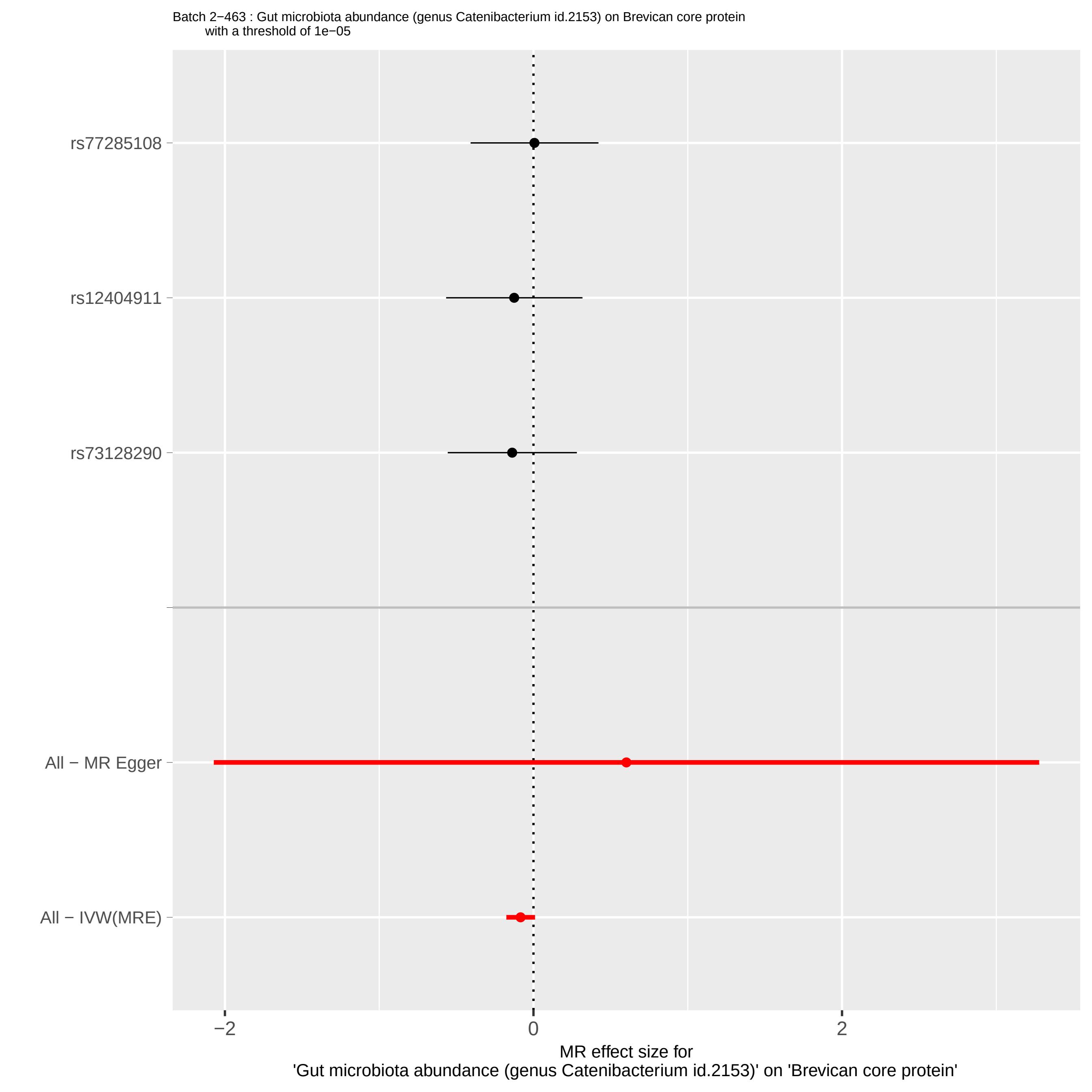

Batch 2−463 : Gut microbiota abundance (genus Catenibacterium id.2153) on Brevican core protein
with a threshold of 1e−05
rs77285108
rs12404911
rs73128290
All − MR Egger
All − IVW(MRE)
−2
0
2
MR effect size for
'Gut microbiota abundance (genus Catenibacterium id.2153)' on 'Brevican core protein'

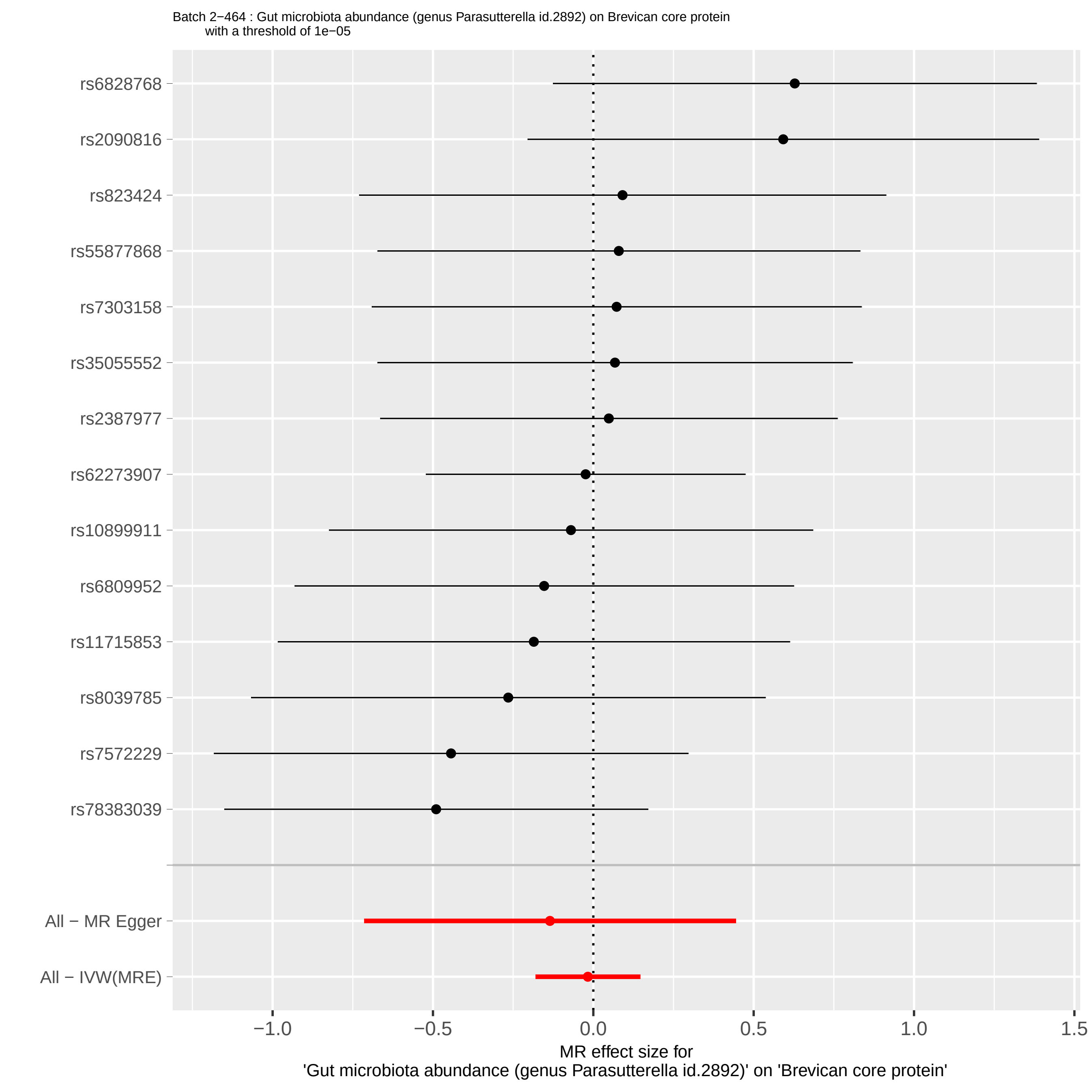

Batch 2−464 : Gut microbiota abundance (genus Parasutterella id.2892) on Brevican core protein
with a threshold of 1e−05
rs6828768
rs2090816
rs823424
rs55877868
rs7303158
rs35055552
rs2387977
rs62273907
rs10899911
rs6809952
rs11715853
rs8039785
rs7572229
rs78383039
All − MR Egger
All − IVW(MRE)
−1.0
−0.5
0.0
0.5
1.0
1.5
MR effect size for
'Gut microbiota abundance (genus Parasutterella id.2892)' on 'Brevican core protein'

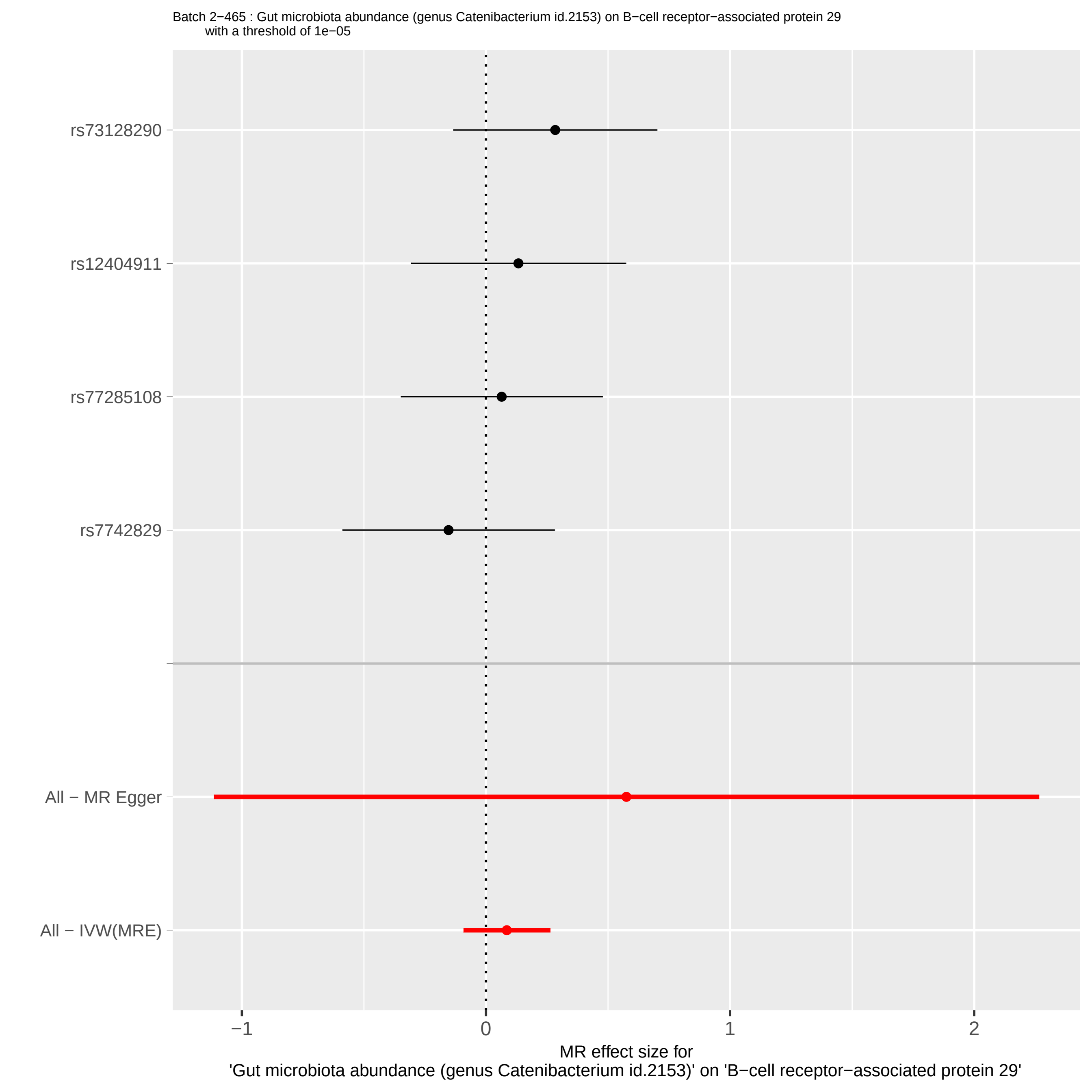
Batch 2−465 : Gut microbiota abundance (genus Catenibacterium id.2153) on B−cell receptor−associated protein 29
with a threshold of 1e−05
rs73128290
rs12404911
rs77285108
rs7742829
All − MR Egger
All − IVW(MRE)
−1
0
1
2
MR effect size for
'Gut microbiota abundance (genus Catenibacterium id.2153)' on 'B−cell receptor−associated protein 29'

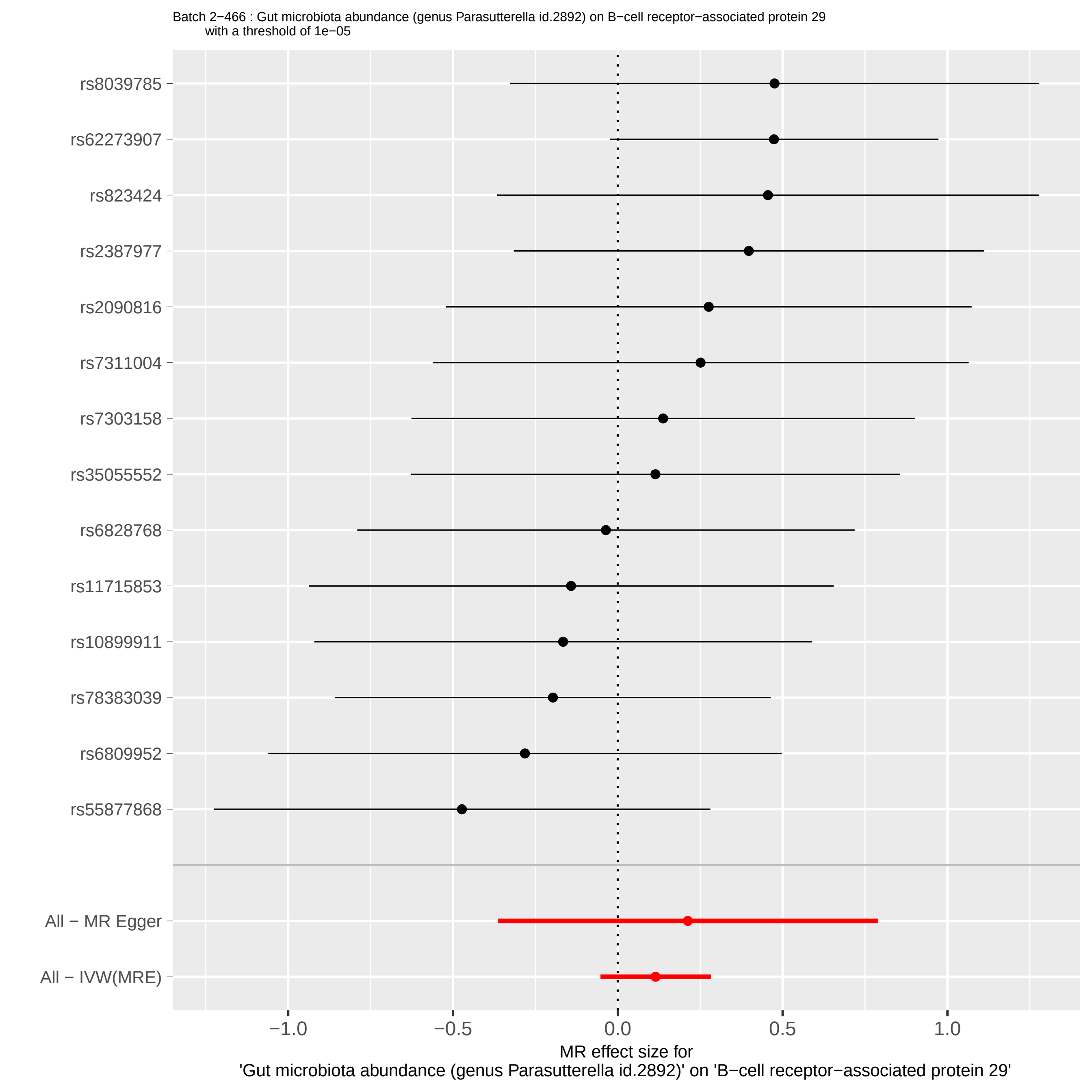
Batch 2−466 : Gut microbiota abundance (genus Parasutterella id.2892) on B−cell receptor−associated protein 29
with a threshold of 1e−05
rs8039785
rs62273907
rs823424
rs2387977
rs2090816
rs7311004
rs7303158
rs35055552
rs6828768
rs11715853
rs10899911
rs78383039
rs6809952
rs55877868
All − MR Egger
All − IVW(MRE)
−1.0
−0.5
0.0
0.5
1.0
MR effect size for
'Gut microbiota abundance (genus Parasutterella id.2892)' on 'B−cell receptor−associated protein 29'

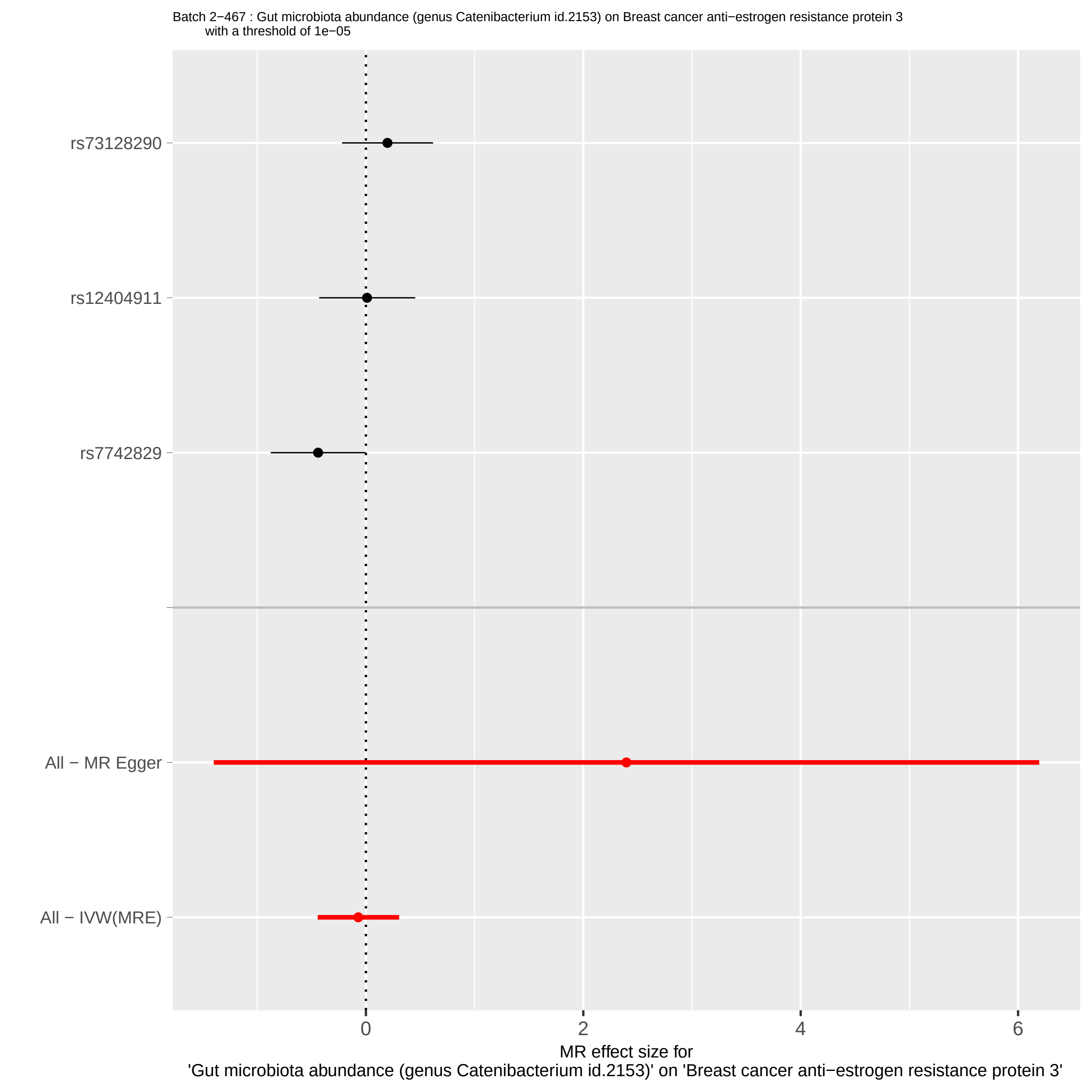

Batch 2−467 : Gut microbiota abundance (genus Catenibacterium id.2153) on Breast cancer anti−estrogen resistance protein 3
with a threshold of 1e−05
rs73128290
rs12404911
rs7742829
All − MR Egger
All − IVW(MRE)
0
2
4
6
MR effect size for
'Gut microbiota abundance (genus Catenibacterium id.2153)' on 'Breast cancer anti−estrogen resistance protein 3'

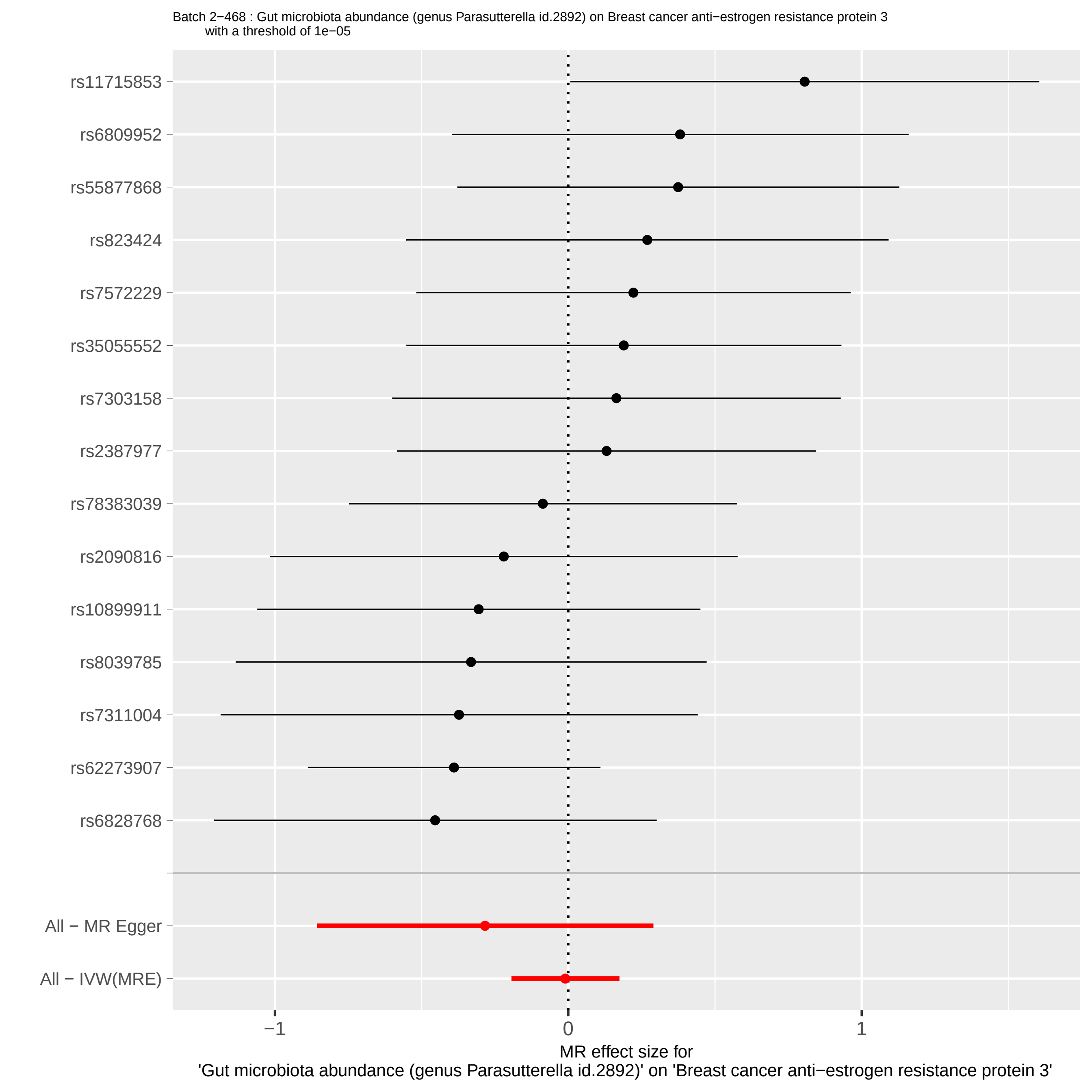
Batch 2−468 : Gut microbiota abundance (genus Parasutterella id.2892) on Breast cancer anti−estrogen resistance protein 3
with a threshold of 1e−05
rs11715853
rs6809952
rs55877868
rs823424
rs7572229
rs35055552
rs7303158
rs2387977
rs78383039
rs2090816
rs10899911
rs8039785
rs7311004
rs62273907
rs6828768
All − MR Egger
All − IVW(MRE)
−1
0
1
MR effect size for
'Gut microbiota abundance (genus Parasutterella id.2892)' on 'Breast cancer anti−estrogen resistance protein 3'

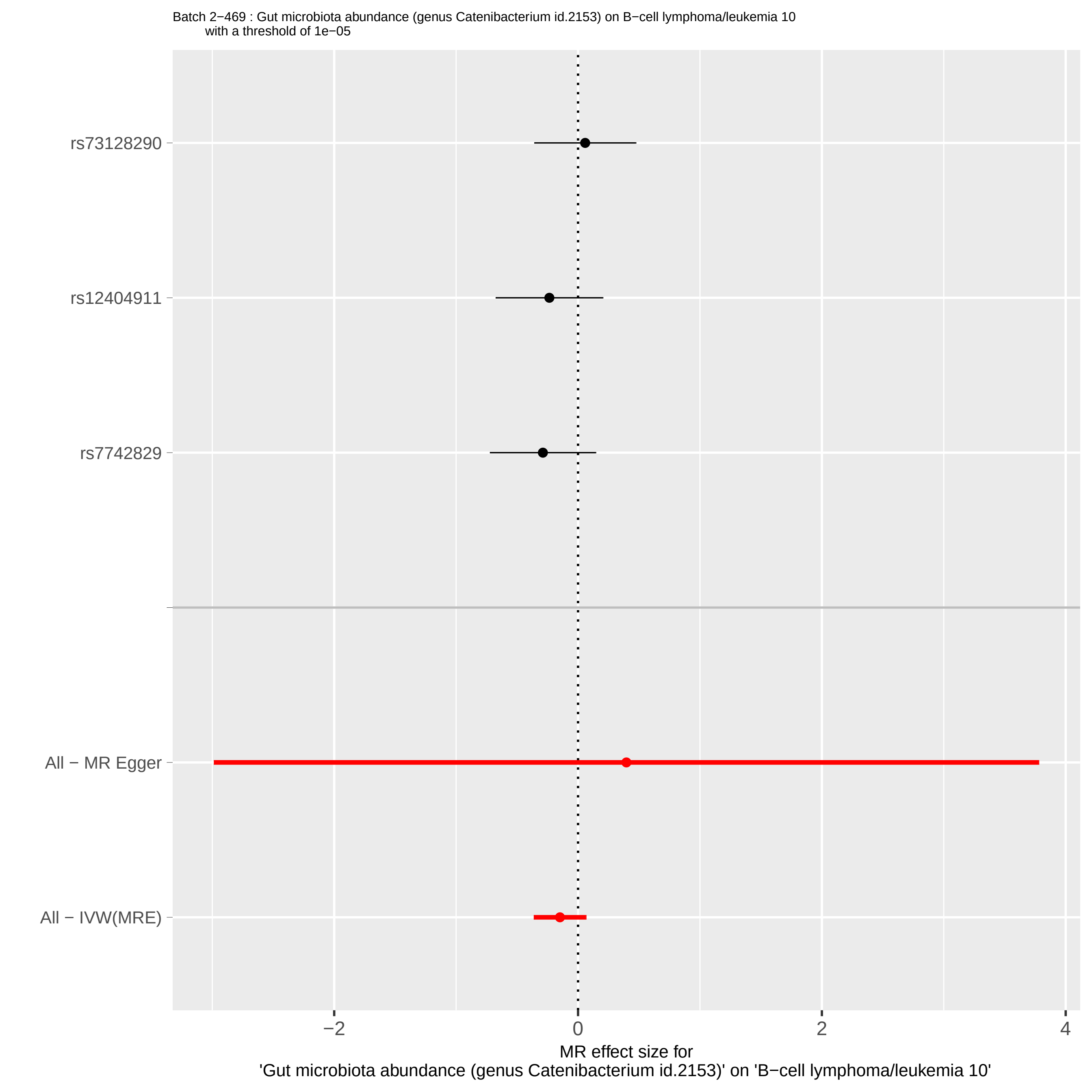

Batch 2−469 : Gut microbiota abundance (genus Catenibacterium id.2153) on B−cell lymphoma/leukemia 10
with a threshold of 1e−05
rs73128290
rs12404911
rs7742829
All − MR Egger
All − IVW(MRE)
−2
0
2
4
MR effect size for
'Gut microbiota abundance (genus Catenibacterium id.2153)' on 'B−cell lymphoma/leukemia 10'

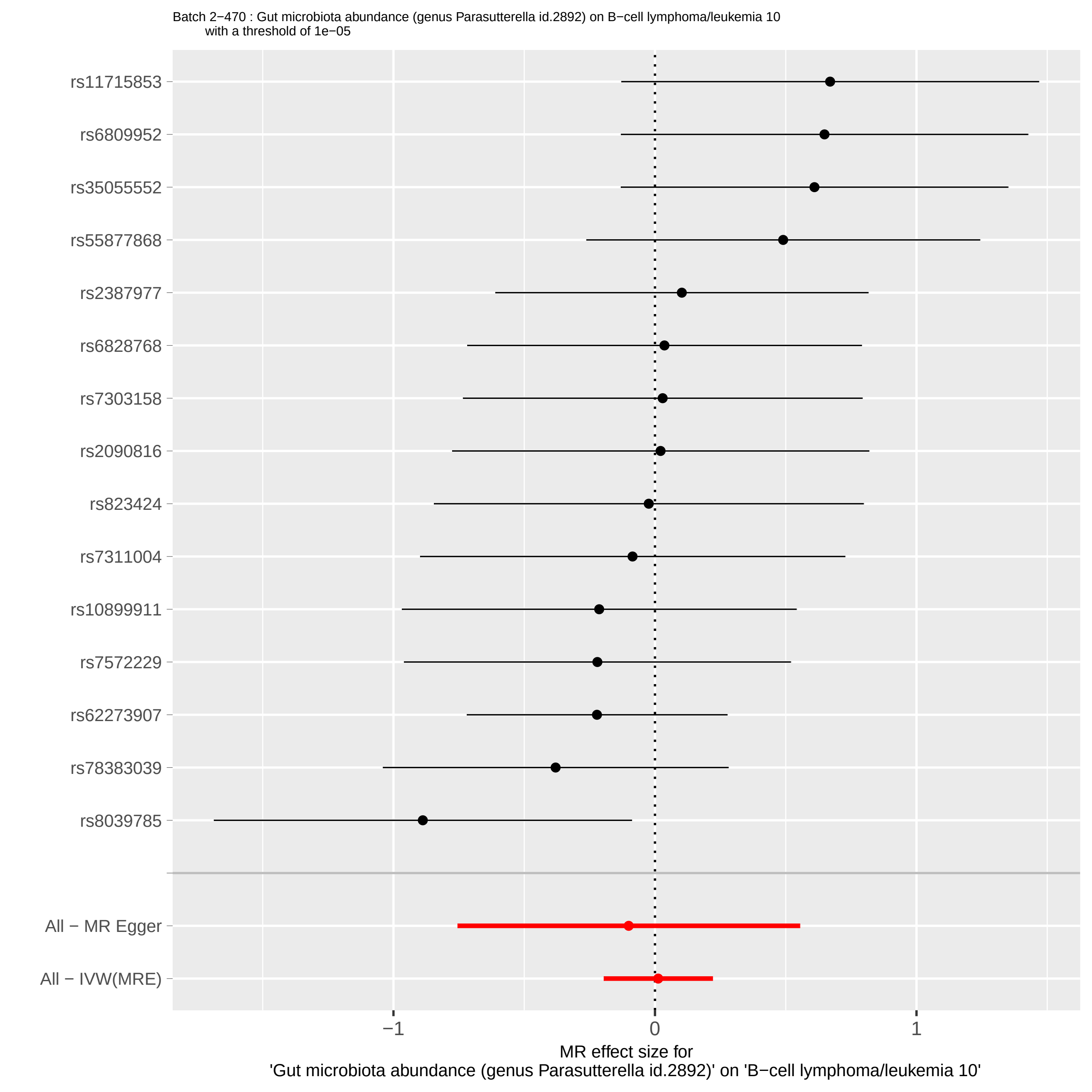

Batch 2−470 : Gut microbiota abundance (genus Parasutterella id.2892) on B−cell lymphoma/leukemia 10
with a threshold of 1e−05
rs11715853
rs6809952
rs35055552
rs55877868
rs2387977
rs6828768
rs7303158
rs2090816
rs823424
rs7311004
rs10899911
rs7572229
rs62273907
rs78383039
rs8039785
All − MR Egger
All − IVW(MRE)
−1
0
1
MR effect size for
'Gut microbiota abundance (genus Parasutterella id.2892)' on 'B−cell lymphoma/leukemia 10'

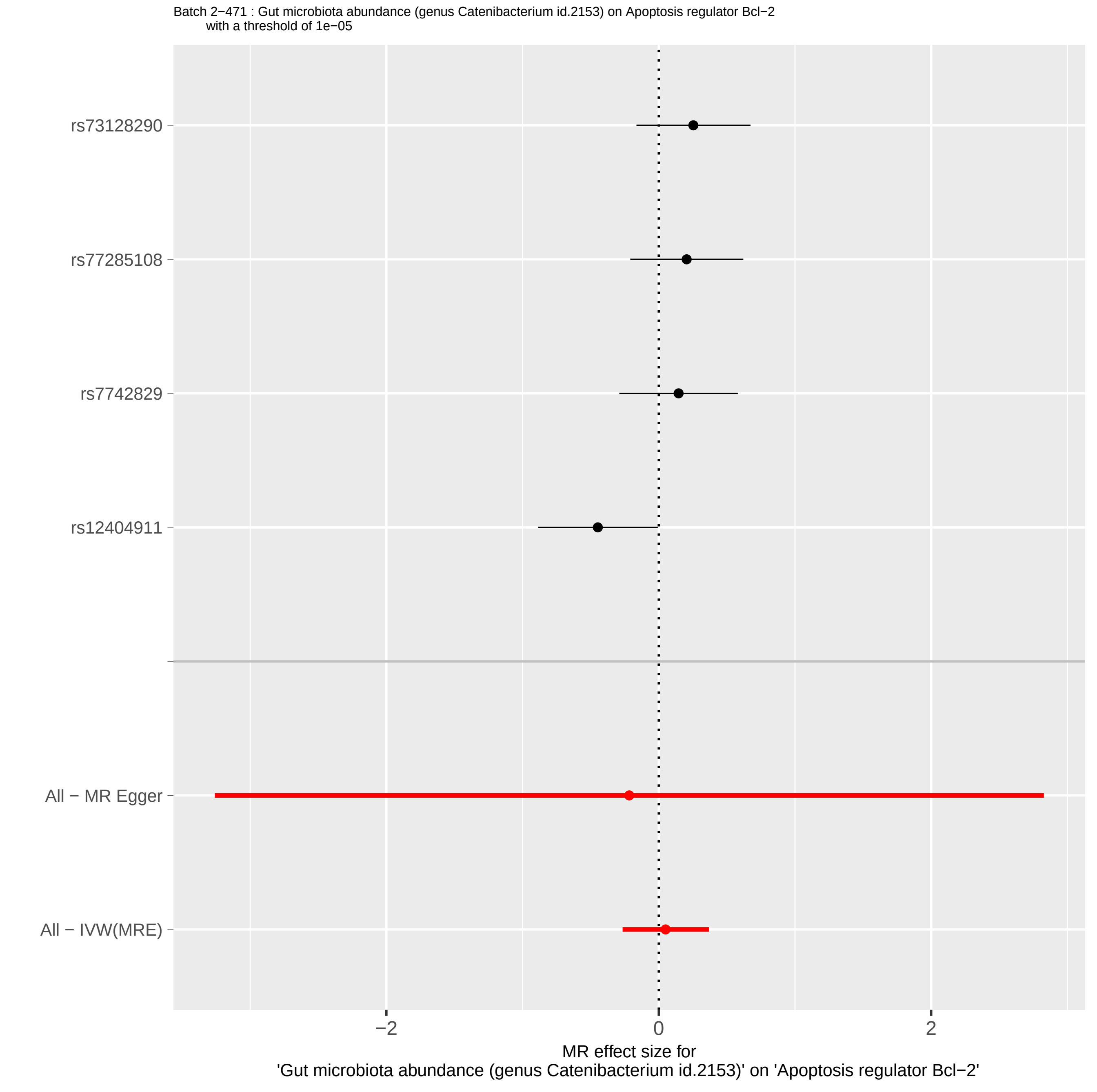

Batch 2−471 : Gut microbiota abundance (genus Catenibacterium id.2153) on Apoptosis regulator Bcl−2
with a threshold of 1e−05
rs73128290
rs77285108
rs7742829
rs12404911
All − MR Egger
All − IVW(MRE)
−2
0
2
MR effect size for
'Gut microbiota abundance (genus Catenibacterium id.2153)' on 'Apoptosis regulator Bcl−2'

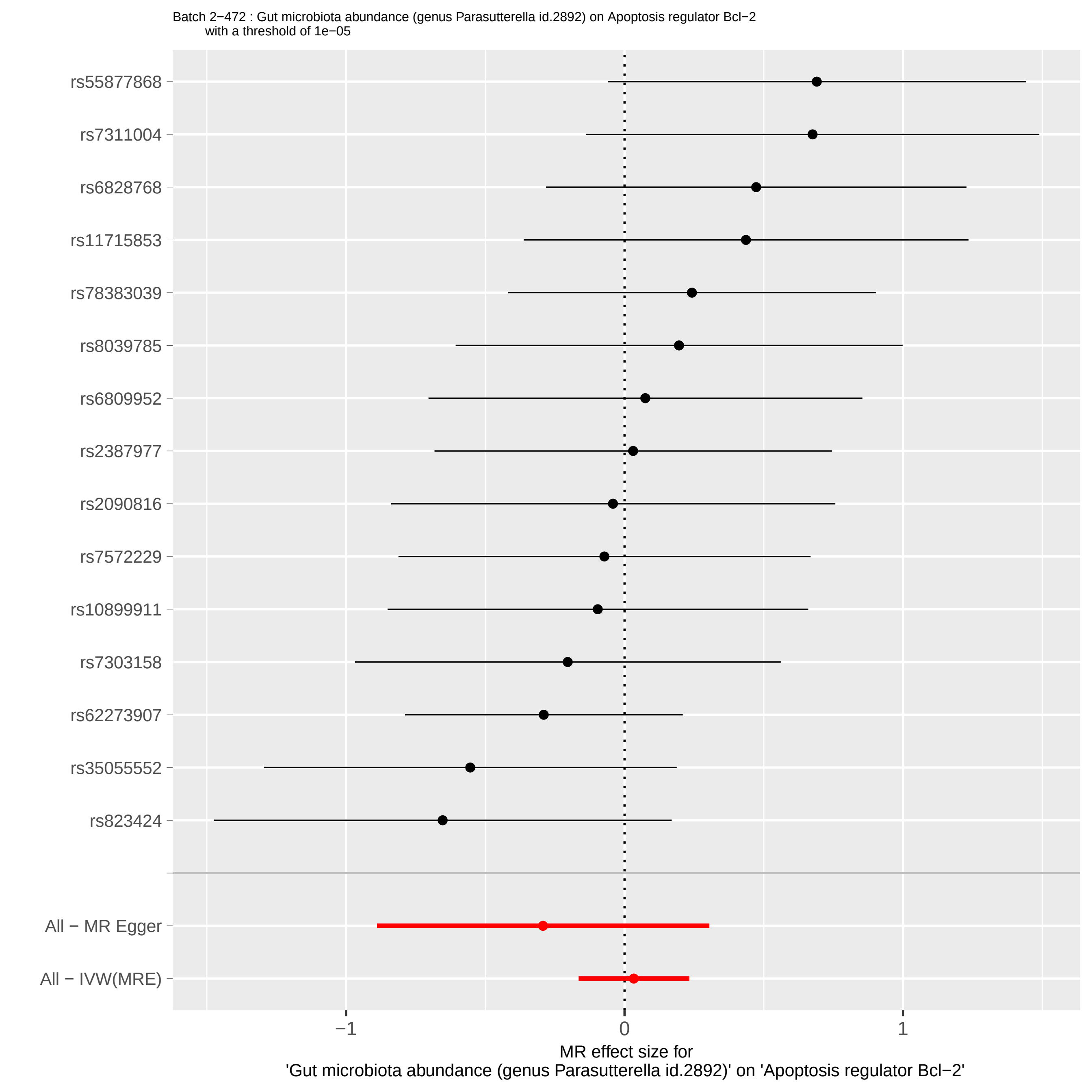

Batch 2−472 : Gut microbiota abundance (genus Parasutterella id.2892) on Apoptosis regulator Bcl−2
with a threshold of 1e−05
rs55877868
rs7311004
rs6828768
rs11715853
rs78383039
rs8039785
rs6809952
rs2387977
rs2090816
rs7572229
rs10899911
rs7303158
rs62273907
rs35055552
rs823424
All − MR Egger
All − IVW(MRE)
−1
0
1
MR effect size for
'Gut microbiota abundance (genus Parasutterella id.2892)' on 'Apoptosis regulator Bcl−2'

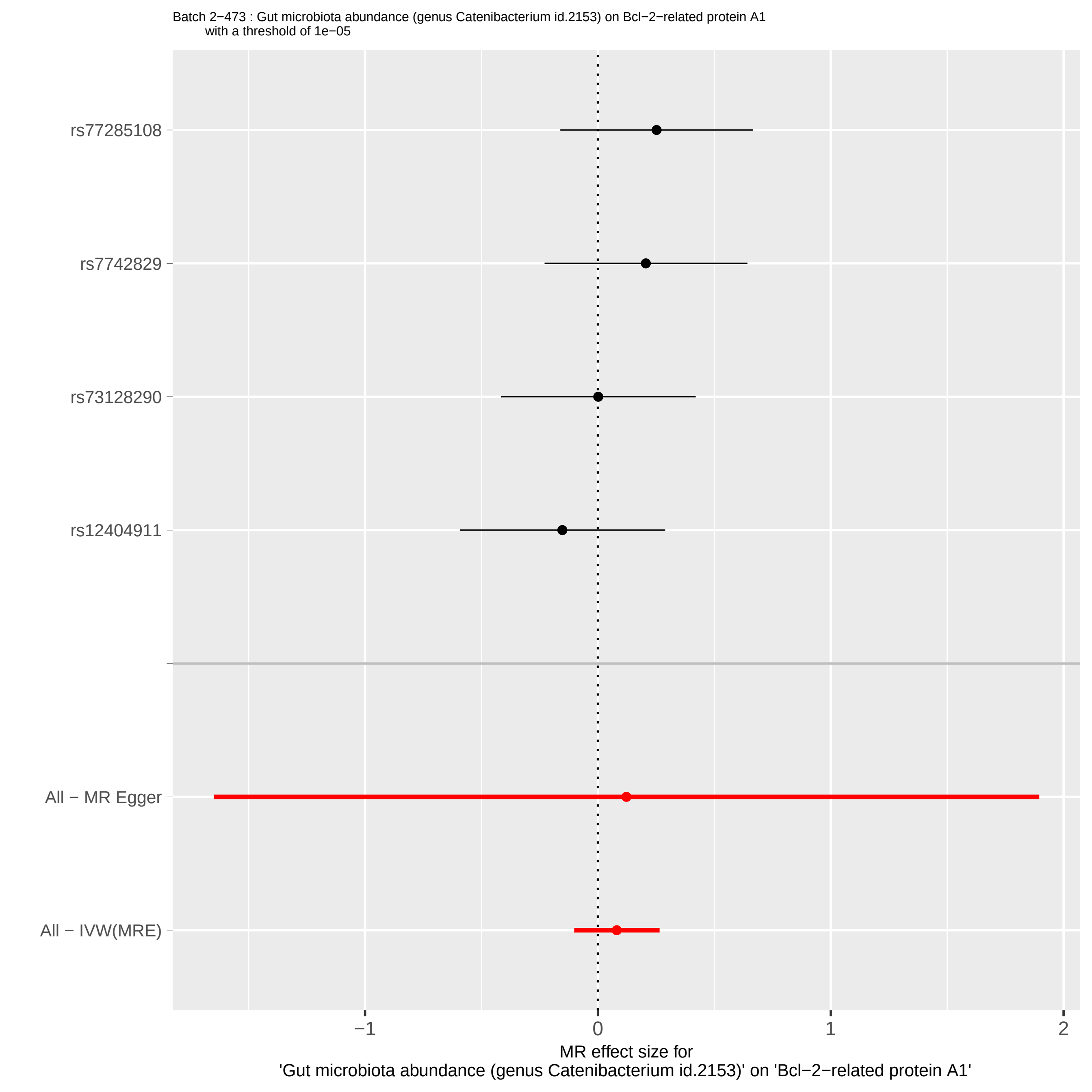

Batch 2−473 : Gut microbiota abundance (genus Catenibacterium id.2153) on Bcl−2−related protein A1
with a threshold of 1e−05
rs77285108
rs7742829
rs73128290
rs12404911
All − MR Egger
All − IVW(MRE)
−1
0
1
2
MR effect size for
'Gut microbiota abundance (genus Catenibacterium id.2153)' on 'Bcl−2−related protein A1'

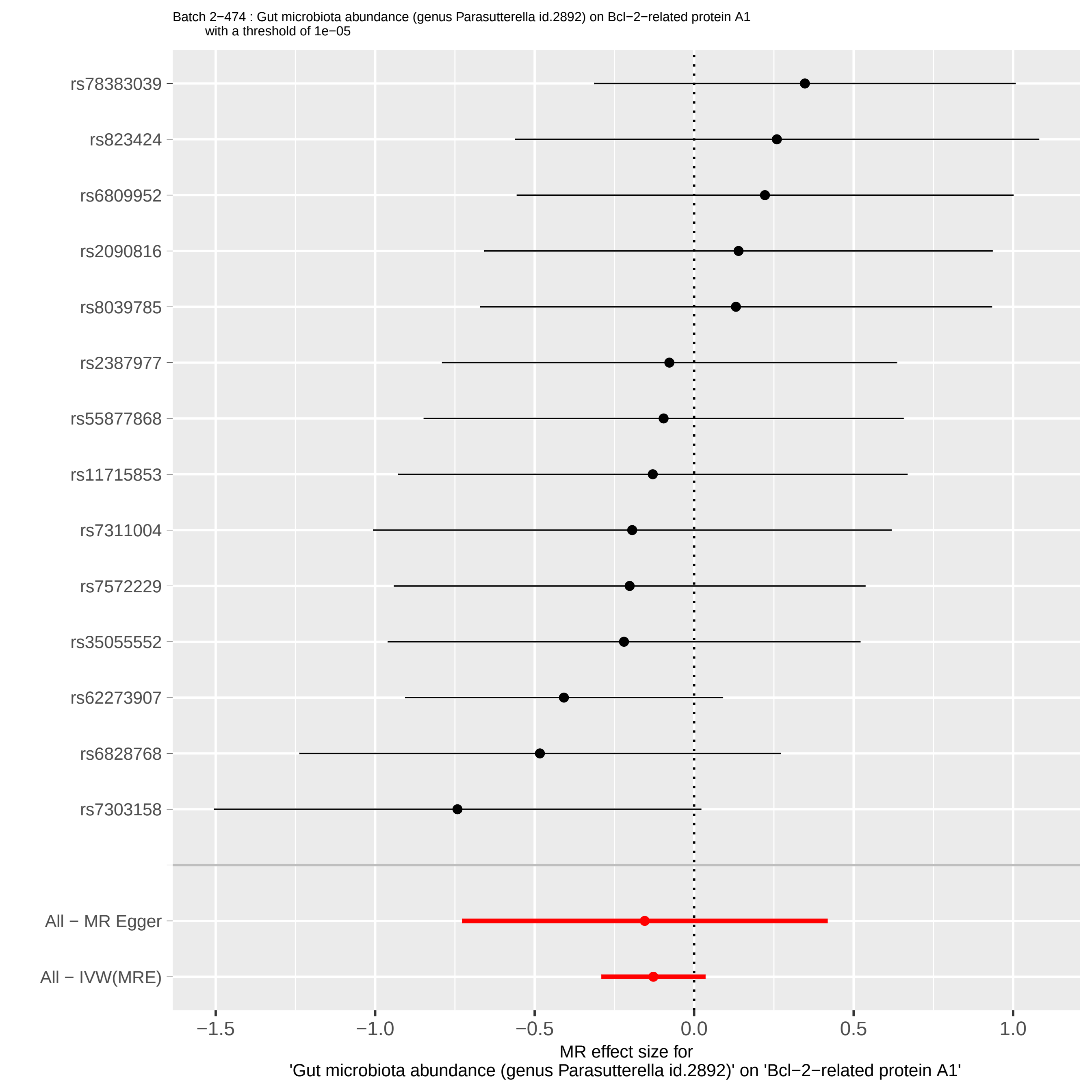
Batch 2−474 : Gut microbiota abundance (genus Parasutterella id.2892) on Bcl−2−related protein A1
with a threshold of 1e−05
rs78383039
rs823424
rs6809952
rs2090816
rs8039785
rs2387977
rs55877868
rs11715853
rs7311004
rs7572229
rs35055552
rs62273907
rs6828768
rs7303158
All − MR Egger
All − IVW(MRE)
−1.5
−1.0
−0.5
0.0
0.5
1.0
MR effect size for
'Gut microbiota abundance (genus Parasutterella id.2892)' on 'Bcl−2−related protein A1'

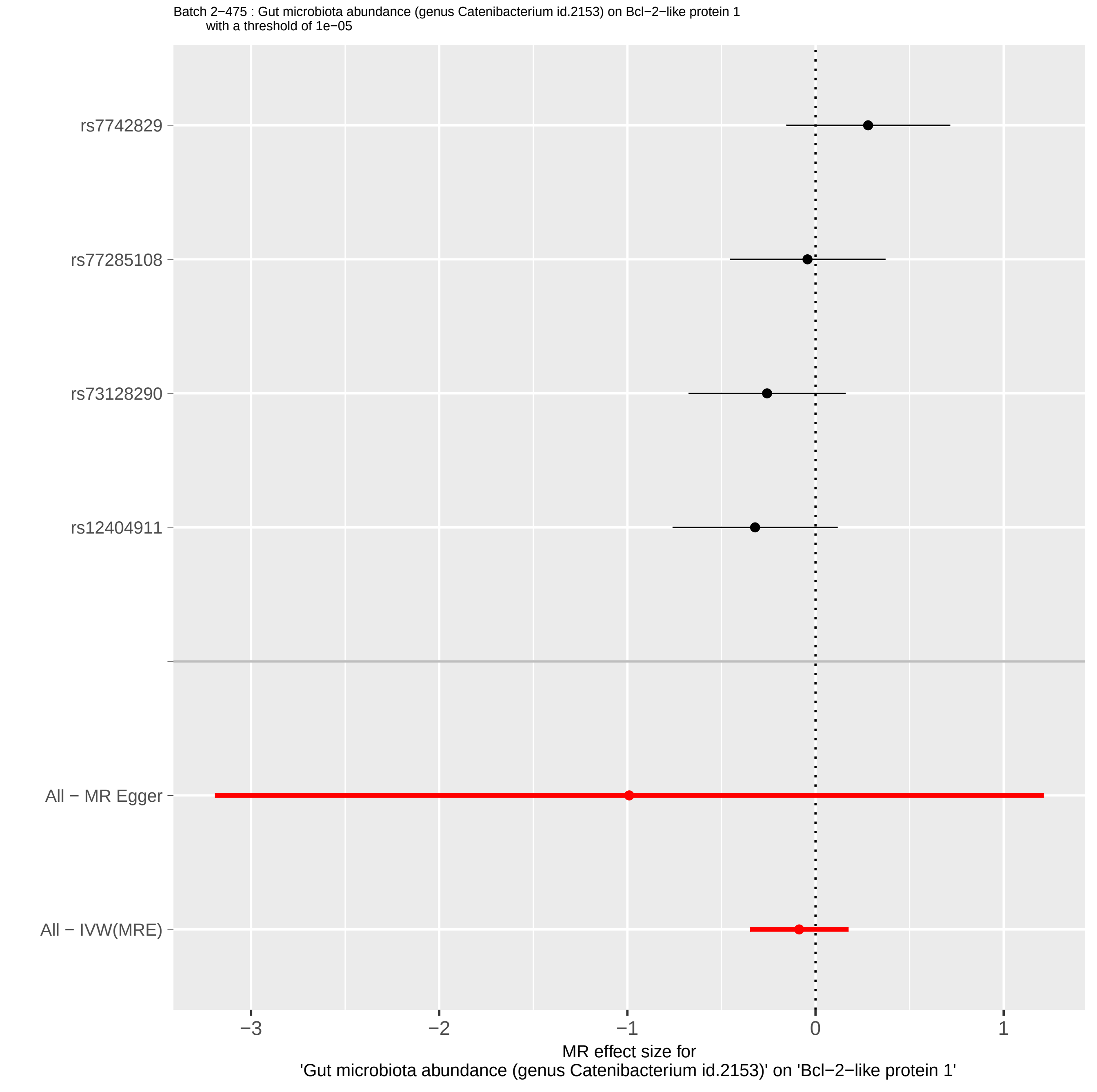

Batch 2−475 : Gut microbiota abundance (genus Catenibacterium id.2153) on Bcl−2−like protein 1
with a threshold of 1e−05
rs7742829
rs77285108
rs73128290
rs12404911
All − MR Egger
All − IVW(MRE)
−3
−2
−1
0
1
MR effect size for
'Gut microbiota abundance (genus Catenibacterium id.2153)' on 'Bcl−2−like protein 1'

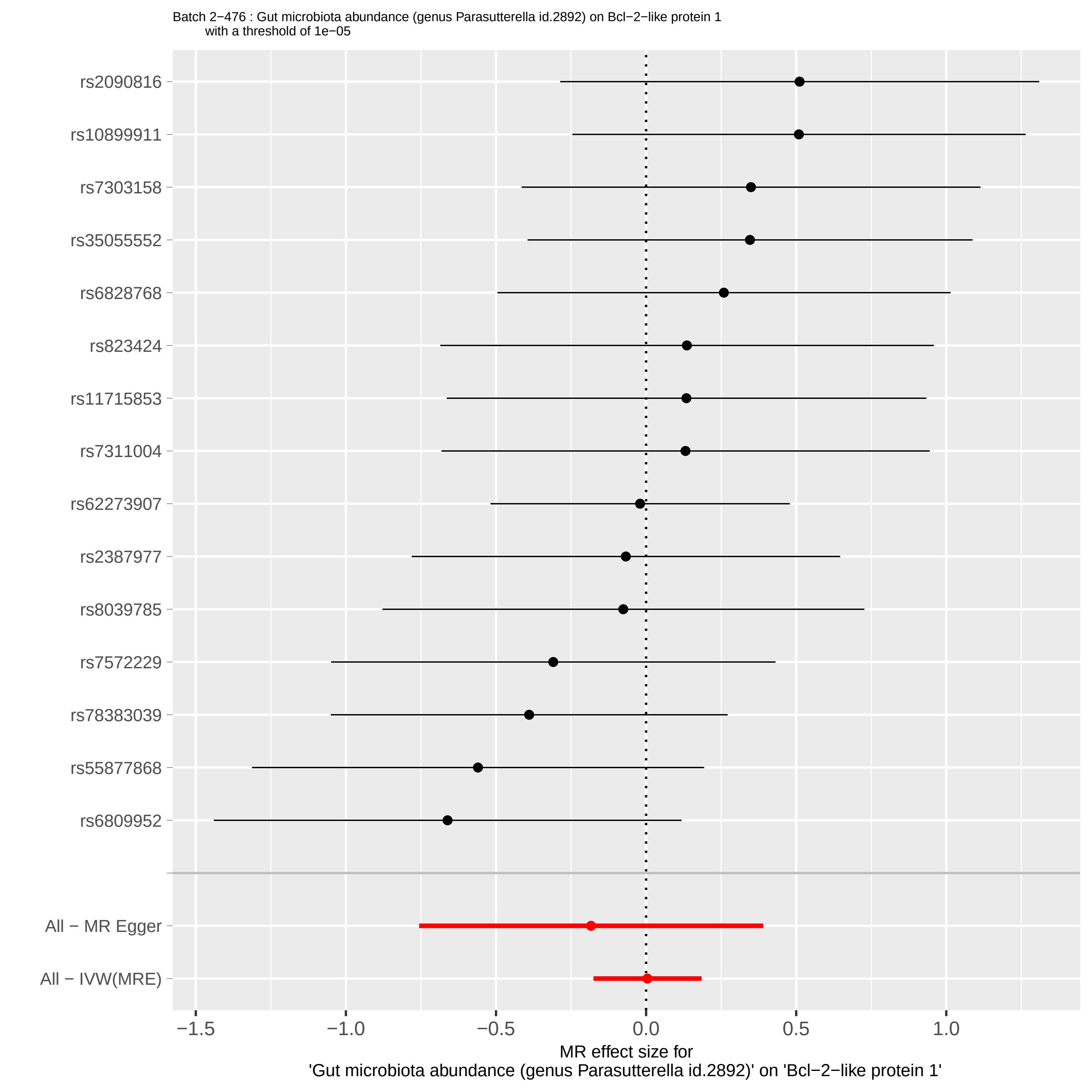
Batch 2−476 : Gut microbiota abundance (genus Parasutterella id.2892) on Bcl−2−like protein 1
with a threshold of 1e−05
rs2090816
rs10899911
rs7303158
rs35055552
rs6828768
rs823424
rs11715853
rs7311004
rs62273907
rs2387977
rs8039785
rs7572229
rs78383039
rs55877868
rs6809952
All − MR Egger
All − IVW(MRE)
−1.5
−1.0
−0.5
0.0
0.5
1.0
MR effect size for
'Gut microbiota abundance (genus Parasutterella id.2892)' on 'Bcl−2−like protein 1'

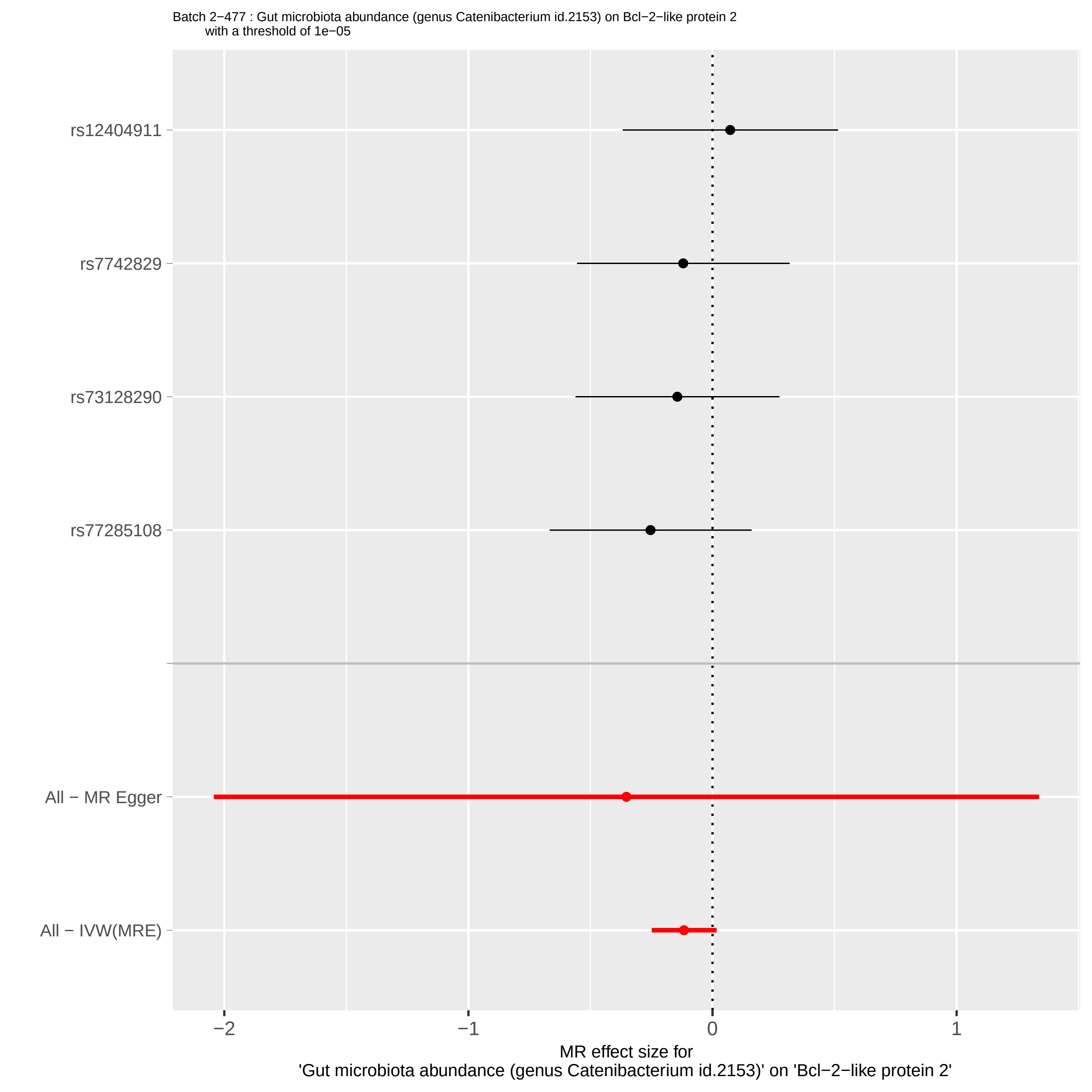
Batch 2−477 : Gut microbiota abundance (genus Catenibacterium id.2153) on Bcl−2−like protein 2
with a threshold of 1e−05
rs12404911
rs7742829
rs73128290
rs77285108
All − MR Egger
All − IVW(MRE)
−2
−1
0
1
MR effect size for
'Gut microbiota abundance (genus Catenibacterium id.2153)' on 'Bcl−2−like protein 2'

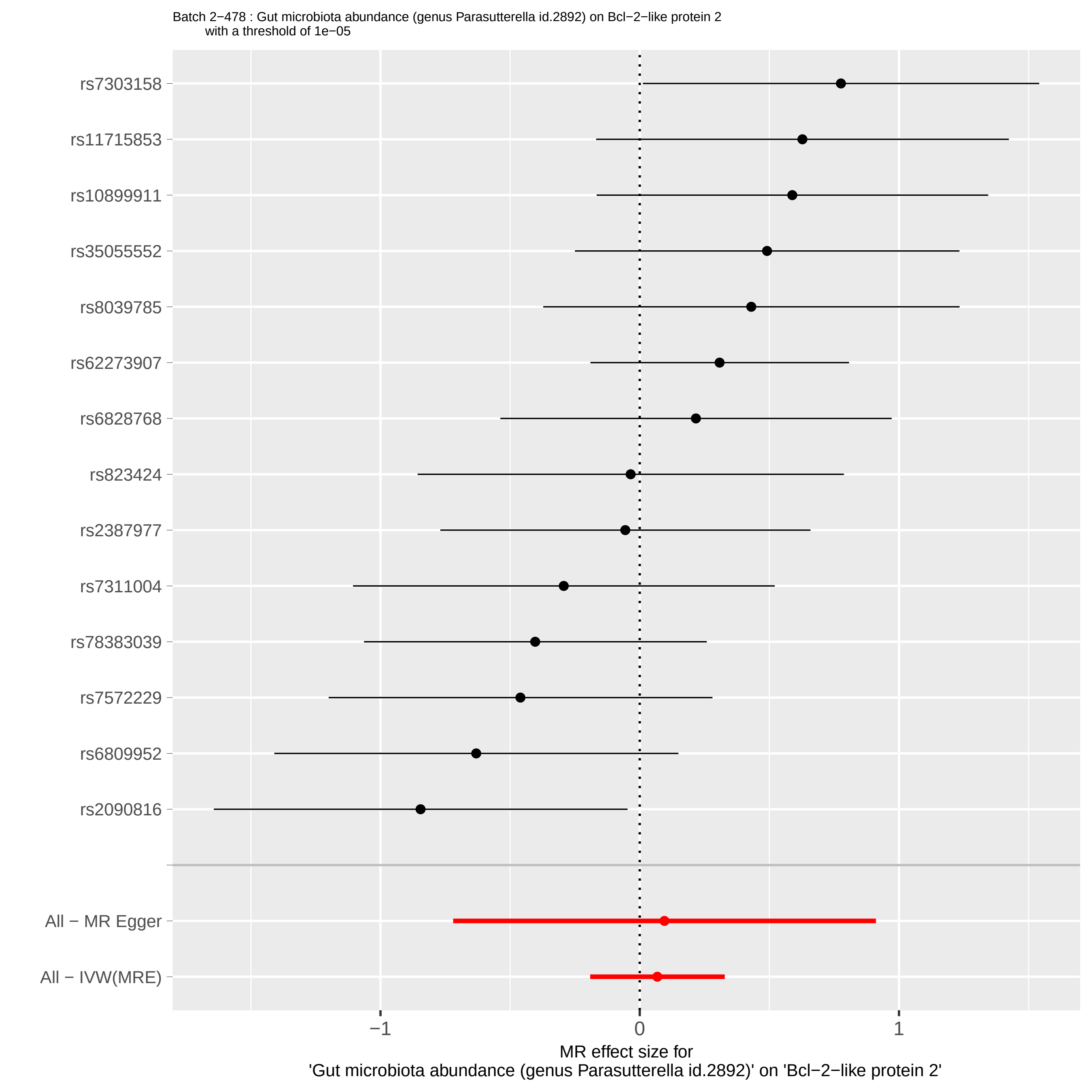

Batch 2−478 : Gut microbiota abundance (genus Parasutterella id.2892) on Bcl−2−like protein 2
with a threshold of 1e−05
rs7303158
rs11715853
rs10899911
rs35055552
rs8039785
rs62273907
rs6828768
rs823424
rs2387977
rs7311004
rs78383039
rs7572229
rs6809952
rs2090816
All − MR Egger
All − IVW(MRE)
−1
0
1
MR effect size for
'Gut microbiota abundance (genus Parasutterella id.2892)' on 'Bcl−2−like protein 2'

Batch 2−479 : Gut microbiota abundance (genus Catenibacterium id.2153) on Bcl−2−like protein 2
with a threshold of 1e−05

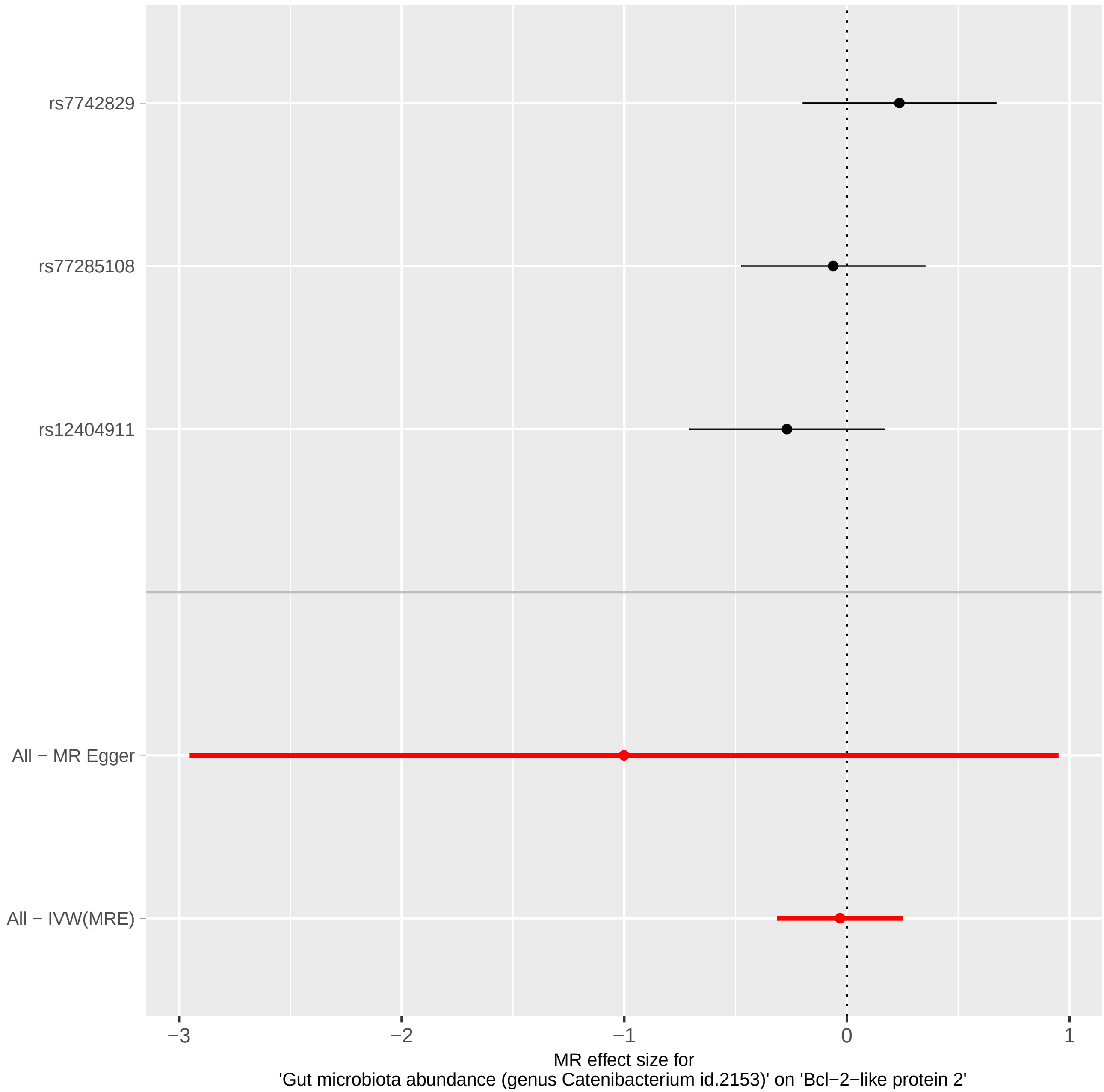

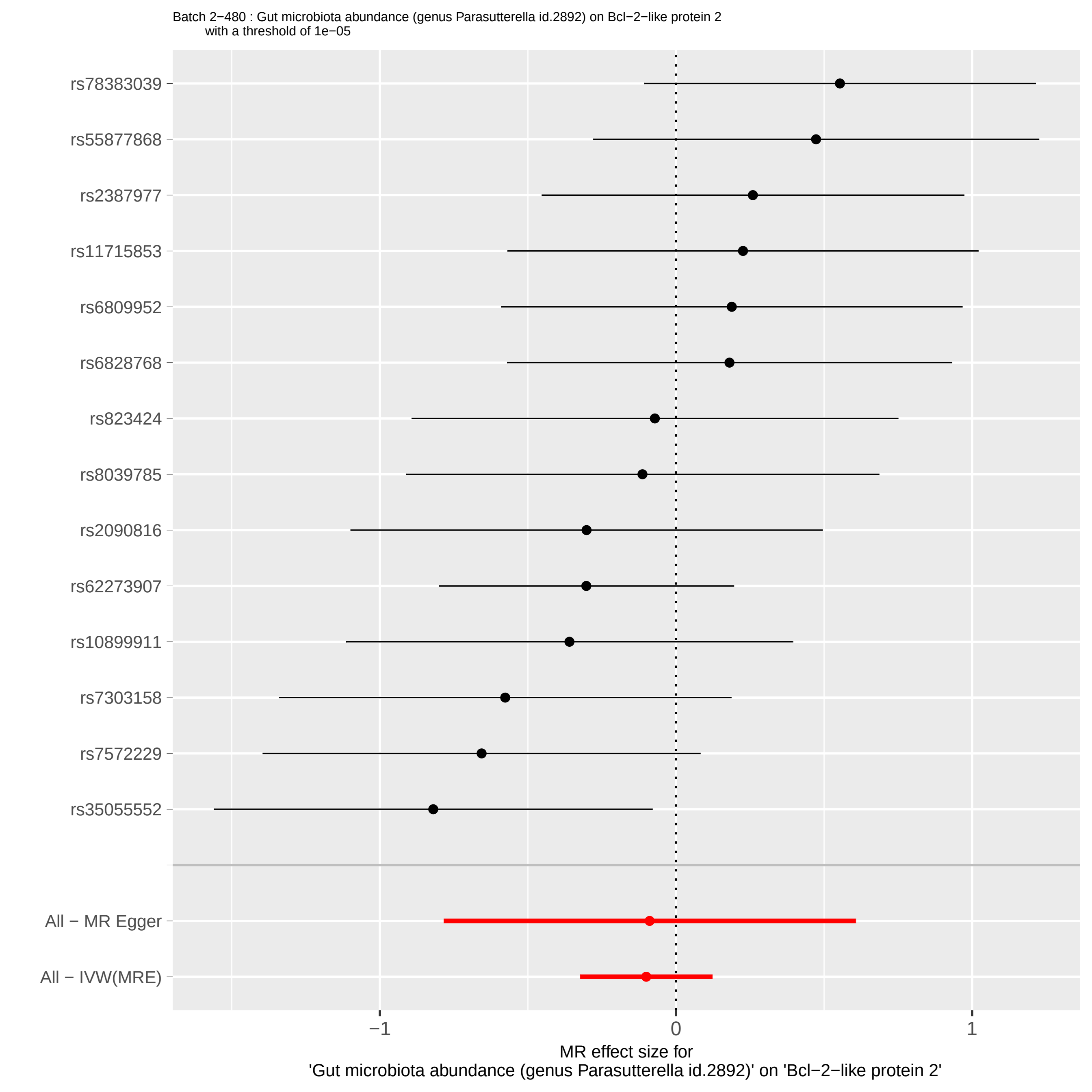

Batch 2−480 : Gut microbiota abundance (genus Parasutterella id.2892) on Bcl−2−like protein 2
with a threshold of 1e−05
rs78383039
rs55877868
rs2387977
rs11715853
rs6809952
rs6828768
rs823424
rs8039785
rs2090816
rs62273907
rs10899911
rs7303158
rs7572229
rs35055552
All − MR Egger
All − IVW(MRE)
−1
0
1
MR effect size for
'Gut microbiota abundance (genus Parasutterella id.2892)' on 'Bcl−2−like protein 2'

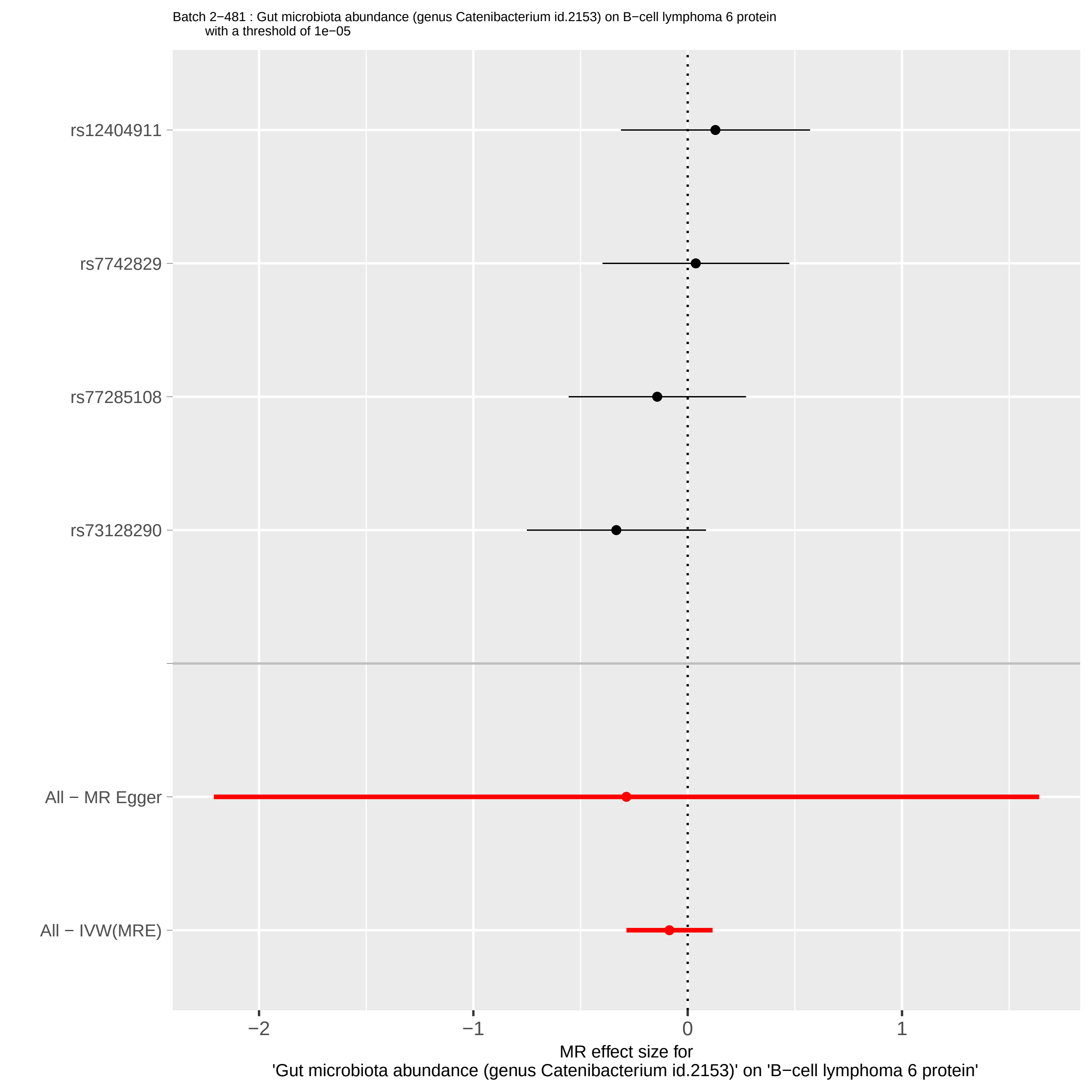
Batch 2−481 : Gut microbiota abundance (genus Catenibacterium id.2153) on B−cell lymphoma 6 protein
with a threshold of 1e−05
rs12404911
rs7742829
rs77285108
rs73128290
All − MR Egger
All − IVW(MRE)
−2
−1
0
1
MR effect size for
'Gut microbiota abundance (genus Catenibacterium id.2153)' on 'B−cell lymphoma 6 protein'

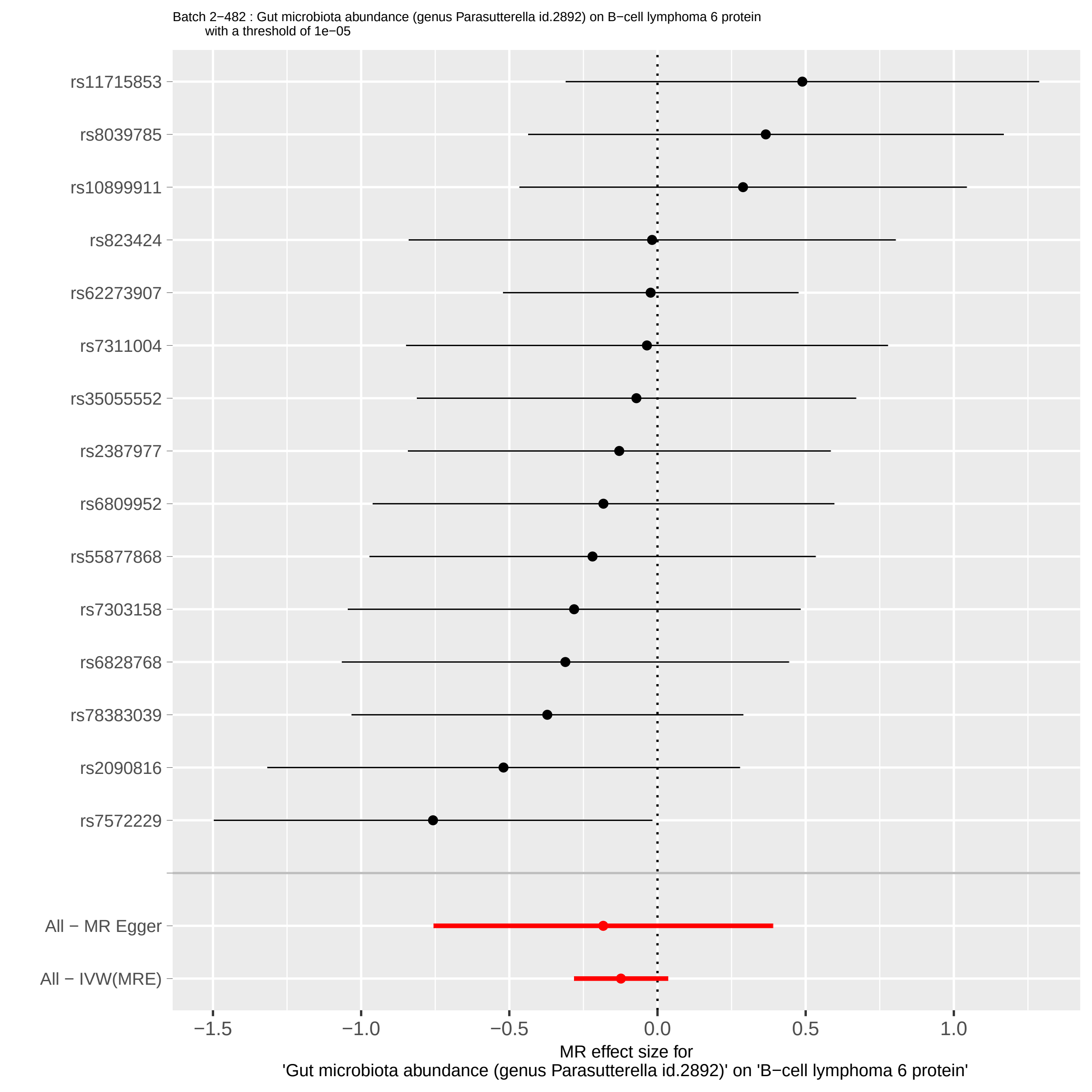

Batch 2−482 : Gut microbiota abundance (genus Parasutterella id.2892) on B−cell lymphoma 6 protein
with a threshold of 1e−05
rs11715853
rs8039785
rs10899911
rs823424
rs62273907
rs7311004
rs35055552
rs2387977
rs6809952
rs55877868
rs7303158
rs6828768
rs78383039
rs2090816
rs7572229
All − MR Egger
All − IVW(MRE)
−1.5
−1.0
−0.5
0.0
0.5
1.0
MR effect size for
'Gut microbiota abundance (genus Parasutterella id.2892)' on 'B−cell lymphoma 6 protein'

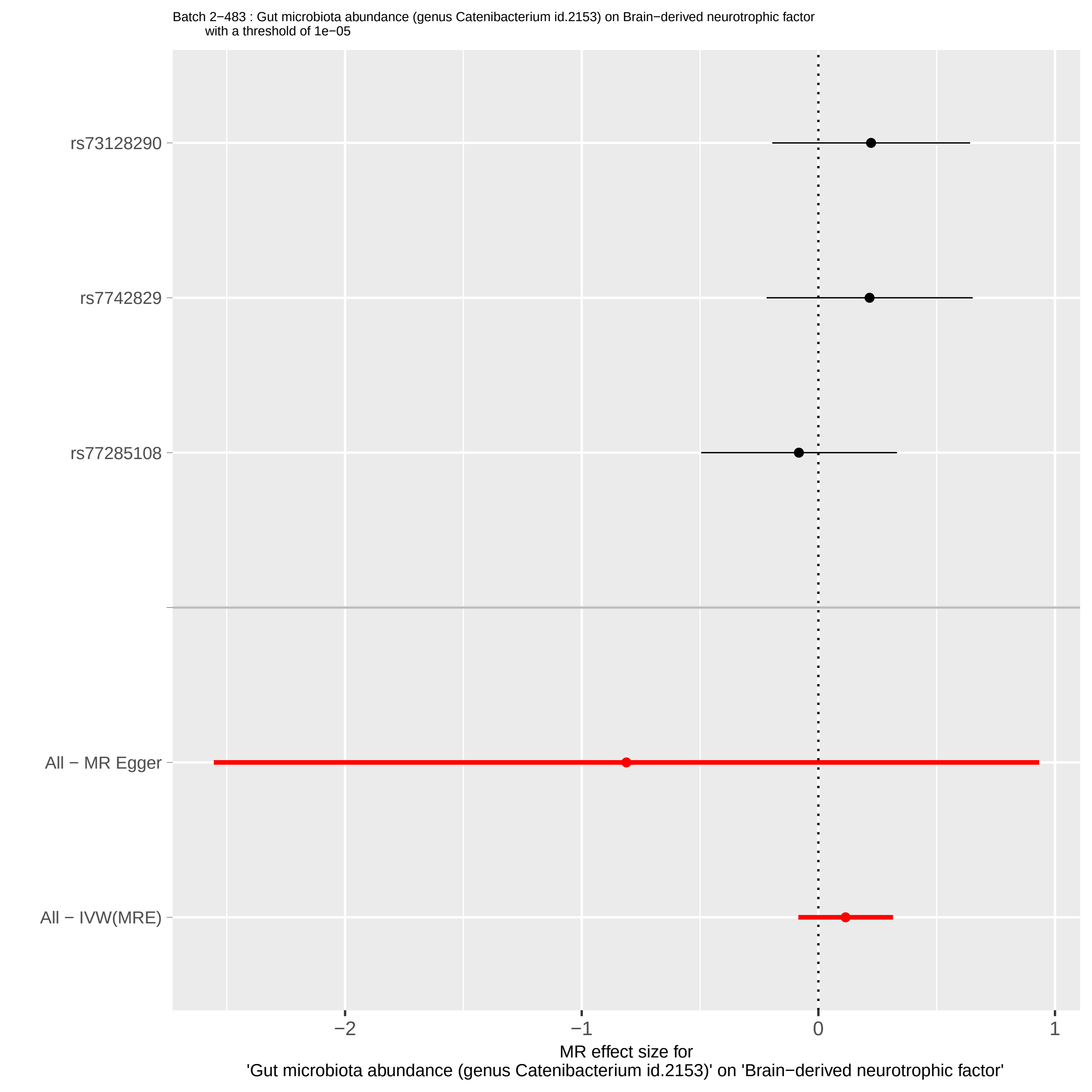

Batch 2−483 : Gut microbiota abundance (genus Catenibacterium id.2153) on Brain−derived neurotrophic factor
with a threshold of 1e−05
rs73128290
rs7742829
rs77285108
All − MR Egger
All − IVW(MRE)
−2
−1
0
1
MR effect size for
'Gut microbiota abundance (genus Catenibacterium id.2153)' on 'Brain−derived neurotrophic factor'

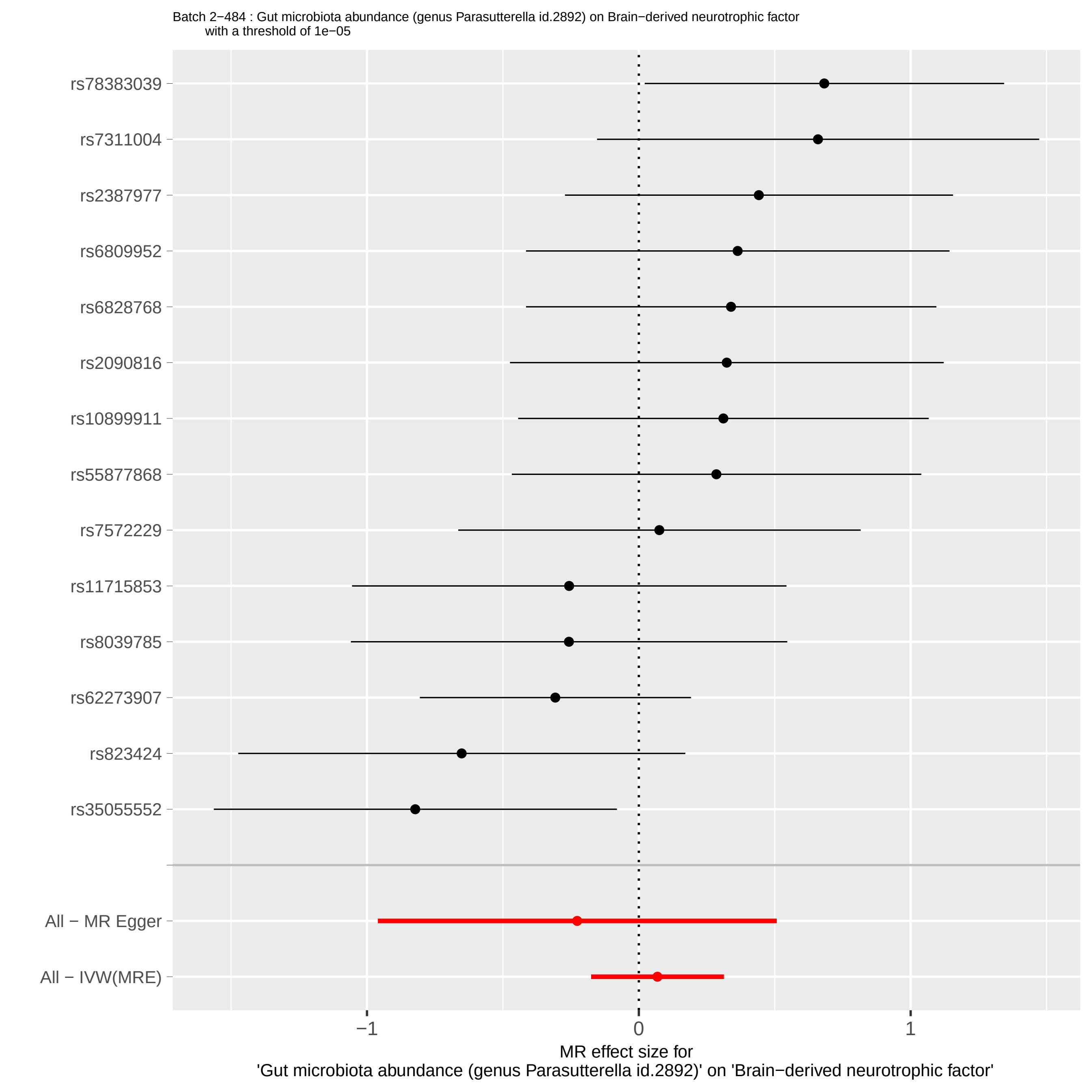
Batch 2−484 : Gut microbiota abundance (genus Parasutterella id.2892) on Brain−derived neurotrophic factor
with a threshold of 1e−05
rs78383039
rs7311004
rs2387977
rs6809952
rs6828768
rs2090816
rs10899911
rs55877868
rs7572229
rs11715853
rs8039785
rs62273907
rs823424
rs35055552
All − MR Egger
All − IVW(MRE)
−1
0
1
MR effect size for
'Gut microbiota abundance (genus Parasutterella id.2892)' on 'Brain−derived neurotrophic factor'

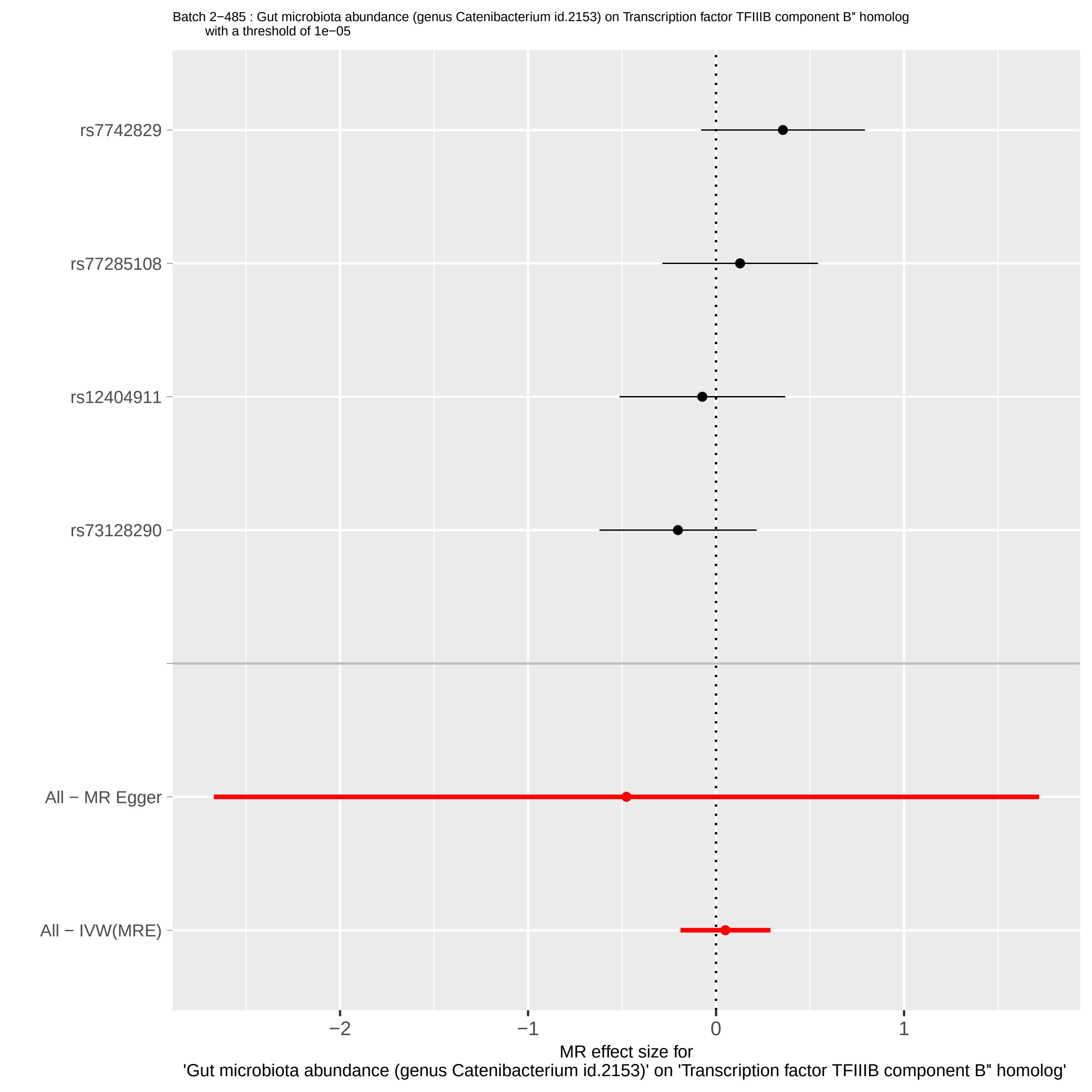

Batch 2−485 : Gut microbiota abundance (genus Catenibacterium id.2153) on Transcription factor TFIIIB component B" homolog with a threshold of 1e−05
rs7742829
rs77285108
rs12404911
rs73128290
All − MR Egger
All − IVW(MRE)
−2
−1
0
1
MR effect size for
'Gut microbiota abundance (genus Catenibacterium id.2153)' on 'Transcription factor TFIIIB component B" homolog'

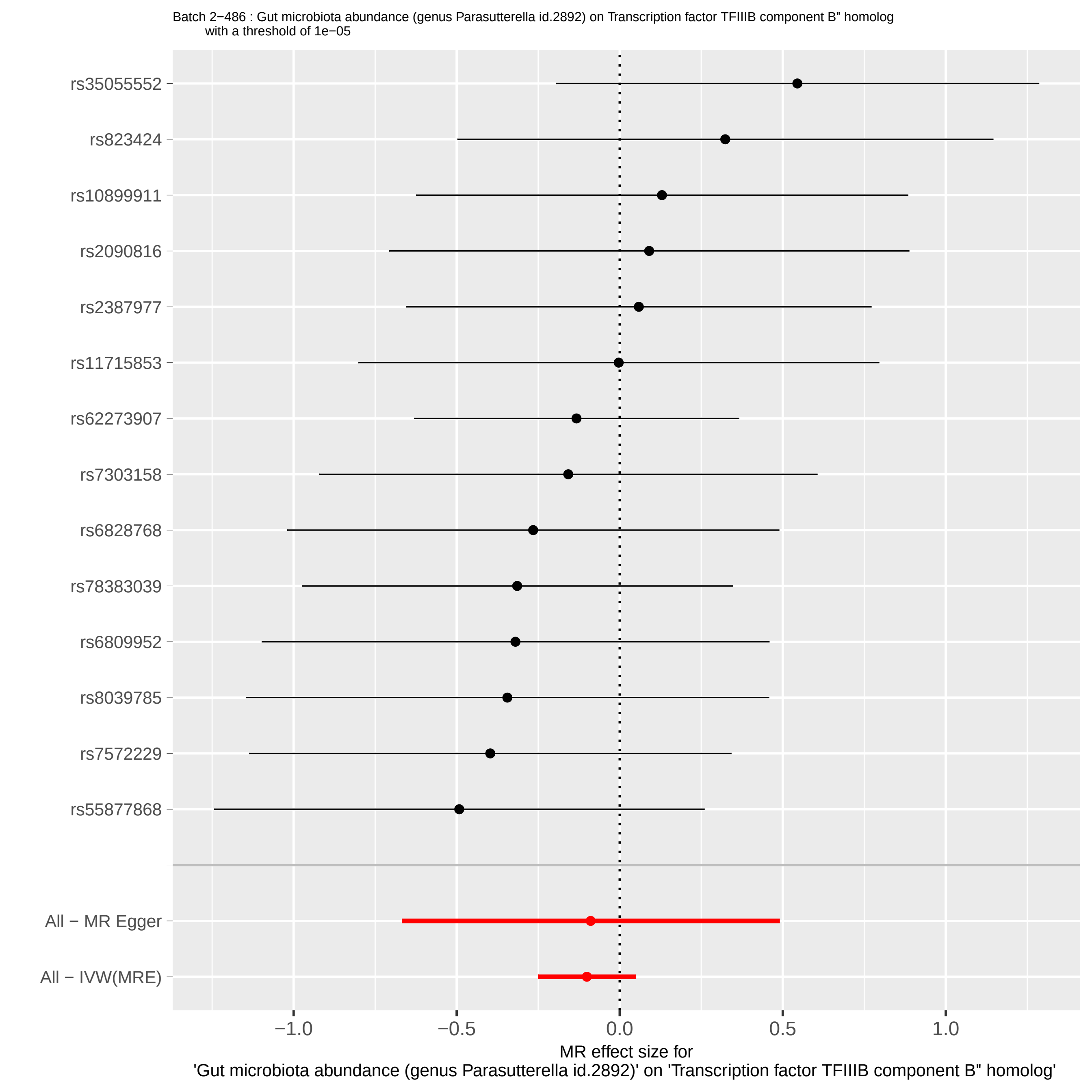

Batch 2−486 : Gut microbiota abundance (genus Parasutterella id.2892) on Transcription factor TFIIIB component B" homolog with a threshold of 1e−05
rs35055552
rs823424
rs10899911
rs2090816
rs2387977
rs11715853
rs62273907
rs7303158
rs6828768
rs78383039
rs6809952
rs8039785
rs7572229
rs55877868
All − MR Egger
All − IVW(MRE)
−1.0
−0.5
0.0
0.5
1.0
MR effect size for
'Gut microbiota abundance (genus Parasutterella id.2892)' on 'Transcription factor TFIIIB component B" homolog'

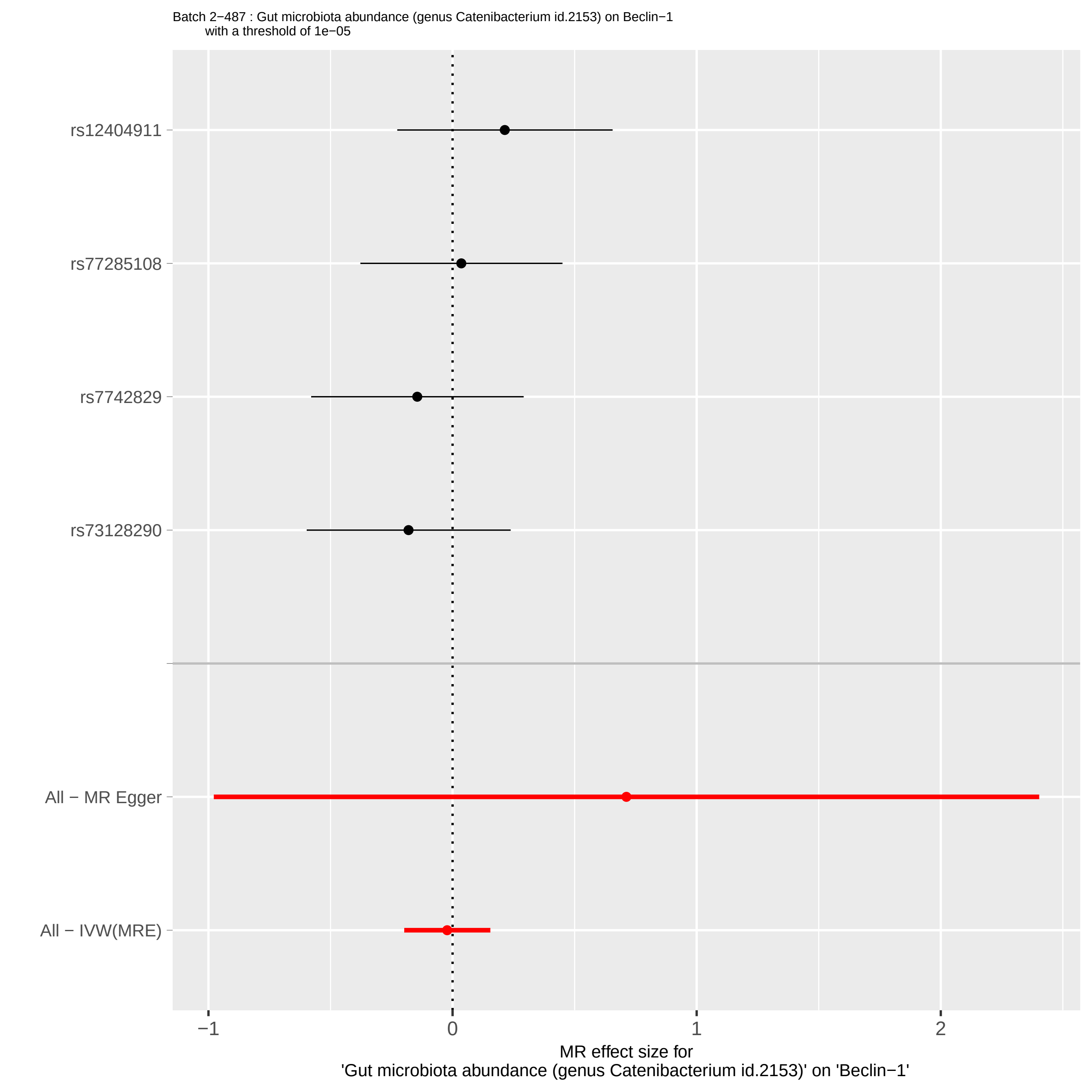

Batch 2−487 : Gut microbiota abundance (genus Catenibacterium id.2153) on Beclin−1
with a threshold of 1e−05
rs12404911
rs77285108
rs7742829
rs73128290
All − MR Egger
All − IVW(MRE)
−1
0
1
2
MR effect size for
'Gut microbiota abundance (genus Catenibacterium id.2153)' on 'Beclin−1'

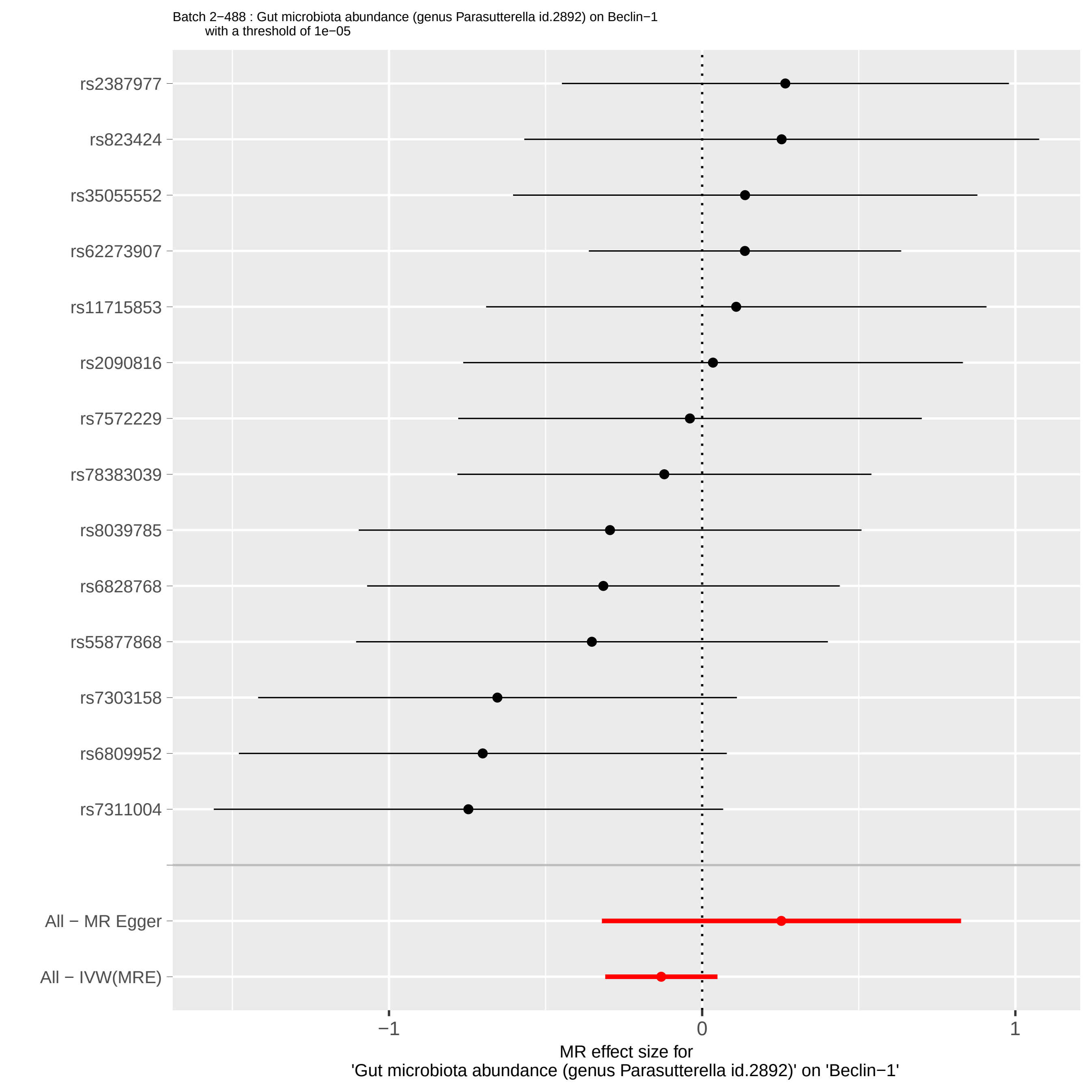

Batch 2−488 : Gut microbiota abundance (genus Parasutterella id.2892) on Beclin−1
with a threshold of 1e−05
rs2387977
rs823424
rs35055552
rs62273907
rs11715853
rs2090816
rs7572229
rs78383039
rs8039785
rs6828768
rs55877868
rs7303158
rs6809952
rs7311004
All − MR Egger
All − IVW(MRE)
−1
0
1
MR effect size for
'Gut microbiota abundance (genus Parasutterella id.2892)' on 'Beclin−1'

Batch 2−489 : Gut microbiota abundance (genus Catenibacterium id.2153) on BET1−like protein
with a threshold of 1e−05

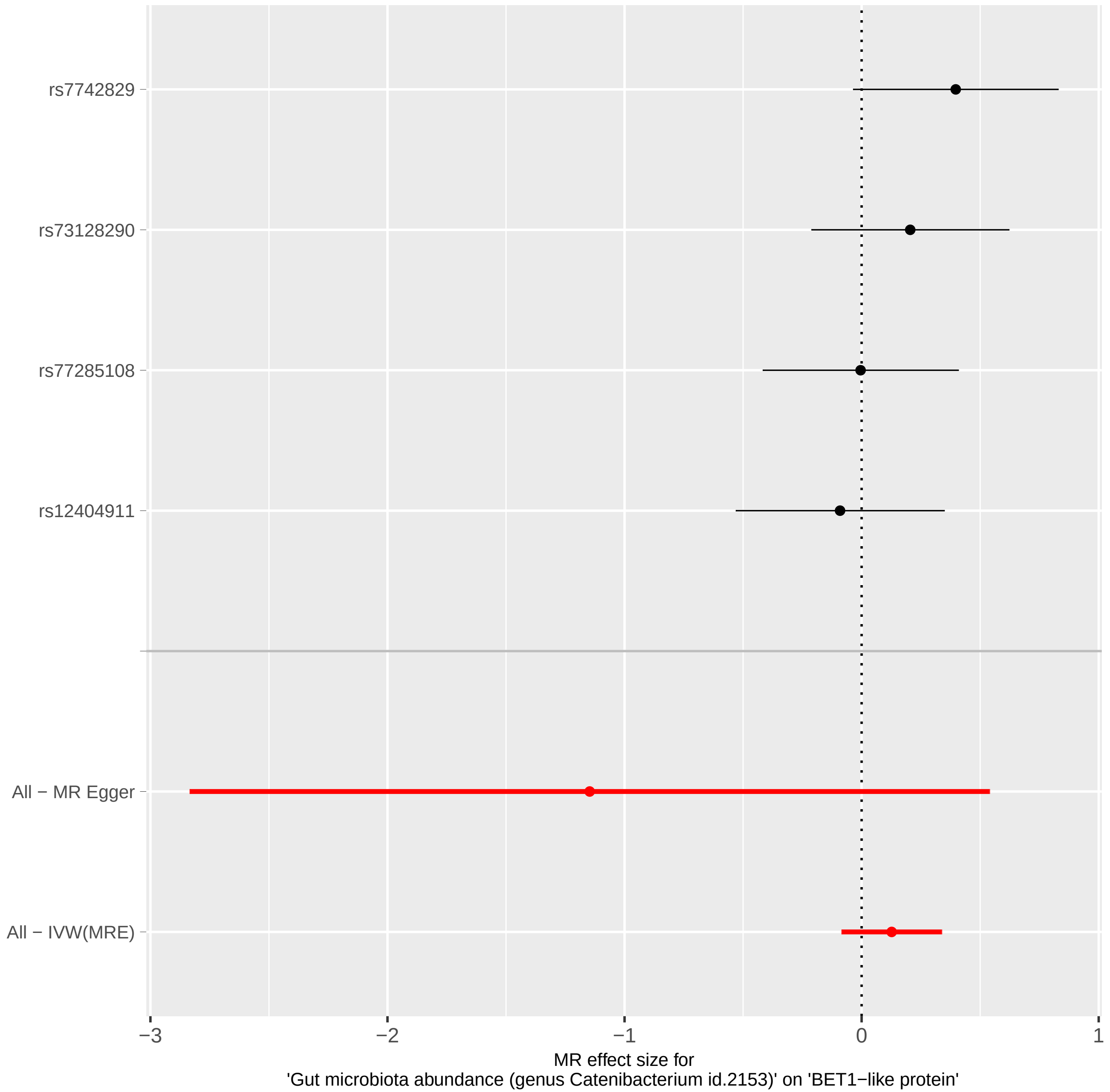

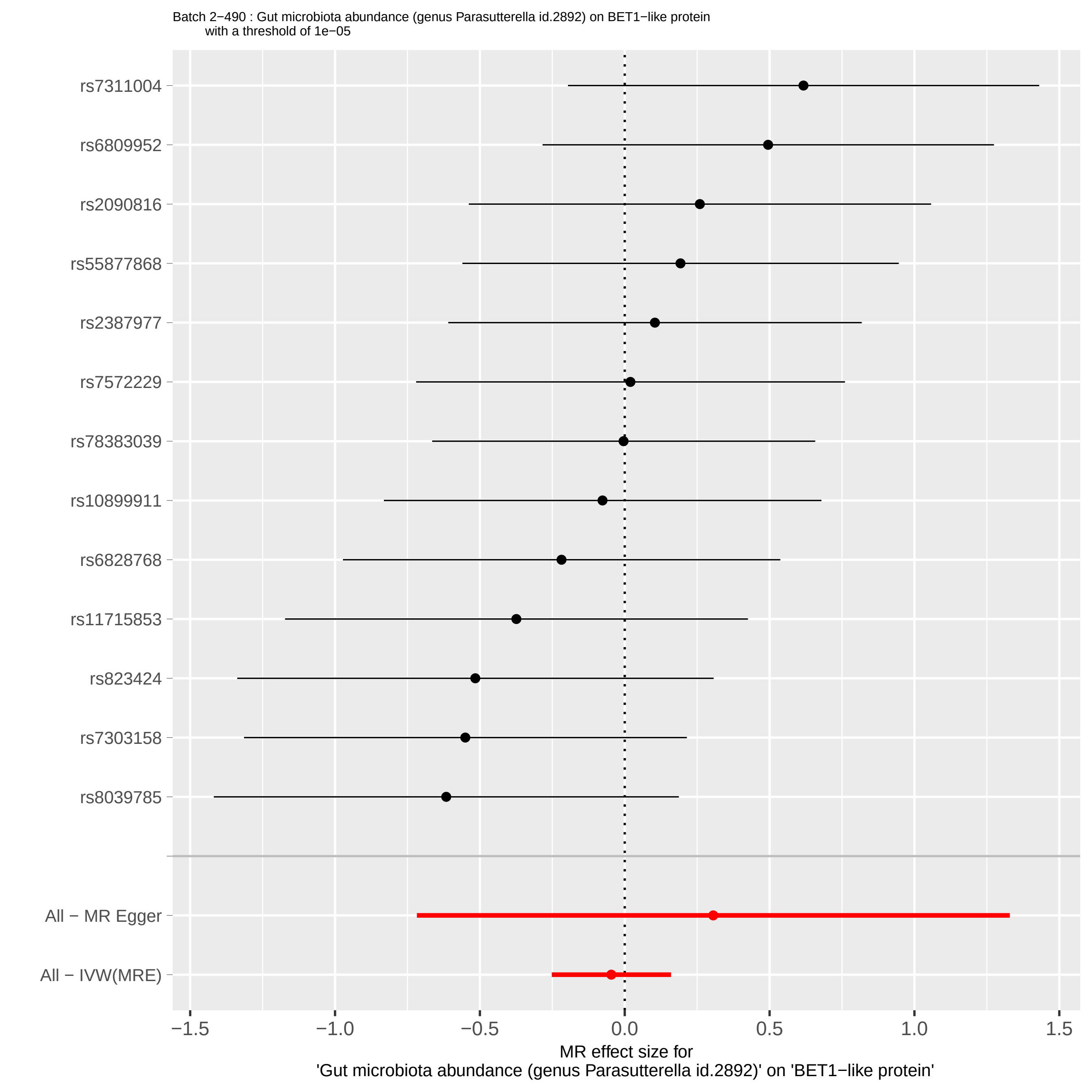
Batch 2−490 : Gut microbiota abundance (genus Parasutterella id.2892) on BET1−like protein
with a threshold of 1e−05
rs7311004
rs6809952
rs2090816
rs55877868
rs2387977
rs7572229
rs78383039
rs10899911
rs6828768
rs11715853
rs823424
rs7303158
rs8039785
All − MR Egger
All − IVW(MRE)
−1.5
−1.0
−0.5
0.0
0.5
1.0
1.5
MR effect size for
'Gut microbiota abundance (genus Parasutterella id.2892)' on 'BET1−like protein'

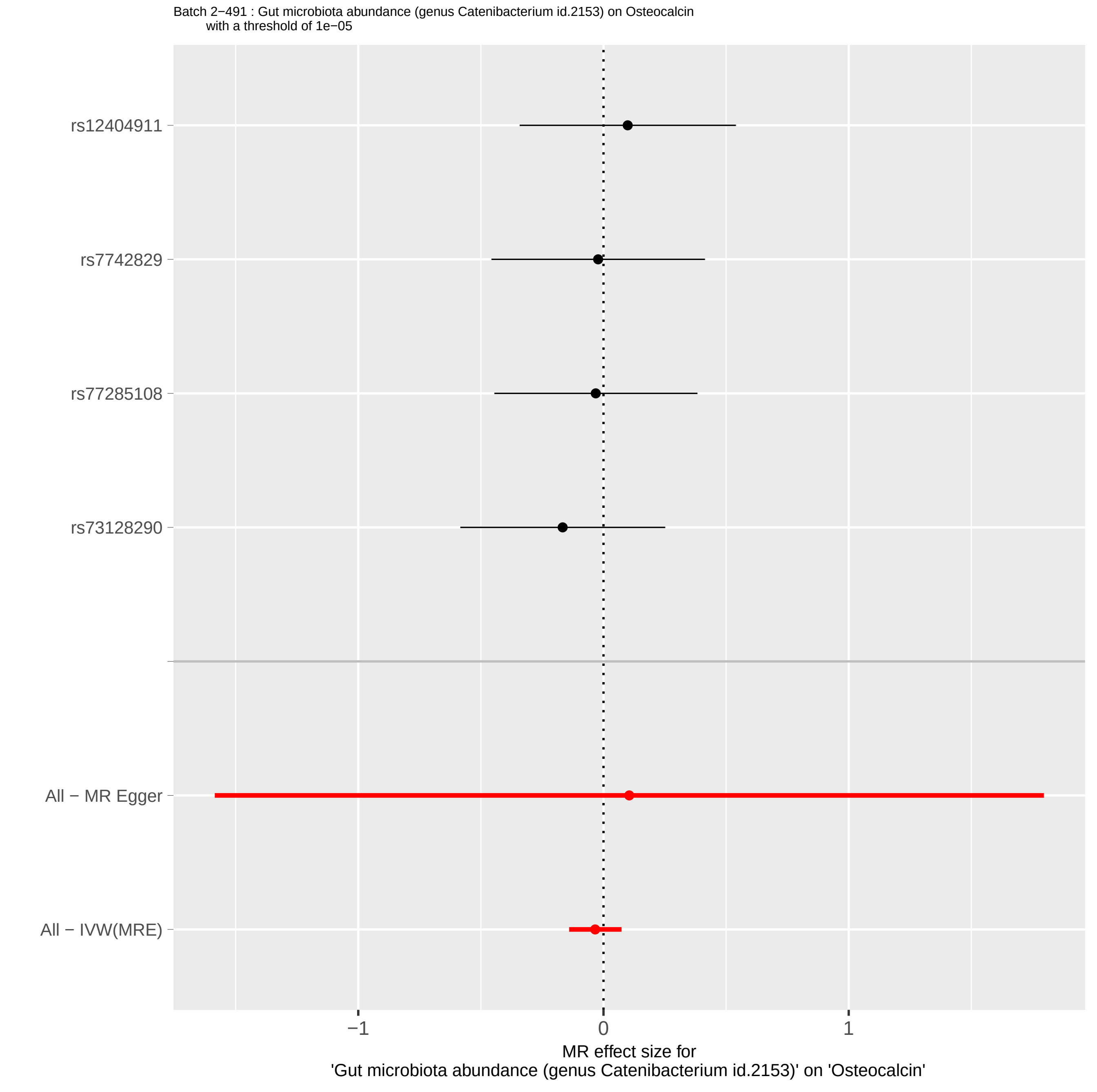

Batch 2−491 : Gut microbiota abundance (genus Catenibacterium id.2153) on Osteocalcin
with a threshold of 1e−05
rs12404911
rs7742829
rs77285108
rs73128290
All − MR Egger
All − IVW(MRE)
−1
0
1
MR effect size for
'Gut microbiota abundance (genus Catenibacterium id.2153)' on 'Osteocalcin'

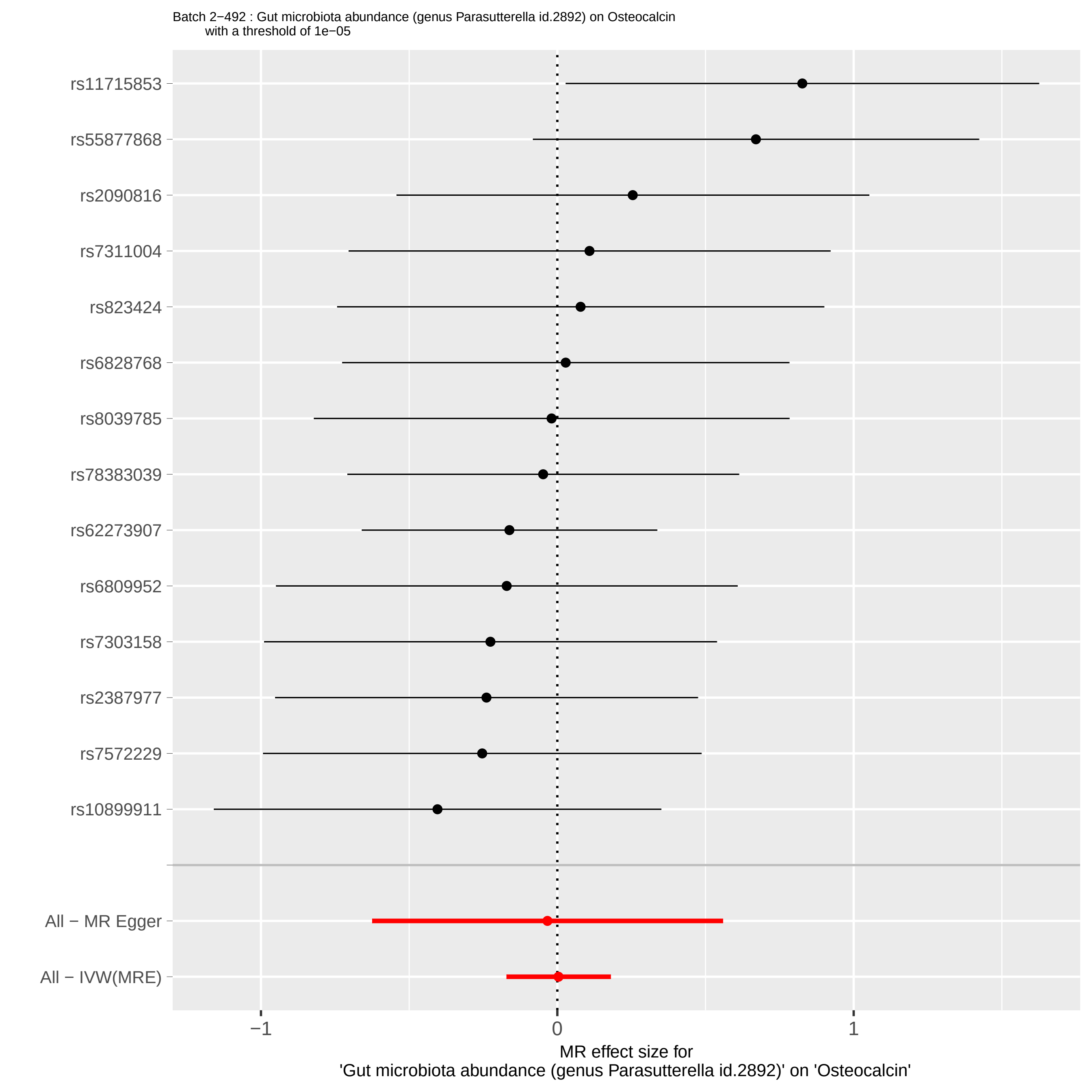

Batch 2−492 : Gut microbiota abundance (genus Parasutterella id.2892) on Osteocalcin
with a threshold of 1e−05
rs11715853
rs55877868
rs2090816
rs7311004
rs823424
rs6828768
rs8039785
rs78383039
rs62273907
rs6809952
rs7303158
rs2387977
rs7572229
rs10899911
All − MR Egger
All − IVW(MRE)
−1
0
1
MR effect size for
'Gut microbiota abundance (genus Parasutterella id.2892)' on 'Osteocalcin'

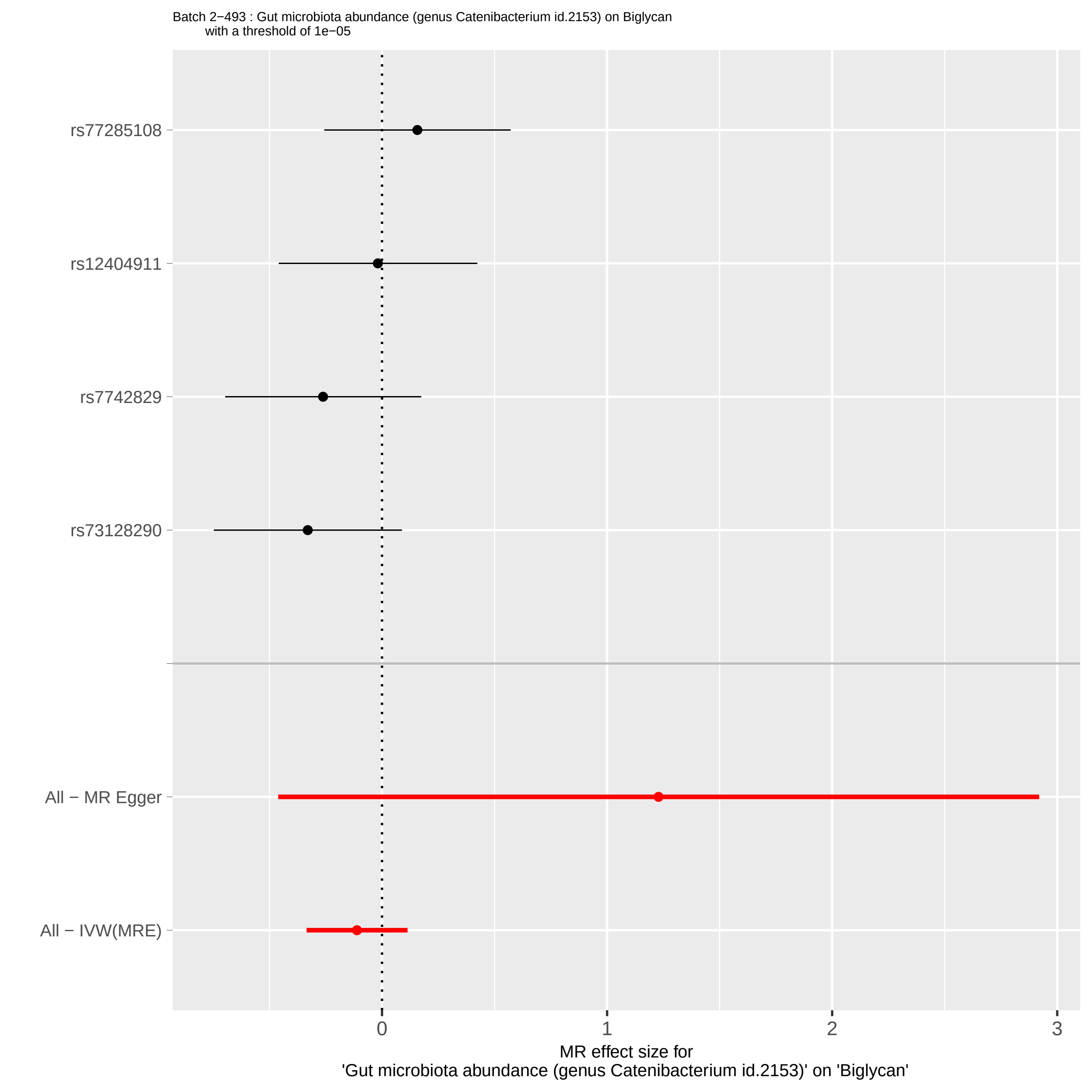

Batch 2−493 : Gut microbiota abundance (genus Catenibacterium id.2153) on Biglycan
with a threshold of 1e−05
rs77285108
rs12404911
rs7742829
rs73128290
All − MR Egger
All − IVW(MRE)
0
1
2
3
MR effect size for
'Gut microbiota abundance (genus Catenibacterium id.2153)' on 'Biglycan'

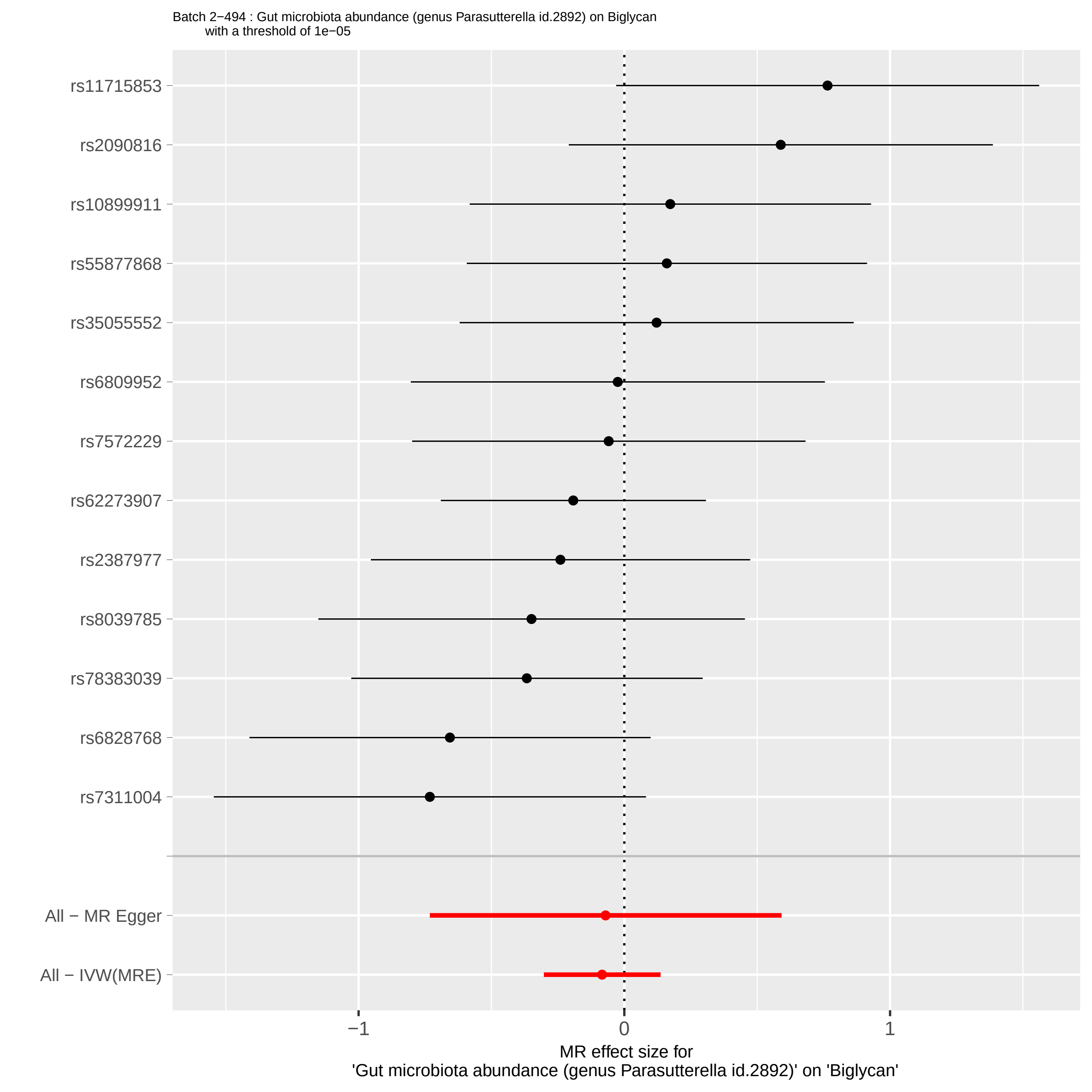
Batch 2−494 : Gut microbiota abundance (genus Parasutterella id.2892) on Biglycan
with a threshold of 1e−05
rs11715853
rs2090816
rs10899911
rs55877868
rs35055552
rs6809952
rs7572229
rs62273907
rs2387977
rs8039785
rs78383039
rs6828768
rs7311004
All − MR Egger
All − IVW(MRE)
−1
0
1
MR effect size for
'Gut microbiota abundance (genus Parasutterella id.2892)' on 'Biglycan'

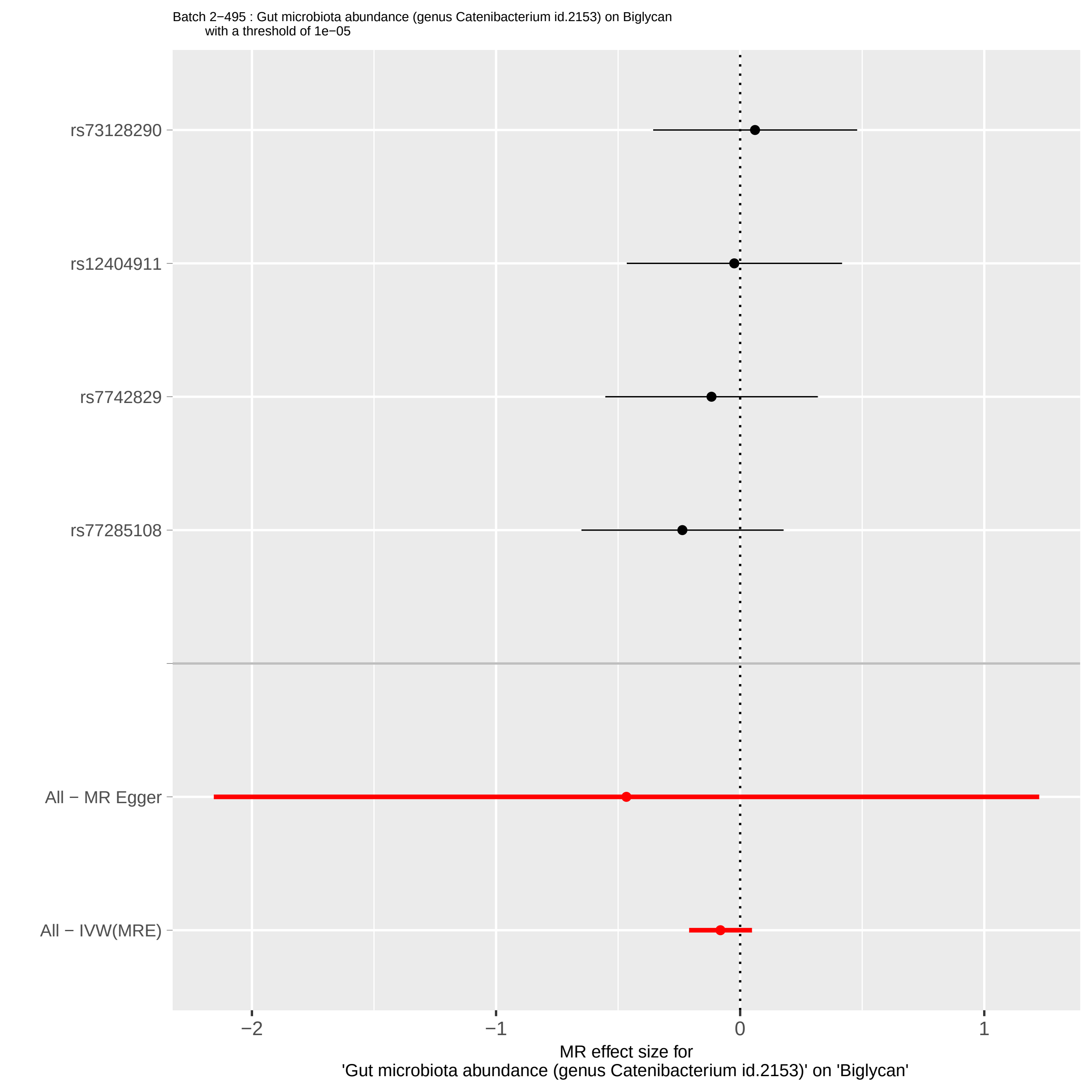
Batch 2−495 : Gut microbiota abundance (genus Catenibacterium id.2153) on Biglycan
with a threshold of 1e−05
rs73128290
rs12404911
rs7742829
rs77285108
All − MR Egger
All − IVW(MRE)
−2
−1
0
1
MR effect size for
'Gut microbiota abundance (genus Catenibacterium id.2153)' on 'Biglycan'

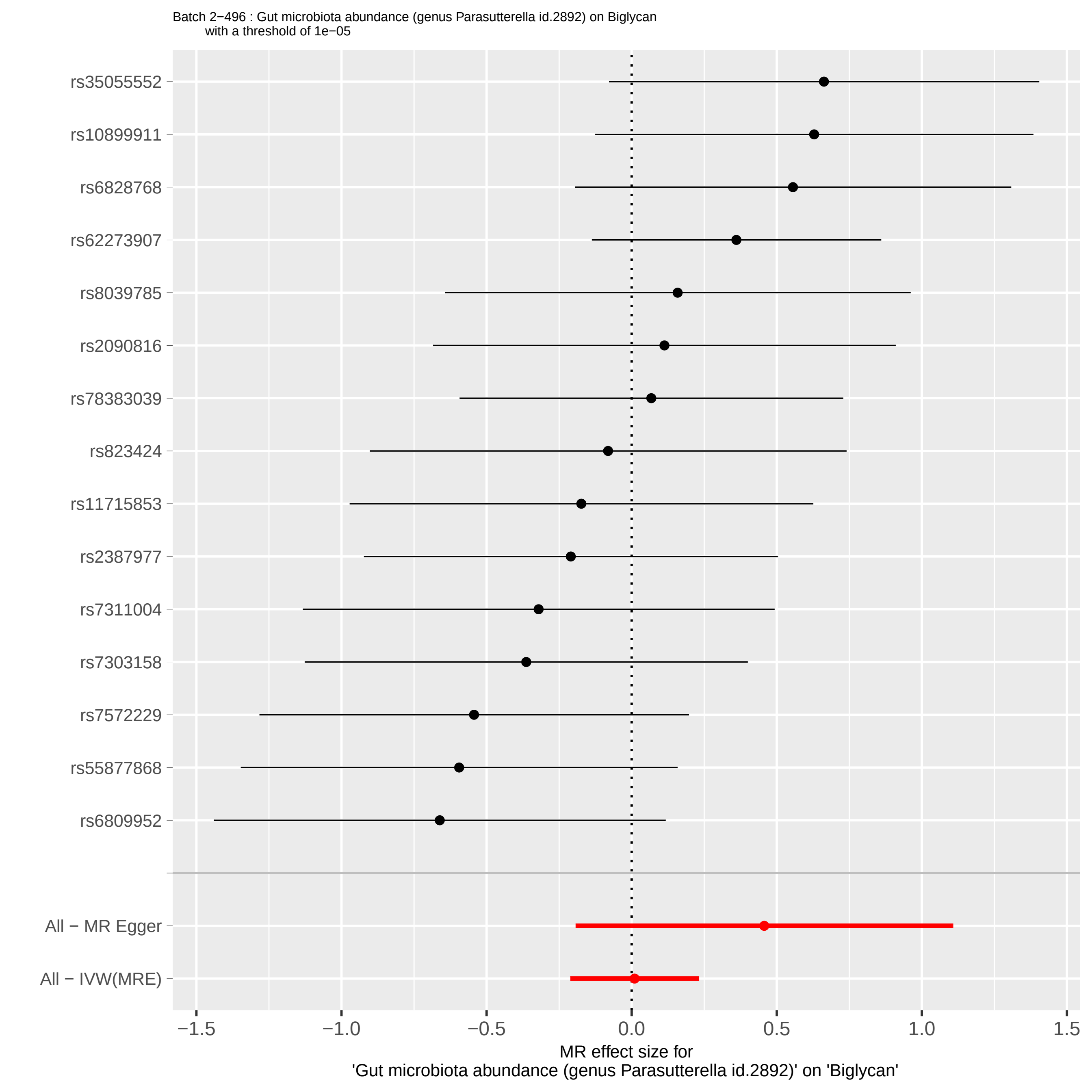

Batch 2−496 : Gut microbiota abundance (genus Parasutterella id.2892) on Biglycan
with a threshold of 1e−05
rs35055552
rs10899911
rs6828768
rs62273907
rs8039785
rs2090816
rs78383039
rs823424
rs11715853
rs2387977
rs7311004
rs7303158
rs7572229
rs55877868
rs6809952
All − MR Egger
All − IVW(MRE)
−1.5
−1.0
−0.5
0.0
0.5
1.0
1.5
MR effect size for
'Gut microbiota abundance (genus Parasutterella id.2892)' on 'Biglycan'

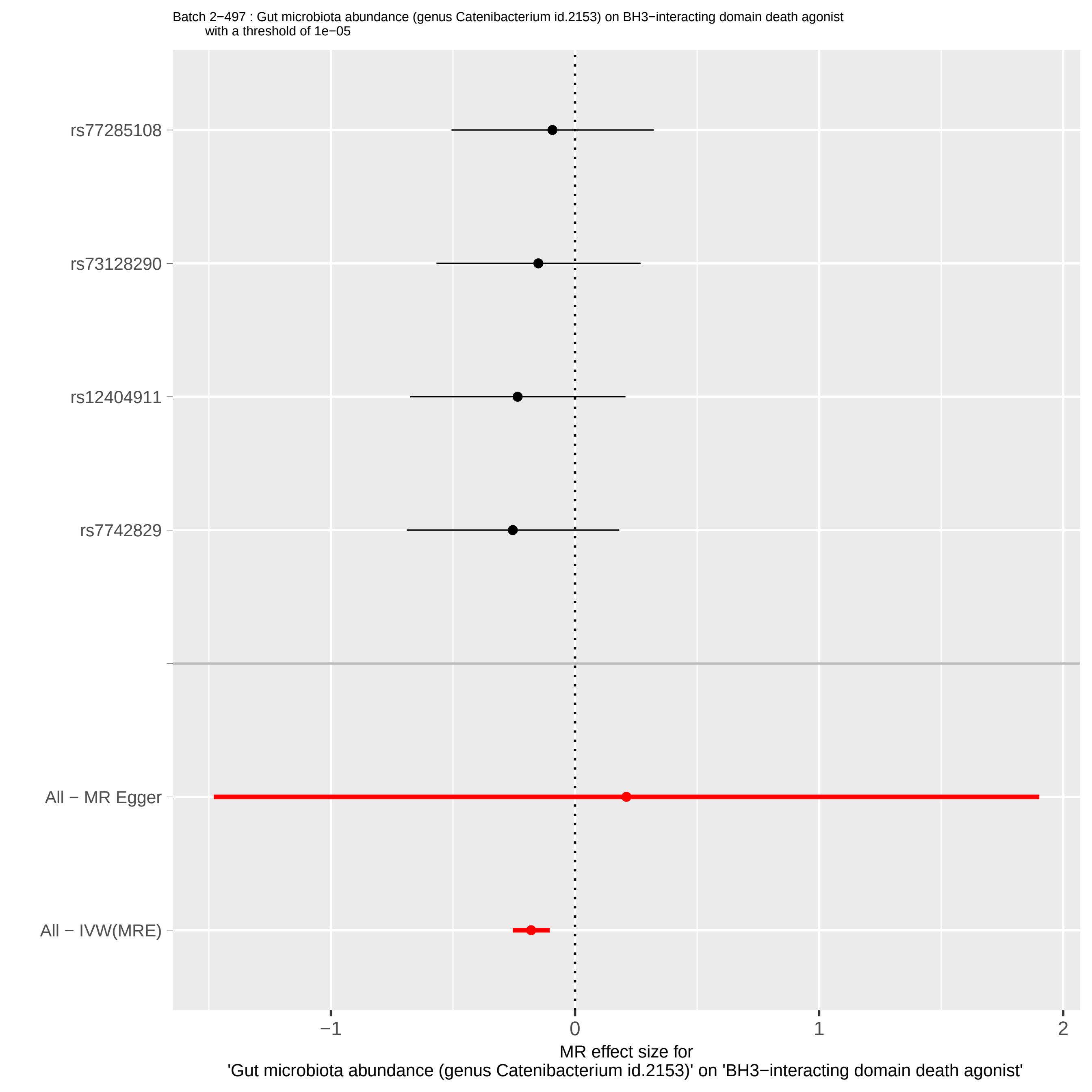
Batch 2−497 : Gut microbiota abundance (genus Catenibacterium id.2153) on BH3−interacting domain death agonist with a threshold of 1e−05
rs77285108
rs73128290
rs12404911
rs7742829
All − MR Egger
All − IVW(MRE)
−1
0
1
2
MR effect size for
'Gut microbiota abundance (genus Catenibacterium id.2153)' on 'BH3−interacting domain death agonist'

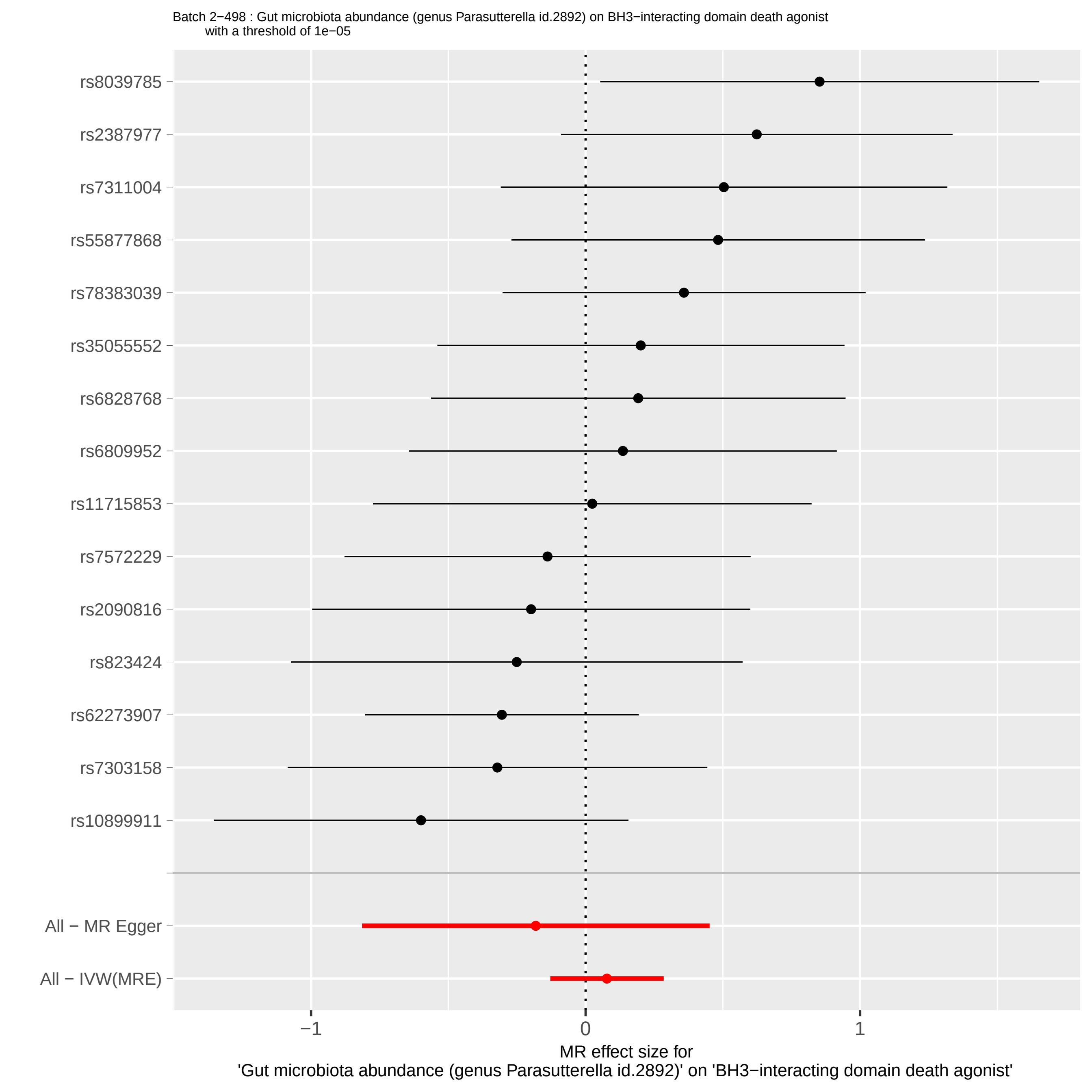
Batch 2−498 : Gut microbiota abundance (genus Parasutterella id.2892) on BH3−interacting domain death agonist with a threshold of 1e−05
rs8039785
rs2387977
rs7311004
rs55877868
rs78383039
rs35055552
rs6828768
rs6809952
rs11715853
rs7572229
rs2090816
rs823424
rs62273907
rs7303158
rs10899911
All − MR Egger
All − IVW(MRE)
−1
0
1
MR effect size for
'Gut microbiota abundance (genus Parasutterella id.2892)' on 'BH3−interacting domain death agonist'

Batch 2−499 : Gut microbiota abundance (genus Catenibacterium id.2153) on Myc box−dependent−interacting protein 1
with a threshold of 1e−05

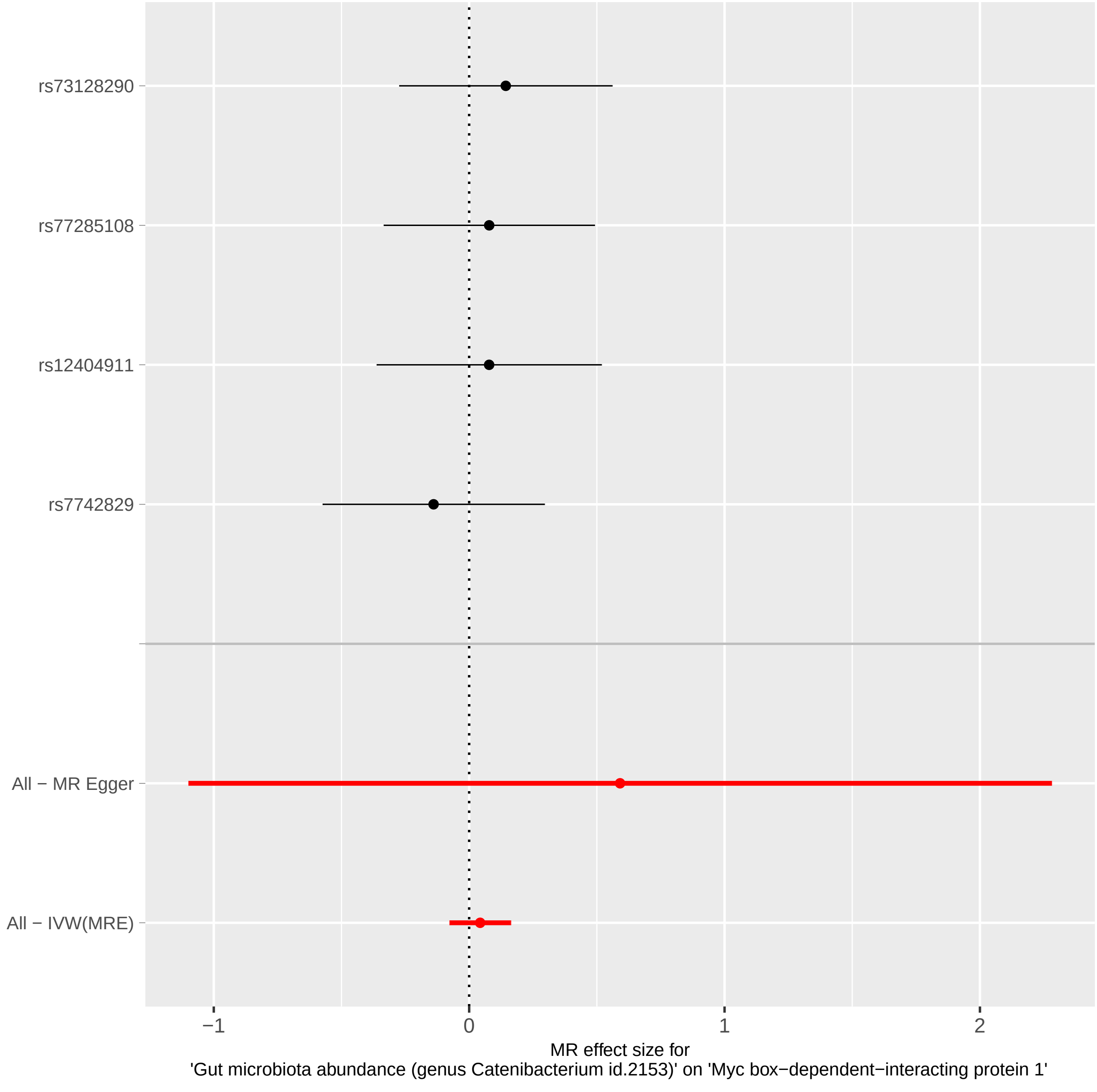

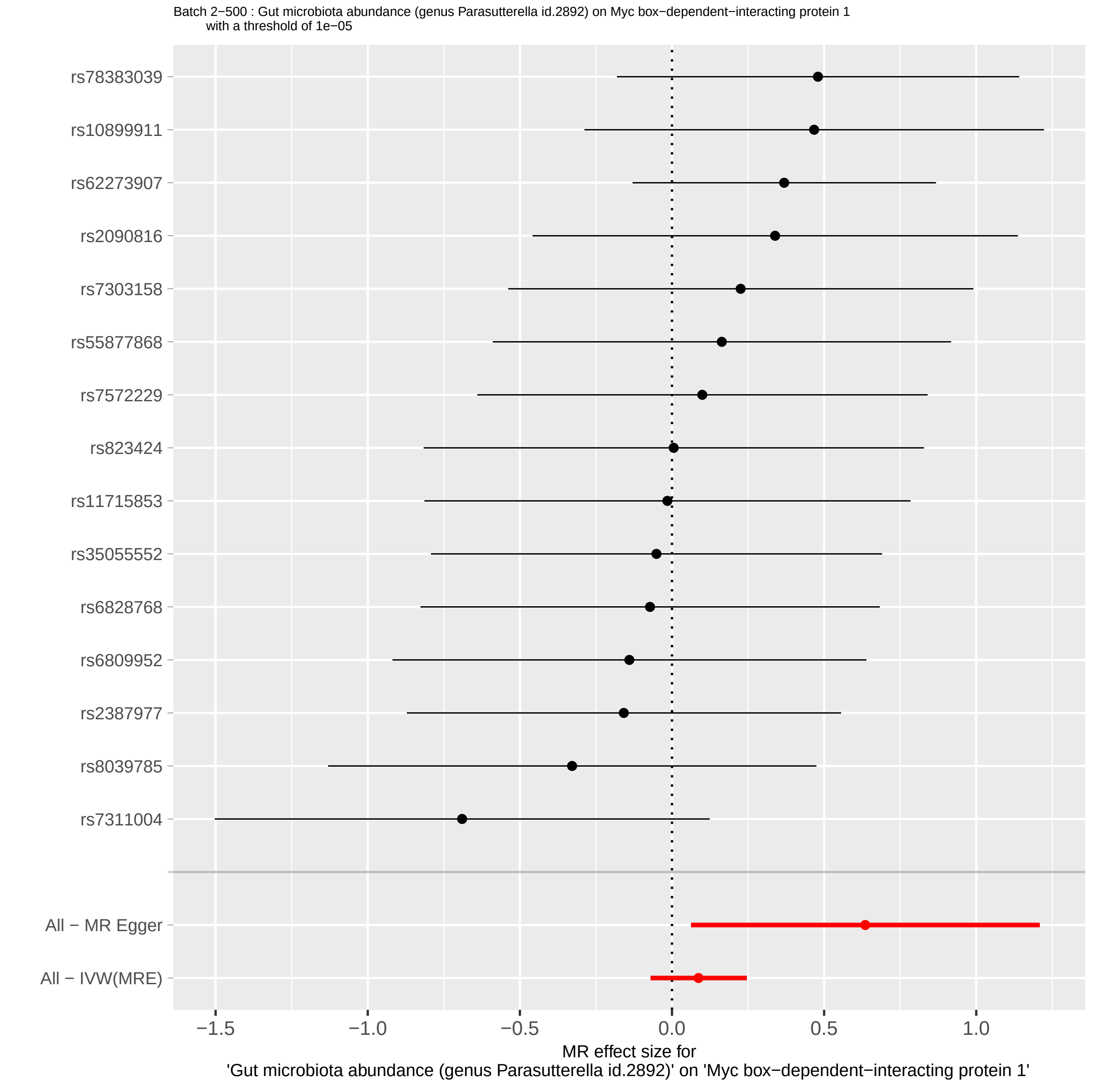

Batch 2−500 : Gut microbiota abundance (genus Parasutterella id.2892) on Myc box−dependent−interacting protein 1
with a threshold of 1e−05
rs78383039
rs10899911
rs62273907
rs2090816
rs7303158
rs55877868
rs7572229
rs823424
rs11715853
rs35055552
rs6828768
rs6809952
rs2387977
rs8039785
rs7311004
All − MR Egger
All − IVW(MRE)
−1.5
−1.0
−0.5
0.0
0.5
1.0
MR effect size for
'Gut microbiota abundance (genus Parasutterella id.2892)' on 'Myc box−dependent−interacting protein 1'

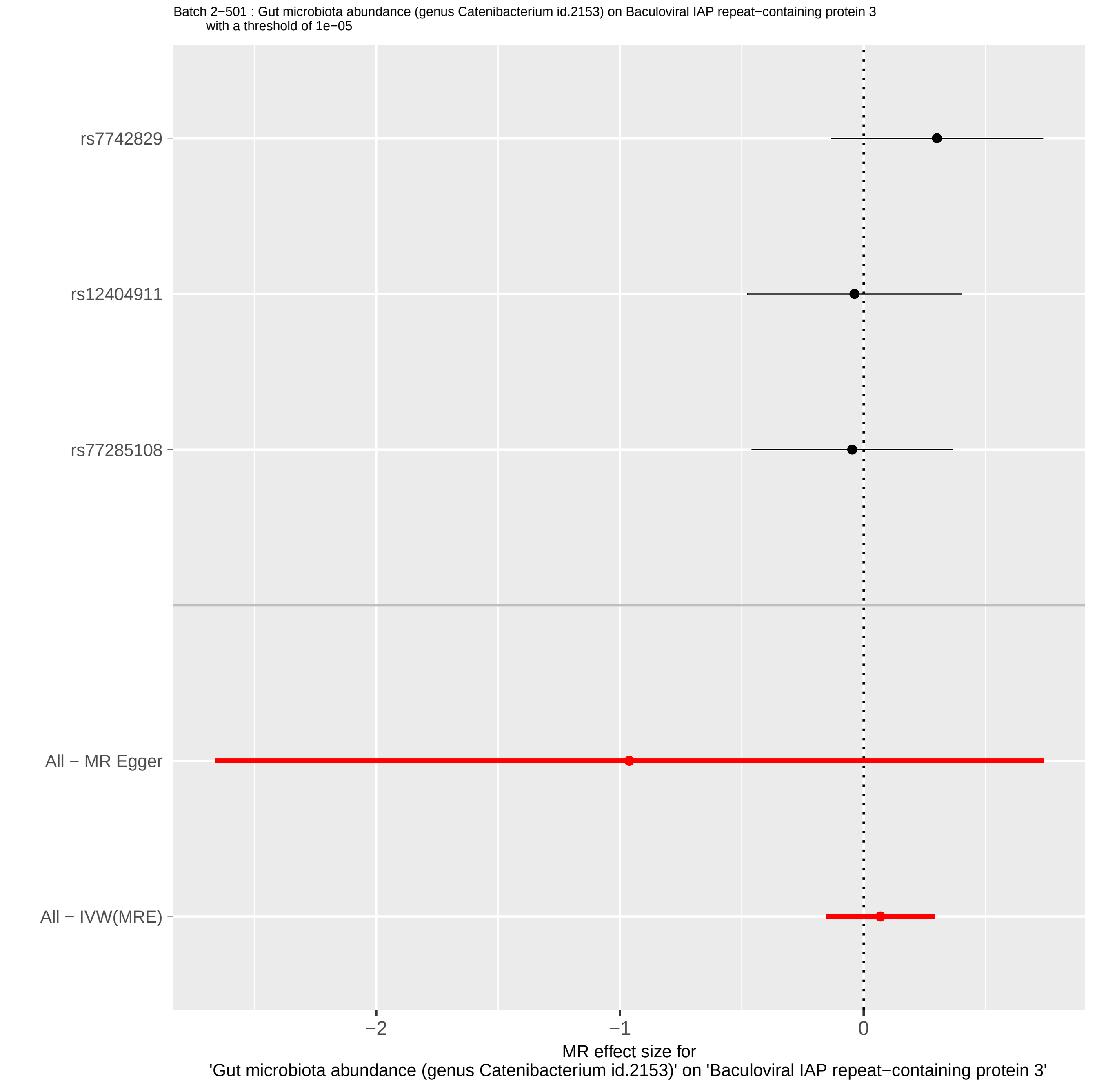

Batch 2−501 : Gut microbiota abundance (genus Catenibacterium id.2153) on Baculoviral IAP repeat−containing protein 3
with a threshold of 1e−05
rs7742829
rs12404911
rs77285108
All − MR Egger
All − IVW(MRE)
−2
−1
0
MR effect size for
'Gut microbiota abundance (genus Catenibacterium id.2153)' on 'Baculoviral IAP repeat−containing protein 3'

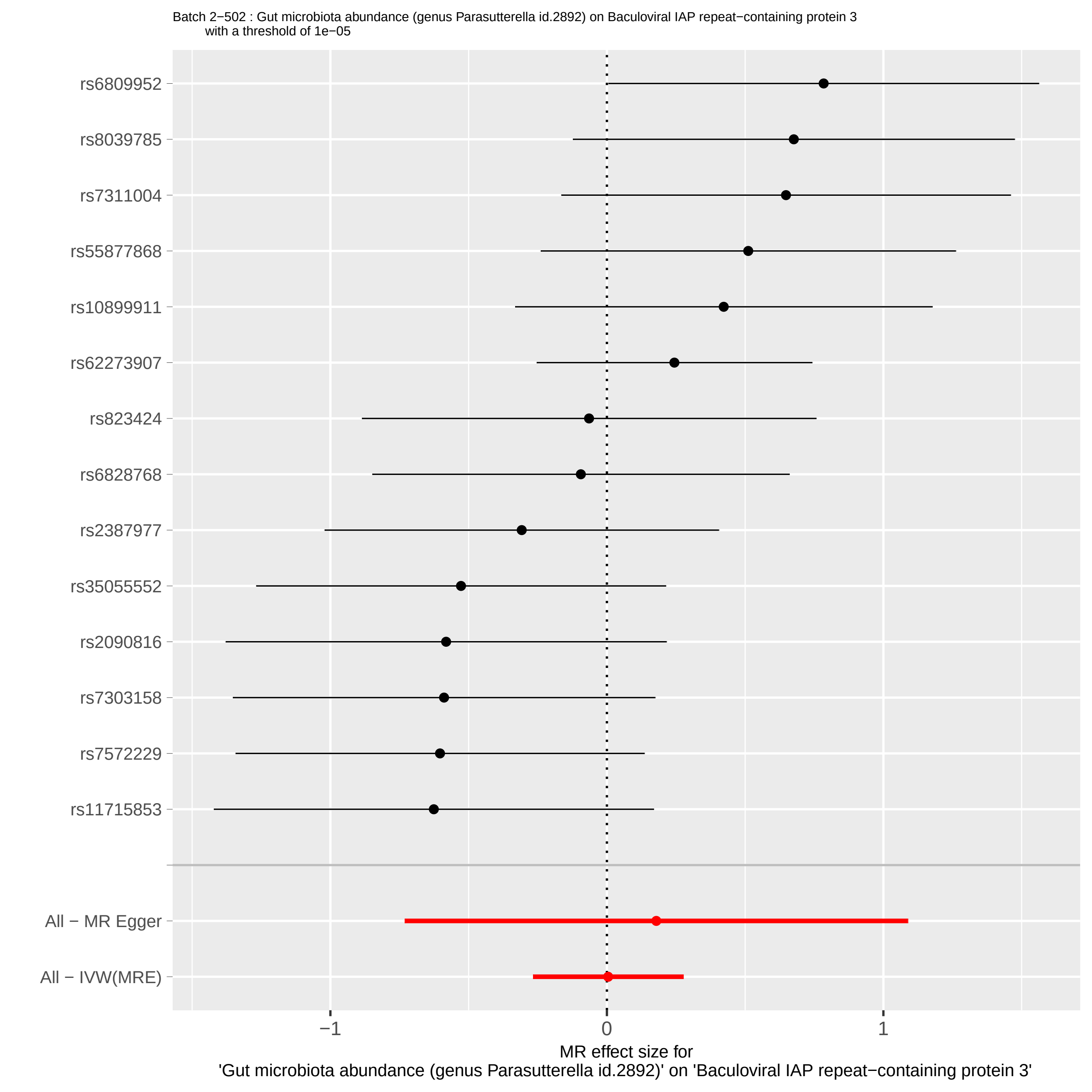
Batch 2−502 : Gut microbiota abundance (genus Parasutterella id.2892) on Baculoviral IAP repeat−containing protein 3
with a threshold of 1e−05
rs6809952
rs8039785
rs7311004
rs55877868
rs10899911
rs62273907
rs823424
rs6828768
rs2387977
rs35055552
rs2090816
rs7303158
rs7572229
rs11715853
All − MR Egger
All − IVW(MRE)
−1
0
1
MR effect size for
'Gut microbiota abundance (genus Parasutterella id.2892)' on 'Baculoviral IAP repeat−containing protein 3'

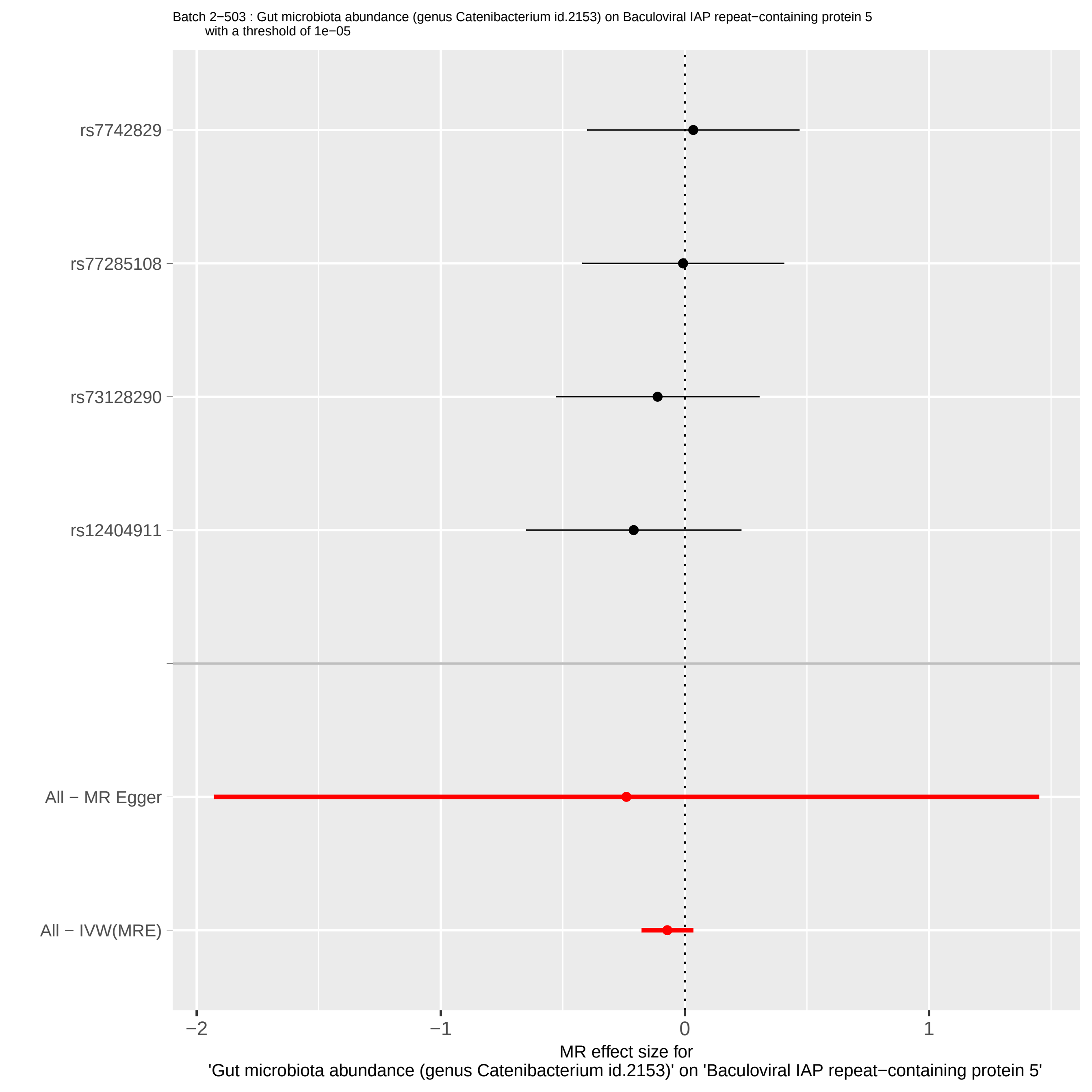
Batch 2−503 : Gut microbiota abundance (genus Catenibacterium id.2153) on Baculoviral IAP repeat−containing protein 5
with a threshold of 1e−05
rs7742829
rs77285108
rs73128290
rs12404911
All − MR Egger
All − IVW(MRE)
−2
−1
0
1
MR effect size for
'Gut microbiota abundance (genus Catenibacterium id.2153)' on 'Baculoviral IAP repeat−containing protein 5'

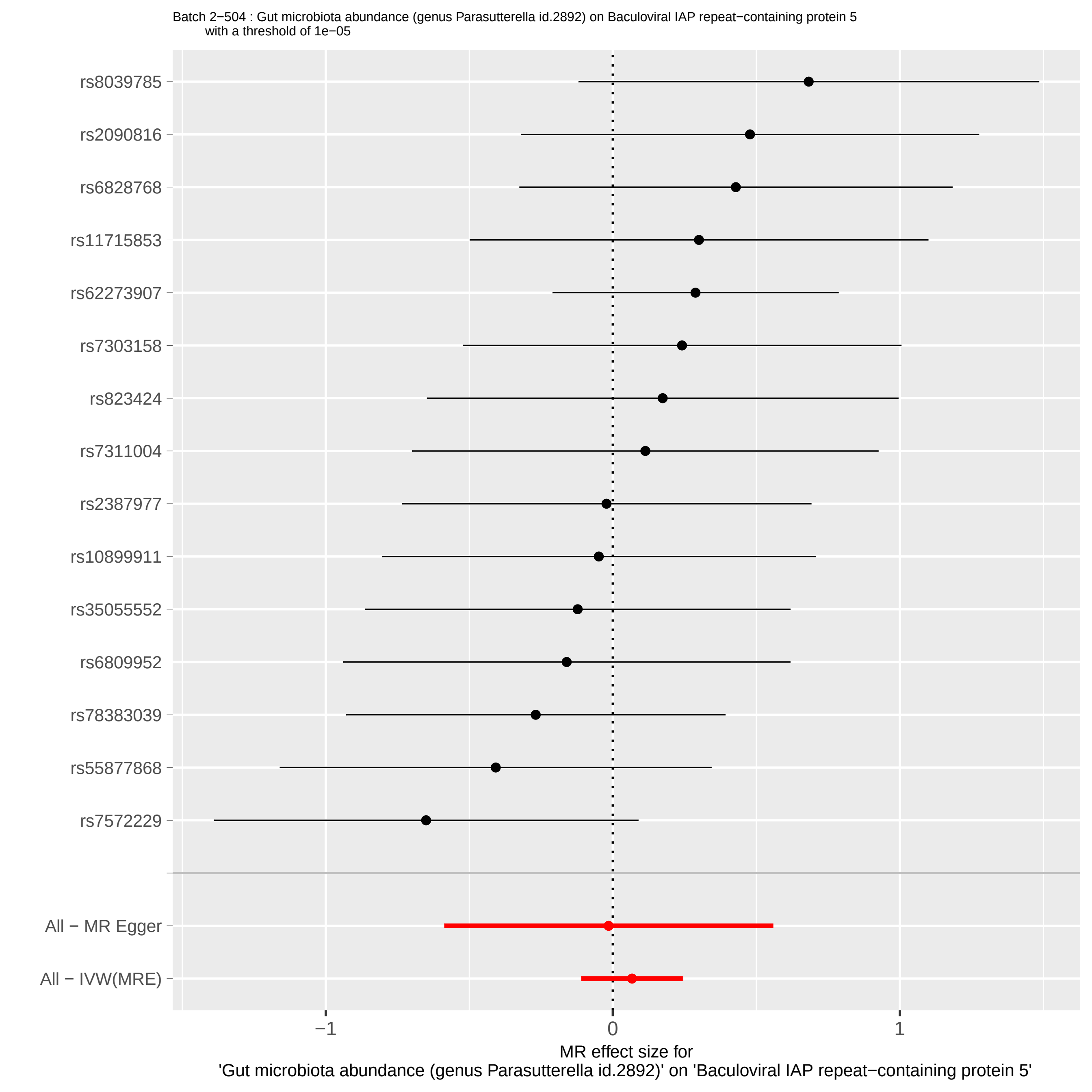

Batch 2−504 : Gut microbiota abundance (genus Parasutterella id.2892) on Baculoviral IAP repeat−containing protein 5
with a threshold of 1e−05
rs8039785
rs2090816
rs6828768
rs11715853
rs62273907
rs7303158
rs823424
rs7311004
rs2387977
rs10899911
rs35055552
rs6809952
rs78383039
rs55877868
rs7572229
All − MR Egger
All − IVW(MRE)
−1
0
1
MR effect size for
'Gut microbiota abundance (genus Parasutterella id.2892)' on 'Baculoviral IAP repeat−containing protein 5'

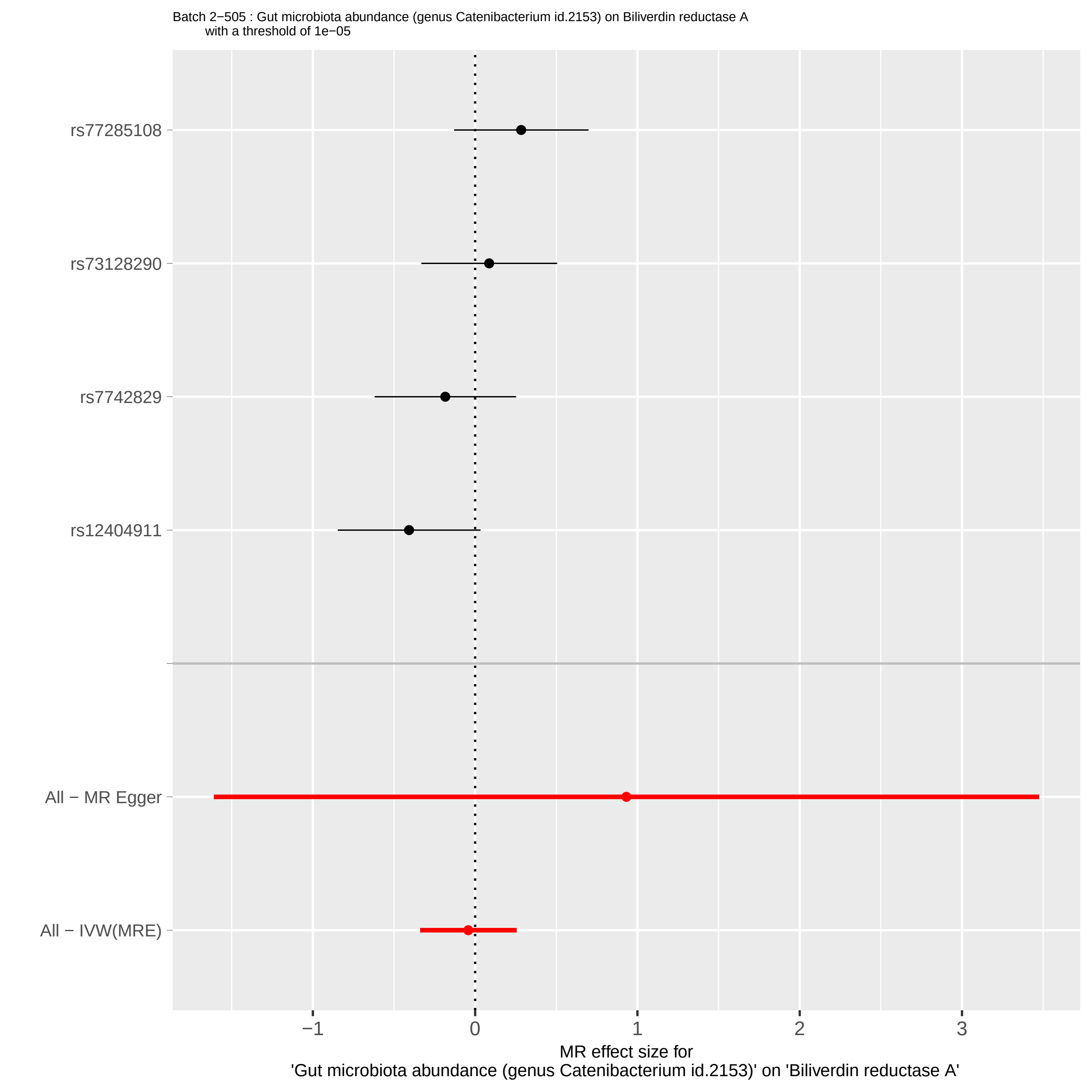
Batch 2−505 : Gut microbiota abundance (genus Catenibacterium id.2153) on Biliverdin reductase A
with a threshold of 1e−05
rs77285108
rs73128290
rs7742829
rs12404911
All − MR Egger
All − IVW(MRE)
−1
0
1
2
3
MR effect size for
'Gut microbiota abundance (genus Catenibacterium id.2153)' on 'Biliverdin reductase A'

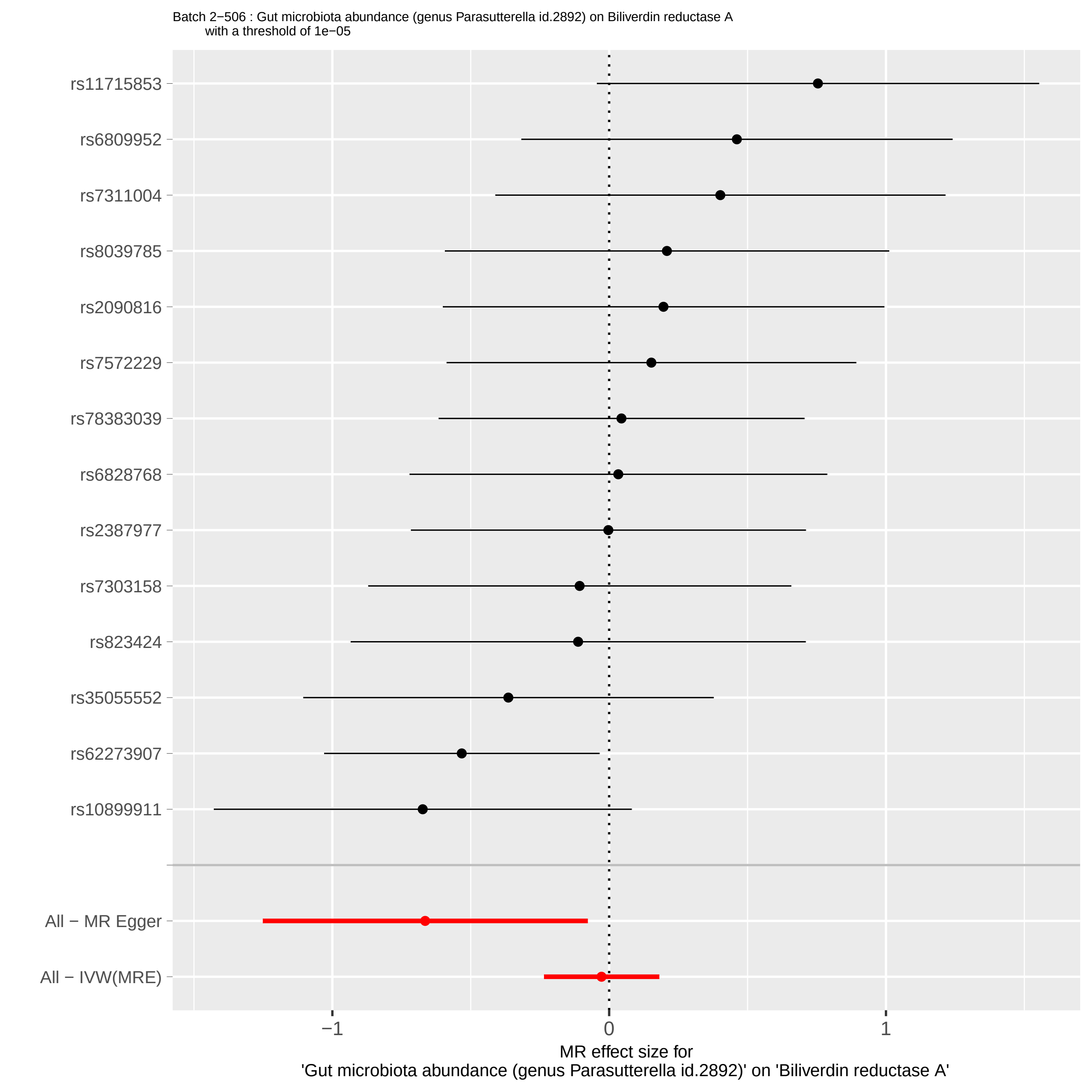

Batch 2−506 : Gut microbiota abundance (genus Parasutterella id.2892) on Biliverdin reductase A
with a threshold of 1e−05
rs11715853
rs6809952
rs7311004
rs8039785
rs2090816
rs7572229
rs78383039
rs6828768
rs2387977
rs7303158
rs823424
rs35055552
rs62273907
rs10899911
All − MR Egger
All − IVW(MRE)
−1
0
1
MR effect size for
'Gut microbiota abundance (genus Parasutterella id.2892)' on 'Biliverdin reductase A'

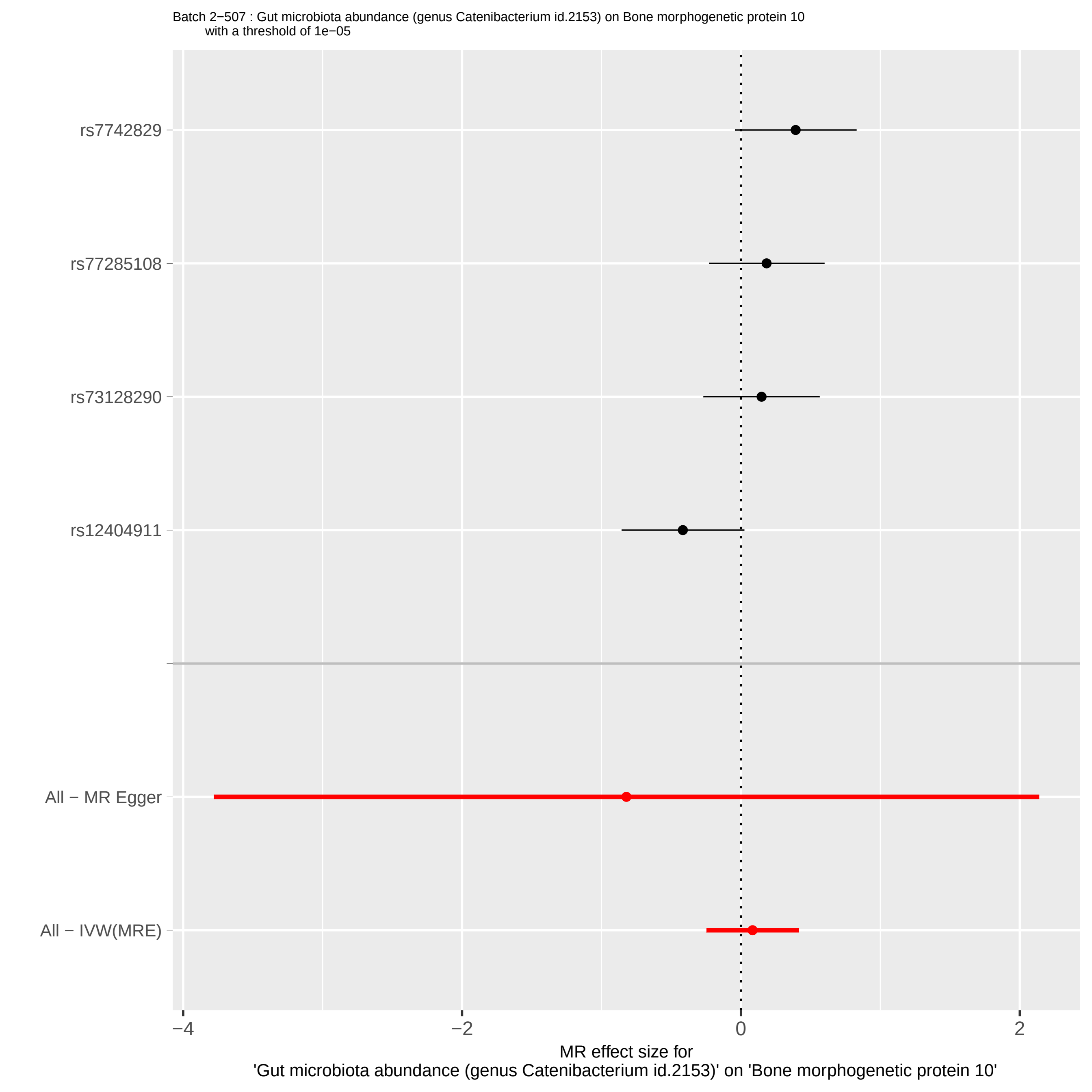
Batch 2−507 : Gut microbiota abundance (genus Catenibacterium id.2153) on Bone morphogenetic protein 10
with a threshold of 1e−05
rs7742829
rs77285108
rs73128290
rs12404911
All − MR Egger
All − IVW(MRE)
−4
−2
0
2
MR effect size for
'Gut microbiota abundance (genus Catenibacterium id.2153)' on 'Bone morphogenetic protein 10'

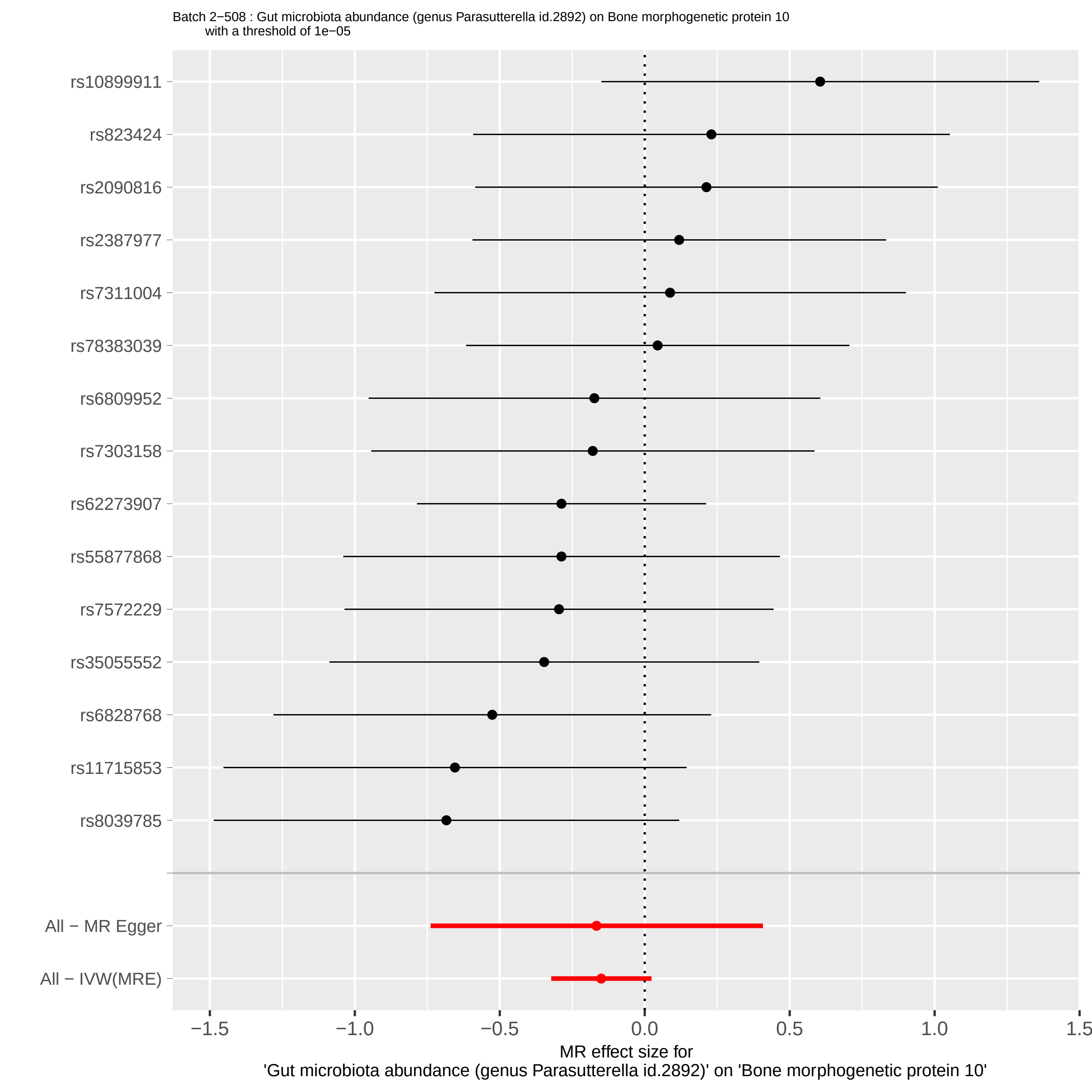

Batch 2−508 : Gut microbiota abundance (genus Parasutterella id.2892) on Bone morphogenetic protein 10
with a threshold of 1e−05
rs10899911
rs823424
rs2090816
rs2387977
rs7311004
rs78383039
rs6809952
rs7303158
rs62273907
rs55877868
rs7572229
rs35055552
rs6828768
rs11715853
rs8039785
All − MR Egger
All − IVW(MRE)
−1.5
−1.0
−0.5
0.0
0.5
1.0
1.5
MR effect size for
'Gut microbiota abundance (genus Parasutterella id.2892)' on 'Bone morphogenetic protein 10'

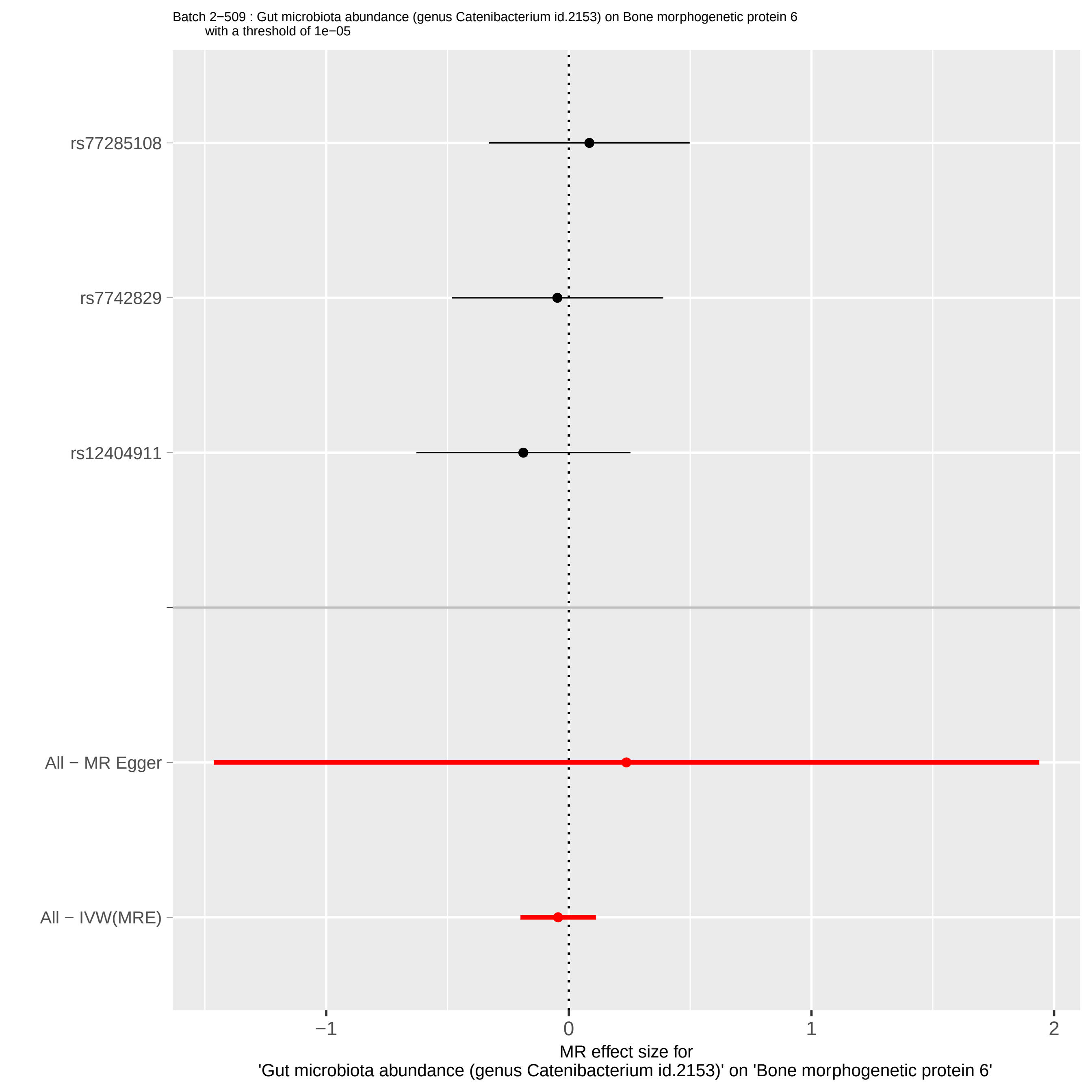
Batch 2−509 : Gut microbiota abundance (genus Catenibacterium id.2153) on Bone morphogenetic protein 6
with a threshold of 1e−05
rs77285108
rs7742829
rs12404911
All − MR Egger
All − IVW(MRE)
−1
0
1
2
MR effect size for
'Gut microbiota abundance (genus Catenibacterium id.2153)' on 'Bone morphogenetic protein 6'

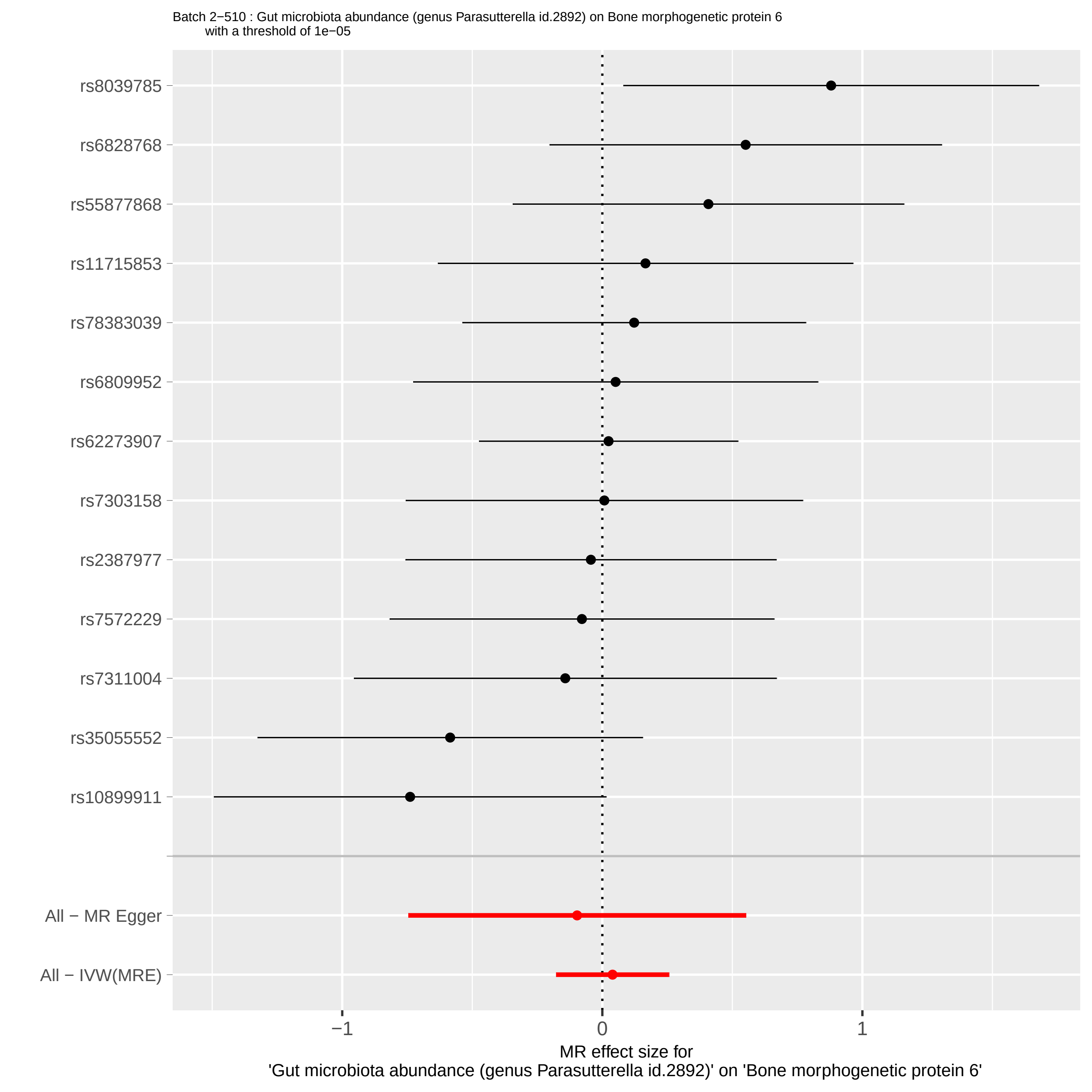

Batch 2−510 : Gut microbiota abundance (genus Parasutterella id.2892) on Bone morphogenetic protein 6
with a threshold of 1e−05
rs8039785
rs6828768
rs55877868
rs11715853
rs78383039
rs6809952
rs62273907
rs7303158
rs2387977
rs7572229
rs7311004
rs35055552
rs10899911
All − MR Egger
All − IVW(MRE)
−1
0
1
MR effect size for
'Gut microbiota abundance (genus Parasutterella id.2892)' on 'Bone morphogenetic protein 6'

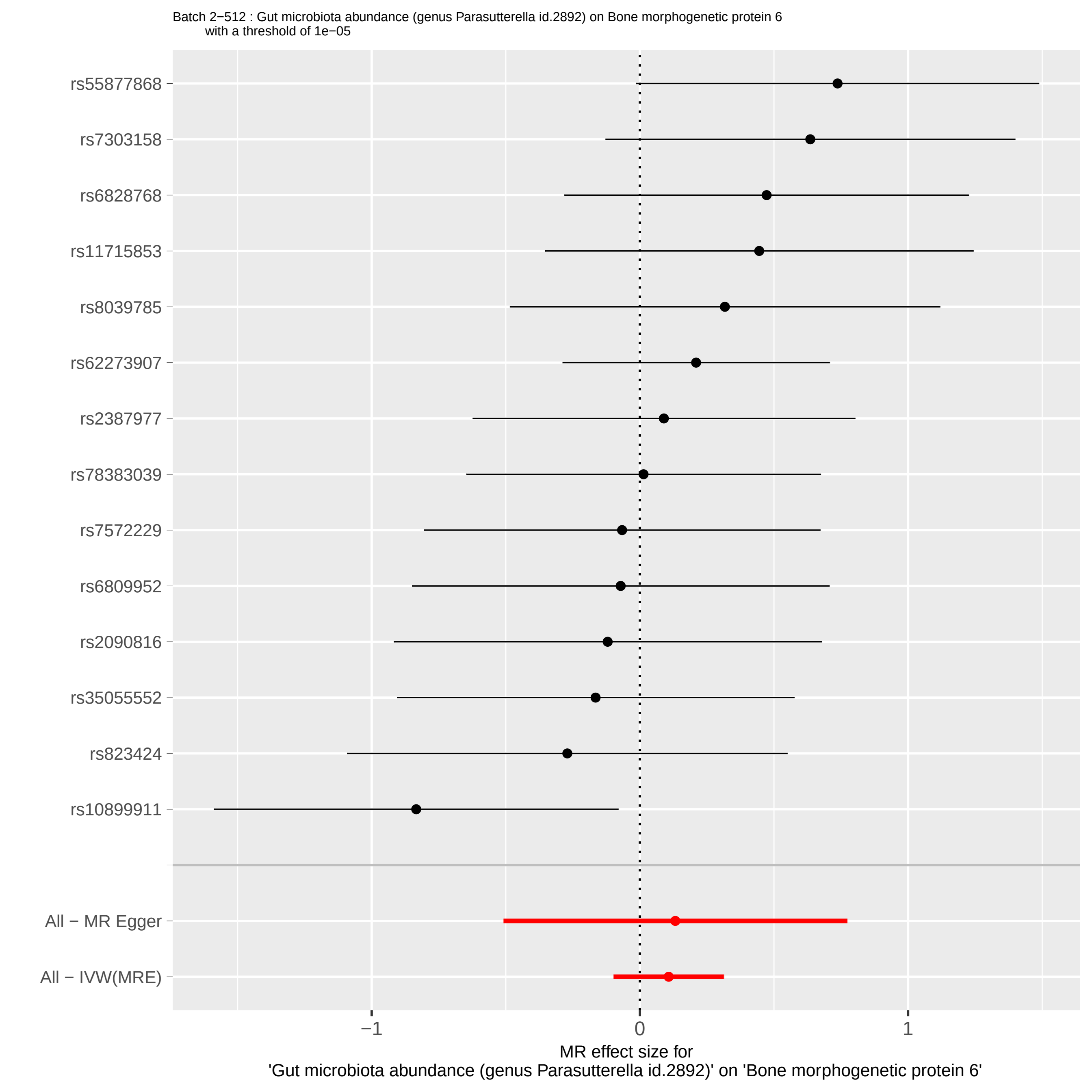
Batch 2−512 : Gut microbiota abundance (genus Parasutterella id.2892) on Bone morphogenetic protein 6
with a threshold of 1e−05
rs55877868
rs7303158
rs6828768
rs11715853
rs8039785
rs62273907
rs2387977
rs78383039
rs7572229
rs6809952
rs2090816
rs35055552
rs823424
rs10899911
All − MR Egger
All − IVW(MRE)
−1
0
1
MR effect size for
'Gut microbiota abundance (genus Parasutterella id.2892)' on 'Bone morphogenetic protein 6'

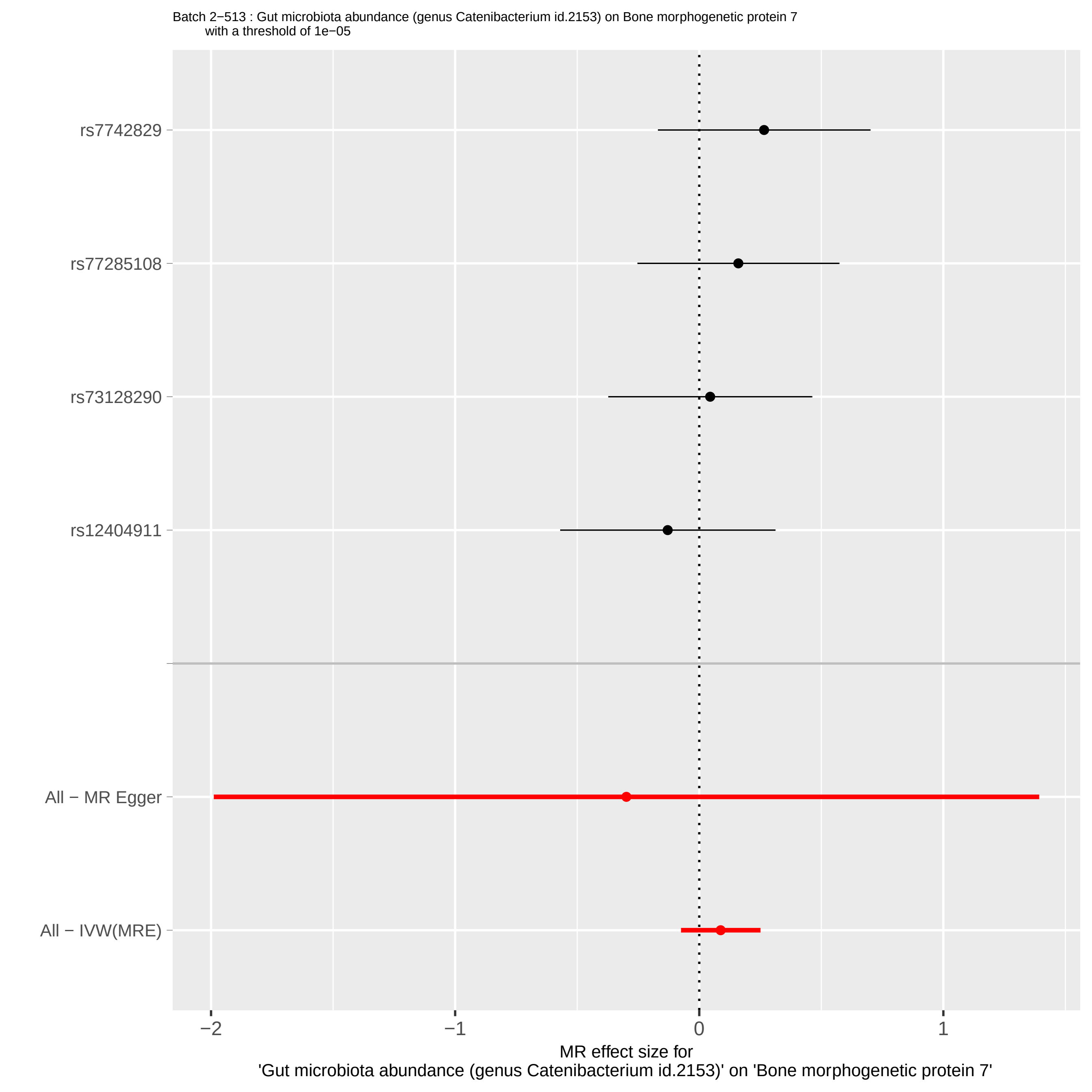

Batch 2−513 : Gut microbiota abundance (genus Catenibacterium id.2153) on Bone morphogenetic protein 7
with a threshold of 1e−05
rs7742829
rs77285108
rs73128290
rs12404911
All − MR Egger
All − IVW(MRE)
−2
−1
0
1
MR effect size for
'Gut microbiota abundance (genus Catenibacterium id.2153)' on 'Bone morphogenetic protein 7'

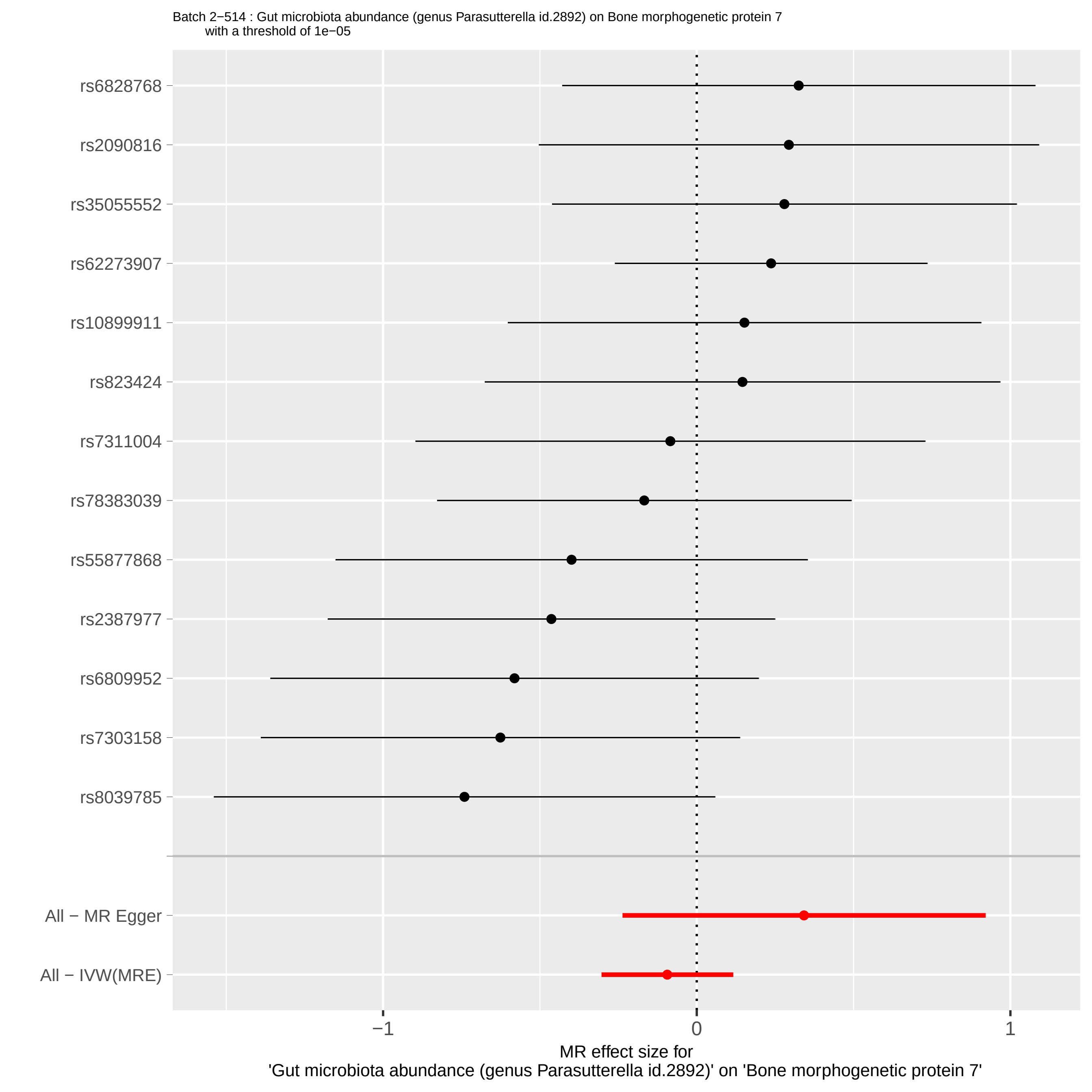

Batch 2−514 : Gut microbiota abundance (genus Parasutterella id.2892) on Bone morphogenetic protein 7
with a threshold of 1e−05
rs6828768
rs2090816
rs35055552
rs62273907
rs10899911
rs823424
rs7311004
rs78383039
rs55877868
rs2387977
rs6809952
rs7303158
rs8039785
All − MR Egger
All − IVW(MRE)
−1
0
1
MR effect size for
'Gut microbiota abundance (genus Parasutterella id.2892)' on 'Bone morphogenetic protein 7'

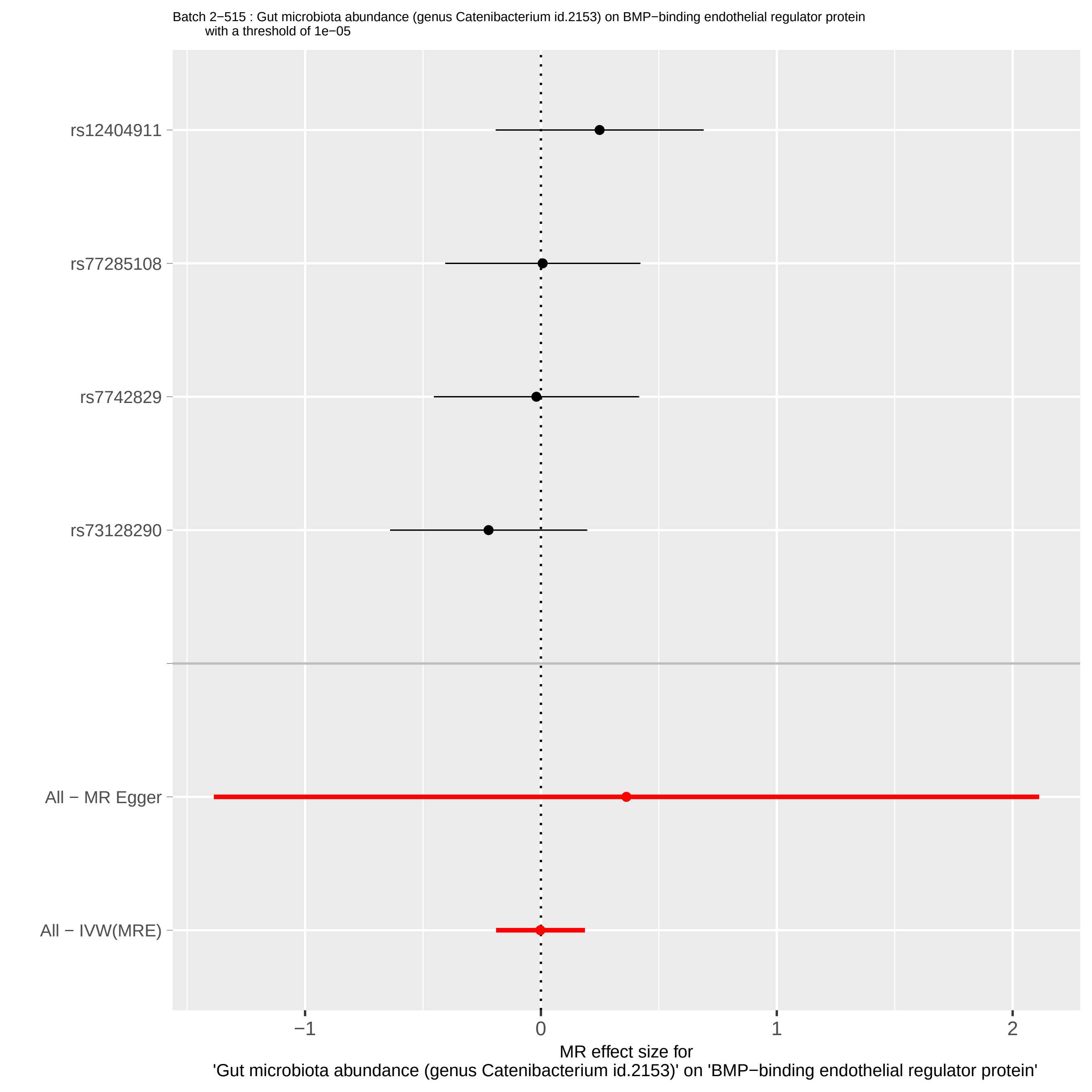

Batch 2−515 : Gut microbiota abundance (genus Catenibacterium id.2153) on BMP−binding endothelial regulator protein
with a threshold of 1e−05
rs12404911
rs77285108
rs7742829
rs73128290
All − MR Egger
All − IVW(MRE)
−1
0
1
2
MR effect size for
'Gut microbiota abundance (genus Catenibacterium id.2153)' on 'BMP−binding endothelial regulator protein'

Batch 2−516 : Gut microbiota abundance (genus Parasutterella id.2892) on BMP−binding endothelial regulator protein
with a threshold of 1e−05

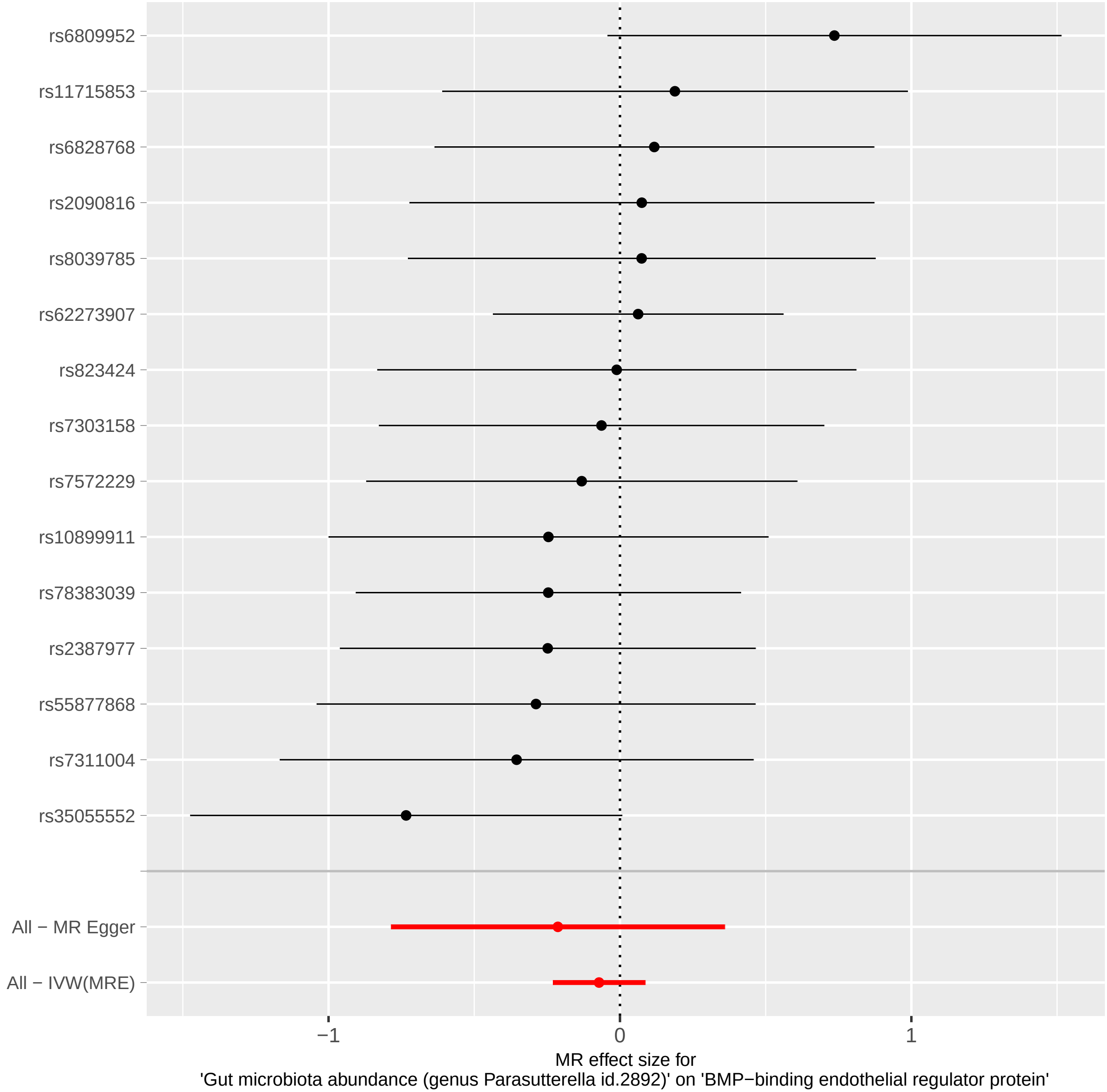

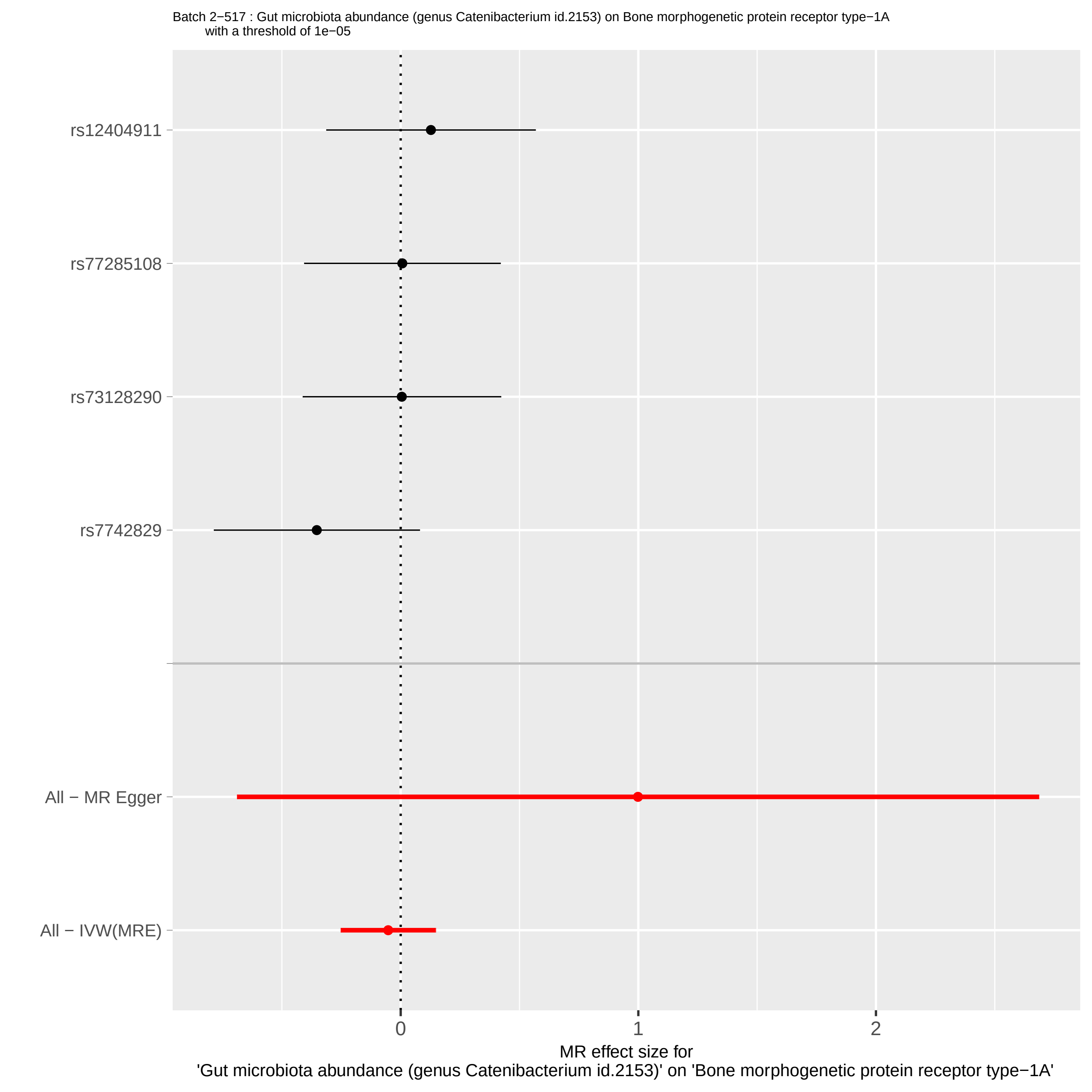

Batch 2−517 : Gut microbiota abundance (genus Catenibacterium id.2153) on Bone morphogenetic protein receptor type−1A
with a threshold of 1e−05
rs12404911
rs77285108
rs73128290
rs7742829
All − MR Egger
All − IVW(MRE)
0
1
2
MR effect size for
'Gut microbiota abundance (genus Catenibacterium id.2153)' on 'Bone morphogenetic protein receptor type−1A'

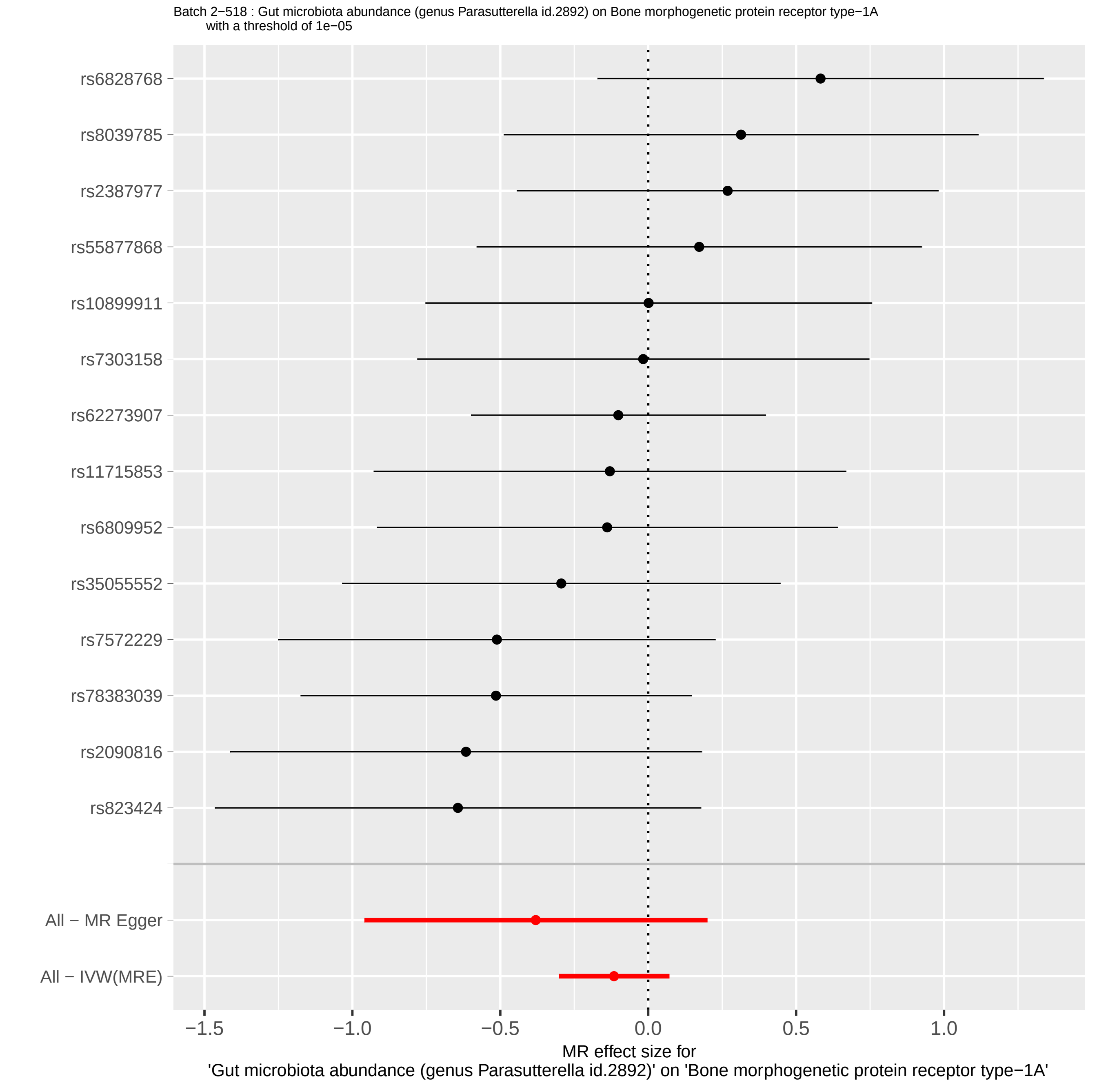

Batch 2−518 : Gut microbiota abundance (genus Parasutterella id.2892) on Bone morphogenetic protein receptor type−1A
with a threshold of 1e−05
rs6828768
rs8039785
rs2387977
rs55877868
rs10899911
rs7303158
rs62273907
rs11715853
rs6809952
rs35055552
rs7572229
rs78383039
rs2090816
rs823424
All − MR Egger
All − IVW(MRE)
−1.5
−1.0
−0.5
0.0
0.5
1.0
MR effect size for
'Gut microbiota abundance (genus Parasutterella id.2892)' on 'Bone morphogenetic protein receptor type−1A'

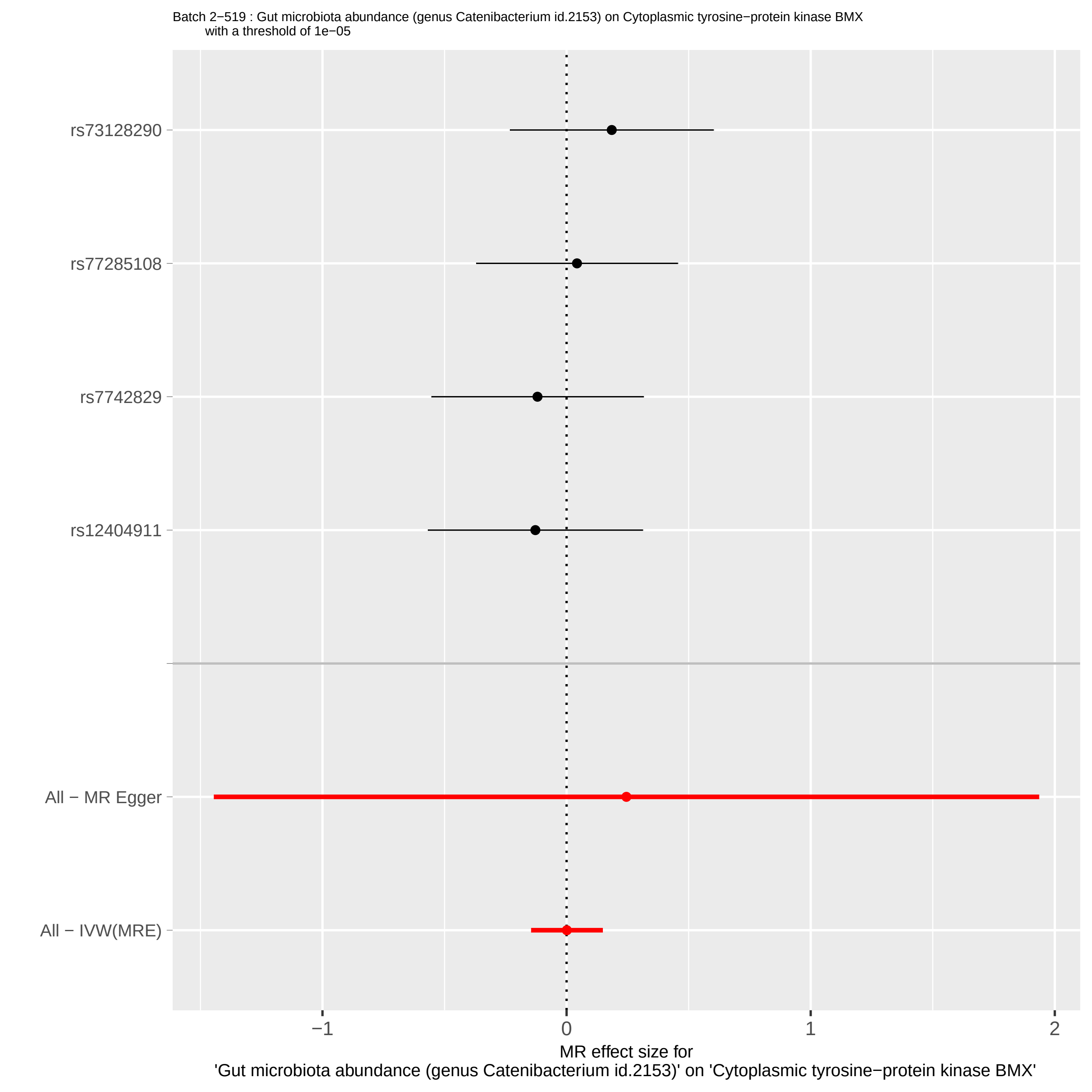

Batch 2−519 : Gut microbiota abundance (genus Catenibacterium id.2153) on Cytoplasmic tyrosine−protein kinase BMX
with a threshold of 1e−05
rs73128290
rs77285108
rs7742829
rs12404911
All − MR Egger
All − IVW(MRE)
−1
0
1
2
MR effect size for
'Gut microbiota abundance (genus Catenibacterium id.2153)' on 'Cytoplasmic tyrosine−protein kinase BMX'

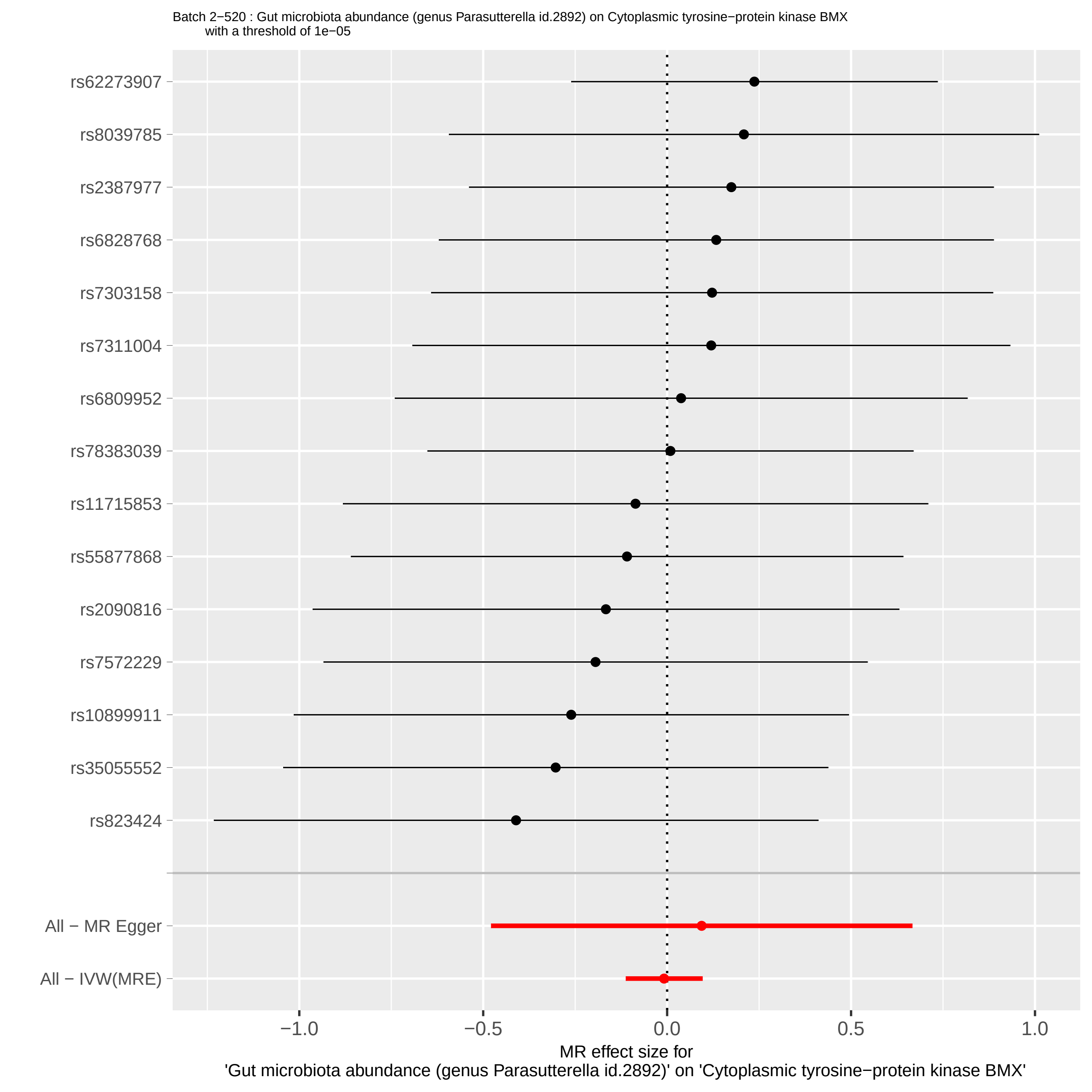
Batch 2−520 : Gut microbiota abundance (genus Parasutterella id.2892) on Cytoplasmic tyrosine−protein kinase BMX
with a threshold of 1e−05
rs62273907
rs8039785
rs2387977
rs6828768
rs7303158
rs7311004
rs6809952
rs78383039
rs11715853
rs55877868
rs2090816
rs7572229
rs10899911
rs35055552
rs823424
All − MR Egger
All − IVW(MRE)
−1.0
−0.5
0.0
0.5
1.0
MR effect size for
'Gut microbiota abundance (genus Parasutterella id.2892)' on 'Cytoplasmic tyrosine−protein kinase BMX'

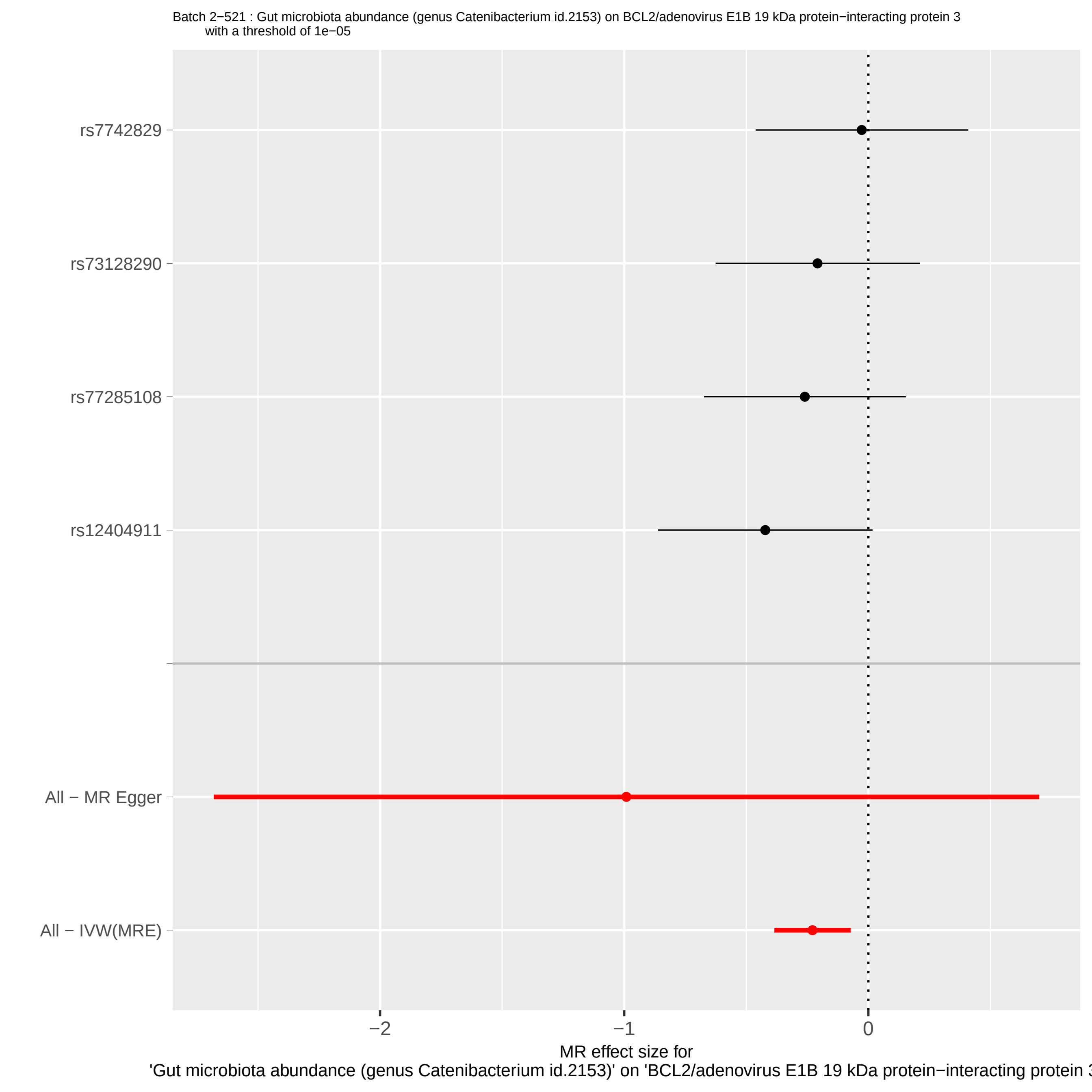

Batch 2−521 : Gut microbiota abundance (genus Catenibacterium id.2153) on BCL2/adenovirus E1B 19 kDa protein−interacting protein 3
with a threshold of 1e−05
rs7742829
rs73128290
rs77285108
rs12404911
All − MR Egger
All − IVW(MRE)
−2
−1
0
MR effect size for
'Gut microbiota abundance (genus Catenibacterium id.2153)' on 'BCL2/adenovirus E1B 19 kDa protein−interacting protein 3

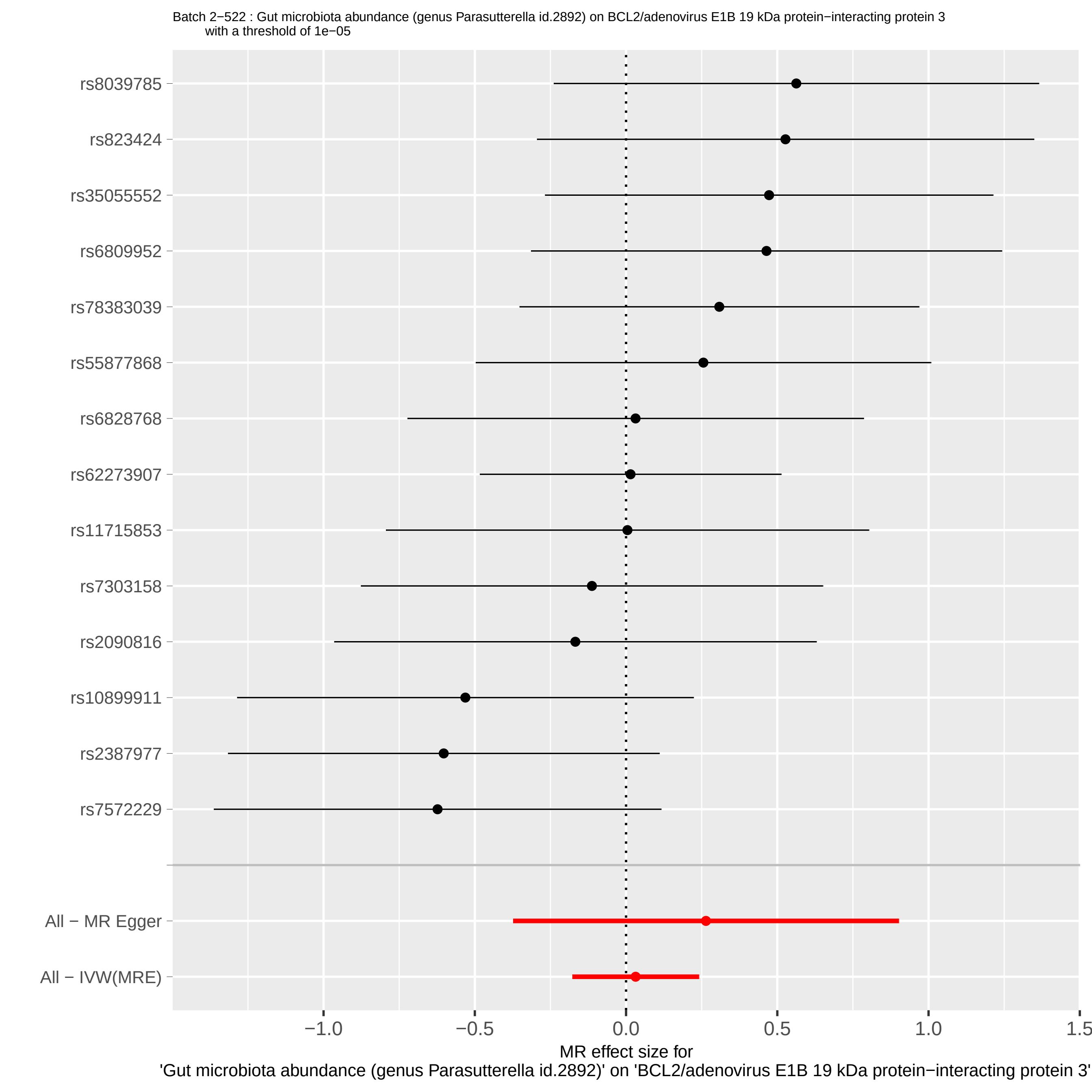
Batch 2−522 : Gut microbiota abundance (genus Parasutterella id.2892) on BCL2/adenovirus E1B 19 kDa protein−interacting protein 3
with a threshold of 1e−05
rs8039785
rs823424
rs35055552
rs6809952
rs78383039
rs55877868
rs6828768
rs62273907
rs11715853
rs7303158
rs2090816
rs10899911
rs2387977
rs7572229
All − MR Egger
All − IVW(MRE)
−1.0
−0.5
0.0
0.5
1.0
1.5
MR effect size for
'Gut microbiota abundance (genus Parasutterella id.2892)' on 'BCL2/adenovirus E1B 19 kDa protein−interacting protein 3'

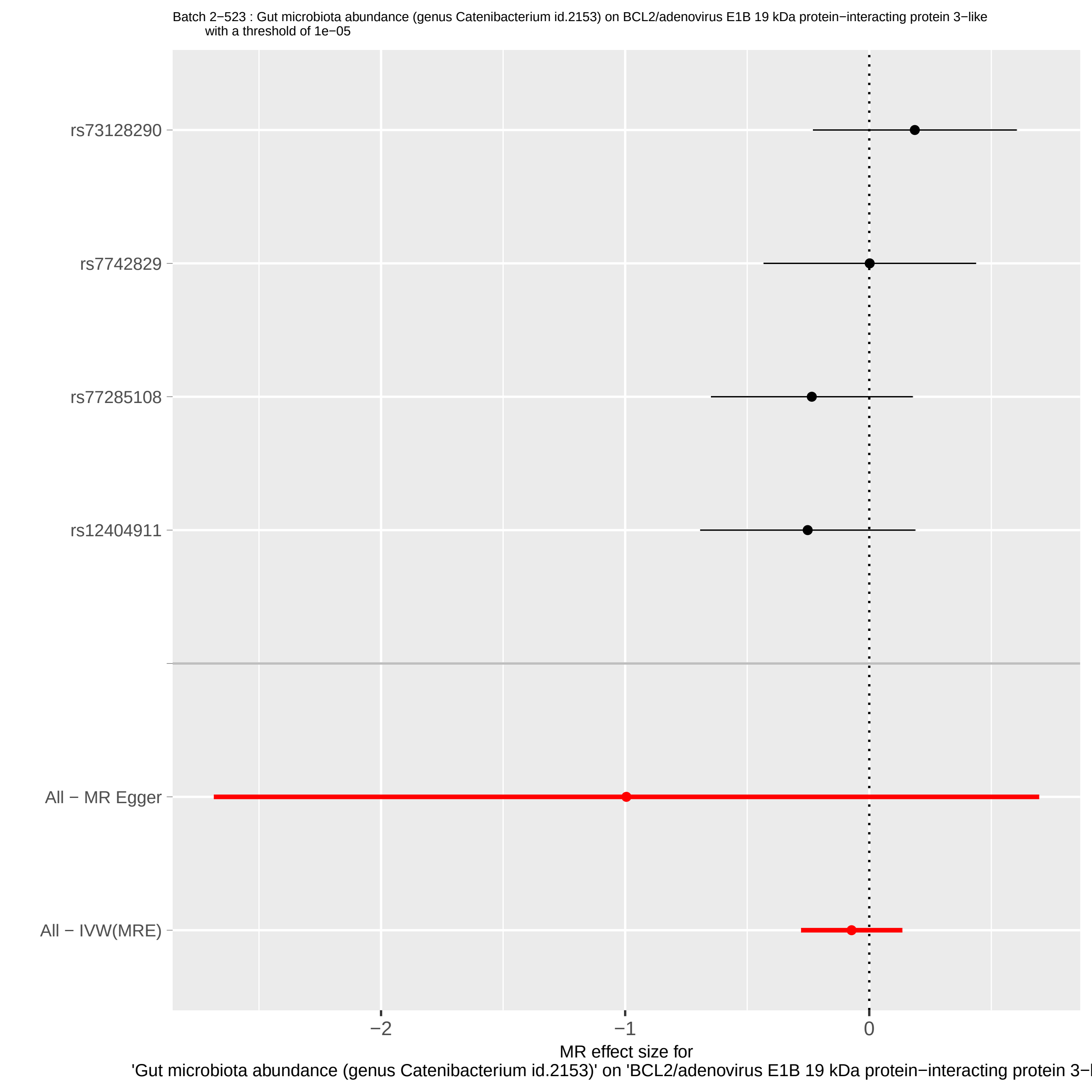

Batch 2−523 : Gut microbiota abundance (genus Catenibacterium id.2153) on BCL2/adenovirus E1B 19 kDa protein−interacting protein 3−like
with a threshold of 1e−05
rs73128290
rs7742829
rs77285108
rs12404911
All − MR Egger
All − IVW(MRE)
−2
−1
0
MR effect size for
'Gut microbiota abundance (genus Catenibacterium id.2153)' on 'BCL2/adenovirus E1B 19 kDa protein−interacting protein 3−l

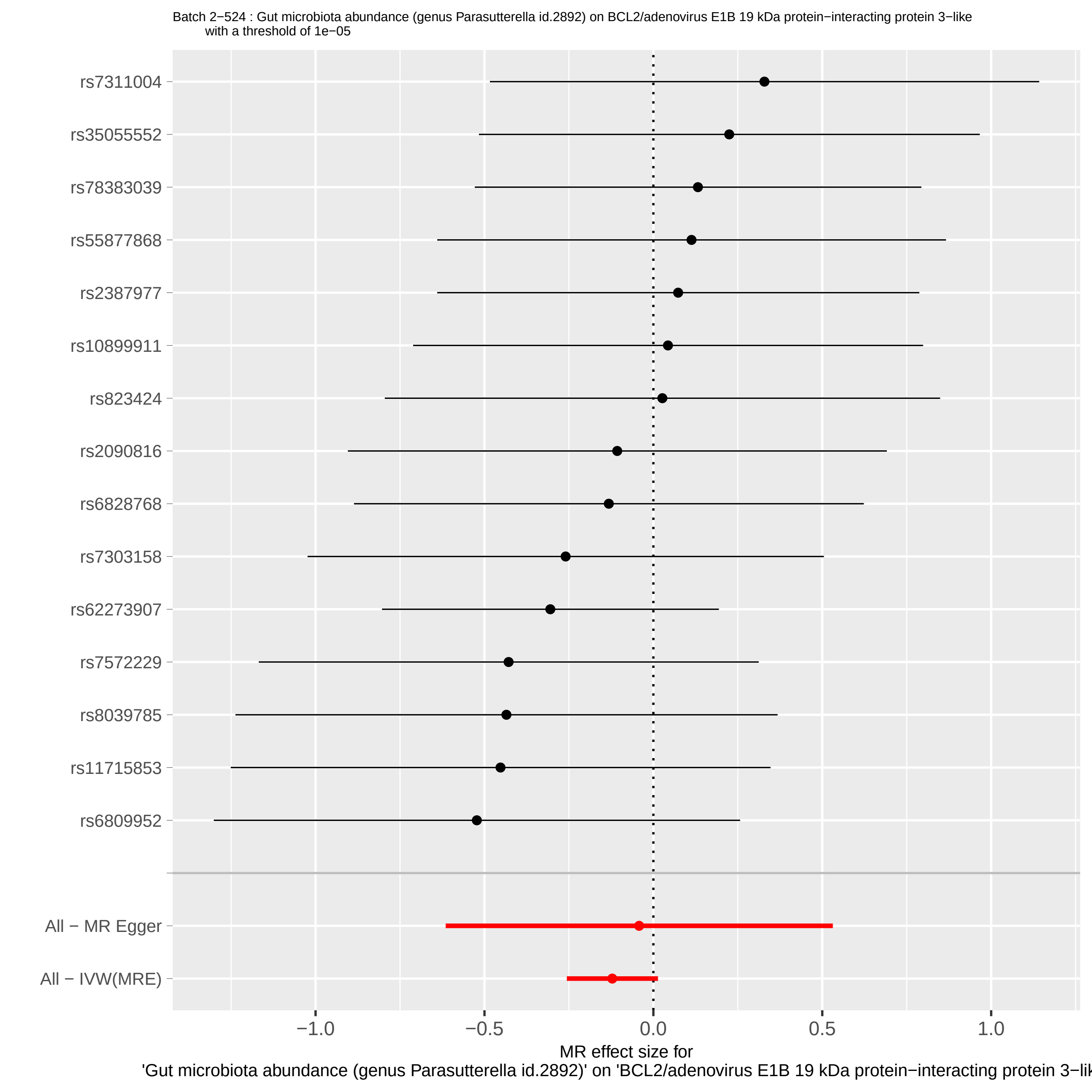

Batch 2−524 : Gut microbiota abundance (genus Parasutterella id.2892) on BCL2/adenovirus E1B 19 kDa protein−interacting protein 3−like with a threshold of 1e−05
rs7311004
rs35055552
rs78383039
rs55877868
rs2387977
rs10899911
rs823424
rs2090816
rs6828768
rs7303158
rs62273907
rs7572229
rs8039785
rs11715853
rs6809952
All − MR Egger
All − IVW(MRE)
−1.0
−0.5
0.0
0.5
1.0
MR effect size for
'Gut microbiota abundance (genus Parasutterella id.2892)' on 'BCL2/adenovirus E1B 19 kDa protein−interacting protein 3−lik

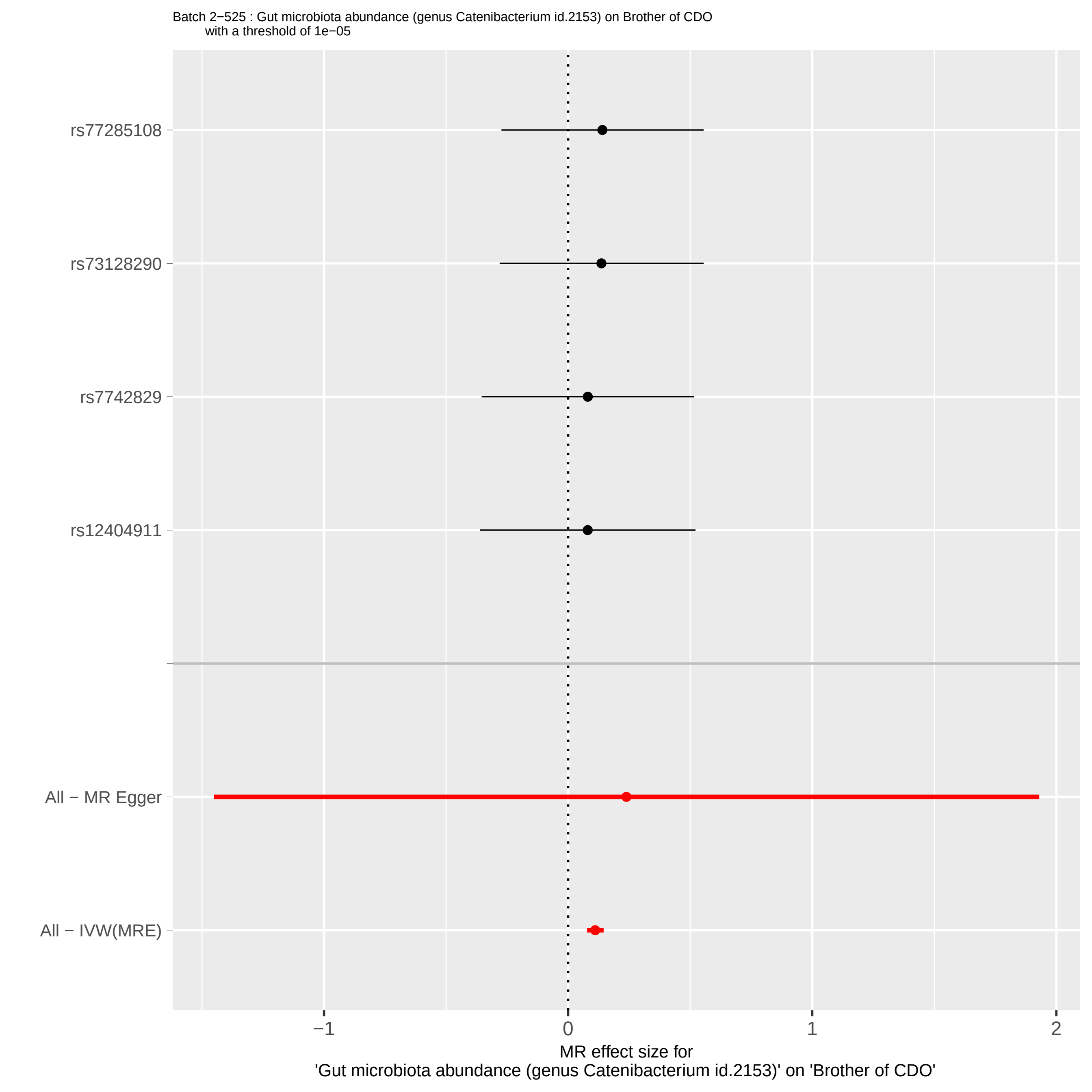

Batch 2−525 : Gut microbiota abundance (genus Catenibacterium id.2153) on Brother of CDO
with a threshold of 1e−05
rs77285108
rs73128290
rs7742829
rs12404911
All − MR Egger
All − IVW(MRE)
−1
0
1
2
MR effect size for
'Gut microbiota abundance (genus Catenibacterium id.2153)' on 'Brother of CDO'

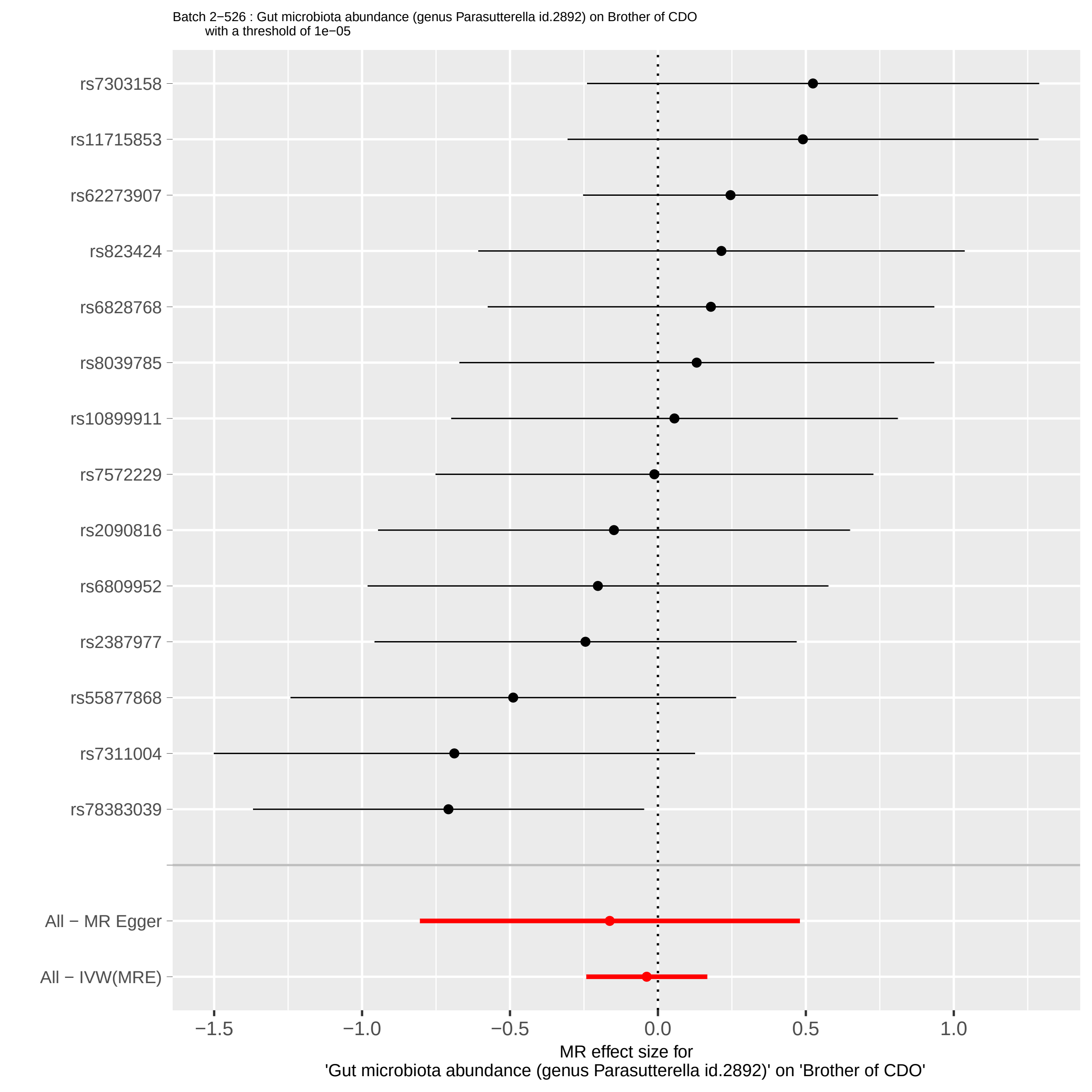

Batch 2−526 : Gut microbiota abundance (genus Parasutterella id.2892) on Brother of CDO
with a threshold of 1e−05
rs7303158
rs11715853
rs62273907
rs823424
rs6828768
rs8039785
rs10899911
rs7572229
rs2090816
rs6809952
rs2387977
rs55877868
rs7311004
rs78383039
All − MR Egger
All − IVW(MRE)
−1.5
−1.0
−0.5
0.0
0.5
1.0
MR effect size for
'Gut microbiota abundance (genus Parasutterella id.2892)' on 'Brother of CDO'

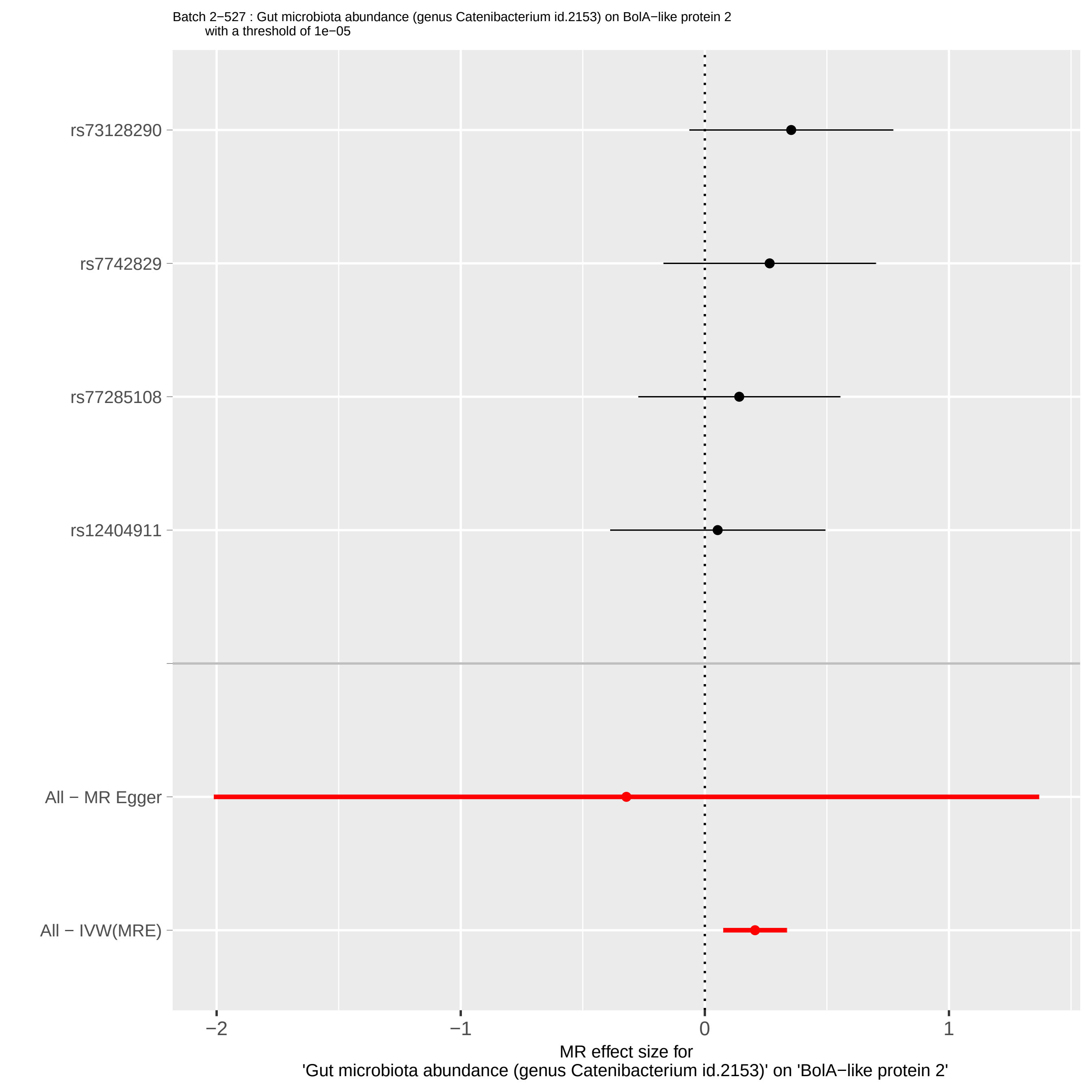

Batch 2−527 : Gut microbiota abundance (genus Catenibacterium id.2153) on BolA−like protein 2
with a threshold of 1e−05
rs73128290
rs7742829
rs77285108
rs12404911
All − MR Egger
All − IVW(MRE)
−2
−1
0
1
MR effect size for
'Gut microbiota abundance (genus Catenibacterium id.2153)' on 'BolA−like protein 2'

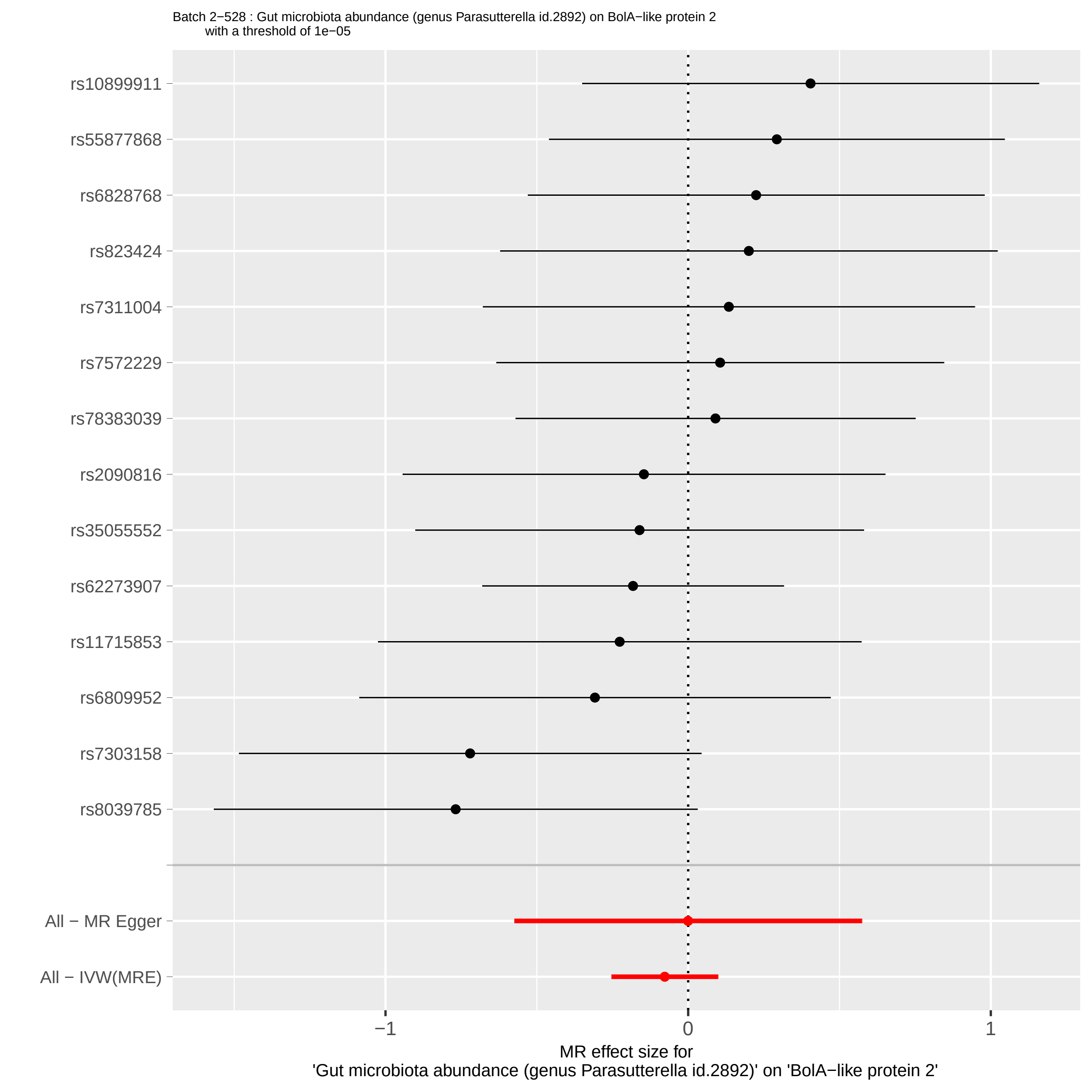

Batch 2−528 : Gut microbiota abundance (genus Parasutterella id.2892) on BolA−like protein 2
with a threshold of 1e−05
rs10899911
rs55877868
rs6828768
rs823424
rs7311004
rs7572229
rs78383039
rs2090816
rs35055552
rs62273907
rs11715853
rs6809952
rs7303158
rs8039785
All − MR Egger
All − IVW(MRE)
−1
0
1
MR effect size for
'Gut microbiota abundance (genus Parasutterella id.2892)' on 'BolA−like protein 2'

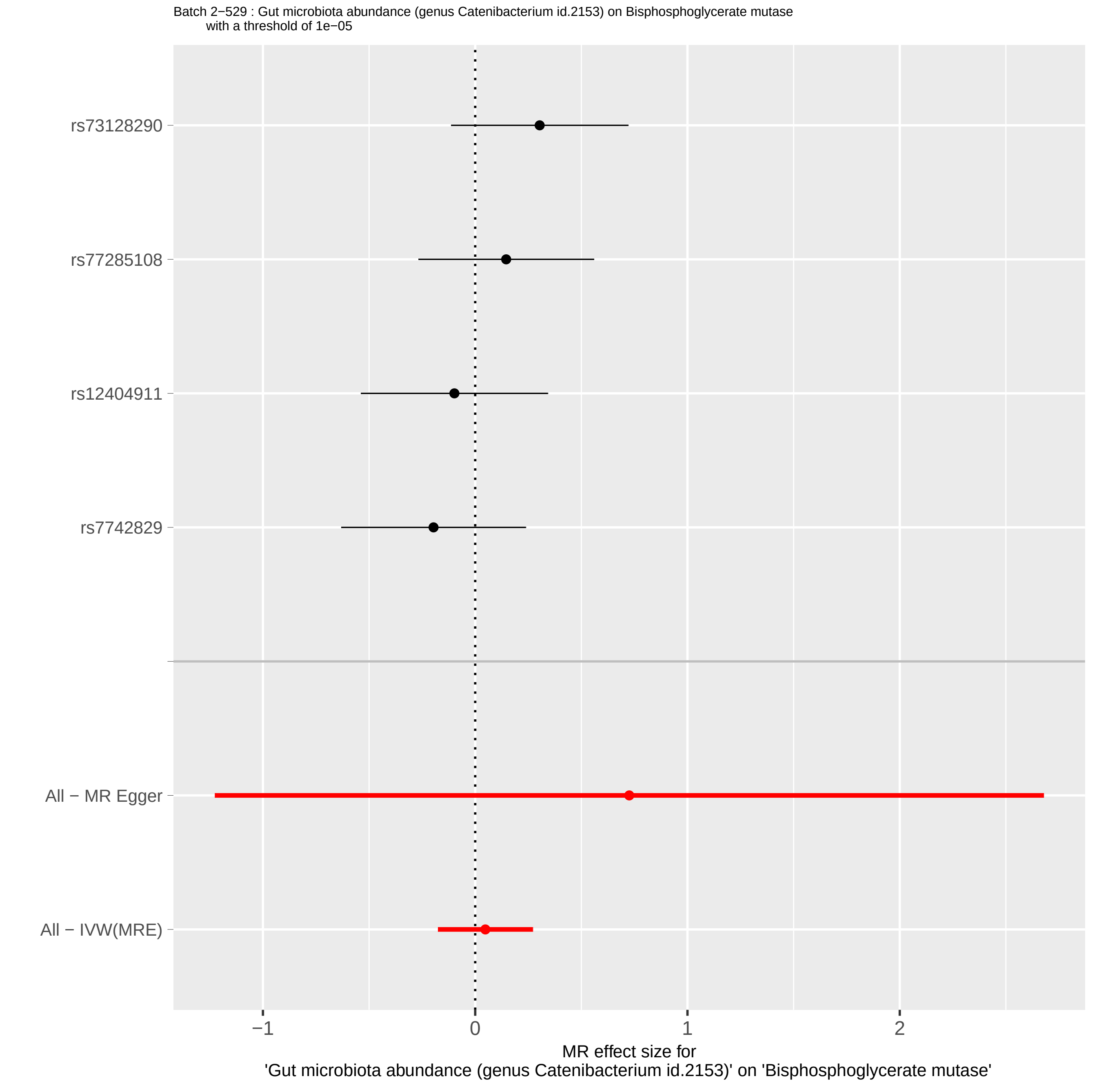

Batch 2−529 : Gut microbiota abundance (genus Catenibacterium id.2153) on Bisphosphoglycerate mutase
with a threshold of 1e−05
rs73128290
rs77285108
rs12404911
rs7742829
All − MR Egger
All − IVW(MRE)
−1
0
1
2
MR effect size for
'Gut microbiota abundance (genus Catenibacterium id.2153)' on 'Bisphosphoglycerate mutase'

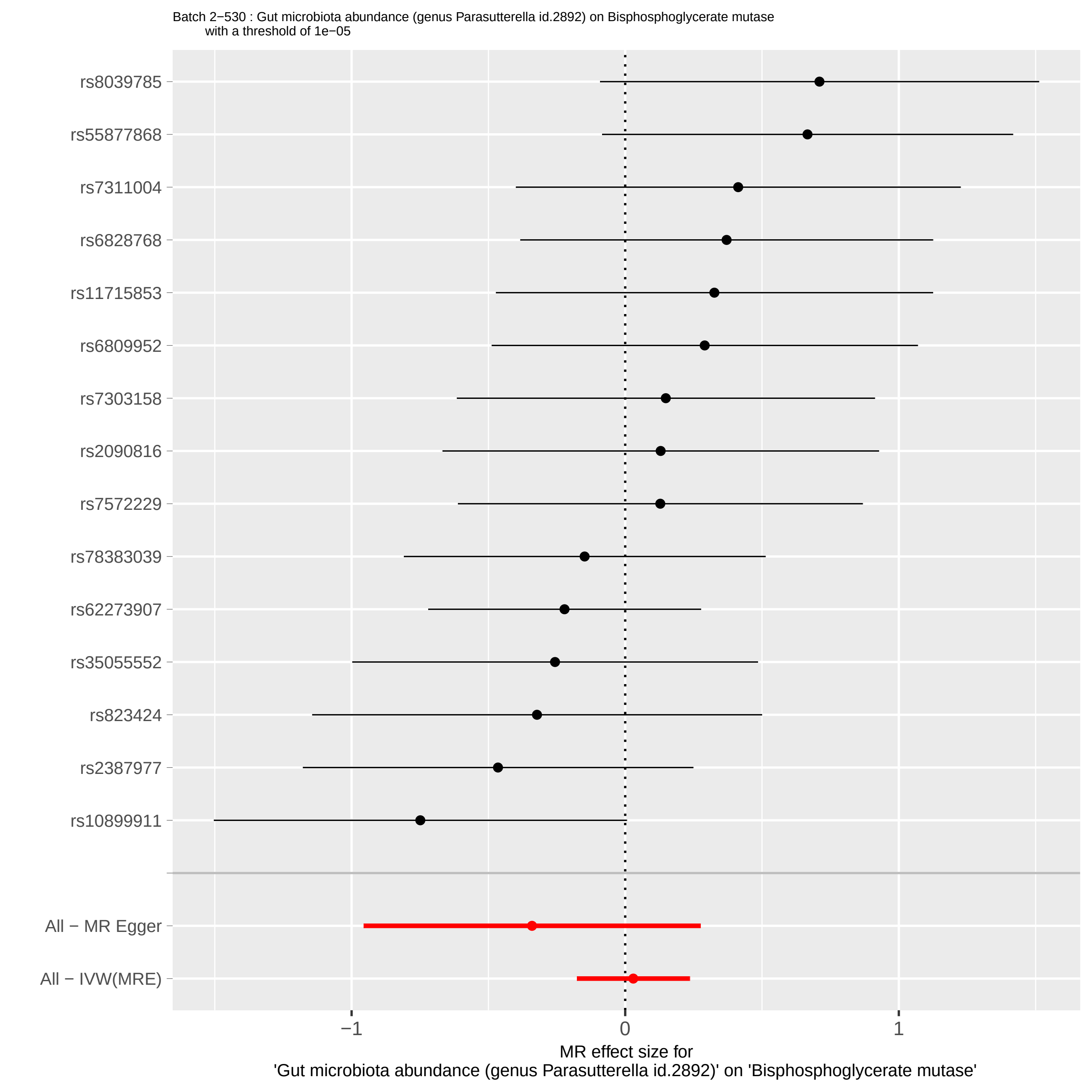

Batch 2−530 : Gut microbiota abundance (genus Parasutterella id.2892) on Bisphosphoglycerate mutase
with a threshold of 1e−05
rs8039785
rs55877868
rs7311004
rs6828768
rs11715853
rs6809952
rs7303158
rs2090816
rs7572229
rs78383039
rs62273907
rs35055552
rs823424
rs2387977
rs10899911
All − MR Egger
All − IVW(MRE)
−1
0
1
MR effect size for
'Gut microbiota abundance (genus Parasutterella id.2892)' on 'Bisphosphoglycerate mutase'

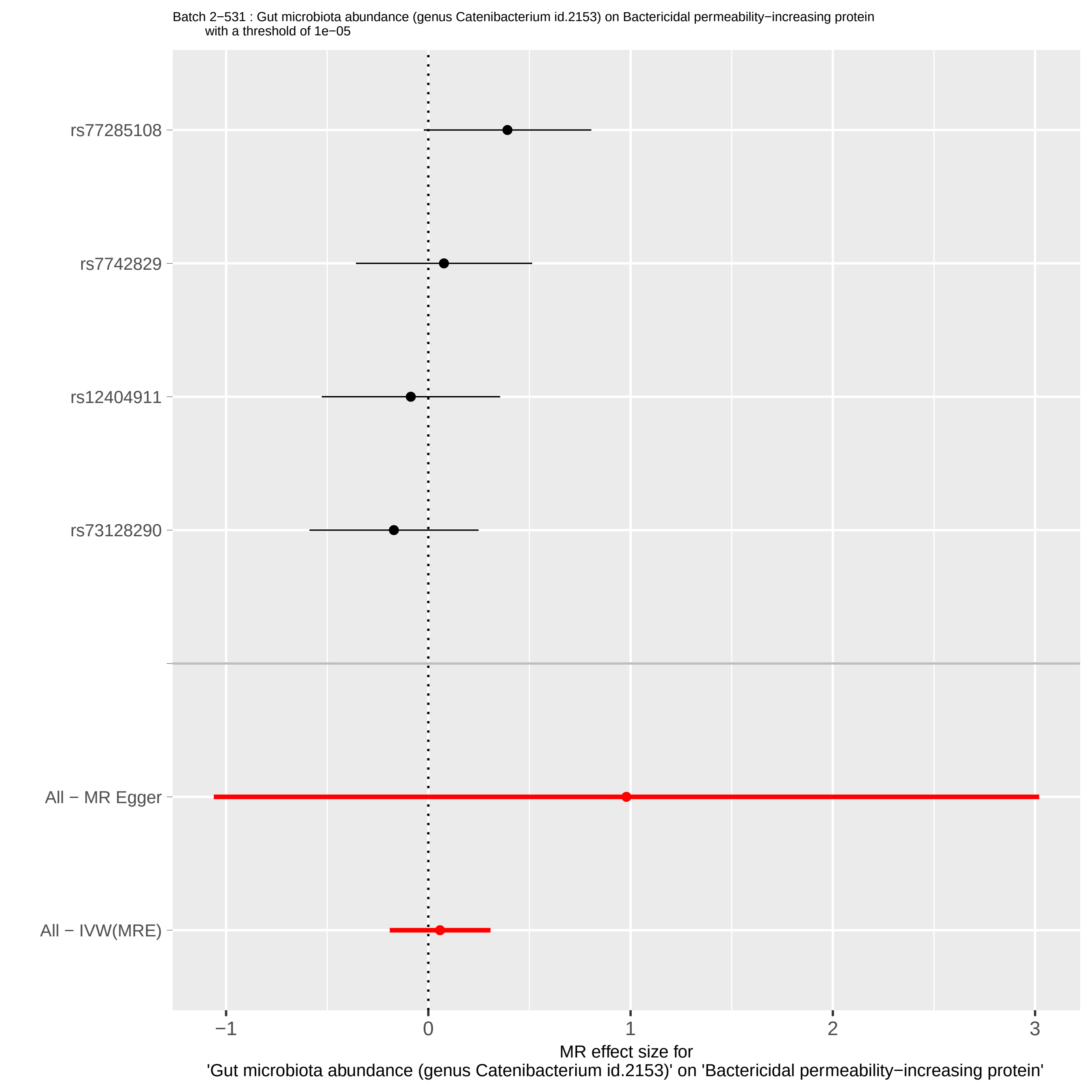

Batch 2−531 : Gut microbiota abundance (genus Catenibacterium id.2153) on Bactericidal permeability−increasing protein
with a threshold of 1e−05
rs77285108
rs7742829
rs12404911
rs73128290
All − MR Egger
All − IVW(MRE)
−1
0
1
2
3
MR effect size for
'Gut microbiota abundance (genus Catenibacterium id.2153)' on 'Bactericidal permeability−increasing protein'

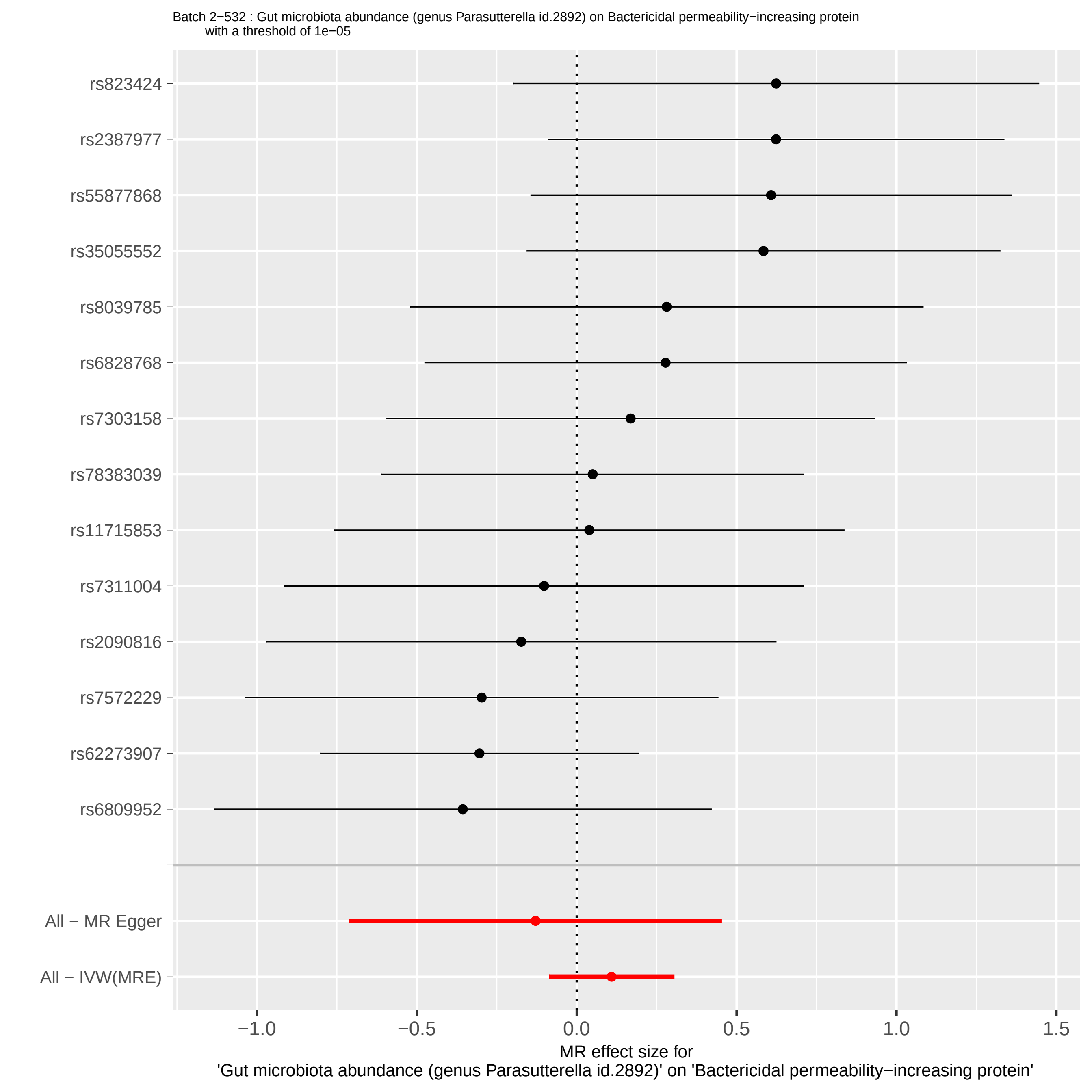

Batch 2−532 : Gut microbiota abundance (genus Parasutterella id.2892) on Bactericidal permeability−increasing protein
with a threshold of 1e−05
rs823424
rs2387977
rs55877868
rs35055552
rs8039785
rs6828768
rs7303158
rs78383039
rs11715853
rs7311004
rs2090816
rs7572229
rs62273907
rs6809952
All − MR Egger
All − IVW(MRE)
−1.0
−0.5
0.0
0.5
1.0
1.5
MR effect size for
'Gut microbiota abundance (genus Parasutterella id.2892)' on 'Bactericidal permeability−increasing protein'

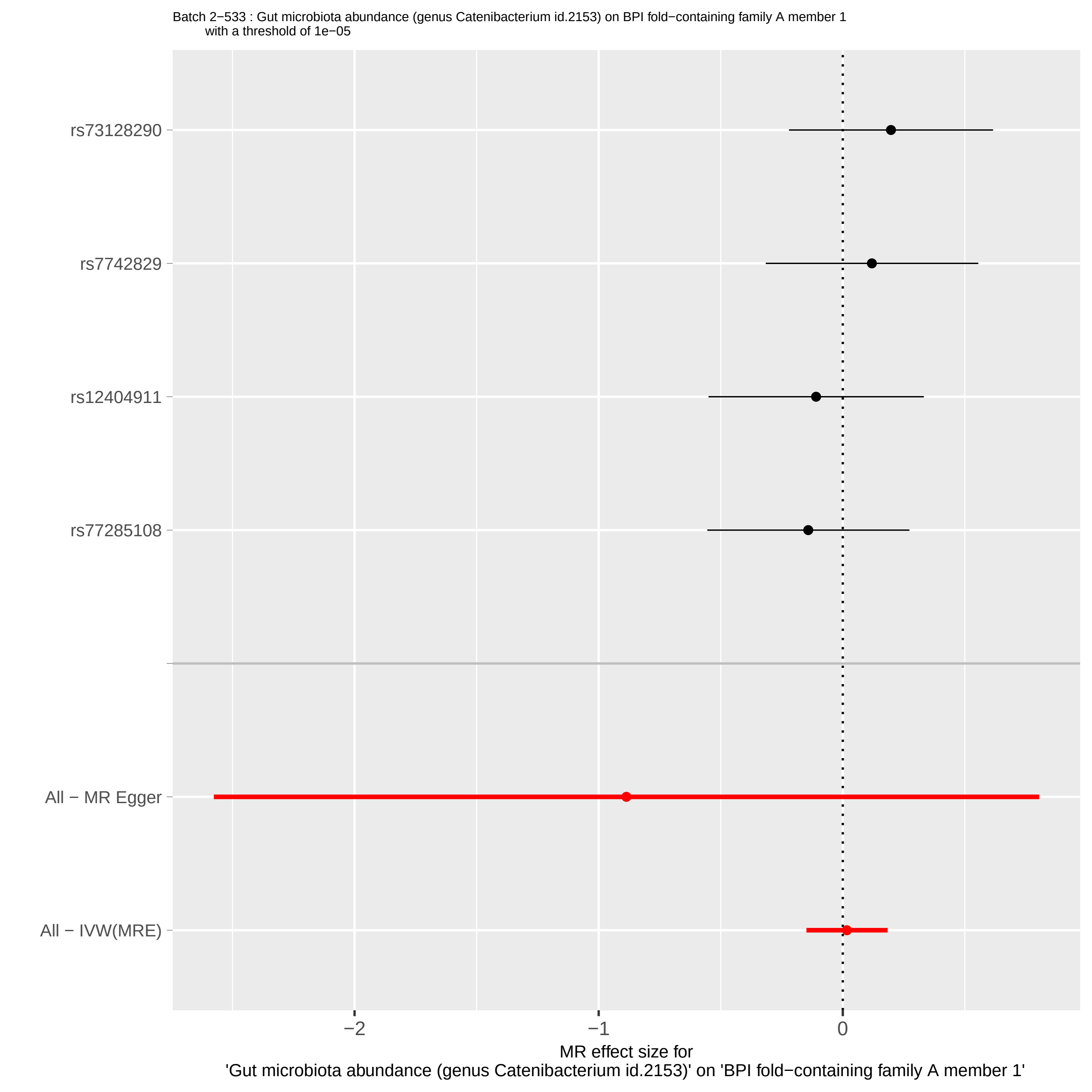

Batch 2−533 : Gut microbiota abundance (genus Catenibacterium id.2153) on BPI fold−containing family A member 1
with a threshold of 1e−05
rs73128290
rs7742829
rs12404911
rs77285108
All − MR Egger
All − IVW(MRE)
−2
−1
0
MR effect size for
'Gut microbiota abundance (genus Catenibacterium id.2153)' on 'BPI fold−containing family A member 1'

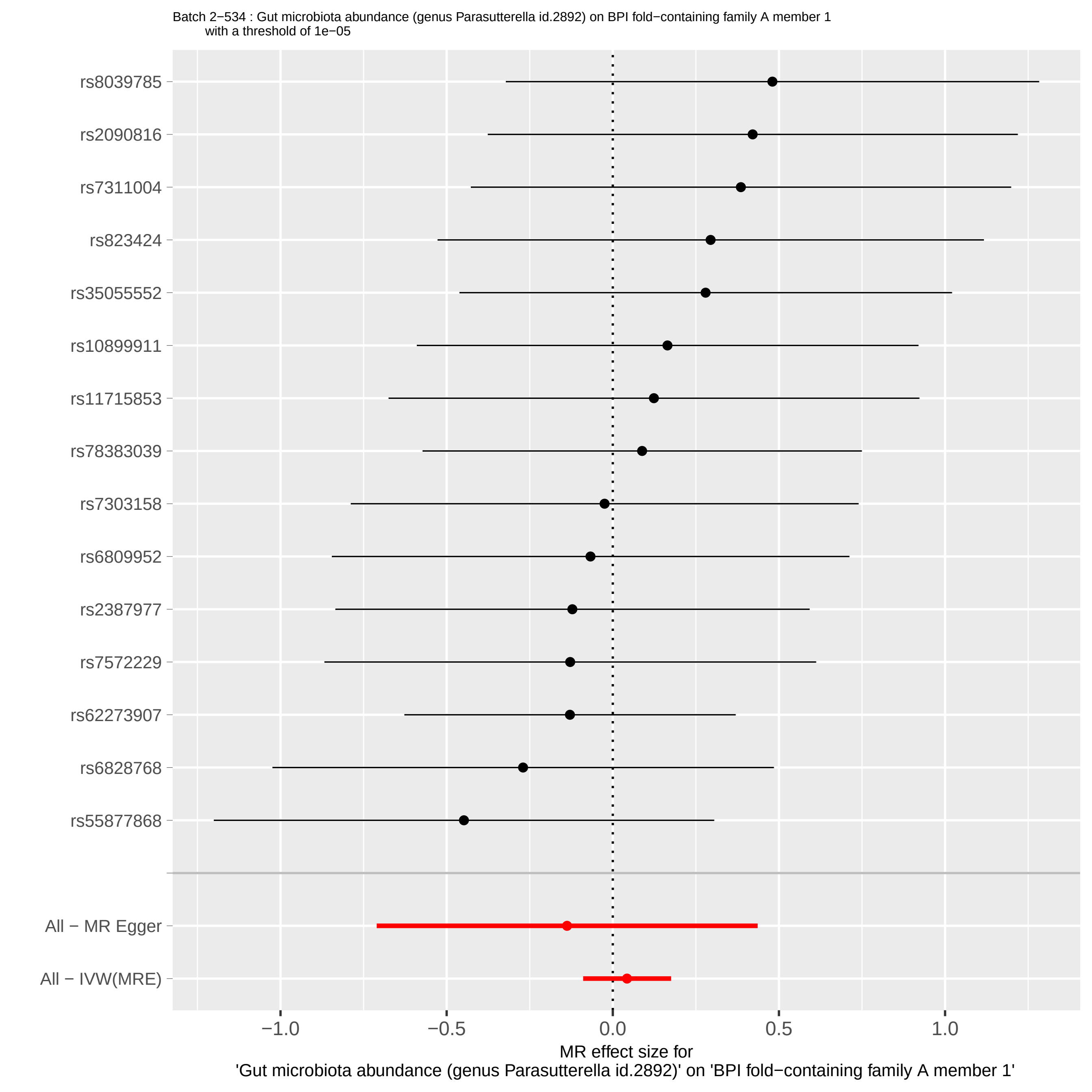

Batch 2−534 : Gut microbiota abundance (genus Parasutterella id.2892) on BPI fold−containing family A member 1
with a threshold of 1e−05
rs8039785
rs2090816
rs7311004
rs823424
rs35055552
rs10899911
rs11715853
rs78383039
rs7303158
rs6809952
rs2387977
rs7572229
rs62273907
rs6828768
rs55877868
All − MR Egger
All − IVW(MRE)
−1.0
−0.5
0.0
0.5
1.0
MR effect size for
'Gut microbiota abundance (genus Parasutterella id.2892)' on 'BPI fold−containing family A member 1'

Batch 2−535 : Gut microbiota abundance (genus Catenibacterium id.2153) on BPI fold−containing family B member 1
with a threshold of 1e−05

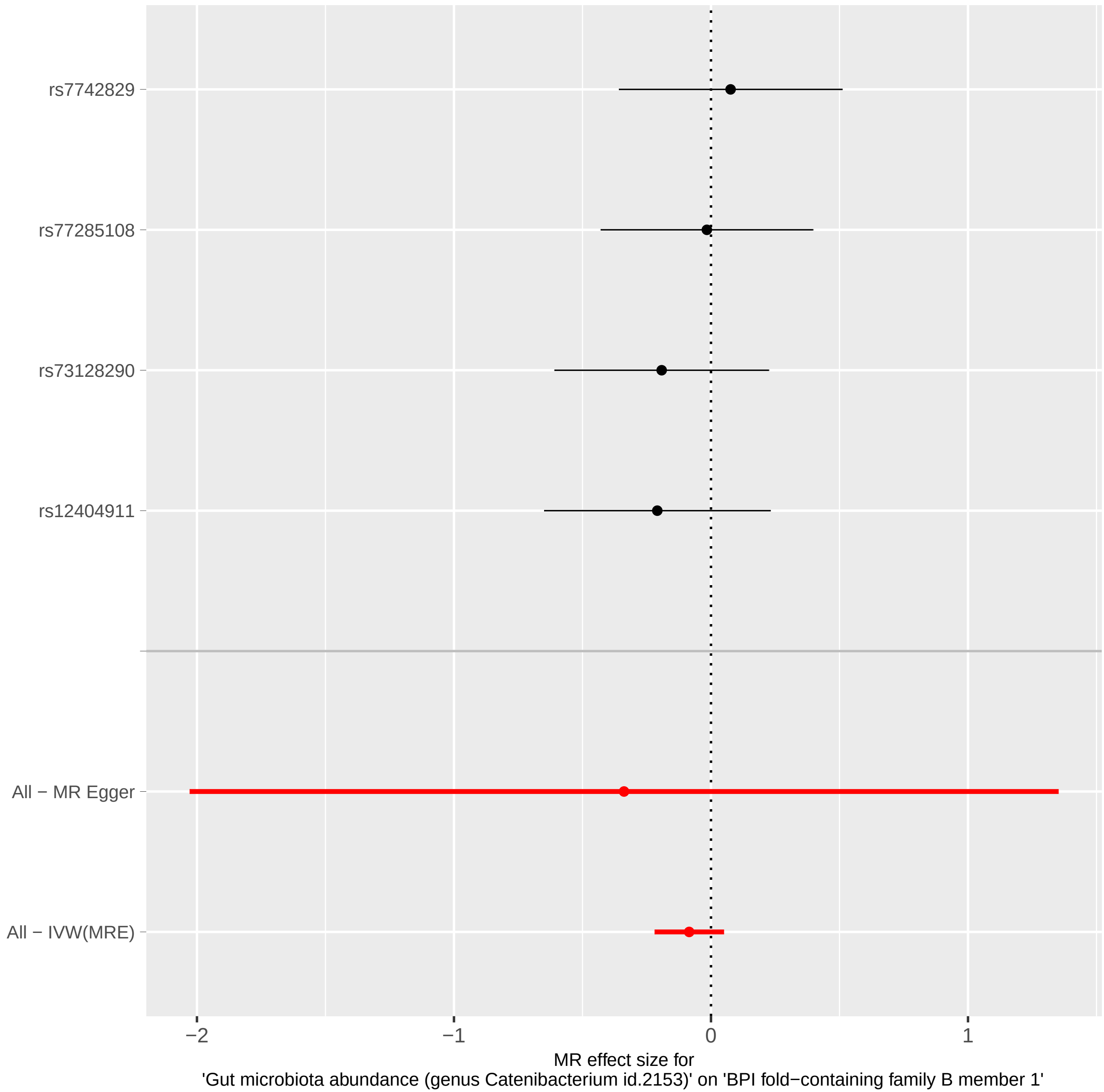

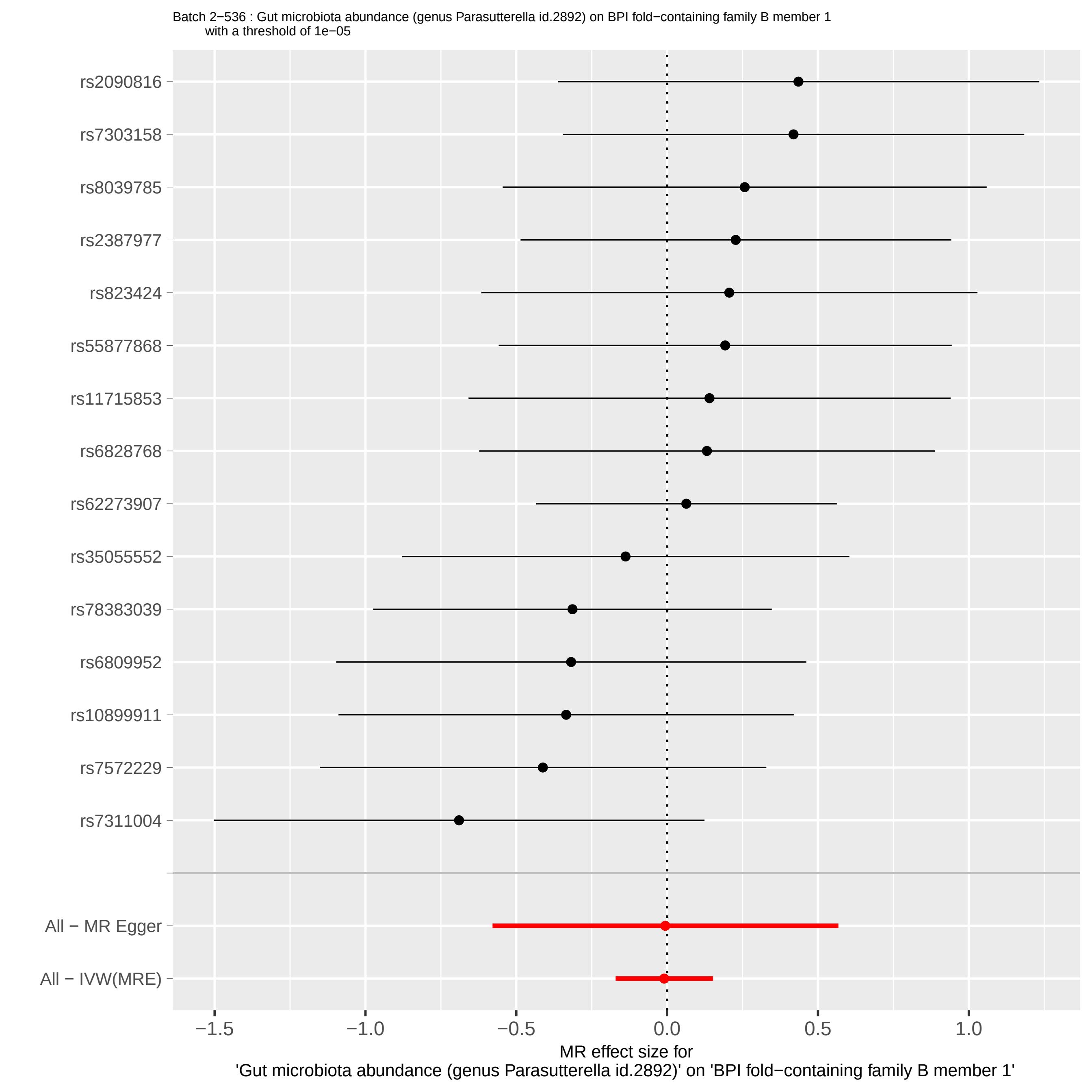

Batch 2−536 : Gut microbiota abundance (genus Parasutterella id.2892) on BPI fold−containing family B member 1
with a threshold of 1e−05
rs2090816
rs7303158
rs8039785
rs2387977
rs823424
rs55877868
rs11715853
rs6828768
rs62273907
rs35055552
rs78383039
rs6809952
rs10899911
rs7572229
rs7311004
All − MR Egger
All − IVW(MRE)
−1.5
−1.0
−0.5
0.0
0.5
1.0
MR effect size for
'Gut microbiota abundance (genus Parasutterella id.2892)' on 'BPI fold−containing family B member 1'

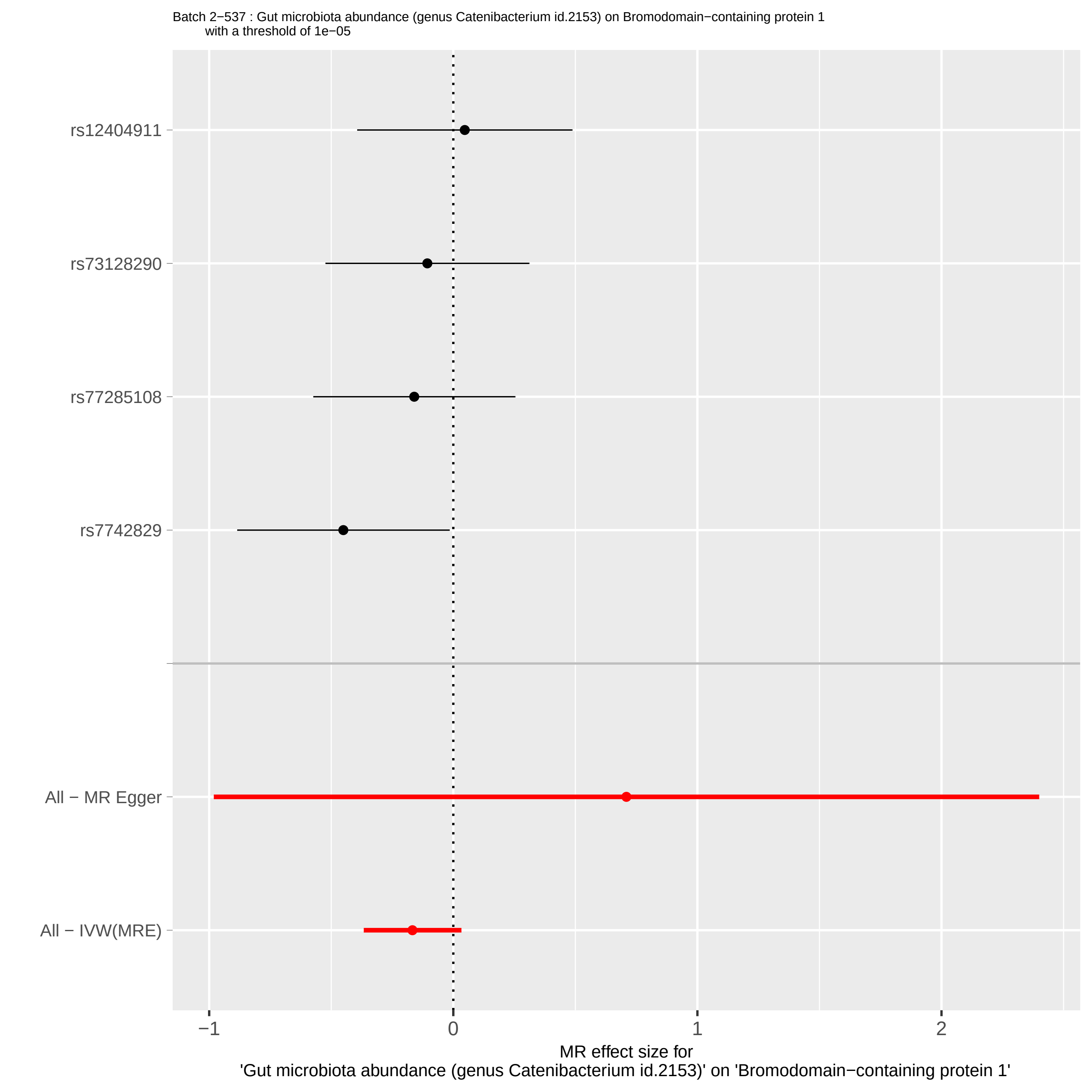
Batch 2−537 : Gut microbiota abundance (genus Catenibacterium id.2153) on Bromodomain−containing protein 1
with a threshold of 1e−05
rs12404911
rs73128290
rs77285108
rs7742829
All − MR Egger
All − IVW(MRE)
−1
0
1
2
MR effect size for
'Gut microbiota abundance (genus Catenibacterium id.2153)' on 'Bromodomain−containing protein 1'

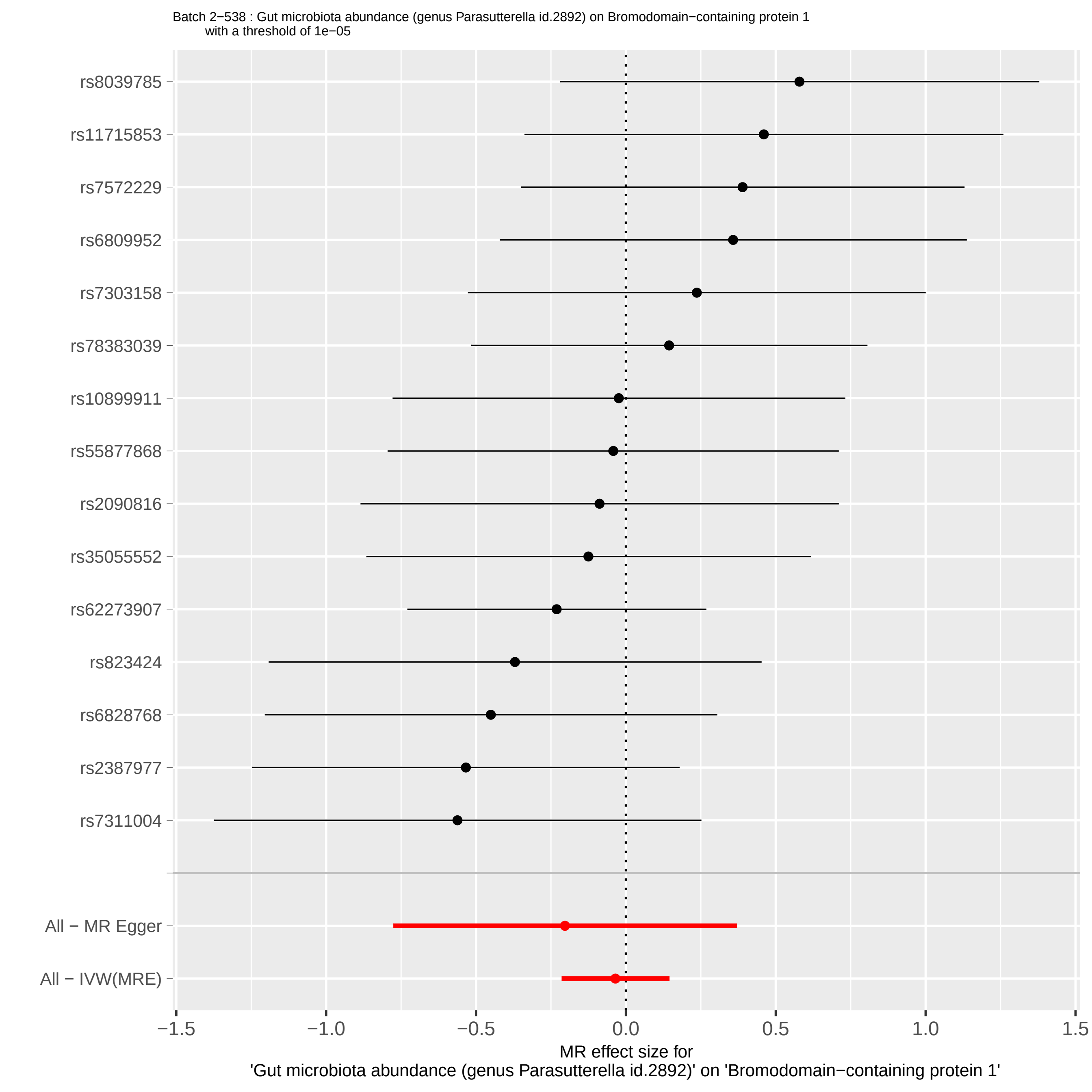

Batch 2−538 : Gut microbiota abundance (genus Parasutterella id.2892) on Bromodomain−containing protein 1
with a threshold of 1e−05
rs8039785
rs11715853
rs7572229
rs6809952
rs7303158
rs78383039
rs10899911
rs55877868
rs2090816
rs35055552
rs62273907
rs823424
rs6828768
rs2387977
rs7311004
All − MR Egger
All − IVW(MRE)
−1.5
−1.0
−0.5
0.0
0.5
1.0
1.5
MR effect size for
'Gut microbiota abundance (genus Parasutterella id.2892)' on 'Bromodomain−containing protein 1'

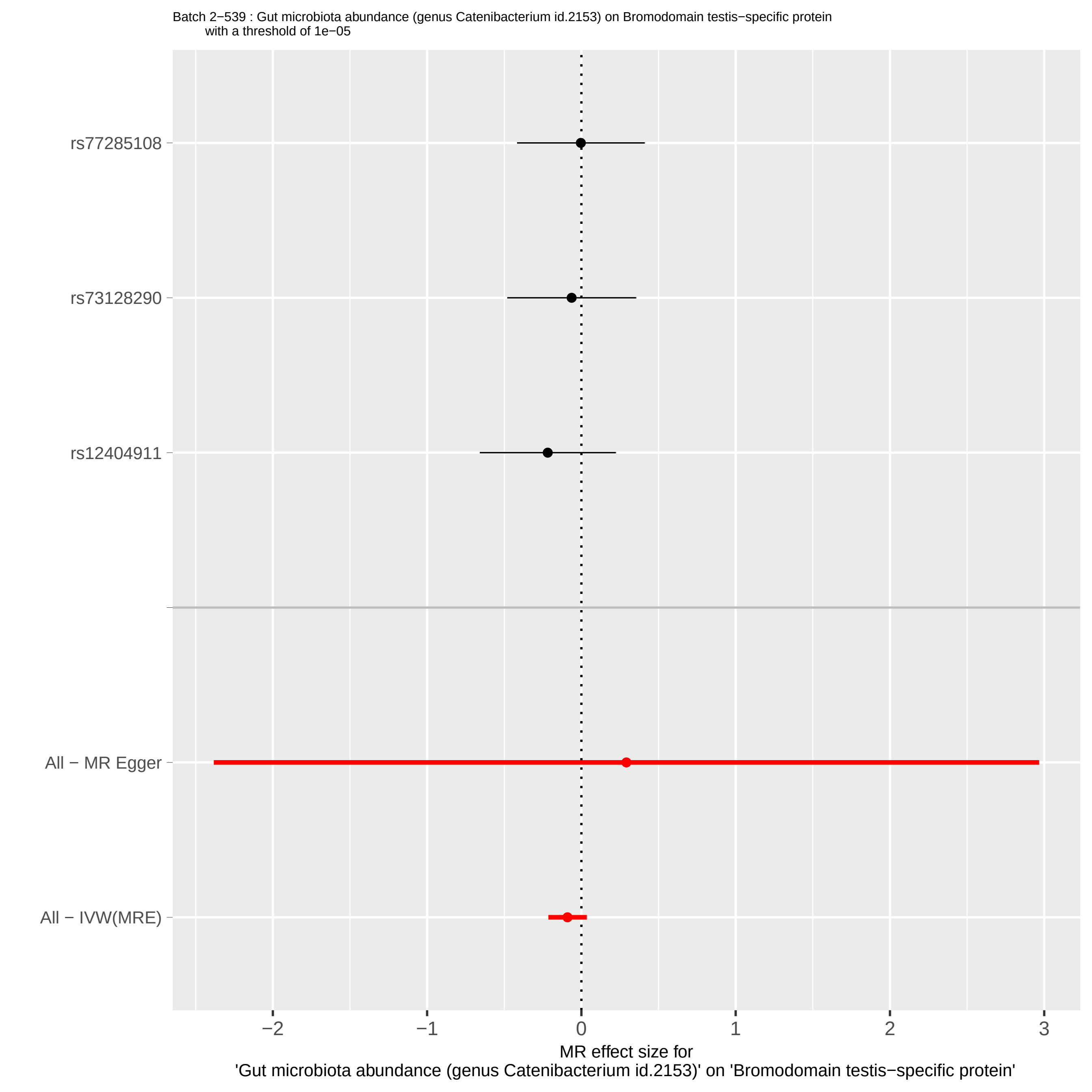

Batch 2−539 : Gut microbiota abundance (genus Catenibacterium id.2153) on Bromodomain testis−specific protein
with a threshold of 1e−05
rs77285108
rs73128290
rs12404911
All − MR Egger
All − IVW(MRE)
−2
−1
0
1
2
3
MR effect size for
'Gut microbiota abundance (genus Catenibacterium id.2153)' on 'Bromodomain testis−specific protein'

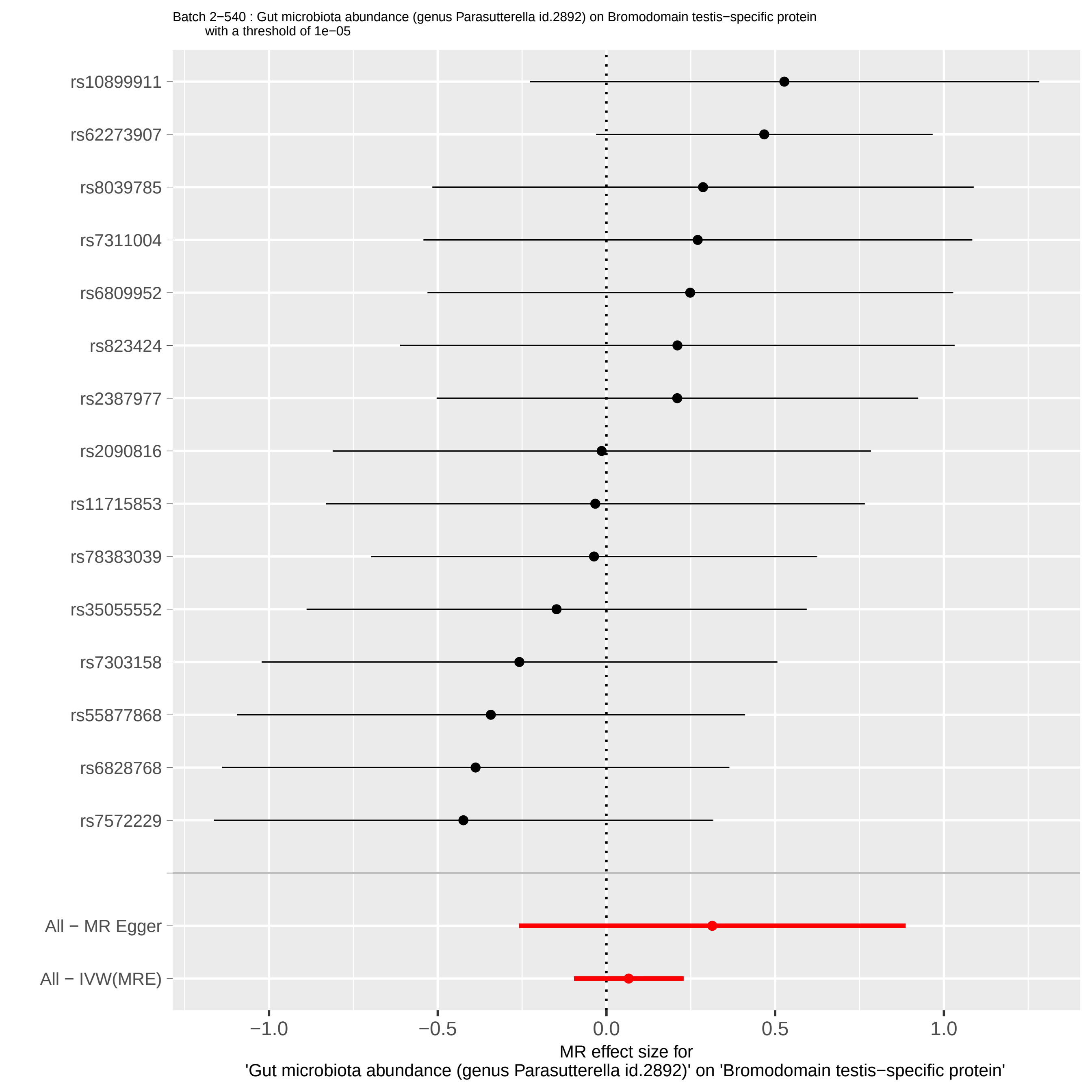
Batch 2−540 : Gut microbiota abundance (genus Parasutterella id.2892) on Bromodomain testis−specific protein with a threshold of 1e−05
rs10899911
rs62273907
rs8039785
rs7311004
rs6809952
rs823424
rs2387977
rs2090816
rs11715853
rs78383039
rs35055552
rs7303158
rs55877868
rs6828768
rs7572229
All − MR Egger
All − IVW(MRE)
−1.0
−0.5
0.0
0.5
1.0
MR effect size for
'Gut microbiota abundance (genus Parasutterella id.2892)' on 'Bromodomain testis−specific protein'

Batch 2−541 : Gut microbiota abundance (genus Catenibacterium id.2153) on Transcription factor IIIB 90 kDa subunit
with a threshold of 1e−05

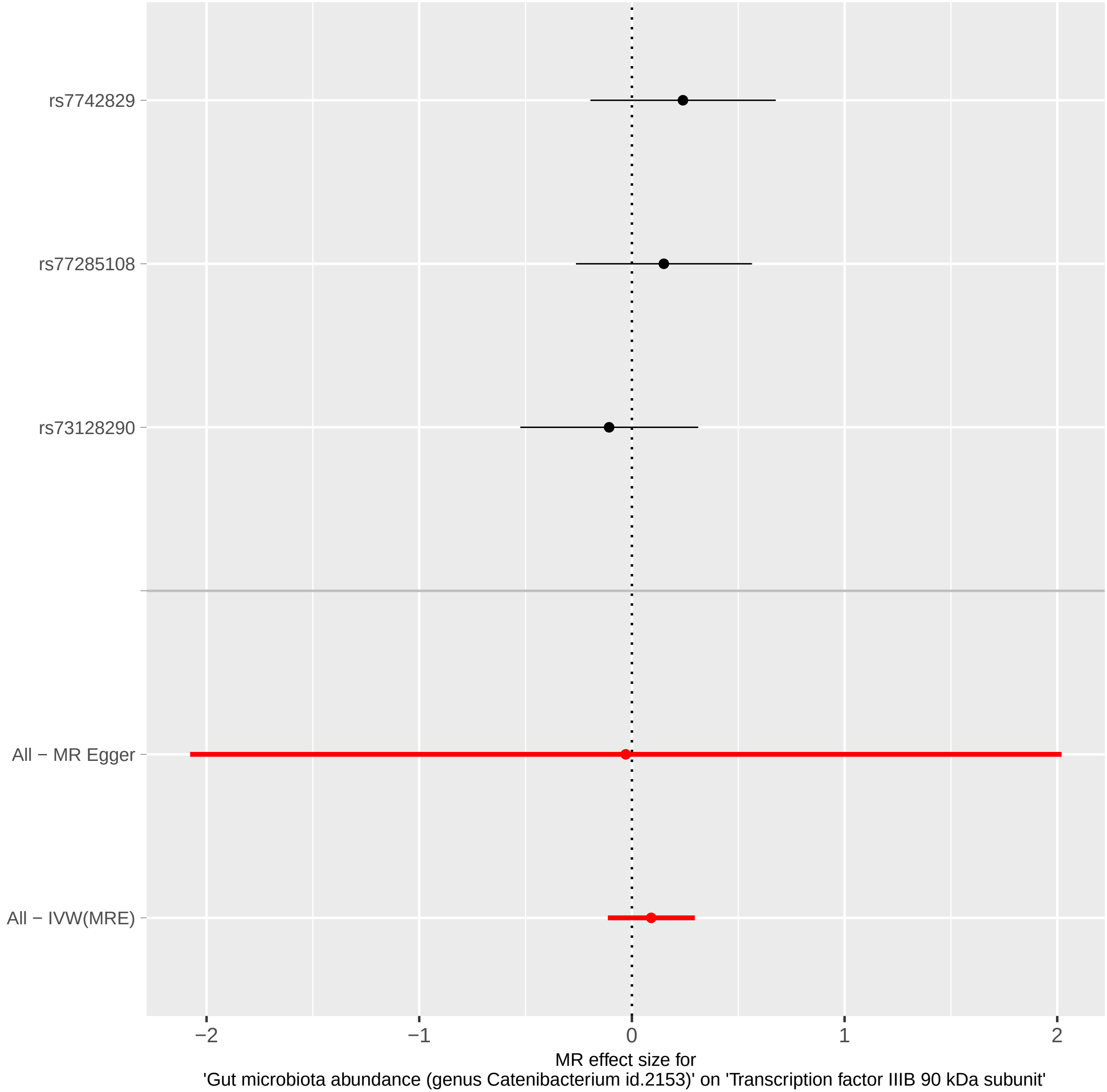

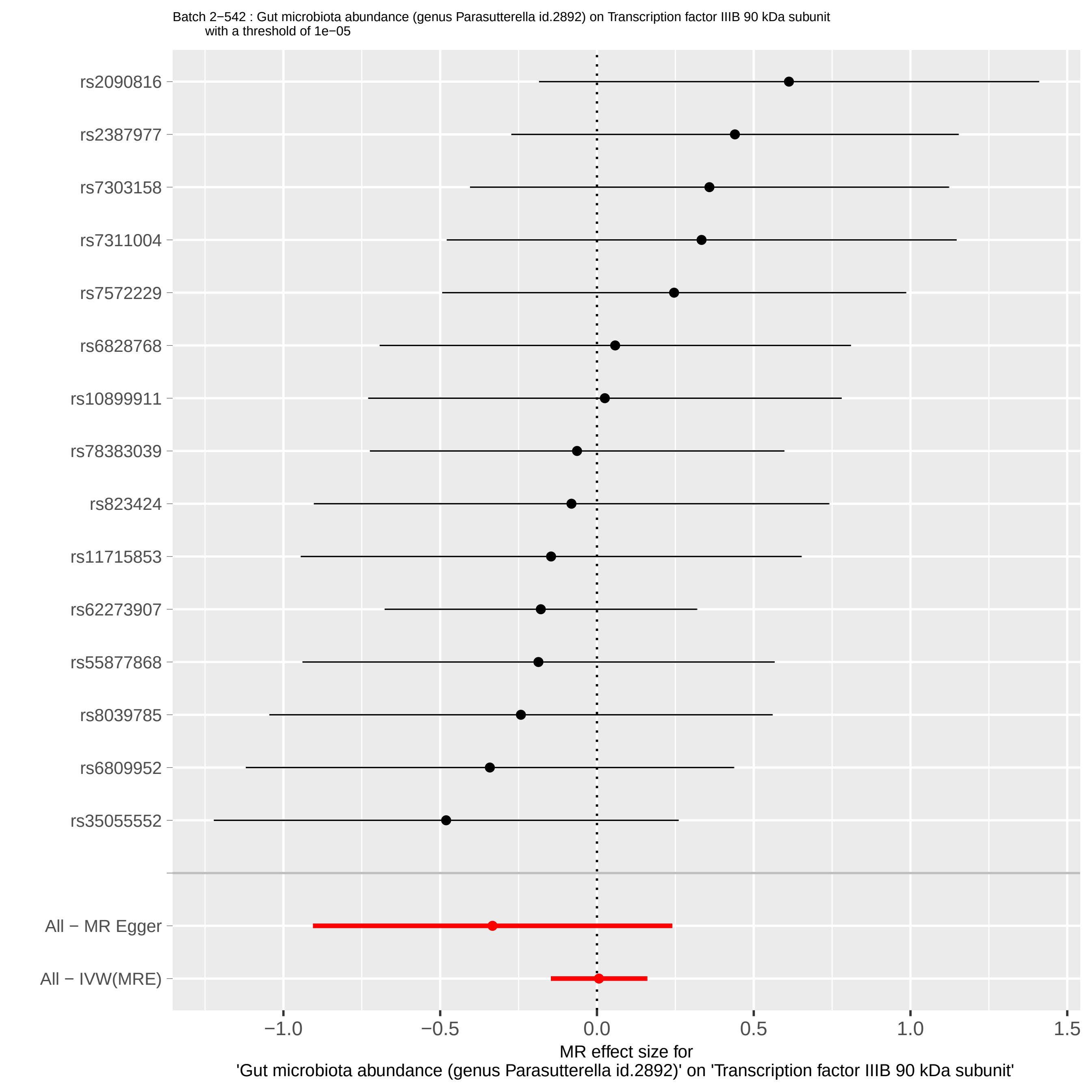

Batch 2−542 : Gut microbiota abundance (genus Parasutterella id.2892) on Transcription factor IIIB 90 kDa subunit with a threshold of 1e−05
rs2090816
rs2387977
rs7303158
rs7311004
rs7572229
rs6828768
rs10899911
rs78383039
rs823424
rs11715853
rs62273907
rs55877868
rs8039785
rs6809952
rs35055552
All − MR Egger
All − IVW(MRE)
−1.0
−0.5
0.0
0.5
1.0
1.5
MR effect size for
'Gut microbiota abundance (genus Parasutterella id.2892)' on 'Transcription factor IIIB 90 kDa subunit'

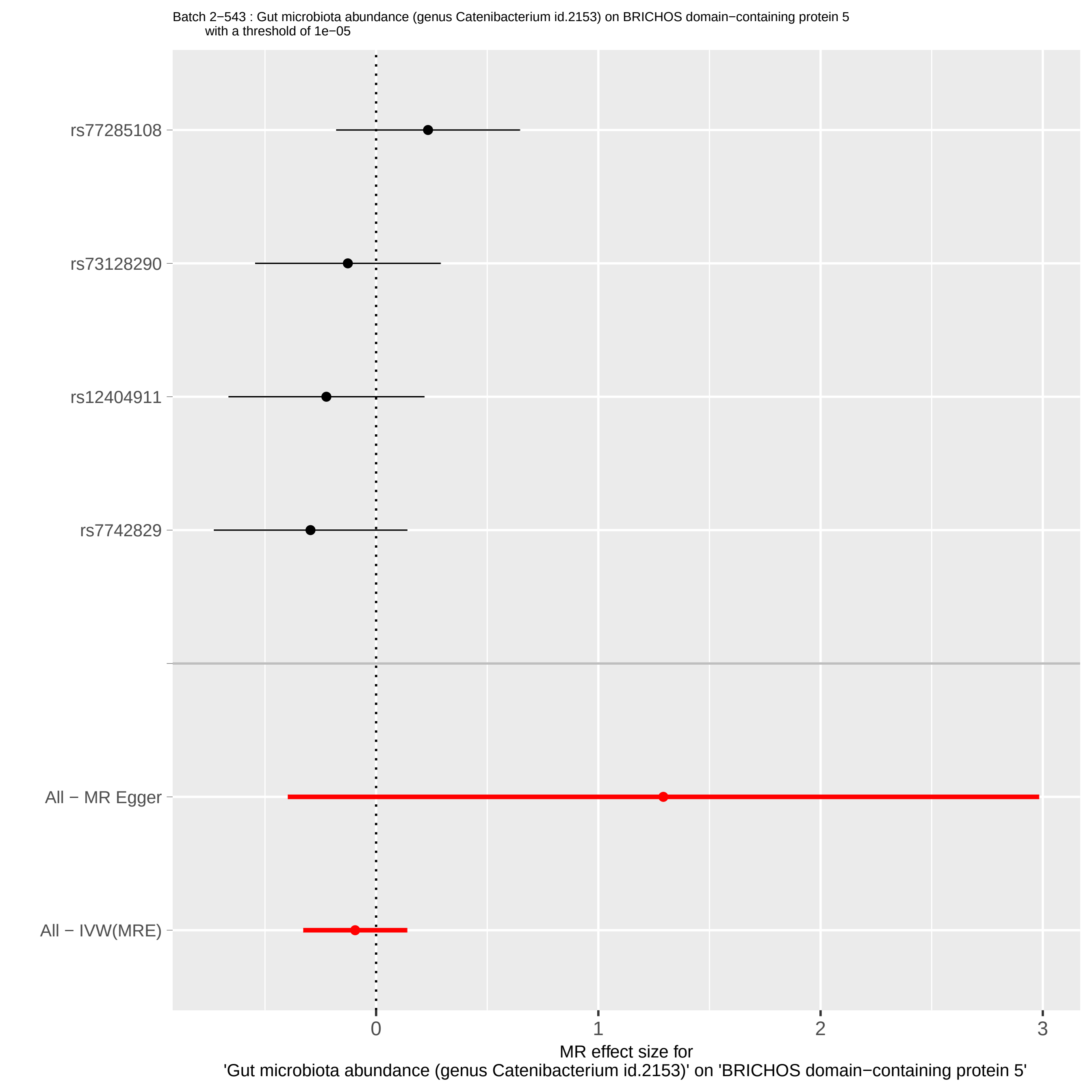

Batch 2−543 : Gut microbiota abundance (genus Catenibacterium id.2153) on BRICHOS domain−containing protein 5
with a threshold of 1e−05
rs77285108
rs73128290
rs12404911
rs7742829
All − MR Egger
All − IVW(MRE)
0
1
2
3
MR effect size for
'Gut microbiota abundance (genus Catenibacterium id.2153)' on 'BRICHOS domain−containing protein 5'

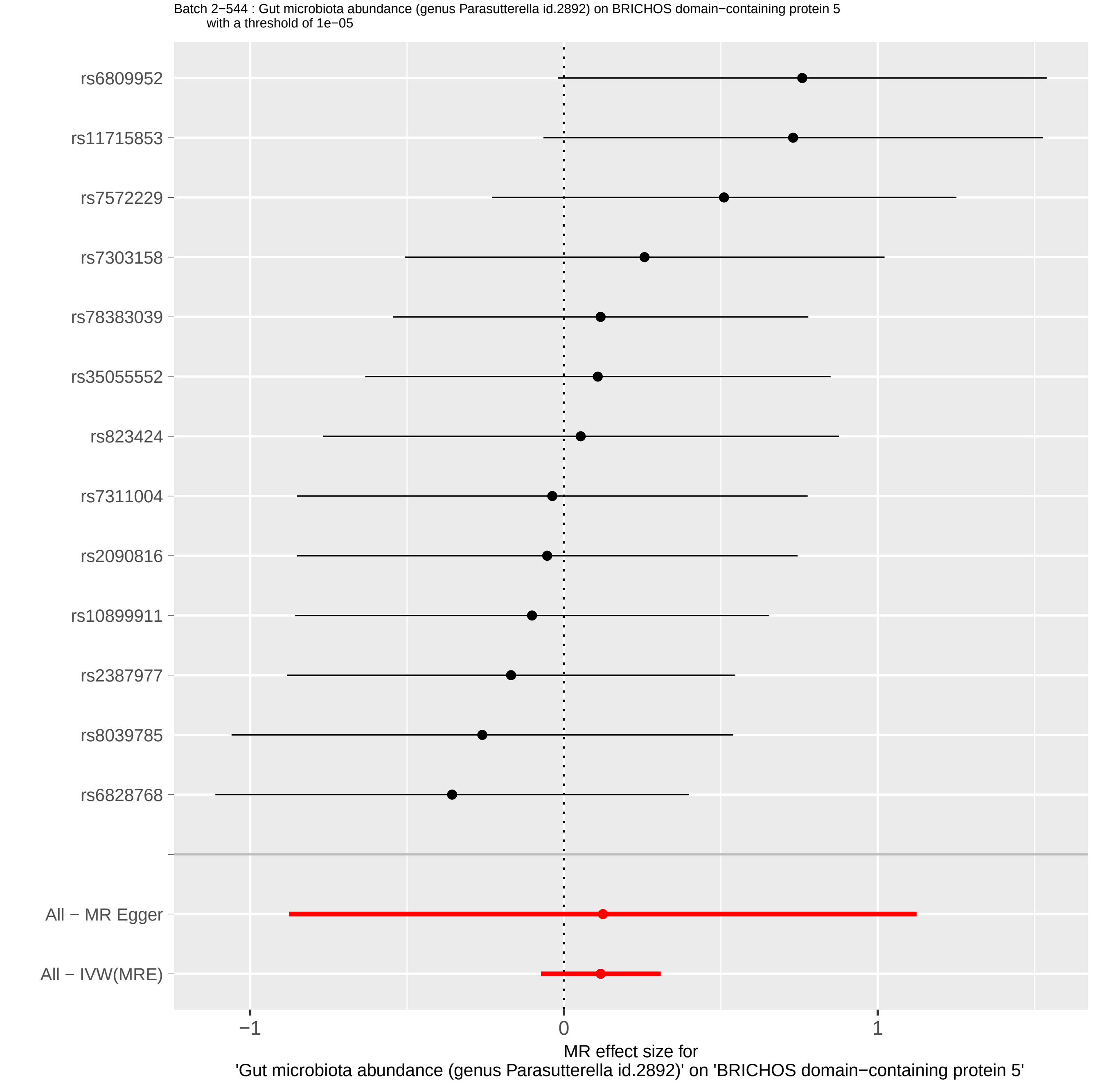

Batch 2−544 : Gut microbiota abundance (genus Parasutterella id.2892) on BRICHOS domain−containing protein 5
with a threshold of 1e−05
rs6809952
rs11715853
rs7572229
rs7303158
rs78383039
rs35055552
rs823424
rs7311004
rs2090816
rs10899911
rs2387977
rs8039785
rs6828768
All − MR Egger
All − IVW(MRE)
−1
0
1
MR effect size for
'Gut microbiota abundance (genus Parasutterella id.2892)' on 'BRICHOS domain−containing protein 5'

Batch 2−545 : Gut microbiota abundance (genus Catenibacterium id.2153) on Peregrin
with a threshold of 1e−05

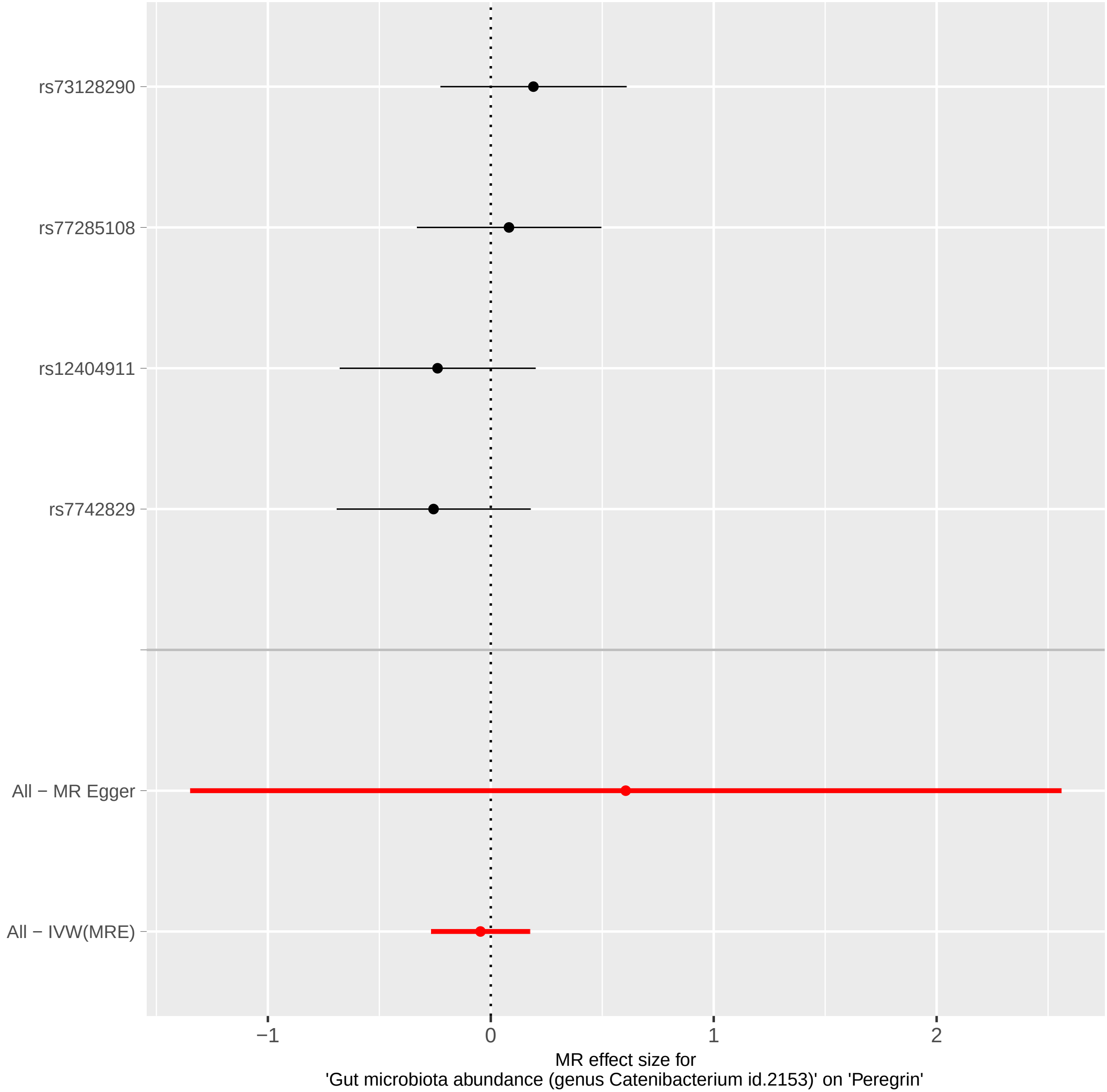

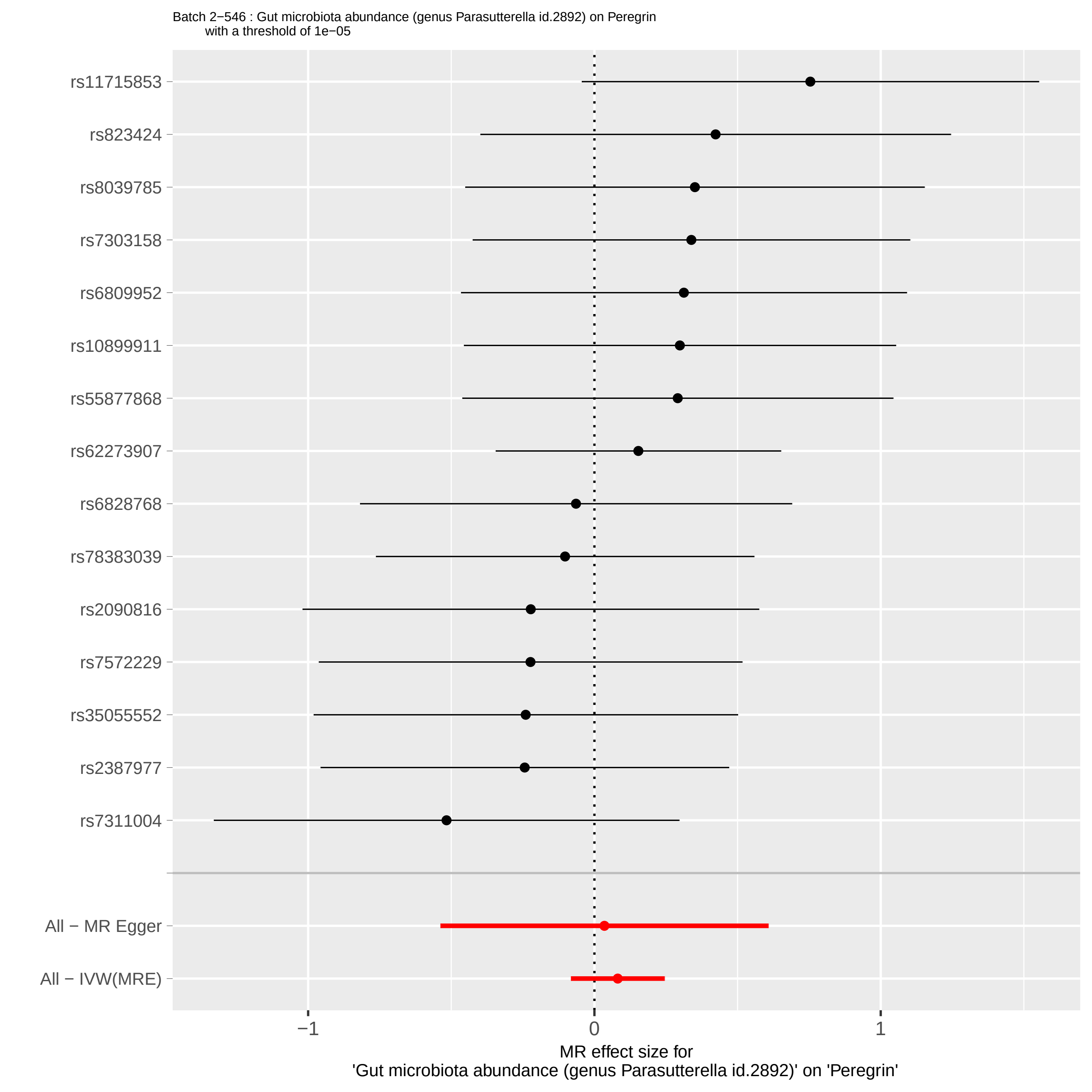

Batch 2−546 : Gut microbiota abundance (genus Parasutterella id.2892) on Peregrin
with a threshold of 1e−05
rs11715853
rs823424
rs8039785
rs7303158
rs6809952
rs10899911
rs55877868
rs62273907
rs6828768
rs78383039
rs2090816
rs7572229
rs35055552
rs2387977
rs7311004
All − MR Egger
All − IVW(MRE)
−1
0
1
MR effect size for
'Gut microbiota abundance (genus Parasutterella id.2892)' on 'Peregrin'

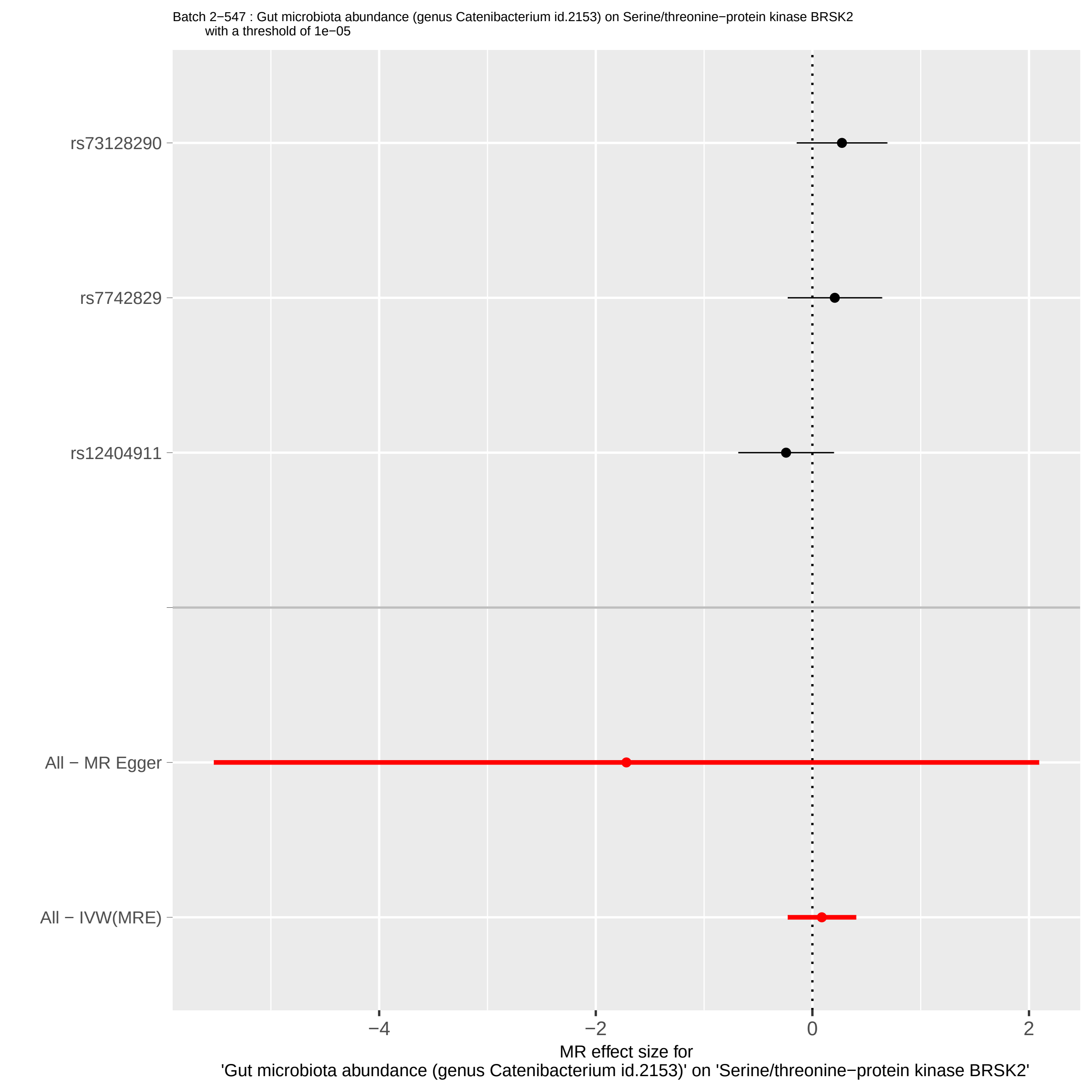

Batch 2−547 : Gut microbiota abundance (genus Catenibacterium id.2153) on Serine/threonine−protein kinase BRSK2
with a threshold of 1e−05
rs73128290
rs7742829
rs12404911
All − MR Egger
All − IVW(MRE)
−4
−2
0
2
MR effect size for
'Gut microbiota abundance (genus Catenibacterium id.2153)' on 'Serine/threonine−protein kinase BRSK2'

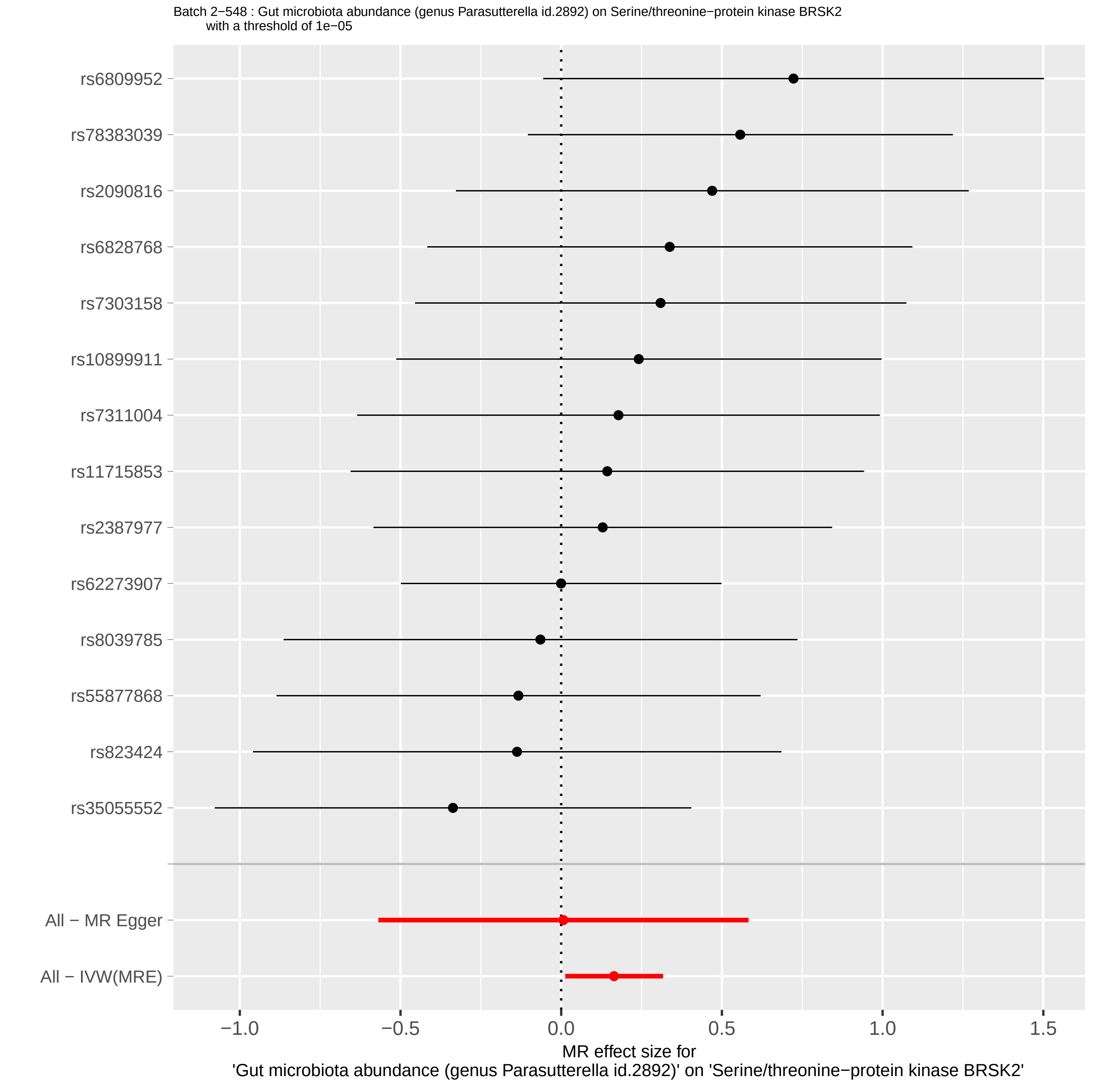

Batch 2−548 : Gut microbiota abundance (genus Parasutterella id.2892) on Serine/threonine−protein kinase BRSK2
with a threshold of 1e−05
rs6809952
rs78383039
rs2090816
rs6828768
rs7303158
rs10899911
rs7311004
rs11715853
rs2387977
rs62273907
rs8039785
rs55877868
rs823424
rs35055552
All − MR Egger
All − IVW(MRE)
−1.0
−0.5
0.0
0.5
1.0
1.5
MR effect size for
'Gut microbiota abundance (genus Parasutterella id.2892)' on 'Serine/threonine−protein kinase BRSK2'

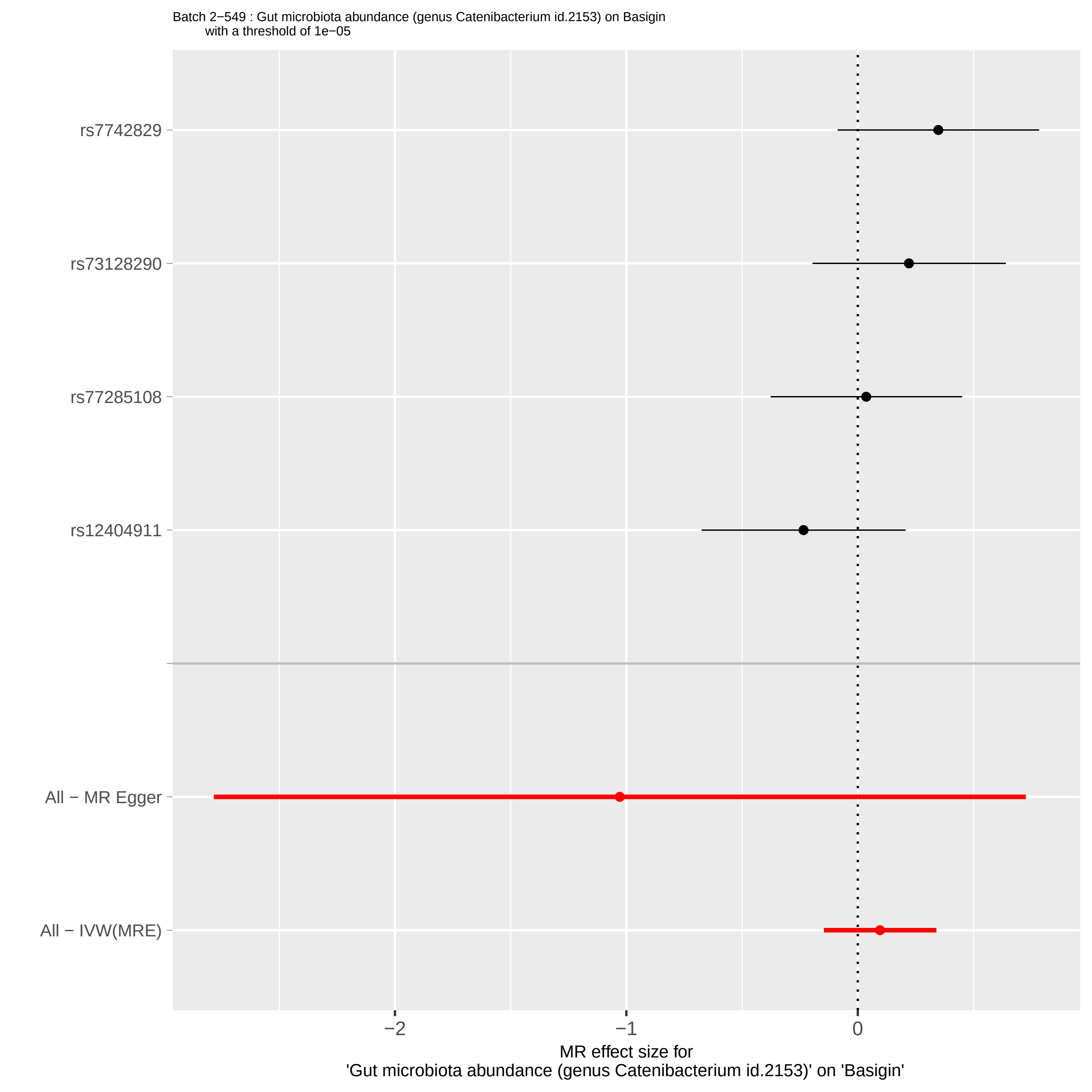
Batch 2−549 : Gut microbiota abundance (genus Catenibacterium id.2153) on Basigin
with a threshold of 1e−05
rs7742829
rs73128290
rs77285108
rs12404911
All − MR Egger
All − IVW(MRE)
−2
−1
0
MR effect size for
'Gut microbiota abundance (genus Catenibacterium id.2153)' on 'Basigin'

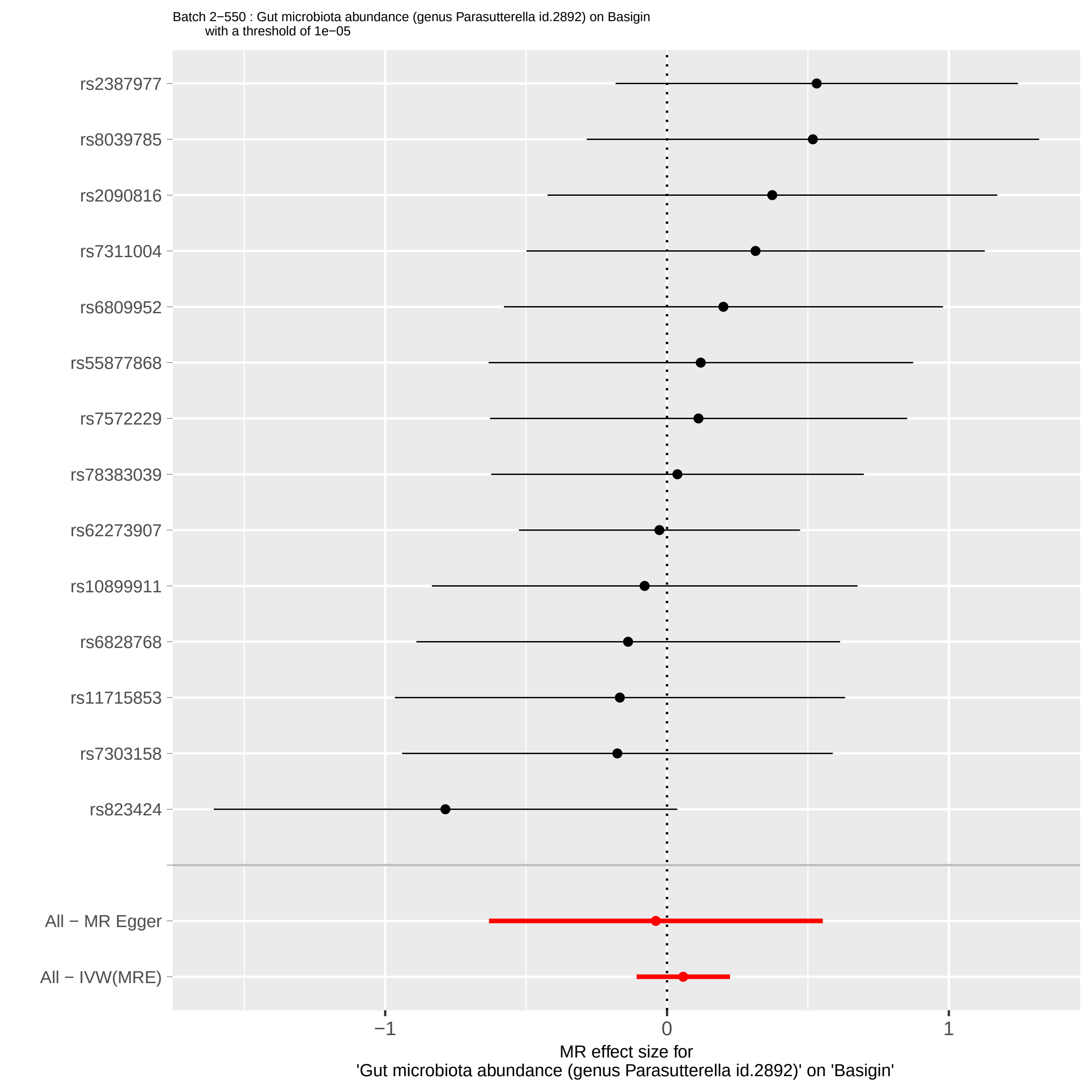

Batch 2−550 : Gut microbiota abundance (genus Parasutterella id.2892) on Basigin
with a threshold of 1e−05
rs2387977
rs8039785
rs2090816
rs7311004
rs6809952
rs55877868
rs7572229
rs78383039
rs62273907
rs10899911
rs6828768
rs11715853
rs7303158
rs823424
All − MR Egger
All − IVW(MRE)
−1
0
1
MR effect size for
'Gut microbiota abundance (genus Parasutterella id.2892)' on 'Basigin'

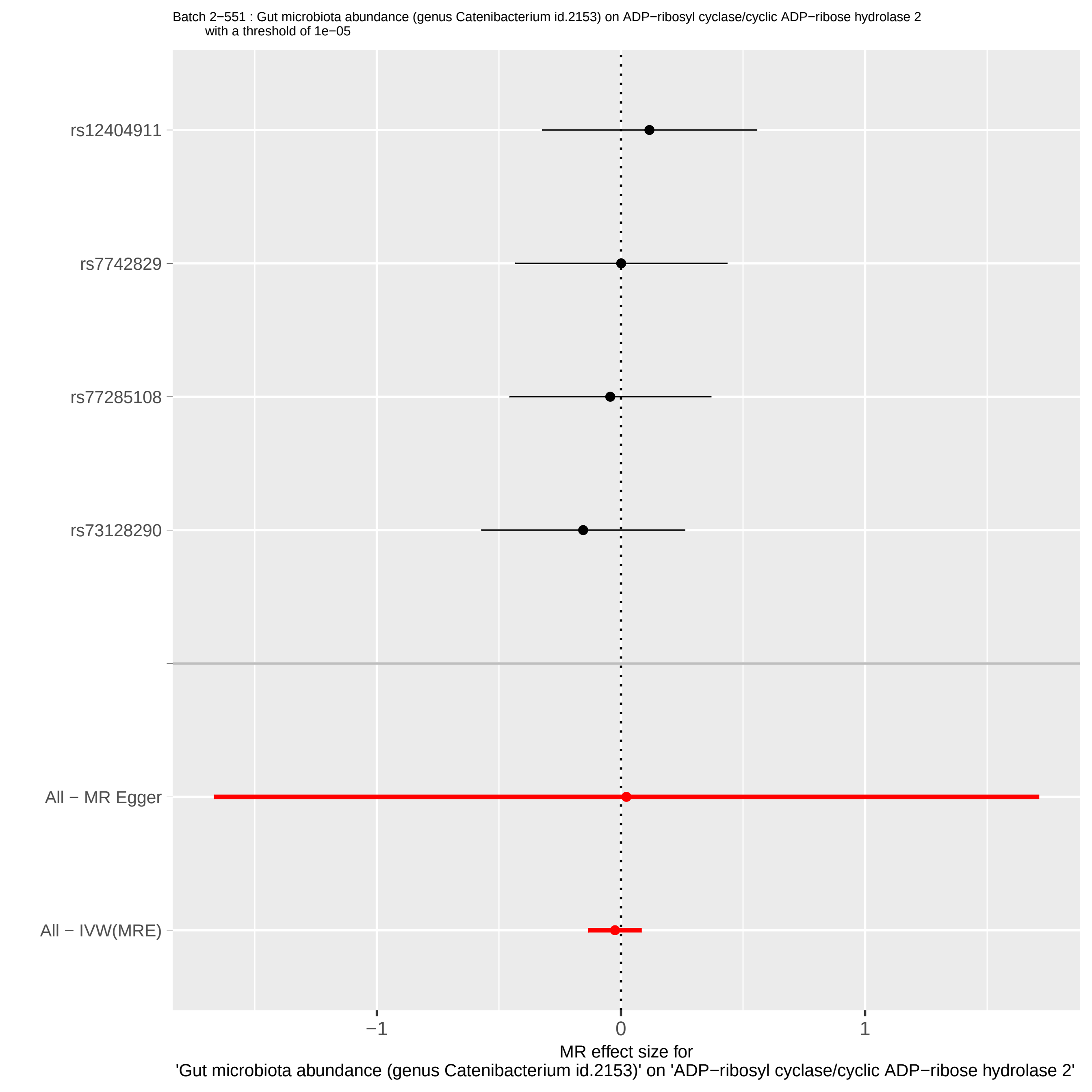

Batch 2−551 : Gut microbiota abundance (genus Catenibacterium id.2153) on ADP−ribosyl cyclase/cyclic ADP−ribose hydrolase 2
with a threshold of 1e−05
rs12404911
rs7742829
rs77285108
rs73128290
All − MR Egger
All − IVW(MRE)
−1
0
1
MR effect size for
'Gut microbiota abundance (genus Catenibacterium id.2153)' on 'ADP−ribosyl cyclase/cyclic ADP−ribose hydrolase 2'

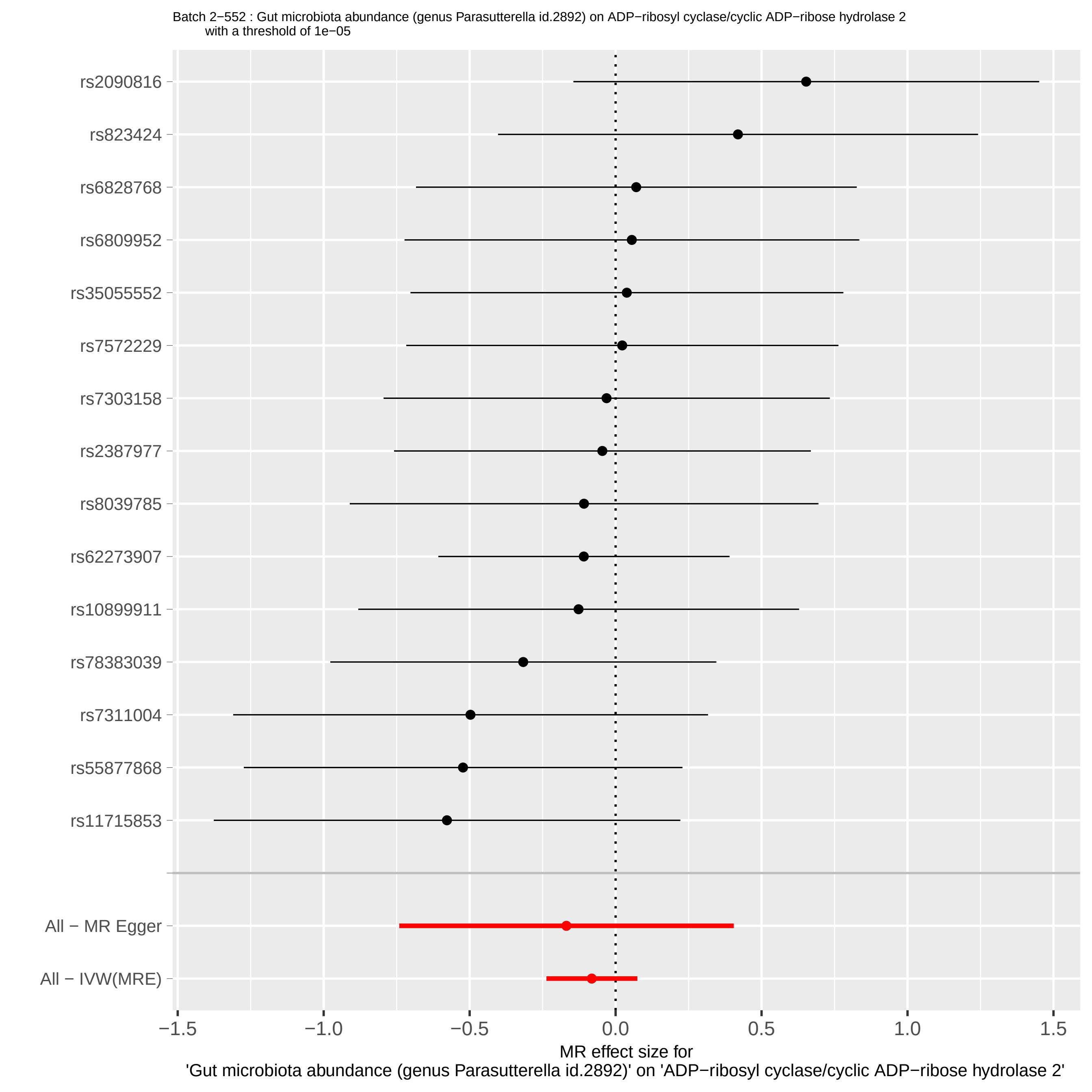

Batch 2−552 : Gut microbiota abundance (genus Parasutterella id.2892) on ADP−ribosyl cyclase/cyclic ADP−ribose hydrolase 2
with a threshold of 1e−05
rs2090816
rs823424
rs6828768
rs6809952
rs35055552
rs7572229
rs7303158
rs2387977
rs8039785
rs62273907
rs10899911
rs78383039
rs7311004
rs55877868
rs11715853
All − MR Egger
All − IVW(MRE)
−1.5
−1.0
−0.5
0.0
0.5
1.0
1.5
MR effect size for
'Gut microbiota abundance (genus Parasutterella id.2892)' on 'ADP−ribosyl cyclase/cyclic ADP−ribose hydrolase 2'

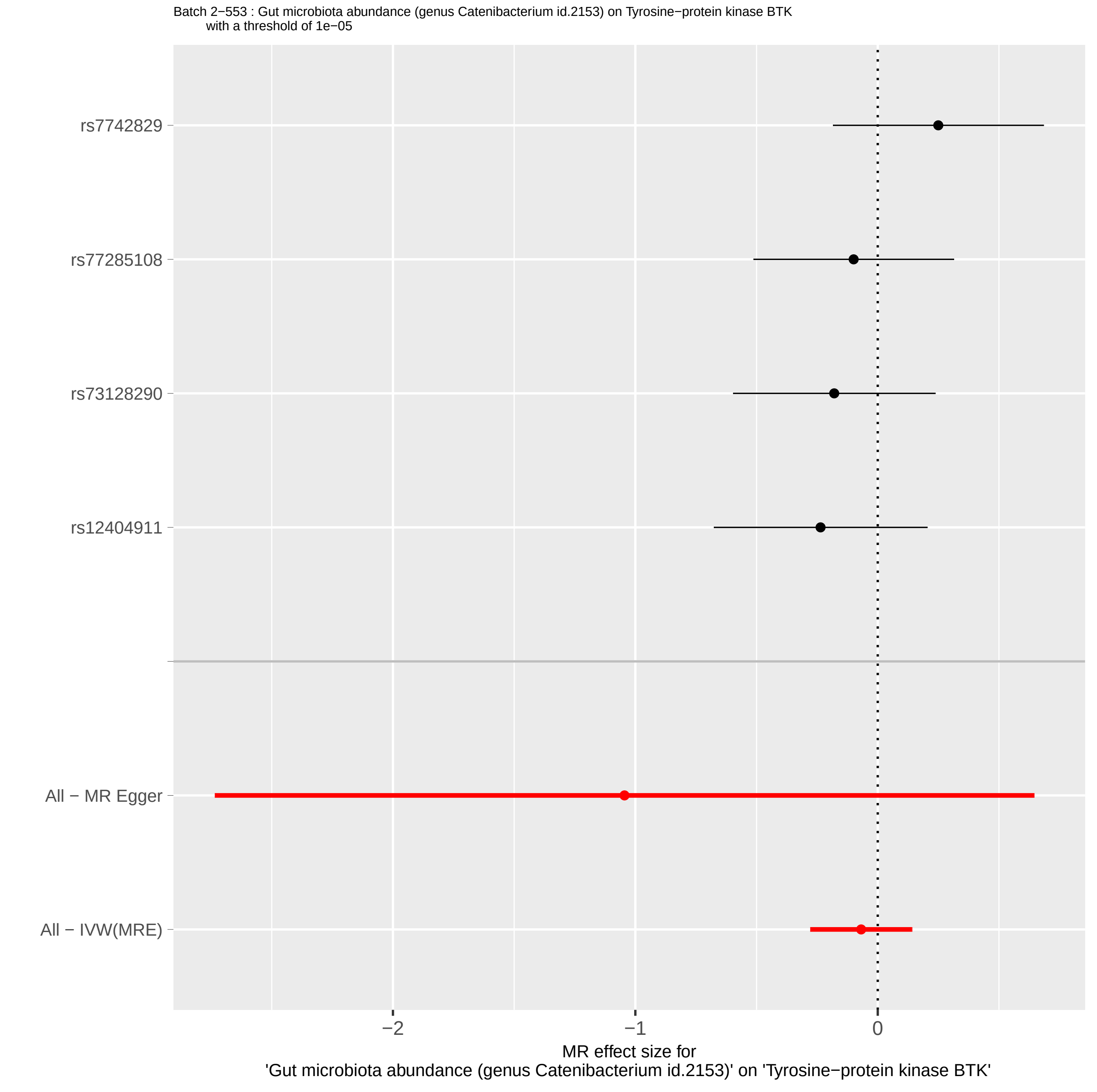
Batch 2−553 : Gut microbiota abundance (genus Catenibacterium id.2153) on Tyrosine−protein kinase BTK
with a threshold of 1e−05
rs7742829
rs77285108
rs73128290
rs12404911
All − MR Egger
All − IVW(MRE)
−2
−1
0
MR effect size for
'Gut microbiota abundance (genus Catenibacterium id.2153)' on 'Tyrosine−protein kinase BTK'

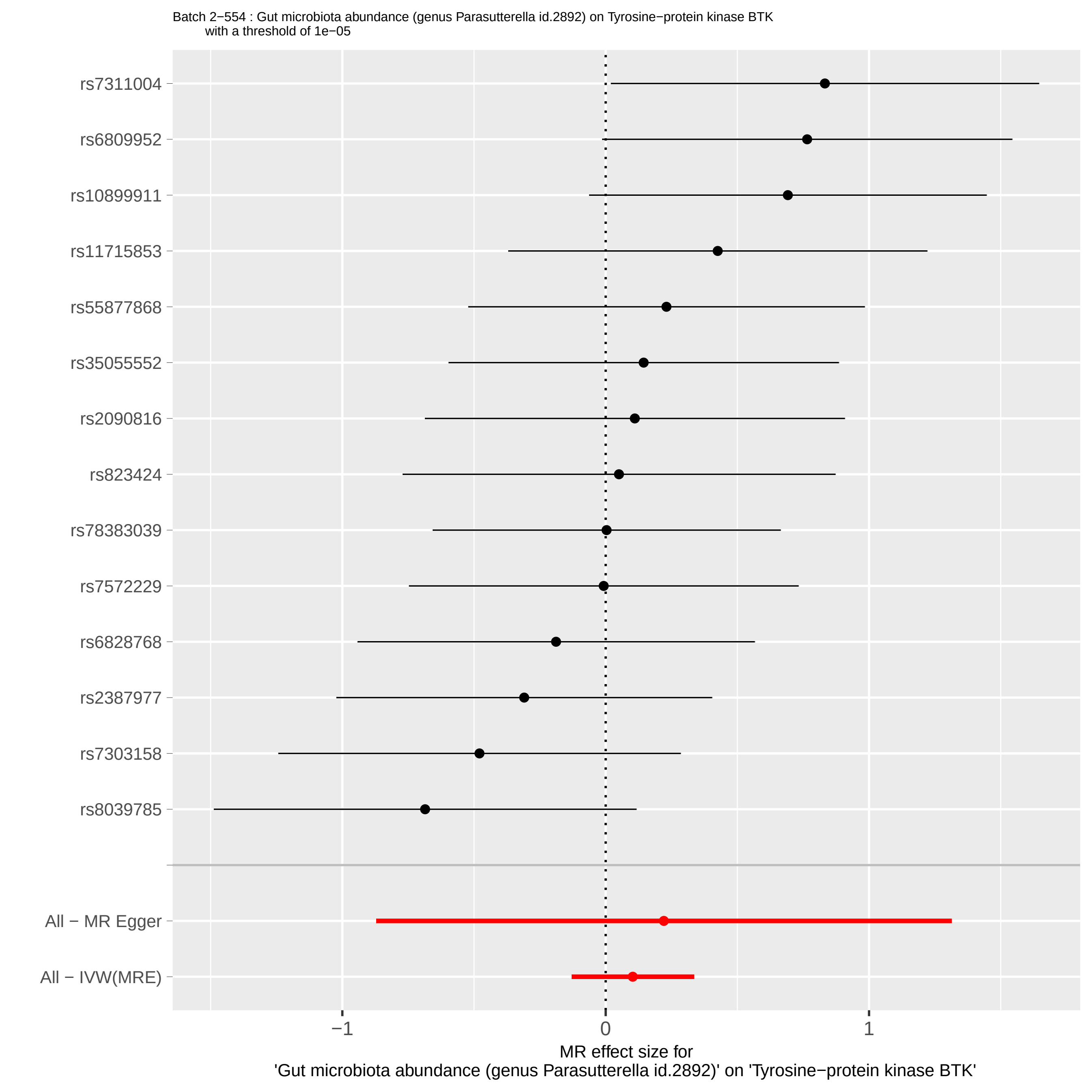

Batch 2−554 : Gut microbiota abundance (genus Parasutterella id.2892) on Tyrosine−protein kinase BTK
with a threshold of 1e−05
rs7311004
rs6809952
rs10899911
rs11715853
rs55877868
rs35055552
rs2090816
rs823424
rs78383039
rs7572229
rs6828768
rs2387977
rs7303158
rs8039785
All − MR Egger
All − IVW(MRE)
−1
0
1
MR effect size for
'Gut microbiota abundance (genus Parasutterella id.2892)' on 'Tyrosine−protein kinase BTK'

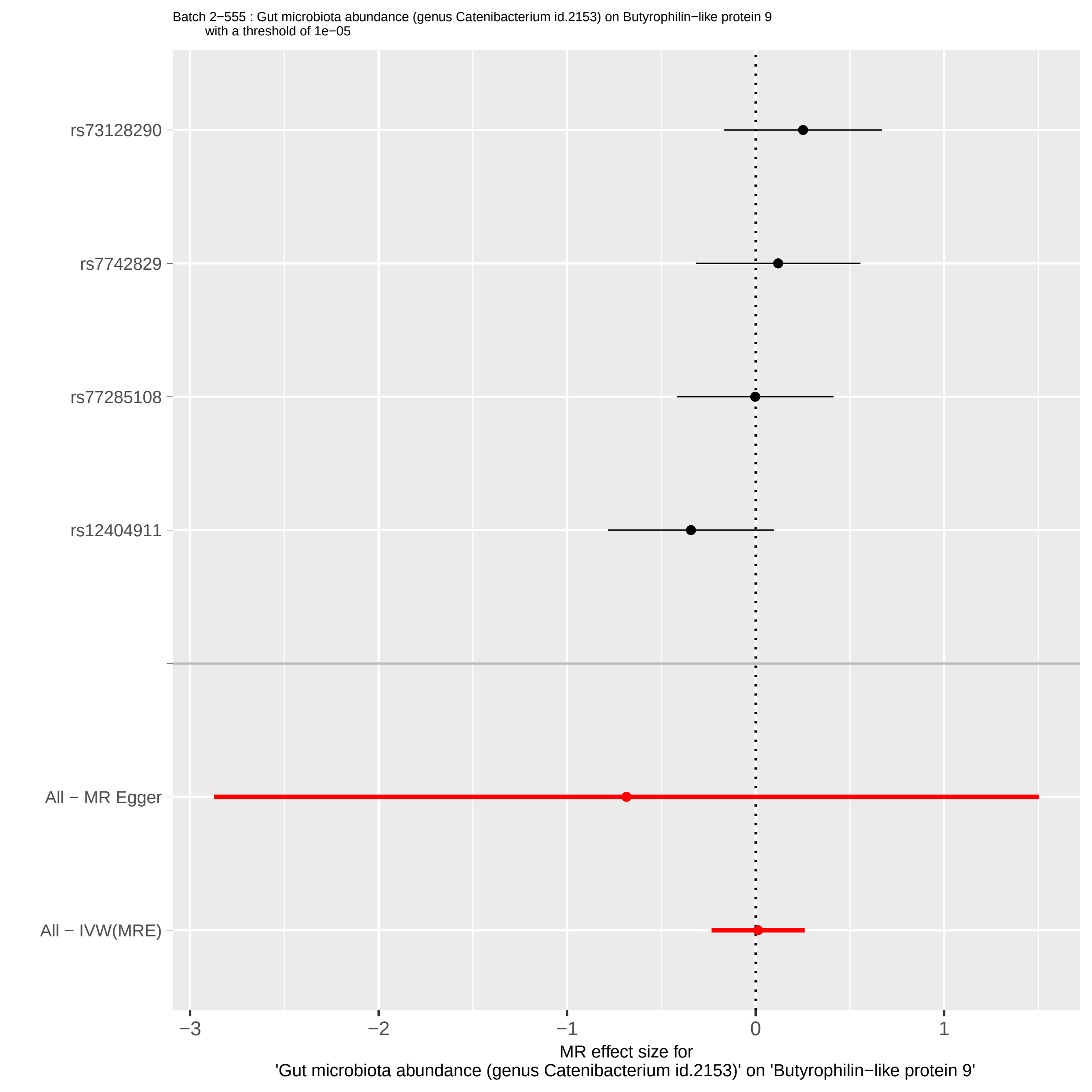
Batch 2−555 : Gut microbiota abundance (genus Catenibacterium id.2153) on Butyrophilin−like protein 9
with a threshold of 1e−05
rs73128290
rs7742829
rs77285108
rs12404911
All − MR Egger
All − IVW(MRE)
−3
−2
−1
0
1
MR effect size for
'Gut microbiota abundance (genus Catenibacterium id.2153)' on 'Butyrophilin−like protein 9'

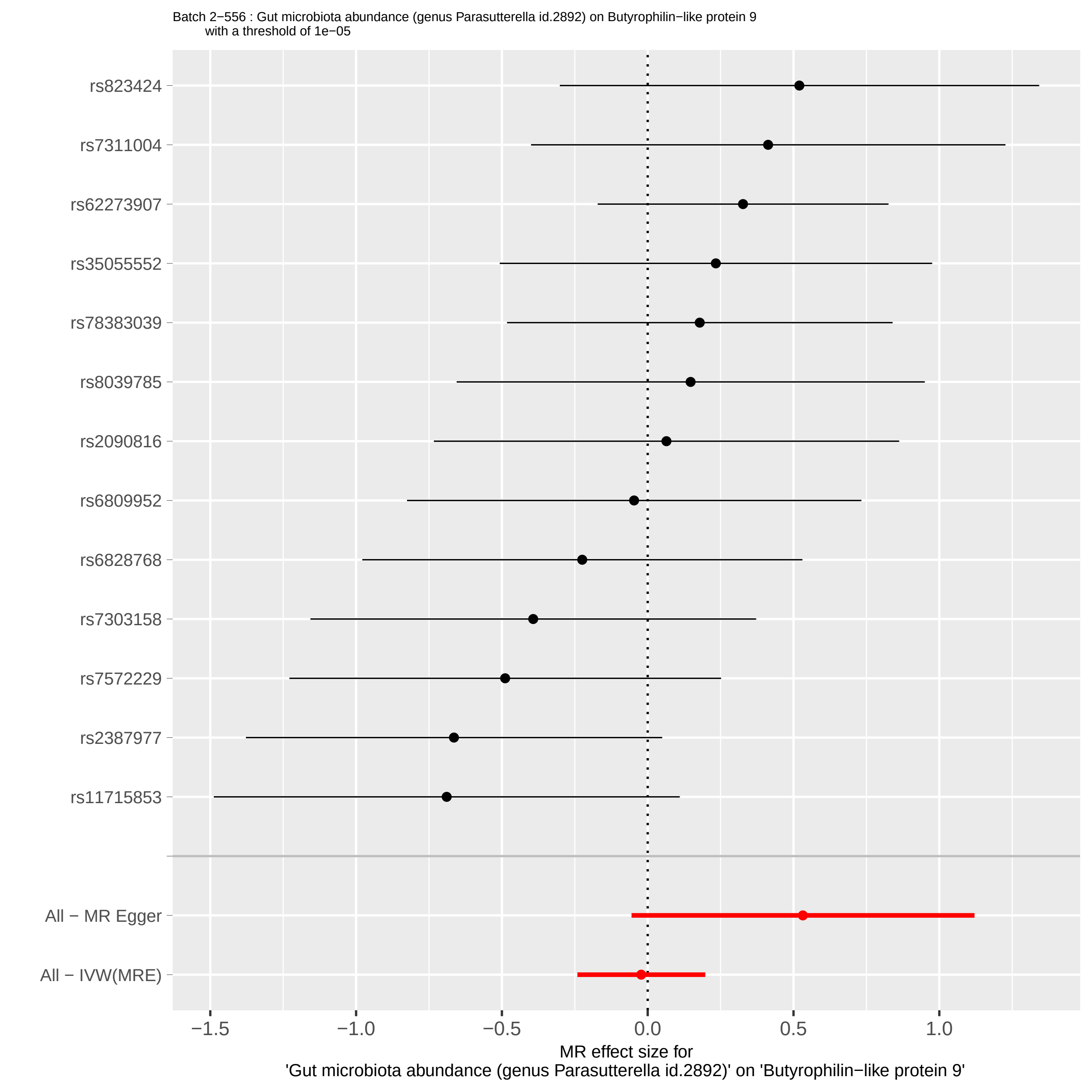

Batch 2−556 : Gut microbiota abundance (genus Parasutterella id.2892) on Butyrophilin−like protein 9
with a threshold of 1e−05
rs823424
rs7311004
rs62273907
rs35055552
rs78383039
rs8039785
rs2090816
rs6809952
rs6828768
rs7303158
rs7572229
rs2387977
rs11715853
All − MR Egger
All − IVW(MRE)
−1.5
−1.0
−0.5
0.0
0.5
1.0
MR effect size for
'Gut microbiota abundance (genus Parasutterella id.2892)' on 'Butyrophilin−like protein 9'

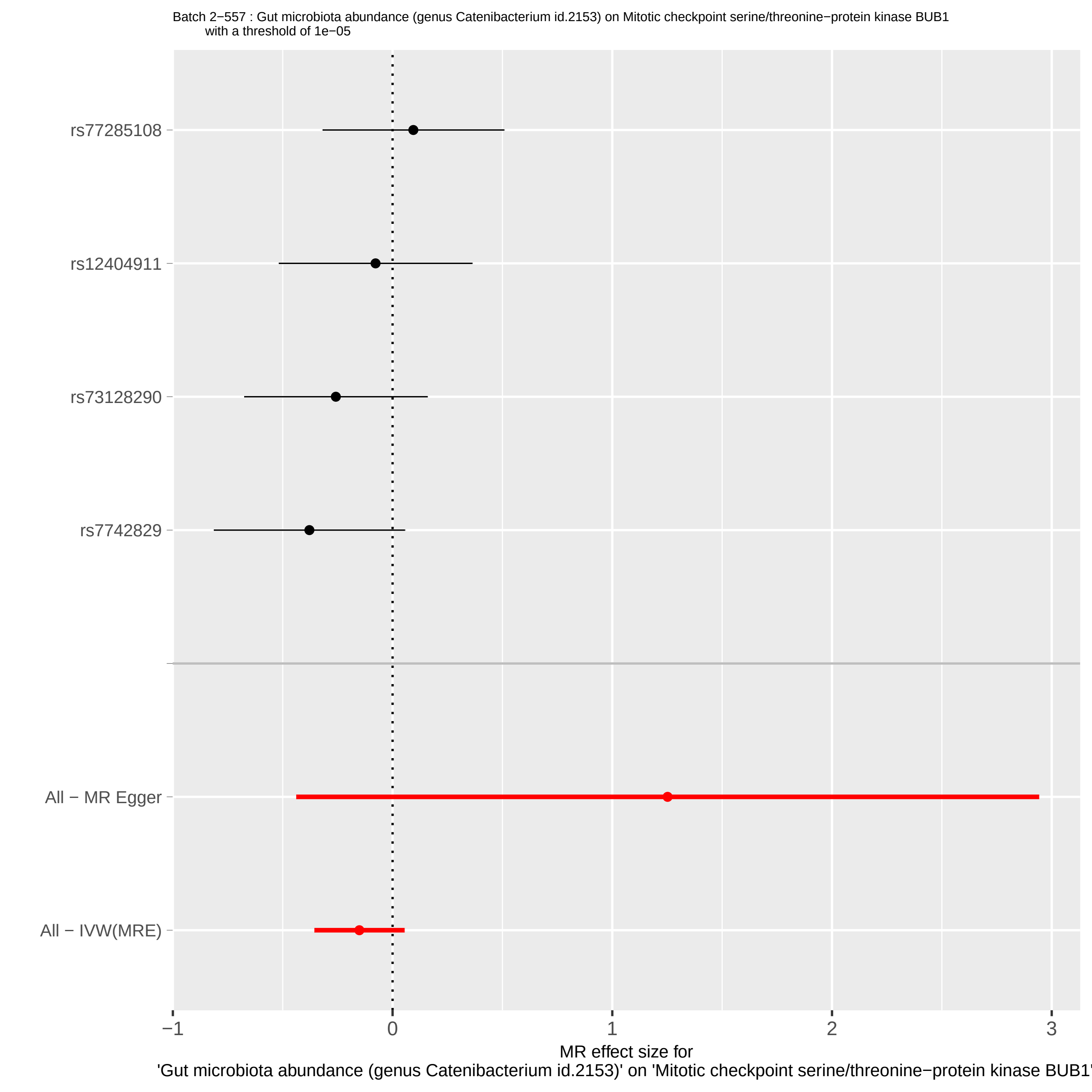
Batch 2−557 : Gut microbiota abundance (genus Catenibacterium id.2153) on Mitotic checkpoint serine/threonine−protein kinase BUB1 with a threshold of 1e−05
rs77285108
rs12404911
rs73128290
rs7742829
All − MR Egger
All − IVW(MRE)
−1
0
1
2
3
MR effect size for
'Gut microbiota abundance (genus Catenibacterium id.2153)' on 'Mitotic checkpoint serine/threonine−protein kinase BUB1'

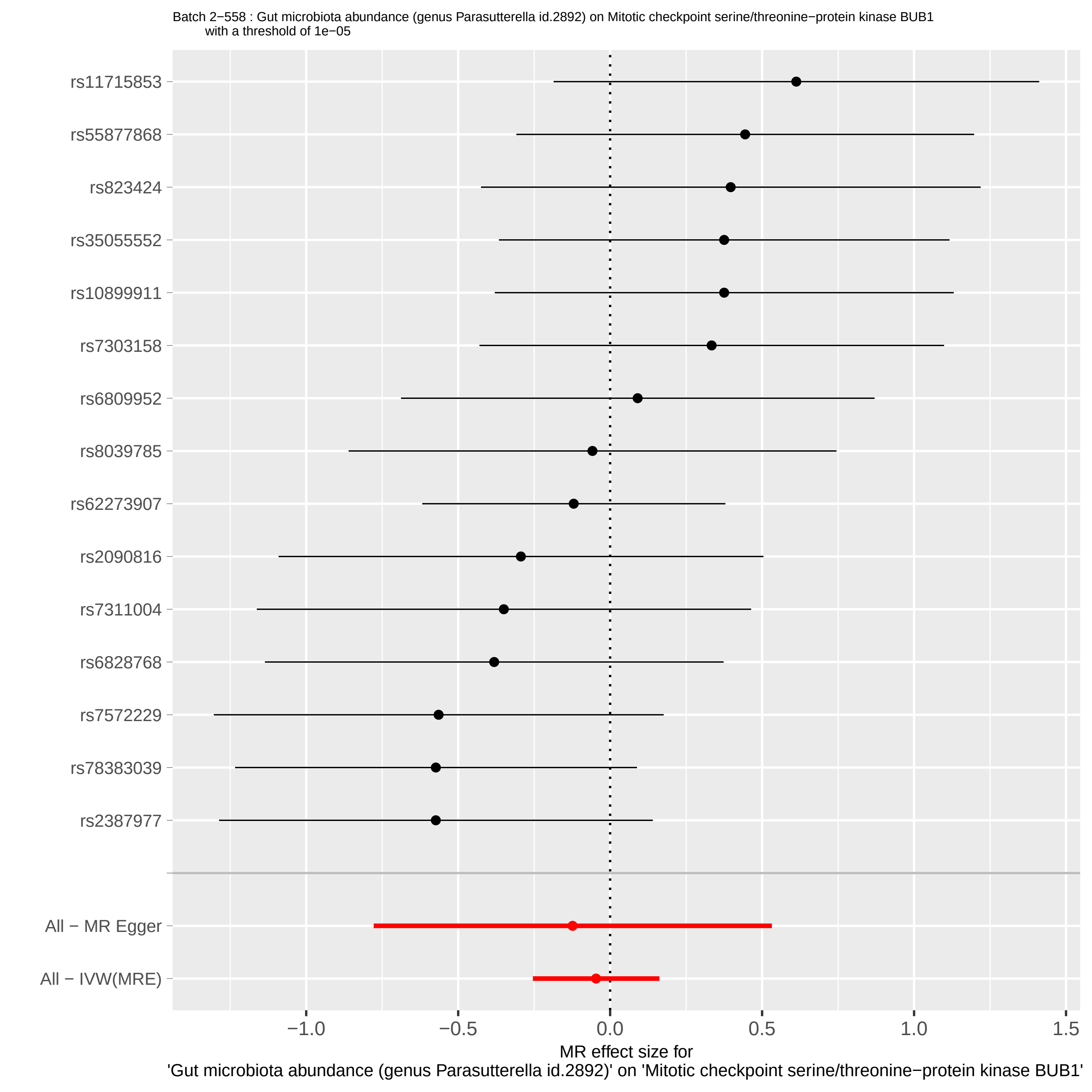

Batch 2−558 : Gut microbiota abundance (genus Parasutterella id.2892) on Mitotic checkpoint serine/threonine−protein kinase BUB1
with a threshold of 1e−05
rs11715853
rs55877868
rs823424
rs35055552
rs10899911
rs7303158
rs6809952
rs8039785
rs62273907
rs2090816
rs7311004
rs6828768
rs7572229
rs78383039
rs2387977
All − MR Egger
All − IVW(MRE)
−1.0
−0.5
0.0
0.5
1.0
1.5
MR effect size for
'Gut microbiota abundance (genus Parasutterella id.2892)' on 'Mitotic checkpoint serine/threonine−protein kinase BUB1'

Batch 2−559 : Gut microbiota abundance (genus Catenibacterium id.2153) on Uncharacterized protein C10orf35
with a threshold of 1e−05

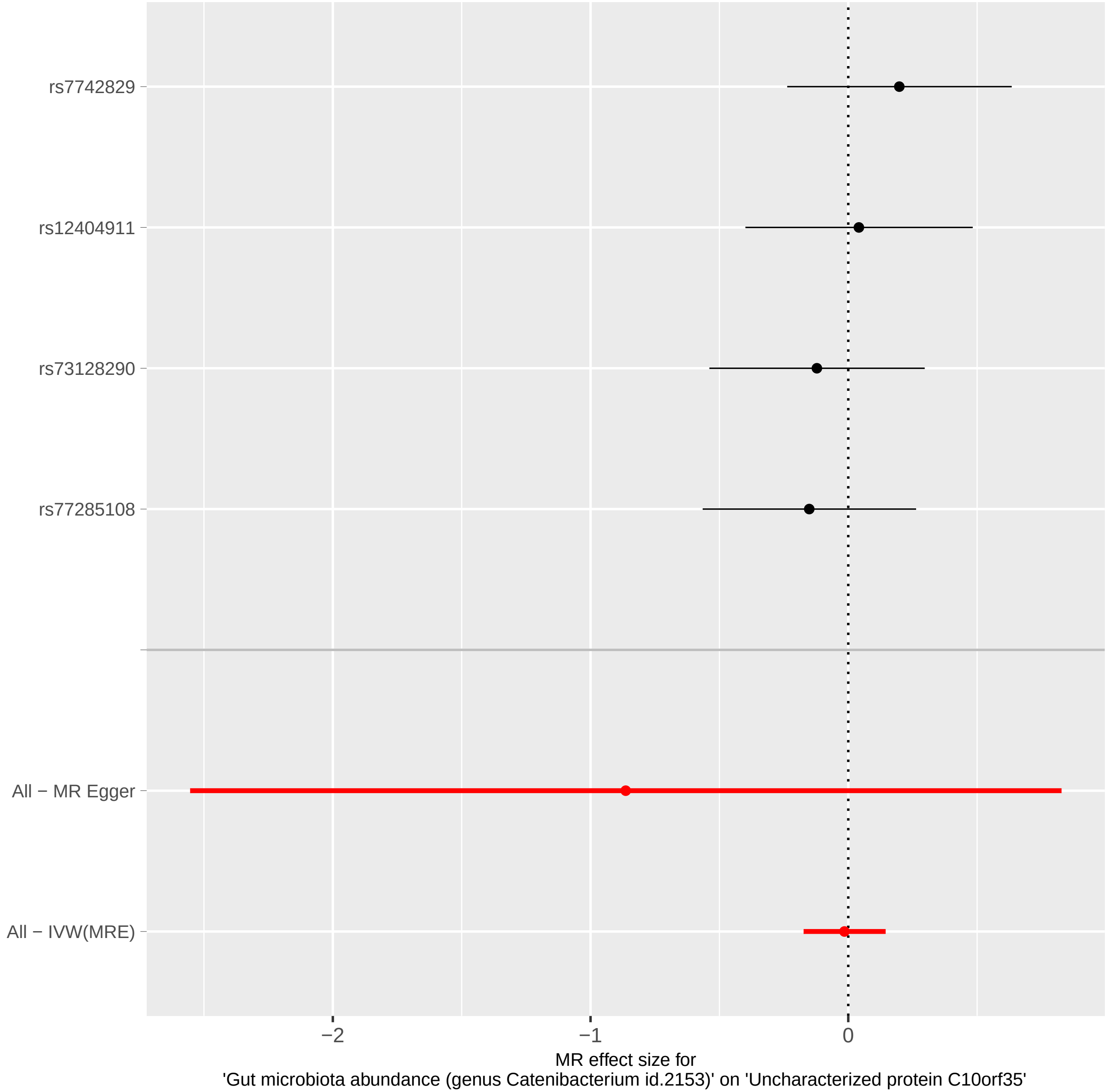

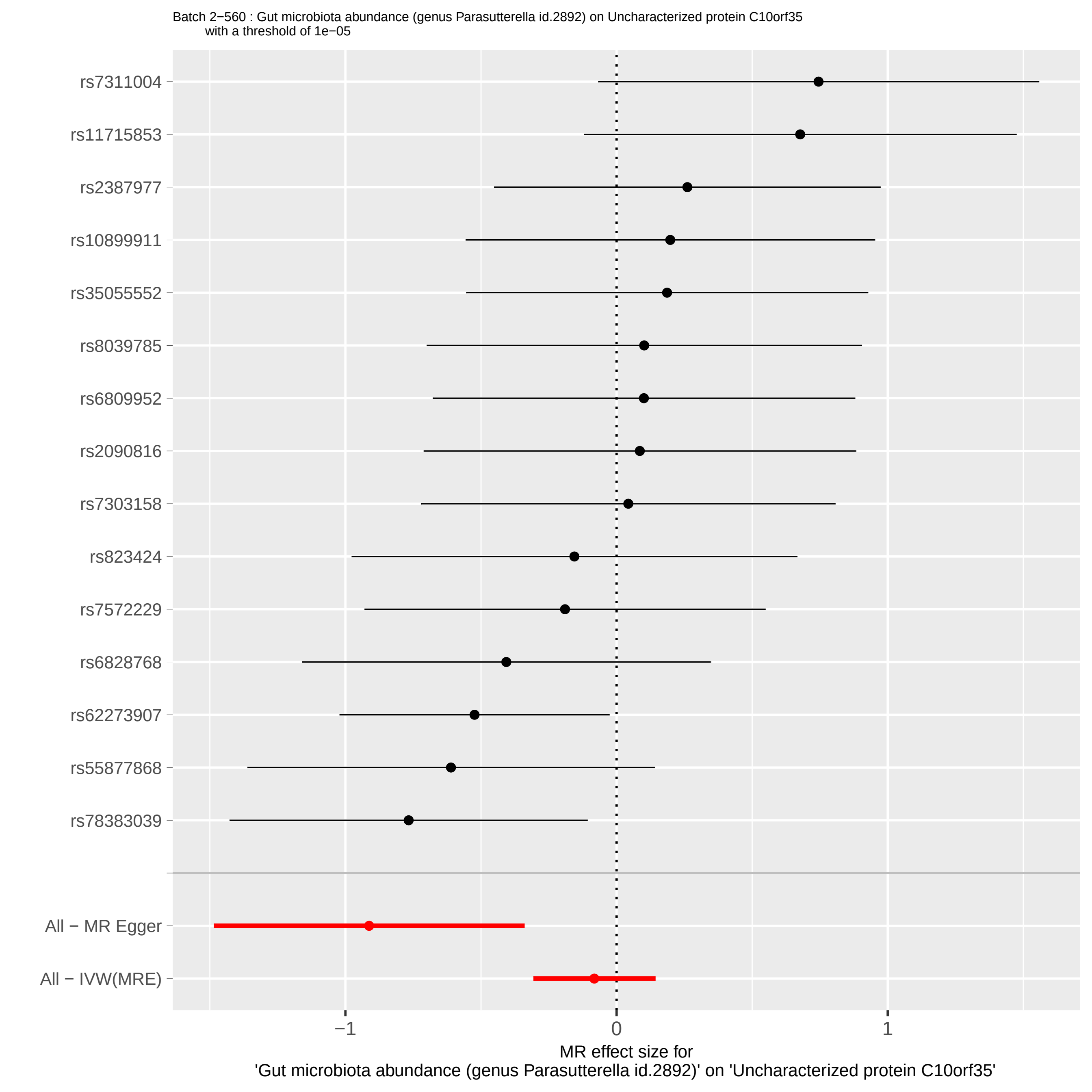

Batch 2−560 : Gut microbiota abundance (genus Parasutterella id.2892) on Uncharacterized protein C10orf35
with a threshold of 1e−05
rs7311004
rs11715853
rs2387977
rs10899911
rs35055552
rs8039785
rs6809952
rs2090816
rs7303158
rs823424
rs7572229
rs6828768
rs62273907
rs55877868
rs78383039
All − MR Egger
All − IVW(MRE)
−1
0
1
MR effect size for
'Gut microbiota abundance (genus Parasutterella id.2892)' on 'Uncharacterized protein C10orf35'

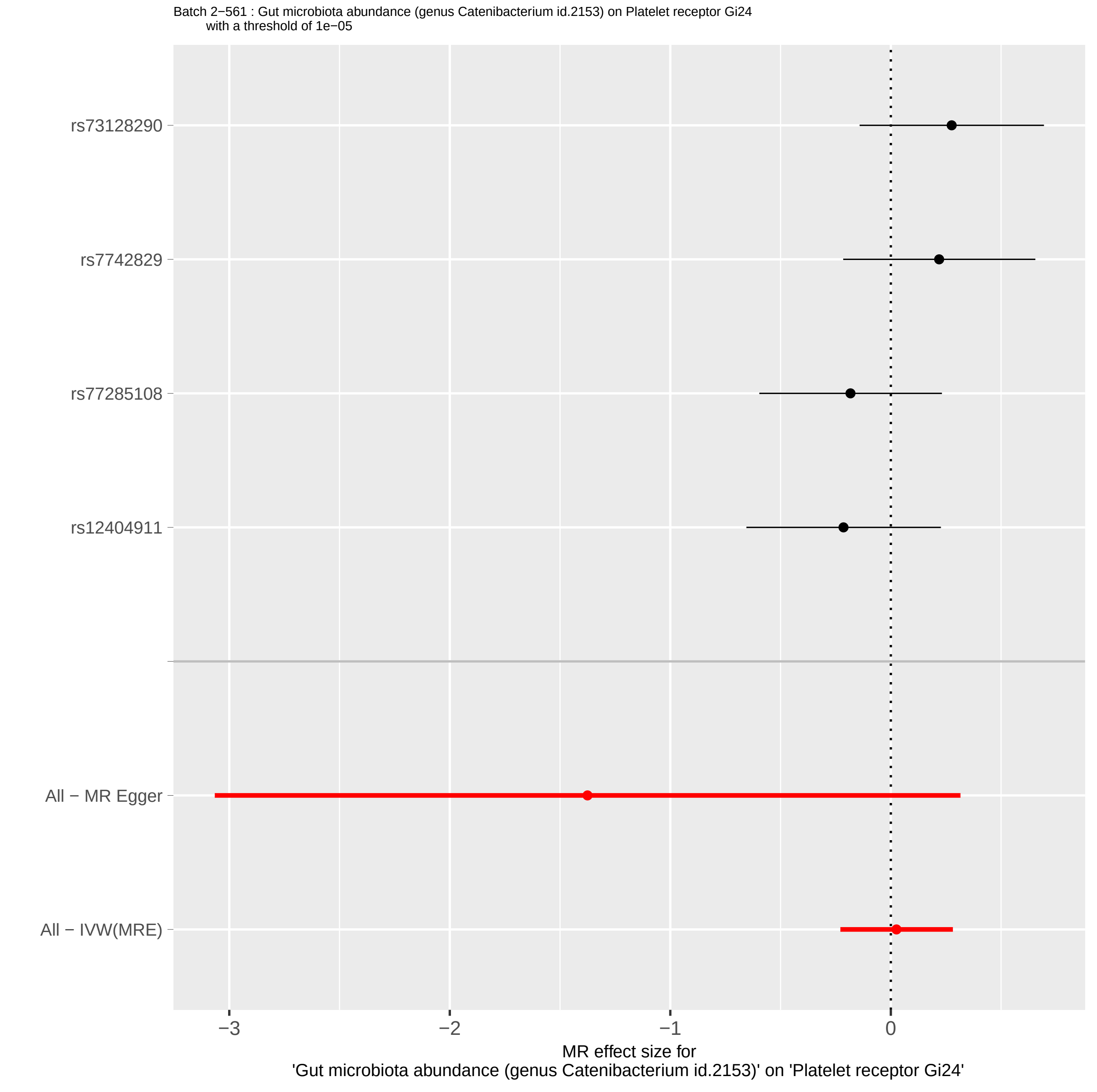

Batch 2−561 : Gut microbiota abundance (genus Catenibacterium id.2153) on Platelet receptor Gi24
with a threshold of 1e−05
rs73128290
rs7742829
rs77285108
rs12404911
All − MR Egger
All − IVW(MRE)
−3
−2
−1
0
MR effect size for
'Gut microbiota abundance (genus Catenibacterium id.2153)' on 'Platelet receptor Gi24'

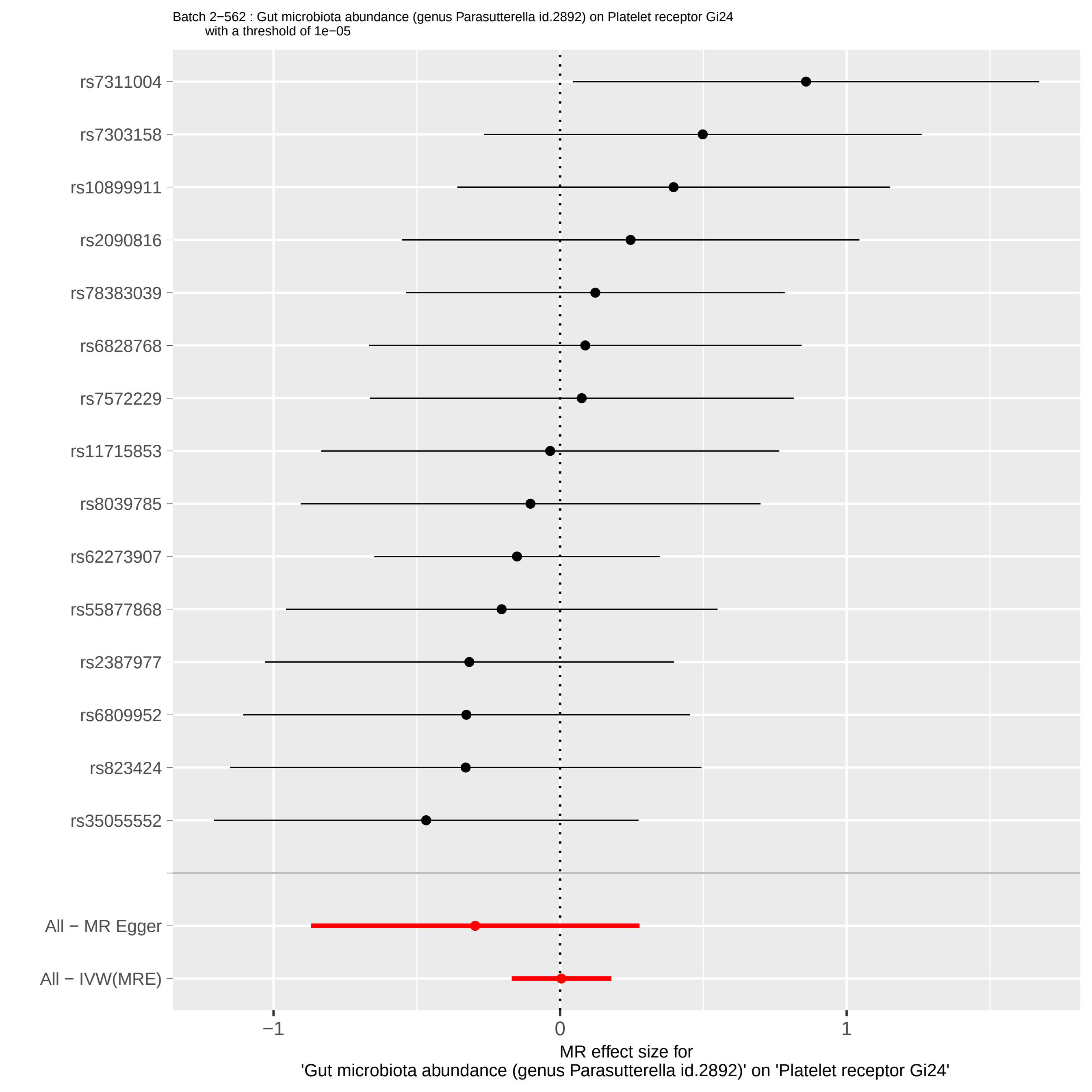

Batch 2−562 : Gut microbiota abundance (genus Parasutterella id.2892) on Platelet receptor Gi24
with a threshold of 1e−05
rs7311004
rs7303158
rs10899911
rs2090816
rs78383039
rs6828768
rs7572229
rs11715853
rs8039785
rs62273907
rs55877868
rs2387977
rs6809952
rs823424
rs35055552
All − MR Egger
All − IVW(MRE)
−1
0
1
MR effect size for
'Gut microbiota abundance (genus Parasutterella id.2892)' on 'Platelet receptor Gi24'

Batch 2−563 : Gut microbiota abundance (genus Catenibacterium id.2153) on UPF0696 protein C11orf68
with a threshold of 1e−05

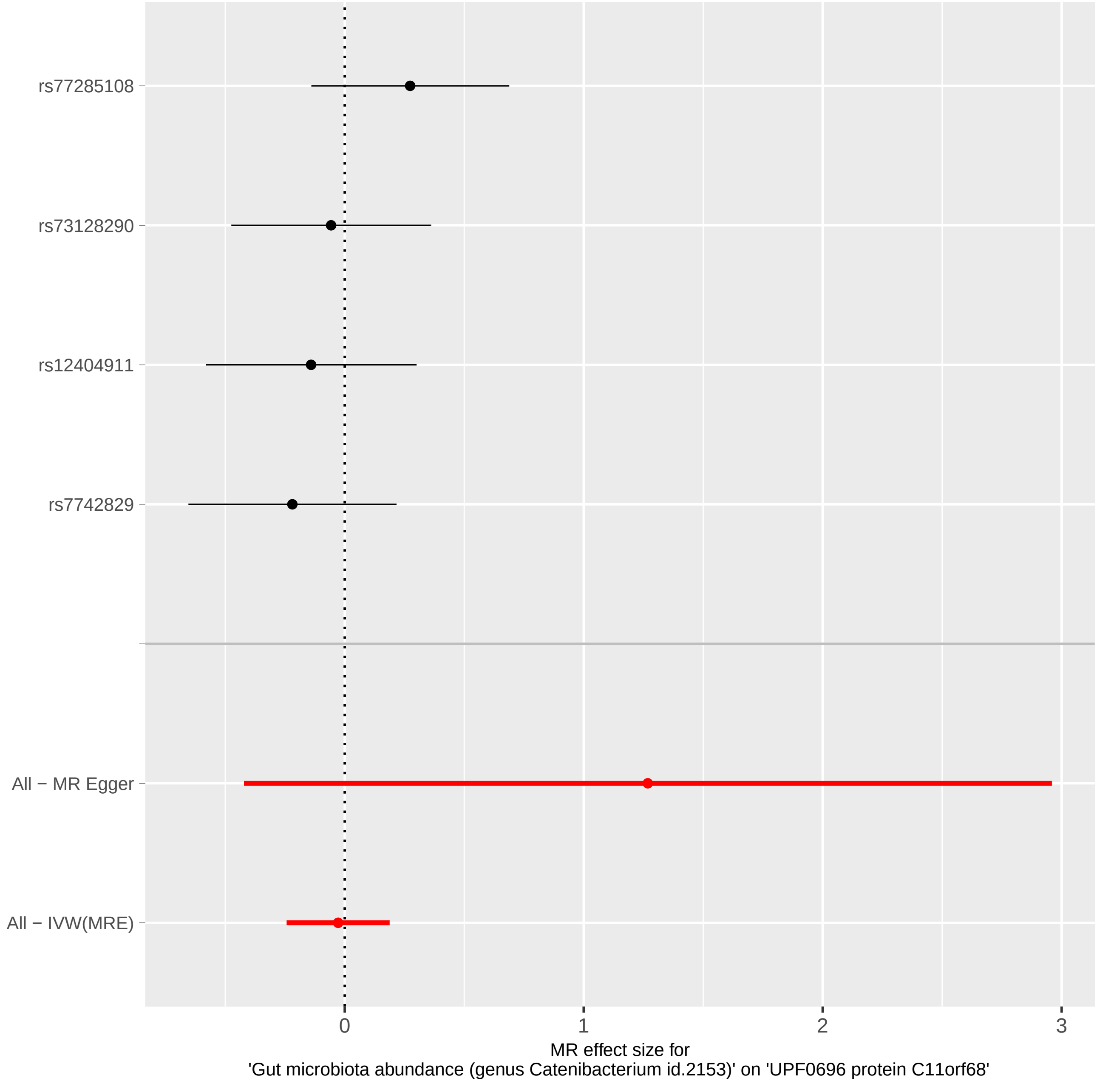

Batch 2−564 : Gut microbiota abundance (genus Parasutterella id.2892) on UPF0696 protein C11orf68
with a threshold of 1e−05

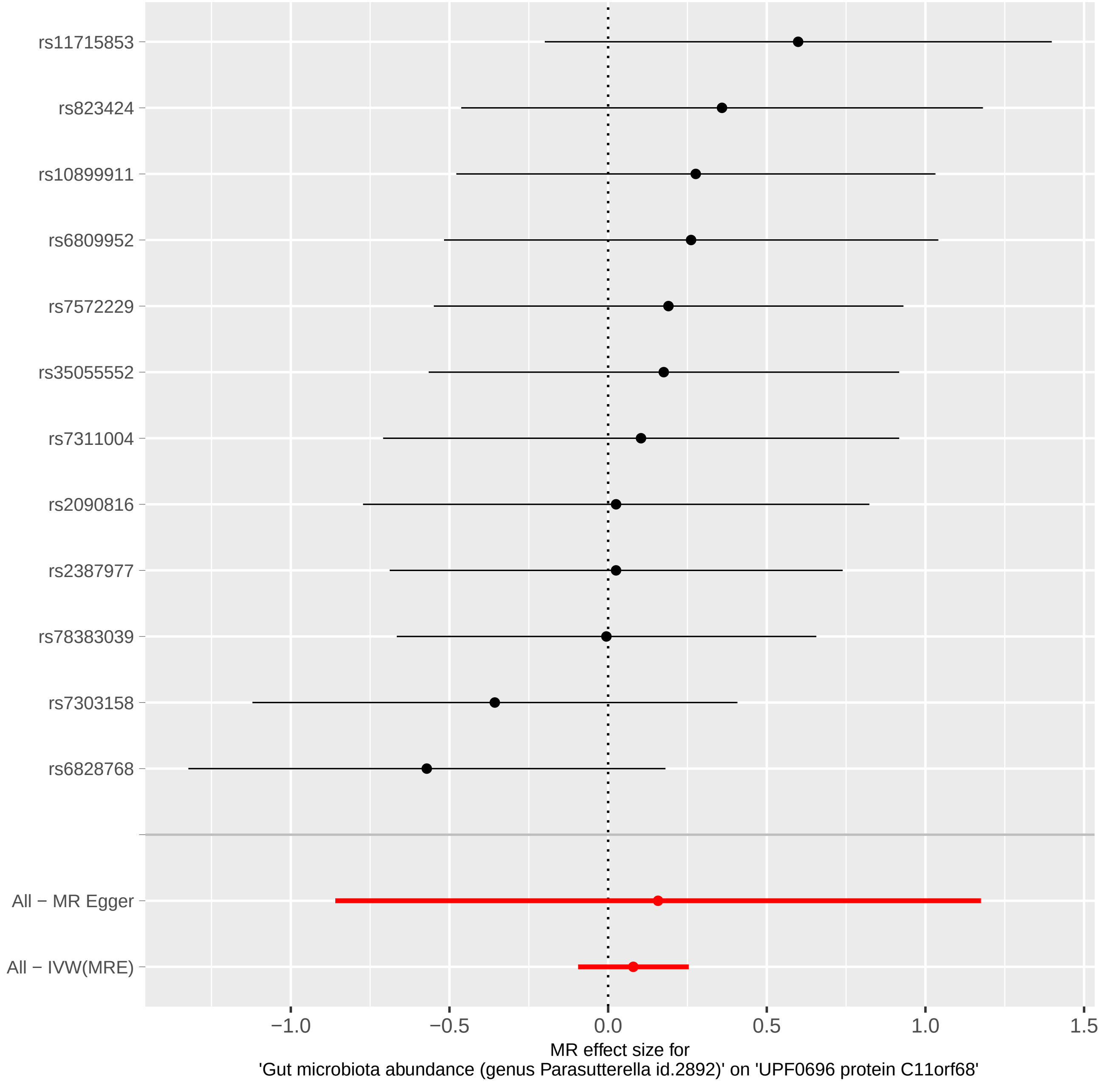

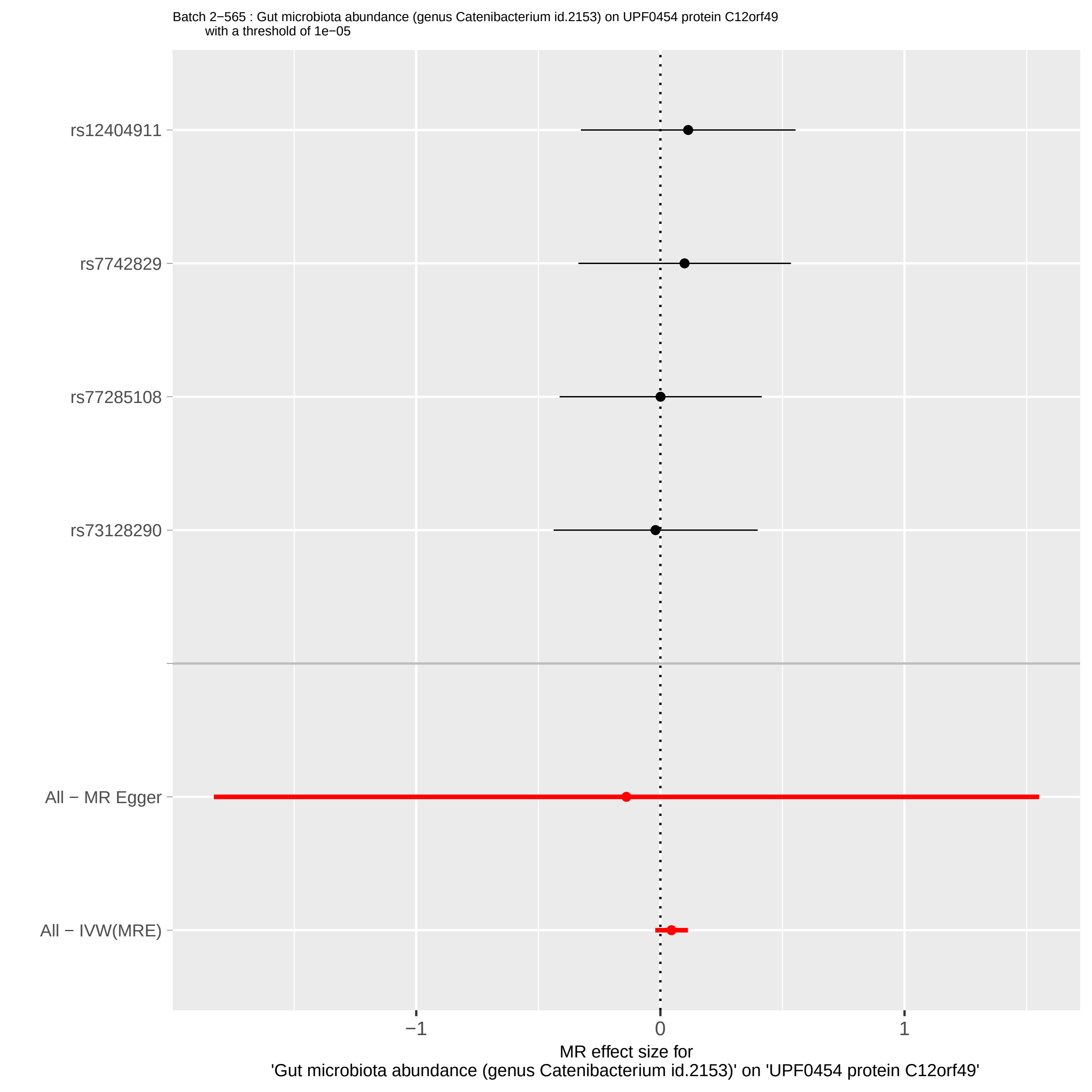
Batch 2−565 : Gut microbiota abundance (genus Catenibacterium id.2153) on UPF0454 protein C12orf49
with a threshold of 1e−05
rs12404911
rs7742829
rs77285108
rs73128290
All − MR Egger
All − IVW(MRE)
−1
0
1
MR effect size for
'Gut microbiota abundance (genus Catenibacterium id.2153)' on 'UPF0454 protein C12orf49'

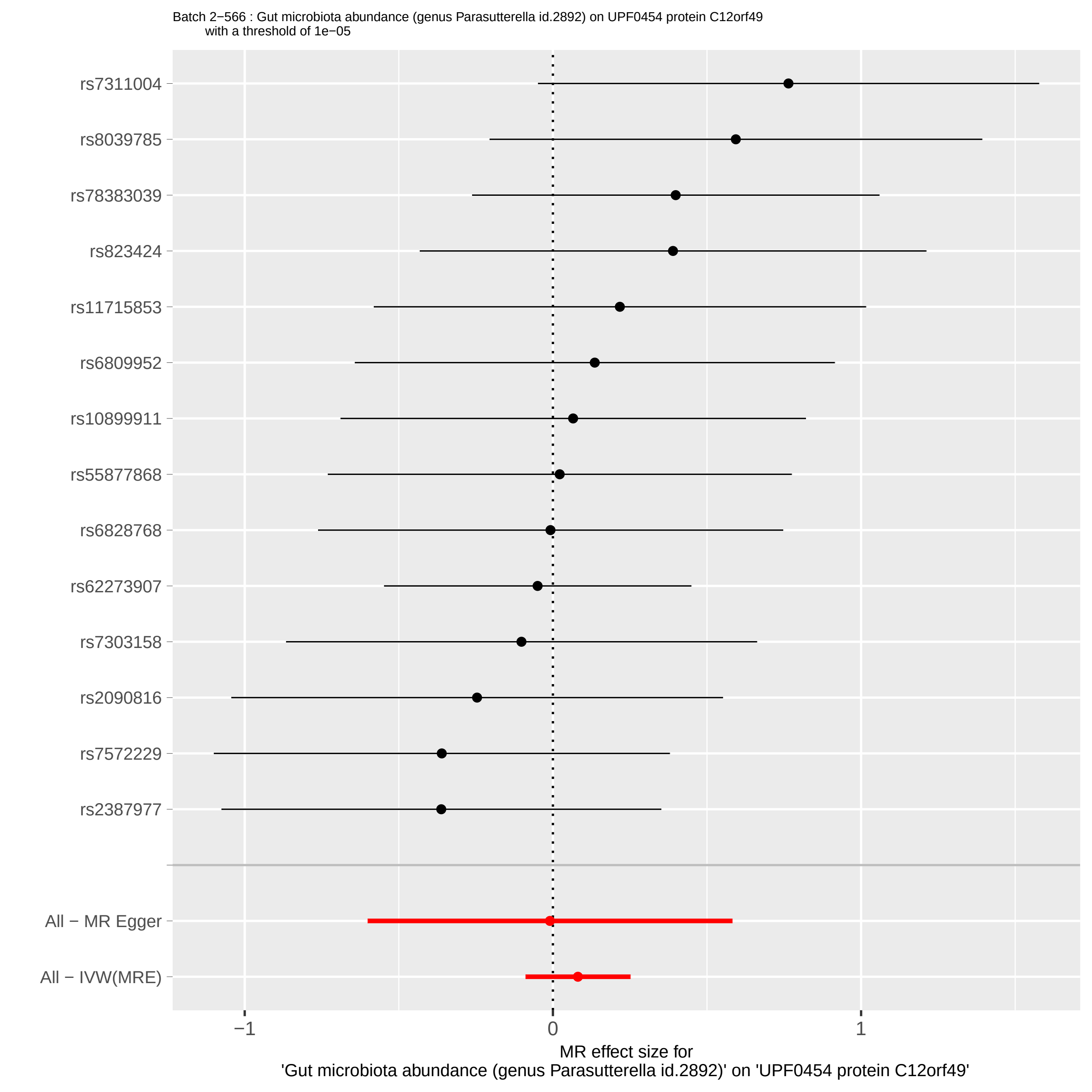

Batch 2−566 : Gut microbiota abundance (genus Parasutterella id.2892) on UPF0454 protein C12orf49
with a threshold of 1e−05
rs7311004
rs8039785
rs78383039
rs823424
rs11715853
rs6809952
rs10899911
rs55877868
rs6828768
rs62273907
rs7303158
rs2090816
rs7572229
rs2387977
All − MR Egger
All − IVW(MRE)
−1
0
1
MR effect size for
'Gut microbiota abundance (genus Parasutterella id.2892)' on 'UPF0454 protein C12orf49'

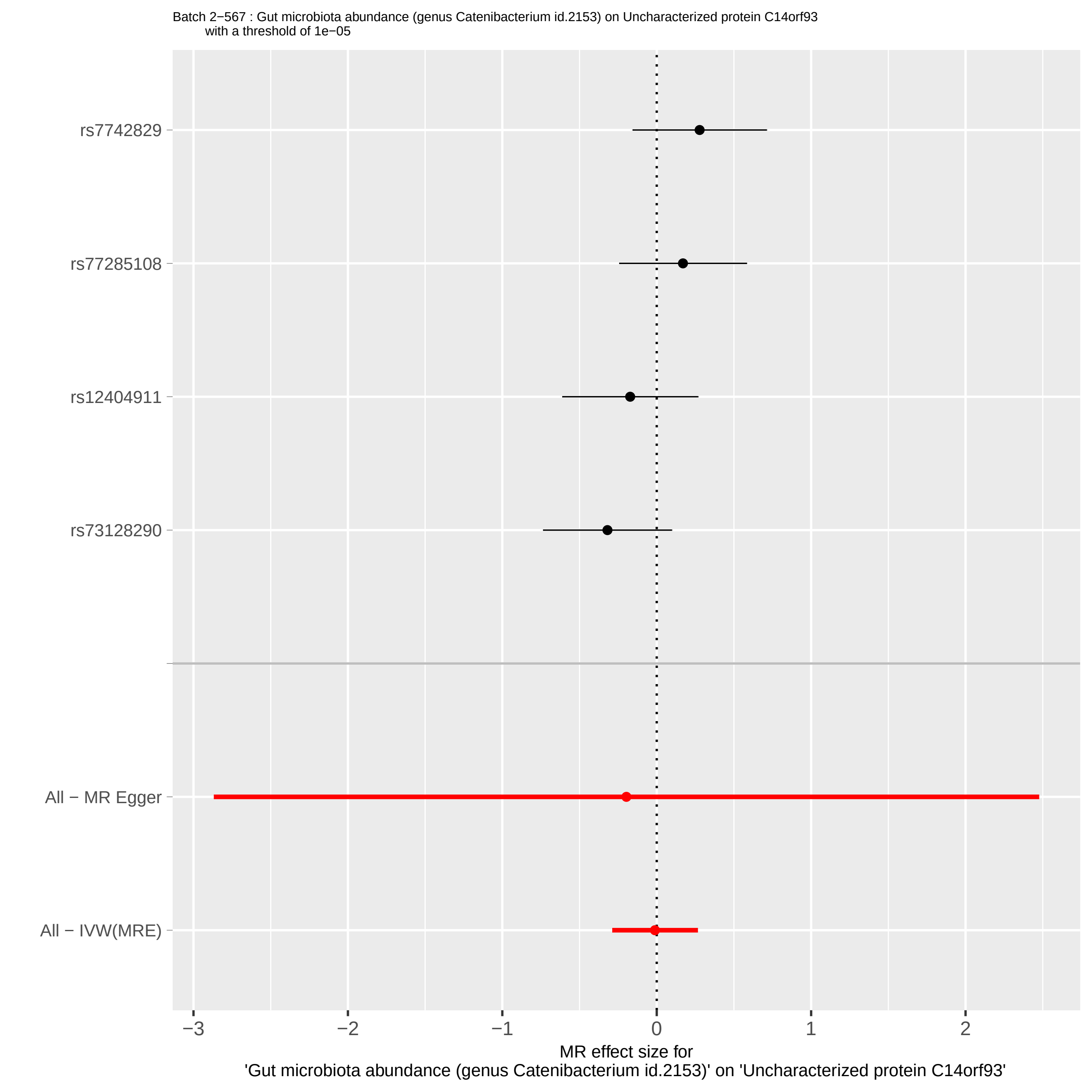
Batch 2−567 : Gut microbiota abundance (genus Catenibacterium id.2153) on Uncharacterized protein C14orf93
with a threshold of 1e−05
rs7742829
rs77285108
rs12404911
rs73128290
All − MR Egger
All − IVW(MRE)
−3
−2
−1
0
1
2
MR effect size for
'Gut microbiota abundance (genus Catenibacterium id.2153)' on 'Uncharacterized protein C14orf93'

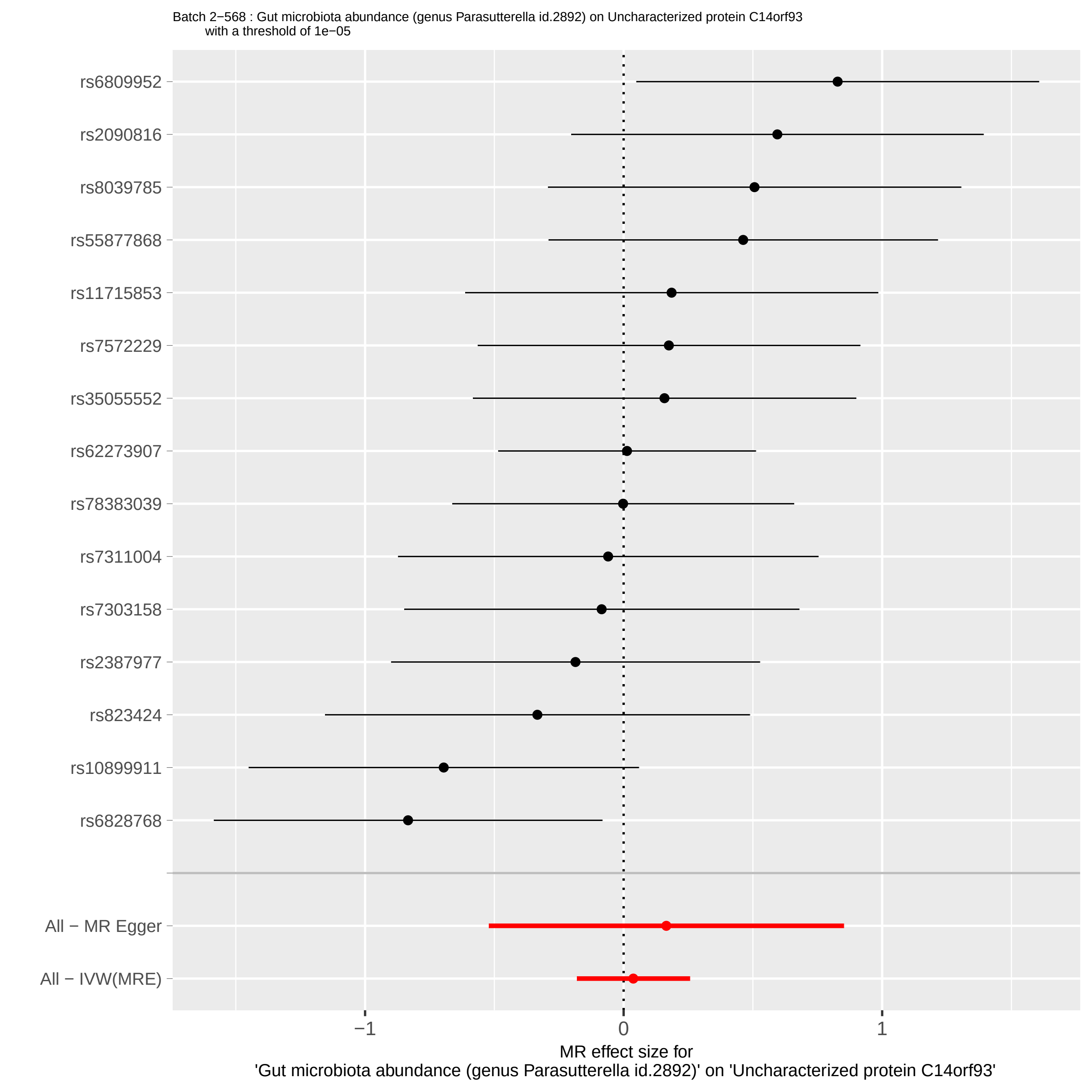

Batch 2−568 : Gut microbiota abundance (genus Parasutterella id.2892) on Uncharacterized protein C14orf93
with a threshold of 1e−05
rs6809952
rs2090816
rs8039785
rs55877868
rs11715853
rs7572229
rs35055552
rs62273907
rs78383039
rs7311004
rs7303158
rs2387977
rs823424
rs10899911
rs6828768
All − MR Egger
All − IVW(MRE)
−1
0
1
MR effect size for
'Gut microbiota abundance (genus Parasutterella id.2892)' on 'Uncharacterized protein C14orf93'

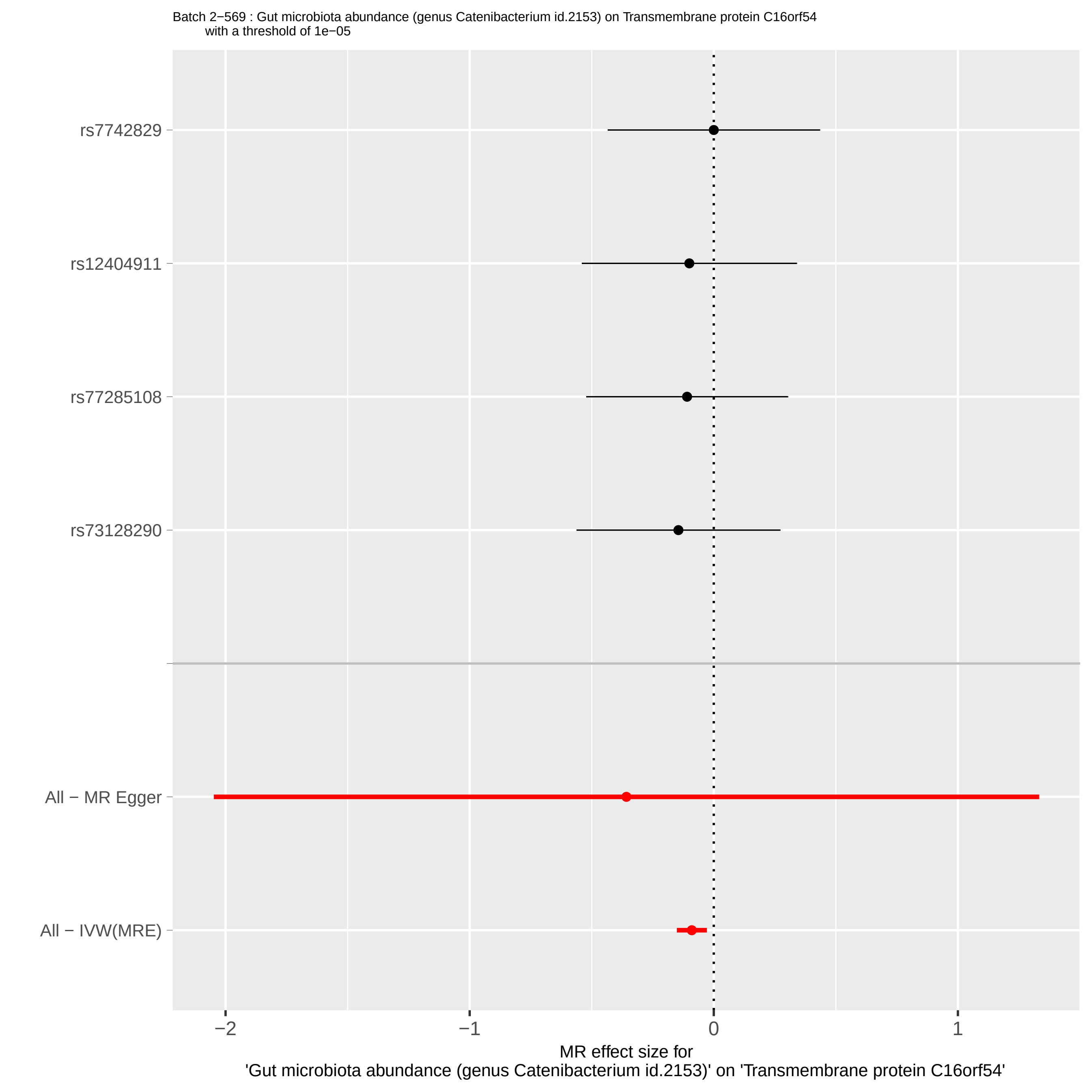

Batch 2−569 : Gut microbiota abundance (genus Catenibacterium id.2153) on Transmembrane protein C16orf54
with a threshold of 1e−05
rs7742829
rs12404911
rs77285108
rs73128290
All − MR Egger
All − IVW(MRE)
−2
−1
0
1
MR effect size for
'Gut microbiota abundance (genus Catenibacterium id.2153)' on 'Transmembrane protein C16orf54'

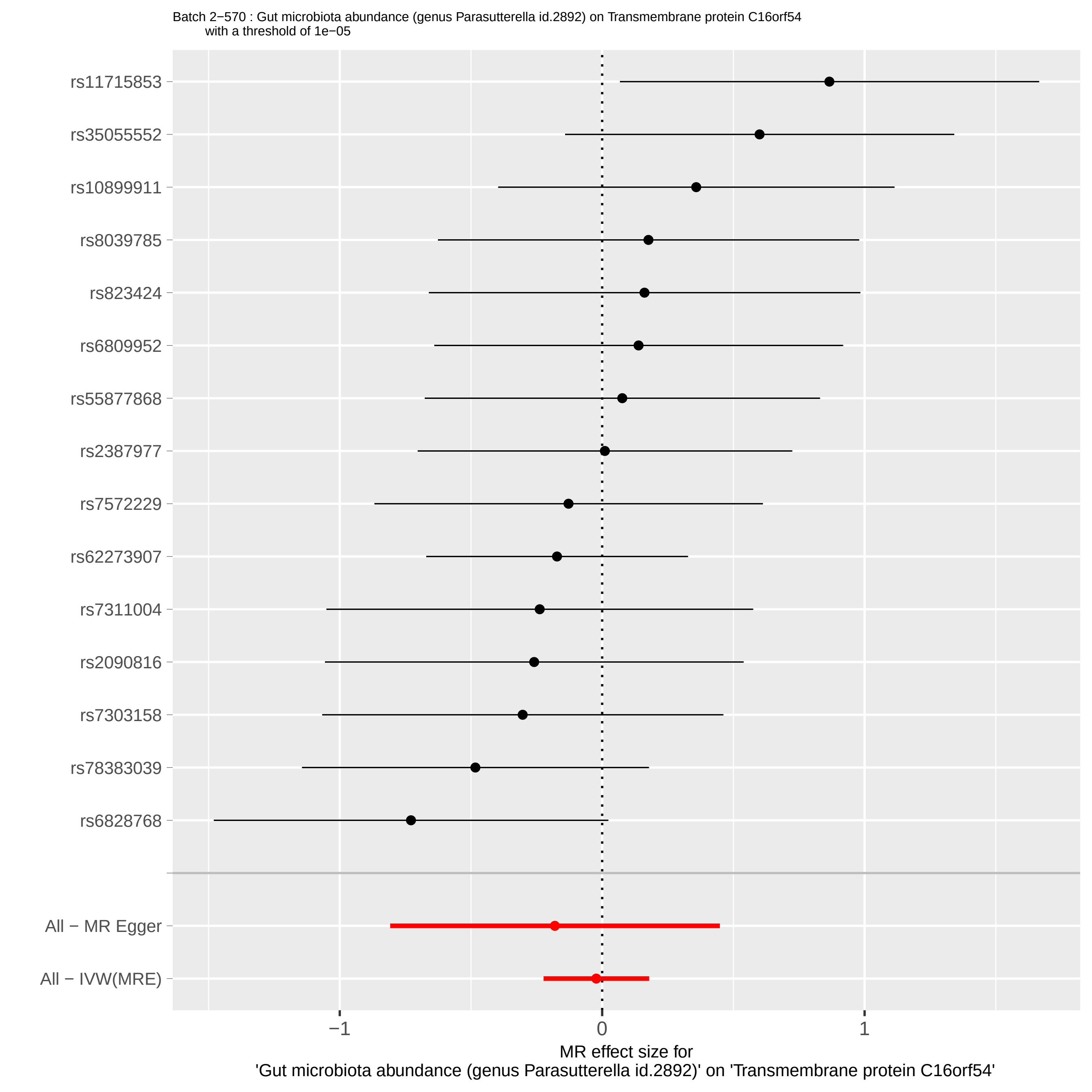
Batch 2−570 : Gut microbiota abundance (genus Parasutterella id.2892) on Transmembrane protein C16orf54
with a threshold of 1e−05
rs11715853
rs35055552
rs10899911
rs8039785
rs823424
rs6809952
rs55877868
rs2387977
rs7572229
rs62273907
rs7311004
rs2090816
rs7303158
rs78383039
rs6828768
All − MR Egger
All − IVW(MRE)
−1
0
1
MR effect size for
'Gut microbiota abundance (genus Parasutterella id.2892)' on 'Transmembrane protein C16orf54'

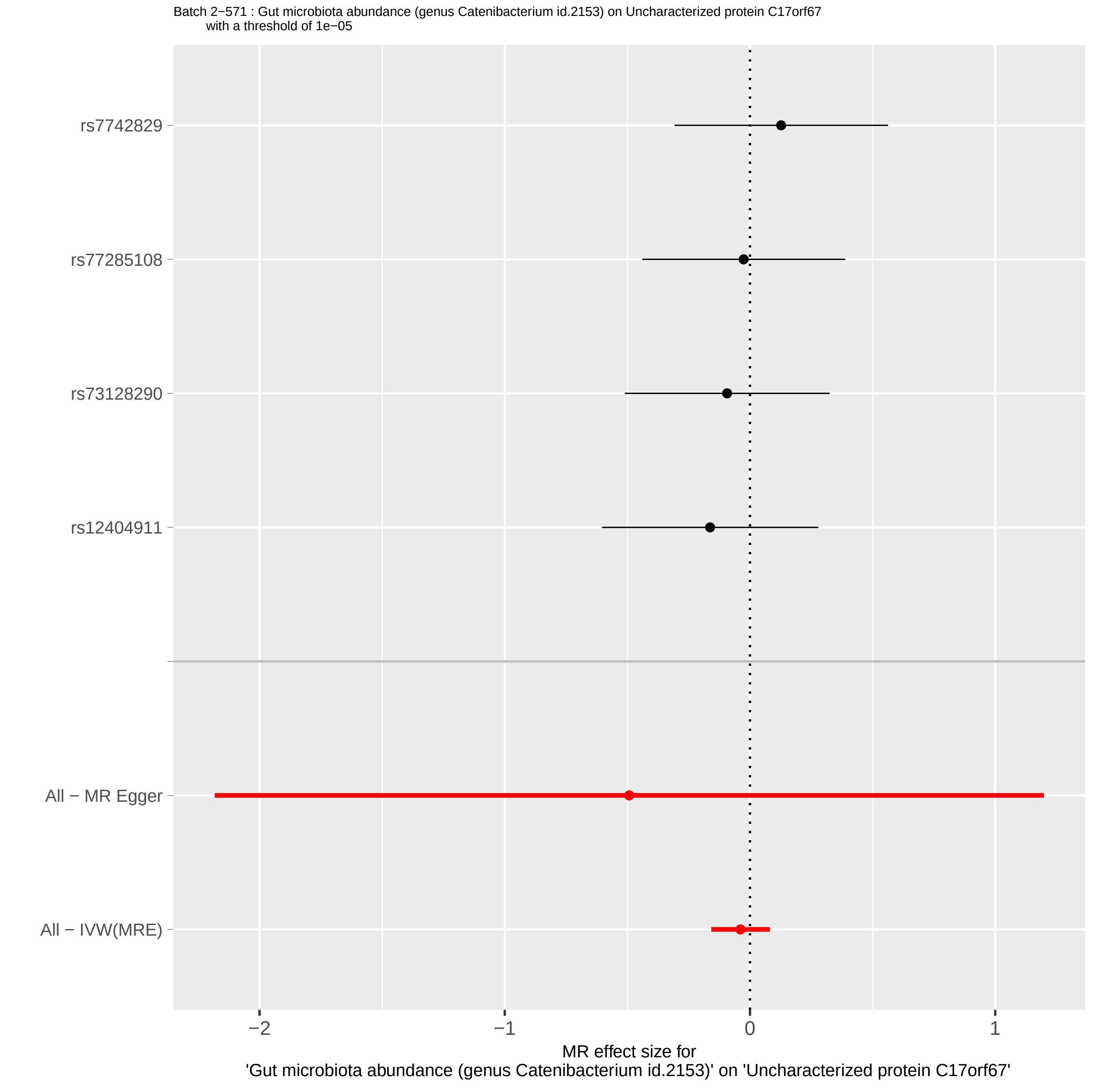

Batch 2−571 : Gut microbiota abundance (genus Catenibacterium id.2153) on Uncharacterized protein C17orf67
with a threshold of 1e−05
rs7742829
rs77285108
rs73128290
rs12404911
All − MR Egger
All − IVW(MRE)
−2
−1
0
1
MR effect size for
'Gut microbiota abundance (genus Catenibacterium id.2153)' on 'Uncharacterized protein C17orf67'

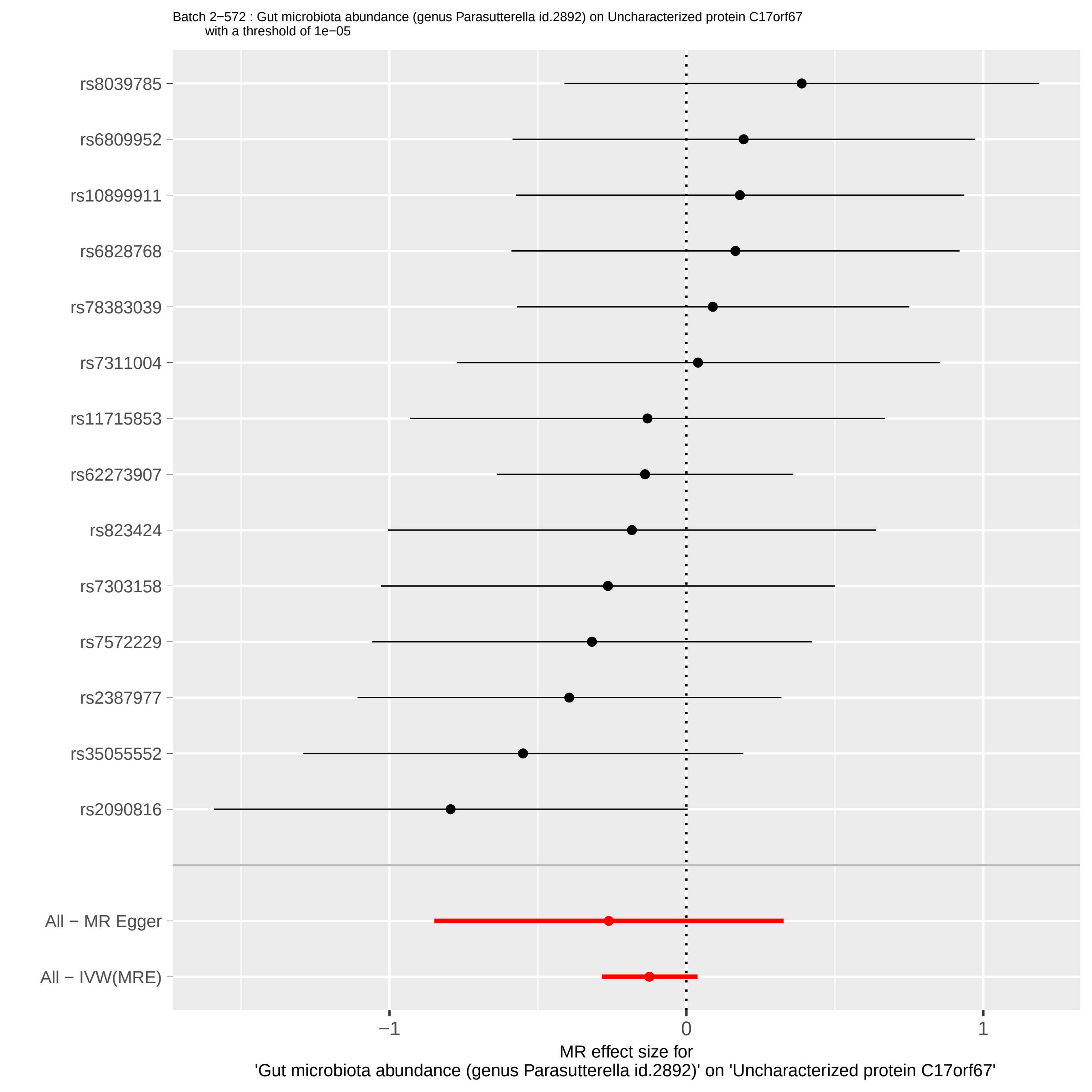

Batch 2−572 : Gut microbiota abundance (genus Parasutterella id.2892) on Uncharacterized protein C17orf67
with a threshold of 1e−05
rs8039785
rs6809952
rs10899911
rs6828768
rs78383039
rs7311004
rs11715853
rs62273907
rs823424
rs7303158
rs7572229
rs2387977
rs35055552
rs2090816
All − MR Egger
All − IVW(MRE)
−1
0
1
MR effect size for
'Gut microbiota abundance (genus Parasutterella id.2892)' on 'Uncharacterized protein C17orf67'

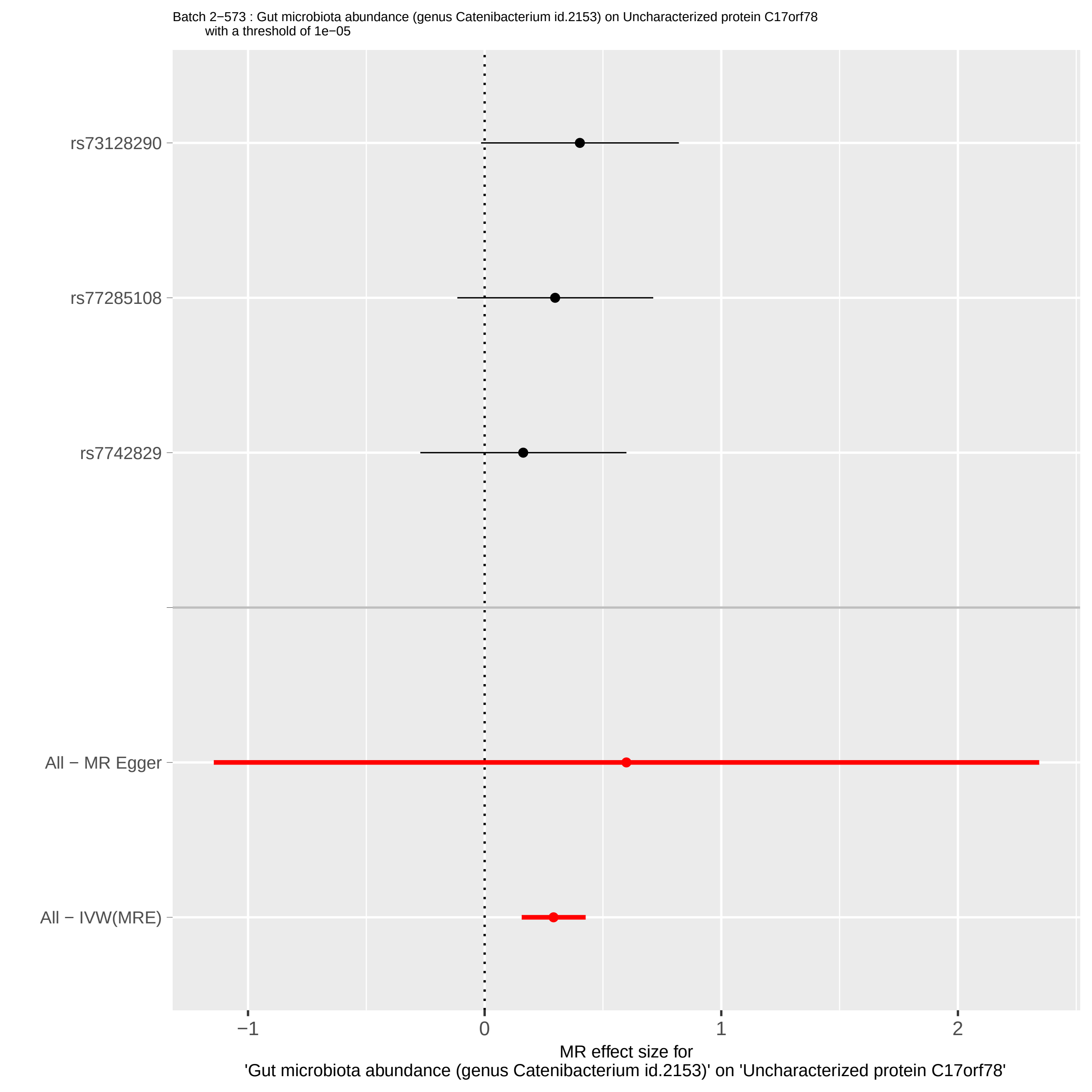
Batch 2−573 : Gut microbiota abundance (genus Catenibacterium id.2153) on Uncharacterized protein C17orf78
with a threshold of 1e−05
rs73128290
rs77285108
rs7742829
All − MR Egger
All − IVW(MRE)
−1
0
1
2
MR effect size for
'Gut microbiota abundance (genus Catenibacterium id.2153)' on 'Uncharacterized protein C17orf78'

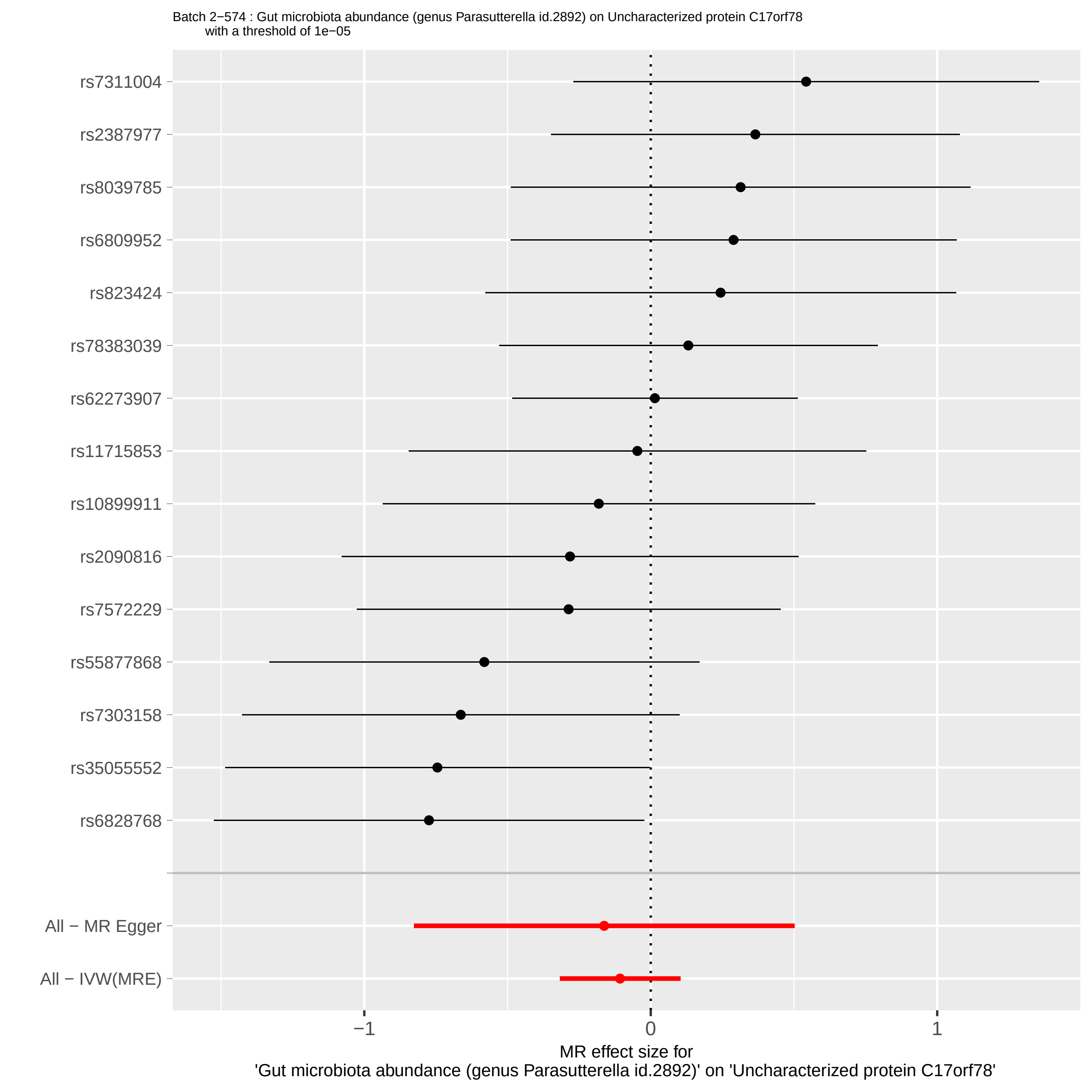

Batch 2−574 : Gut microbiota abundance (genus Parasutterella id.2892) on Uncharacterized protein C17orf78
with a threshold of 1e−05
rs7311004
rs2387977
rs8039785
rs6809952
rs823424
rs78383039
rs62273907
rs11715853
rs10899911
rs2090816
rs7572229
rs55877868
rs7303158
rs35055552
rs6828768
All − MR Egger
All − IVW(MRE)
−1
0
1
MR effect size for
'Gut microbiota abundance (genus Parasutterella id.2892)' on 'Uncharacterized protein C17orf78'

Batch 2−575 : Gut microbiota abundance (genus Catenibacterium id.2153) on Uncharacterized protein C17orf78
with a threshold of 1e−05

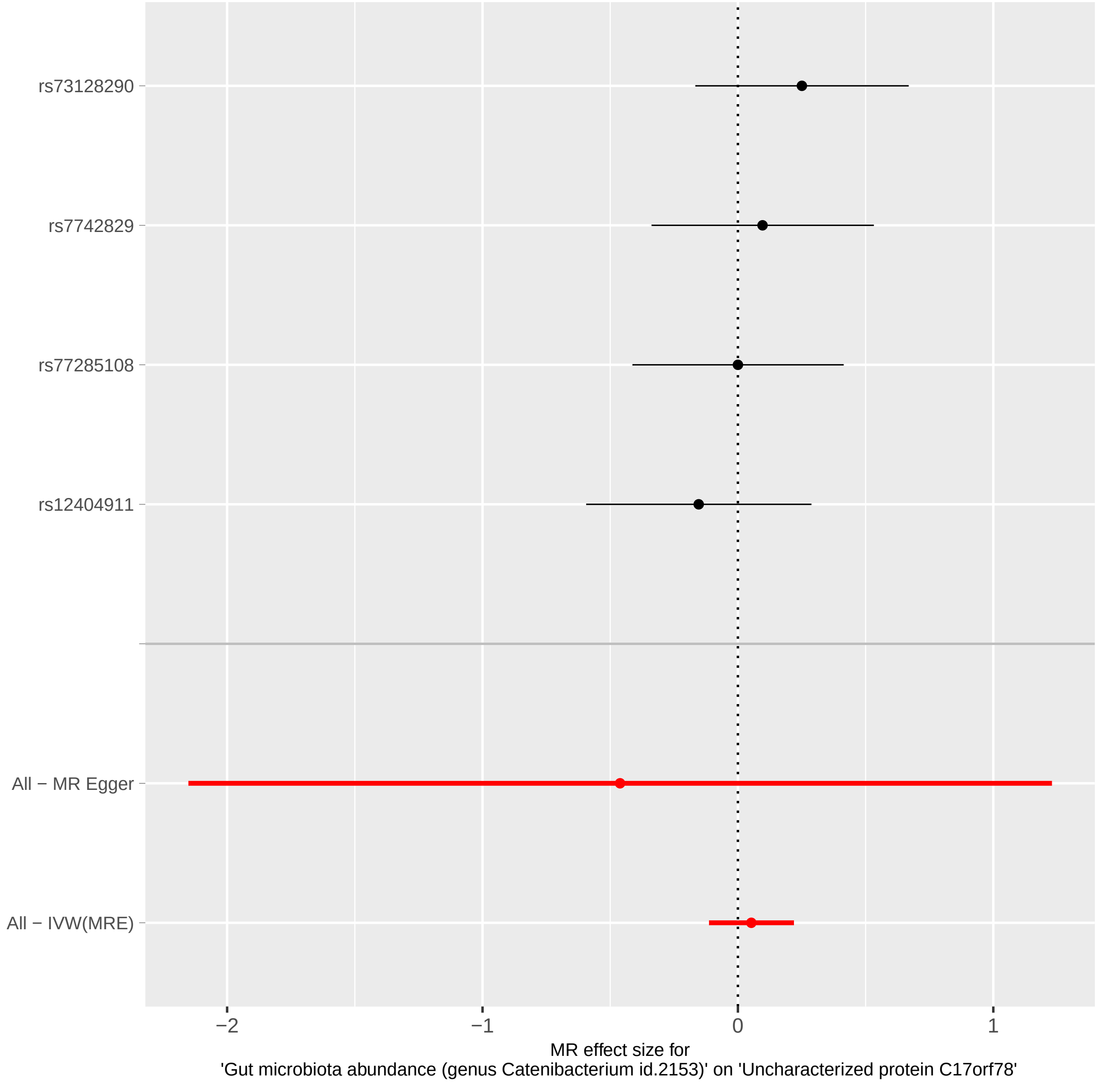

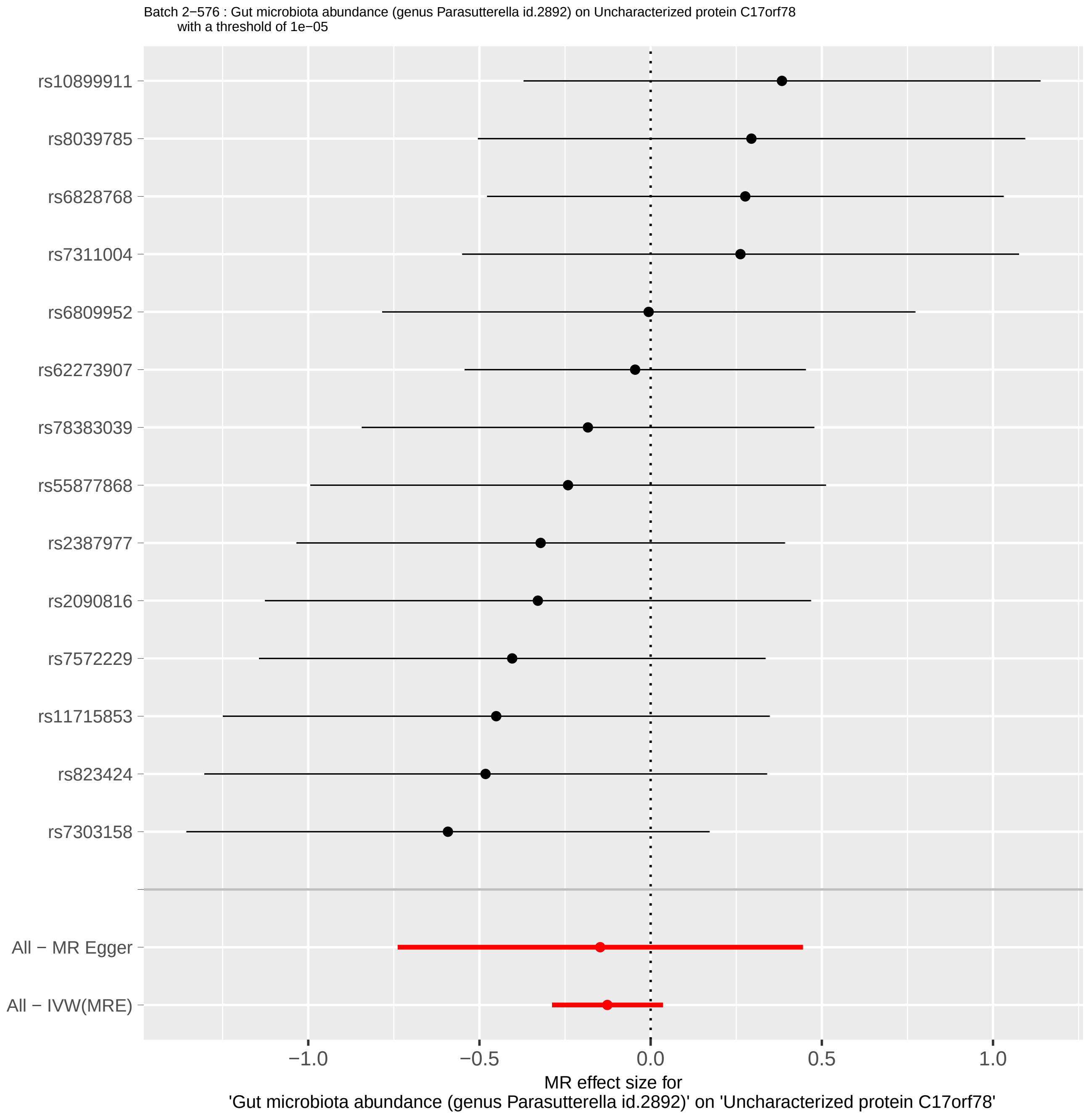

Batch 2−576 : Gut microbiota abundance (genus Parasutterella id.2892) on Uncharacterized protein C17orf78
with a threshold of 1e−05
rs10899911
rs8039785
rs6828768
rs7311004
rs6809952
rs62273907
rs78383039
rs55877868
rs2387977
rs2090816
rs7572229
rs11715853
rs823424
rs7303158
All − MR Egger
All − IVW(MRE)
−1.0
−0.5
0.0
0.5
1.0
MR effect size for
'Gut microbiota abundance (genus Parasutterella id.2892)' on 'Uncharacterized protein C17orf78'

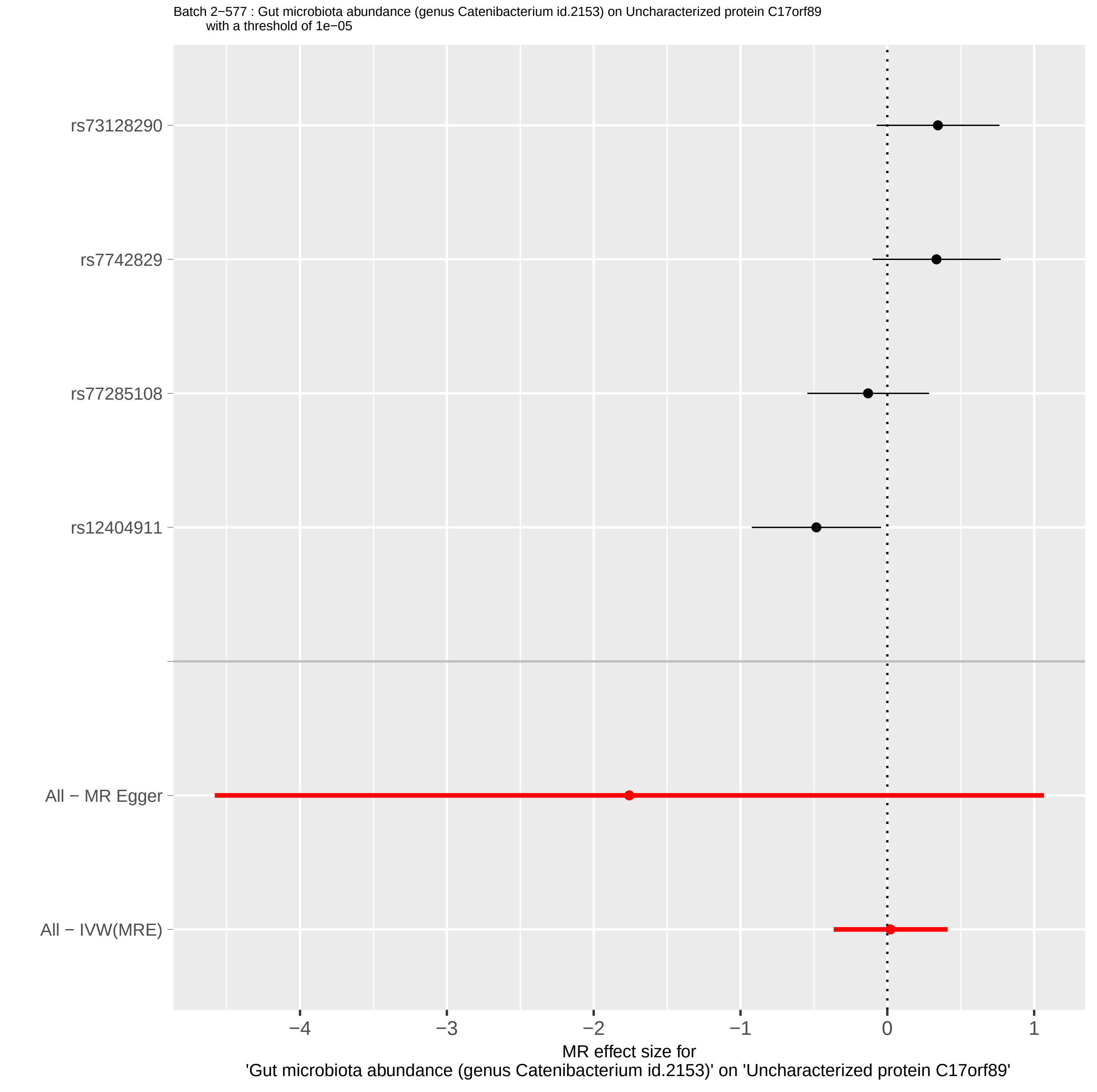

Batch 2−577 : Gut microbiota abundance (genus Catenibacterium id.2153) on Uncharacterized protein C17orf89
with a threshold of 1e−05
rs73128290
rs7742829
rs77285108
rs12404911
All − MR Egger
All − IVW(MRE)
−4
−3
−2
−1
0
1
MR effect size for
'Gut microbiota abundance (genus Catenibacterium id.2153)' on 'Uncharacterized protein C17orf89'

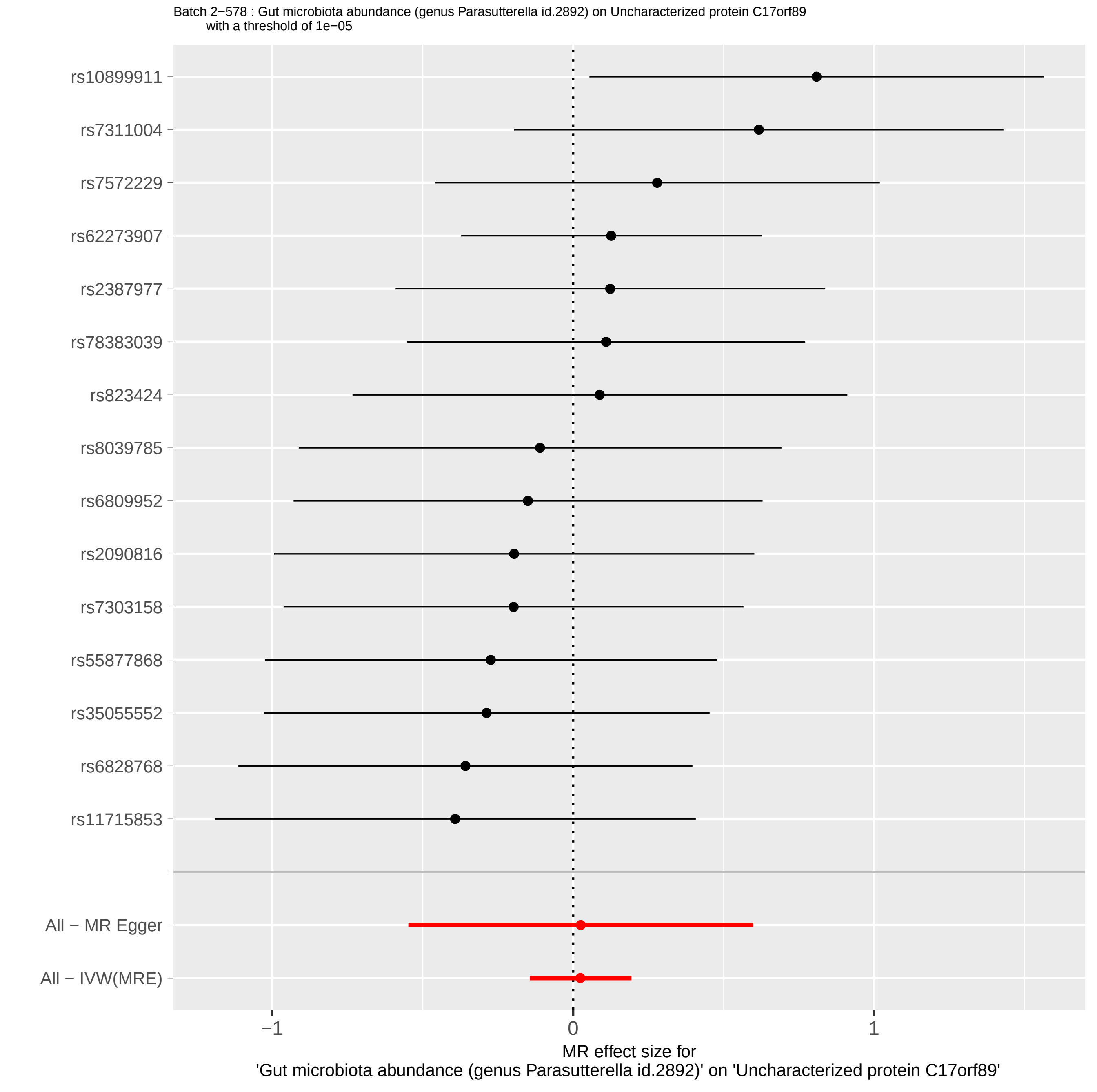

Batch 2−578 : Gut microbiota abundance (genus Parasutterella id.2892) on Uncharacterized protein C17orf89
with a threshold of 1e−05
rs10899911
rs7311004
rs7572229
rs62273907
rs2387977
rs78383039
rs823424
rs8039785
rs6809952
rs2090816
rs7303158
rs55877868
rs35055552
rs6828768
rs11715853
All − MR Egger
All − IVW(MRE)
−1
0
1
MR effect size for
'Gut microbiota abundance (genus Parasutterella id.2892)' on 'Uncharacterized protein C17orf89'

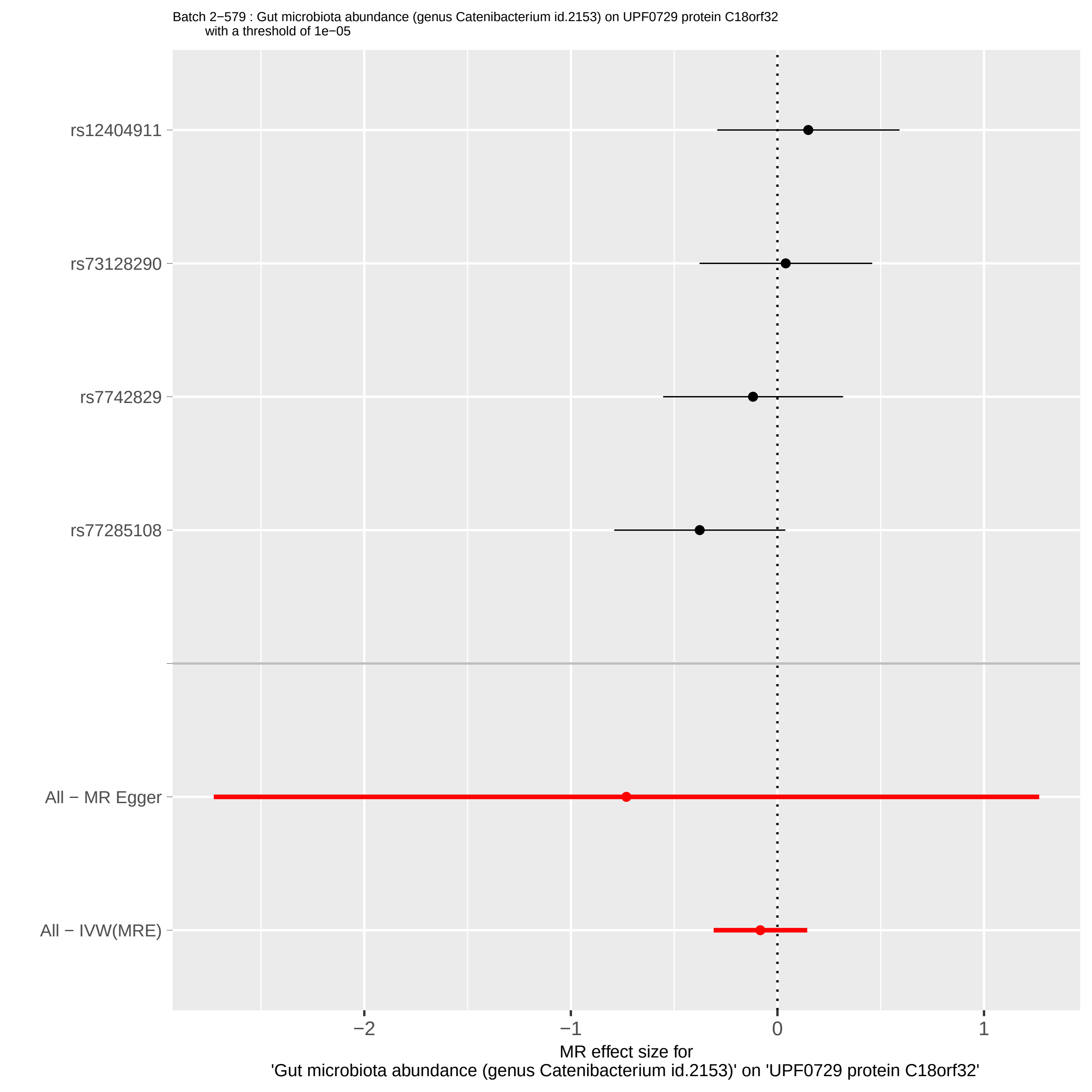

Batch 2−579 : Gut microbiota abundance (genus Catenibacterium id.2153) on UPF0729 protein C18orf32
with a threshold of 1e−05
rs12404911
rs73128290
rs7742829
rs77285108
All − MR Egger
All − IVW(MRE)
−2
−1
0
1
MR effect size for
'Gut microbiota abundance (genus Catenibacterium id.2153)' on 'UPF0729 protein C18orf32'

Batch 2−580 : Gut microbiota abundance (genus Parasutterella id.2892) on UPF0729 protein C18orf32
with a threshold of 1e−05

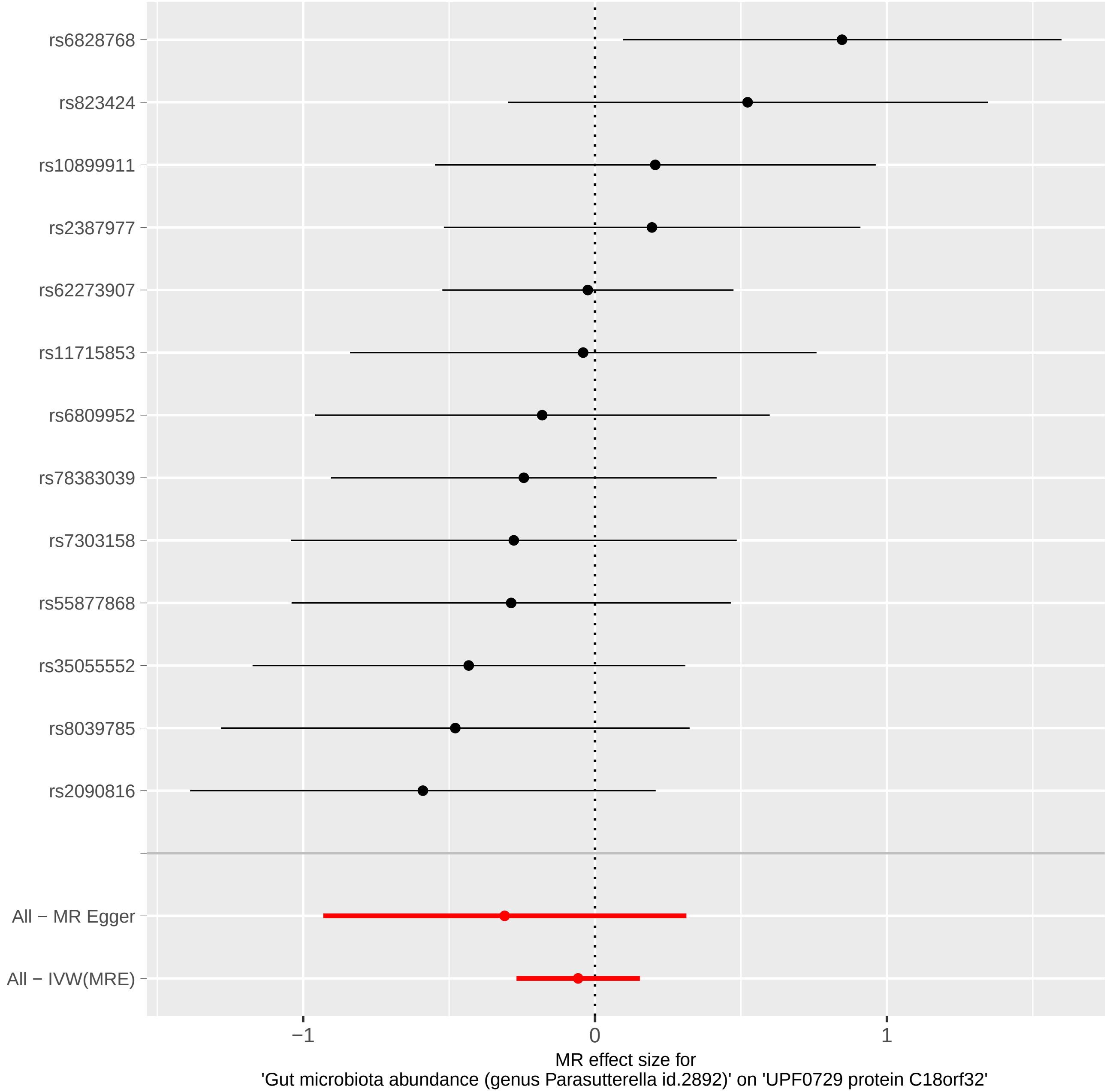

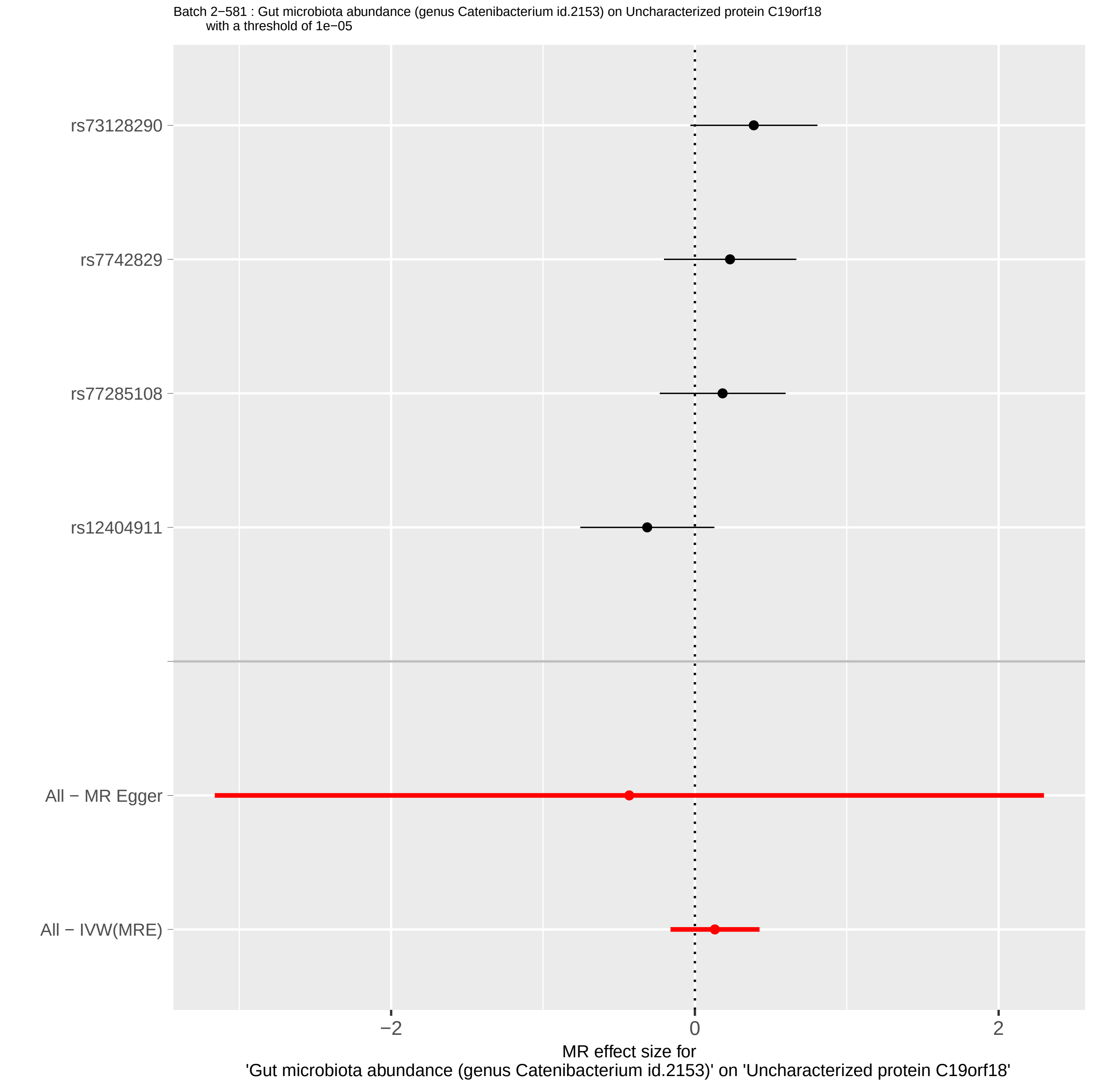

Batch 2−581 : Gut microbiota abundance (genus Catenibacterium id.2153) on Uncharacterized protein C19orf18
with a threshold of 1e−05
rs73128290
rs7742829
rs77285108
rs12404911
All − MR Egger
All − IVW(MRE)
−2
0
2
MR effect size for
'Gut microbiota abundance (genus Catenibacterium id.2153)' on 'Uncharacterized protein C19orf18'

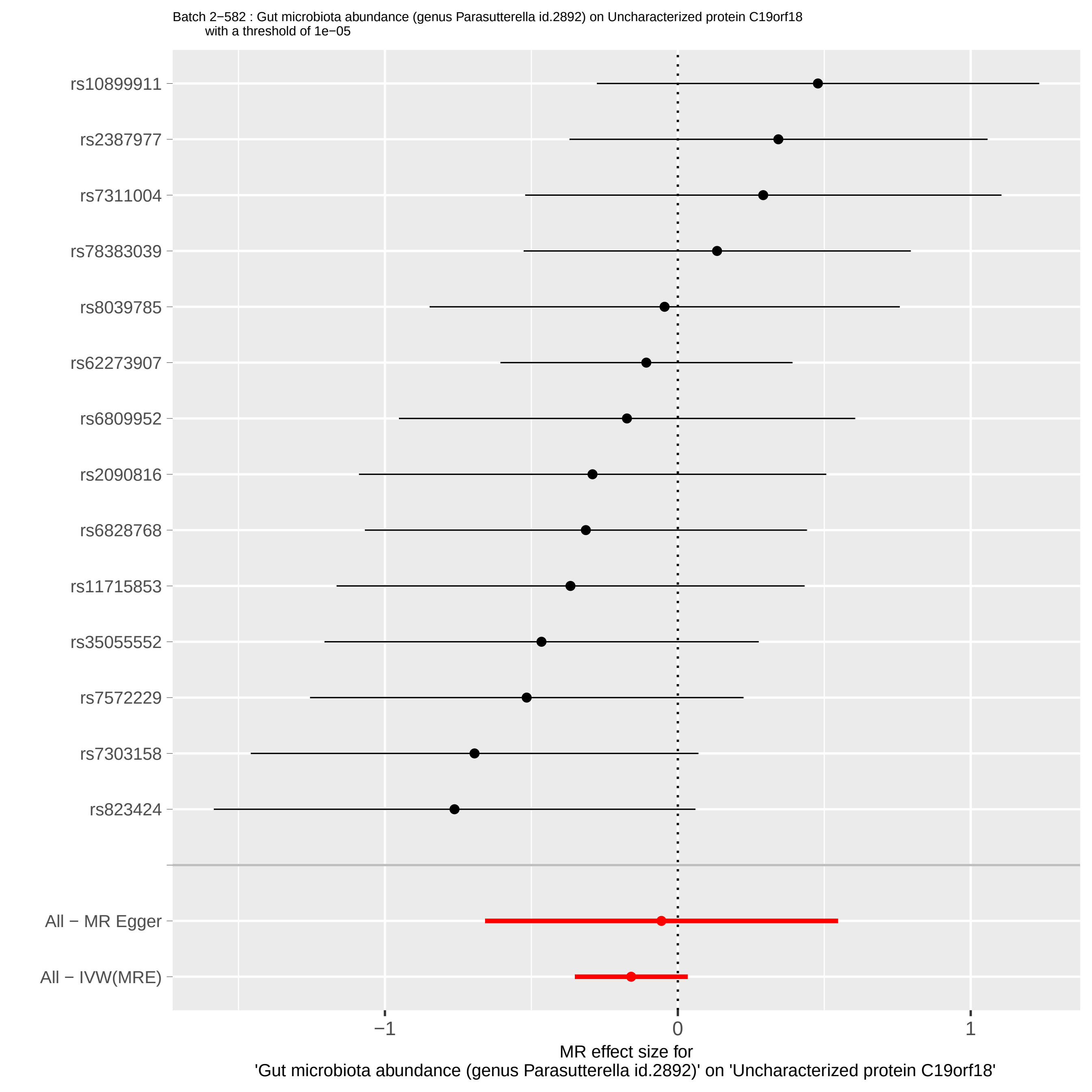
Batch 2−582 : Gut microbiota abundance (genus Parasutterella id.2892) on Uncharacterized protein C19orf18
with a threshold of 1e−05
rs10899911
rs2387977
rs7311004
rs78383039
rs8039785
rs62273907
rs6809952
rs2090816
rs6828768
rs11715853
rs35055552
rs7572229
rs7303158
rs823424
All − MR Egger
All − IVW(MRE)
−1
0
1
MR effect size for
'Gut microbiota abundance (genus Parasutterella id.2892)' on 'Uncharacterized protein C19orf18'

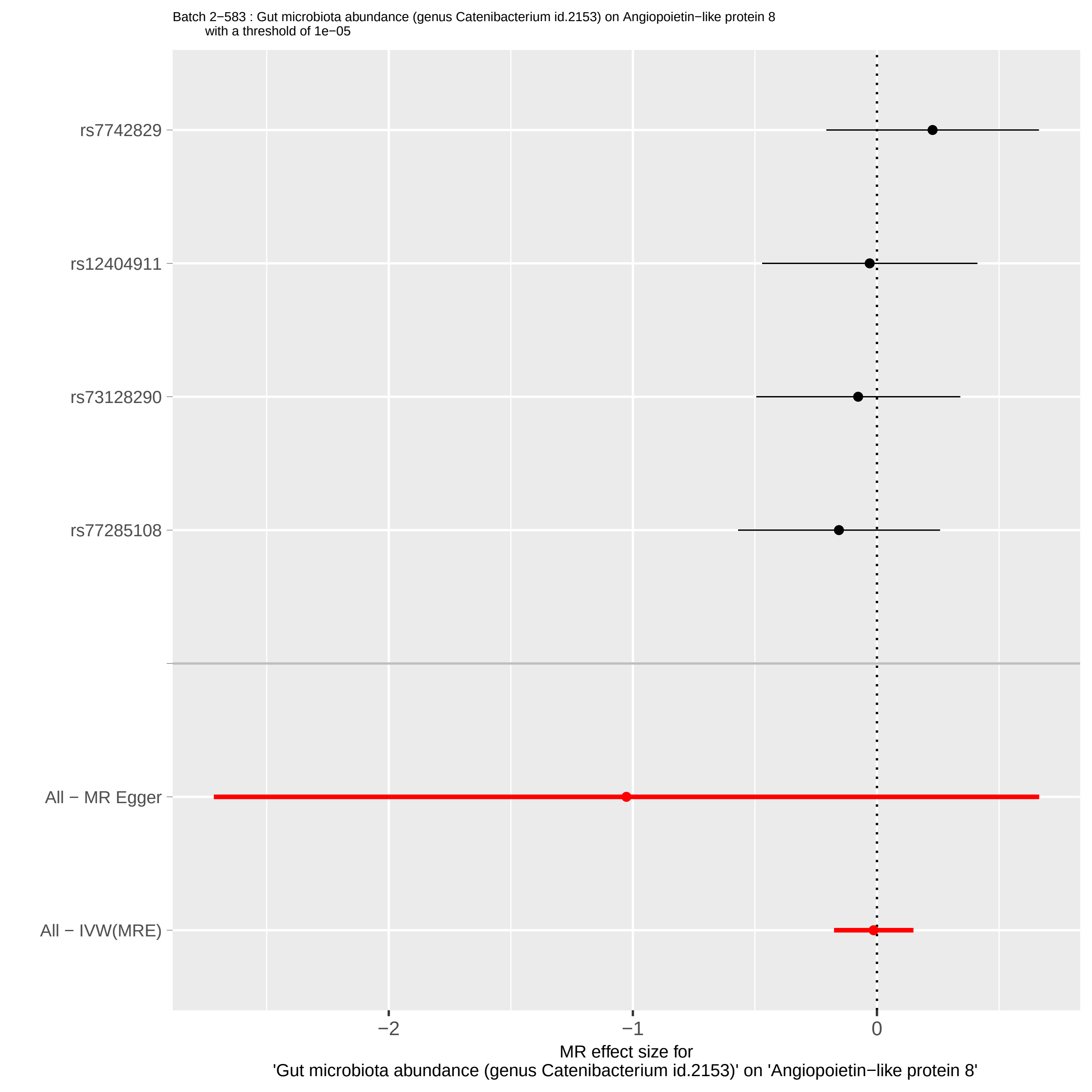
Batch 2−583 : Gut microbiota abundance (genus Catenibacterium id.2153) on Angiopoietin−like protein 8
with a threshold of 1e−05
rs7742829
rs12404911
rs73128290
rs77285108
All − MR Egger
All − IVW(MRE)
−2
−1
0
MR effect size for
'Gut microbiota abundance (genus Catenibacterium id.2153)' on 'Angiopoietin−like protein 8'

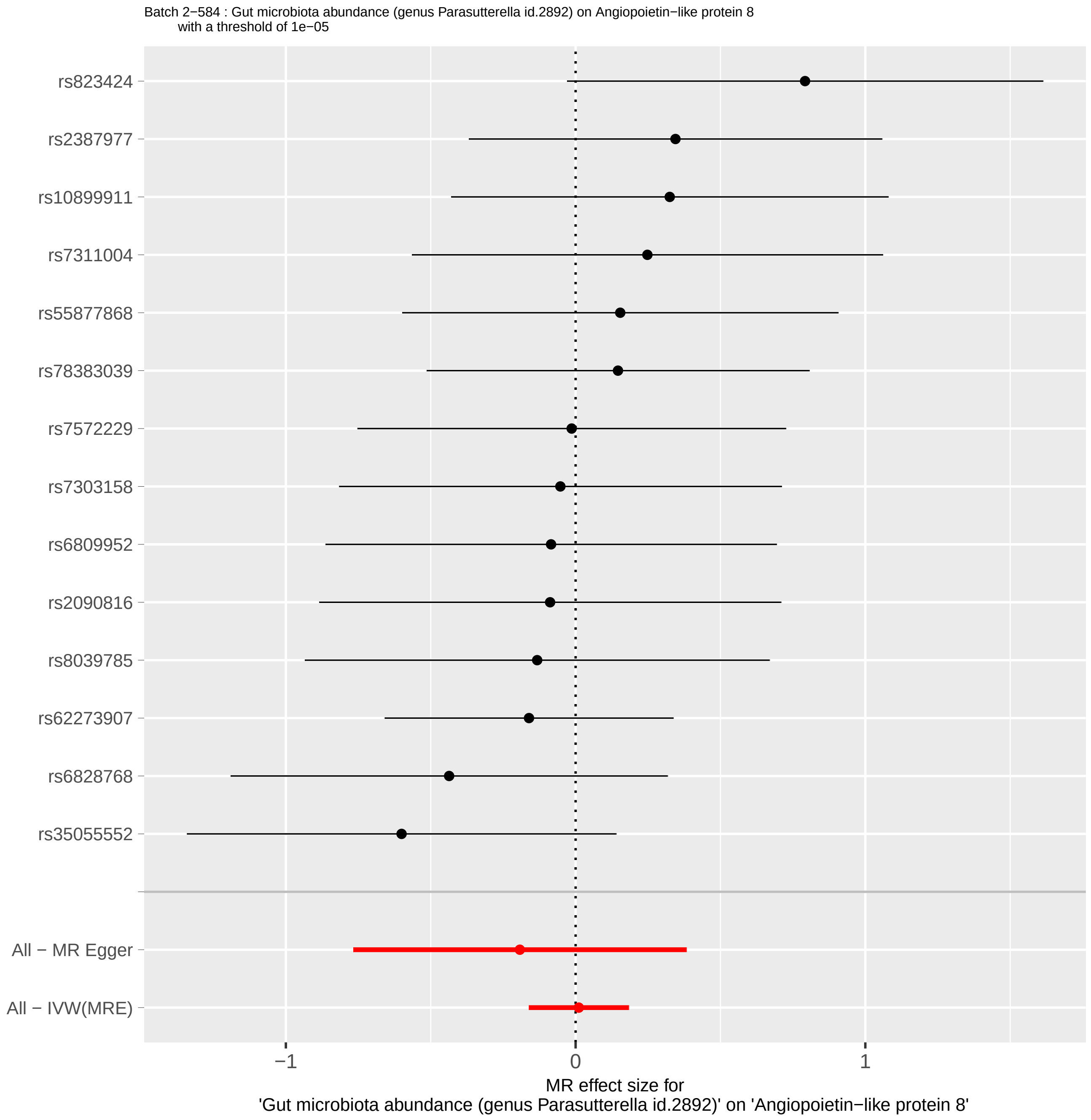
Batch 2−584 : Gut microbiota abundance (genus Parasutterella id.2892) on Angiopoietin−like protein 8
with a threshold of 1e−05
rs823424
rs2387977
rs10899911
rs7311004
rs55877868
rs78383039
rs7572229
rs7303158
rs6809952
rs2090816
rs8039785
rs62273907
rs6828768
rs35055552
All − MR Egger
All − IVW(MRE)
−1
0
1
MR effect size for
'Gut microbiota abundance (genus Parasutterella id.2892)' on 'Angiopoietin−like protein 8'

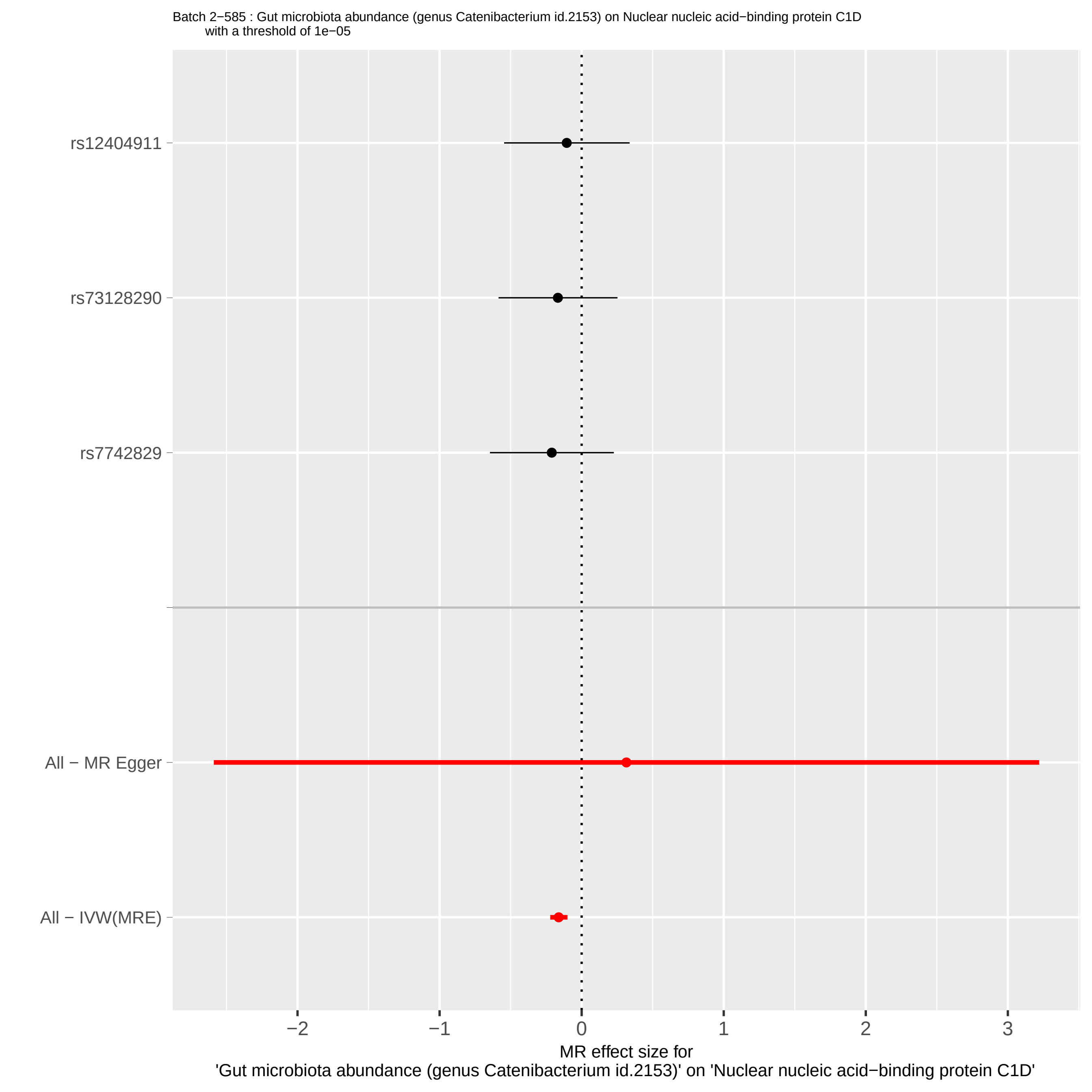

Batch 2−585 : Gut microbiota abundance (genus Catenibacterium id.2153) on Nuclear nucleic acid−binding protein C1D
with a threshold of 1e−05
rs12404911
rs73128290
rs7742829
All − MR Egger
All − IVW(MRE)
−2
−1
0
1
2
3
MR effect size for
'Gut microbiota abundance (genus Catenibacterium id.2153)' on 'Nuclear nucleic acid−binding protein C1D'

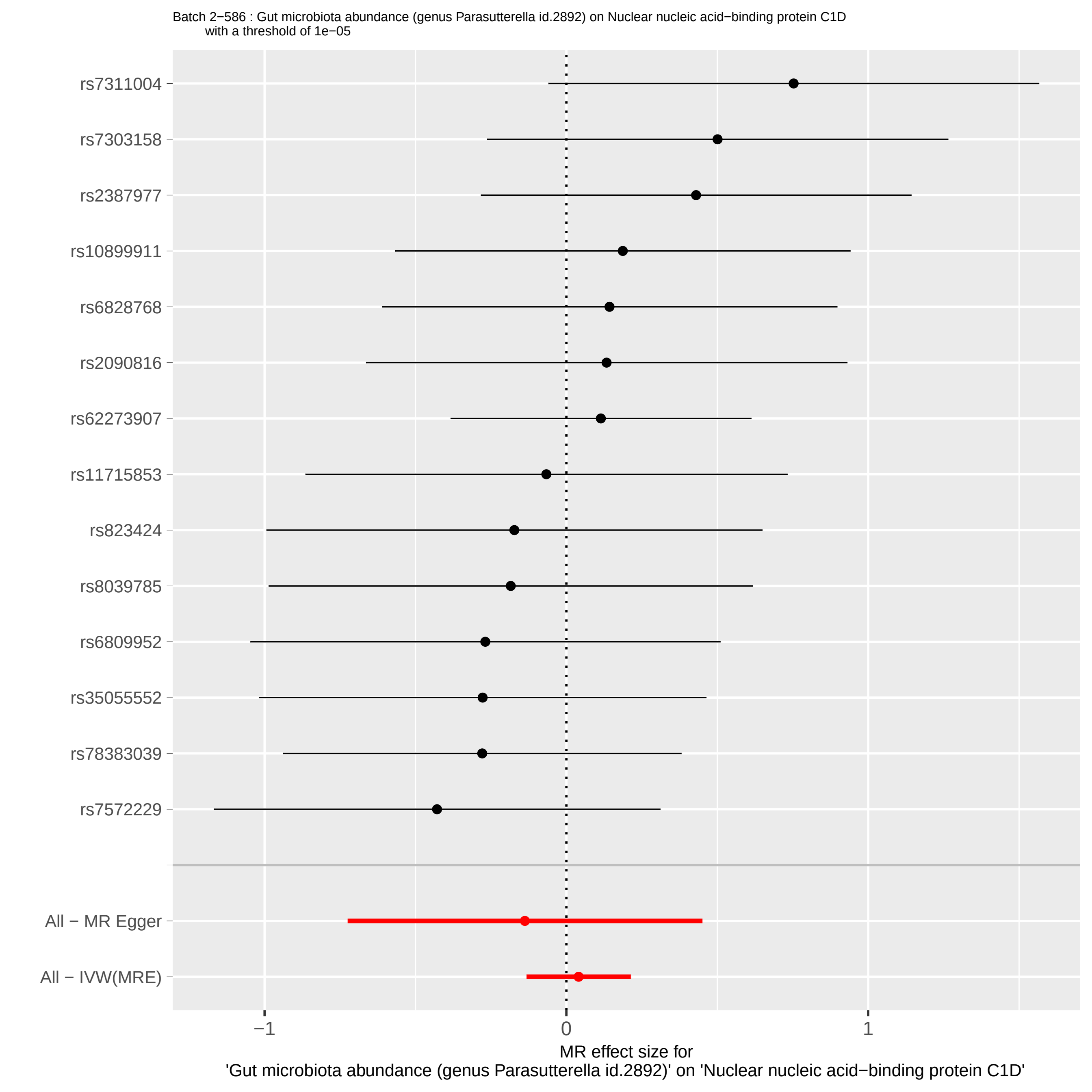

Batch 2−586 : Gut microbiota abundance (genus Parasutterella id.2892) on Nuclear nucleic acid−binding protein C1D
with a threshold of 1e−05
rs7311004
rs7303158
rs2387977
rs10899911
rs6828768
rs2090816
rs62273907
rs11715853
rs823424
rs8039785
rs6809952
rs35055552
rs78383039
rs7572229
All − MR Egger
All − IVW(MRE)
−1
0
1
MR effect size for
'Gut microbiota abundance (genus Parasutterella id.2892)' on 'Nuclear nucleic acid−binding protein C1D'

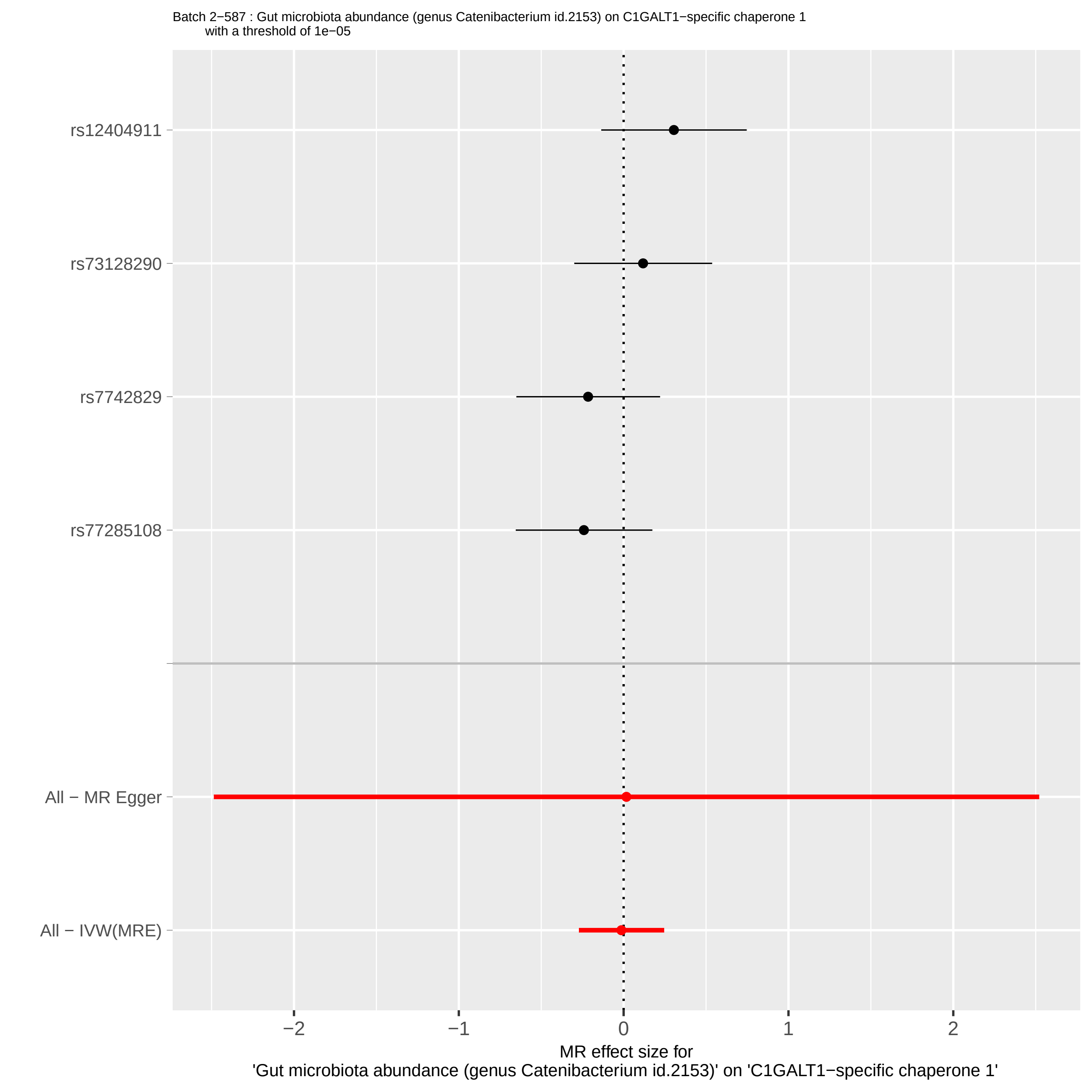

Batch 2−587 : Gut microbiota abundance (genus Catenibacterium id.2153) on C1GALT1−specific chaperone 1
with a threshold of 1e−05
rs12404911
rs73128290
rs7742829
rs77285108
All − MR Egger
All − IVW(MRE)
−2
−1
0
1
2
MR effect size for
'Gut microbiota abundance (genus Catenibacterium id.2153)' on 'C1GALT1−specific chaperone 1'

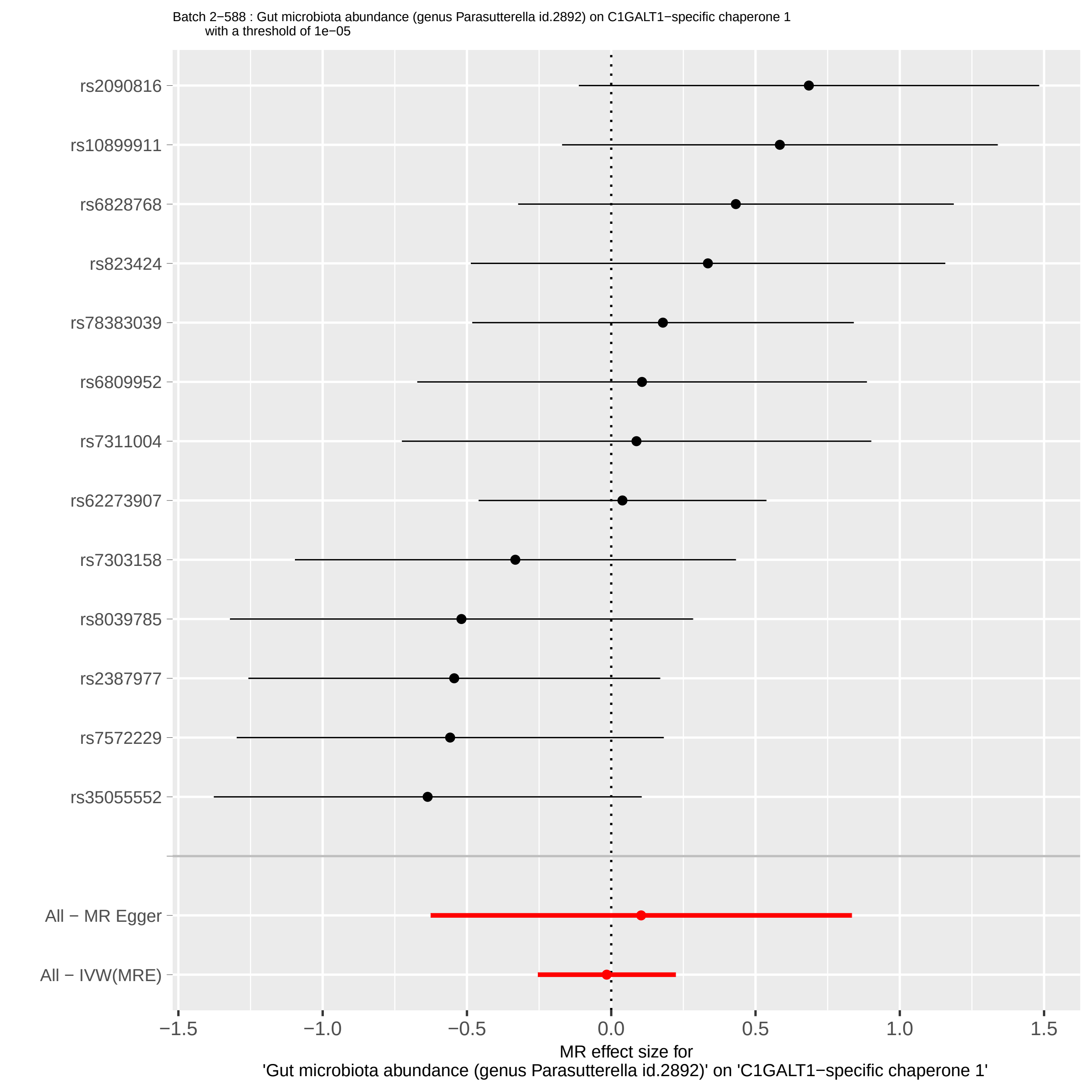

Batch 2−588 : Gut microbiota abundance (genus Parasutterella id.2892) on C1GALT1−specific chaperone 1
with a threshold of 1e−05
rs2090816
rs10899911
rs6828768
rs823424
rs78383039
rs6809952
rs7311004
rs62273907
rs7303158
rs8039785
rs2387977
rs7572229
rs35055552
All − MR Egger
All − IVW(MRE)
−1.5
−1.0
−0.5
0.0
0.5
1.0
1.5
MR effect size for
'Gut microbiota abundance (genus Parasutterella id.2892)' on 'C1GALT1−specific chaperone 1'

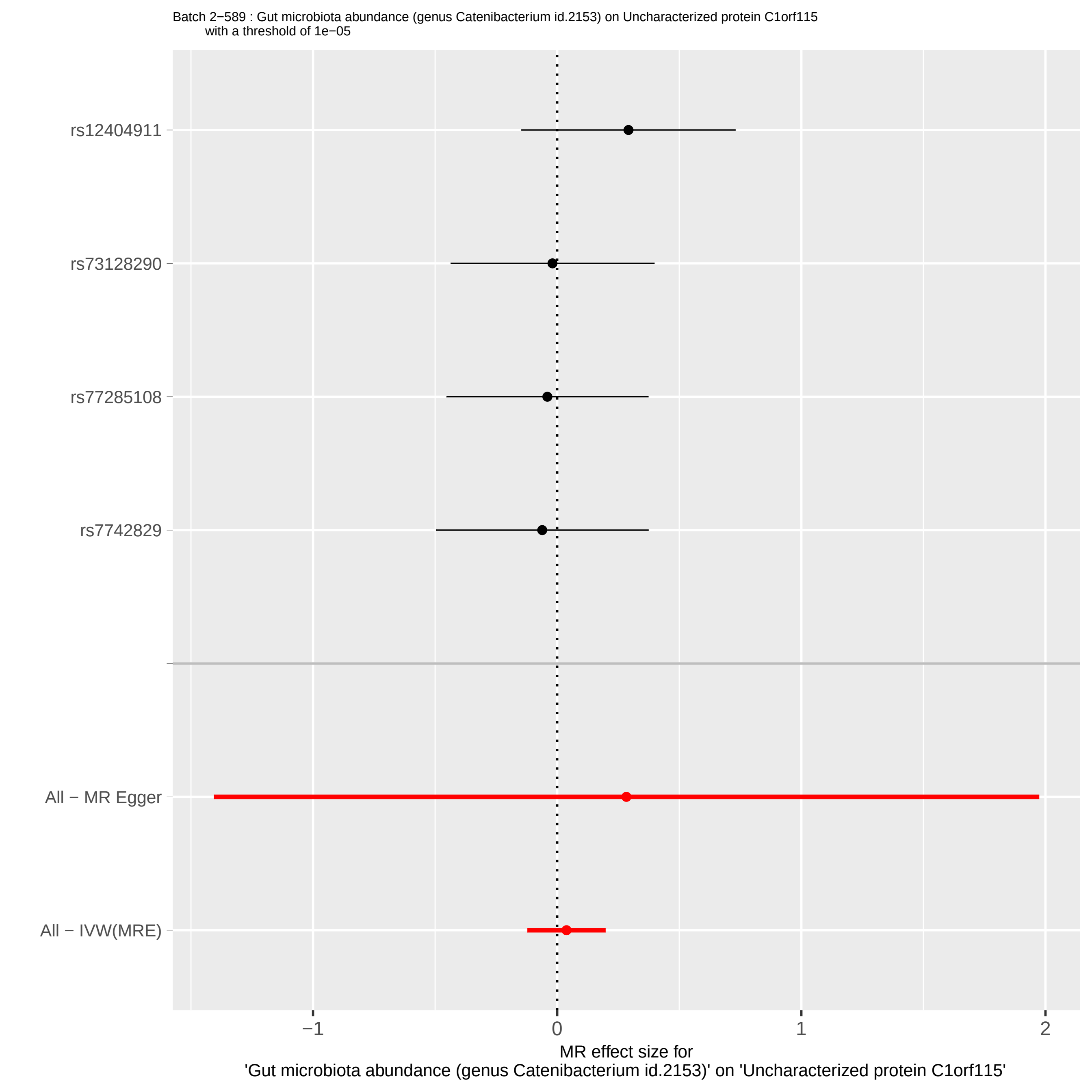
Batch 2−589 : Gut microbiota abundance (genus Catenibacterium id.2153) on Uncharacterized protein C1orf115
with a threshold of 1e−05
rs12404911
rs73128290
rs77285108
rs7742829
All − MR Egger
All − IVW(MRE)
−1
0
1
2
MR effect size for
'Gut microbiota abundance (genus Catenibacterium id.2153)' on 'Uncharacterized protein C1orf115'

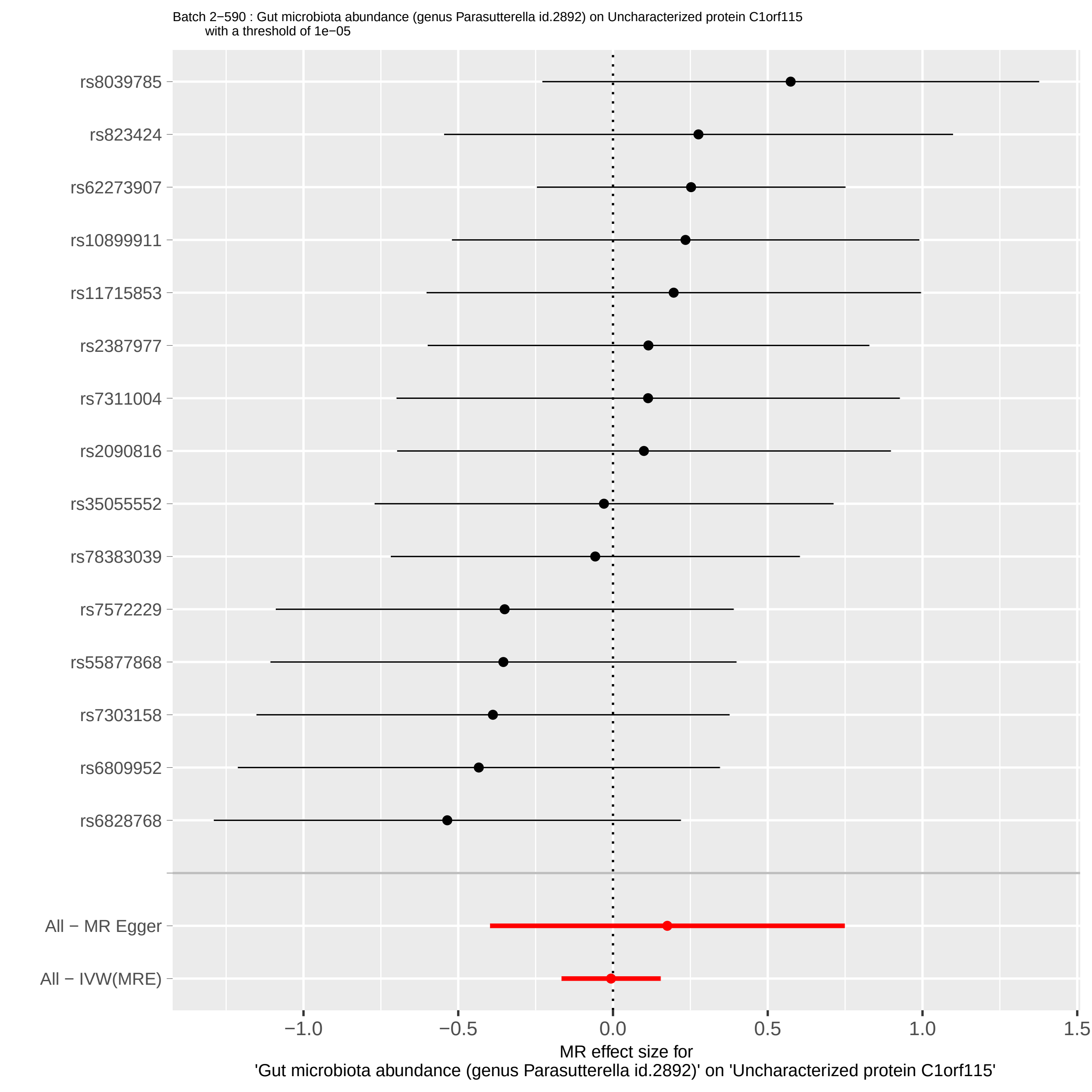

Batch 2−590 : Gut microbiota abundance (genus Parasutterella id.2892) on Uncharacterized protein C1orf115
with a threshold of 1e−05
rs8039785
rs823424
rs62273907
rs10899911
rs11715853
rs2387977
rs7311004
rs2090816
rs35055552
rs78383039
rs7572229
rs55877868
rs7303158
rs6809952
rs6828768
All − MR Egger
All − IVW(MRE)
−1.0
−0.5
0.0
0.5
1.0
1.5
MR effect size for
'Gut microbiota abundance (genus Parasutterella id.2892)' on 'Uncharacterized protein C1orf115'

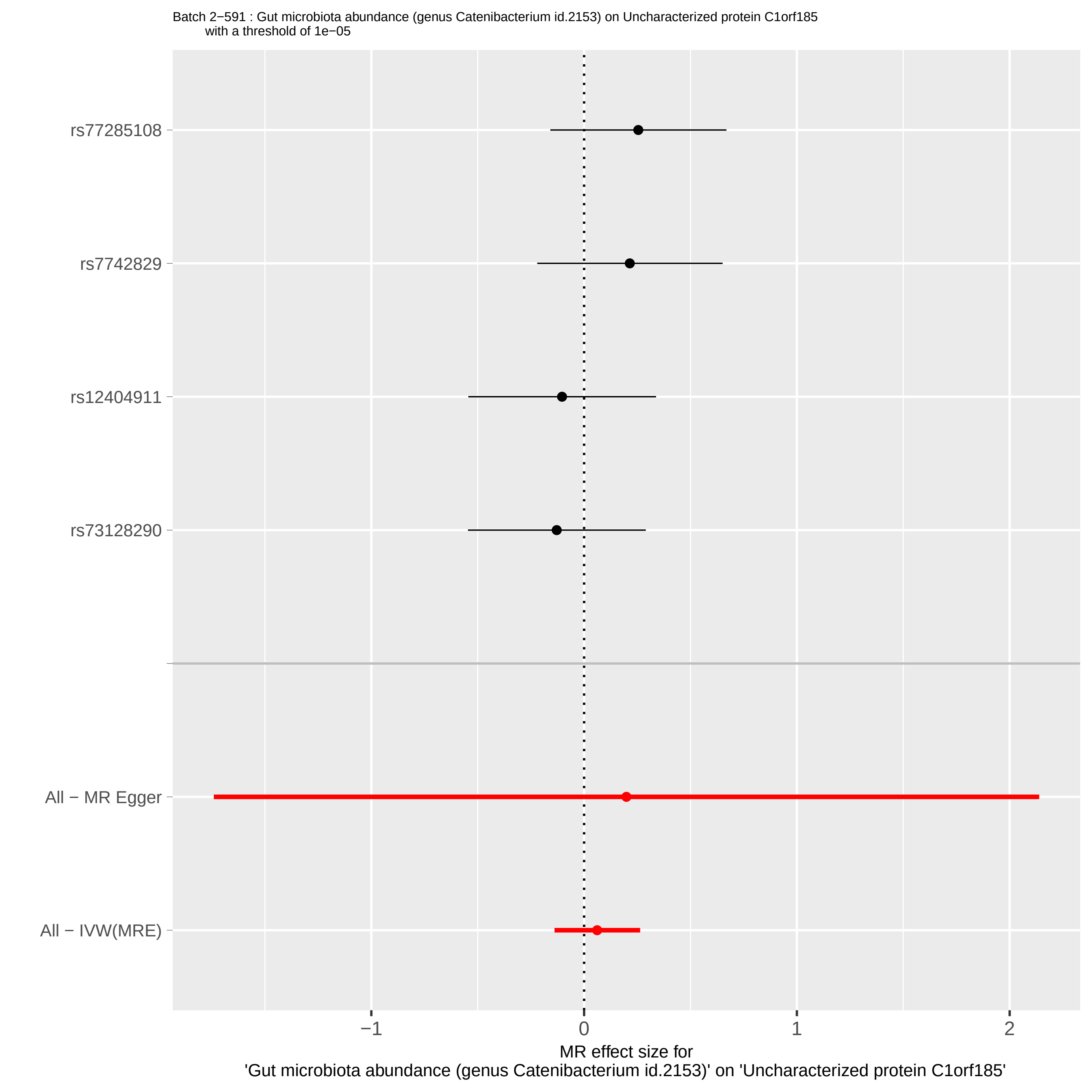
Batch 2−591 : Gut microbiota abundance (genus Catenibacterium id.2153) on Uncharacterized protein C1orf185
with a threshold of 1e−05
rs77285108
rs7742829
rs12404911
rs73128290
All − MR Egger
All − IVW(MRE)
−1
0
1
2
MR effect size for
'Gut microbiota abundance (genus Catenibacterium id.2153)' on 'Uncharacterized protein C1orf185'

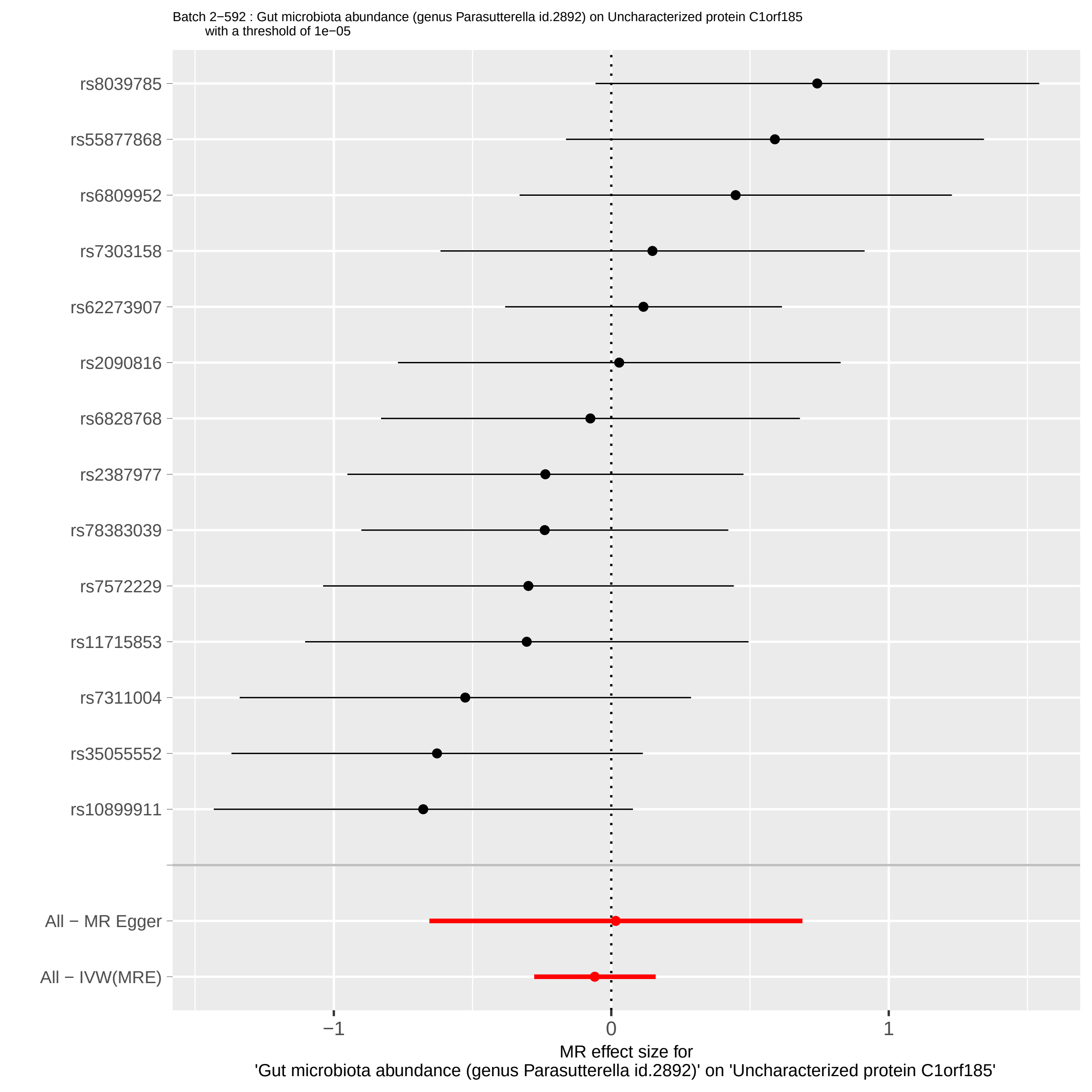

Batch 2−592 : Gut microbiota abundance (genus Parasutterella id.2892) on Uncharacterized protein C1orf185
with a threshold of 1e−05
rs8039785
rs55877868
rs6809952
rs7303158
rs62273907
rs2090816
rs6828768
rs2387977
rs78383039
rs7572229
rs11715853
rs7311004
rs35055552
rs10899911
All − MR Egger
All − IVW(MRE)
−1
0
1
MR effect size for
'Gut microbiota abundance (genus Parasutterella id.2892)' on 'Uncharacterized protein C1orf185'

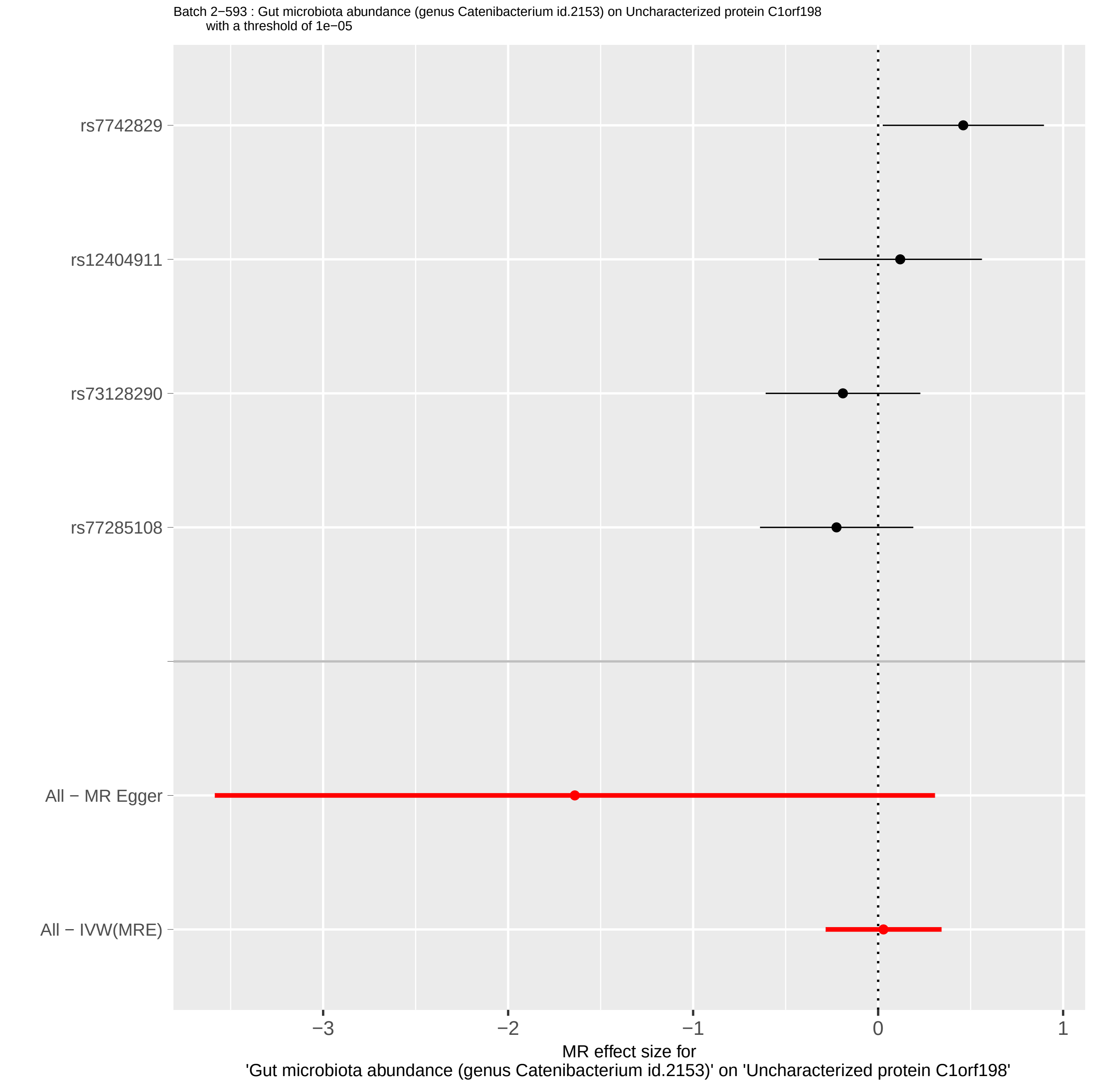

Batch 2−593 : Gut microbiota abundance (genus Catenibacterium id.2153) on Uncharacterized protein C1orf198
with a threshold of 1e−05
rs7742829
rs12404911
rs73128290
rs77285108
All − MR Egger
All − IVW(MRE)
−3
−2
−1
0
1
MR effect size for
'Gut microbiota abundance (genus Catenibacterium id.2153)' on 'Uncharacterized protein C1orf198'

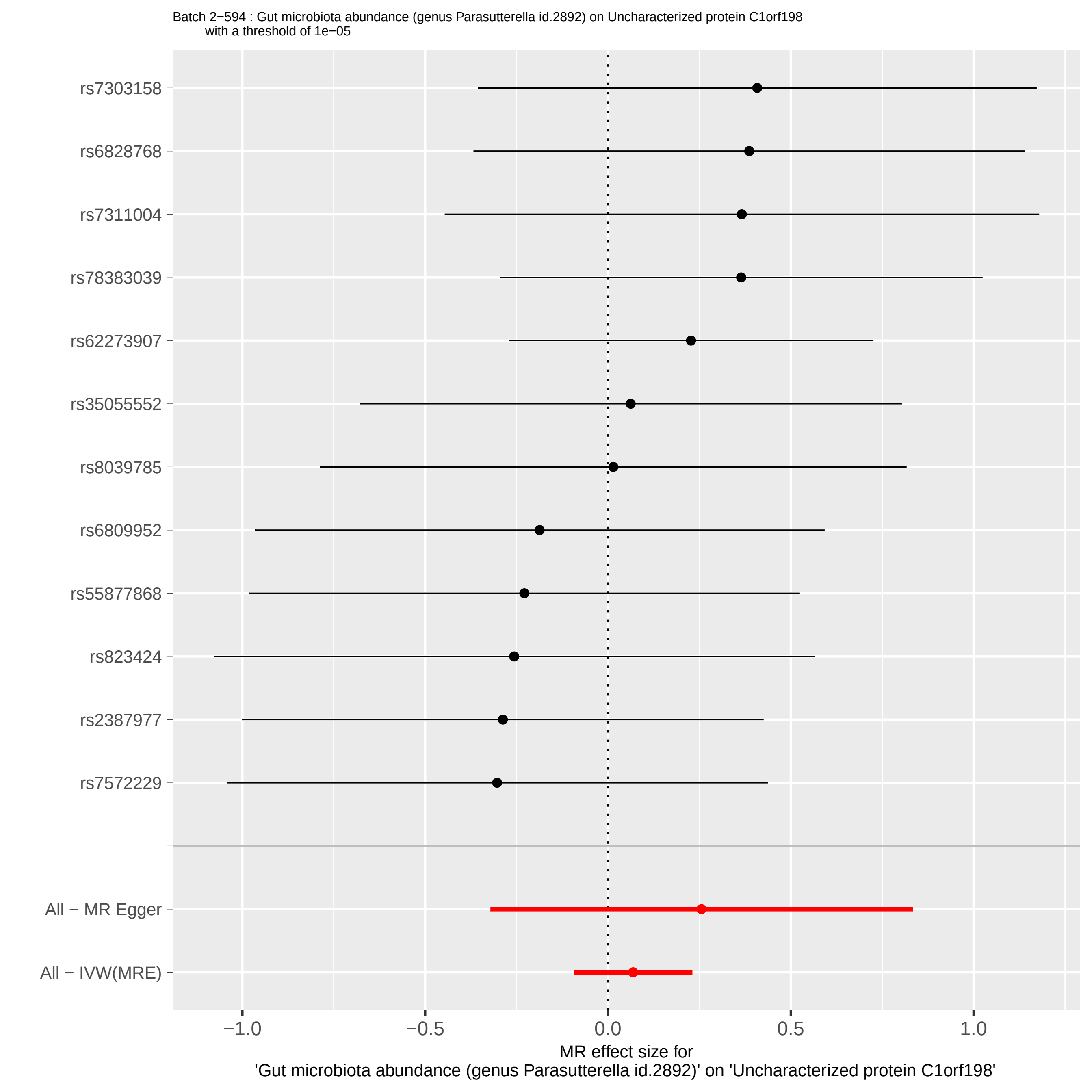

Batch 2−594 : Gut microbiota abundance (genus Parasutterella id.2892) on Uncharacterized protein C1orf198
with a threshold of 1e−05
rs7303158
rs6828768
rs7311004
rs78383039
rs62273907
rs35055552
rs8039785
rs6809952
rs55877868
rs823424
rs2387977
rs7572229
All − MR Egger
All − IVW(MRE)
−1.0
−0.5
0.0
0.5
1.0
MR effect size for
'Gut microbiota abundance (genus Parasutterella id.2892)' on 'Uncharacterized protein C1orf198'

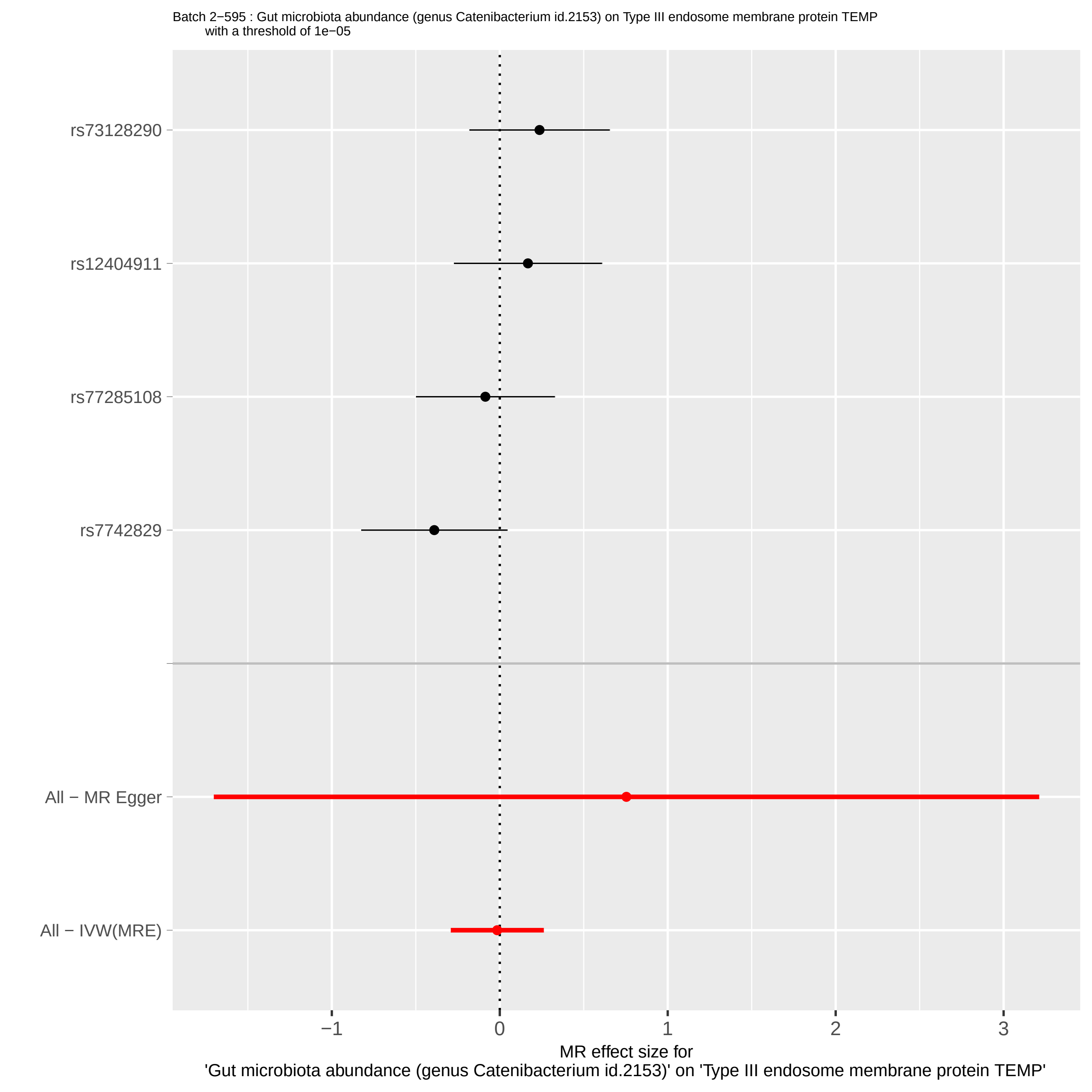
Batch 2−595 : Gut microbiota abundance (genus Catenibacterium id.2153) on Type III endosome membrane protein TEMP
with a threshold of 1e−05
rs73128290
rs12404911
rs77285108
rs7742829
All − MR Egger
All − IVW(MRE)
−1
0
1
2
3
MR effect size for
'Gut microbiota abundance (genus Catenibacterium id.2153)' on 'Type III endosome membrane protein TEMP'

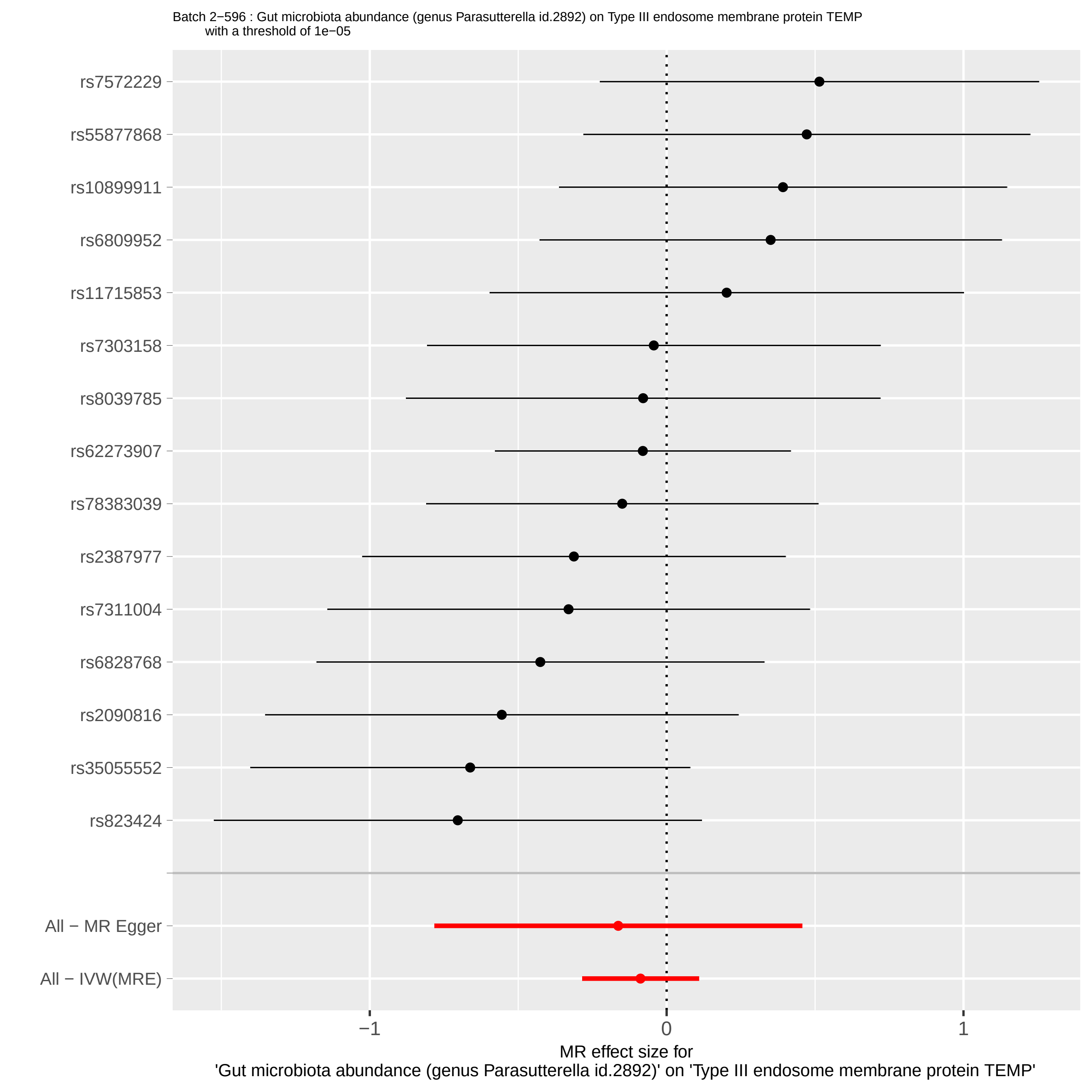

Batch 2−596 : Gut microbiota abundance (genus Parasutterella id.2892) on Type III endosome membrane protein TEMP
with a threshold of 1e−05
rs7572229
rs55877868
rs10899911
rs6809952
rs11715853
rs7303158
rs8039785
rs62273907
rs78383039
rs2387977
rs7311004
rs6828768
rs2090816
rs35055552
rs823424
All − MR Egger
All − IVW(MRE)
−1
0
1
MR effect size for
'Gut microbiota abundance (genus Parasutterella id.2892)' on 'Type III endosome membrane protein TEMP'

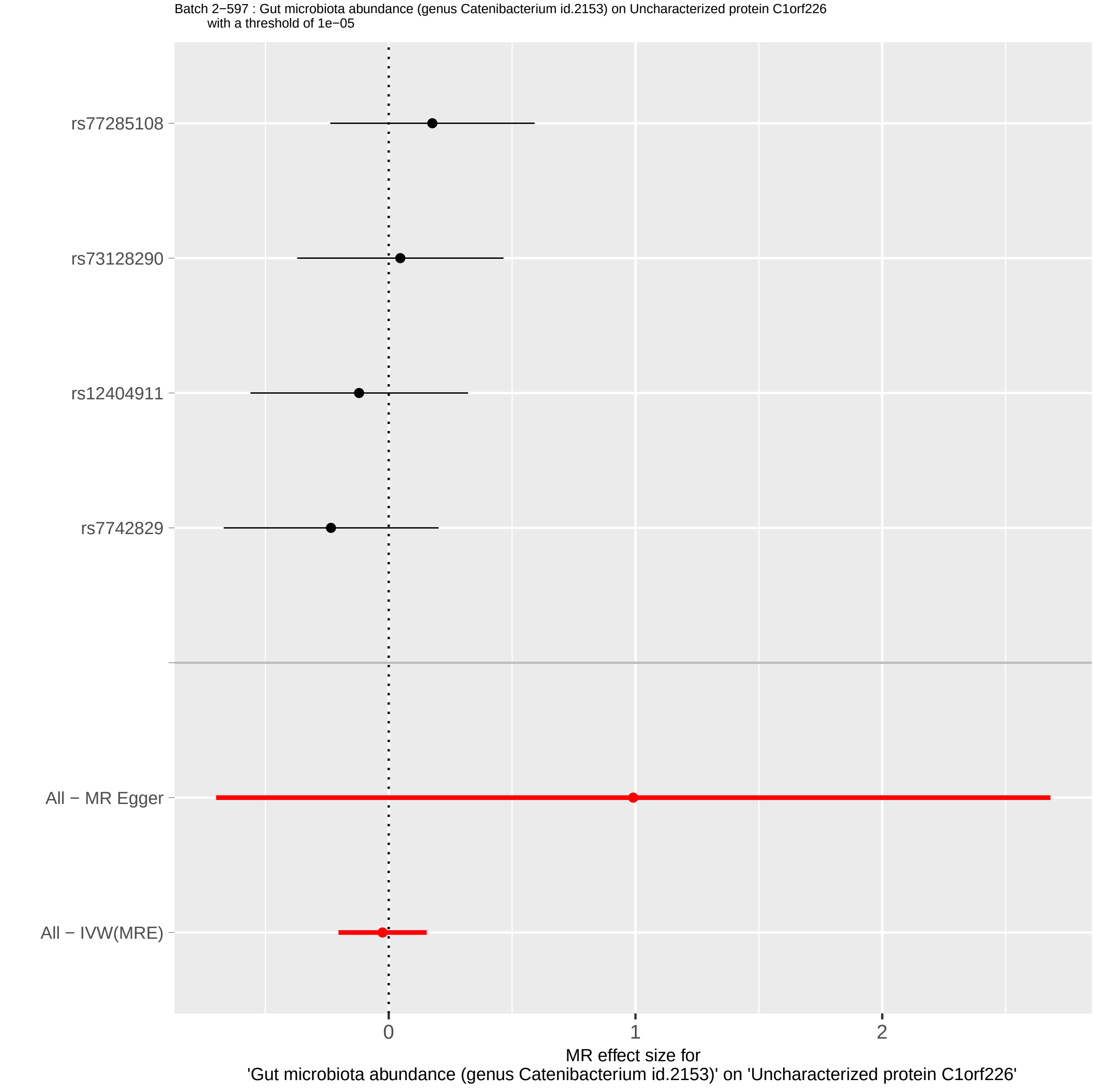

Batch 2−597 : Gut microbiota abundance (genus Catenibacterium id.2153) on Uncharacterized protein C1orf226
with a threshold of 1e−05
rs77285108
rs73128290
rs12404911
rs7742829
All − MR Egger
All − IVW(MRE)
0
1
2
MR effect size for
'Gut microbiota abundance (genus Catenibacterium id.2153)' on 'Uncharacterized protein C1orf226'

Batch 2−598 : Gut microbiota abundance (genus Parasutterella id.2892) on Uncharacterized protein C1orf226
with a threshold of 1e−05

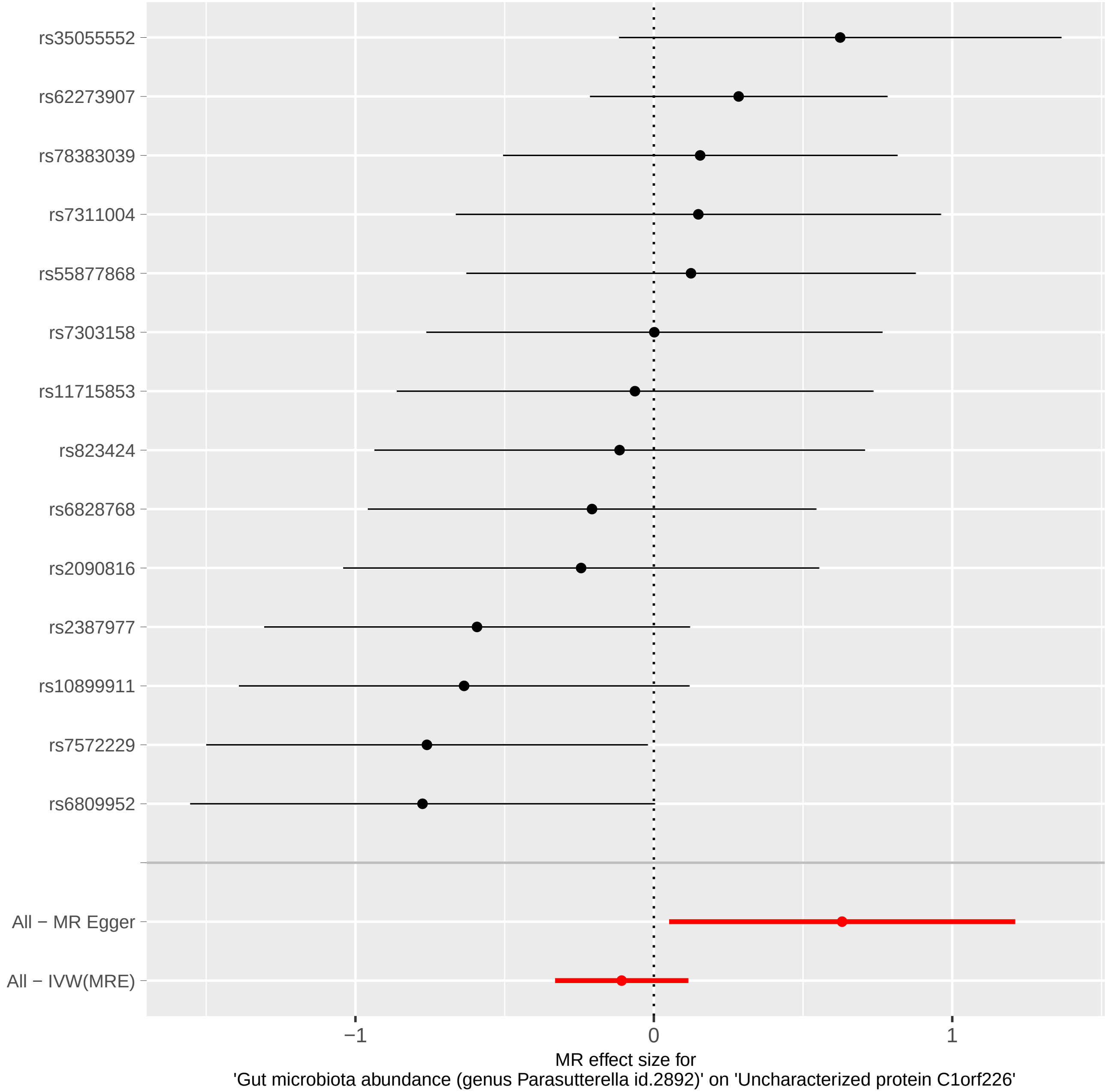

Batch 2−599 : Gut microbiota abundance (genus Catenibacterium id.2153) on Complement component 1 Q subcomponent−binding protein, mitochondrial with a threshold of 1e−05

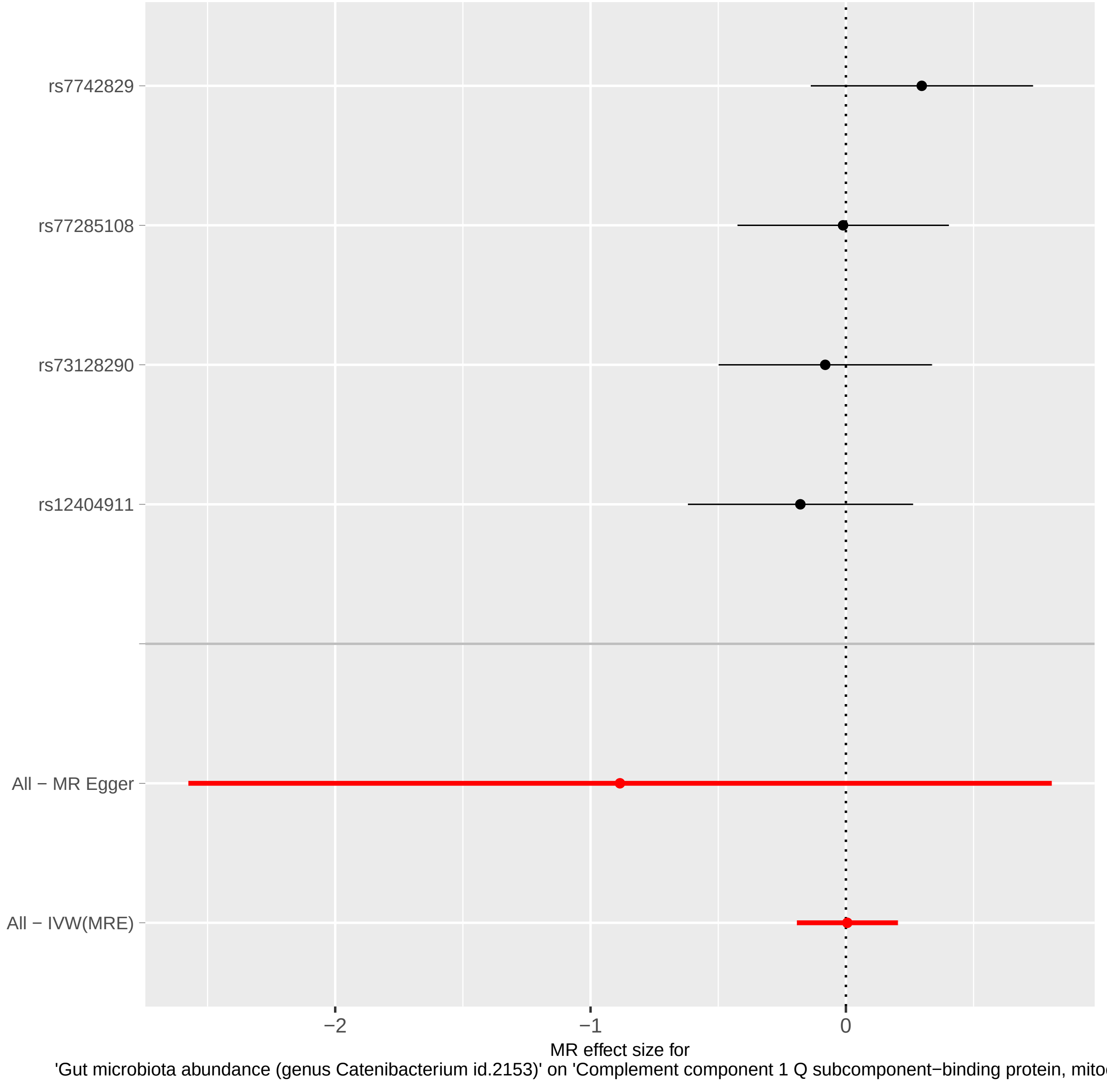

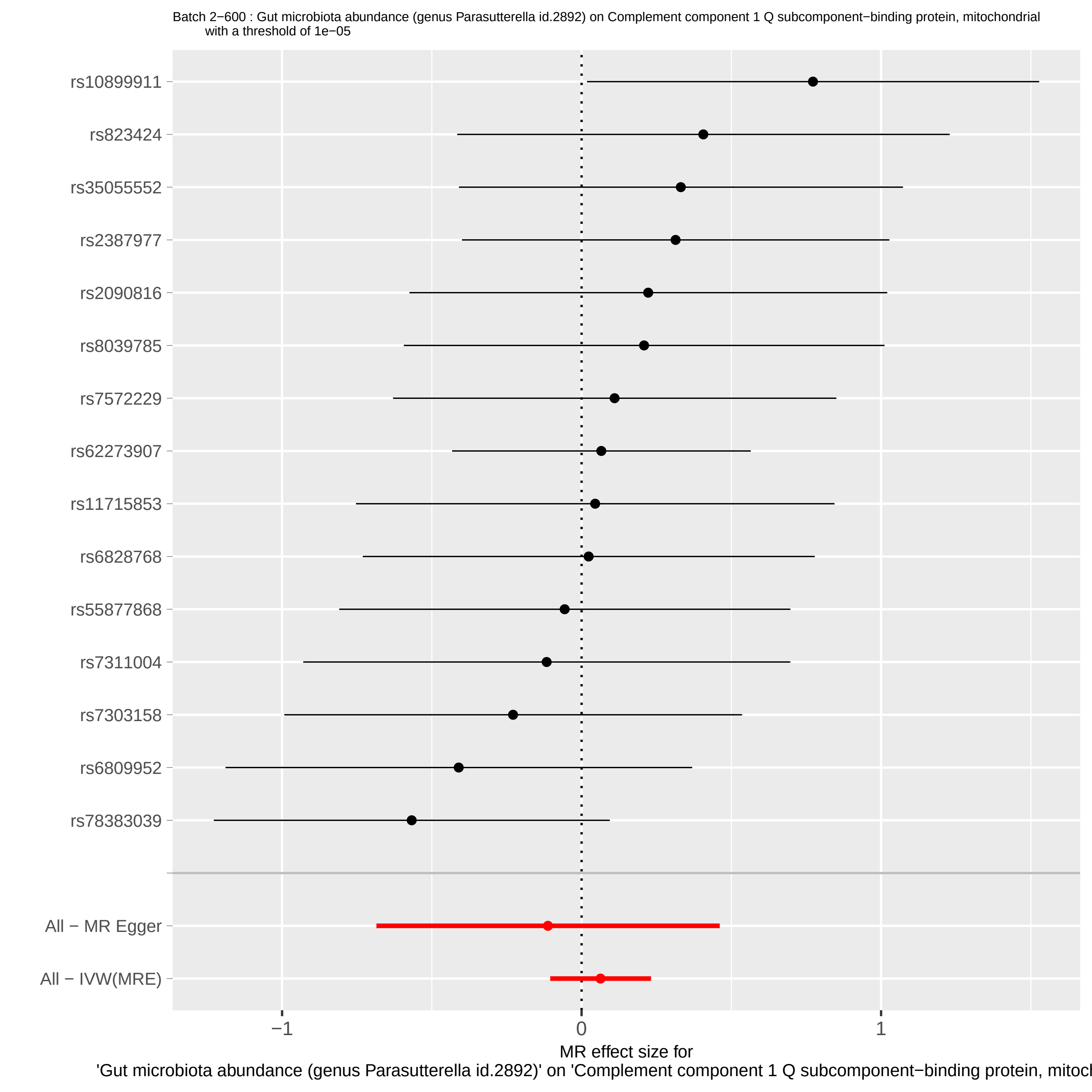
Batch 2−600 : Gut microbiota abundance (genus Parasutterella id.2892) on Complement component 1 Q subcomponent−binding protein, mitochondrial with a threshold of 1e−05
rs10899911
rs823424
rs35055552
rs2387977
rs2090816
rs8039785
rs7572229
rs62273907
rs11715853
rs6828768
rs55877868
rs7311004
rs7303158
rs6809952
rs78383039
All − MR Egger
All − IVW(MRE)
−1
0
1
MR effect size for
'Gut microbiota abundance (genus Parasutterella id.2892)' on 'Complement component 1 Q subcomponent−binding protein, mitoch

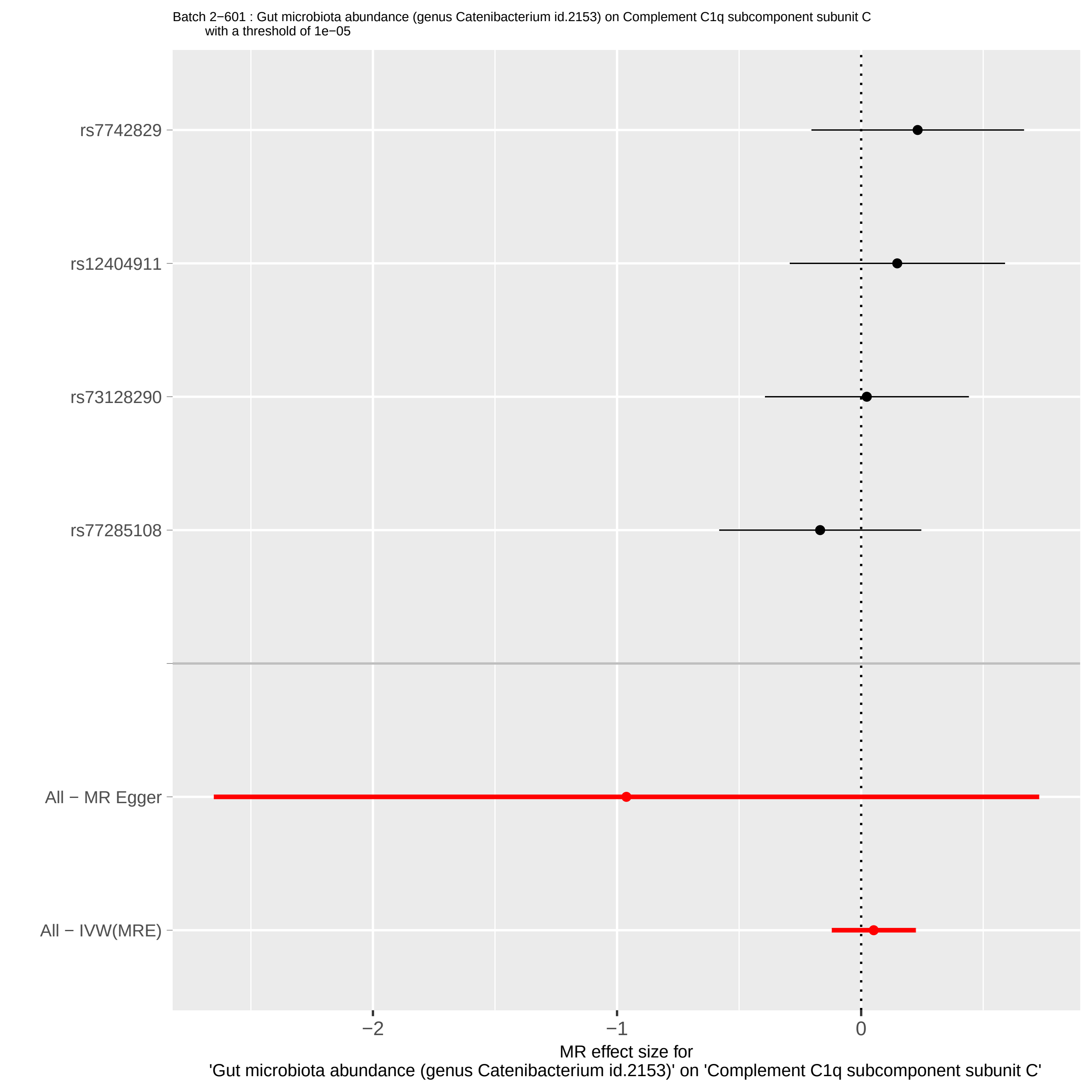
Batch 2−601 : Gut microbiota abundance (genus Catenibacterium id.2153) on Complement C1q subcomponent subunit C
with a threshold of 1e−05
rs7742829
rs12404911
rs73128290
rs77285108
All − MR Egger
All − IVW(MRE)
−2
−1
0
MR effect size for
'Gut microbiota abundance (genus Catenibacterium id.2153)' on 'Complement C1q subcomponent subunit C'

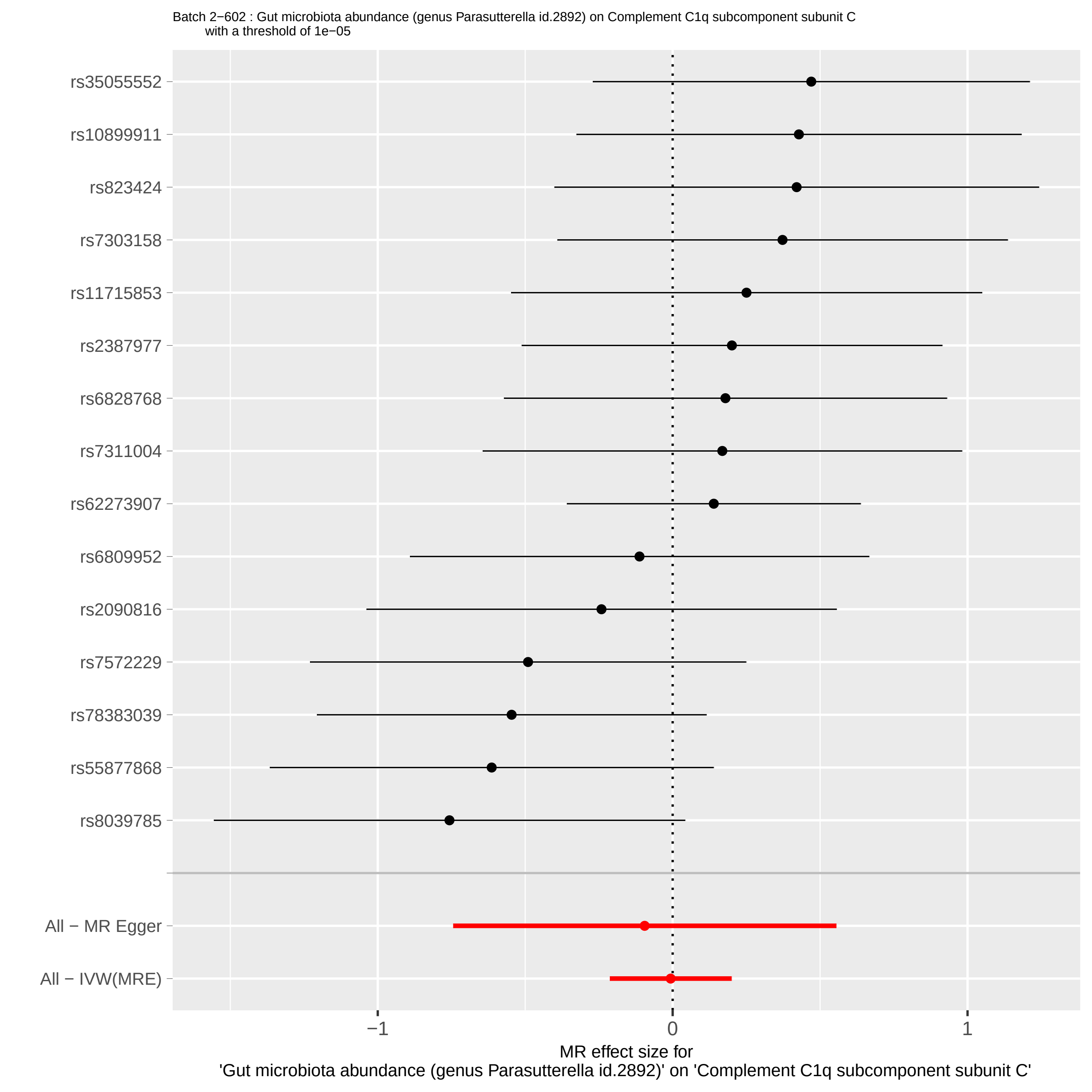

Batch 2−602 : Gut microbiota abundance (genus Parasutterella id.2892) on Complement C1q subcomponent subunit C
with a threshold of 1e−05
rs35055552
rs10899911
rs823424
rs7303158
rs11715853
rs2387977
rs6828768
rs7311004
rs62273907
rs6809952
rs2090816
rs7572229
rs78383039
rs55877868
rs8039785
All − MR Egger
All − IVW(MRE)
−1
0
1
MR effect size for
'Gut microbiota abundance (genus Parasutterella id.2892)' on 'Complement C1q subcomponent subunit C'

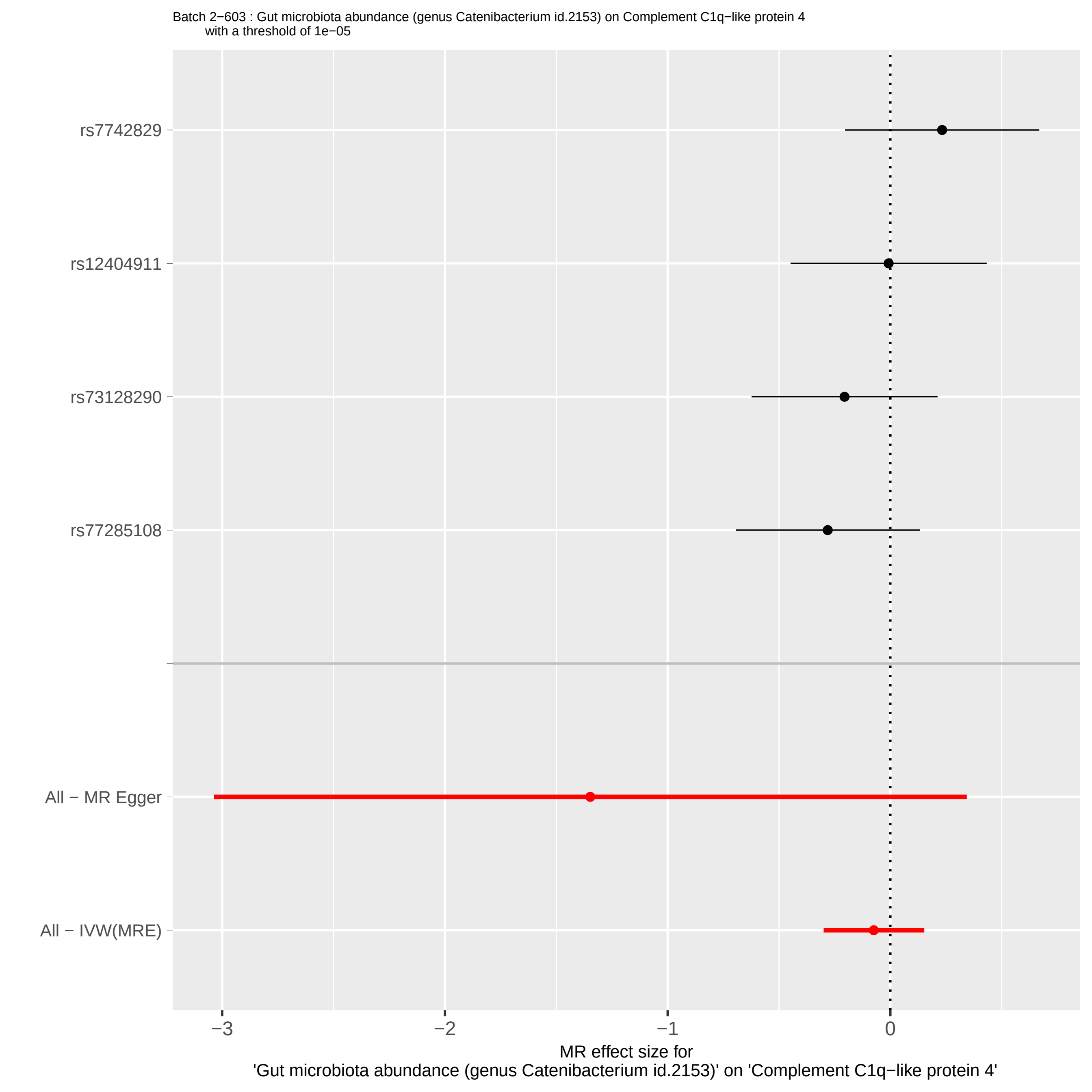

Batch 2−603 : Gut microbiota abundance (genus Catenibacterium id.2153) on Complement C1q−like protein 4
with a threshold of 1e−05
rs7742829
rs12404911
rs73128290
rs77285108
All − MR Egger
All − IVW(MRE)
−3
−2
−1
0
MR effect size for
'Gut microbiota abundance (genus Catenibacterium id.2153)' on 'Complement C1q−like protein 4'

Batch 2−604 : Gut microbiota abundance (genus Parasutterella id.2892) on Complement C1q−like protein 4
with a threshold of 1e−05

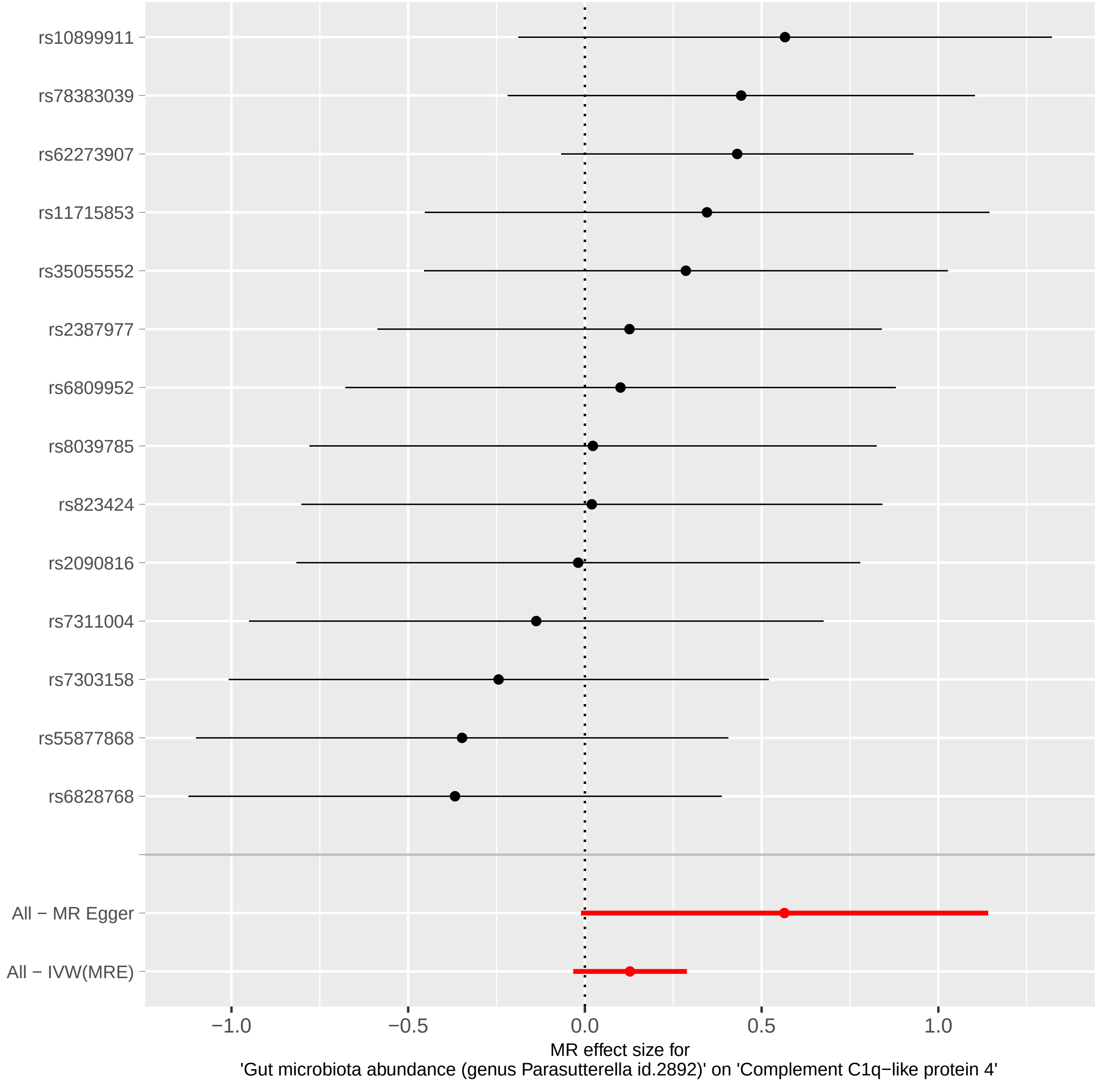

Batch 2−605 : Gut microbiota abundance (genus Catenibacterium id.2153) on Complement C1q tumor necrosis factor−related protein 1 with a threshold of 1e−05

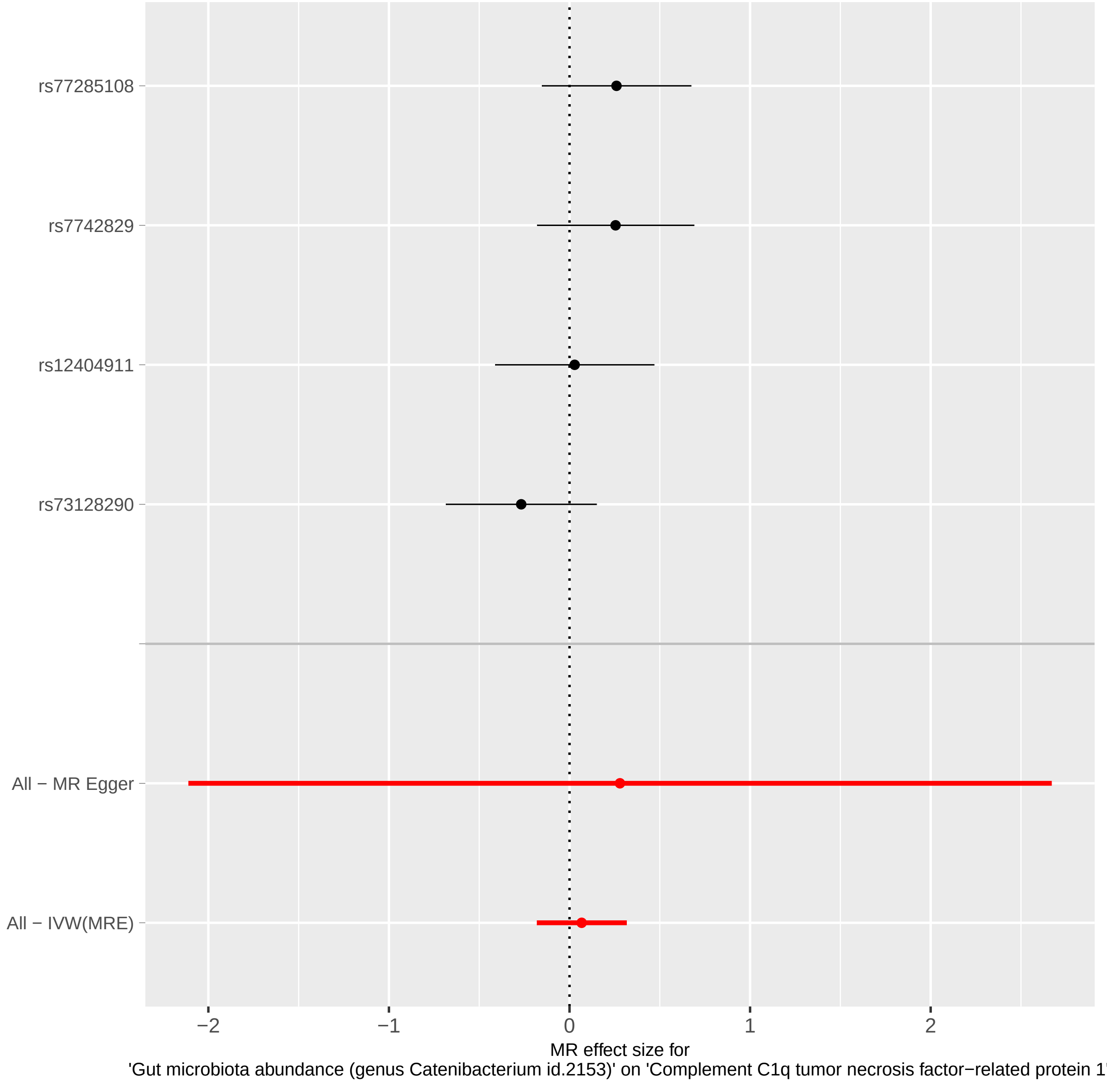

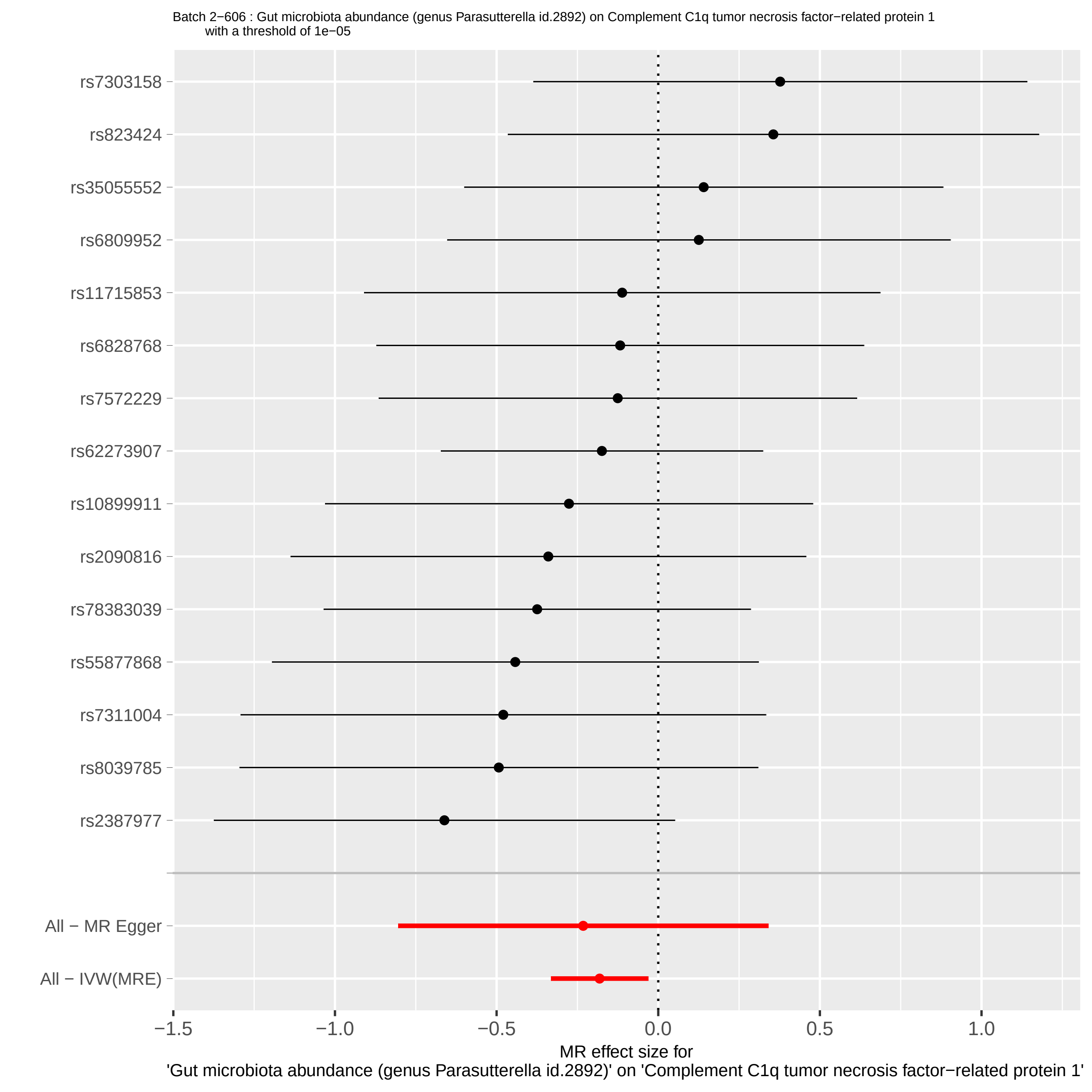

Batch 2−606 : Gut microbiota abundance (genus Parasutterella id.2892) on Complement C1q tumor necrosis factor−related protein 1 with a threshold of 1e−05
rs7303158
rs823424
rs35055552
rs6809952
rs11715853
rs6828768
rs7572229
rs62273907
rs10899911
rs2090816
rs78383039
rs55877868
rs7311004
rs8039785
rs2387977
All − MR Egger
All − IVW(MRE)
−1.5
−1.0
−0.5
0.0
0.5
1.0
MR effect size for
'Gut microbiota abundance (genus Parasutterella id.2892)' on 'Complement C1q tumor necrosis factor−related protein 1'

Batch 2−607 : Gut microbiota abundance (genus Catenibacterium id.2153) on Complement C1q tumor necrosis factor−related protein 3 with a threshold of 1e−05

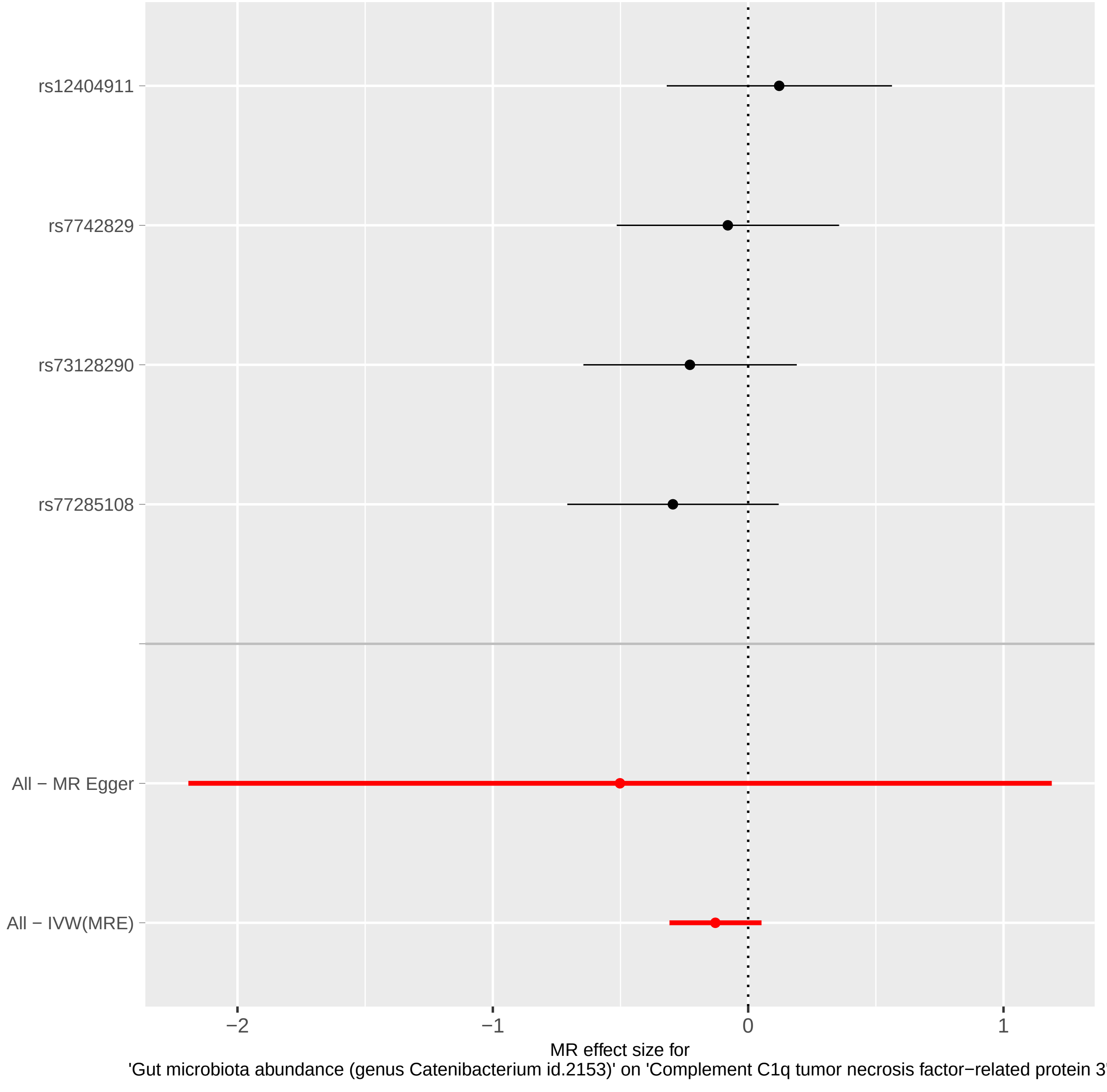

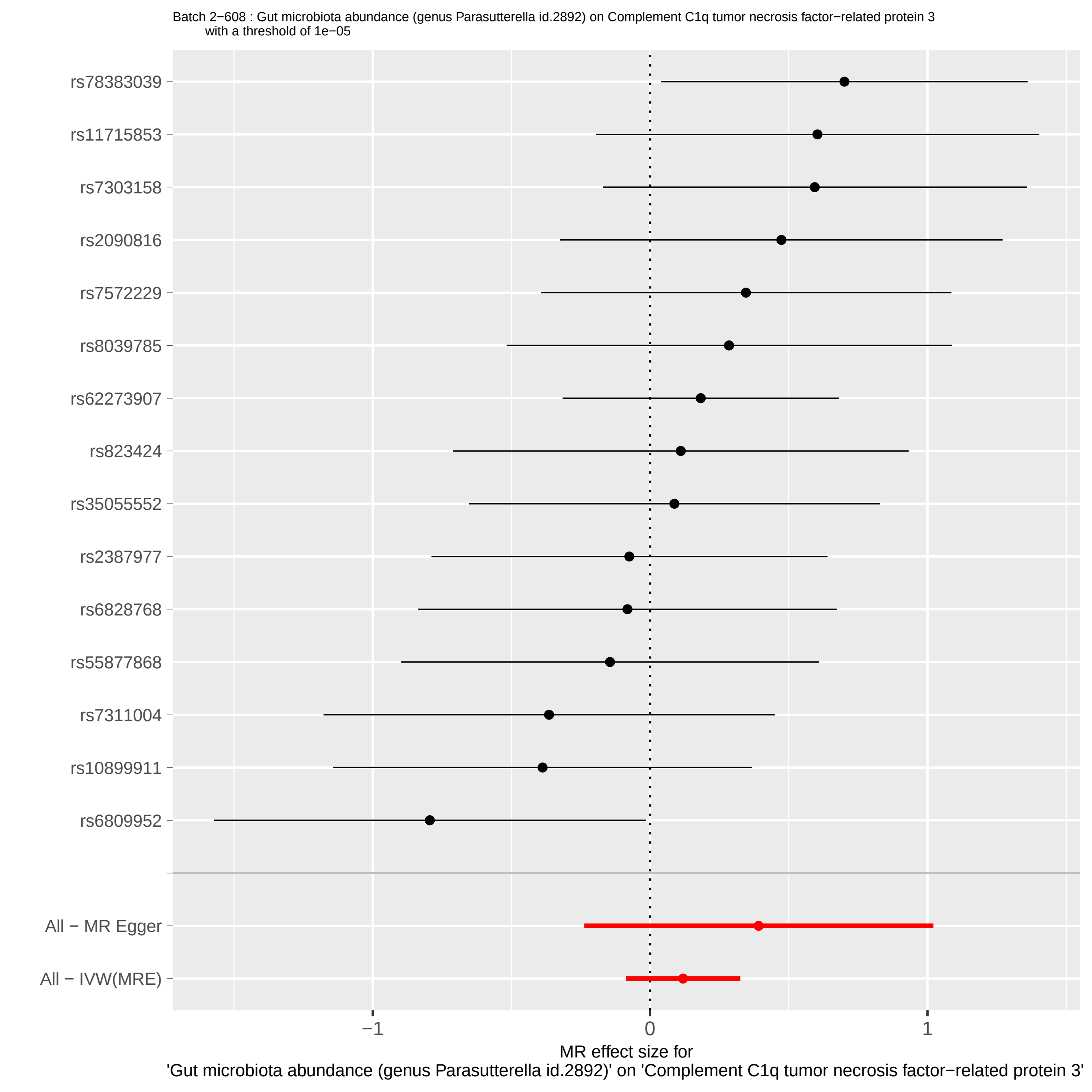
Batch 2−608 : Gut microbiota abundance (genus Parasutterella id.2892) on Complement C1q tumor necrosis factor−related protein 3
with a threshold of 1e−05
rs78383039
rs11715853
rs7303158
rs2090816
rs7572229
rs8039785
rs62273907
rs823424
rs35055552
rs2387977
rs6828768
rs55877868
rs7311004
rs10899911
rs6809952
All − MR Egger
All − IVW(MRE)
−1
0
1
MR effect size for
'Gut microbiota abundance (genus Parasutterella id.2892)' on 'Complement C1q tumor necrosis factor−related protein 3'

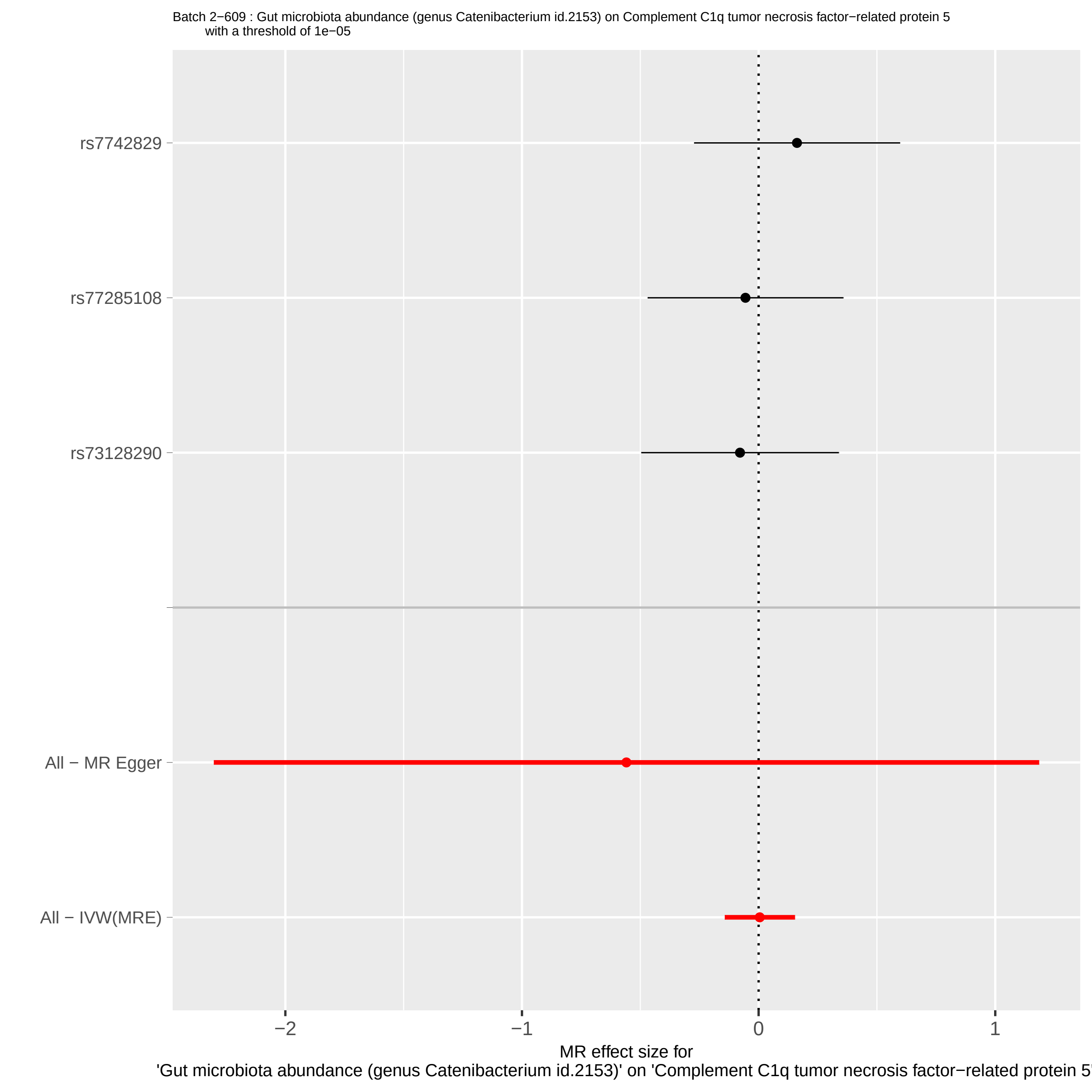

Batch 2−609 : Gut microbiota abundance (genus Catenibacterium id.2153) on Complement C1q tumor necrosis factor−related protein 5
with a threshold of 1e−05
rs7742829
rs77285108
rs73128290
All − MR Egger
All − IVW(MRE)
−2
−1
0
1
MR effect size for
'Gut microbiota abundance (genus Catenibacterium id.2153)' on 'Complement C1q tumor necrosis factor−related protein 5'

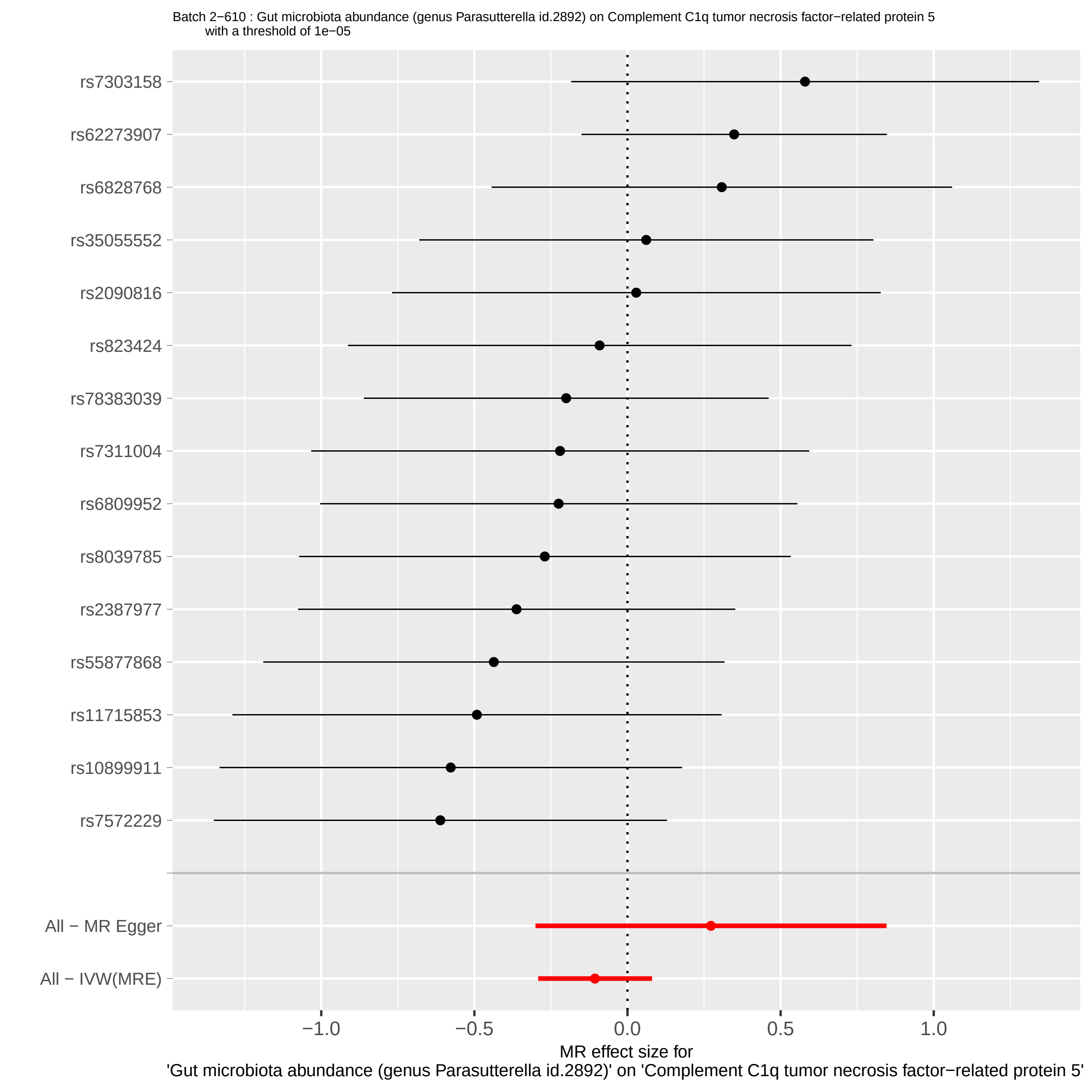

Batch 2−610 : Gut microbiota abundance (genus Parasutterella id.2892) on Complement C1q tumor necrosis factor−related protein 5
with a threshold of 1e−05
rs7303158
rs62273907
rs6828768
rs35055552
rs2090816
rs823424
rs78383039
rs7311004
rs6809952
rs8039785
rs2387977
rs55877868
rs11715853
rs10899911
rs7572229
All − MR Egger
All − IVW(MRE)
−1.0
−0.5
0.0
0.5
1.0
MR effect size for
'Gut microbiota abundance (genus Parasutterella id.2892)' on 'Complement C1q tumor necrosis factor−related protein 5'

Batch 2−611 : Gut microbiota abundance (genus Catenibacterium id.2153) on Complement C1q and tumor necrosis factor−related protein 9A with a threshold of 1e−05

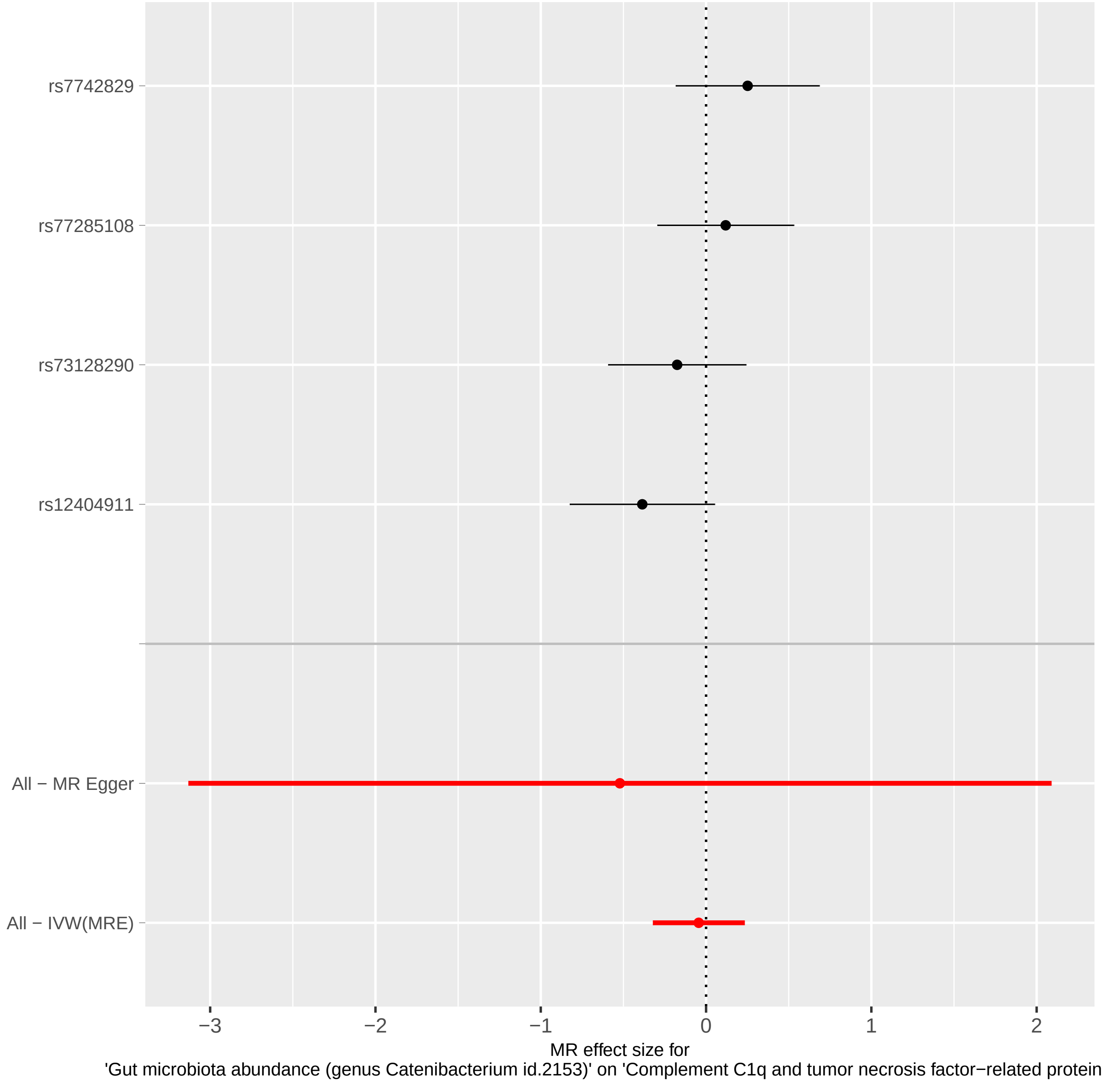

Batch 2−612 : Gut microbiota abundance (genus Parasutterella id.2892) on Complement C1q and tumor necrosis factor−related protein 9A with a threshold of 1e−05

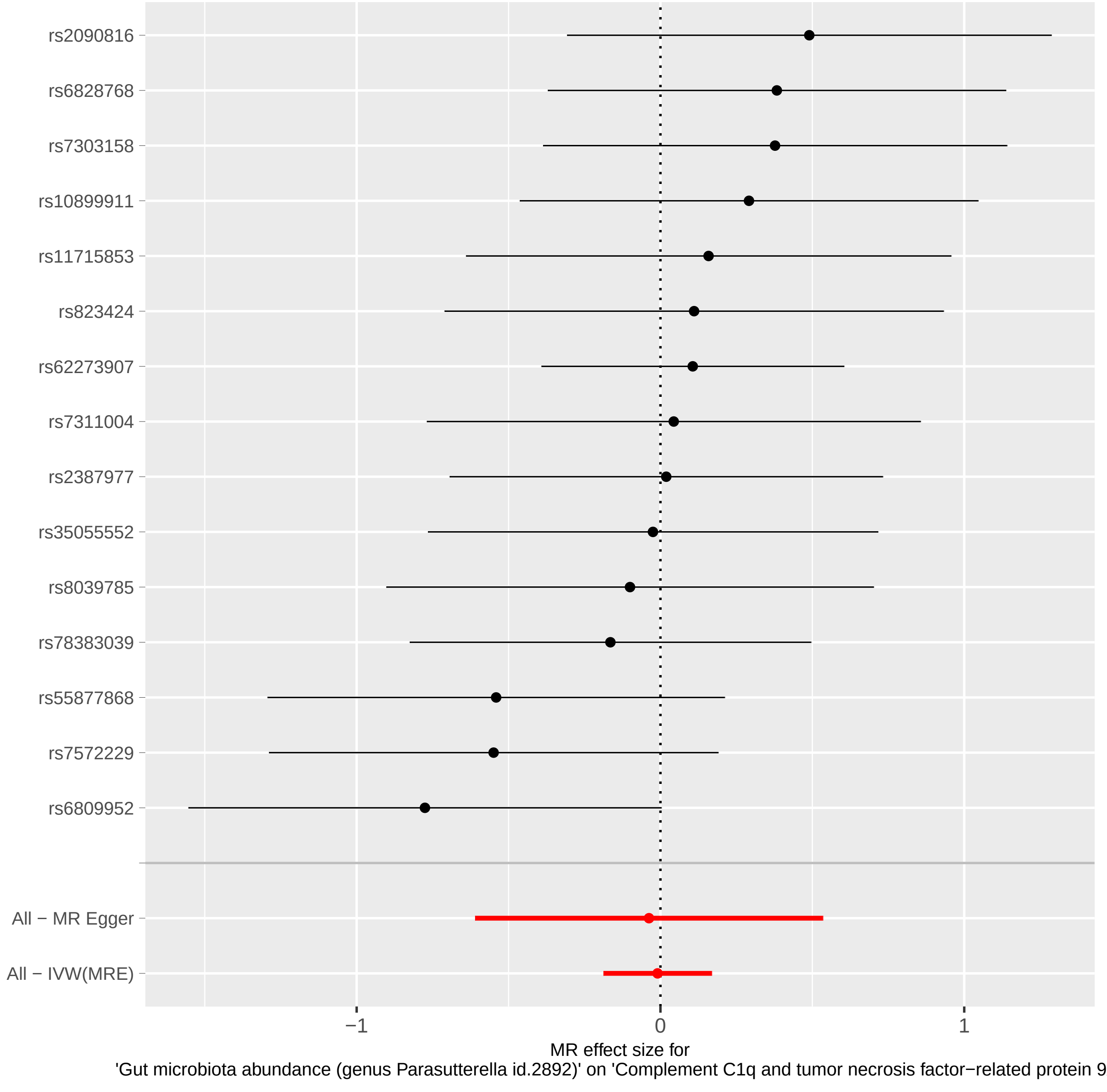

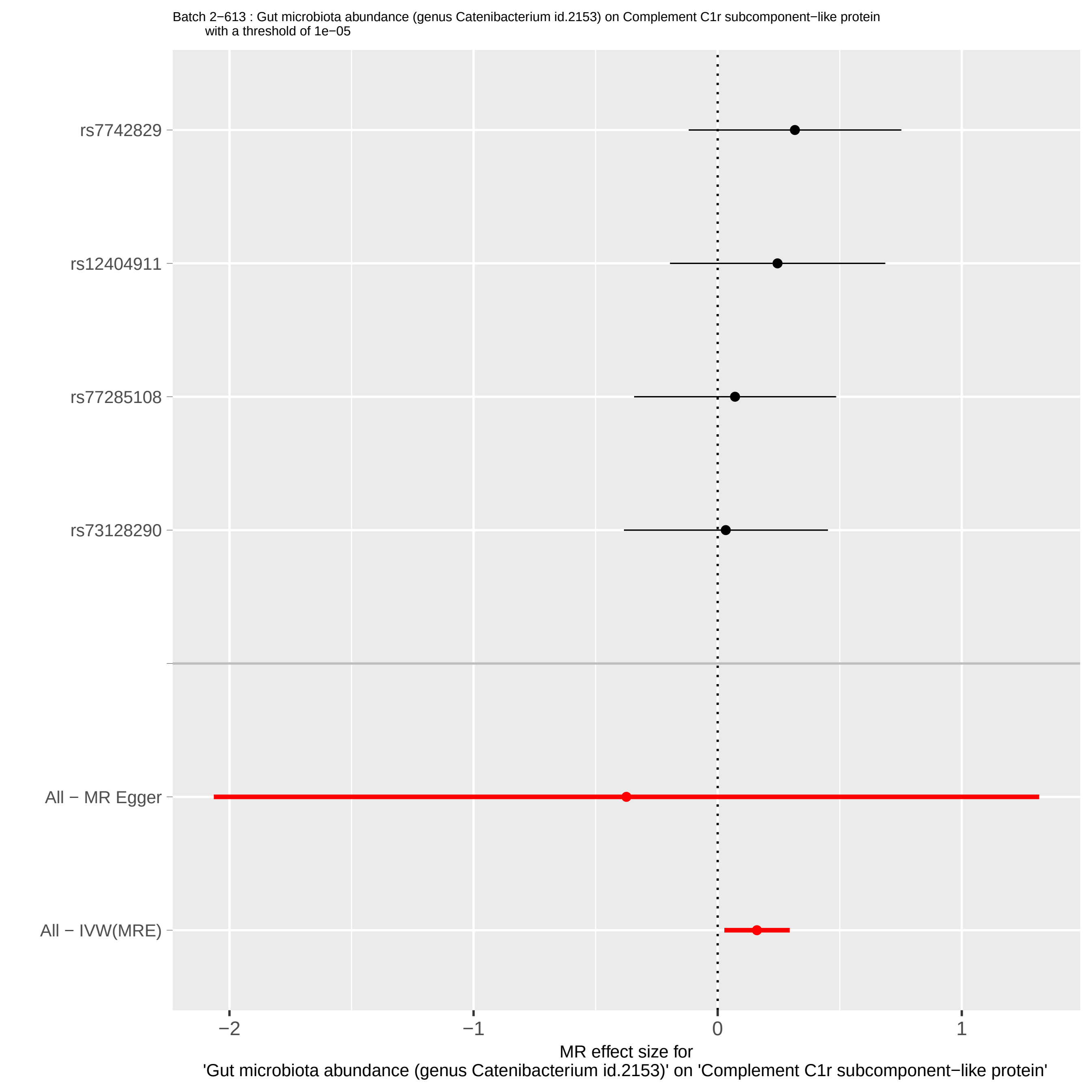
Batch 2−613 : Gut microbiota abundance (genus Catenibacterium id.2153) on Complement C1r subcomponent−like protein
with a threshold of 1e−05
rs7742829
rs12404911
rs77285108
rs73128290
All − MR Egger
All − IVW(MRE)
−2
−1
0
1
MR effect size for
'Gut microbiota abundance (genus Catenibacterium id.2153)' on 'Complement C1r subcomponent−like protein'

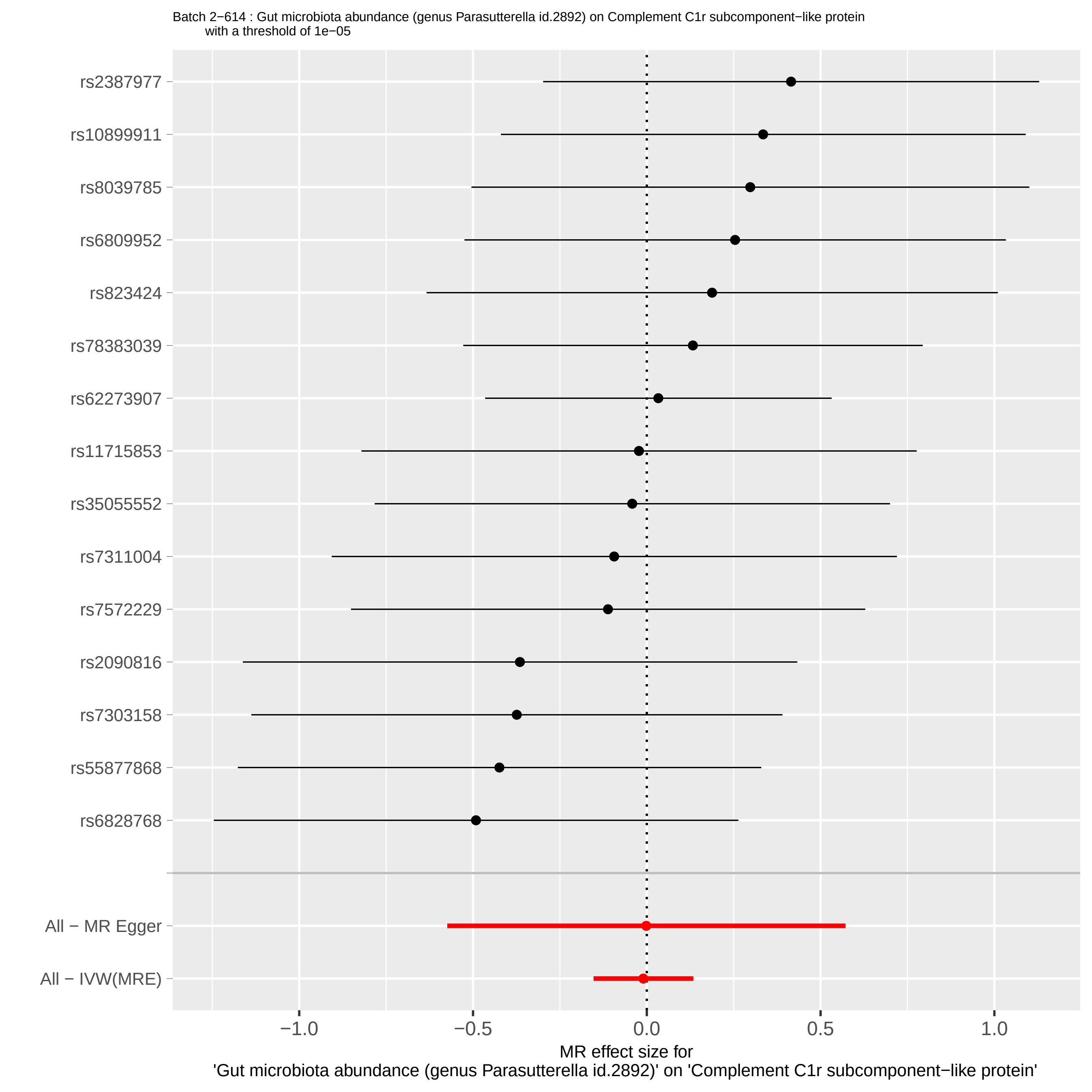
Batch 2−614 : Gut microbiota abundance (genus Parasutterella id.2892) on Complement C1r subcomponent−like protein
with a threshold of 1e−05
rs2387977
rs10899911
rs8039785
rs6809952
rs823424
rs78383039
rs62273907
rs11715853
rs35055552
rs7311004
rs7572229
rs2090816
rs7303158
rs55877868
rs6828768
All − MR Egger
All − IVW(MRE)
−1.0
−0.5
0.0
0.5
1.0
MR effect size for
'Gut microbiota abundance (genus Parasutterella id.2892)' on 'Complement C1r subcomponent−like protein'

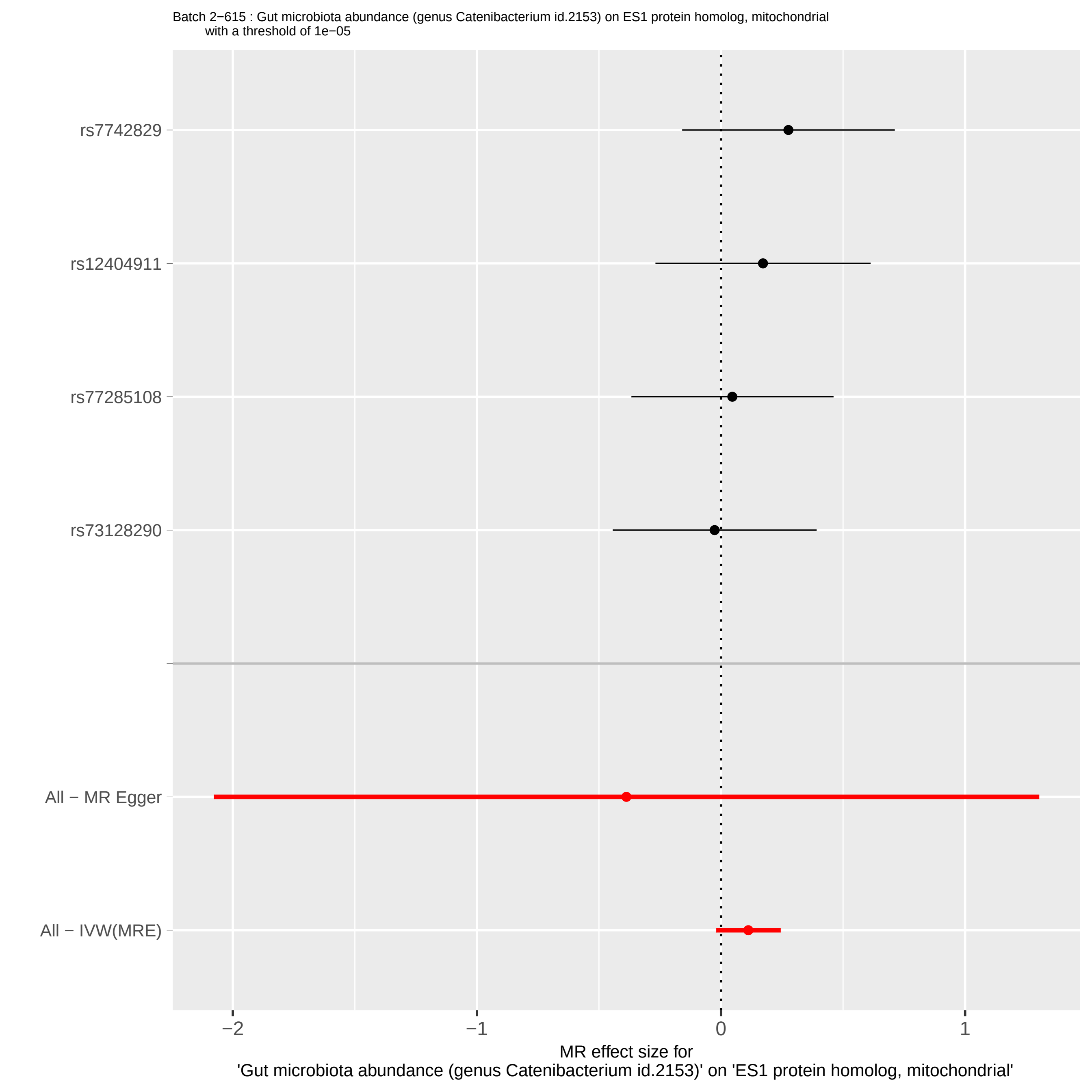

Batch 2−615 : Gut microbiota abundance (genus Catenibacterium id.2153) on ES1 protein homolog, mitochondrial
with a threshold of 1e−05
rs7742829
rs12404911
rs77285108
rs73128290
All − MR Egger
All − IVW(MRE)
−2
−1
0
1
MR effect size for
'Gut microbiota abundance (genus Catenibacterium id.2153)' on 'ES1 protein homolog, mitochondrial'

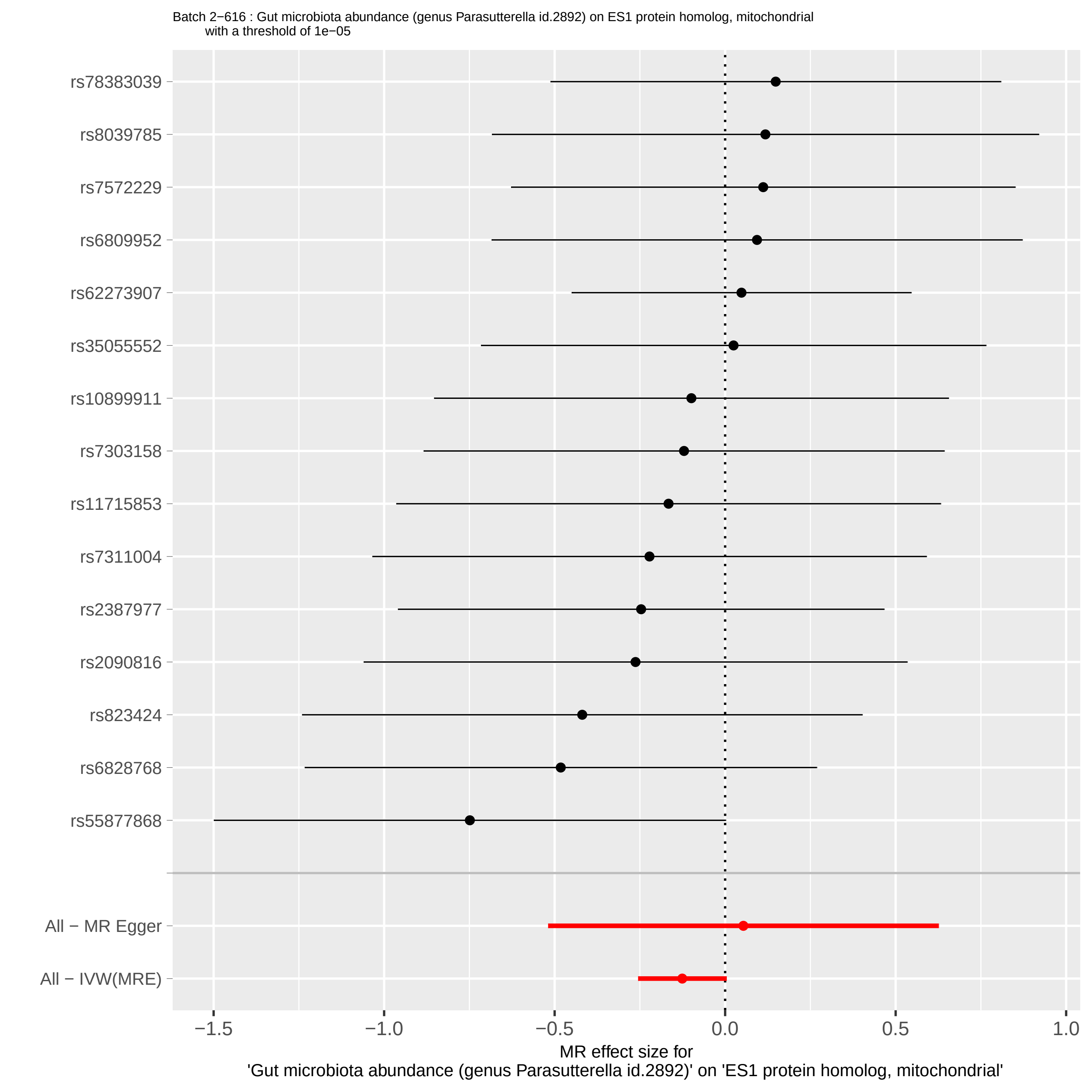

Batch 2−616 : Gut microbiota abundance (genus Parasutterella id.2892) on ES1 protein homolog, mitochondrial
with a threshold of 1e−05
rs78383039
rs8039785
rs7572229
rs6809952
rs62273907
rs35055552
rs10899911
rs7303158
rs11715853
rs7311004
rs2387977
rs2090816
rs823424
rs6828768
rs55877868
All − MR Egger
All − IVW(MRE)
−1.5
−1.0
−0.5
0.0
0.5
1.0
MR effect size for
'Gut microbiota abundance (genus Parasutterella id.2892)' on 'ES1 protein homolog, mitochondrial'

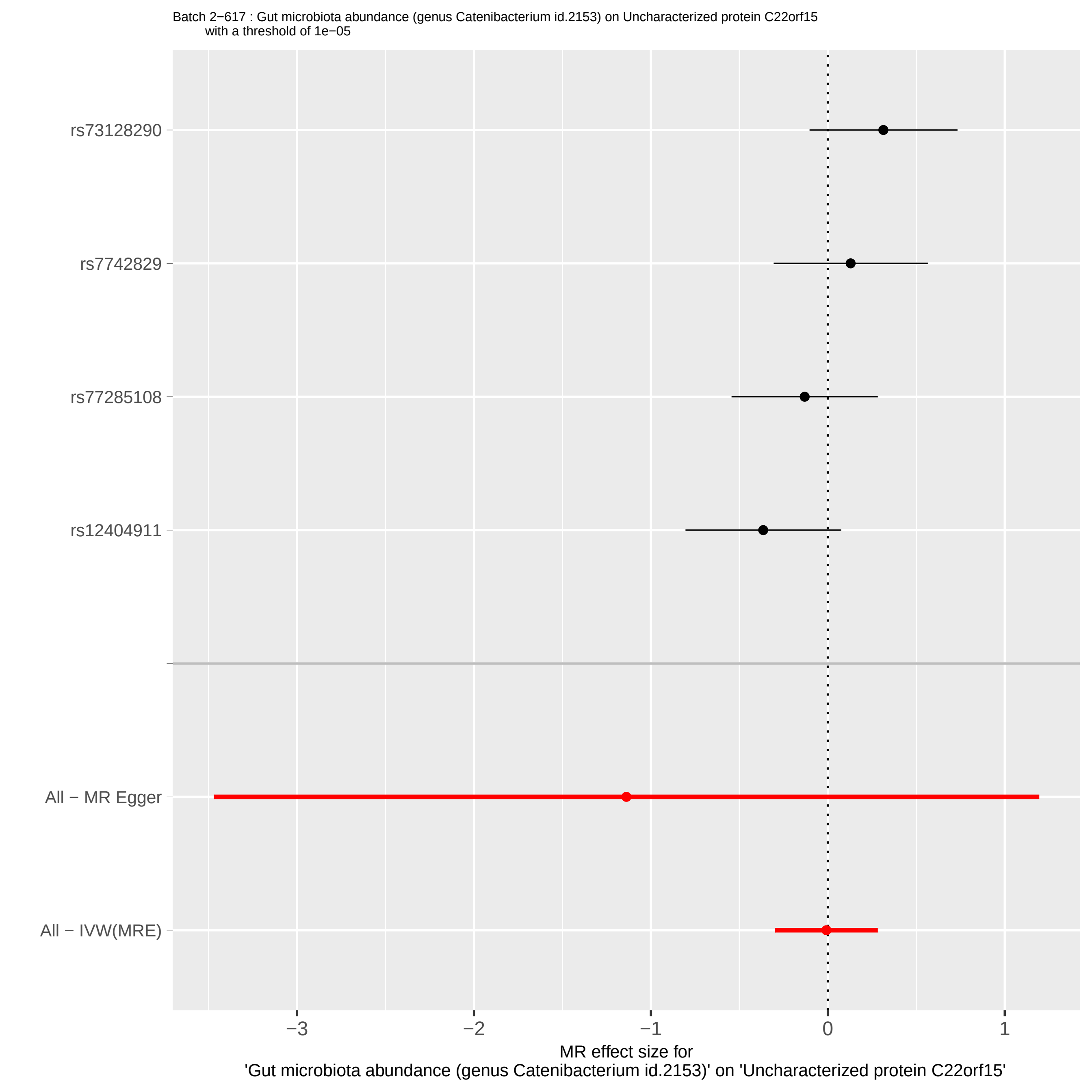
Batch 2−617 : Gut microbiota abundance (genus Catenibacterium id.2153) on Uncharacterized protein C22orf15
with a threshold of 1e−05
rs73128290
rs7742829
rs77285108
rs12404911
All − MR Egger
All − IVW(MRE)
−3
−2
−1
0
1
MR effect size for
'Gut microbiota abundance (genus Catenibacterium id.2153)' on 'Uncharacterized protein C22orf15'

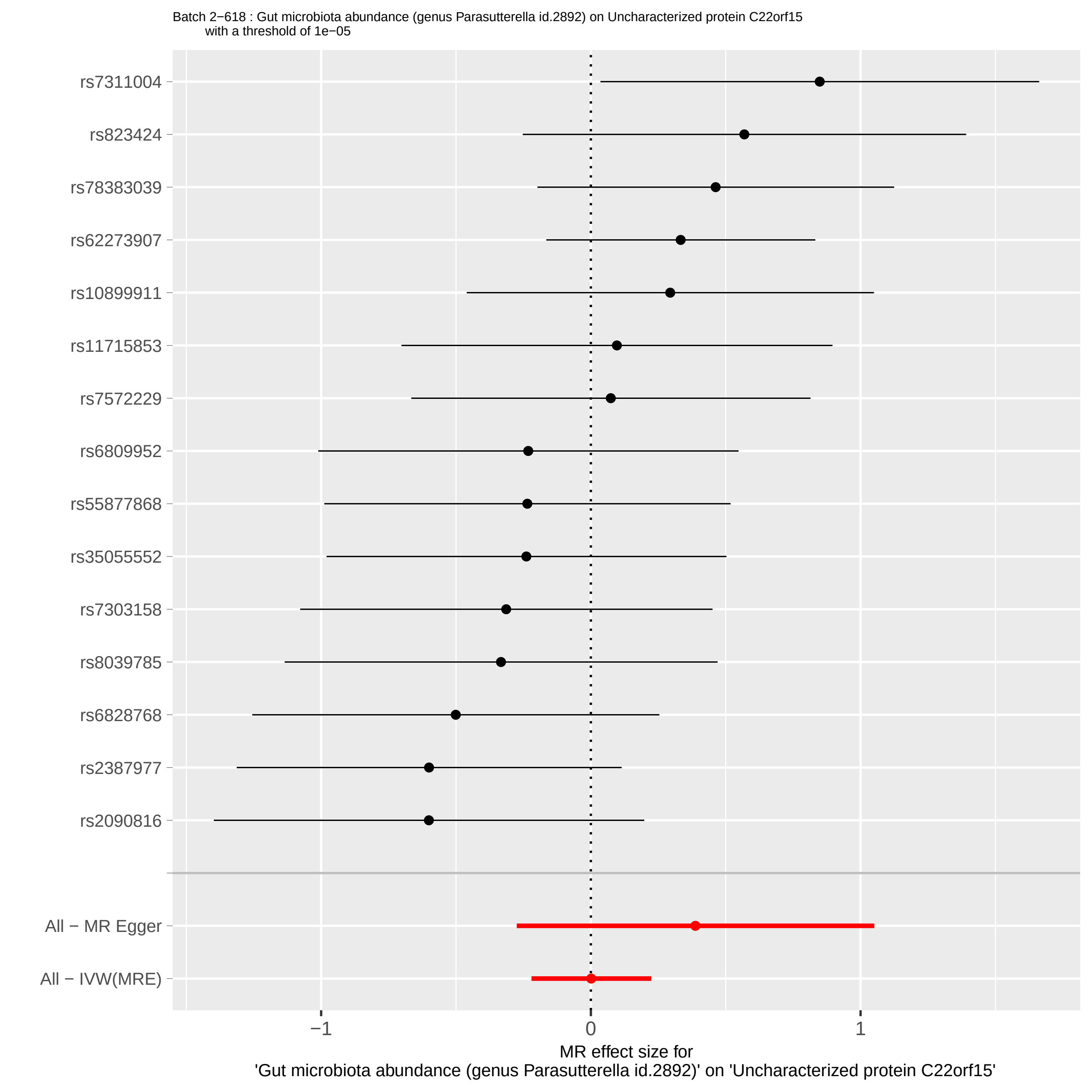

Batch 2−618 : Gut microbiota abundance (genus Parasutterella id.2892) on Uncharacterized protein C22orf15
with a threshold of 1e−05
rs7311004
rs823424
rs78383039
rs62273907
rs10899911
rs11715853
rs7572229
rs6809952
rs55877868
rs35055552
rs7303158
rs8039785
rs6828768
rs2387977
rs2090816
All − MR Egger
All − IVW(MRE)
−1
0
1
MR effect size for
'Gut microbiota abundance (genus Parasutterella id.2892)' on 'Uncharacterized protein C22orf15'

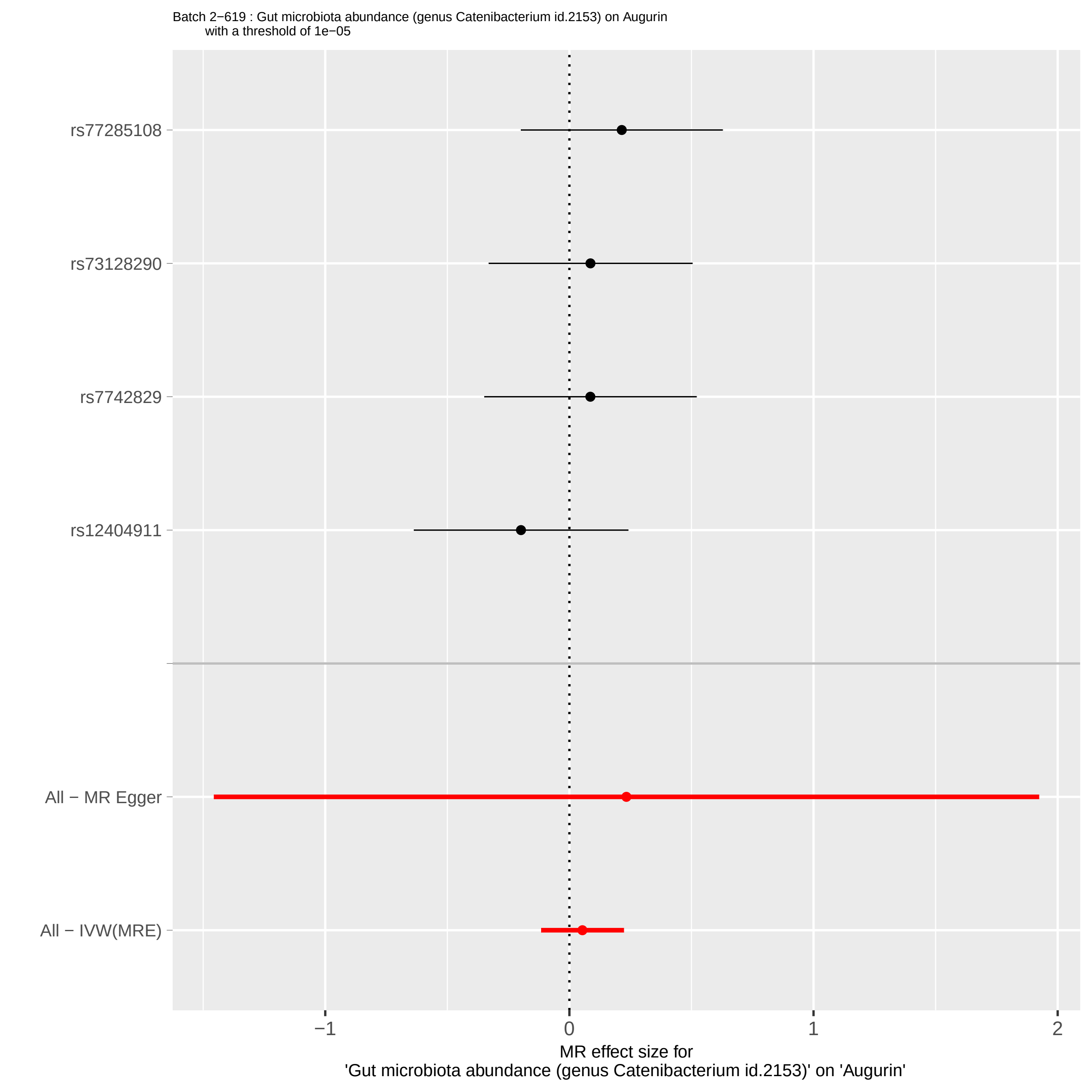

Batch 2−619 : Gut microbiota abundance (genus Catenibacterium id.2153) on Augurin
with a threshold of 1e−05
rs77285108
rs73128290
rs7742829
rs12404911
All − MR Egger
All − IVW(MRE)
−1
0
1
2
MR effect size for
'Gut microbiota abundance (genus Catenibacterium id.2153)' on 'Augurin'

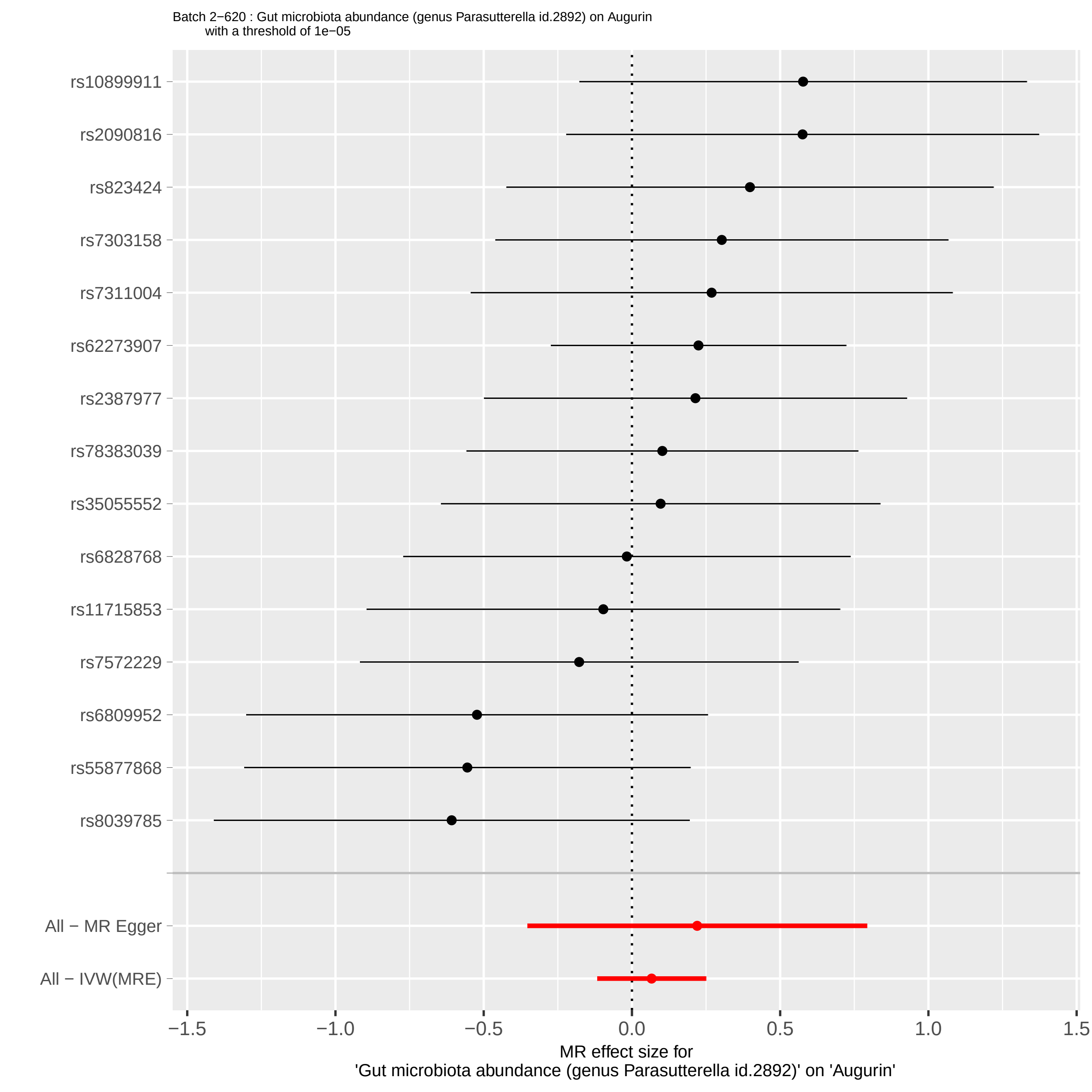

Batch 2−620 : Gut microbiota abundance (genus Parasutterella id.2892) on Augurin
with a threshold of 1e−05
rs10899911
rs2090816
rs823424
rs7303158
rs7311004
rs62273907
rs2387977
rs78383039
rs35055552
rs6828768
rs11715853
rs7572229
rs6809952
rs55877868
rs8039785
All − MR Egger
All − IVW(MRE)
−1.5
−1.0
−0.5
0.0
0.5
1.0
1.5
MR effect size for
'Gut microbiota abundance (genus Parasutterella id.2892)' on 'Augurin'

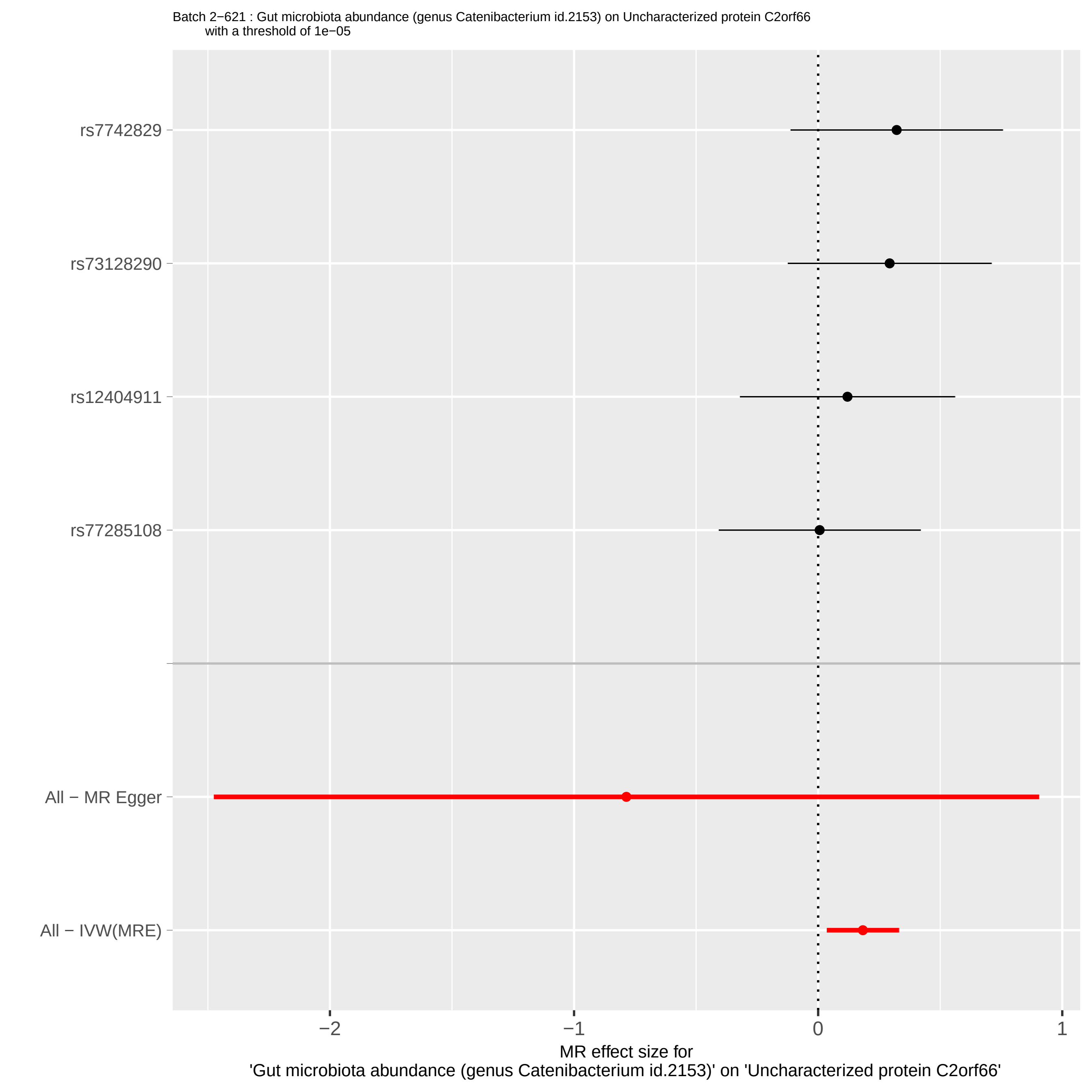
Batch 2−621 : Gut microbiota abundance (genus Catenibacterium id.2153) on Uncharacterized protein C2orf66
with a threshold of 1e−05
rs7742829
rs73128290
rs12404911
rs77285108
All − MR Egger
All − IVW(MRE)
−2
−1
0
1
MR effect size for
'Gut microbiota abundance (genus Catenibacterium id.2153)' on 'Uncharacterized protein C2orf66'

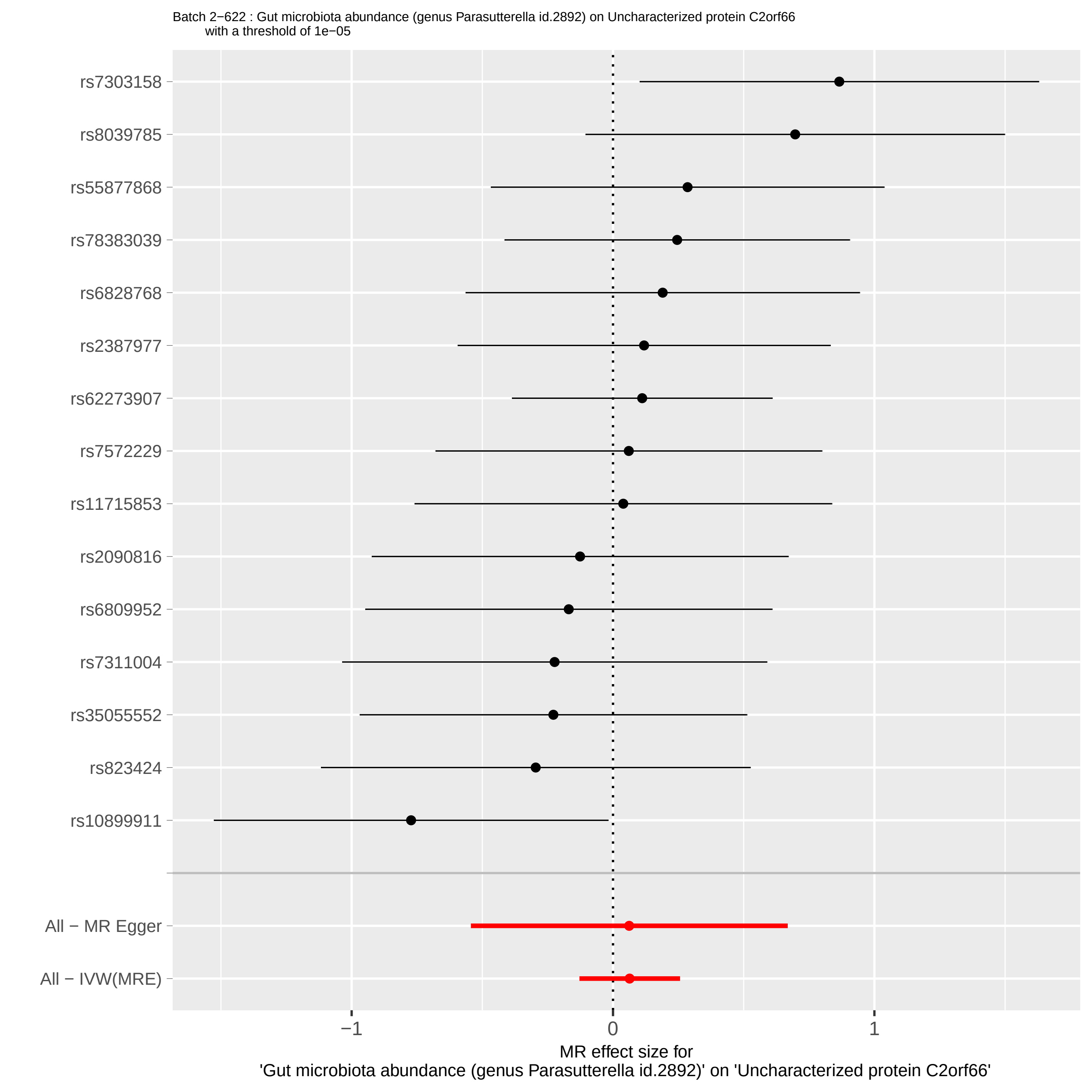

Batch 2−622 : Gut microbiota abundance (genus Parasutterella id.2892) on Uncharacterized protein C2orf66
with a threshold of 1e−05
rs7303158
rs8039785
rs55877868
rs78383039
rs6828768
rs2387977
rs62273907
rs7572229
rs11715853
rs2090816
rs6809952
rs7311004
rs35055552
rs823424
rs10899911
All − MR Egger
All − IVW(MRE)
−1
0
1
MR effect size for
'Gut microbiota abundance (genus Parasutterella id.2892)' on 'Uncharacterized protein C2orf66'

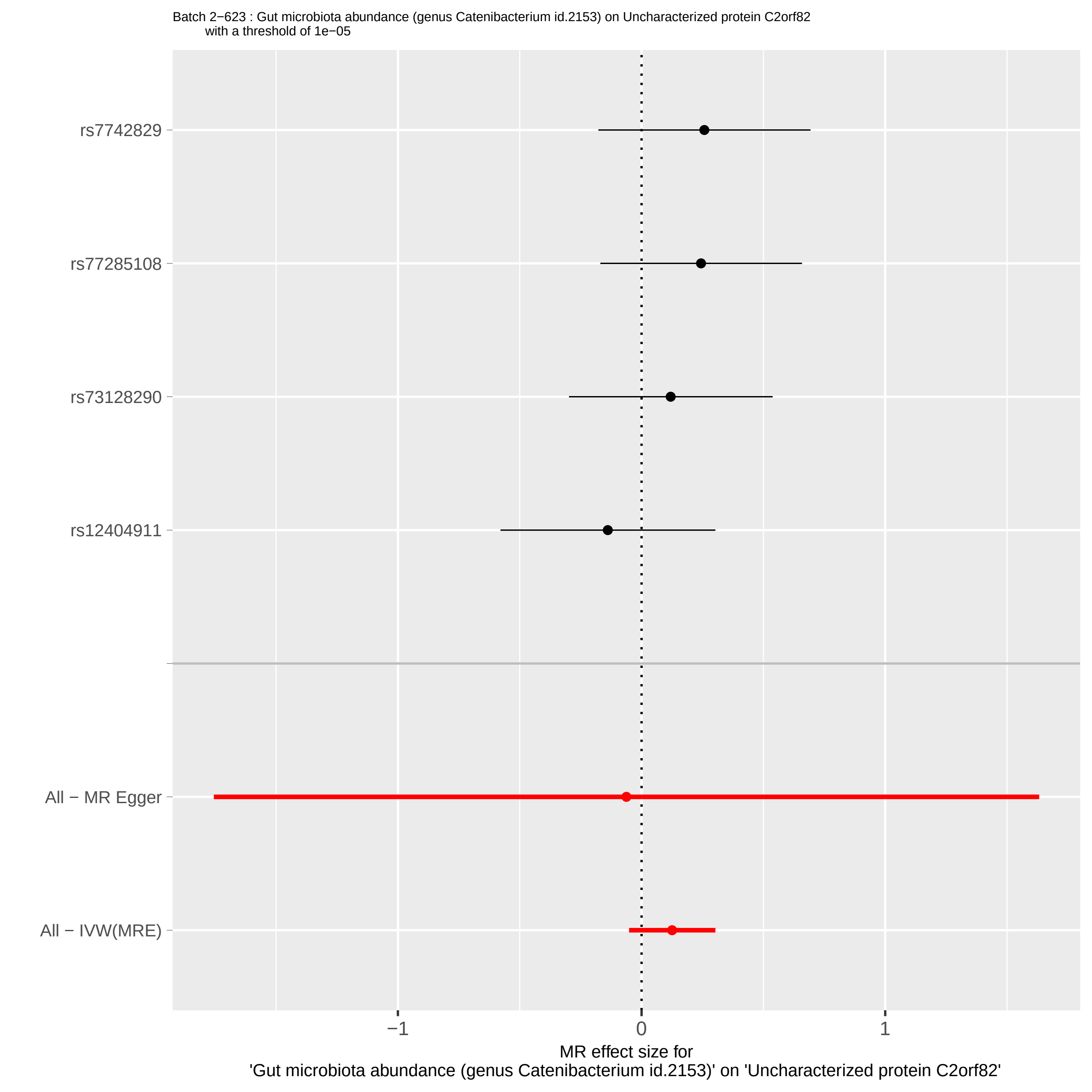
Batch 2−623 : Gut microbiota abundance (genus Catenibacterium id.2153) on Uncharacterized protein C2orf82
with a threshold of 1e−05
rs7742829
rs77285108
rs73128290
rs12404911
All − MR Egger
All − IVW(MRE)
−1
0
1
MR effect size for
'Gut microbiota abundance (genus Catenibacterium id.2153)' on 'Uncharacterized protein C2orf82'

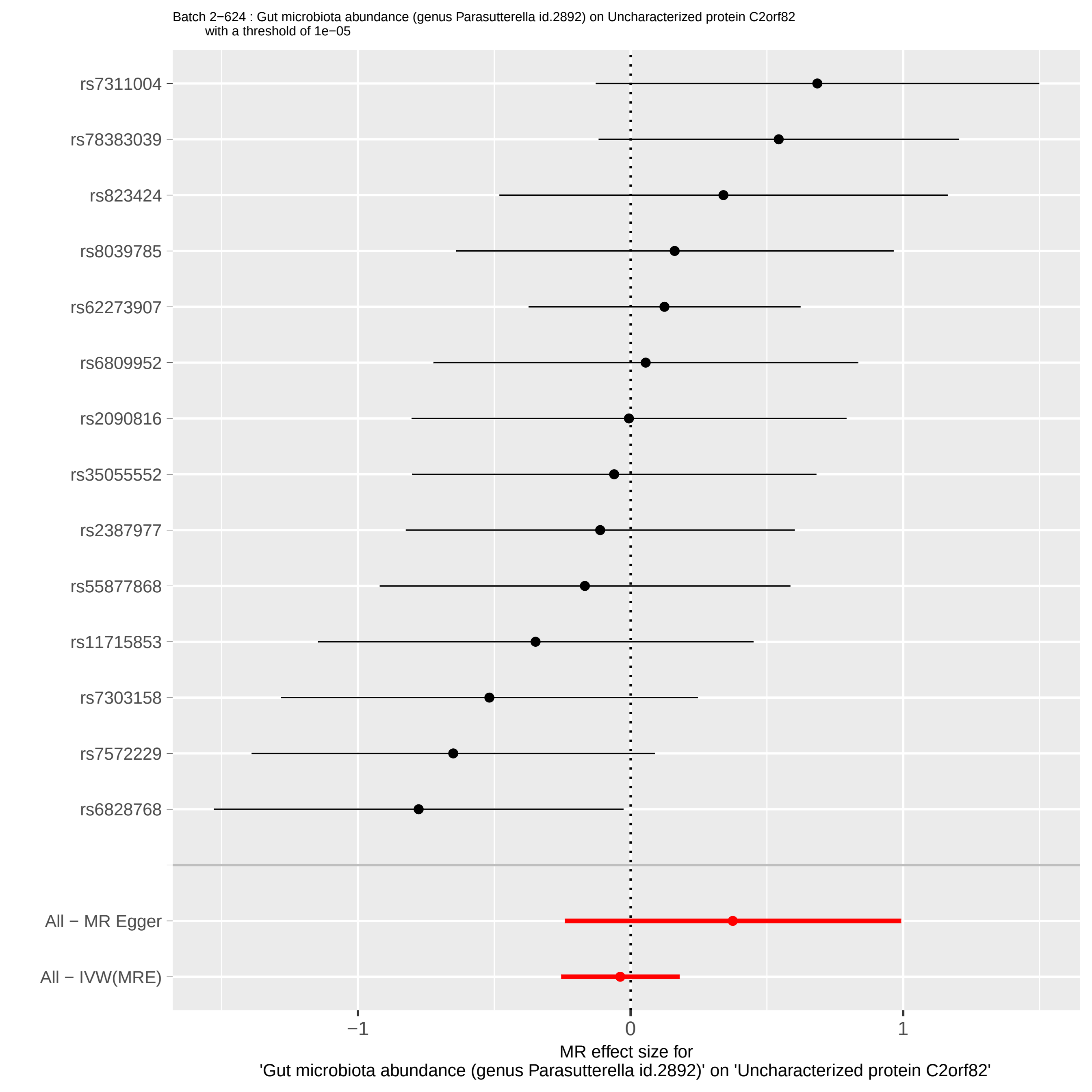

Batch 2−624 : Gut microbiota abundance (genus Parasutterella id.2892) on Uncharacterized protein C2orf82
with a threshold of 1e−05
rs7311004
rs78383039
rs823424
rs8039785
rs62273907
rs6809952
rs2090816
rs35055552
rs2387977
rs55877868
rs11715853
rs7303158
rs7572229
rs6828768
All − MR Egger
All − IVW(MRE)
−1
0
1
MR effect size for
'Gut microbiota abundance (genus Parasutterella id.2892)' on 'Uncharacterized protein C2orf82'

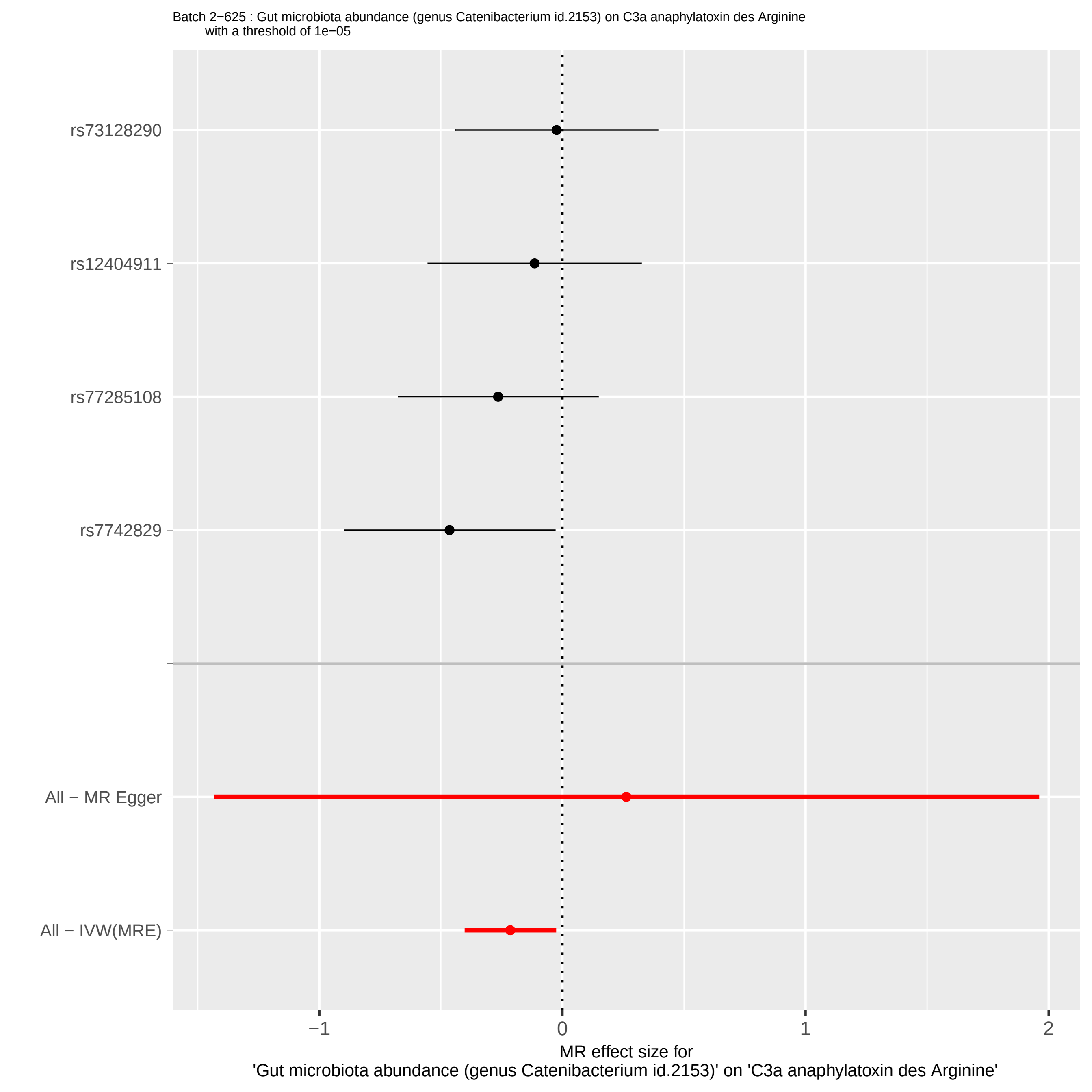

Batch 2−625 : Gut microbiota abundance (genus Catenibacterium id.2153) on C3a anaphylatoxin des Arginine
with a threshold of 1e−05
rs73128290
rs12404911
rs77285108
rs7742829
All − MR Egger
All − IVW(MRE)
−1
0
1
2
MR effect size for
'Gut microbiota abundance (genus Catenibacterium id.2153)' on 'C3a anaphylatoxin des Arginine'

Batch 2−626 : Gut microbiota abundance (genus Parasutterella id.2892) on C3a anaphylatoxin des Arginine
with a threshold of 1e−05

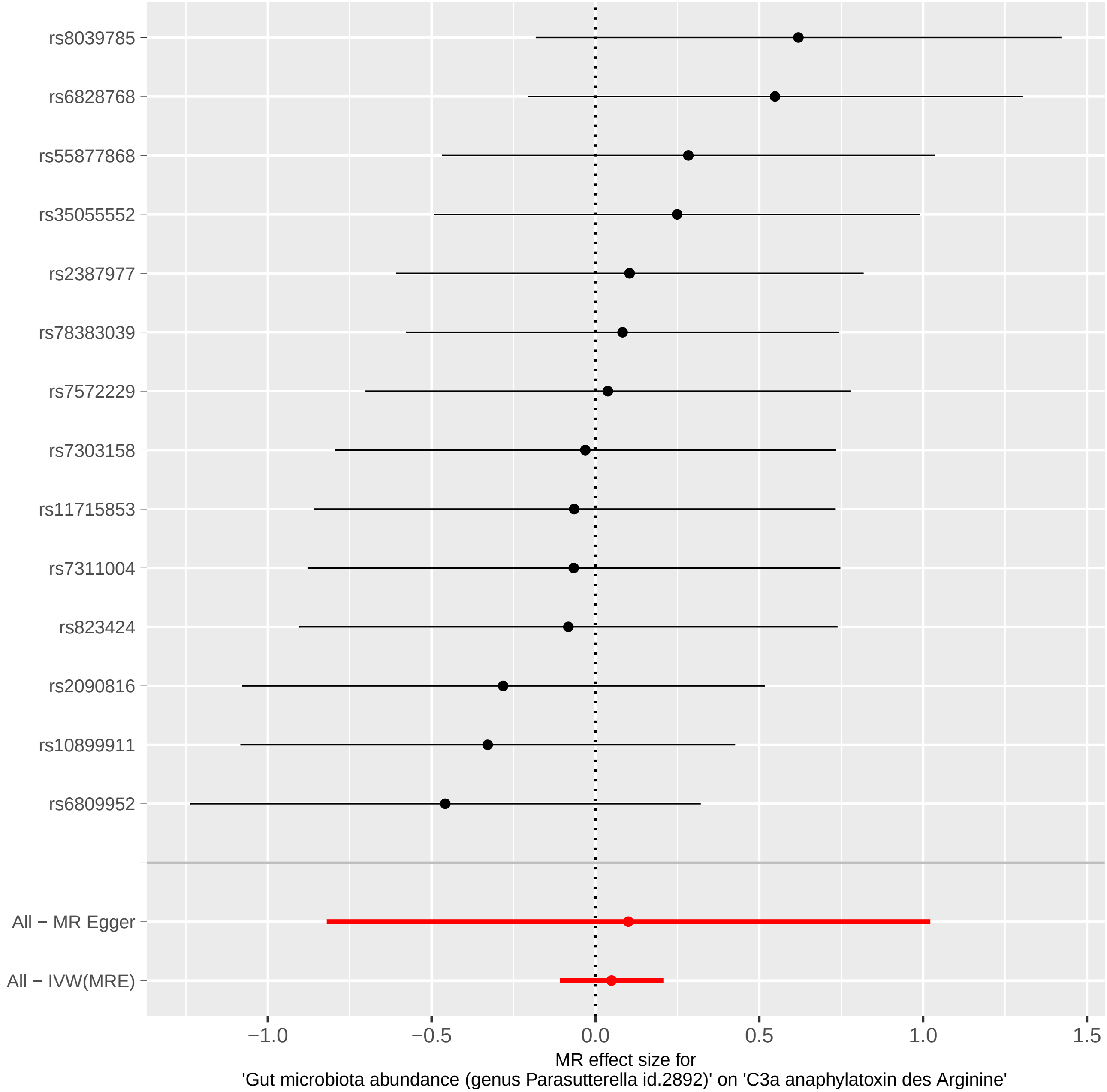

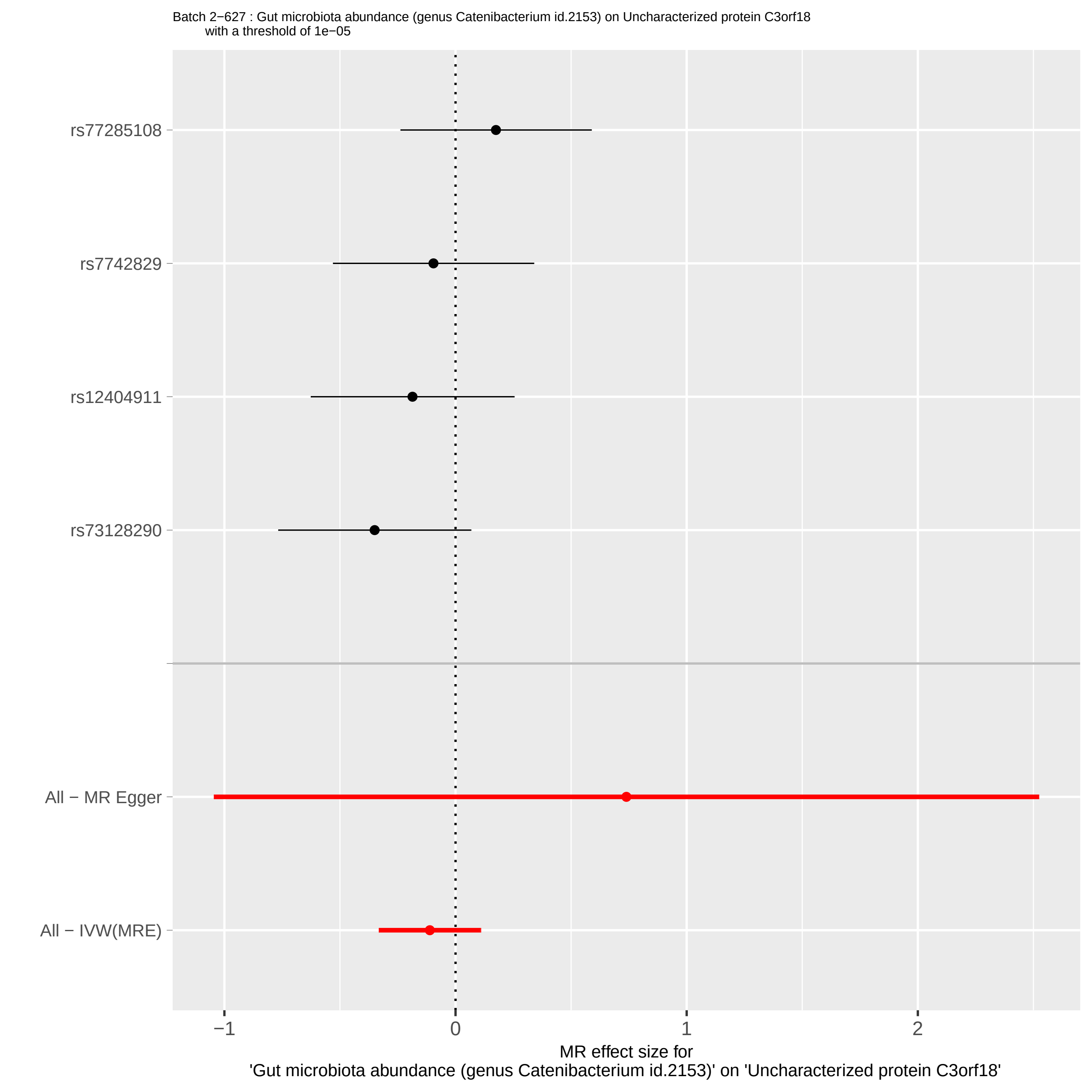
Batch 2−627 : Gut microbiota abundance (genus Catenibacterium id.2153) on Uncharacterized protein C3orf18
with a threshold of 1e−05
rs77285108
rs7742829
rs12404911
rs73128290
All − MR Egger
All − IVW(MRE)
−1
0
1
2
MR effect size for
'Gut microbiota abundance (genus Catenibacterium id.2153)' on 'Uncharacterized protein C3orf18'

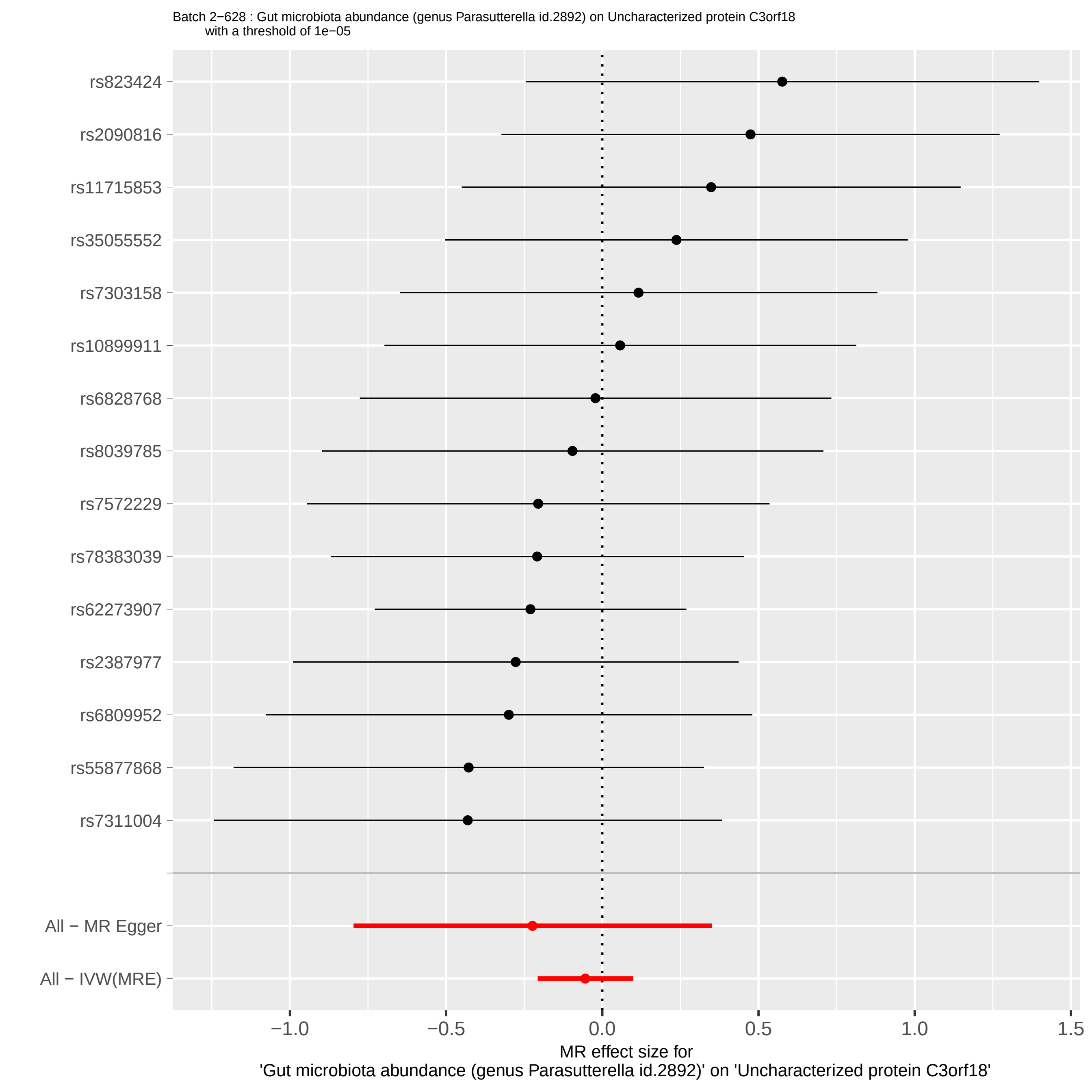

Batch 2−628 : Gut microbiota abundance (genus Parasutterella id.2892) on Uncharacterized protein C3orf18
with a threshold of 1e−05
rs823424
rs2090816
rs11715853
rs35055552
rs7303158
rs10899911
rs6828768
rs8039785
rs7572229
rs78383039
rs62273907
rs2387977
rs6809952
rs55877868
rs7311004
All − MR Egger
All − IVW(MRE)
−1.0
−0.5
0.0
0.5
1.0
1.5
MR effect size for
'Gut microbiota abundance (genus Parasutterella id.2892)' on 'Uncharacterized protein C3orf18'

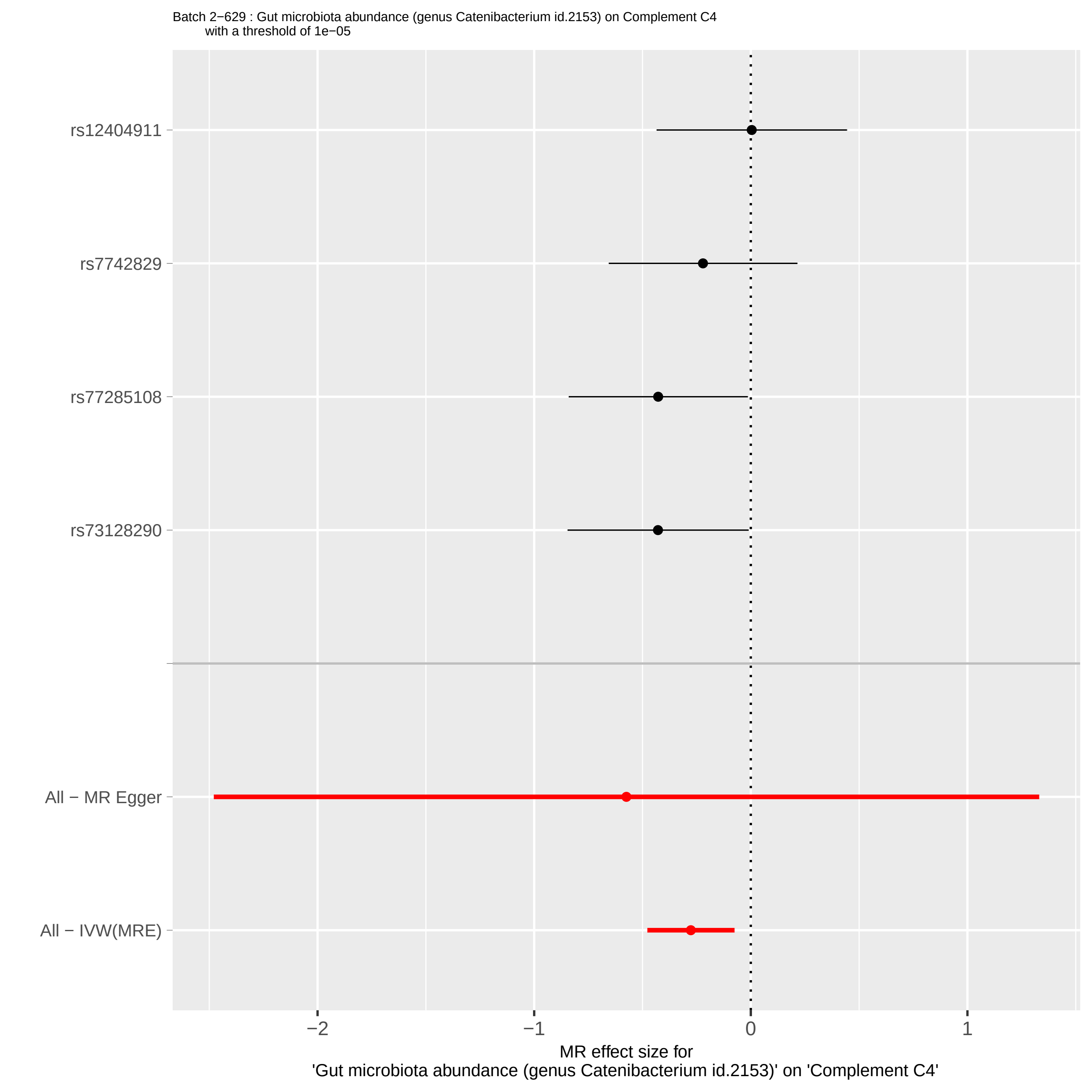

Batch 2−629 : Gut microbiota abundance (genus Catenibacterium id.2153) on Complement C4
with a threshold of 1e−05
rs12404911
rs7742829
rs77285108
rs73128290
All − MR Egger
All − IVW(MRE)
−2
−1
0
1
MR effect size for
'Gut microbiota abundance (genus Catenibacterium id.2153)' on 'Complement C4'

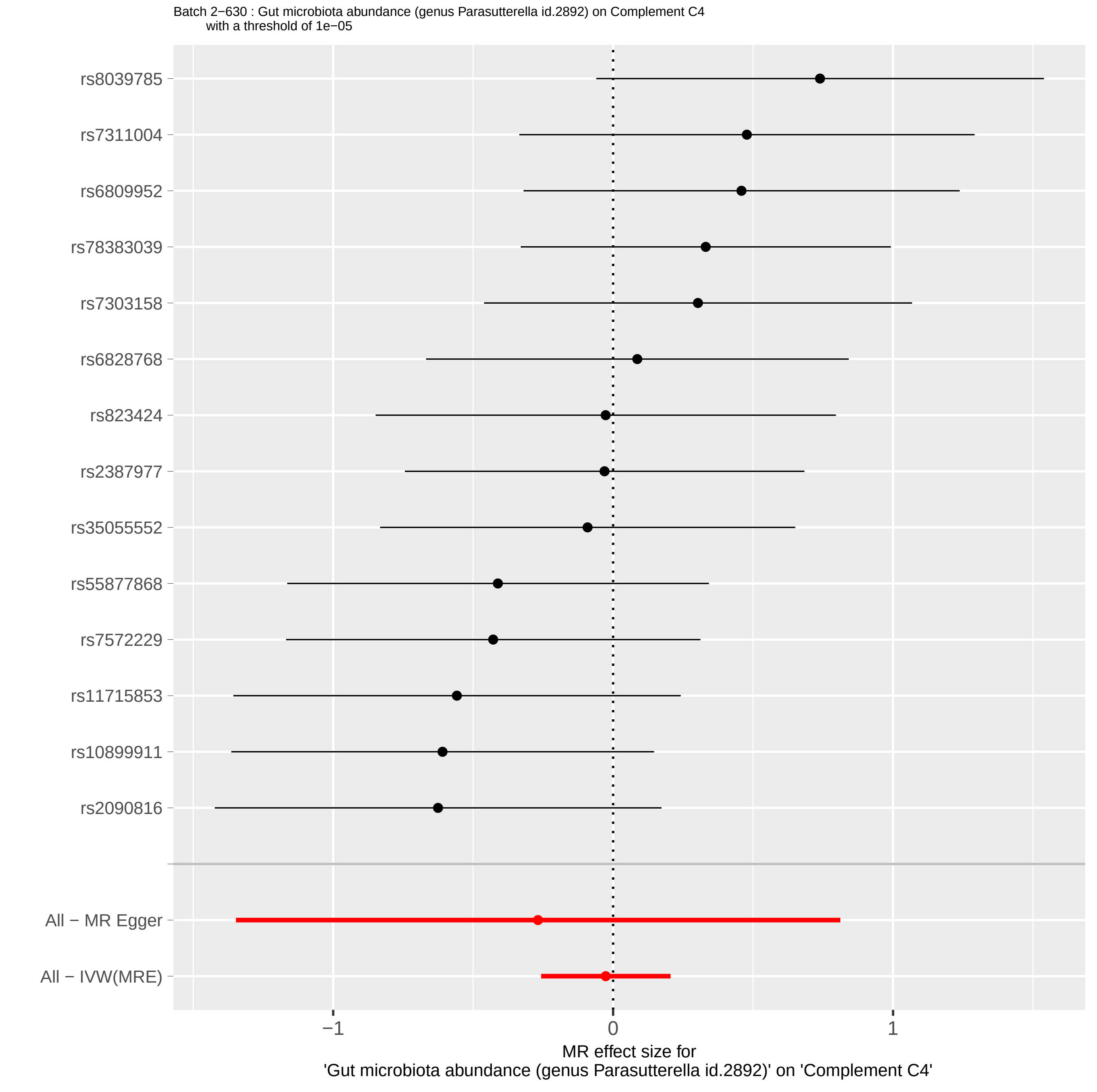

Batch 2−630 : Gut microbiota abundance (genus Parasutterella id.2892) on Complement C4
with a threshold of 1e−05
rs8039785
rs7311004
rs6809952
rs78383039
rs7303158
rs6828768
rs823424
rs2387977
rs35055552
rs55877868
rs7572229
rs11715853
rs10899911
rs2090816
All − MR Egger
All − IVW(MRE)
−1
0
1
MR effect size for
'Gut microbiota abundance (genus Parasutterella id.2892)' on 'Complement C4'

Batch 2−631 : Gut microbiota abundance (genus Catenibacterium id.2153) on Protein CEI
with a threshold of 1e−05

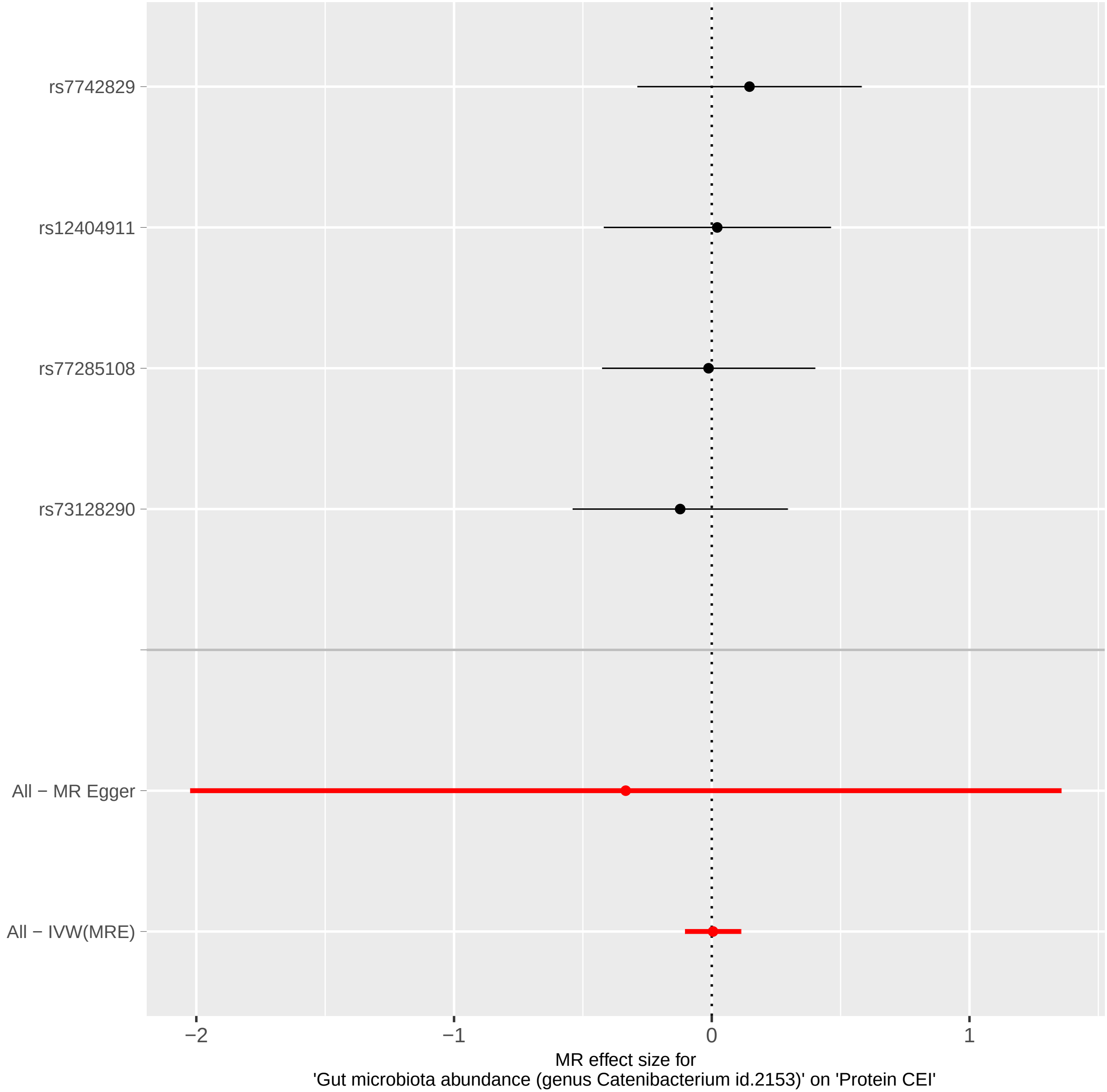

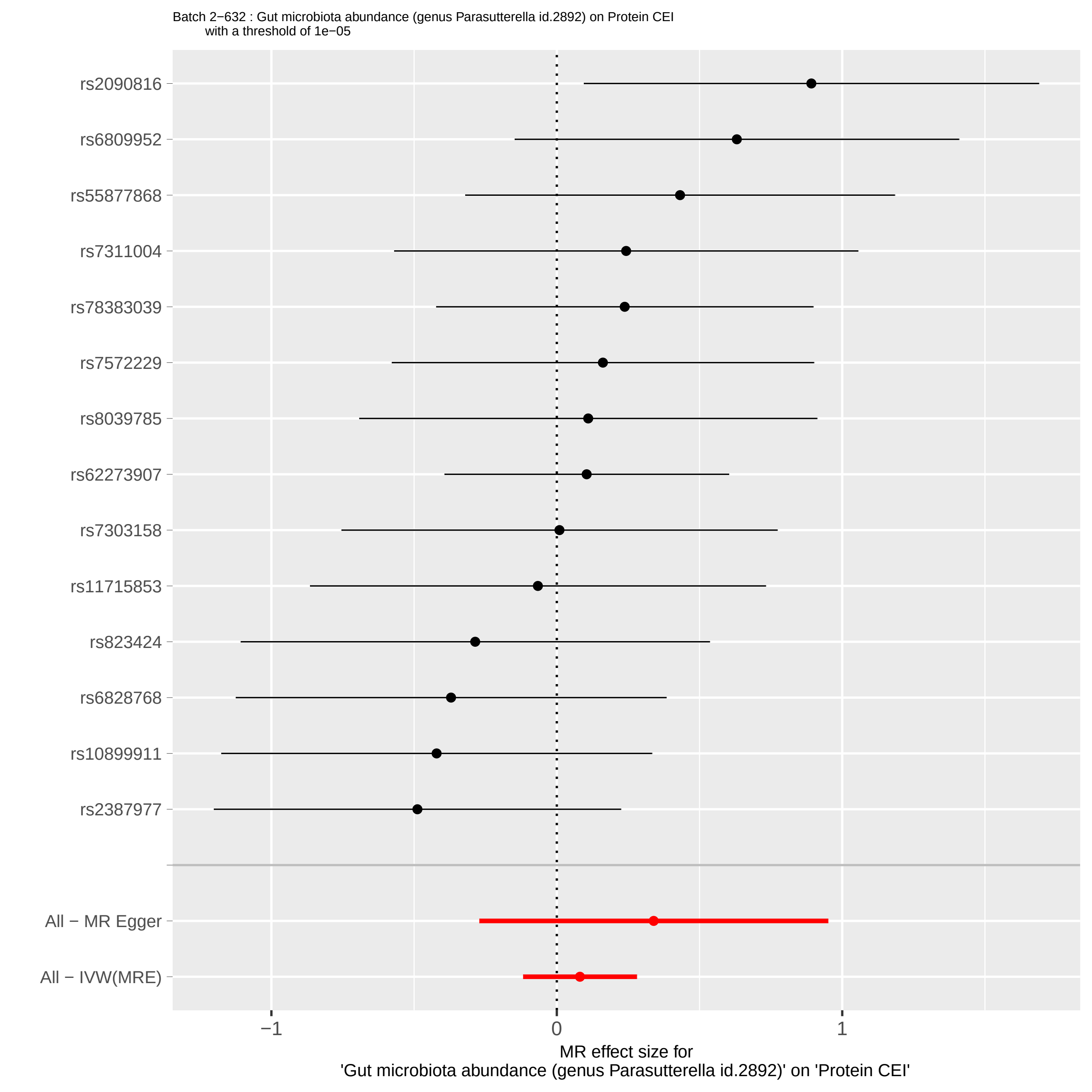

Batch 2−632 : Gut microbiota abundance (genus Parasutterella id.2892) on Protein CEI
with a threshold of 1e−05
rs2090816
rs6809952
rs55877868
rs7311004
rs78383039
rs7572229
rs8039785
rs62273907
rs7303158
rs11715853
rs823424
rs6828768
rs10899911
rs2387977
All − MR Egger
All − IVW(MRE)
−1
0
1
MR effect size for
'Gut microbiota abundance (genus Parasutterella id.2892)' on 'Protein CEI'

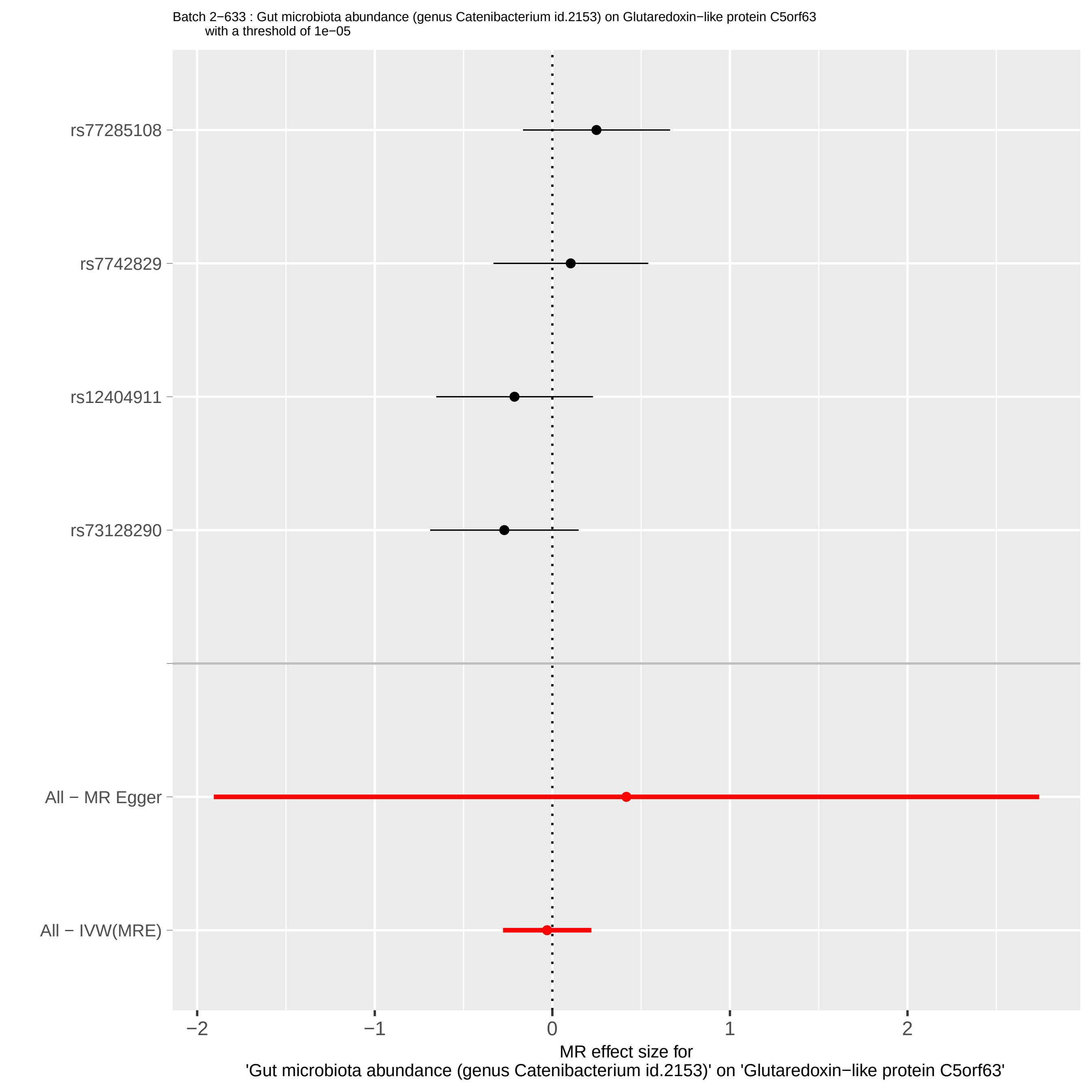

Batch 2−633 : Gut microbiota abundance (genus Catenibacterium id.2153) on Glutaredoxin−like protein C5orf63
with a threshold of 1e−05
rs77285108
rs7742829
rs12404911
rs73128290
All − MR Egger
All − IVW(MRE)
−2
−1
0
1
2
MR effect size for
'Gut microbiota abundance (genus Catenibacterium id.2153)' on 'Glutaredoxin−like protein C5orf63'

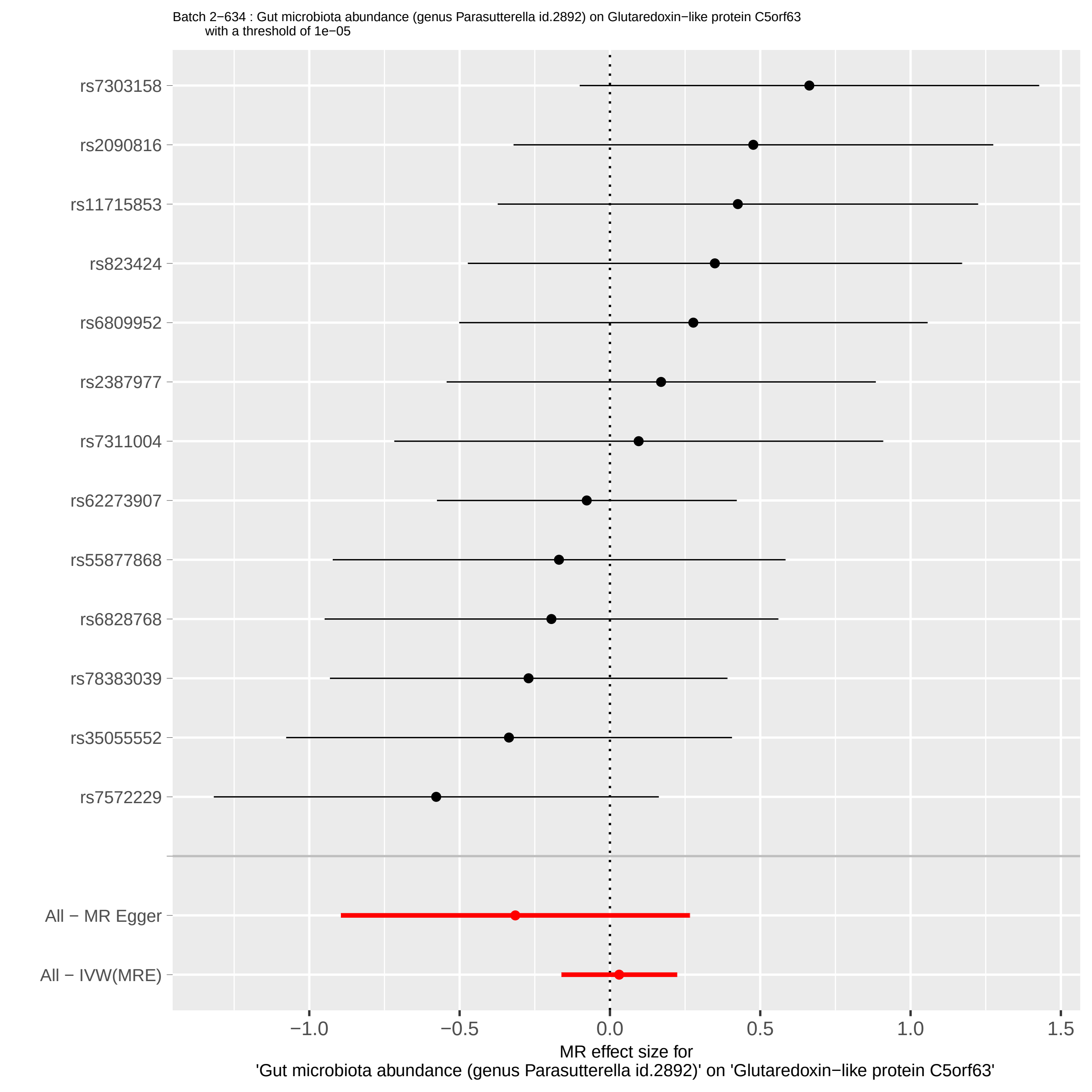

Batch 2−634 : Gut microbiota abundance (genus Parasutterella id.2892) on Glutaredoxin−like protein C5orf63
with a threshold of 1e−05
rs7303158
rs2090816
rs11715853
rs823424
rs6809952
rs2387977
rs7311004
rs62273907
rs55877868
rs6828768
rs78383039
rs35055552
rs7572229
All − MR Egger
All − IVW(MRE)
−1.0
−0.5
0.0
0.5
1.0
1.5
MR effect size for
'Gut microbiota abundance (genus Parasutterella id.2892)' on 'Glutaredoxin−like protein C5orf63'

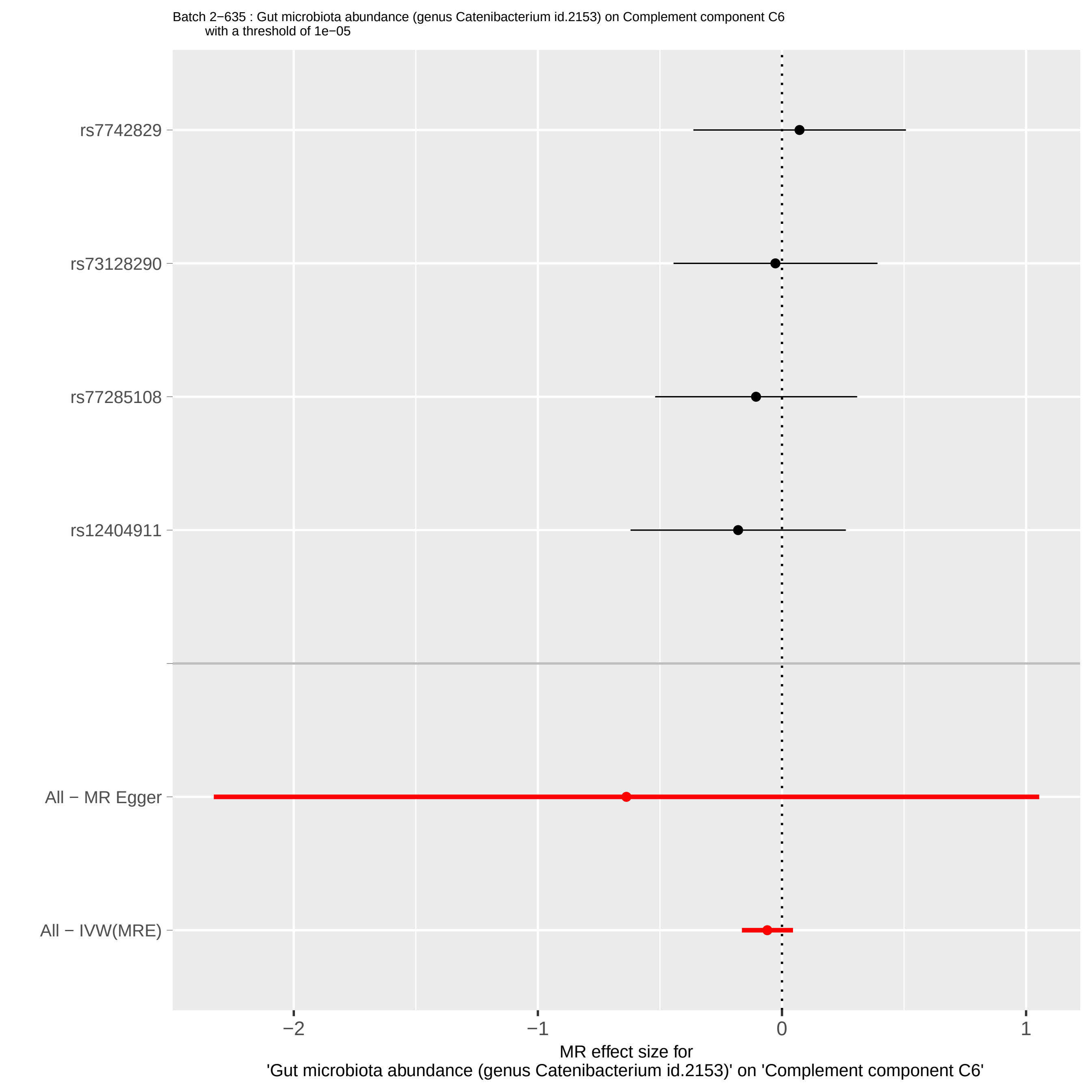

Batch 2−635 : Gut microbiota abundance (genus Catenibacterium id.2153) on Complement component C6
with a threshold of 1e−05
rs7742829
rs73128290
rs77285108
rs12404911
All − MR Egger
All − IVW(MRE)
−2
−1
0
1
MR effect size for
'Gut microbiota abundance (genus Catenibacterium id.2153)' on 'Complement component C6'

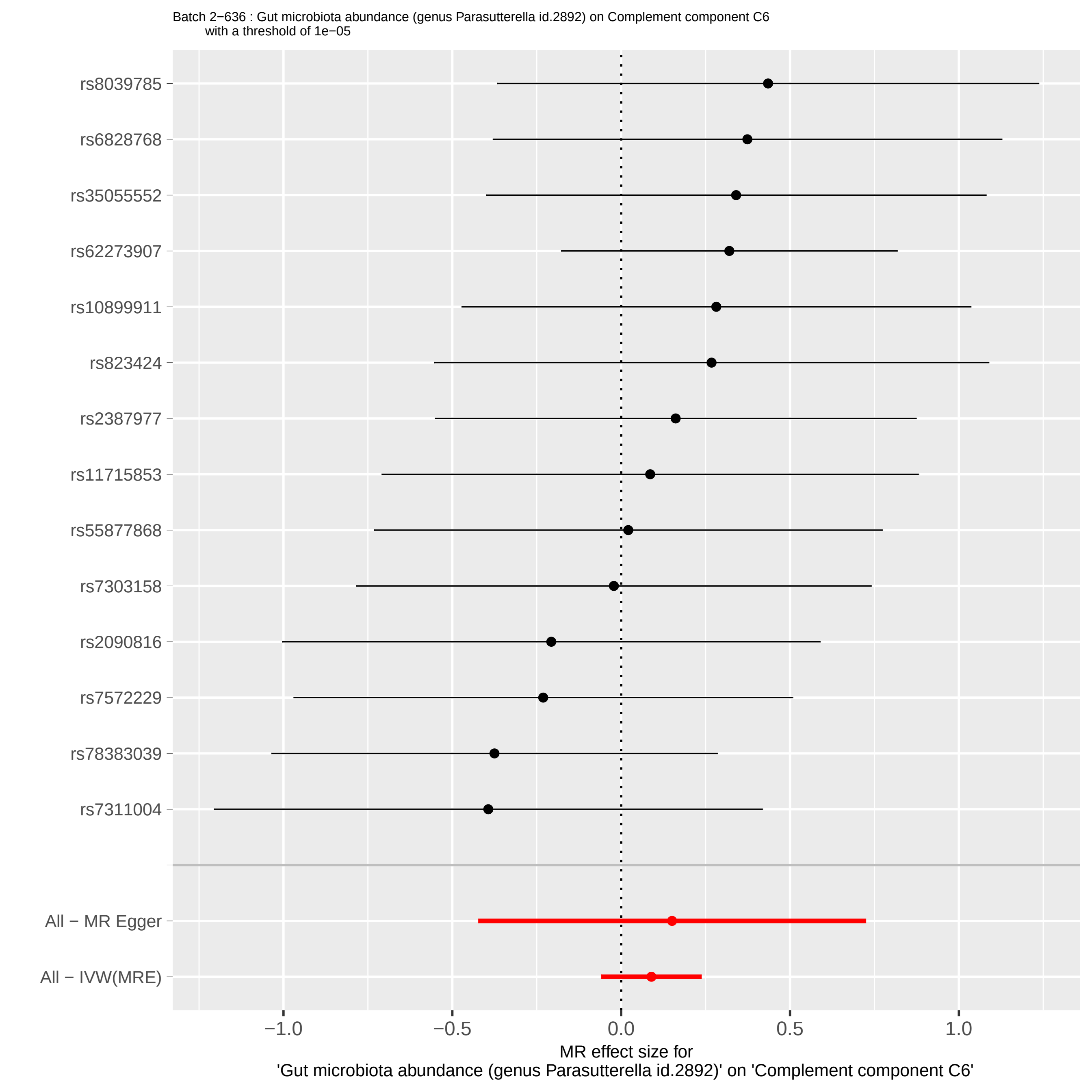

Batch 2−636 : Gut microbiota abundance (genus Parasutterella id.2892) on Complement component C6
with a threshold of 1e−05
rs8039785
rs6828768
rs35055552
rs62273907
rs10899911
rs823424
rs2387977
rs11715853
rs55877868
rs7303158
rs2090816
rs7572229
rs78383039
rs7311004
All − MR Egger
All − IVW(MRE)
−1.0
−0.5
0.0
0.5
1.0
MR effect size for
'Gut microbiota abundance (genus Parasutterella id.2892)' on 'Complement component C6'

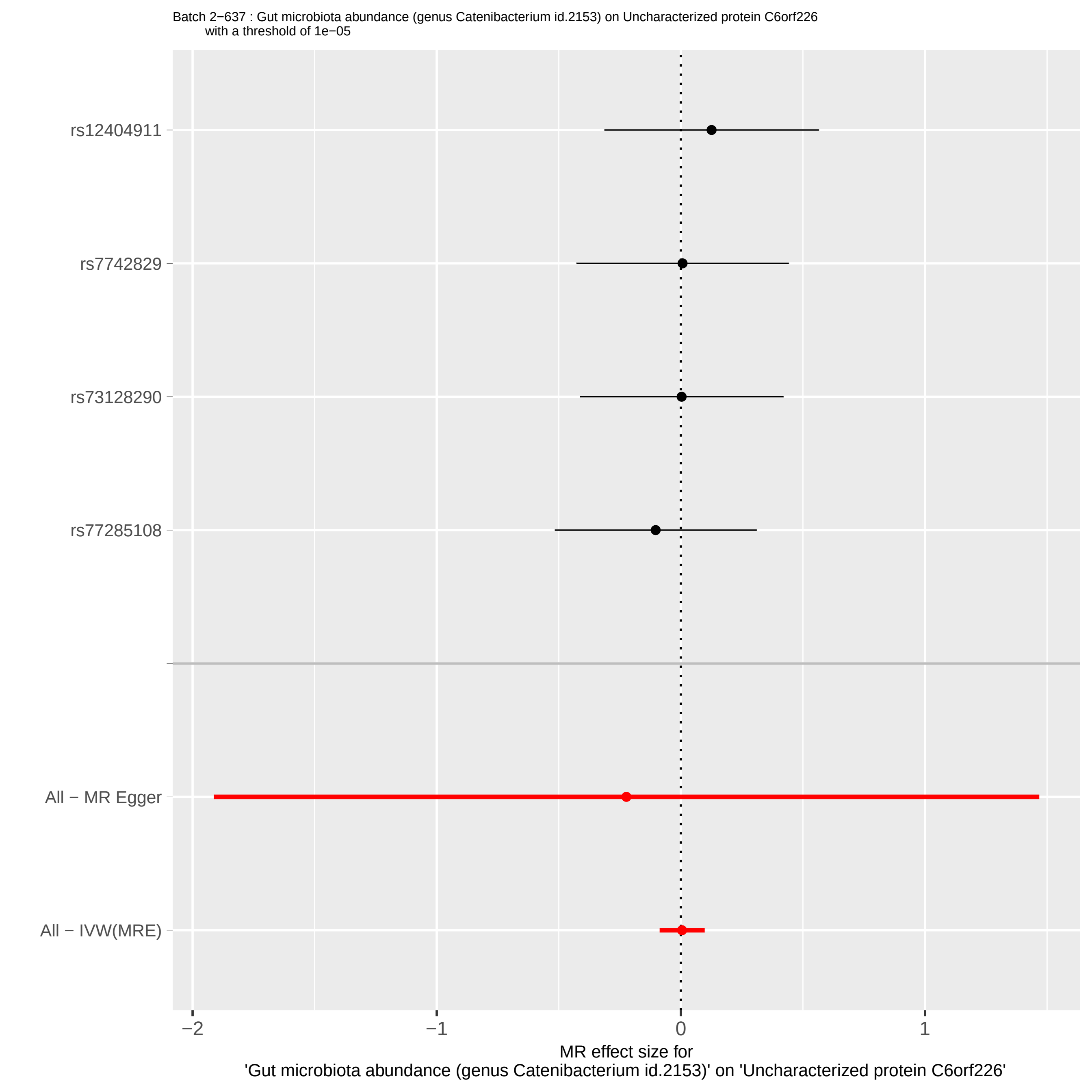
Batch 2−637 : Gut microbiota abundance (genus Catenibacterium id.2153) on Uncharacterized protein C6orf226
with a threshold of 1e−05
rs12404911
rs7742829
rs73128290
rs77285108
All − MR Egger
All − IVW(MRE)
−2
−1
0
1
MR effect size for
'Gut microbiota abundance (genus Catenibacterium id.2153)' on 'Uncharacterized protein C6orf226'

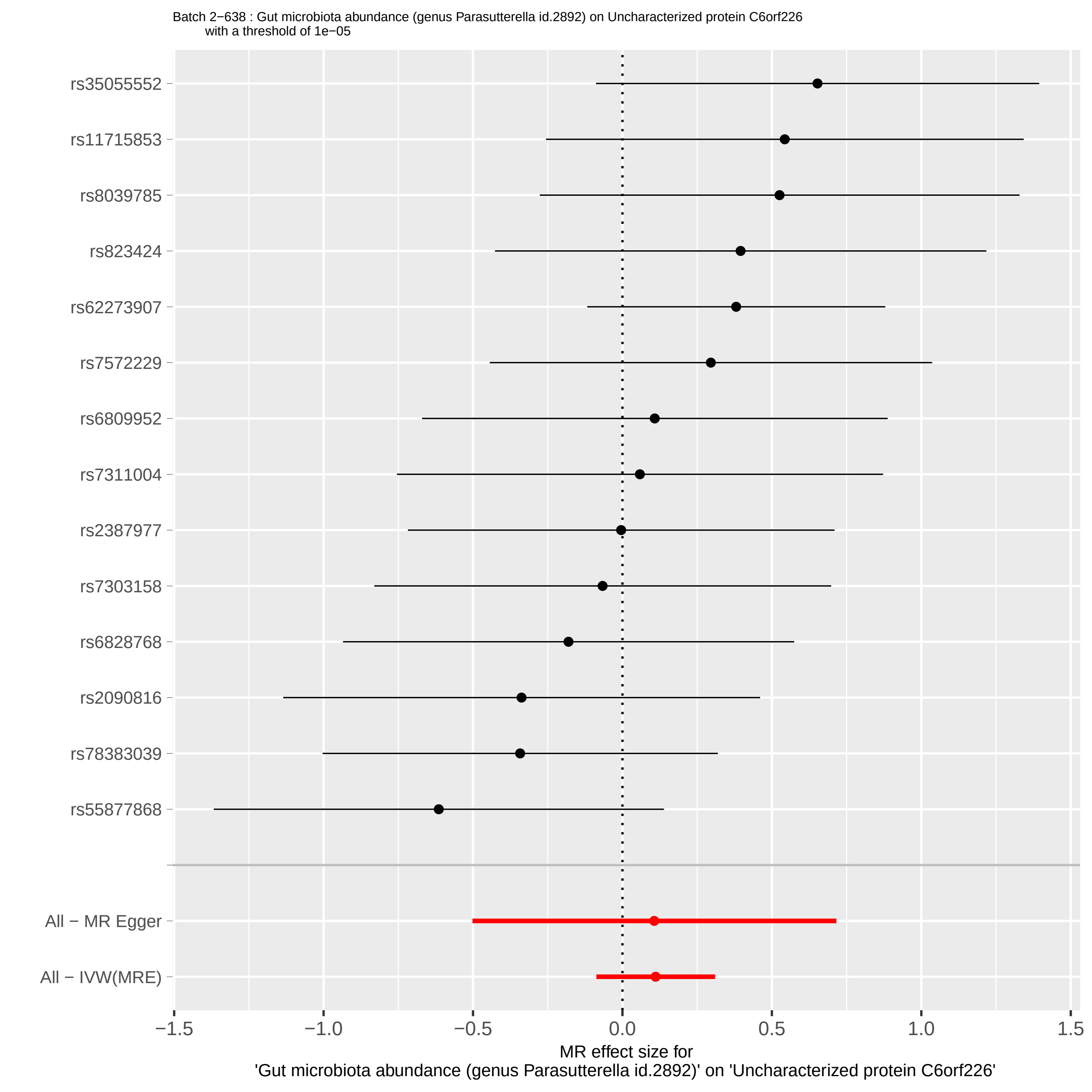

Batch 2−638 : Gut microbiota abundance (genus Parasutterella id.2892) on Uncharacterized protein C6orf226
with a threshold of 1e−05
rs35055552
rs11715853
rs8039785
rs823424
rs62273907
rs7572229
rs6809952
rs7311004
rs2387977
rs7303158
rs6828768
rs2090816
rs78383039
rs55877868
All − MR Egger
All − IVW(MRE)
−1.5
−1.0
−0.5
0.0
0.5
1.0
1.5
MR effect size for
'Gut microbiota abundance (genus Parasutterella id.2892)' on 'Uncharacterized protein C6orf226'

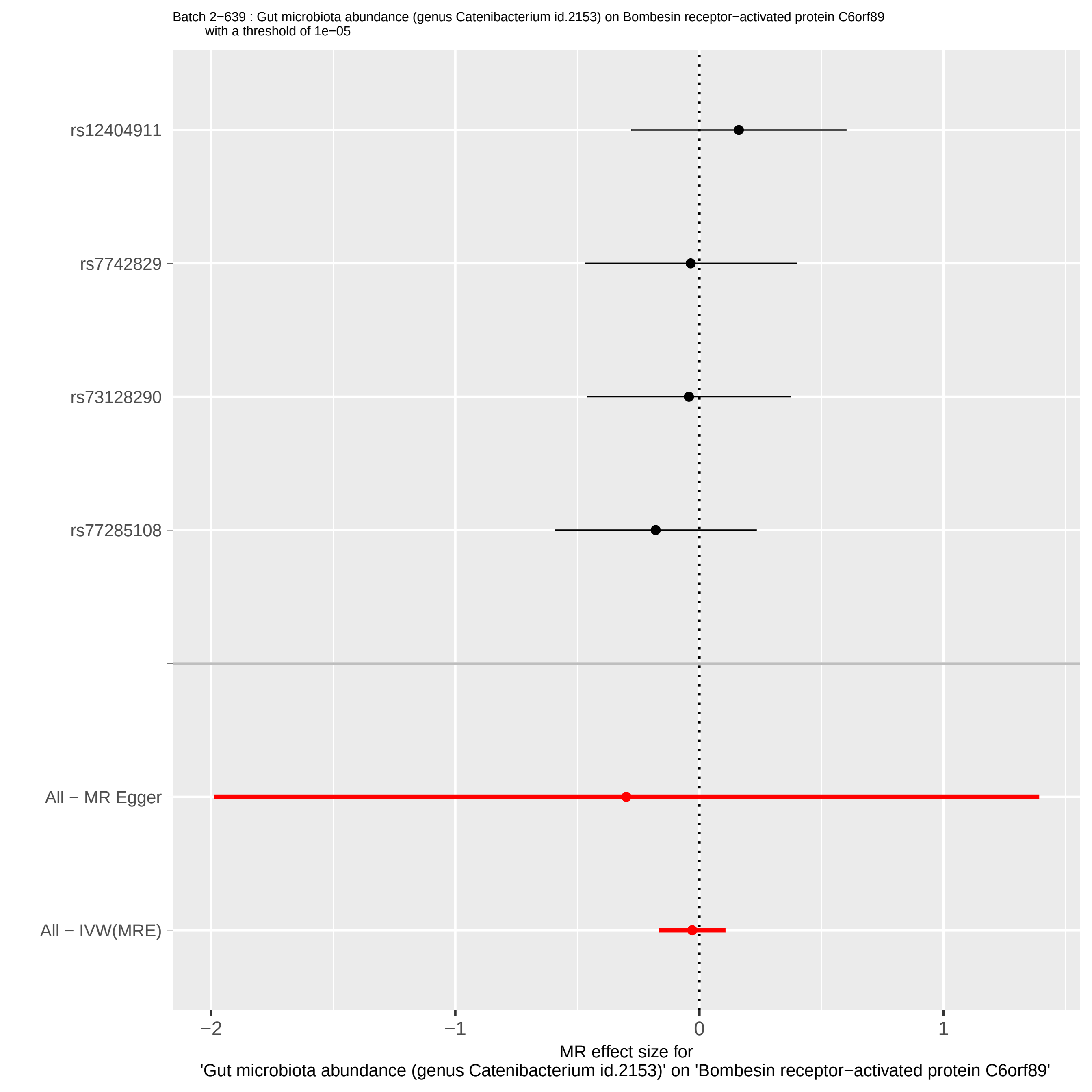

Batch 2−639 : Gut microbiota abundance (genus Catenibacterium id.2153) on Bombesin receptor−activated protein C6orf89
with a threshold of 1e−05
rs12404911
rs7742829
rs73128290
rs77285108
All − MR Egger
All − IVW(MRE)
−2
−1
0
1
MR effect size for
'Gut microbiota abundance (genus Catenibacterium id.2153)' on 'Bombesin receptor−activated protein C6orf89'

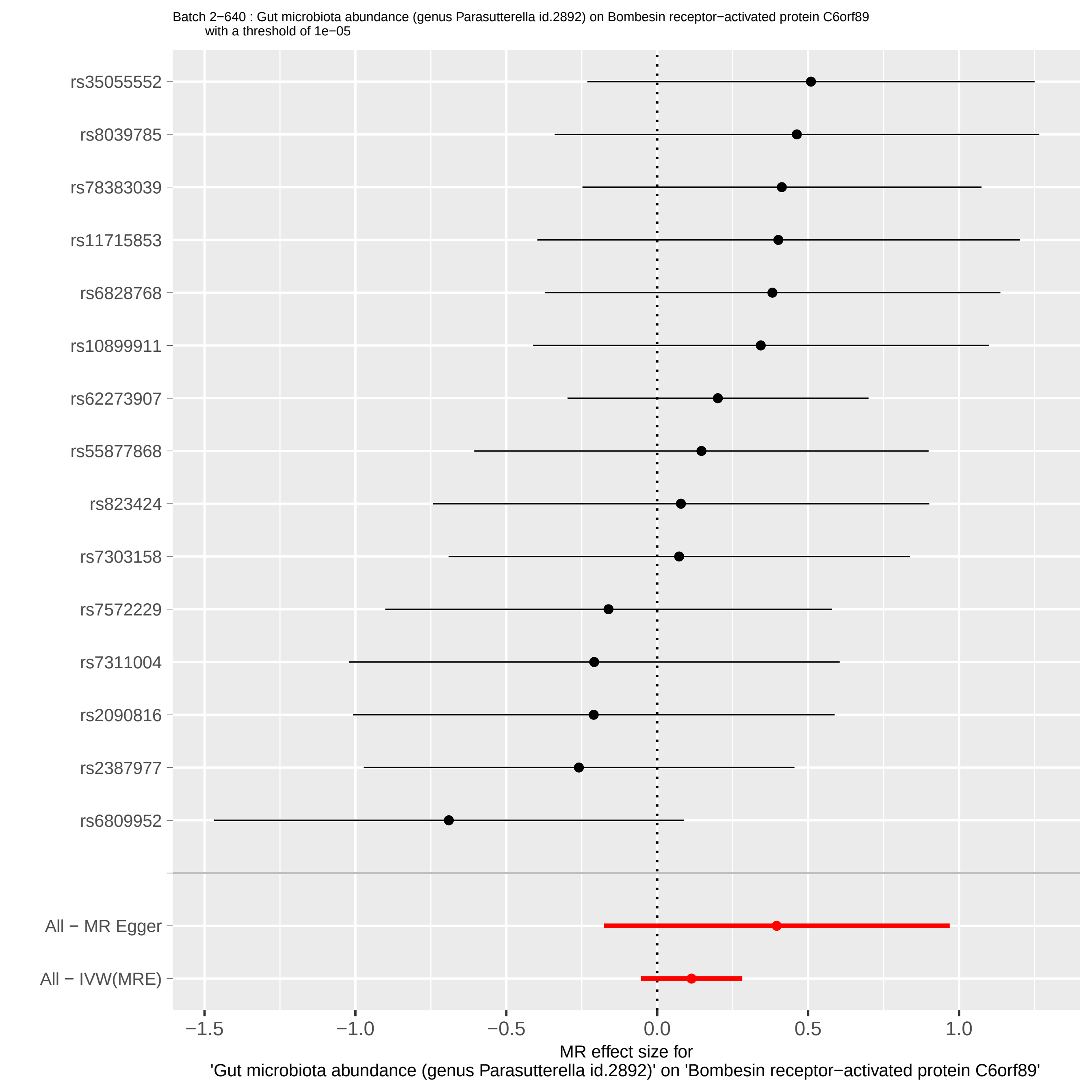

Batch 2−640 : Gut microbiota abundance (genus Parasutterella id.2892) on Bombesin receptor−activated protein C6orf89
with a threshold of 1e−05
rs35055552
rs8039785
rs78383039
rs11715853
rs6828768
rs10899911
rs62273907
rs55877868
rs823424
rs7303158
rs7572229
rs7311004
rs2090816
rs2387977
rs6809952
All − MR Egger
All − IVW(MRE)
−1.5
−1.0
−0.5
0.0
0.5
1.0
MR effect size for
'Gut microbiota abundance (genus Parasutterella id.2892)' on 'Bombesin receptor−activated protein C6orf89'

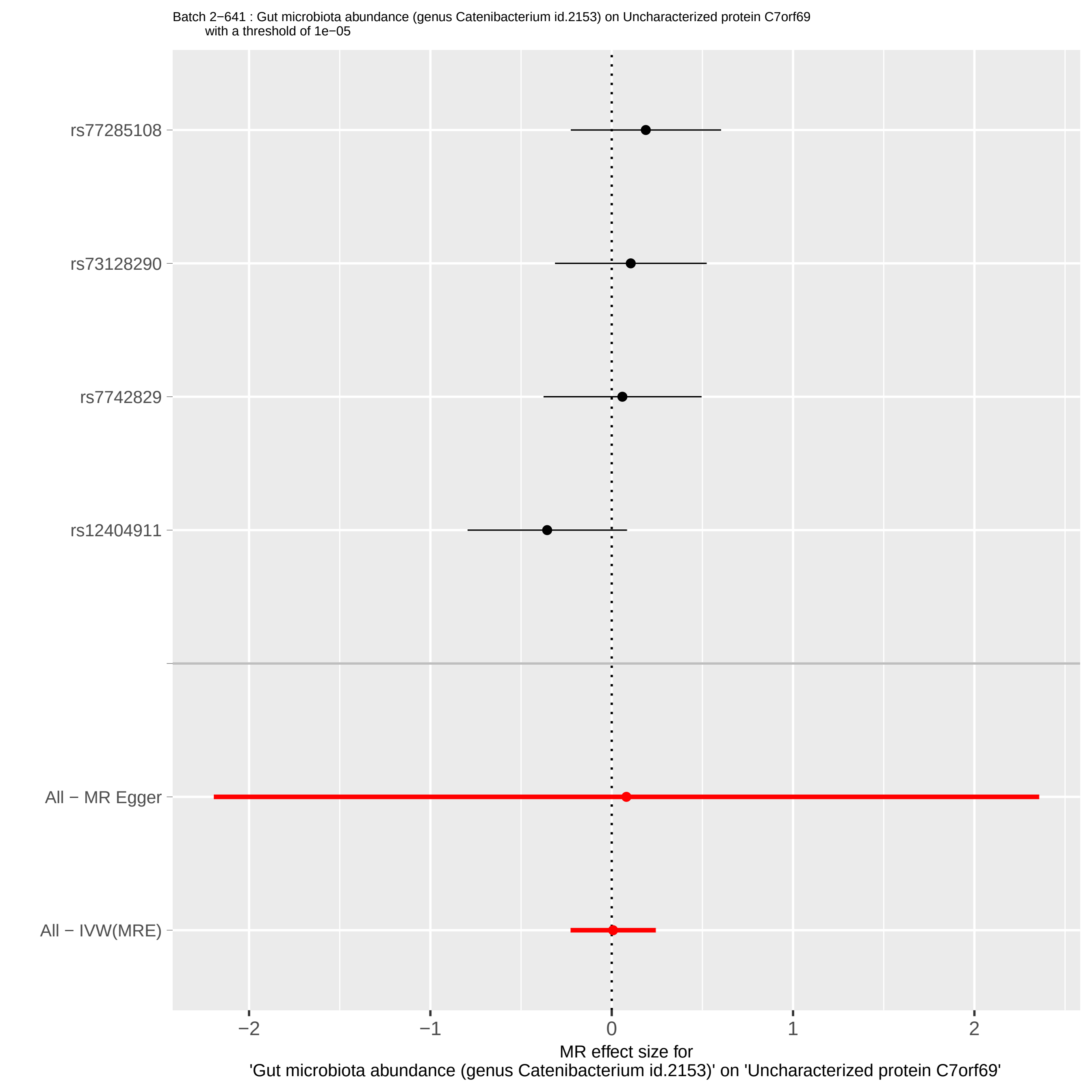

Batch 2−641 : Gut microbiota abundance (genus Catenibacterium id.2153) on Uncharacterized protein C7orf69
with a threshold of 1e−05
rs77285108
rs73128290
rs7742829
rs12404911
All − MR Egger
All − IVW(MRE)
−2
−1
0
1
2
MR effect size for
'Gut microbiota abundance (genus Catenibacterium id.2153)' on 'Uncharacterized protein C7orf69'

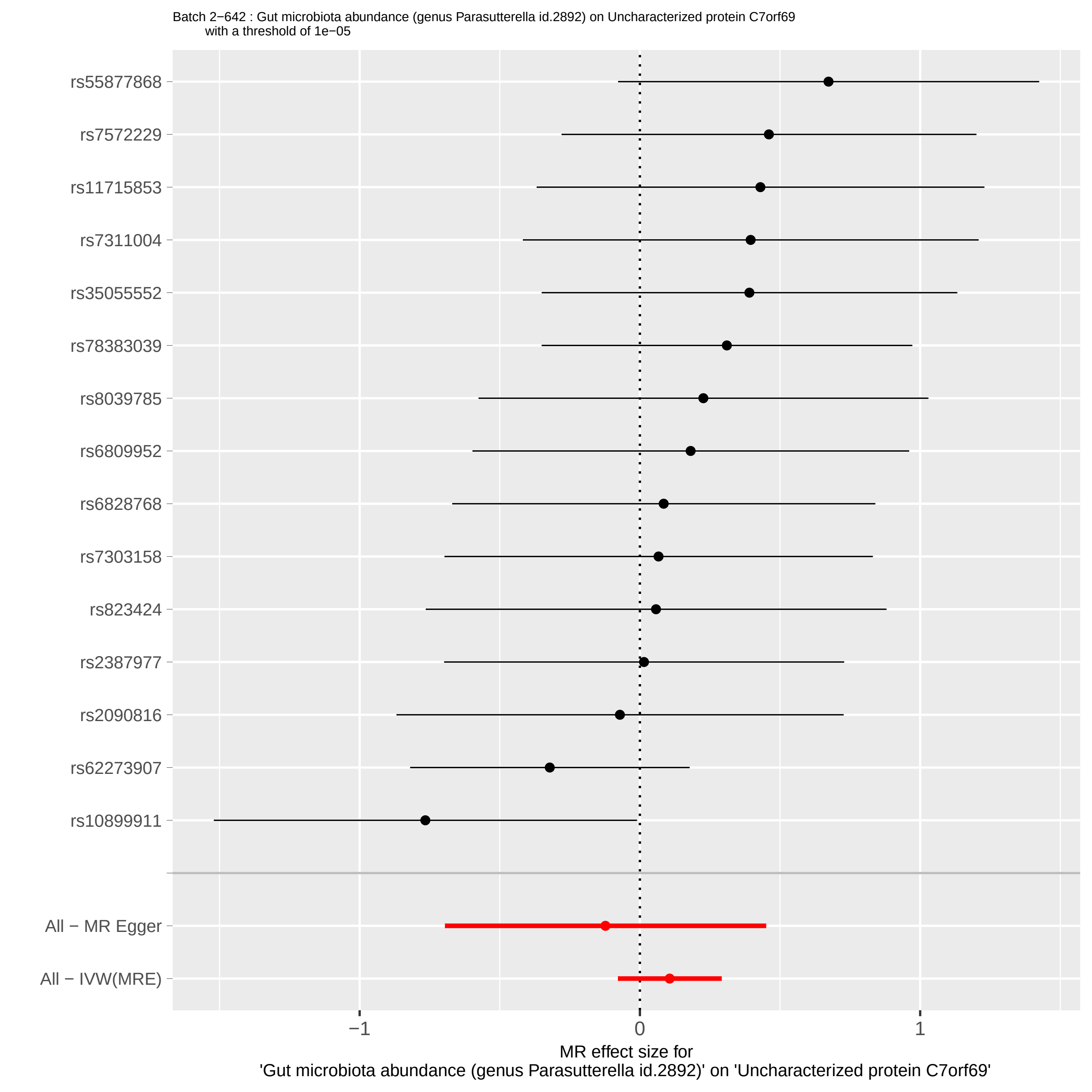
Batch 2−642 : Gut microbiota abundance (genus Parasutterella id.2892) on Uncharacterized protein C7orf69
with a threshold of 1e−05
rs55877868
rs7572229
rs11715853
rs7311004
rs35055552
rs78383039
rs8039785
rs6809952
rs6828768
rs7303158
rs823424
rs2387977
rs2090816
rs62273907
rs10899911
All − MR Egger
All − IVW(MRE)
−1
0
1
MR effect size for
'Gut microbiota abundance (genus Parasutterella id.2892)' on 'Uncharacterized protein C7orf69'

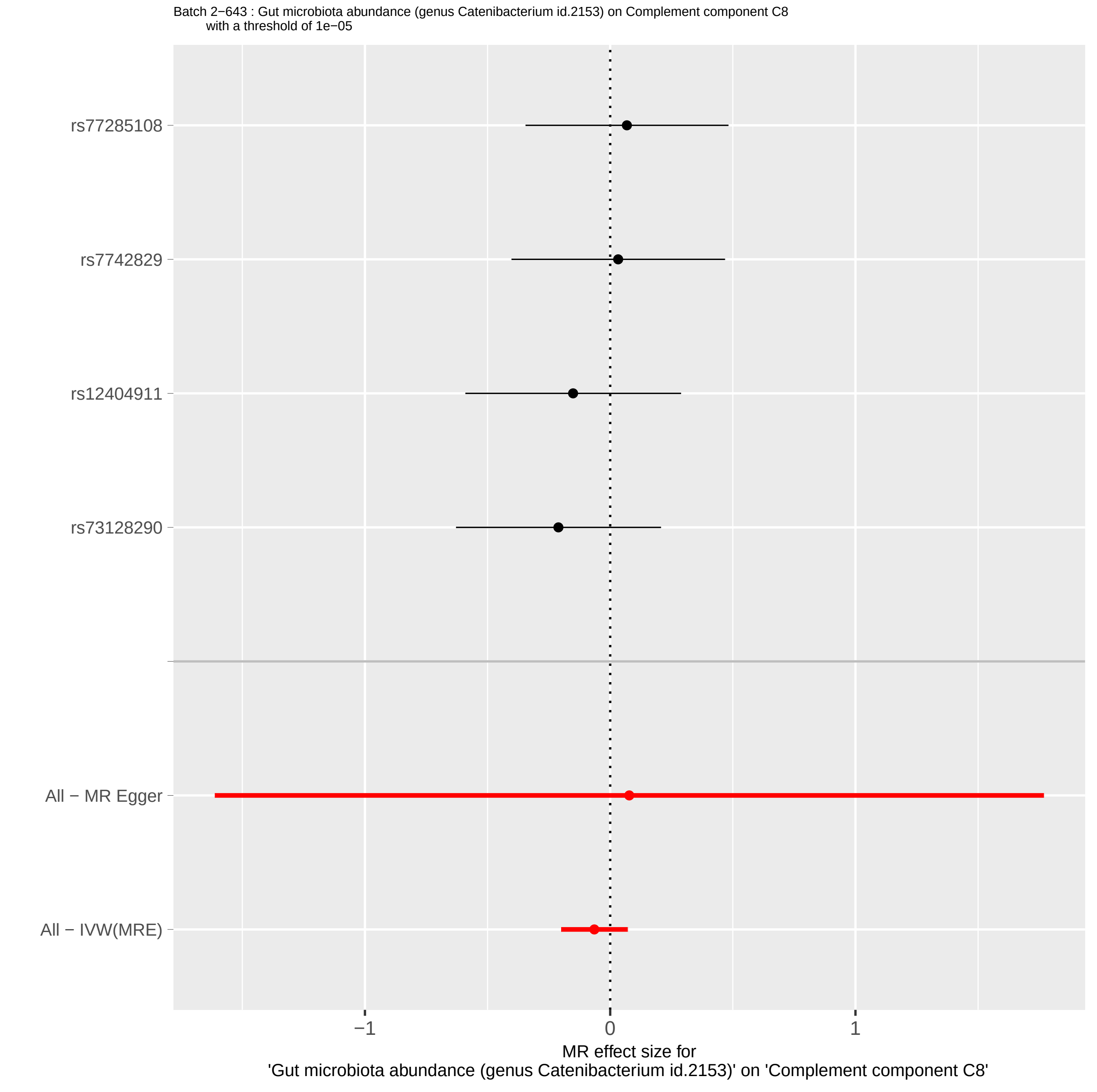

Batch 2−643 : Gut microbiota abundance (genus Catenibacterium id.2153) on Complement component C8
with a threshold of 1e−05
rs77285108
rs7742829
rs12404911
rs73128290
All − MR Egger
All − IVW(MRE)
−1
0
1
MR effect size for
'Gut microbiota abundance (genus Catenibacterium id.2153)' on 'Complement component C8'

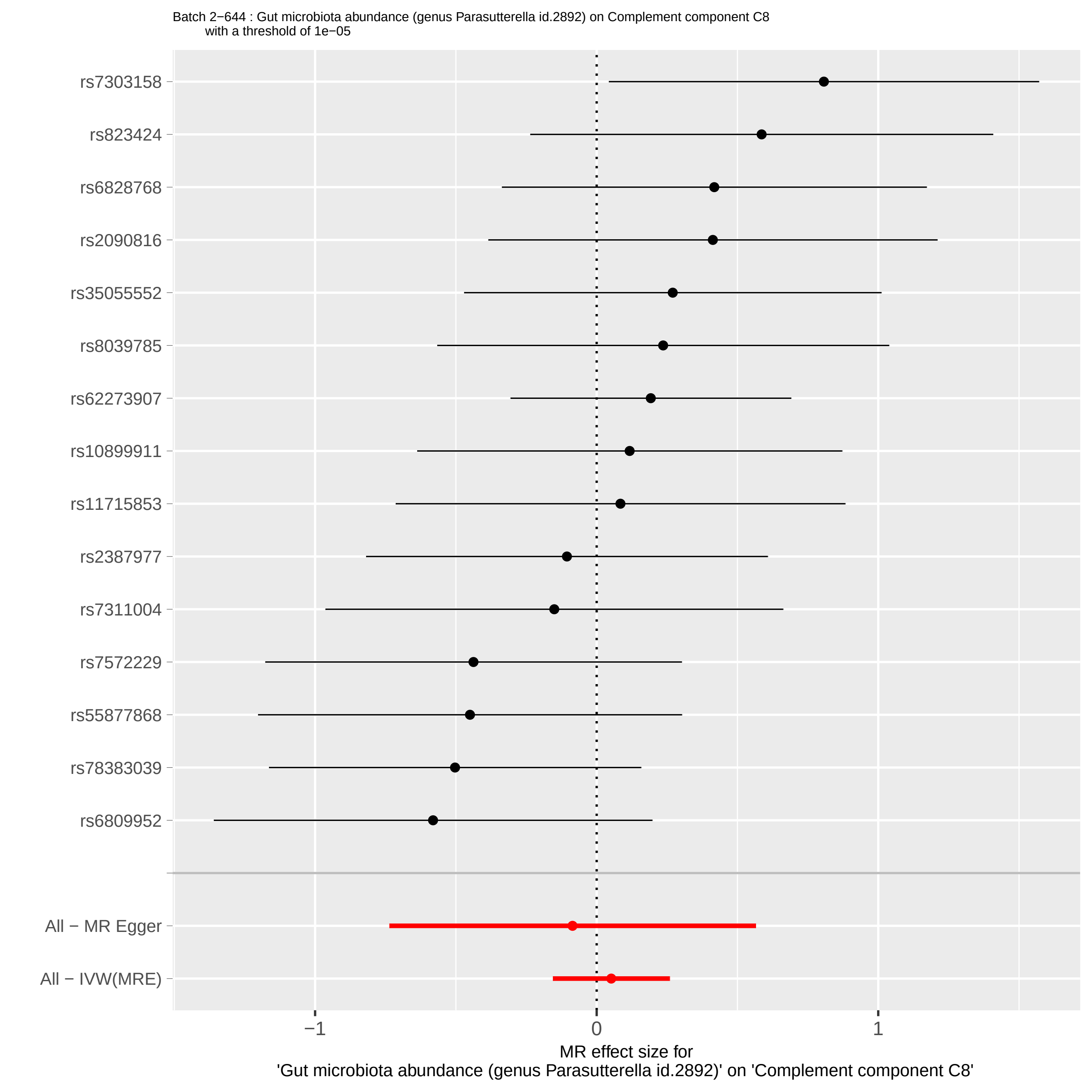

Batch 2−644 : Gut microbiota abundance (genus Parasutterella id.2892) on Complement component C8
with a threshold of 1e−05
rs7303158
rs823424
rs6828768
rs2090816
rs35055552
rs8039785
rs62273907
rs10899911
rs11715853
rs2387977
rs7311004
rs7572229
rs55877868
rs78383039
rs6809952
All − MR Egger
All − IVW(MRE)
−1
0
1
MR effect size for
'Gut microbiota abundance (genus Parasutterella id.2892)' on 'Complement component C8'

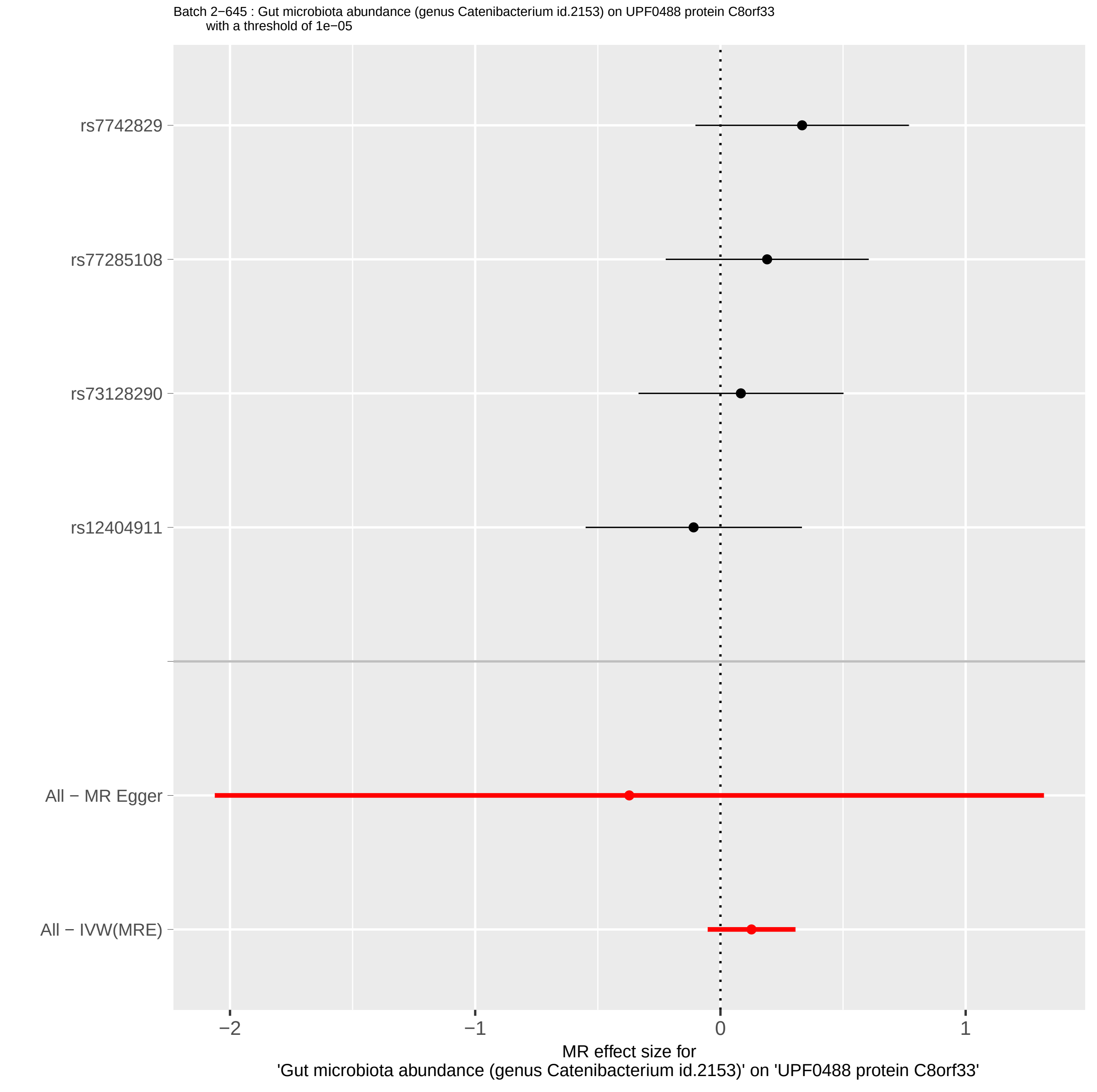
Batch 2−645 : Gut microbiota abundance (genus Catenibacterium id.2153) on UPF0488 protein C8orf33
with a threshold of 1e−05
rs7742829
rs77285108
rs73128290
rs12404911
All − MR Egger
All − IVW(MRE)
−2
−1
0
1
MR effect size for
'Gut microbiota abundance (genus Catenibacterium id.2153)' on 'UPF0488 protein C8orf33'

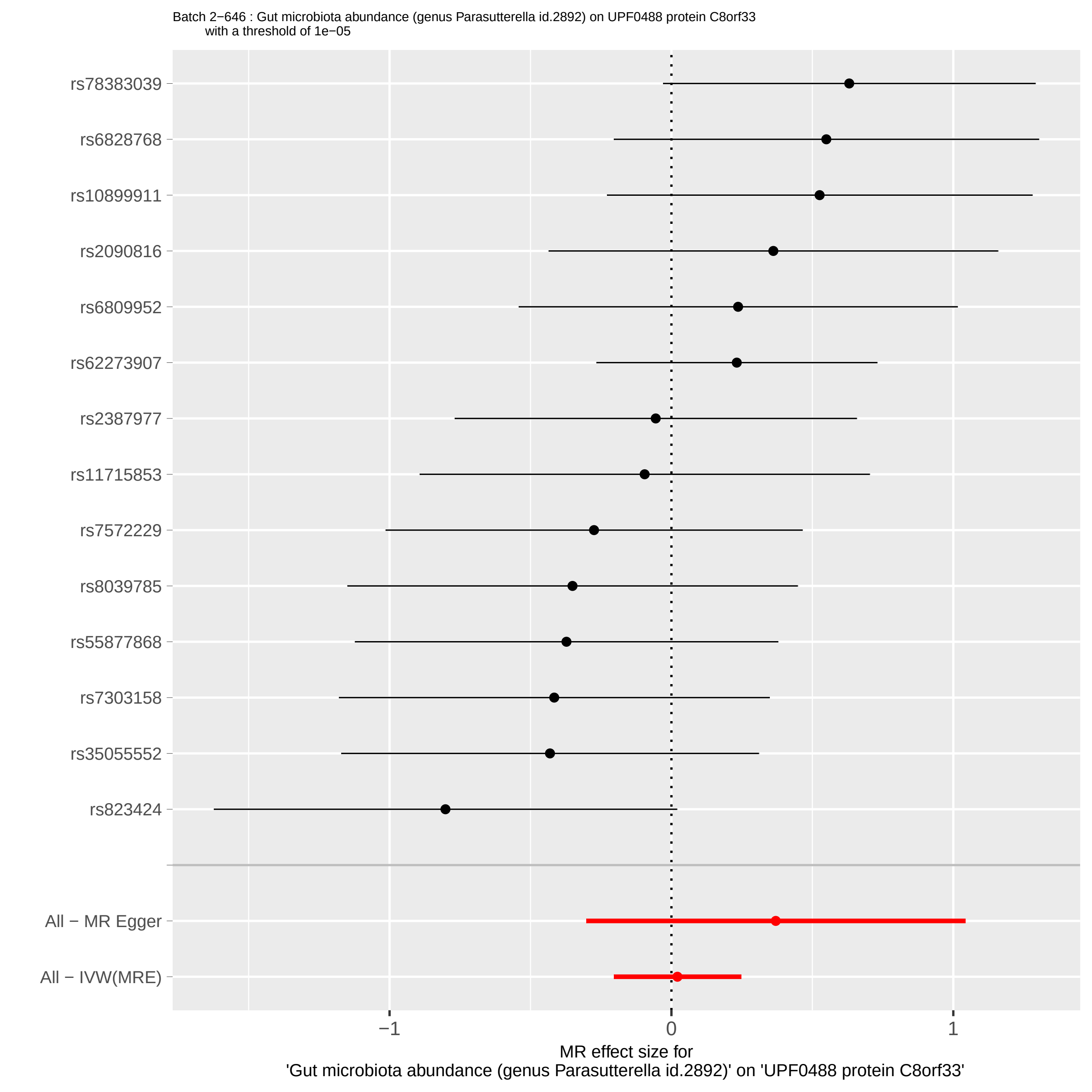

Batch 2−646 : Gut microbiota abundance (genus Parasutterella id.2892) on UPF0488 protein C8orf33
with a threshold of 1e−05
rs78383039
rs6828768
rs10899911
rs2090816
rs6809952
rs62273907
rs2387977
rs11715853
rs7572229
rs8039785
rs55877868
rs7303158
rs35055552
rs823424
All − MR Egger
All − IVW(MRE)
−1
0
1
MR effect size for
'Gut microbiota abundance (genus Parasutterella id.2892)' on 'UPF0488 protein C8orf33'

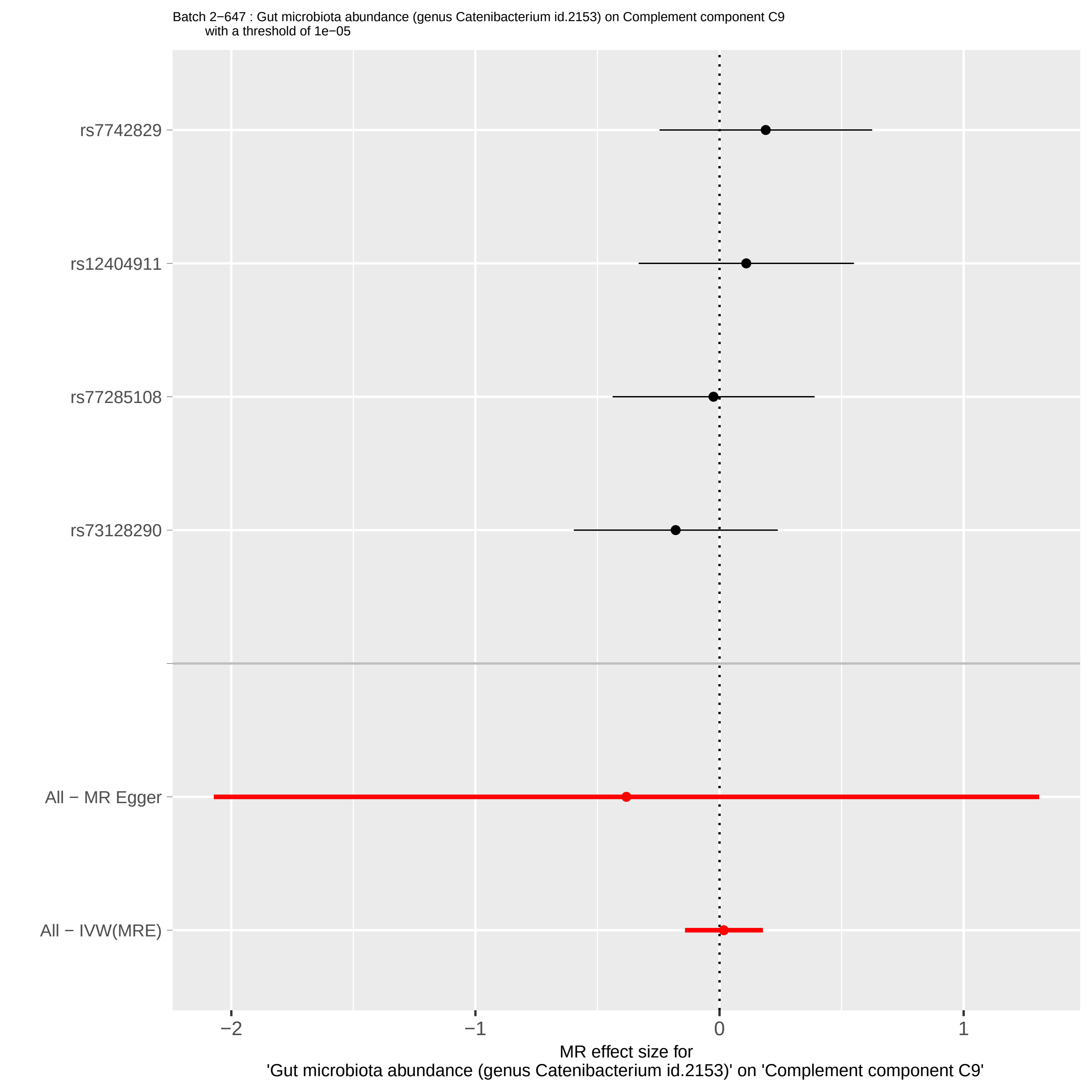
Batch 2−647 : Gut microbiota abundance (genus Catenibacterium id.2153) on Complement component C9
with a threshold of 1e−05
rs7742829
rs12404911
rs77285108
rs73128290
All − MR Egger
All − IVW(MRE)
−2
−1
0
1
MR effect size for
'Gut microbiota abundance (genus Catenibacterium id.2153)' on 'Complement component C9'

Batch 2−648 : Gut microbiota abundance (genus Parasutterella id.2892) on Complement component C9
with a threshold of 1e−05

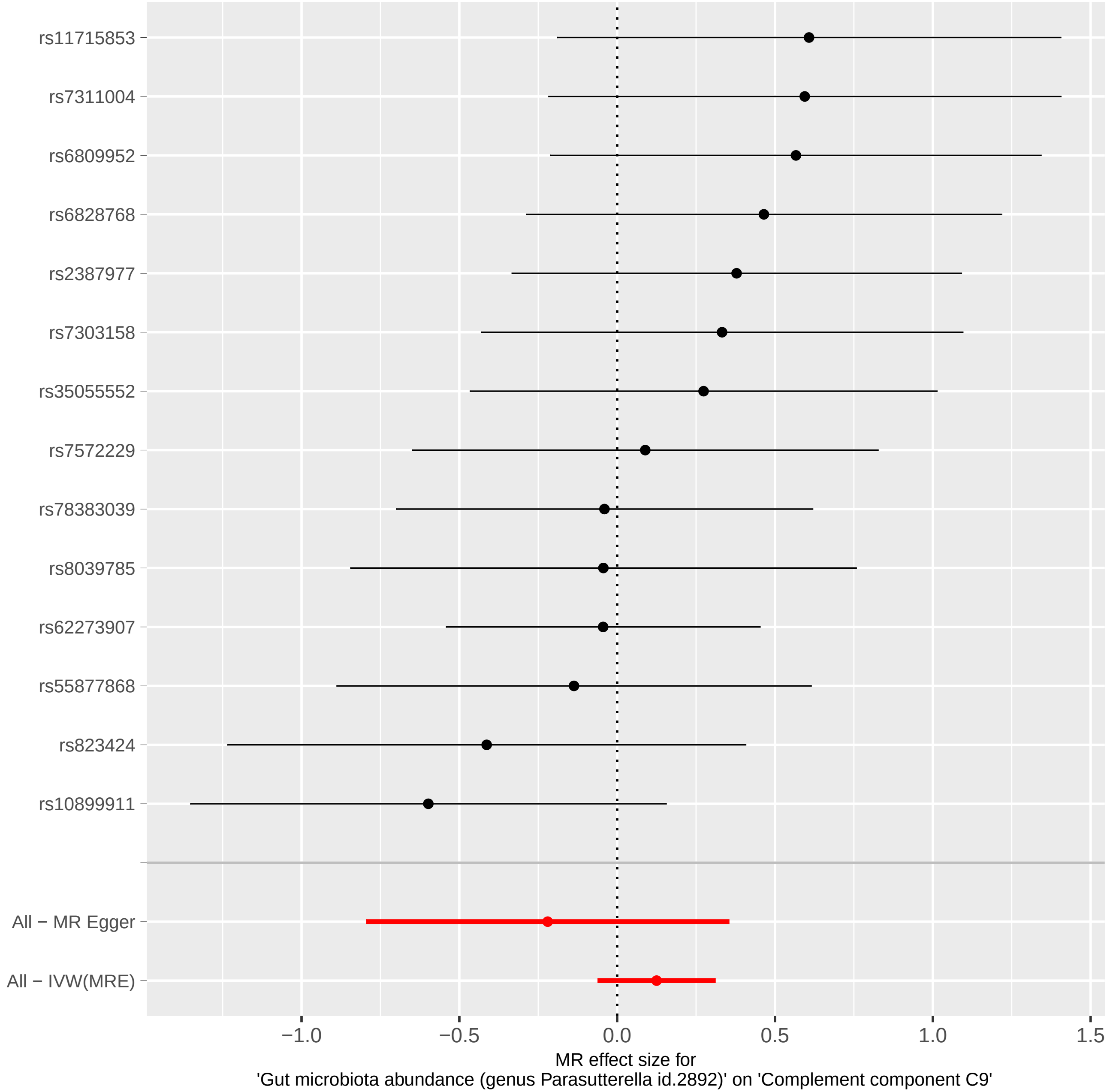

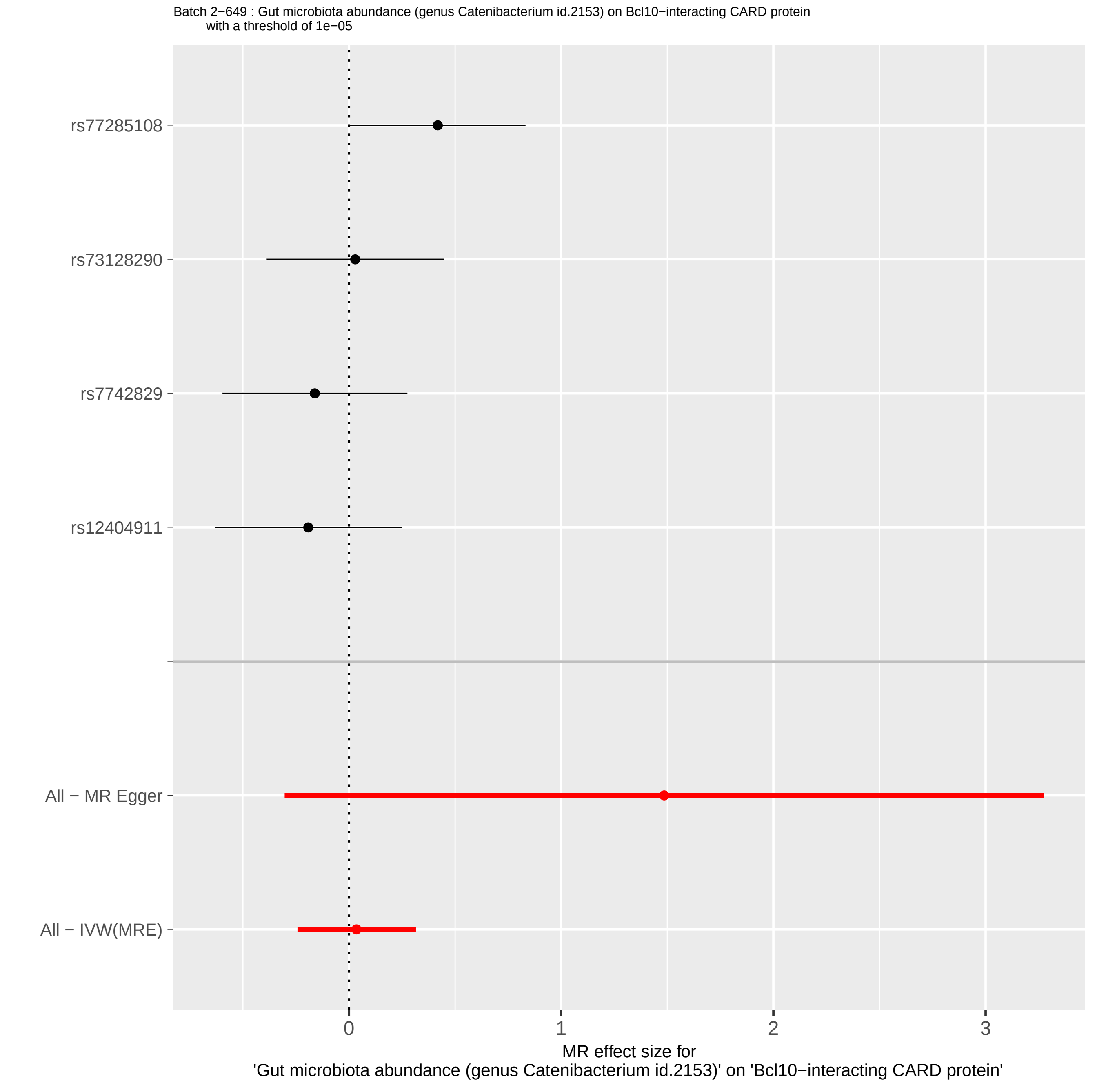

Batch 2−649 : Gut microbiota abundance (genus Catenibacterium id.2153) on Bcl10−interacting CARD protein
with a threshold of 1e−05
rs77285108
rs73128290
rs7742829
rs12404911
All − MR Egger
All − IVW(MRE)
0
1
2
3
MR effect size for
'Gut microbiota abundance (genus Catenibacterium id.2153)' on 'Bcl10−interacting CARD protein'

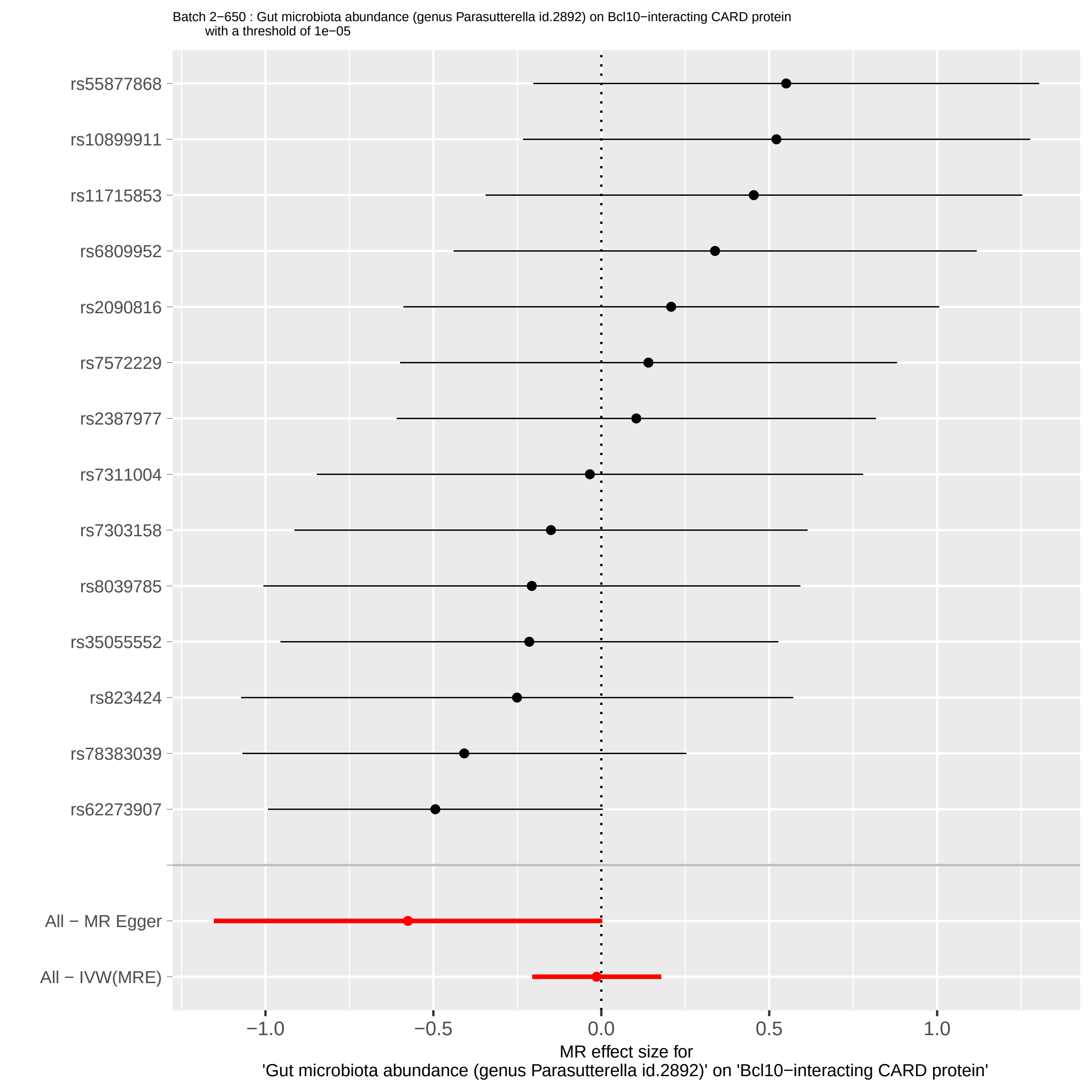

Batch 2−650 : Gut microbiota abundance (genus Parasutterella id.2892) on Bcl10−interacting CARD protein with a threshold of 1e−05
rs55877868
rs10899911
rs11715853
rs6809952
rs2090816
rs7572229
rs2387977
rs7311004
rs7303158
rs8039785
rs35055552
rs823424
rs78383039
rs62273907
All − MR Egger
All − IVW(MRE)
−1.0
−0.5
0.0
0.5
1.0
MR effect size for
'Gut microbiota abundance (genus Parasutterella id.2892)' on 'Bcl10−interacting CARD protein'

Batch 2−651 : Gut microbiota abundance (genus Catenibacterium id.2153) on Carbonic anhydrase−related protein 10
with a threshold of 1e−05

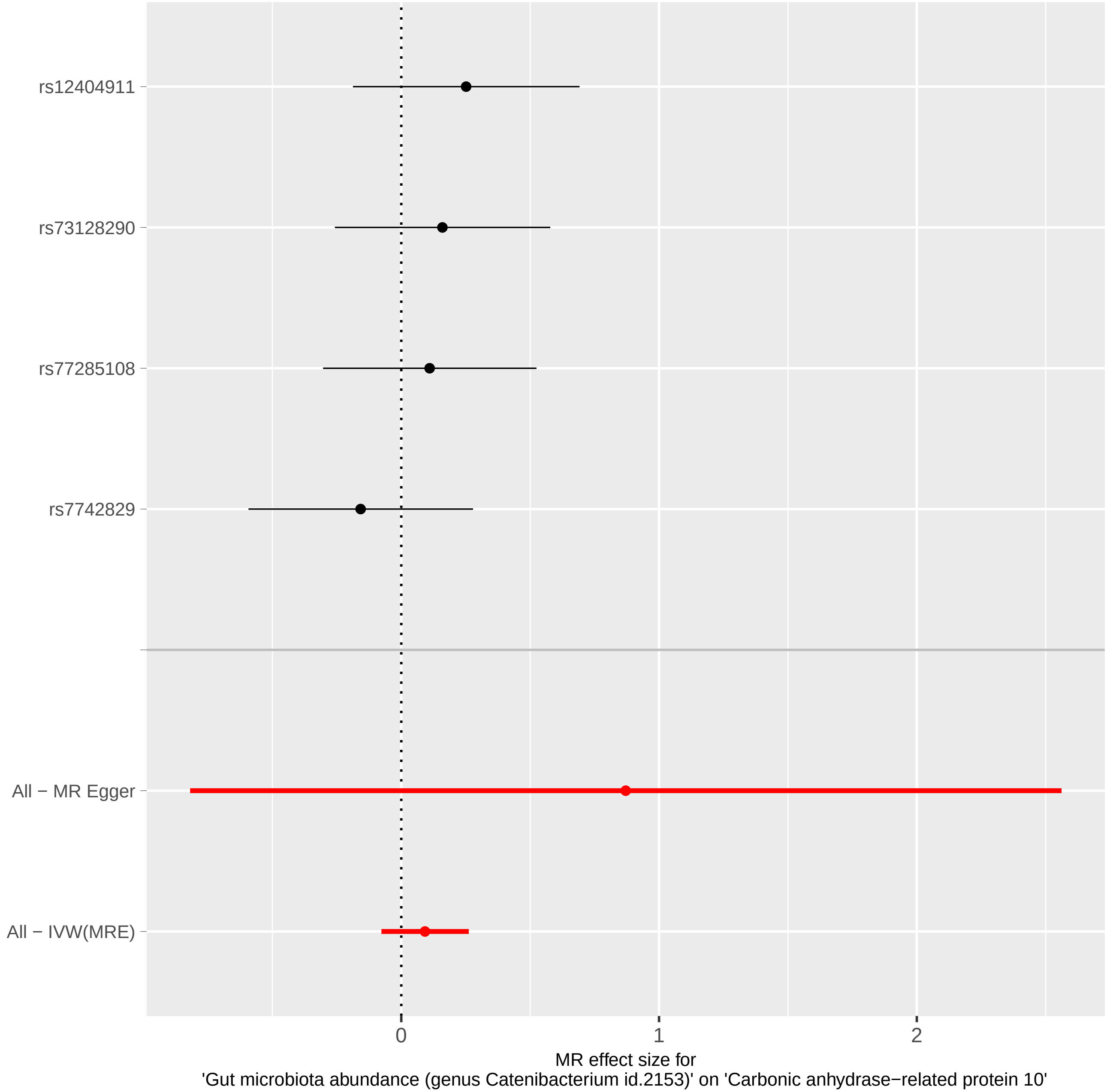

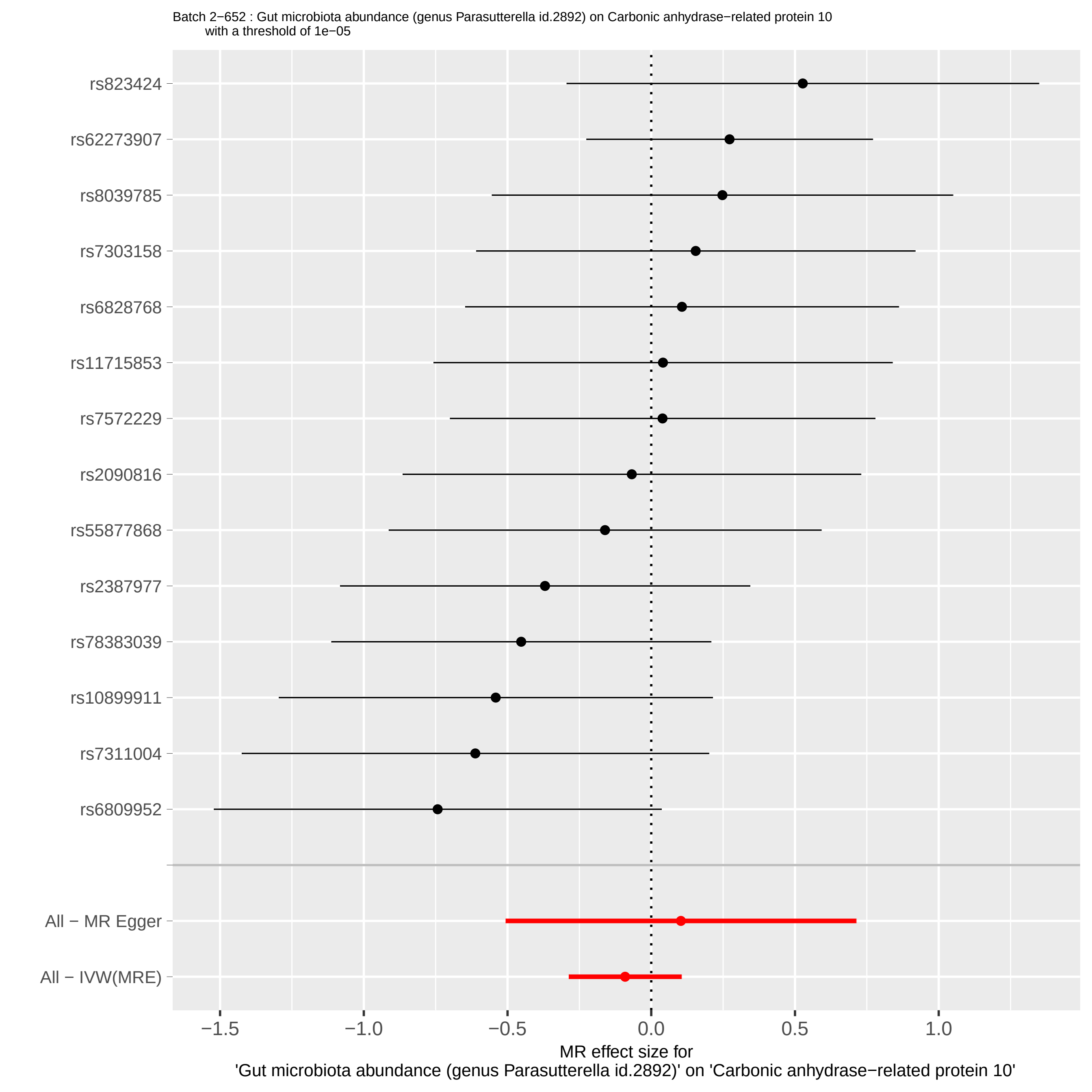

Batch 2−652 : Gut microbiota abundance (genus Parasutterella id.2892) on Carbonic anhydrase−related protein 10
with a threshold of 1e−05
rs823424
rs62273907
rs8039785
rs7303158
rs6828768
rs11715853
rs7572229
rs2090816
rs55877868
rs2387977
rs78383039
rs10899911
rs7311004
rs6809952
All − MR Egger
All − IVW(MRE)
−1.5
−1.0
−0.5
0.0
0.5
1.0
MR effect size for
'Gut microbiota abundance (genus Parasutterella id.2892)' on 'Carbonic anhydrase−related protein 10'

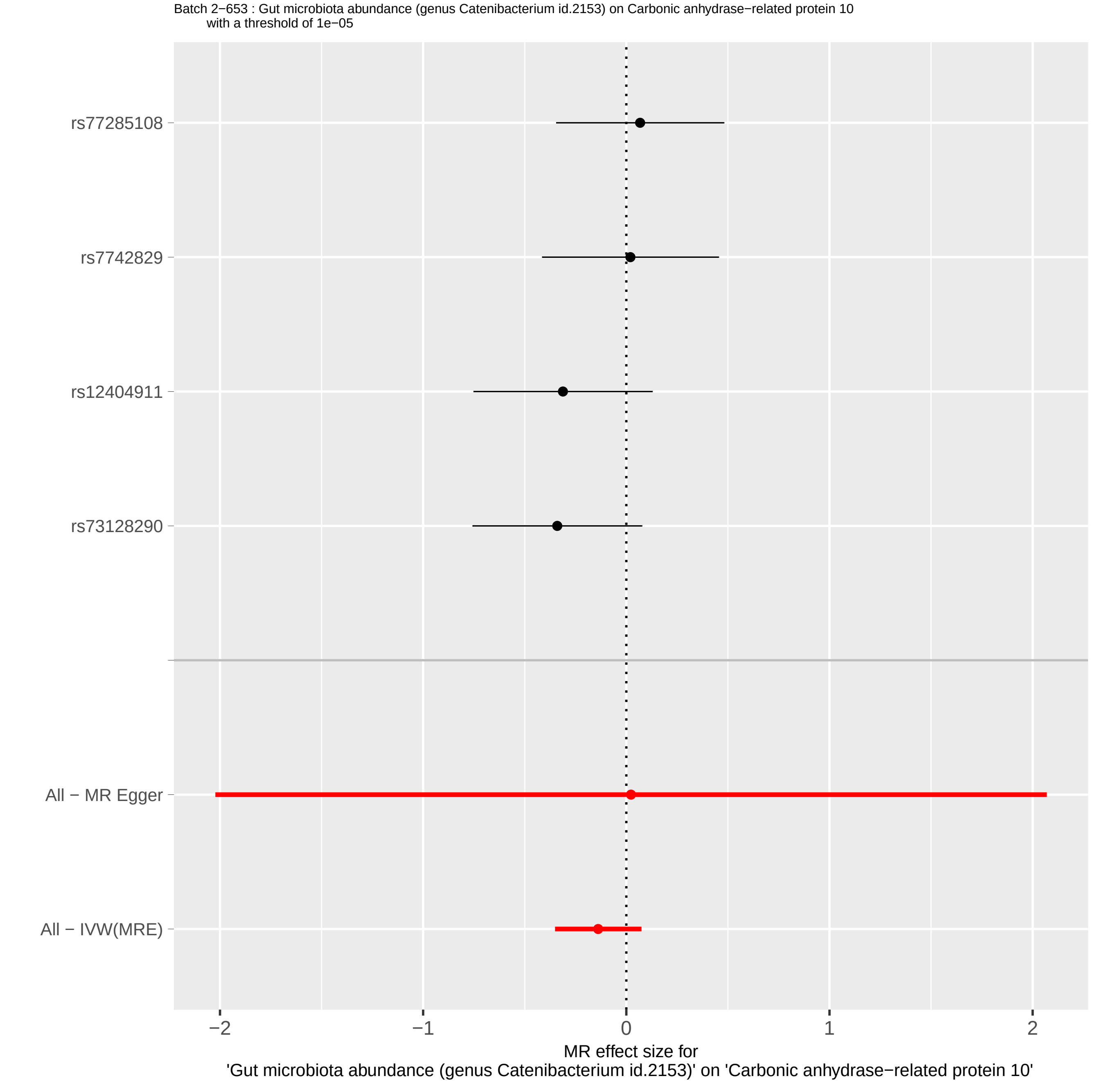
Batch 2−653 : Gut microbiota abundance (genus Catenibacterium id.2153) on Carbonic anhydrase−related protein 10
with a threshold of 1e−05
rs77285108
rs7742829
rs12404911
rs73128290
All − MR Egger
All − IVW(MRE)
−2
−1
0
1
2
MR effect size for
'Gut microbiota abundance (genus Catenibacterium id.2153)' on 'Carbonic anhydrase−related protein 10'

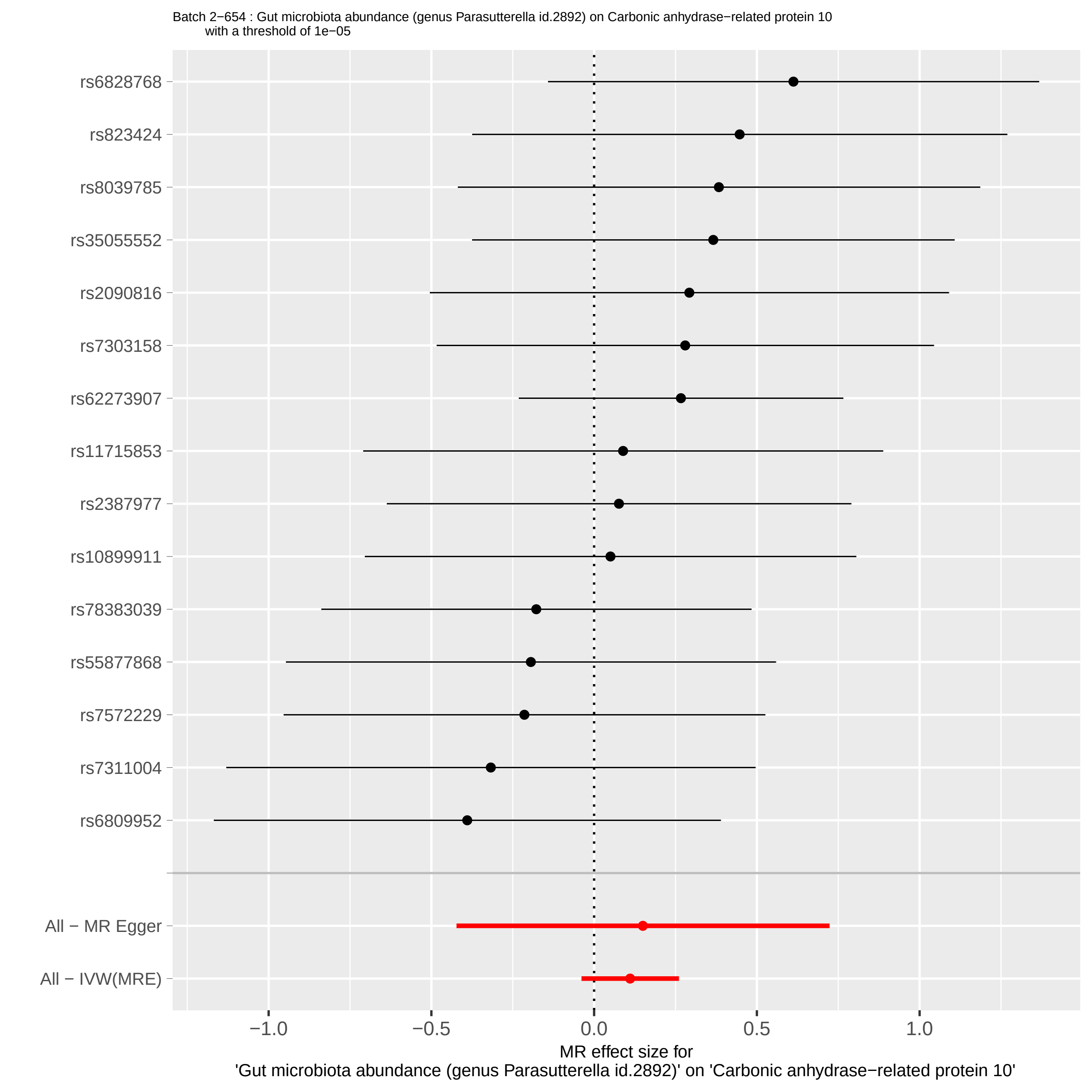

Batch 2−654 : Gut microbiota abundance (genus Parasutterella id.2892) on Carbonic anhydrase−related protein 10
with a threshold of 1e−05
rs6828768
rs823424
rs8039785
rs35055552
rs2090816
rs7303158
rs62273907
rs11715853
rs2387977
rs10899911
rs78383039
rs55877868
rs7572229
rs7311004
rs6809952
All − MR Egger
All − IVW(MRE)
−1.0
−0.5
0.0
0.5
1.0
MR effect size for
'Gut microbiota abundance (genus Parasutterella id.2892)' on 'Carbonic anhydrase−related protein 10'

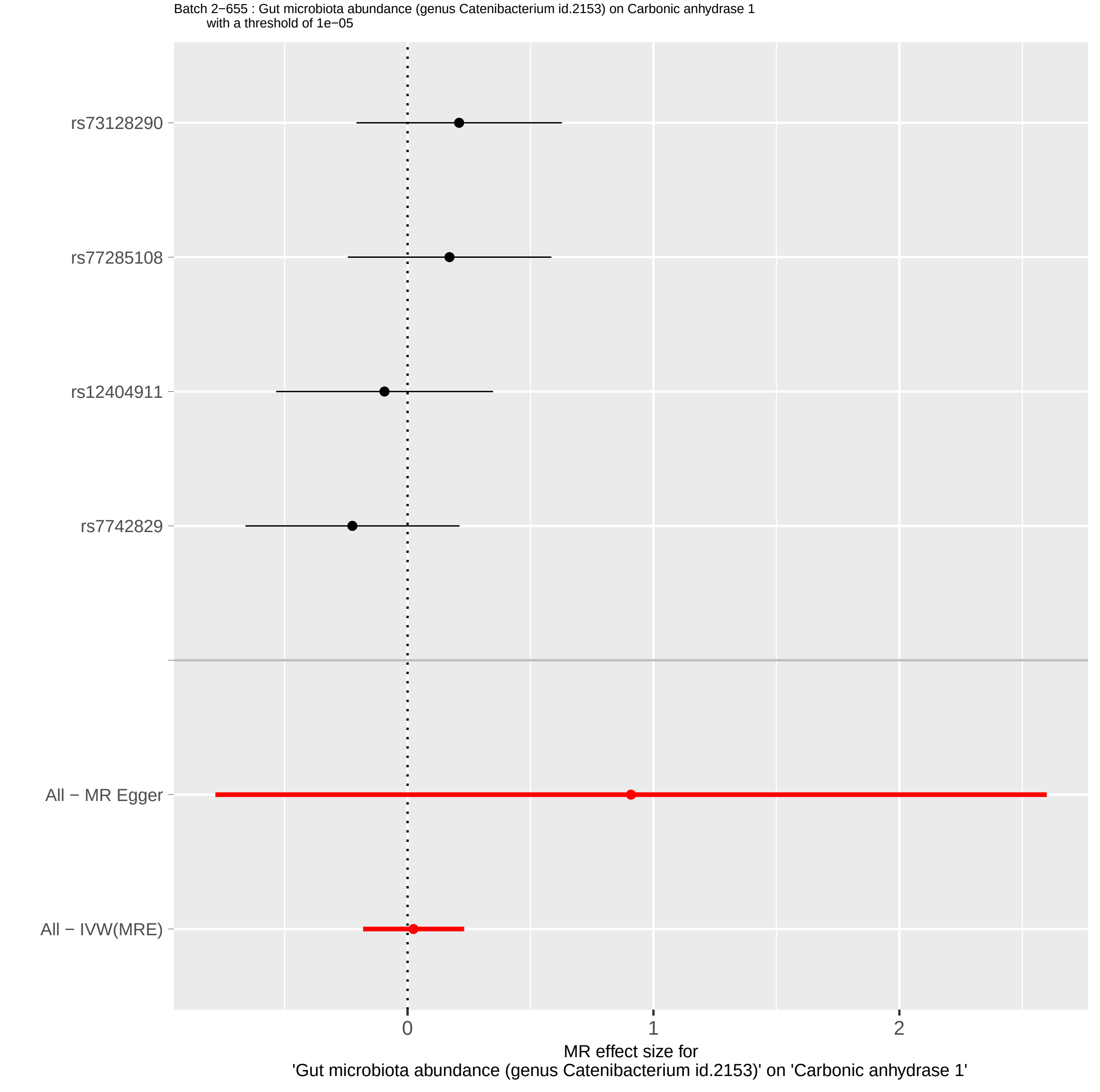
Batch 2−655 : Gut microbiota abundance (genus Catenibacterium id.2153) on Carbonic anhydrase 1
with a threshold of 1e−05
rs73128290
rs77285108
rs12404911
rs7742829
All − MR Egger
All − IVW(MRE)
0
1
2
MR effect size for
'Gut microbiota abundance (genus Catenibacterium id.2153)' on 'Carbonic anhydrase 1'

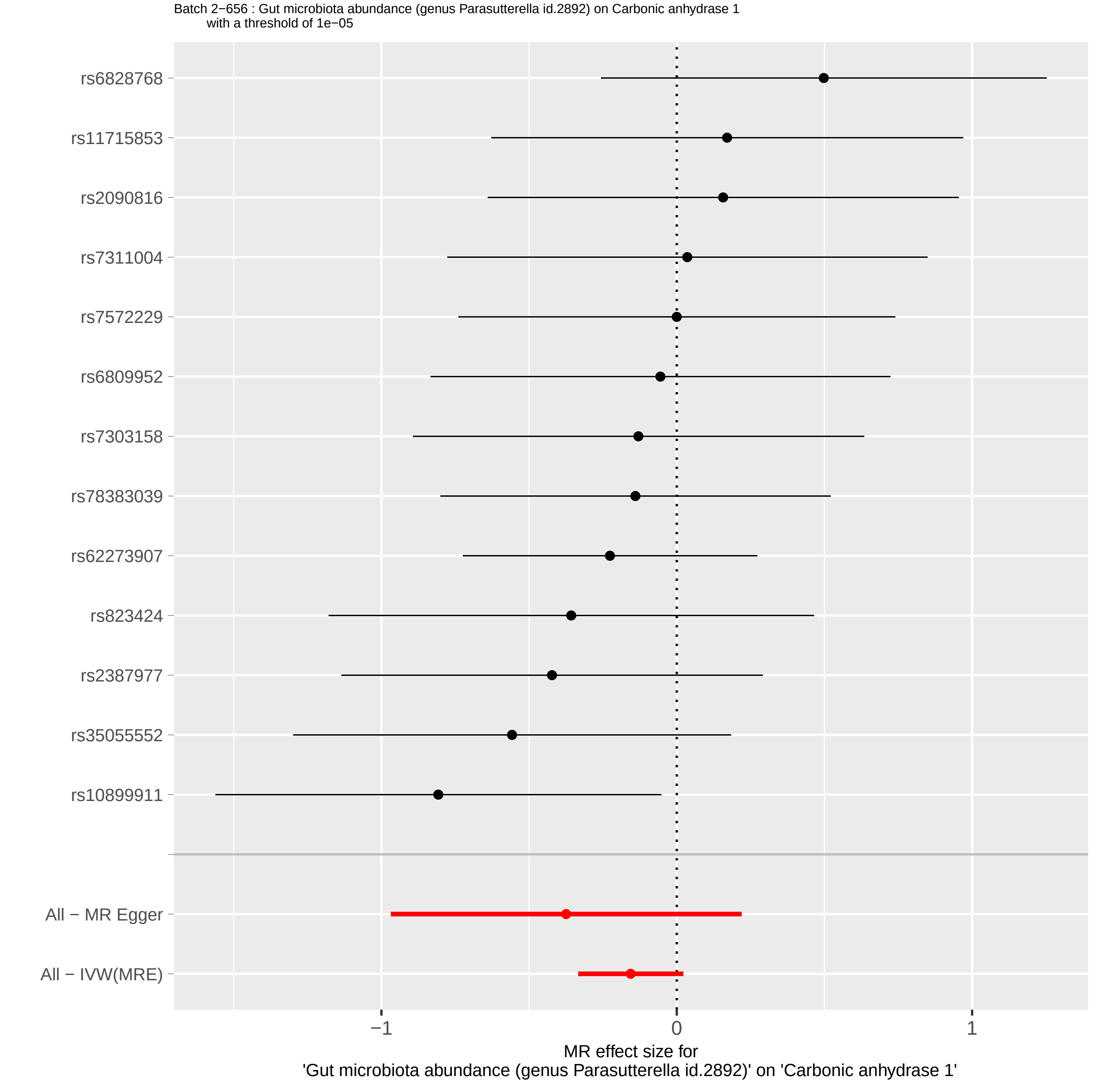

Batch 2−656 : Gut microbiota abundance (genus Parasutterella id.2892) on Carbonic anhydrase 1
with a threshold of 1e−05
rs6828768
rs11715853
rs2090816
rs7311004
rs7572229
rs6809952
rs7303158
rs78383039
rs62273907
rs823424
rs2387977
rs35055552
rs10899911
All − MR Egger
All − IVW(MRE)
−1
0
1
MR effect size for
'Gut microbiota abundance (genus Parasutterella id.2892)' on 'Carbonic anhydrase 1'

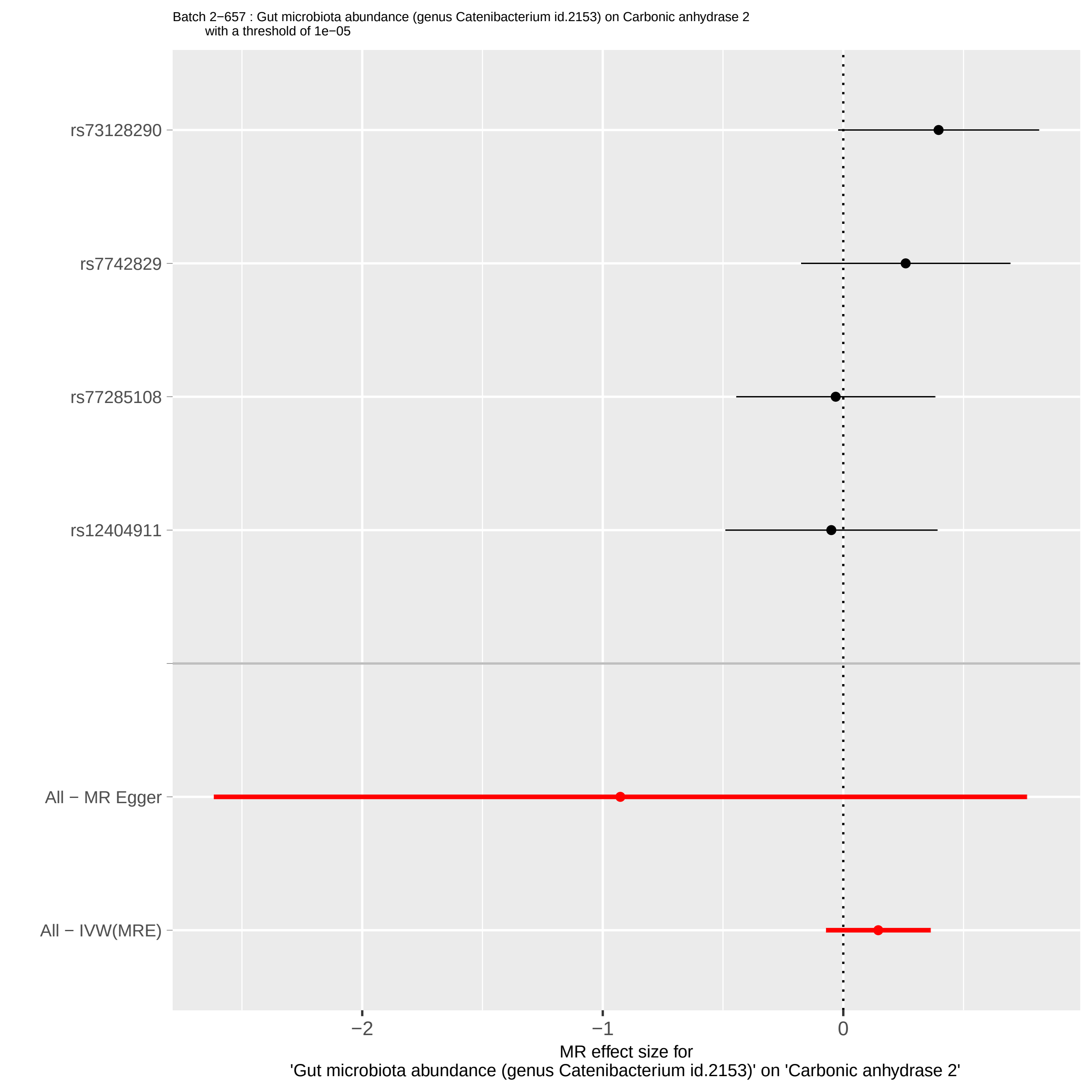
Batch 2−657 : Gut microbiota abundance (genus Catenibacterium id.2153) on Carbonic anhydrase 2
with a threshold of 1e−05
rs73128290
rs7742829
rs77285108
rs12404911
All − MR Egger
All − IVW(MRE)
−2
−1
0
MR effect size for
'Gut microbiota abundance (genus Catenibacterium id.2153)' on 'Carbonic anhydrase 2'

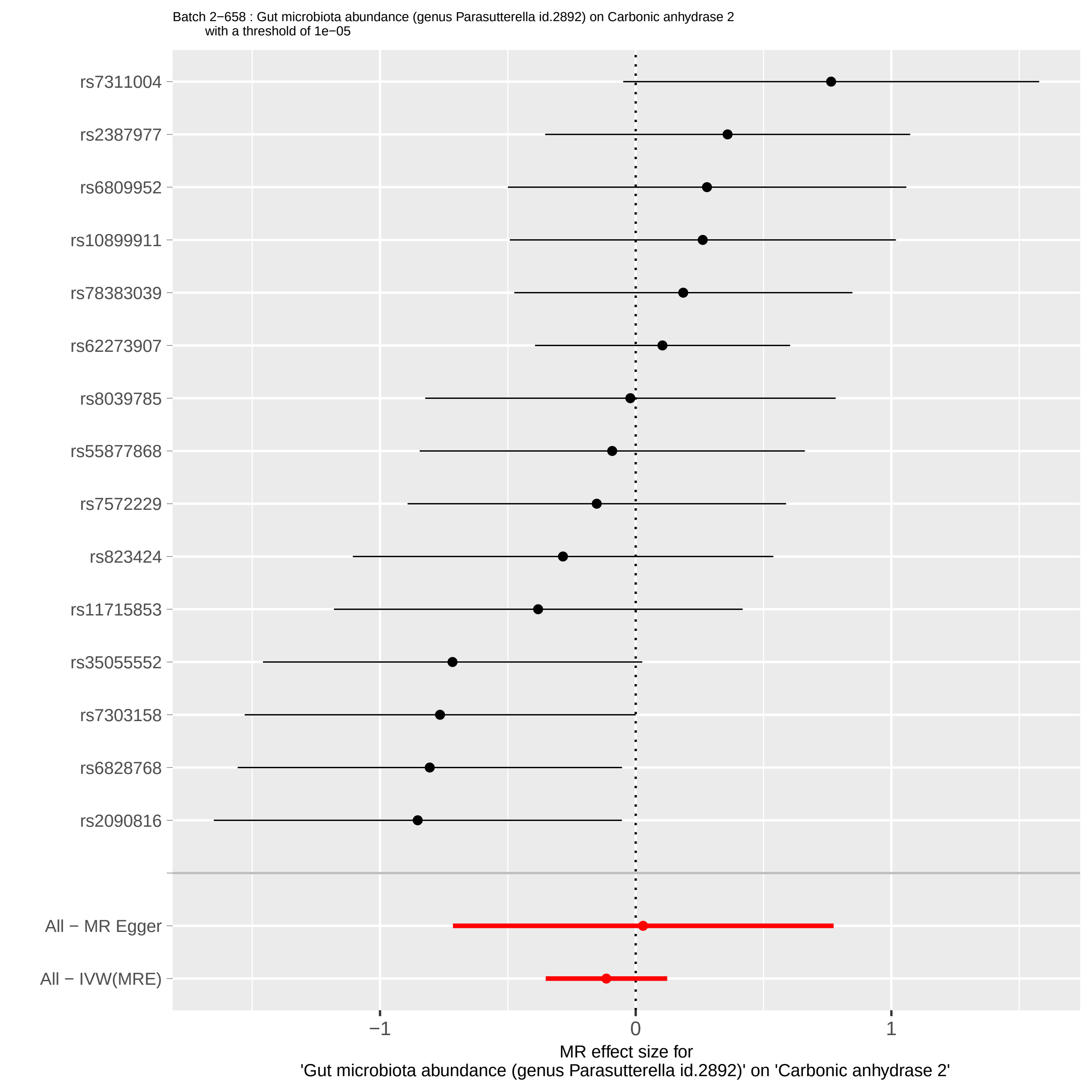
Batch 2−658 : Gut microbiota abundance (genus Parasutterella id.2892) on Carbonic anhydrase 2
with a threshold of 1e−05
rs7311004
rs2387977
rs6809952
rs10899911
rs78383039
rs62273907
rs8039785
rs55877868
rs7572229
rs823424
rs11715853
rs35055552
rs7303158
rs6828768
rs2090816
All − MR Egger
All − IVW(MRE)
−1
0
1
MR effect size for
'Gut microbiota abundance (genus Parasutterella id.2892)' on 'Carbonic anhydrase 2'

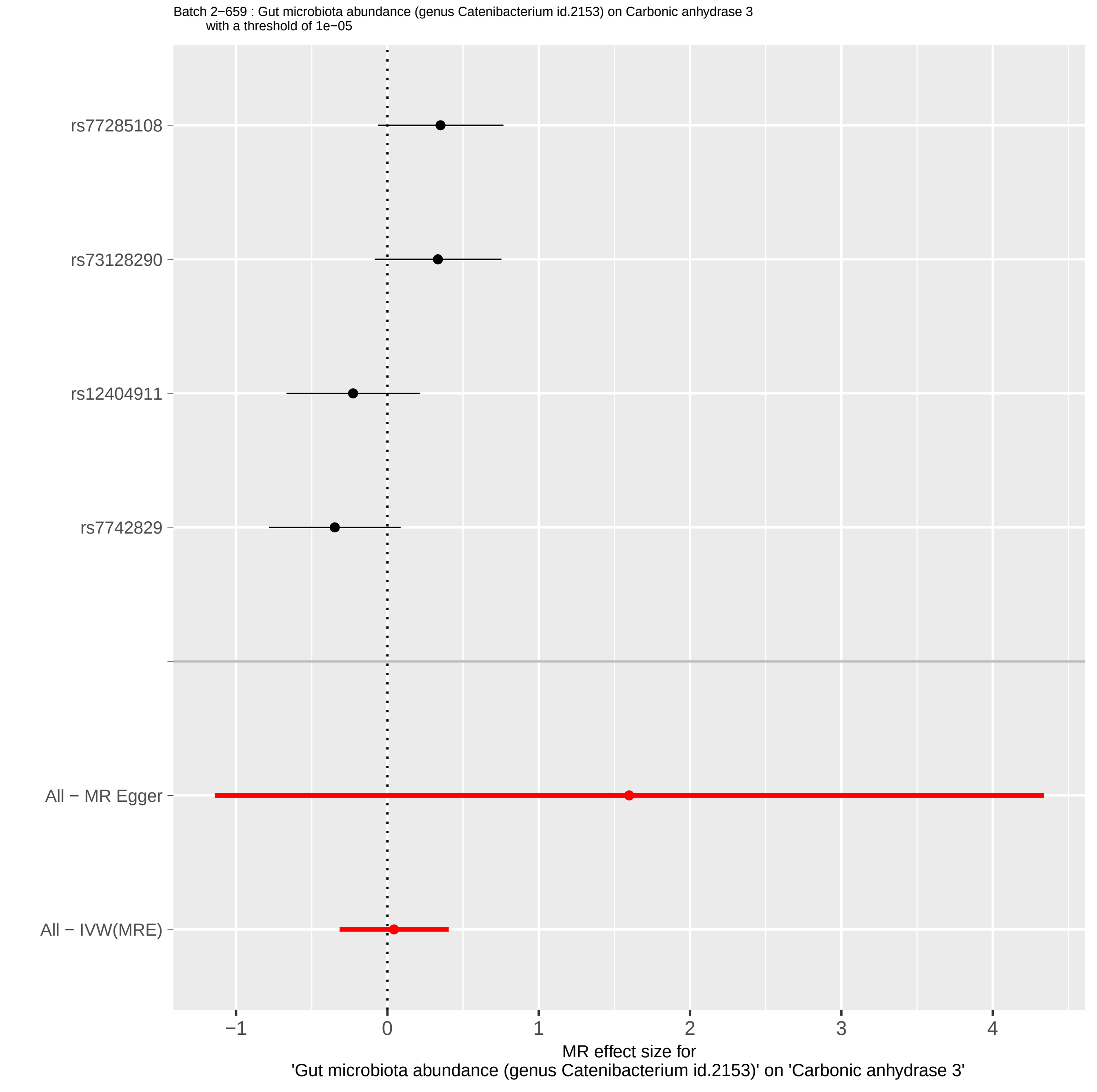
Batch 2−659 : Gut microbiota abundance (genus Catenibacterium id.2153) on Carbonic anhydrase 3
with a threshold of 1e−05
rs77285108
rs73128290
rs12404911
rs7742829
All − MR Egger
All − IVW(MRE)
−1
0
1
2
3
4
MR effect size for
'Gut microbiota abundance (genus Catenibacterium id.2153)' on 'Carbonic anhydrase 3'

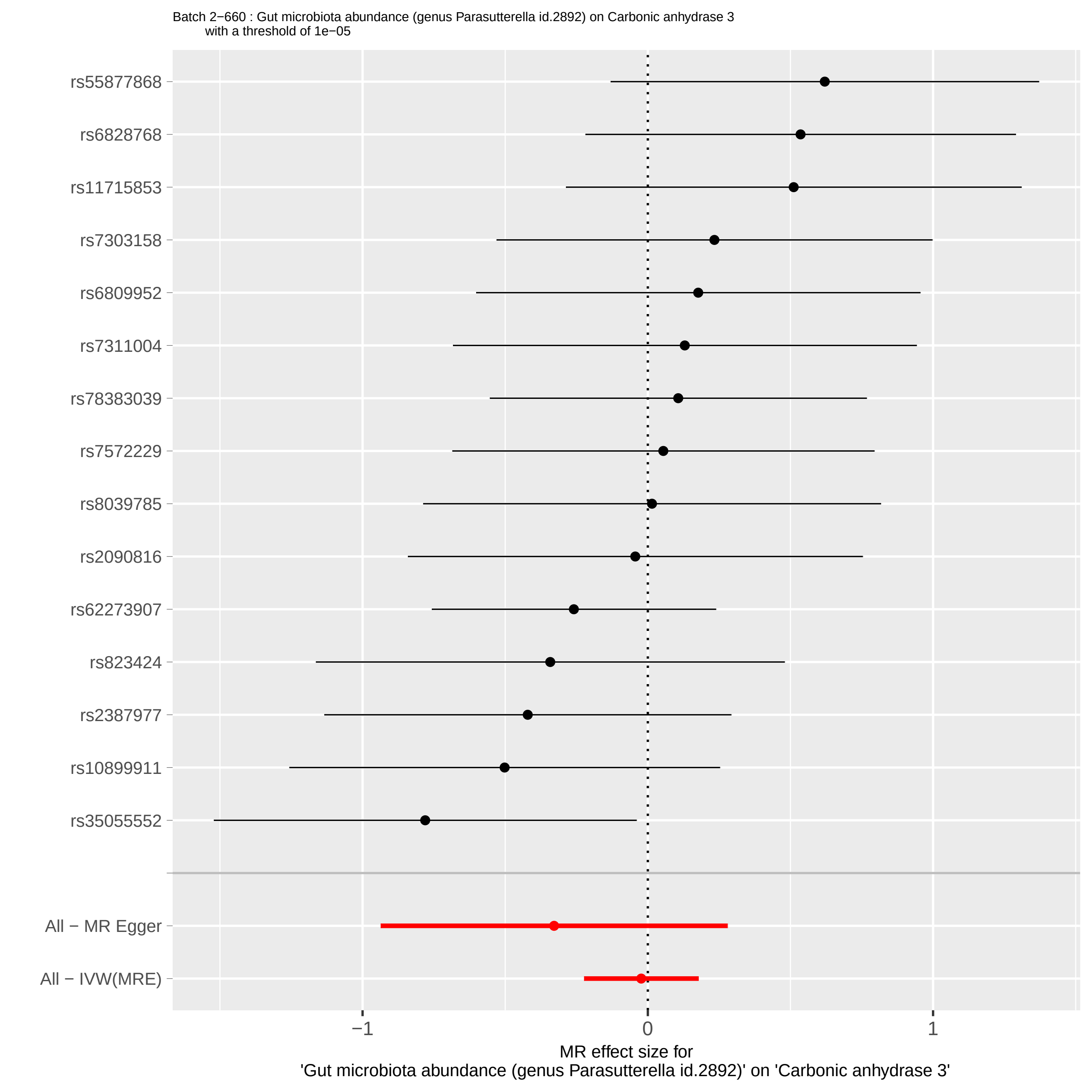

Batch 2−660 : Gut microbiota abundance (genus Parasutterella id.2892) on Carbonic anhydrase 3
with a threshold of 1e−05
rs55877868
rs6828768
rs11715853
rs7303158
rs6809952
rs7311004
rs78383039
rs7572229
rs8039785
rs2090816
rs62273907
rs823424
rs2387977
rs10899911
rs35055552
All − MR Egger
All − IVW(MRE)
−1
0
1
MR effect size for
'Gut microbiota abundance (genus Parasutterella id.2892)' on 'Carbonic anhydrase 3'

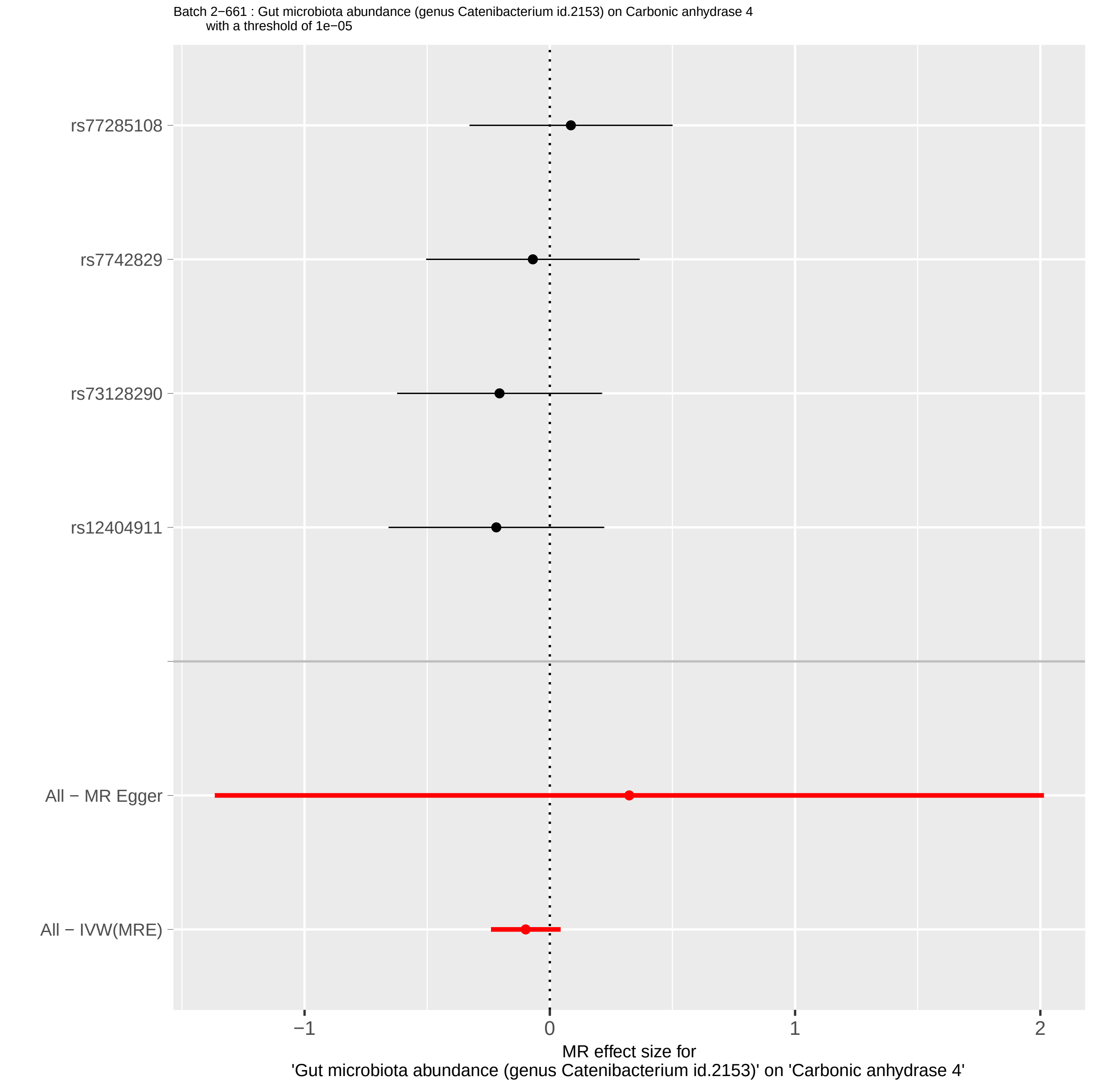

Batch 2−661 : Gut microbiota abundance (genus Catenibacterium id.2153) on Carbonic anhydrase 4
with a threshold of 1e−05
rs77285108
rs7742829
rs73128290
rs12404911
All − MR Egger
All − IVW(MRE)
−1
0
1
2
MR effect size for
'Gut microbiota abundance (genus Catenibacterium id.2153)' on 'Carbonic anhydrase 4'

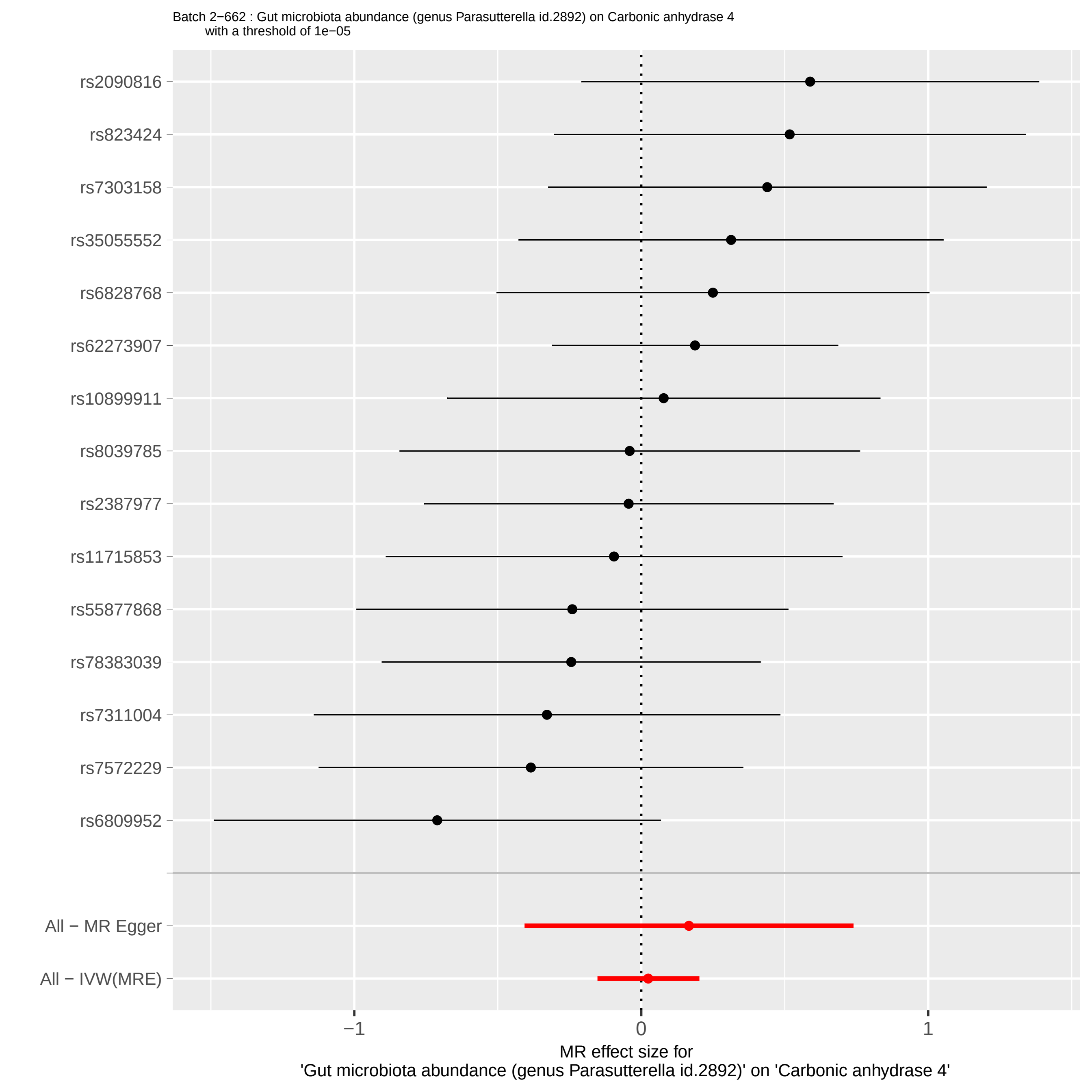
Batch 2−662 : Gut microbiota abundance (genus Parasutterella id.2892) on Carbonic anhydrase 4
with a threshold of 1e−05
rs2090816
rs823424
rs7303158
rs35055552
rs6828768
rs62273907
rs10899911
rs8039785
rs2387977
rs11715853
rs55877868
rs78383039
rs7311004
rs7572229
rs6809952
All − MR Egger
All − IVW(MRE)
−1
0
1
MR effect size for
'Gut microbiota abundance (genus Parasutterella id.2892)' on 'Carbonic anhydrase 4'

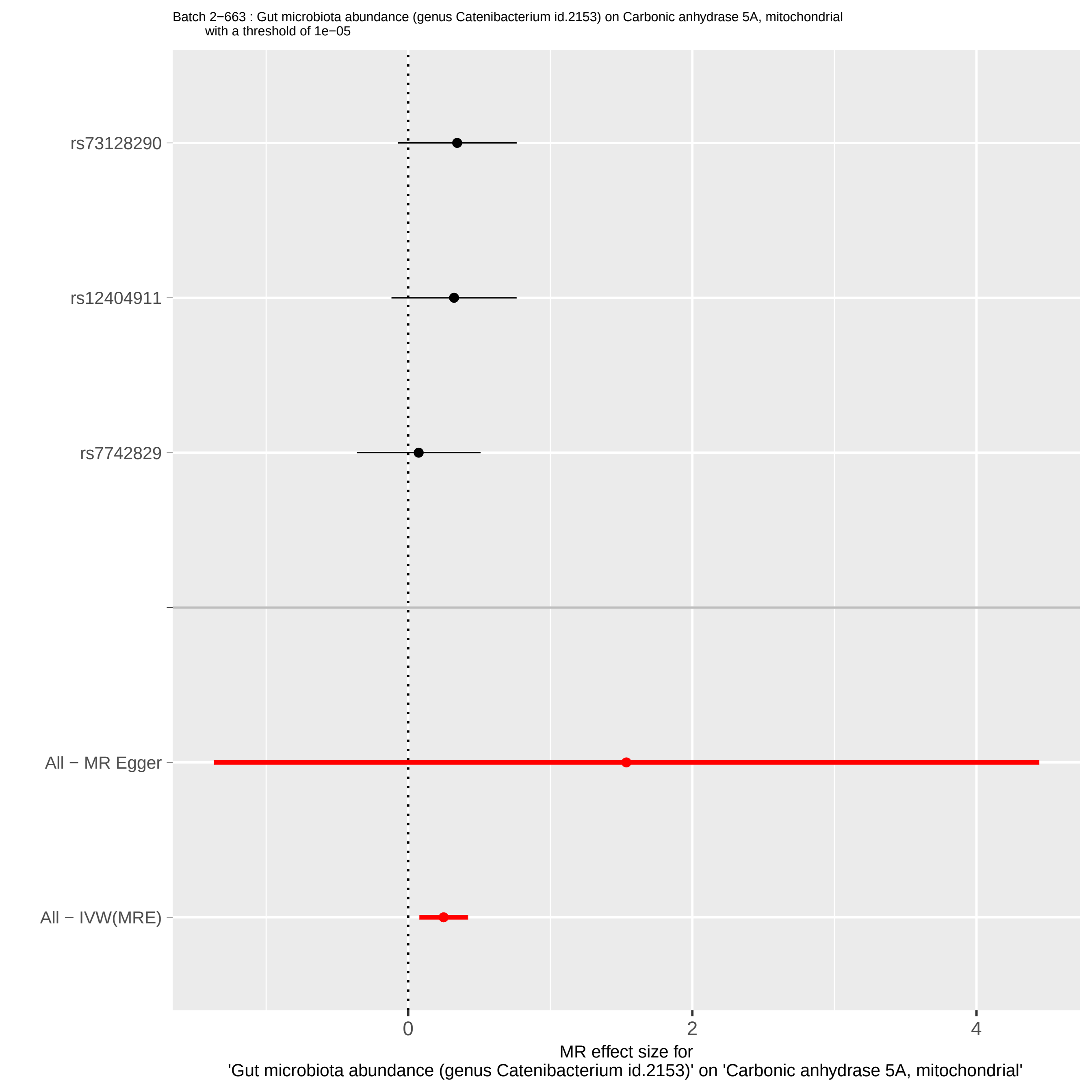

Batch 2−663 : Gut microbiota abundance (genus Catenibacterium id.2153) on Carbonic anhydrase 5A, mitochondrial
with a threshold of 1e−05
rs73128290
rs12404911
rs7742829
All − MR Egger
All − IVW(MRE)
0
2
4
MR effect size for
'Gut microbiota abundance (genus Catenibacterium id.2153)' on 'Carbonic anhydrase 5A, mitochondrial'

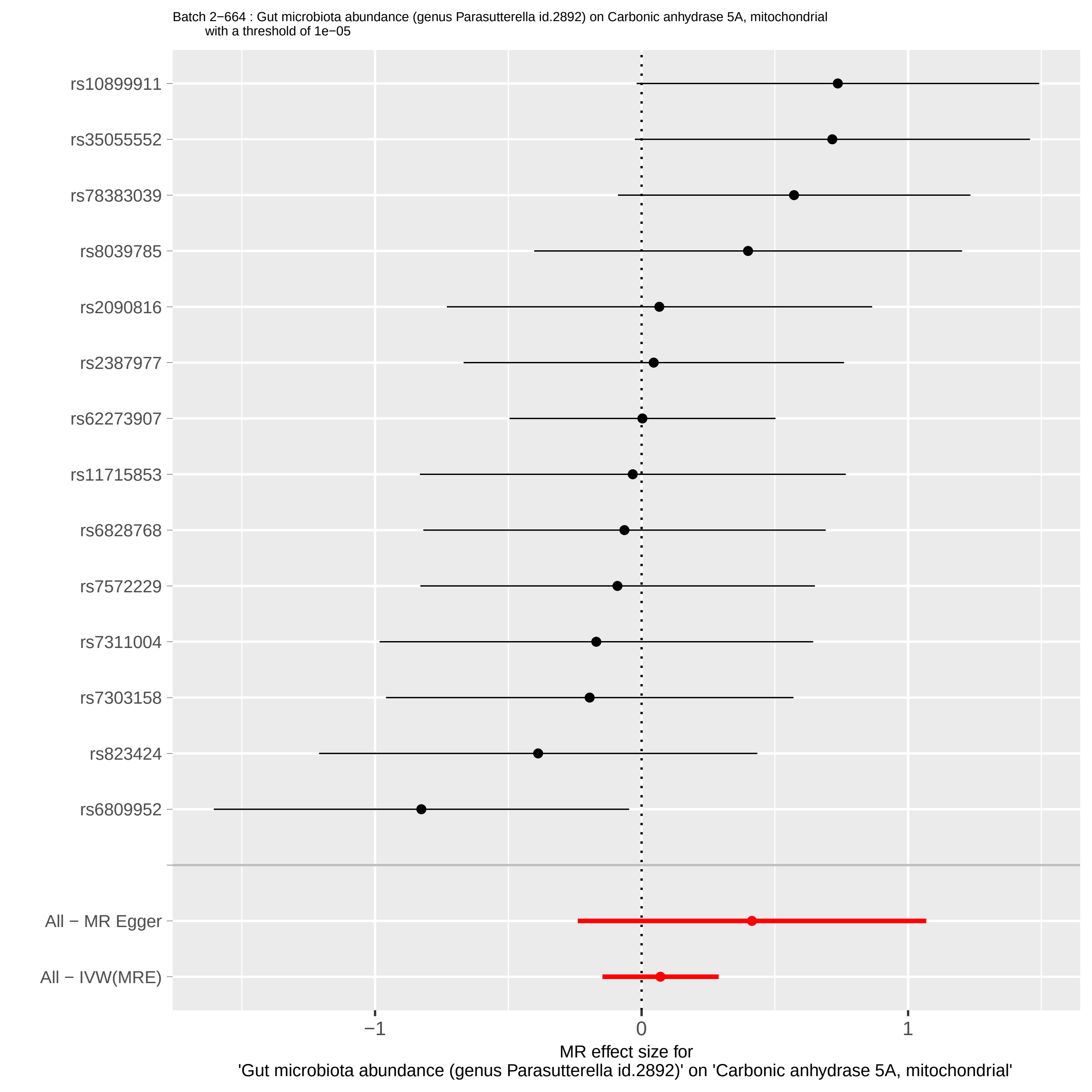

Batch 2−664 : Gut microbiota abundance (genus Parasutterella id.2892) on Carbonic anhydrase 5A, mitochondrial
with a threshold of 1e−05
rs10899911
rs35055552
rs78383039
rs8039785
rs2090816
rs2387977
rs62273907
rs11715853
rs6828768
rs7572229
rs7311004
rs7303158
rs823424
rs6809952
All − MR Egger
All − IVW(MRE)
−1
0
1
MR effect size for
'Gut microbiota abundance (genus Parasutterella id.2892)' on 'Carbonic anhydrase 5A, mitochondrial'

Batch 2−665 : Gut microbiota abundance (genus Catenibacterium id.2153) on Carbonic anhydrase 6
with a threshold of 1e−05

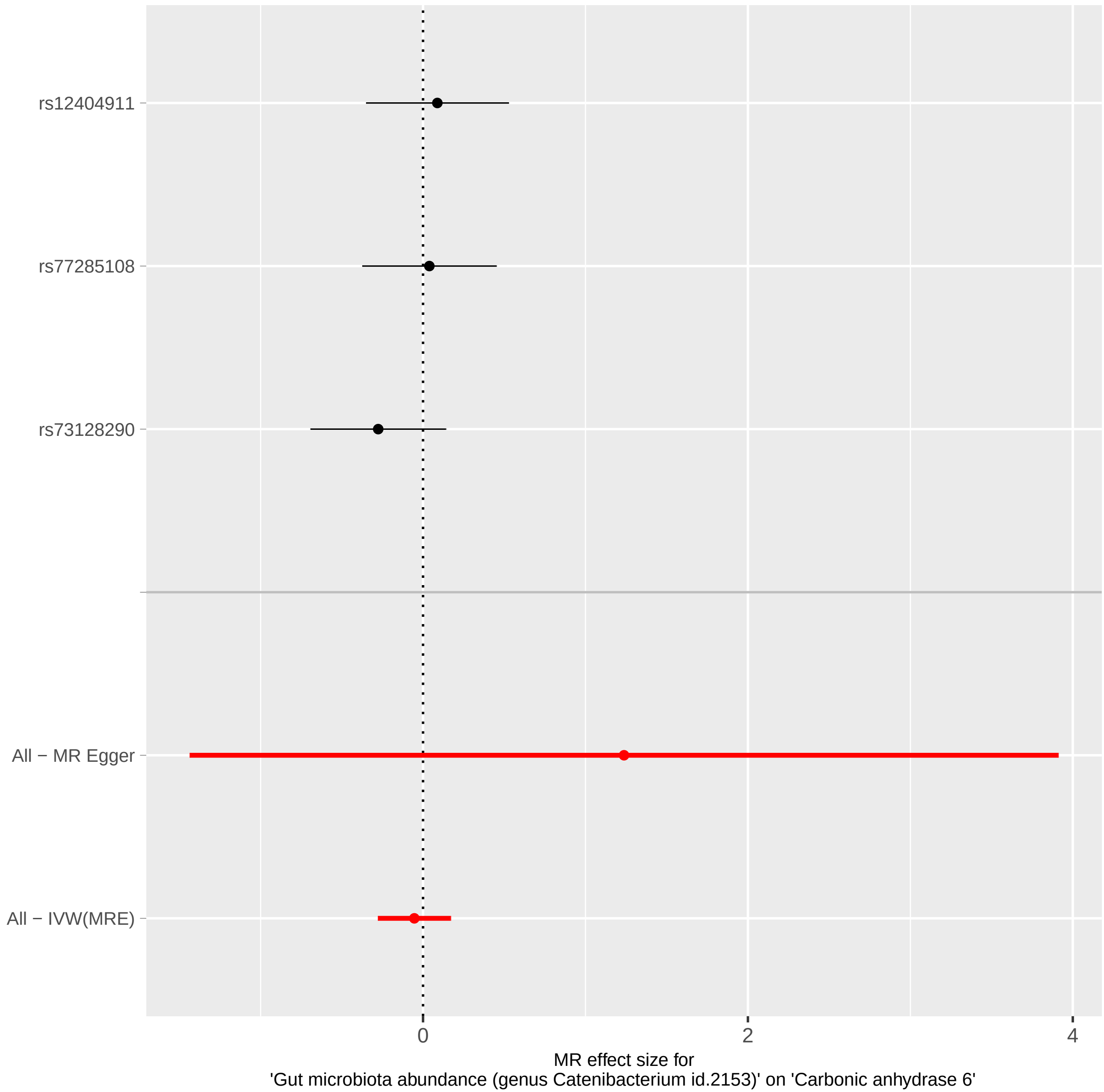

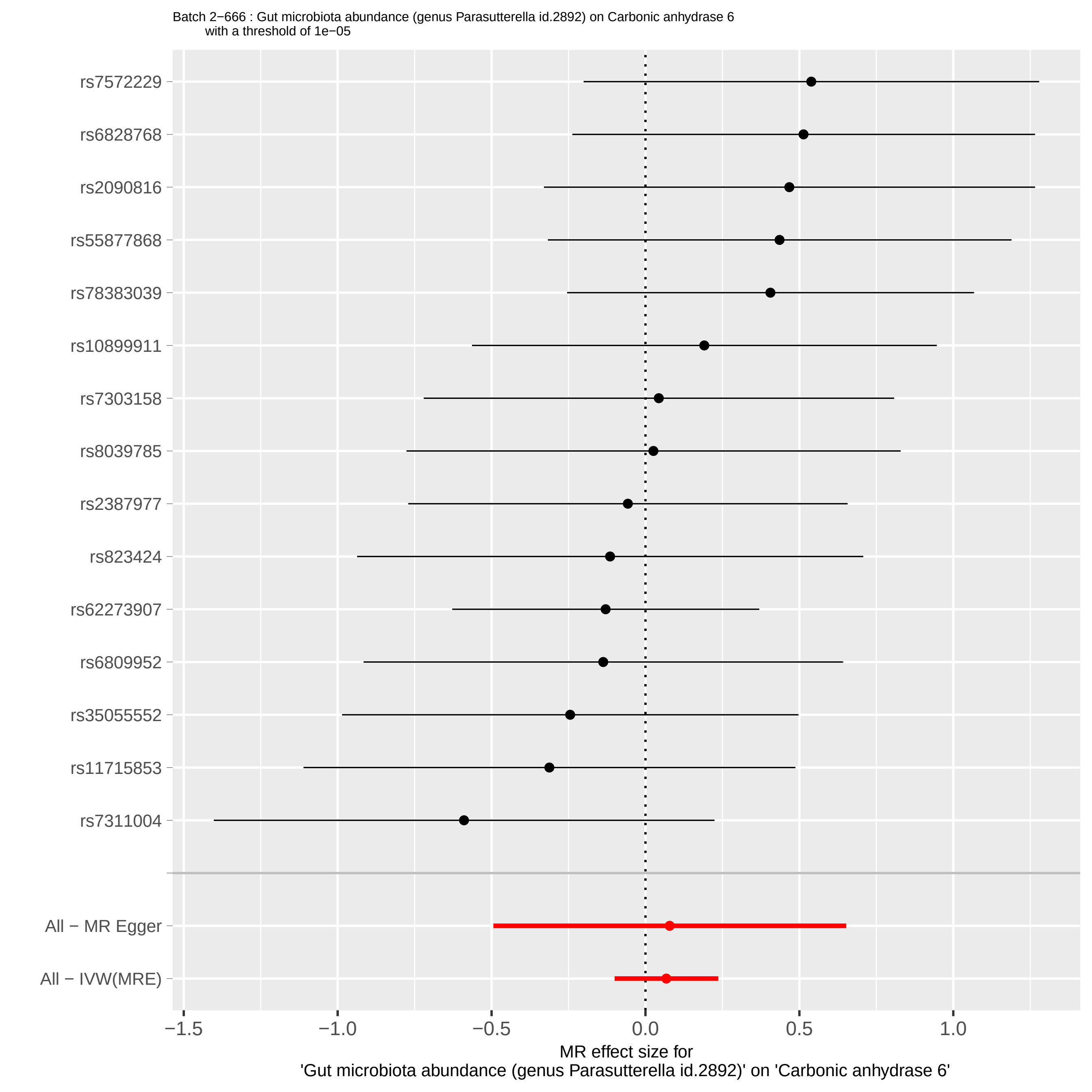
Batch 2−666 : Gut microbiota abundance (genus Parasutterella id.2892) on Carbonic anhydrase 6
with a threshold of 1e−05
rs7572229
rs6828768
rs2090816
rs55877868
rs78383039
rs10899911
rs7303158
rs8039785
rs2387977
rs823424
rs62273907
rs6809952
rs35055552
rs11715853
rs7311004
All − MR Egger
All − IVW(MRE)
−1.5
−1.0
−0.5
0.0
0.5
1.0
MR effect size for
'Gut microbiota abundance (genus Parasutterella id.2892)' on 'Carbonic anhydrase 6'

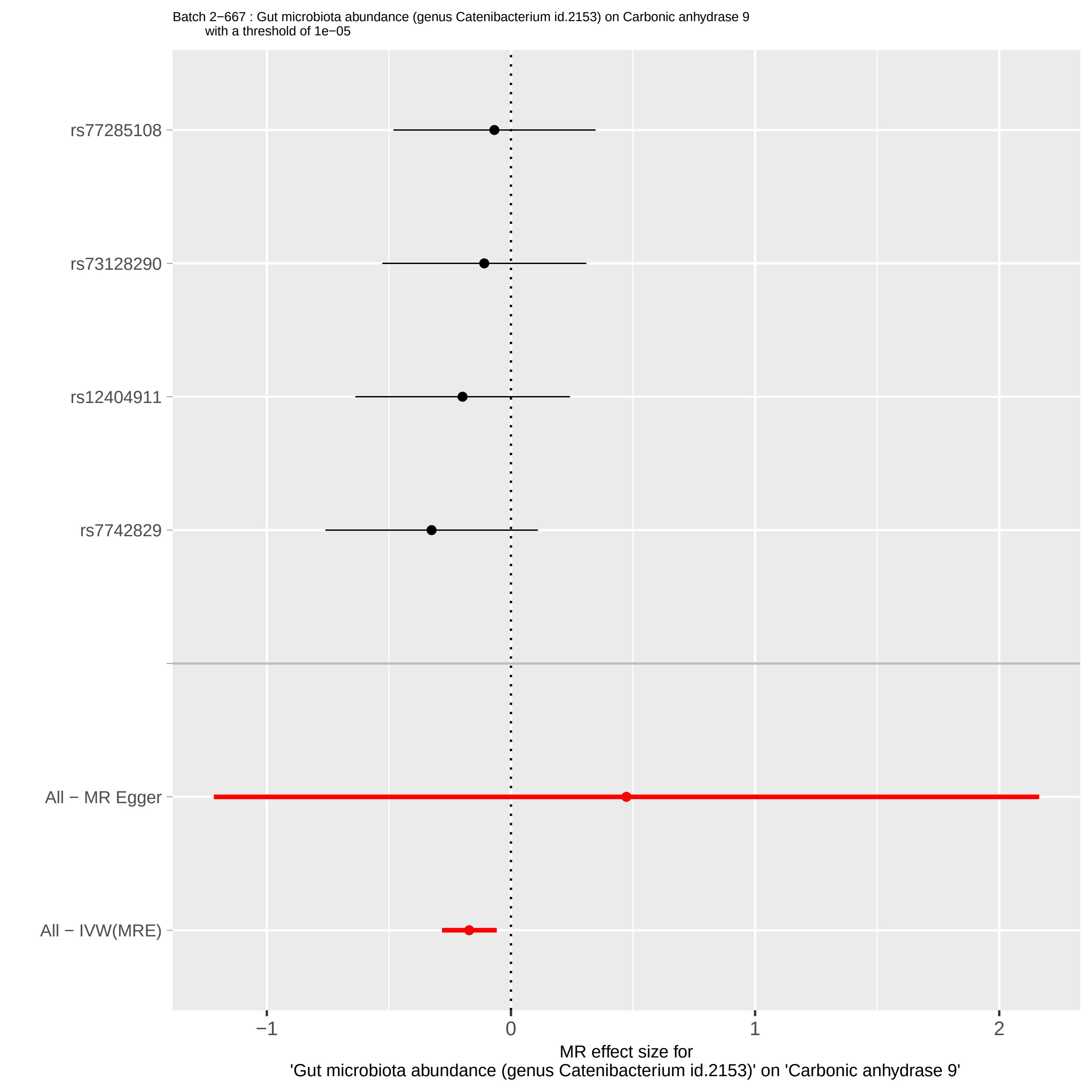

Batch 2−667 : Gut microbiota abundance (genus Catenibacterium id.2153) on Carbonic anhydrase 9
with a threshold of 1e−05
rs77285108
rs73128290
rs12404911
rs7742829
All − MR Egger
All − IVW(MRE)
−1
0
1
2
MR effect size for
'Gut microbiota abundance (genus Catenibacterium id.2153)' on 'Carbonic anhydrase 9'

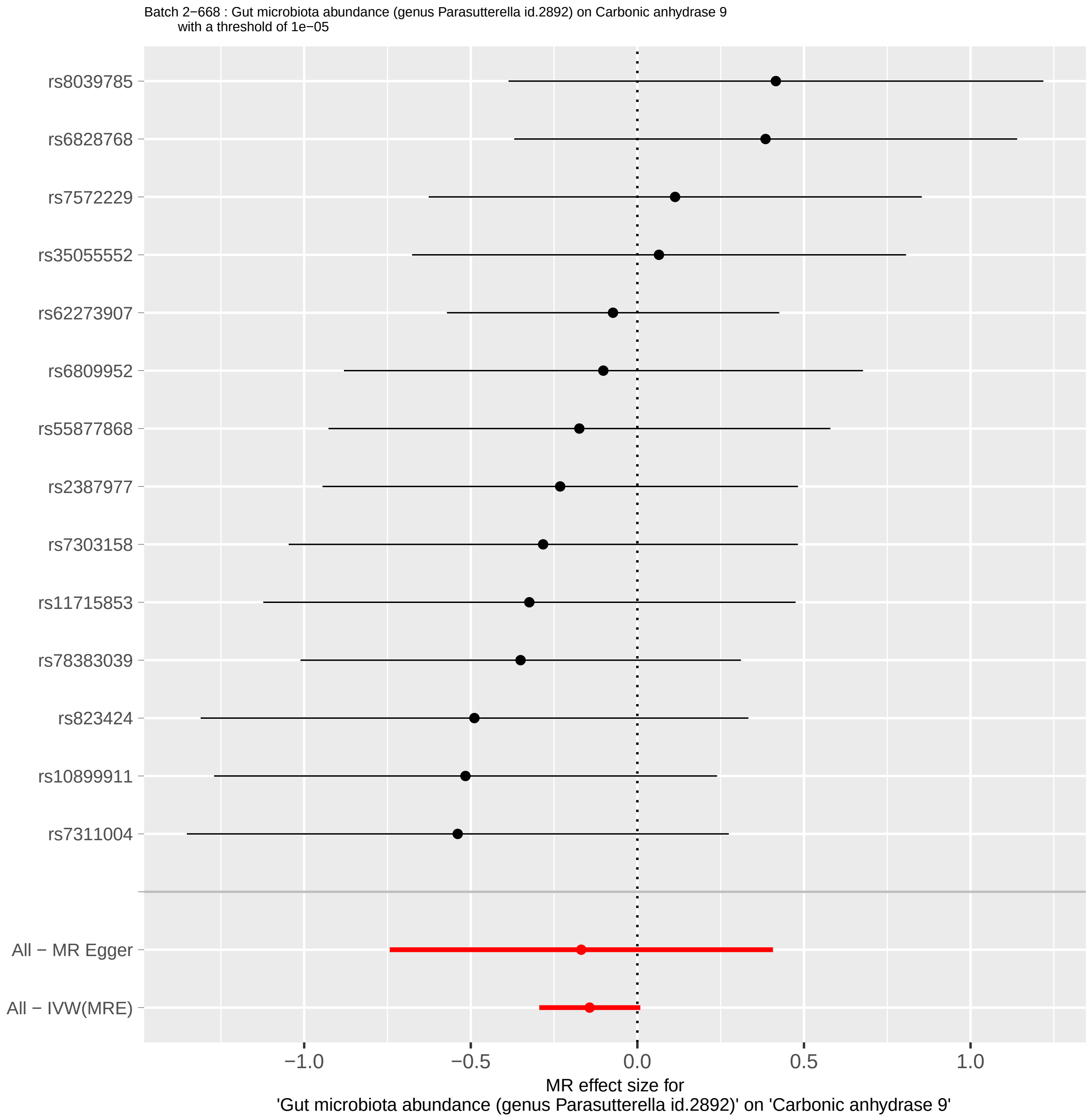

Batch 2−668 : Gut microbiota abundance (genus Parasutterella id.2892) on Carbonic anhydrase 9
with a threshold of 1e−05
rs8039785
rs6828768
rs7572229
rs35055552
rs62273907
rs6809952
rs55877868
rs2387977
rs7303158
rs11715853
rs78383039
rs823424
rs10899911
rs7311004
All − MR Egger
All − IVW(MRE)
−1.0
−0.5
0.0
0.5
1.0
MR effect size for
'Gut microbiota abundance (genus Parasutterella id.2892)' on 'Carbonic anhydrase 9'

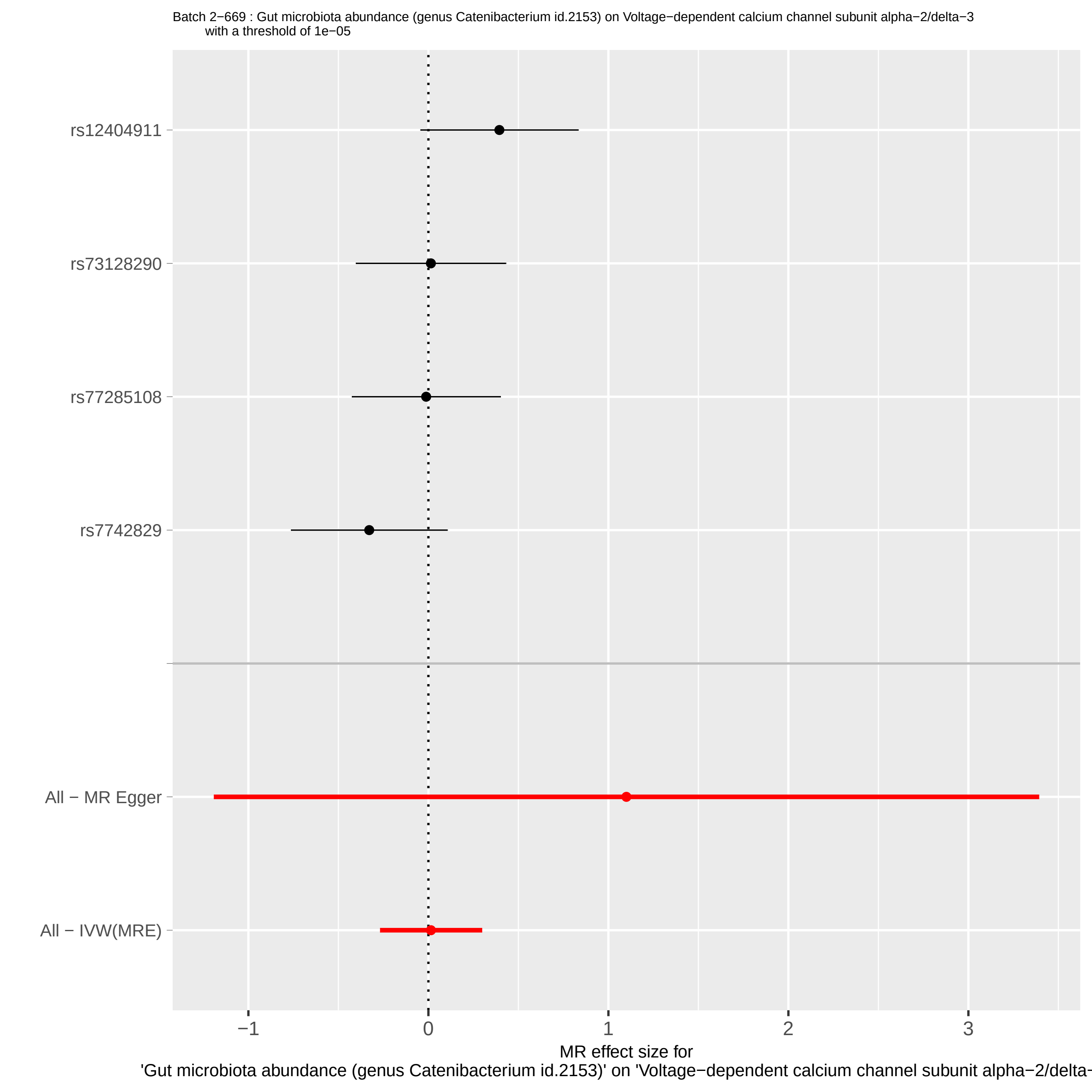
Batch 2−669 : Gut microbiota abundance (genus Catenibacterium id.2153) on Voltage−dependent calcium channel subunit alpha−2/delta−3
with a threshold of 1e−05
rs12404911
rs73128290
rs77285108
rs7742829
All − MR Egger
All − IVW(MRE)
−1
0
1
2
3
MR effect size for
'Gut microbiota abundance (genus Catenibacterium id.2153)' on 'Voltage−dependent calcium channel subunit alpha−2/delta−

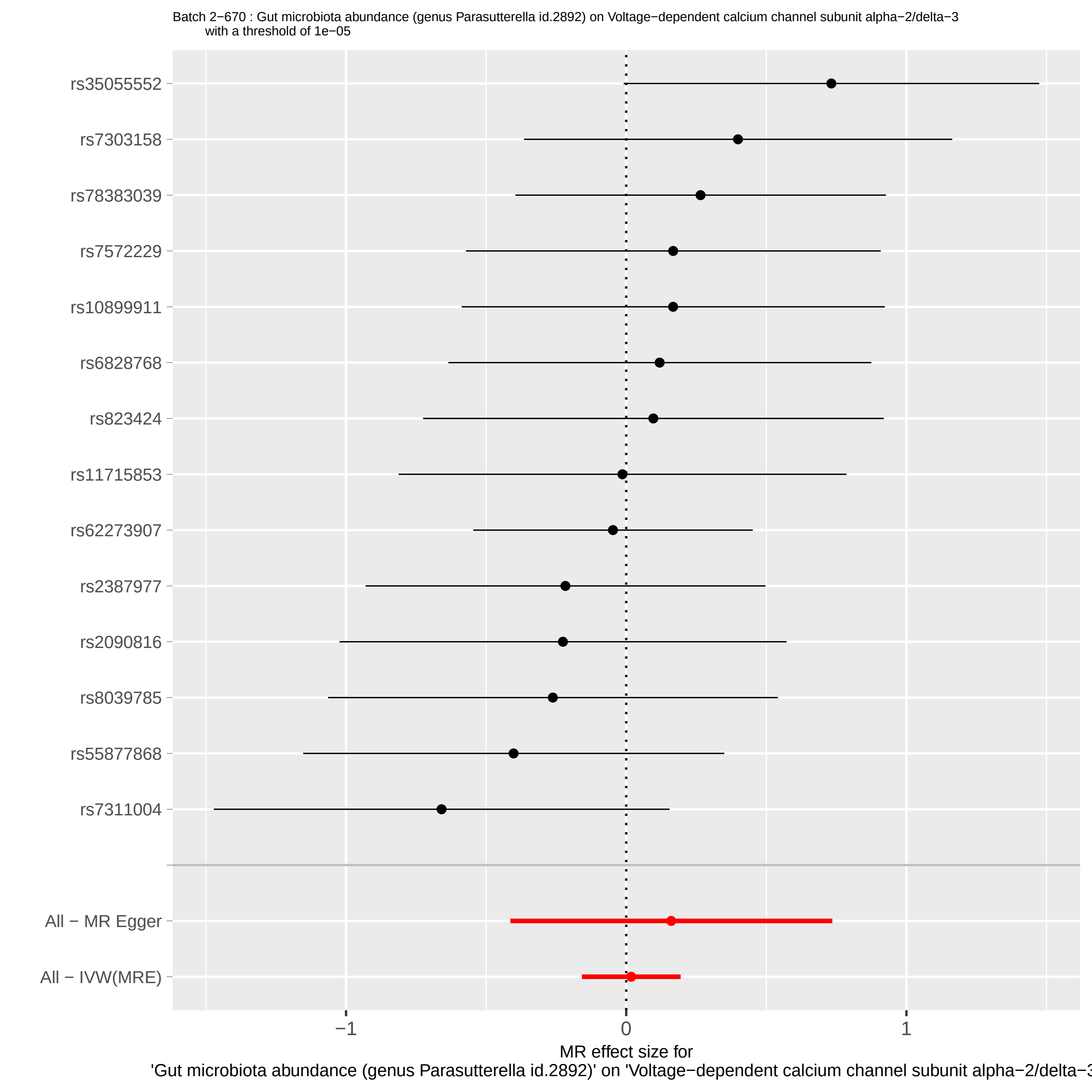

Batch 2−670 : Gut microbiota abundance (genus Parasutterella id.2892) on Voltage−dependent calcium channel subunit alpha−2/delta−3
with a threshold of 1e−05
rs35055552
rs7303158
rs78383039
rs7572229
rs10899911
rs6828768
rs823424
rs11715853
rs62273907
rs2387977
rs2090816
rs8039785
rs55877868
rs7311004
All − MR Egger
All − IVW(MRE)
−1
0
1
MR effect size for
'Gut microbiota abundance (genus Parasutterella id.2892)' on 'Voltage−dependent calcium channel subunit alpha−2/delta−3

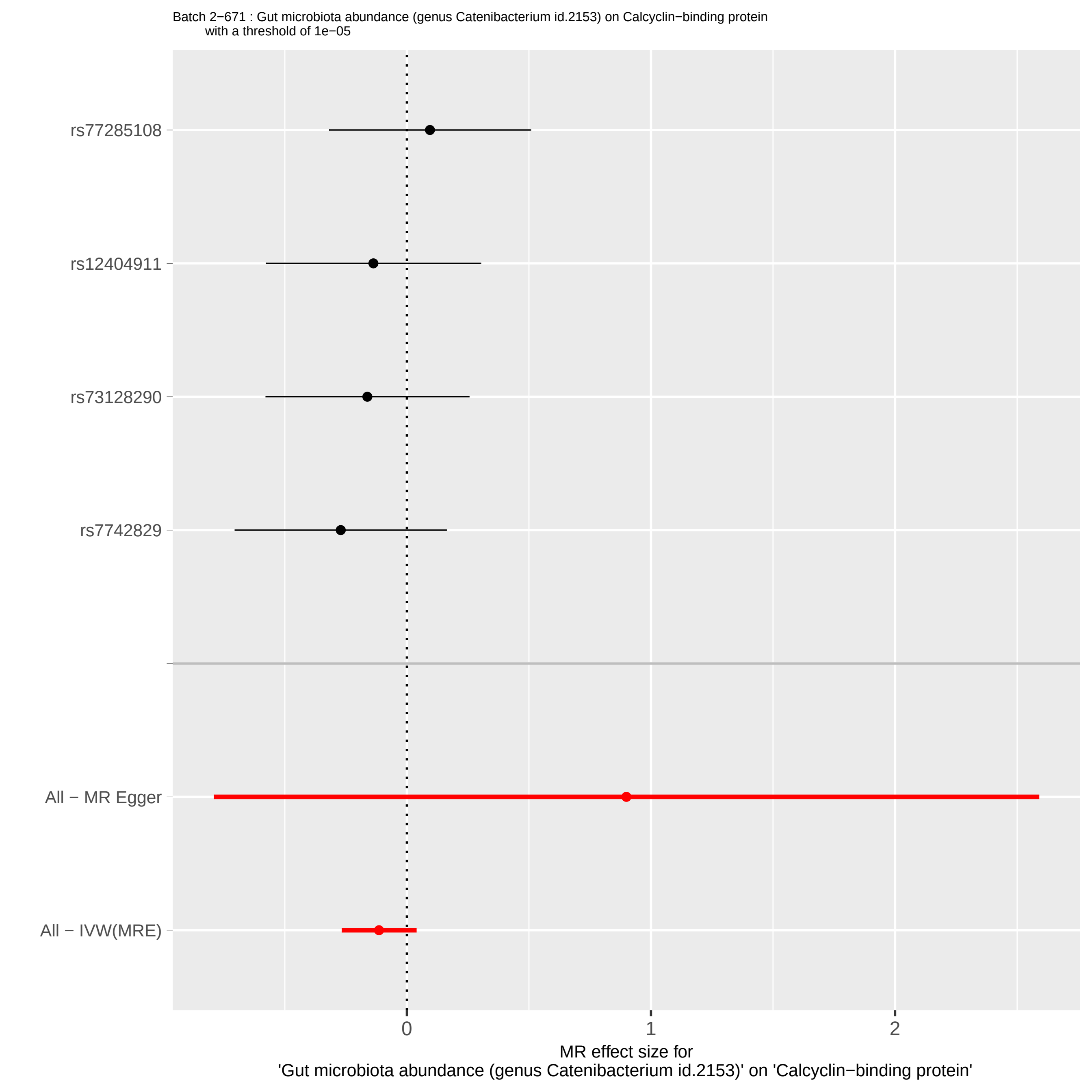

Batch 2−671 : Gut microbiota abundance (genus Catenibacterium id.2153) on Calcyclin−binding protein
with a threshold of 1e−05
rs77285108
rs12404911
rs73128290
rs7742829
All − MR Egger
All − IVW(MRE)
0
1
2
MR effect size for
'Gut microbiota abundance (genus Catenibacterium id.2153)' on 'Calcyclin−binding protein'

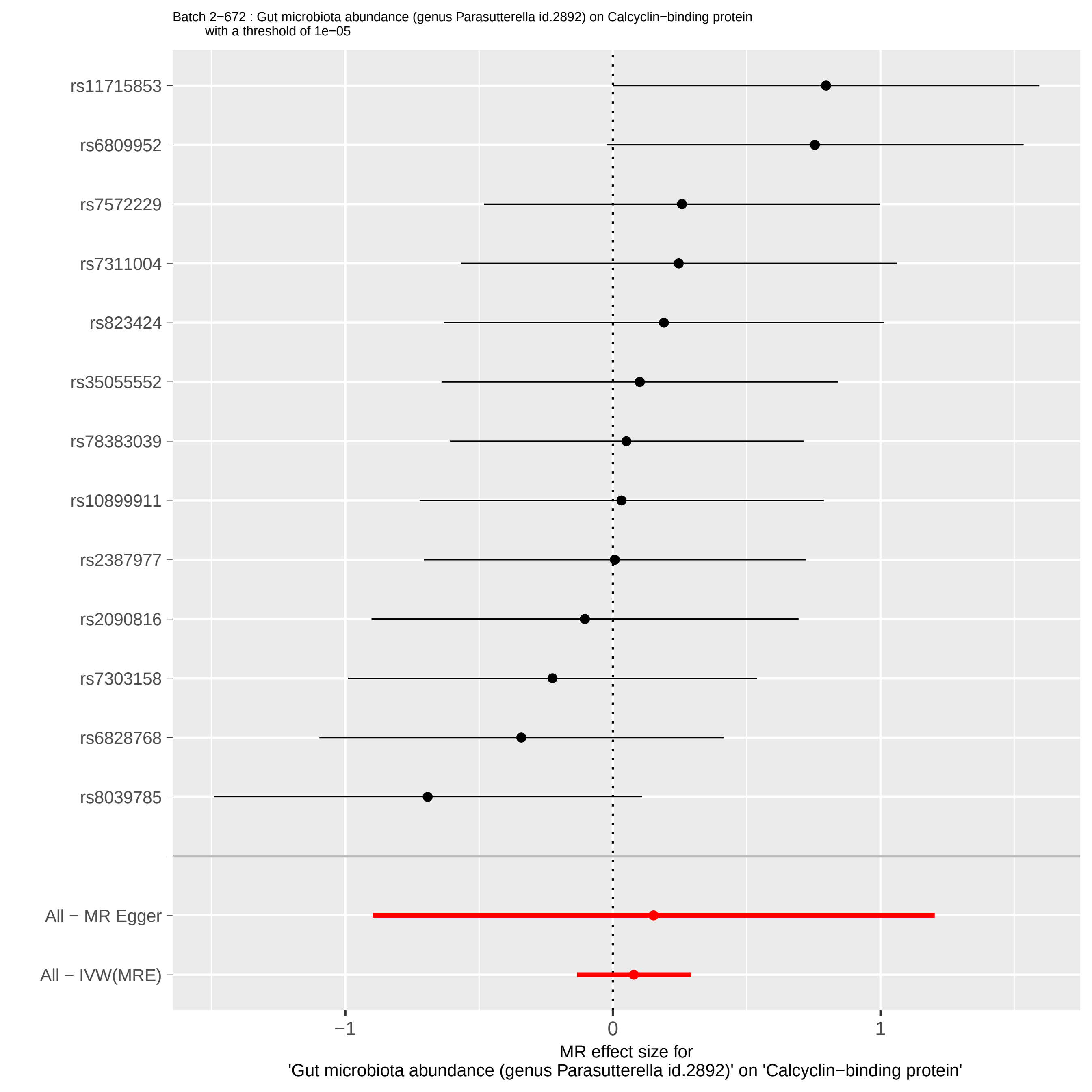
Batch 2−672 : Gut microbiota abundance (genus Parasutterella id.2892) on Calcyclin−binding protein
with a threshold of 1e−05
rs11715853
rs6809952
rs7572229
rs7311004
rs823424
rs35055552
rs78383039
rs10899911
rs2387977
rs2090816
rs7303158
rs6828768
rs8039785
All − MR Egger
All − IVW(MRE)
−1
0
1
MR effect size for
'Gut microbiota abundance (genus Parasutterella id.2892)' on 'Calcyclin−binding protein'

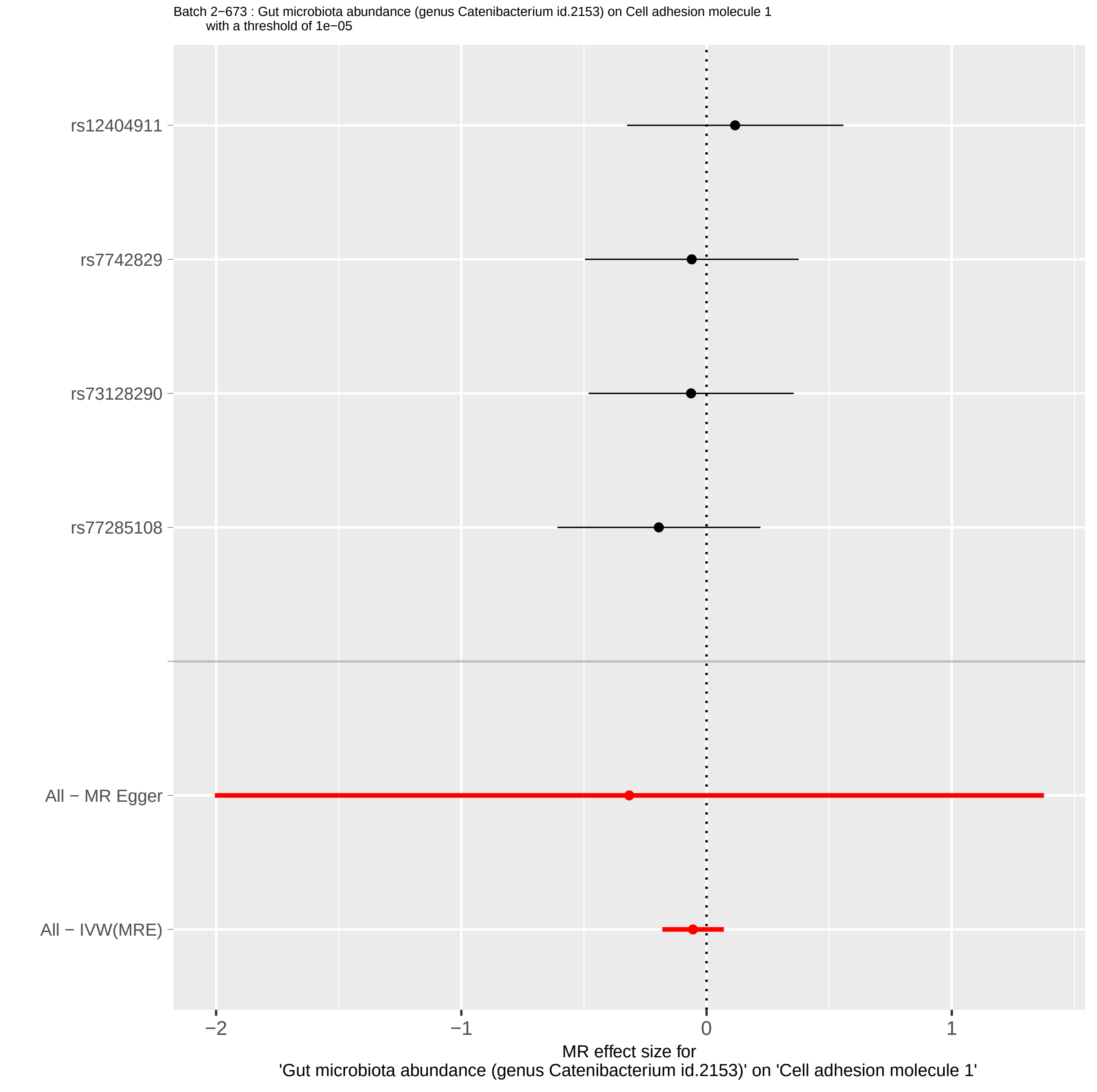
Batch 2−673 : Gut microbiota abundance (genus Catenibacterium id.2153) on Cell adhesion molecule 1
with a threshold of 1e−05
rs12404911
rs7742829
rs73128290
rs77285108
All − MR Egger
All − IVW(MRE)
−2
−1
0
1
MR effect size for
'Gut microbiota abundance (genus Catenibacterium id.2153)' on 'Cell adhesion molecule 1'

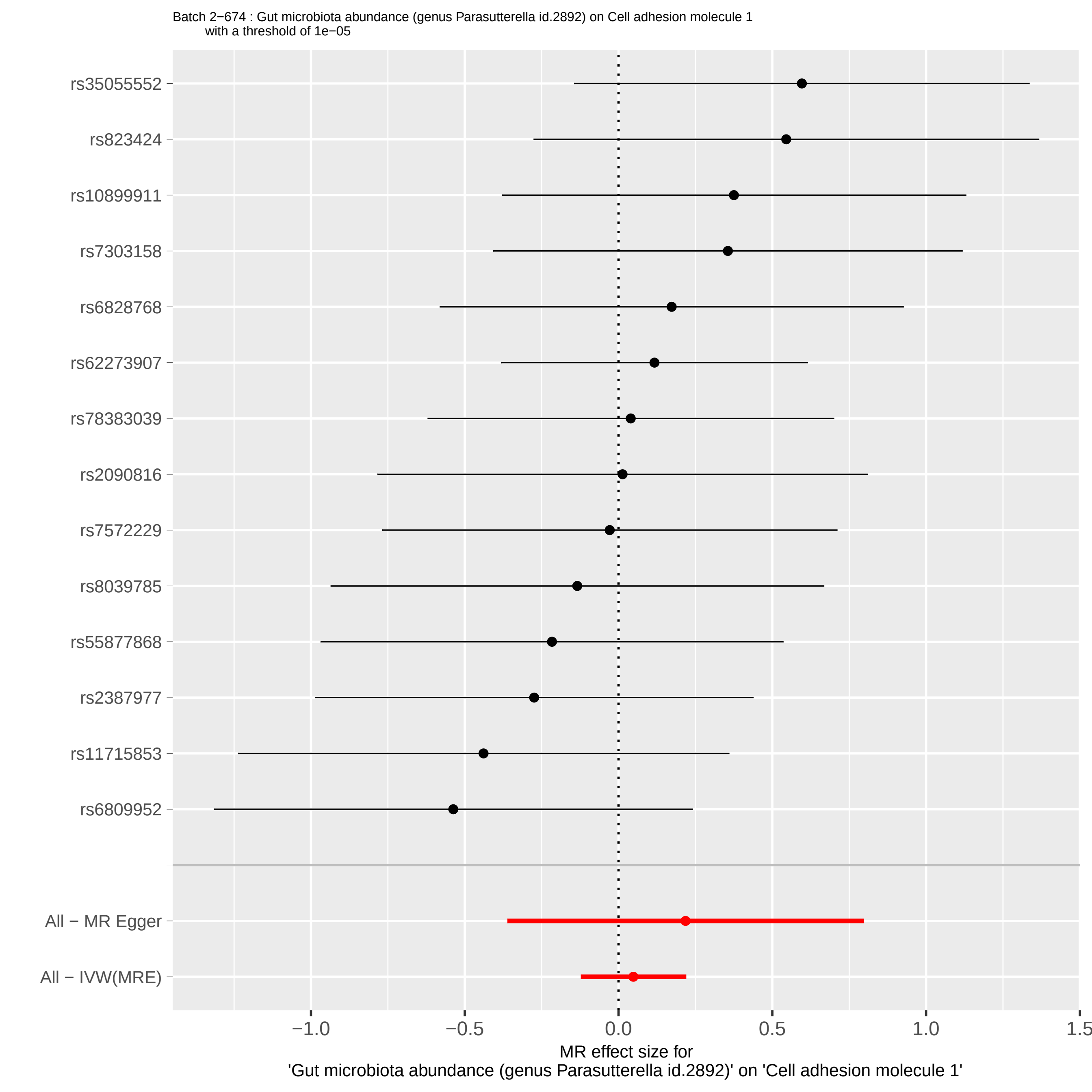

Batch 2−674 : Gut microbiota abundance (genus Parasutterella id.2892) on Cell adhesion molecule 1
with a threshold of 1e−05
rs35055552
rs823424
rs10899911
rs7303158
rs6828768
rs62273907
rs78383039
rs2090816
rs7572229
rs8039785
rs55877868
rs2387977
rs11715853
rs6809952
All − MR Egger
All − IVW(MRE)
−1.0
−0.5
0.0
0.5
1.0
1.5
MR effect size for
'Gut microbiota abundance (genus Parasutterella id.2892)' on 'Cell adhesion molecule 1'

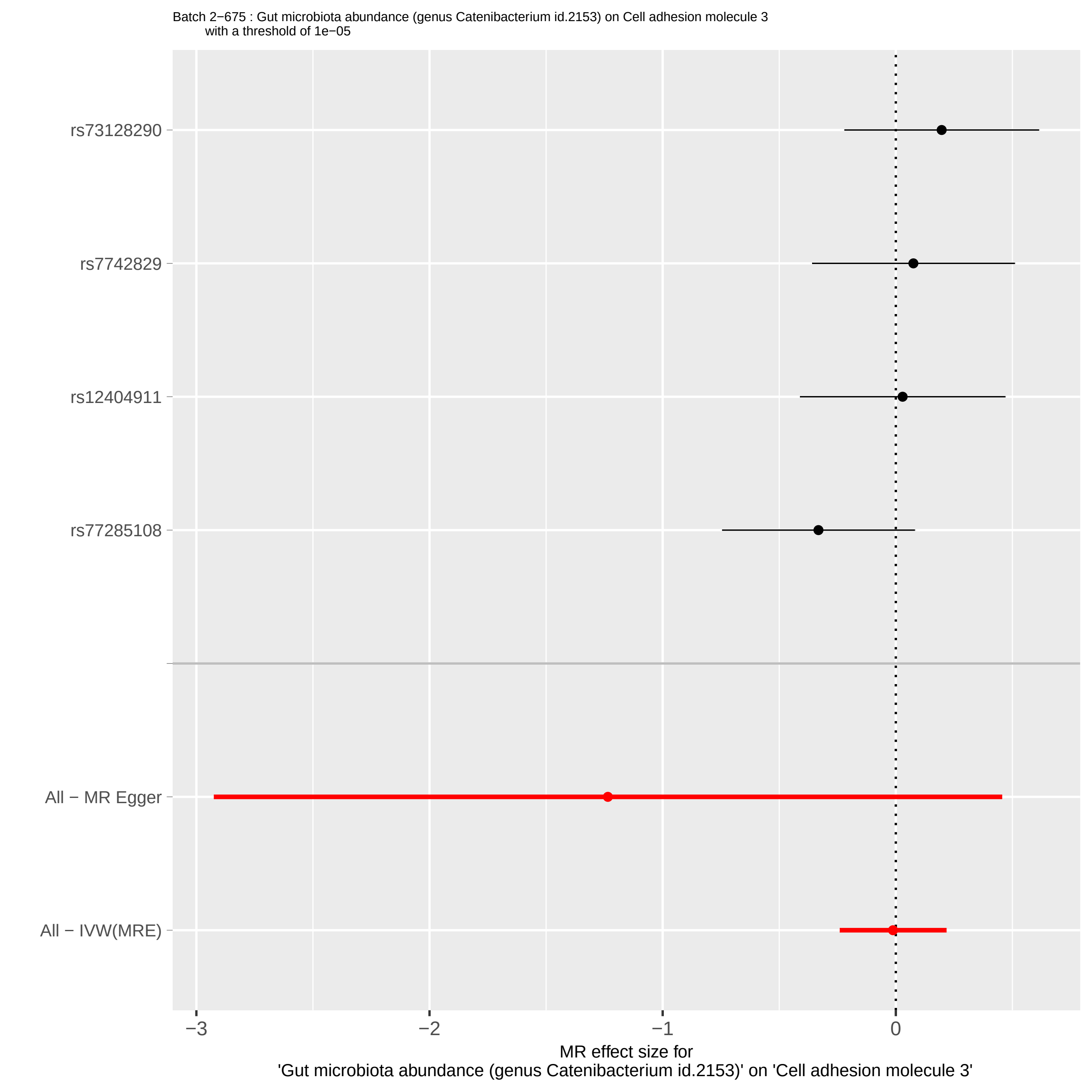

Batch 2−675 : Gut microbiota abundance (genus Catenibacterium id.2153) on Cell adhesion molecule 3
with a threshold of 1e−05
rs73128290
rs7742829
rs12404911
rs77285108
All − MR Egger
All − IVW(MRE)
−3
−2
−1
0
MR effect size for
'Gut microbiota abundance (genus Catenibacterium id.2153)' on 'Cell adhesion molecule 3'

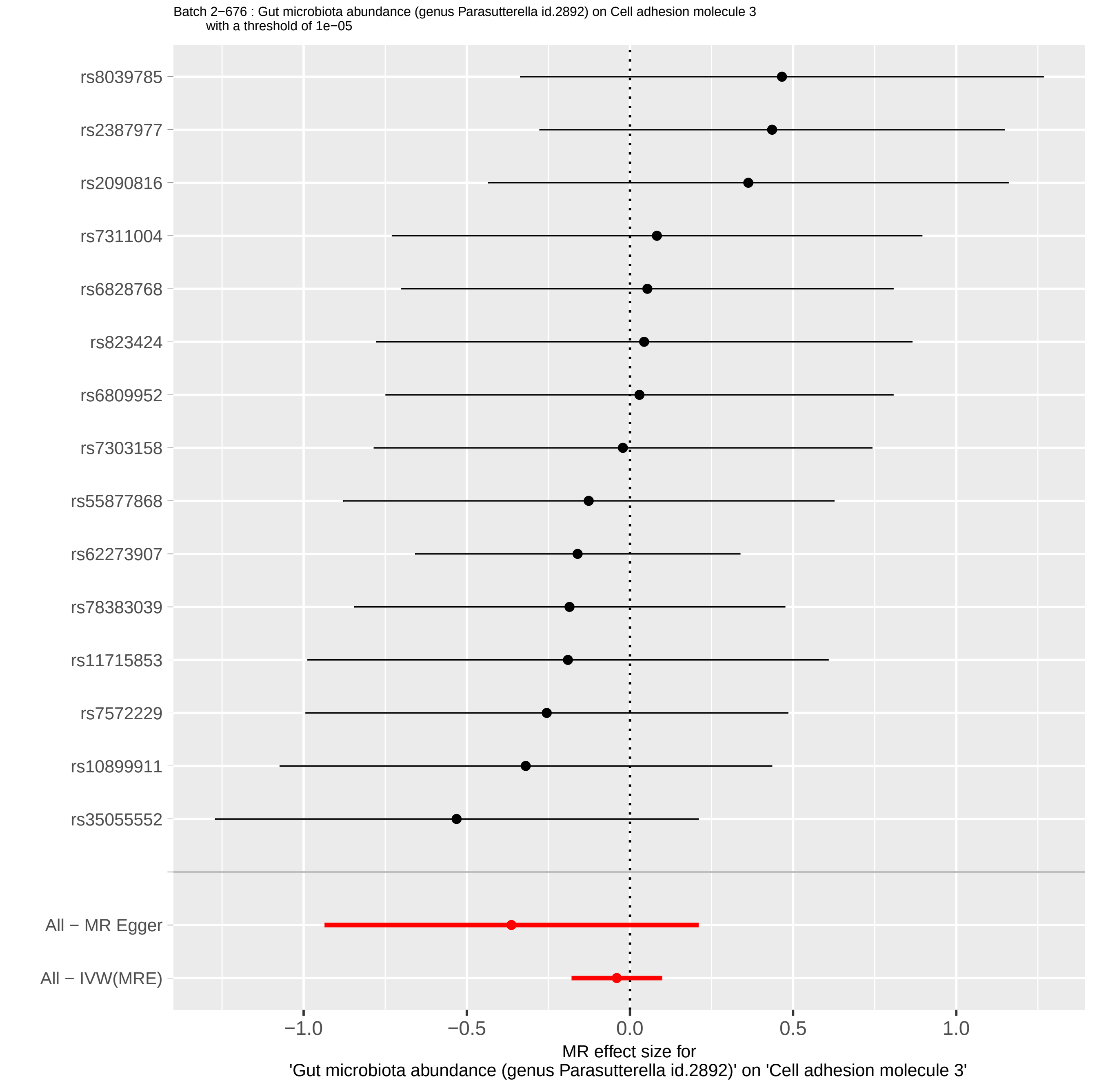

Batch 2−676 : Gut microbiota abundance (genus Parasutterella id.2892) on Cell adhesion molecule 3
with a threshold of 1e−05
rs8039785
rs2387977
rs2090816
rs7311004
rs6828768
rs823424
rs6809952
rs7303158
rs55877868
rs62273907
rs78383039
rs11715853
rs7572229
rs10899911
rs35055552
All − MR Egger
All − IVW(MRE)
−1.0
−0.5
0.0
0.5
1.0
MR effect size for
'Gut microbiota abundance (genus Parasutterella id.2892)' on 'Cell adhesion molecule 3'

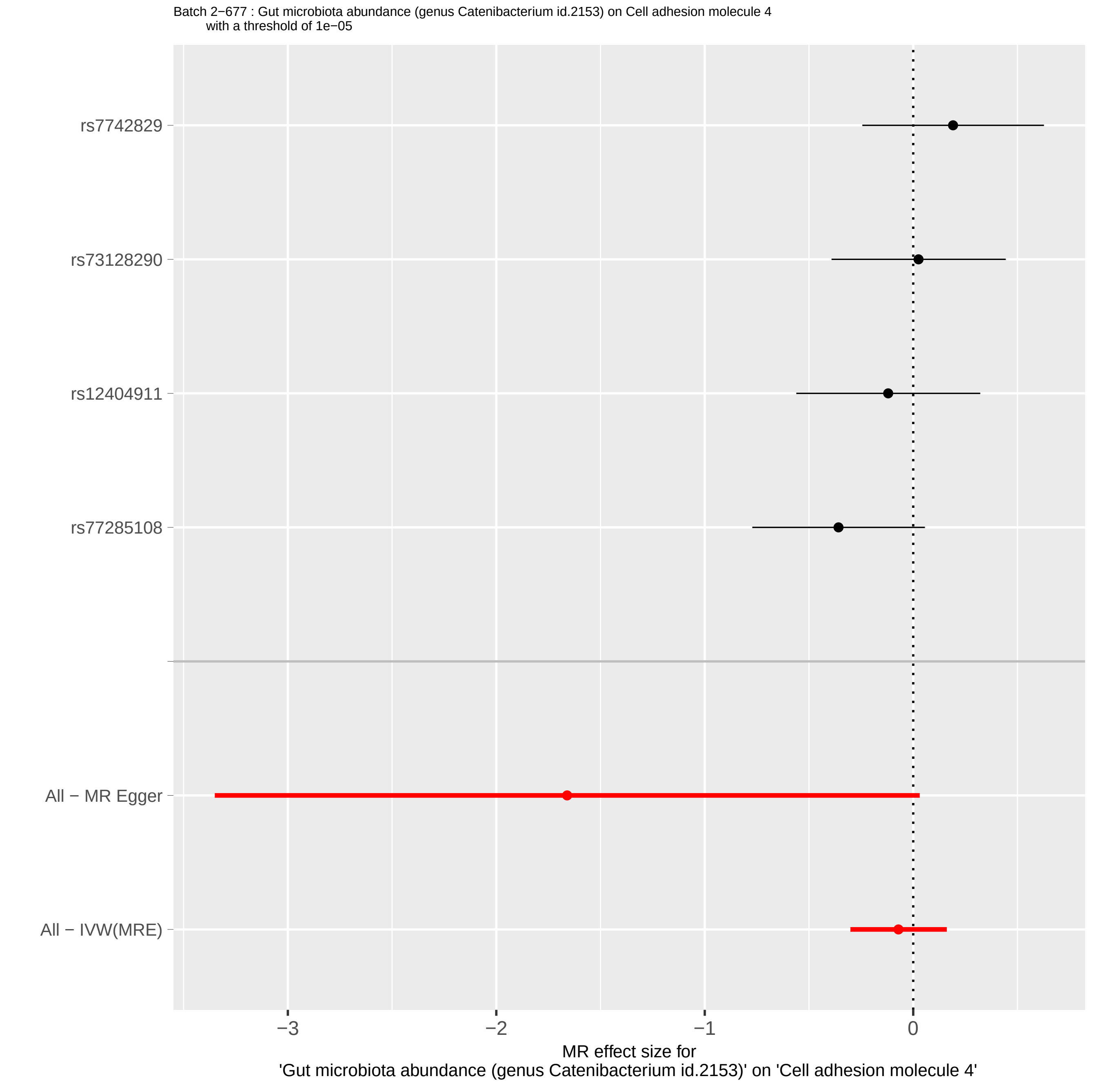

Batch 2−677 : Gut microbiota abundance (genus Catenibacterium id.2153) on Cell adhesion molecule 4
with a threshold of 1e−05
rs7742829
rs73128290
rs12404911
rs77285108
All − MR Egger
All − IVW(MRE)
−3
−2
−1
0
MR effect size for
'Gut microbiota abundance (genus Catenibacterium id.2153)' on 'Cell adhesion molecule 4'

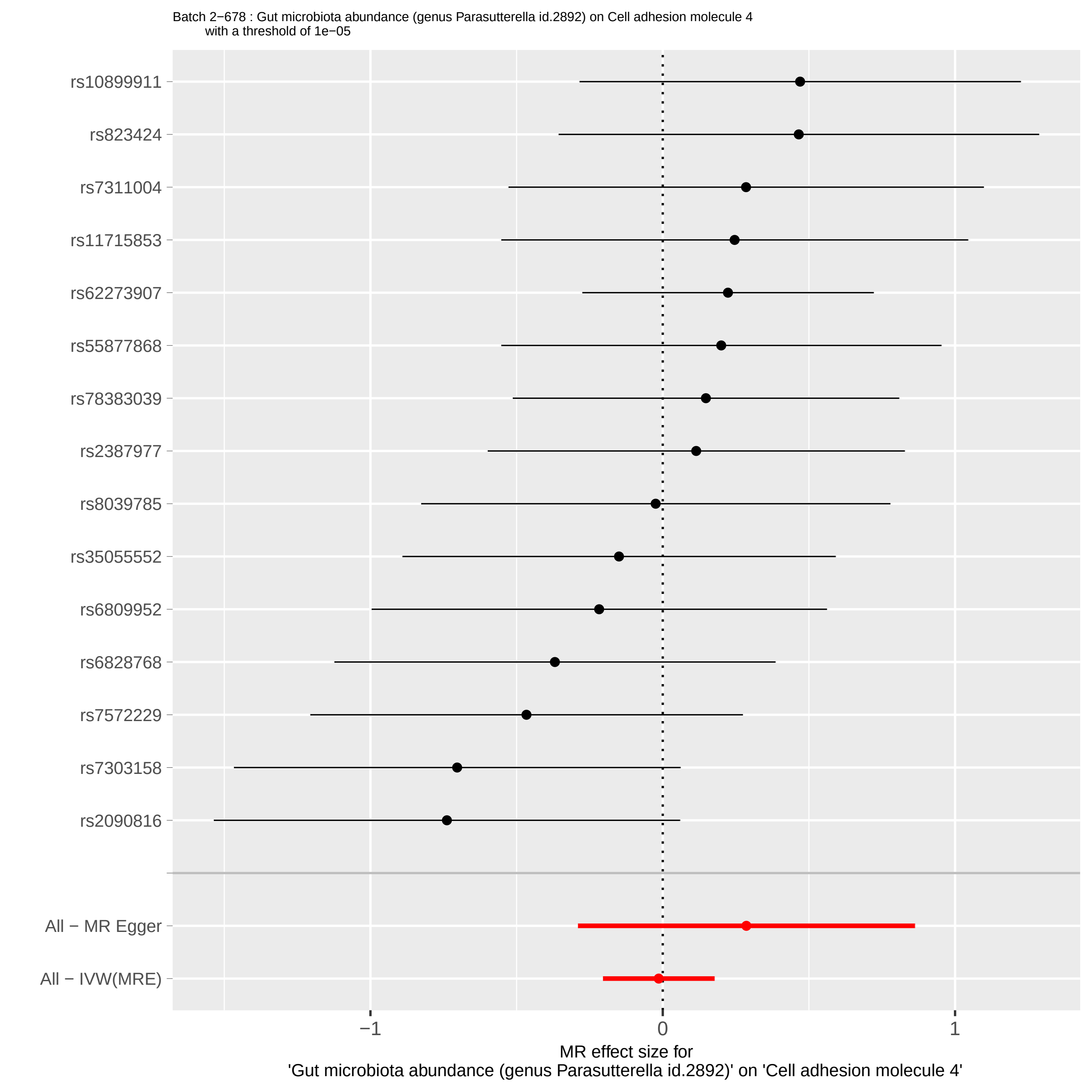

Batch 2−678 : Gut microbiota abundance (genus Parasutterella id.2892) on Cell adhesion molecule 4
with a threshold of 1e−05
rs10899911
rs823424
rs7311004
rs11715853
rs62273907
rs55877868
rs78383039
rs2387977
rs8039785
rs35055552
rs6809952
rs6828768
rs7572229
rs7303158
rs2090816
All − MR Egger
All − IVW(MRE)
−1
0
1
MR effect size for
'Gut microbiota abundance (genus Parasutterella id.2892)' on 'Cell adhesion molecule 4'

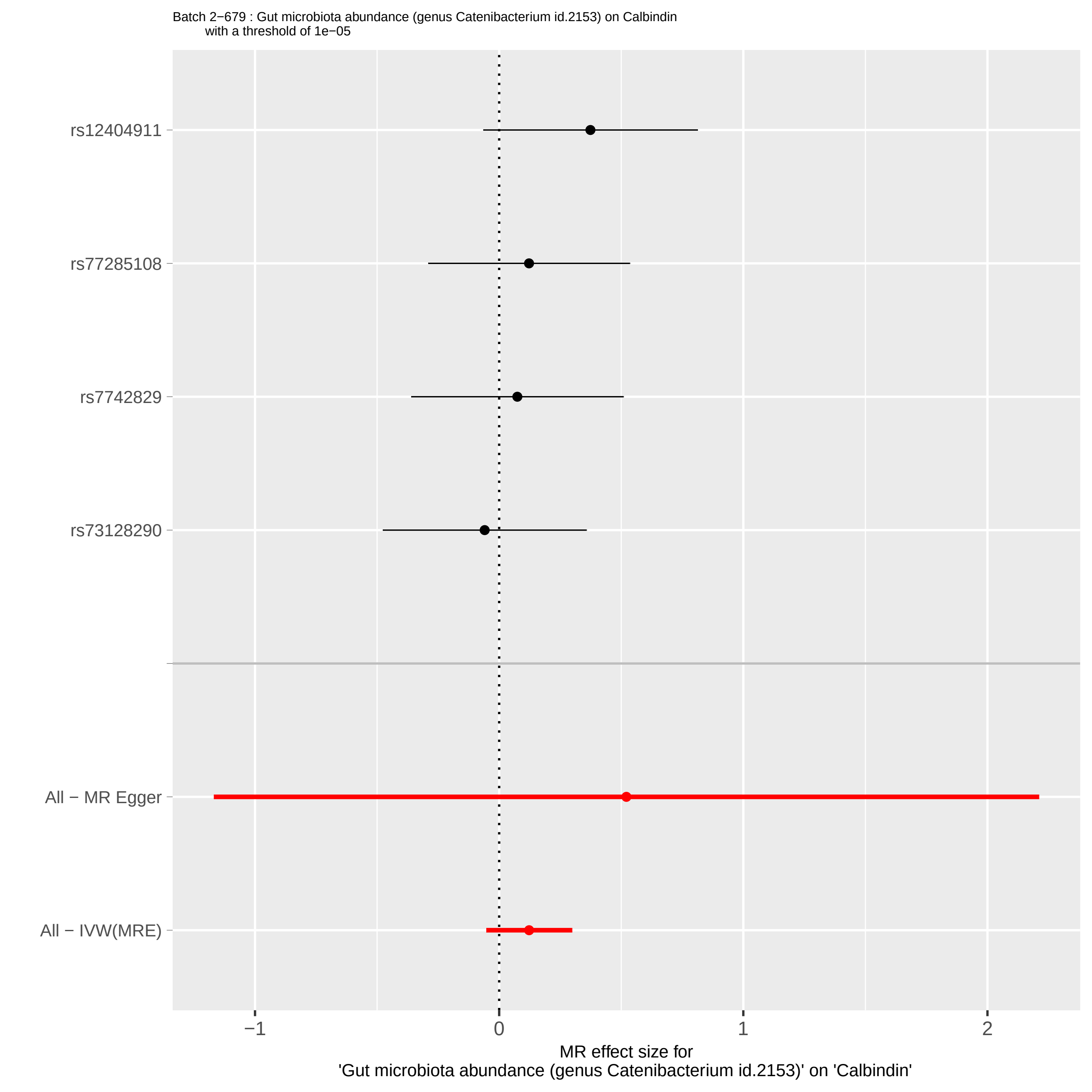
Batch 2−679 : Gut microbiota abundance (genus Catenibacterium id.2153) on Calbindin
with a threshold of 1e−05
rs12404911
rs77285108
rs7742829
rs73128290
All − MR Egger
All − IVW(MRE)
−1
0
1
2
MR effect size for
'Gut microbiota abundance (genus Catenibacterium id.2153)' on 'Calbindin'

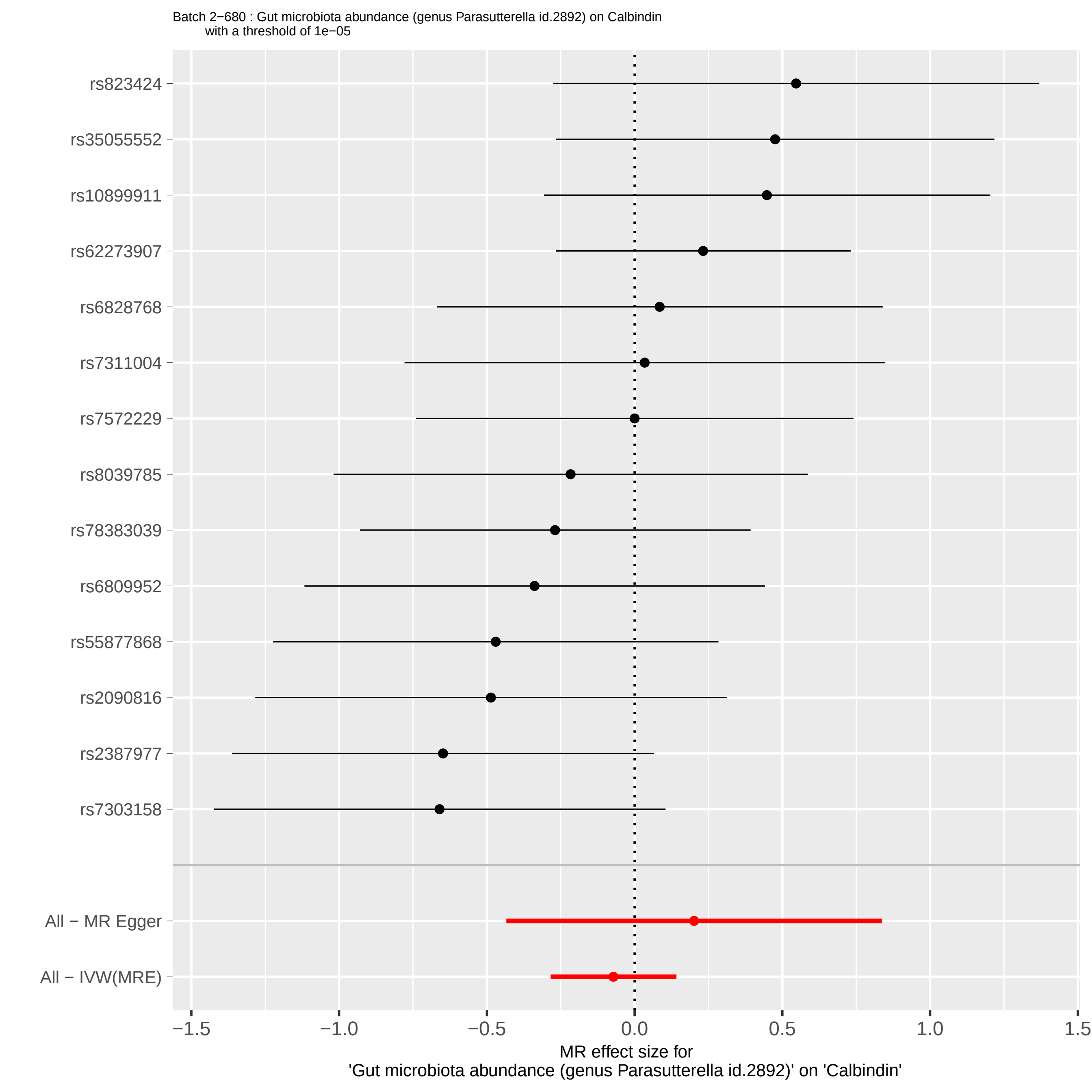

Batch 2−680 : Gut microbiota abundance (genus Parasutterella id.2892) on Calbindin
with a threshold of 1e−05
rs823424
rs35055552
rs10899911
rs62273907
rs6828768
rs7311004
rs7572229
rs8039785
rs78383039
rs6809952
rs55877868
rs2090816
rs2387977
rs7303158
All − MR Egger
All − IVW(MRE)
−1.5
−1.0
−0.5
0.0
0.5
1.0
1.5
MR effect size for
'Gut microbiota abundance (genus Parasutterella id.2892)' on 'Calbindin'

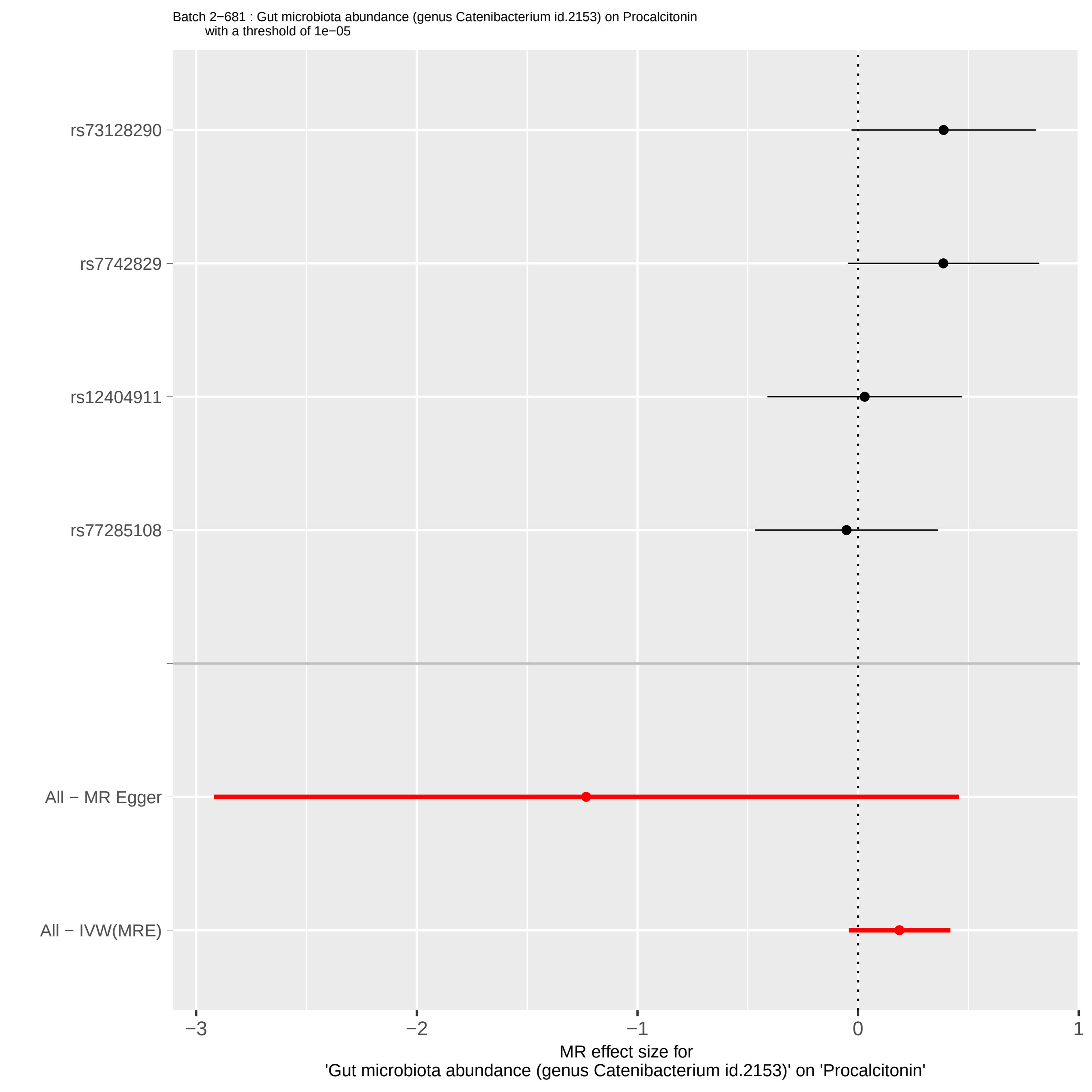
Batch 2−681 : Gut microbiota abundance (genus Catenibacterium id.2153) on Procalcitonin
with a threshold of 1e−05
rs73128290
rs7742829
rs12404911
rs77285108
All − MR Egger
All − IVW(MRE)
−3
−2
−1
0
1
MR effect size for
'Gut microbiota abundance (genus Catenibacterium id.2153)' on 'Procalcitonin'

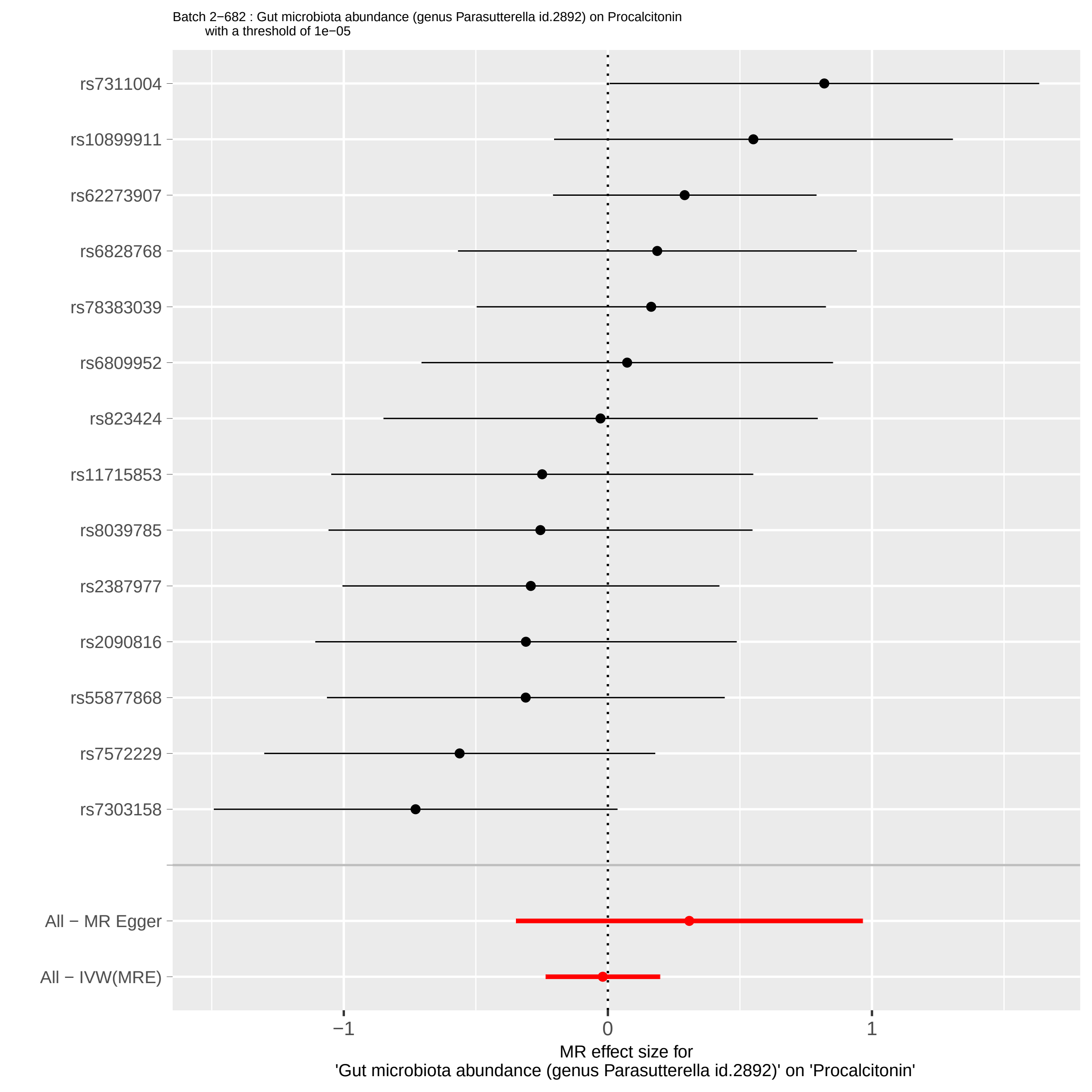
Batch 2−682 : Gut microbiota abundance (genus Parasutterella id.2892) on Procalcitonin
with a threshold of 1e−05
rs7311004
rs10899911
rs62273907
rs6828768
rs78383039
rs6809952
rs823424
rs11715853
rs8039785
rs2387977
rs2090816
rs55877868
rs7572229
rs7303158
All − MR Egger
All − IVW(MRE)
−1
0
1
MR effect size for
'Gut microbiota abundance (genus Parasutterella id.2892)' on 'Procalcitonin'

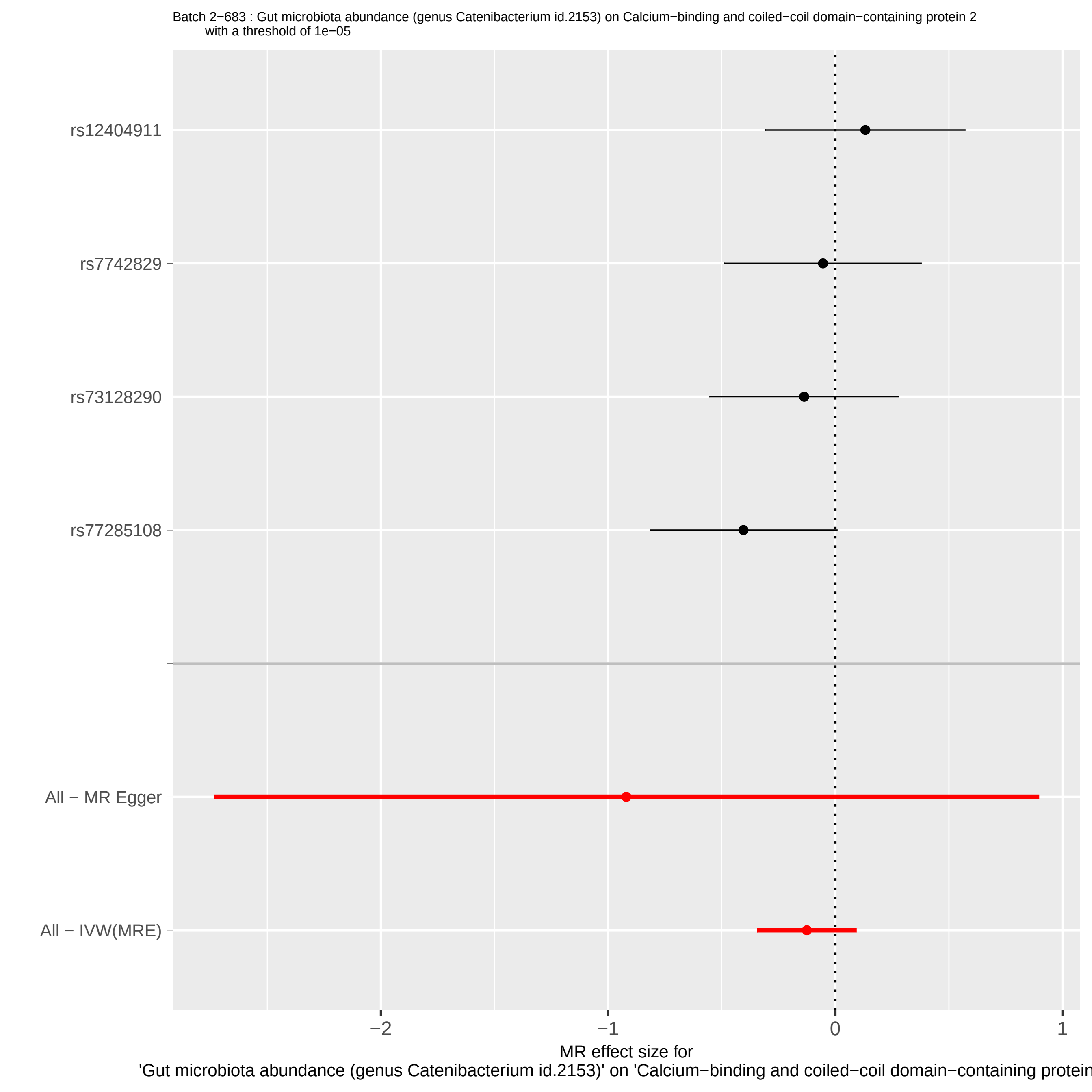
Batch 2−683 : Gut microbiota abundance (genus Catenibacterium id.2153) on Calcium−binding and coiled−coil domain−containing protein 2
with a threshold of 1e−05
rs12404911
rs7742829
rs73128290
rs77285108
All − MR Egger
All − IVW(MRE)
−2
−1
0
1
MR effect size for
'Gut microbiota abundance (genus Catenibacterium id.2153)' on 'Calcium−binding and coiled−coil domain−containing protein

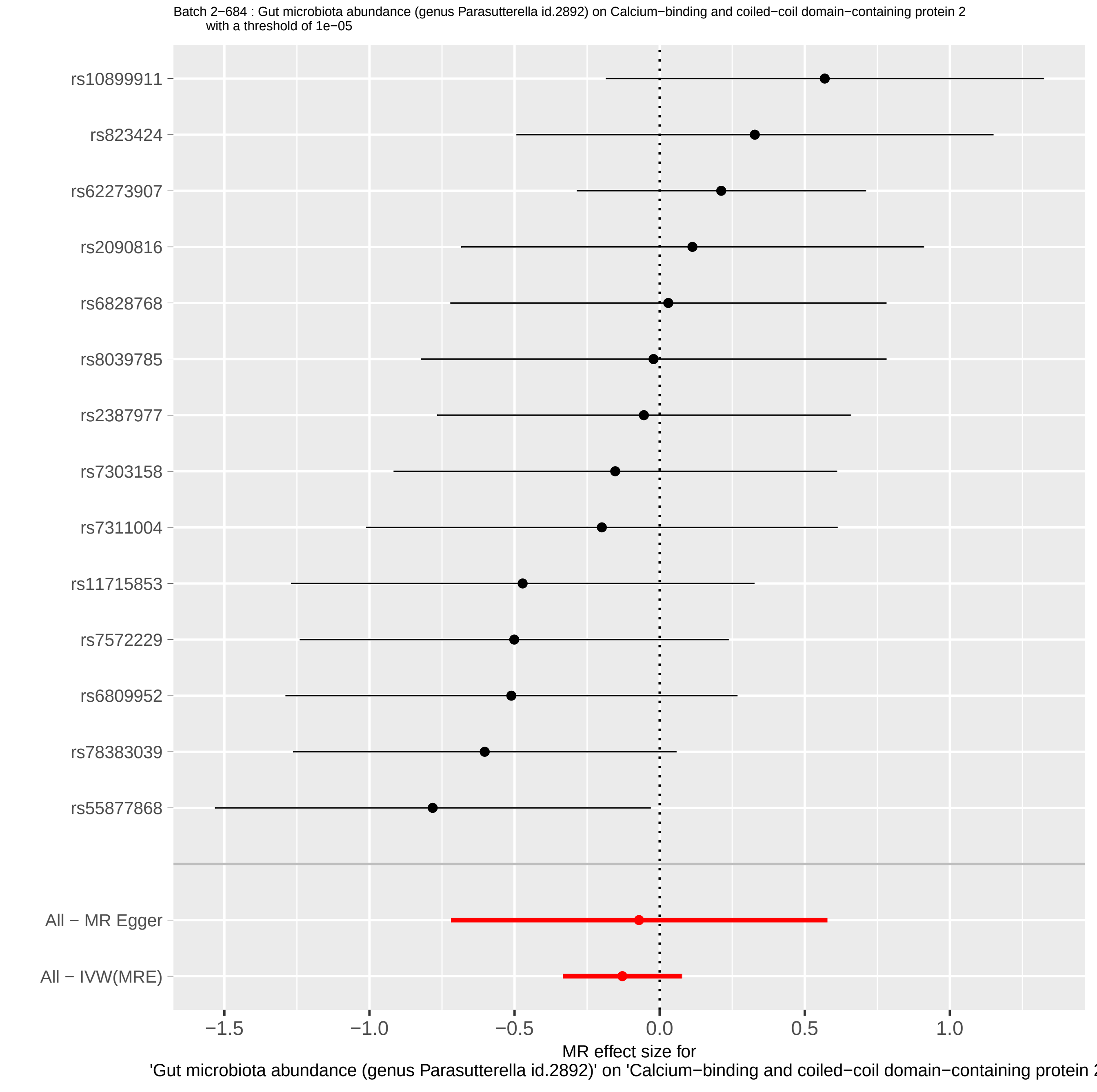

Batch 2−684 : Gut microbiota abundance (genus Parasutterella id.2892) on Calcium−binding and coiled−coil domain−containing protein 2
with a threshold of 1e−05
rs10899911
rs823424
rs62273907
rs2090816
rs6828768
rs8039785
rs2387977
rs7303158
rs7311004
rs11715853
rs7572229
rs6809952
rs78383039
rs55877868
All − MR Egger
All − IVW(MRE)
−1.5
−1.0
−0.5
0.0
0.5
1.0
MR effect size for
'Gut microbiota abundance (genus Parasutterella id.2892)' on 'Calcium−binding and coiled−coil domain−containing protein 2

Batch 2−685 : Gut microbiota abundance (genus Catenibacterium id.2153) on Calcium−binding protein 8
with a threshold of 1e−05

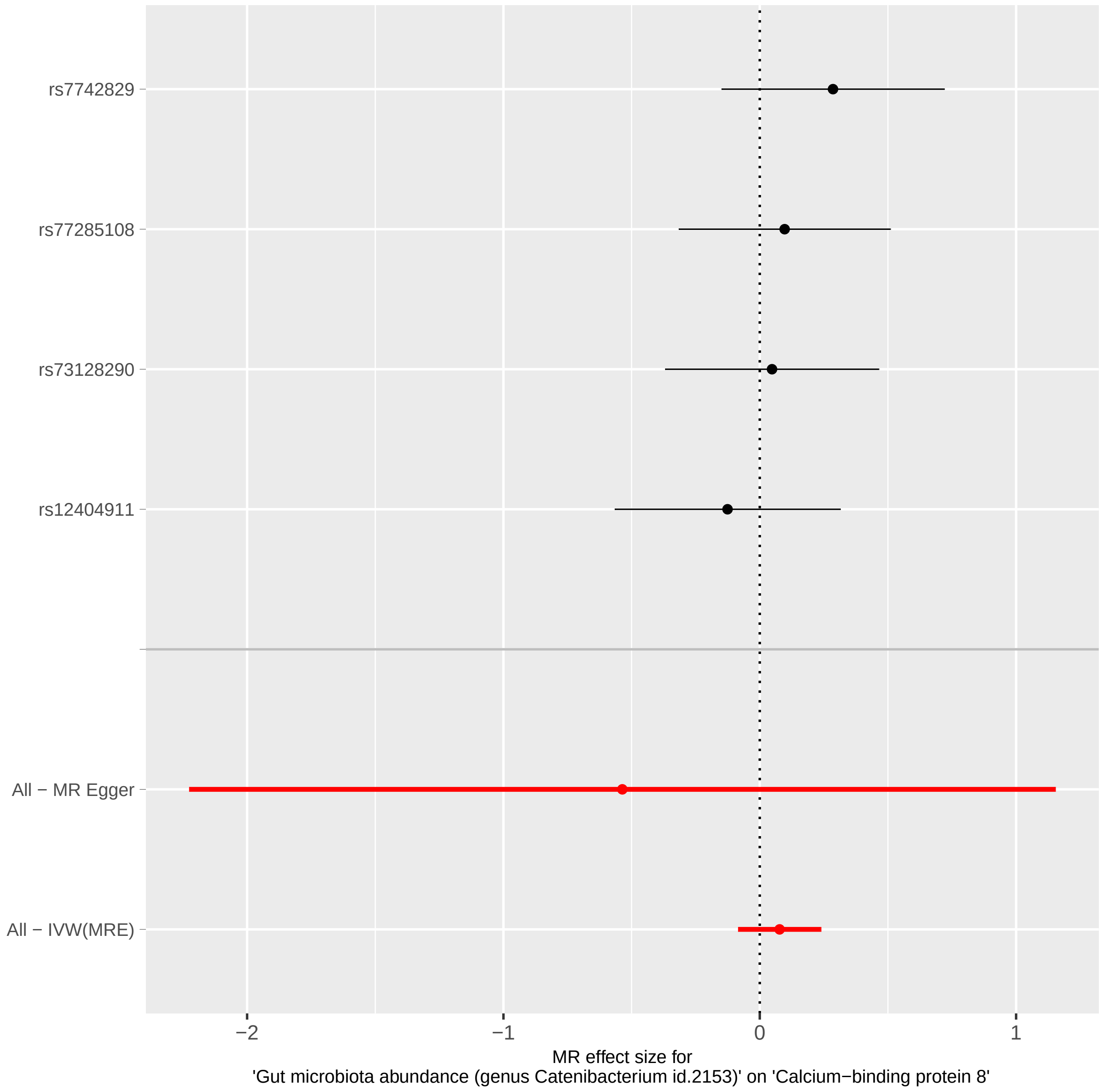

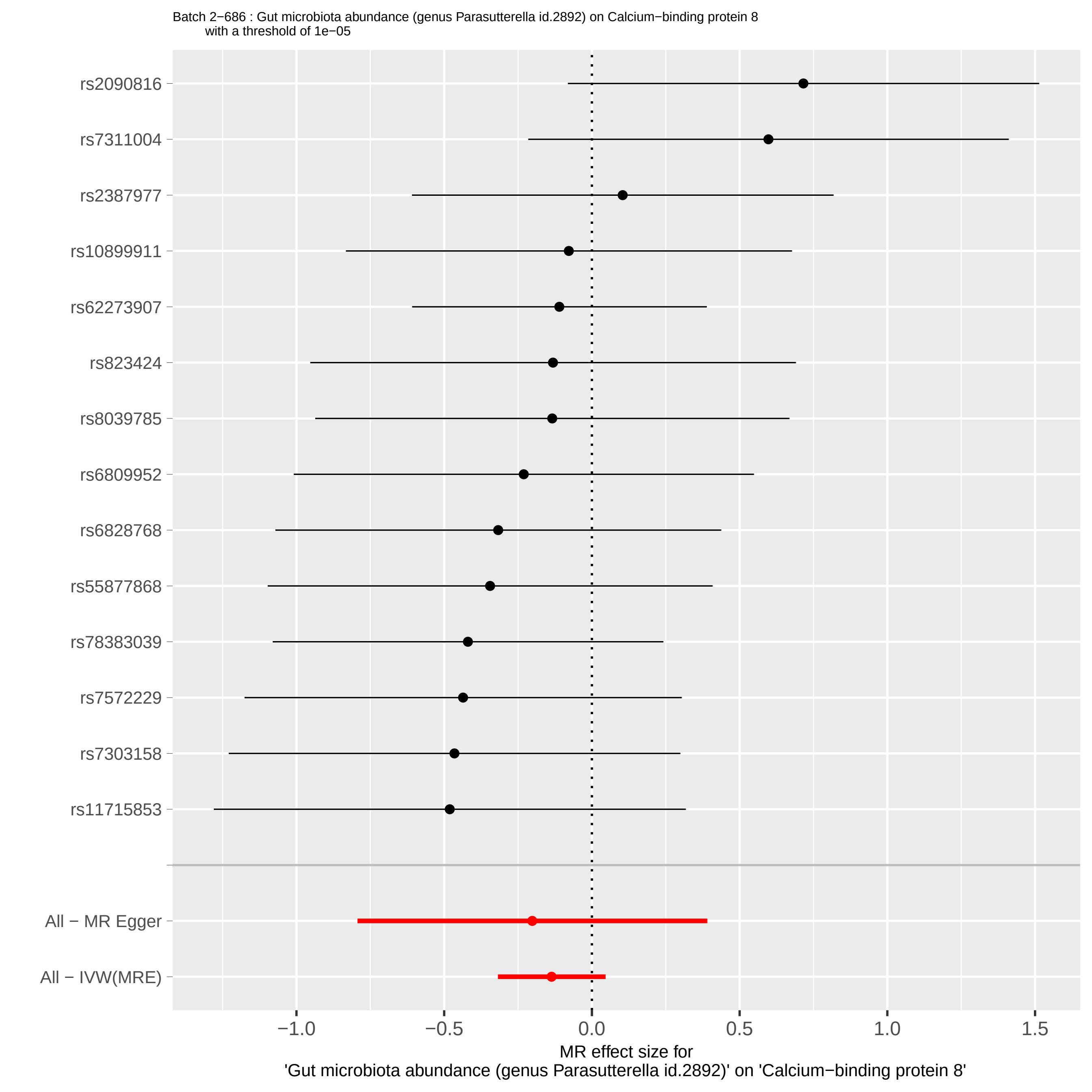

Batch 2−686 : Gut microbiota abundance (genus Parasutterella id.2892) on Calcium−binding protein 8
with a threshold of 1e−05
rs2090816
rs7311004
rs2387977
rs10899911
rs62273907
rs823424
rs8039785
rs6809952
rs6828768
rs55877868
rs78383039
rs7572229
rs7303158
rs11715853
All − MR Egger
All − IVW(MRE)
−1.0
−0.5
0.0
0.5
1.0
1.5
MR effect size for
'Gut microbiota abundance (genus Parasutterella id.2892)' on 'Calcium−binding protein 8'

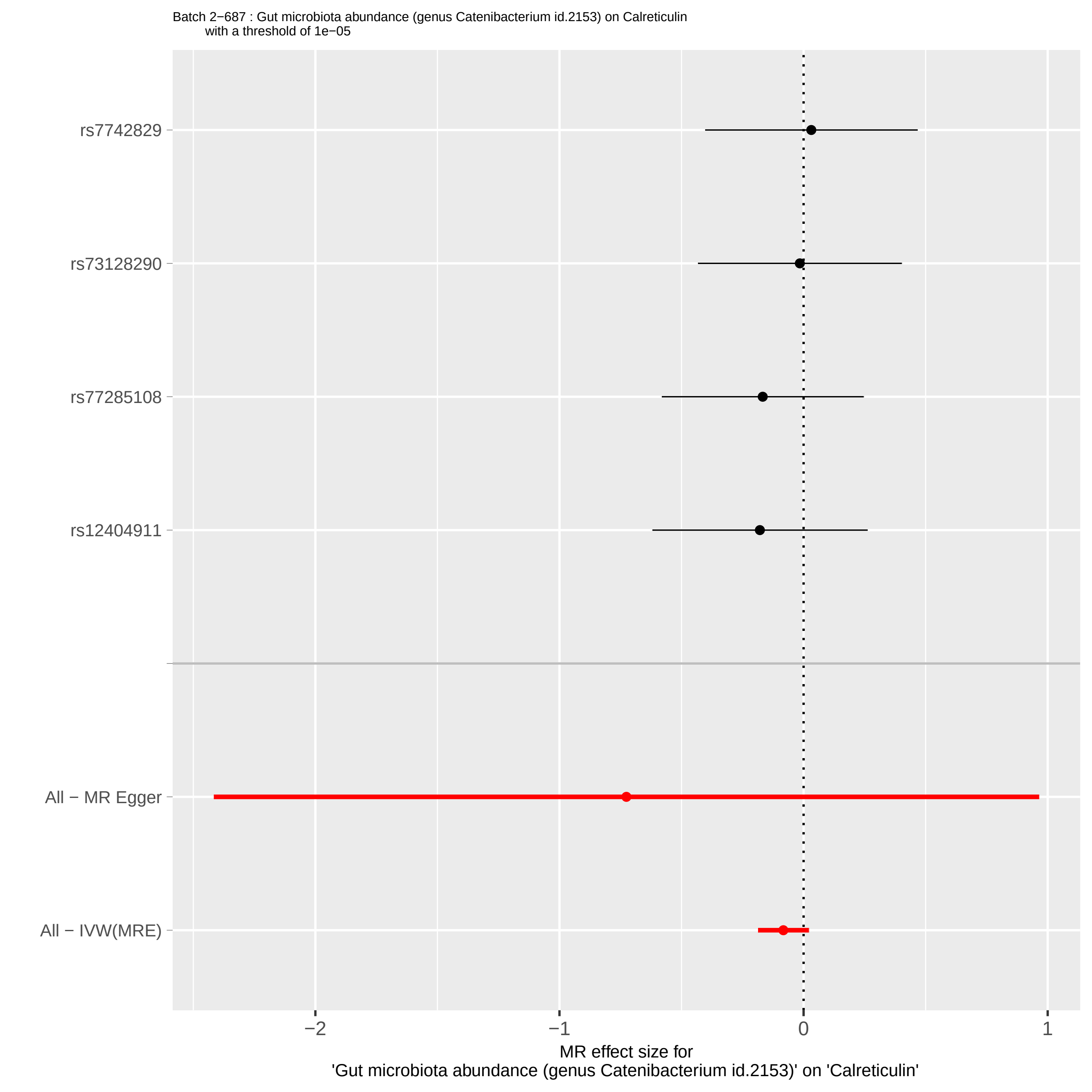
Batch 2−687 : Gut microbiota abundance (genus Catenibacterium id.2153) on Calreticulin
with a threshold of 1e−05
rs7742829
rs73128290
rs77285108
rs12404911
All − MR Egger
All − IVW(MRE)
−2
−1
0
1
MR effect size for
'Gut microbiota abundance (genus Catenibacterium id.2153)' on 'Calreticulin'

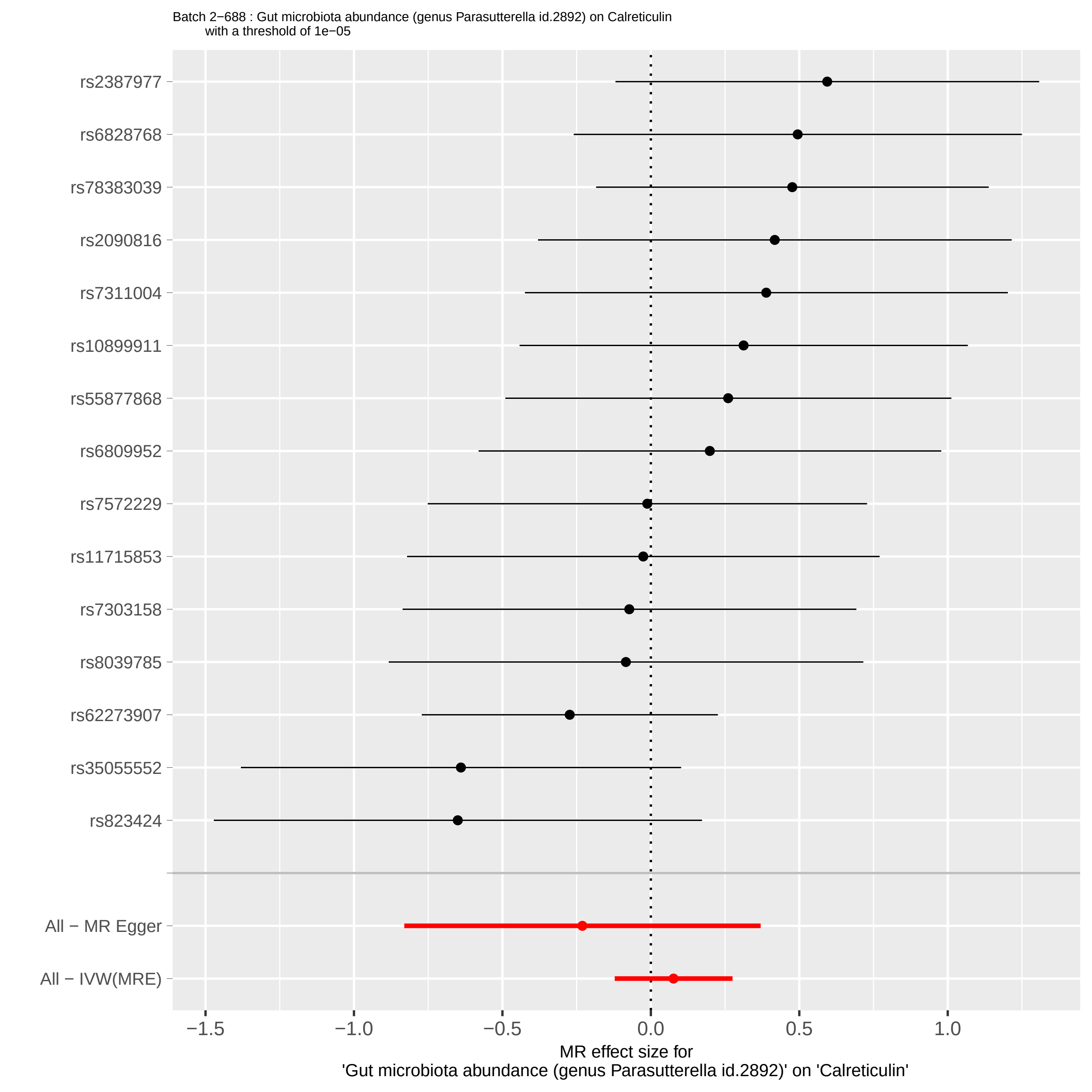
Batch 2−688 : Gut microbiota abundance (genus Parasutterella id.2892) on Calreticulin
with a threshold of 1e−05
rs2387977
rs6828768
rs78383039
rs2090816
rs7311004
rs10899911
rs55877868
rs6809952
rs7572229
rs11715853
rs7303158
rs8039785
rs62273907
rs35055552
rs823424
All − MR Egger
All − IVW(MRE)
−1.5
−1.0
−0.5
0.0
0.5
1.0
MR effect size for
'Gut microbiota abundance (genus Parasutterella id.2892)' on 'Calreticulin'

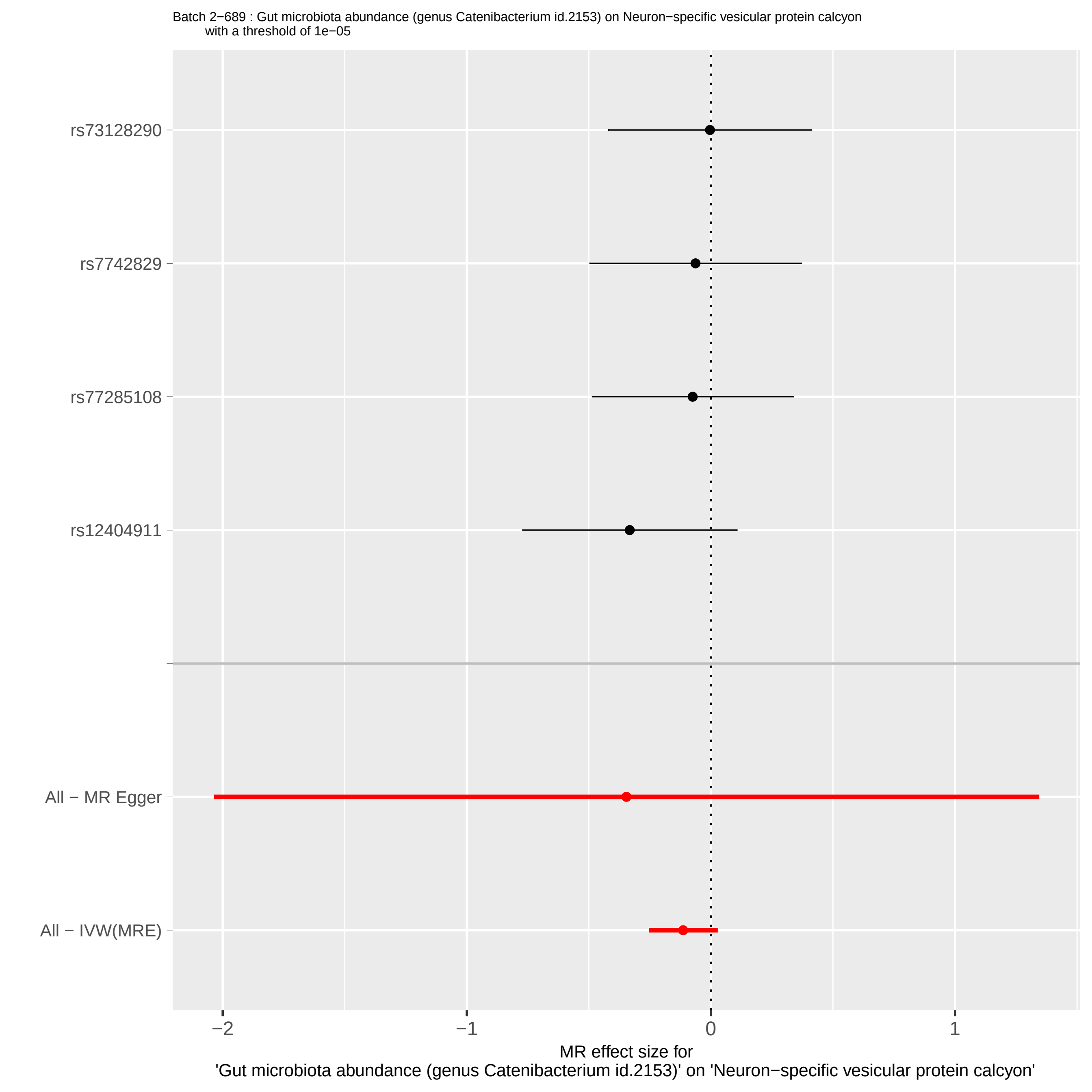

Batch 2−689 : Gut microbiota abundance (genus Catenibacterium id.2153) on Neuron−specific vesicular protein calcyon
with a threshold of 1e−05
rs73128290
rs7742829
rs77285108
rs12404911
All − MR Egger
All − IVW(MRE)
−2
−1
0
1
MR effect size for
'Gut microbiota abundance (genus Catenibacterium id.2153)' on 'Neuron−specific vesicular protein calcyon'

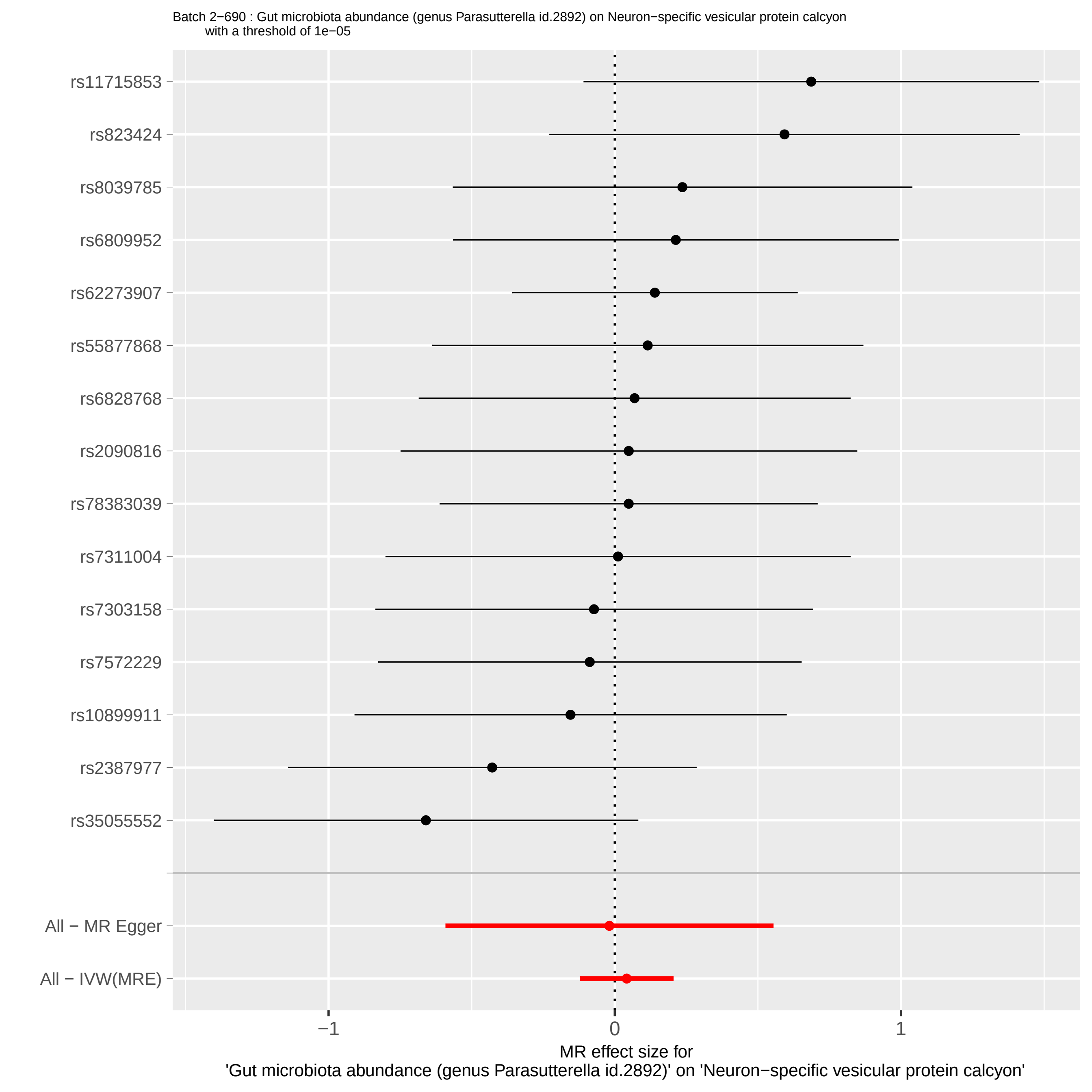
Batch 2−690 : Gut microbiota abundance (genus Parasutterella id.2892) on Neuron−specific vesicular protein calcyon with a threshold of 1e−05
rs11715853
rs823424
rs8039785
rs6809952
rs62273907
rs55877868
rs6828768
rs2090816
rs78383039
rs7311004
rs7303158
rs7572229
rs10899911
rs2387977
rs35055552
All − MR Egger
All − IVW(MRE)
−1
0
1
MR effect size for
'Gut microbiota abundance (genus Parasutterella id.2892)' on 'Neuron−specific vesicular protein calcyon'

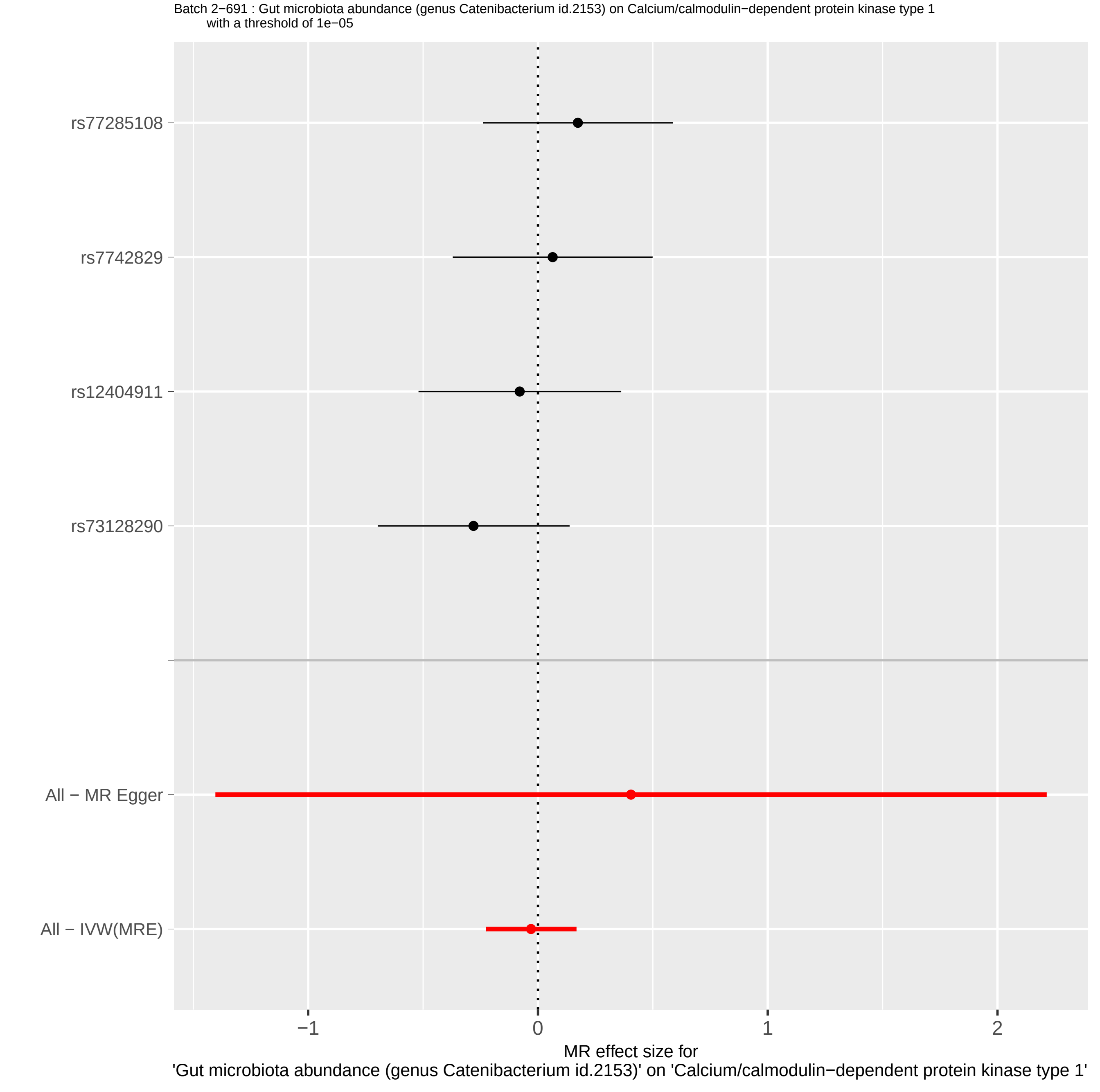

Batch 2−691 : Gut microbiota abundance (genus Catenibacterium id.2153) on Calcium/calmodulin−dependent protein kinase type 1
with a threshold of 1e−05
rs77285108
rs7742829
rs12404911
rs73128290
All − MR Egger
All − IVW(MRE)
−1
0
1
2
MR effect size for
'Gut microbiota abundance (genus Catenibacterium id.2153)' on 'Calcium/calmodulin−dependent protein kinase type 1'

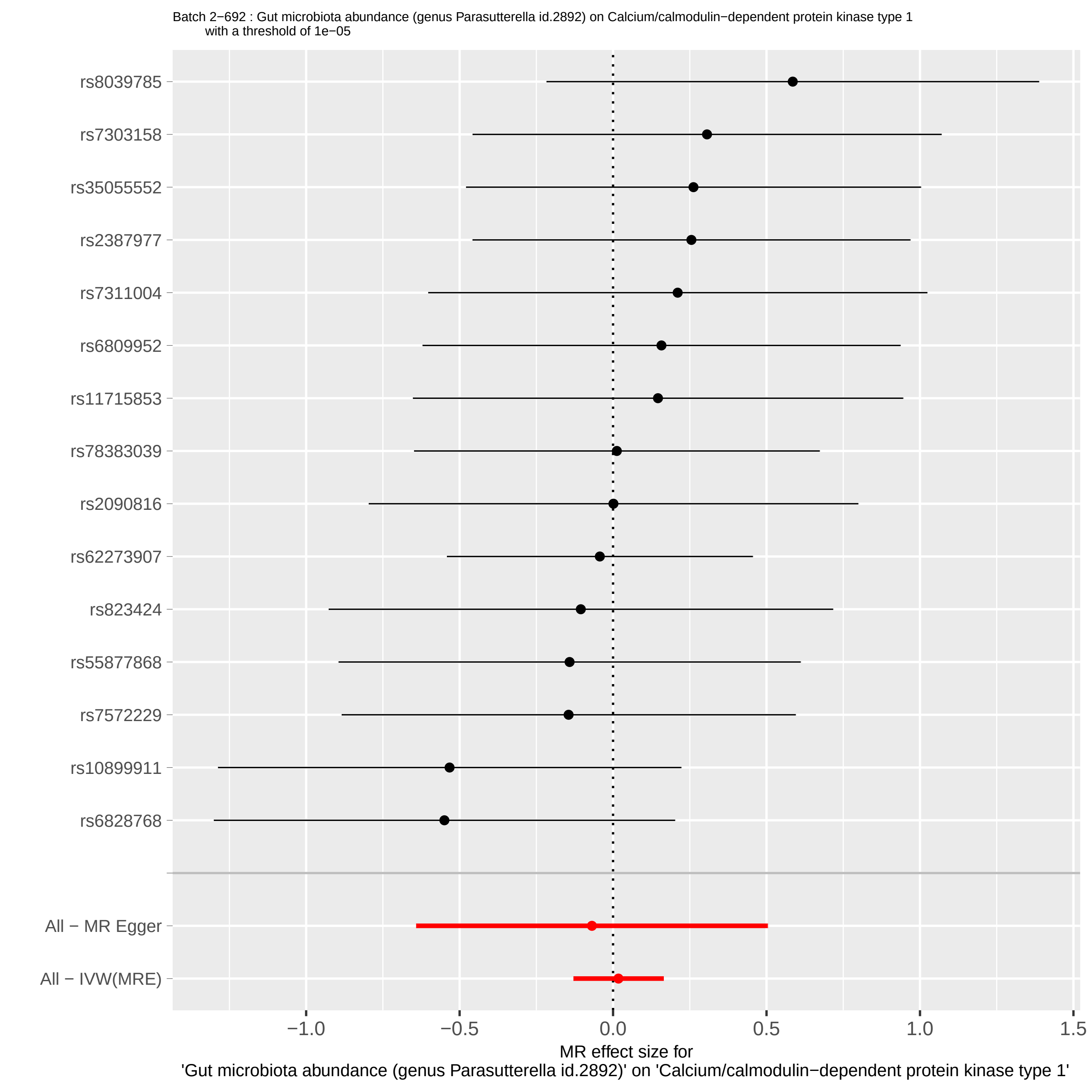

Batch 2−692 : Gut microbiota abundance (genus Parasutterella id.2892) on Calcium/calmodulin−dependent protein kinase type 1
with a threshold of 1e−05
rs8039785
rs7303158
rs35055552
rs2387977
rs7311004
rs6809952
rs11715853
rs78383039
rs2090816
rs62273907
rs823424
rs55877868
rs7572229
rs10899911
rs6828768
All − MR Egger
All − IVW(MRE)
−1.0
−0.5
0.0
0.5
1.0
1.5
MR effect size for
'Gut microbiota abundance (genus Parasutterella id.2892)' on 'Calcium/calmodulin−dependent protein kinase type 1'

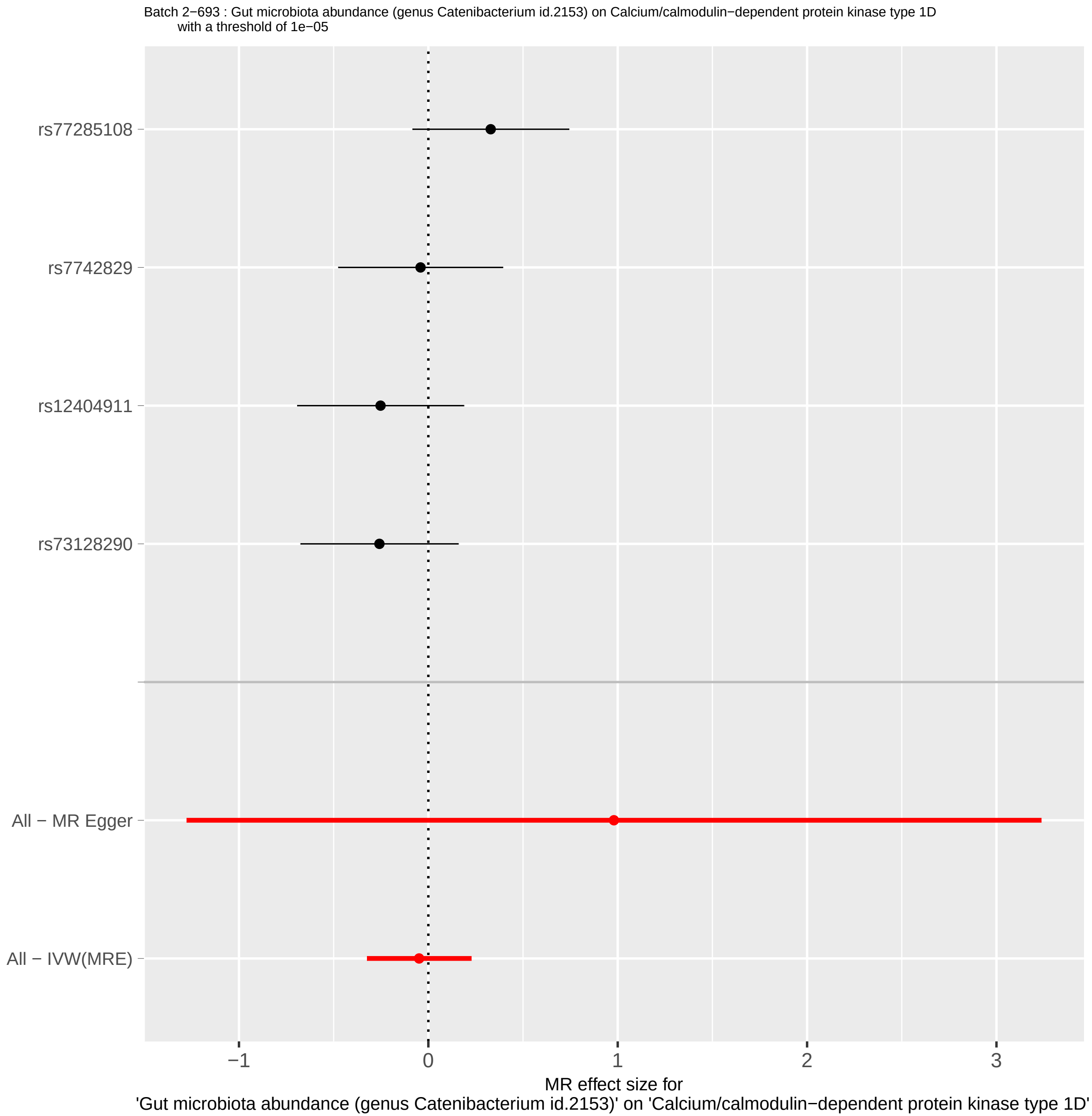
Batch 2−693 : Gut microbiota abundance (genus Catenibacterium id.2153) on Calcium/calmodulin−dependent protein kinase type 1D
with a threshold of 1e−05
rs77285108
rs7742829
rs12404911
rs73128290
All − MR Egger
All − IVW(MRE)
−1
0
1
2
3
MR effect size for
'Gut microbiota abundance (genus Catenibacterium id.2153)' on 'Calcium/calmodulin−dependent protein kinase type 1D'

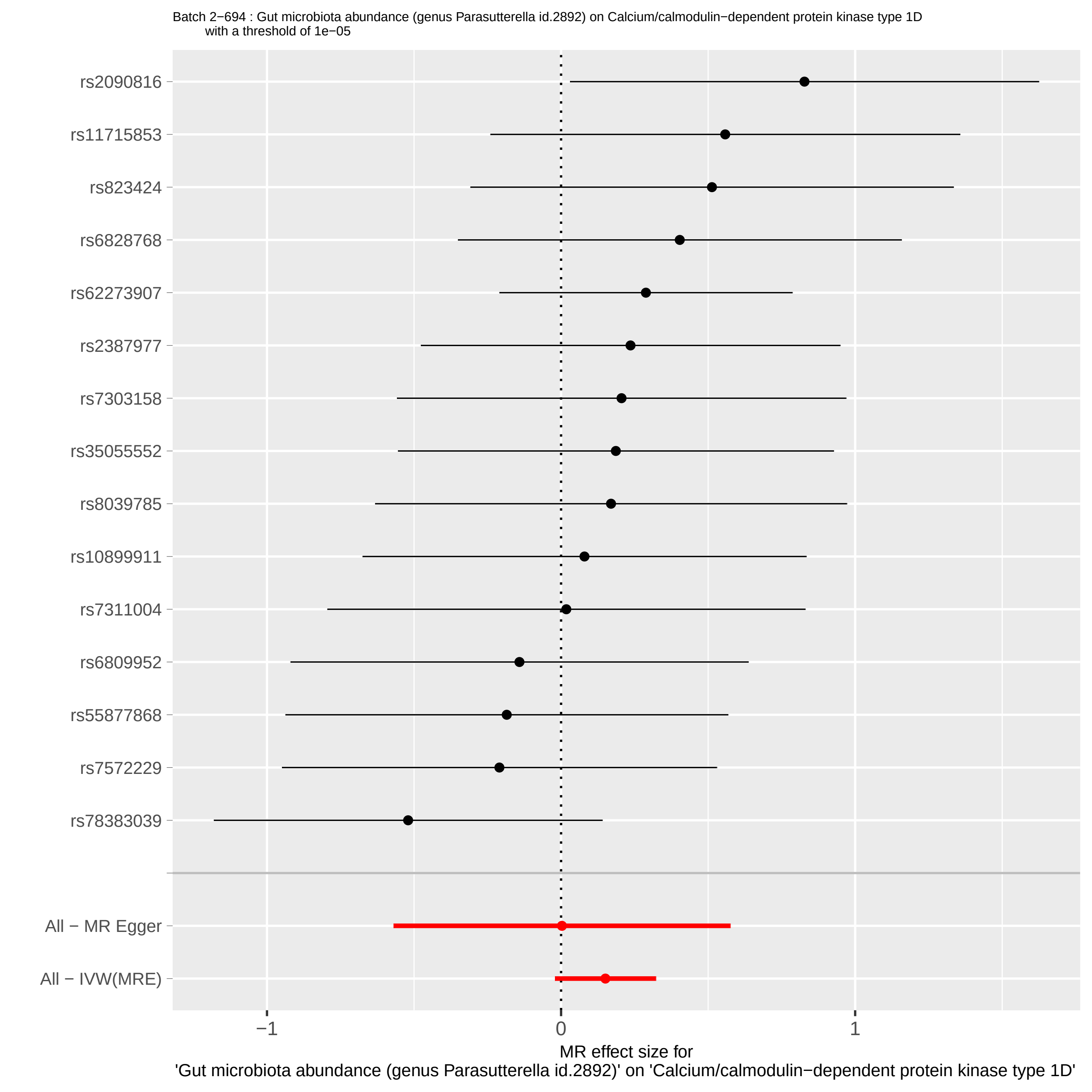

Batch 2−694 : Gut microbiota abundance (genus Parasutterella id.2892) on Calcium/calmodulin−dependent protein kinase type 1D
with a threshold of 1e−05
rs2090816
rs11715853
rs823424
rs6828768
rs62273907
rs2387977
rs7303158
rs35055552
rs8039785
rs10899911
rs7311004
rs6809952
rs55877868
rs7572229
rs78383039
All − MR Egger
All − IVW(MRE)
−1
0
1
MR effect size for
'Gut microbiota abundance (genus Parasutterella id.2892)' on 'Calcium/calmodulin−dependent protein kinase type 1D'

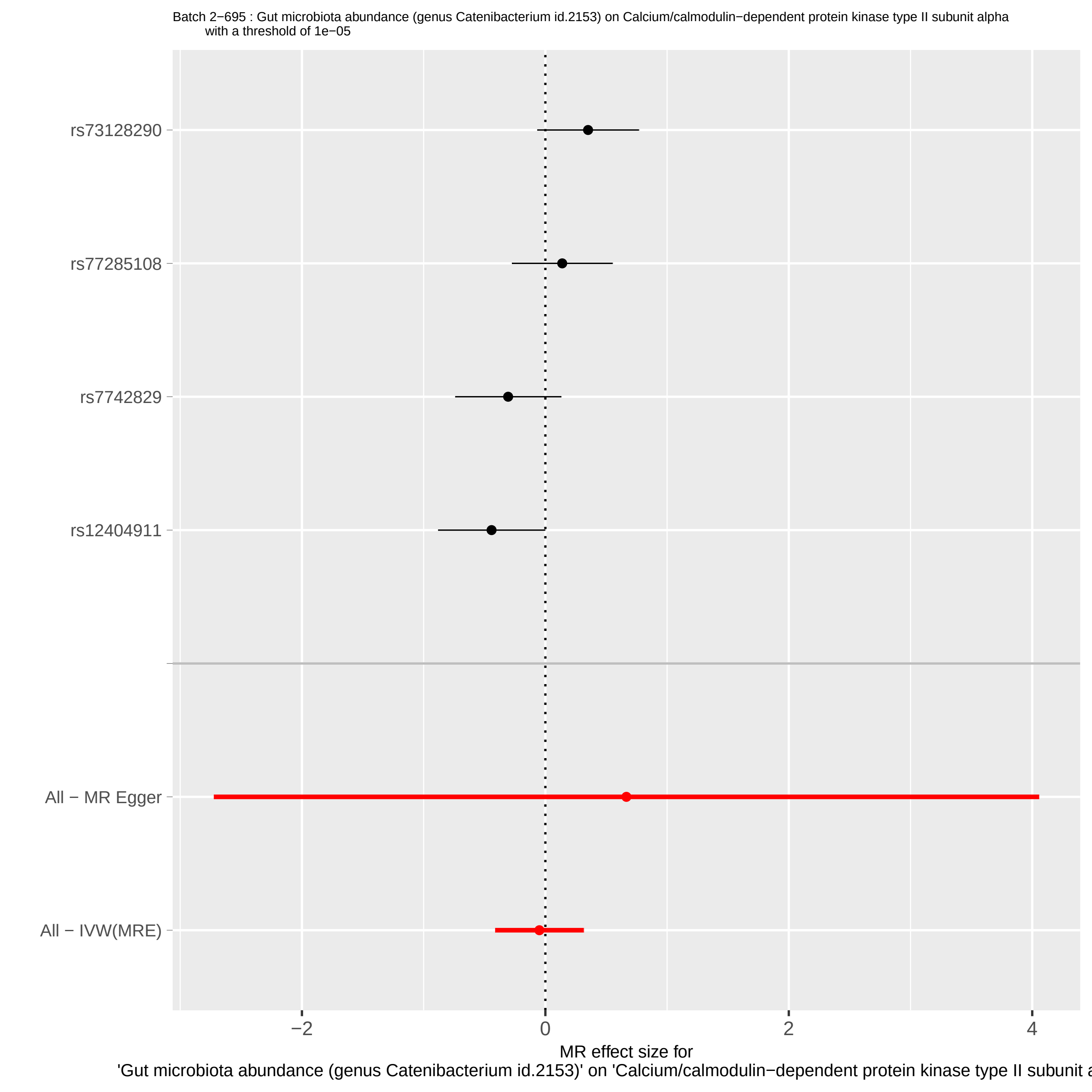

Batch 2−695 : Gut microbiota abundance (genus Catenibacterium id.2153) on Calcium/calmodulin−dependent protein kinase type II subunit alpha
with a threshold of 1e−05
rs73128290
rs77285108
rs7742829
rs12404911
All − MR Egger
All − IVW(MRE)
−2
0
2
4
MR effect size for
'Gut microbiota abundance (genus Catenibacterium id.2153)' on 'Calcium/calmodulin−dependent protein kinase type II subunit a

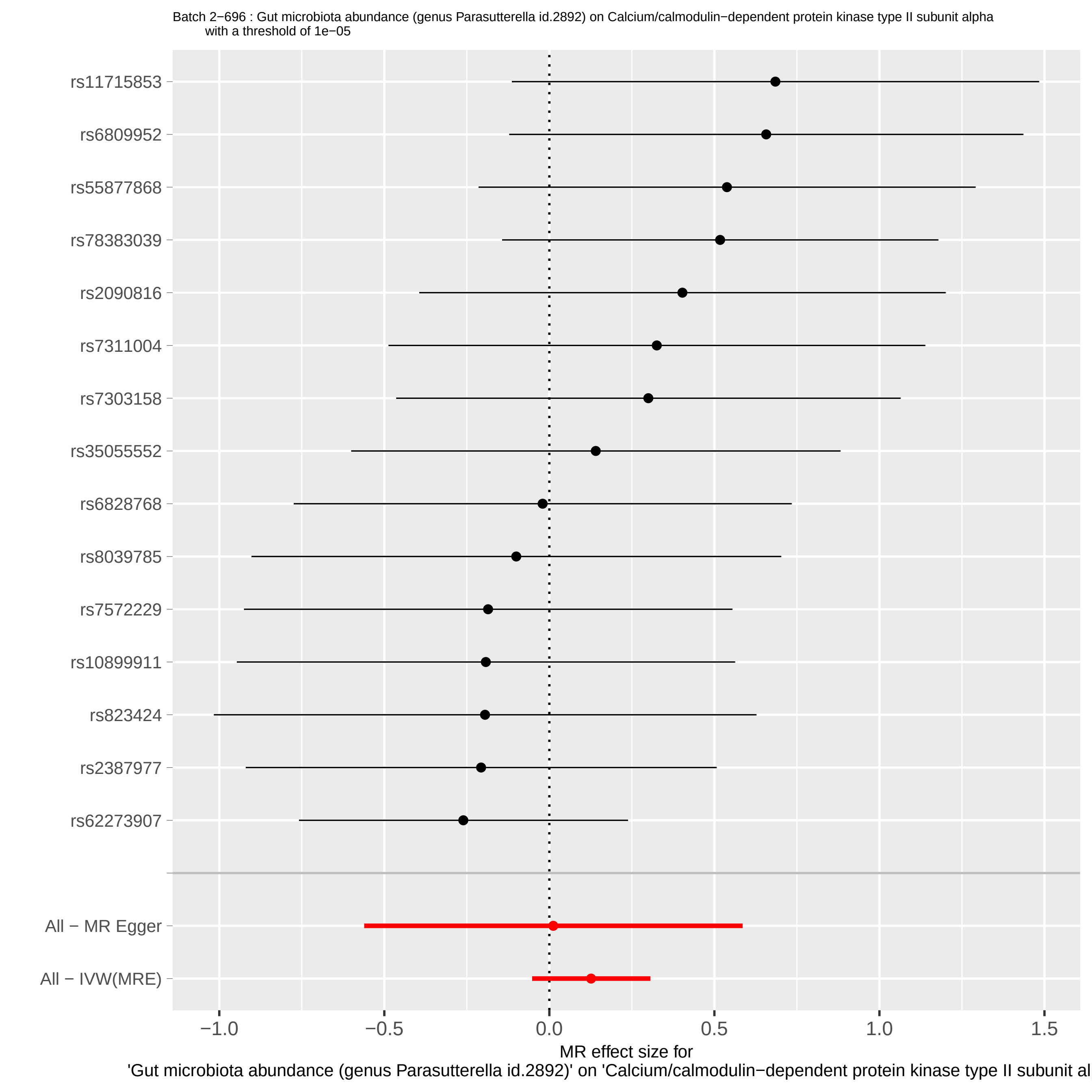

Batch 2−696 : Gut microbiota abundance (genus Parasutterella id.2892) on Calcium/calmodulin−dependent protein kinase type II subunit alpha
with a threshold of 1e−05
rs11715853
rs6809952
rs55877868
rs78383039
rs2090816
rs7311004
rs7303158
rs35055552
rs6828768
rs8039785
rs7572229
rs10899911
rs823424
rs2387977
rs62273907
All − MR Egger
All − IVW(MRE)
−1.0
−0.5
0.0
0.5
1.0
1.5
MR effect size for
'Gut microbiota abundance (genus Parasutterella id.2892)' on 'Calcium/calmodulin−dependent protein kinase type II subunit al

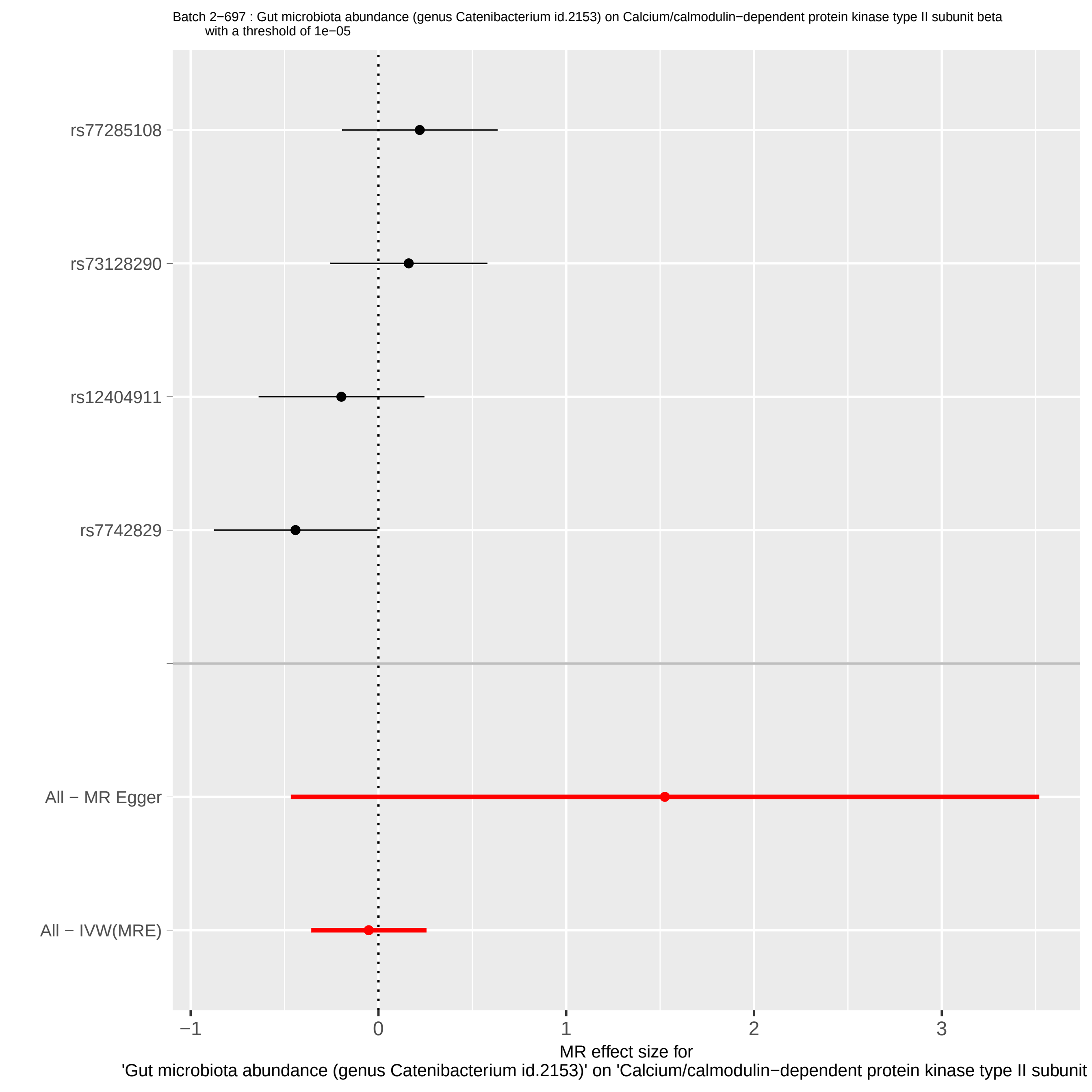
Batch 2−697 : Gut microbiota abundance (genus Catenibacterium id.2153) on Calcium/calmodulin−dependent protein kinase type II subunit beta
with a threshold of 1e−05
rs77285108
rs73128290
rs12404911
rs7742829
All − MR Egger
All − IVW(MRE)
−1
0
1
2
3
MR effect size for
'Gut microbiota abundance (genus Catenibacterium id.2153)' on 'Calcium/calmodulin−dependent protein kinase type II subunit

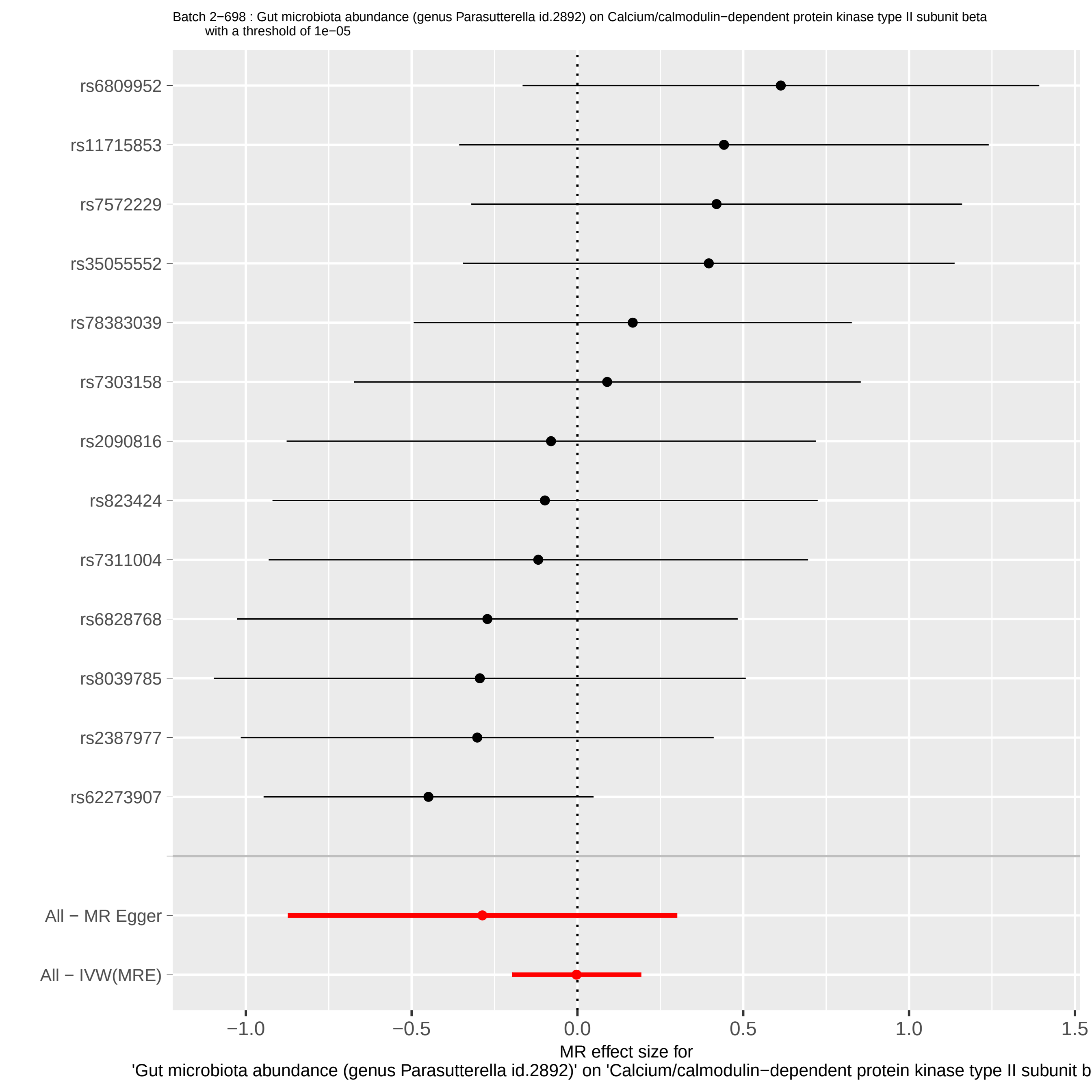

Batch 2−698 : Gut microbiota abundance (genus Parasutterella id.2892) on Calcium/calmodulin−dependent protein kinase type II subunit beta
with a threshold of 1e−05
rs6809952
rs11715853
rs7572229
rs35055552
rs78383039
rs7303158
rs2090816
rs823424
rs7311004
rs6828768
rs8039785
rs2387977
rs62273907
All − MR Egger
All − IVW(MRE)
−1.0
−0.5
0.0
0.5
1.0
1.5
MR effect size for
'Gut microbiota abundance (genus Parasutterella id.2892)' on 'Calcium/calmodulin−dependent protein kinase type II subunit b

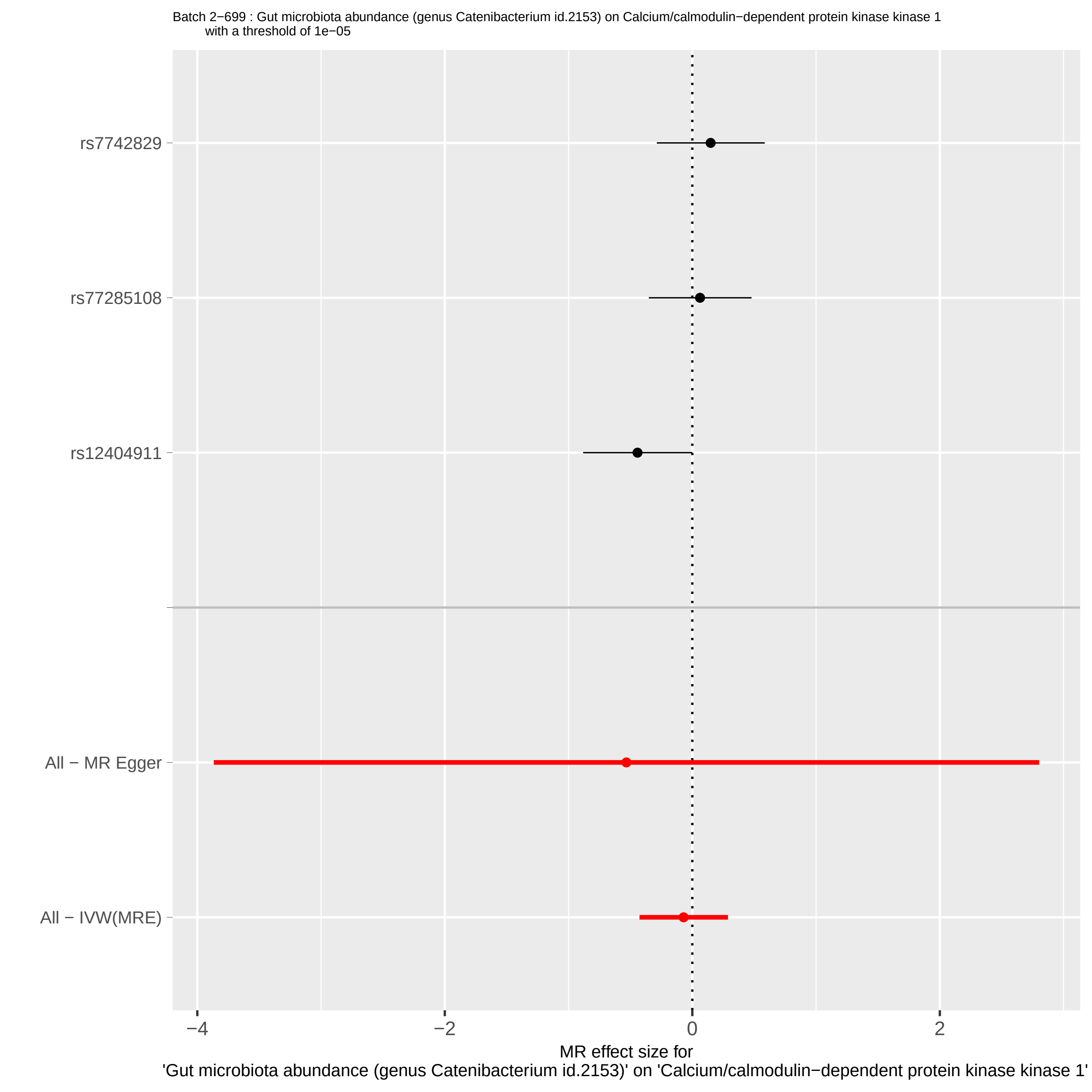

Batch 2−699 : Gut microbiota abundance (genus Catenibacterium id.2153) on Calcium/calmodulin−dependent protein kinase kinase 1
with a threshold of 1e−05
rs7742829
rs77285108
rs12404911
All − MR Egger
All − IVW(MRE)
−4
−2
0
2
MR effect size for
'Gut microbiota abundance (genus Catenibacterium id.2153)' on 'Calcium/calmodulin−dependent protein kinase kinase 1'

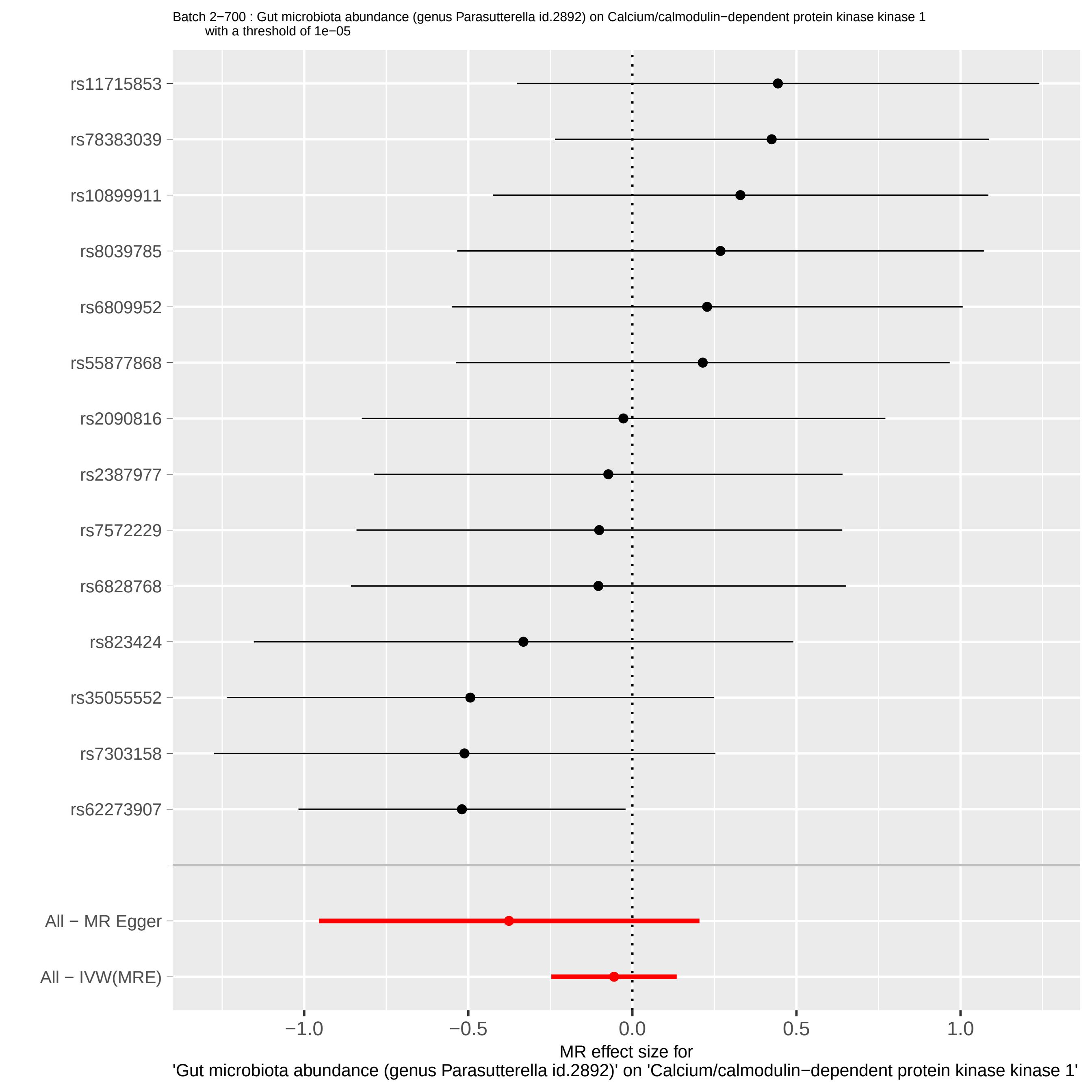
Batch 2−700 : Gut microbiota abundance (genus Parasutterella id.2892) on Calcium/calmodulin−dependent protein kinase kinase 1
with a threshold of 1e−05
rs11715853
rs78383039
rs10899911
rs8039785
rs6809952
rs55877868
rs2090816
rs2387977
rs7572229
rs6828768
rs823424
rs35055552
rs7303158
rs62273907
All − MR Egger
All − IVW(MRE)
−1.0
−0.5
0.0
0.5
1.0
MR effect size for
'Gut microbiota abundance (genus Parasutterella id.2892)' on 'Calcium/calmodulin−dependent protein kinase kinase 1'

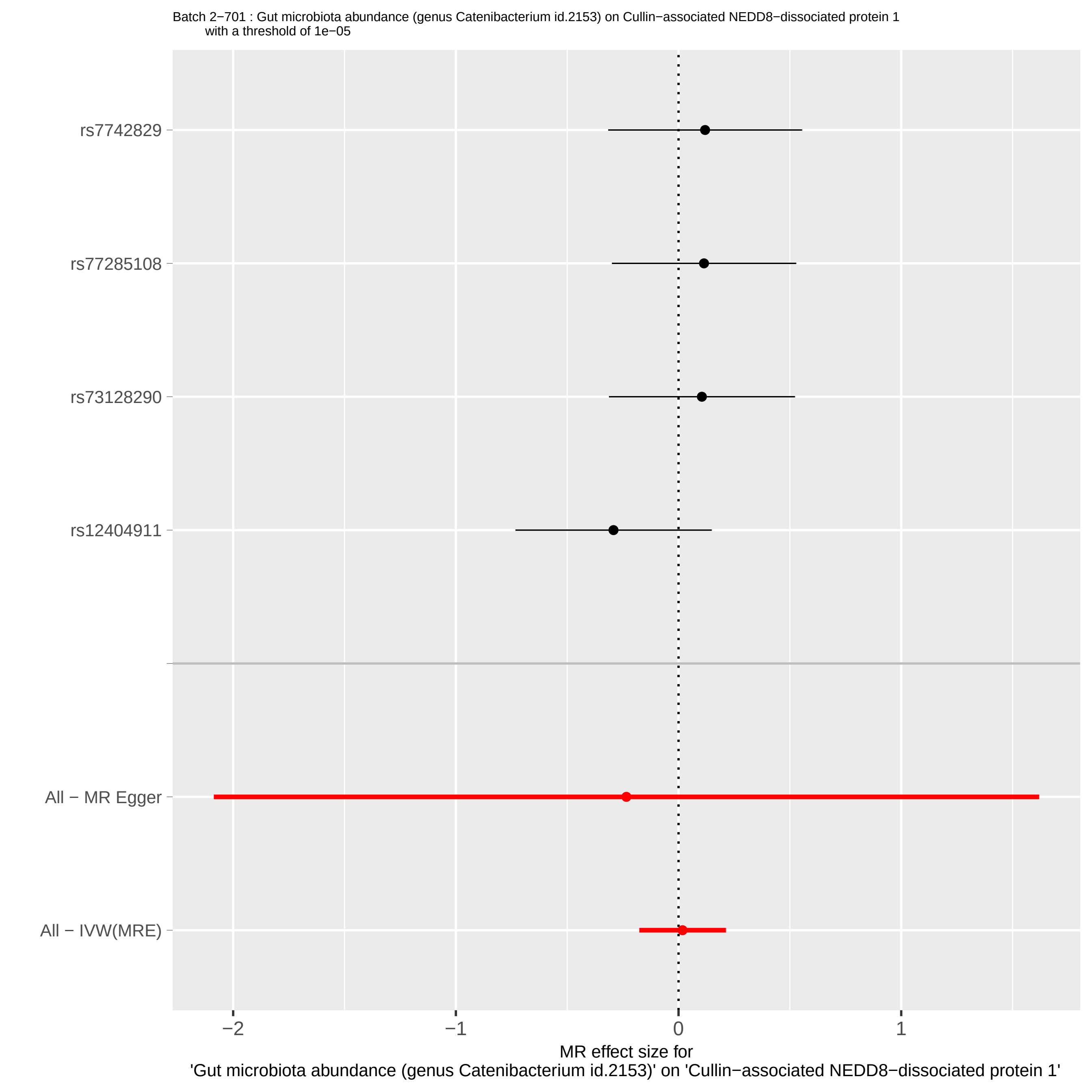

Batch 2−701 : Gut microbiota abundance (genus Catenibacterium id.2153) on Cullin−associated NEDD8−dissociated protein 1
with a threshold of 1e−05
rs7742829
rs77285108
rs73128290
rs12404911
All − MR Egger
All − IVW(MRE)
−2
−1
0
1
MR effect size for
'Gut microbiota abundance (genus Catenibacterium id.2153)' on 'Cullin−associated NEDD8−dissociated protein 1'

Batch 2−702 : Gut microbiota abundance (genus Parasutterella id.2892) on Cullin−associated NEDD8−dissociated protein 1 with a threshold of 1e−05

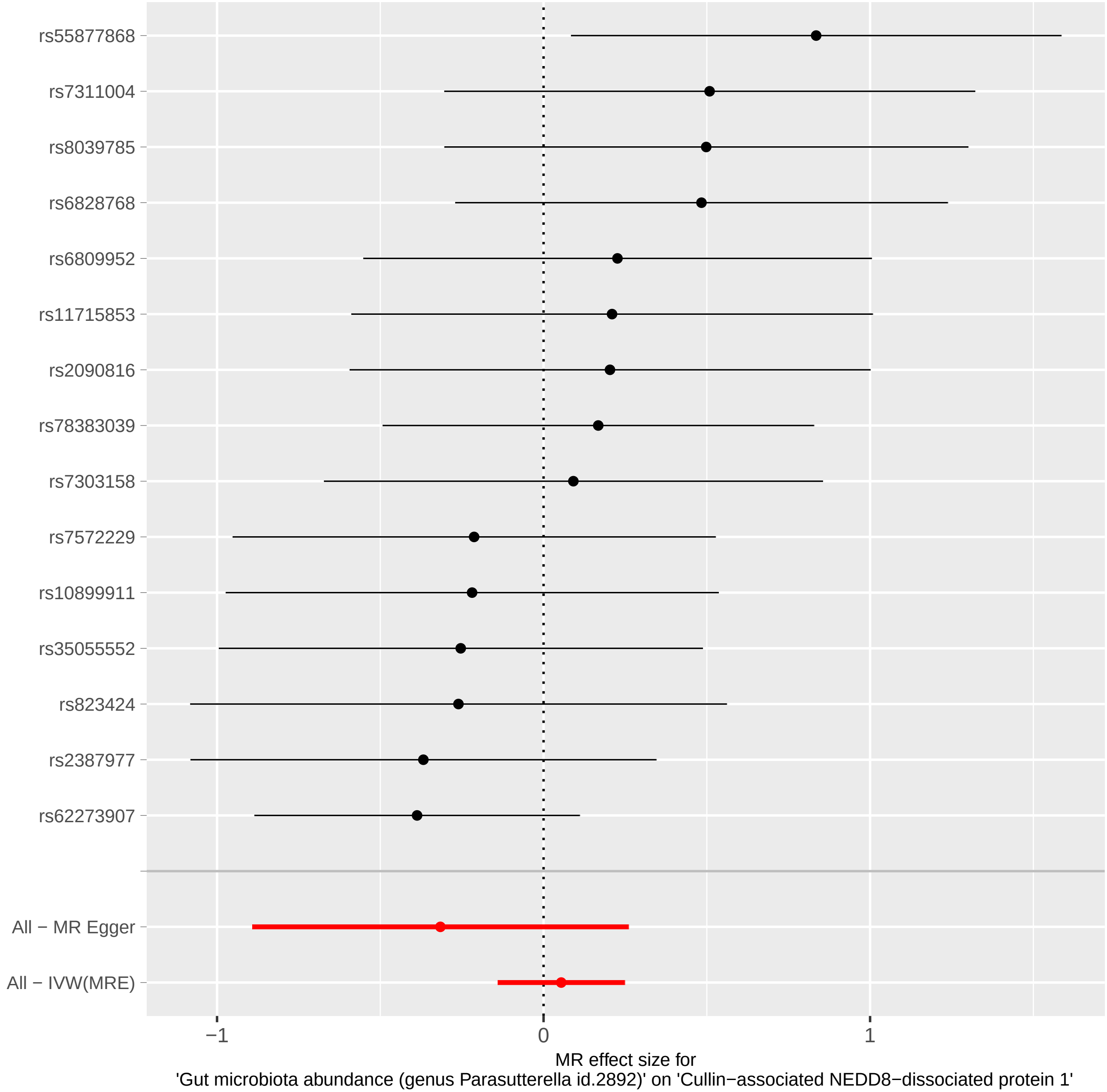

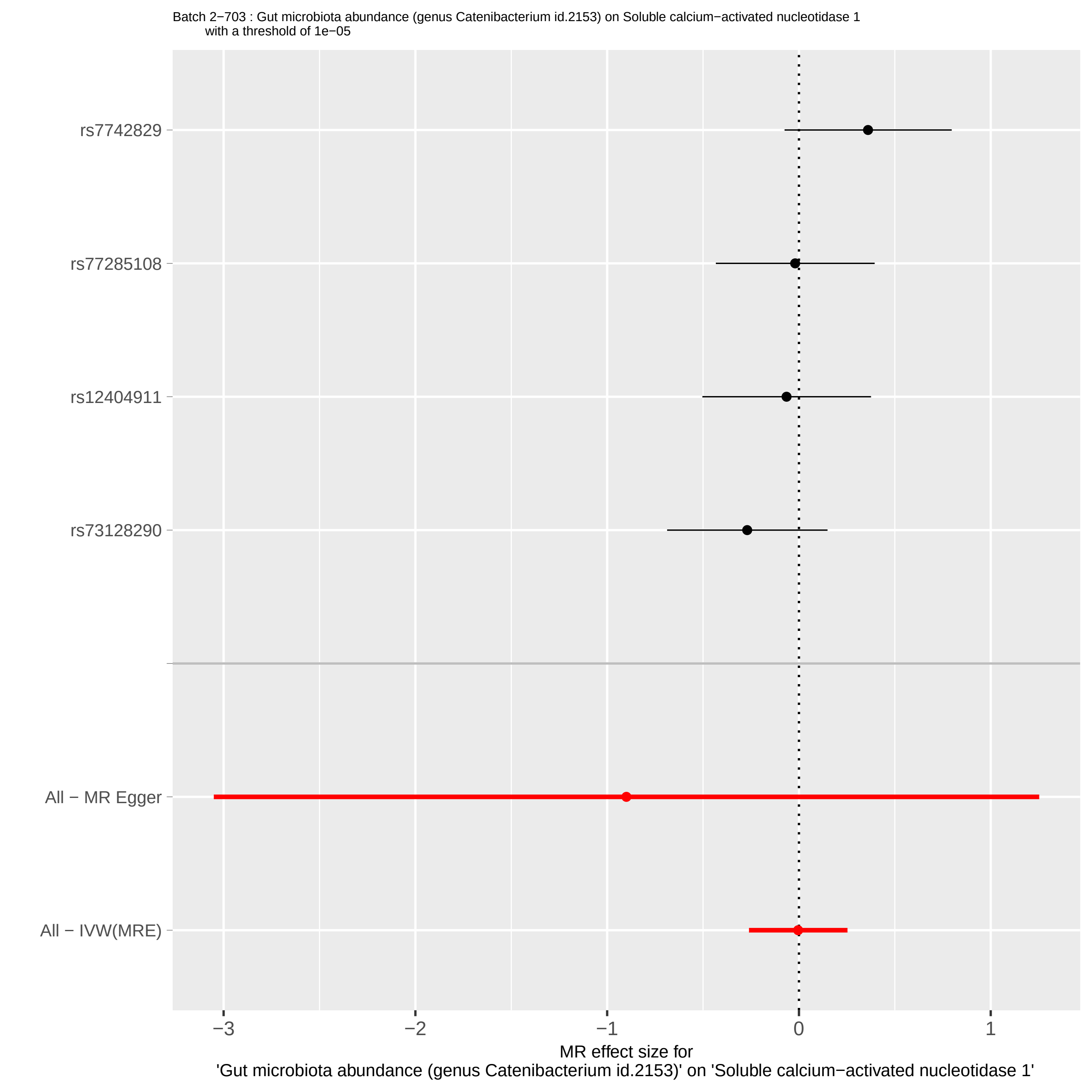

Batch 2−703 : Gut microbiota abundance (genus Catenibacterium id.2153) on Soluble calcium−activated nucleotidase 1
with a threshold of 1e−05
rs7742829
rs77285108
rs12404911
rs73128290
All − MR Egger
All − IVW(MRE)
−3
−2
−1
0
1
MR effect size for
'Gut microbiota abundance (genus Catenibacterium id.2153)' on 'Soluble calcium−activated nucleotidase 1'

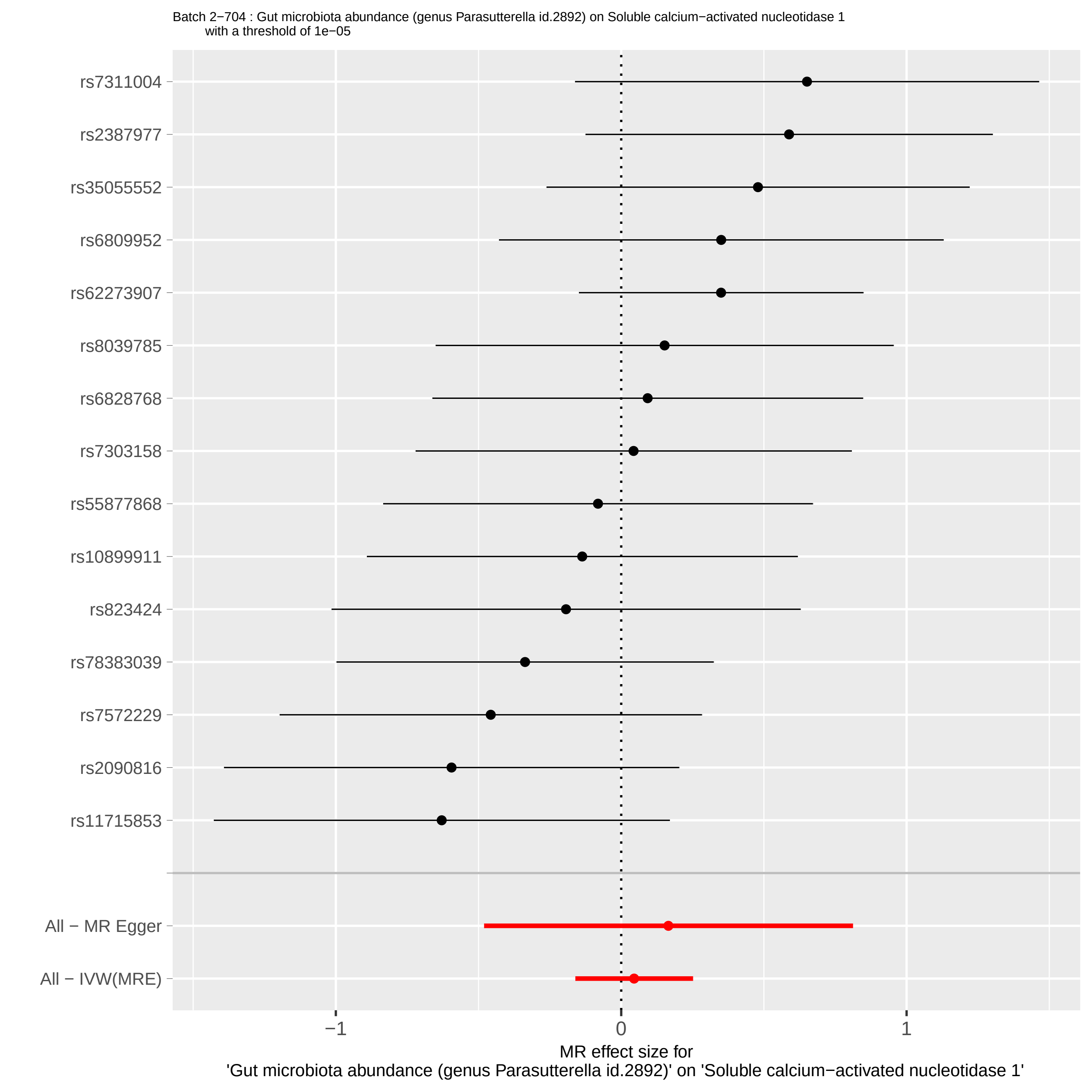

Batch 2−704 : Gut microbiota abundance (genus Parasutterella id.2892) on Soluble calcium−activated nucleotidase 1
with a threshold of 1e−05
rs7311004
rs2387977
rs35055552
rs6809952
rs62273907
rs8039785
rs6828768
rs7303158
rs55877868
rs10899911
rs823424
rs78383039
rs7572229
rs2090816
rs11715853
All − MR Egger
All − IVW(MRE)
−1
0
1
MR effect size for
'Gut microbiota abundance (genus Parasutterella id.2892)' on 'Soluble calcium−activated nucleotidase 1'

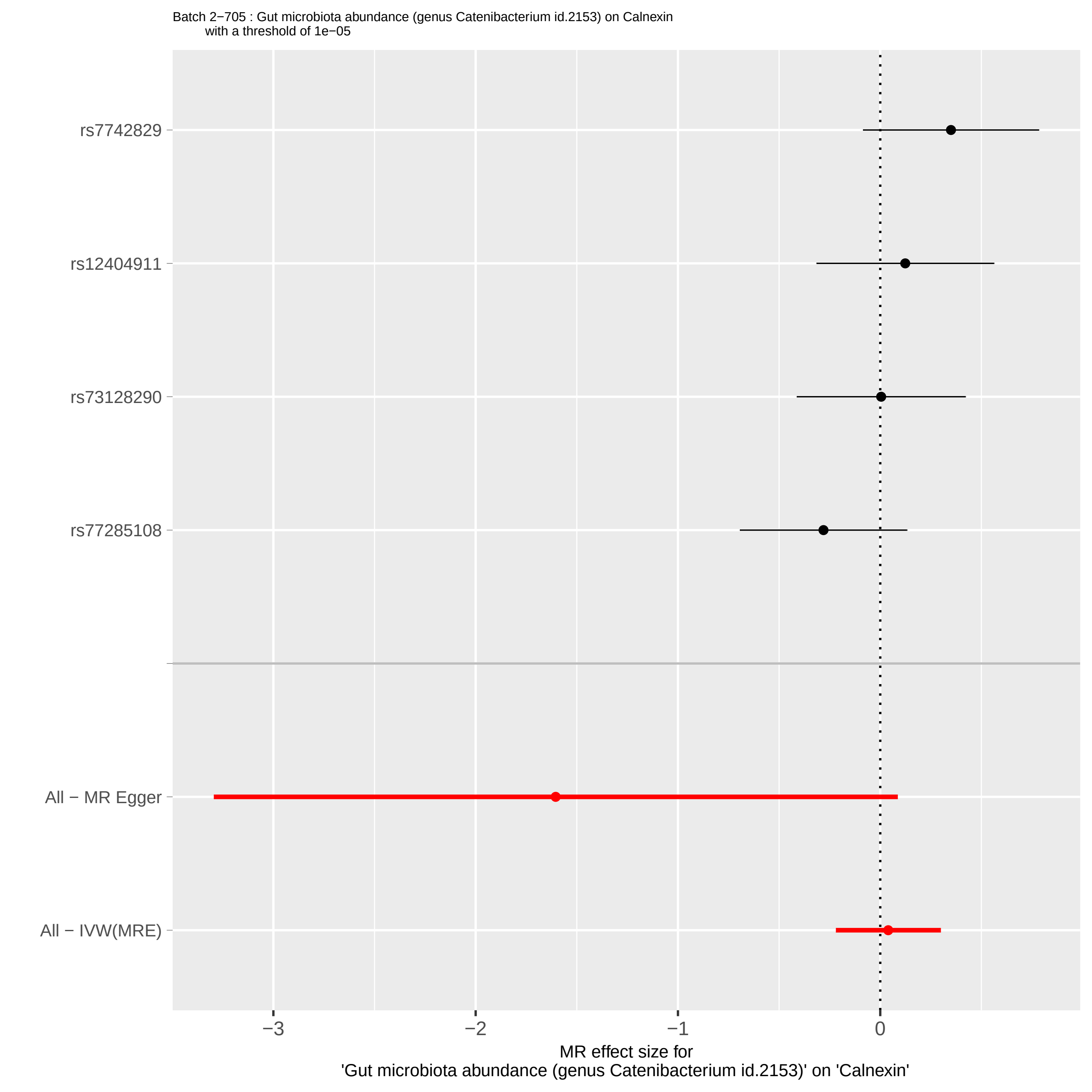

Batch 2−705 : Gut microbiota abundance (genus Catenibacterium id.2153) on Calnexin
with a threshold of 1e−05
rs7742829
rs12404911
rs73128290
rs77285108
All − MR Egger
All − IVW(MRE)
−3
−2
−1
0
MR effect size for
'Gut microbiota abundance (genus Catenibacterium id.2153)' on 'Calnexin'

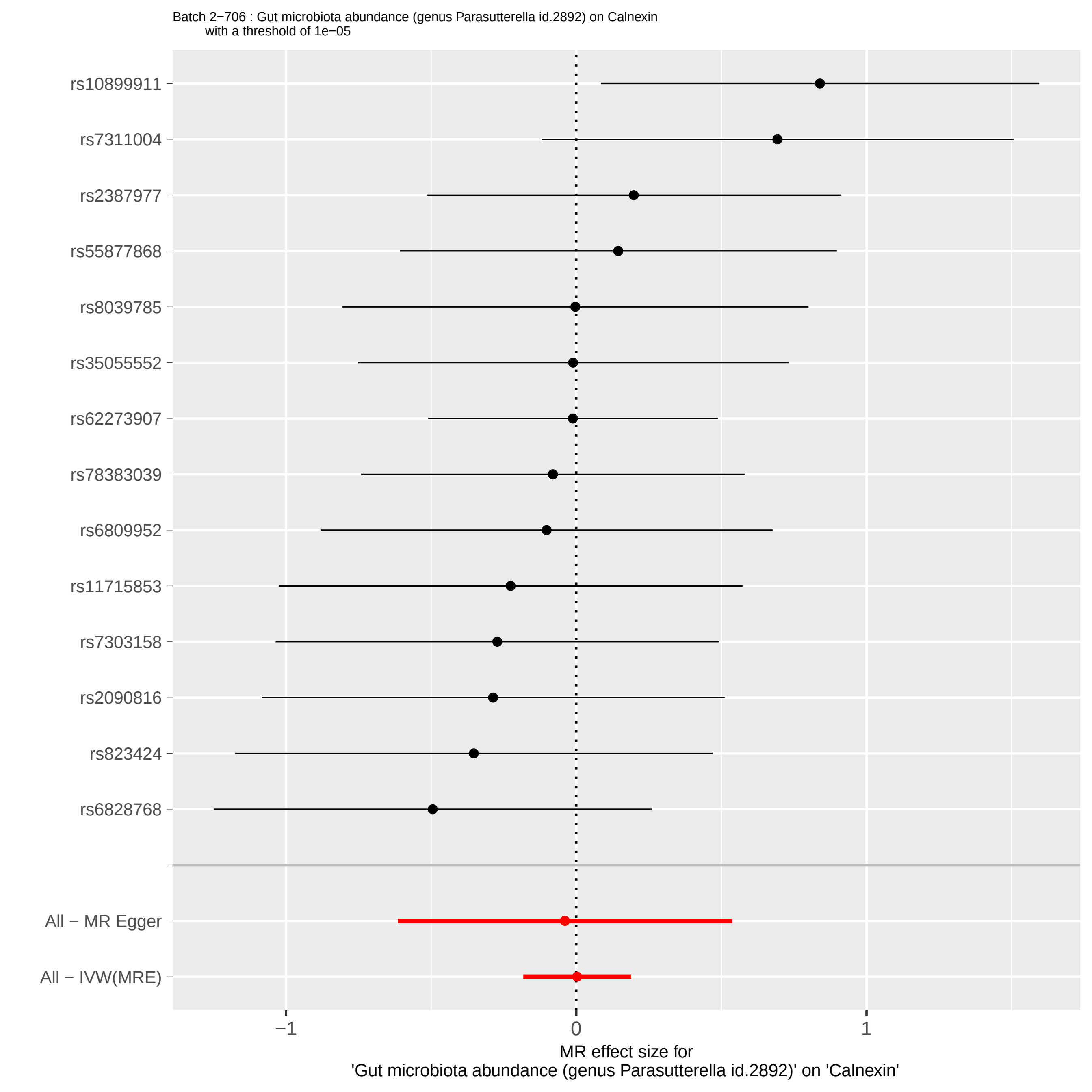

Batch 2−706 : Gut microbiota abundance (genus Parasutterella id.2892) on Calnexin
with a threshold of 1e−05
rs10899911
rs7311004
rs2387977
rs55877868
rs8039785
rs35055552
rs62273907
rs78383039
rs6809952
rs11715853
rs7303158
rs2090816
rs823424
rs6828768
All − MR Egger
All − IVW(MRE)
−1
0
1
MR effect size for
'Gut microbiota abundance (genus Parasutterella id.2892)' on 'Calnexin'

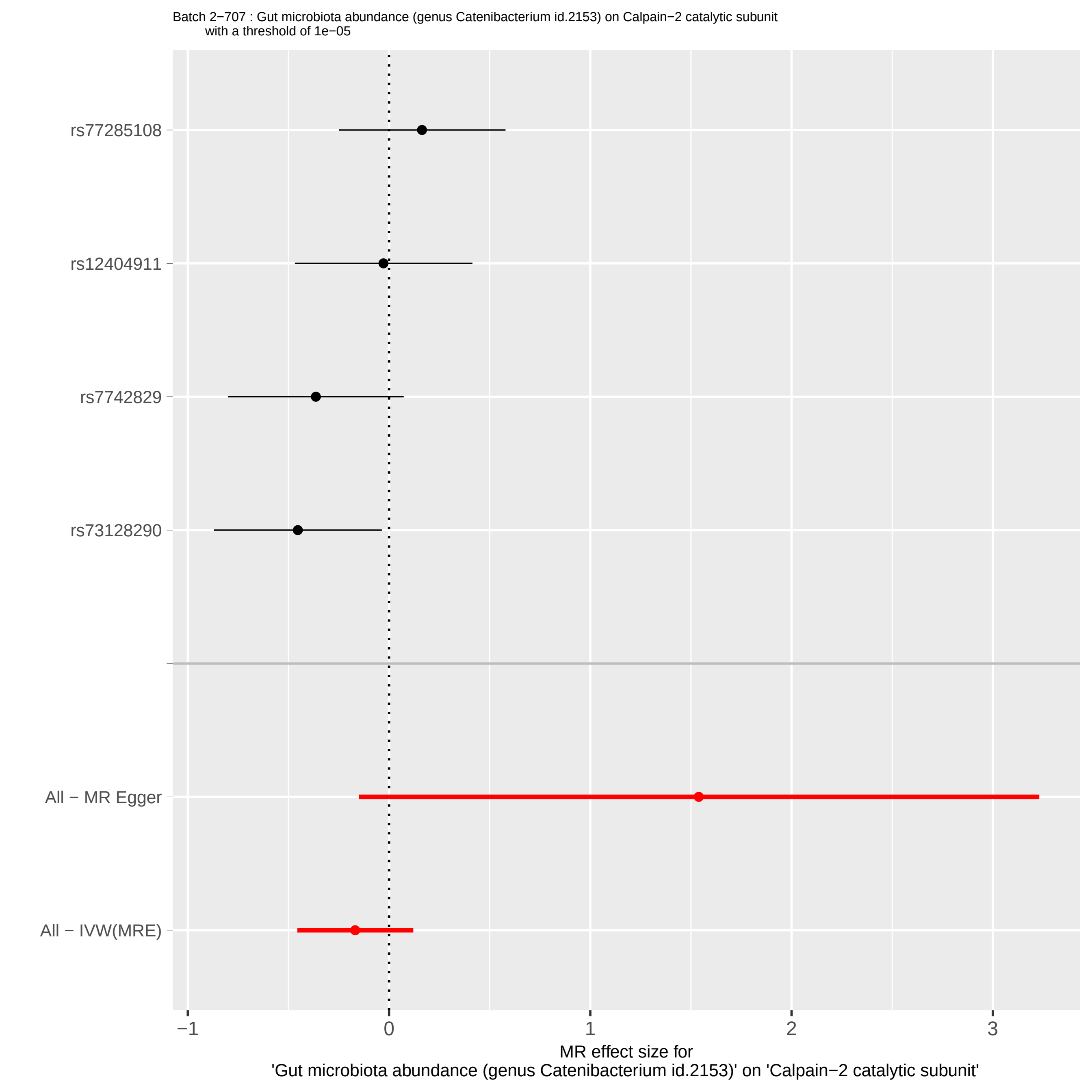

Batch 2−707 : Gut microbiota abundance (genus Catenibacterium id.2153) on Calpain−2 catalytic subunit
with a threshold of 1e−05
rs77285108
rs12404911
rs7742829
rs73128290
All − MR Egger
All − IVW(MRE)
−1
0
1
2
3
MR effect size for
'Gut microbiota abundance (genus Catenibacterium id.2153)' on 'Calpain−2 catalytic subunit'

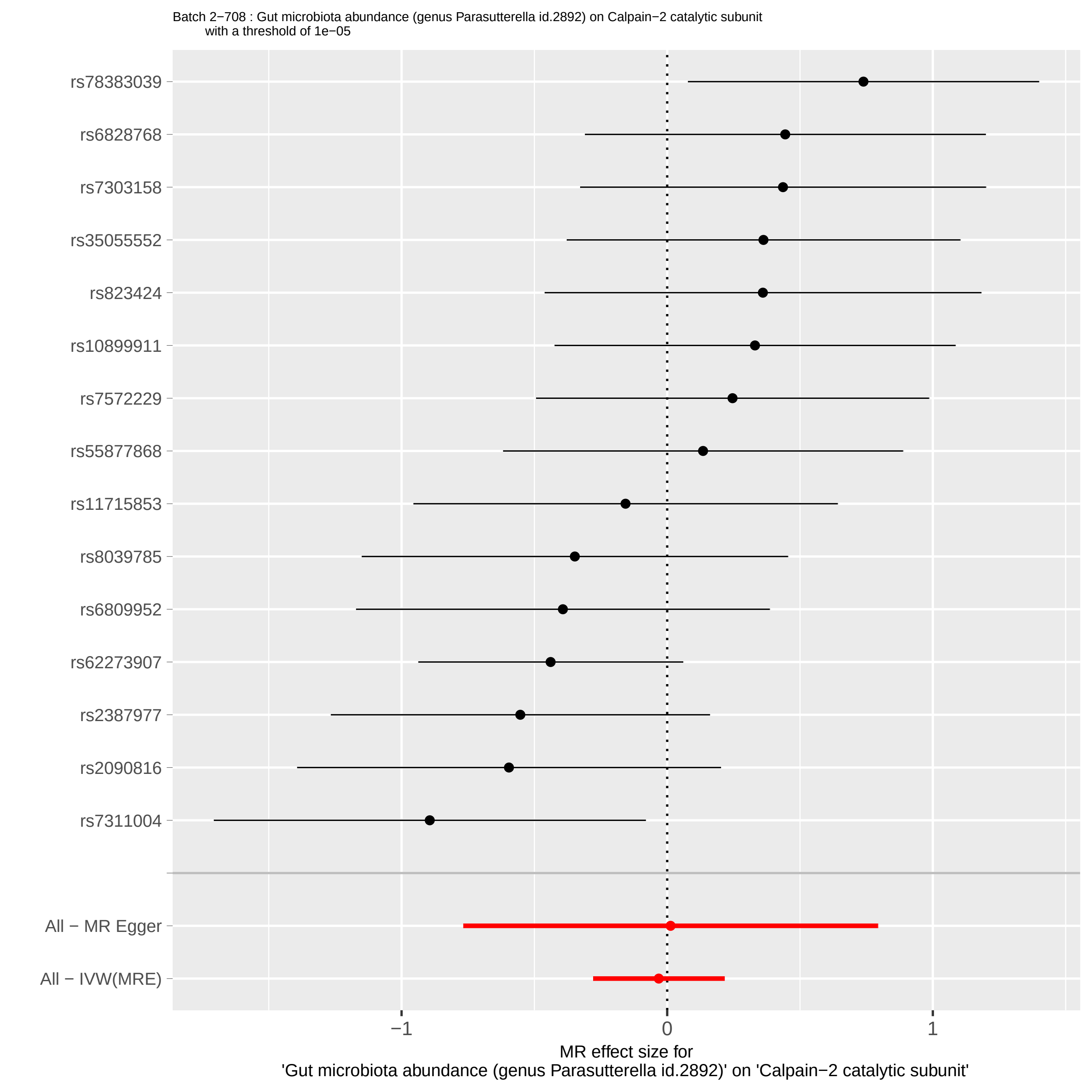

Batch 2−708 : Gut microbiota abundance (genus Parasutterella id.2892) on Calpain−2 catalytic subunit
with a threshold of 1e−05
rs78383039
rs6828768
rs7303158
rs35055552
rs823424
rs10899911
rs7572229
rs55877868
rs11715853
rs8039785
rs6809952
rs62273907
rs2387977
rs2090816
rs7311004
All − MR Egger
All − IVW(MRE)
−1
0
1
MR effect size for
'Gut microbiota abundance (genus Parasutterella id.2892)' on 'Calpain−2 catalytic subunit'

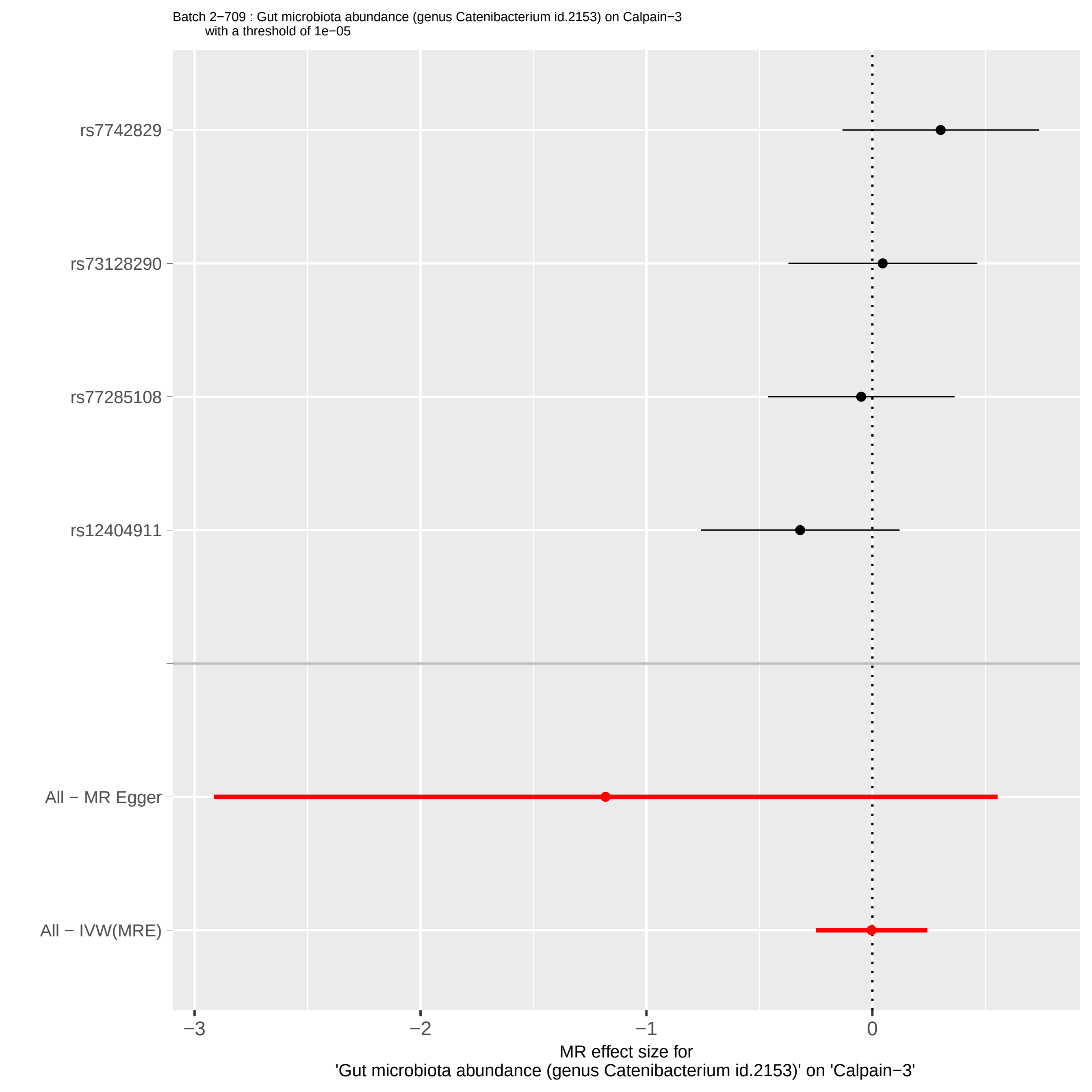

Batch 2−709 : Gut microbiota abundance (genus Catenibacterium id.2153) on Calpain−3
with a threshold of 1e−05
rs7742829
rs73128290
rs77285108
rs12404911
All − MR Egger
All − IVW(MRE)
−3
−2
−1
0
MR effect size for
'Gut microbiota abundance (genus Catenibacterium id.2153)' on 'Calpain−3'

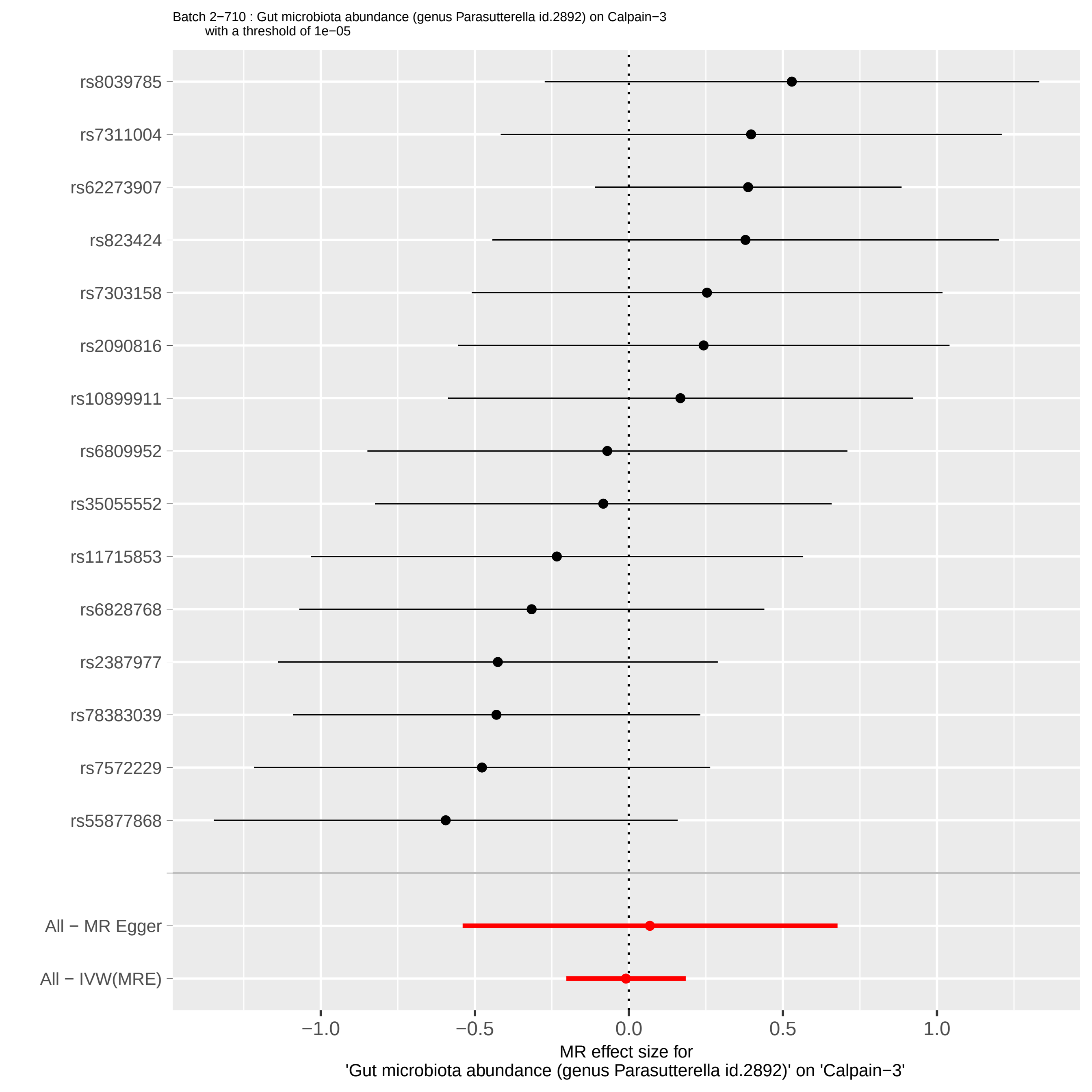

Batch 2−710 : Gut microbiota abundance (genus Parasutterella id.2892) on Calpain−3
with a threshold of 1e−05
rs8039785
rs7311004
rs62273907
rs823424
rs7303158
rs2090816
rs10899911
rs6809952
rs35055552
rs11715853
rs6828768
rs2387977
rs78383039
rs7572229
rs55877868
All − MR Egger
All − IVW(MRE)
−1.0
−0.5
0.0
0.5
1.0
MR effect size for
'Gut microbiota abundance (genus Parasutterella id.2892)' on 'Calpain−3'

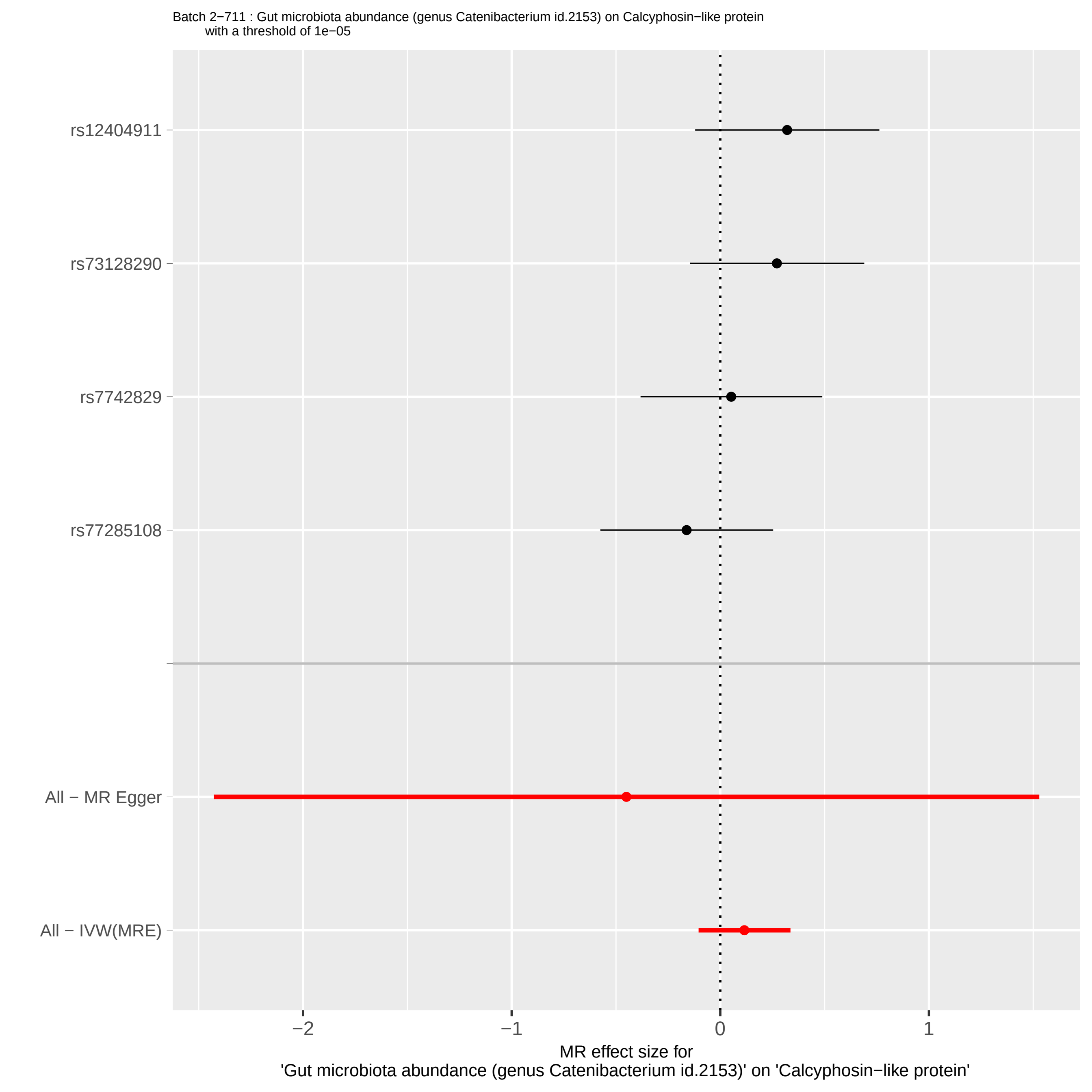
Batch 2−711 : Gut microbiota abundance (genus Catenibacterium id.2153) on Calcyphosin−like protein
with a threshold of 1e−05
rs12404911
rs73128290
rs7742829
rs77285108
All − MR Egger
All − IVW(MRE)
−2
−1
0
1
MR effect size for
'Gut microbiota abundance (genus Catenibacterium id.2153)' on 'Calcyphosin−like protein'

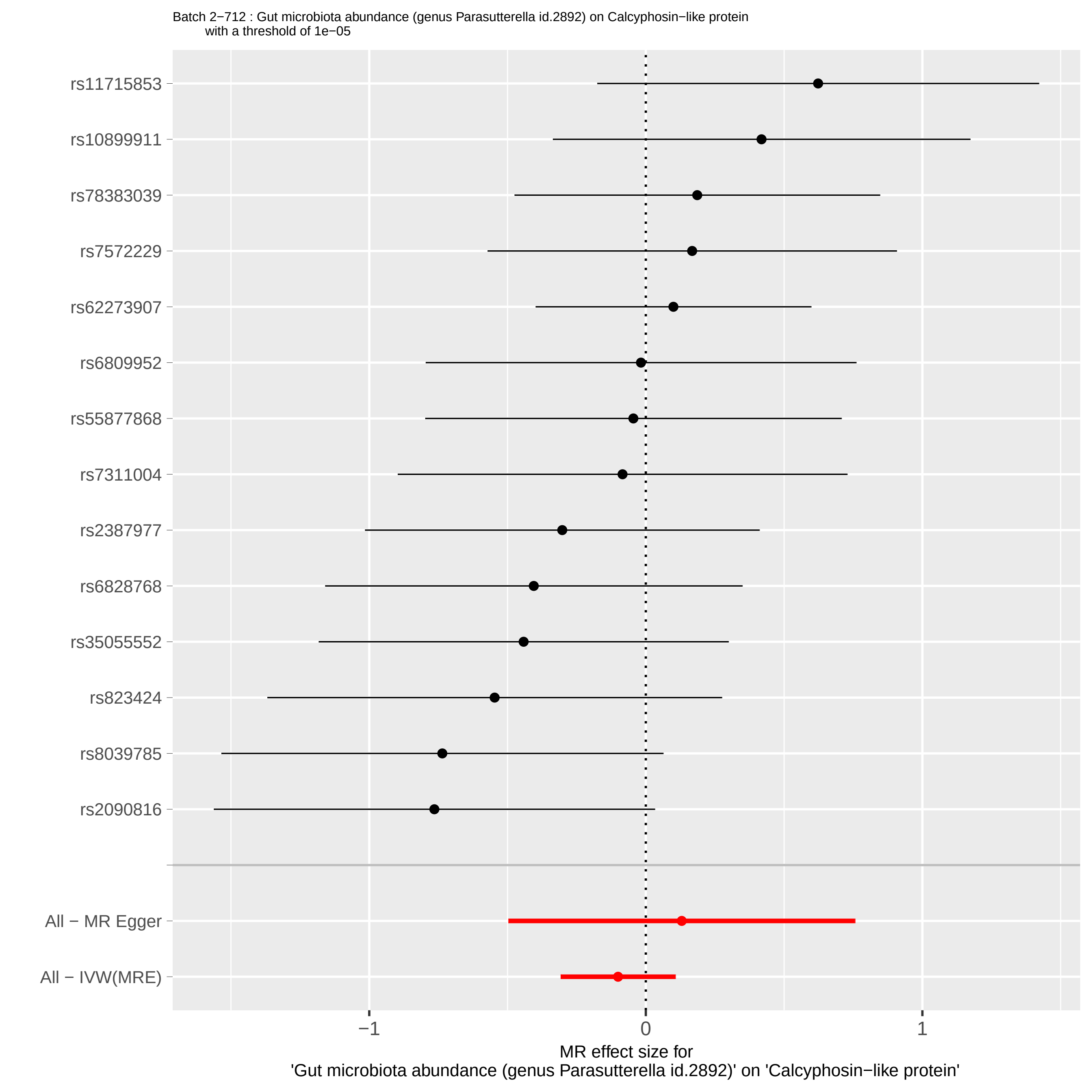

Batch 2−712 : Gut microbiota abundance (genus Parasutterella id.2892) on Calcyphosin−like protein
with a threshold of 1e−05
rs11715853
rs10899911
rs78383039
rs7572229
rs62273907
rs6809952
rs55877868
rs7311004
rs2387977
rs6828768
rs35055552
rs823424
rs8039785
rs2090816
All − MR Egger
All − IVW(MRE)
−1
0
1
MR effect size for
'Gut microbiota abundance (genus Parasutterella id.2892)' on 'Calcyphosin−like protein'

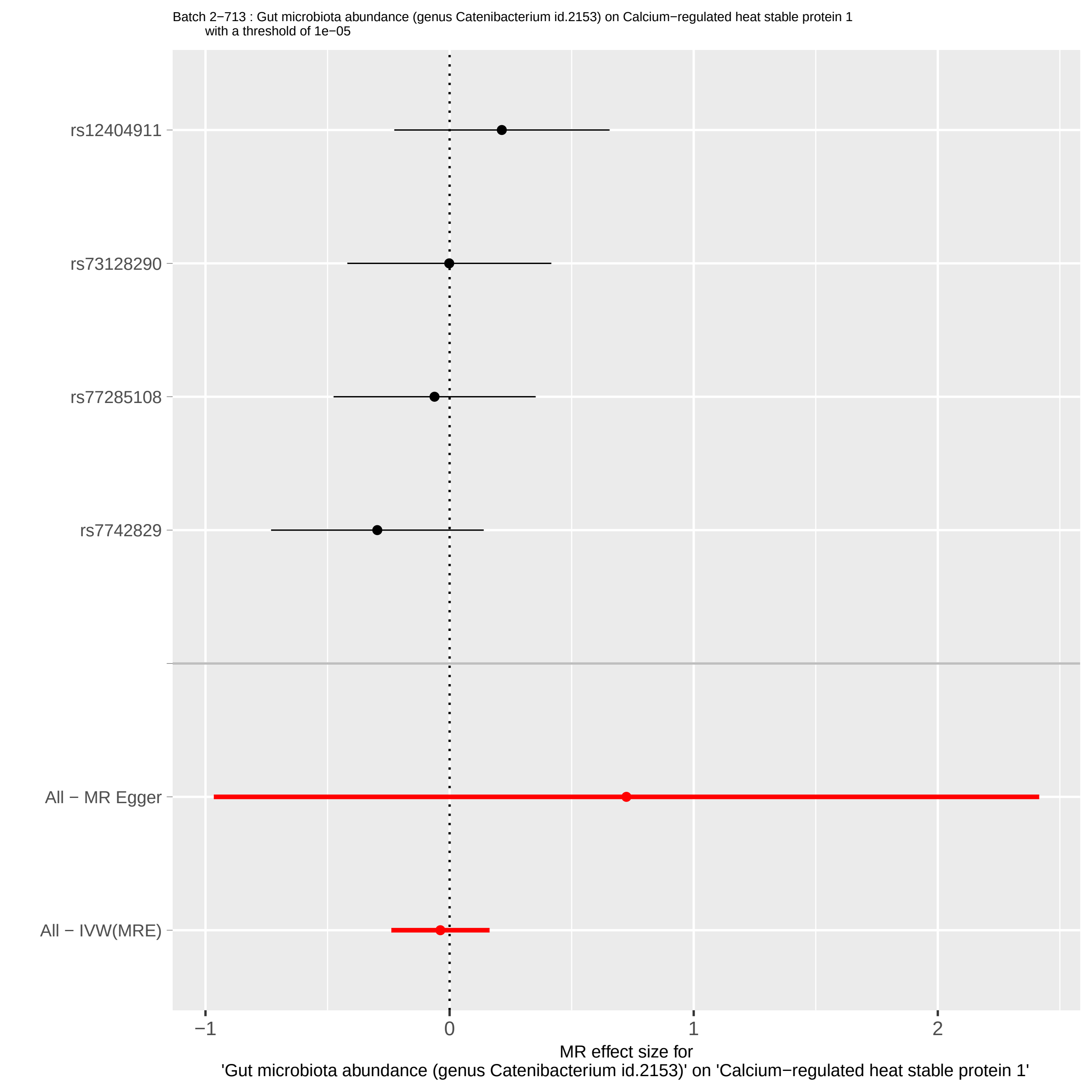
Batch 2−713 : Gut microbiota abundance (genus Catenibacterium id.2153) on Calcium−regulated heat stable protein 1
with a threshold of 1e−05
rs12404911
rs73128290
rs77285108
rs7742829
All − MR Egger
All − IVW(MRE)
−1
0
1
2
MR effect size for
'Gut microbiota abundance (genus Catenibacterium id.2153)' on 'Calcium−regulated heat stable protein 1'

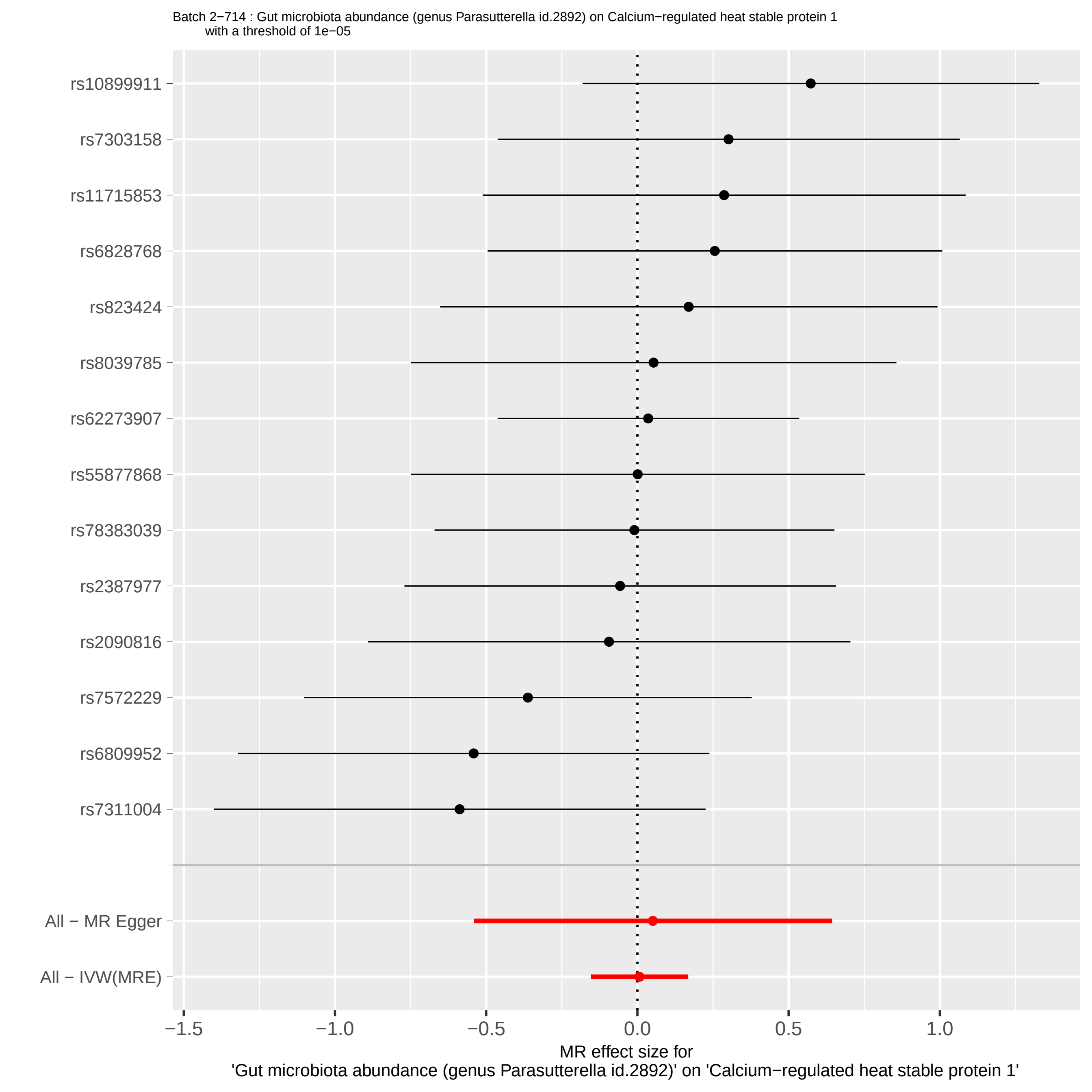

Batch 2−714 : Gut microbiota abundance (genus Parasutterella id.2892) on Calcium−regulated heat stable protein 1
with a threshold of 1e−05
rs10899911
rs7303158
rs11715853
rs6828768
rs823424
rs8039785
rs62273907
rs55877868
rs78383039
rs2387977
rs2090816
rs7572229
rs6809952
rs7311004
All − MR Egger
All − IVW(MRE)
−1.5
−1.0
−0.5
0.0
0.5
1.0
MR effect size for
'Gut microbiota abundance (genus Parasutterella id.2892)' on 'Calcium−regulated heat stable protein 1'

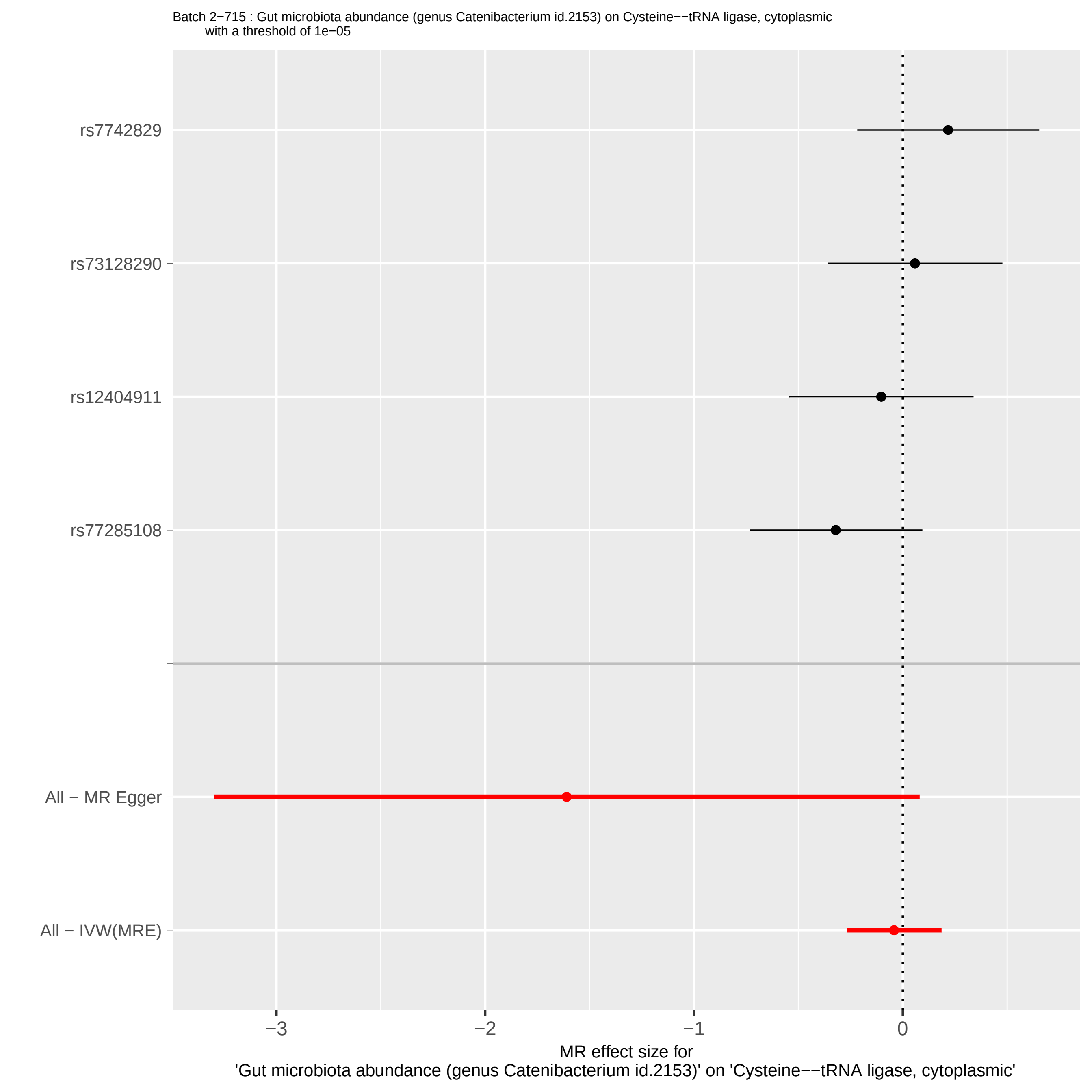
Batch 2−715 : Gut microbiota abundance (genus Catenibacterium id.2153) on Cysteine−−tRNA ligase, cytoplasmic
with a threshold of 1e−05
rs7742829
rs73128290
rs12404911
rs77285108
All − MR Egger
All − IVW(MRE)
−3
−2
−1
0
MR effect size for
'Gut microbiota abundance (genus Catenibacterium id.2153)' on 'Cysteine−−tRNA ligase, cytoplasmic'

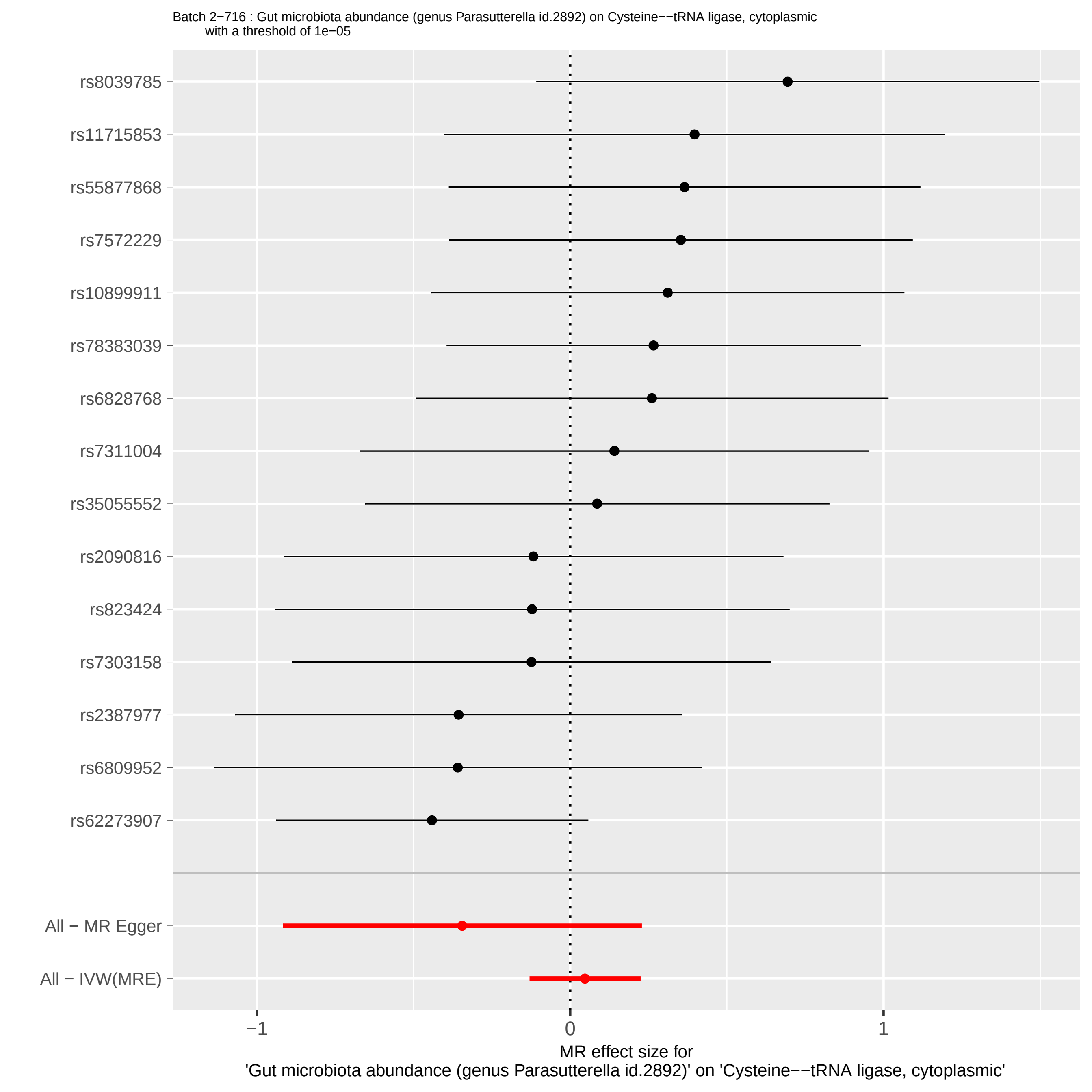

Batch 2−716 : Gut microbiota abundance (genus Parasutterella id.2892) on Cysteine−−tRNA ligase, cytoplasmic
with a threshold of 1e−05
rs8039785
rs11715853
rs55877868
rs7572229
rs10899911
rs78383039
rs6828768
rs7311004
rs35055552
rs2090816
rs823424
rs7303158
rs2387977
rs6809952
rs62273907
All − MR Egger
All − IVW(MRE)
−1
0
1
MR effect size for
'Gut microbiota abundance (genus Parasutterella id.2892)' on 'Cysteine−−tRNA ligase, cytoplasmic'

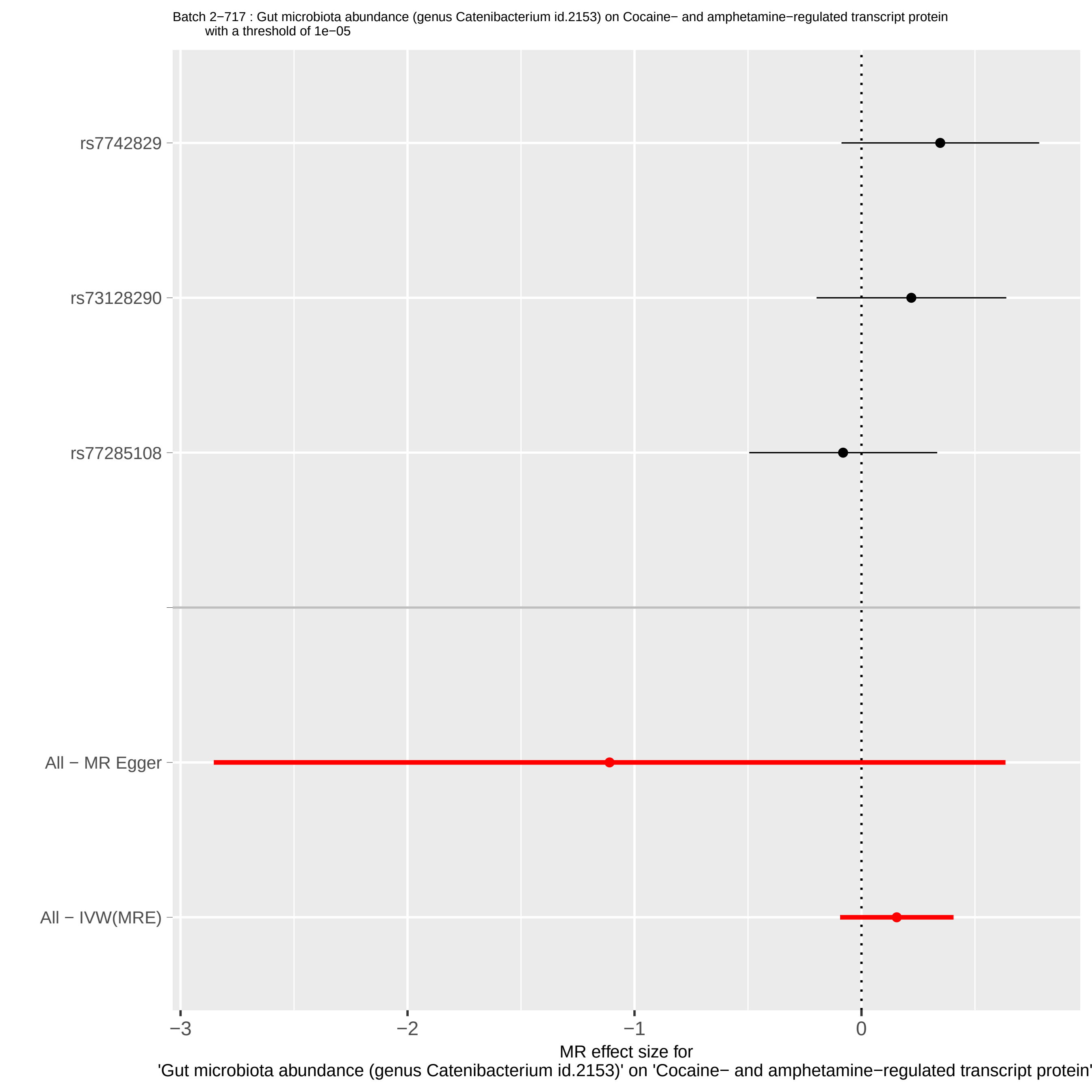

Batch 2−717 : Gut microbiota abundance (genus Catenibacterium id.2153) on Cocaine− and amphetamine−regulated transcript protein
with a threshold of 1e−05
rs7742829
rs73128290
rs77285108
All − MR Egger
All − IVW(MRE)
−3
−2
−1
0
MR effect size for
'Gut microbiota abundance (genus Catenibacterium id.2153)' on 'Cocaine− and amphetamine−regulated transcript protein'

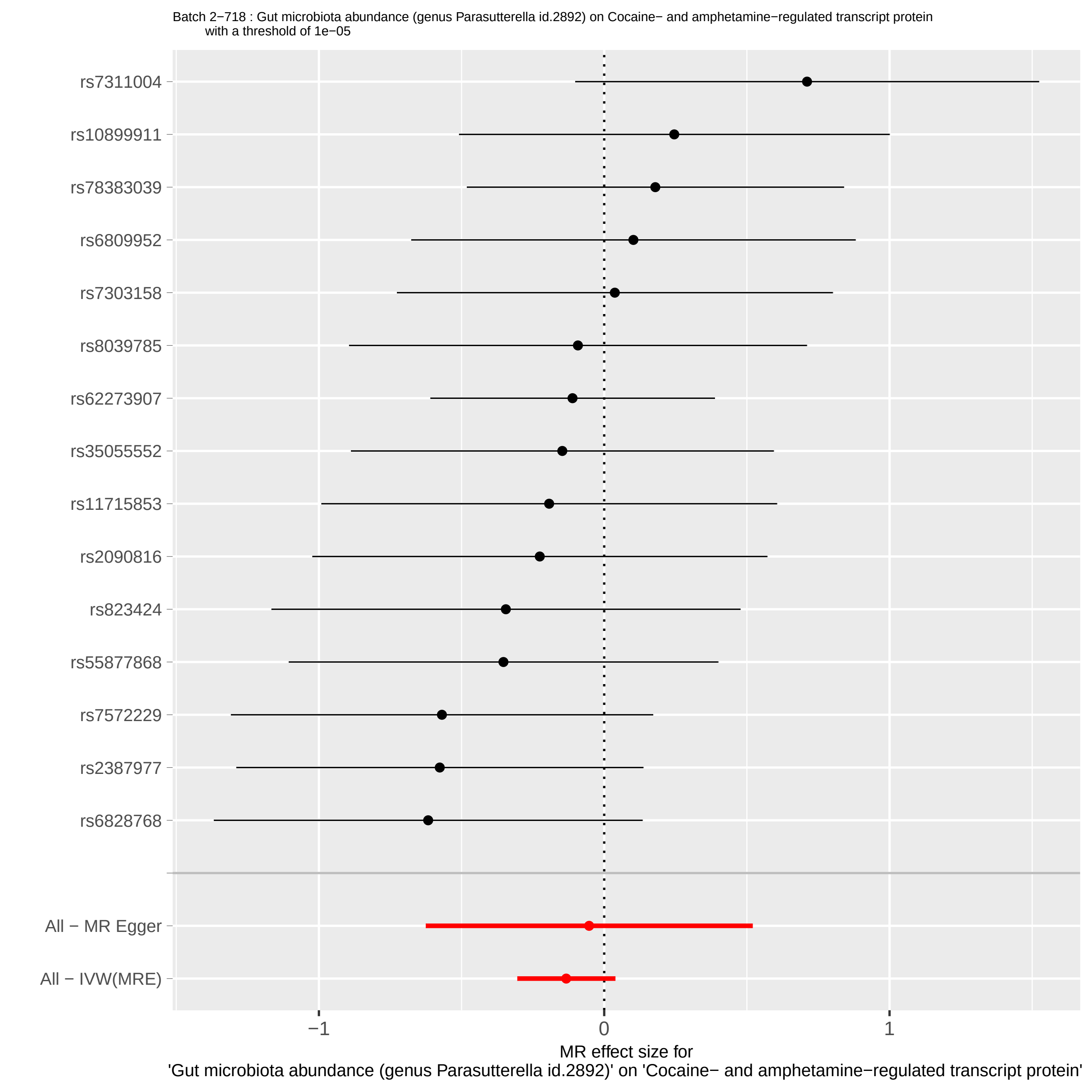

Batch 2−718 : Gut microbiota abundance (genus Parasutterella id.2892) on Cocaine− and amphetamine−regulated transcript protein
with a threshold of 1e−05
rs7311004
rs10899911
rs78383039
rs6809952
rs7303158
rs8039785
rs62273907
rs35055552
rs11715853
rs2090816
rs823424
rs55877868
rs7572229
rs2387977
rs6828768
All − MR Egger
All − IVW(MRE)
−1
0
1
MR effect size for
'Gut microbiota abundance (genus Parasutterella id.2892)' on 'Cocaine− and amphetamine−regulated transcript protein'

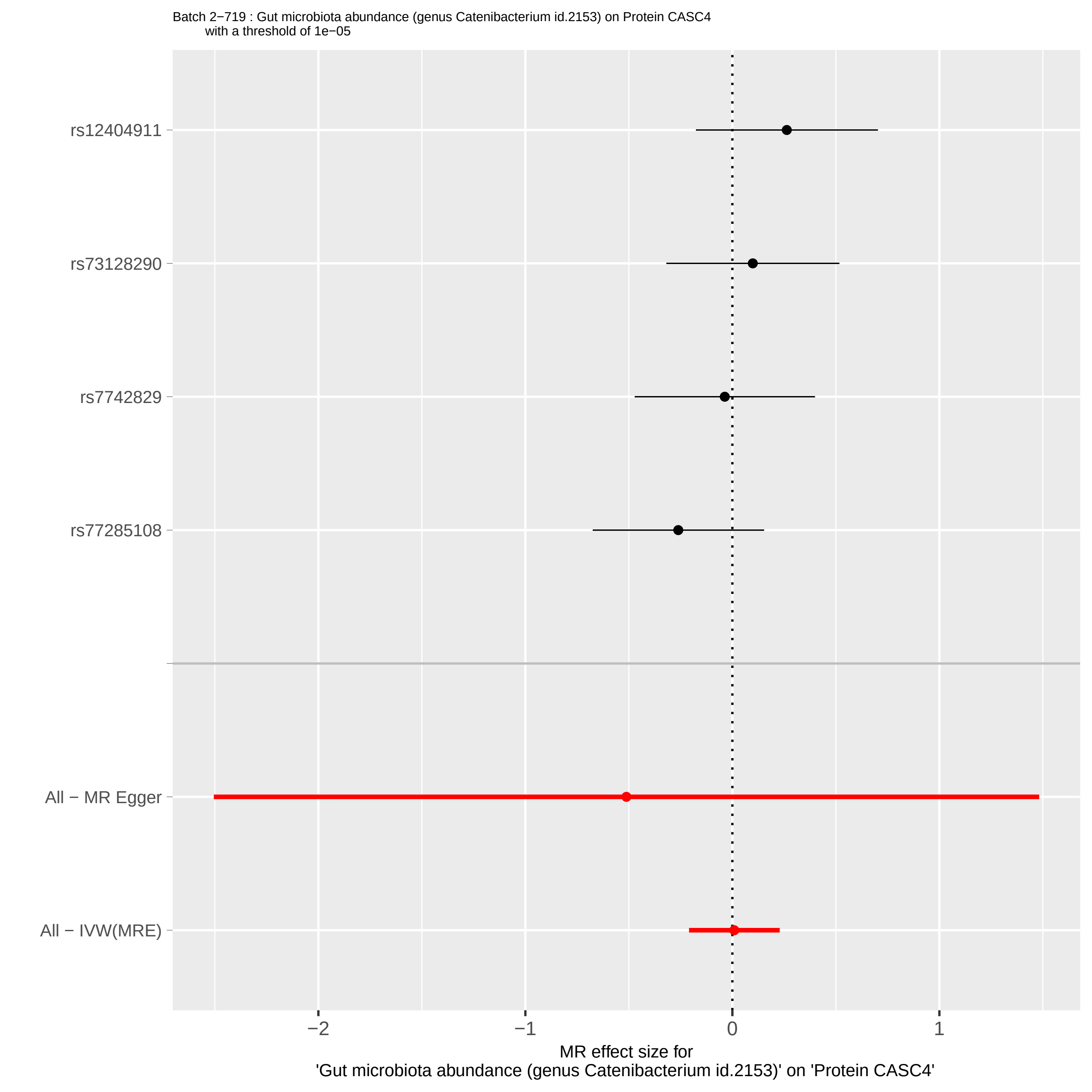
Batch 2−719 : Gut microbiota abundance (genus Catenibacterium id.2153) on Protein CASC4
with a threshold of 1e−05
rs12404911
rs73128290
rs7742829
rs77285108
All − MR Egger
All − IVW(MRE)
−2
−1
0
1
MR effect size for
'Gut microbiota abundance (genus Catenibacterium id.2153)' on 'Protein CASC4'

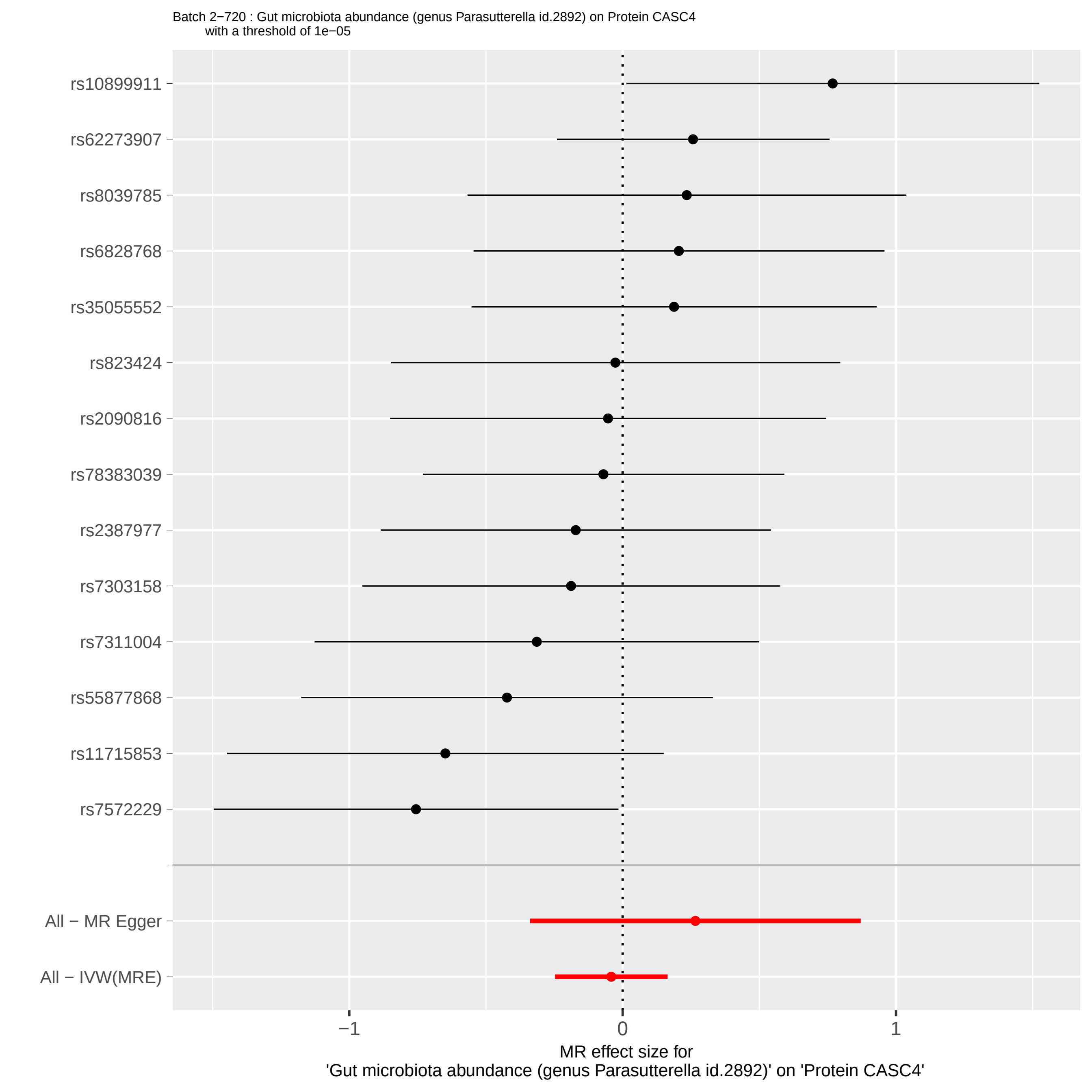
Batch 2−720 : Gut microbiota abundance (genus Parasutterella id.2892) on Protein CASC4
with a threshold of 1e−05
rs10899911
rs62273907
rs8039785
rs6828768
rs35055552
rs823424
rs2090816
rs78383039
rs2387977
rs7303158
rs7311004
rs55877868
rs11715853
rs7572229
All − MR Egger
All − IVW(MRE)
−1
0
1
MR effect size for
'Gut microbiota abundance (genus Parasutterella id.2892)' on 'Protein CASC4'

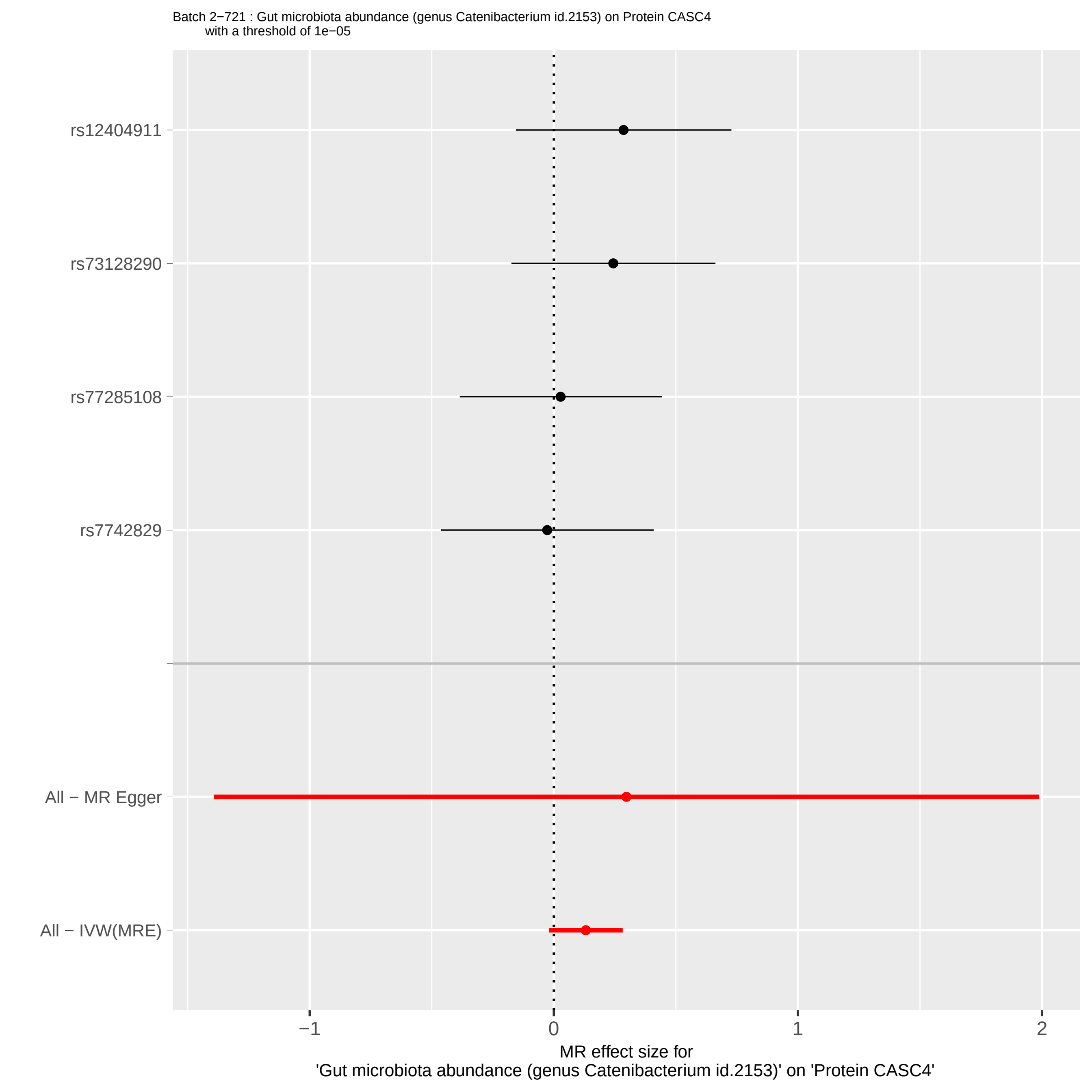

Batch 2−721 : Gut microbiota abundance (genus Catenibacterium id.2153) on Protein CASC4
with a threshold of 1e−05
rs12404911
rs73128290
rs77285108
rs7742829
All − MR Egger
All − IVW(MRE)
−1
0
1
2
MR effect size for
'Gut microbiota abundance (genus Catenibacterium id.2153)' on 'Protein CASC4'

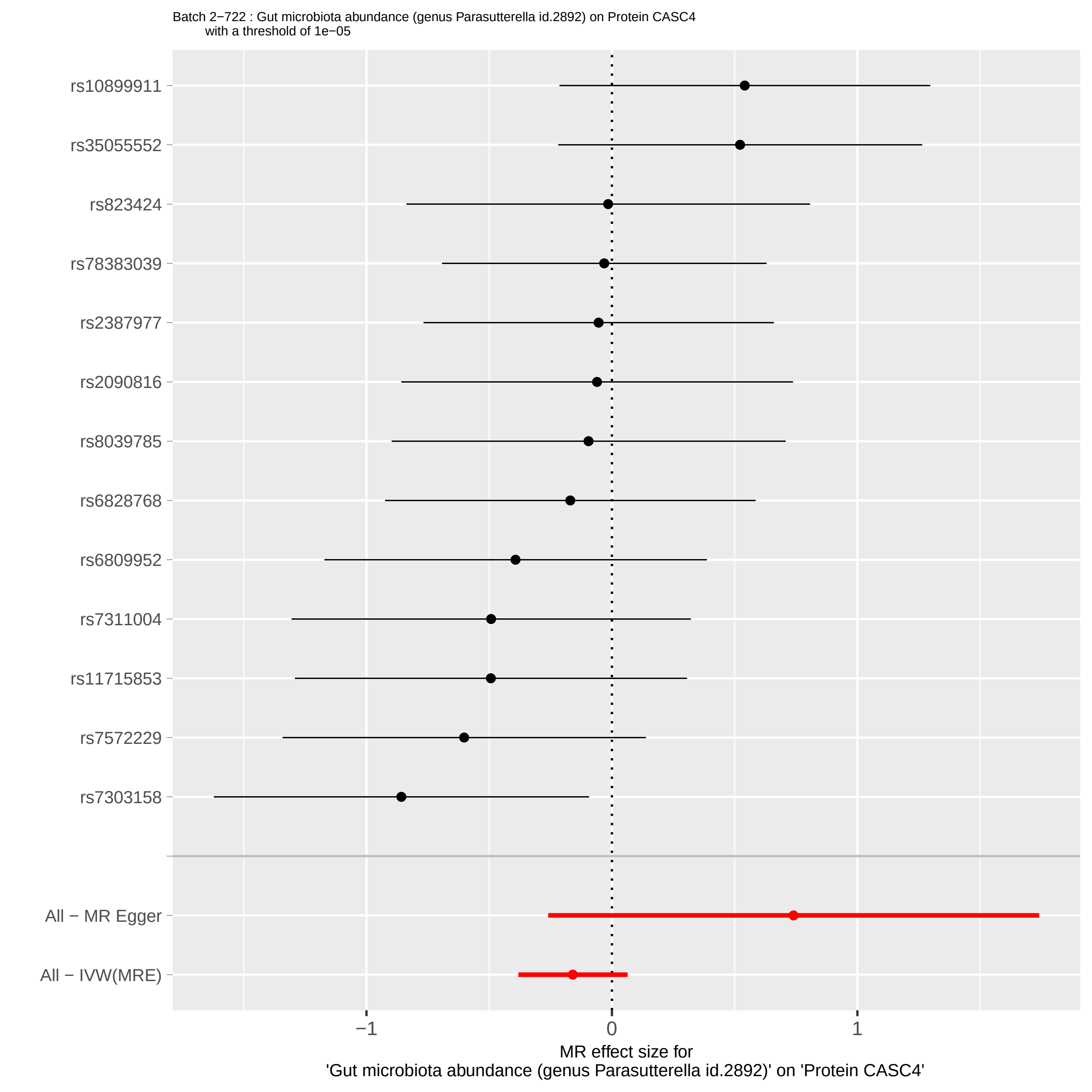
Batch 2−722 : Gut microbiota abundance (genus Parasutterella id.2892) on Protein CASC4
with a threshold of 1e−05
rs10899911
rs35055552
rs823424
rs78383039
rs2387977
rs2090816
rs8039785
rs6828768
rs6809952
rs7311004
rs11715853
rs7572229
rs7303158
All − MR Egger
All − IVW(MRE)
−1
0
1
MR effect size for
'Gut microbiota abundance (genus Parasutterella id.2892)' on 'Protein CASC4'

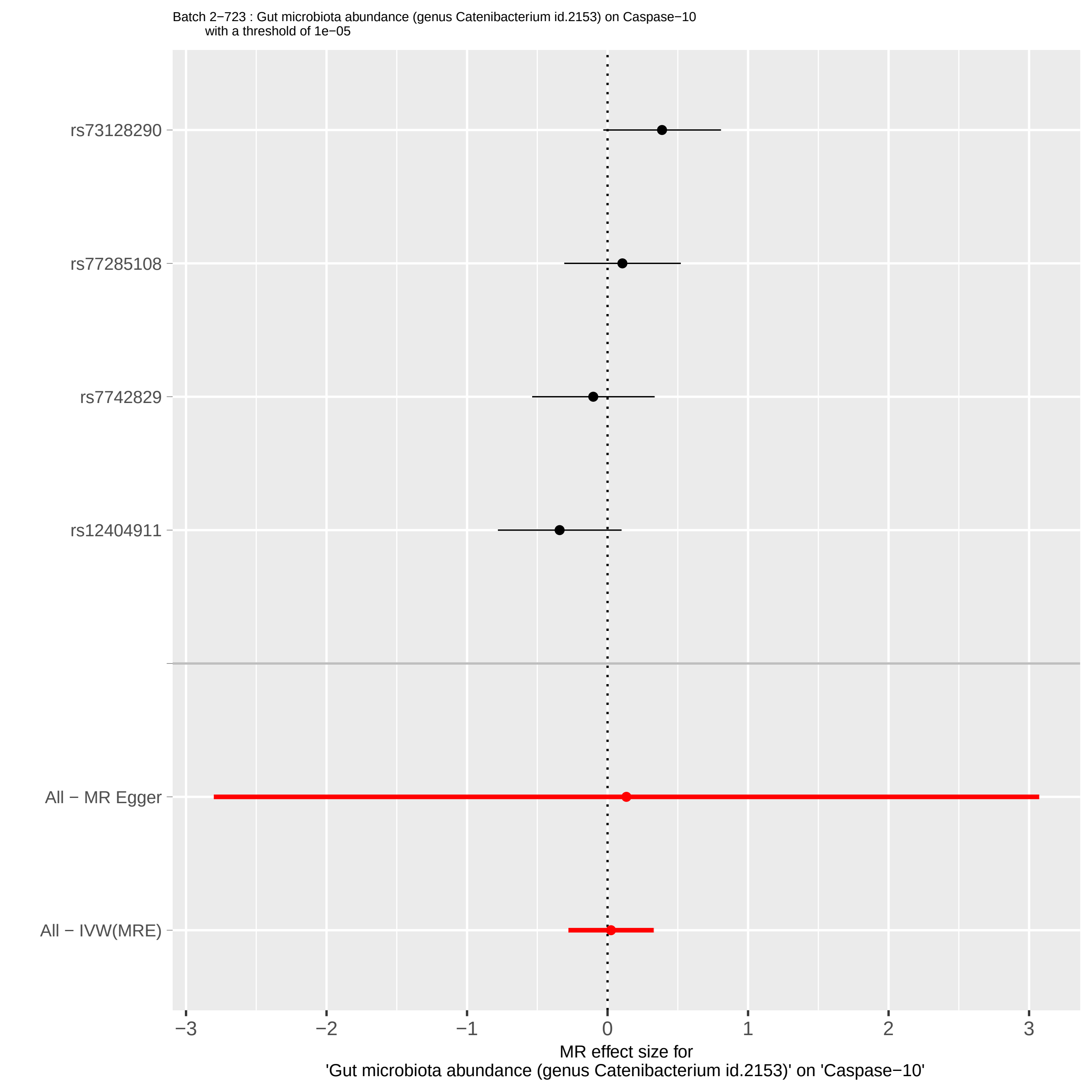

Batch 2−723 : Gut microbiota abundance (genus Catenibacterium id.2153) on Caspase−10
with a threshold of 1e−05
rs73128290
rs77285108
rs7742829
rs12404911
All − MR Egger
All − IVW(MRE)
−3
−2
−1
0
1
2
3
MR effect size for
'Gut microbiota abundance (genus Catenibacterium id.2153)' on 'Caspase−10'

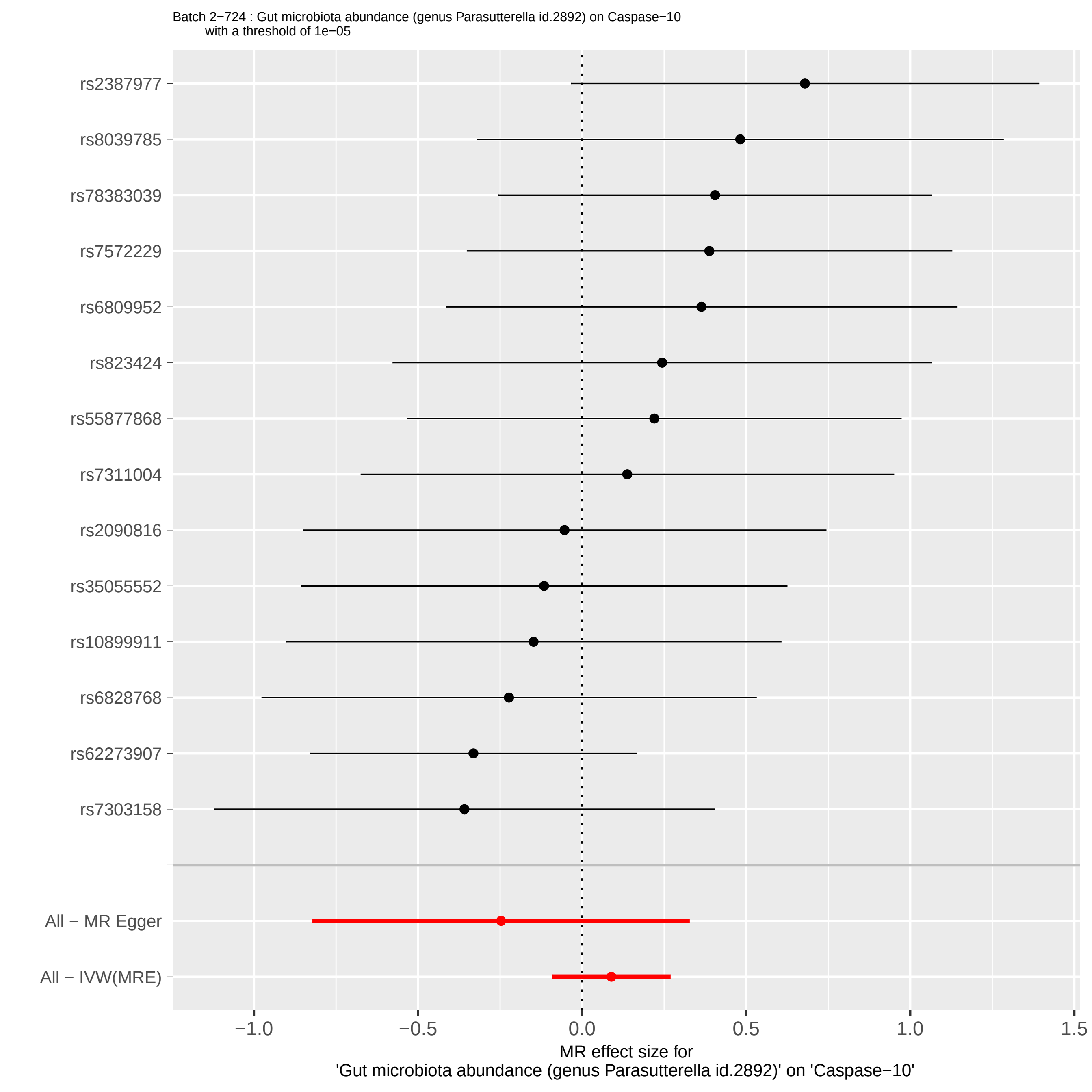
Batch 2−724 : Gut microbiota abundance (genus Parasutterella id.2892) on Caspase−10
with a threshold of 1e−05
rs2387977
rs8039785
rs78383039
rs7572229
rs6809952
rs823424
rs55877868
rs7311004
rs2090816
rs35055552
rs10899911
rs6828768
rs62273907
rs7303158
All − MR Egger
All − IVW(MRE)
−1.0
−0.5
0.0
0.5
1.0
1.5
MR effect size for
'Gut microbiota abundance (genus Parasutterella id.2892)' on 'Caspase−10'

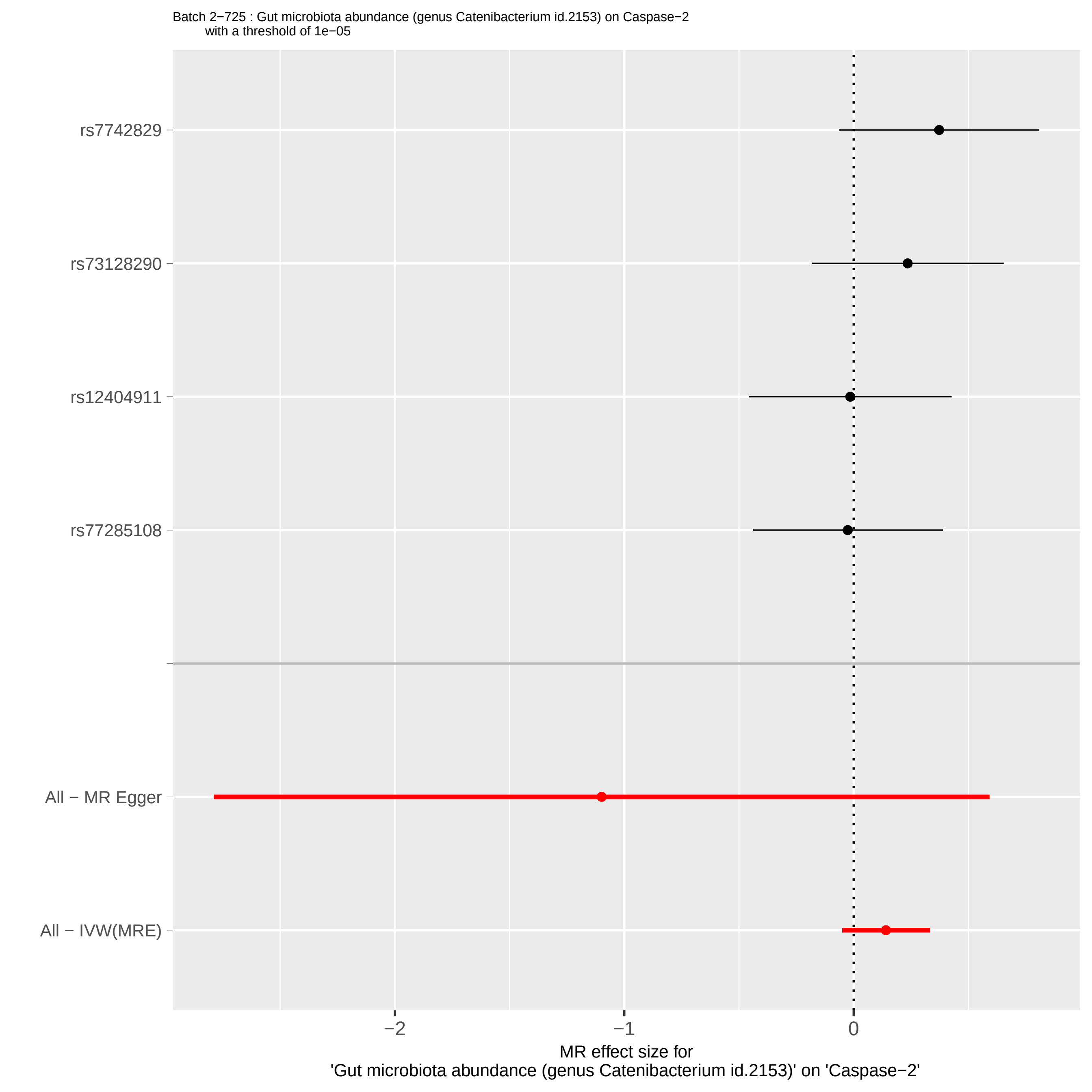
Batch 2−725 : Gut microbiota abundance (genus Catenibacterium id.2153) on Caspase−2
with a threshold of 1e−05
rs7742829
rs73128290
rs12404911
rs77285108
All − MR Egger
All − IVW(MRE)
−2
−1
0
MR effect size for
'Gut microbiota abundance (genus Catenibacterium id.2153)' on 'Caspase−2'

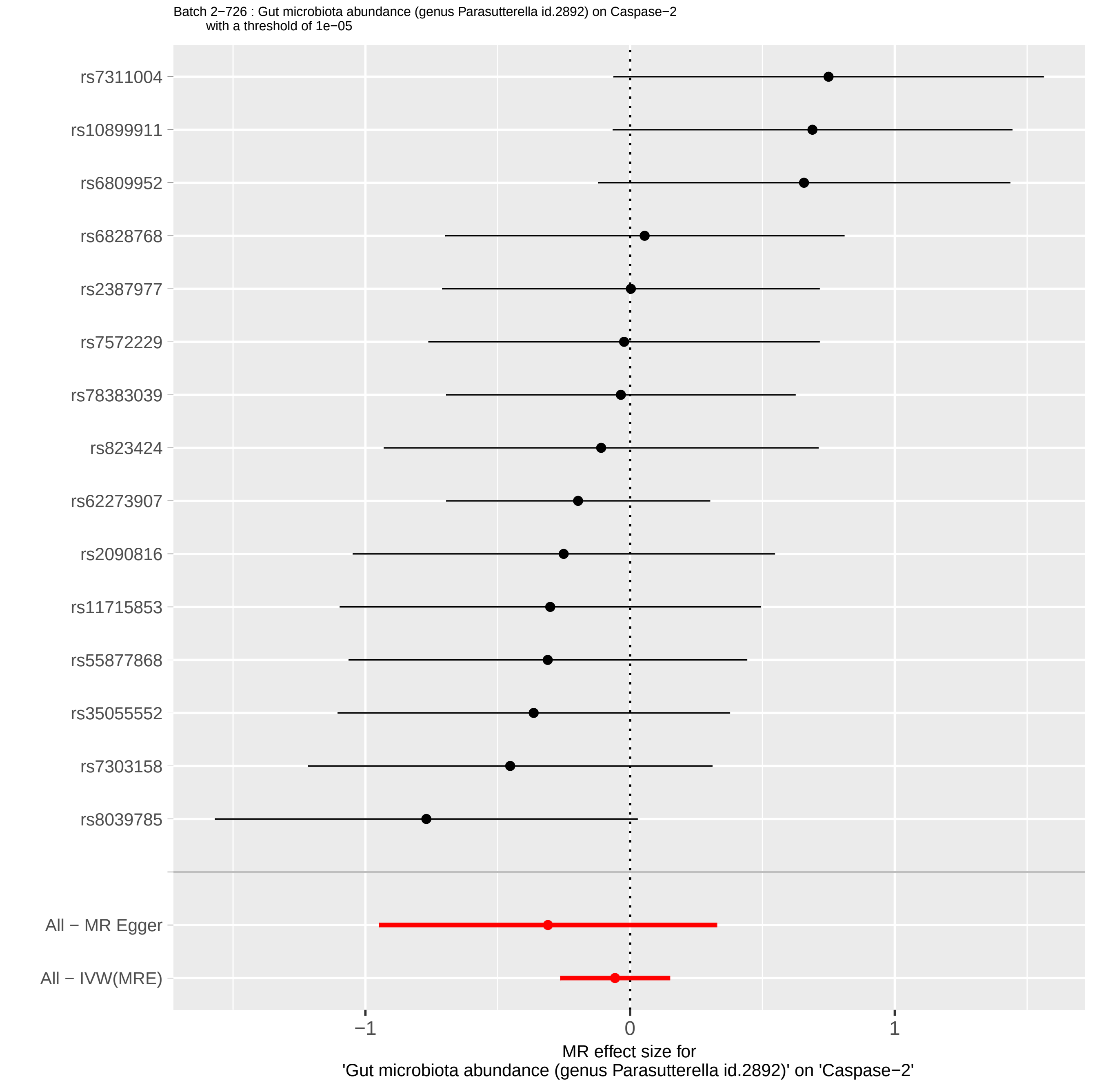

Batch 2−726 : Gut microbiota abundance (genus Parasutterella id.2892) on Caspase−2
with a threshold of 1e−05
rs7311004
rs10899911
rs6809952
rs6828768
rs2387977
rs7572229
rs78383039
rs823424
rs62273907
rs2090816
rs11715853
rs55877868
rs35055552
rs7303158
rs8039785
All − MR Egger
All − IVW(MRE)
−1
0
1
MR effect size for
'Gut microbiota abundance (genus Parasutterella id.2892)' on 'Caspase−2'

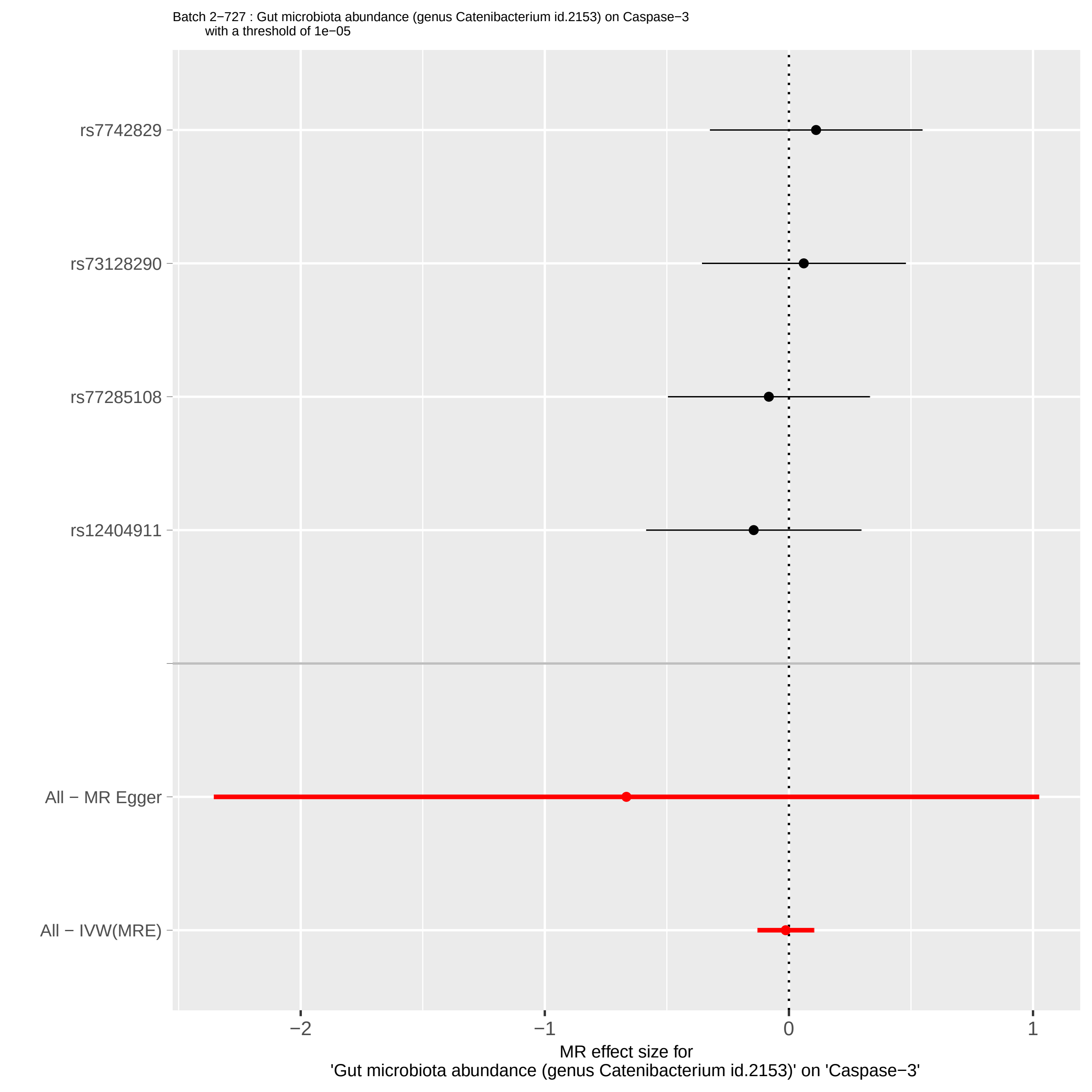

Batch 2−727 : Gut microbiota abundance (genus Catenibacterium id.2153) on Caspase−3
with a threshold of 1e−05
rs7742829
rs73128290
rs77285108
rs12404911
All − MR Egger
All − IVW(MRE)
−2
−1
0
1
MR effect size for
'Gut microbiota abundance (genus Catenibacterium id.2153)' on 'Caspase−3'

Batch 2−728 : Gut microbiota abundance (genus Parasutterella id.2892) on Caspase−3
with a threshold of 1e−05

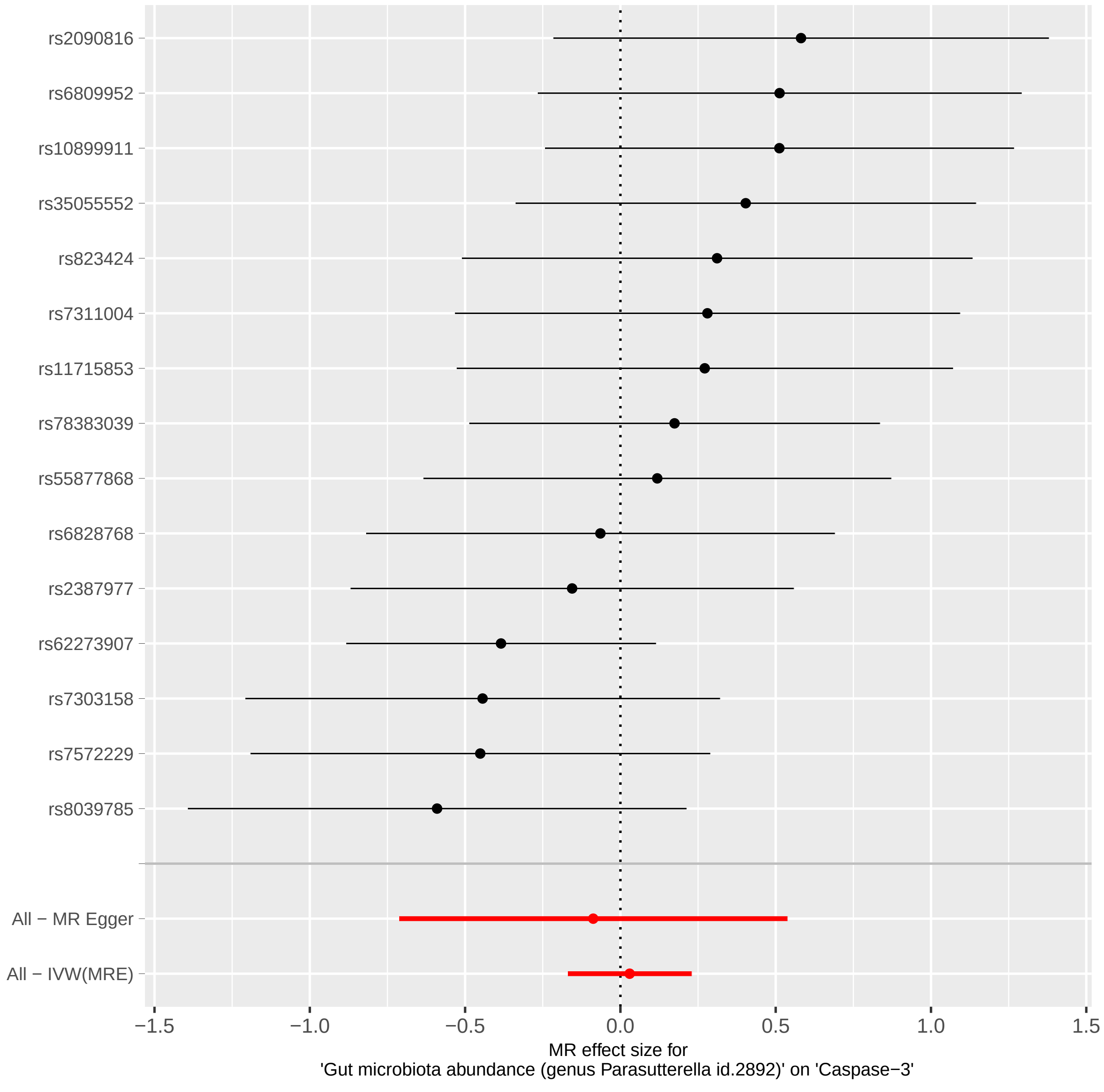

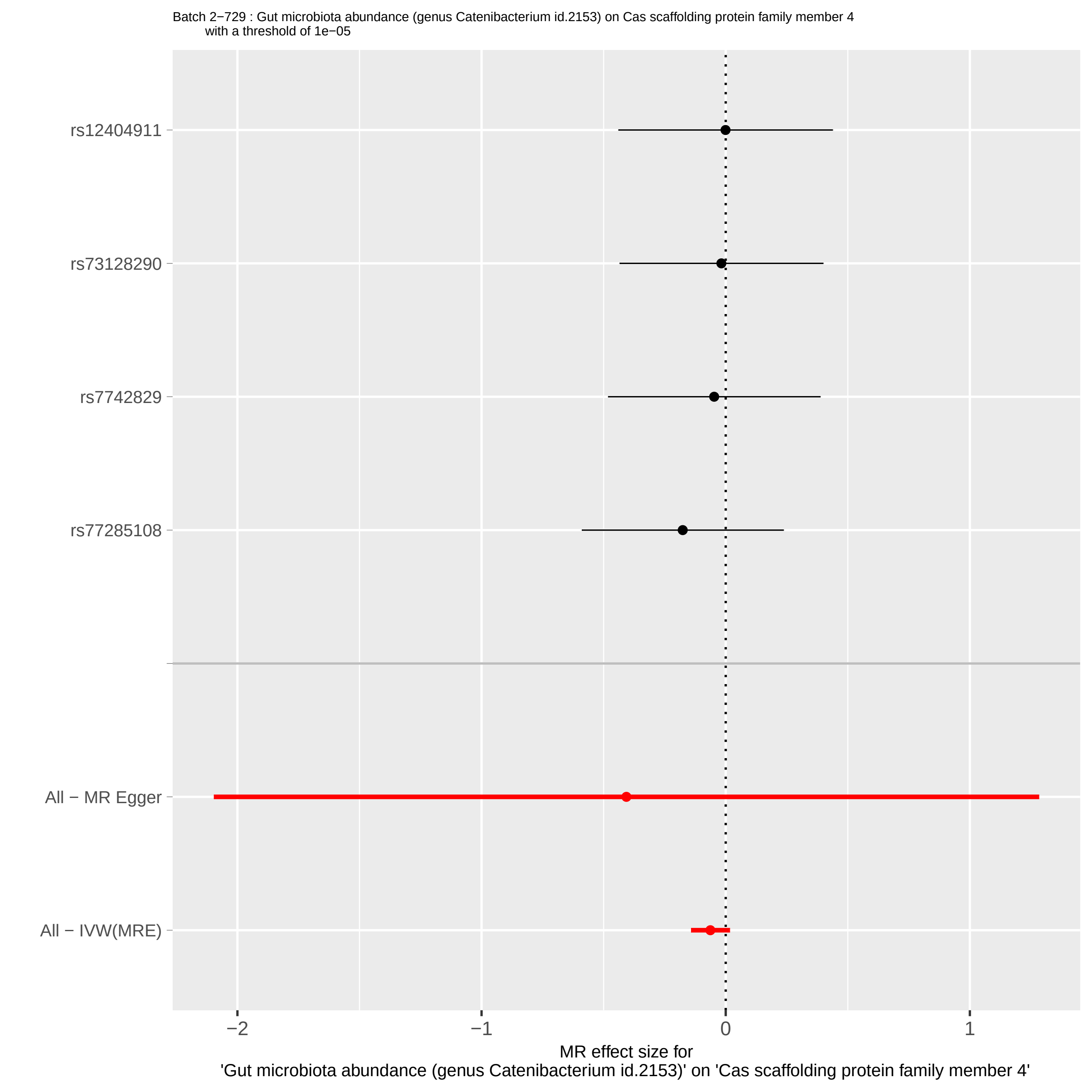
Batch 2−729 : Gut microbiota abundance (genus Catenibacterium id.2153) on Cas scaffolding protein family member 4
with a threshold of 1e−05
rs12404911
rs73128290
rs7742829
rs77285108
All − MR Egger
All − IVW(MRE)
−2
−1
0
1
MR effect size for
'Gut microbiota abundance (genus Catenibacterium id.2153)' on 'Cas scaffolding protein family member 4'

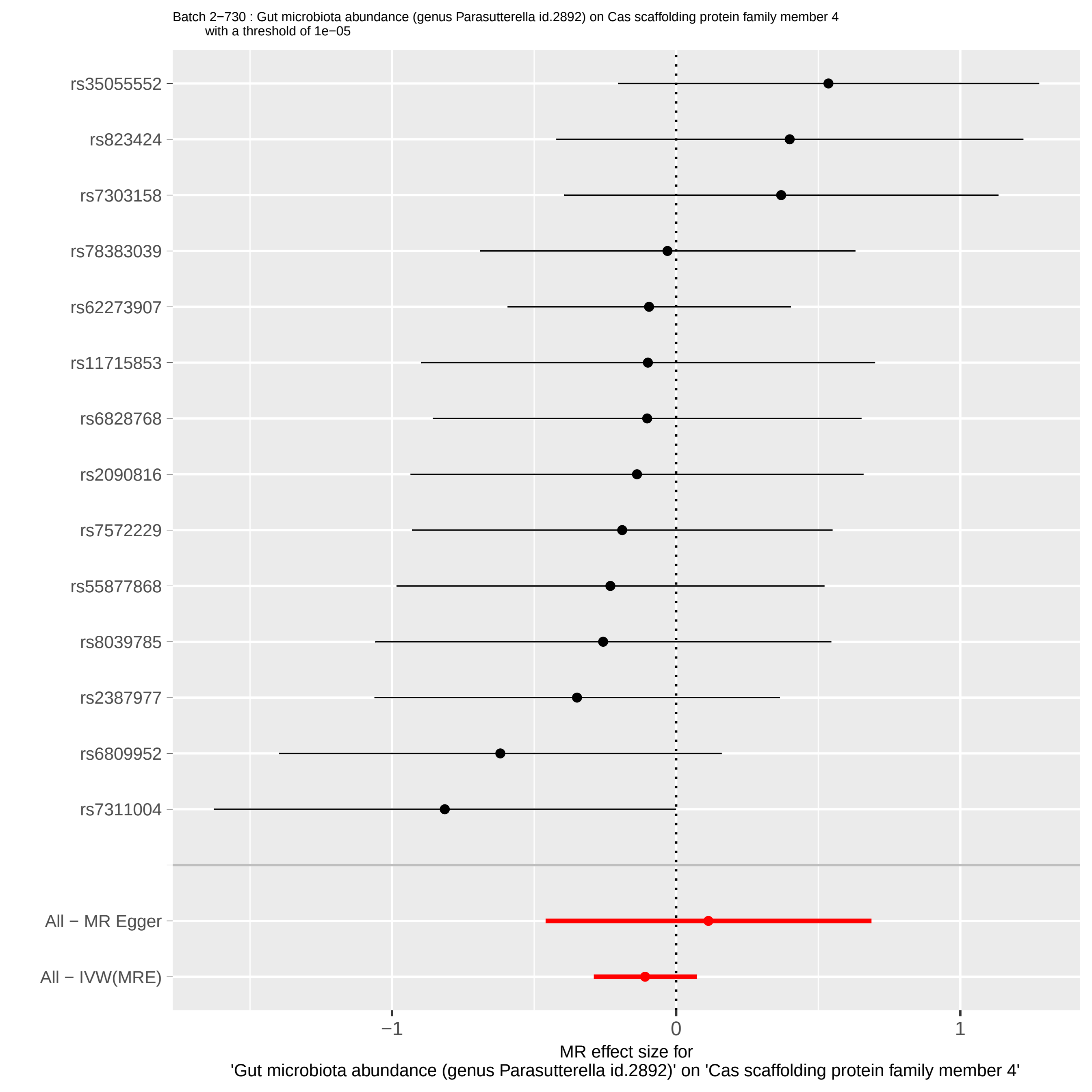

Batch 2−730 : Gut microbiota abundance (genus Parasutterella id.2892) on Cas scaffolding protein family member 4
with a threshold of 1e−05
rs35055552
rs823424
rs7303158
rs78383039
rs62273907
rs11715853
rs6828768
rs2090816
rs7572229
rs55877868
rs8039785
rs2387977
rs6809952
rs7311004
All − MR Egger
All − IVW(MRE)
−1
0
1
MR effect size for
'Gut microbiota abundance (genus Parasutterella id.2892)' on 'Cas scaffolding protein family member 4'

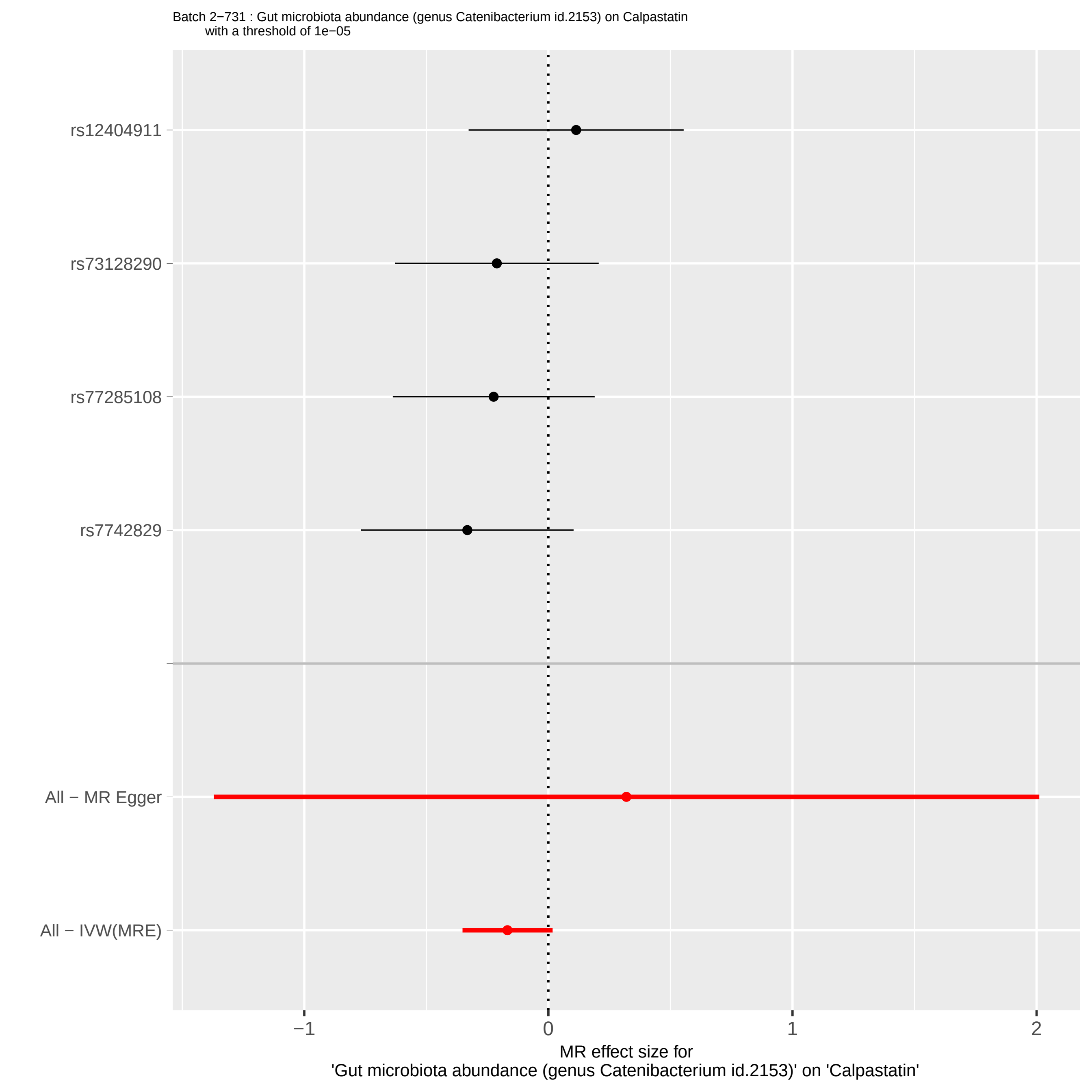

Batch 2−731 : Gut microbiota abundance (genus Catenibacterium id.2153) on Calpastatin
with a threshold of 1e−05
rs12404911
rs73128290
rs77285108
rs7742829
All − MR Egger
All − IVW(MRE)
−1
0
1
2
MR effect size for
'Gut microbiota abundance (genus Catenibacterium id.2153)' on 'Calpastatin'

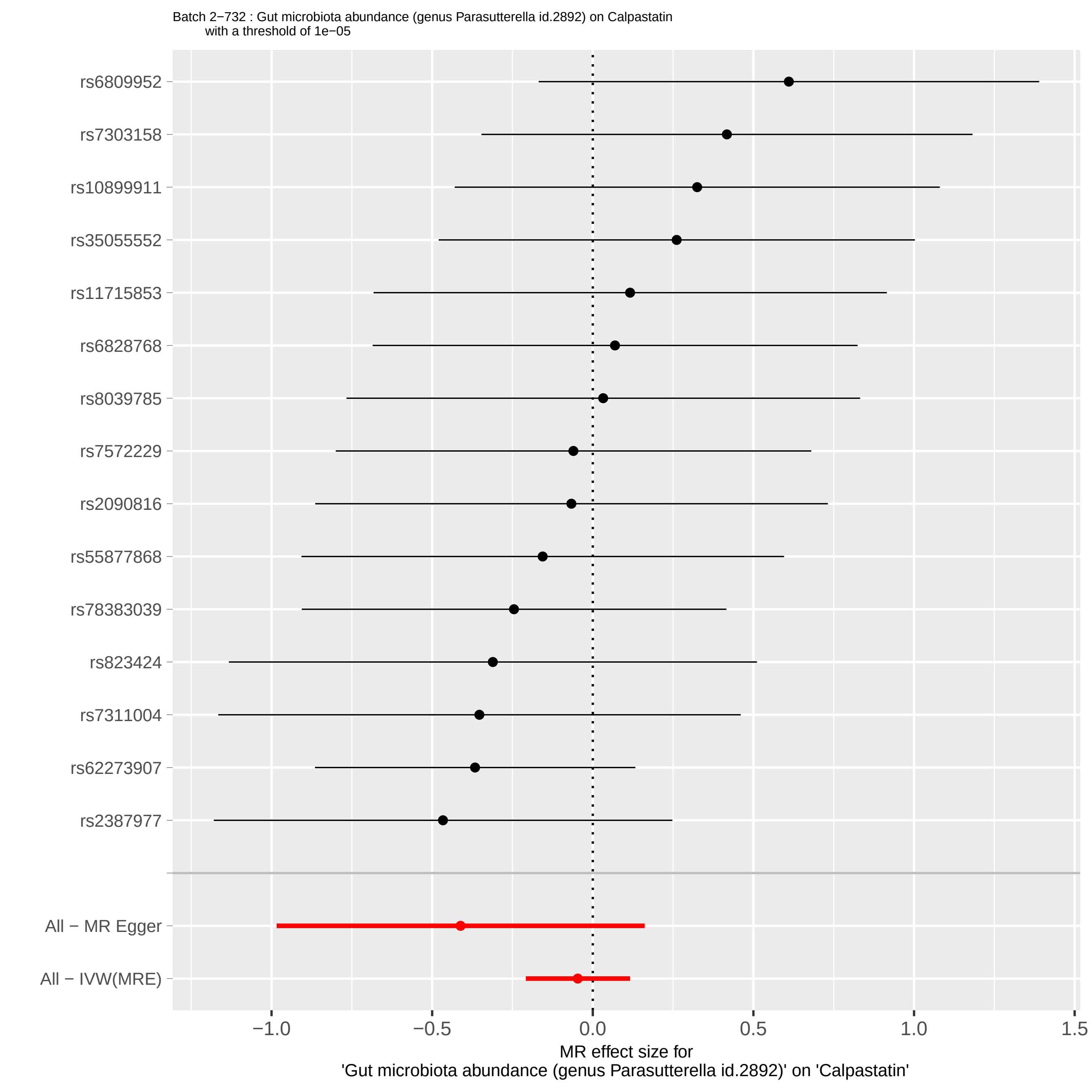

Batch 2−732 : Gut microbiota abundance (genus Parasutterella id.2892) on Calpastatin
with a threshold of 1e−05
rs6809952
rs7303158
rs10899911
rs35055552
rs11715853
rs6828768
rs8039785
rs7572229
rs2090816
rs55877868
rs78383039
rs823424
rs7311004
rs62273907
rs2387977
All − MR Egger
All − IVW(MRE)
−1.0
−0.5
0.0
0.5
1.0
1.5
MR effect size for
'Gut microbiota abundance (genus Parasutterella id.2892)' on 'Calpastatin'

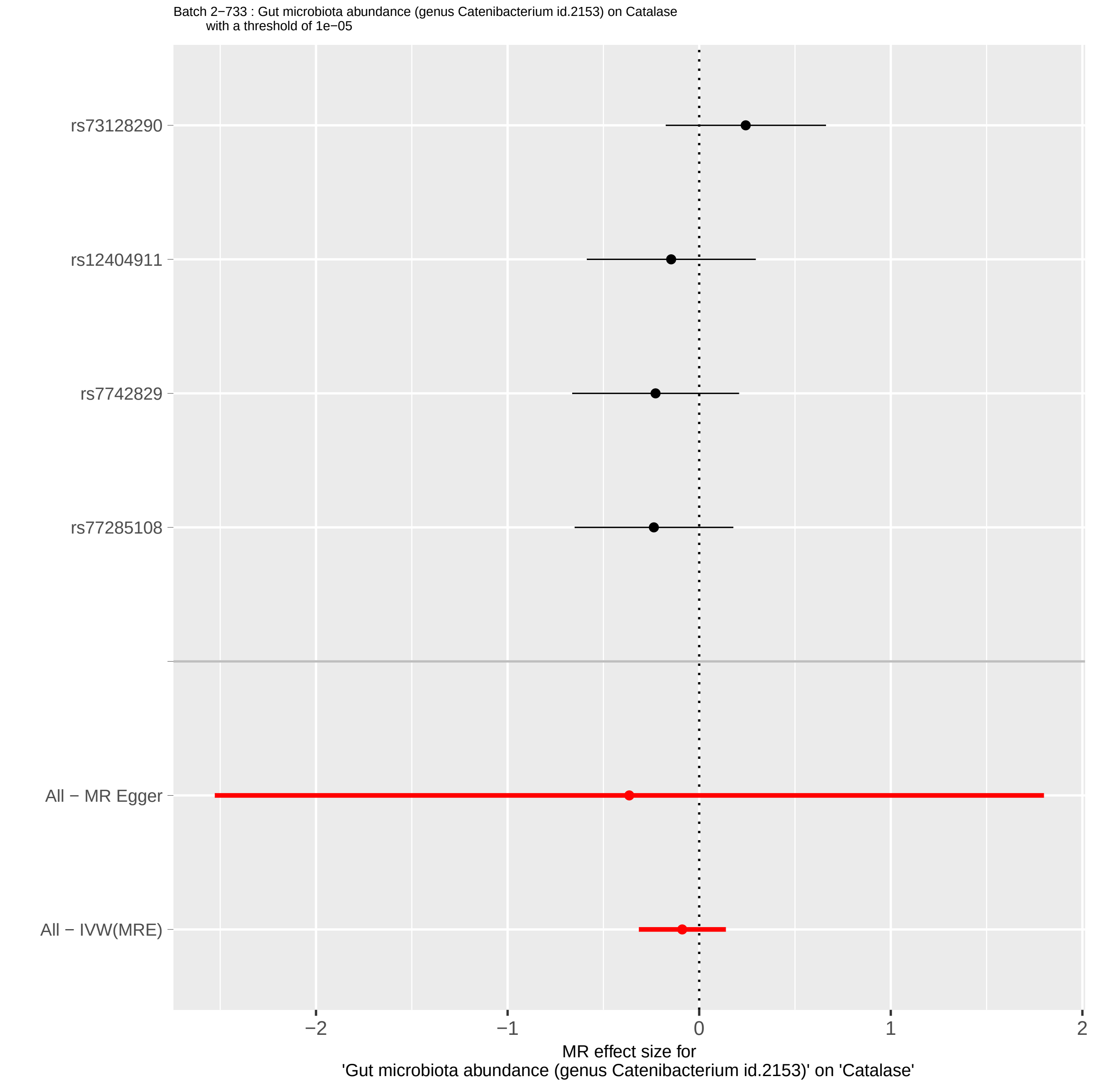

Batch 2−733 : Gut microbiota abundance (genus Catenibacterium id.2153) on Catalase
with a threshold of 1e−05
rs73128290
rs12404911
rs7742829
rs77285108
All − MR Egger
All − IVW(MRE)
−2
−1
0
1
2
MR effect size for
'Gut microbiota abundance (genus Catenibacterium id.2153)' on 'Catalase'

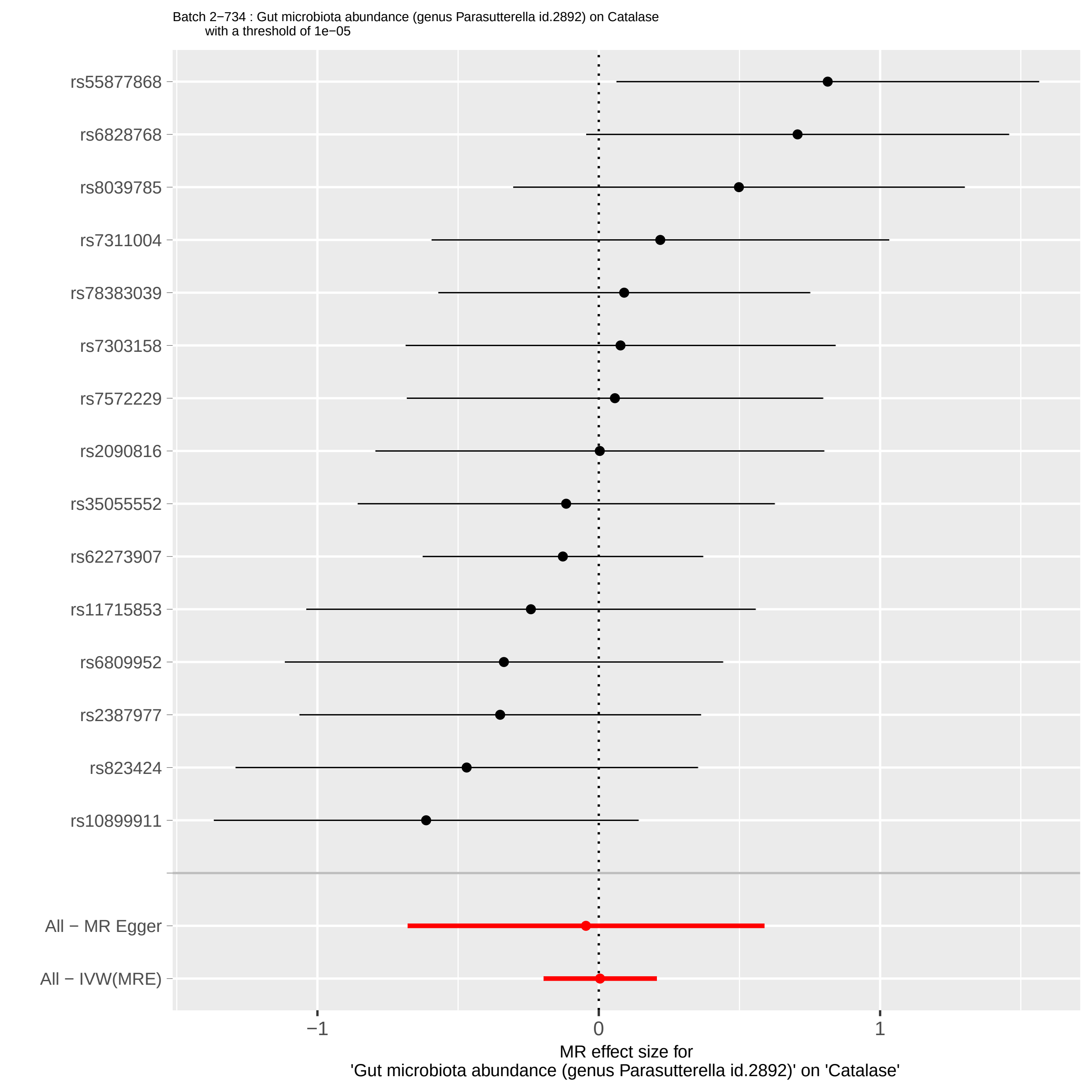
Batch 2−734 : Gut microbiota abundance (genus Parasutterella id.2892) on Catalase
with a threshold of 1e−05
rs55877868
rs6828768
rs8039785
rs7311004
rs78383039
rs7303158
rs7572229
rs2090816
rs35055552
rs62273907
rs11715853
rs6809952
rs2387977
rs823424
rs10899911
All − MR Egger
All − IVW(MRE)
−1
0
1
MR effect size for
'Gut microbiota abundance (genus Parasutterella id.2892)' on 'Catalase'

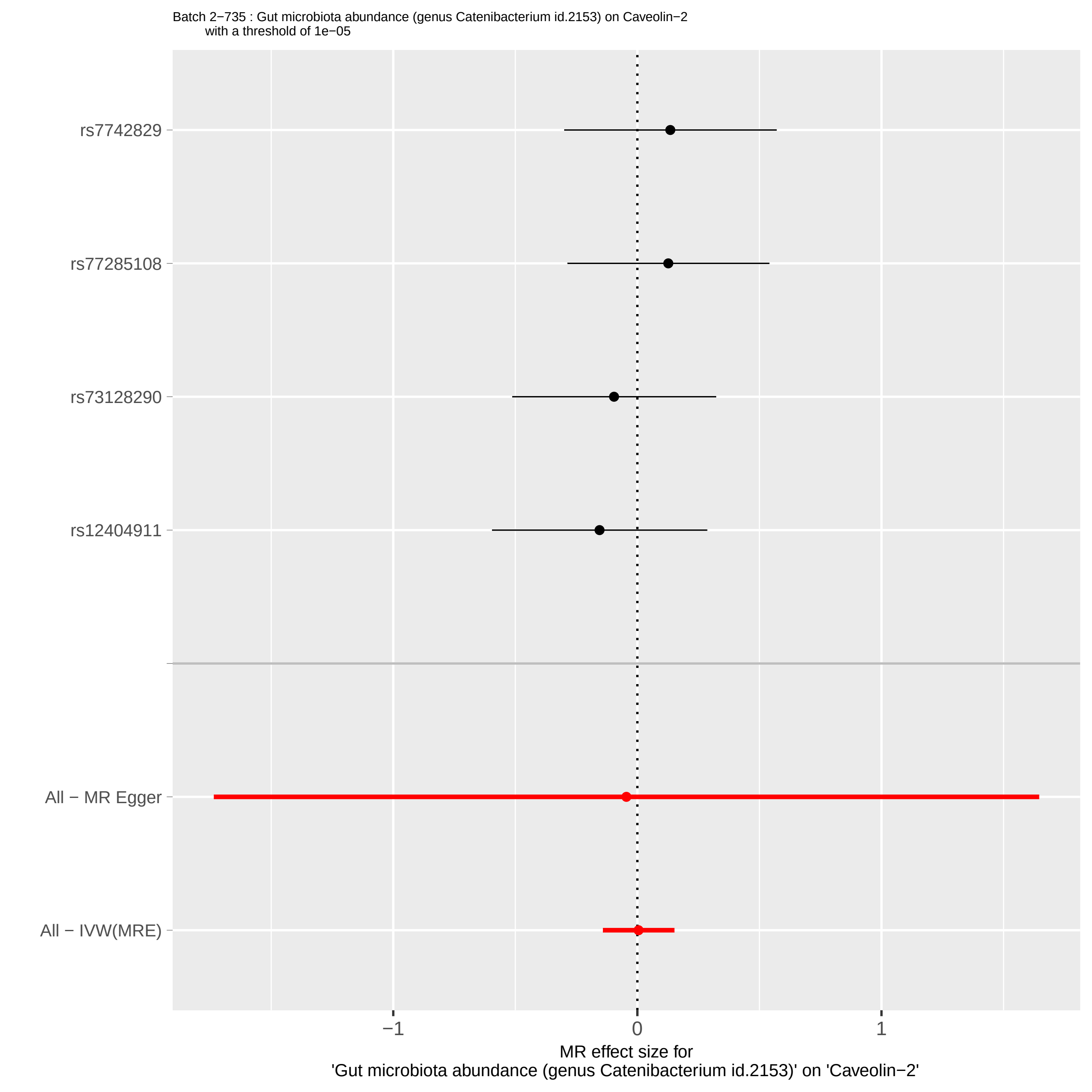

Batch 2−735 : Gut microbiota abundance (genus Catenibacterium id.2153) on Caveolin−2
with a threshold of 1e−05
rs7742829
rs77285108
rs73128290
rs12404911
All − MR Egger
All − IVW(MRE)
−1
0
1
MR effect size for
'Gut microbiota abundance (genus Catenibacterium id.2153)' on 'Caveolin−2'

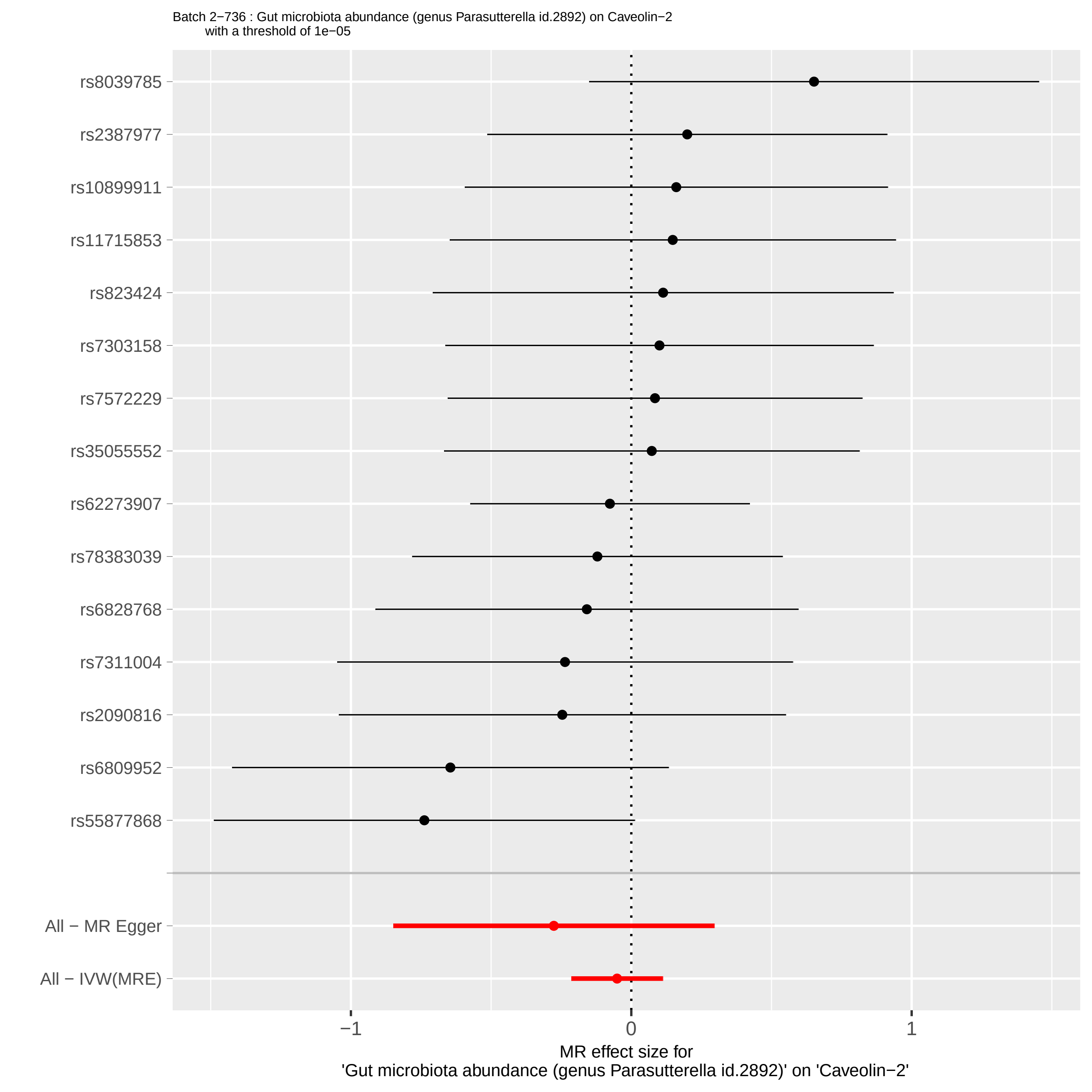

Batch 2−736 : Gut microbiota abundance (genus Parasutterella id.2892) on Caveolin−2
with a threshold of 1e−05
rs8039785
rs2387977
rs10899911
rs11715853
rs823424
rs7303158
rs7572229
rs35055552
rs62273907
rs78383039
rs6828768
rs7311004
rs2090816
rs6809952
rs55877868
All − MR Egger
All − IVW(MRE)
−1
0
1
MR effect size for
'Gut microbiota abundance (genus Parasutterella id.2892)' on 'Caveolin−2'

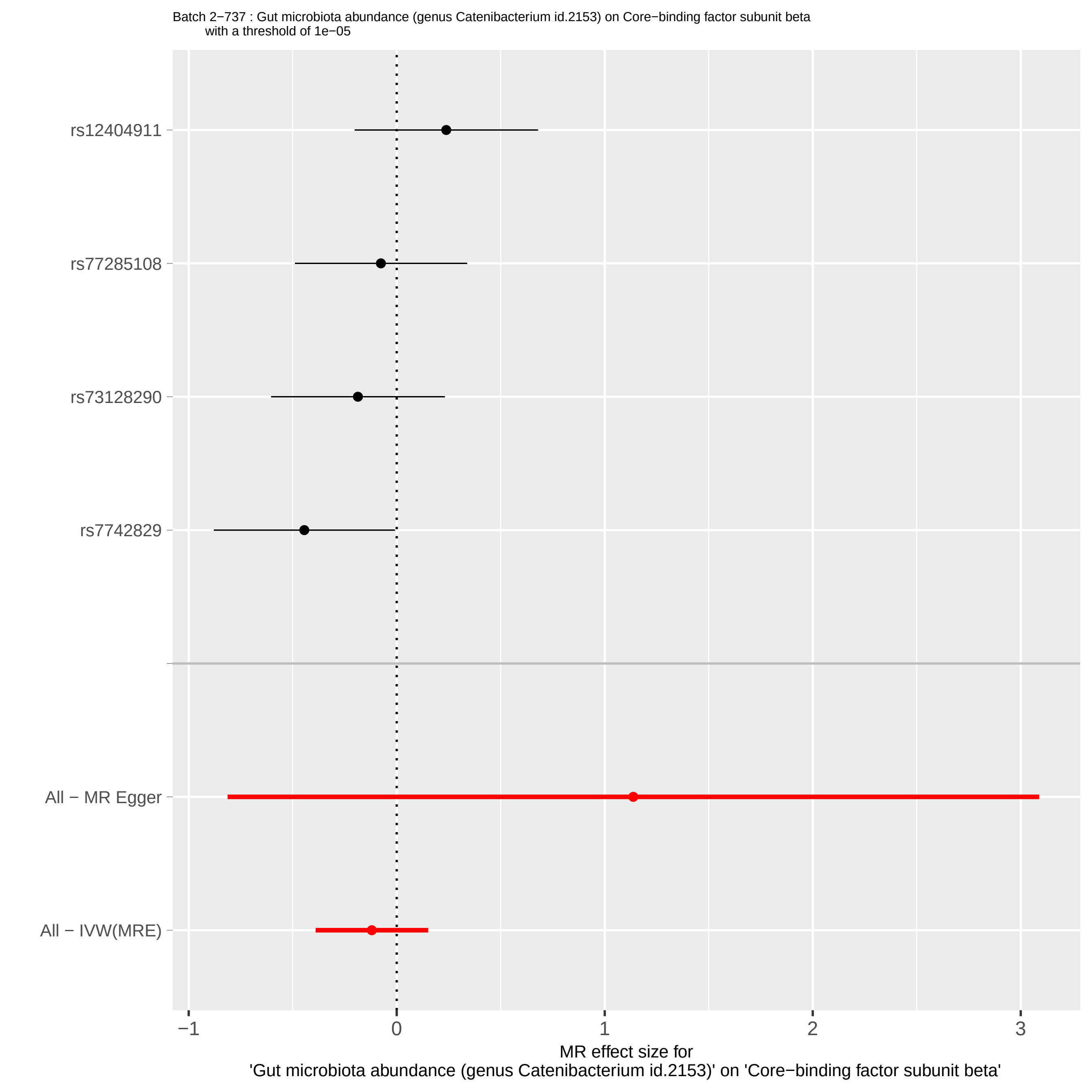

Batch 2−737 : Gut microbiota abundance (genus Catenibacterium id.2153) on Core−binding factor subunit beta
with a threshold of 1e−05
rs12404911
rs77285108
rs73128290
rs7742829
All − MR Egger
All − IVW(MRE)
−1
0
1
2
3
MR effect size for
'Gut microbiota abundance (genus Catenibacterium id.2153)' on 'Core−binding factor subunit beta'

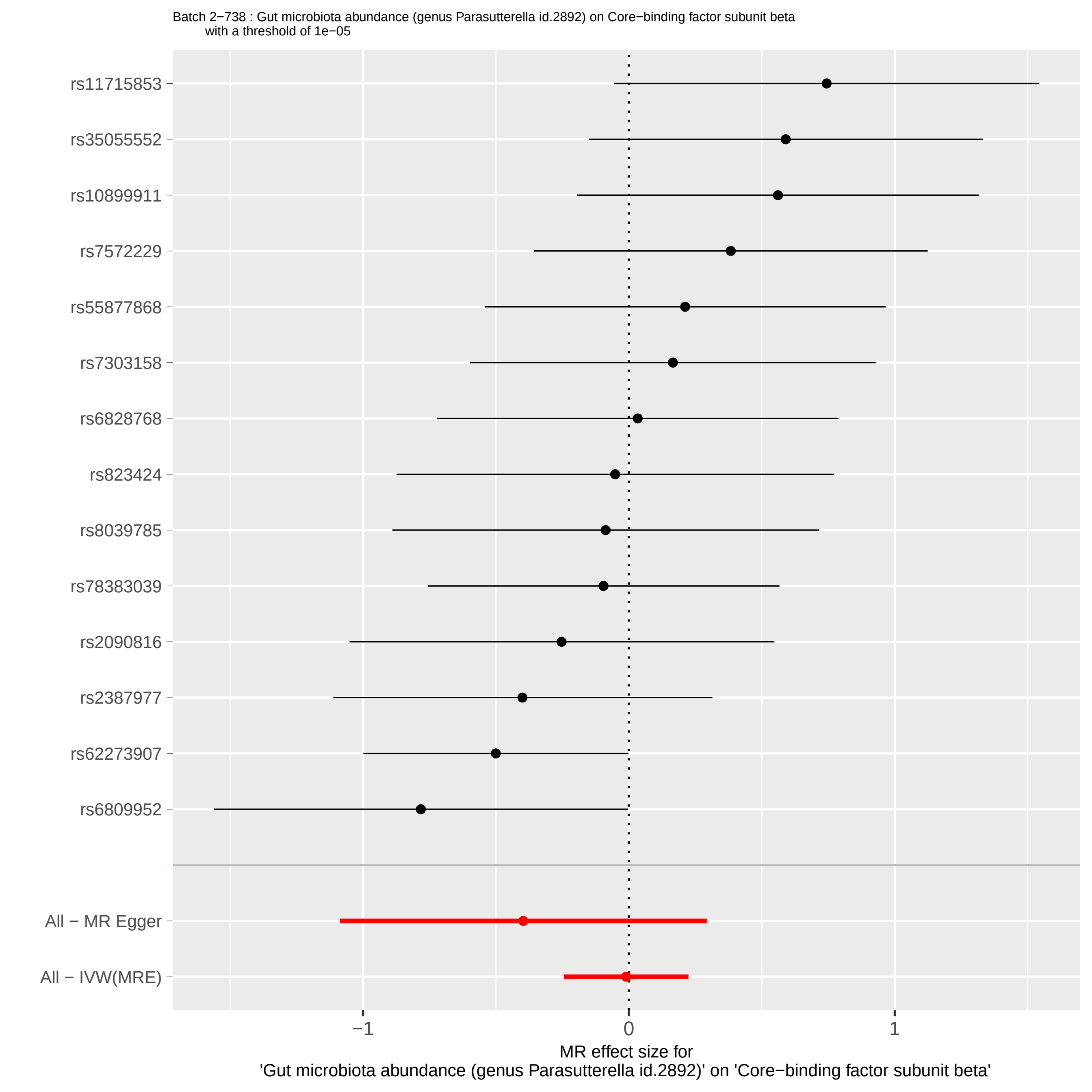

Batch 2−738 : Gut microbiota abundance (genus Parasutterella id.2892) on Core−binding factor subunit beta
with a threshold of 1e−05
rs11715853
rs35055552
rs10899911
rs7572229
rs55877868
rs7303158
rs6828768
rs823424
rs8039785
rs78383039
rs2090816
rs2387977
rs62273907
rs6809952
All − MR Egger
All − IVW(MRE)
−1
0
1
MR effect size for
'Gut microbiota abundance (genus Parasutterella id.2892)' on 'Core−binding factor subunit beta'

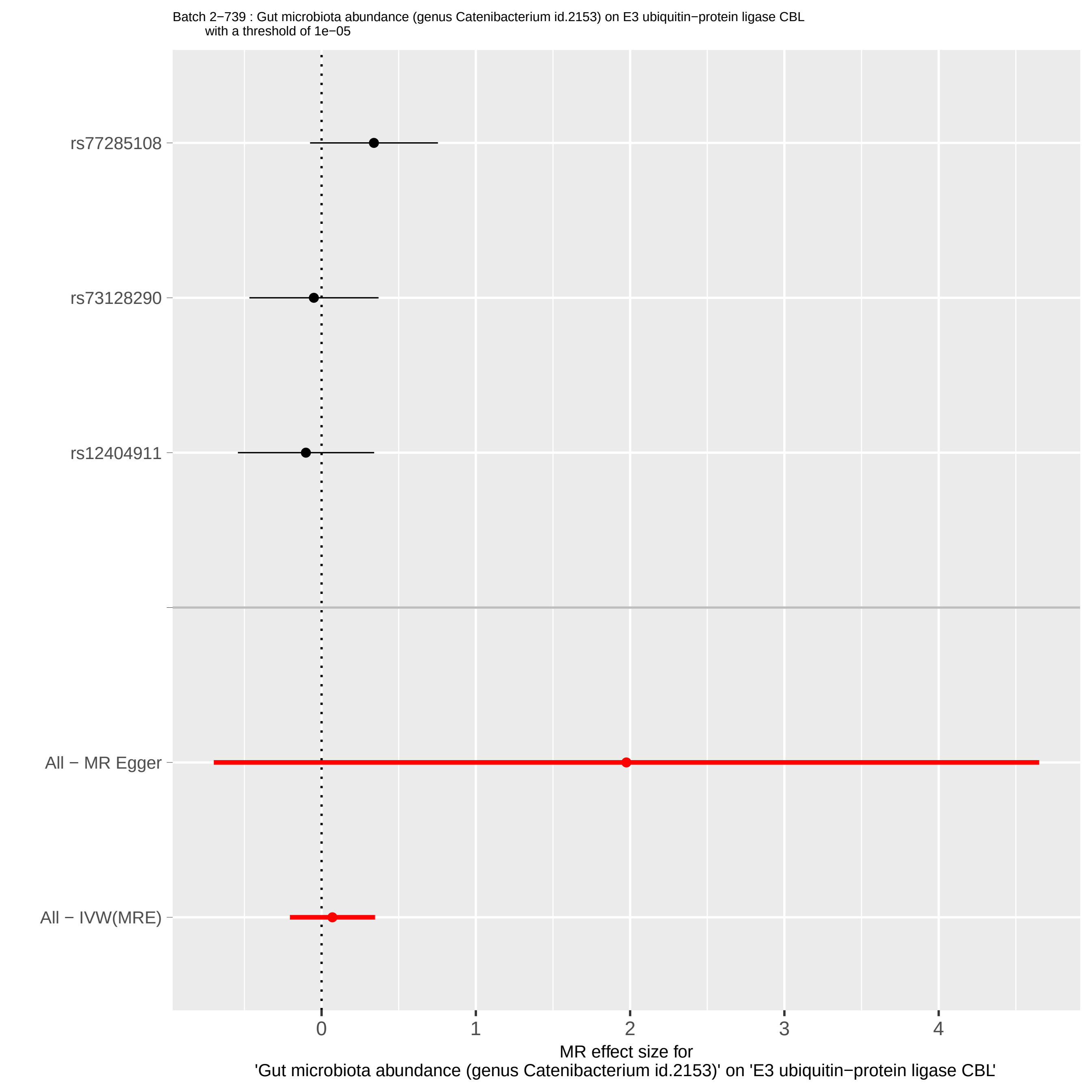

Batch 2−739 : Gut microbiota abundance (genus Catenibacterium id.2153) on E3 ubiquitin−protein ligase CBL
with a threshold of 1e−05
rs77285108
rs73128290
rs12404911
All − MR Egger
All − IVW(MRE)
0
1
2
3
4
MR effect size for
'Gut microbiota abundance (genus Catenibacterium id.2153)' on 'E3 ubiquitin−protein ligase CBL'

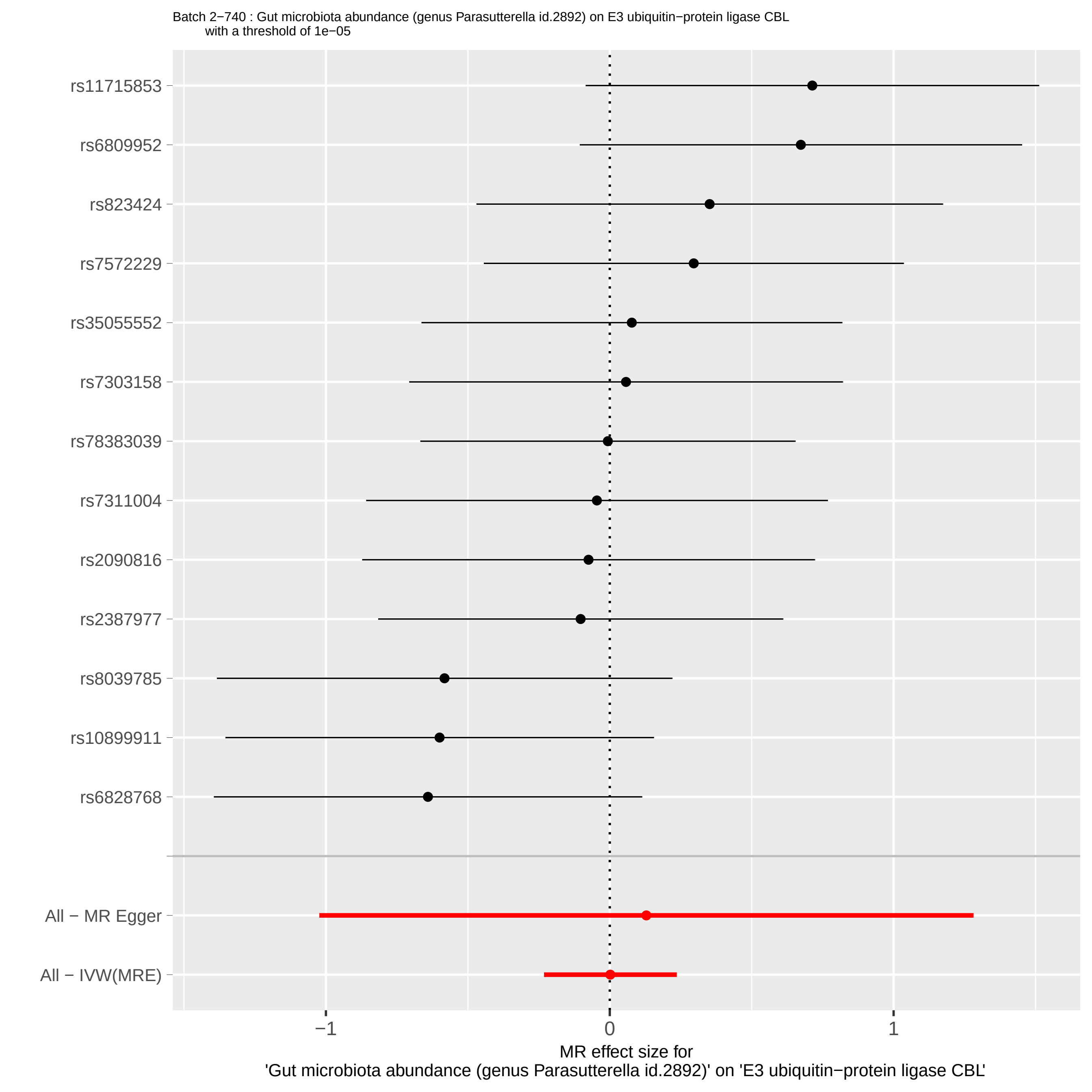

Batch 2−740 : Gut microbiota abundance (genus Parasutterella id.2892) on E3 ubiquitin−protein ligase CBL
with a threshold of 1e−05
rs11715853
rs6809952
rs823424
rs7572229
rs35055552
rs7303158
rs78383039
rs7311004
rs2090816
rs2387977
rs8039785
rs10899911
rs6828768
All − MR Egger
All − IVW(MRE)
−1
0
1
MR effect size for
'Gut microbiota abundance (genus Parasutterella id.2892)' on 'E3 ubiquitin−protein ligase CBL'

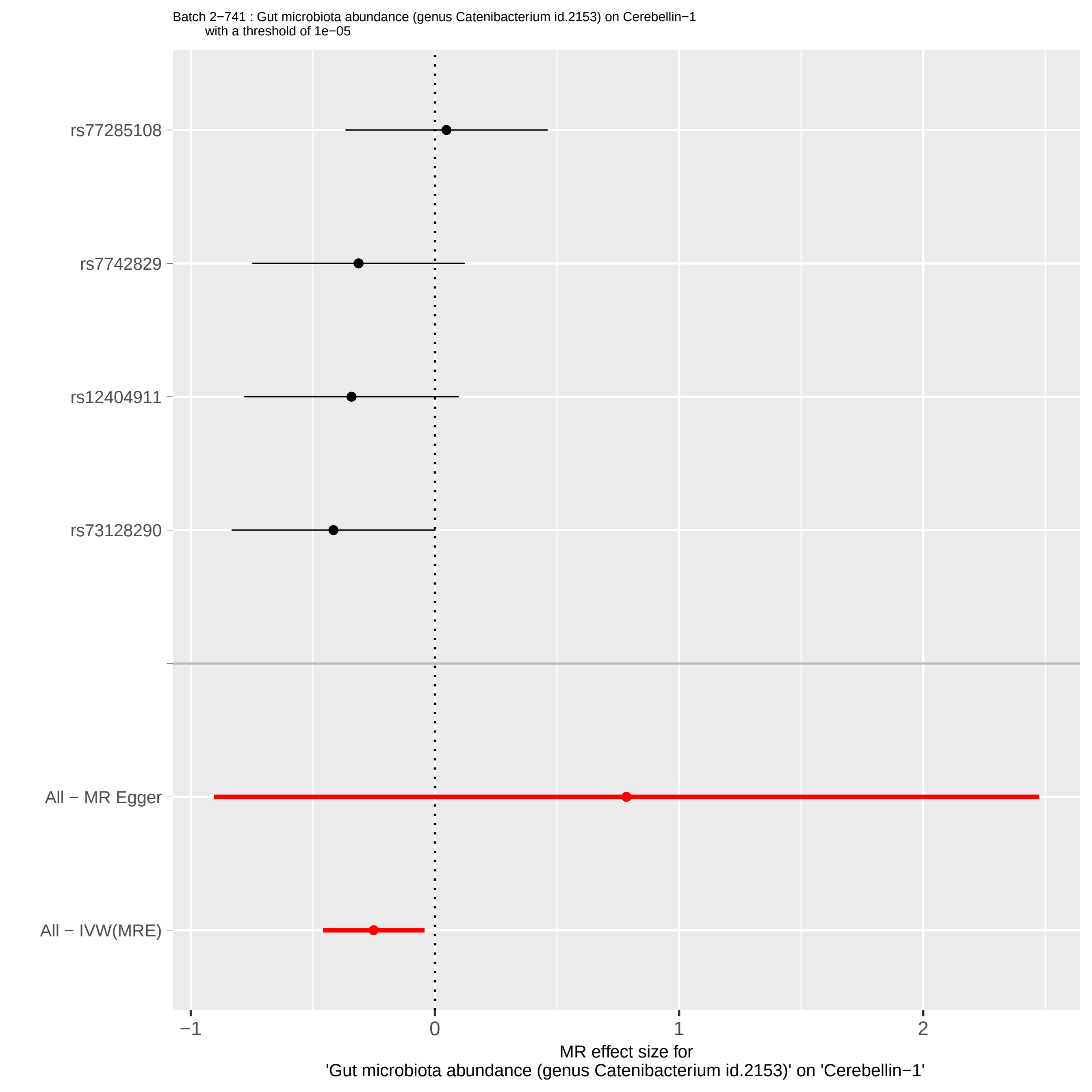

Batch 2−741 : Gut microbiota abundance (genus Catenibacterium id.2153) on Cerebellin−1
with a threshold of 1e−05
rs77285108
rs7742829
rs12404911
rs73128290
All − MR Egger
All − IVW(MRE)
−1
0
1
2
MR effect size for
'Gut microbiota abundance (genus Catenibacterium id.2153)' on 'Cerebellin−1'

Batch 2−742 : Gut microbiota abundance (genus Parasutterella id.2892) on Cerebellin−1
with a threshold of 1e−05

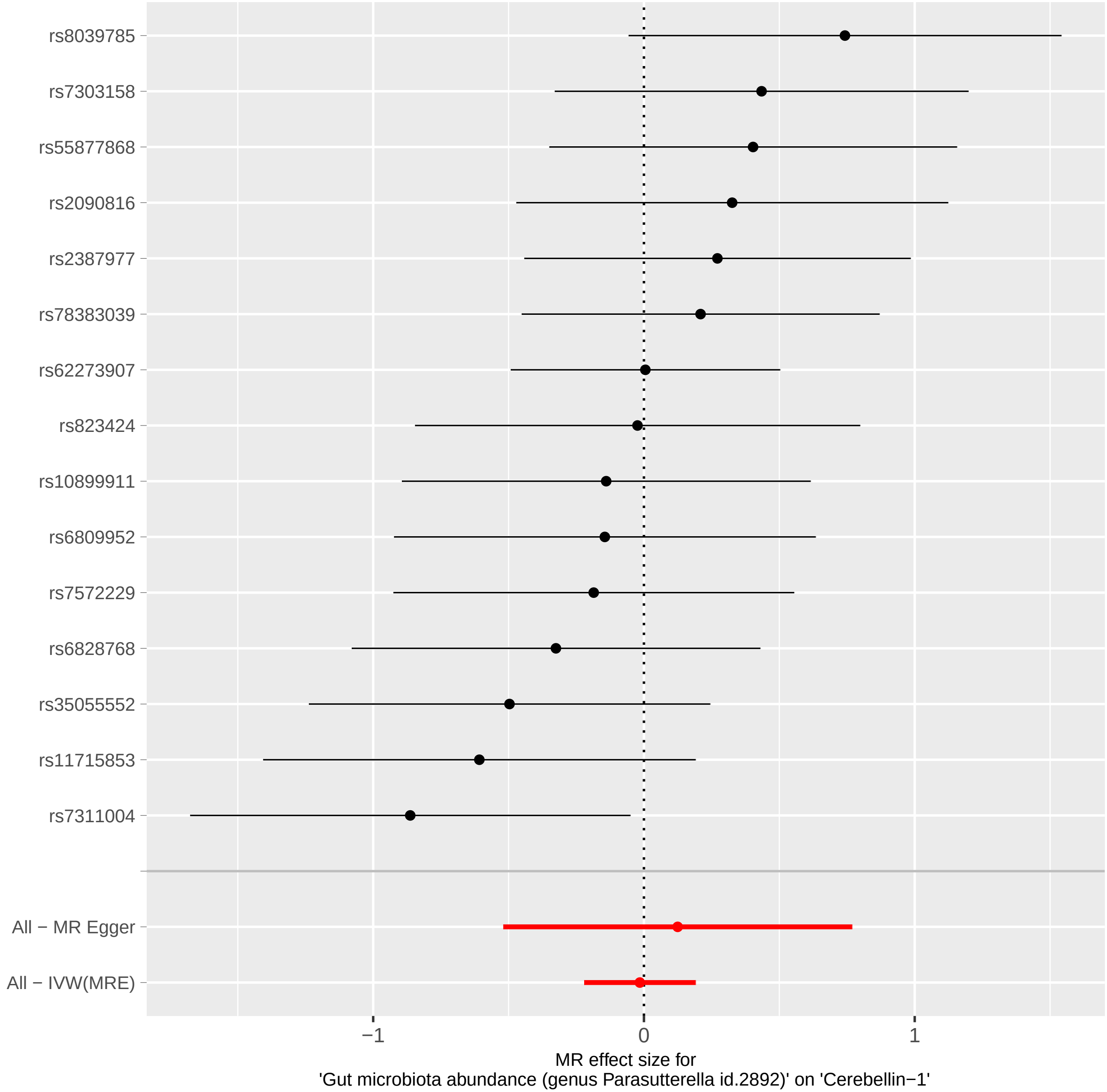

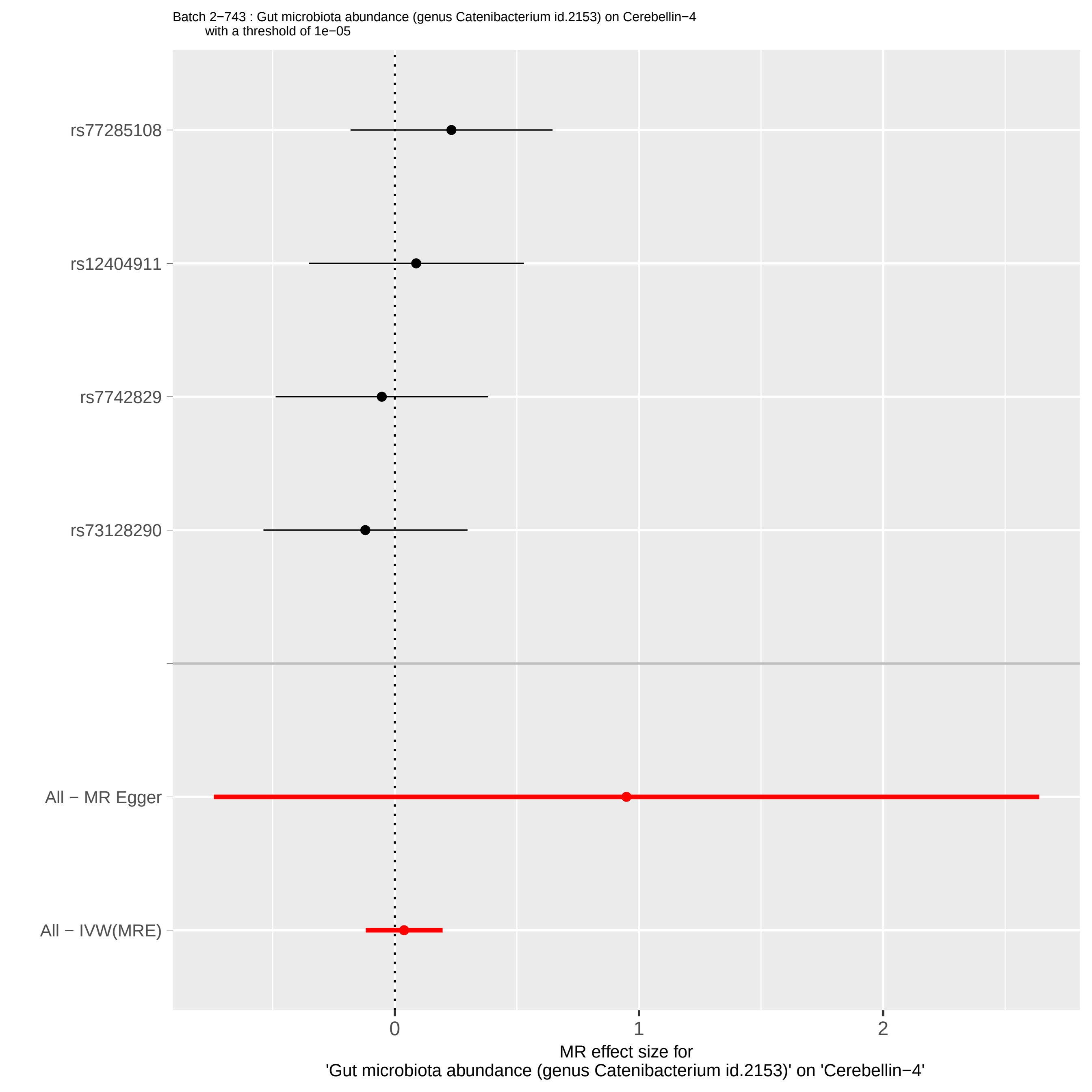

Batch 2−743 : Gut microbiota abundance (genus Catenibacterium id.2153) on Cerebellin−4
with a threshold of 1e−05
rs77285108
rs12404911
rs7742829
rs73128290
All − MR Egger
All − IVW(MRE)
0
1
2
MR effect size for
'Gut microbiota abundance (genus Catenibacterium id.2153)' on 'Cerebellin−4'

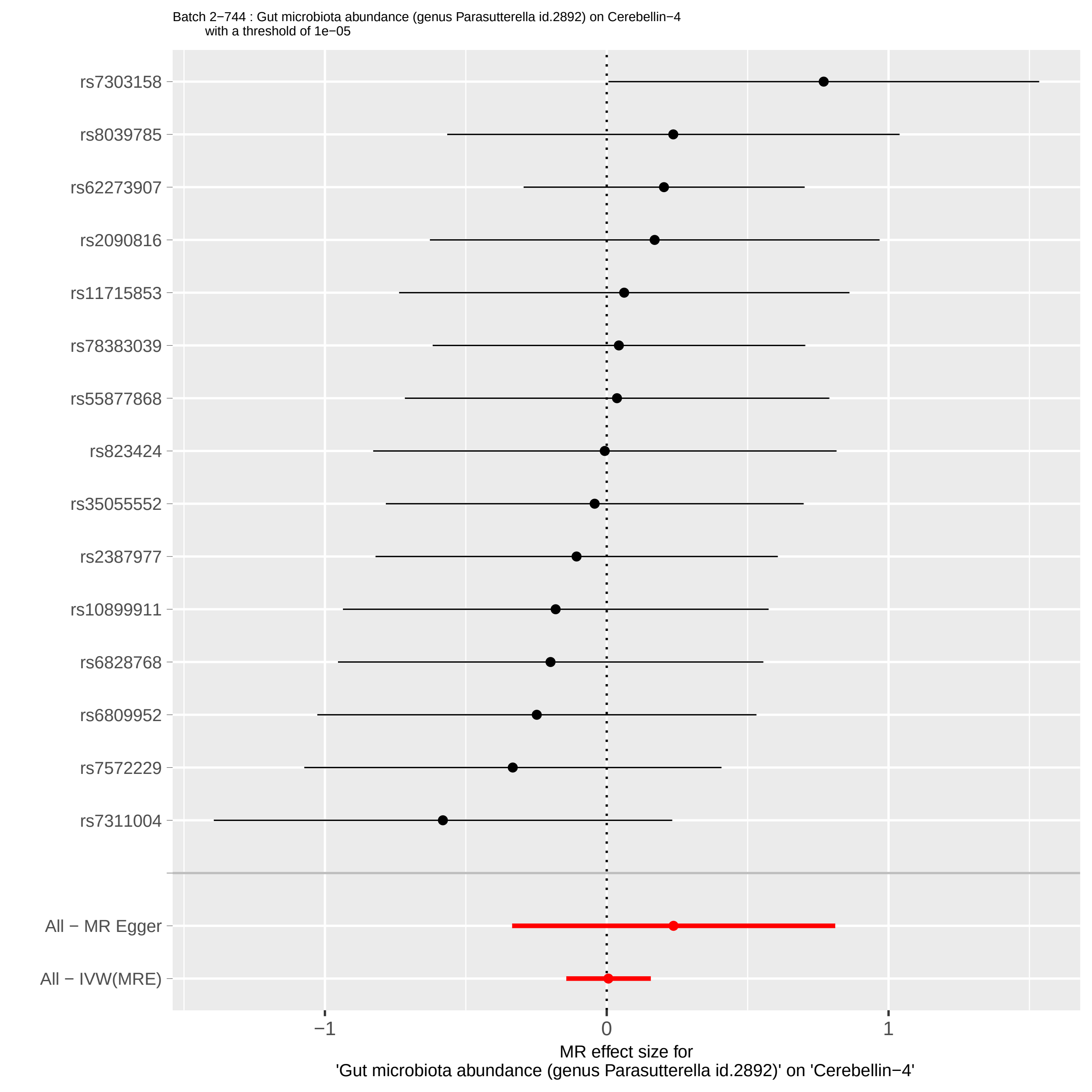

Batch 2−744 : Gut microbiota abundance (genus Parasutterella id.2892) on Cerebellin−4
with a threshold of 1e−05
rs7303158
rs8039785
rs62273907
rs2090816
rs11715853
rs78383039
rs55877868
rs823424
rs35055552
rs2387977
rs10899911
rs6828768
rs6809952
rs7572229
rs7311004
All − MR Egger
All − IVW(MRE)
−1
0
1
MR effect size for
'Gut microbiota abundance (genus Parasutterella id.2892)' on 'Cerebellin−4'

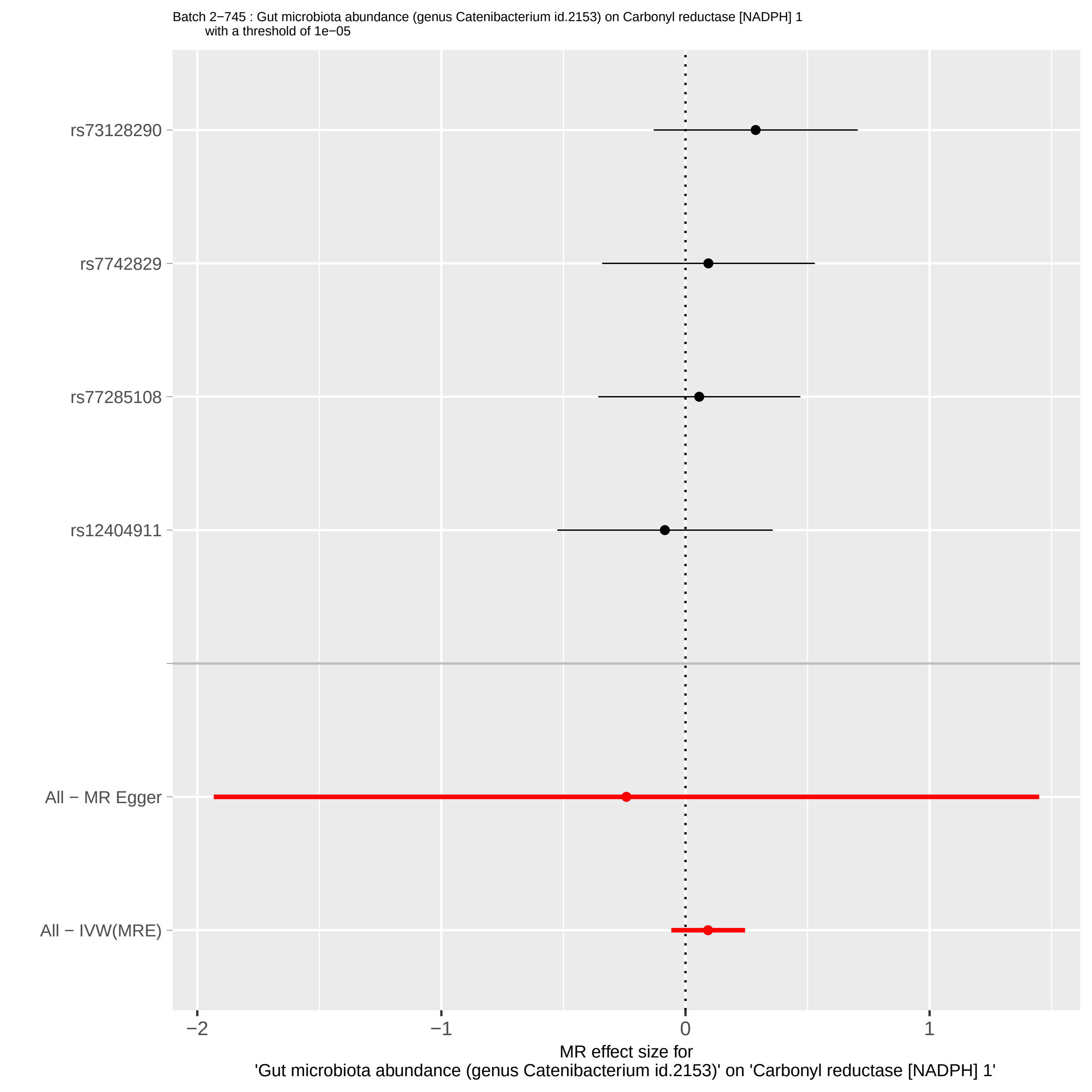
Batch 2−745 : Gut microbiota abundance (genus Catenibacterium id.2153) on Carbonyl reductase [NADPH] 1
with a threshold of 1e−05
rs73128290
rs7742829
rs77285108
rs12404911
All − MR Egger
All − IVW(MRE)
−2
−1
0
1
MR effect size for
'Gut microbiota abundance (genus Catenibacterium id.2153)' on 'Carbonyl reductase [NADPH] 1'

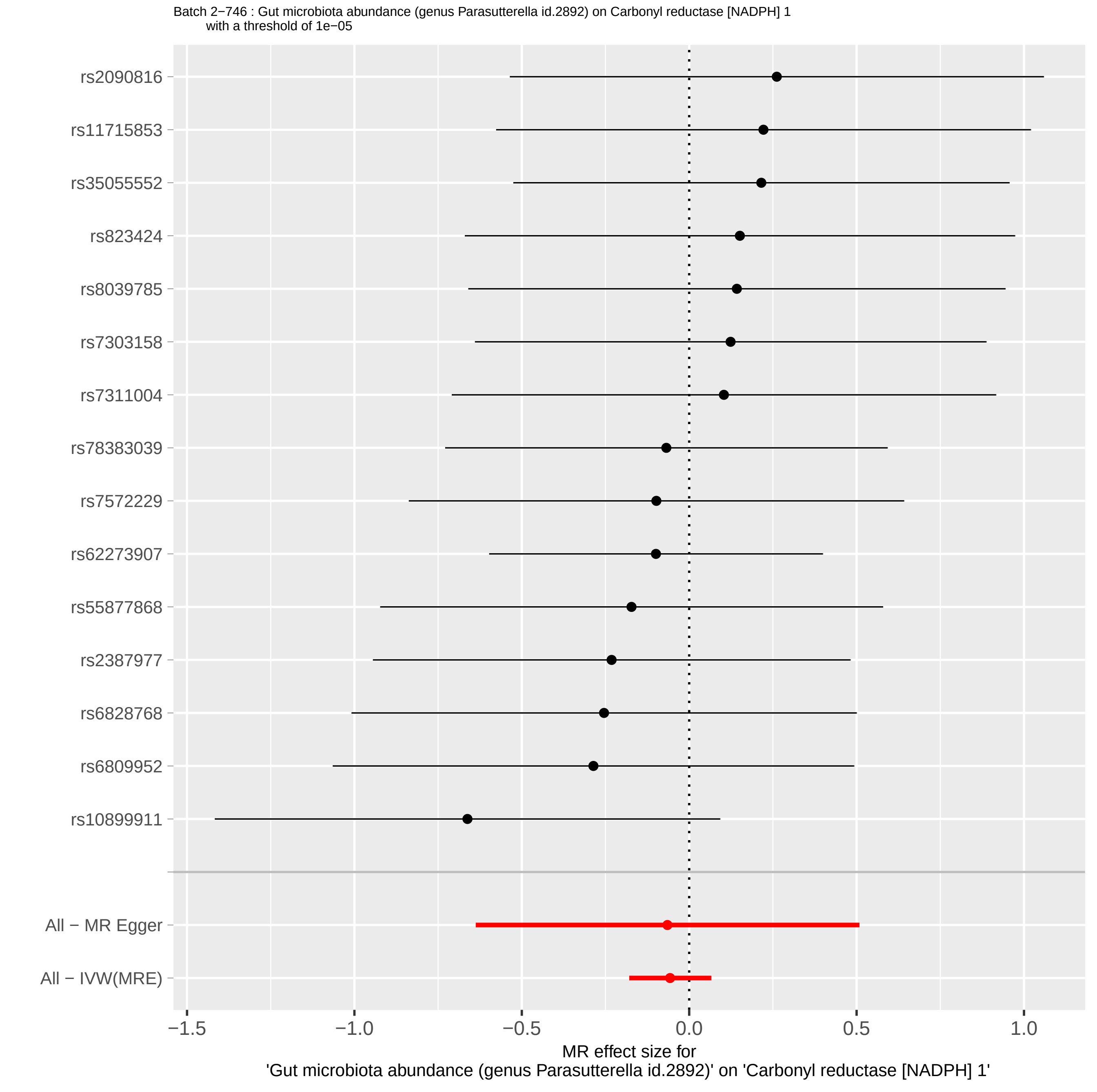

Batch 2−746 : Gut microbiota abundance (genus Parasutterella id.2892) on Carbonyl reductase [NADPH] 1
with a threshold of 1e−05
rs2090816
rs11715853
rs35055552
rs823424
rs8039785
rs7303158
rs7311004
rs78383039
rs7572229
rs62273907
rs55877868
rs2387977
rs6828768
rs6809952
rs10899911
All − MR Egger
All − IVW(MRE)
−1.5
−1.0
−0.5
0.0
0.5
1.0
MR effect size for
'Gut microbiota abundance (genus Parasutterella id.2892)' on 'Carbonyl reductase [NADPH] 1'

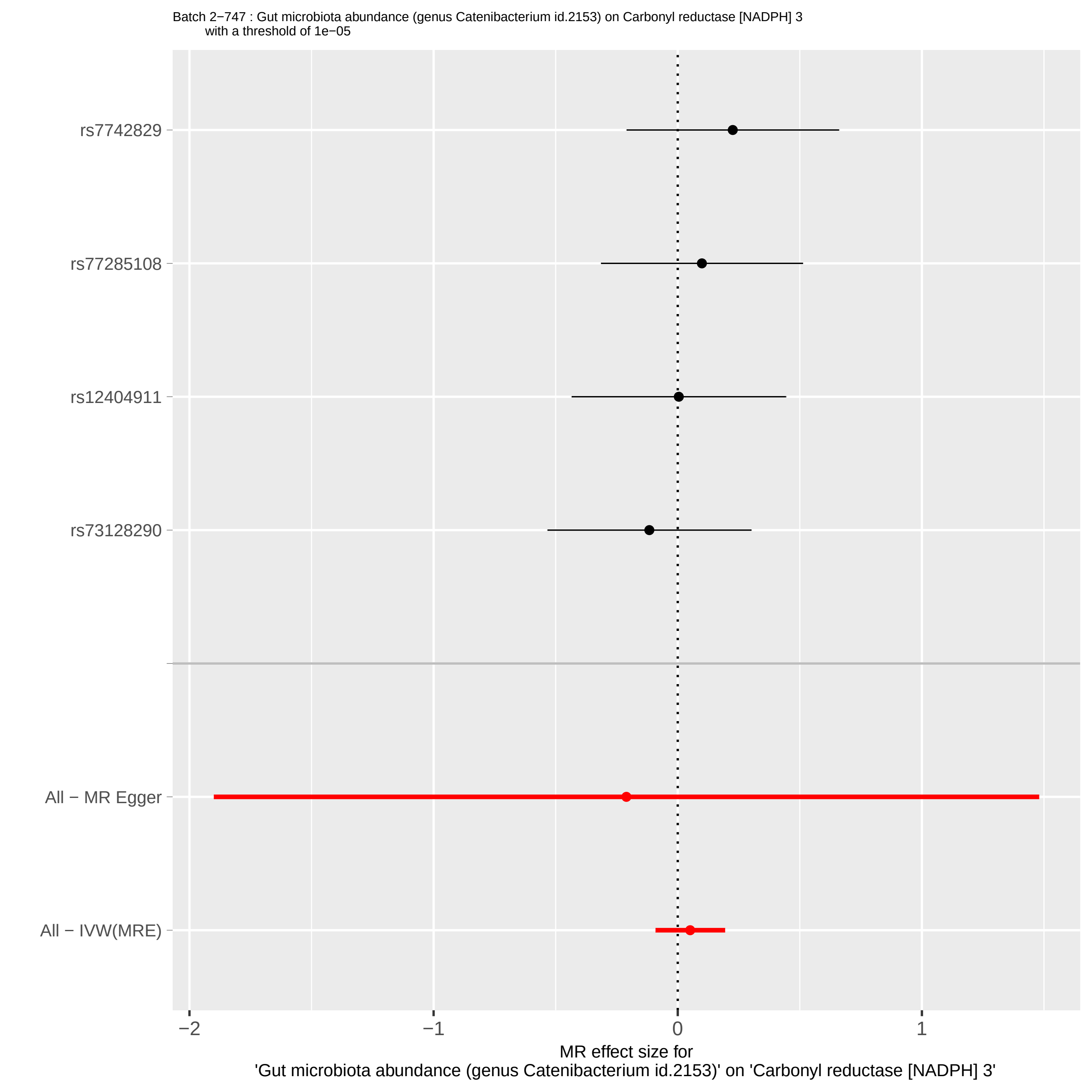

Batch 2−747 : Gut microbiota abundance (genus Catenibacterium id.2153) on Carbonyl reductase [NADPH] 3
with a threshold of 1e−05
rs7742829
rs77285108
rs12404911
rs73128290
All − MR Egger
All − IVW(MRE)
−2
−1
0
1
MR effect size for
'Gut microbiota abundance (genus Catenibacterium id.2153)' on 'Carbonyl reductase [NADPH] 3'

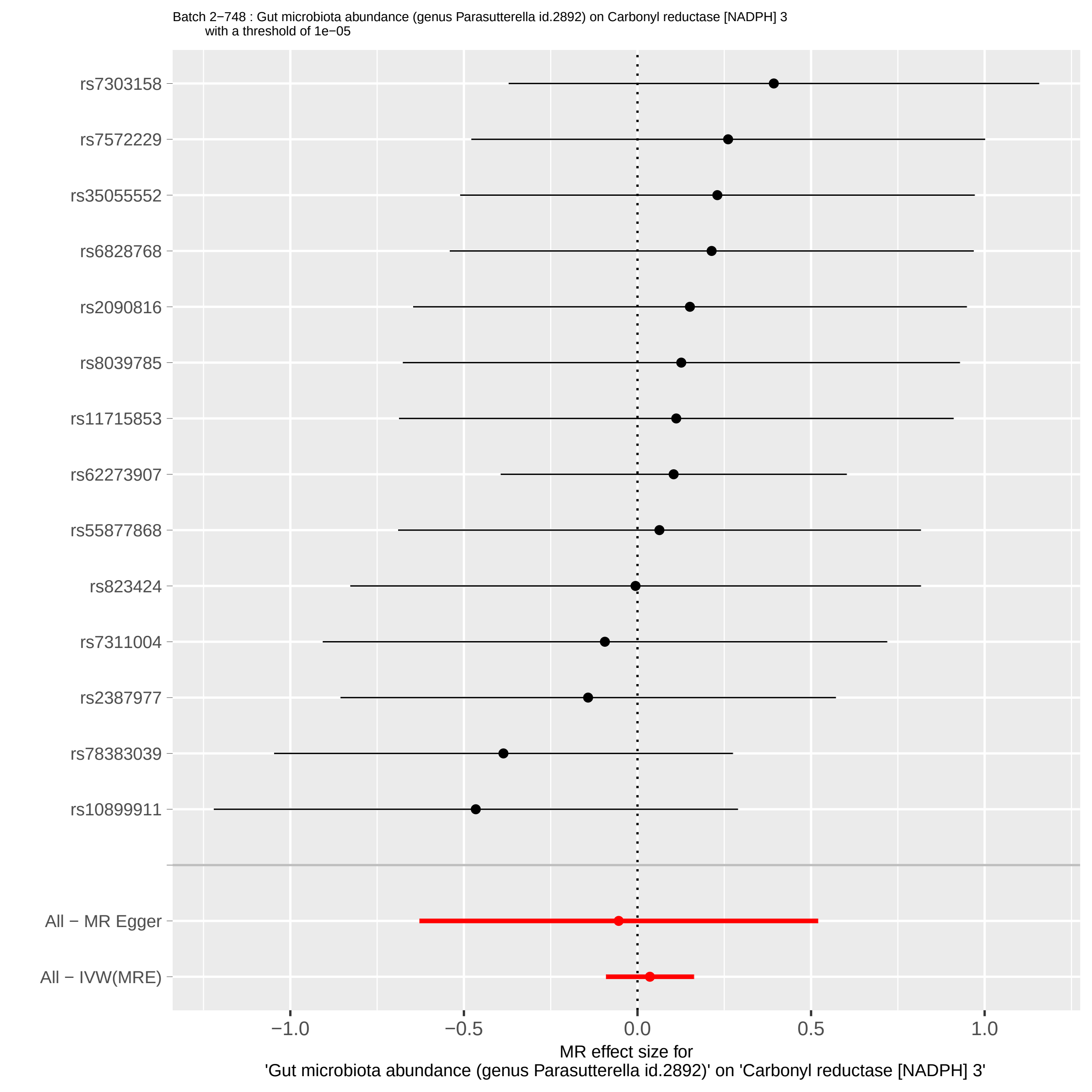

Batch 2−748 : Gut microbiota abundance (genus Parasutterella id.2892) on Carbonyl reductase [NADPH] 3
with a threshold of 1e−05
rs7303158
rs7572229
rs35055552
rs6828768
rs2090816
rs8039785
rs11715853
rs62273907
rs55877868
rs823424
rs7311004
rs2387977
rs78383039
rs10899911
All − MR Egger
All − IVW(MRE)
−1.0
−0.5
0.0
0.5
1.0
MR effect size for
'Gut microbiota abundance (genus Parasutterella id.2892)' on 'Carbonyl reductase [NADPH] 3'

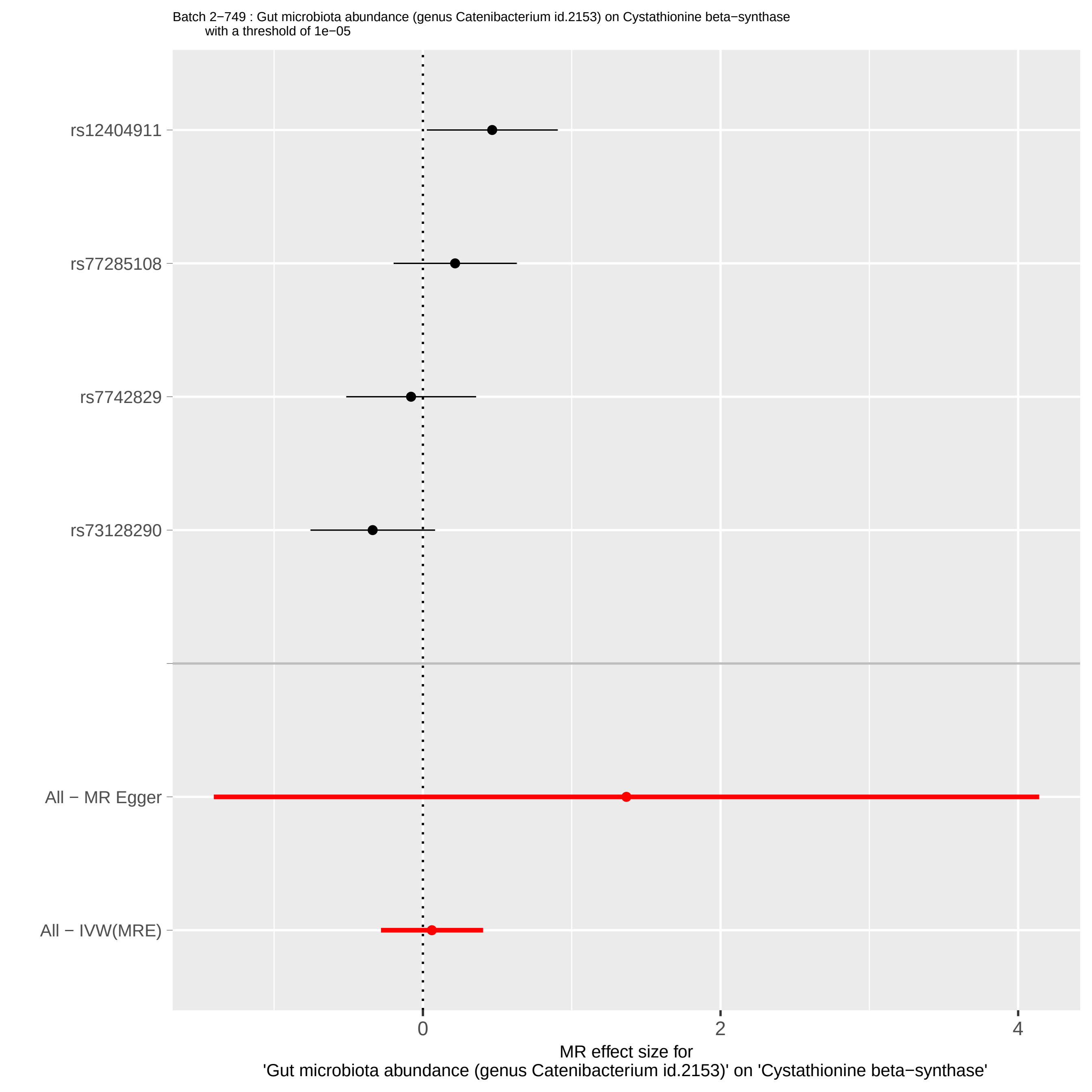

Batch 2−749 : Gut microbiota abundance (genus Catenibacterium id.2153) on Cystathionine beta−synthase
with a threshold of 1e−05
rs12404911
rs77285108
rs7742829
rs73128290
All − MR Egger
All − IVW(MRE)
0
2
4
MR effect size for
'Gut microbiota abundance (genus Catenibacterium id.2153)' on 'Cystathionine beta−synthase'

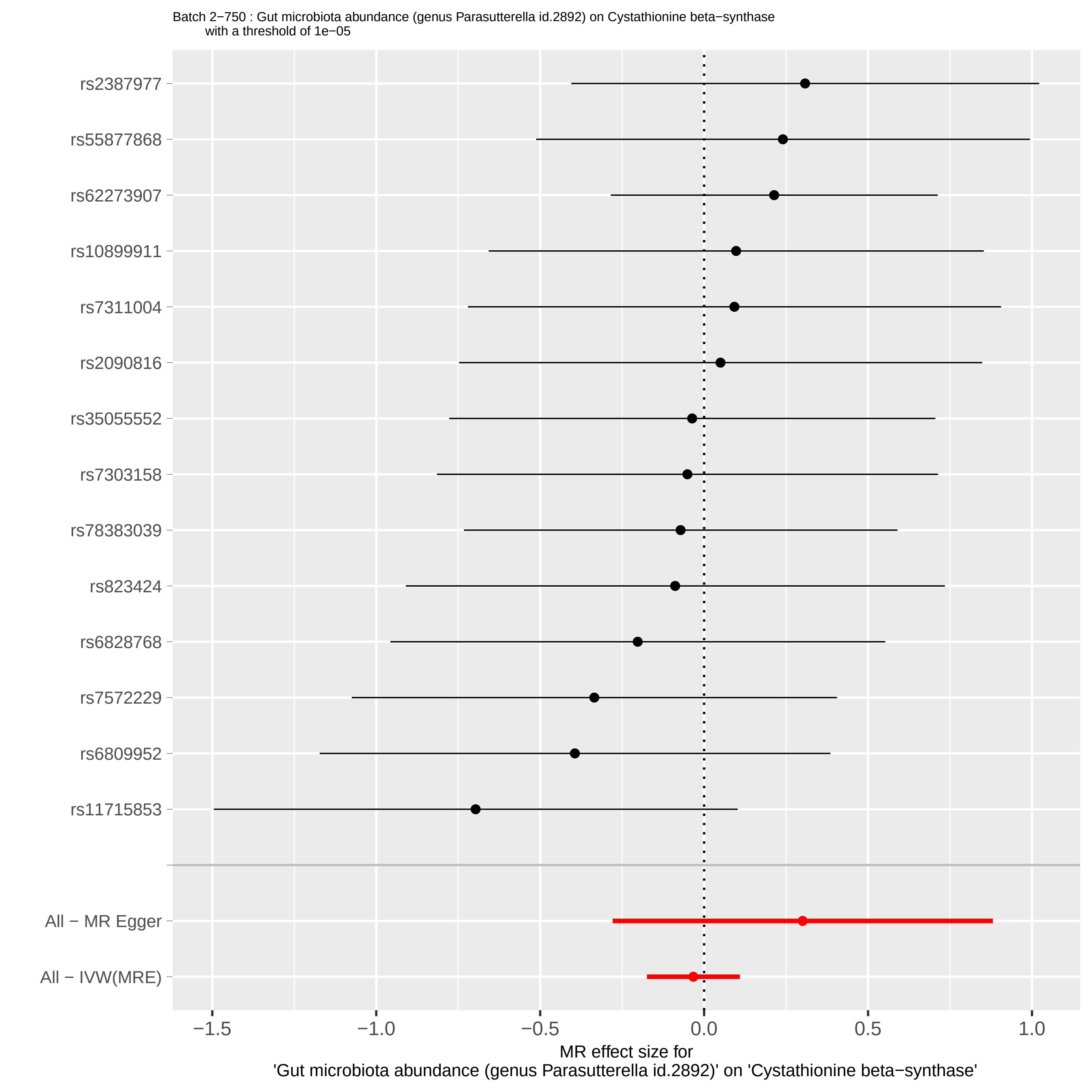

Batch 2−750 : Gut microbiota abundance (genus Parasutterella id.2892) on Cystathionine beta−synthase
with a threshold of 1e−05
rs2387977
rs55877868
rs62273907
rs10899911
rs7311004
rs2090816
rs35055552
rs7303158
rs78383039
rs823424
rs6828768
rs7572229
rs6809952
rs11715853
All − MR Egger
All − IVW(MRE)
−1.5
−1.0
−0.5
0.0
0.5
1.0
MR effect size for
'Gut microbiota abundance (genus Parasutterella id.2892)' on 'Cystathionine beta−synthase'

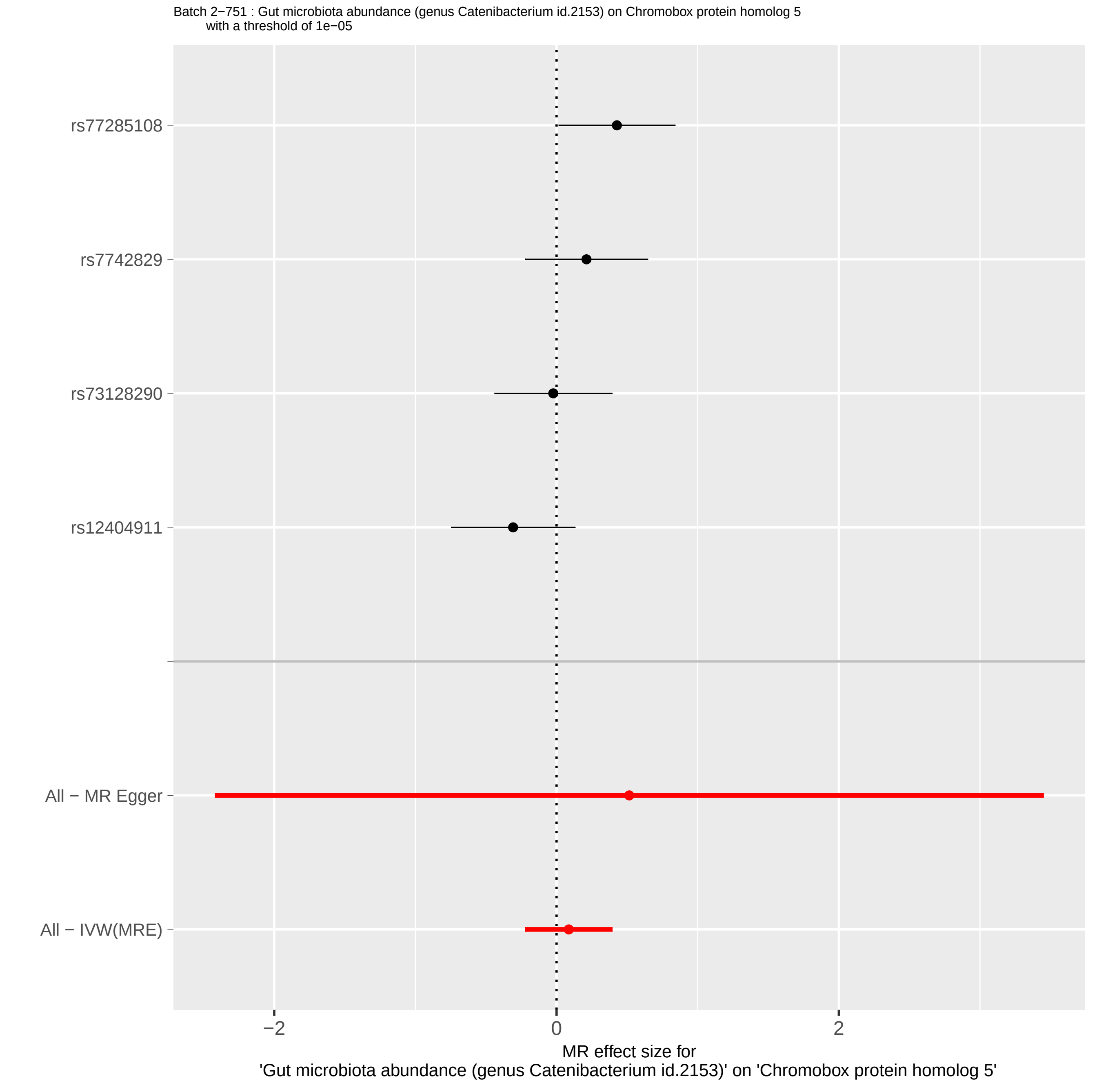

Batch 2−751 : Gut microbiota abundance (genus Catenibacterium id.2153) on Chromobox protein homolog 5
with a threshold of 1e−05
rs77285108
rs7742829
rs73128290
rs12404911
All − MR Egger
All − IVW(MRE)
−2
0
2
MR effect size for
'Gut microbiota abundance (genus Catenibacterium id.2153)' on 'Chromobox protein homolog 5'

Batch 2−752 : Gut microbiota abundance (genus Parasutterella id.2892) on Chromobox protein homolog 5 with a threshold of 1e−05

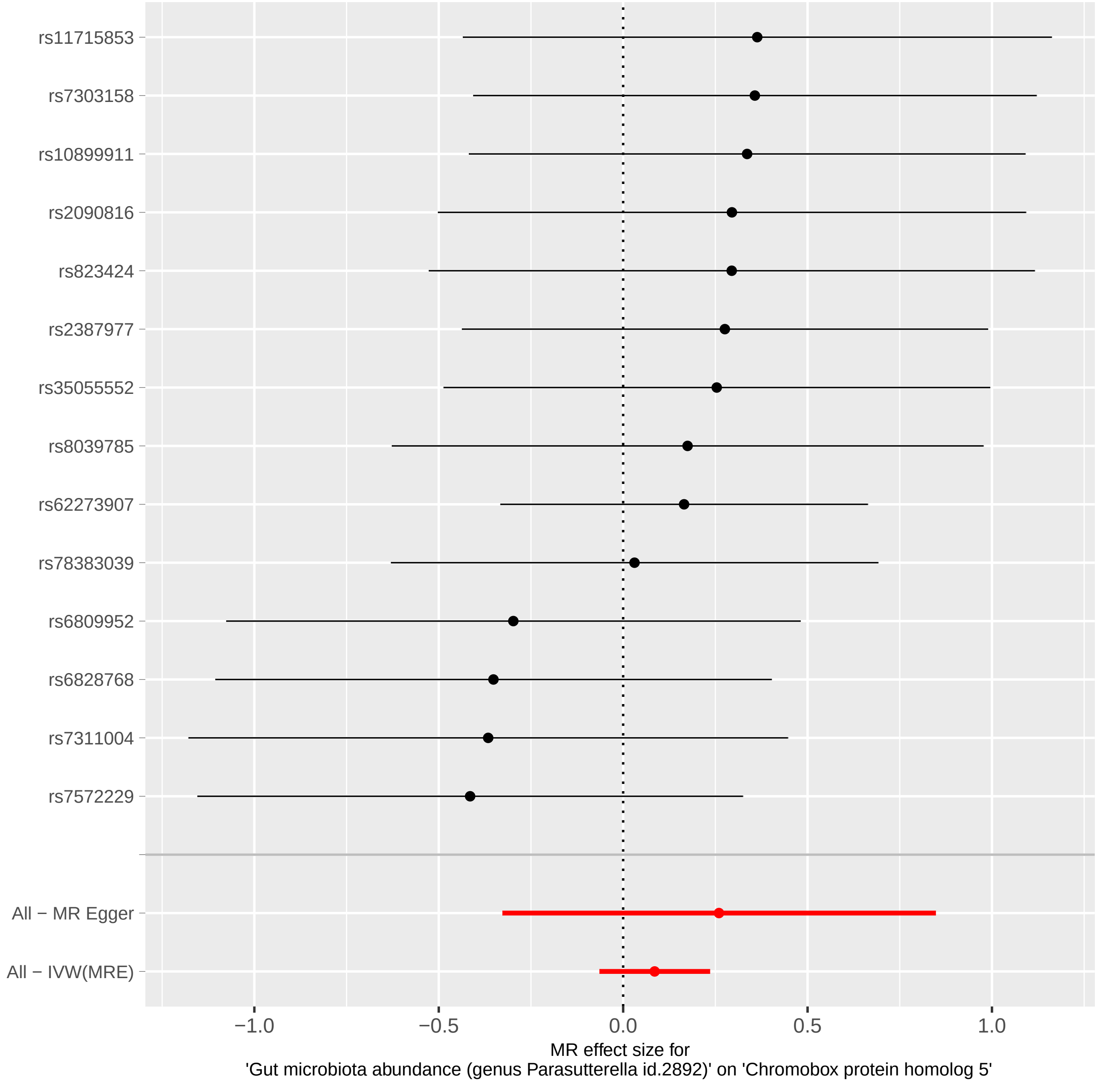

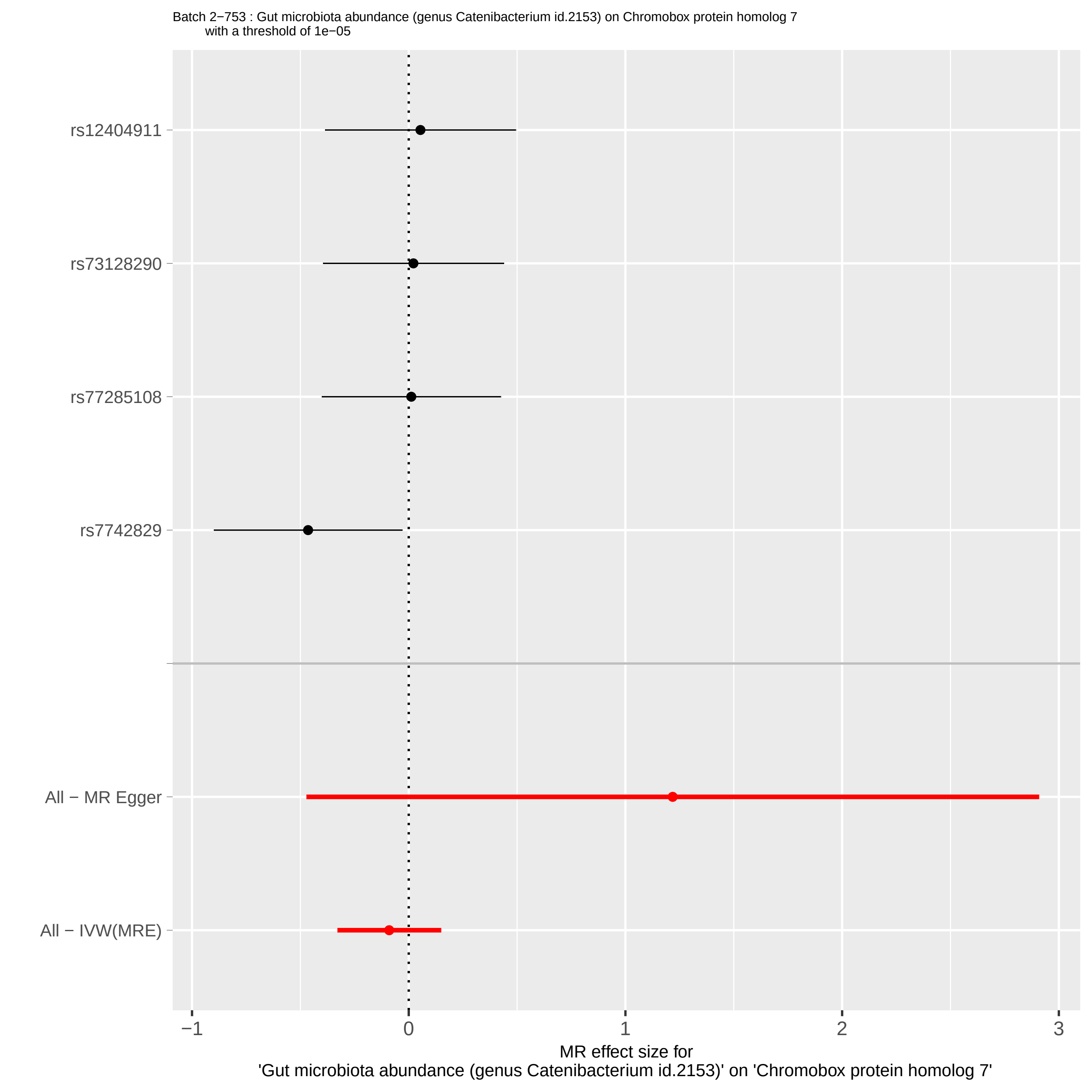

Batch 2−753 : Gut microbiota abundance (genus Catenibacterium id.2153) on Chromobox protein homolog 7
with a threshold of 1e−05
rs12404911
rs73128290
rs77285108
rs7742829
All − MR Egger
All − IVW(MRE)
−1
0
1
2
3
MR effect size for
'Gut microbiota abundance (genus Catenibacterium id.2153)' on 'Chromobox protein homolog 7'

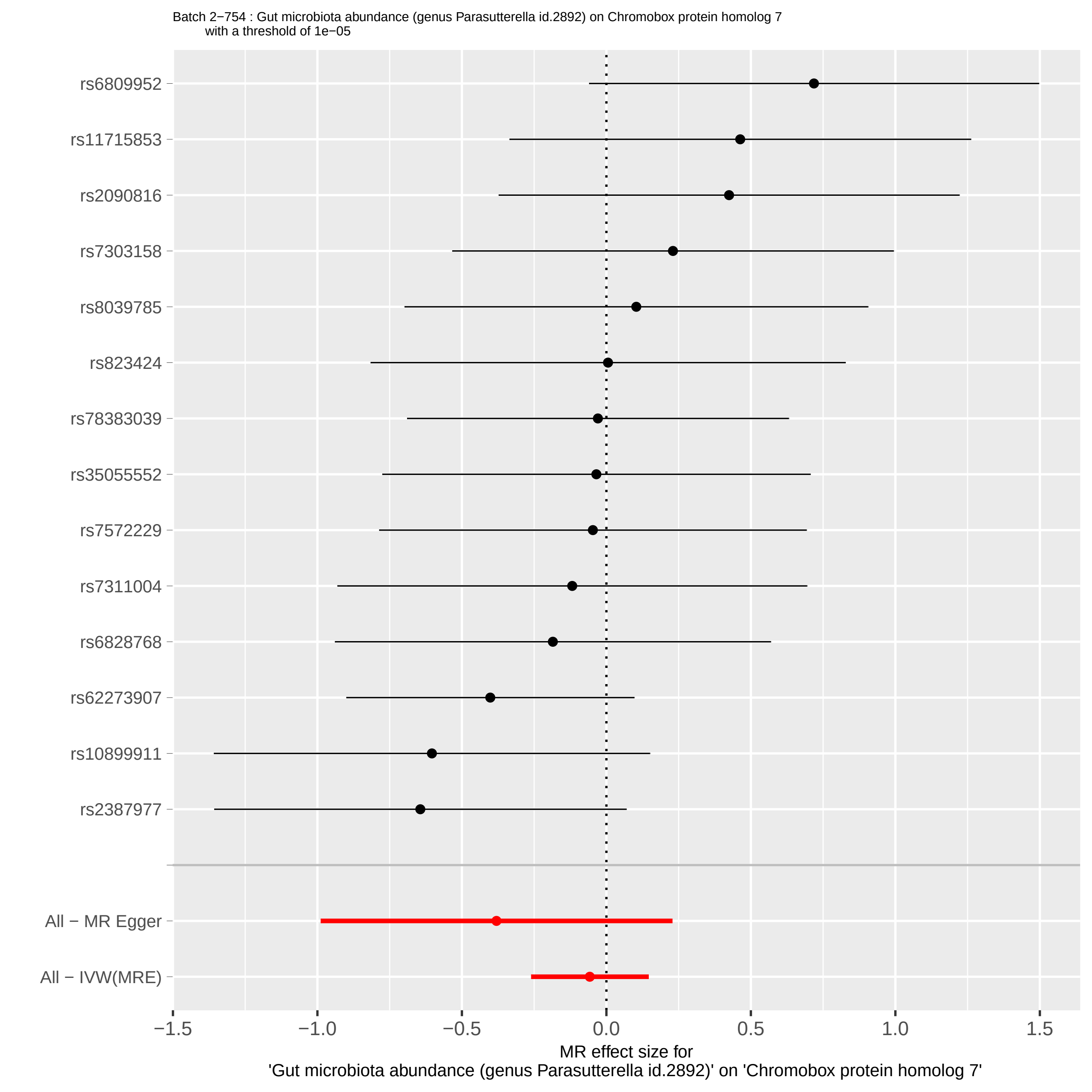
Batch 2−754 : Gut microbiota abundance (genus Parasutterella id.2892) on Chromobox protein homolog 7
with a threshold of 1e−05
rs6809952
rs11715853
rs2090816
rs7303158
rs8039785
rs823424
rs78383039
rs35055552
rs7572229
rs7311004
rs6828768
rs62273907
rs10899911
rs2387977
All − MR Egger
All − IVW(MRE)
−1.5
−1.0
−0.5
0.0
0.5
1.0
1.5
MR effect size for
'Gut microbiota abundance (genus Parasutterella id.2892)' on 'Chromobox protein homolog 7'

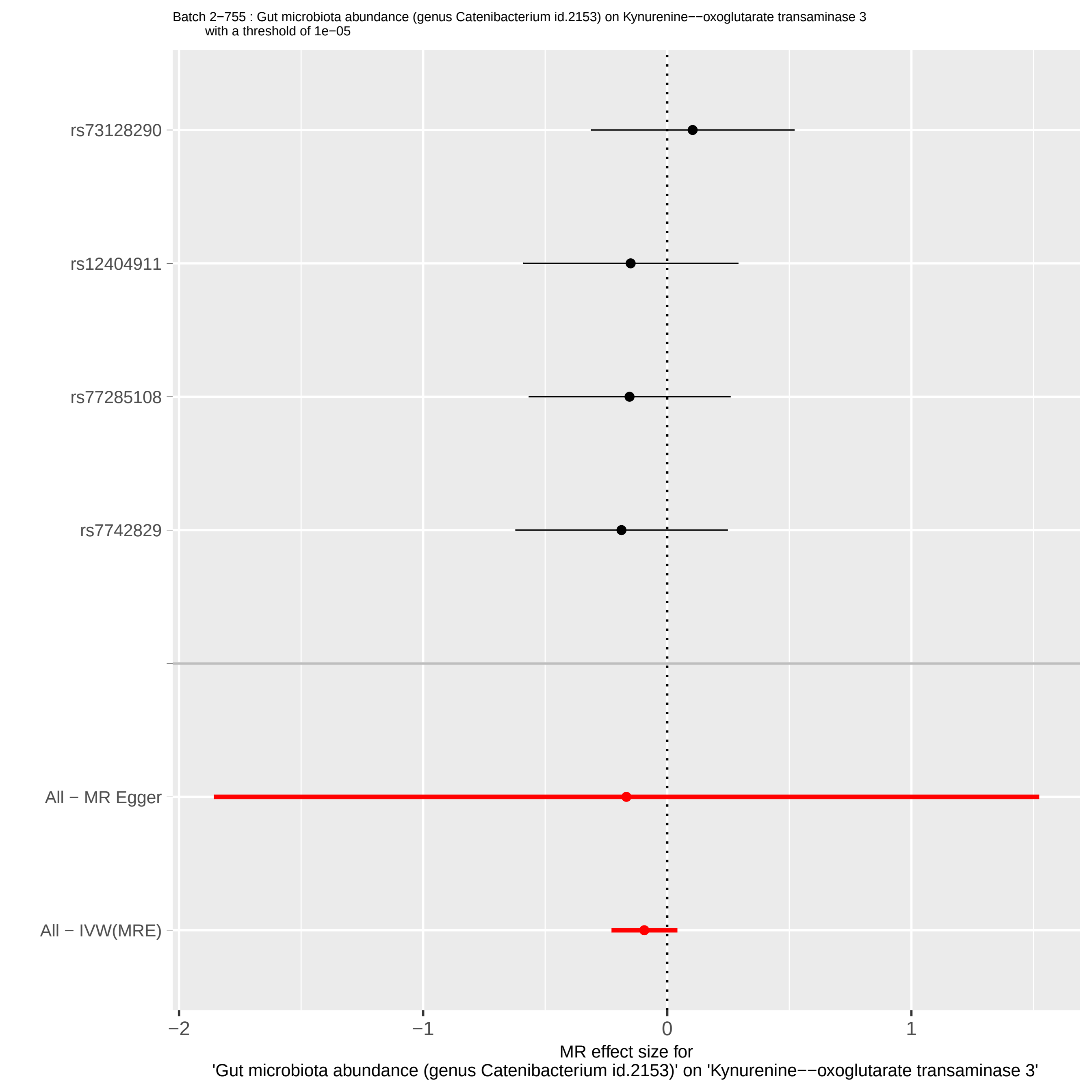

Batch 2−755 : Gut microbiota abundance (genus Catenibacterium id.2153) on Kynurenine−−oxoglutarate transaminase 3
with a threshold of 1e−05
rs73128290
rs12404911
rs77285108
rs7742829
All − MR Egger
All − IVW(MRE)
−2
−1
0
1
MR effect size for
'Gut microbiota abundance (genus Catenibacterium id.2153)' on 'Kynurenine−−oxoglutarate transaminase 3'

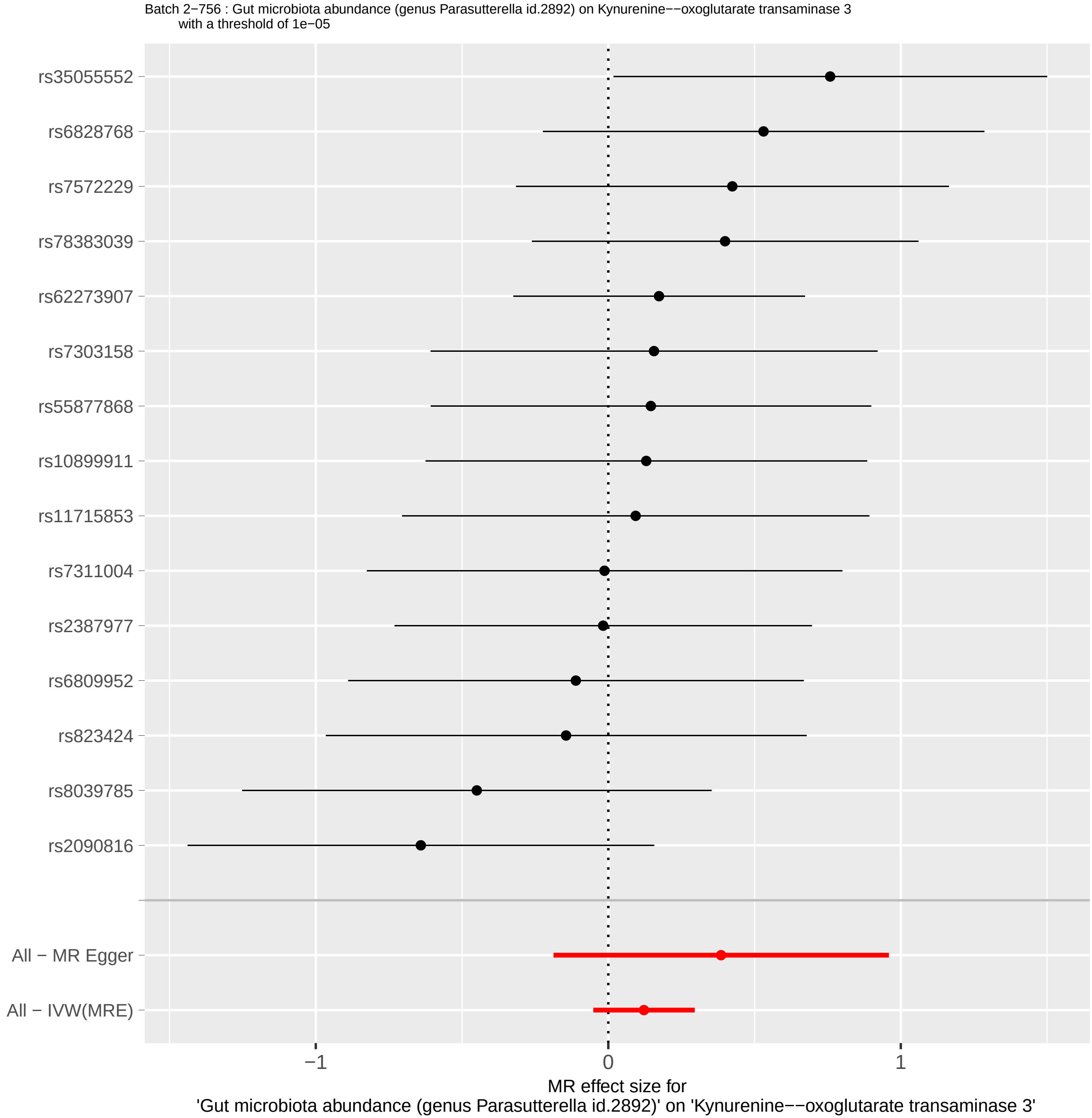

Batch 2−756 : Gut microbiota abundance (genus Parasutterella id.2892) on Kynurenine−−oxoglutarate transaminase 3
with a threshold of 1e−05
rs35055552
rs6828768
rs7572229
rs78383039
rs62273907
rs7303158
rs55877868
rs10899911
rs11715853
rs7311004
rs2387977
rs6809952
rs823424
rs8039785
rs2090816
All − MR Egger
All − IVW(MRE)
−1
0
1
MR effect size for
'Gut microbiota abundance (genus Parasutterella id.2892)' on 'Kynurenine−−oxoglutarate transaminase 3'

Batch 2−757 : Gut microbiota abundance (genus Catenibacterium id.2153) on SAGA−associated factor 29 homolog with a threshold of 1e−05

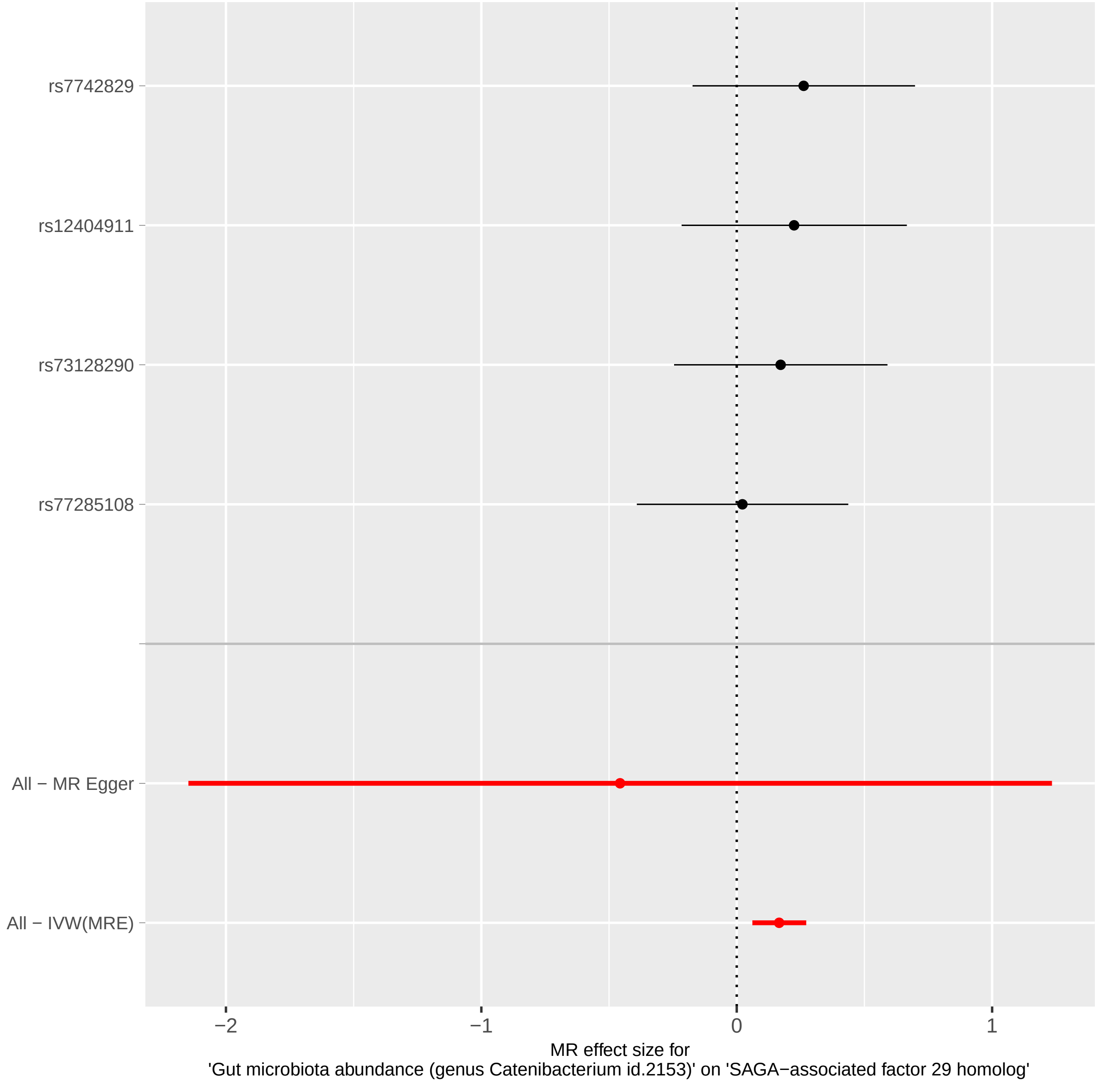

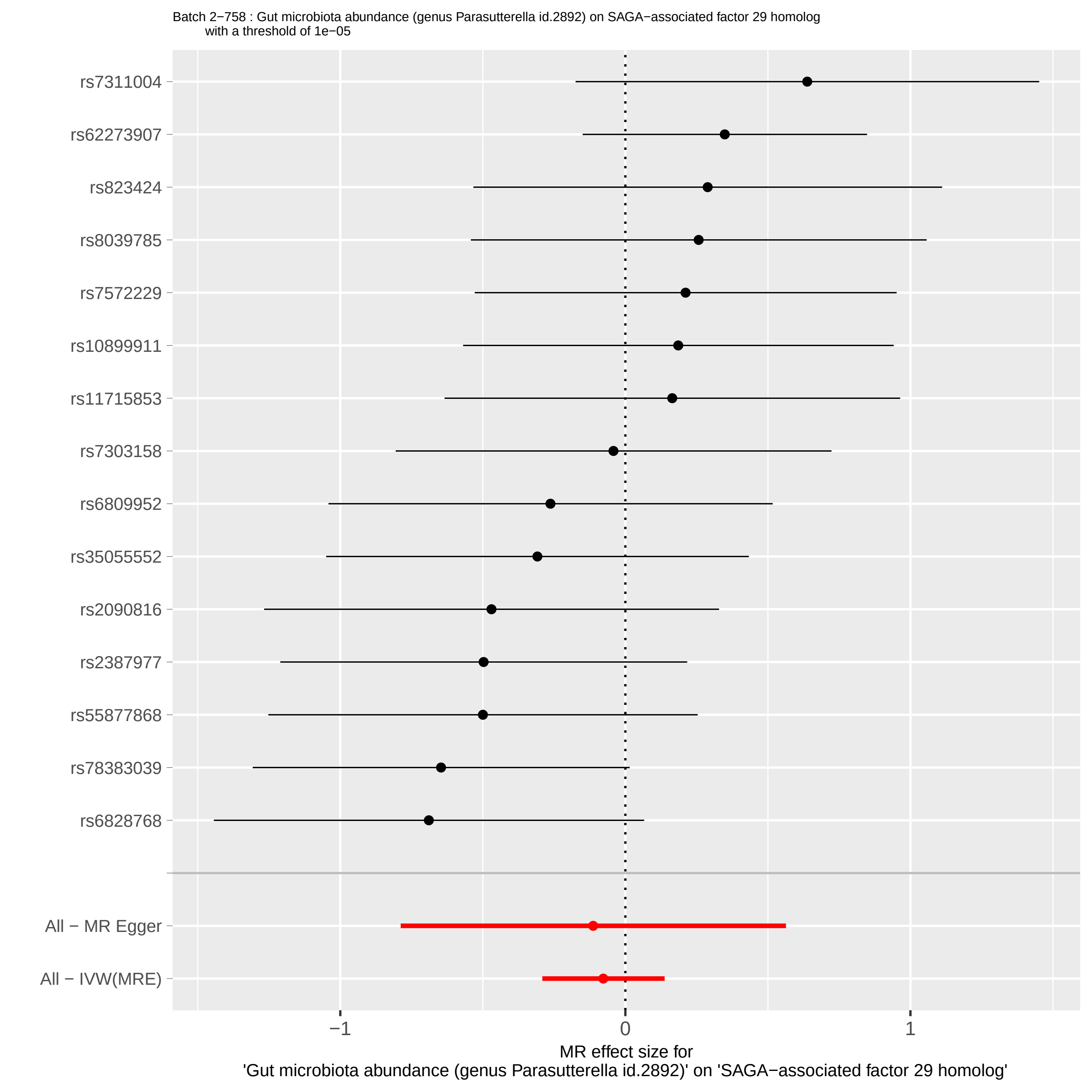

Batch 2−758 : Gut microbiota abundance (genus Parasutterella id.2892) on SAGA−associated factor 29 homolog
with a threshold of 1e−05
rs7311004
rs62273907
rs823424
rs8039785
rs7572229
rs10899911
rs11715853
rs7303158
rs6809952
rs35055552
rs2090816
rs2387977
rs55877868
rs78383039
rs6828768
All − MR Egger
All − IVW(MRE)
−1
0
1
MR effect size for
'Gut microbiota abundance (genus Parasutterella id.2892)' on 'SAGA−associated factor 29 homolog'

Batch 2−759 : Gut microbiota abundance (genus Catenibacterium id.2153) on Coiled−coil domain−containing protein 126
with a threshold of 1e−05

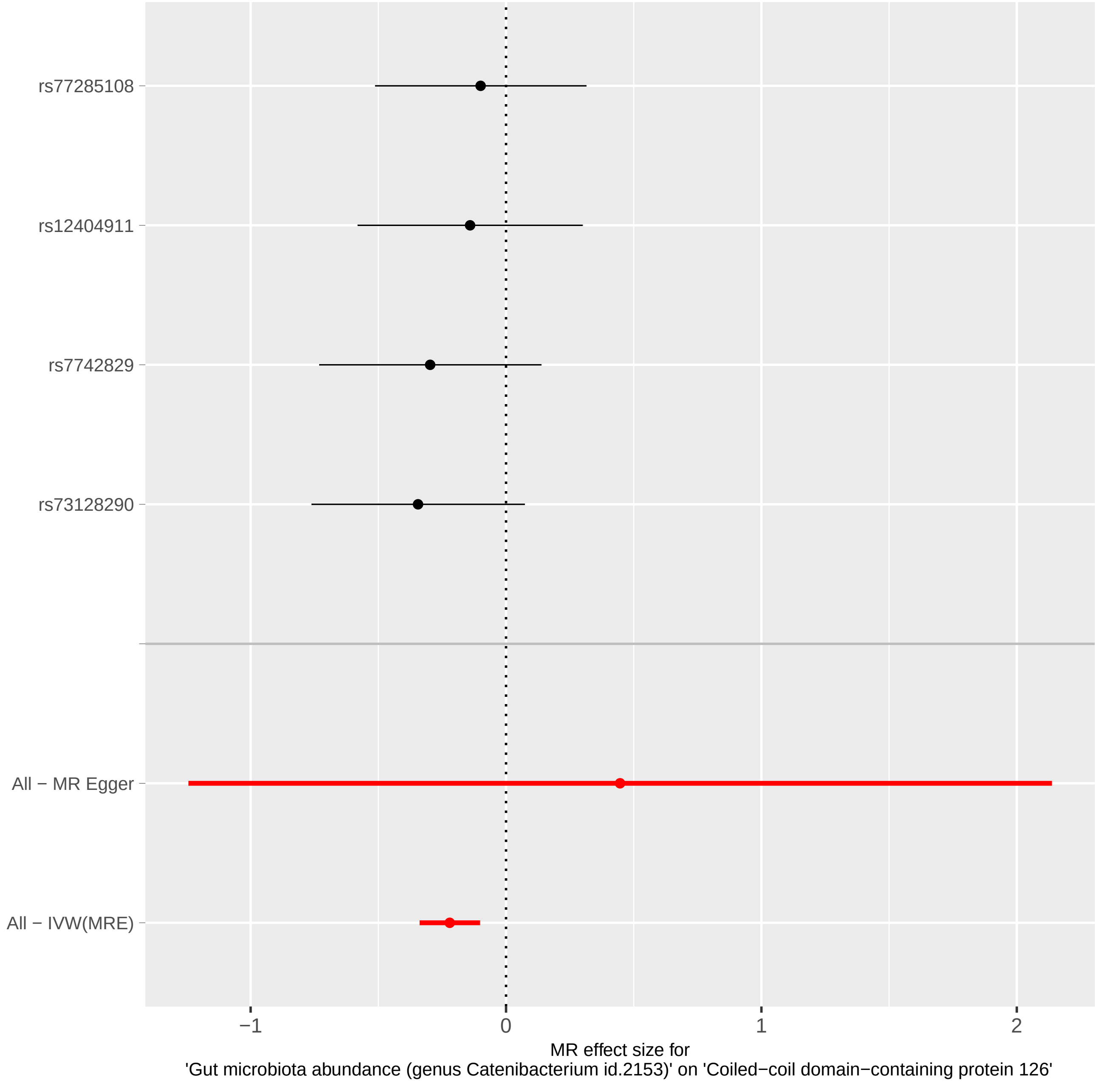

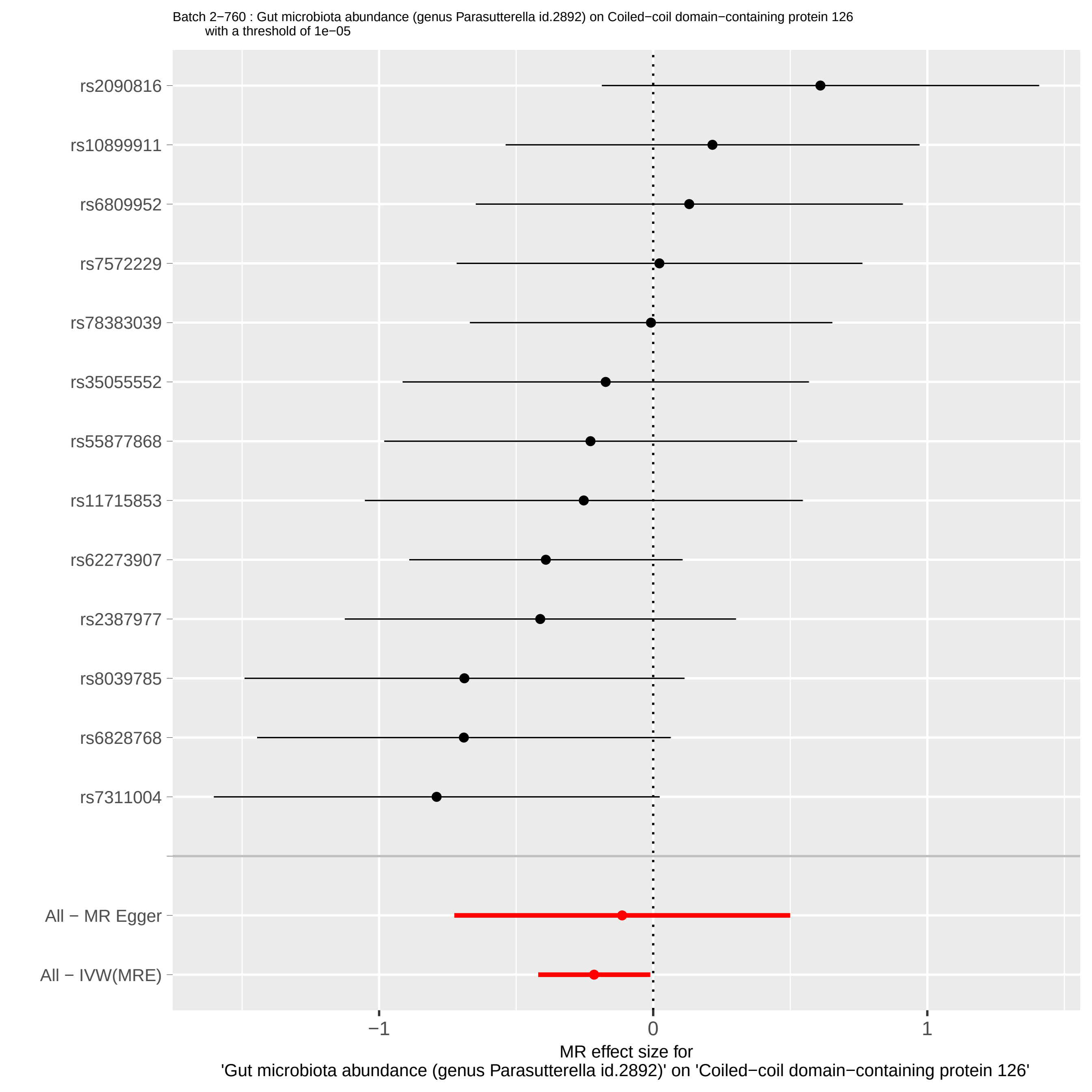

Batch 2−760 : Gut microbiota abundance (genus Parasutterella id.2892) on Coiled−coil domain−containing protein 126
with a threshold of 1e−05
rs2090816
rs10899911
rs6809952
rs7572229
rs78383039
rs35055552
rs55877868
rs11715853
rs62273907
rs2387977
rs8039785
rs6828768
rs7311004
All − MR Egger
All − IVW(MRE)
−1
0
1
MR effect size for
'Gut microbiota abundance (genus Parasutterella id.2892)' on 'Coiled−coil domain−containing protein 126'

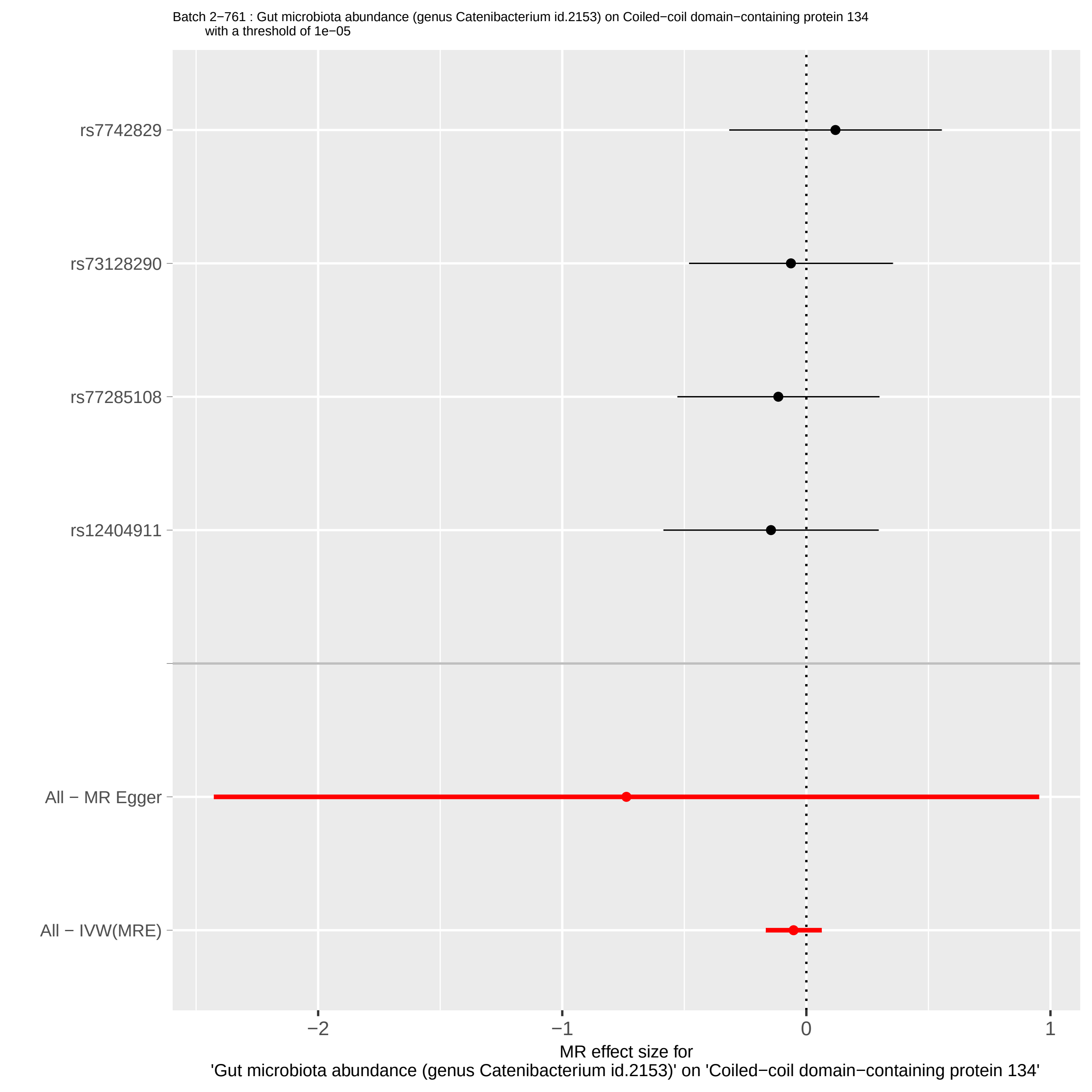
Batch 2−761 : Gut microbiota abundance (genus Catenibacterium id.2153) on Coiled−coil domain−containing protein 134
with a threshold of 1e−05
rs7742829
rs73128290
rs77285108
rs12404911
All − MR Egger
All − IVW(MRE)
−2
−1
0
1
MR effect size for
'Gut microbiota abundance (genus Catenibacterium id.2153)' on 'Coiled−coil domain−containing protein 134'

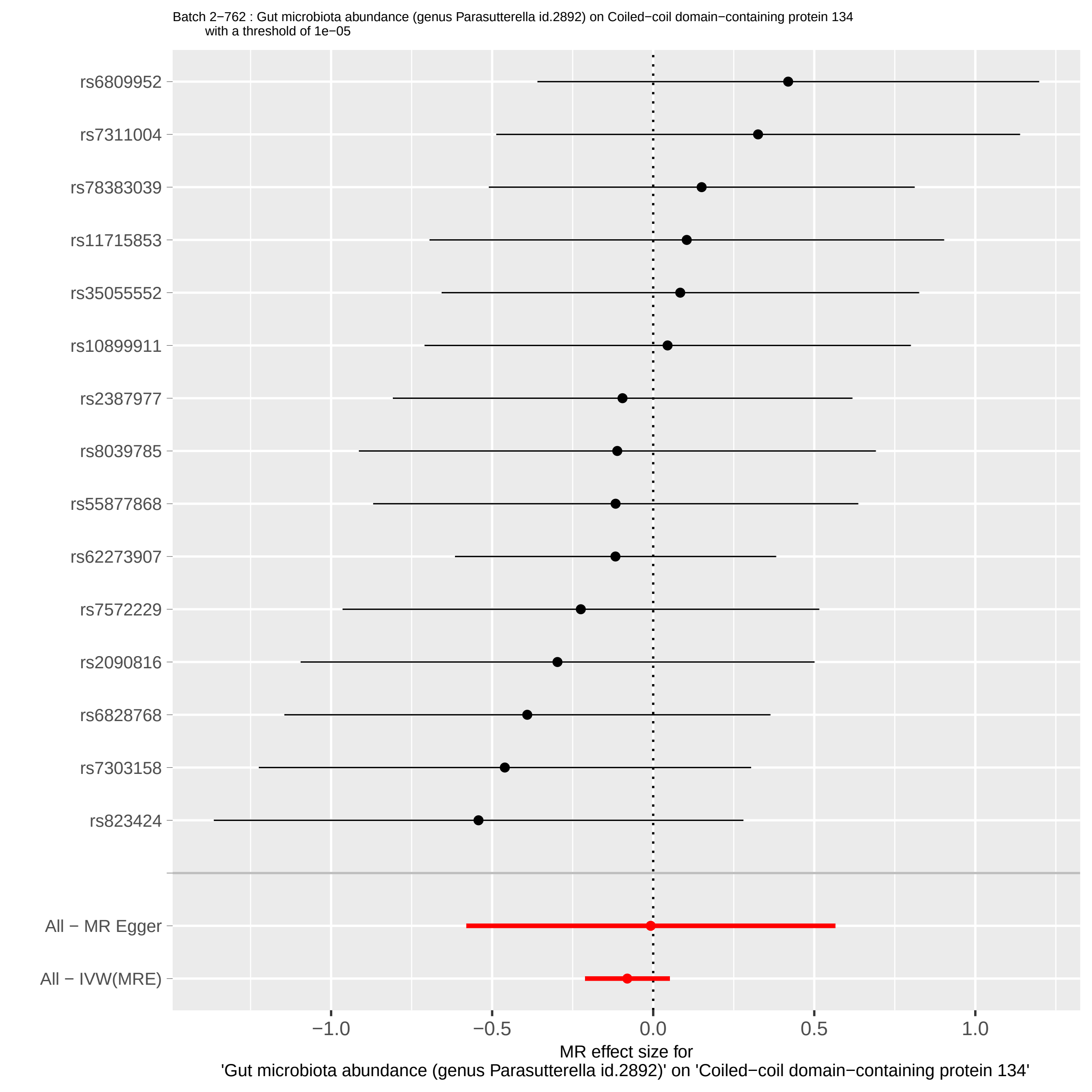

Batch 2−762 : Gut microbiota abundance (genus Parasutterella id.2892) on Coiled−coil domain−containing protein 134
with a threshold of 1e−05
rs6809952
rs7311004
rs78383039
rs11715853
rs35055552
rs10899911
rs2387977
rs8039785
rs55877868
rs62273907
rs7572229
rs2090816
rs6828768
rs7303158
rs823424
All − MR Egger
All − IVW(MRE)
−1.0
−0.5
0.0
0.5
1.0
MR effect size for
'Gut microbiota abundance (genus Parasutterella id.2892)' on 'Coiled−coil domain−containing protein 134'

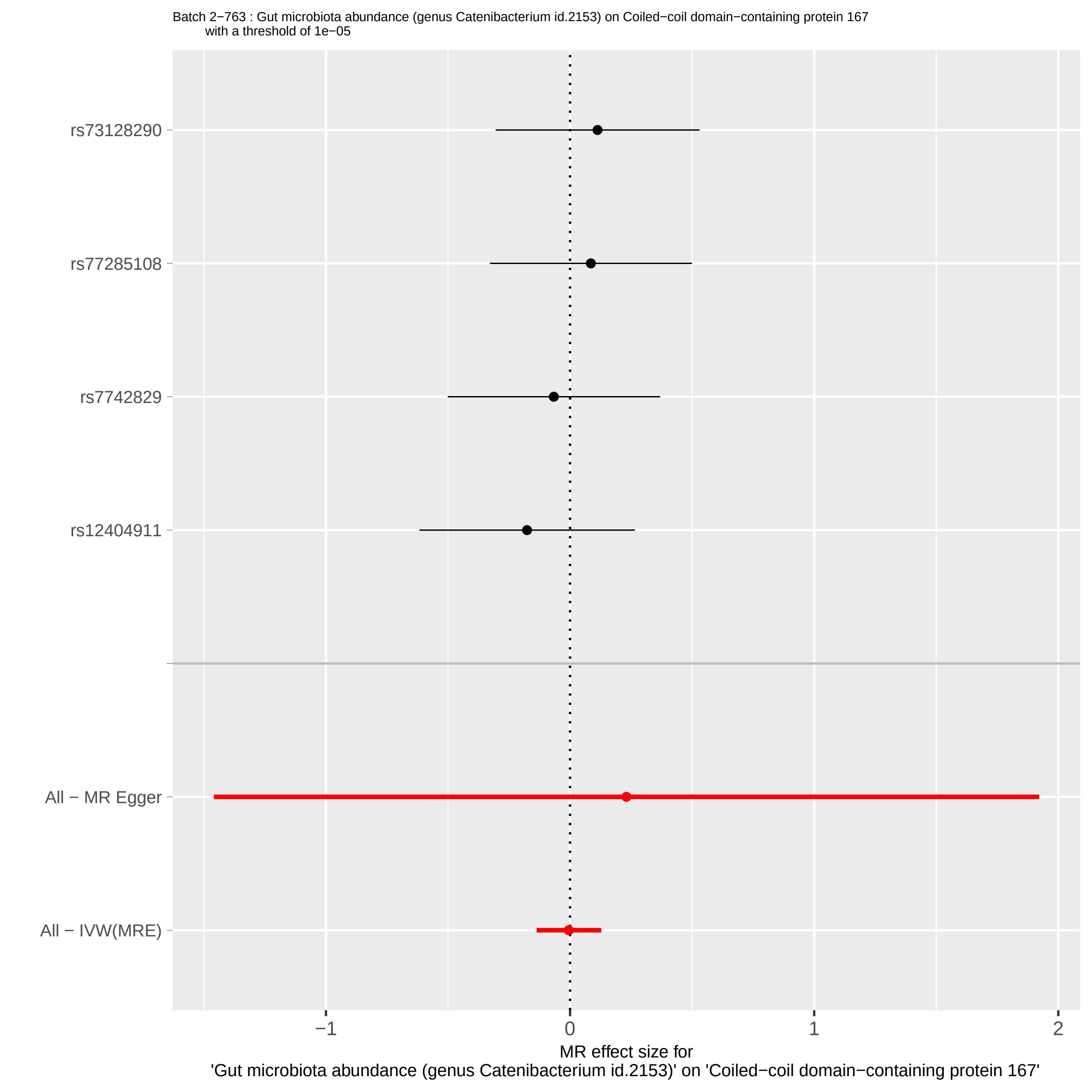

Batch 2−763 : Gut microbiota abundance (genus Catenibacterium id.2153) on Coiled−coil domain−containing protein 167
with a threshold of 1e−05
rs73128290
rs77285108
rs7742829
rs12404911
All − MR Egger
All − IVW(MRE)
−1
0
1
2
MR effect size for
'Gut microbiota abundance (genus Catenibacterium id.2153)' on 'Coiled−coil domain−containing protein 167'

Batch 2−764 : Gut microbiota abundance (genus Parasutterella id.2892) on Coiled−coil domain−containing protein 167
with a threshold of 1e−05

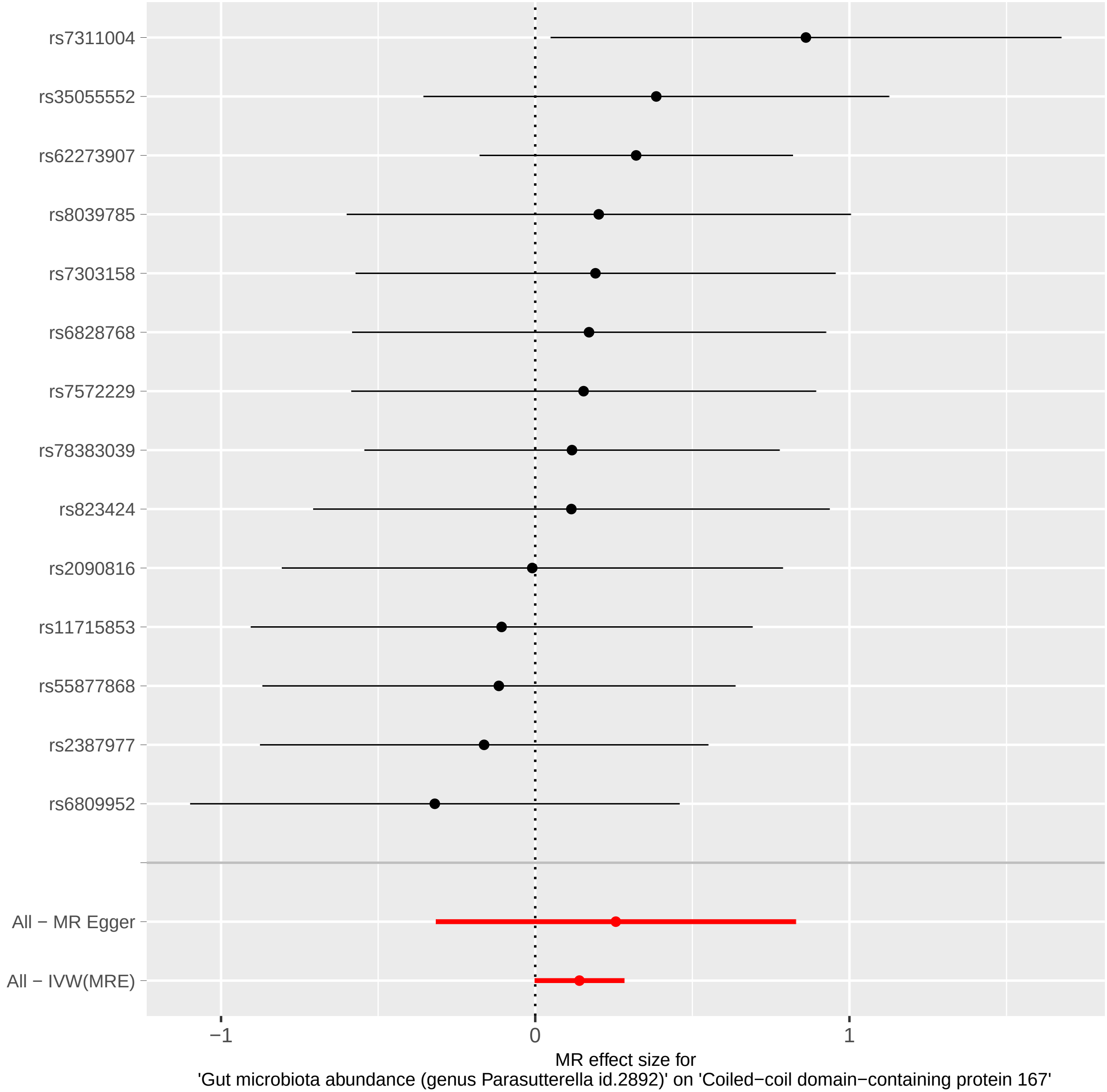

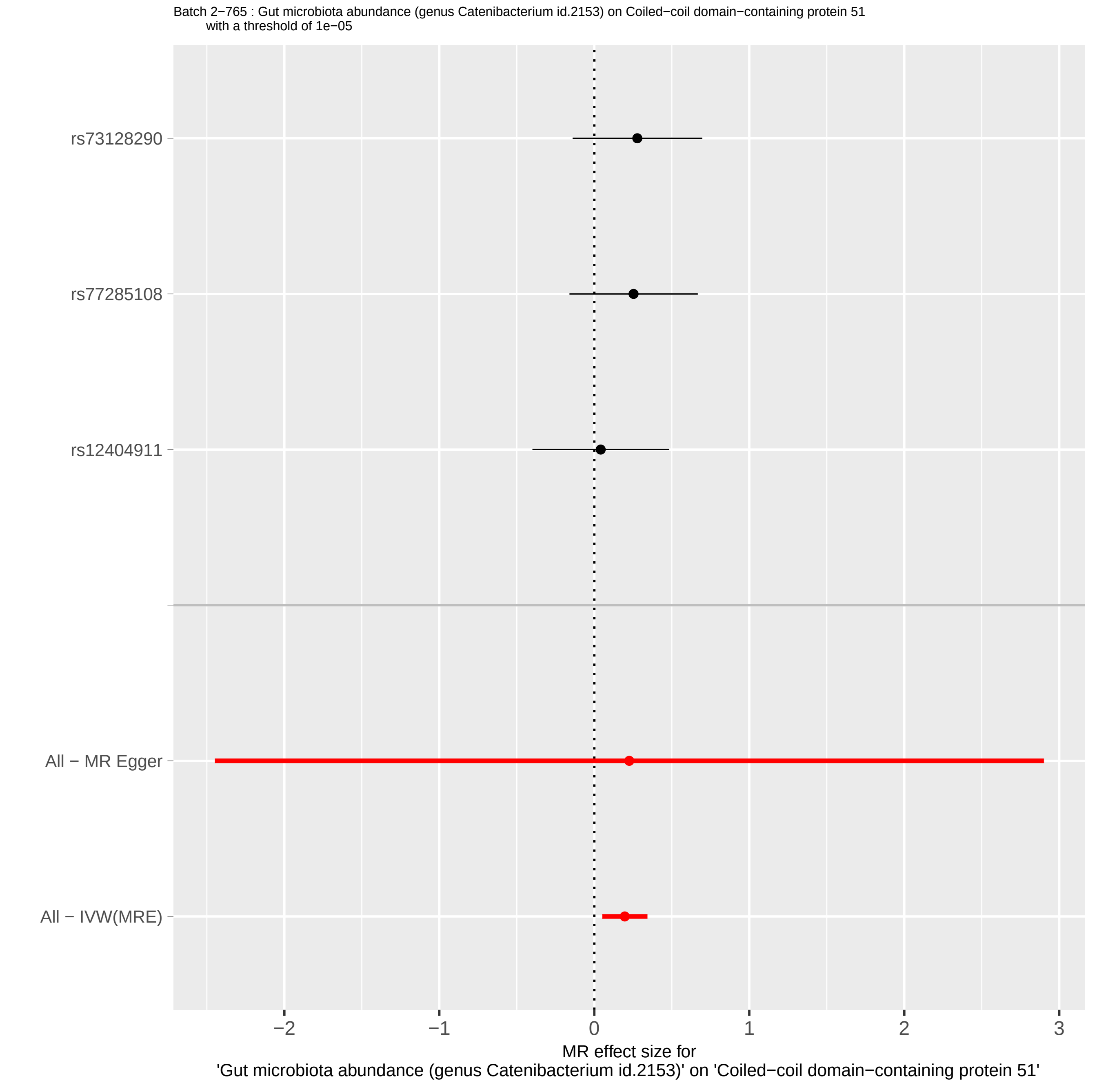
Batch 2−765 : Gut microbiota abundance (genus Catenibacterium id.2153) on Coiled−coil domain−containing protein 51
with a threshold of 1e−05
rs73128290
rs77285108
rs12404911
All − MR Egger
All − IVW(MRE)
−2
−1
0
1
2
3
MR effect size for
'Gut microbiota abundance (genus Catenibacterium id.2153)' on 'Coiled−coil domain−containing protein 51'

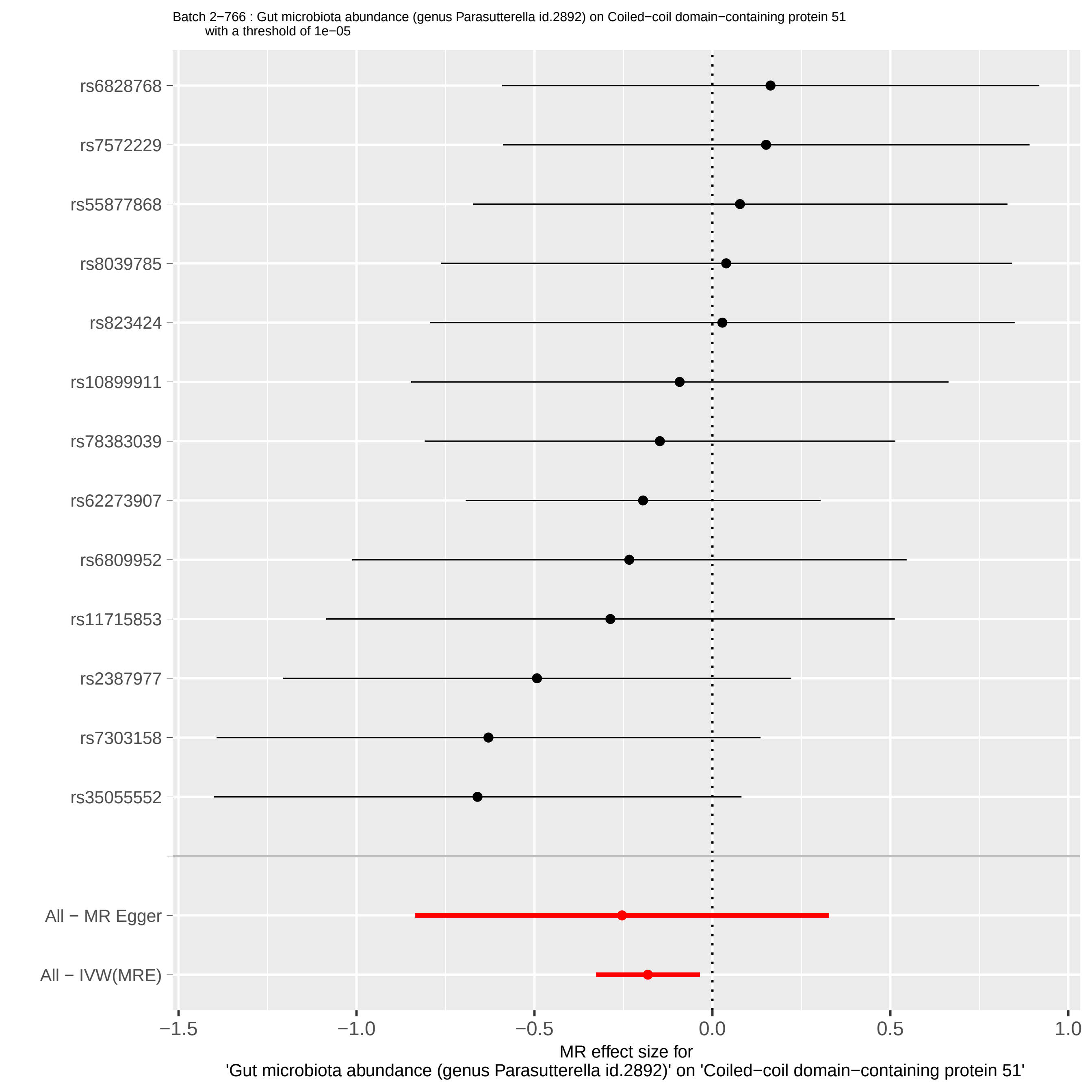
Batch 2−766 : Gut microbiota abundance (genus Parasutterella id.2892) on Coiled−coil domain−containing protein 51
with a threshold of 1e−05
rs6828768
rs7572229
rs55877868
rs8039785
rs823424
rs10899911
rs78383039
rs62273907
rs6809952
rs11715853
rs2387977
rs7303158
rs35055552
All − MR Egger
All − IVW(MRE)
−1.5
−1.0
−0.5
0.0
0.5
1.0
MR effect size for
'Gut microbiota abundance (genus Parasutterella id.2892)' on 'Coiled−coil domain−containing protein 51'

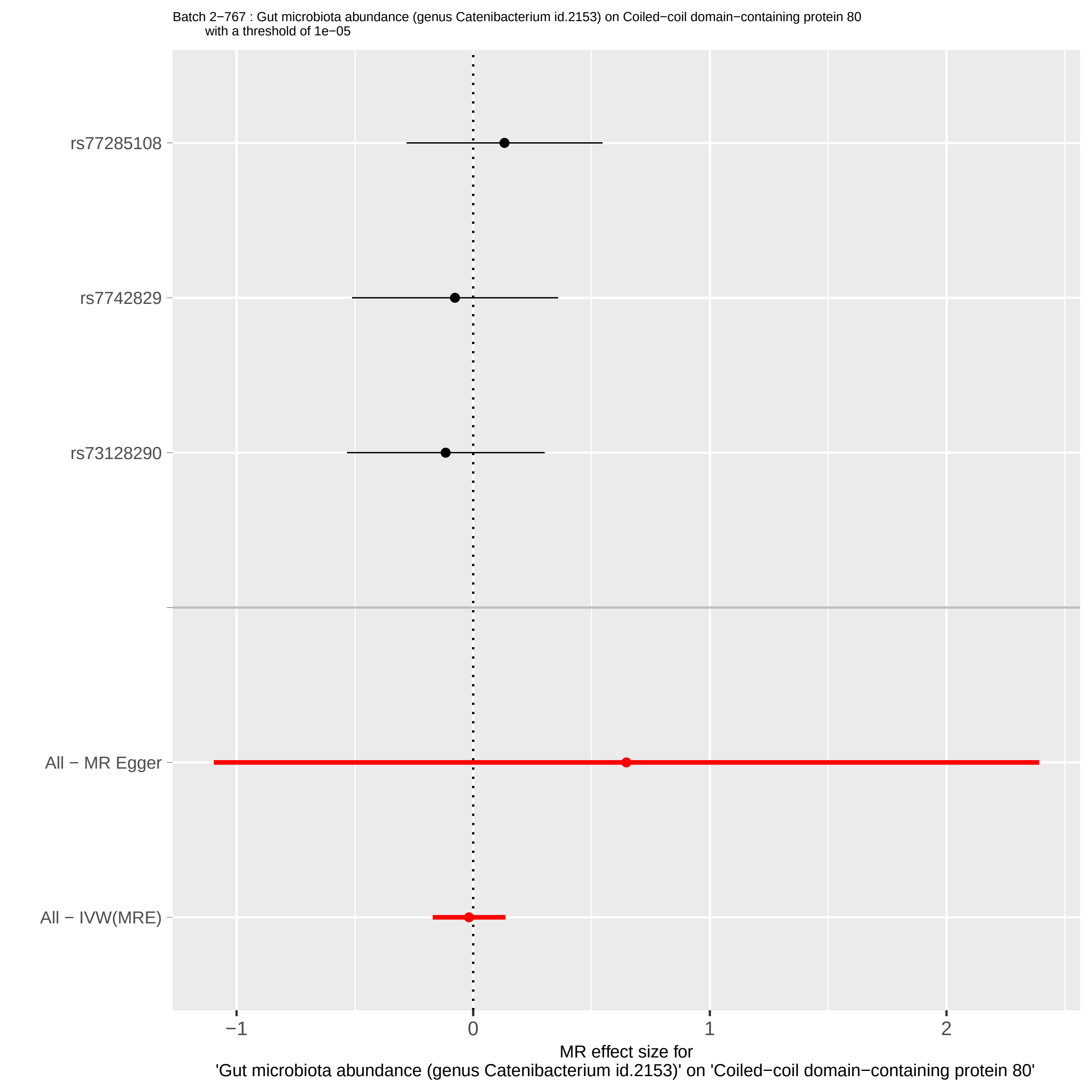

Batch 2−767 : Gut microbiota abundance (genus Catenibacterium id.2153) on Coiled−coil domain−containing protein 80
with a threshold of 1e−05
rs77285108
rs7742829
rs73128290
All − MR Egger
All − IVW(MRE)
−1
0
1
2
MR effect size for
'Gut microbiota abundance (genus Catenibacterium id.2153)' on 'Coiled−coil domain−containing protein 80'

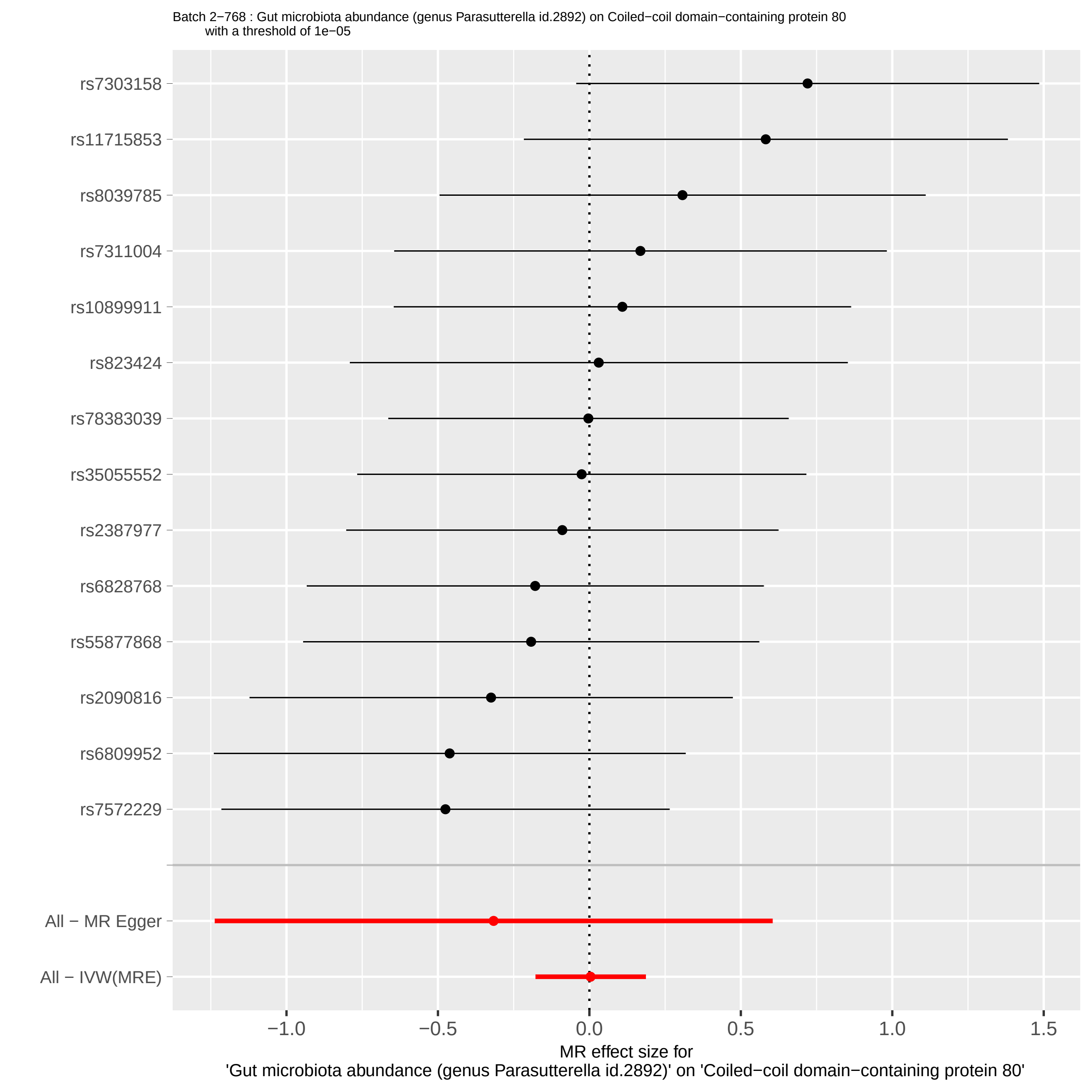

Batch 2−768 : Gut microbiota abundance (genus Parasutterella id.2892) on Coiled−coil domain−containing protein 80
with a threshold of 1e−05
rs7303158
rs11715853
rs8039785
rs7311004
rs10899911
rs823424
rs78383039
rs35055552
rs2387977
rs6828768
rs55877868
rs2090816
rs6809952
rs7572229
All − MR Egger
All − IVW(MRE)
−1.0
−0.5
0.0
0.5
1.0
1.5
MR effect size for
'Gut microbiota abundance (genus Parasutterella id.2892)' on 'Coiled−coil domain−containing protein 80'

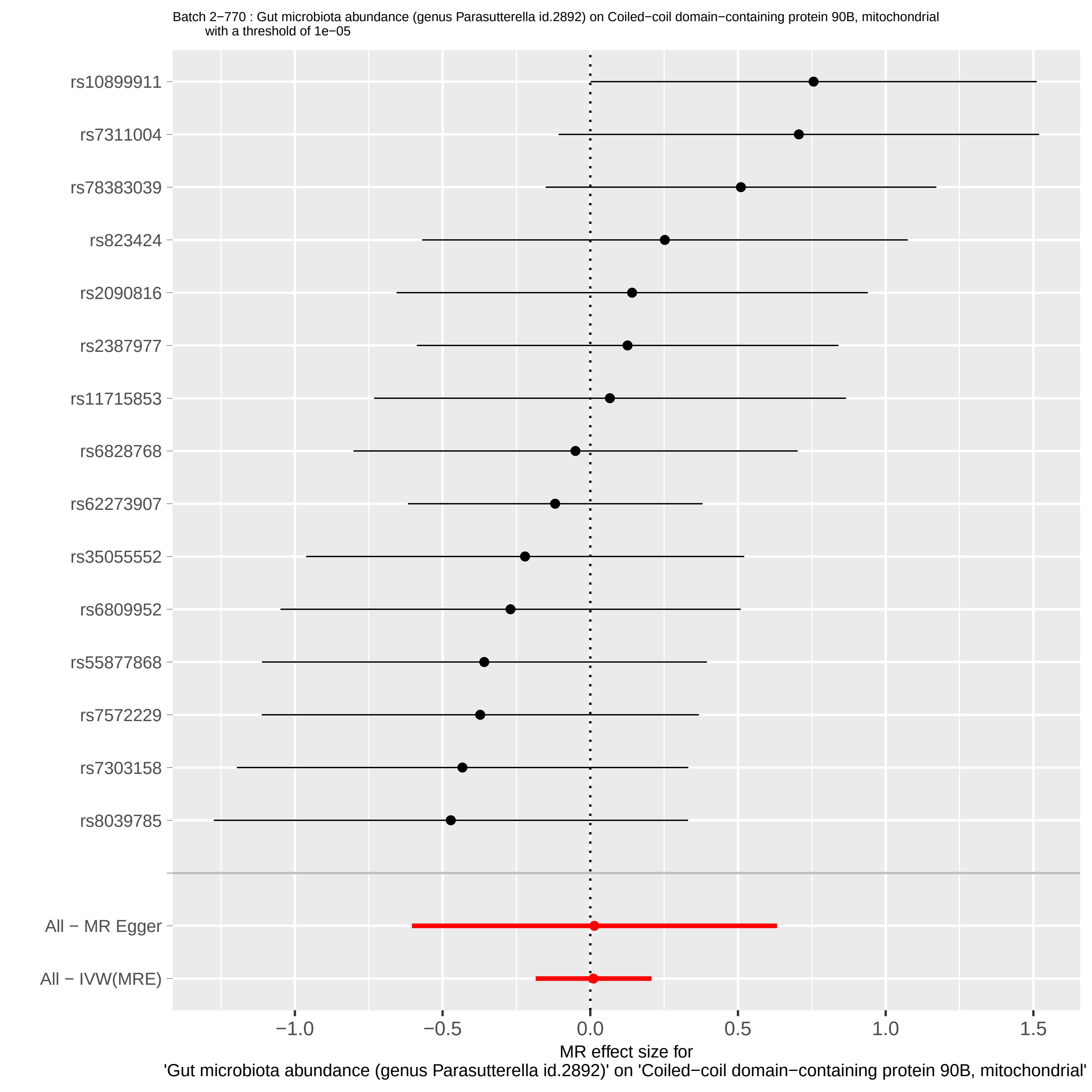
Batch 2−770 : Gut microbiota abundance (genus Parasutterella id.2892) on Coiled−coil domain−containing protein 90B, mitochondrial
with a threshold of 1e−05
rs10899911
rs7311004
rs78383039
rs823424
rs2090816
rs2387977
rs11715853
rs6828768
rs62273907
rs35055552
rs6809952
rs55877868
rs7572229
rs7303158
rs8039785
All − MR Egger
All − IVW(MRE)
−1.0
−0.5
0.0
0.5
1.0
1.5
MR effect size for
'Gut microbiota abundance (genus Parasutterella id.2892)' on 'Coiled−coil domain−containing protein 90B, mitochondrial'

Batch 2−771 : Gut microbiota abundance (genus Catenibacterium id.2153) on C−C motif chemokine 1
with a threshold of 1e−05

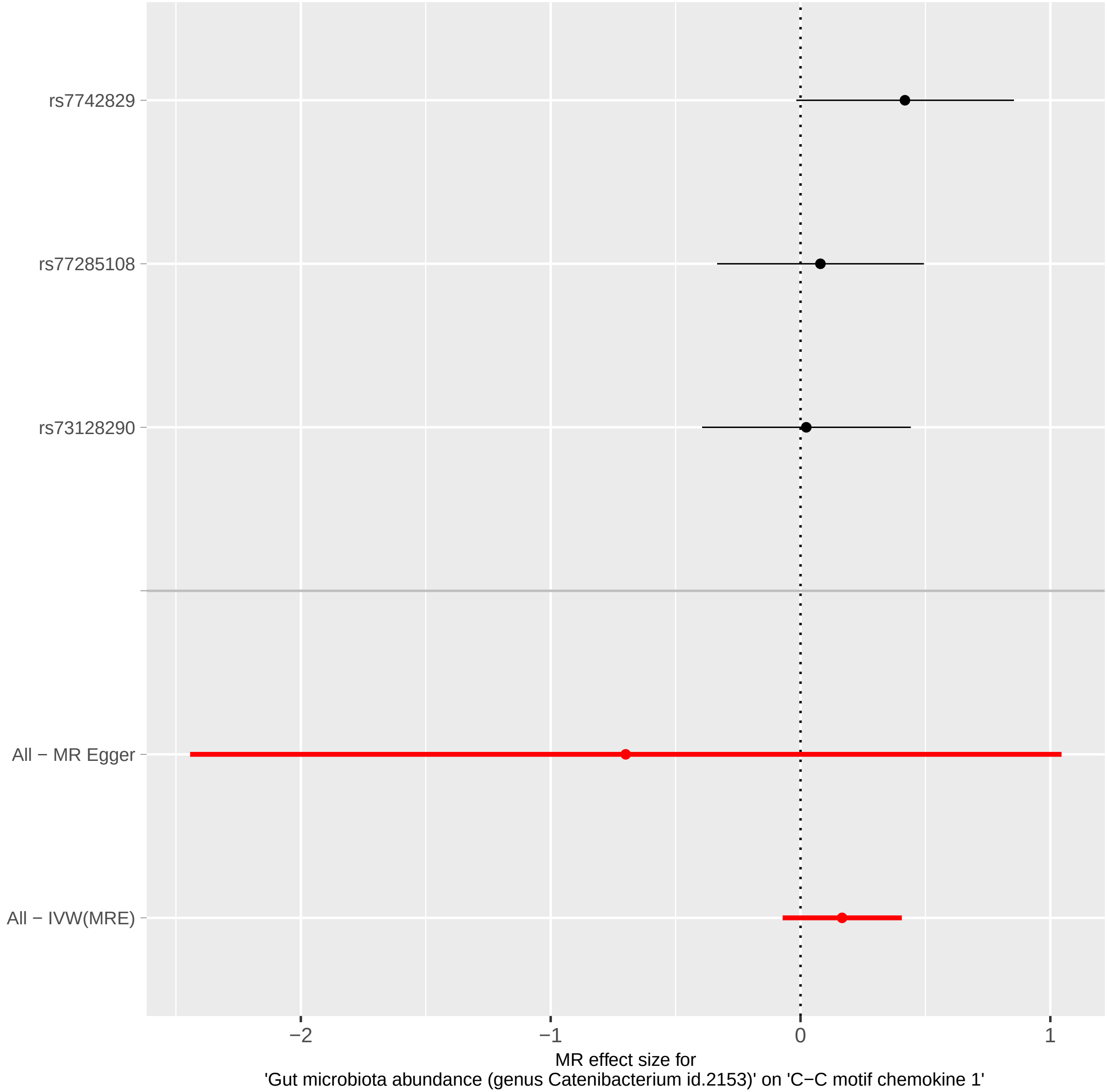

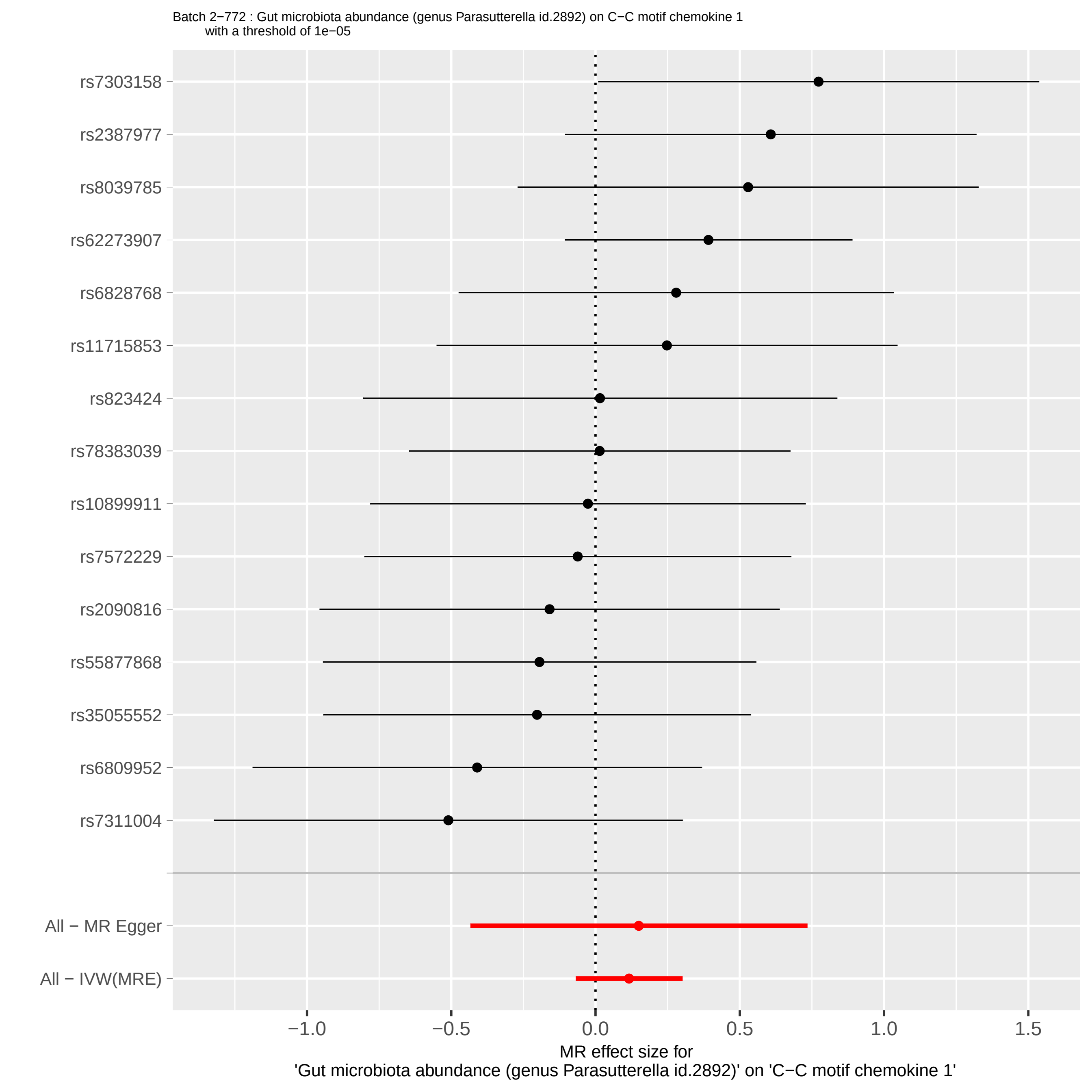

Batch 2−772 : Gut microbiota abundance (genus Parasutterella id.2892) on C−C motif chemokine 1
with a threshold of 1e−05
rs7303158
rs2387977
rs8039785
rs62273907
rs6828768
rs11715853
rs823424
rs78383039
rs10899911
rs7572229
rs2090816
rs55877868
rs35055552
rs6809952
rs7311004
All − MR Egger
All − IVW(MRE)
−1.0
−0.5
0.0
0.5
1.0
1.5
MR effect size for
'Gut microbiota abundance (genus Parasutterella id.2892)' on 'C−C motif chemokine 1'

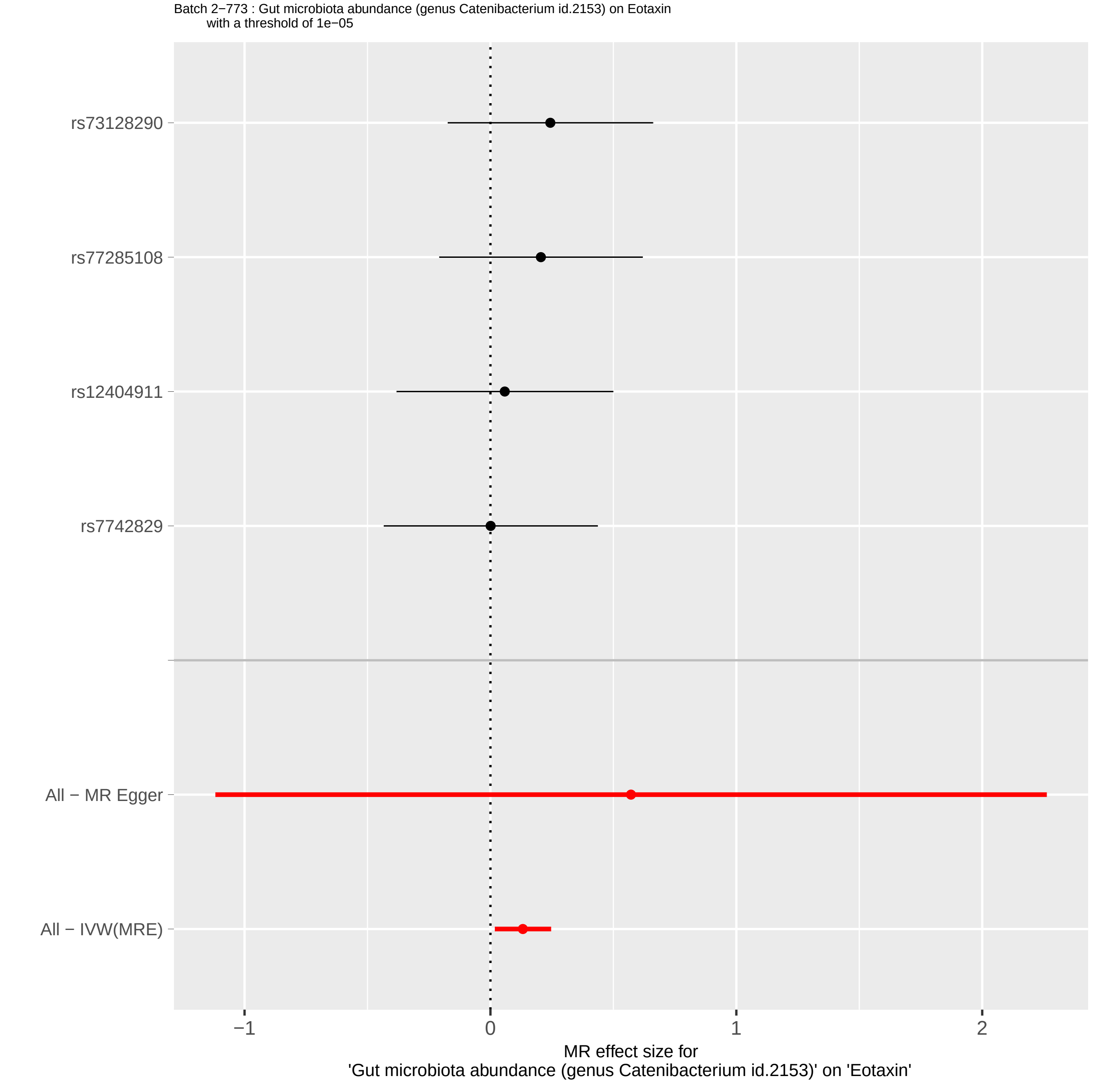

Batch 2−773 : Gut microbiota abundance (genus Catenibacterium id.2153) on Eotaxin
with a threshold of 1e−05
rs73128290
rs77285108
rs12404911
rs7742829
All − MR Egger
All − IVW(MRE)
−1
0
1
2
MR effect size for
'Gut microbiota abundance (genus Catenibacterium id.2153)' on 'Eotaxin'

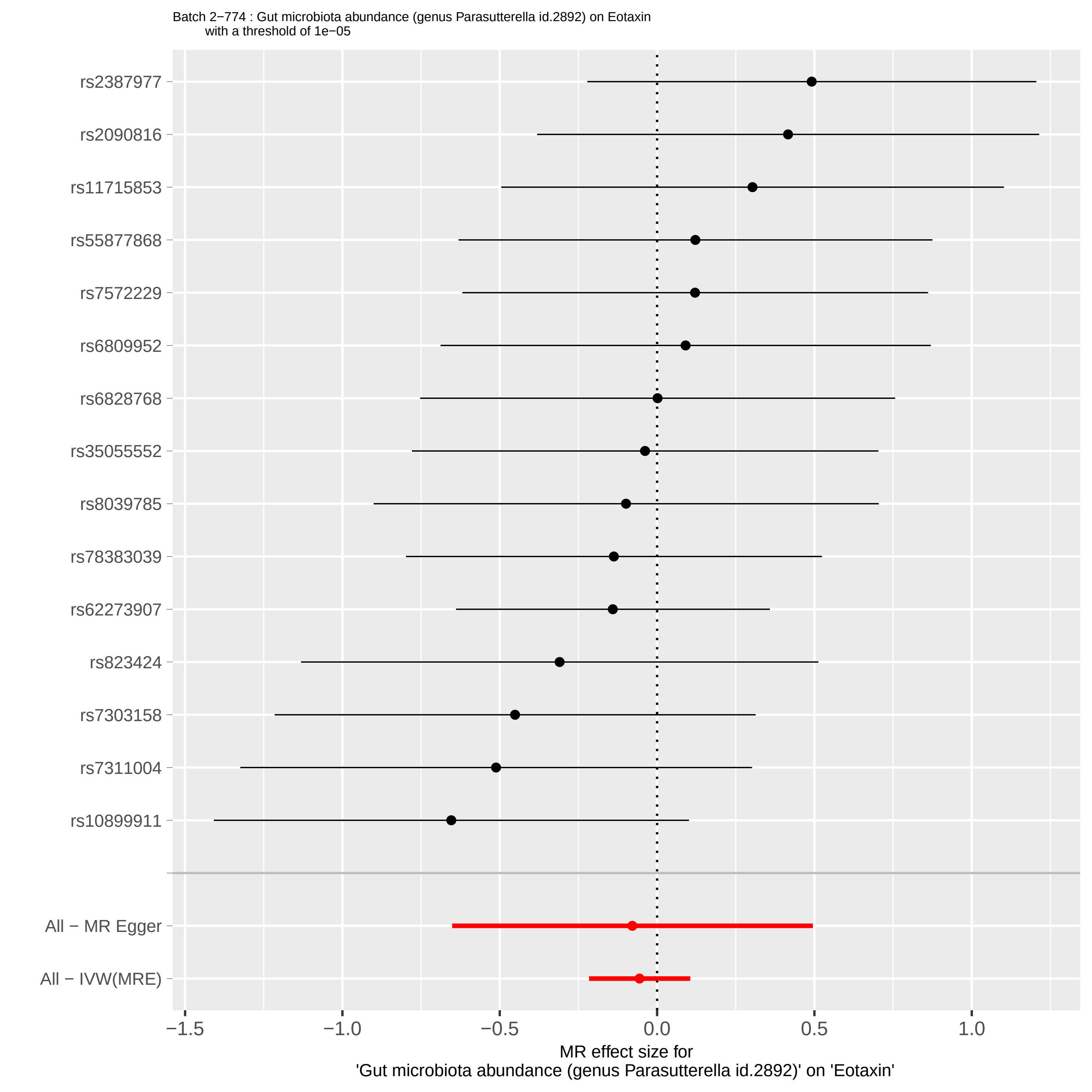

Batch 2−774 : Gut microbiota abundance (genus Parasutterella id.2892) on Eotaxin
with a threshold of 1e−05
rs2387977
rs2090816
rs11715853
rs55877868
rs7572229
rs6809952
rs6828768
rs35055552
rs8039785
rs78383039
rs62273907
rs823424
rs7303158
rs7311004
rs10899911
All − MR Egger
All − IVW(MRE)
−1.5
−1.0
−0.5
0.0
0.5
1.0
MR effect size for
'Gut microbiota abundance (genus Parasutterella id.2892)' on 'Eotaxin'

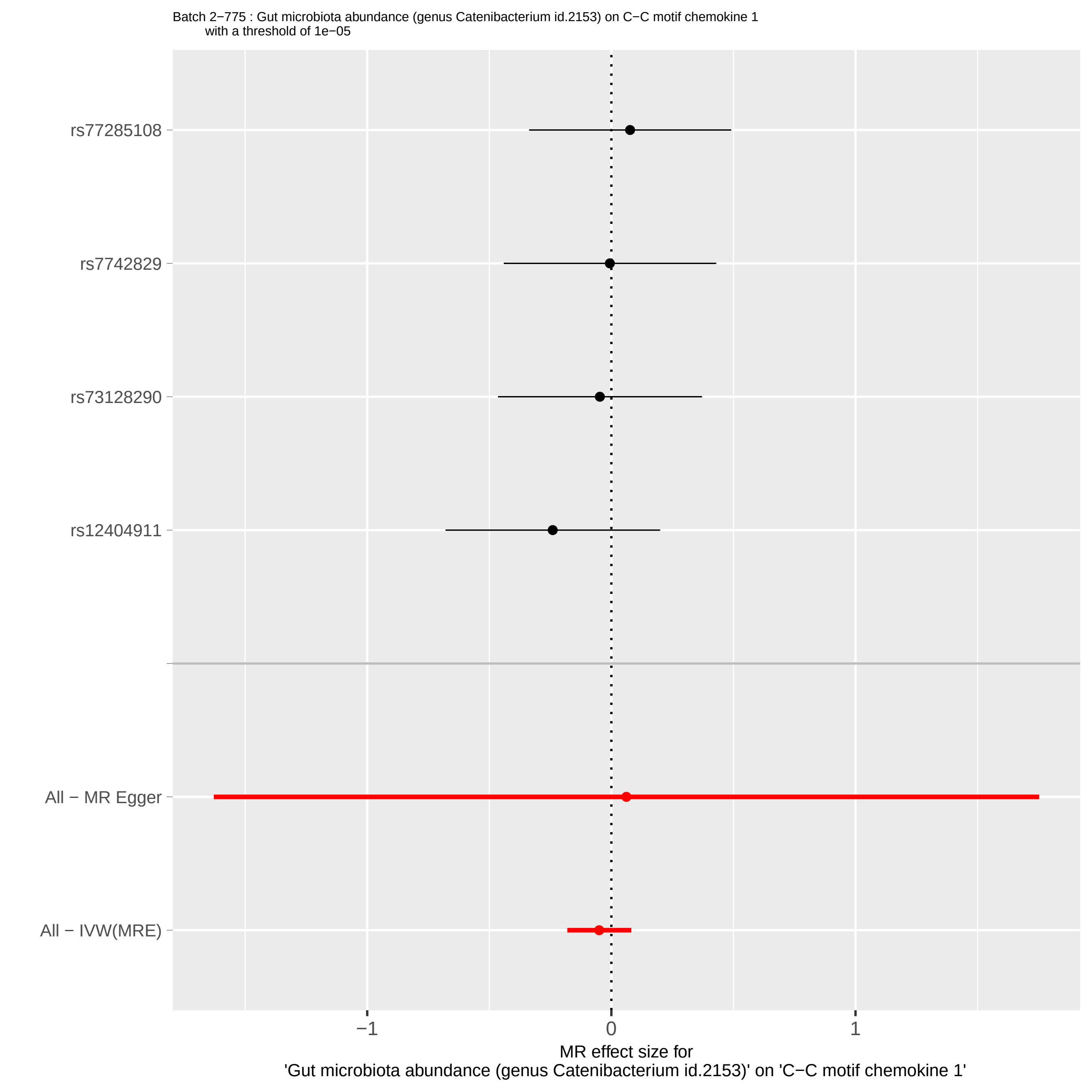

Batch 2−775 : Gut microbiota abundance (genus Catenibacterium id.2153) on C−C motif chemokine 1
with a threshold of 1e−05
rs77285108
rs7742829
rs73128290
rs12404911
All − MR Egger
All − IVW(MRE)
−1
0
1
MR effect size for
'Gut microbiota abundance (genus Catenibacterium id.2153)' on 'C−C motif chemokine 1'

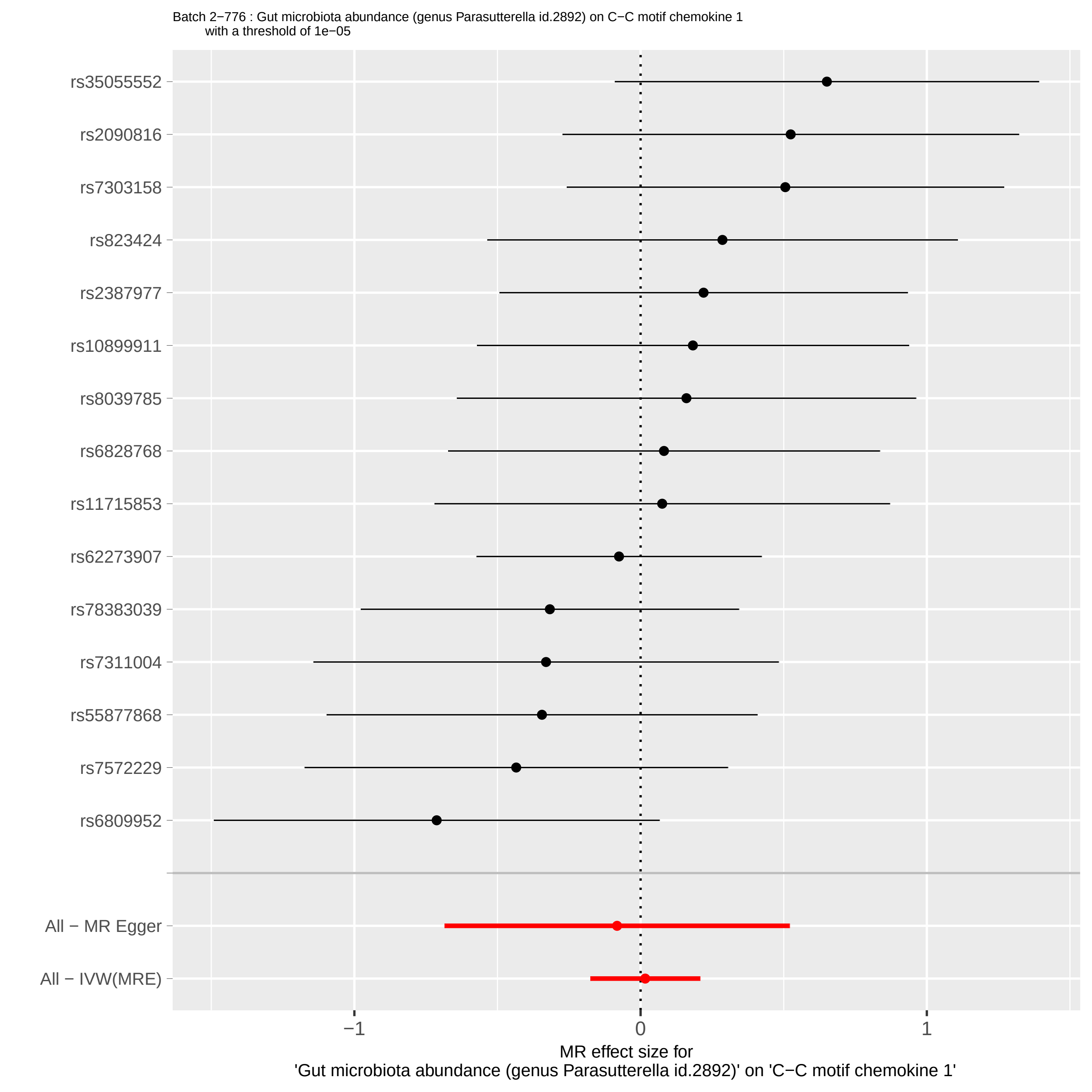
Batch 2−776 : Gut microbiota abundance (genus Parasutterella id.2892) on C−C motif chemokine 1
with a threshold of 1e−05
rs35055552
rs2090816
rs7303158
rs823424
rs2387977
rs10899911
rs8039785
rs6828768
rs11715853
rs62273907
rs78383039
rs7311004
rs55877868
rs7572229
rs6809952
All − MR Egger
All − IVW(MRE)
−1
0
1
MR effect size for
'Gut microbiota abundance (genus Parasutterella id.2892)' on 'C−C motif chemokine 1'

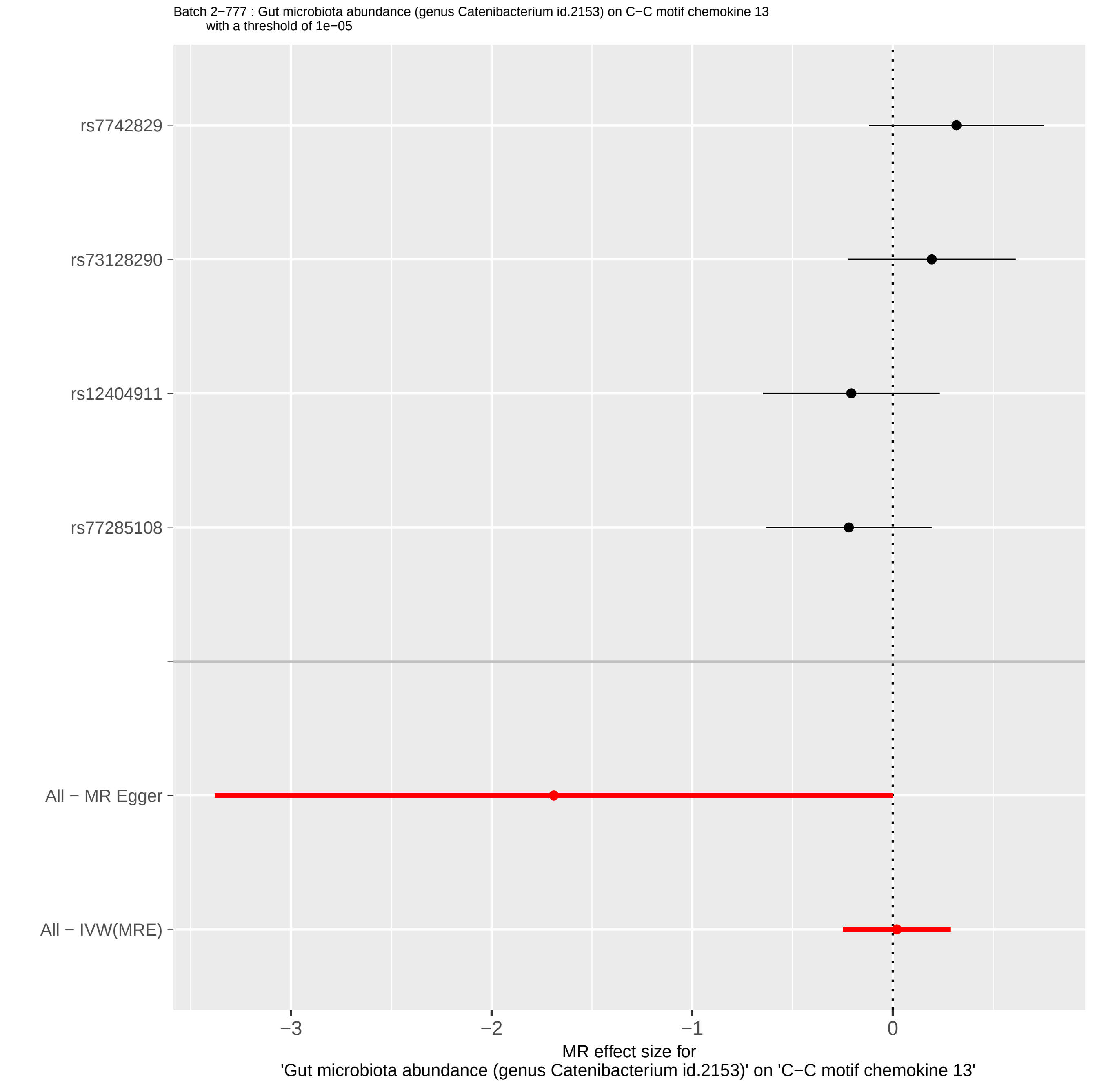
Batch 2−777 : Gut microbiota abundance (genus Catenibacterium id.2153) on C−C motif chemokine 13
with a threshold of 1e−05
rs7742829
rs73128290
rs12404911
rs77285108
All − MR Egger
All − IVW(MRE)
−3
−2
−1
0
MR effect size for
'Gut microbiota abundance (genus Catenibacterium id.2153)' on 'C−C motif chemokine 13'

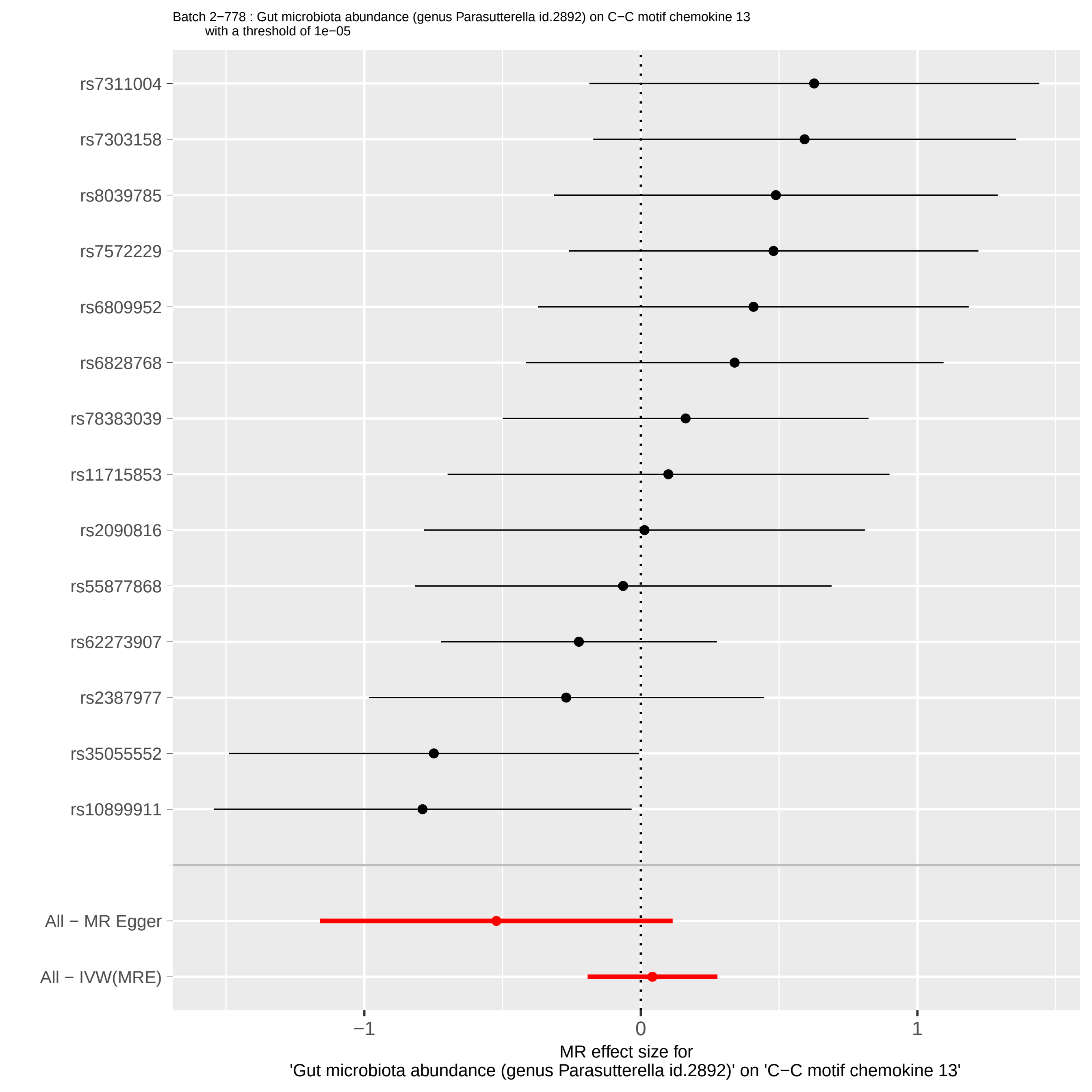

Batch 2−778 : Gut microbiota abundance (genus Parasutterella id.2892) on C−C motif chemokine 13
with a threshold of 1e−05
rs7311004
rs7303158
rs8039785
rs7572229
rs6809952
rs6828768
rs78383039
rs11715853
rs2090816
rs55877868
rs62273907
rs2387977
rs35055552
rs10899911
All − MR Egger
All − IVW(MRE)
−1
0
1
MR effect size for
'Gut microbiota abundance (genus Parasutterella id.2892)' on 'C−C motif chemokine 13'

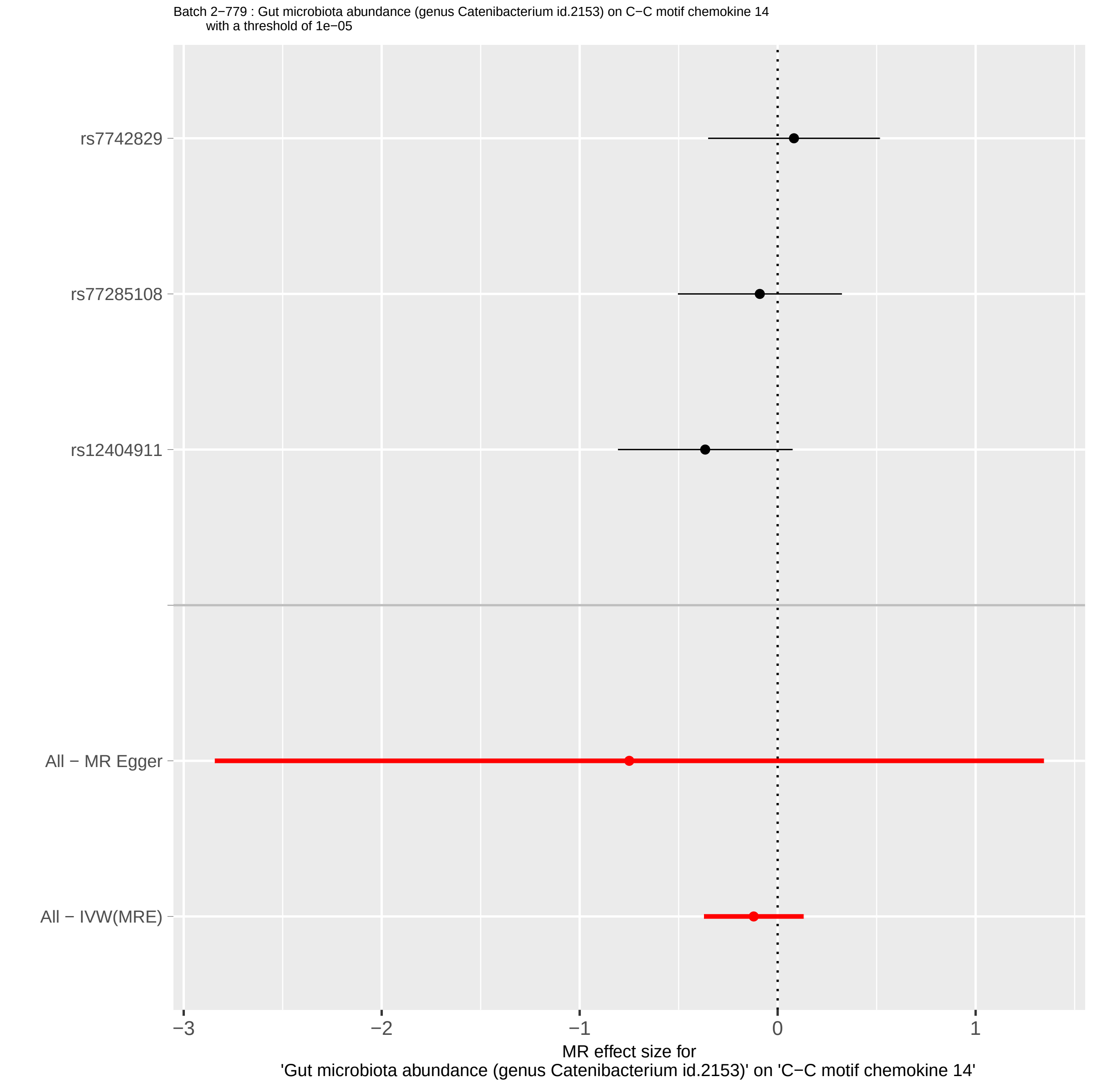
Batch 2−779 : Gut microbiota abundance (genus Catenibacterium id.2153) on C−C motif chemokine 14
with a threshold of 1e−05
rs7742829
rs77285108
rs12404911
All − MR Egger
All − IVW(MRE)
−3
−2
−1
0
1
MR effect size for
'Gut microbiota abundance (genus Catenibacterium id.2153)' on 'C−C motif chemokine 14'

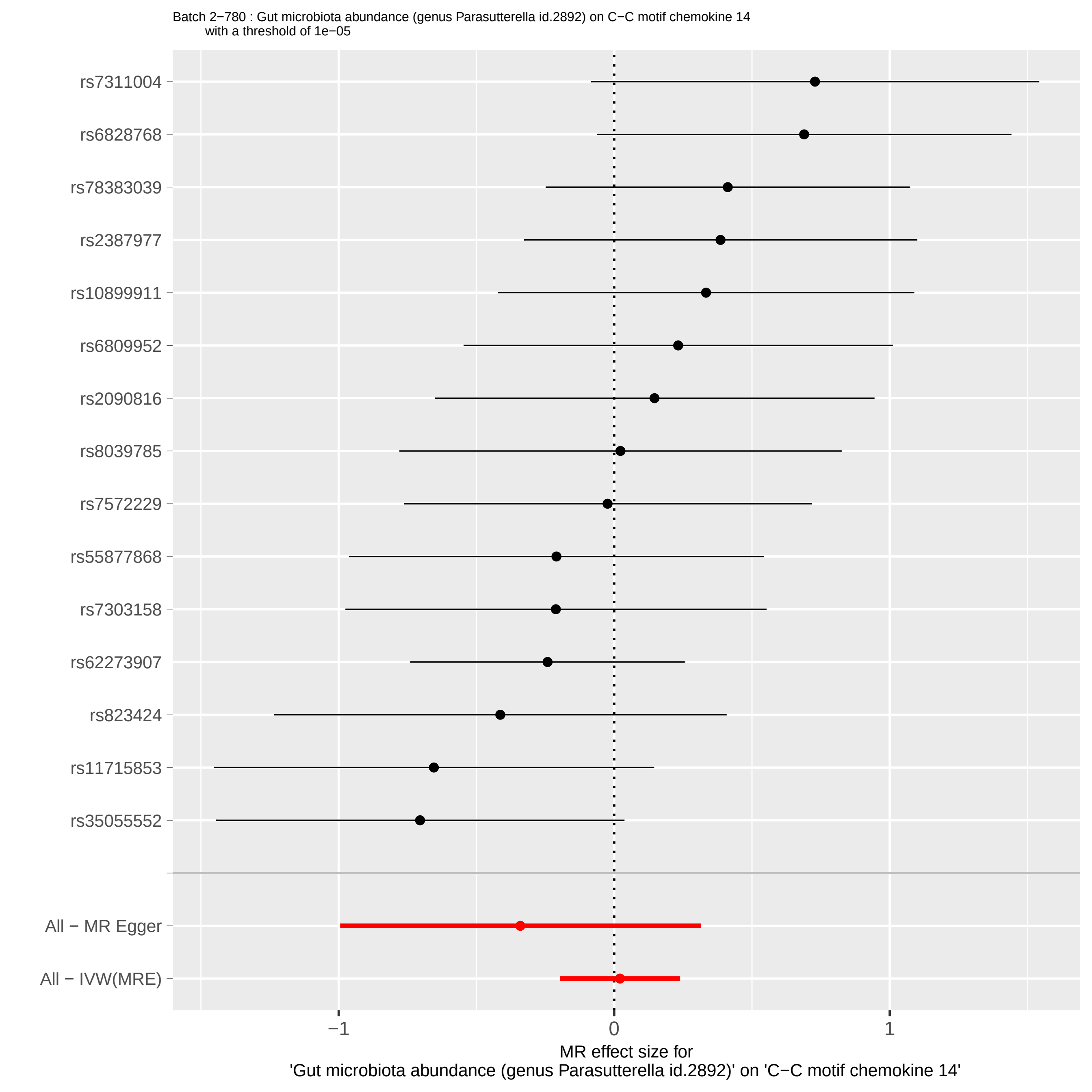
Batch 2−780 : Gut microbiota abundance (genus Parasutterella id.2892) on C−C motif chemokine 14
with a threshold of 1e−05
rs7311004
rs6828768
rs78383039
rs2387977
rs10899911
rs6809952
rs2090816
rs8039785
rs7572229
rs55877868
rs7303158
rs62273907
rs823424
rs11715853
rs35055552
All − MR Egger
All − IVW(MRE)
−1
0
1
MR effect size for
'Gut microbiota abundance (genus Parasutterella id.2892)' on 'C−C motif chemokine 14'

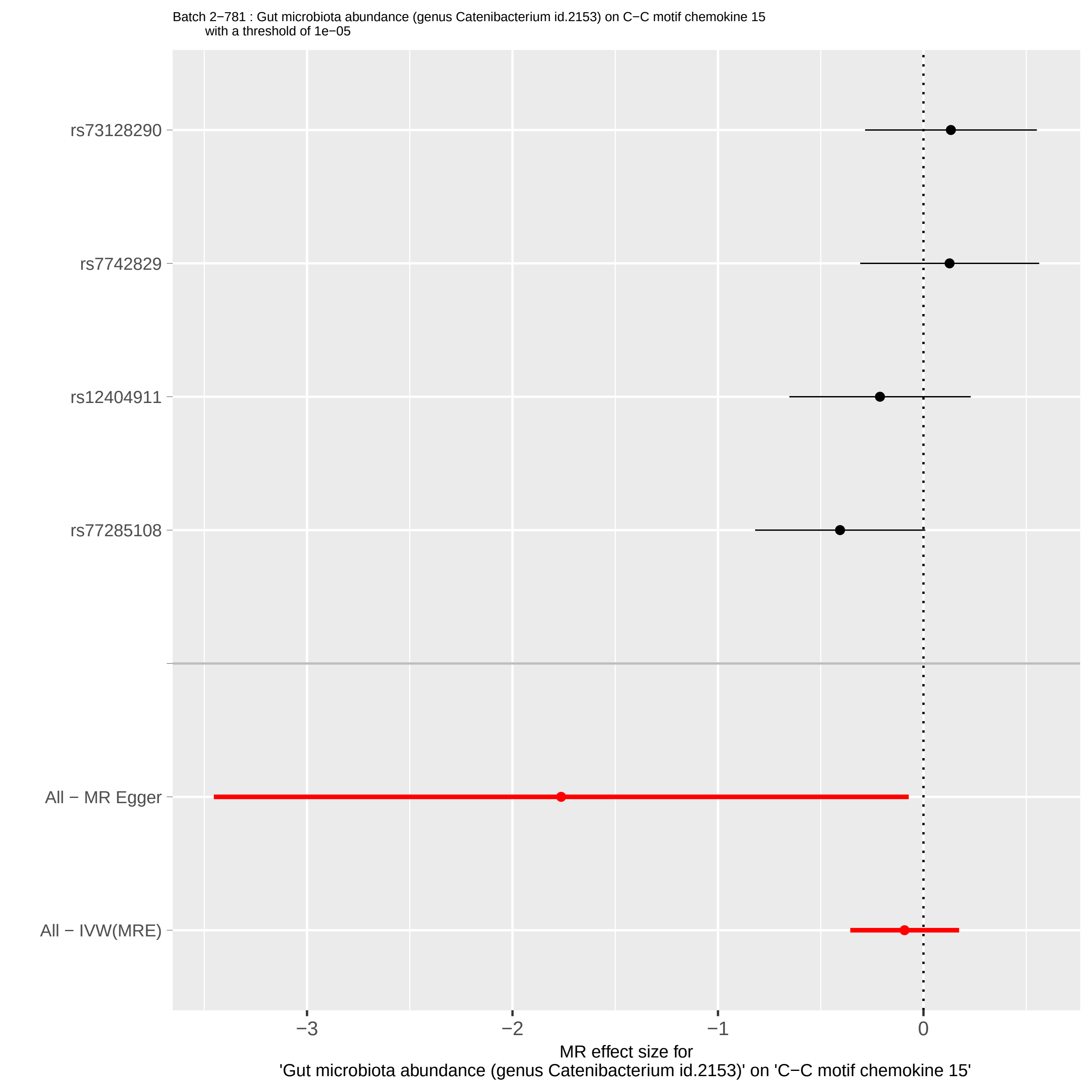

Batch 2−781 : Gut microbiota abundance (genus Catenibacterium id.2153) on C−C motif chemokine 15
with a threshold of 1e−05
rs73128290
rs7742829
rs12404911
rs77285108
All − MR Egger
All − IVW(MRE)
−3
−2
−1
0
MR effect size for
'Gut microbiota abundance (genus Catenibacterium id.2153)' on 'C−C motif chemokine 15'

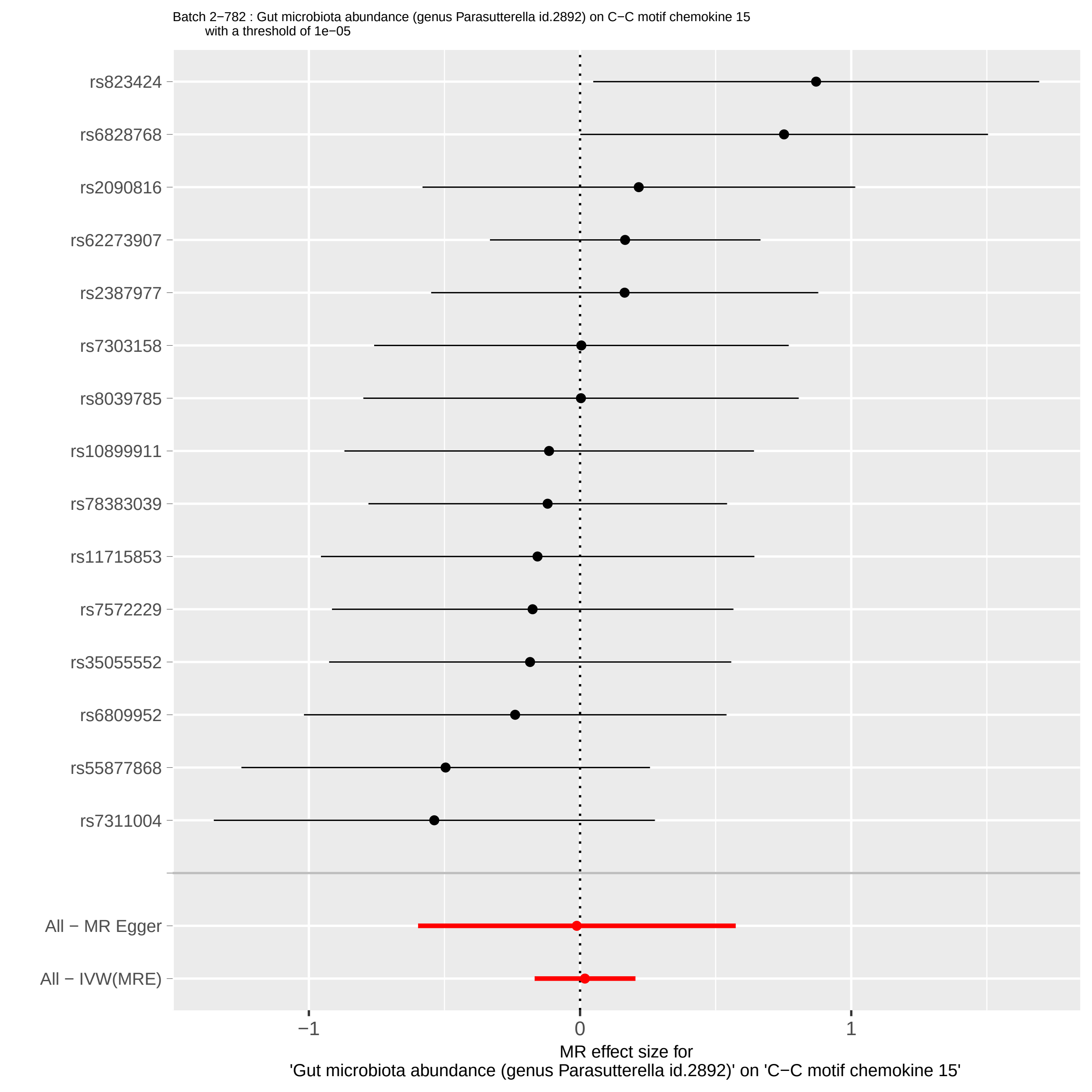
Batch 2−782 : Gut microbiota abundance (genus Parasutterella id.2892) on C−C motif chemokine 15
with a threshold of 1e−05
rs823424
rs6828768
rs2090816
rs62273907
rs2387977
rs7303158
rs8039785
rs10899911
rs78383039
rs11715853
rs7572229
rs35055552
rs6809952
rs55877868
rs7311004
All − MR Egger
All − IVW(MRE)
−1
0
1
MR effect size for
'Gut microbiota abundance (genus Parasutterella id.2892)' on 'C−C motif chemokine 15'

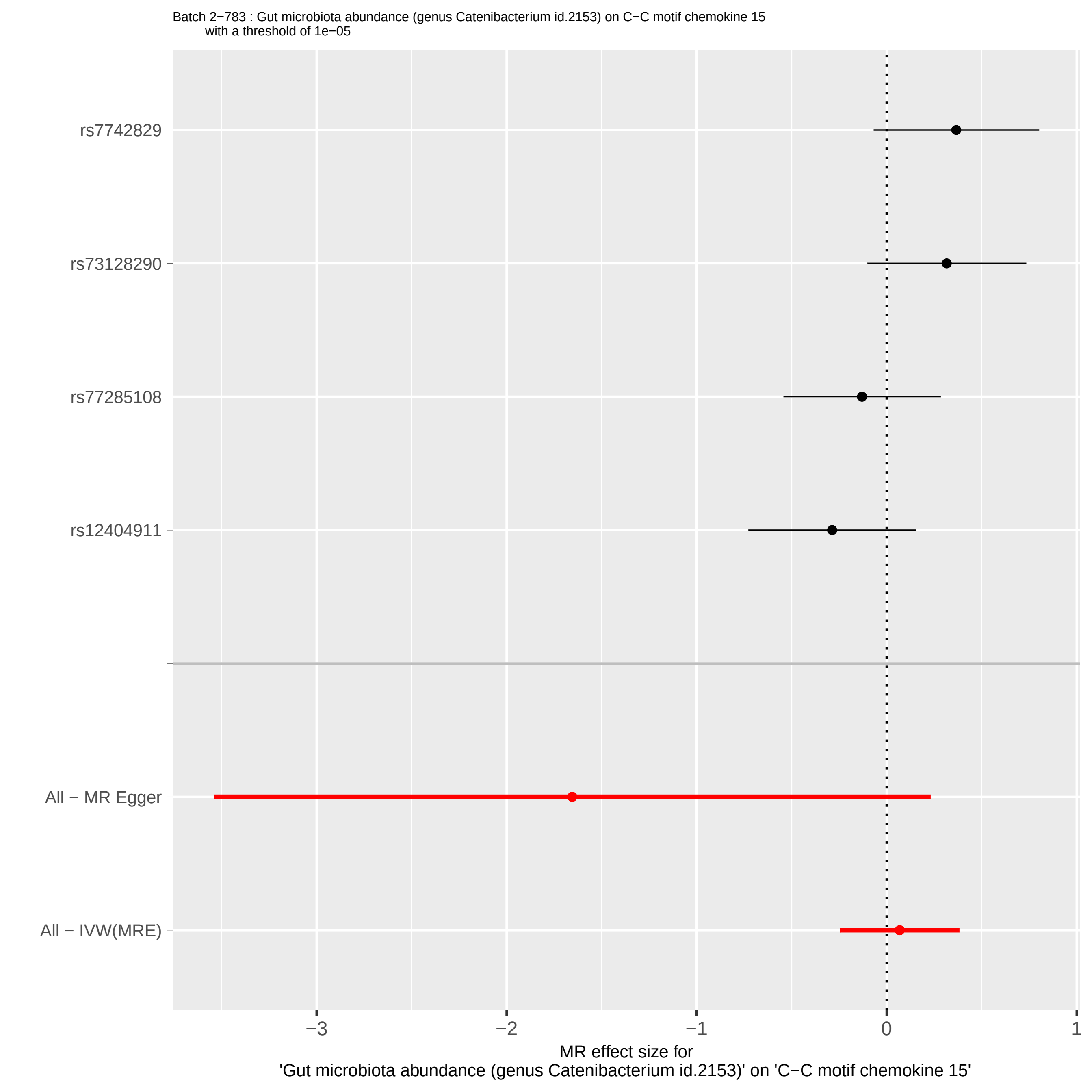
Batch 2−783 : Gut microbiota abundance (genus Catenibacterium id.2153) on C−C motif chemokine 15
with a threshold of 1e−05
rs7742829
rs73128290
rs77285108
rs12404911
All − MR Egger
All − IVW(MRE)
−3
−2
−1
0
1
MR effect size for
'Gut microbiota abundance (genus Catenibacterium id.2153)' on 'C−C motif chemokine 15'

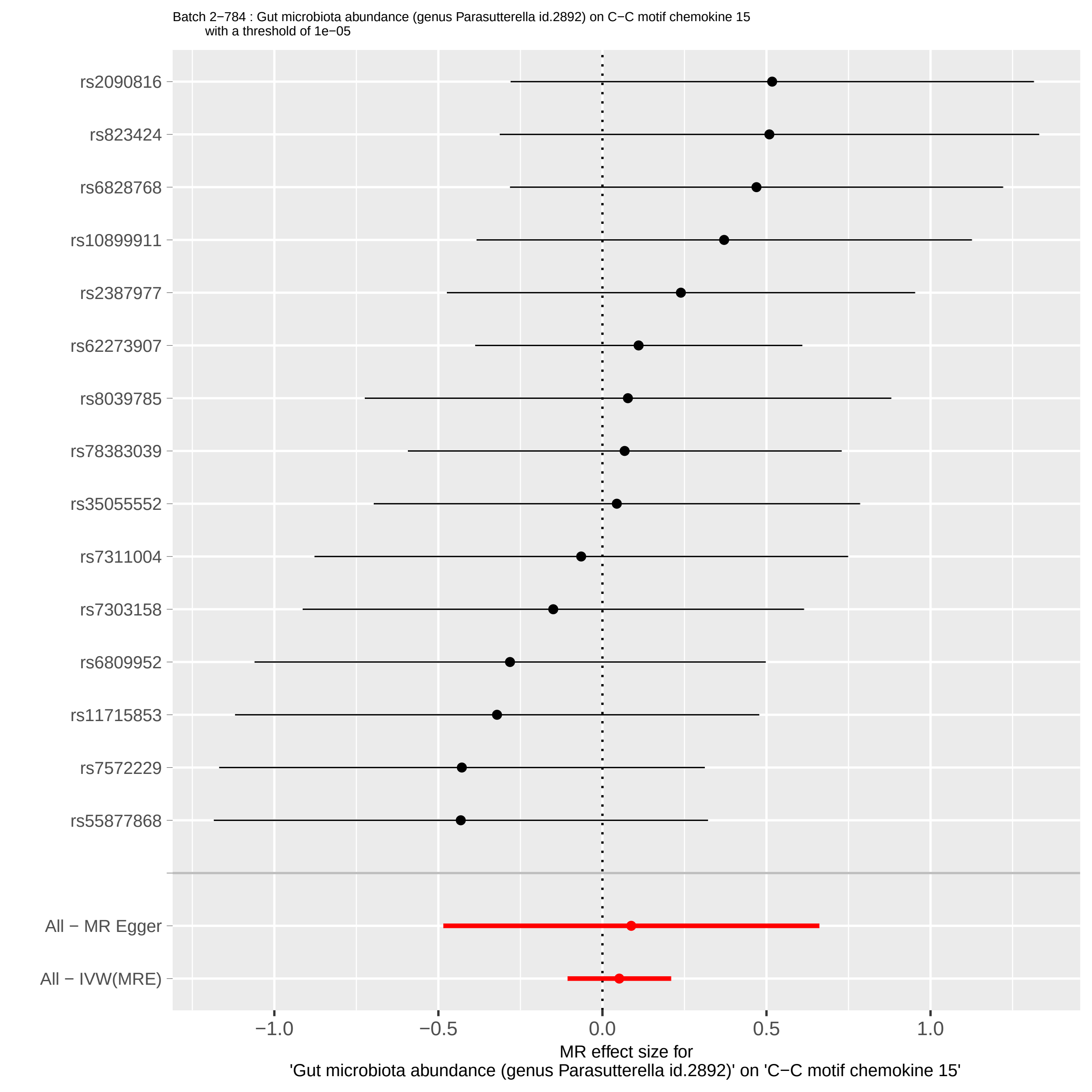

Batch 2−784 : Gut microbiota abundance (genus Parasutterella id.2892) on C−C motif chemokine 15
with a threshold of 1e−05
rs2090816
rs823424
rs6828768
rs10899911
rs2387977
rs62273907
rs8039785
rs78383039
rs35055552
rs7311004
rs7303158
rs6809952
rs11715853
rs7572229
rs55877868
All − MR Egger
All − IVW(MRE)
−1.0
−0.5
0.0
0.5
1.0
MR effect size for
'Gut microbiota abundance (genus Parasutterella id.2892)' on 'C−C motif chemokine 15'

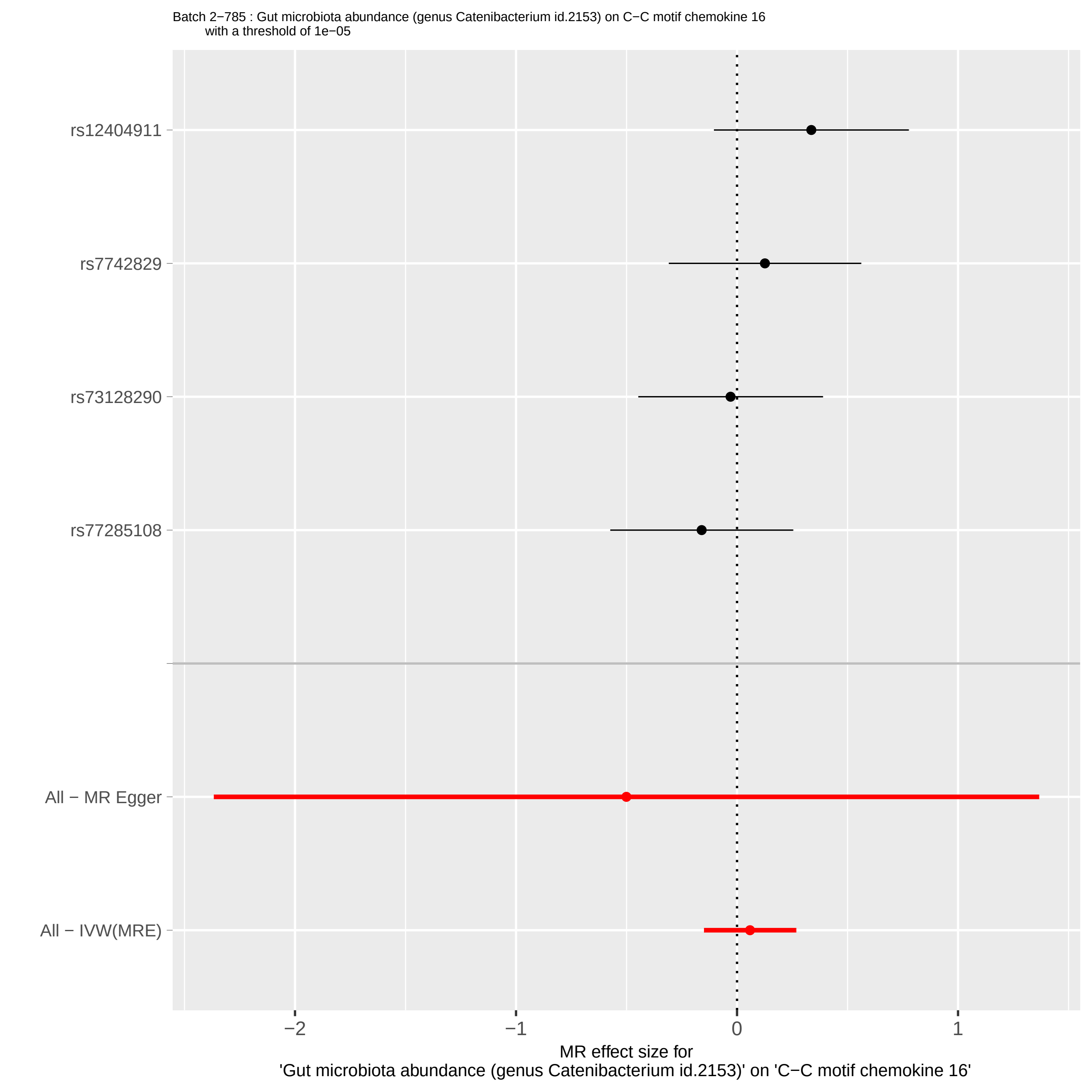
Batch 2−785 : Gut microbiota abundance (genus Catenibacterium id.2153) on C−C motif chemokine 16
with a threshold of 1e−05
rs12404911
rs7742829
rs73128290
rs77285108
All − MR Egger
All − IVW(MRE)
−2
−1
0
1
MR effect size for
'Gut microbiota abundance (genus Catenibacterium id.2153)' on 'C−C motif chemokine 16'

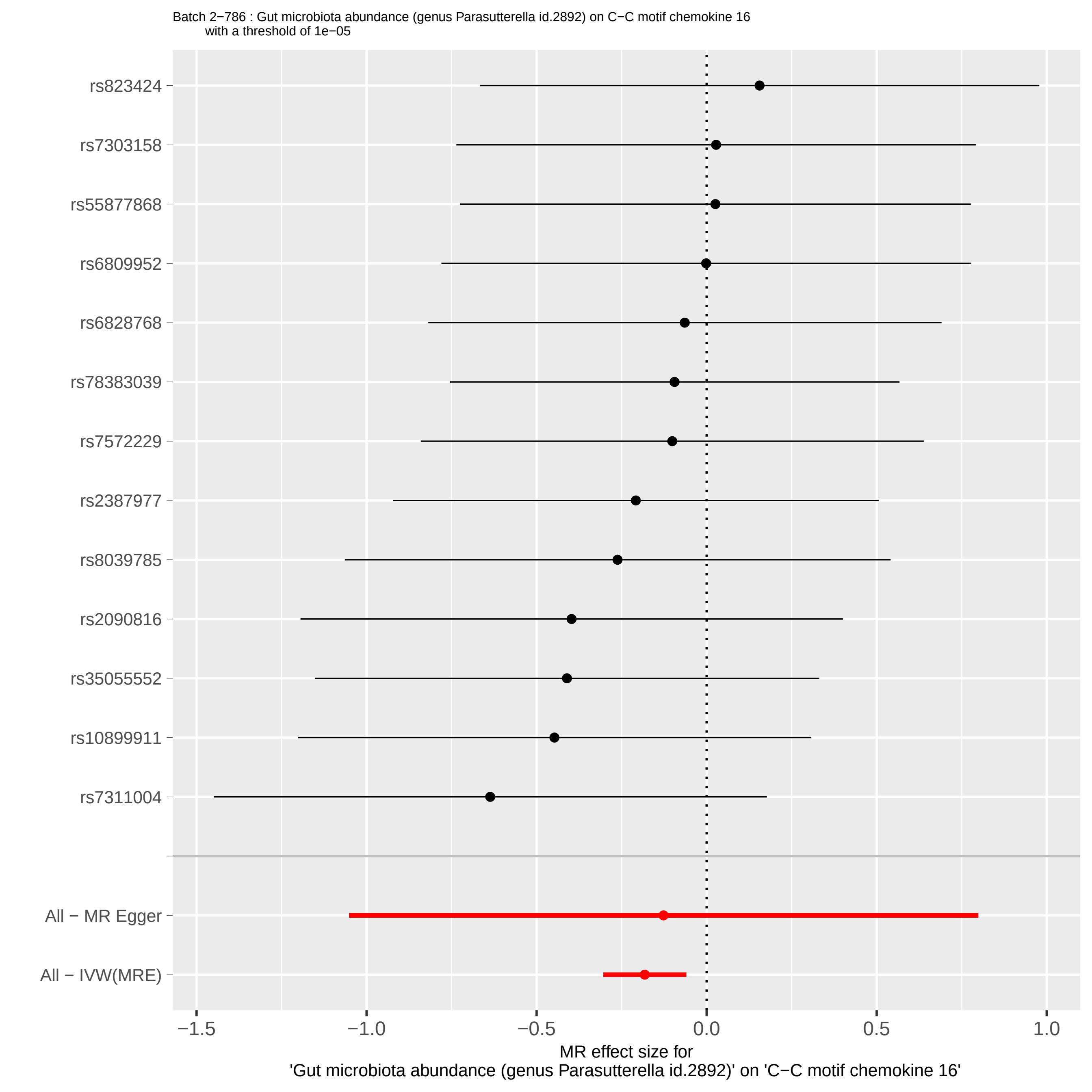

Batch 2−786 : Gut microbiota abundance (genus Parasutterella id.2892) on C−C motif chemokine 16
with a threshold of 1e−05
rs823424
rs7303158
rs55877868
rs6809952
rs6828768
rs78383039
rs7572229
rs2387977
rs8039785
rs2090816
rs35055552
rs10899911
rs7311004
All − MR Egger
All − IVW(MRE)
−1.5
−1.0
−0.5
0.0
0.5
1.0
MR effect size for
'Gut microbiota abundance (genus Parasutterella id.2892)' on 'C−C motif chemokine 16'

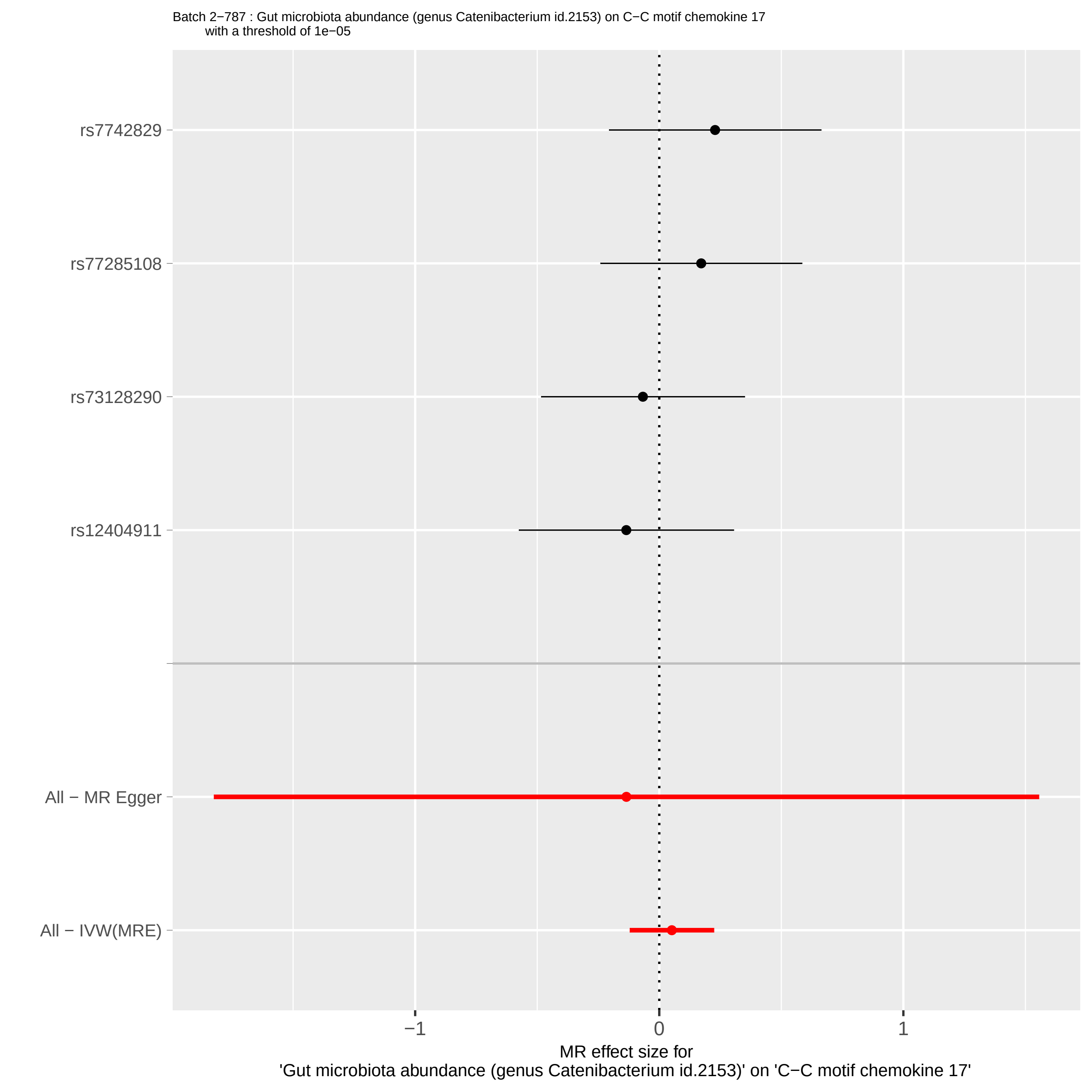
Batch 2−787 : Gut microbiota abundance (genus Catenibacterium id.2153) on C−C motif chemokine 17
with a threshold of 1e−05
rs7742829
rs77285108
rs73128290
rs12404911
All − MR Egger
All − IVW(MRE)
−1
0
1
MR effect size for
'Gut microbiota abundance (genus Catenibacterium id.2153)' on 'C−C motif chemokine 17'

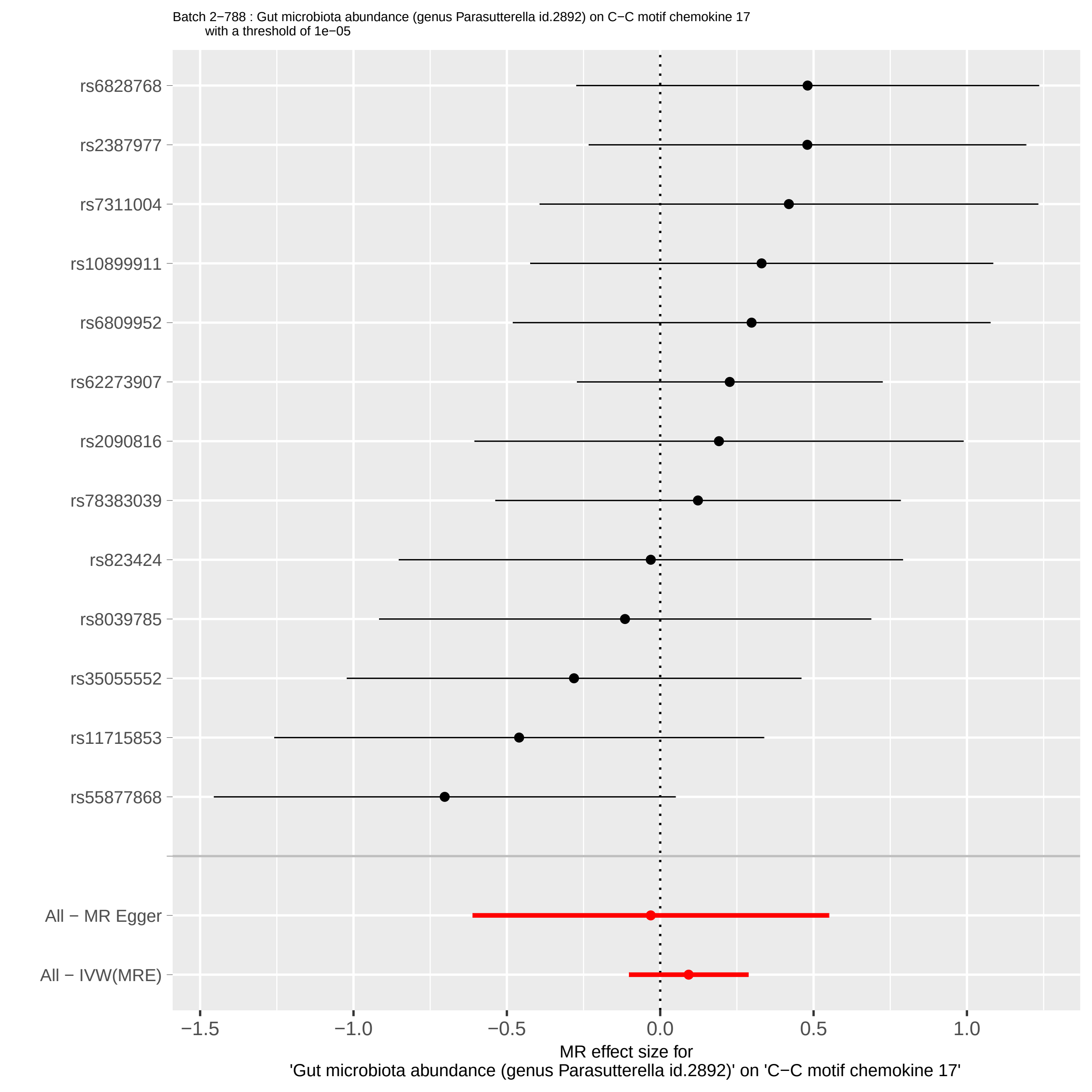
Batch 2−788 : Gut microbiota abundance (genus Parasutterella id.2892) on C−C motif chemokine 17
with a threshold of 1e−05
rs6828768
rs2387977
rs7311004
rs10899911
rs6809952
rs62273907
rs2090816
rs78383039
rs823424
rs8039785
rs35055552
rs11715853
rs55877868
All − MR Egger
All − IVW(MRE)
−1.5
−1.0
−0.5
0.0
0.5
1.0
MR effect size for
'Gut microbiota abundance (genus Parasutterella id.2892)' on 'C−C motif chemokine 17'

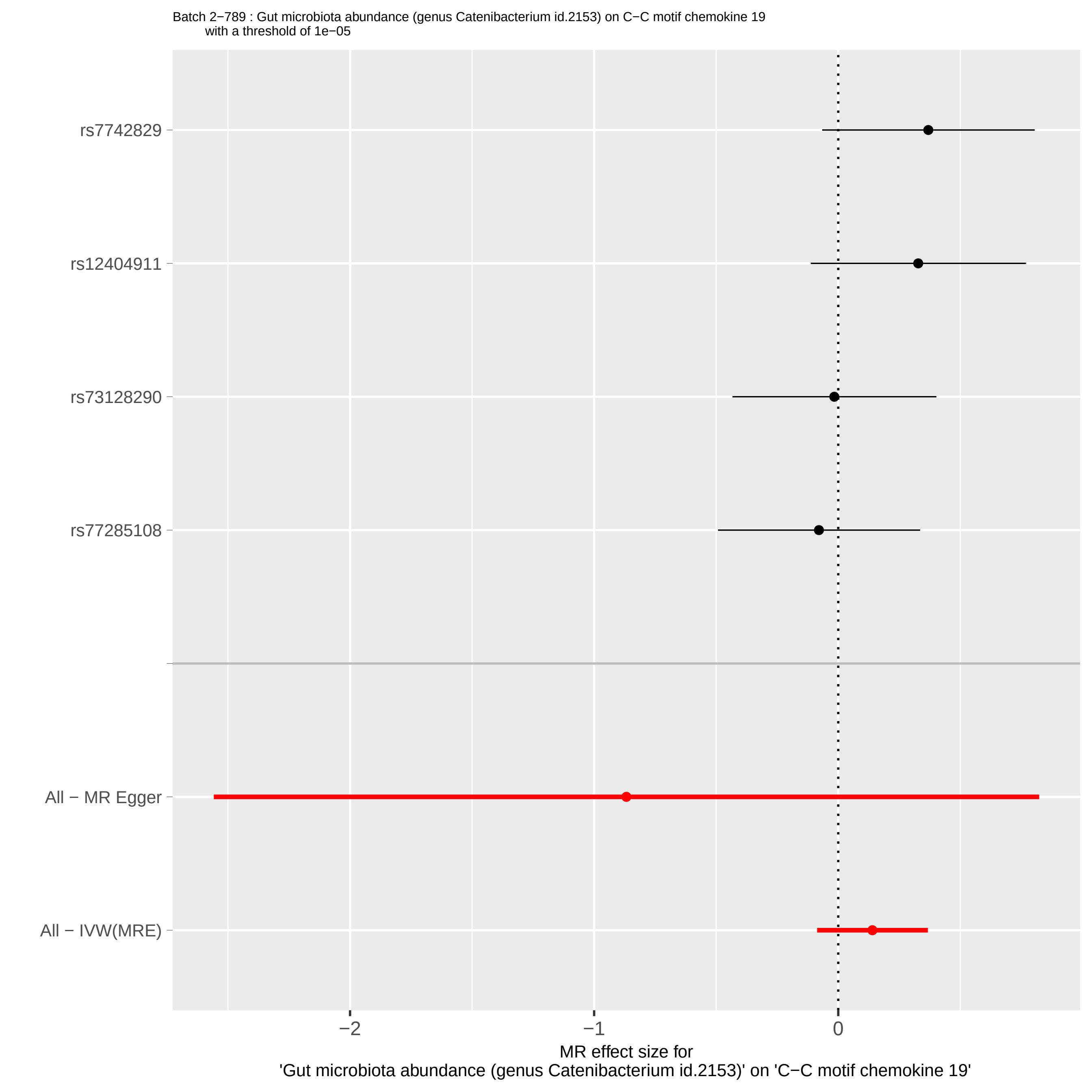
Batch 2−789 : Gut microbiota abundance (genus Catenibacterium id.2153) on C−C motif chemokine 19
with a threshold of 1e−05
rs7742829
rs12404911
rs73128290
rs77285108
All − MR Egger
All − IVW(MRE)
−2
−1
0
MR effect size for
'Gut microbiota abundance (genus Catenibacterium id.2153)' on 'C−C motif chemokine 19'

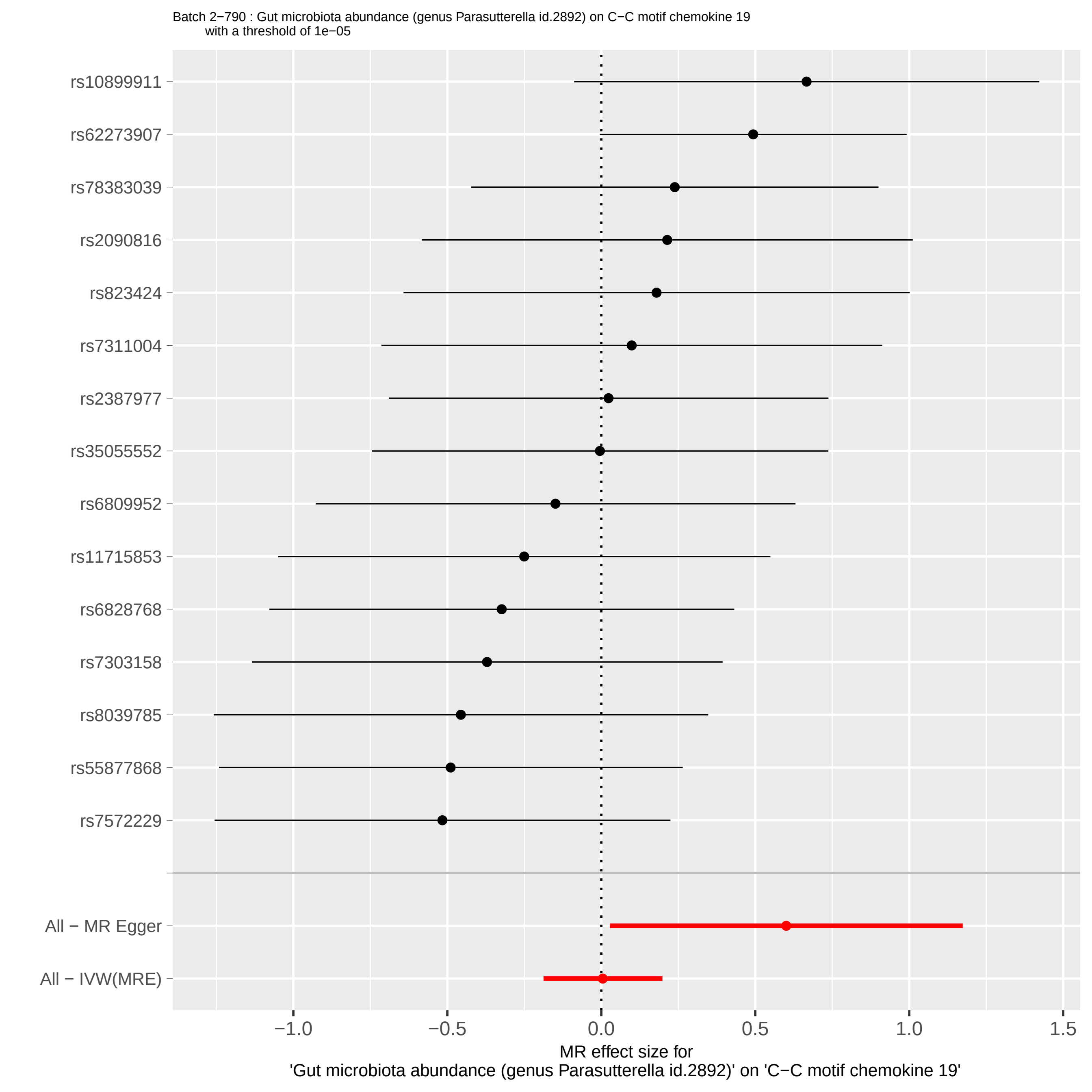

Batch 2−790 : Gut microbiota abundance (genus Parasutterella id.2892) on C−C motif chemokine 19
with a threshold of 1e−05
rs10899911
rs62273907
rs78383039
rs2090816
rs823424
rs7311004
rs2387977
rs35055552
rs6809952
rs11715853
rs6828768
rs7303158
rs8039785
rs55877868
rs7572229
All − MR Egger
All − IVW(MRE)
−1.0
−0.5
0.0
0.5
1.0
1.5
MR effect size for
'Gut microbiota abundance (genus Parasutterella id.2892)' on 'C−C motif chemokine 19'

Batch 2−791 : Gut microbiota abundance (genus Catenibacterium id.2153) on C−C motif chemokine 20
with a threshold of 1e−05

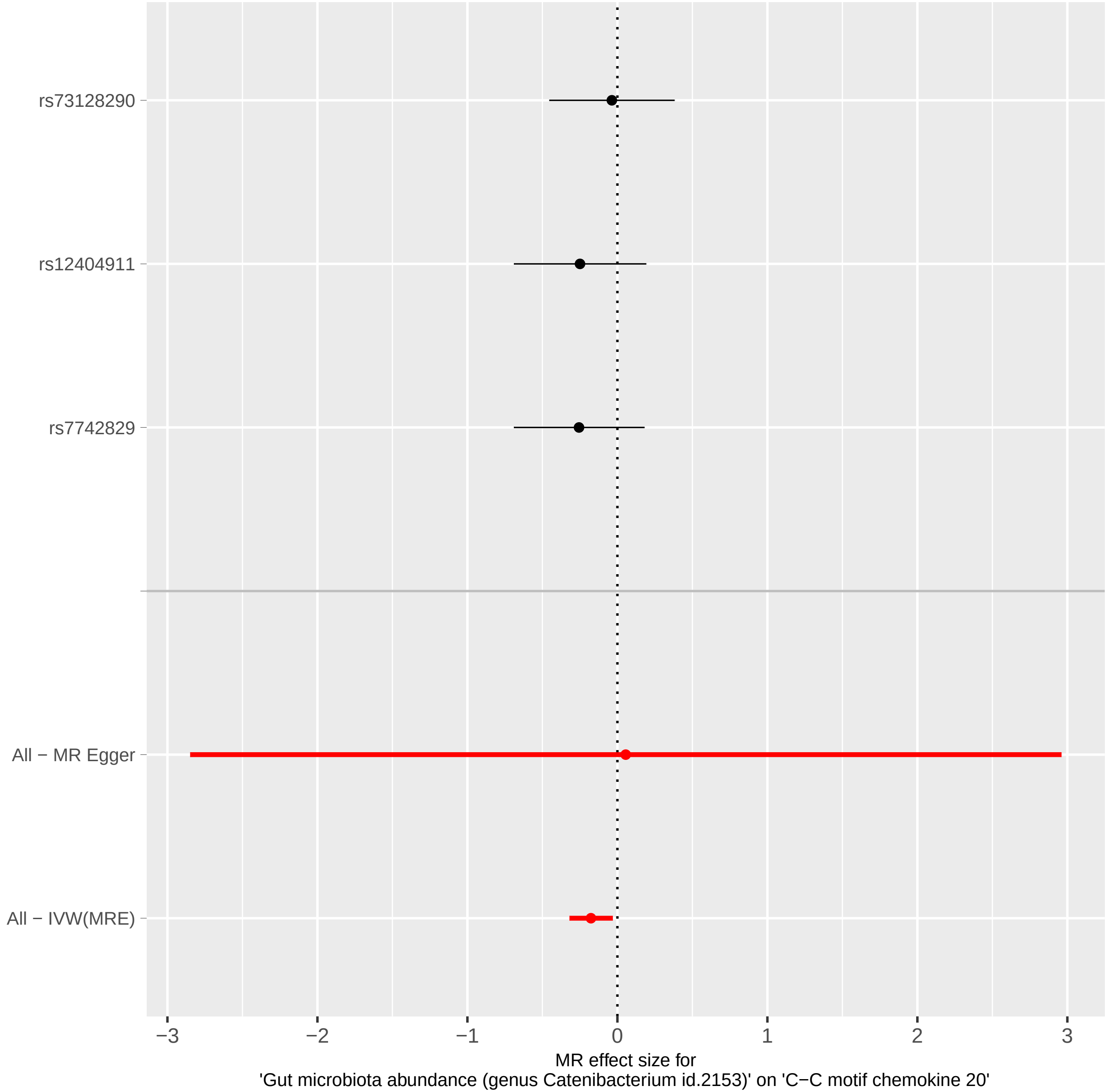

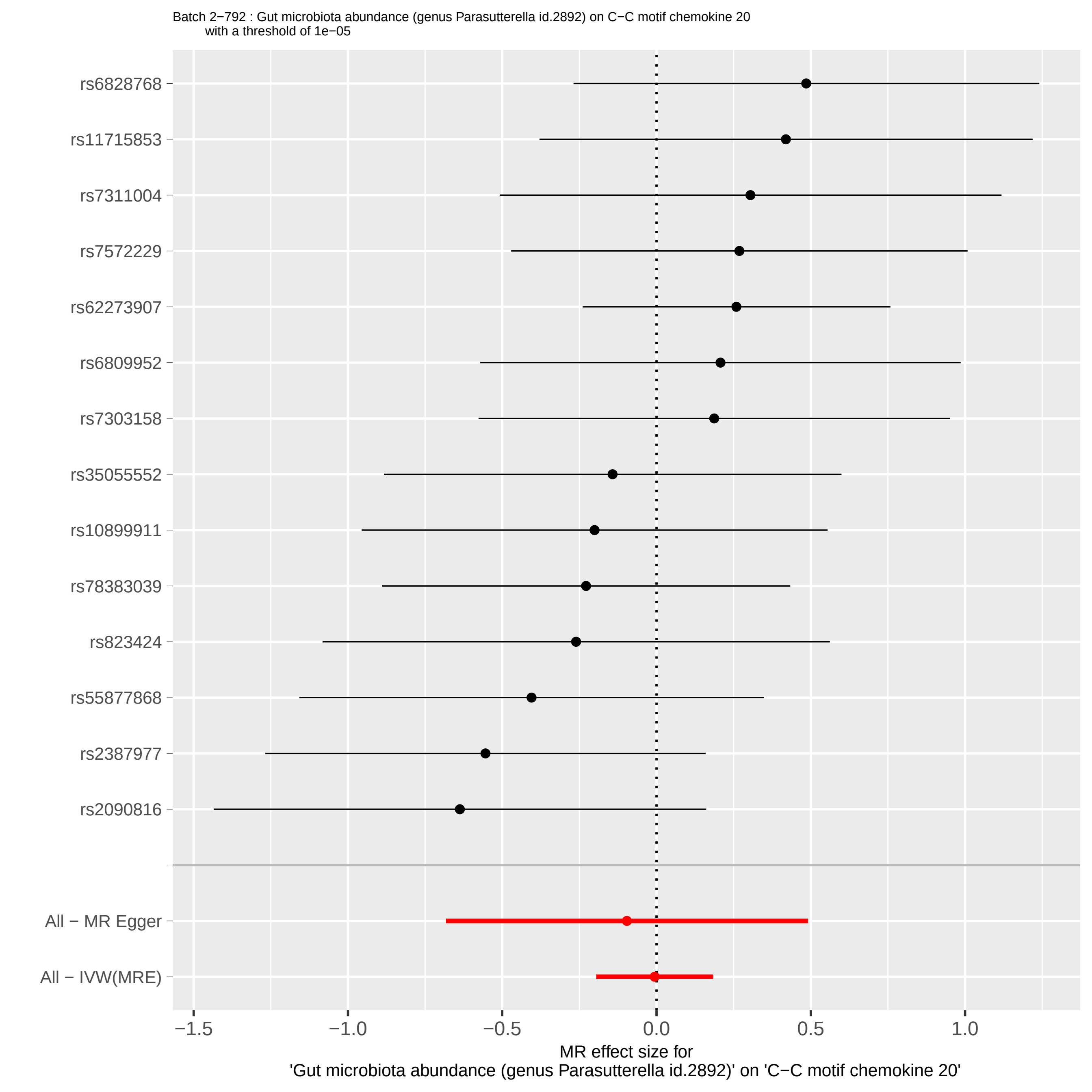

Batch 2−792 : Gut microbiota abundance (genus Parasutterella id.2892) on C−C motif chemokine 20
with a threshold of 1e−05
rs6828768
rs11715853
rs7311004
rs7572229
rs62273907
rs6809952
rs7303158
rs35055552
rs10899911
rs78383039
rs823424
rs55877868
rs2387977
rs2090816
All − MR Egger
All − IVW(MRE)
−1.5
−1.0
−0.5
0.0
0.5
1.0
MR effect size for
'Gut microbiota abundance (genus Parasutterella id.2892)' on 'C−C motif chemokine 20'

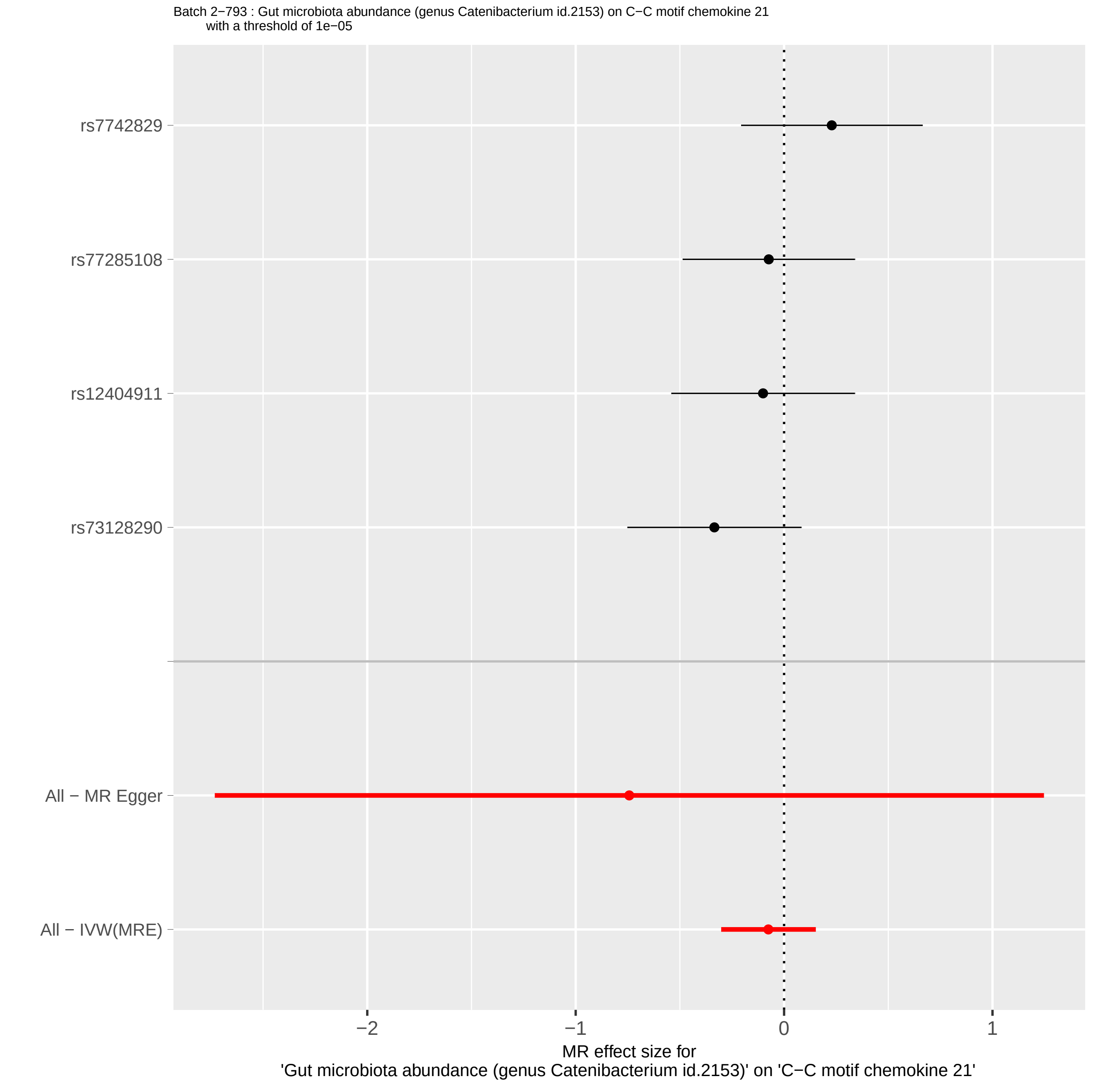

Batch 2−793 : Gut microbiota abundance (genus Catenibacterium id.2153) on C−C motif chemokine 21
with a threshold of 1e−05
rs7742829
rs77285108
rs12404911
rs73128290
All − MR Egger
All − IVW(MRE)
−2
−1
0
1
MR effect size for
'Gut microbiota abundance (genus Catenibacterium id.2153)' on 'C−C motif chemokine 21'

Batch 2−794 : Gut microbiota abundance (genus Parasutterella id.2892) on C−C motif chemokine 21
with a threshold of 1e−05

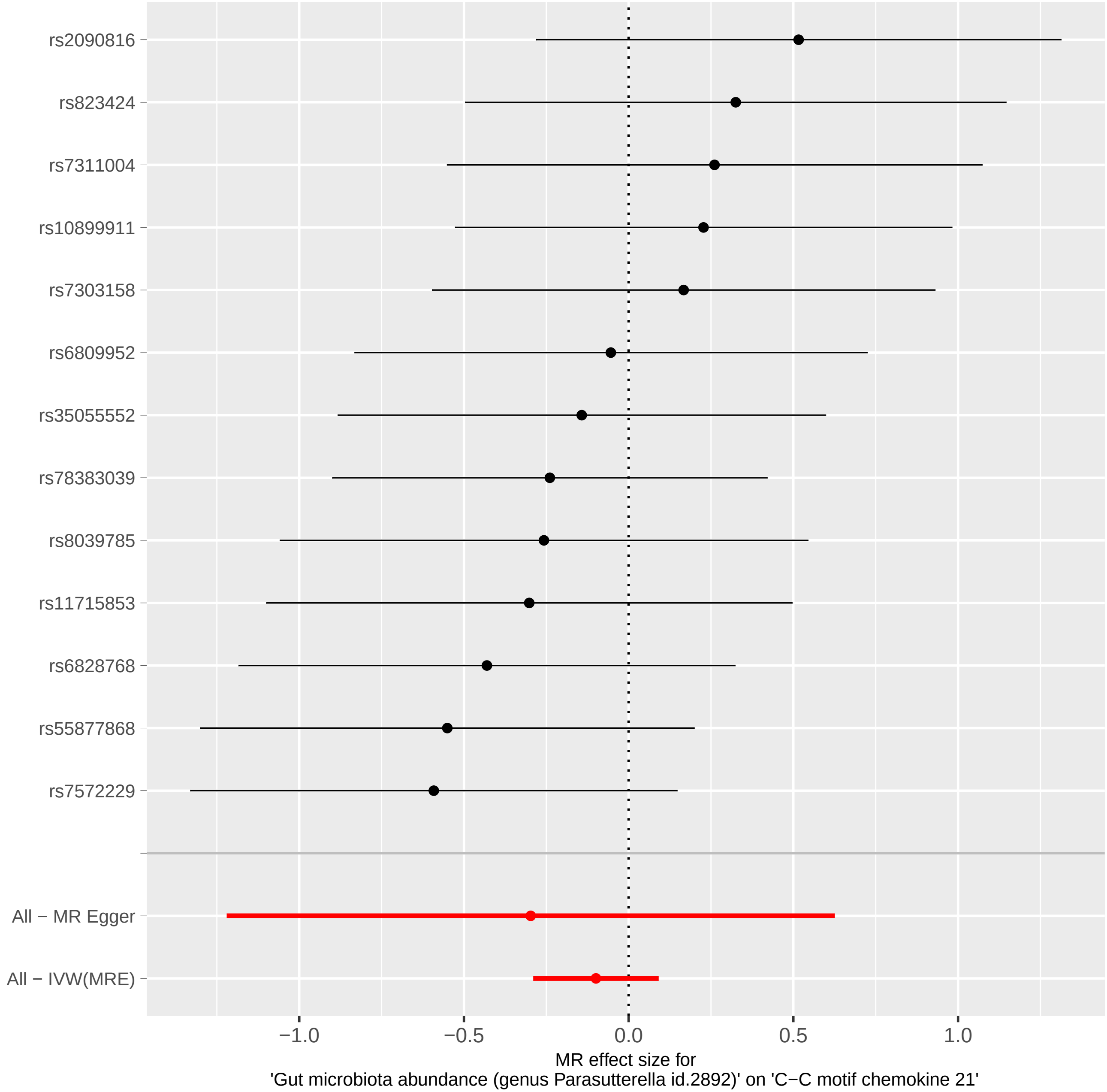

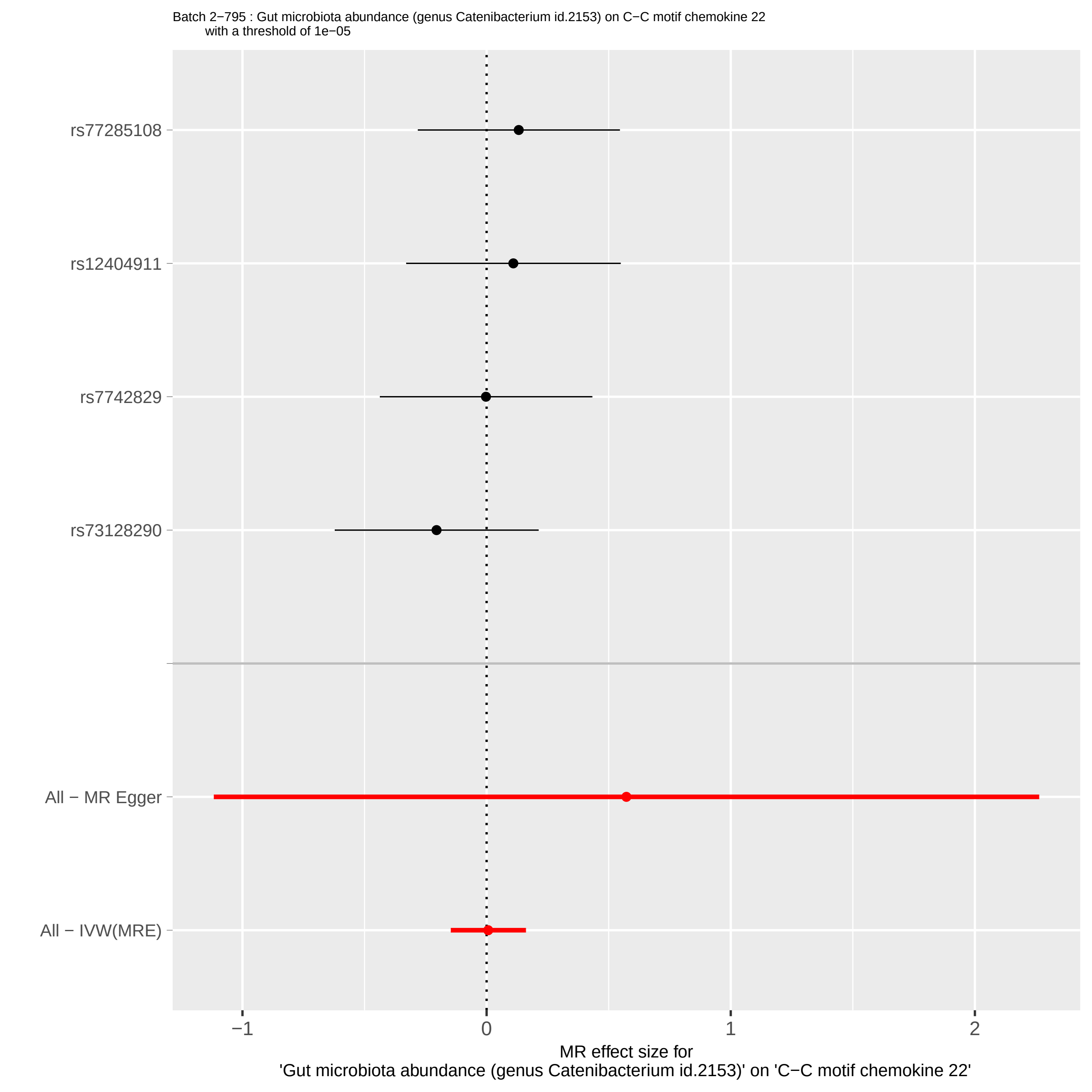

Batch 2−795 : Gut microbiota abundance (genus Catenibacterium id.2153) on C−C motif chemokine 22
with a threshold of 1e−05
rs77285108
rs12404911
rs7742829
rs73128290
All − MR Egger
All − IVW(MRE)
−1
0
1
2
MR effect size for
'Gut microbiota abundance (genus Catenibacterium id.2153)' on 'C−C motif chemokine 22'

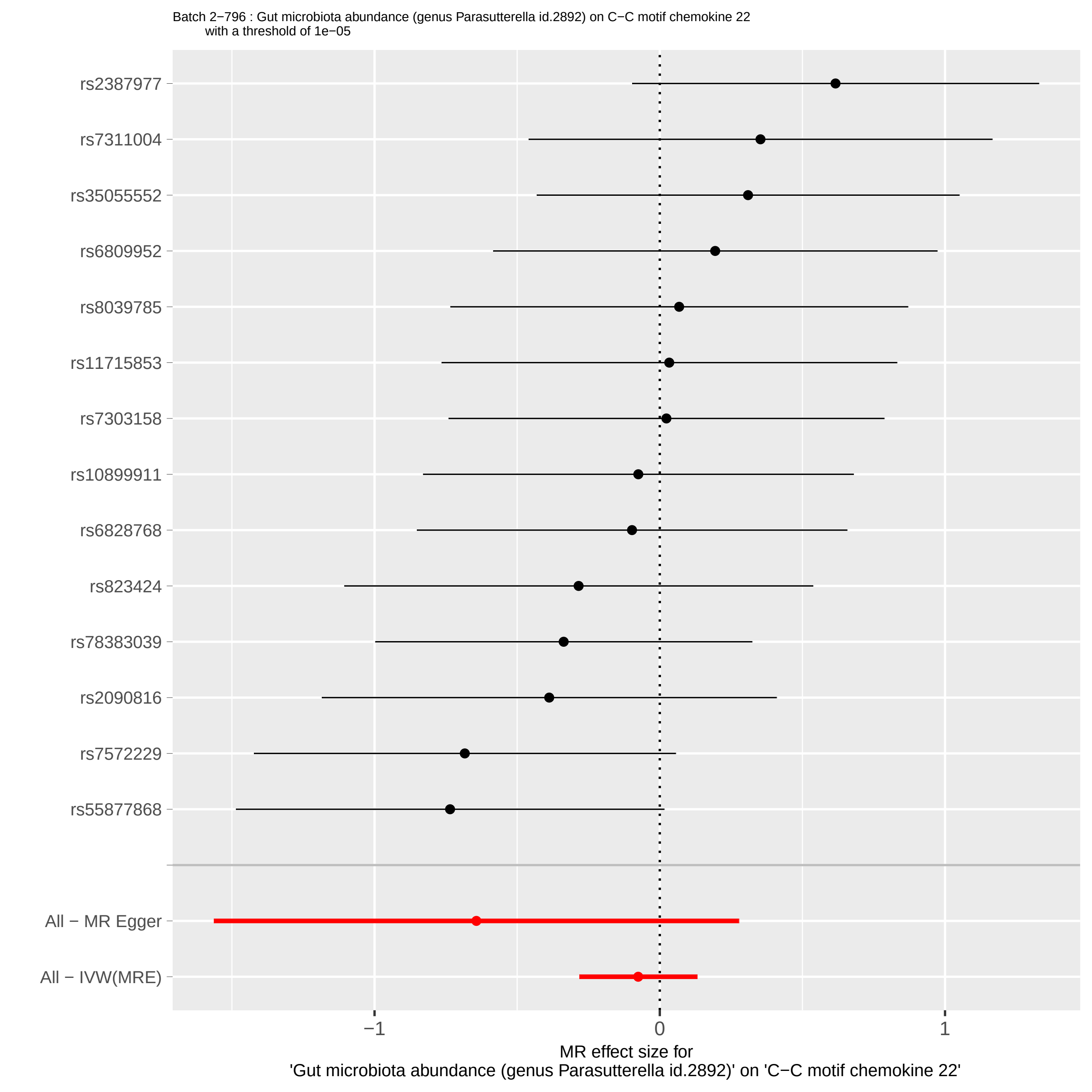
Batch 2−796 : Gut microbiota abundance (genus Parasutterella id.2892) on C−C motif chemokine 22
with a threshold of 1e−05
rs2387977
rs7311004
rs35055552
rs6809952
rs8039785
rs11715853
rs7303158
rs10899911
rs6828768
rs823424
rs78383039
rs2090816
rs7572229
rs55877868
All − MR Egger
All − IVW(MRE)
−1
0
1
MR effect size for
'Gut microbiota abundance (genus Parasutterella id.2892)' on 'C−C motif chemokine 22'

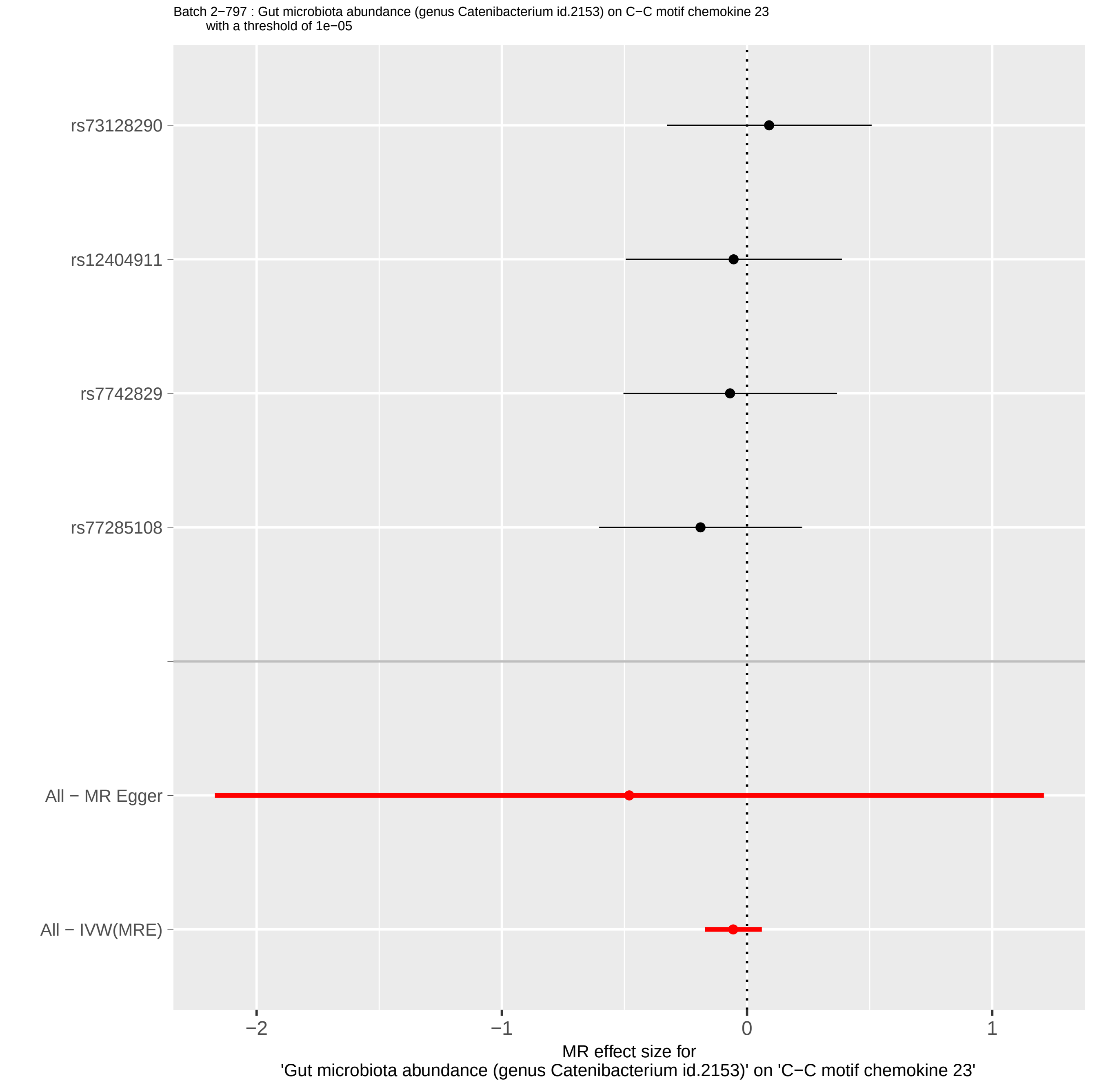

Batch 2−797 : Gut microbiota abundance (genus Catenibacterium id.2153) on C−C motif chemokine 23
with a threshold of 1e−05
rs73128290
rs12404911
rs7742829
rs77285108
All − MR Egger
All − IVW(MRE)
−2
−1
0
1
MR effect size for
'Gut microbiota abundance (genus Catenibacterium id.2153)' on 'C−C motif chemokine 23'

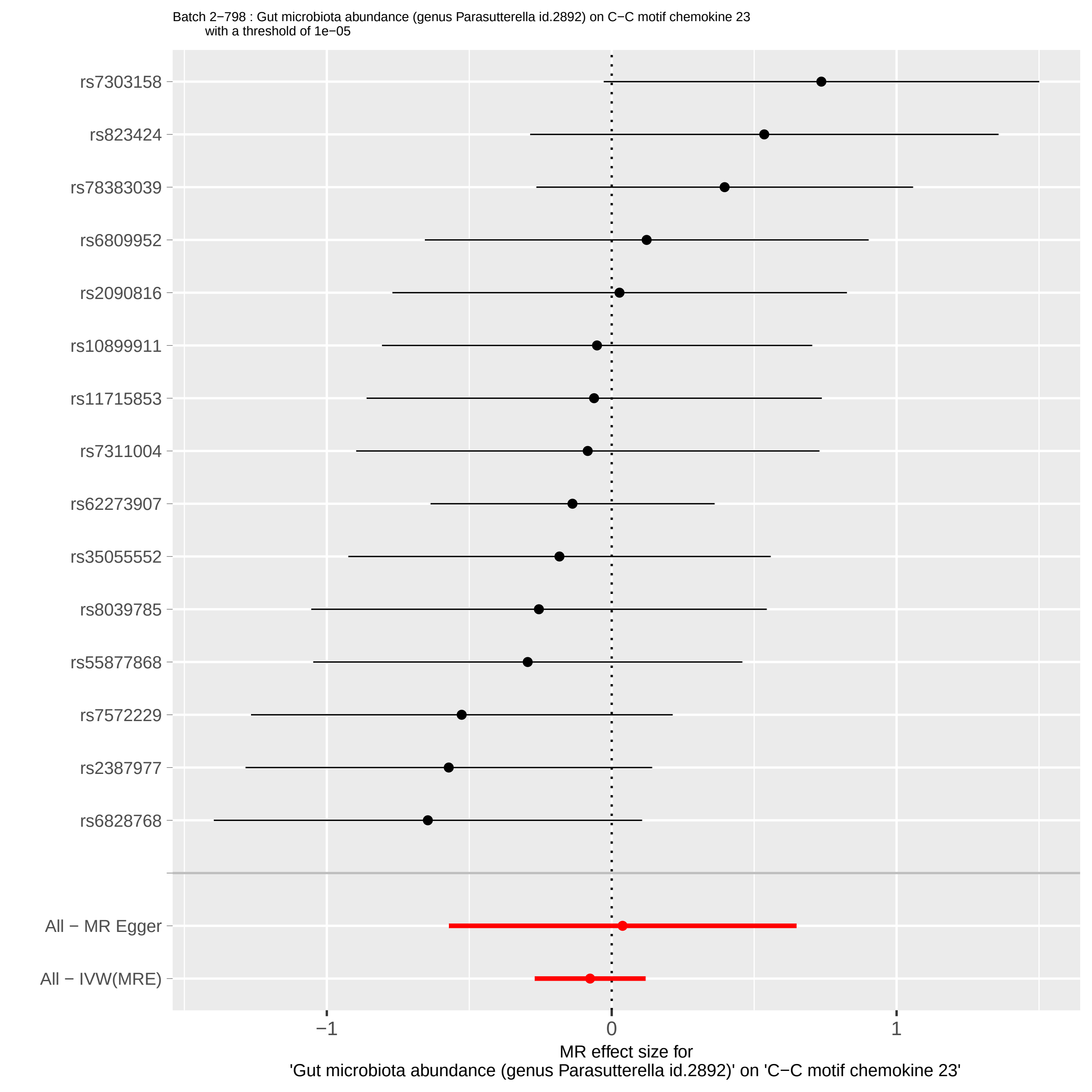

Batch 2−798 : Gut microbiota abundance (genus Parasutterella id.2892) on C−C motif chemokine 23
with a threshold of 1e−05
rs7303158
rs823424
rs78383039
rs6809952
rs2090816
rs10899911
rs11715853
rs7311004
rs62273907
rs35055552
rs8039785
rs55877868
rs7572229
rs2387977
rs6828768
All − MR Egger
All − IVW(MRE)
−1
0
1
MR effect size for
'Gut microbiota abundance (genus Parasutterella id.2892)' on 'C−C motif chemokine 23'

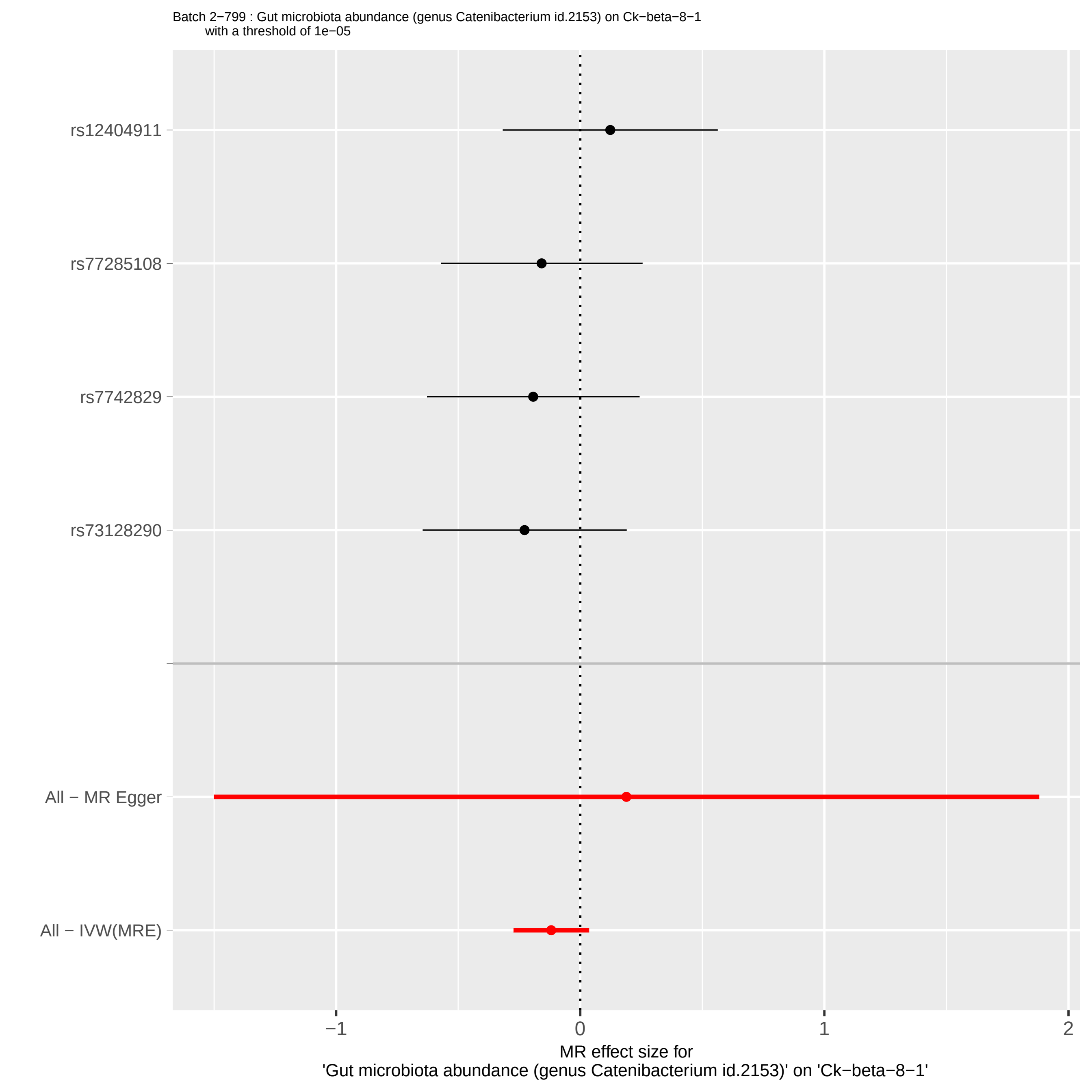

Batch 2−799 : Gut microbiota abundance (genus Catenibacterium id.2153) on Ck−beta−8−1
with a threshold of 1e−05
rs12404911
rs77285108
rs7742829
rs73128290
All − MR Egger
All − IVW(MRE)
−1
0
1
2
MR effect size for
'Gut microbiota abundance (genus Catenibacterium id.2153)' on 'Ck−beta−8−1'

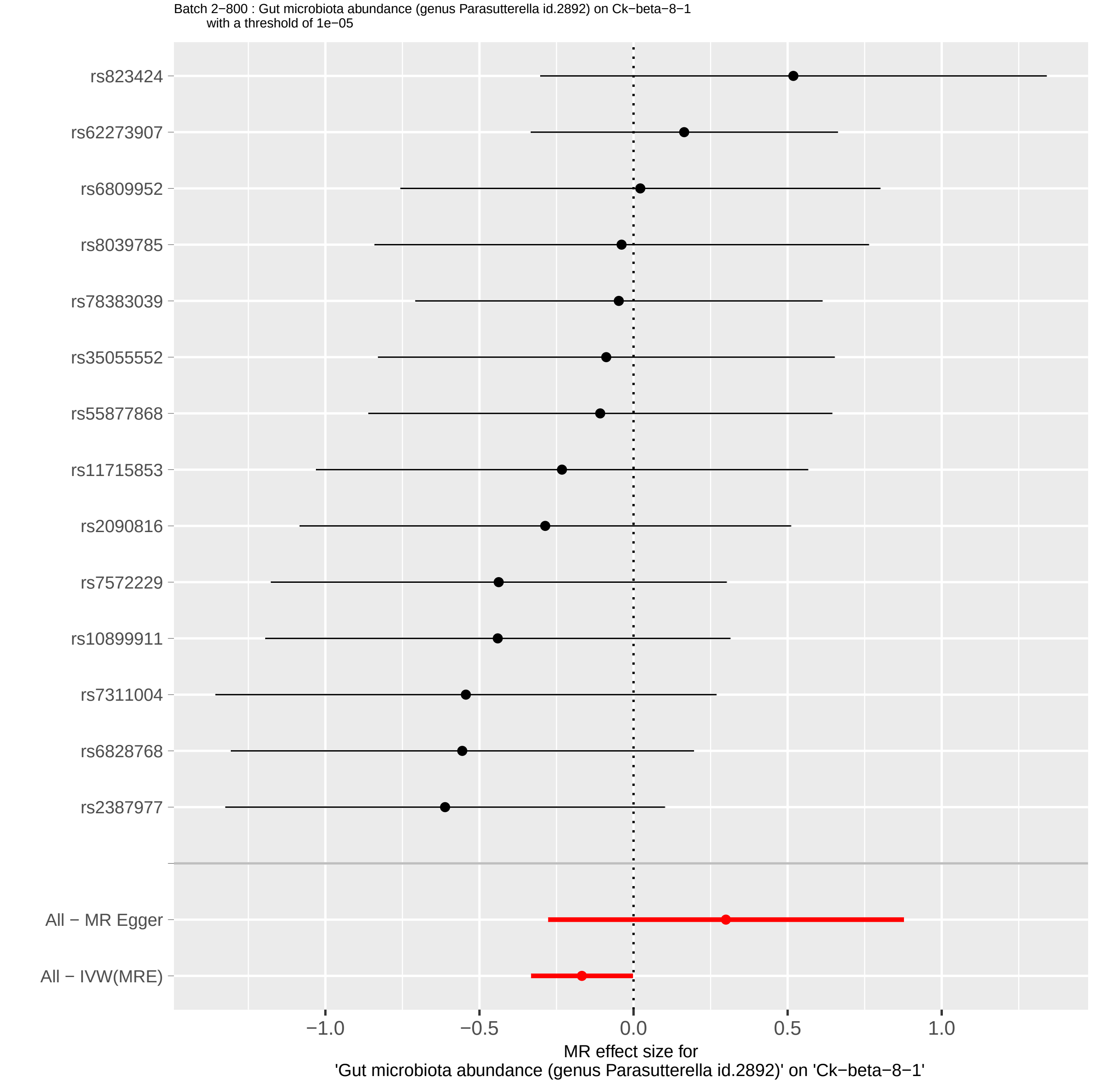
Batch 2−800 : Gut microbiota abundance (genus Parasutterella id.2892) on Ck−beta−8−1
with a threshold of 1e−05
rs823424
rs62273907
rs6809952
rs8039785
rs78383039
rs35055552
rs55877868
rs11715853
rs2090816
rs7572229
rs10899911
rs7311004
rs6828768
rs2387977
All − MR Egger
All − IVW(MRE)
−1.0
−0.5
0.0
0.5
1.0
MR effect size for
'Gut microbiota abundance (genus Parasutterella id.2892)' on 'Ck−beta−8−1'

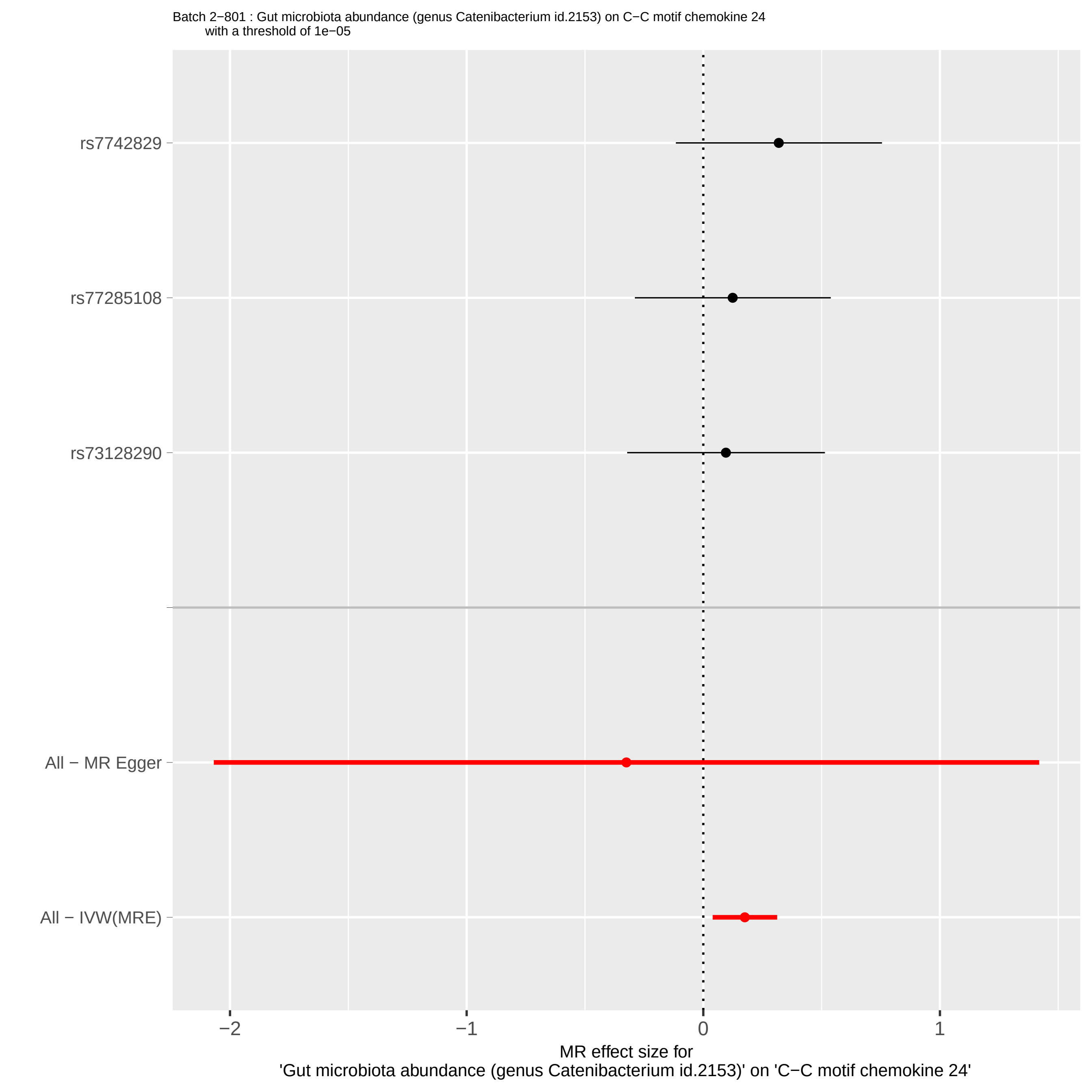
Batch 2−801 : Gut microbiota abundance (genus Catenibacterium id.2153) on C−C motif chemokine 24
with a threshold of 1e−05
rs7742829
rs77285108
rs73128290
All − MR Egger
All − IVW(MRE)
−2
−1
0
1
MR effect size for
'Gut microbiota abundance (genus Catenibacterium id.2153)' on 'C−C motif chemokine 24'

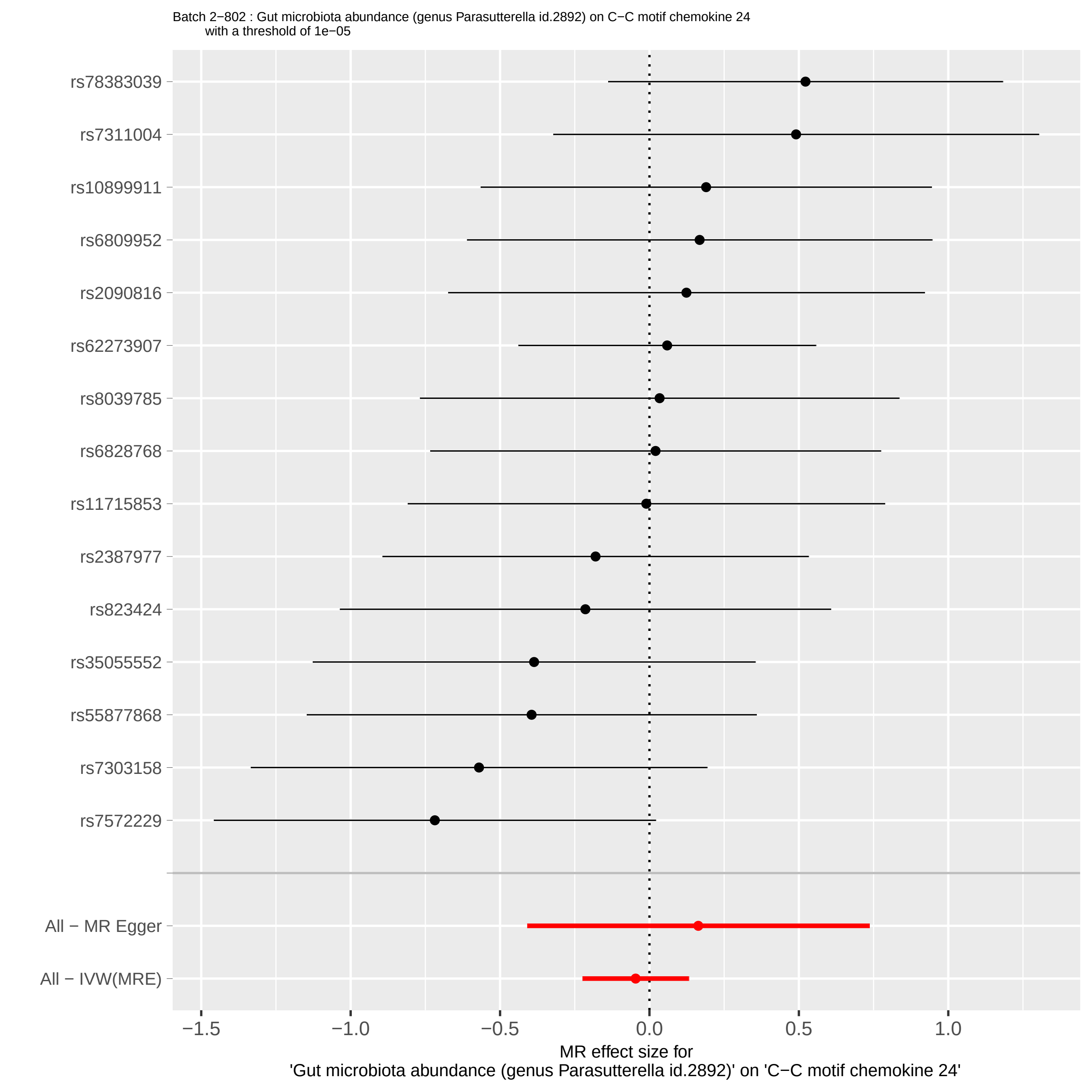
Batch 2−802 : Gut microbiota abundance (genus Parasutterella id.2892) on C−C motif chemokine 24
with a threshold of 1e−05
rs78383039
rs7311004
rs10899911
rs6809952
rs2090816
rs62273907
rs8039785
rs6828768
rs11715853
rs2387977
rs823424
rs35055552
rs55877868
rs7303158
rs7572229
All − MR Egger
All − IVW(MRE)
−1.5
−1.0
−0.5
0.0
0.5
1.0
MR effect size for
'Gut microbiota abundance (genus Parasutterella id.2892)' on 'C−C motif chemokine 24'

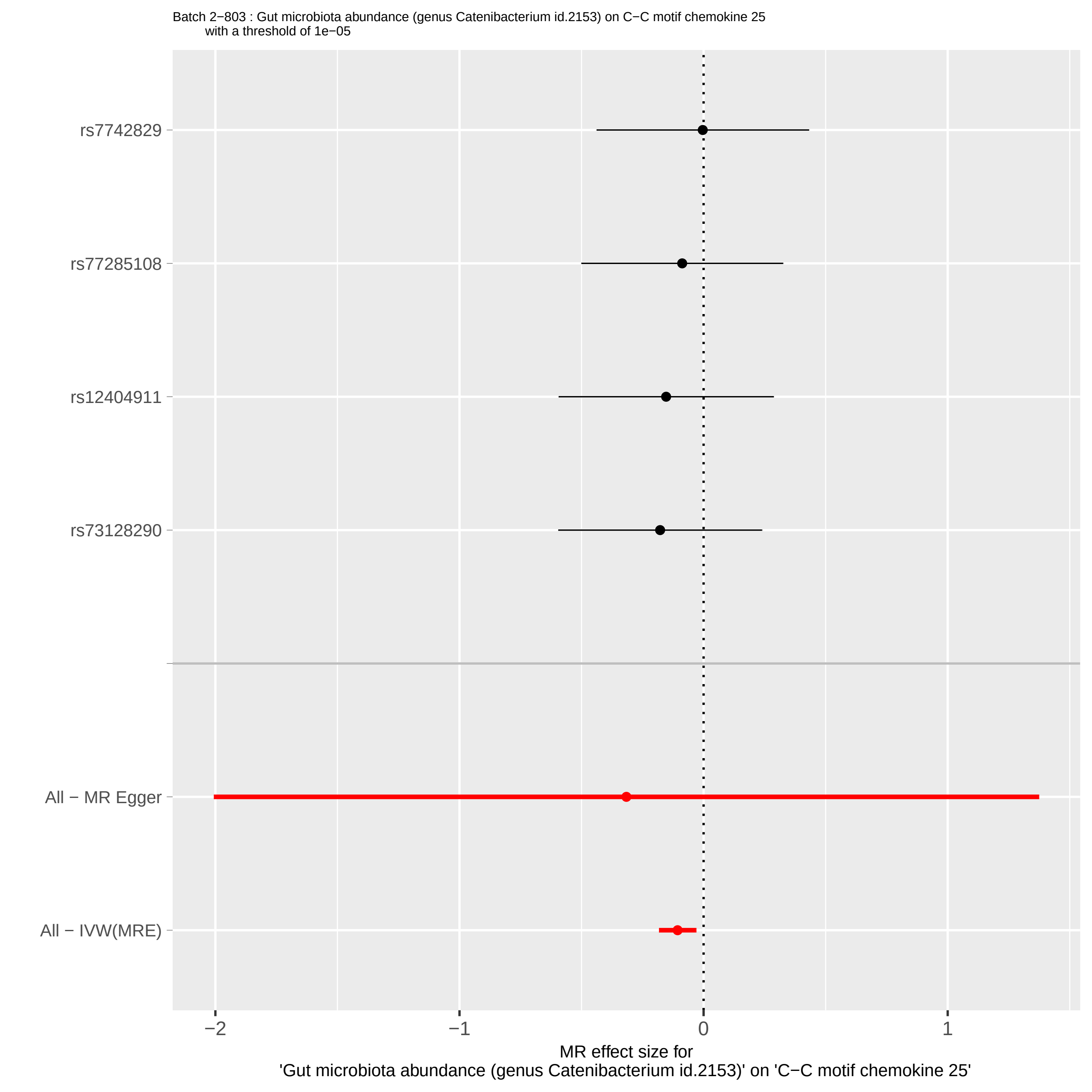

Batch 2−803 : Gut microbiota abundance (genus Catenibacterium id.2153) on C−C motif chemokine 25
with a threshold of 1e−05
rs7742829
rs77285108
rs12404911
rs73128290
All − MR Egger
All − IVW(MRE)
−2
−1
0
1
MR effect size for
'Gut microbiota abundance (genus Catenibacterium id.2153)' on 'C−C motif chemokine 25'

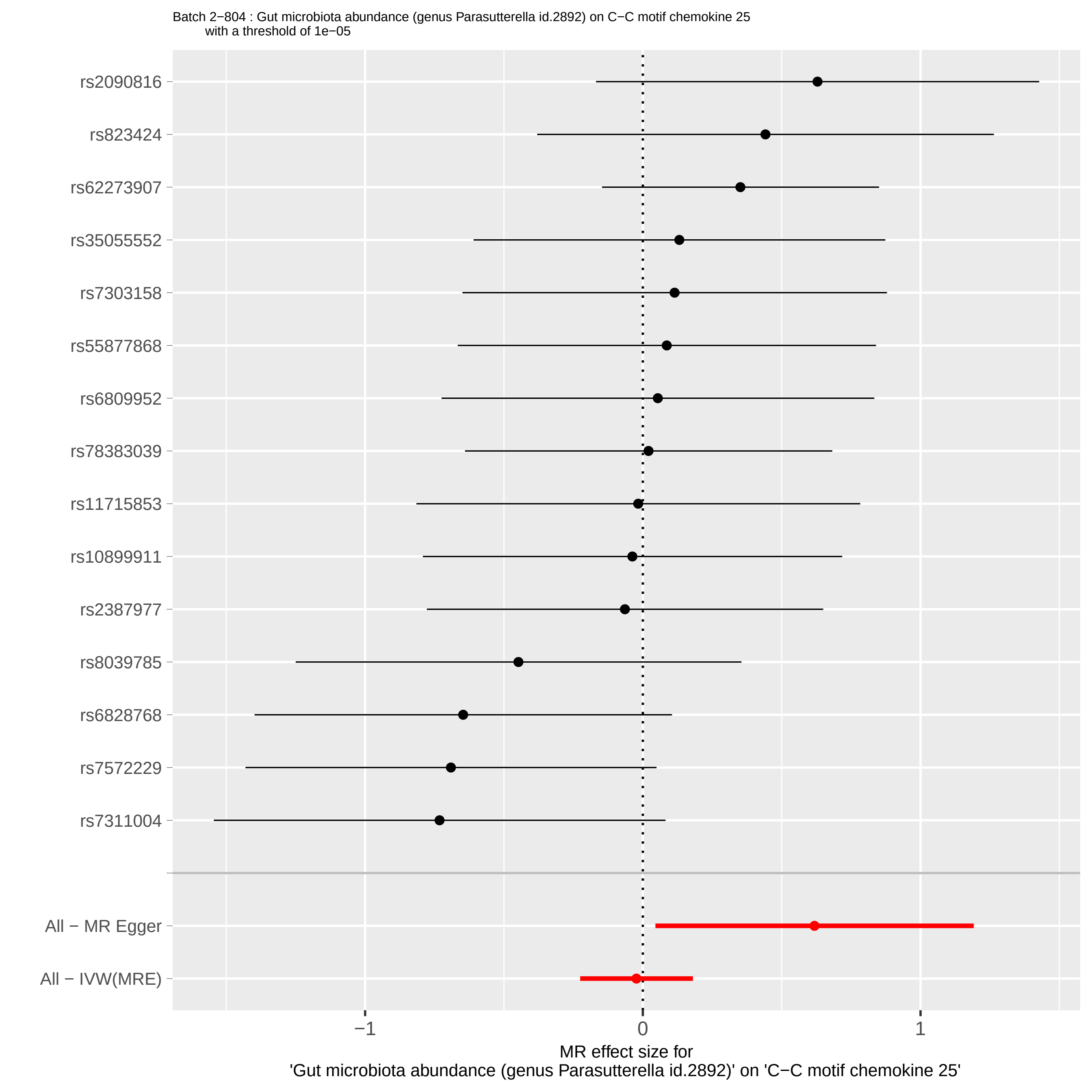

Batch 2−804 : Gut microbiota abundance (genus Parasutterella id.2892) on C−C motif chemokine 25 with a threshold of 1e−05
rs2090816
rs823424
rs62273907
rs35055552
rs7303158
rs55877868
rs6809952
rs78383039
rs11715853
rs10899911
rs2387977
rs8039785
rs6828768
rs7572229
rs7311004
All − MR Egger
All − IVW(MRE)
−1
0
1
MR effect size for
'Gut microbiota abundance (genus Parasutterella id.2892)' on 'C−C motif chemokine 25'

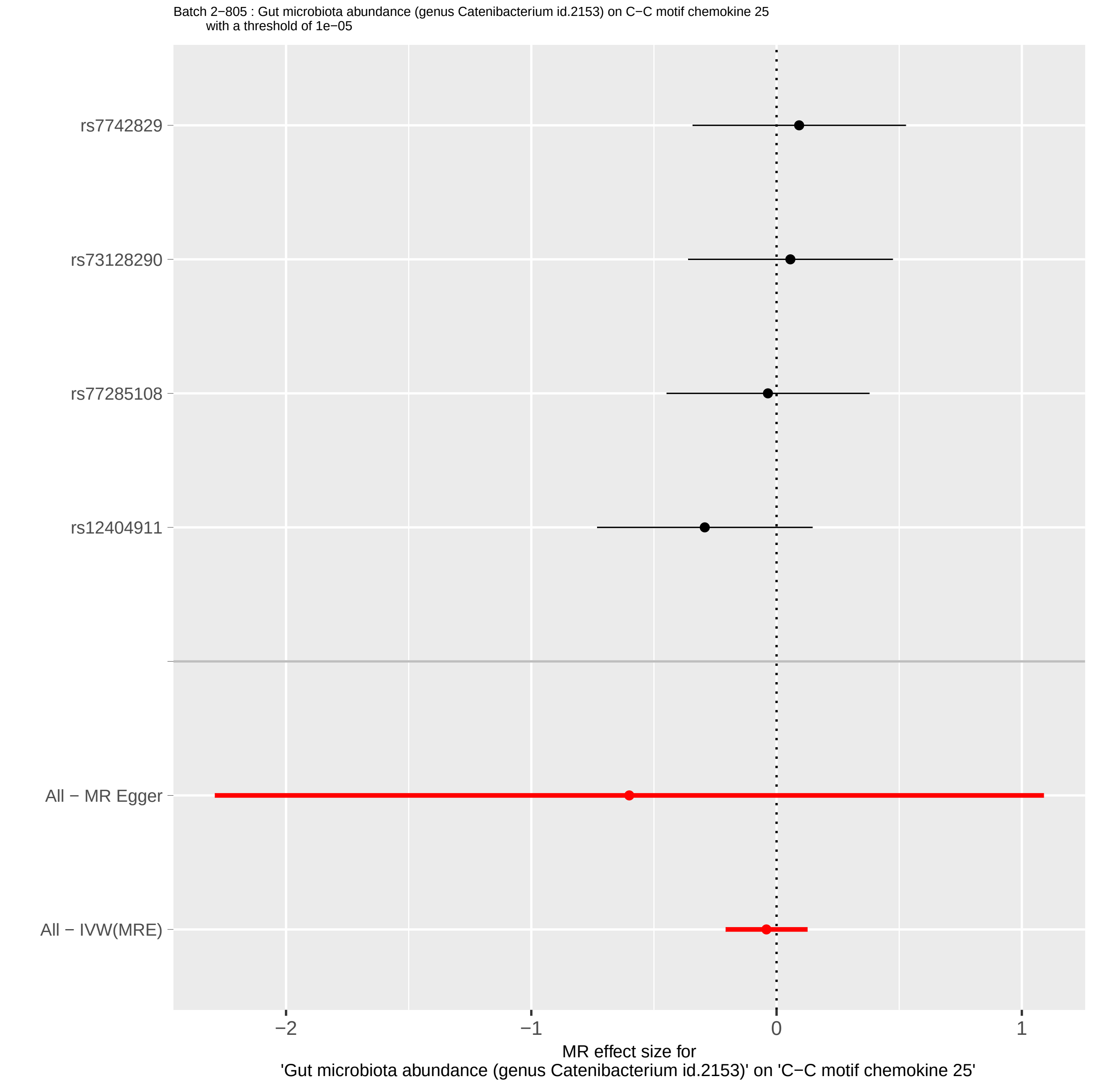

Batch 2−805 : Gut microbiota abundance (genus Catenibacterium id.2153) on C−C motif chemokine 25
with a threshold of 1e−05
rs7742829
rs73128290
rs77285108
rs12404911
All − MR Egger
All − IVW(MRE)
−2
−1
0
1
MR effect size for
'Gut microbiota abundance (genus Catenibacterium id.2153)' on 'C−C motif chemokine 25'

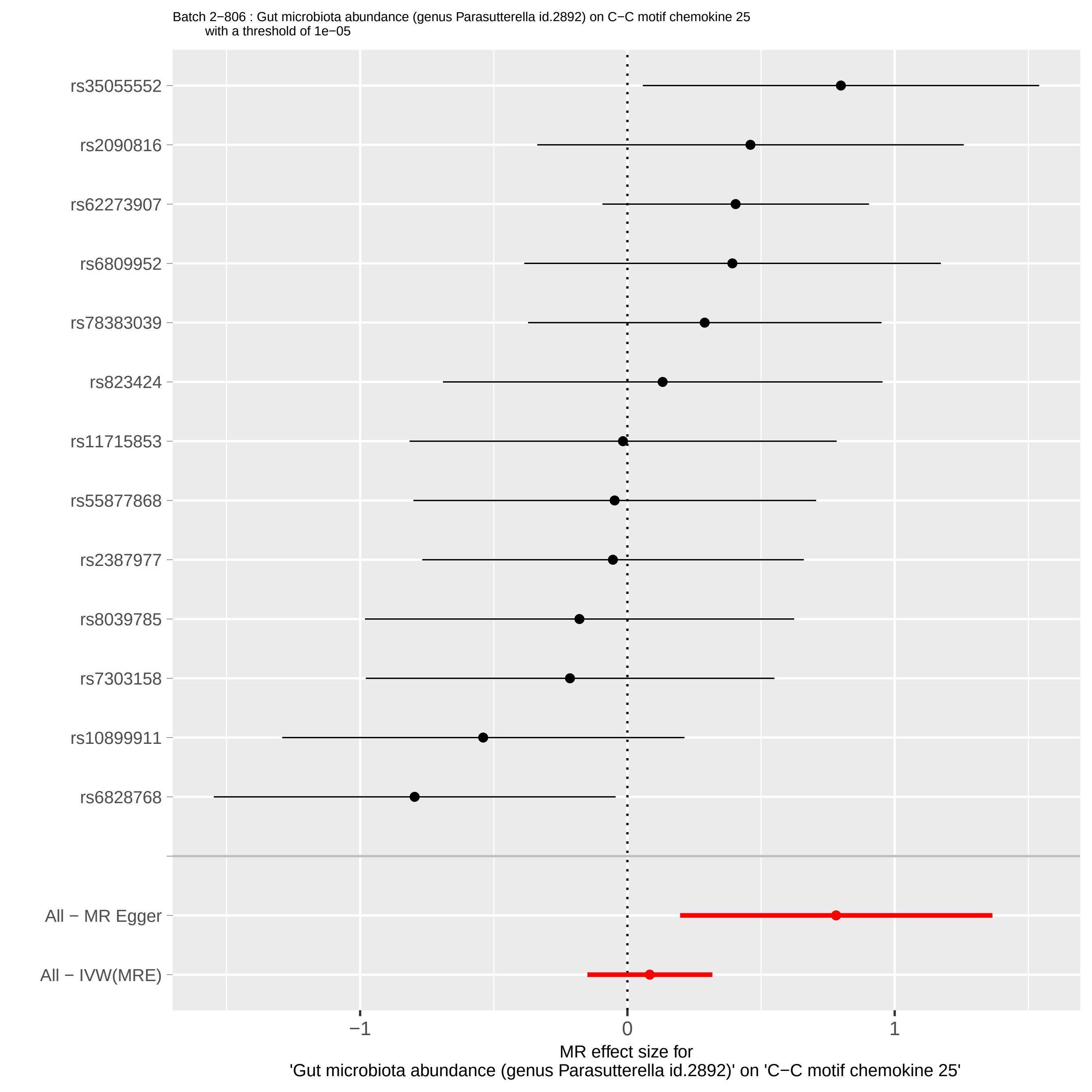
Batch 2−806 : Gut microbiota abundance (genus Parasutterella id.2892) on C−C motif chemokine 25
with a threshold of 1e−05
rs35055552
rs2090816
rs62273907
rs6809952
rs78383039
rs823424
rs11715853
rs55877868
rs2387977
rs8039785
rs7303158
rs10899911
rs6828768
All − MR Egger
All − IVW(MRE)
−1
0
1
MR effect size for
'Gut microbiota abundance (genus Parasutterella id.2892)' on 'C−C motif chemokine 25'

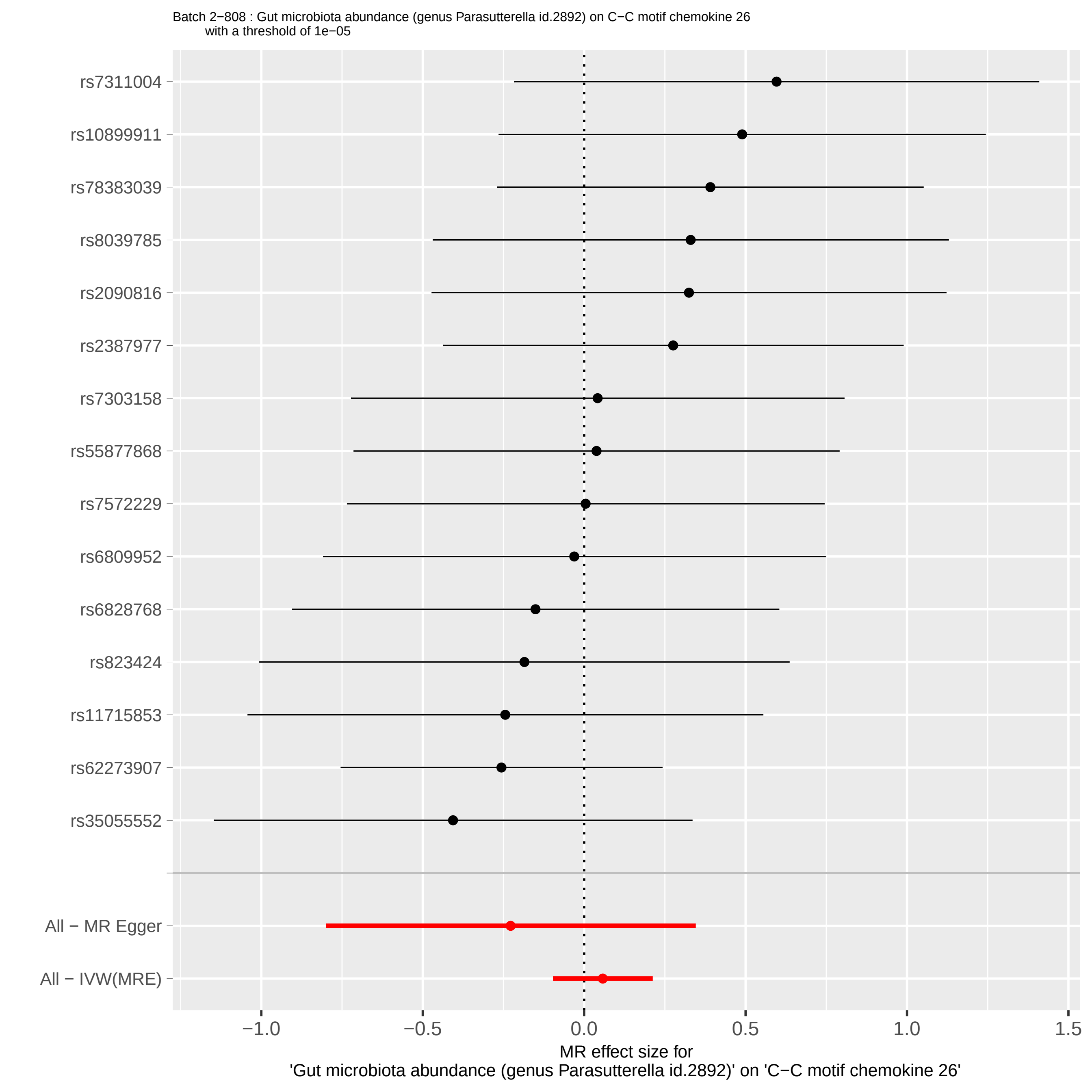
Batch 2−808 : Gut microbiota abundance (genus Parasutterella id.2892) on C−C motif chemokine 26
with a threshold of 1e−05
rs7311004
rs10899911
rs78383039
rs8039785
rs2090816
rs2387977
rs7303158
rs55877868
rs7572229
rs6809952
rs6828768
rs823424
rs11715853
rs62273907
rs35055552
All − MR Egger
All − IVW(MRE)
−1.0
−0.5
0.0
0.5
1.0
1.5
MR effect size for
'Gut microbiota abundance (genus Parasutterella id.2892)' on 'C−C motif chemokine 26'

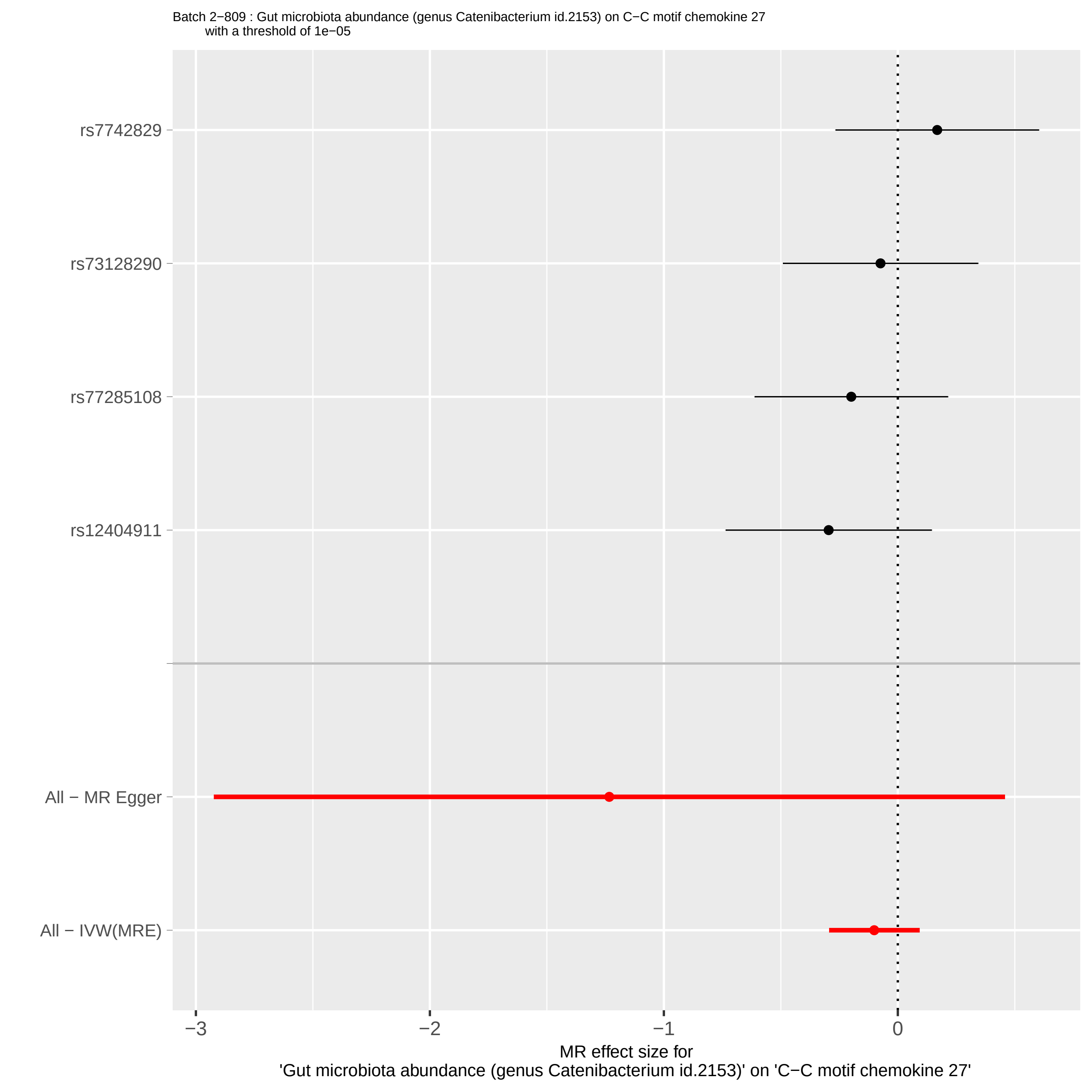

Batch 2−809 : Gut microbiota abundance (genus Catenibacterium id.2153) on C−C motif chemokine 27
with a threshold of 1e−05
rs7742829
rs73128290
rs77285108
rs12404911
All − MR Egger
All − IVW(MRE)
−3
−2
−1
0
MR effect size for
'Gut microbiota abundance (genus Catenibacterium id.2153)' on 'C−C motif chemokine 27'

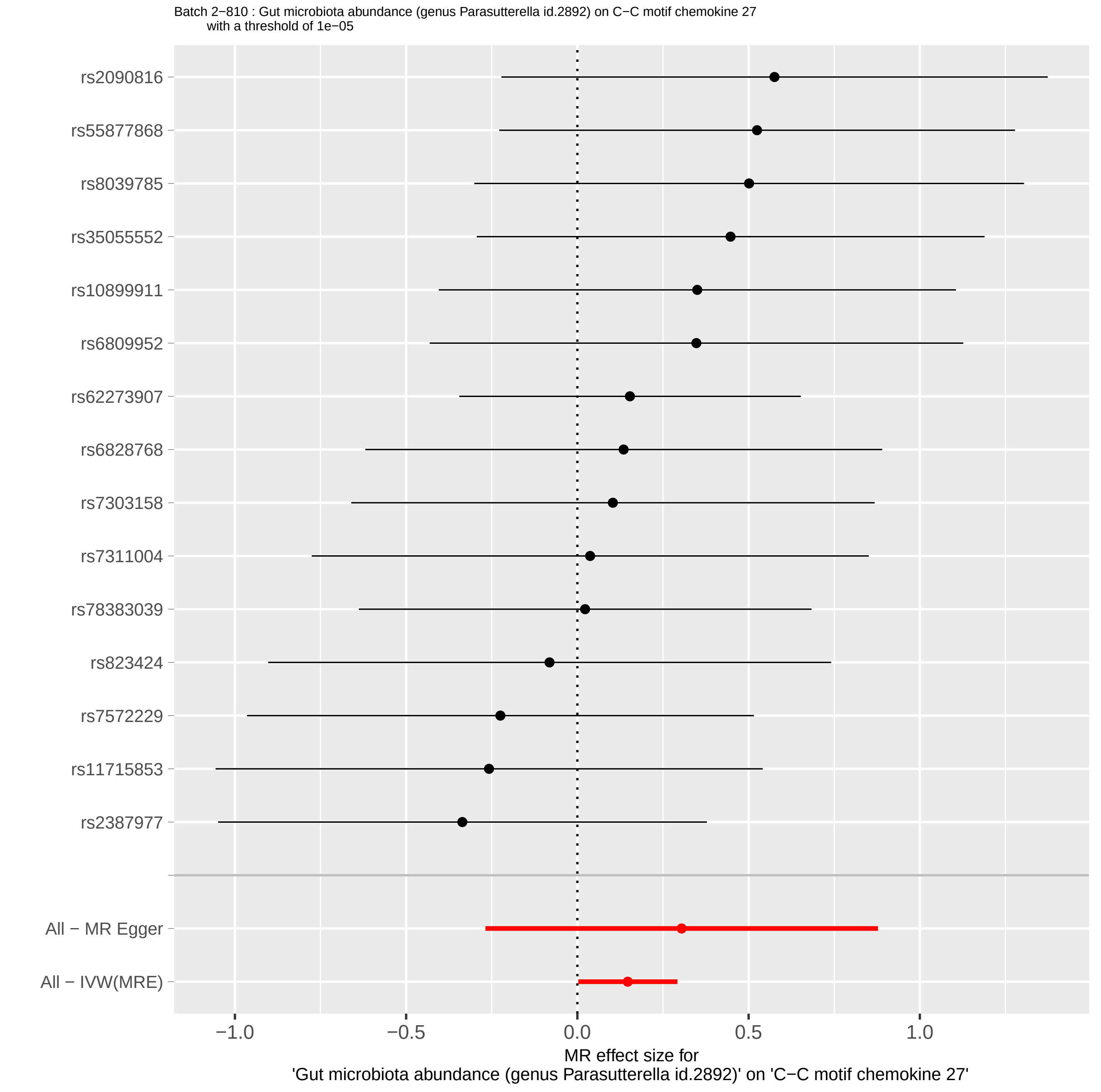
Batch 2−810 : Gut microbiota abundance (genus Parasutterella id.2892) on C−C motif chemokine 27
with a threshold of 1e−05
rs2090816
rs55877868
rs8039785
rs35055552
rs10899911
rs6809952
rs62273907
rs6828768
rs7303158
rs7311004
rs78383039
rs823424
rs7572229
rs11715853
rs2387977
All − MR Egger
All − IVW(MRE)
−1.0
−0.5
0.0
0.5
1.0
MR effect size for
'Gut microbiota abundance (genus Parasutterella id.2892)' on 'C−C motif chemokine 27'

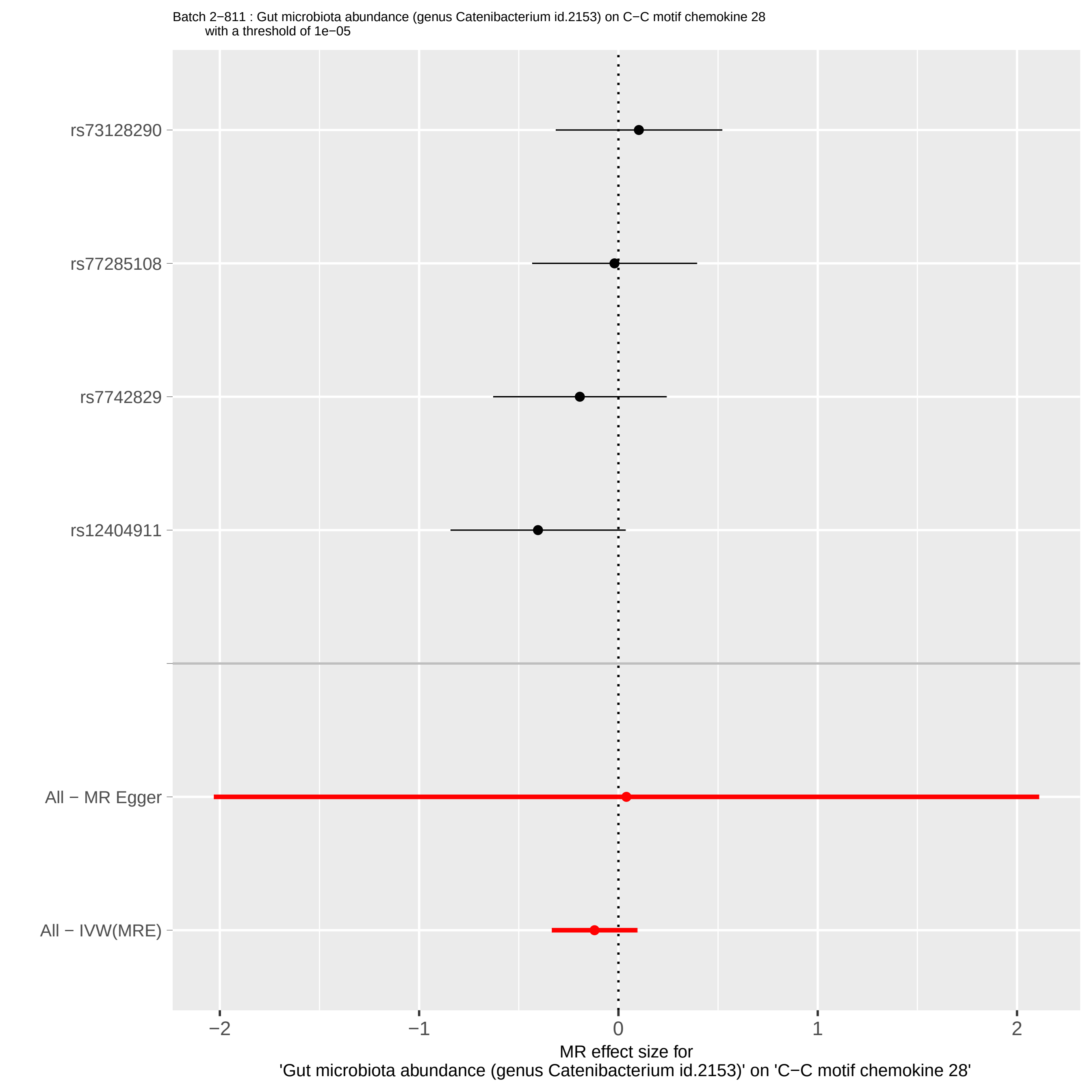

Batch 2−811 : Gut microbiota abundance (genus Catenibacterium id.2153) on C−C motif chemokine 28
with a threshold of 1e−05
rs73128290
rs77285108
rs7742829
rs12404911
All − MR Egger
All − IVW(MRE)
−2
−1
0
1
2
MR effect size for
'Gut microbiota abundance (genus Catenibacterium id.2153)' on 'C−C motif chemokine 28'

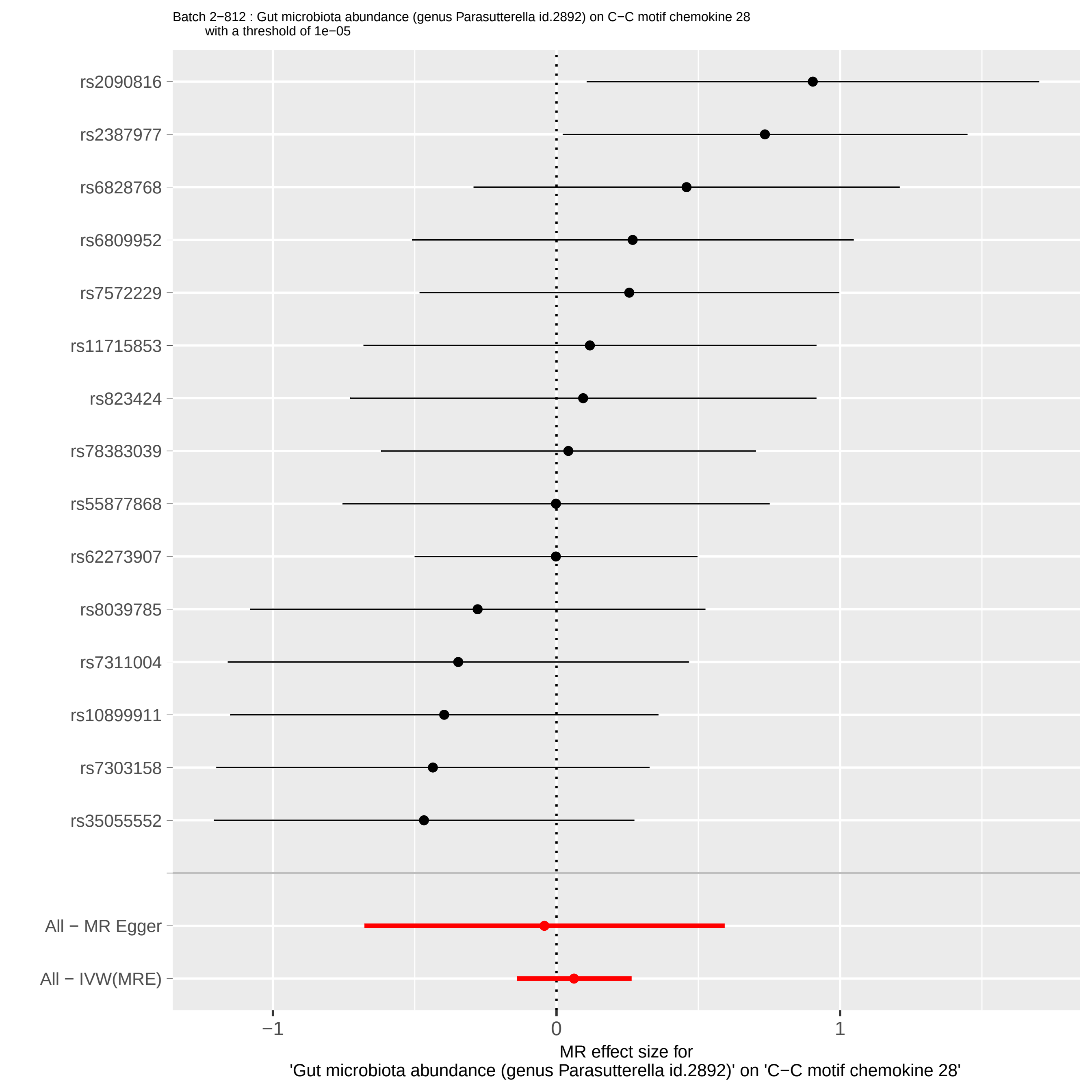

Batch 2−812 : Gut microbiota abundance (genus Parasutterella id.2892) on C−C motif chemokine 28
with a threshold of 1e−05
rs2090816
rs2387977
rs6828768
rs6809952
rs7572229
rs11715853
rs823424
rs78383039
rs55877868
rs62273907
rs8039785
rs7311004
rs10899911
rs7303158
rs35055552
All − MR Egger
All − IVW(MRE)
−1
0
1
MR effect size for
'Gut microbiota abundance (genus Parasutterella id.2892)' on 'C−C motif chemokine 28'

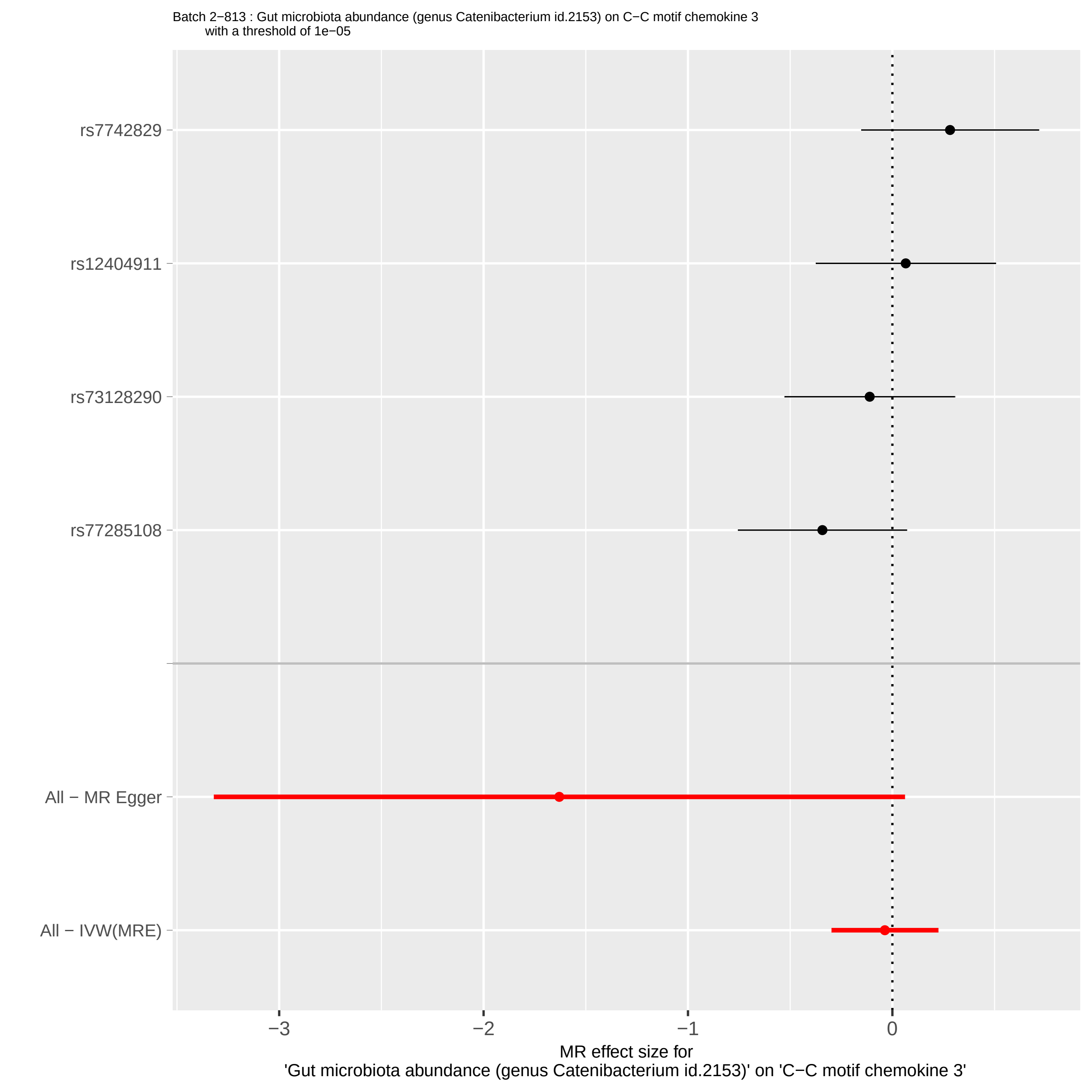
Batch 2−813 : Gut microbiota abundance (genus Catenibacterium id.2153) on C−C motif chemokine 3
with a threshold of 1e−05
rs7742829
rs12404911
rs73128290
rs77285108
All − MR Egger
All − IVW(MRE)
−3
−2
−1
0
MR effect size for
'Gut microbiota abundance (genus Catenibacterium id.2153)' on 'C−C motif chemokine 3'

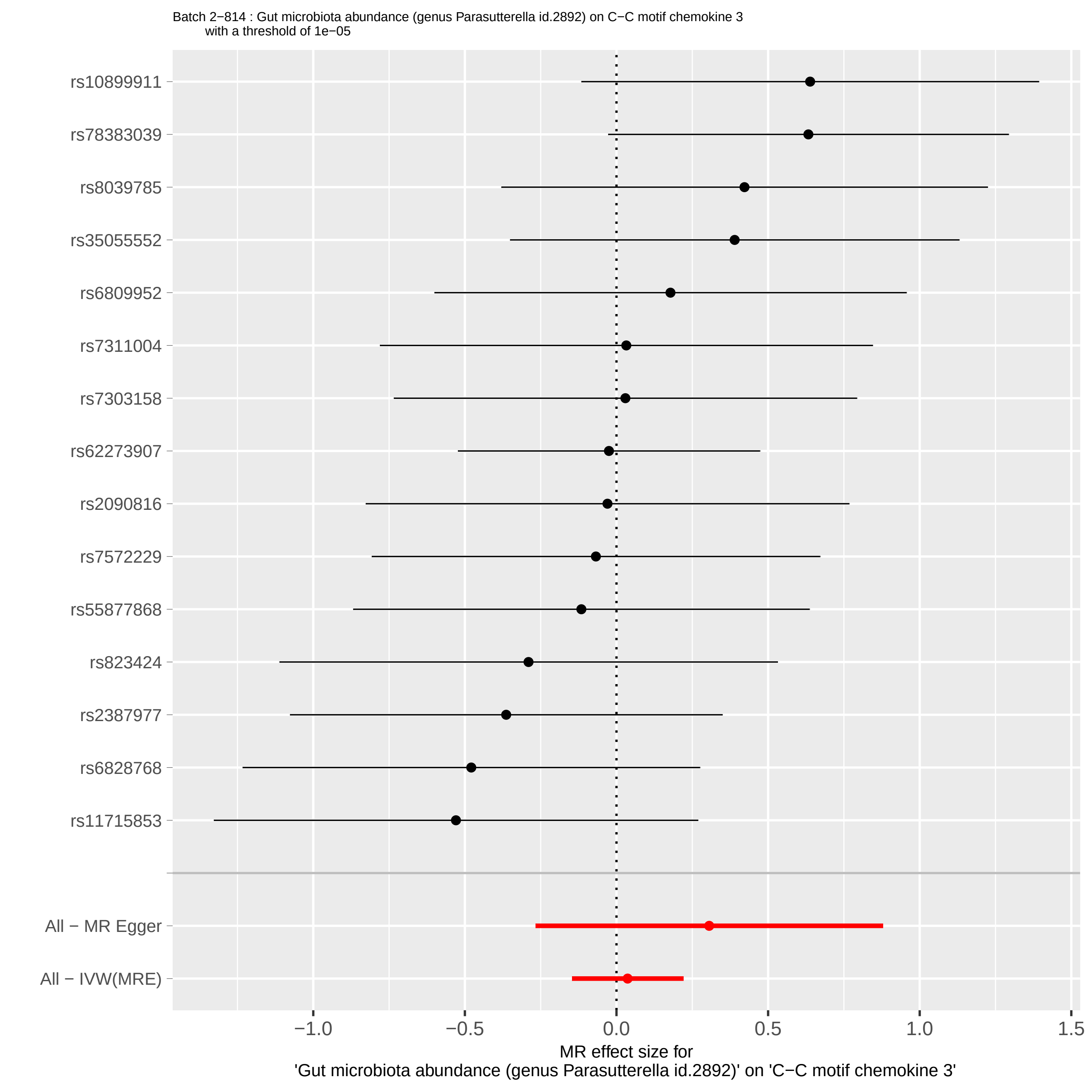
Batch 2−814 : Gut microbiota abundance (genus Parasutterella id.2892) on C−C motif chemokine 3
with a threshold of 1e−05
rs10899911
rs78383039
rs8039785
rs35055552
rs6809952
rs7311004
rs7303158
rs62273907
rs2090816
rs7572229
rs55877868
rs823424
rs2387977
rs6828768
rs11715853
All − MR Egger
All − IVW(MRE)
−1.0
−0.5
0.0
0.5
1.0
1.5
MR effect size for
'Gut microbiota abundance (genus Parasutterella id.2892)' on 'C−C motif chemokine 3'

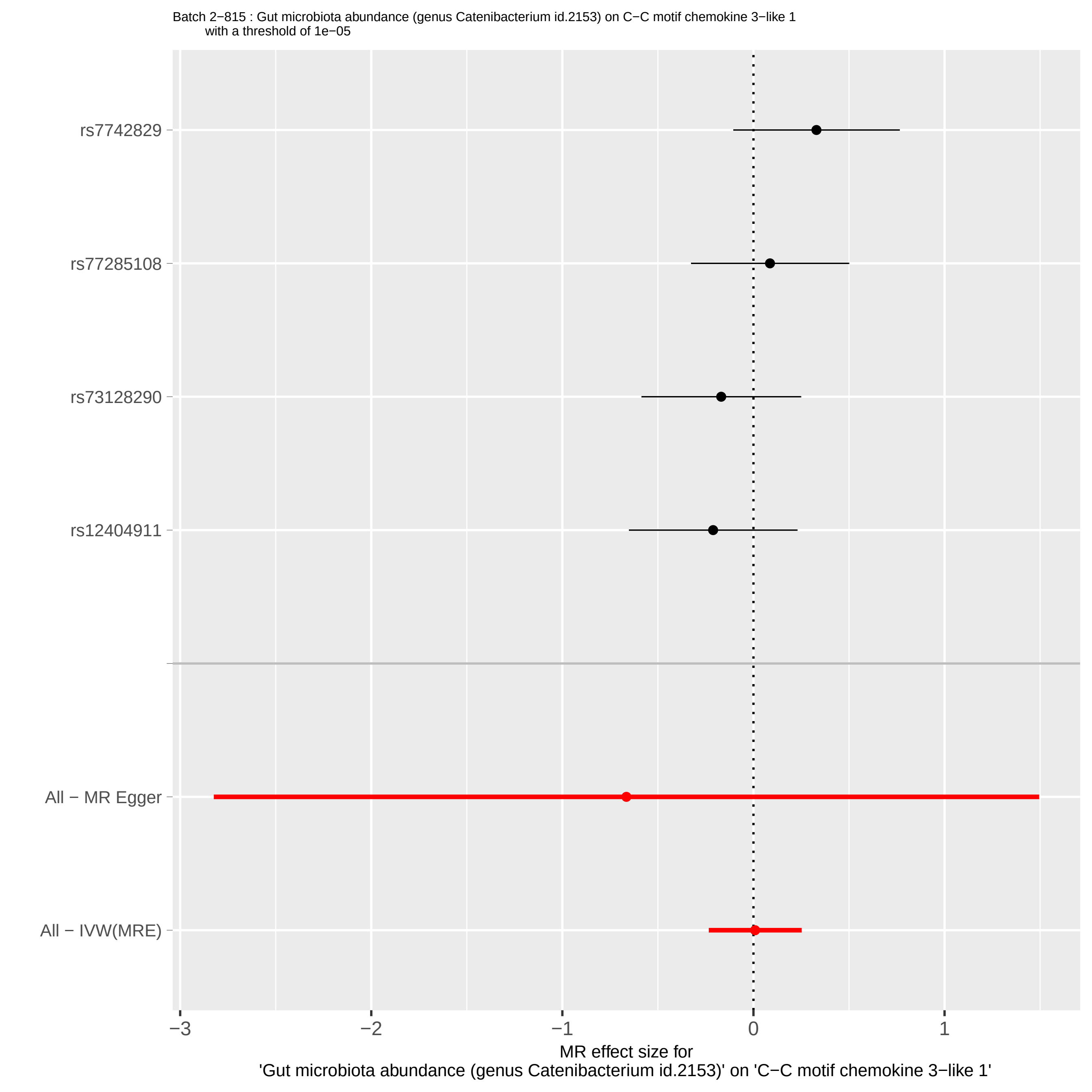

Batch 2−815 : Gut microbiota abundance (genus Catenibacterium id.2153) on C−C motif chemokine 3−like 1
with a threshold of 1e−05
rs7742829
rs77285108
rs73128290
rs12404911
All − MR Egger
All − IVW(MRE)
−3
−2
−1
0
1
MR effect size for
'Gut microbiota abundance (genus Catenibacterium id.2153)' on 'C−C motif chemokine 3−like 1'

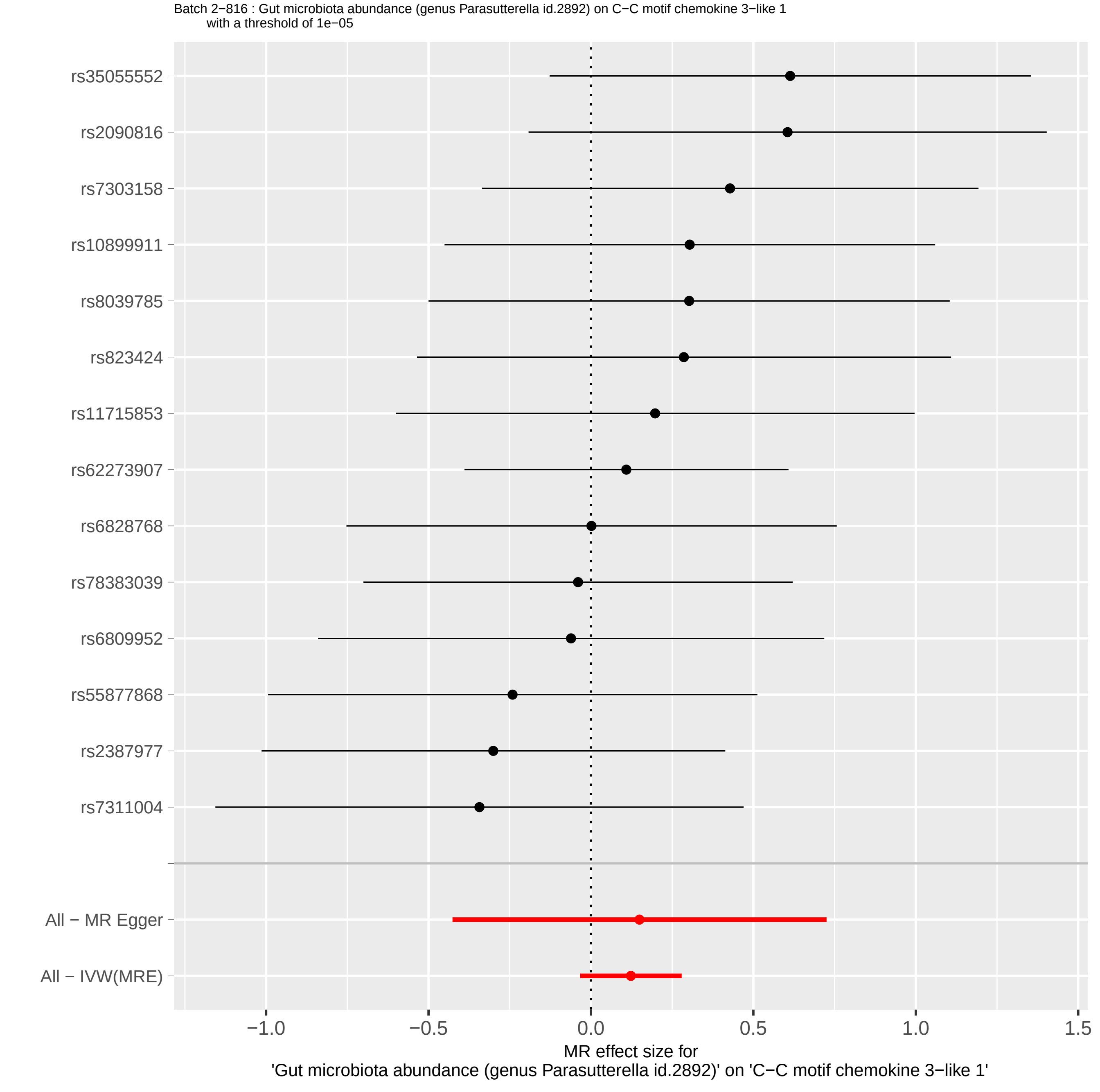

Batch 2−816 : Gut microbiota abundance (genus Parasutterella id.2892) on C−C motif chemokine 3−like 1
with a threshold of 1e−05
rs35055552
rs2090816
rs7303158
rs10899911
rs8039785
rs823424
rs11715853
rs62273907
rs6828768
rs78383039
rs6809952
rs55877868
rs2387977
rs7311004
All − MR Egger
All − IVW(MRE)
−1.0
−0.5
0.0
0.5
1.0
1.5
MR effect size for
'Gut microbiota abundance (genus Parasutterella id.2892)' on 'C−C motif chemokine 3−like 1'

Batch 2−817 : Gut microbiota abundance (genus Catenibacterium id.2153) on C−C motif chemokine 5
with a threshold of 1e−05

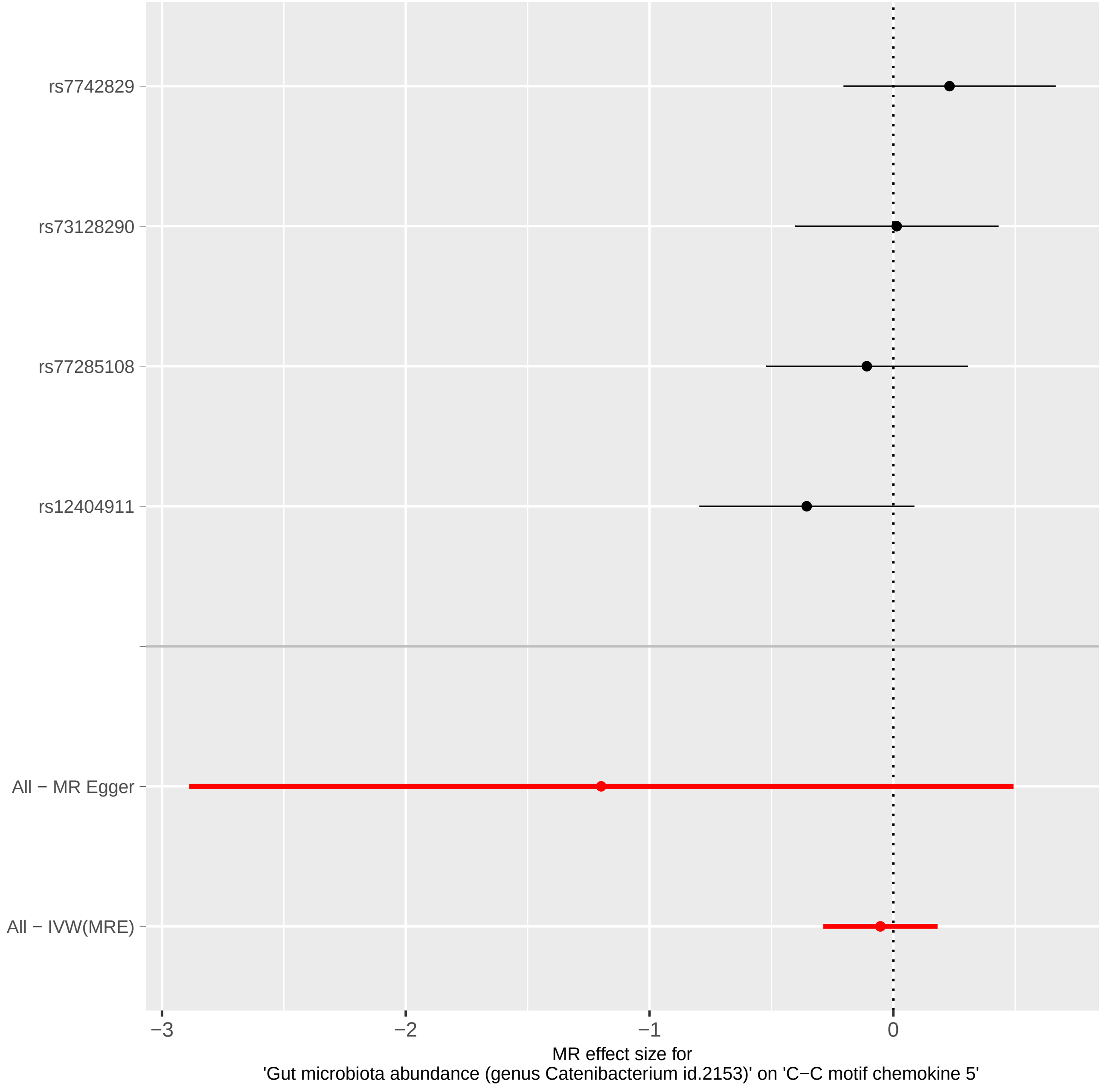

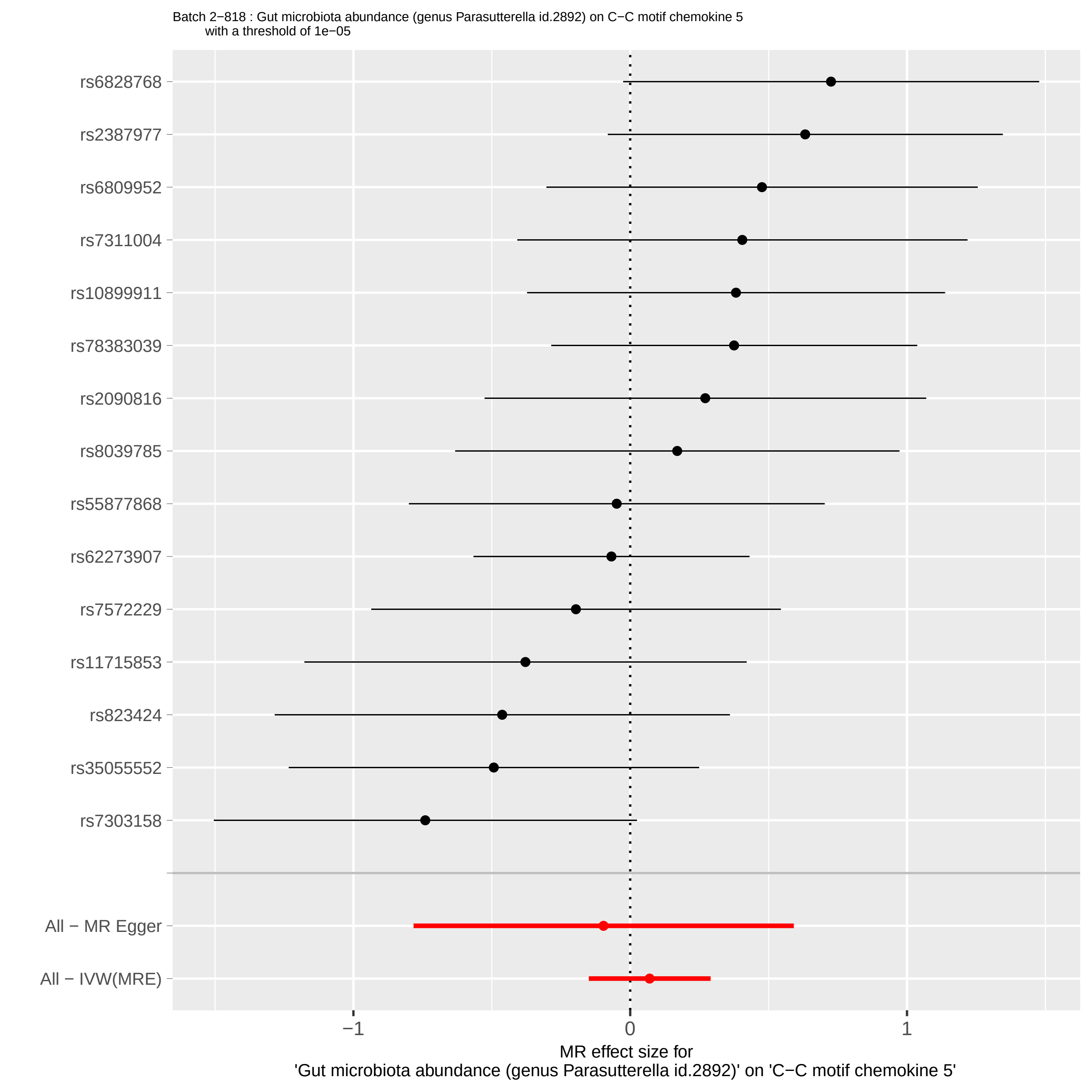

Batch 2−818 : Gut microbiota abundance (genus Parasutterella id.2892) on C−C motif chemokine 5
with a threshold of 1e−05
rs6828768
rs2387977
rs6809952
rs7311004
rs10899911
rs78383039
rs2090816
rs8039785
rs55877868
rs62273907
rs7572229
rs11715853
rs823424
rs35055552
rs7303158
All − MR Egger
All − IVW(MRE)
−1
0
1
MR effect size for
'Gut microbiota abundance (genus Parasutterella id.2892)' on 'C−C motif chemokine 5'

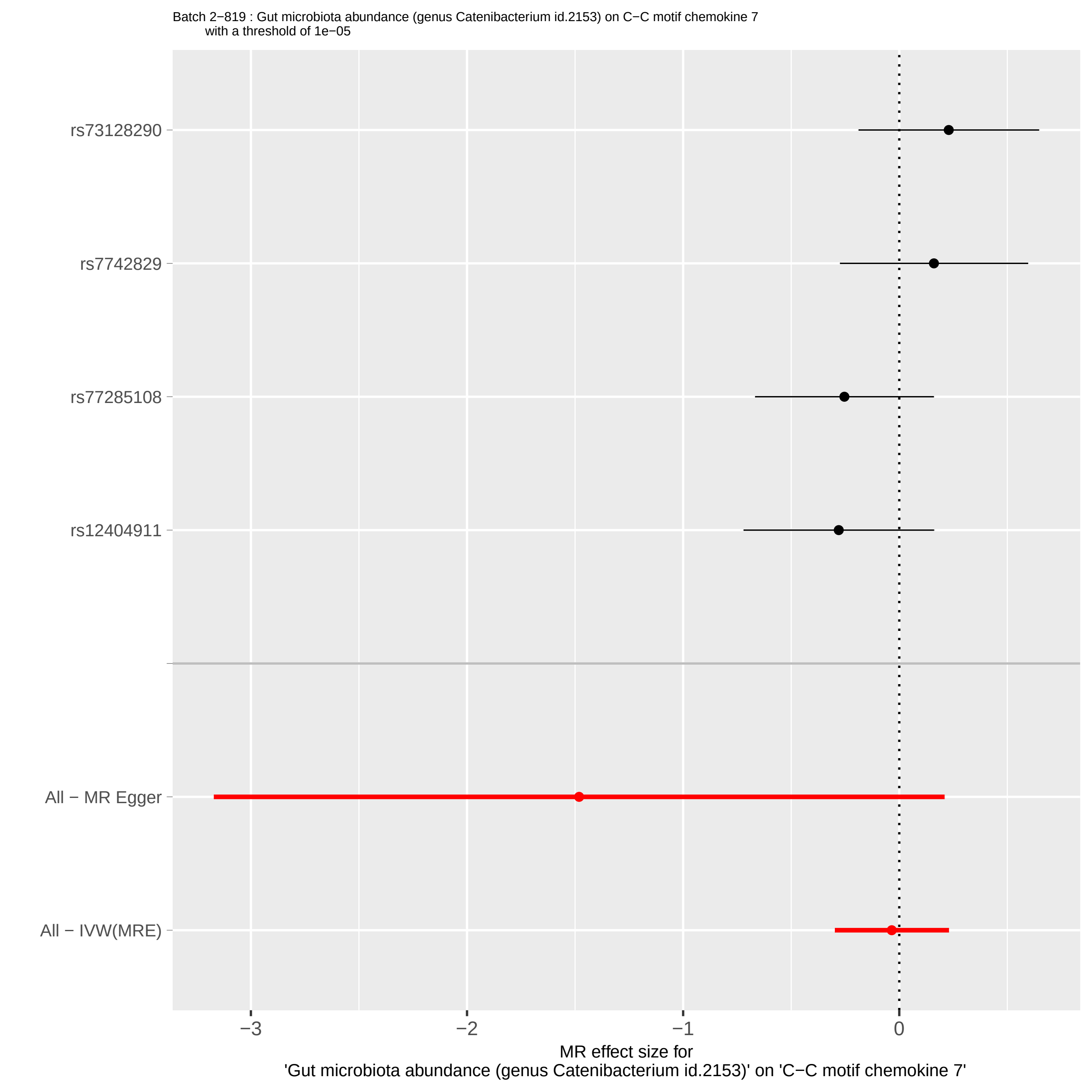
Batch 2−819 : Gut microbiota abundance (genus Catenibacterium id.2153) on C−C motif chemokine 7
with a threshold of 1e−05
rs73128290
rs7742829
rs77285108
rs12404911
All − MR Egger
All − IVW(MRE)
−3
−2
−1
0
MR effect size for
'Gut microbiota abundance (genus Catenibacterium id.2153)' on 'C−C motif chemokine 7'

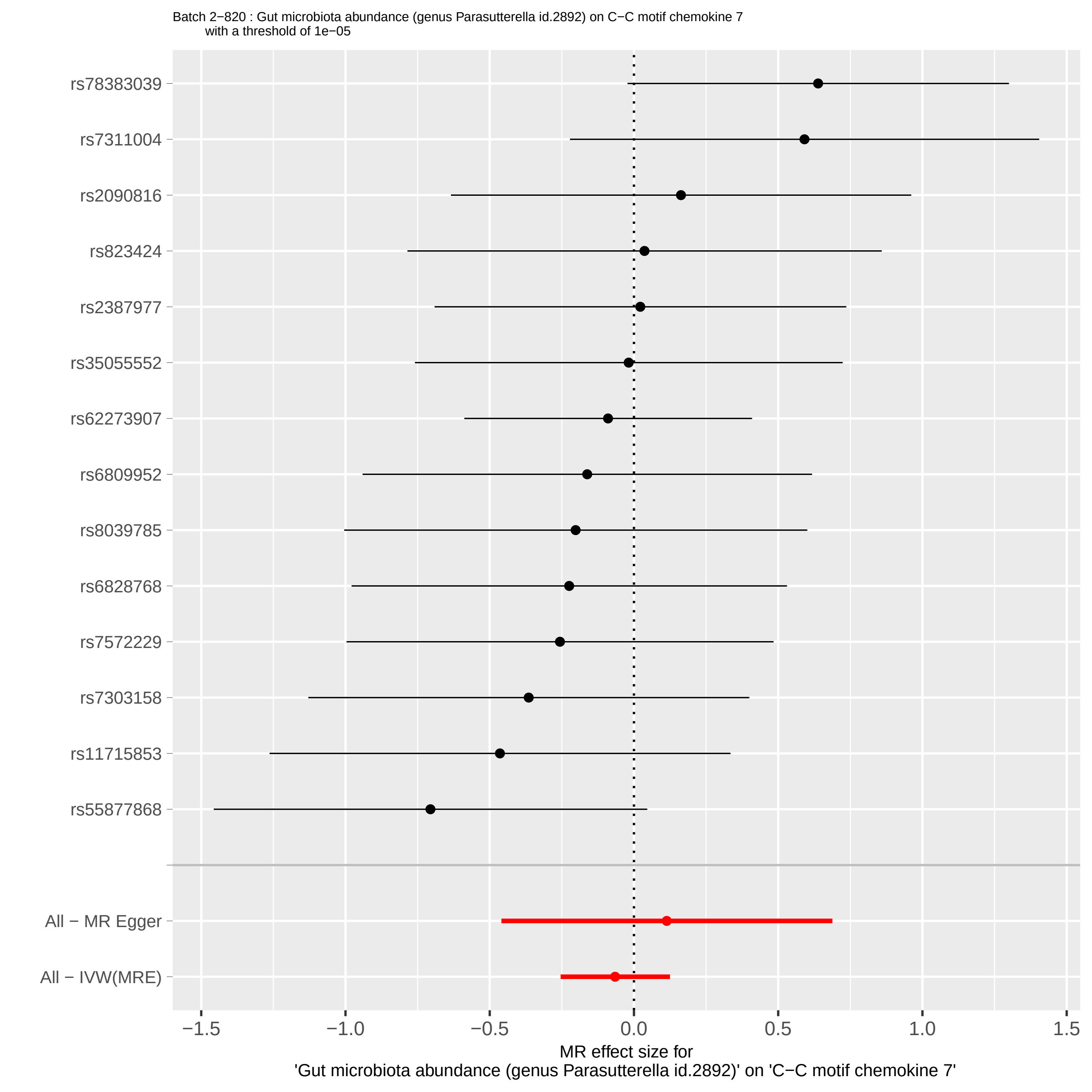
Batch 2−820 : Gut microbiota abundance (genus Parasutterella id.2892) on C−C motif chemokine 7
with a threshold of 1e−05
rs78383039
rs7311004
rs2090816
rs823424
rs2387977
rs35055552
rs62273907
rs6809952
rs8039785
rs6828768
rs7572229
rs7303158
rs11715853
rs55877868
All − MR Egger
All − IVW(MRE)
−1.5
−1.0
−0.5
0.0
0.5
1.0
1.5
MR effect size for
'Gut microbiota abundance (genus Parasutterella id.2892)' on 'C−C motif chemokine 7'

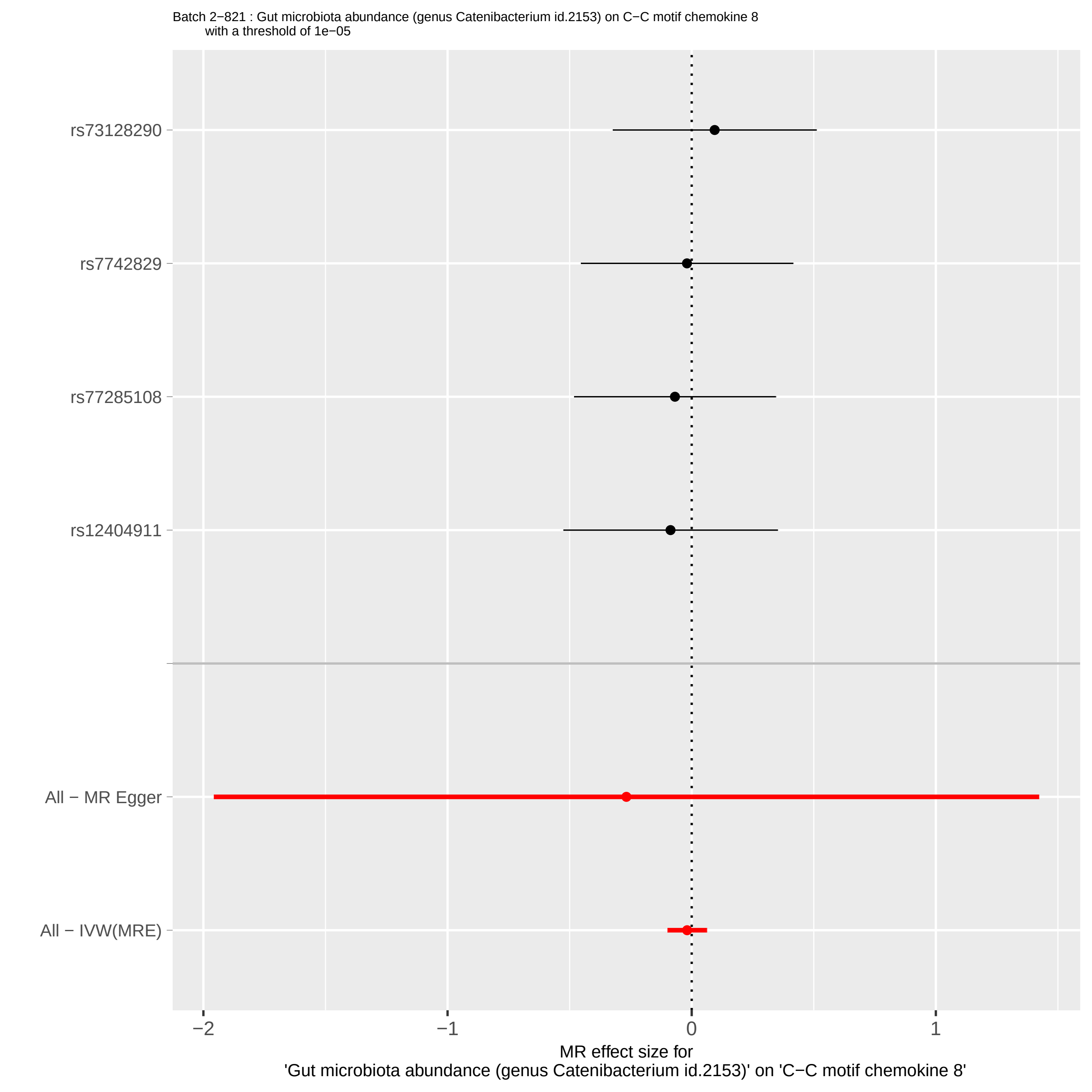
Batch 2−821 : Gut microbiota abundance (genus Catenibacterium id.2153) on C−C motif chemokine 8
with a threshold of 1e−05
rs73128290
rs7742829
rs77285108
rs12404911
All − MR Egger
All − IVW(MRE)
−2
−1
0
1
MR effect size for
'Gut microbiota abundance (genus Catenibacterium id.2153)' on 'C−C motif chemokine 8'

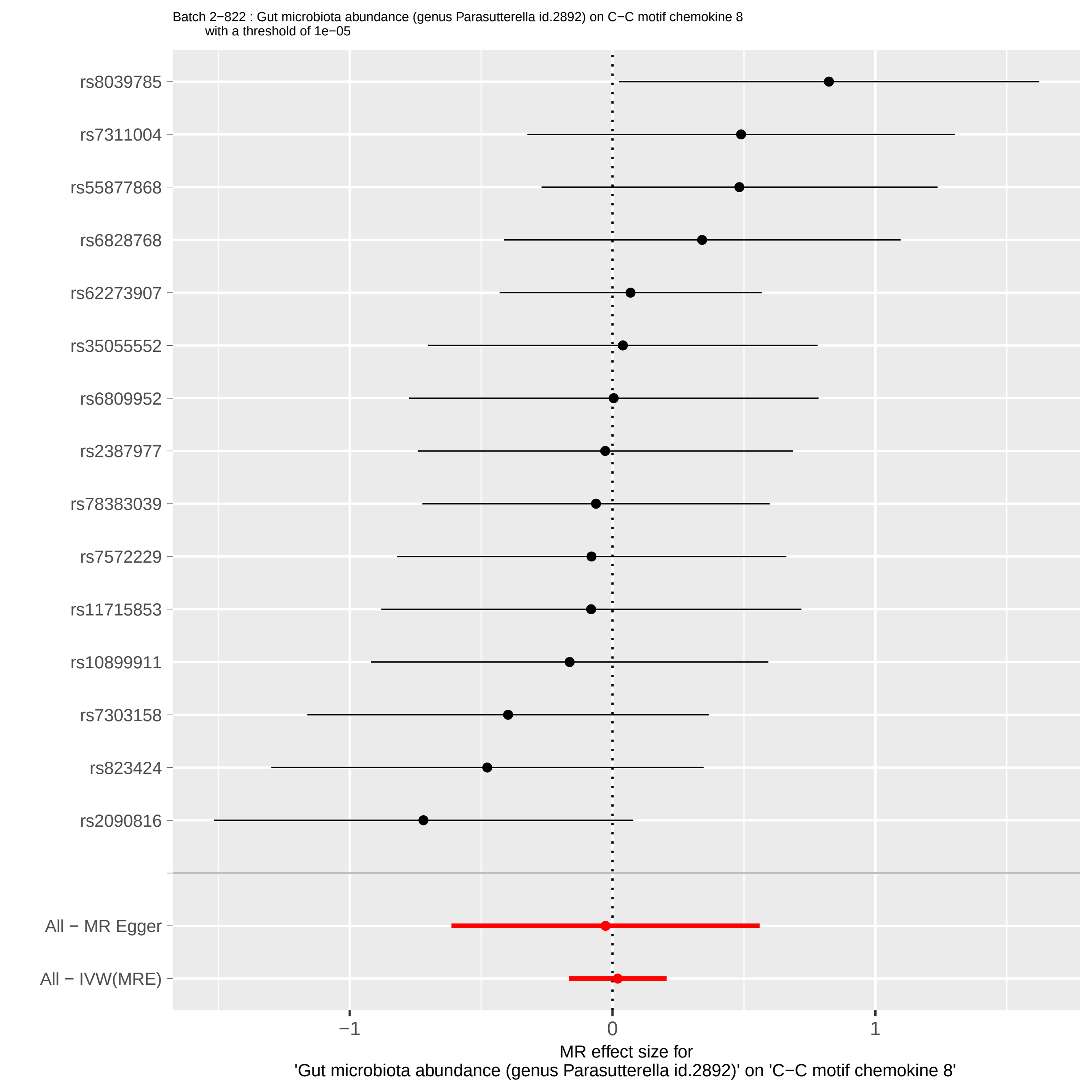
Batch 2−822 : Gut microbiota abundance (genus Parasutterella id.2892) on C−C motif chemokine 8
with a threshold of 1e−05
rs8039785
rs7311004
rs55877868
rs6828768
rs62273907
rs35055552
rs6809952
rs2387977
rs78383039
rs7572229
rs11715853
rs10899911
rs7303158
rs823424
rs2090816
All − MR Egger
All − IVW(MRE)
−1
0
1
MR effect size for
'Gut microbiota abundance (genus Parasutterella id.2892)' on 'C−C motif chemokine 8'

Batch 2−823 : Gut microbiota abundance (genus Catenibacterium id.2153) on Cerebral cavernous malformations 2 protein with a threshold of 1e−05

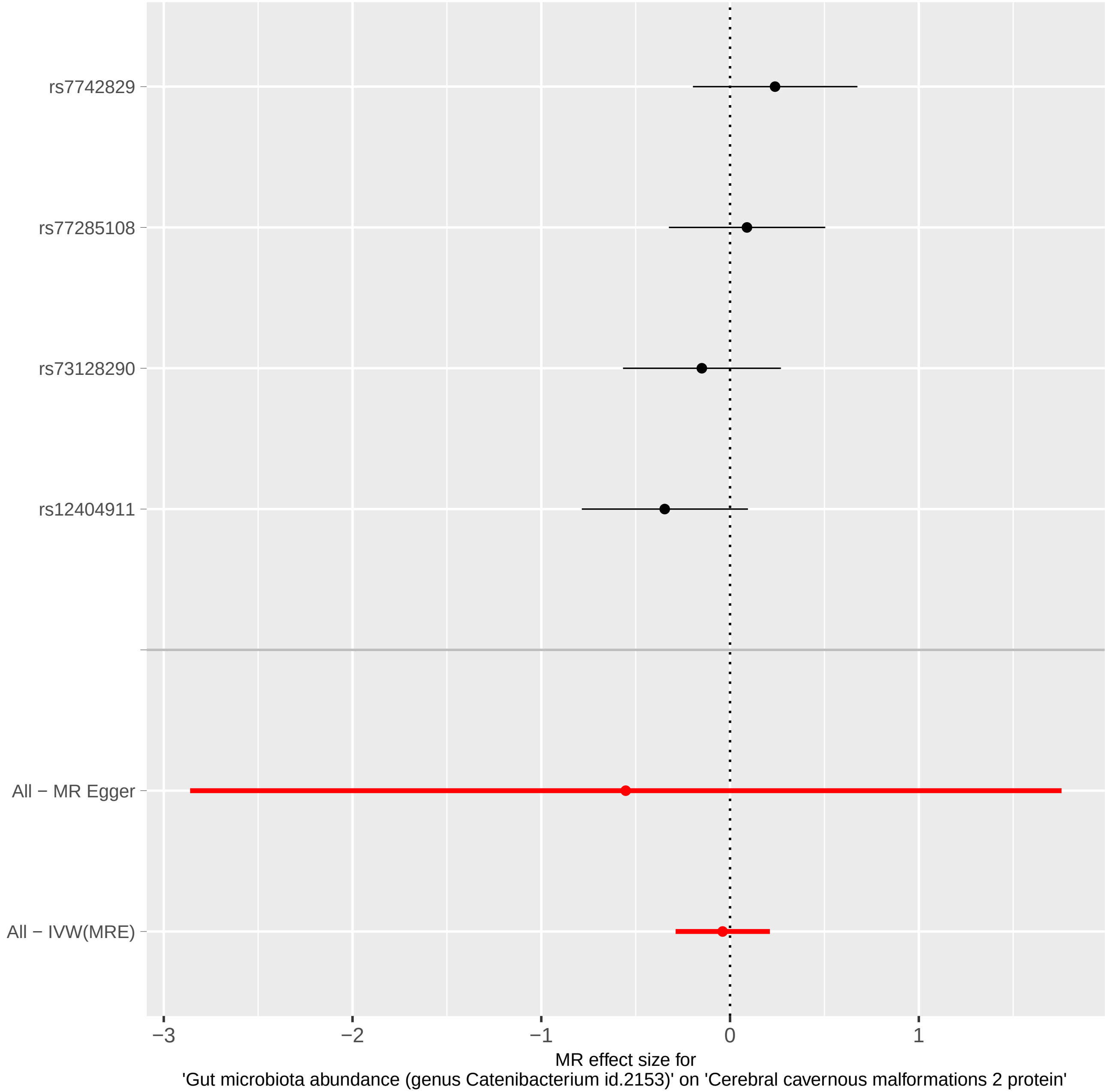

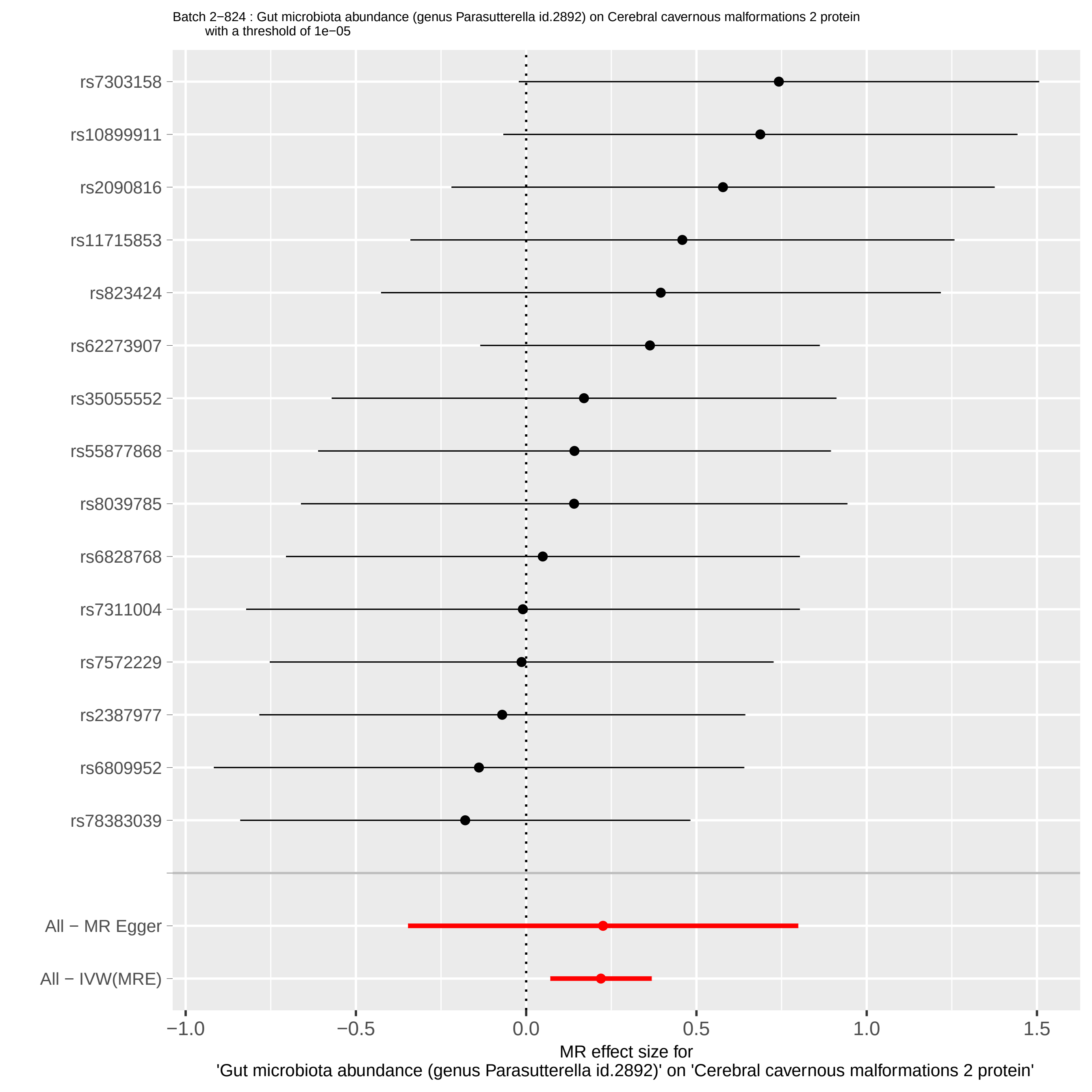

Batch 2−824 : Gut microbiota abundance (genus Parasutterella id.2892) on Cerebral cavernous malformations 2 protein with a threshold of 1e−05
rs7303158
rs10899911
rs2090816
rs11715853
rs823424
rs62273907
rs35055552
rs55877868
rs8039785
rs6828768
rs7311004
rs7572229
rs2387977
rs6809952
rs78383039
All − MR Egger
All − IVW(MRE)
−1.0
−0.5
0.0
0.5
1.0
1.5
MR effect size for
'Gut microbiota abundance (genus Parasutterella id.2892)' on 'Cerebral cavernous malformations 2 protein'

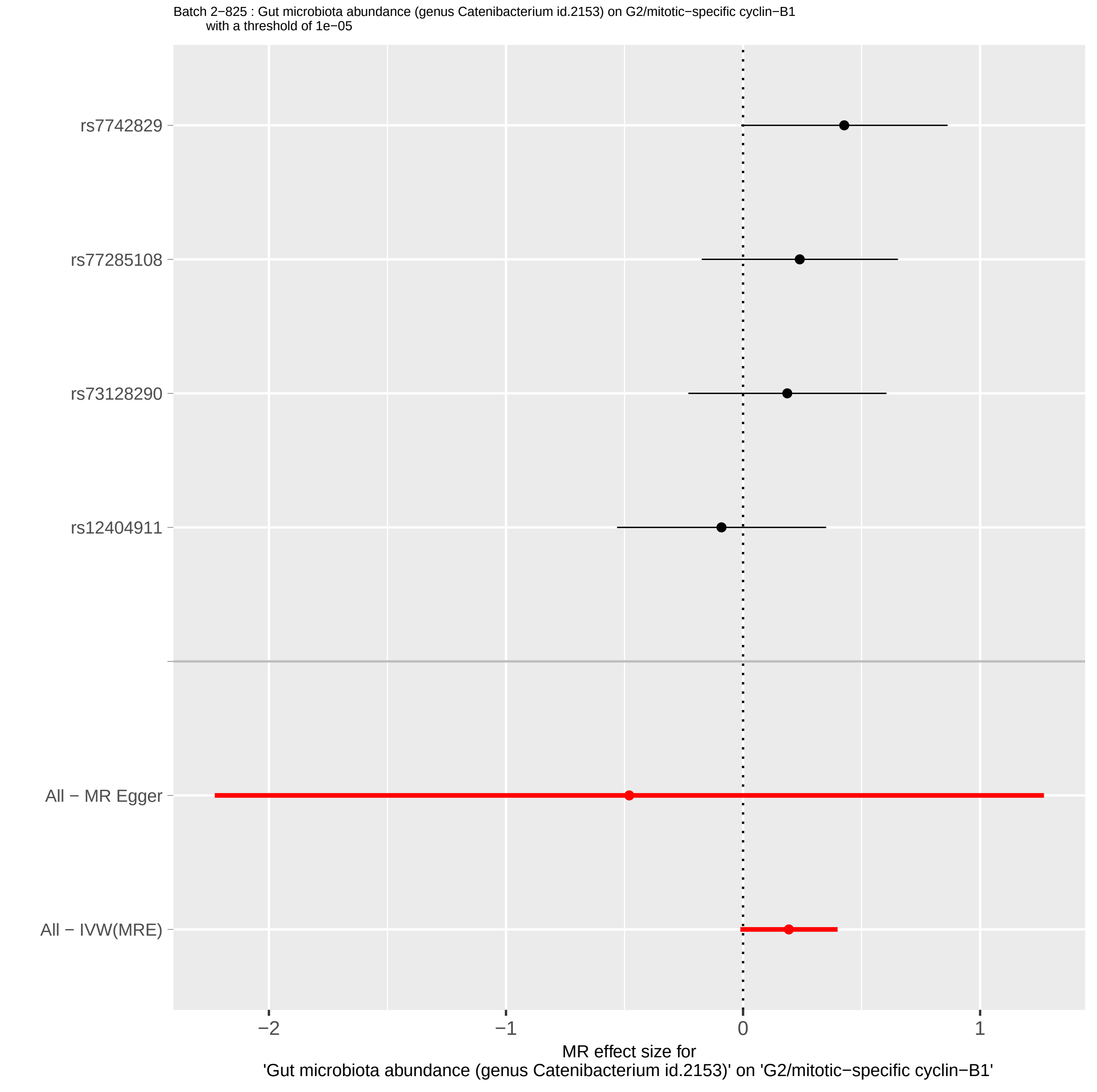

Batch 2−825 : Gut microbiota abundance (genus Catenibacterium id.2153) on G2/mitotic−specific cyclin−B1
with a threshold of 1e−05
rs7742829
rs77285108
rs73128290
rs12404911
All − MR Egger
All − IVW(MRE)
−2
−1
0
1
MR effect size for
'Gut microbiota abundance (genus Catenibacterium id.2153)' on 'G2/mitotic−specific cyclin−B1'

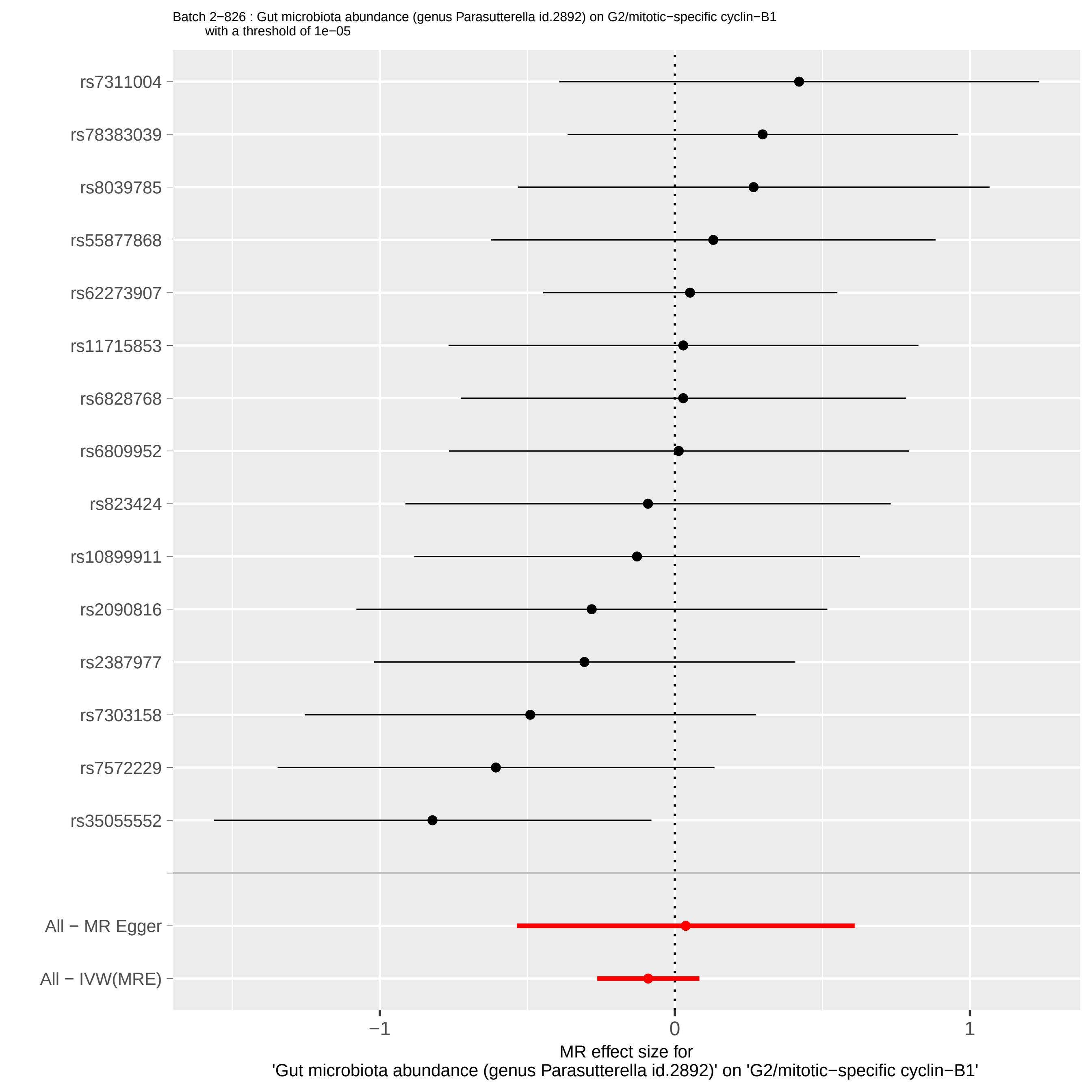

Batch 2−826 : Gut microbiota abundance (genus Parasutterella id.2892) on G2/mitotic−specific cyclin−B1
with a threshold of 1e−05
rs7311004
rs78383039
rs8039785
rs55877868
rs62273907
rs11715853
rs6828768
rs6809952
rs823424
rs10899911
rs2090816
rs2387977
rs7303158
rs7572229
rs35055552
All − MR Egger
All − IVW(MRE)
−1
0
1
MR effect size for
'Gut microbiota abundance (genus Parasutterella id.2892)' on 'G2/mitotic−specific cyclin−B1'

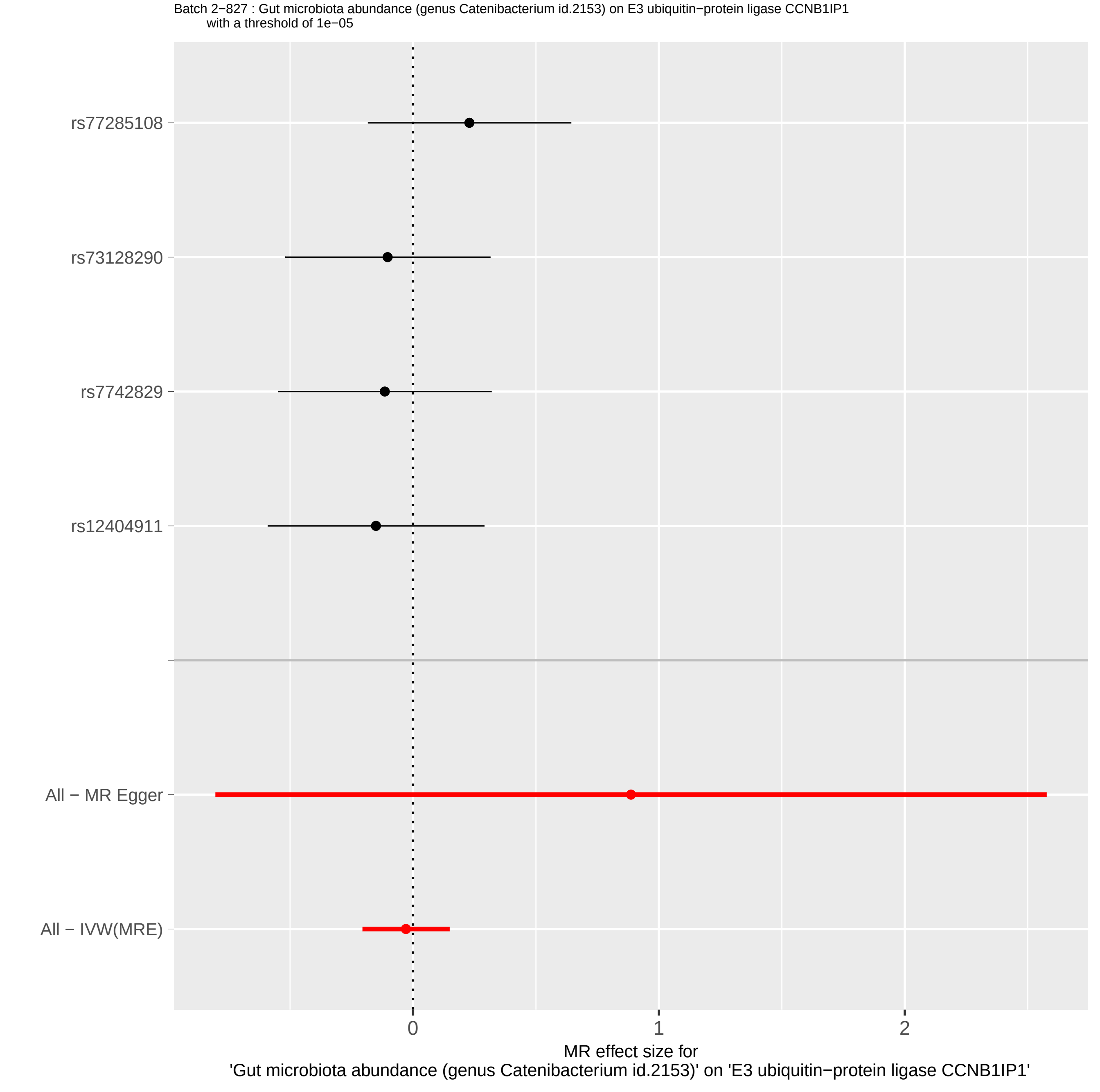

Batch 2−827 : Gut microbiota abundance (genus Catenibacterium id.2153) on E3 ubiquitin−protein ligase CCNB1IP1
with a threshold of 1e−05
rs77285108
rs73128290
rs7742829
rs12404911
All − MR Egger
All − IVW(MRE)
0
1
2
MR effect size for
'Gut microbiota abundance (genus Catenibacterium id.2153)' on 'E3 ubiquitin−protein ligase CCNB1IP1'

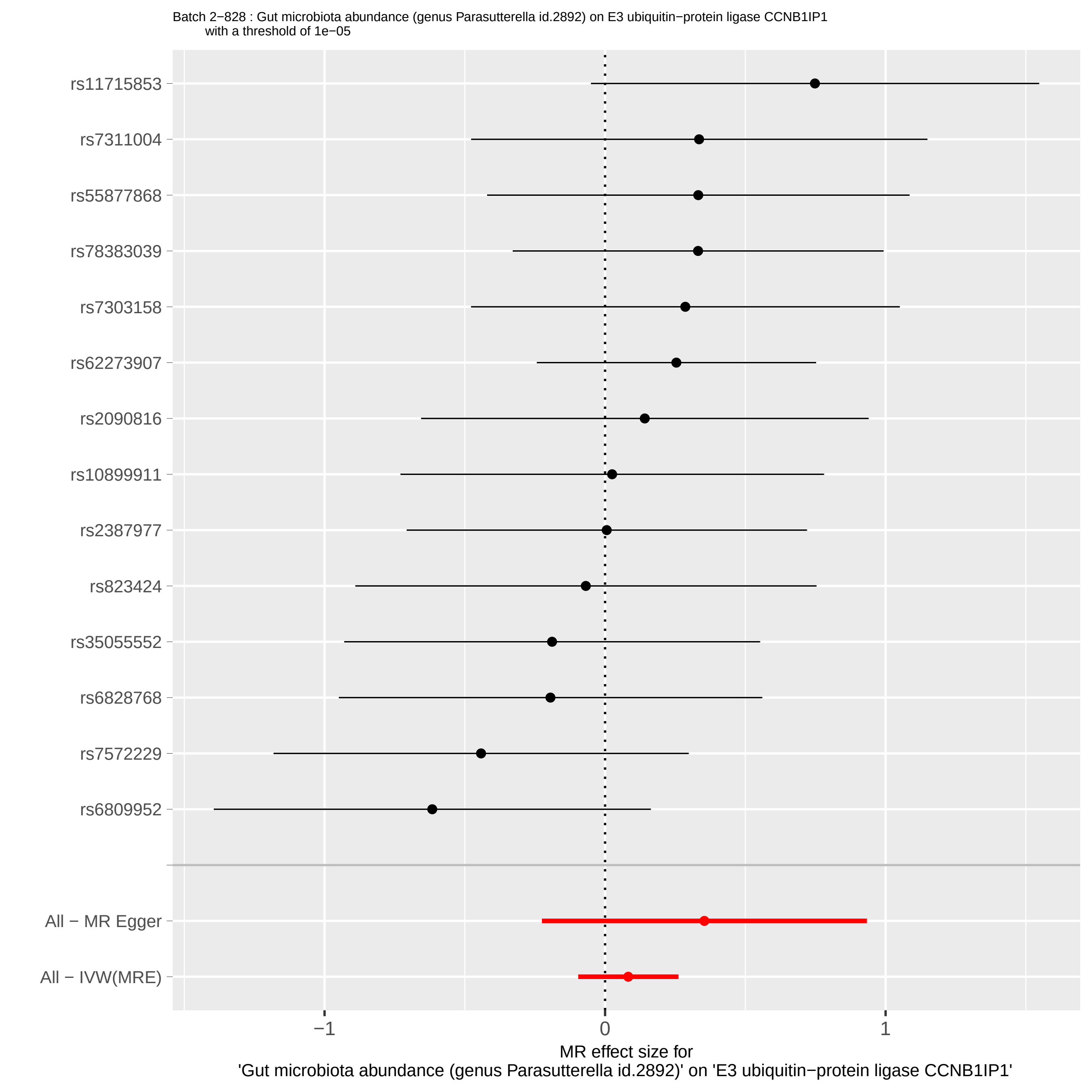

Batch 2−828 : Gut microbiota abundance (genus Parasutterella id.2892) on E3 ubiquitin−protein ligase CCNB1IP1
with a threshold of 1e−05
rs11715853
rs7311004
rs55877868
rs78383039
rs7303158
rs62273907
rs2090816
rs10899911
rs2387977
rs823424
rs35055552
rs6828768
rs7572229
rs6809952
All − MR Egger
All − IVW(MRE)
−1
0
1
MR effect size for
'Gut microbiota abundance (genus Parasutterella id.2892)' on 'E3 ubiquitin−protein ligase CCNB1IP1'

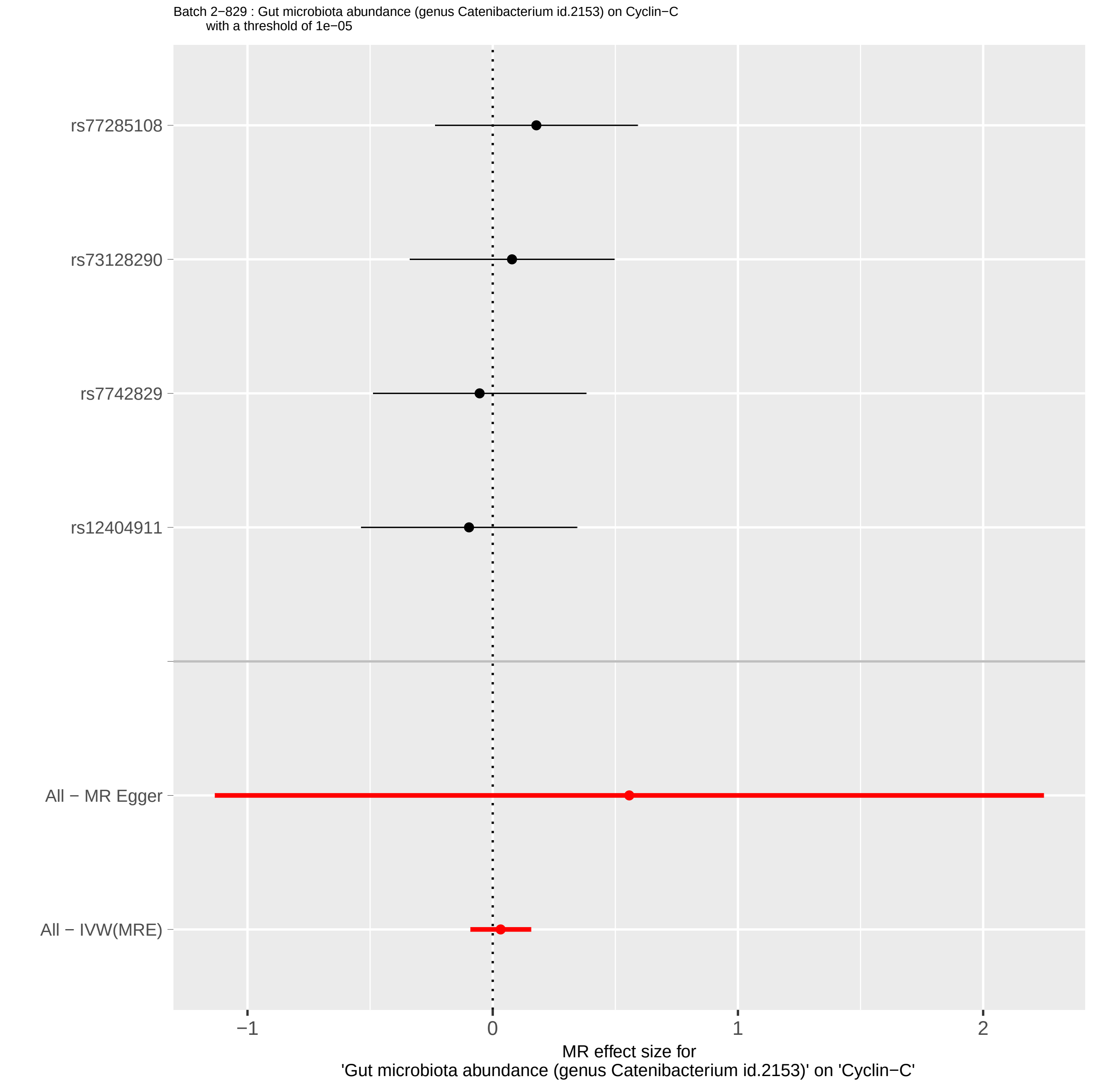

Batch 2−829 : Gut microbiota abundance (genus Catenibacterium id.2153) on Cyclin−C
with a threshold of 1e−05
rs77285108
rs73128290
rs7742829
rs12404911
All − MR Egger
All − IVW(MRE)
−1
0
1
2
MR effect size for
'Gut microbiota abundance (genus Catenibacterium id.2153)' on 'Cyclin−C'

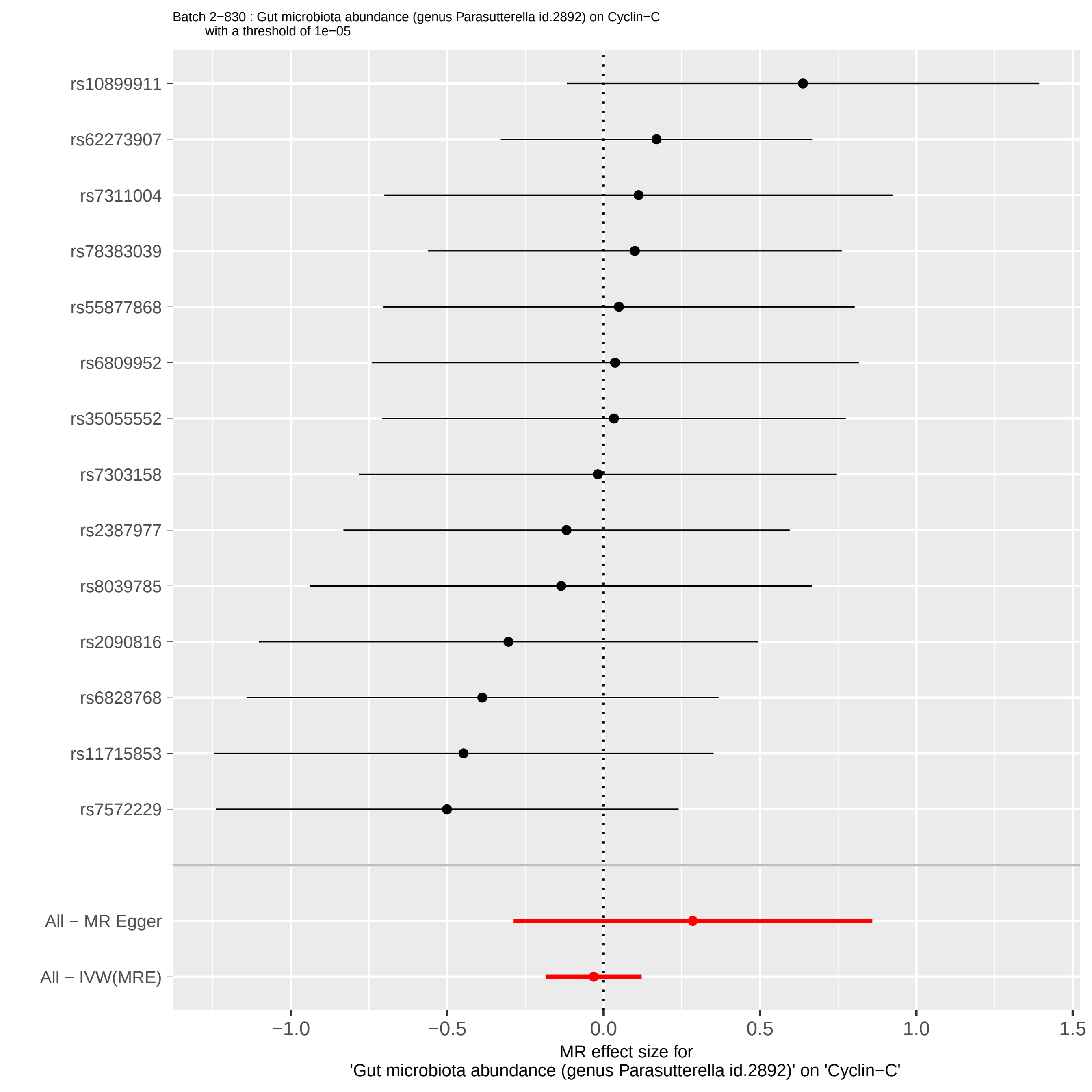

Batch 2−830 : Gut microbiota abundance (genus Parasutterella id.2892) on Cyclin−C
with a threshold of 1e−05
rs10899911
rs62273907
rs7311004
rs78383039
rs55877868
rs6809952
rs35055552
rs7303158
rs2387977
rs8039785
rs2090816
rs6828768
rs11715853
rs7572229
All − MR Egger
All − IVW(MRE)
−1.0
−0.5
0.0
0.5
1.0
1.5
MR effect size for
'Gut microbiota abundance (genus Parasutterella id.2892)' on 'Cyclin−C'

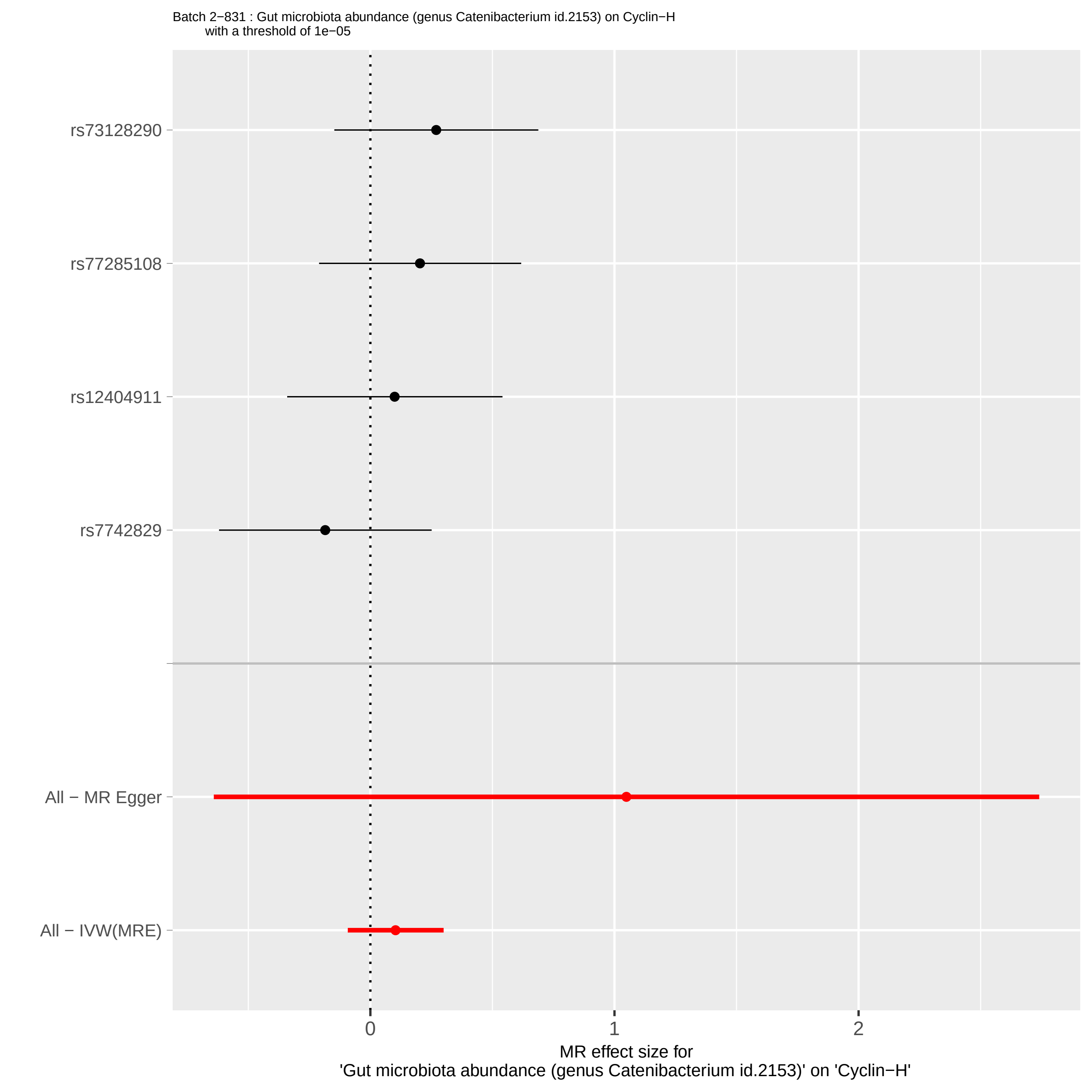

Batch 2−831 : Gut microbiota abundance (genus Catenibacterium id.2153) on Cyclin−H
with a threshold of 1e−05
rs73128290
rs77285108
rs12404911
rs7742829
All − MR Egger
All − IVW(MRE)
0
1
2
MR effect size for
'Gut microbiota abundance (genus Catenibacterium id.2153)' on 'Cyclin−H'

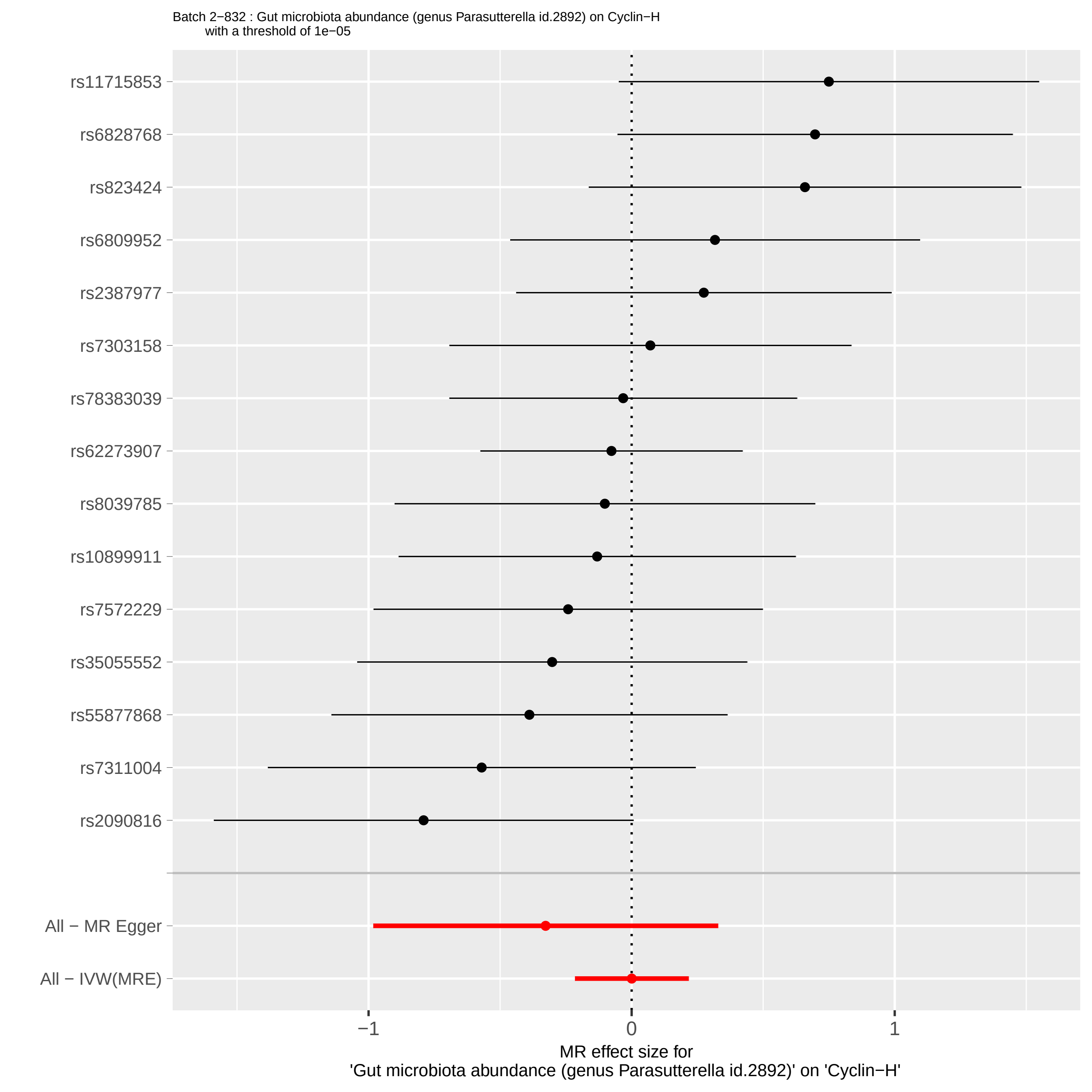

Batch 2−832 : Gut microbiota abundance (genus Parasutterella id.2892) on Cyclin−H
with a threshold of 1e−05
rs11715853
rs6828768
rs823424
rs6809952
rs2387977
rs7303158
rs78383039
rs62273907
rs8039785
rs10899911
rs7572229
rs35055552
rs55877868
rs7311004
rs2090816
All − MR Egger
All − IVW(MRE)
−1
0
1
MR effect size for
'Gut microbiota abundance (genus Parasutterella id.2892)' on 'Cyclin−H'

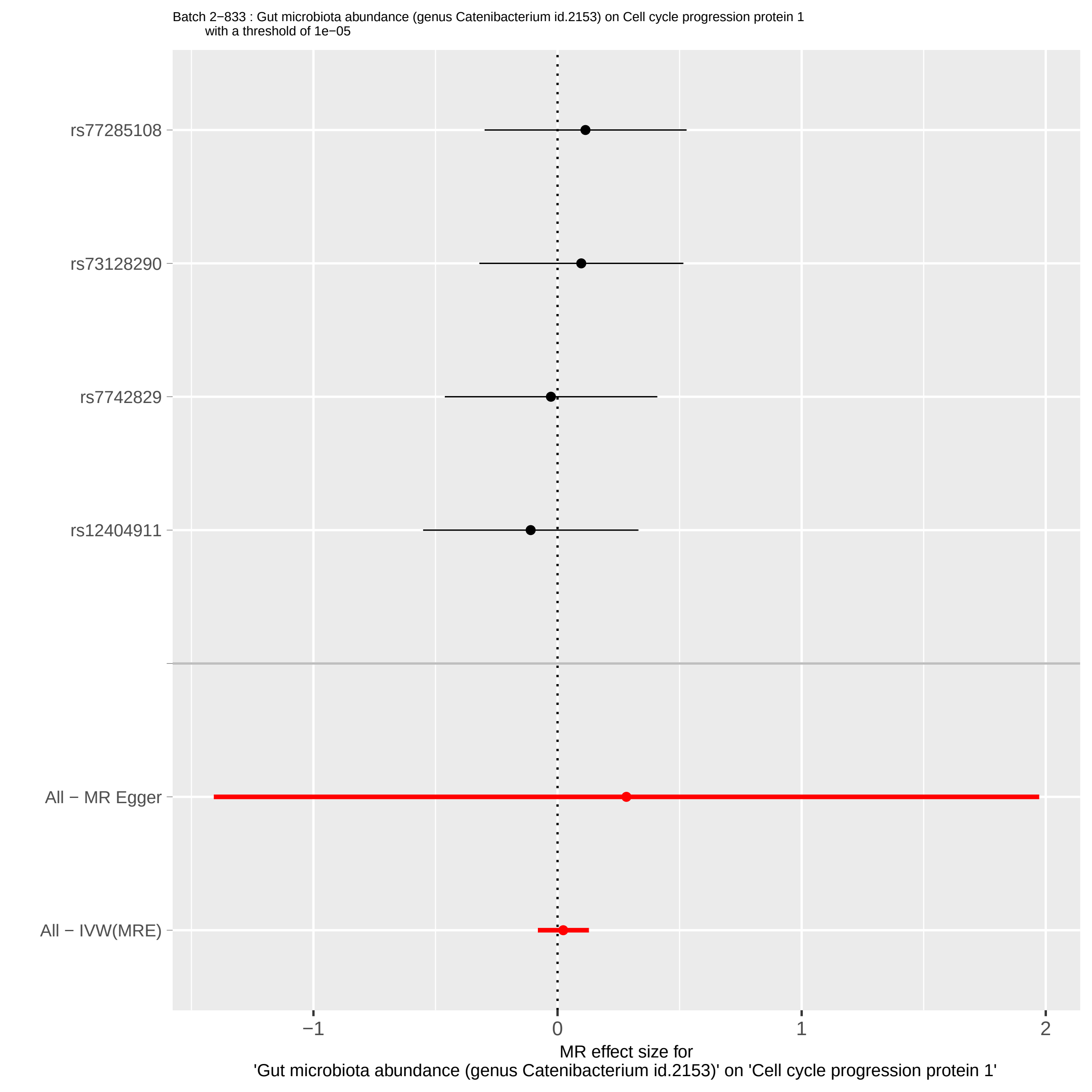

Batch 2−833 : Gut microbiota abundance (genus Catenibacterium id.2153) on Cell cycle progression protein 1
with a threshold of 1e−05
rs77285108
rs73128290
rs7742829
rs12404911
All − MR Egger
All − IVW(MRE)
−1
0
1
2
MR effect size for
'Gut microbiota abundance (genus Catenibacterium id.2153)' on 'Cell cycle progression protein 1'

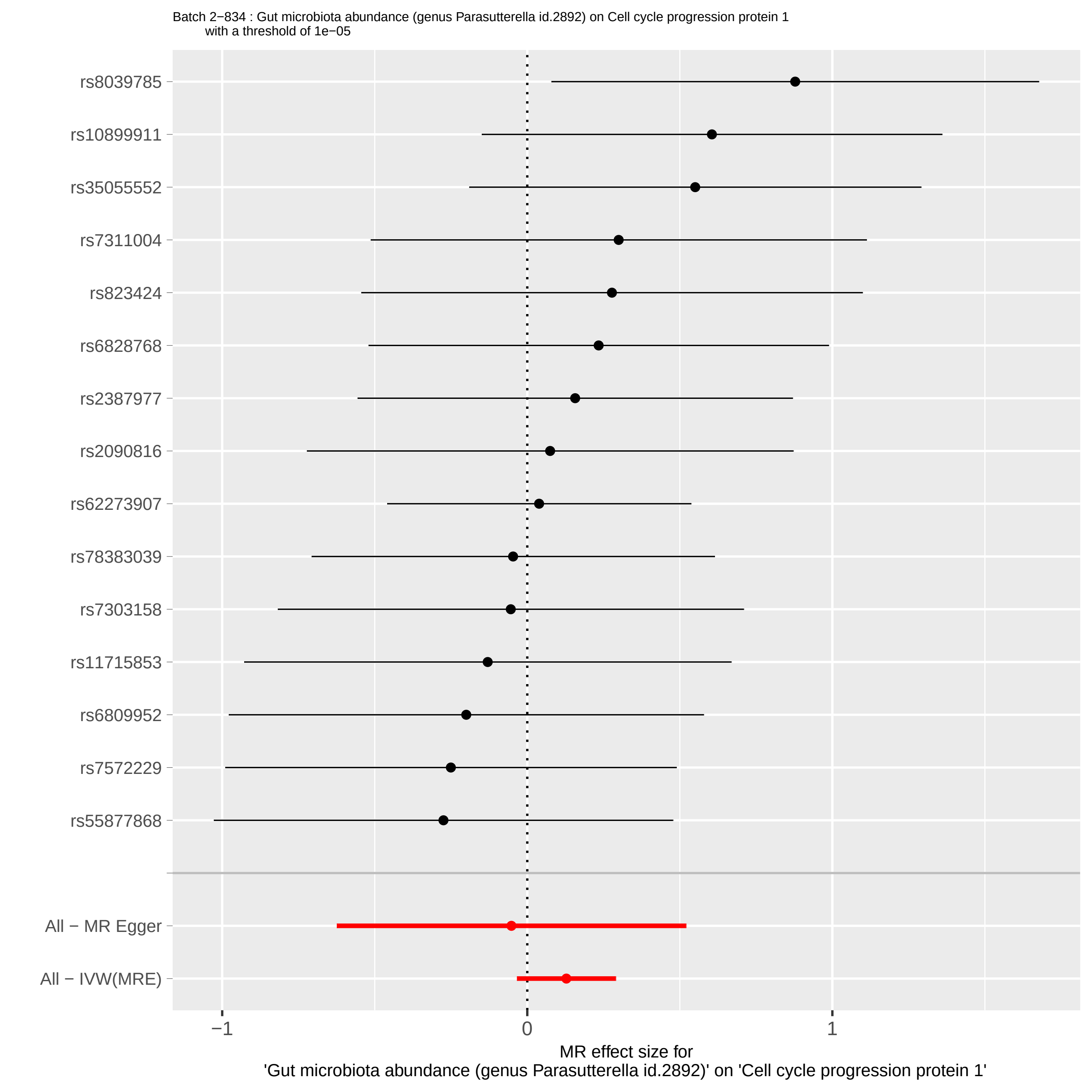

Batch 2−834 : Gut microbiota abundance (genus Parasutterella id.2892) on Cell cycle progression protein 1
with a threshold of 1e−05
rs8039785
rs10899911
rs35055552
rs7311004
rs823424
rs6828768
rs2387977
rs2090816
rs62273907
rs78383039
rs7303158
rs11715853
rs6809952
rs7572229
rs55877868
All − MR Egger
All − IVW(MRE)
−1
0
1
MR effect size for
'Gut microbiota abundance (genus Parasutterella id.2892)' on 'Cell cycle progression protein 1'

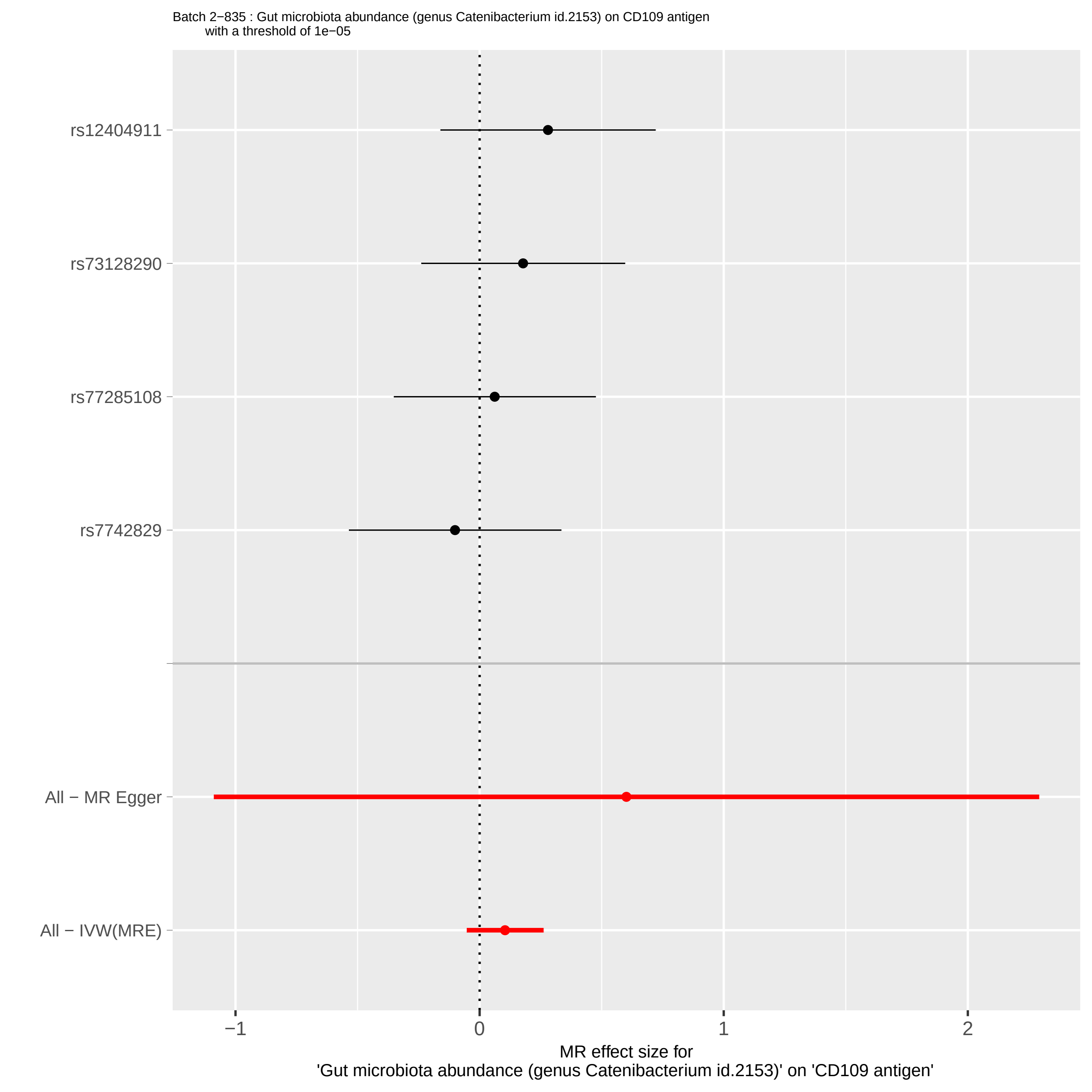

Batch 2−835 : Gut microbiota abundance (genus Catenibacterium id.2153) on CD109 antigen
with a threshold of 1e−05
rs12404911
rs73128290
rs77285108
rs7742829
All − MR Egger
All − IVW(MRE)
−1
0
1
2
MR effect size for
'Gut microbiota abundance (genus Catenibacterium id.2153)' on 'CD109 antigen'

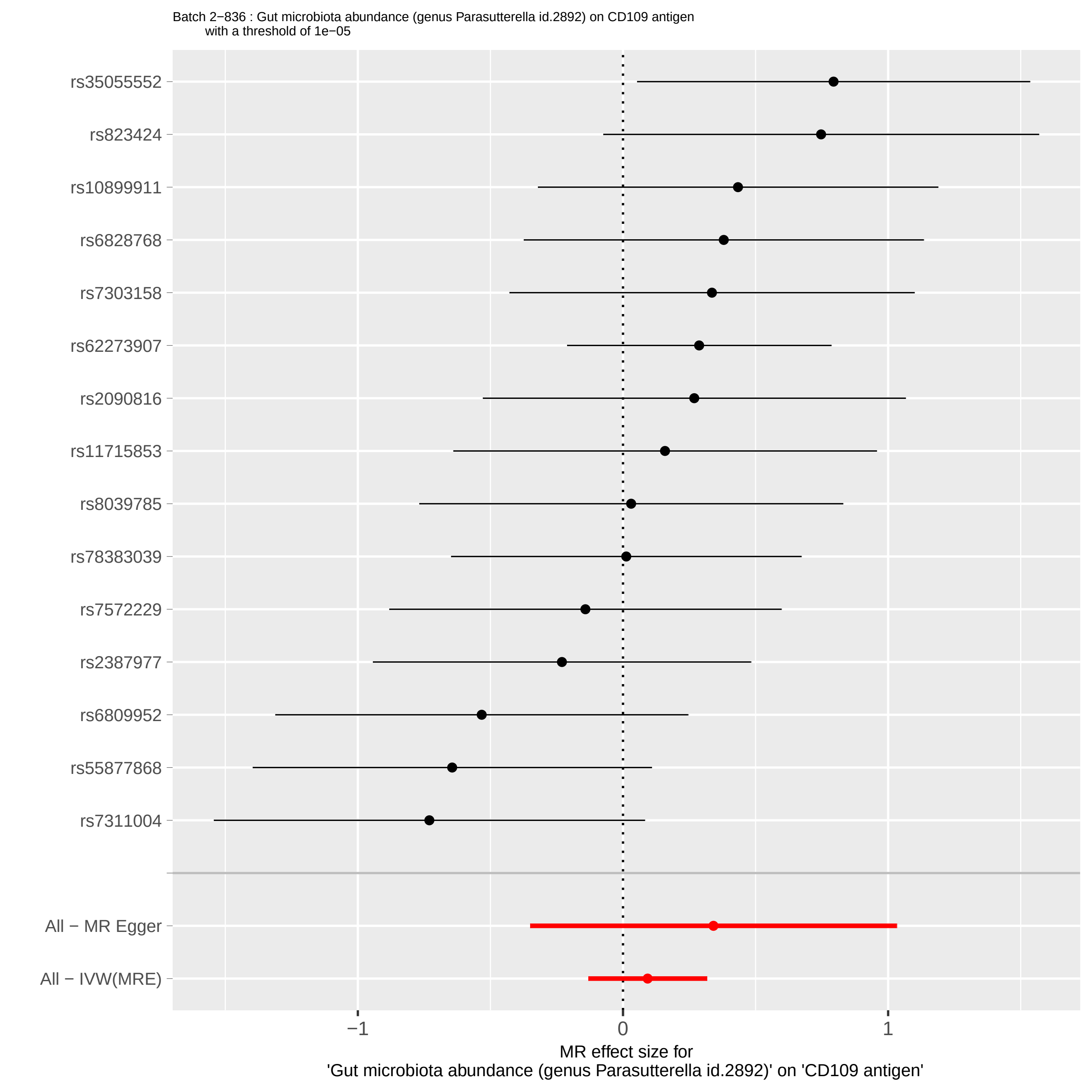

Batch 2−836 : Gut microbiota abundance (genus Parasutterella id.2892) on CD109 antigen
with a threshold of 1e−05
rs35055552
rs823424
rs10899911
rs6828768
rs7303158
rs62273907
rs2090816
rs11715853
rs8039785
rs78383039
rs7572229
rs2387977
rs6809952
rs55877868
rs7311004
All − MR Egger
All − IVW(MRE)
−1
0
1
MR effect size for
'Gut microbiota abundance (genus Parasutterella id.2892)' on 'CD109 antigen'

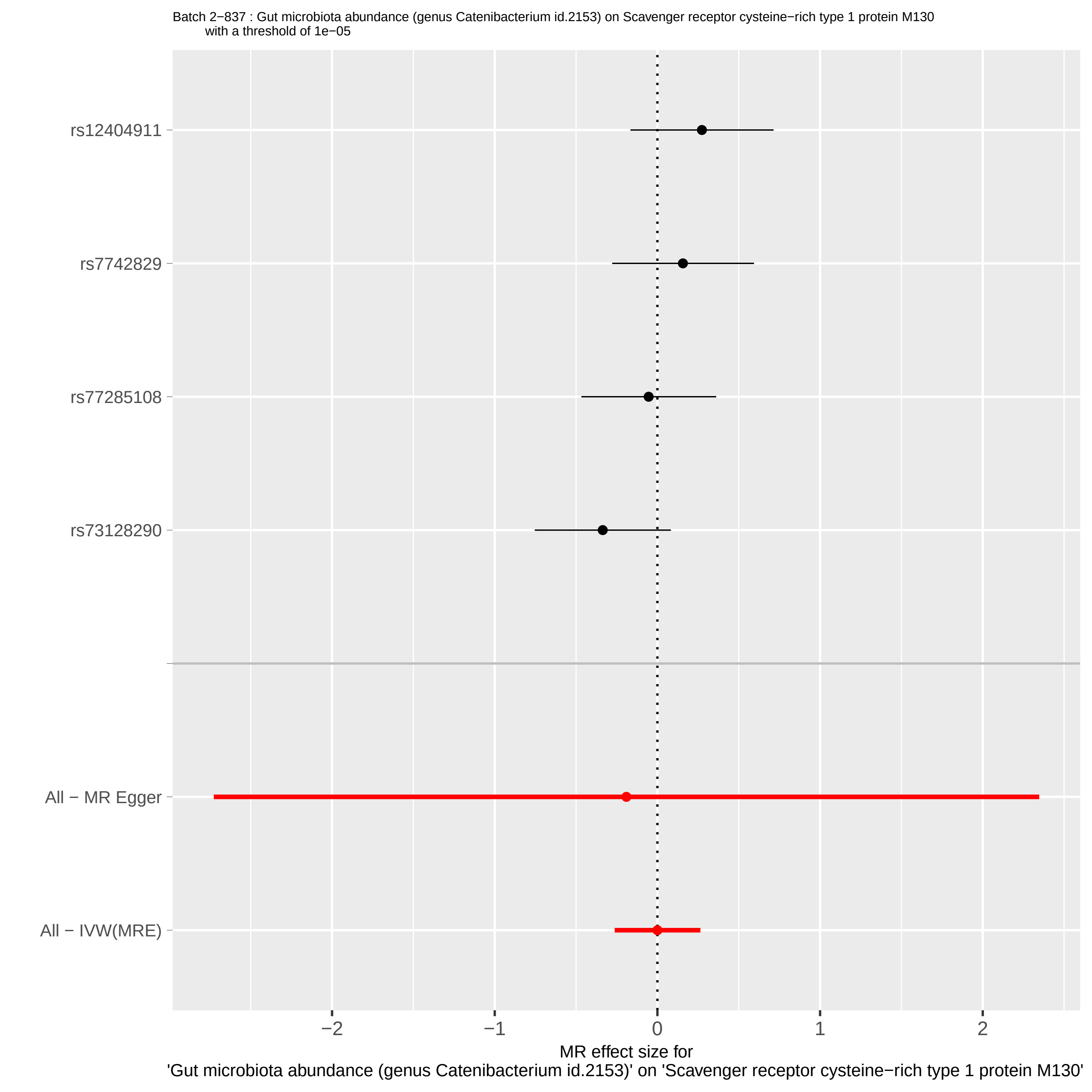

Batch 2−837 : Gut microbiota abundance (genus Catenibacterium id.2153) on Scavenger receptor cysteine−rich type 1 protein M130
with a threshold of 1e−05
rs12404911
rs7742829
rs77285108
rs73128290
All − MR Egger
All − IVW(MRE)
−2
−1
0
1
2
MR effect size for
'Gut microbiota abundance (genus Catenibacterium id.2153)' on 'Scavenger receptor cysteine−rich type 1 protein M130'

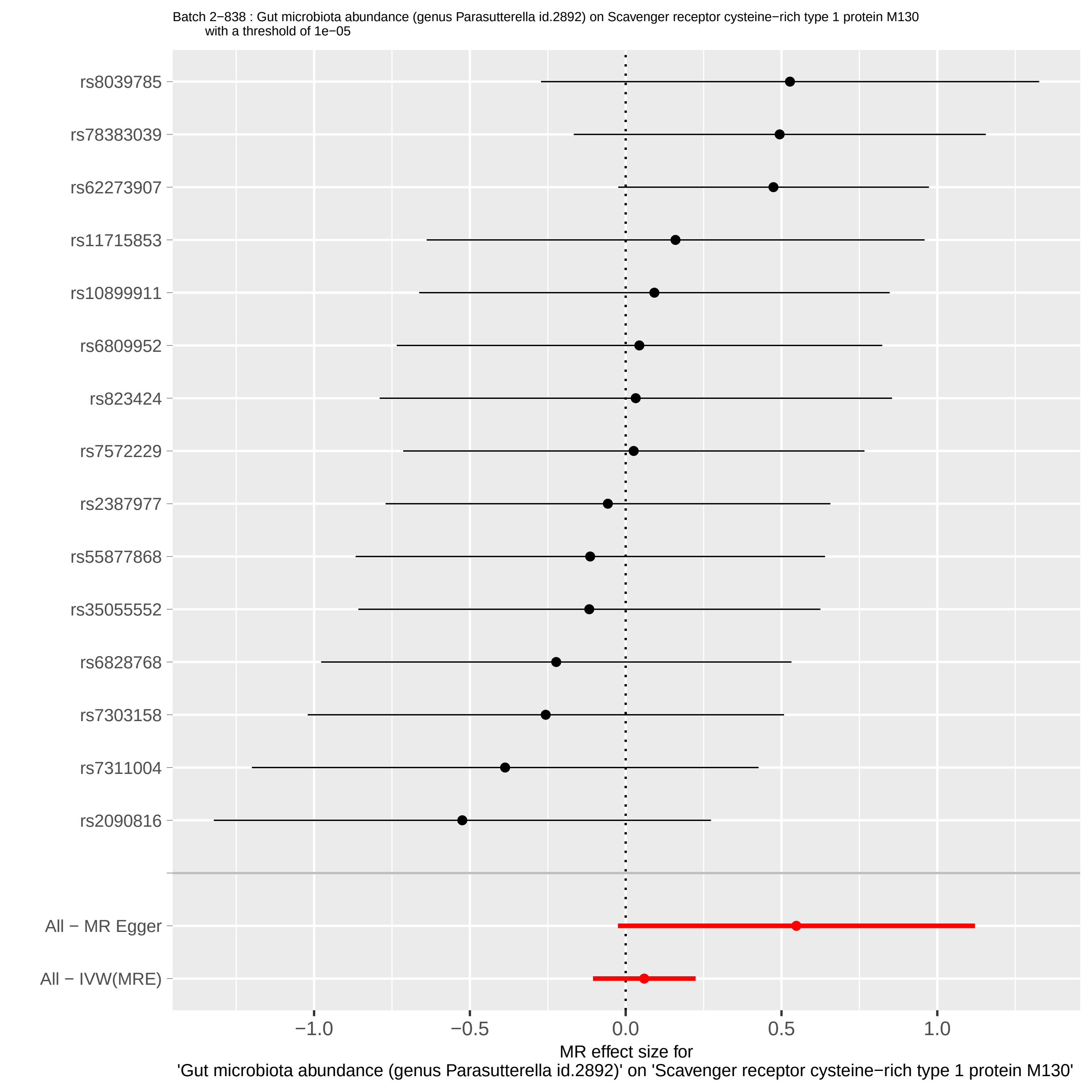
Batch 2−838 : Gut microbiota abundance (genus Parasutterella id.2892) on Scavenger receptor cysteine−rich type 1 protein M130
with a threshold of 1e−05
rs8039785
rs78383039
rs62273907
rs11715853
rs10899911
rs6809952
rs823424
rs7572229
rs2387977
rs55877868
rs35055552
rs6828768
rs7303158
rs7311004
rs2090816
All − MR Egger
All − IVW(MRE)
−1.0
−0.5
0.0
0.5
1.0
MR effect size for
'Gut microbiota abundance (genus Parasutterella id.2892)' on 'Scavenger receptor cysteine−rich type 1 protein M130'

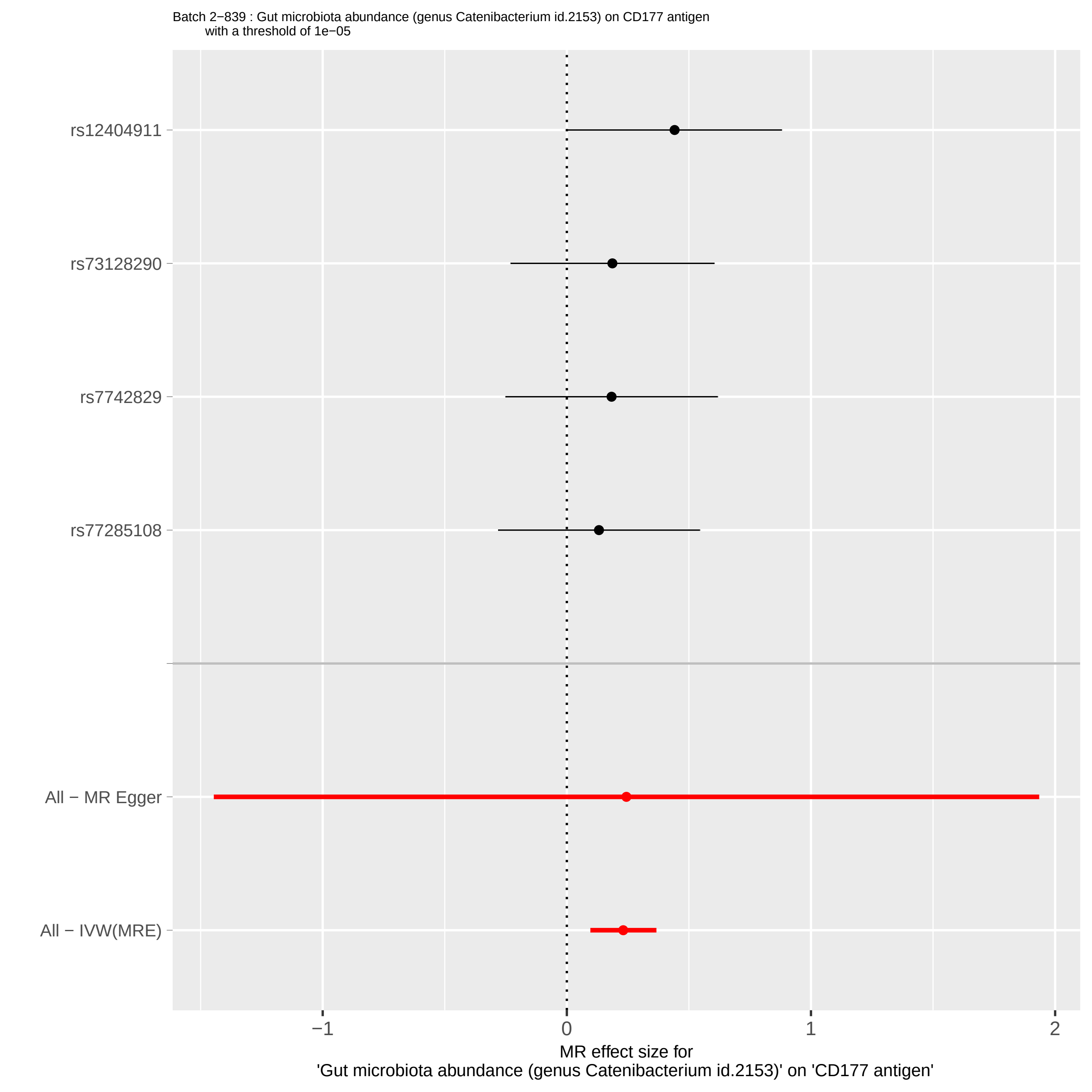
Batch 2−839 : Gut microbiota abundance (genus Catenibacterium id.2153) on CD177 antigen
with a threshold of 1e−05
rs12404911
rs73128290
rs7742829
rs77285108
All − MR Egger
All − IVW(MRE)
−1
0
1
2
MR effect size for
'Gut microbiota abundance (genus Catenibacterium id.2153)' on 'CD177 antigen'

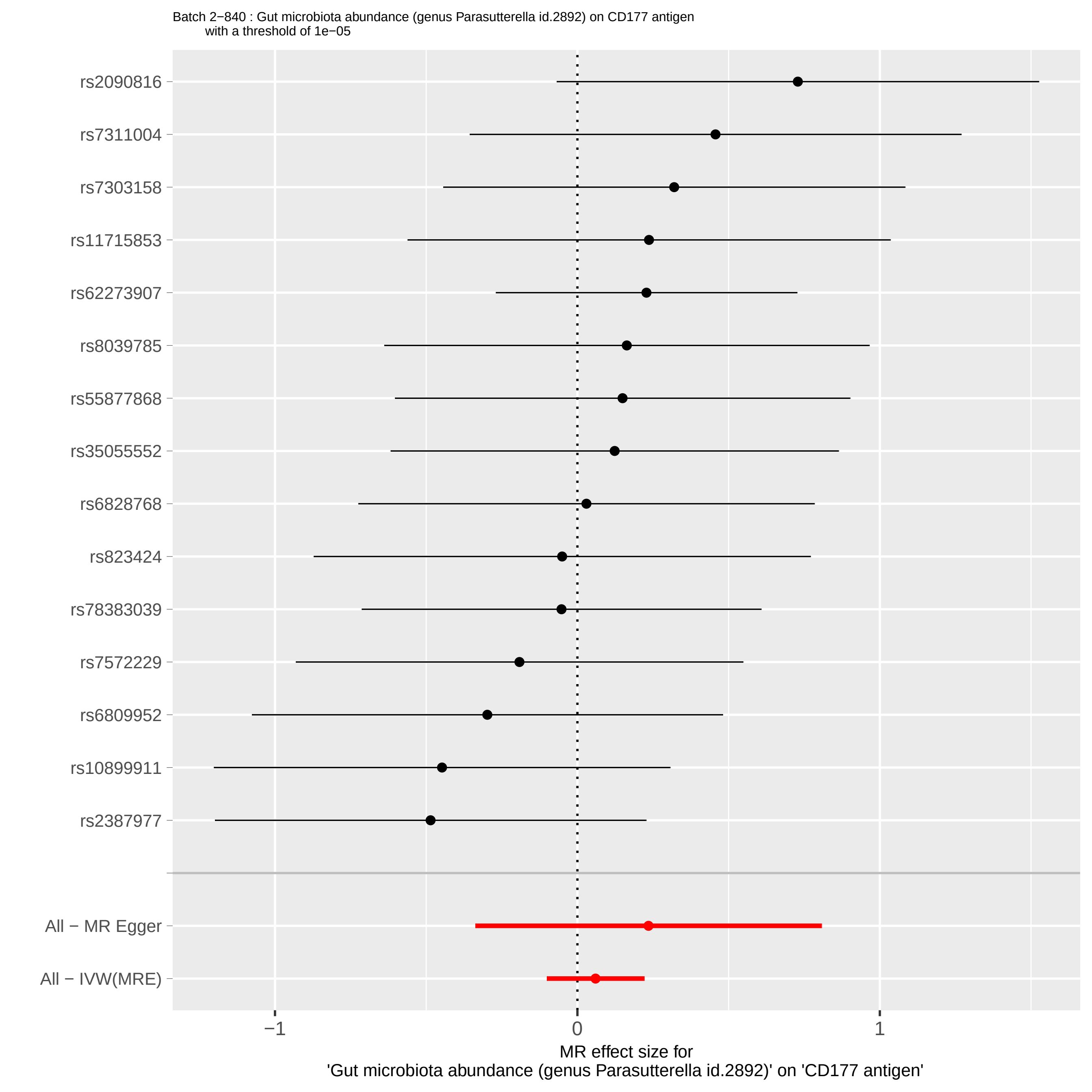

Batch 2−840 : Gut microbiota abundance (genus Parasutterella id.2892) on CD177 antigen
with a threshold of 1e−05
rs2090816
rs7311004
rs7303158
rs11715853
rs62273907
rs8039785
rs55877868
rs35055552
rs6828768
rs823424
rs78383039
rs7572229
rs6809952
rs10899911
rs2387977
All − MR Egger
All − IVW(MRE)
−1
0
1
MR effect size for
'Gut microbiota abundance (genus Parasutterella id.2892)' on 'CD177 antigen'

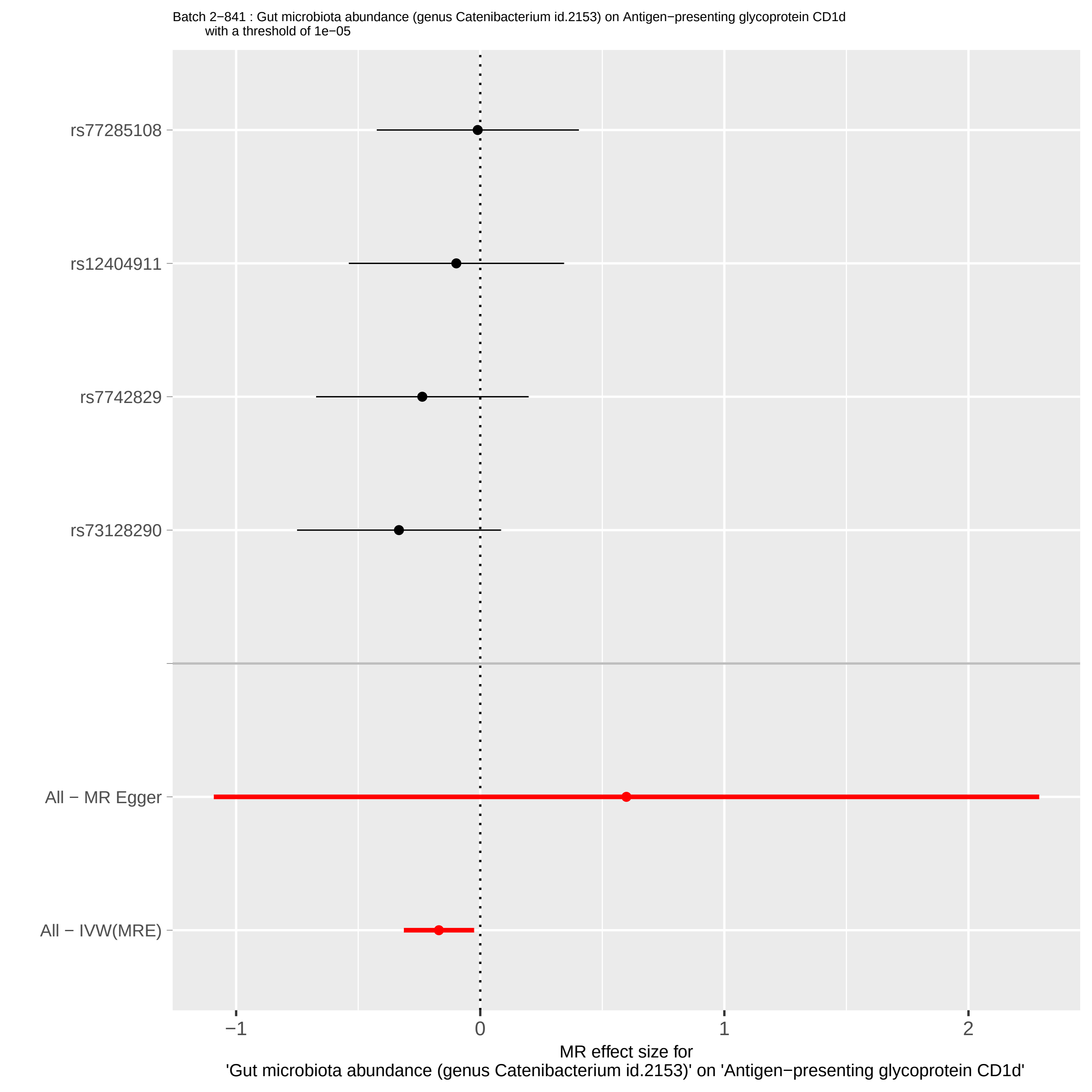

Batch 2−841 : Gut microbiota abundance (genus Catenibacterium id.2153) on Antigen−presenting glycoprotein CD1d
with a threshold of 1e−05
rs77285108
rs12404911
rs7742829
rs73128290
All − MR Egger
All − IVW(MRE)
−1
0
1
2
MR effect size for
'Gut microbiota abundance (genus Catenibacterium id.2153)' on 'Antigen−presenting glycoprotein CD1d'

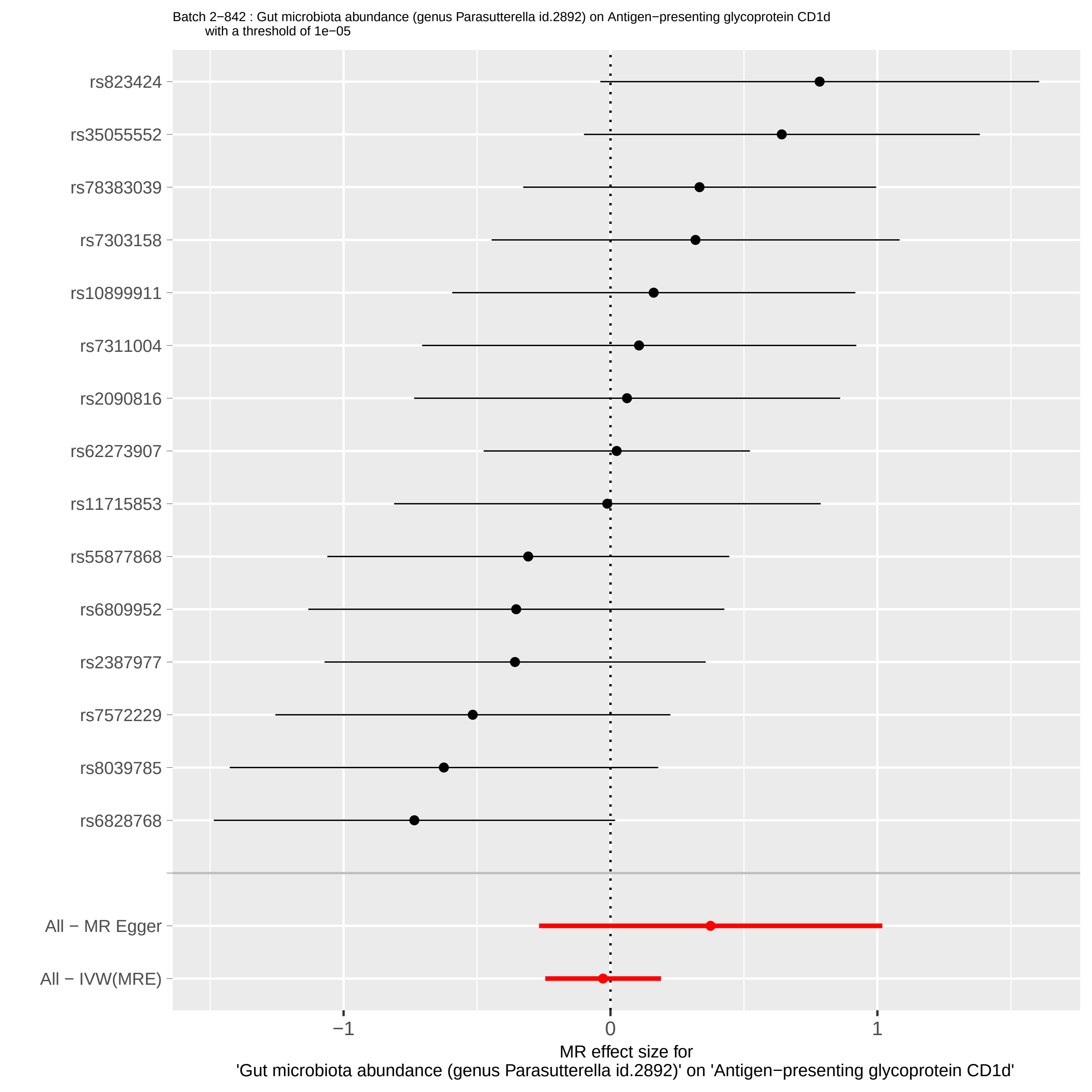
Batch 2−842 : Gut microbiota abundance (genus Parasutterella id.2892) on Antigen−presenting glycoprotein CD1d
with a threshold of 1e−05
rs823424
rs35055552
rs78383039
rs7303158
rs10899911
rs7311004
rs2090816
rs62273907
rs11715853
rs55877868
rs6809952
rs2387977
rs7572229
rs8039785
rs6828768
All − MR Egger
All − IVW(MRE)
−1
0
1
MR effect size for
'Gut microbiota abundance (genus Parasutterella id.2892)' on 'Antigen−presenting glycoprotein CD1d'

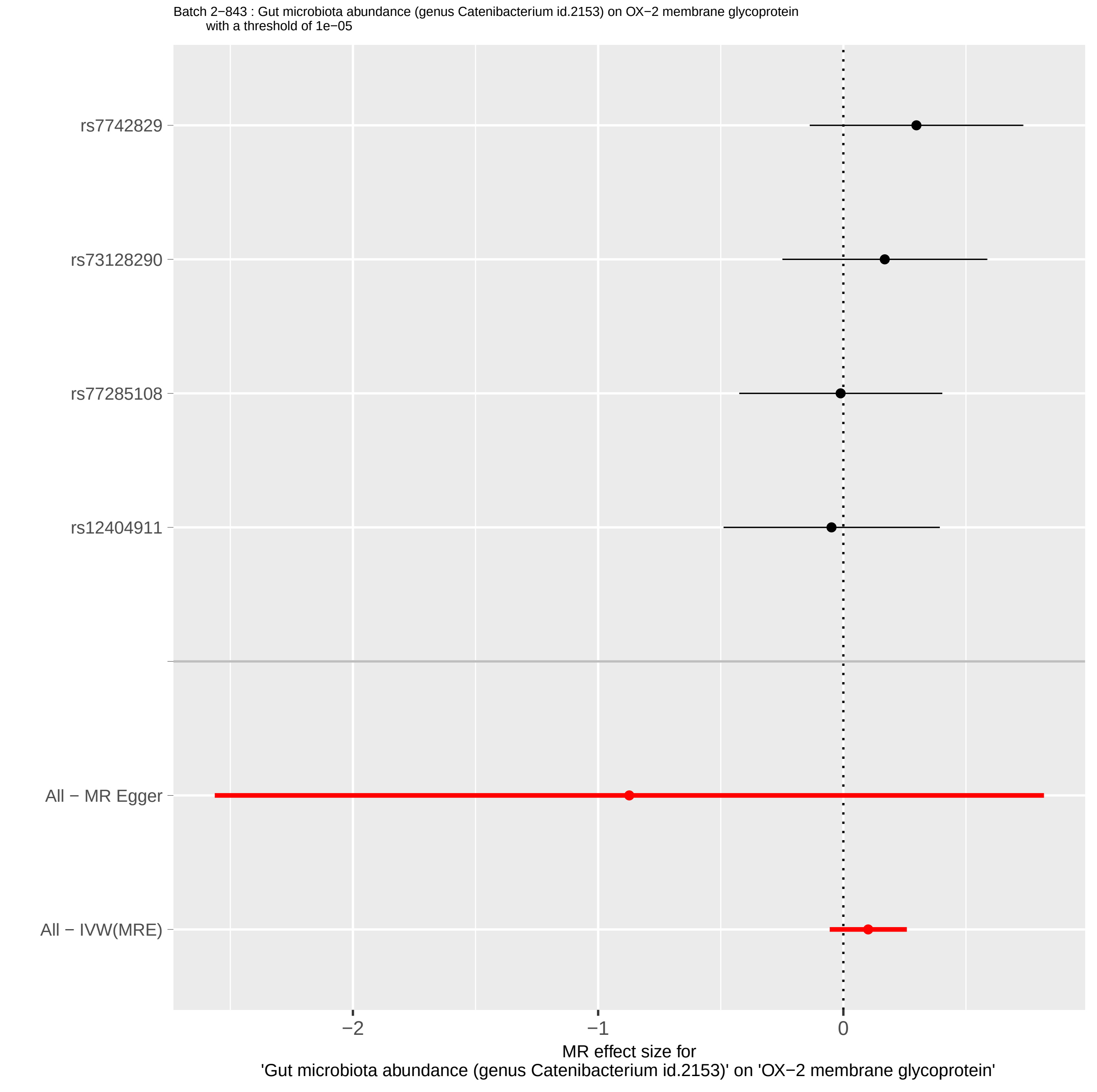

Batch 2−843 : Gut microbiota abundance (genus Catenibacterium id.2153) on OX−2 membrane glycoprotein
with a threshold of 1e−05
rs7742829
rs73128290
rs77285108
rs12404911
All − MR Egger
All − IVW(MRE)
−2
−1
0
MR effect size for
'Gut microbiota abundance (genus Catenibacterium id.2153)' on 'OX−2 membrane glycoprotein'

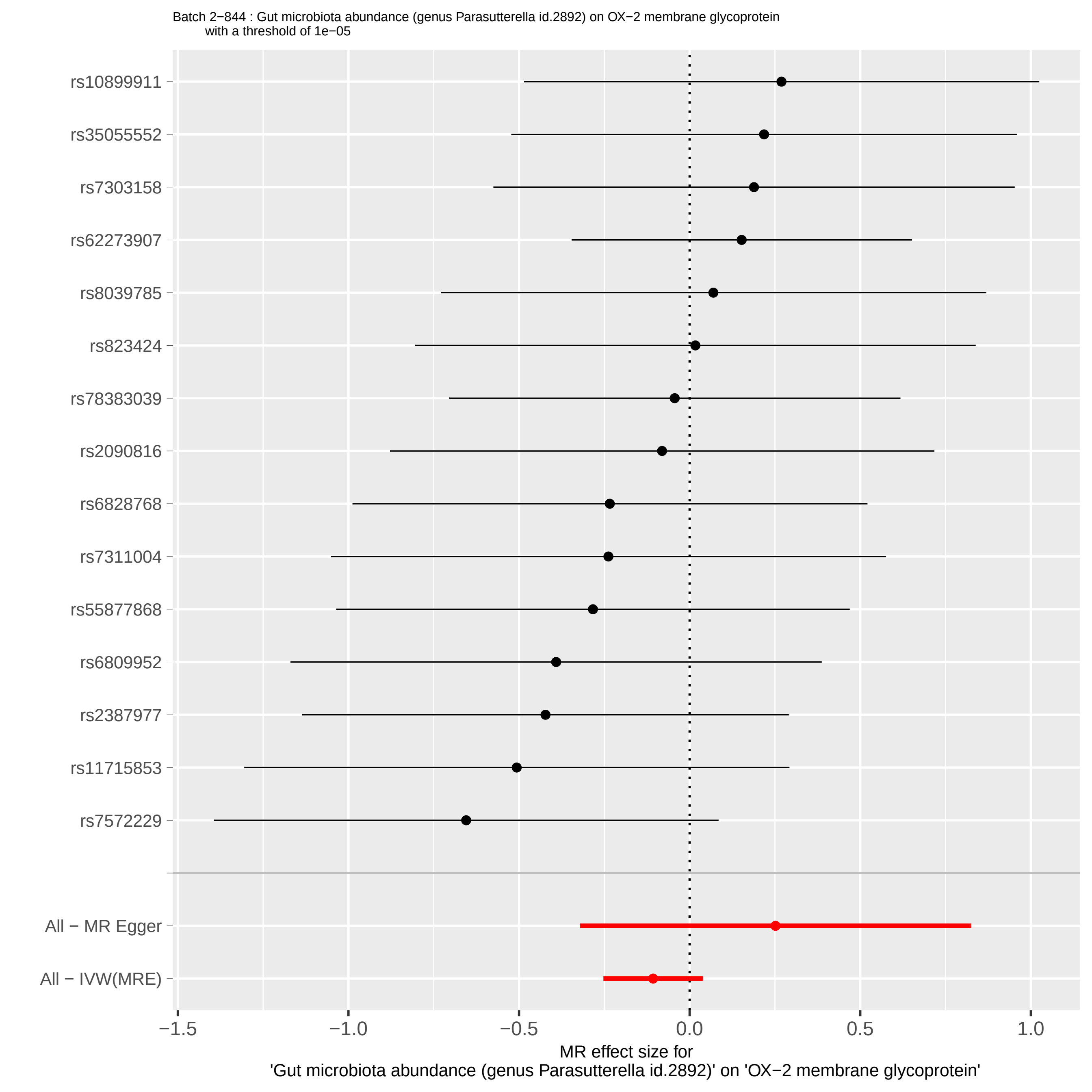

Batch 2−844 : Gut microbiota abundance (genus Parasutterella id.2892) on OX−2 membrane glycoprotein
with a threshold of 1e−05
rs10899911
rs35055552
rs7303158
rs62273907
rs8039785
rs823424
rs78383039
rs2090816
rs6828768
rs7311004
rs55877868
rs6809952
rs2387977
rs11715853
rs7572229
All − MR Egger
All − IVW(MRE)
−1.5
−1.0
−0.5
0.0
0.5
1.0
MR effect size for
'Gut microbiota abundance (genus Parasutterella id.2892)' on 'OX−2 membrane glycoprotein'

Batch 2−845 : Gut microbiota abundance (genus Catenibacterium id.2153) on Cell surface glycoprotein CD200 receptor 1
with a threshold of 1e−05

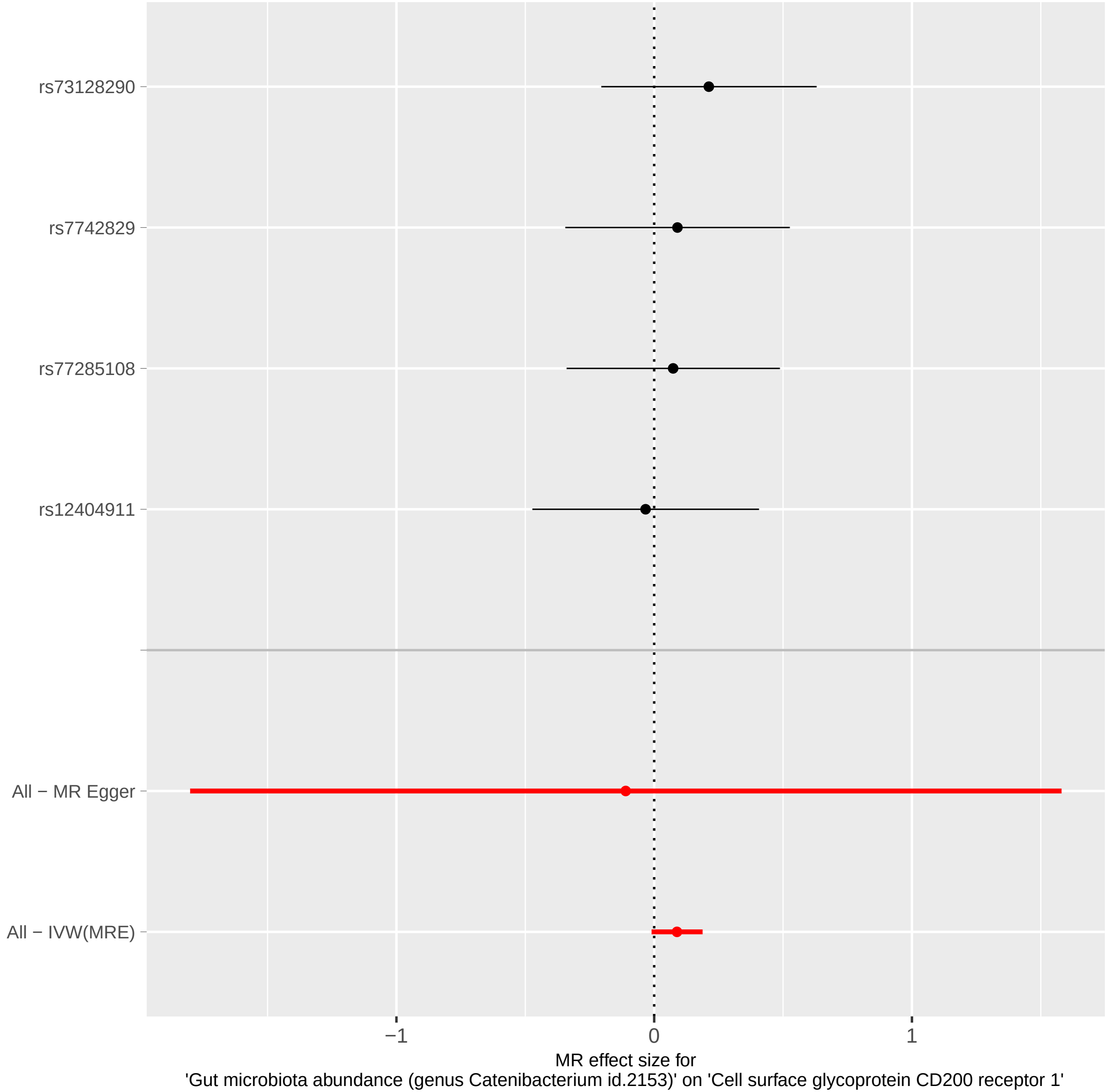

Batch 2−846 : Gut microbiota abundance (genus Parasutterella id.2892) on Cell surface glycoprotein CD200 receptor 1
with a threshold of 1e−05

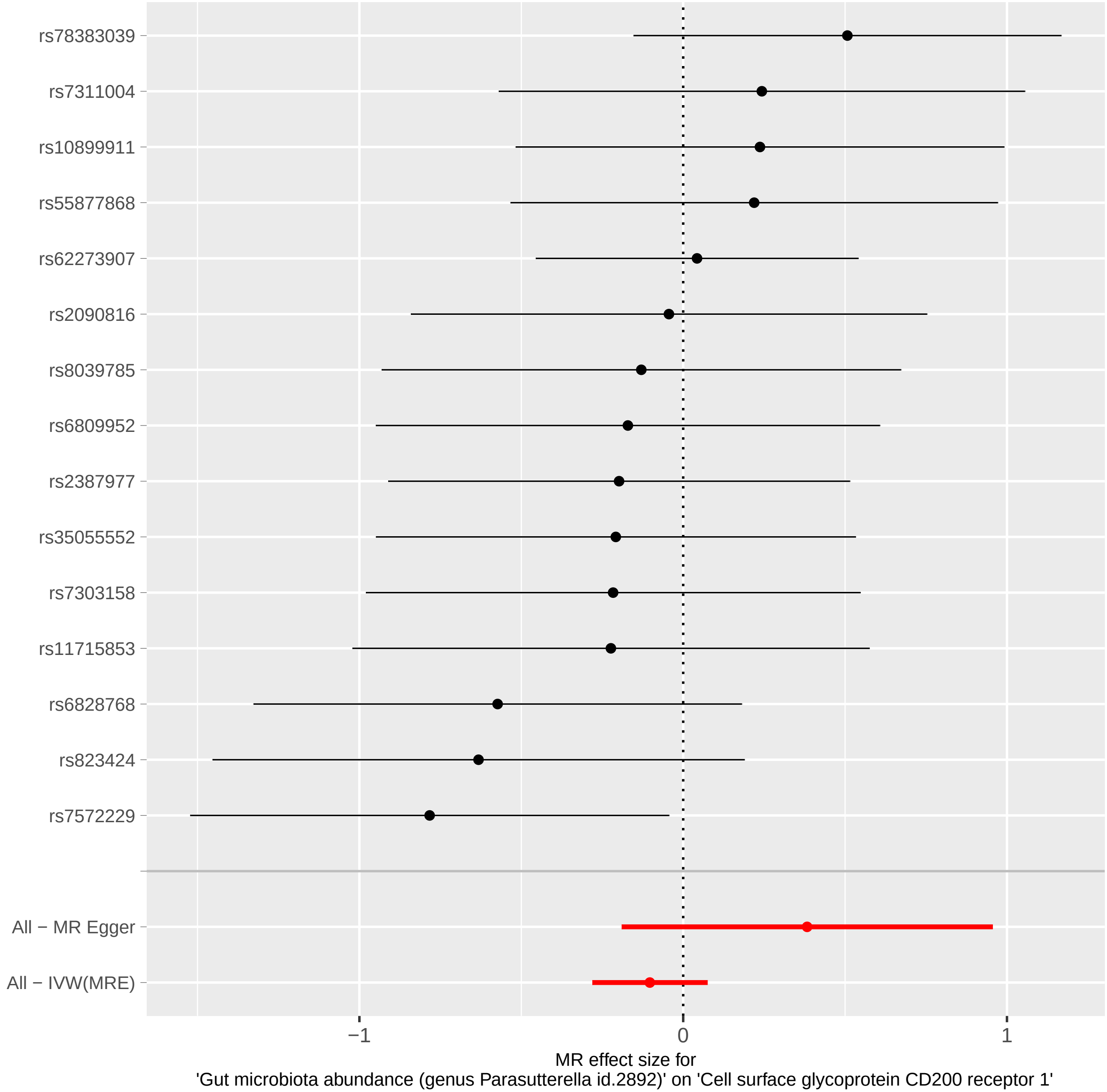

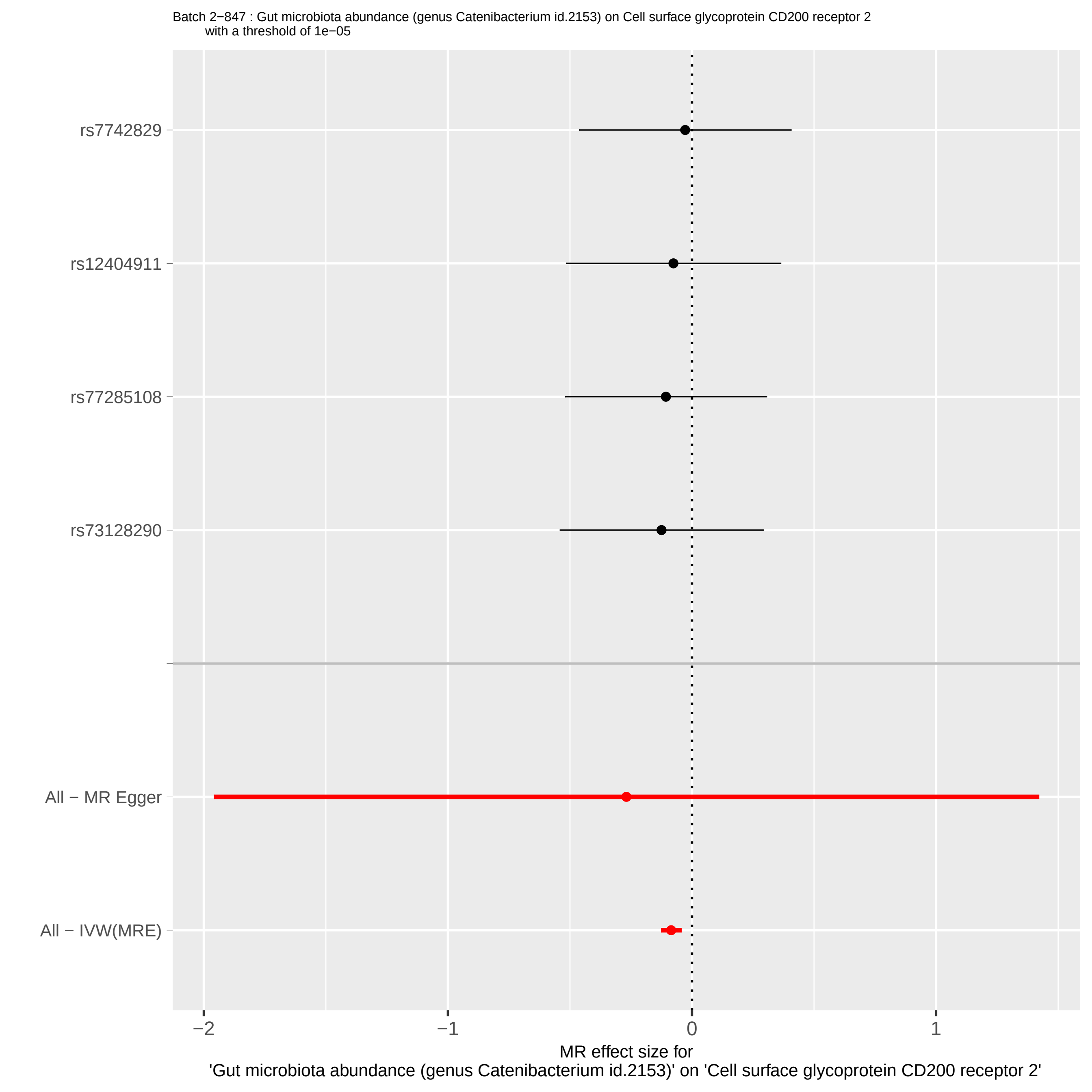

Batch 2−847 : Gut microbiota abundance (genus Catenibacterium id.2153) on Cell surface glycoprotein CD200 receptor 2
with a threshold of 1e−05
rs7742829
rs12404911
rs77285108
rs73128290
All − MR Egger
All − IVW(MRE)
−2
−1
0
1
MR effect size for
'Gut microbiota abundance (genus Catenibacterium id.2153)' on 'Cell surface glycoprotein CD200 receptor 2'

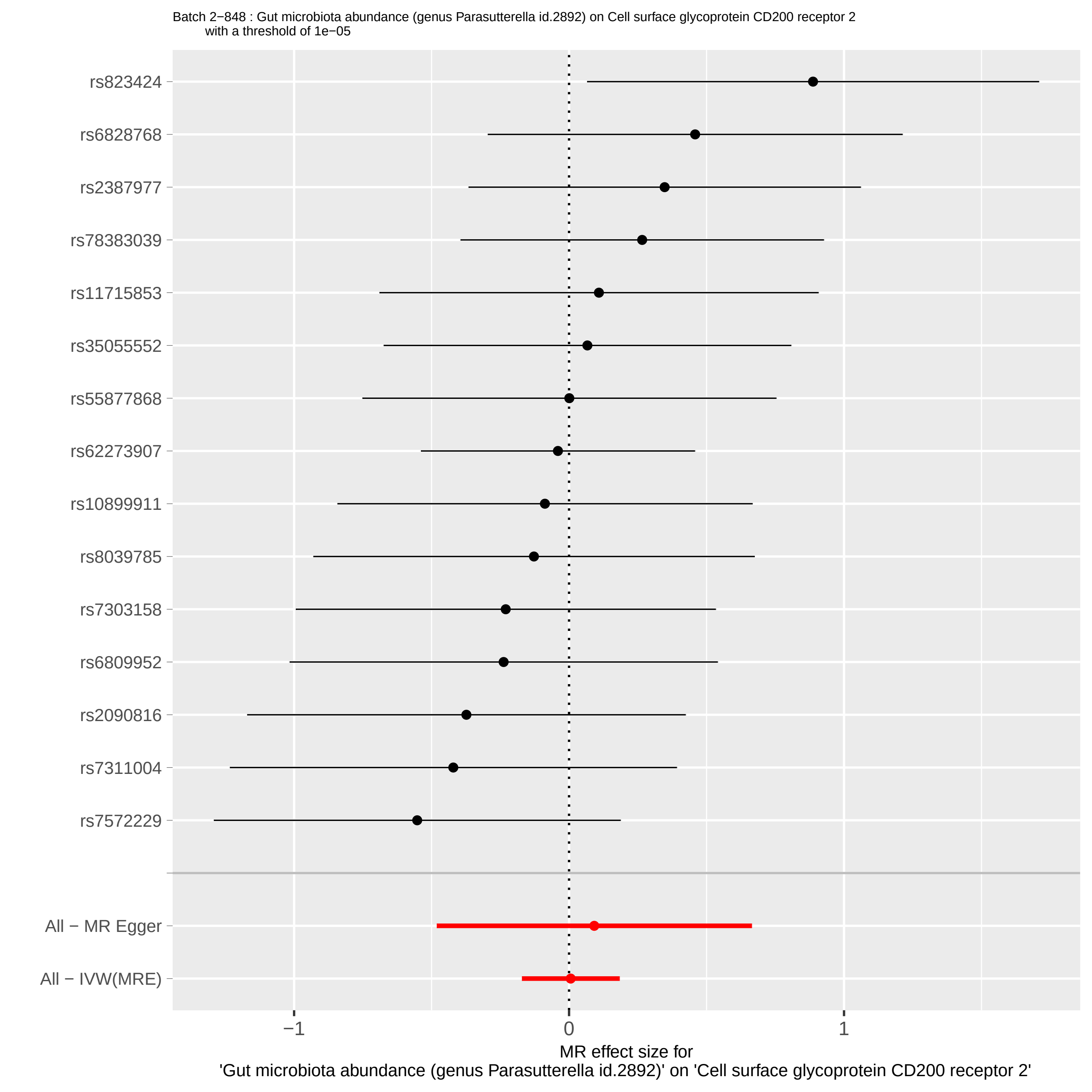
Batch 2−848 : Gut microbiota abundance (genus Parasutterella id.2892) on Cell surface glycoprotein CD200 receptor 2
with a threshold of 1e−05
rs823424
rs6828768
rs2387977
rs78383039
rs11715853
rs35055552
rs55877868
rs62273907
rs10899911
rs8039785
rs7303158
rs6809952
rs2090816
rs7311004
rs7572229
All − MR Egger
All − IVW(MRE)
−1
0
1
MR effect size for
'Gut microbiota abundance (genus Parasutterella id.2892)' on 'Cell surface glycoprotein CD200 receptor 2'

Batch 2−849 : Gut microbiota abundance (genus Catenibacterium id.2153) on C−type lectin domain family 4 member K
with a threshold of 1e−05

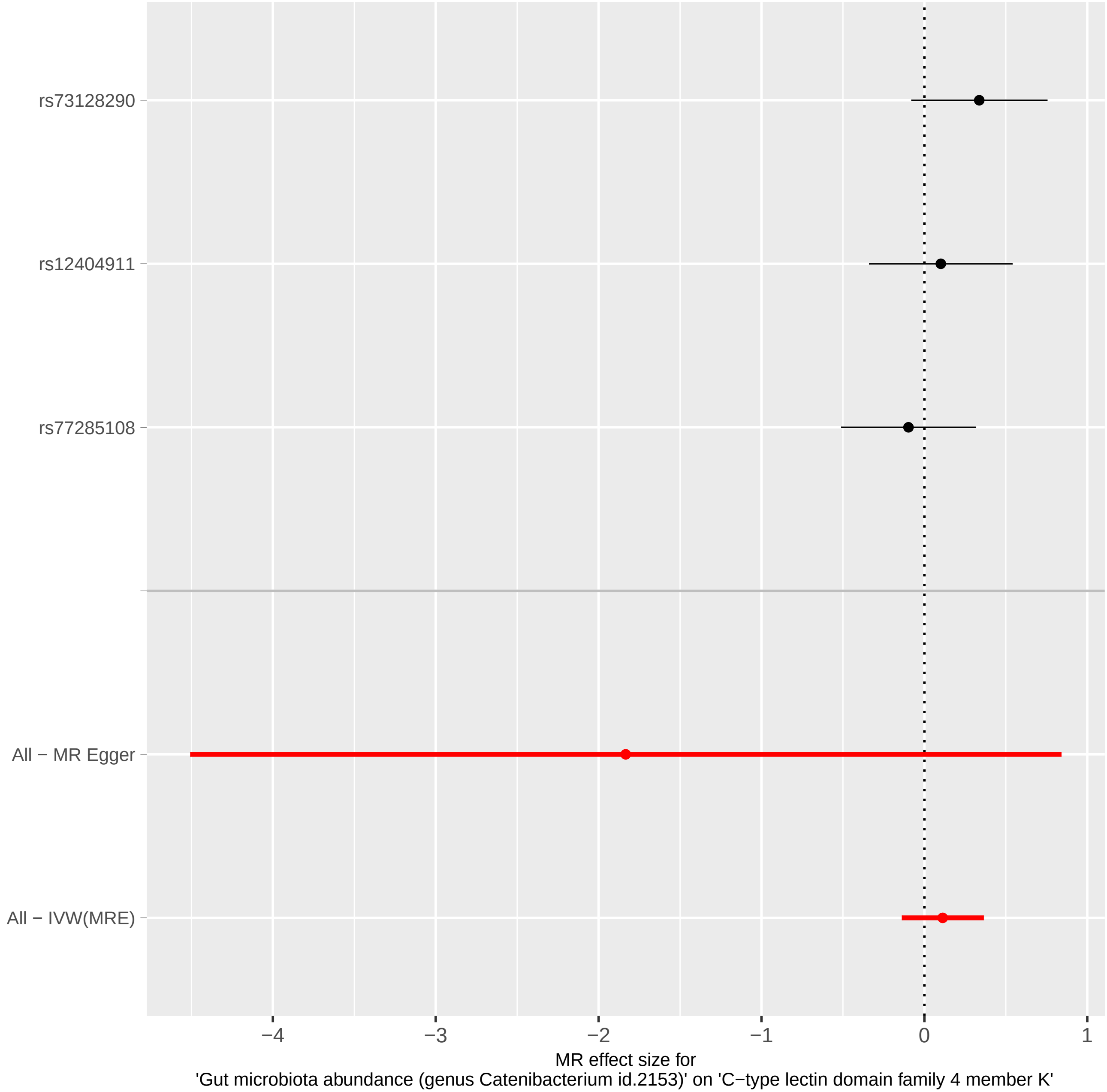

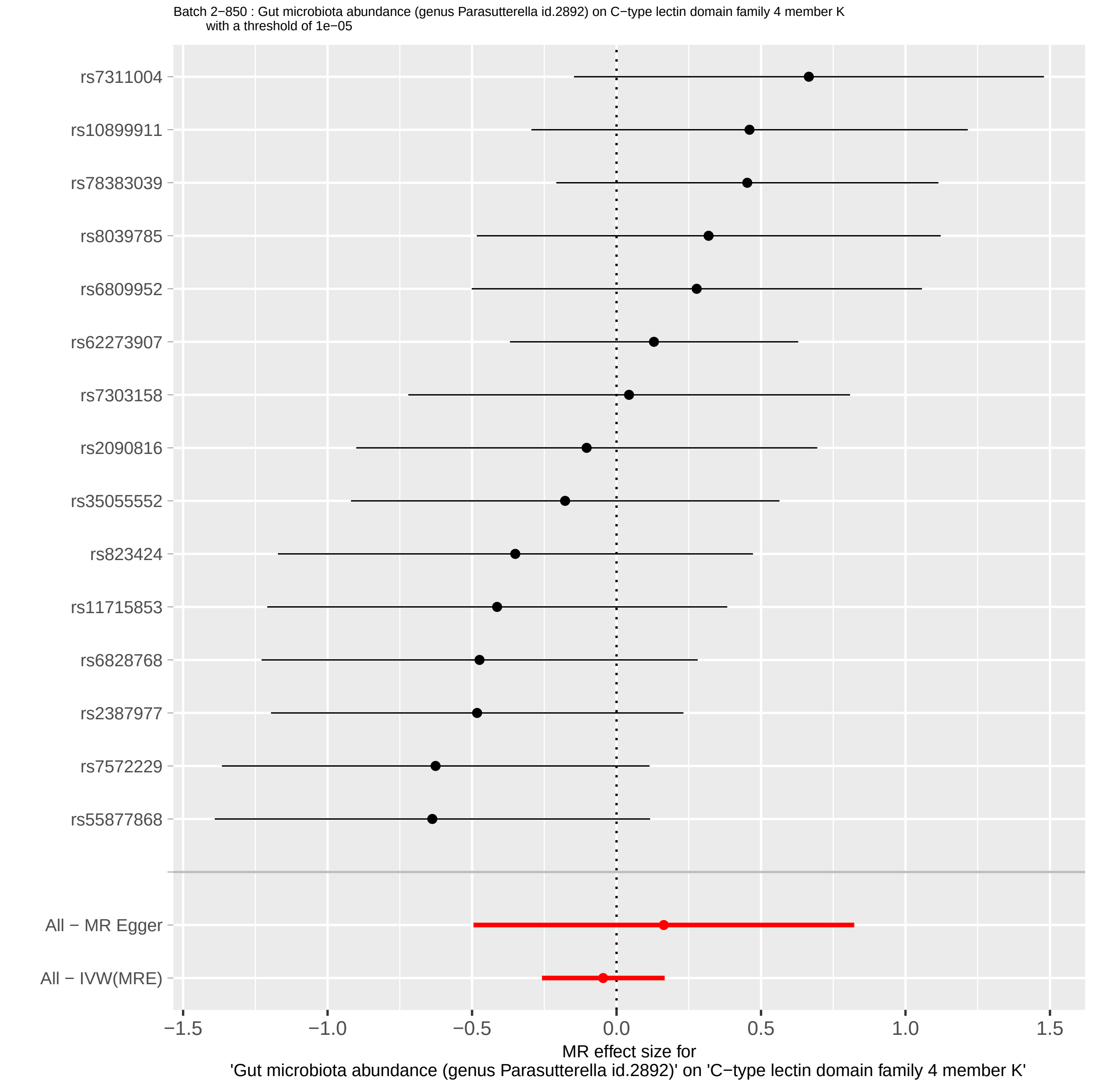
Batch 2−850 : Gut microbiota abundance (genus Parasutterella id.2892) on C−type lectin domain family 4 member K
with a threshold of 1e−05
rs7311004
rs10899911
rs78383039
rs8039785
rs6809952
rs62273907
rs7303158
rs2090816
rs35055552
rs823424
rs11715853
rs6828768
rs2387977
rs7572229
rs55877868
All − MR Egger
All − IVW(MRE)
−1.5
−1.0
−0.5
0.0
0.5
1.0
1.5
MR effect size for
'Gut microbiota abundance (genus Parasutterella id.2892)' on 'C−type lectin domain family 4 member K'

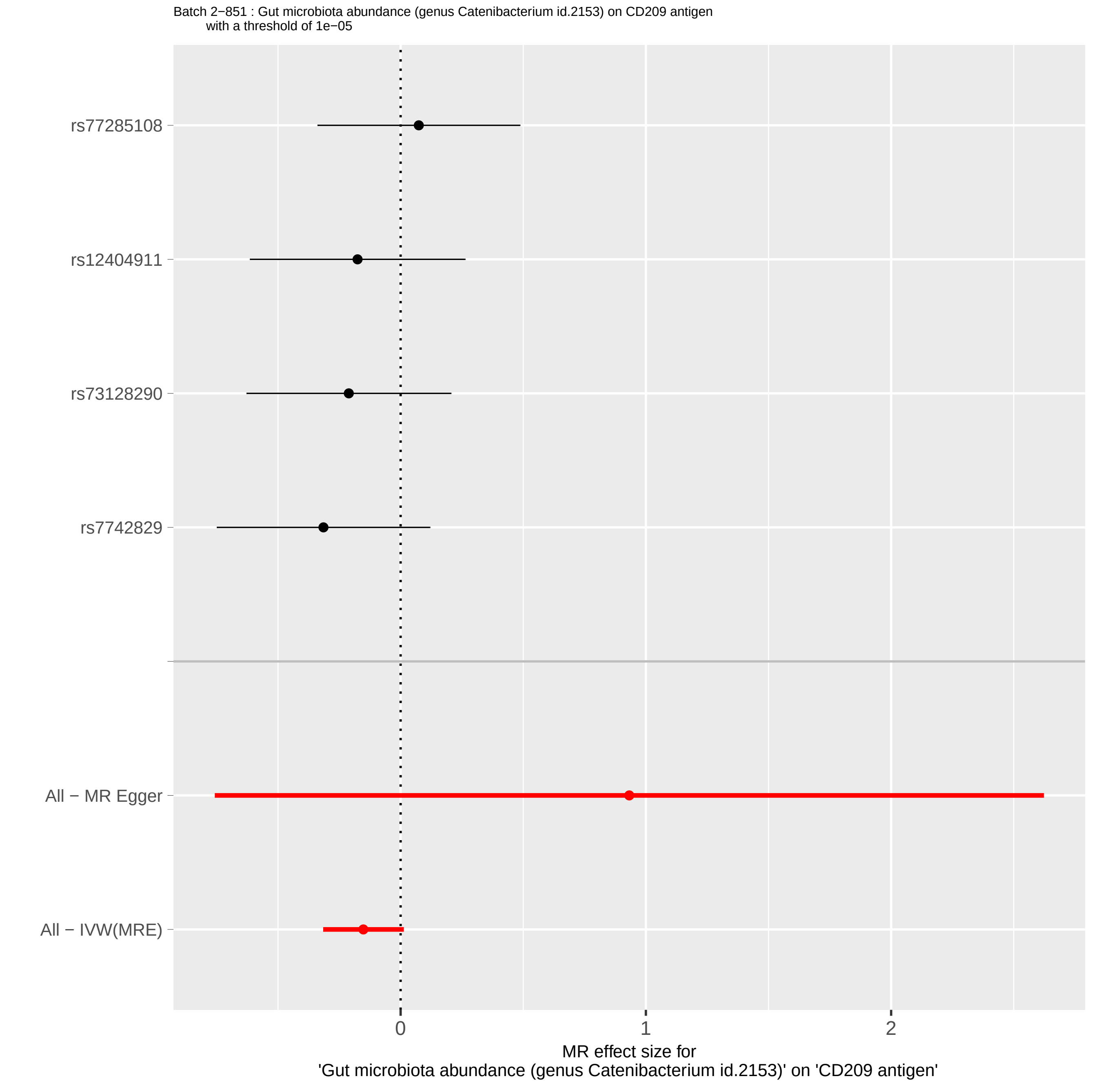
Batch 2−851 : Gut microbiota abundance (genus Catenibacterium id.2153) on CD209 antigen
with a threshold of 1e−05
rs77285108
rs12404911
rs73128290
rs7742829
All − MR Egger
All − IVW(MRE)
0
1
2
MR effect size for
'Gut microbiota abundance (genus Catenibacterium id.2153)' on 'CD209 antigen'

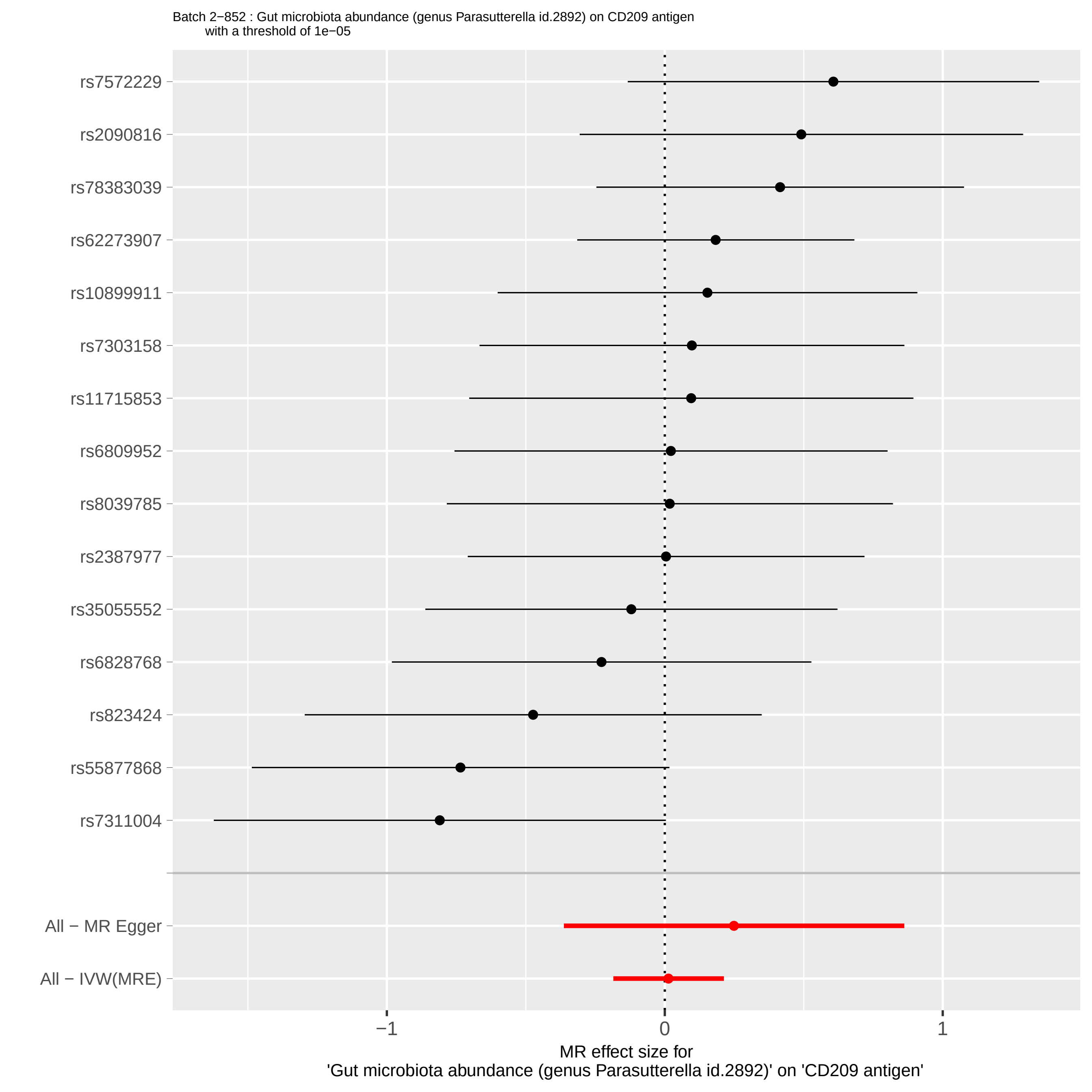

Batch 2−852 : Gut microbiota abundance (genus Parasutterella id.2892) on CD209 antigen
with a threshold of 1e−05
rs7572229
rs2090816
rs78383039
rs62273907
rs10899911
rs7303158
rs11715853
rs6809952
rs8039785
rs2387977
rs35055552
rs6828768
rs823424
rs55877868
rs7311004
All − MR Egger
All − IVW(MRE)
−1
0
1
MR effect size for
'Gut microbiota abundance (genus Parasutterella id.2892)' on 'CD209 antigen'

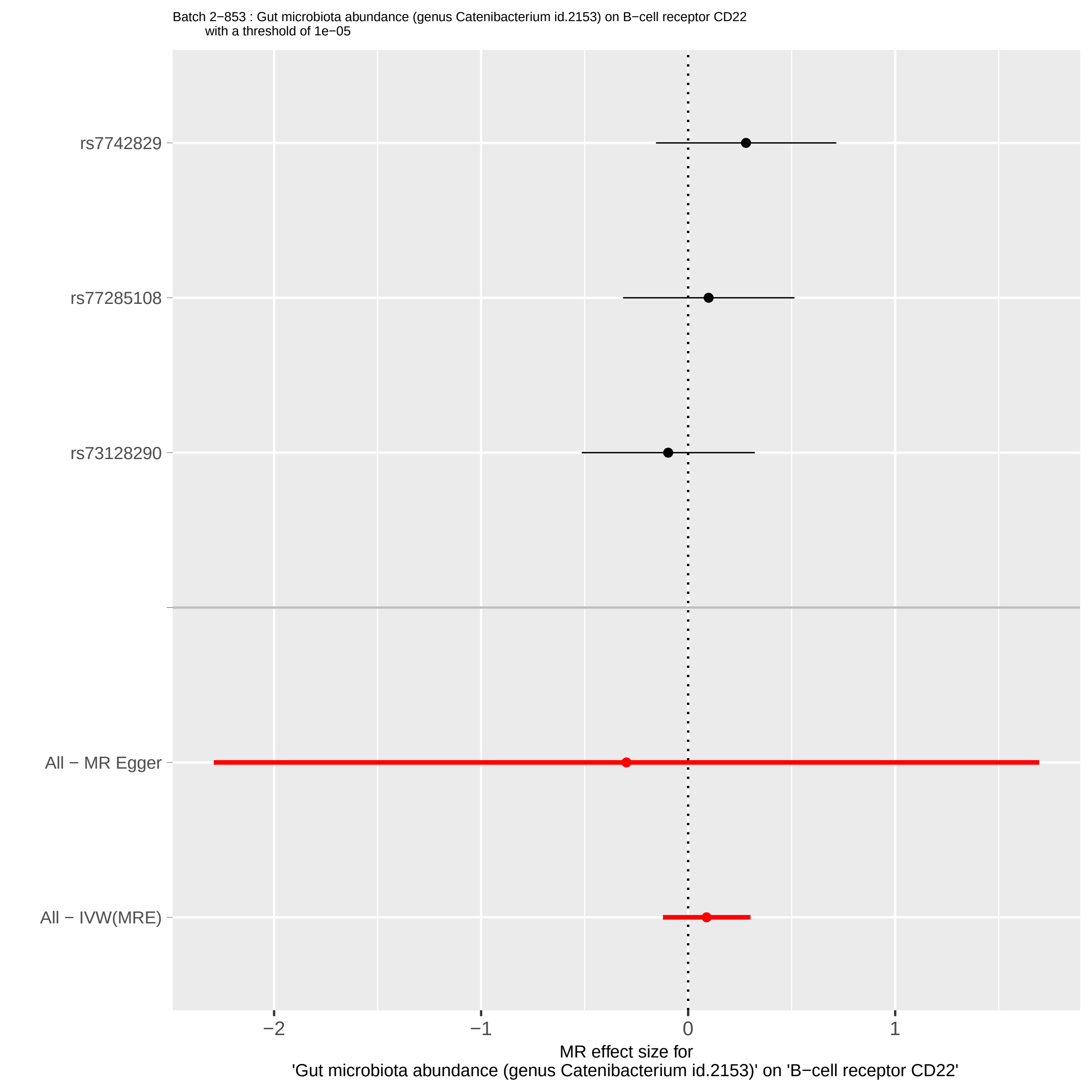

Batch 2−853 : Gut microbiota abundance (genus Catenibacterium id.2153) on B−cell receptor CD22
with a threshold of 1e−05
rs7742829
rs77285108
rs73128290
All − MR Egger
All − IVW(MRE)
−2
−1
0
1
MR effect size for
'Gut microbiota abundance (genus Catenibacterium id.2153)' on 'B−cell receptor CD22'

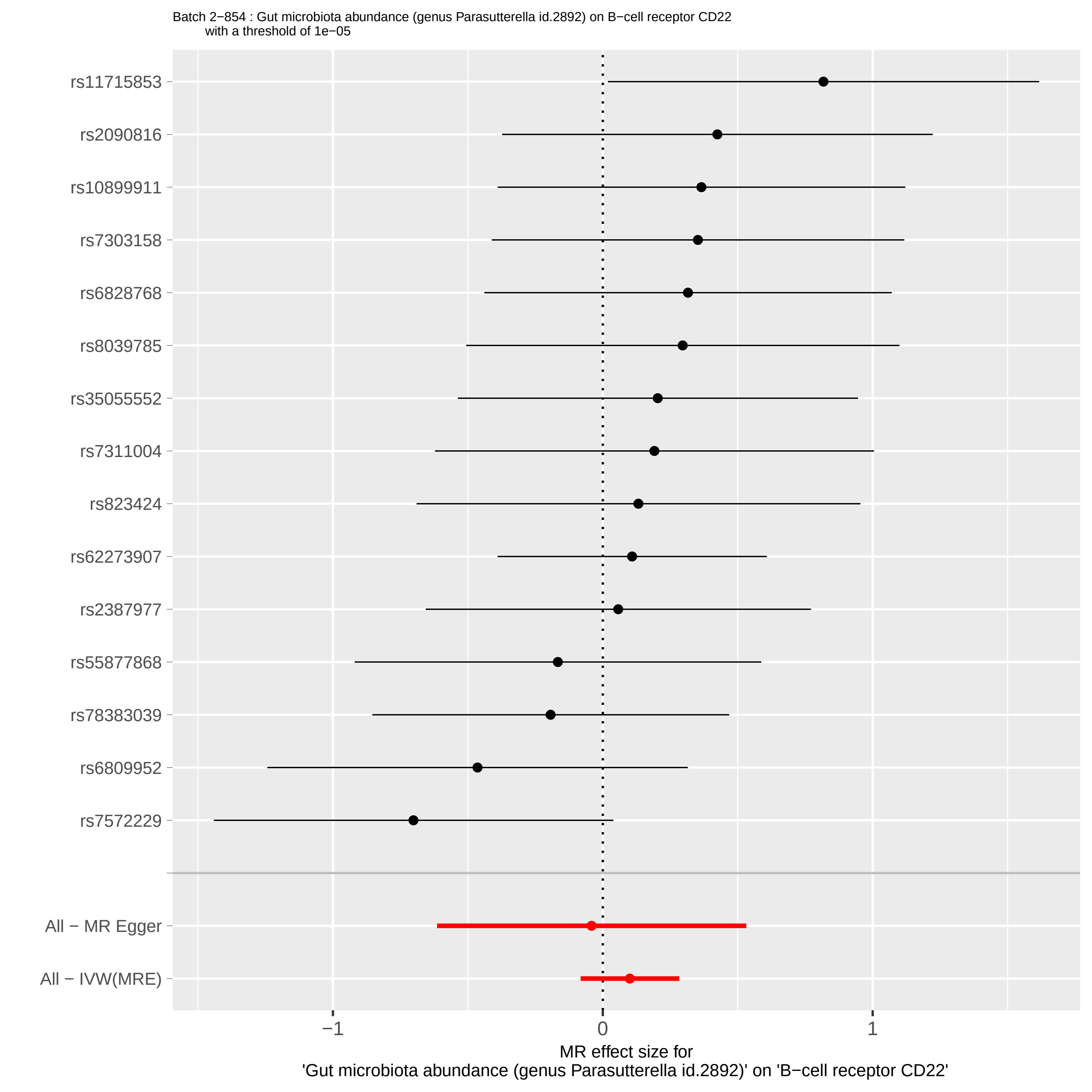

Batch 2−854 : Gut microbiota abundance (genus Parasutterella id.2892) on B−cell receptor CD22
with a threshold of 1e−05
rs11715853
rs2090816
rs10899911
rs7303158
rs6828768
rs8039785
rs35055552
rs7311004
rs823424
rs62273907
rs2387977
rs55877868
rs78383039
rs6809952
rs7572229
All − MR Egger
All − IVW(MRE)
−1
0
1
MR effect size for
'Gut microbiota abundance (genus Parasutterella id.2892)' on 'B−cell receptor CD22'

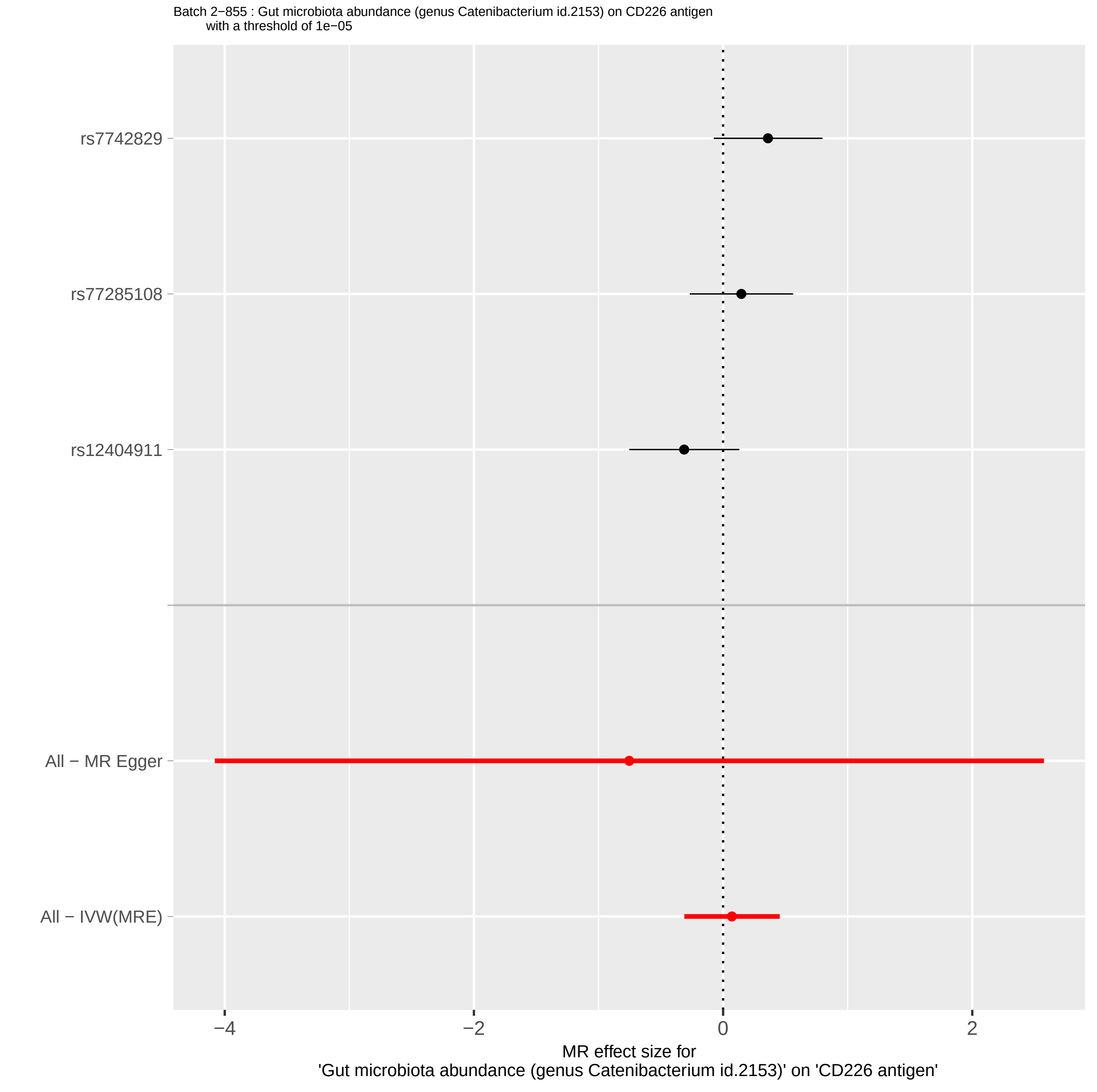
Batch 2−855 : Gut microbiota abundance (genus Catenibacterium id.2153) on CD226 antigen
with a threshold of 1e−05
rs7742829
rs77285108
rs12404911
All − MR Egger
All − IVW(MRE)
−4
−2
0
2
MR effect size for
'Gut microbiota abundance (genus Catenibacterium id.2153)' on 'CD226 antigen'

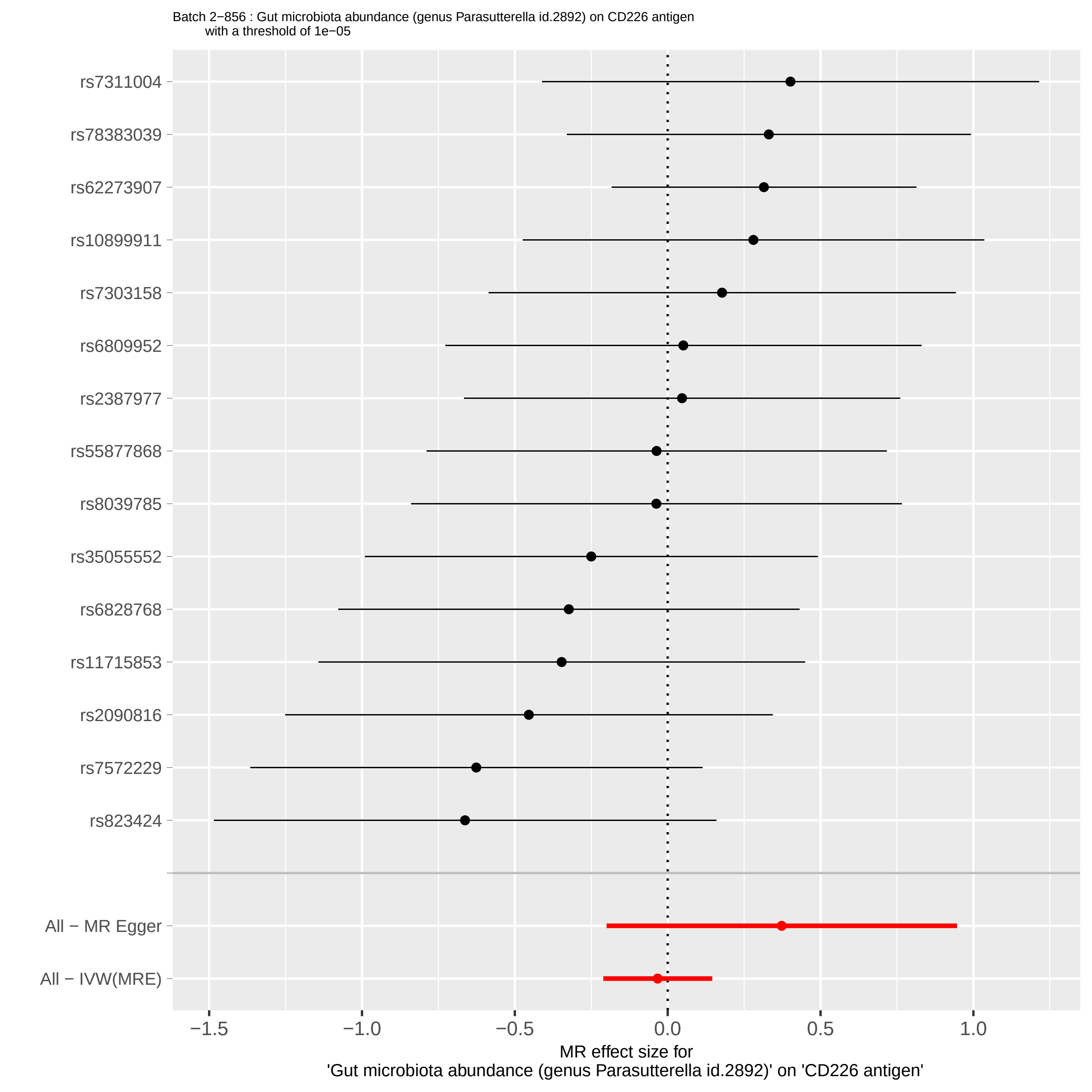

Batch 2−856 : Gut microbiota abundance (genus Parasutterella id.2892) on CD226 antigen
with a threshold of 1e−05
rs7311004
rs78383039
rs62273907
rs10899911
rs7303158
rs6809952
rs2387977
rs55877868
rs8039785
rs35055552
rs6828768
rs11715853
rs2090816
rs7572229
rs823424
All − MR Egger
All − IVW(MRE)
−1.5
−1.0
−0.5
0.0
0.5
1.0
MR effect size for
'Gut microbiota abundance (genus Parasutterella id.2892)' on 'CD226 antigen'

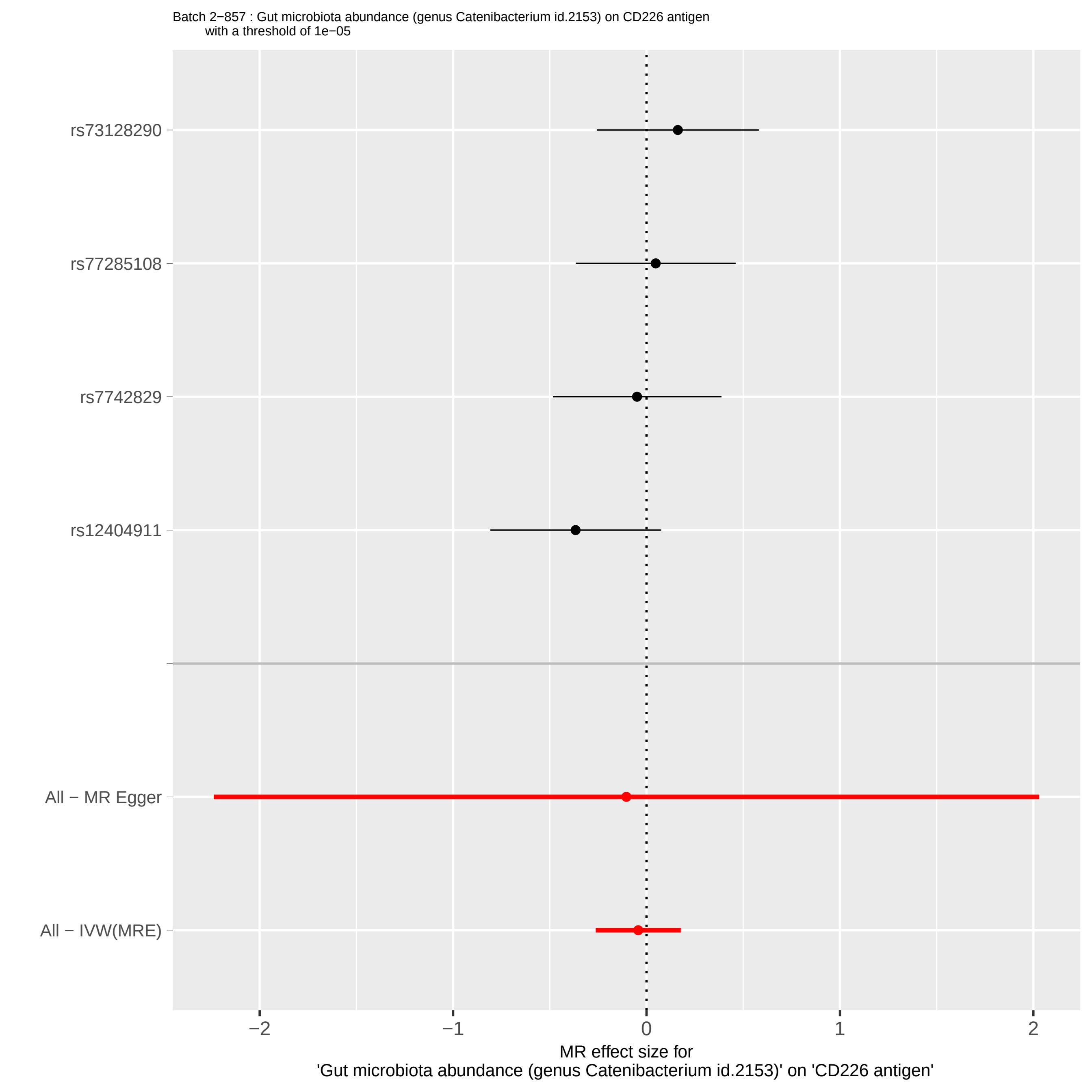

Batch 2−857 : Gut microbiota abundance (genus Catenibacterium id.2153) on CD226 antigen
with a threshold of 1e−05
rs73128290
rs77285108
rs7742829
rs12404911
All − MR Egger
All − IVW(MRE)
−2
−1
0
1
2
MR effect size for
'Gut microbiota abundance (genus Catenibacterium id.2153)' on 'CD226 antigen'

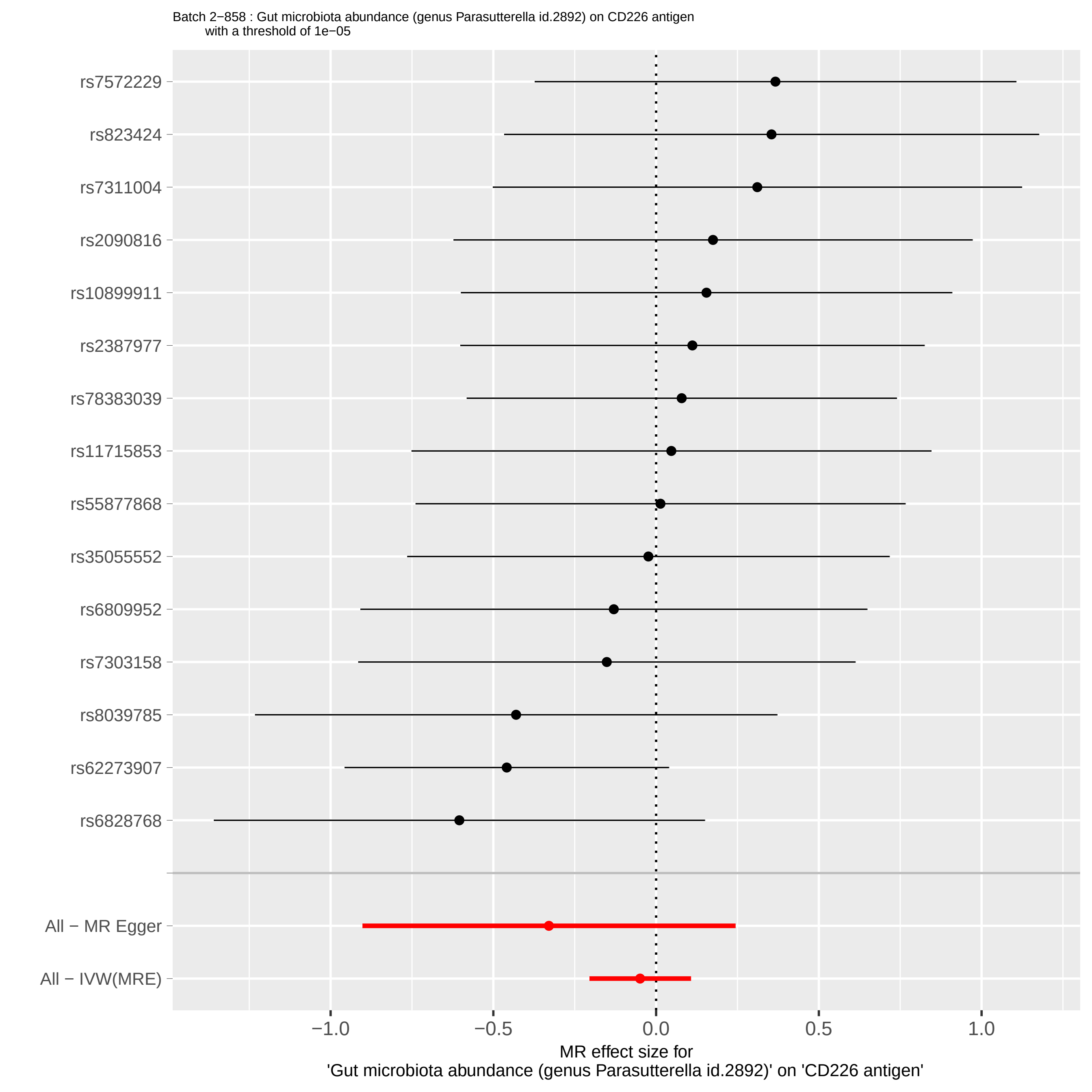

Batch 2−858 : Gut microbiota abundance (genus Parasutterella id.2892) on CD226 antigen
with a threshold of 1e−05
rs7572229
rs823424
rs7311004
rs2090816
rs10899911
rs2387977
rs78383039
rs11715853
rs55877868
rs35055552
rs6809952
rs7303158
rs8039785
rs62273907
rs6828768
All − MR Egger
All − IVW(MRE)
−1.0
−0.5
0.0
0.5
1.0
MR effect size for
'Gut microbiota abundance (genus Parasutterella id.2892)' on 'CD226 antigen'

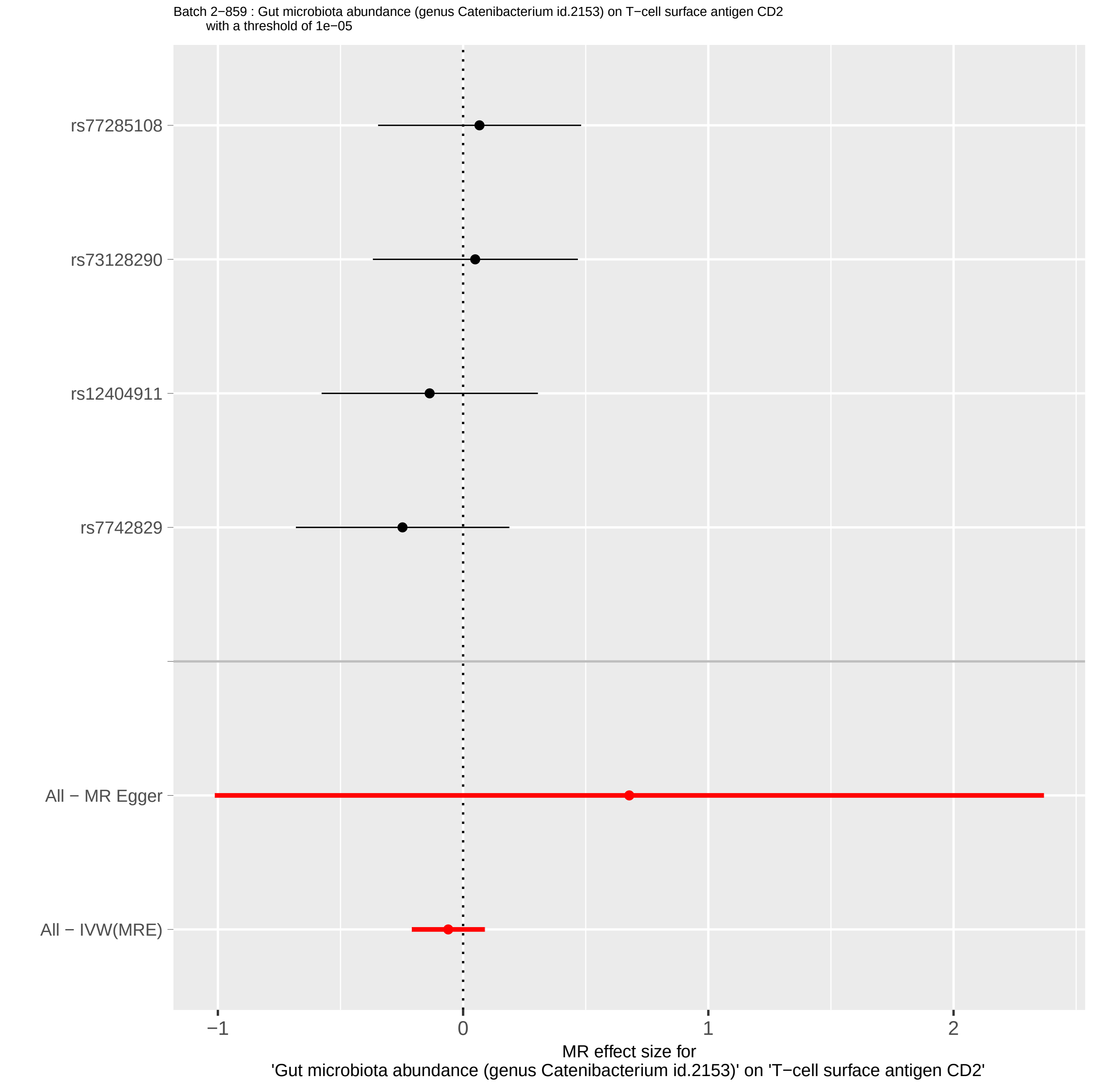

Batch 2−859 : Gut microbiota abundance (genus Catenibacterium id.2153) on T−cell surface antigen CD2
with a threshold of 1e−05
rs77285108
rs73128290
rs12404911
rs7742829
All − MR Egger
All − IVW(MRE)
−1
0
1
2
MR effect size for
'Gut microbiota abundance (genus Catenibacterium id.2153)' on 'T−cell surface antigen CD2'

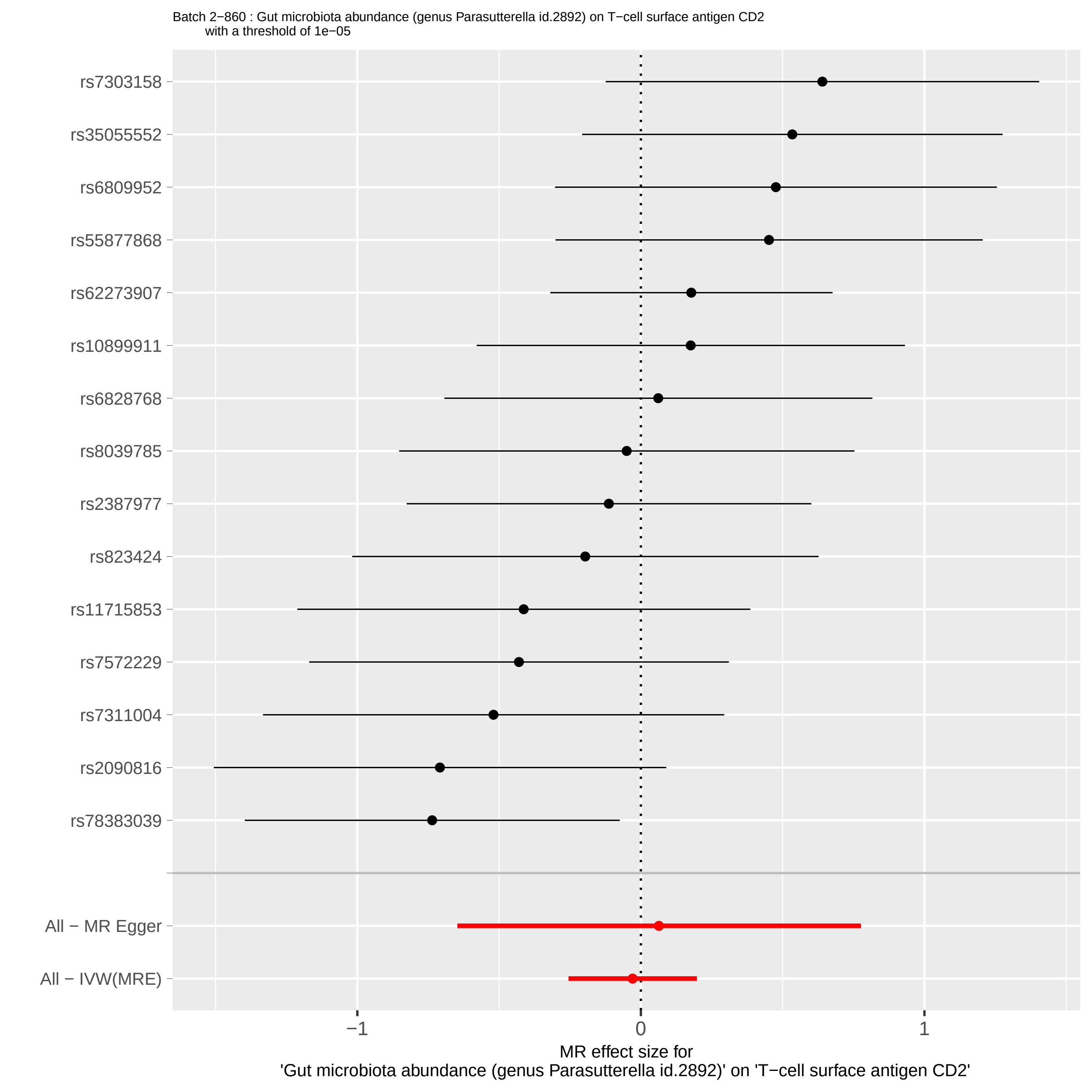

Batch 2−860 : Gut microbiota abundance (genus Parasutterella id.2892) on T−cell surface antigen CD2
with a threshold of 1e−05
rs7303158
rs35055552
rs6809952
rs55877868
rs62273907
rs10899911
rs6828768
rs8039785
rs2387977
rs823424
rs11715853
rs7572229
rs7311004
rs2090816
rs78383039
All − MR Egger
All − IVW(MRE)
−1
0
1
MR effect size for
'Gut microbiota abundance (genus Parasutterella id.2892)' on 'T−cell surface antigen CD2'

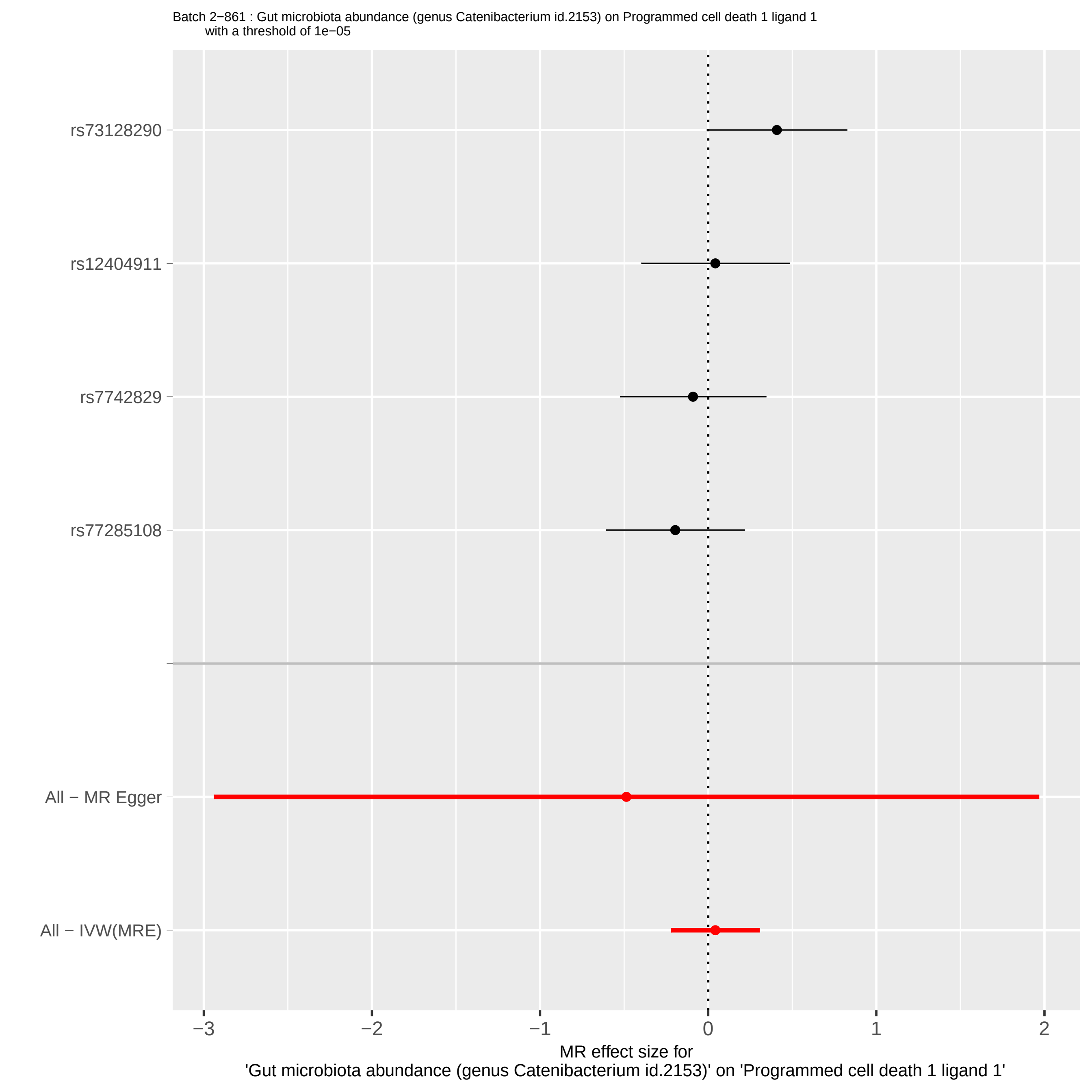
Batch 2−861 : Gut microbiota abundance (genus Catenibacterium id.2153) on Programmed cell death 1 ligand 1
with a threshold of 1e−05
rs73128290
rs12404911
rs7742829
rs77285108
All − MR Egger
All − IVW(MRE)
−3
−2
−1
0
1
2
MR effect size for
'Gut microbiota abundance (genus Catenibacterium id.2153)' on 'Programmed cell death 1 ligand 1'

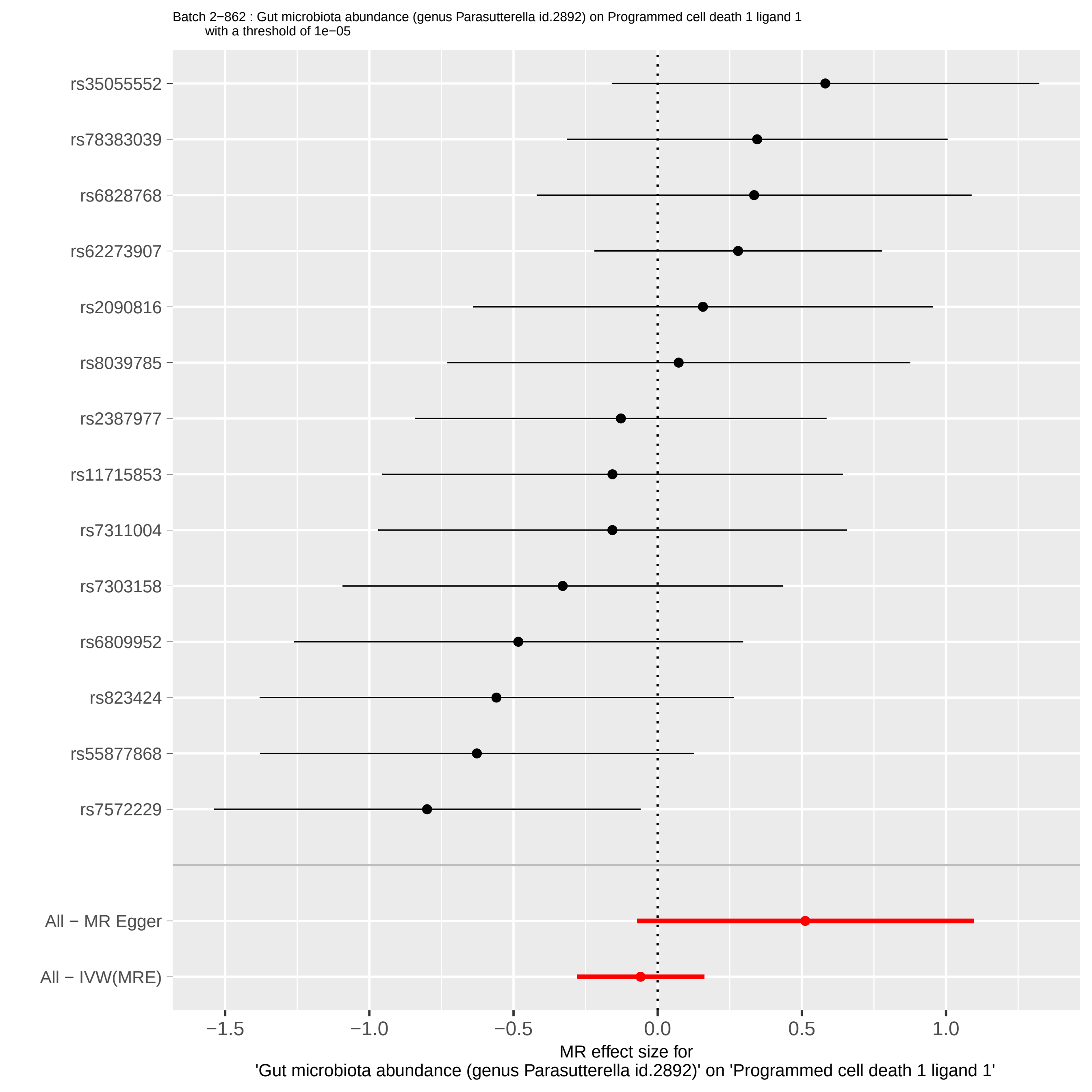

Batch 2−862 : Gut microbiota abundance (genus Parasutterella id.2892) on Programmed cell death 1 ligand 1
with a threshold of 1e−05
rs35055552
rs78383039
rs6828768
rs62273907
rs2090816
rs8039785
rs2387977
rs11715853
rs7311004
rs7303158
rs6809952
rs823424
rs55877868
rs7572229
All − MR Egger
All − IVW(MRE)
−1.5
−1.0
−0.5
0.0
0.5
1.0
MR effect size for
'Gut microbiota abundance (genus Parasutterella id.2892)' on 'Programmed cell death 1 ligand 1'

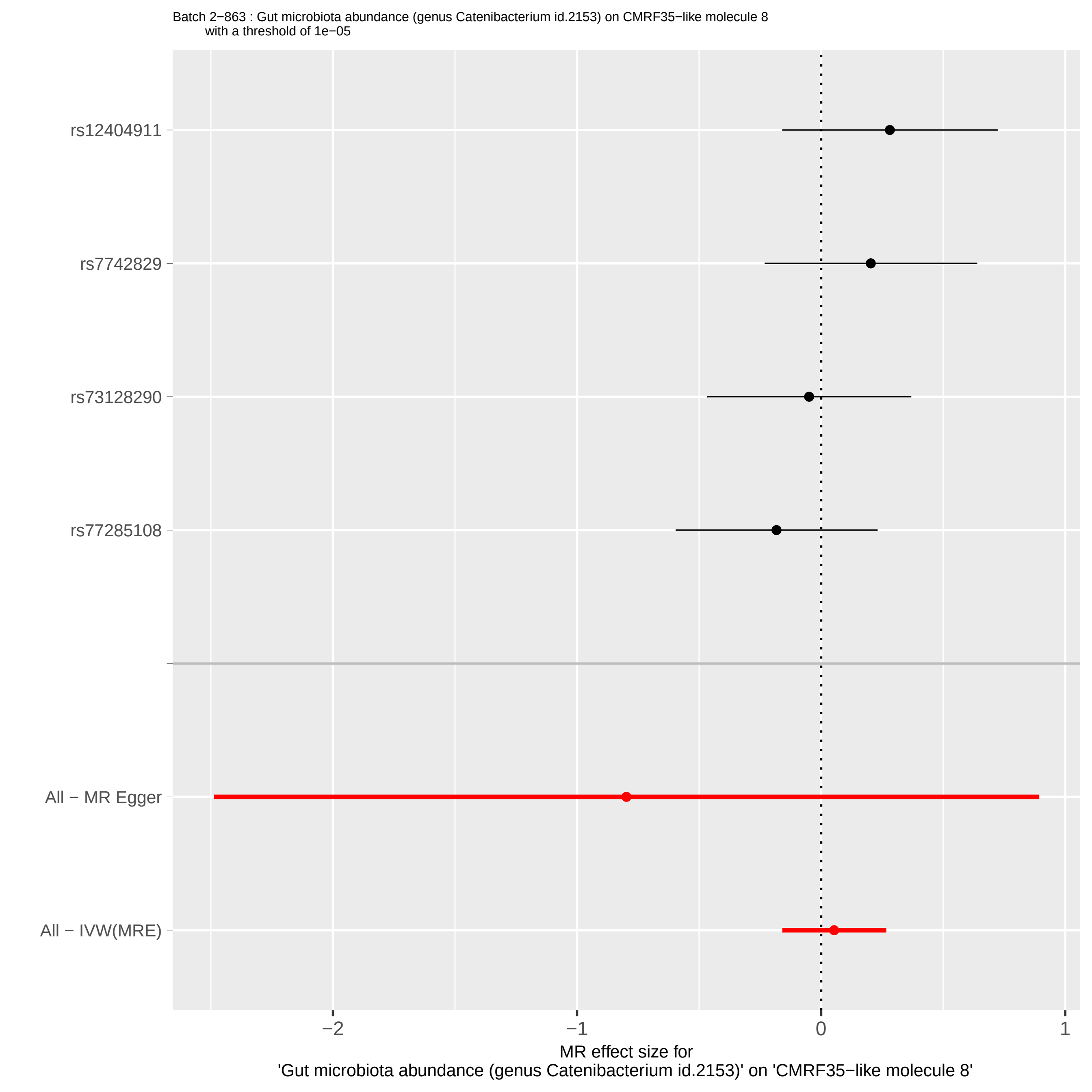
Batch 2−863 : Gut microbiota abundance (genus Catenibacterium id.2153) on CMRF35−like molecule 8
with a threshold of 1e−05
rs12404911
rs7742829
rs73128290
rs77285108
All − MR Egger
All − IVW(MRE)
−2
−1
0
1
MR effect size for
'Gut microbiota abundance (genus Catenibacterium id.2153)' on 'CMRF35−like molecule 8'

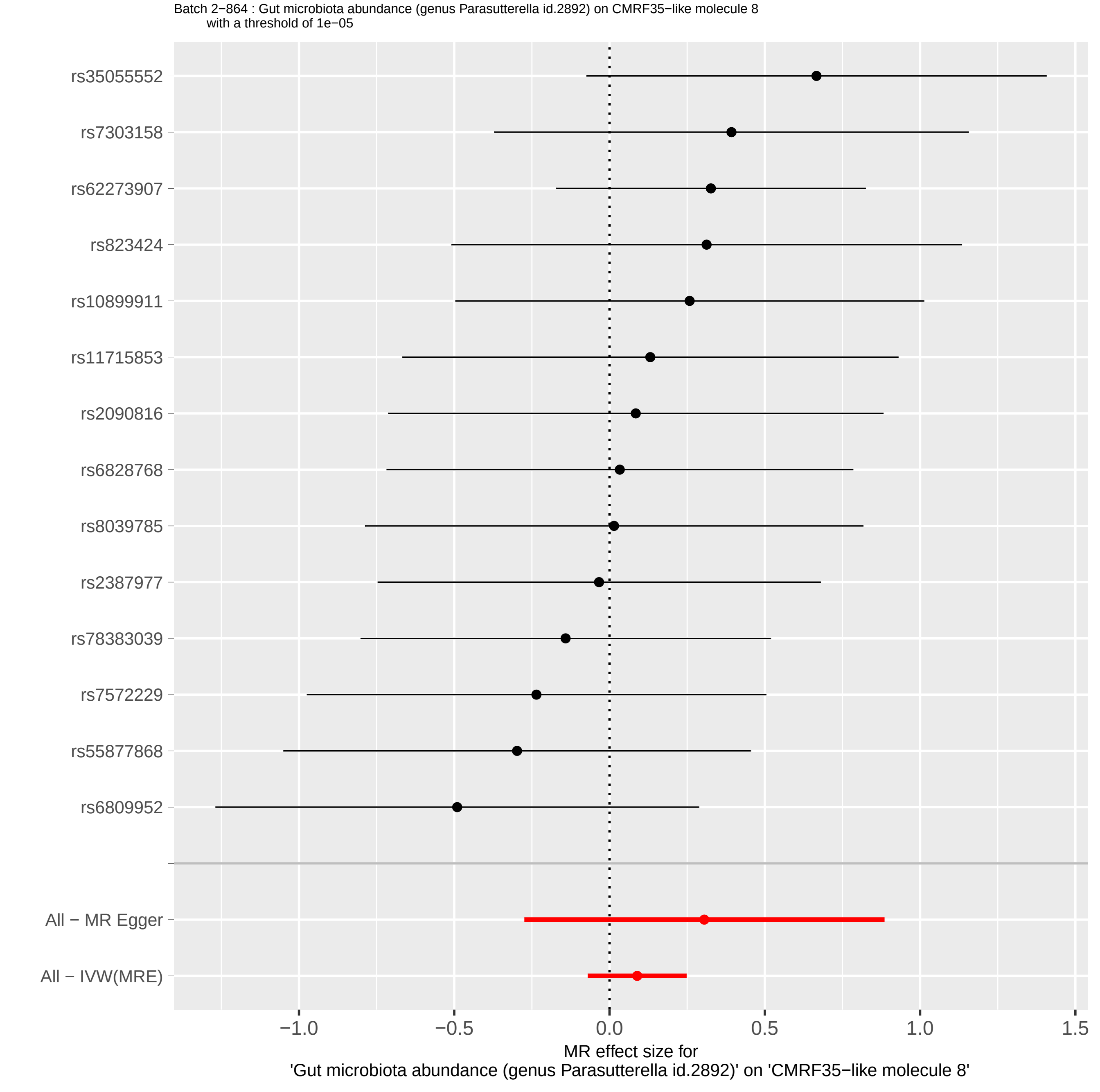

Batch 2−864 : Gut microbiota abundance (genus Parasutterella id.2892) on CMRF35−like molecule 8
with a threshold of 1e−05
rs35055552
rs7303158
rs62273907
rs823424
rs10899911
rs11715853
rs2090816
rs6828768
rs8039785
rs2387977
rs78383039
rs7572229
rs55877868
rs6809952
All − MR Egger
All − IVW(MRE)
−1.0
−0.5
0.0
0.5
1.0
1.5
MR effect size for
'Gut microbiota abundance (genus Parasutterella id.2892)' on 'CMRF35−like molecule 8'

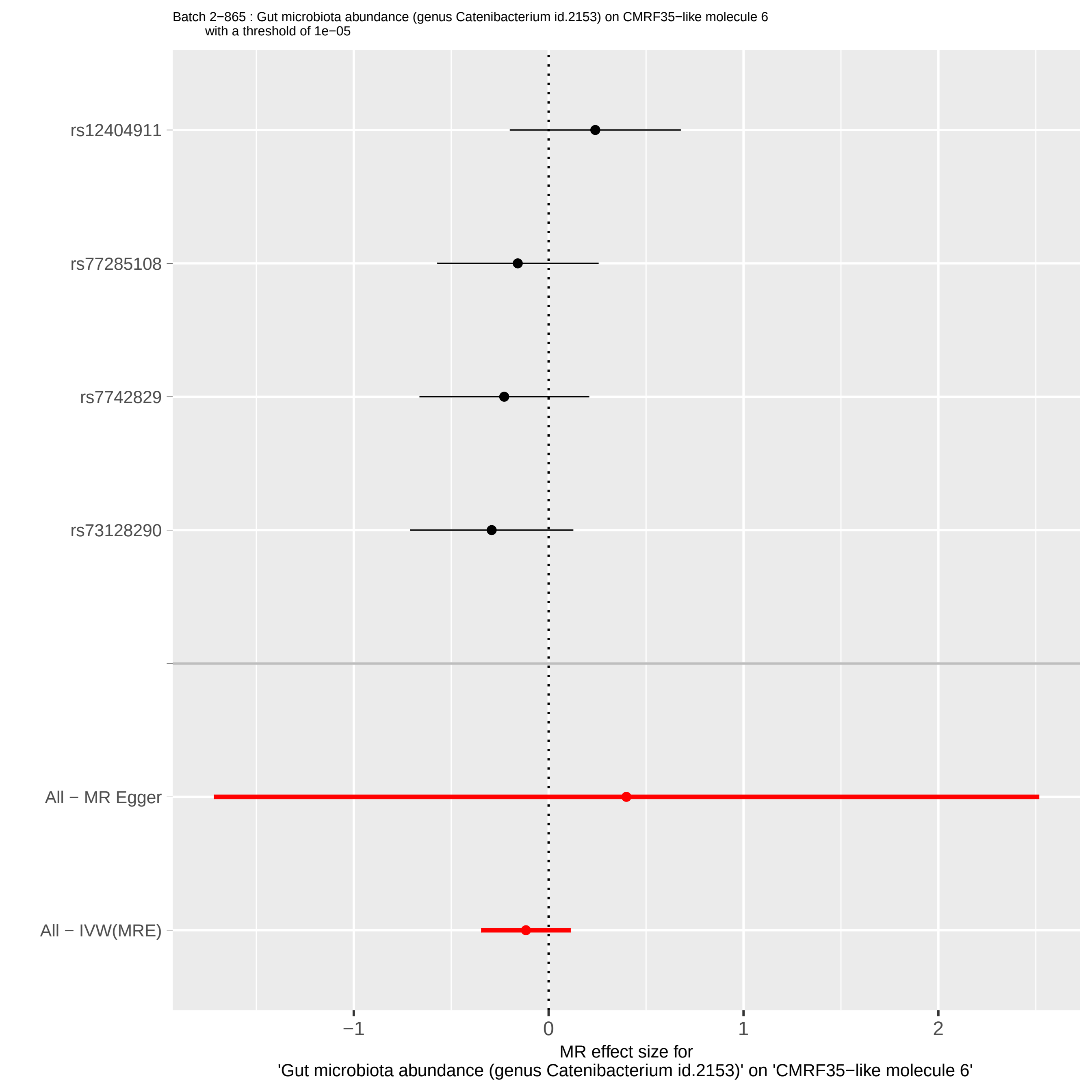

Batch 2−865 : Gut microbiota abundance (genus Catenibacterium id.2153) on CMRF35−like molecule 6
with a threshold of 1e−05
rs12404911
rs77285108
rs7742829
rs73128290
All − MR Egger
All − IVW(MRE)
−1
0
1
2
MR effect size for
'Gut microbiota abundance (genus Catenibacterium id.2153)' on 'CMRF35−like molecule 6'

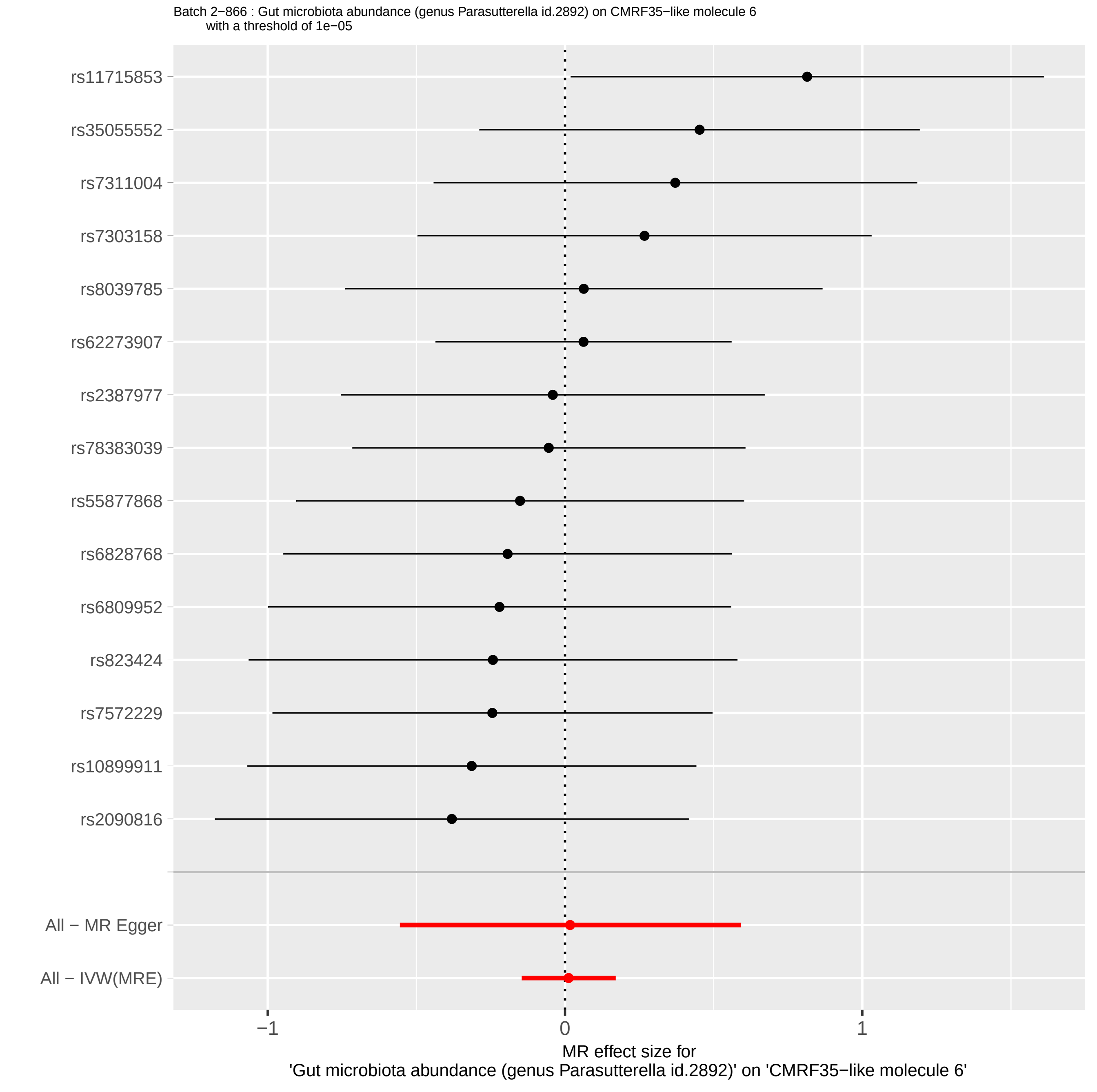

Batch 2−866 : Gut microbiota abundance (genus Parasutterella id.2892) on CMRF35−like molecule 6
with a threshold of 1e−05
rs11715853
rs35055552
rs7311004
rs7303158
rs8039785
rs62273907
rs2387977
rs78383039
rs55877868
rs6828768
rs6809952
rs823424
rs7572229
rs10899911
rs2090816
All − MR Egger
All − IVW(MRE)
−1
0
1
MR effect size for
'Gut microbiota abundance (genus Parasutterella id.2892)' on 'CMRF35−like molecule 6'

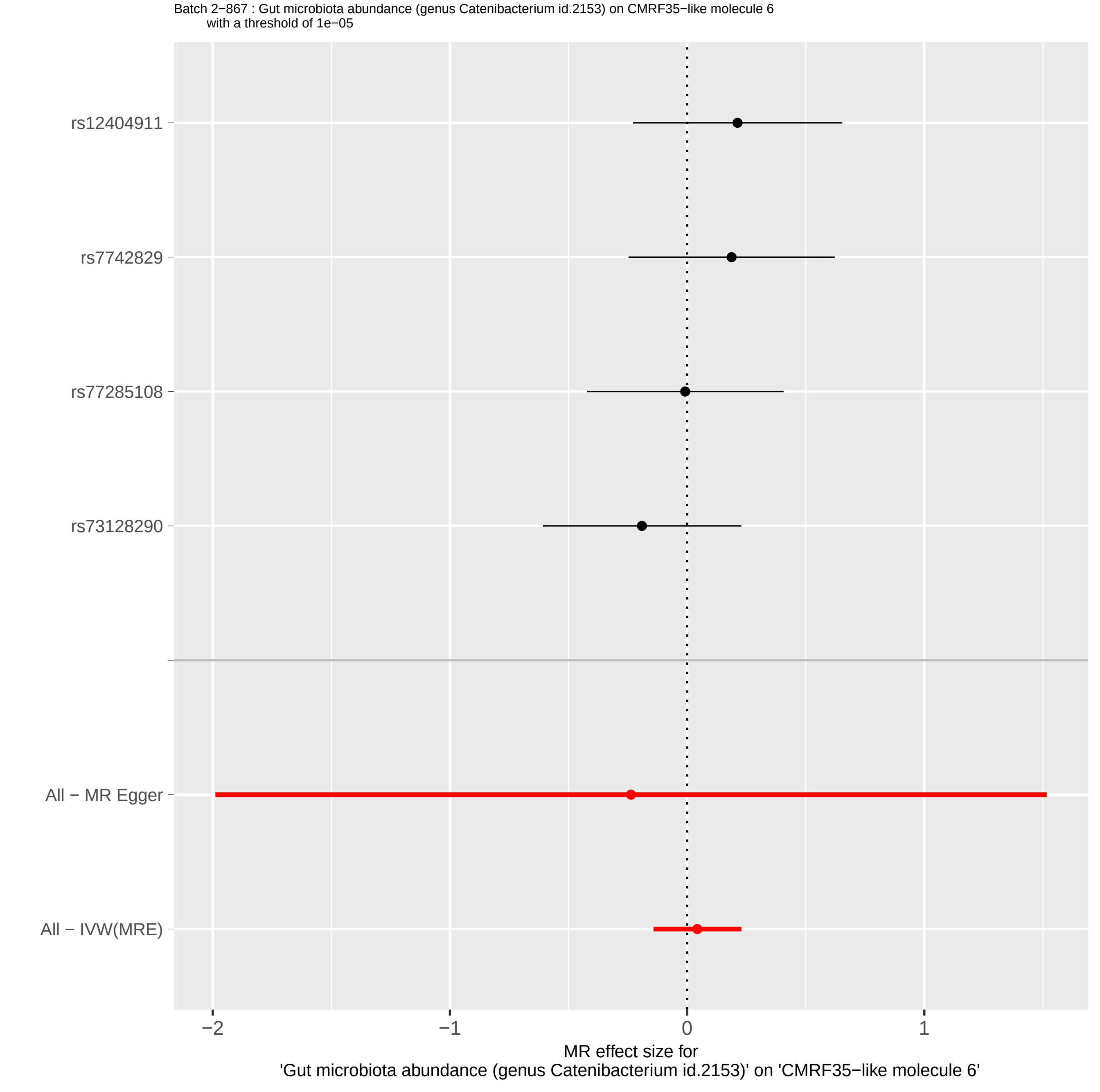

Batch 2−867 : Gut microbiota abundance (genus Catenibacterium id.2153) on CMRF35−like molecule 6
with a threshold of 1e−05
rs12404911
rs7742829
rs77285108
rs73128290
All − MR Egger
All − IVW(MRE)
−2
−1
0
1
MR effect size for
'Gut microbiota abundance (genus Catenibacterium id.2153)' on 'CMRF35−like molecule 6'

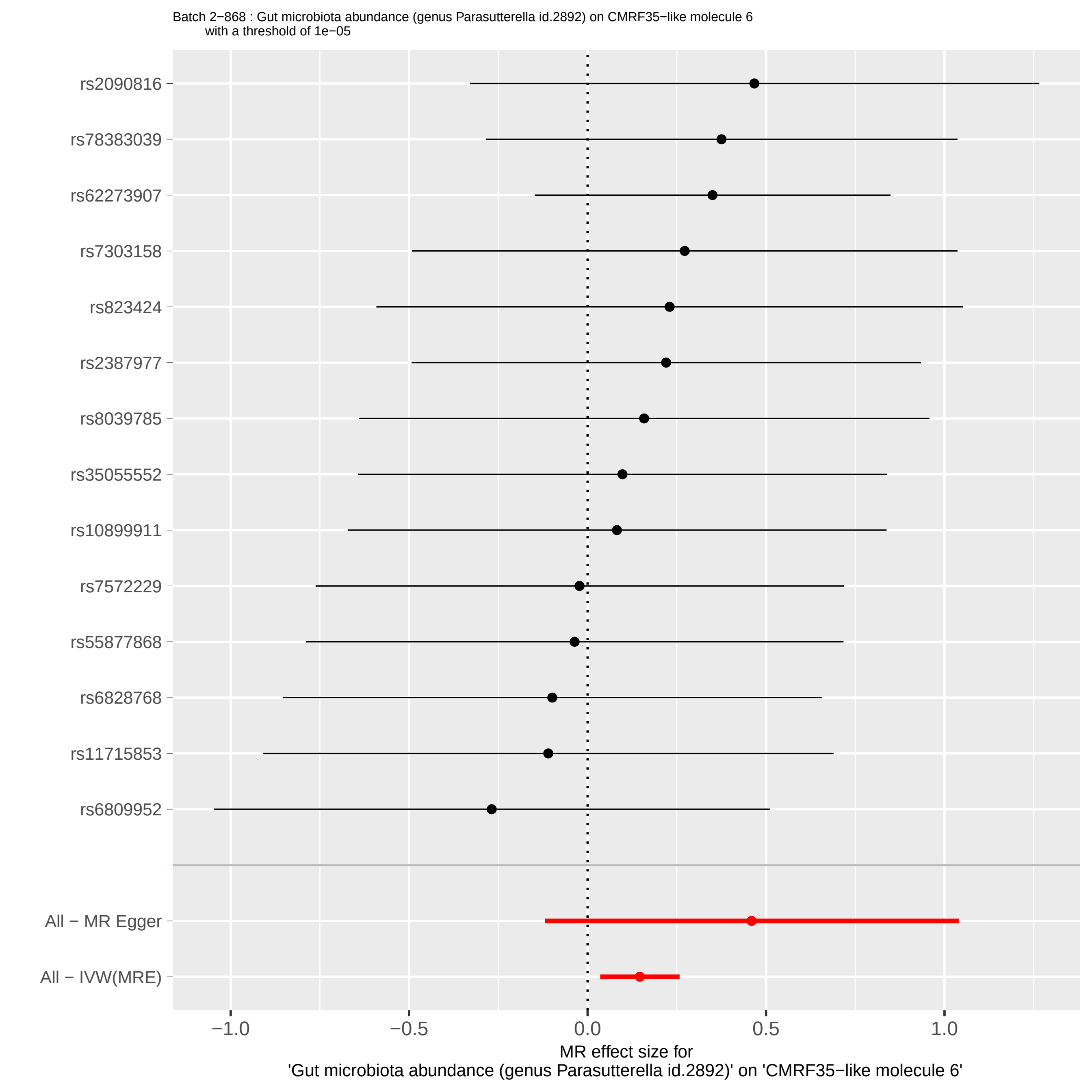

Batch 2−868 : Gut microbiota abundance (genus Parasutterella id.2892) on CMRF35−like molecule 6
with a threshold of 1e−05
rs2090816
rs78383039
rs62273907
rs7303158
rs823424
rs2387977
rs8039785
rs35055552
rs10899911
rs7572229
rs55877868
rs6828768
rs11715853
rs6809952
All − MR Egger
All − IVW(MRE)
−1.0
−0.5
0.0
0.5
1.0
MR effect size for
'Gut microbiota abundance (genus Parasutterella id.2892)' on 'CMRF35−like molecule 6'

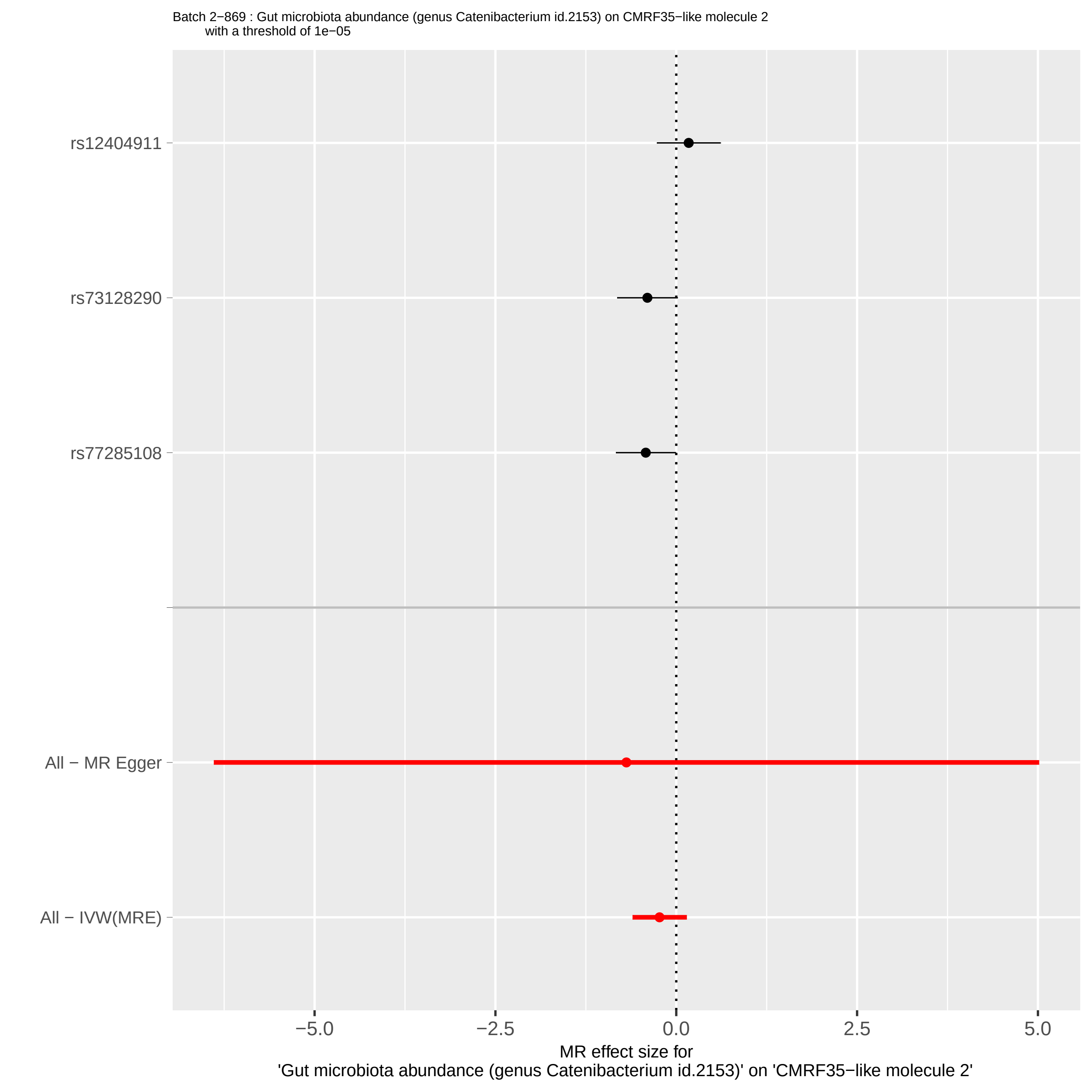

Batch 2−869 : Gut microbiota abundance (genus Catenibacterium id.2153) on CMRF35−like molecule 2
with a threshold of 1e−05
rs12404911
rs73128290
rs77285108
All − MR Egger
All − IVW(MRE)
−5.0
−2.5
0.0
2.5
5.0
MR effect size for
'Gut microbiota abundance (genus Catenibacterium id.2153)' on 'CMRF35−like molecule 2'

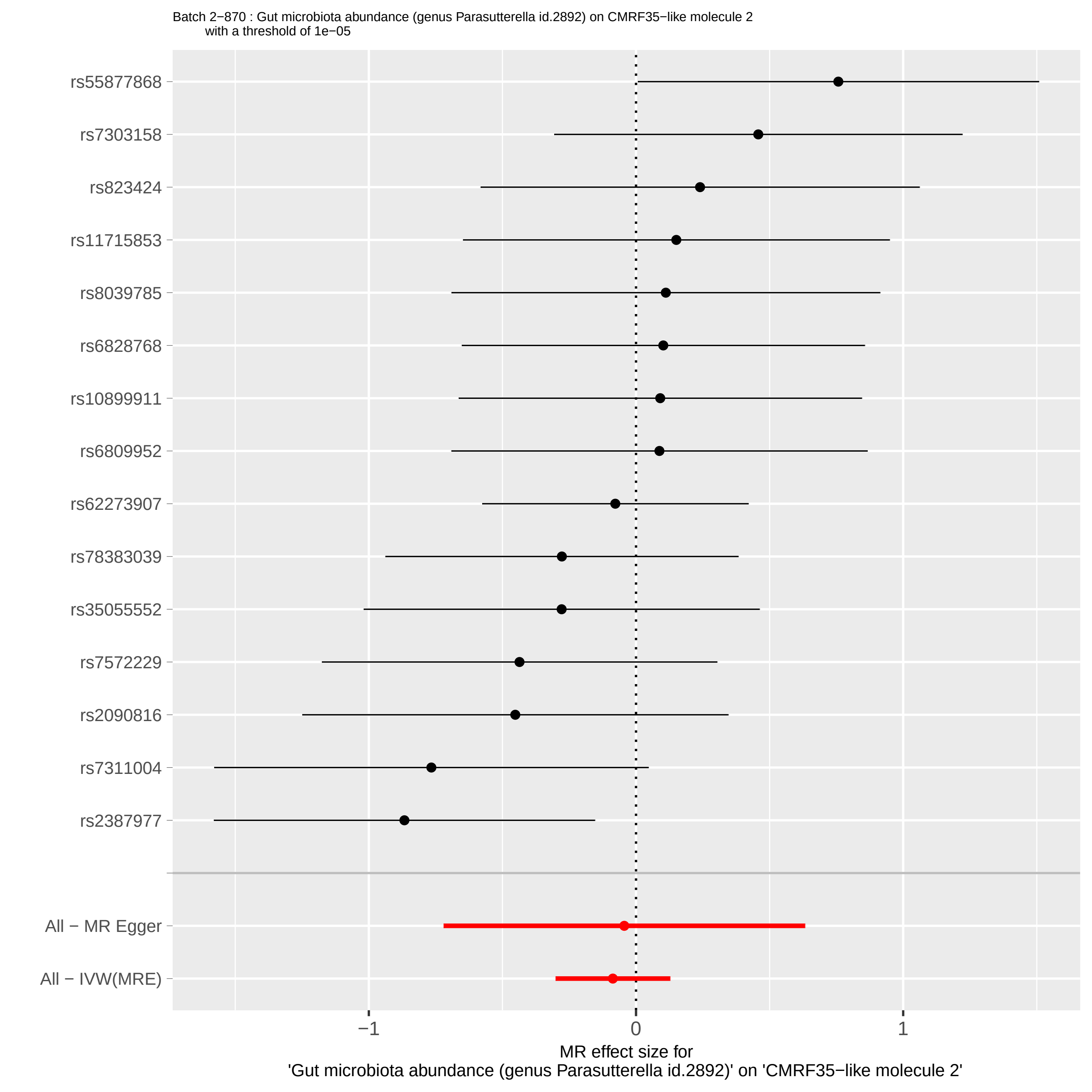

Batch 2−870 : Gut microbiota abundance (genus Parasutterella id.2892) on CMRF35−like molecule 2
with a threshold of 1e−05
rs55877868
rs7303158
rs823424
rs11715853
rs8039785
rs6828768
rs10899911
rs6809952
rs62273907
rs78383039
rs35055552
rs7572229
rs2090816
rs7311004
rs2387977
All − MR Egger
All − IVW(MRE)
−1
0
1
MR effect size for
'Gut microbiota abundance (genus Parasutterella id.2892)' on 'CMRF35−like molecule 2'

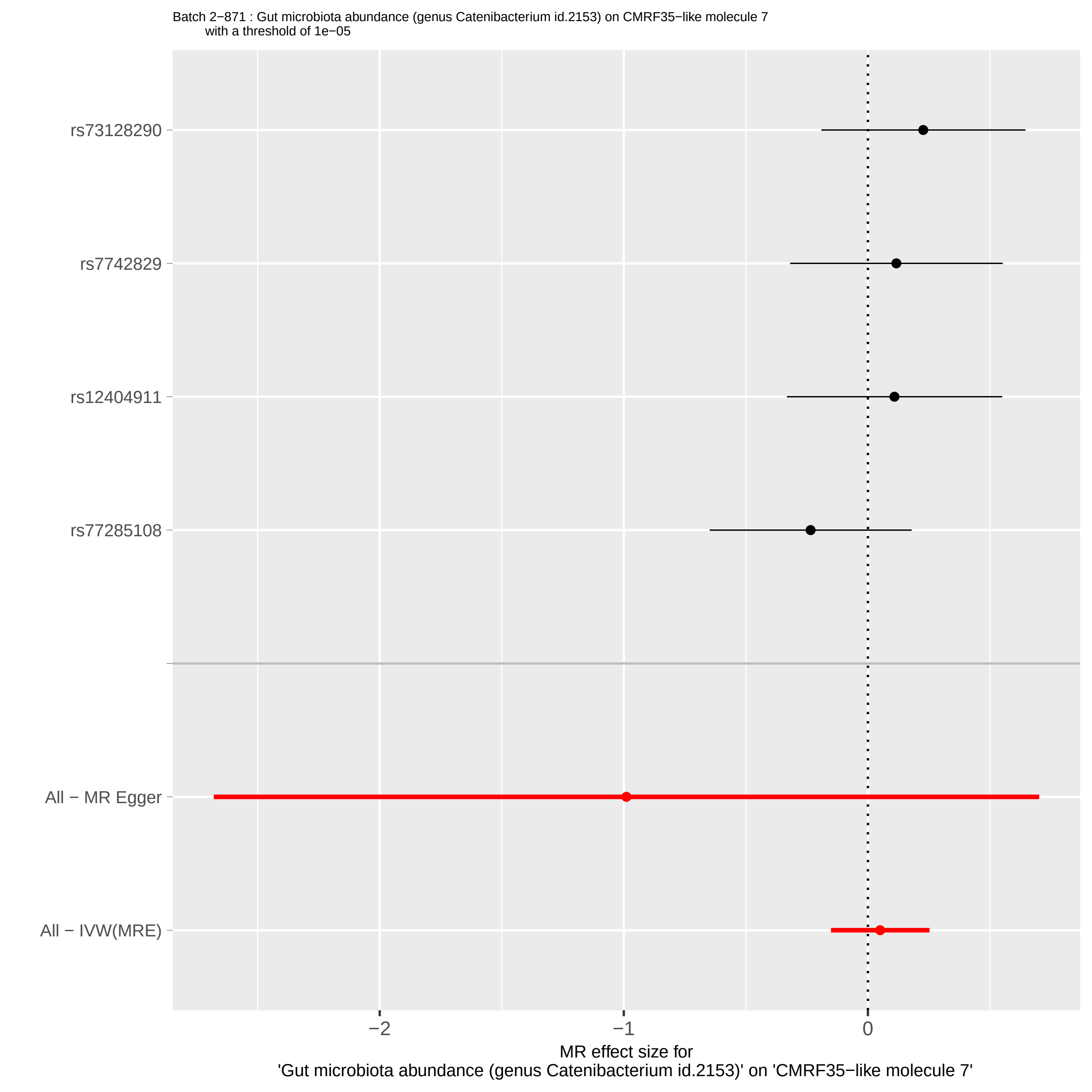

Batch 2−871 : Gut microbiota abundance (genus Catenibacterium id.2153) on CMRF35−like molecule 7
with a threshold of 1e−05
rs73128290
rs7742829
rs12404911
rs77285108
All − MR Egger
All − IVW(MRE)
−2
−1
0
MR effect size for
'Gut microbiota abundance (genus Catenibacterium id.2153)' on 'CMRF35−like molecule 7'

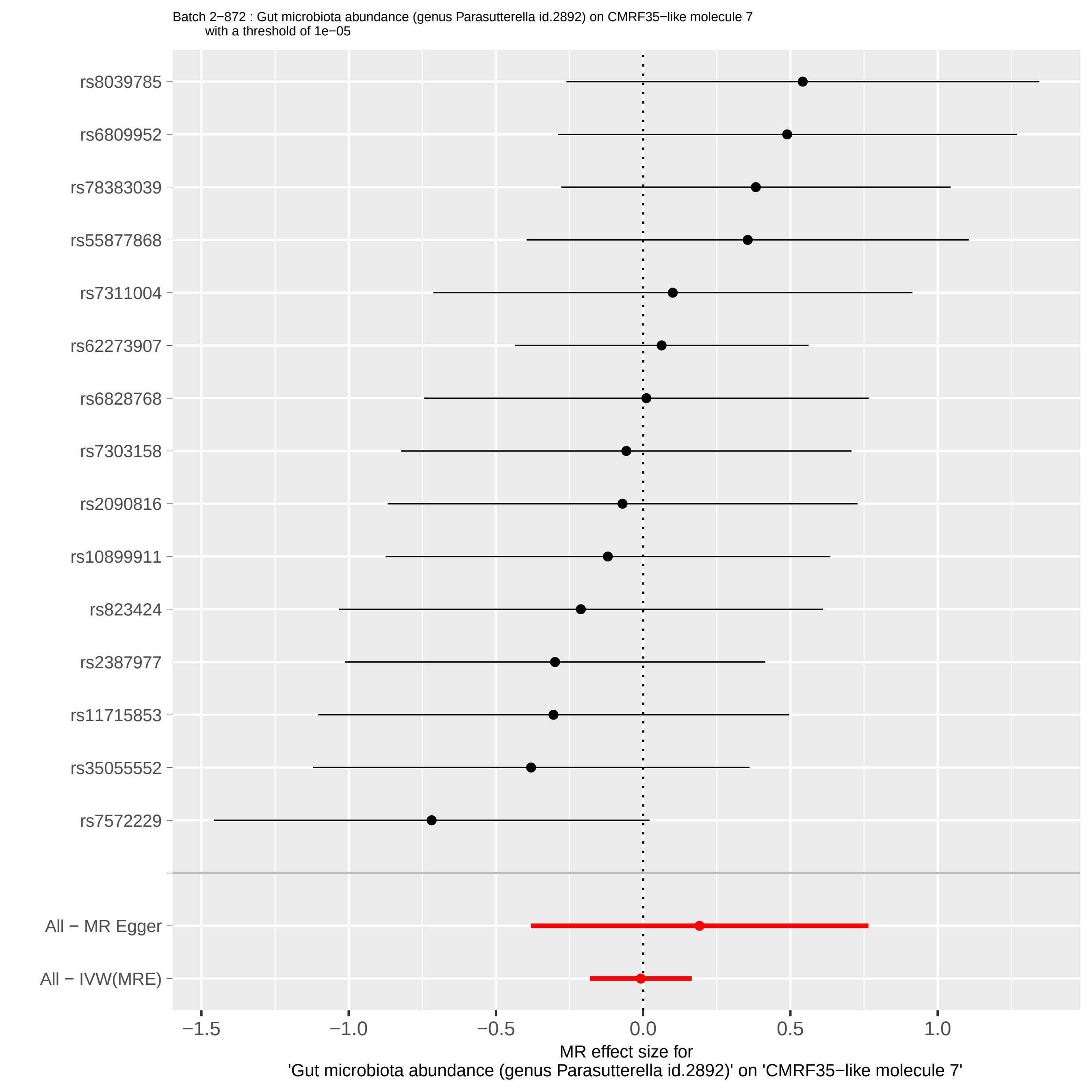

Batch 2−872 : Gut microbiota abundance (genus Parasutterella id.2892) on CMRF35−like molecule 7
with a threshold of 1e−05
rs8039785
rs6809952
rs78383039
rs55877868
rs7311004
rs62273907
rs6828768
rs7303158
rs2090816
rs10899911
rs823424
rs2387977
rs11715853
rs35055552
rs7572229
All − MR Egger
All − IVW(MRE)
−1.5
−1.0
−0.5
0.0
0.5
1.0
MR effect size for
'Gut microbiota abundance (genus Parasutterella id.2892)' on 'CMRF35−like molecule 7'

Batch 2−873 : Gut microbiota abundance (genus Catenibacterium id.2153) on CMRF35−like molecule 1
with a threshold of 1e−05

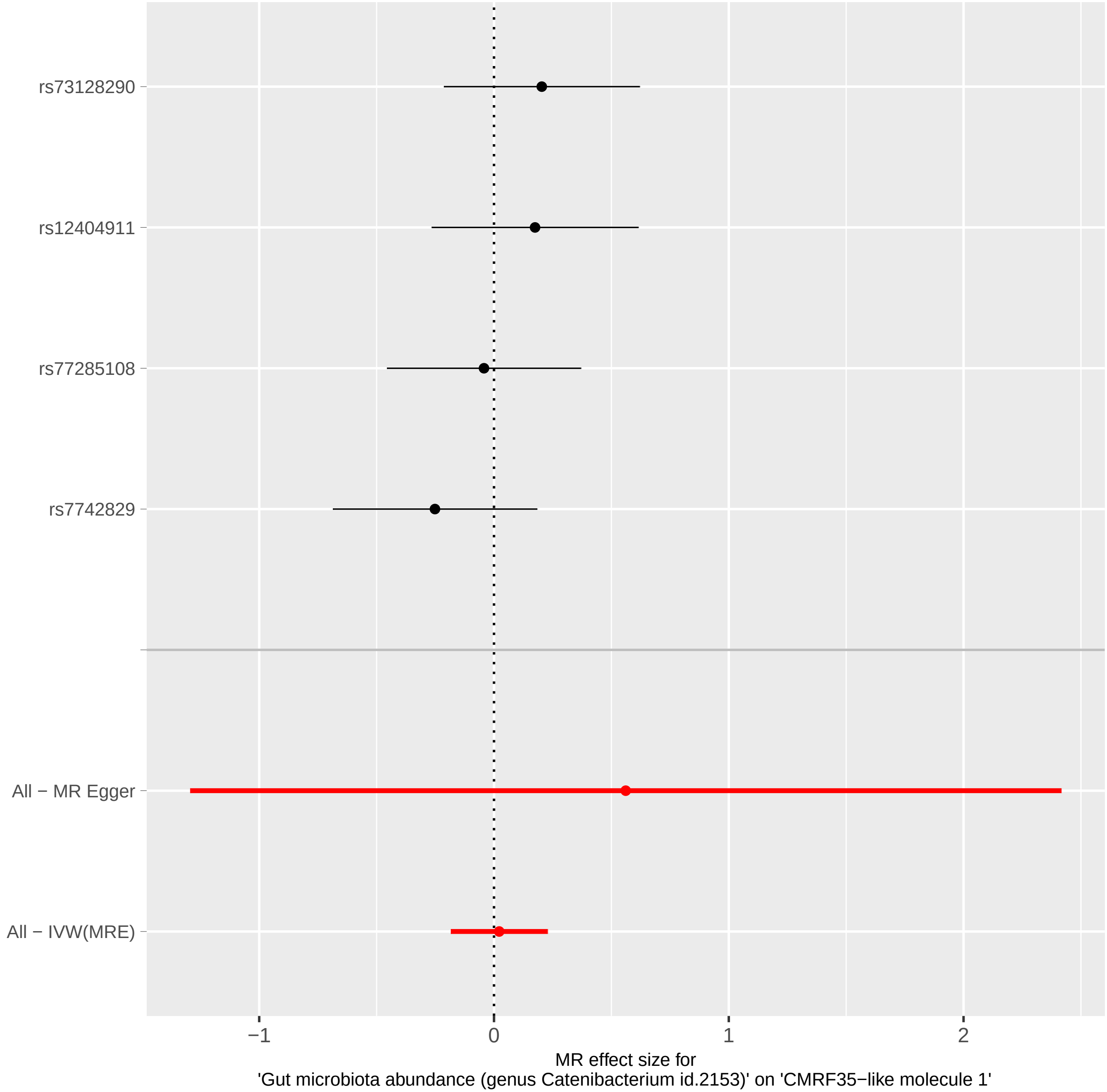

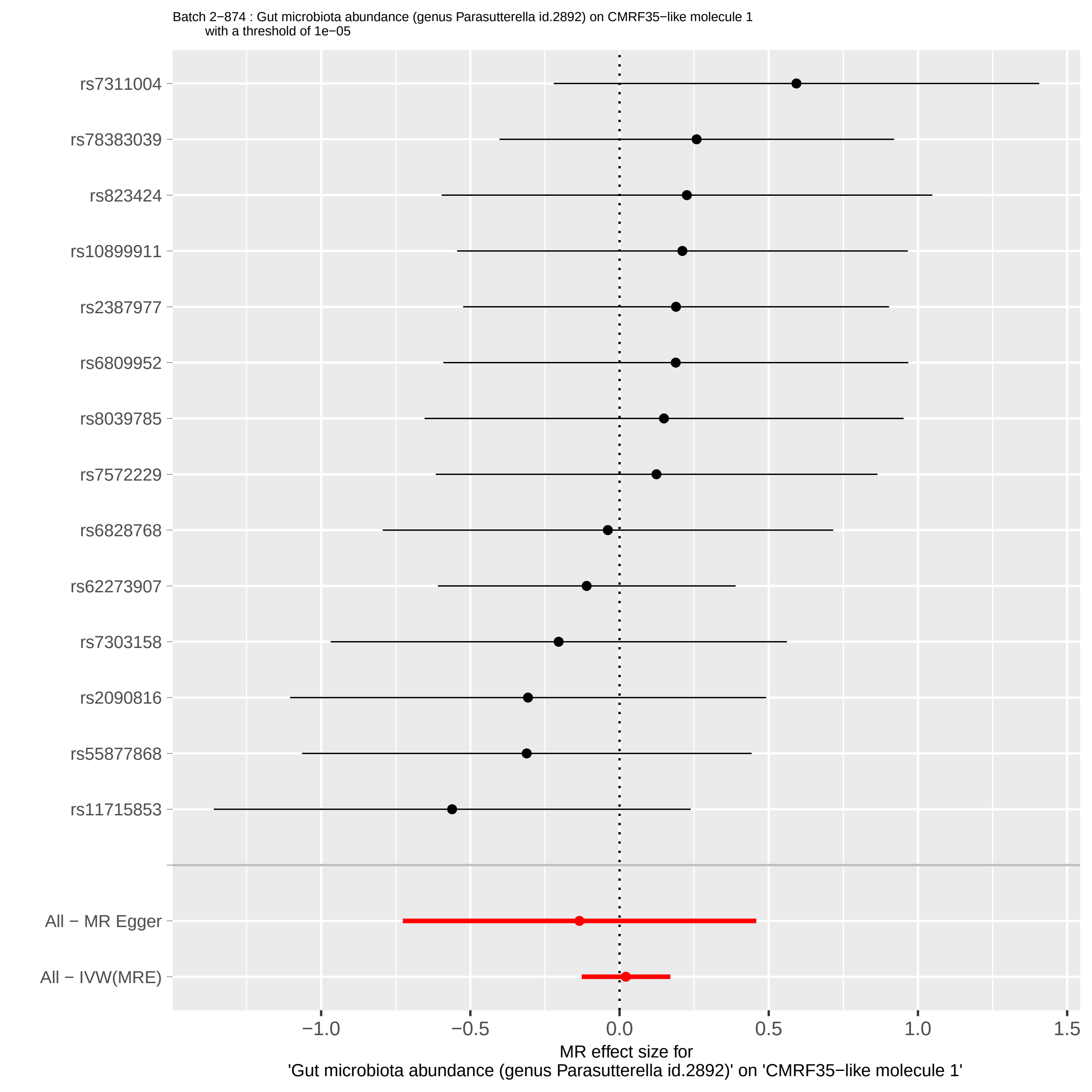

Batch 2−874 : Gut microbiota abundance (genus Parasutterella id.2892) on CMRF35−like molecule 1
with a threshold of 1e−05
rs7311004
rs78383039
rs823424
rs10899911
rs2387977
rs6809952
rs8039785
rs7572229
rs6828768
rs62273907
rs7303158
rs2090816
rs55877868
rs11715853
All − MR Egger
All − IVW(MRE)
−1.0
−0.5
0.0
0.5
1.0
1.5
MR effect size for
'Gut microbiota abundance (genus Parasutterella id.2892)' on 'CMRF35−like molecule 1'

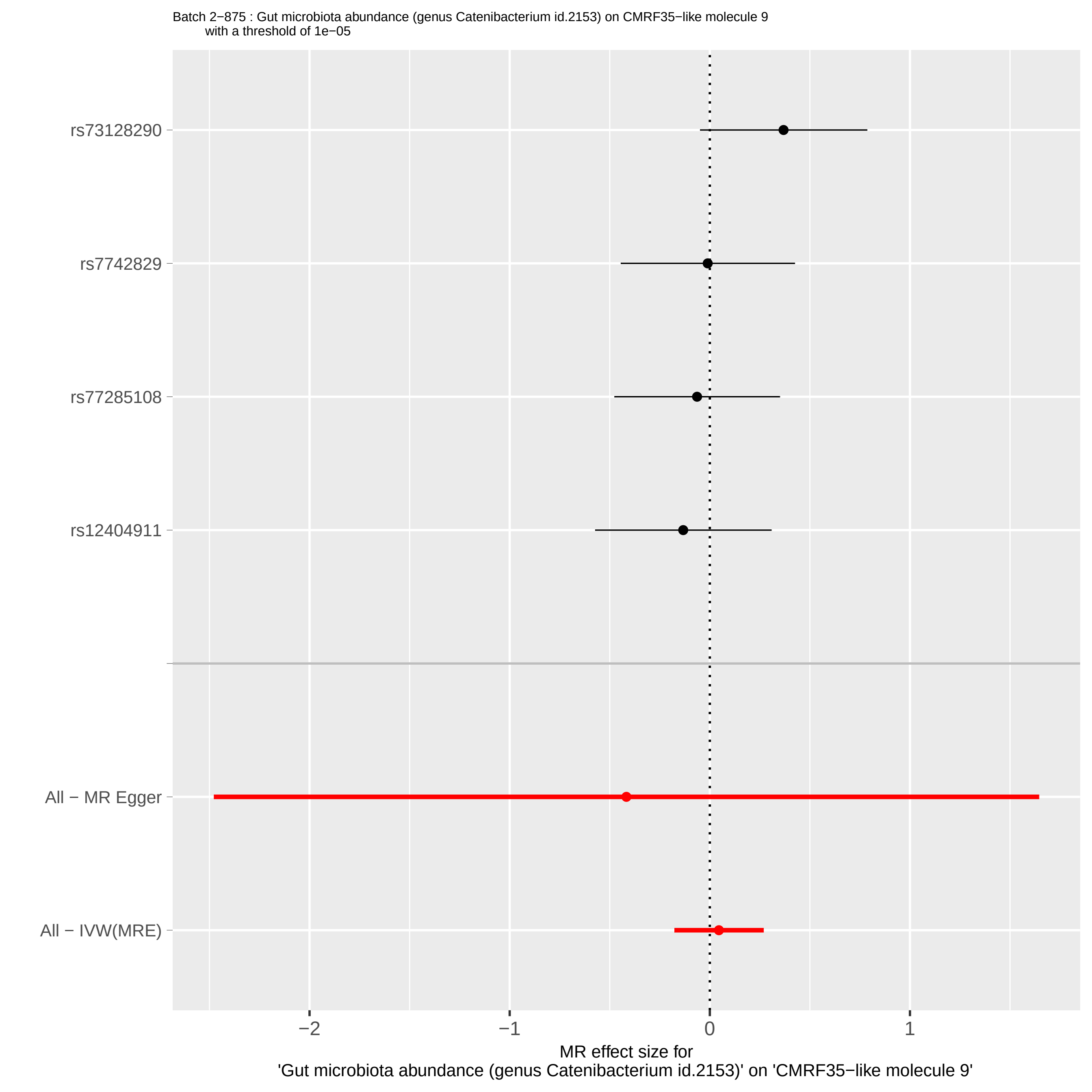
Batch 2−875 : Gut microbiota abundance (genus Catenibacterium id.2153) on CMRF35−like molecule 9
with a threshold of 1e−05
rs73128290
rs7742829
rs77285108
rs12404911
All − MR Egger
All − IVW(MRE)
−2
−1
0
1
MR effect size for
'Gut microbiota abundance (genus Catenibacterium id.2153)' on 'CMRF35−like molecule 9'

Batch 2−876 : Gut microbiota abundance (genus Parasutterella id.2892) on CMRF35−like molecule 9 with a threshold of 1e−05

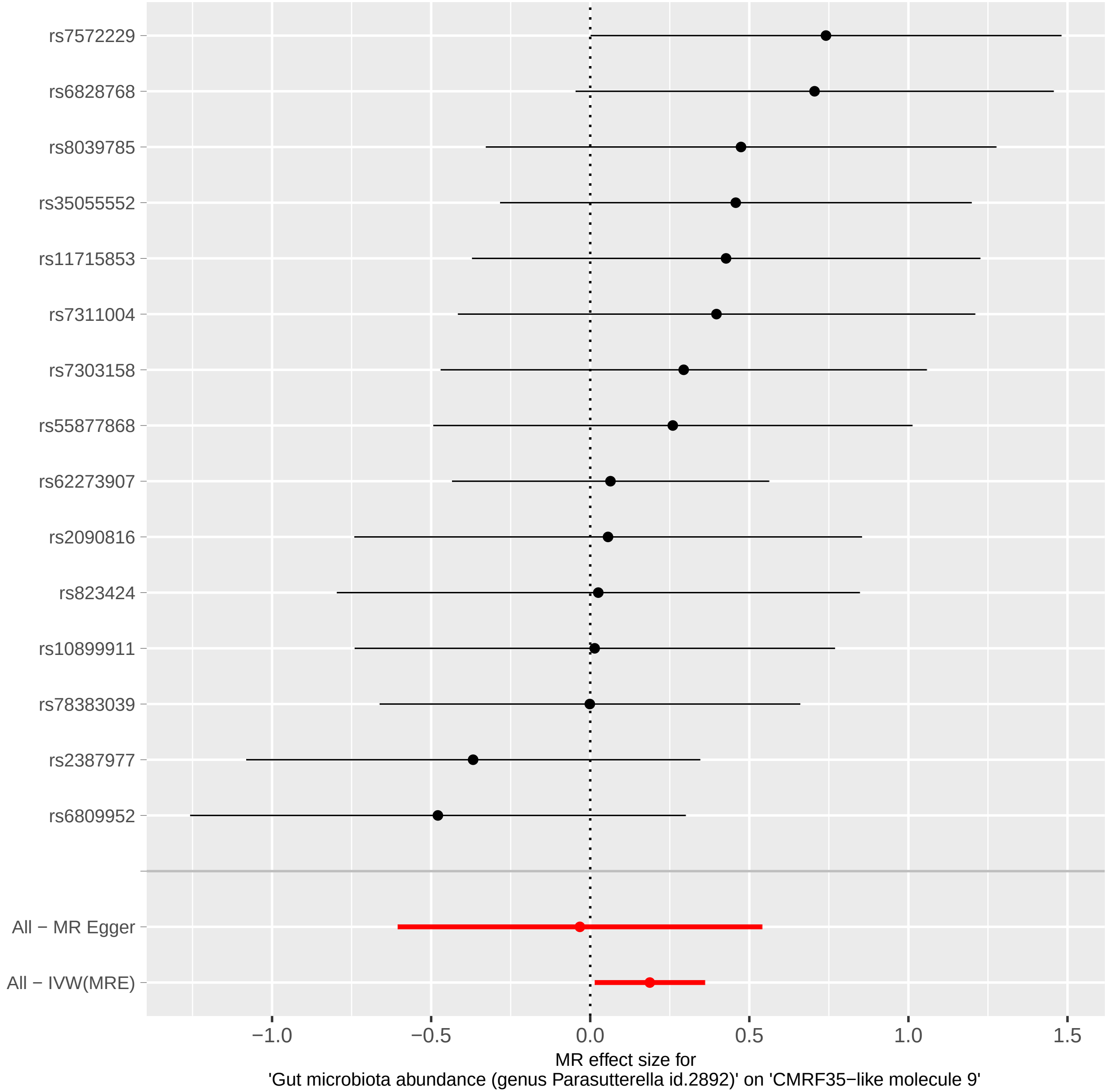

Batch 2−877 : Gut microbiota abundance (genus Catenibacterium id.2153) on Myeloid cell surface antigen CD33
with a threshold of 1e−05

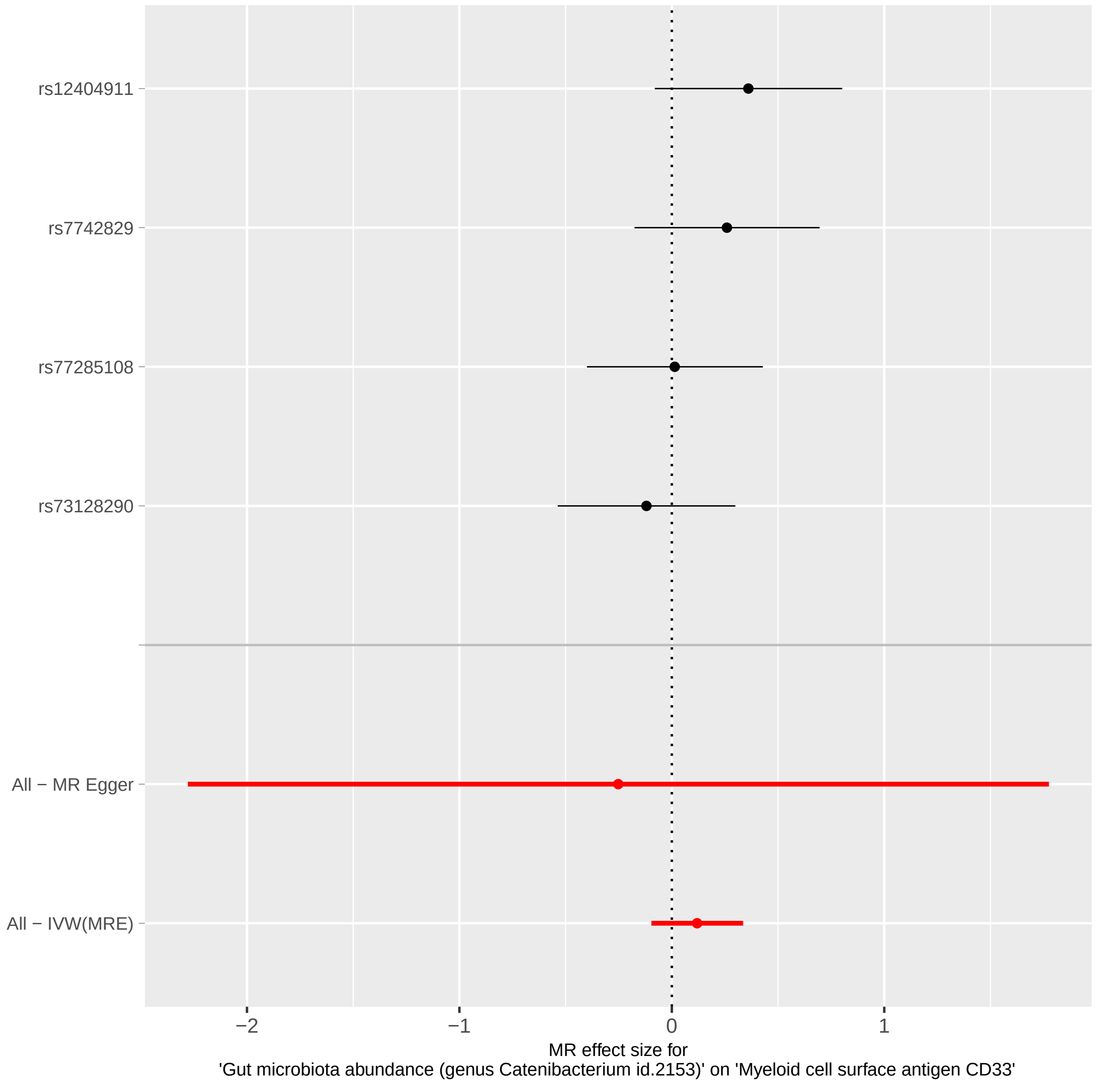

Batch 2−878 : Gut microbiota abundance (genus Parasutterella id.2892) on Myeloid cell surface antigen CD33
with a threshold of 1e−05

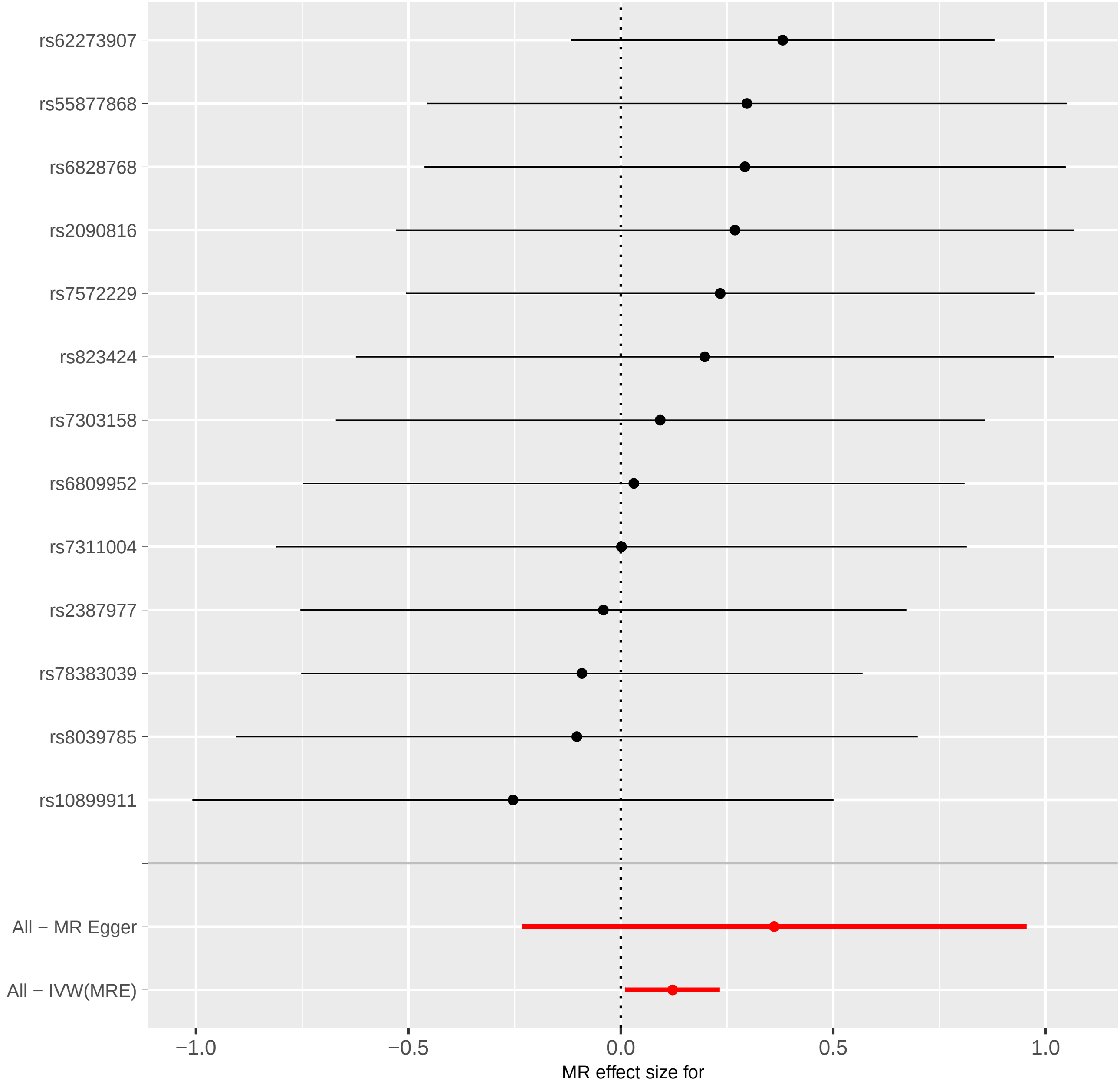

MR effect size for
'Gut microbiota abundance (genus Parasutterella id.2892)' on 'Myeloid cell surface antigen CD33'

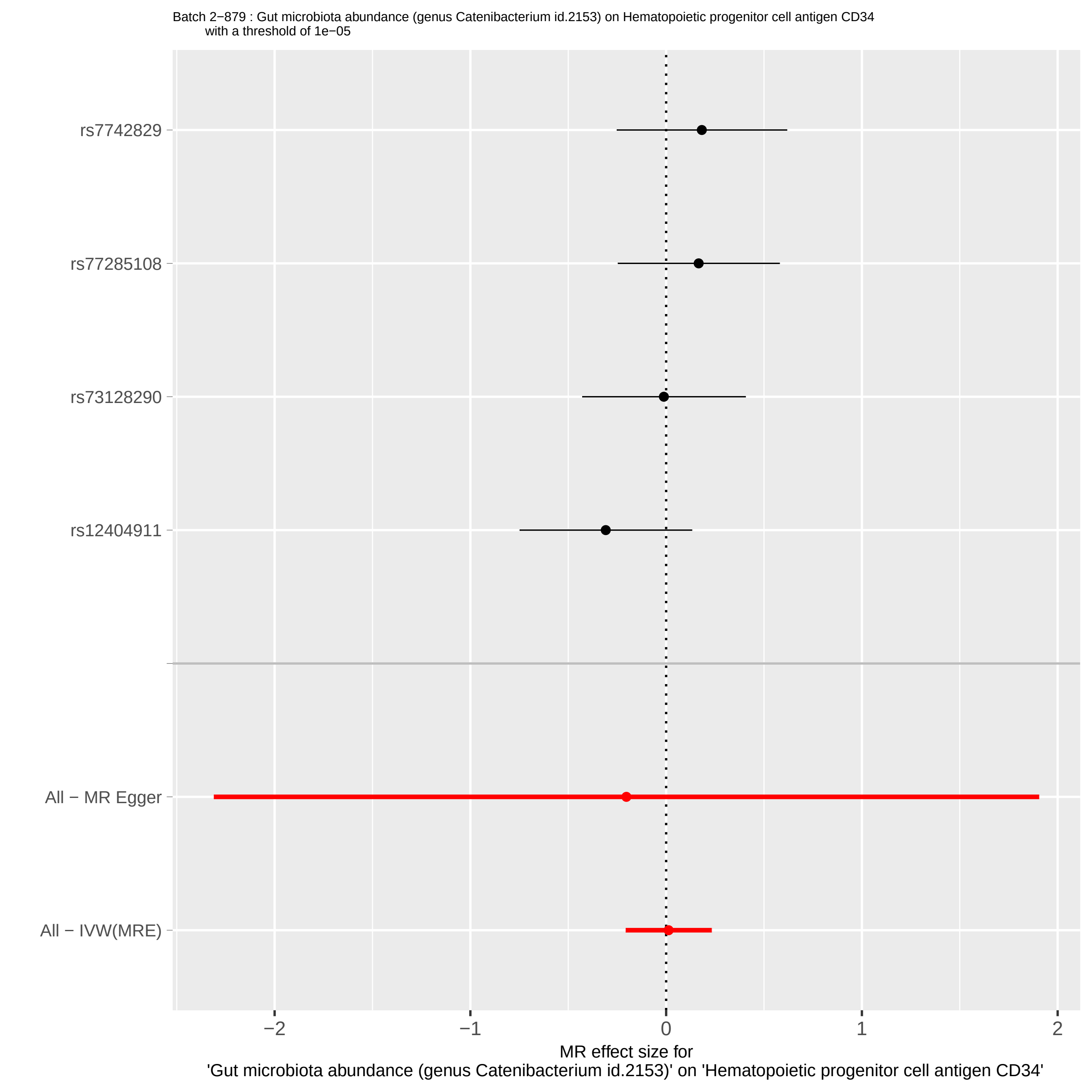

Batch 2−879 : Gut microbiota abundance (genus Catenibacterium id.2153) on Hematopoietic progenitor cell antigen CD34
with a threshold of 1e−05
rs7742829
rs77285108
rs73128290
rs12404911
All − MR Egger
All − IVW(MRE)
−2
−1
0
1
2
MR effect size for
'Gut microbiota abundance (genus Catenibacterium id.2153)' on 'Hematopoietic progenitor cell antigen CD34'

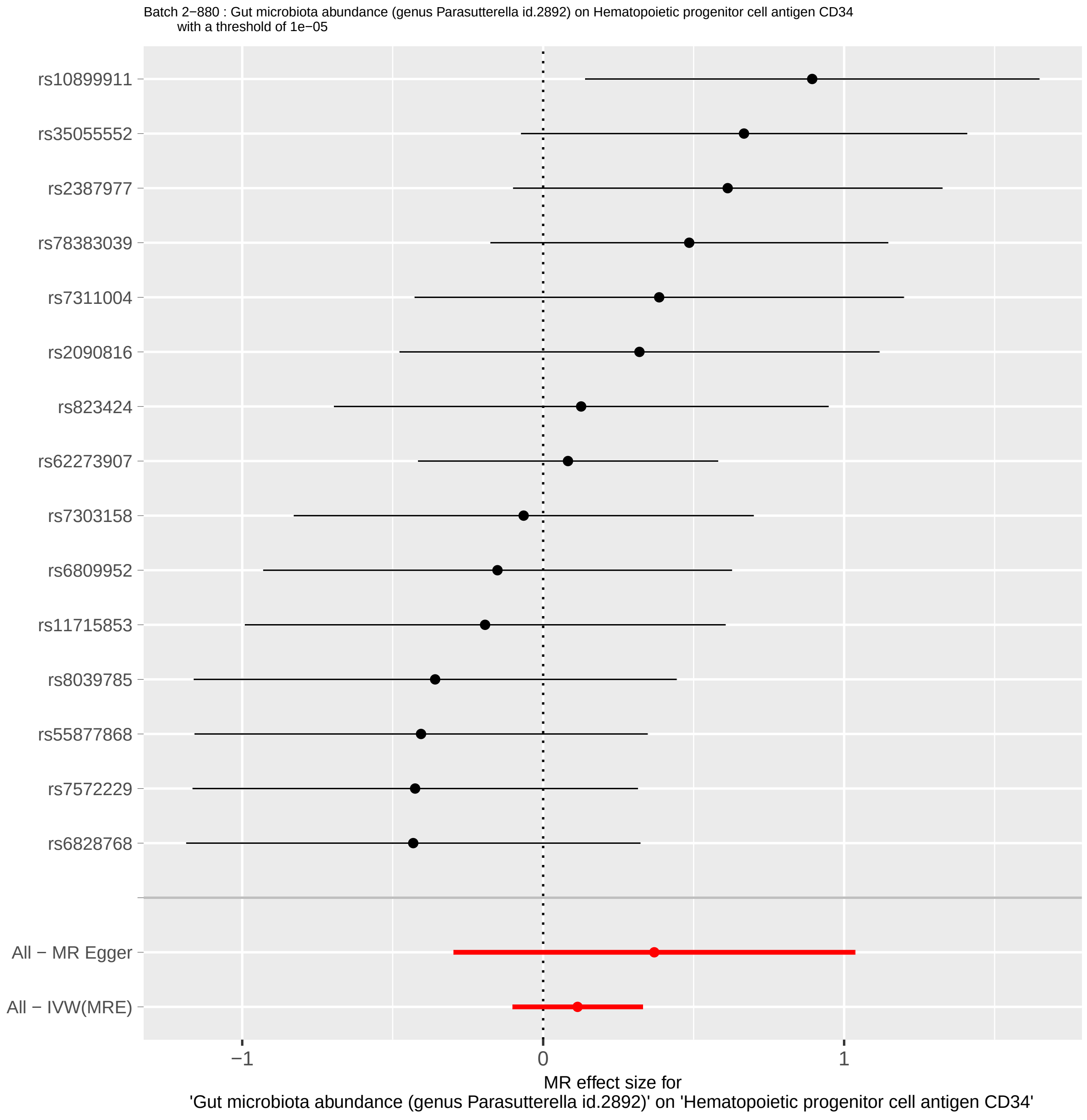

Batch 2−880 : Gut microbiota abundance (genus Parasutterella id.2892) on Hematopoietic progenitor cell antigen CD34
with a threshold of 1e−05
rs10899911
rs35055552
rs2387977
rs78383039
rs7311004
rs2090816
rs823424
rs62273907
rs7303158
rs6809952
rs11715853
rs8039785
rs55877868
rs7572229
rs6828768
All − MR Egger
All − IVW(MRE)
−1
0
1
MR effect size for
'Gut microbiota abundance (genus Parasutterella id.2892)' on 'Hematopoietic progenitor cell antigen CD34'

Batch 2−881 : Gut microbiota abundance (genus Catenibacterium id.2153) on Hematopoietic progenitor cell antigen CD34
with a threshold of 1e−05

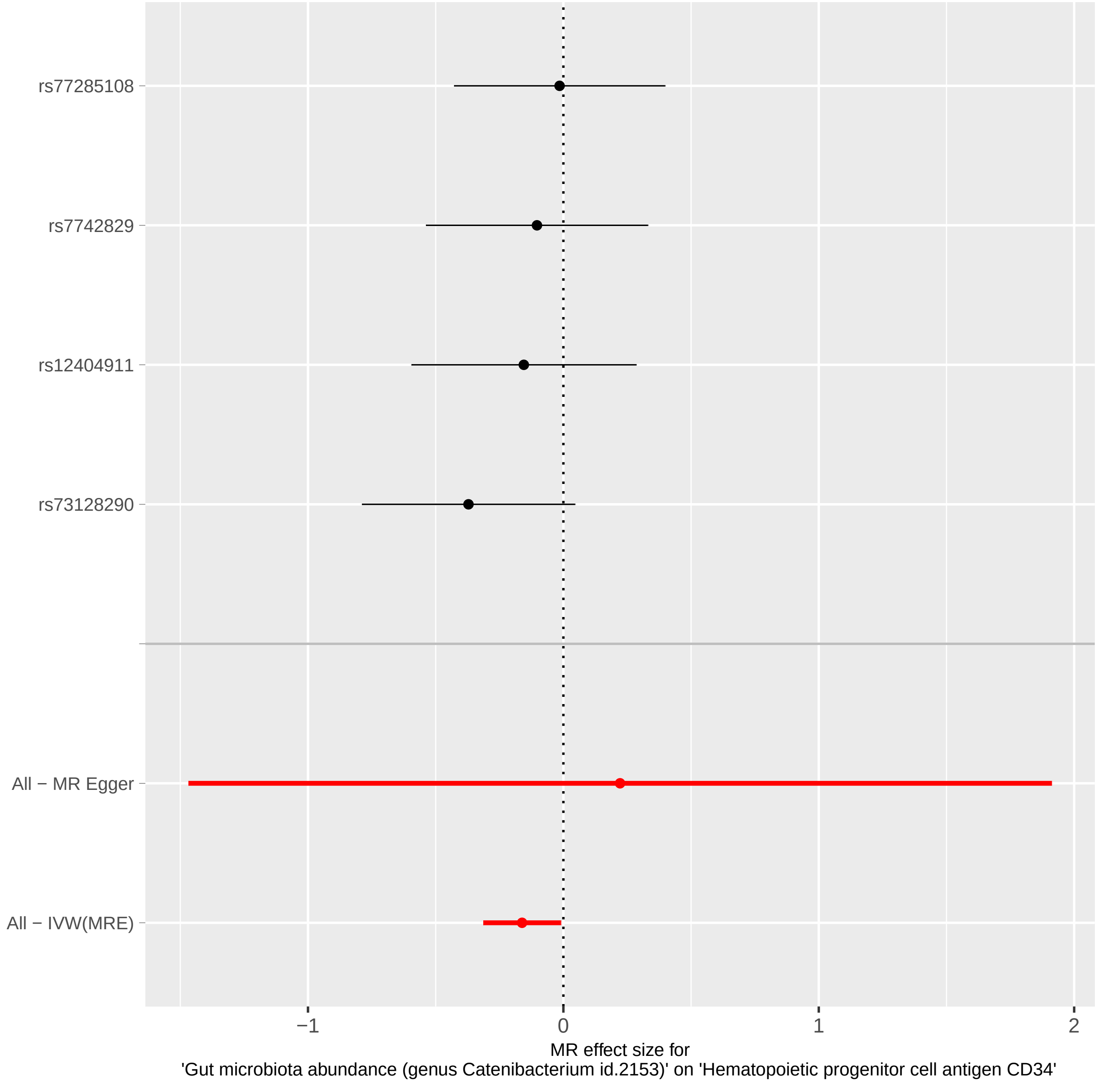

Batch 2−882 : Gut microbiota abundance (genus Parasutterella id.2892) on Hematopoietic progenitor cell antigen CD34
with a threshold of 1e−05

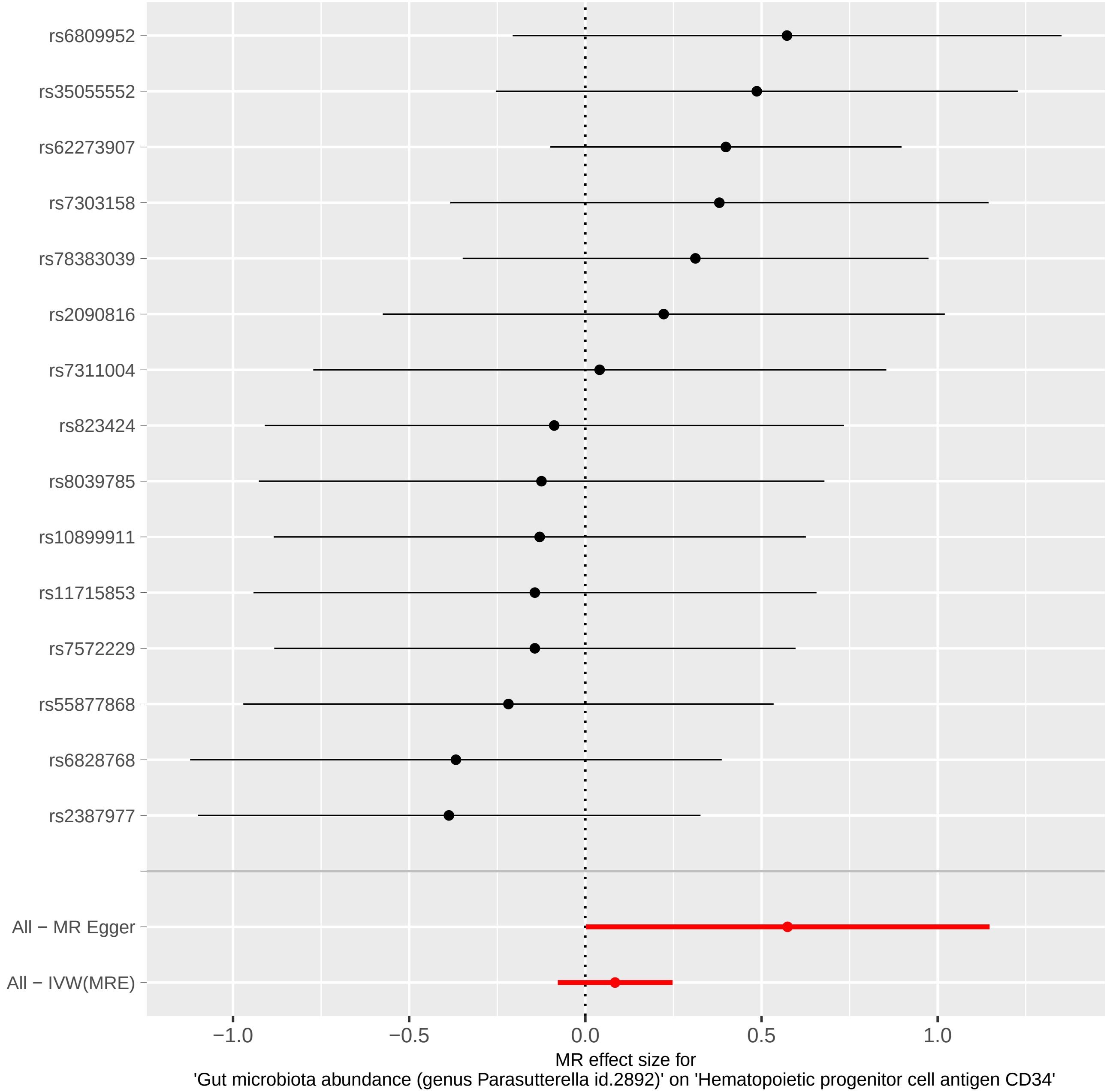

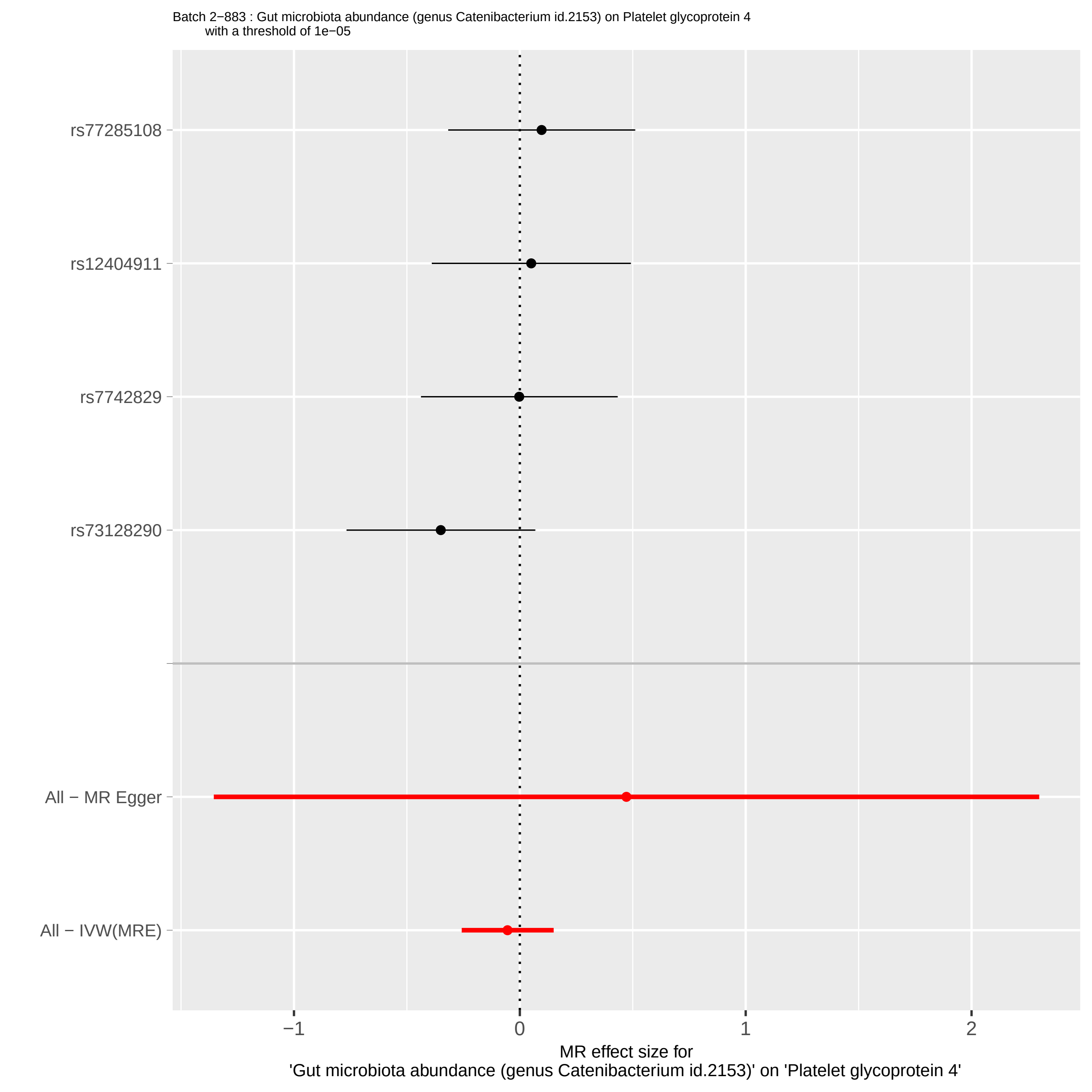

Batch 2−883 : Gut microbiota abundance (genus Catenibacterium id.2153) on Platelet glycoprotein 4
with a threshold of 1e−05
rs77285108
rs12404911
rs7742829
rs73128290
All − MR Egger
All − IVW(MRE)
−1
0
1
2
MR effect size for
'Gut microbiota abundance (genus Catenibacterium id.2153)' on 'Platelet glycoprotein 4'

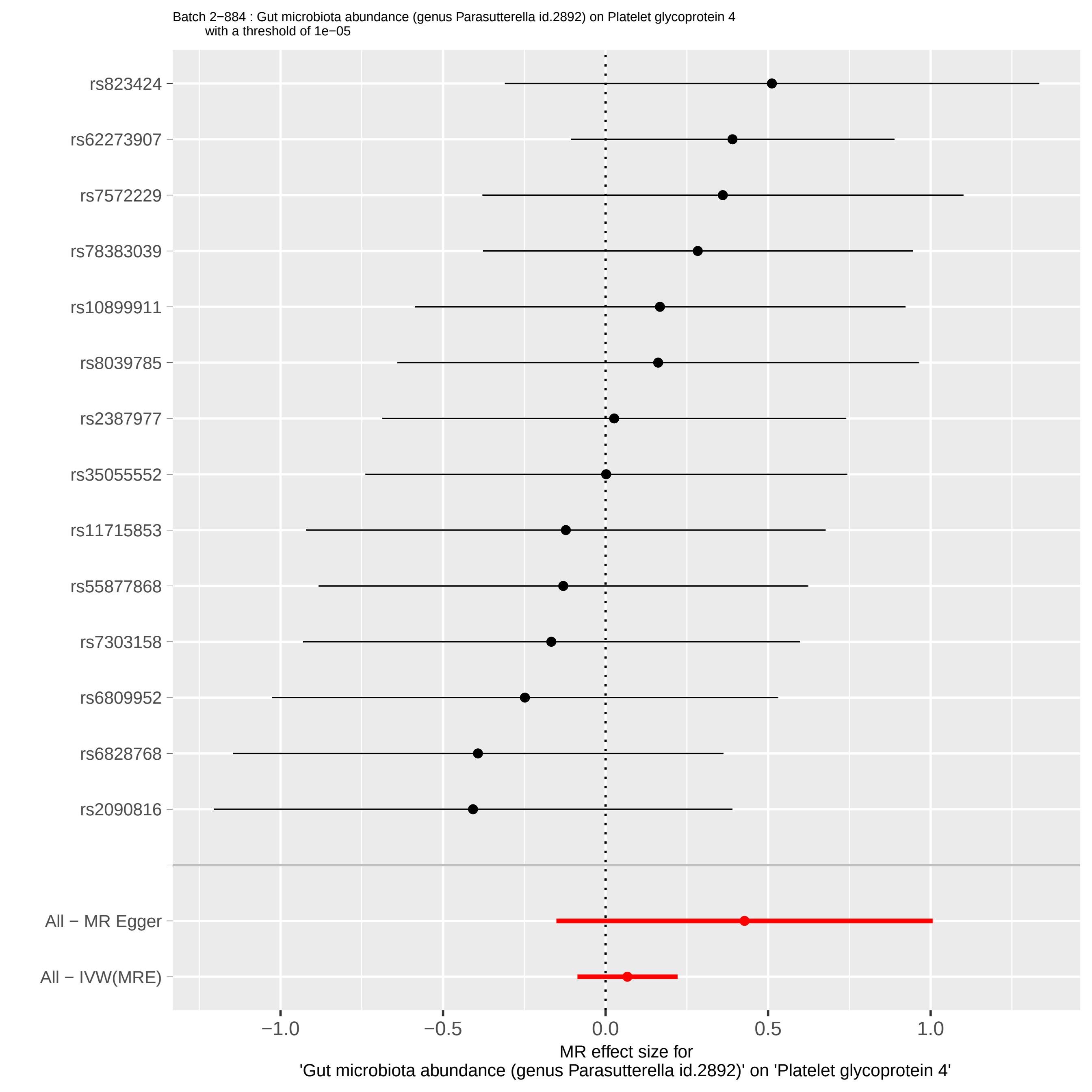
Batch 2−884 : Gut microbiota abundance (genus Parasutterella id.2892) on Platelet glycoprotein 4
with a threshold of 1e−05
rs823424
rs62273907
rs7572229
rs78383039
rs10899911
rs8039785
rs2387977
rs35055552
rs11715853
rs55877868
rs7303158
rs6809952
rs6828768
rs2090816
All − MR Egger
All − IVW(MRE)
−1.0
−0.5
0.0
0.5
1.0
MR effect size for
'Gut microbiota abundance (genus Parasutterella id.2892)' on 'Platelet glycoprotein 4'

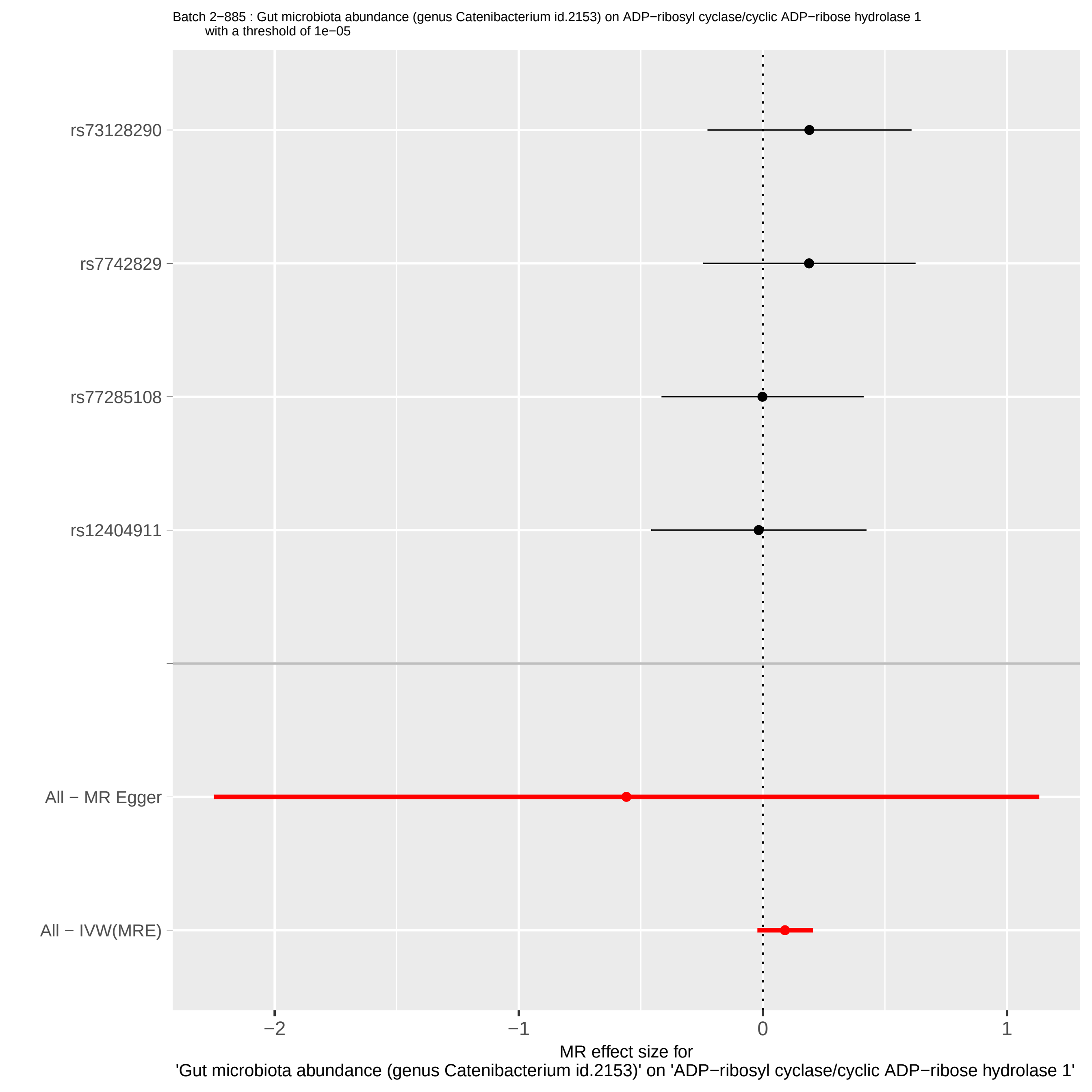
Batch 2−885 : Gut microbiota abundance (genus Catenibacterium id.2153) on ADP−ribosyl cyclase/cyclic ADP−ribose hydrolase 1
with a threshold of 1e−05
rs73128290
rs7742829
rs77285108
rs12404911
All − MR Egger
All − IVW(MRE)
−2
−1
0
1
MR effect size for
'Gut microbiota abundance (genus Catenibacterium id.2153)' on 'ADP−ribosyl cyclase/cyclic ADP−ribose hydrolase 1'

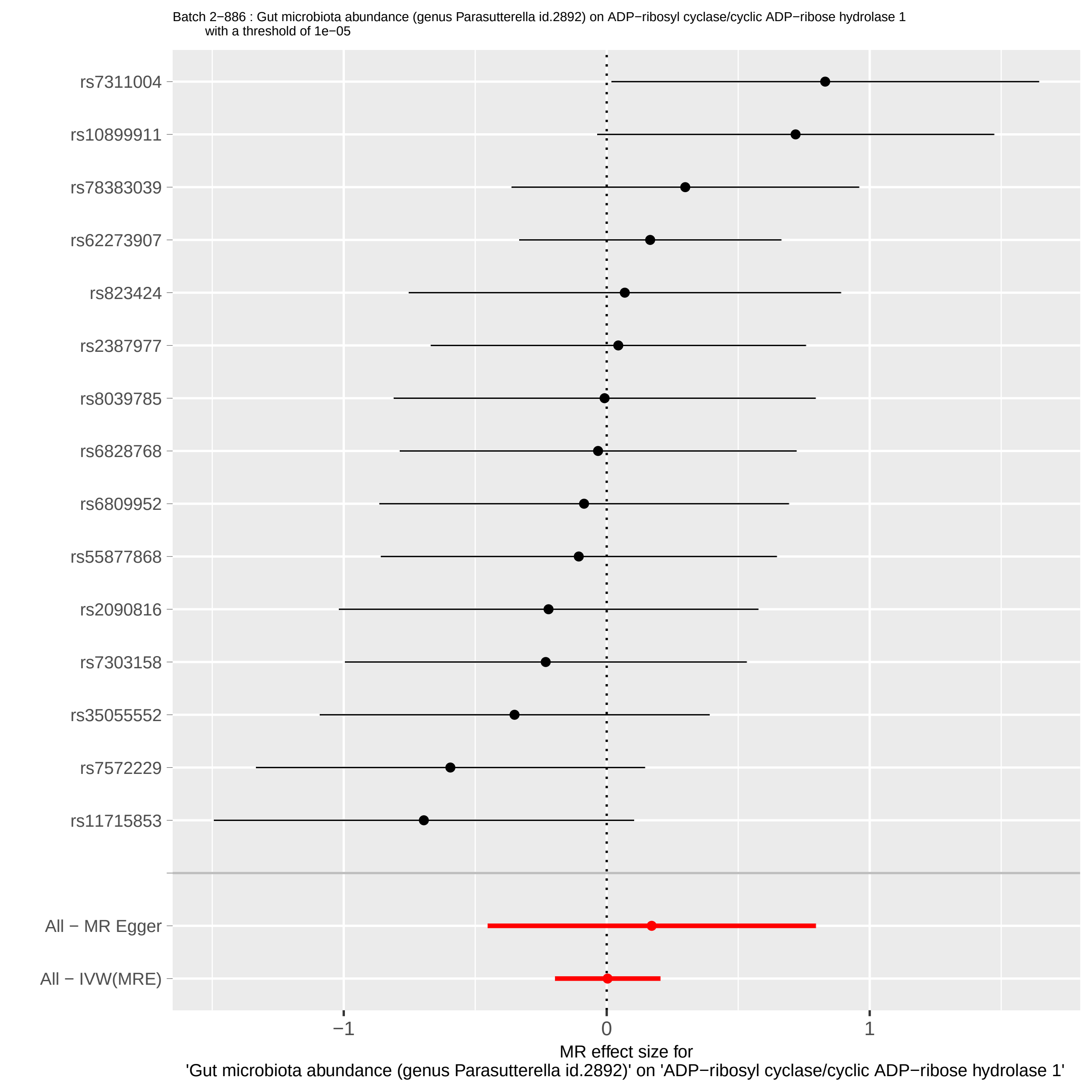

Batch 2−886 : Gut microbiota abundance (genus Parasutterella id.2892) on ADP−ribosyl cyclase/cyclic ADP−ribose hydrolase 1
with a threshold of 1e−05
rs7311004
rs10899911
rs78383039
rs62273907
rs823424
rs2387977
rs8039785
rs6828768
rs6809952
rs55877868
rs2090816
rs7303158
rs35055552
rs7572229
rs11715853
All − MR Egger
All − IVW(MRE)
−1
0
1
MR effect size for
'Gut microbiota abundance (genus Parasutterella id.2892)' on 'ADP−ribosyl cyclase/cyclic ADP−ribose hydrolase 1'

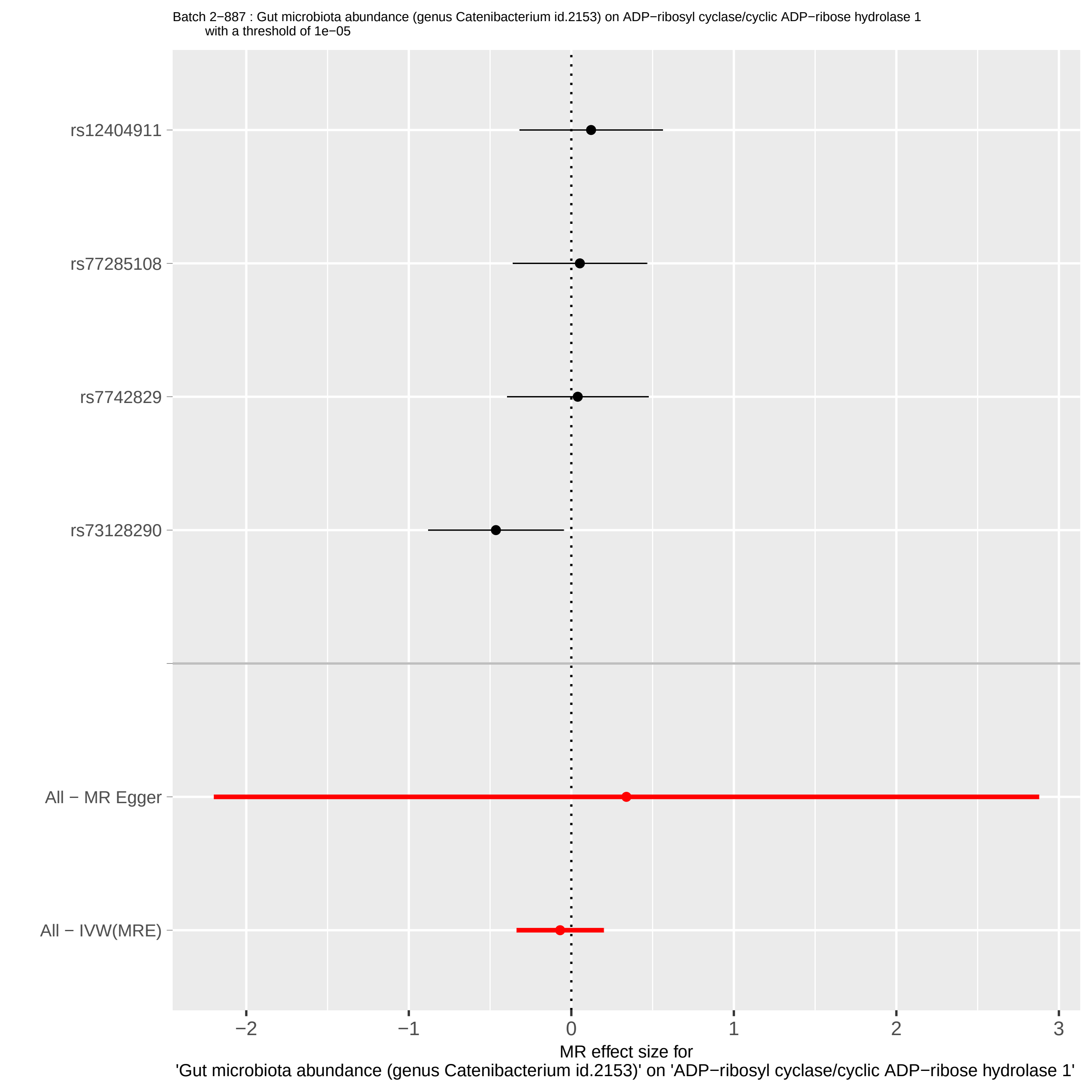

Batch 2−887 : Gut microbiota abundance (genus Catenibacterium id.2153) on ADP−ribosyl cyclase/cyclic ADP−ribose hydrolase 1
with a threshold of 1e−05
rs12404911
rs77285108
rs7742829
rs73128290
All − MR Egger
All − IVW(MRE)
−2
−1
0
1
2
3
MR effect size for
'Gut microbiota abundance (genus Catenibacterium id.2153)' on 'ADP−ribosyl cyclase/cyclic ADP−ribose hydrolase 1'

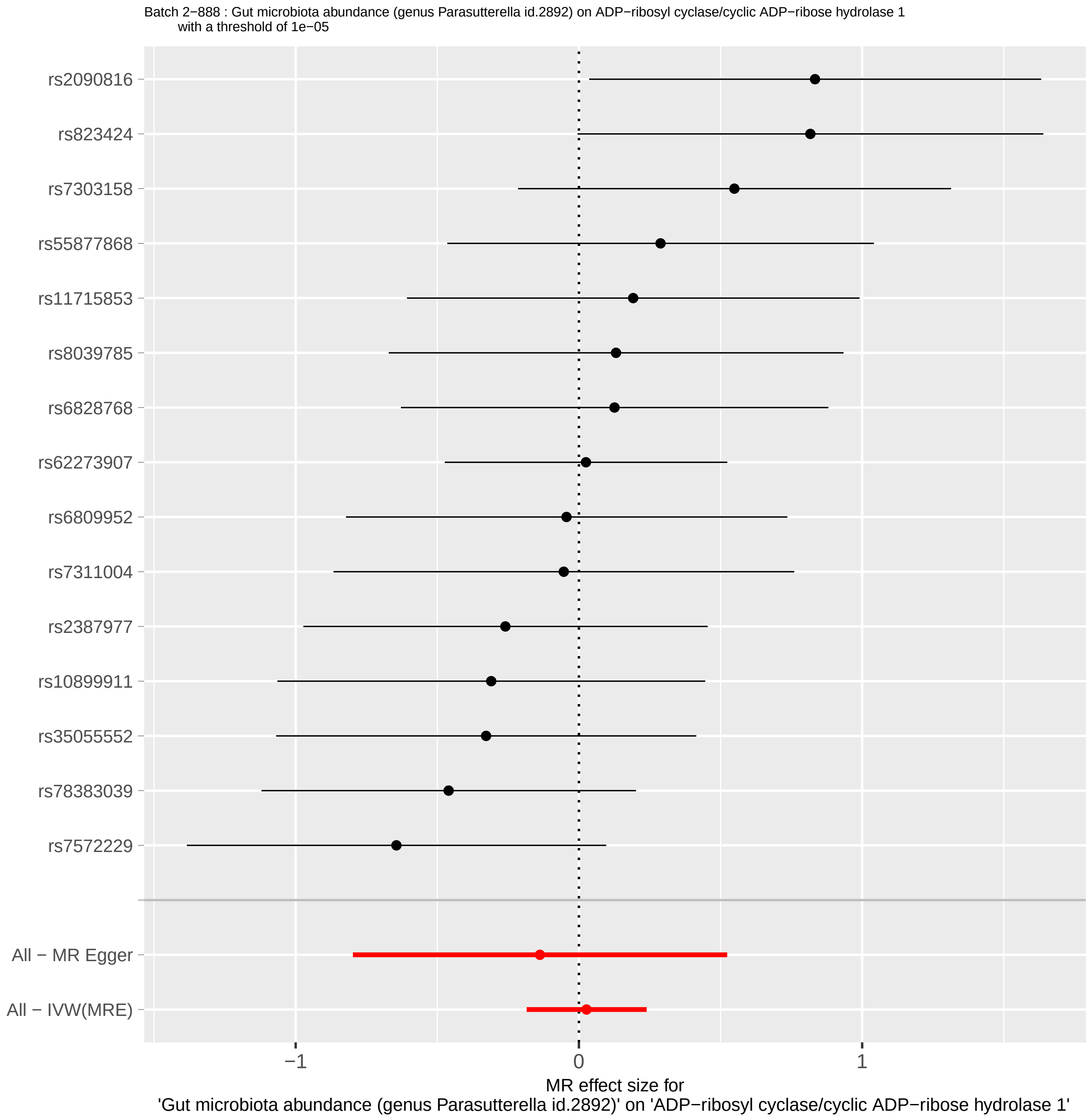

Batch 2−888 : Gut microbiota abundance (genus Parasutterella id.2892) on ADP−ribosyl cyclase/cyclic ADP−ribose hydrolase 1
with a threshold of 1e−05
rs2090816
rs823424
rs7303158
rs55877868
rs11715853
rs8039785
rs6828768
rs62273907
rs6809952
rs7311004
rs2387977
rs10899911
rs35055552
rs78383039
rs7572229
All − MR Egger
All − IVW(MRE)
−1
0
1
MR effect size for
'Gut microbiota abundance (genus Parasutterella id.2892)' on 'ADP−ribosyl cyclase/cyclic ADP−ribose hydrolase 1'

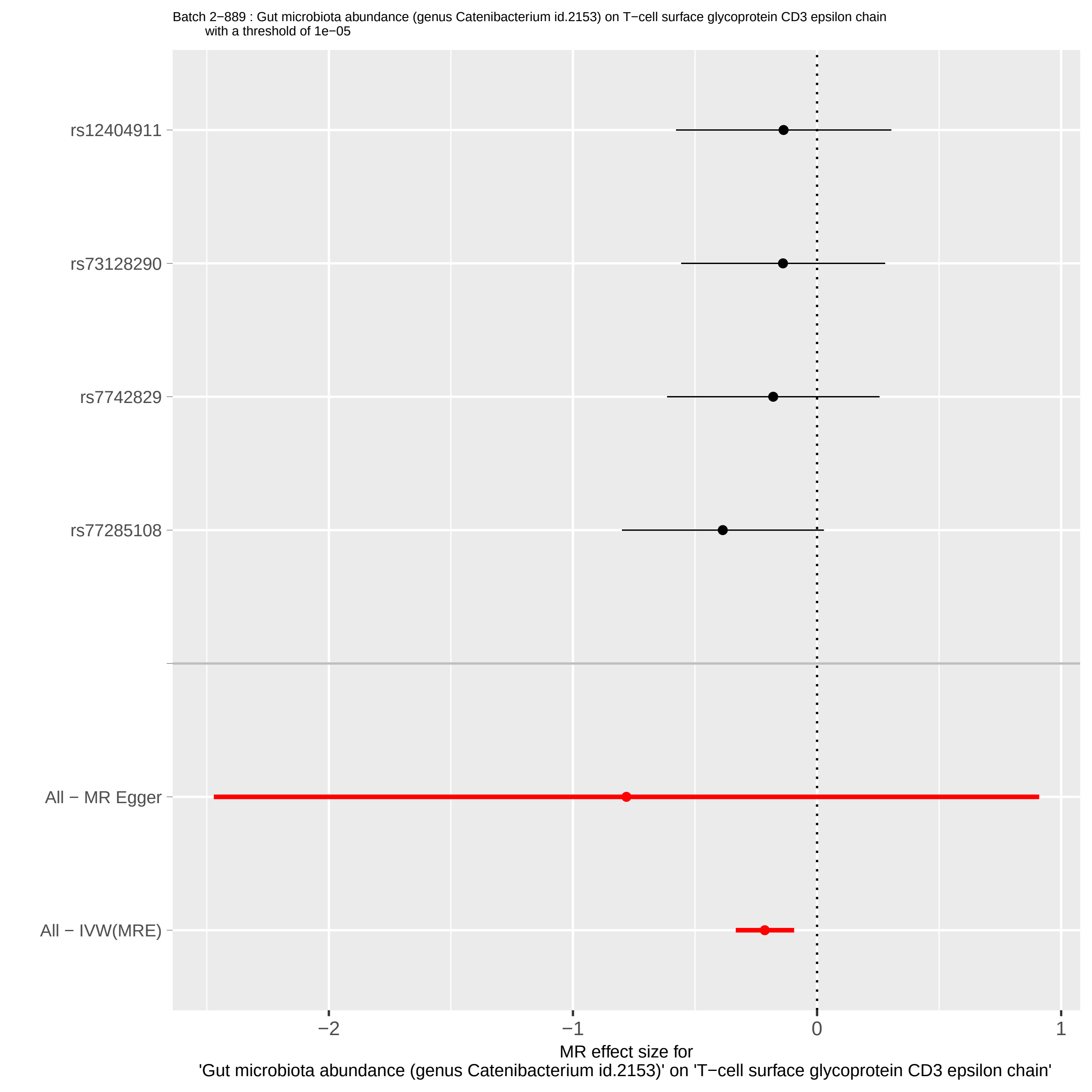
Batch 2−889 : Gut microbiota abundance (genus Catenibacterium id.2153) on T−cell surface glycoprotein CD3 epsilon chain
with a threshold of 1e−05
rs12404911
rs73128290
rs7742829
rs77285108
All − MR Egger
All − IVW(MRE)
−2
−1
0
1
MR effect size for
'Gut microbiota abundance (genus Catenibacterium id.2153)' on 'T−cell surface glycoprotein CD3 epsilon chain'

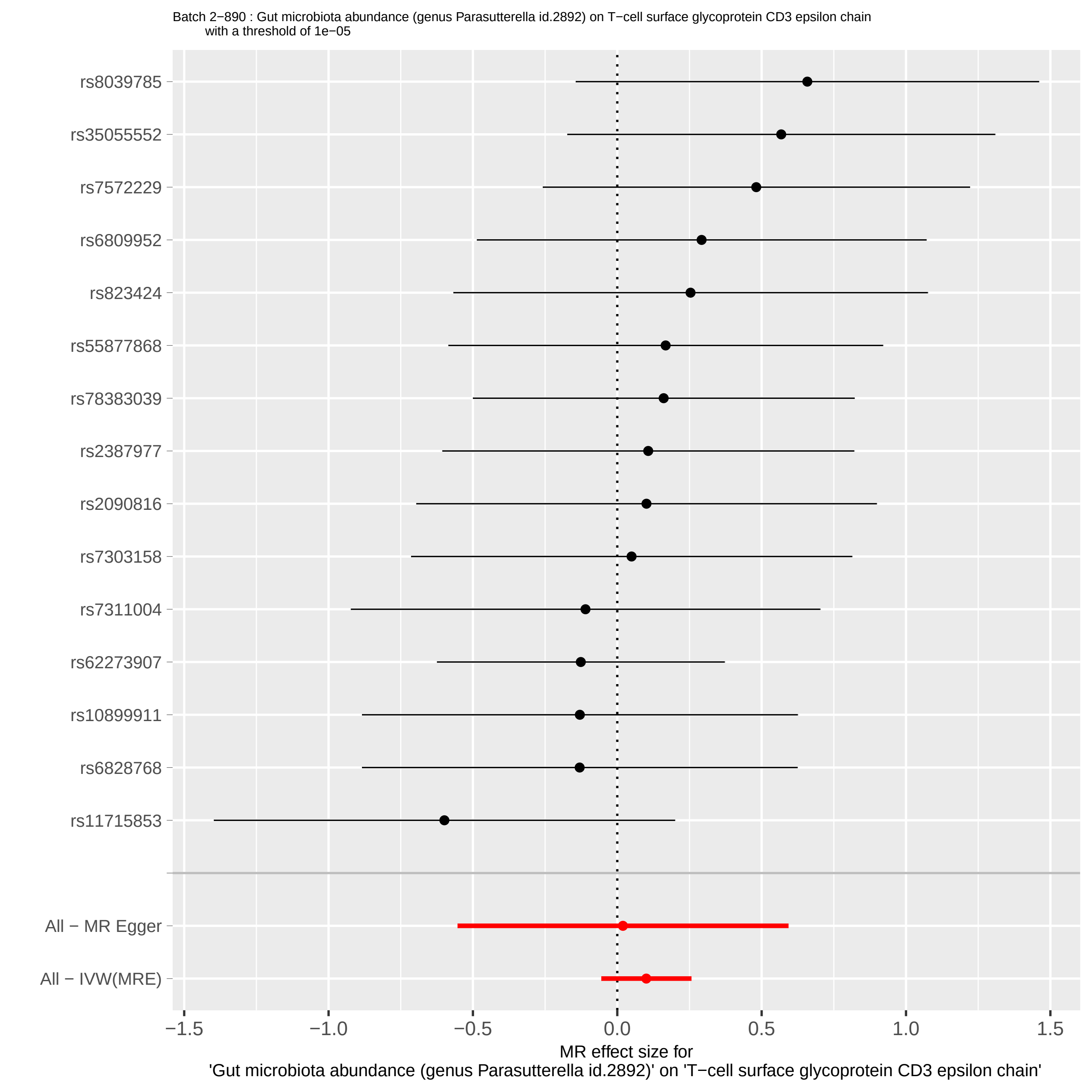
Batch 2−890 : Gut microbiota abundance (genus Parasutterella id.2892) on T−cell surface glycoprotein CD3 epsilon chain
with a threshold of 1e−05
rs8039785
rs35055552
rs7572229
rs6809952
rs823424
rs55877868
rs78383039
rs2387977
rs2090816
rs7303158
rs7311004
rs62273907
rs10899911
rs6828768
rs11715853
All − MR Egger
All − IVW(MRE)
−1.5
−1.0
−0.5
0.0
0.5
1.0
1.5
MR effect size for
'Gut microbiota abundance (genus Parasutterella id.2892)' on 'T−cell surface glycoprotein CD3 epsilon chain'

Batch 2−891 : Gut microbiota abundance (genus Catenibacterium id.2153) on T−cell surface glycoprotein CD3 gamma chain with a threshold of 1e−05

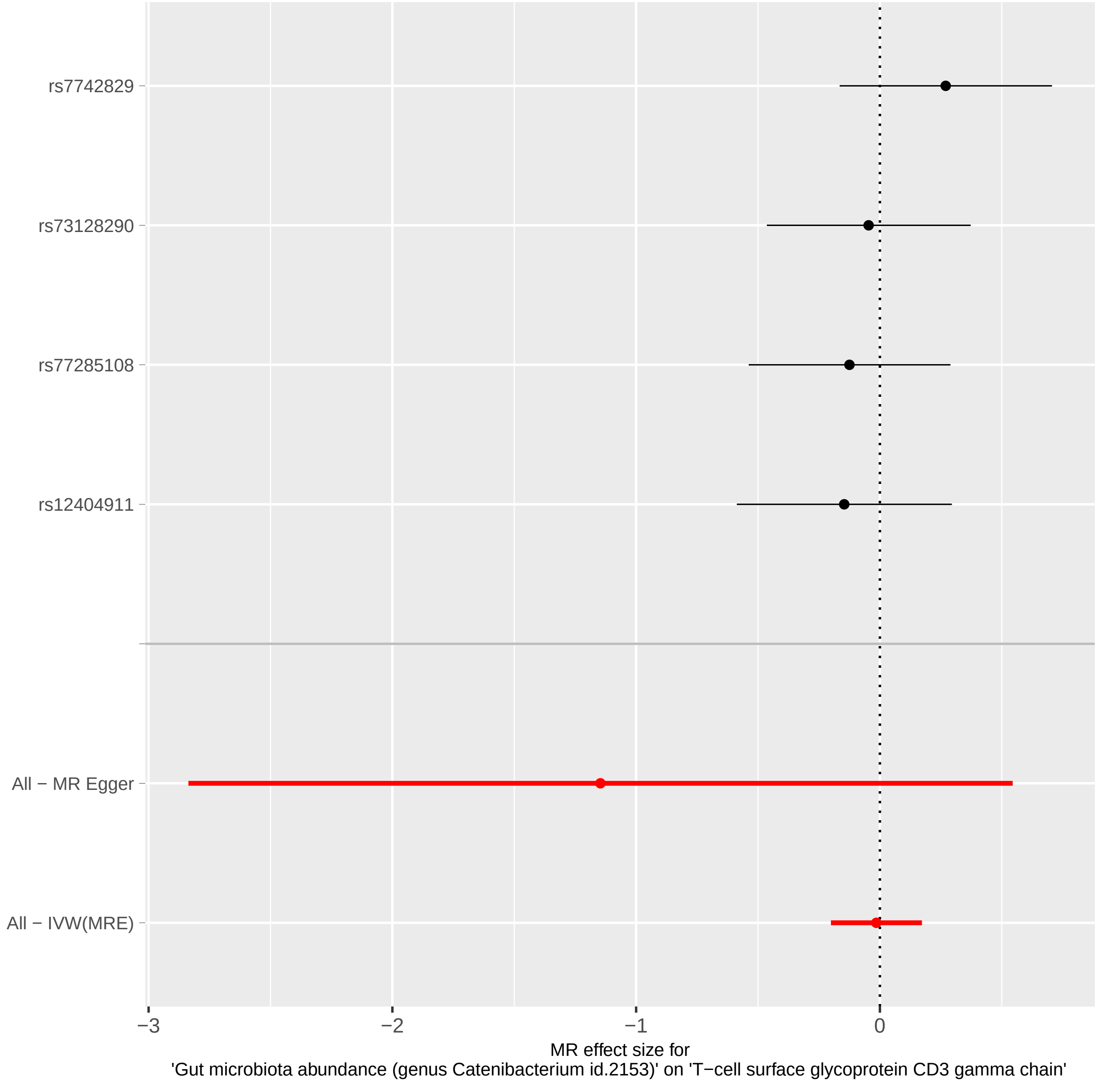

Batch 2−892 : Gut microbiota abundance (genus Parasutterella id.2892) on T−cell surface glycoprotein CD3 gamma chain
with a threshold of 1e−05

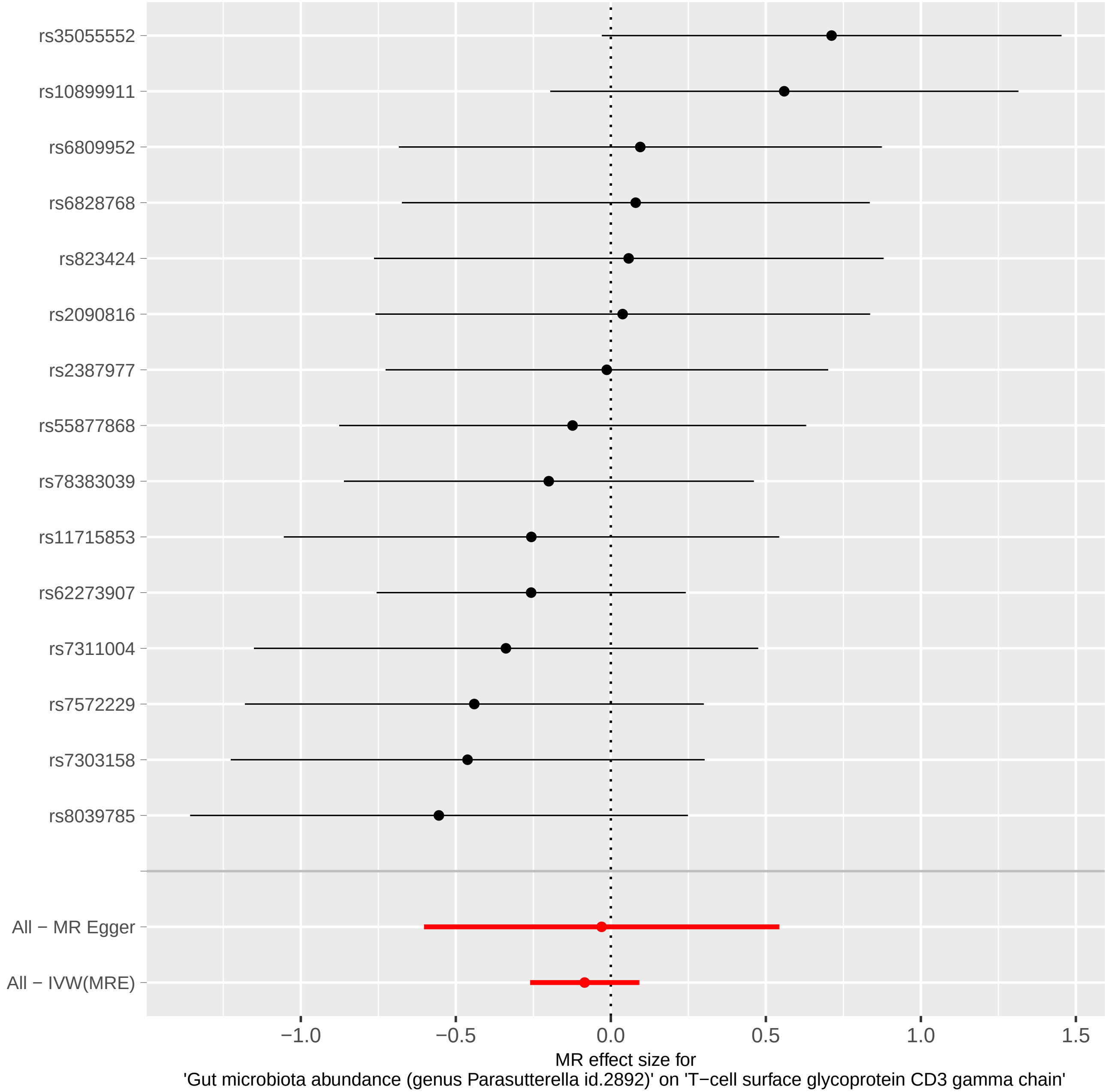

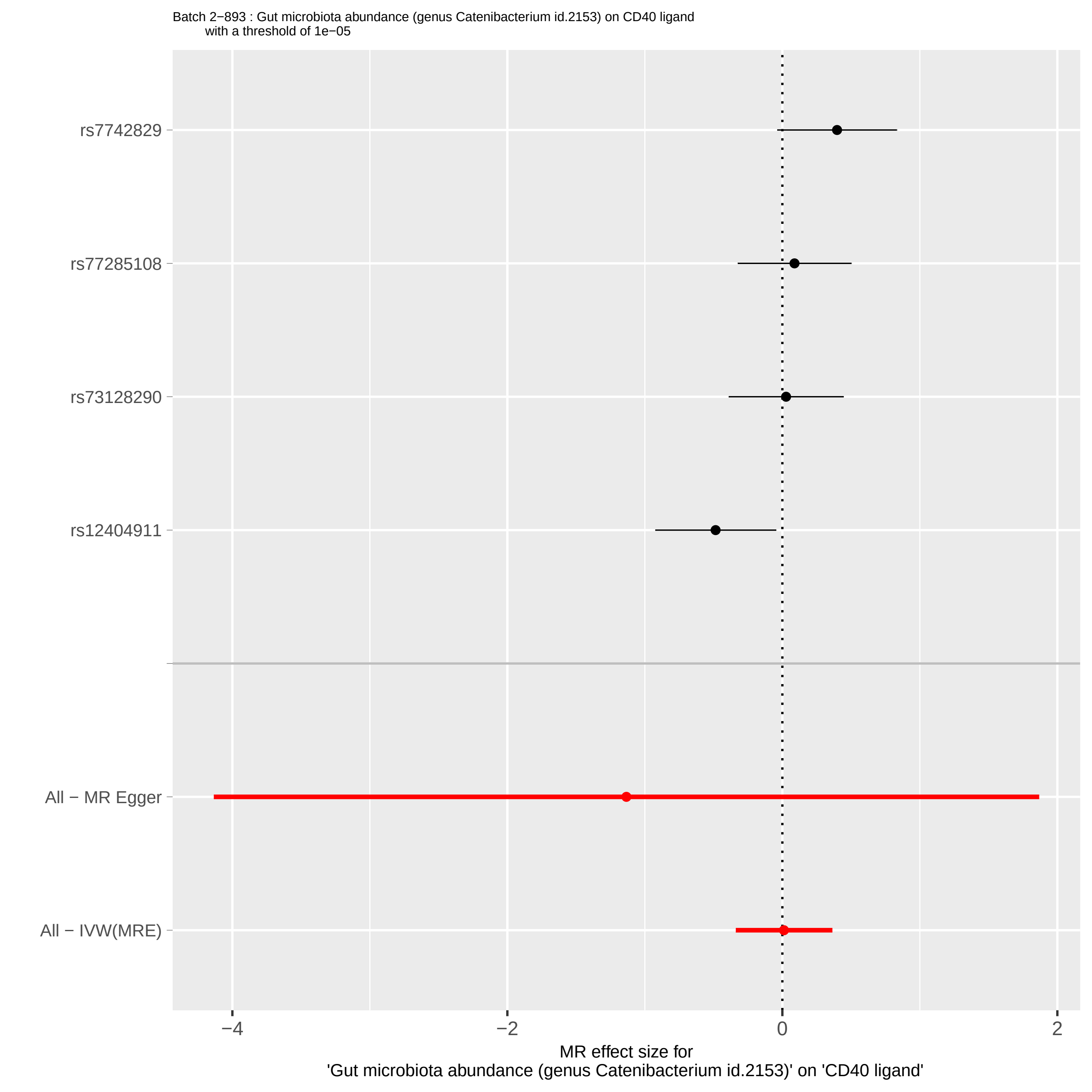
Batch 2−893 : Gut microbiota abundance (genus Catenibacterium id.2153) on CD40 ligand
with a threshold of 1e−05
rs7742829
rs77285108
rs73128290
rs12404911
All − MR Egger
All − IVW(MRE)
−4
−2
0
2
MR effect size for
'Gut microbiota abundance (genus Catenibacterium id.2153)' on 'CD40 ligand'

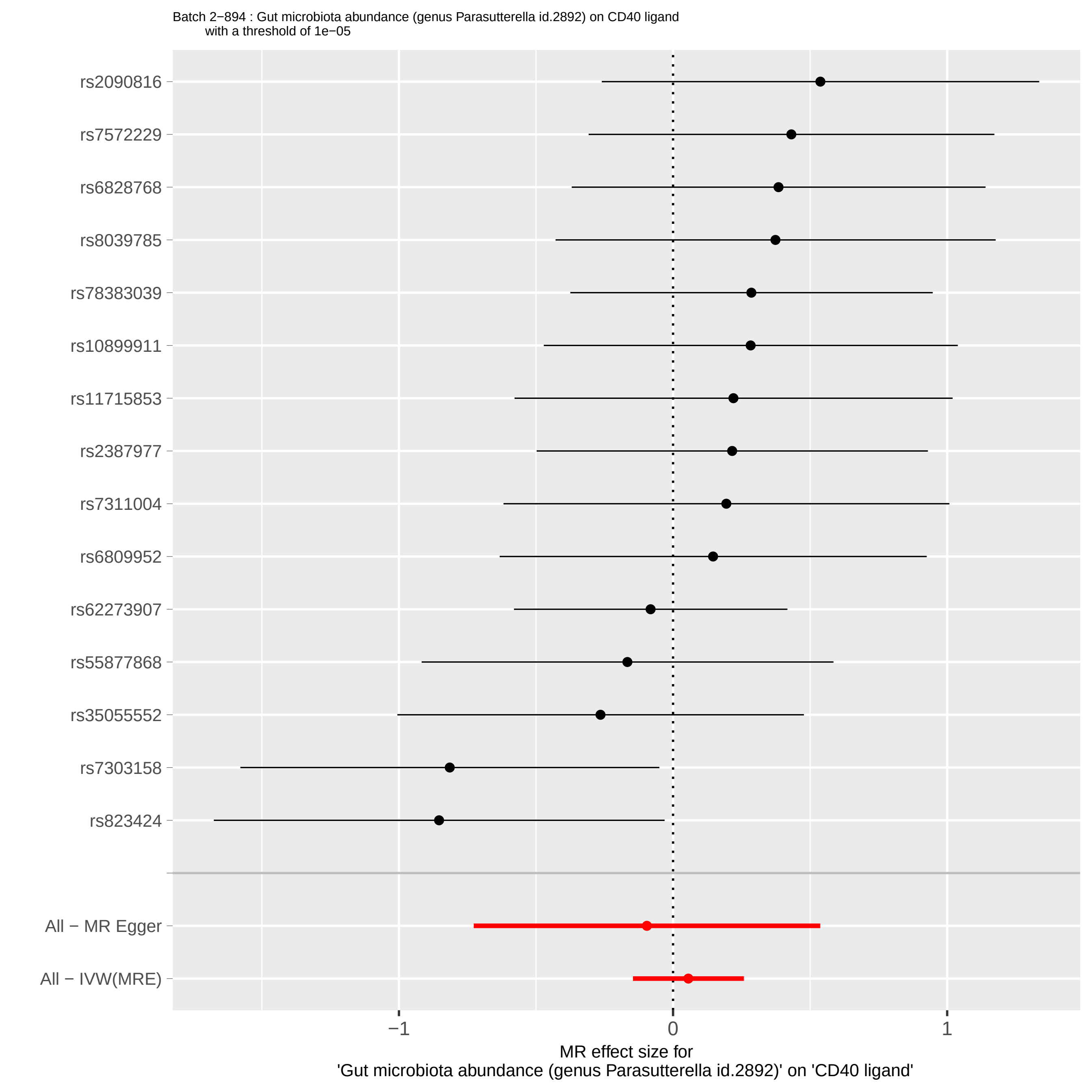
Batch 2−894 : Gut microbiota abundance (genus Parasutterella id.2892) on CD40 ligand
with a threshold of 1e−05
rs2090816
rs7572229
rs6828768
rs8039785
rs78383039
rs10899911
rs11715853
rs2387977
rs7311004
rs6809952
rs62273907
rs55877868
rs35055552
rs7303158
rs823424
All − MR Egger
All − IVW(MRE)
−1
0
1
MR effect size for
'Gut microbiota abundance (genus Parasutterella id.2892)' on 'CD40 ligand'

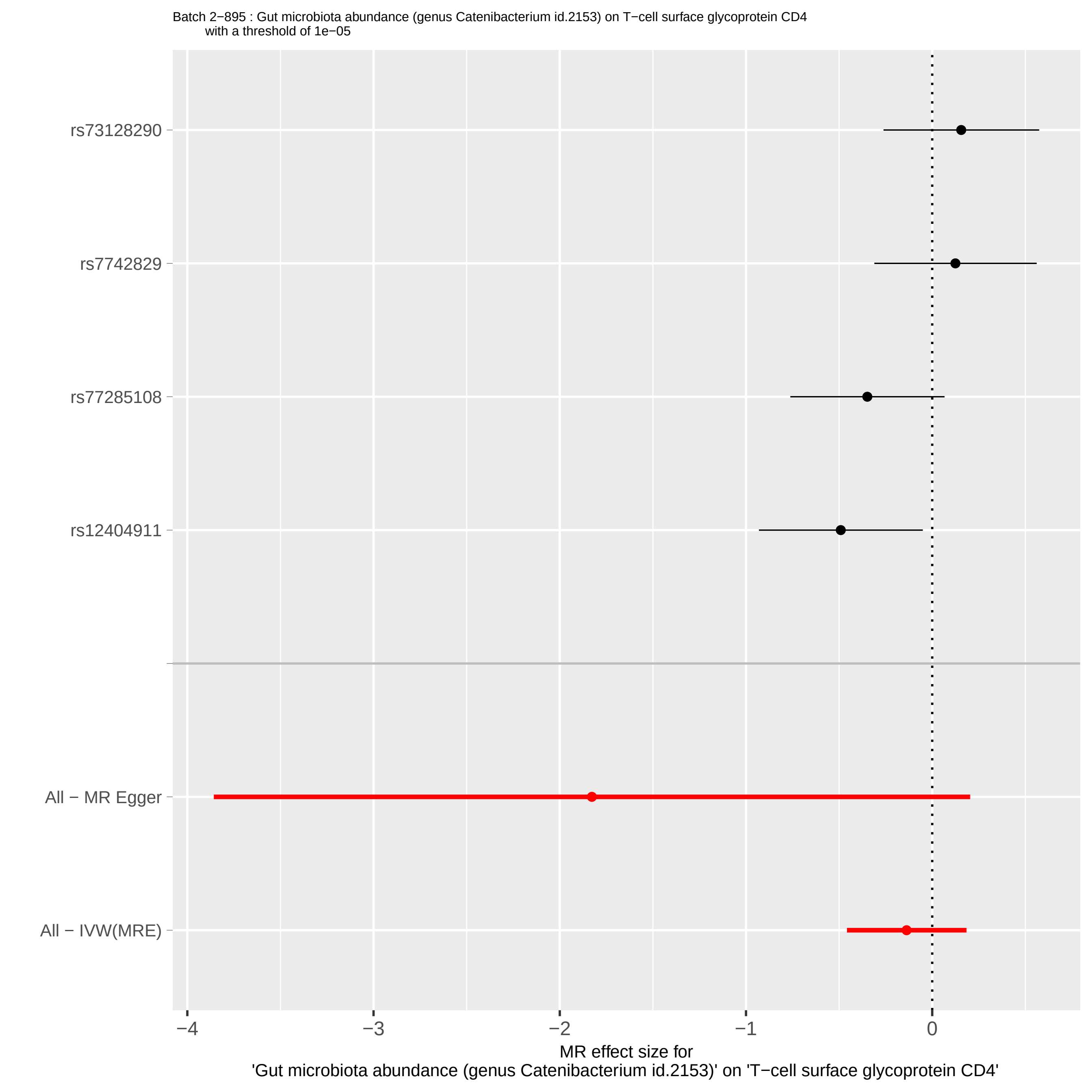

Batch 2−895 : Gut microbiota abundance (genus Catenibacterium id.2153) on T−cell surface glycoprotein CD4
with a threshold of 1e−05
rs73128290
rs7742829
rs77285108
rs12404911
All − MR Egger
All − IVW(MRE)
−4
−3
−2
−1
0
MR effect size for
'Gut microbiota abundance (genus Catenibacterium id.2153)' on 'T−cell surface glycoprotein CD4'

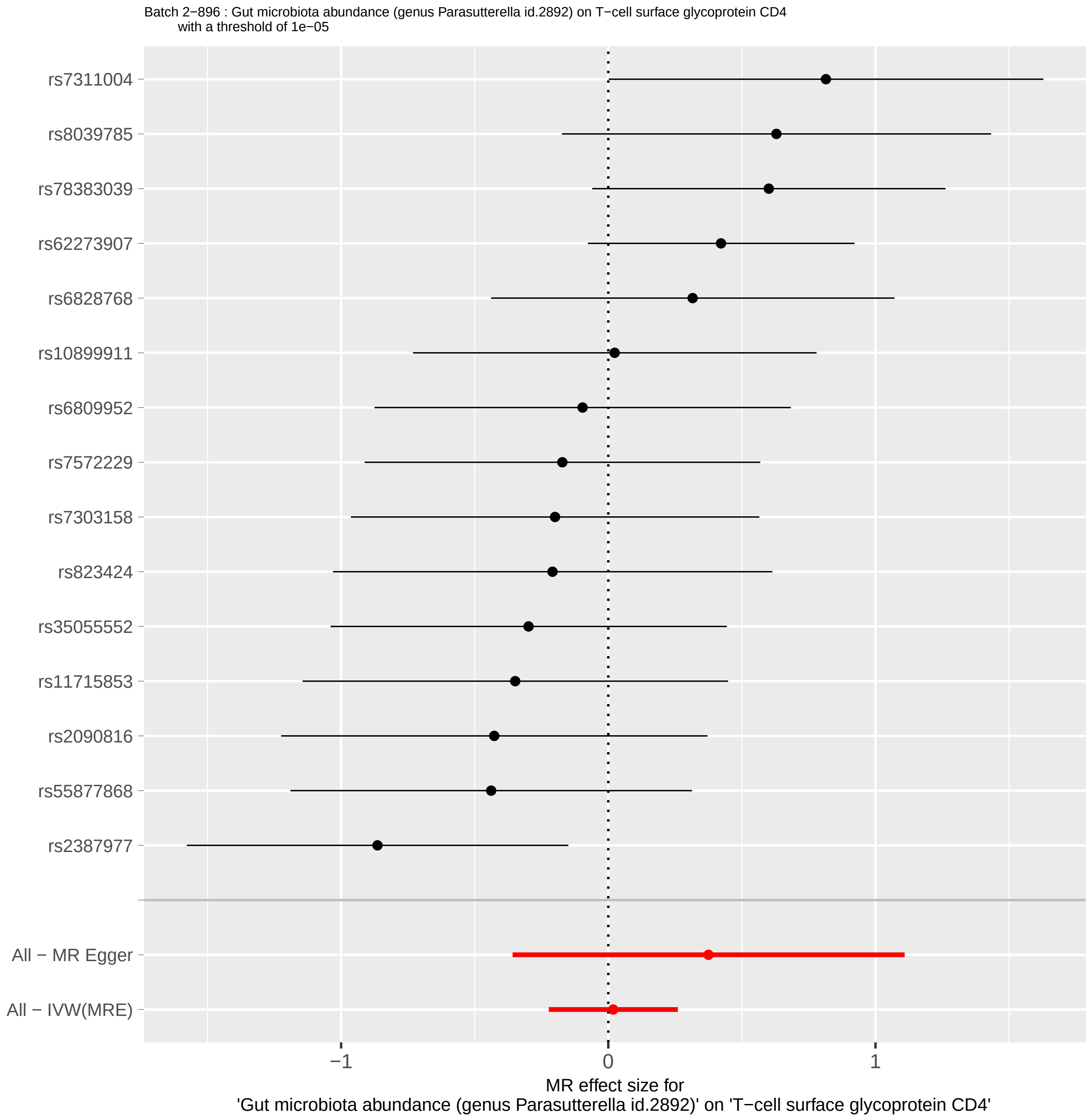

Batch 2−896 : Gut microbiota abundance (genus Parasutterella id.2892) on T−cell surface glycoprotein CD4
with a threshold of 1e−05
rs7311004
rs8039785
rs78383039
rs62273907
rs6828768
rs10899911
rs6809952
rs7572229
rs7303158
rs823424
rs35055552
rs11715853
rs2090816
rs55877868
rs2387977
All − MR Egger
All − IVW(MRE)
−1
0
1
MR effect size for
'Gut microbiota abundance (genus Parasutterella id.2892)' on 'T−cell surface glycoprotein CD4'

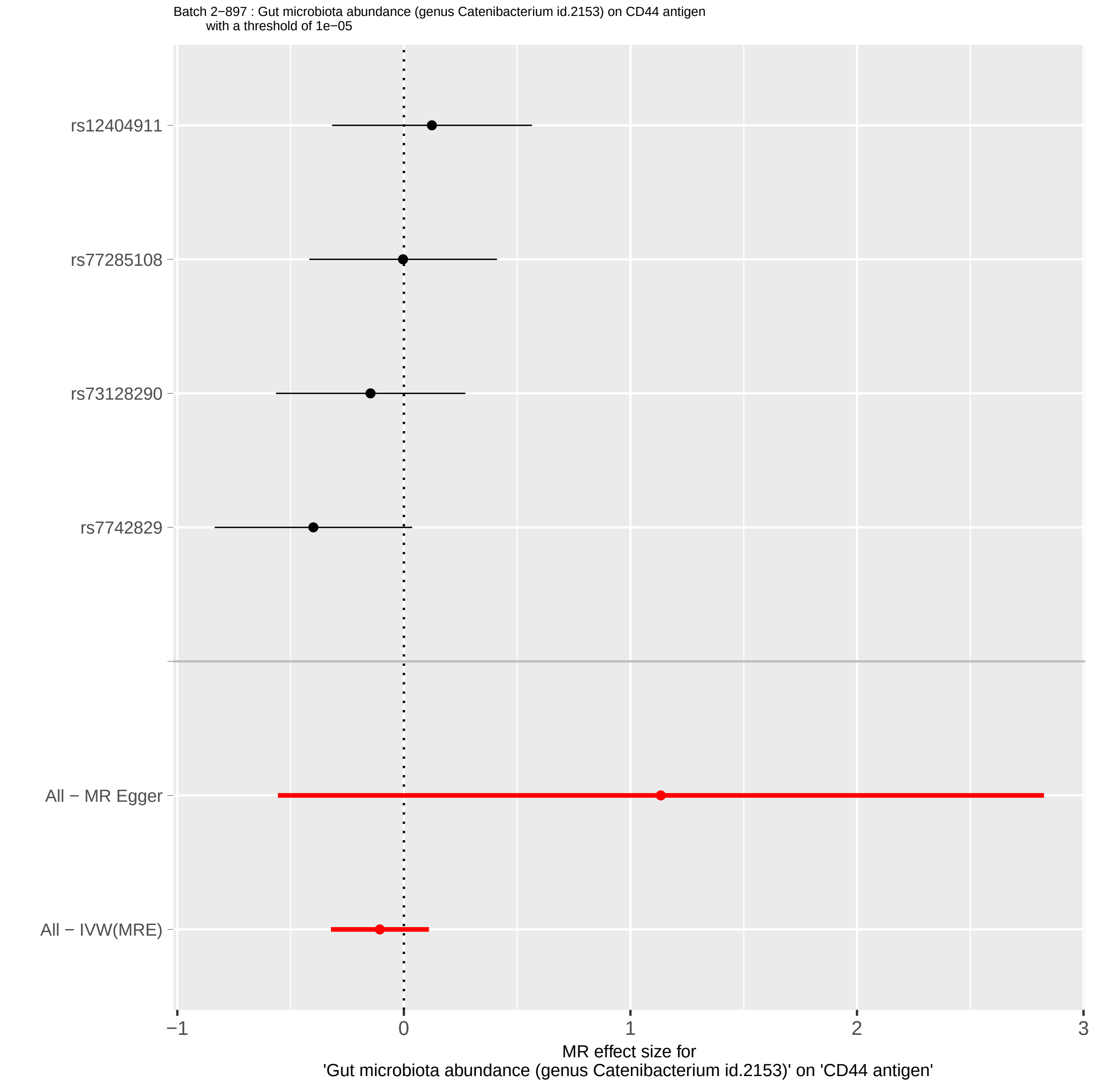
Batch 2−897 : Gut microbiota abundance (genus Catenibacterium id.2153) on CD44 antigen
with a threshold of 1e−05
rs12404911
rs77285108
rs73128290
rs7742829
All − MR Egger
All − IVW(MRE)
−1
0
1
2
3
MR effect size for
'Gut microbiota abundance (genus Catenibacterium id.2153)' on 'CD44 antigen'

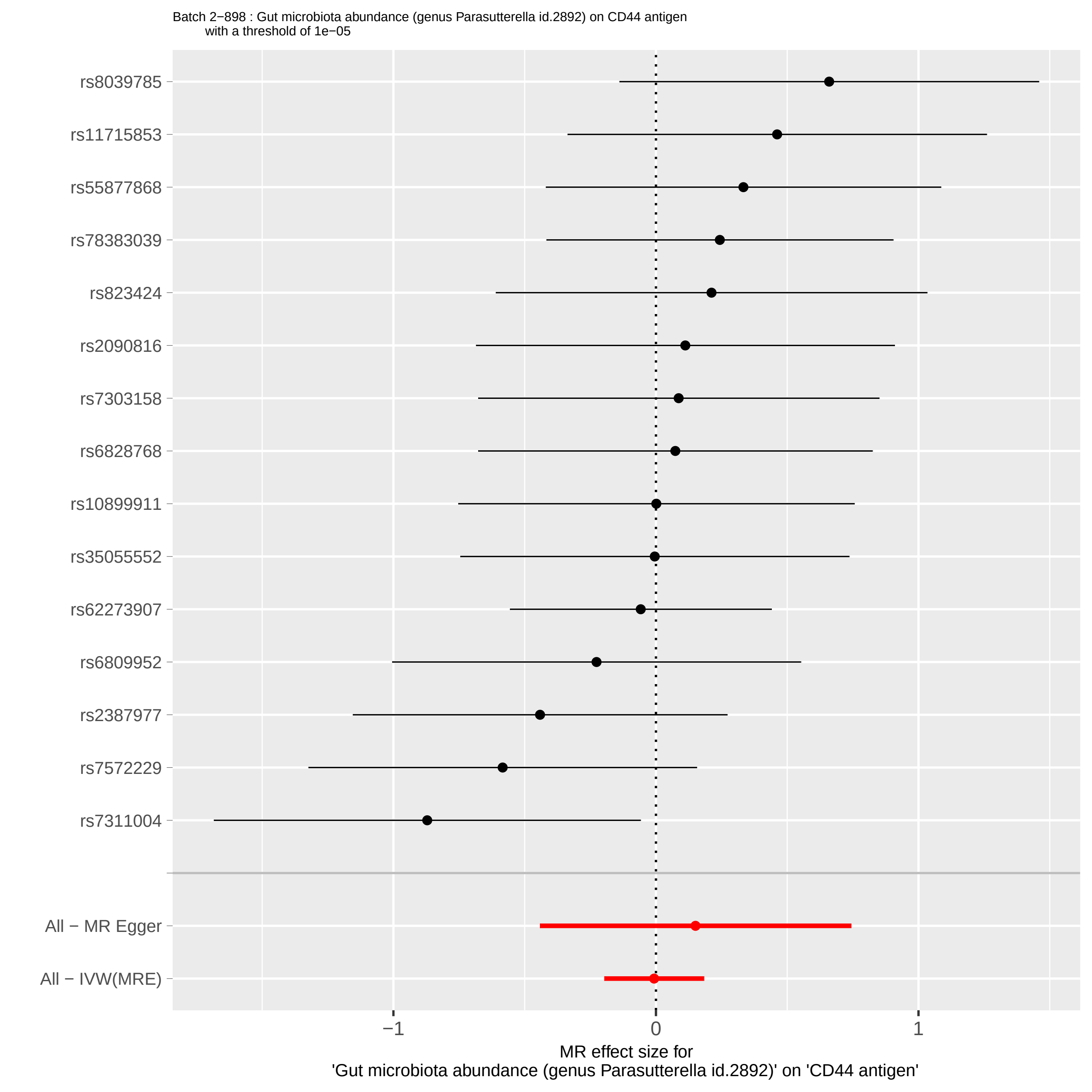

Batch 2−898 : Gut microbiota abundance (genus Parasutterella id.2892) on CD44 antigen
with a threshold of 1e−05
rs8039785
rs11715853
rs55877868
rs78383039
rs823424
rs2090816
rs7303158
rs6828768
rs10899911
rs35055552
rs62273907
rs6809952
rs2387977
rs7572229
rs7311004
All − MR Egger
All − IVW(MRE)
−1
0
1
MR effect size for
'Gut microbiota abundance (genus Parasutterella id.2892)' on 'CD44 antigen'

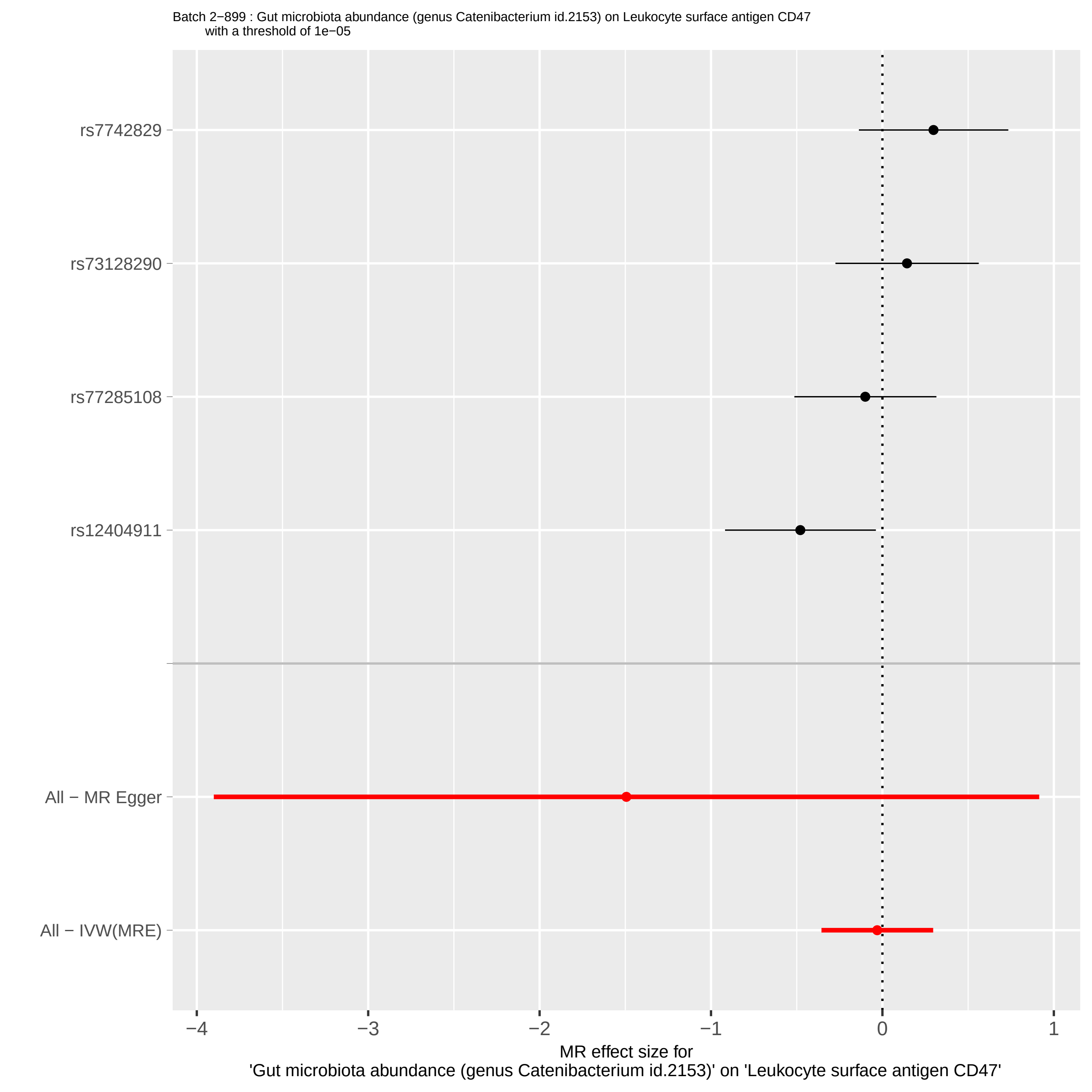

Batch 2−899 : Gut microbiota abundance (genus Catenibacterium id.2153) on Leukocyte surface antigen CD47
with a threshold of 1e−05
rs7742829
rs73128290
rs77285108
rs12404911
All − MR Egger
All − IVW(MRE)
−4
−3
−2
−1
0
1
MR effect size for
'Gut microbiota abundance (genus Catenibacterium id.2153)' on 'Leukocyte surface antigen CD47'

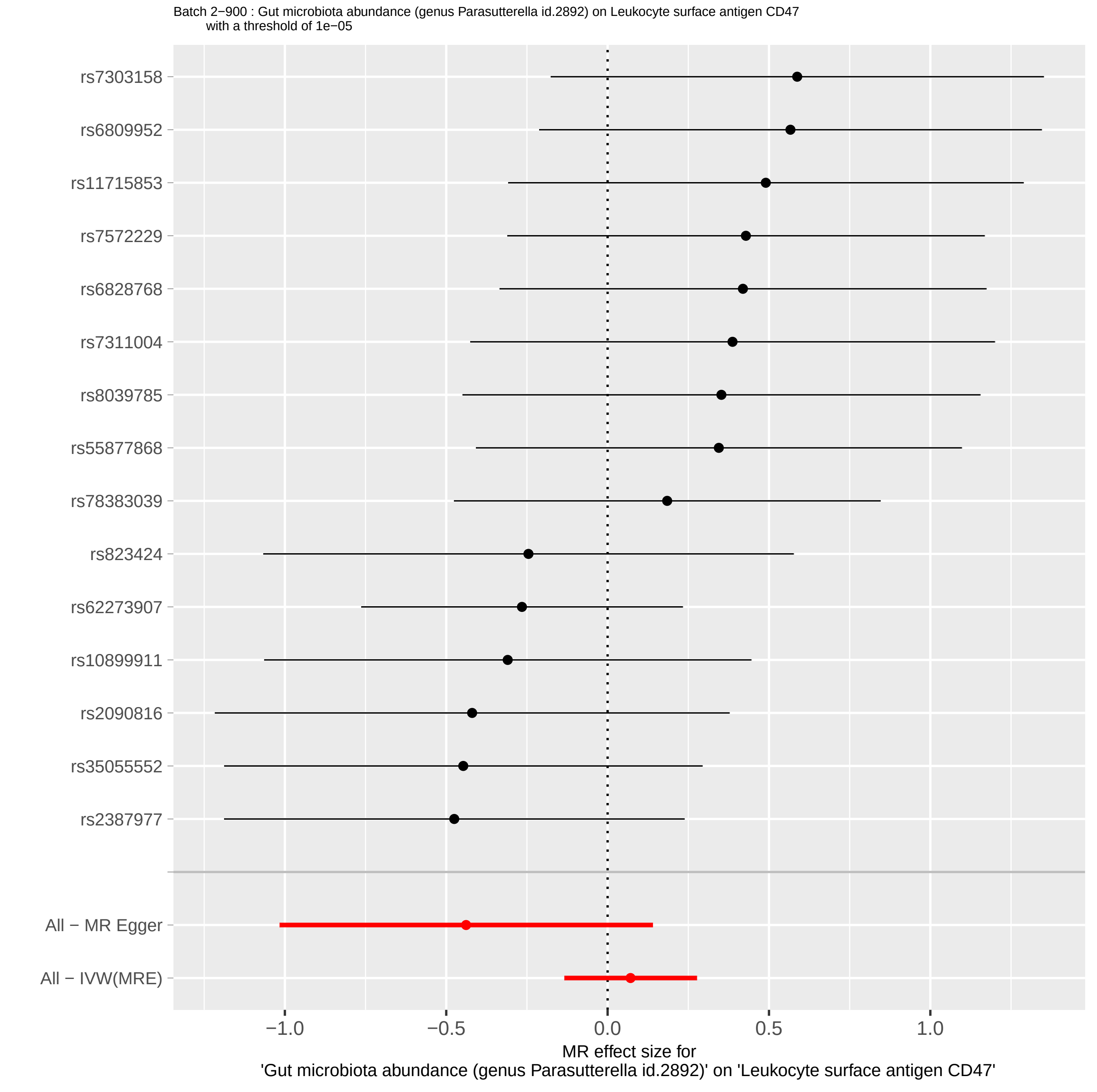
Batch 2−900 : Gut microbiota abundance (genus Parasutterella id.2892) on Leukocyte surface antigen CD47
with a threshold of 1e−05
rs7303158
rs6809952
rs11715853
rs7572229
rs6828768
rs7311004
rs8039785
rs55877868
rs78383039
rs823424
rs62273907
rs10899911
rs2090816
rs35055552
rs2387977
All − MR Egger
All − IVW(MRE)
−1.0
−0.5
0.0
0.5
1.0
MR effect size for
'Gut microbiota abundance (genus Parasutterella id.2892)' on 'Leukocyte surface antigen CD47'

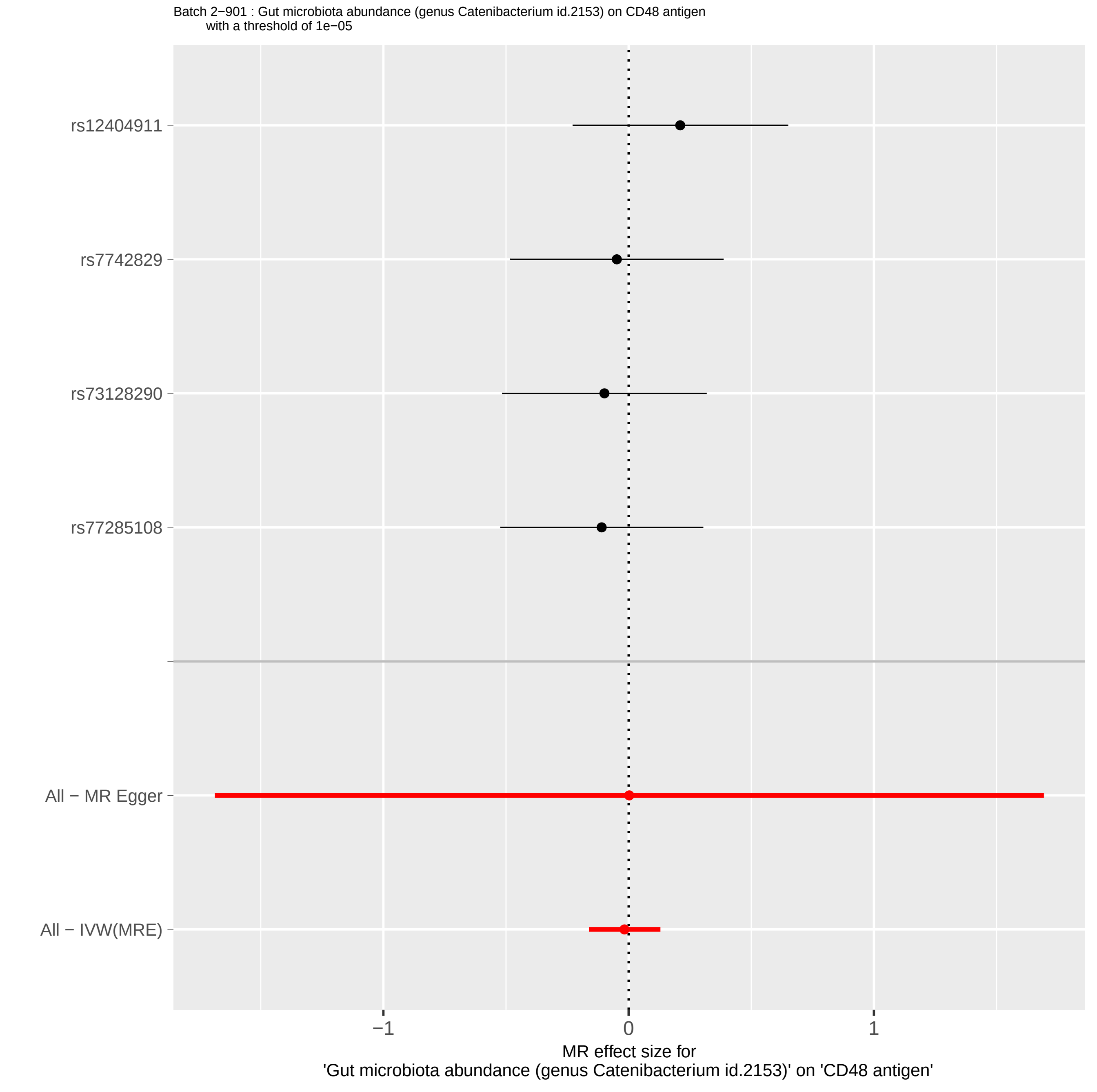

Batch 2−901 : Gut microbiota abundance (genus Catenibacterium id.2153) on CD48 antigen
with a threshold of 1e−05
rs12404911
rs7742829
rs73128290
rs77285108
All − MR Egger
All − IVW(MRE)
−1
0
1
MR effect size for
'Gut microbiota abundance (genus Catenibacterium id.2153)' on 'CD48 antigen'

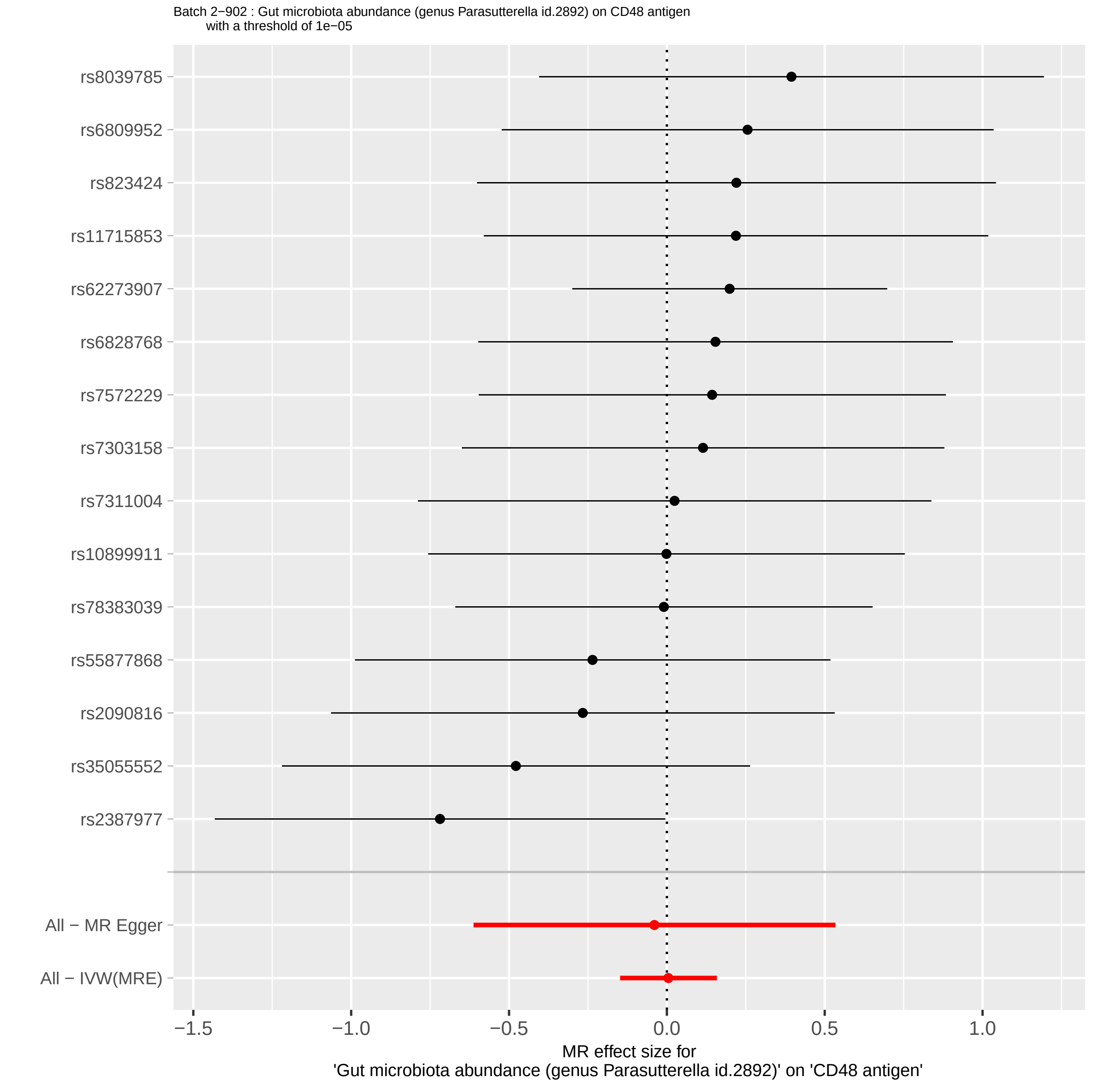

Batch 2−902 : Gut microbiota abundance (genus Parasutterella id.2892) on CD48 antigen
with a threshold of 1e−05
rs8039785
rs6809952
rs823424
rs11715853
rs62273907
rs6828768
rs7572229
rs7303158
rs7311004
rs10899911
rs78383039
rs55877868
rs2090816
rs35055552
rs2387977
All − MR Egger
All − IVW(MRE)
−1.5
−1.0
−0.5
0.0
0.5
1.0
MR effect size for
'Gut microbiota abundance (genus Parasutterella id.2892)' on 'CD48 antigen'

Batch 2−903 : Gut microbiota abundance (genus Catenibacterium id.2153) on T−cell surface glycoprotein CD5 with a threshold of 1e−05

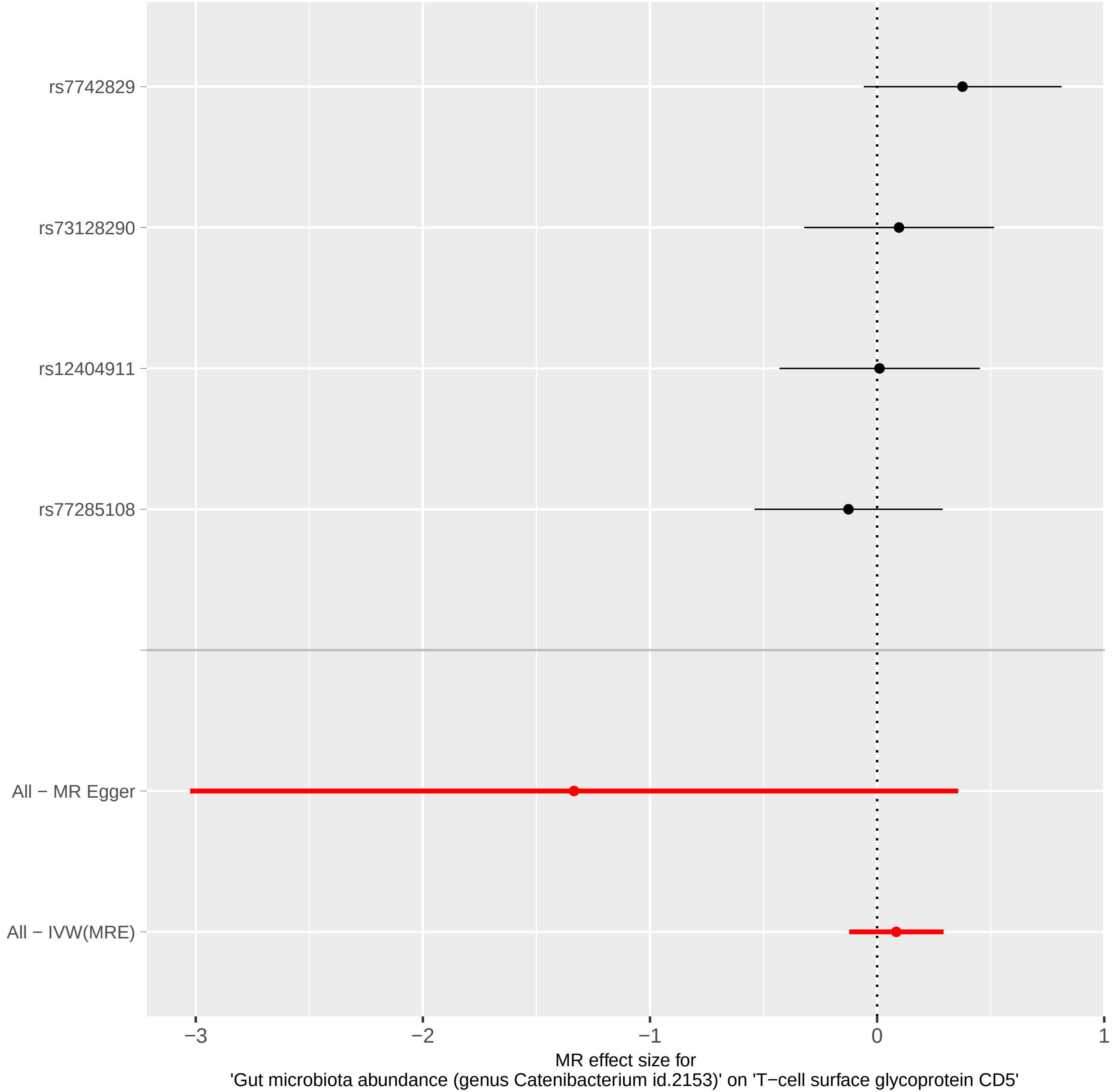

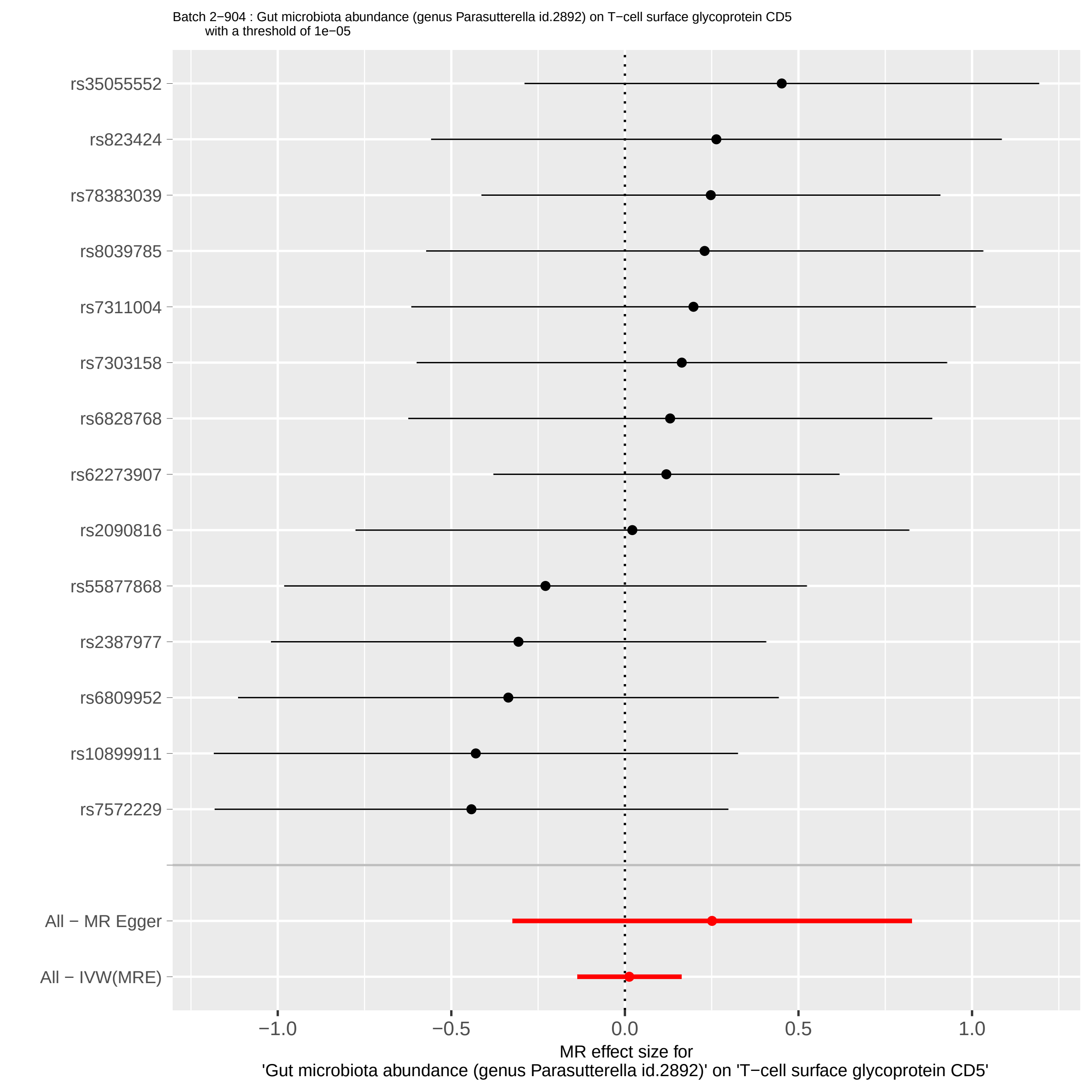

Batch 2−904 : Gut microbiota abundance (genus Parasutterella id.2892) on T−cell surface glycoprotein CD5
with a threshold of 1e−05
rs35055552
rs823424
rs78383039
rs8039785
rs7311004
rs7303158
rs6828768
rs62273907
rs2090816
rs55877868
rs2387977
rs6809952
rs10899911
rs7572229
All − MR Egger
All − IVW(MRE)
−1.0
−0.5
0.0
0.5
1.0
MR effect size for
'Gut microbiota abundance (genus Parasutterella id.2892)' on 'T−cell surface glycoprotein CD5'

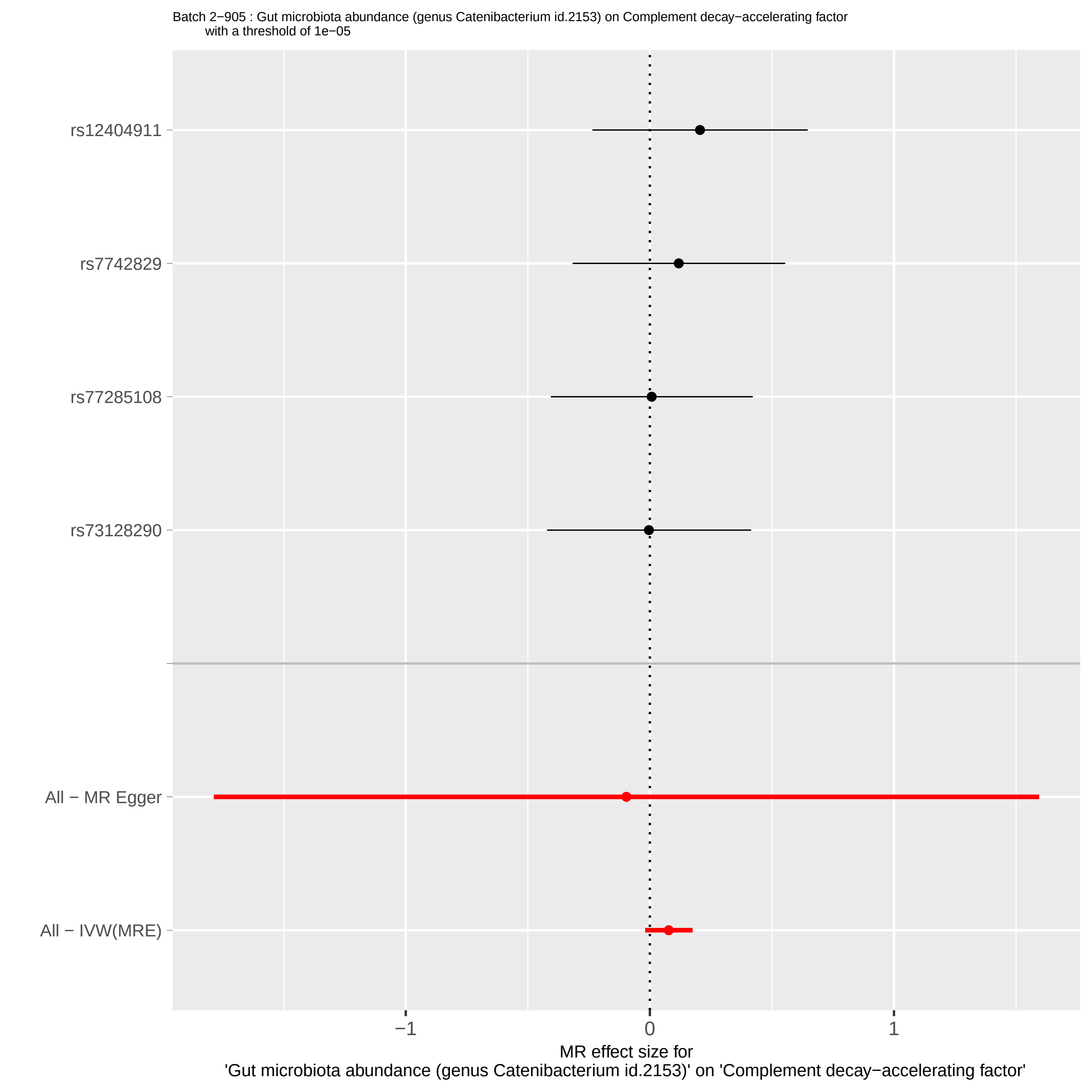
Batch 2−905 : Gut microbiota abundance (genus Catenibacterium id.2153) on Complement decay−accelerating factor
with a threshold of 1e−05
rs12404911
rs7742829
rs77285108
rs73128290
All − MR Egger
All − IVW(MRE)
−1
0
1
MR effect size for
'Gut microbiota abundance (genus Catenibacterium id.2153)' on 'Complement decay−accelerating factor'

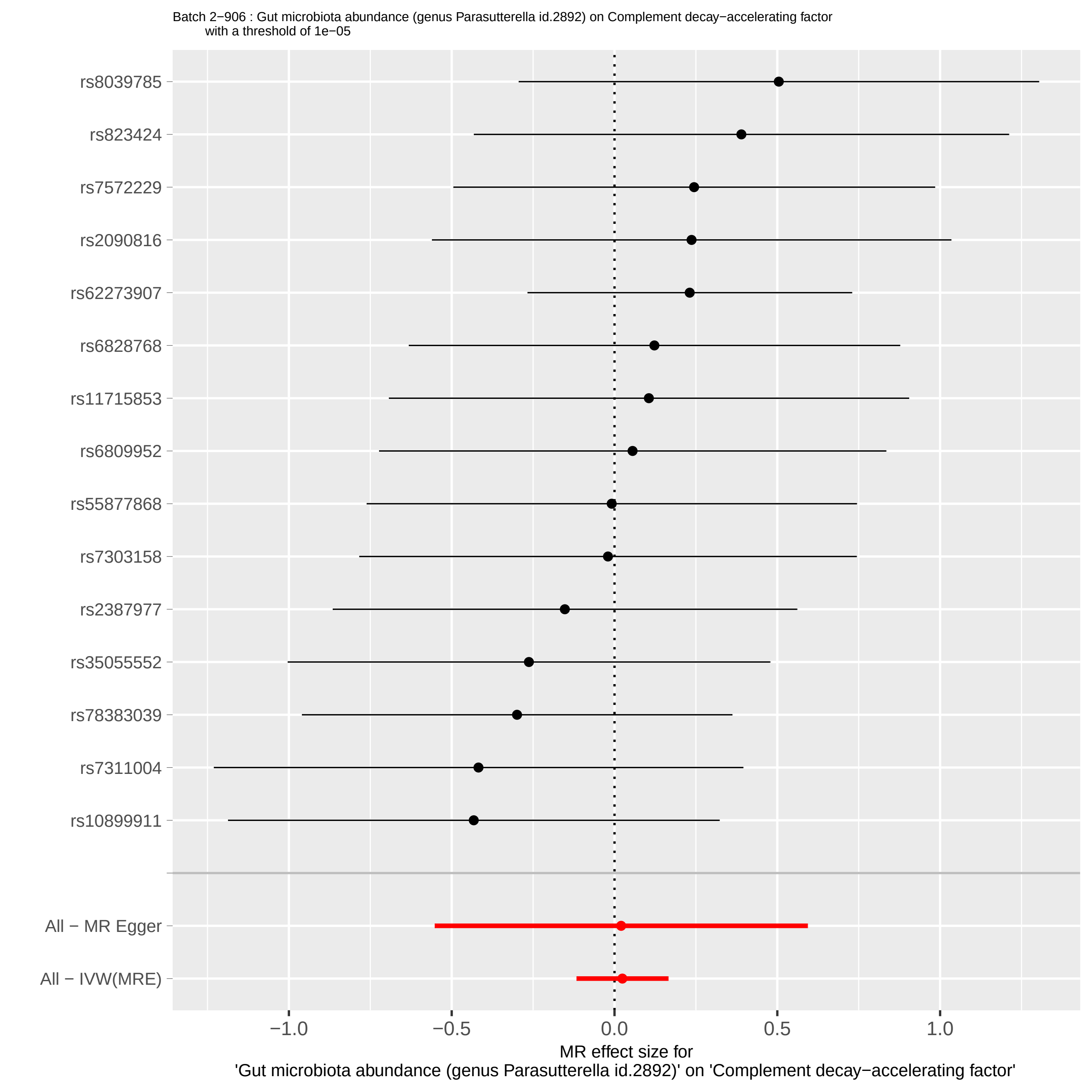
Batch 2−906 : Gut microbiota abundance (genus Parasutterella id.2892) on Complement decay−accelerating factor
with a threshold of 1e−05
rs8039785
rs823424
rs7572229
rs2090816
rs62273907
rs6828768
rs11715853
rs6809952
rs55877868
rs7303158
rs2387977
rs35055552
rs78383039
rs7311004
rs10899911
All − MR Egger
All − IVW(MRE)
−1.0
−0.5
0.0
0.5
1.0
MR effect size for
'Gut microbiota abundance (genus Parasutterella id.2892)' on 'Complement decay−accelerating factor'

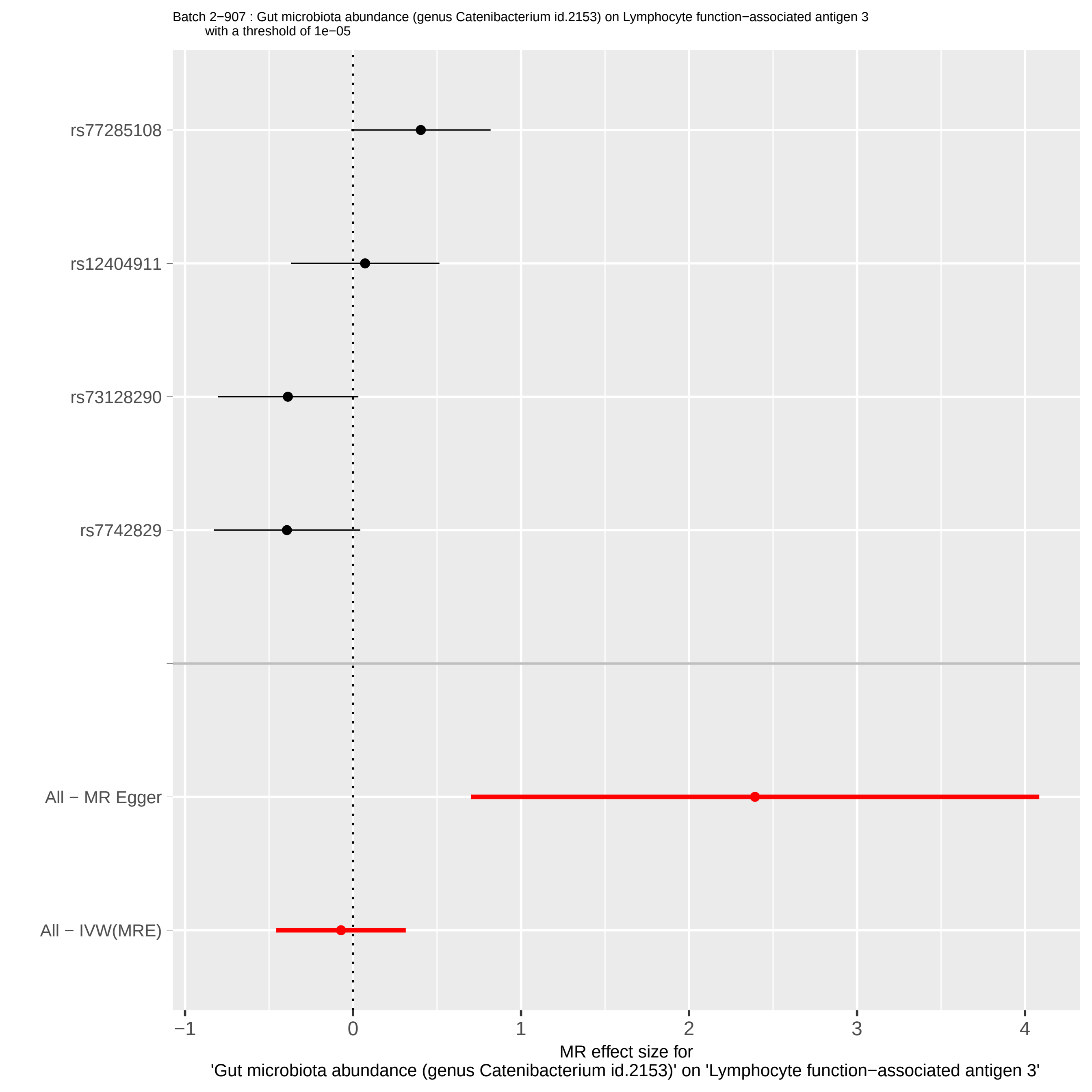

Batch 2−907 : Gut microbiota abundance (genus Catenibacterium id.2153) on Lymphocyte function−associated antigen 3
with a threshold of 1e−05
rs77285108
rs12404911
rs73128290
rs7742829
All − MR Egger
All − IVW(MRE)
−1
0
1
2
3
4
MR effect size for
'Gut microbiota abundance (genus Catenibacterium id.2153)' on 'Lymphocyte function−associated antigen 3'

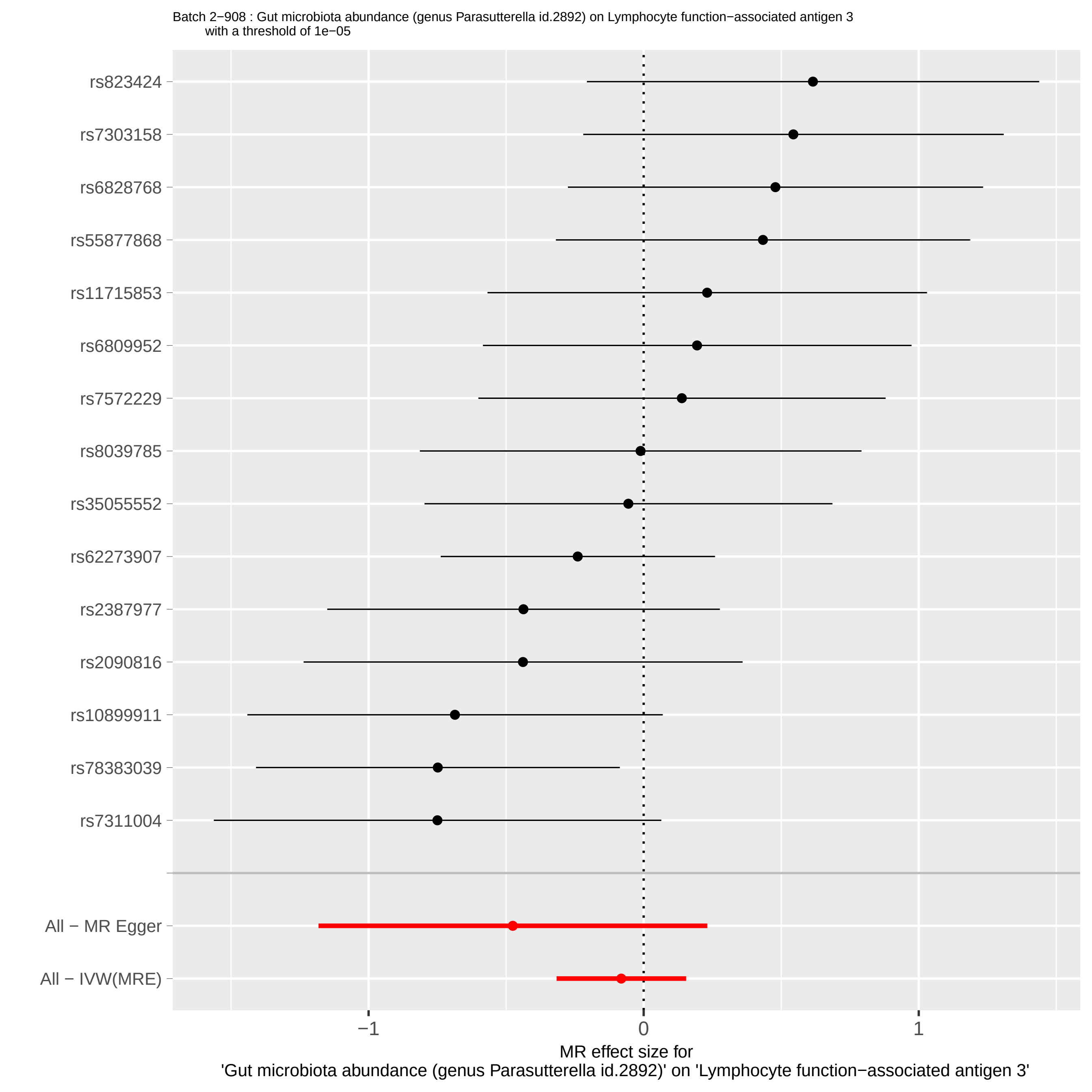

Batch 2−908 : Gut microbiota abundance (genus Parasutterella id.2892) on Lymphocyte function−associated antigen 3 with a threshold of 1e−05
rs823424
rs7303158
rs6828768
rs55877868
rs11715853
rs6809952
rs7572229
rs8039785
rs35055552
rs62273907
rs2387977
rs2090816
rs10899911
rs78383039
rs7311004
All − MR Egger
All − IVW(MRE)
−1
0
1
MR effect size for
'Gut microbiota abundance (genus Parasutterella id.2892)' on 'Lymphocyte function−associated antigen 3'

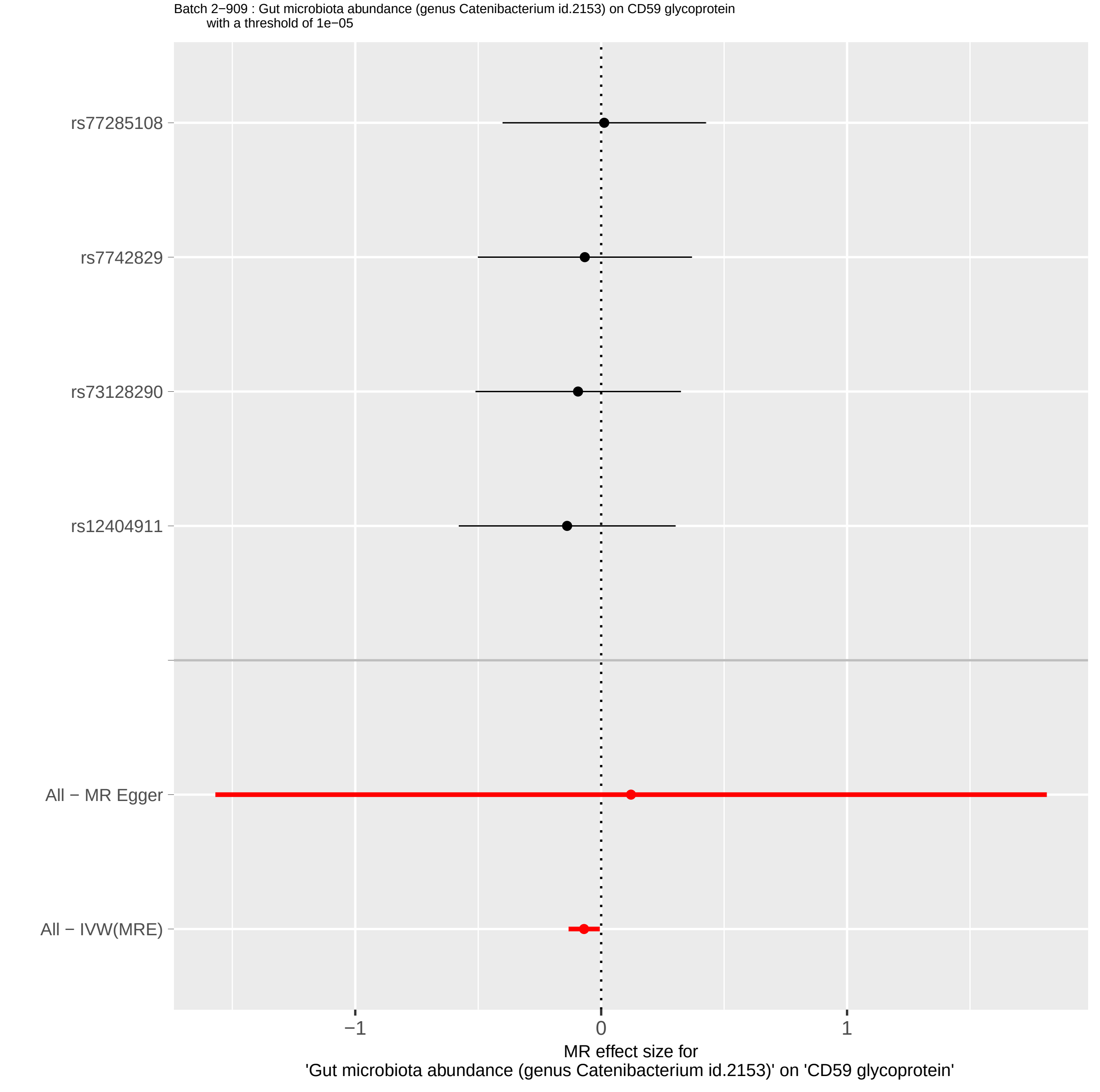
Batch 2−909 : Gut microbiota abundance (genus Catenibacterium id.2153) on CD59 glycoprotein
with a threshold of 1e−05
rs77285108
rs7742829
rs73128290
rs12404911
All − MR Egger
All − IVW(MRE)
−1
0
1
MR effect size for
'Gut microbiota abundance (genus Catenibacterium id.2153)' on 'CD59 glycoprotein'

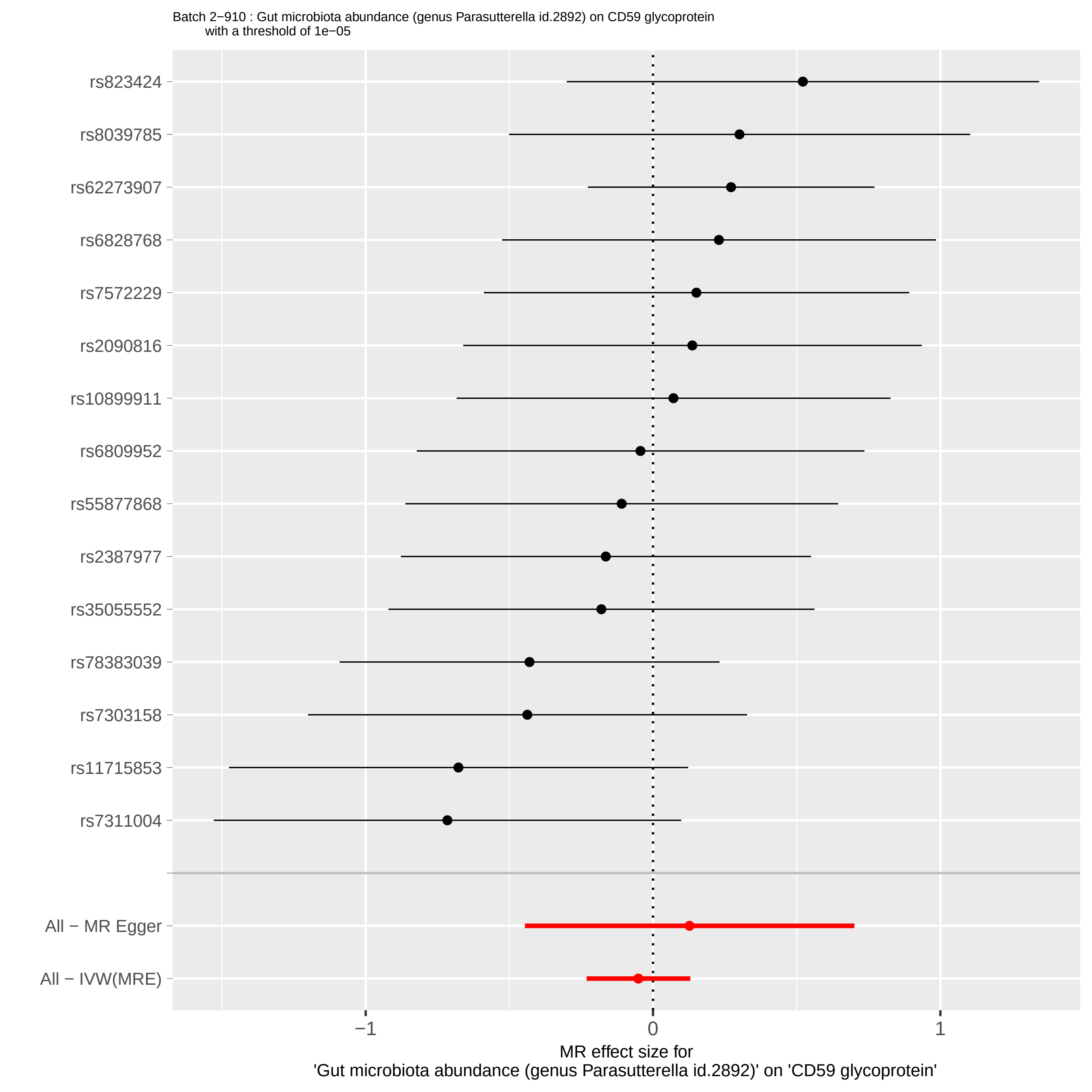

Batch 2−910 : Gut microbiota abundance (genus Parasutterella id.2892) on CD59 glycoprotein
with a threshold of 1e−05
rs823424
rs8039785
rs62273907
rs6828768
rs7572229
rs2090816
rs10899911
rs6809952
rs55877868
rs2387977
rs35055552
rs78383039
rs7303158
rs11715853
rs7311004
All − MR Egger
All − IVW(MRE)
−1
0
1
MR effect size for
'Gut microbiota abundance (genus Parasutterella id.2892)' on 'CD59 glycoprotein'

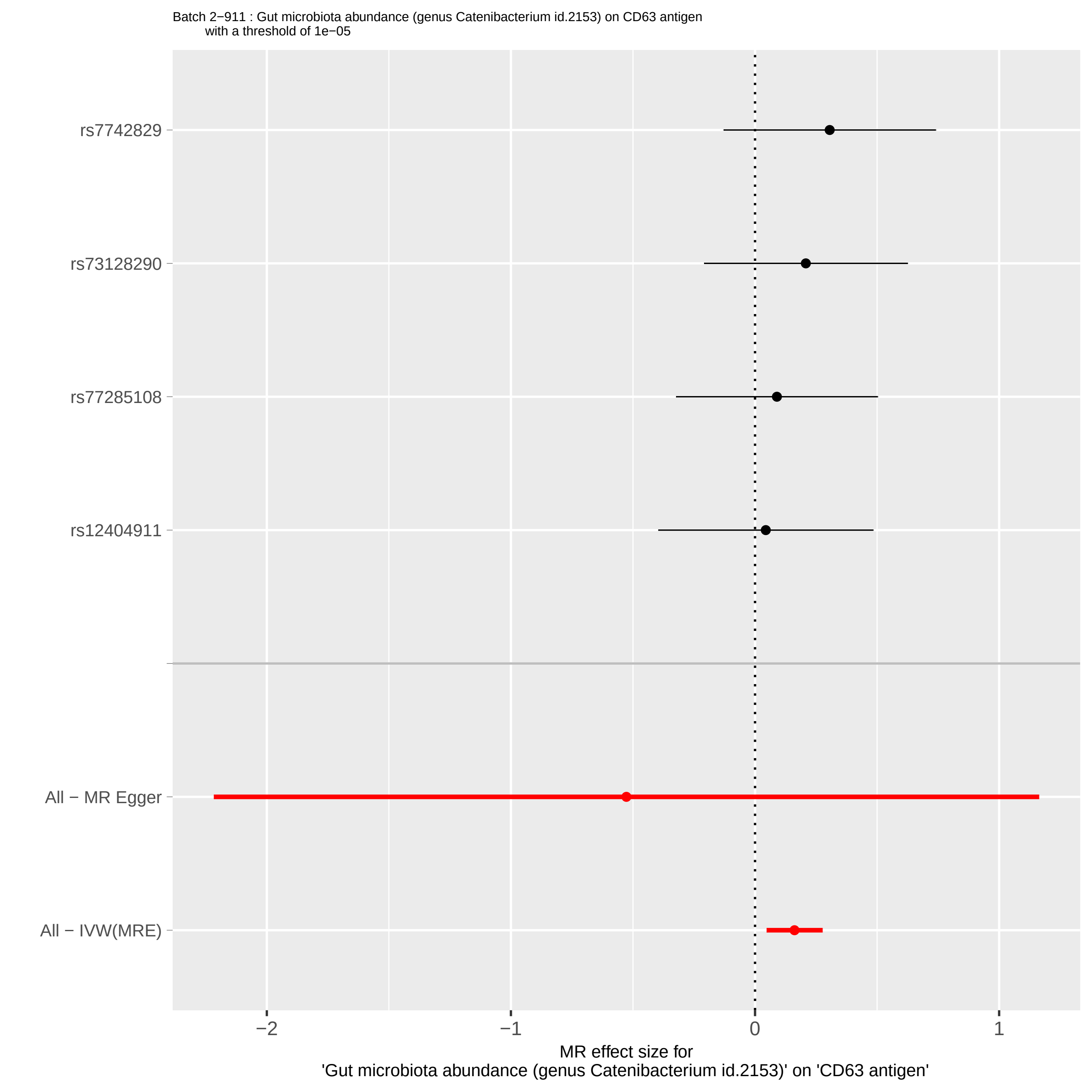

Batch 2−911 : Gut microbiota abundance (genus Catenibacterium id.2153) on CD63 antigen
with a threshold of 1e−05
rs7742829
rs73128290
rs77285108
rs12404911
All − MR Egger
All − IVW(MRE)
−2
−1
0
1
MR effect size for
'Gut microbiota abundance (genus Catenibacterium id.2153)' on 'CD63 antigen'

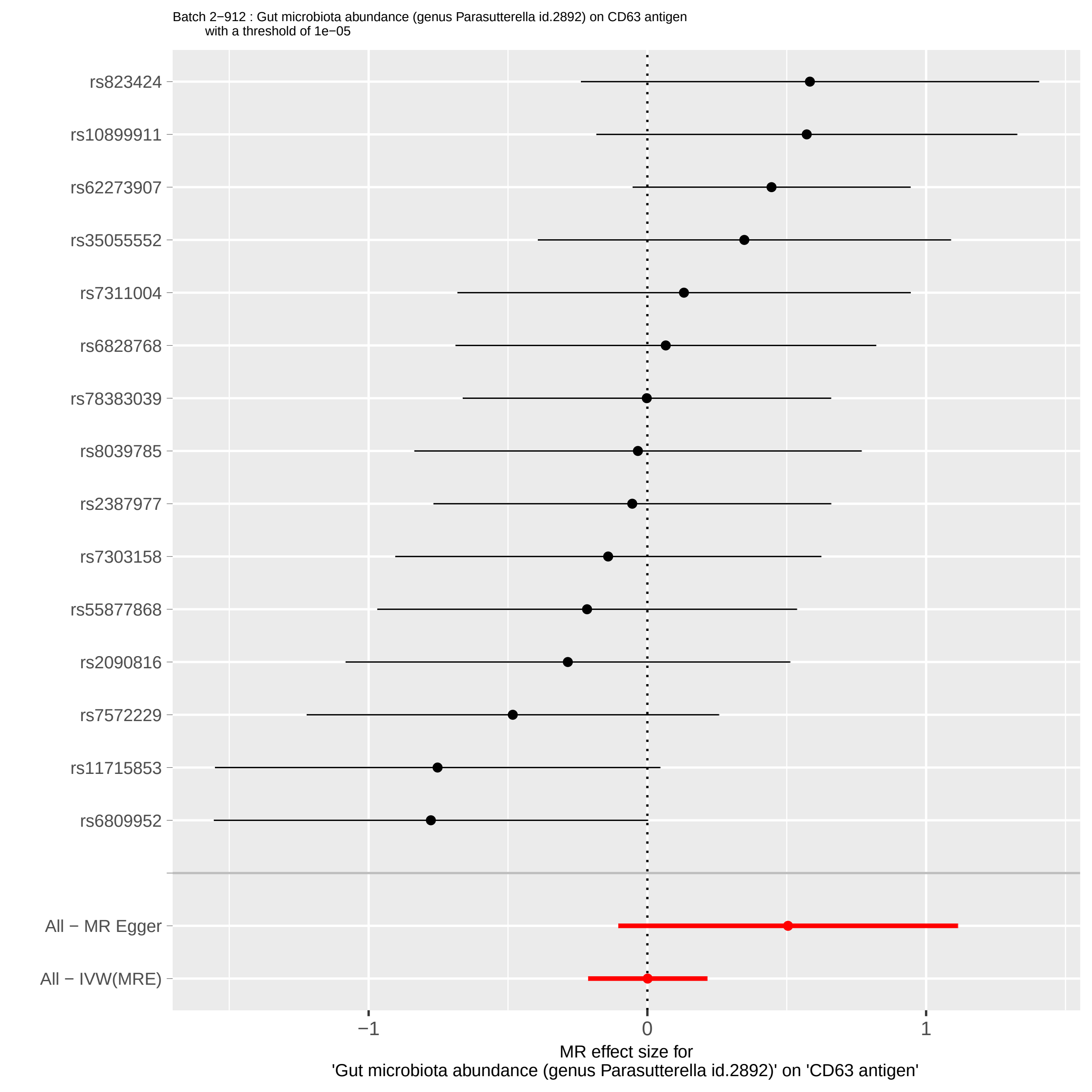
Batch 2−912 : Gut microbiota abundance (genus Parasutterella id.2892) on CD63 antigen
with a threshold of 1e−05
rs823424
rs10899911
rs62273907
rs35055552
rs7311004
rs6828768
rs78383039
rs8039785
rs2387977
rs7303158
rs55877868
rs2090816
rs7572229
rs11715853
rs6809952
All − MR Egger
All − IVW(MRE)
−1
0
1
MR effect size for
'Gut microbiota abundance (genus Parasutterella id.2892)' on 'CD63 antigen'

Batch 2−913 : Gut microbiota abundance (genus Catenibacterium id.2153) on CD70 antigen
with a threshold of 1e−05

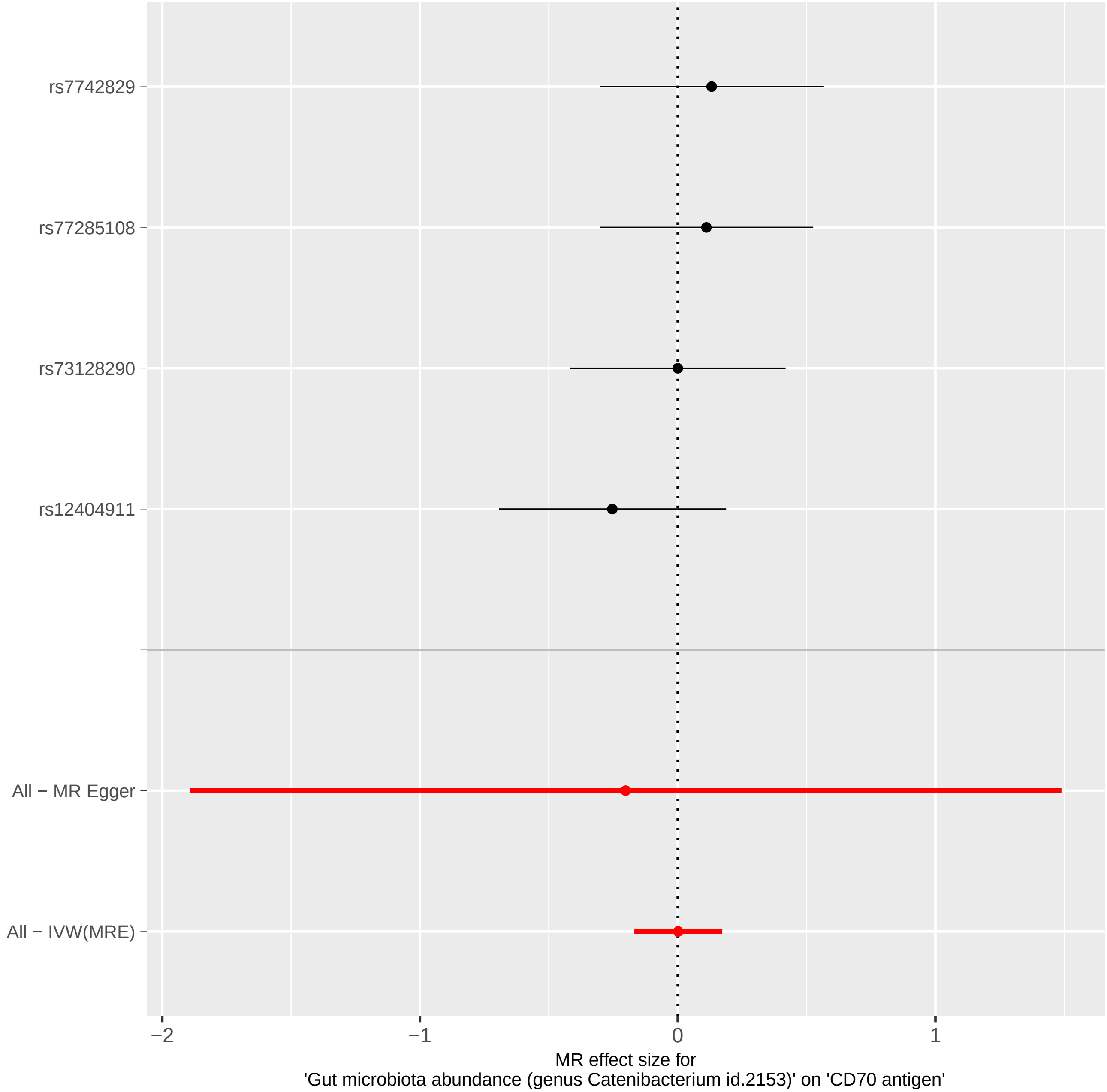

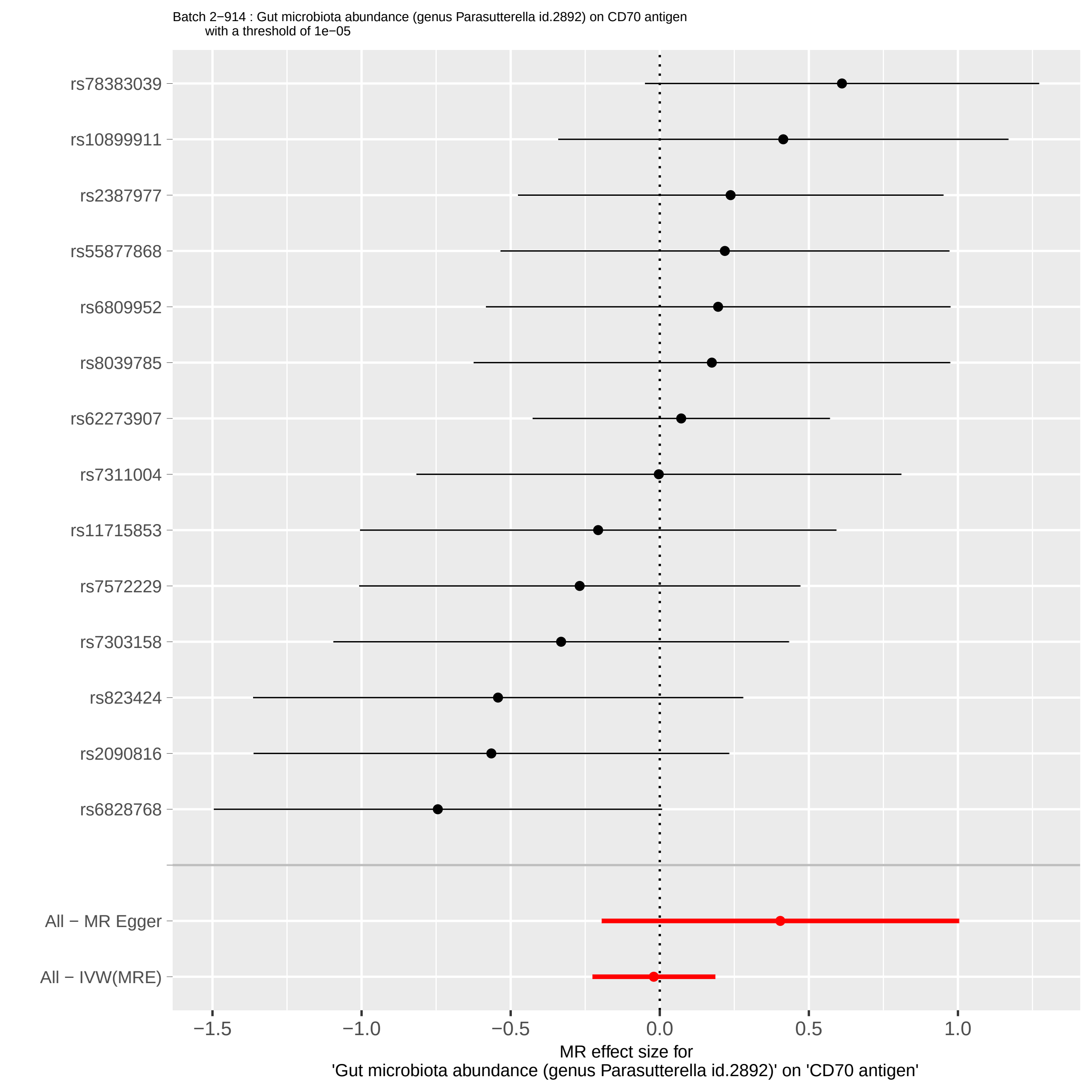

Batch 2−914 : Gut microbiota abundance (genus Parasutterella id.2892) on CD70 antigen
with a threshold of 1e−05
rs78383039
rs10899911
rs2387977
rs55877868
rs6809952
rs8039785
rs62273907
rs7311004
rs11715853
rs7572229
rs7303158
rs823424
rs2090816
rs6828768
All − MR Egger
All − IVW(MRE)
−1.5
−1.0
−0.5
0.0
0.5
1.0
MR effect size for
'Gut microbiota abundance (genus Parasutterella id.2892)' on 'CD70 antigen'

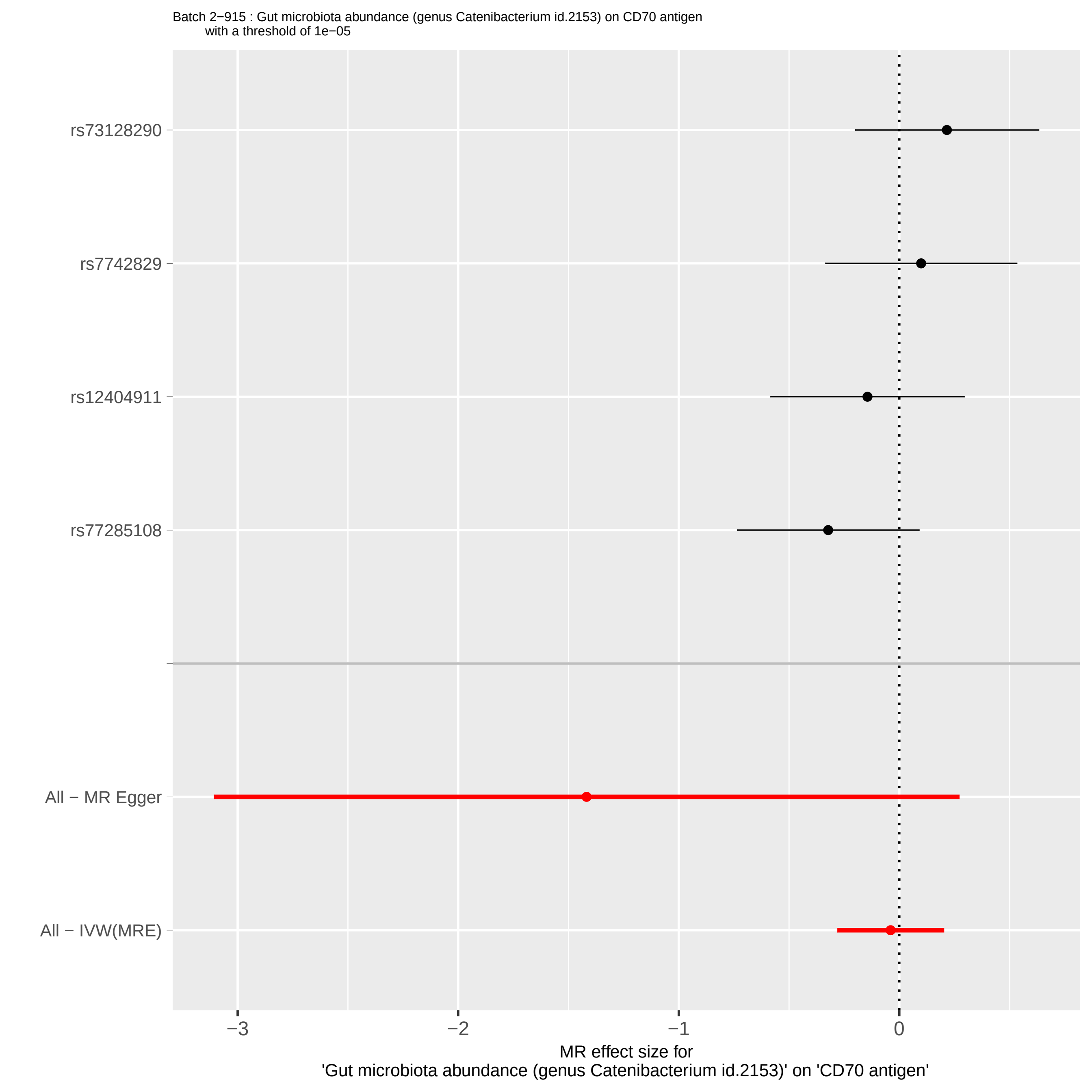

Batch 2−915 : Gut microbiota abundance (genus Catenibacterium id.2153) on CD70 antigen
with a threshold of 1e−05
rs73128290
rs7742829
rs12404911
rs77285108
All − MR Egger
All − IVW(MRE)
−3
−2
−1
0
MR effect size for
'Gut microbiota abundance (genus Catenibacterium id.2153)' on 'CD70 antigen'

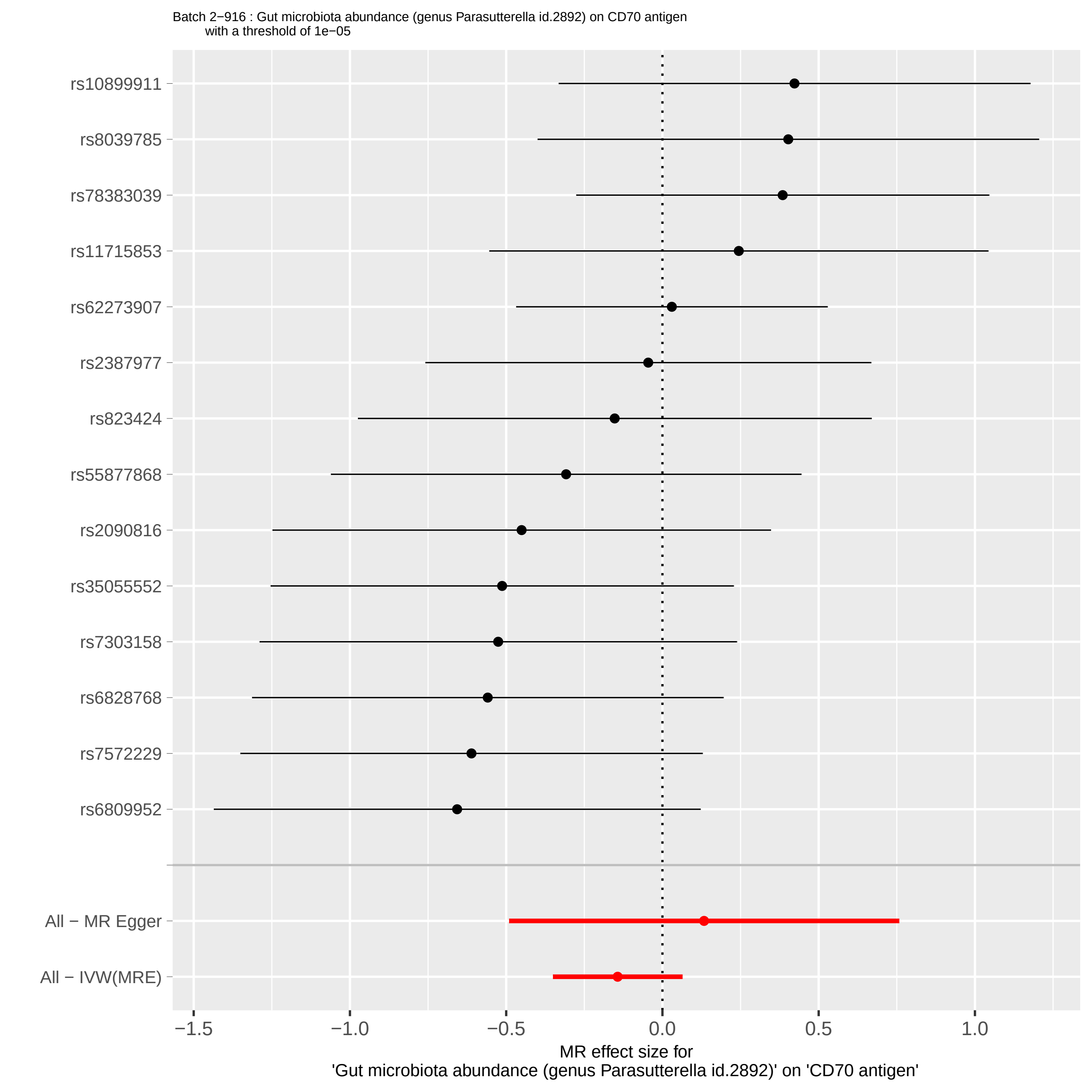

Batch 2−916 : Gut microbiota abundance (genus Parasutterella id.2892) on CD70 antigen
with a threshold of 1e−05
rs10899911
rs8039785
rs78383039
rs11715853
rs62273907
rs2387977
rs823424
rs55877868
rs2090816
rs35055552
rs7303158
rs6828768
rs7572229
rs6809952
All − MR Egger
All − IVW(MRE)
−1.5
−1.0
−0.5
0.0
0.5
1.0
MR effect size for
'Gut microbiota abundance (genus Parasutterella id.2892)' on 'CD70 antigen'

Batch 2−917 : Gut microbiota abundance (genus Catenibacterium id.2153) on HLA class II histocompatibility antigen gamma chain
with a threshold of 1e−05

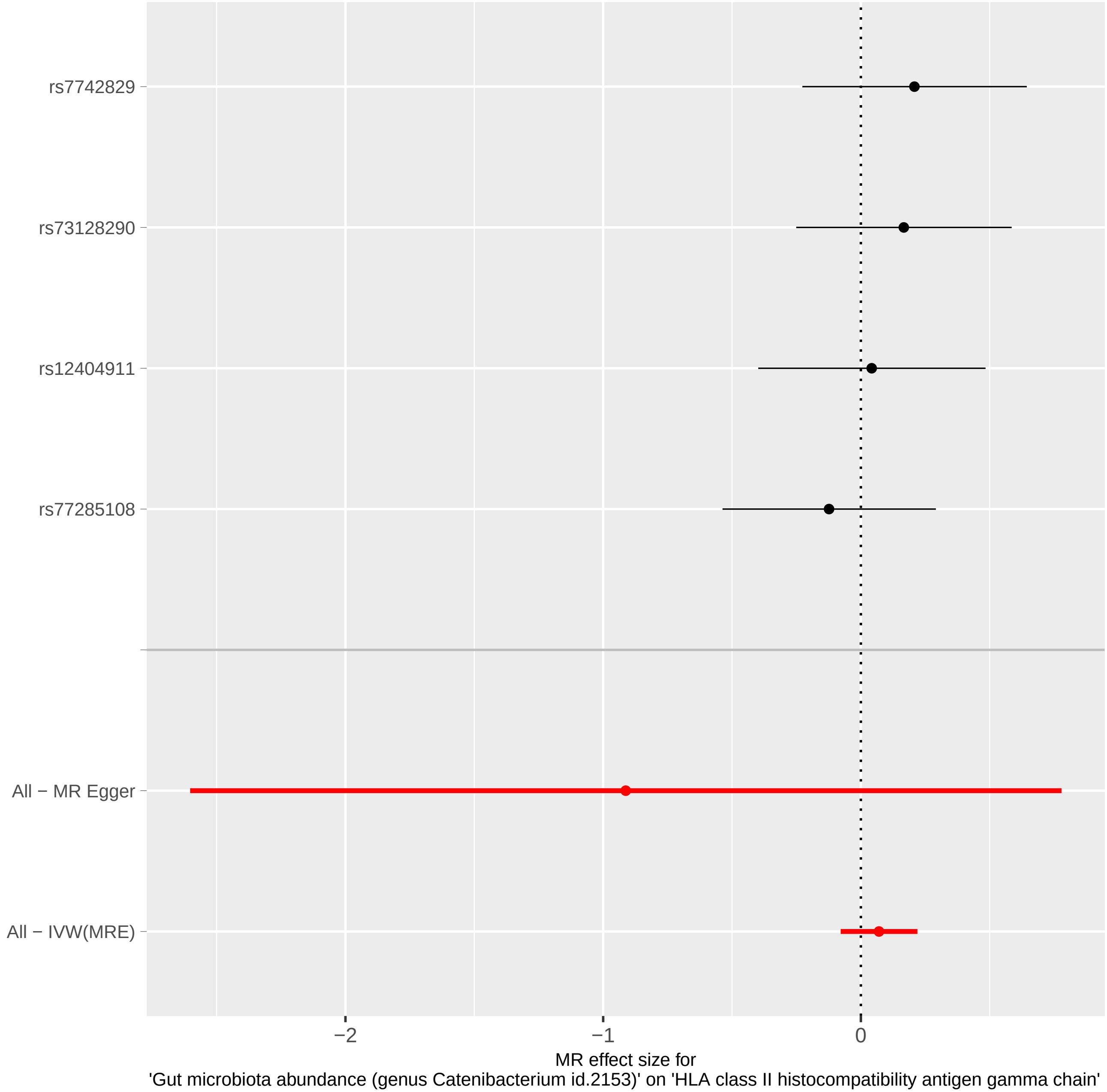

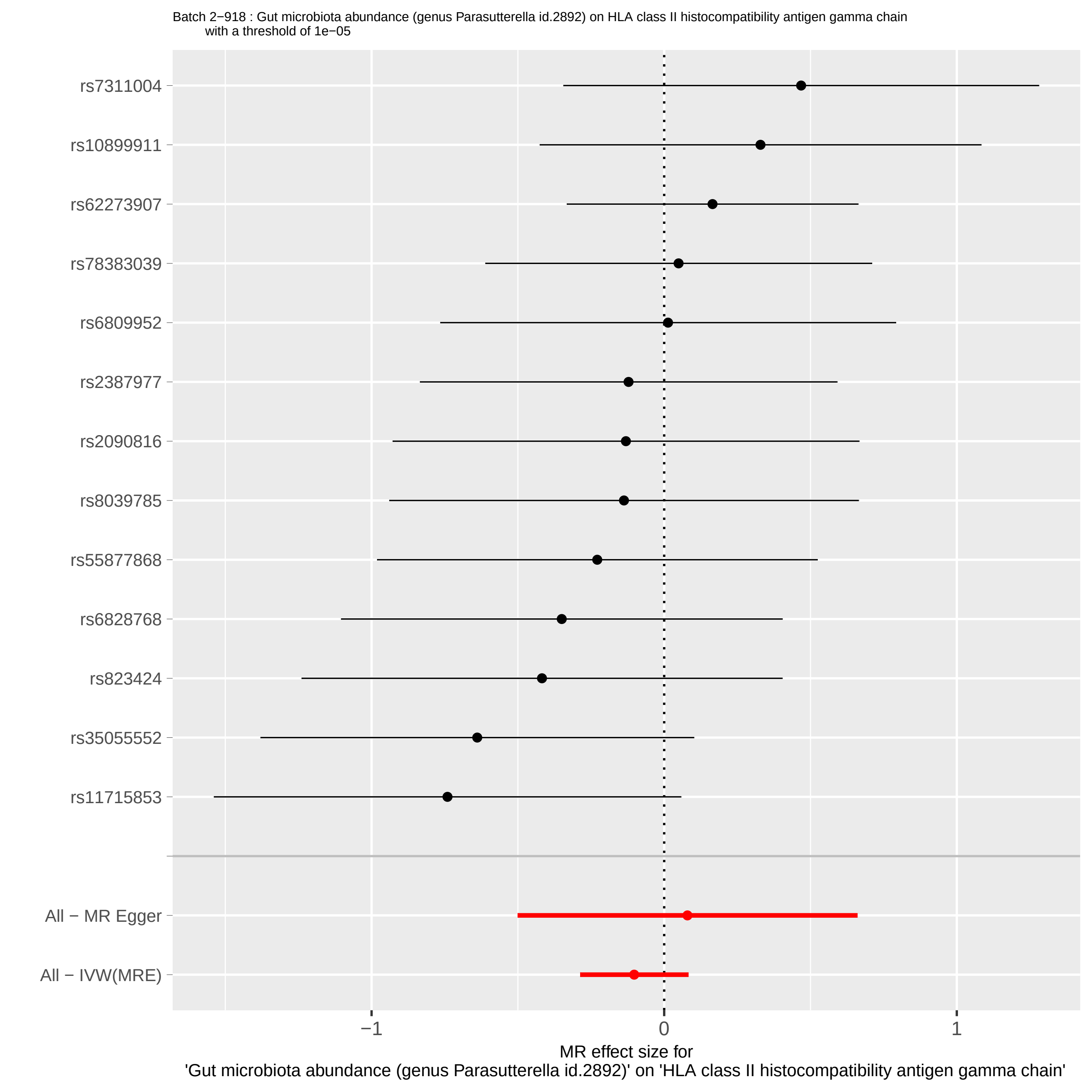

Batch 2−918 : Gut microbiota abundance (genus Parasutterella id.2892) on HLA class II histocompatibility antigen gamma chain with a threshold of 1e−05
rs7311004
rs10899911
rs62273907
rs78383039
rs6809952
rs2387977
rs2090816
rs8039785
rs55877868
rs6828768
rs823424
rs35055552
rs11715853
All − MR Egger
All − IVW(MRE)
−1
0
1
MR effect size for
'Gut microbiota abundance (genus Parasutterella id.2892)' on 'HLA class II histocompatibility antigen gamma chain'

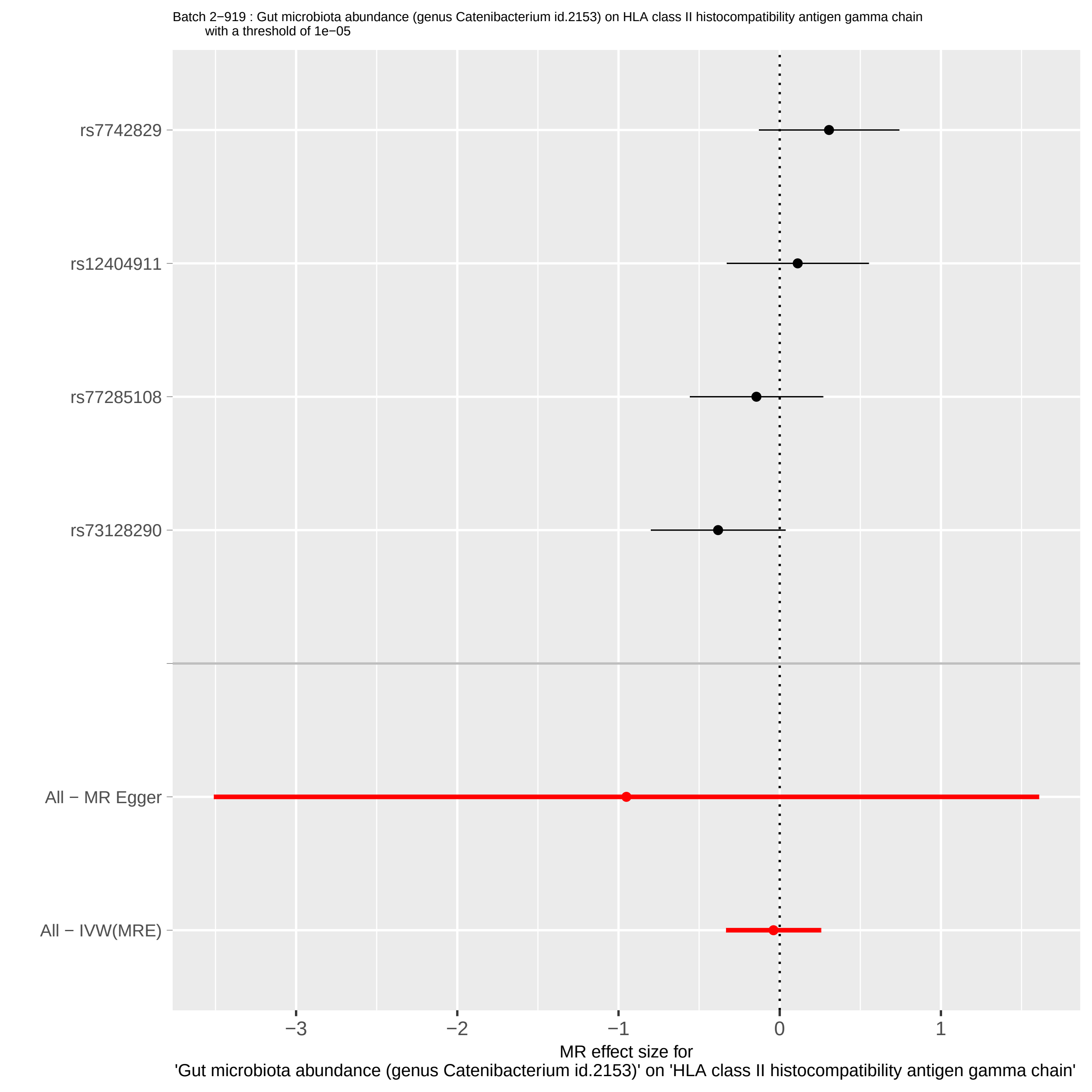

Batch 2−919 : Gut microbiota abundance (genus Catenibacterium id.2153) on HLA class II histocompatibility antigen gamma chain
with a threshold of 1e−05
rs7742829
rs12404911
rs77285108
rs73128290
All − MR Egger
All − IVW(MRE)
−3
−2
−1
0
1
MR effect size for
'Gut microbiota abundance (genus Catenibacterium id.2153)' on 'HLA class II histocompatibility antigen gamma chain'

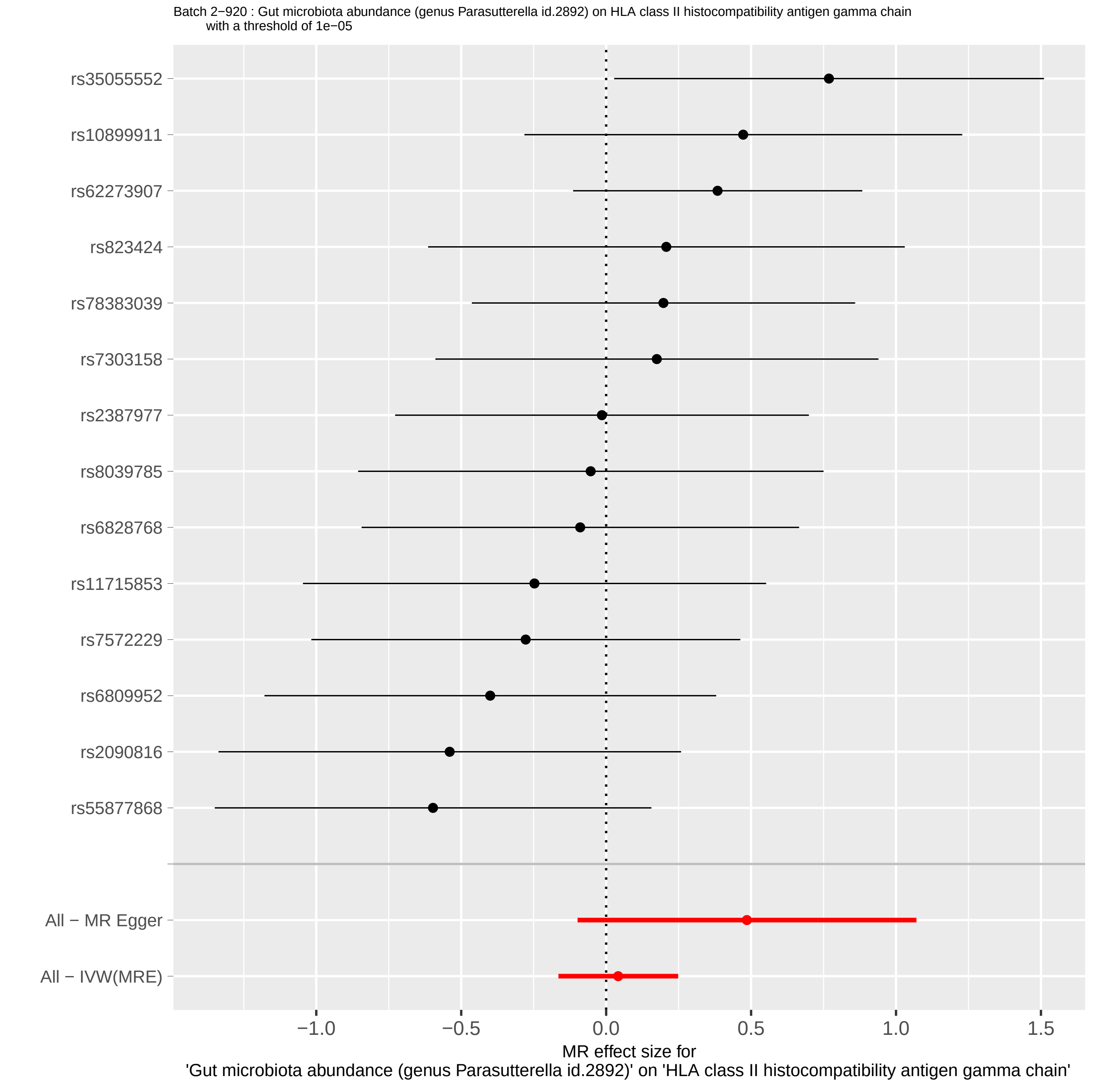

Batch 2−920 : Gut microbiota abundance (genus Parasutterella id.2892) on HLA class II histocompatibility antigen gamma chain
with a threshold of 1e−05
rs35055552
rs10899911
rs62273907
rs823424
rs78383039
rs7303158
rs2387977
rs8039785
rs6828768
rs11715853
rs7572229
rs6809952
rs2090816
rs55877868
All − MR Egger
All − IVW(MRE)
−1.0
−0.5
0.0
0.5
1.0
1.5
MR effect size for
'Gut microbiota abundance (genus Parasutterella id.2892)' on 'HLA class II histocompatibility antigen gamma chain'

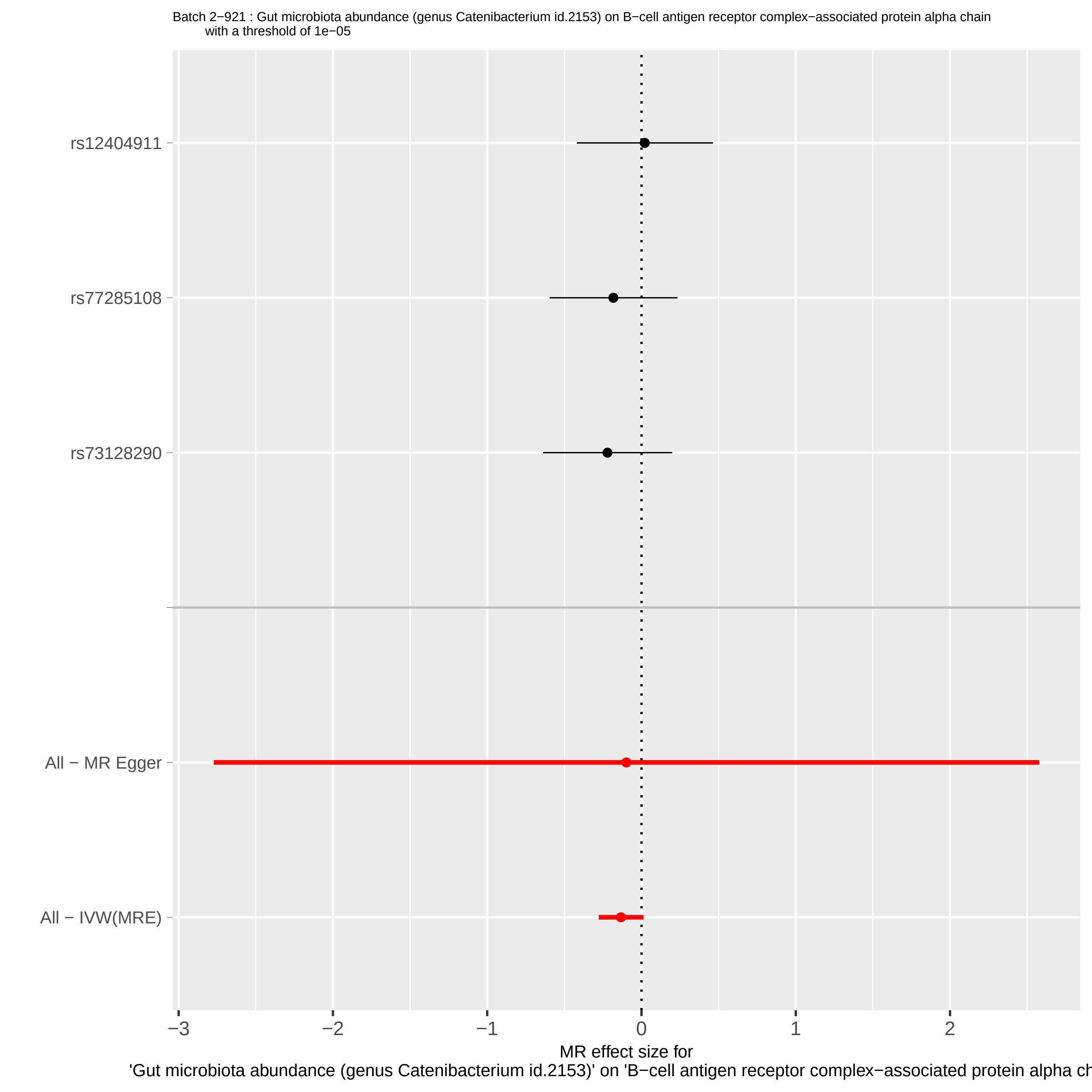
Batch 2−921 : Gut microbiota abundance (genus Catenibacterium id.2153) on B−cell antigen receptor complex−associated protein alpha chain
with a threshold of 1e−05
rs12404911
rs77285108
rs73128290
All − MR Egger
All − IVW(MRE)
−3
−2
−1
0
1
2
MR effect size for
'Gut microbiota abundance (genus Catenibacterium id.2153)' on 'B−cell antigen receptor complex−associated protein alpha ch

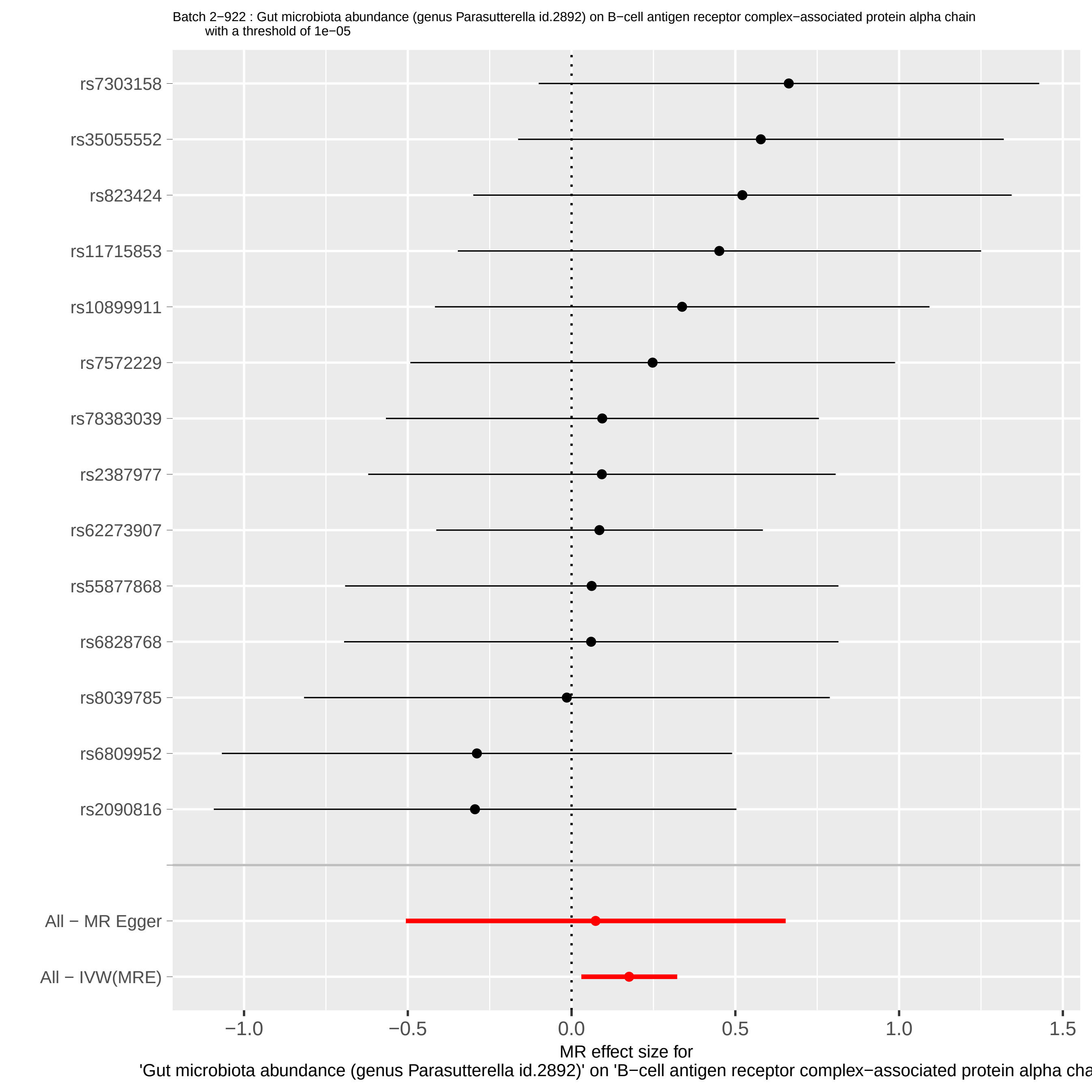
Batch 2−922 : Gut microbiota abundance (genus Parasutterella id.2892) on B−cell antigen receptor complex−associated protein alpha chain
with a threshold of 1e−05
rs7303158
rs35055552
rs823424
rs11715853
rs10899911
rs7572229
rs78383039
rs2387977
rs62273907
rs55877868
rs6828768
rs8039785
rs6809952
rs2090816
All − MR Egger
All − IVW(MRE)
−1.0
−0.5
0.0
0.5
1.0
1.5
MR effect size for
'Gut microbiota abundance (genus Parasutterella id.2892)' on 'B−cell antigen receptor complex−associated protein alpha cha

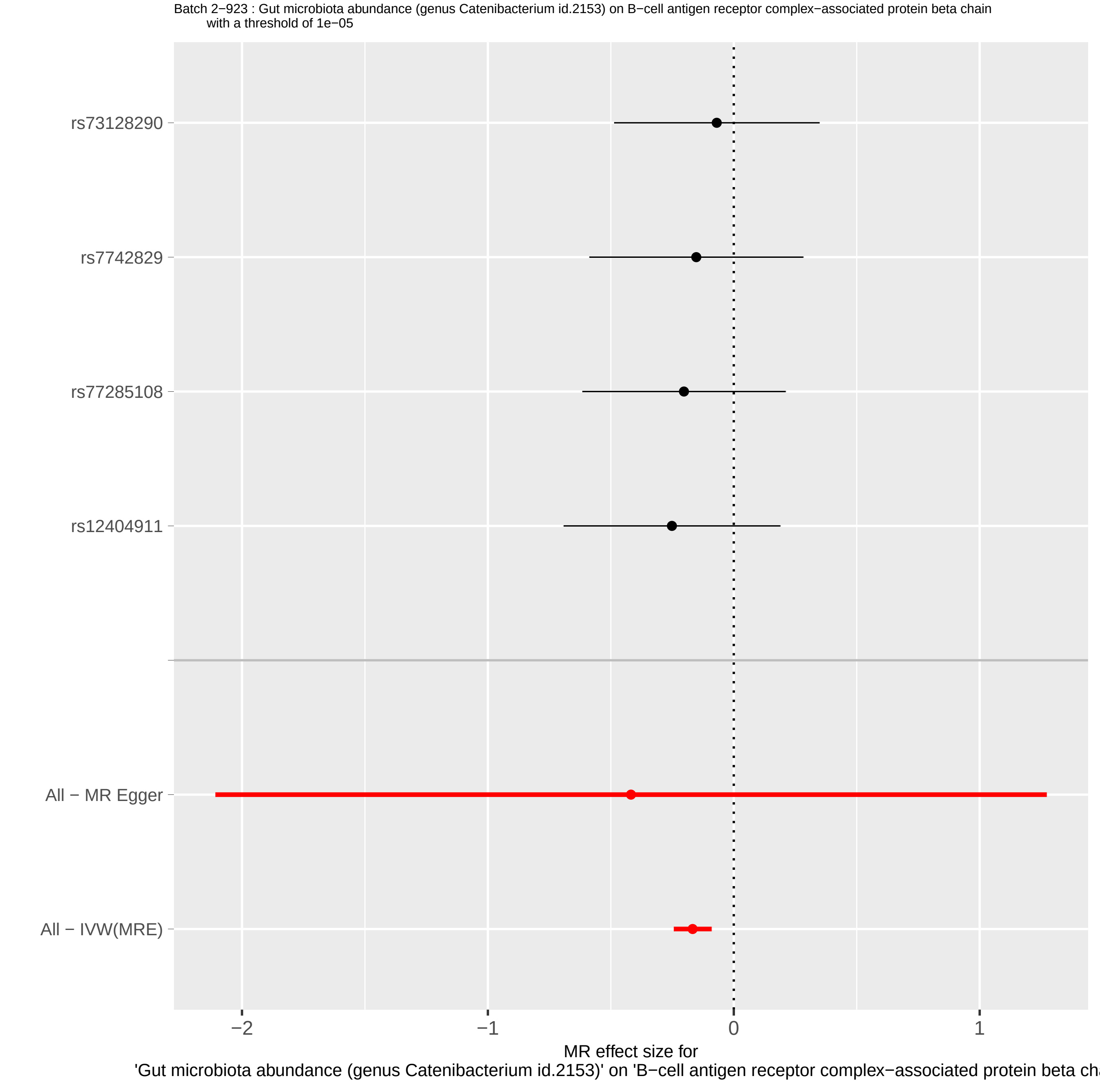
Batch 2−923 : Gut microbiota abundance (genus Catenibacterium id.2153) on B−cell antigen receptor complex−associated protein beta chain
with a threshold of 1e−05
rs73128290
rs7742829
rs77285108
rs12404911
All − MR Egger
All − IVW(MRE)
−2
−1
0
1
MR effect size for
'Gut microbiota abundance (genus Catenibacterium id.2153)' on 'B−cell antigen receptor complex−associated protein beta cha

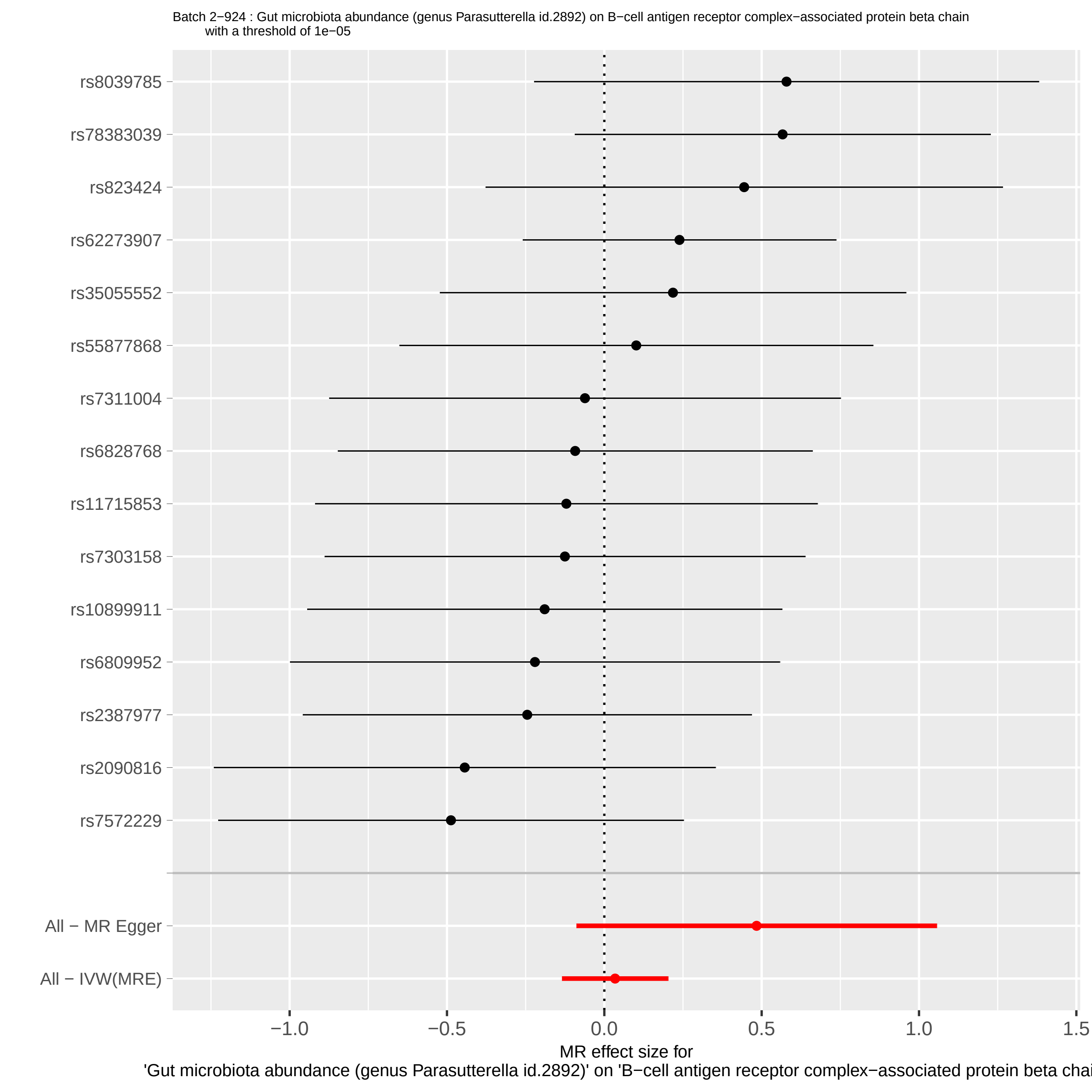

Batch 2−924 : Gut microbiota abundance (genus Parasutterella id.2892) on B−cell antigen receptor complex−associated protein beta chain with a threshold of 1e−05
rs8039785
rs78383039
rs823424
rs62273907
rs35055552
rs55877868
rs7311004
rs6828768
rs11715853
rs7303158
rs10899911
rs6809952
rs2387977
rs2090816
rs7572229
All − MR Egger
All − IVW(MRE)
−1.0
−0.5
0.0
0.5
1.0
1.5
MR effect size for
'Gut microbiota abundance (genus Parasutterella id.2892)' on 'B−cell antigen receptor complex−associated protein beta chai

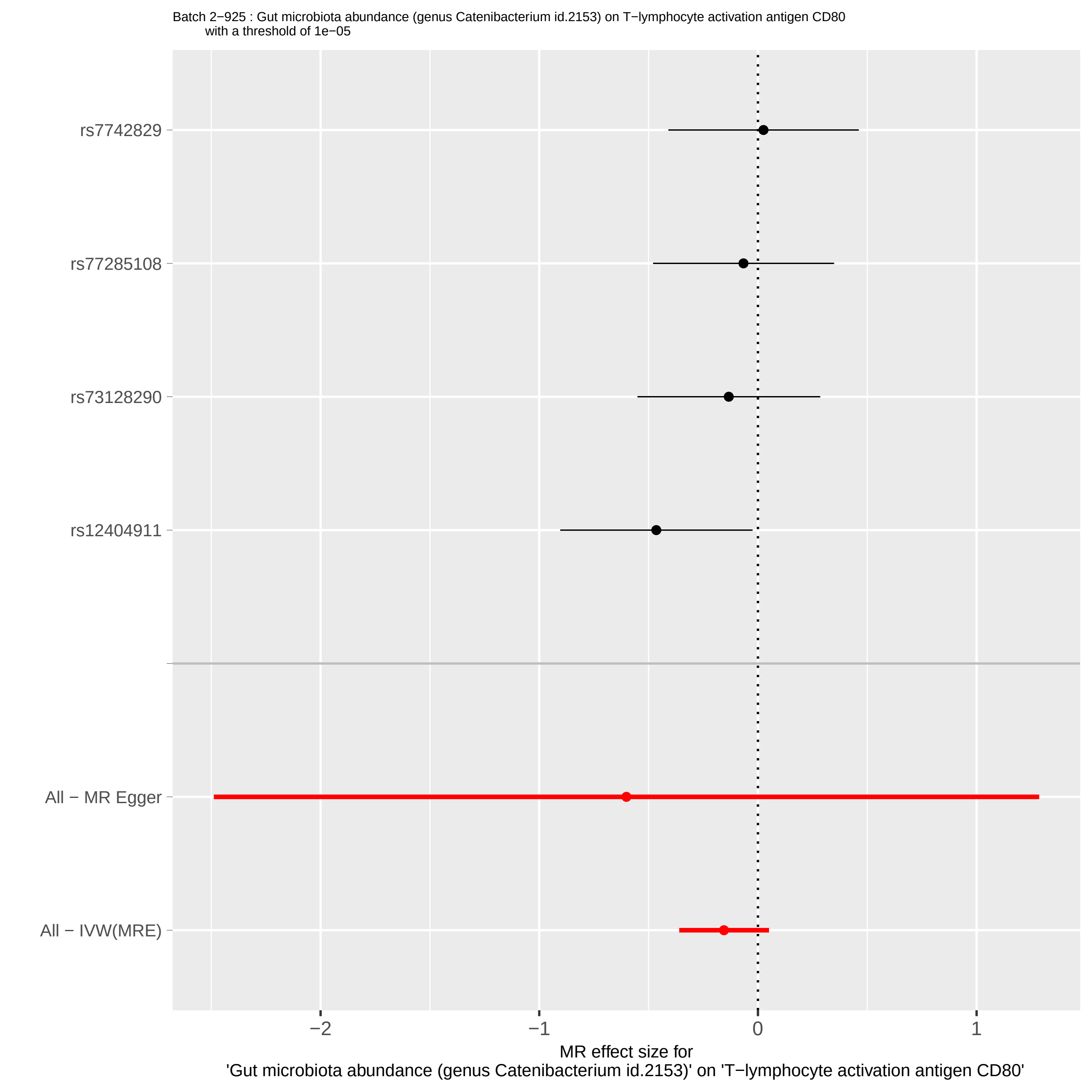

Batch 2−925 : Gut microbiota abundance (genus Catenibacterium id.2153) on T−lymphocyte activation antigen CD80
with a threshold of 1e−05
rs7742829
rs77285108
rs73128290
rs12404911
All − MR Egger
All − IVW(MRE)
−2
−1
0
1
MR effect size for
'Gut microbiota abundance (genus Catenibacterium id.2153)' on 'T−lymphocyte activation antigen CD80'

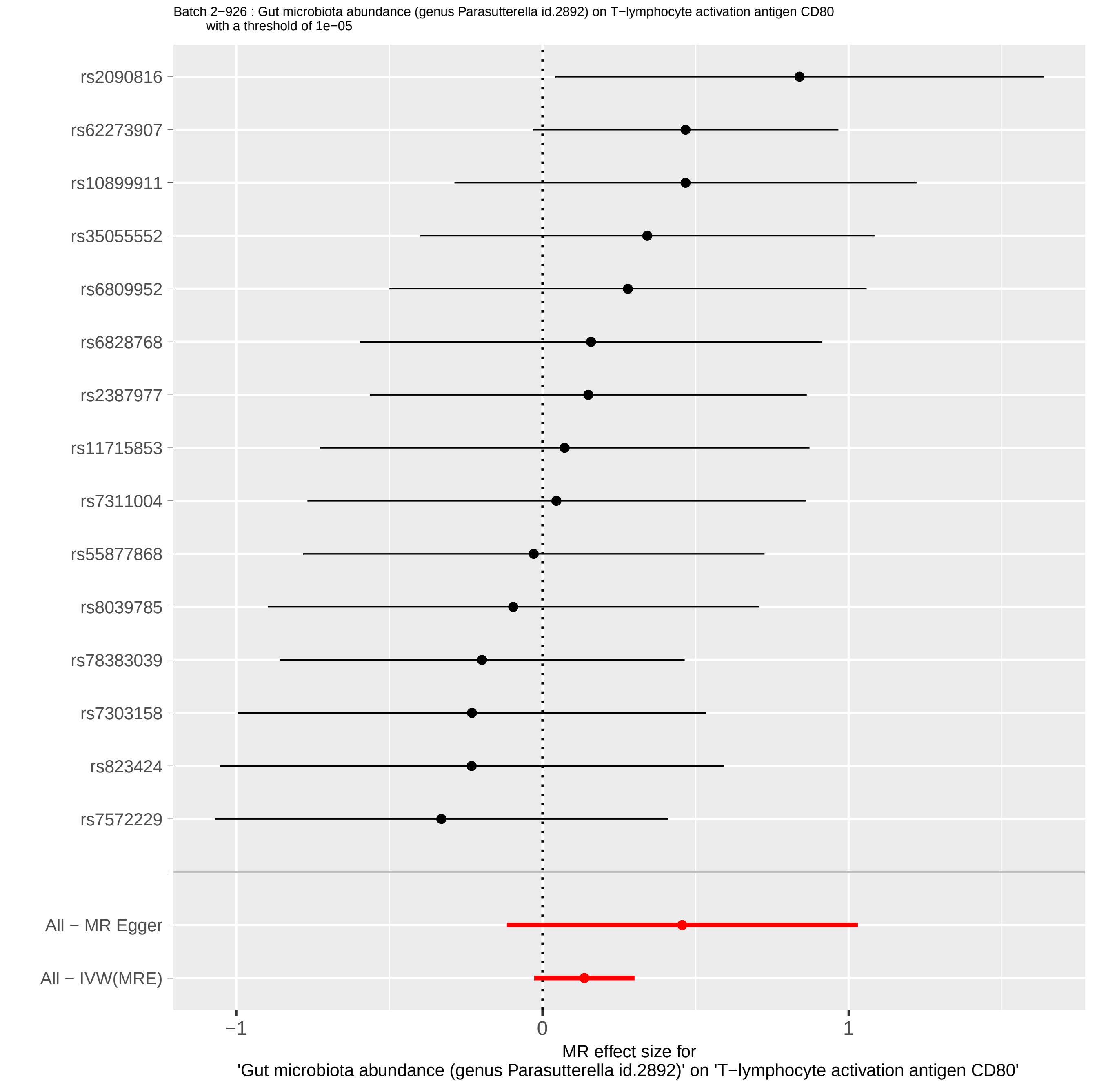

Batch 2−926 : Gut microbiota abundance (genus Parasutterella id.2892) on T−lymphocyte activation antigen CD80
with a threshold of 1e−05
rs2090816
rs62273907
rs10899911
rs35055552
rs6809952
rs6828768
rs2387977
rs11715853
rs7311004
rs55877868
rs8039785
rs78383039
rs7303158
rs823424
rs7572229
All − MR Egger
All − IVW(MRE)
−1
0
1
MR effect size for
'Gut microbiota abundance (genus Parasutterella id.2892)' on 'T−lymphocyte activation antigen CD80'

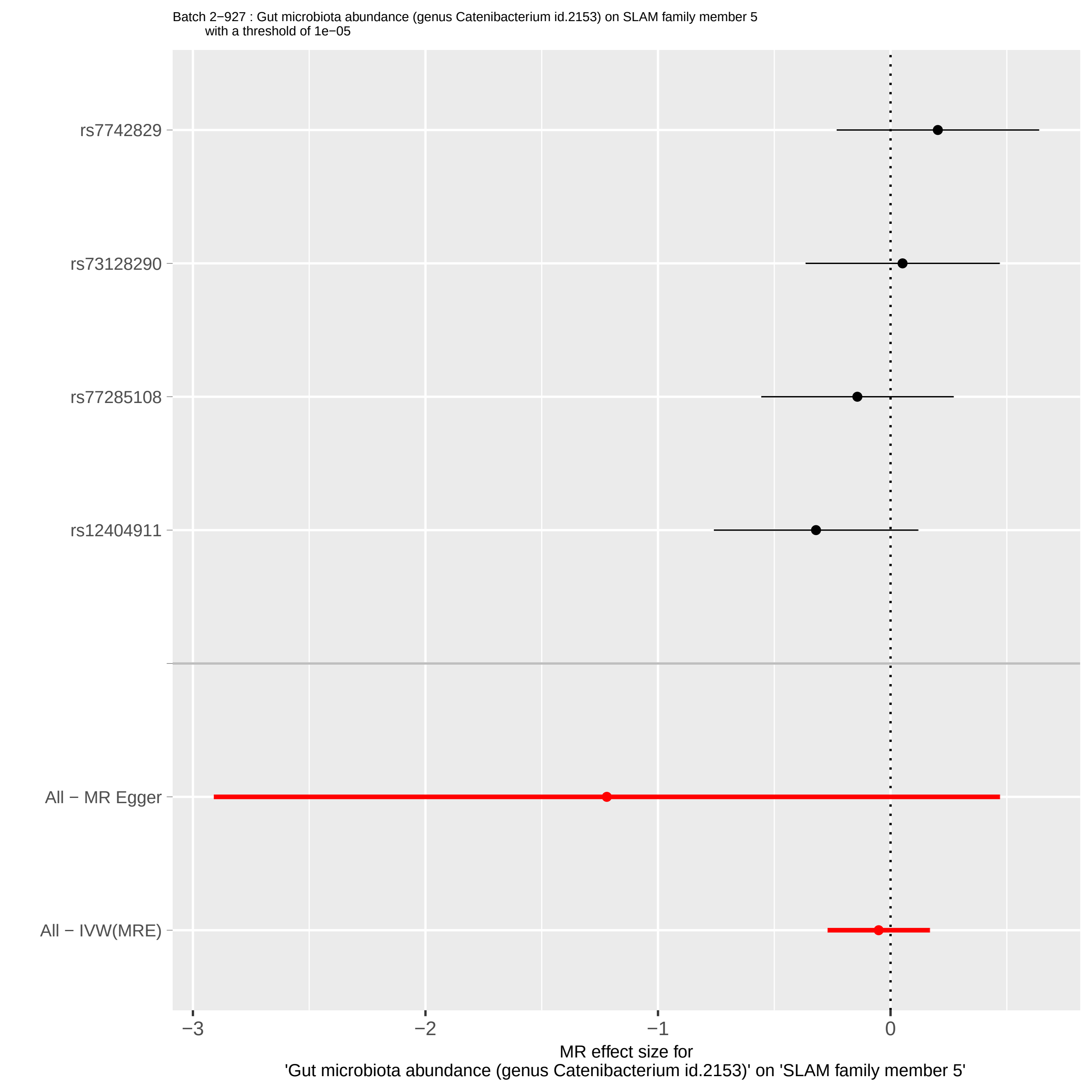

Batch 2−927 : Gut microbiota abundance (genus Catenibacterium id.2153) on SLAM family member 5
with a threshold of 1e−05
rs7742829
rs73128290
rs77285108
rs12404911
All − MR Egger
All − IVW(MRE)
−3
−2
−1
0
MR effect size for
'Gut microbiota abundance (genus Catenibacterium id.2153)' on 'SLAM family member 5'

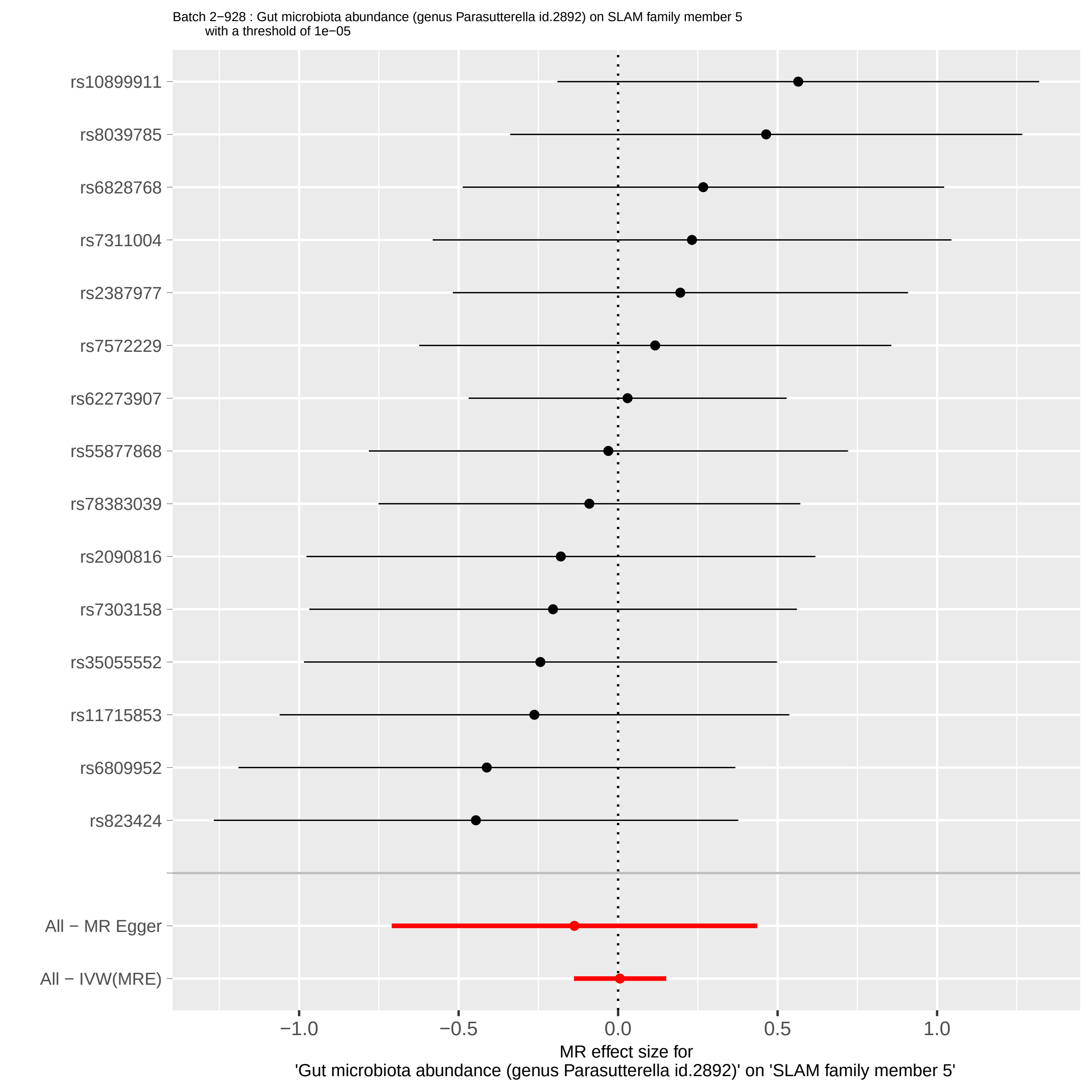
Batch 2−928 : Gut microbiota abundance (genus Parasutterella id.2892) on SLAM family member 5
with a threshold of 1e−05
rs10899911
rs8039785
rs6828768
rs7311004
rs2387977
rs7572229
rs62273907
rs55877868
rs78383039
rs2090816
rs7303158
rs35055552
rs11715853
rs6809952
rs823424
All − MR Egger
All − IVW(MRE)
−1.0
−0.5
0.0
0.5
1.0
MR effect size for
'Gut microbiota abundance (genus Parasutterella id.2892)' on 'SLAM family member 5'

Batch 2−929 : Gut microbiota abundance (genus Catenibacterium id.2153) on SLAM family member 5
with a threshold of 1e−05

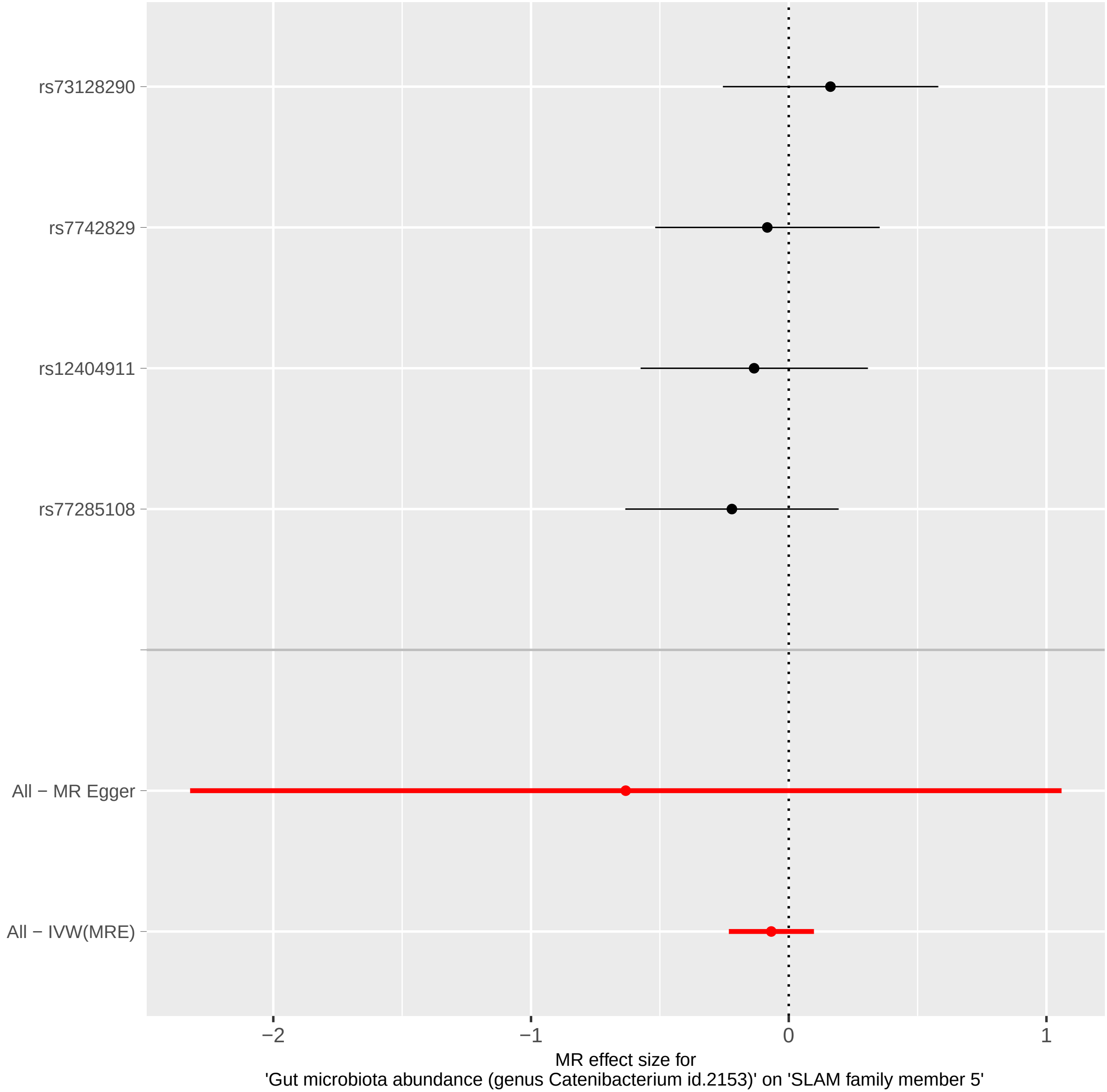

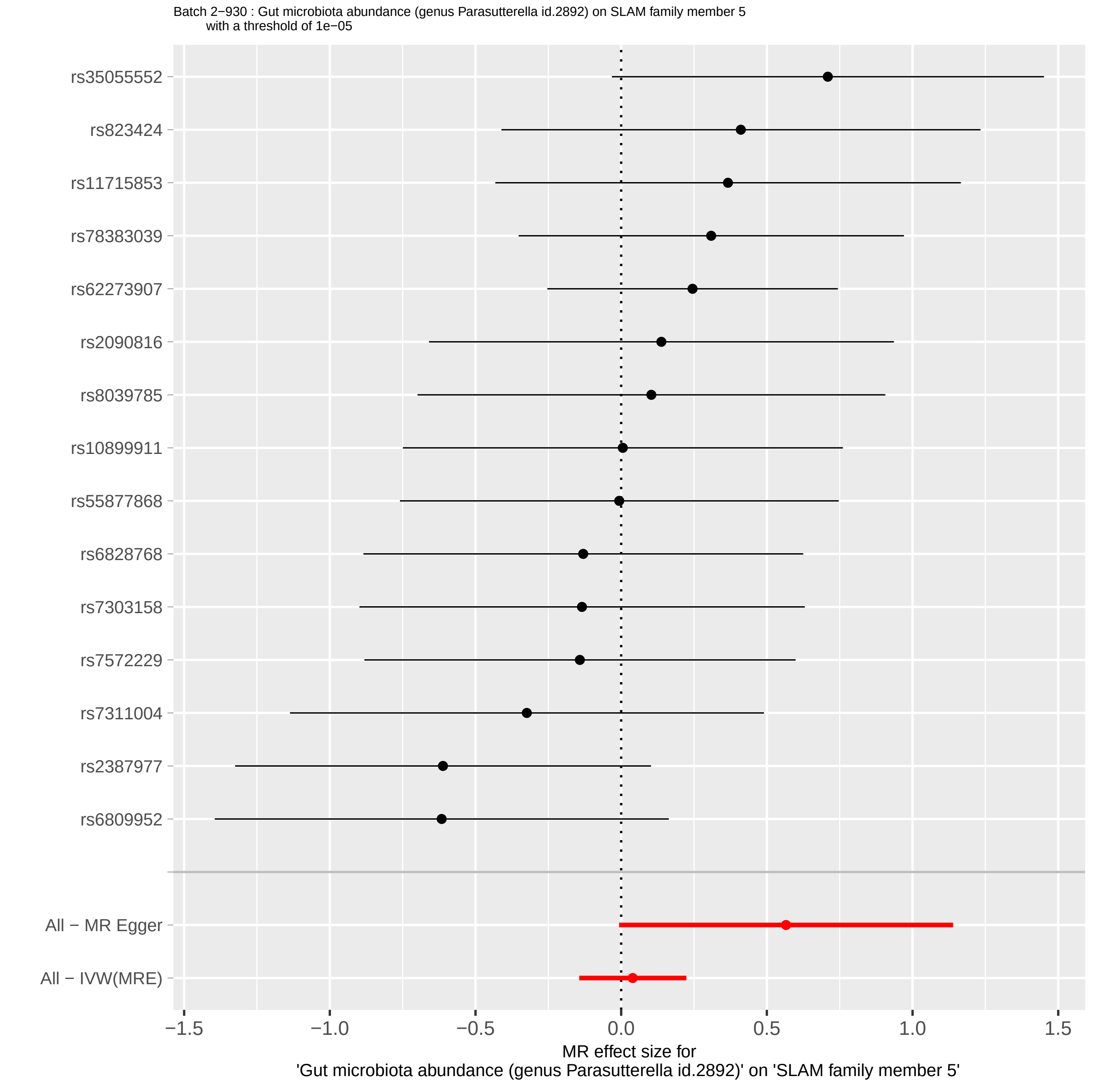

Batch 2−930 : Gut microbiota abundance (genus Parasutterella id.2892) on SLAM family member 5
with a threshold of 1e−05
rs35055552
rs823424
rs11715853
rs78383039
rs62273907
rs2090816
rs8039785
rs10899911
rs55877868
rs6828768
rs7303158
rs7572229
rs7311004
rs2387977
rs6809952
All − MR Egger
All − IVW(MRE)
−1.5
−1.0
−0.5
0.0
0.5
1.0
1.5
MR effect size for
'Gut microbiota abundance (genus Parasutterella id.2892)' on 'SLAM family member 5'

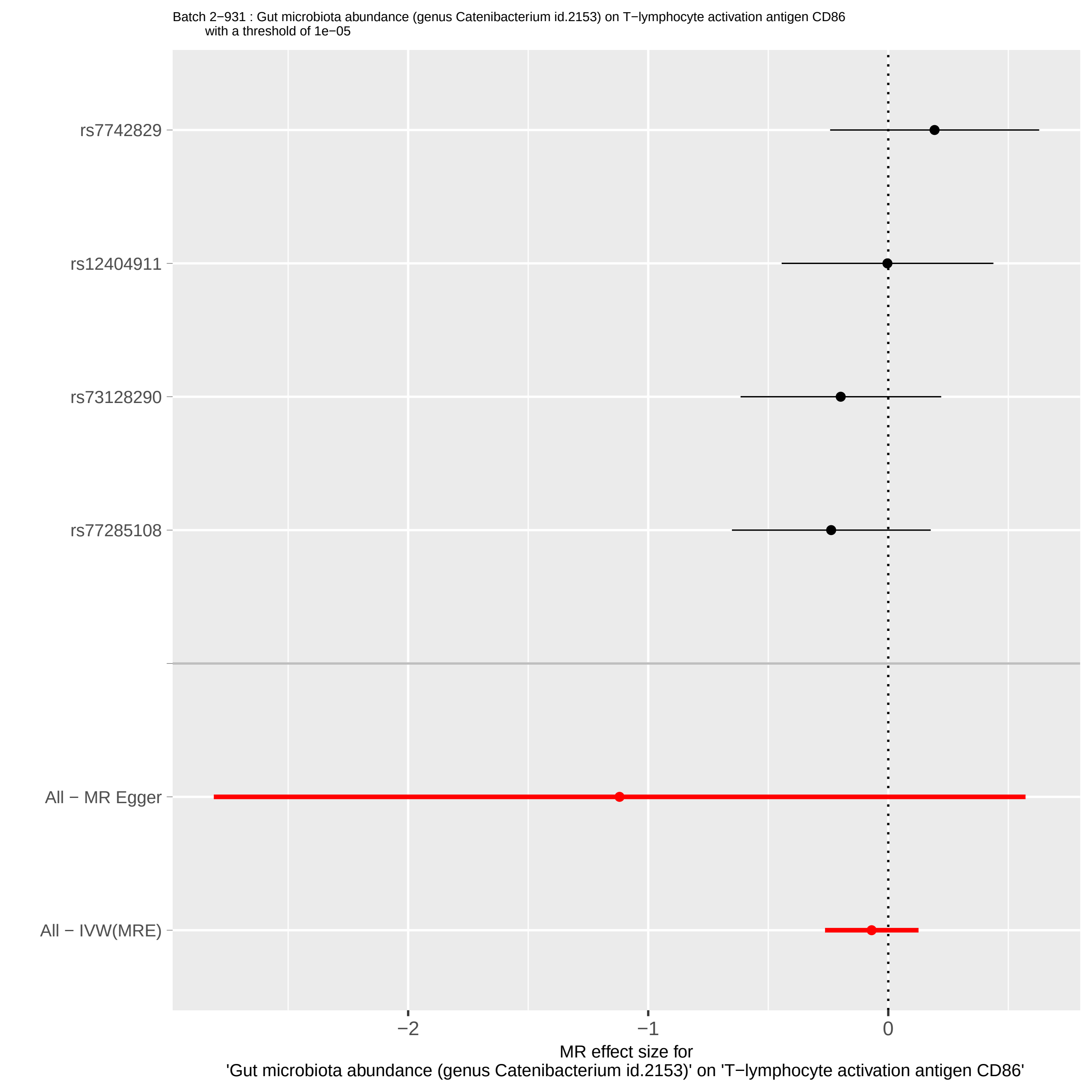
Batch 2−931 : Gut microbiota abundance (genus Catenibacterium id.2153) on T−lymphocyte activation antigen CD86
with a threshold of 1e−05
rs7742829
rs12404911
rs73128290
rs77285108
All − MR Egger
All − IVW(MRE)
−2
−1
0
MR effect size for
'Gut microbiota abundance (genus Catenibacterium id.2153)' on 'T−lymphocyte activation antigen CD86'

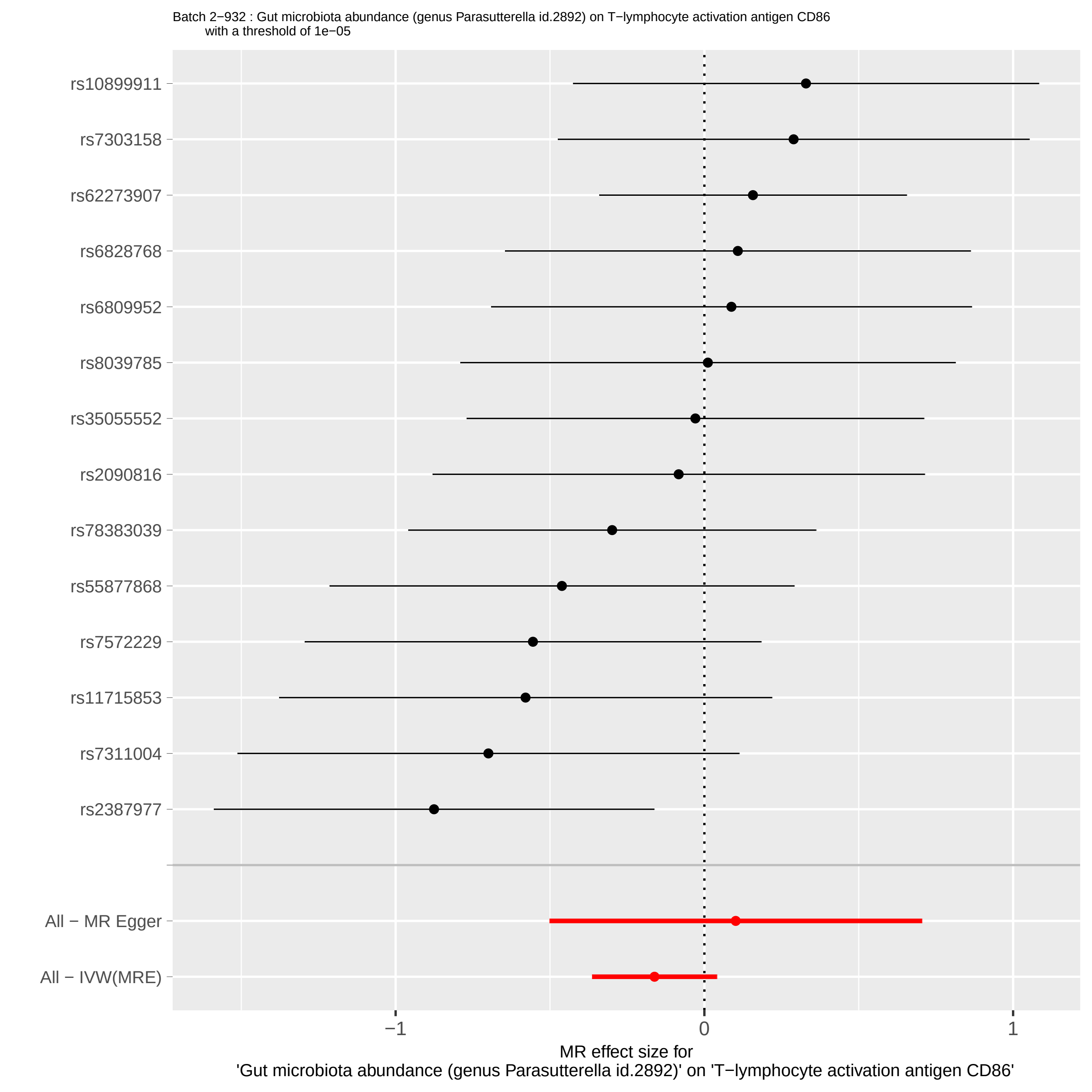

Batch 2−932 : Gut microbiota abundance (genus Parasutterella id.2892) on T−lymphocyte activation antigen CD86
with a threshold of 1e−05
rs10899911
rs7303158
rs62273907
rs6828768
rs6809952
rs8039785
rs35055552
rs2090816
rs78383039
rs55877868
rs7572229
rs11715853
rs7311004
rs2387977
All − MR Egger
All − IVW(MRE)
−1
0
1
MR effect size for
'Gut microbiota abundance (genus Parasutterella id.2892)' on 'T−lymphocyte activation antigen CD86'

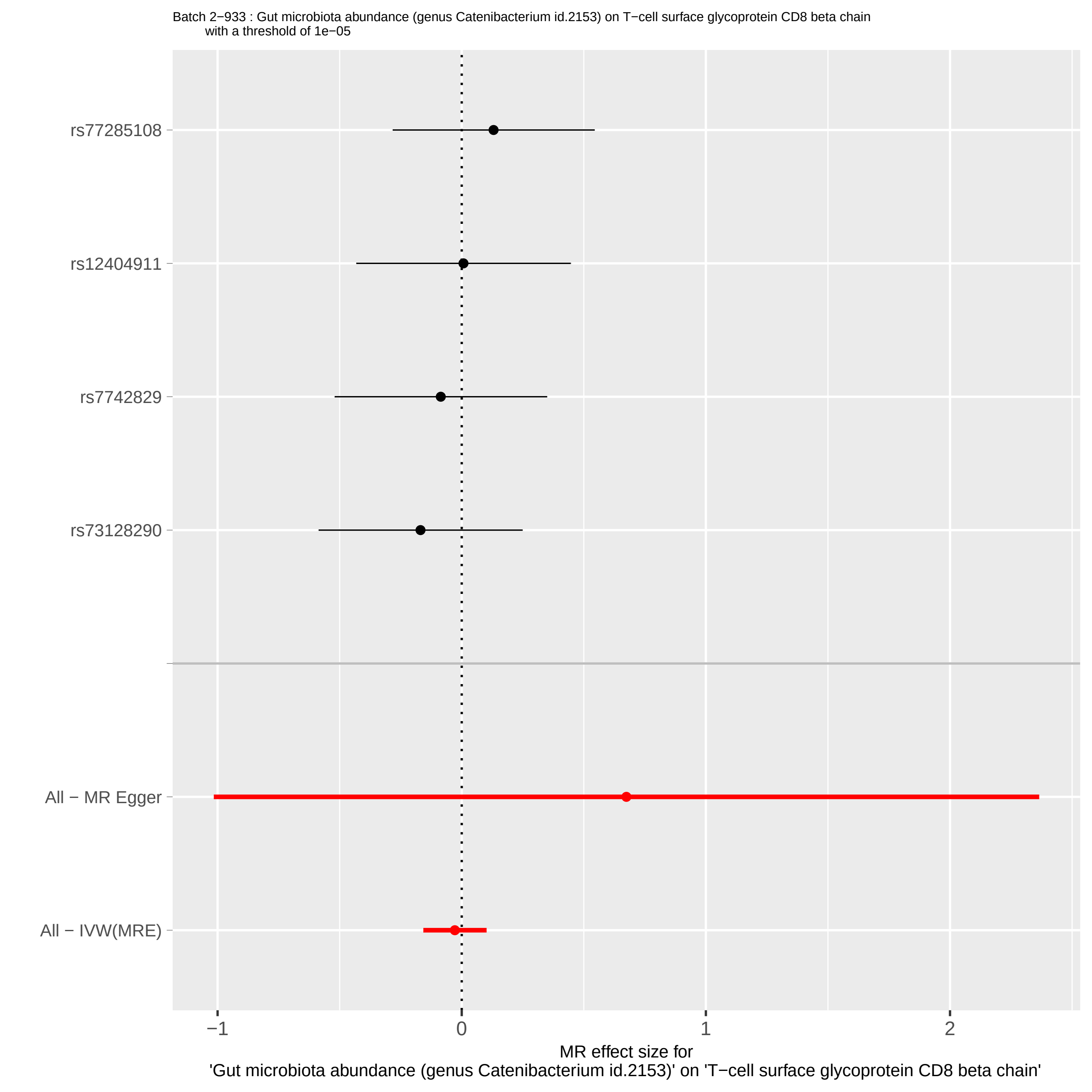

Batch 2−933 : Gut microbiota abundance (genus Catenibacterium id.2153) on T−cell surface glycoprotein CD8 beta chain
with a threshold of 1e−05
rs77285108
rs12404911
rs7742829
rs73128290
All − MR Egger
All − IVW(MRE)
−1
0
1
2
MR effect size for
'Gut microbiota abundance (genus Catenibacterium id.2153)' on 'T−cell surface glycoprotein CD8 beta chain'

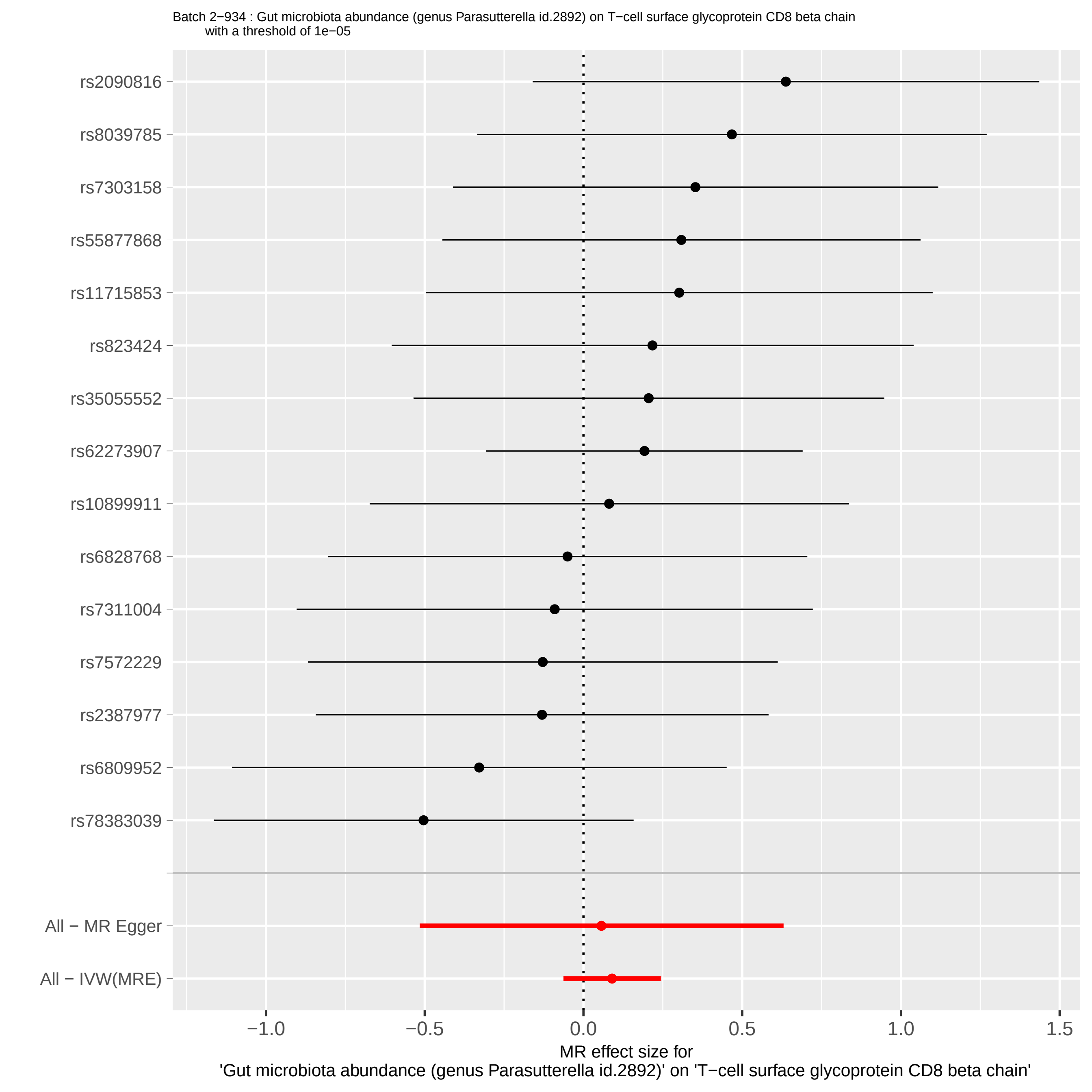

Batch 2−934 : Gut microbiota abundance (genus Parasutterella id.2892) on T−cell surface glycoprotein CD8 beta chain
with a threshold of 1e−05
rs2090816
rs8039785
rs7303158
rs55877868
rs11715853
rs823424
rs35055552
rs62273907
rs10899911
rs6828768
rs7311004
rs7572229
rs2387977
rs6809952
rs78383039
All − MR Egger
All − IVW(MRE)
−1.0
−0.5
0.0
0.5
1.0
1.5
MR effect size for
'Gut microbiota abundance (genus Parasutterella id.2892)' on 'T−cell surface glycoprotein CD8 beta chain'

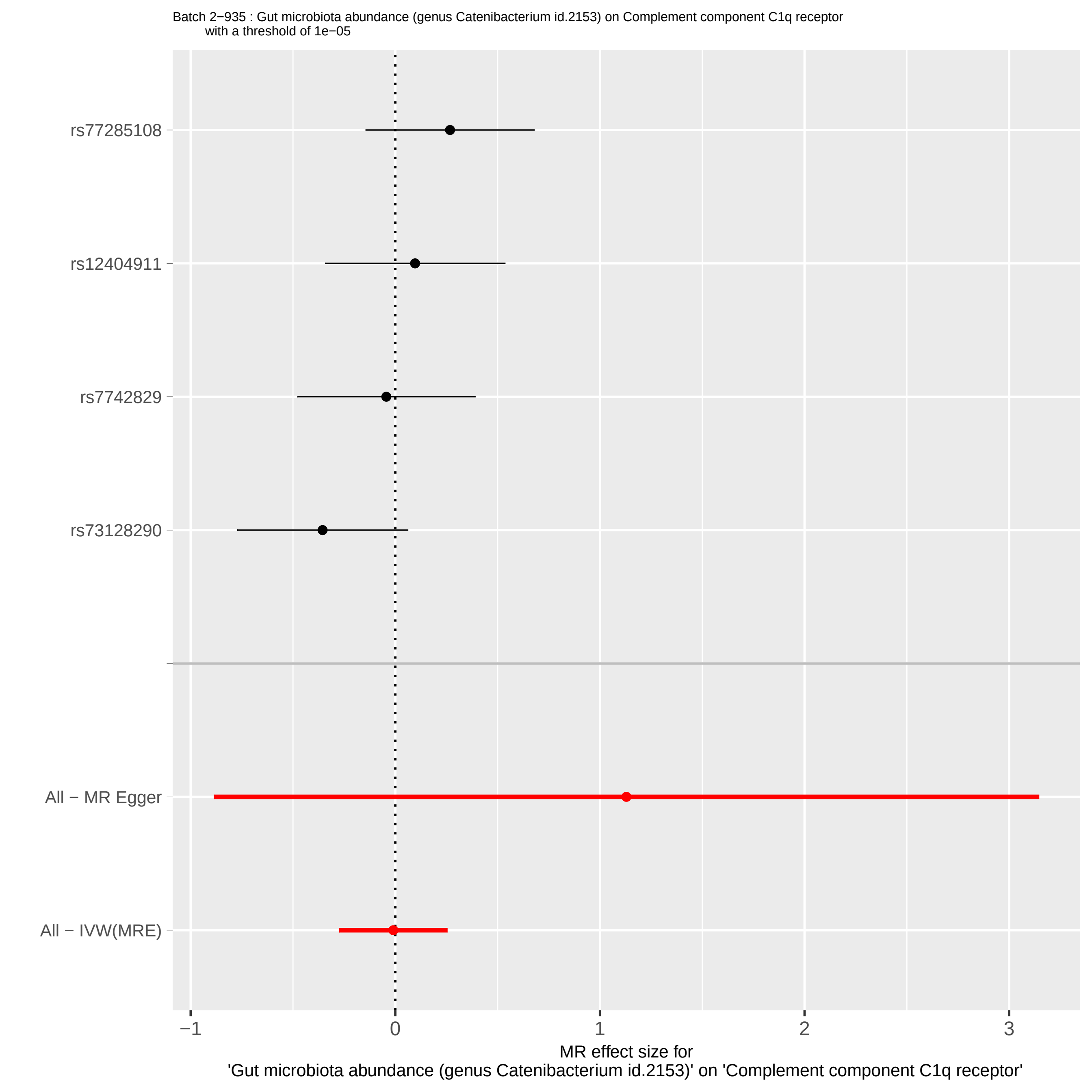

Batch 2−935 : Gut microbiota abundance (genus Catenibacterium id.2153) on Complement component C1q receptor
with a threshold of 1e−05
rs77285108
rs12404911
rs7742829
rs73128290
All − MR Egger
All − IVW(MRE)
−1
0
1
2
3
MR effect size for
'Gut microbiota abundance (genus Catenibacterium id.2153)' on 'Complement component C1q receptor'

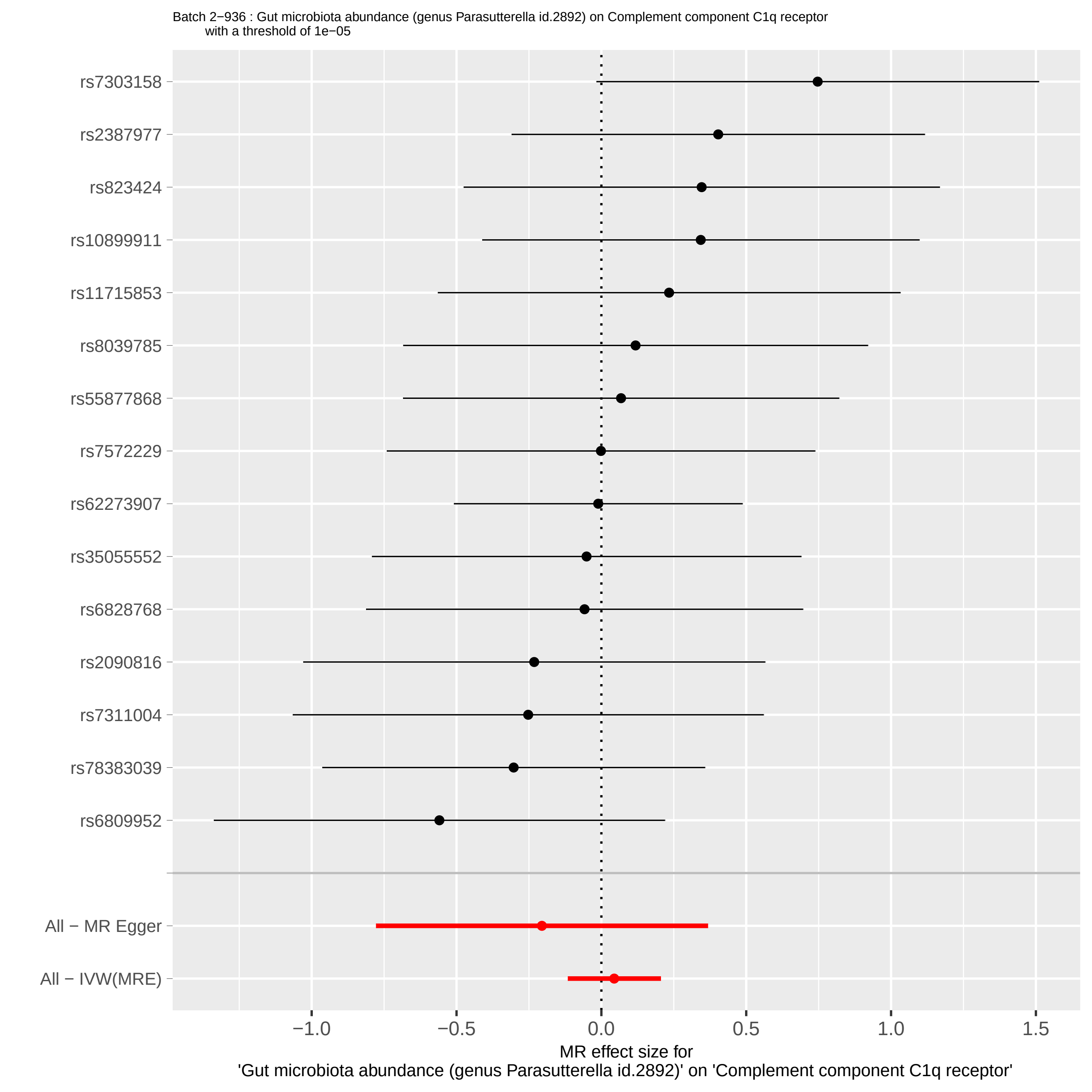

Batch 2−936 : Gut microbiota abundance (genus Parasutterella id.2892) on Complement component C1q receptor
with a threshold of 1e−05
rs7303158
rs2387977
rs823424
rs10899911
rs11715853
rs8039785
rs55877868
rs7572229
rs62273907
rs35055552
rs6828768
rs2090816
rs7311004
rs78383039
rs6809952
All − MR Egger
All − IVW(MRE)
−1.0
−0.5
0.0
0.5
1.0
1.5
MR effect size for
'Gut microbiota abundance (genus Parasutterella id.2892)' on 'Complement component C1q receptor'

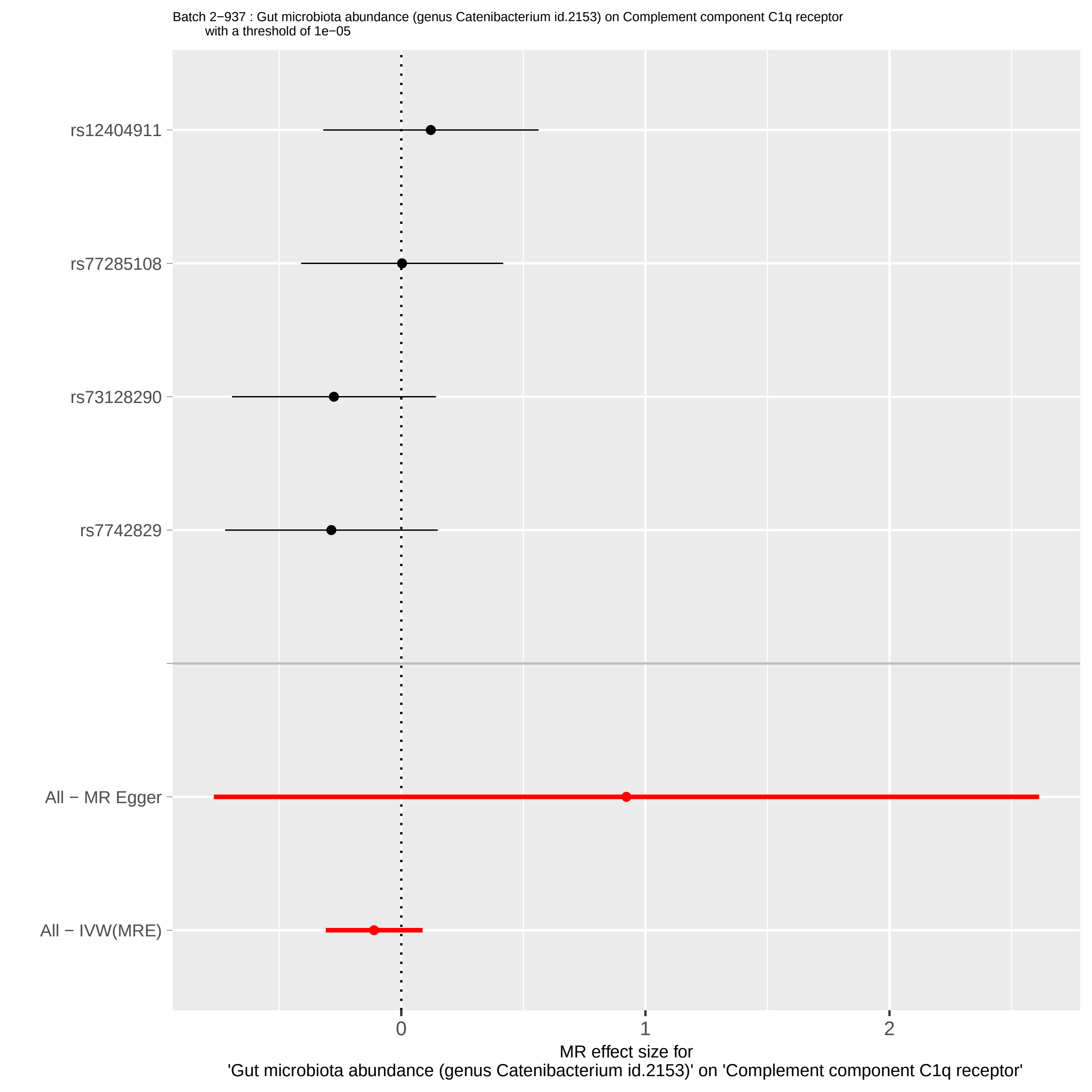

Batch 2−937 : Gut microbiota abundance (genus Catenibacterium id.2153) on Complement component C1q receptor
with a threshold of 1e−05
rs12404911
rs77285108
rs73128290
rs7742829
All − MR Egger
All − IVW(MRE)
0
1
2
MR effect size for
'Gut microbiota abundance (genus Catenibacterium id.2153)' on 'Complement component C1q receptor'

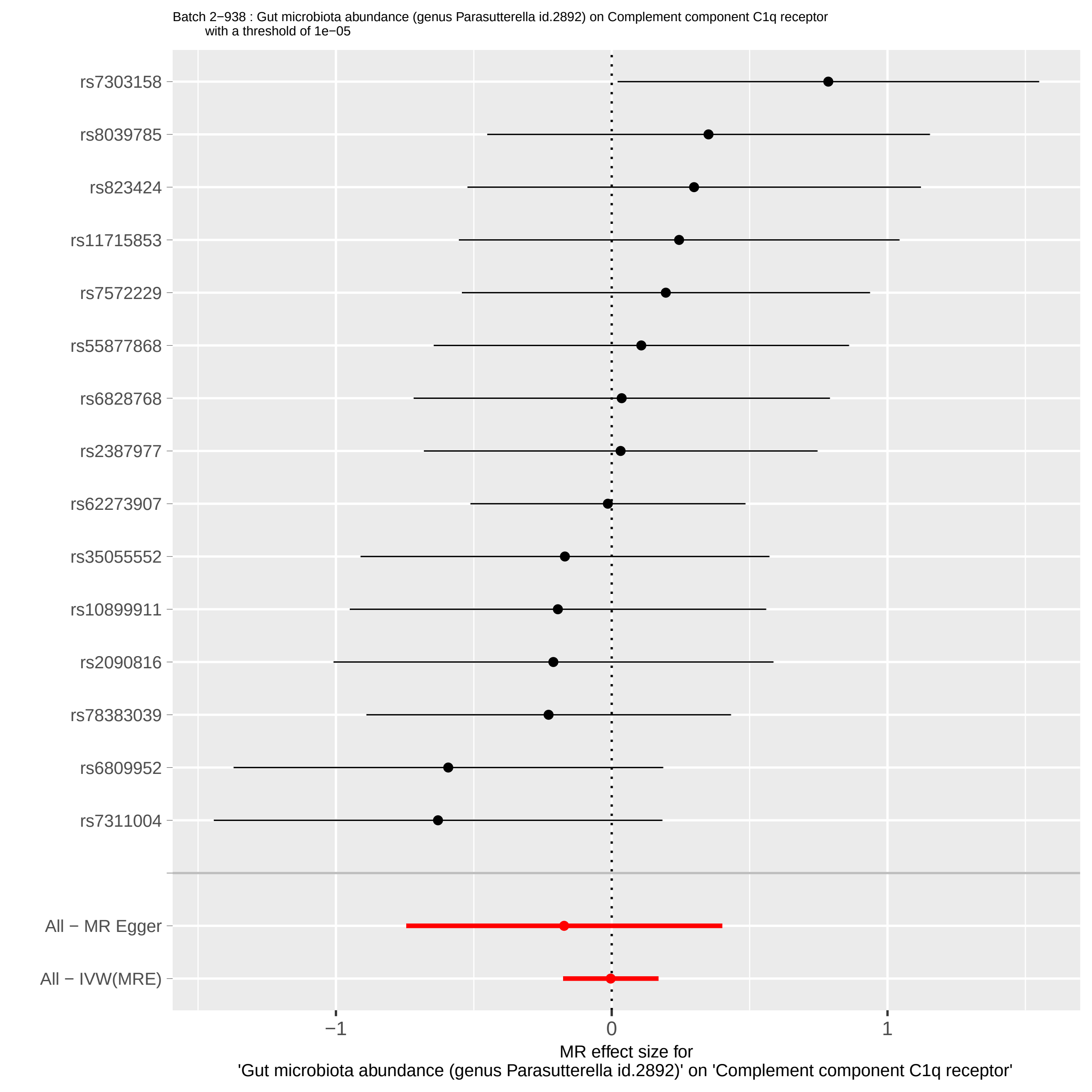
Batch 2−938 : Gut microbiota abundance (genus Parasutterella id.2892) on Complement component C1q receptor
with a threshold of 1e−05
rs7303158
rs8039785
rs823424
rs11715853
rs7572229
rs55877868
rs6828768
rs2387977
rs62273907
rs35055552
rs10899911
rs2090816
rs78383039
rs6809952
rs7311004
All − MR Egger
All − IVW(MRE)
−1
0
1
MR effect size for
'Gut microbiota abundance (genus Parasutterella id.2892)' on 'Complement component C1q receptor'

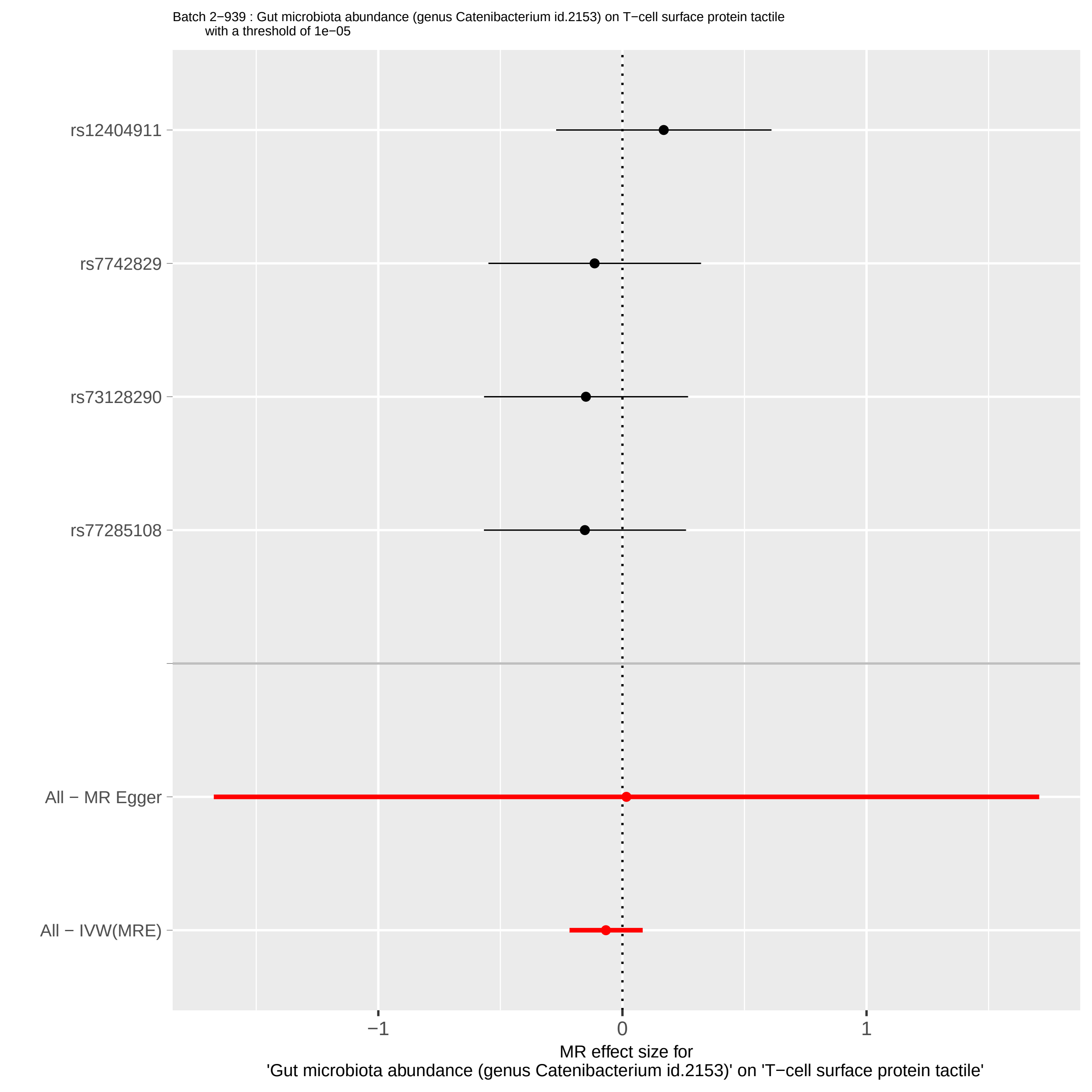
Batch 2−939 : Gut microbiota abundance (genus Catenibacterium id.2153) on T−cell surface protein tactile
with a threshold of 1e−05
rs12404911
rs7742829
rs73128290
rs77285108
All − MR Egger
All − IVW(MRE)
−1
0
1
MR effect size for
'Gut microbiota abundance (genus Catenibacterium id.2153)' on 'T−cell surface protein tactile'

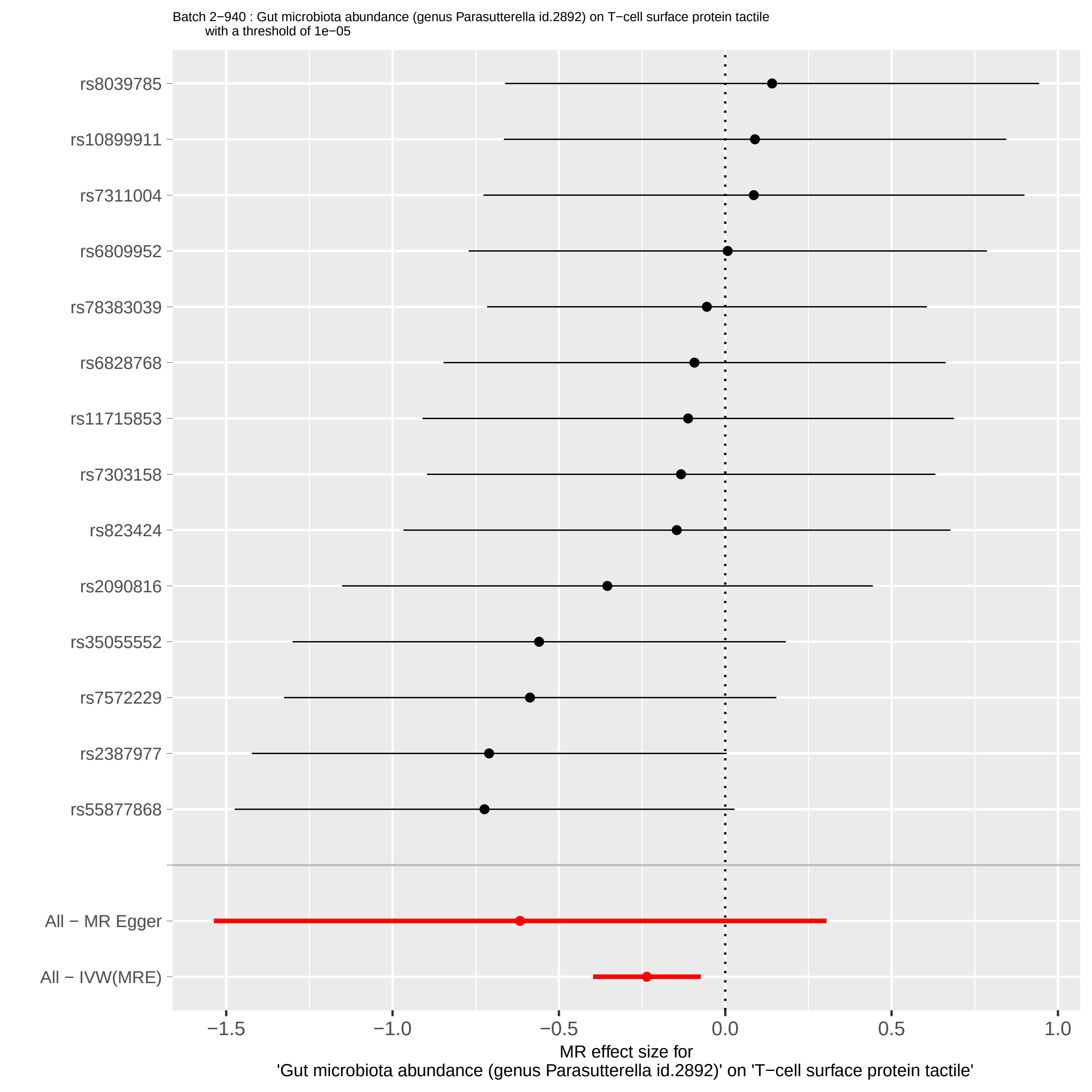

Batch 2−940 : Gut microbiota abundance (genus Parasutterella id.2892) on T−cell surface protein tactile
with a threshold of 1e−05
rs8039785
rs10899911
rs7311004
rs6809952
rs78383039
rs6828768
rs11715853
rs7303158
rs823424
rs2090816
rs35055552
rs7572229
rs2387977
rs55877868
All − MR Egger
All − IVW(MRE)
−1.5
−1.0
−0.5
0.0
0.5
1.0
MR effect size for
'Gut microbiota abundance (genus Parasutterella id.2892)' on 'T−cell surface protein tactile'

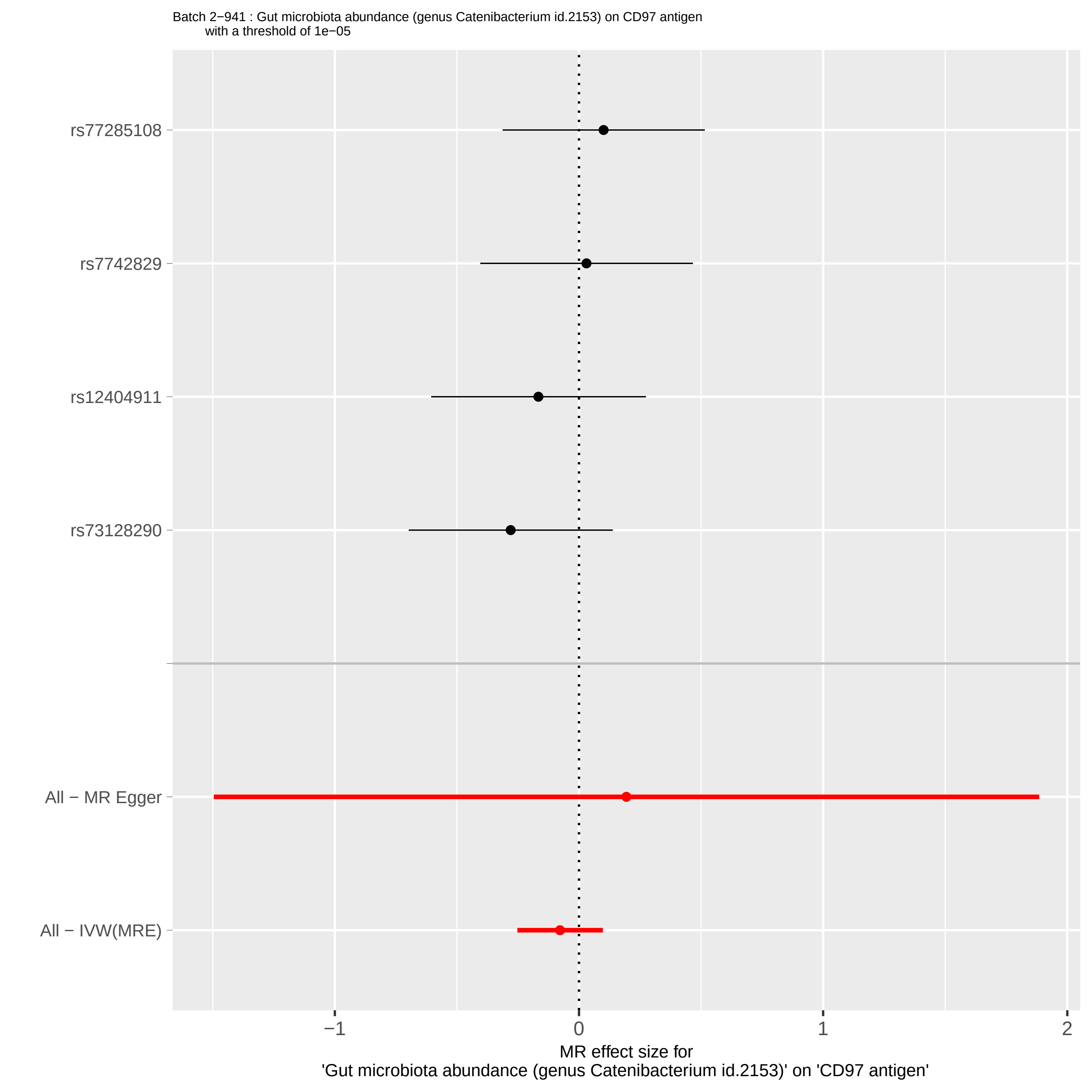

Batch 2−941 : Gut microbiota abundance (genus Catenibacterium id.2153) on CD97 antigen
with a threshold of 1e−05
rs77285108
rs7742829
rs12404911
rs73128290
All − MR Egger
All − IVW(MRE)
−1
0
1
2
MR effect size for
'Gut microbiota abundance (genus Catenibacterium id.2153)' on 'CD97 antigen'

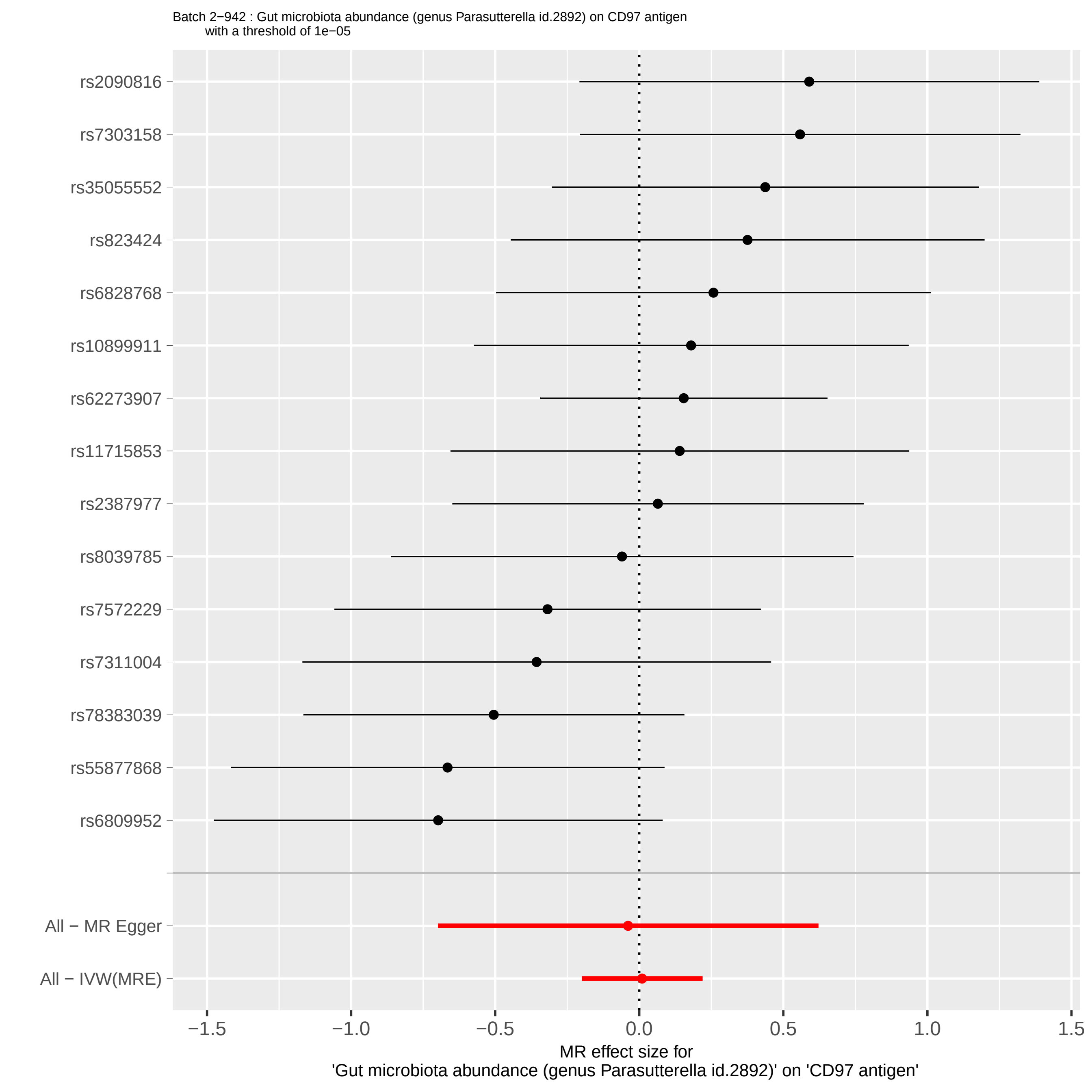

Batch 2−942 : Gut microbiota abundance (genus Parasutterella id.2892) on CD97 antigen
with a threshold of 1e−05
rs2090816
rs7303158
rs35055552
rs823424
rs6828768
rs10899911
rs62273907
rs11715853
rs2387977
rs8039785
rs7572229
rs7311004
rs78383039
rs55877868
rs6809952
All − MR Egger
All − IVW(MRE)
−1.5
−1.0
−0.5
0.0
0.5
1.0
1.5
MR effect size for
'Gut microbiota abundance (genus Parasutterella id.2892)' on 'CD97 antigen'

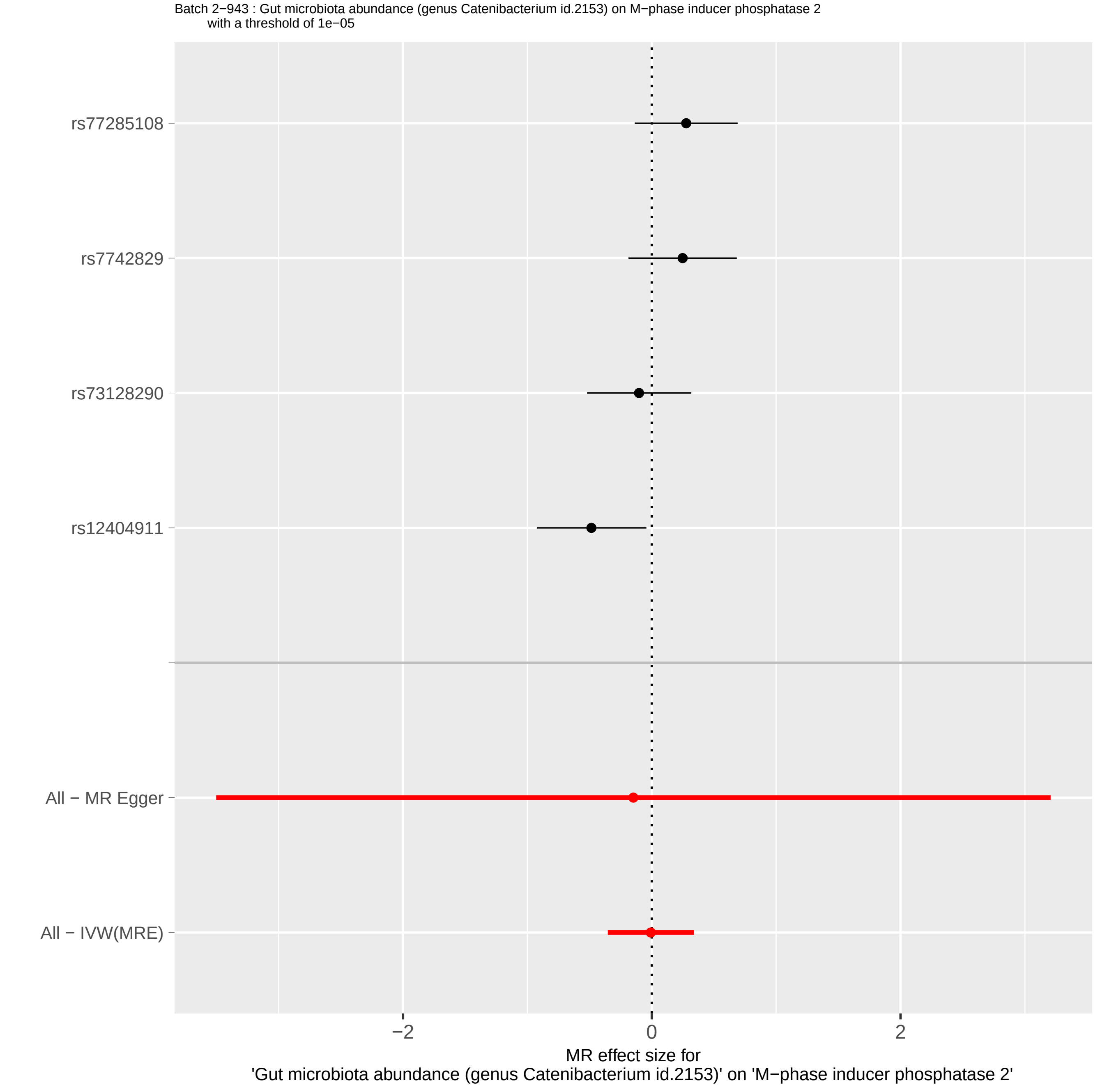

Batch 2−943 : Gut microbiota abundance (genus Catenibacterium id.2153) on M−phase inducer phosphatase 2
with a threshold of 1e−05
rs77285108
rs7742829
rs73128290
rs12404911
All − MR Egger
All − IVW(MRE)
−2
0
2
MR effect size for
'Gut microbiota abundance (genus Catenibacterium id.2153)' on 'M−phase inducer phosphatase 2'

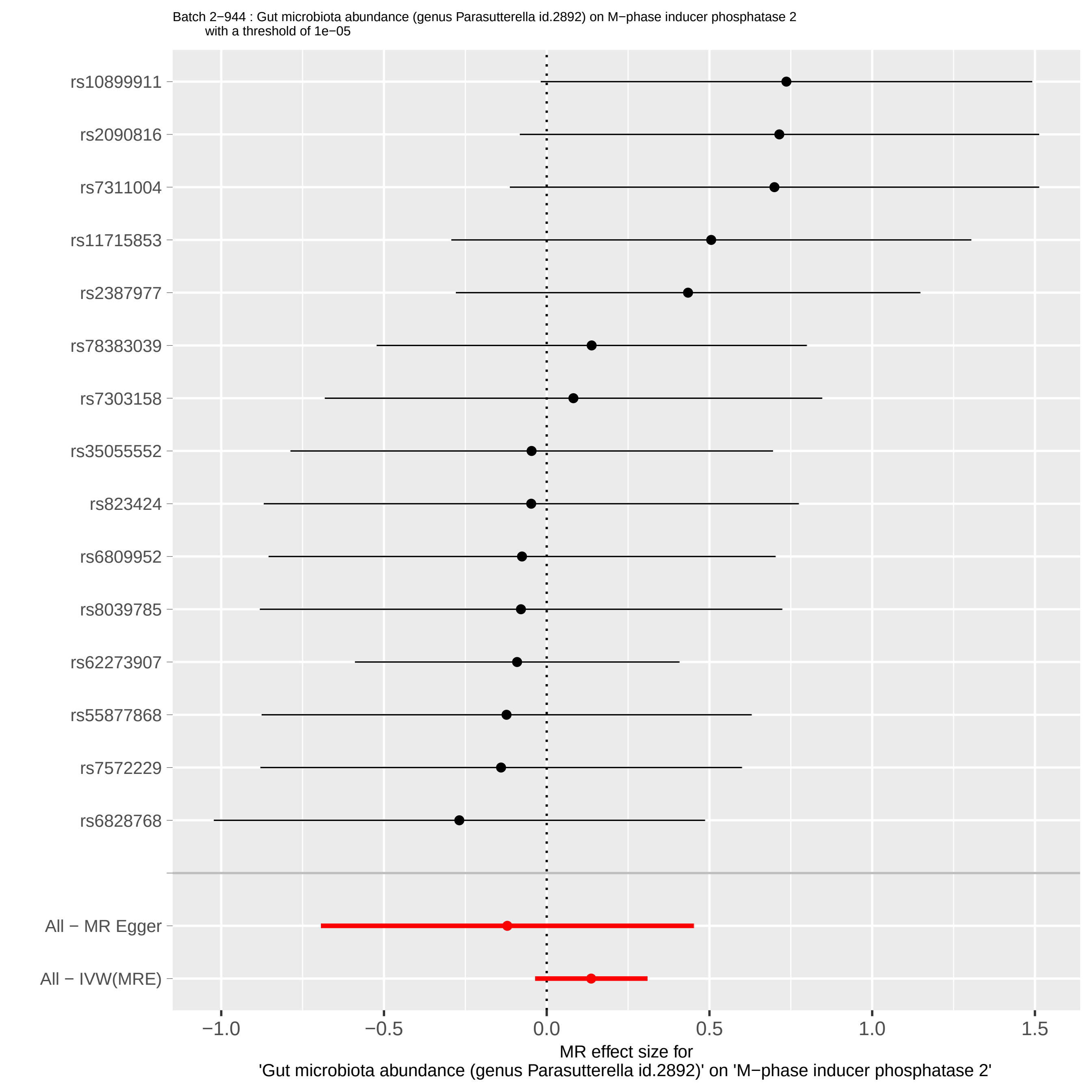
Batch 2−944 : Gut microbiota abundance (genus Parasutterella id.2892) on M−phase inducer phosphatase 2
with a threshold of 1e−05
rs10899911
rs2090816
rs7311004
rs11715853
rs2387977
rs78383039
rs7303158
rs35055552
rs823424
rs6809952
rs8039785
rs62273907
rs55877868
rs7572229
rs6828768
All − MR Egger
All − IVW(MRE)
−1.0
−0.5
0.0
0.5
1.0
1.5
MR effect size for
'Gut microbiota abundance (genus Parasutterella id.2892)' on 'M−phase inducer phosphatase 2'

Batch 2−945 : Gut microbiota abundance (genus Catenibacterium id.2153) on Cyclin−dependent kinase 1:G2/mitotic−specific cyclin−B1 complex with a threshold of 1e−05

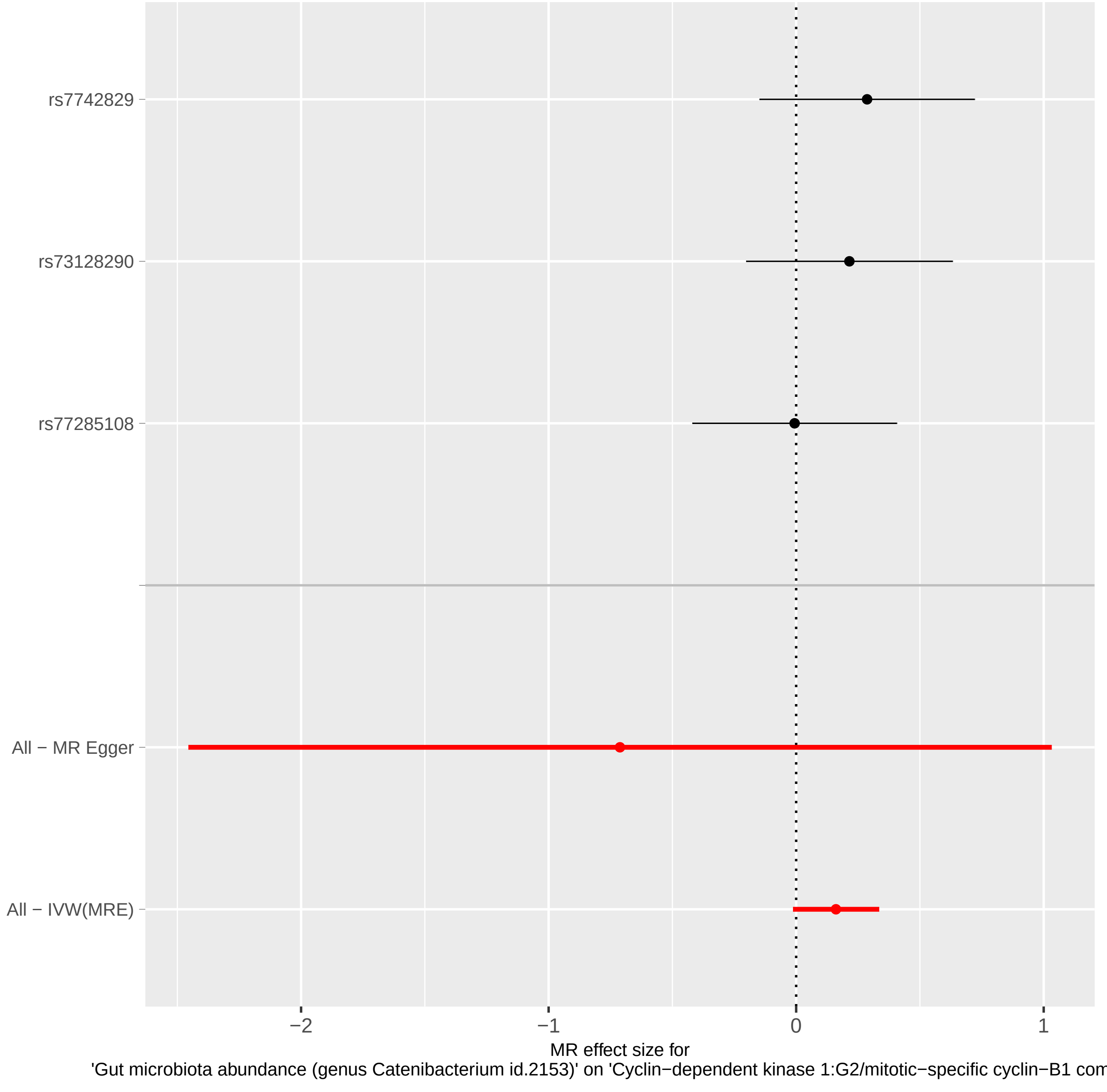

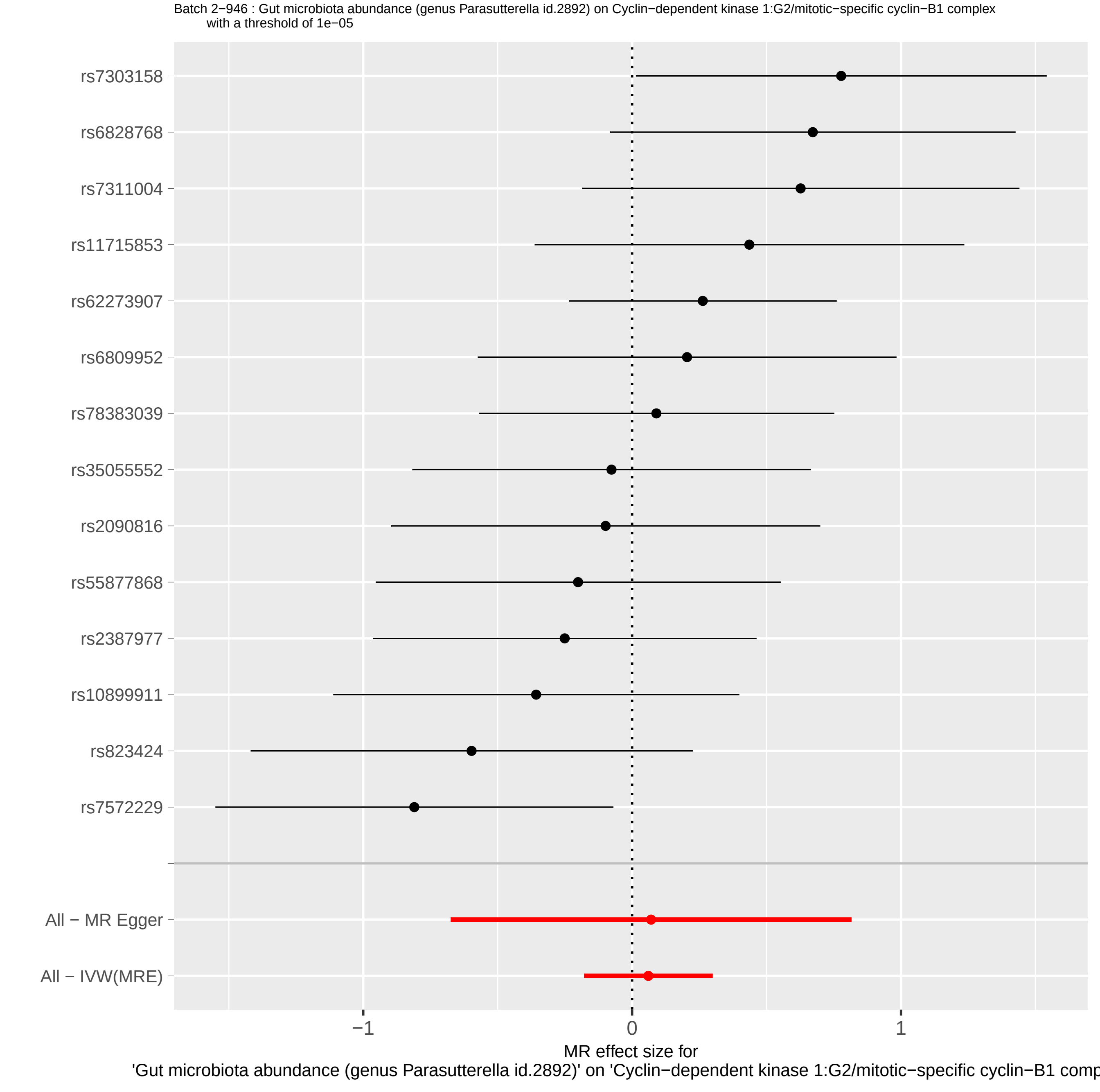
Batch 2−946 : Gut microbiota abundance (genus Parasutterella id.2892) on Cyclin−dependent kinase 1:G2/mitotic−specific cyclin−B1 complex
with a threshold of 1e−05
rs7303158
rs6828768
rs7311004
rs11715853
rs62273907
rs6809952
rs78383039
rs35055552
rs2090816
rs55877868
rs2387977
rs10899911
rs823424
rs7572229
All − MR Egger
All − IVW(MRE)
−1
0
1
MR effect size for
'Gut microbiota abundance (genus Parasutterella id.2892)' on 'Cyclin−dependent kinase 1:G2/mitotic−specific cyclin−B1 comp

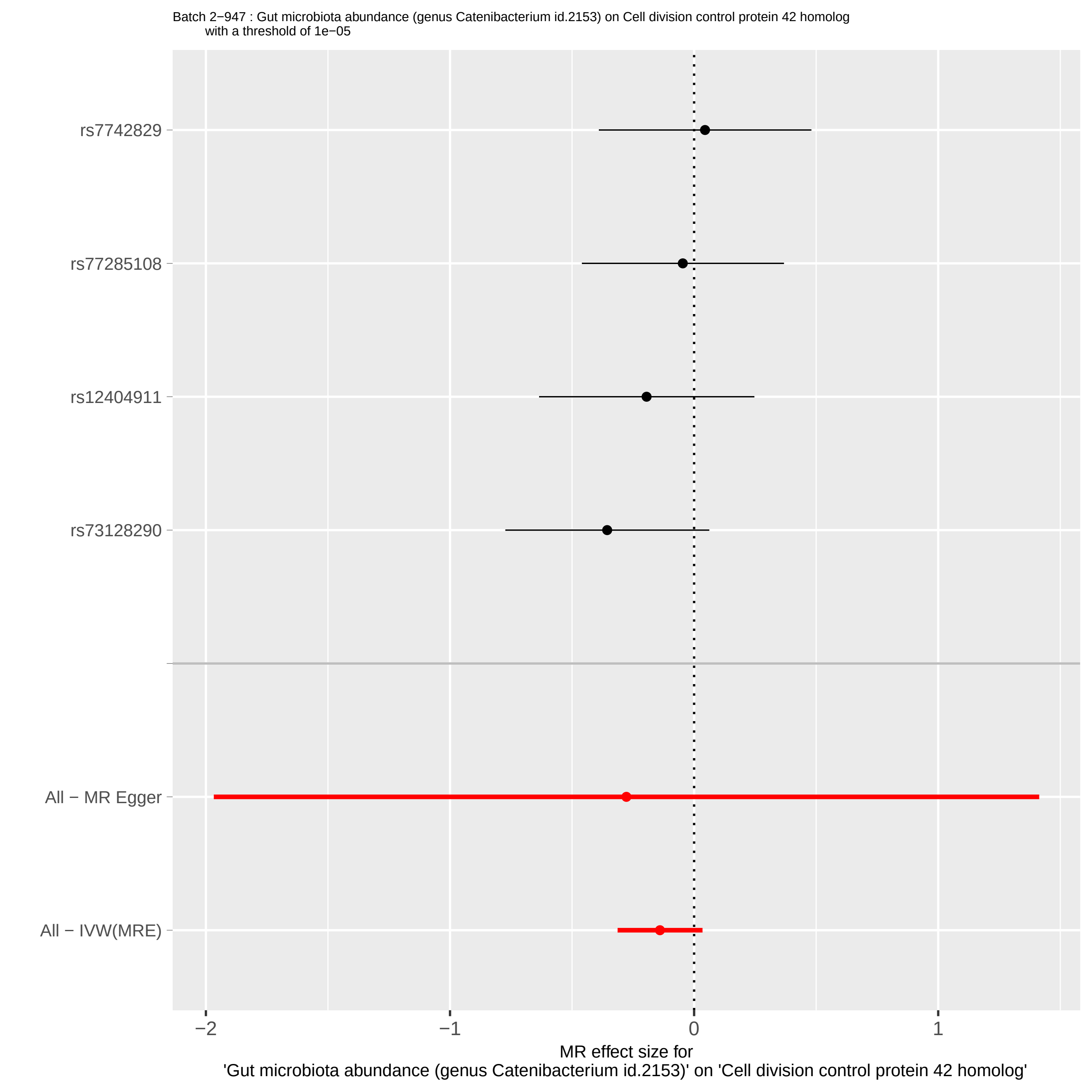
Batch 2−947 : Gut microbiota abundance (genus Catenibacterium id.2153) on Cell division control protein 42 homolog
with a threshold of 1e−05
rs7742829
rs77285108
rs12404911
rs73128290
All − MR Egger
All − IVW(MRE)
−2
−1
0
1
MR effect size for
'Gut microbiota abundance (genus Catenibacterium id.2153)' on 'Cell division control protein 42 homolog'

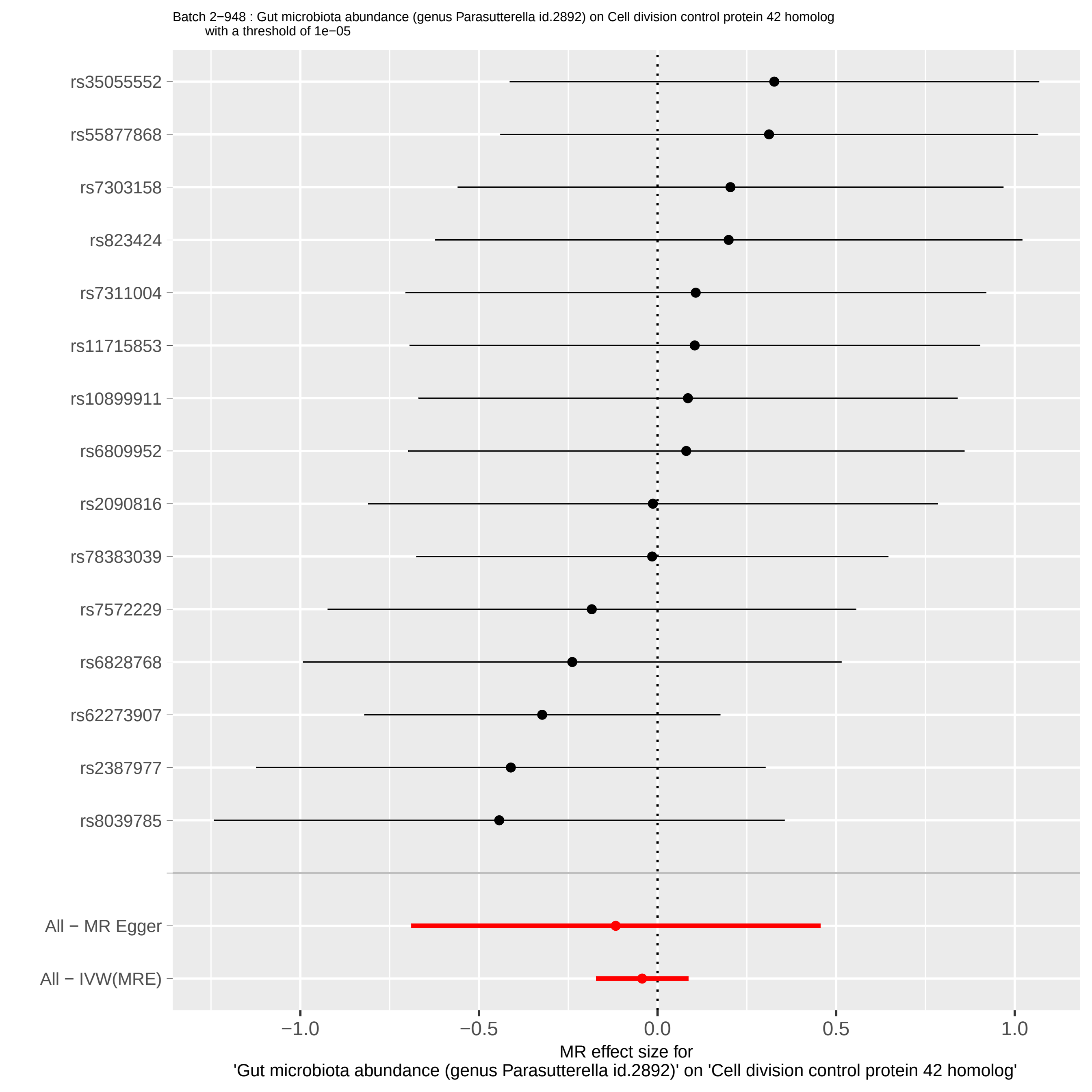

Batch 2−948 : Gut microbiota abundance (genus Parasutterella id.2892) on Cell division control protein 42 homolog
with a threshold of 1e−05
rs35055552
rs55877868
rs7303158
rs823424
rs7311004
rs11715853
rs10899911
rs6809952
rs2090816
rs78383039
rs7572229
rs6828768
rs62273907
rs2387977
rs8039785
All − MR Egger
All − IVW(MRE)
−1.0
−0.5
0.0
0.5
1.0
MR effect size for
'Gut microbiota abundance (genus Parasutterella id.2892)' on 'Cell division control protein 42 homolog'

Batch 2−949 : Gut microbiota abundance (genus Catenibacterium id.2153) on Serine/threonine−protein kinase MRCK alpha
with a threshold of 1e−05

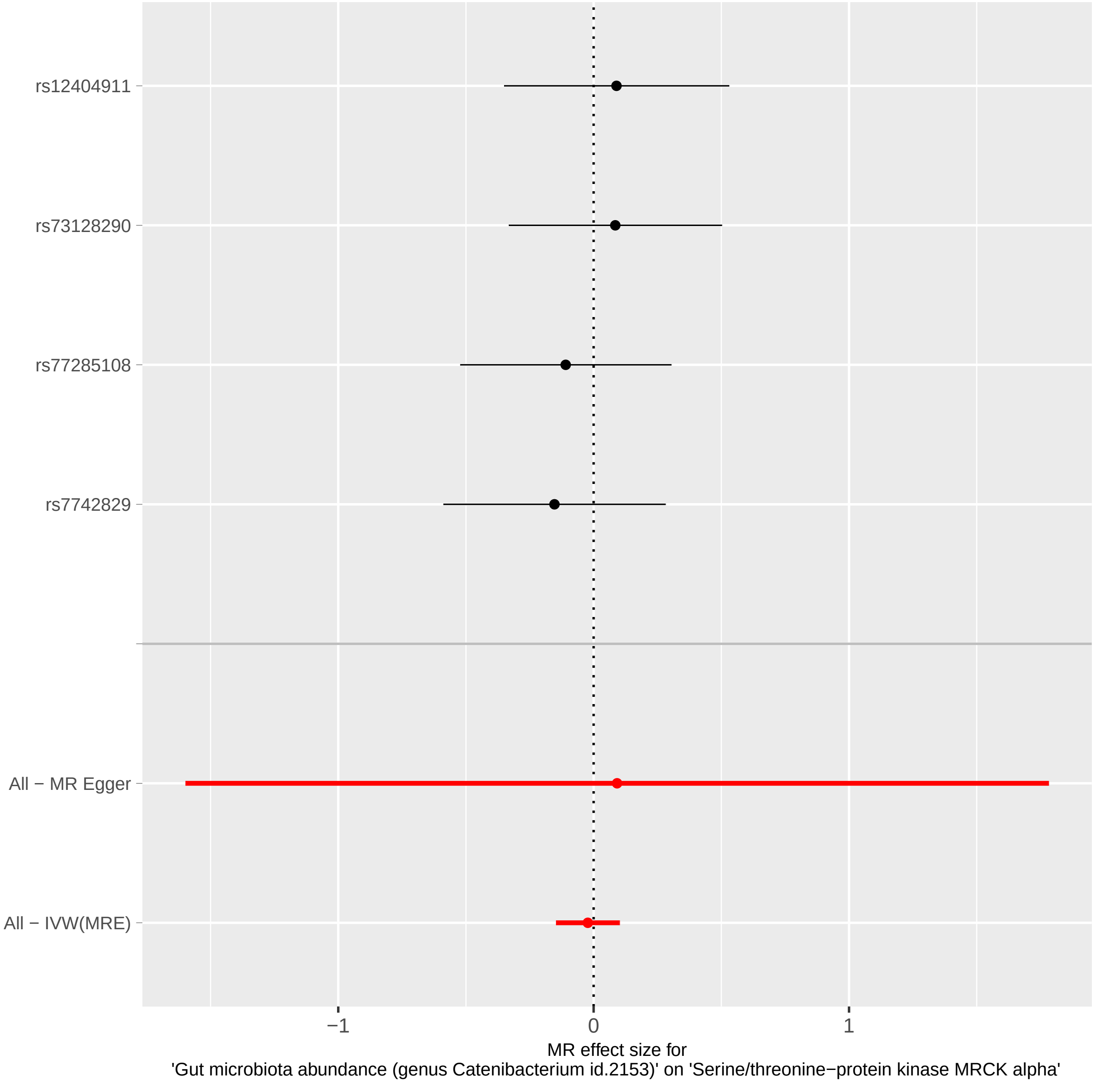

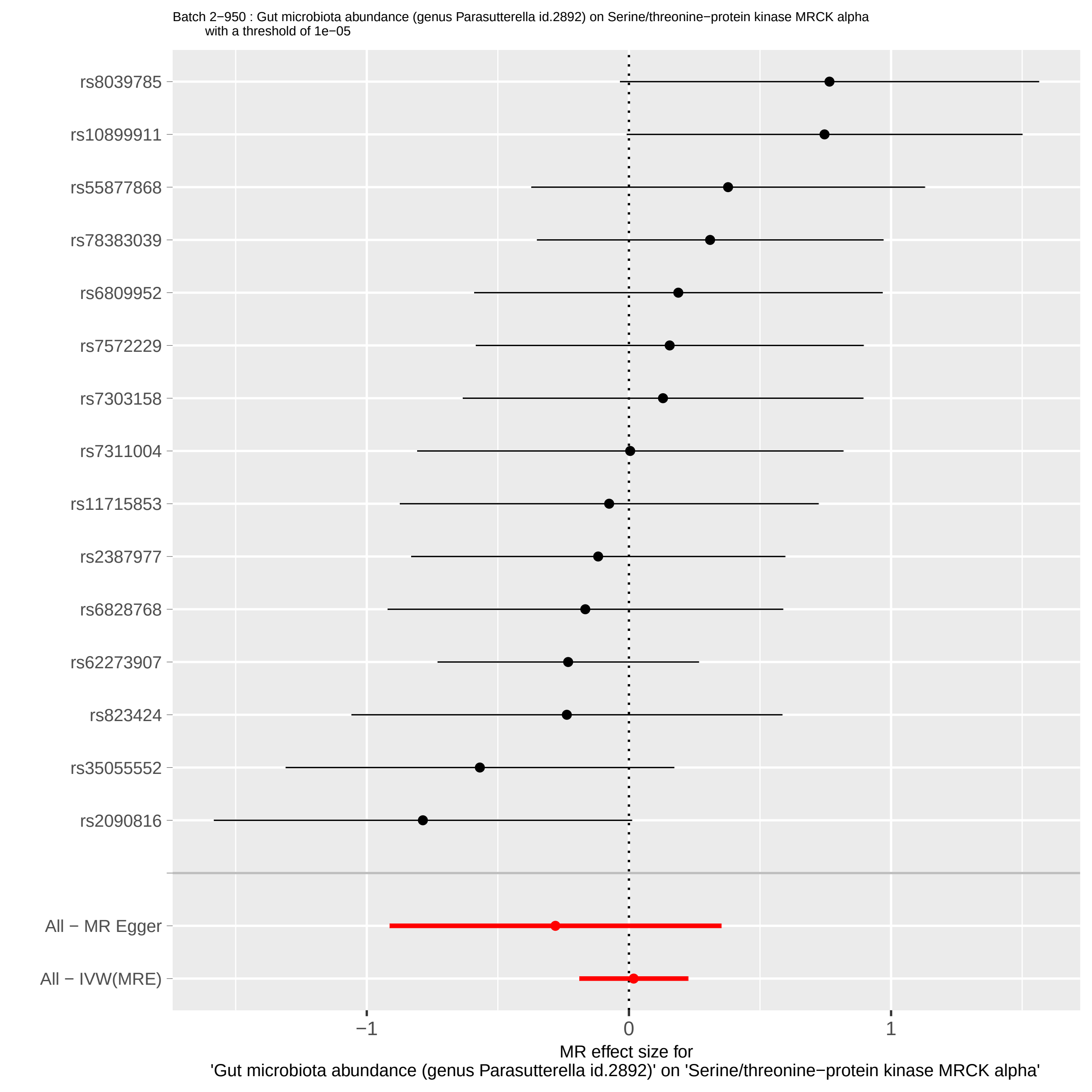

Batch 2−950 : Gut microbiota abundance (genus Parasutterella id.2892) on Serine/threonine−protein kinase MRCK alpha
with a threshold of 1e−05
rs8039785
rs10899911
rs55877868
rs78383039
rs6809952
rs7572229
rs7303158
rs7311004
rs11715853
rs2387977
rs6828768
rs62273907
rs823424
rs35055552
rs2090816
All − MR Egger
All − IVW(MRE)
−1
0
1
MR effect size for
'Gut microbiota abundance (genus Parasutterella id.2892)' on 'Serine/threonine−protein kinase MRCK alpha'

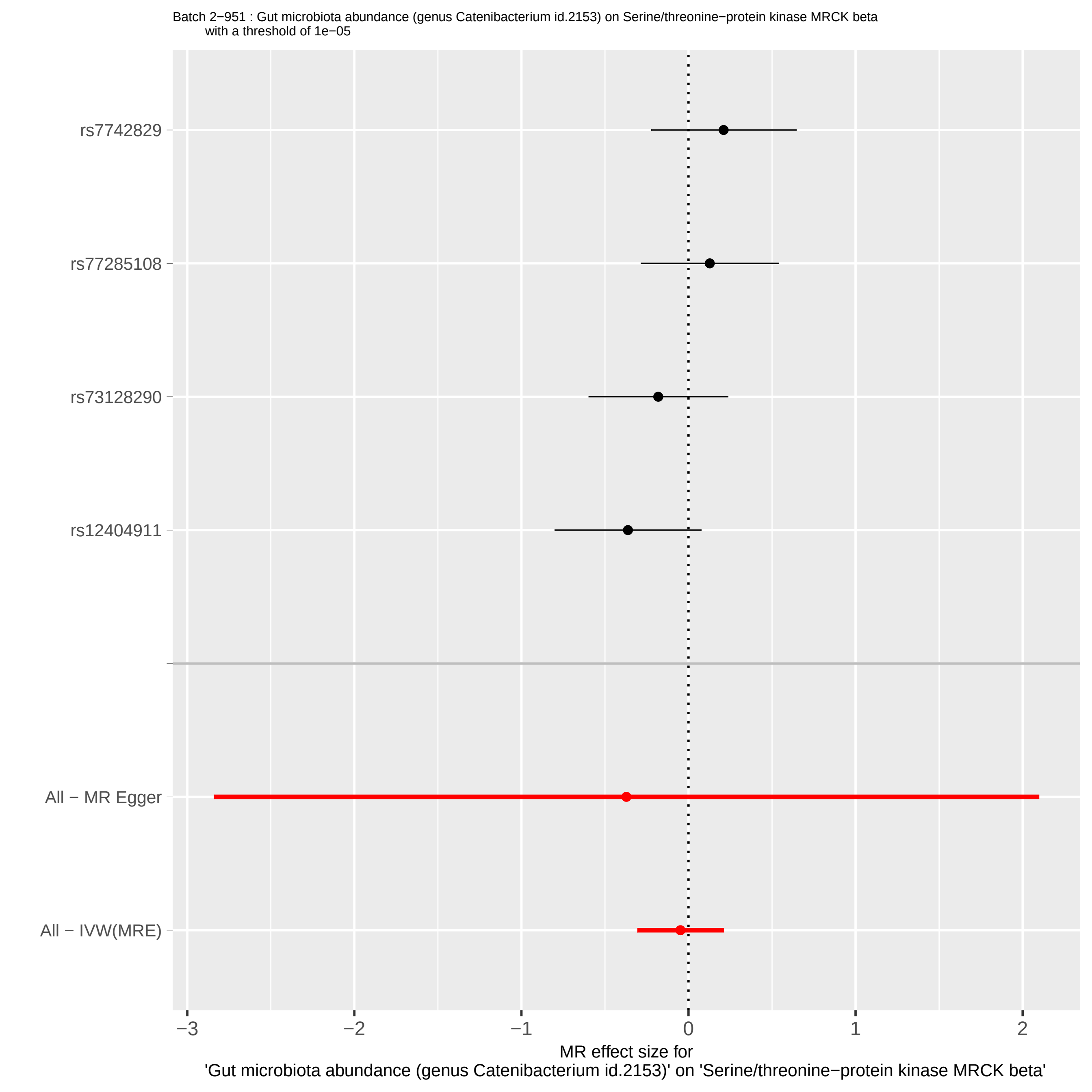
Batch 2−951 : Gut microbiota abundance (genus Catenibacterium id.2153) on Serine/threonine−protein kinase MRCK beta
with a threshold of 1e−05
rs7742829
rs77285108
rs73128290
rs12404911
All − MR Egger
All − IVW(MRE)
−3
−2
−1
0
1
2
MR effect size for
'Gut microbiota abundance (genus Catenibacterium id.2153)' on 'Serine/threonine−protein kinase MRCK beta'

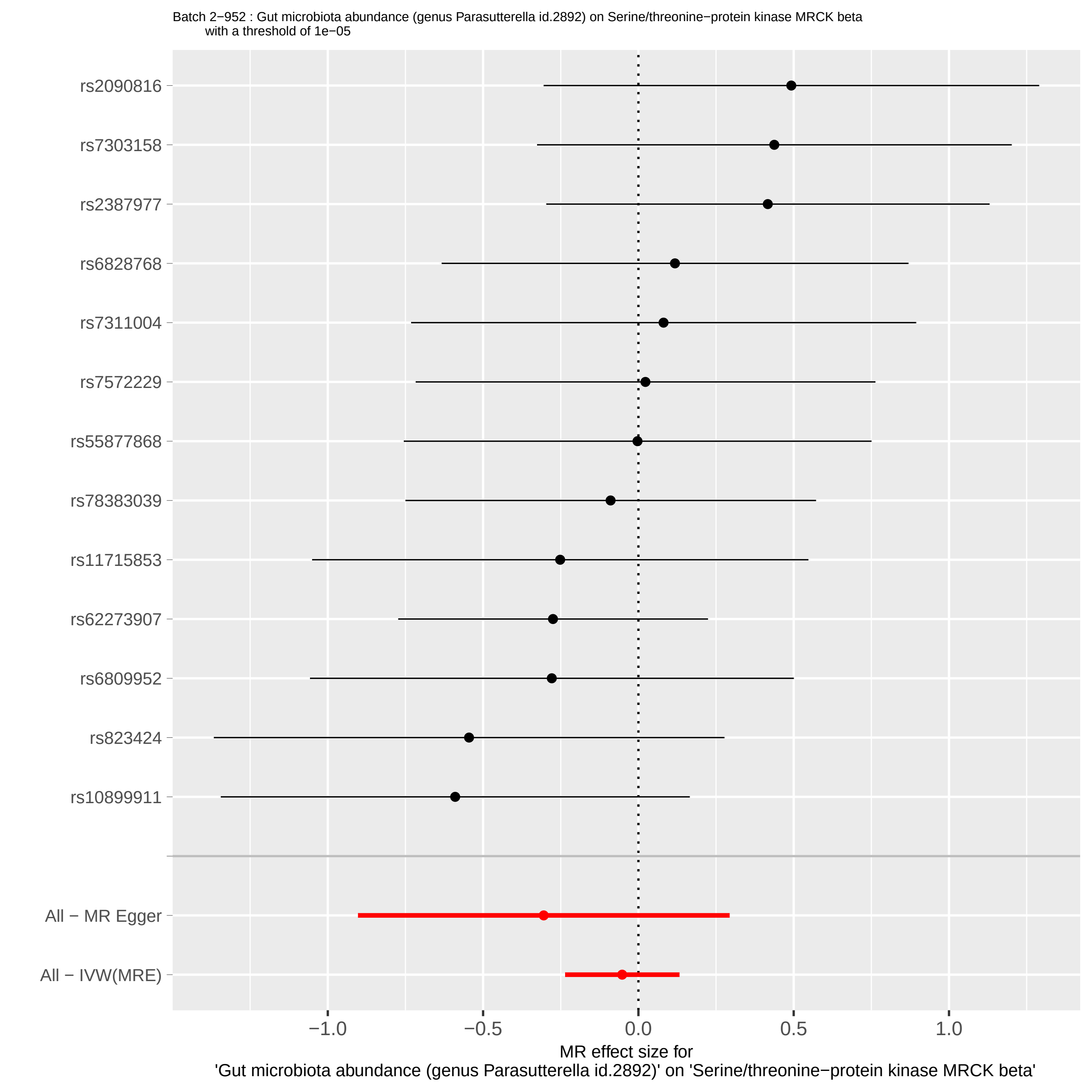
Batch 2−952 : Gut microbiota abundance (genus Parasutterella id.2892) on Serine/threonine−protein kinase MRCK beta
with a threshold of 1e−05
rs2090816
rs7303158
rs2387977
rs6828768
rs7311004
rs7572229
rs55877868
rs78383039
rs11715853
rs62273907
rs6809952
rs823424
rs10899911
All − MR Egger
All − IVW(MRE)
−1.0
−0.5
0.0
0.5
1.0
MR effect size for
'Gut microbiota abundance (genus Parasutterella id.2892)' on 'Serine/threonine−protein kinase MRCK beta'

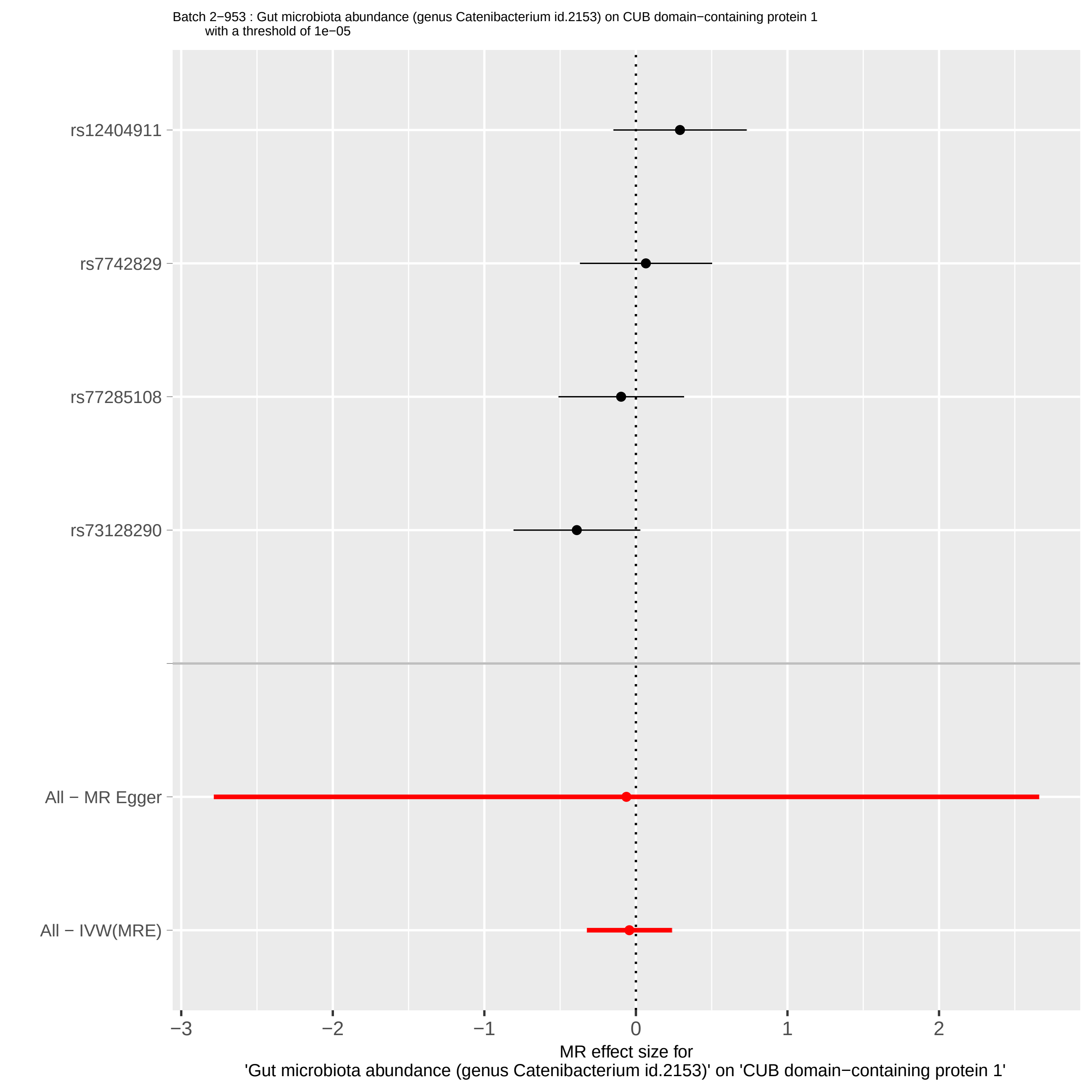

Batch 2−953 : Gut microbiota abundance (genus Catenibacterium id.2153) on CUB domain−containing protein 1
with a threshold of 1e−05
rs12404911
rs7742829
rs77285108
rs73128290
All − MR Egger
All − IVW(MRE)
−3
−2
−1
0
1
2
MR effect size for
'Gut microbiota abundance (genus Catenibacterium id.2153)' on 'CUB domain−containing protein 1'

Batch 2−954 : Gut microbiota abundance (genus Parasutterella id.2892) on CUB domain−containing protein 1
with a threshold of 1e−05

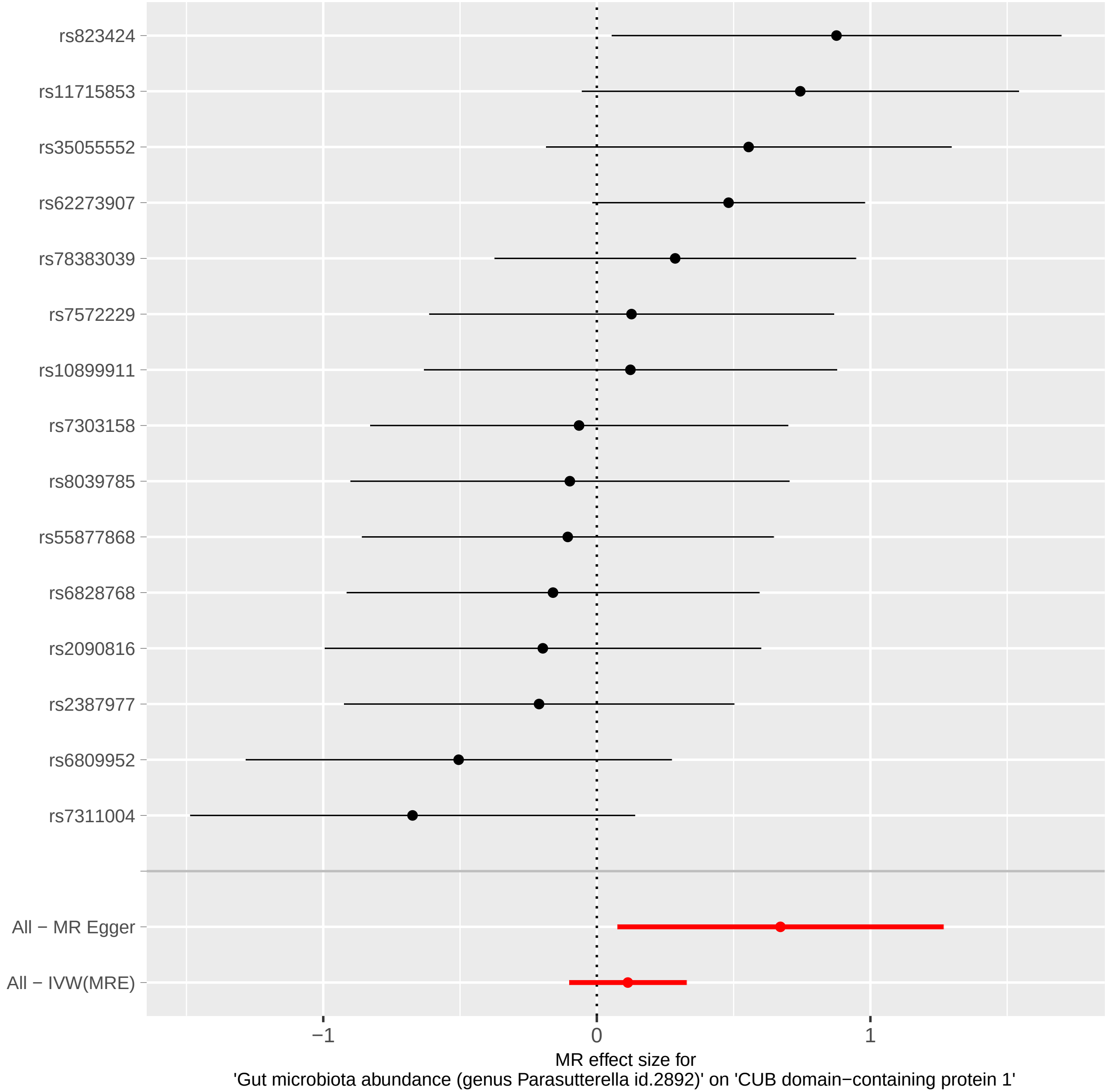

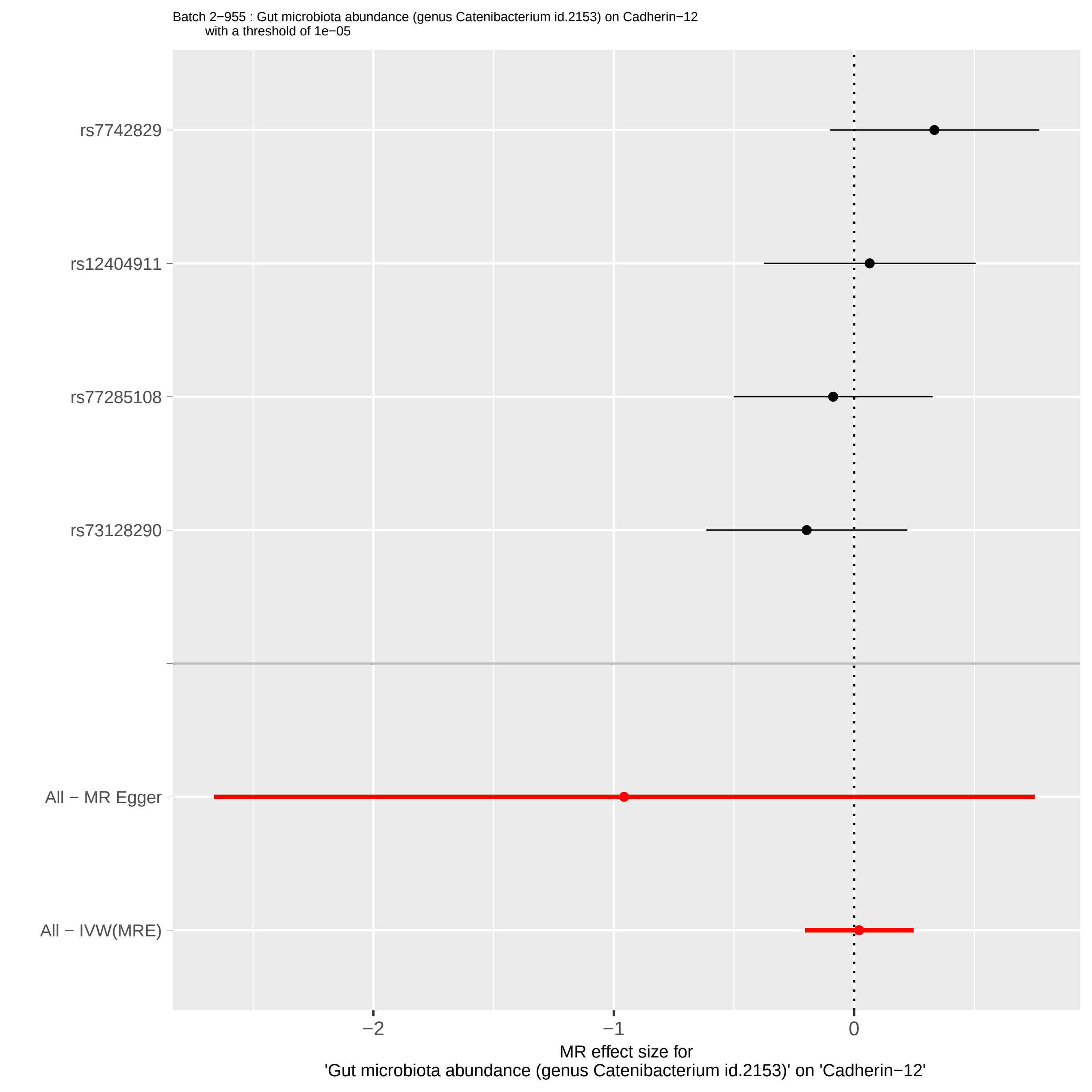
Batch 2−955 : Gut microbiota abundance (genus Catenibacterium id.2153) on Cadherin−12
with a threshold of 1e−05
rs7742829
rs12404911
rs77285108
rs73128290
All − MR Egger
All − IVW(MRE)
−2
−1
0
MR effect size for
'Gut microbiota abundance (genus Catenibacterium id.2153)' on 'Cadherin−12'

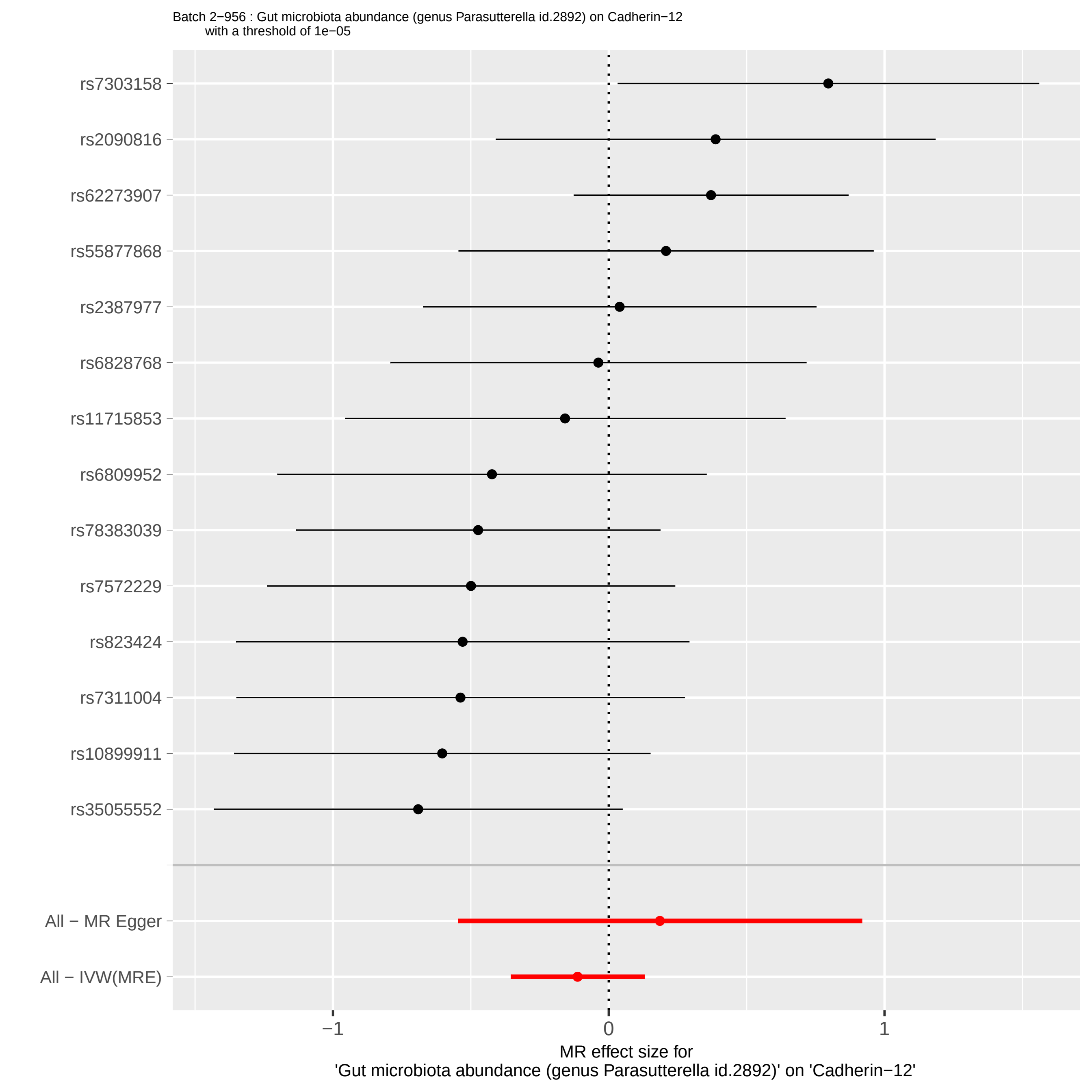

Batch 2−956 : Gut microbiota abundance (genus Parasutterella id.2892) on Cadherin−12
with a threshold of 1e−05
rs7303158
rs2090816
rs62273907
rs55877868
rs2387977
rs6828768
rs11715853
rs6809952
rs78383039
rs7572229
rs823424
rs7311004
rs10899911
rs35055552
All − MR Egger
All − IVW(MRE)
−1
0
1
MR effect size for
'Gut microbiota abundance (genus Parasutterella id.2892)' on 'Cadherin−12'

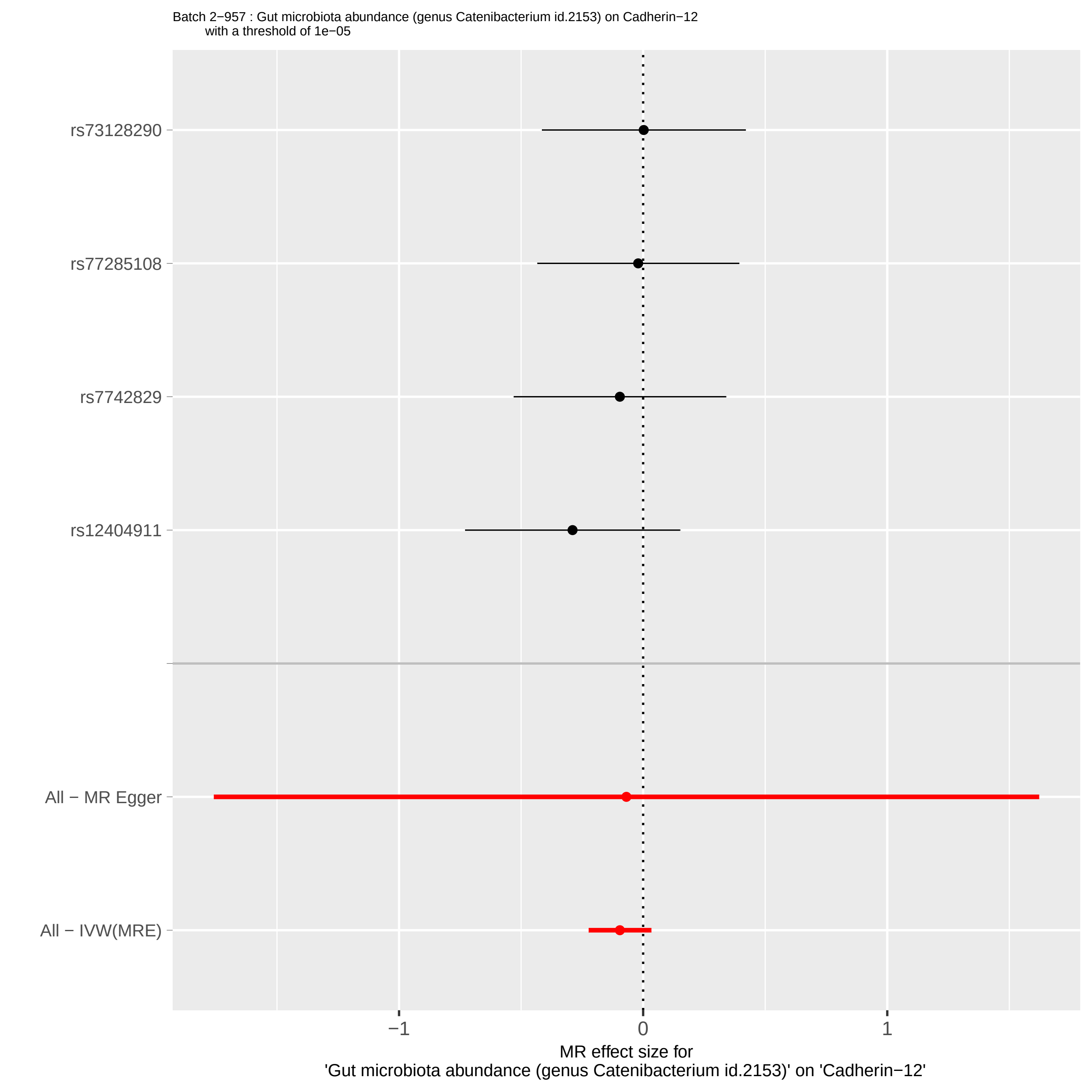

Batch 2−957 : Gut microbiota abundance (genus Catenibacterium id.2153) on Cadherin−12
with a threshold of 1e−05
rs73128290
rs77285108
rs7742829
rs12404911
All − MR Egger
All − IVW(MRE)
−1
0
1
MR effect size for
'Gut microbiota abundance (genus Catenibacterium id.2153)' on 'Cadherin−12'

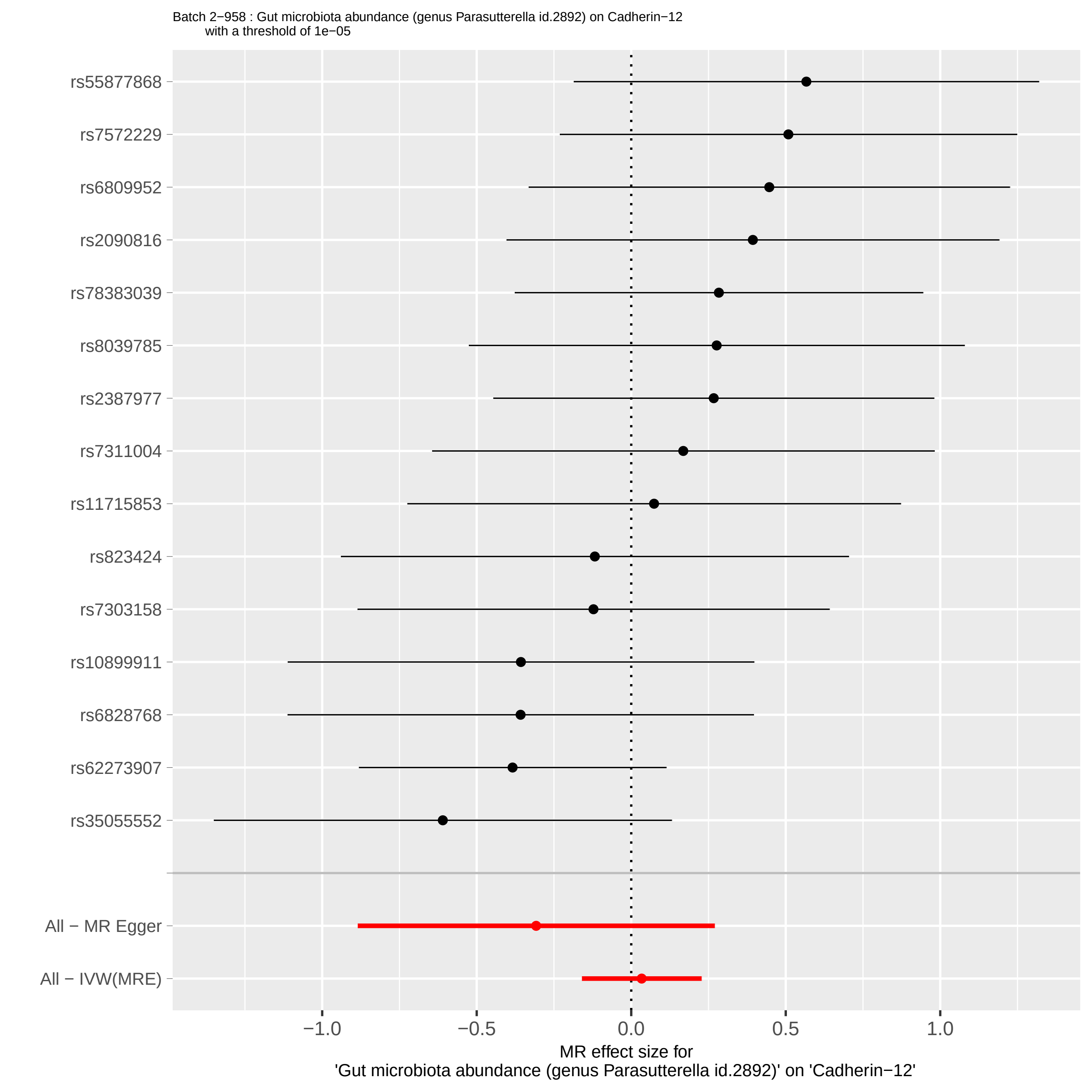
Batch 2−958 : Gut microbiota abundance (genus Parasutterella id.2892) on Cadherin−12
with a threshold of 1e−05
rs55877868
rs7572229
rs6809952
rs2090816
rs78383039
rs8039785
rs2387977
rs7311004
rs11715853
rs823424
rs7303158
rs10899911
rs6828768
rs62273907
rs35055552
All − MR Egger
All − IVW(MRE)
−1.0
−0.5
0.0
0.5
1.0
MR effect size for
'Gut microbiota abundance (genus Parasutterella id.2892)' on 'Cadherin−12'

Batch 2−959 : Gut microbiota abundance (genus Catenibacterium id.2153) on Cadherin−1
with a threshold of 1e−05

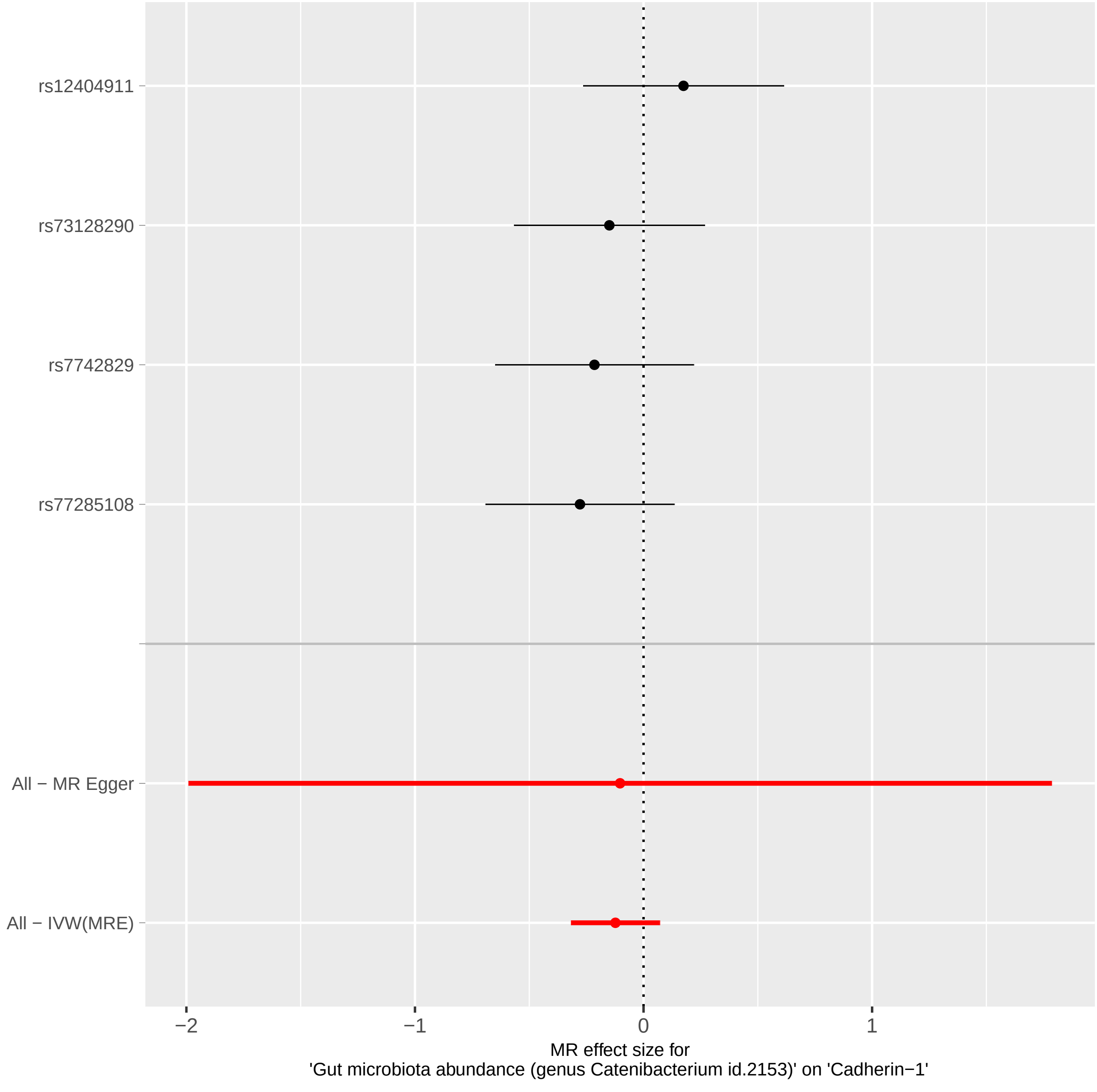

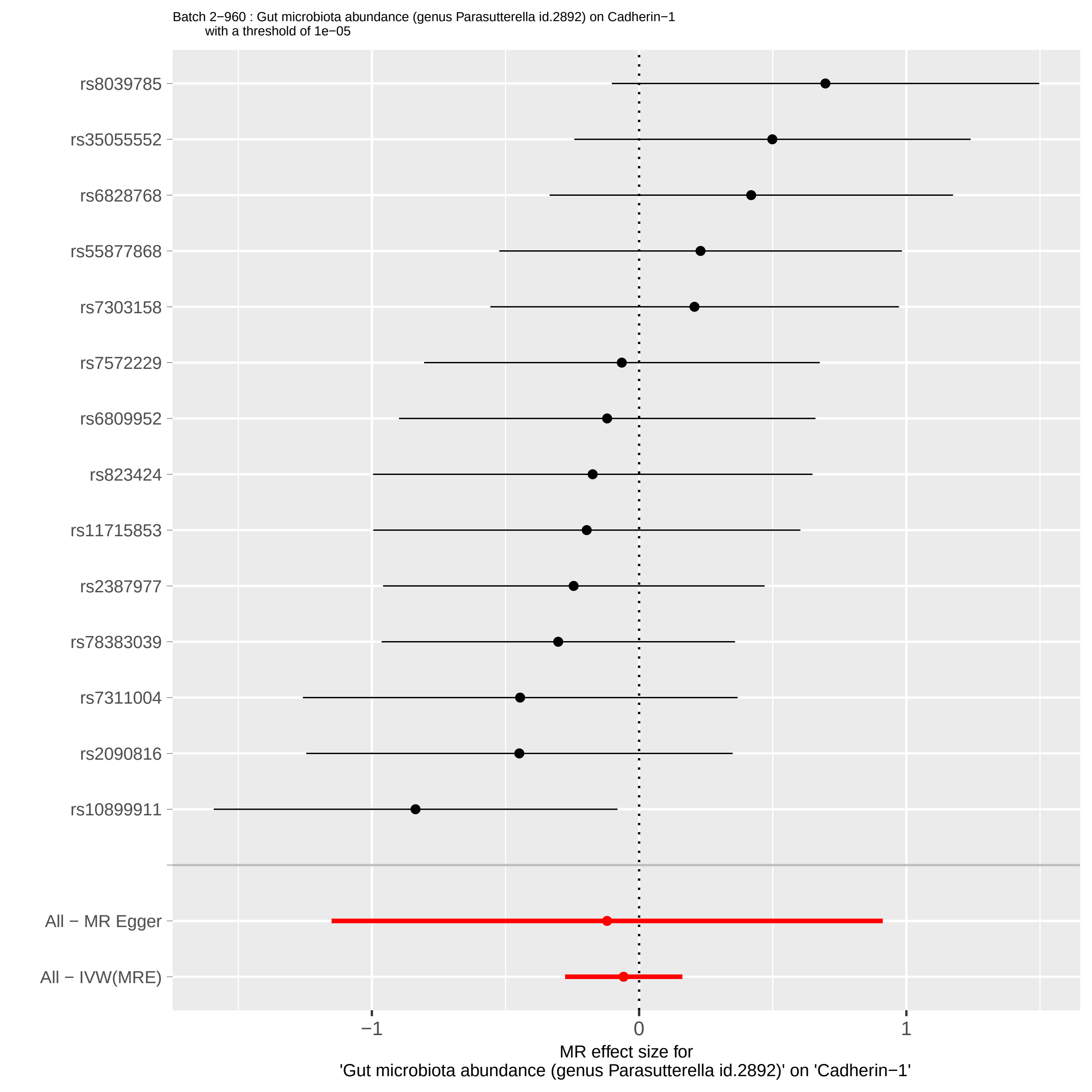

Batch 2−960 : Gut microbiota abundance (genus Parasutterella id.2892) on Cadherin−1
with a threshold of 1e−05
rs8039785
rs35055552
rs6828768
rs55877868
rs7303158
rs7572229
rs6809952
rs823424
rs11715853
rs2387977
rs78383039
rs7311004
rs2090816
rs10899911
All − MR Egger
All − IVW(MRE)
−1
0
1
MR effect size for
'Gut microbiota abundance (genus Parasutterella id.2892)' on 'Cadherin−1'

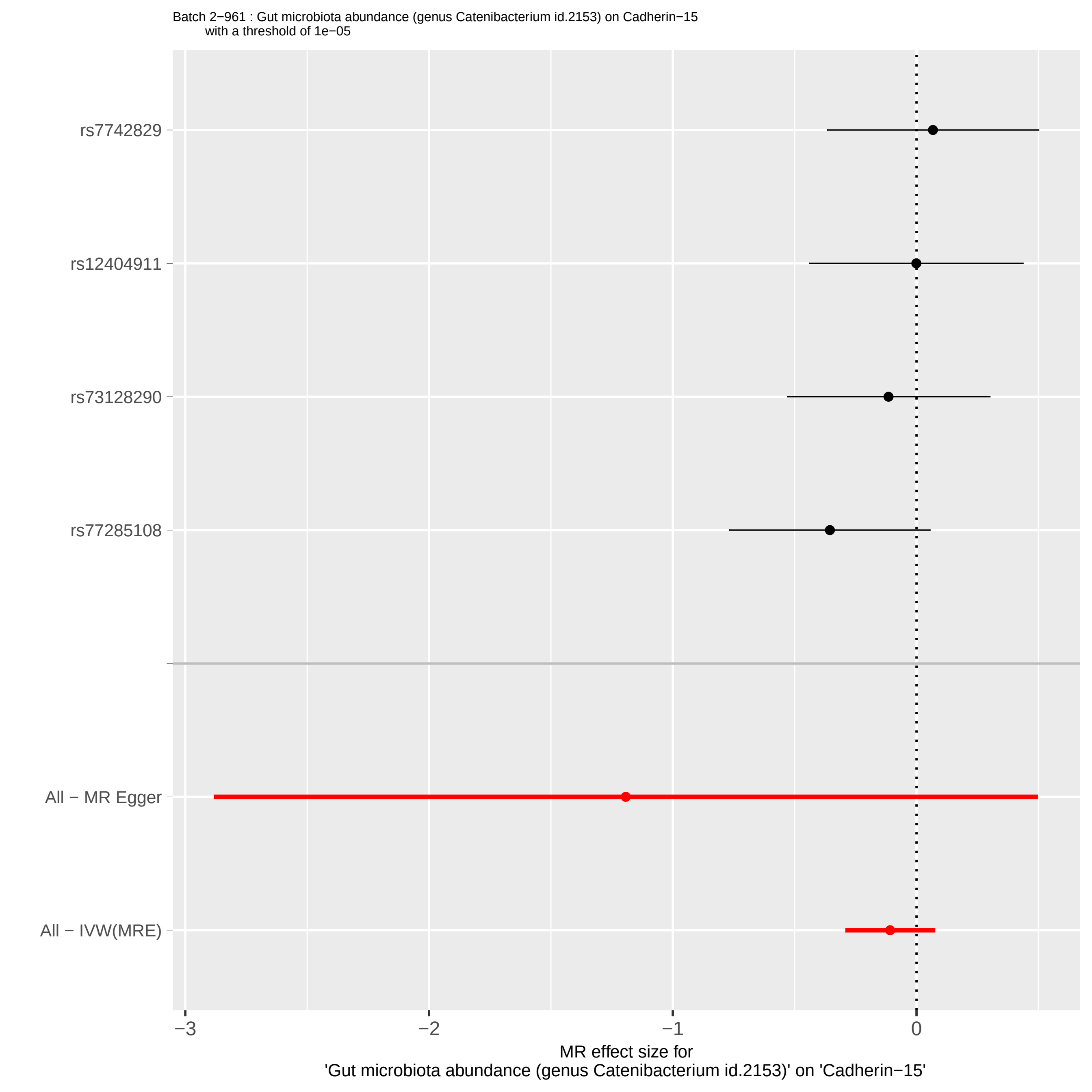
Batch 2−961 : Gut microbiota abundance (genus Catenibacterium id.2153) on Cadherin−15
with a threshold of 1e−05
rs7742829
rs12404911
rs73128290
rs77285108
All − MR Egger
All − IVW(MRE)
−3
−2
−1
0
MR effect size for
'Gut microbiota abundance (genus Catenibacterium id.2153)' on 'Cadherin−15'

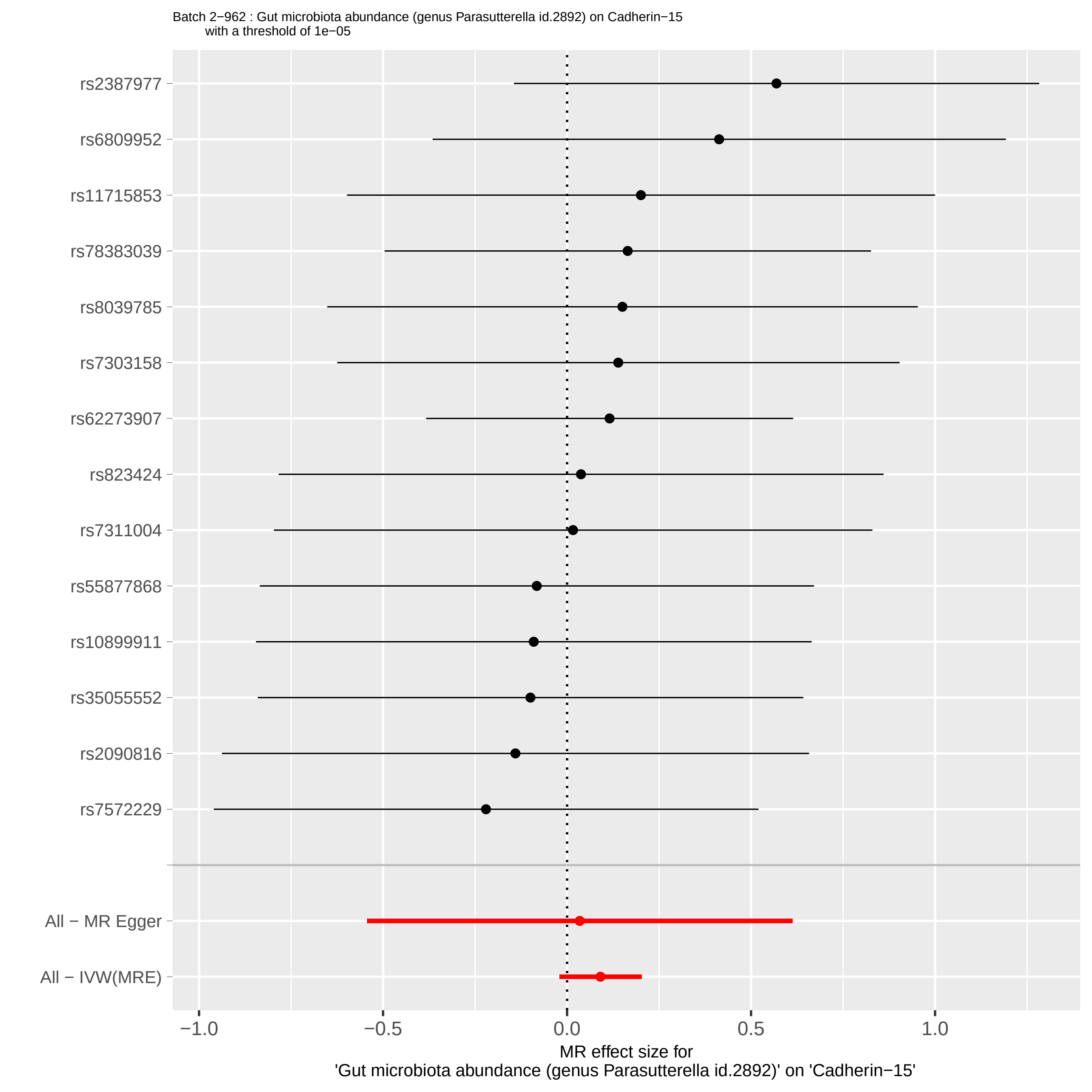
Batch 2−962 : Gut microbiota abundance (genus Parasutterella id.2892) on Cadherin−15
with a threshold of 1e−05
rs2387977
rs6809952
rs11715853
rs78383039
rs8039785
rs7303158
rs62273907
rs823424
rs7311004
rs55877868
rs10899911
rs35055552
rs2090816
rs7572229
All − MR Egger
All − IVW(MRE)
−1.0
−0.5
0.0
0.5
1.0
MR effect size for
'Gut microbiota abundance (genus Parasutterella id.2892)' on 'Cadherin−15'

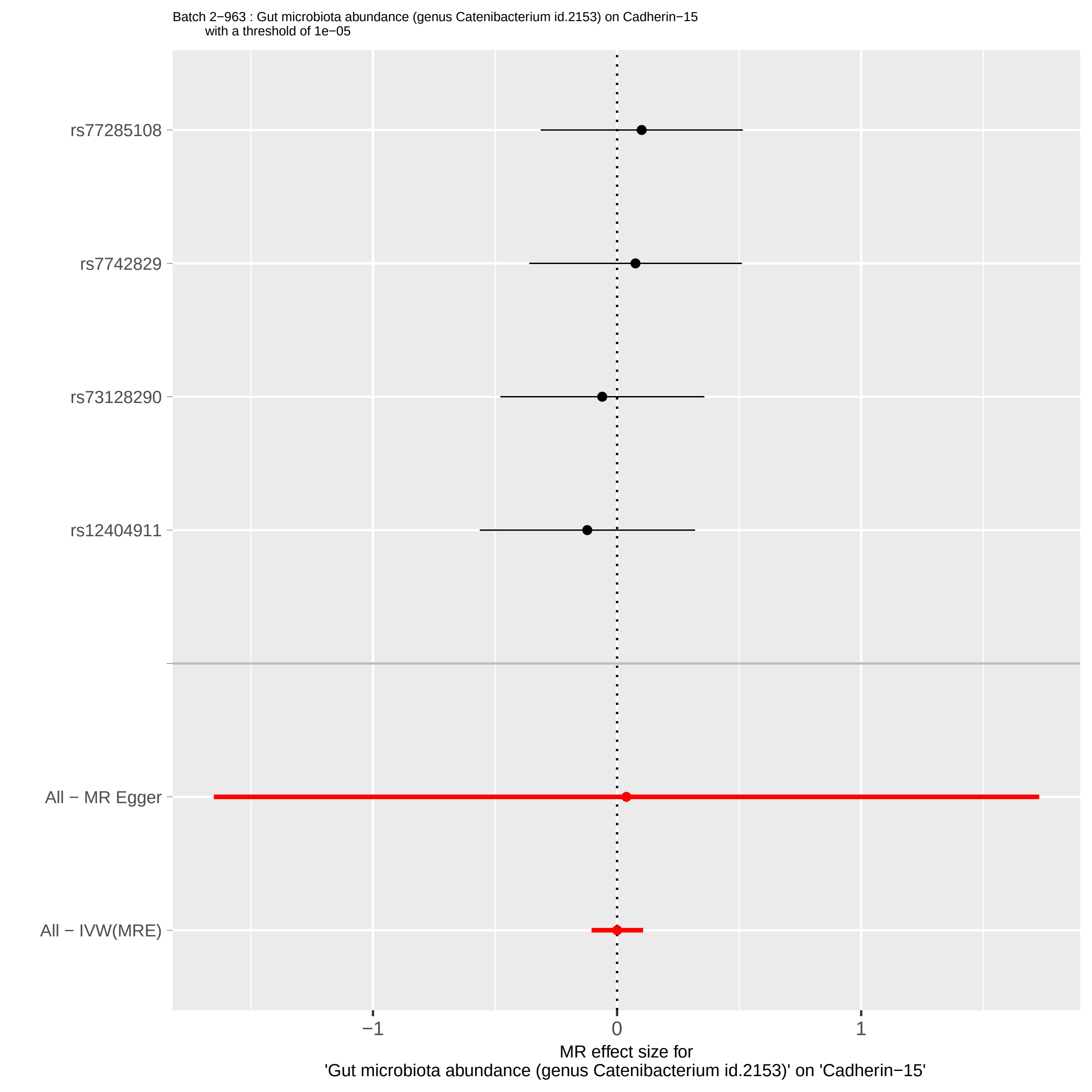

Batch 2−963 : Gut microbiota abundance (genus Catenibacterium id.2153) on Cadherin−15
with a threshold of 1e−05
rs77285108
rs7742829
rs73128290
rs12404911
All − MR Egger
All − IVW(MRE)
−1
0
1
MR effect size for
'Gut microbiota abundance (genus Catenibacterium id.2153)' on 'Cadherin−15'

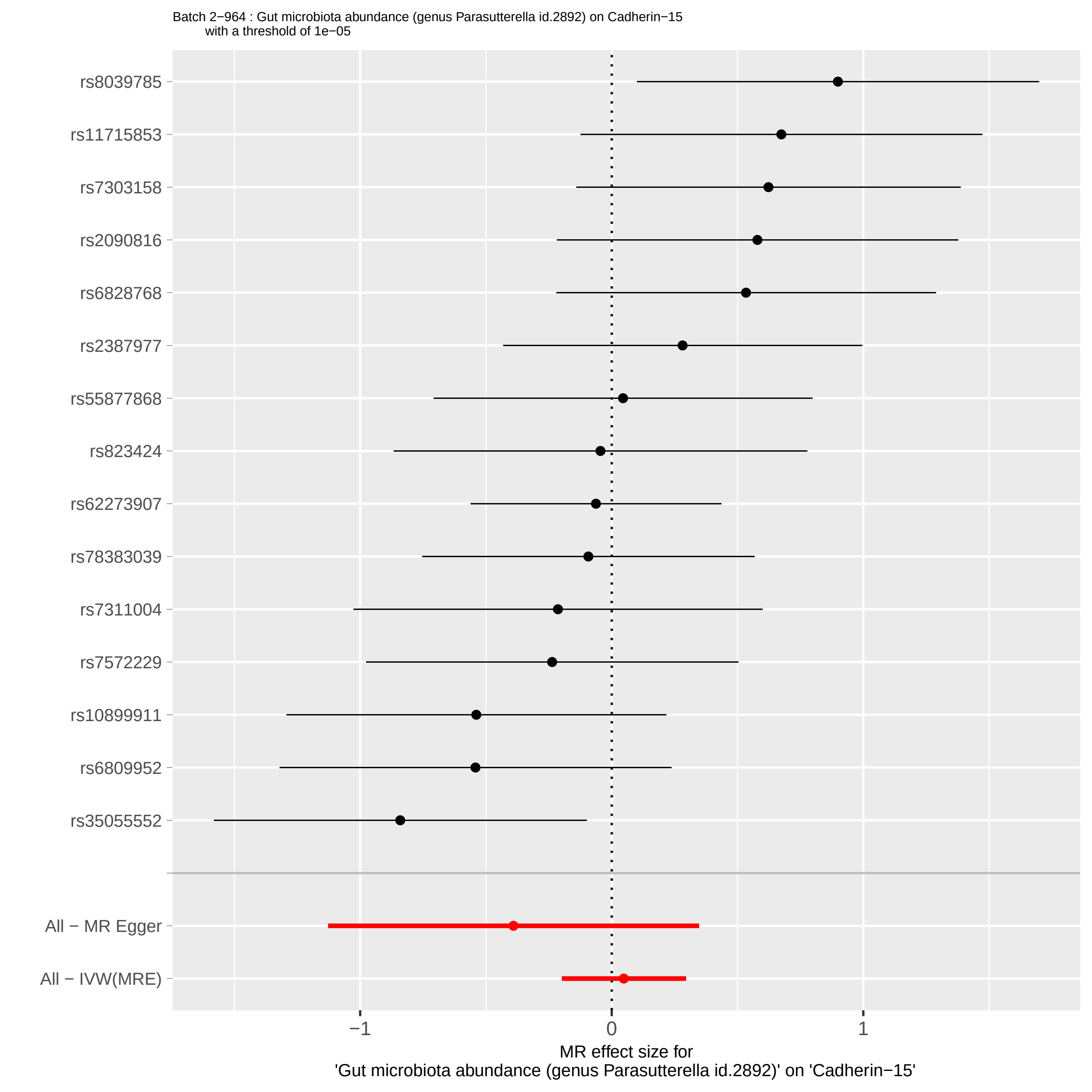

Batch 2−964 : Gut microbiota abundance (genus Parasutterella id.2892) on Cadherin−15
with a threshold of 1e−05
rs8039785
rs11715853
rs7303158
rs2090816
rs6828768
rs2387977
rs55877868
rs823424
rs62273907
rs78383039
rs7311004
rs7572229
rs10899911
rs6809952
rs35055552
All − MR Egger
All − IVW(MRE)
−1
0
1
MR effect size for
'Gut microbiota abundance (genus Parasutterella id.2892)' on 'Cadherin−15'

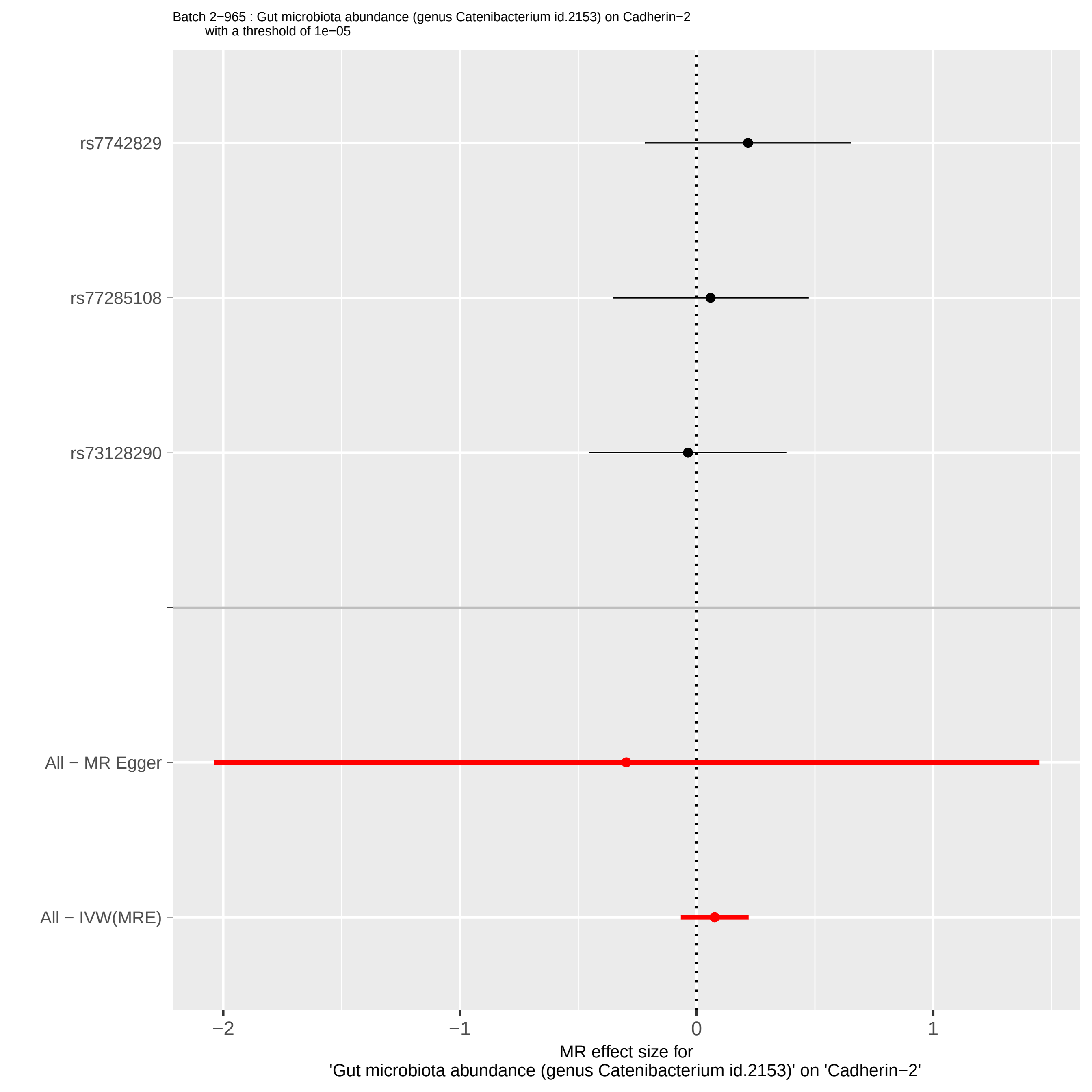
Batch 2−965 : Gut microbiota abundance (genus Catenibacterium id.2153) on Cadherin−2
with a threshold of 1e−05
rs7742829
rs77285108
rs73128290
All − MR Egger
All − IVW(MRE)
−2
−1
0
1
MR effect size for
'Gut microbiota abundance (genus Catenibacterium id.2153)' on 'Cadherin−2'

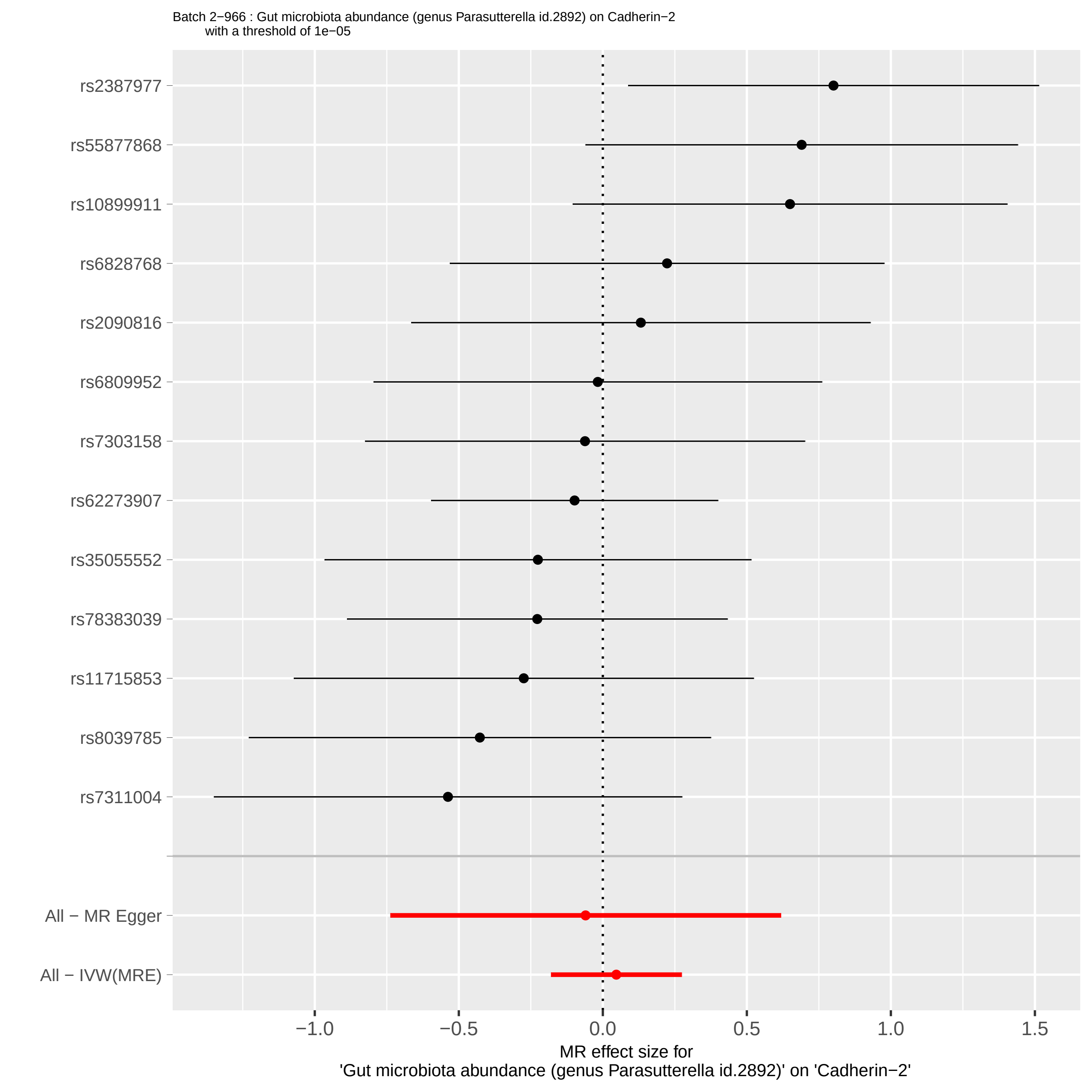

Batch 2−966 : Gut microbiota abundance (genus Parasutterella id.2892) on Cadherin−2
with a threshold of 1e−05
rs2387977
rs55877868
rs10899911
rs6828768
rs2090816
rs6809952
rs7303158
rs62273907
rs35055552
rs78383039
rs11715853
rs8039785
rs7311004
All − MR Egger
All − IVW(MRE)
−1.0
−0.5
0.0
0.5
1.0
1.5
MR effect size for
'Gut microbiota abundance (genus Parasutterella id.2892)' on 'Cadherin−2'

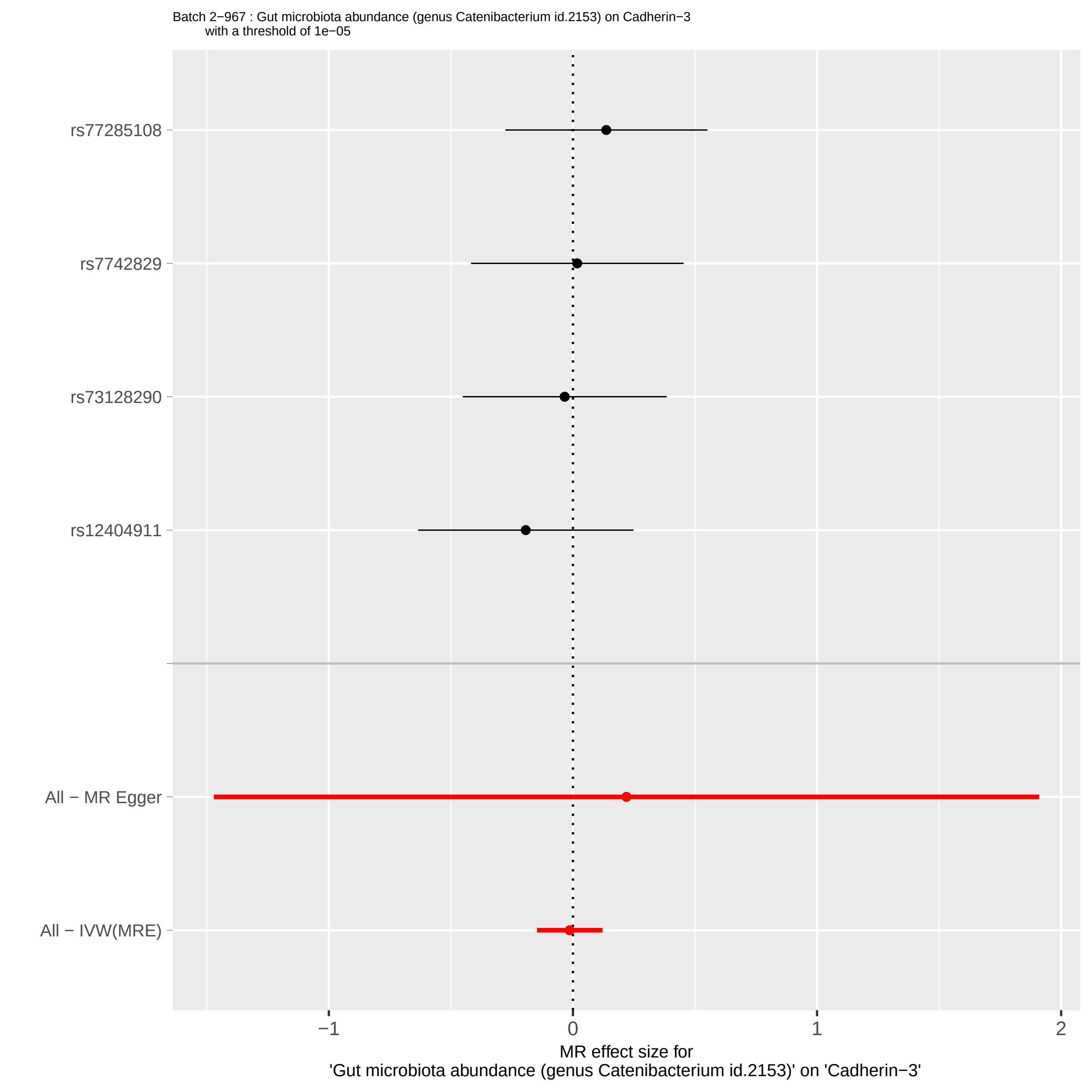

Batch 2−967 : Gut microbiota abundance (genus Catenibacterium id.2153) on Cadherin−3
with a threshold of 1e−05
rs77285108
rs7742829
rs73128290
rs12404911
All − MR Egger
All − IVW(MRE)
−1
0
1
2
MR effect size for
'Gut microbiota abundance (genus Catenibacterium id.2153)' on 'Cadherin−3'

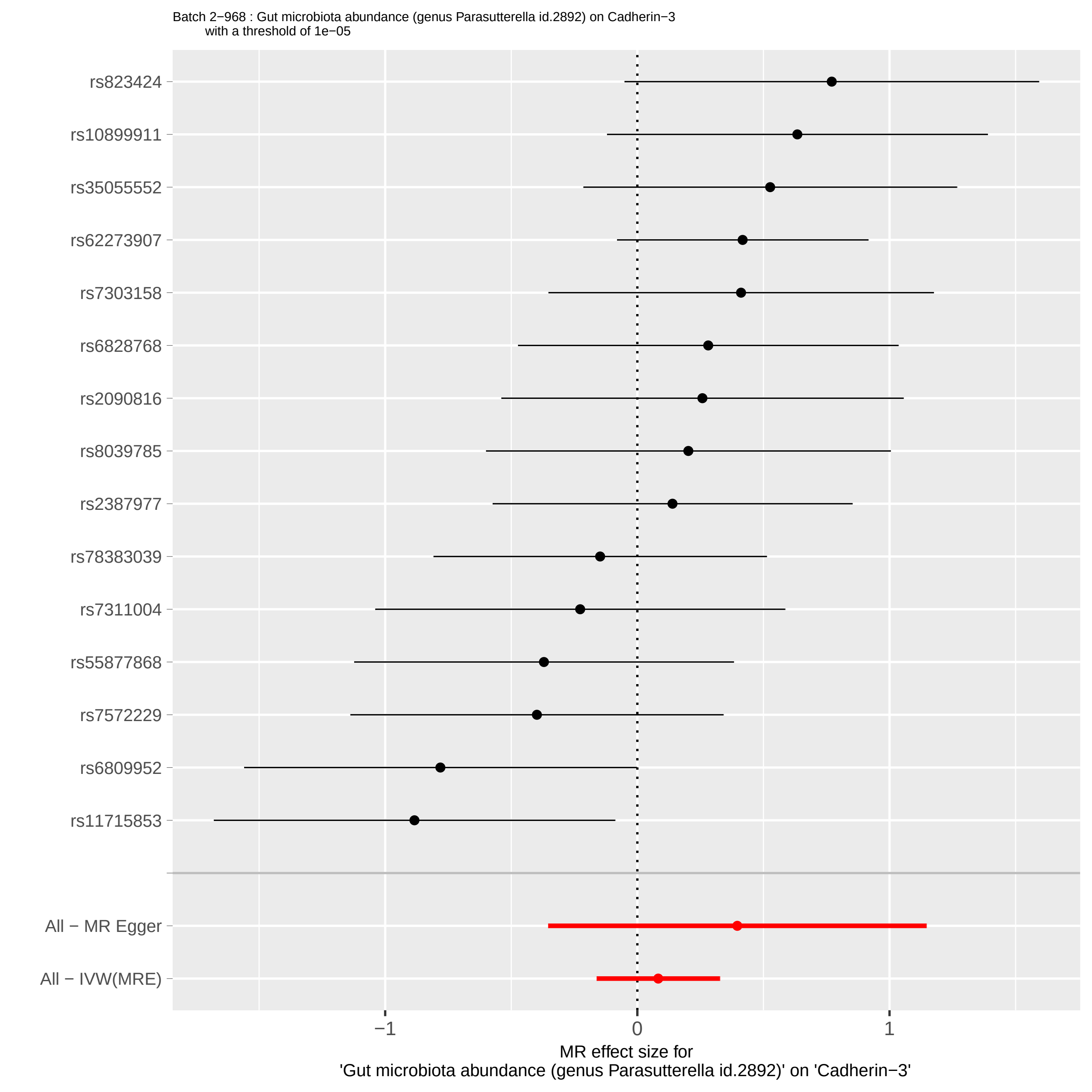
Batch 2−968 : Gut microbiota abundance (genus Parasutterella id.2892) on Cadherin−3
with a threshold of 1e−05
rs823424
rs10899911
rs35055552
rs62273907
rs7303158
rs6828768
rs2090816
rs8039785
rs2387977
rs78383039
rs7311004
rs55877868
rs7572229
rs6809952
rs11715853
All − MR Egger
All − IVW(MRE)
−1
0
1
MR effect size for
'Gut microbiota abundance (genus Parasutterella id.2892)' on 'Cadherin−3'

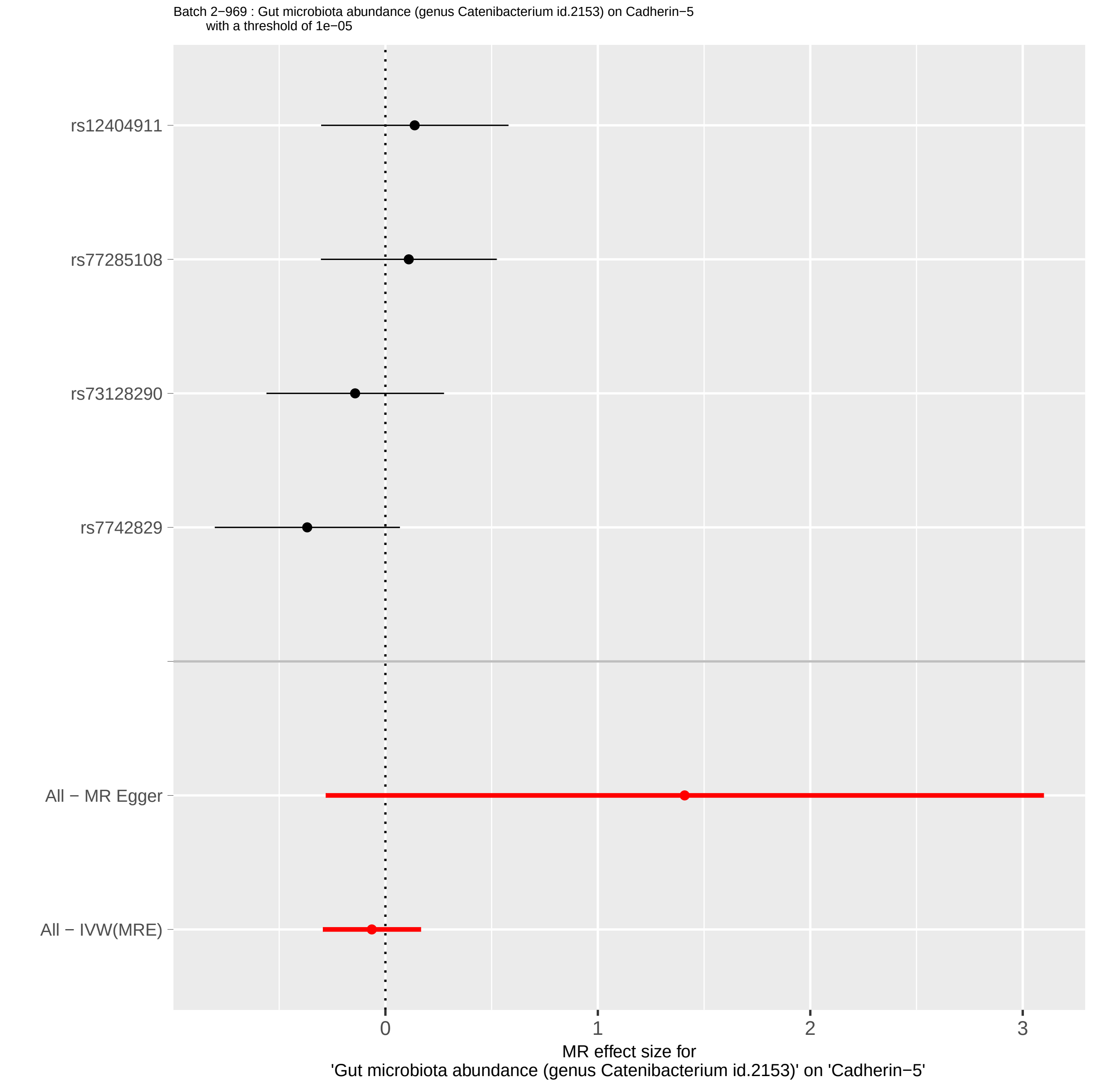

Batch 2−969 : Gut microbiota abundance (genus Catenibacterium id.2153) on Cadherin−5
with a threshold of 1e−05
rs12404911
rs77285108
rs73128290
rs7742829
All − MR Egger
All − IVW(MRE)
0
1
2
3
MR effect size for
'Gut microbiota abundance (genus Catenibacterium id.2153)' on 'Cadherin−5'

Batch 2−970 : Gut microbiota abundance (genus Parasutterella id.2892) on Cadherin−5
with a threshold of 1e−05

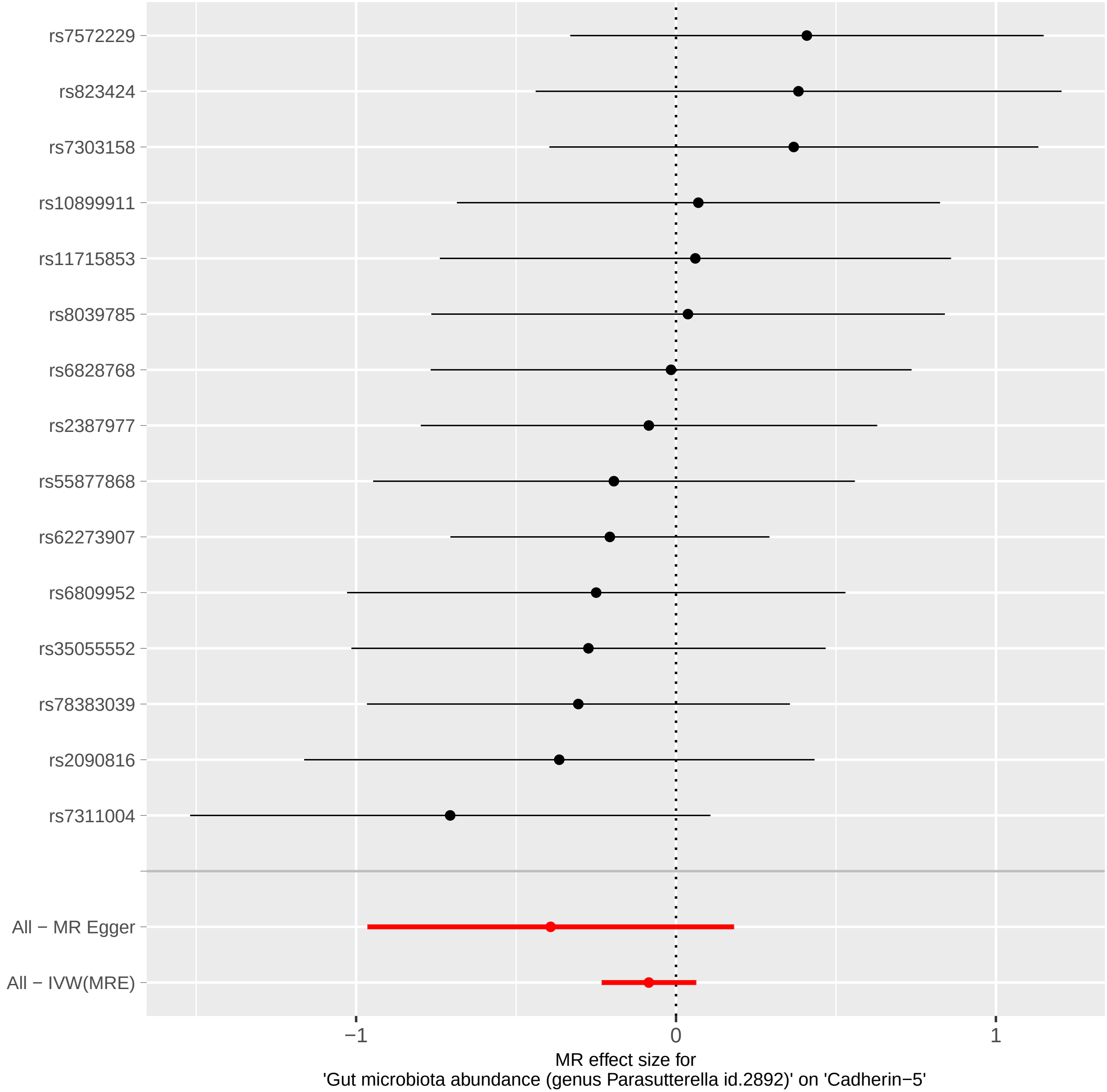

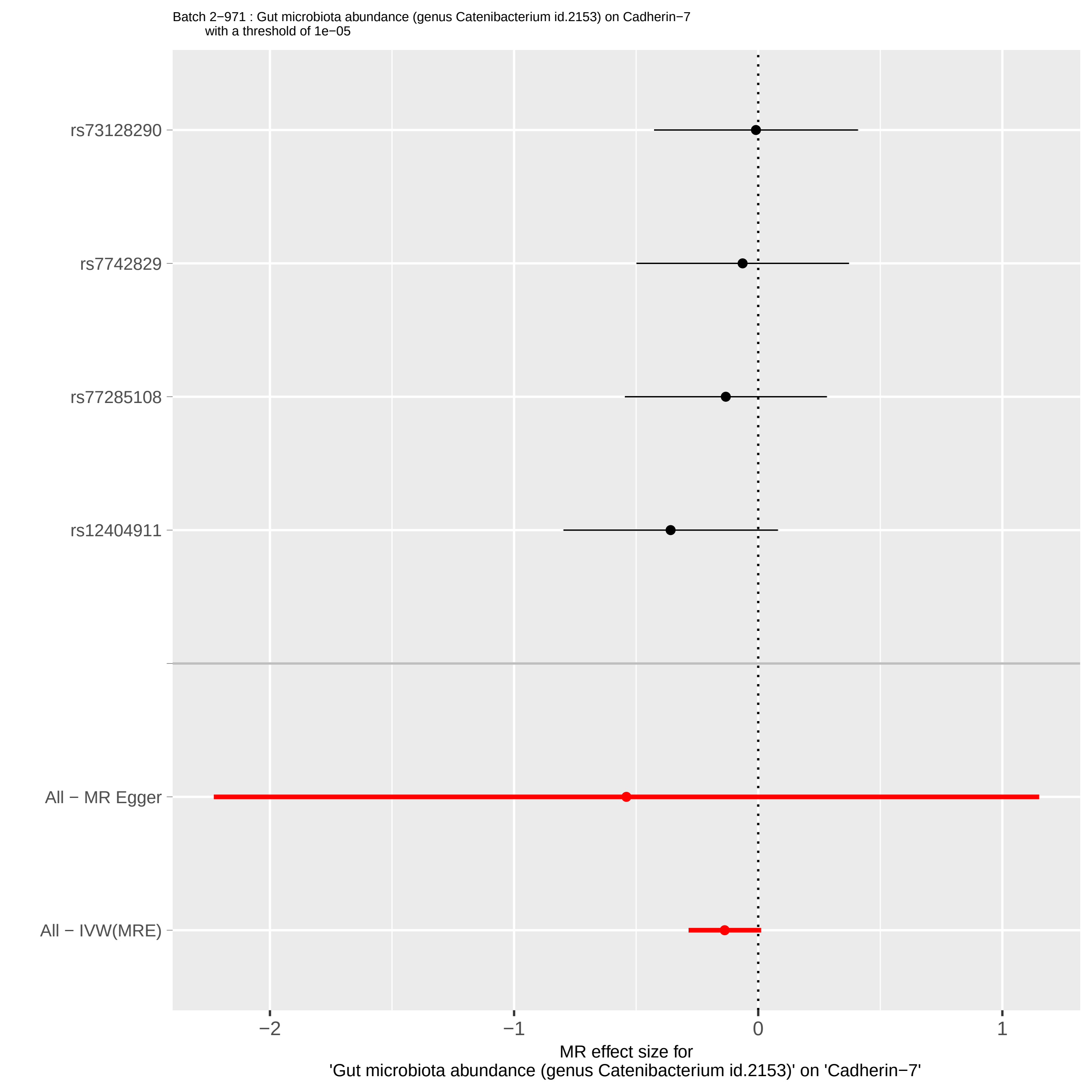
Batch 2−971 : Gut microbiota abundance (genus Catenibacterium id.2153) on Cadherin−7
with a threshold of 1e−05
rs73128290
rs7742829
rs77285108
rs12404911
All − MR Egger
All − IVW(MRE)
−2
−1
0
1
MR effect size for
'Gut microbiota abundance (genus Catenibacterium id.2153)' on 'Cadherin−7'

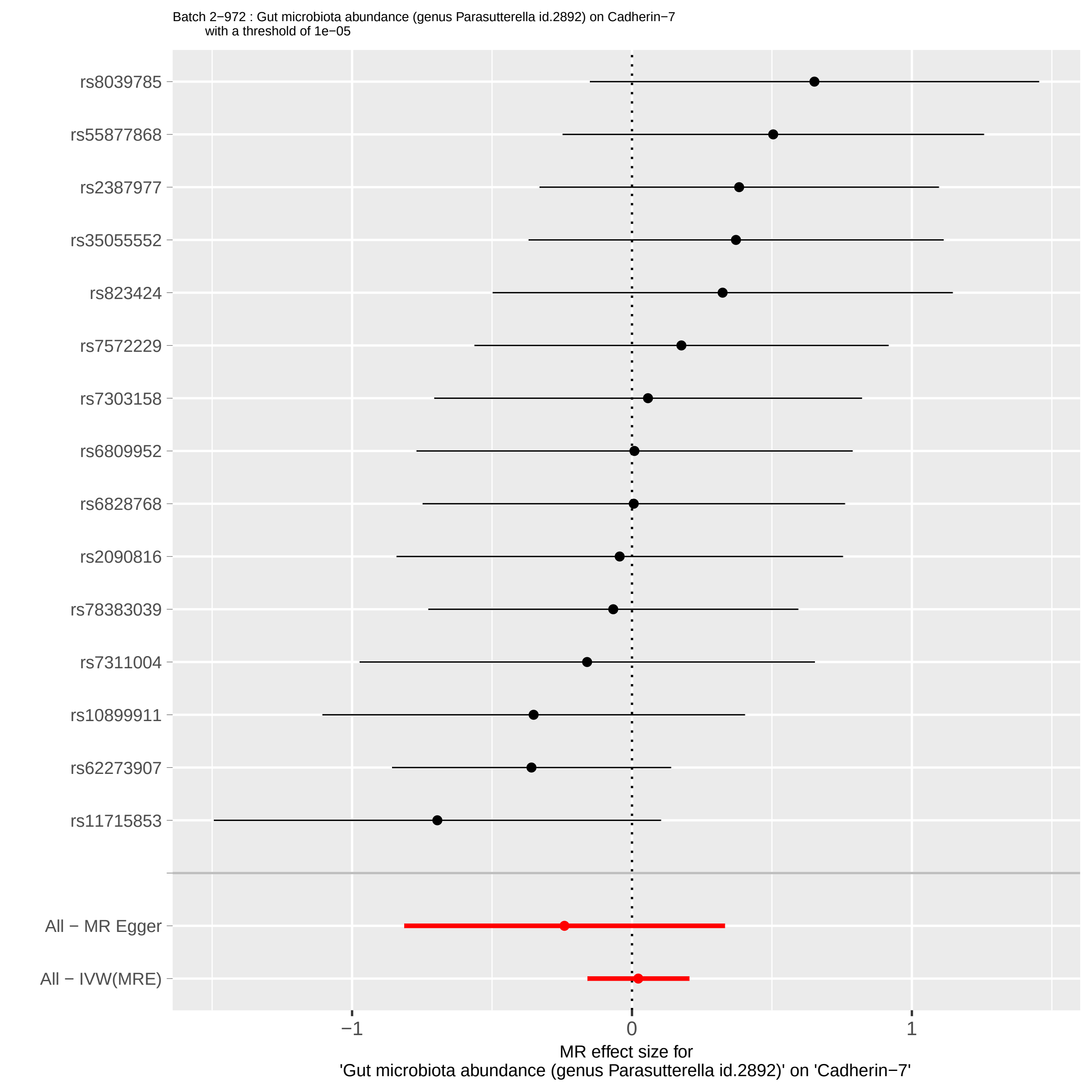

Batch 2−972 : Gut microbiota abundance (genus Parasutterella id.2892) on Cadherin−7
with a threshold of 1e−05
rs8039785
rs55877868
rs2387977
rs35055552
rs823424
rs7572229
rs7303158
rs6809952
rs6828768
rs2090816
rs78383039
rs7311004
rs10899911
rs62273907
rs11715853
All − MR Egger
All − IVW(MRE)
−1
0
1
MR effect size for
'Gut microbiota abundance (genus Parasutterella id.2892)' on 'Cadherin−7'

Batch 2−973 : Gut microbiota abundance (genus Catenibacterium id.2153) on Cadherin−related family member 1
with a threshold of 1e−05

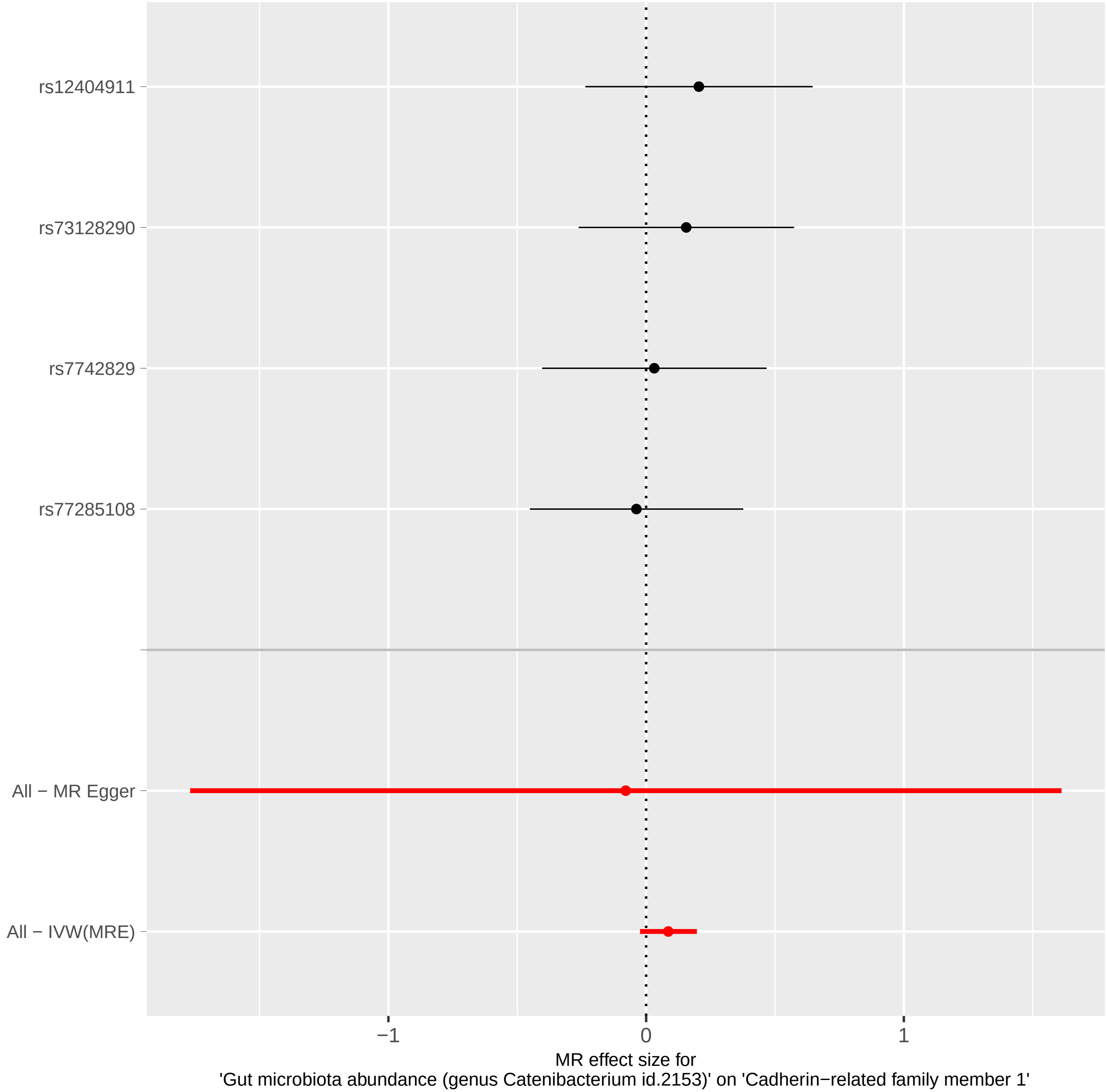

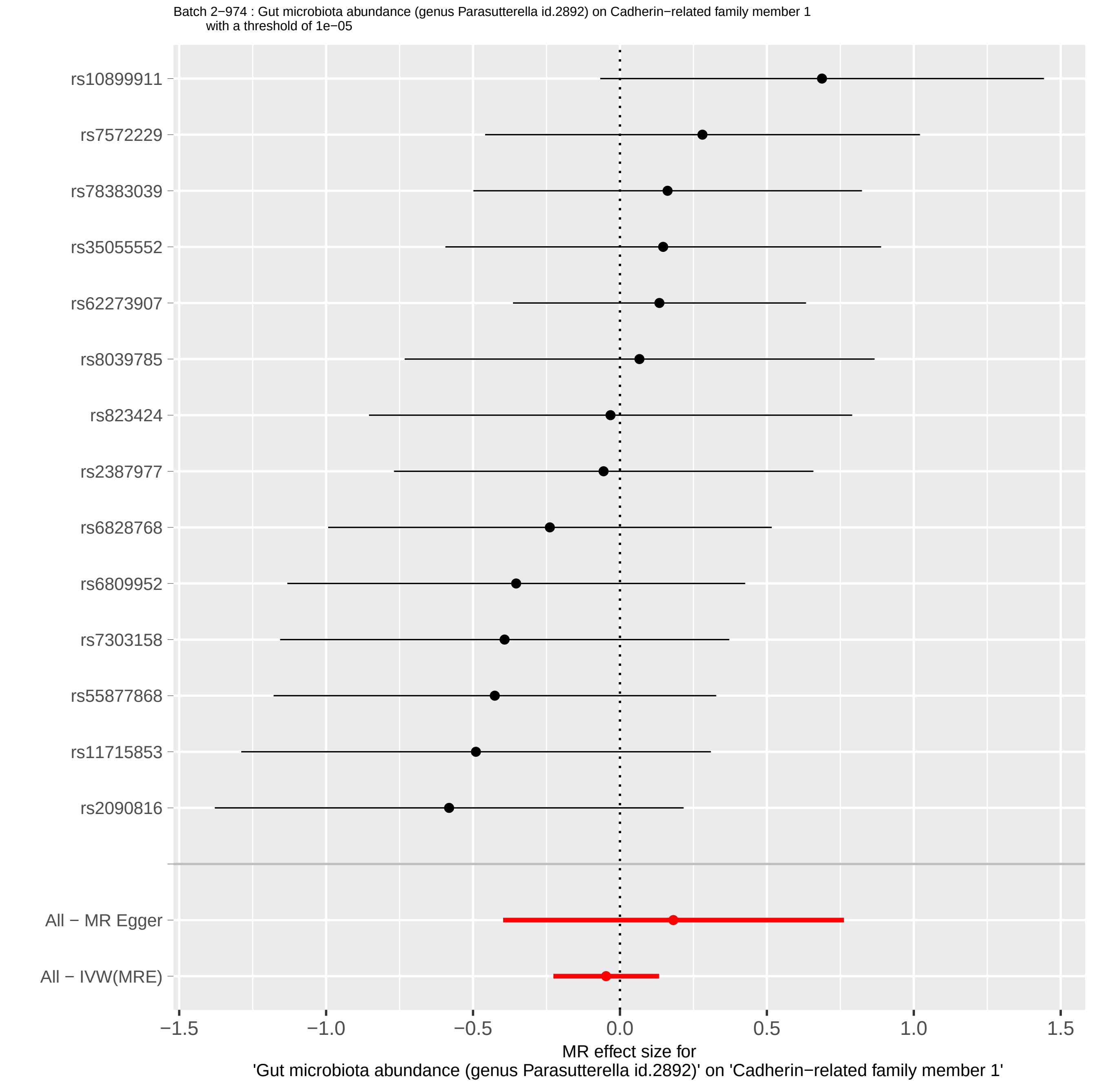

Batch 2−974 : Gut microbiota abundance (genus Parasutterella id.2892) on Cadherin−related family member 1
with a threshold of 1e−05
rs10899911
rs7572229
rs78383039
rs35055552
rs62273907
rs8039785
rs823424
rs2387977
rs6828768
rs6809952
rs7303158
rs55877868
rs11715853
rs2090816
All − MR Egger
All − IVW(MRE)
−1.5
−1.0
−0.5
0.0
0.5
1.0
1.5
MR effect size for
'Gut microbiota abundance (genus Parasutterella id.2892)' on 'Cadherin−related family member 1'

Batch 2−975 : Gut microbiota abundance (genus Catenibacterium id.2153) on Cadherin−related family member 3
with a threshold of 1e−05

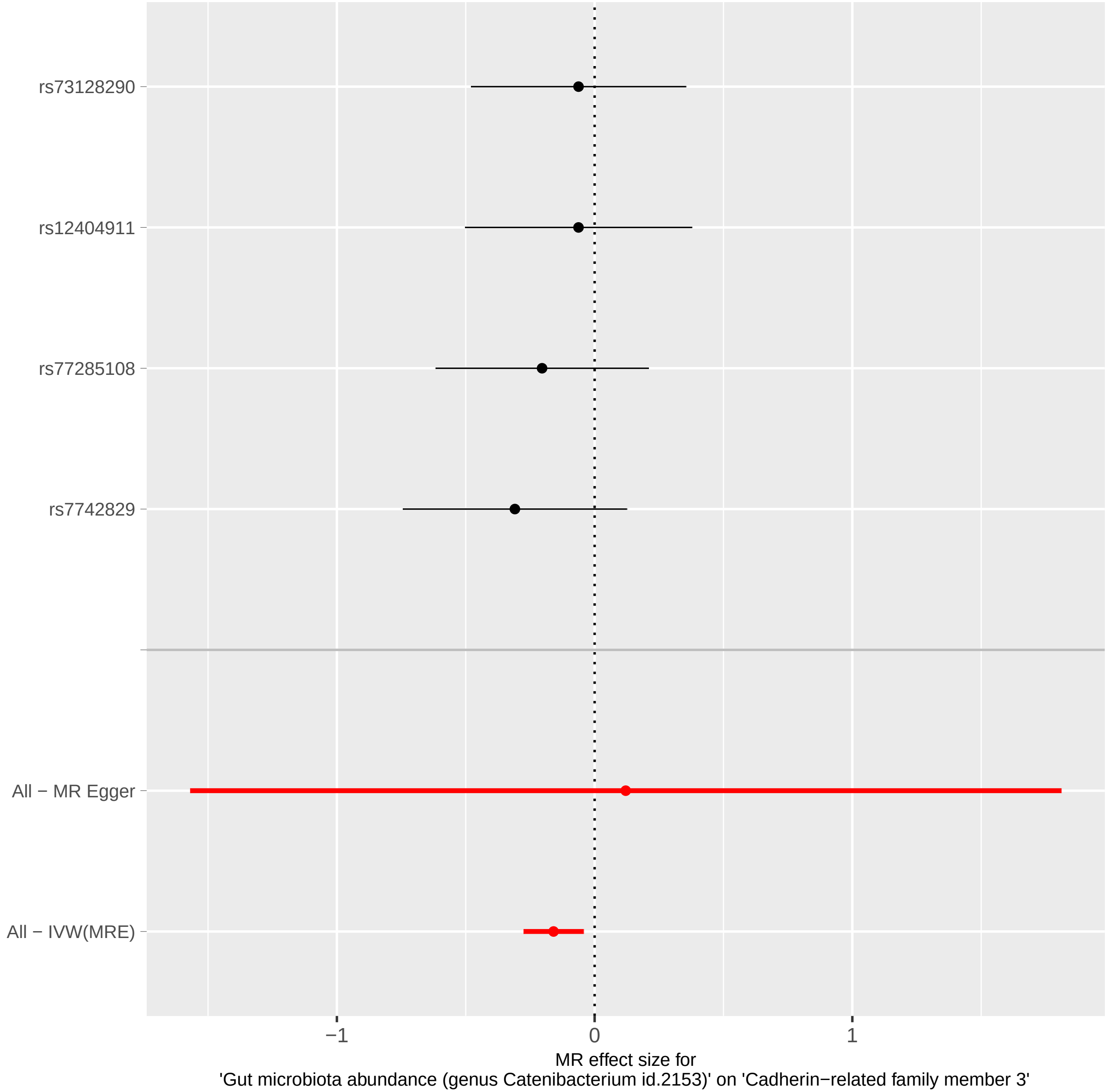

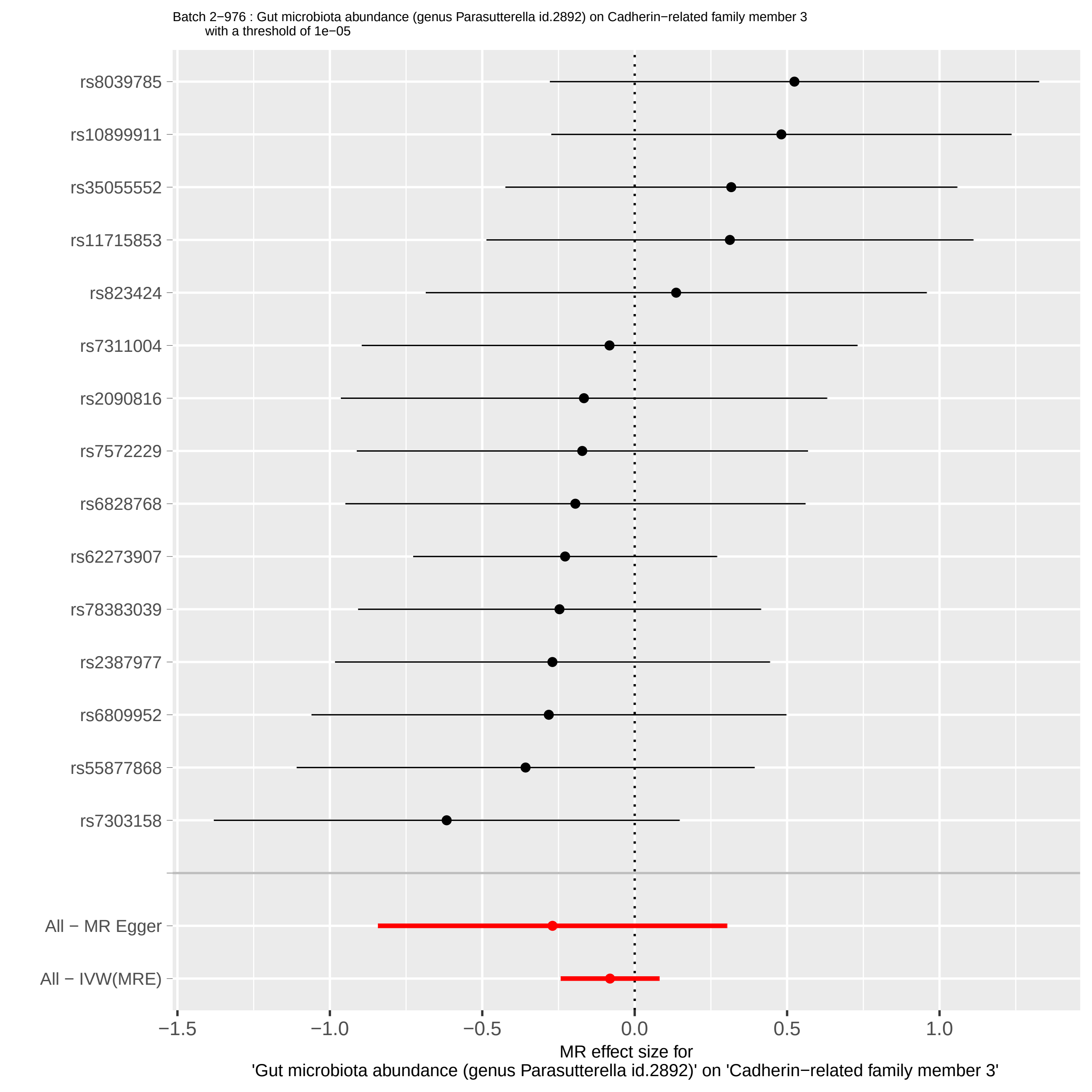
Batch 2−976 : Gut microbiota abundance (genus Parasutterella id.2892) on Cadherin−related family member 3
with a threshold of 1e−05
rs8039785
rs10899911
rs35055552
rs11715853
rs823424
rs7311004
rs2090816
rs7572229
rs6828768
rs62273907
rs78383039
rs2387977
rs6809952
rs55877868
rs7303158
All − MR Egger
All − IVW(MRE)
−1.5
−1.0
−0.5
0.0
0.5
1.0
MR effect size for
'Gut microbiota abundance (genus Parasutterella id.2892)' on 'Cadherin−related family member 3'

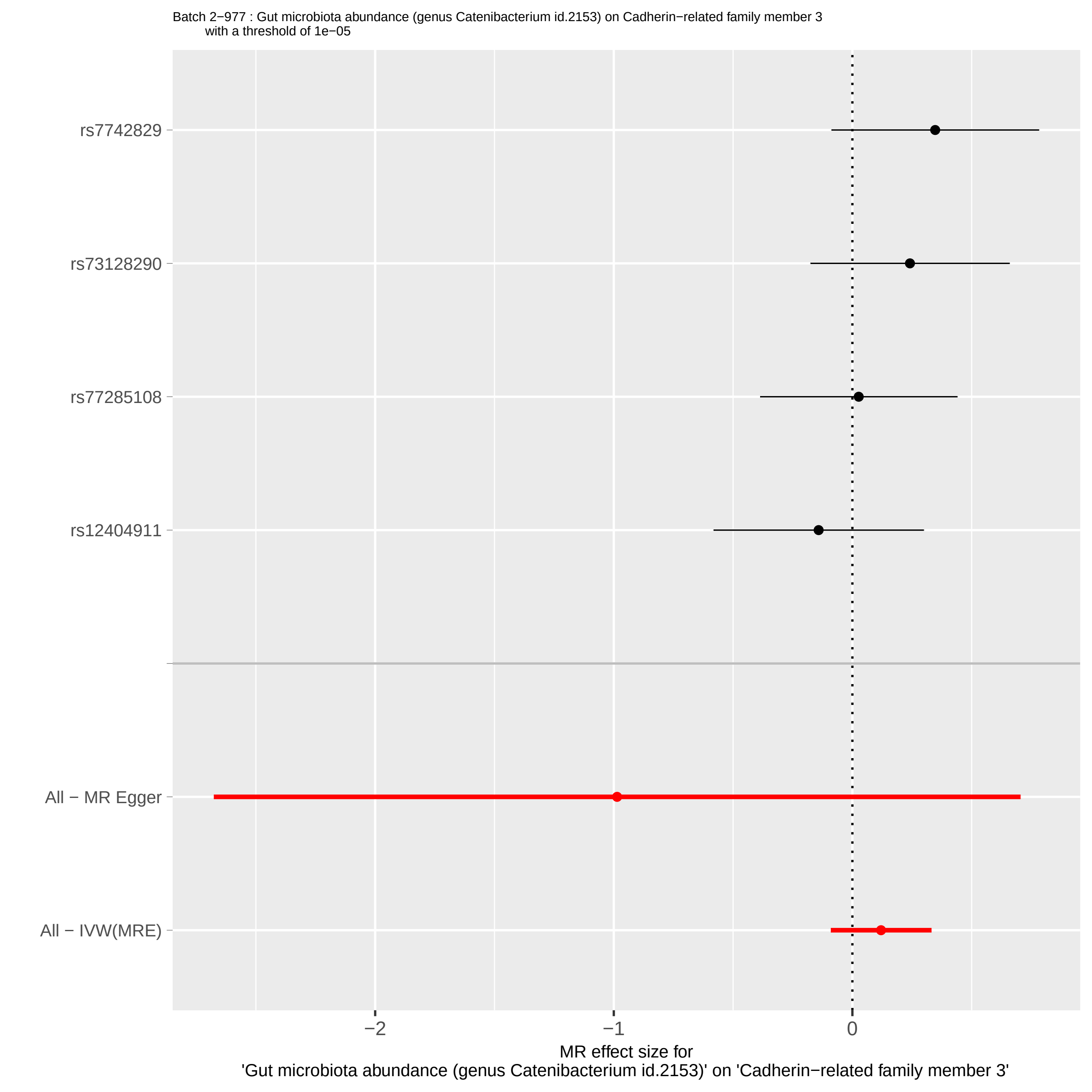
Batch 2−977 : Gut microbiota abundance (genus Catenibacterium id.2153) on Cadherin−related family member 3
with a threshold of 1e−05
rs7742829
rs73128290
rs77285108
rs12404911
All − MR Egger
All − IVW(MRE)
−2
−1
0
MR effect size for
'Gut microbiota abundance (genus Catenibacterium id.2153)' on 'Cadherin−related family member 3'

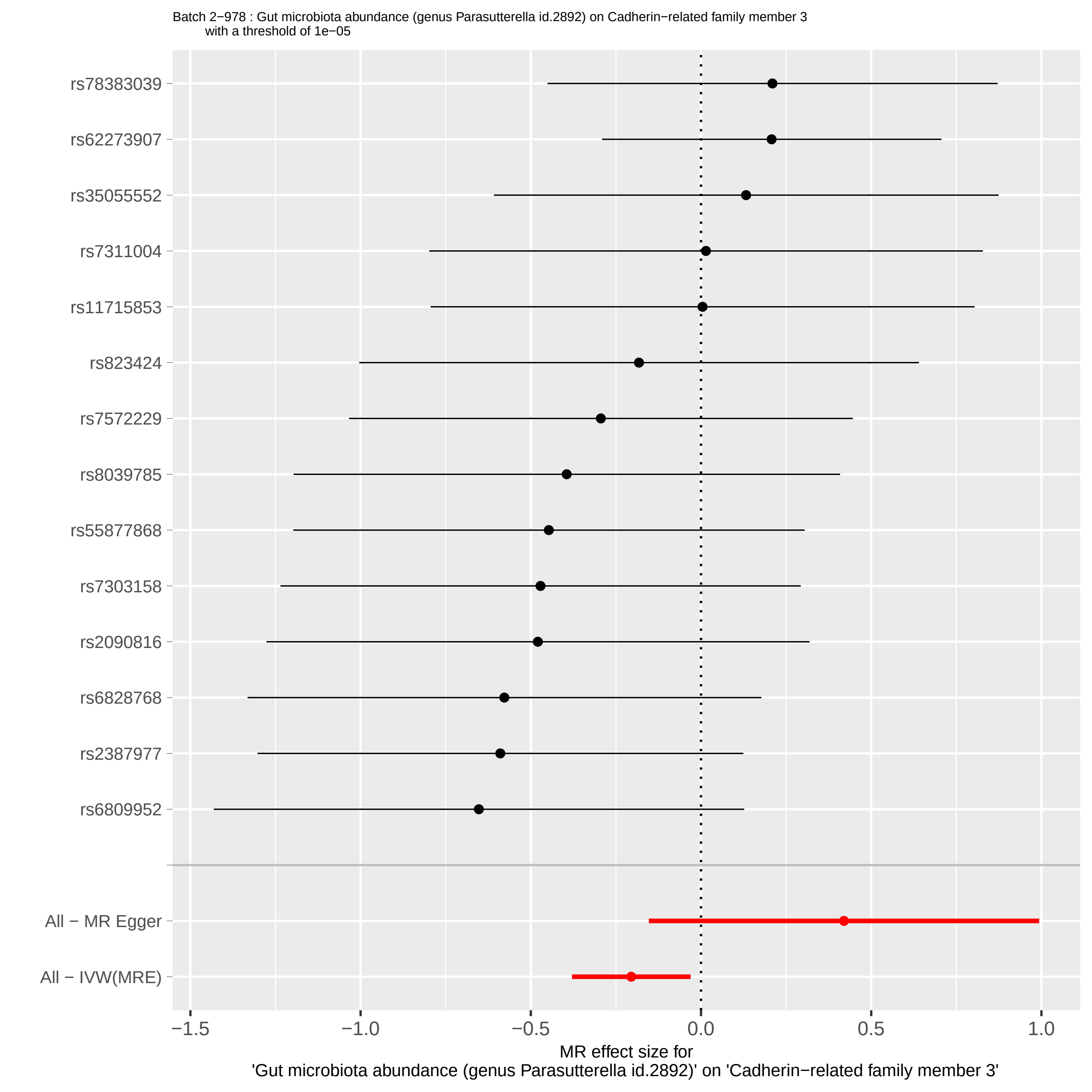

Batch 2−978 : Gut microbiota abundance (genus Parasutterella id.2892) on Cadherin−related family member 3
with a threshold of 1e−05
rs78383039
rs62273907
rs35055552
rs7311004
rs11715853
rs823424
rs7572229
rs8039785
rs55877868
rs7303158
rs2090816
rs6828768
rs2387977
rs6809952
All − MR Egger
All − IVW(MRE)
−1.5
−1.0
−0.5
0.0
0.5
1.0
MR effect size for
'Gut microbiota abundance (genus Parasutterella id.2892)' on 'Cadherin−related family member 3'

Batch 2−979 : Gut microbiota abundance (genus Catenibacterium id.2153) on Cadherin−related family member 5
with a threshold of 1e−05

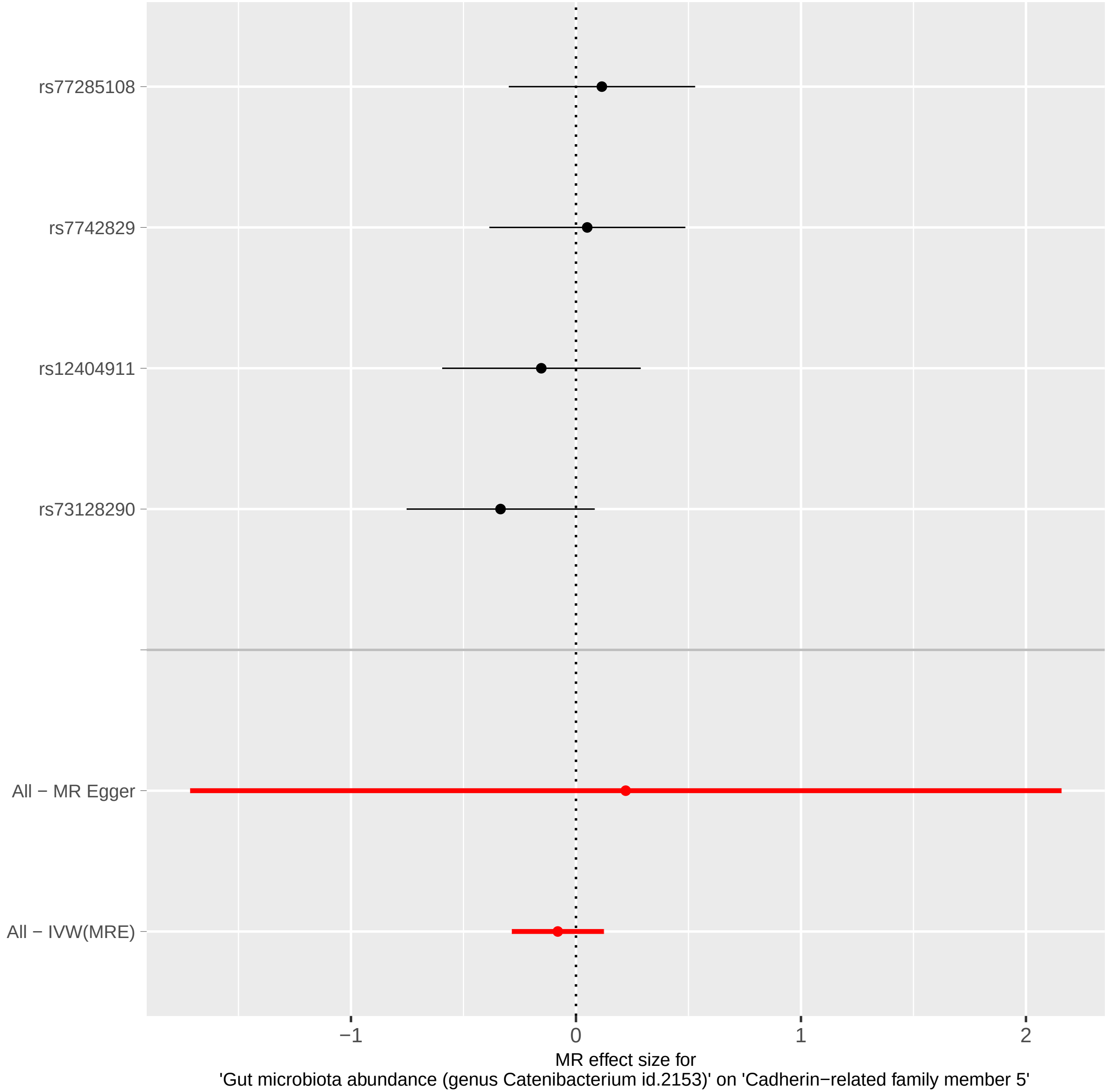

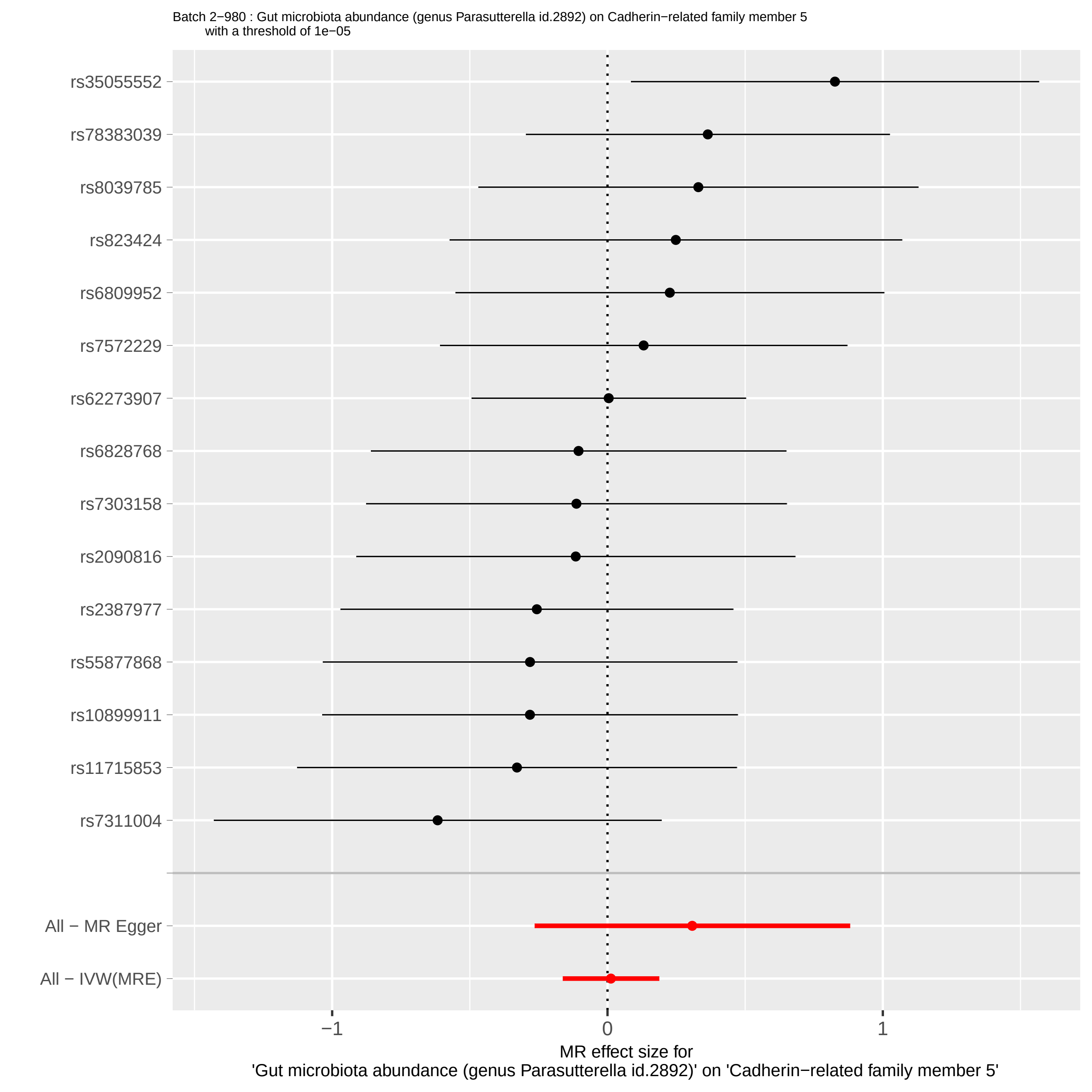

Batch 2−980 : Gut microbiota abundance (genus Parasutterella id.2892) on Cadherin−related family member 5
with a threshold of 1e−05
rs35055552
rs78383039
rs8039785
rs823424
rs6809952
rs7572229
rs62273907
rs6828768
rs7303158
rs2090816
rs2387977
rs55877868
rs10899911
rs11715853
rs7311004
All − MR Egger
All − IVW(MRE)
−1
0
1
MR effect size for
'Gut microbiota abundance (genus Parasutterella id.2892)' on 'Cadherin−related family member 5'

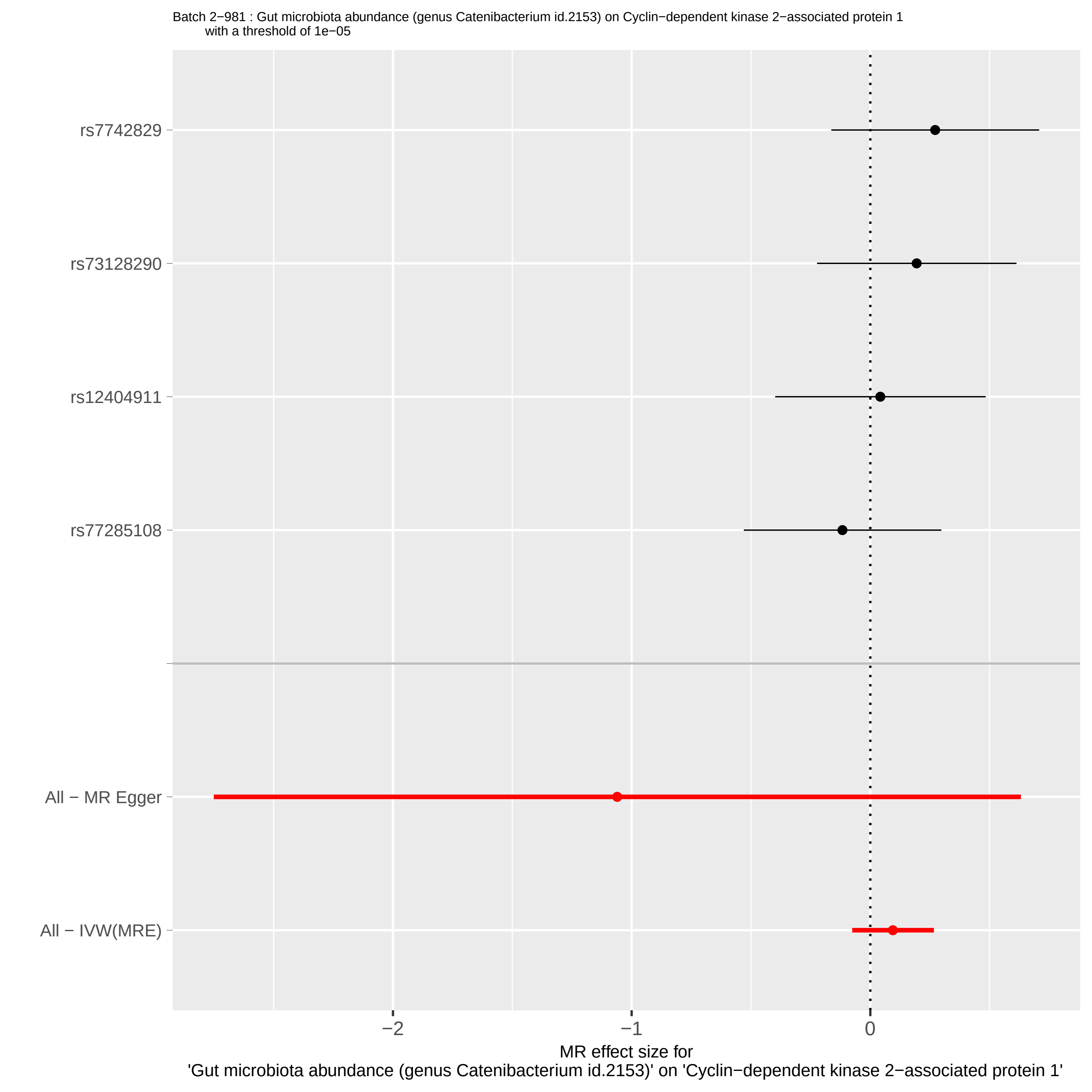

Batch 2−981 : Gut microbiota abundance (genus Catenibacterium id.2153) on Cyclin−dependent kinase 2−associated protein 1
with a threshold of 1e−05
rs7742829
rs73128290
rs12404911
rs77285108
All − MR Egger
All − IVW(MRE)
−2
−1
0
MR effect size for
'Gut microbiota abundance (genus Catenibacterium id.2153)' on 'Cyclin−dependent kinase 2−associated protein 1'

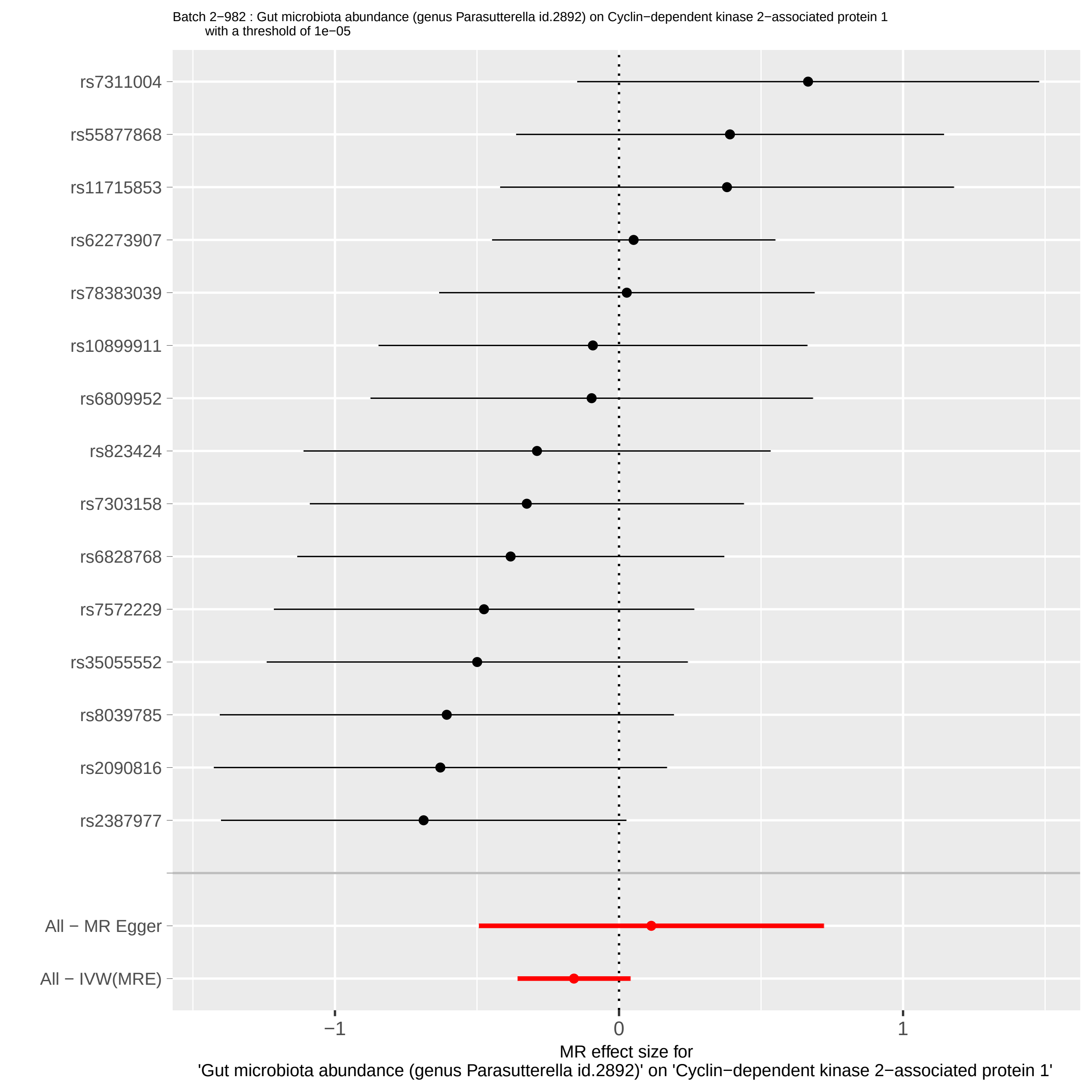

Batch 2−982 : Gut microbiota abundance (genus Parasutterella id.2892) on Cyclin−dependent kinase 2−associated protein 1
with a threshold of 1e−05
rs7311004
rs55877868
rs11715853
rs62273907
rs78383039
rs10899911
rs6809952
rs823424
rs7303158
rs6828768
rs7572229
rs35055552
rs8039785
rs2090816
rs2387977
All − MR Egger
All − IVW(MRE)
−1
0
1
MR effect size for
'Gut microbiota abundance (genus Parasutterella id.2892)' on 'Cyclin−dependent kinase 2−associated protein 1'

Batch 2−983 : Gut microbiota abundance (genus Catenibacterium id.2153) on Cyclin−dependent kinase 2:Cyclin−A2 complex
with a threshold of 1e−05

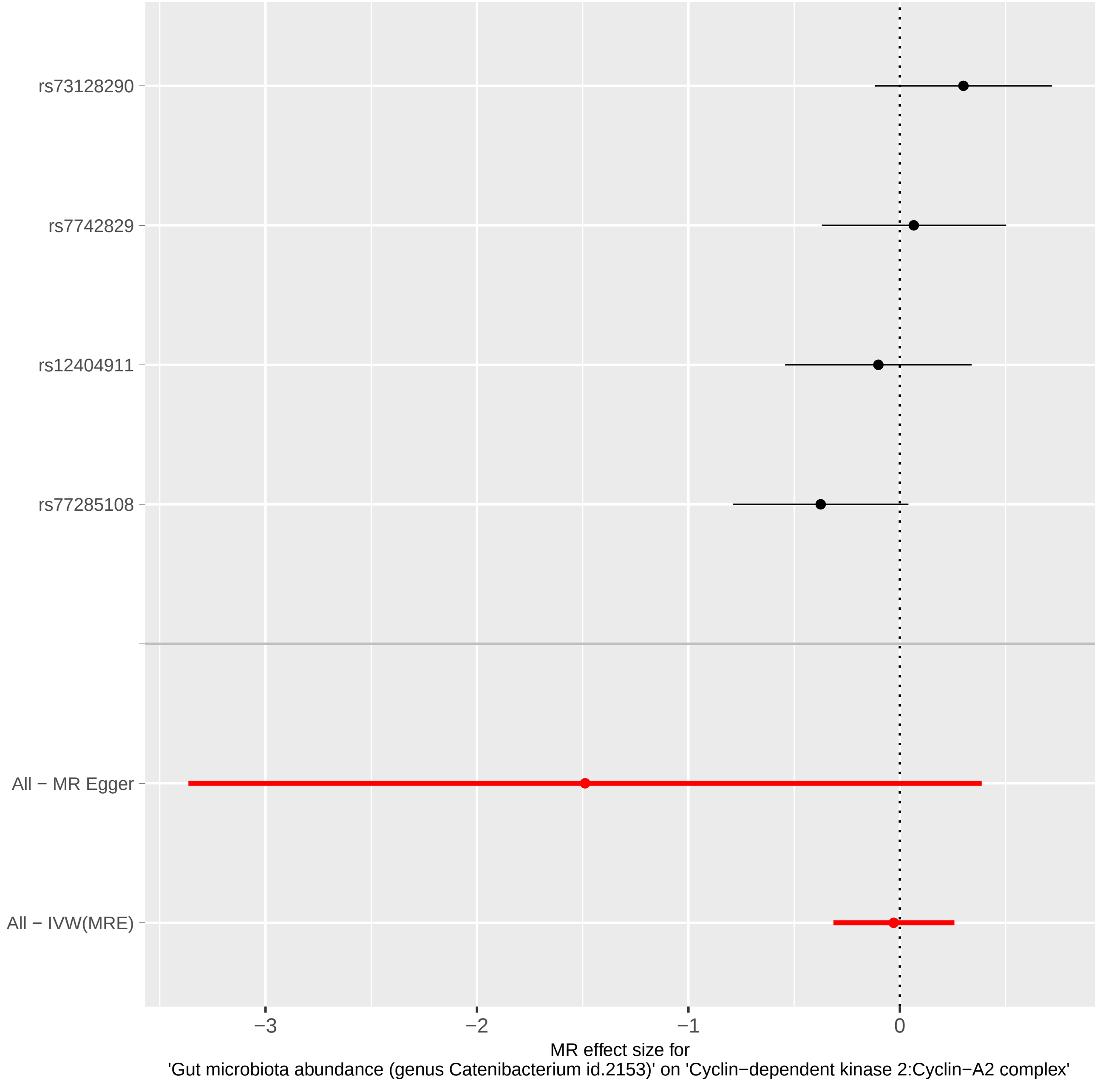

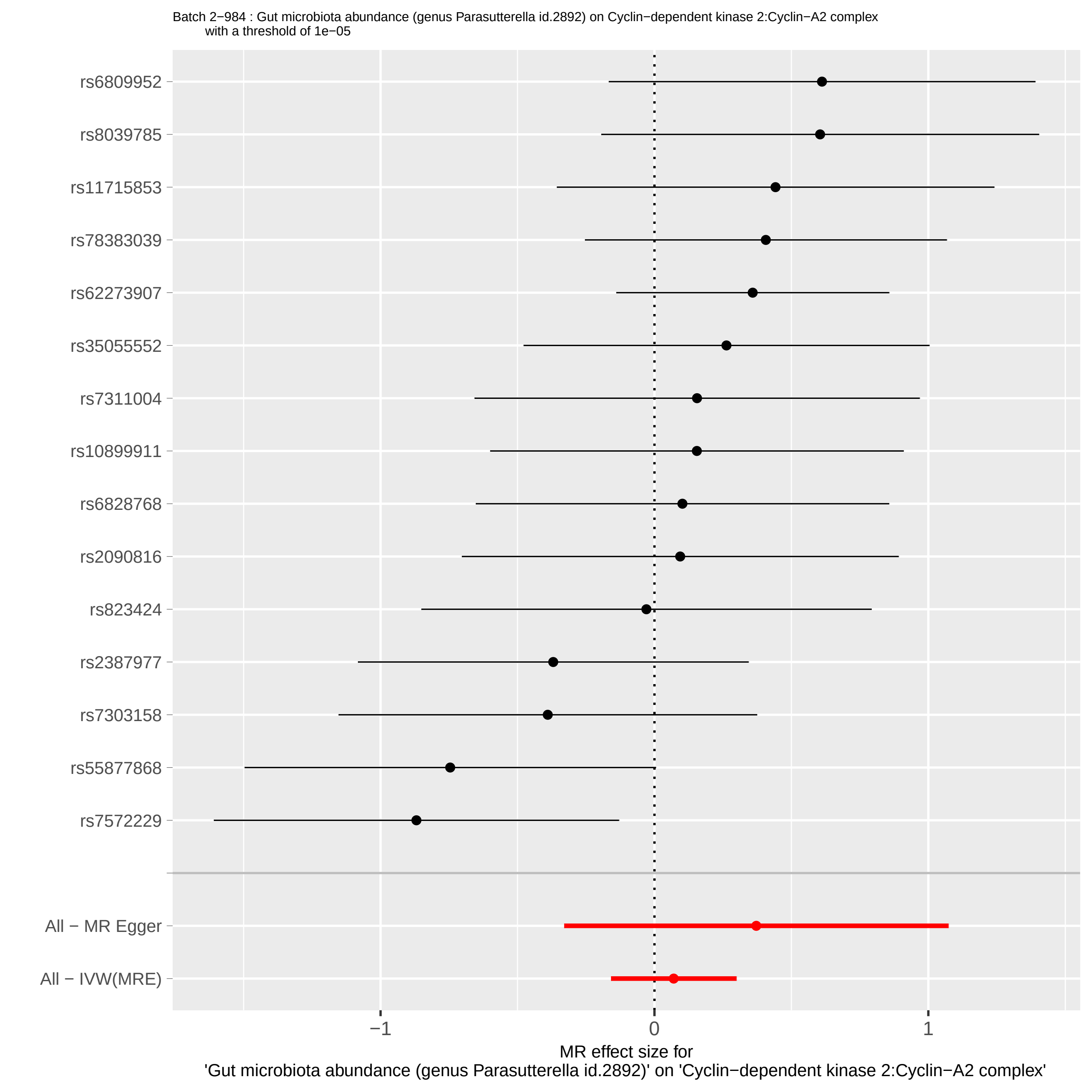

Batch 2−984 : Gut microbiota abundance (genus Parasutterella id.2892) on Cyclin−dependent kinase 2:Cyclin−A2 complex
with a threshold of 1e−05
rs6809952
rs8039785
rs11715853
rs78383039
rs62273907
rs35055552
rs7311004
rs10899911
rs6828768
rs2090816
rs823424
rs2387977
rs7303158
rs55877868
rs7572229
All − MR Egger
All − IVW(MRE)
−1
0
1
MR effect size for
'Gut microbiota abundance (genus Parasutterella id.2892)' on 'Cyclin−dependent kinase 2:Cyclin−A2 complex'

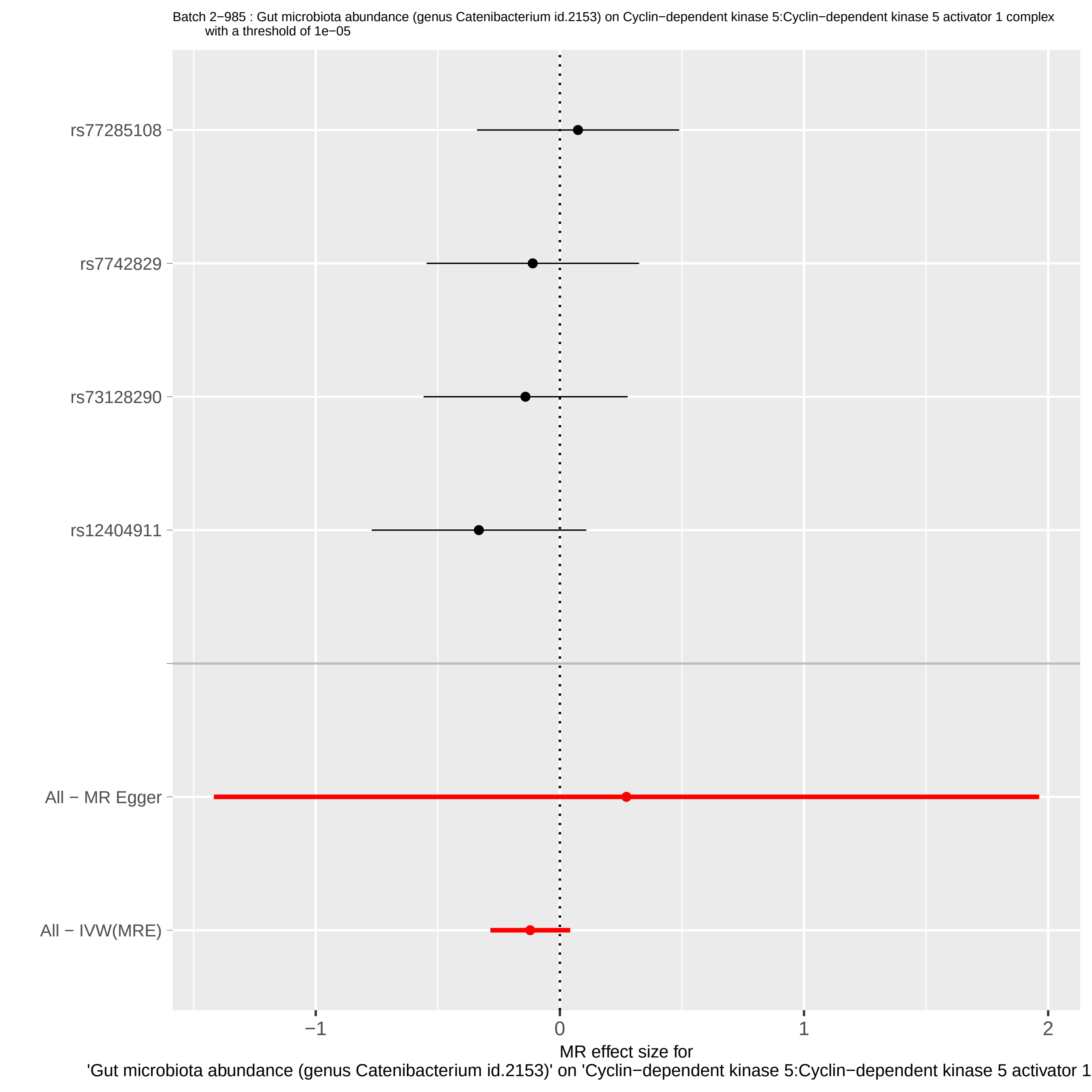

Batch 2−985 : Gut microbiota abundance (genus Catenibacterium id.2153) on Cyclin−dependent kinase 5:Cyclin−dependent kinase 5 activator 1 complex
with a threshold of 1e−05
rs77285108
rs7742829
rs73128290
rs12404911
All − MR Egger
All − IVW(MRE)
−1
0
1
2
MR effect size for
'Gut microbiota abundance (genus Catenibacterium id.2153)' on 'Cyclin−dependent kinase 5:Cyclin−dependent kinase 5 activator 1

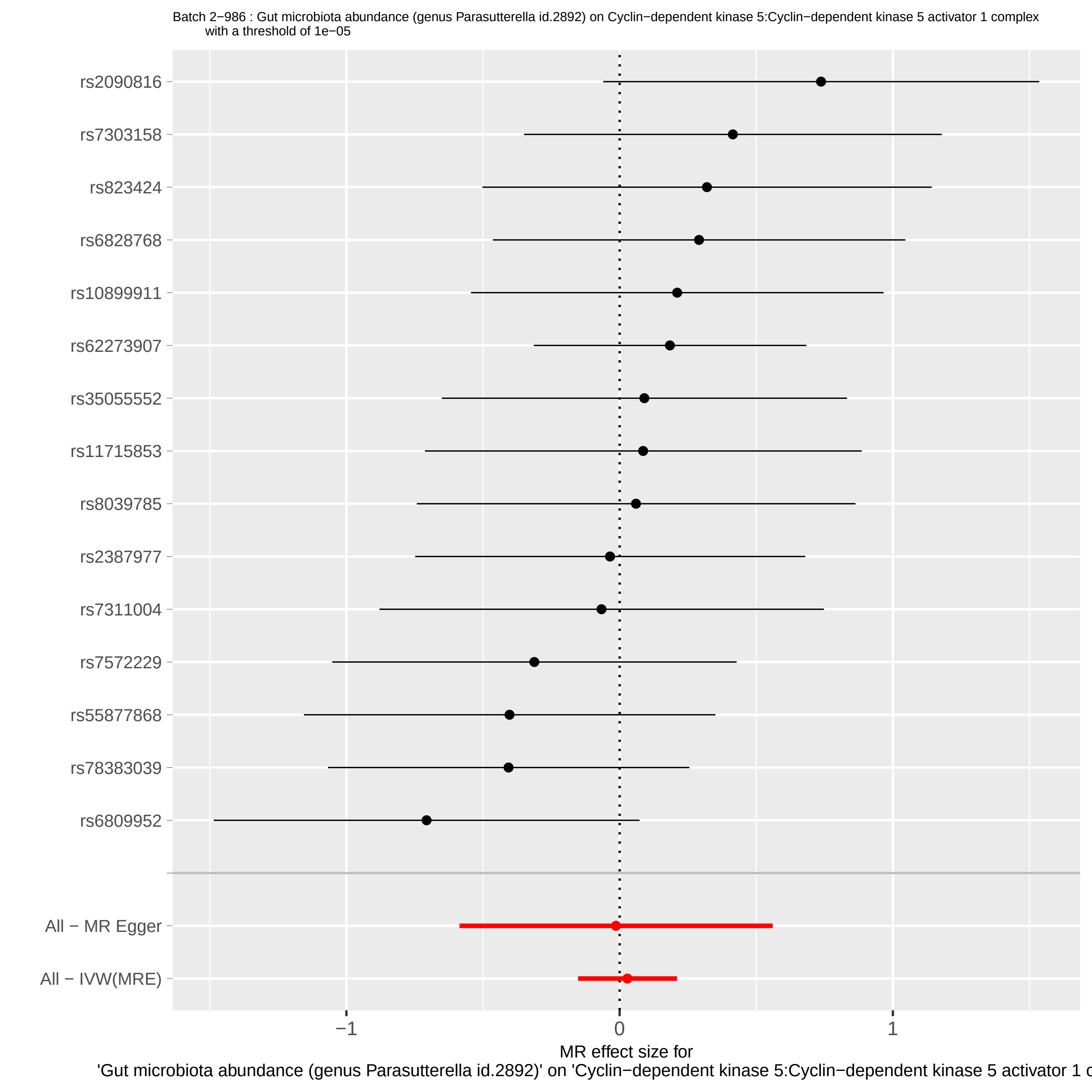

Batch 2−986 : Gut microbiota abundance (genus Parasutterella id.2892) on Cyclin−dependent kinase 5:Cyclin−dependent kinase 5 activator 1 complex with a threshold of 1e−05
rs2090816
rs7303158
rs823424
rs6828768
rs10899911
rs62273907
rs35055552
rs11715853
rs8039785
rs2387977
rs7311004
rs7572229
rs55877868
rs78383039
rs6809952
All − MR Egger
All − IVW(MRE)
−1
0
1
MR effect size for
'Gut microbiota abundance (genus Parasutterella id.2892)' on 'Cyclin−dependent kinase 5:Cyclin−dependent kinase 5 activator 1 c

Batch 2−987 : Gut microbiota abundance (genus Catenibacterium id.2153) on Cyclin−dependent kinase 8:Cyclin−C complex
with a threshold of 1e−05

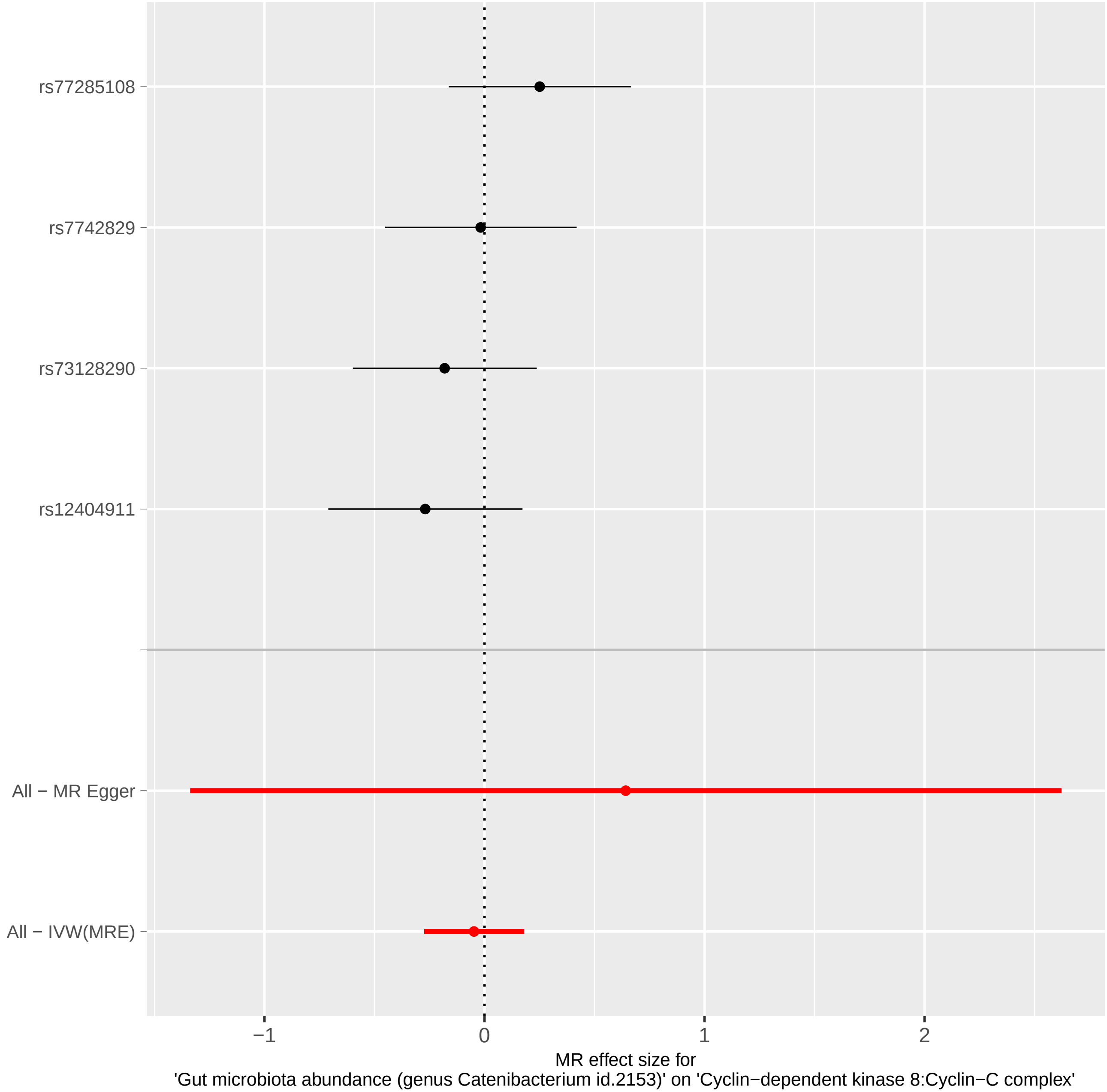

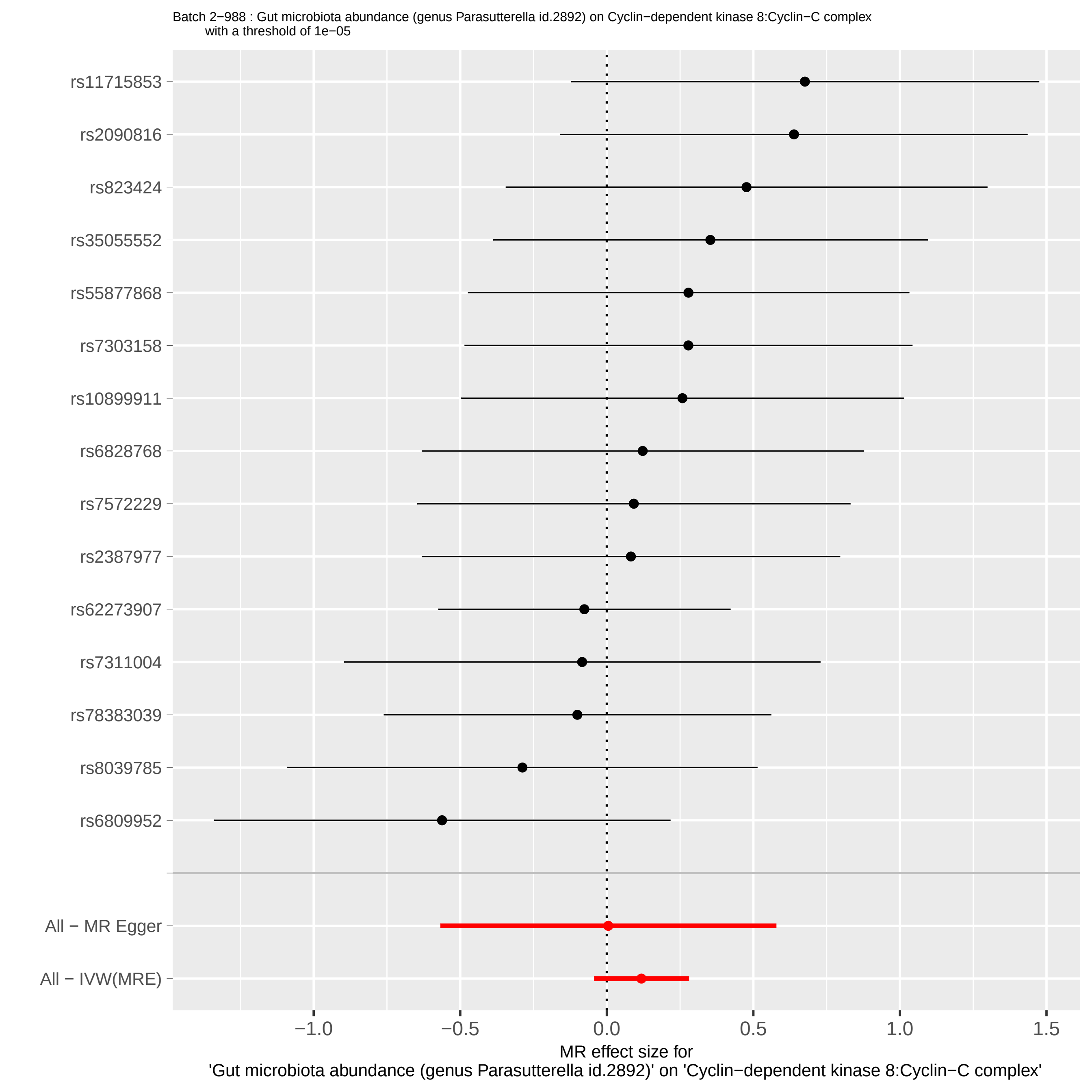

Batch 2−988 : Gut microbiota abundance (genus Parasutterella id.2892) on Cyclin−dependent kinase 8:Cyclin−C complex
with a threshold of 1e−05
rs11715853
rs2090816
rs823424
rs35055552
rs55877868
rs7303158
rs10899911
rs6828768
rs7572229
rs2387977
rs62273907
rs7311004
rs78383039
rs8039785
rs6809952
All − MR Egger
All − IVW(MRE)
−1.0
−0.5
0.0
0.5
1.0
1.5
MR effect size for
'Gut microbiota abundance (genus Parasutterella id.2892)' on 'Cyclin−dependent kinase 8:Cyclin−C complex'

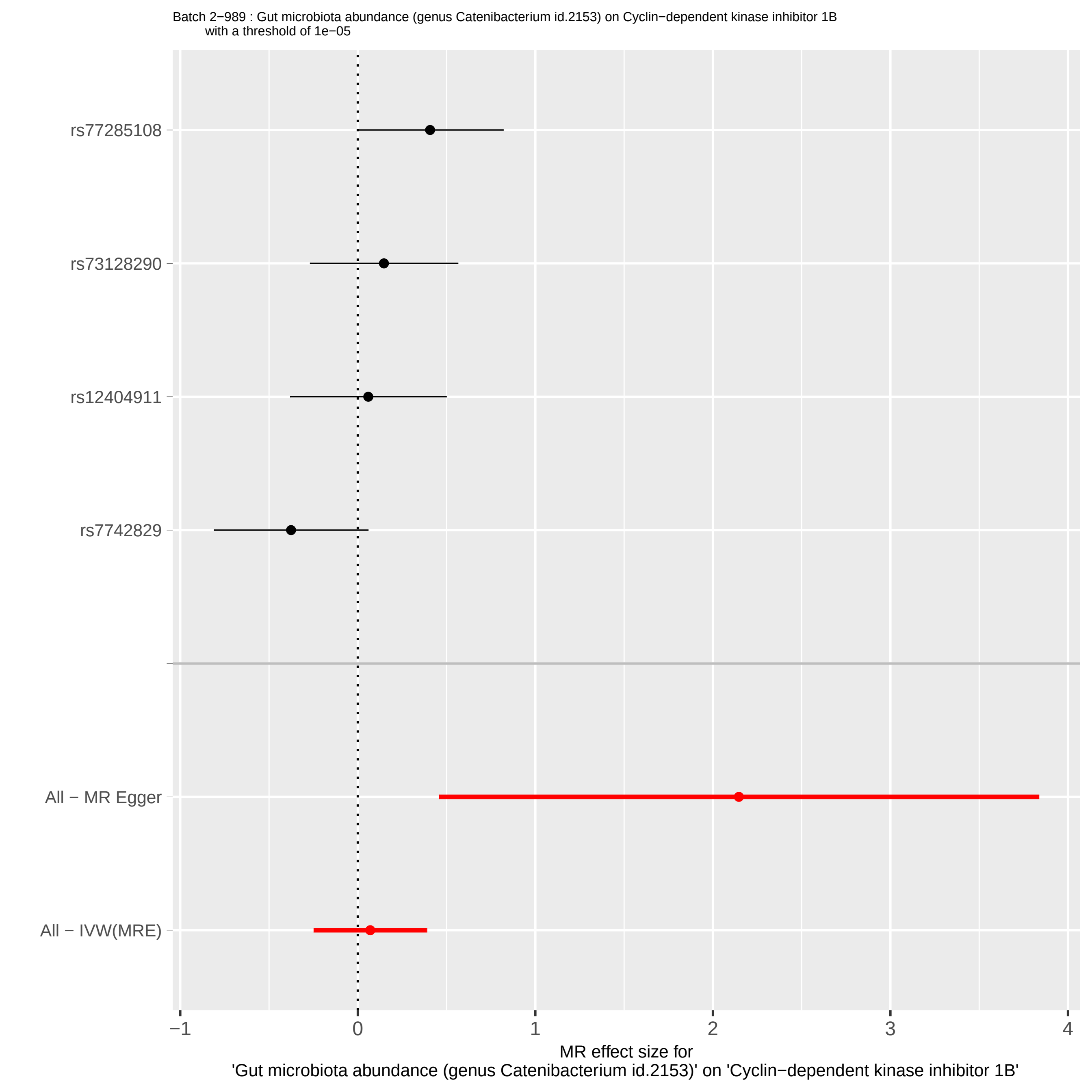

Batch 2−989 : Gut microbiota abundance (genus Catenibacterium id.2153) on Cyclin−dependent kinase inhibitor 1B
with a threshold of 1e−05
rs77285108
rs73128290
rs12404911
rs7742829
All − MR Egger
All − IVW(MRE)
−1
0
1
2
3
4
MR effect size for
'Gut microbiota abundance (genus Catenibacterium id.2153)' on 'Cyclin−dependent kinase inhibitor 1B'

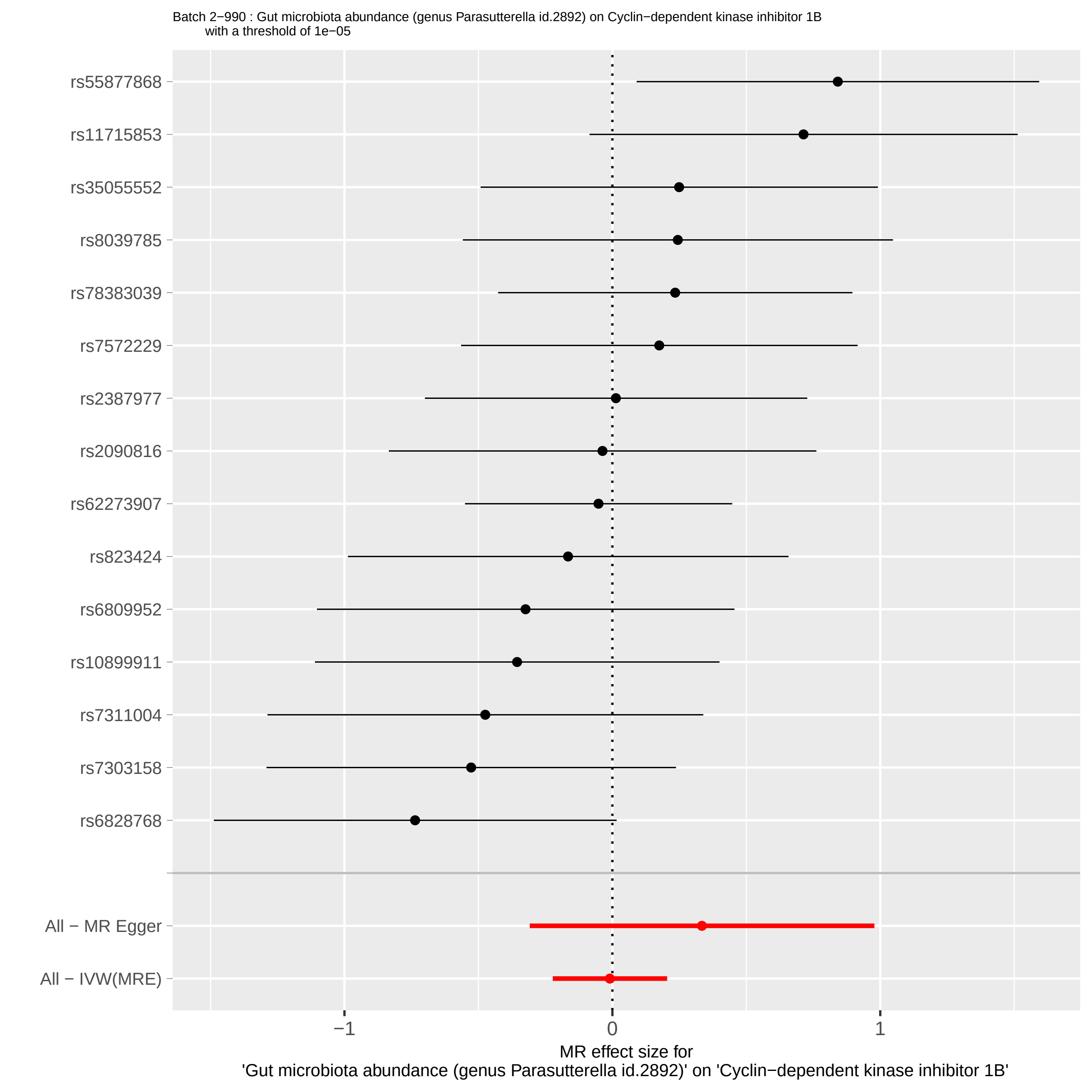

Batch 2−990 : Gut microbiota abundance (genus Parasutterella id.2892) on Cyclin−dependent kinase inhibitor 1B
with a threshold of 1e−05
rs55877868
rs11715853
rs35055552
rs8039785
rs78383039
rs7572229
rs2387977
rs2090816
rs62273907
rs823424
rs6809952
rs10899911
rs7311004
rs7303158
rs6828768
All − MR Egger
All − IVW(MRE)
−1
0
1
MR effect size for
'Gut microbiota abundance (genus Parasutterella id.2892)' on 'Cyclin−dependent kinase inhibitor 1B'

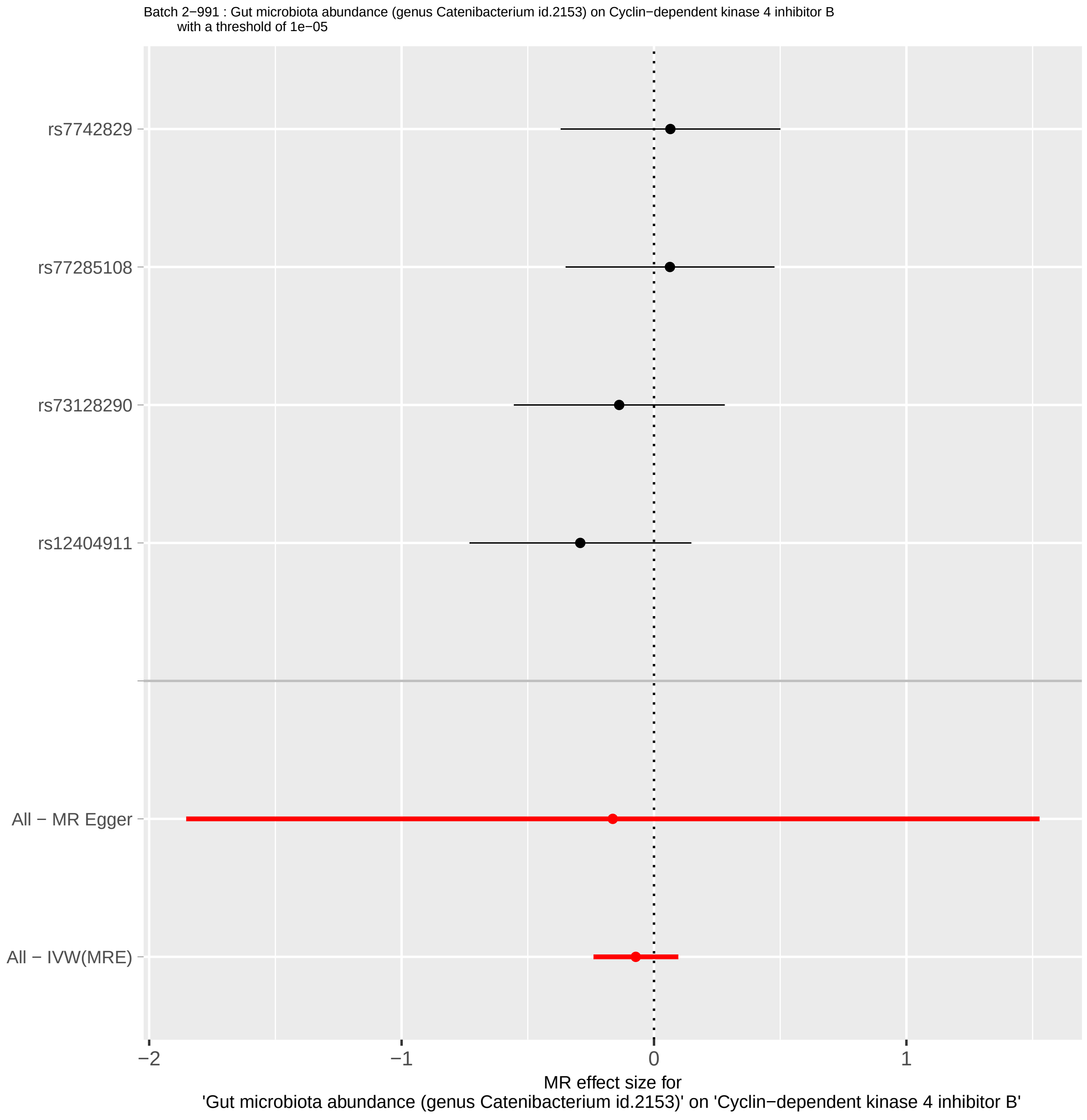
Batch 2−991 : Gut microbiota abundance (genus Catenibacterium id.2153) on Cyclin−dependent kinase 4 inhibitor B
with a threshold of 1e−05
rs7742829
rs77285108
rs73128290
rs12404911
All − MR Egger
All − IVW(MRE)
−2
−1
0
1
MR effect size for
'Gut microbiota abundance (genus Catenibacterium id.2153)' on 'Cyclin−dependent kinase 4 inhibitor B'

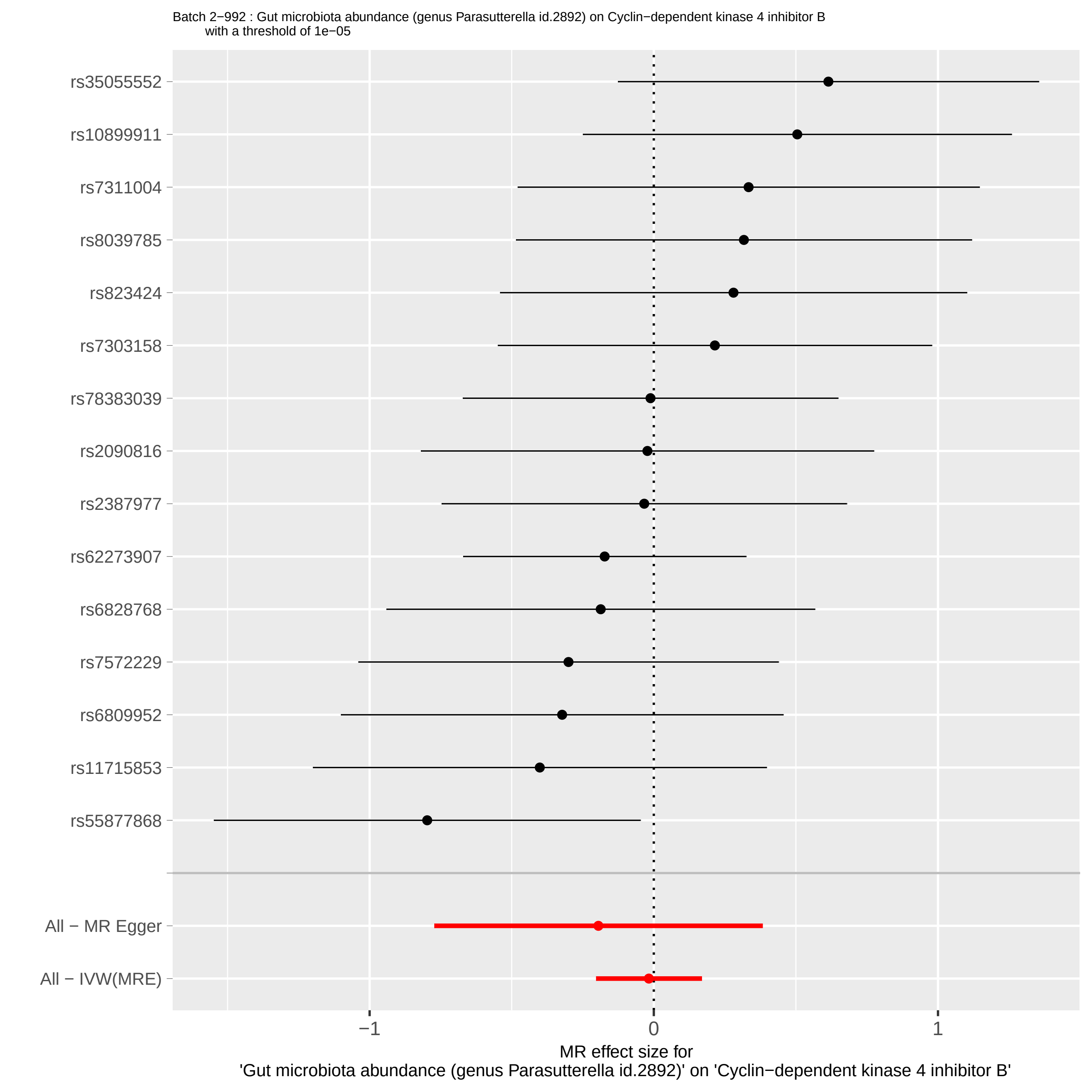

Batch 2−992 : Gut microbiota abundance (genus Parasutterella id.2892) on Cyclin−dependent kinase 4 inhibitor B
with a threshold of 1e−05
rs35055552
rs10899911
rs7311004
rs8039785
rs823424
rs7303158
rs78383039
rs2090816
rs2387977
rs62273907
rs6828768
rs7572229
rs6809952
rs11715853
rs55877868
All − MR Egger
All − IVW(MRE)
−1
0
1
MR effect size for
'Gut microbiota abundance (genus Parasutterella id.2892)' on 'Cyclin−dependent kinase 4 inhibitor B'

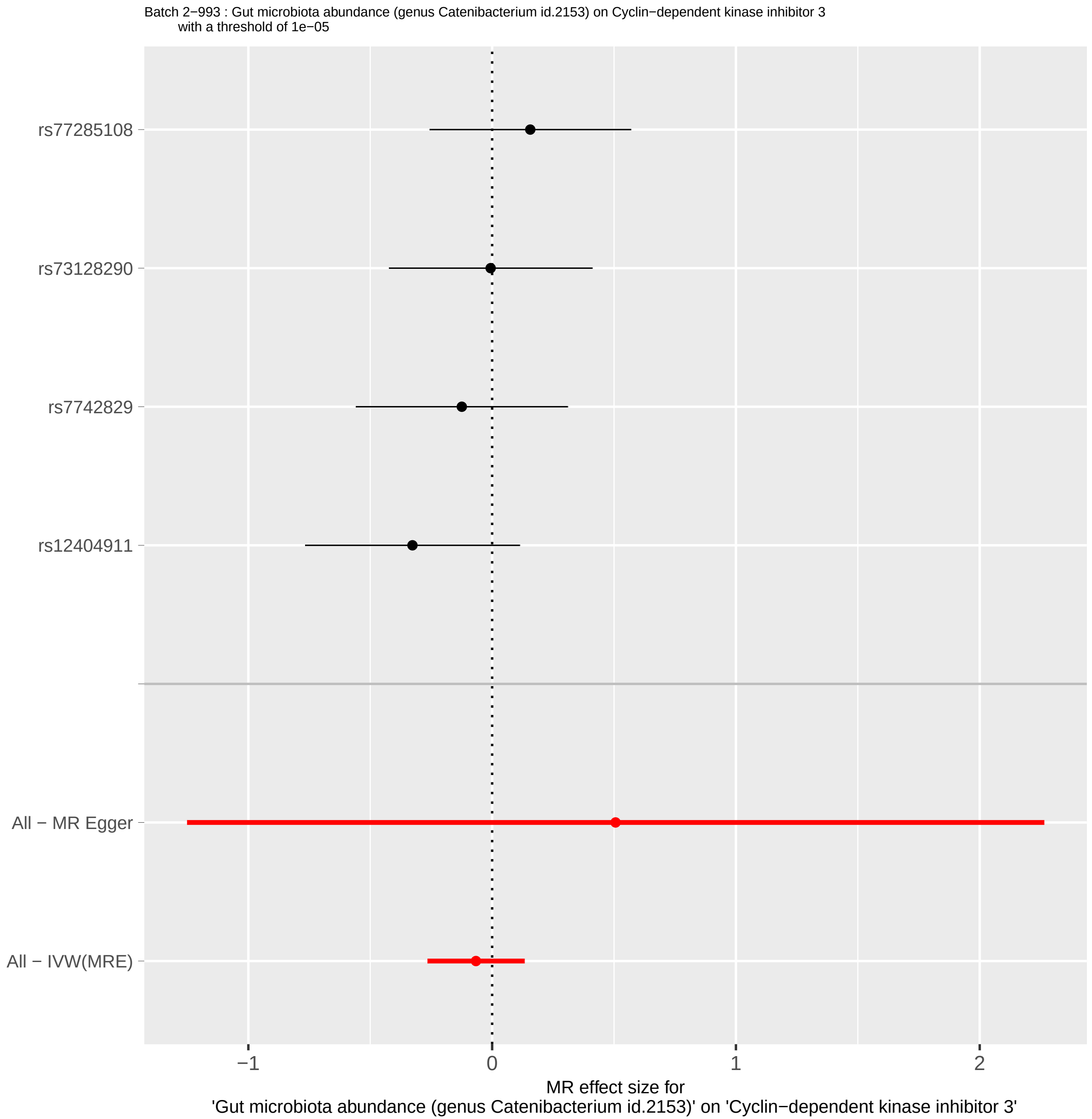

Batch 2−993 : Gut microbiota abundance (genus Catenibacterium id.2153) on Cyclin−dependent kinase inhibitor 3
with a threshold of 1e−05
rs77285108
rs73128290
rs7742829
rs12404911
All − MR Egger
All − IVW(MRE)
−1
0
1
2
MR effect size for
'Gut microbiota abundance (genus Catenibacterium id.2153)' on 'Cyclin−dependent kinase inhibitor 3'

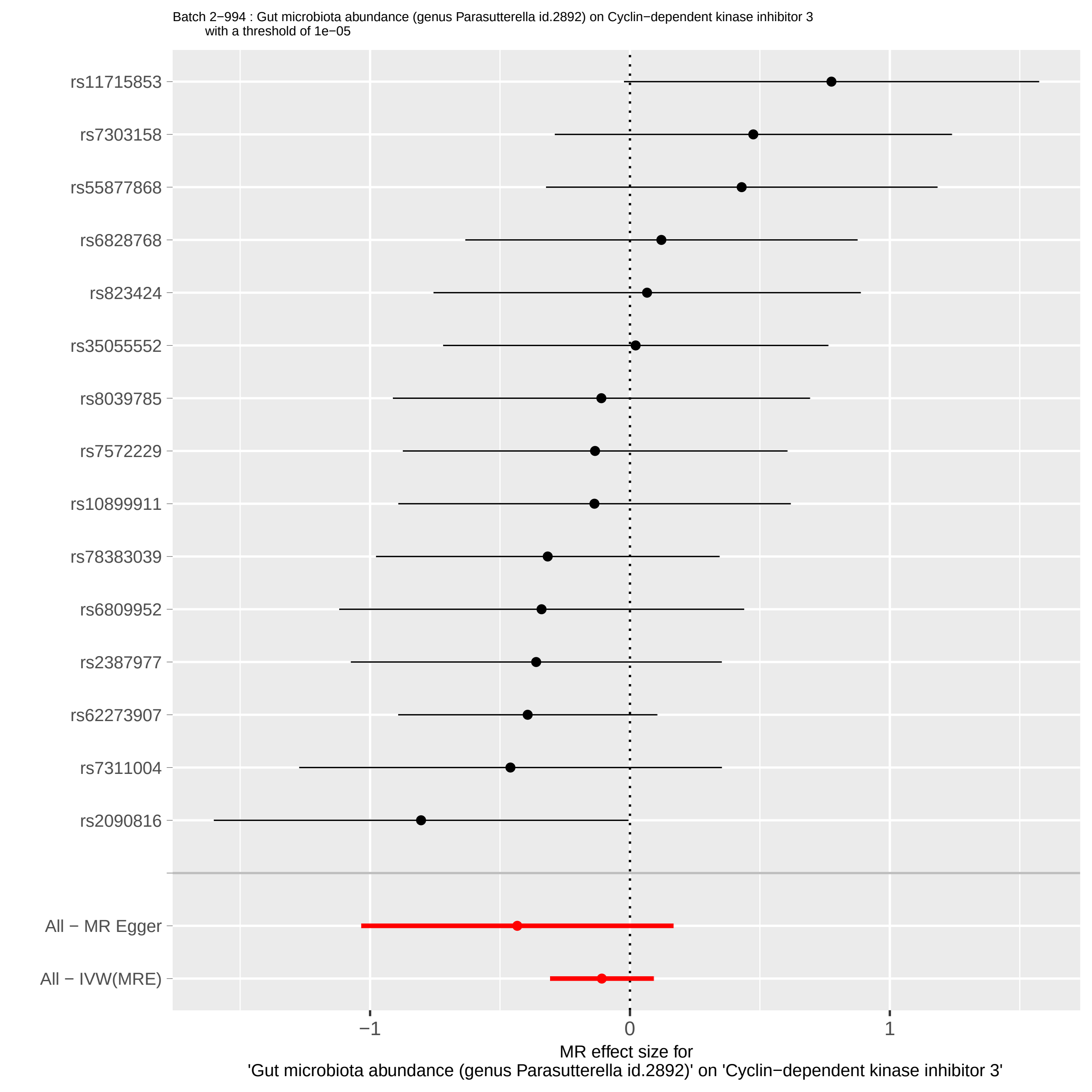
Batch 2−994 : Gut microbiota abundance (genus Parasutterella id.2892) on Cyclin−dependent kinase inhibitor 3
with a threshold of 1e−05
rs11715853
rs7303158
rs55877868
rs6828768
rs823424
rs35055552
rs8039785
rs7572229
rs10899911
rs78383039
rs6809952
rs2387977
rs62273907
rs7311004
rs2090816
All − MR Egger
All − IVW(MRE)
−1
0
1
MR effect size for
'Gut microbiota abundance (genus Parasutterella id.2892)' on 'Cyclin−dependent kinase inhibitor 3'

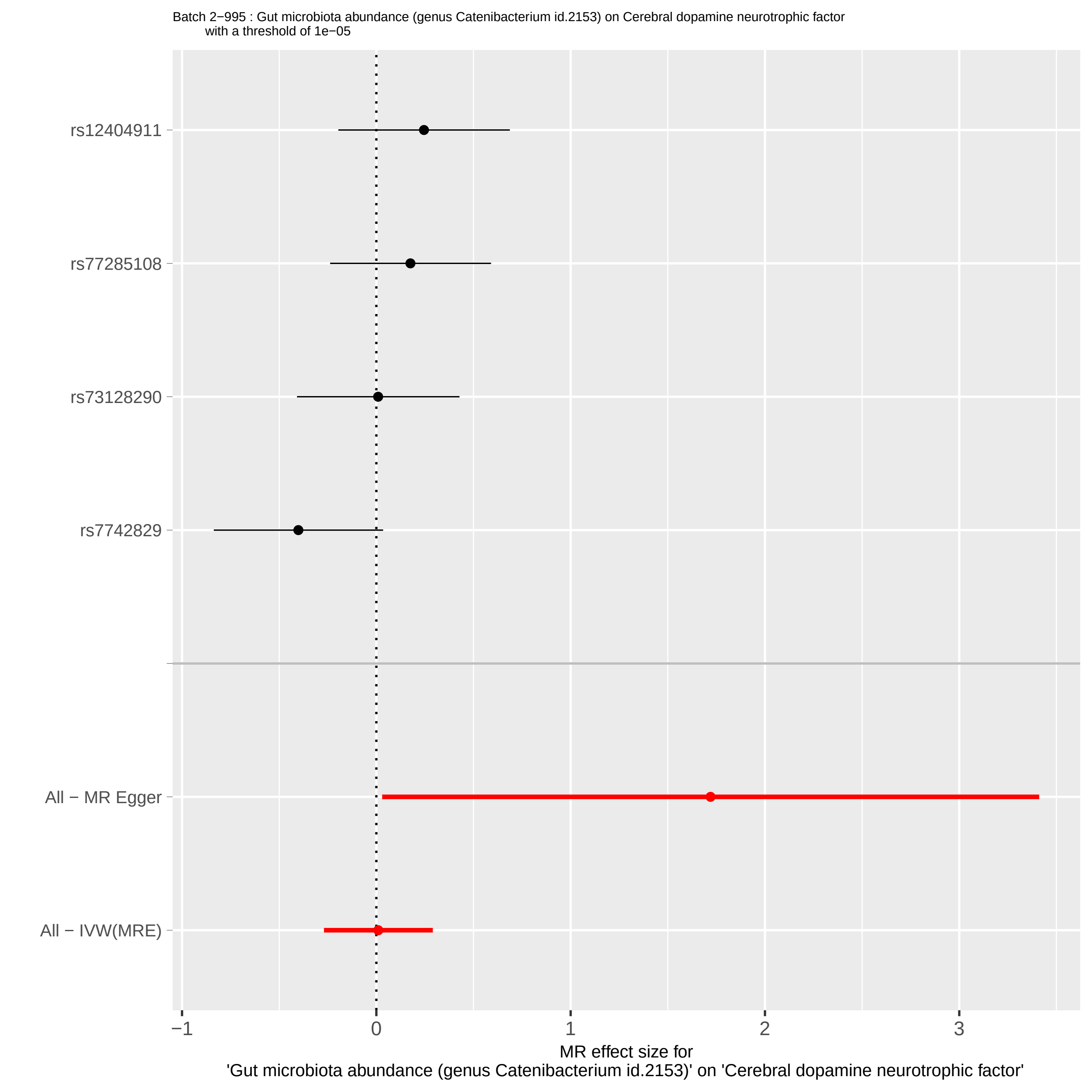

Batch 2−995 : Gut microbiota abundance (genus Catenibacterium id.2153) on Cerebral dopamine neurotrophic factor
with a threshold of 1e−05
rs12404911
rs77285108
rs73128290
rs7742829
All − MR Egger
All − IVW(MRE)
−1
0
1
2
3
MR effect size for
'Gut microbiota abundance (genus Catenibacterium id.2153)' on 'Cerebral dopamine neurotrophic factor'

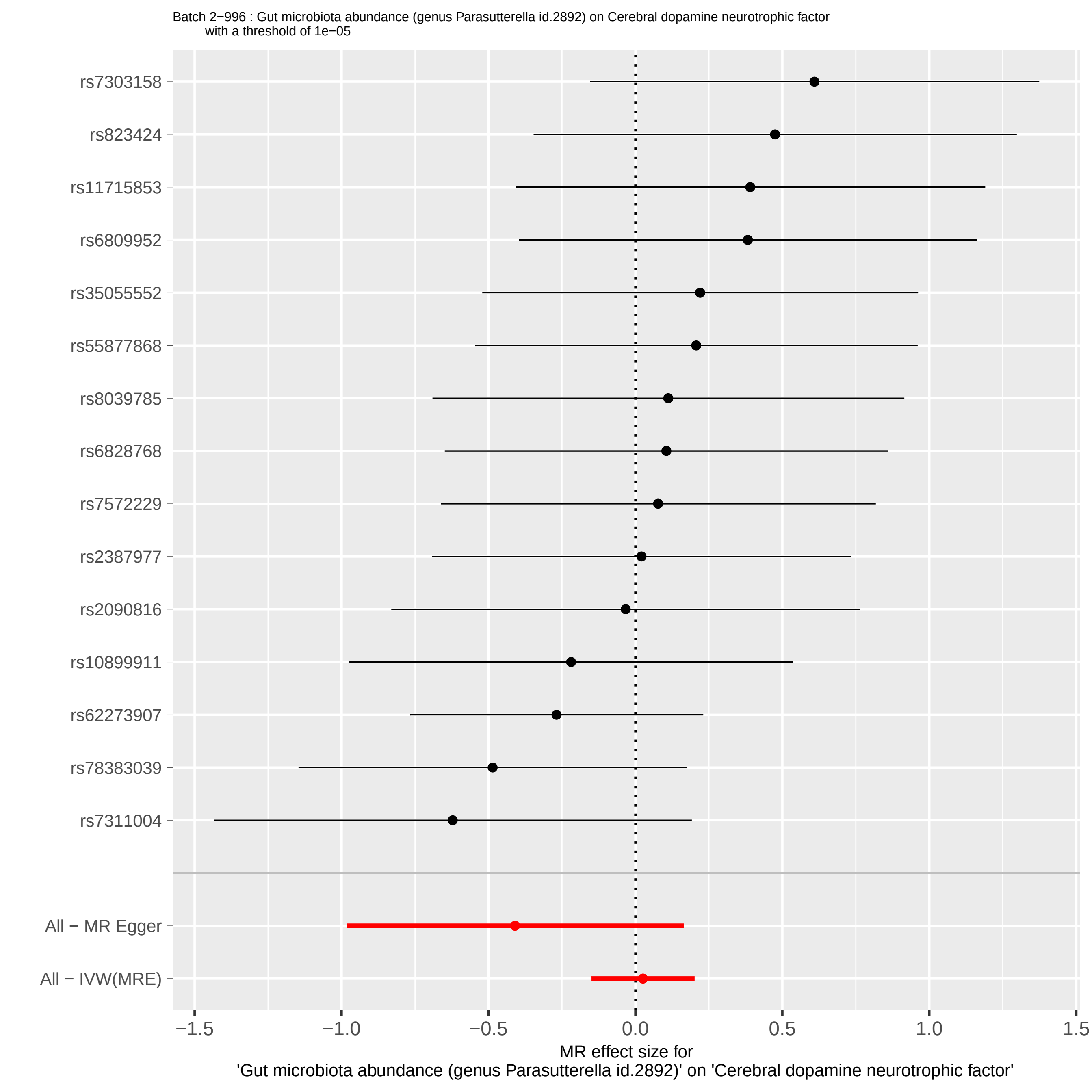
Batch 2−996 : Gut microbiota abundance (genus Parasutterella id.2892) on Cerebral dopamine neurotrophic factor
with a threshold of 1e−05
rs7303158
rs823424
rs11715853
rs6809952
rs35055552
rs55877868
rs8039785
rs6828768
rs7572229
rs2387977
rs2090816
rs10899911
rs62273907
rs78383039
rs7311004
All − MR Egger
All − IVW(MRE)
−1.5
−1.0
−0.5
0.0
0.5
1.0
1.5
MR effect size for
'Gut microbiota abundance (genus Parasutterella id.2892)' on 'Cerebral dopamine neurotrophic factor'

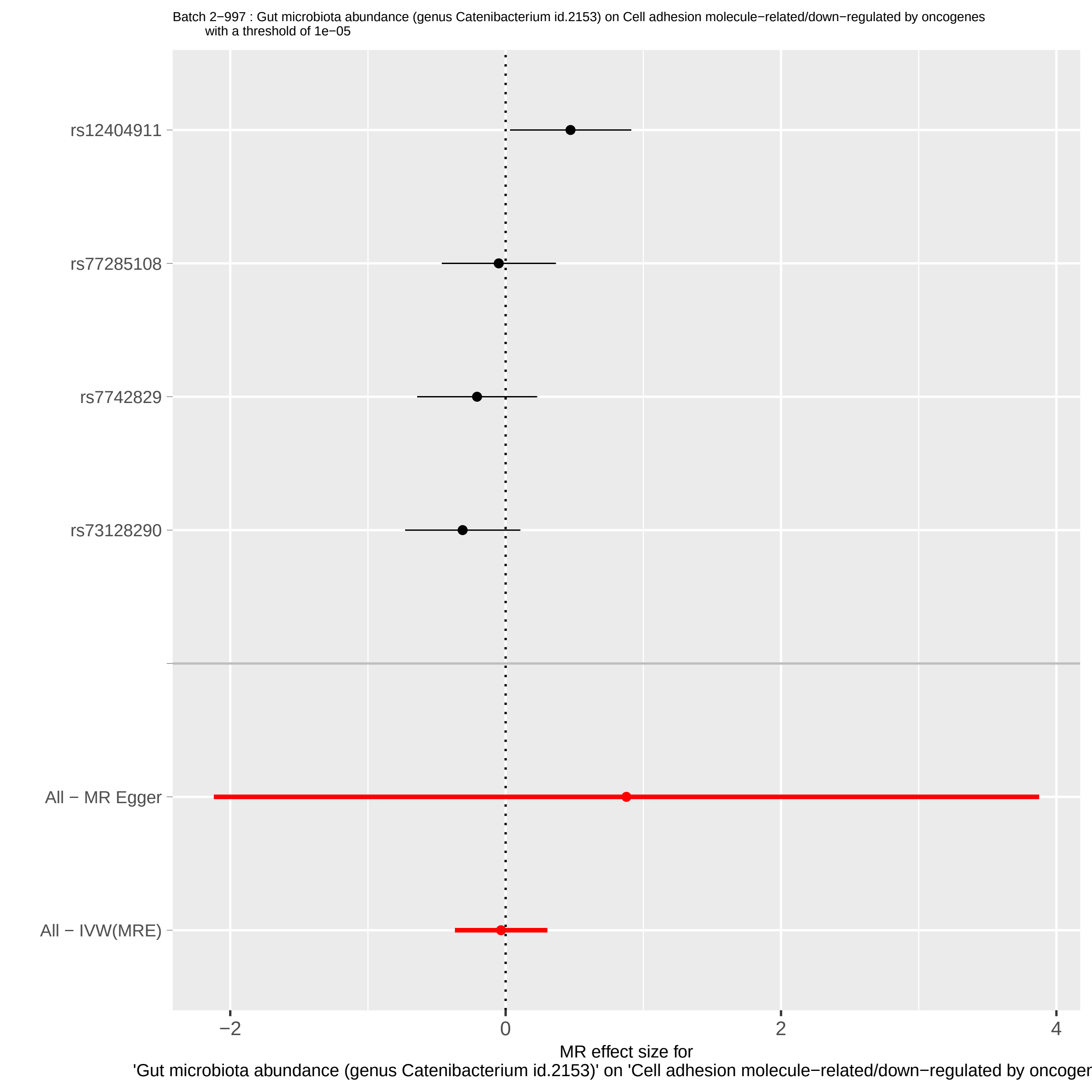
Batch 2−997 : Gut microbiota abundance (genus Catenibacterium id.2153) on Cell adhesion molecule−related/down−regulated by oncogenes
with a threshold of 1e−05
rs12404911
rs77285108
rs7742829
rs73128290
All − MR Egger
All − IVW(MRE)
−2
0
2
4
MR effect size for
'Gut microbiota abundance (genus Catenibacterium id.2153)' on 'Cell adhesion molecule−related/down−regulated by oncoger

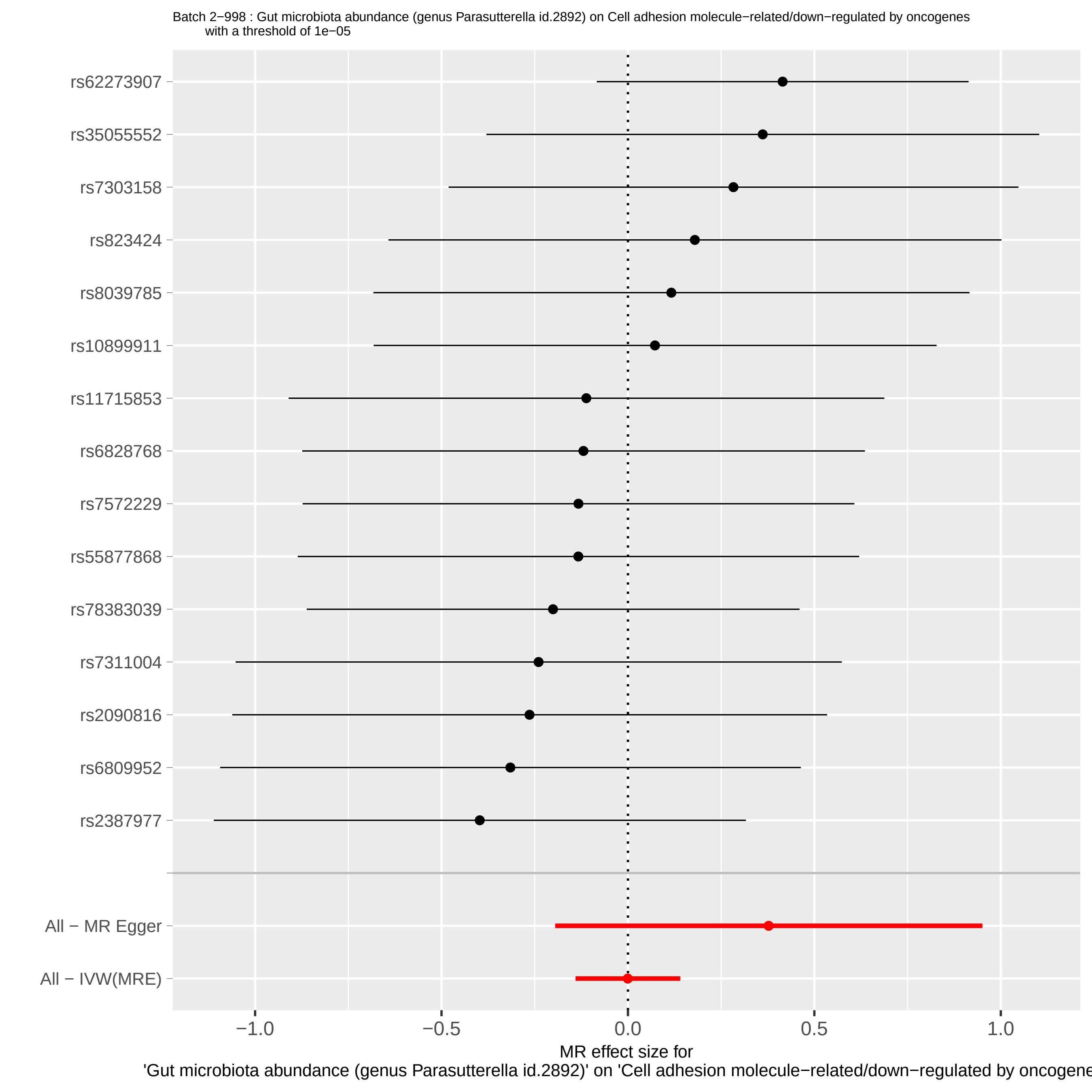

Batch 2−998 : Gut microbiota abundance (genus Parasutterella id.2892) on Cell adhesion molecule−related/down−regulated by oncogenes
with a threshold of 1e−05
rs62273907
rs35055552
rs7303158
rs823424
rs8039785
rs10899911
rs11715853
rs6828768
rs7572229
rs55877868
rs78383039
rs7311004
rs2090816
rs6809952
rs2387977
All − MR Egger
All − IVW(MRE)
−1.0
−0.5
0.0
0.5
1.0
MR effect size for
'Gut microbiota abundance (genus Parasutterella id.2892)' on 'Cell adhesion molecule−related/down−regulated by oncogene

Batch 2−999 : Gut microbiota abundance (genus Catenibacterium id.2153) on Corneodesmosin
with a threshold of 1e−05

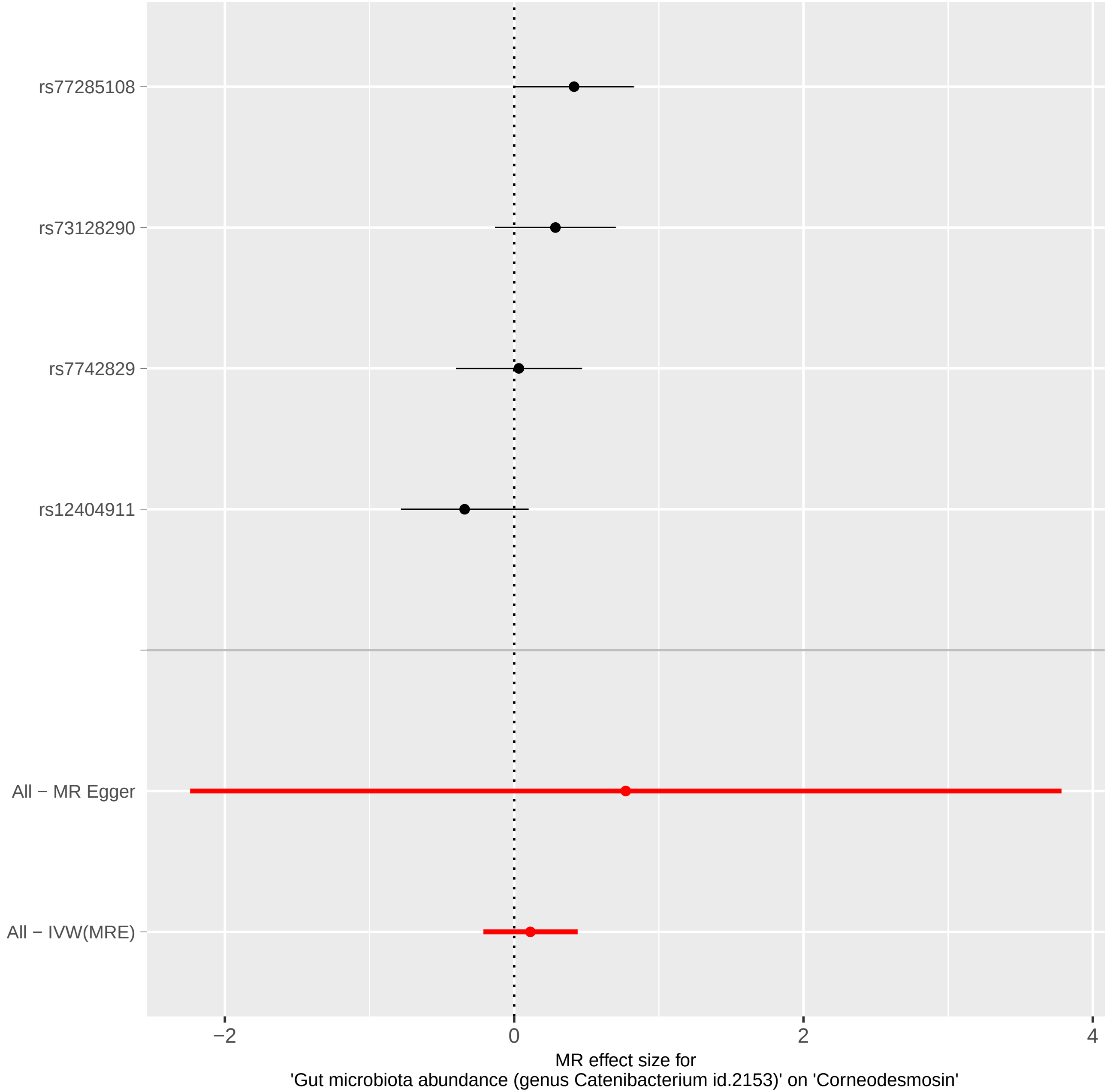

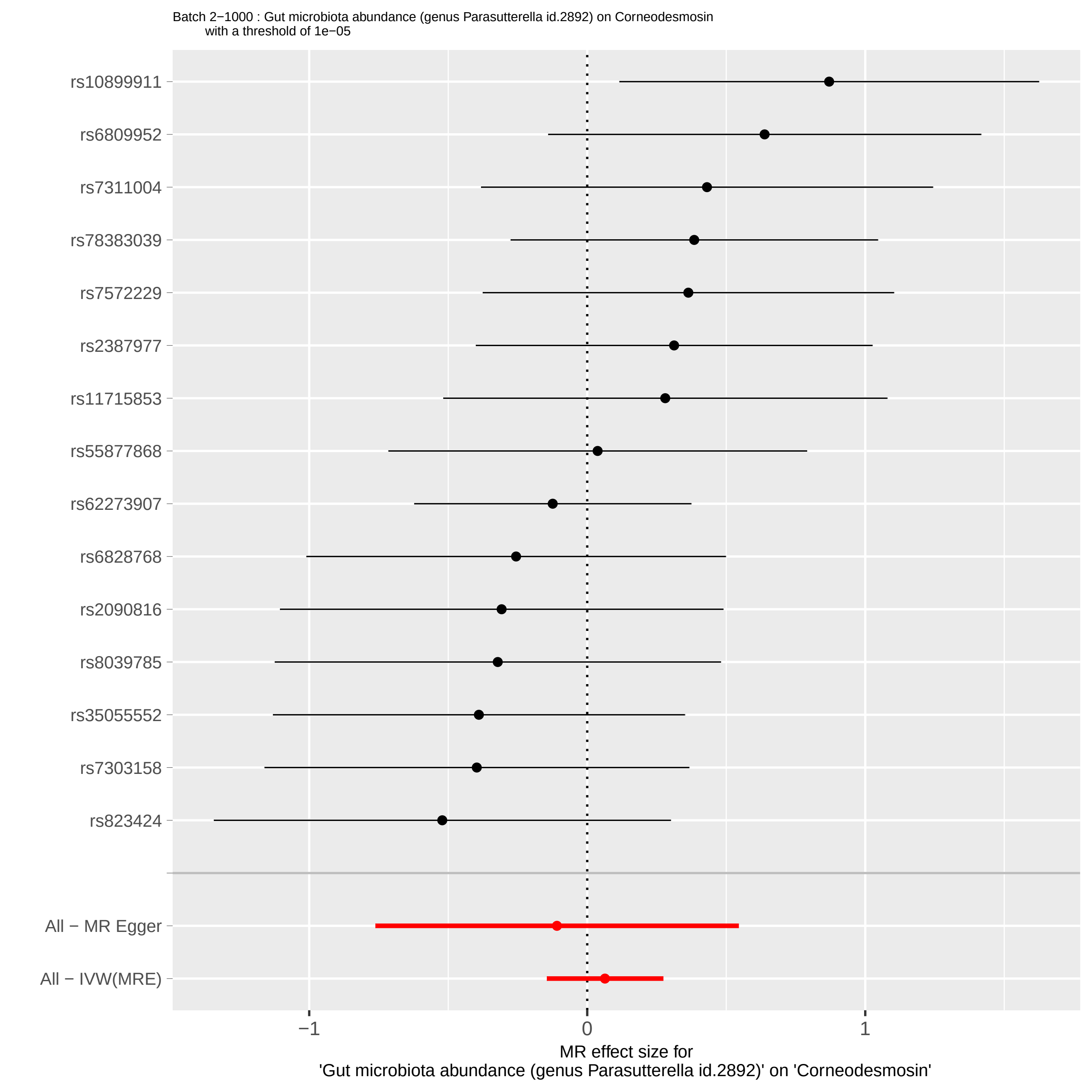

Batch 2−1000 : Gut microbiota abundance (genus Parasutterella id.2892) on Corneodesmosin
with a threshold of 1e−05
rs10899911
rs6809952
rs7311004
rs78383039
rs7572229
rs2387977
rs11715853
rs55877868
rs62273907
rs6828768
rs2090816
rs8039785
rs35055552
rs7303158
rs823424
All − MR Egger
All − IVW(MRE)
−1
0
1
MR effect size for
'Gut microbiota abundance (genus Parasutterella id.2892)' on 'Corneodesmosin'

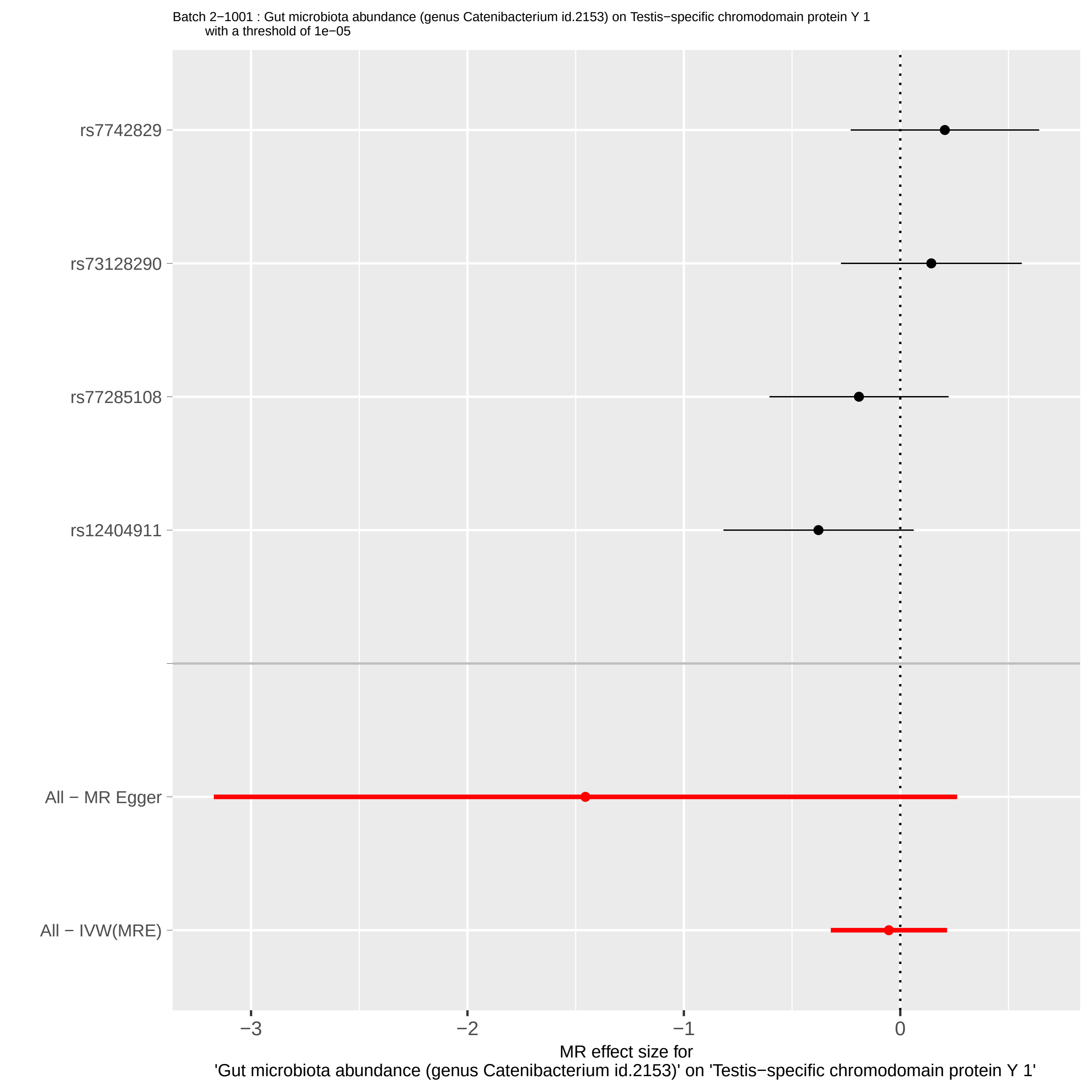

Batch 2−1001 : Gut microbiota abundance (genus Catenibacterium id.2153) on Testis−specific chromodomain protein Y 1
with a threshold of 1e−05
rs7742829
rs73128290
rs77285108
rs12404911
All − MR Egger
All − IVW(MRE)
−3
−2
−1
0
MR effect size for
'Gut microbiota abundance (genus Catenibacterium id.2153)' on 'Testis−specific chromodomain protein Y 1'

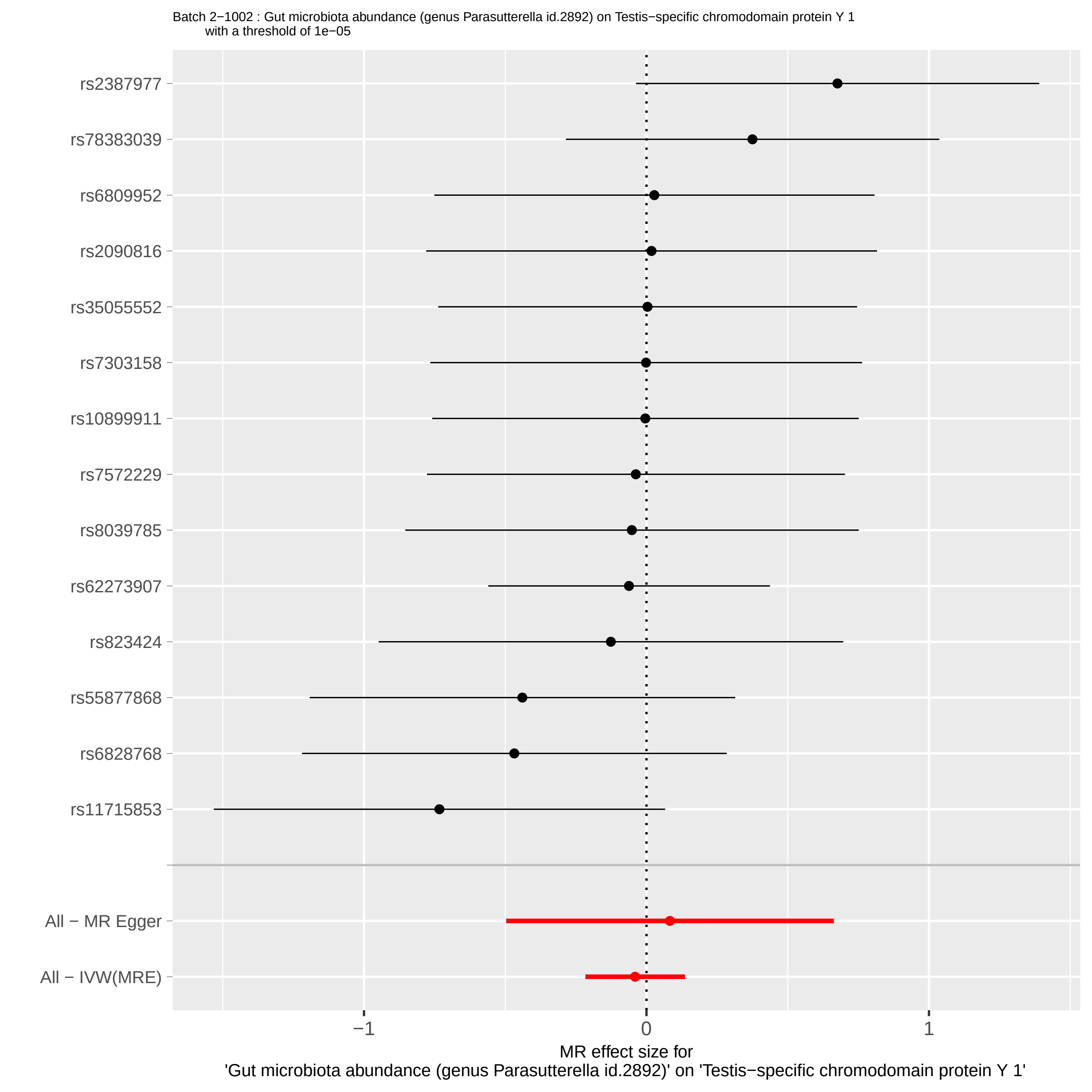
Batch 2−1002 : Gut microbiota abundance (genus Parasutterella id.2892) on Testis−specific chromodomain protein Y 1
with a threshold of 1e−05
rs2387977
rs78383039
rs6809952
rs2090816
rs35055552
rs7303158
rs10899911
rs7572229
rs8039785
rs62273907
rs823424
rs55877868
rs6828768
rs11715853
All − MR Egger
All − IVW(MRE)
−1
0
1
MR effect size for
'Gut microbiota abundance (genus Parasutterella id.2892)' on 'Testis−specific chromodomain protein Y 1'

Batch 2−1003 : Gut microbiota abundance (genus Catenibacterium id.2153) on Carcinoembryonic antigen−related cell adhesion molecule 1
with a threshold of 1e−05

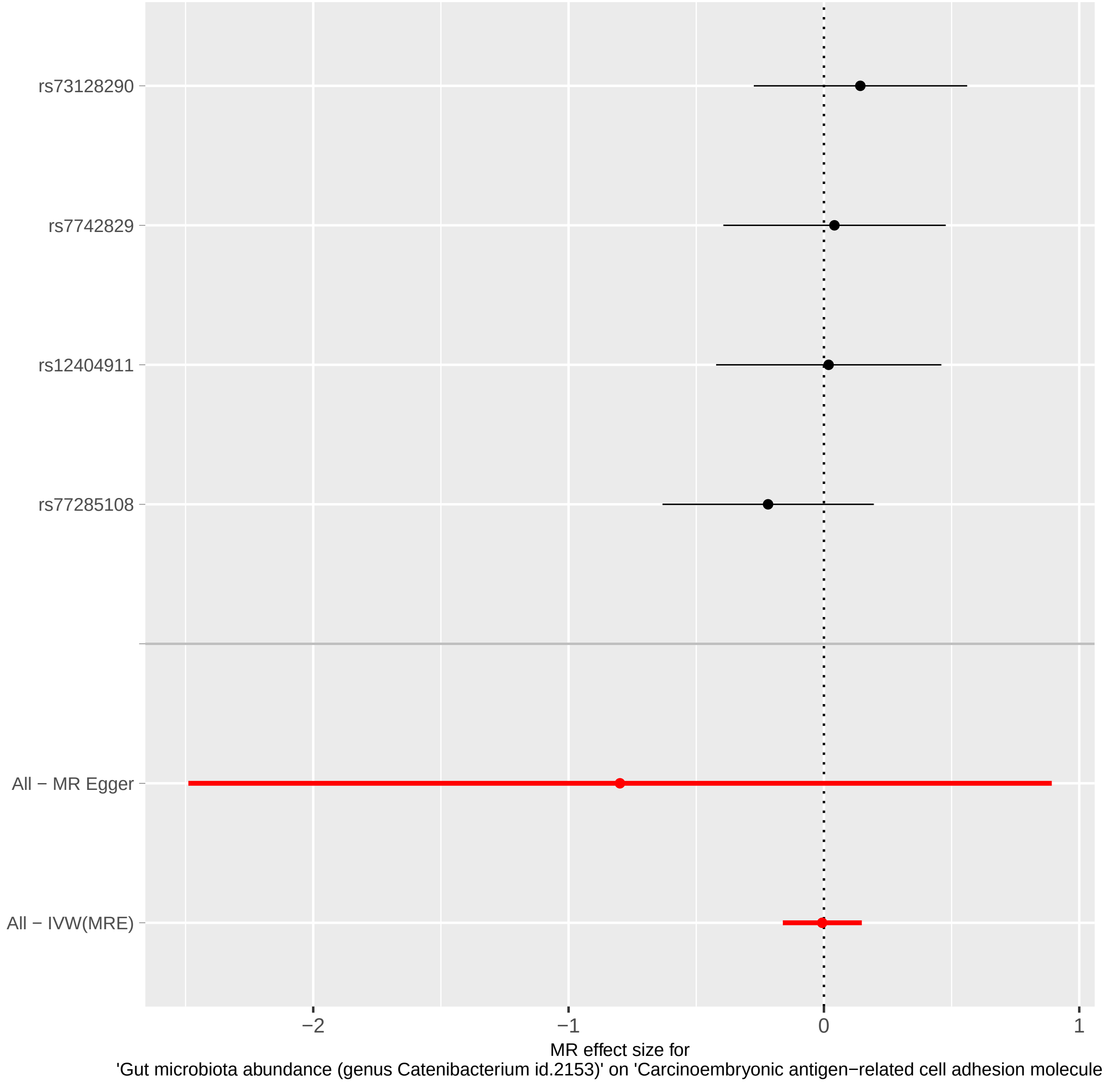

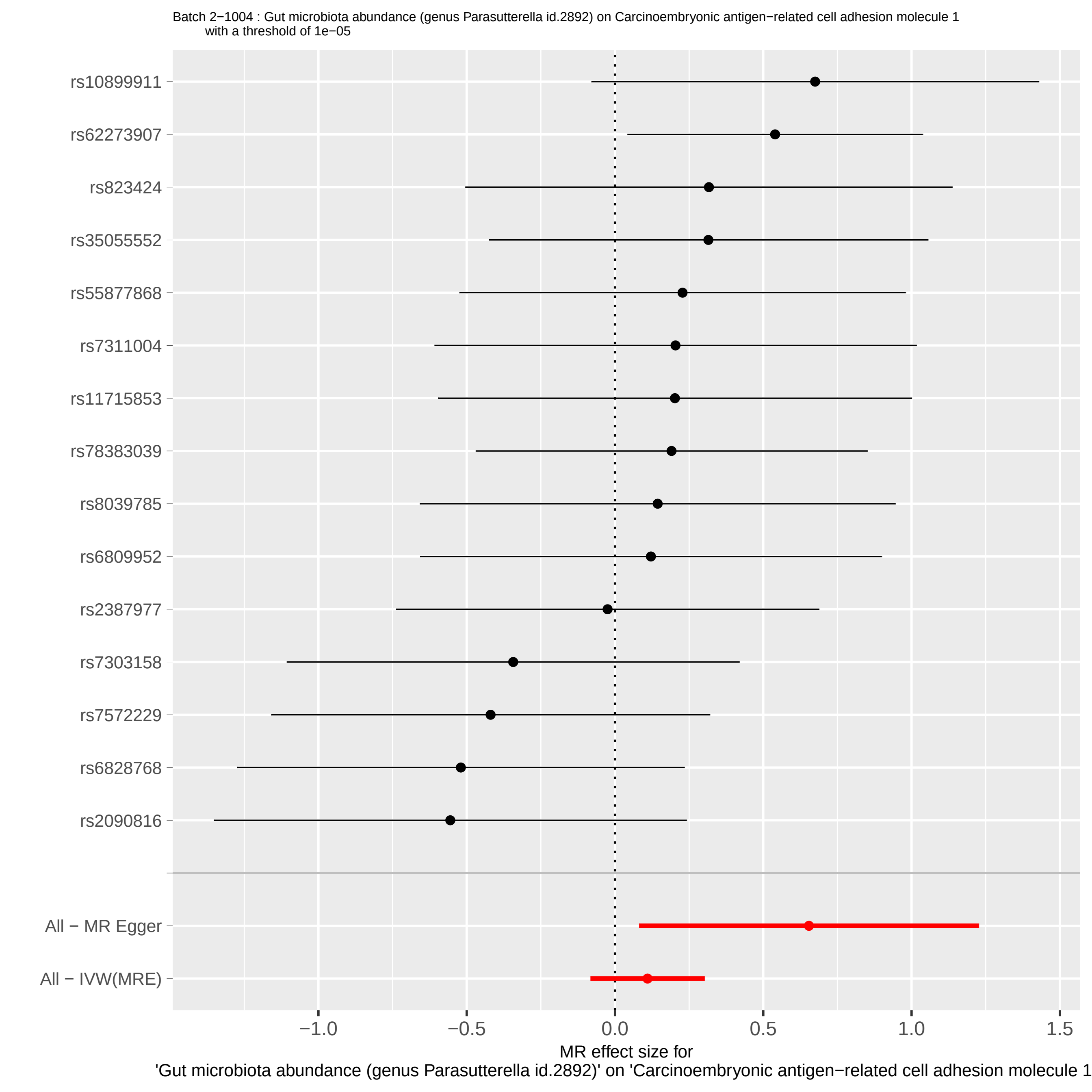

Batch 2−1004 : Gut microbiota abundance (genus Parasutterella id.2892) on Carcinoembryonic antigen−related cell adhesion molecule 1
with a threshold of 1e−05
rs10899911
rs62273907
rs823424
rs35055552
rs55877868
rs7311004
rs11715853
rs78383039
rs8039785
rs6809952
rs2387977
rs7303158
rs7572229
rs6828768
rs2090816
All − MR Egger
All − IVW(MRE)
−1.0
−0.5
0.0
0.5
1.0
1.5
MR effect size for
'Gut microbiota abundance (genus Parasutterella id.2892)' on 'Carcinoembryonic antigen−related cell adhesion molecule 1

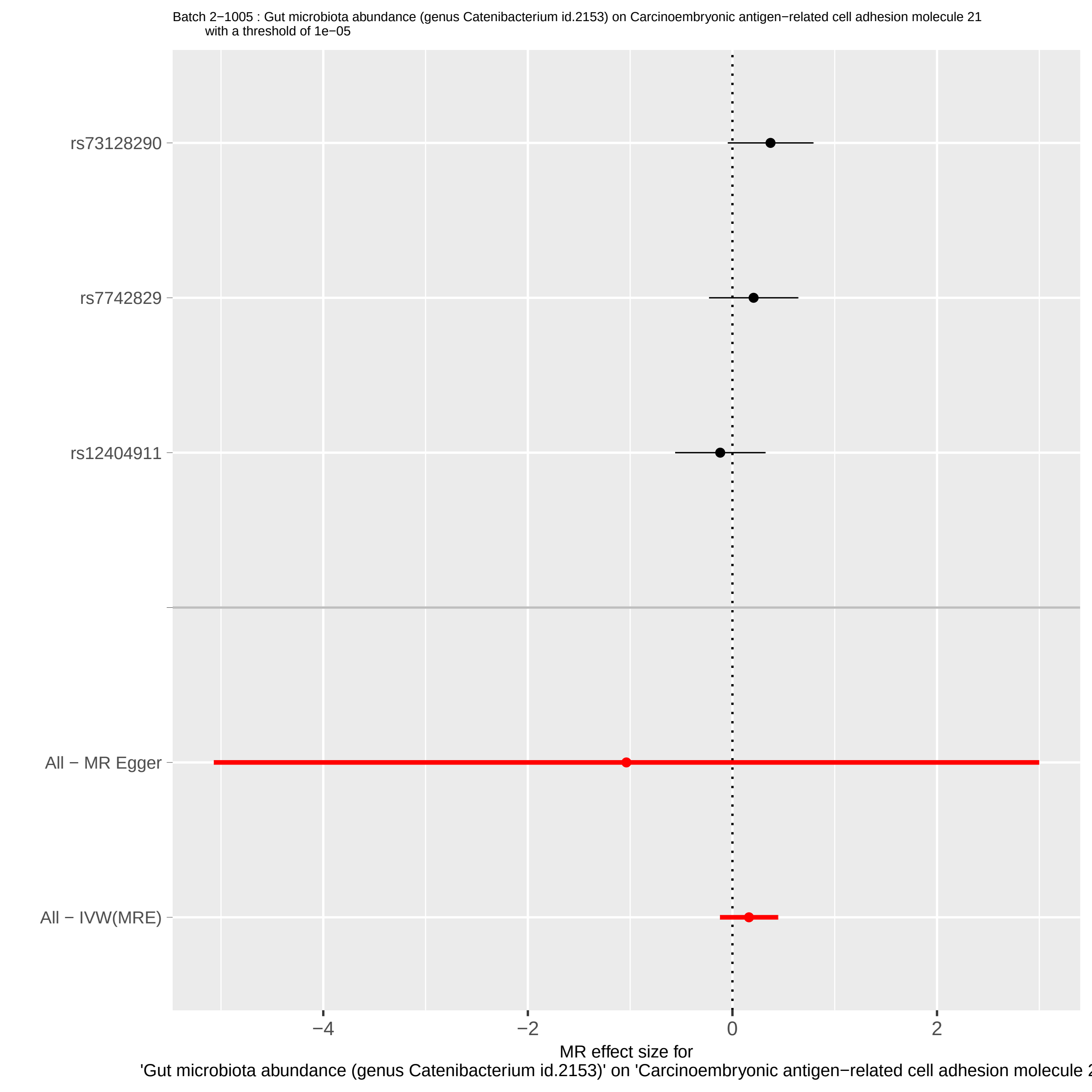
Batch 2−1005 : Gut microbiota abundance (genus Catenibacterium id.2153) on Carcinoembryonic antigen−related cell adhesion molecule 21
with a threshold of 1e−05
rs73128290
rs7742829
rs12404911
All − MR Egger
All − IVW(MRE)
−4
−2
0
2
MR effect size for
'Gut microbiota abundance (genus Catenibacterium id.2153)' on 'Carcinoembryonic antigen−related cell adhesion molecule 2

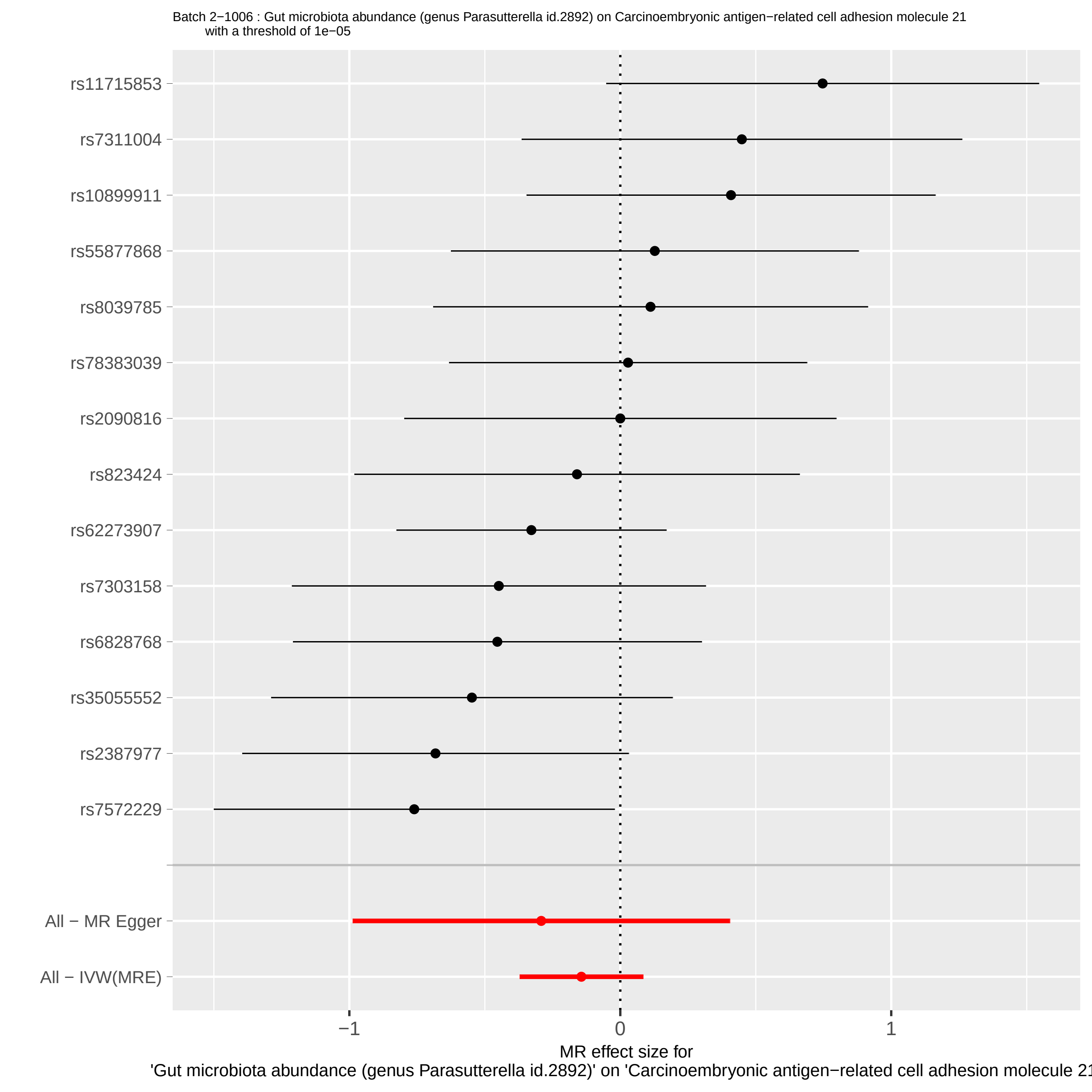
Batch 2−1006 : Gut microbiota abundance (genus Parasutterella id.2892) on Carcinoembryonic antigen−related cell adhesion molecule 21
with a threshold of 1e−05
rs11715853
rs7311004
rs10899911
rs55877868
rs8039785
rs78383039
rs2090816
rs823424
rs62273907
rs7303158
rs6828768
rs35055552
rs2387977
rs7572229
All − MR Egger
All − IVW(MRE)
−1
0
1
MR effect size for
'Gut microbiota abundance (genus Parasutterella id.2892)' on 'Carcinoembryonic antigen−related cell adhesion molecule 21

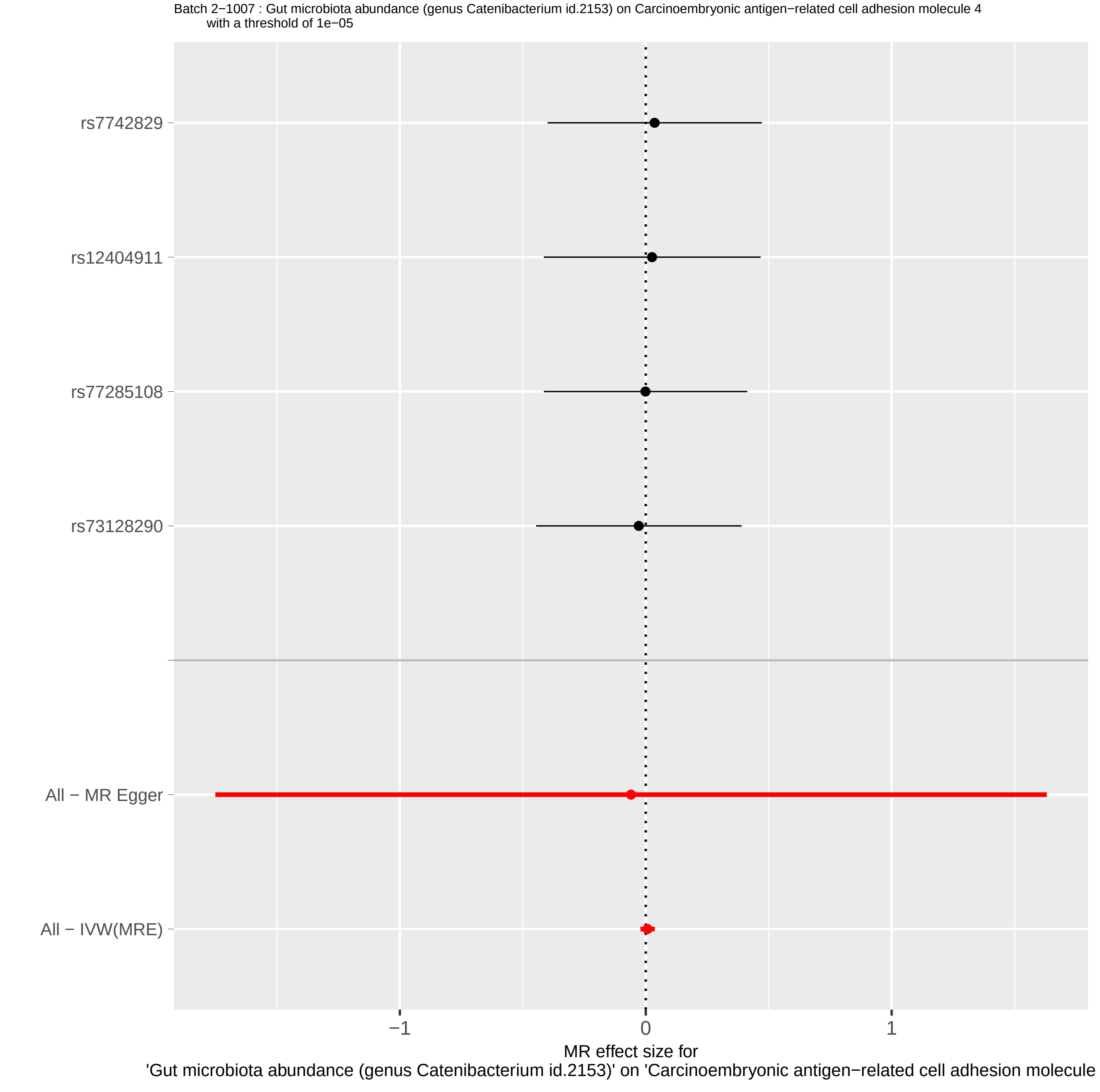

Batch 2−1007 : Gut microbiota abundance (genus Catenibacterium id.2153) on Carcinoembryonic antigen−related cell adhesion molecule 4
with a threshold of 1e−05
rs7742829
rs12404911
rs77285108
rs73128290
All − MR Egger
All − IVW(MRE)
−1
0
1
MR effect size for
'Gut microbiota abundance (genus Catenibacterium id.2153)' on 'Carcinoembryonic antigen−related cell adhesion molecule

Batch 2−1008 : Gut microbiota abundance (genus Parasutterella id.2892) on Carcinoembryonic antigen−related cell adhesion molecule 4 with a threshold of 1e−05

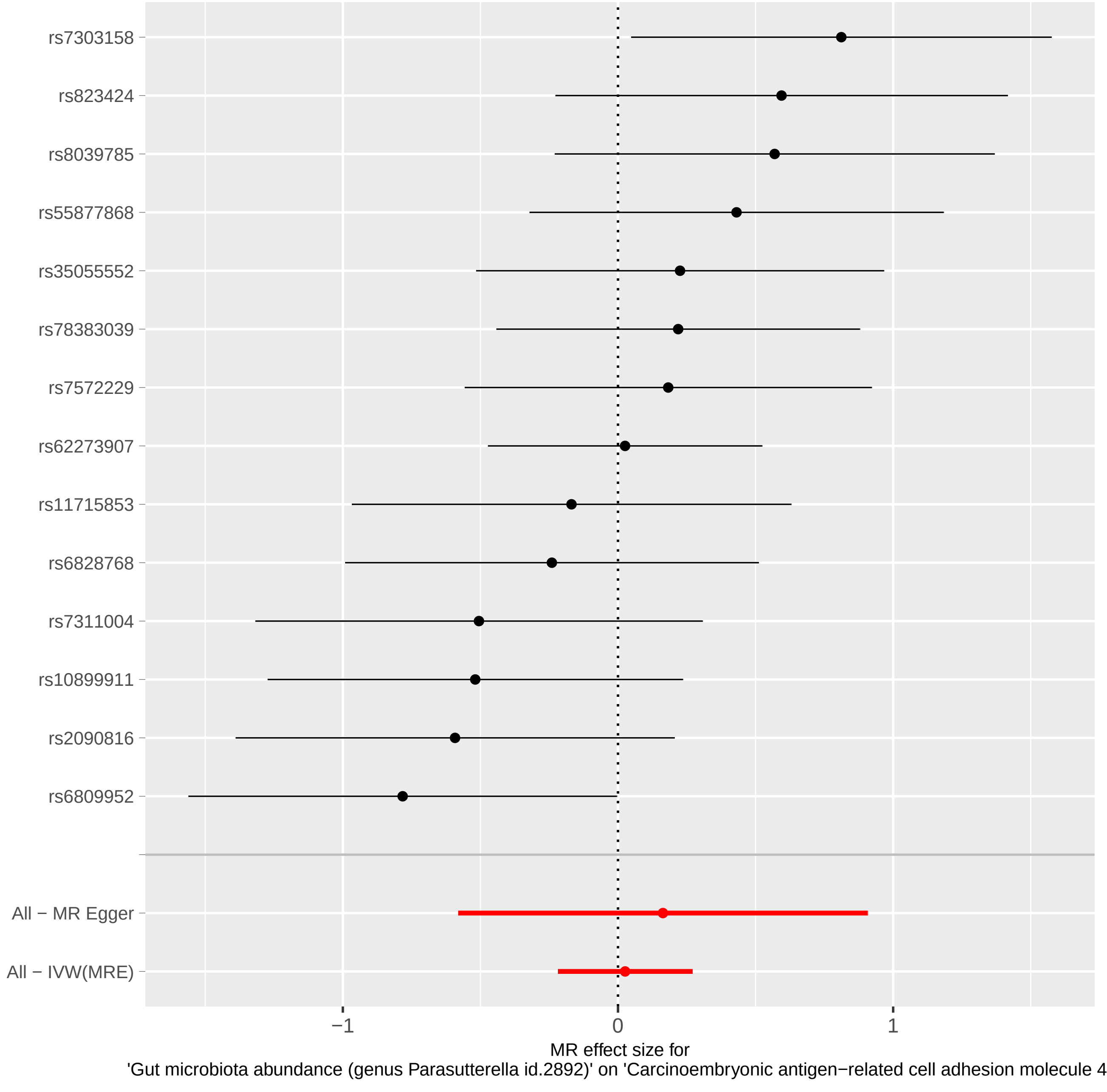

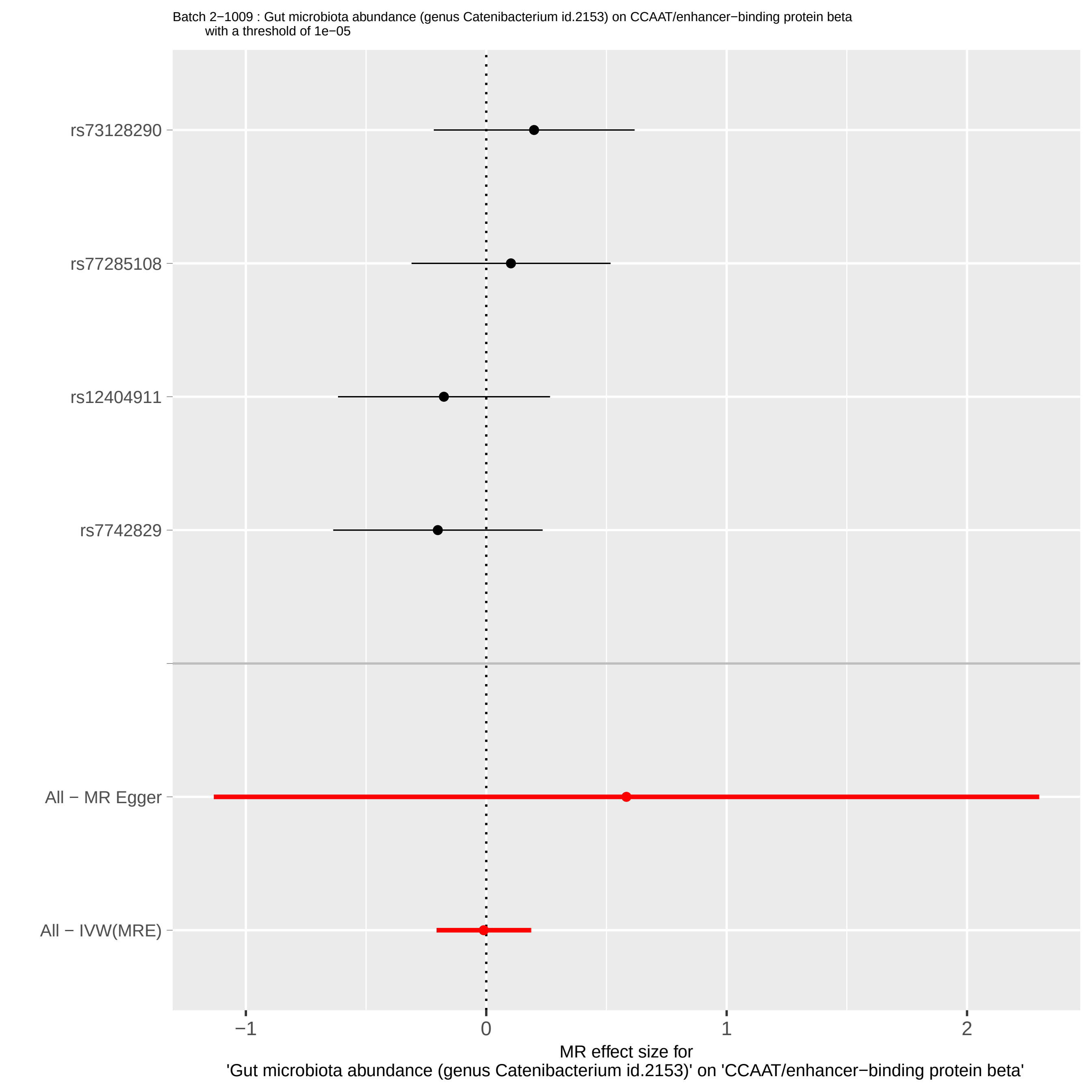

Batch 2−1009 : Gut microbiota abundance (genus Catenibacterium id.2153) on CCAAT/enhancer−binding protein beta
with a threshold of 1e−05
rs73128290
rs77285108
rs12404911
rs7742829
All − MR Egger
All − IVW(MRE)
−1
0
1
2
MR effect size for
'Gut microbiota abundance (genus Catenibacterium id.2153)' on 'CCAAT/enhancer−binding protein beta'

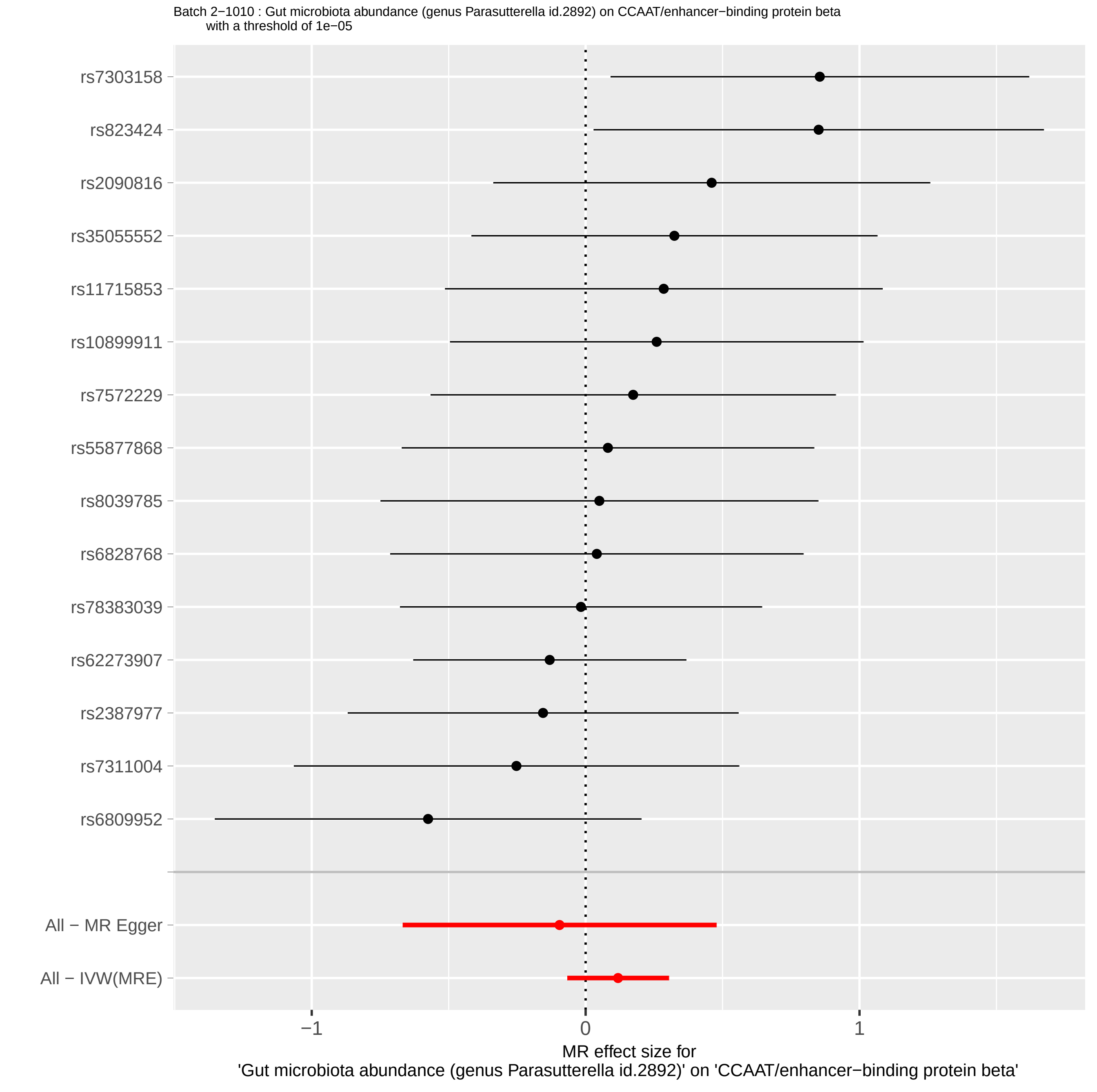

Batch 2−1010 : Gut microbiota abundance (genus Parasutterella id.2892) on CCAAT/enhancer−binding protein beta
with a threshold of 1e−05
rs7303158
rs823424
rs2090816
rs35055552
rs11715853
rs10899911
rs7572229
rs55877868
rs8039785
rs6828768
rs78383039
rs62273907
rs2387977
rs7311004
rs6809952
All − MR Egger
All − IVW(MRE)
−1
0
1
MR effect size for
'Gut microbiota abundance (genus Parasutterella id.2892)' on 'CCAAT/enhancer−binding protein beta'

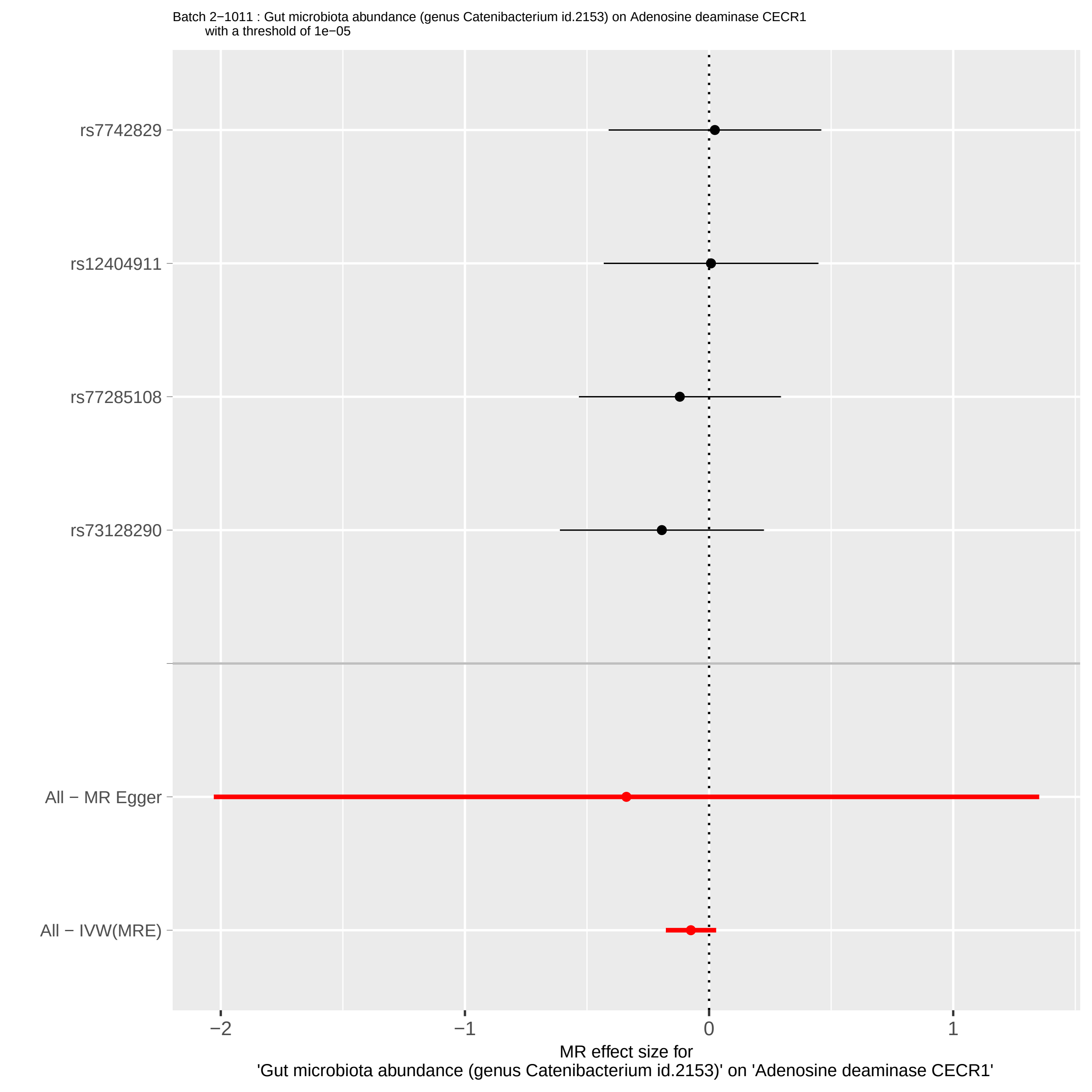

Batch 2−1011 : Gut microbiota abundance (genus Catenibacterium id.2153) on Adenosine deaminase CECR1
with a threshold of 1e−05
rs7742829
rs12404911
rs77285108
rs73128290
All − MR Egger
All − IVW(MRE)
−2
−1
0
1
MR effect size for
'Gut microbiota abundance (genus Catenibacterium id.2153)' on 'Adenosine deaminase CECR1'

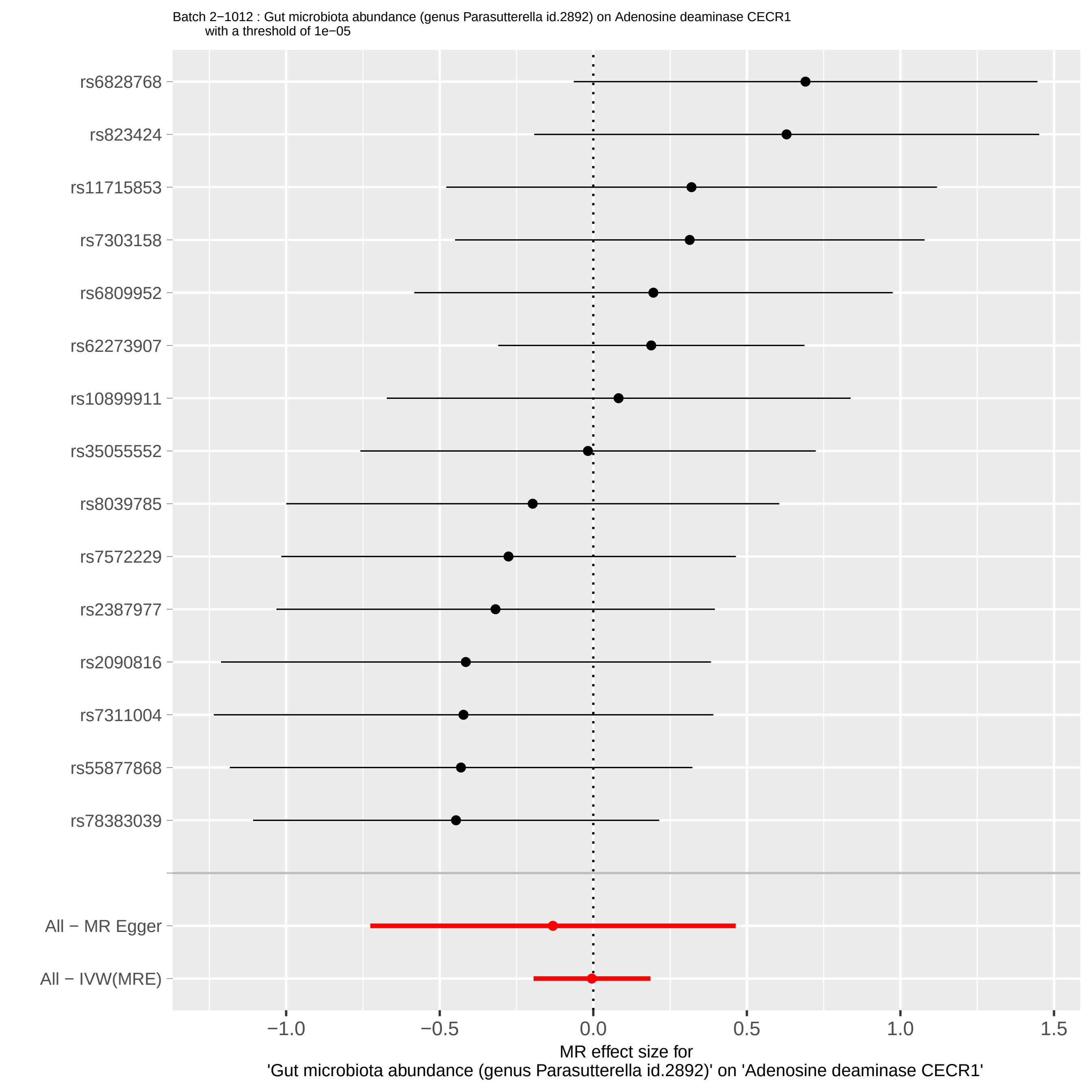

Batch 2−1012 : Gut microbiota abundance (genus Parasutterella id.2892) on Adenosine deaminase CECR1
with a threshold of 1e−05
rs6828768
rs823424
rs11715853
rs7303158
rs6809952
rs62273907
rs10899911
rs35055552
rs8039785
rs7572229
rs2387977
rs2090816
rs7311004
rs55877868
rs78383039
All − MR Egger
All − IVW(MRE)
−1.0
−0.5
0.0
0.5
1.0
1.5
MR effect size for
'Gut microbiota abundance (genus Parasutterella id.2892)' on 'Adenosine deaminase CECR1'

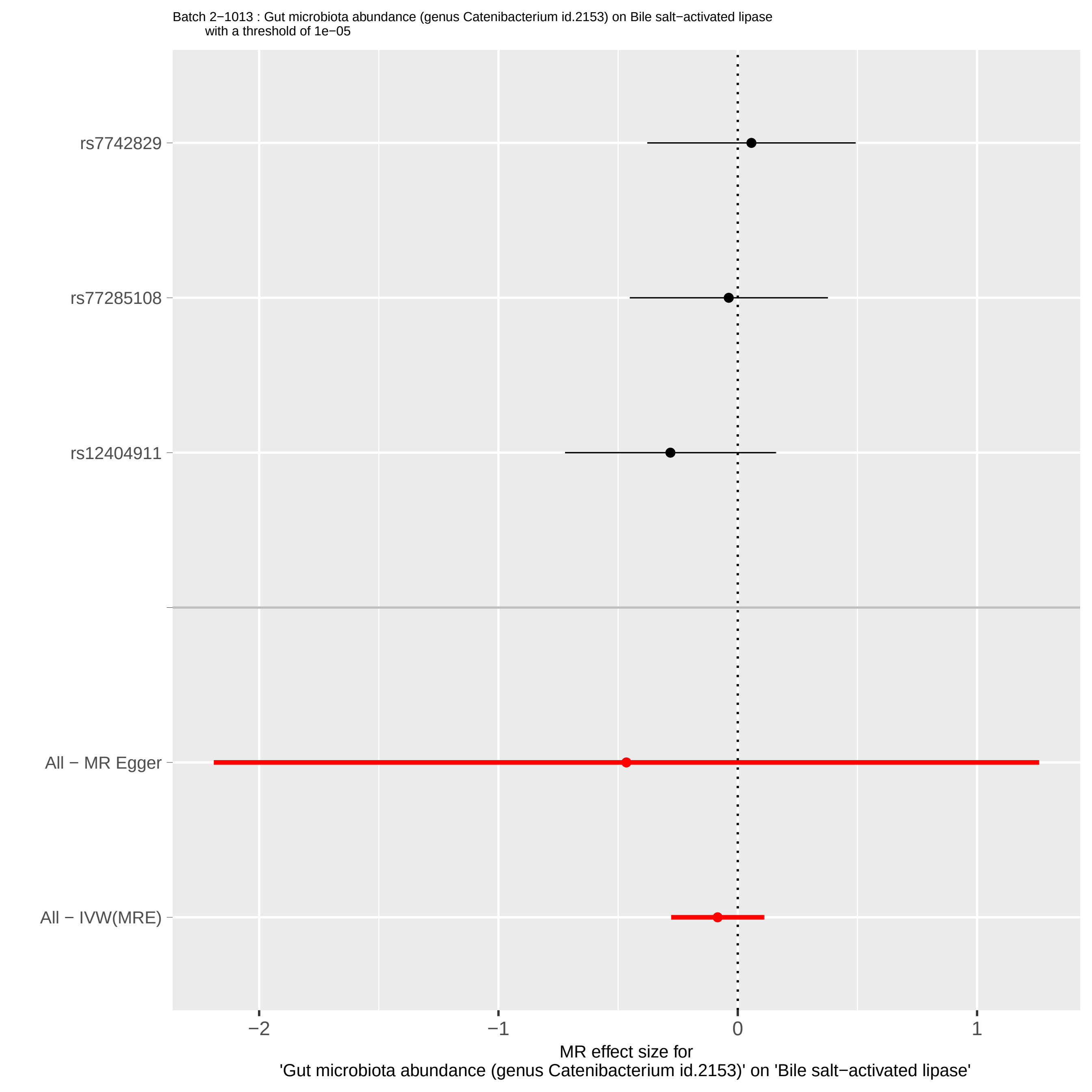
Batch 2−1013 : Gut microbiota abundance (genus Catenibacterium id.2153) on Bile salt−activated lipase
with a threshold of 1e−05
rs7742829
rs77285108
rs12404911
All − MR Egger
All − IVW(MRE)
−2
−1
0
1
MR effect size for
'Gut microbiota abundance (genus Catenibacterium id.2153)' on 'Bile salt−activated lipase'

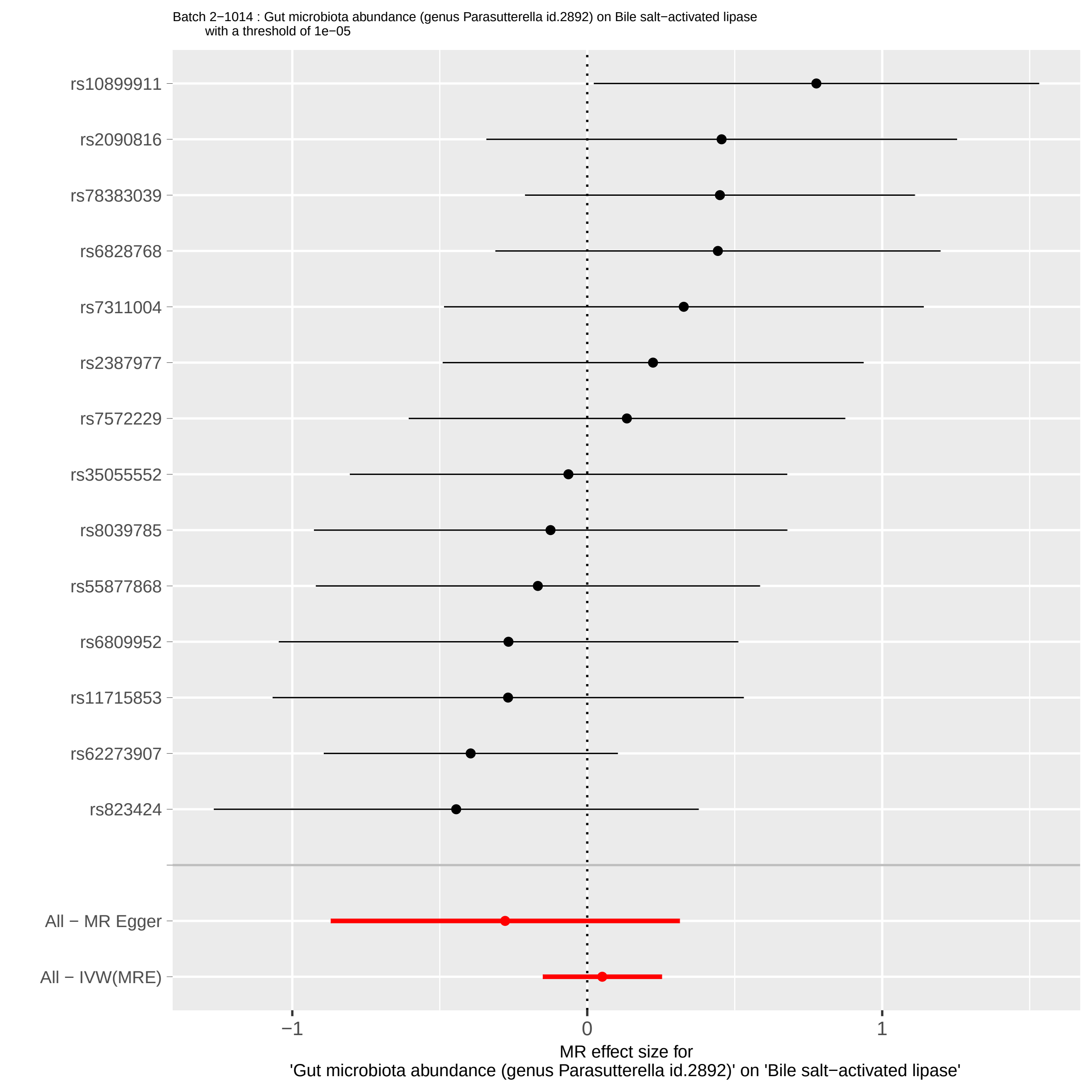

Batch 2−1014 : Gut microbiota abundance (genus Parasutterella id.2892) on Bile salt−activated lipase
with a threshold of 1e−05
rs10899911
rs2090816
rs78383039
rs6828768
rs7311004
rs2387977
rs7572229
rs35055552
rs8039785
rs55877868
rs6809952
rs11715853
rs62273907
rs823424
All − MR Egger
All − IVW(MRE)
−1
0
1
MR effect size for
'Gut microbiota abundance (genus Parasutterella id.2892)' on 'Bile salt−activated lipase'

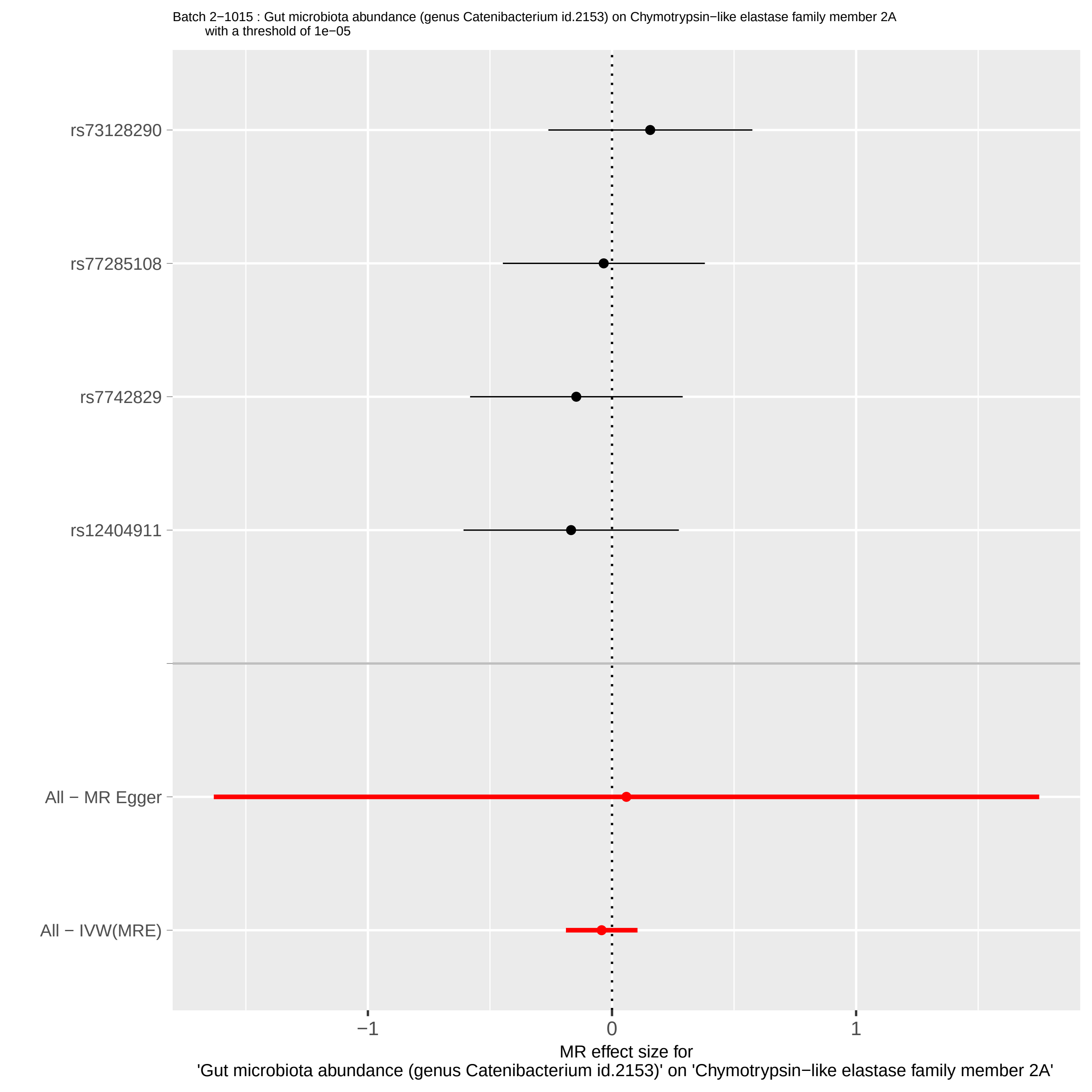
Batch 2−1015 : Gut microbiota abundance (genus Catenibacterium id.2153) on Chymotrypsin−like elastase family member 2A
with a threshold of 1e−05
rs73128290
rs77285108
rs7742829
rs12404911
All − MR Egger
All − IVW(MRE)
−1
0
1
MR effect size for
'Gut microbiota abundance (genus Catenibacterium id.2153)' on 'Chymotrypsin−like elastase family member 2A'

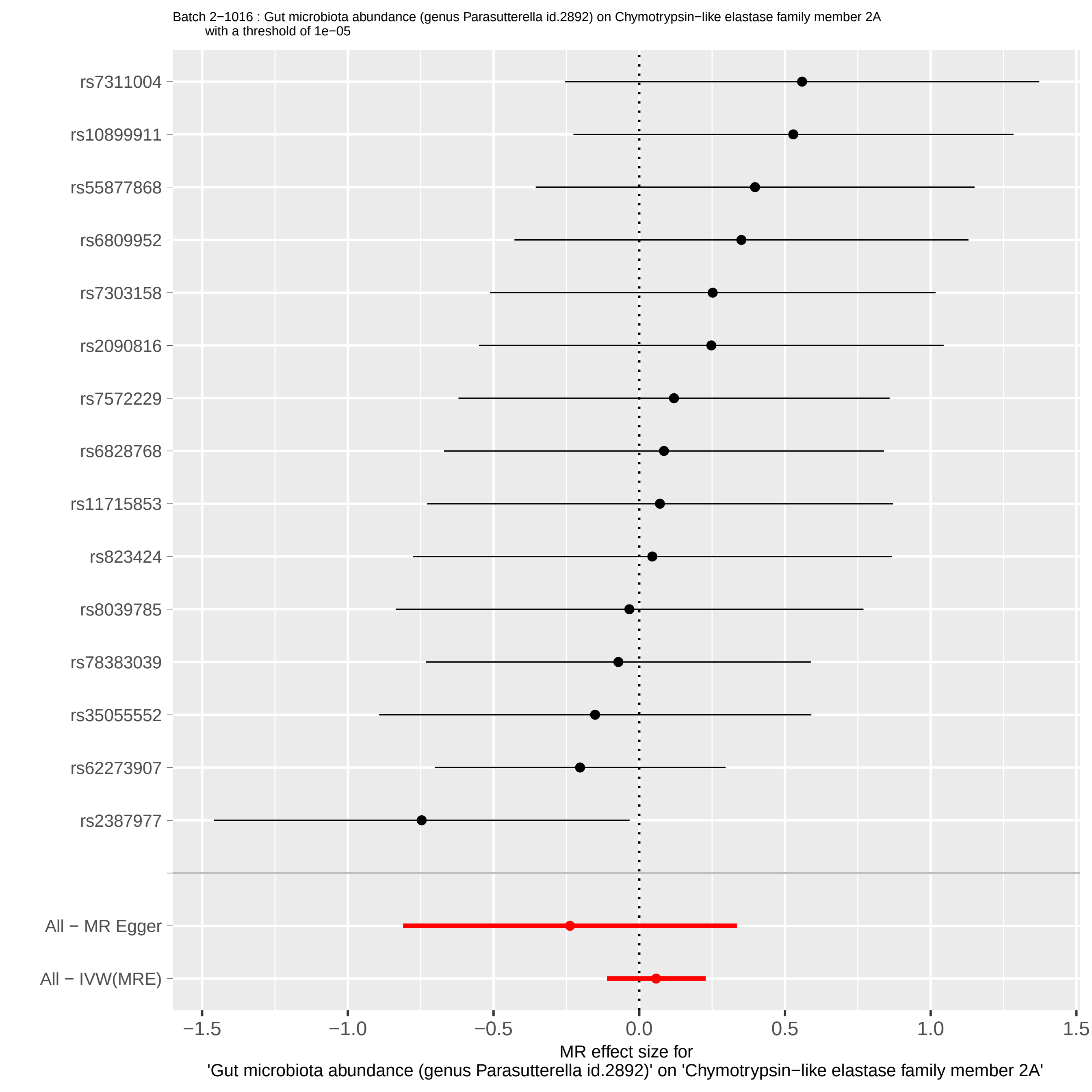
Batch 2−1016 : Gut microbiota abundance (genus Parasutterella id.2892) on Chymotrypsin−like elastase family member 2A
with a threshold of 1e−05
rs7311004
rs10899911
rs55877868
rs6809952
rs7303158
rs2090816
rs7572229
rs6828768
rs11715853
rs823424
rs8039785
rs78383039
rs35055552
rs62273907
rs2387977
All − MR Egger
All − IVW(MRE)
−1.5
−1.0
−0.5
0.0
0.5
1.0
1.5
MR effect size for
'Gut microbiota abundance (genus Parasutterella id.2892)' on 'Chymotrypsin−like elastase family member 2A'

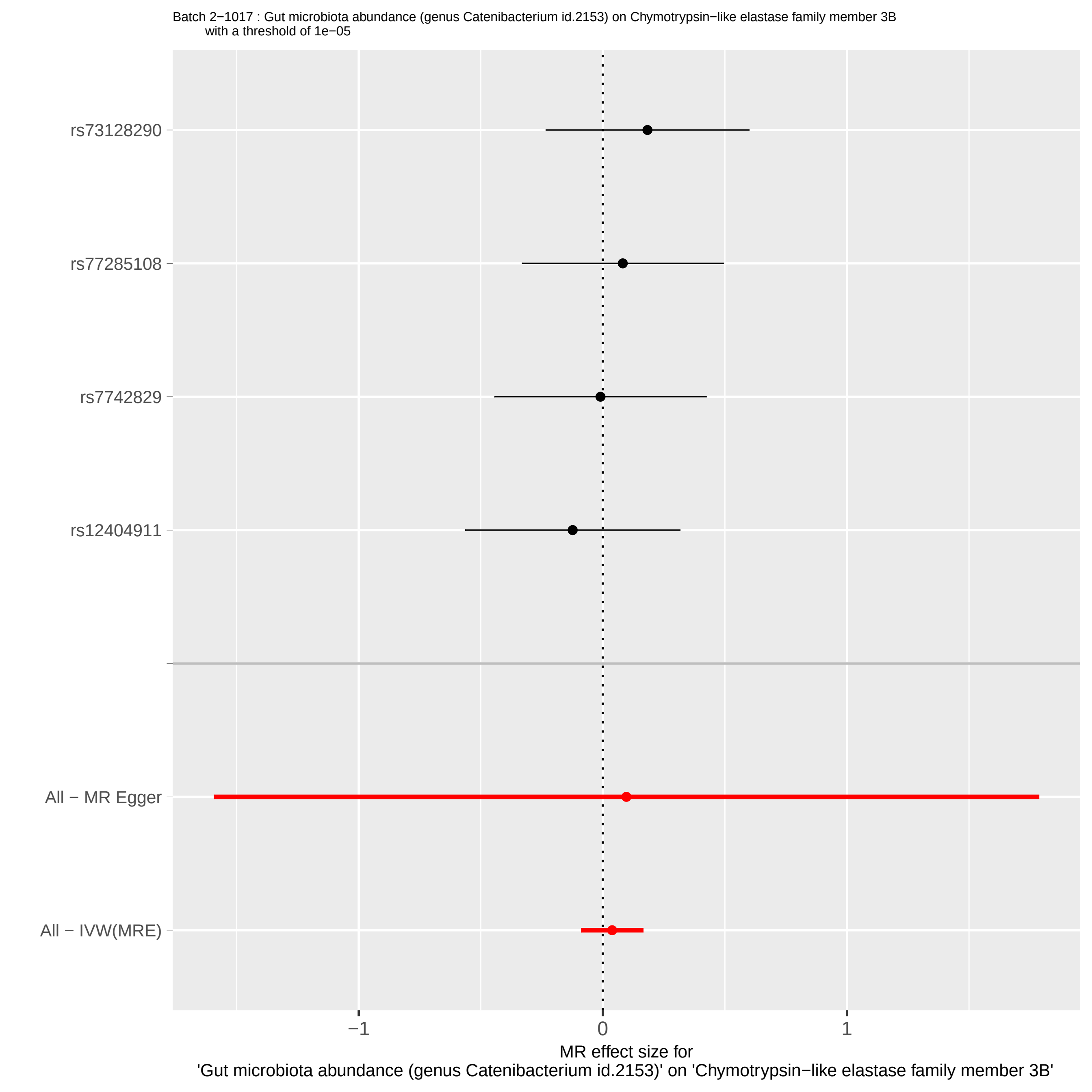

Batch 2−1017 : Gut microbiota abundance (genus Catenibacterium id.2153) on Chymotrypsin−like elastase family member 3B
with a threshold of 1e−05
rs73128290
rs77285108
rs7742829
rs12404911
All − MR Egger
All − IVW(MRE)
−1
0
1
MR effect size for
'Gut microbiota abundance (genus Catenibacterium id.2153)' on 'Chymotrypsin−like elastase family member 3B'

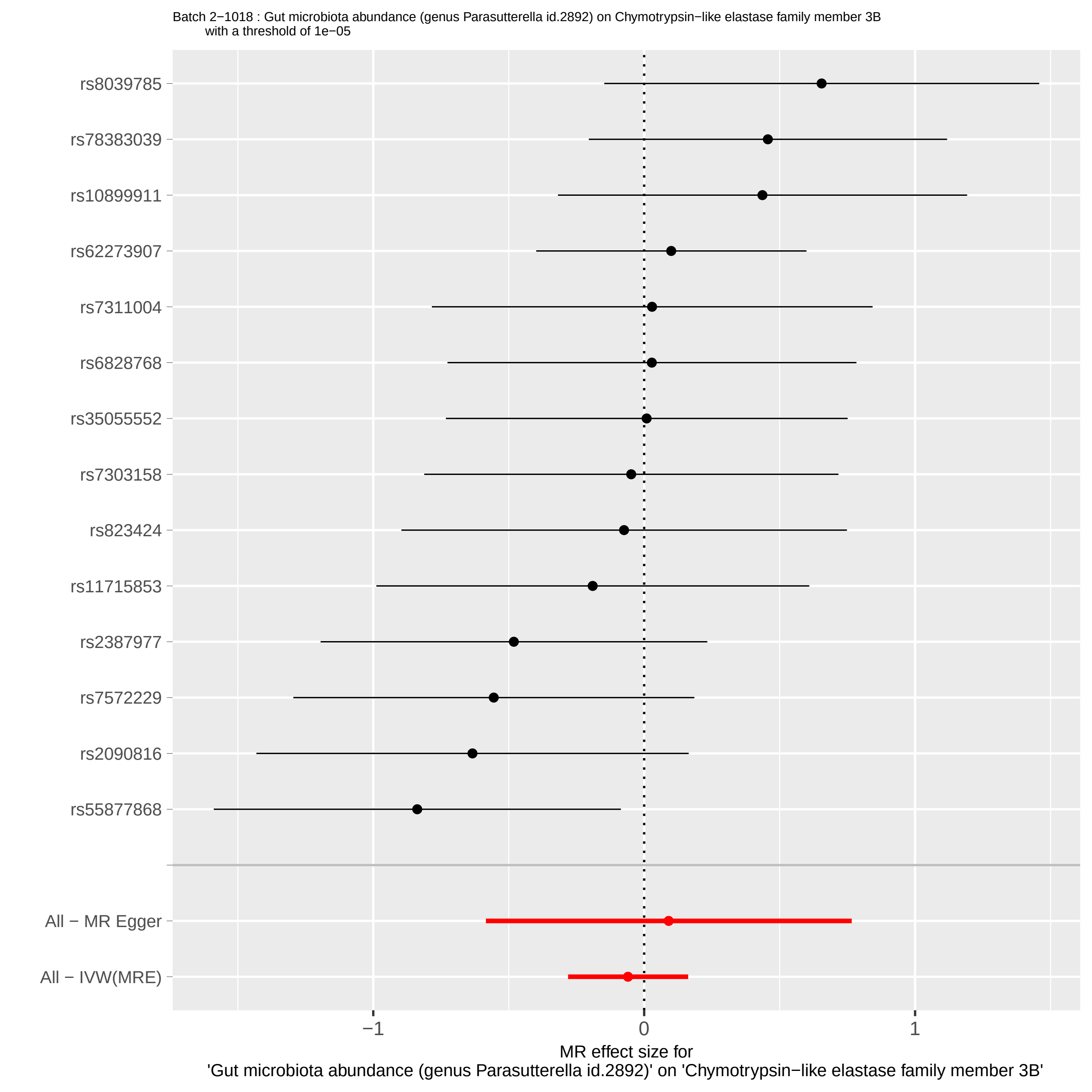
Batch 2−1018 : Gut microbiota abundance (genus Parasutterella id.2892) on Chymotrypsin−like elastase family member 3B
with a threshold of 1e−05
rs8039785
rs78383039
rs10899911
rs62273907
rs7311004
rs6828768
rs35055552
rs7303158
rs823424
rs11715853
rs2387977
rs7572229
rs2090816
rs55877868
All − MR Egger
All − IVW(MRE)
−1
0
1
MR effect size for
'Gut microbiota abundance (genus Parasutterella id.2892)' on 'Chymotrypsin−like elastase family member 3B'

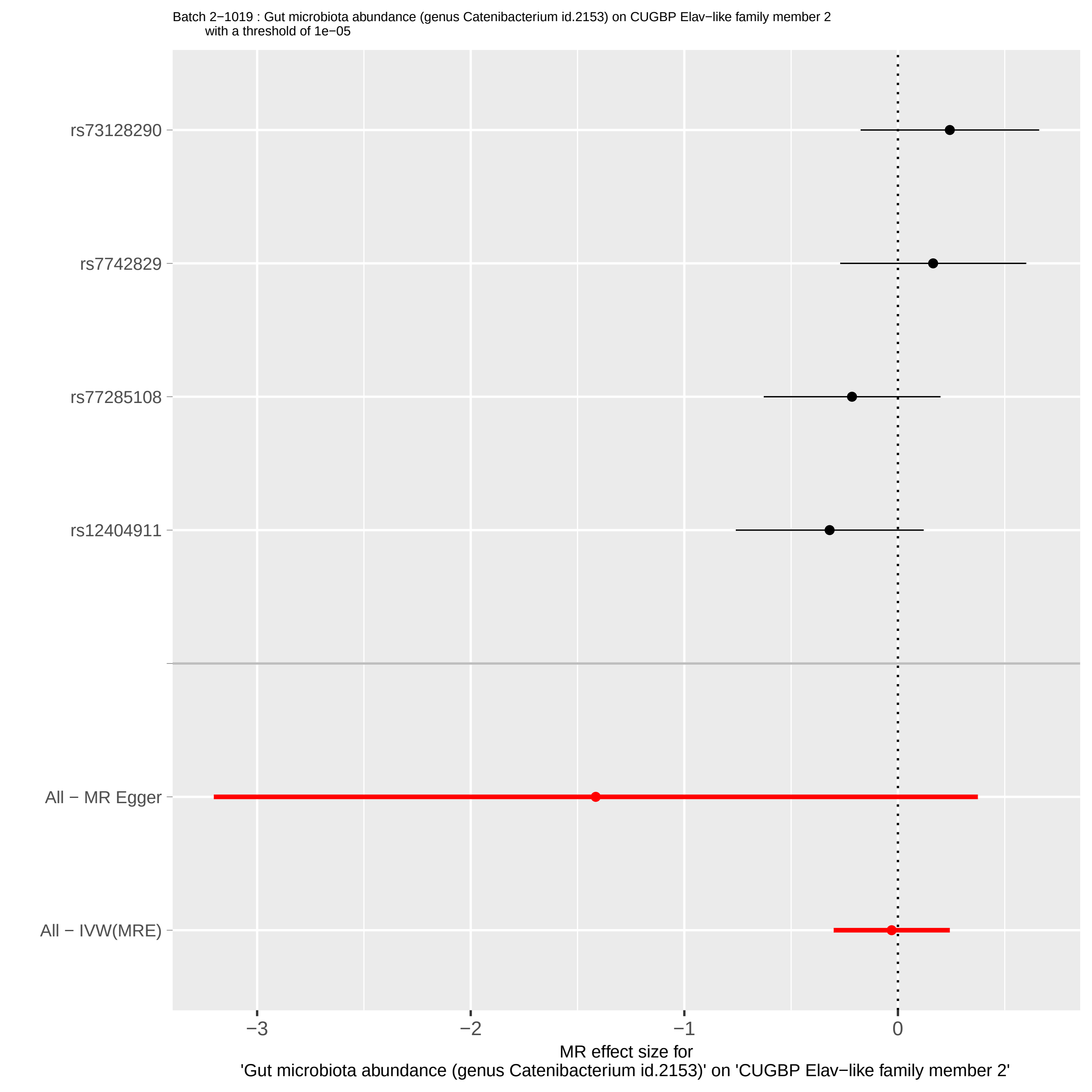

Batch 2−1019 : Gut microbiota abundance (genus Catenibacterium id.2153) on CUGBP Elav−like family member 2
with a threshold of 1e−05
rs73128290
rs7742829
rs77285108
rs12404911
All − MR Egger
All − IVW(MRE)
−3
−2
−1
0
MR effect size for
'Gut microbiota abundance (genus Catenibacterium id.2153)' on 'CUGBP Elav−like family member 2'

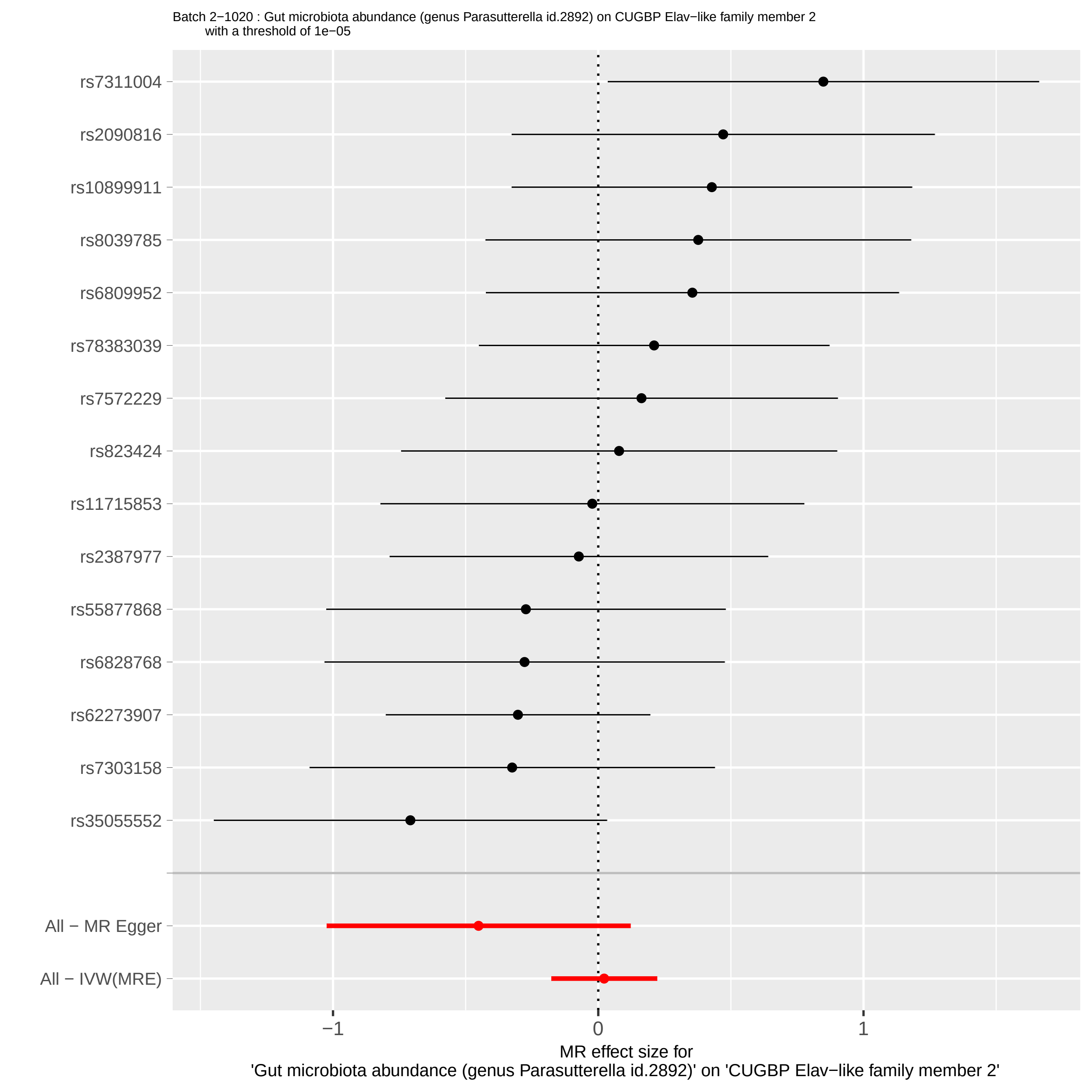

Batch 2−1020 : Gut microbiota abundance (genus Parasutterella id.2892) on CUGBP Elav−like family member 2
with a threshold of 1e−05
rs7311004
rs2090816
rs10899911
rs8039785
rs6809952
rs78383039
rs7572229
rs823424
rs11715853
rs2387977
rs55877868
rs6828768
rs62273907
rs7303158
rs35055552
All − MR Egger
All − IVW(MRE)
−1
0
1
MR effect size for
'Gut microbiota abundance (genus Parasutterella id.2892)' on 'CUGBP Elav−like family member 2'

Batch 2−1021 : Gut microbiota abundance (genus Catenibacterium id.2153) on Centromere protein W
with a threshold of 1e−05

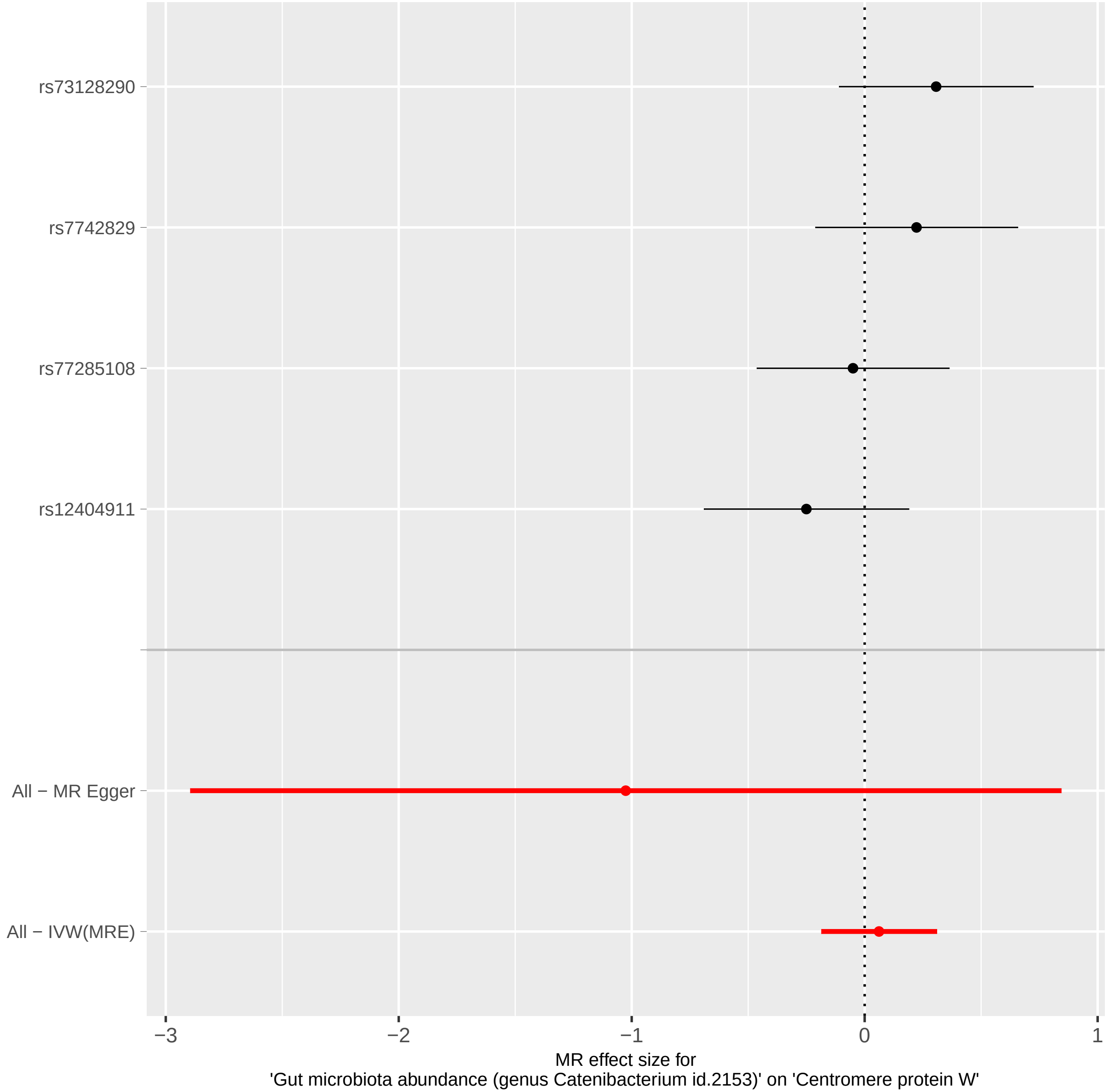

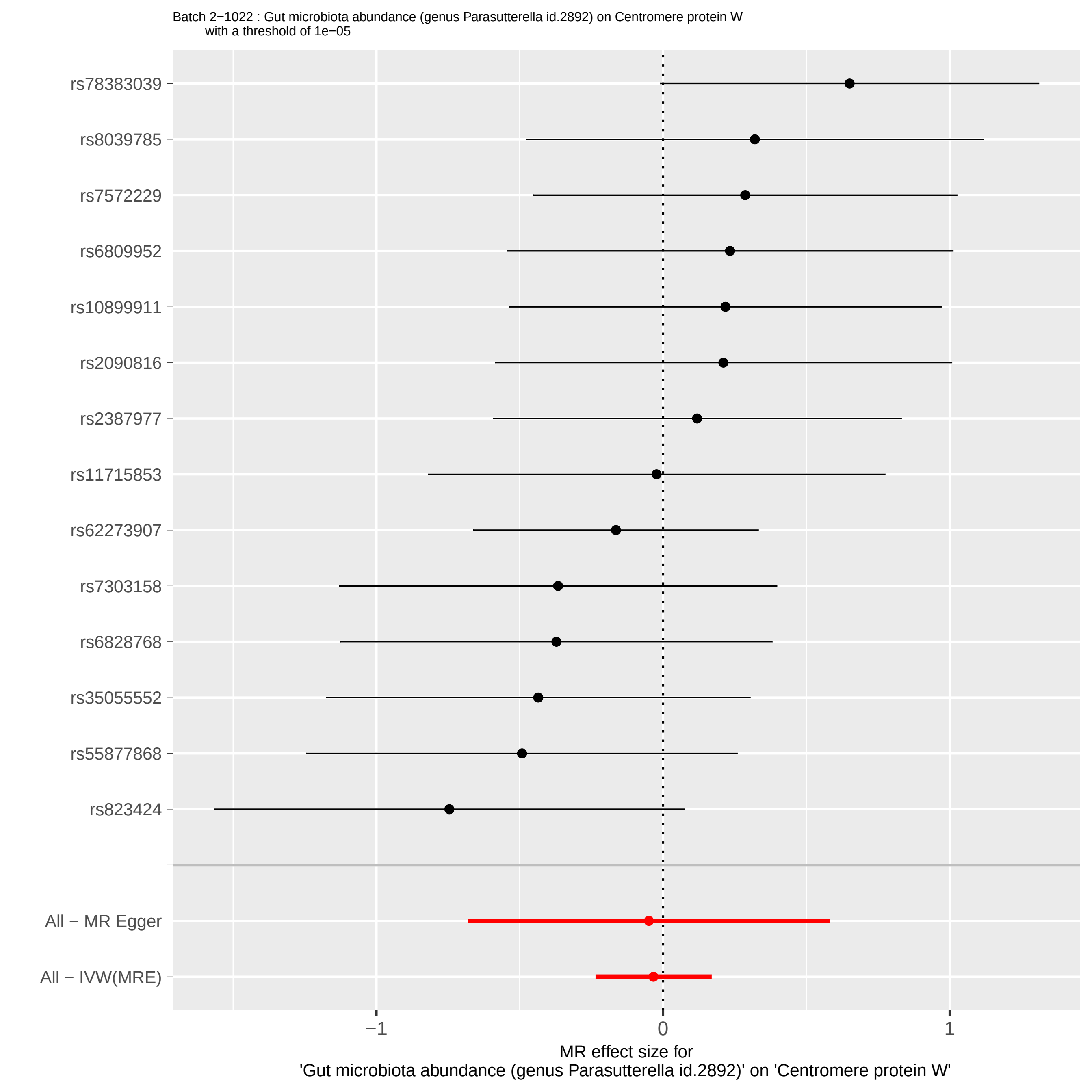

Batch 2−1022 : Gut microbiota abundance (genus Parasutterella id.2892) on Centromere protein W
with a threshold of 1e−05
rs78383039
rs8039785
rs7572229
rs6809952
rs10899911
rs2090816
rs2387977
rs11715853
rs62273907
rs7303158
rs6828768
rs35055552
rs55877868
rs823424
All − MR Egger
All − IVW(MRE)
−1
0
1
MR effect size for
'Gut microbiota abundance (genus Parasutterella id.2892)' on 'Centromere protein W'

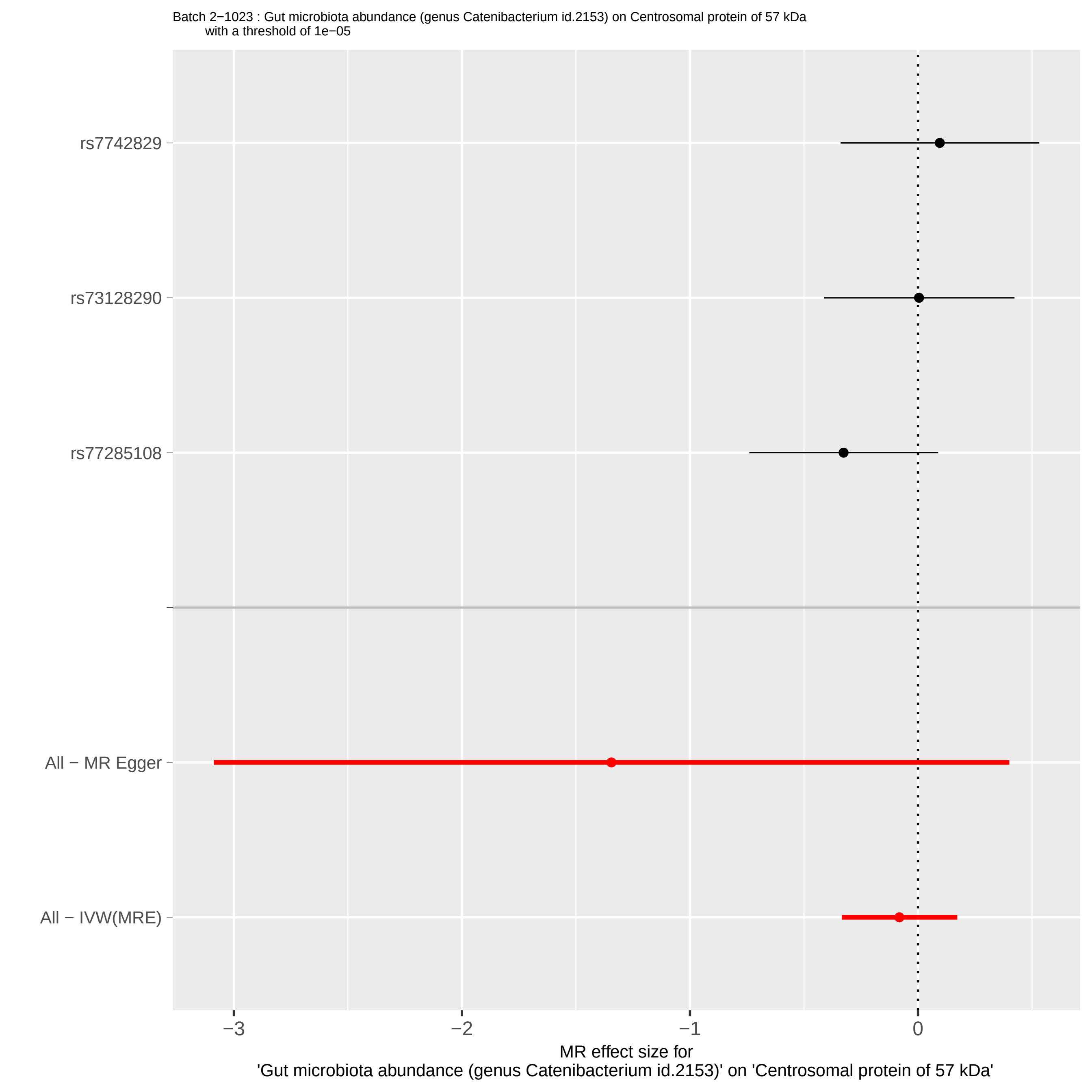

Batch 2−1023 : Gut microbiota abundance (genus Catenibacterium id.2153) on Centrosomal protein of 57 kDa
with a threshold of 1e−05
rs7742829
rs73128290
rs77285108
All − MR Egger
All − IVW(MRE)
−3
−2
−1
0
MR effect size for
'Gut microbiota abundance (genus Catenibacterium id.2153)' on 'Centrosomal protein of 57 kDa'

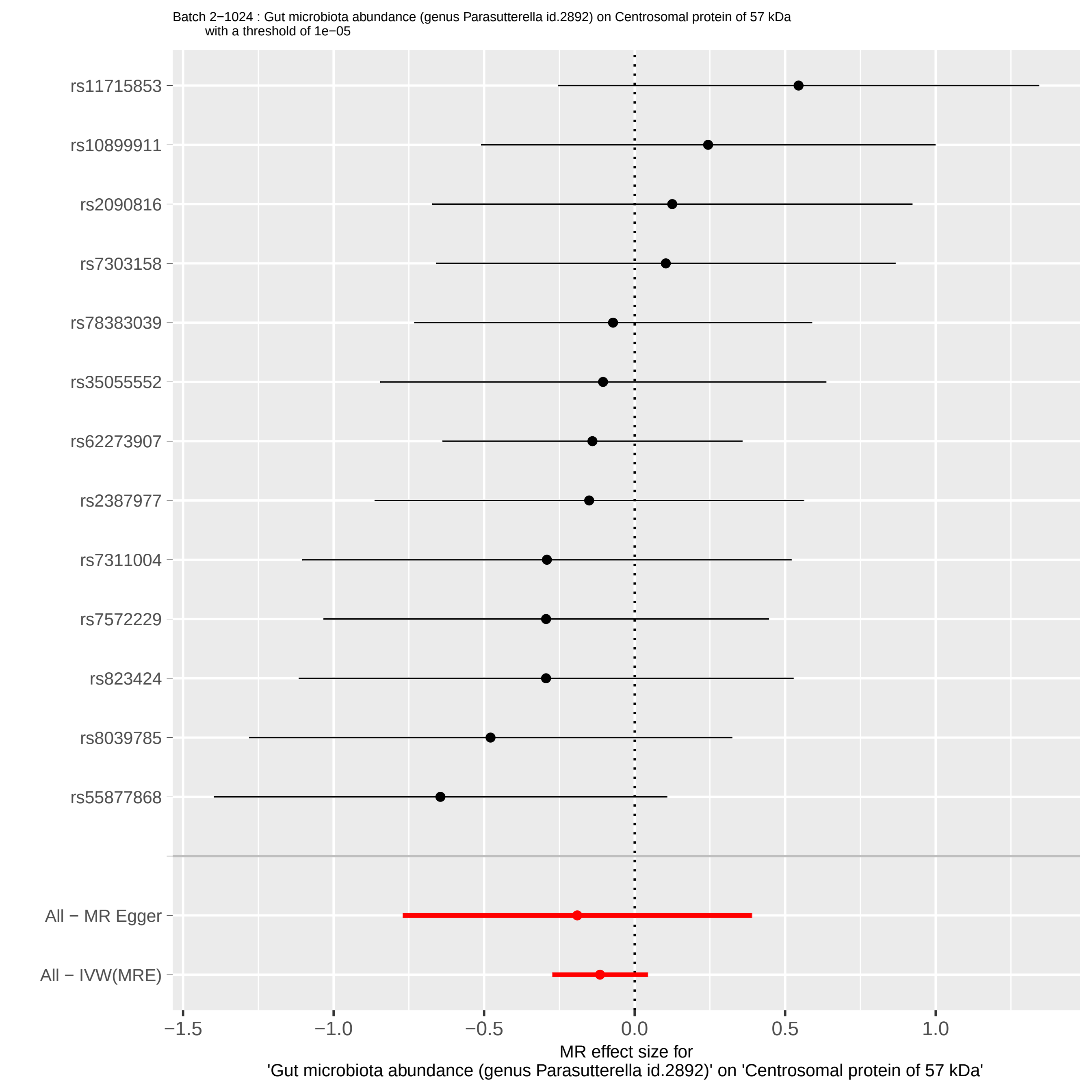

Batch 2−1024 : Gut microbiota abundance (genus Parasutterella id.2892) on Centrosomal protein of 57 kDa
with a threshold of 1e−05
rs11715853
rs10899911
rs2090816
rs7303158
rs78383039
rs35055552
rs62273907
rs2387977
rs7311004
rs7572229
rs823424
rs8039785
rs55877868
All − MR Egger
All − IVW(MRE)
−1.5
−1.0
−0.5
0.0
0.5
1.0
MR effect size for
'Gut microbiota abundance (genus Parasutterella id.2892)' on 'Centrosomal protein of 57 kDa'

Batch 2−1025 : Gut microbiota abundance (genus Catenibacterium id.2153) on Centrosomal protein of 57 kDa
with a threshold of 1e−05

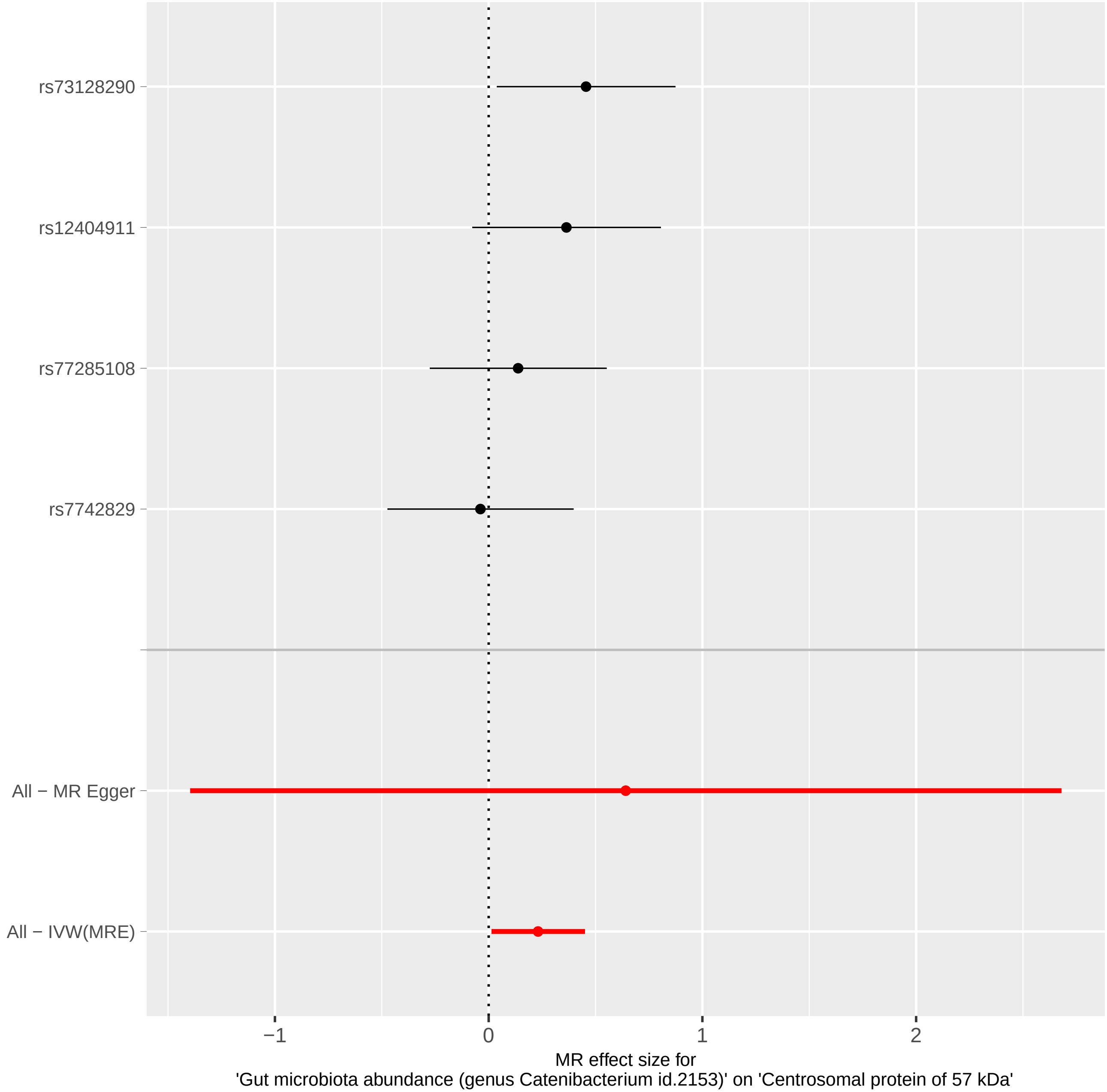

Batch 2−1026 : Gut microbiota abundance (genus Parasutterella id.2892) on Centrosomal protein of 57 kDa
with a threshold of 1e−05

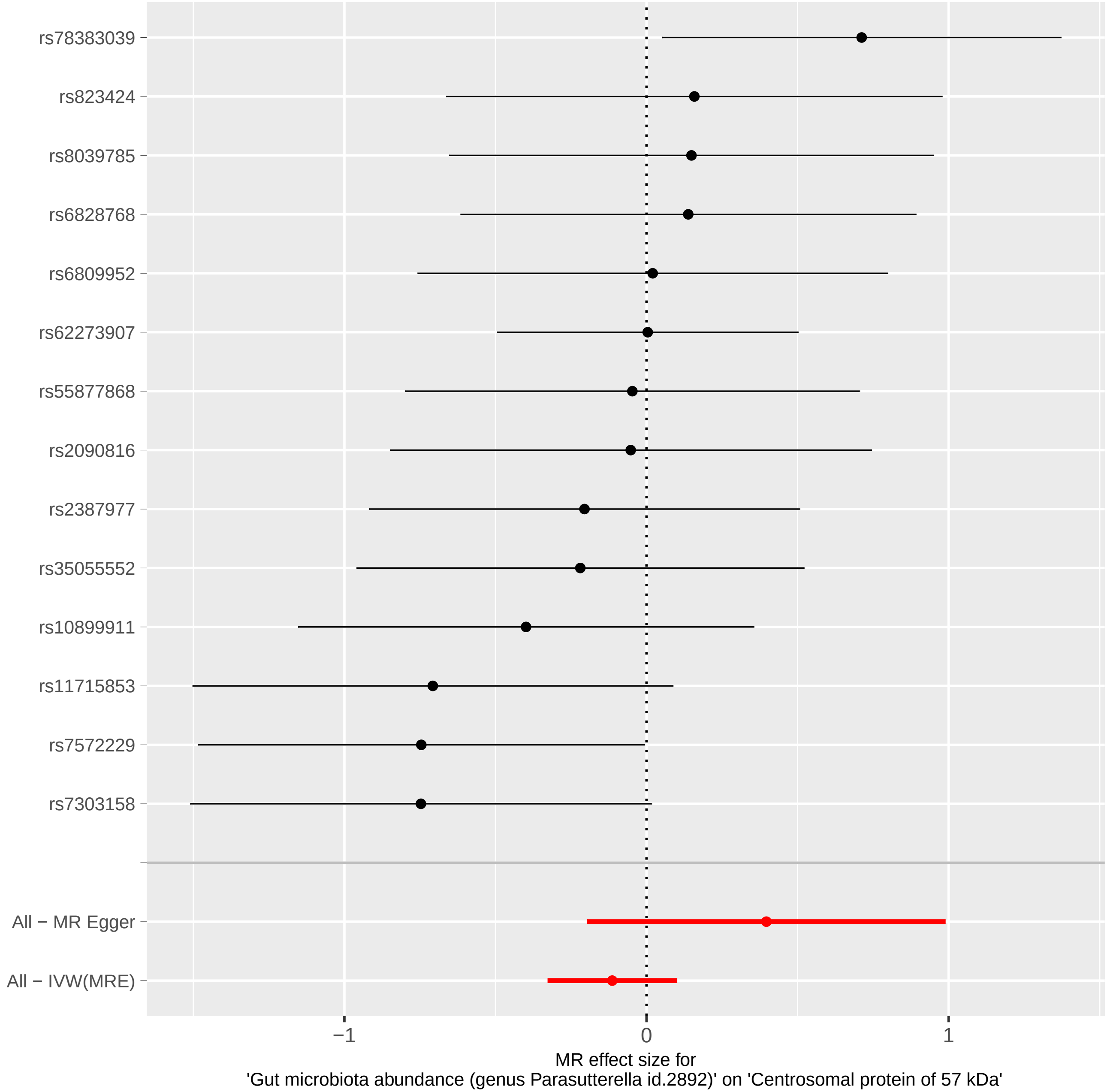

Batch 2−1027 : Gut microbiota abundance (genus Catenibacterium id.2153) on Centrosomal protein of 57 kDa
with a threshold of 1e−05

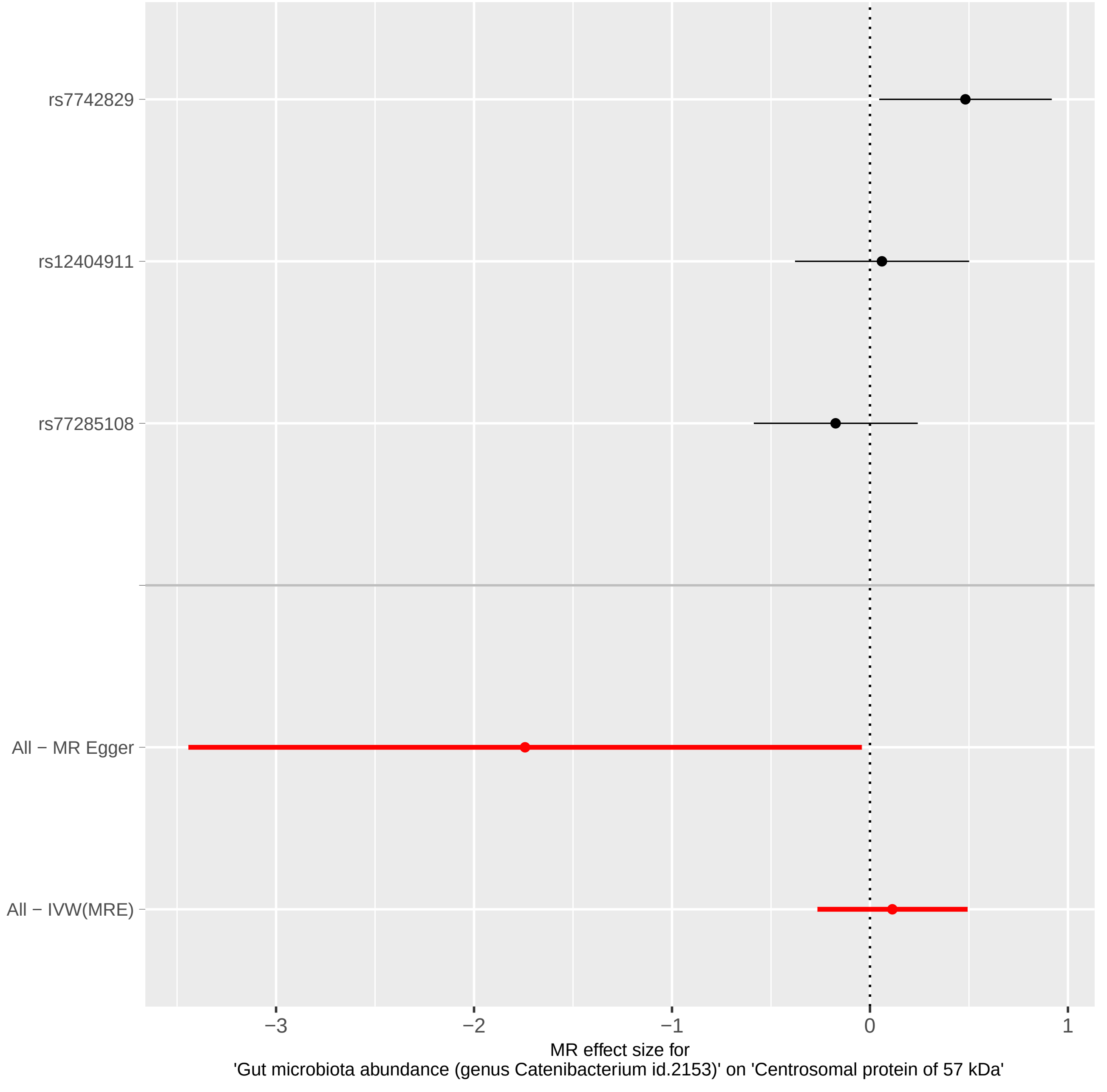

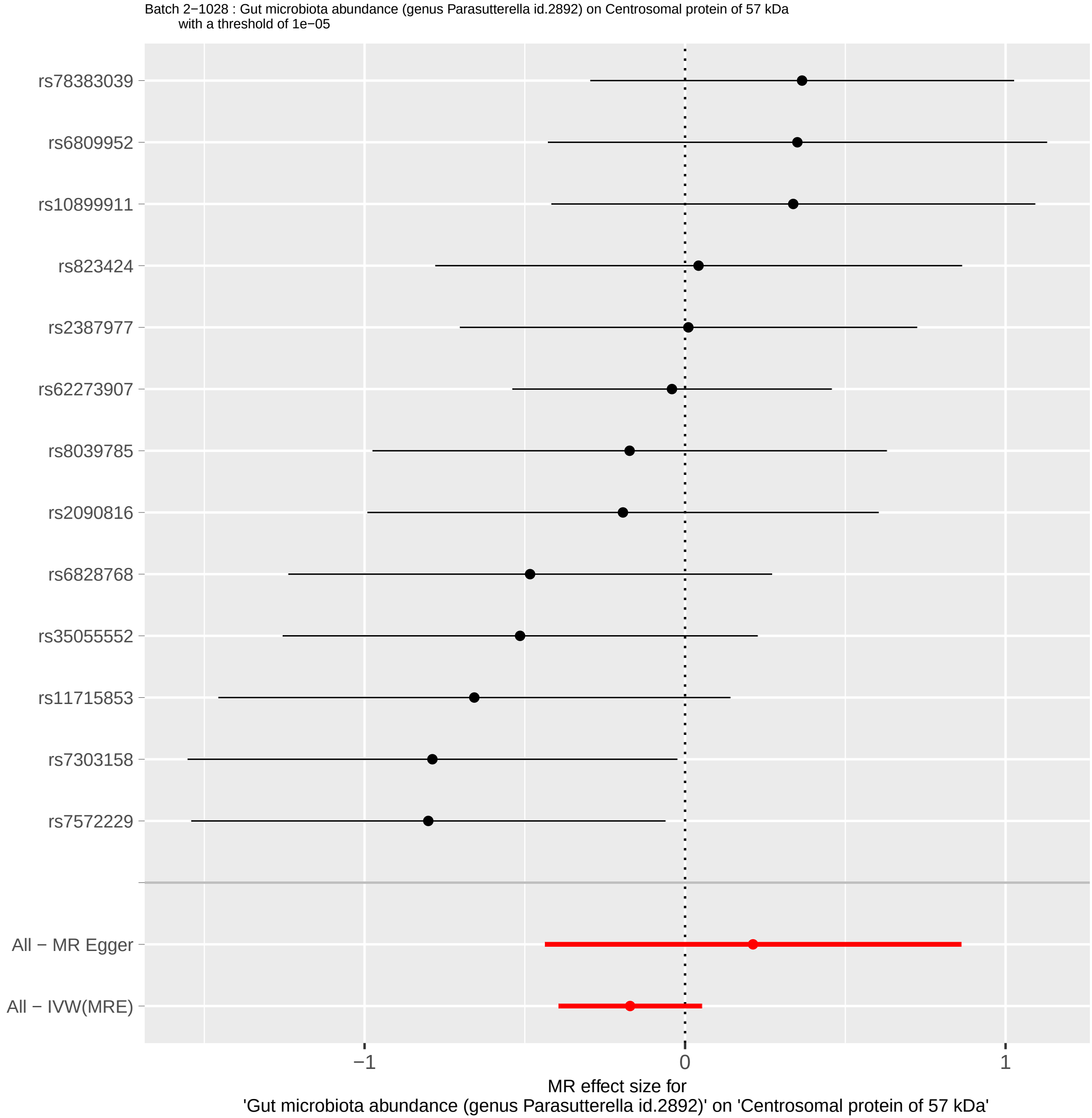

Batch 2−1028 : Gut microbiota abundance (genus Parasutterella id.2892) on Centrosomal protein of 57 kDa
with a threshold of 1e−05
rs78383039
rs6809952
rs10899911
rs823424
rs2387977
rs62273907
rs8039785
rs2090816
rs6828768
rs35055552
rs11715853
rs7303158
rs7572229
All − MR Egger
All − IVW(MRE)
−1
0
1
MR effect size for
'Gut microbiota abundance (genus Parasutterella id.2892)' on 'Centrosomal protein of 57 kDa'

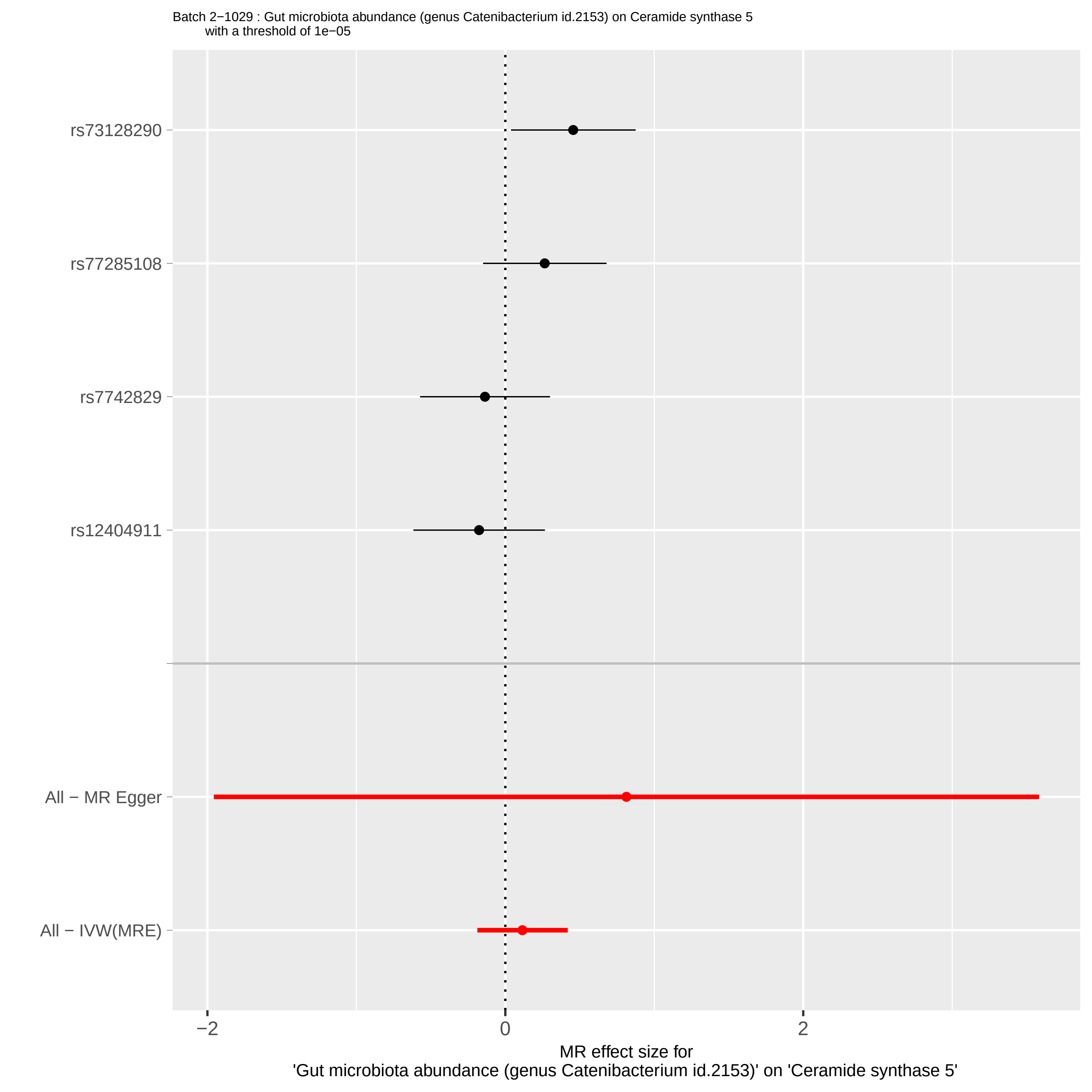
Batch 2−1029 : Gut microbiota abundance (genus Catenibacterium id.2153) on Ceramide synthase 5
with a threshold of 1e−05
rs73128290
rs77285108
rs7742829
rs12404911
All − MR Egger
All − IVW(MRE)
−2
0
2
MR effect size for
'Gut microbiota abundance (genus Catenibacterium id.2153)' on 'Ceramide synthase 5'

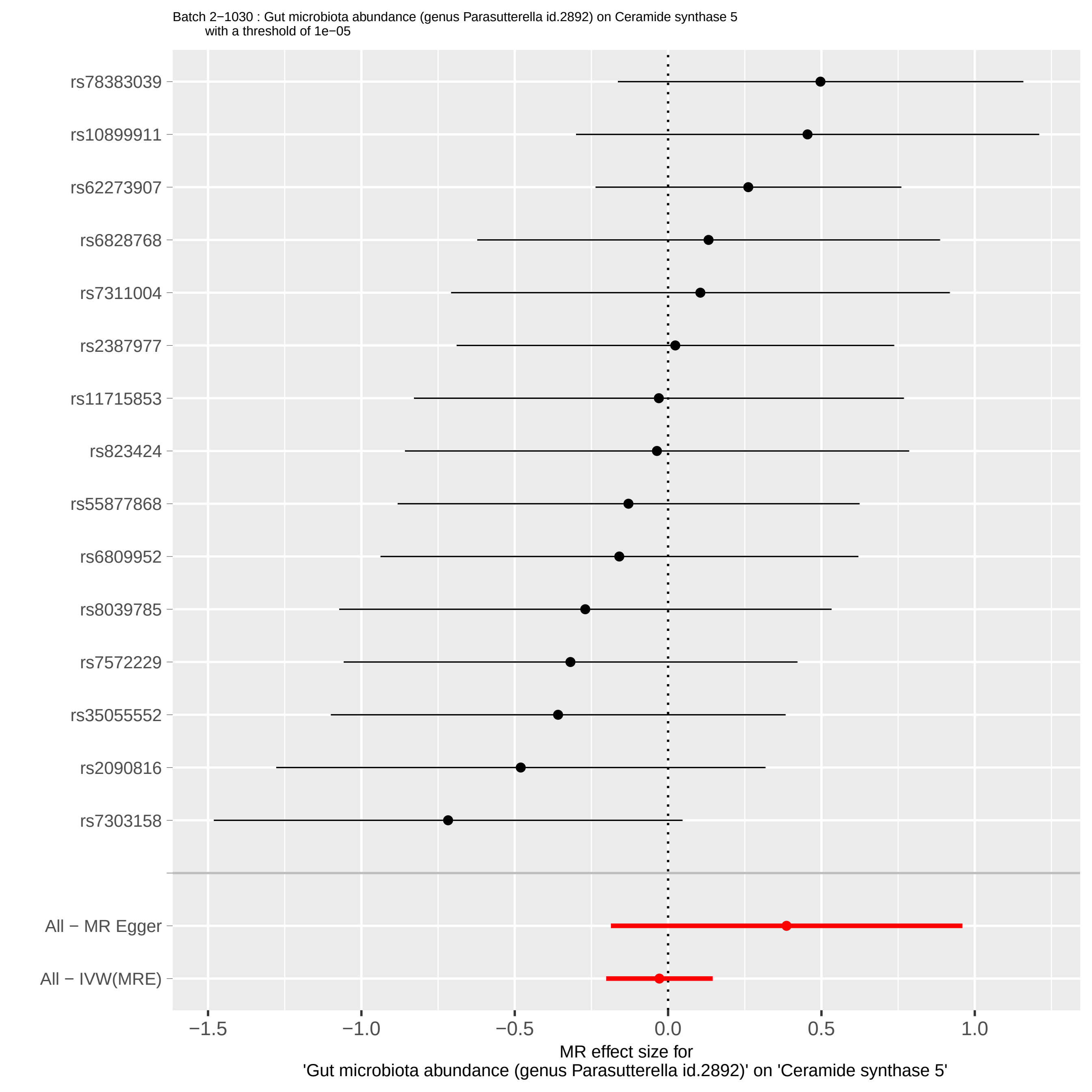

Batch 2−1030 : Gut microbiota abundance (genus Parasutterella id.2892) on Ceramide synthase 5
with a threshold of 1e−05
rs78383039
rs10899911
rs62273907
rs6828768
rs7311004
rs2387977
rs11715853
rs823424
rs55877868
rs6809952
rs8039785
rs7572229
rs35055552
rs2090816
rs7303158
All − MR Egger
All − IVW(MRE)
−1.5
−1.0
−0.5
0.0
0.5
1.0
MR effect size for
'Gut microbiota abundance (genus Parasutterella id.2892)' on 'Ceramide synthase 5'

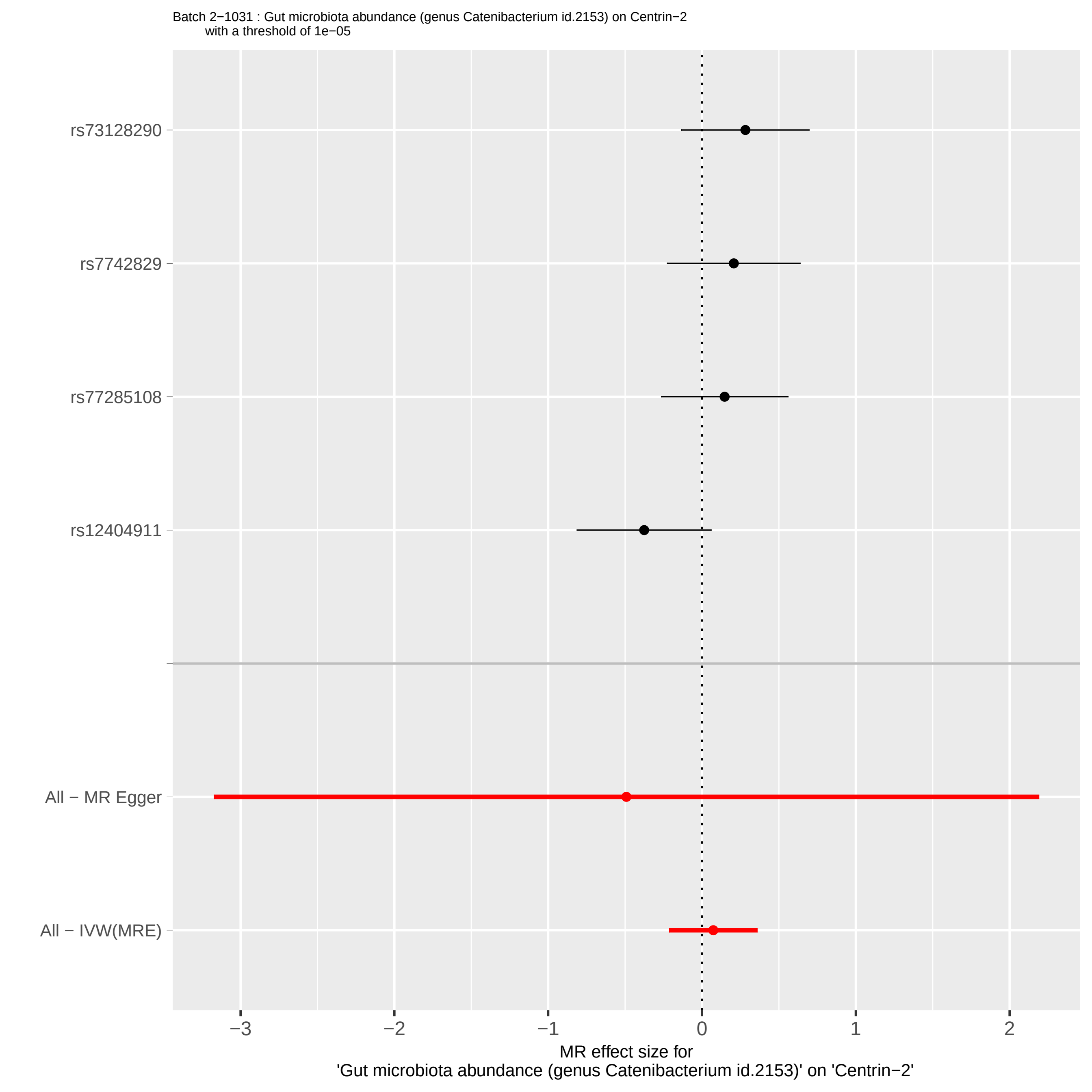
Batch 2−1031 : Gut microbiota abundance (genus Catenibacterium id.2153) on Centrin−2
with a threshold of 1e−05
rs73128290
rs7742829
rs77285108
rs12404911
All − MR Egger
All − IVW(MRE)
−3
−2
−1
0
1
2
MR effect size for
'Gut microbiota abundance (genus Catenibacterium id.2153)' on 'Centrin−2'

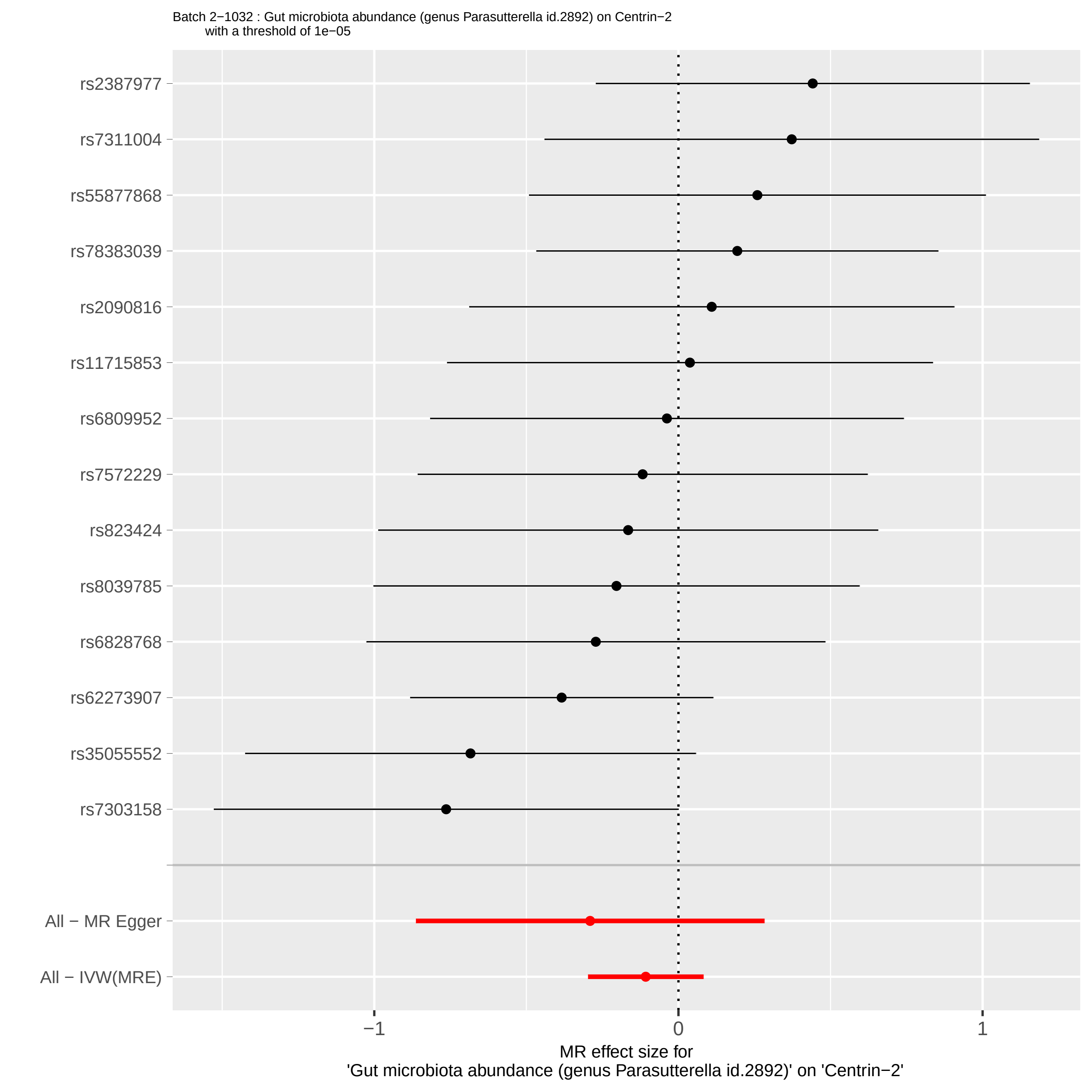

Batch 2−1032 : Gut microbiota abundance (genus Parasutterella id.2892) on Centrin−2
with a threshold of 1e−05
rs2387977
rs7311004
rs55877868
rs78383039
rs2090816
rs11715853
rs6809952
rs7572229
rs823424
rs8039785
rs6828768
rs62273907
rs35055552
rs7303158
All − MR Egger
All − IVW(MRE)
−1
0
1
MR effect size for
'Gut microbiota abundance (genus Parasutterella id.2892)' on 'Centrin−2'

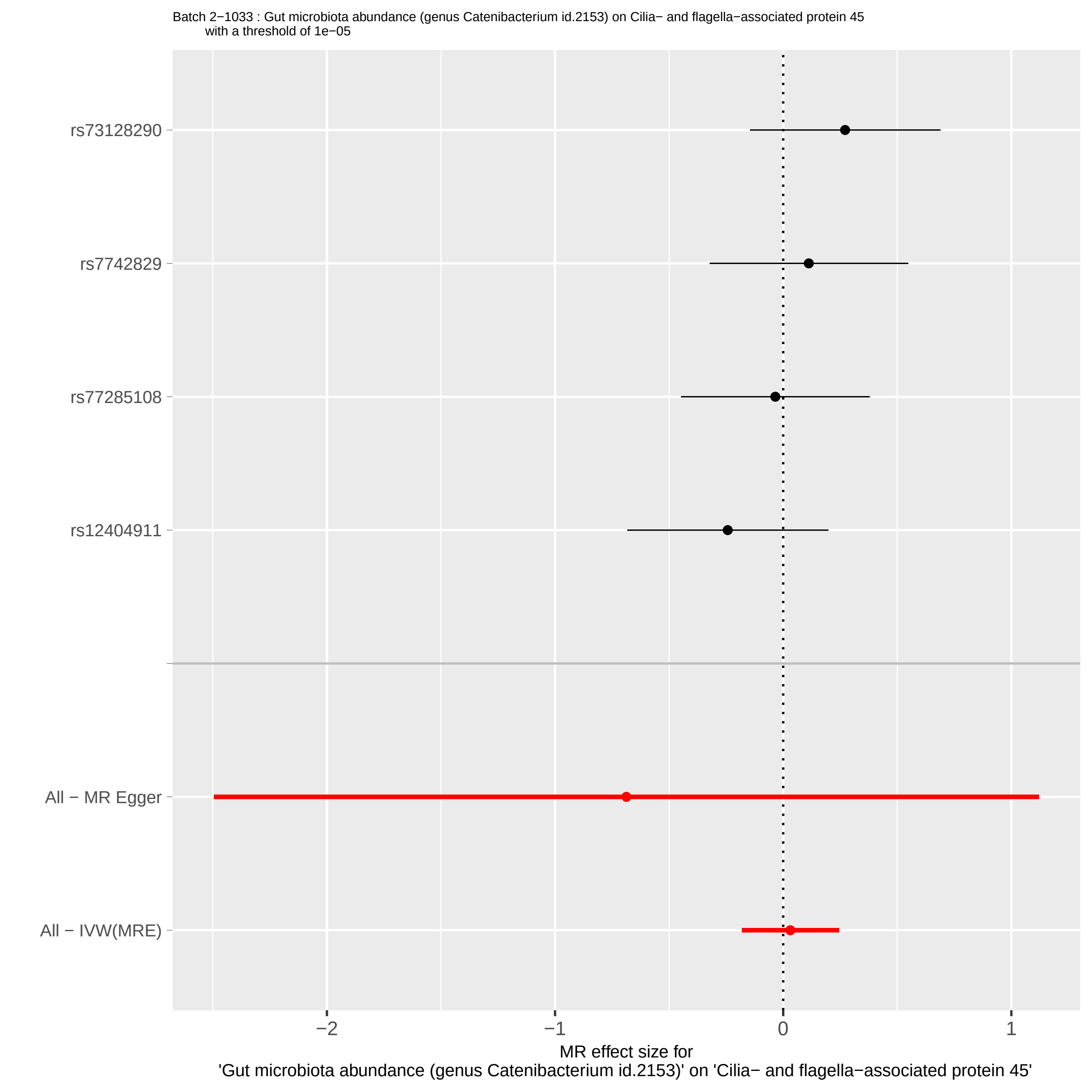

Batch 2−1033 : Gut microbiota abundance (genus Catenibacterium id.2153) on Cilia− and flagella−associated protein 45
with a threshold of 1e−05
rs73128290
rs7742829
rs77285108
rs12404911
All − MR Egger
All − IVW(MRE)
−2
−1
0
1
MR effect size for
'Gut microbiota abundance (genus Catenibacterium id.2153)' on 'Cilia− and flagella−associated protein 45'

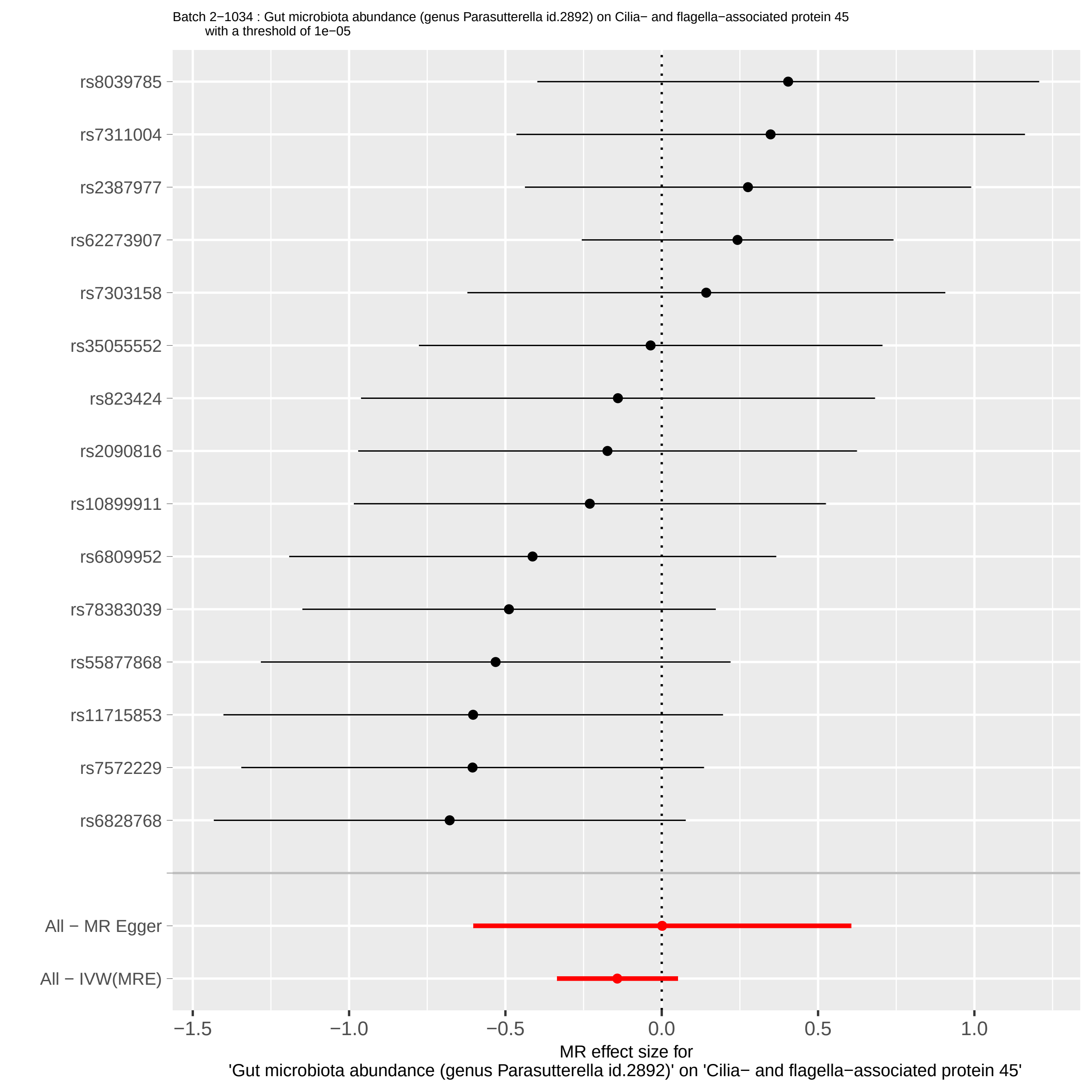
Batch 2−1034 : Gut microbiota abundance (genus Parasutterella id.2892) on Cilia− and flagella−associated protein 45
with a threshold of 1e−05
rs8039785
rs7311004
rs2387977
rs62273907
rs7303158
rs35055552
rs823424
rs2090816
rs10899911
rs6809952
rs78383039
rs55877868
rs11715853
rs7572229
rs6828768
All − MR Egger
All − IVW(MRE)
−1.5
−1.0
−0.5
0.0
0.5
1.0
MR effect size for
'Gut microbiota abundance (genus Parasutterella id.2892)' on 'Cilia− and flagella−associated protein 45'

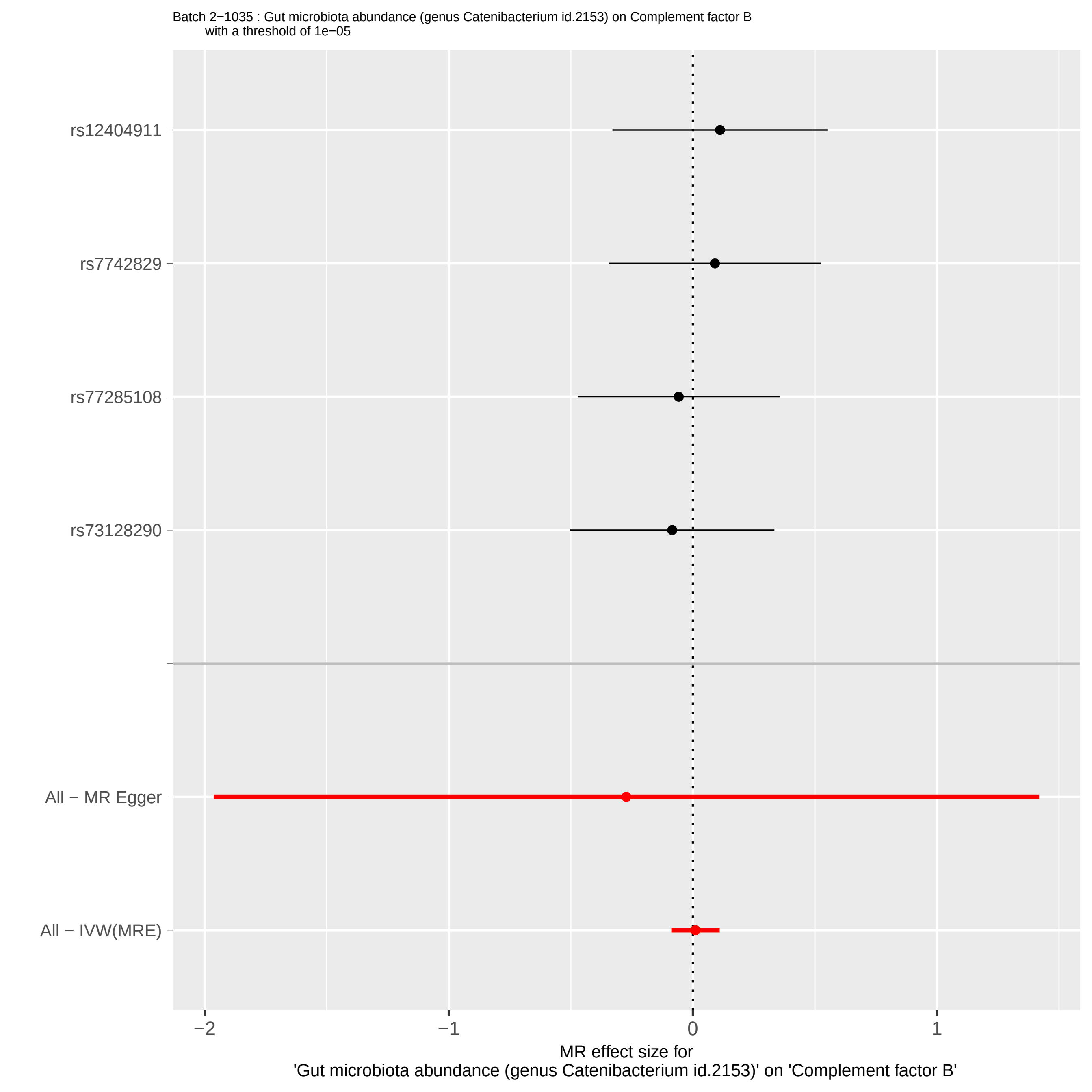
Batch 2−1035 : Gut microbiota abundance (genus Catenibacterium id.2153) on Complement factor B
with a threshold of 1e−05
rs12404911
rs7742829
rs77285108
rs73128290
All − MR Egger
All − IVW(MRE)
−2
−1
0
1
MR effect size for
'Gut microbiota abundance (genus Catenibacterium id.2153)' on 'Complement factor B'

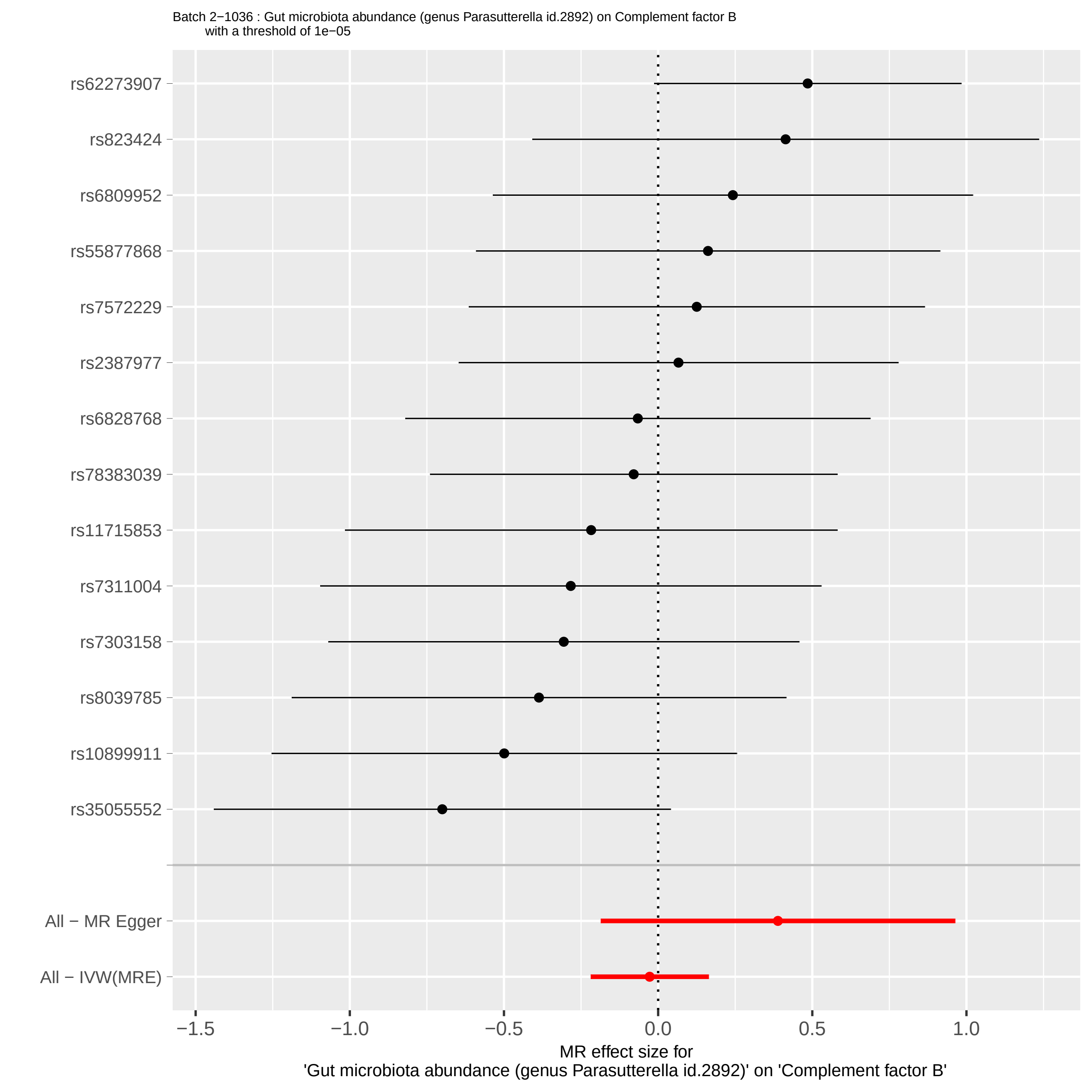

Batch 2−1036 : Gut microbiota abundance (genus Parasutterella id.2892) on Complement factor B
with a threshold of 1e−05
rs62273907
rs823424
rs6809952
rs55877868
rs7572229
rs2387977
rs6828768
rs78383039
rs11715853
rs7311004
rs7303158
rs8039785
rs10899911
rs35055552
All − MR Egger
All − IVW(MRE)
−1.5
−1.0
−0.5
0.0
0.5
1.0
MR effect size for
'Gut microbiota abundance (genus Parasutterella id.2892)' on 'Complement factor B'

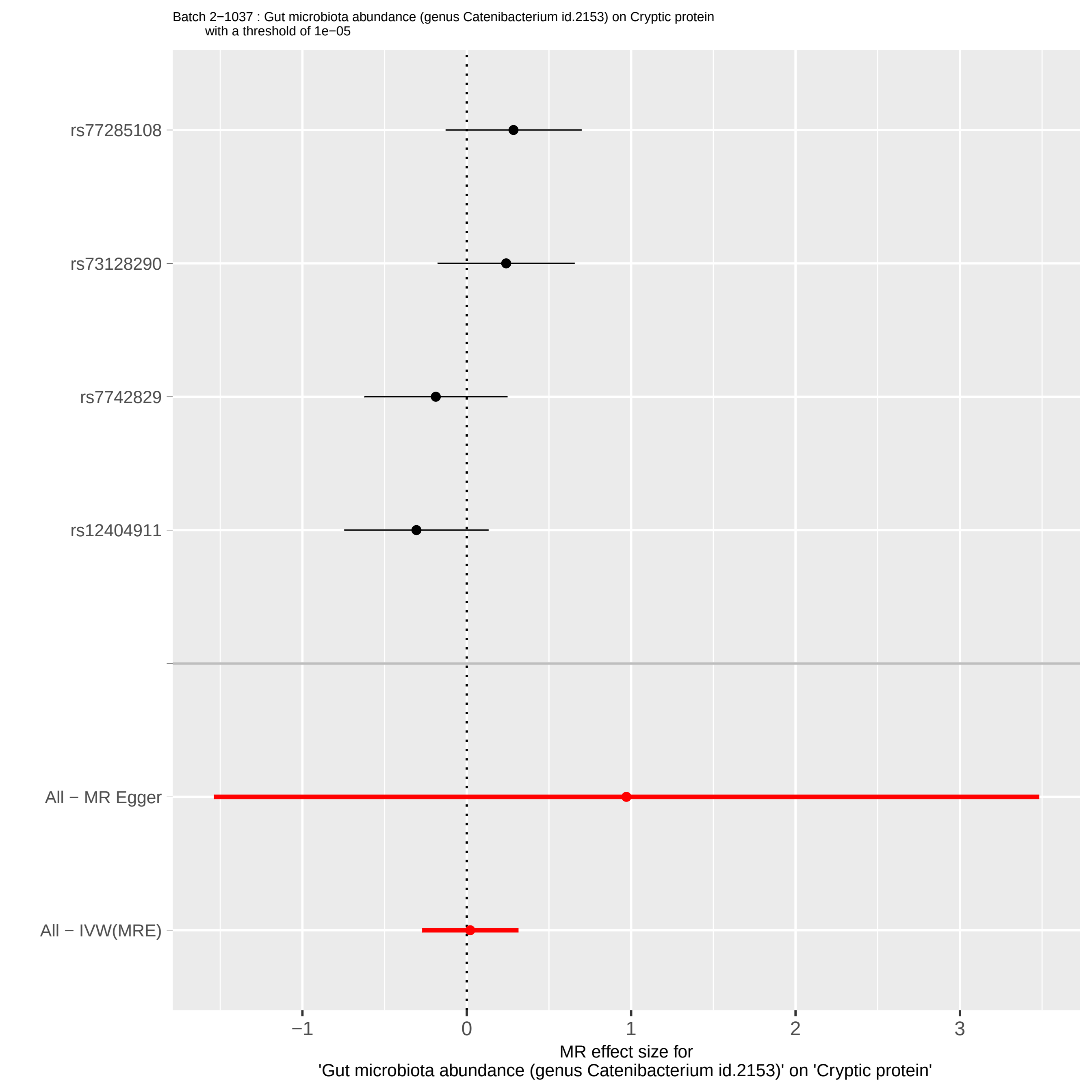
Batch 2−1037 : Gut microbiota abundance (genus Catenibacterium id.2153) on Cryptic protein
with a threshold of 1e−05
rs77285108
rs73128290
rs7742829
rs12404911
All − MR Egger
All − IVW(MRE)
−1
0
1
2
3
MR effect size for
'Gut microbiota abundance (genus Catenibacterium id.2153)' on 'Cryptic protein'

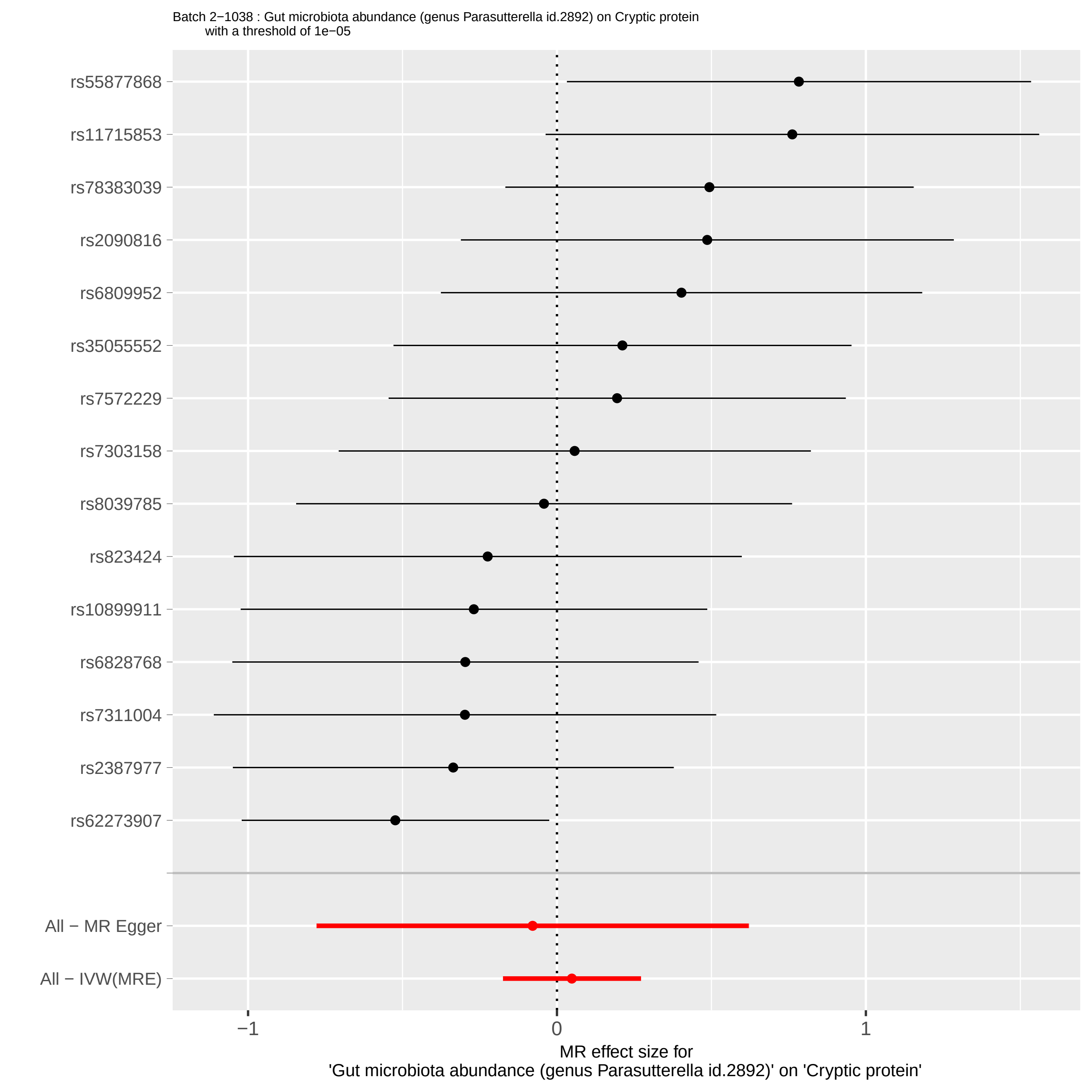
Batch 2−1038 : Gut microbiota abundance (genus Parasutterella id.2892) on Cryptic protein
with a threshold of 1e−05
rs55877868
rs11715853
rs78383039
rs2090816
rs6809952
rs35055552
rs7572229
rs7303158
rs8039785
rs823424
rs10899911
rs6828768
rs7311004
rs2387977
rs62273907
All − MR Egger
All − IVW(MRE)
−1
0
1
MR effect size for
'Gut microbiota abundance (genus Parasutterella id.2892)' on 'Cryptic protein'

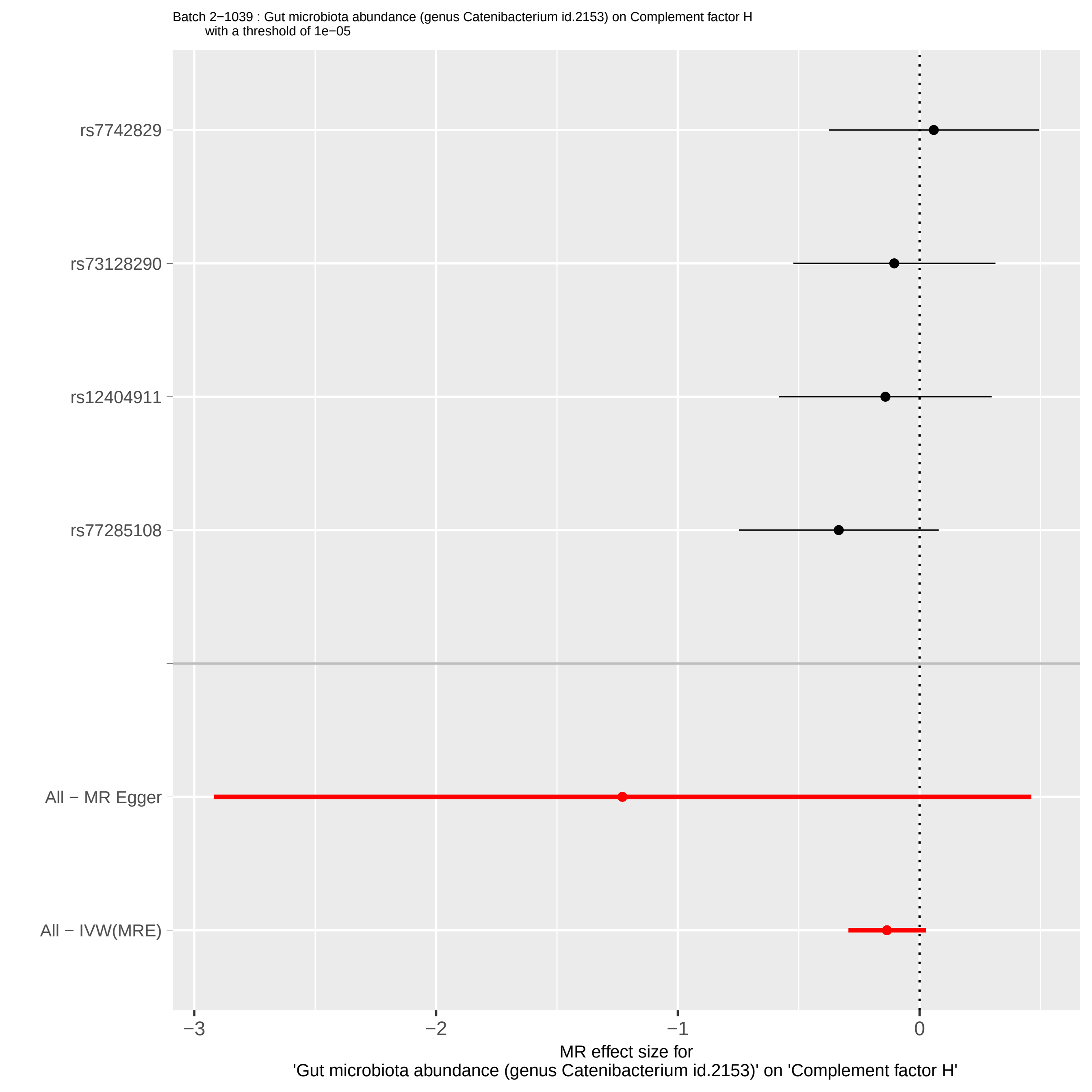

Batch 2−1039 : Gut microbiota abundance (genus Catenibacterium id.2153) on Complement factor H
with a threshold of 1e−05
rs7742829
rs73128290
rs12404911
rs77285108
All − MR Egger
All − IVW(MRE)
−3
−2
−1
0
MR effect size for
'Gut microbiota abundance (genus Catenibacterium id.2153)' on 'Complement factor H'

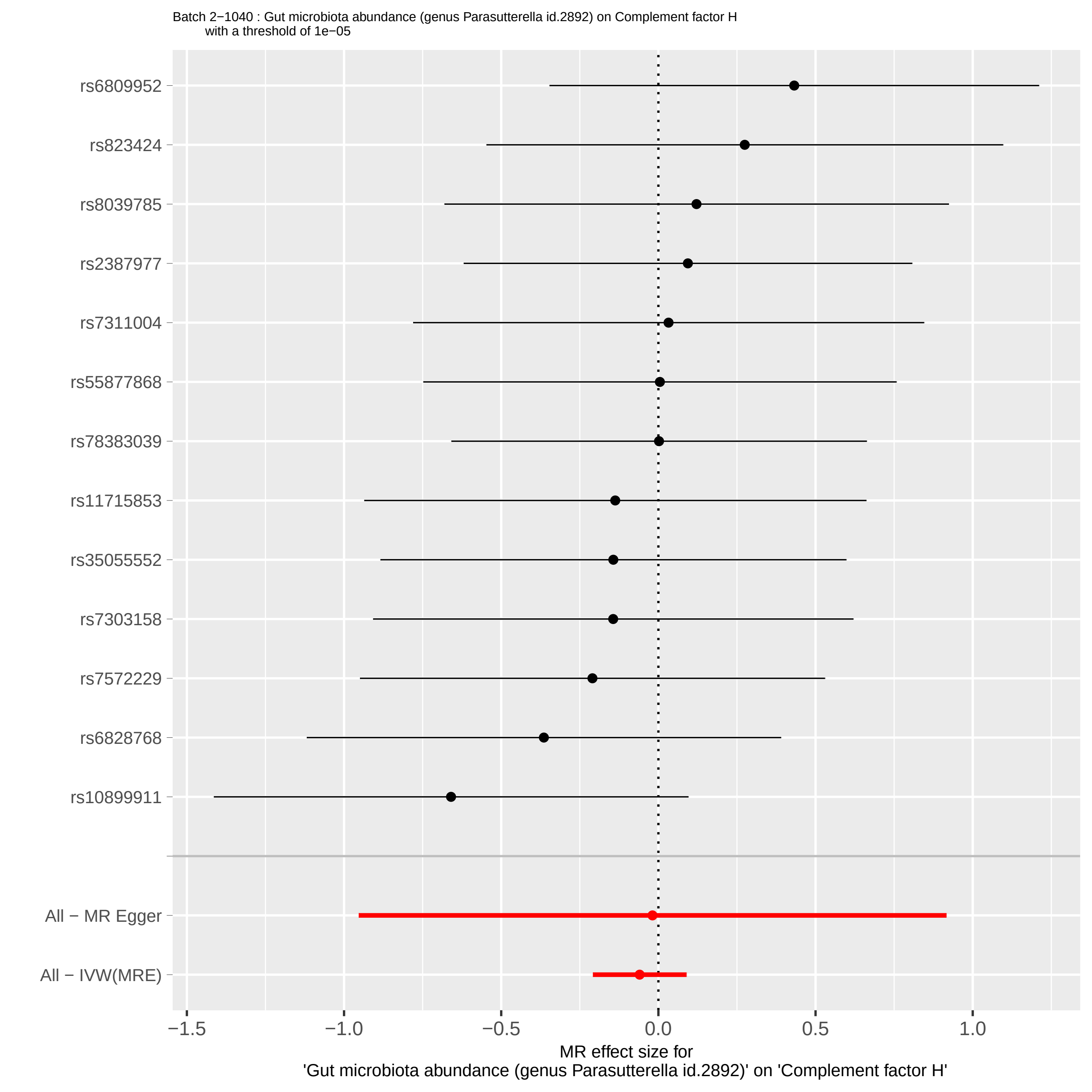
Batch 2−1040 : Gut microbiota abundance (genus Parasutterella id.2892) on Complement factor H
with a threshold of 1e−05
rs6809952
rs823424
rs8039785
rs2387977
rs7311004
rs55877868
rs78383039
rs11715853
rs35055552
rs7303158
rs7572229
rs6828768
rs10899911
All − MR Egger
All − IVW(MRE)
−1.5
−1.0
−0.5
0.0
0.5
1.0
MR effect size for
'Gut microbiota abundance (genus Parasutterella id.2892)' on 'Complement factor H'

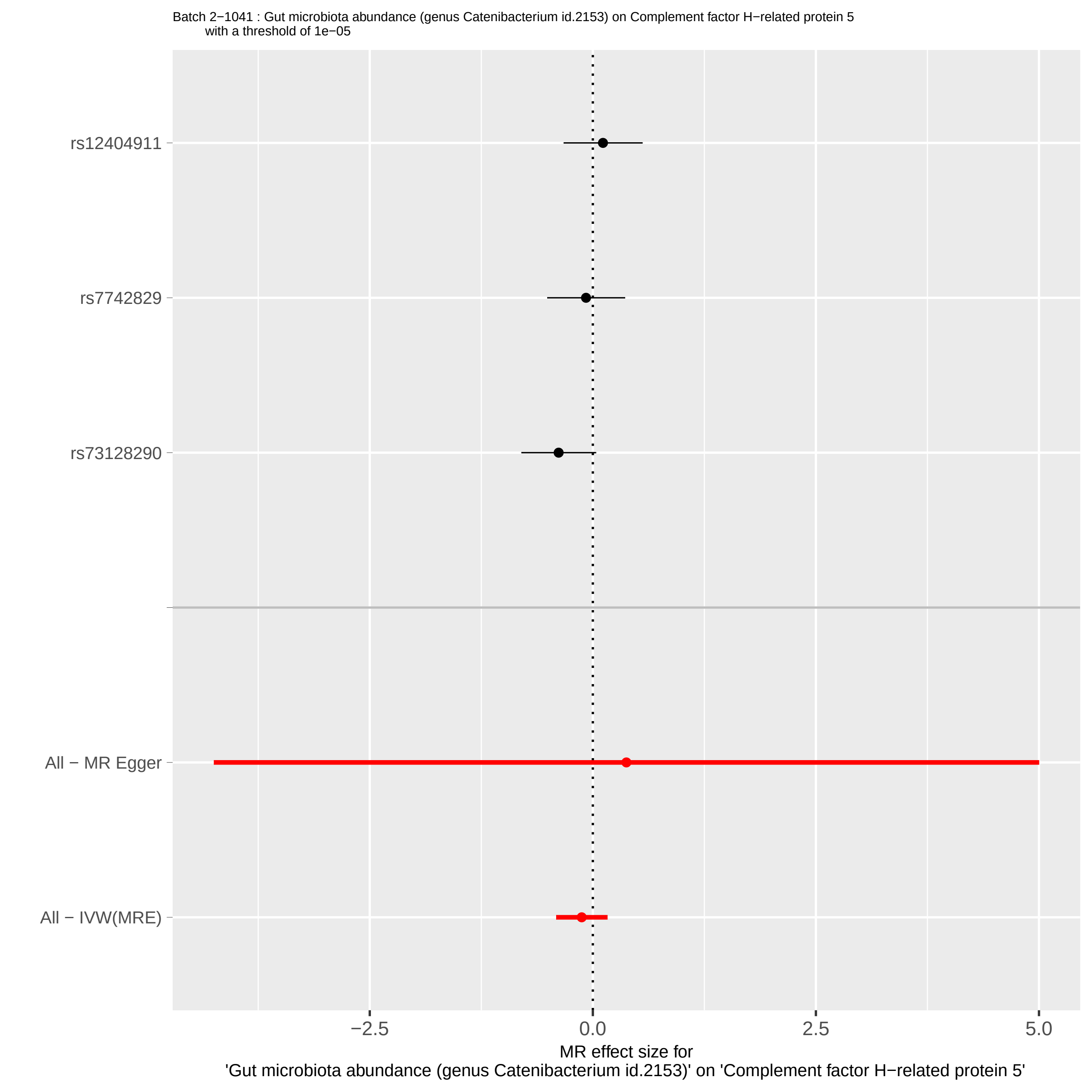
Batch 2−1041 : Gut microbiota abundance (genus Catenibacterium id.2153) on Complement factor H−related protein 5
with a threshold of 1e−05
rs12404911
rs7742829
rs73128290
All − MR Egger
All − IVW(MRE)
−2.5
0.0
2.5
5.0
MR effect size for
'Gut microbiota abundance (genus Catenibacterium id.2153)' on 'Complement factor H−related protein 5'

Batch 2−1042 : Gut microbiota abundance (genus Parasutterella id.2892) on Complement factor H−related protein 5
with a threshold of 1e−05

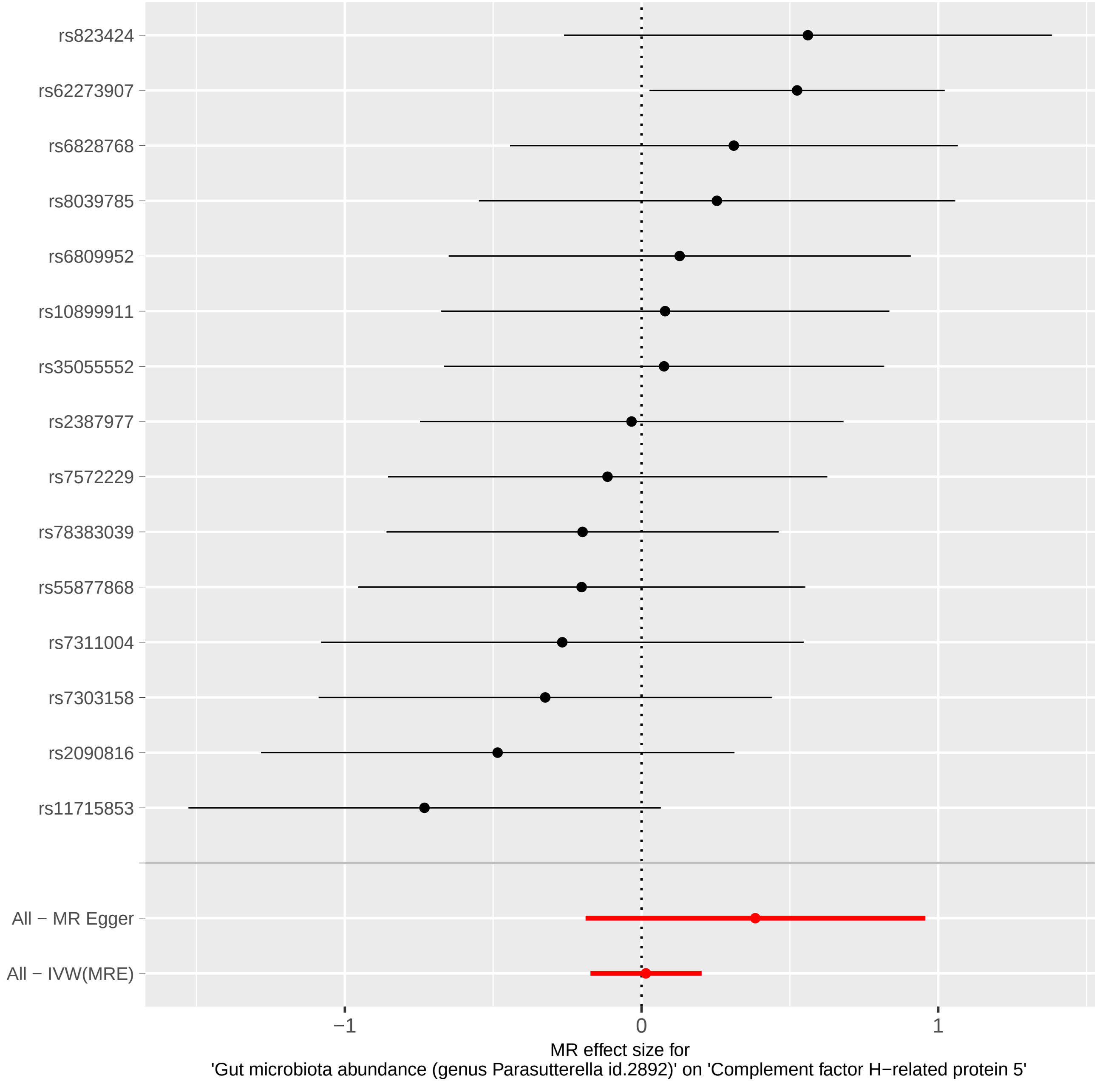

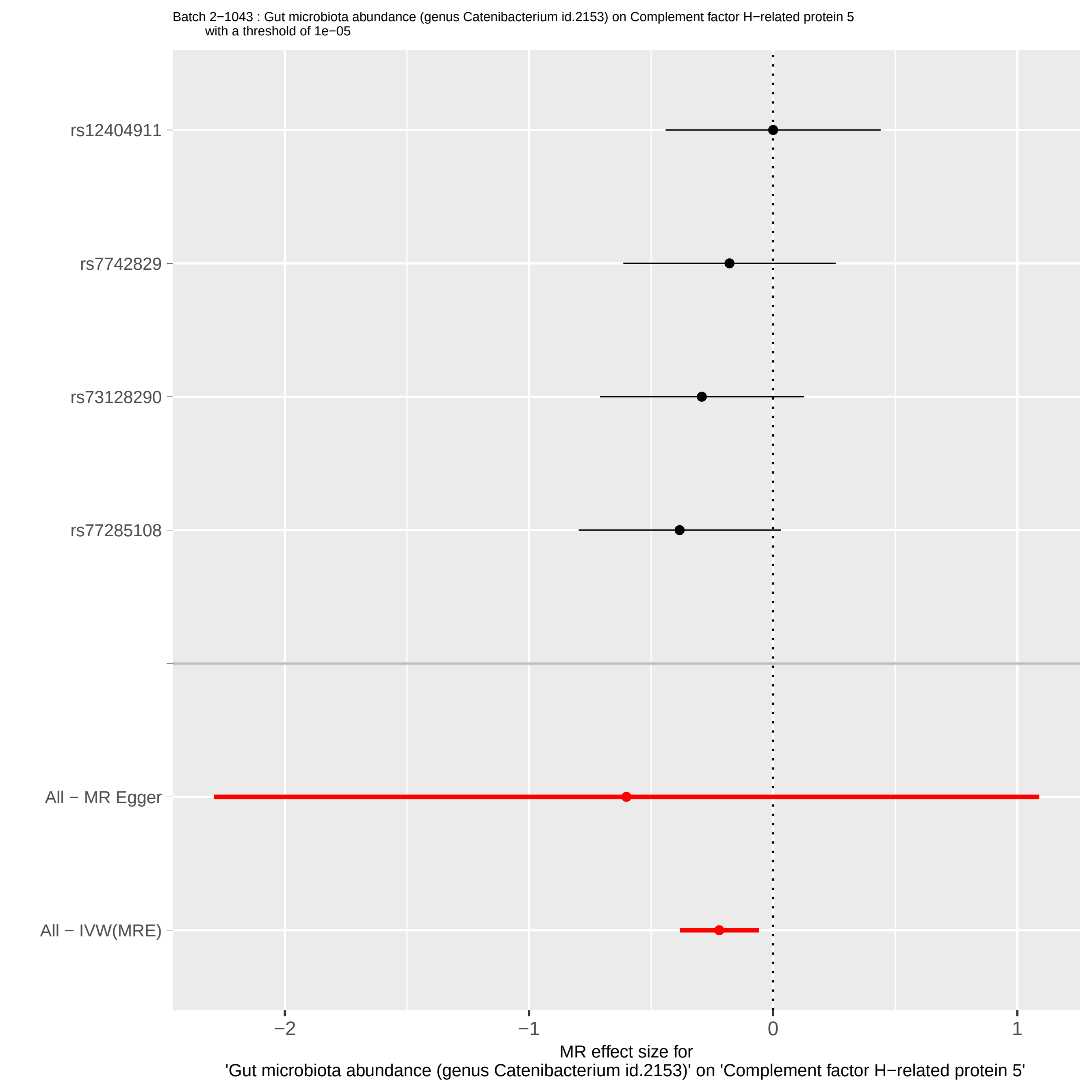
Batch 2−1043 : Gut microbiota abundance (genus Catenibacterium id.2153) on Complement factor H−related protein 5
with a threshold of 1e−05
rs12404911
rs7742829
rs73128290
rs77285108
All − MR Egger
All − IVW(MRE)
−2
−1
0
1
MR effect size for
'Gut microbiota abundance (genus Catenibacterium id.2153)' on 'Complement factor H−related protein 5'

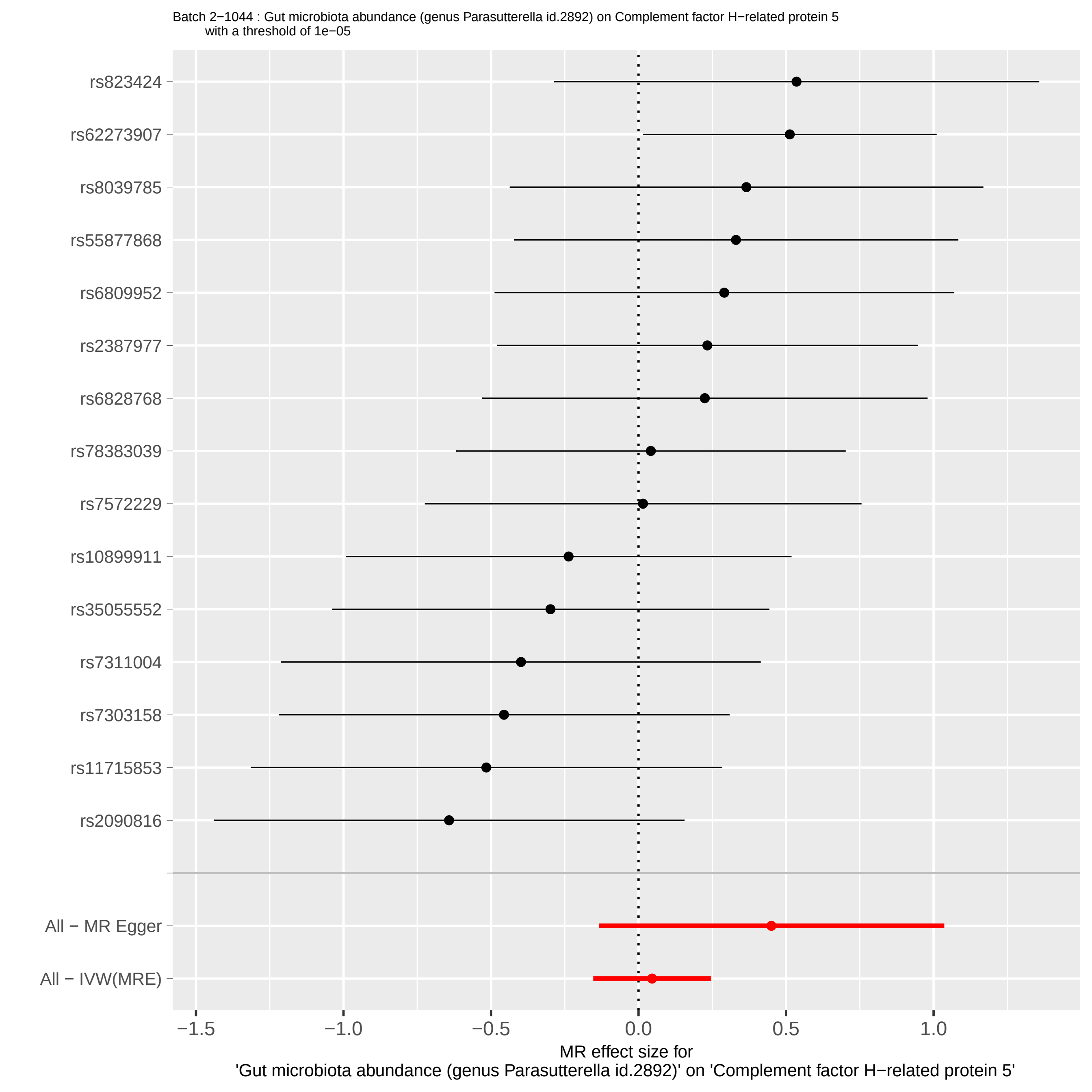

Batch 2−1044 : Gut microbiota abundance (genus Parasutterella id.2892) on Complement factor H−related protein 5
with a threshold of 1e−05
rs823424
rs62273907
rs8039785
rs55877868
rs6809952
rs2387977
rs6828768
rs78383039
rs7572229
rs10899911
rs35055552
rs7311004
rs7303158
rs11715853
rs2090816
All − MR Egger
All − IVW(MRE)
−1.5
−1.0
−0.5
0.0
0.5
1.0
MR effect size for
'Gut microbiota abundance (genus Parasutterella id.2892)' on 'Complement factor H−related protein 5'

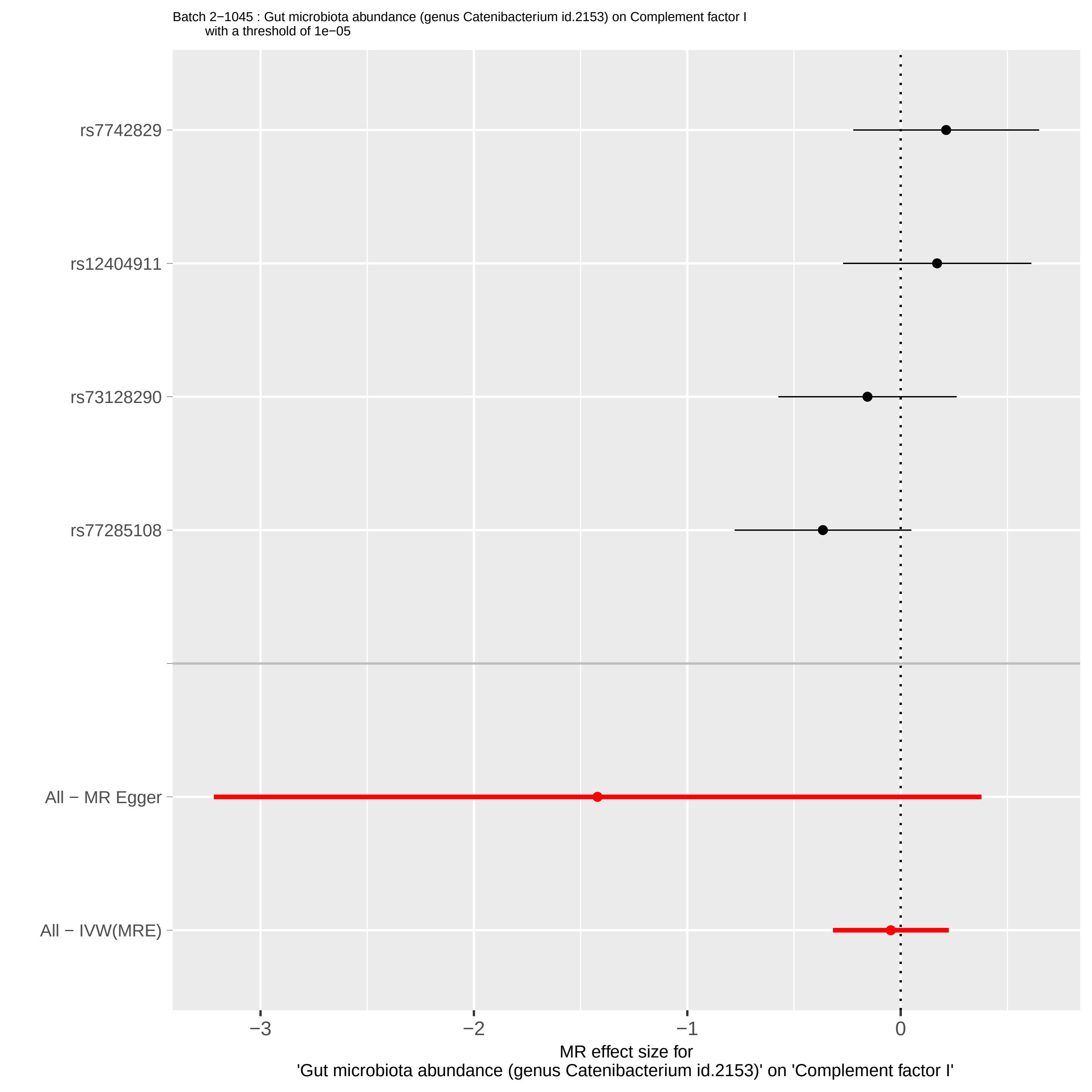

Batch 2−1045 : Gut microbiota abundance (genus Catenibacterium id.2153) on Complement factor I
with a threshold of 1e−05
rs7742829
rs12404911
rs73128290
rs77285108
All − MR Egger
All − IVW(MRE)
−3
−2
−1
0
MR effect size for
'Gut microbiota abundance (genus Catenibacterium id.2153)' on 'Complement factor I'

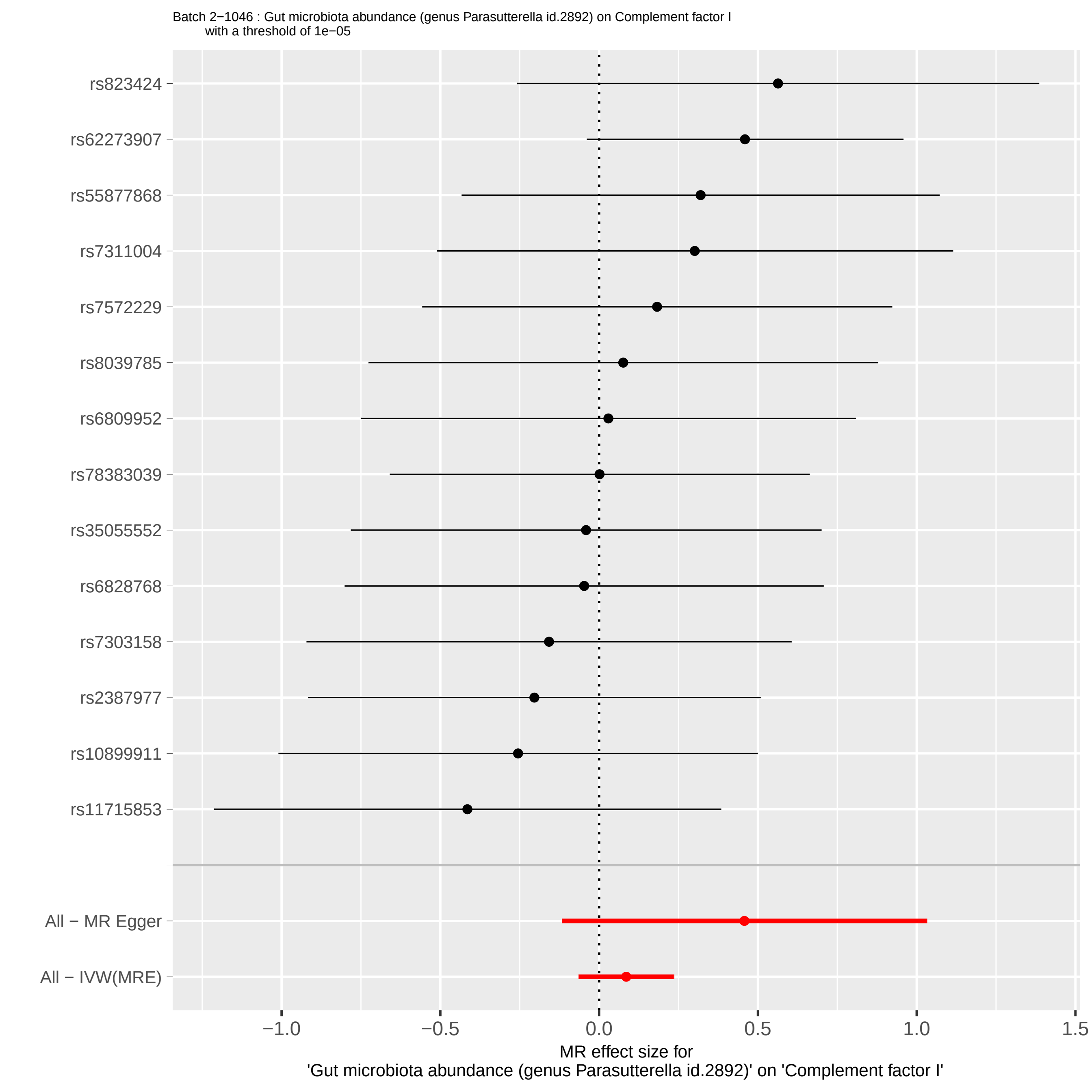

Batch 2−1046 : Gut microbiota abundance (genus Parasutterella id.2892) on Complement factor I
with a threshold of 1e−05
rs823424
rs62273907
rs55877868
rs7311004
rs7572229
rs8039785
rs6809952
rs78383039
rs35055552
rs6828768
rs7303158
rs2387977
rs10899911
rs11715853
All − MR Egger
All − IVW(MRE)
−1.0
−0.5
0.0
0.5
1.0
1.5
MR effect size for
'Gut microbiota abundance (genus Parasutterella id.2892)' on 'Complement factor I'

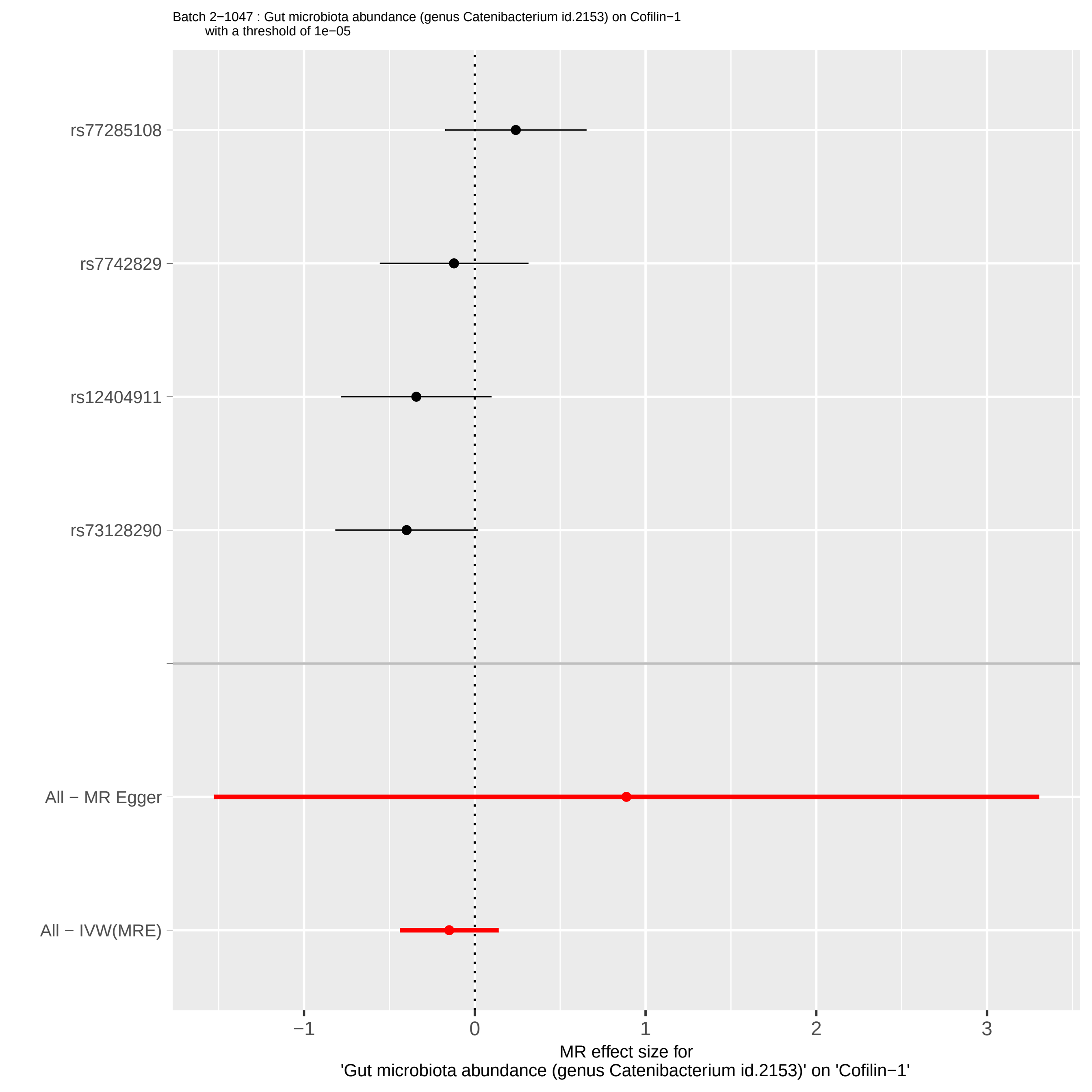

Batch 2−1047 : Gut microbiota abundance (genus Catenibacterium id.2153) on Cofilin−1
with a threshold of 1e−05
rs77285108
rs7742829
rs12404911
rs73128290
All − MR Egger
All − IVW(MRE)
−1
0
1
2
3
MR effect size for
'Gut microbiota abundance (genus Catenibacterium id.2153)' on 'Cofilin−1'

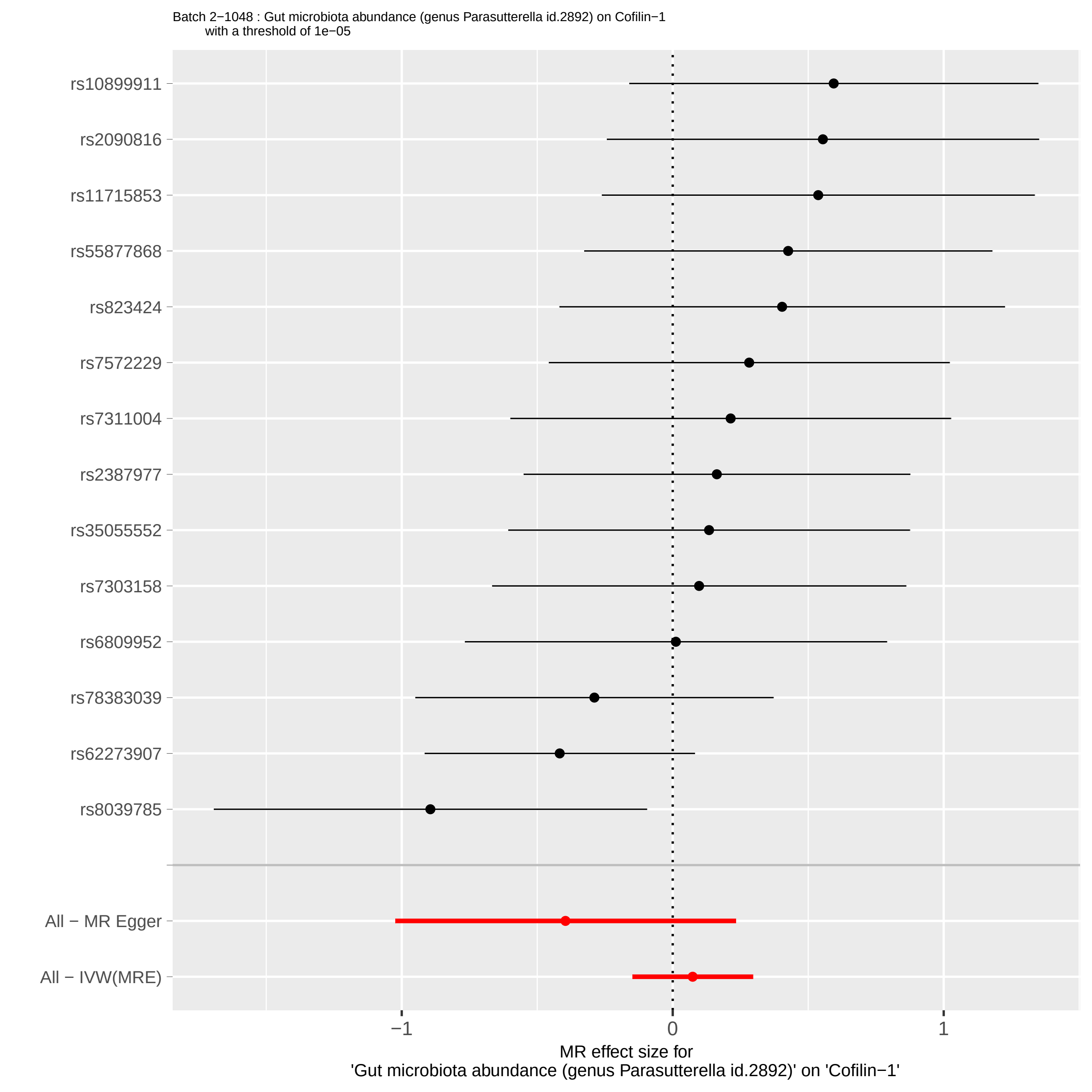
Batch 2−1048 : Gut microbiota abundance (genus Parasutterella id.2892) on Cofilin−1
with a threshold of 1e−05
rs10899911
rs2090816
rs11715853
rs55877868
rs823424
rs7572229
rs7311004
rs2387977
rs35055552
rs7303158
rs6809952
rs78383039
rs62273907
rs8039785
All − MR Egger
All − IVW(MRE)
−1
0
1
MR effect size for
'Gut microbiota abundance (genus Parasutterella id.2892)' on 'Cofilin−1'

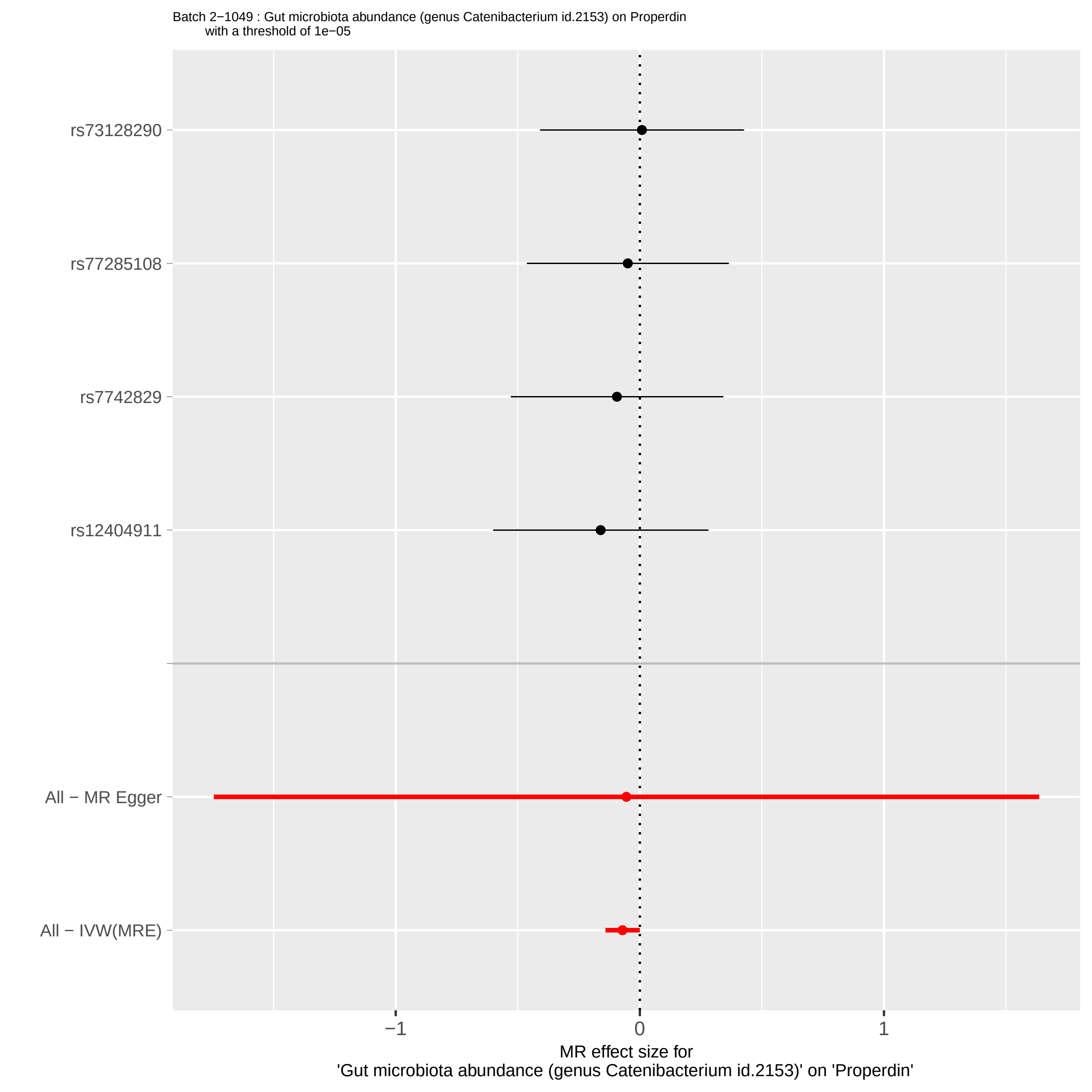

Batch 2−1049 : Gut microbiota abundance (genus Catenibacterium id.2153) on Properdin
with a threshold of 1e−05
rs73128290
rs77285108
rs7742829
rs12404911
All − MR Egger
All − IVW(MRE)
−1
0
1
MR effect size for
'Gut microbiota abundance (genus Catenibacterium id.2153)' on 'Properdin'

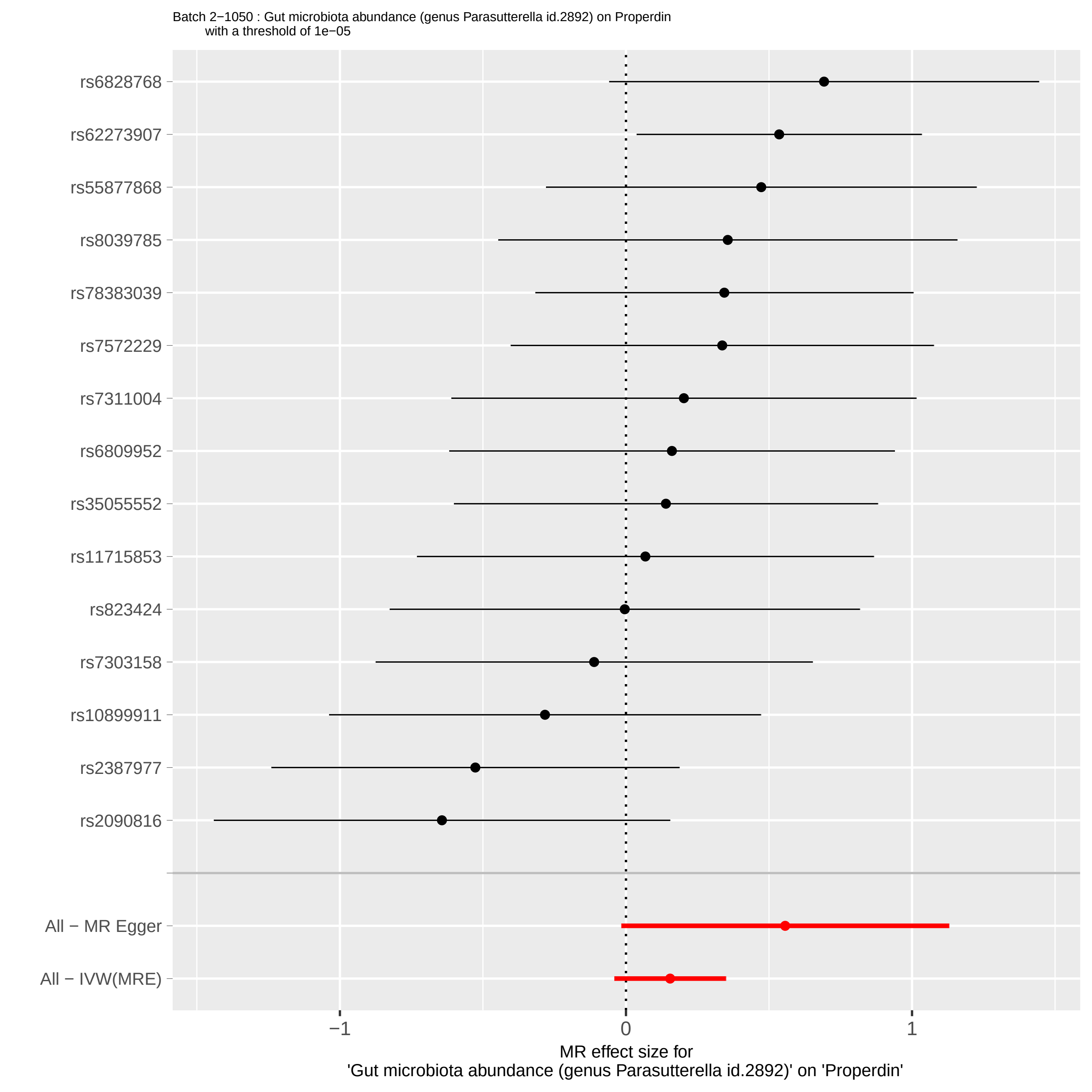

Batch 2−1050 : Gut microbiota abundance (genus Parasutterella id.2892) on Properdin
with a threshold of 1e−05
rs6828768
rs62273907
rs55877868
rs8039785
rs78383039
rs7572229
rs7311004
rs6809952
rs35055552
rs11715853
rs823424
rs7303158
rs10899911
rs2387977
rs2090816
All − MR Egger
All − IVW(MRE)
−1
0
1
MR effect size for
'Gut microbiota abundance (genus Parasutterella id.2892)' on 'Properdin'

Batch 2−1051 : Gut microbiota abundance (genus Catenibacterium id.2153) on Glycoprotein hormones alpha chain
with a threshold of 1e−05

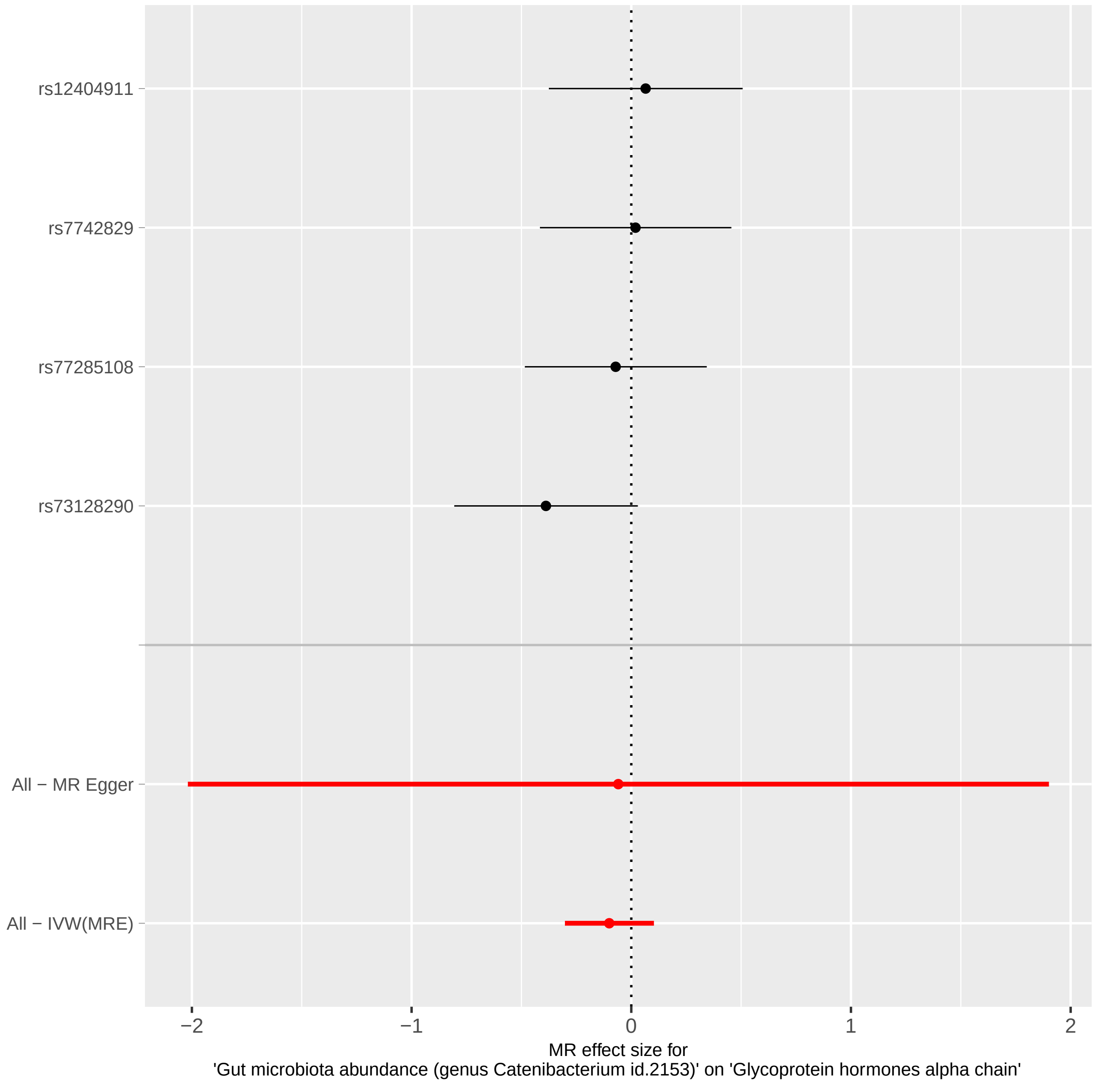

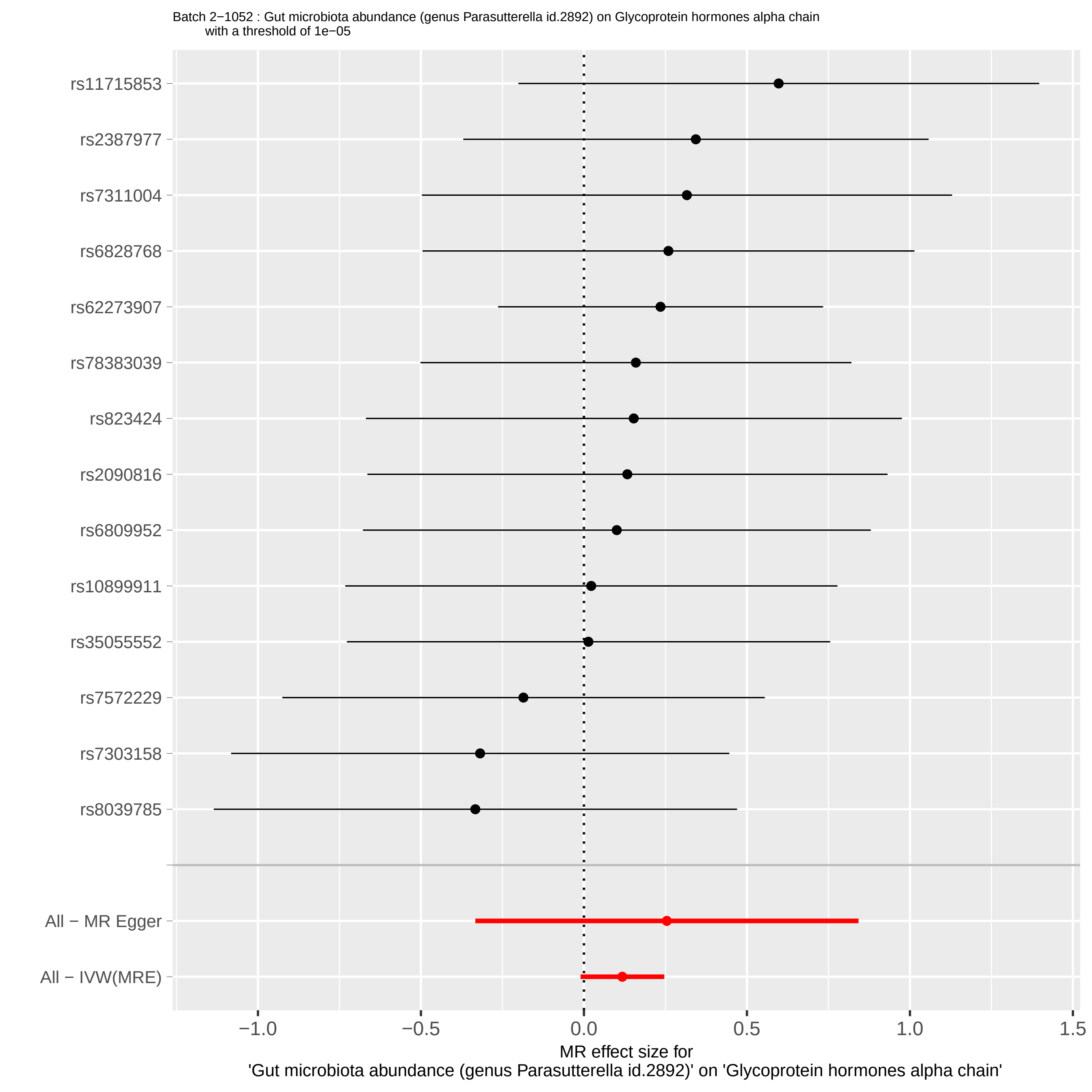

Batch 2−1052 : Gut microbiota abundance (genus Parasutterella id.2892) on Glycoprotein hormones alpha chain
with a threshold of 1e−05
rs11715853
rs2387977
rs7311004
rs6828768
rs62273907
rs78383039
rs823424
rs2090816
rs6809952
rs10899911
rs35055552
rs7572229
rs7303158
rs8039785
All − MR Egger
All − IVW(MRE)
−1.0
−0.5
0.0
0.5
1.0
1.5
MR effect size for
'Gut microbiota abundance (genus Parasutterella id.2892)' on 'Glycoprotein hormones alpha chain'

Batch 2−1053 : Gut microbiota abundance (genus Catenibacterium id.2153) on Human Chorionic Gonadotropin
with a threshold of 1e−05

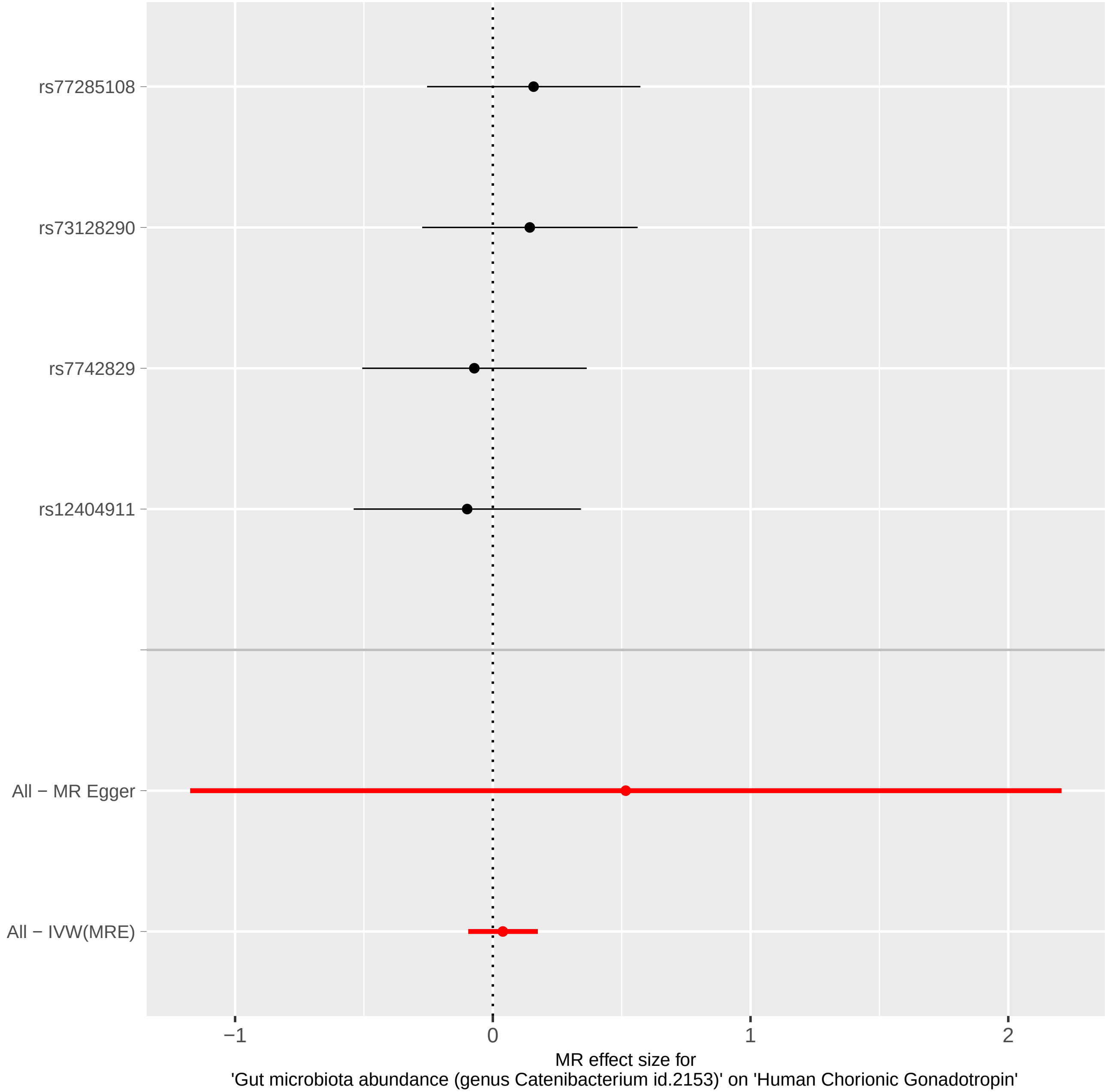

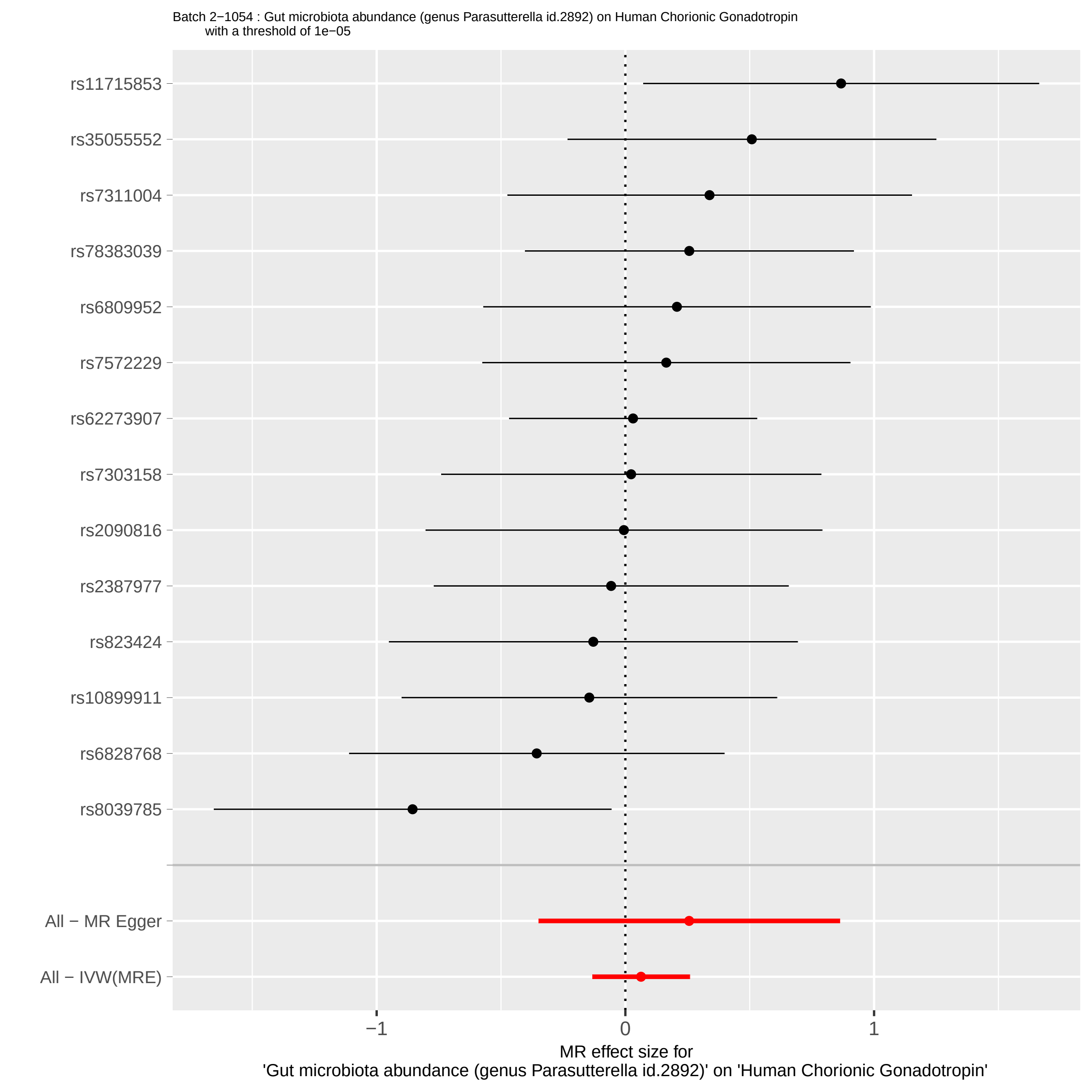

Batch 2−1054 : Gut microbiota abundance (genus Parasutterella id.2892) on Human Chorionic Gonadotropin
with a threshold of 1e−05
rs11715853
rs35055552
rs7311004
rs78383039
rs6809952
rs7572229
rs62273907
rs7303158
rs2090816
rs2387977
rs823424
rs10899911
rs6828768
rs8039785
All − MR Egger
All − IVW(MRE)
−1
0
1
MR effect size for
'Gut microbiota abundance (genus Parasutterella id.2892)' on 'Human Chorionic Gonadotropin'

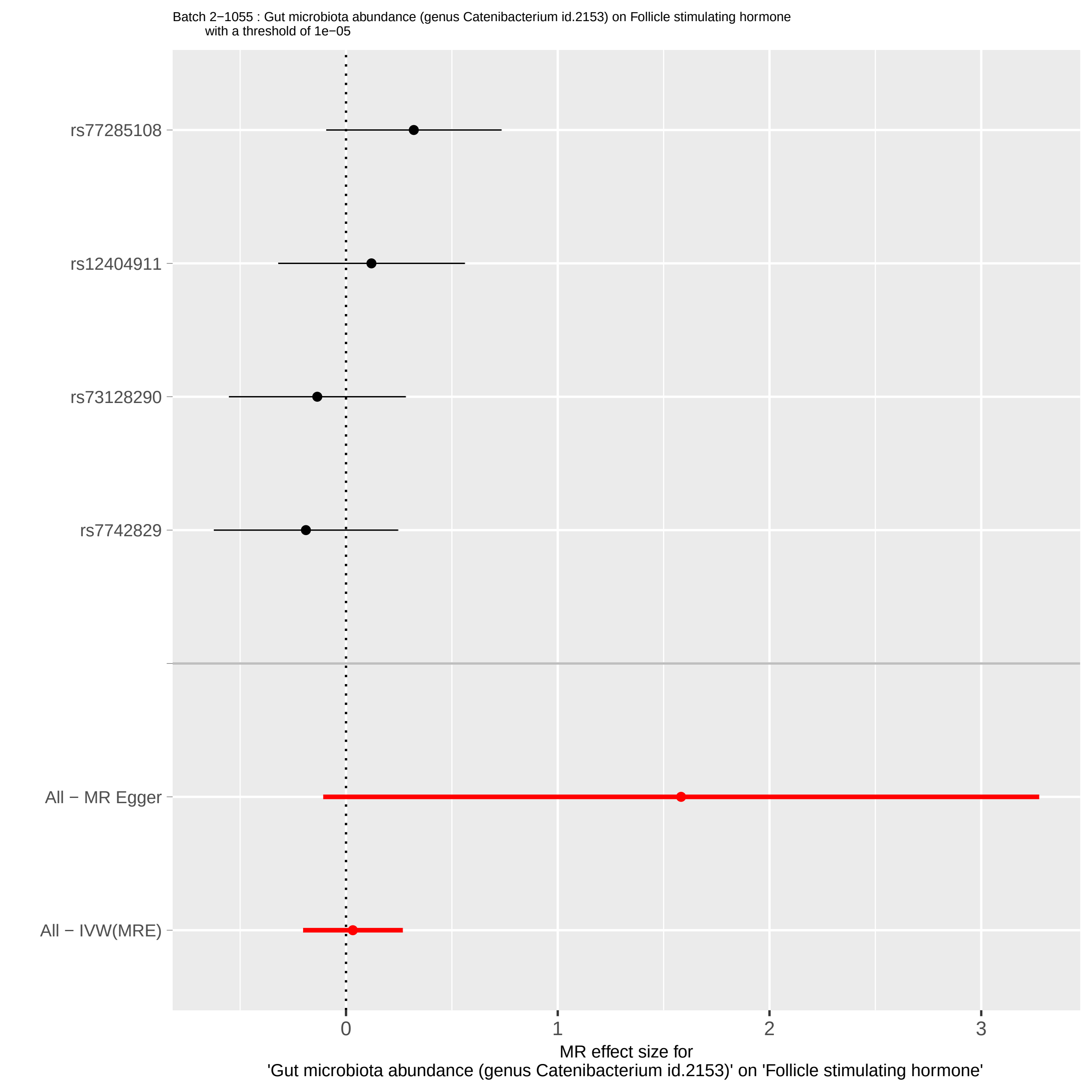

Batch 2−1055 : Gut microbiota abundance (genus Catenibacterium id.2153) on Follicle stimulating hormone
with a threshold of 1e−05
rs77285108
rs12404911
rs73128290
rs7742829
All − MR Egger
All − IVW(MRE)
0
1
2
3
MR effect size for
'Gut microbiota abundance (genus Catenibacterium id.2153)' on 'Follicle stimulating hormone'

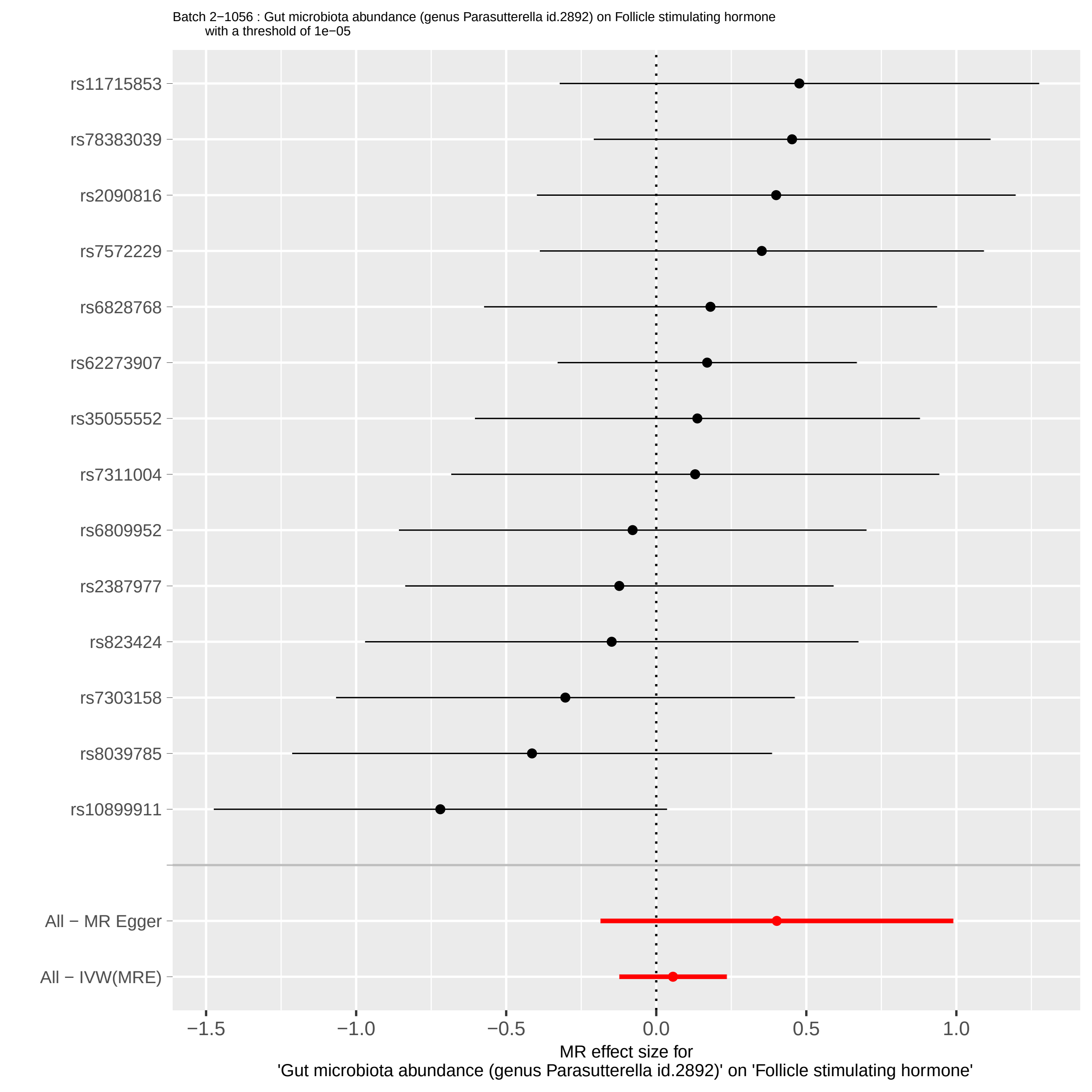
Batch 2−1056 : Gut microbiota abundance (genus Parasutterella id.2892) on Follicle stimulating hormone
with a threshold of 1e−05
rs11715853
rs78383039
rs2090816
rs7572229
rs6828768
rs62273907
rs35055552
rs7311004
rs6809952
rs2387977
rs823424
rs7303158
rs8039785
rs10899911
All − MR Egger
All − IVW(MRE)
−1.5
−1.0
−0.5
0.0
0.5
1.0
MR effect size for
'Gut microbiota abundance (genus Parasutterella id.2892)' on 'Follicle stimulating hormone'

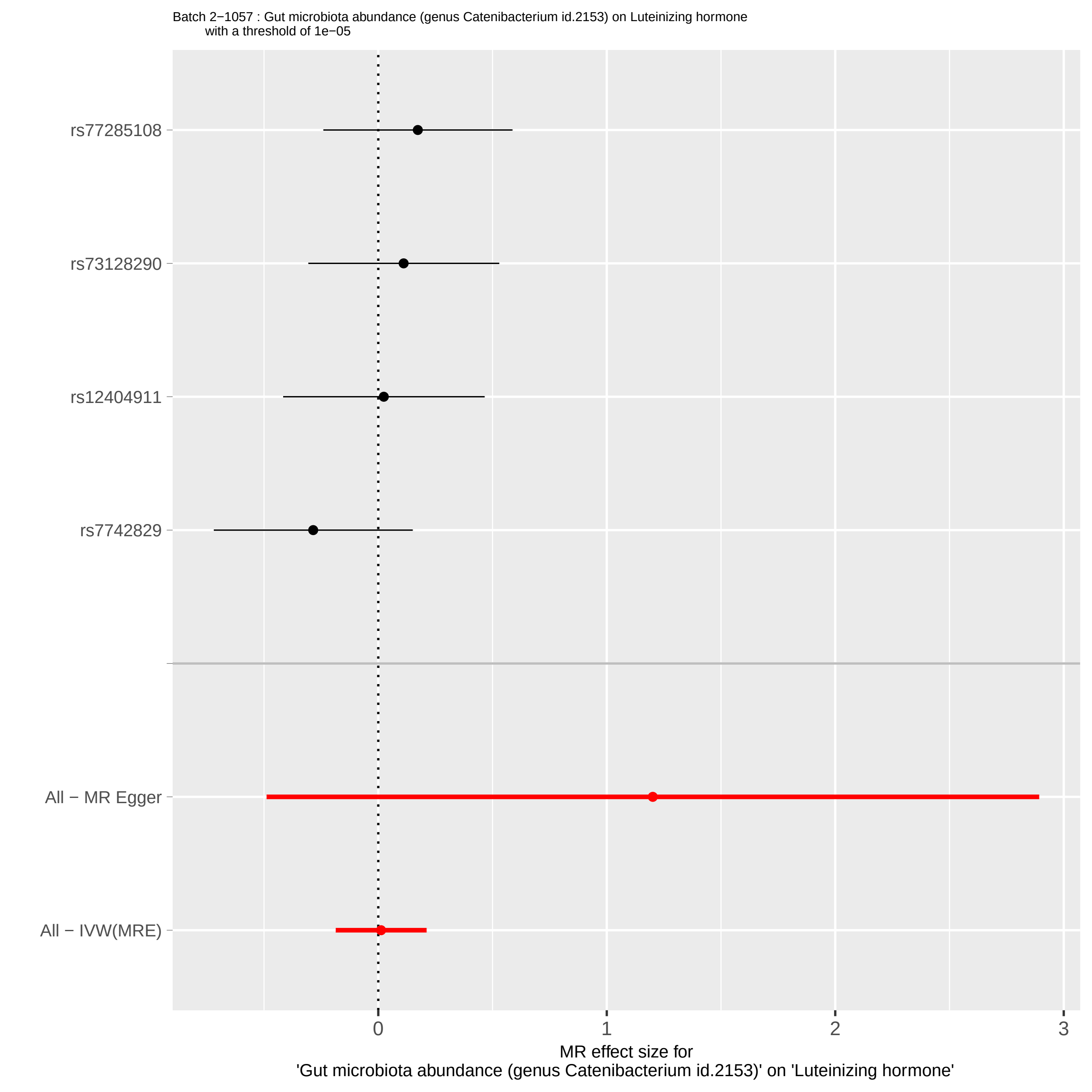

Batch 2−1057 : Gut microbiota abundance (genus Catenibacterium id.2153) on Luteinizing hormone
with a threshold of 1e−05
rs77285108
rs73128290
rs12404911
rs7742829
All − MR Egger
All − IVW(MRE)
0
1
2
3
MR effect size for
'Gut microbiota abundance (genus Catenibacterium id.2153)' on 'Luteinizing hormone'

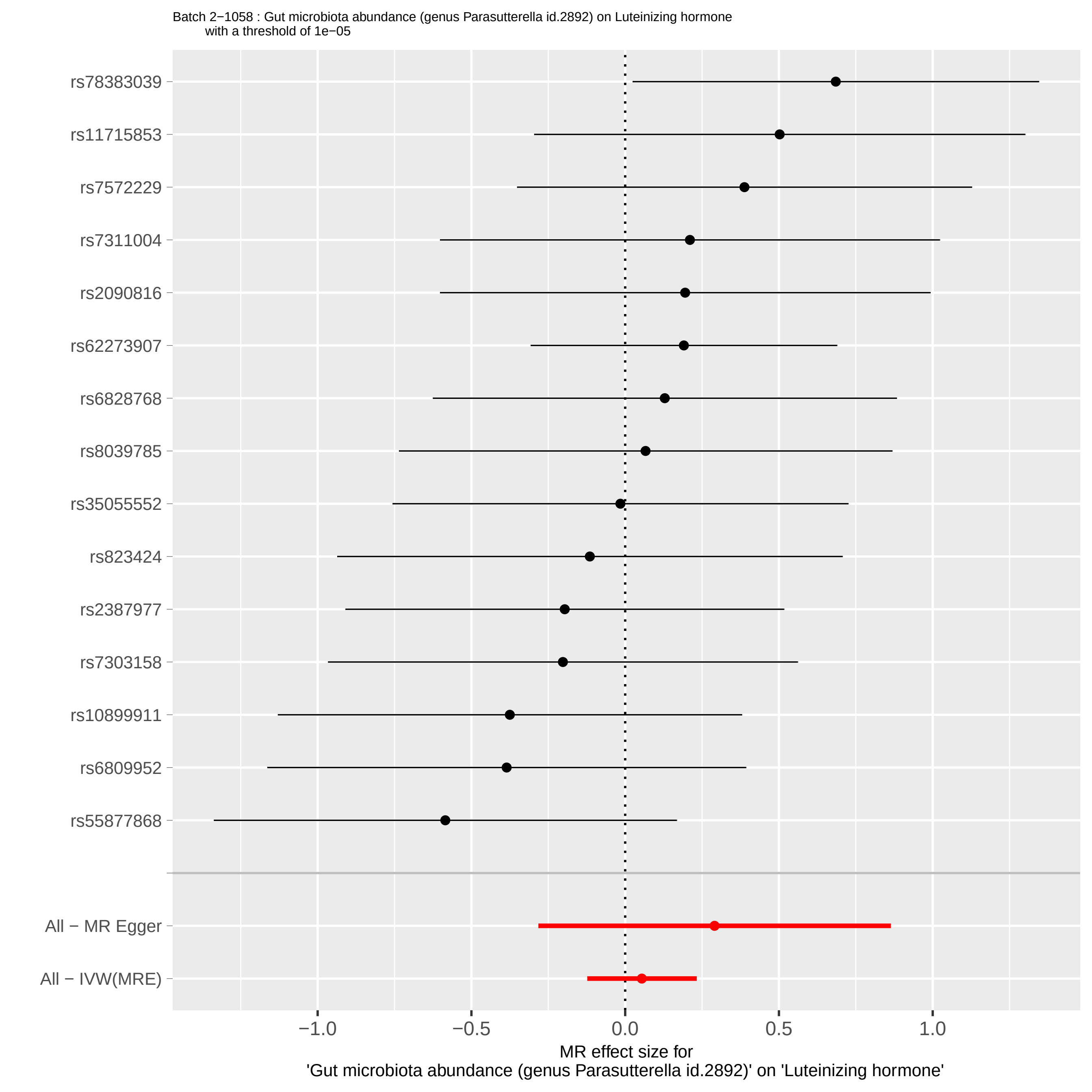
Batch 2−1058 : Gut microbiota abundance (genus Parasutterella id.2892) on Luteinizing hormone
with a threshold of 1e−05
rs78383039
rs11715853
rs7572229
rs7311004
rs2090816
rs62273907
rs6828768
rs8039785
rs35055552
rs823424
rs2387977
rs7303158
rs10899911
rs6809952
rs55877868
All − MR Egger
All − IVW(MRE)
−1.0
−0.5
0.0
0.5
1.0
MR effect size for
'Gut microbiota abundance (genus Parasutterella id.2892)' on 'Luteinizing hormone'

Batch 2−1059 : Gut microbiota abundance (genus Catenibacterium id.2153) on Thyroid Stimulating Hormone
with a threshold of 1e−05

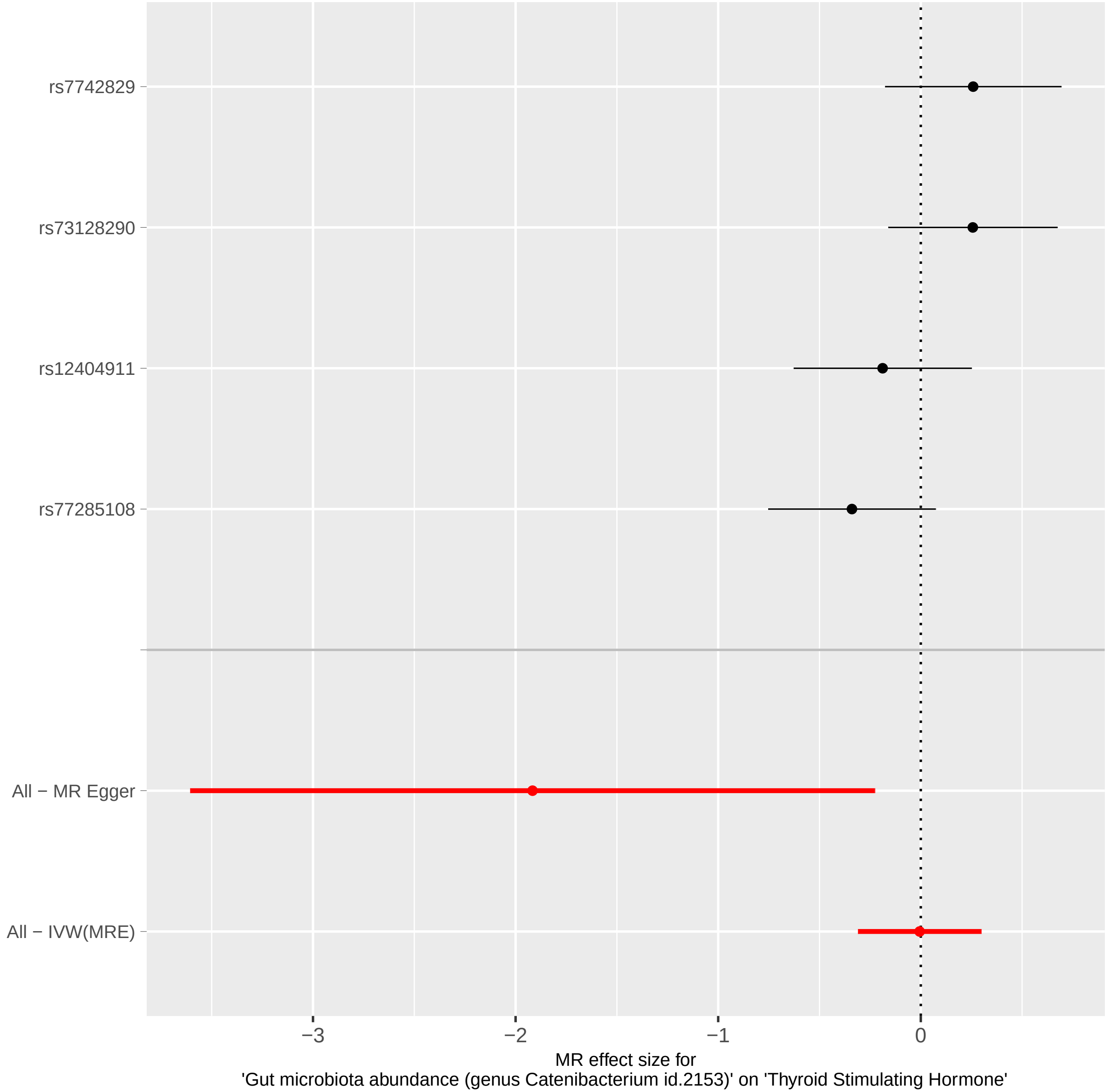

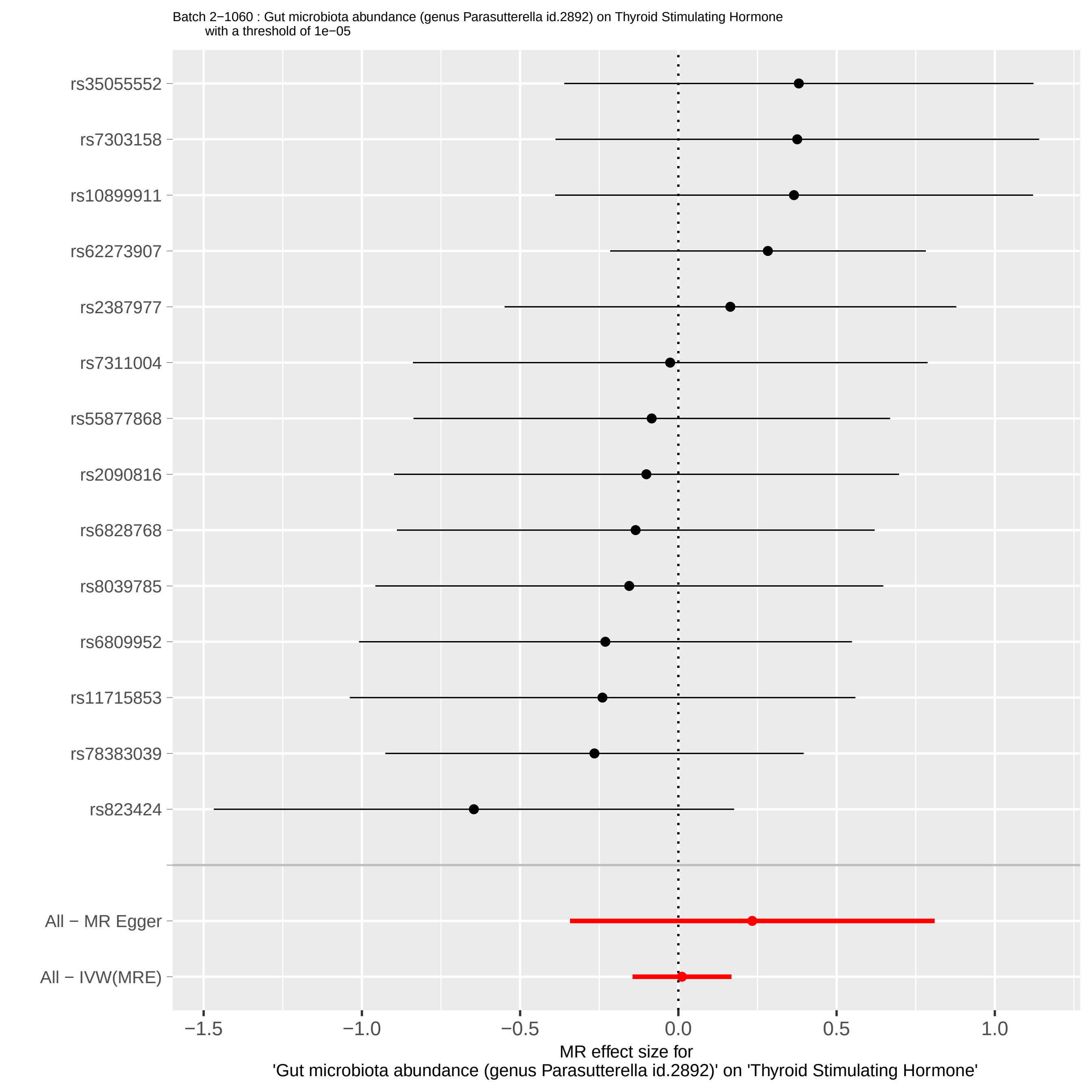

Batch 2−1060 : Gut microbiota abundance (genus Parasutterella id.2892) on Thyroid Stimulating Hormone
with a threshold of 1e−05
rs35055552
rs7303158
rs10899911
rs62273907
rs2387977
rs7311004
rs55877868
rs2090816
rs6828768
rs8039785
rs6809952
rs11715853
rs78383039
rs823424
All − MR Egger
All − IVW(MRE)
−1.5
−1.0
−0.5
0.0
0.5
1.0
MR effect size for
'Gut microbiota abundance (genus Parasutterella id.2892)' on 'Thyroid Stimulating Hormone'

Batch 2−1061 : Gut microbiota abundance (genus Catenibacterium id.2153) on Choriogonadotropin subunit beta variant 2
with a threshold of 1e−05

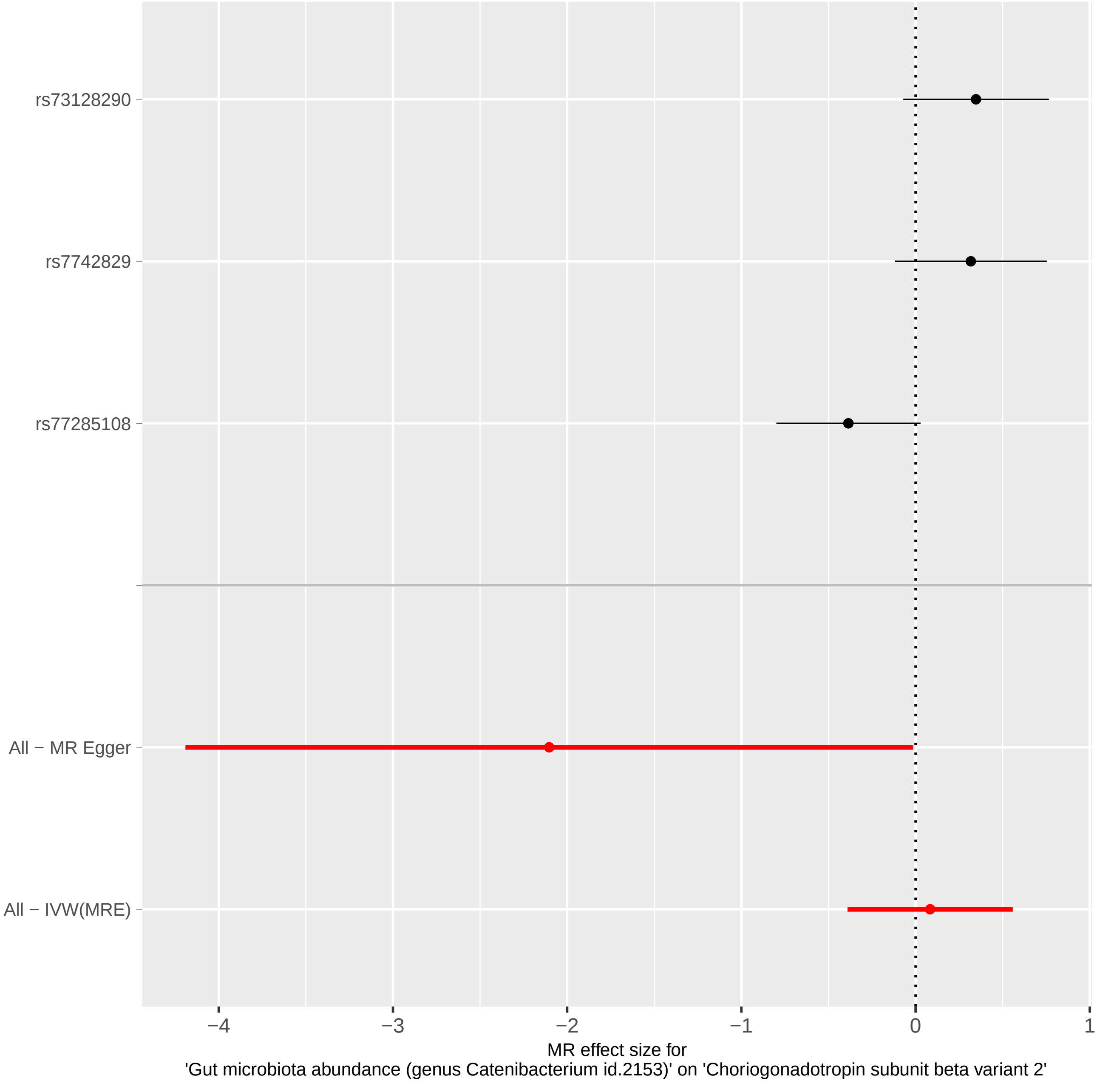

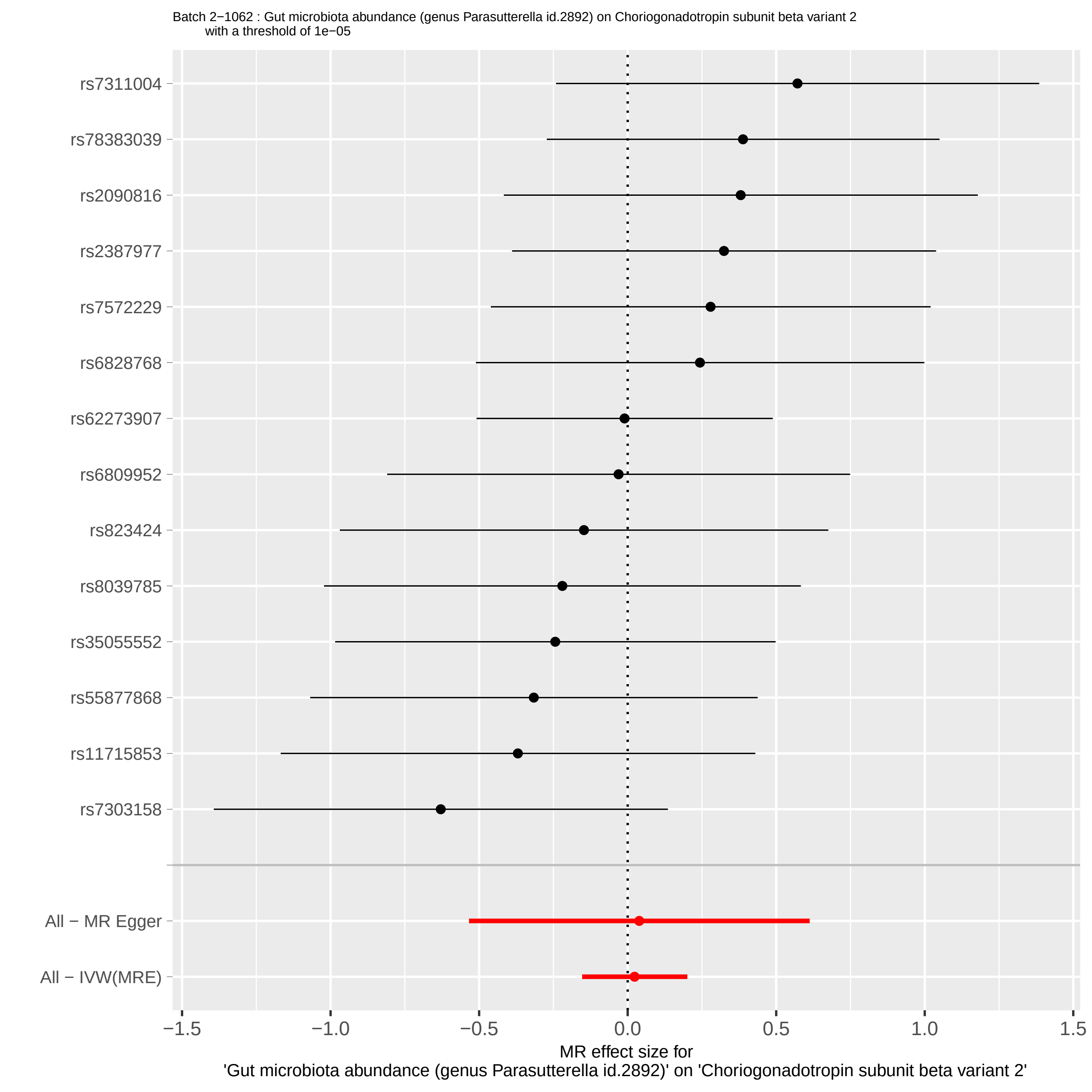

Batch 2−1062 : Gut microbiota abundance (genus Parasutterella id.2892) on Choriogonadotropin subunit beta variant 2
with a threshold of 1e−05
rs7311004
rs78383039
rs2090816
rs2387977
rs7572229
rs6828768
rs62273907
rs6809952
rs823424
rs8039785
rs35055552
rs55877868
rs11715853
rs7303158
All − MR Egger
All − IVW(MRE)
−1.5
−1.0
−0.5
0.0
0.5
1.0
1.5
MR effect size for
'Gut microbiota abundance (genus Parasutterella id.2892)' on 'Choriogonadotropin subunit beta variant 2'

Batch 2−1063 : Gut microbiota abundance (genus Catenibacterium id.2153) on Cell growth regulator with EF hand domain protein 1
with a threshold of 1e−05

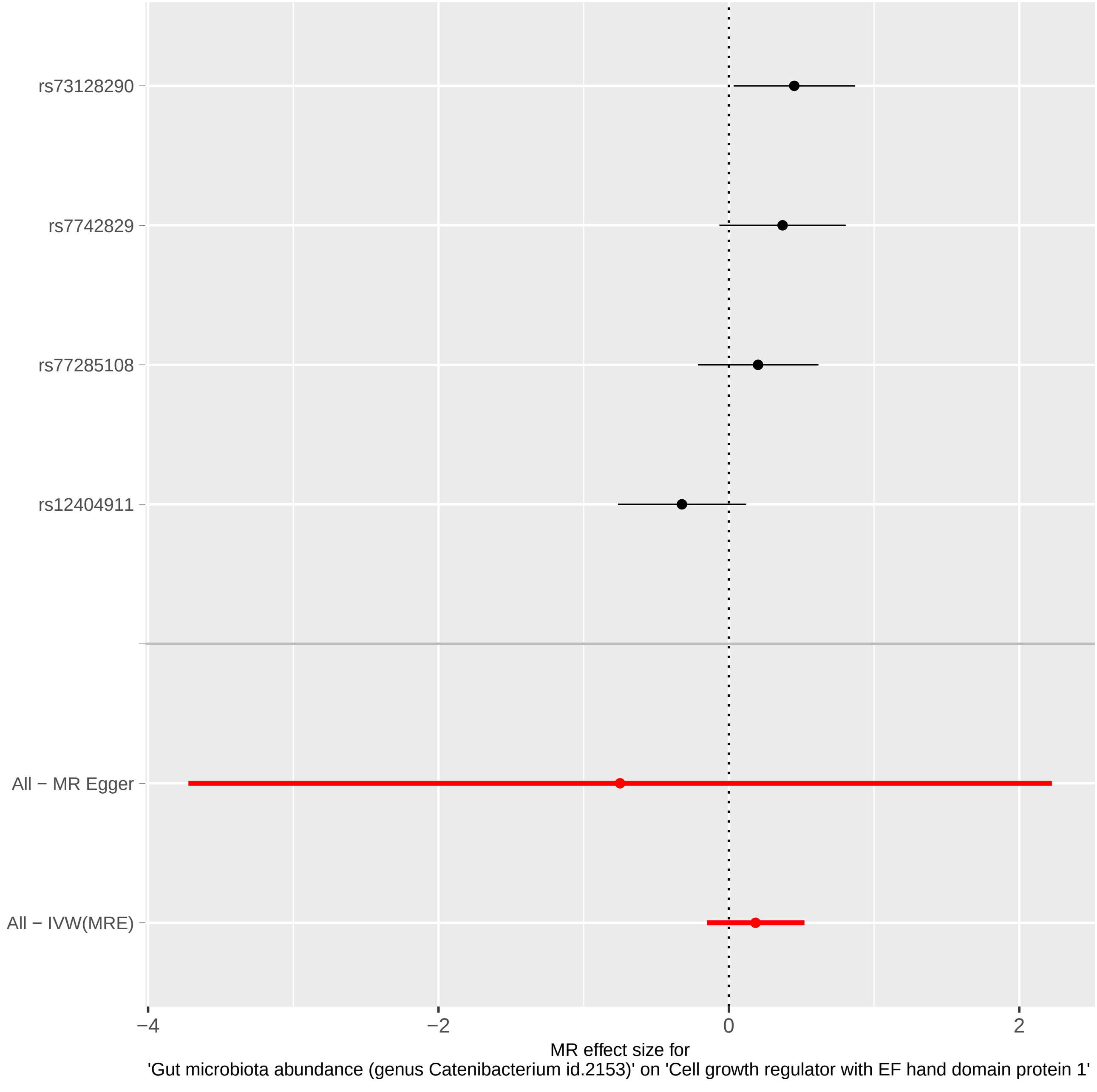

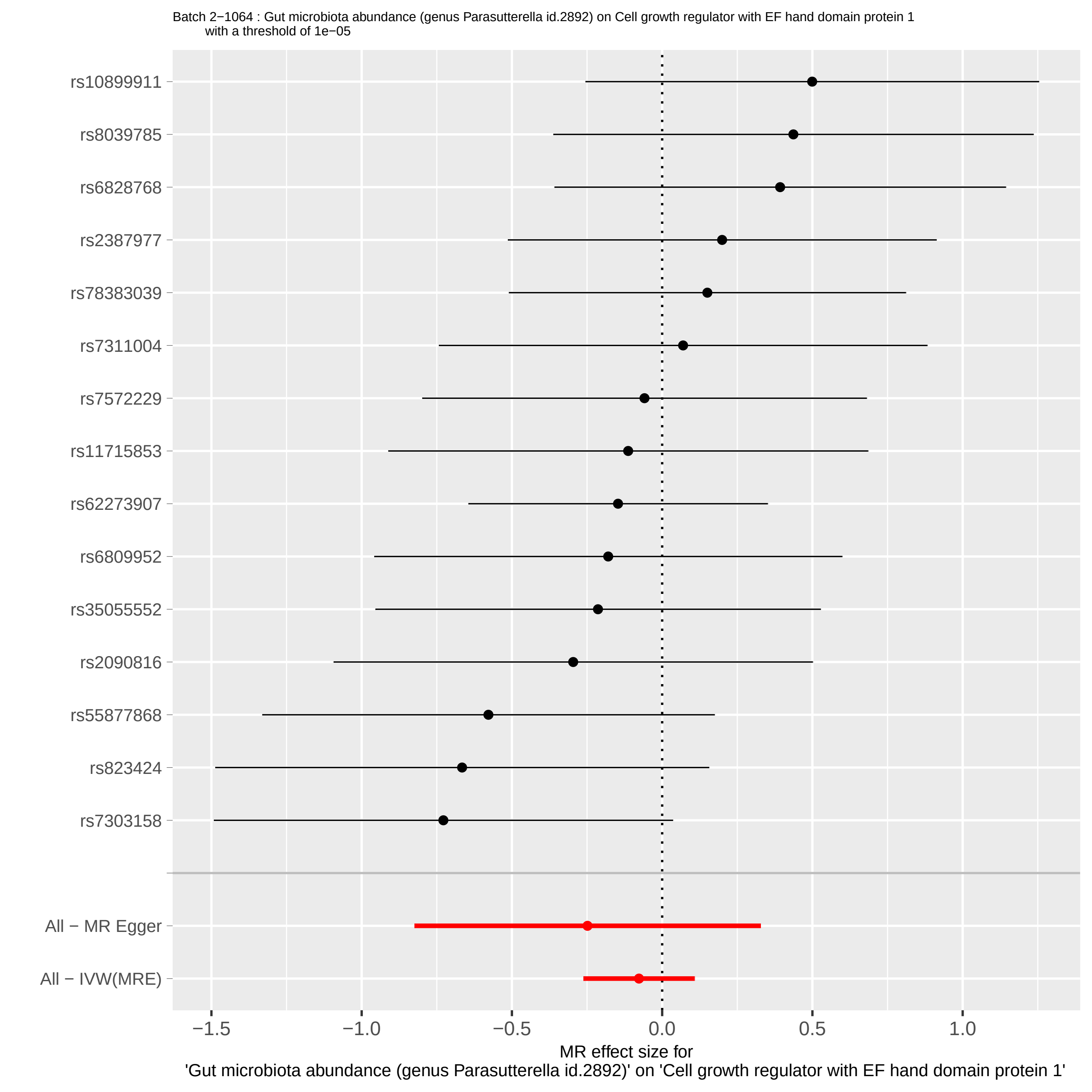
Batch 2−1064 : Gut microbiota abundance (genus Parasutterella id.2892) on Cell growth regulator with EF hand domain protein 1 with a threshold of 1e−05
rs10899911
rs8039785
rs6828768
rs2387977
rs78383039
rs7311004
rs7572229
rs11715853
rs62273907
rs6809952
rs35055552
rs2090816
rs55877868
rs823424
rs7303158
All − MR Egger
All − IVW(MRE)
−1.5
−1.0
−0.5
0.0
0.5
1.0
MR effect size for
'Gut microbiota abundance (genus Parasutterella id.2892)' on 'Cell growth regulator with EF hand domain protein 1'

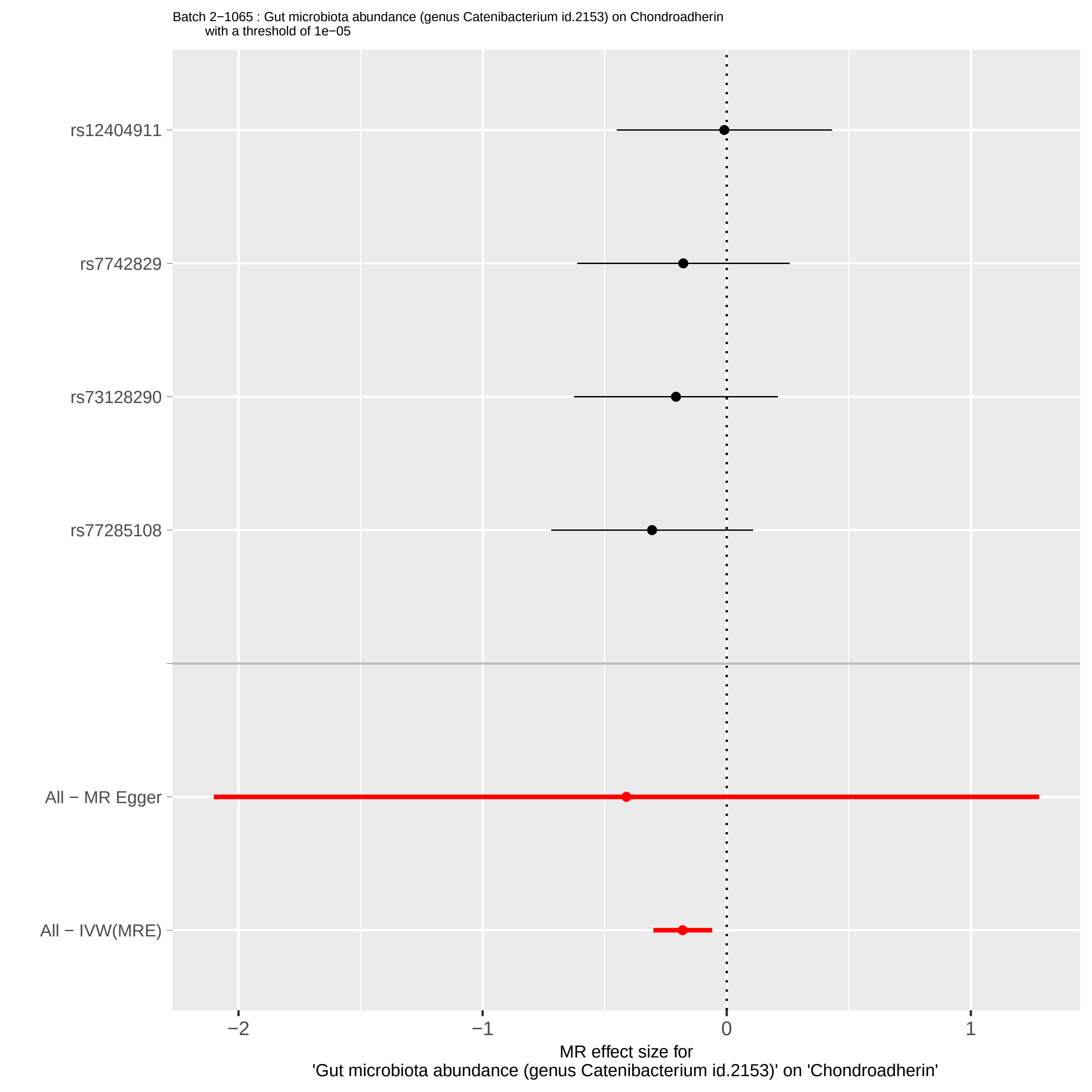

Batch 2−1065 : Gut microbiota abundance (genus Catenibacterium id.2153) on Chondroadherin
with a threshold of 1e−05
rs12404911
rs7742829
rs73128290
rs77285108
All − MR Egger
All − IVW(MRE)
−2
−1
0
1
MR effect size for
'Gut microbiota abundance (genus Catenibacterium id.2153)' on 'Chondroadherin'

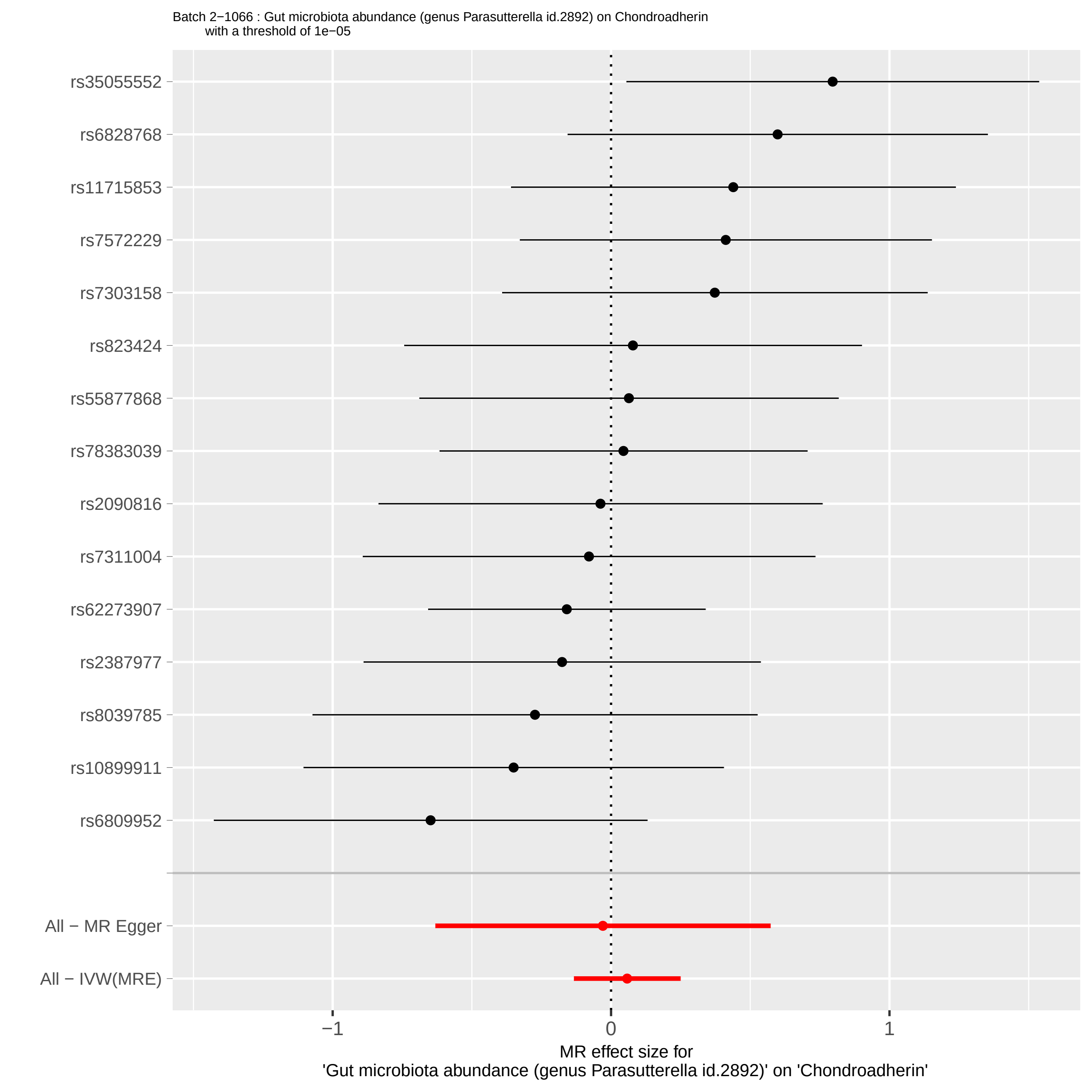
Batch 2−1066 : Gut microbiota abundance (genus Parasutterella id.2892) on Chondroadherin
with a threshold of 1e−05
rs35055552
rs6828768
rs11715853
rs7572229
rs7303158
rs823424
rs55877868
rs78383039
rs2090816
rs7311004
rs62273907
rs2387977
rs8039785
rs10899911
rs6809952
All − MR Egger
All − IVW(MRE)
−1
0
1
MR effect size for
'Gut microbiota abundance (genus Parasutterella id.2892)' on 'Chondroadherin'

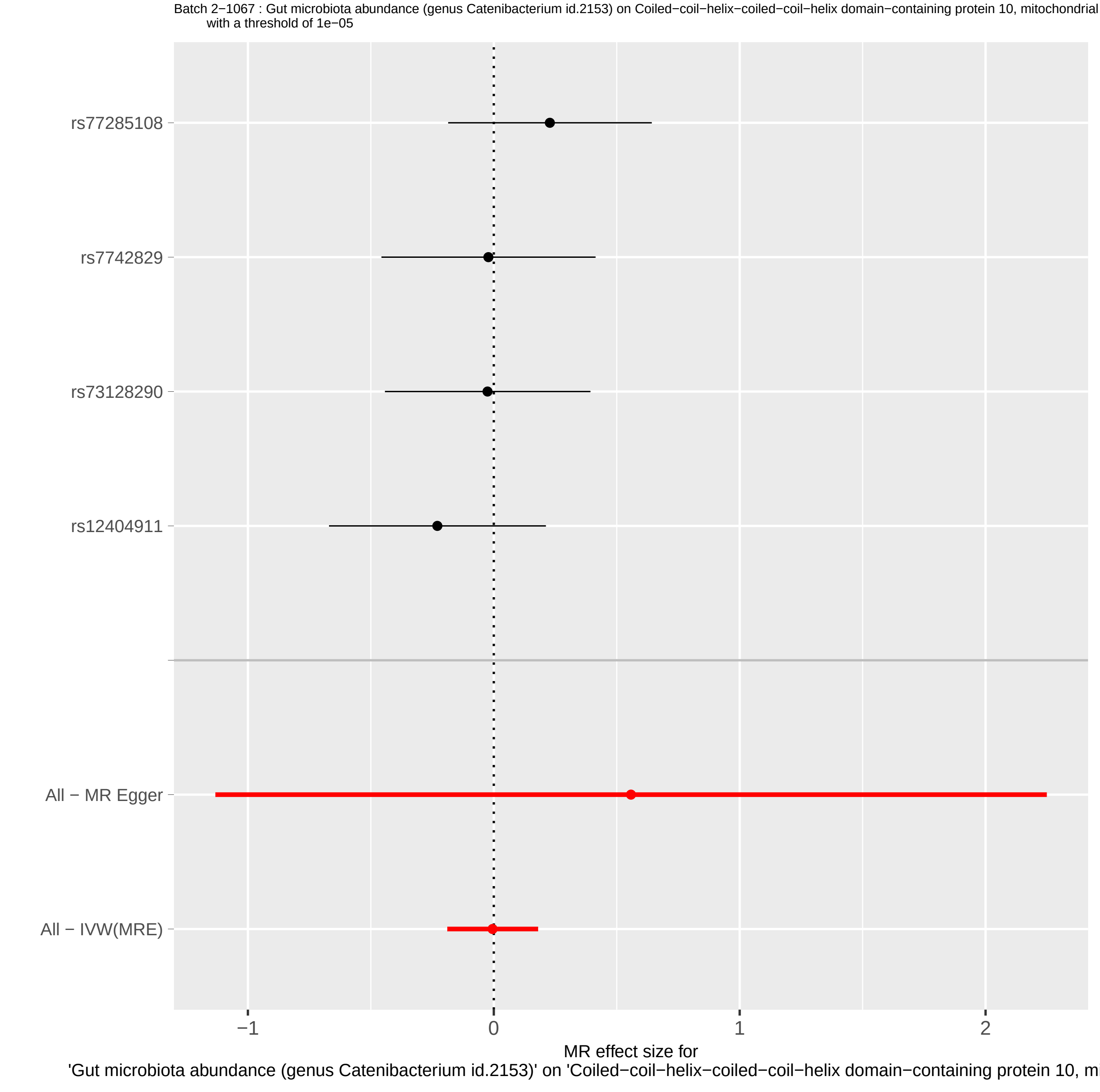
Batch 2−1067 : Gut microbiota abundance (genus Catenibacterium id.2153) on Coiled−coil−helix−coiled−coil−helix domain−containing protein 10, mitochondrial
with a threshold of 1e−05
rs77285108
rs7742829
rs73128290
rs12404911
All − MR Egger
All − IVW(MRE)
−1
0
1
2
MR effect size for
'Gut microbiota abundance (genus Catenibacterium id.2153)' on 'Coiled−coil−helix−coiled−coil−helix domain−containing protein 10, m

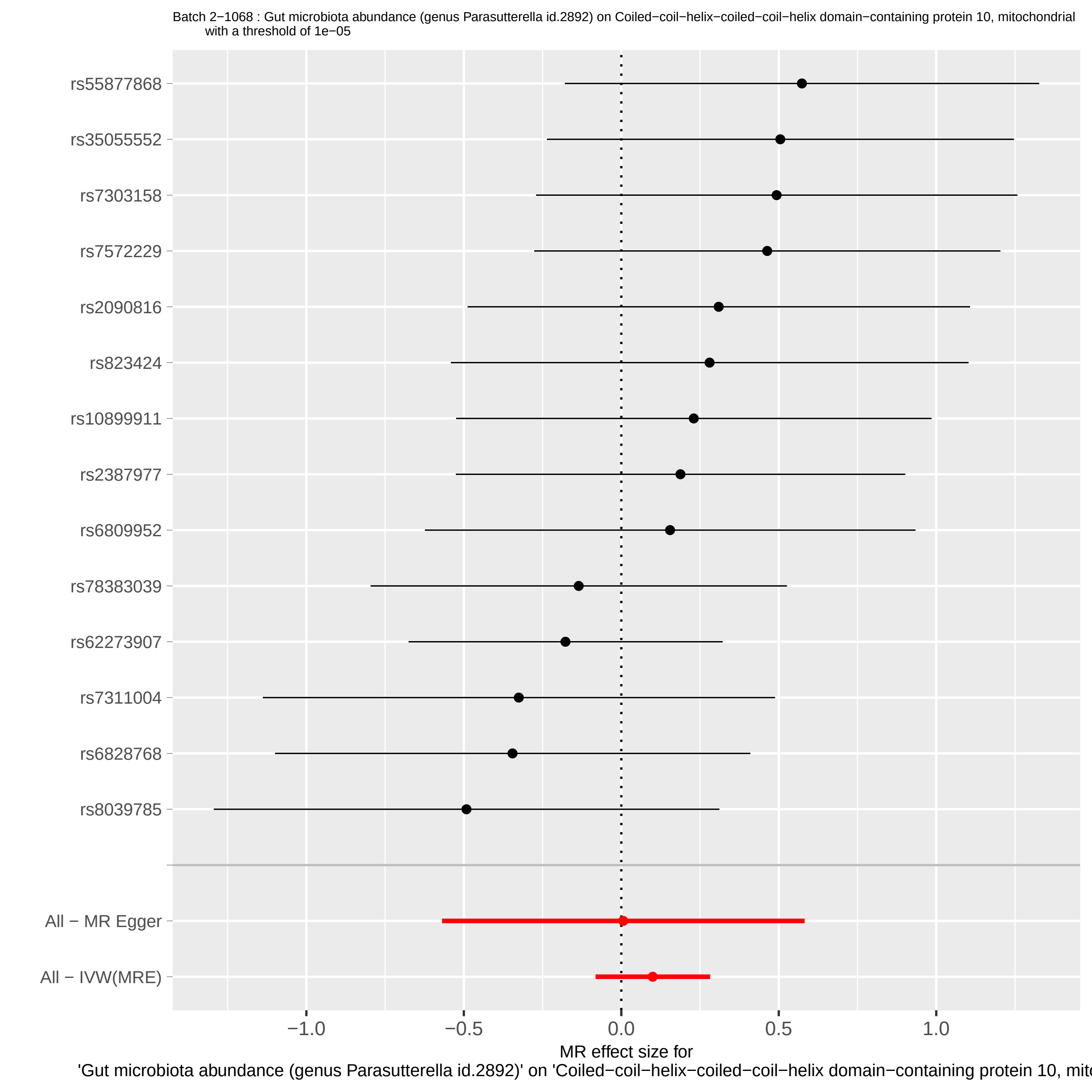
Batch 2−1068 : Gut microbiota abundance (genus Parasutterella id.2892) on Coiled−coil−helix−coiled−coil−helix domain−containing protein 10, mitochondrial
with a threshold of 1e−05
rs55877868
rs35055552
rs7303158
rs7572229
rs2090816
rs823424
rs10899911
rs2387977
rs6809952
rs78383039
rs62273907
rs7311004
rs6828768
rs8039785
All − MR Egger
All − IVW(MRE)
−1.0
−0.5
0.0
0.5
1.0
MR effect size for
'Gut microbiota abundance (genus Parasutterella id.2892)' on 'Coiled−coil−helix−coiled−coil−helix domain−containing protein 10, mito

Batch 2−1069 : Gut microbiota abundance (genus Catenibacterium id.2153) on Coiled−coil−helix−coiled−coil−helix domain−containing protein 2
with a threshold of 1e−05

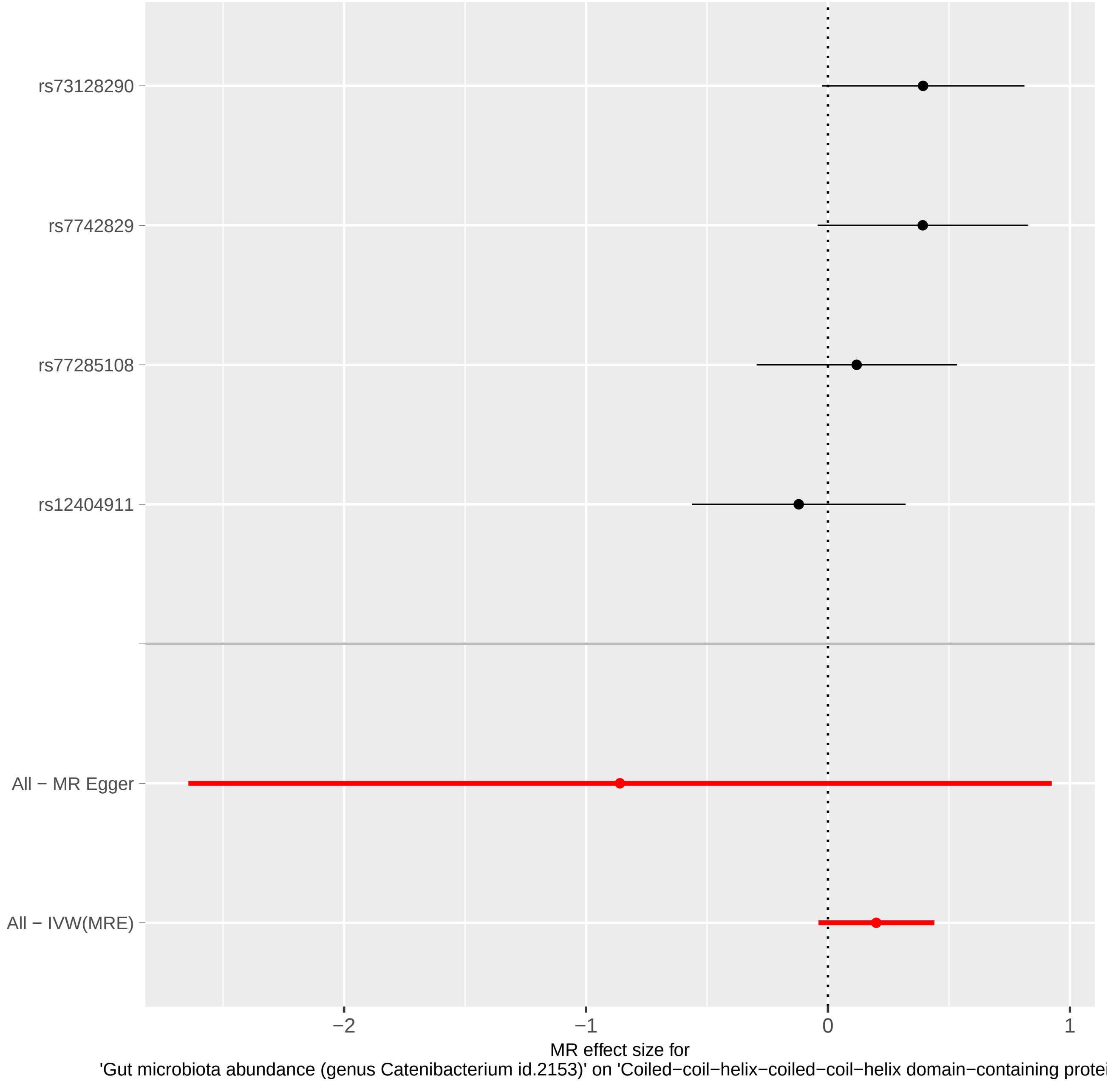

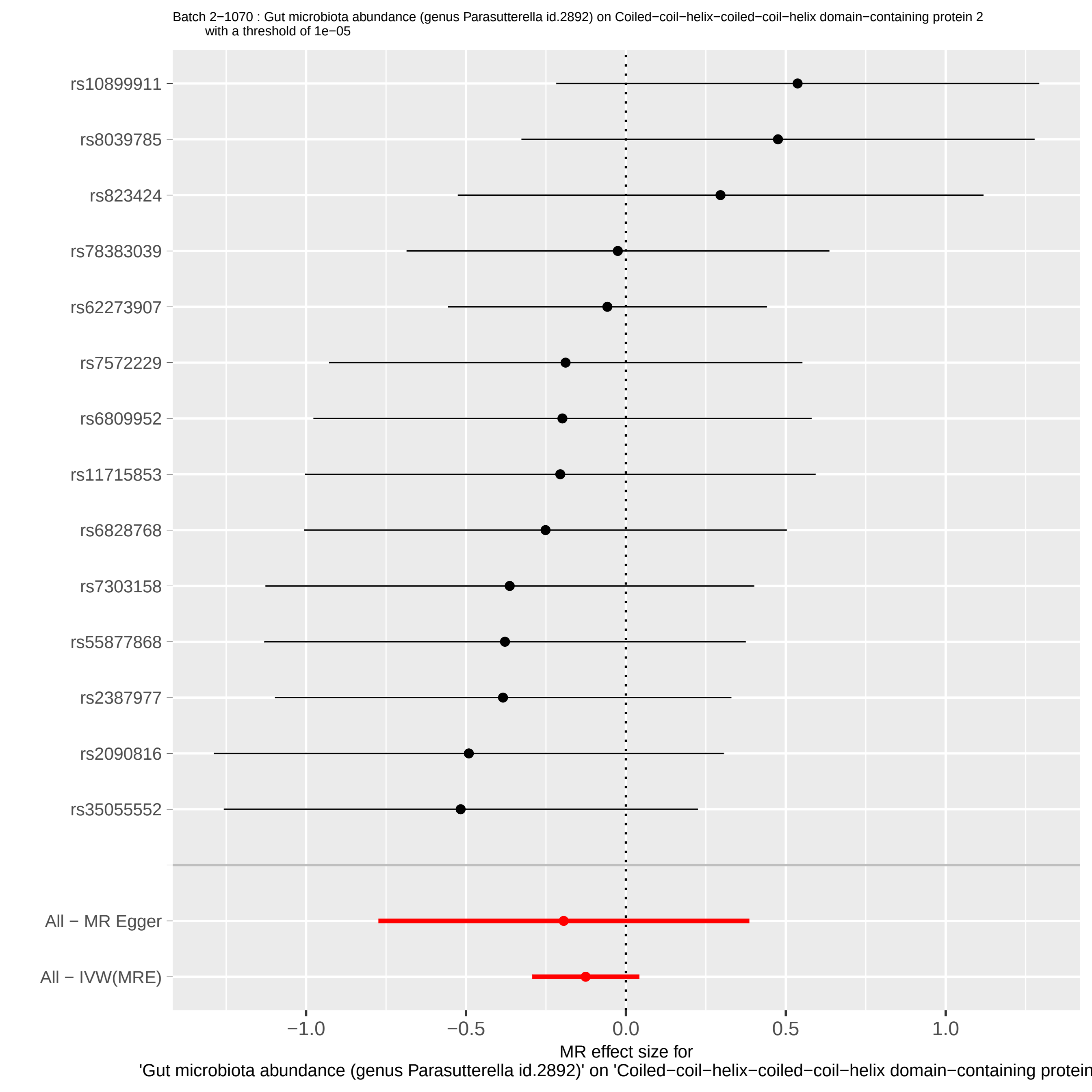

Batch 2−1070 : Gut microbiota abundance (genus Parasutterella id.2892) on Coiled−coil−helix−coiled−coil−helix domain−containing protein 2
with a threshold of 1e−05
rs10899911
rs8039785
rs823424
rs78383039
rs62273907
rs7572229
rs6809952
rs11715853
rs6828768
rs7303158
rs55877868
rs2387977
rs2090816
rs35055552
All − MR Egger
All − IVW(MRE)
−1.0
−0.5
0.0
0.5
1.0
MR effect size for
'Gut microbiota abundance (genus Parasutterella id.2892)' on 'Coiled−coil−helix−coiled−coil−helix domain−containing protein

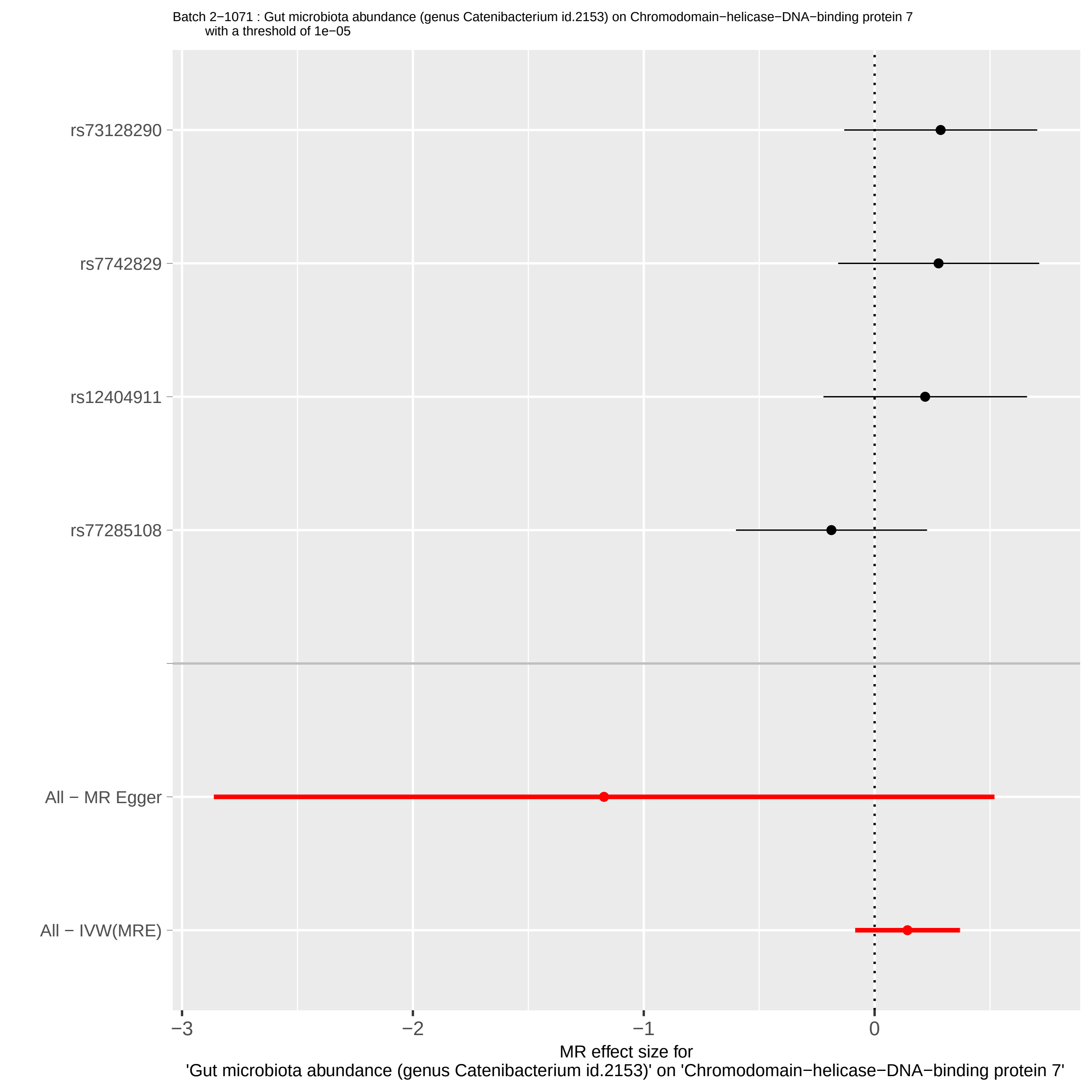

Batch 2−1071 : Gut microbiota abundance (genus Catenibacterium id.2153) on Chromodomain−helicase−DNA−binding protein 7
with a threshold of 1e−05
rs73128290
rs7742829
rs12404911
rs77285108
All − MR Egger
All − IVW(MRE)
−3
−2
−1
0
MR effect size for
'Gut microbiota abundance (genus Catenibacterium id.2153)' on 'Chromodomain−helicase−DNA−binding protein 7'

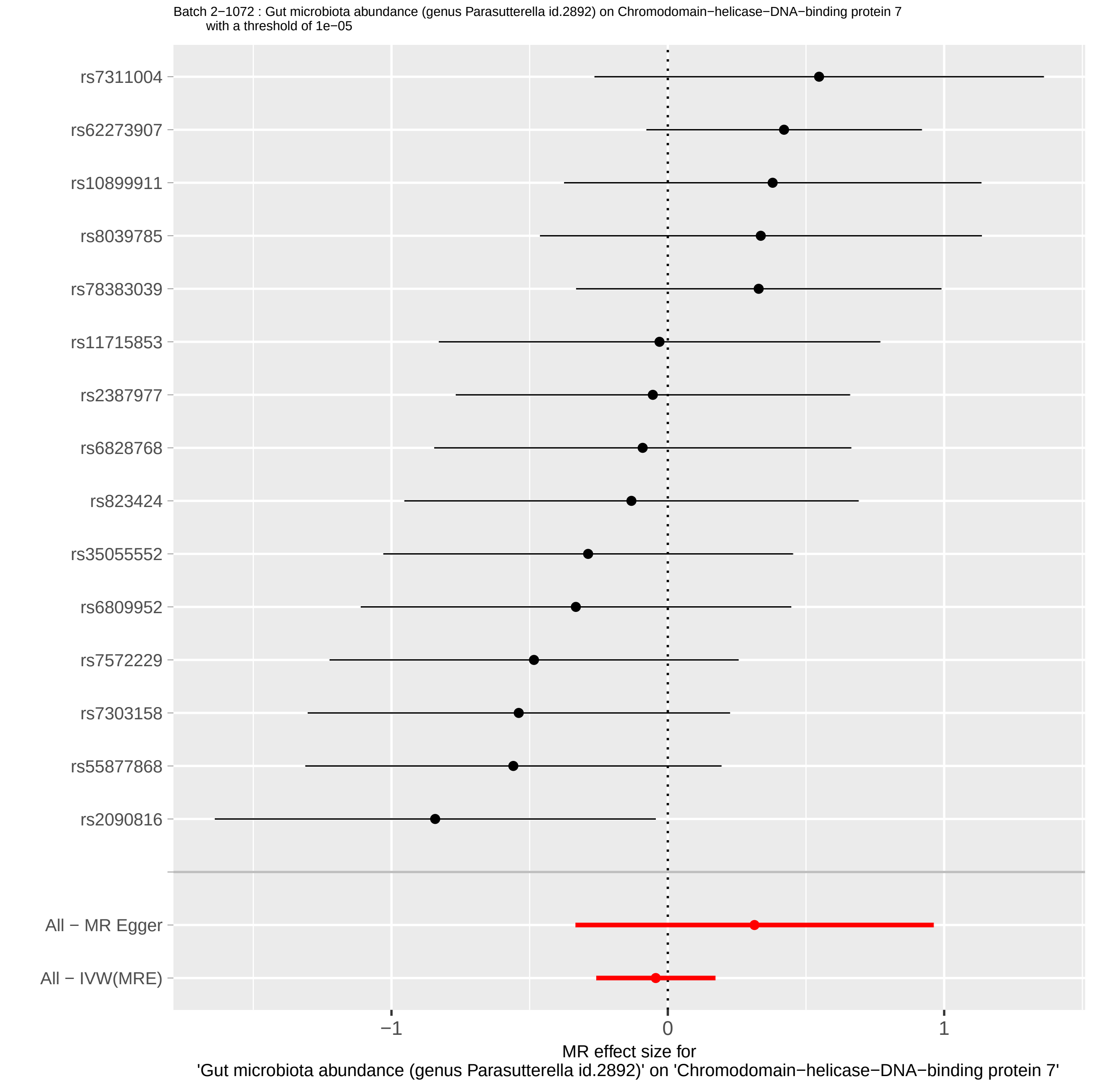
Batch 2−1072 : Gut microbiota abundance (genus Parasutterella id.2892) on Chromodomain−helicase−DNA−binding protein 7
with a threshold of 1e−05
rs7311004
rs62273907
rs10899911
rs8039785
rs78383039
rs11715853
rs2387977
rs6828768
rs823424
rs35055552
rs6809952
rs7572229
rs7303158
rs55877868
rs2090816
All − MR Egger
All − IVW(MRE)
−1
0
1
MR effect size for
'Gut microbiota abundance (genus Parasutterella id.2892)' on 'Chromodomain−helicase−DNA−binding protein 7'

Batch 2−1073 : Gut microbiota abundance (genus Catenibacterium id.2153) on Serine/threonine−protein kinase Chk1
with a threshold of 1e−05

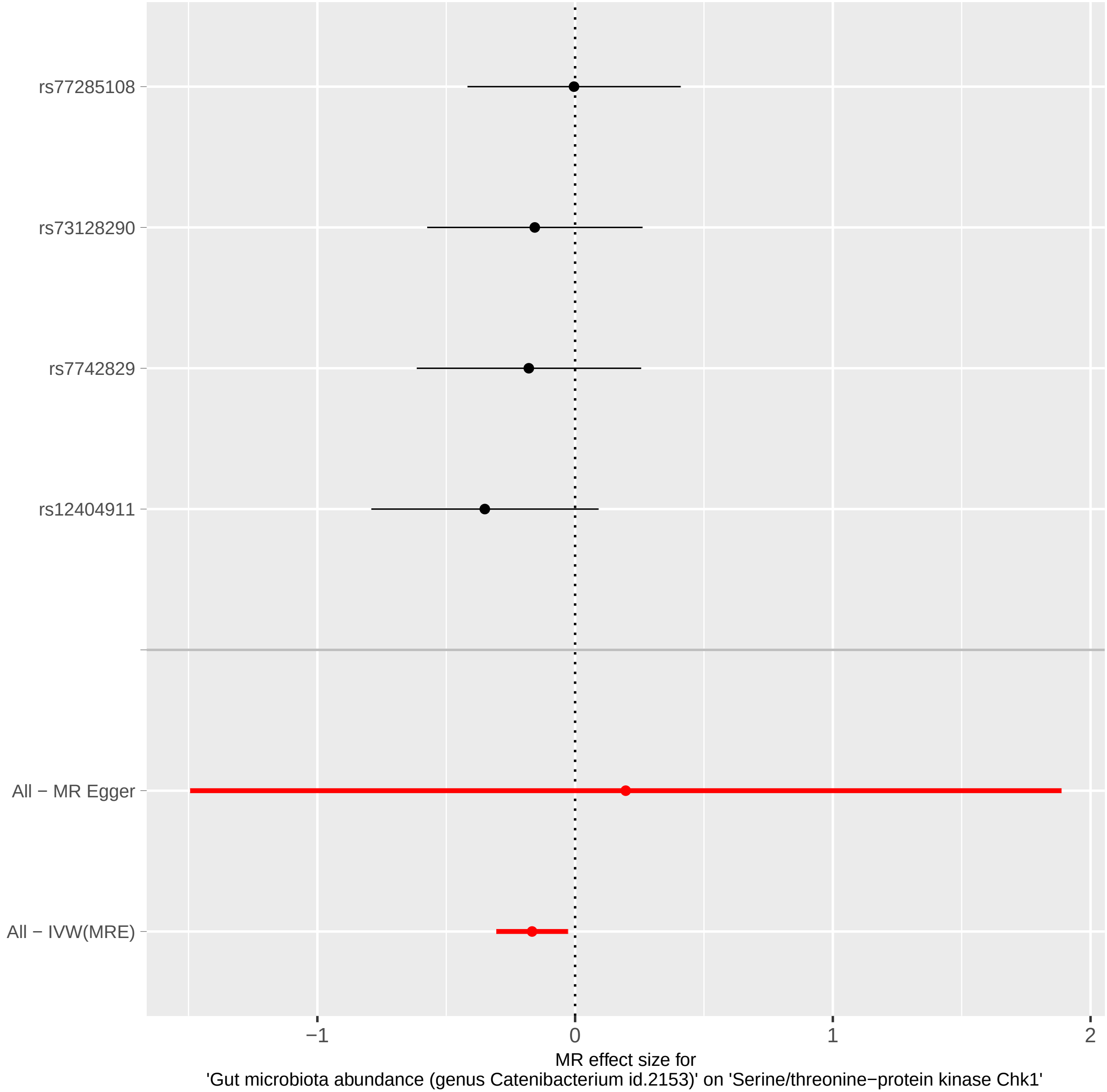

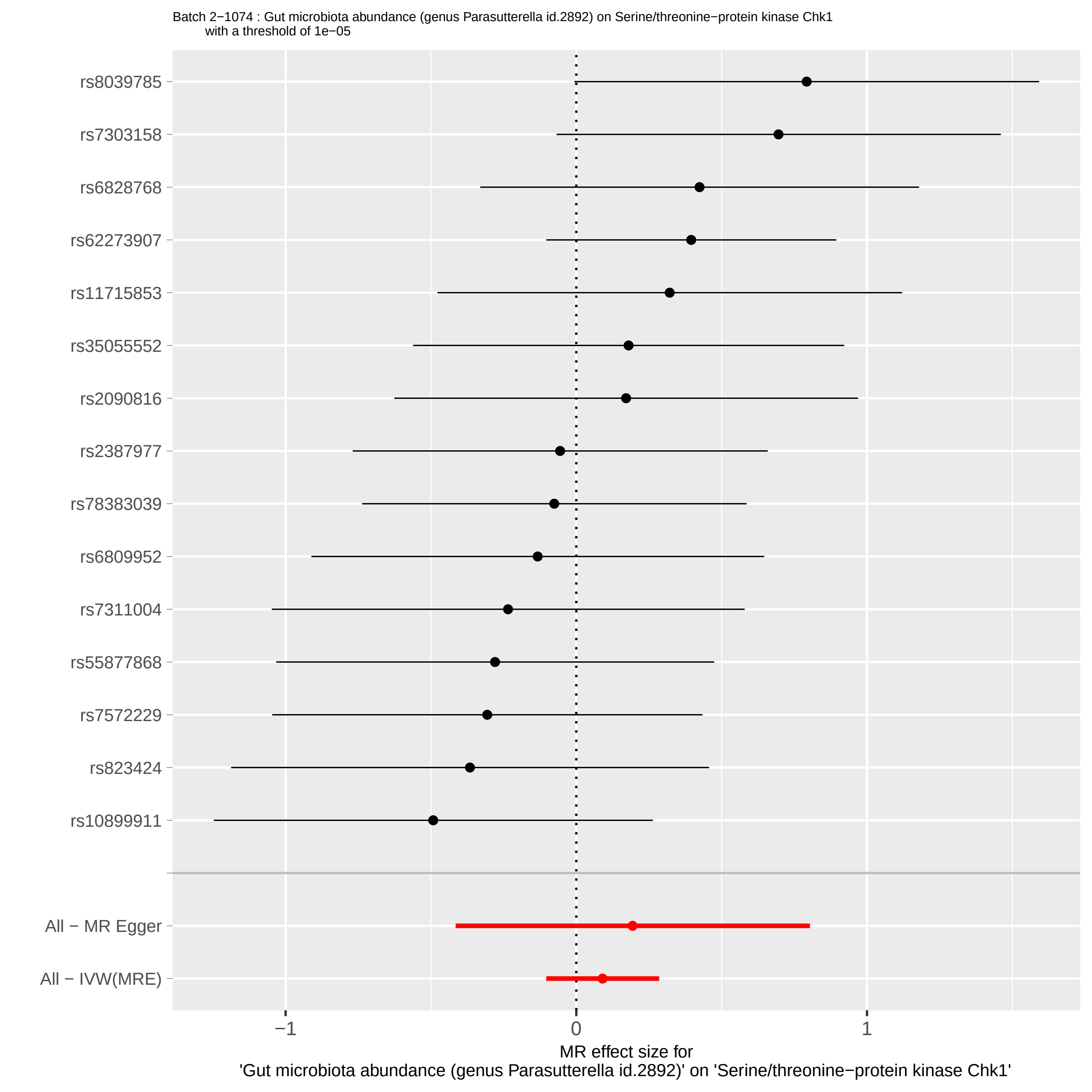
Batch 2−1074 : Gut microbiota abundance (genus Parasutterella id.2892) on Serine/threonine−protein kinase Chk1
with a threshold of 1e−05
rs8039785
rs7303158
rs6828768
rs62273907
rs11715853
rs35055552
rs2090816
rs2387977
rs78383039
rs6809952
rs7311004
rs55877868
rs7572229
rs823424
rs10899911
All − MR Egger
All − IVW(MRE)
−1
0
1
MR effect size for
'Gut microbiota abundance (genus Parasutterella id.2892)' on 'Serine/threonine−protein kinase Chk1'

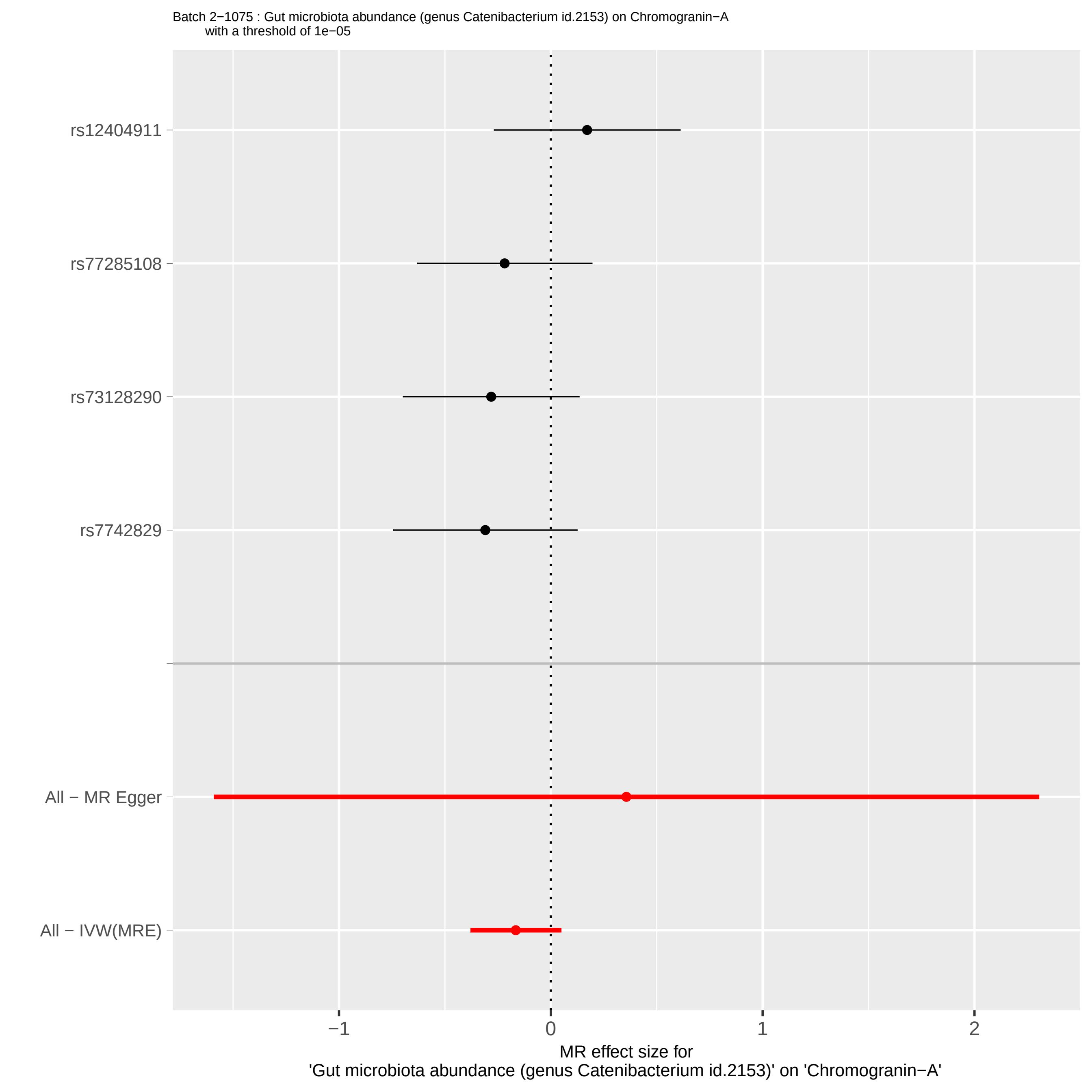
Batch 2−1075 : Gut microbiota abundance (genus Catenibacterium id.2153) on Chromogranin−A
with a threshold of 1e−05
rs12404911
rs77285108
rs73128290
rs7742829
All − MR Egger
All − IVW(MRE)
−1
0
1
2
MR effect size for
'Gut microbiota abundance (genus Catenibacterium id.2153)' on 'Chromogranin−A'

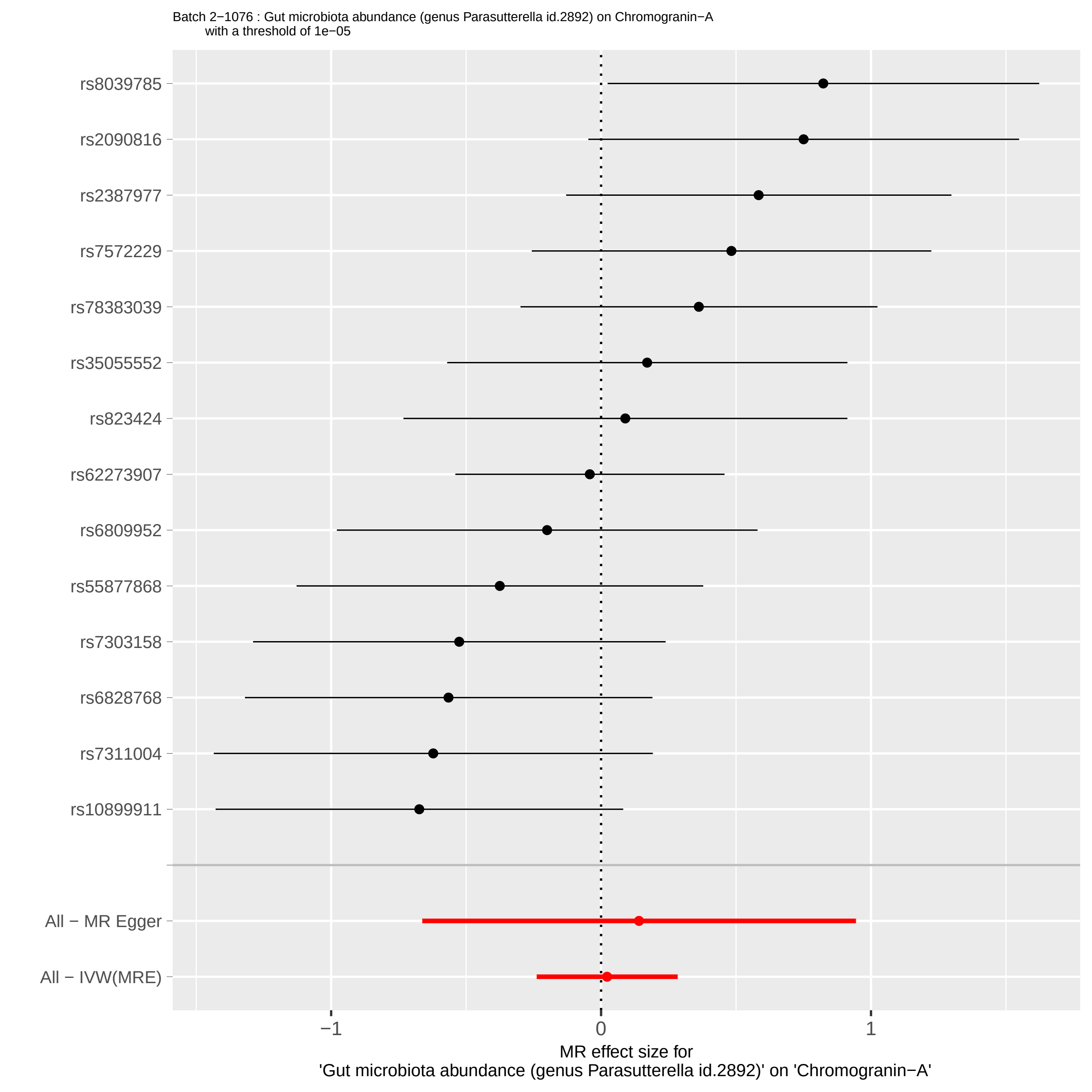
Batch 2−1076 : Gut microbiota abundance (genus Parasutterella id.2892) on Chromogranin−A
with a threshold of 1e−05
rs8039785
rs2090816
rs2387977
rs7572229
rs78383039
rs35055552
rs823424
rs62273907
rs6809952
rs55877868
rs7303158
rs6828768
rs7311004
rs10899911
All − MR Egger
All − IVW(MRE)
−1
0
1
MR effect size for
'Gut microbiota abundance (genus Parasutterella id.2892)' on 'Chromogranin−A'

Batch 2−1077 : Gut microbiota abundance (genus Catenibacterium id.2153) on Secretogranin−1
with a threshold of 1e−05

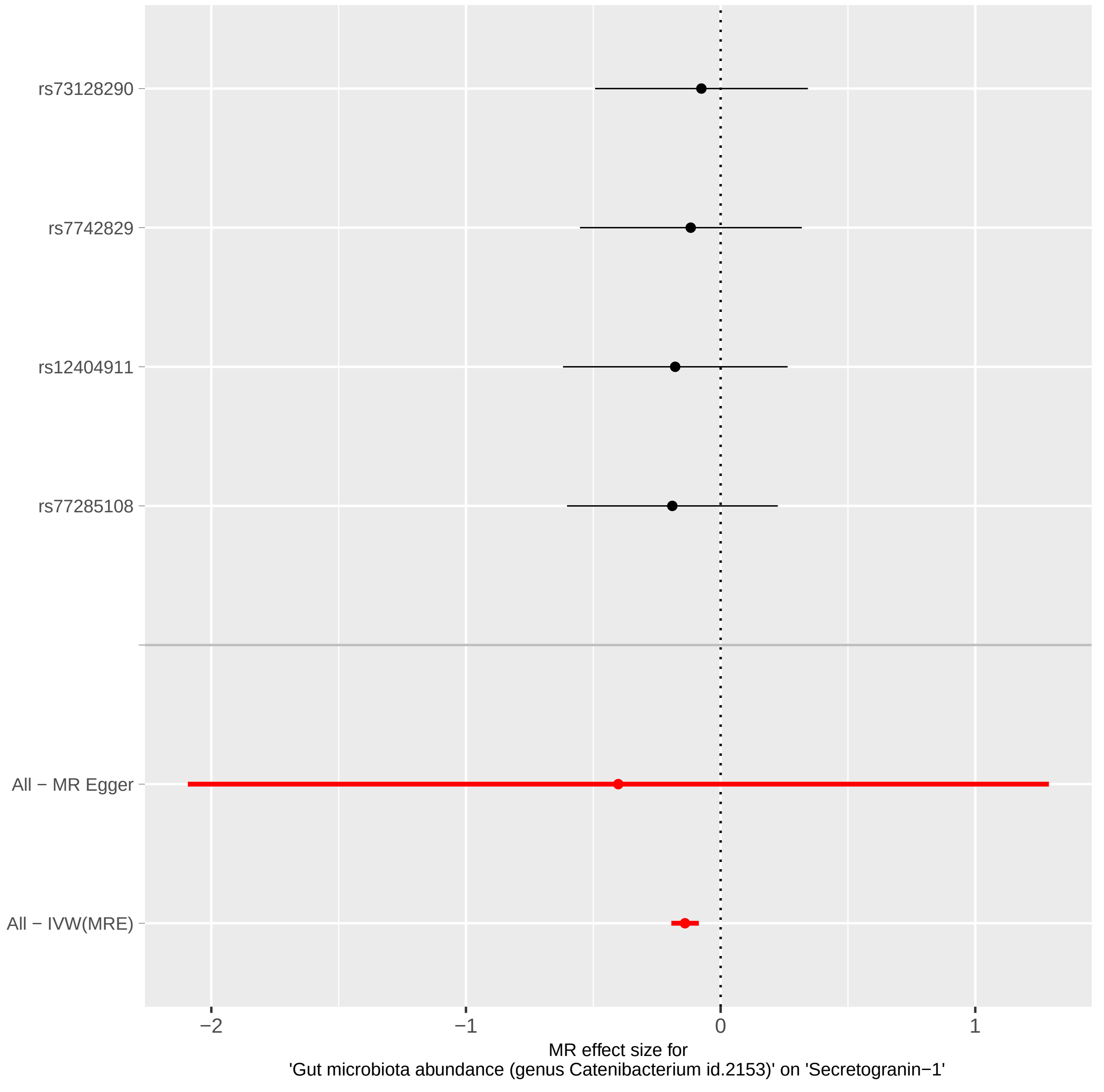

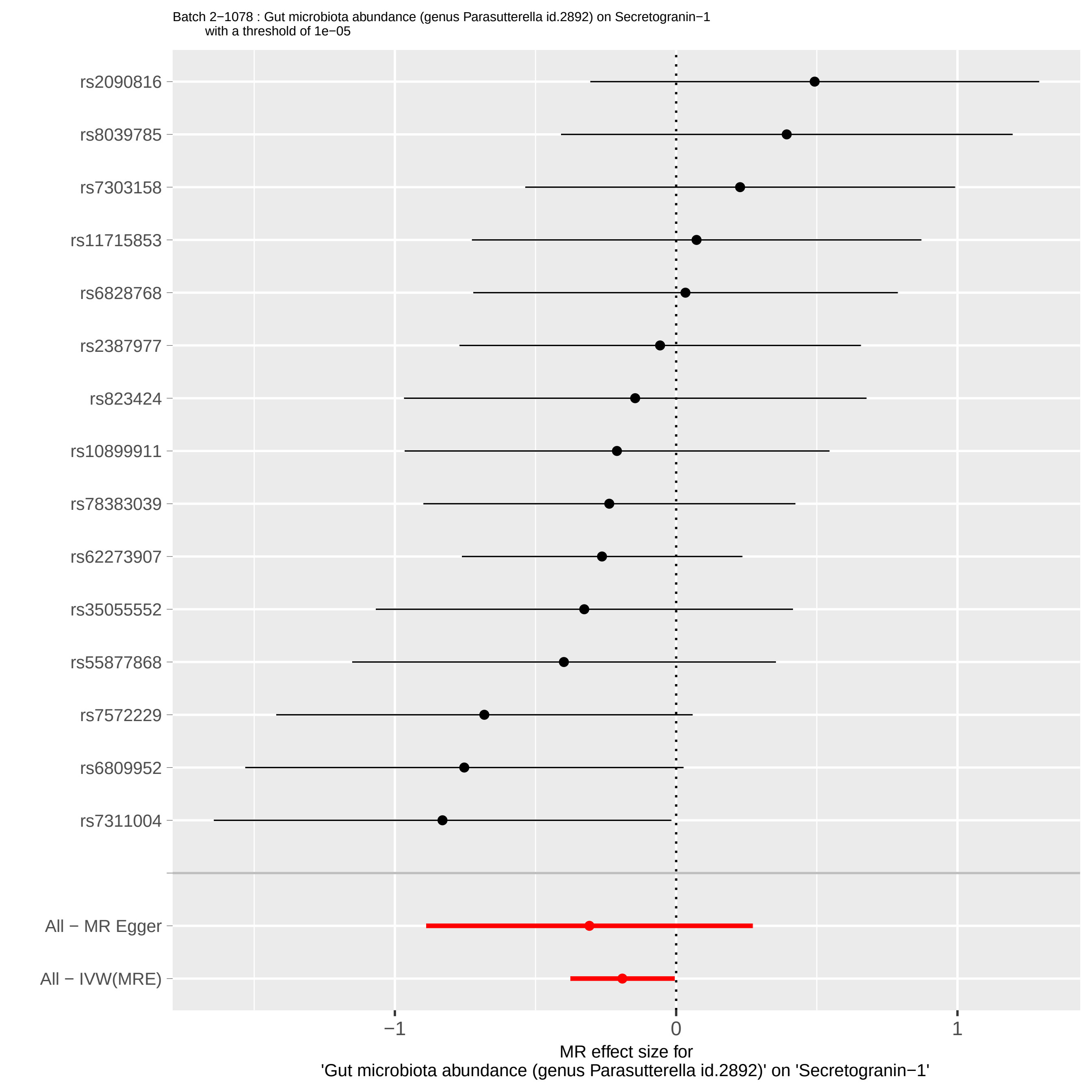
Batch 2−1078 : Gut microbiota abundance (genus Parasutterella id.2892) on Secretogranin−1
with a threshold of 1e−05
rs2090816
rs8039785
rs7303158
rs11715853
rs6828768
rs2387977
rs823424
rs10899911
rs78383039
rs62273907
rs35055552
rs55877868
rs7572229
rs6809952
rs7311004
All − MR Egger
All − IVW(MRE)
−1
0
1
MR effect size for
'Gut microbiota abundance (genus Parasutterella id.2892)' on 'Secretogranin−1'

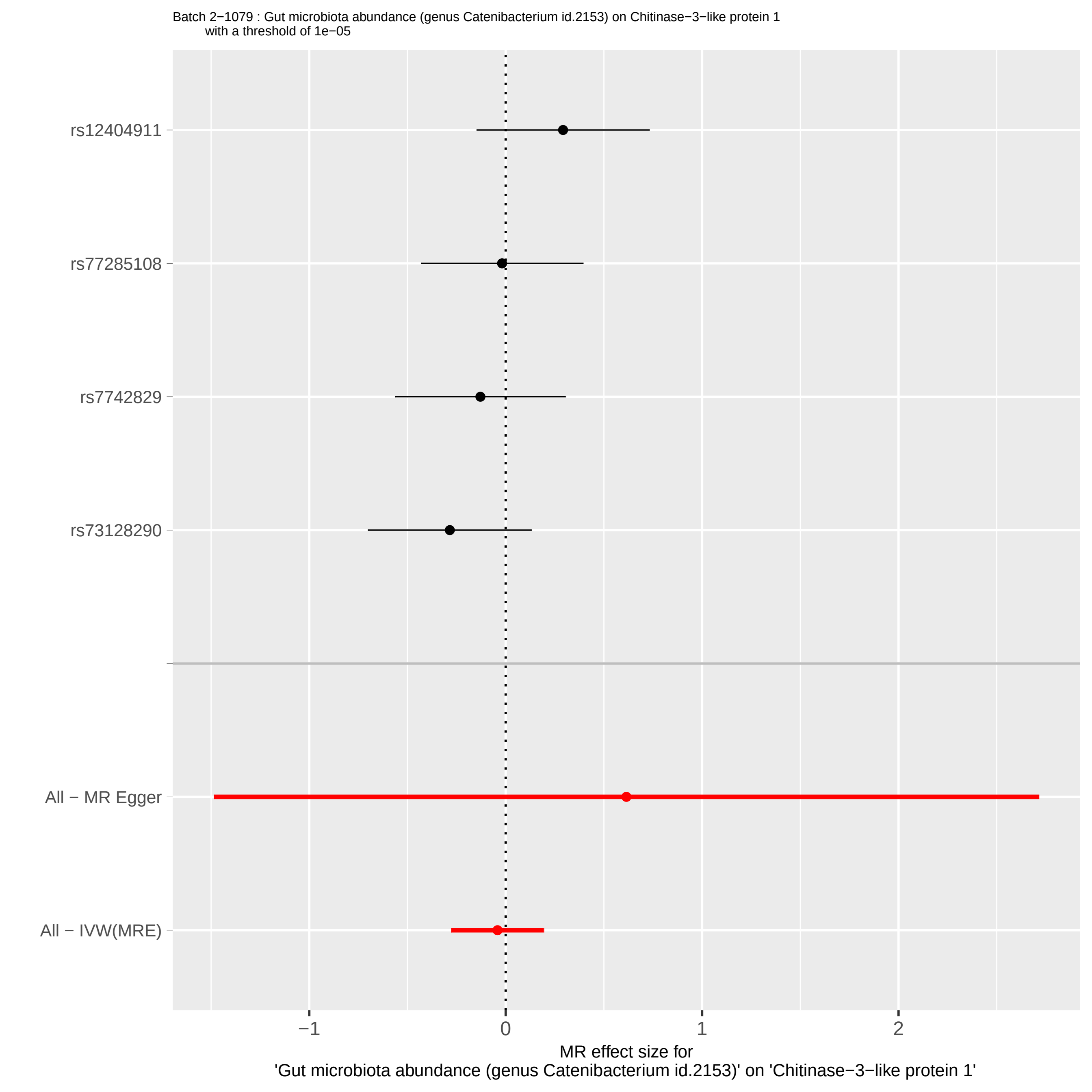

Batch 2−1079 : Gut microbiota abundance (genus Catenibacterium id.2153) on Chitinase−3−like protein 1
with a threshold of 1e−05
rs12404911
rs77285108
rs7742829
rs73128290
All − MR Egger
All − IVW(MRE)
−1
0
1
2
MR effect size for
'Gut microbiota abundance (genus Catenibacterium id.2153)' on 'Chitinase−3−like protein 1'

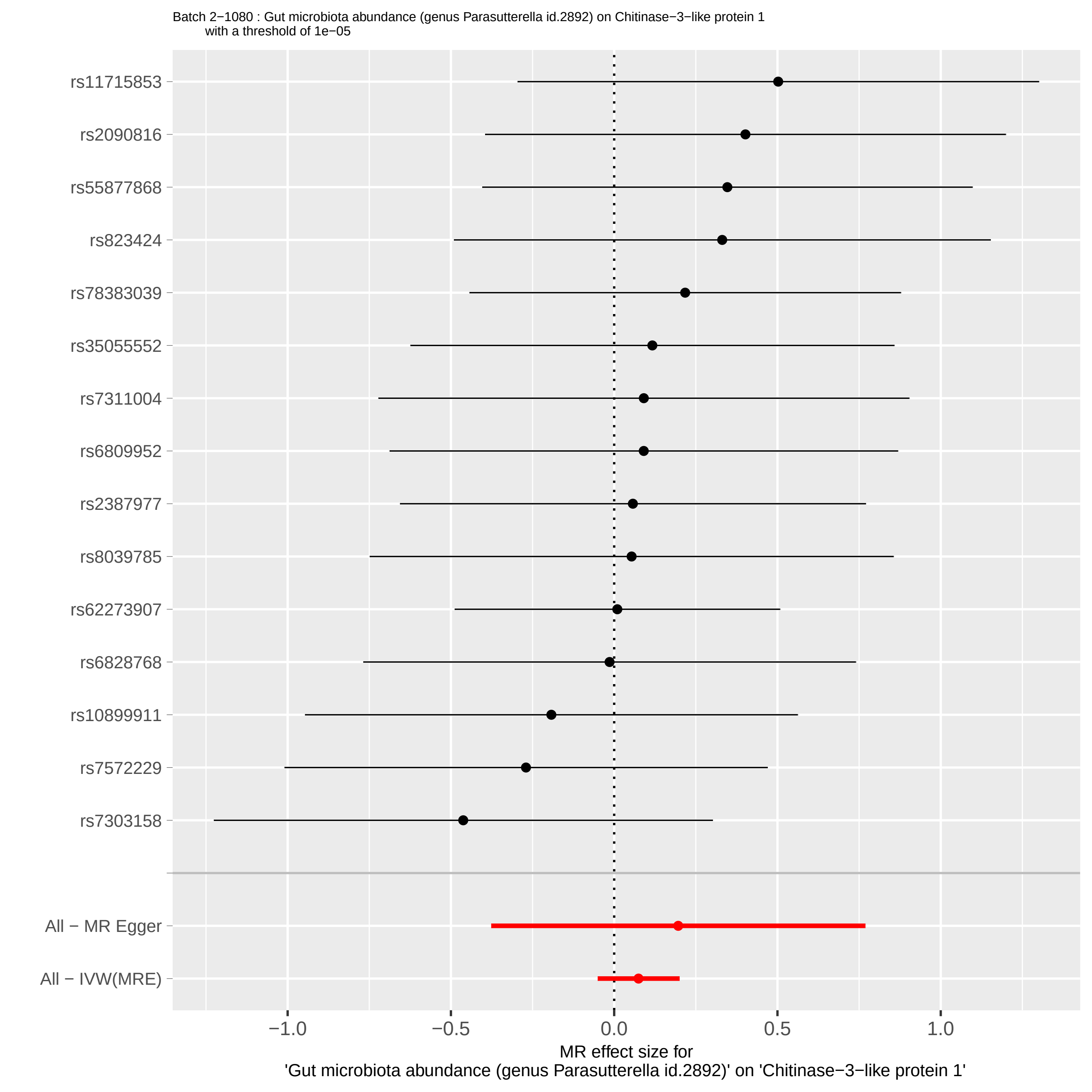

Batch 2−1080 : Gut microbiota abundance (genus Parasutterella id.2892) on Chitinase−3−like protein 1
with a threshold of 1e−05
rs11715853
rs2090816
rs55877868
rs823424
rs78383039
rs35055552
rs7311004
rs6809952
rs2387977
rs8039785
rs62273907
rs6828768
rs10899911
rs7572229
rs7303158
All − MR Egger
All − IVW(MRE)
−1.0
−0.5
0.0
0.5
1.0
MR effect size for
'Gut microbiota abundance (genus Parasutterella id.2892)' on 'Chitinase−3−like protein 1'

Batch 2−1081 : Gut microbiota abundance (genus Catenibacterium id.2153) on Chitinase−3−like protein 2
with a threshold of 1e−05

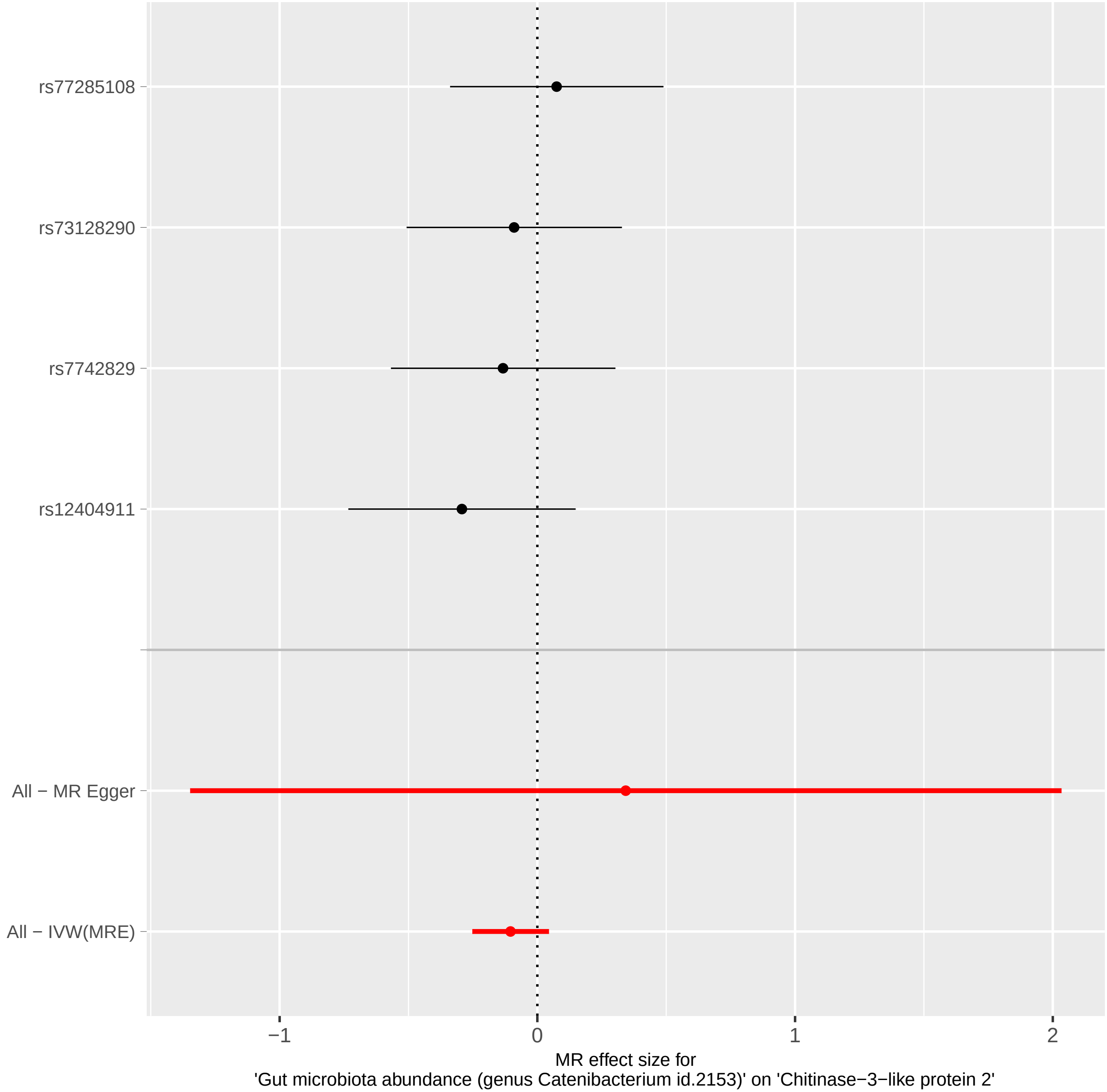

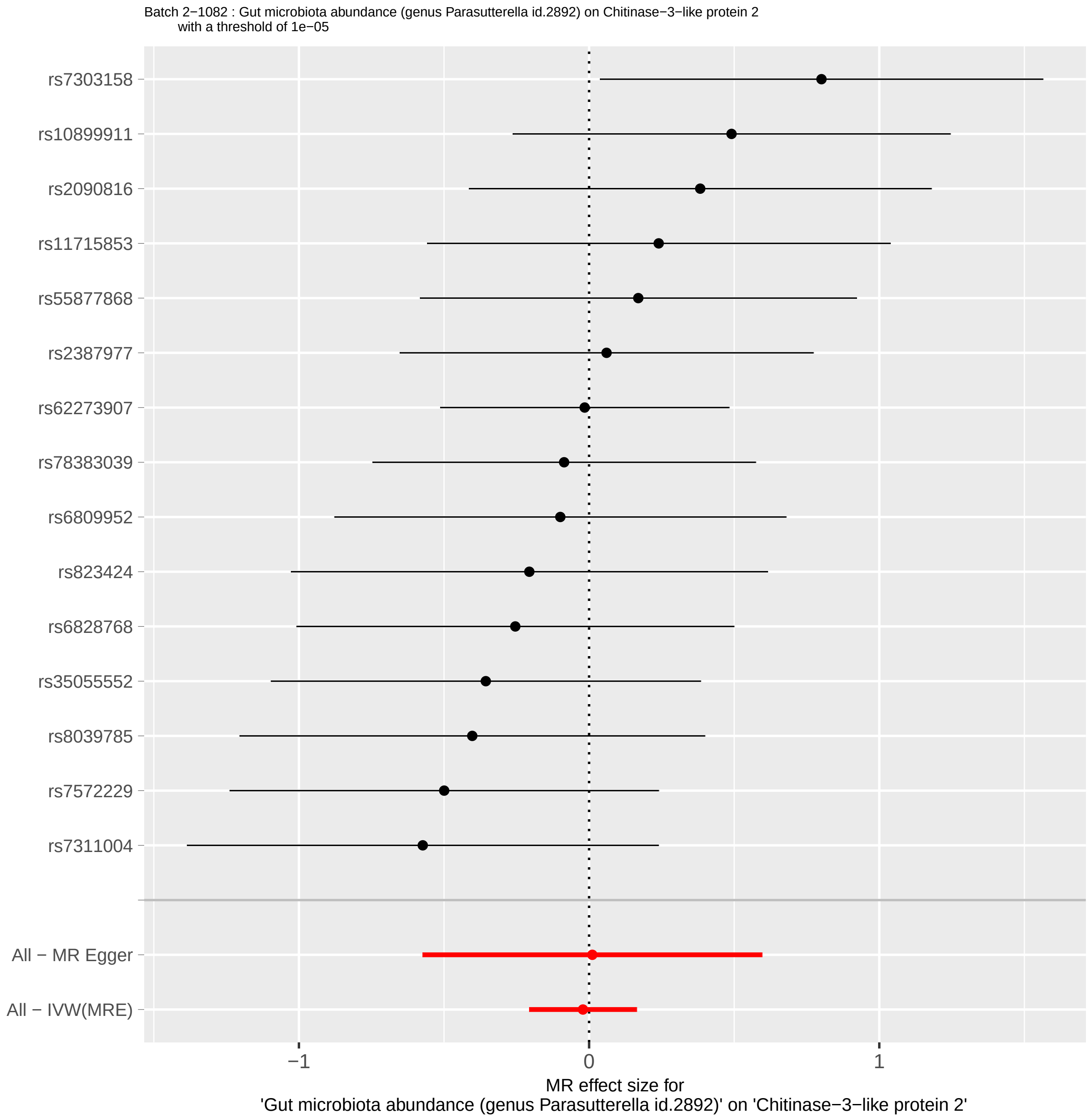

Batch 2−1082 : Gut microbiota abundance (genus Parasutterella id.2892) on Chitinase−3−like protein 2
with a threshold of 1e−05
rs7303158
rs10899911
rs2090816
rs11715853
rs55877868
rs2387977
rs62273907
rs78383039
rs6809952
rs823424
rs6828768
rs35055552
rs8039785
rs7572229
rs7311004
All − MR Egger
All − IVW(MRE)
−1
0
1
MR effect size for
'Gut microbiota abundance (genus Parasutterella id.2892)' on 'Chitinase−3−like protein 2'

Batch 2−1083 : Gut microbiota abundance (genus Catenibacterium id.2153) on Cysteine−rich hydrophobic domain−containing protein 2
with a threshold of 1e−05

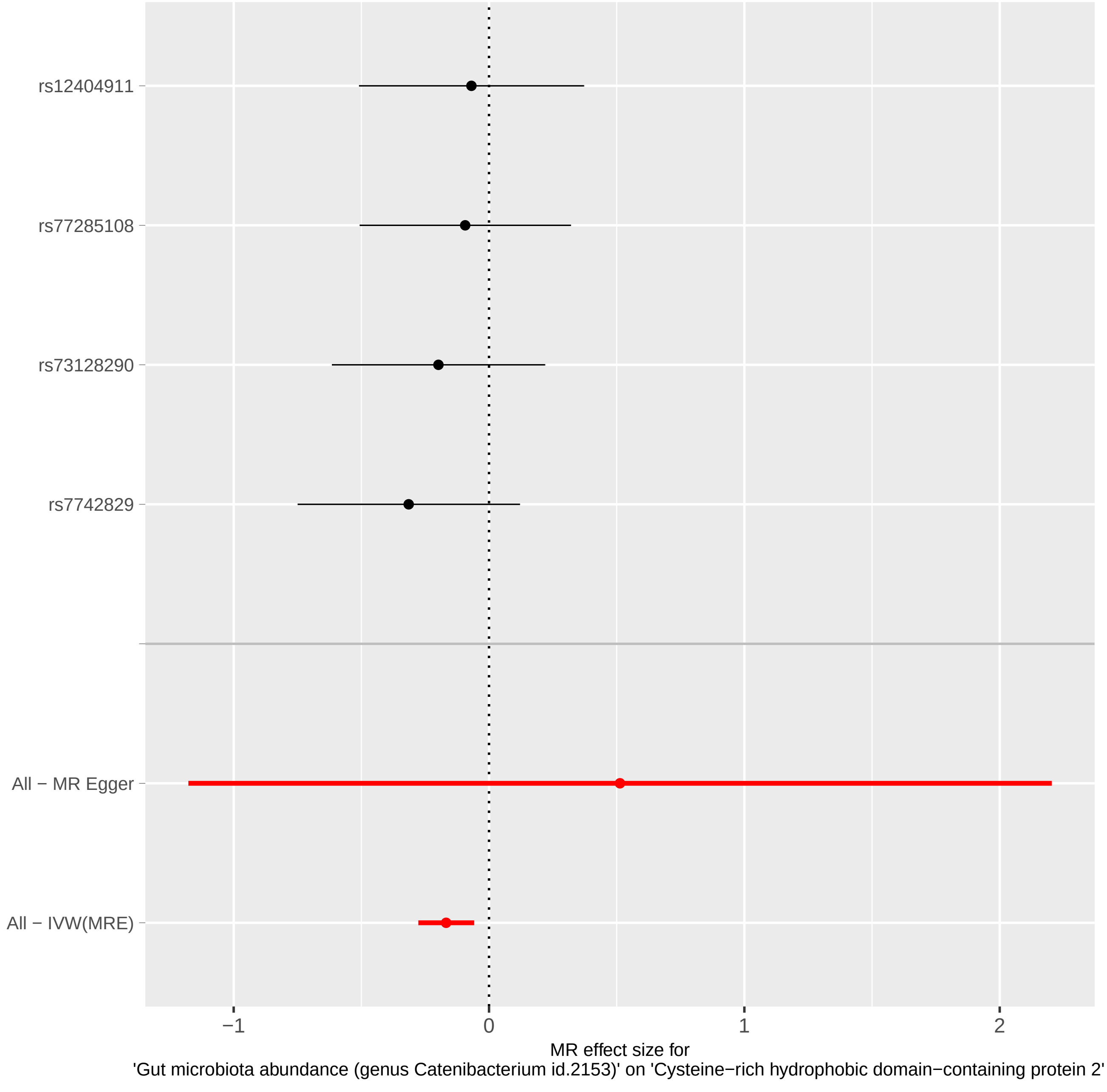

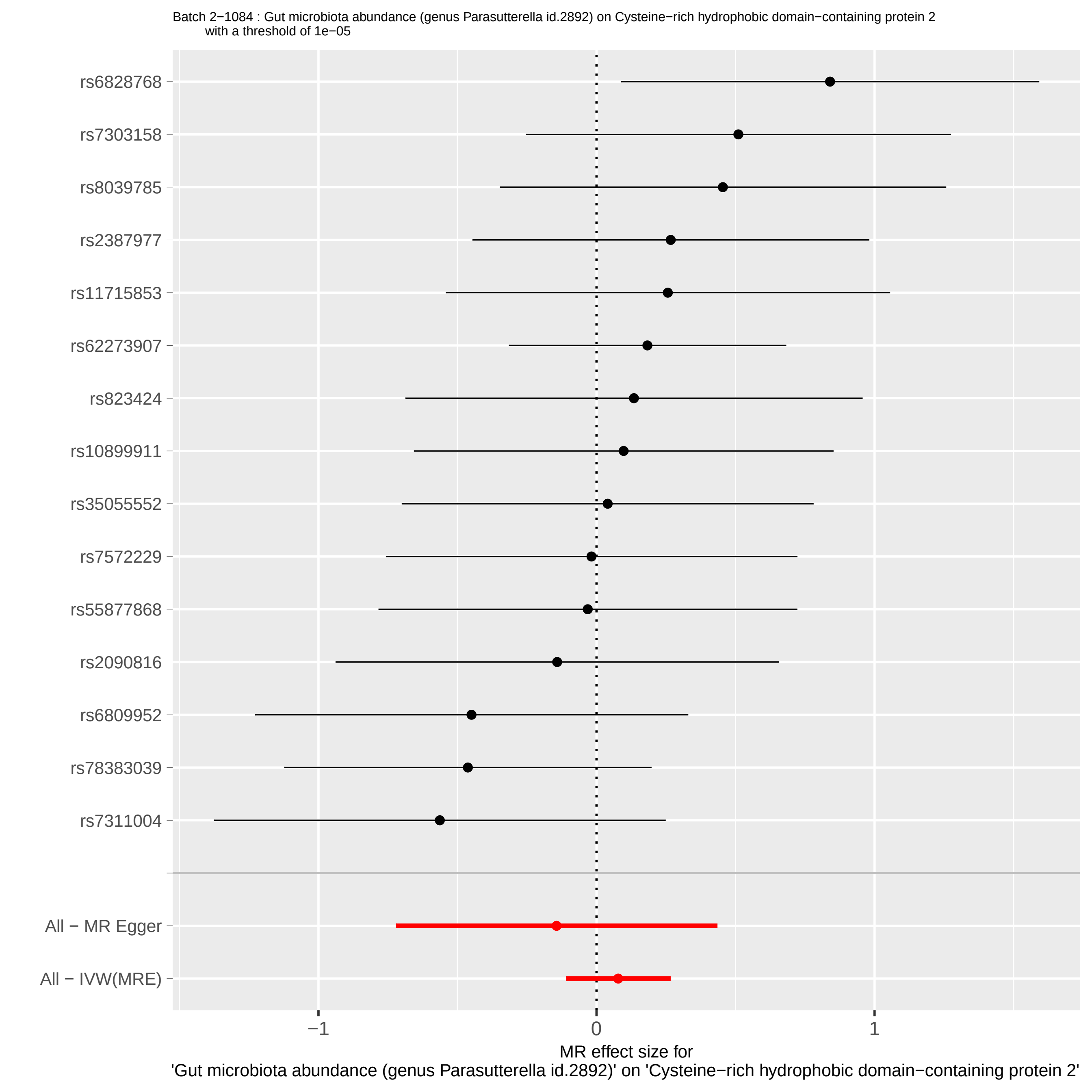

Batch 2−1084 : Gut microbiota abundance (genus Parasutterella id.2892) on Cysteine−rich hydrophobic domain−containing protein 2 with a threshold of 1e−05
rs6828768
rs7303158
rs8039785
rs2387977
rs11715853
rs62273907
rs823424
rs10899911
rs35055552
rs7572229
rs55877868
rs2090816
rs6809952
rs78383039
rs7311004
All − MR Egger
All − IVW(MRE)
−1
0
1
MR effect size for
'Gut microbiota abundance (genus Parasutterella id.2892)' on 'Cysteine−rich hydrophobic domain−containing protein 2'

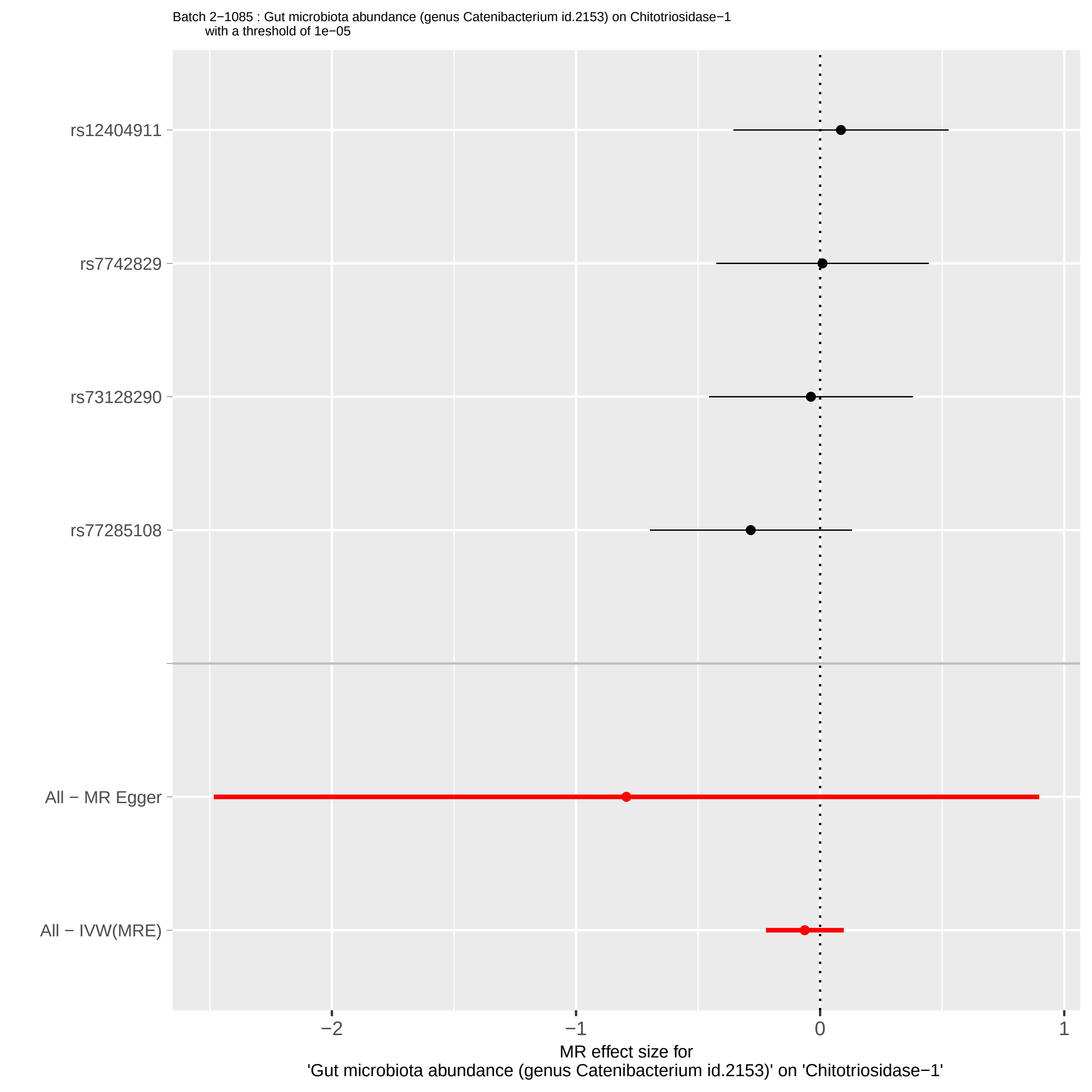

Batch 2−1085 : Gut microbiota abundance (genus Catenibacterium id.2153) on Chitotriosidase−1
with a threshold of 1e−05
rs12404911
rs7742829
rs73128290
rs77285108
All − MR Egger
All − IVW(MRE)
−2
−1
0
1
MR effect size for
'Gut microbiota abundance (genus Catenibacterium id.2153)' on 'Chitotriosidase−1'

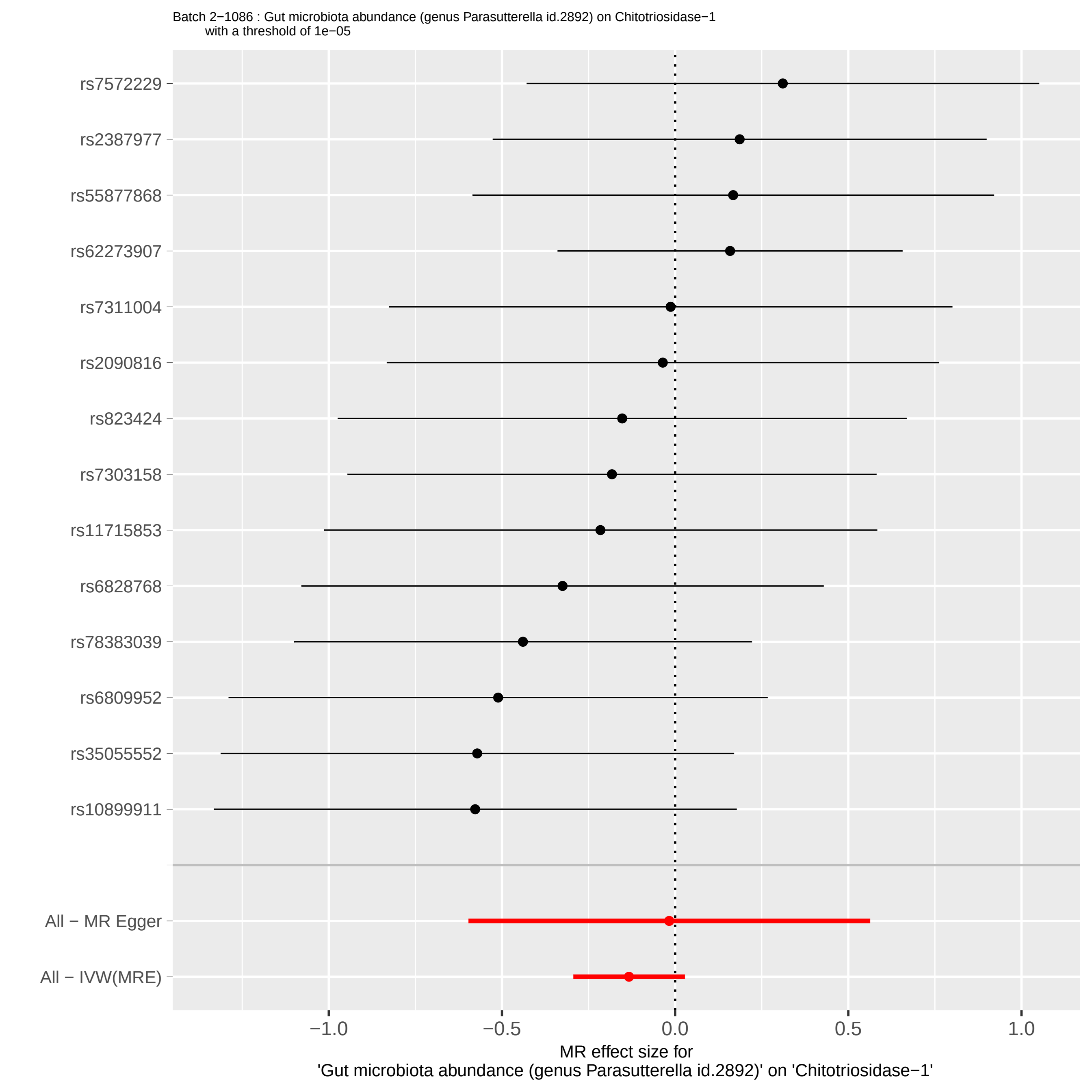

Batch 2−1086 : Gut microbiota abundance (genus Parasutterella id.2892) on Chitotriosidase−1
with a threshold of 1e−05
rs7572229
rs2387977
rs55877868
rs62273907
rs7311004
rs2090816
rs823424
rs7303158
rs11715853
rs6828768
rs78383039
rs6809952
rs35055552
rs10899911
All − MR Egger
All − IVW(MRE)
−1.0
−0.5
0.0
0.5
1.0
MR effect size for
'Gut microbiota abundance (genus Parasutterella id.2892)' on 'Chitotriosidase−1'

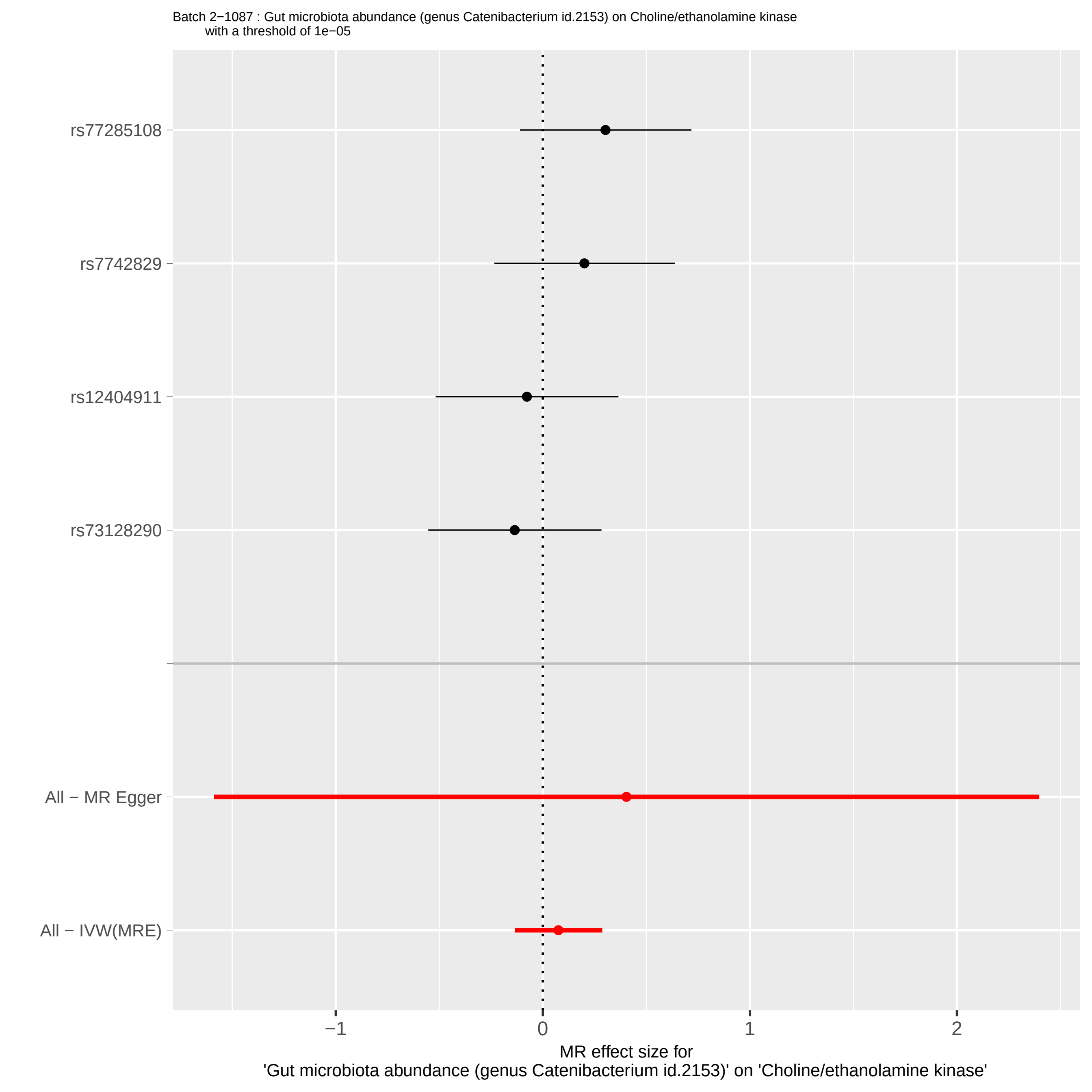
Batch 2−1087 : Gut microbiota abundance (genus Catenibacterium id.2153) on Choline/ethanolamine kinase
with a threshold of 1e−05
rs77285108
rs7742829
rs12404911
rs73128290
All − MR Egger
All − IVW(MRE)
−1
0
1
2
MR effect size for
'Gut microbiota abundance (genus Catenibacterium id.2153)' on 'Choline/ethanolamine kinase'

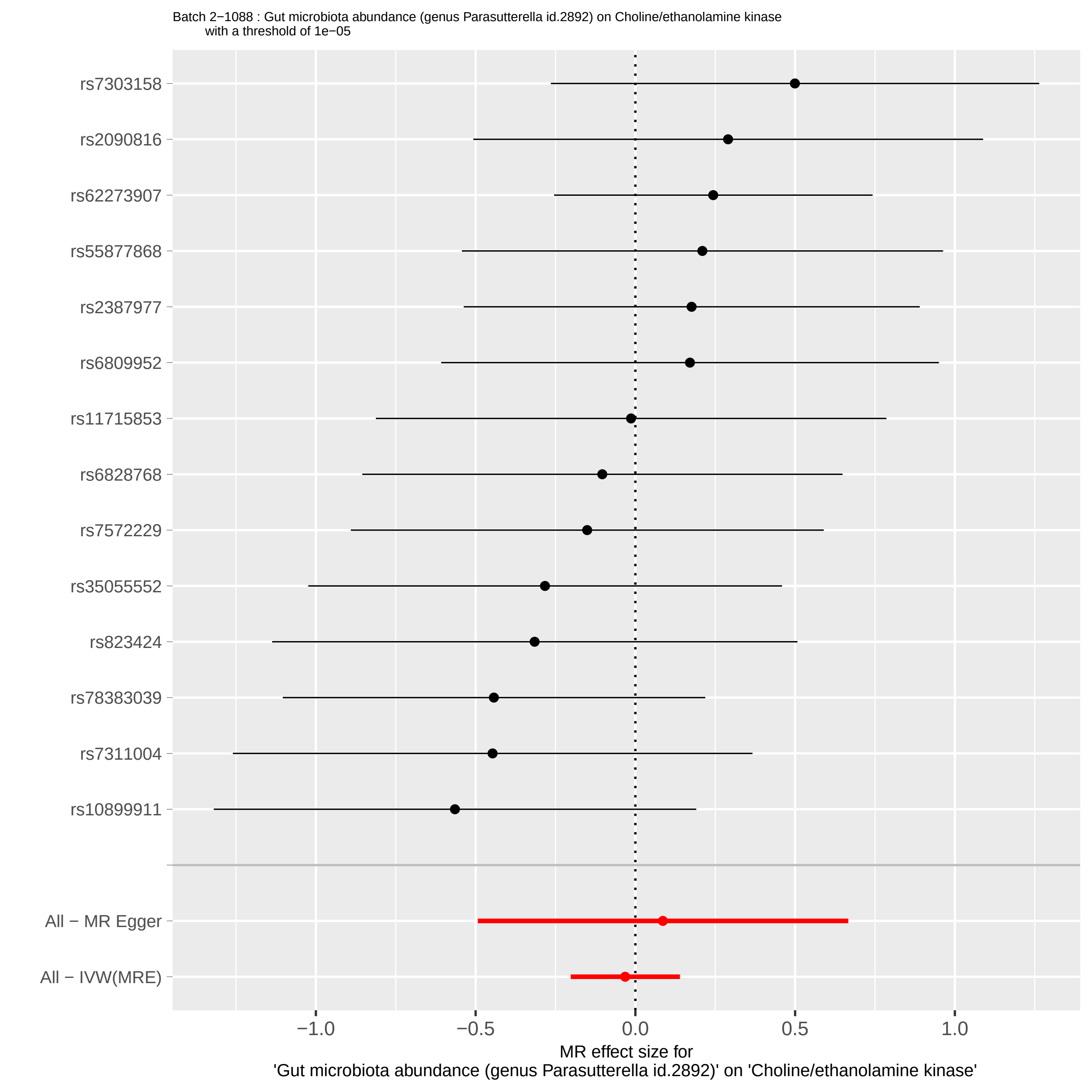

Batch 2−1088 : Gut microbiota abundance (genus Parasutterella id.2892) on Choline/ethanolamine kinase
with a threshold of 1e−05
rs7303158
rs2090816
rs62273907
rs55877868
rs2387977
rs6809952
rs11715853
rs6828768
rs7572229
rs35055552
rs823424
rs78383039
rs7311004
rs10899911
All − MR Egger
All − IVW(MRE)
−1.0
−0.5
0.0
0.5
1.0
MR effect size for
'Gut microbiota abundance (genus Parasutterella id.2892)' on 'Choline/ethanolamine kinase'

Batch 2−1089 : Gut microbiota abundance (genus Catenibacterium id.2153) on Neural cell adhesion molecule L1−like protein with a threshold of 1e−05

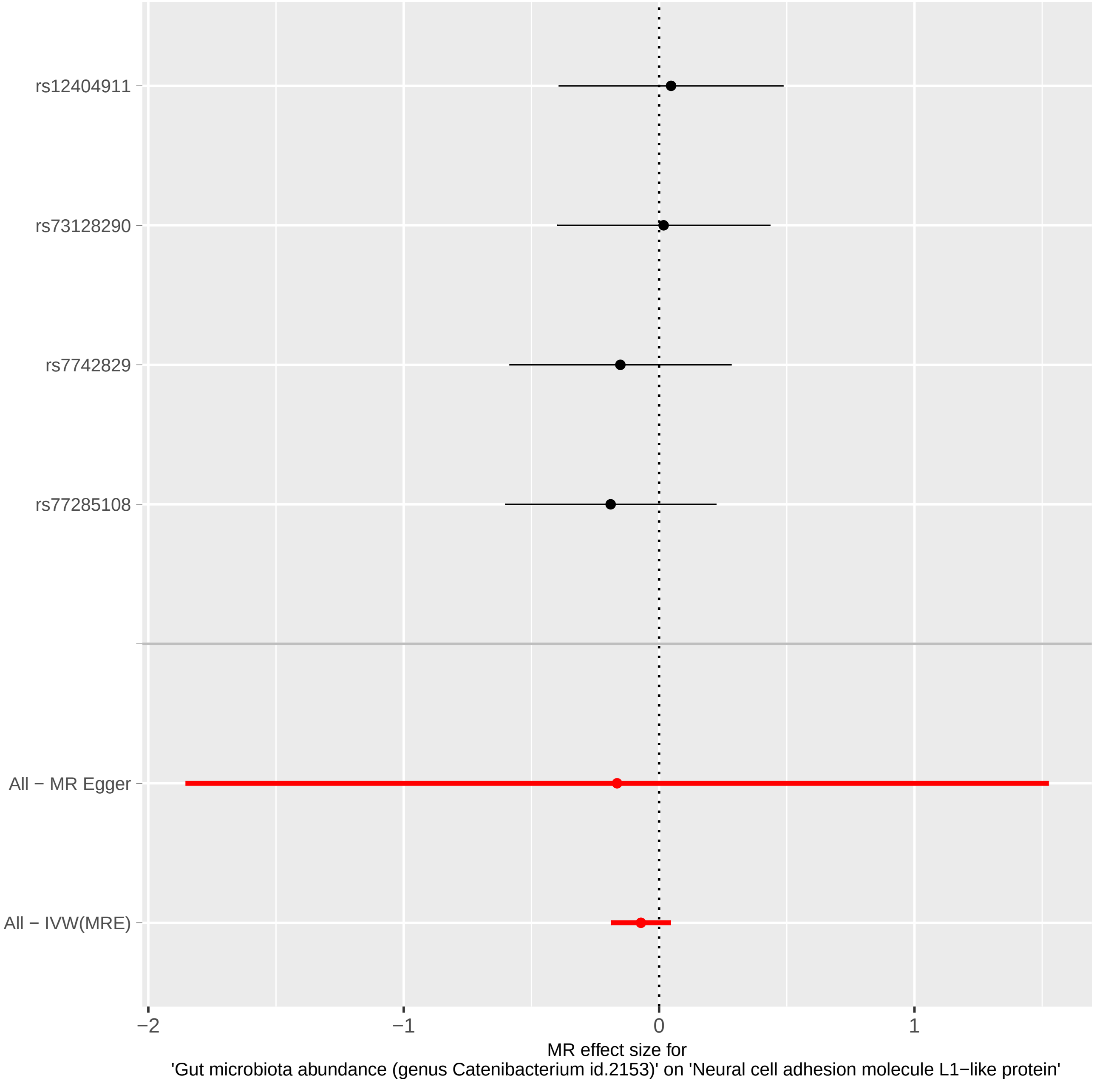

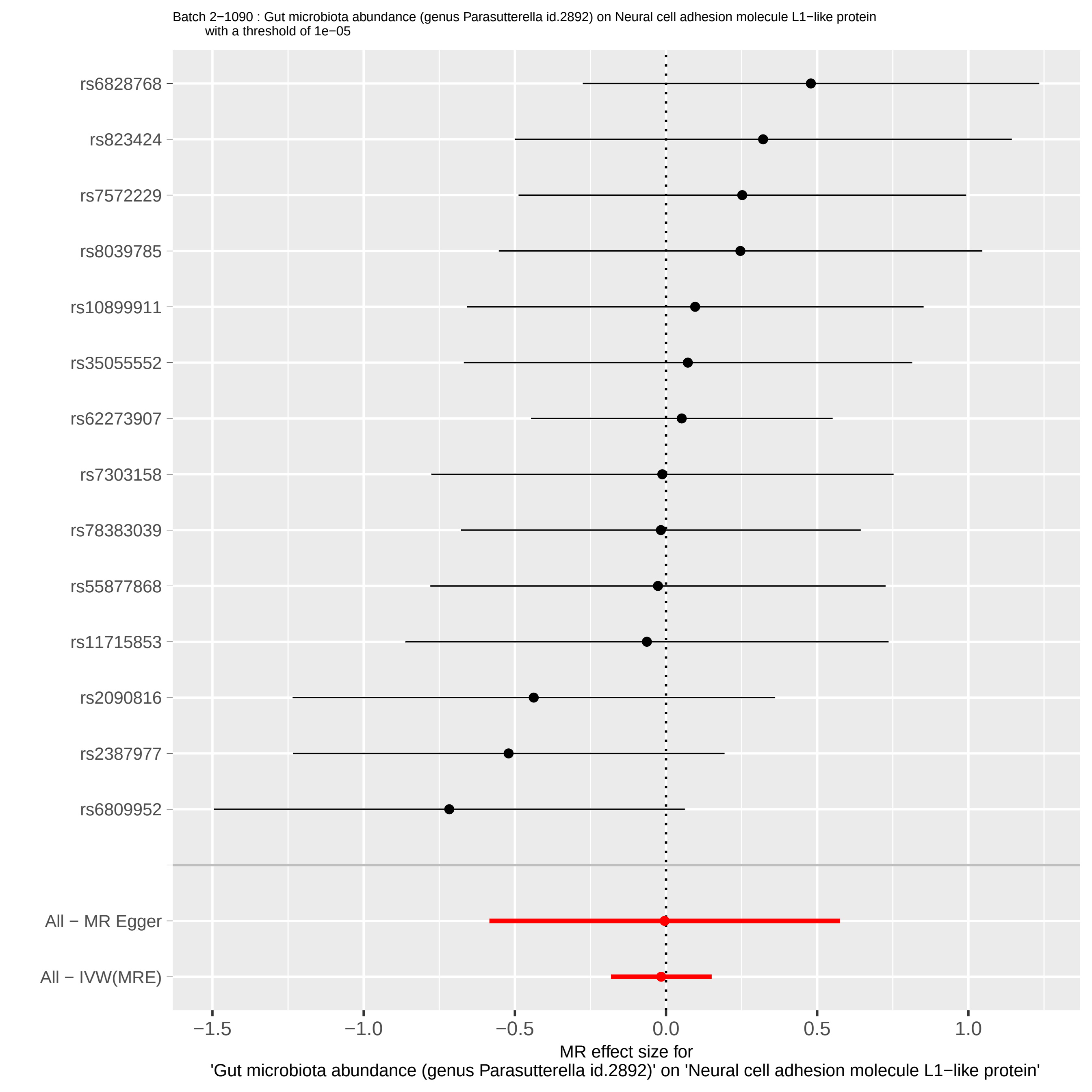
Batch 2−1090 : Gut microbiota abundance (genus Parasutterella id.2892) on Neural cell adhesion molecule L1−like protein
with a threshold of 1e−05
rs6828768
rs823424
rs7572229
rs8039785
rs10899911
rs35055552
rs62273907
rs7303158
rs78383039
rs55877868
rs11715853
rs2090816
rs2387977
rs6809952
All − MR Egger
All − IVW(MRE)
−1.5
−1.0
−0.5
0.0
0.5
1.0
MR effect size for
'Gut microbiota abundance (genus Parasutterella id.2892)' on 'Neural cell adhesion molecule L1−like protein'

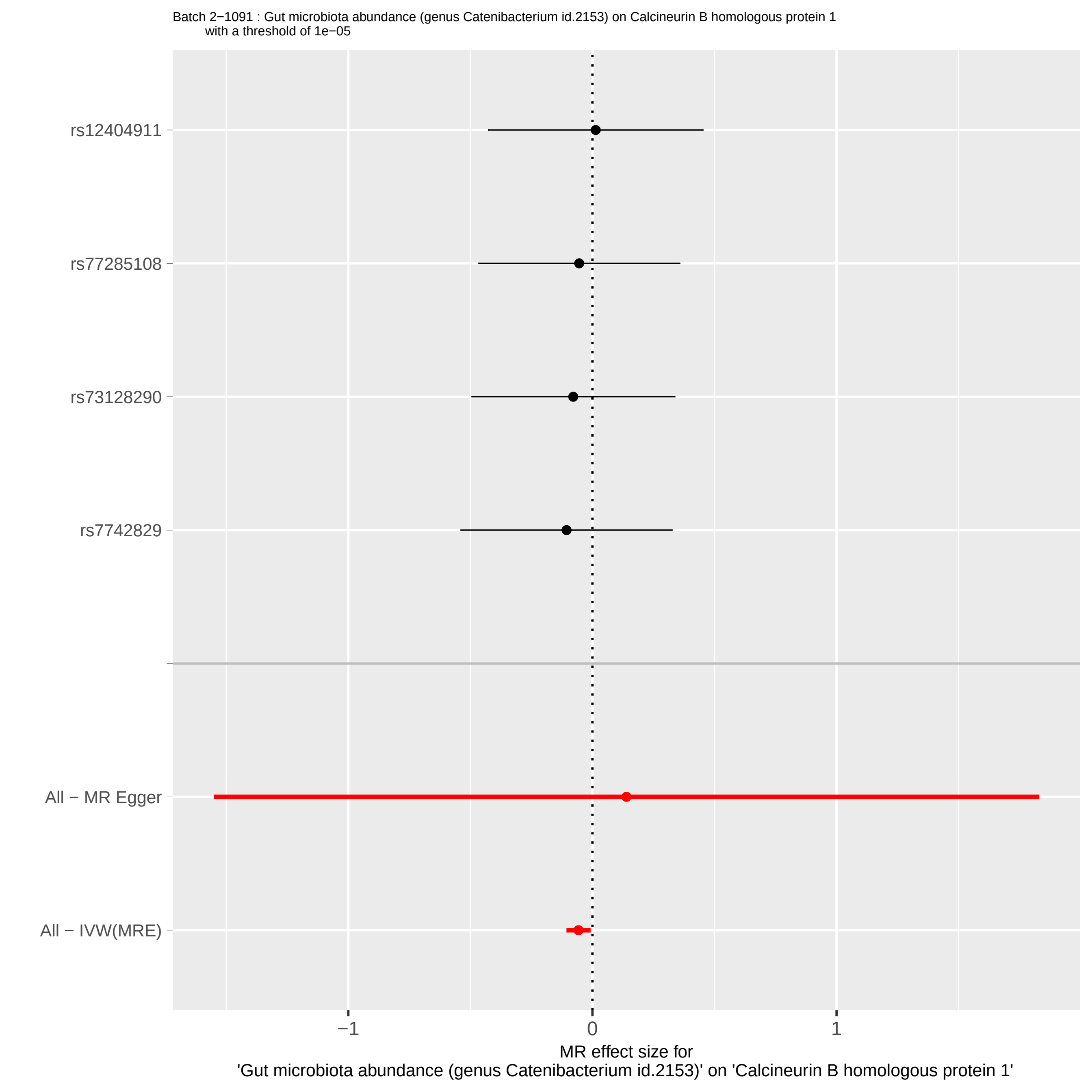
Batch 2−1091 : Gut microbiota abundance (genus Catenibacterium id.2153) on Calcineurin B homologous protein 1
with a threshold of 1e−05
rs12404911
rs77285108
rs73128290
rs7742829
All − MR Egger
All − IVW(MRE)
−1
0
1
MR effect size for
'Gut microbiota abundance (genus Catenibacterium id.2153)' on 'Calcineurin B homologous protein 1'

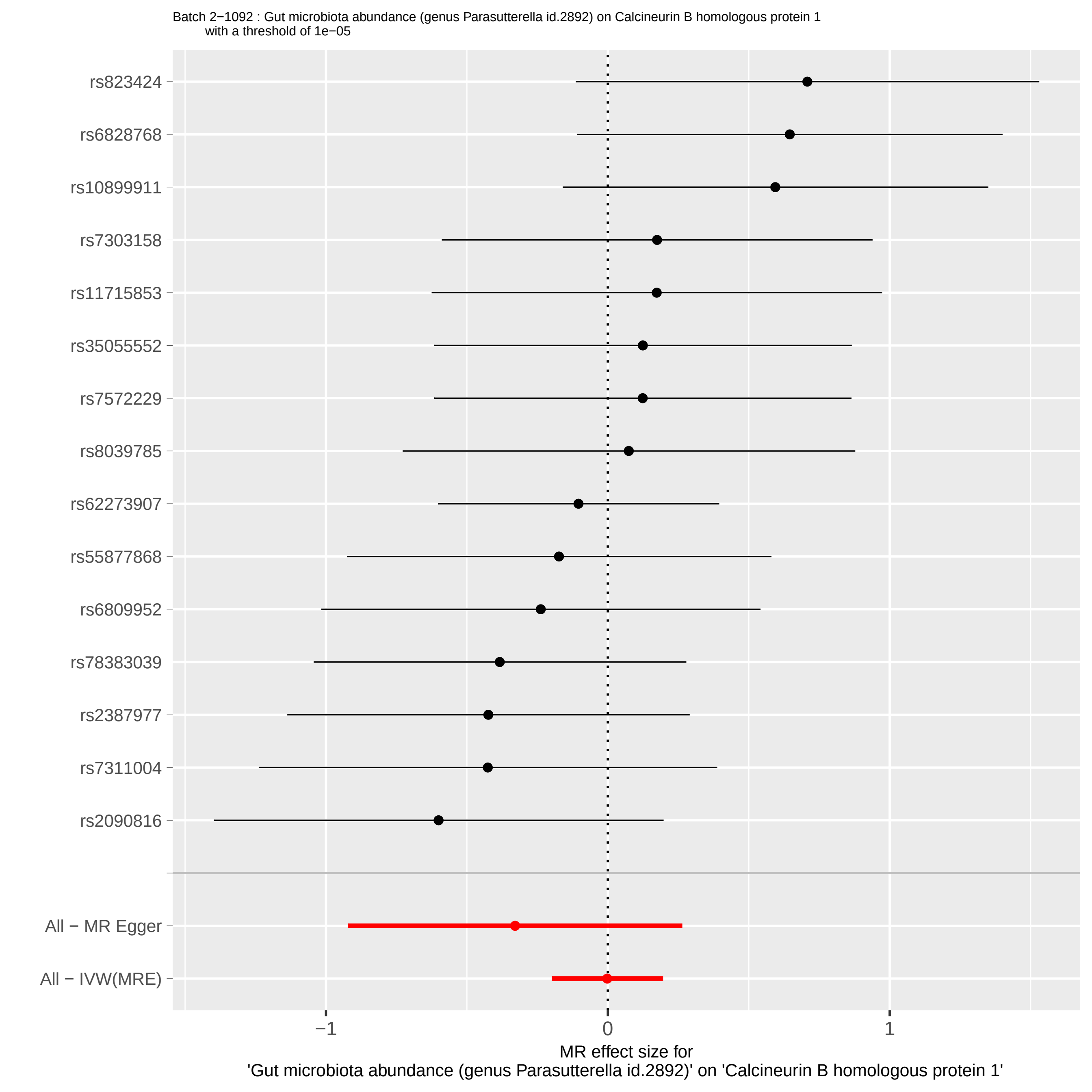
Batch 2−1092 : Gut microbiota abundance (genus Parasutterella id.2892) on Calcineurin B homologous protein 1
with a threshold of 1e−05
rs823424
rs6828768
rs10899911
rs7303158
rs11715853
rs35055552
rs7572229
rs8039785
rs62273907
rs55877868
rs6809952
rs78383039
rs2387977
rs7311004
rs2090816
All − MR Egger
All − IVW(MRE)
−1
0
1
MR effect size for
'Gut microbiota abundance (genus Parasutterella id.2892)' on 'Calcineurin B homologous protein 1'

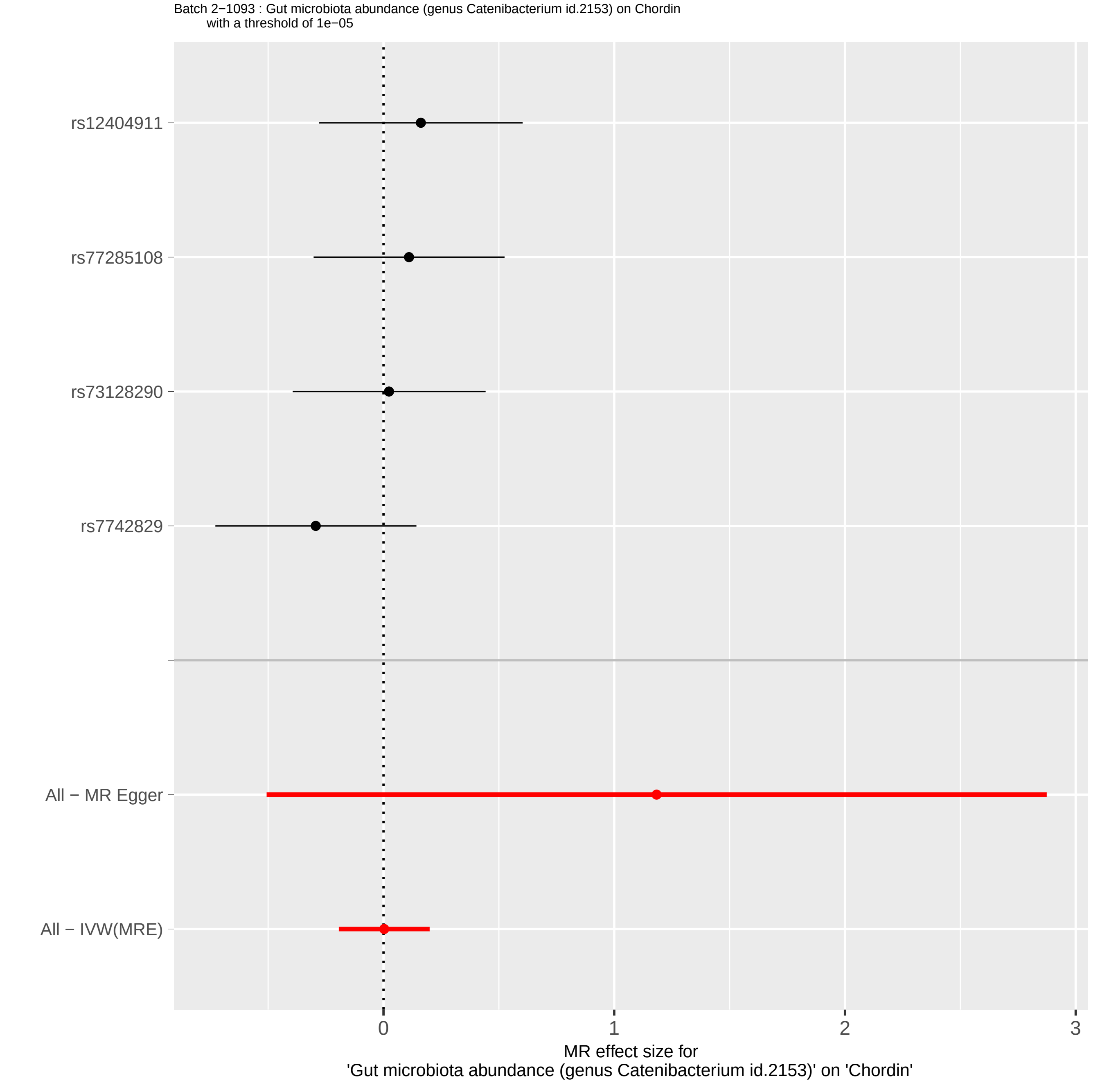

Batch 2−1093 : Gut microbiota abundance (genus Catenibacterium id.2153) on Chordin
with a threshold of 1e−05
rs12404911
rs77285108
rs73128290
rs7742829
All − MR Egger
All − IVW(MRE)
0
1
2
3
MR effect size for
'Gut microbiota abundance (genus Catenibacterium id.2153)' on 'Chordin'

Batch 2−1094 : Gut microbiota abundance (genus Parasutterella id.2892) on Chordin
with a threshold of 1e−05

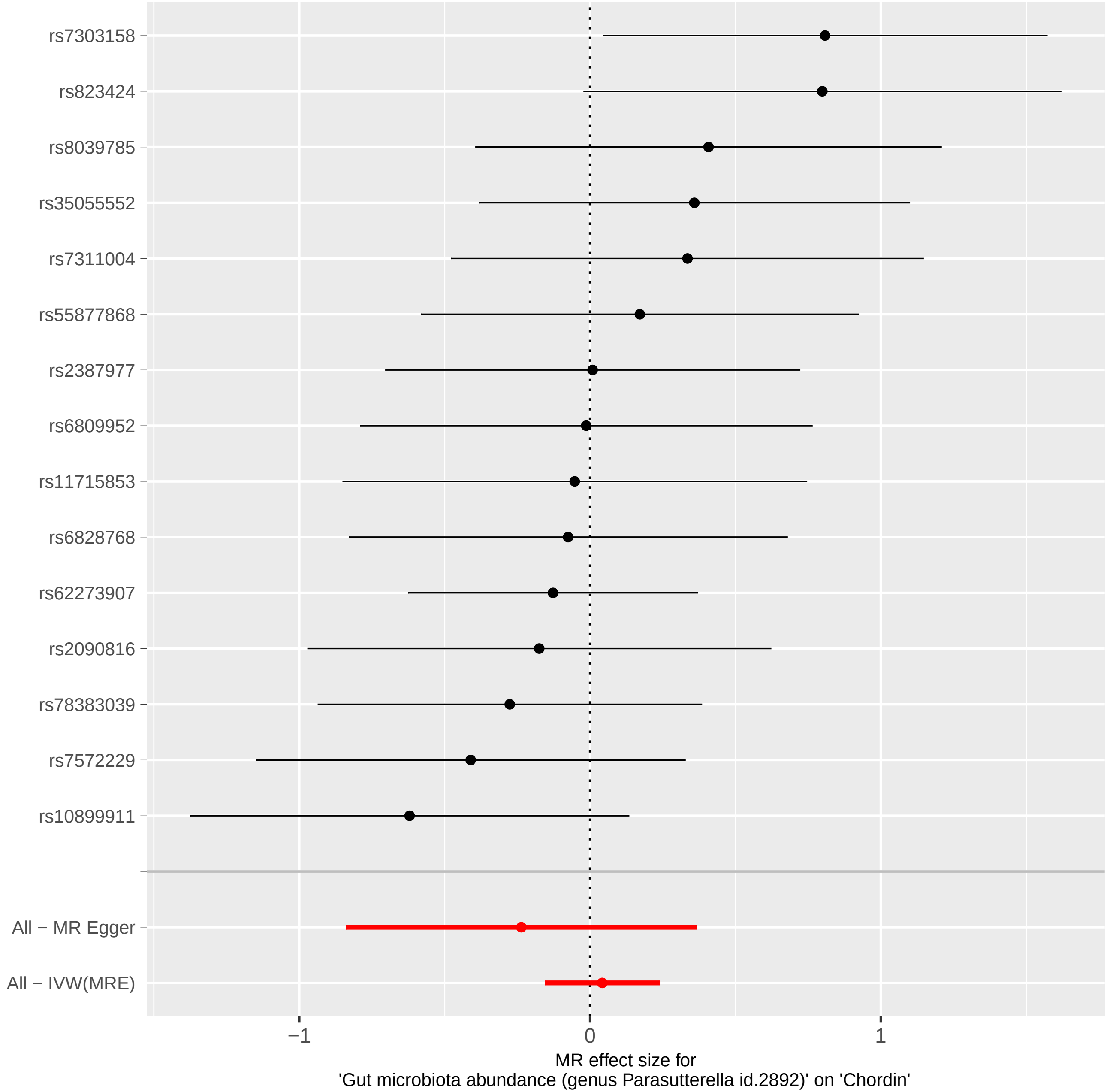

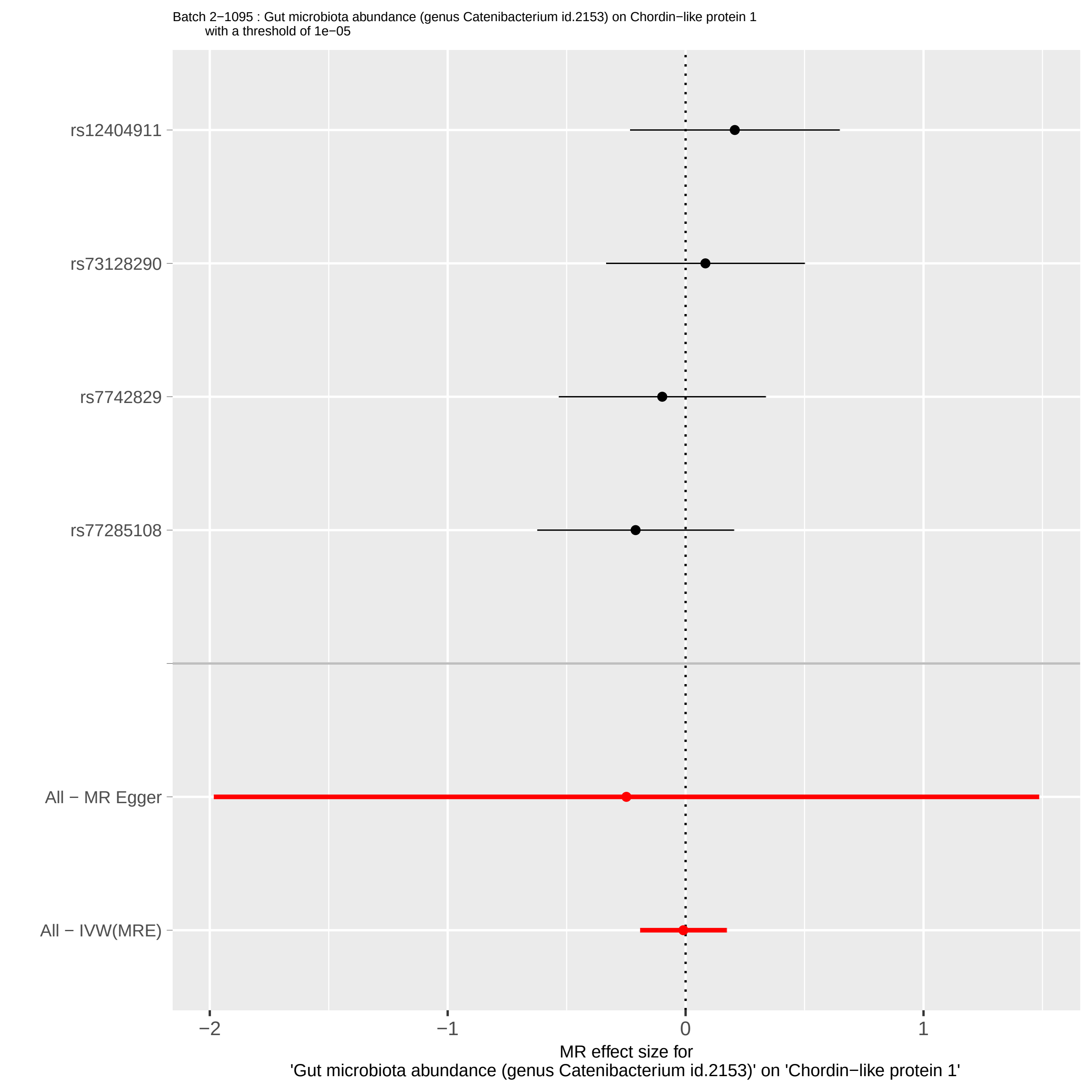

Batch 2−1095 : Gut microbiota abundance (genus Catenibacterium id.2153) on Chordin−like protein 1
with a threshold of 1e−05
rs12404911
rs73128290
rs7742829
rs77285108
All − MR Egger
All − IVW(MRE)
−2
−1
0
1
MR effect size for
'Gut microbiota abundance (genus Catenibacterium id.2153)' on 'Chordin−like protein 1'

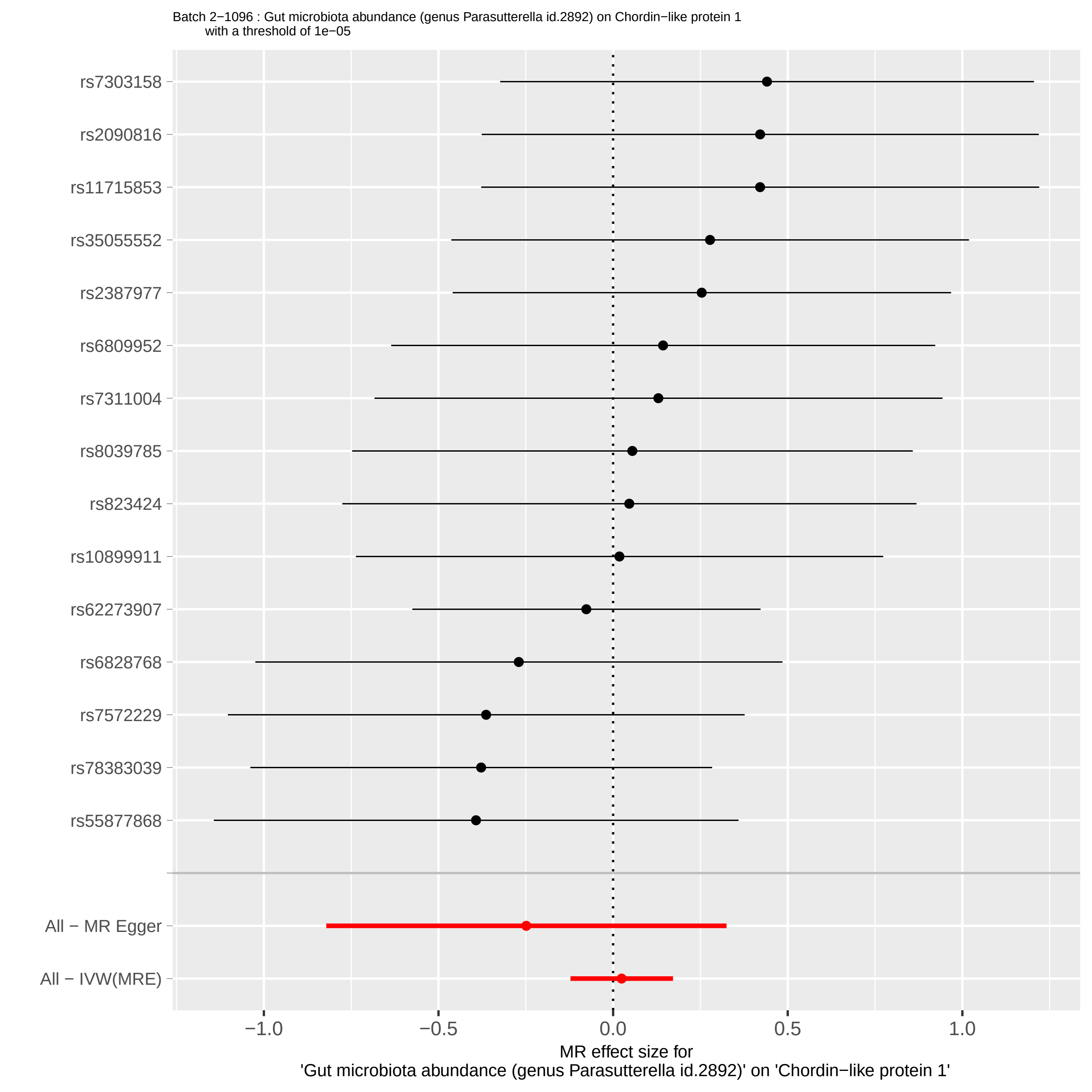

Batch 2−1096 : Gut microbiota abundance (genus Parasutterella id.2892) on Chordin−like protein 1
with a threshold of 1e−05
rs7303158
rs2090816
rs11715853
rs35055552
rs2387977
rs6809952
rs7311004
rs8039785
rs823424
rs10899911
rs62273907
rs6828768
rs7572229
rs78383039
rs55877868
All − MR Egger
All − IVW(MRE)
−1.0
−0.5
0.0
0.5
1.0
MR effect size for
'Gut microbiota abundance (genus Parasutterella id.2892)' on 'Chordin−like protein 1'

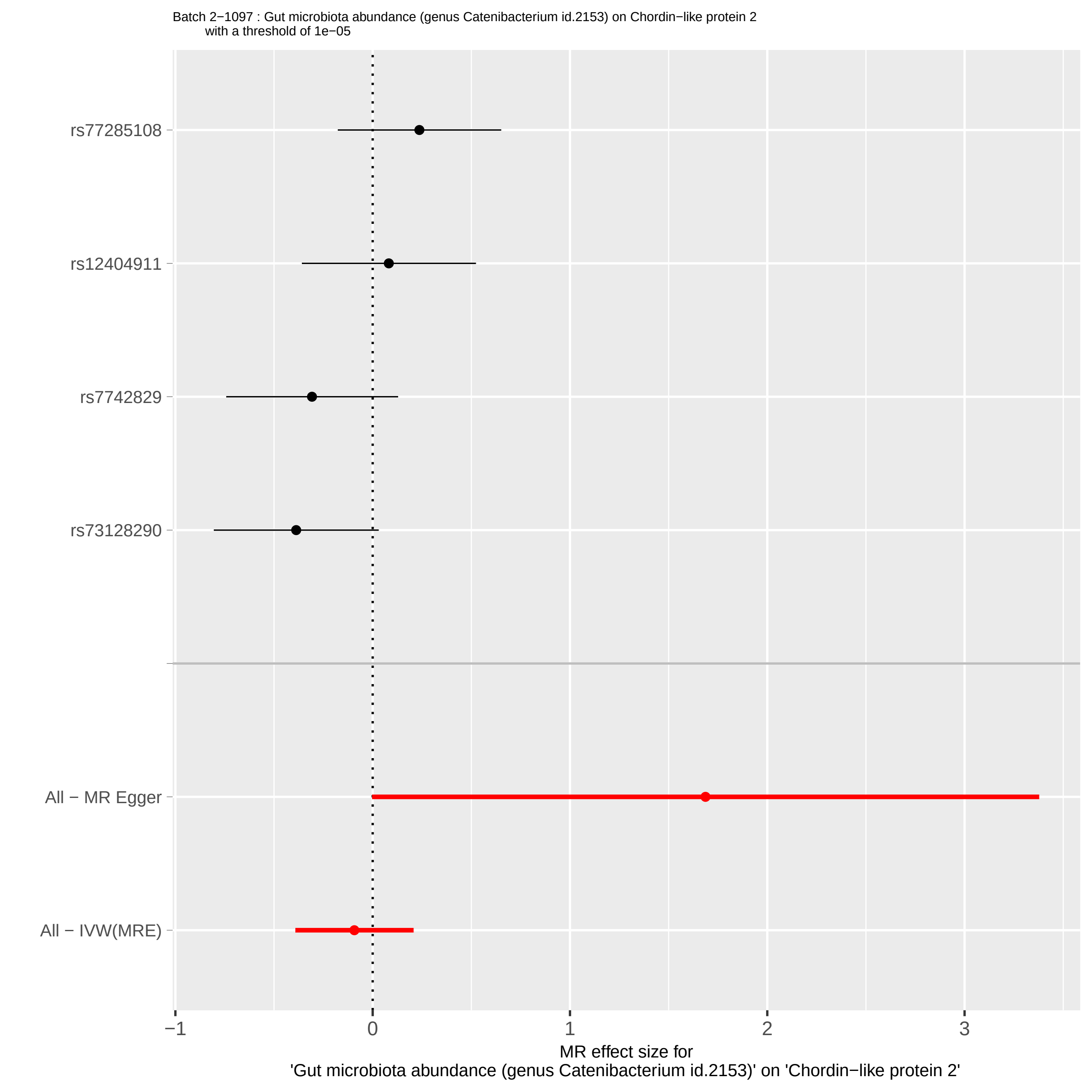

Batch 2−1097 : Gut microbiota abundance (genus Catenibacterium id.2153) on Chordin−like protein 2
with a threshold of 1e−05
rs77285108
rs12404911
rs7742829
rs73128290
All − MR Egger
All − IVW(MRE)
−1
0
1
2
3
MR effect size for
'Gut microbiota abundance (genus Catenibacterium id.2153)' on 'Chordin−like protein 2'

Batch 2−1098 : Gut microbiota abundance (genus Parasutterella id.2892) on Chordin−like protein 2
with a threshold of 1e−05

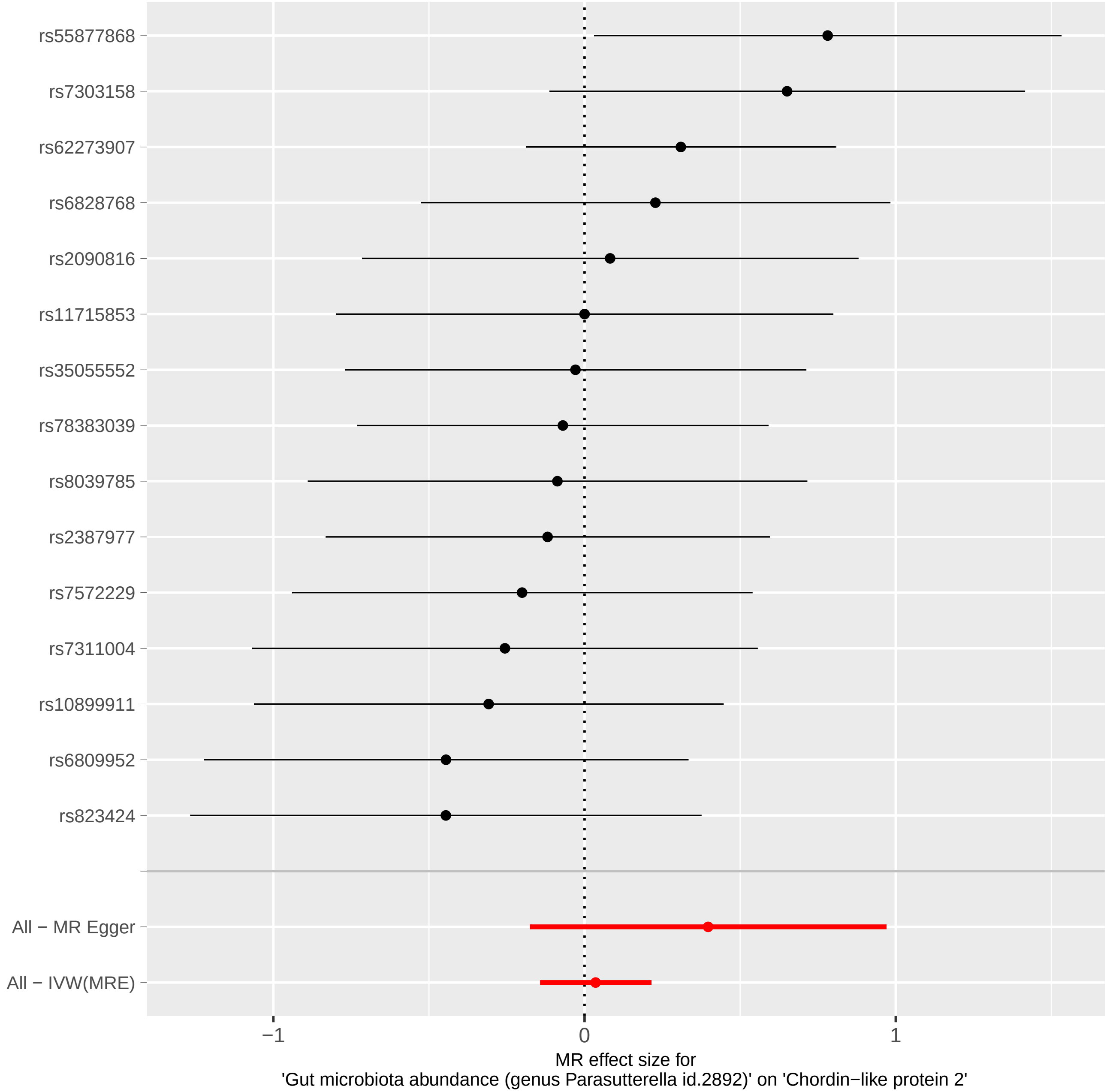

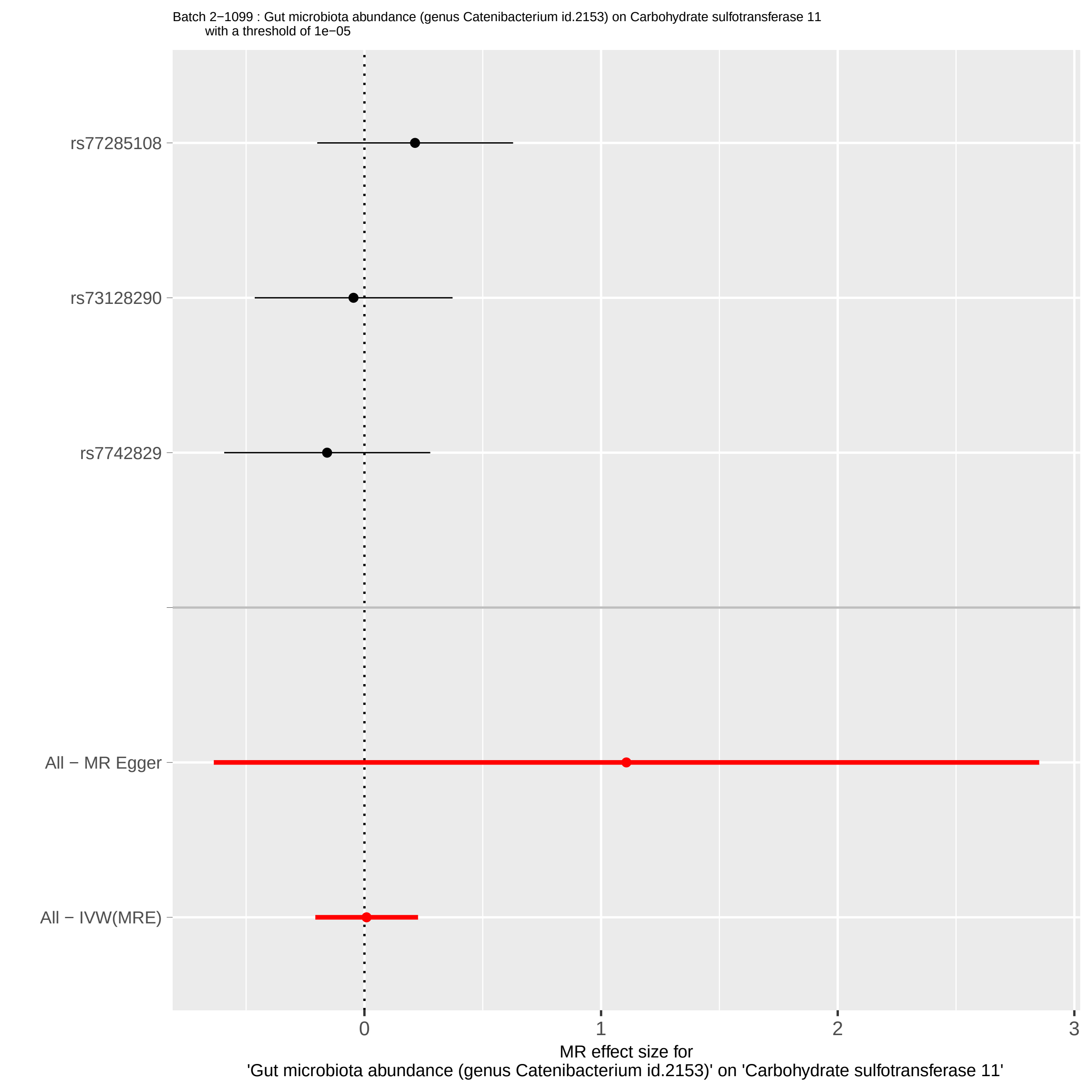

Batch 2−1099 : Gut microbiota abundance (genus Catenibacterium id.2153) on Carbohydrate sulfotransferase 11
with a threshold of 1e−05
rs77285108
rs73128290
rs7742829
All − MR Egger
All − IVW(MRE)
0
1
2
3
MR effect size for
'Gut microbiota abundance (genus Catenibacterium id.2153)' on 'Carbohydrate sulfotransferase 11'

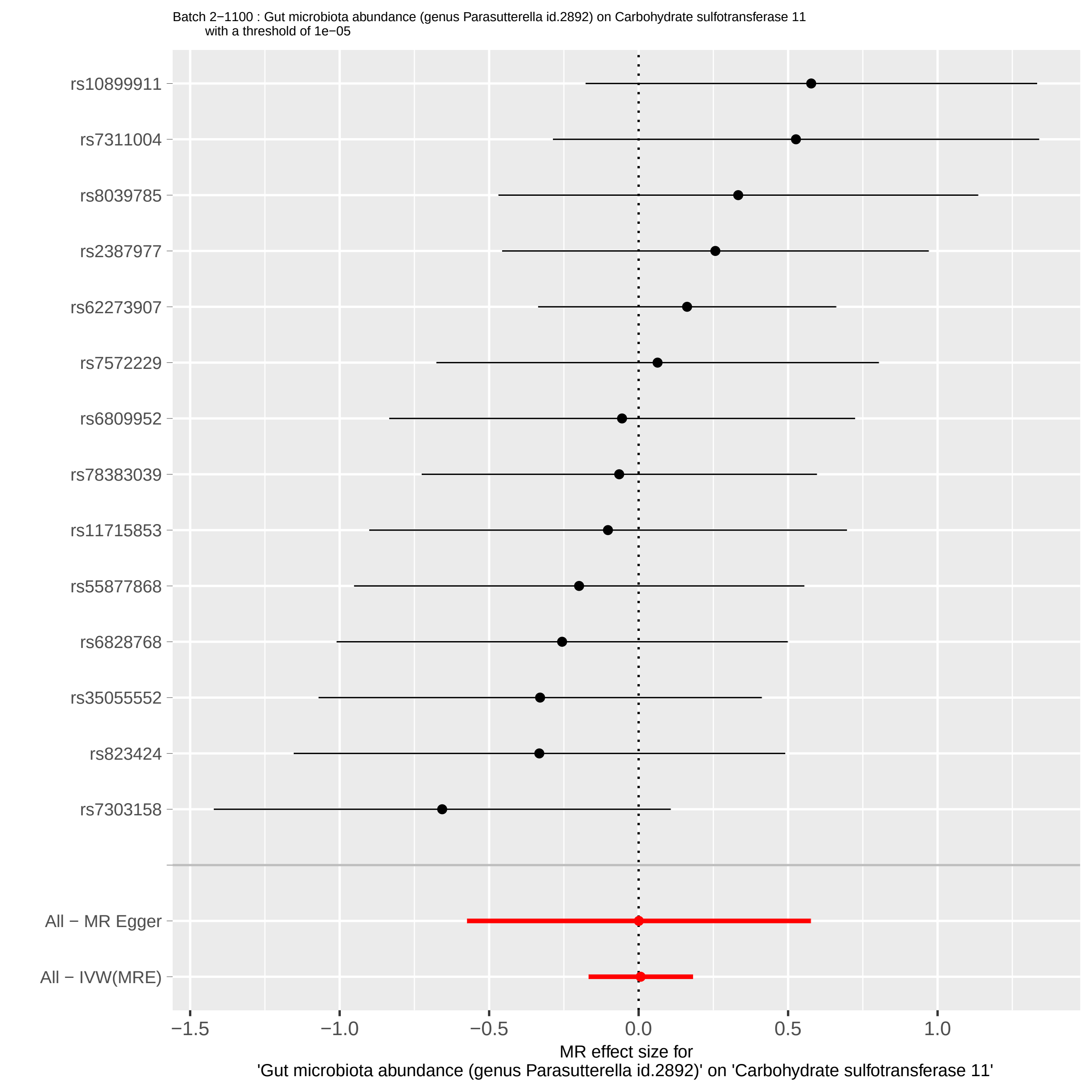

Batch 2−1100 : Gut microbiota abundance (genus Parasutterella id.2892) on Carbohydrate sulfotransferase 11
with a threshold of 1e−05
rs10899911
rs7311004
rs8039785
rs2387977
rs62273907
rs7572229
rs6809952
rs78383039
rs11715853
rs55877868
rs6828768
rs35055552
rs823424
rs7303158
All − MR Egger
All − IVW(MRE)
−1.5
−1.0
−0.5
0.0
0.5
1.0
MR effect size for
'Gut microbiota abundance (genus Parasutterella id.2892)' on 'Carbohydrate sulfotransferase 11'

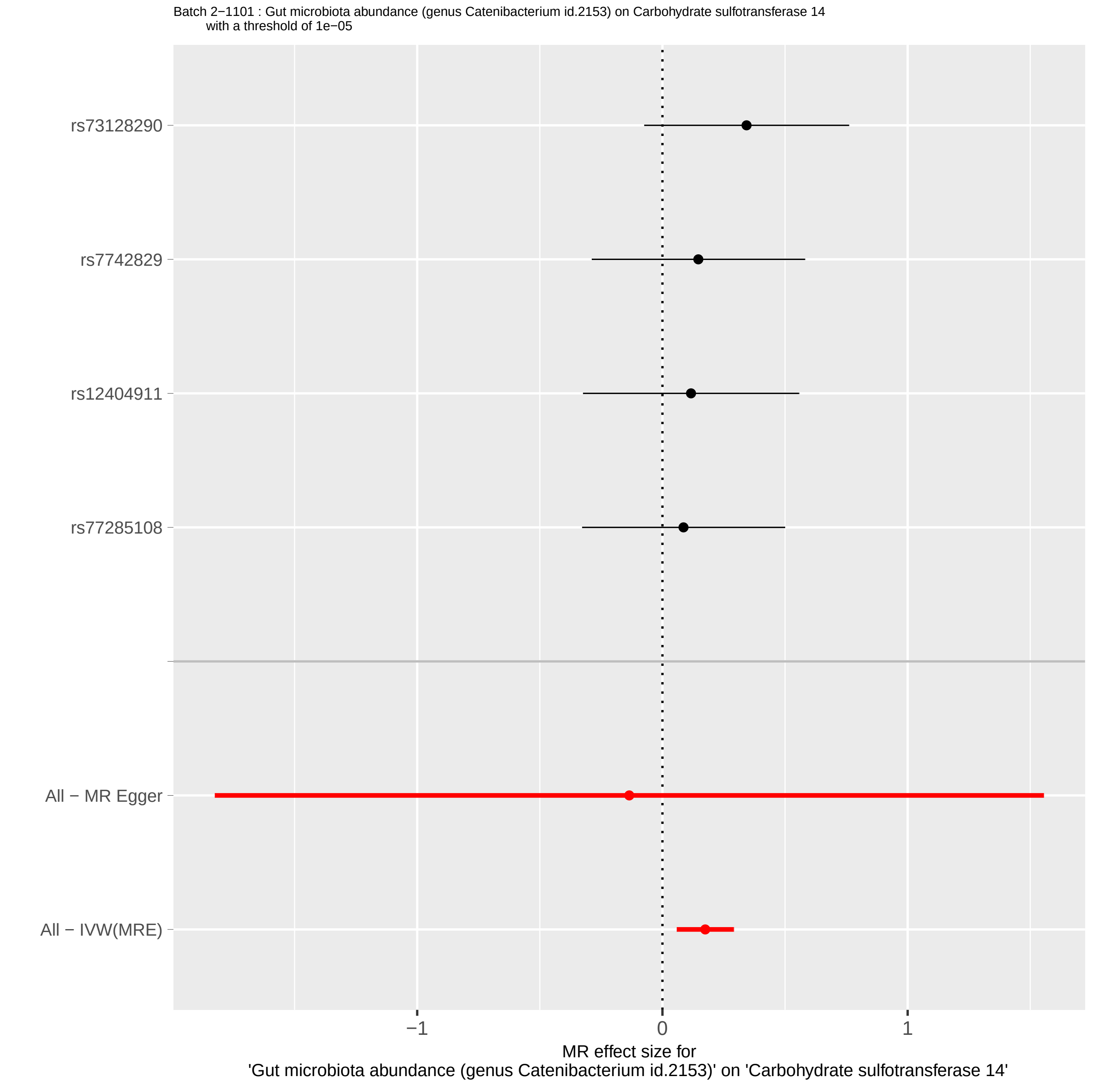

Batch 2−1101 : Gut microbiota abundance (genus Catenibacterium id.2153) on Carbohydrate sulfotransferase 14
with a threshold of 1e−05
rs73128290
rs7742829
rs12404911
rs77285108
All − MR Egger
All − IVW(MRE)
−1
0
1
MR effect size for
'Gut microbiota abundance (genus Catenibacterium id.2153)' on 'Carbohydrate sulfotransferase 14'

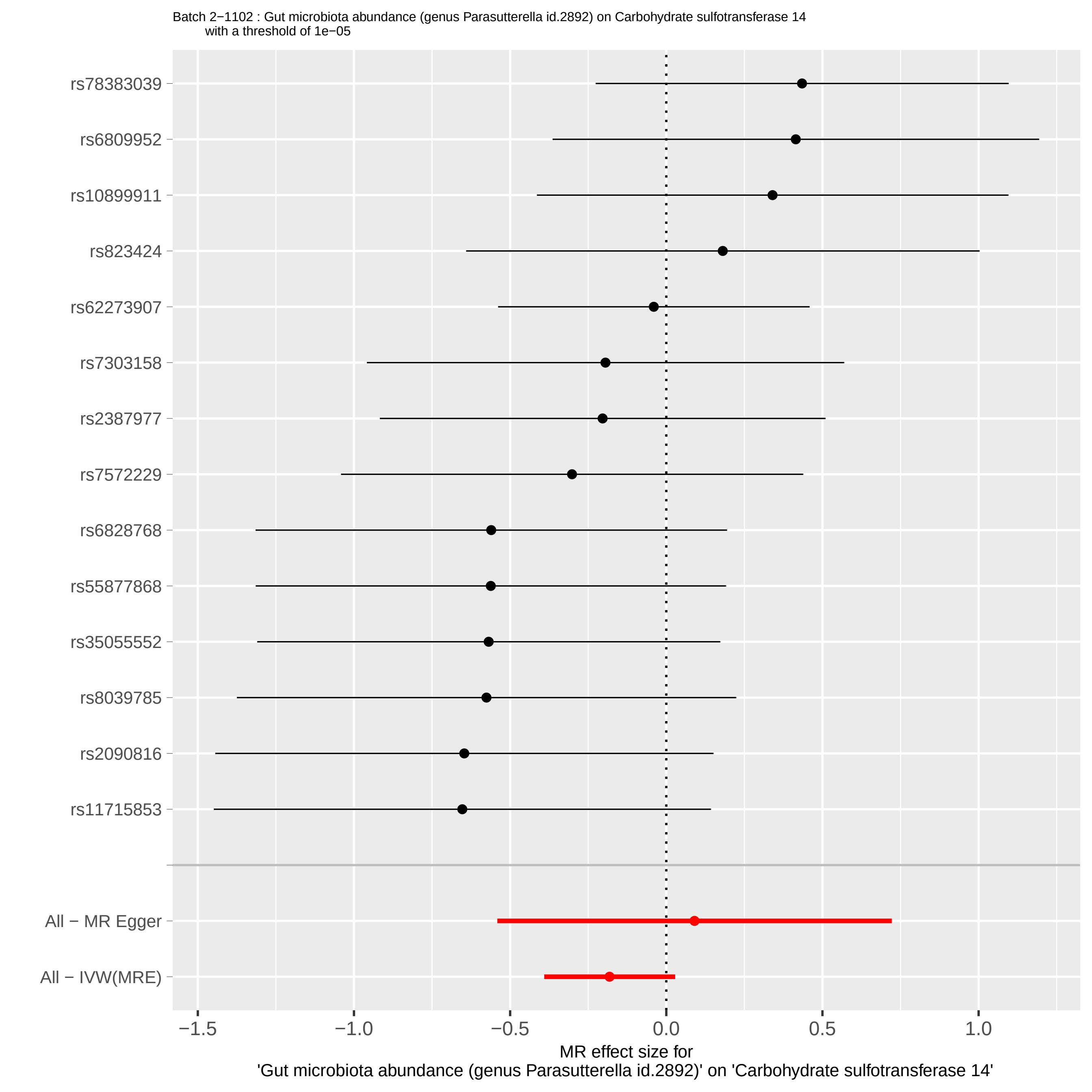

Batch 2−1102 : Gut microbiota abundance (genus Parasutterella id.2892) on Carbohydrate sulfotransferase 14
with a threshold of 1e−05
rs78383039
rs6809952
rs10899911
rs823424
rs62273907
rs7303158
rs2387977
rs7572229
rs6828768
rs55877868
rs35055552
rs8039785
rs2090816
rs11715853
All − MR Egger
All − IVW(MRE)
−1.5
−1.0
−0.5
0.0
0.5
1.0
MR effect size for
'Gut microbiota abundance (genus Parasutterella id.2892)' on 'Carbohydrate sulfotransferase 14'

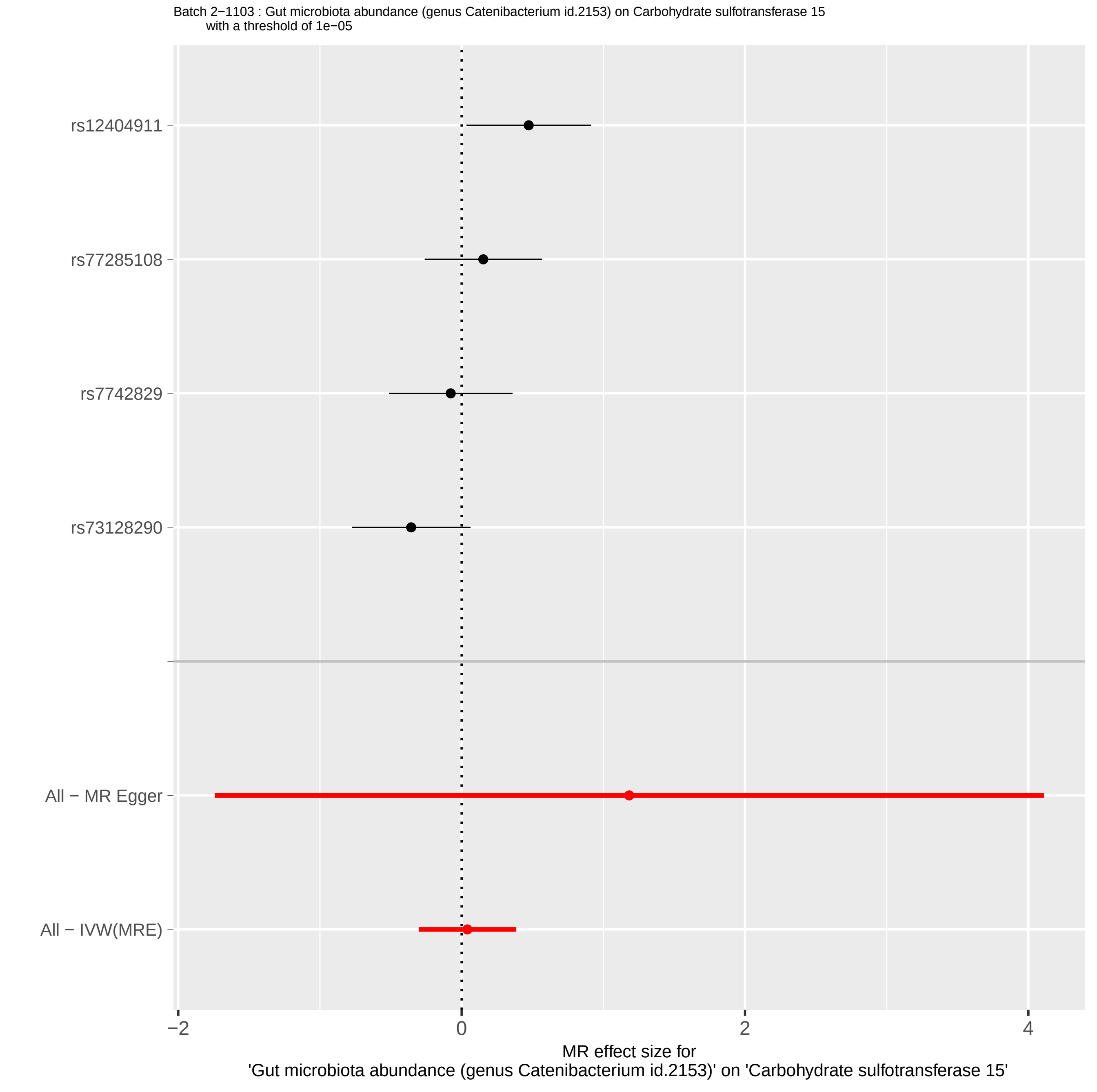

Batch 2−1103 : Gut microbiota abundance (genus Catenibacterium id.2153) on Carbohydrate sulfotransferase 15
with a threshold of 1e−05
rs12404911
rs77285108
rs7742829
rs73128290
All − MR Egger
All − IVW(MRE)
−2
0
2
4
MR effect size for
'Gut microbiota abundance (genus Catenibacterium id.2153)' on 'Carbohydrate sulfotransferase 15'

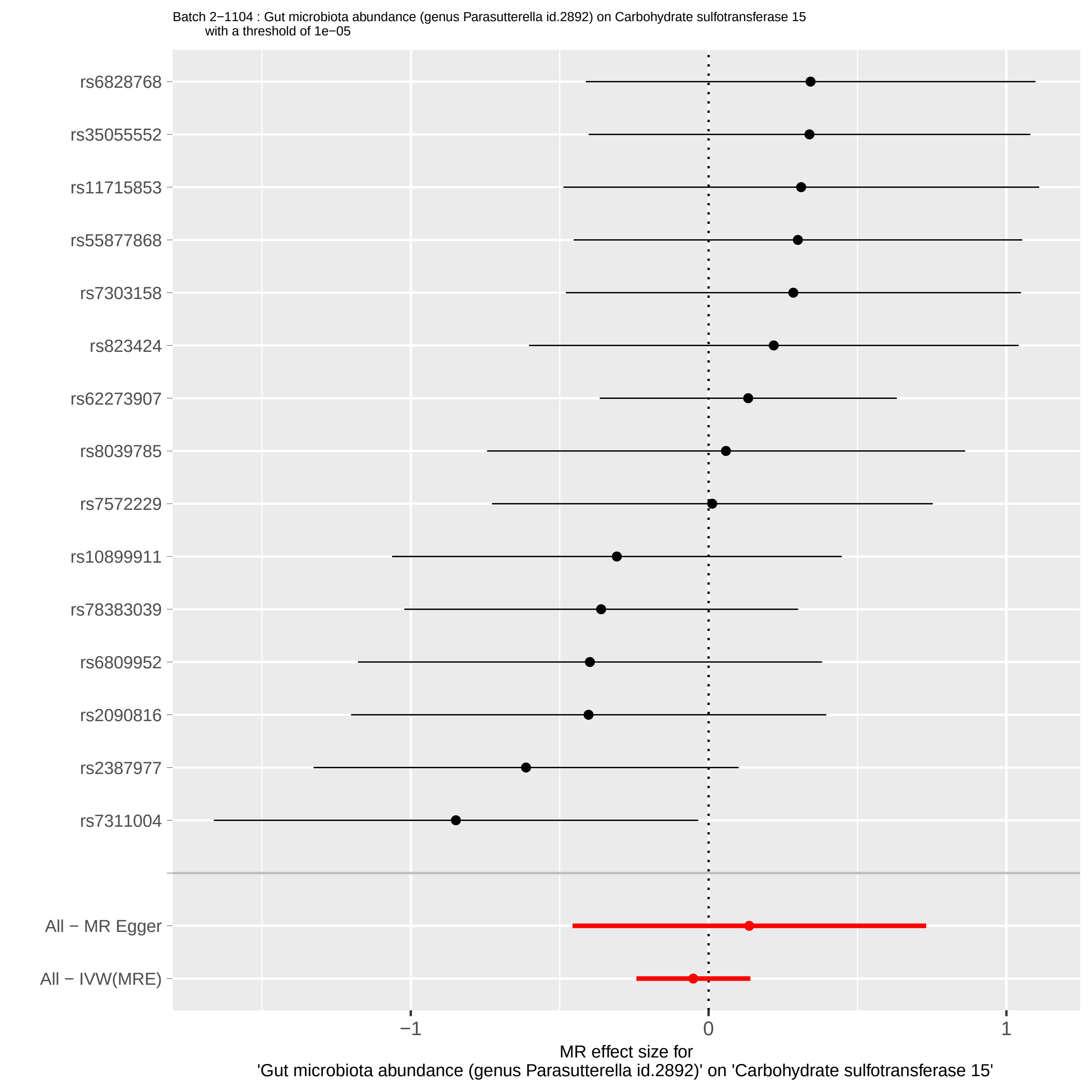

Batch 2−1104 : Gut microbiota abundance (genus Parasutterella id.2892) on Carbohydrate sulfotransferase 15
with a threshold of 1e−05
rs6828768
rs35055552
rs11715853
rs55877868
rs7303158
rs823424
rs62273907
rs8039785
rs7572229
rs10899911
rs78383039
rs6809952
rs2090816
rs2387977
rs7311004
All − MR Egger
All − IVW(MRE)
−1
0
1
MR effect size for
'Gut microbiota abundance (genus Parasutterella id.2892)' on 'Carbohydrate sulfotransferase 15'

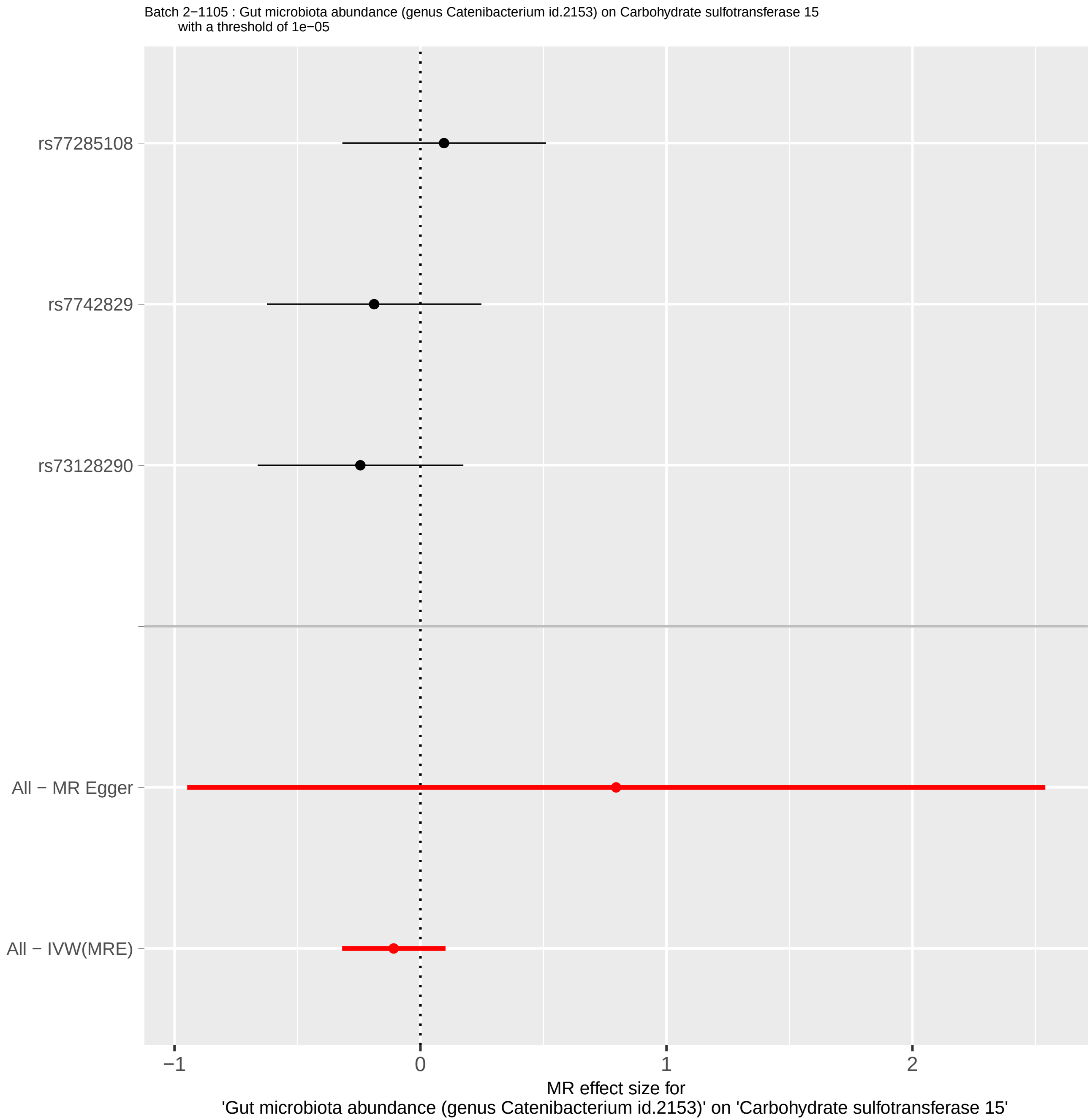
Batch 2−1105 : Gut microbiota abundance (genus Catenibacterium id.2153) on Carbohydrate sulfotransferase 15
with a threshold of 1e−05
rs77285108
rs7742829
rs73128290
All − MR Egger
All − IVW(MRE)
−1
0
1
2
MR effect size for
'Gut microbiota abundance (genus Catenibacterium id.2153)' on 'Carbohydrate sulfotransferase 15'

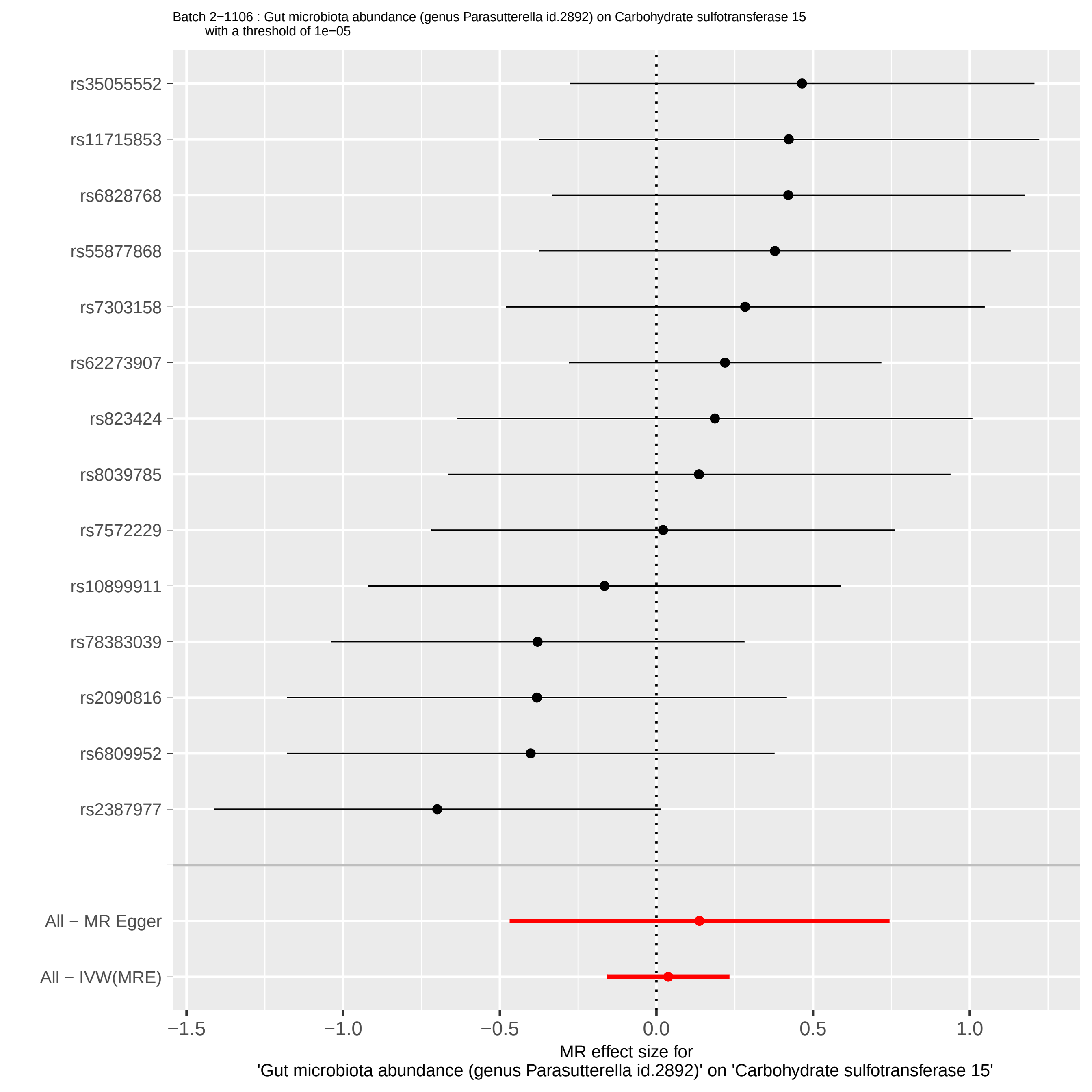
Batch 2−1106 : Gut microbiota abundance (genus Parasutterella id.2892) on Carbohydrate sulfotransferase 15
with a threshold of 1e−05
rs35055552
rs11715853
rs6828768
rs55877868
rs7303158
rs62273907
rs823424
rs8039785
rs7572229
rs10899911
rs78383039
rs2090816
rs6809952
rs2387977
All − MR Egger
All − IVW(MRE)
−1.5
−1.0
−0.5
0.0
0.5
1.0
MR effect size for
'Gut microbiota abundance (genus Parasutterella id.2892)' on 'Carbohydrate sulfotransferase 15'

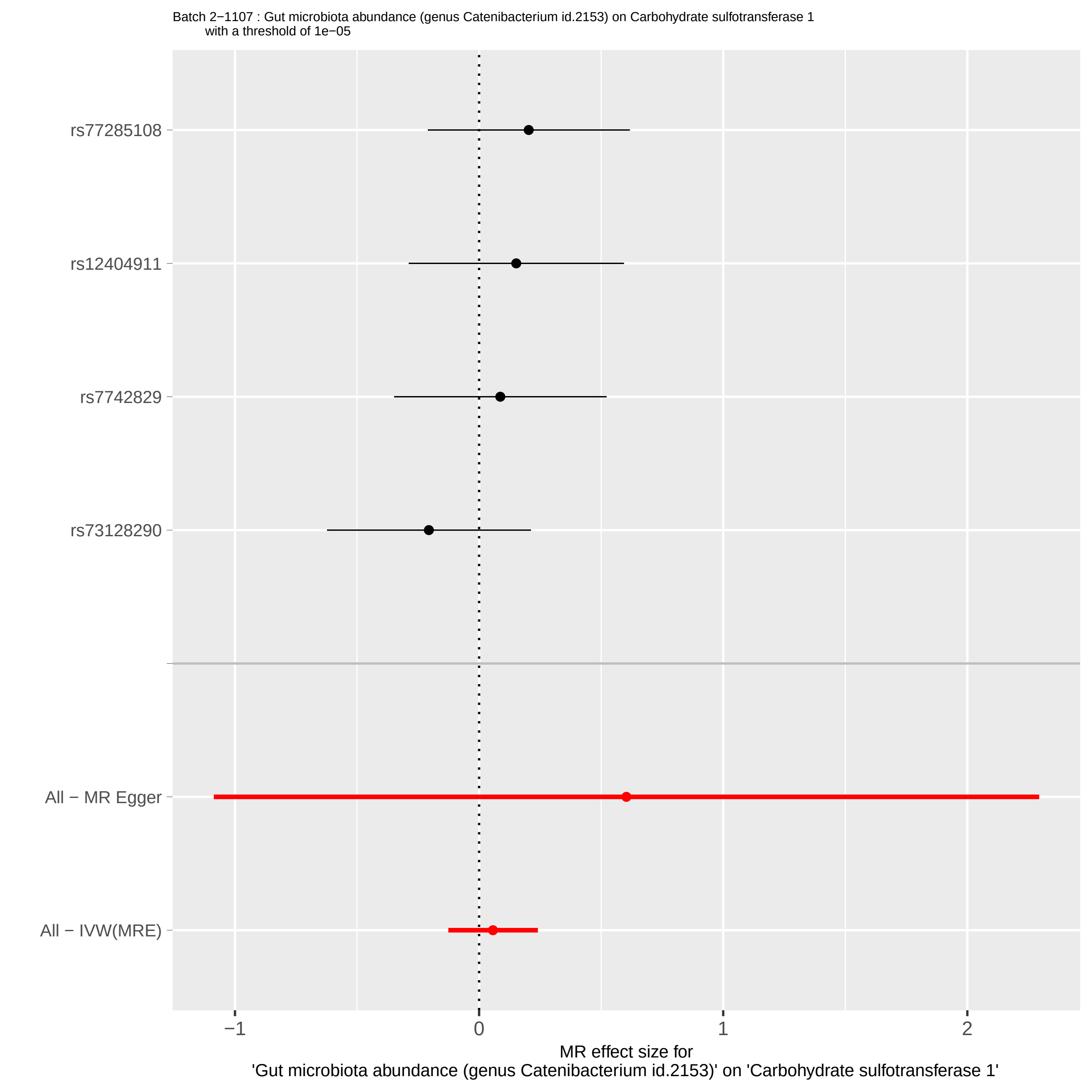

Batch 2−1107 : Gut microbiota abundance (genus Catenibacterium id.2153) on Carbohydrate sulfotransferase 1
with a threshold of 1e−05
rs77285108
rs12404911
rs7742829
rs73128290
All − MR Egger
All − IVW(MRE)
−1
0
1
2
MR effect size for
'Gut microbiota abundance (genus Catenibacterium id.2153)' on 'Carbohydrate sulfotransferase 1'

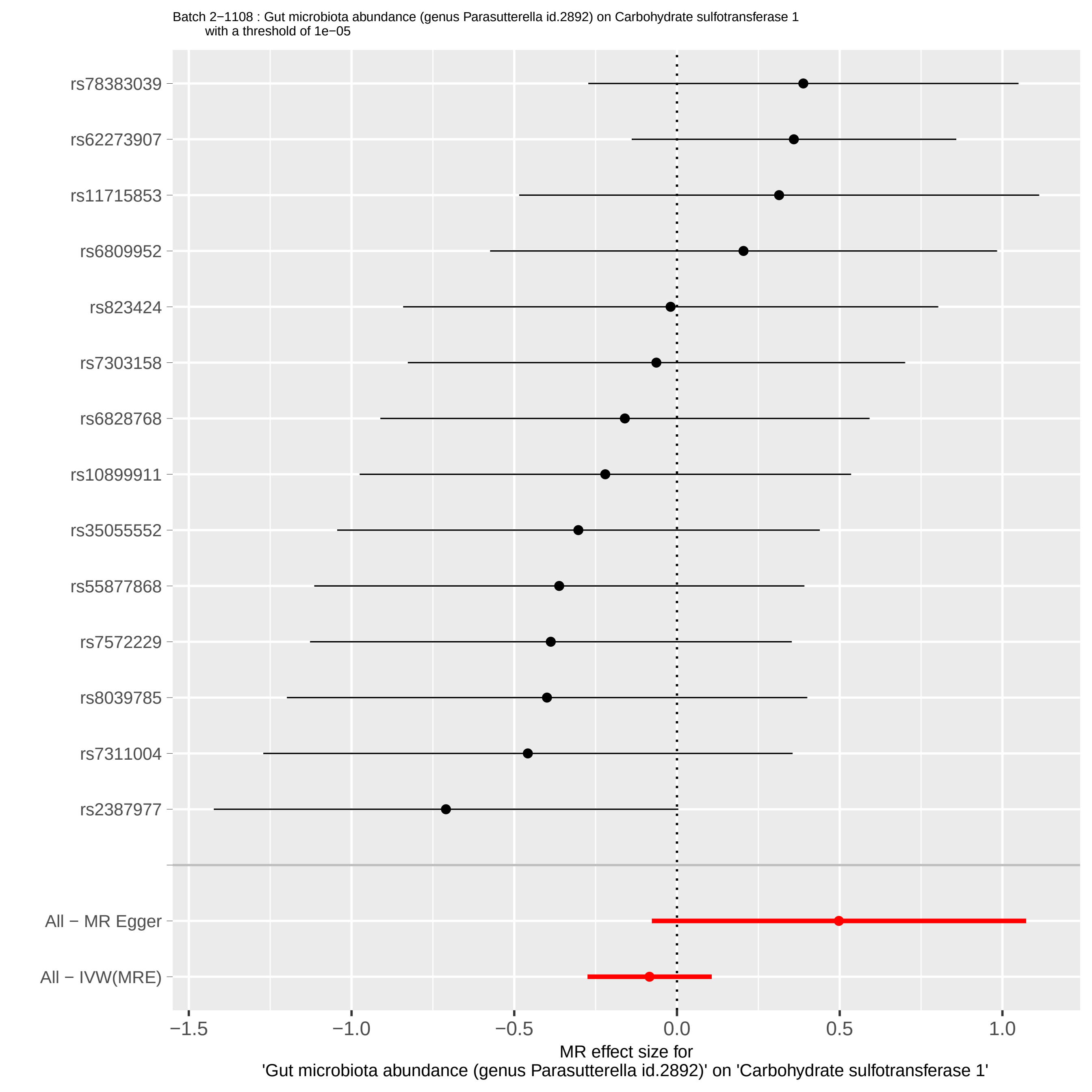

Batch 2−1108 : Gut microbiota abundance (genus Parasutterella id.2892) on Carbohydrate sulfotransferase 1
with a threshold of 1e−05
rs78383039
rs62273907
rs11715853
rs6809952
rs823424
rs7303158
rs6828768
rs10899911
rs35055552
rs55877868
rs7572229
rs8039785
rs7311004
rs2387977
All − MR Egger
All − IVW(MRE)
−1.5
−1.0
−0.5
0.0
0.5
1.0
MR effect size for
'Gut microbiota abundance (genus Parasutterella id.2892)' on 'Carbohydrate sulfotransferase 1'

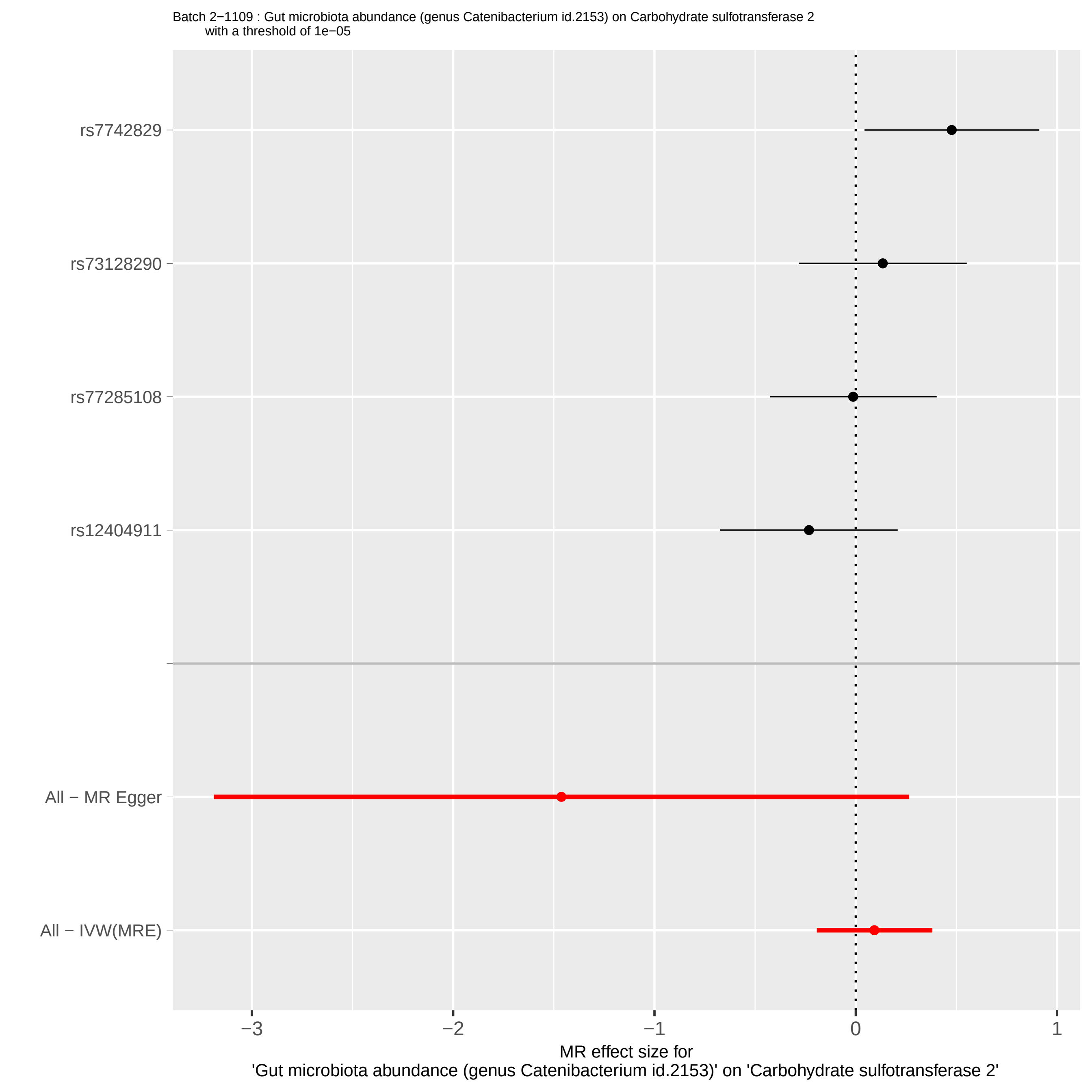

Batch 2−1109 : Gut microbiota abundance (genus Catenibacterium id.2153) on Carbohydrate sulfotransferase 2
with a threshold of 1e−05
rs7742829
rs73128290
rs77285108
rs12404911
All − MR Egger
All − IVW(MRE)
−3
−2
−1
0
1
MR effect size for
'Gut microbiota abundance (genus Catenibacterium id.2153)' on 'Carbohydrate sulfotransferase 2'

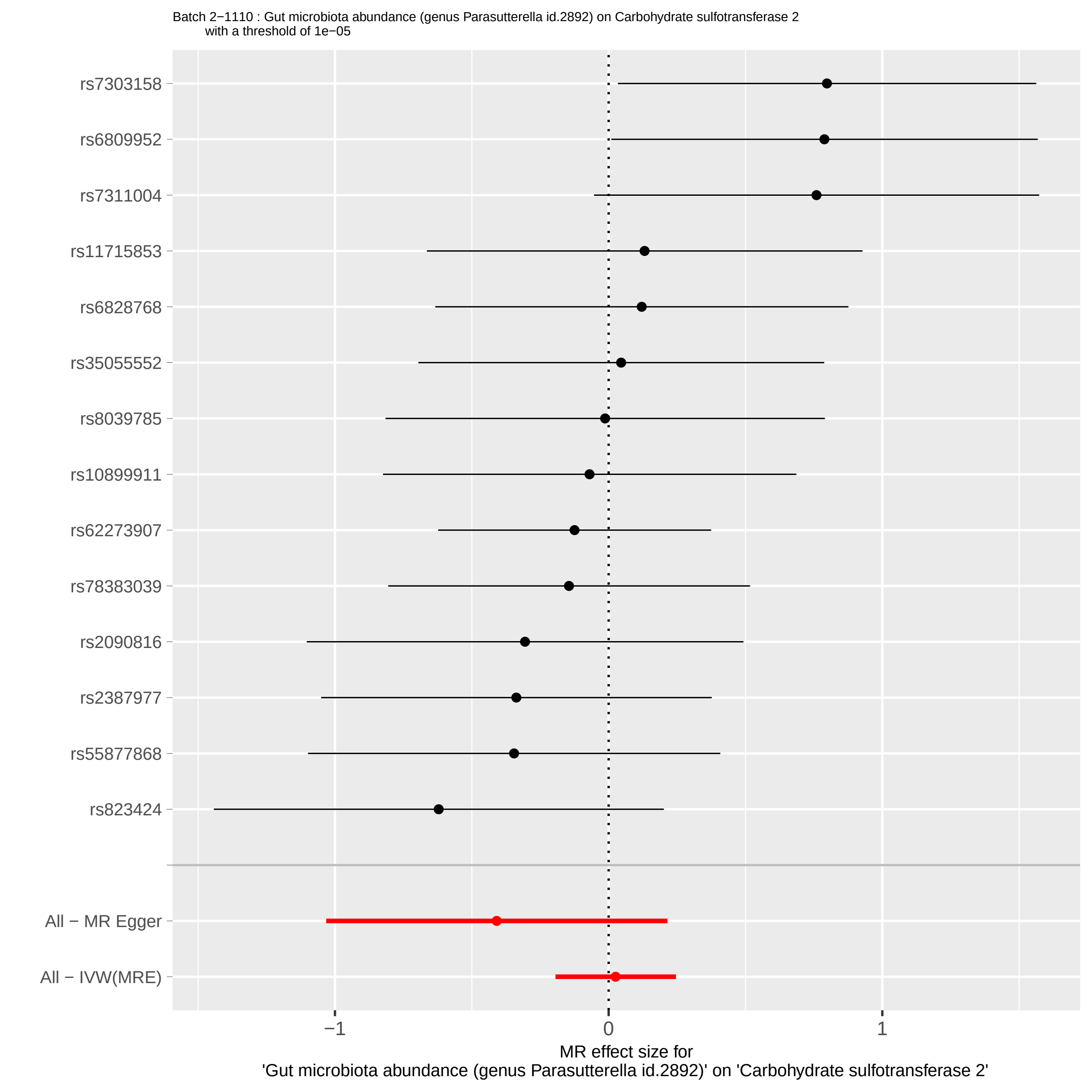

Batch 2−1110 : Gut microbiota abundance (genus Parasutterella id.2892) on Carbohydrate sulfotransferase 2
with a threshold of 1e−05
rs7303158
rs6809952
rs7311004
rs11715853
rs6828768
rs35055552
rs8039785
rs10899911
rs62273907
rs78383039
rs2090816
rs2387977
rs55877868
rs823424
All − MR Egger
All − IVW(MRE)
−1
0
1
MR effect size for
'Gut microbiota abundance (genus Parasutterella id.2892)' on 'Carbohydrate sulfotransferase 2'

Batch 2−1111 : Gut microbiota abundance (genus Catenibacterium id.2153) on Carbohydrate sulfotransferase 3
with a threshold of 1e−05

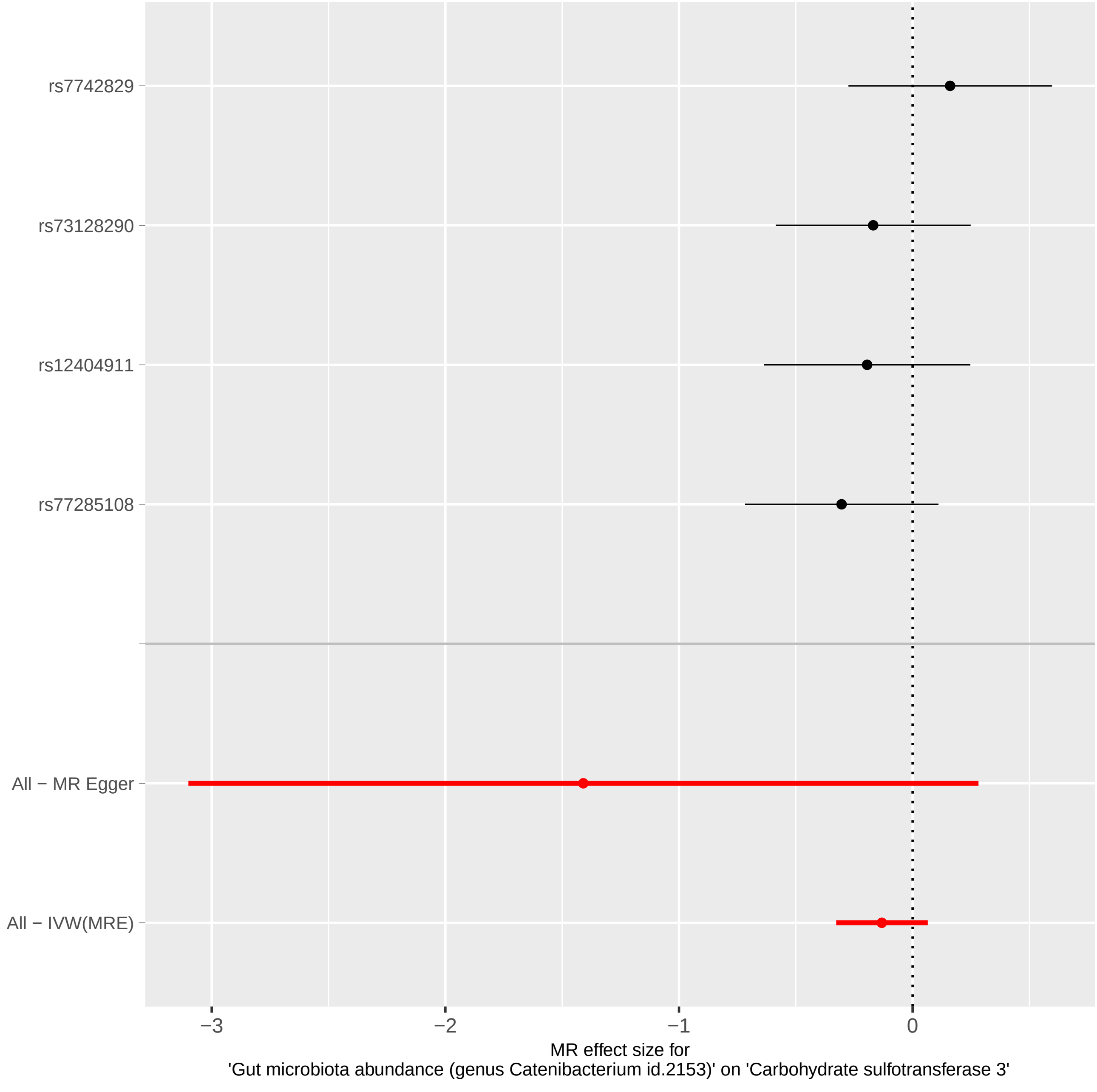

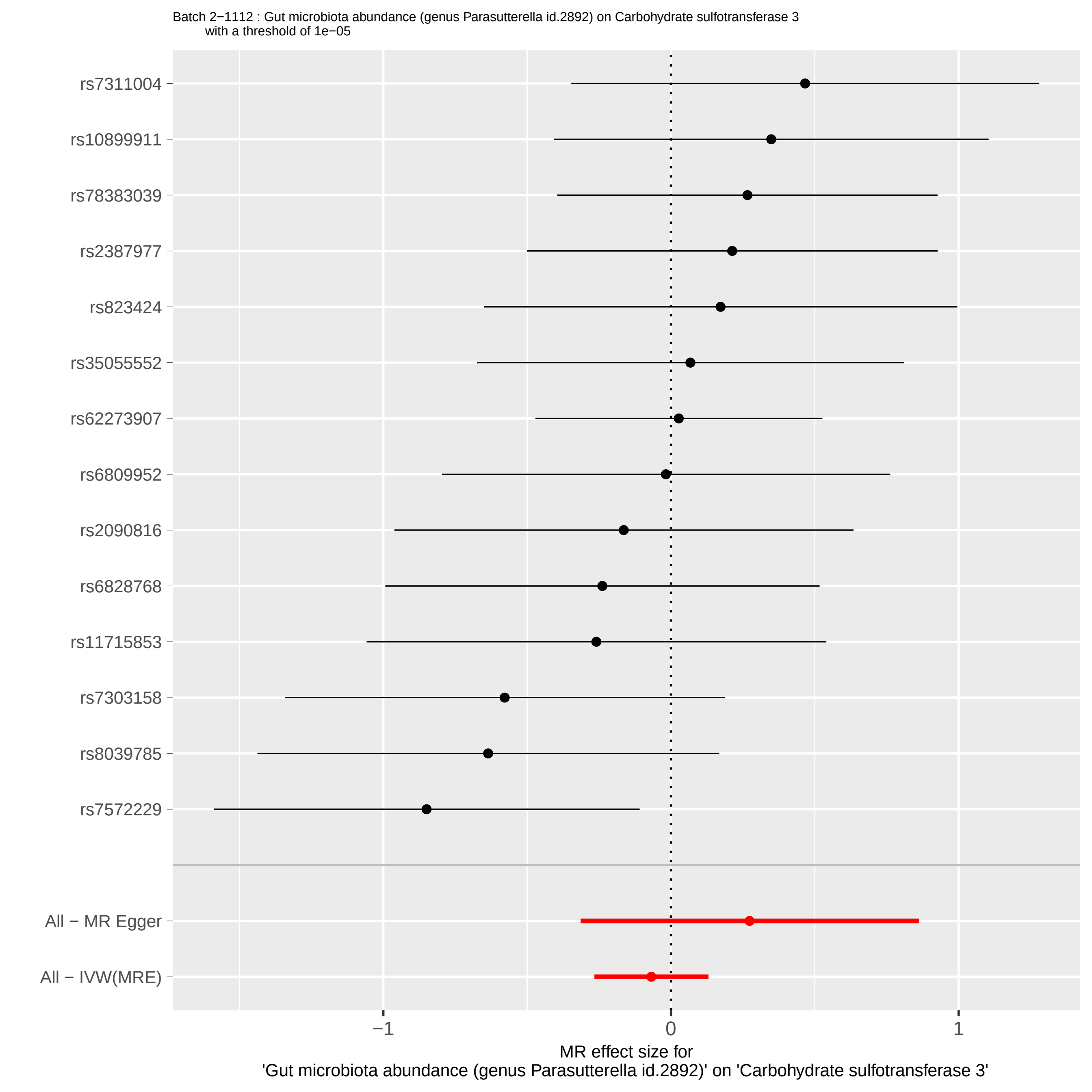
Batch 2−1112 : Gut microbiota abundance (genus Parasutterella id.2892) on Carbohydrate sulfotransferase 3
with a threshold of 1e−05
rs7311004
rs10899911
rs78383039
rs2387977
rs823424
rs35055552
rs62273907
rs6809952
rs2090816
rs6828768
rs11715853
rs7303158
rs8039785
rs7572229
All − MR Egger
All − IVW(MRE)
−1
0
1
MR effect size for
'Gut microbiota abundance (genus Parasutterella id.2892)' on 'Carbohydrate sulfotransferase 3'

Batch 2−1113 : Gut microbiota abundance (genus Catenibacterium id.2153) on Carbohydrate sulfotransferase 5
with a threshold of 1e−05

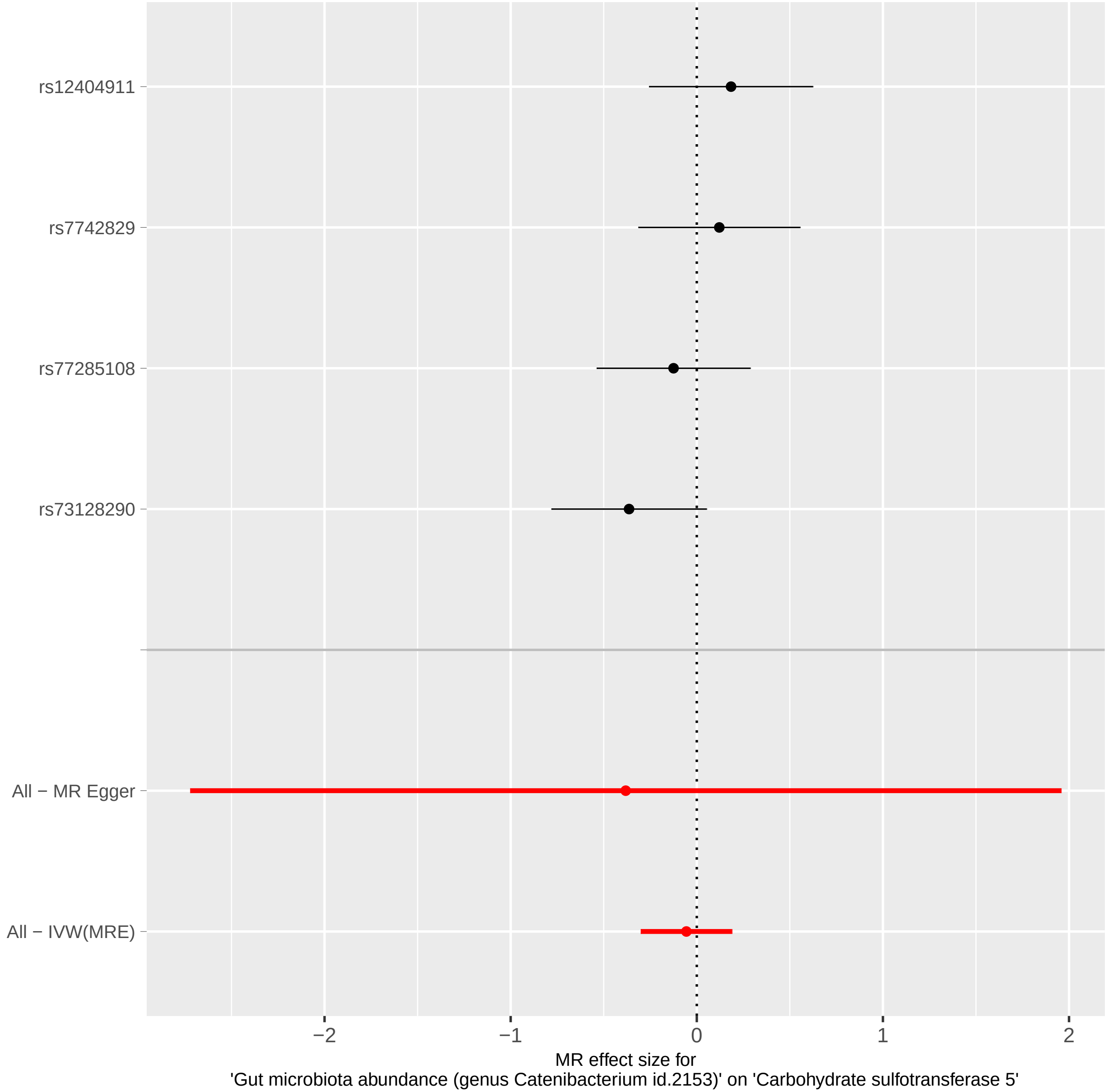

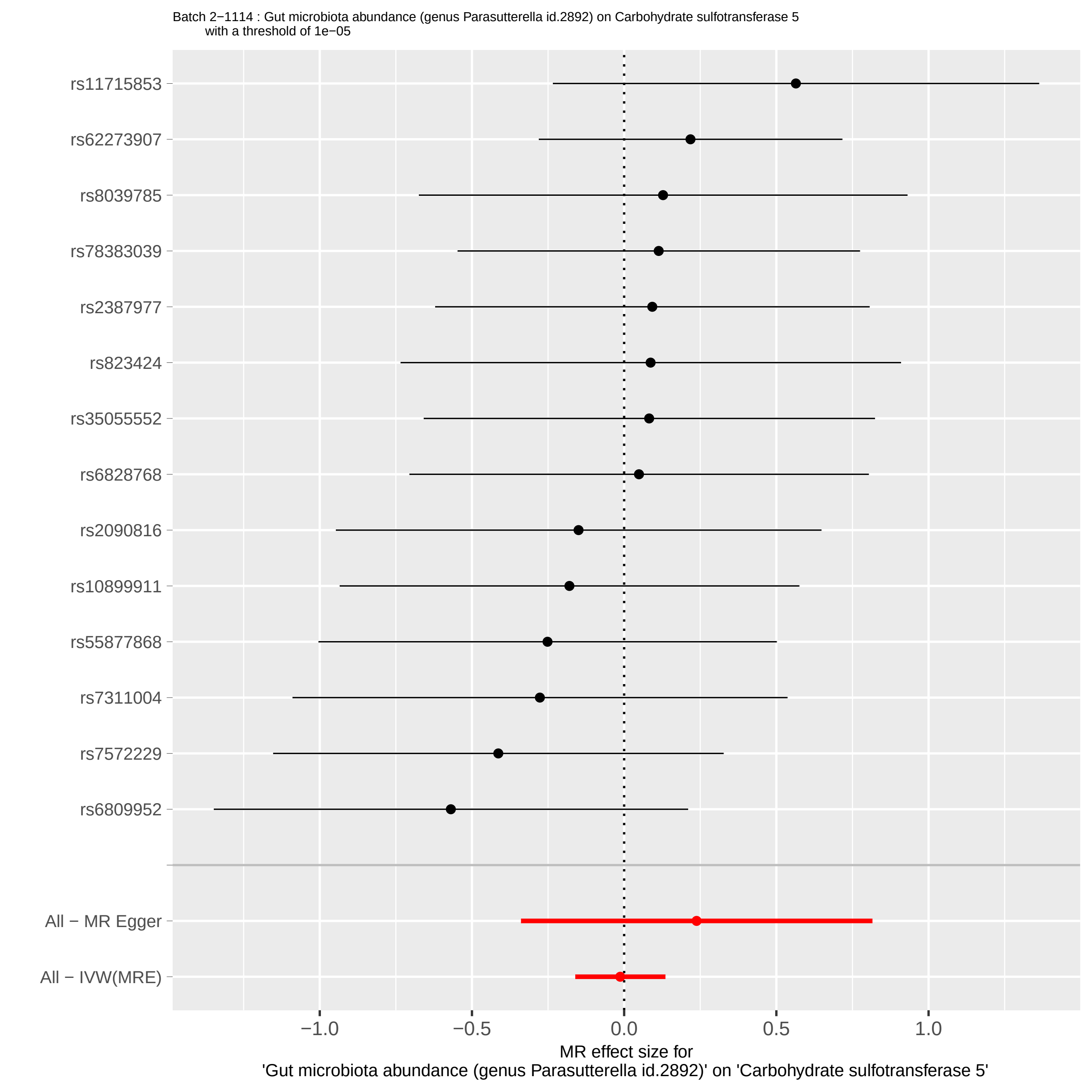

Batch 2−1114 : Gut microbiota abundance (genus Parasutterella id.2892) on Carbohydrate sulfotransferase 5
with a threshold of 1e−05
rs11715853
rs62273907
rs8039785
rs78383039
rs2387977
rs823424
rs35055552
rs6828768
rs2090816
rs10899911
rs55877868
rs7311004
rs7572229
rs6809952
All − MR Egger
All − IVW(MRE)
−1.0
−0.5
0.0
0.5
1.0
MR effect size for
'Gut microbiota abundance (genus Parasutterella id.2892)' on 'Carbohydrate sulfotransferase 5'

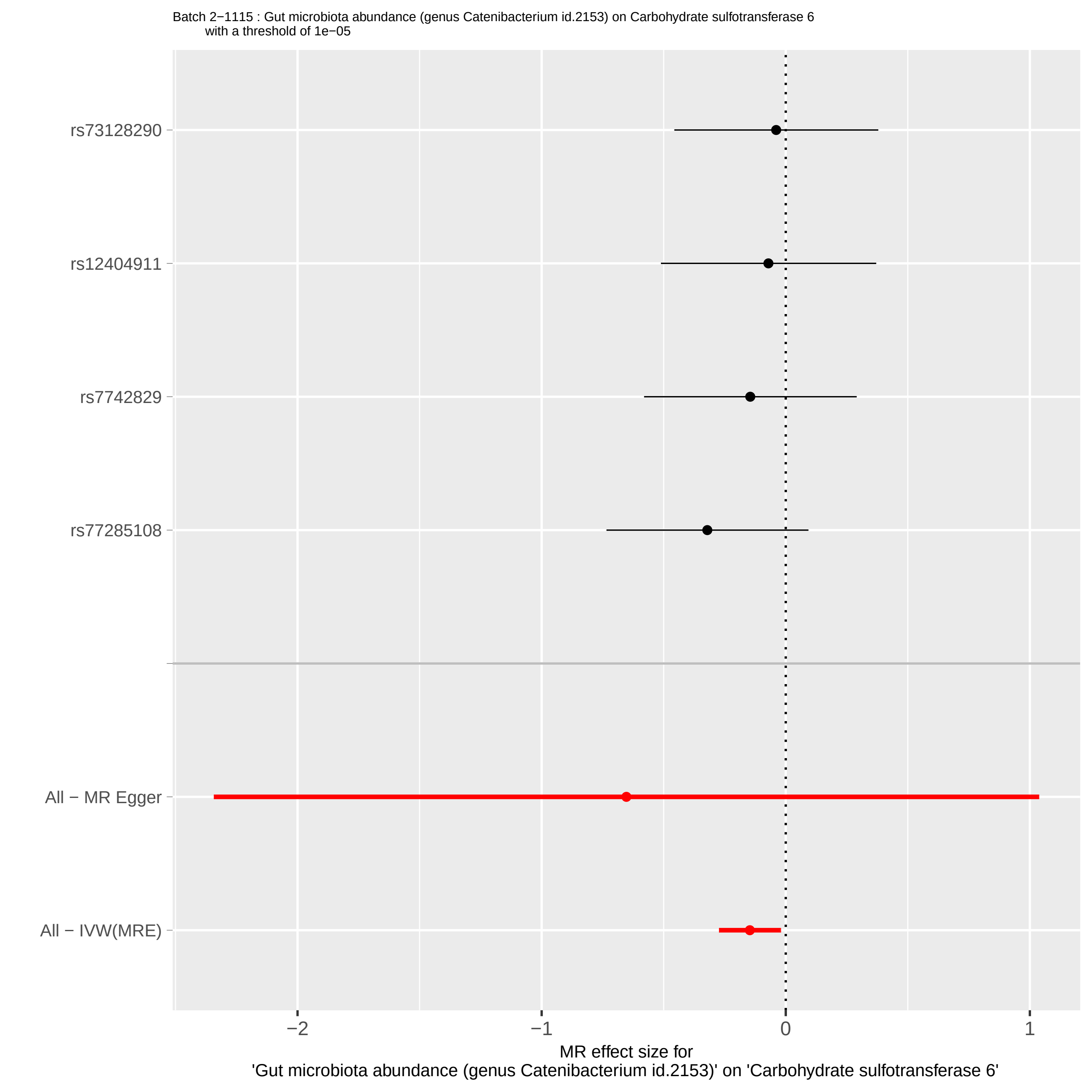
Batch 2−1115 : Gut microbiota abundance (genus Catenibacterium id.2153) on Carbohydrate sulfotransferase 6
with a threshold of 1e−05
rs73128290
rs12404911
rs7742829
rs77285108
All − MR Egger
All − IVW(MRE)
−2
−1
0
1
MR effect size for
'Gut microbiota abundance (genus Catenibacterium id.2153)' on 'Carbohydrate sulfotransferase 6'

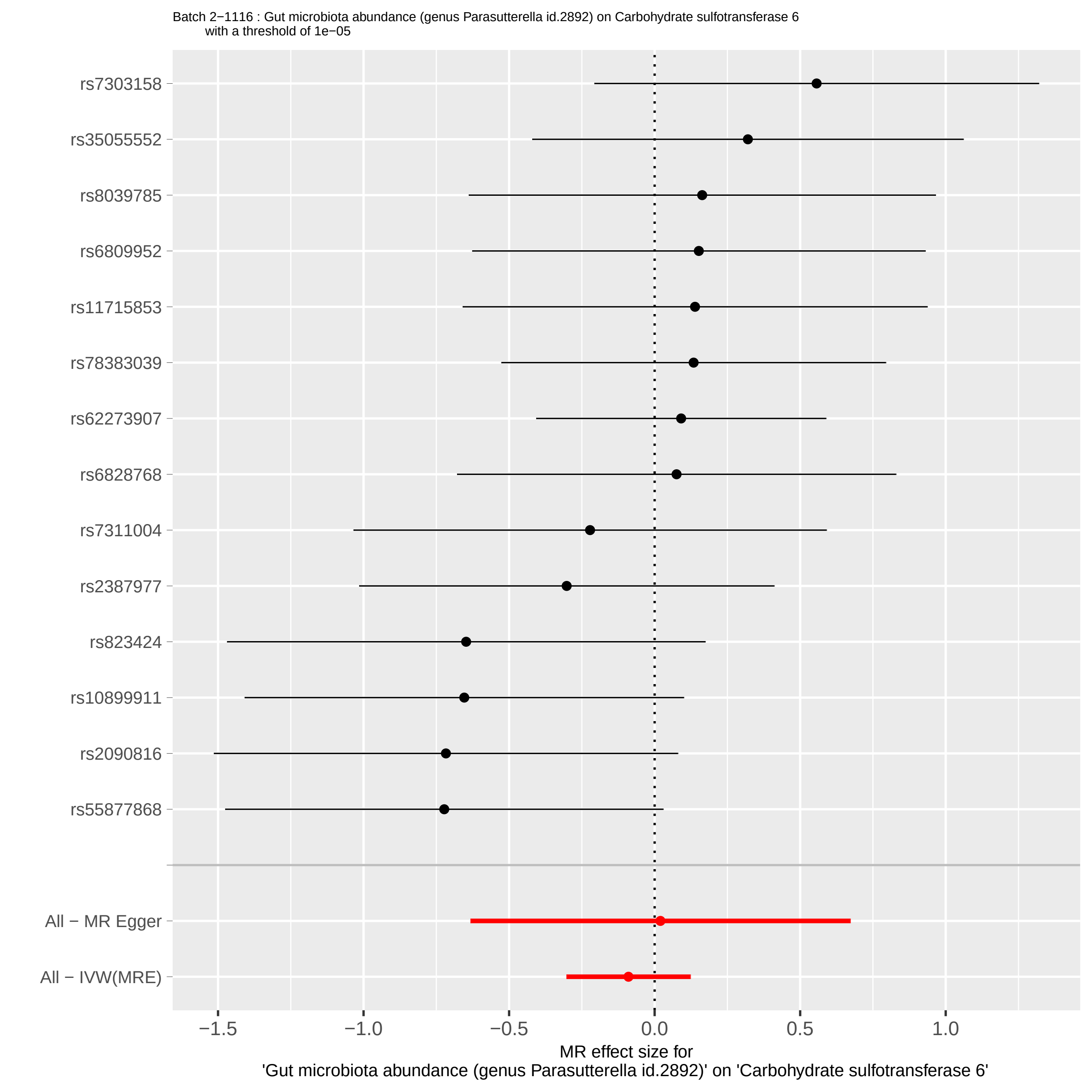

Batch 2−1116 : Gut microbiota abundance (genus Parasutterella id.2892) on Carbohydrate sulfotransferase 6
with a threshold of 1e−05
rs7303158
rs35055552
rs8039785
rs6809952
rs11715853
rs78383039
rs62273907
rs6828768
rs7311004
rs2387977
rs823424
rs10899911
rs2090816
rs55877868
All − MR Egger
All − IVW(MRE)
−1.5
−1.0
−0.5
0.0
0.5
1.0
MR effect size for
'Gut microbiota abundance (genus Parasutterella id.2892)' on 'Carbohydrate sulfotransferase 6'

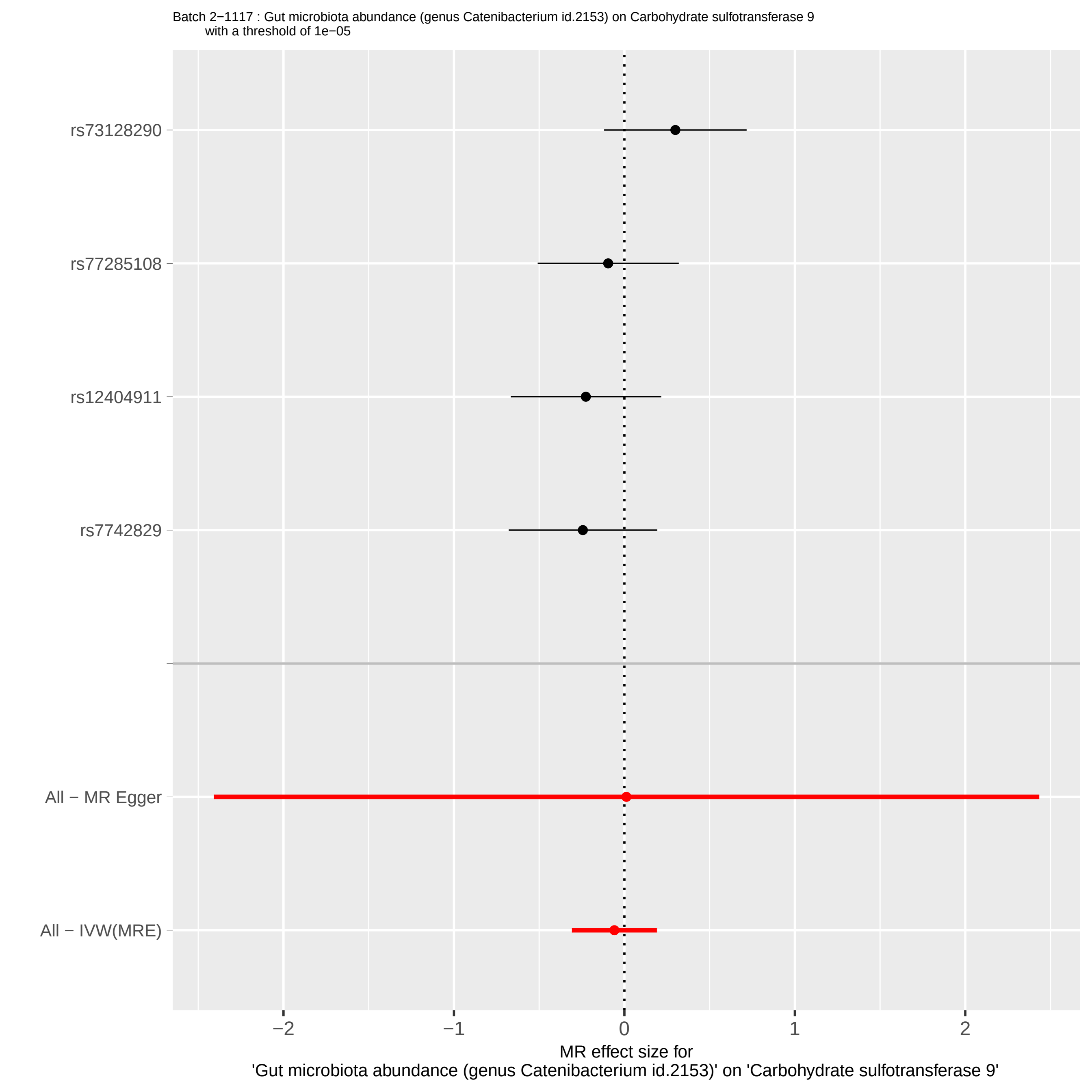

Batch 2−1117 : Gut microbiota abundance (genus Catenibacterium id.2153) on Carbohydrate sulfotransferase 9
with a threshold of 1e−05
rs73128290
rs77285108
rs12404911
rs7742829
All − MR Egger
All − IVW(MRE)
−2
−1
0
1
2
MR effect size for
'Gut microbiota abundance (genus Catenibacterium id.2153)' on 'Carbohydrate sulfotransferase 9'

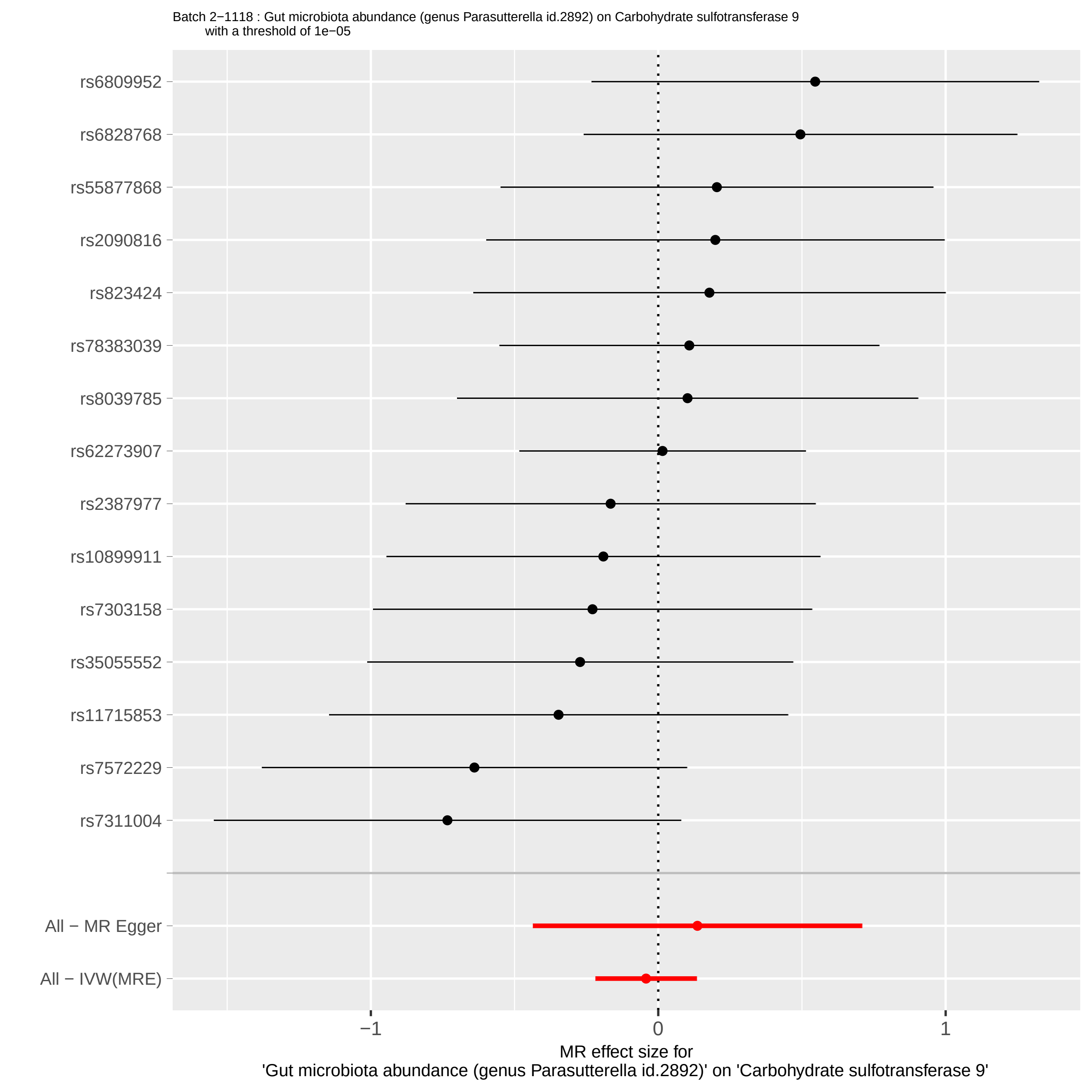

Batch 2−1118 : Gut microbiota abundance (genus Parasutterella id.2892) on Carbohydrate sulfotransferase 9
with a threshold of 1e−05
rs6809952
rs6828768
rs55877868
rs2090816
rs823424
rs78383039
rs8039785
rs62273907
rs2387977
rs10899911
rs7303158
rs35055552
rs11715853
rs7572229
rs7311004
All − MR Egger
All − IVW(MRE)
−1
0
1
MR effect size for
'Gut microbiota abundance (genus Parasutterella id.2892)' on 'Carbohydrate sulfotransferase 9'

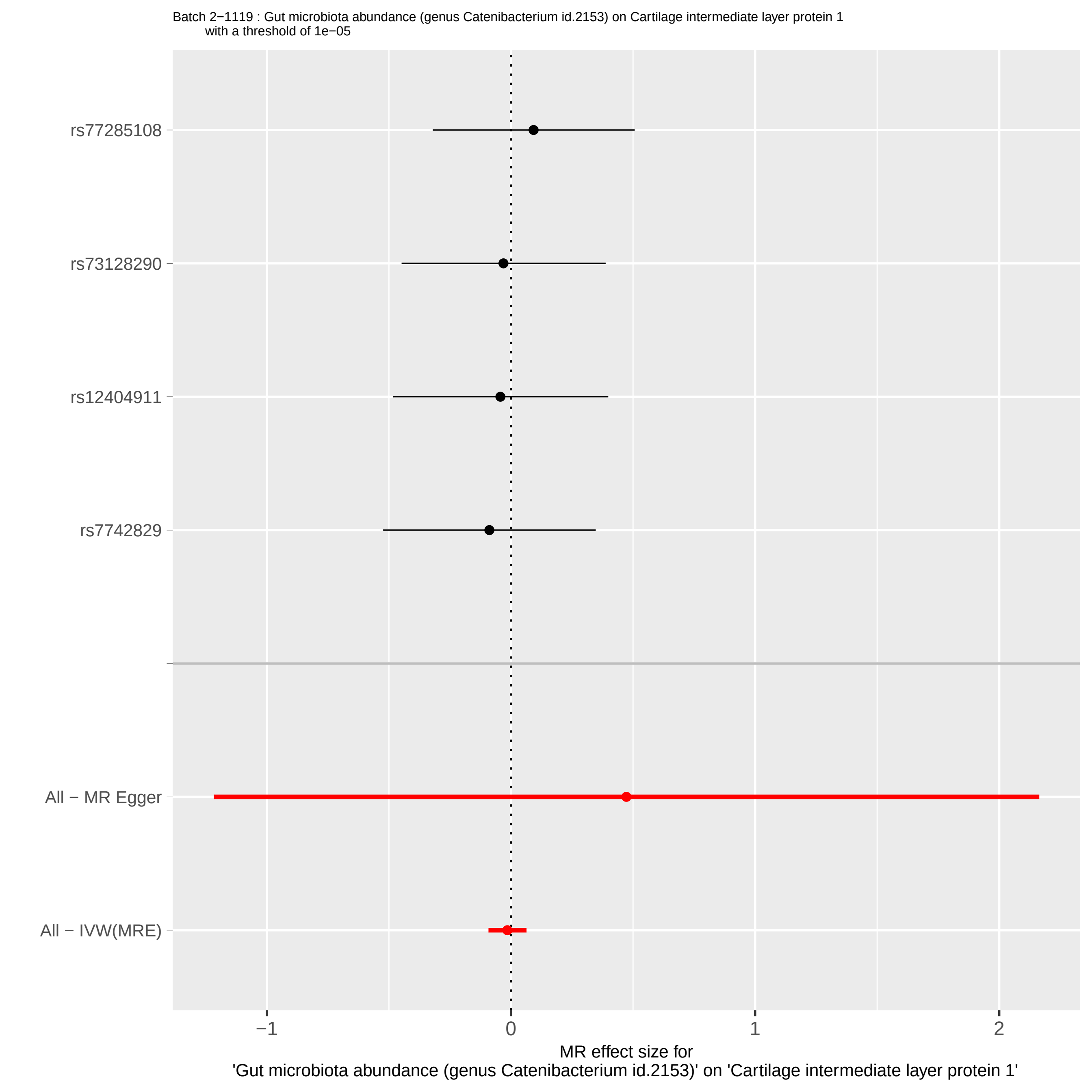

Batch 2−1119 : Gut microbiota abundance (genus Catenibacterium id.2153) on Cartilage intermediate layer protein 1
with a threshold of 1e−05
rs77285108
rs73128290
rs12404911
rs7742829
All − MR Egger
All − IVW(MRE)
−1
0
1
2
MR effect size for
'Gut microbiota abundance (genus Catenibacterium id.2153)' on 'Cartilage intermediate layer protein 1'

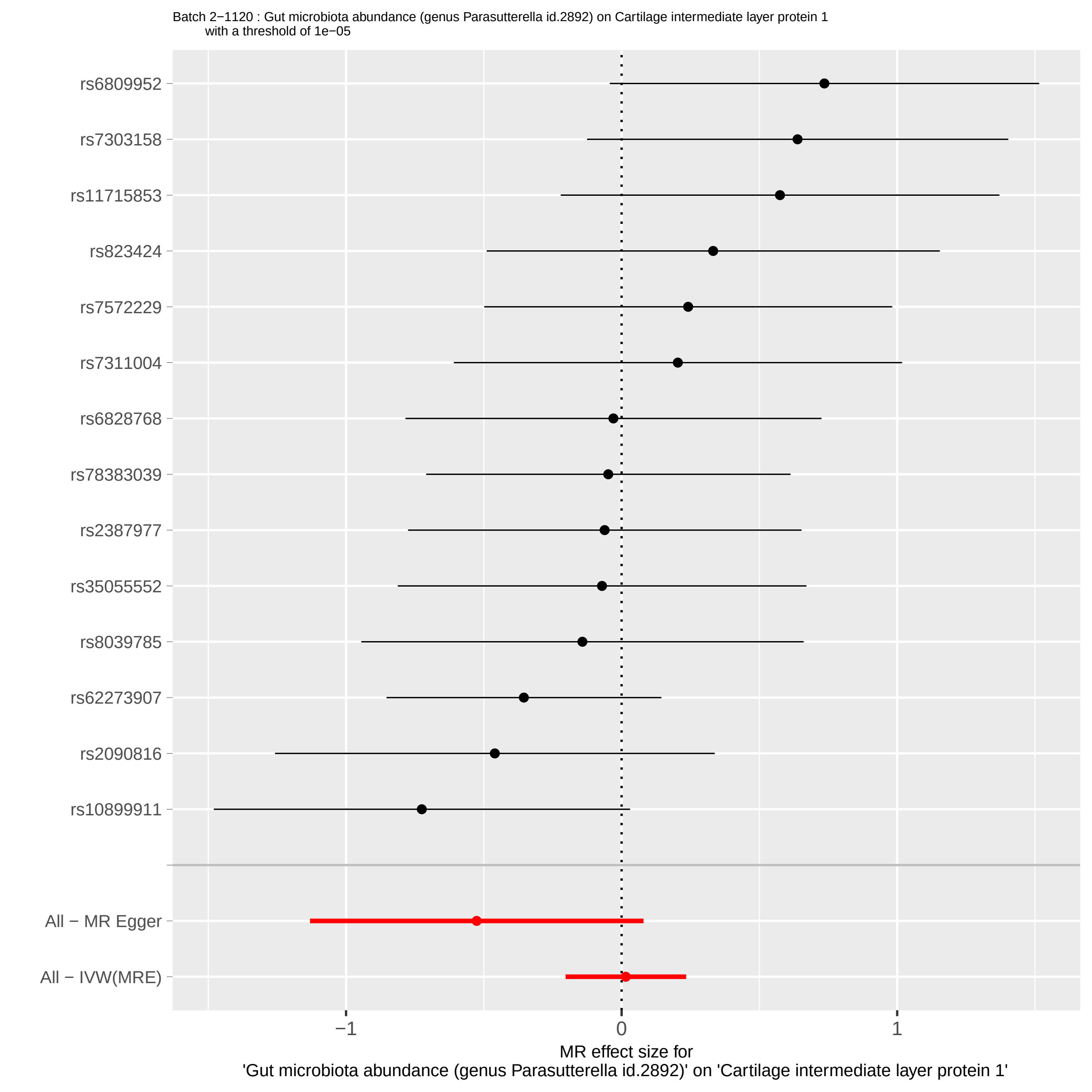

Batch 2−1120 : Gut microbiota abundance (genus Parasutterella id.2892) on Cartilage intermediate layer protein 1
with a threshold of 1e−05
rs6809952
rs7303158
rs11715853
rs823424
rs7572229
rs7311004
rs6828768
rs78383039
rs2387977
rs35055552
rs8039785
rs62273907
rs2090816
rs10899911
All − MR Egger
All − IVW(MRE)
−1
0
1
MR effect size for
'Gut microbiota abundance (genus Parasutterella id.2892)' on 'Cartilage intermediate layer protein 1'

Batch 2−1121 : Gut microbiota abundance (genus Catenibacterium id.2153) on Cold−inducible RNA−binding protein
with a threshold of 1e−05

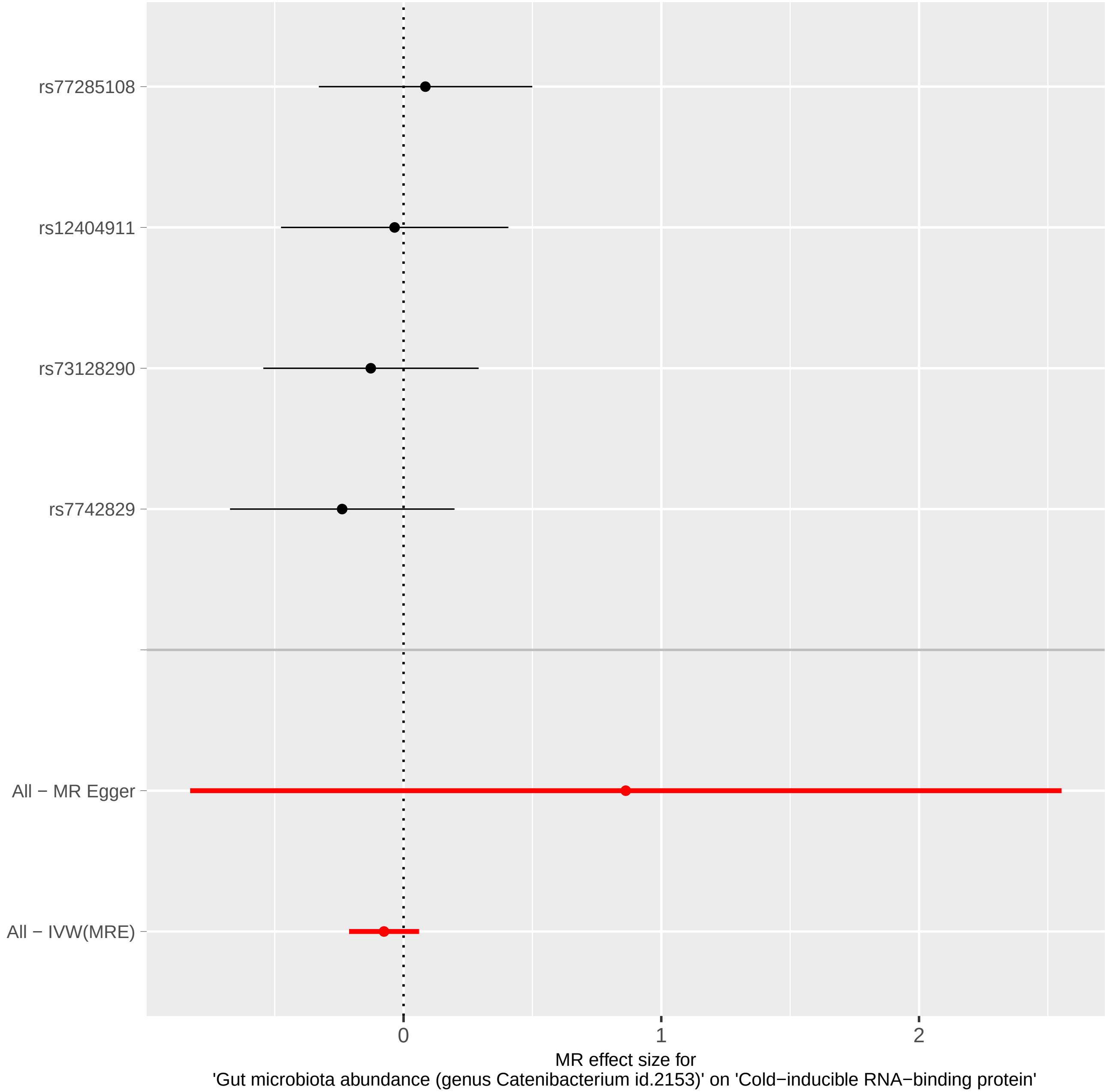

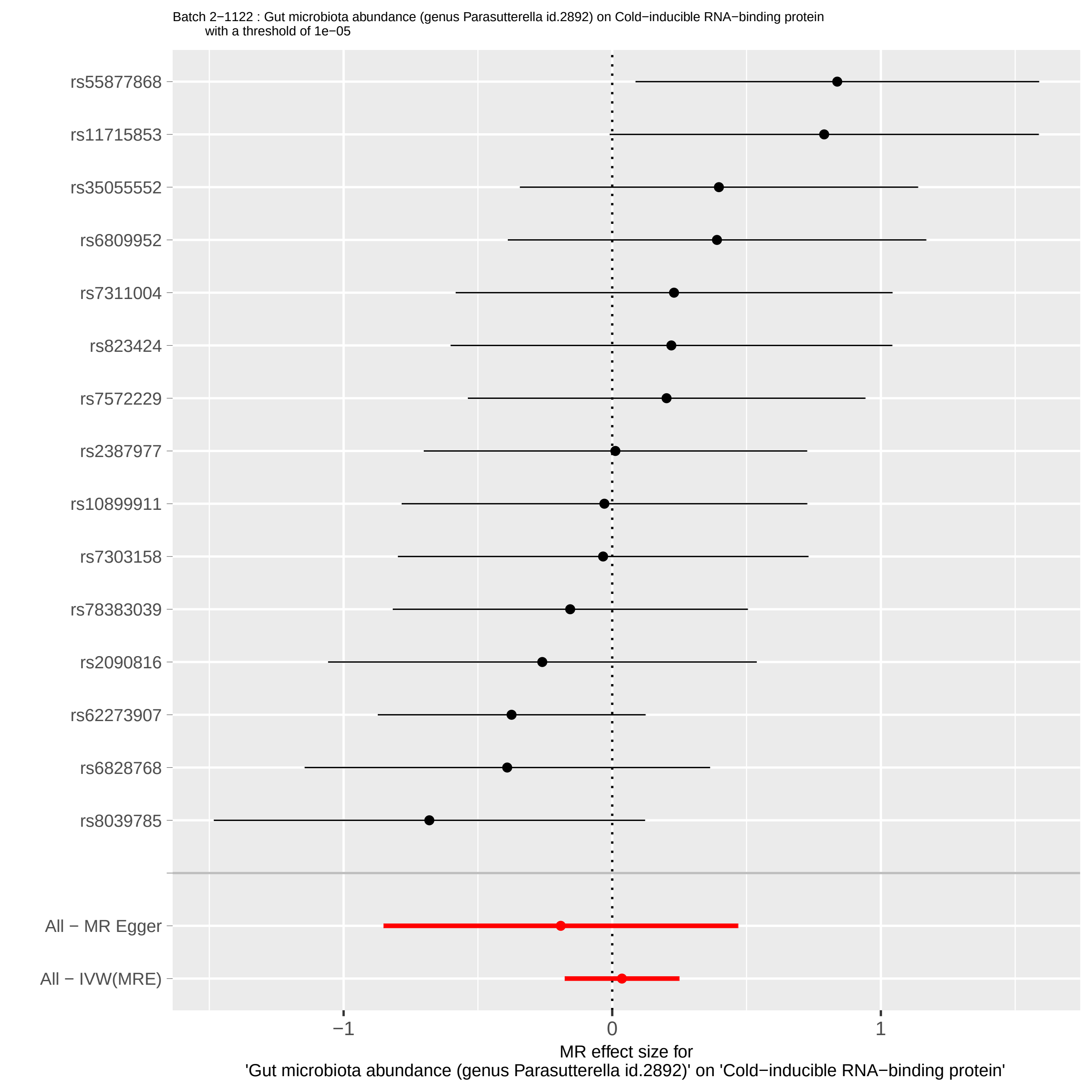

Batch 2−1122 : Gut microbiota abundance (genus Parasutterella id.2892) on Cold−inducible RNA−binding protein
with a threshold of 1e−05
rs55877868
rs11715853
rs35055552
rs6809952
rs7311004
rs823424
rs7572229
rs2387977
rs10899911
rs7303158
rs78383039
rs2090816
rs62273907
rs6828768
rs8039785
All − MR Egger
All − IVW(MRE)
−1
0
1
MR effect size for
'Gut microbiota abundance (genus Parasutterella id.2892)' on 'Cold−inducible RNA−binding protein'

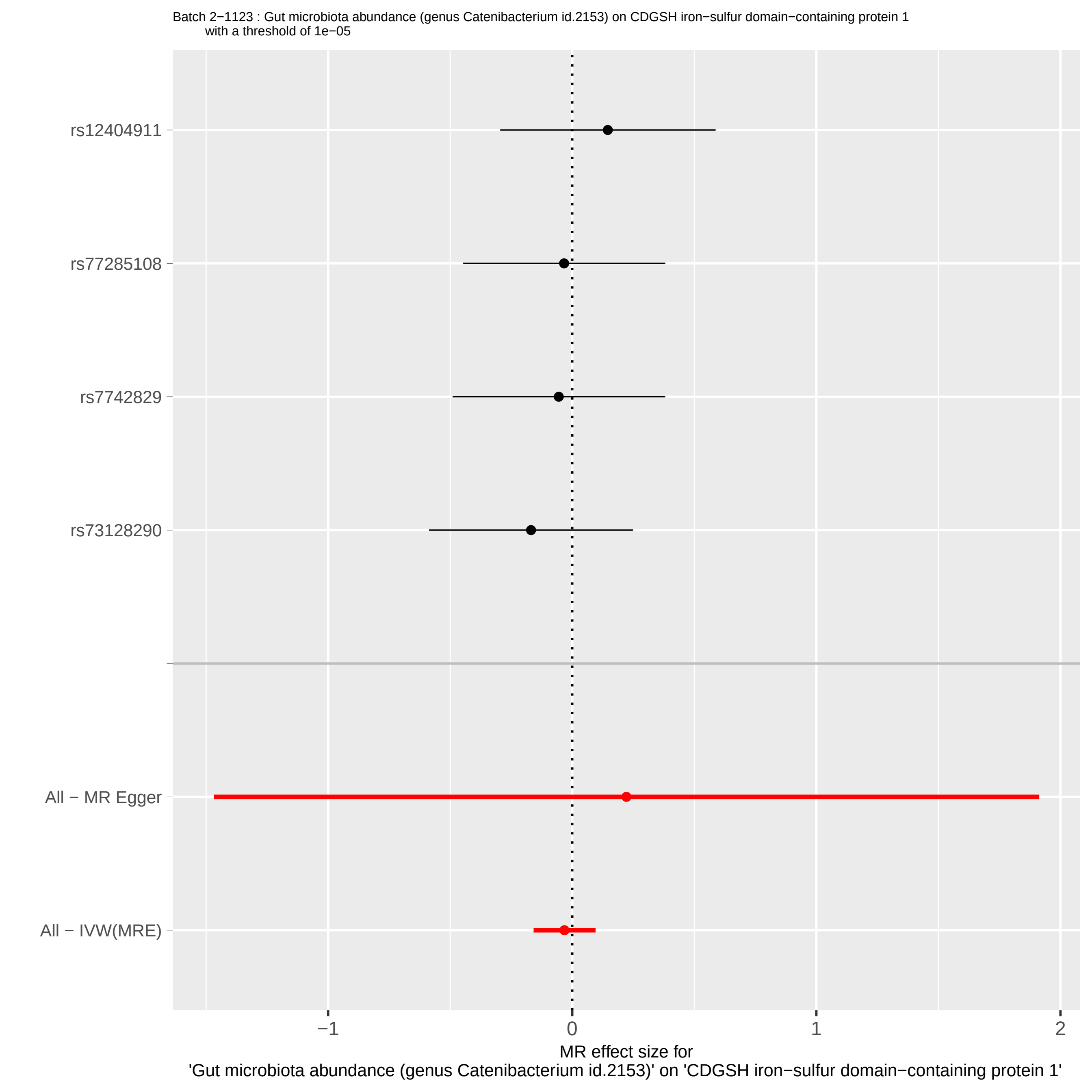

Batch 2−1123 : Gut microbiota abundance (genus Catenibacterium id.2153) on CDGSH iron−sulfur domain−containing protein 1
with a threshold of 1e−05
rs12404911
rs77285108
rs7742829
rs73128290
All − MR Egger
All − IVW(MRE)
−1
0
1
2
MR effect size for
'Gut microbiota abundance (genus Catenibacterium id.2153)' on 'CDGSH iron−sulfur domain−containing protein 1'

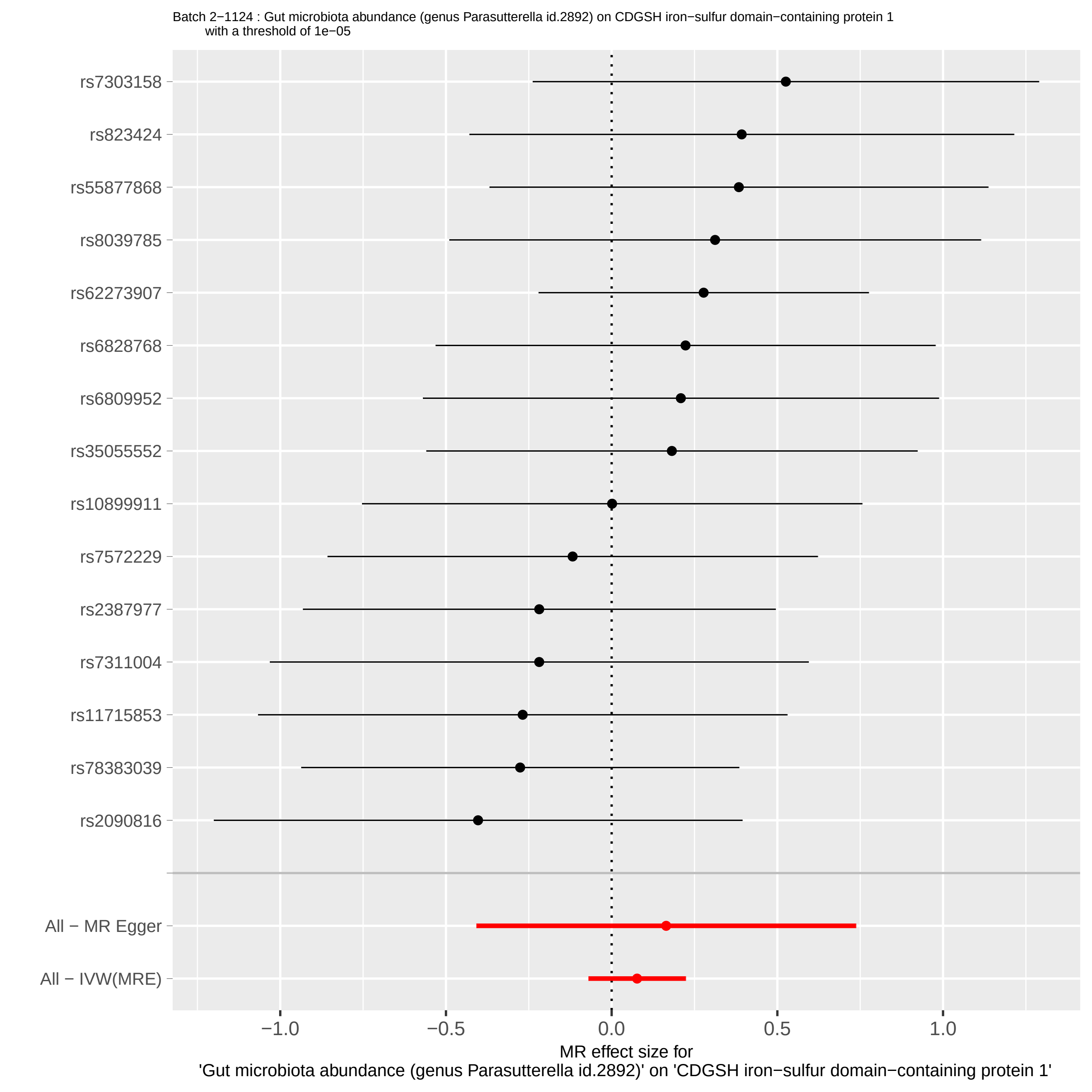

Batch 2−1124 : Gut microbiota abundance (genus Parasutterella id.2892) on CDGSH iron−sulfur domain−containing protein 1
with a threshold of 1e−05
rs7303158
rs823424
rs55877868
rs8039785
rs62273907
rs6828768
rs6809952
rs35055552
rs10899911
rs7572229
rs2387977
rs7311004
rs11715853
rs78383039
rs2090816
All − MR Egger
All − IVW(MRE)
−1.0
−0.5
0.0
0.5
1.0
MR effect size for
'Gut microbiota abundance (genus Parasutterella id.2892)' on 'CDGSH iron−sulfur domain−containing protein 1'

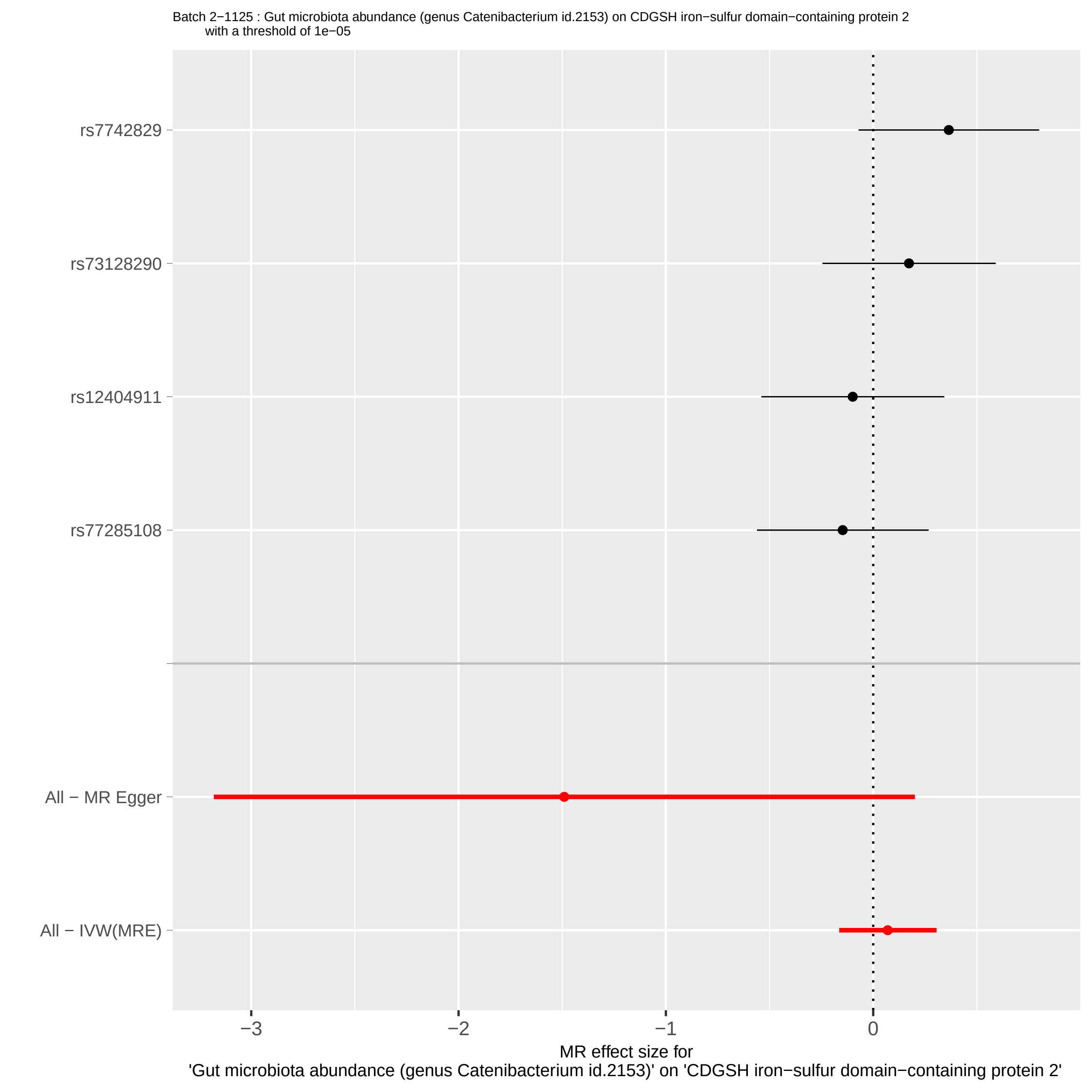
Batch 2−1125 : Gut microbiota abundance (genus Catenibacterium id.2153) on CDGSH iron−sulfur domain−containing protein 2
with a threshold of 1e−05
rs7742829
rs73128290
rs12404911
rs77285108
All − MR Egger
All − IVW(MRE)
−3
−2
−1
0
MR effect size for
'Gut microbiota abundance (genus Catenibacterium id.2153)' on 'CDGSH iron−sulfur domain−containing protein 2'

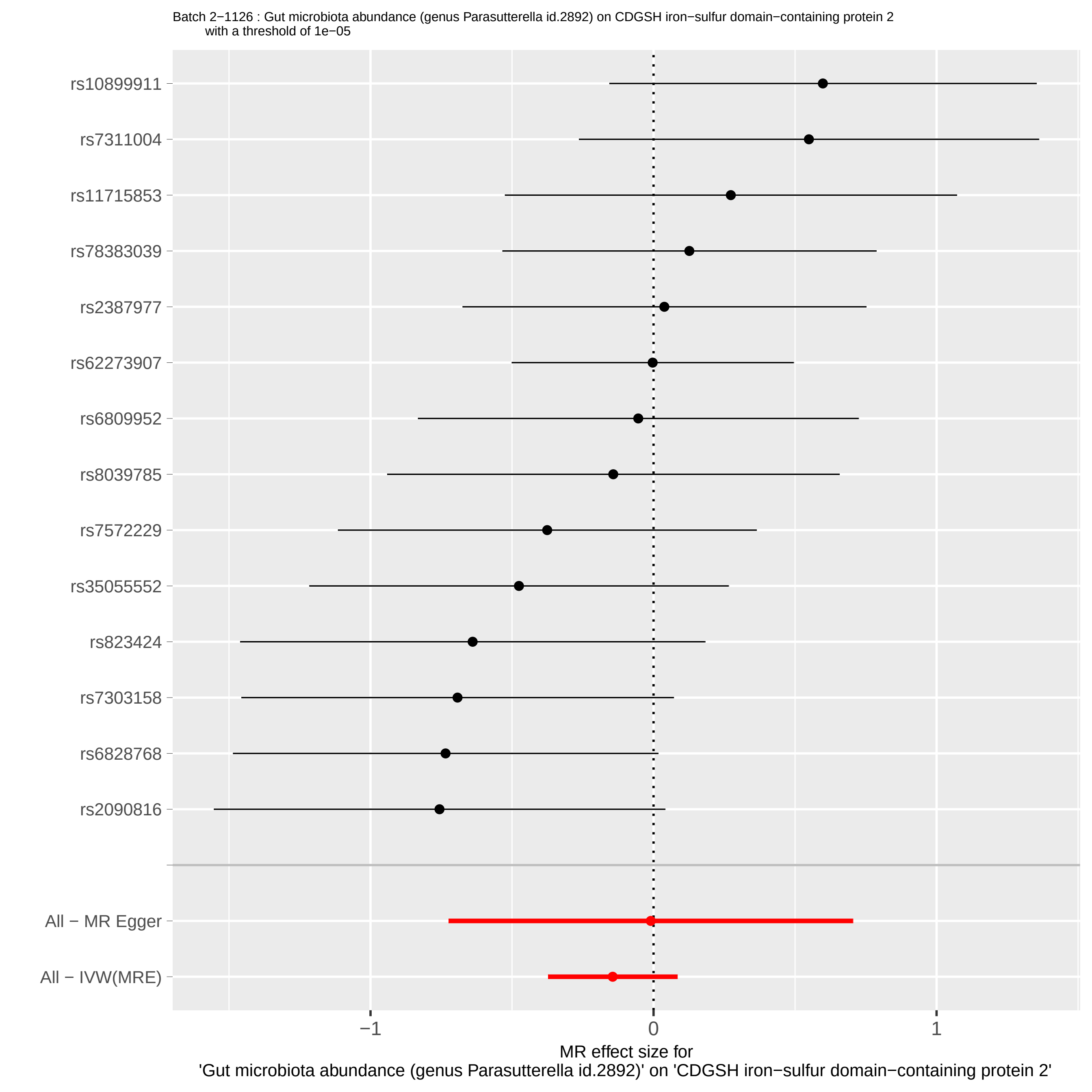

Batch 2−1126 : Gut microbiota abundance (genus Parasutterella id.2892) on CDGSH iron−sulfur domain−containing protein 2
with a threshold of 1e−05
rs10899911
rs7311004
rs11715853
rs78383039
rs2387977
rs62273907
rs6809952
rs8039785
rs7572229
rs35055552
rs823424
rs7303158
rs6828768
rs2090816
All − MR Egger
All − IVW(MRE)
−1
0
1
MR effect size for
'Gut microbiota abundance (genus Parasutterella id.2892)' on 'CDGSH iron−sulfur domain−containing protein 2'

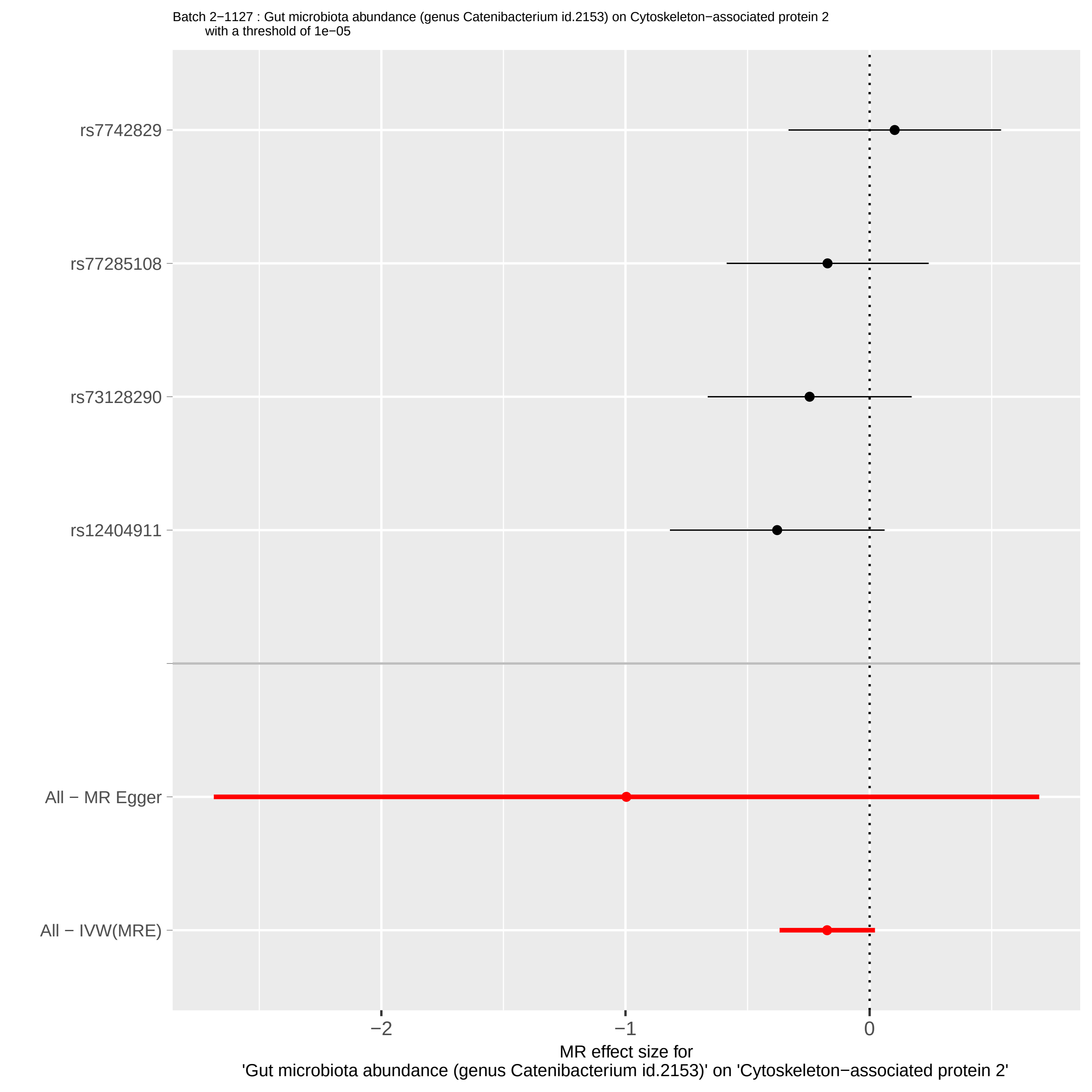
Batch 2−1127 : Gut microbiota abundance (genus Catenibacterium id.2153) on Cytoskeleton−associated protein 2
with a threshold of 1e−05
rs7742829
rs77285108
rs73128290
rs12404911
All − MR Egger
All − IVW(MRE)
−2
−1
0
MR effect size for
'Gut microbiota abundance (genus Catenibacterium id.2153)' on 'Cytoskeleton−associated protein 2'

Batch 2−1128 : Gut microbiota abundance (genus Parasutterella id.2892) on Cytoskeleton−associated protein 2 with a threshold of 1e−05

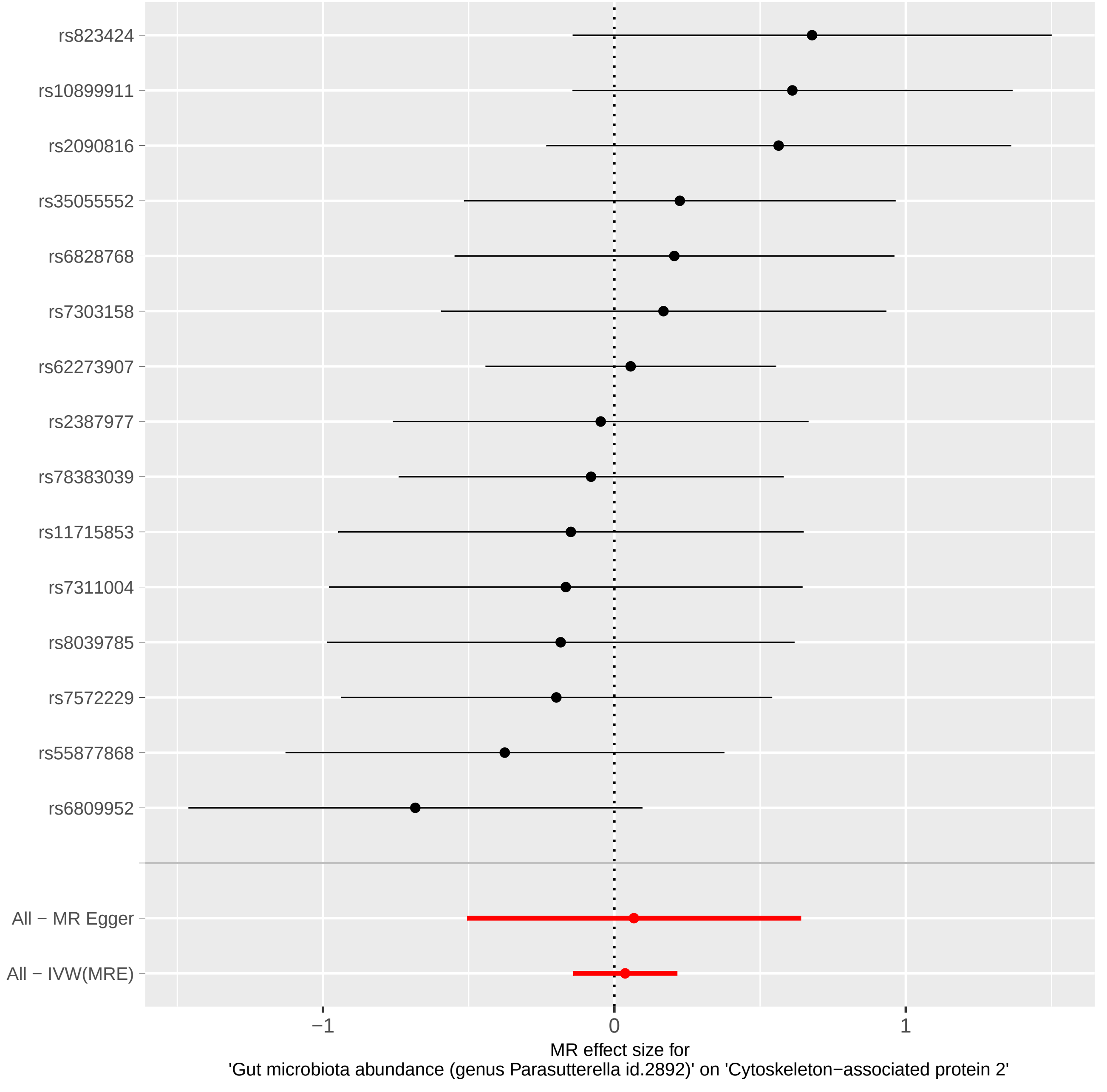

Batch 2−1129 : Gut microbiota abundance (genus Catenibacterium id.2153) on Creatine kinase B−type
with a threshold of 1e−05

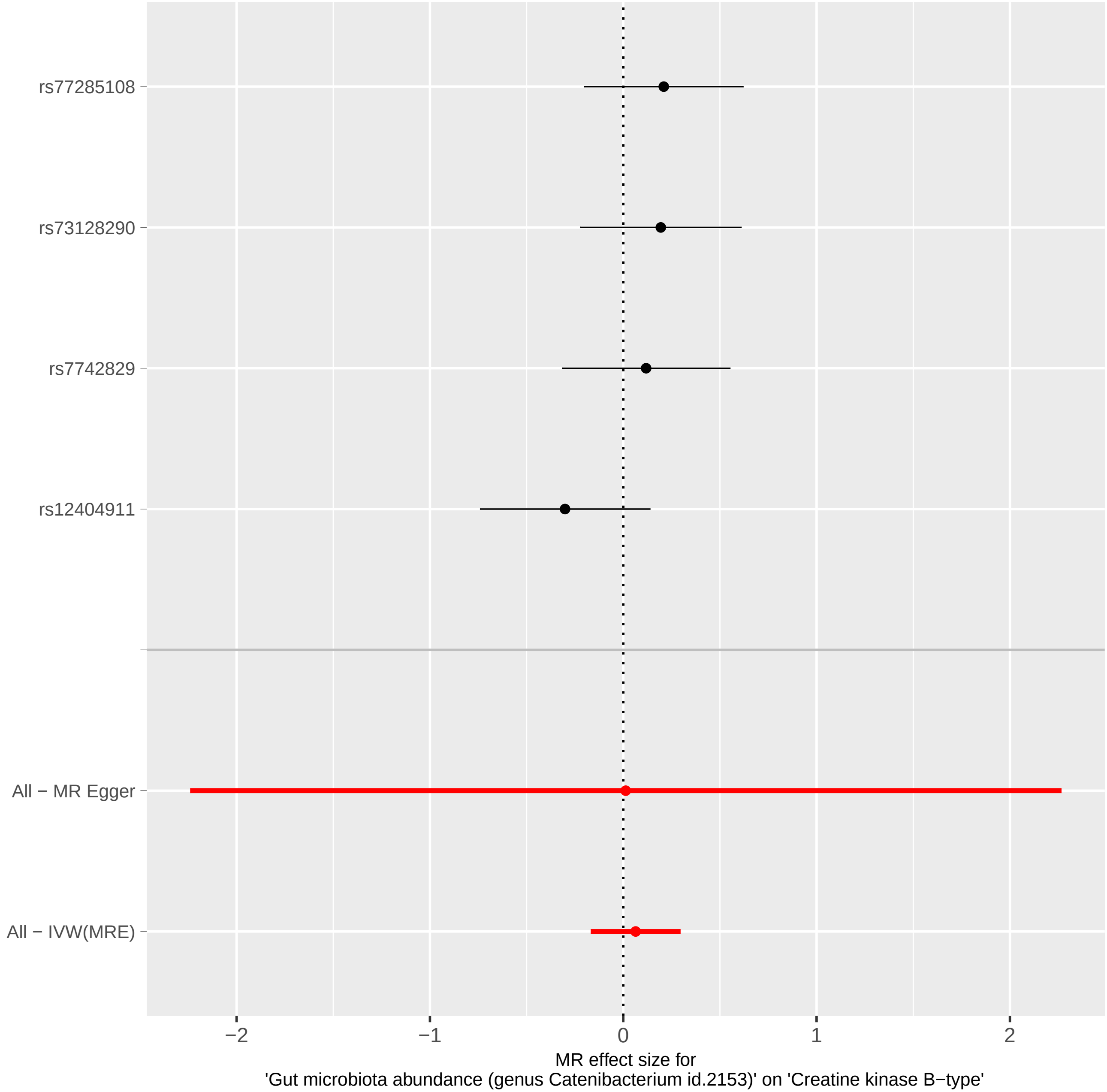

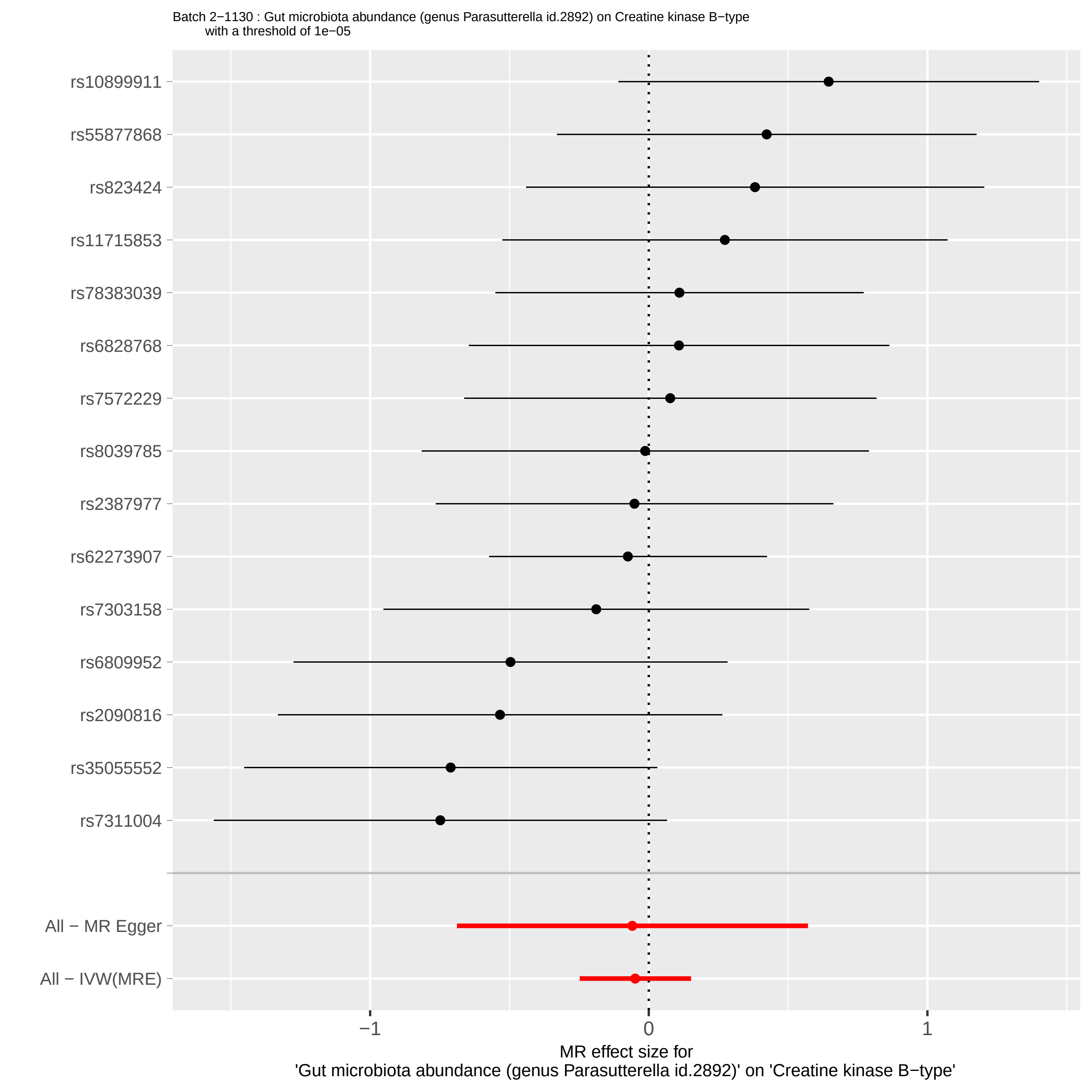
Batch 2−1130 : Gut microbiota abundance (genus Parasutterella id.2892) on Creatine kinase B−type
with a threshold of 1e−05
rs10899911
rs55877868
rs823424
rs11715853
rs78383039
rs6828768
rs7572229
rs8039785
rs2387977
rs62273907
rs7303158
rs6809952
rs2090816
rs35055552
rs7311004
All − MR Egger
All − IVW(MRE)
−1
0
1
MR effect size for
'Gut microbiota abundance (genus Parasutterella id.2892)' on 'Creatine kinase B−type'

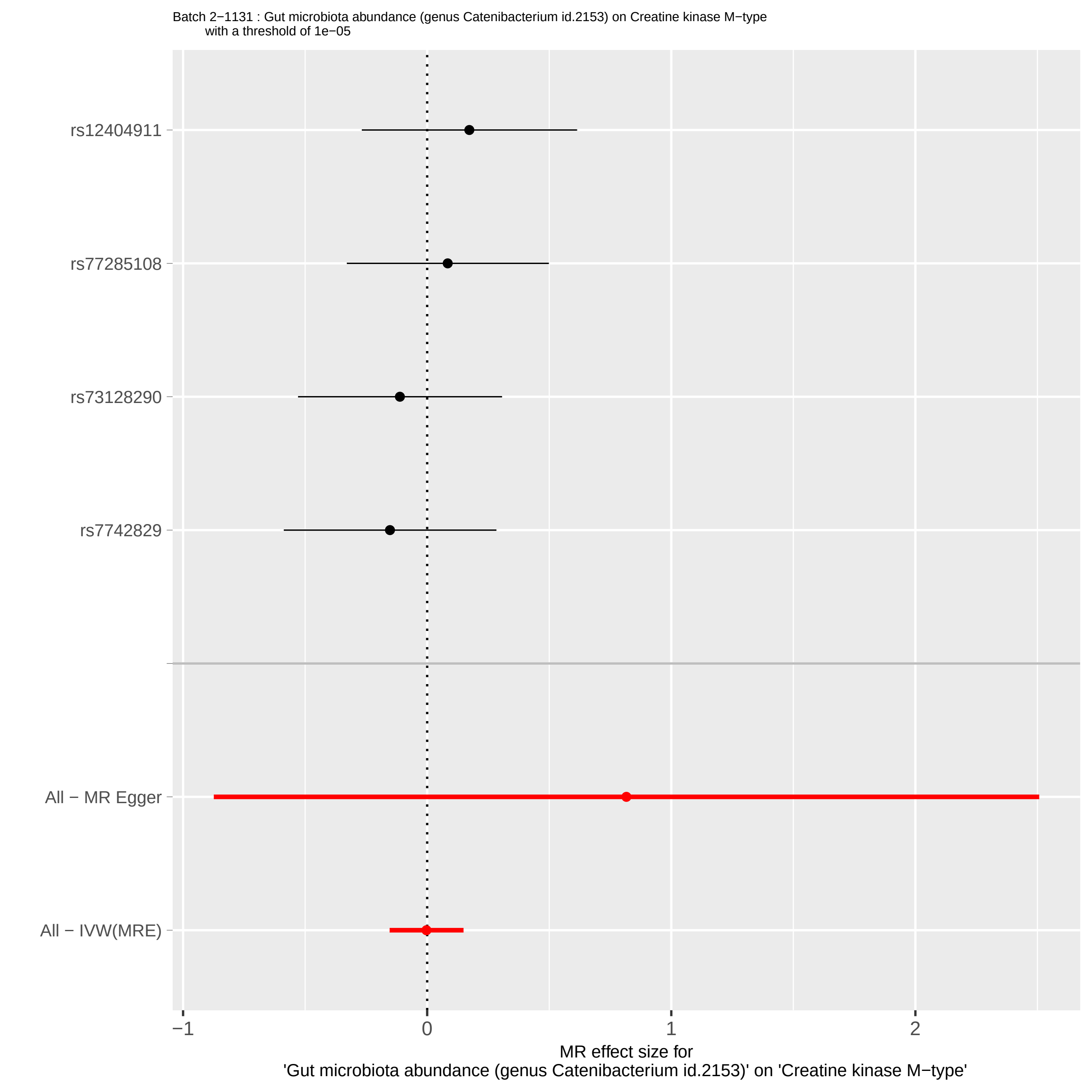
Batch 2−1131 : Gut microbiota abundance (genus Catenibacterium id.2153) on Creatine kinase M−type
with a threshold of 1e−05
rs12404911
rs77285108
rs73128290
rs7742829
All − MR Egger
All − IVW(MRE)
−1
0
1
2
MR effect size for
'Gut microbiota abundance (genus Catenibacterium id.2153)' on 'Creatine kinase M−type'

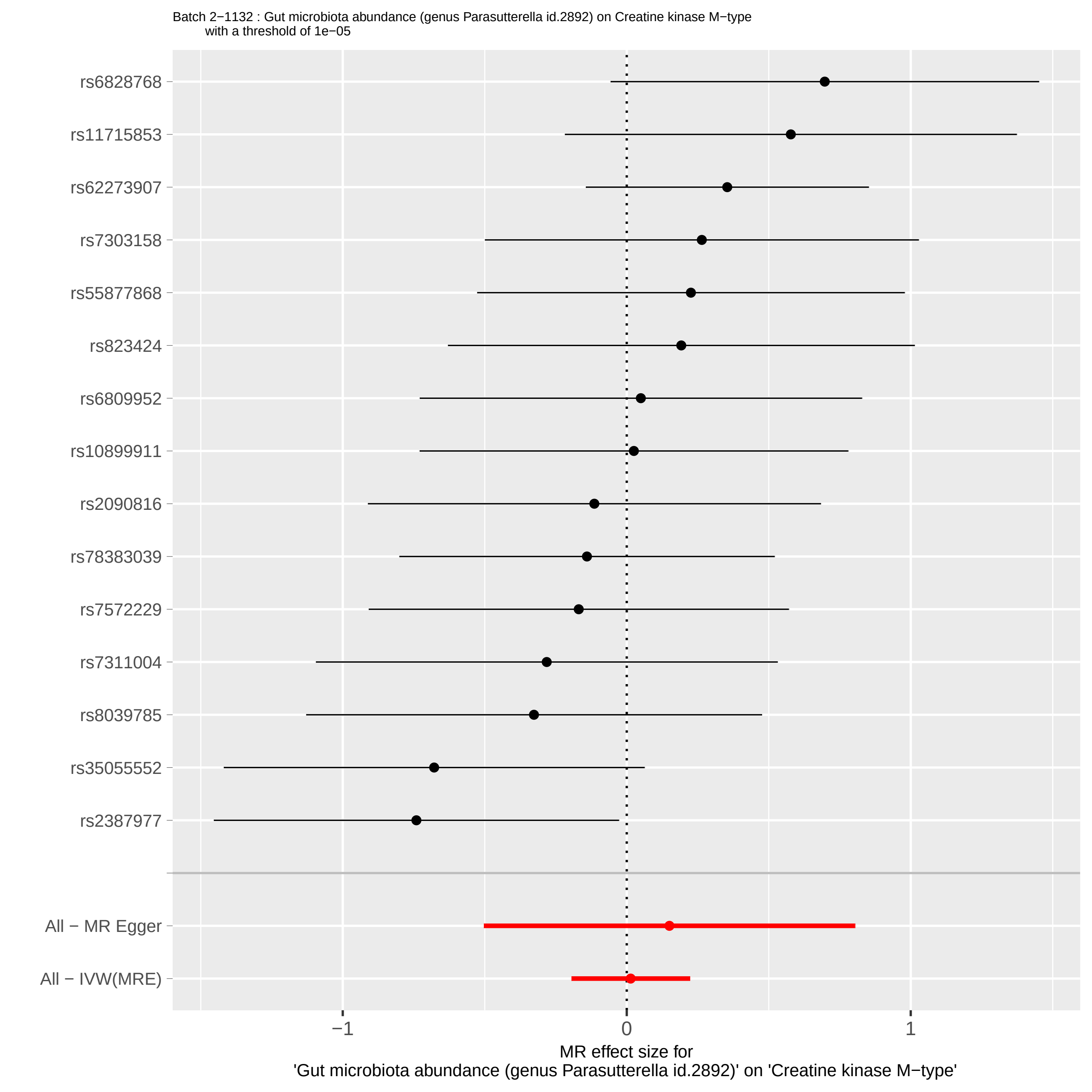
Batch 2−1132 : Gut microbiota abundance (genus Parasutterella id.2892) on Creatine kinase M−type
with a threshold of 1e−05
rs6828768
rs11715853
rs62273907
rs7303158
rs55877868
rs823424
rs6809952
rs10899911
rs2090816
rs78383039
rs7572229
rs7311004
rs8039785
rs35055552
rs2387977
All − MR Egger
All − IVW(MRE)
−1
0
1
MR effect size for
'Gut microbiota abundance (genus Parasutterella id.2892)' on 'Creatine kinase M−type'

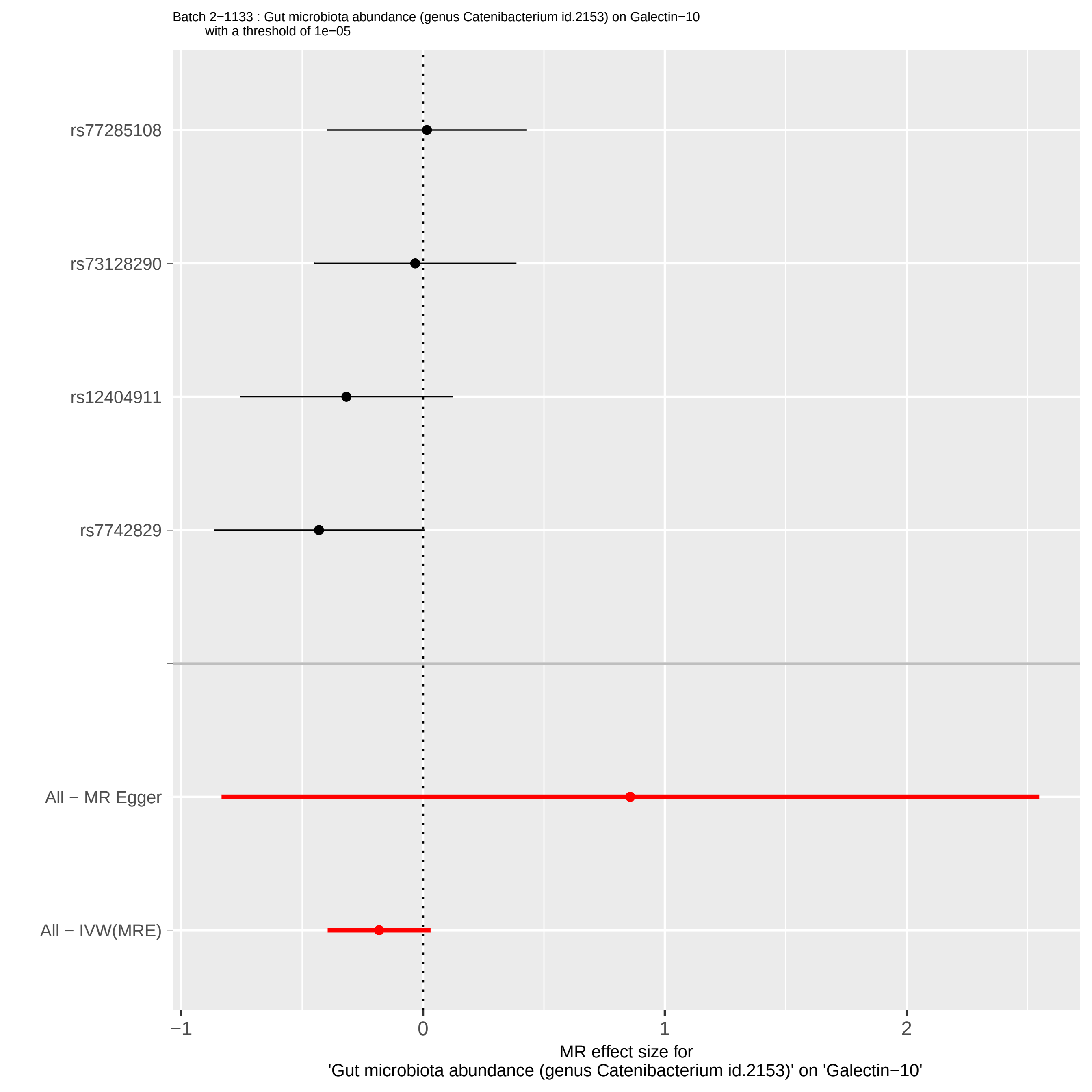

Batch 2−1133 : Gut microbiota abundance (genus Catenibacterium id.2153) on Galectin−10
with a threshold of 1e−05
rs77285108
rs73128290
rs12404911
rs7742829
All − MR Egger
All − IVW(MRE)
−1
0
1
2
MR effect size for
'Gut microbiota abundance (genus Catenibacterium id.2153)' on 'Galectin−10'

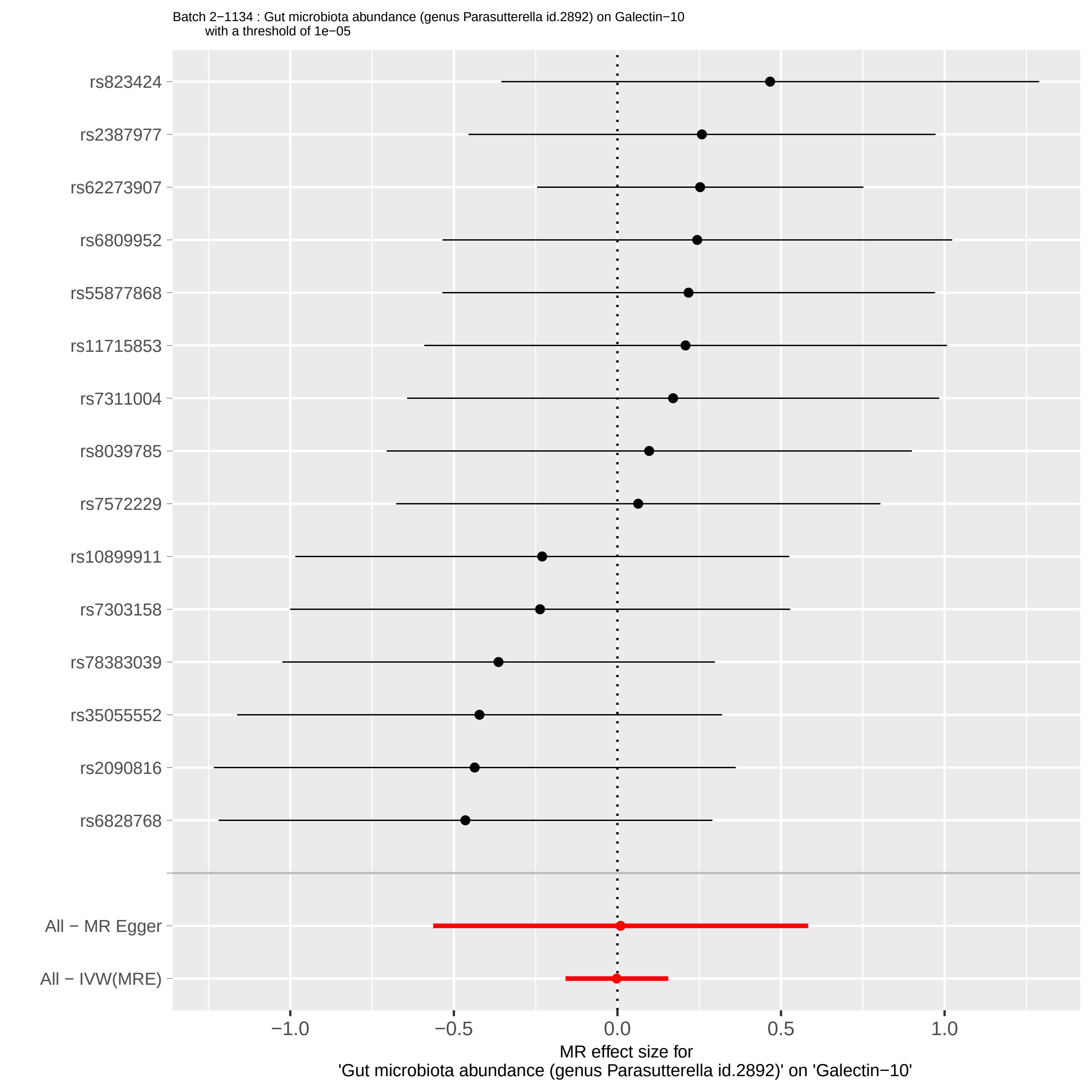
Batch 2−1134 : Gut microbiota abundance (genus Parasutterella id.2892) on Galectin−10
with a threshold of 1e−05
rs823424
rs2387977
rs62273907
rs6809952
rs55877868
rs11715853
rs7311004
rs8039785
rs7572229
rs10899911
rs7303158
rs78383039
rs35055552
rs2090816
rs6828768
All − MR Egger
All − IVW(MRE)
−1.0
−0.5
0.0
0.5
1.0
MR effect size for
'Gut microbiota abundance (genus Parasutterella id.2892)' on 'Galectin−10'

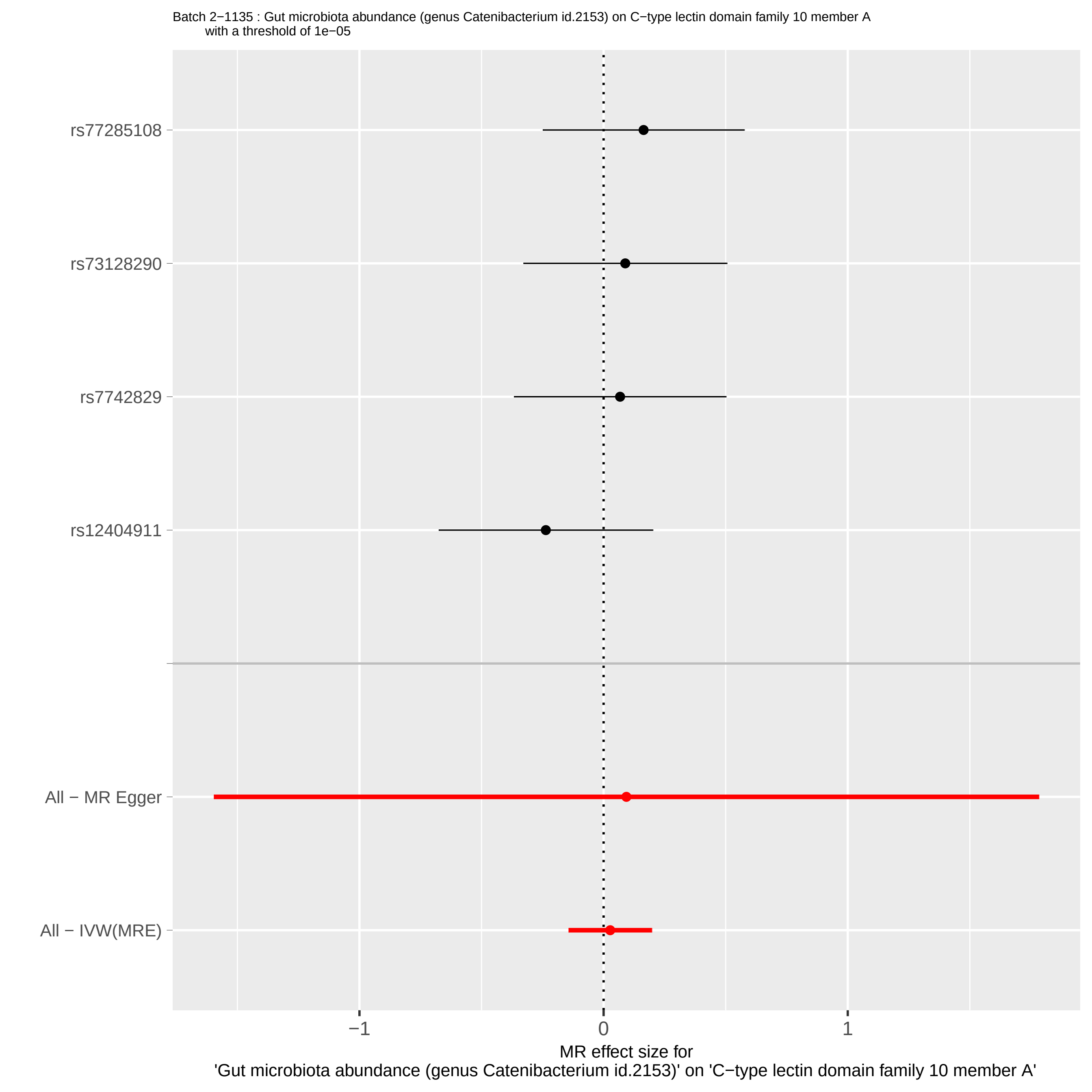

Batch 2−1135 : Gut microbiota abundance (genus Catenibacterium id.2153) on C−type lectin domain family 10 member A
with a threshold of 1e−05
rs77285108
rs73128290
rs7742829
rs12404911
All − MR Egger
All − IVW(MRE)
−1
0
1
MR effect size for
'Gut microbiota abundance (genus Catenibacterium id.2153)' on 'C−type lectin domain family 10 member A'

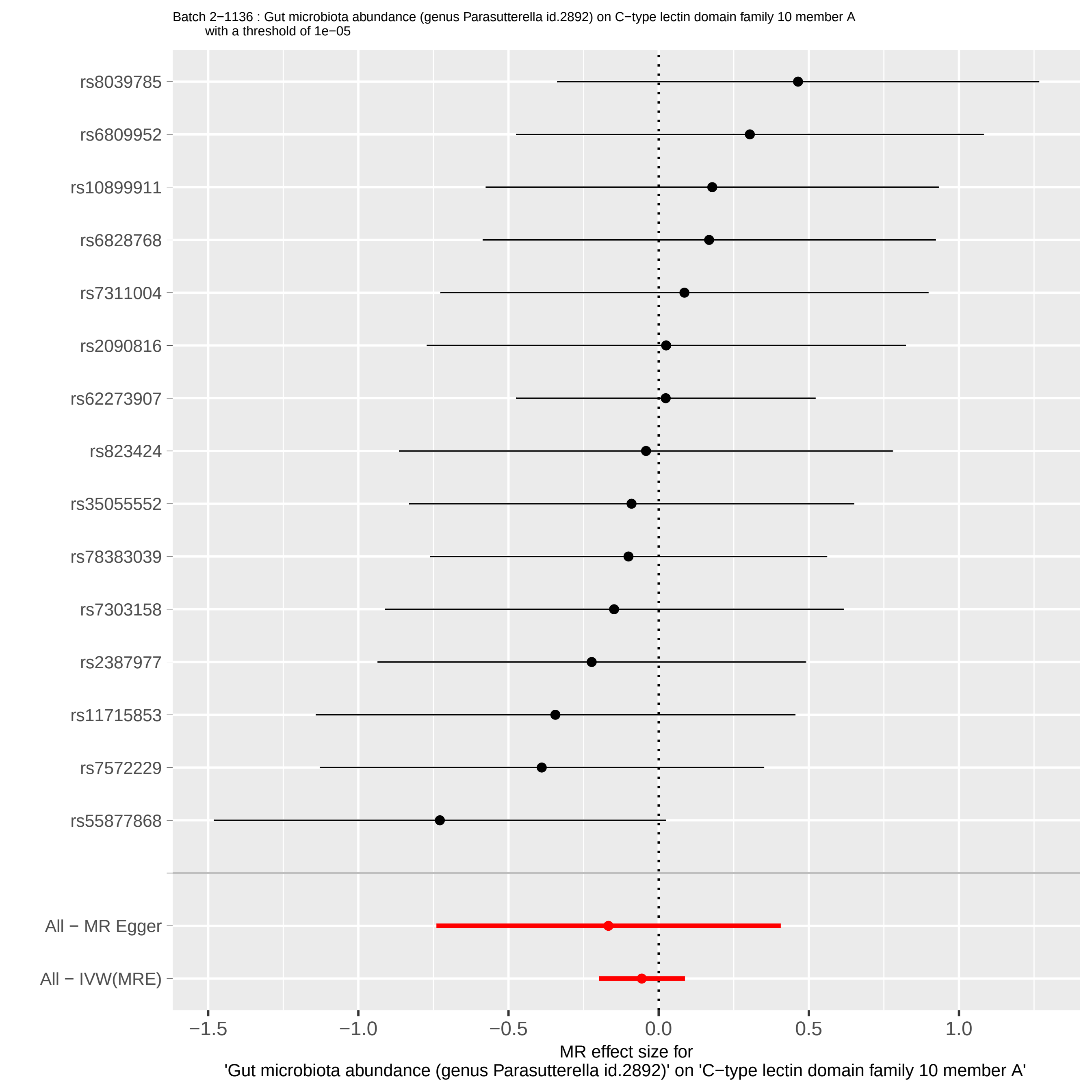

Batch 2−1136 : Gut microbiota abundance (genus Parasutterella id.2892) on C−type lectin domain family 10 member A
with a threshold of 1e−05
rs8039785
rs6809952
rs10899911
rs6828768
rs7311004
rs2090816
rs62273907
rs823424
rs35055552
rs78383039
rs7303158
rs2387977
rs11715853
rs7572229
rs55877868
All − MR Egger
All − IVW(MRE)
−1.5
−1.0
−0.5
0.0
0.5
1.0
MR effect size for
'Gut microbiota abundance (genus Parasutterella id.2892)' on 'C−type lectin domain family 10 member A'

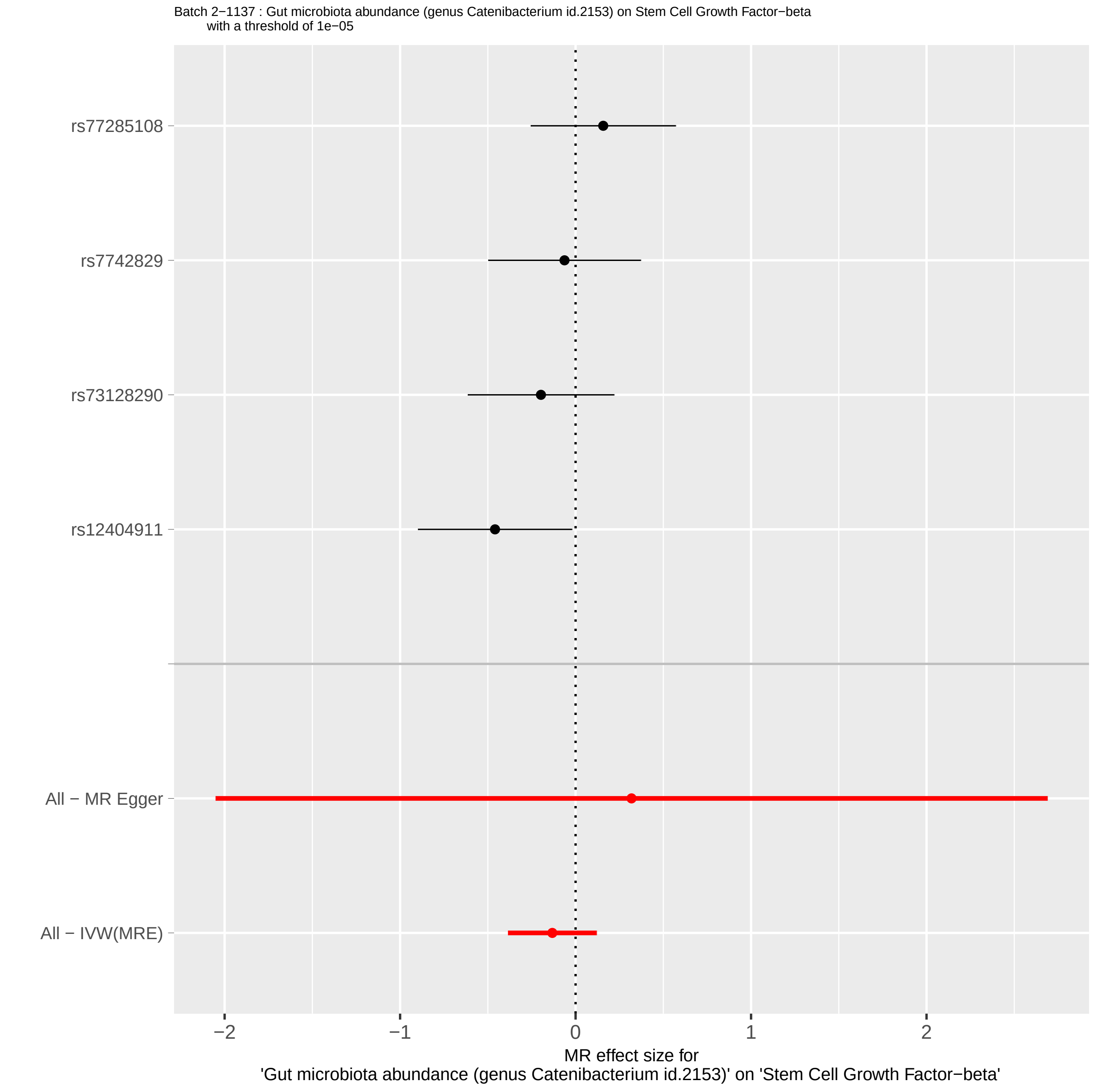
Batch 2−1137 : Gut microbiota abundance (genus Catenibacterium id.2153) on Stem Cell Growth Factor−beta
with a threshold of 1e−05
rs77285108
rs7742829
rs73128290
rs12404911
All − MR Egger
All − IVW(MRE)
−2
−1
0
1
2
MR effect size for
'Gut microbiota abundance (genus Catenibacterium id.2153)' on 'Stem Cell Growth Factor−beta'

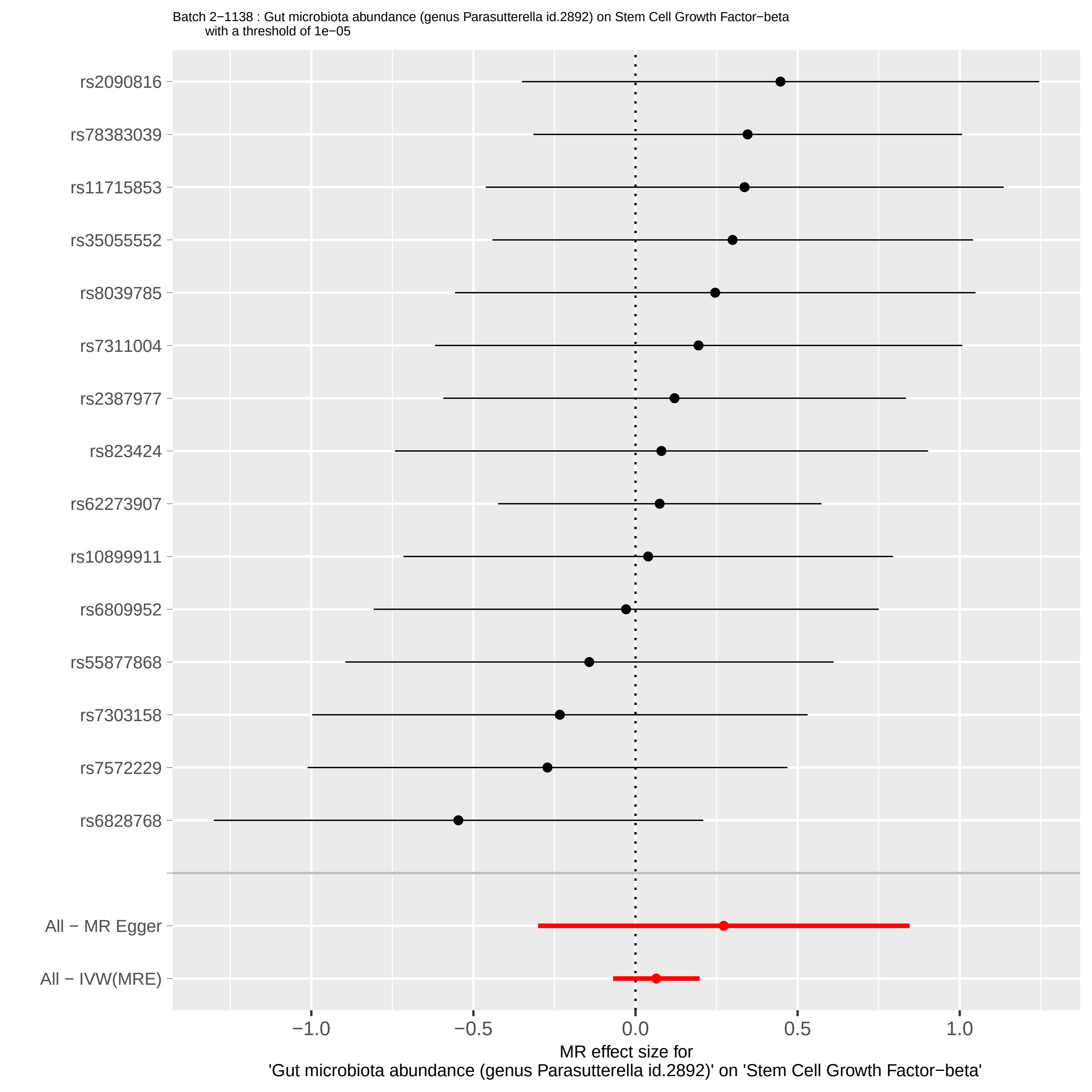

Batch 2−1138 : Gut microbiota abundance (genus Parasutterella id.2892) on Stem Cell Growth Factor−beta
with a threshold of 1e−05
rs2090816
rs78383039
rs11715853
rs35055552
rs8039785
rs7311004
rs2387977
rs823424
rs62273907
rs10899911
rs6809952
rs55877868
rs7303158
rs7572229
rs6828768
All − MR Egger
All − IVW(MRE)
−1.0
−0.5
0.0
0.5
1.0
MR effect size for
'Gut microbiota abundance (genus Parasutterella id.2892)' on 'Stem Cell Growth Factor−beta'

Batch 2−1139 : Gut microbiota abundance (genus Catenibacterium id.2153) on C−type lectin domain family 12 member A
with a threshold of 1e−05

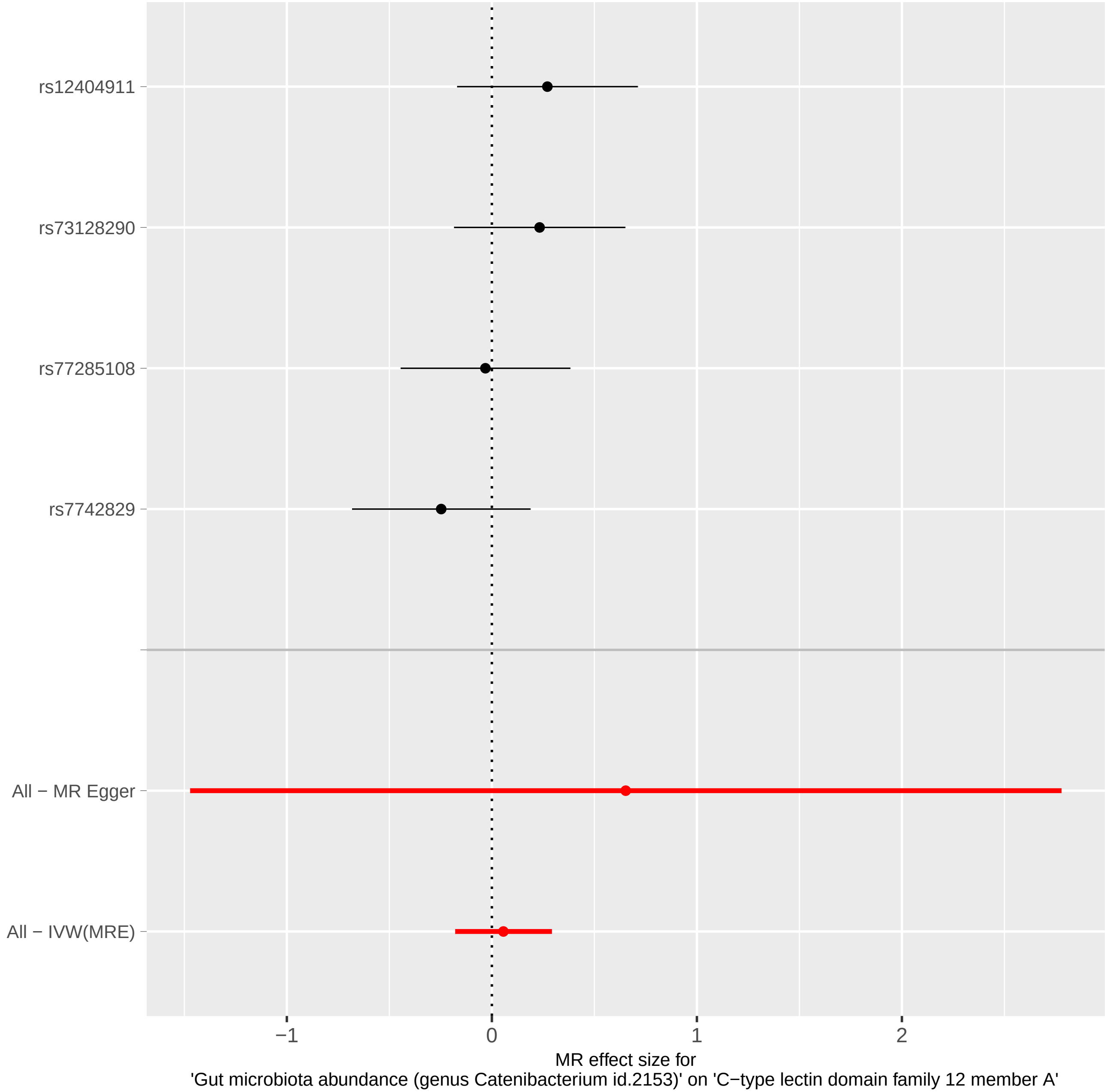

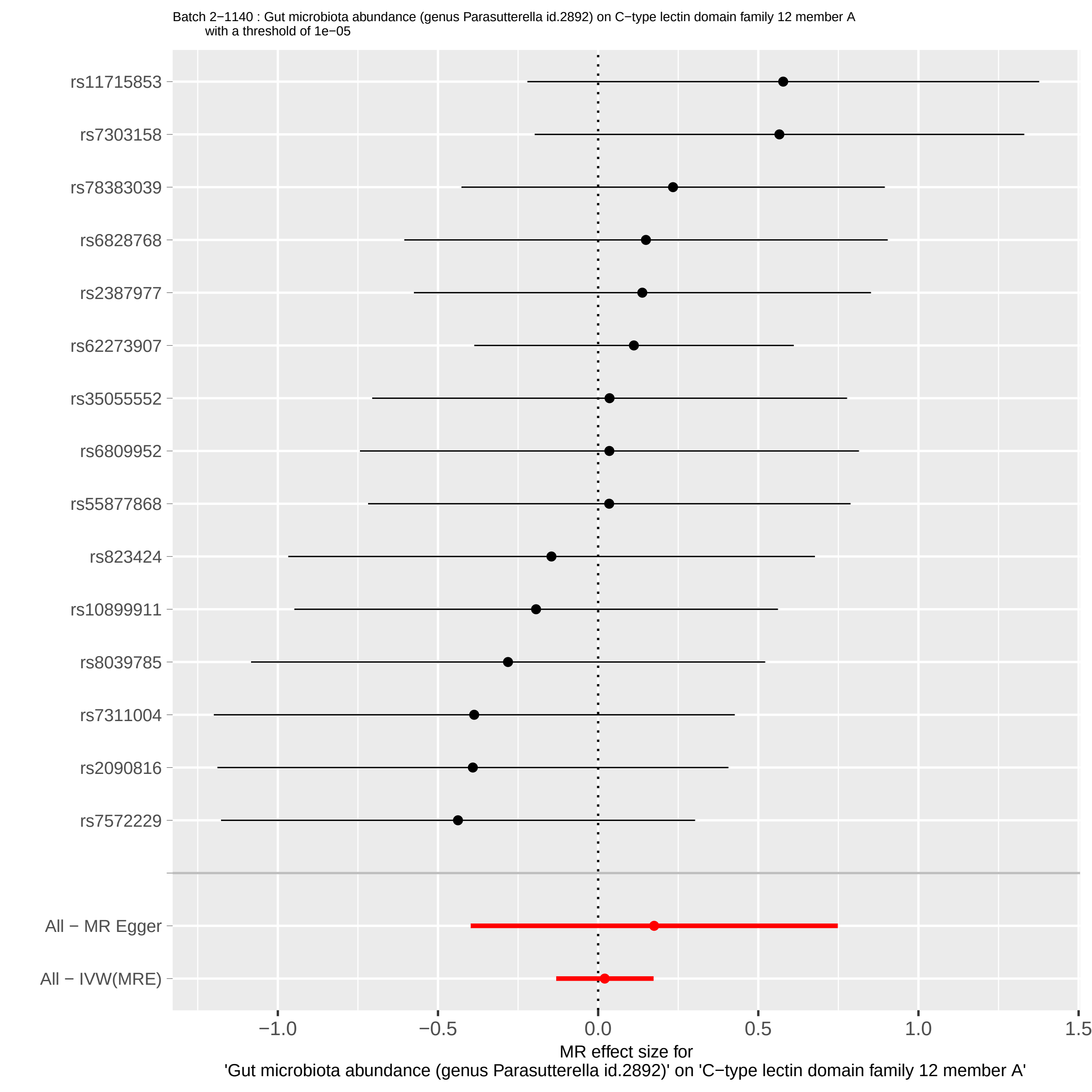

Batch 2−1140 : Gut microbiota abundance (genus Parasutterella id.2892) on C−type lectin domain family 12 member A
with a threshold of 1e−05
rs11715853
rs7303158
rs78383039
rs6828768
rs2387977
rs62273907
rs35055552
rs6809952
rs55877868
rs823424
rs10899911
rs8039785
rs7311004
rs2090816
rs7572229
All − MR Egger
All − IVW(MRE)
−1.0
−0.5
0.0
0.5
1.0
1.5
MR effect size for
'Gut microbiota abundance (genus Parasutterella id.2892)' on 'C−type lectin domain family 12 member A'

Batch 2−1141 : Gut microbiota abundance (genus Catenibacterium id.2153) on C−type lectin domain family 14 member A
with a threshold of 1e−05

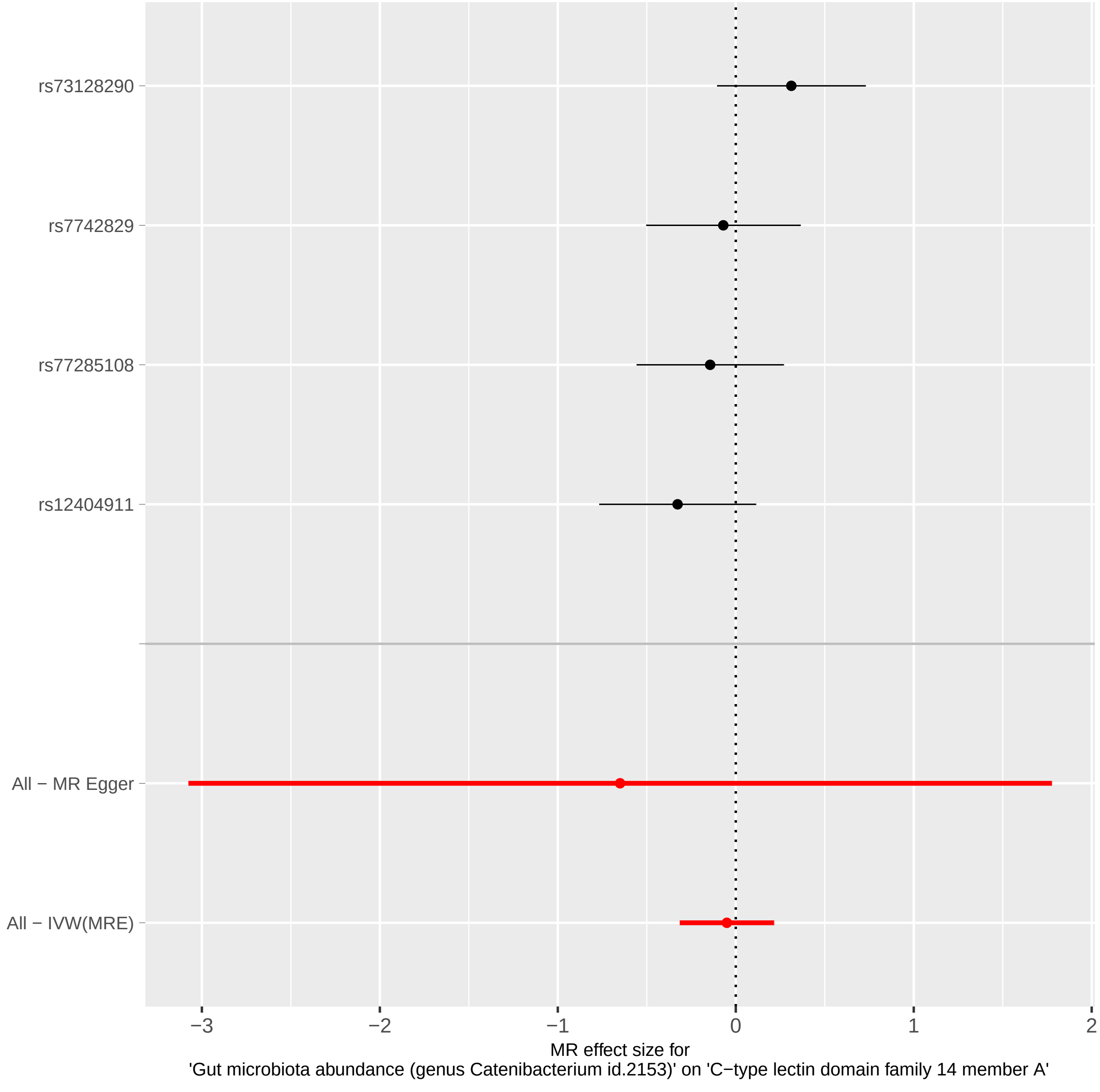

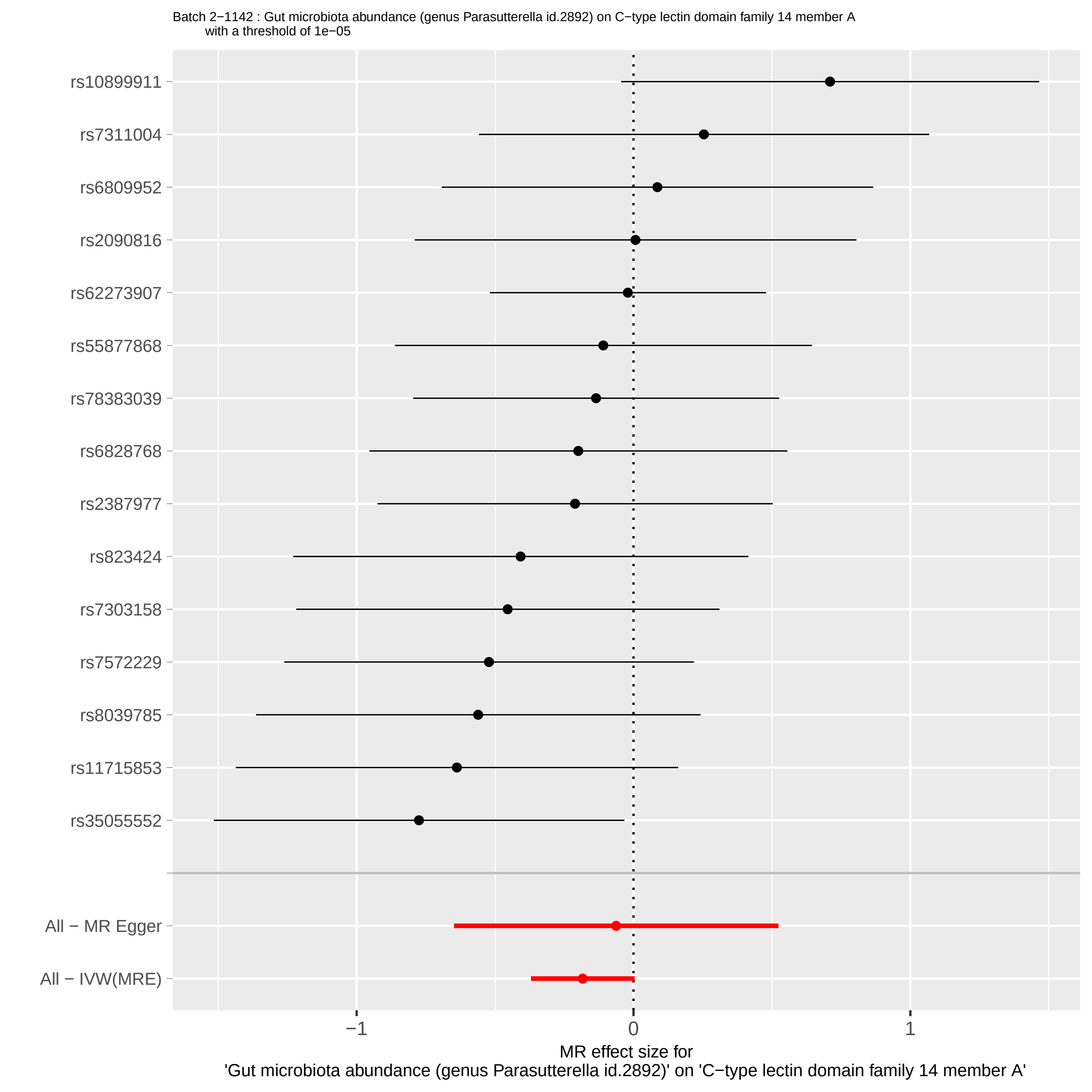
Batch 2−1142 : Gut microbiota abundance (genus Parasutterella id.2892) on C−type lectin domain family 14 member A
with a threshold of 1e−05
rs10899911
rs7311004
rs6809952
rs2090816
rs62273907
rs55877868
rs78383039
rs6828768
rs2387977
rs823424
rs7303158
rs7572229
rs8039785
rs11715853
rs35055552
All − MR Egger
All − IVW(MRE)
−1
0
1
MR effect size for
'Gut microbiota abundance (genus Parasutterella id.2892)' on 'C−type lectin domain family 14 member A'

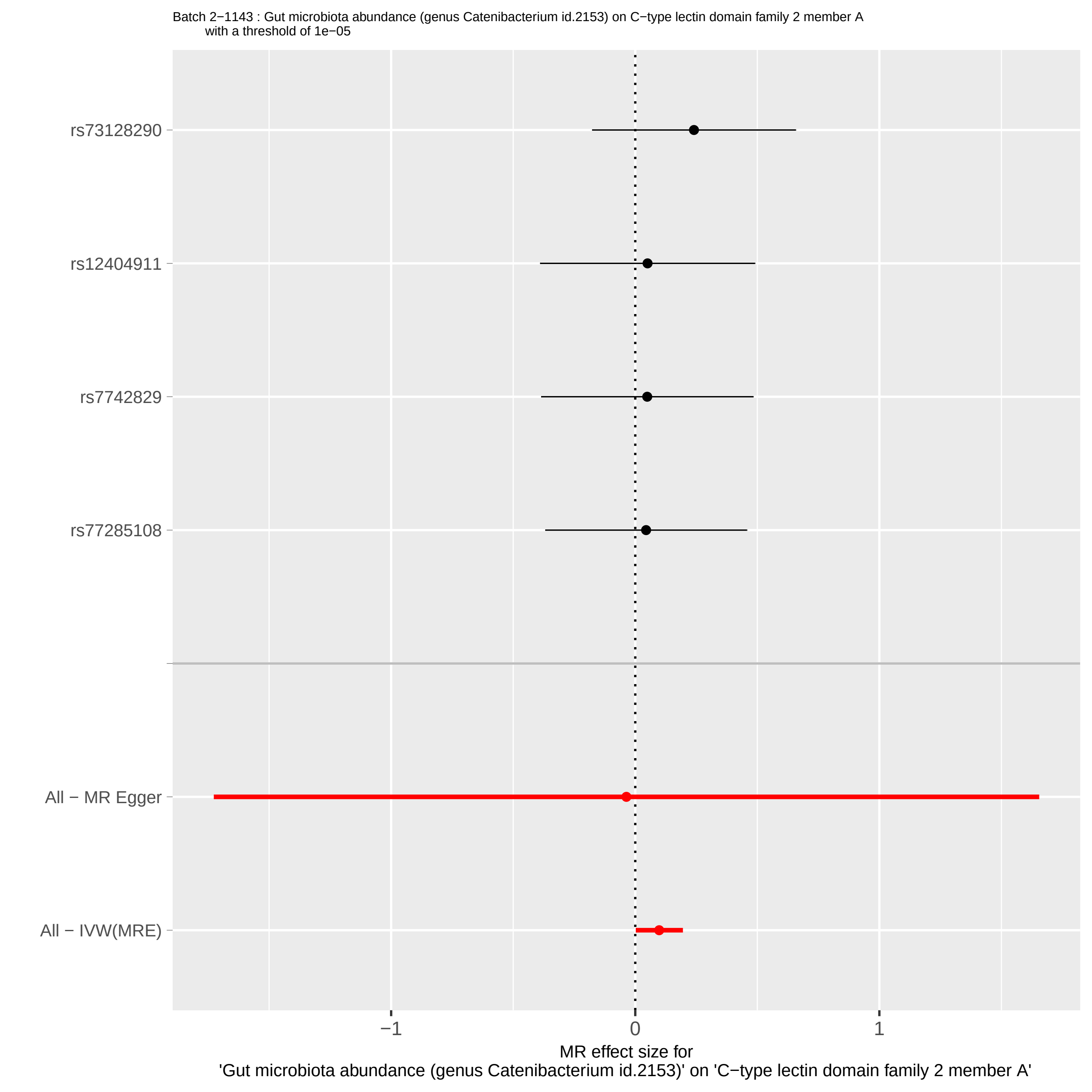

Batch 2−1143 : Gut microbiota abundance (genus Catenibacterium id.2153) on C−type lectin domain family 2 member A
with a threshold of 1e−05
rs73128290
rs12404911
rs7742829
rs77285108
All − MR Egger
All − IVW(MRE)
−1
0
1
MR effect size for
'Gut microbiota abundance (genus Catenibacterium id.2153)' on 'C−type lectin domain family 2 member A'

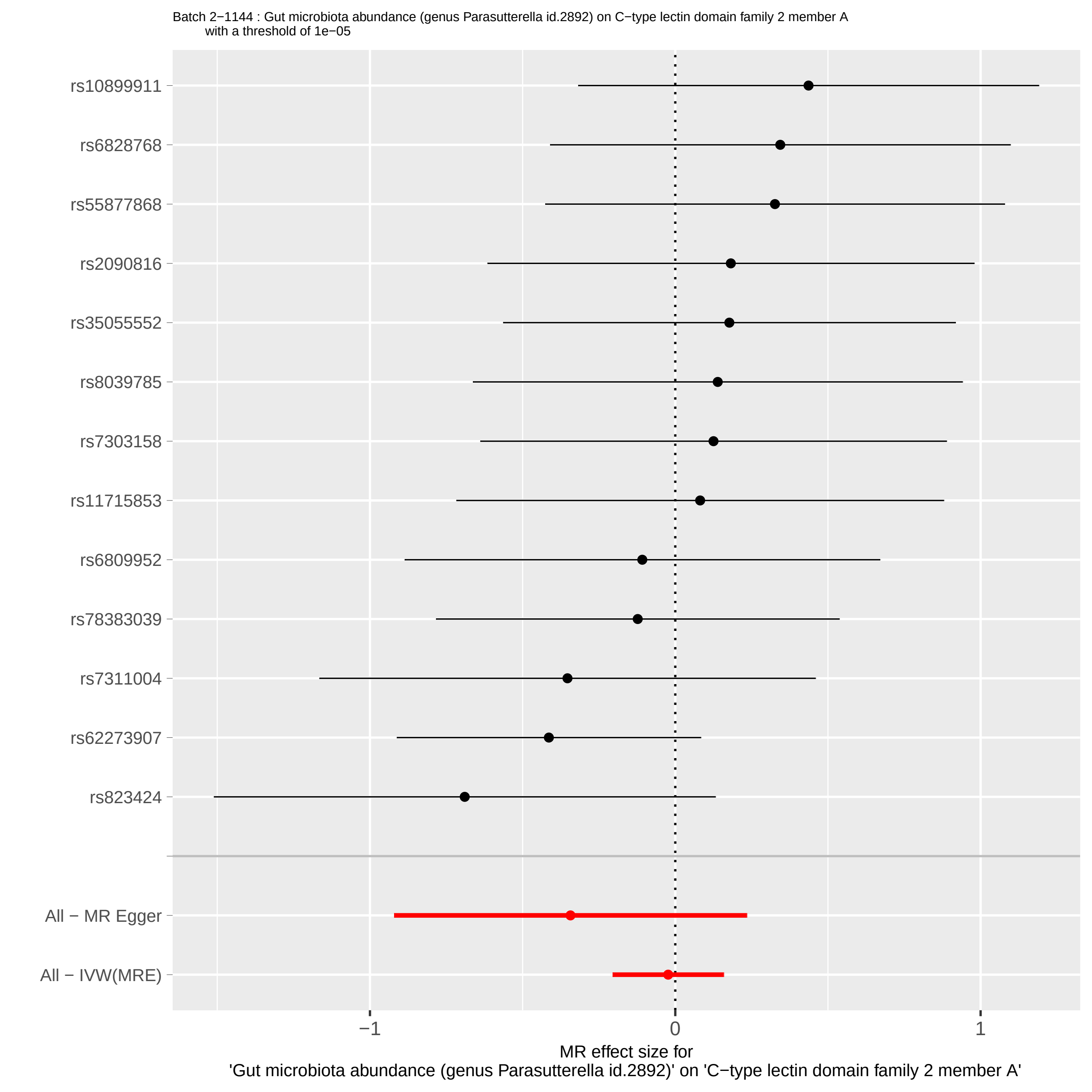

Batch 2−1144 : Gut microbiota abundance (genus Parasutterella id.2892) on C−type lectin domain family 2 member A
with a threshold of 1e−05
rs10899911
rs6828768
rs55877868
rs2090816
rs35055552
rs8039785
rs7303158
rs11715853
rs6809952
rs78383039
rs7311004
rs62273907
rs823424
All − MR Egger
All − IVW(MRE)
−1
0
1
MR effect size for
'Gut microbiota abundance (genus Parasutterella id.2892)' on 'C−type lectin domain family 2 member A'

Batch 2−1145 : Gut microbiota abundance (genus Catenibacterium id.2153) on C−type lectin domain family 2 member B
with a threshold of 1e−05

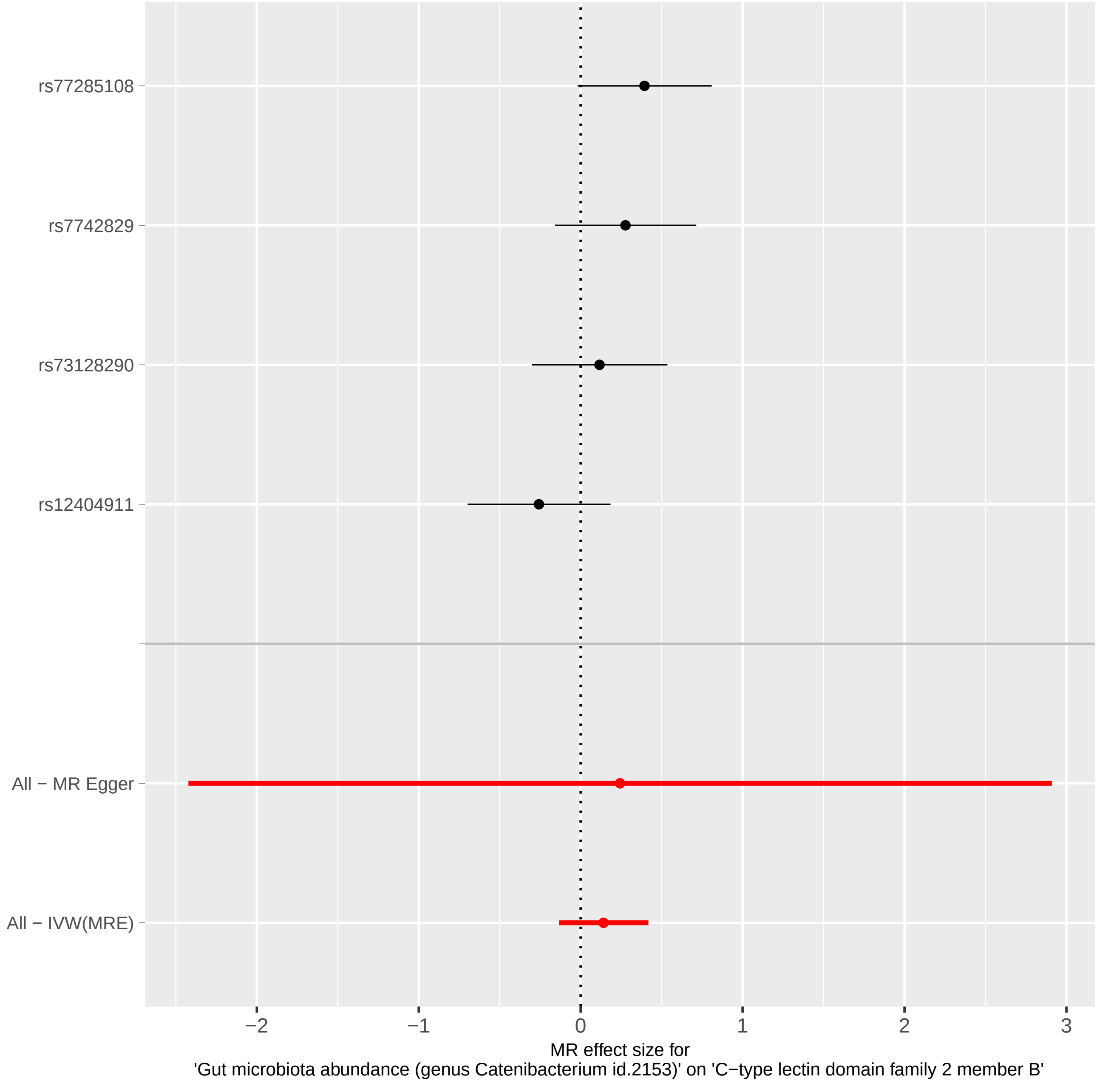

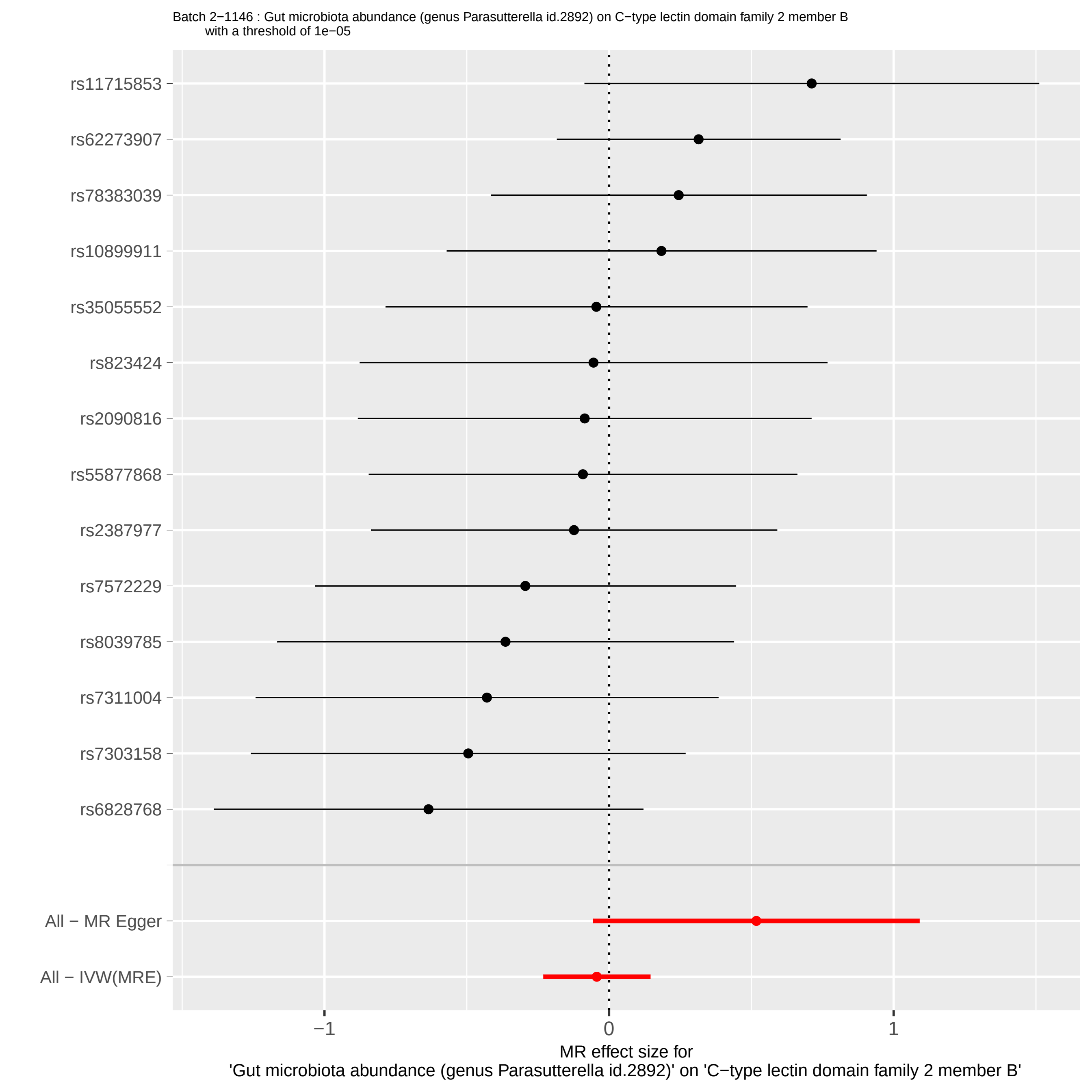

Batch 2−1146 : Gut microbiota abundance (genus Parasutterella id.2892) on C−type lectin domain family 2 member B
with a threshold of 1e−05
rs11715853
rs62273907
rs78383039
rs10899911
rs35055552
rs823424
rs2090816
rs55877868
rs2387977
rs7572229
rs8039785
rs7311004
rs7303158
rs6828768
All − MR Egger
All − IVW(MRE)
−1
0
1
MR effect size for
'Gut microbiota abundance (genus Parasutterella id.2892)' on 'C−type lectin domain family 2 member B'

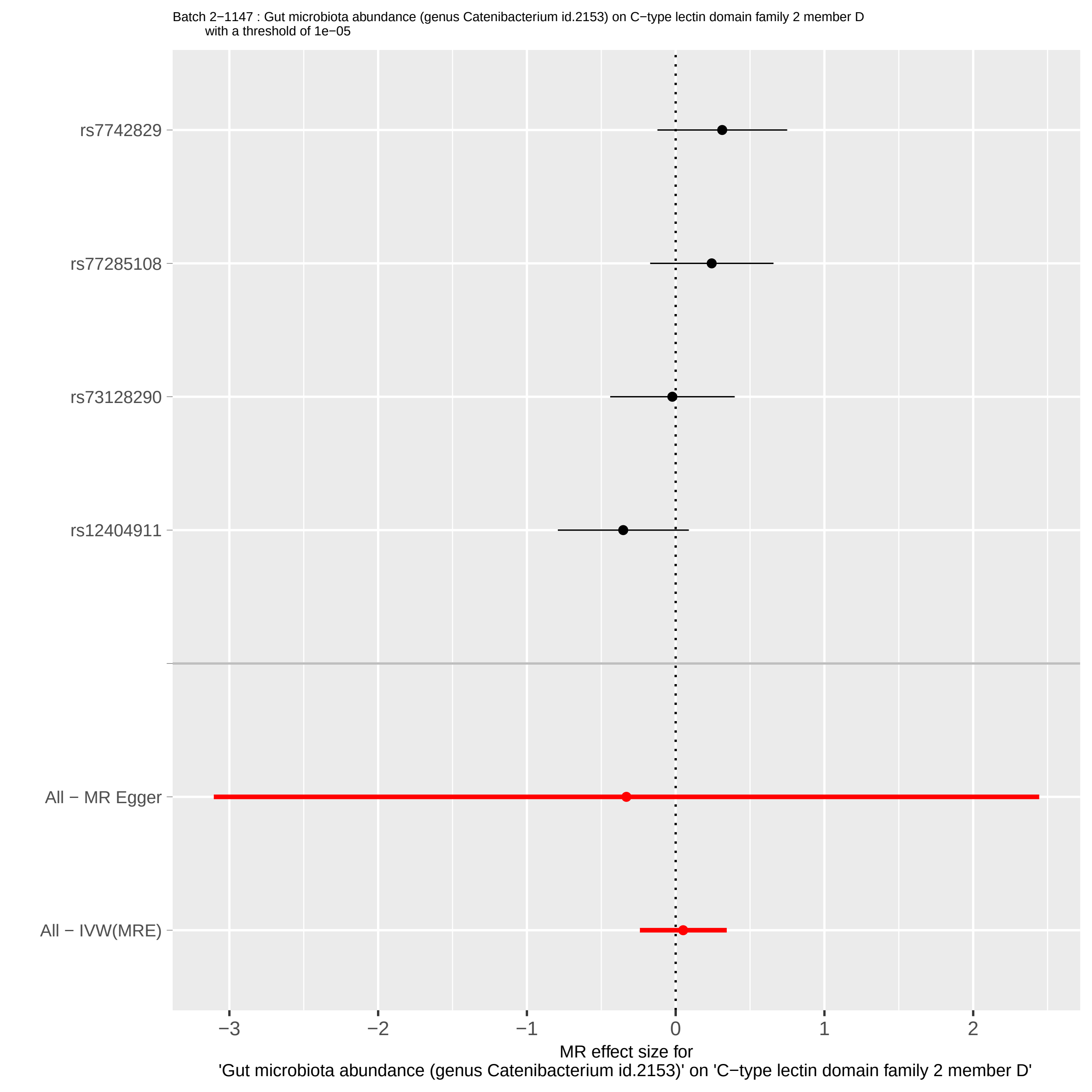

Batch 2−1147 : Gut microbiota abundance (genus Catenibacterium id.2153) on C−type lectin domain family 2 member D
with a threshold of 1e−05
rs7742829
rs77285108
rs73128290
rs12404911
All − MR Egger
All − IVW(MRE)
−3
−2
−1
0
1
2
MR effect size for
'Gut microbiota abundance (genus Catenibacterium id.2153)' on 'C−type lectin domain family 2 member D'

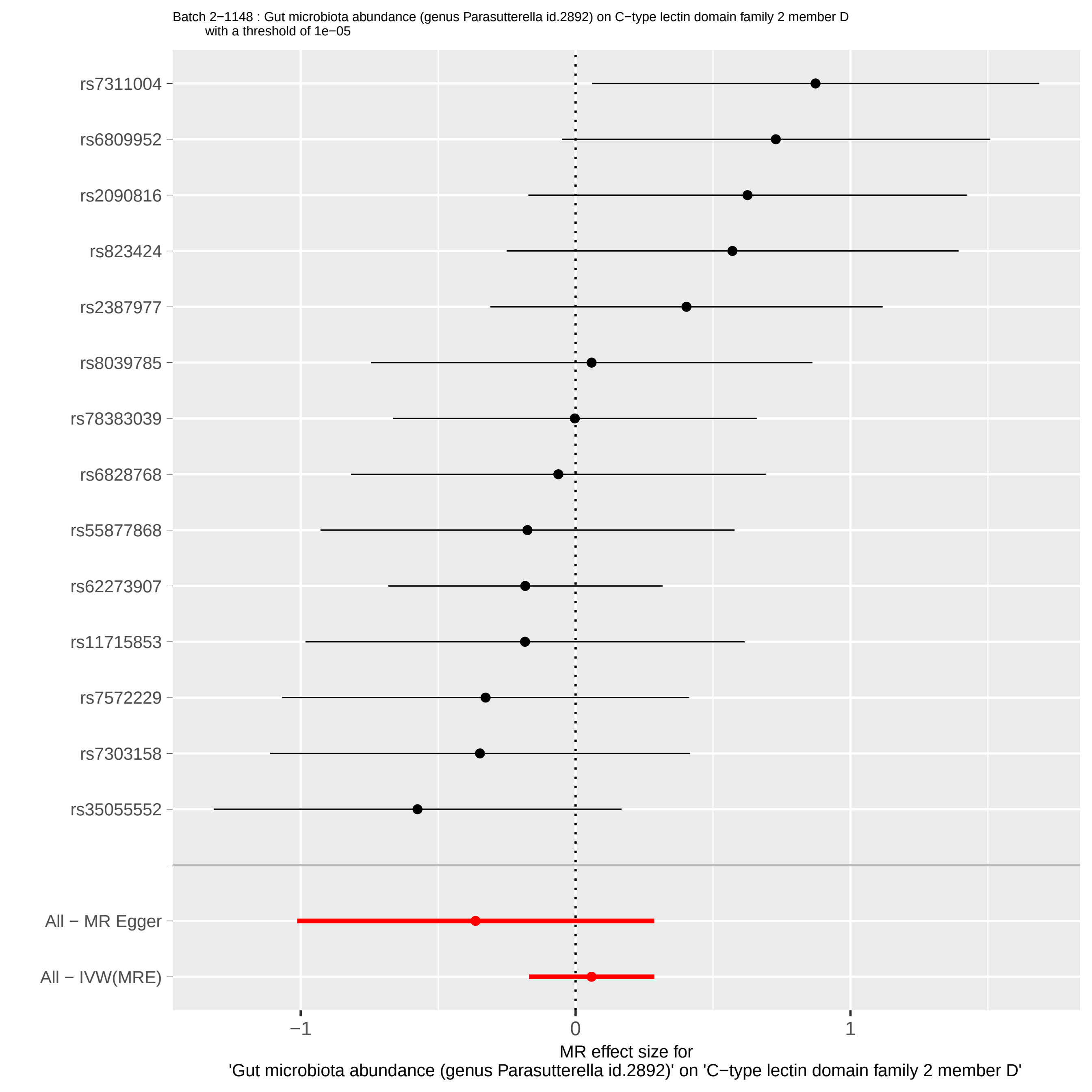

Batch 2−1148 : Gut microbiota abundance (genus Parasutterella id.2892) on C−type lectin domain family 2 member D
with a threshold of 1e−05
rs7311004
rs6809952
rs2090816
rs823424
rs2387977
rs8039785
rs78383039
rs6828768
rs55877868
rs62273907
rs11715853
rs7572229
rs7303158
rs35055552
All − MR Egger
All − IVW(MRE)
−1
0
1
MR effect size for
'Gut microbiota abundance (genus Parasutterella id.2892)' on 'C−type lectin domain family 2 member D'

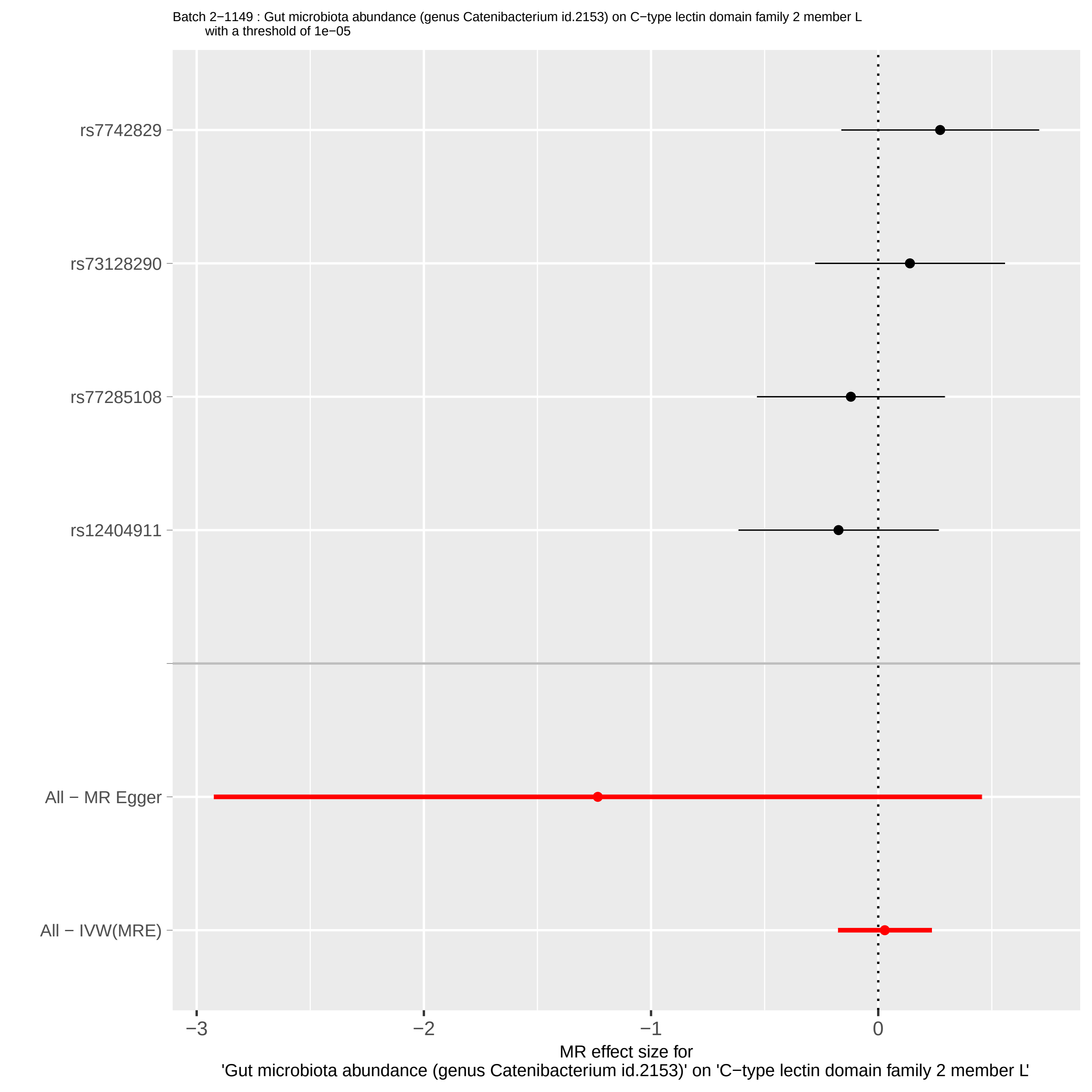

Batch 2−1149 : Gut microbiota abundance (genus Catenibacterium id.2153) on C−type lectin domain family 2 member L
with a threshold of 1e−05
rs7742829
rs73128290
rs77285108
rs12404911
All − MR Egger
All − IVW(MRE)
−3
−2
−1
0
MR effect size for
'Gut microbiota abundance (genus Catenibacterium id.2153)' on 'C−type lectin domain family 2 member L'

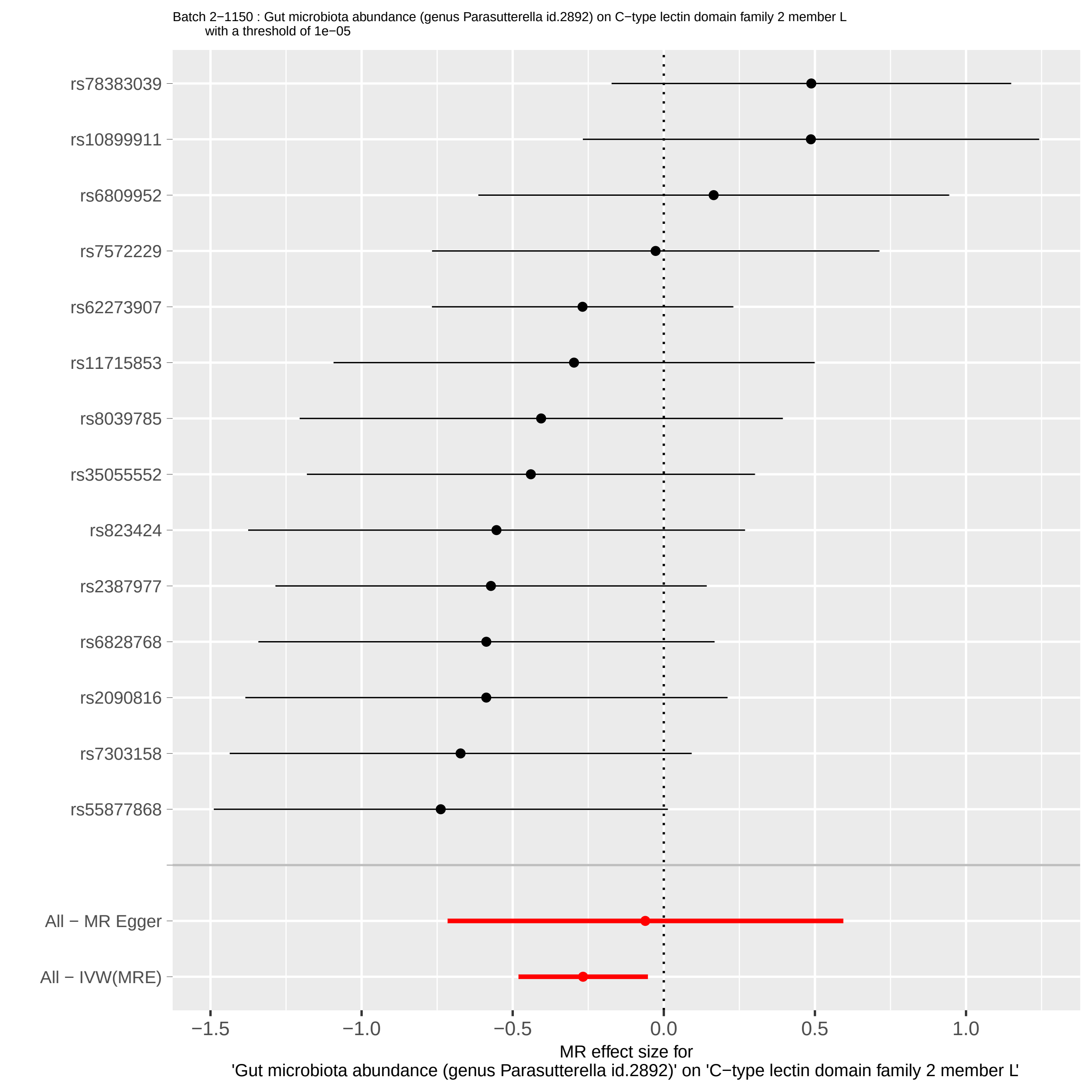

Batch 2−1150 : Gut microbiota abundance (genus Parasutterella id.2892) on C−type lectin domain family 2 member L
with a threshold of 1e−05
rs78383039
rs10899911
rs6809952
rs7572229
rs62273907
rs11715853
rs8039785
rs35055552
rs823424
rs2387977
rs6828768
rs2090816
rs7303158
rs55877868
All − MR Egger
All − IVW(MRE)
−1.5
−1.0
−0.5
0.0
0.5
1.0
MR effect size for
'Gut microbiota abundance (genus Parasutterella id.2892)' on 'C−type lectin domain family 2 member L'

Batch 2−1151 : Gut microbiota abundance (genus Catenibacterium id.2153) on C−type lectin domain family 4 member D
with a threshold of 1e−05

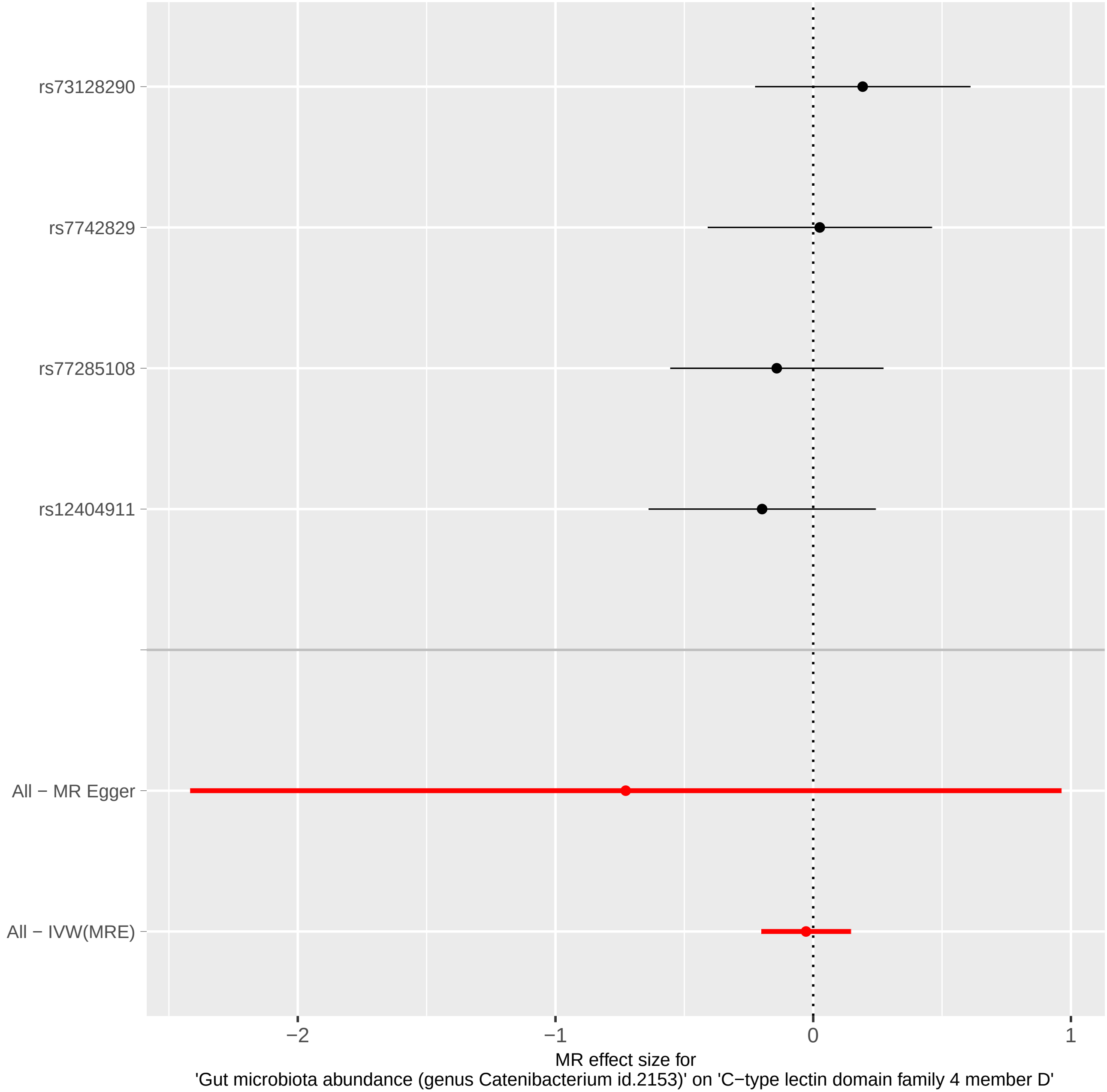

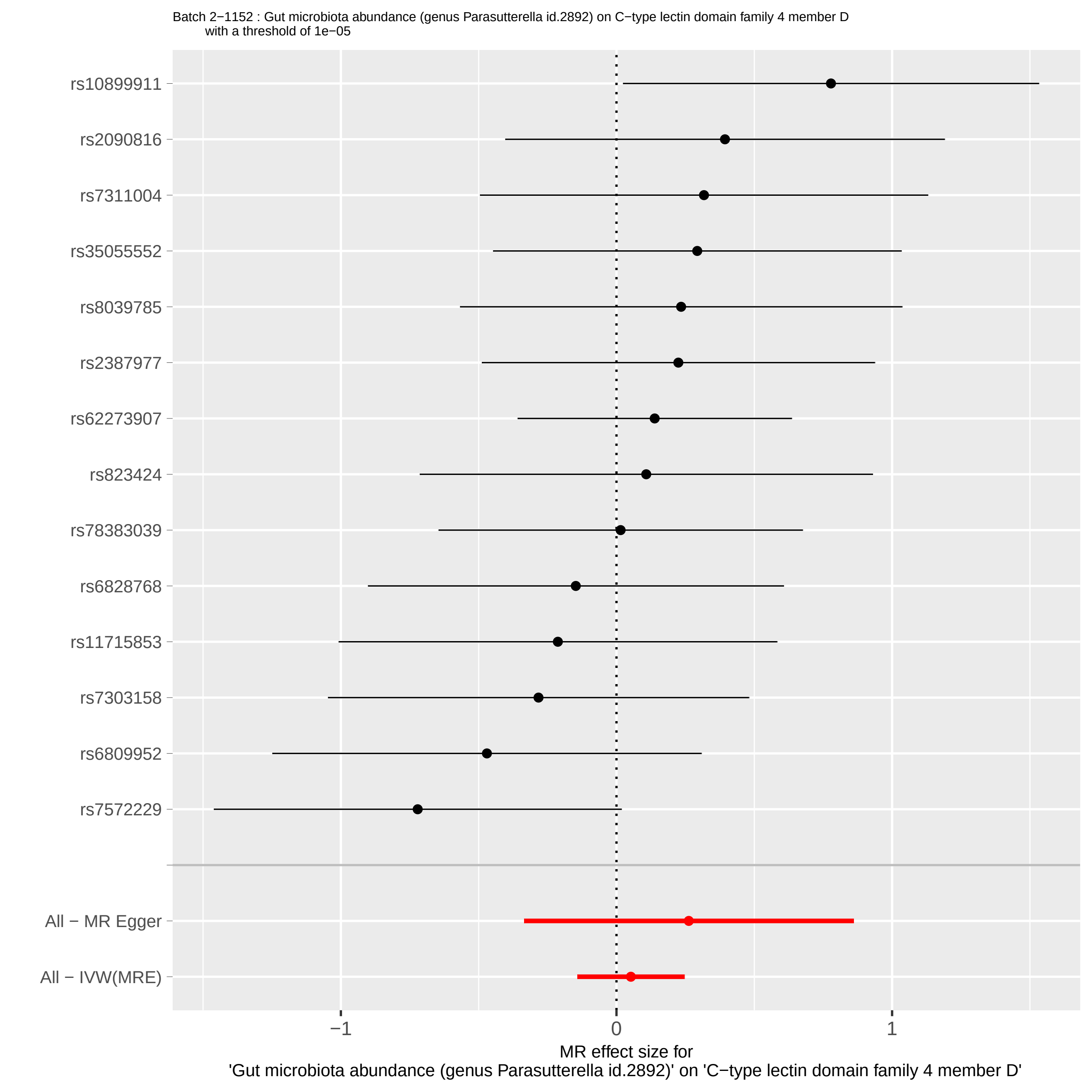
Batch 2−1152 : Gut microbiota abundance (genus Parasutterella id.2892) on C−type lectin domain family 4 member D
with a threshold of 1e−05
rs10899911
rs2090816
rs7311004
rs35055552
rs8039785
rs2387977
rs62273907
rs823424
rs78383039
rs6828768
rs11715853
rs7303158
rs6809952
rs7572229
All − MR Egger
All − IVW(MRE)
−1
0
1
MR effect size for
'Gut microbiota abundance (genus Parasutterella id.2892)' on 'C−type lectin domain family 4 member D'

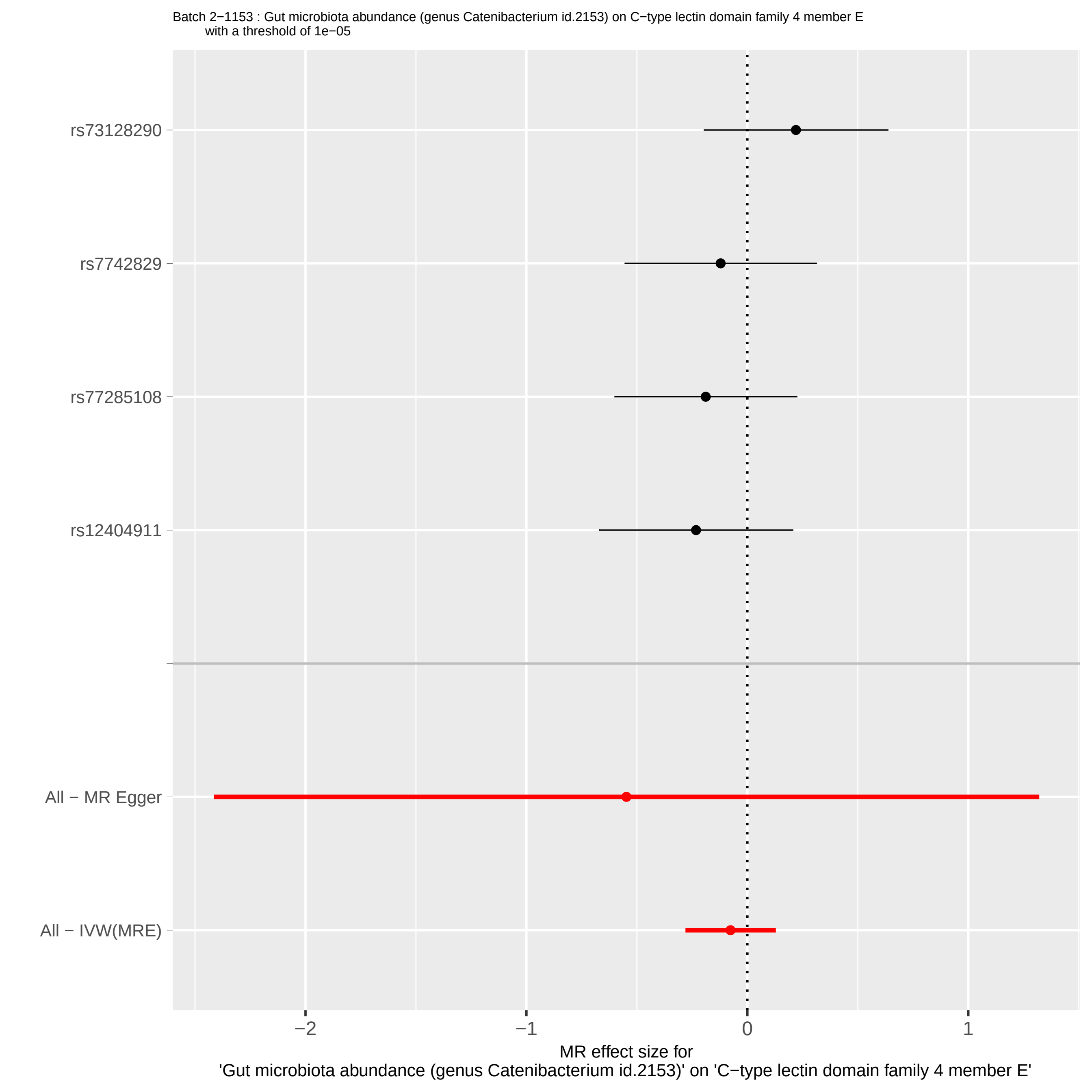

Batch 2−1153 : Gut microbiota abundance (genus Catenibacterium id.2153) on C−type lectin domain family 4 member E
with a threshold of 1e−05
rs73128290
rs7742829
rs77285108
rs12404911
All − MR Egger
All − IVW(MRE)
−2
−1
0
1
MR effect size for
'Gut microbiota abundance (genus Catenibacterium id.2153)' on 'C−type lectin domain family 4 member E'

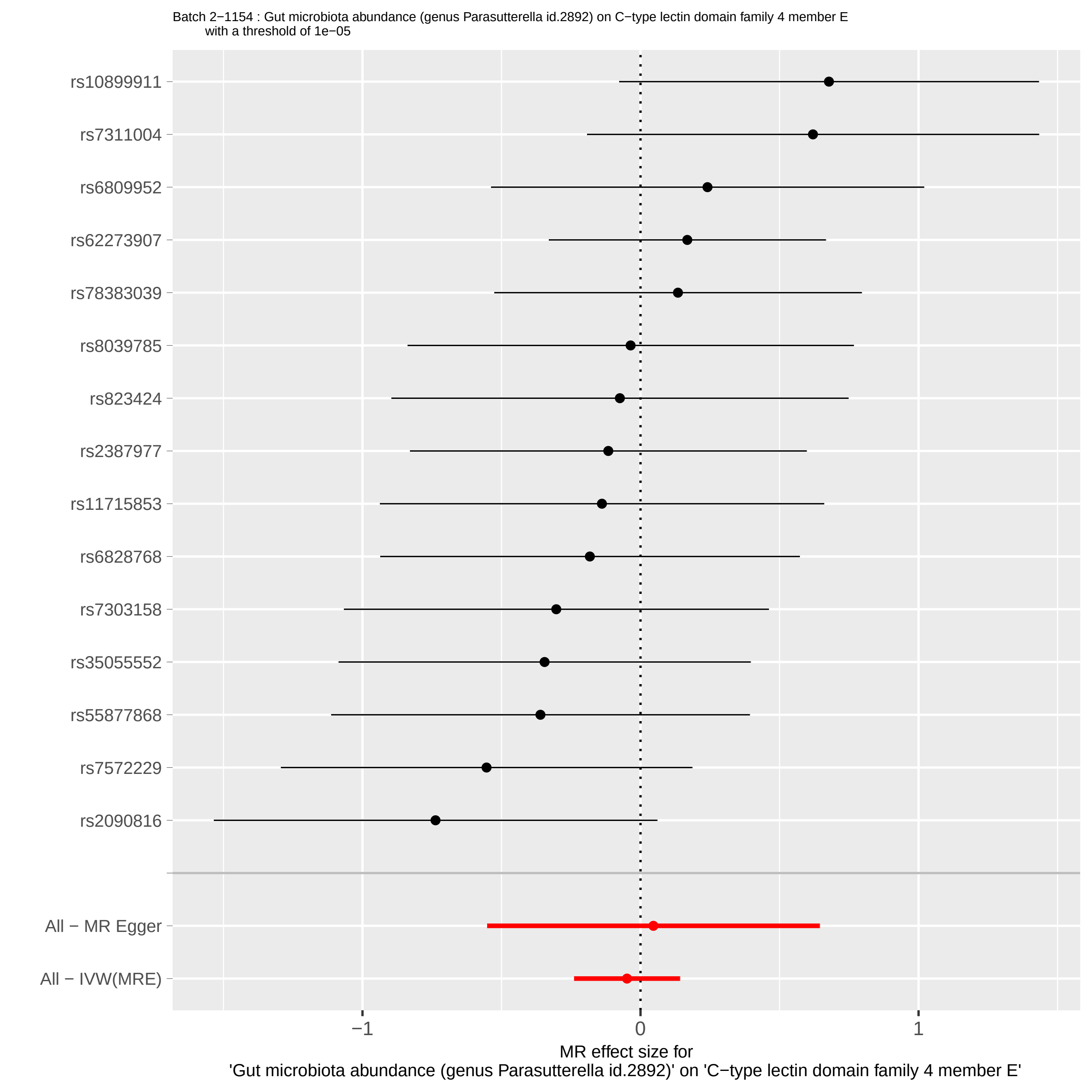

Batch 2−1154 : Gut microbiota abundance (genus Parasutterella id.2892) on C−type lectin domain family 4 member E
with a threshold of 1e−05
rs10899911
rs7311004
rs6809952
rs62273907
rs78383039
rs8039785
rs823424
rs2387977
rs11715853
rs6828768
rs7303158
rs35055552
rs55877868
rs7572229
rs2090816
All − MR Egger
All − IVW(MRE)
−1
0
1
MR effect size for
'Gut microbiota abundance (genus Parasutterella id.2892)' on 'C−type lectin domain family 4 member E'

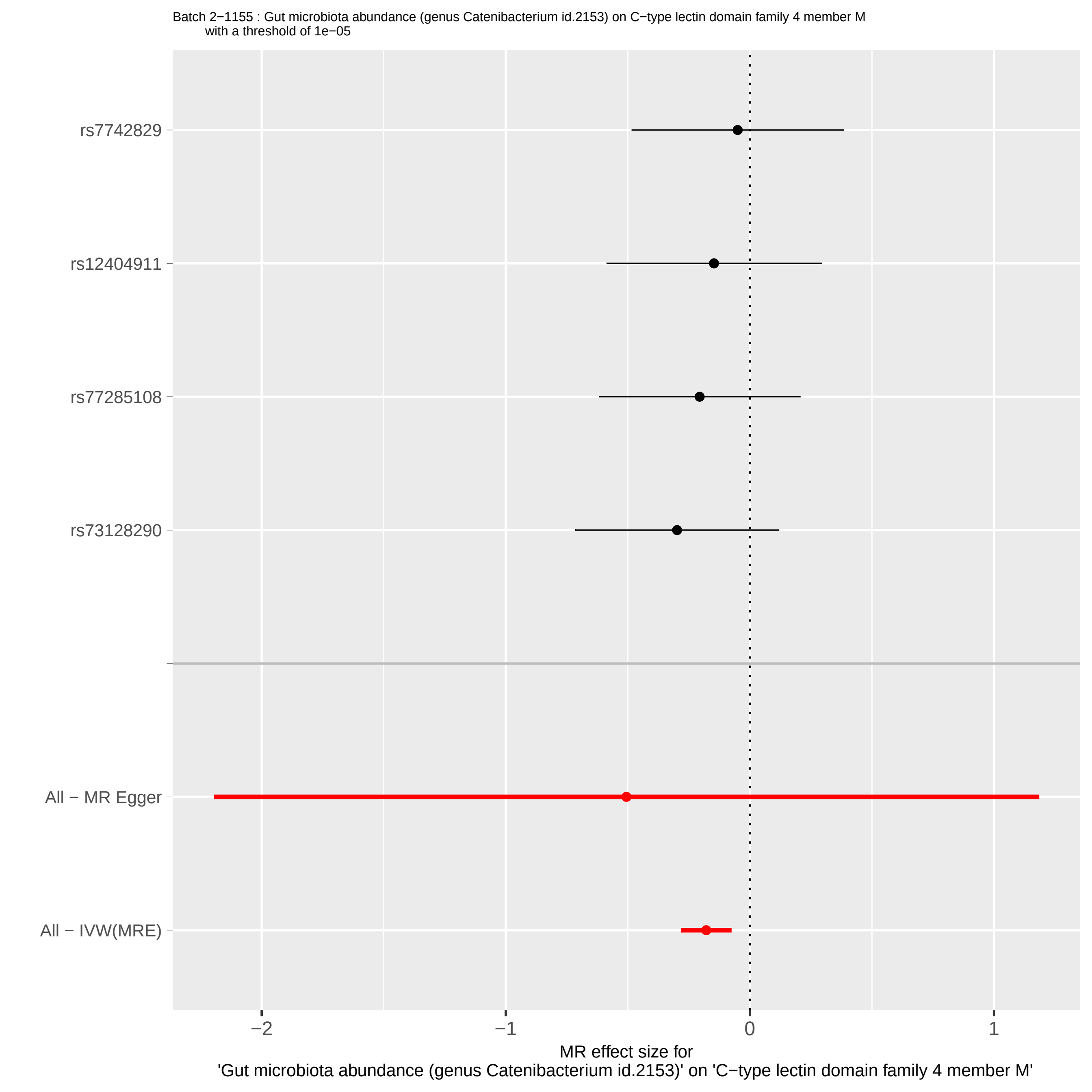
Batch 2−1155 : Gut microbiota abundance (genus Catenibacterium id.2153) on C−type lectin domain family 4 member M
with a threshold of 1e−05
rs7742829
rs12404911
rs77285108
rs73128290
All − MR Egger
All − IVW(MRE)
−2
−1
0
1
MR effect size for
'Gut microbiota abundance (genus Catenibacterium id.2153)' on 'C−type lectin domain family 4 member M'

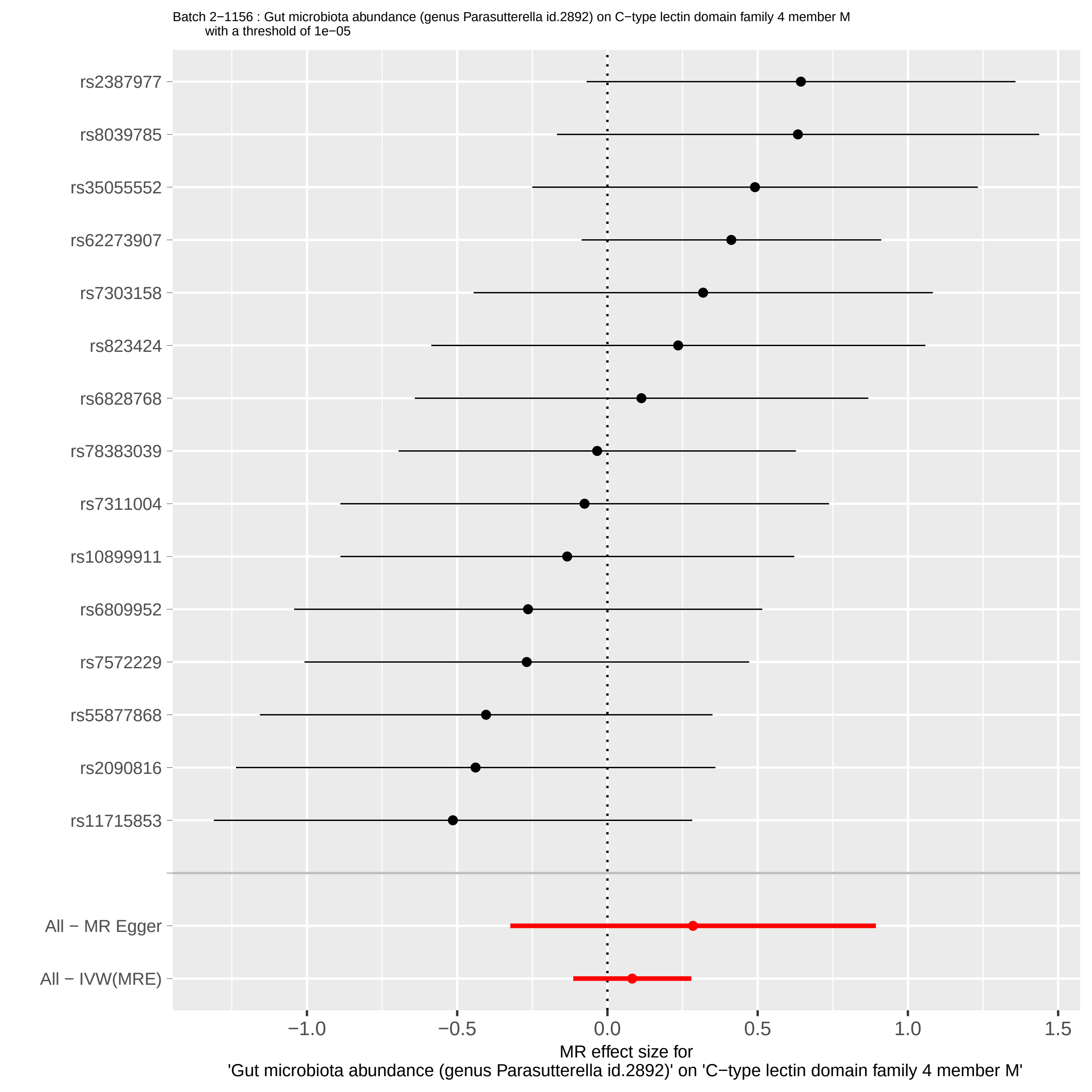

Batch 2−1156 : Gut microbiota abundance (genus Parasutterella id.2892) on C−type lectin domain family 4 member M
with a threshold of 1e−05
rs2387977
rs8039785
rs35055552
rs62273907
rs7303158
rs823424
rs6828768
rs78383039
rs7311004
rs10899911
rs6809952
rs7572229
rs55877868
rs2090816
rs11715853
All − MR Egger
All − IVW(MRE)
−1.0
−0.5
0.0
0.5
1.0
1.5
MR effect size for
'Gut microbiota abundance (genus Parasutterella id.2892)' on 'C−type lectin domain family 4 member M'

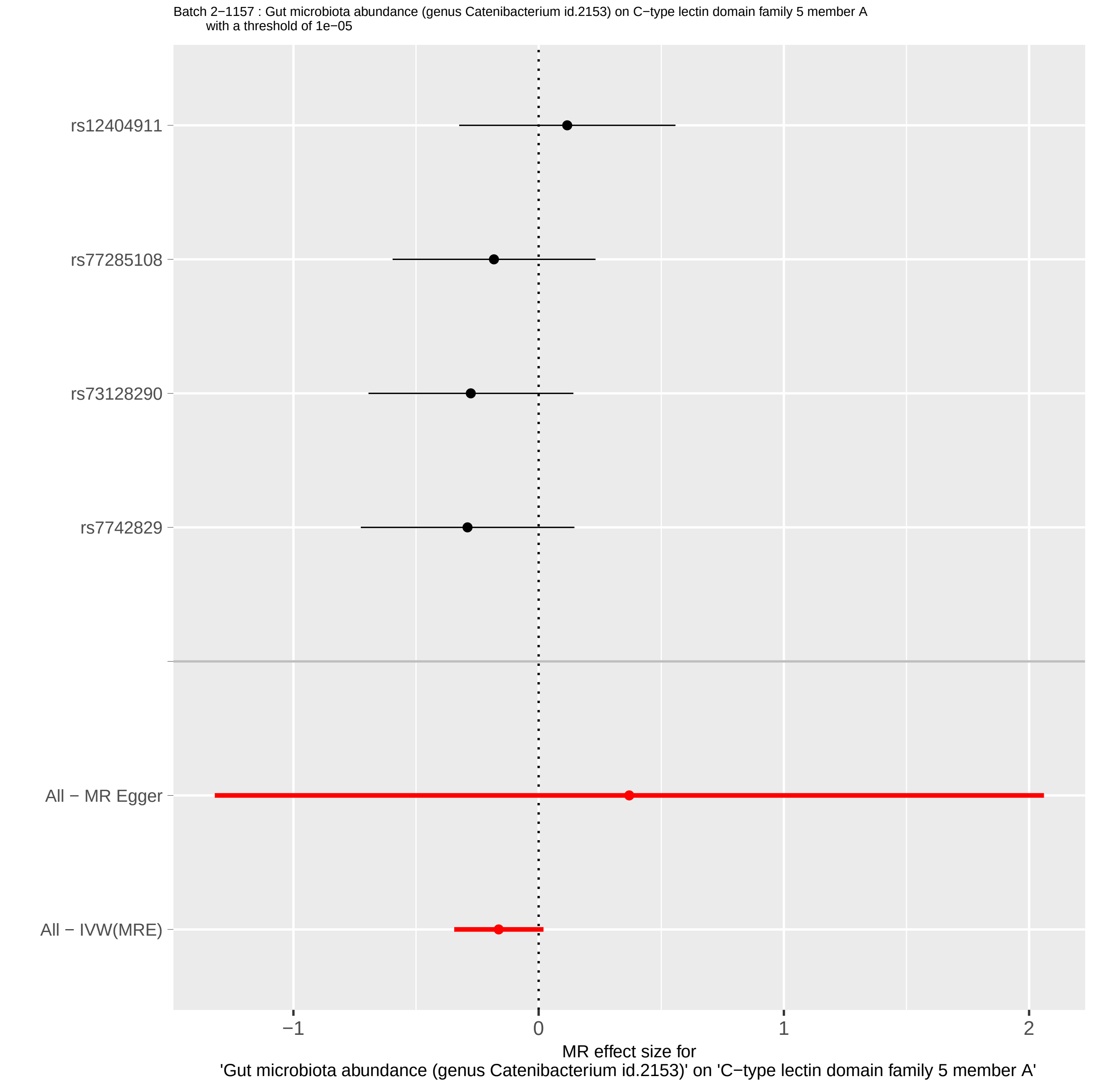

Batch 2−1157 : Gut microbiota abundance (genus Catenibacterium id.2153) on C−type lectin domain family 5 member A
with a threshold of 1e−05
rs12404911
rs77285108
rs73128290
rs7742829
All − MR Egger
All − IVW(MRE)
−1
0
1
2
MR effect size for
'Gut microbiota abundance (genus Catenibacterium id.2153)' on 'C−type lectin domain family 5 member A'

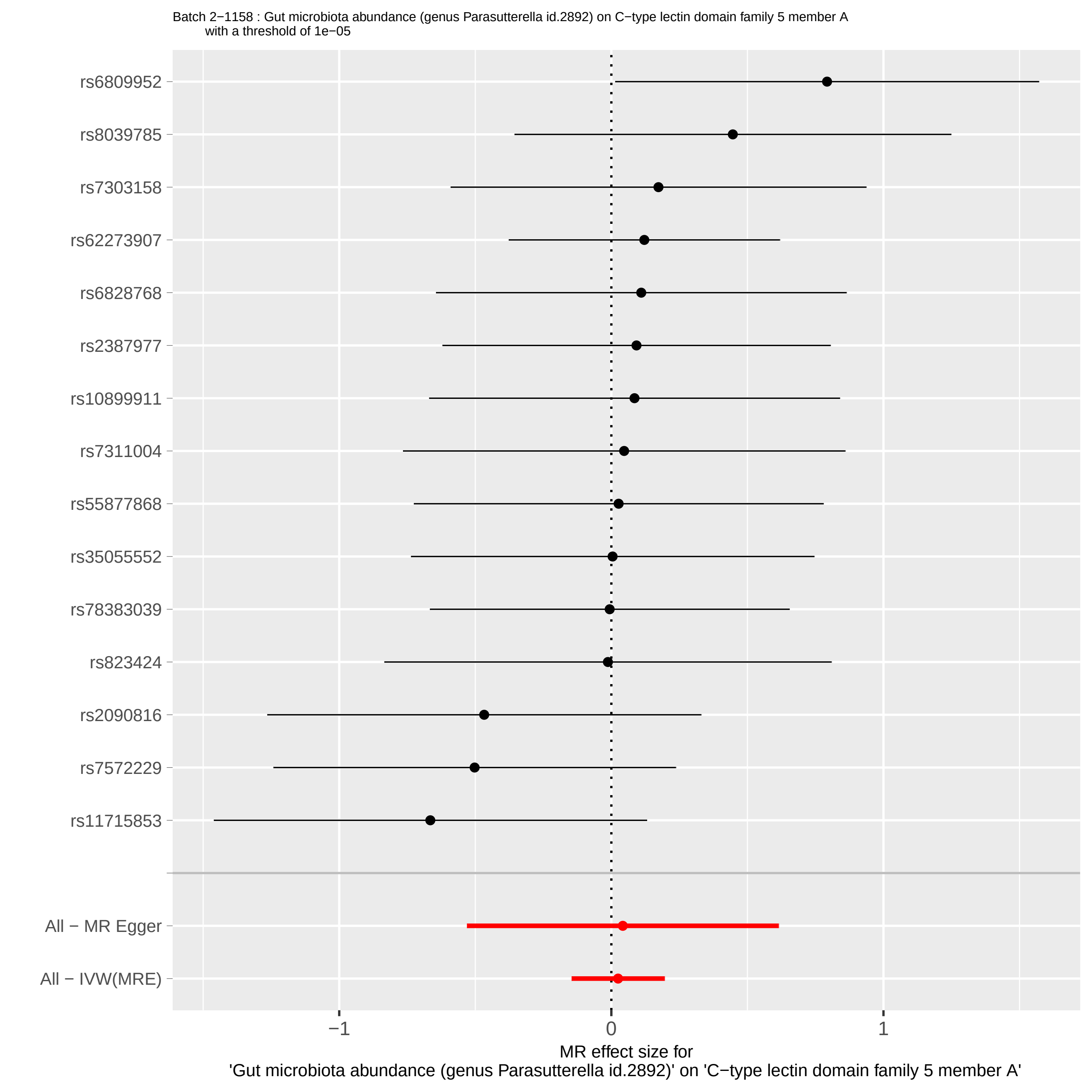
Batch 2−1158 : Gut microbiota abundance (genus Parasutterella id.2892) on C−type lectin domain family 5 member A
with a threshold of 1e−05
rs6809952
rs8039785
rs7303158
rs62273907
rs6828768
rs2387977
rs10899911
rs7311004
rs55877868
rs35055552
rs78383039
rs823424
rs2090816
rs7572229
rs11715853
All − MR Egger
All − IVW(MRE)
−1
0
1
MR effect size for
'Gut microbiota abundance (genus Parasutterella id.2892)' on 'C−type lectin domain family 5 member A'

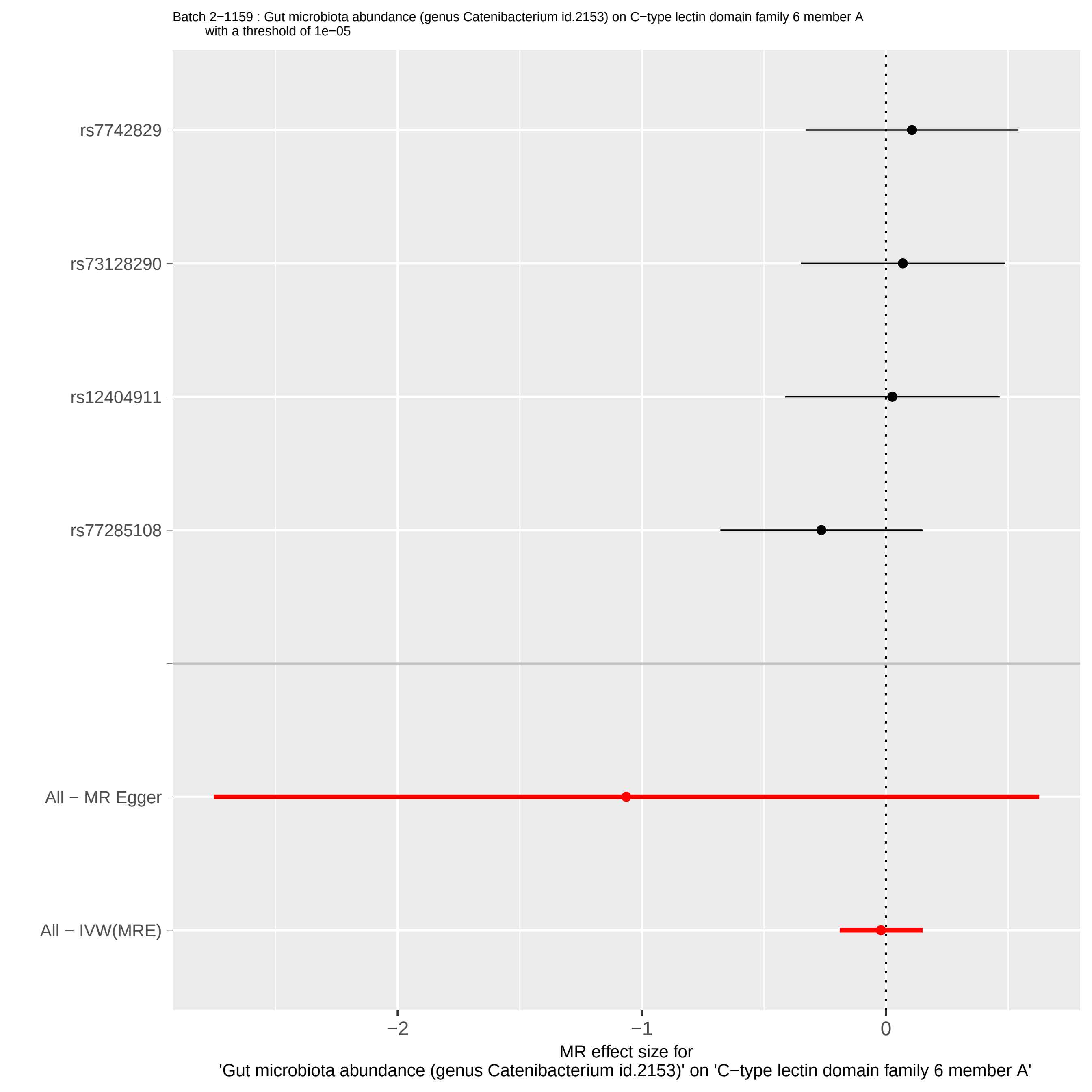

Batch 2−1159 : Gut microbiota abundance (genus Catenibacterium id.2153) on C−type lectin domain family 6 member A
with a threshold of 1e−05
rs7742829
rs73128290
rs12404911
rs77285108
All − MR Egger
All − IVW(MRE)
−2
−1
0
MR effect size for
'Gut microbiota abundance (genus Catenibacterium id.2153)' on 'C−type lectin domain family 6 member A'

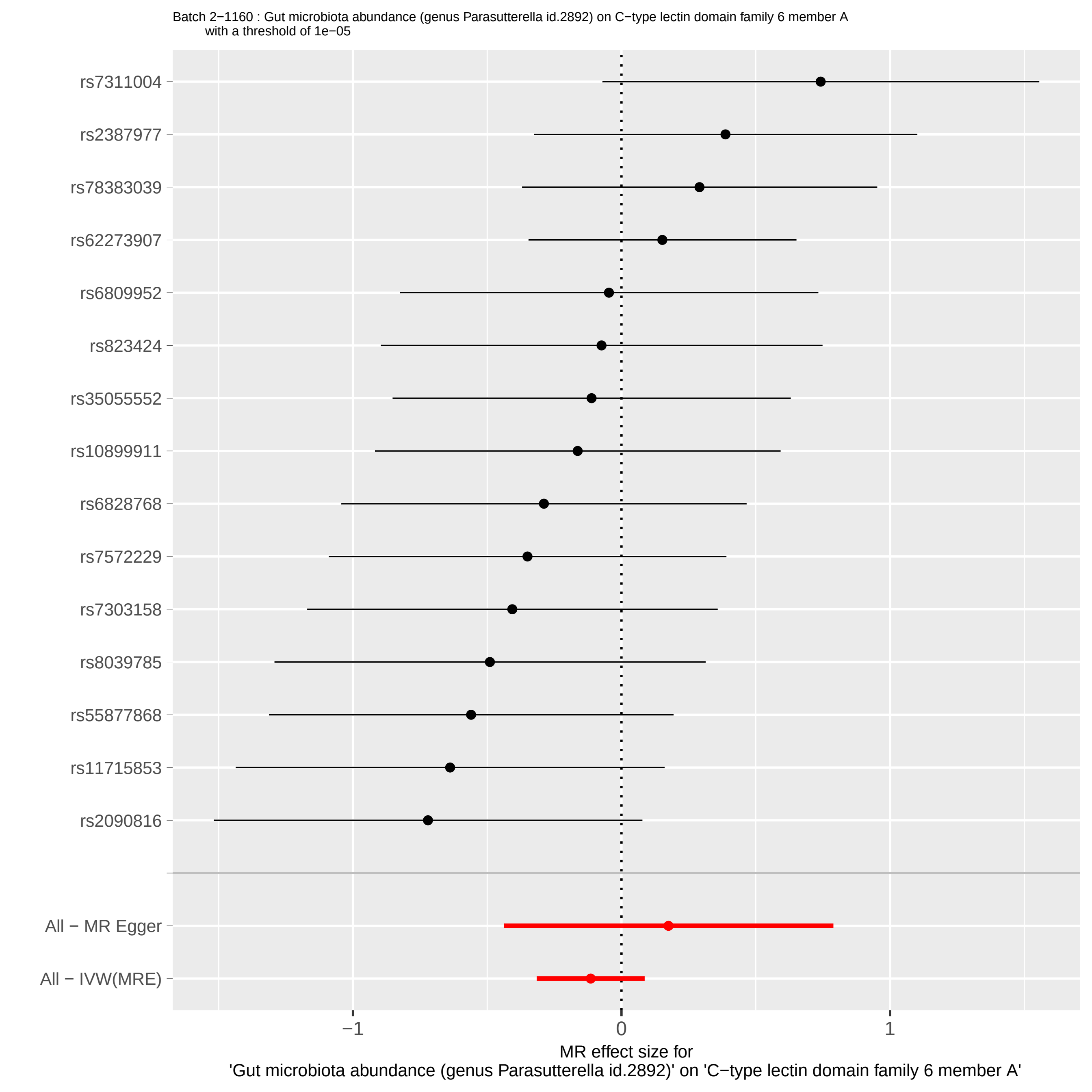
Batch 2−1160 : Gut microbiota abundance (genus Parasutterella id.2892) on C−type lectin domain family 6 member A
with a threshold of 1e−05
rs7311004
rs2387977
rs78383039
rs62273907
rs6809952
rs823424
rs35055552
rs10899911
rs6828768
rs7572229
rs7303158
rs8039785
rs55877868
rs11715853
rs2090816
All − MR Egger
All − IVW(MRE)
−1
0
1
MR effect size for
'Gut microbiota abundance (genus Parasutterella id.2892)' on 'C−type lectin domain family 6 member A'

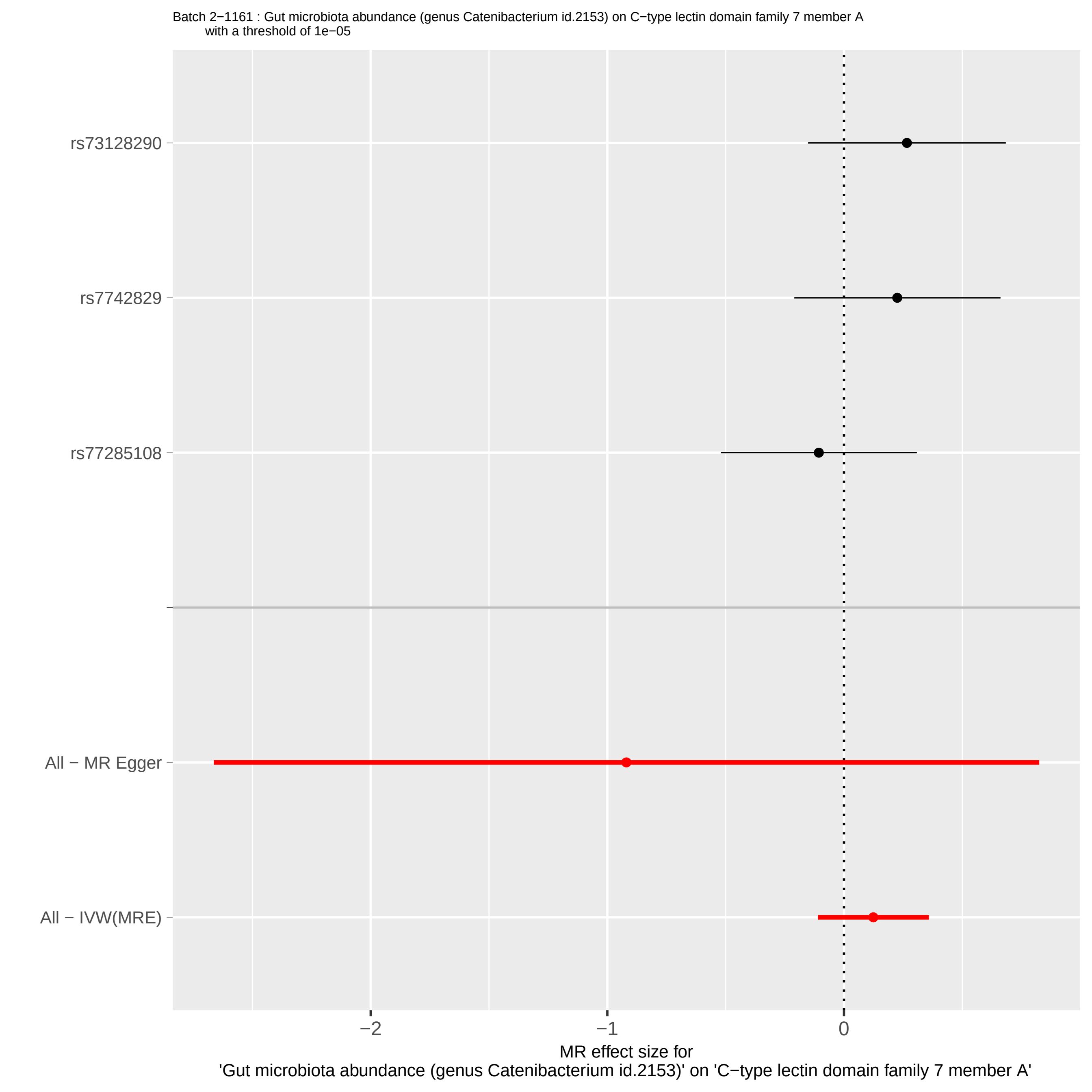
Batch 2−1161 : Gut microbiota abundance (genus Catenibacterium id.2153) on C−type lectin domain family 7 member A
with a threshold of 1e−05
rs73128290
rs7742829
rs77285108
All − MR Egger
All − IVW(MRE)
−2
−1
0
MR effect size for
'Gut microbiota abundance (genus Catenibacterium id.2153)' on 'C−type lectin domain family 7 member A'

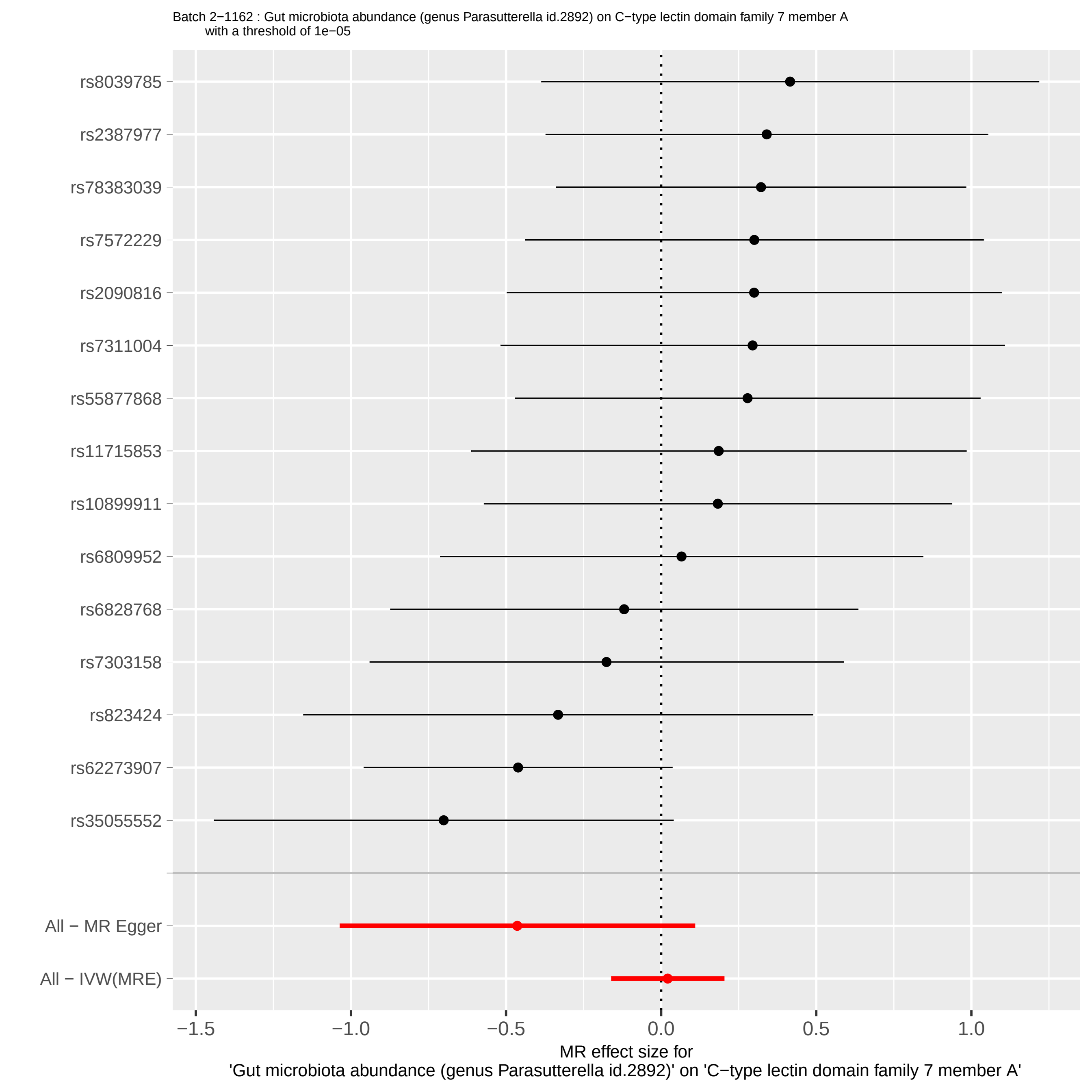

Batch 2−1162 : Gut microbiota abundance (genus Parasutterella id.2892) on C−type lectin domain family 7 member A
with a threshold of 1e−05
rs8039785
rs2387977
rs78383039
rs7572229
rs2090816
rs7311004
rs55877868
rs11715853
rs10899911
rs6809952
rs6828768
rs7303158
rs823424
rs62273907
rs35055552
All − MR Egger
All − IVW(MRE)
−1.5
−1.0
−0.5
0.0
0.5
1.0
MR effect size for
'Gut microbiota abundance (genus Parasutterella id.2892)' on 'C−type lectin domain family 7 member A'

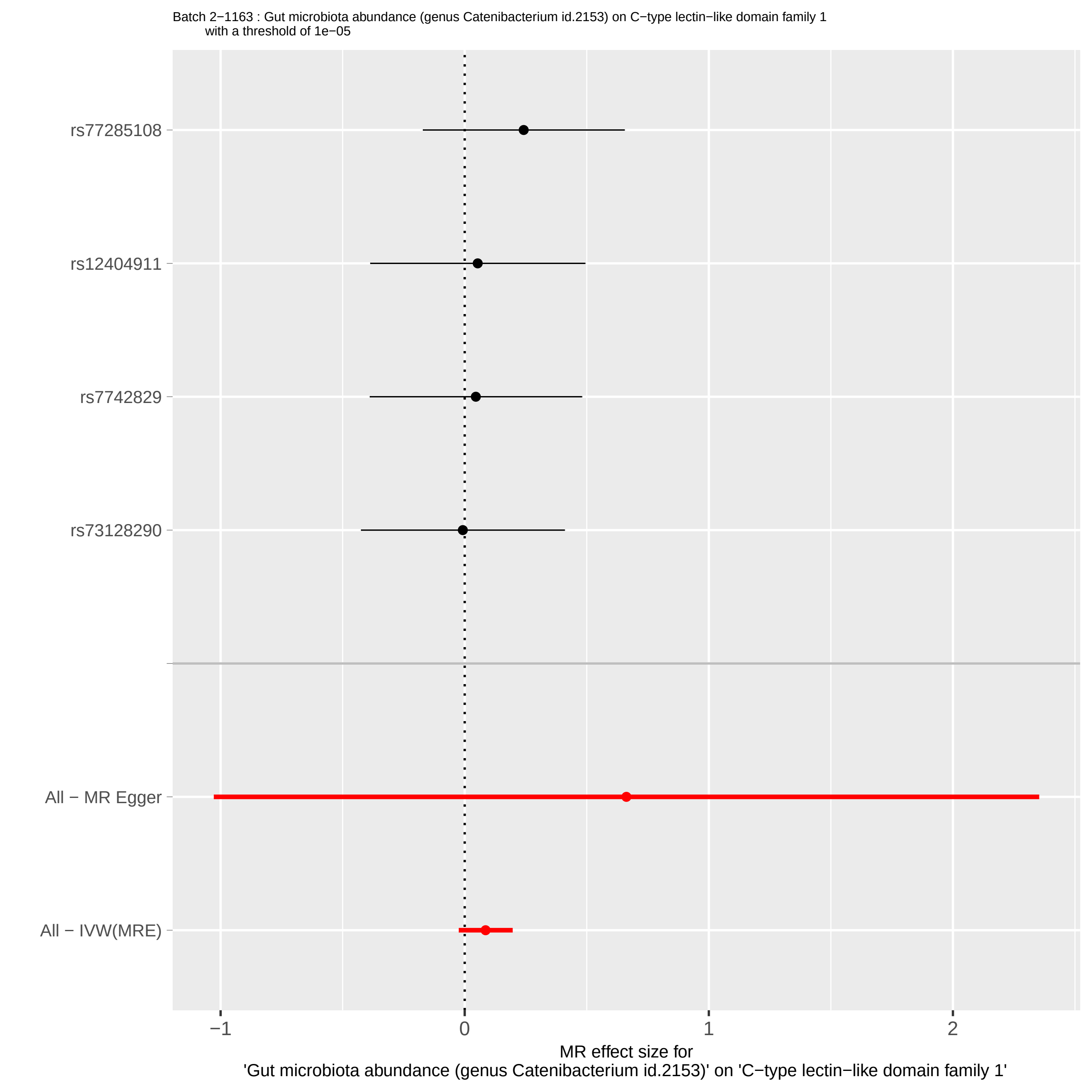

Batch 2−1163 : Gut microbiota abundance (genus Catenibacterium id.2153) on C−type lectin−like domain family 1
with a threshold of 1e−05
rs77285108
rs12404911
rs7742829
rs73128290
All − MR Egger
All − IVW(MRE)
−1
0
1
2
MR effect size for
'Gut microbiota abundance (genus Catenibacterium id.2153)' on 'C−type lectin−like domain family 1'

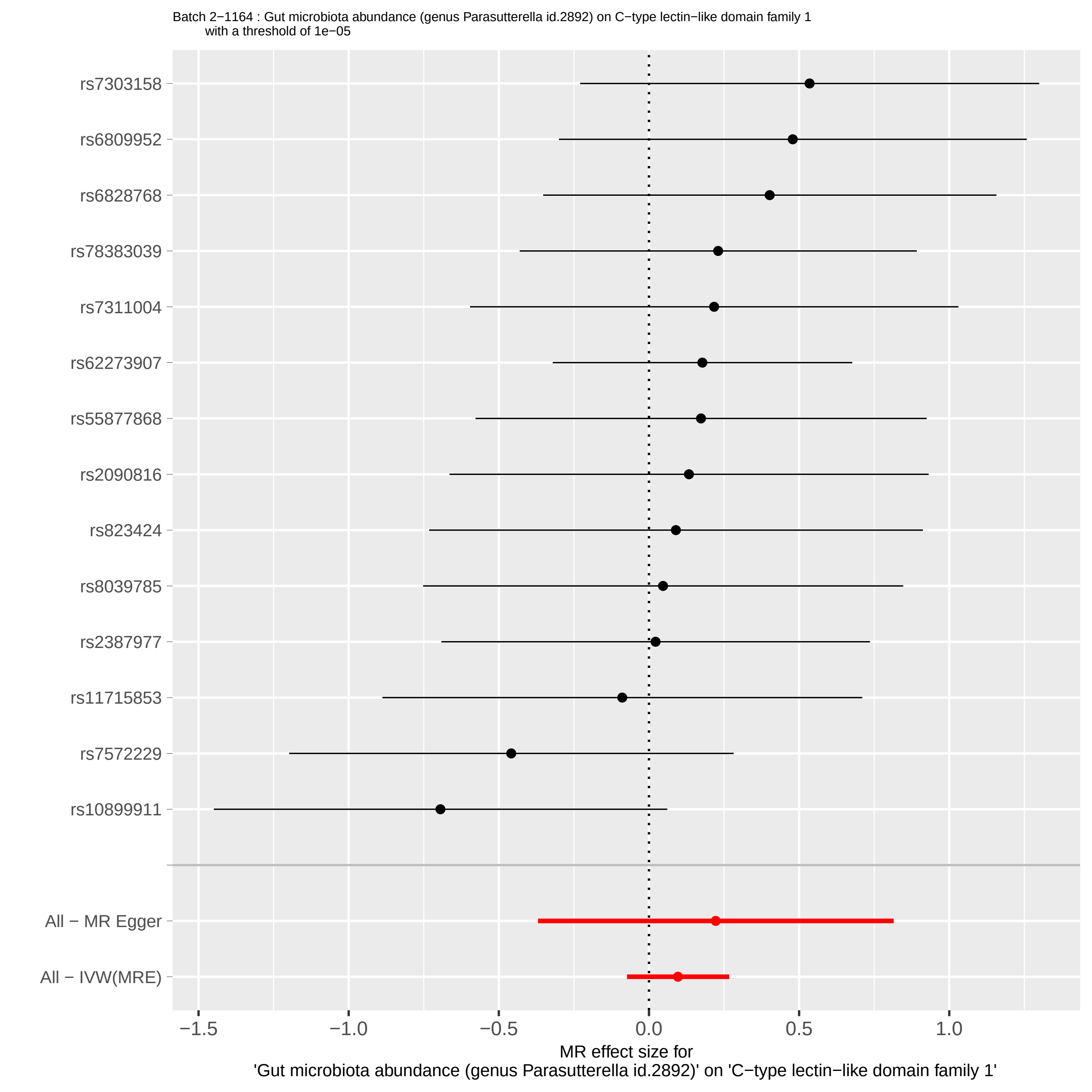
Batch 2−1164 : Gut microbiota abundance (genus Parasutterella id.2892) on C−type lectin−like domain family 1
with a threshold of 1e−05
rs7303158
rs6809952
rs6828768
rs78383039
rs7311004
rs62273907
rs55877868
rs2090816
rs823424
rs8039785
rs2387977
rs11715853
rs7572229
rs10899911
All − MR Egger
All − IVW(MRE)
−1.5
−1.0
−0.5
0.0
0.5
1.0
MR effect size for
'Gut microbiota abundance (genus Parasutterella id.2892)' on 'C−type lectin−like domain family 1'

Batch 2−1165 : Gut microbiota abundance (genus Catenibacterium id.2153) on Chloride intracellular channel protein 4
with a threshold of 1e−05

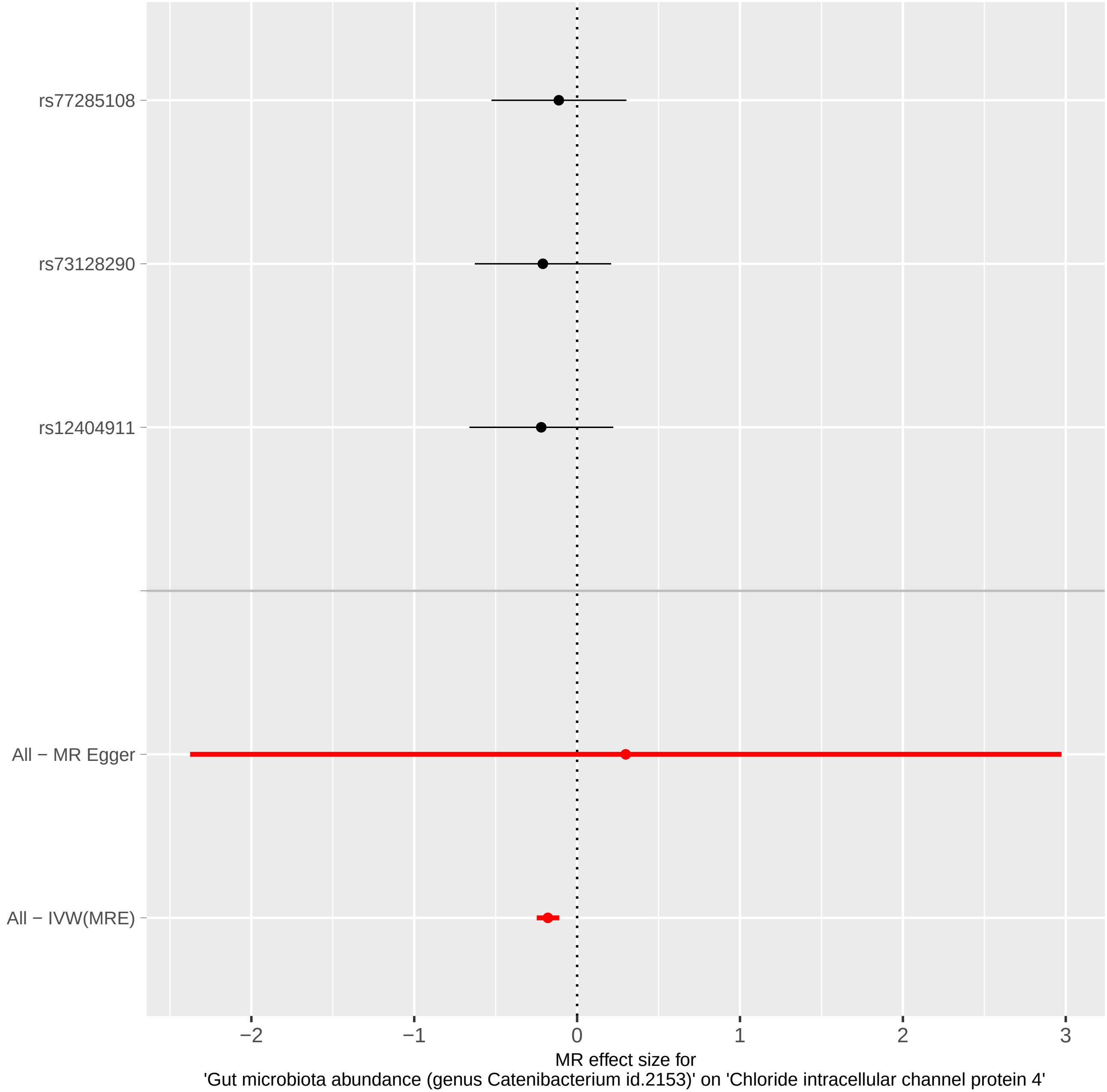

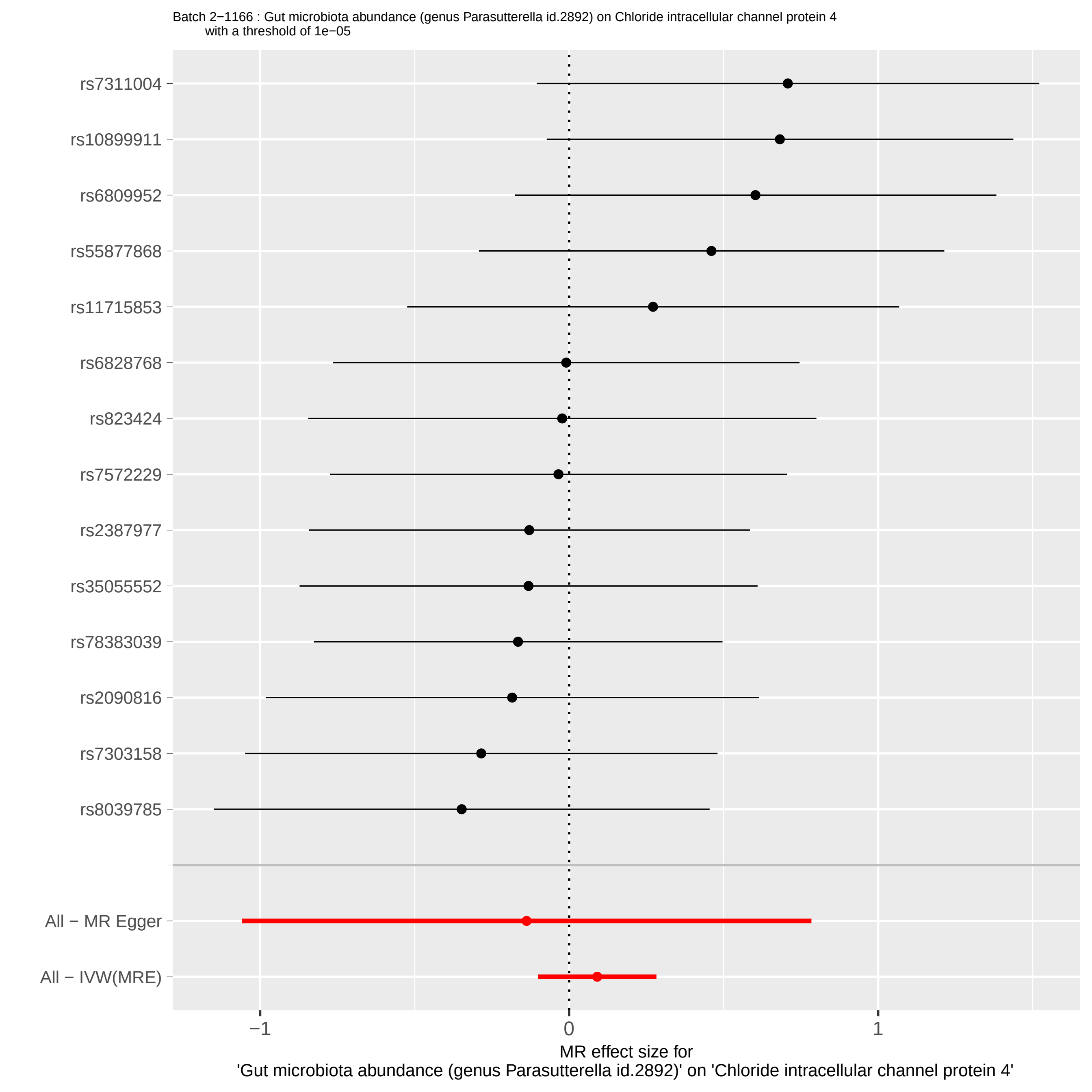
Batch 2−1166 : Gut microbiota abundance (genus Parasutterella id.2892) on Chloride intracellular channel protein 4
with a threshold of 1e−05
rs7311004
rs10899911
rs6809952
rs55877868
rs11715853
rs6828768
rs823424
rs7572229
rs2387977
rs35055552
rs78383039
rs2090816
rs7303158
rs8039785
All − MR Egger
All − IVW(MRE)
−1
0
1
MR effect size for
'Gut microbiota abundance (genus Parasutterella id.2892)' on 'Chloride intracellular channel protein 4'

Batch 2−1167 : Gut microbiota abundance (genus Catenibacterium id.2153) on Chloride intracellular channel protein 5
with a threshold of 1e−05

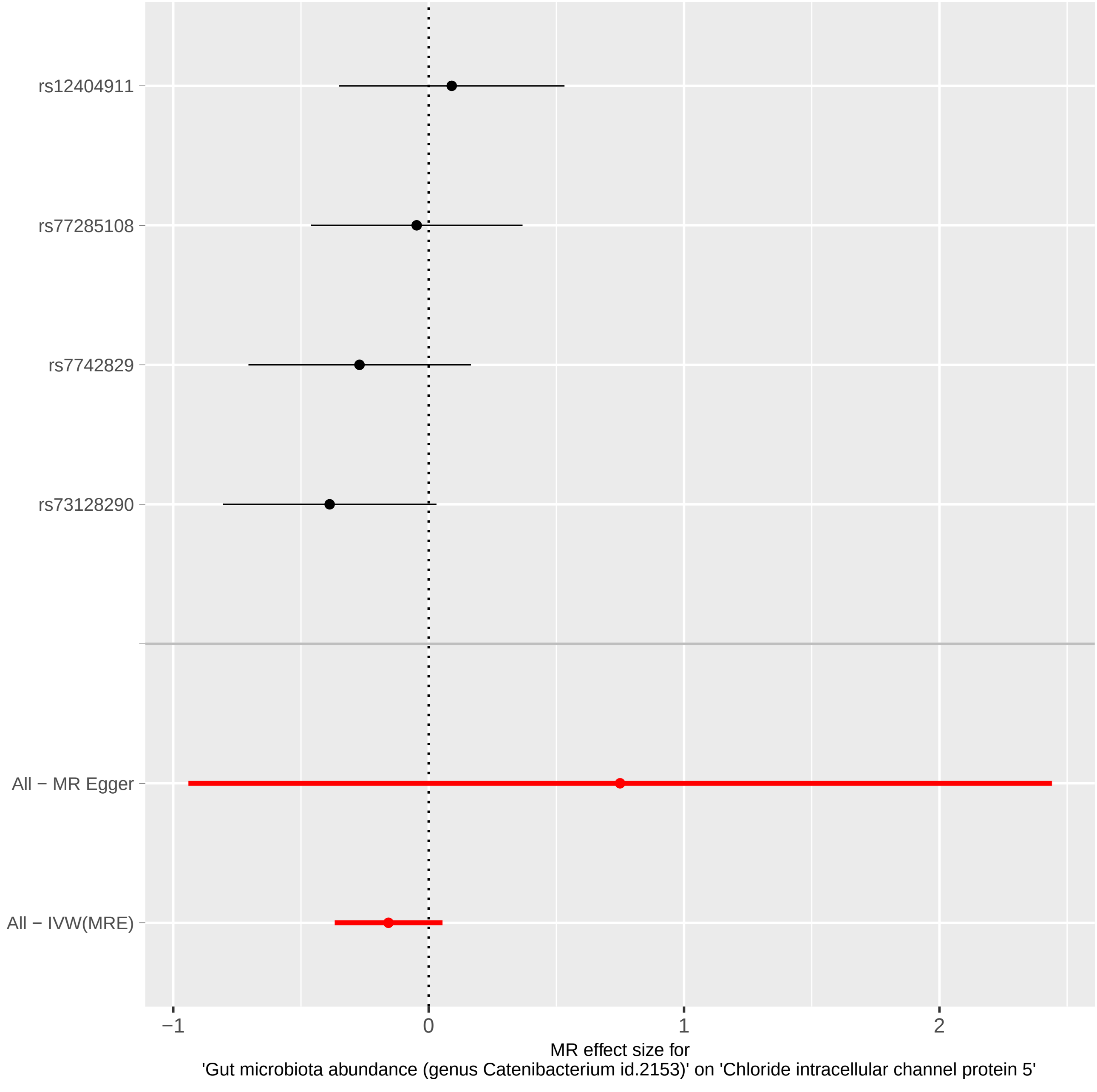

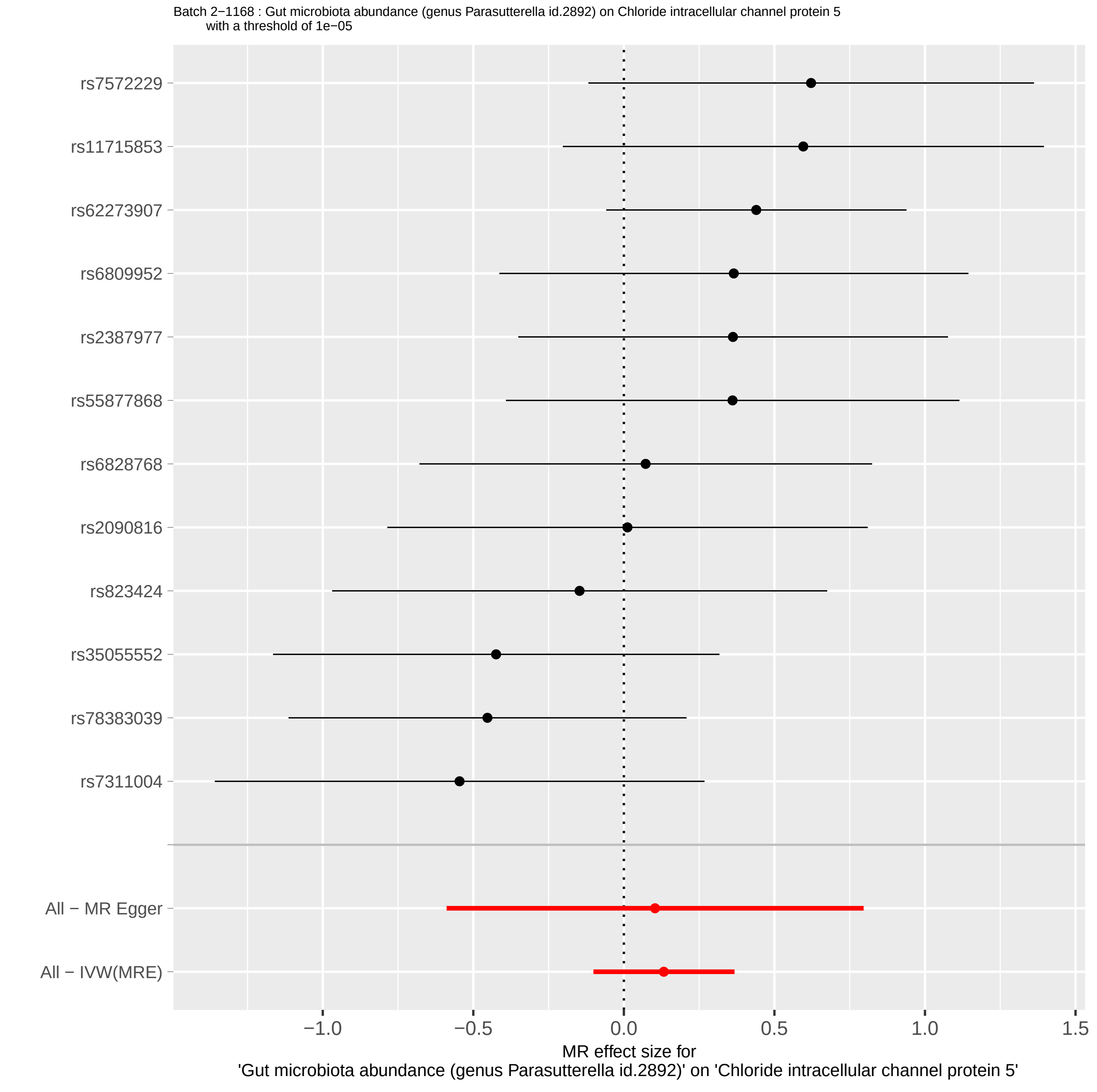

Batch 2−1168 : Gut microbiota abundance (genus Parasutterella id.2892) on Chloride intracellular channel protein 5
with a threshold of 1e−05
rs7572229
rs11715853
rs62273907
rs6809952
rs2387977
rs55877868
rs6828768
rs2090816
rs823424
rs35055552
rs78383039
rs7311004
All − MR Egger
All − IVW(MRE)
−1.0
−0.5
0.0
0.5
1.0
1.5
MR effect size for
'Gut microbiota abundance (genus Parasutterella id.2892)' on 'Chloride intracellular channel protein 5'

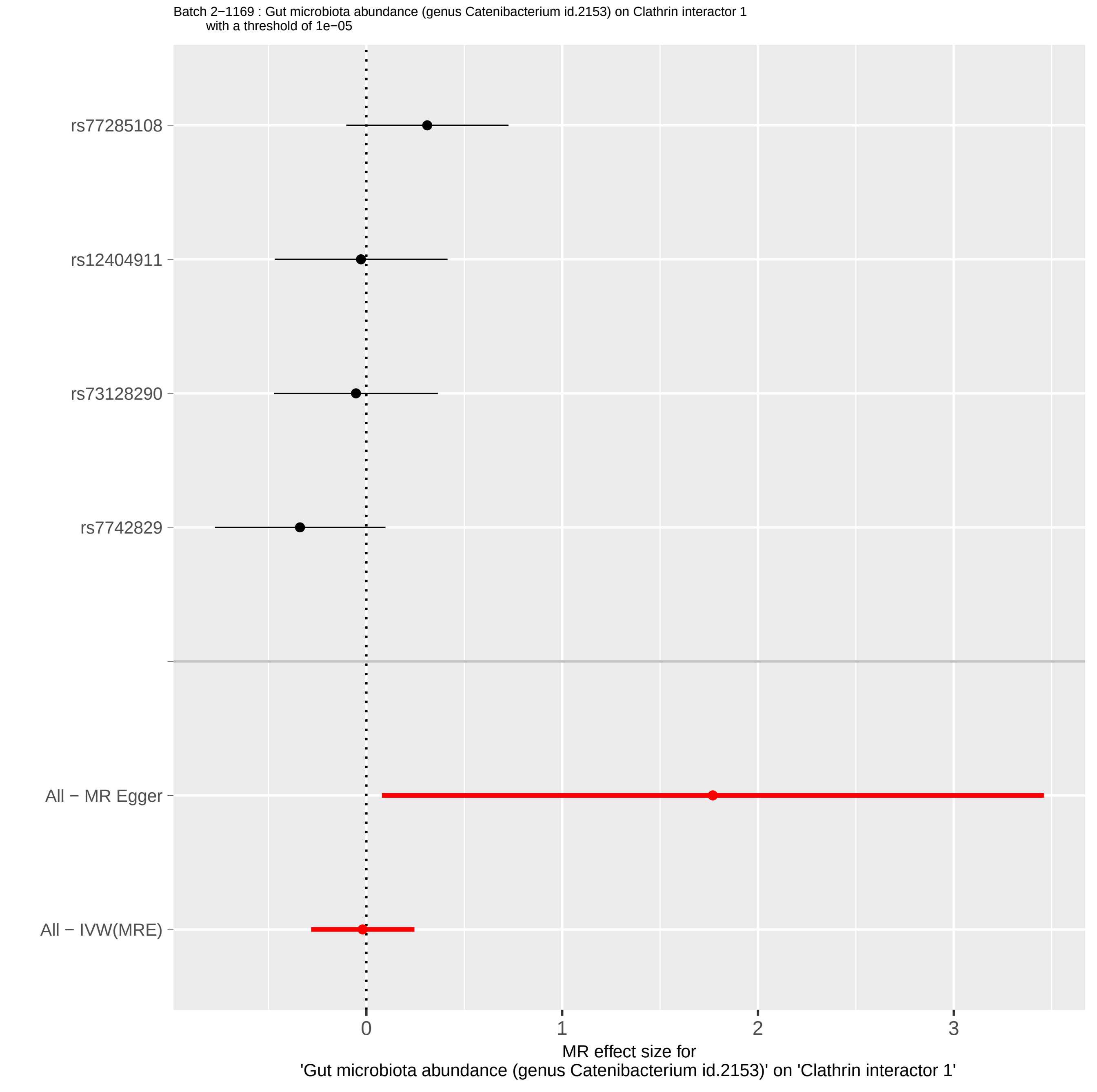

Batch 2−1169 : Gut microbiota abundance (genus Catenibacterium id.2153) on Clathrin interactor 1
with a threshold of 1e−05
rs77285108
rs12404911
rs73128290
rs7742829
All − MR Egger
All − IVW(MRE)
0
1
2
3
MR effect size for
'Gut microbiota abundance (genus Catenibacterium id.2153)' on 'Clathrin interactor 1'

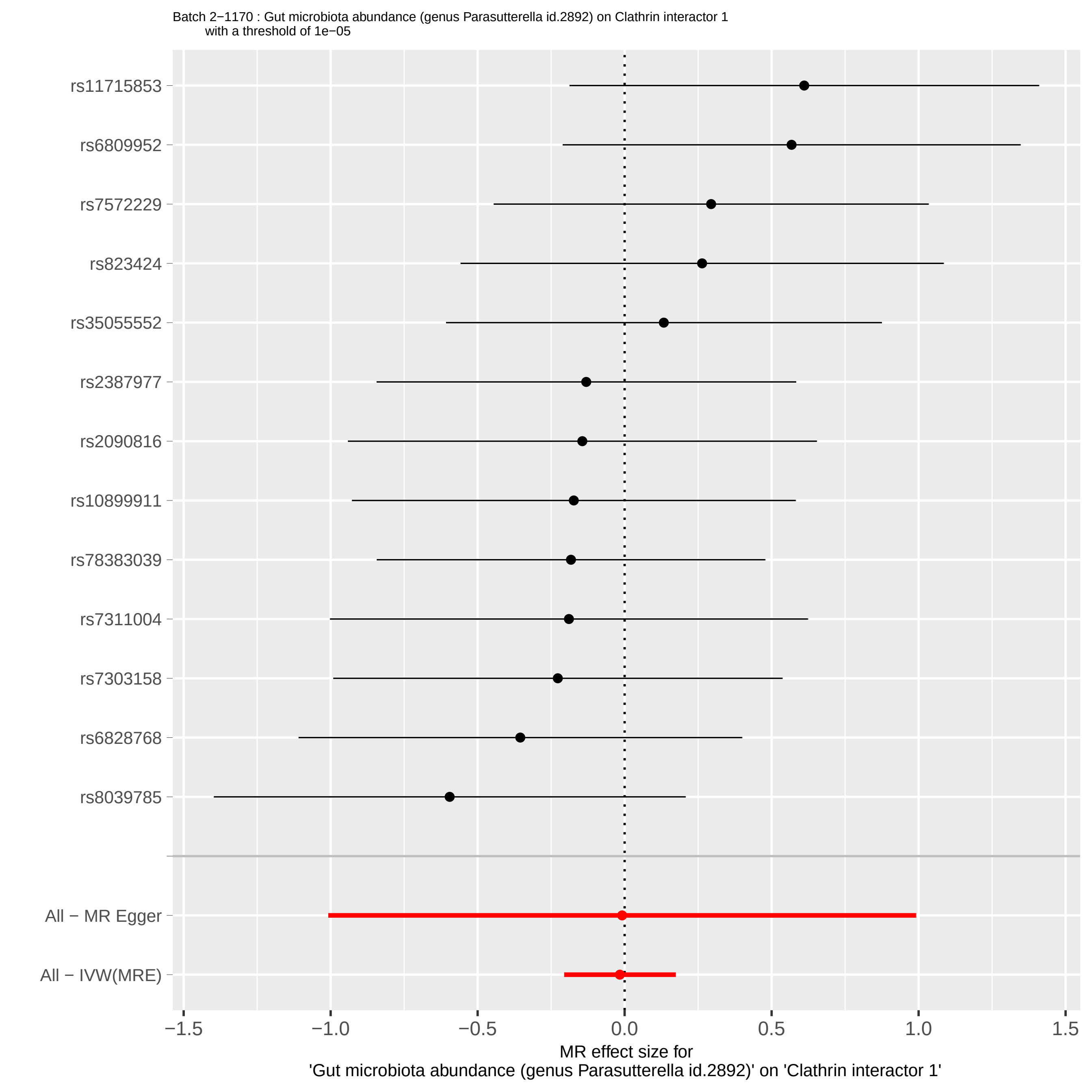
Batch 2−1170 : Gut microbiota abundance (genus Parasutterella id.2892) on Clathrin interactor 1
with a threshold of 1e−05
rs11715853
rs6809952
rs7572229
rs823424
rs35055552
rs2387977
rs2090816
rs10899911
rs78383039
rs7311004
rs7303158
rs6828768
rs8039785
All − MR Egger
All − IVW(MRE)
−1.5
−1.0
−0.5
0.0
0.5
1.0
1.5
MR effect size for
'Gut microbiota abundance (genus Parasutterella id.2892)' on 'Clathrin interactor 1'

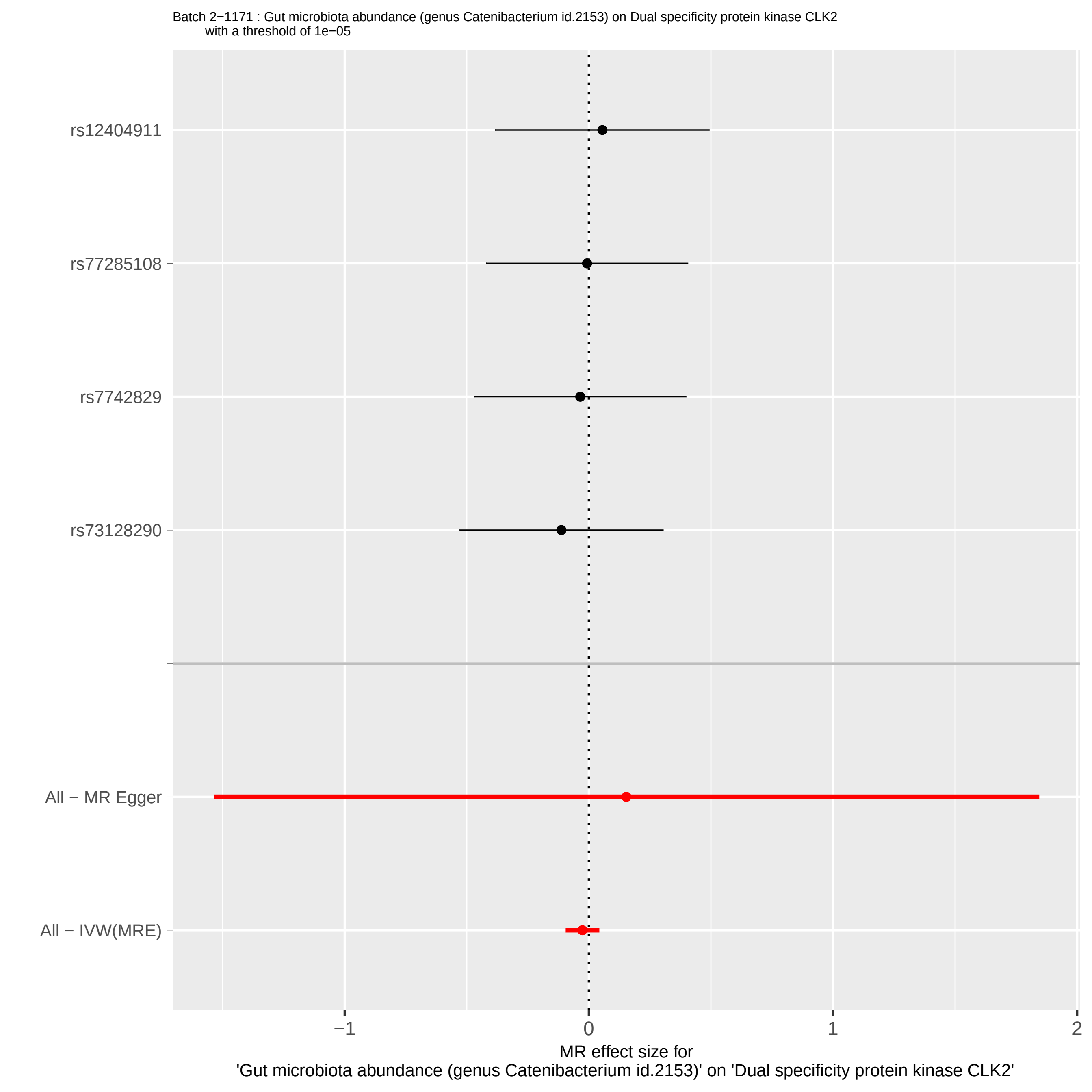
Batch 2−1171 : Gut microbiota abundance (genus Catenibacterium id.2153) on Dual specificity protein kinase CLK2
with a threshold of 1e−05
rs12404911
rs77285108
rs7742829
rs73128290
All − MR Egger
All − IVW(MRE)
−1
0
1
2
MR effect size for
'Gut microbiota abundance (genus Catenibacterium id.2153)' on 'Dual specificity protein kinase CLK2'

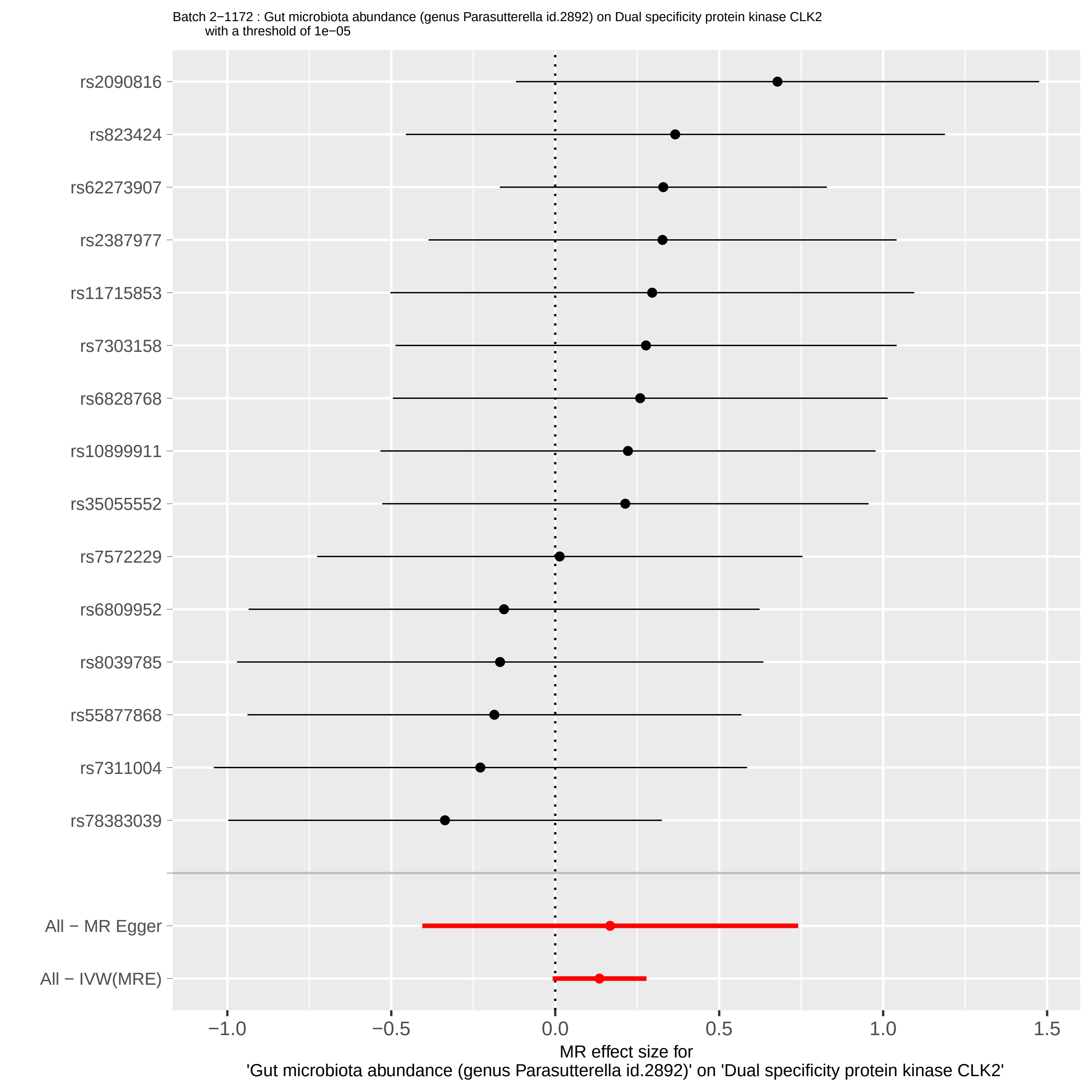
Batch 2−1172 : Gut microbiota abundance (genus Parasutterella id.2892) on Dual specificity protein kinase CLK2
with a threshold of 1e−05
rs2090816
rs823424
rs62273907
rs2387977
rs11715853
rs7303158
rs6828768
rs10899911
rs35055552
rs7572229
rs6809952
rs8039785
rs55877868
rs7311004
rs78383039
All − MR Egger
All − IVW(MRE)
−1.0
−0.5
0.0
0.5
1.0
1.5
MR effect size for
'Gut microbiota abundance (genus Parasutterella id.2892)' on 'Dual specificity protein kinase CLK2'

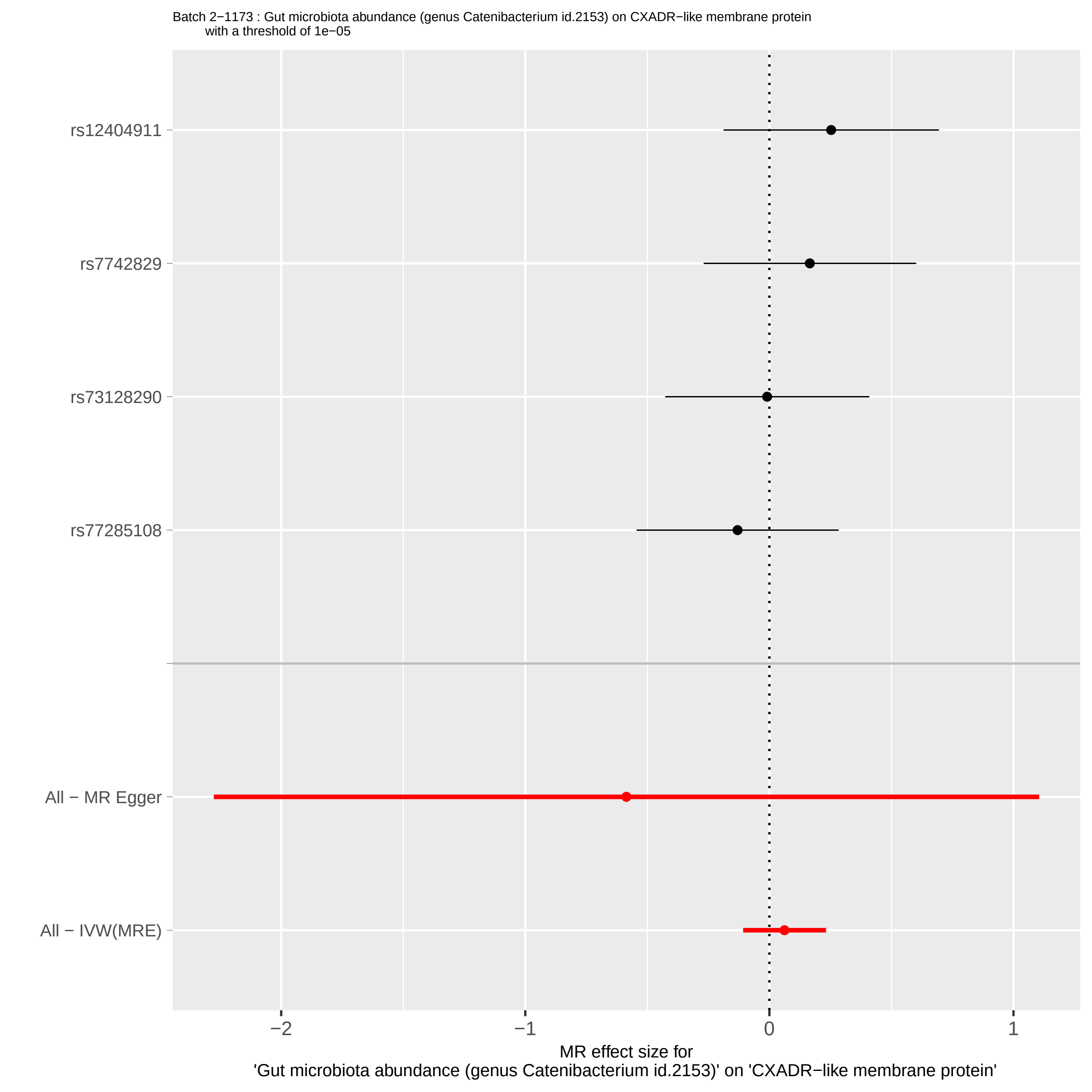

Batch 2−1173 : Gut microbiota abundance (genus Catenibacterium id.2153) on CXADR−like membrane protein
with a threshold of 1e−05
rs12404911
rs7742829
rs73128290
rs77285108
All − MR Egger
All − IVW(MRE)
−2
−1
0
1
MR effect size for
'Gut microbiota abundance (genus Catenibacterium id.2153)' on 'CXADR−like membrane protein'

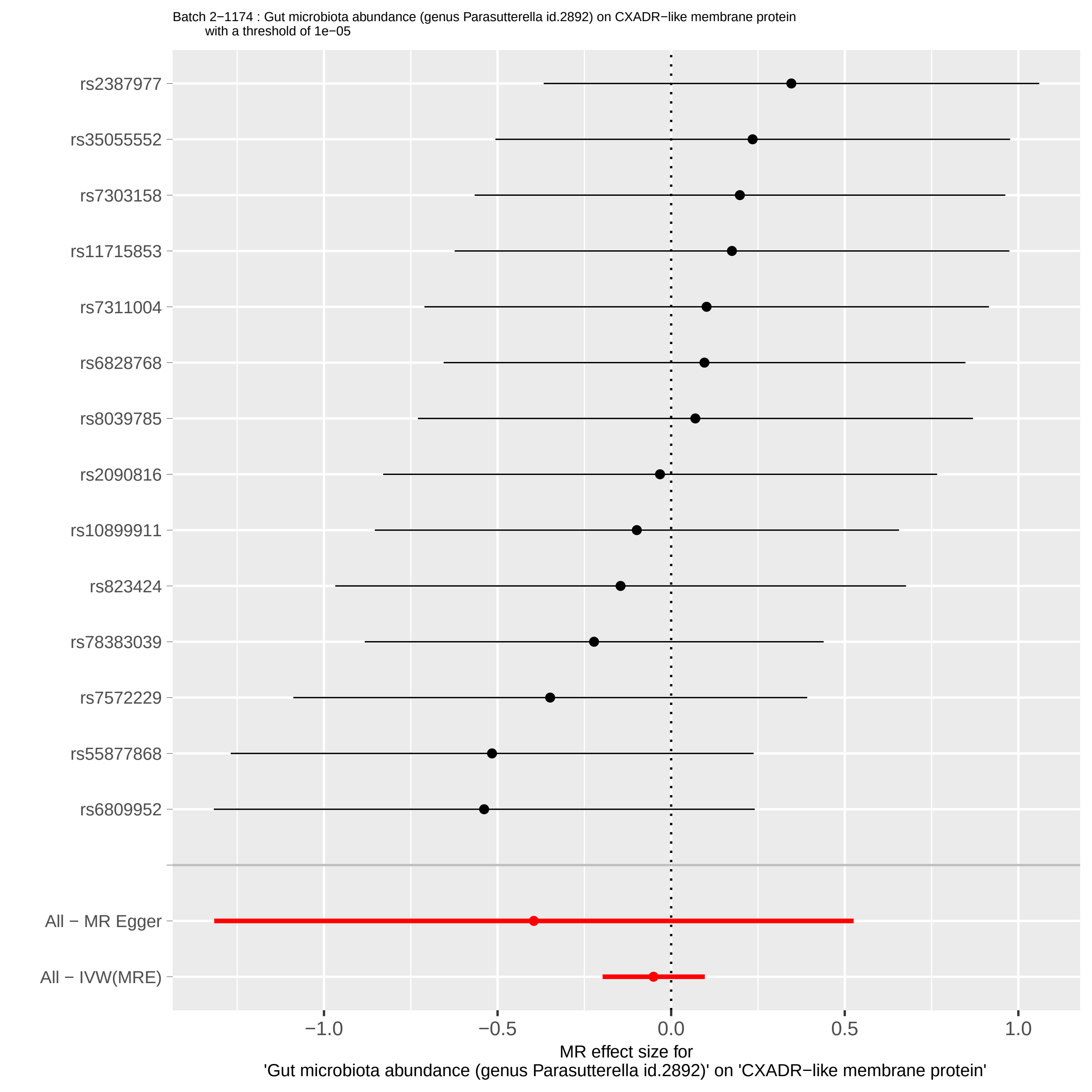

Batch 2−1174 : Gut microbiota abundance (genus Parasutterella id.2892) on CXADR−like membrane protein with a threshold of 1e−05
rs2387977
rs35055552
rs7303158
rs11715853
rs7311004
rs6828768
rs8039785
rs2090816
rs10899911
rs823424
rs78383039
rs7572229
rs55877868
rs6809952
All − MR Egger
All − IVW(MRE)
−1.0
−0.5
0.0
0.5
1.0
MR effect size for
'Gut microbiota abundance (genus Parasutterella id.2892)' on 'CXADR−like membrane protein'

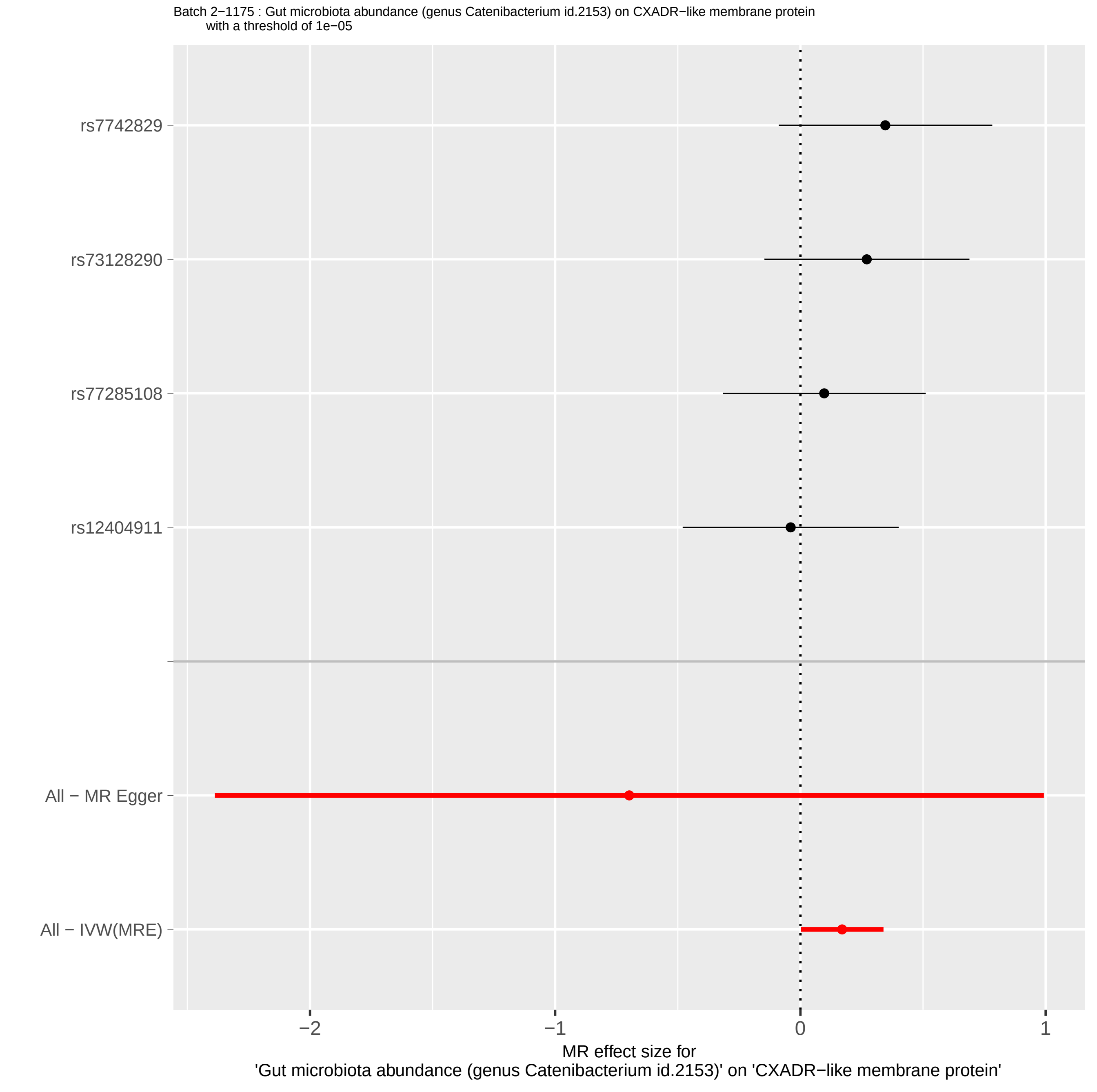

Batch 2−1175 : Gut microbiota abundance (genus Catenibacterium id.2153) on CXADR−like membrane protein
with a threshold of 1e−05
rs7742829
rs73128290
rs77285108
rs12404911
All − MR Egger
All − IVW(MRE)
−2
−1
0
1
MR effect size for
'Gut microbiota abundance (genus Catenibacterium id.2153)' on 'CXADR−like membrane protein'

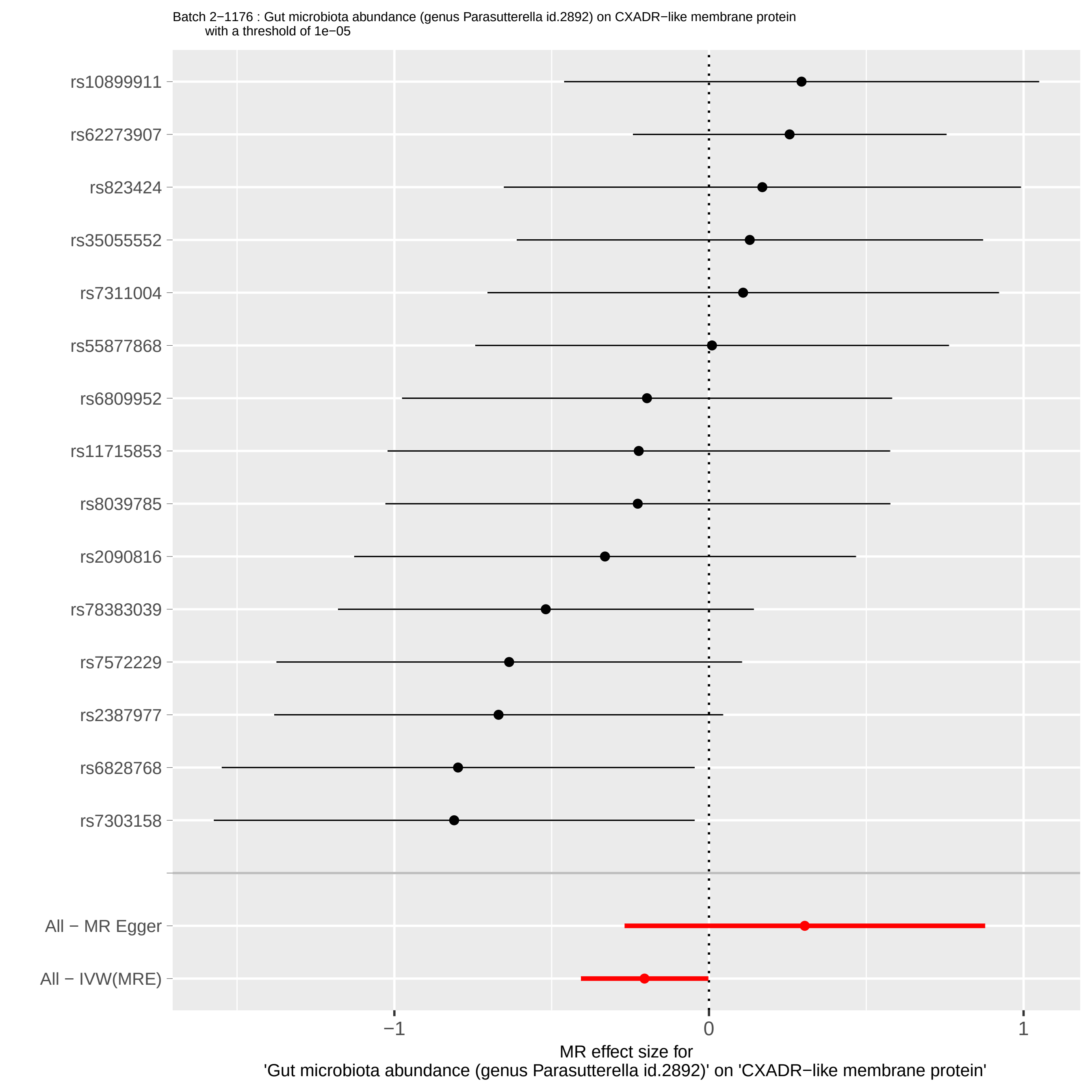
Batch 2−1176 : Gut microbiota abundance (genus Parasutterella id.2892) on CXADR−like membrane protein
with a threshold of 1e−05
rs10899911
rs62273907
rs823424
rs35055552
rs7311004
rs55877868
rs6809952
rs11715853
rs8039785
rs2090816
rs78383039
rs7572229
rs2387977
rs6828768
rs7303158
All − MR Egger
All − IVW(MRE)
−1
0
1
MR effect size for
'Gut microbiota abundance (genus Parasutterella id.2892)' on 'CXADR−like membrane protein'

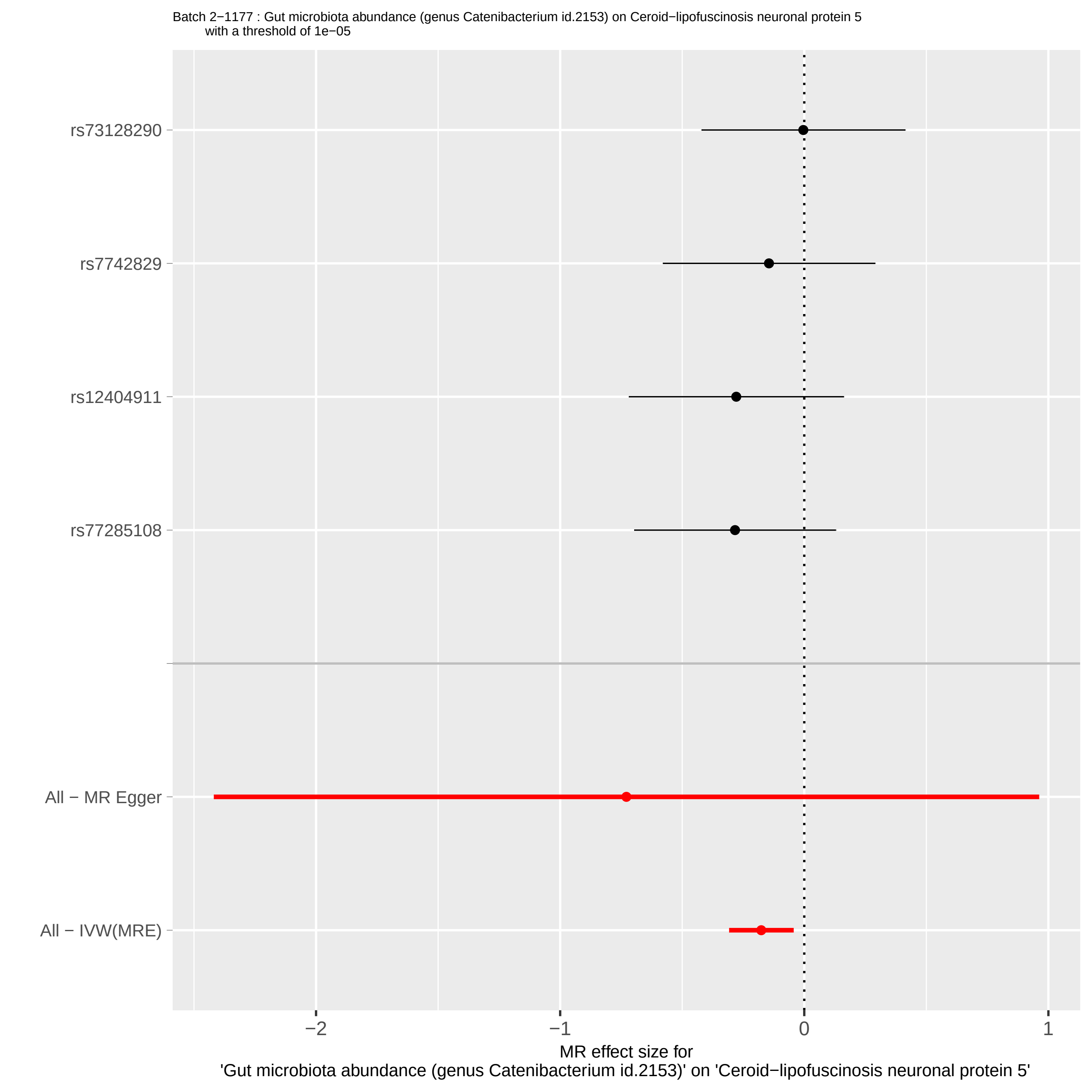
Batch 2−1177 : Gut microbiota abundance (genus Catenibacterium id.2153) on Ceroid−lipofuscinosis neuronal protein 5
with a threshold of 1e−05
rs73128290
rs7742829
rs12404911
rs77285108
All − MR Egger
All − IVW(MRE)
−2
−1
0
1
MR effect size for
'Gut microbiota abundance (genus Catenibacterium id.2153)' on 'Ceroid−lipofuscinosis neuronal protein 5'

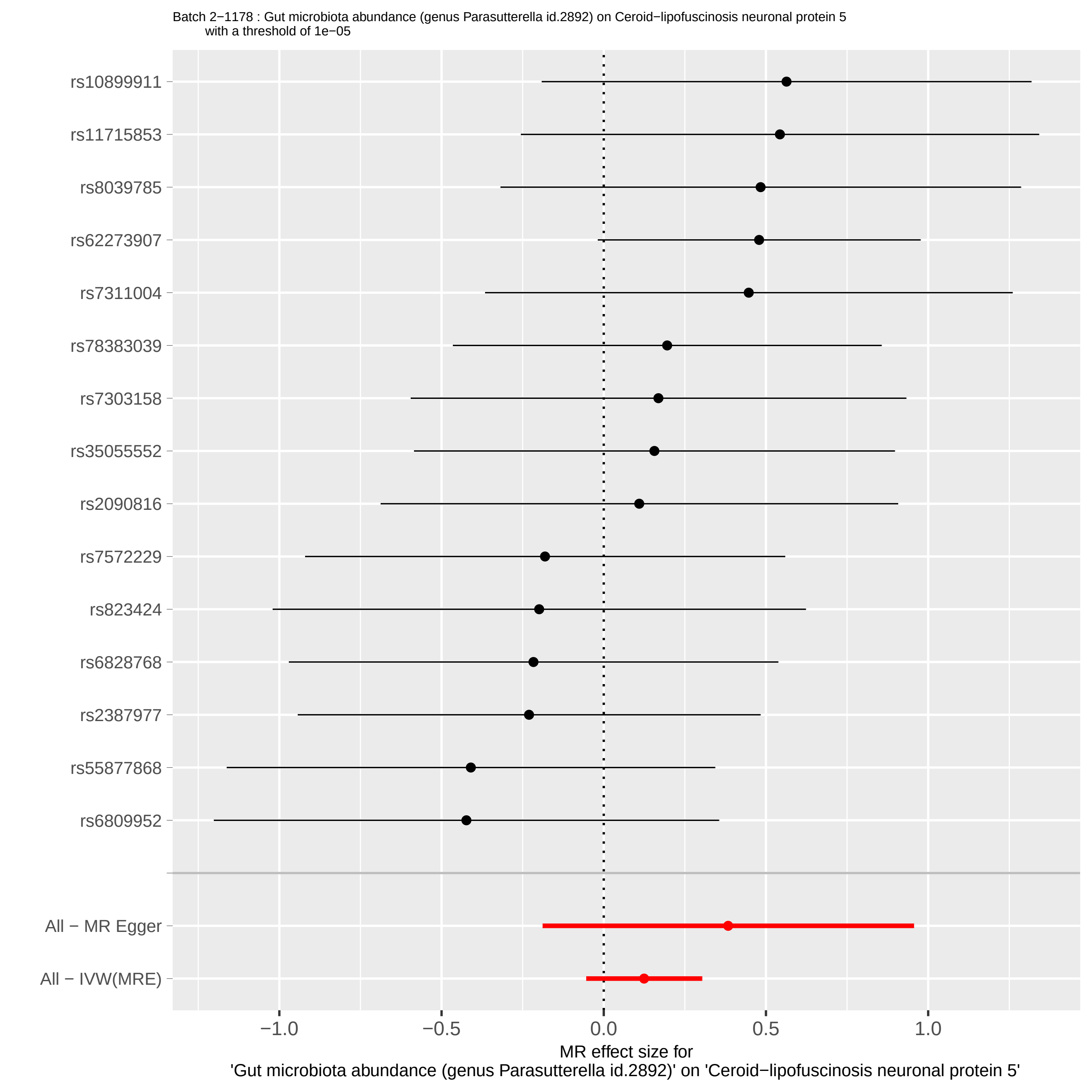

Batch 2−1178 : Gut microbiota abundance (genus Parasutterella id.2892) on Ceroid−lipofuscinosis neuronal protein 5
with a threshold of 1e−05
rs10899911
rs11715853
rs8039785
rs62273907
rs7311004
rs78383039
rs7303158
rs35055552
rs2090816
rs7572229
rs823424
rs6828768
rs2387977
rs55877868
rs6809952
All − MR Egger
All − IVW(MRE)
−1.0
−0.5
0.0
0.5
1.0
MR effect size for
'Gut microbiota abundance (genus Parasutterella id.2892)' on 'Ceroid−lipofuscinosis neuronal protein 5'

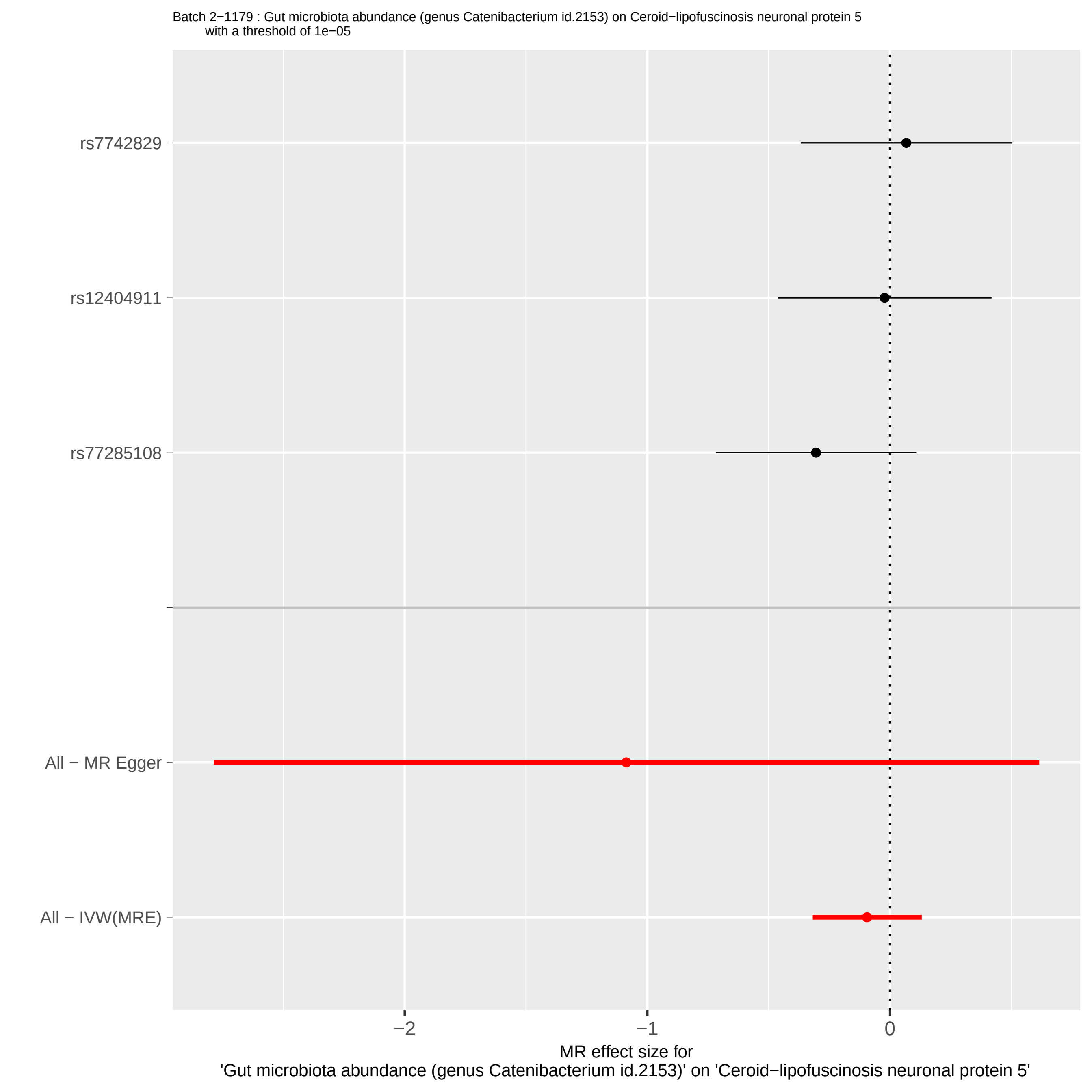
Batch 2−1179 : Gut microbiota abundance (genus Catenibacterium id.2153) on Ceroid−lipofuscinosis neuronal protein 5
with a threshold of 1e−05
rs7742829
rs12404911
rs77285108
All − MR Egger
All − IVW(MRE)
−2
−1
0
MR effect size for
'Gut microbiota abundance (genus Catenibacterium id.2153)' on 'Ceroid−lipofuscinosis neuronal protein 5'

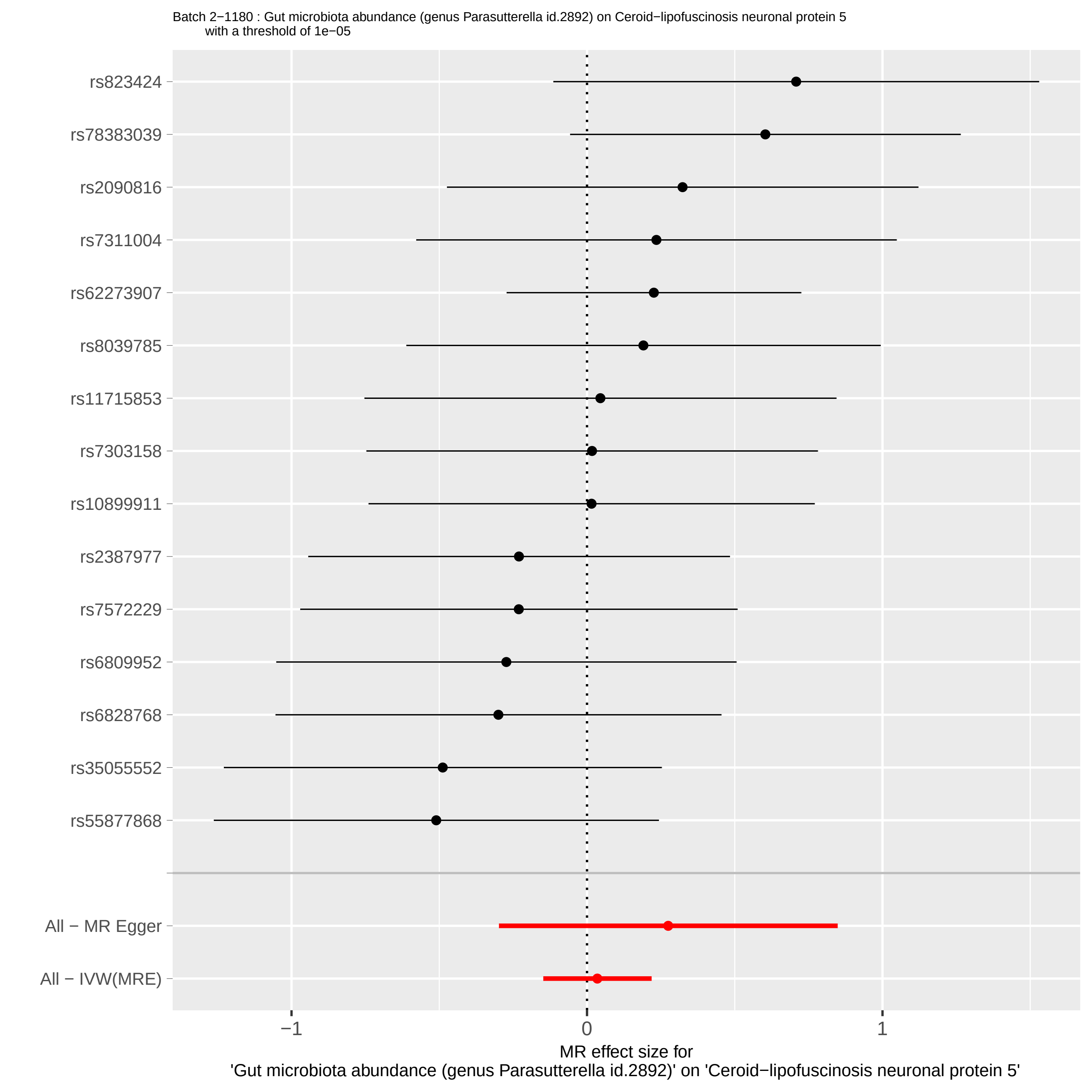
Batch 2−1180 : Gut microbiota abundance (genus Parasutterella id.2892) on Ceroid−lipofuscinosis neuronal protein 5
with a threshold of 1e−05
rs823424
rs78383039
rs2090816
rs7311004
rs62273907
rs8039785
rs11715853
rs7303158
rs10899911
rs2387977
rs7572229
rs6809952
rs6828768
rs35055552
rs55877868
All − MR Egger
All − IVW(MRE)
−1
0
1
MR effect size for
'Gut microbiota abundance (genus Parasutterella id.2892)' on 'Ceroid−lipofuscinosis neuronal protein 5'

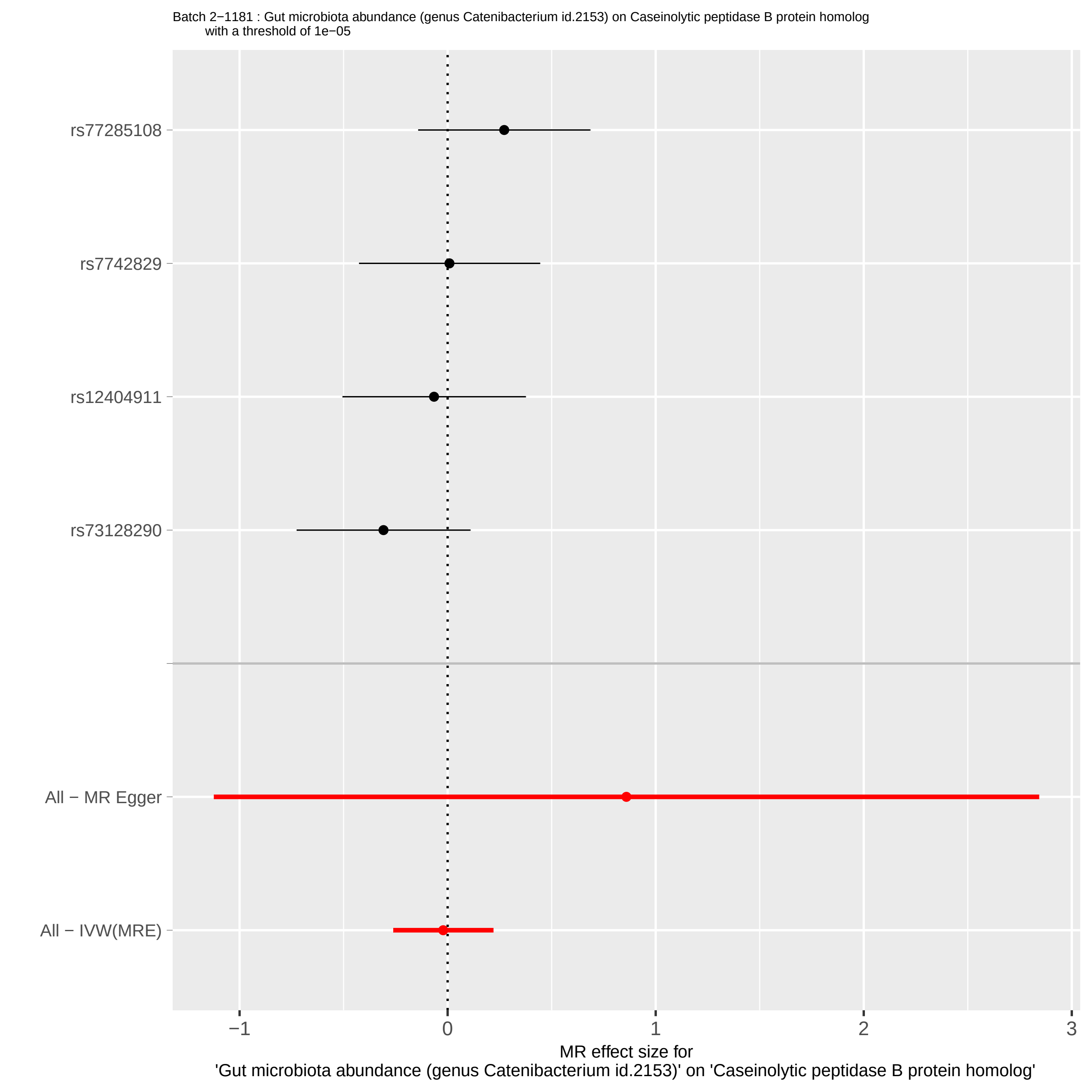

Batch 2−1181 : Gut microbiota abundance (genus Catenibacterium id.2153) on Caseinolytic peptidase B protein homolog
with a threshold of 1e−05
rs77285108
rs7742829
rs12404911
rs73128290
All − MR Egger
All − IVW(MRE)
−1
0
1
2
3
MR effect size for
'Gut microbiota abundance (genus Catenibacterium id.2153)' on 'Caseinolytic peptidase B protein homolog'

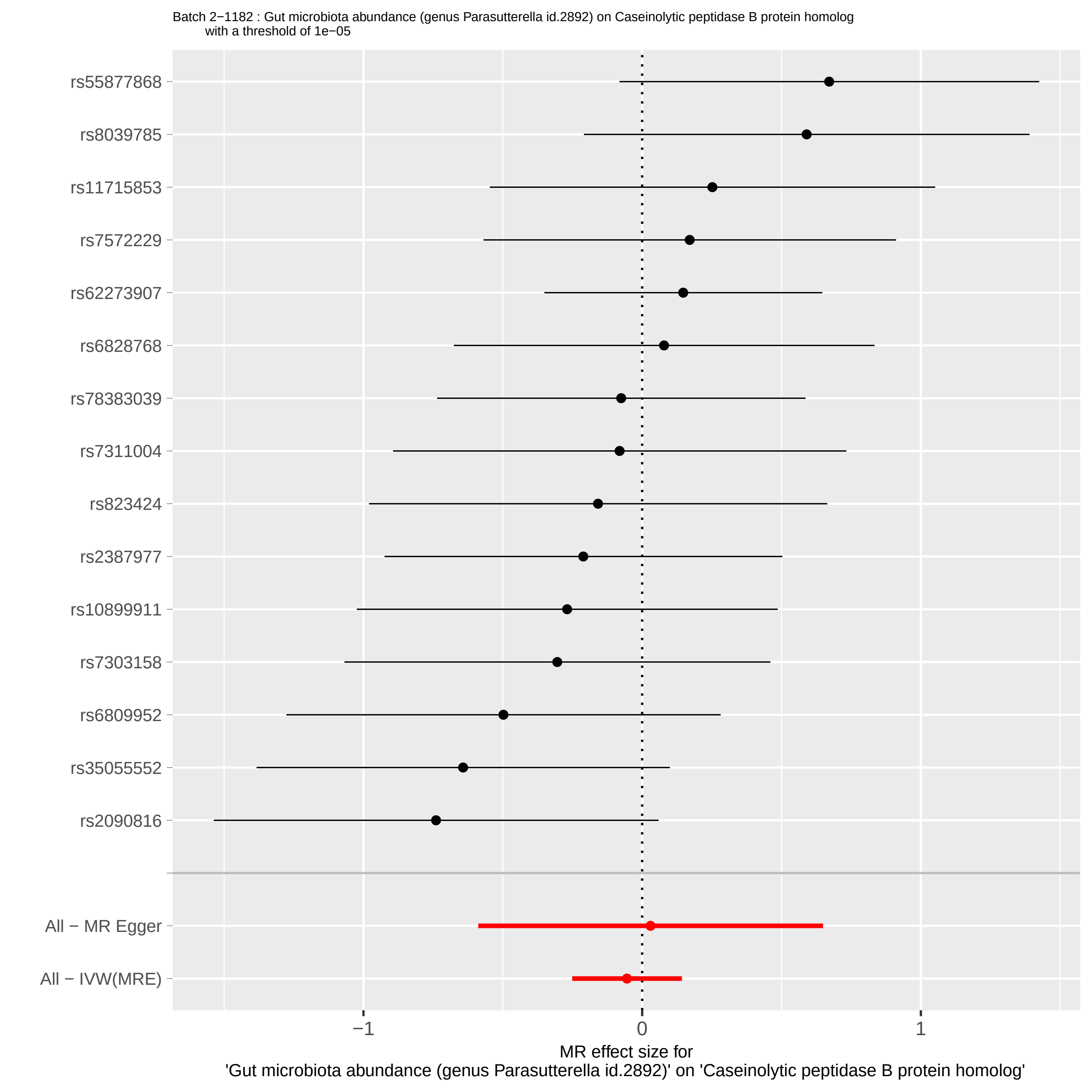

Batch 2−1182 : Gut microbiota abundance (genus Parasutterella id.2892) on Caseinolytic peptidase B protein homolog
with a threshold of 1e−05
rs55877868
rs8039785
rs11715853
rs7572229
rs62273907
rs6828768
rs78383039
rs7311004
rs823424
rs2387977
rs10899911
rs7303158
rs6809952
rs35055552
rs2090816
All − MR Egger
All − IVW(MRE)
−1
0
1
MR effect size for
'Gut microbiota abundance (genus Parasutterella id.2892)' on 'Caseinolytic peptidase B protein homolog'

Batch 2−1183 : Gut microbiota abundance (genus Catenibacterium id.2153) on Colipase
with a threshold of 1e−05

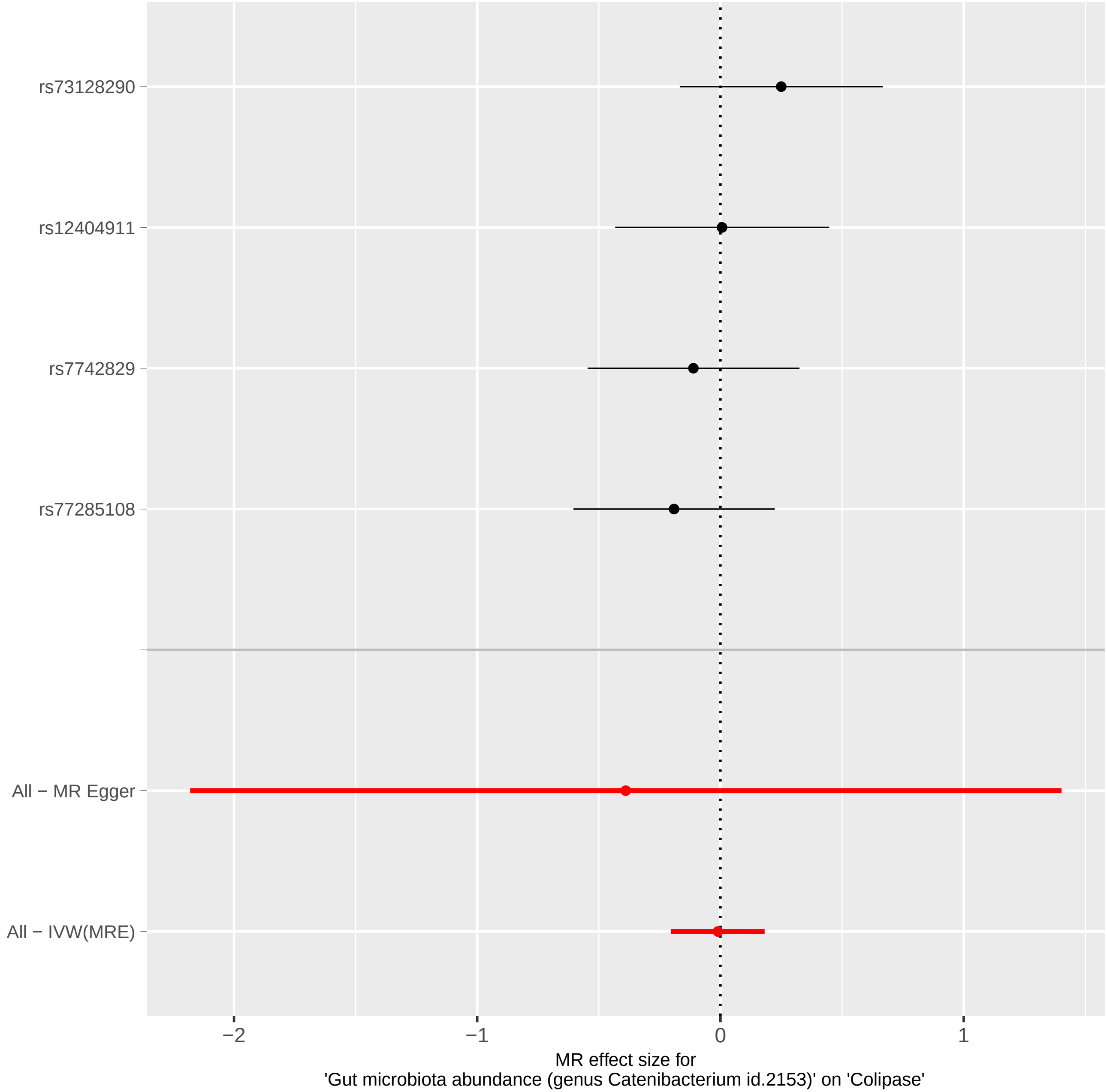

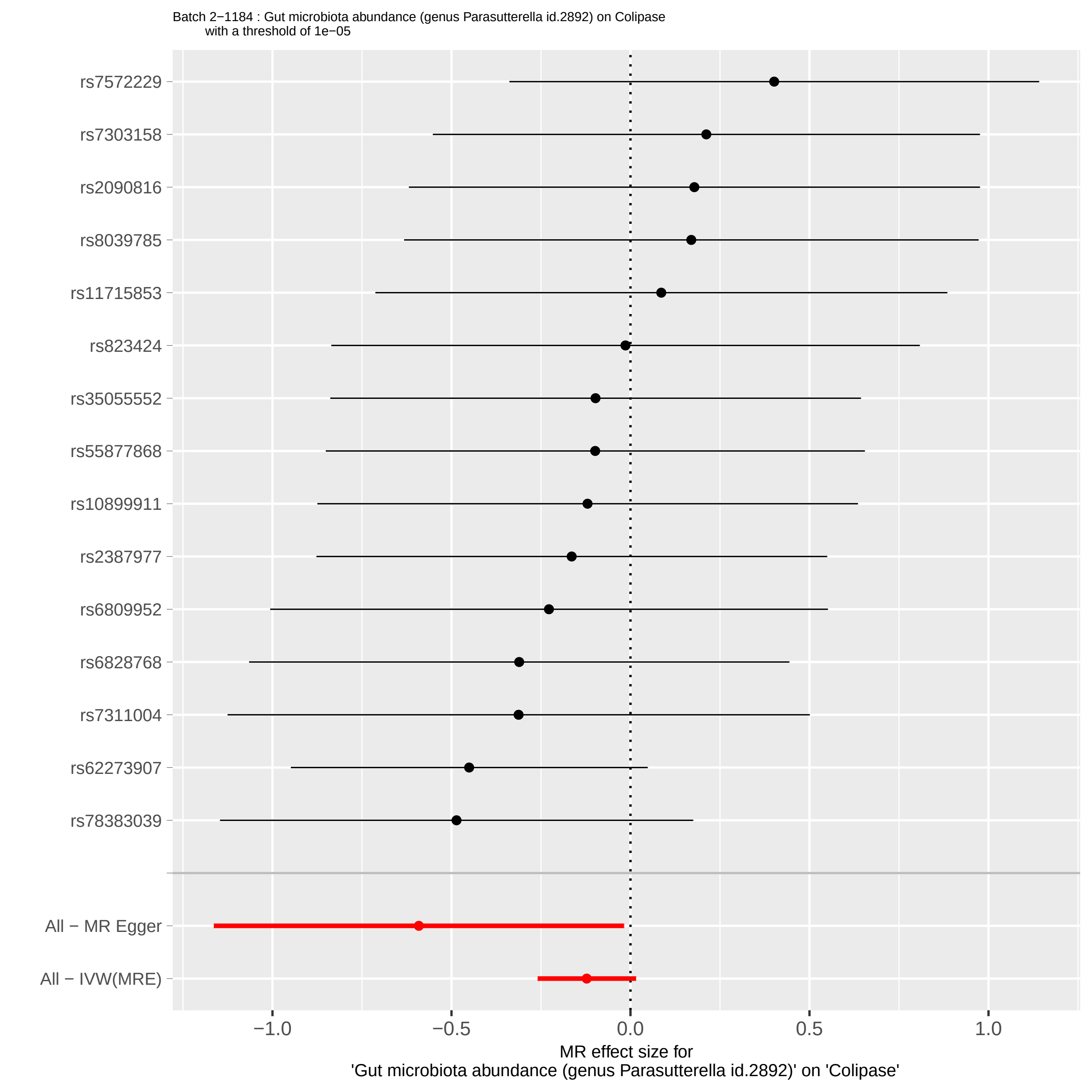

Batch 2−1184 : Gut microbiota abundance (genus Parasutterella id.2892) on Colipase
with a threshold of 1e−05
rs7572229
rs7303158
rs2090816
rs8039785
rs11715853
rs823424
rs35055552
rs55877868
rs10899911
rs2387977
rs6809952
rs6828768
rs7311004
rs62273907
rs78383039
All − MR Egger
All − IVW(MRE)
−1.0
−0.5
0.0
0.5
1.0
MR effect size for
'Gut microbiota abundance (genus Parasutterella id.2892)' on 'Colipase'

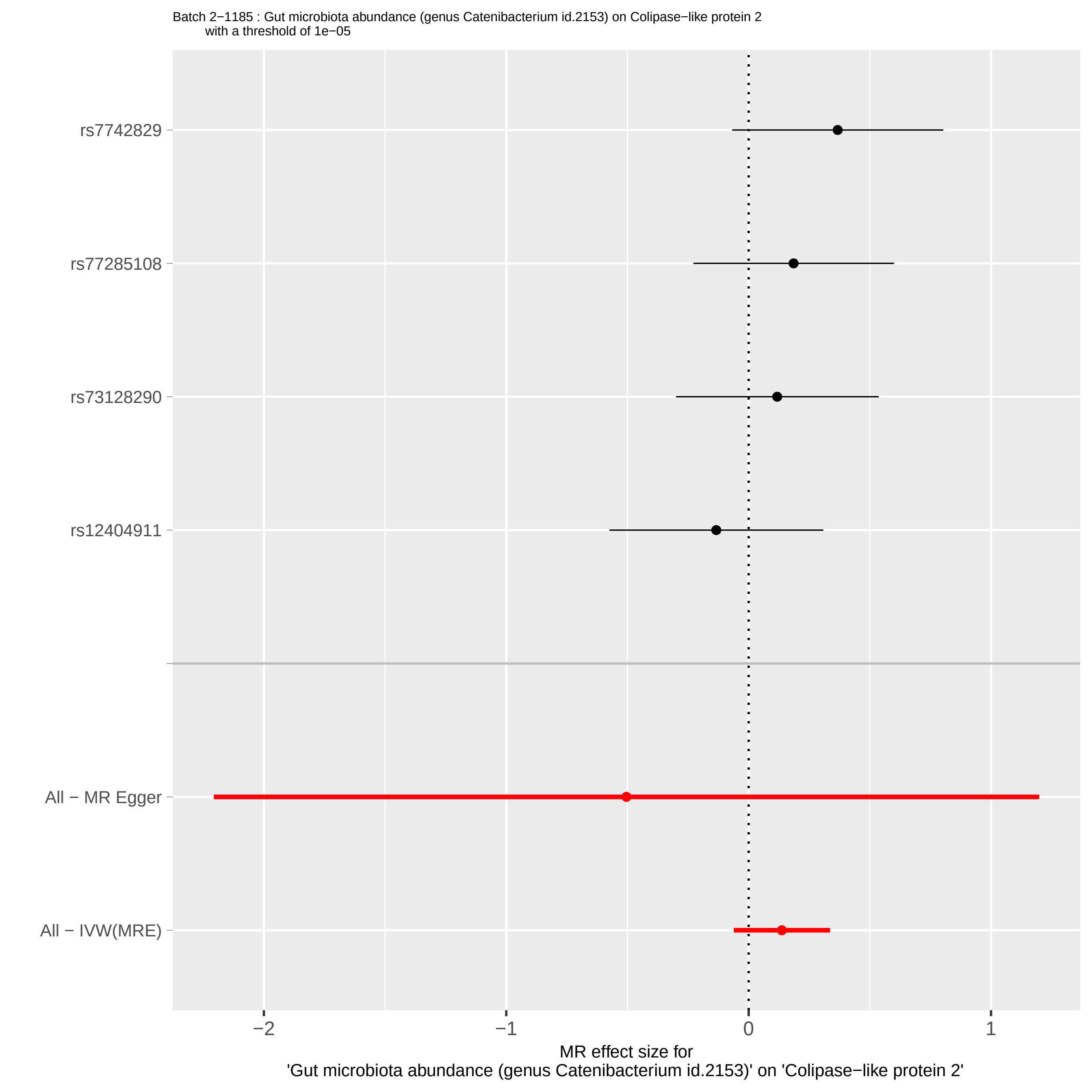

Batch 2−1185 : Gut microbiota abundance (genus Catenibacterium id.2153) on Colipase−like protein 2
with a threshold of 1e−05
rs7742829
rs77285108
rs73128290
rs12404911
All − MR Egger
All − IVW(MRE)
−2
−1
0
1
MR effect size for
'Gut microbiota abundance (genus Catenibacterium id.2153)' on 'Colipase−like protein 2'

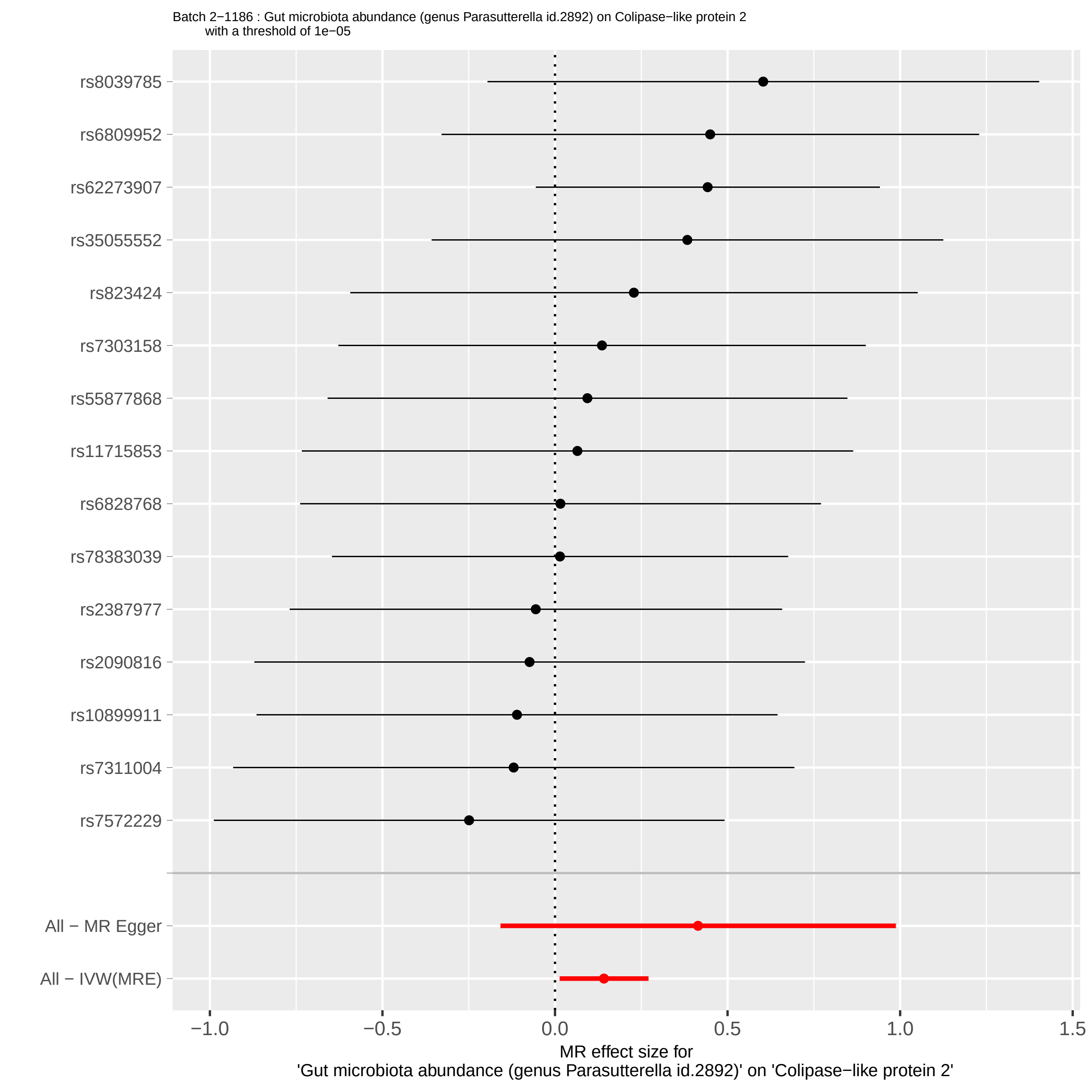

Batch 2−1186 : Gut microbiota abundance (genus Parasutterella id.2892) on Colipase−like protein 2
with a threshold of 1e−05
rs8039785
rs6809952
rs62273907
rs35055552
rs823424
rs7303158
rs55877868
rs11715853
rs6828768
rs78383039
rs2387977
rs2090816
rs10899911
rs7311004
rs7572229
All − MR Egger
All − IVW(MRE)
−1.0
−0.5
0.0
0.5
1.0
1.5
MR effect size for
'Gut microbiota abundance (genus Parasutterella id.2892)' on 'Colipase−like protein 2'

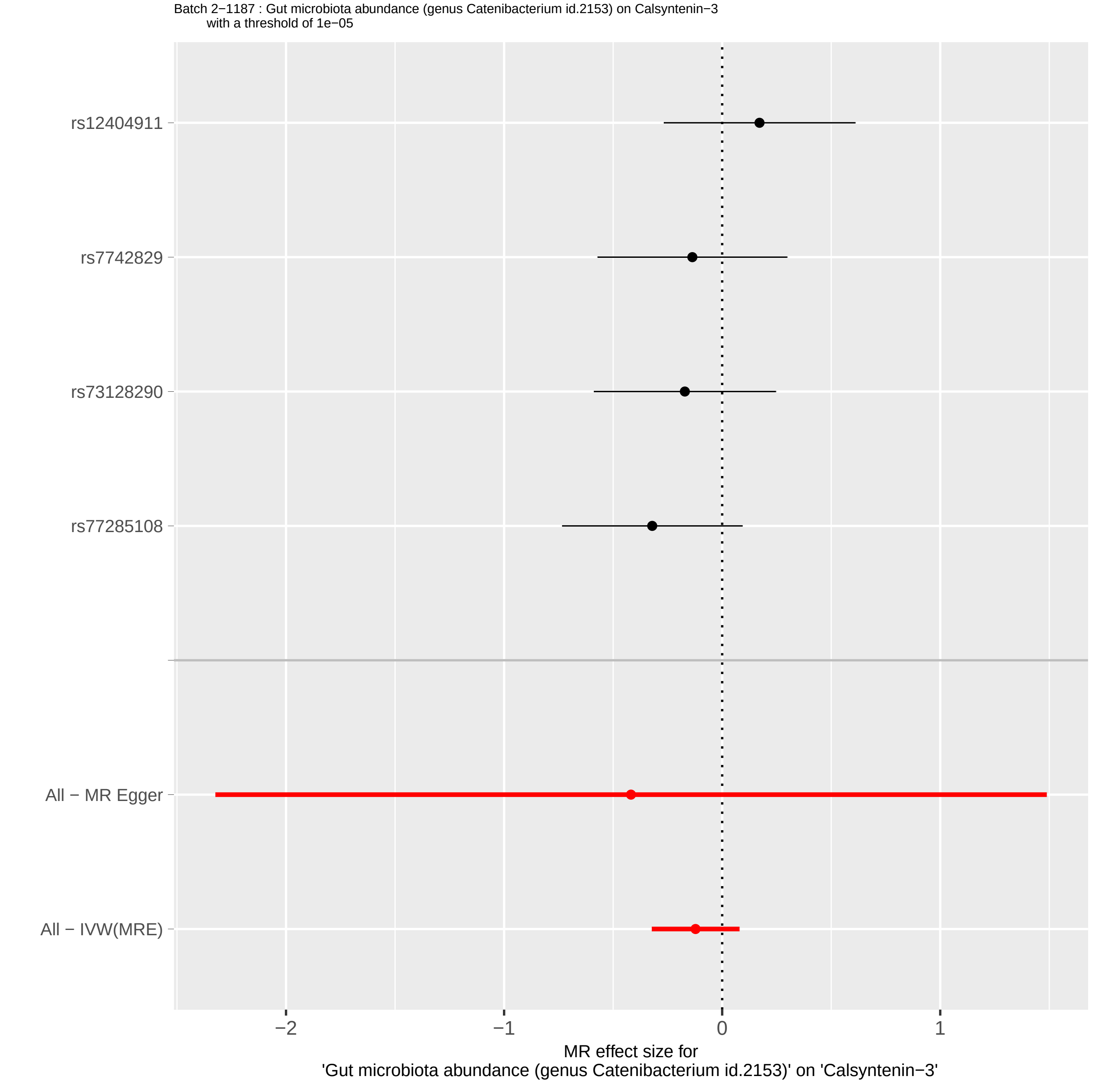

Batch 2−1187 : Gut microbiota abundance (genus Catenibacterium id.2153) on Calsyntenin−3
with a threshold of 1e−05
rs12404911
rs7742829
rs73128290
rs77285108
All − MR Egger
All − IVW(MRE)
−2
−1
0
1
MR effect size for
'Gut microbiota abundance (genus Catenibacterium id.2153)' on 'Calsyntenin−3'

Batch 2−1188 : Gut microbiota abundance (genus Parasutterella id.2892) on Calsyntenin−3
with a threshold of 1e−05

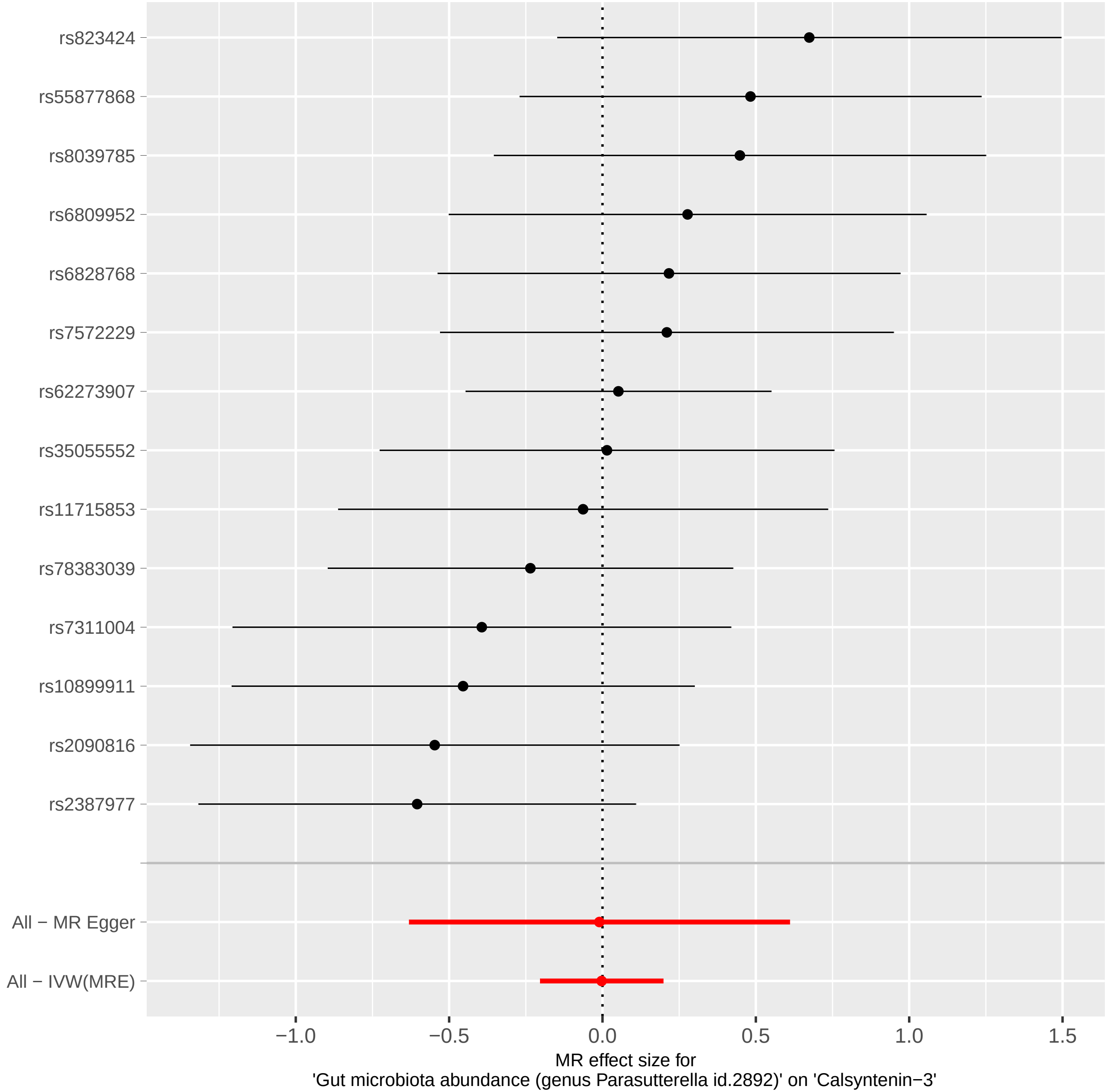

Batch 2−1189 : Gut microbiota abundance (genus Catenibacterium id.2153) on Clathrin heavy chain 1
with a threshold of 1e−05

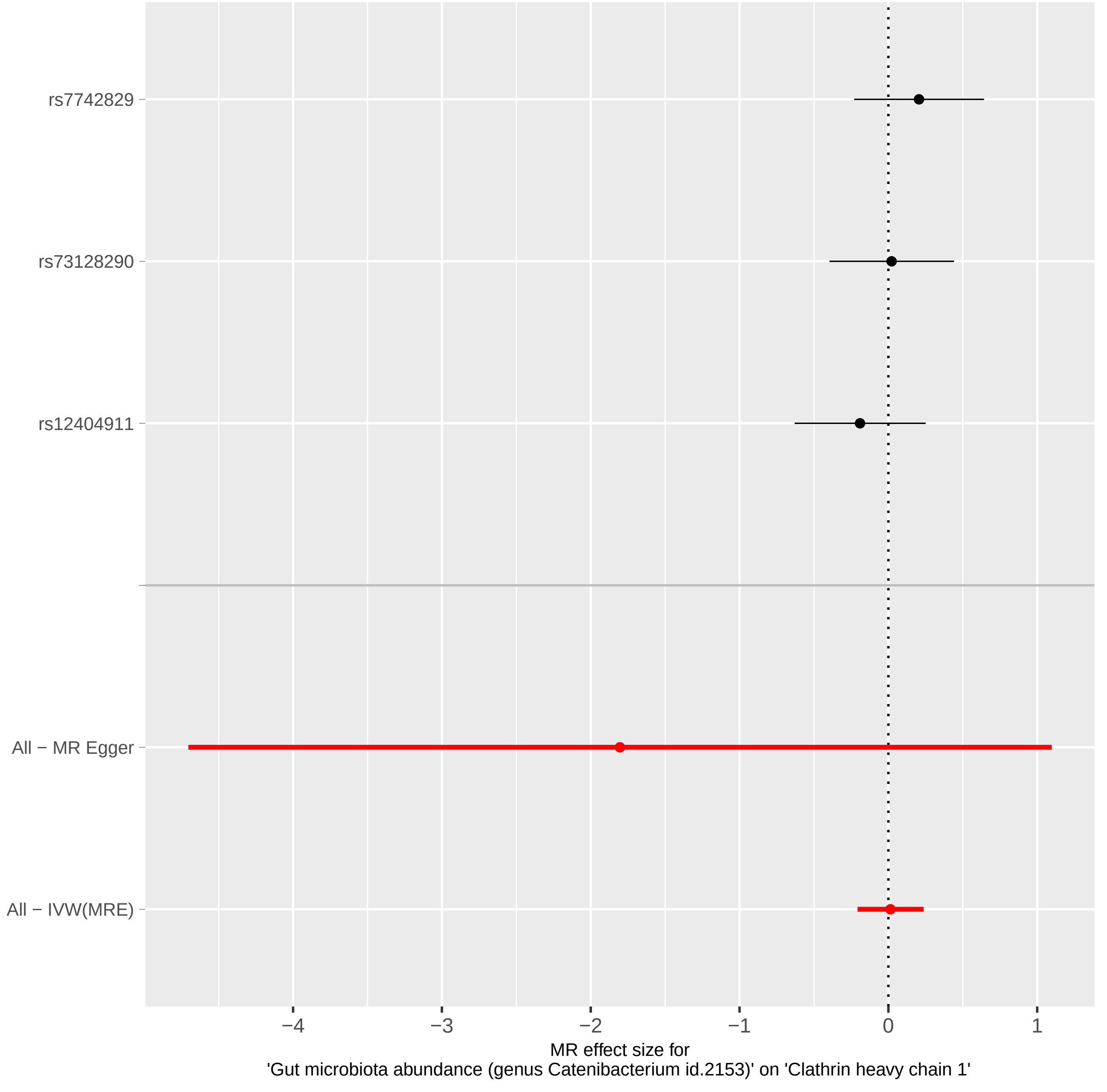

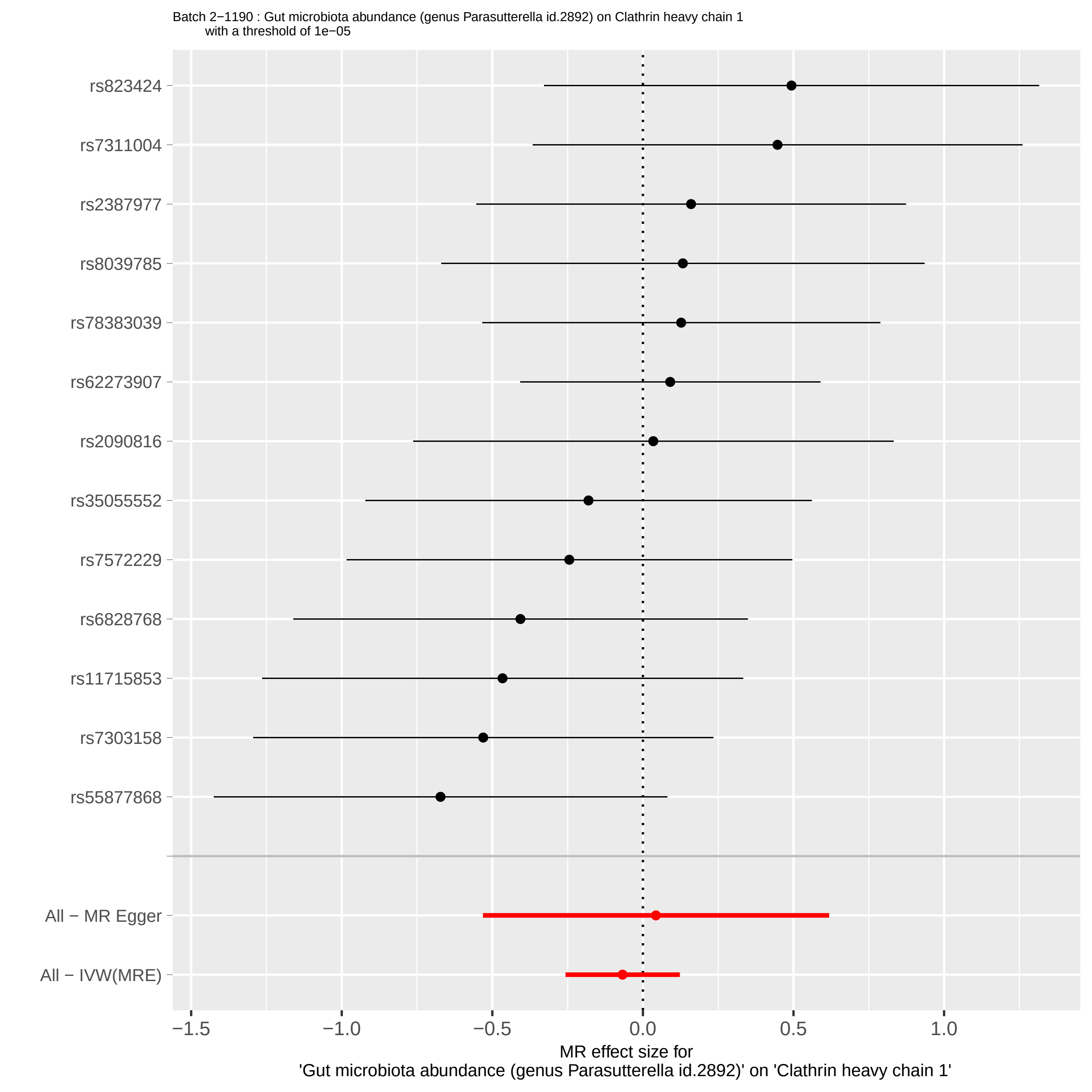

Batch 2−1190 : Gut microbiota abundance (genus Parasutterella id.2892) on Clathrin heavy chain 1
with a threshold of 1e−05
rs823424
rs7311004
rs2387977
rs8039785
rs78383039
rs62273907
rs2090816
rs35055552
rs7572229
rs6828768
rs11715853
rs7303158
rs55877868
All − MR Egger
All − IVW(MRE)
−1.5
−1.0
−0.5
0.0
0.5
1.0
MR effect size for
'Gut microbiota abundance (genus Parasutterella id.2892)' on 'Clathrin heavy chain 1'

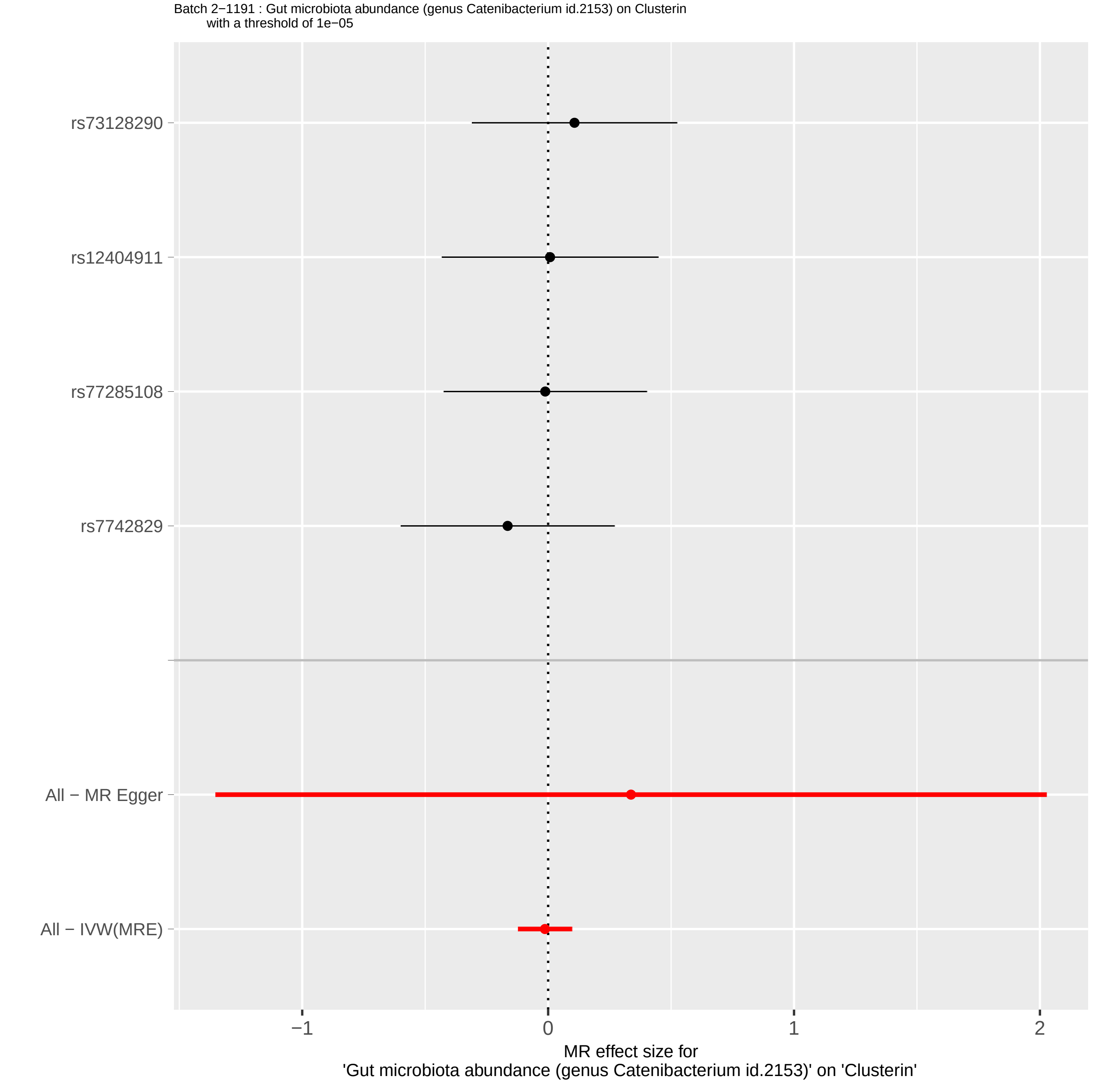
Batch 2−1191 : Gut microbiota abundance (genus Catenibacterium id.2153) on Clusterin
with a threshold of 1e−05
rs73128290
rs12404911
rs77285108
rs7742829
All − MR Egger
All − IVW(MRE)
−1
0
1
2
MR effect size for
'Gut microbiota abundance (genus Catenibacterium id.2153)' on 'Clusterin'

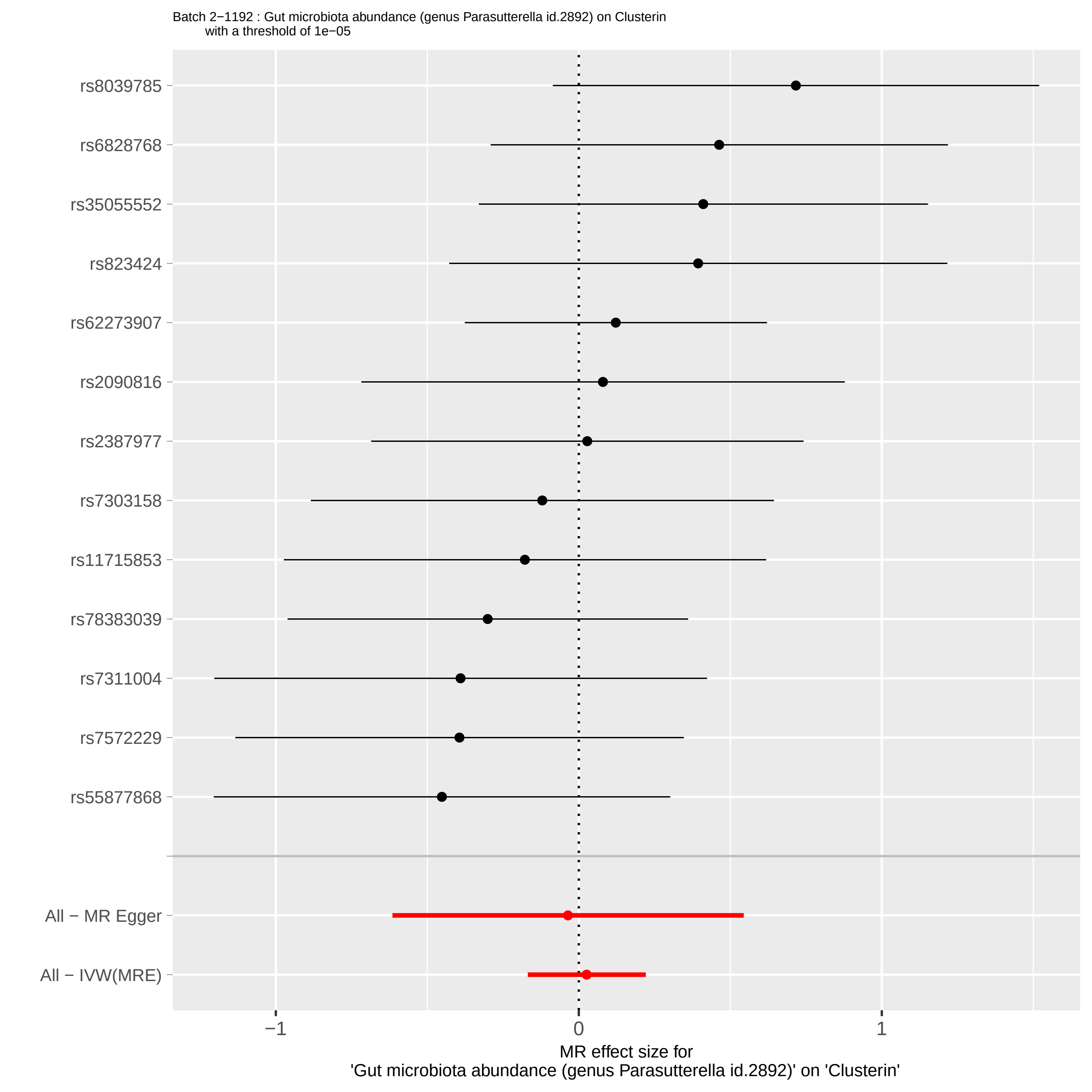
Batch 2−1192 : Gut microbiota abundance (genus Parasutterella id.2892) on Clusterin
with a threshold of 1e−05
rs8039785
rs6828768
rs35055552
rs823424
rs62273907
rs2090816
rs2387977
rs7303158
rs11715853
rs78383039
rs7311004
rs7572229
rs55877868
All − MR Egger
All − IVW(MRE)
−1
0
1
MR effect size for
'Gut microbiota abundance (genus Parasutterella id.2892)' on 'Clusterin'

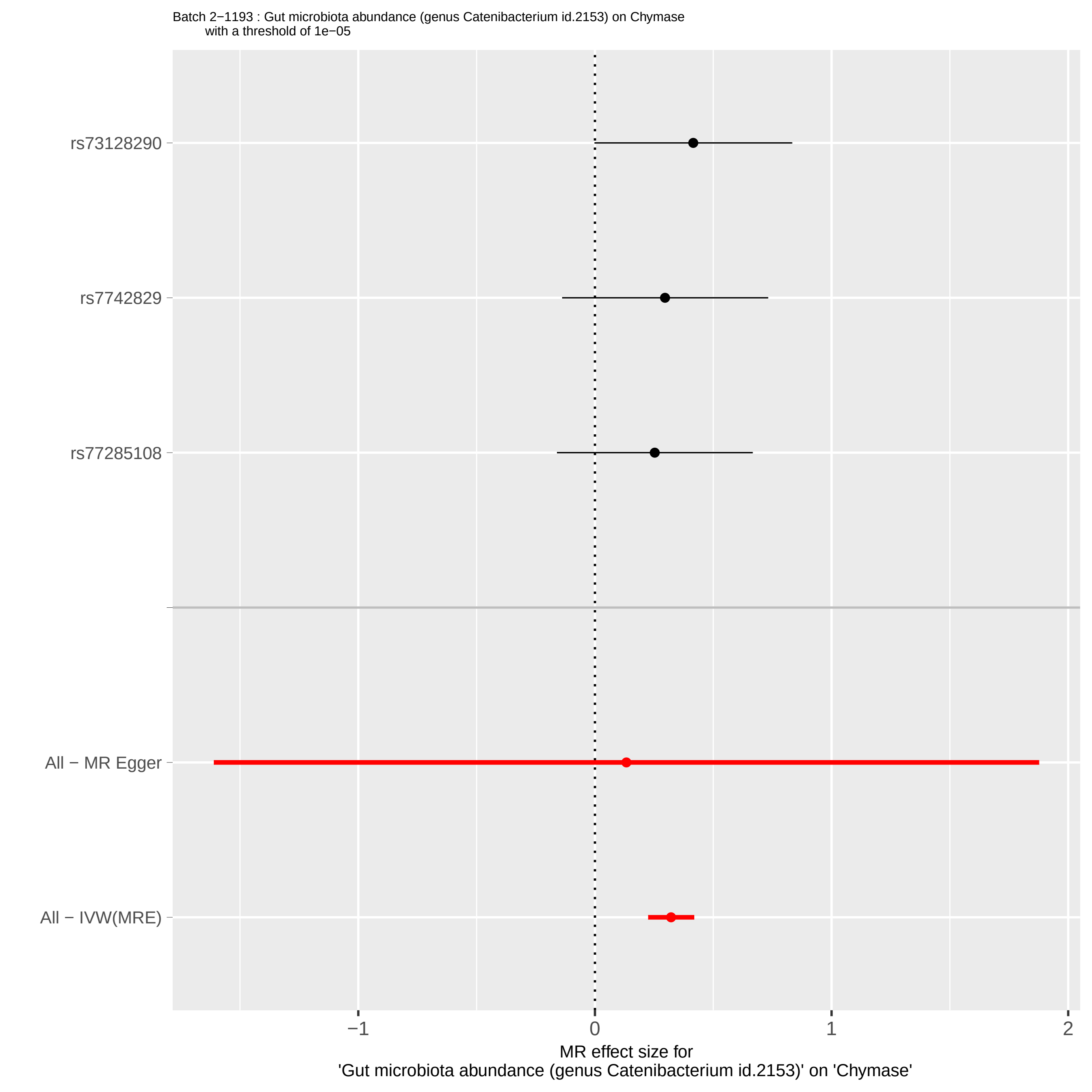
Batch 2−1193 : Gut microbiota abundance (genus Catenibacterium id.2153) on Chymase
with a threshold of 1e−05
rs73128290
rs7742829
rs77285108
All − MR Egger
All − IVW(MRE)
−1
0
1
2
MR effect size for
'Gut microbiota abundance (genus Catenibacterium id.2153)' on 'Chymase'

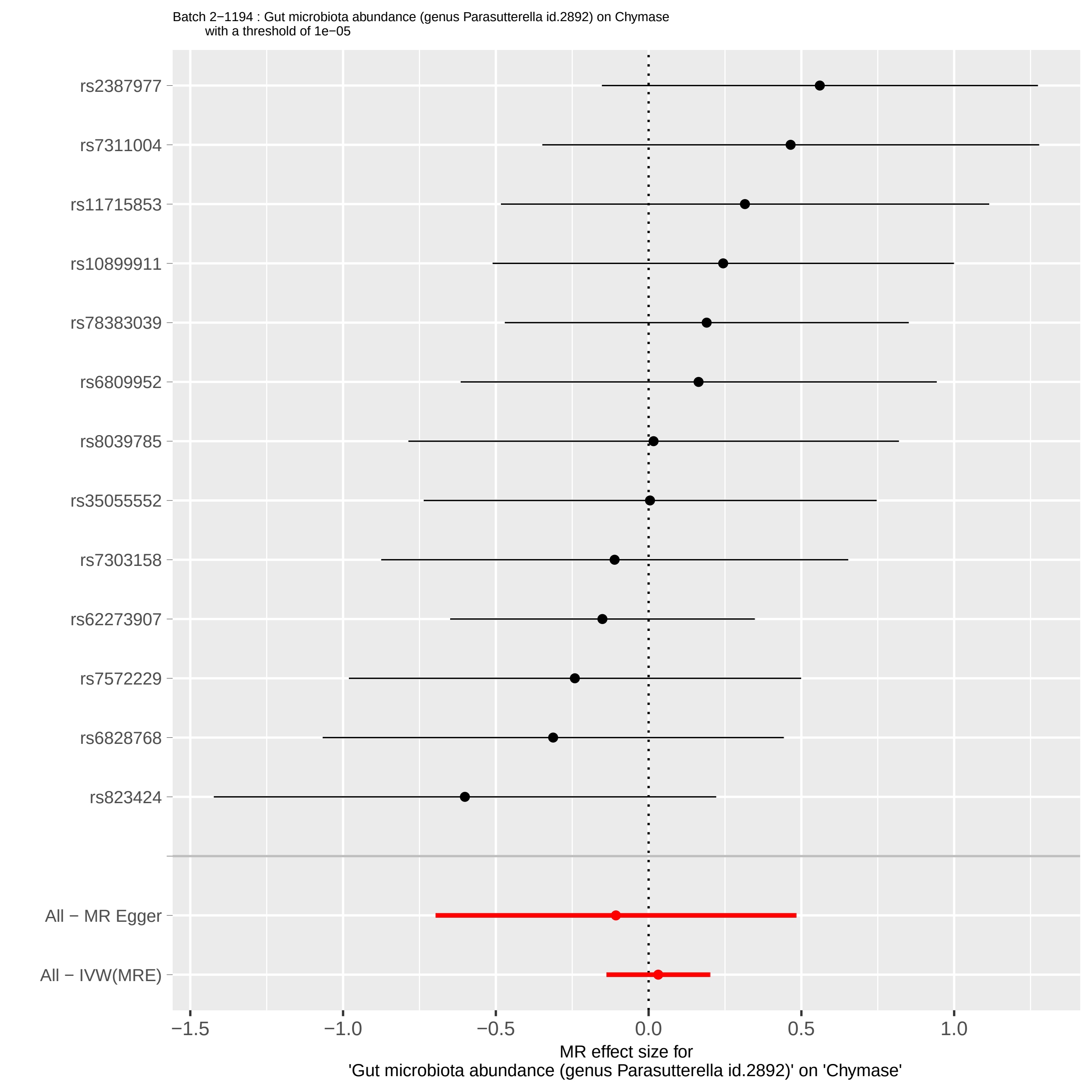
Batch 2−1194 : Gut microbiota abundance (genus Parasutterella id.2892) on Chymase
with a threshold of 1e−05
rs2387977
rs7311004
rs11715853
rs10899911
rs78383039
rs6809952
rs8039785
rs35055552
rs7303158
rs62273907
rs7572229
rs6828768
rs823424
All − MR Egger
All − IVW(MRE)
−1.5
−1.0
−0.5
0.0
0.5
1.0
MR effect size for
'Gut microbiota abundance (genus Parasutterella id.2892)' on 'Chymase'

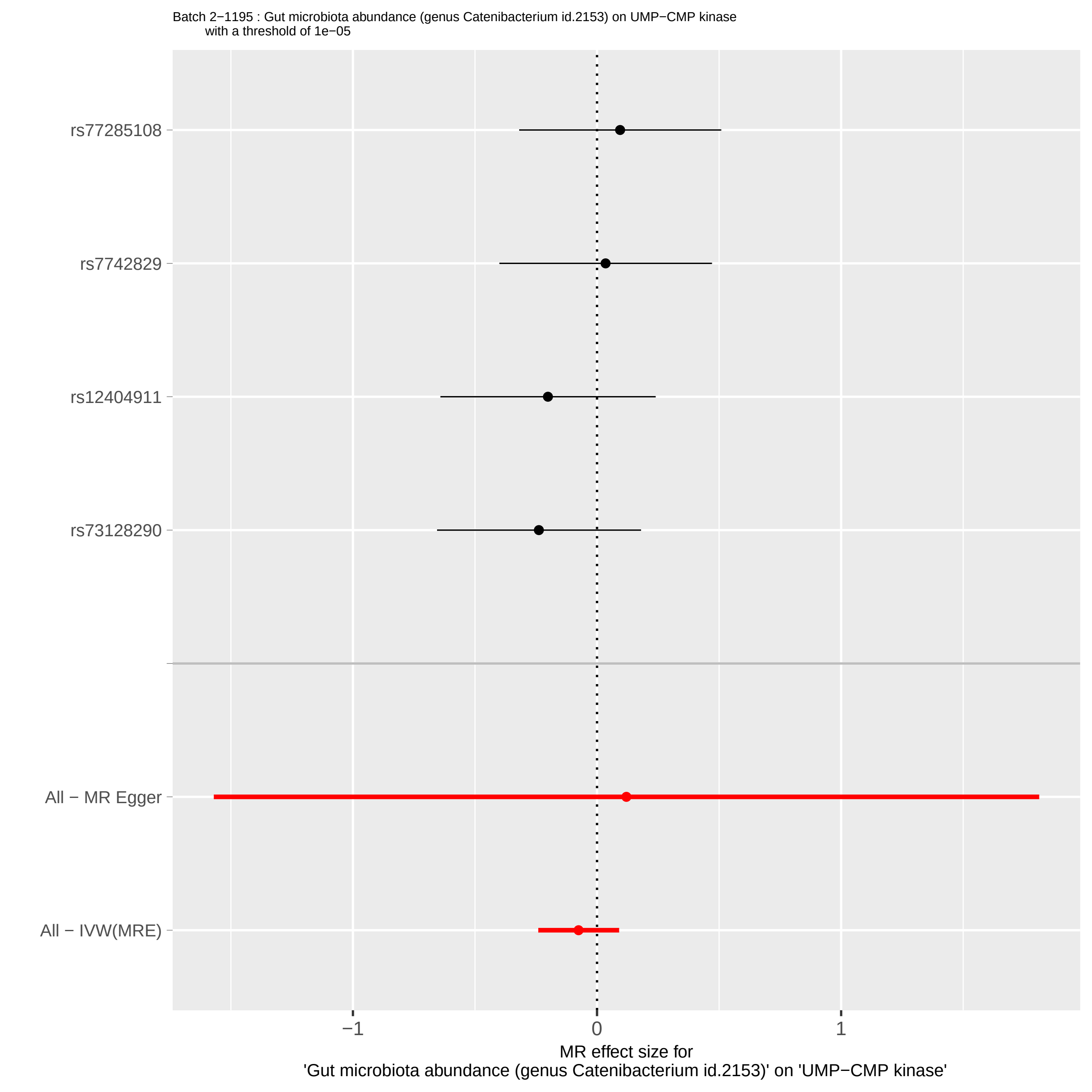
Batch 2−1195 : Gut microbiota abundance (genus Catenibacterium id.2153) on UMP−CMP kinase
with a threshold of 1e−05
rs77285108
rs7742829
rs12404911
rs73128290
All − MR Egger
All − IVW(MRE)
−1
0
1
MR effect size for
'Gut microbiota abundance (genus Catenibacterium id.2153)' on 'UMP−CMP kinase'

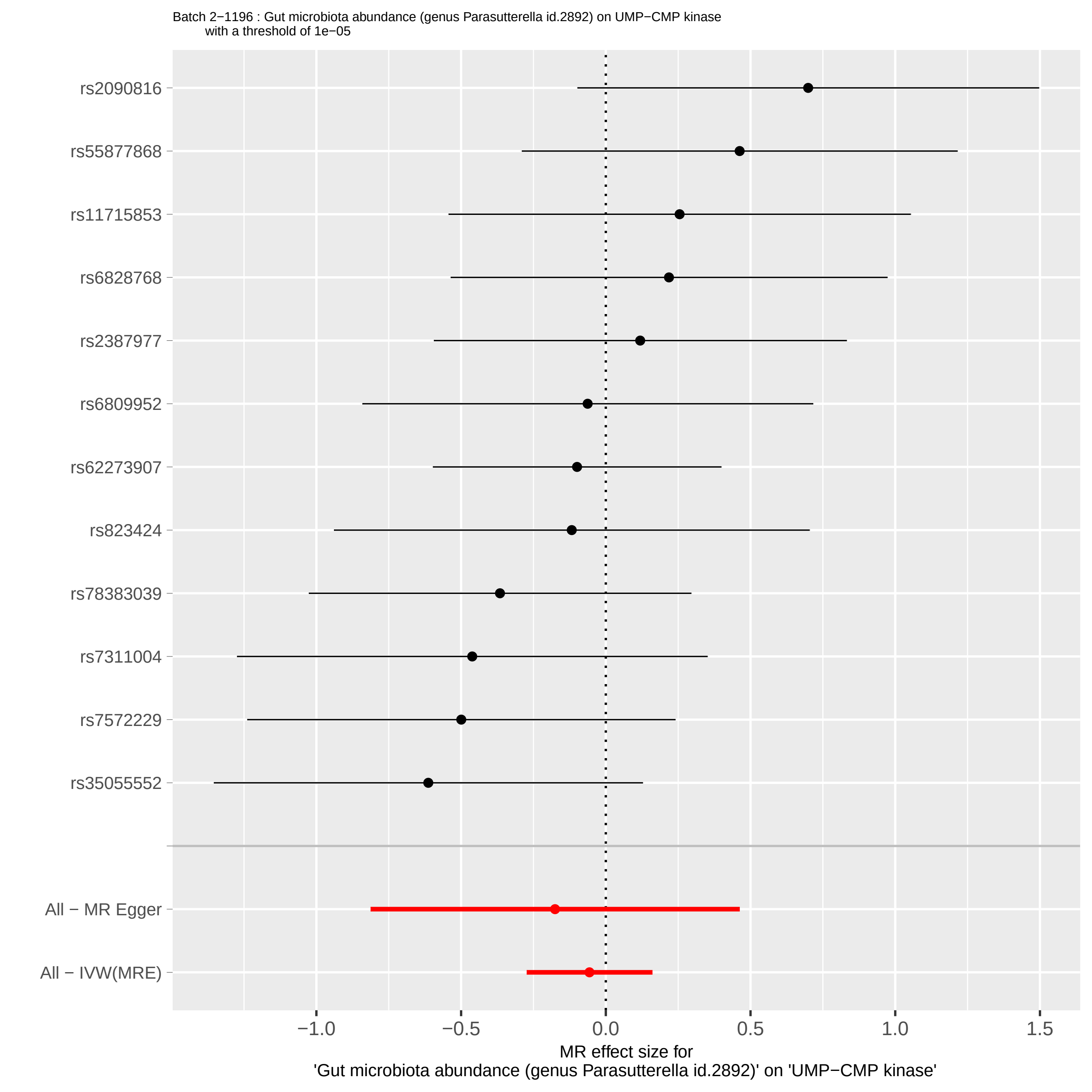

Batch 2−1196 : Gut microbiota abundance (genus Parasutterella id.2892) on UMP−CMP kinase
with a threshold of 1e−05
rs2090816
rs55877868
rs11715853
rs6828768
rs2387977
rs6809952
rs62273907
rs823424
rs78383039
rs7311004
rs7572229
rs35055552
All − MR Egger
All − IVW(MRE)
−1.0
−0.5
0.0
0.5
1.0
1.5
MR effect size for
'Gut microbiota abundance (genus Parasutterella id.2892)' on 'UMP−CMP kinase'

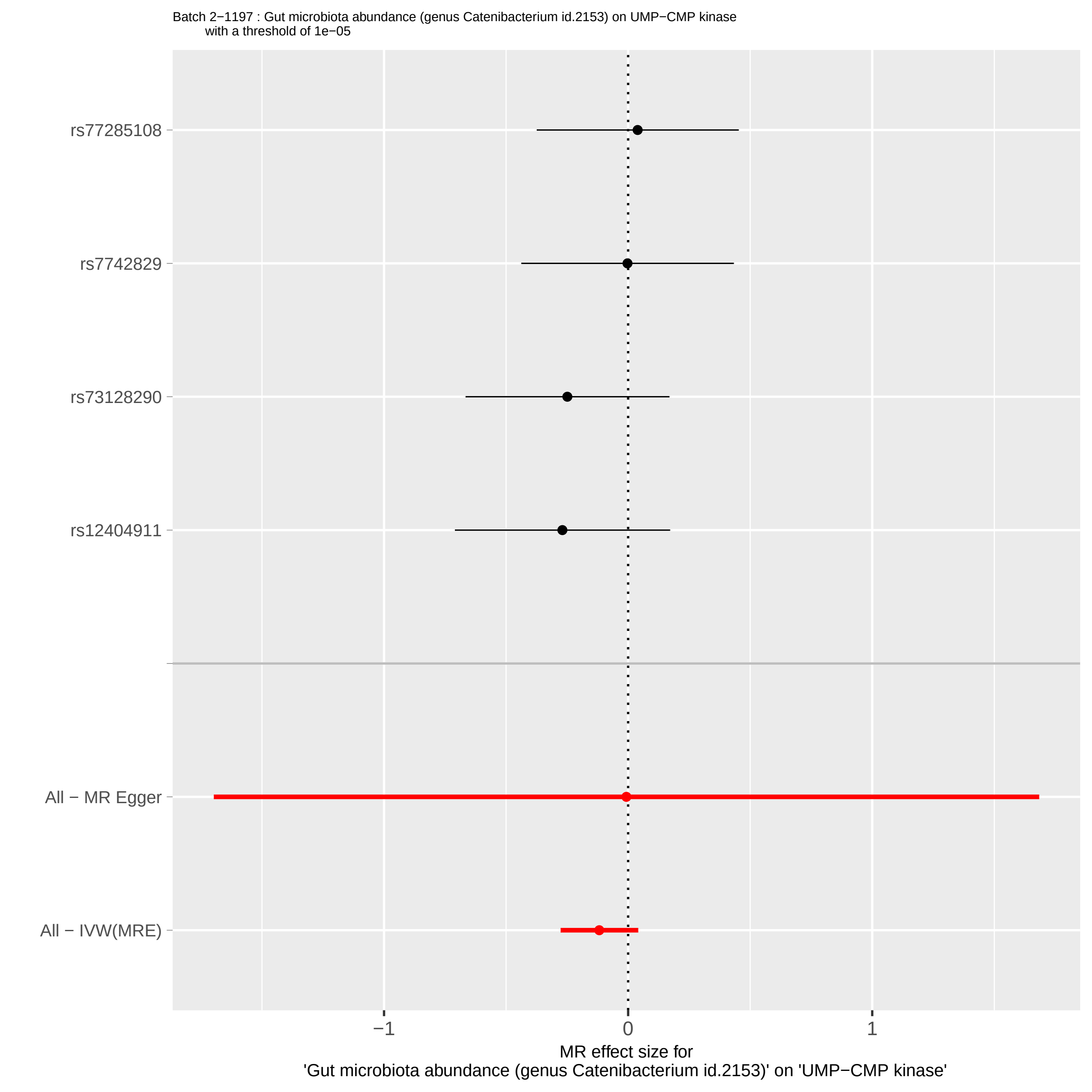
Batch 2−1197 : Gut microbiota abundance (genus Catenibacterium id.2153) on UMP−CMP kinase
with a threshold of 1e−05
rs77285108
rs7742829
rs73128290
rs12404911
All − MR Egger
All − IVW(MRE)
−1
0
1
MR effect size for
'Gut microbiota abundance (genus Catenibacterium id.2153)' on 'UMP−CMP kinase'

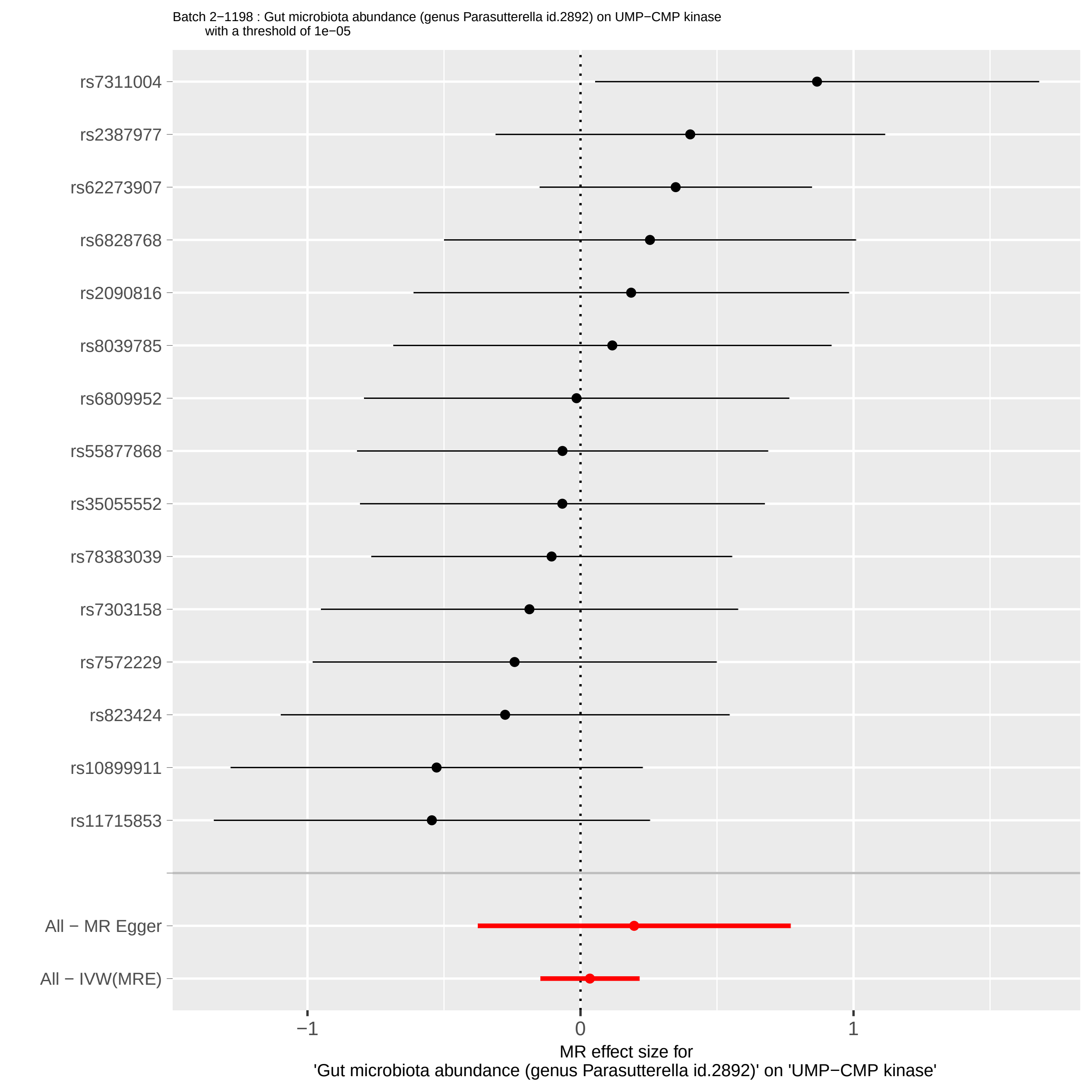

Batch 2−1198 : Gut microbiota abundance (genus Parasutterella id.2892) on UMP−CMP kinase
with a threshold of 1e−05
rs7311004
rs2387977
rs62273907
rs6828768
rs2090816
rs8039785
rs6809952
rs55877868
rs35055552
rs78383039
rs7303158
rs7572229
rs823424
rs10899911
rs11715853
All − MR Egger
All − IVW(MRE)
−1
0
1
MR effect size for
'Gut microbiota abundance (genus Parasutterella id.2892)' on 'UMP−CMP kinase'

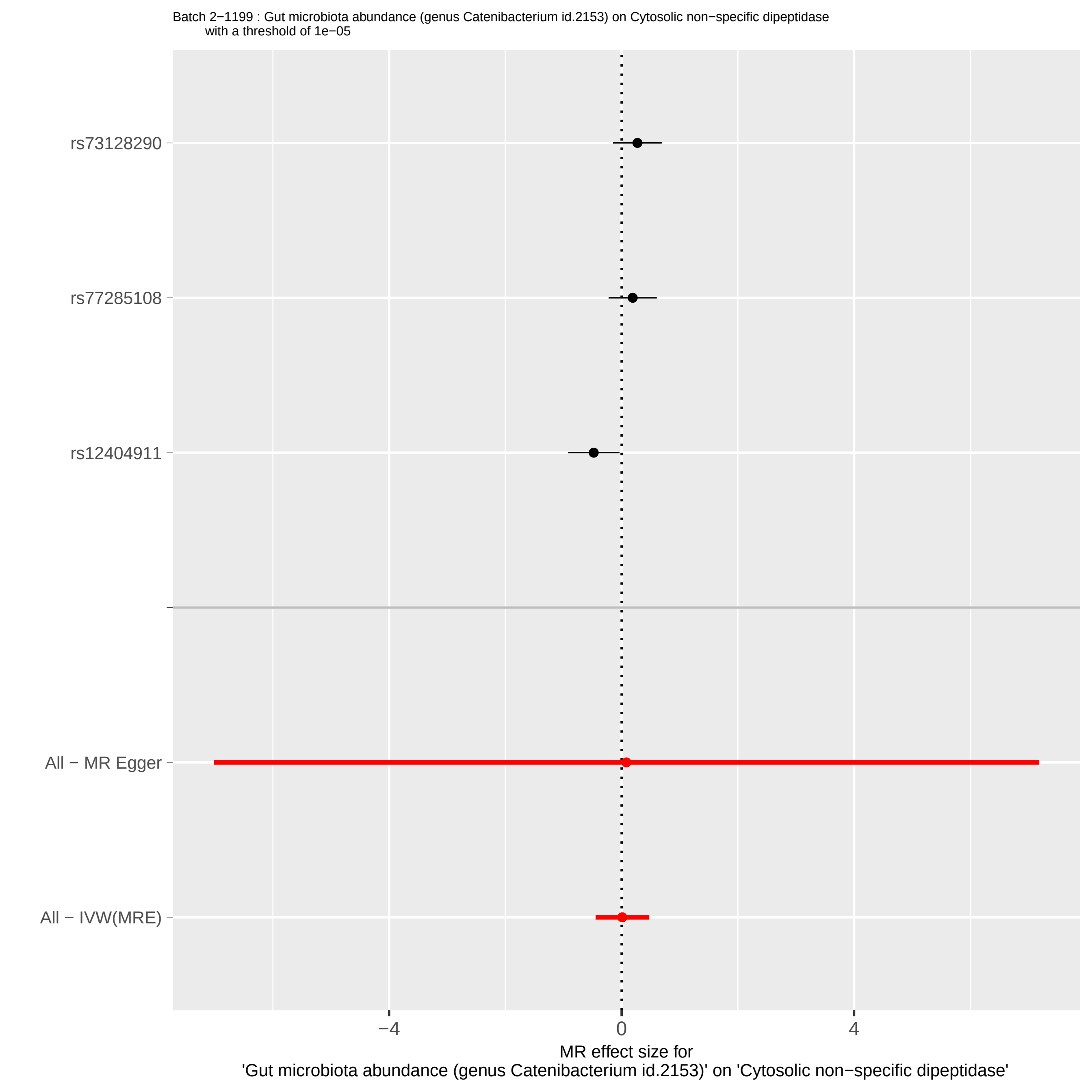

Batch 2−1199 : Gut microbiota abundance (genus Catenibacterium id.2153) on Cytosolic non−specific dipeptidase
with a threshold of 1e−05
rs73128290
rs77285108
rs12404911
All − MR Egger
All − IVW(MRE)
−4
0
4
MR effect size for
'Gut microbiota abundance (genus Catenibacterium id.2153)' on 'Cytosolic non−specific dipeptidase'

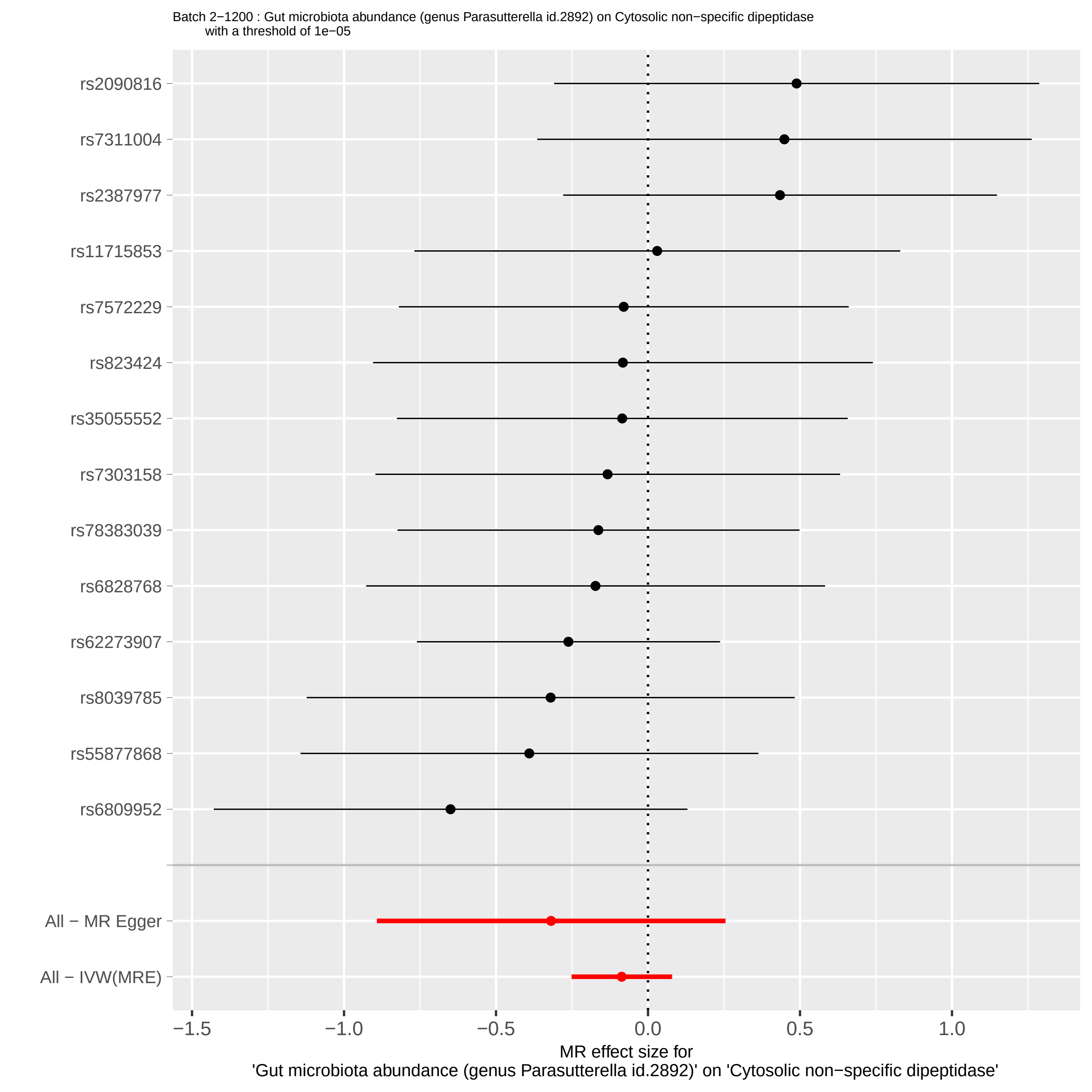
Batch 2−1200 : Gut microbiota abundance (genus Parasutterella id.2892) on Cytosolic non−specific dipeptidase
with a threshold of 1e−05
rs2090816
rs7311004
rs2387977
rs11715853
rs7572229
rs823424
rs35055552
rs7303158
rs78383039
rs6828768
rs62273907
rs8039785
rs55877868
rs6809952
All − MR Egger
All − IVW(MRE)
−1.5
−1.0
−0.5
0.0
0.5
1.0
MR effect size for
'Gut microbiota abundance (genus Parasutterella id.2892)' on 'Cytosolic non−specific dipeptidase'

Batch 2−1201 : Gut microbiota abundance (genus Catenibacterium id.2153) on Nuclear envelope phosphatase−regulatory subunit 1
with a threshold of 1e−05

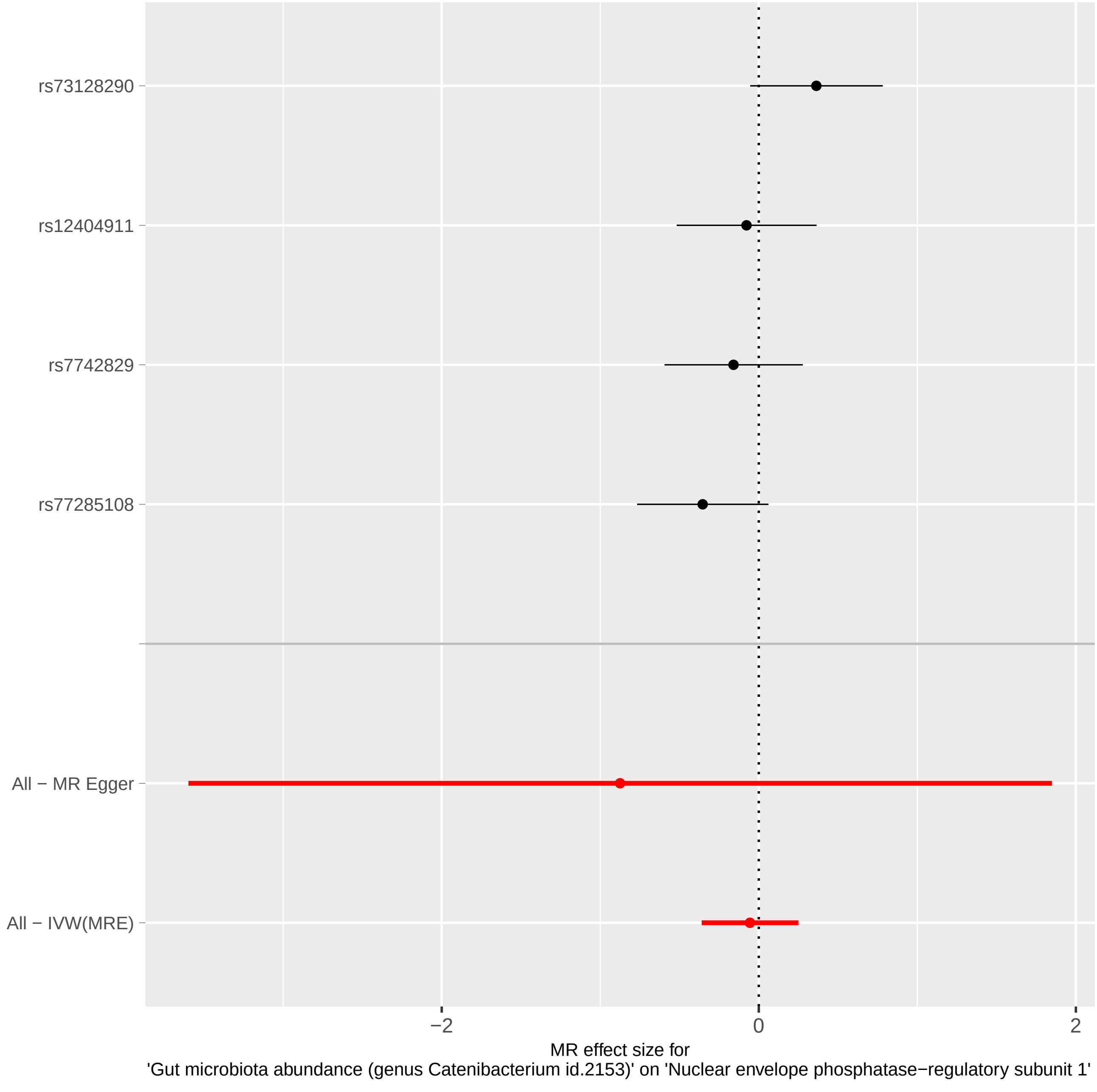

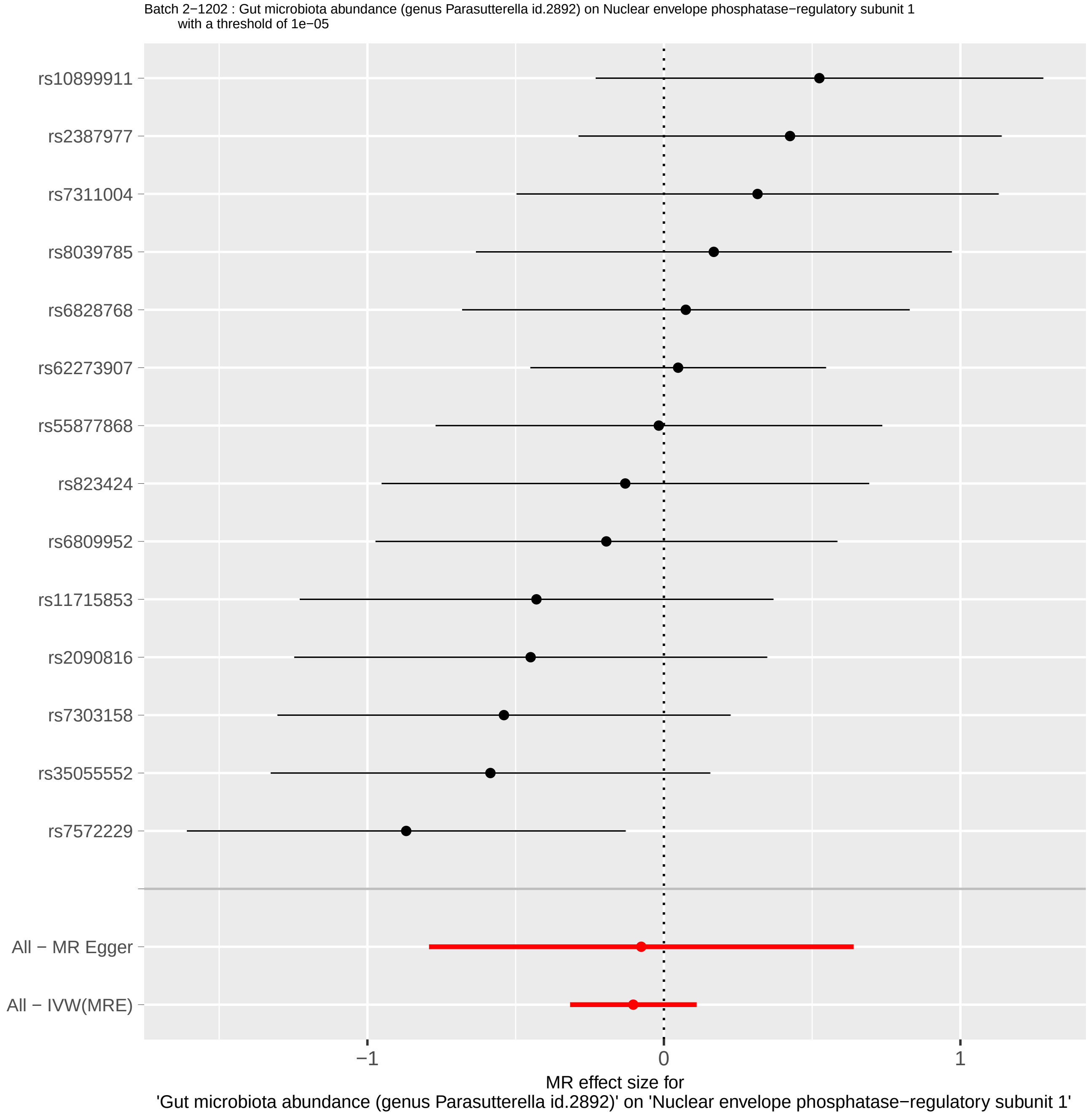

Batch 2−1202 : Gut microbiota abundance (genus Parasutterella id.2892) on Nuclear envelope phosphatase−regulatory subunit 1
with a threshold of 1e−05
rs10899911
rs2387977
rs7311004
rs8039785
rs6828768
rs62273907
rs55877868
rs823424
rs6809952
rs11715853
rs2090816
rs7303158
rs35055552
rs7572229
All − MR Egger
All − IVW(MRE)
−1
0
1
MR effect size for
'Gut microbiota abundance (genus Parasutterella id.2892)' on 'Nuclear envelope phosphatase−regulatory subunit 1'

Batch 2−1203 : Gut microbiota abundance (genus Catenibacterium id.2153) on CCR4−NOT transcription complex subunit 1
with a threshold of 1e−05

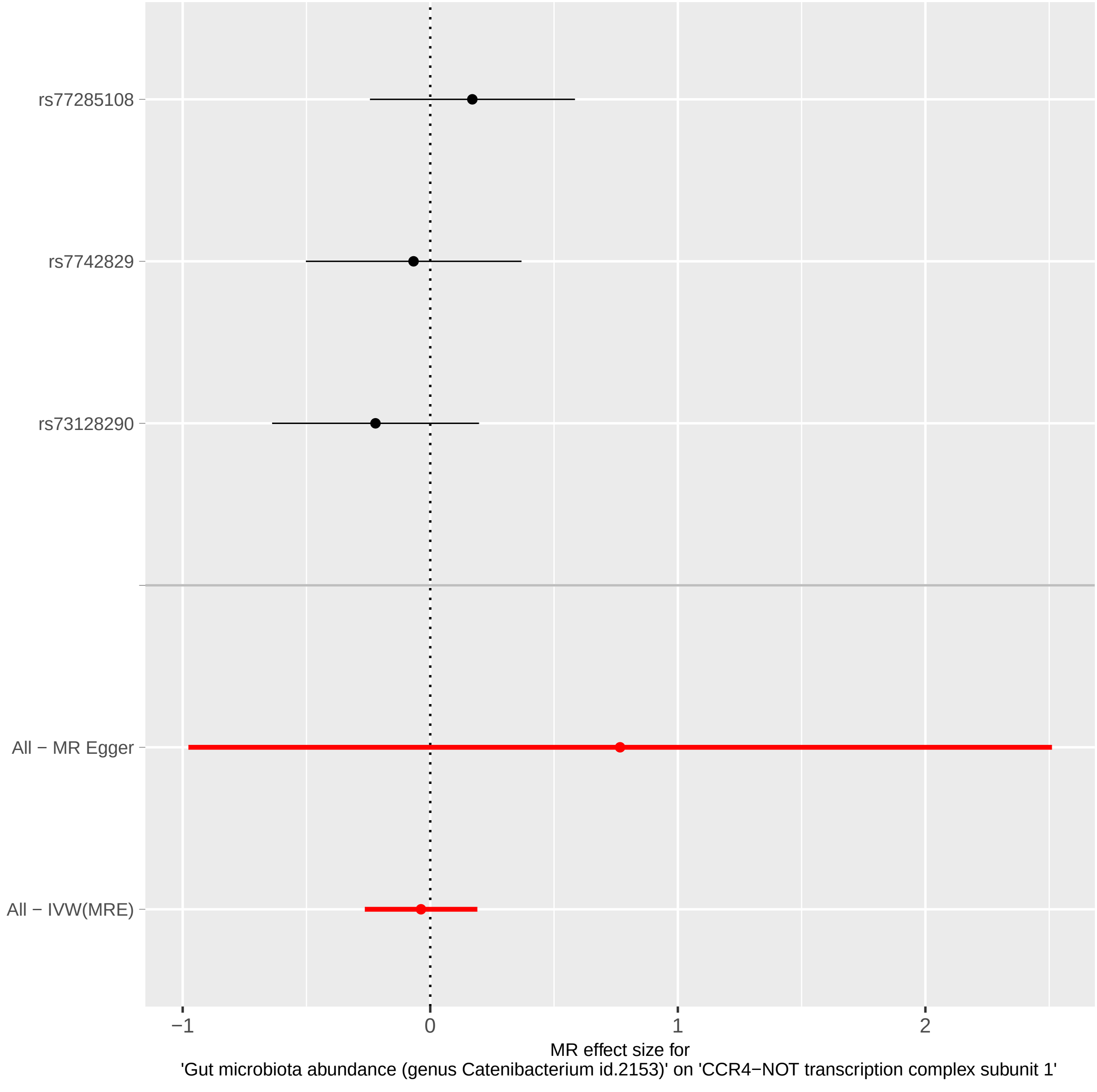

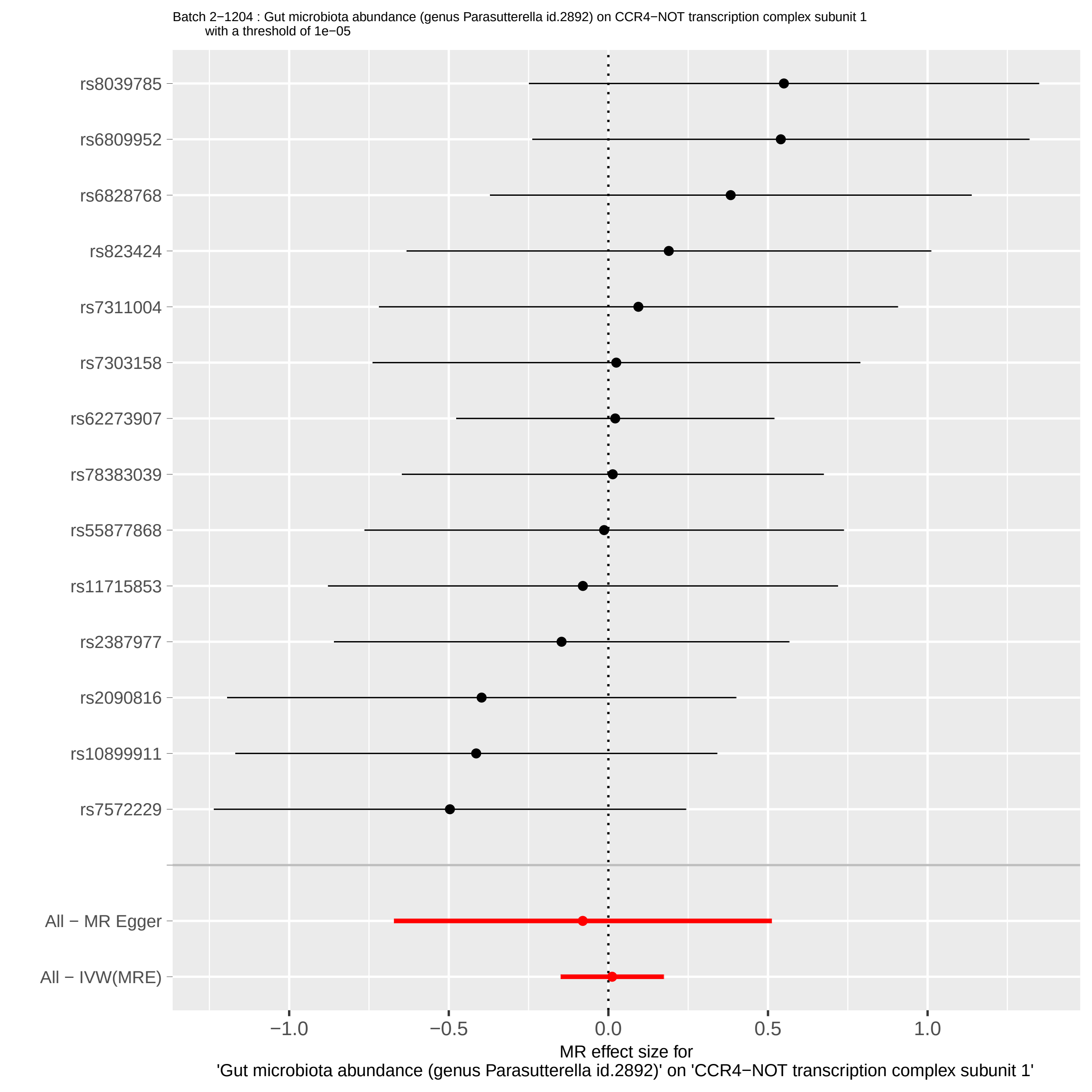
Batch 2−1204 : Gut microbiota abundance (genus Parasutterella id.2892) on CCR4−NOT transcription complex subunit 1
with a threshold of 1e−05
rs8039785
rs6809952
rs6828768
rs823424
rs7311004
rs7303158
rs62273907
rs78383039
rs55877868
rs11715853
rs2387977
rs2090816
rs10899911
rs7572229
All − MR Egger
All − IVW(MRE)
−1.0
−0.5
0.0
0.5
1.0
MR effect size for
'Gut microbiota abundance (genus Parasutterella id.2892)' on 'CCR4−NOT transcription complex subunit 1'

Batch 2−1205 : Gut microbiota abundance (genus Catenibacterium id.2153) on 2',3'−cyclic−nucleotide 3'−phosphodiesterase
with a threshold of 1e−05

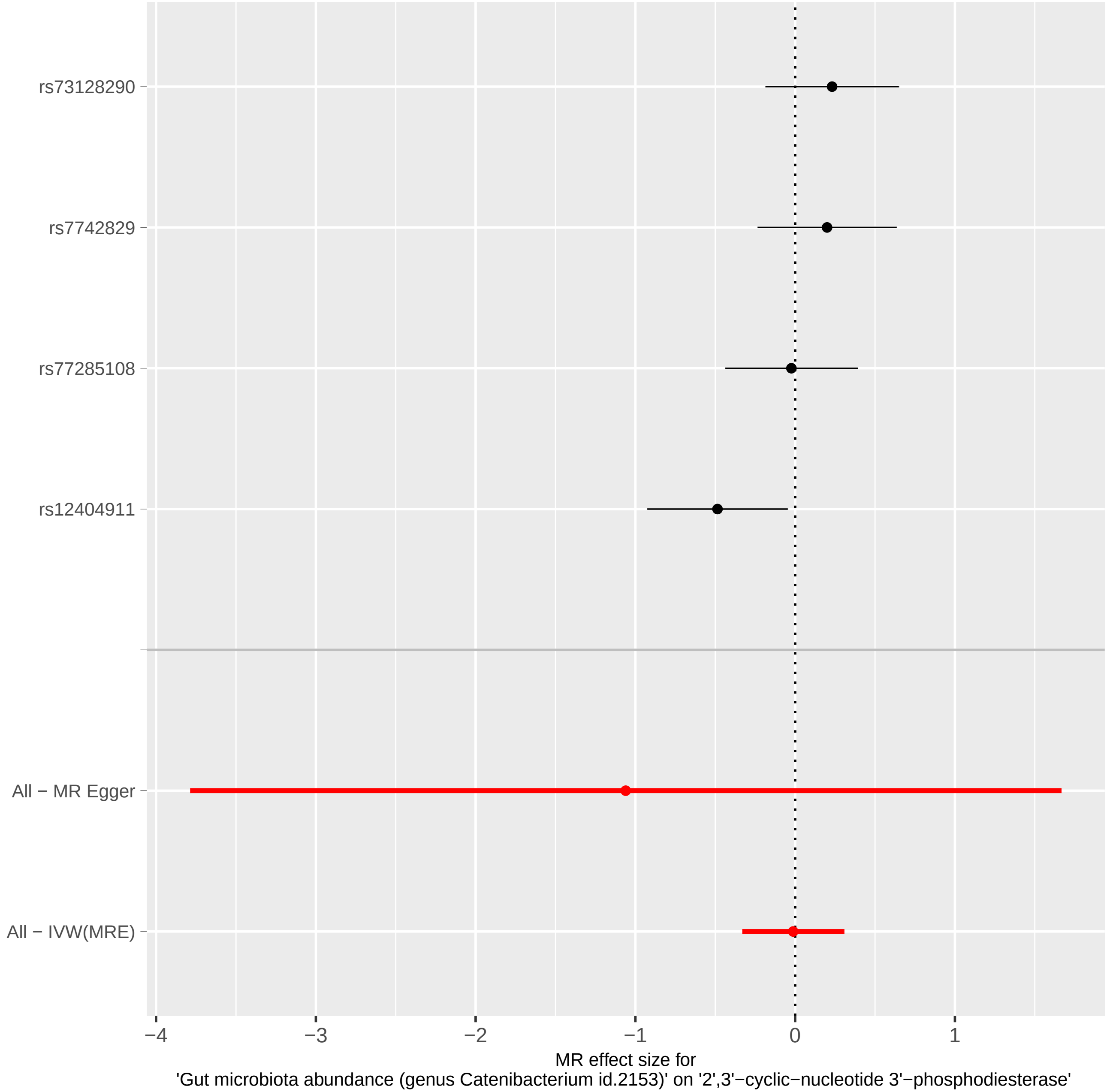

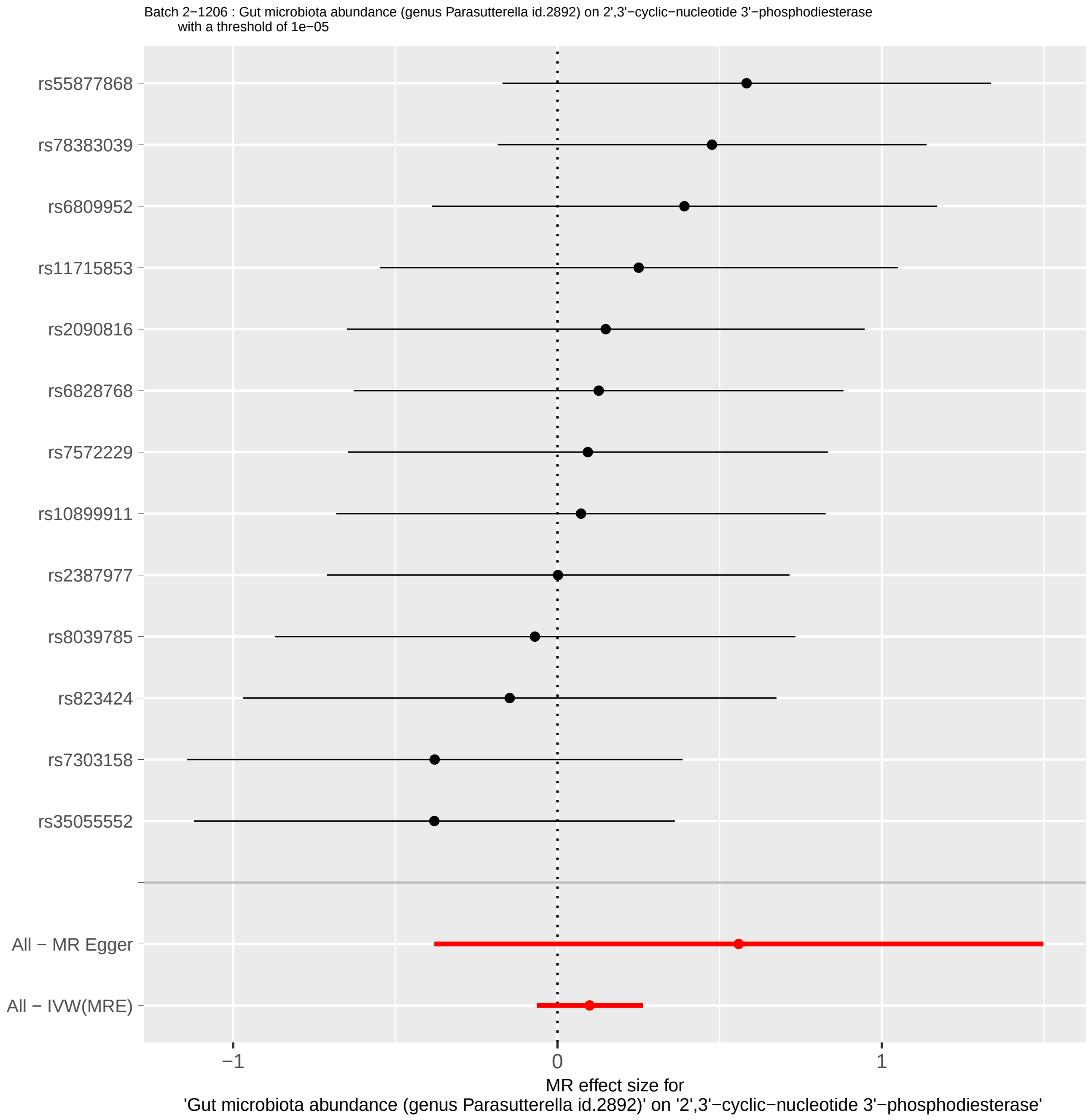

Batch 2−1206 : Gut microbiota abundance (genus Parasutterella id.2892) on 2',3'−cyclic−nucleotide 3'−phosphodiesterase
with a threshold of 1e−05
rs55877868
rs78383039
rs6809952
rs11715853
rs2090816
rs6828768
rs7572229
rs10899911
rs2387977
rs8039785
rs823424
rs7303158
rs35055552
All − MR Egger
All − IVW(MRE)
−1
0
1
MR effect size for
'Gut microbiota abundance (genus Parasutterella id.2892)' on '2',3'−cyclic−nucleotide 3'−phosphodiesterase'

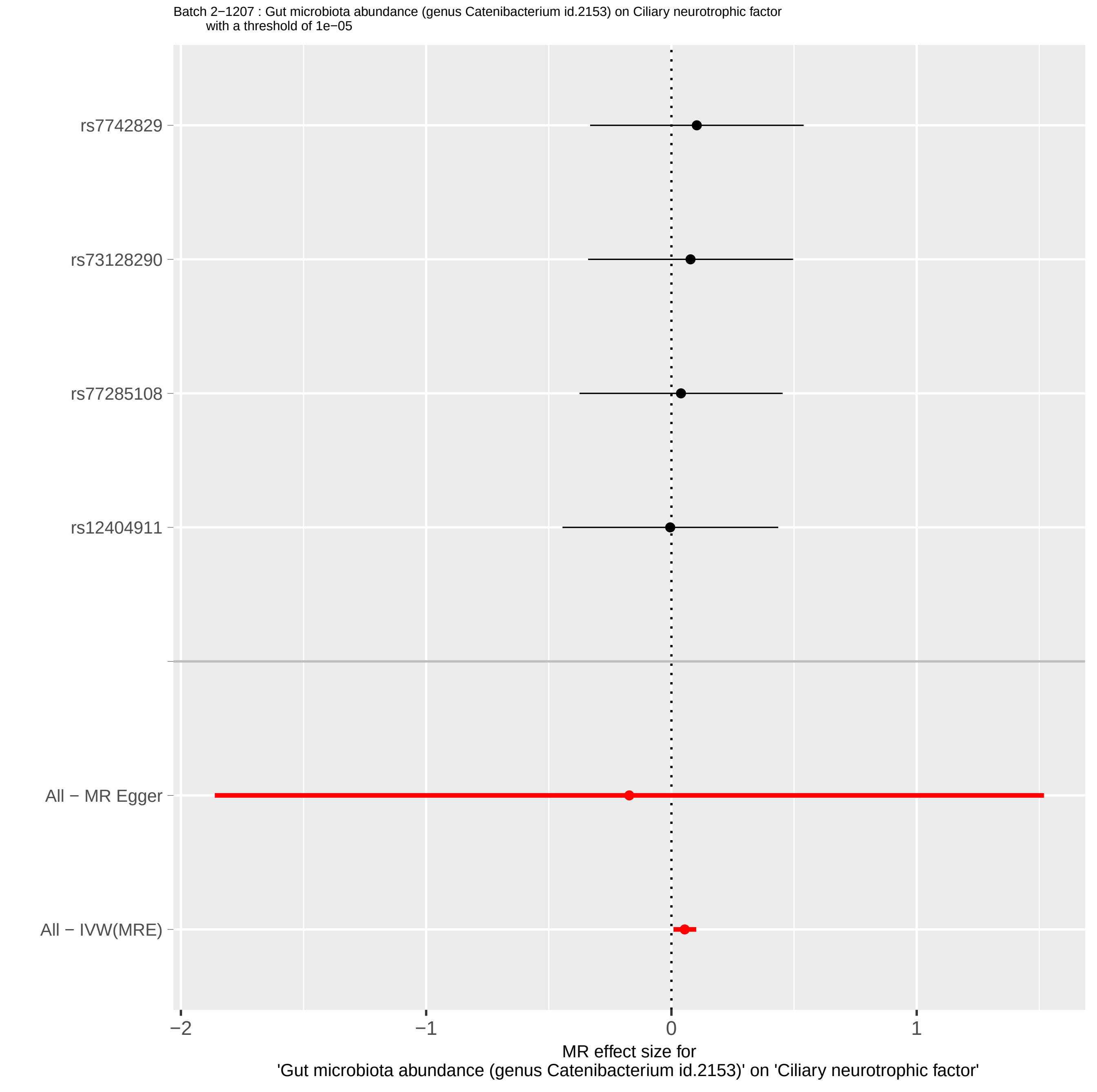

Batch 2−1207 : Gut microbiota abundance (genus Catenibacterium id.2153) on Ciliary neurotrophic factor
with a threshold of 1e−05
rs7742829
rs73128290
rs77285108
rs12404911
All − MR Egger
All − IVW(MRE)
−2
−1
0
1
MR effect size for
'Gut microbiota abundance (genus Catenibacterium id.2153)' on 'Ciliary neurotrophic factor'

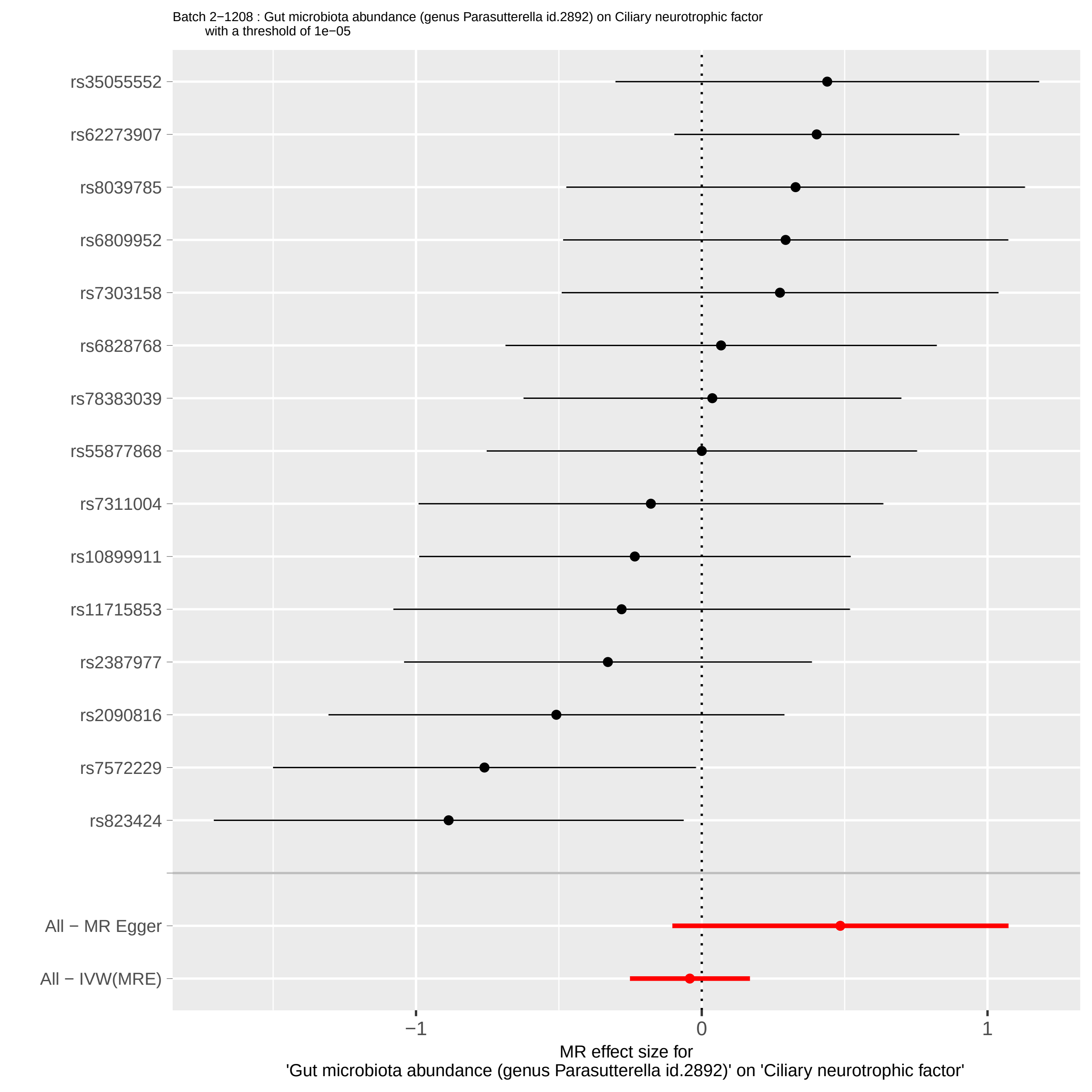

Batch 2−1208 : Gut microbiota abundance (genus Parasutterella id.2892) on Ciliary neurotrophic factor
with a threshold of 1e−05
rs35055552
rs62273907
rs8039785
rs6809952
rs7303158
rs6828768
rs78383039
rs55877868
rs7311004
rs10899911
rs11715853
rs2387977
rs2090816
rs7572229
rs823424
All − MR Egger
All − IVW(MRE)
−1
0
1
MR effect size for
'Gut microbiota abundance (genus Parasutterella id.2892)' on 'Ciliary neurotrophic factor'

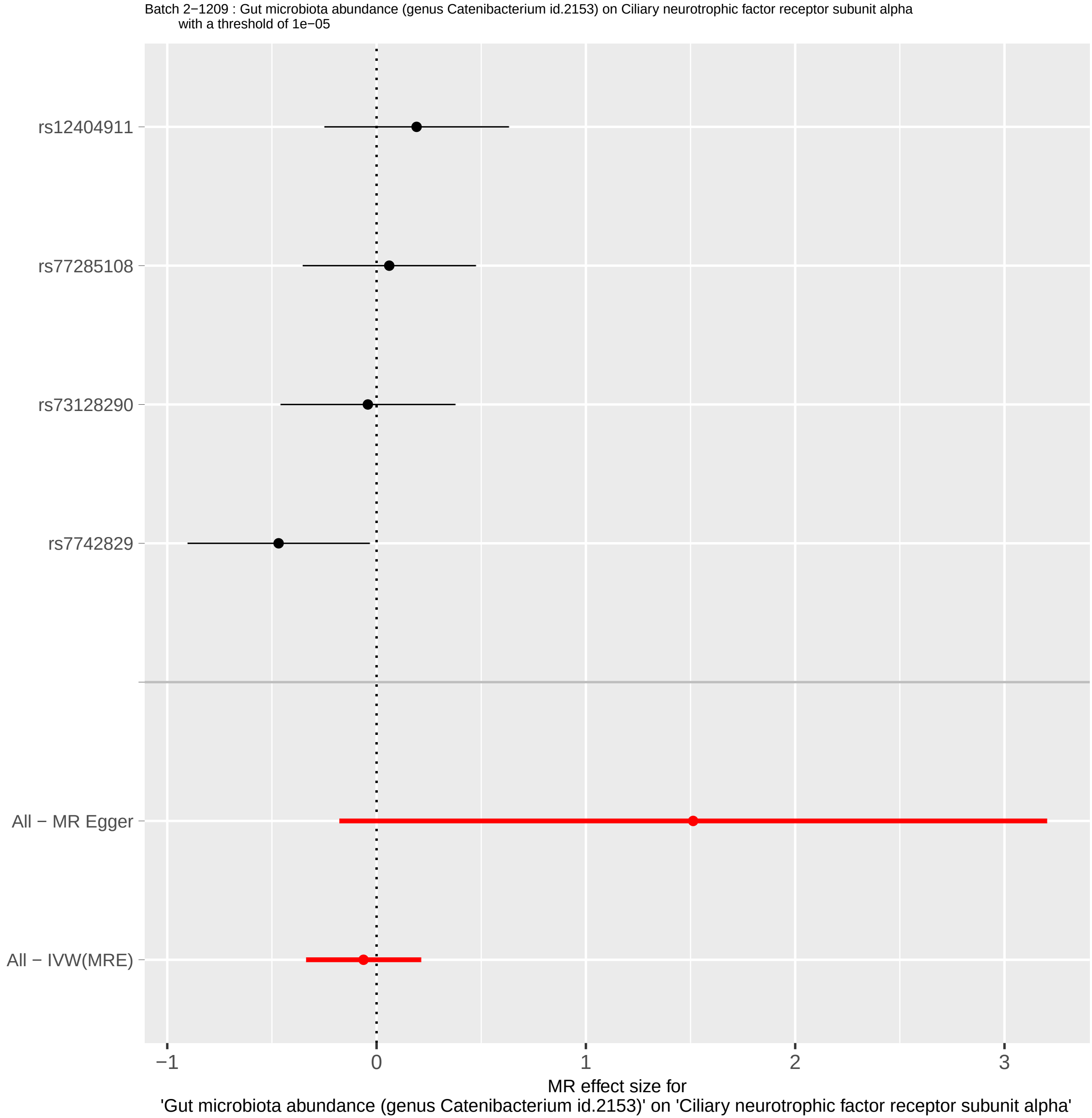

Batch 2−1209 : Gut microbiota abundance (genus Catenibacterium id.2153) on Ciliary neurotrophic factor receptor subunit alpha
with a threshold of 1e−05
rs12404911
rs77285108
rs73128290
rs7742829
All − MR Egger
All − IVW(MRE)
−1
0
1
2
3
MR effect size for
'Gut microbiota abundance (genus Catenibacterium id.2153)' on 'Ciliary neurotrophic factor receptor subunit alpha'

Batch 2−1210 : Gut microbiota abundance (genus Parasutterella id.2892) on Ciliary neurotrophic factor receptor subunit alpha with a threshold of 1e−05

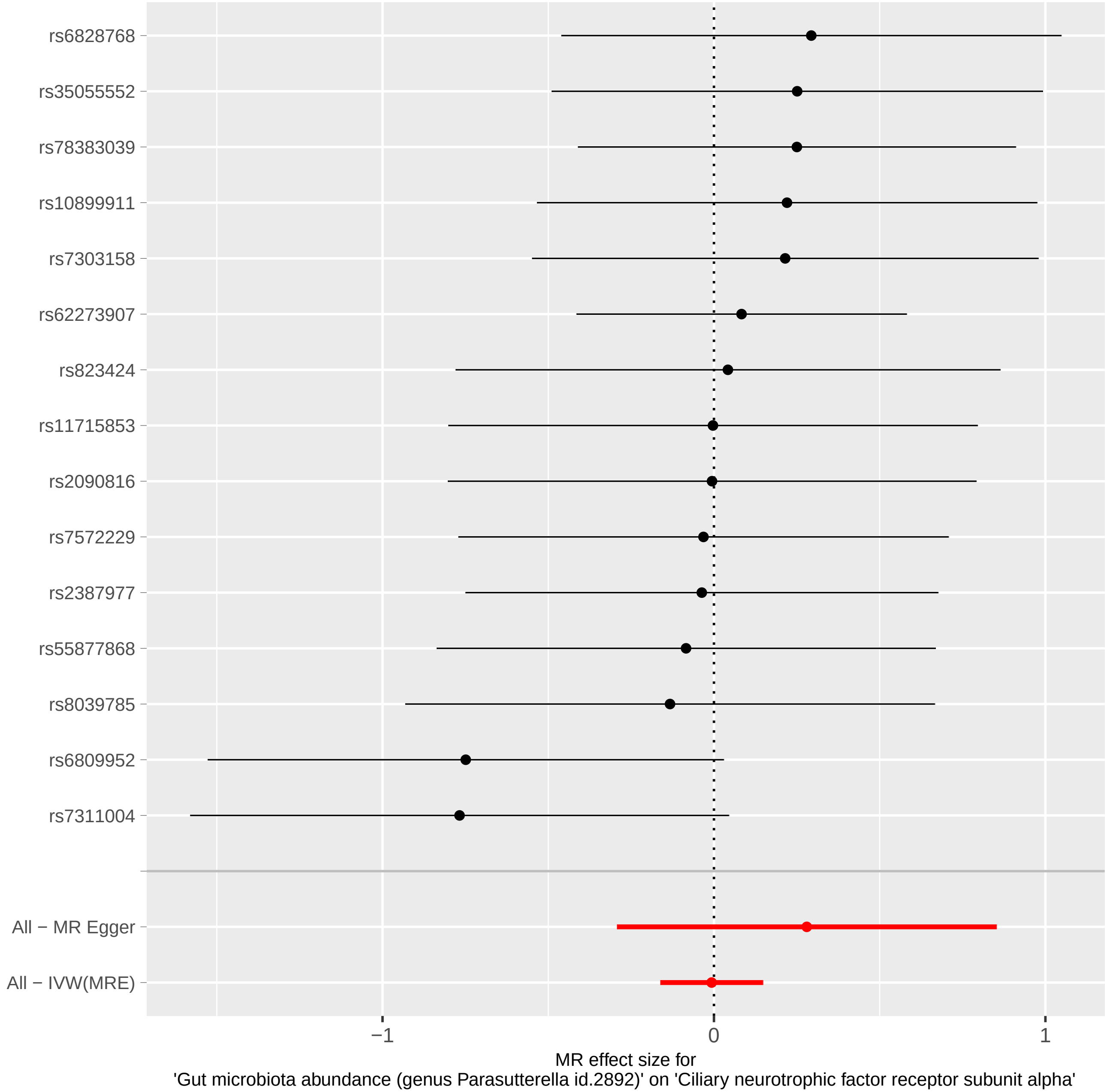

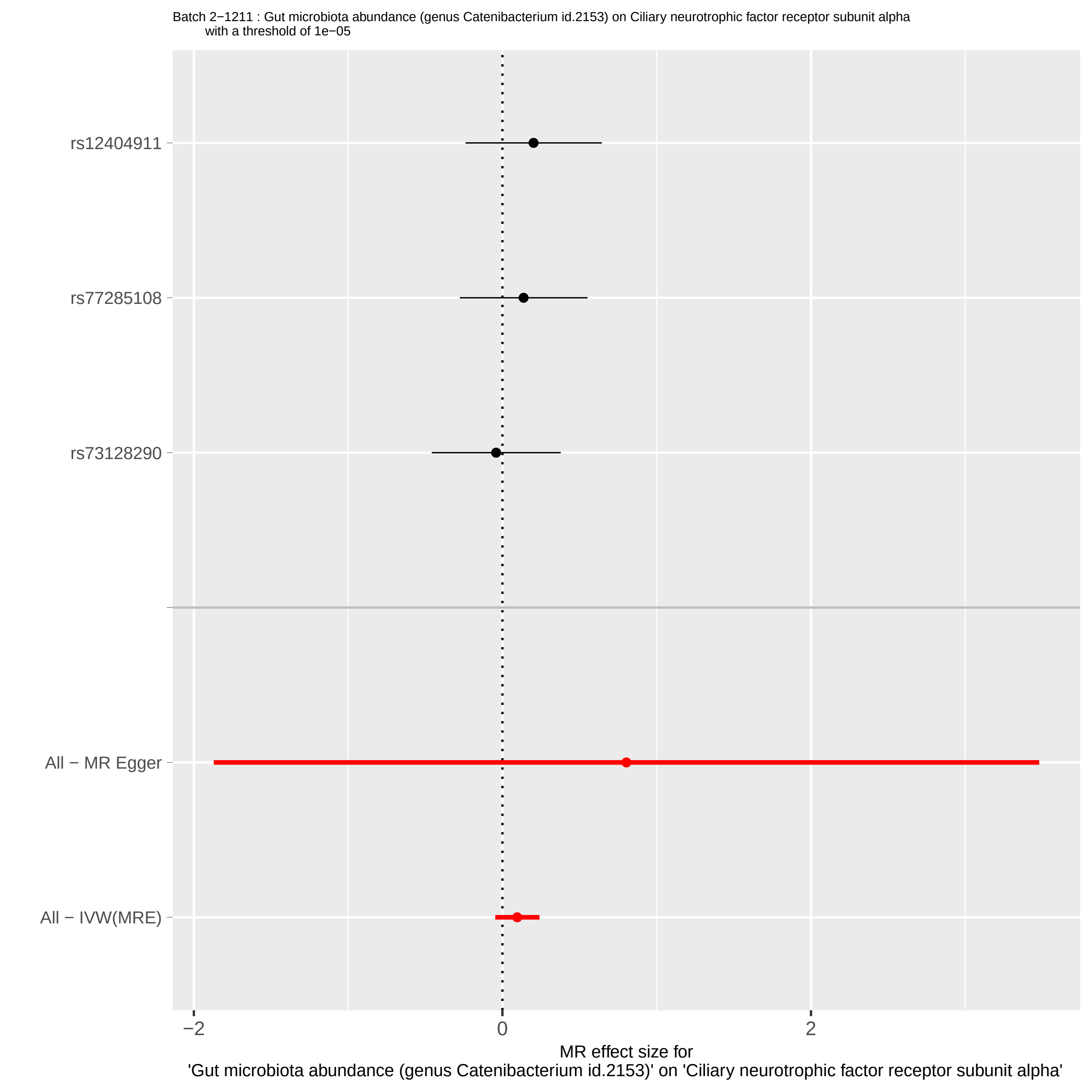
Batch 2−1211 : Gut microbiota abundance (genus Catenibacterium id.2153) on Ciliary neurotrophic factor receptor subunit alpha
with a threshold of 1e−05
rs12404911
rs77285108
rs73128290
All − MR Egger
All − IVW(MRE)
−2
0
2
MR effect size for
'Gut microbiota abundance (genus Catenibacterium id.2153)' on 'Ciliary neurotrophic factor receptor subunit alpha'

Batch 2−1212 : Gut microbiota abundance (genus Parasutterella id.2892) on Ciliary neurotrophic factor receptor subunit alpha
with a threshold of 1e−05

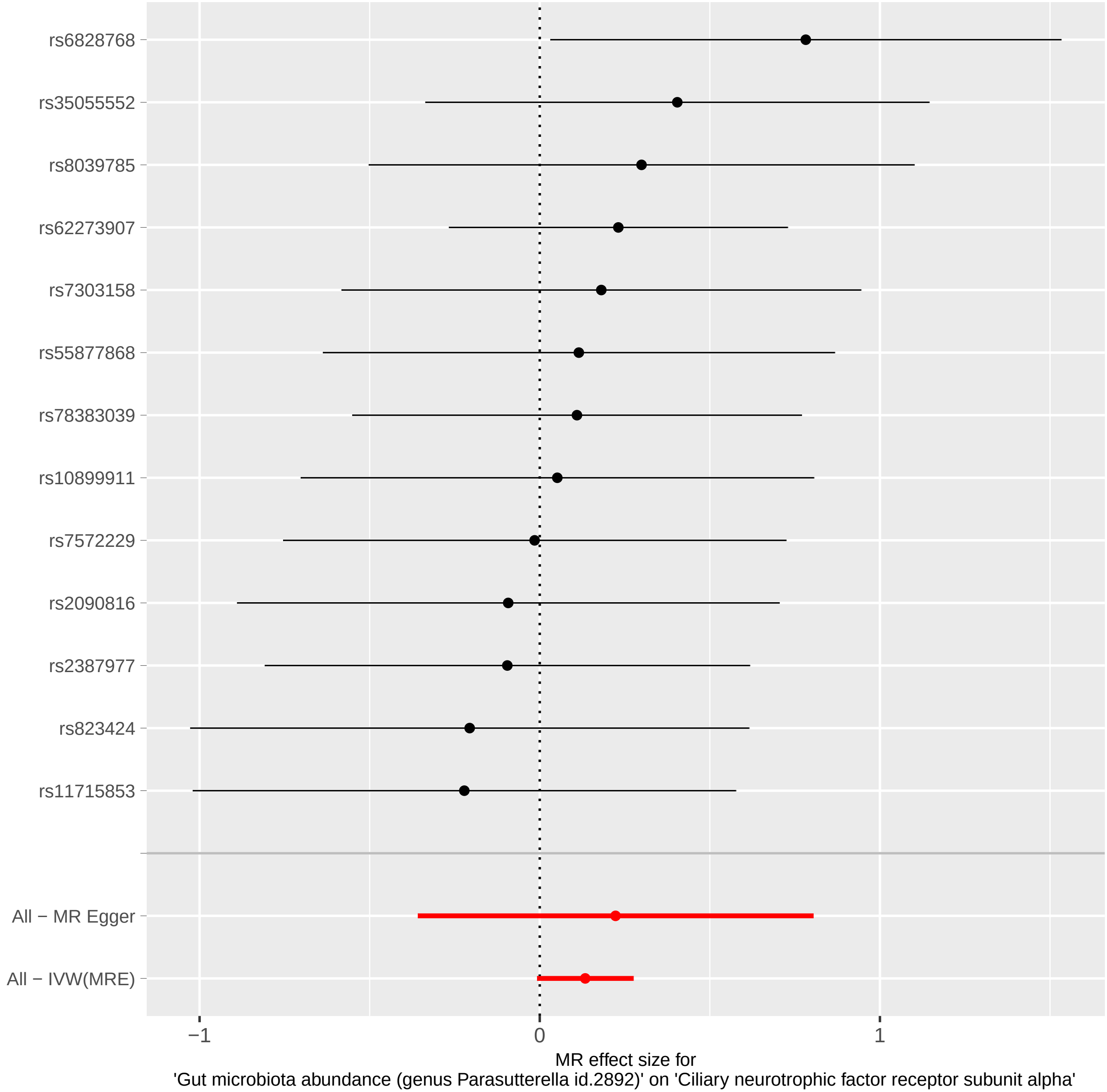

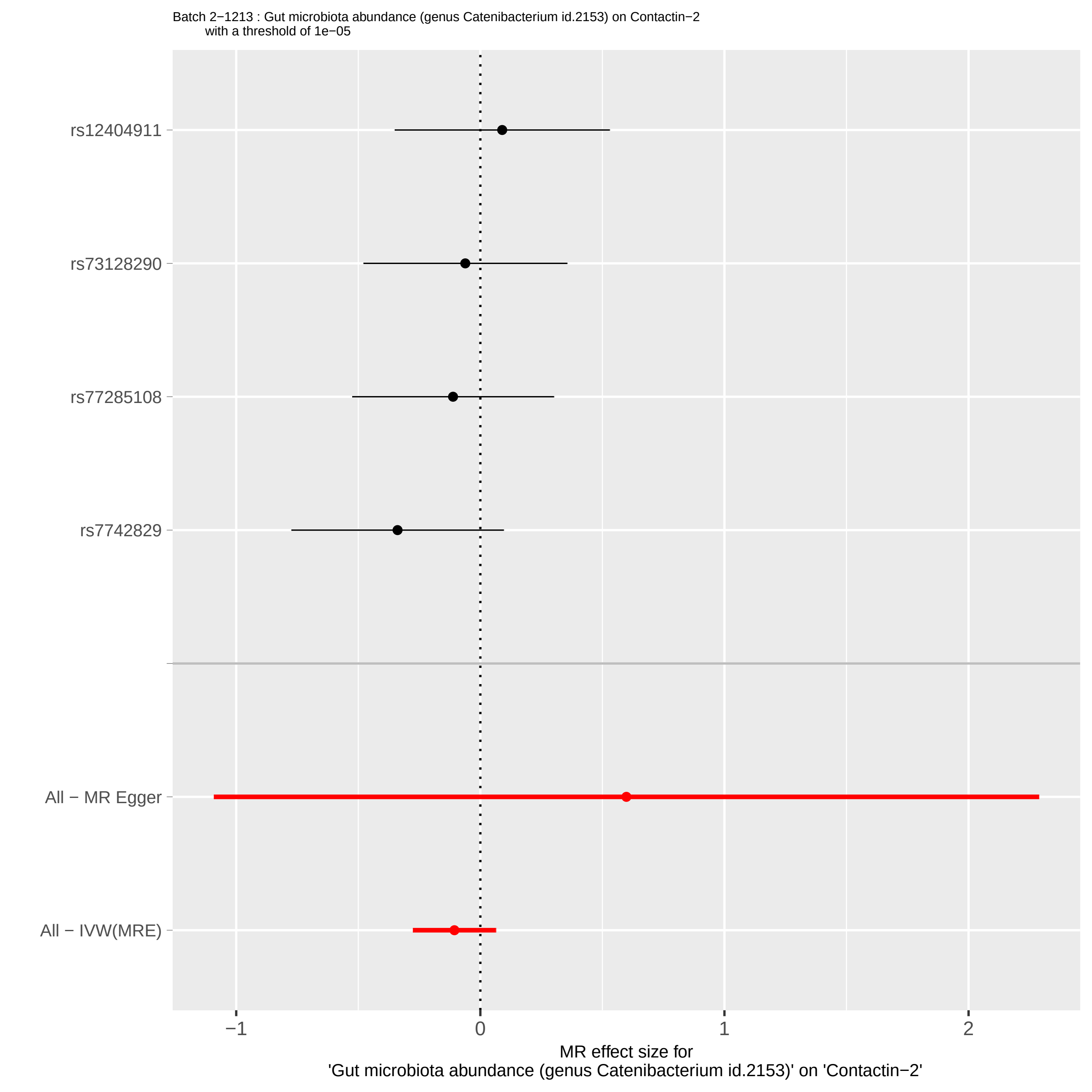

Batch 2−1213 : Gut microbiota abundance (genus Catenibacterium id.2153) on Contactin−2
with a threshold of 1e−05
rs12404911
rs73128290
rs77285108
rs7742829
All − MR Egger
All − IVW(MRE)
−1
0
1
2
MR effect size for
'Gut microbiota abundance (genus Catenibacterium id.2153)' on 'Contactin−2'

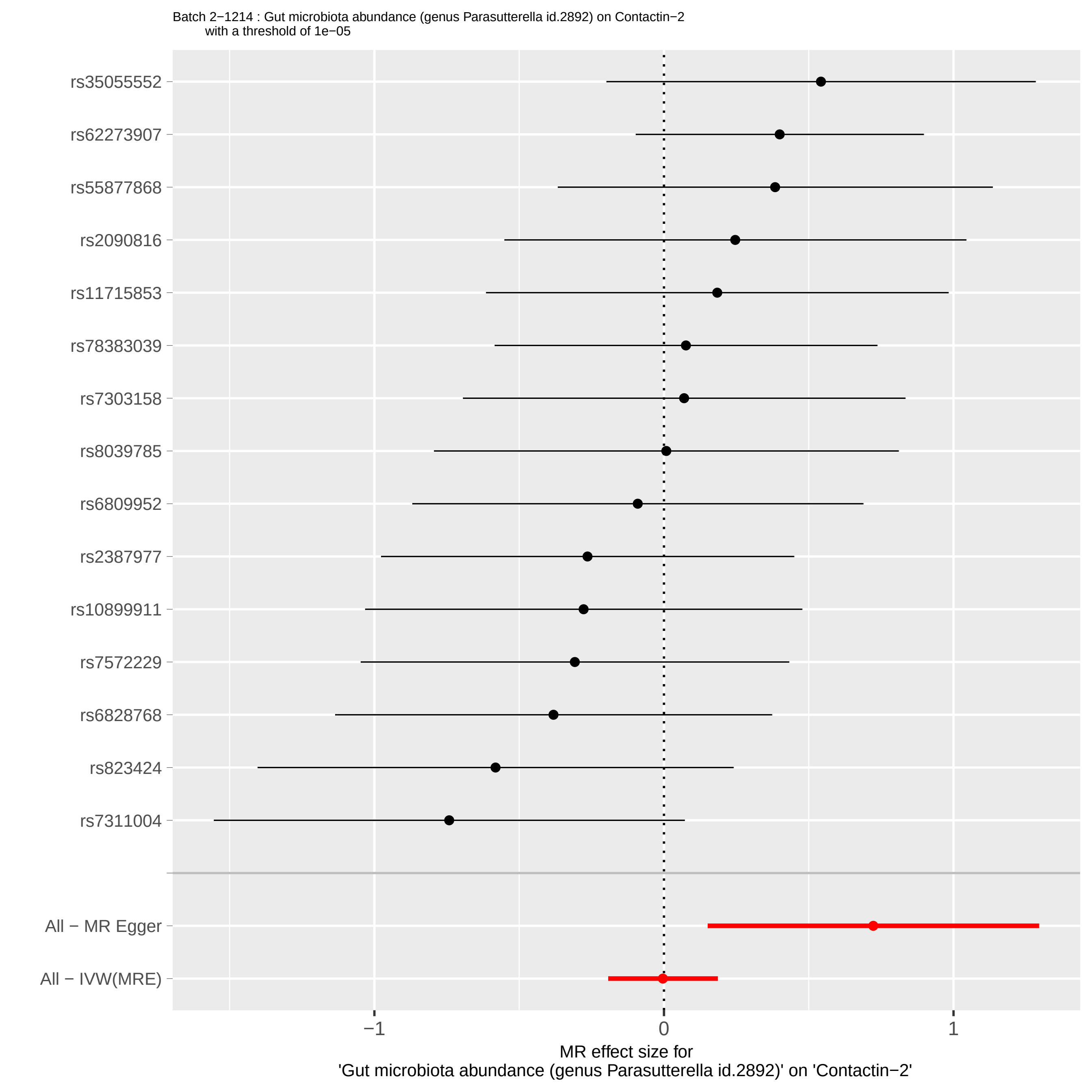
Batch 2−1214 : Gut microbiota abundance (genus Parasutterella id.2892) on Contactin−2
with a threshold of 1e−05
rs35055552
rs62273907
rs55877868
rs2090816
rs11715853
rs78383039
rs7303158
rs8039785
rs6809952
rs2387977
rs10899911
rs7572229
rs6828768
rs823424
rs7311004
All − MR Egger
All − IVW(MRE)
−1
0
1
MR effect size for
'Gut microbiota abundance (genus Parasutterella id.2892)' on 'Contactin−2'

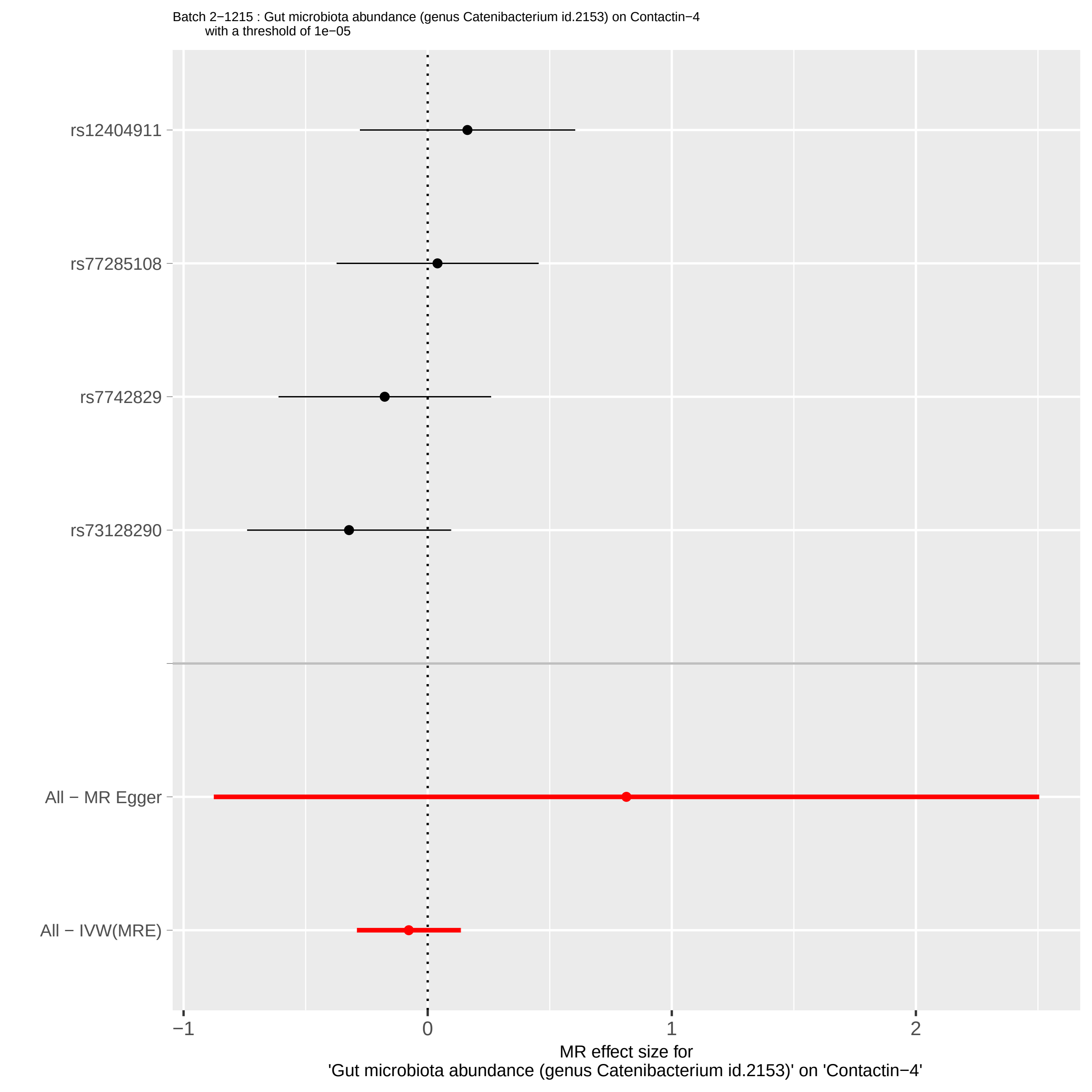

Batch 2−1215 : Gut microbiota abundance (genus Catenibacterium id.2153) on Contactin−4
with a threshold of 1e−05
rs12404911
rs77285108
rs7742829
rs73128290
All − MR Egger
All − IVW(MRE)
−1
0
1
2
MR effect size for
'Gut microbiota abundance (genus Catenibacterium id.2153)' on 'Contactin−4'

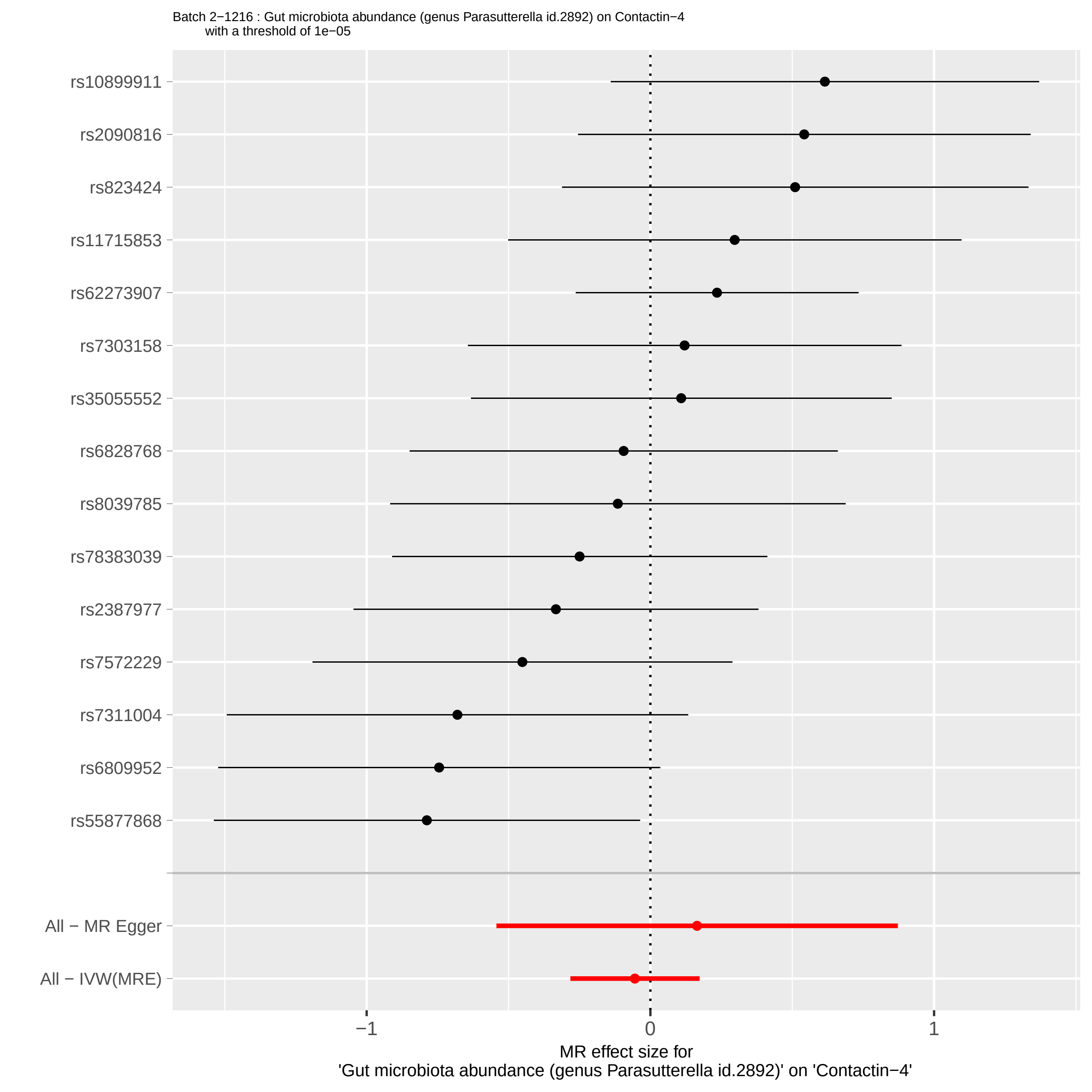

Batch 2−1216 : Gut microbiota abundance (genus Parasutterella id.2892) on Contactin−4
with a threshold of 1e−05
rs10899911
rs2090816
rs823424
rs11715853
rs62273907
rs7303158
rs35055552
rs6828768
rs8039785
rs78383039
rs2387977
rs7572229
rs7311004
rs6809952
rs55877868
All − MR Egger
All − IVW(MRE)
−1
0
1
MR effect size for
'Gut microbiota abundance (genus Parasutterella id.2892)' on 'Contactin−4'

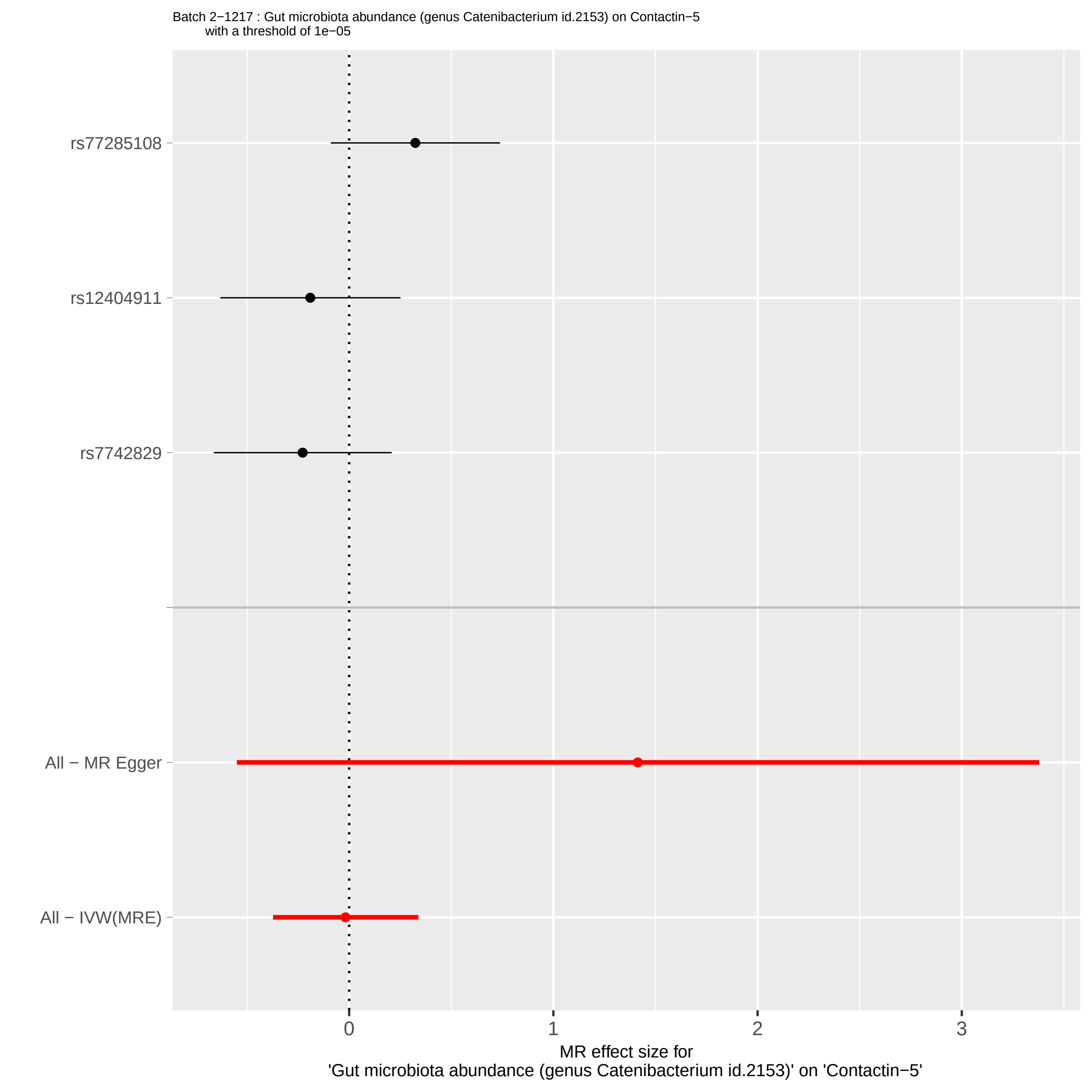

Batch 2−1217 : Gut microbiota abundance (genus Catenibacterium id.2153) on Contactin−5
with a threshold of 1e−05
rs77285108
rs12404911
rs7742829
All − MR Egger
All − IVW(MRE)
0
1
2
3
MR effect size for
'Gut microbiota abundance (genus Catenibacterium id.2153)' on 'Contactin−5'

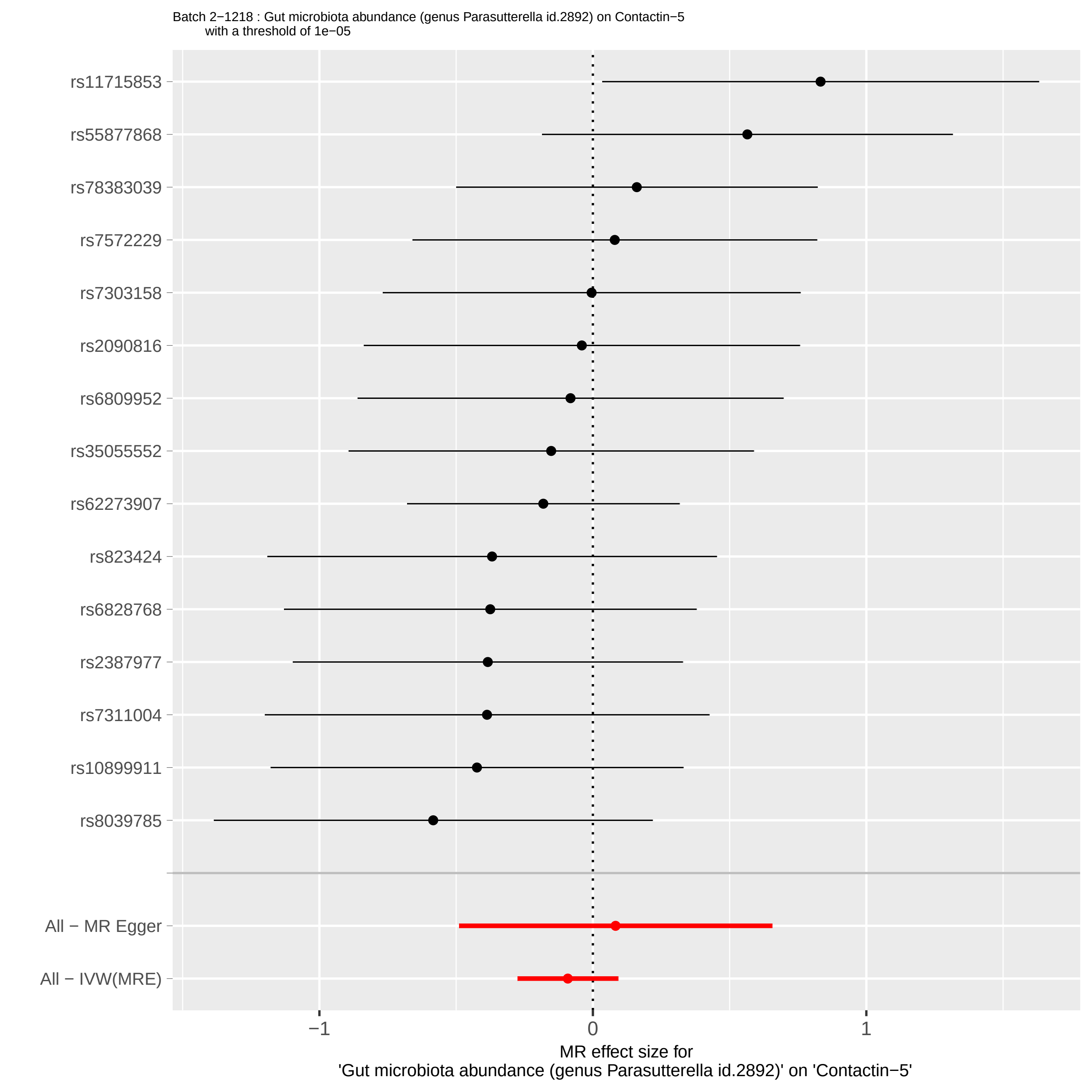
Batch 2−1218 : Gut microbiota abundance (genus Parasutterella id.2892) on Contactin−5
with a threshold of 1e−05
rs11715853
rs55877868
rs78383039
rs7572229
rs7303158
rs2090816
rs6809952
rs35055552
rs62273907
rs823424
rs6828768
rs2387977
rs7311004
rs10899911
rs8039785
All − MR Egger
All − IVW(MRE)
−1
0
1
MR effect size for
'Gut microbiota abundance (genus Parasutterella id.2892)' on 'Contactin−5'

Batch 2−1219 : Gut microbiota abundance (genus Catenibacterium id.2153) on Contactin−associated protein 1
with a threshold of 1e−05

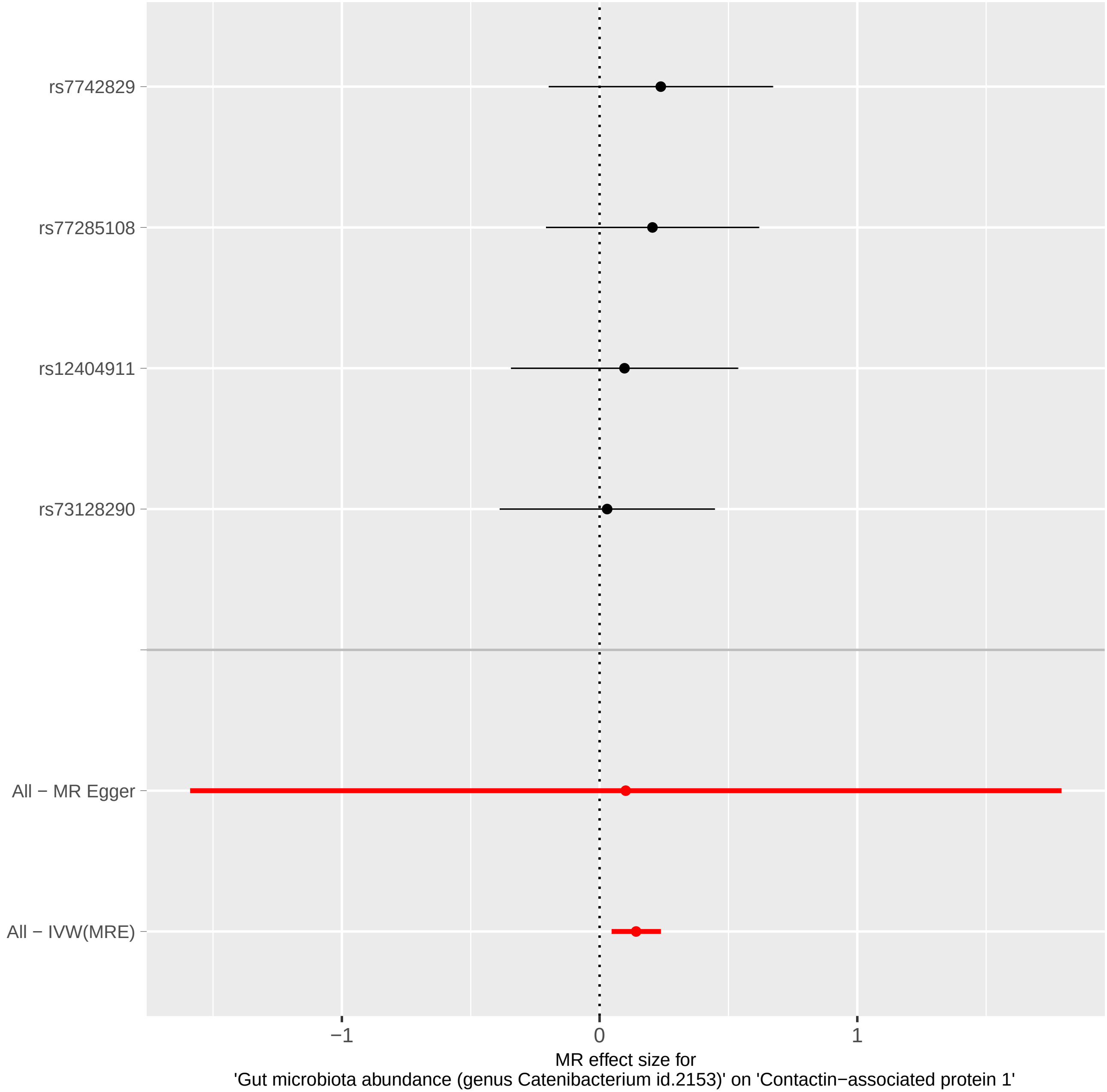

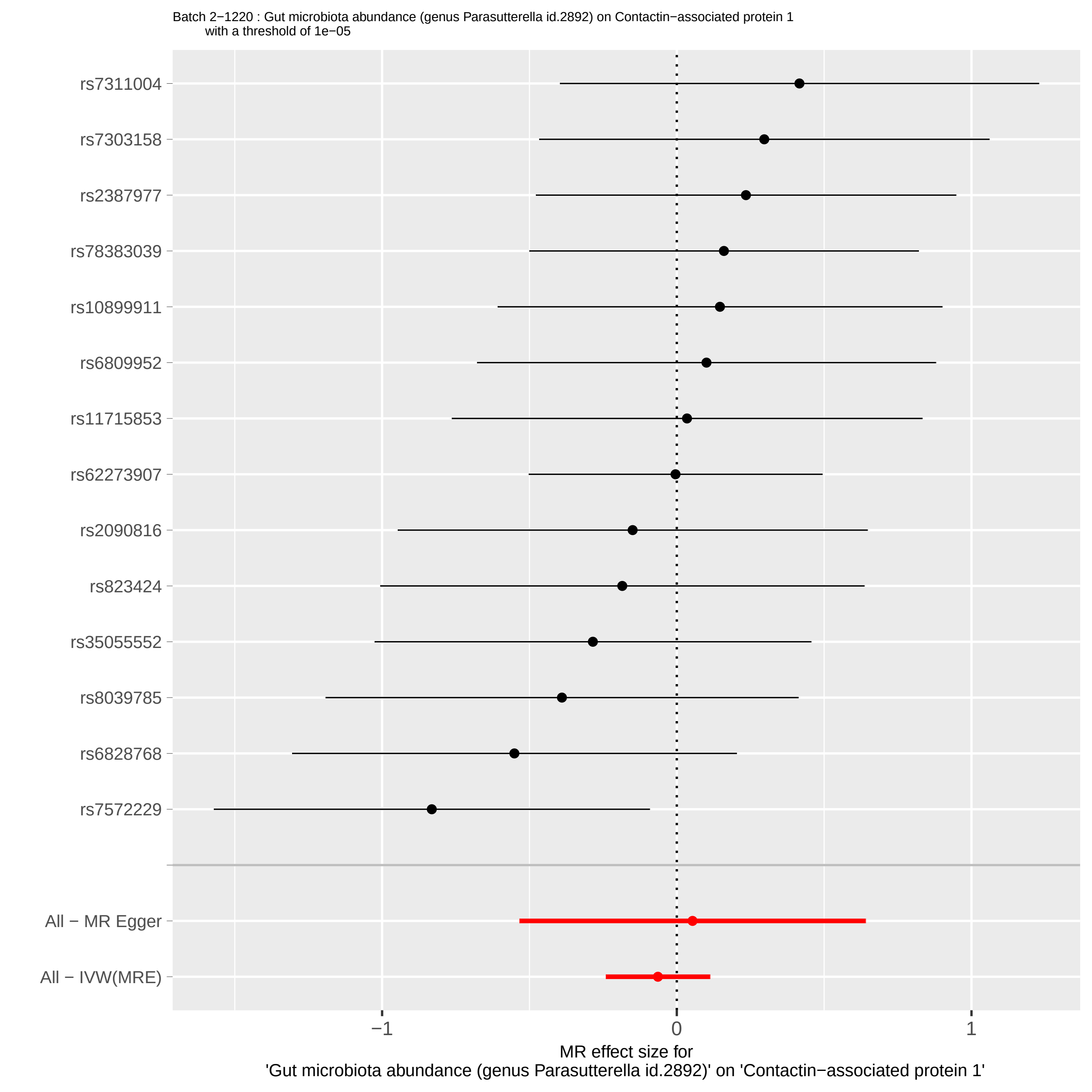

Batch 2−1220 : Gut microbiota abundance (genus Parasutterella id.2892) on Contactin−associated protein 1
with a threshold of 1e−05
rs7311004
rs7303158
rs2387977
rs78383039
rs10899911
rs6809952
rs11715853
rs62273907
rs2090816
rs823424
rs35055552
rs8039785
rs6828768
rs7572229
All − MR Egger
All − IVW(MRE)
−1
0
1
MR effect size for
'Gut microbiota abundance (genus Parasutterella id.2892)' on 'Contactin−associated protein 1'

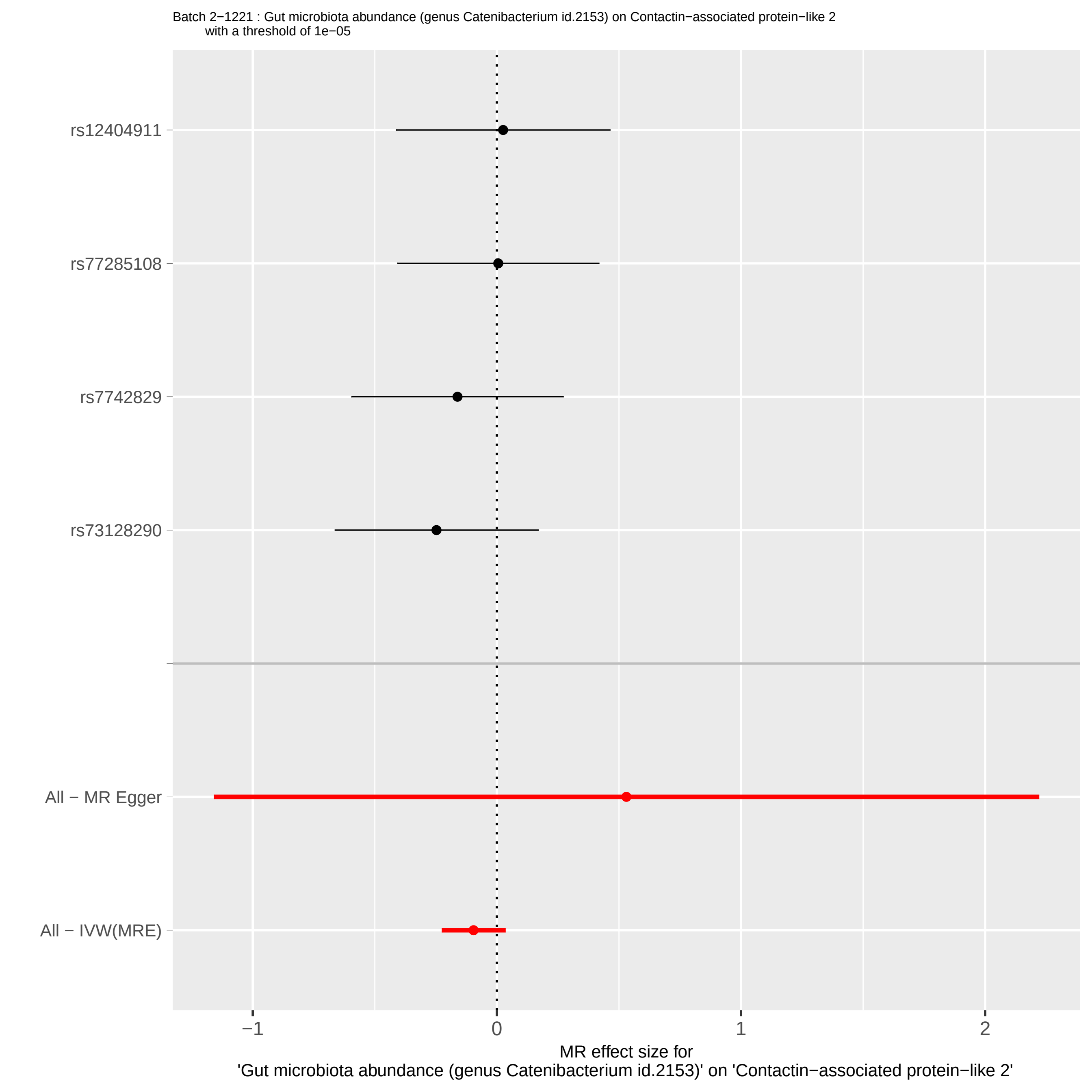
Batch 2−1221 : Gut microbiota abundance (genus Catenibacterium id.2153) on Contactin−associated protein−like 2
with a threshold of 1e−05
rs12404911
rs77285108
rs7742829
rs73128290
All − MR Egger
All − IVW(MRE)
−1
0
1
2
MR effect size for
'Gut microbiota abundance (genus Catenibacterium id.2153)' on 'Contactin−associated protein−like 2'

Batch 2−1222 : Gut microbiota abundance (genus Parasutterella id.2892) on Contactin−associated protein−like 2 with a threshold of 1e−05

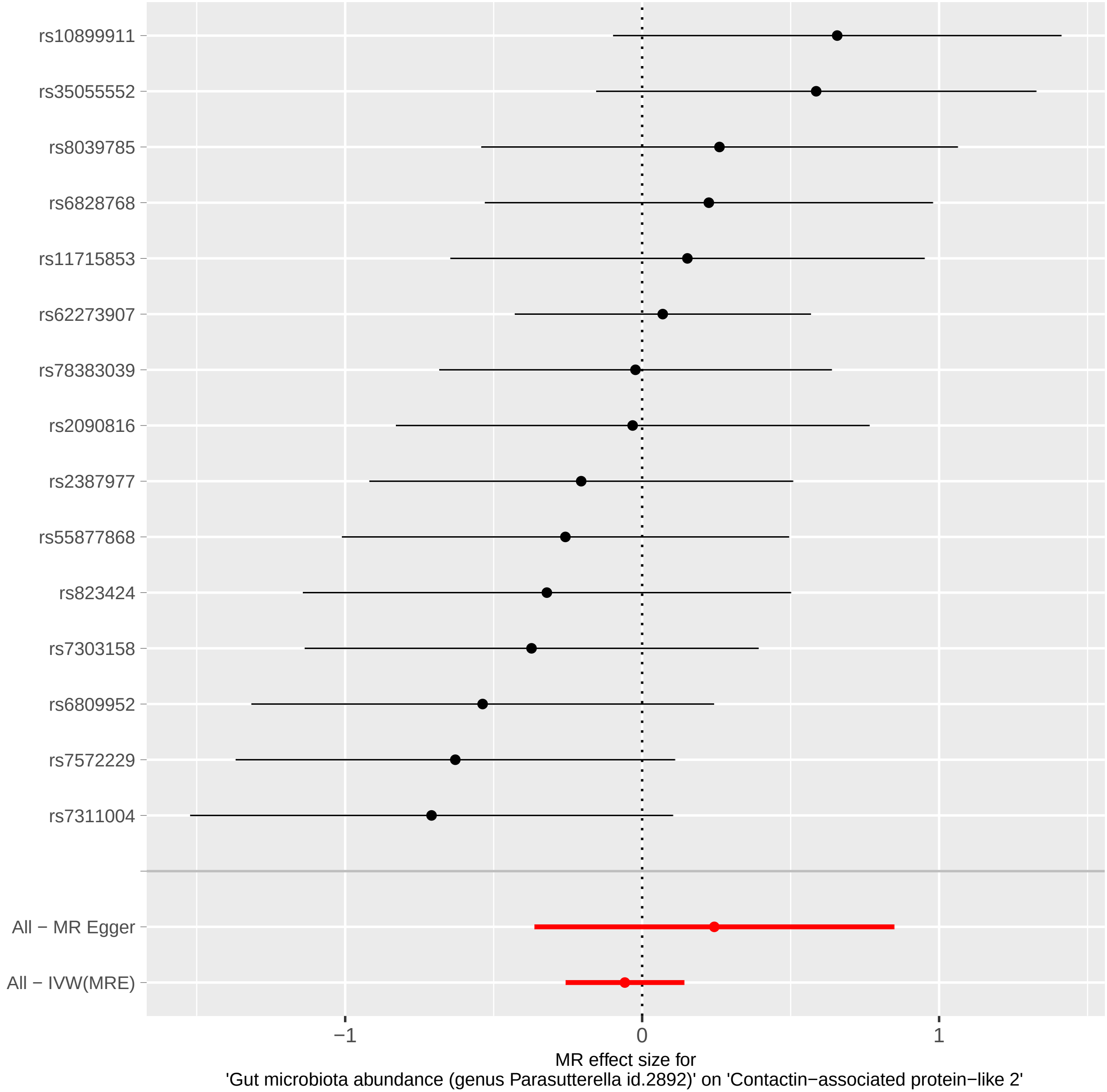

Batch 2−1223 : Gut microbiota abundance (genus Catenibacterium id.2153) on Cytochrome c oxidase assembly factor 3 homolog, mitochondrial
with a threshold of 1e−05

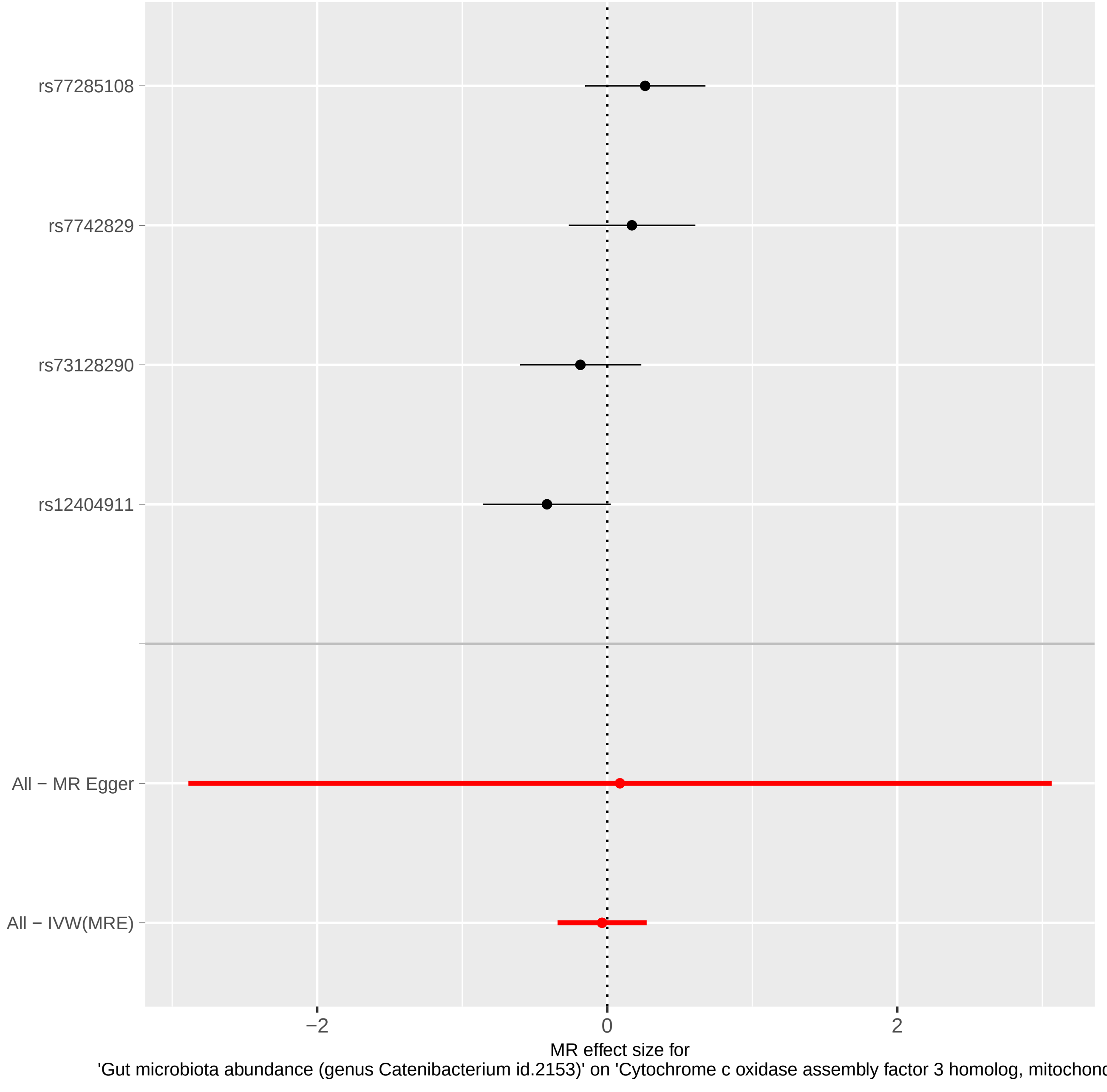

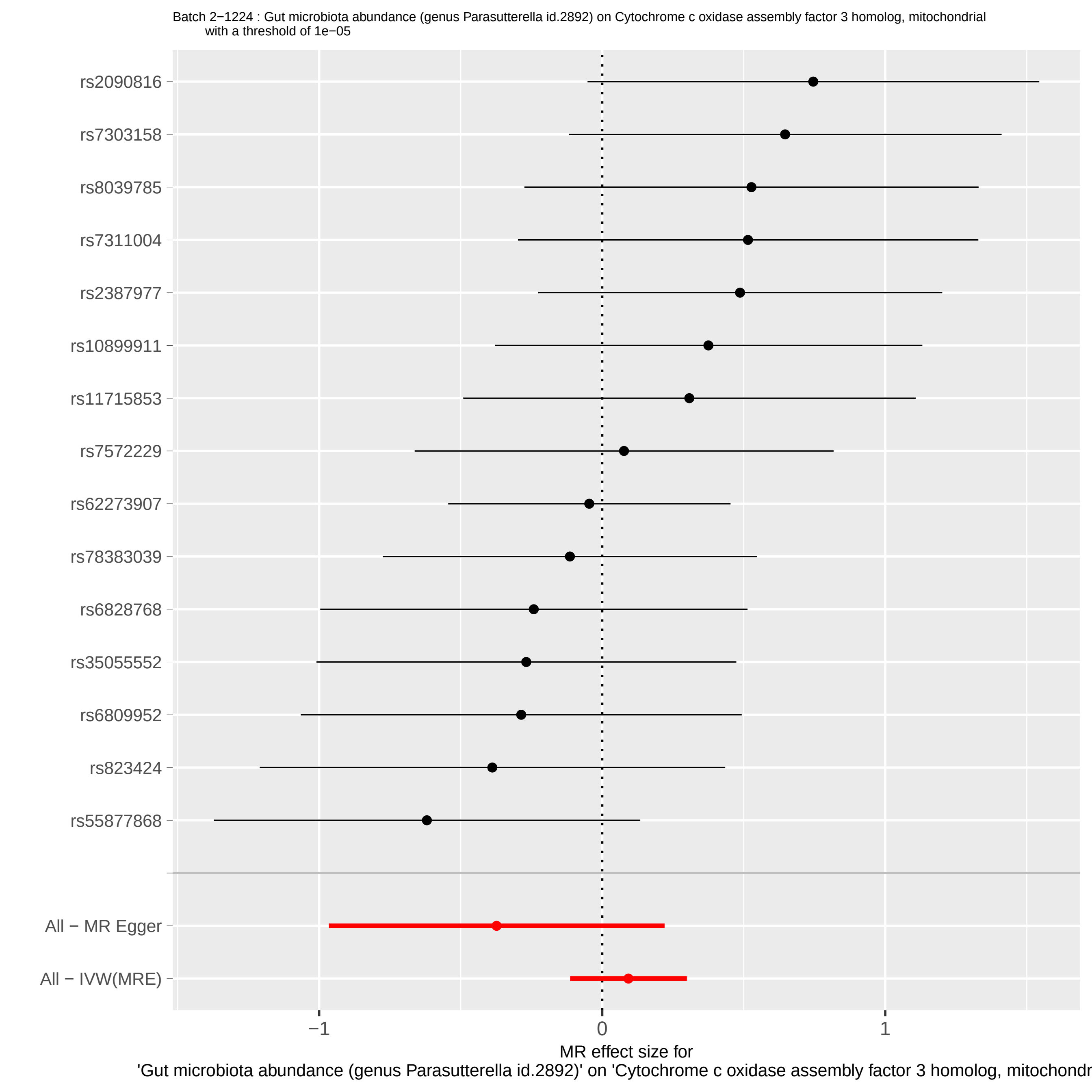

Batch 2−1224 : Gut microbiota abundance (genus Parasutterella id.2892) on Cytochrome c oxidase assembly factor 3 homolog, mitochondrial
with a threshold of 1e−05
rs2090816
rs7303158
rs8039785
rs7311004
rs2387977
rs10899911
rs11715853
rs7572229
rs62273907
rs78383039
rs6828768
rs35055552
rs6809952
rs823424
rs55877868
All − MR Egger
All − IVW(MRE)
−1
0
1
MR effect size for
'Gut microbiota abundance (genus Parasutterella id.2892)' on 'Cytochrome c oxidase assembly factor 3 homolog, mitochondr

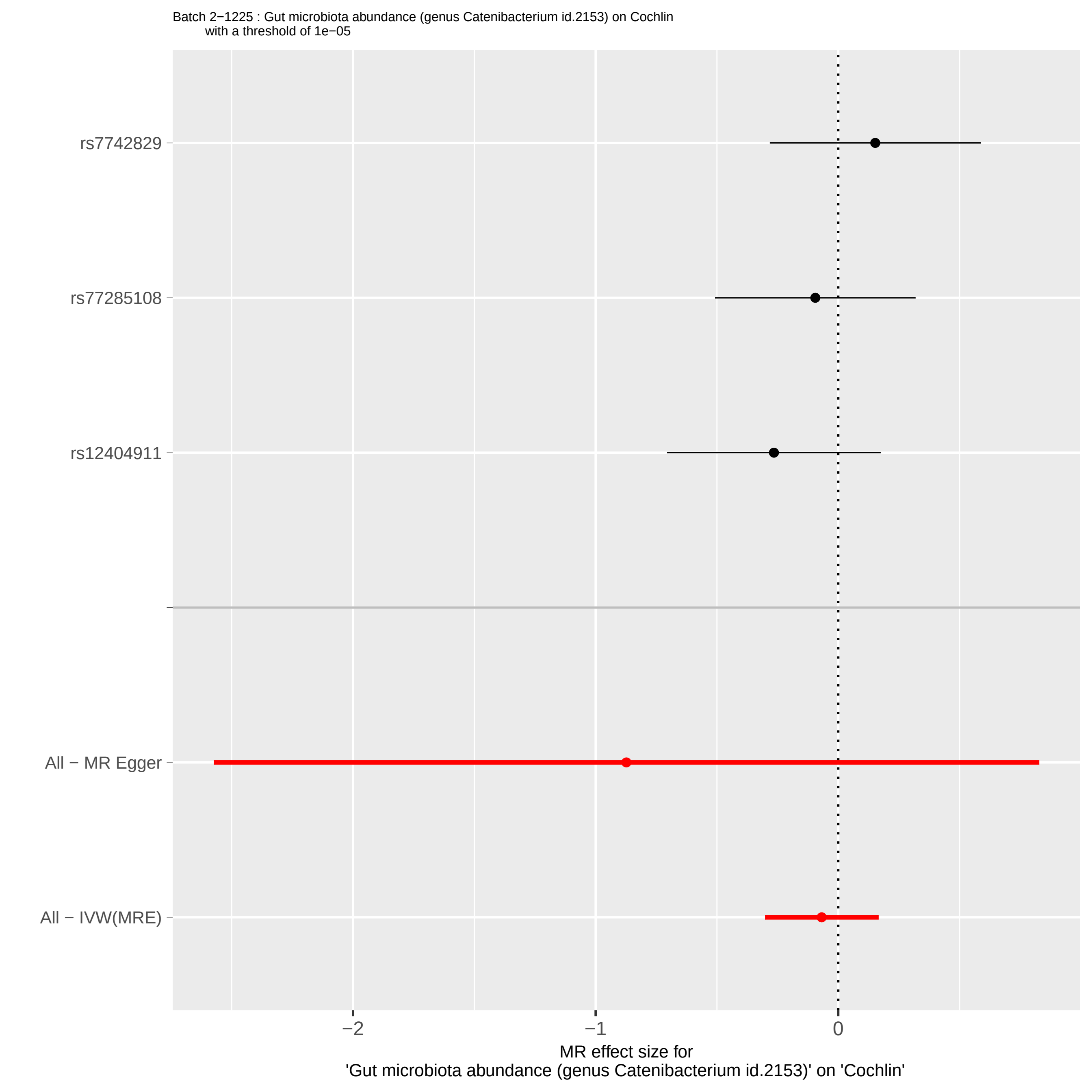

Batch 2−1225 : Gut microbiota abundance (genus Catenibacterium id.2153) on Cochlin
with a threshold of 1e−05
rs7742829
rs77285108
rs12404911
All − MR Egger
All − IVW(MRE)
−2
−1
0
MR effect size for
'Gut microbiota abundance (genus Catenibacterium id.2153)' on 'Cochlin'

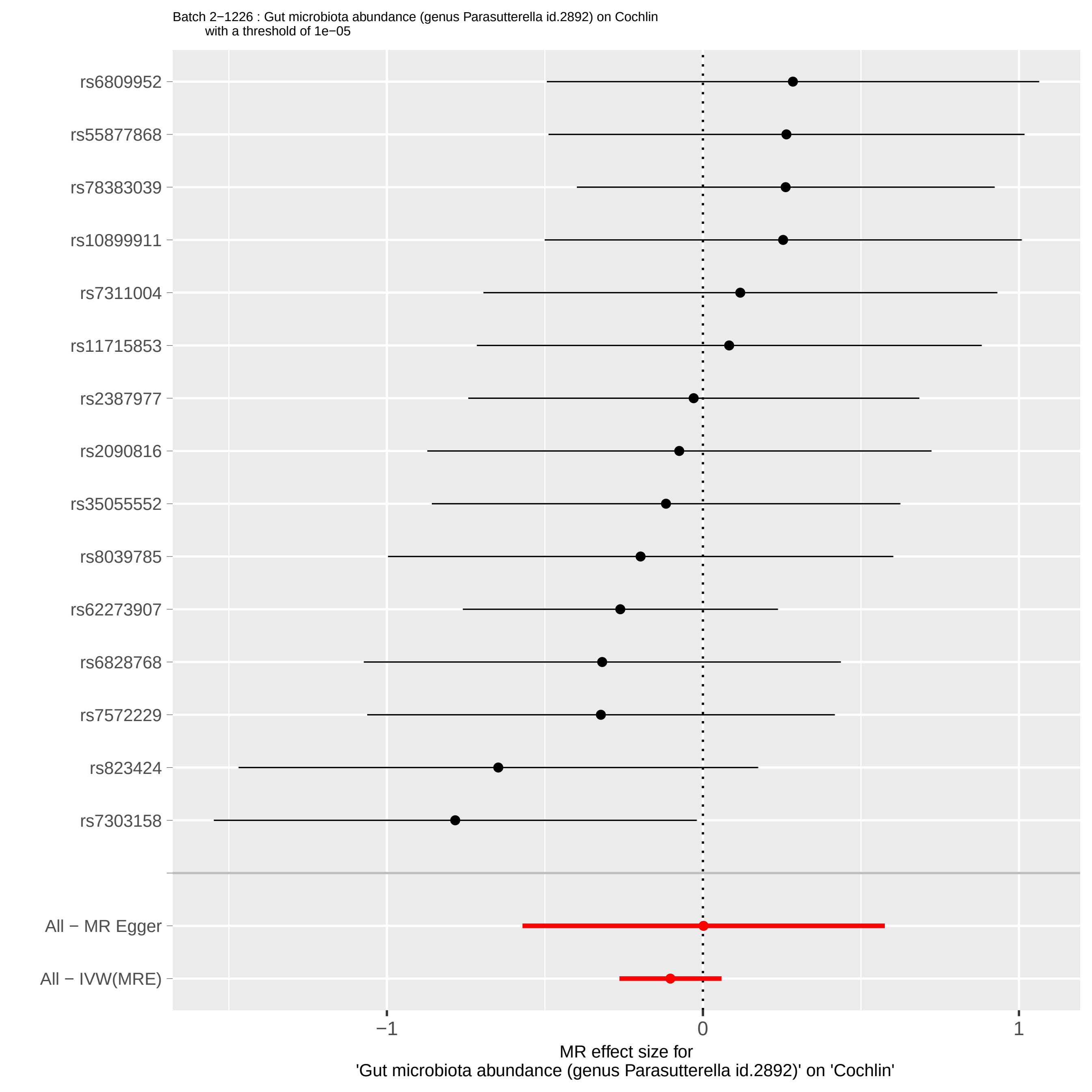

Batch 2−1226 : Gut microbiota abundance (genus Parasutterella id.2892) on Cochlin
with a threshold of 1e−05
rs6809952
rs55877868
rs78383039
rs10899911
rs7311004
rs11715853
rs2387977
rs2090816
rs35055552
rs8039785
rs62273907
rs6828768
rs7572229
rs823424
rs7303158
All − MR Egger
All − IVW(MRE)
−1
0
1
MR effect size for
'Gut microbiota abundance (genus Parasutterella id.2892)' on 'Cochlin'

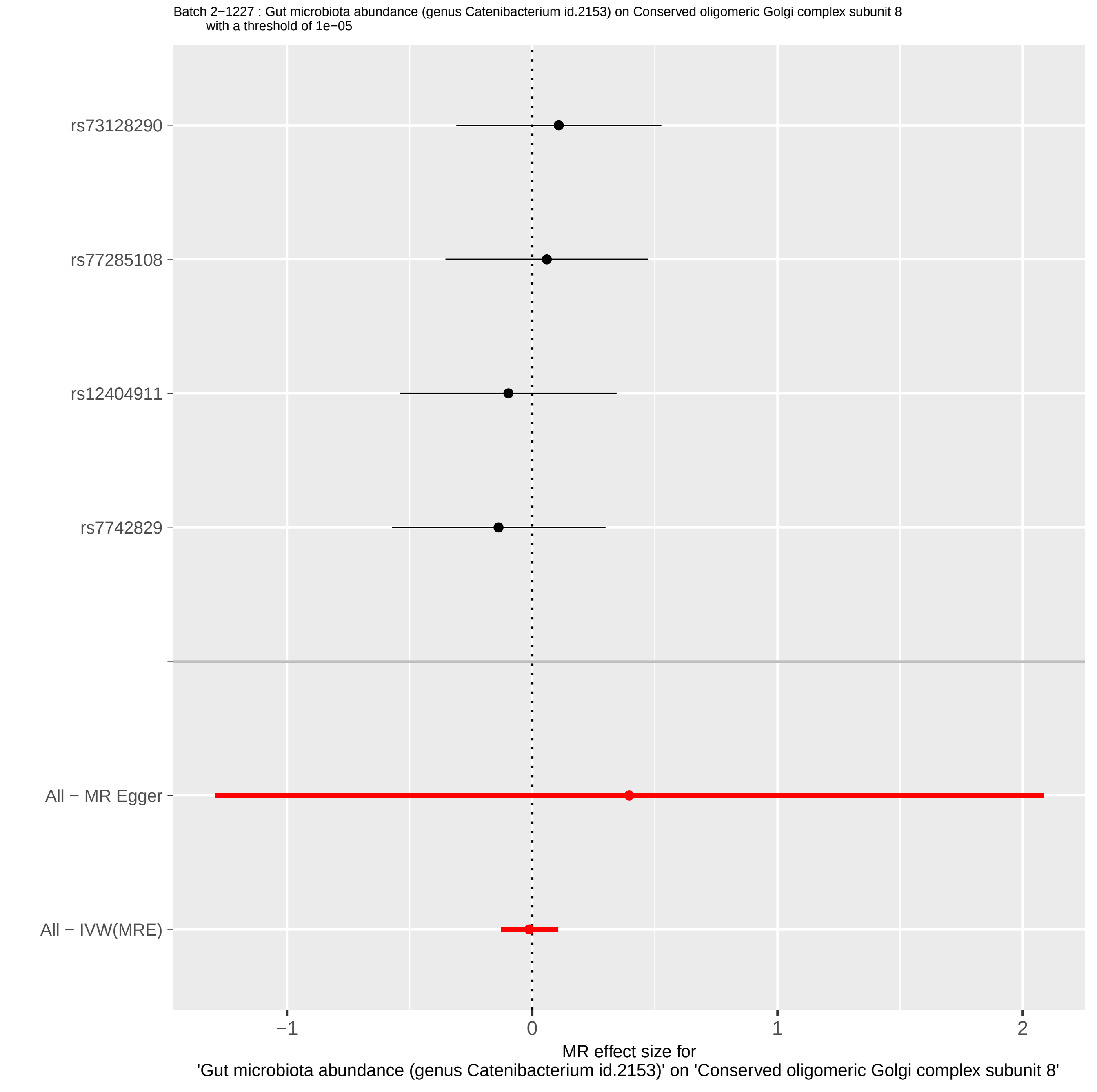

Batch 2−1227 : Gut microbiota abundance (genus Catenibacterium id.2153) on Conserved oligomeric Golgi complex subunit 8
with a threshold of 1e−05
rs73128290
rs77285108
rs12404911
rs7742829
All − MR Egger
All − IVW(MRE)
−1
0
1
2
MR effect size for
'Gut microbiota abundance (genus Catenibacterium id.2153)' on 'Conserved oligomeric Golgi complex subunit 8'

Batch 2−1228 : Gut microbiota abundance (genus Parasutterella id.2892) on Conserved oligomeric Golgi complex subunit 8
with a threshold of 1e−05

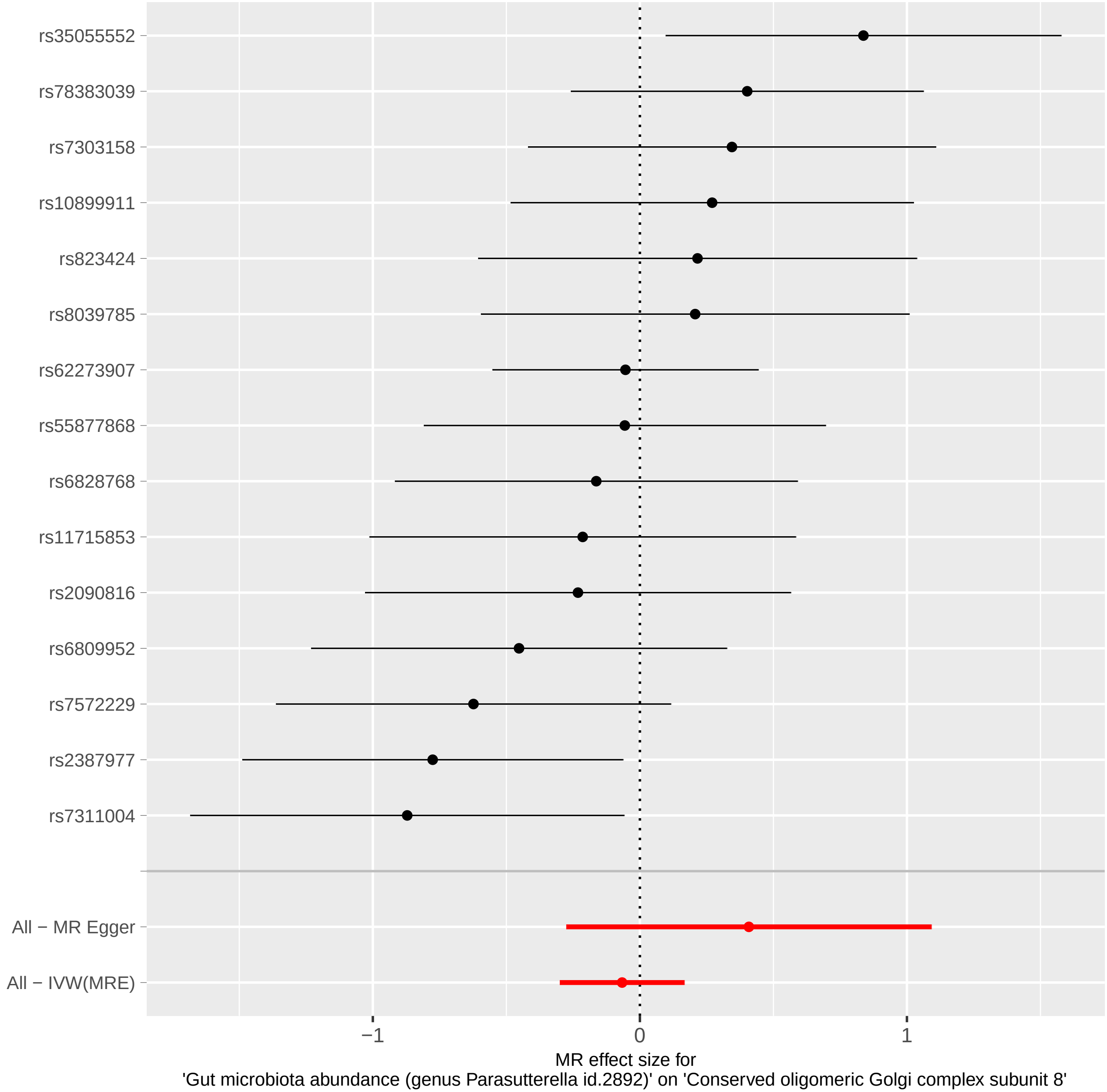

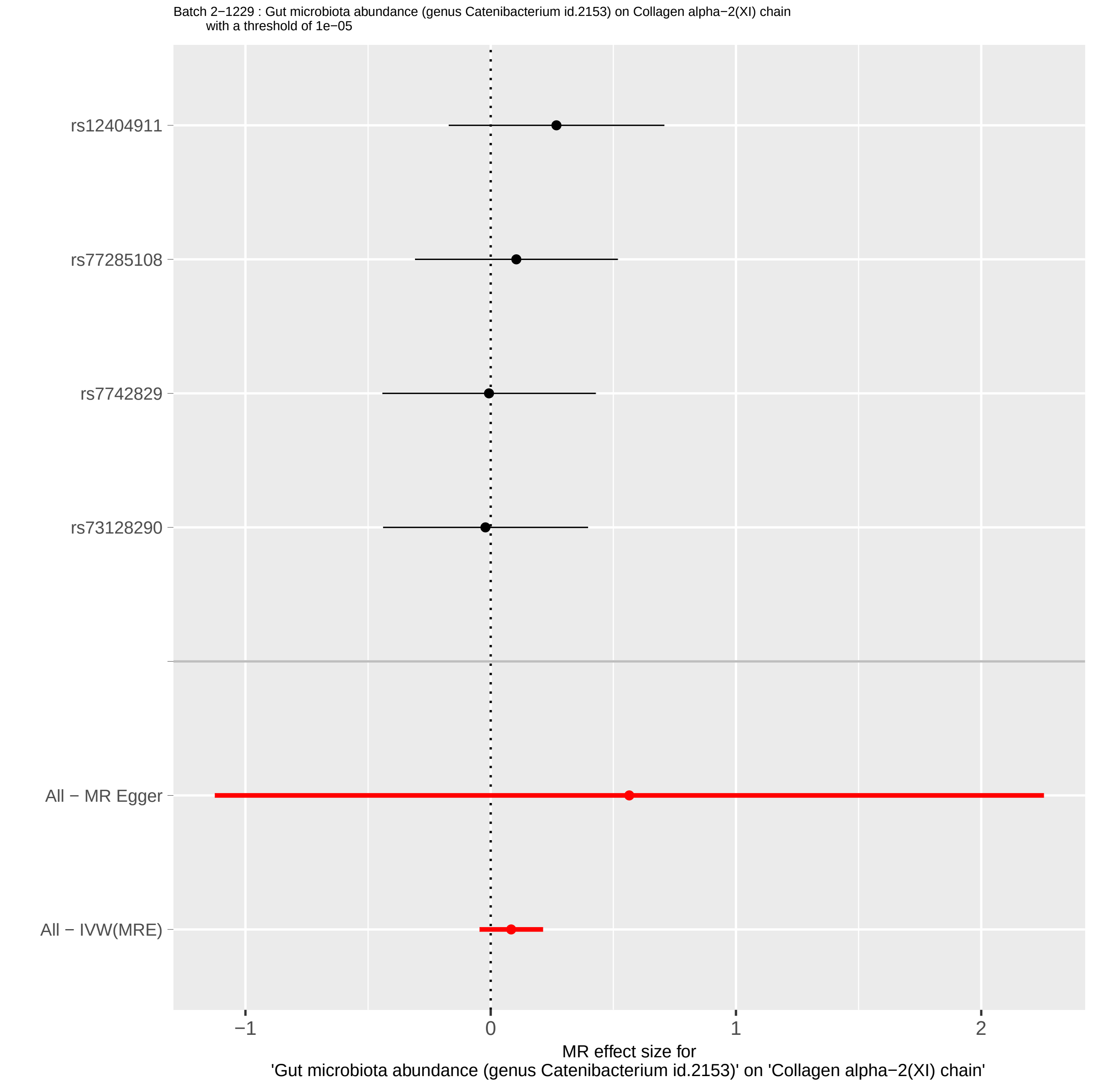
Batch 2−1229 : Gut microbiota abundance (genus Catenibacterium id.2153) on Collagen alpha−2(XI) chain
with a threshold of 1e−05
rs12404911
rs77285108
rs7742829
rs73128290
All − MR Egger
All − IVW(MRE)
−1
0
1
2
MR effect size for
'Gut microbiota abundance (genus Catenibacterium id.2153)' on 'Collagen alpha−2(XI) chain'

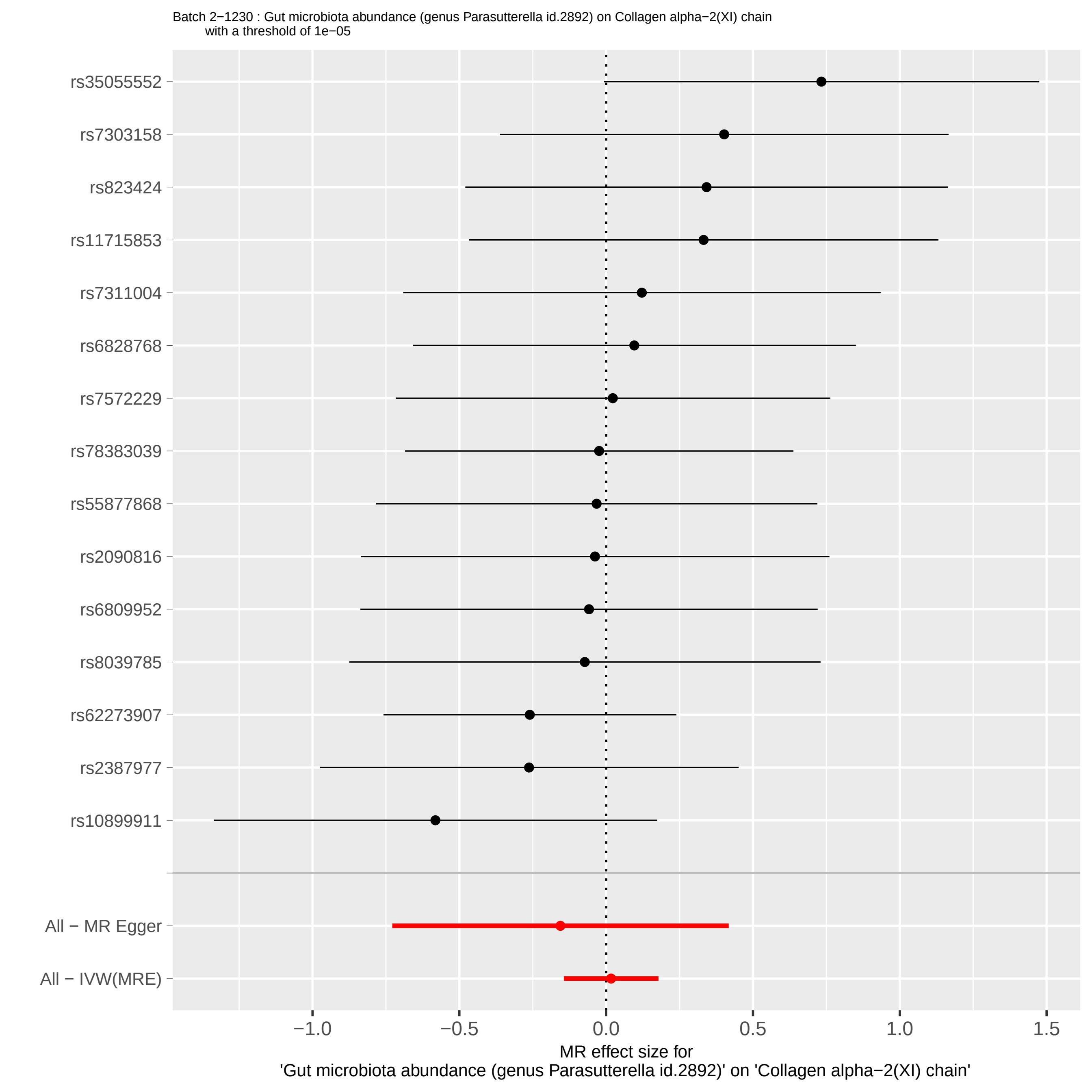

Batch 2−1230 : Gut microbiota abundance (genus Parasutterella id.2892) on Collagen alpha−2(XI) chain
with a threshold of 1e−05
rs35055552
rs7303158
rs823424
rs11715853
rs7311004
rs6828768
rs7572229
rs78383039
rs55877868
rs2090816
rs6809952
rs8039785
rs62273907
rs2387977
rs10899911
All − MR Egger
All − IVW(MRE)
−1.0
−0.5
0.0
0.5
1.0
1.5
MR effect size for
'Gut microbiota abundance (genus Parasutterella id.2892)' on 'Collagen alpha−2(XI) chain'

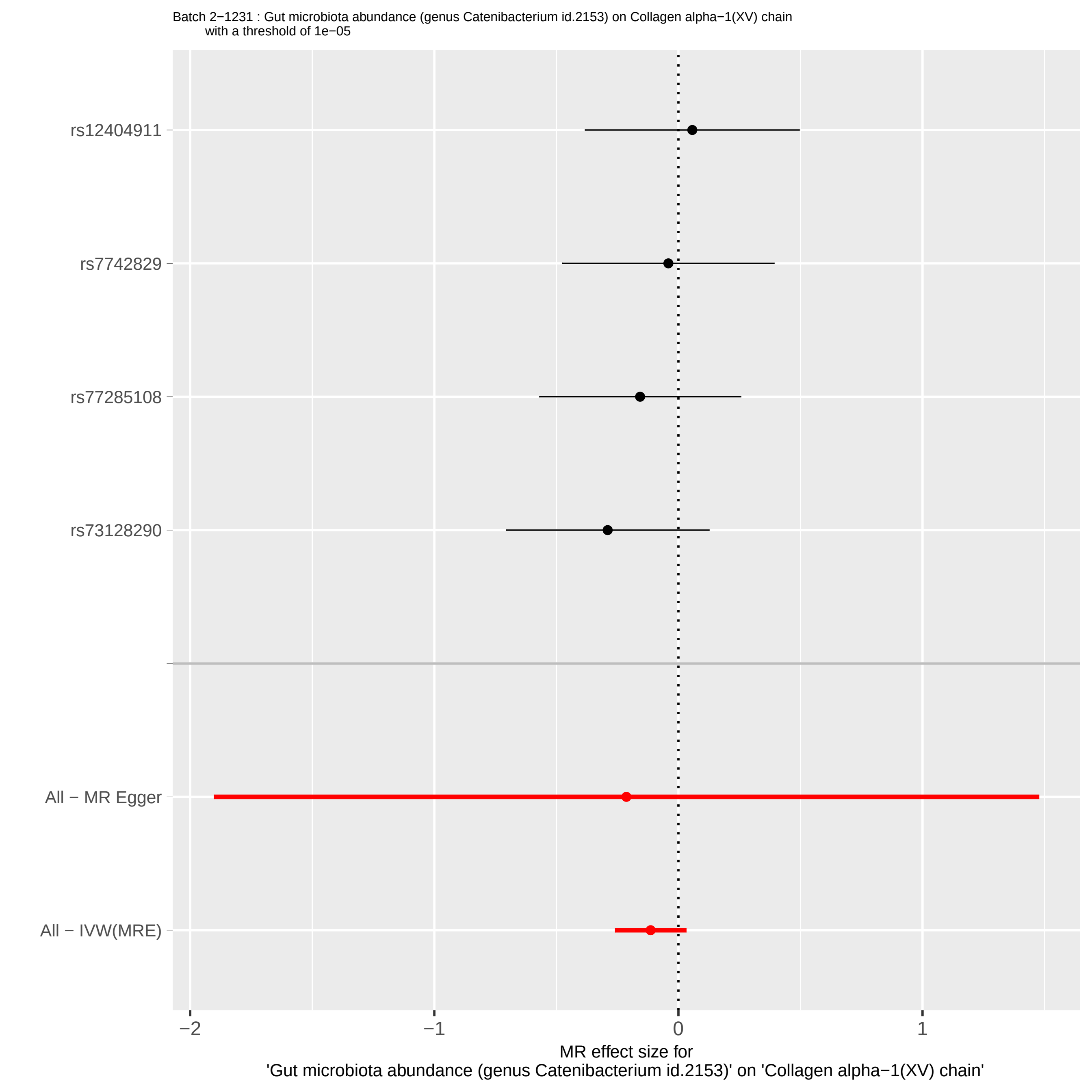

Batch 2−1231 : Gut microbiota abundance (genus Catenibacterium id.2153) on Collagen alpha−1(XV) chain
with a threshold of 1e−05
rs12404911
rs7742829
rs77285108
rs73128290
All − MR Egger
All − IVW(MRE)
−2
−1
0
1
MR effect size for
'Gut microbiota abundance (genus Catenibacterium id.2153)' on 'Collagen alpha−1(XV) chain'

Batch 2−1232 : Gut microbiota abundance (genus Parasutterella id.2892) on Collagen alpha−1(XV) chain
with a threshold of 1e−05

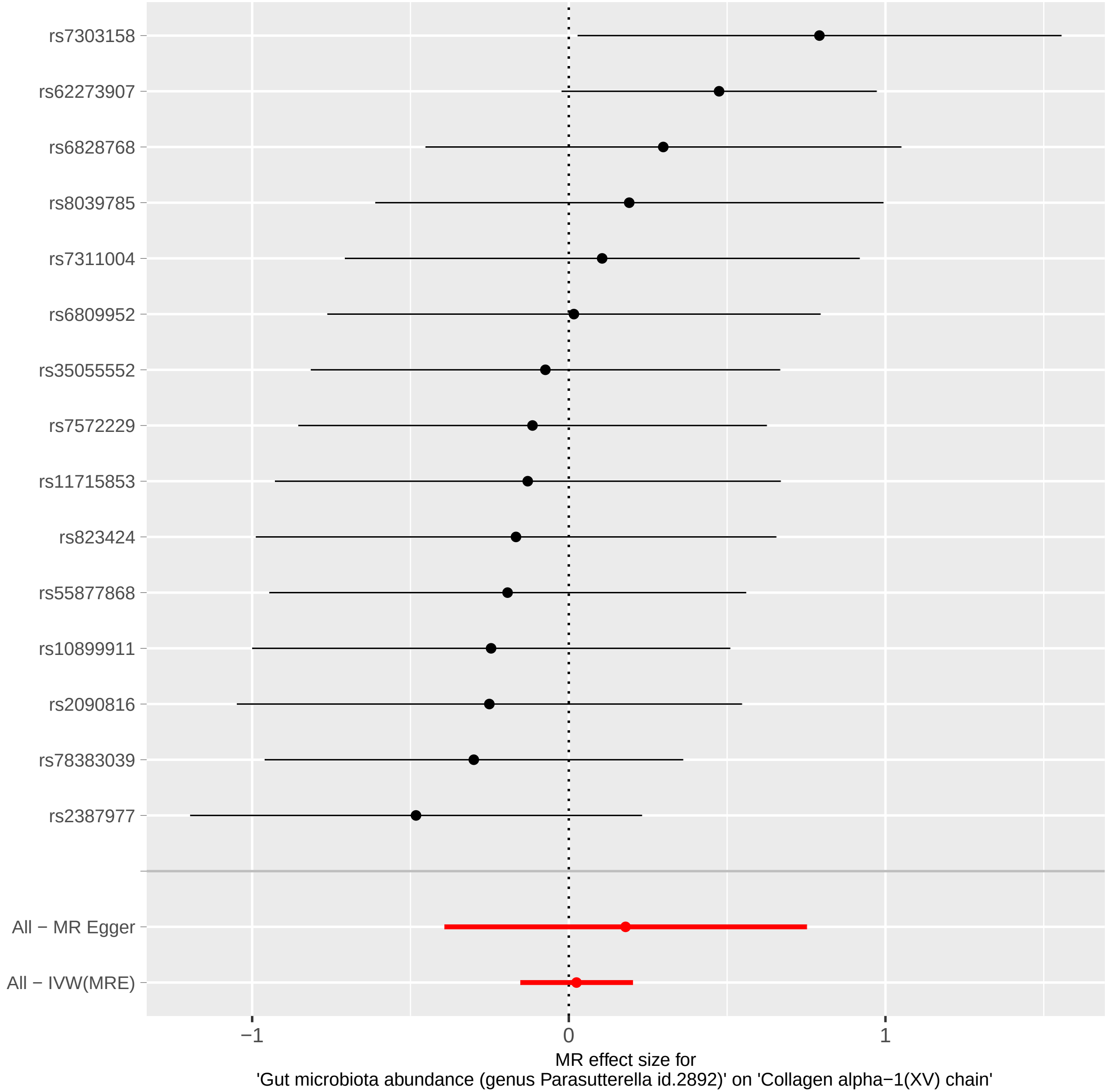

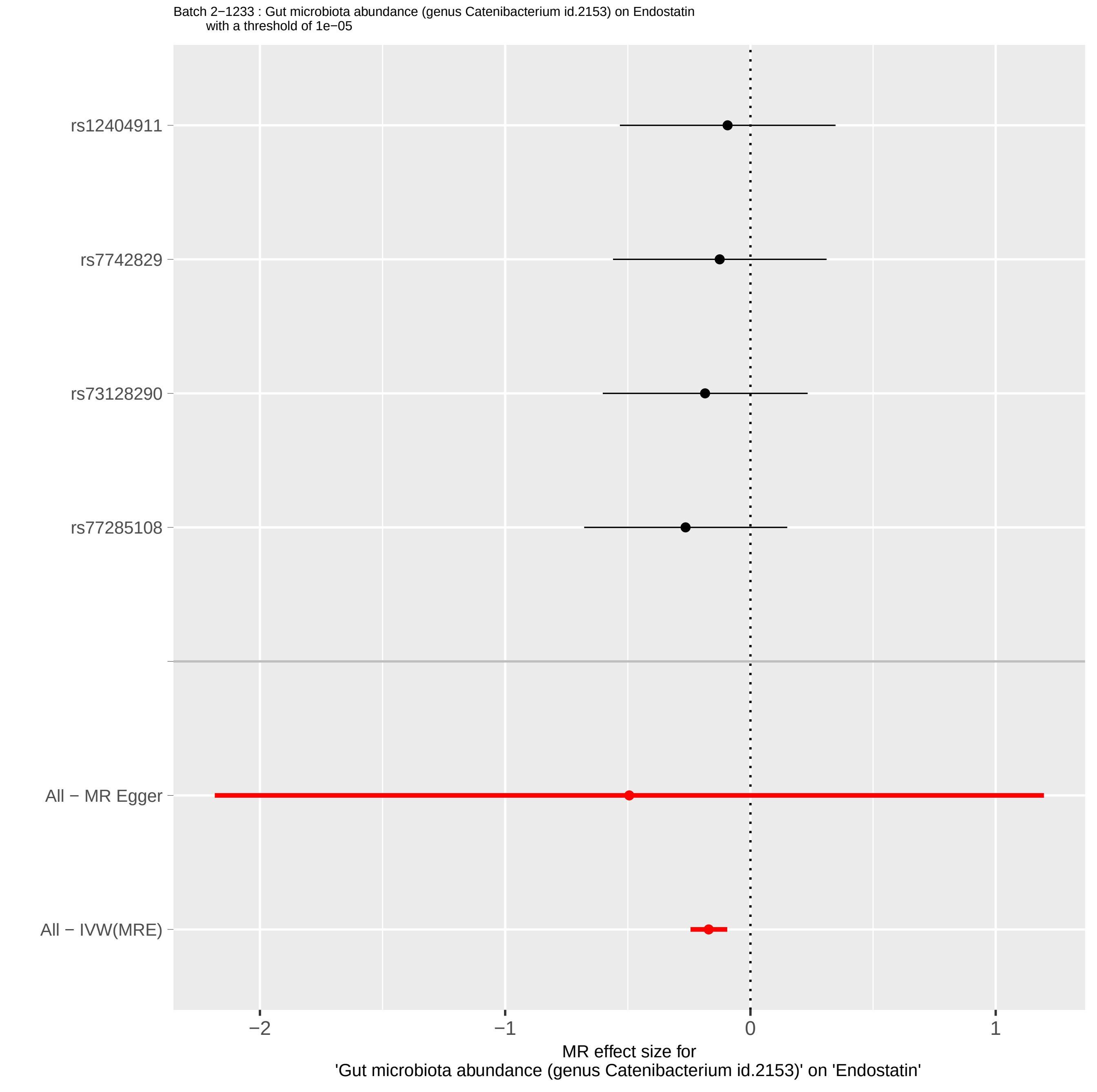

Batch 2−1233 : Gut microbiota abundance (genus Catenibacterium id.2153) on Endostatin
with a threshold of 1e−05
rs12404911
rs7742829
rs73128290
rs77285108
All − MR Egger
All − IVW(MRE)
−2
−1
0
1
MR effect size for
'Gut microbiota abundance (genus Catenibacterium id.2153)' on 'Endostatin'

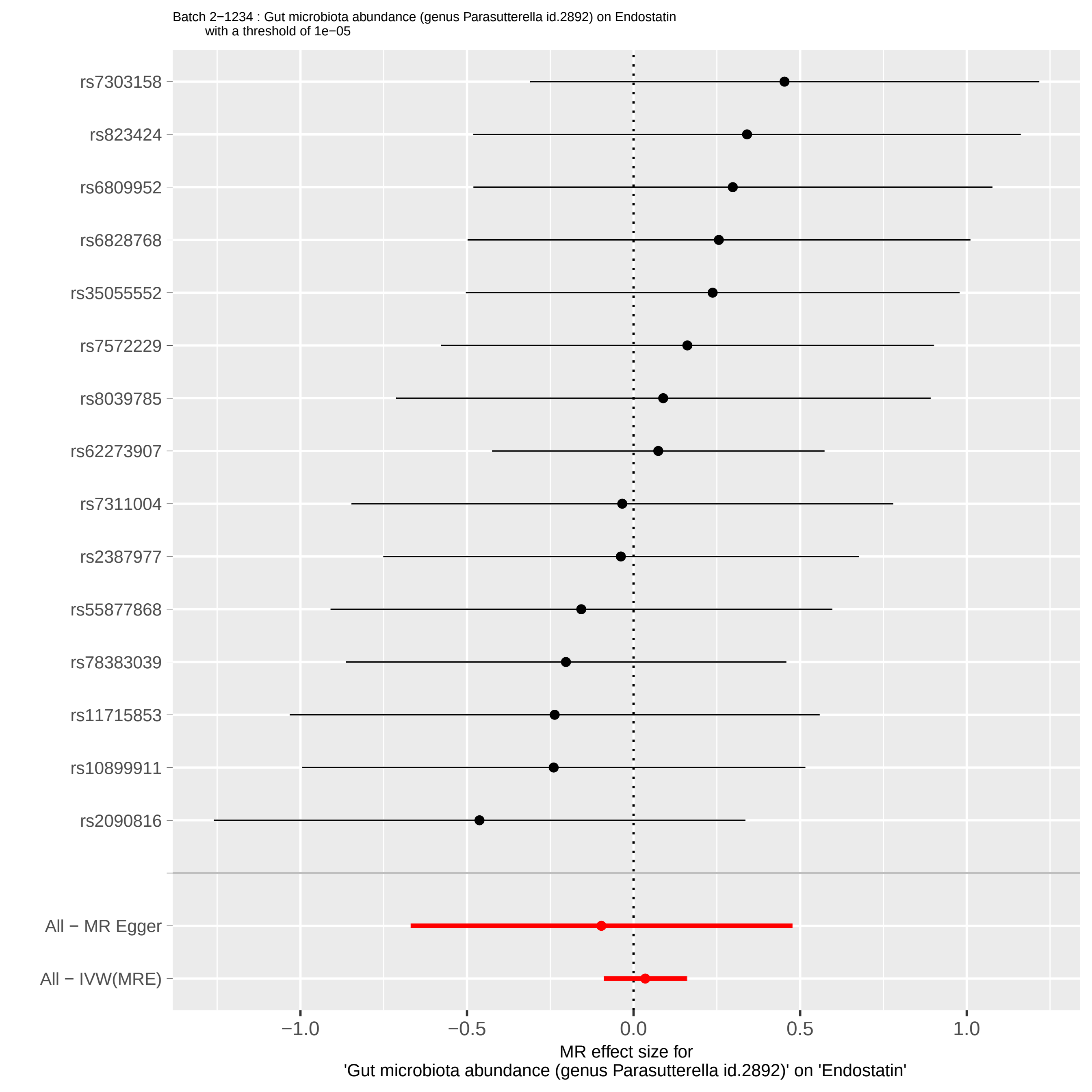
Batch 2−1234 : Gut microbiota abundance (genus Parasutterella id.2892) on Endostatin
with a threshold of 1e−05
rs7303158
rs823424
rs6809952
rs6828768
rs35055552
rs7572229
rs8039785
rs62273907
rs7311004
rs2387977
rs55877868
rs78383039
rs11715853
rs10899911
rs2090816
All − MR Egger
All − IVW(MRE)
−1.0
−0.5
0.0
0.5
1.0
MR effect size for
'Gut microbiota abundance (genus Parasutterella id.2892)' on 'Endostatin'

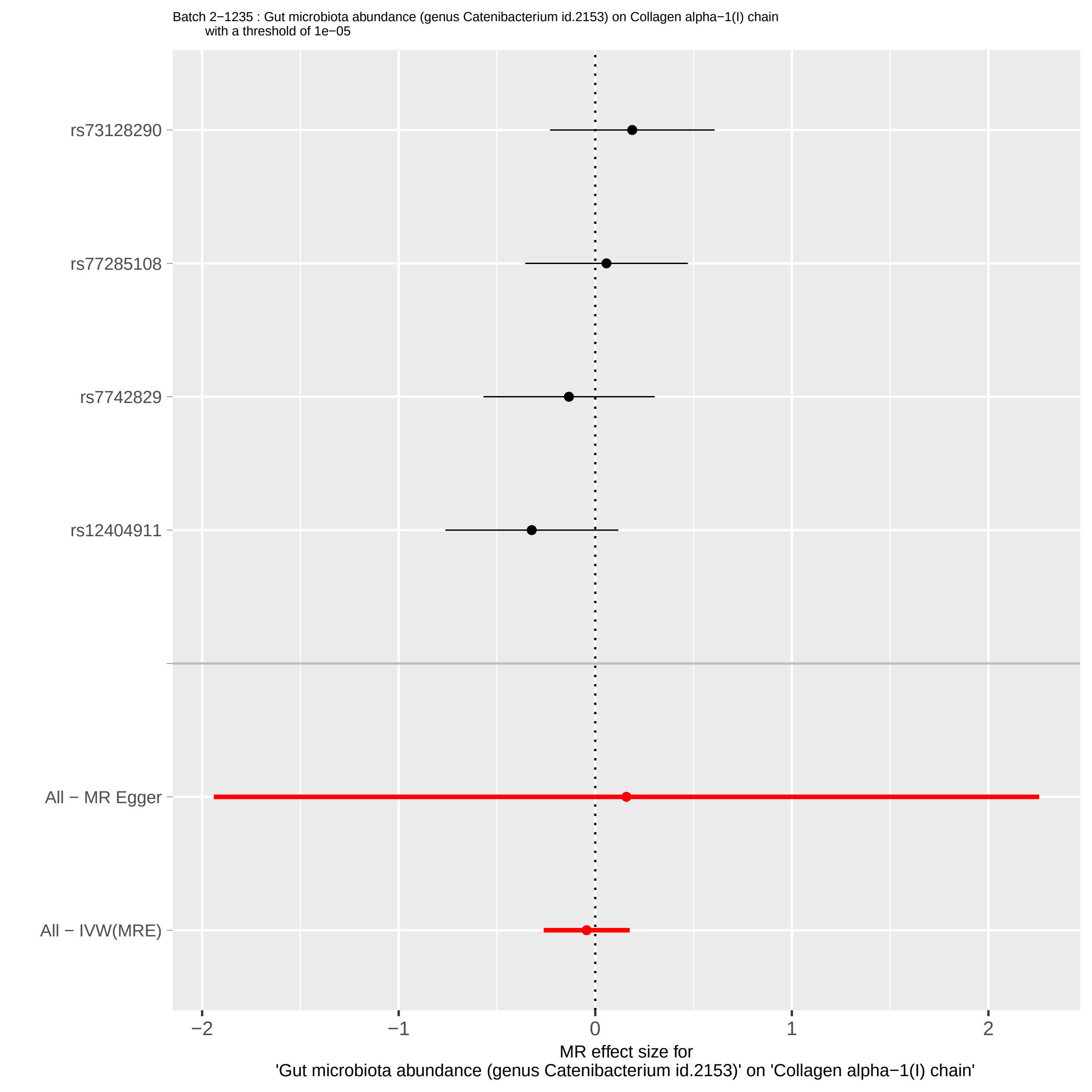

Batch 2−1235 : Gut microbiota abundance (genus Catenibacterium id.2153) on Collagen alpha−1(I) chain
with a threshold of 1e−05
rs73128290
rs77285108
rs7742829
rs12404911
All − MR Egger
All − IVW(MRE)
−2
−1
0
1
2
MR effect size for
'Gut microbiota abundance (genus Catenibacterium id.2153)' on 'Collagen alpha−1(I) chain'

Batch 2−1236 : Gut microbiota abundance (genus Parasutterella id.2892) on Collagen alpha−1(I) chain
with a threshold of 1e−05

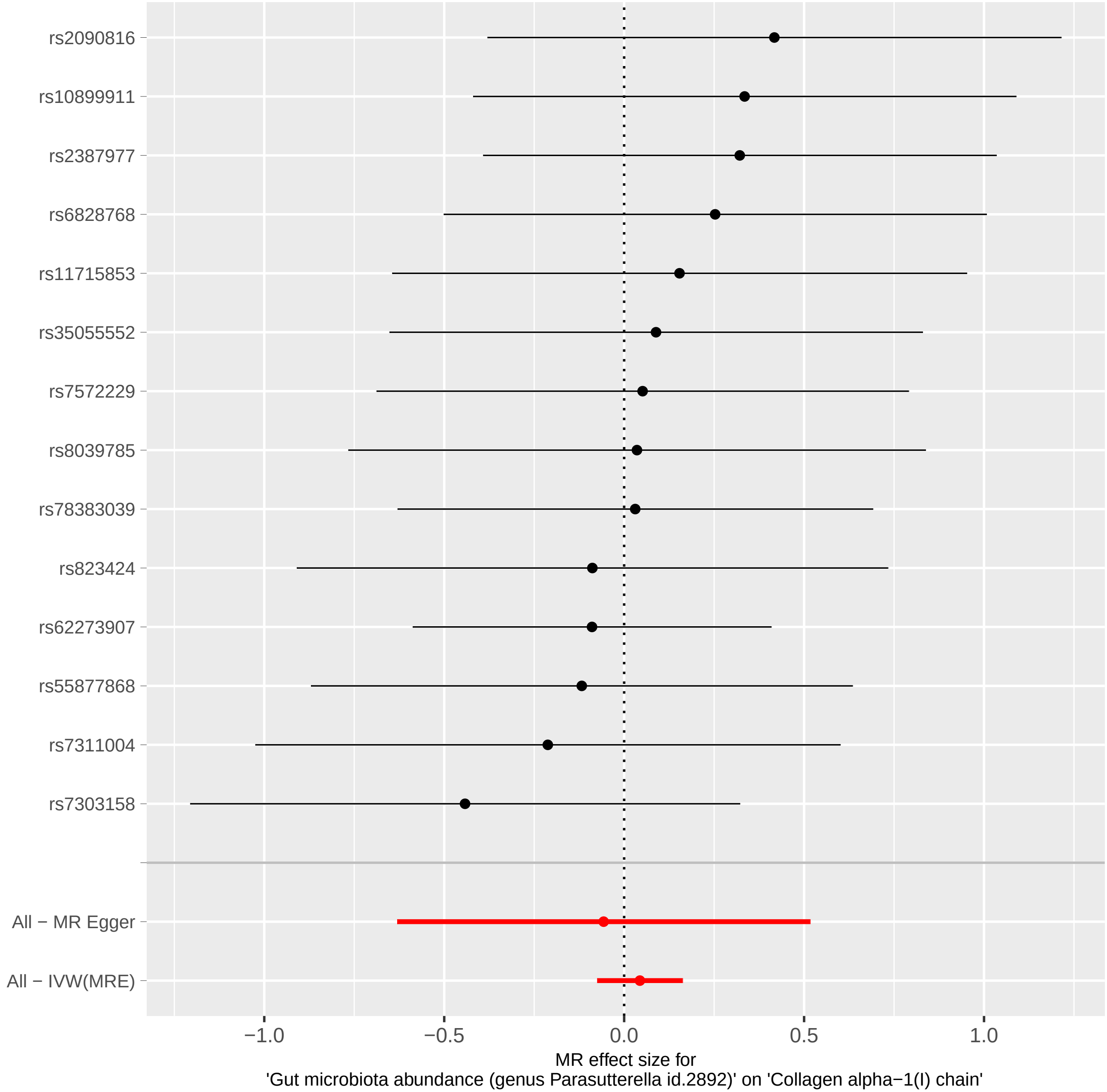

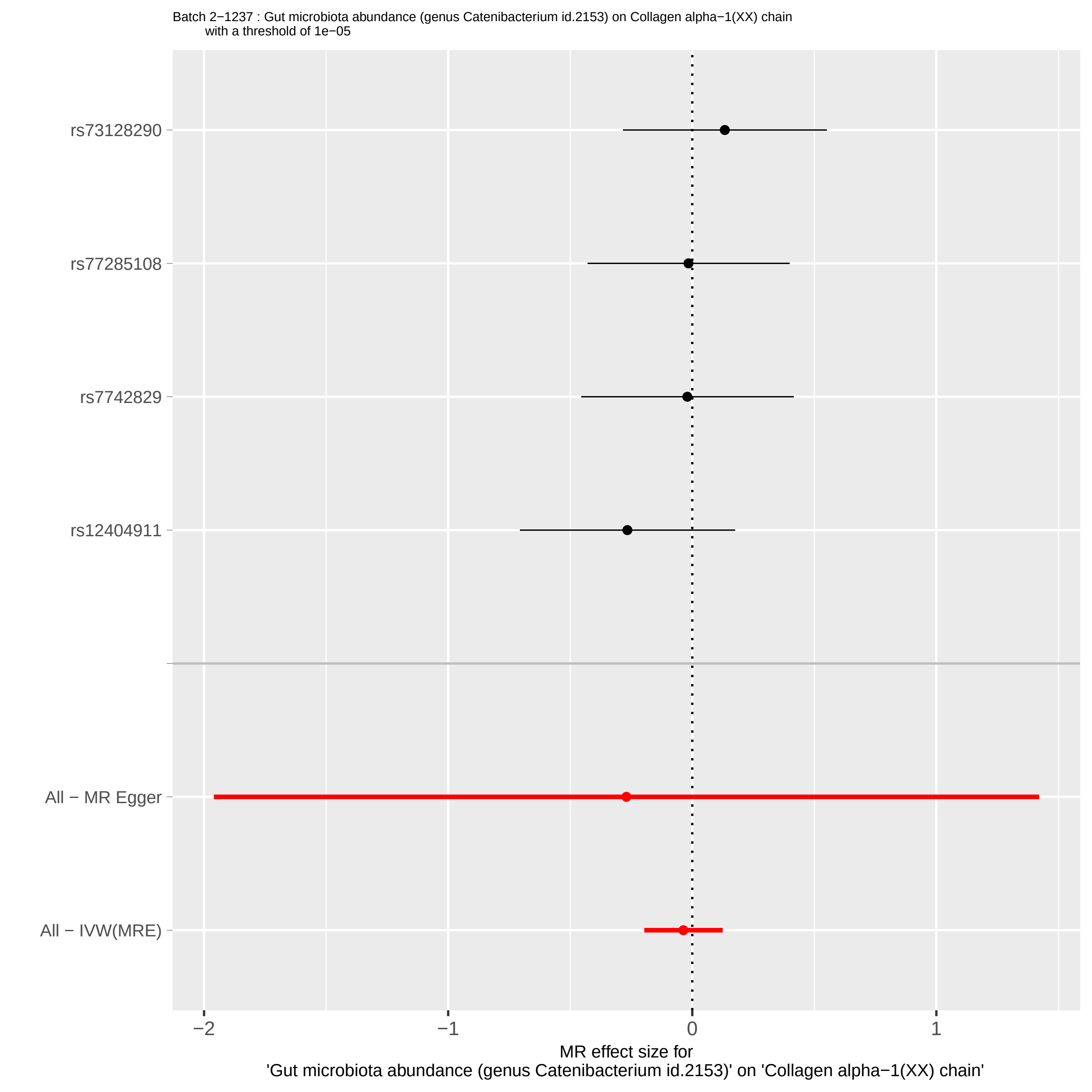
Batch 2−1237 : Gut microbiota abundance (genus Catenibacterium id.2153) on Collagen alpha−1(XX) chain
with a threshold of 1e−05
rs73128290
rs77285108
rs7742829
rs12404911
All − MR Egger
All − IVW(MRE)
−2
−1
0
1
MR effect size for
'Gut microbiota abundance (genus Catenibacterium id.2153)' on 'Collagen alpha−1(XX) chain'

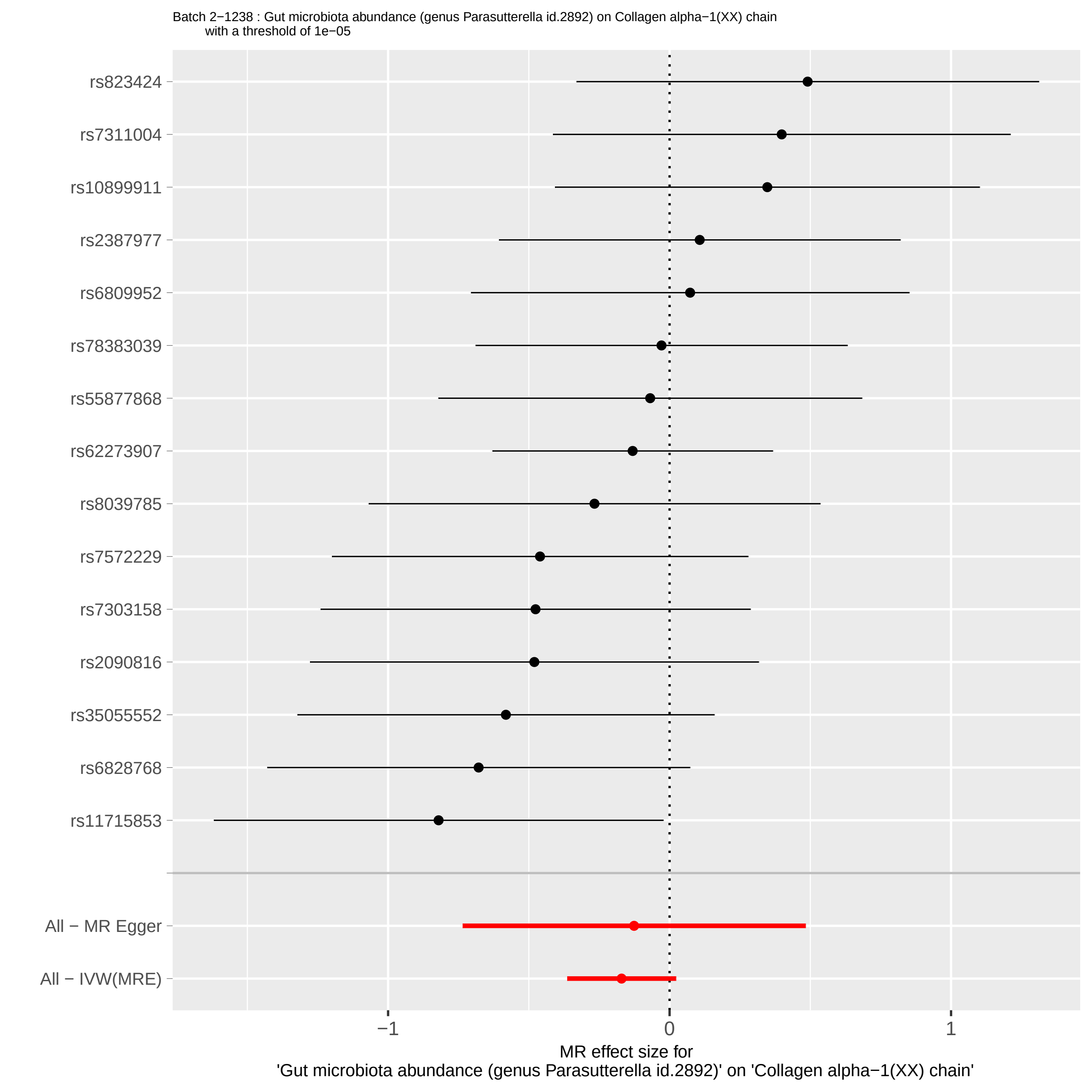
Batch 2−1238 : Gut microbiota abundance (genus Parasutterella id.2892) on Collagen alpha−1(XX) chain
with a threshold of 1e−05
rs823424
rs7311004
rs10899911
rs2387977
rs6809952
rs78383039
rs55877868
rs62273907
rs8039785
rs7572229
rs7303158
rs2090816
rs35055552
rs6828768
rs11715853
All − MR Egger
All − IVW(MRE)
−1
0
1
MR effect size for
'Gut microbiota abundance (genus Parasutterella id.2892)' on 'Collagen alpha−1(XX) chain'

Batch 2−1239 : Gut microbiota abundance (genus Catenibacterium id.2153) on Collagen alpha−1(XXVI) chain
with a threshold of 1e−05

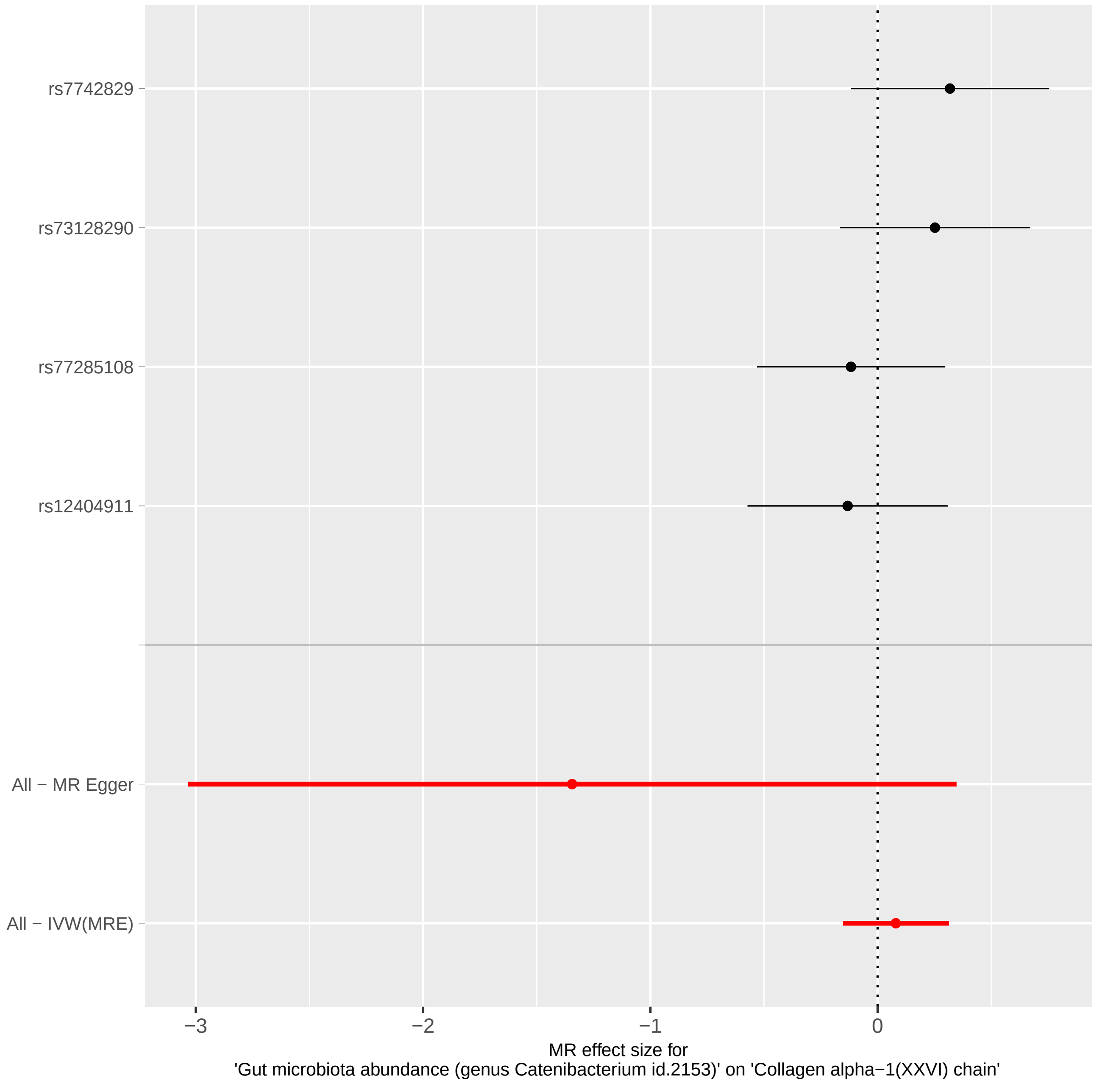

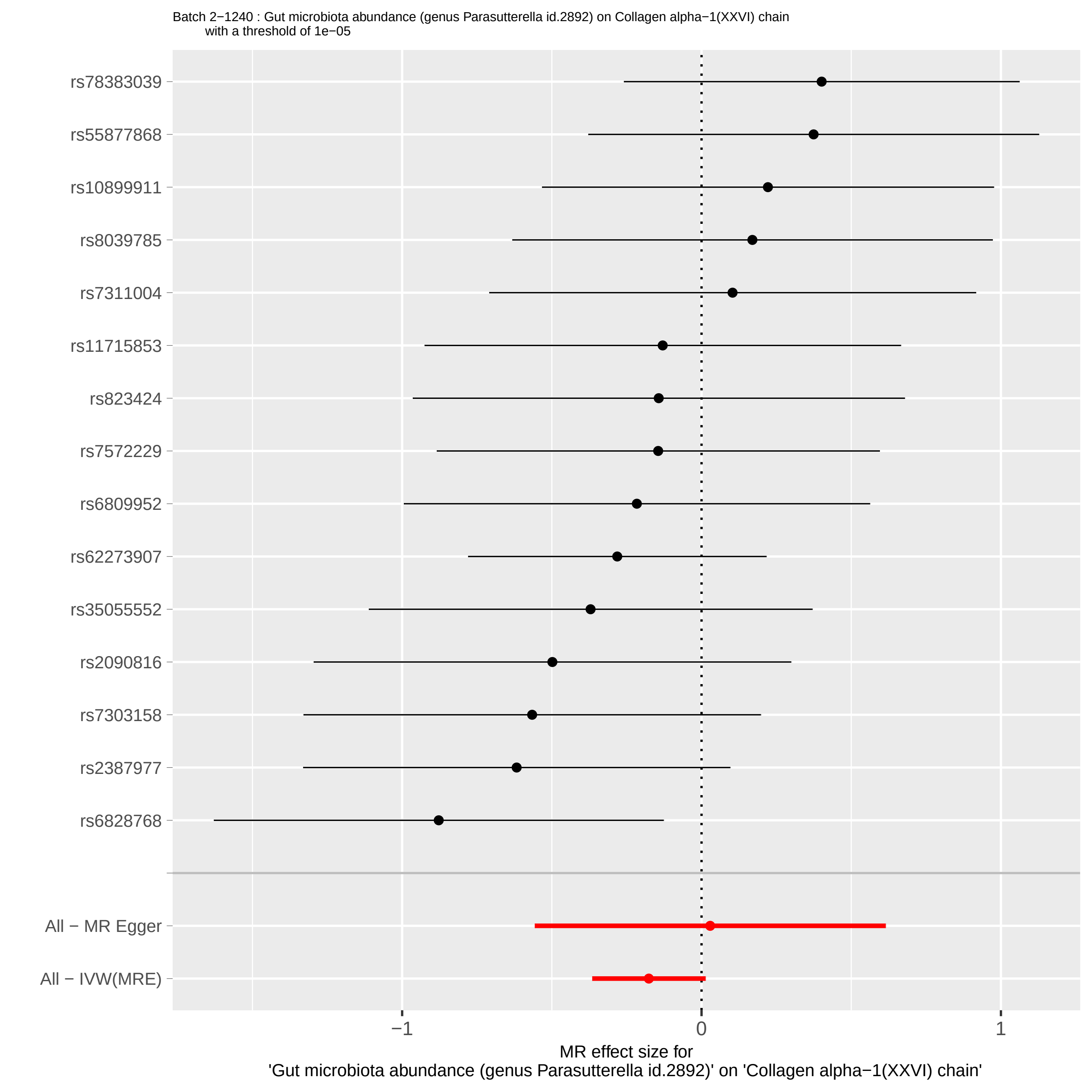
Batch 2−1240 : Gut microbiota abundance (genus Parasutterella id.2892) on Collagen alpha−1(XXVI) chain
with a threshold of 1e−05
rs78383039
rs55877868
rs10899911
rs8039785
rs7311004
rs11715853
rs823424
rs7572229
rs6809952
rs62273907
rs35055552
rs2090816
rs7303158
rs2387977
rs6828768
All − MR Egger
All − IVW(MRE)
−1
0
1
MR effect size for
'Gut microbiota abundance (genus Parasutterella id.2892)' on 'Collagen alpha−1(XXVI) chain'

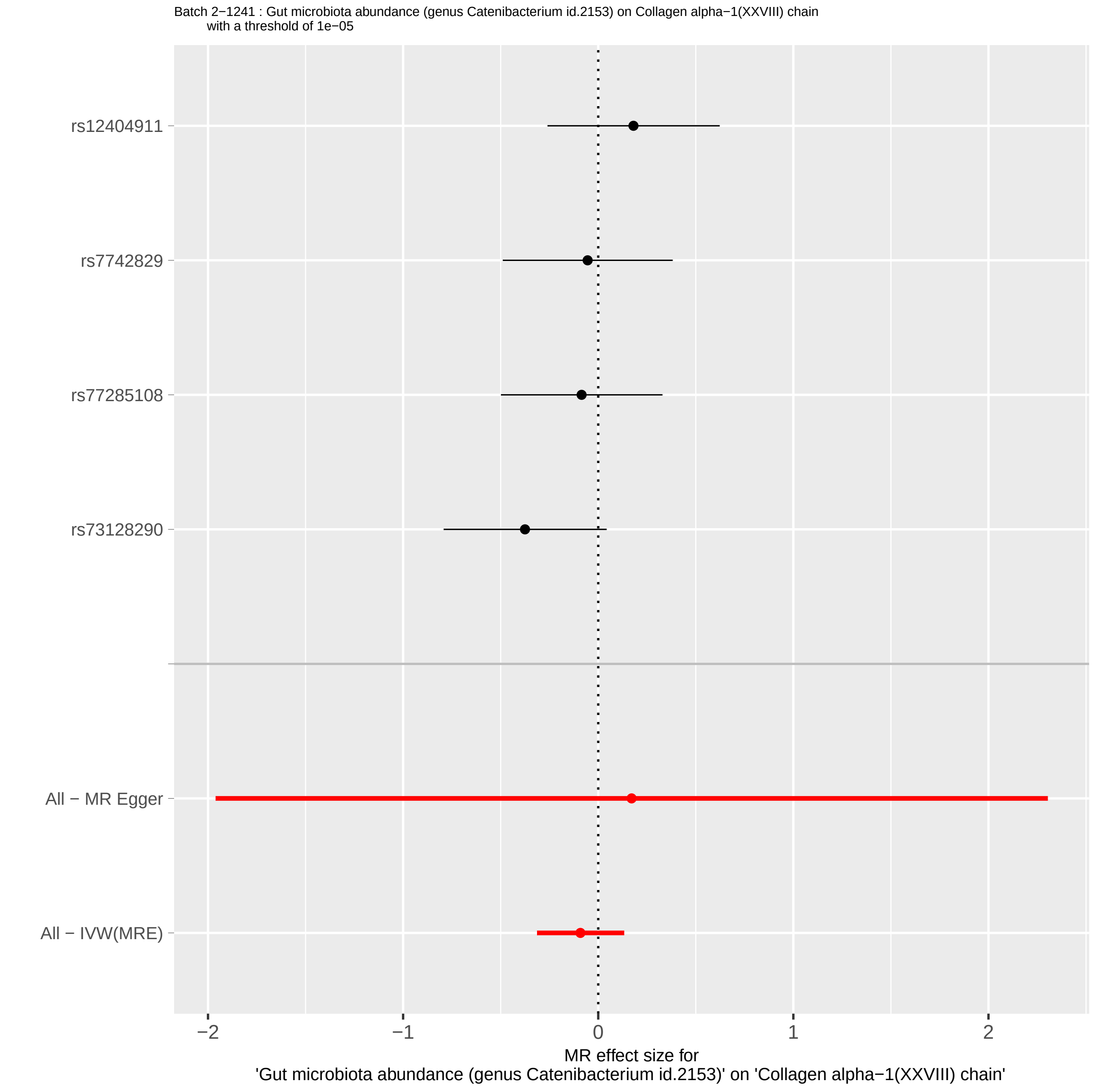

Batch 2−1241 : Gut microbiota abundance (genus Catenibacterium id.2153) on Collagen alpha−1(XXVIII) chain
with a threshold of 1e−05
rs12404911
rs7742829
rs77285108
rs73128290
All − MR Egger
All − IVW(MRE)
−2
−1
0
1
2
MR effect size for
'Gut microbiota abundance (genus Catenibacterium id.2153)' on 'Collagen alpha−1(XXVIII) chain'

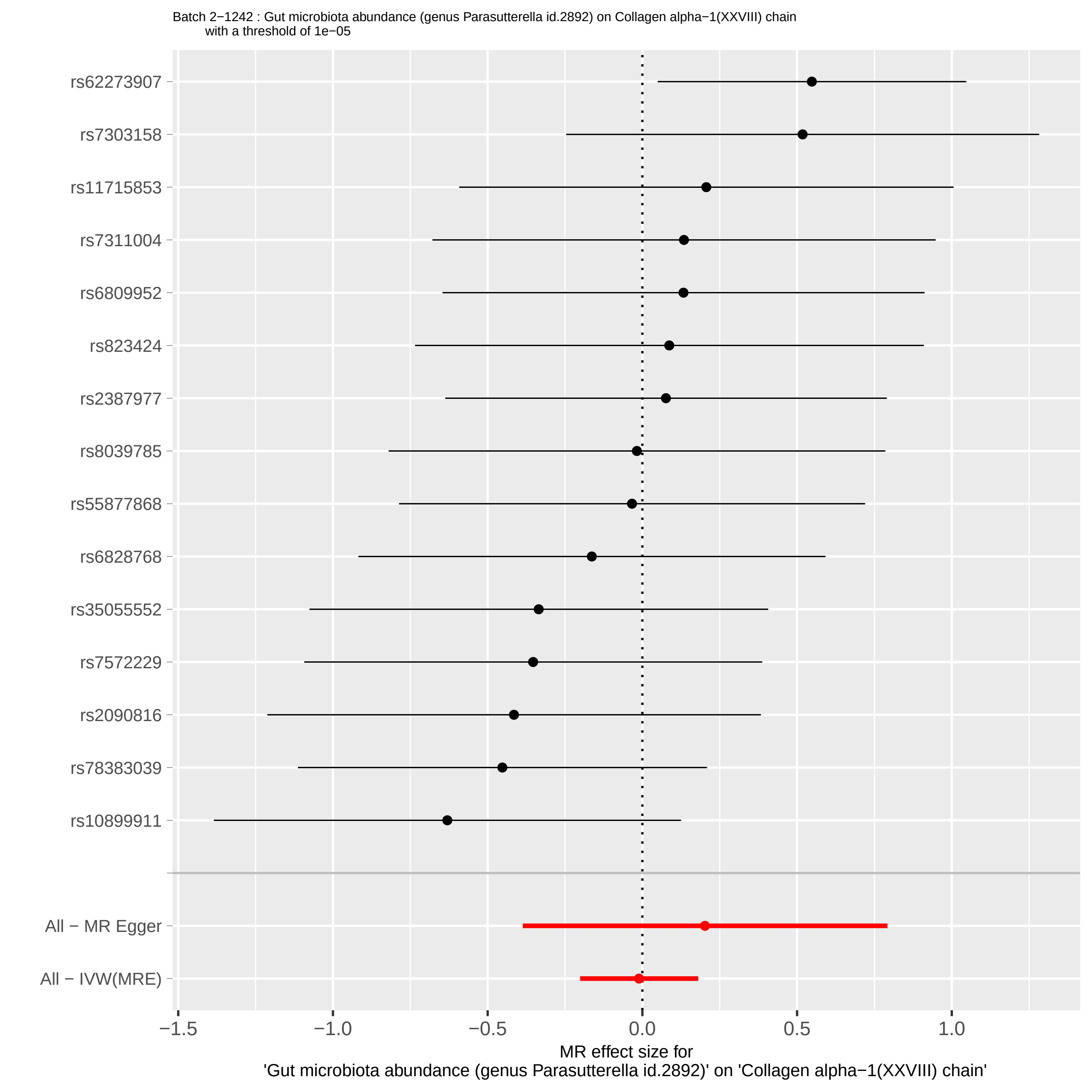
Batch 2−1242 : Gut microbiota abundance (genus Parasutterella id.2892) on Collagen alpha−1(XXVIII) chain
with a threshold of 1e−05
rs62273907
rs7303158
rs11715853
rs7311004
rs6809952
rs823424
rs2387977
rs8039785
rs55877868
rs6828768
rs35055552
rs7572229
rs2090816
rs78383039
rs10899911
All − MR Egger
All − IVW(MRE)
−1.5
−1.0
−0.5
0.0
0.5
1.0
MR effect size for
'Gut microbiota abundance (genus Parasutterella id.2892)' on 'Collagen alpha−1(XXVIII) chain'

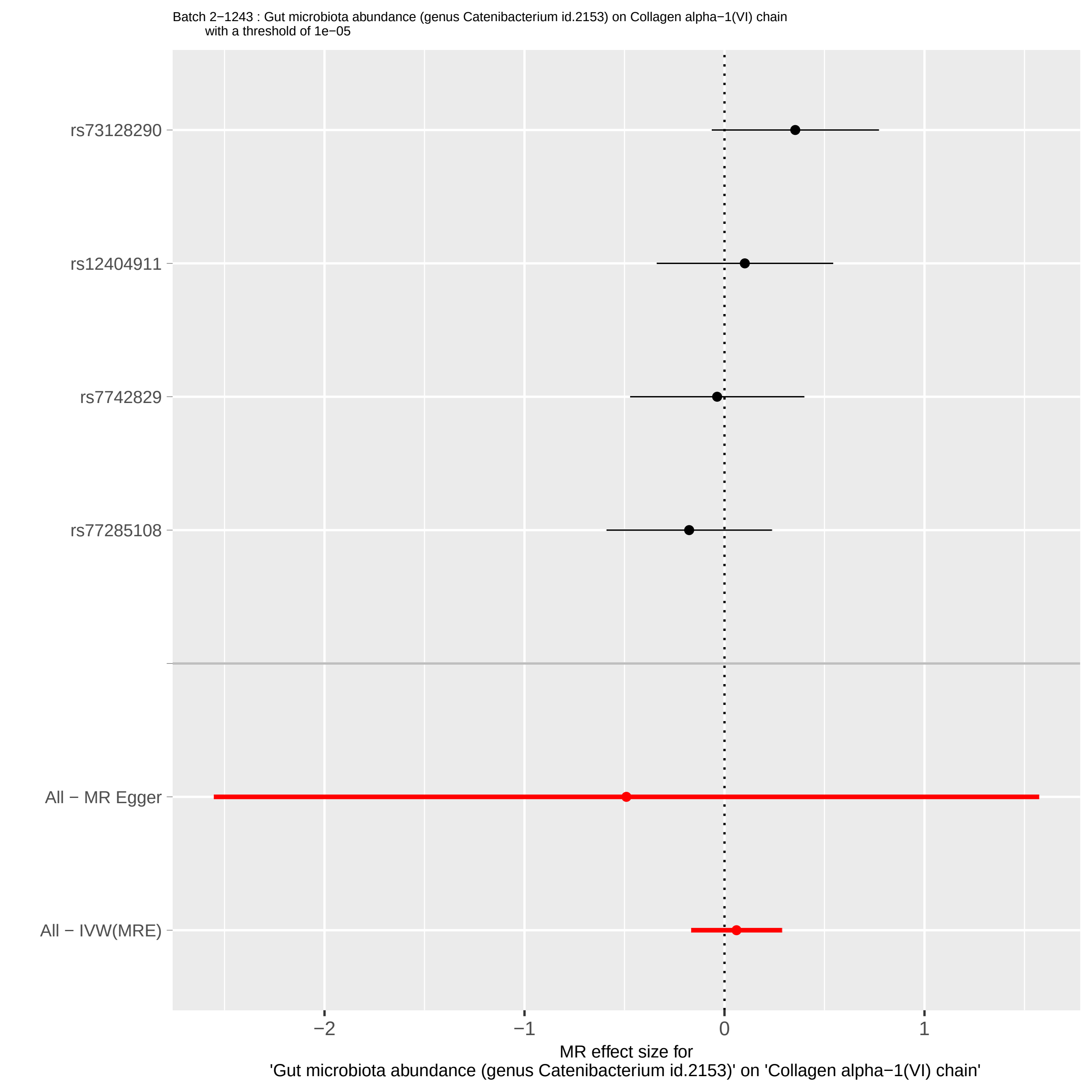
Batch 2−1243 : Gut microbiota abundance (genus Catenibacterium id.2153) on Collagen alpha−1(VI) chain
with a threshold of 1e−05
rs73128290
rs12404911
rs7742829
rs77285108
All − MR Egger
All − IVW(MRE)
−2
−1
0
1
MR effect size for
'Gut microbiota abundance (genus Catenibacterium id.2153)' on 'Collagen alpha−1(VI) chain'

Batch 2−1244 : Gut microbiota abundance (genus Parasutterella id.2892) on Collagen alpha−1(VI) chain
with a threshold of 1e−05

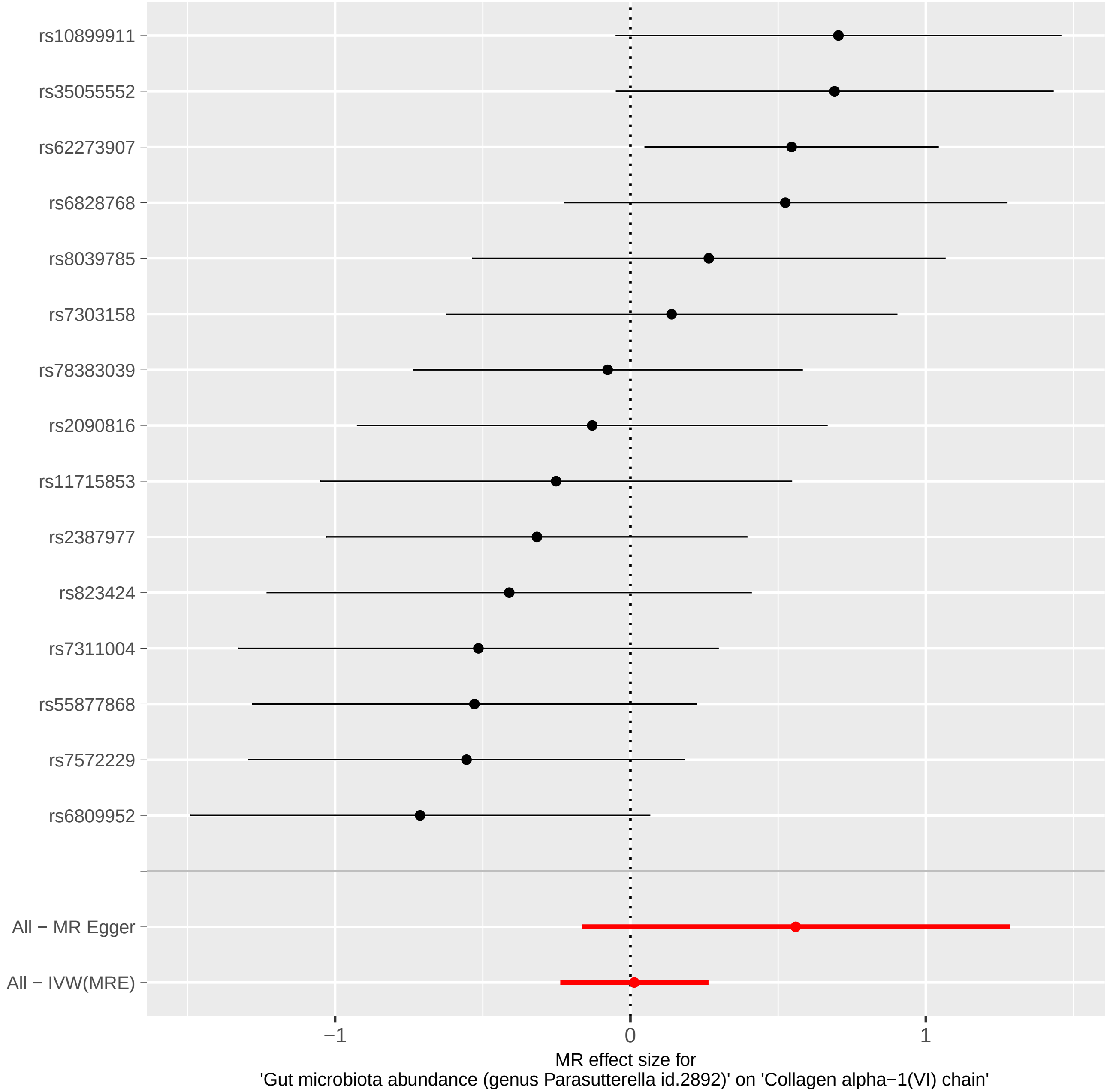

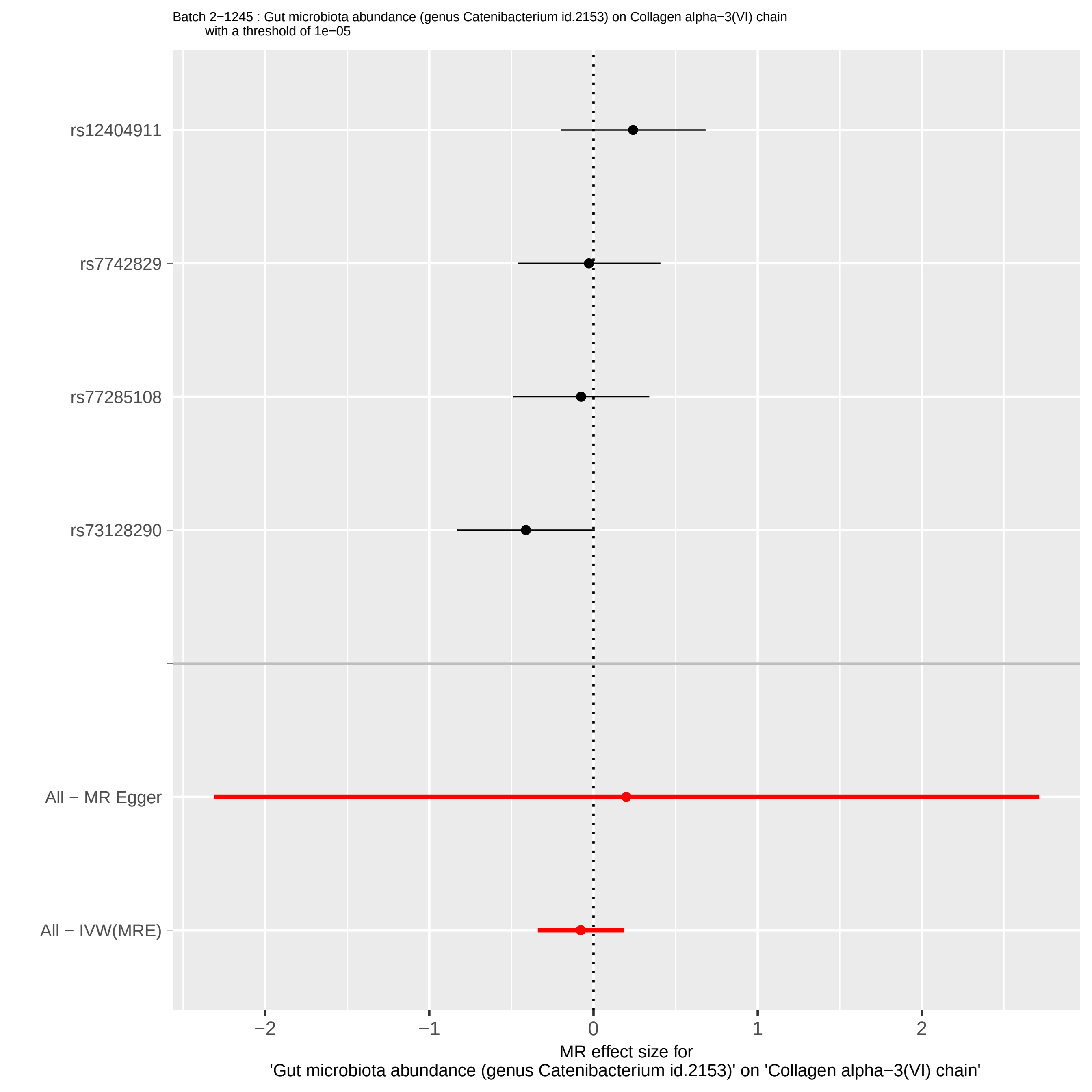
Batch 2−1245 : Gut microbiota abundance (genus Catenibacterium id.2153) on Collagen alpha−3(VI) chain
with a threshold of 1e−05
rs12404911
rs7742829
rs77285108
rs73128290
All − MR Egger
All − IVW(MRE)
−2
−1
0
1
2
MR effect size for
'Gut microbiota abundance (genus Catenibacterium id.2153)' on 'Collagen alpha−3(VI) chain'

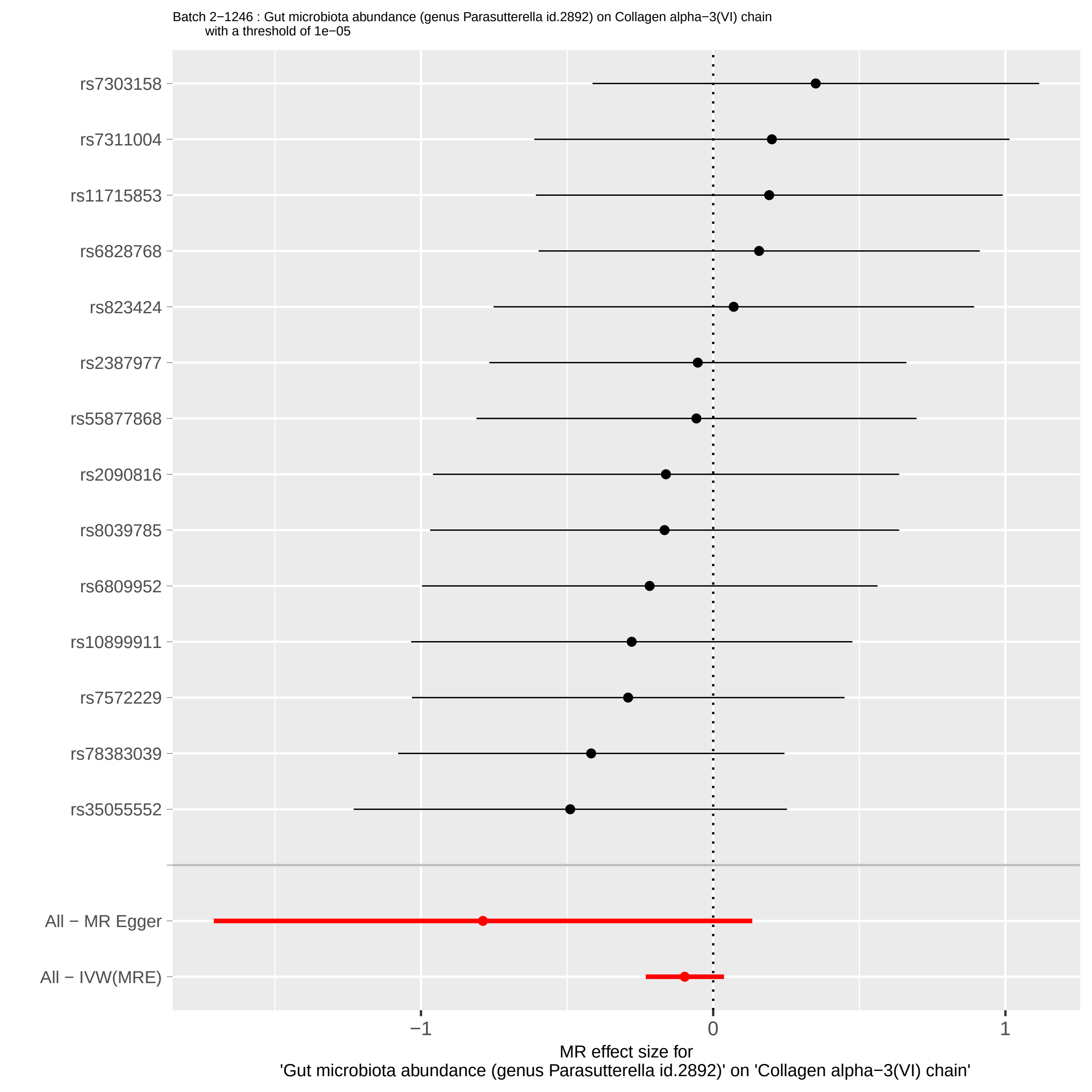

Batch 2−1246 : Gut microbiota abundance (genus Parasutterella id.2892) on Collagen alpha−3(VI) chain
with a threshold of 1e−05
rs7303158
rs7311004
rs11715853
rs6828768
rs823424
rs2387977
rs55877868
rs2090816
rs8039785
rs6809952
rs10899911
rs7572229
rs78383039
rs35055552
All − MR Egger
All − IVW(MRE)
−1
0
1
MR effect size for
'Gut microbiota abundance (genus Parasutterella id.2892)' on 'Collagen alpha−3(VI) chain'

Batch 2−1247 : Gut microbiota abundance (genus Catenibacterium id.2153) on Collagen alpha−5(VI) chain
with a threshold of 1e−05

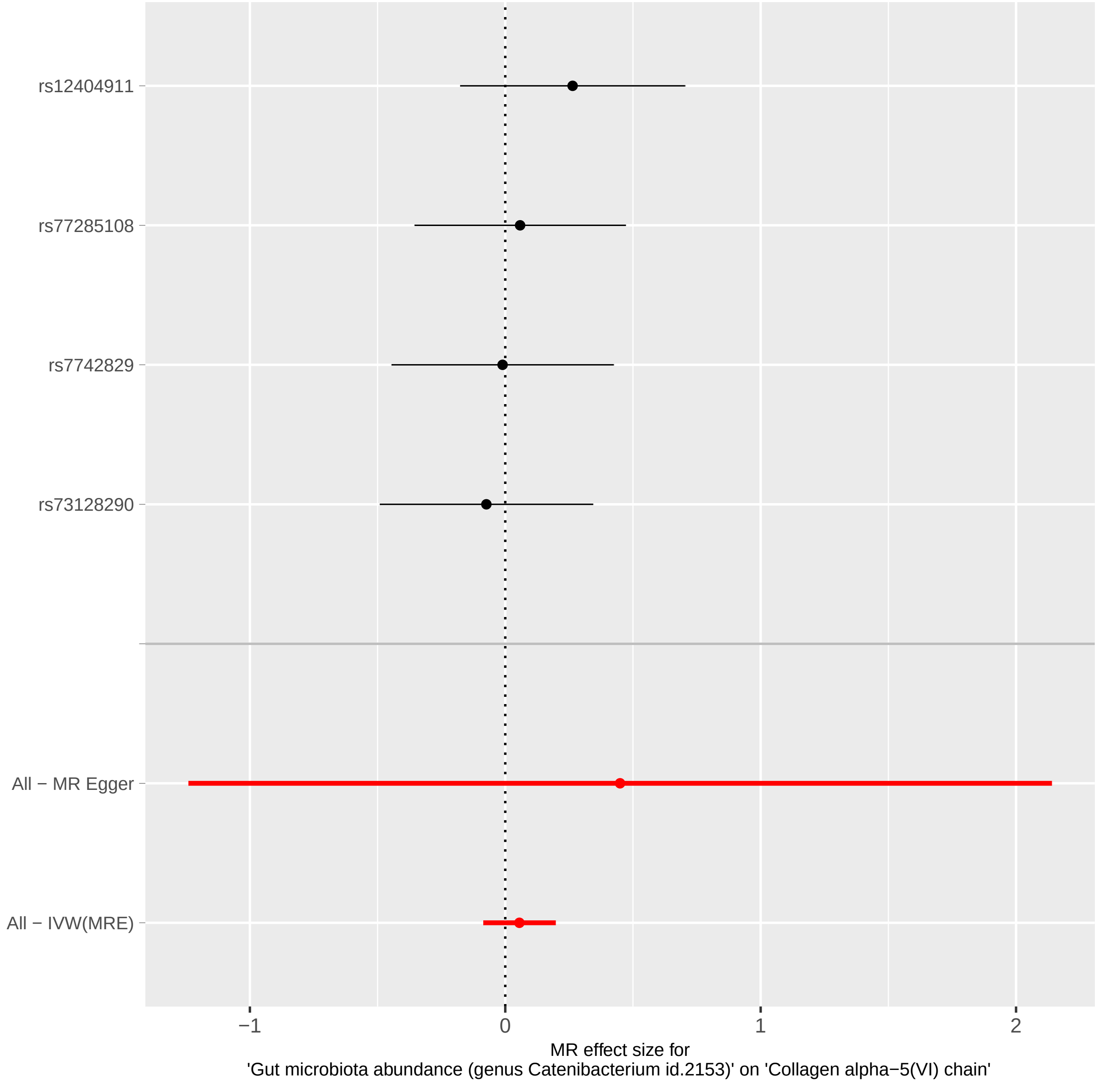

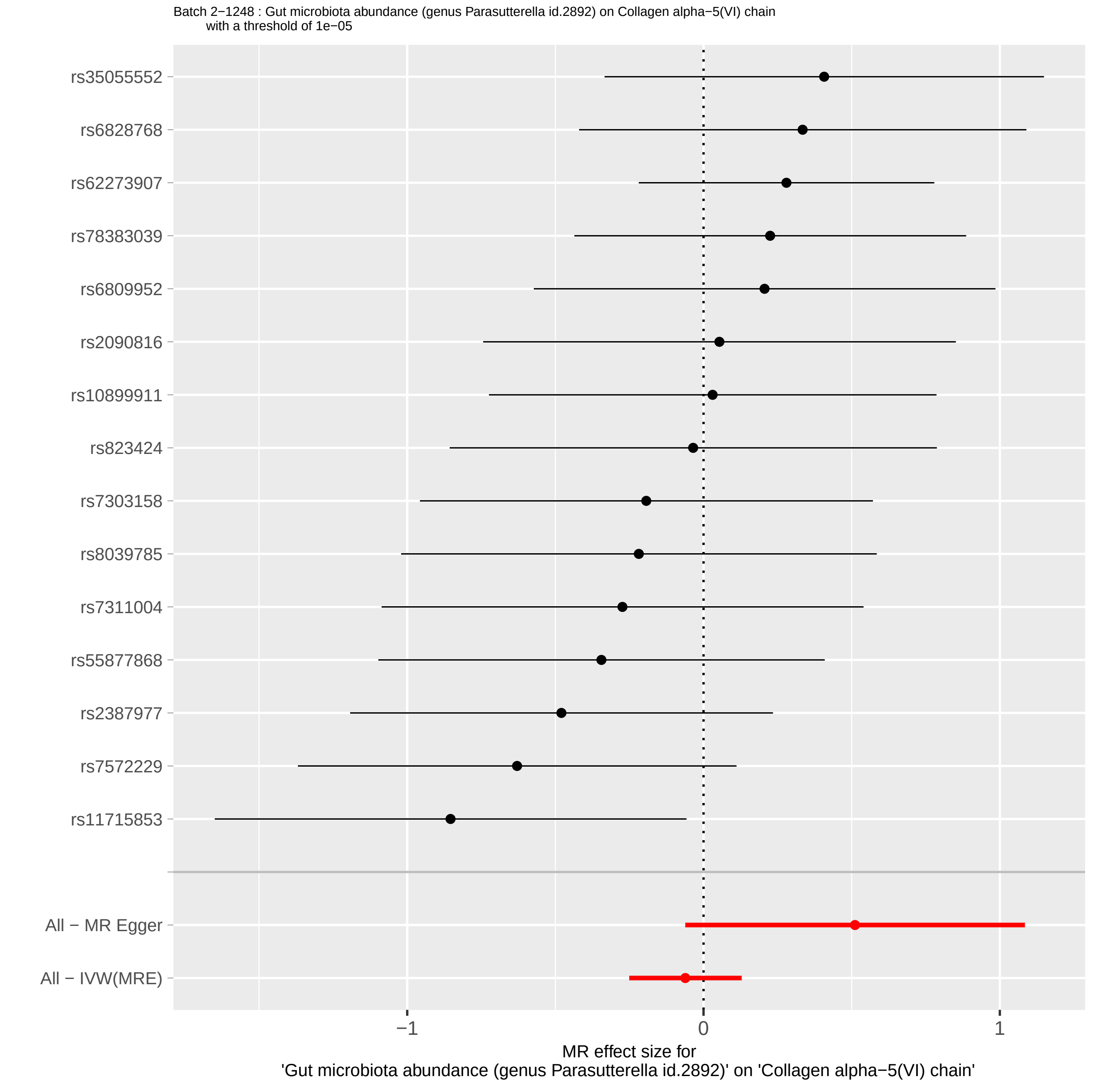

Batch 2−1248 : Gut microbiota abundance (genus Parasutterella id.2892) on Collagen alpha−5(VI) chain
with a threshold of 1e−05
rs35055552
rs6828768
rs62273907
rs78383039
rs6809952
rs2090816
rs10899911
rs823424
rs7303158
rs8039785
rs7311004
rs55877868
rs2387977
rs7572229
rs11715853
All − MR Egger
All − IVW(MRE)
−1
0
1
MR effect size for
'Gut microbiota abundance (genus Parasutterella id.2892)' on 'Collagen alpha−5(VI) chain'

Batch 2−1249 : Gut microbiota abundance (genus Catenibacterium id.2153) on Collagen alpha−1(VIII) chain
with a threshold of 1e−05

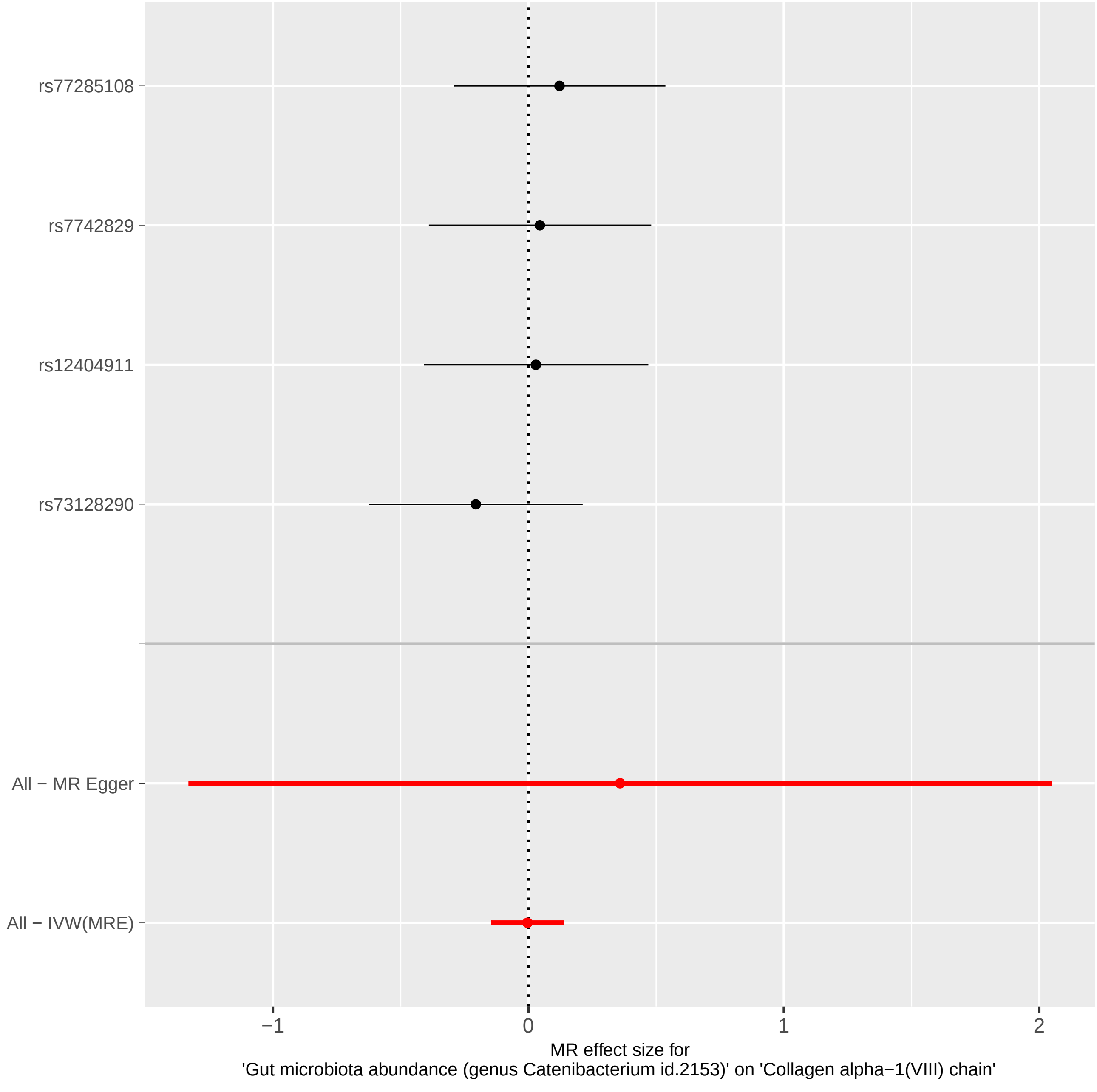

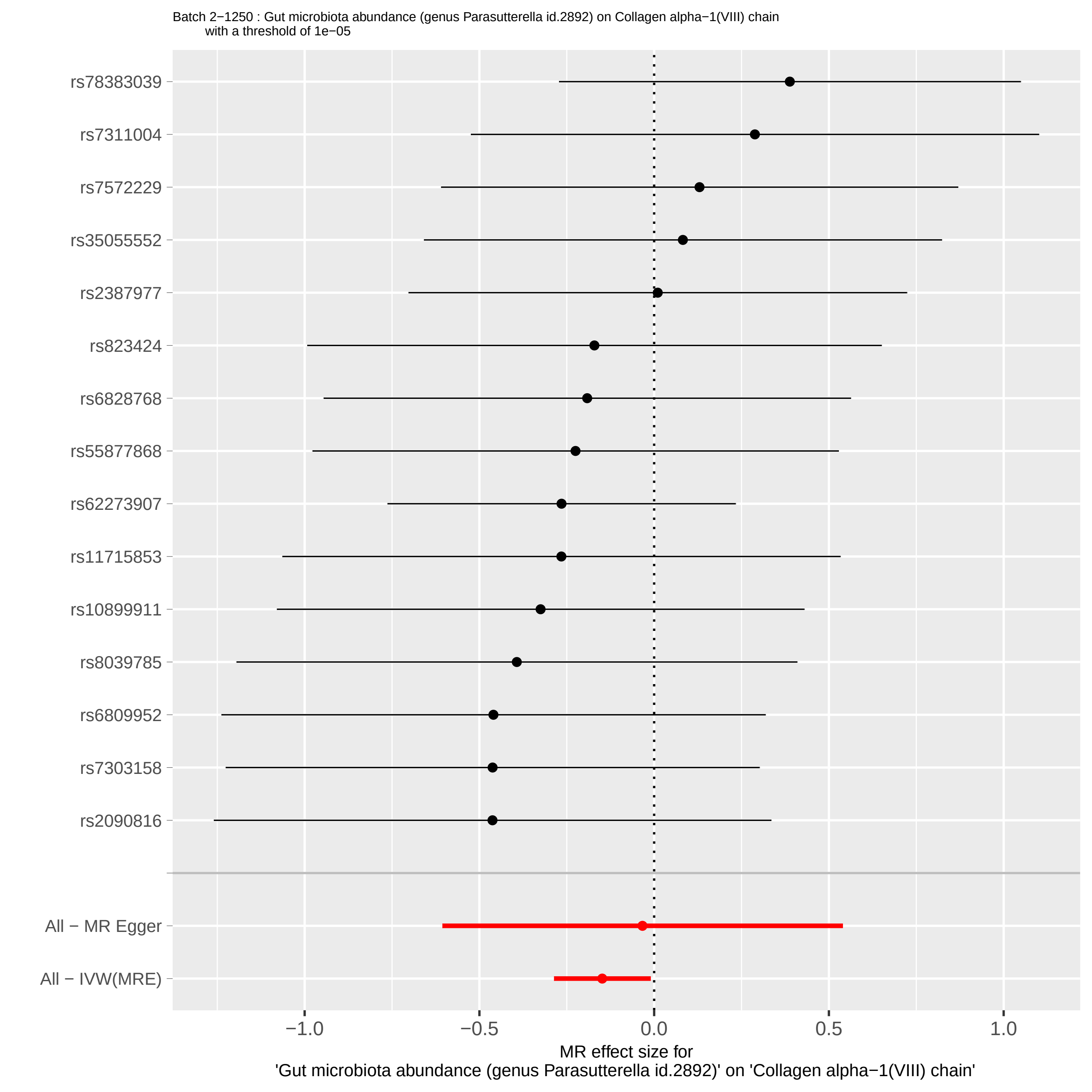
Batch 2−1250 : Gut microbiota abundance (genus Parasutterella id.2892) on Collagen alpha−1(VIII) chain
with a threshold of 1e−05
rs78383039
rs7311004
rs7572229
rs35055552
rs2387977
rs823424
rs6828768
rs55877868
rs62273907
rs11715853
rs10899911
rs8039785
rs6809952
rs7303158
rs2090816
All − MR Egger
All − IVW(MRE)
−1.0
−0.5
0.0
0.5
1.0
MR effect size for
'Gut microbiota abundance (genus Parasutterella id.2892)' on 'Collagen alpha−1(VIII) chain'

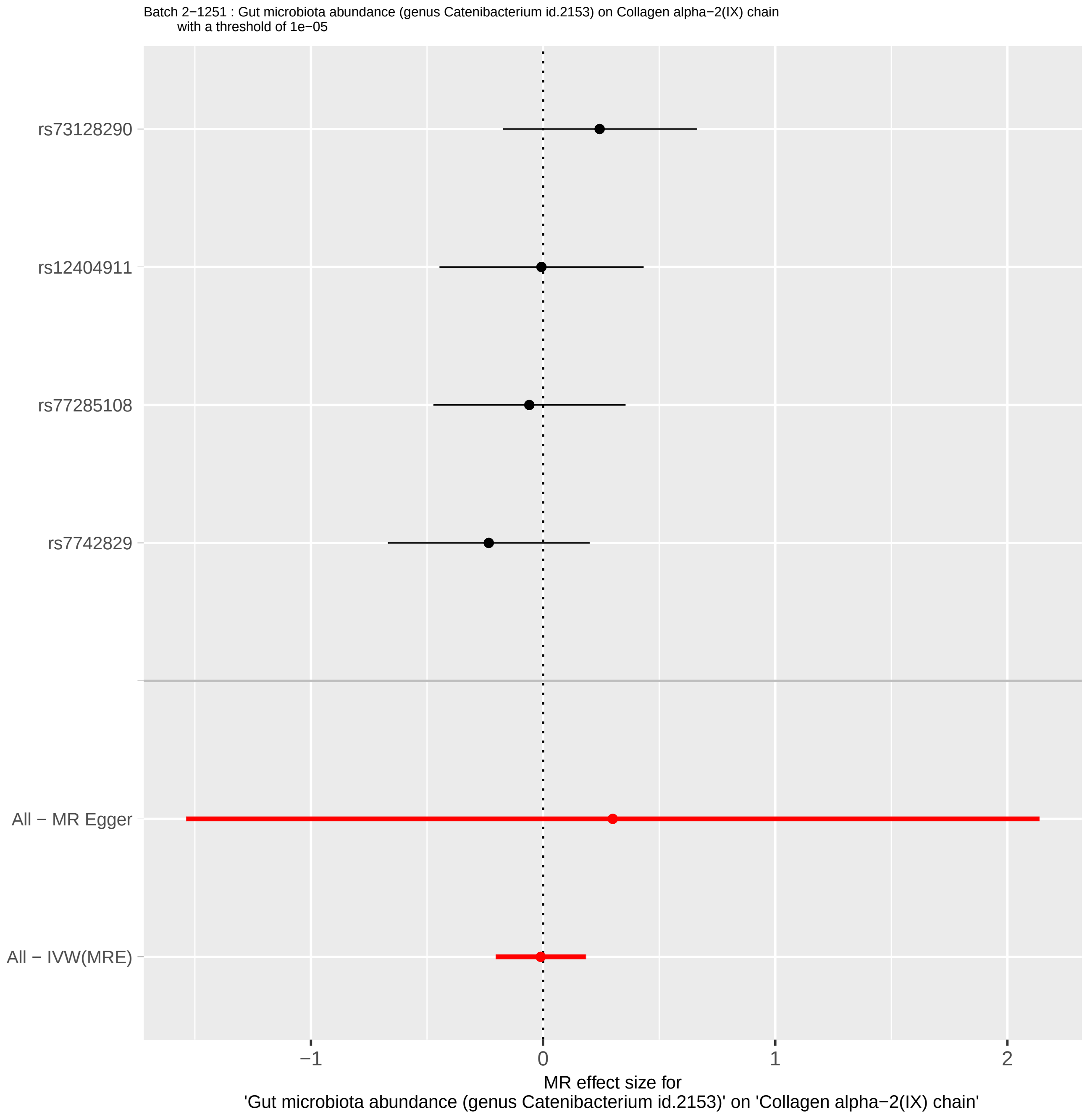

Batch 2−1251 : Gut microbiota abundance (genus Catenibacterium id.2153) on Collagen alpha−2(IX) chain
with a threshold of 1e−05
rs73128290
rs12404911
rs77285108
rs7742829
All − MR Egger
All − IVW(MRE)
−1
0
1
2
MR effect size for
'Gut microbiota abundance (genus Catenibacterium id.2153)' on 'Collagen alpha−2(IX) chain'

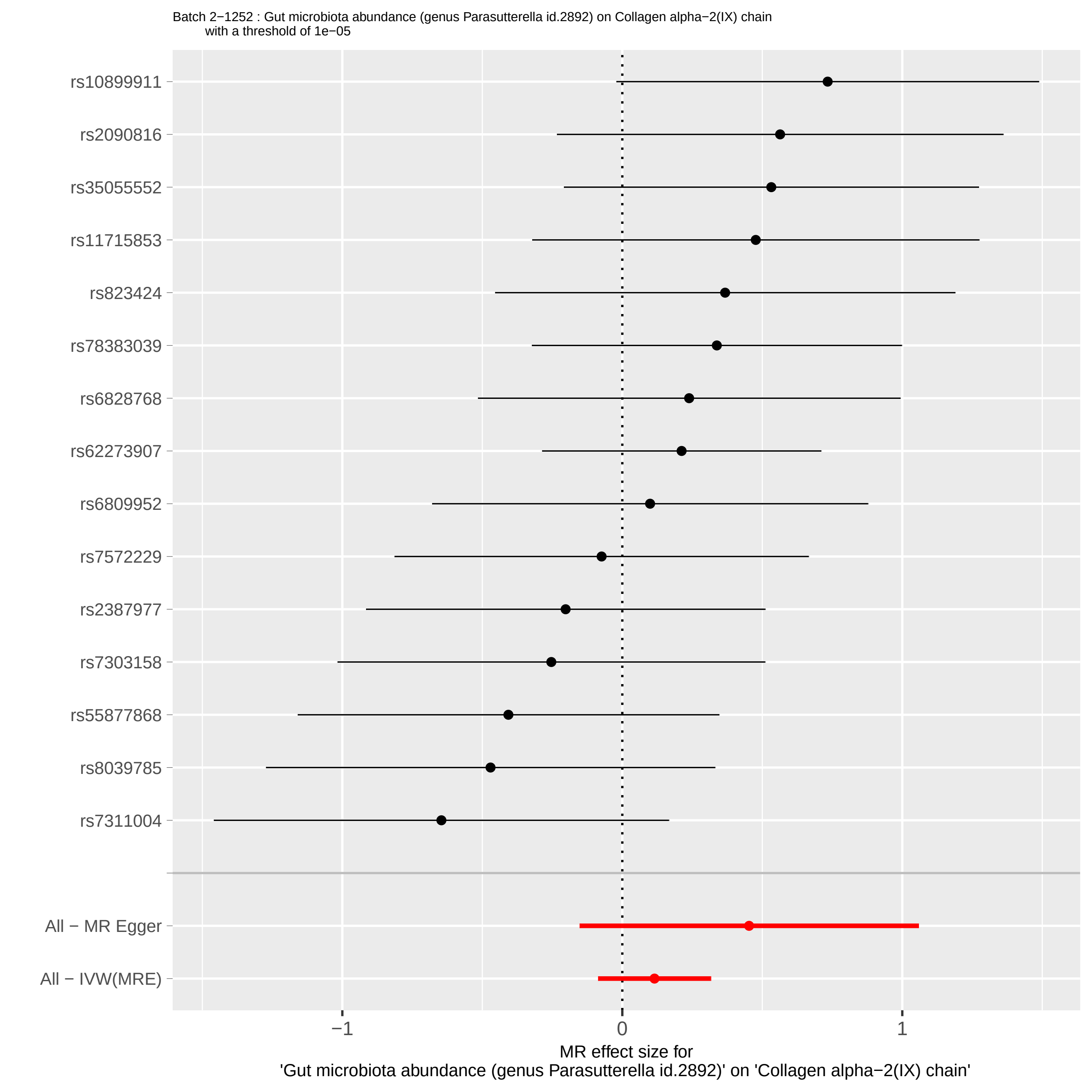

Batch 2−1252 : Gut microbiota abundance (genus Parasutterella id.2892) on Collagen alpha−2(IX) chain
with a threshold of 1e−05
rs10899911
rs2090816
rs35055552
rs11715853
rs823424
rs78383039
rs6828768
rs62273907
rs6809952
rs7572229
rs2387977
rs7303158
rs55877868
rs8039785
rs7311004
All − MR Egger
All − IVW(MRE)
−1
0
1
MR effect size for
'Gut microbiota abundance (genus Parasutterella id.2892)' on 'Collagen alpha−2(IX) chain'

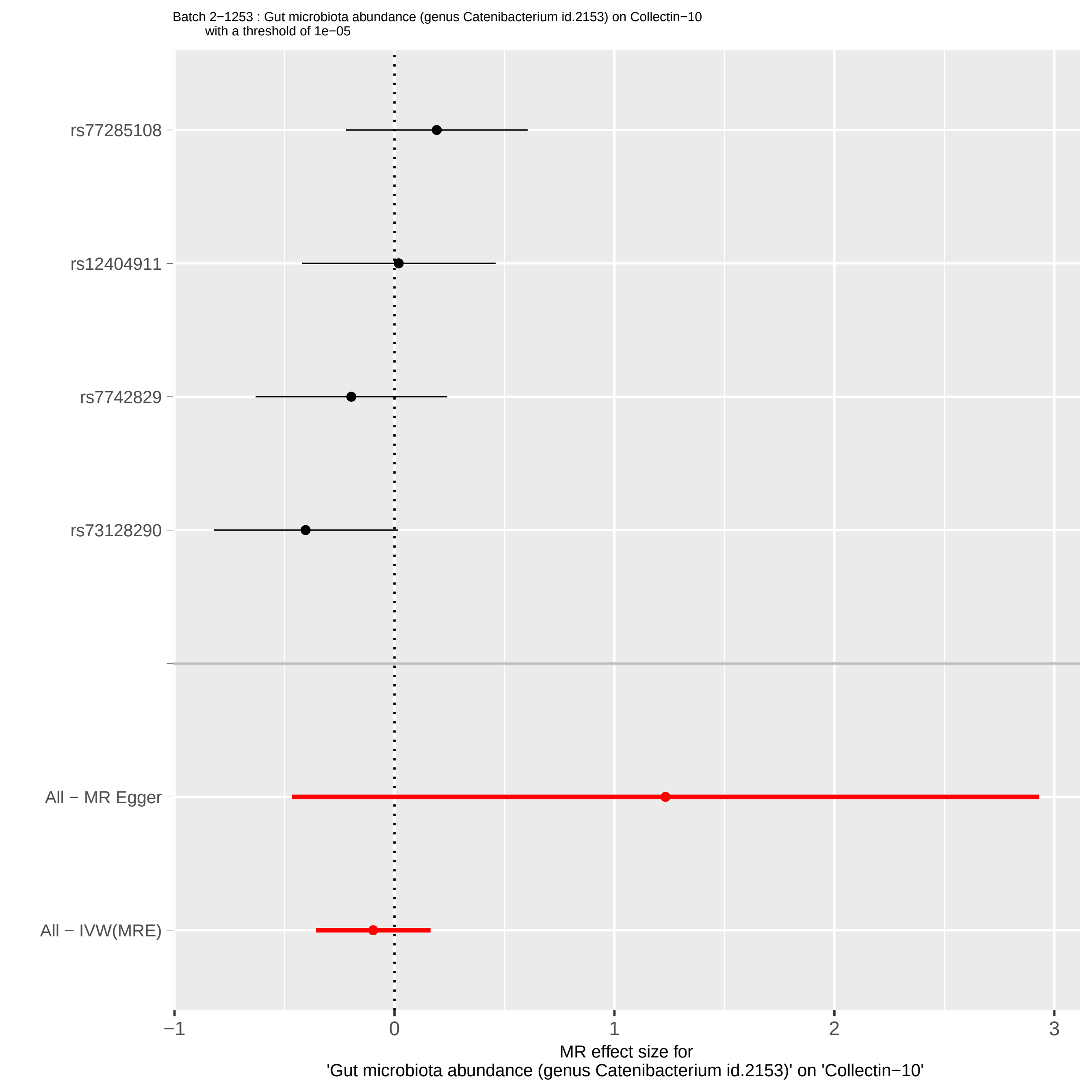

Batch 2−1253 : Gut microbiota abundance (genus Catenibacterium id.2153) on Collectin−10
with a threshold of 1e−05
rs77285108
rs12404911
rs7742829
rs73128290
All − MR Egger
All − IVW(MRE)
−1
0
1
2
3
MR effect size for
'Gut microbiota abundance (genus Catenibacterium id.2153)' on 'Collectin−10'

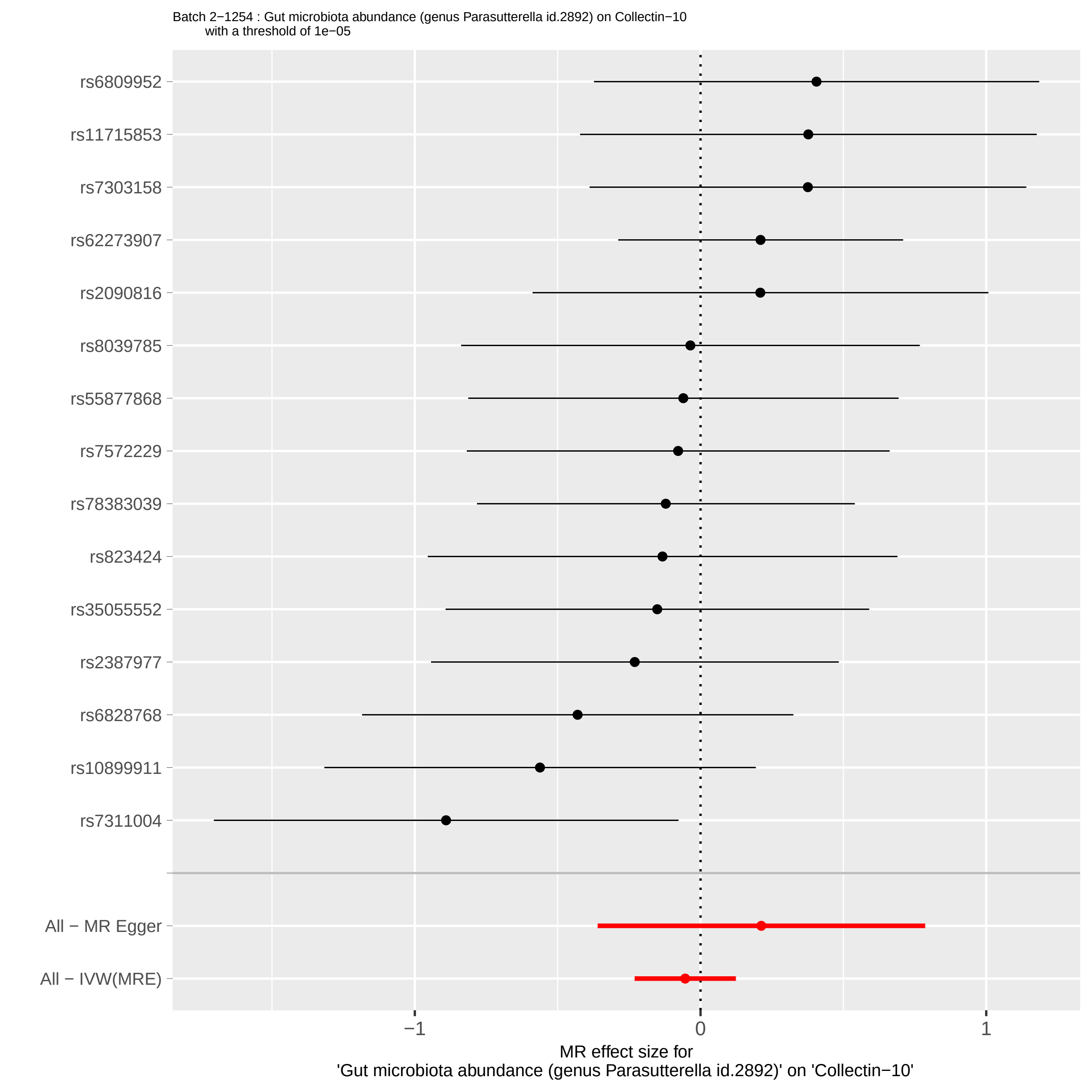

Batch 2−1254 : Gut microbiota abundance (genus Parasutterella id.2892) on Collectin−10
with a threshold of 1e−05
rs6809952
rs11715853
rs7303158
rs62273907
rs2090816
rs8039785
rs55877868
rs7572229
rs78383039
rs823424
rs35055552
rs2387977
rs6828768
rs10899911
rs7311004
All − MR Egger
All − IVW(MRE)
−1
0
1
MR effect size for
'Gut microbiota abundance (genus Parasutterella id.2892)' on 'Collectin−10'

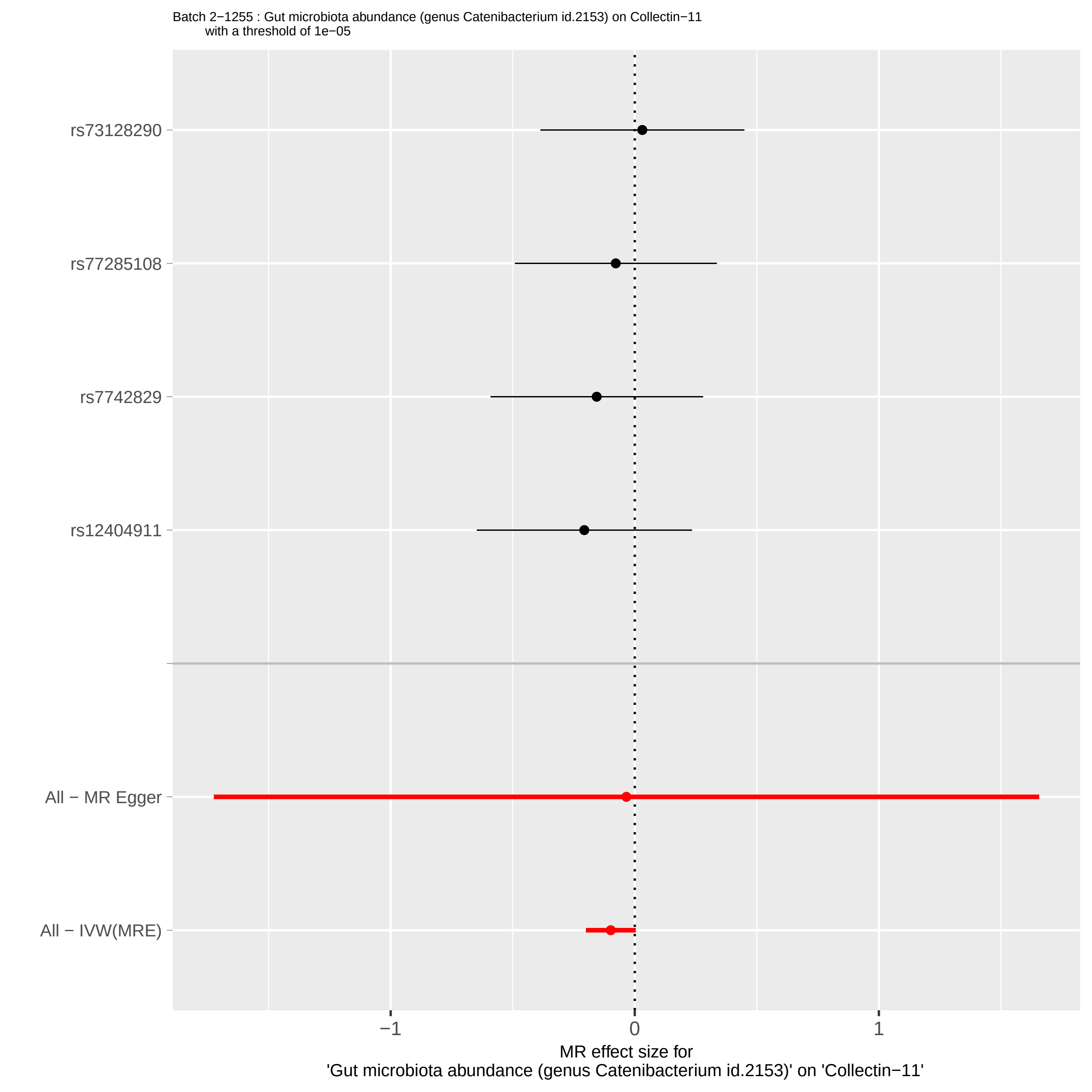

Batch 2−1255 : Gut microbiota abundance (genus Catenibacterium id.2153) on Collectin−11
with a threshold of 1e−05
rs73128290
rs77285108
rs7742829
rs12404911
All − MR Egger
All − IVW(MRE)
−1
0
1
MR effect size for
'Gut microbiota abundance (genus Catenibacterium id.2153)' on 'Collectin−11'

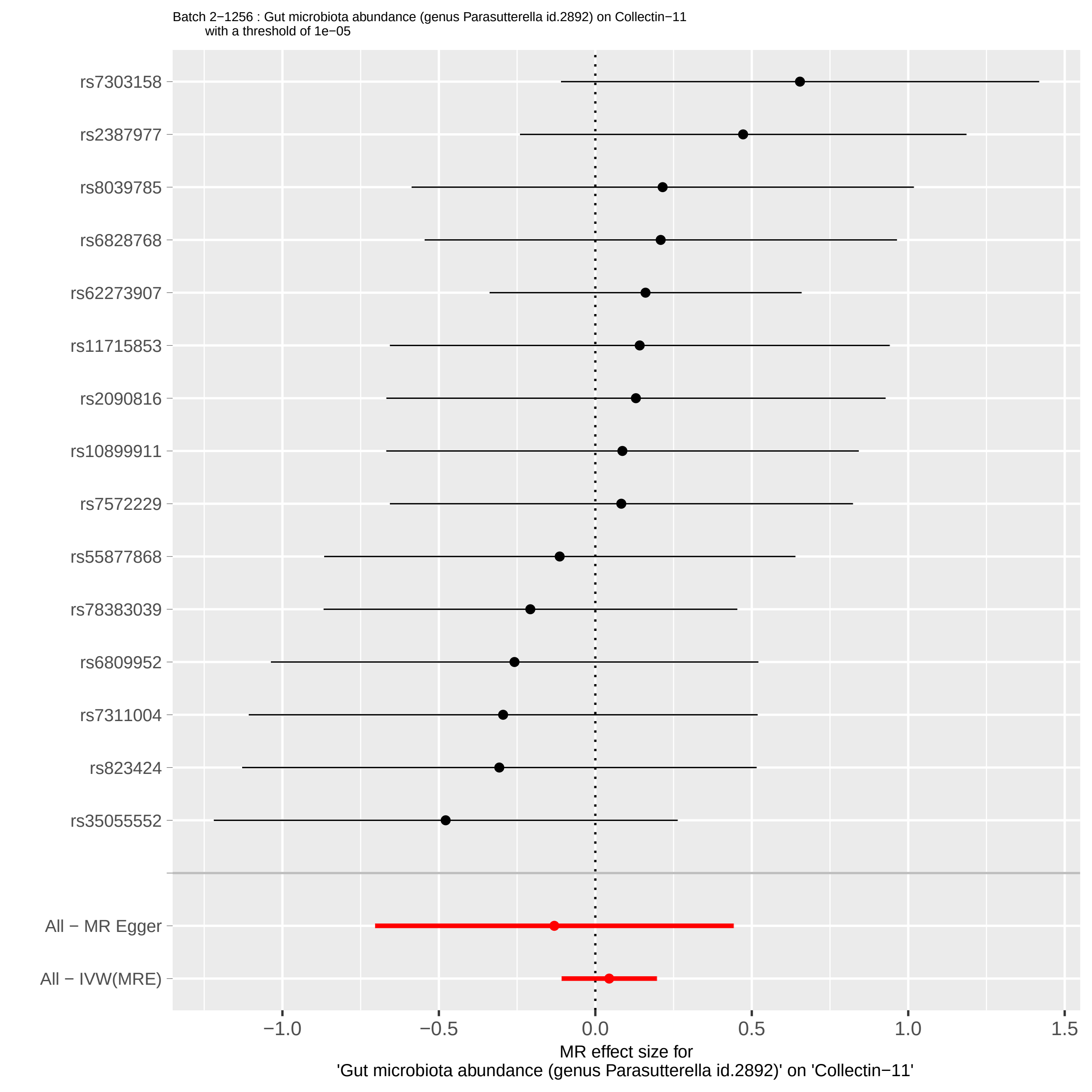
Batch 2−1256 : Gut microbiota abundance (genus Parasutterella id.2892) on Collectin−11
with a threshold of 1e−05
rs7303158
rs2387977
rs8039785
rs6828768
rs62273907
rs11715853
rs2090816
rs10899911
rs7572229
rs55877868
rs78383039
rs6809952
rs7311004
rs823424
rs35055552
All − MR Egger
All − IVW(MRE)
−1.0
−0.5
0.0
0.5
1.0
1.5
MR effect size for
'Gut microbiota abundance (genus Parasutterella id.2892)' on 'Collectin−11'

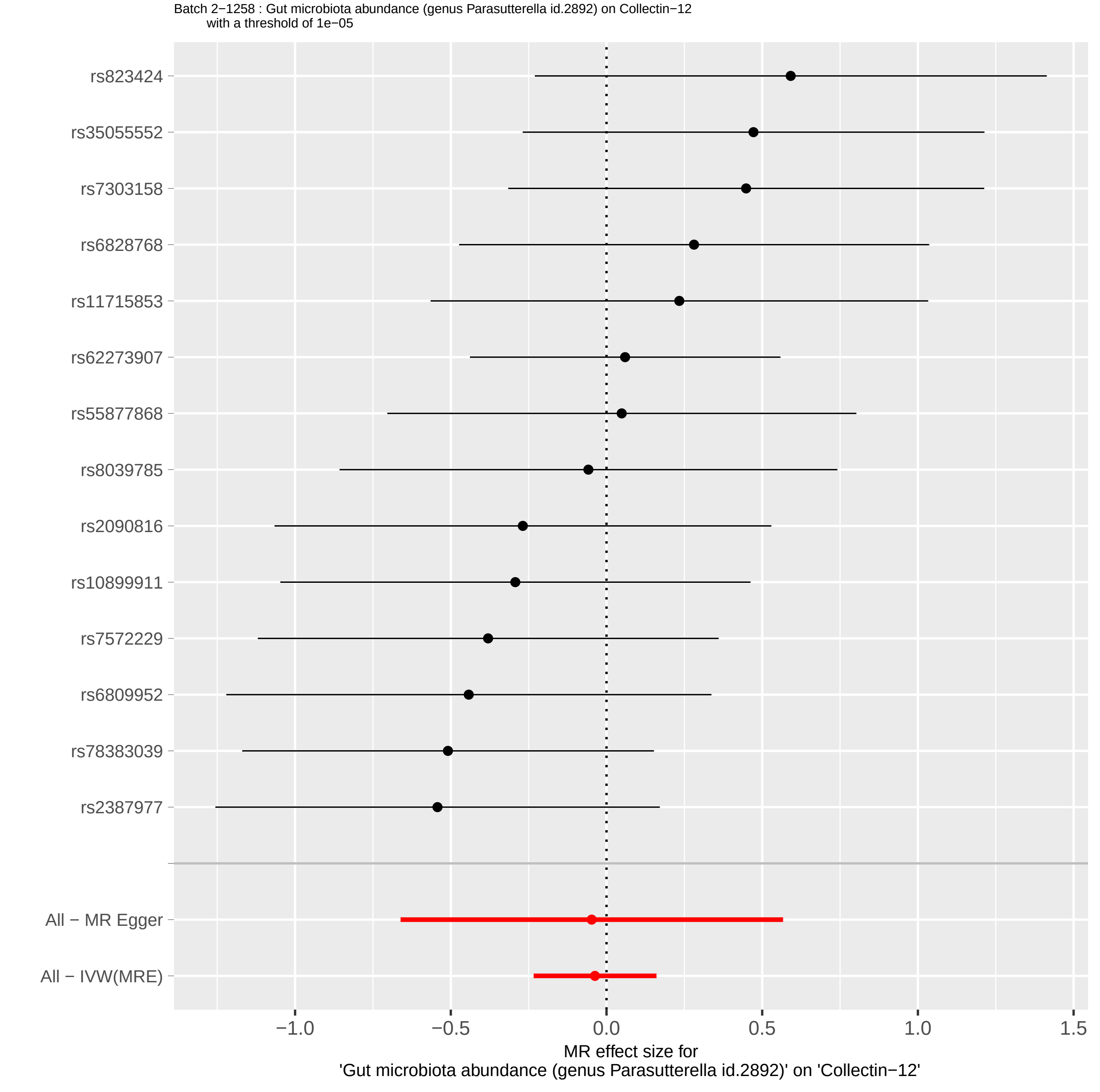

Batch 2−1258 : Gut microbiota abundance (genus Parasutterella id.2892) on Collectin−12
with a threshold of 1e−05
rs823424
rs35055552
rs7303158
rs6828768
rs11715853
rs62273907
rs55877868
rs8039785
rs2090816
rs10899911
rs7572229
rs6809952
rs78383039
rs2387977
All − MR Egger
All − IVW(MRE)
−1.0
−0.5
0.0
0.5
1.0
1.5
MR effect size for
'Gut microbiota abundance (genus Parasutterella id.2892)' on 'Collectin−12'

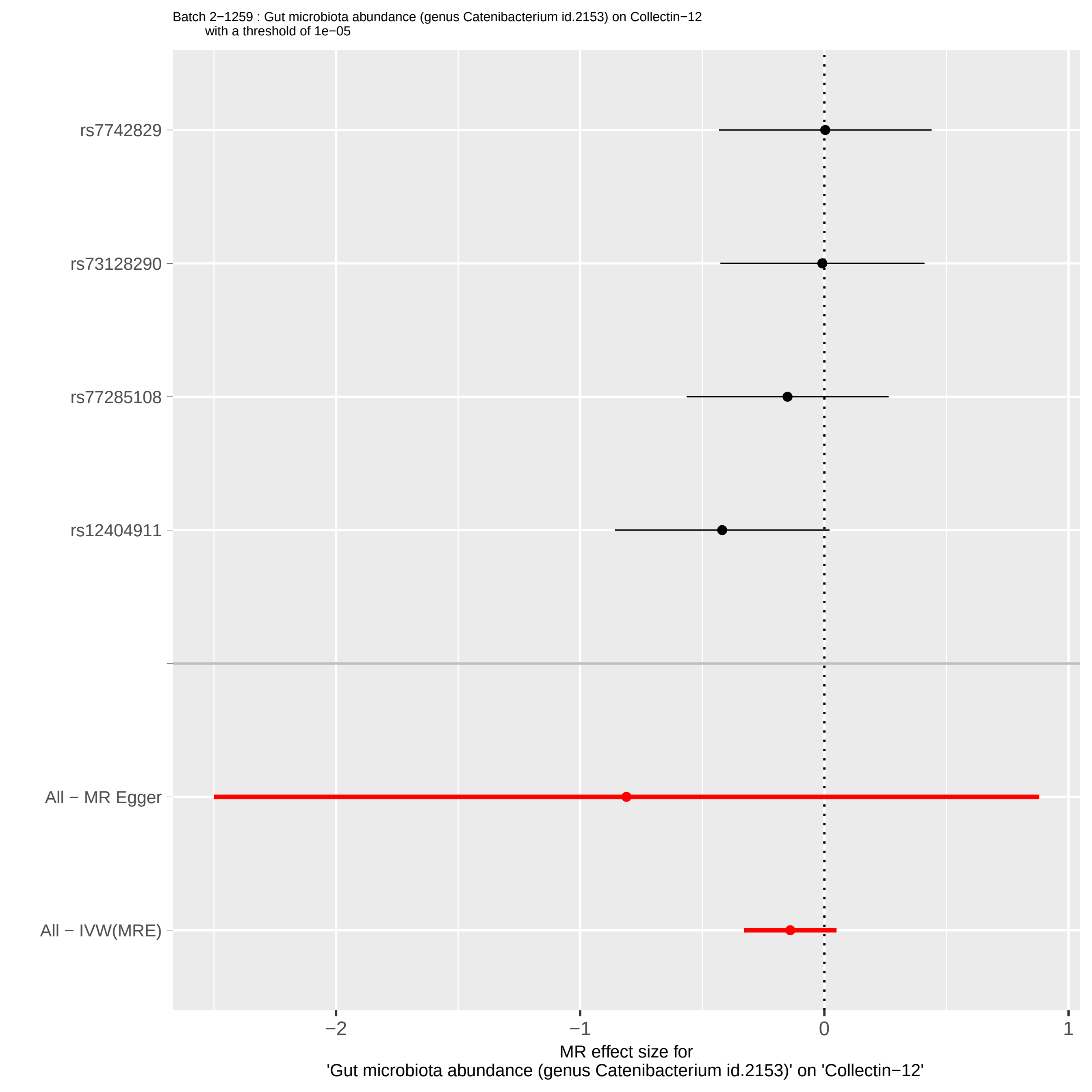

Batch 2−1259 : Gut microbiota abundance (genus Catenibacterium id.2153) on Collectin−12
with a threshold of 1e−05
rs7742829
rs73128290
rs77285108
rs12404911
All − MR Egger
All − IVW(MRE)
−2
−1
0
1
MR effect size for
'Gut microbiota abundance (genus Catenibacterium id.2153)' on 'Collectin−12'

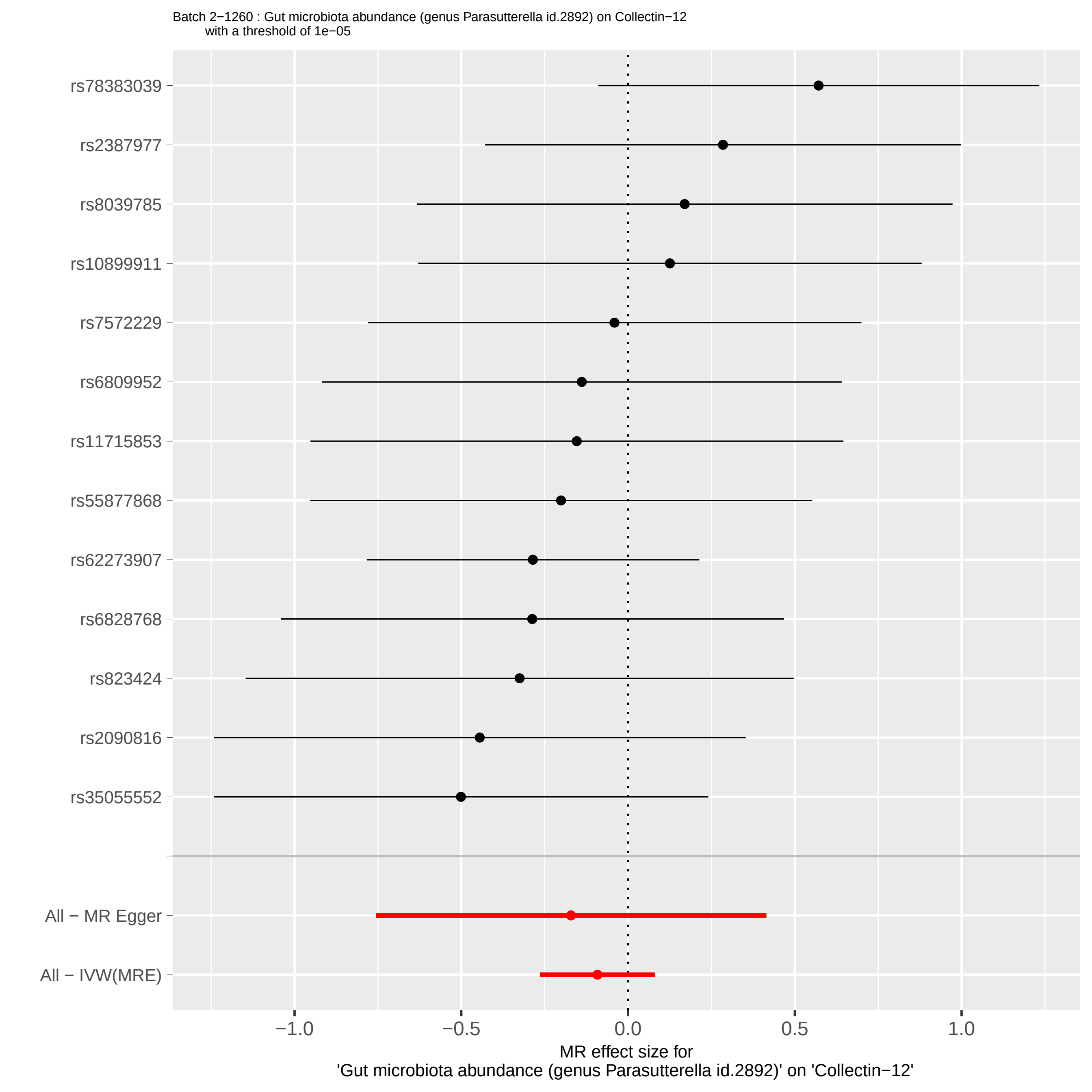

Batch 2−1260 : Gut microbiota abundance (genus Parasutterella id.2892) on Collectin−12
with a threshold of 1e−05
rs78383039
rs2387977
rs8039785
rs10899911
rs7572229
rs6809952
rs11715853
rs55877868
rs62273907
rs6828768
rs823424
rs2090816
rs35055552
All − MR Egger
All − IVW(MRE)
−1.0
−0.5
0.0
0.5
1.0
MR effect size for
'Gut microbiota abundance (genus Parasutterella id.2892)' on 'Collectin−12'

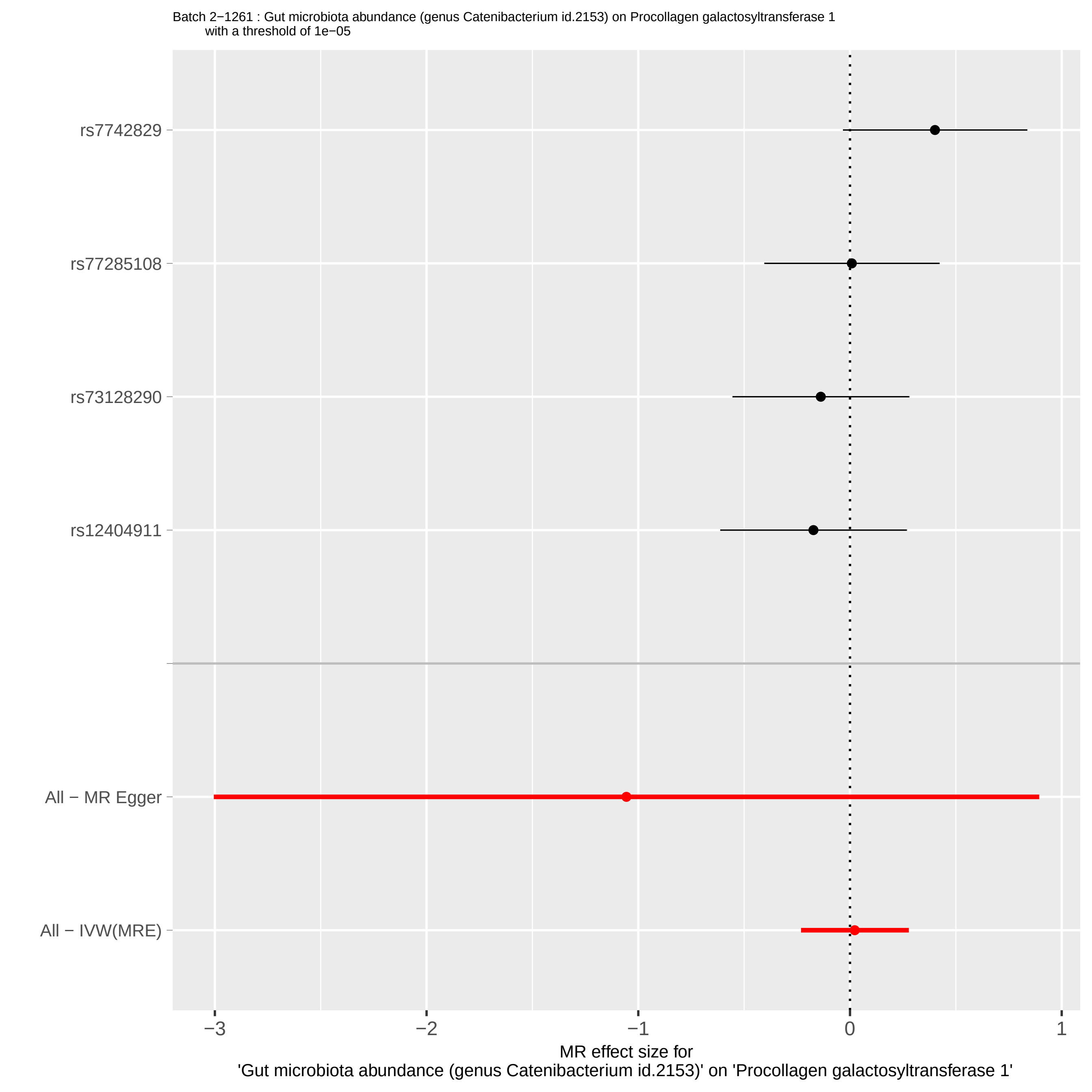
Batch 2−1261 : Gut microbiota abundance (genus Catenibacterium id.2153) on Procollagen galactosyltransferase 1
with a threshold of 1e−05
rs7742829
rs77285108
rs73128290
rs12404911
All − MR Egger
All − IVW(MRE)
−3
−2
−1
0
1
MR effect size for
'Gut microbiota abundance (genus Catenibacterium id.2153)' on 'Procollagen galactosyltransferase 1'

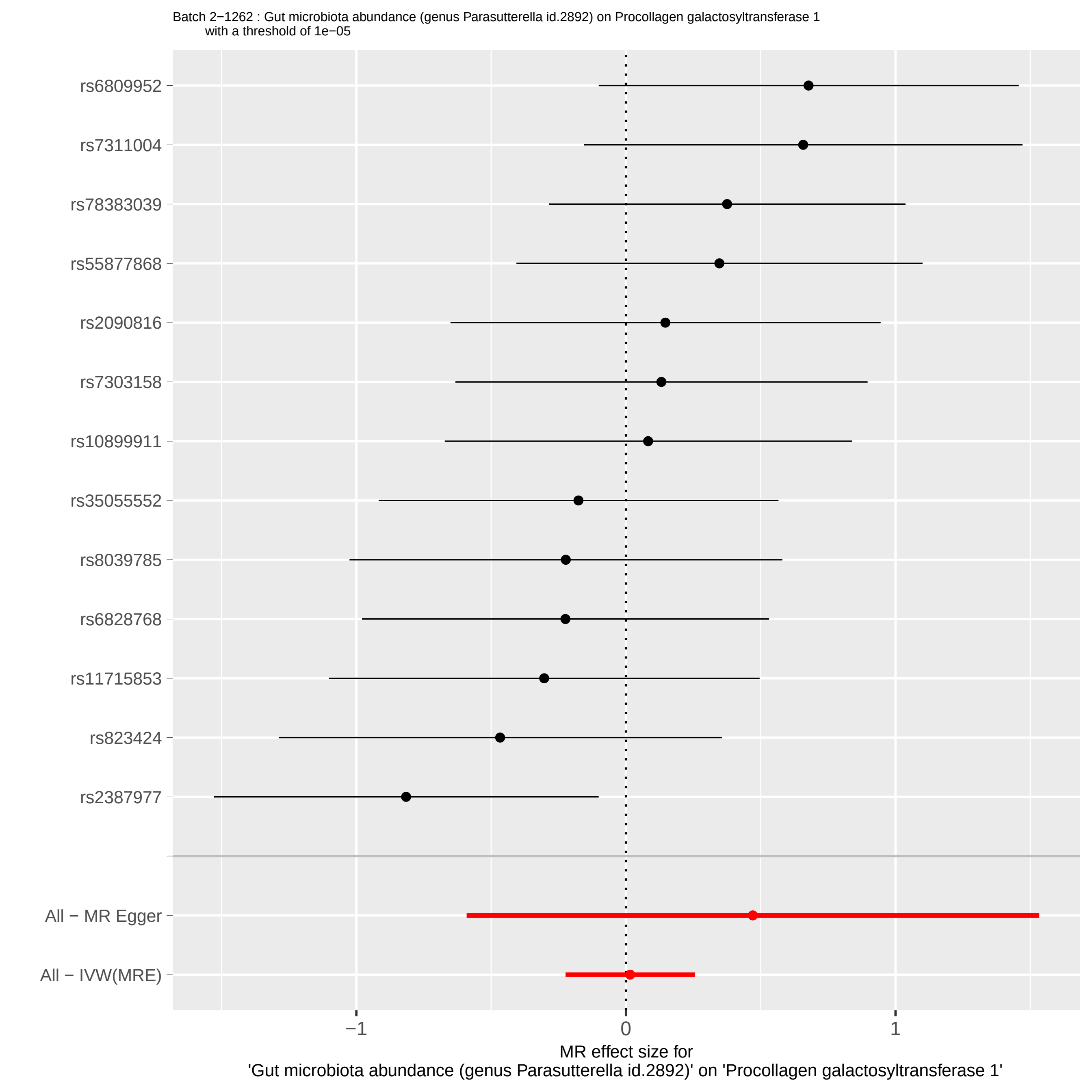

Batch 2−1262 : Gut microbiota abundance (genus Parasutterella id.2892) on Procollagen galactosyltransferase 1
with a threshold of 1e−05
rs6809952
rs7311004
rs78383039
rs55877868
rs2090816
rs7303158
rs10899911
rs35055552
rs8039785
rs6828768
rs11715853
rs823424
rs2387977
All − MR Egger
All − IVW(MRE)
−1
0
1
MR effect size for
'Gut microbiota abundance (genus Parasutterella id.2892)' on 'Procollagen galactosyltransferase 1'

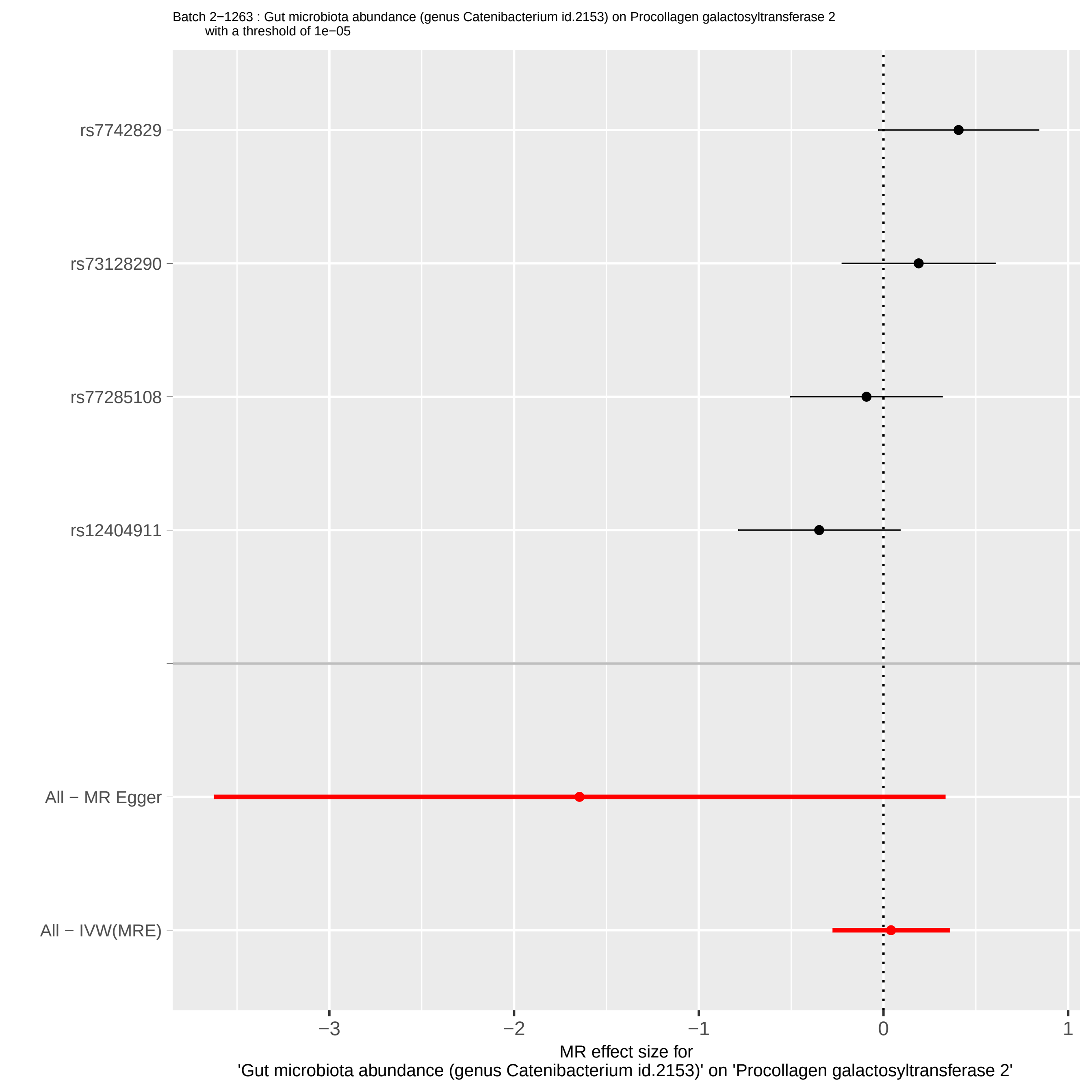

Batch 2−1263 : Gut microbiota abundance (genus Catenibacterium id.2153) on Procollagen galactosyltransferase 2
with a threshold of 1e−05
rs7742829
rs73128290
rs77285108
rs12404911
All − MR Egger
All − IVW(MRE)
−3
−2
−1
0
1
MR effect size for
'Gut microbiota abundance (genus Catenibacterium id.2153)' on 'Procollagen galactosyltransferase 2'

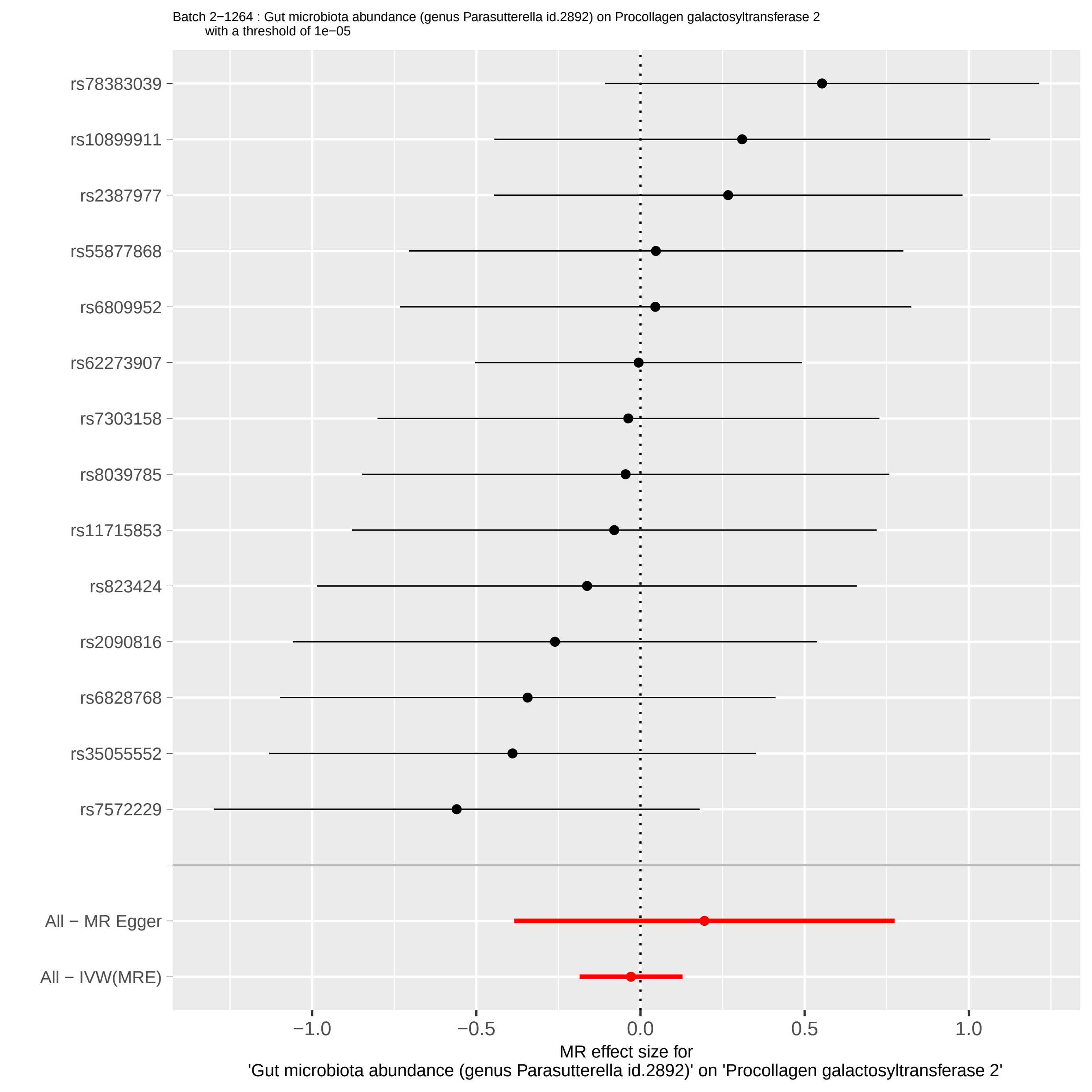

Batch 2−1264 : Gut microbiota abundance (genus Parasutterella id.2892) on Procollagen galactosyltransferase 2
with a threshold of 1e−05
rs78383039
rs10899911
rs2387977
rs55877868
rs6809952
rs62273907
rs7303158
rs8039785
rs11715853
rs823424
rs2090816
rs6828768
rs35055552
rs7572229
All − MR Egger
All − IVW(MRE)
−1.0
−0.5
0.0
0.5
1.0
MR effect size for
'Gut microbiota abundance (genus Parasutterella id.2892)' on 'Procollagen galactosyltransferase 2'

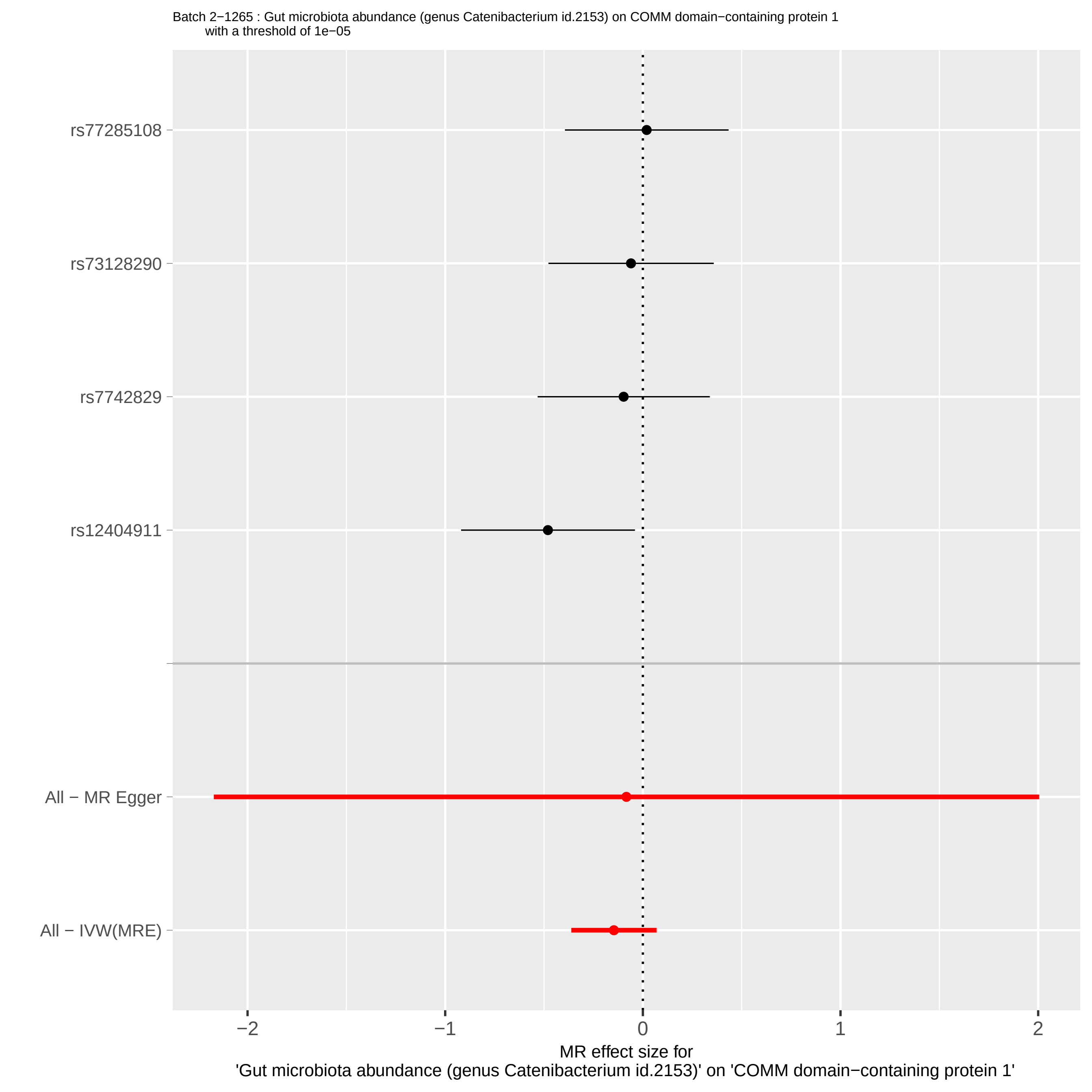

Batch 2−1265 : Gut microbiota abundance (genus Catenibacterium id.2153) on COMM domain−containing protein 1
with a threshold of 1e−05
rs77285108
rs73128290
rs7742829
rs12404911
All − MR Egger
All − IVW(MRE)
−2
−1
0
1
2
MR effect size for
'Gut microbiota abundance (genus Catenibacterium id.2153)' on 'COMM domain−containing protein 1'

Batch 2−1266 : Gut microbiota abundance (genus Parasutterella id.2892) on COMM domain−containing protein 1
with a threshold of 1e−05

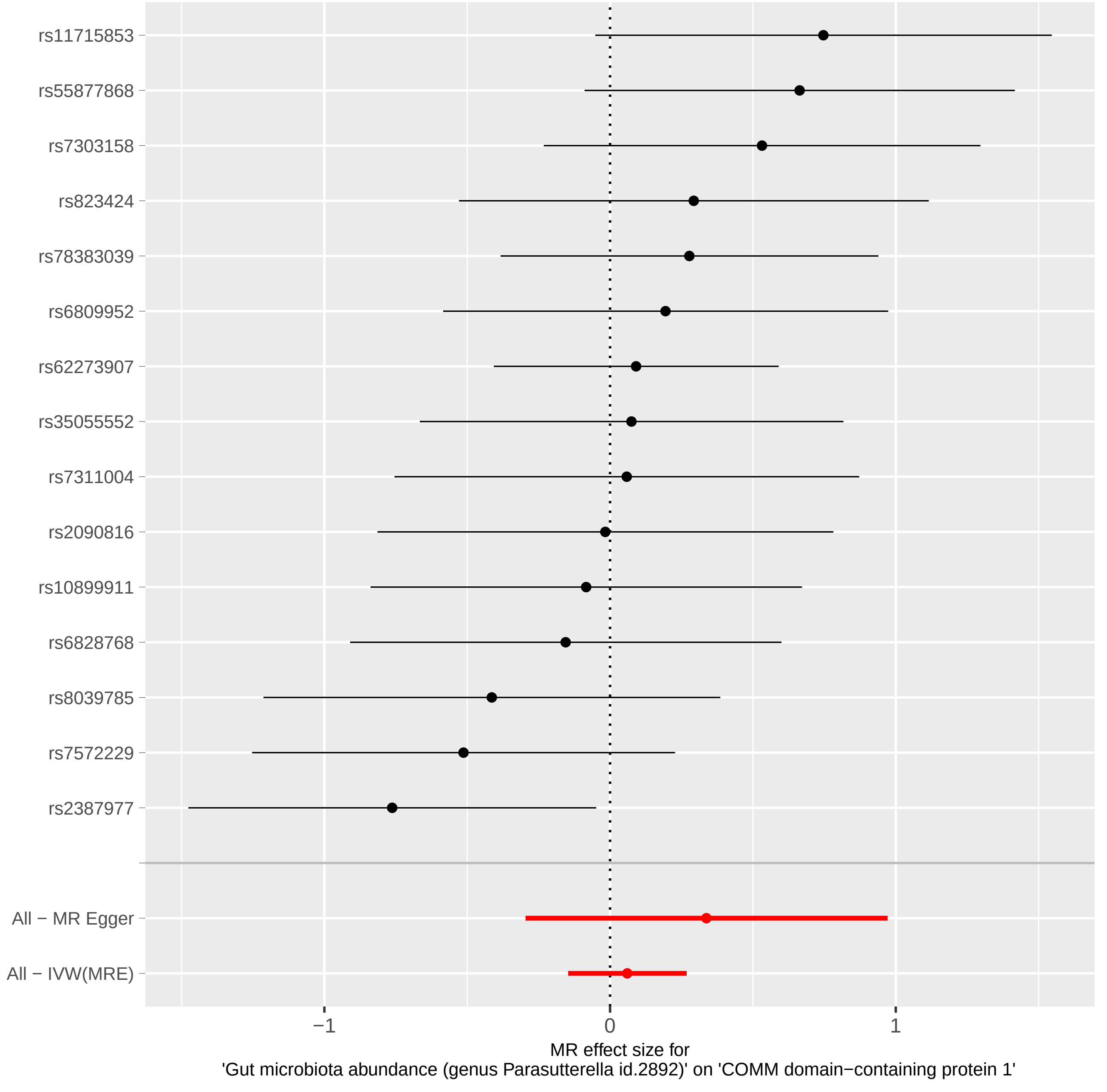

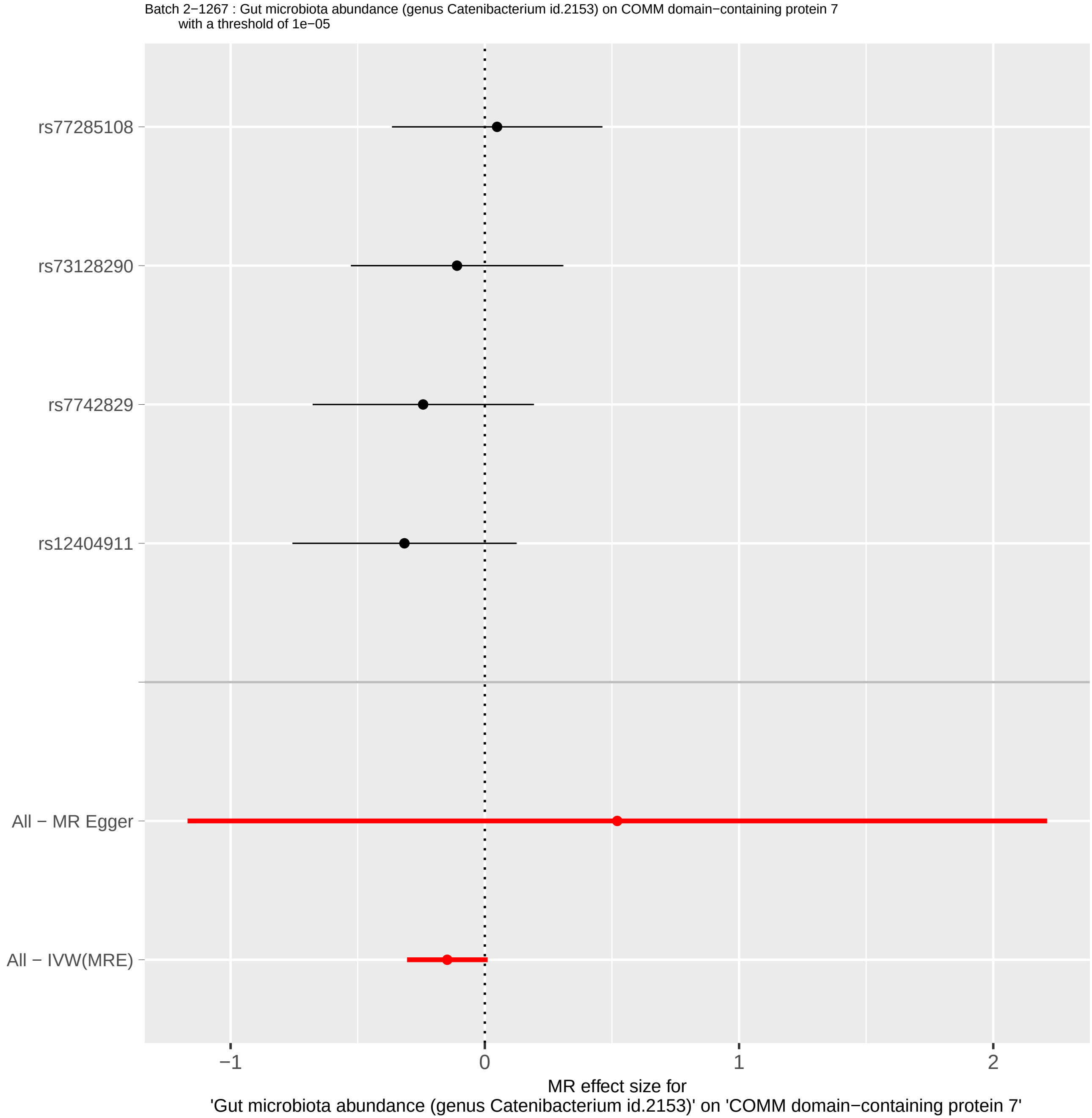

Batch 2−1267 : Gut microbiota abundance (genus Catenibacterium id.2153) on COMM domain−containing protein 7
with a threshold of 1e−05
rs77285108
rs73128290
rs7742829
rs12404911
All − MR Egger
All − IVW(MRE)
−1
0
1
2
MR effect size for
'Gut microbiota abundance (genus Catenibacterium id.2153)' on 'COMM domain−containing protein 7'

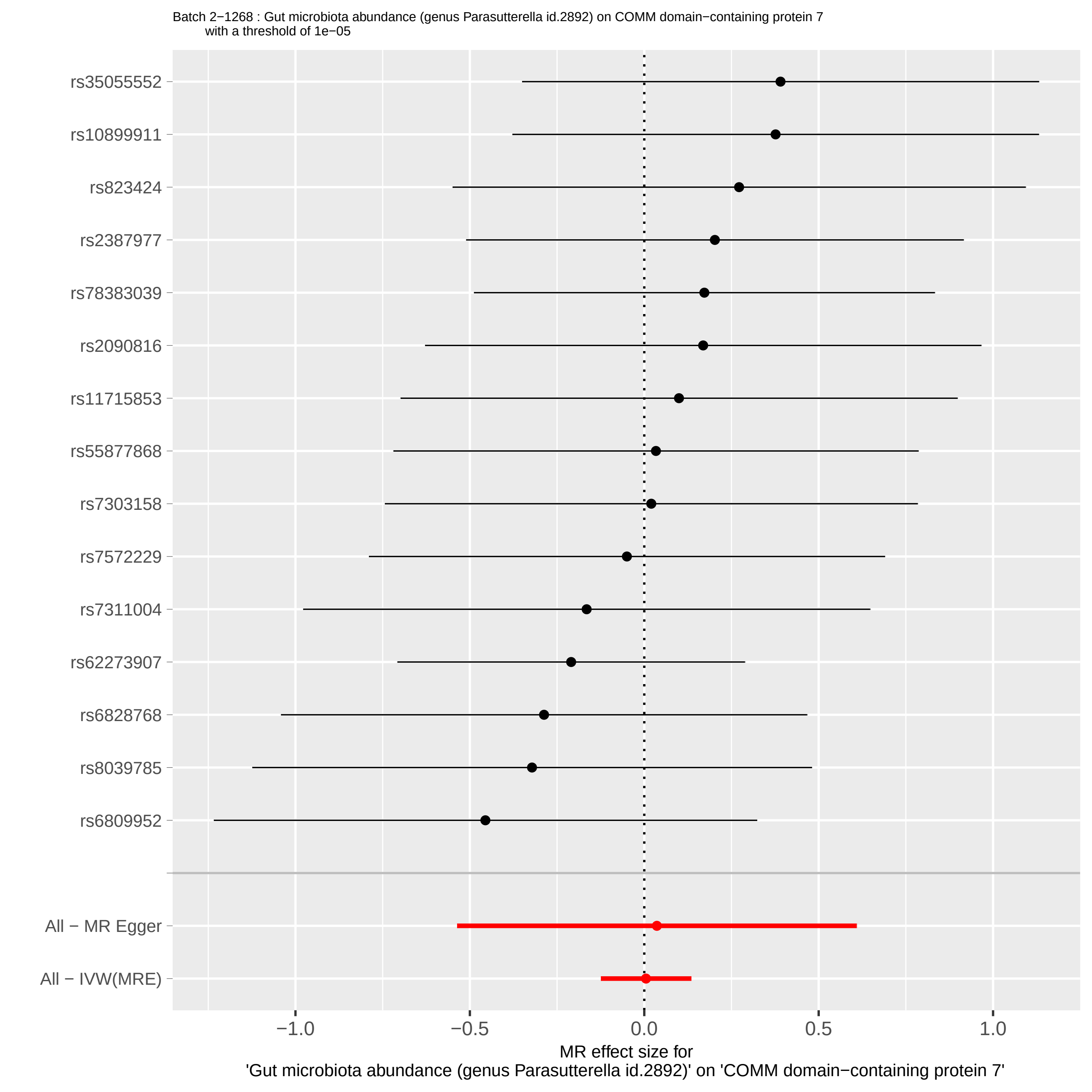

Batch 2−1268 : Gut microbiota abundance (genus Parasutterella id.2892) on COMM domain−containing protein 7
with a threshold of 1e−05
rs35055552
rs10899911
rs823424
rs2387977
rs78383039
rs2090816
rs11715853
rs55877868
rs7303158
rs7572229
rs7311004
rs62273907
rs6828768
rs8039785
rs6809952
All − MR Egger
All − IVW(MRE)
−1.0
−0.5
0.0
0.5
1.0
MR effect size for
'Gut microbiota abundance (genus Parasutterella id.2892)' on 'COMM domain−containing protein 7'

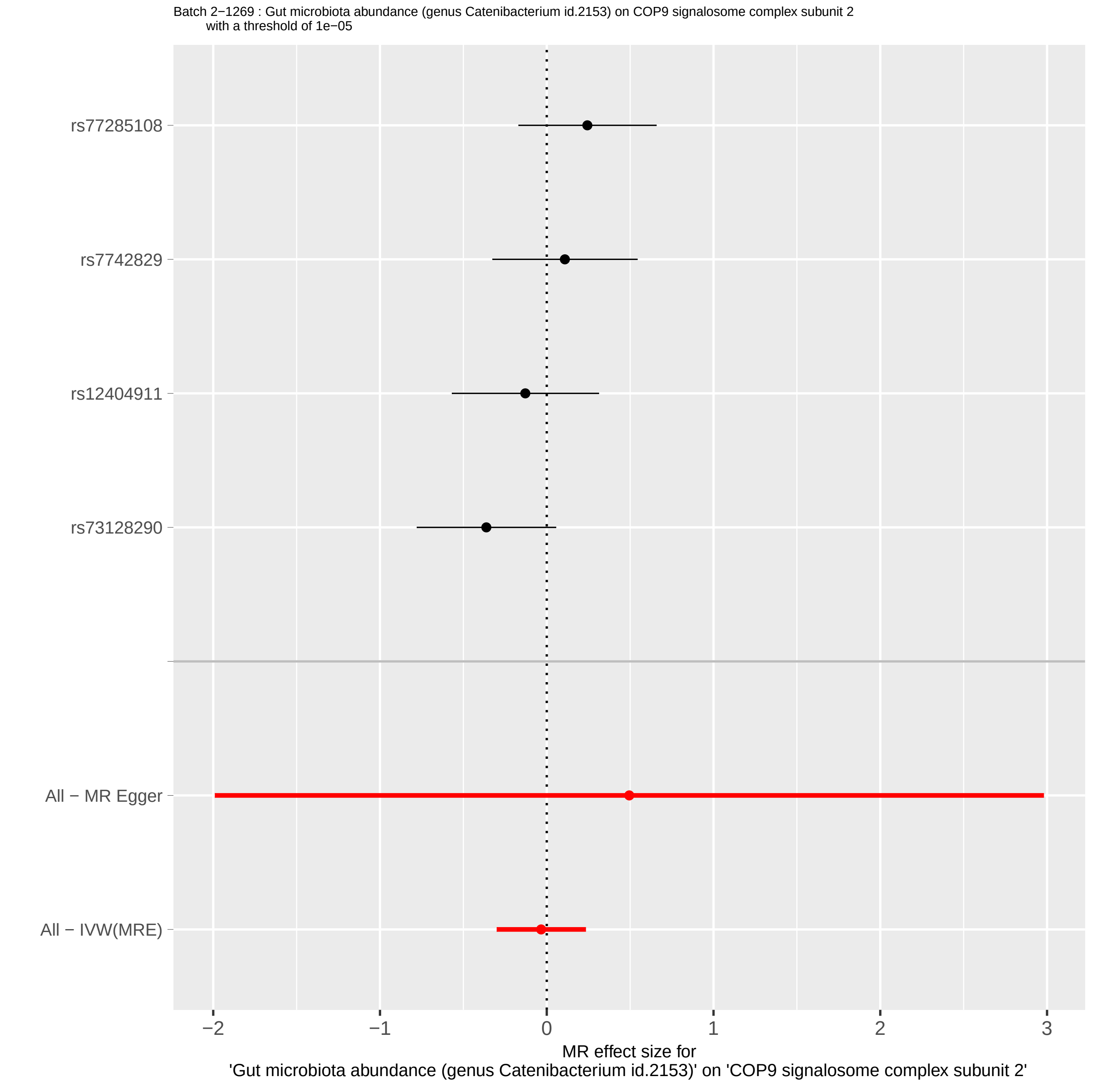
Batch 2−1269 : Gut microbiota abundance (genus Catenibacterium id.2153) on COP9 signalosome complex subunit 2
with a threshold of 1e−05
rs77285108
rs7742829
rs12404911
rs73128290
All − MR Egger
All − IVW(MRE)
−2
−1
0
1
2
3
MR effect size for
'Gut microbiota abundance (genus Catenibacterium id.2153)' on 'COP9 signalosome complex subunit 2'

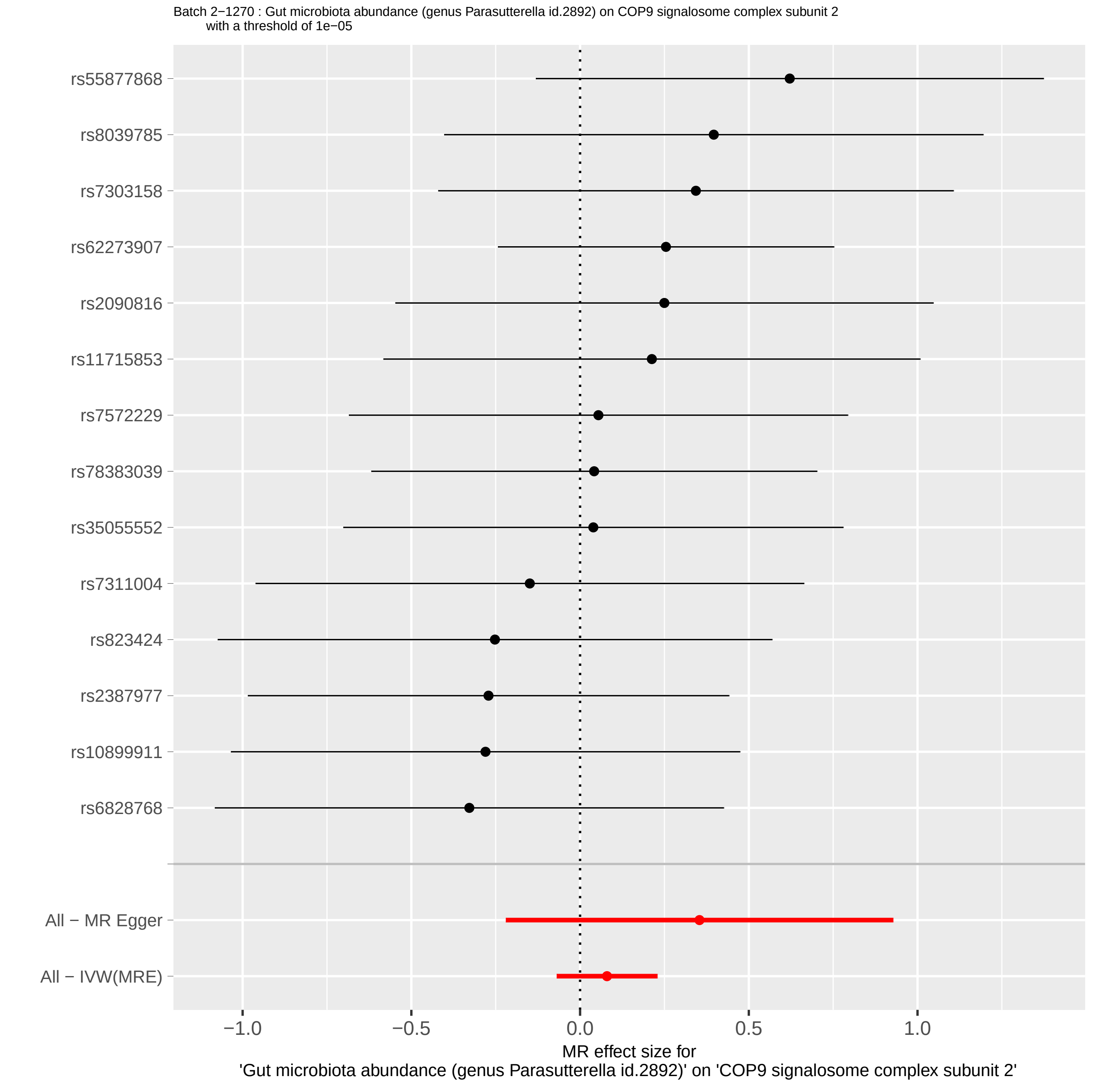

Batch 2−1270 : Gut microbiota abundance (genus Parasutterella id.2892) on COP9 signalosome complex subunit 2
with a threshold of 1e−05
rs55877868
rs8039785
rs7303158
rs62273907
rs2090816
rs11715853
rs7572229
rs78383039
rs35055552
rs7311004
rs823424
rs2387977
rs10899911
rs6828768
All − MR Egger
All − IVW(MRE)
−1.0
−0.5
0.0
0.5
1.0
MR effect size for
'Gut microbiota abundance (genus Parasutterella id.2892)' on 'COP9 signalosome complex subunit 2'

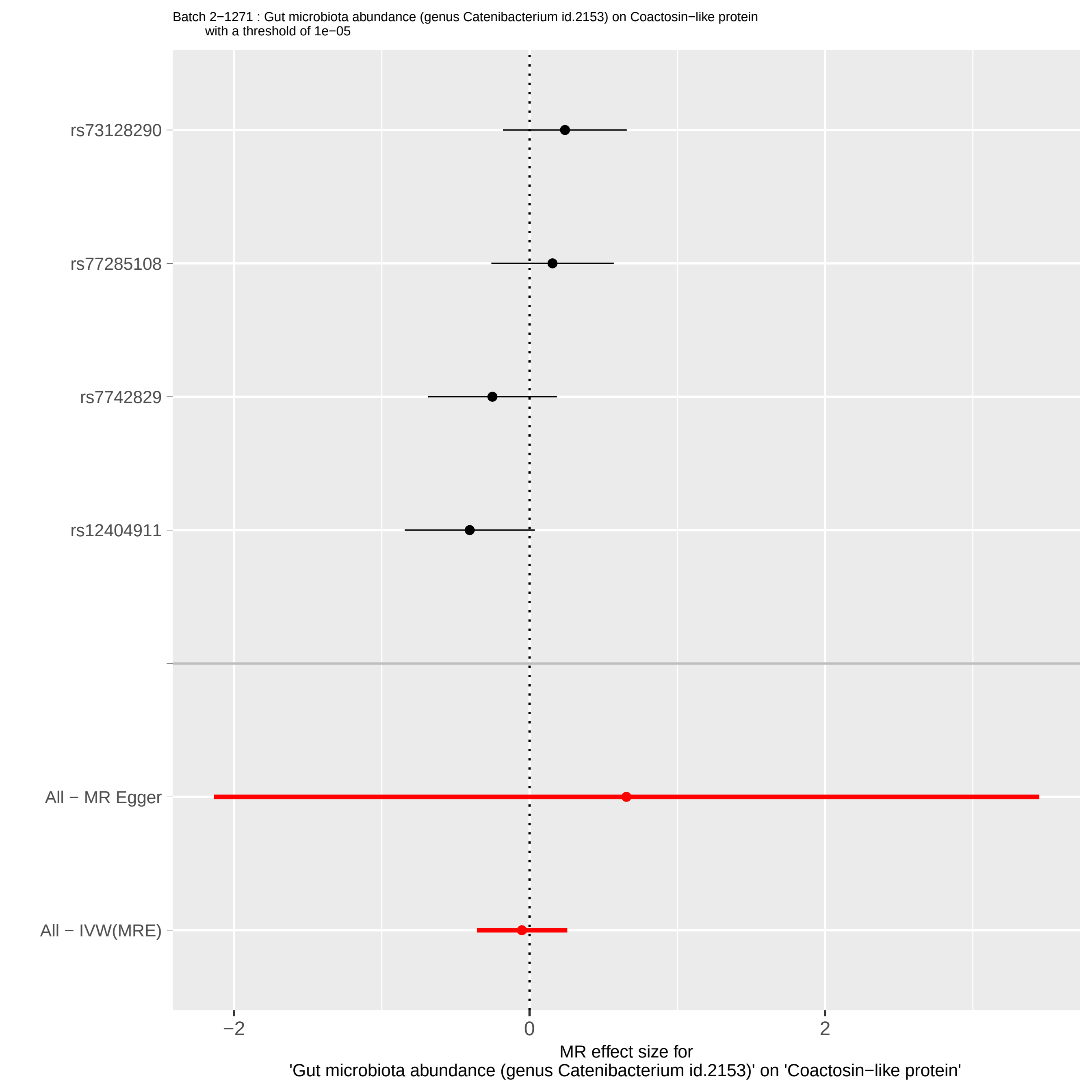
Batch 2−1271 : Gut microbiota abundance (genus Catenibacterium id.2153) on Coactosin−like protein
with a threshold of 1e−05
rs73128290
rs77285108
rs7742829
rs12404911
All − MR Egger
All − IVW(MRE)
−2
0
2
MR effect size for
'Gut microbiota abundance (genus Catenibacterium id.2153)' on 'Coactosin−like protein'

Batch 2−1272 : Gut microbiota abundance (genus Parasutterella id.2892) on Coactosin−like protein
with a threshold of 1e−05

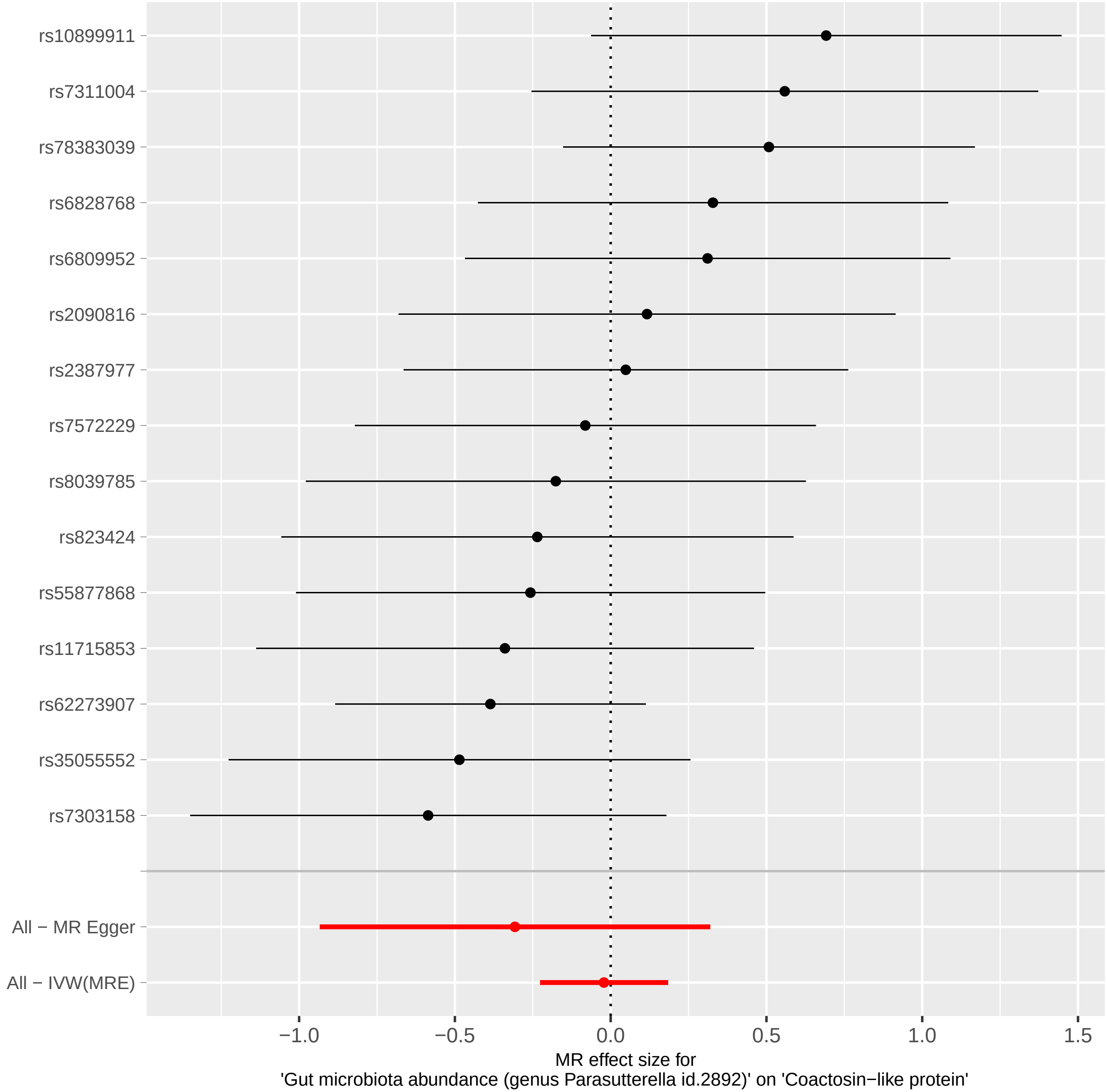

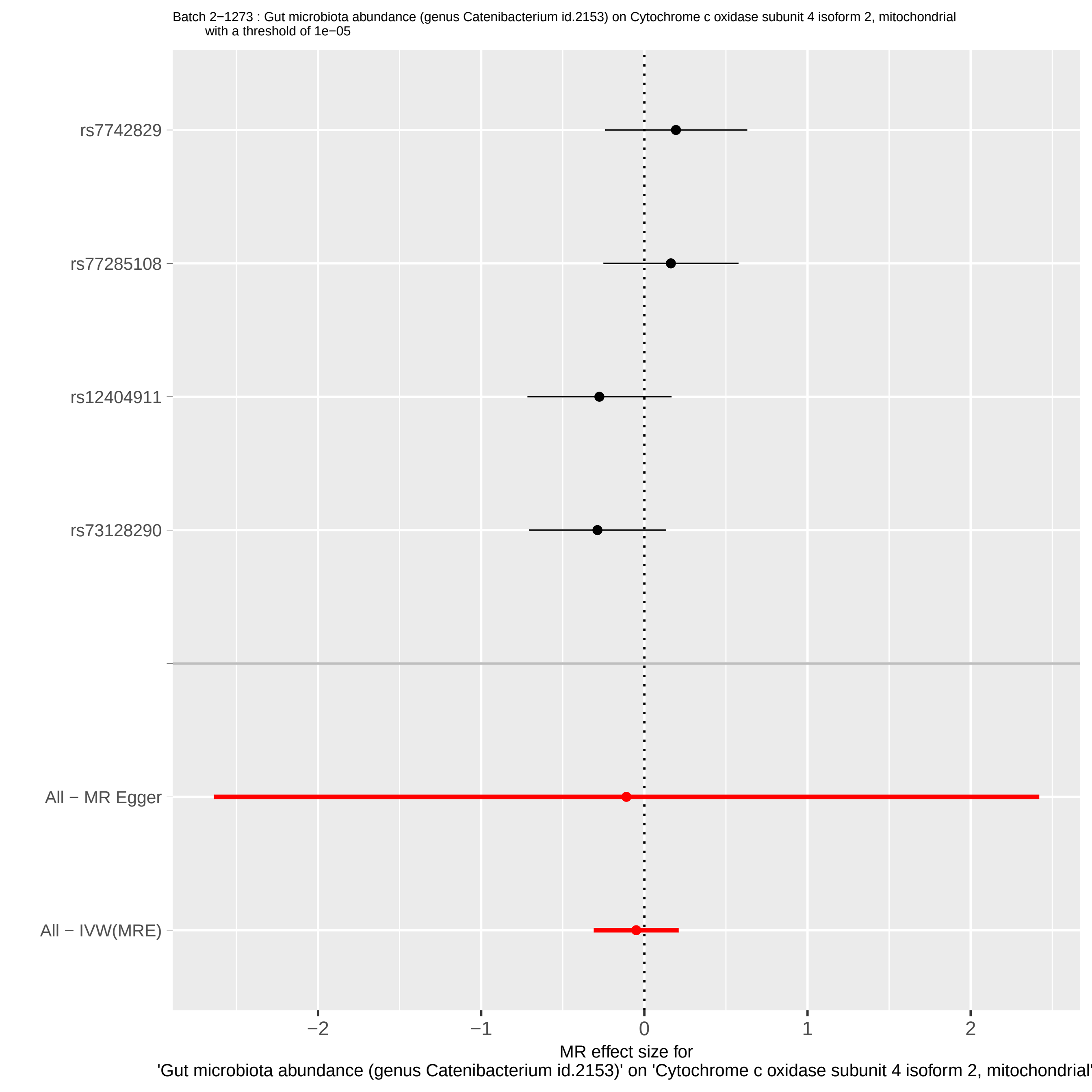

Batch 2−1273 : Gut microbiota abundance (genus Catenibacterium id.2153) on Cytochrome c oxidase subunit 4 isoform 2, mitochondrial
with a threshold of 1e−05
rs7742829
rs77285108
rs12404911
rs73128290
All − MR Egger
All − IVW(MRE)
−2
−1
0
1
2
MR effect size for
'Gut microbiota abundance (genus Catenibacterium id.2153)' on 'Cytochrome c oxidase subunit 4 isoform 2, mitochondrial'

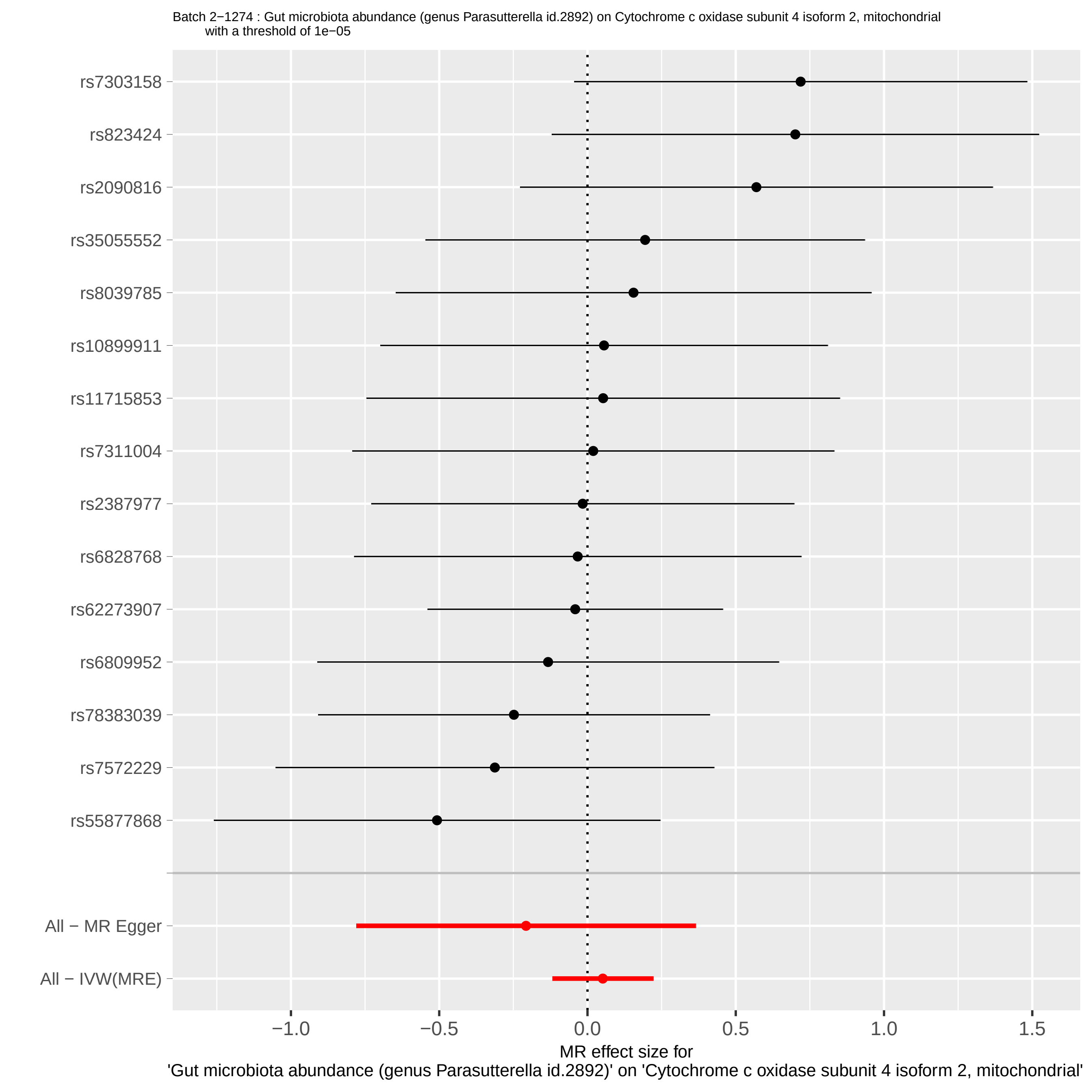
Batch 2−1274 : Gut microbiota abundance (genus Parasutterella id.2892) on Cytochrome c oxidase subunit 4 isoform 2, mitochondrial
with a threshold of 1e−05
rs7303158
rs823424
rs2090816
rs35055552
rs8039785
rs10899911
rs11715853
rs7311004
rs2387977
rs6828768
rs62273907
rs6809952
rs78383039
rs7572229
rs55877868
All − MR Egger
All − IVW(MRE)
−1.0
−0.5
0.0
0.5
1.0
1.5
MR effect size for
'Gut microbiota abundance (genus Parasutterella id.2892)' on 'Cytochrome c oxidase subunit 4 isoform 2, mitochondrial'

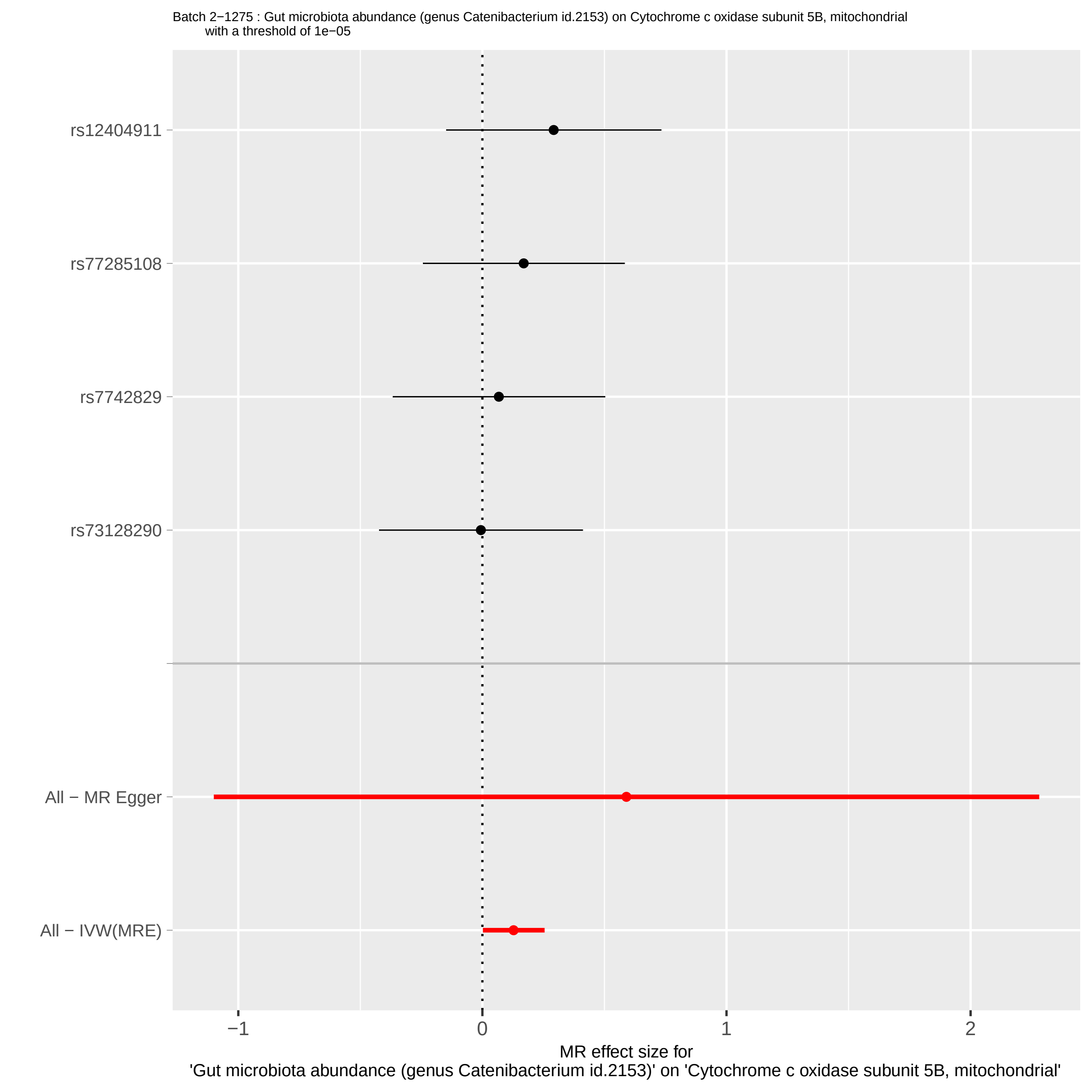

Batch 2−1275 : Gut microbiota abundance (genus Catenibacterium id.2153) on Cytochrome c oxidase subunit 5B, mitochondrial
with a threshold of 1e−05
rs12404911
rs77285108
rs7742829
rs73128290
All − MR Egger
All − IVW(MRE)
−1
0
1
2
MR effect size for
'Gut microbiota abundance (genus Catenibacterium id.2153)' on 'Cytochrome c oxidase subunit 5B, mitochondrial'

Batch 2−1276 : Gut microbiota abundance (genus Parasutterella id.2892) on Cytochrome c oxidase subunit 5B, mitochondrial
with a threshold of 1e−05

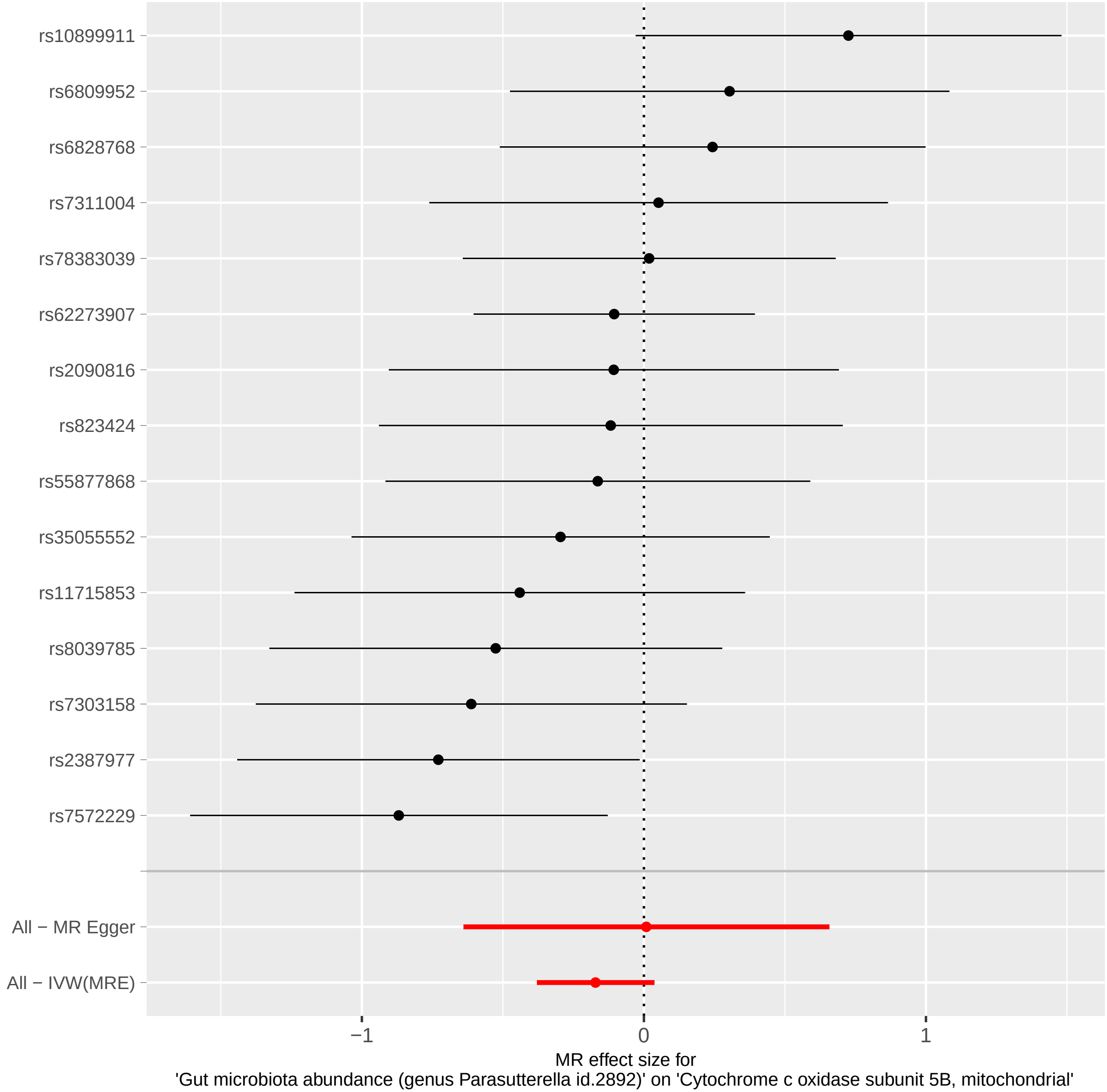

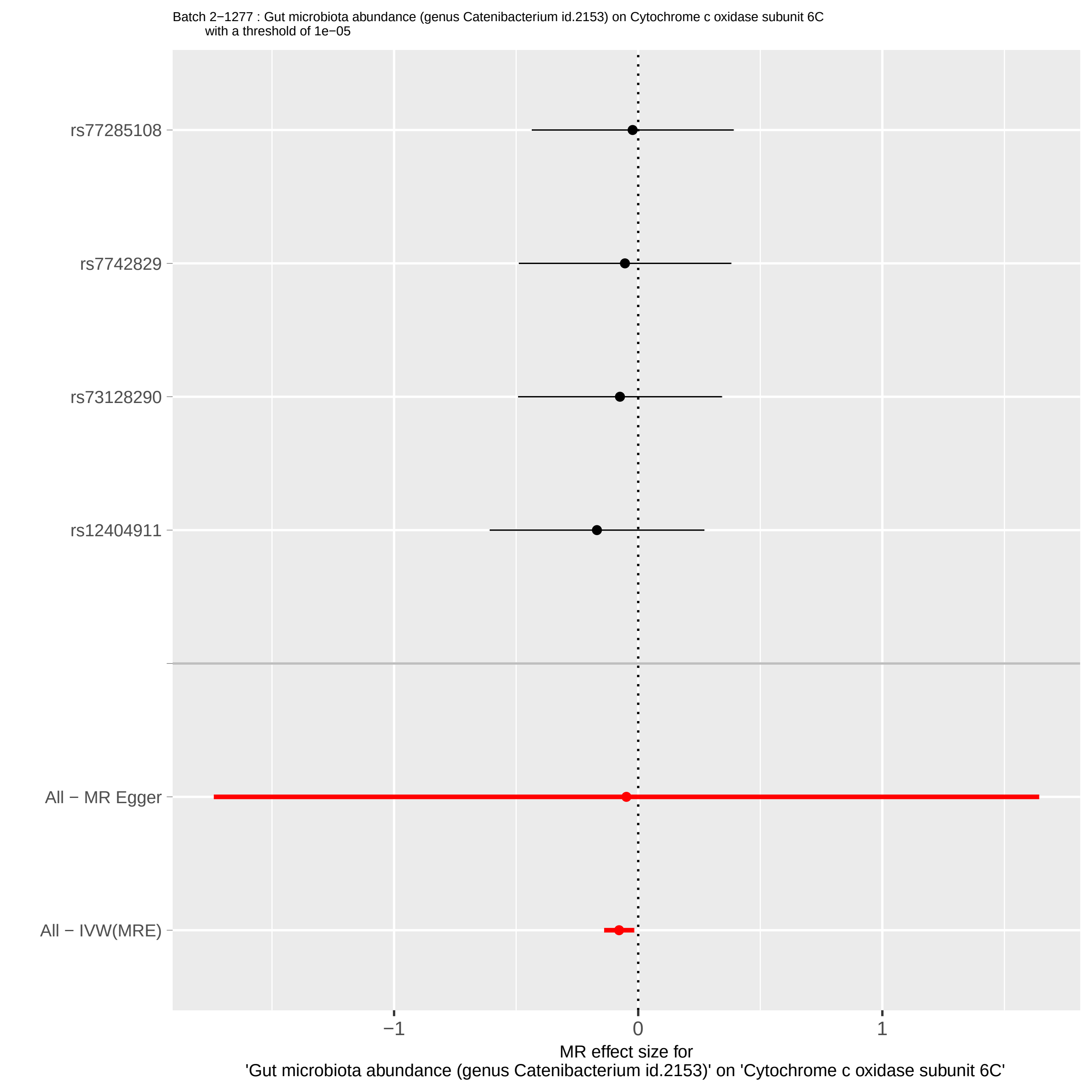

Batch 2−1277 : Gut microbiota abundance (genus Catenibacterium id.2153) on Cytochrome c oxidase subunit 6C
with a threshold of 1e−05
rs77285108
rs7742829
rs73128290
rs12404911
All − MR Egger
All − IVW(MRE)
−1
0
1
MR effect size for
'Gut microbiota abundance (genus Catenibacterium id.2153)' on 'Cytochrome c oxidase subunit 6C'

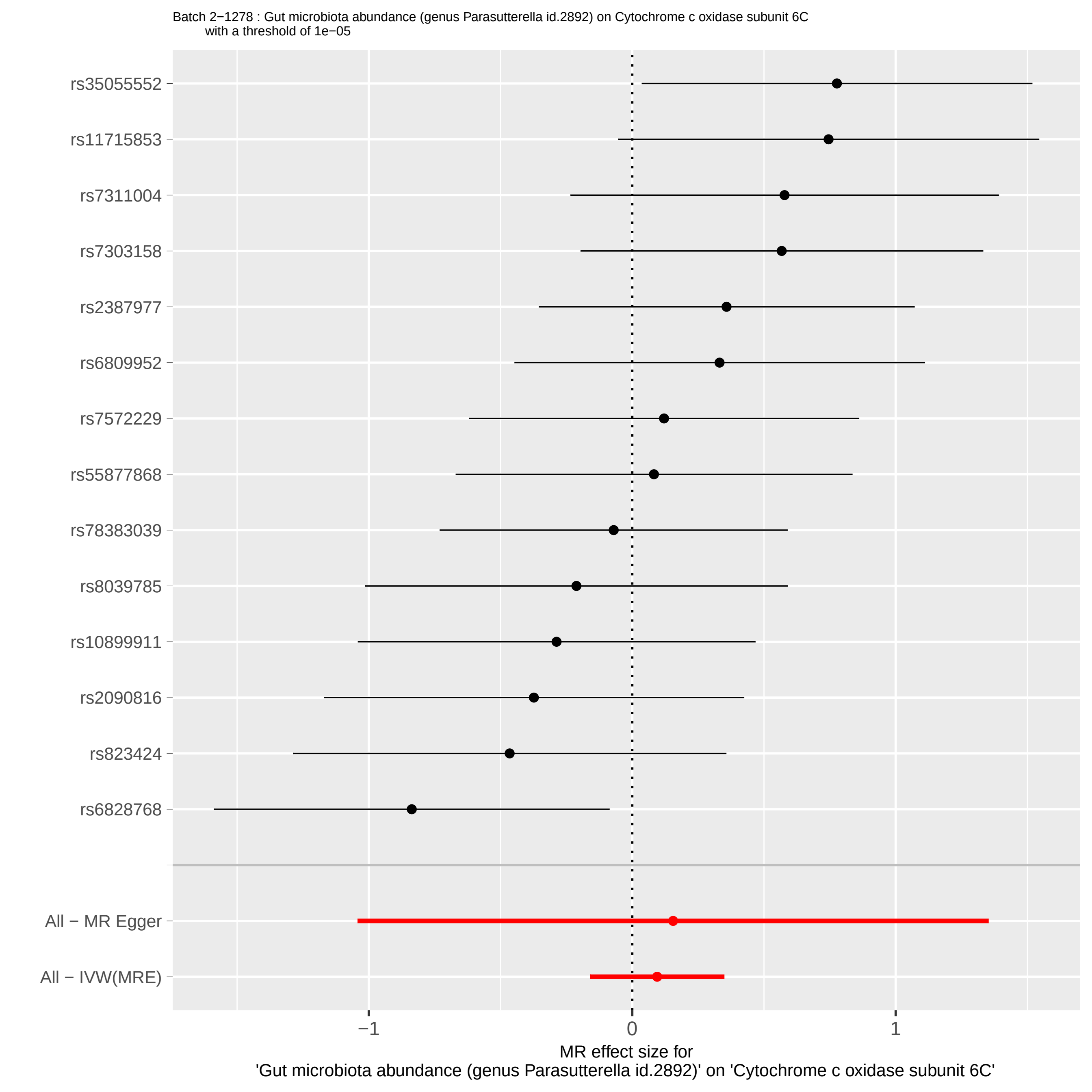

Batch 2−1278 : Gut microbiota abundance (genus Parasutterella id.2892) on Cytochrome c oxidase subunit 6C
with a threshold of 1e−05
rs35055552
rs11715853
rs7311004
rs7303158
rs2387977
rs6809952
rs7572229
rs55877868
rs78383039
rs8039785
rs10899911
rs2090816
rs823424
rs6828768
All − MR Egger
All − IVW(MRE)
−1
0
1
MR effect size for
'Gut microbiota abundance (genus Parasutterella id.2892)' on 'Cytochrome c oxidase subunit 6C'

Batch 2−1279 : Gut microbiota abundance (genus Catenibacterium id.2153) on Cytochrome c oxidase subunit 7A1, mitochondrial
with a threshold of 1e−05

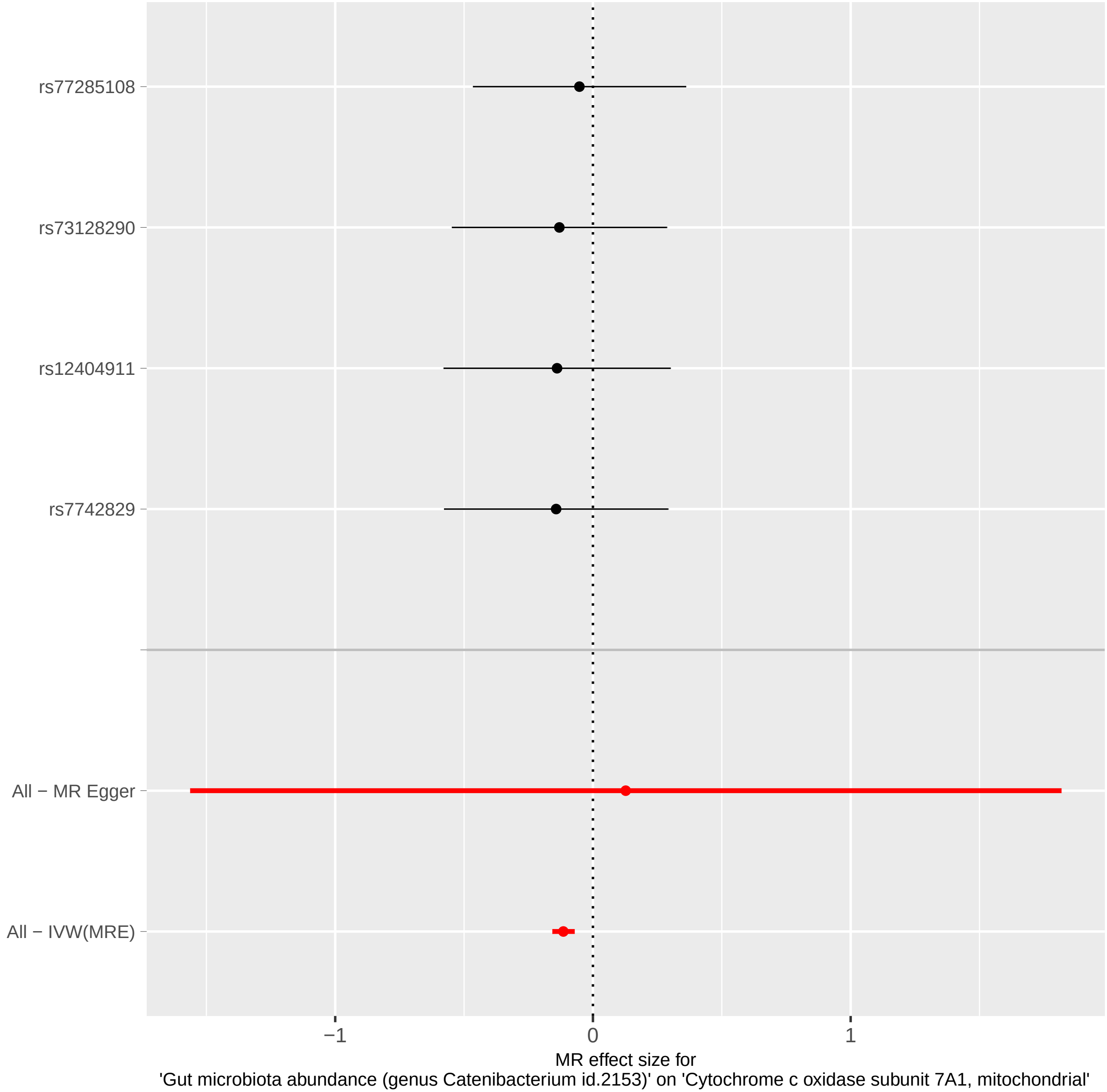

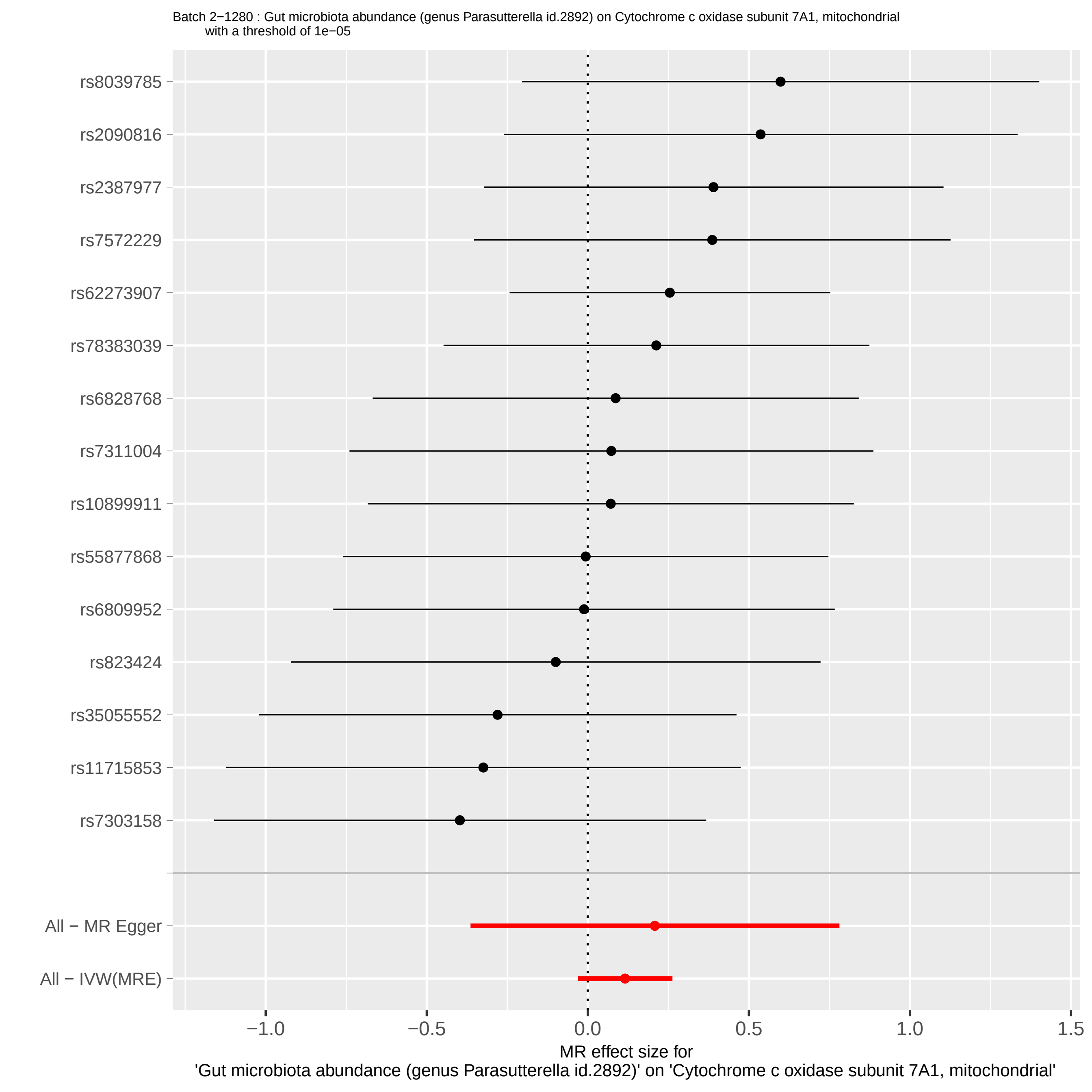

Batch 2−1280 : Gut microbiota abundance (genus Parasutterella id.2892) on Cytochrome c oxidase subunit 7A1, mitochondrial
with a threshold of 1e−05
rs8039785
rs2090816
rs2387977
rs7572229
rs62273907
rs78383039
rs6828768
rs7311004
rs10899911
rs55877868
rs6809952
rs823424
rs35055552
rs11715853
rs7303158
All − MR Egger
All − IVW(MRE)
−1.0
−0.5
0.0
0.5
1.0
1.5
MR effect size for
'Gut microbiota abundance (genus Parasutterella id.2892)' on 'Cytochrome c oxidase subunit 7A1, mitochondrial'

Batch 2−1281 : Gut microbiota abundance (genus Catenibacterium id.2153) on Cytochrome c oxidase subunit 8A, mitochondrial
with a threshold of 1e−05

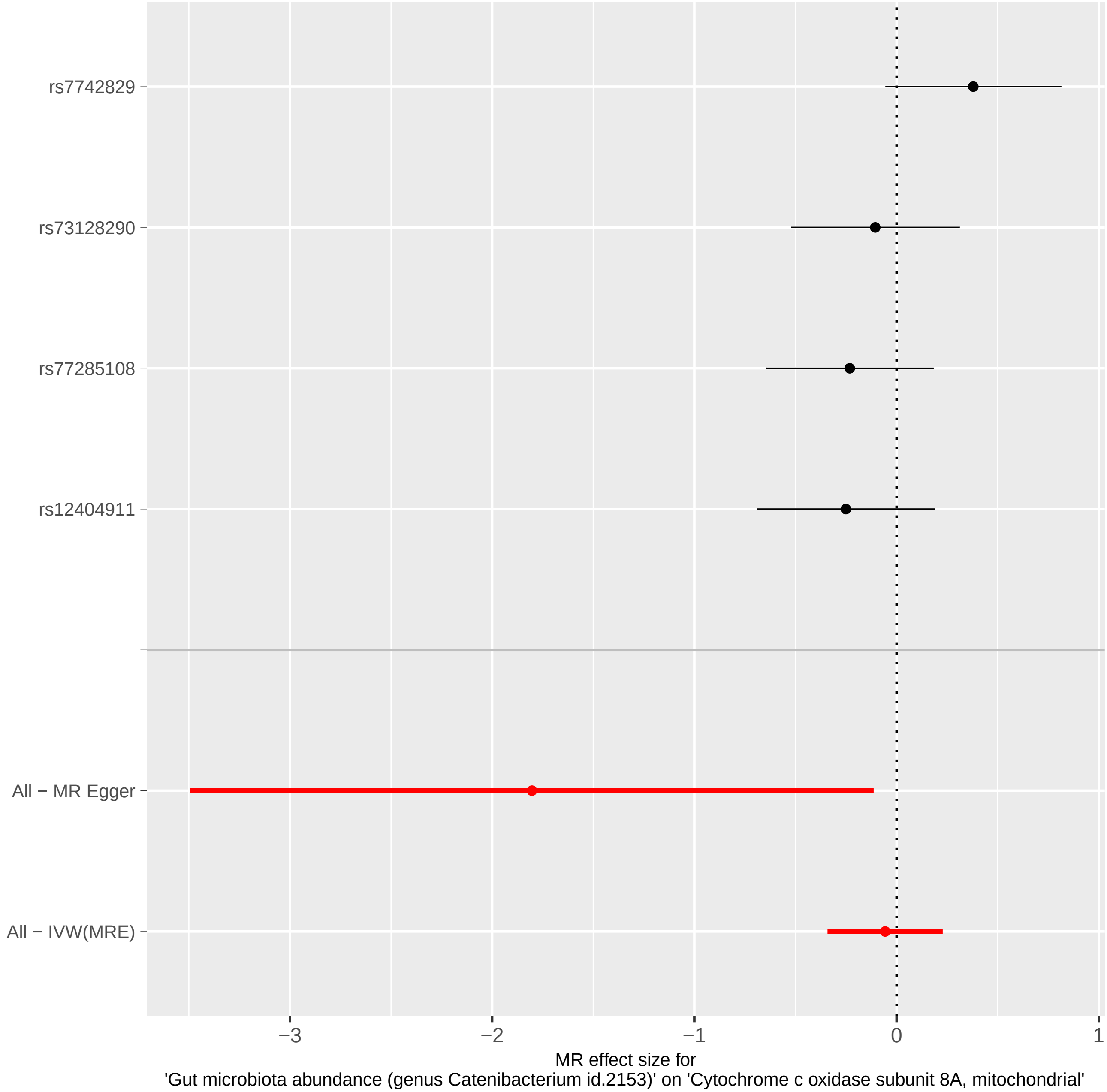

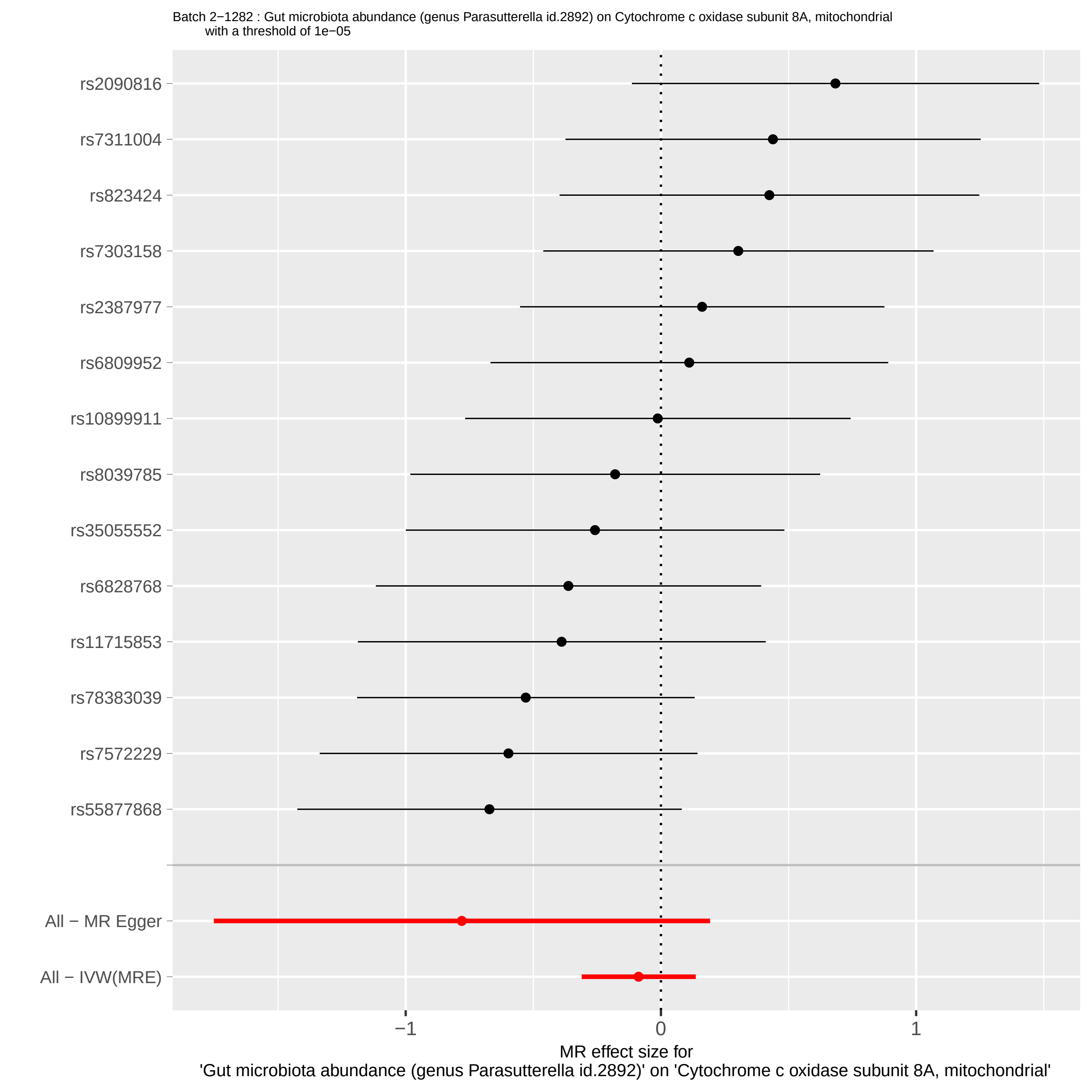

Batch 2−1282 : Gut microbiota abundance (genus Parasutterella id.2892) on Cytochrome c oxidase subunit 8A, mitochondrial
with a threshold of 1e−05
rs2090816
rs7311004
rs823424
rs7303158
rs2387977
rs6809952
rs10899911
rs8039785
rs35055552
rs6828768
rs11715853
rs78383039
rs7572229
rs55877868
All − MR Egger
All − IVW(MRE)
−1
0
1
MR effect size for
'Gut microbiota abundance (genus Parasutterella id.2892)' on 'Cytochrome c oxidase subunit 8A, mitochondrial'

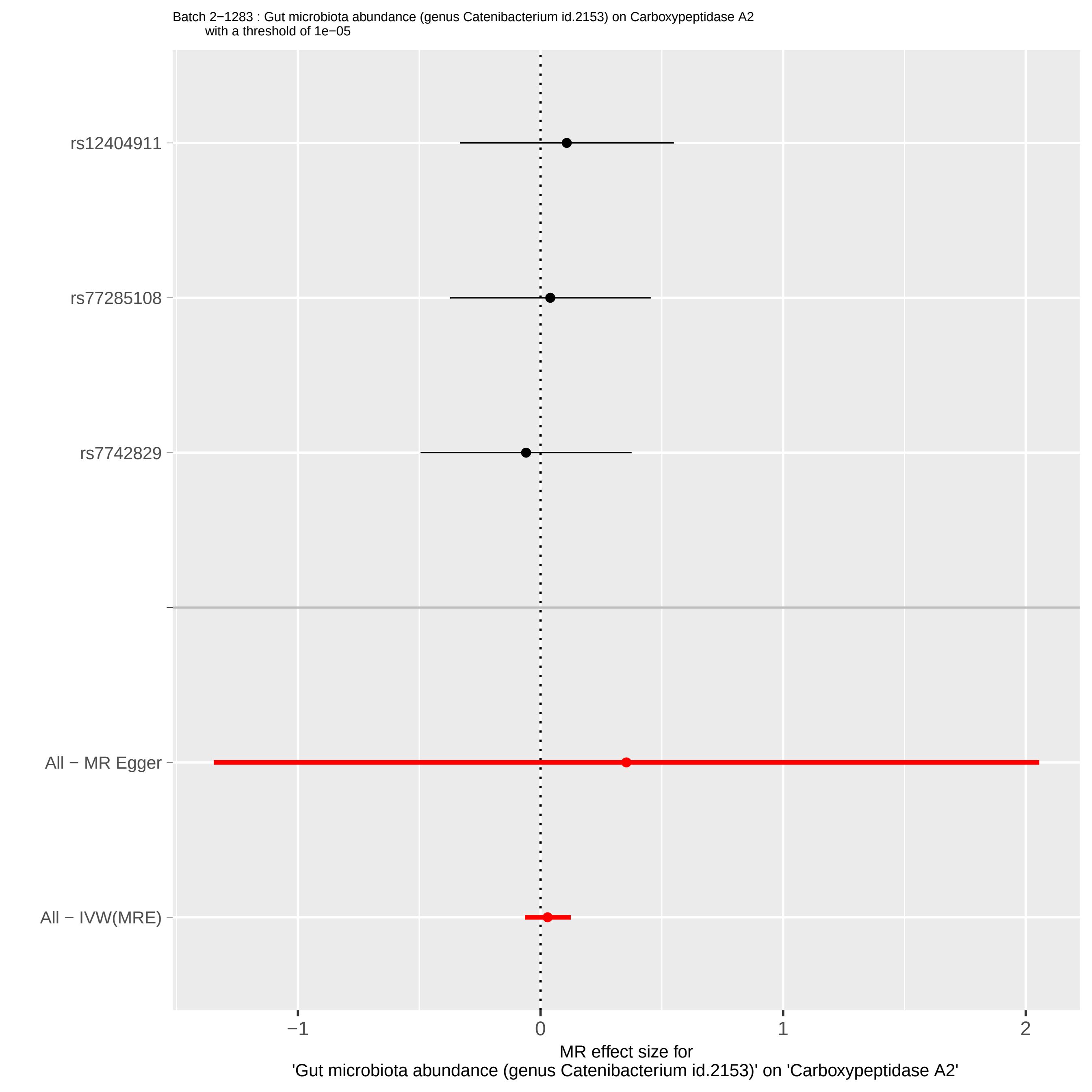

Batch 2−1283 : Gut microbiota abundance (genus Catenibacterium id.2153) on Carboxypeptidase A2
with a threshold of 1e−05
rs12404911
rs77285108
rs7742829
All − MR Egger
All − IVW(MRE)
−1
0
1
2
MR effect size for
'Gut microbiota abundance (genus Catenibacterium id.2153)' on 'Carboxypeptidase A2'

Batch 2−1284 : Gut microbiota abundance (genus Parasutterella id.2892) on Carboxypeptidase A2
with a threshold of 1e−05

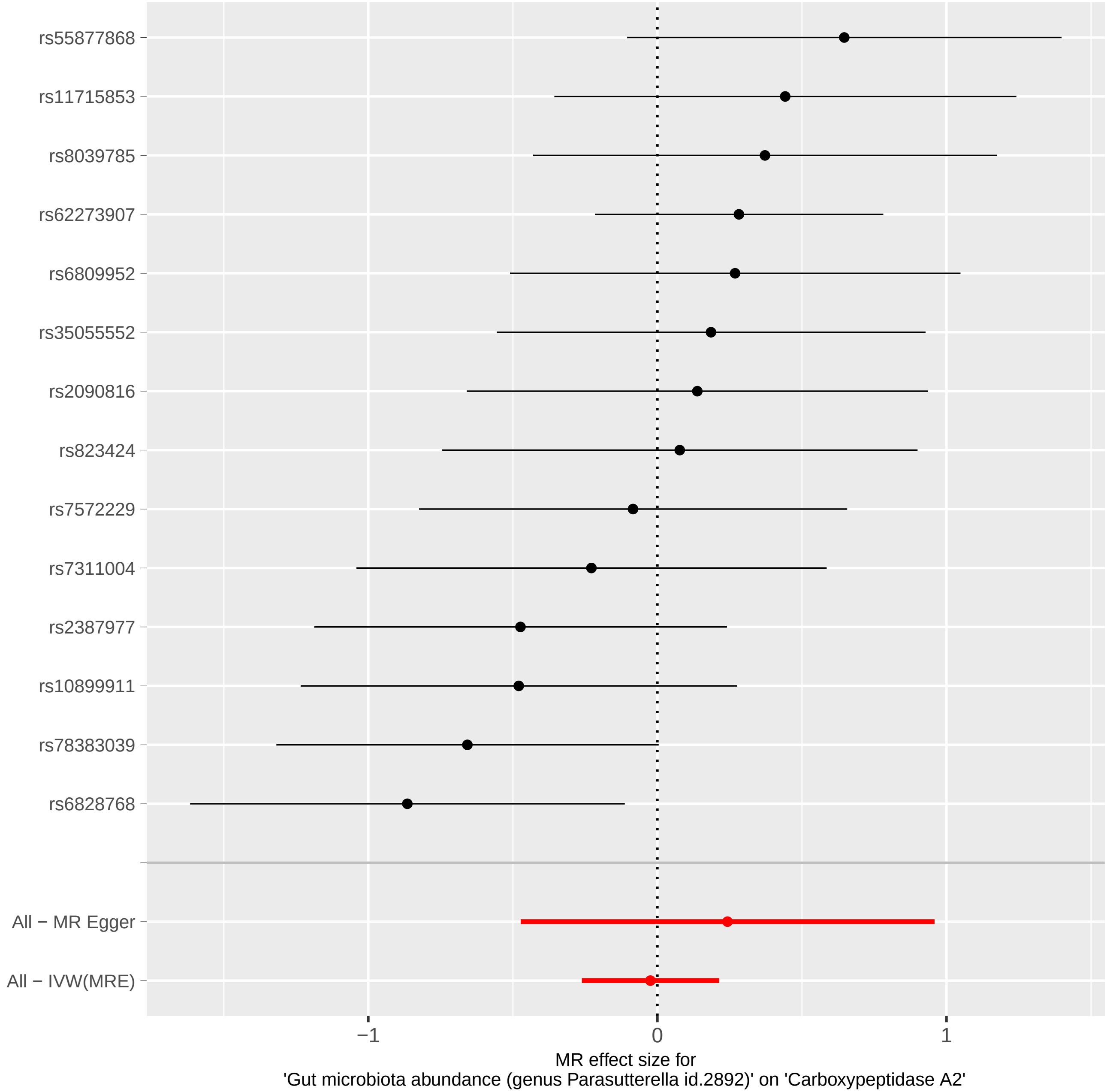

Batch 2−1285 : Gut microbiota abundance (genus Catenibacterium id.2153) on Carboxypeptidase A4
with a threshold of 1e−05

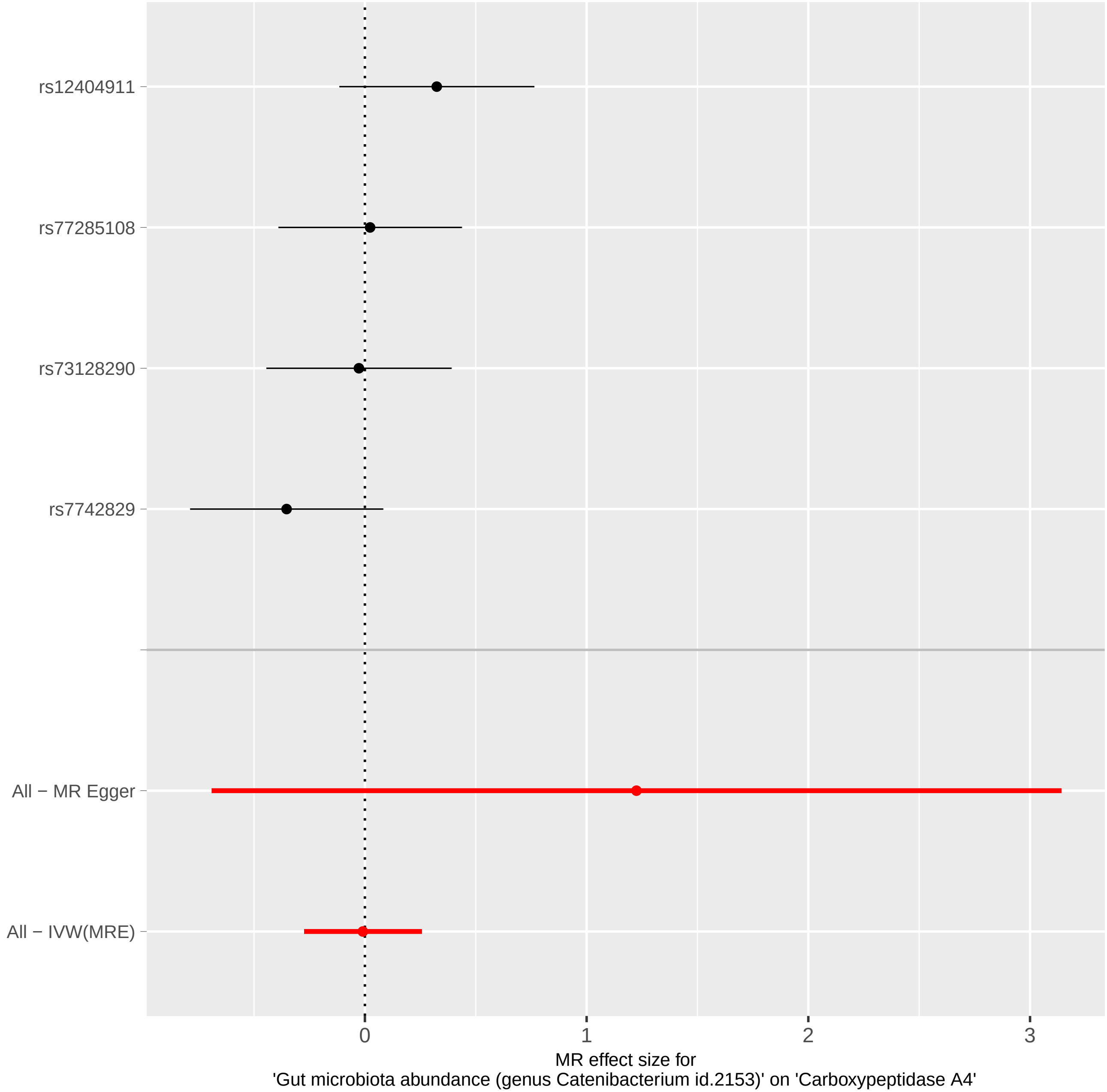

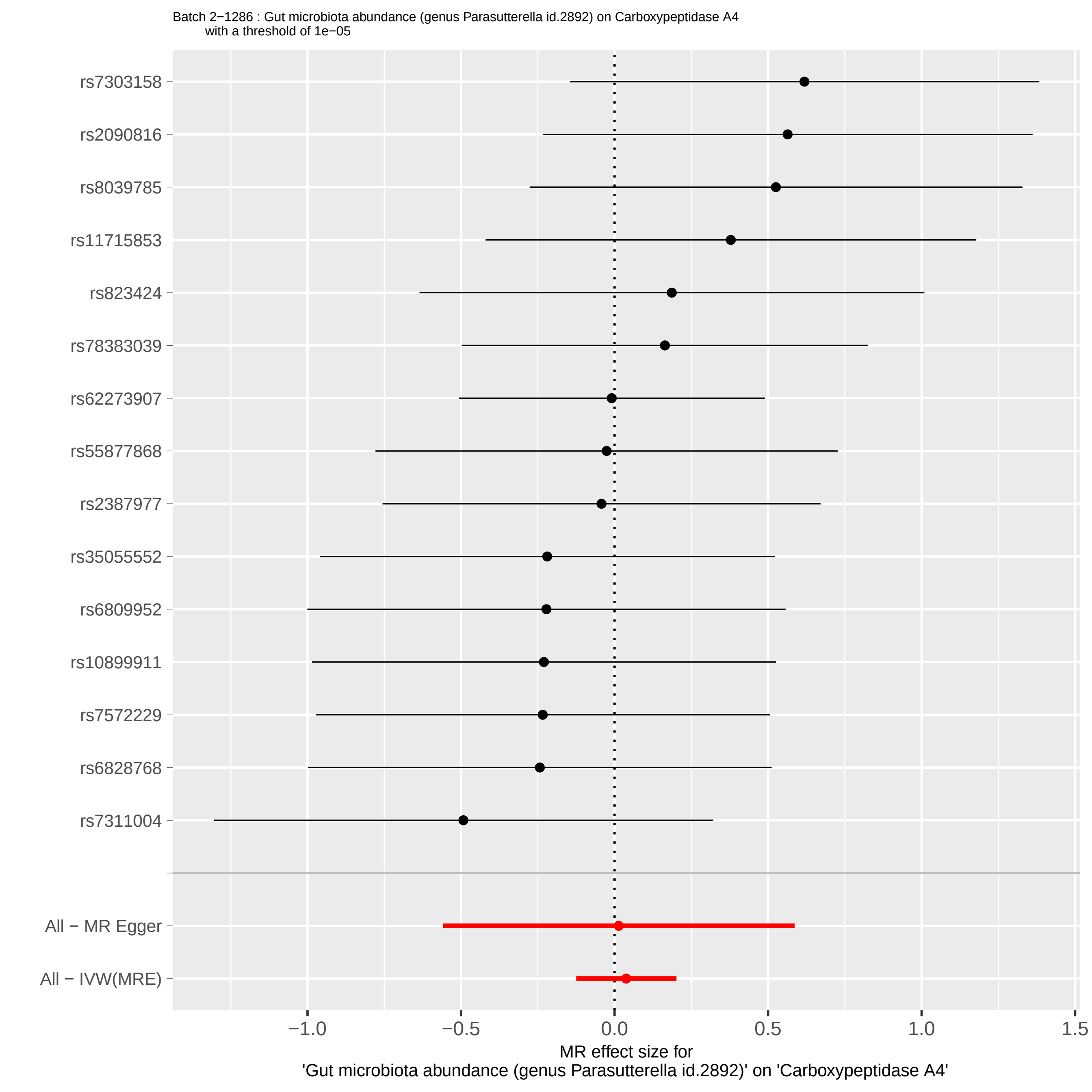
Batch 2−1286 : Gut microbiota abundance (genus Parasutterella id.2892) on Carboxypeptidase A4
with a threshold of 1e−05
rs7303158
rs2090816
rs8039785
rs11715853
rs823424
rs78383039
rs62273907
rs55877868
rs2387977
rs35055552
rs6809952
rs10899911
rs7572229
rs6828768
rs7311004
All − MR Egger
All − IVW(MRE)
−1.0
−0.5
0.0
0.5
1.0
1.5
MR effect size for
'Gut microbiota abundance (genus Parasutterella id.2892)' on 'Carboxypeptidase A4'

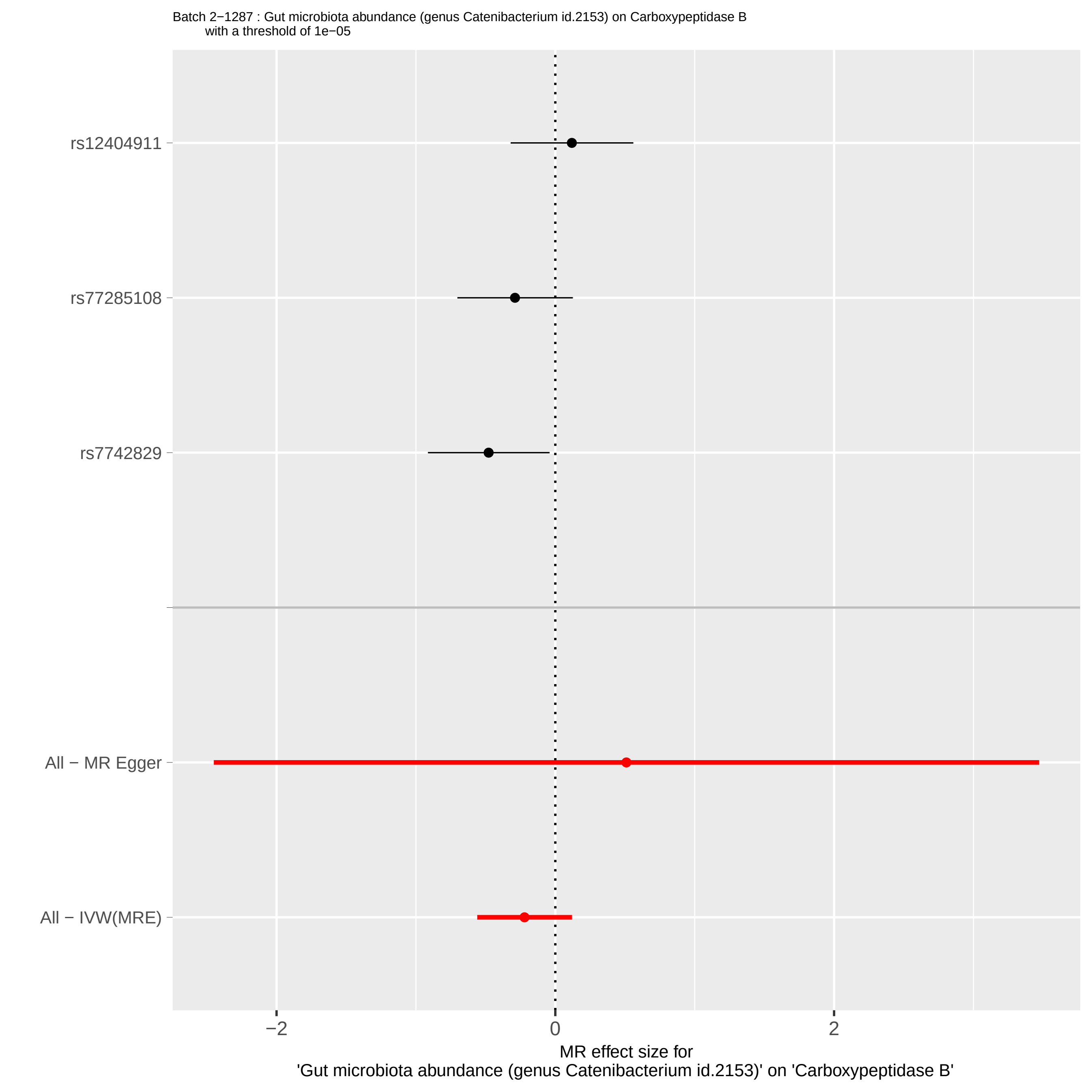

Batch 2−1287 : Gut microbiota abundance (genus Catenibacterium id.2153) on Carboxypeptidase B
with a threshold of 1e−05
rs12404911
rs77285108
rs7742829
All − MR Egger
All − IVW(MRE)
−2
0
2
MR effect size for
'Gut microbiota abundance (genus Catenibacterium id.2153)' on 'Carboxypeptidase B'

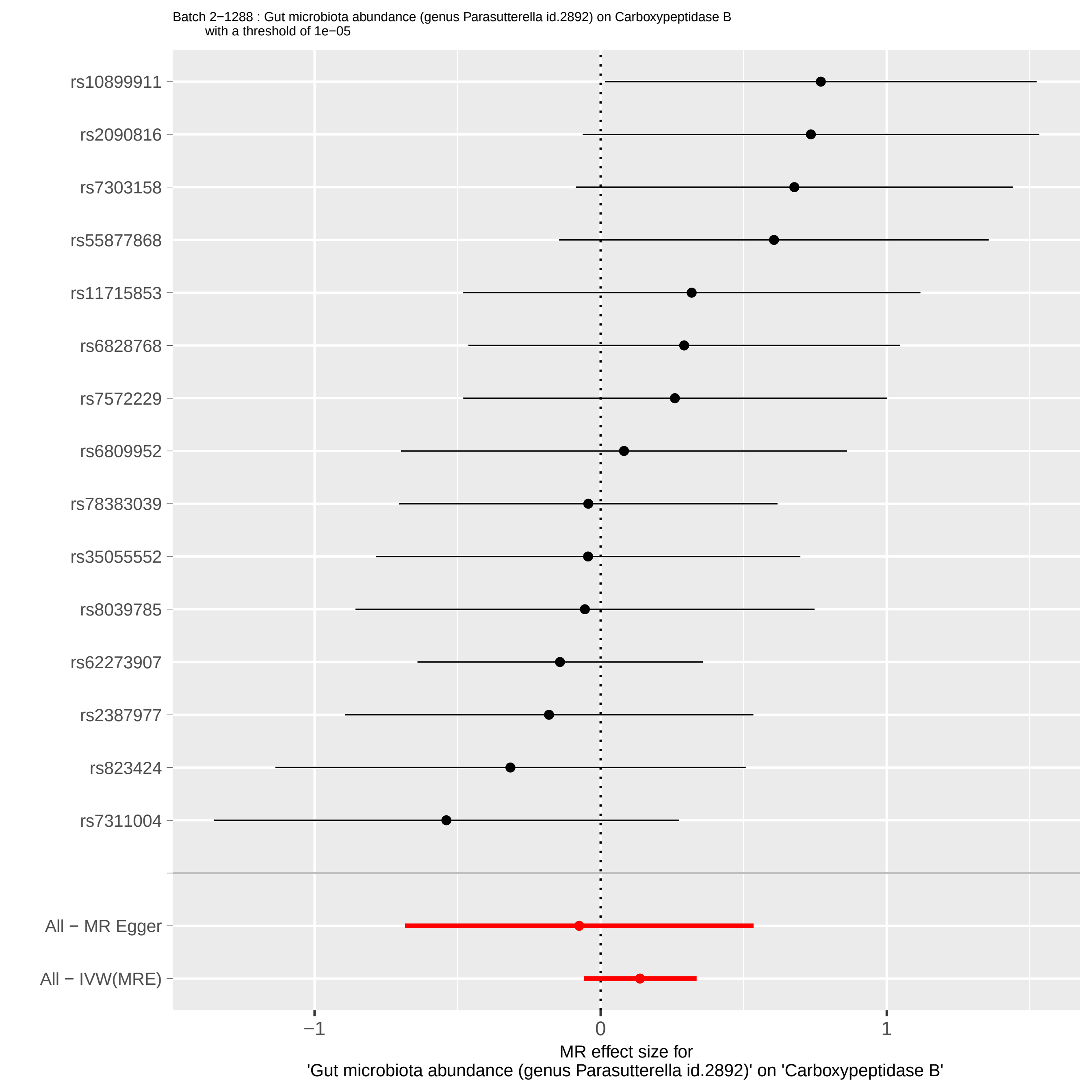

Batch 2−1288 : Gut microbiota abundance (genus Parasutterella id.2892) on Carboxypeptidase B
with a threshold of 1e−05
rs10899911
rs2090816
rs7303158
rs55877868
rs11715853
rs6828768
rs7572229
rs6809952
rs78383039
rs35055552
rs8039785
rs62273907
rs2387977
rs823424
rs7311004
All − MR Egger
All − IVW(MRE)
−1
0
1
MR effect size for
'Gut microbiota abundance (genus Parasutterella id.2892)' on 'Carboxypeptidase B'

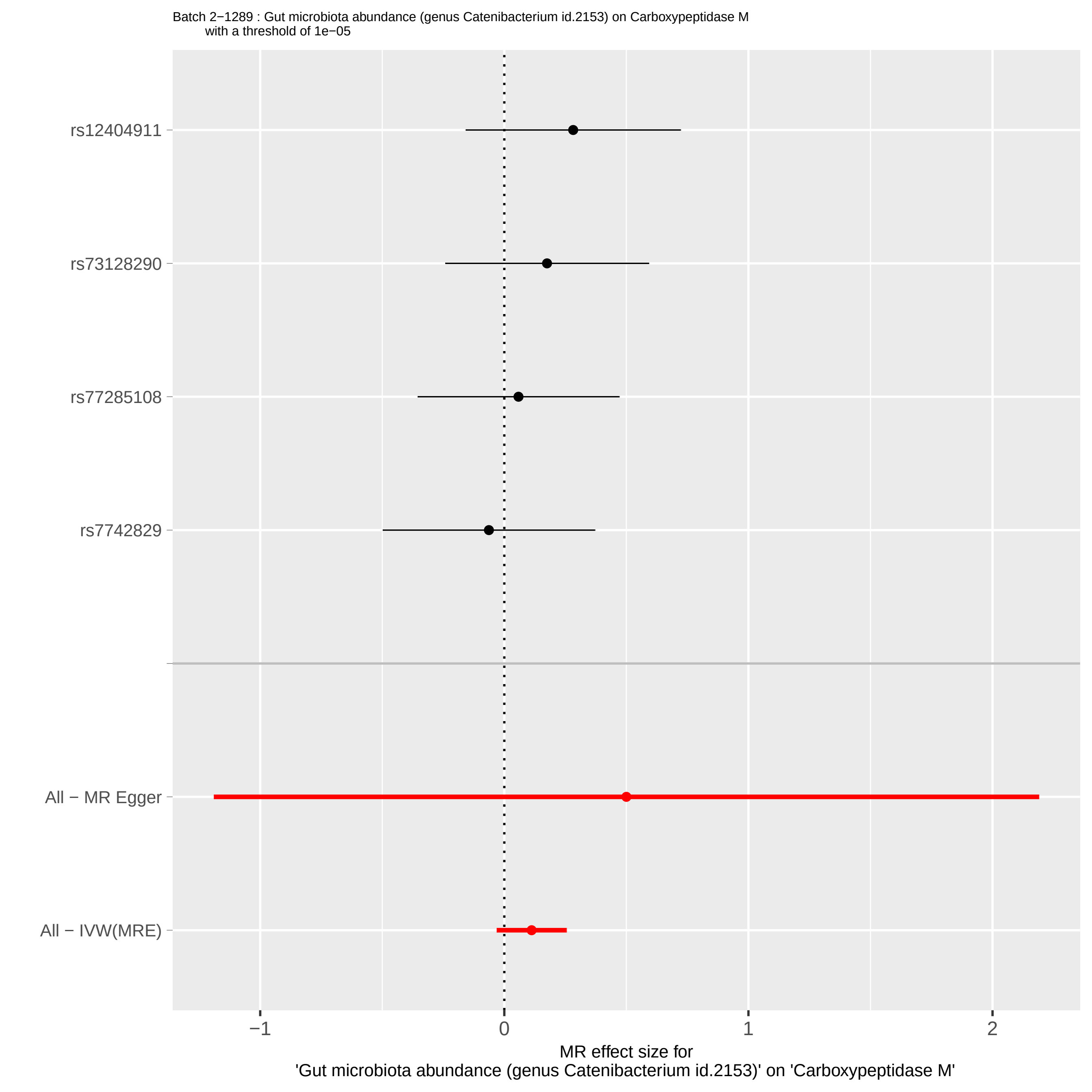
Batch 2−1289 : Gut microbiota abundance (genus Catenibacterium id.2153) on Carboxypeptidase M
with a threshold of 1e−05
rs12404911
rs73128290
rs77285108
rs7742829
All − MR Egger
All − IVW(MRE)
−1
0
1
2
MR effect size for
'Gut microbiota abundance (genus Catenibacterium id.2153)' on 'Carboxypeptidase M'

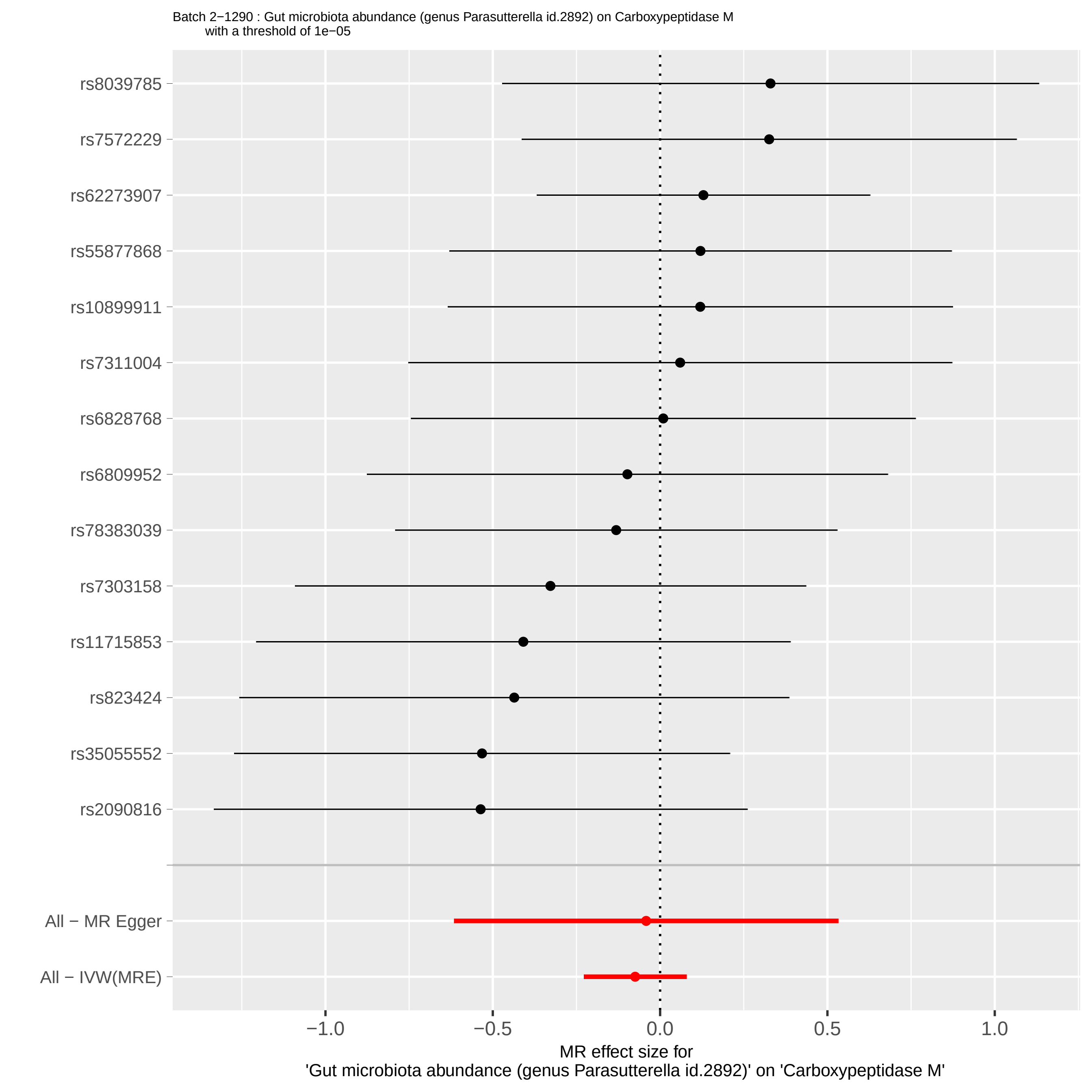

Batch 2−1290 : Gut microbiota abundance (genus Parasutterella id.2892) on Carboxypeptidase M
with a threshold of 1e−05
rs8039785
rs7572229
rs62273907
rs55877868
rs10899911
rs7311004
rs6828768
rs6809952
rs78383039
rs7303158
rs11715853
rs823424
rs35055552
rs2090816
All − MR Egger
All − IVW(MRE)
−1.0
−0.5
0.0
0.5
1.0
MR effect size for
'Gut microbiota abundance (genus Parasutterella id.2892)' on 'Carboxypeptidase M'

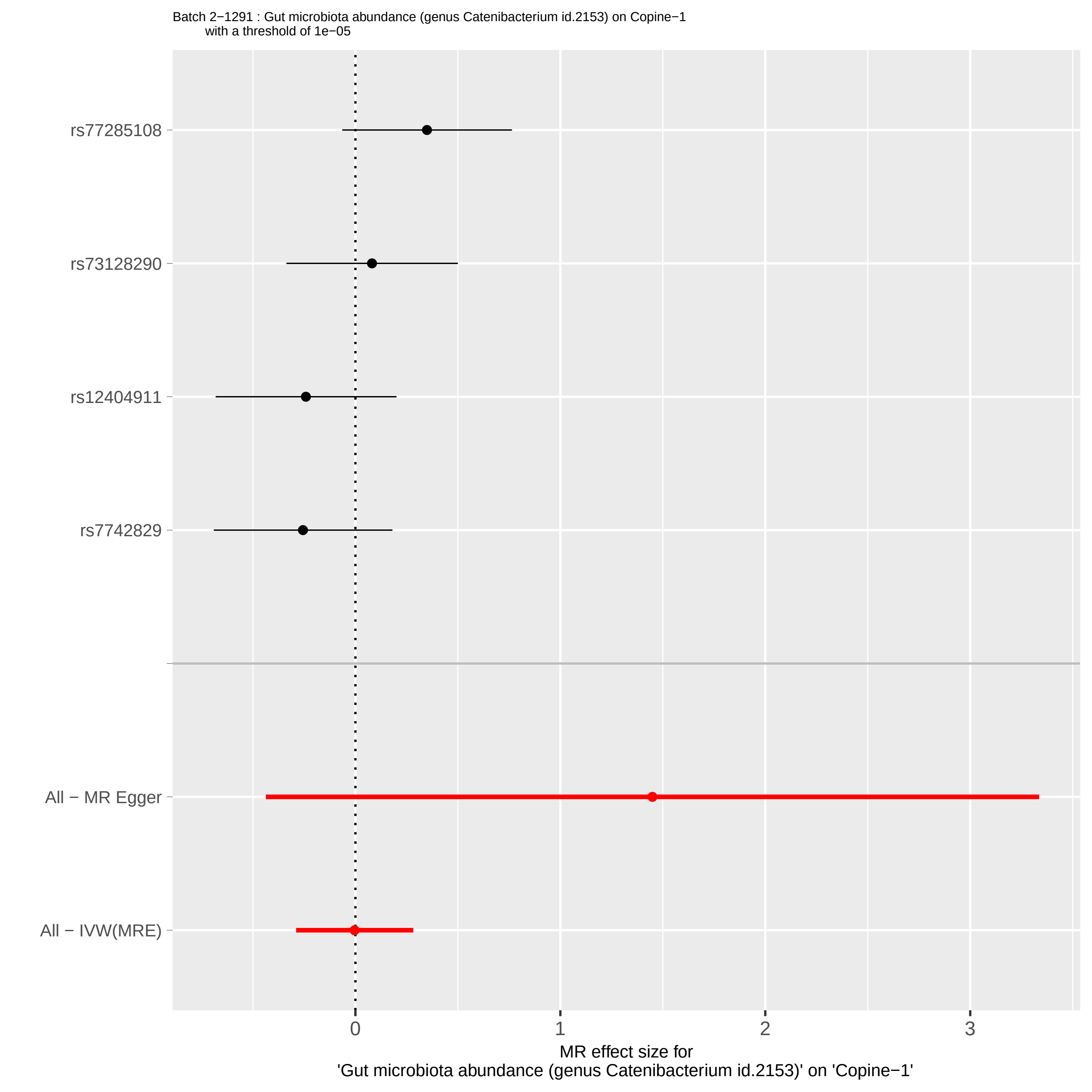

Batch 2−1291 : Gut microbiota abundance (genus Catenibacterium id.2153) on Copine−1
with a threshold of 1e−05
rs77285108
rs73128290
rs12404911
rs7742829
All − MR Egger
All − IVW(MRE)
0
1
2
3
MR effect size for
'Gut microbiota abundance (genus Catenibacterium id.2153)' on 'Copine−1'

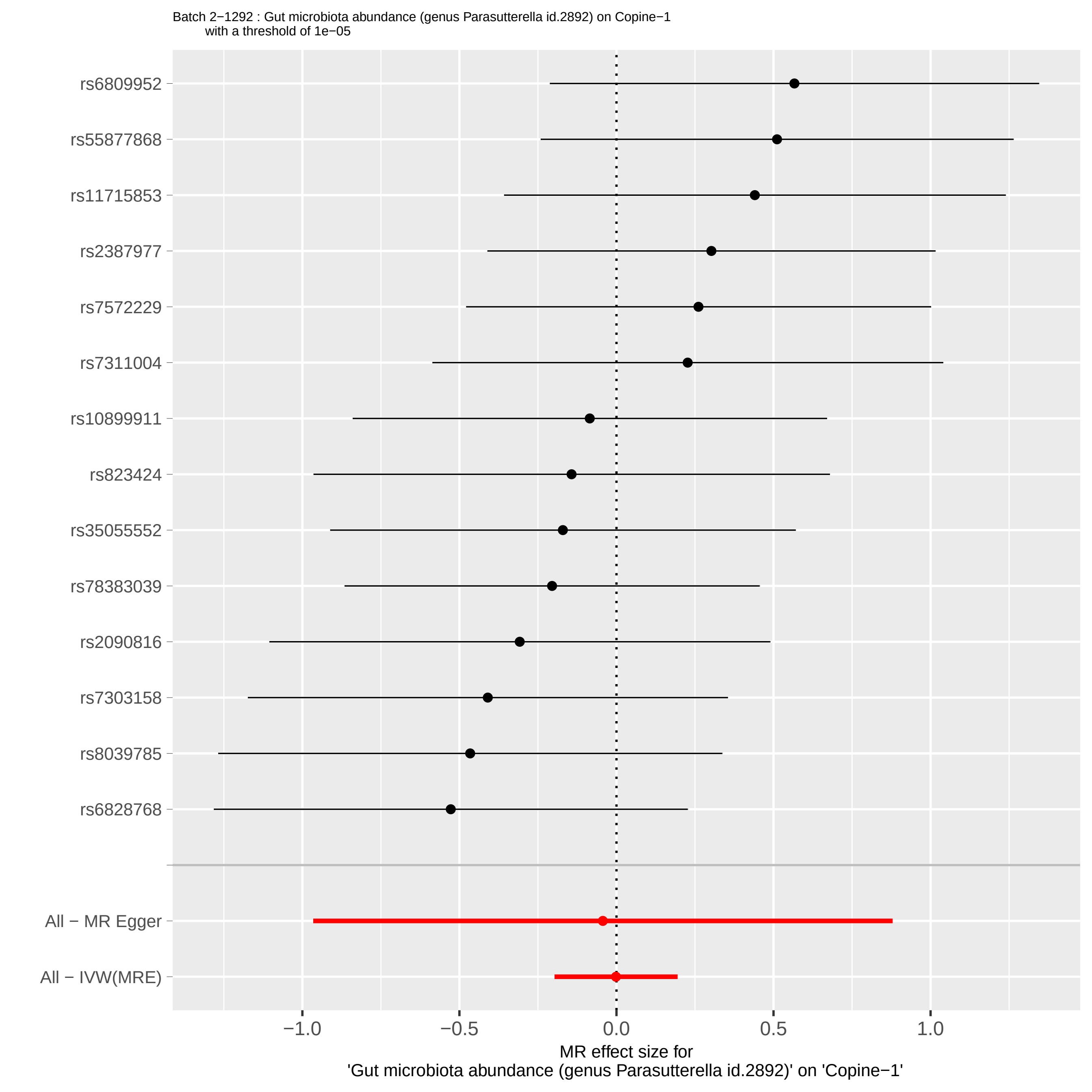

Batch 2−1292 : Gut microbiota abundance (genus Parasutterella id.2892) on Copine−1
with a threshold of 1e−05
rs6809952
rs55877868
rs11715853
rs2387977
rs7572229
rs7311004
rs10899911
rs823424
rs35055552
rs78383039
rs2090816
rs7303158
rs8039785
rs6828768
All − MR Egger
All − IVW(MRE)
−1.0
−0.5
0.0
0.5
1.0
MR effect size for
'Gut microbiota abundance (genus Parasutterella id.2892)' on 'Copine−1'

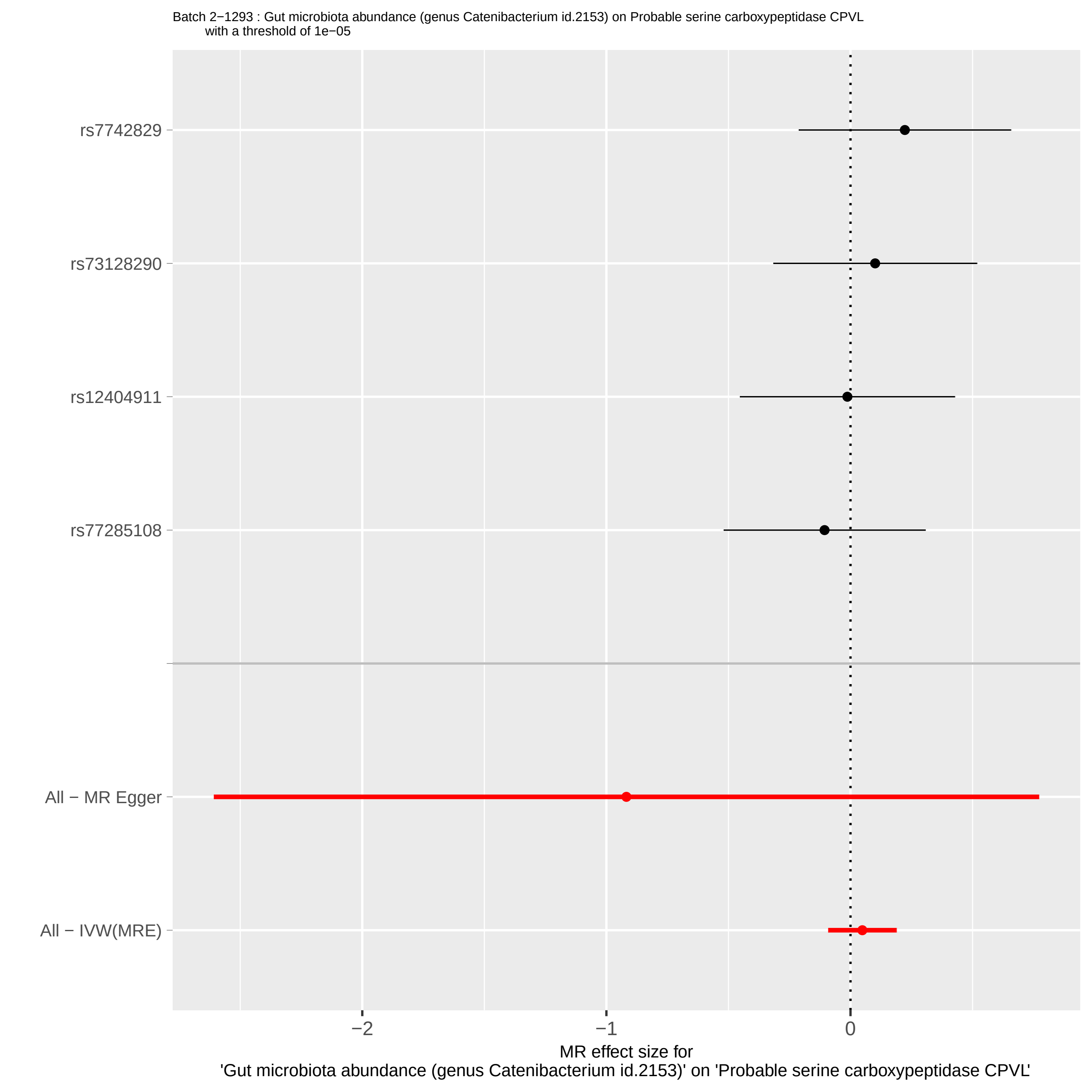
Batch 2−1293 : Gut microbiota abundance (genus Catenibacterium id.2153) on Probable serine carboxypeptidase CPVL
with a threshold of 1e−05
rs7742829
rs73128290
rs12404911
rs77285108
All − MR Egger
All − IVW(MRE)
−2
−1
0
MR effect size for
'Gut microbiota abundance (genus Catenibacterium id.2153)' on 'Probable serine carboxypeptidase CPVL'

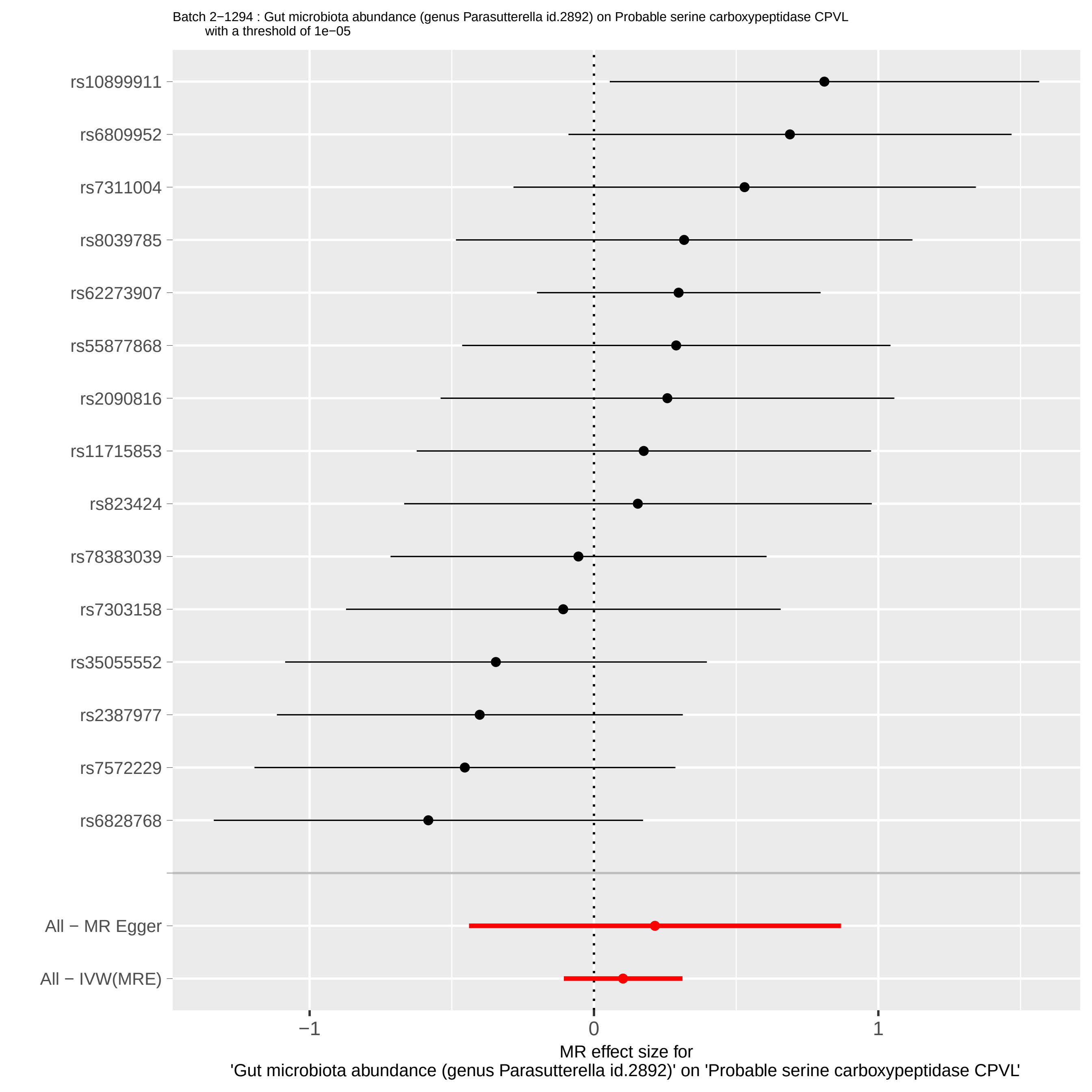
Batch 2−1294 : Gut microbiota abundance (genus Parasutterella id.2892) on Probable serine carboxypeptidase CPVL
with a threshold of 1e−05
rs10899911
rs6809952
rs7311004
rs8039785
rs62273907
rs55877868
rs2090816
rs11715853
rs823424
rs78383039
rs7303158
rs35055552
rs2387977
rs7572229
rs6828768
All − MR Egger
All − IVW(MRE)
−1
0
1
MR effect size for
'Gut microbiota abundance (genus Parasutterella id.2892)' on 'Probable serine carboxypeptidase CPVL'

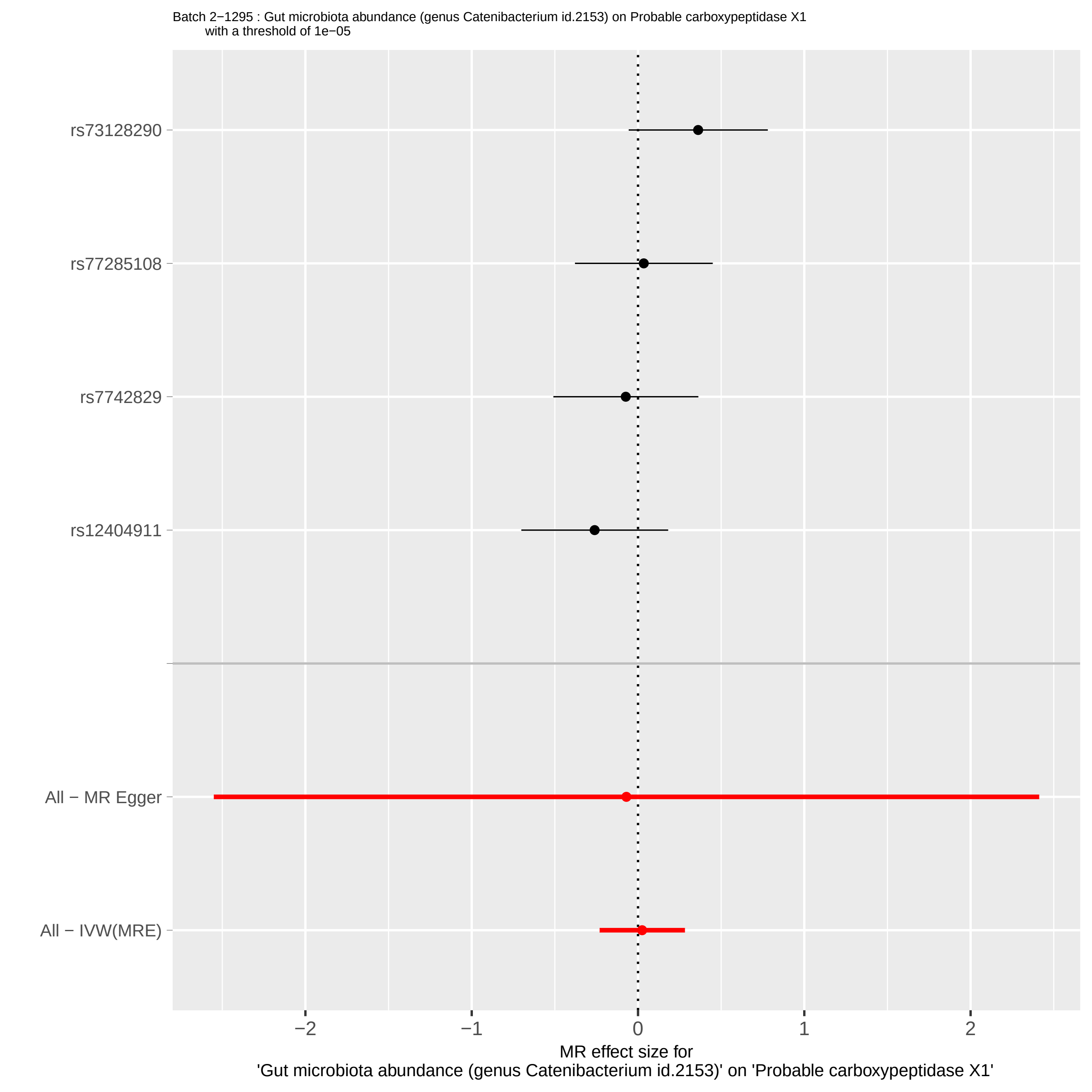
Batch 2−1295 : Gut microbiota abundance (genus Catenibacterium id.2153) on Probable carboxypeptidase X1
with a threshold of 1e−05
rs73128290
rs77285108
rs7742829
rs12404911
All − MR Egger
All − IVW(MRE)
−2
−1
0
1
2
MR effect size for
'Gut microbiota abundance (genus Catenibacterium id.2153)' on 'Probable carboxypeptidase X1'

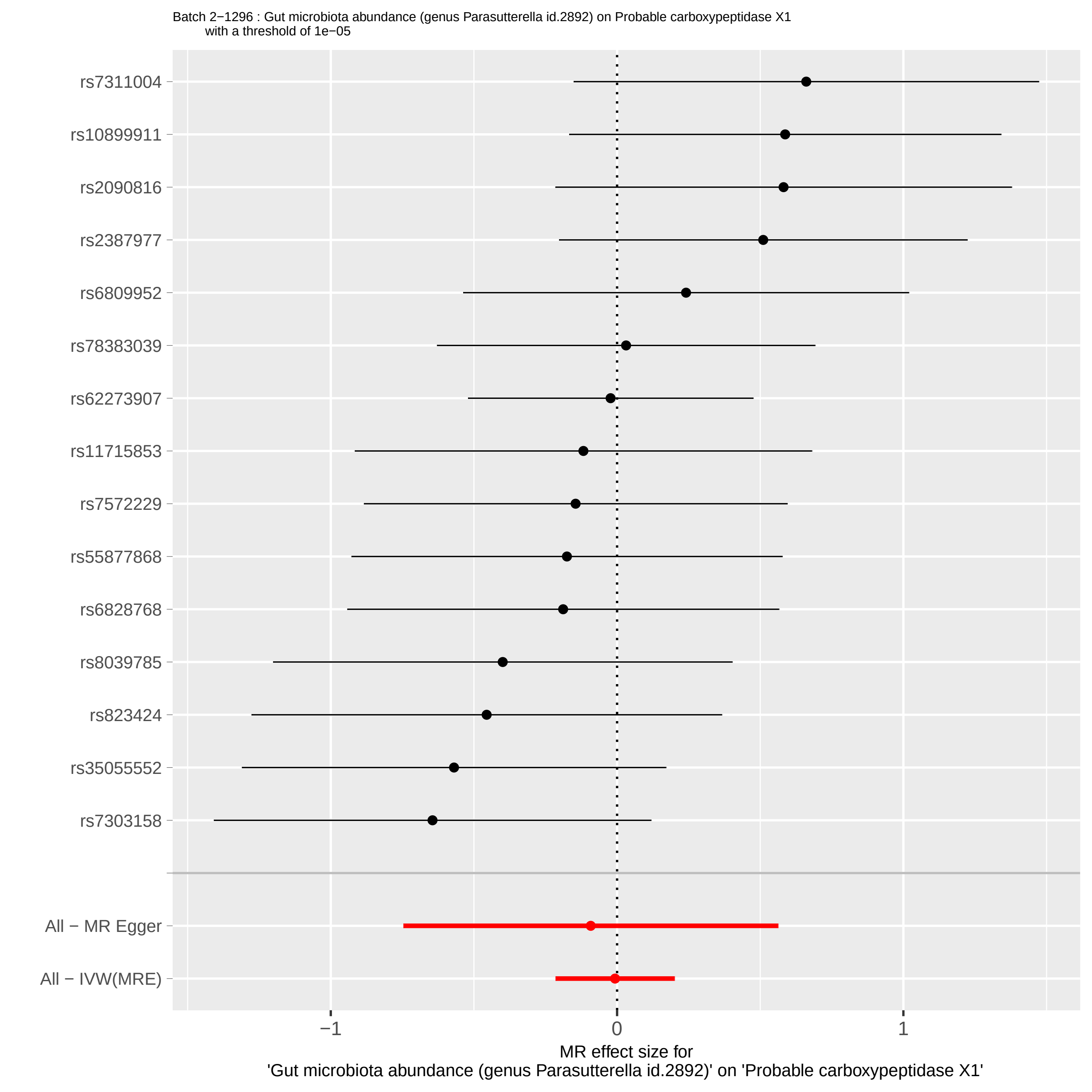

Batch 2−1296 : Gut microbiota abundance (genus Parasutterella id.2892) on Probable carboxypeptidase X1
with a threshold of 1e−05
rs7311004
rs10899911
rs2090816
rs2387977
rs6809952
rs78383039
rs62273907
rs11715853
rs7572229
rs55877868
rs6828768
rs8039785
rs823424
rs35055552
rs7303158
All − MR Egger
All − IVW(MRE)
−1
0
1
MR effect size for
'Gut microbiota abundance (genus Parasutterella id.2892)' on 'Probable carboxypeptidase X1'

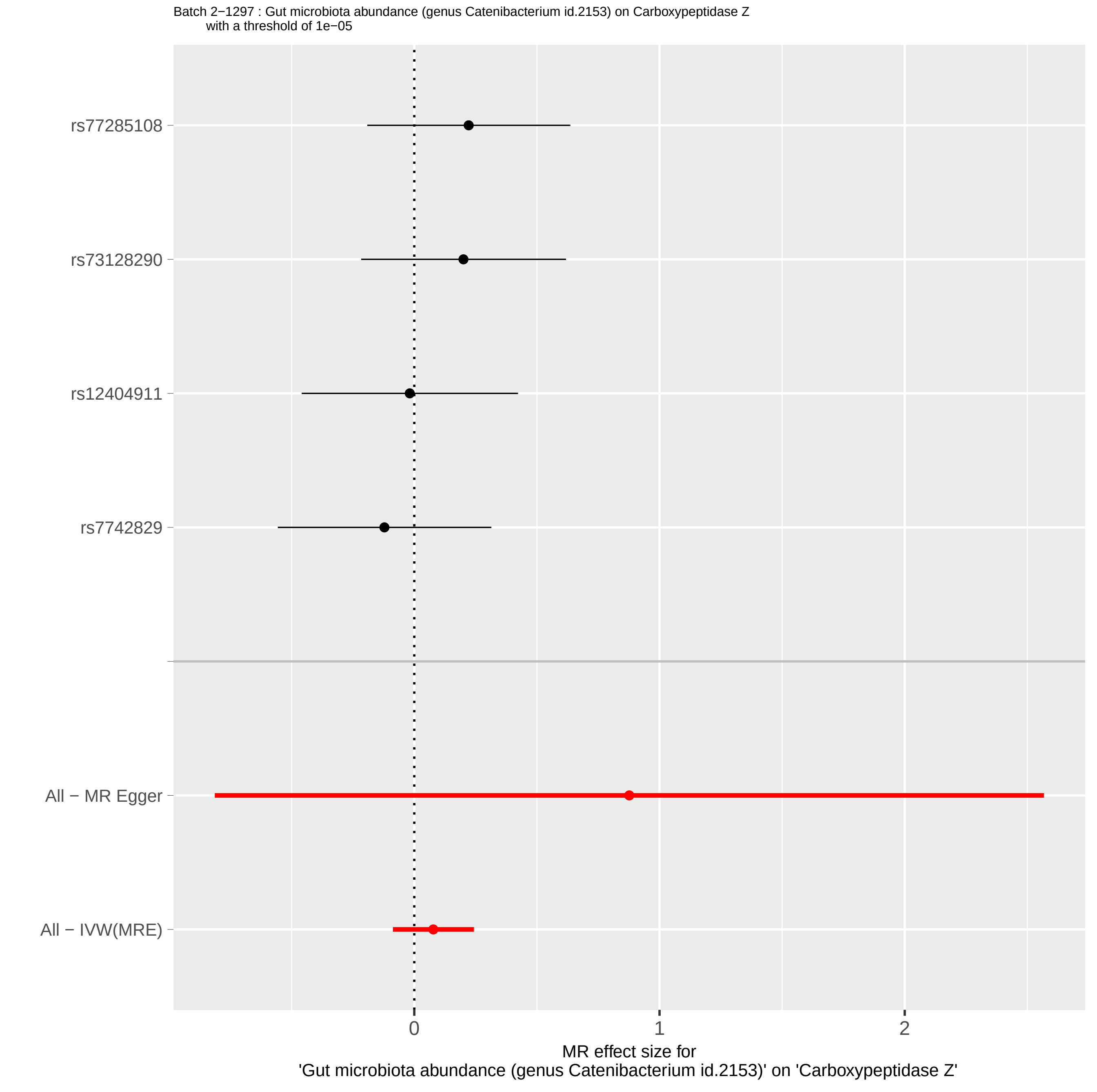

Batch 2−1297 : Gut microbiota abundance (genus Catenibacterium id.2153) on Carboxypeptidase Z
with a threshold of 1e−05
rs77285108
rs73128290
rs12404911
rs7742829
All − MR Egger
All − IVW(MRE)
0
1
2
MR effect size for
'Gut microbiota abundance (genus Catenibacterium id.2153)' on 'Carboxypeptidase Z'

Batch 2−1298 : Gut microbiota abundance (genus Parasutterella id.2892) on Carboxypeptidase Z
with a threshold of 1e−05

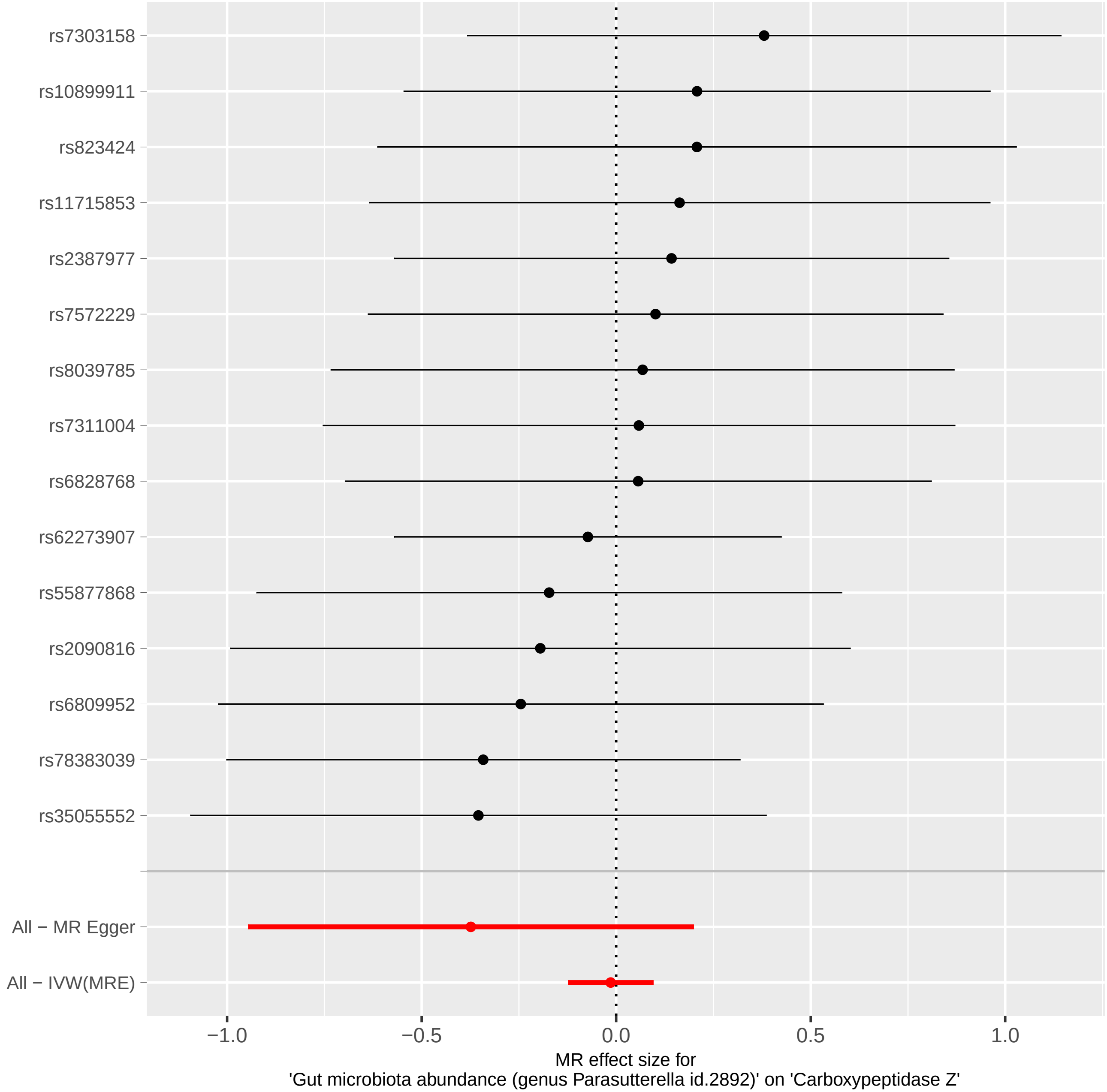

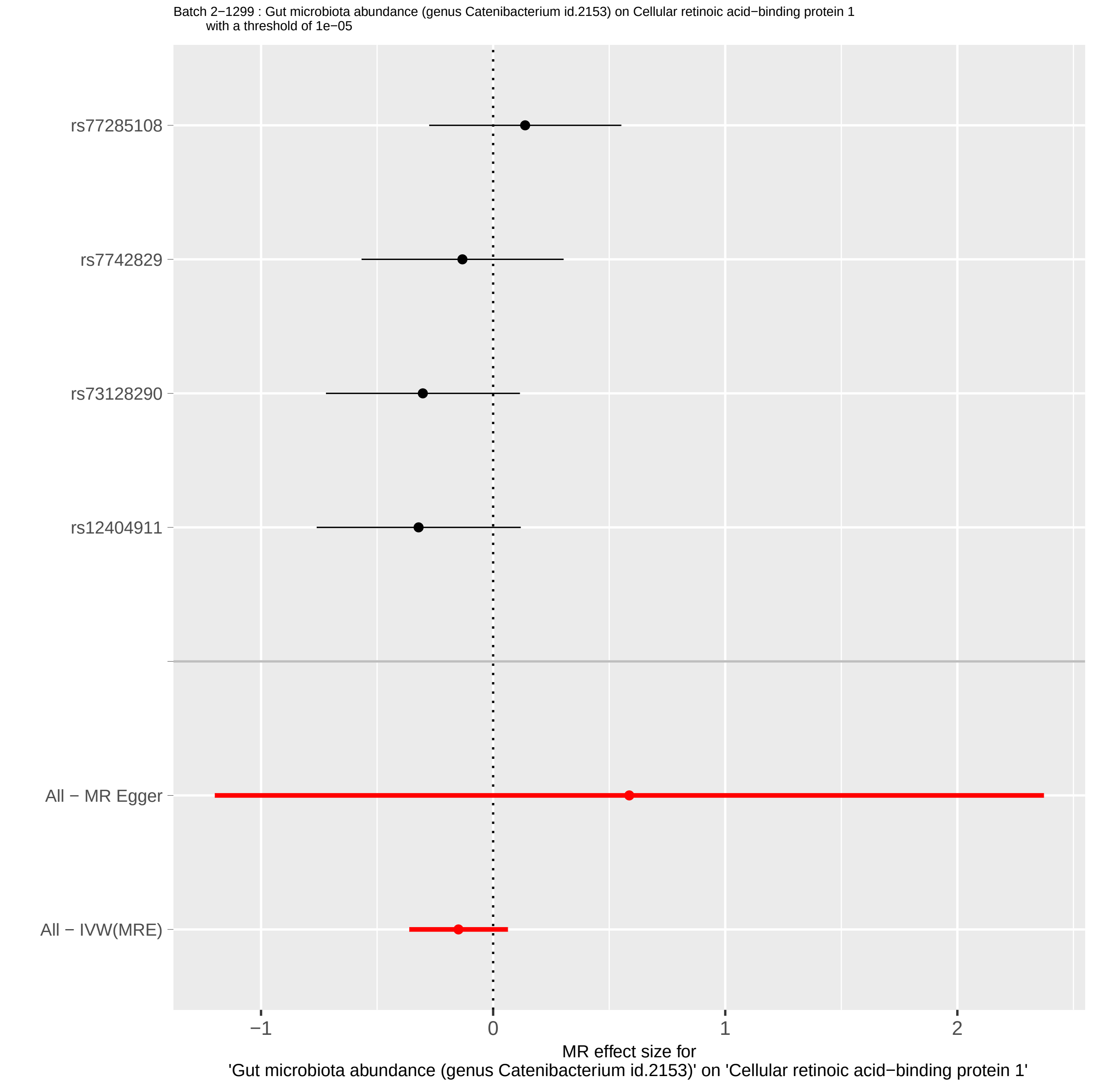

Batch 2−1299 : Gut microbiota abundance (genus Catenibacterium id.2153) on Cellular retinoic acid−binding protein 1
with a threshold of 1e−05
rs77285108
rs7742829
rs73128290
rs12404911
All − MR Egger
All − IVW(MRE)
−1
0
1
2
MR effect size for
'Gut microbiota abundance (genus Catenibacterium id.2153)' on 'Cellular retinoic acid−binding protein 1'

Batch 2−1300 : Gut microbiota abundance (genus Parasutterella id.2892) on Cellular retinoic acid−binding protein 1
with a threshold of 1e−05

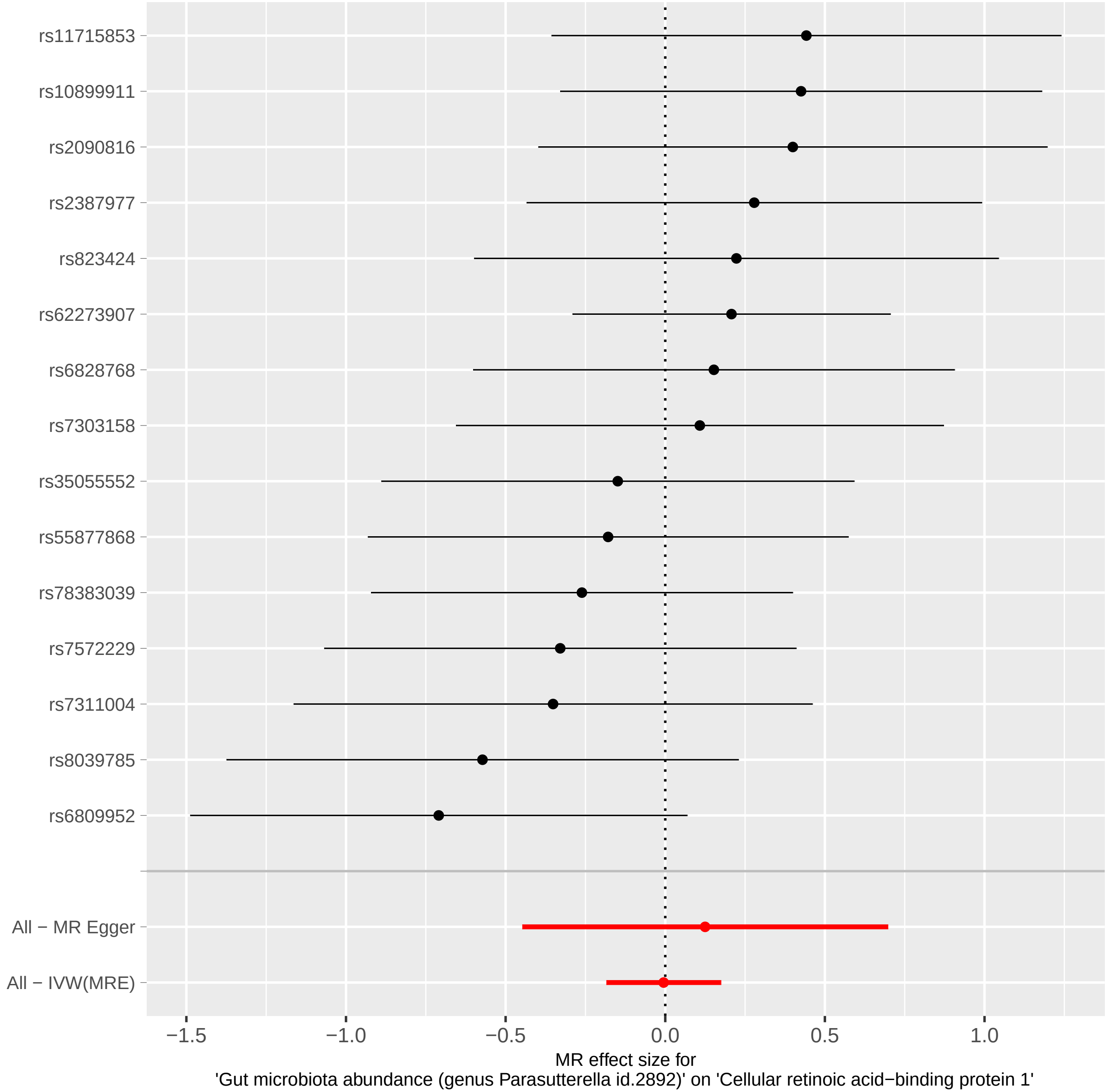

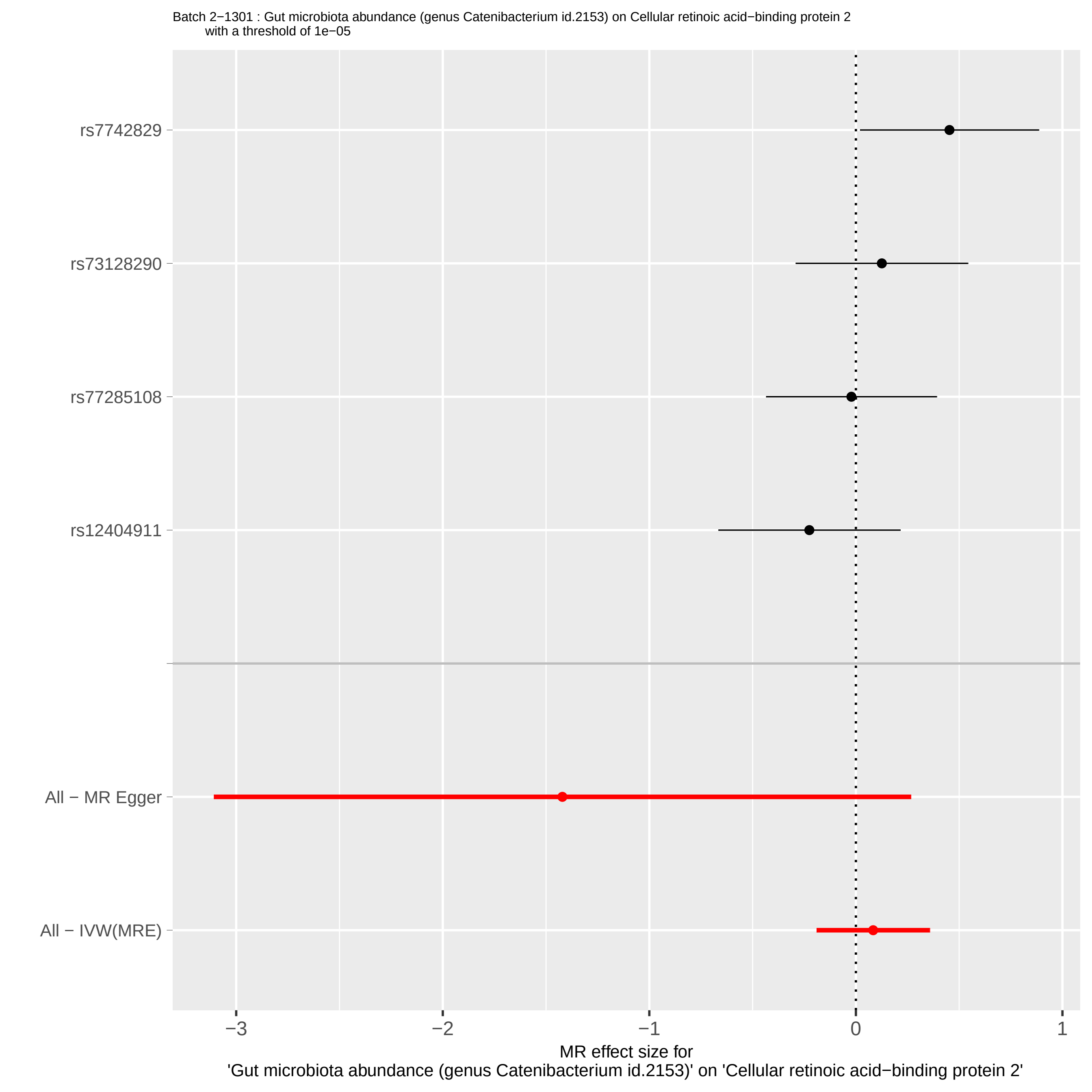

Batch 2−1301 : Gut microbiota abundance (genus Catenibacterium id.2153) on Cellular retinoic acid−binding protein 2
with a threshold of 1e−05
rs7742829
rs73128290
rs77285108
rs12404911
All − MR Egger
All − IVW(MRE)
−3
−2
−1
0
1
MR effect size for
'Gut microbiota abundance (genus Catenibacterium id.2153)' on 'Cellular retinoic acid−binding protein 2'

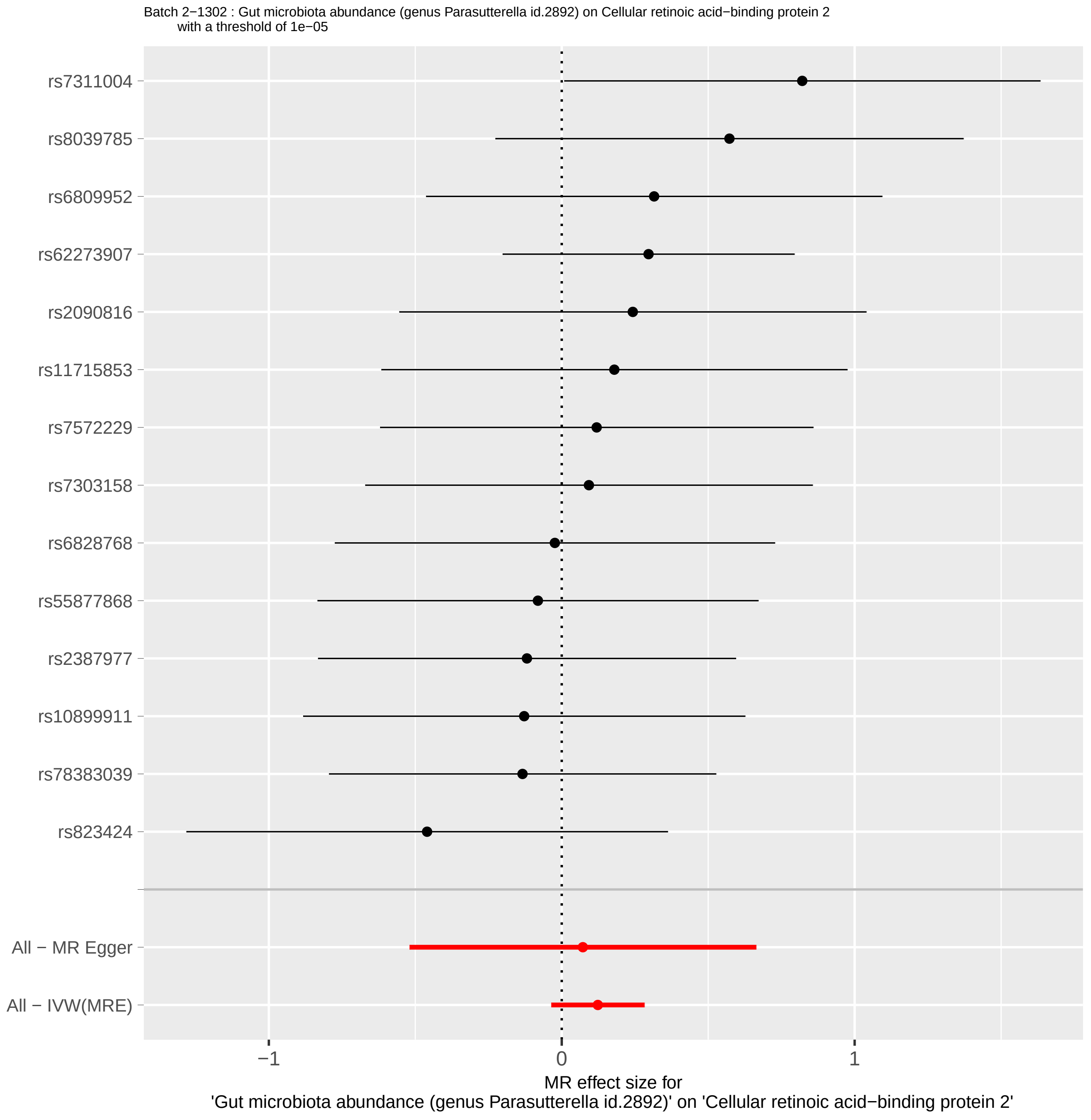
Batch 2−1302 : Gut microbiota abundance (genus Parasutterella id.2892) on Cellular retinoic acid−binding protein 2
with a threshold of 1e−05
rs7311004
rs8039785
rs6809952
rs62273907
rs2090816
rs11715853
rs7572229
rs7303158
rs6828768
rs55877868
rs2387977
rs10899911
rs78383039
rs823424
All − MR Egger
All − IVW(MRE)
−1
0
1
MR effect size for
'Gut microbiota abundance (genus Parasutterella id.2892)' on 'Cellular retinoic acid−binding protein 2'

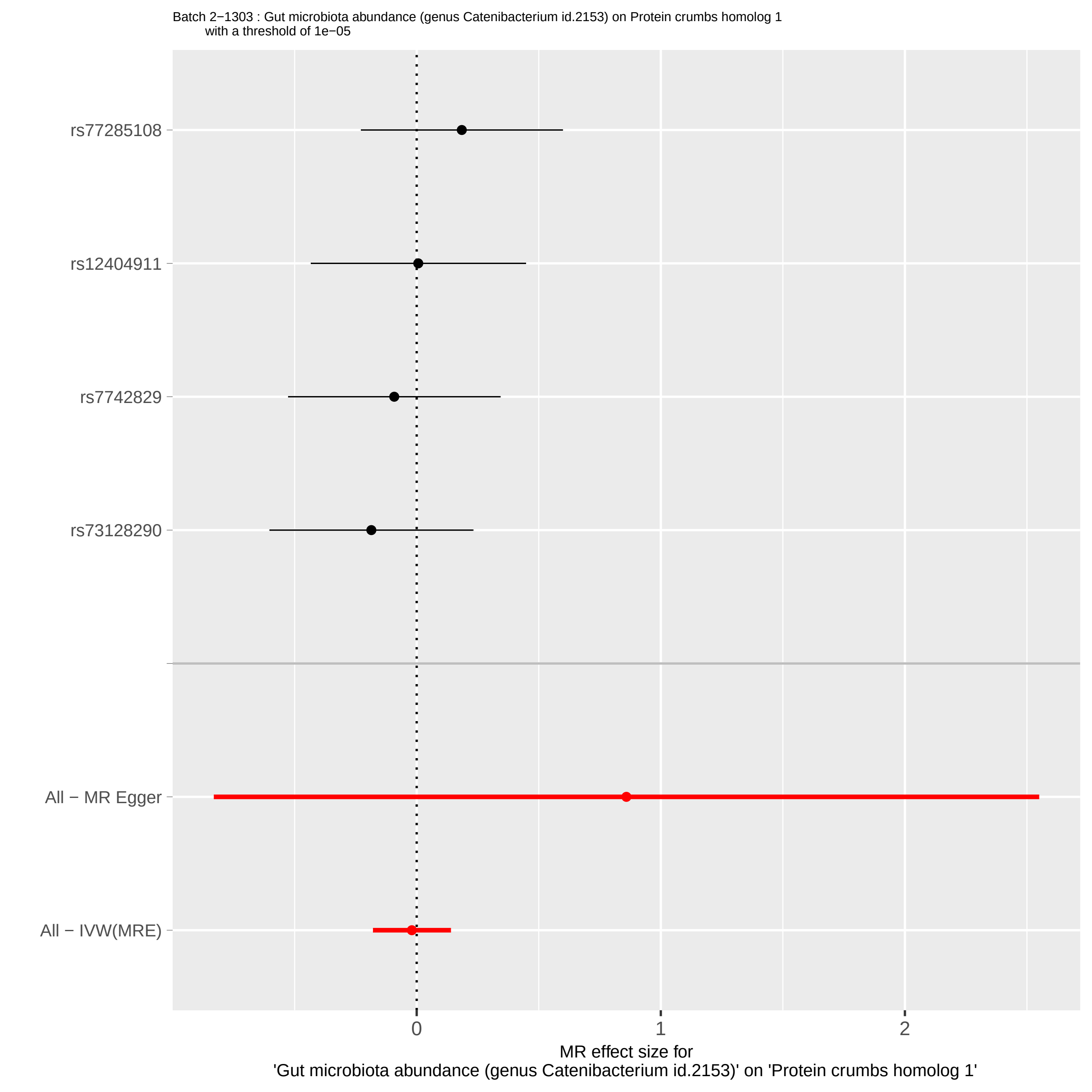

Batch 2−1303 : Gut microbiota abundance (genus Catenibacterium id.2153) on Protein crumbs homolog 1
with a threshold of 1e−05
rs77285108
rs12404911
rs7742829
rs73128290
All − MR Egger
All − IVW(MRE)
0
1
2
MR effect size for
'Gut microbiota abundance (genus Catenibacterium id.2153)' on 'Protein crumbs homolog 1'

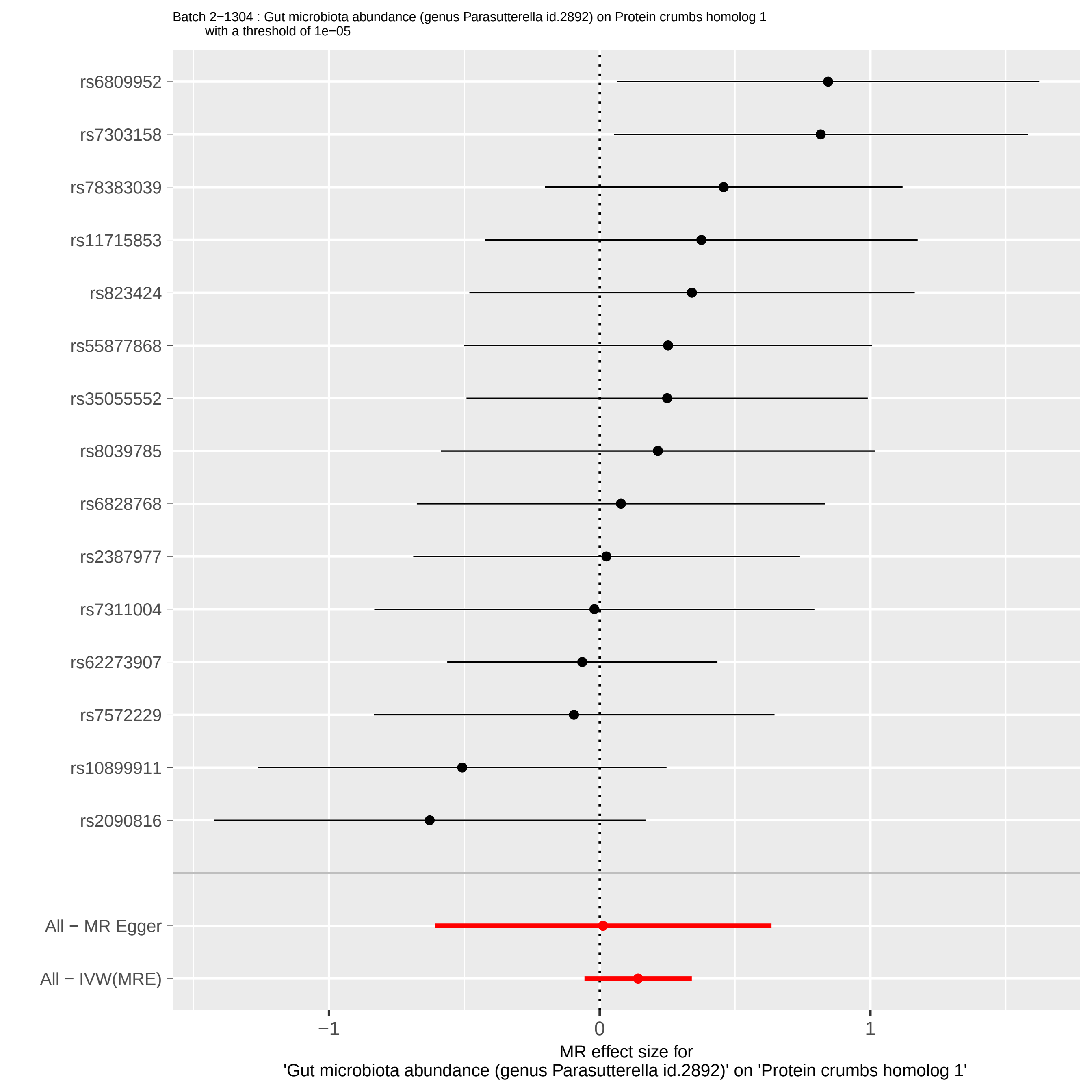
Batch 2−1304 : Gut microbiota abundance (genus Parasutterella id.2892) on Protein crumbs homolog 1
with a threshold of 1e−05
rs6809952
rs7303158
rs78383039
rs11715853
rs823424
rs55877868
rs35055552
rs8039785
rs6828768
rs2387977
rs7311004
rs62273907
rs7572229
rs10899911
rs2090816
All − MR Egger
All − IVW(MRE)
−1
0
1
MR effect size for
'Gut microbiota abundance (genus Parasutterella id.2892)' on 'Protein crumbs homolog 1'

Batch 2−1305 : Gut microbiota abundance (genus Catenibacterium id.2153) on Cyclic AMP−responsive element−binding protein 3−like protein 1 with a threshold of 1e−05

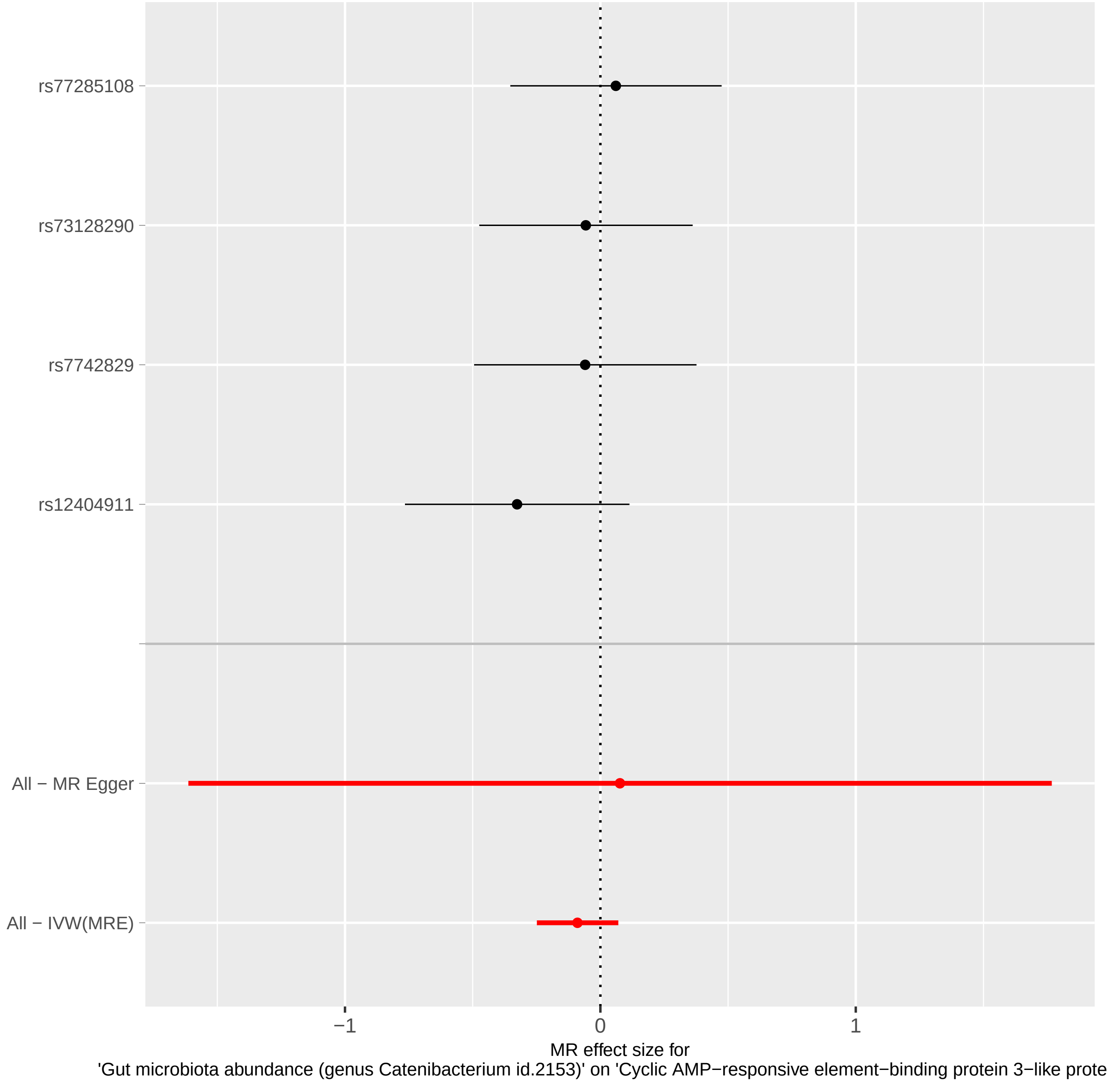

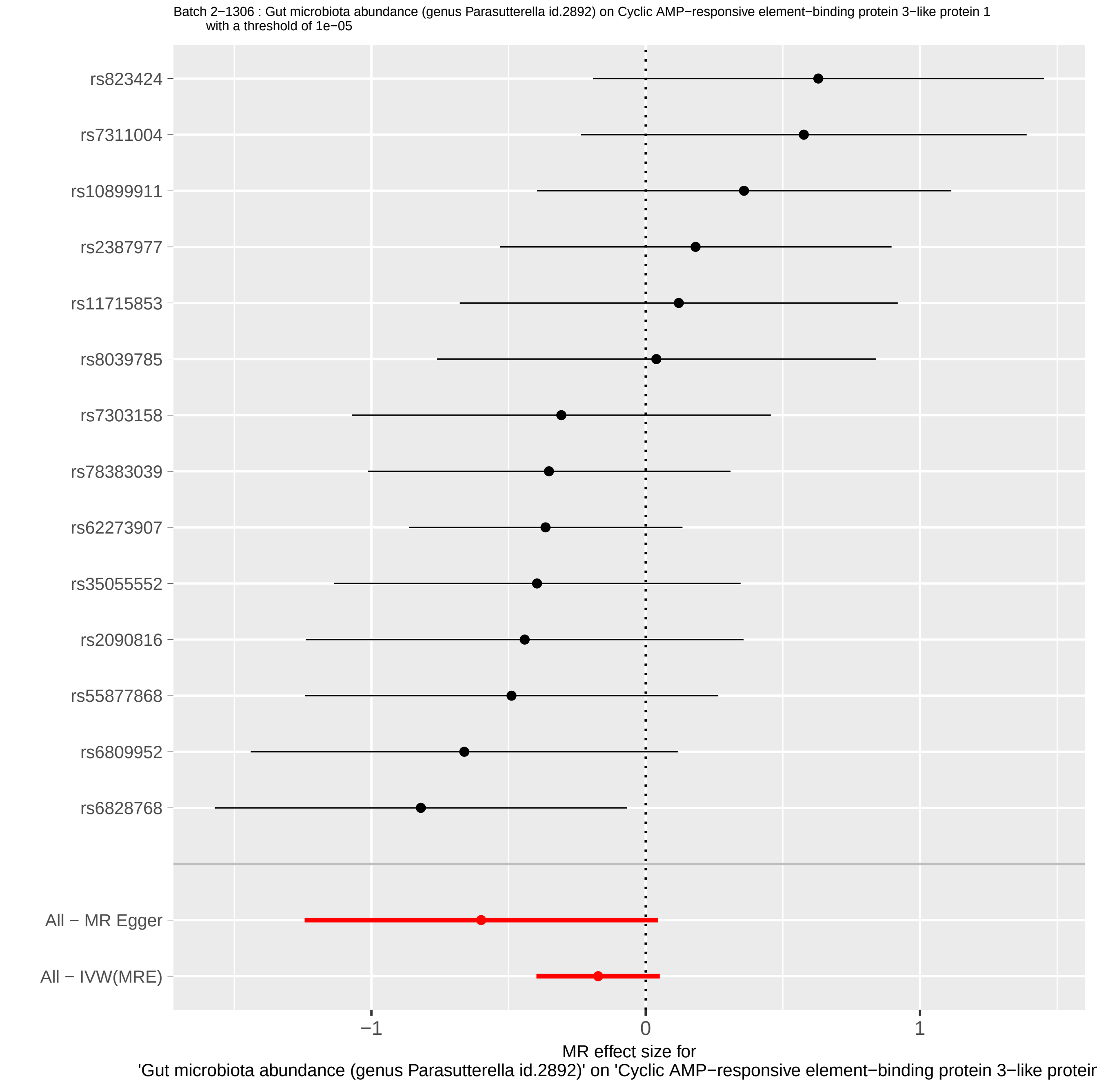

Batch 2−1306 : Gut microbiota abundance (genus Parasutterella id.2892) on Cyclic AMP−responsive element−binding protein 3−like protein 1
with a threshold of 1e−05
rs823424
rs7311004
rs10899911
rs2387977
rs11715853
rs8039785
rs7303158
rs78383039
rs62273907
rs35055552
rs2090816
rs55877868
rs6809952
rs6828768
All − MR Egger
All − IVW(MRE)
−1
0
1
MR effect size for
'Gut microbiota abundance (genus Parasutterella id.2892)' on 'Cyclic AMP−responsive element−binding protein 3−like protein

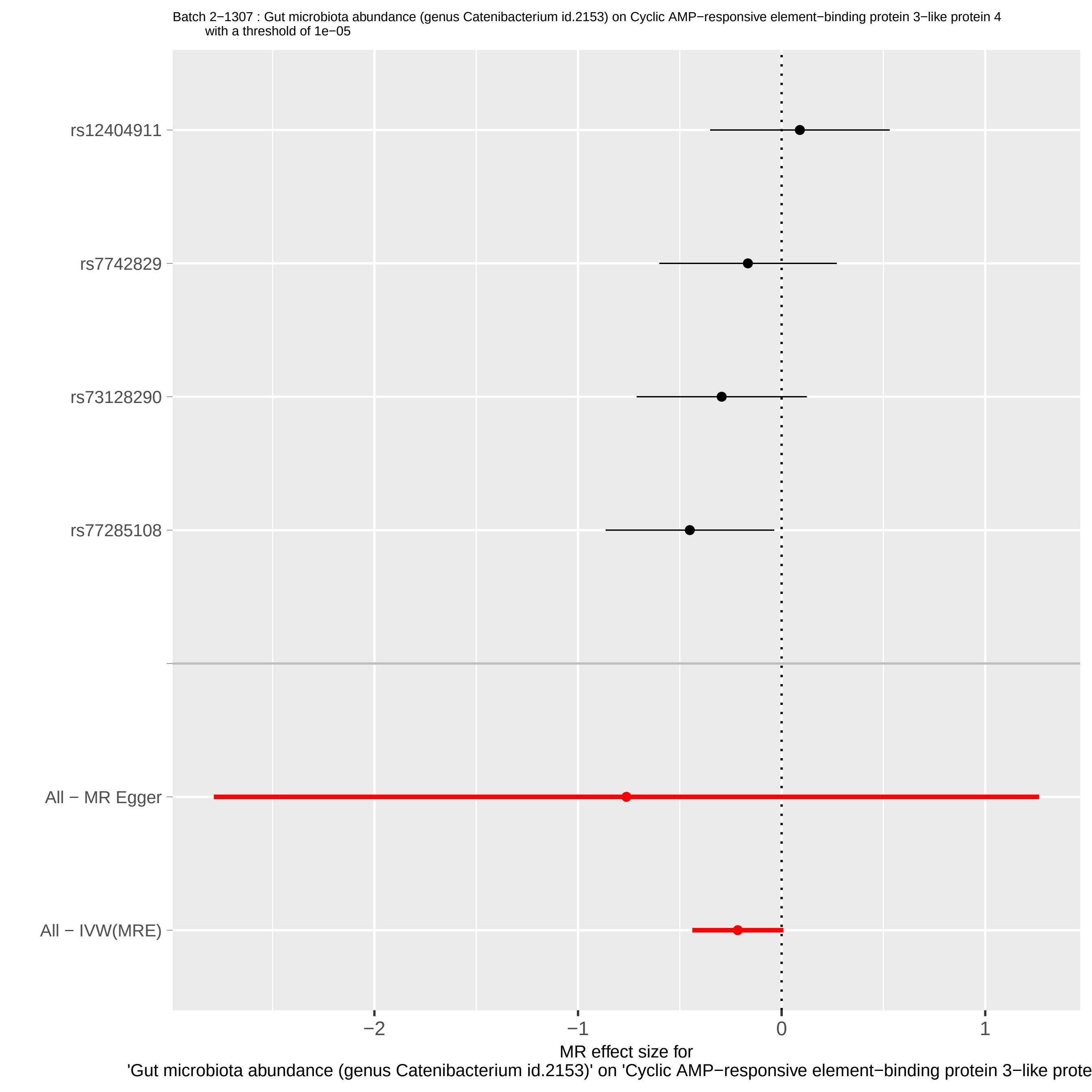
Batch 2−1307 : Gut microbiota abundance (genus Catenibacterium id.2153) on Cyclic AMP−responsive element−binding protein 3−like protein 4
with a threshold of 1e−05
rs12404911
rs7742829
rs73128290
rs77285108
All − MR Egger
All − IVW(MRE)
−2
−1
0
1
MR effect size for
'Gut microbiota abundance (genus Catenibacterium id.2153)' on 'Cyclic AMP−responsive element−binding protein 3−like prote

Batch 2−1308 : Gut microbiota abundance (genus Parasutterella id.2892) on Cyclic AMP−responsive element−binding protein 3−like protein 4 with a threshold of 1e−05

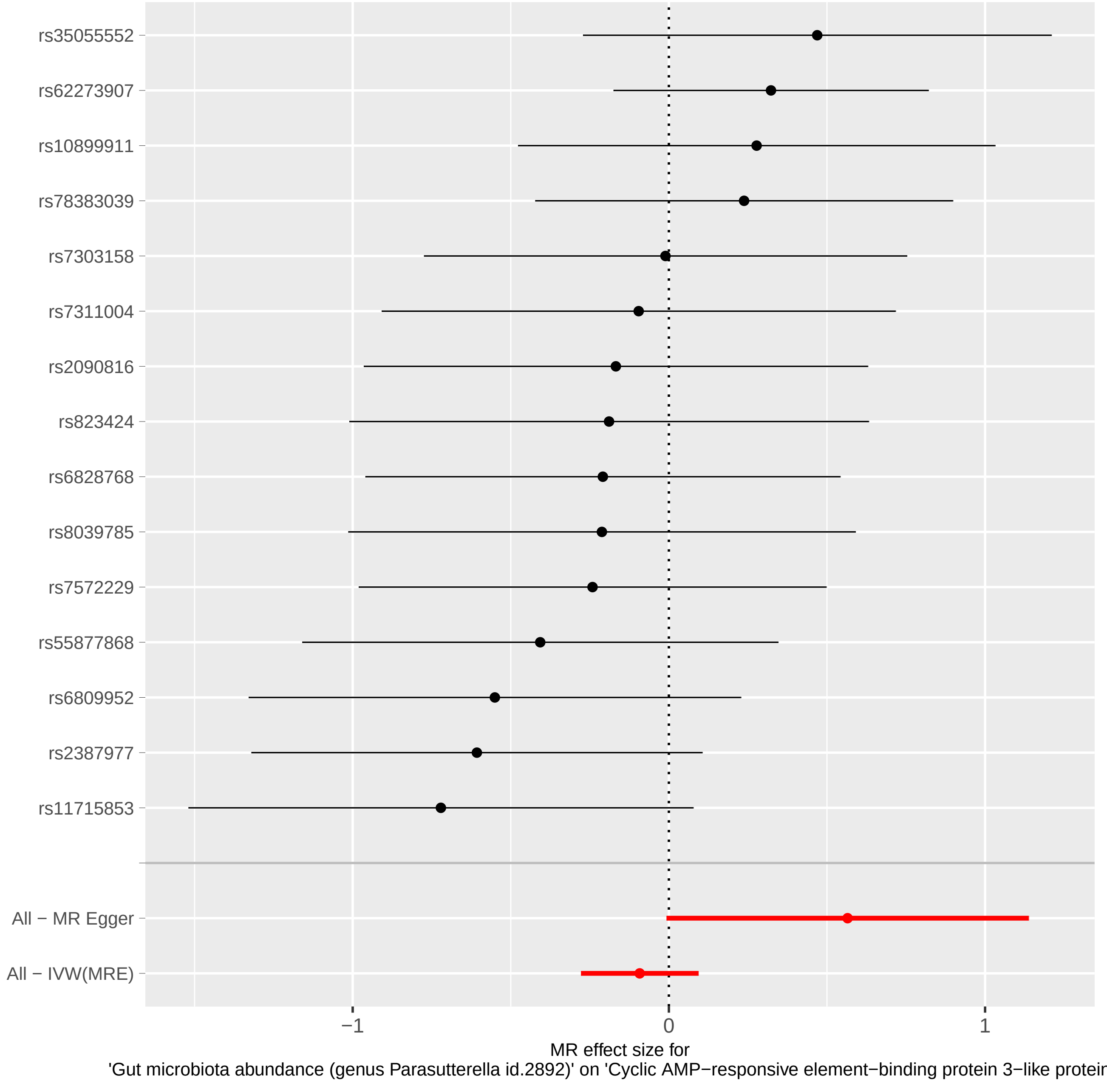

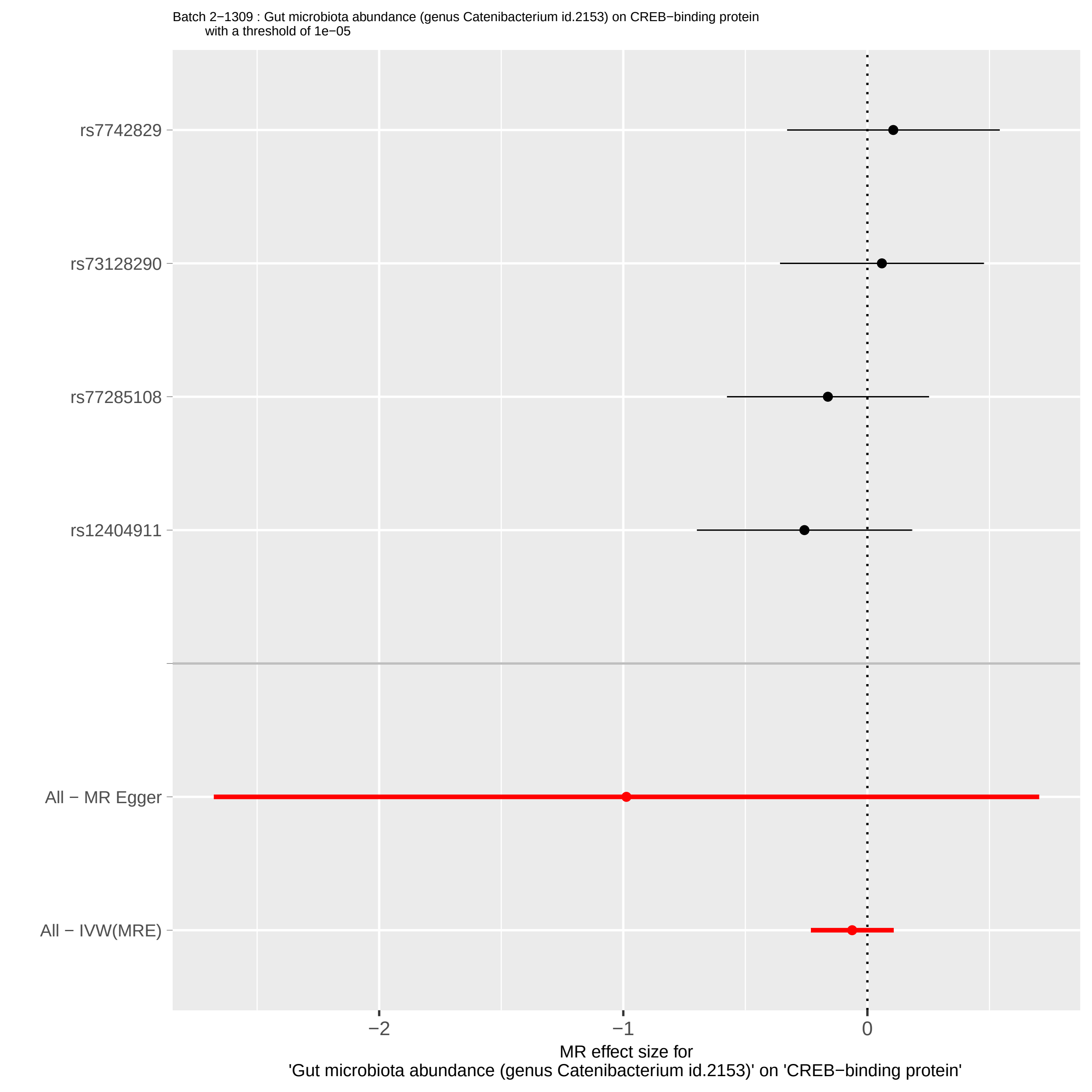

Batch 2−1309 : Gut microbiota abundance (genus Catenibacterium id.2153) on CREB−binding protein
with a threshold of 1e−05
rs7742829
rs73128290
rs77285108
rs12404911
All − MR Egger
All − IVW(MRE)
−2
−1
0
MR effect size for
'Gut microbiota abundance (genus Catenibacterium id.2153)' on 'CREB−binding protein'

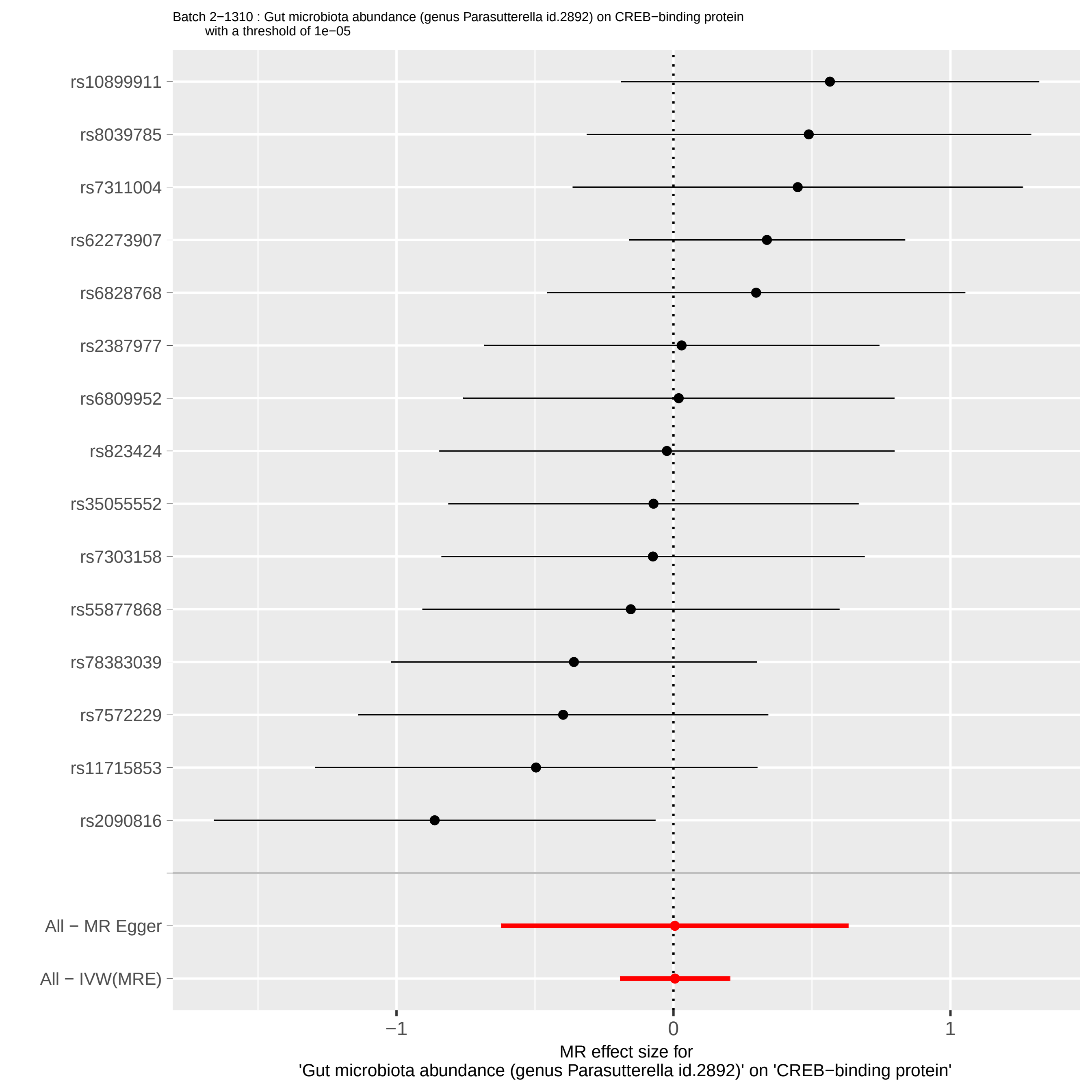
Batch 2−1310 : Gut microbiota abundance (genus Parasutterella id.2892) on CREB−binding protein
with a threshold of 1e−05
rs10899911
rs8039785
rs7311004
rs62273907
rs6828768
rs2387977
rs6809952
rs823424
rs35055552
rs7303158
rs55877868
rs78383039
rs7572229
rs11715853
rs2090816
All − MR Egger
All − IVW(MRE)
−1
0
1
MR effect size for
'Gut microbiota abundance (genus Parasutterella id.2892)' on 'CREB−binding protein'

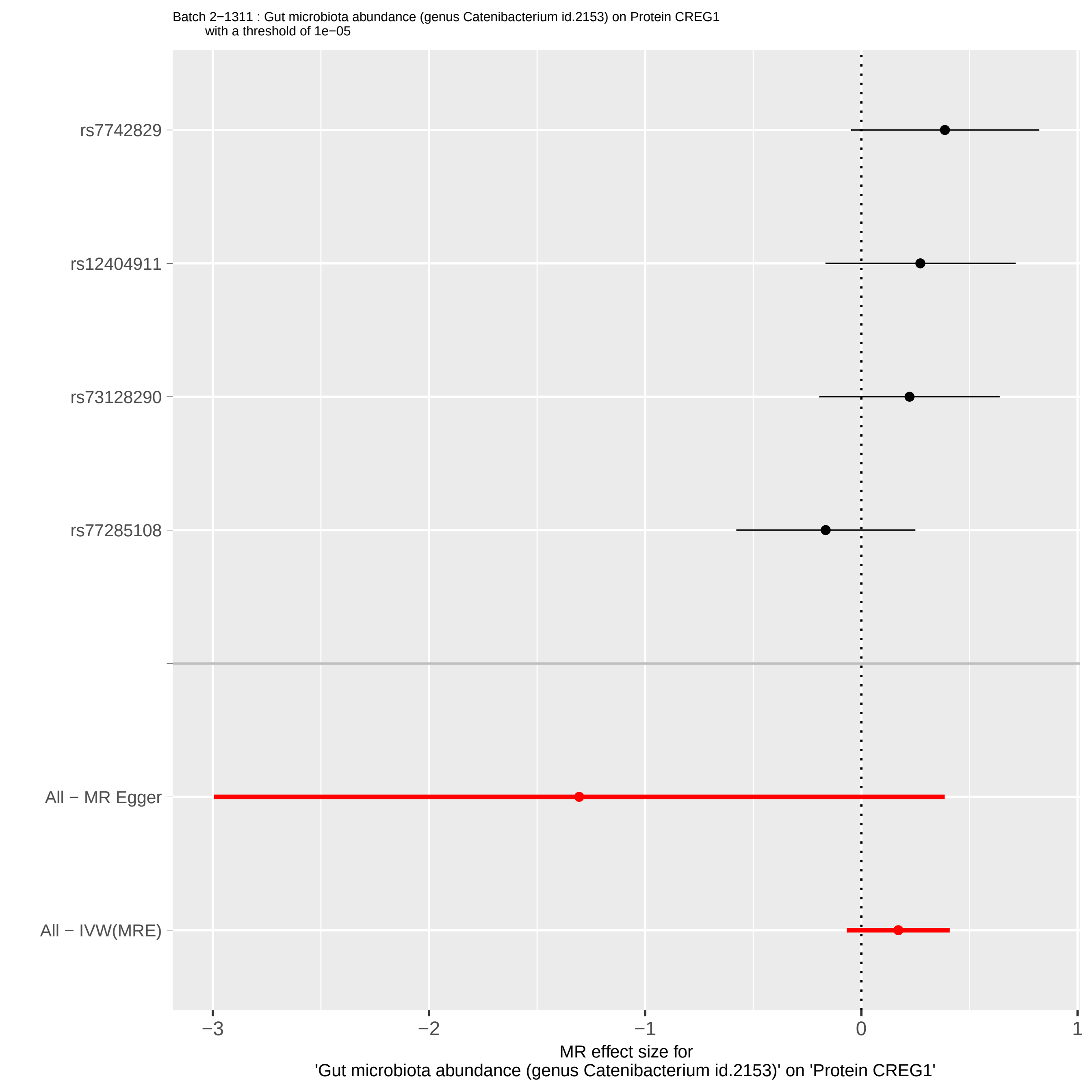

Batch 2−1311 : Gut microbiota abundance (genus Catenibacterium id.2153) on Protein CREG1
with a threshold of 1e−05
rs7742829
rs12404911
rs73128290
rs77285108
All − MR Egger
All − IVW(MRE)
−3
−2
−1
0
1
MR effect size for
'Gut microbiota abundance (genus Catenibacterium id.2153)' on 'Protein CREG1'

Batch 2−1312 : Gut microbiota abundance (genus Parasutterella id.2892) on Protein CREG1
with a threshold of 1e−05

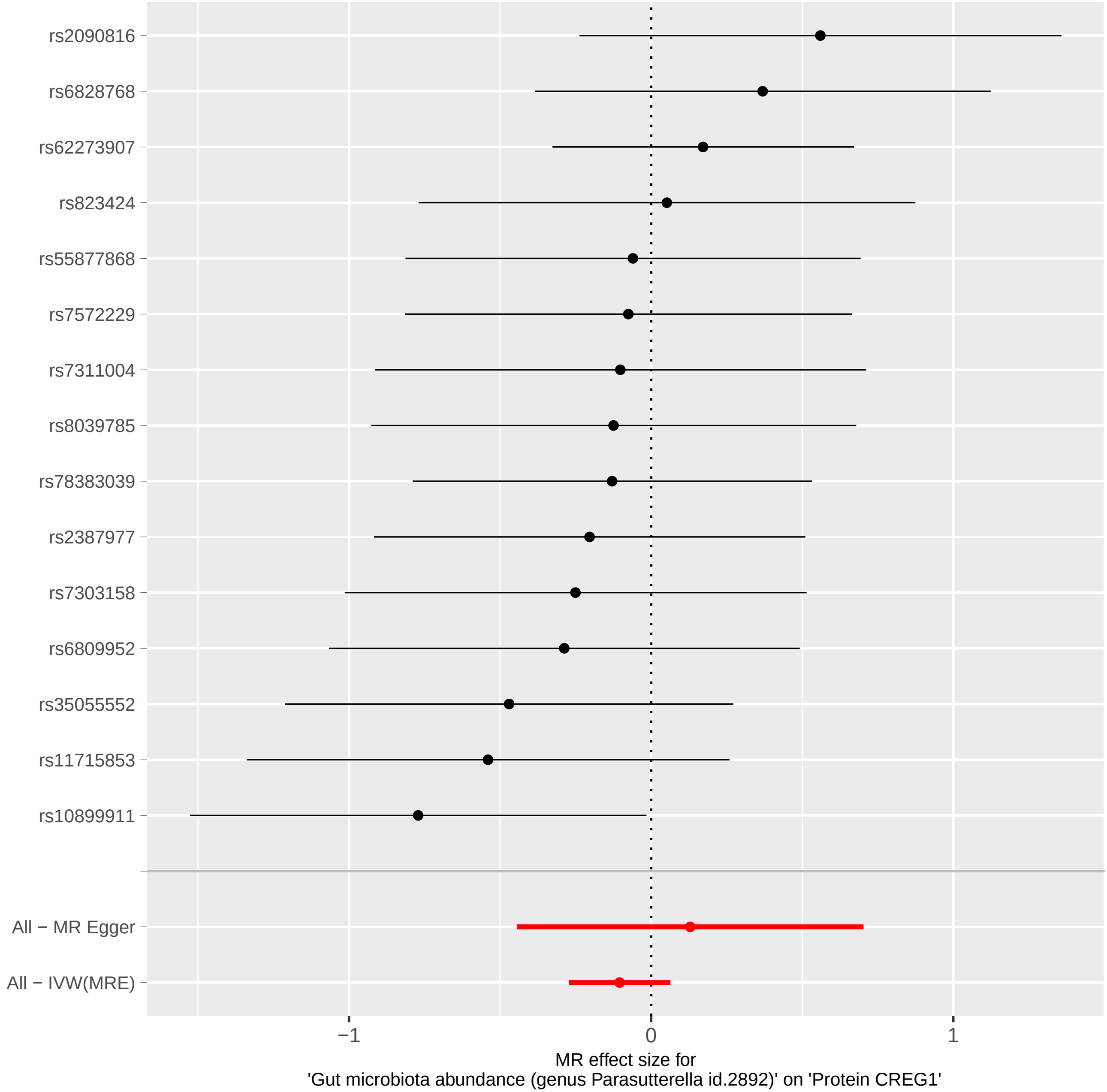

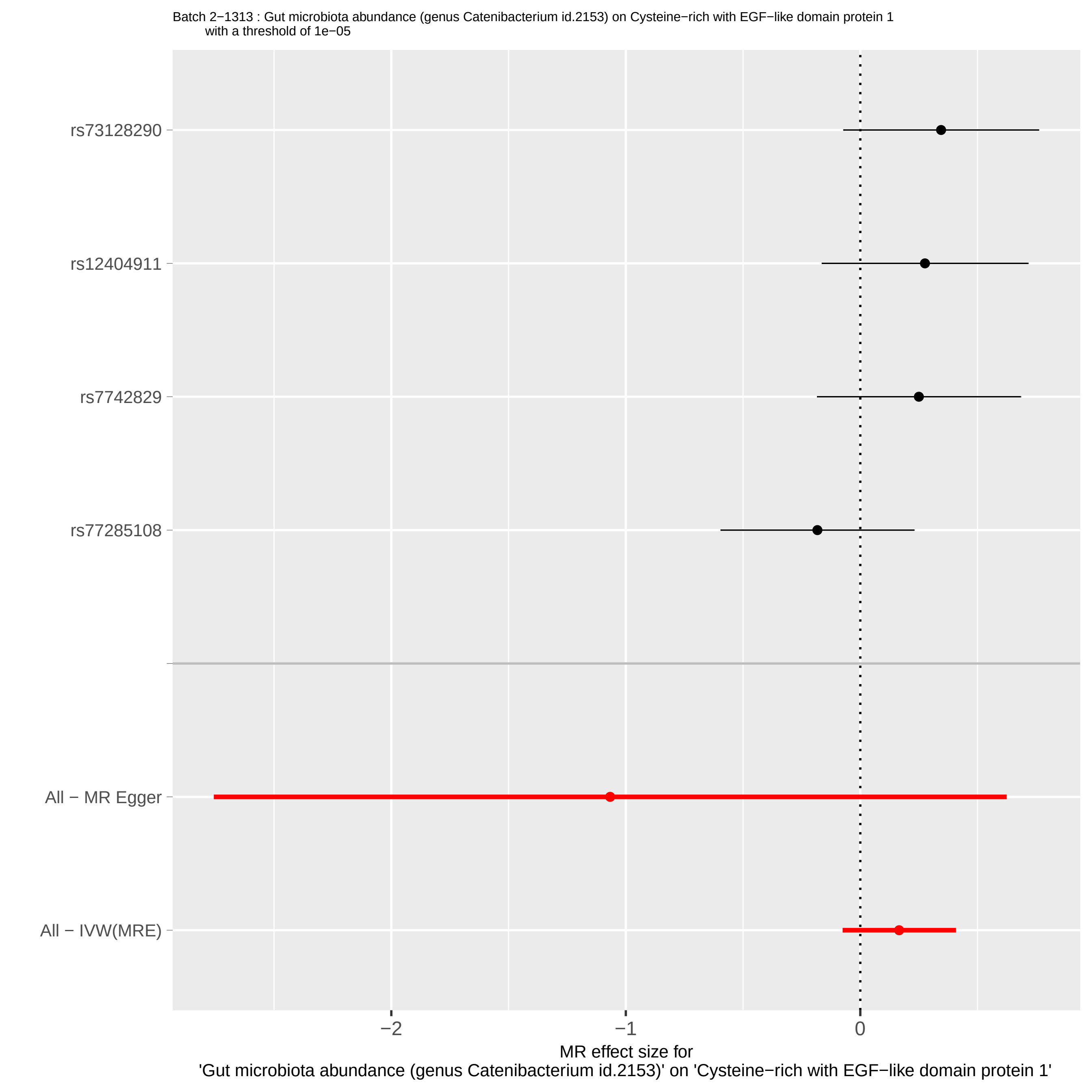

Batch 2−1313 : Gut microbiota abundance (genus Catenibacterium id.2153) on Cysteine−rich with EGF−like domain protein 1
with a threshold of 1e−05
rs73128290
rs12404911
rs7742829
rs77285108
All − MR Egger
All − IVW(MRE)
−2
−1
0
MR effect size for
'Gut microbiota abundance (genus Catenibacterium id.2153)' on 'Cysteine−rich with EGF−like domain protein 1'

Batch 2−1314 : Gut microbiota abundance (genus Parasutterella id.2892) on Cysteine−rich with EGF−like domain protein 1 with a threshold of 1e−05

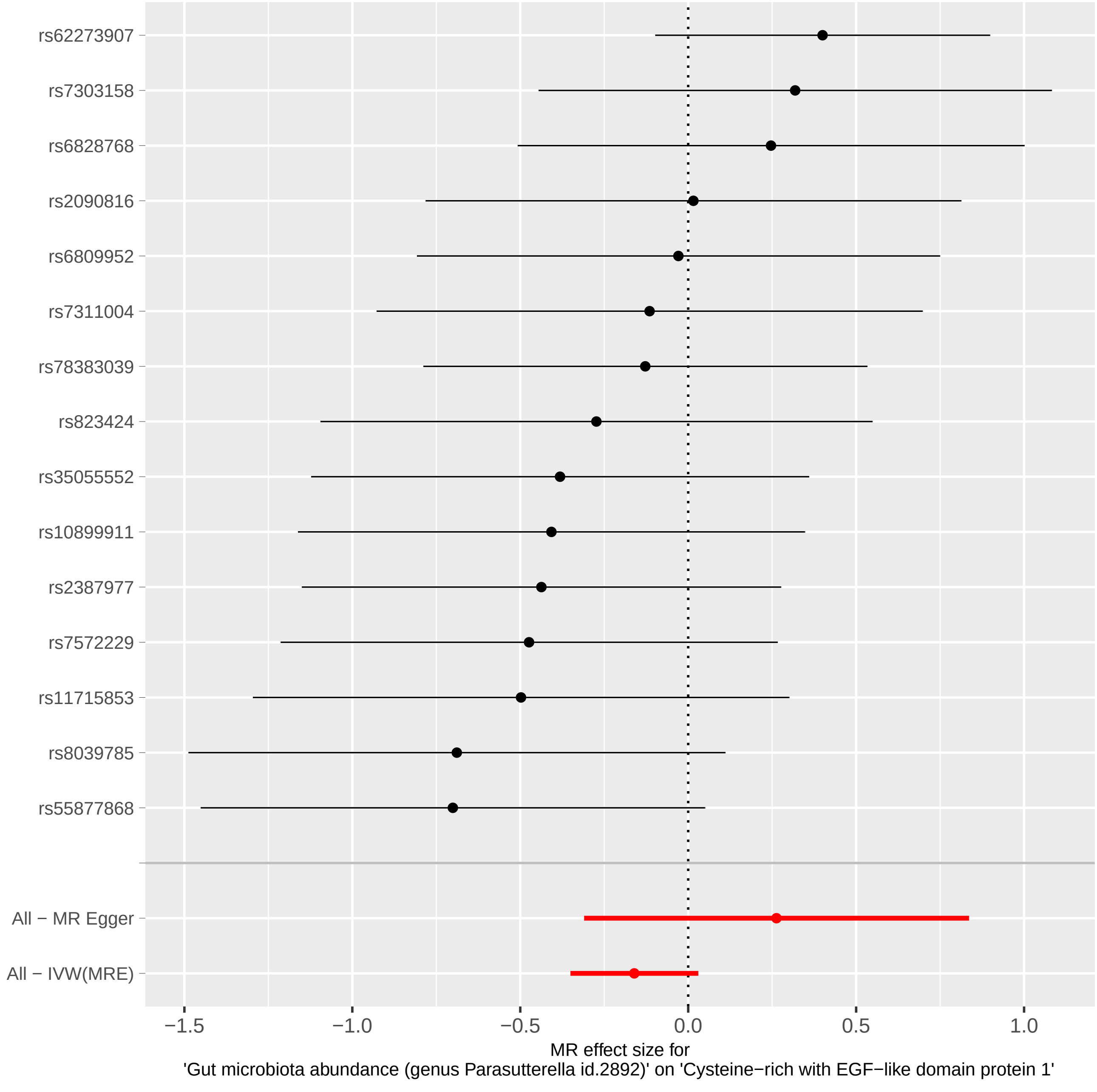

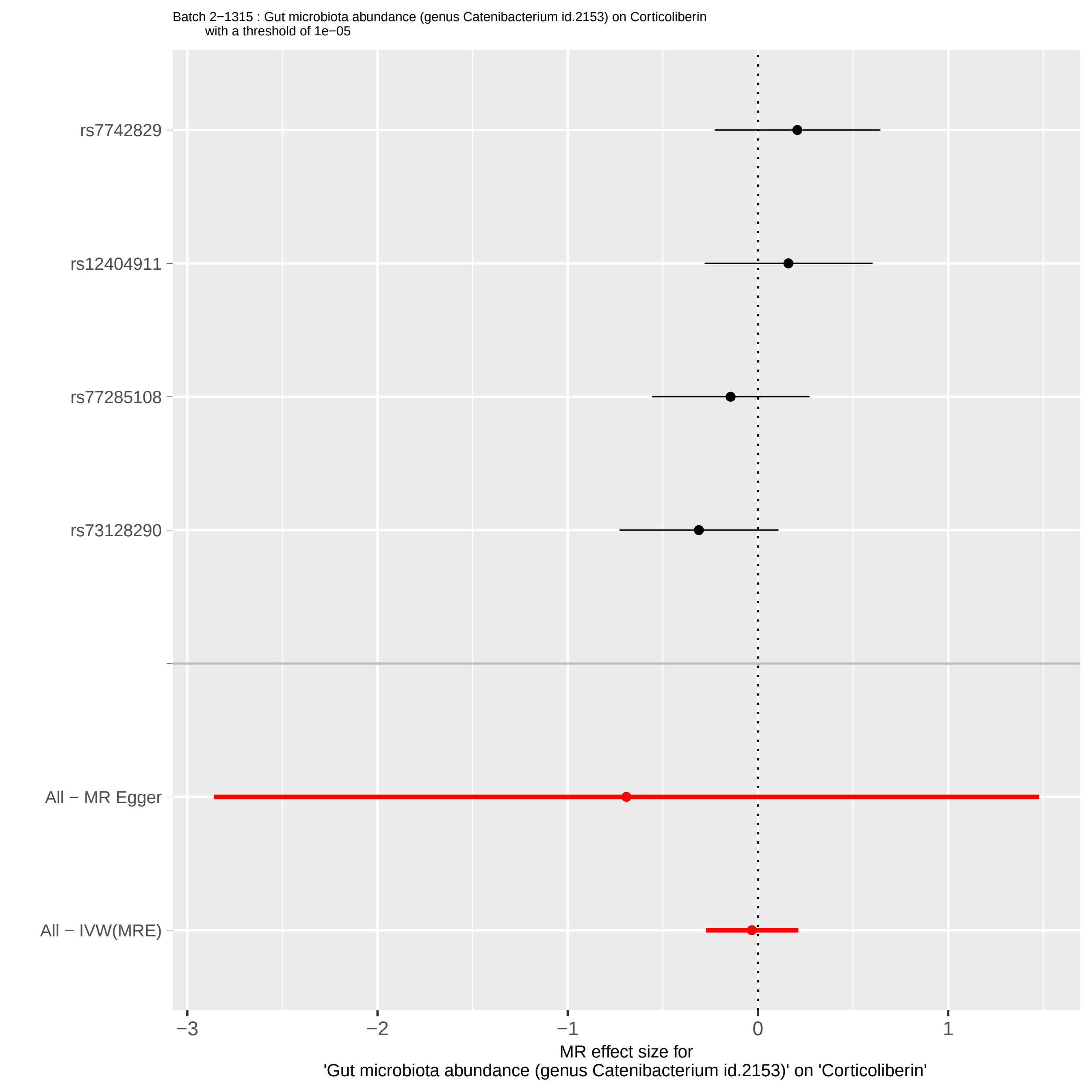

Batch 2−1315 : Gut microbiota abundance (genus Catenibacterium id.2153) on Corticoliberin
with a threshold of 1e−05
rs7742829
rs12404911
rs77285108
rs73128290
All − MR Egger
All − IVW(MRE)
−3
−2
−1
0
1
MR effect size for
'Gut microbiota abundance (genus Catenibacterium id.2153)' on 'Corticoliberin'

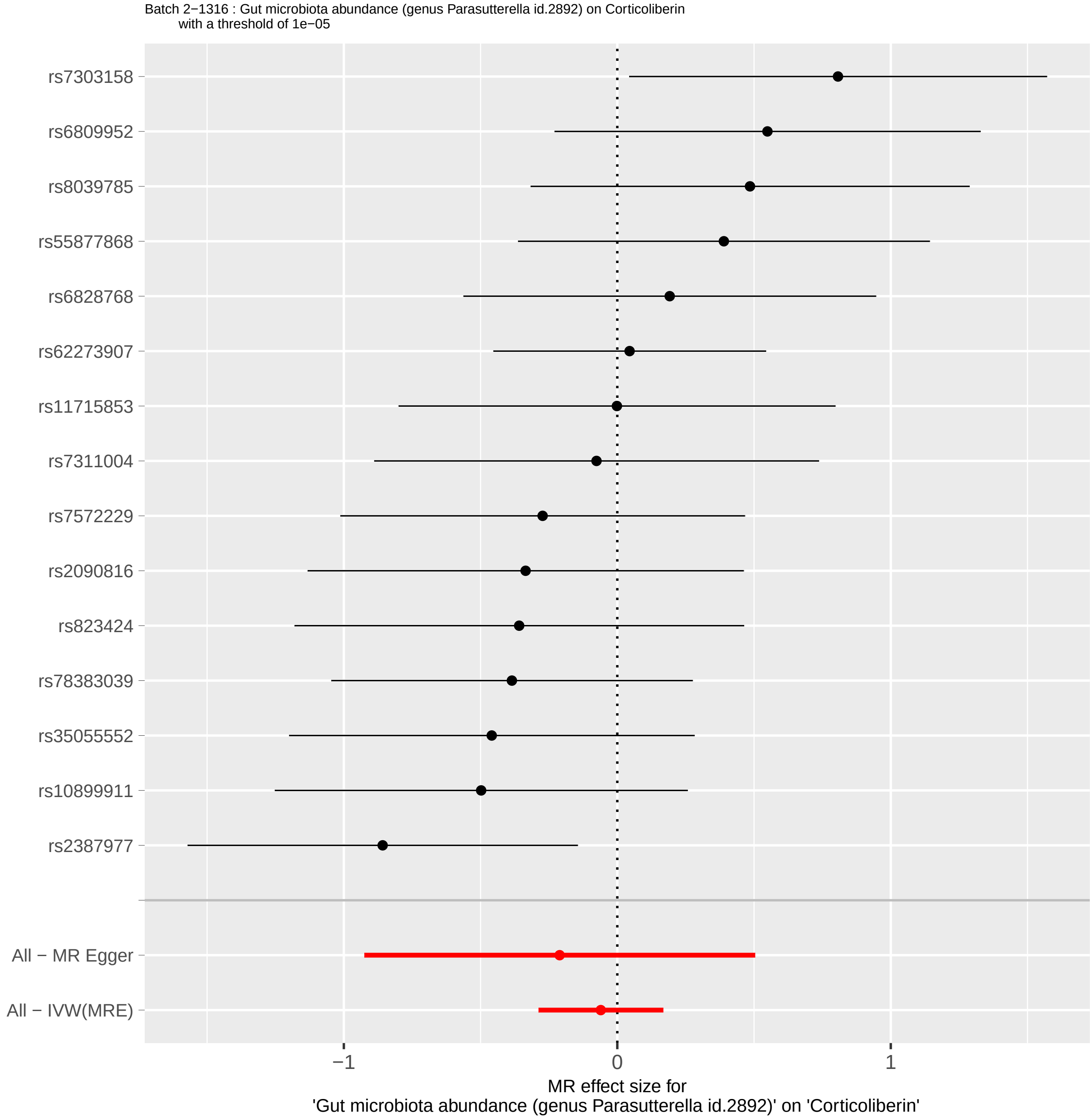
Batch 2−1316 : Gut microbiota abundance (genus Parasutterella id.2892) on Corticoliberin
with a threshold of 1e−05
rs7303158
rs6809952
rs8039785
rs55877868
rs6828768
rs62273907
rs11715853
rs7311004
rs7572229
rs2090816
rs823424
rs78383039
rs35055552
rs10899911
rs2387977
All − MR Egger
All − IVW(MRE)
−1
0
1
MR effect size for
'Gut microbiota abundance (genus Parasutterella id.2892)' on 'Corticoliberin'

Batch 2−1317 : Gut microbiota abundance (genus Catenibacterium id.2153) on Corticotropin−releasing factor−binding protein with a threshold of 1e−05

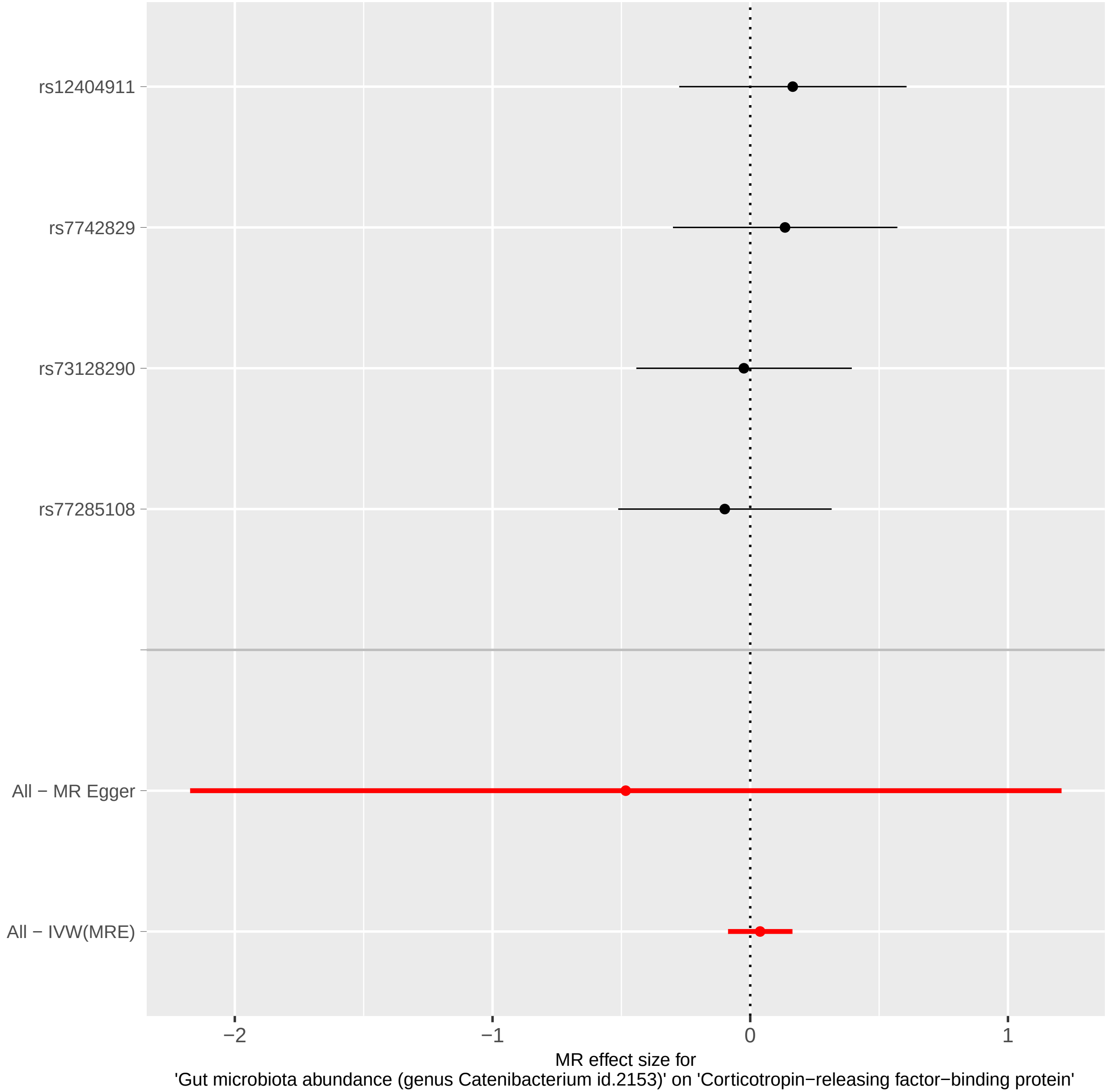

Batch 2−1318 : Gut microbiota abundance (genus Parasutterella id.2892) on Corticotropin−releasing factor−binding protein
with a threshold of 1e−05

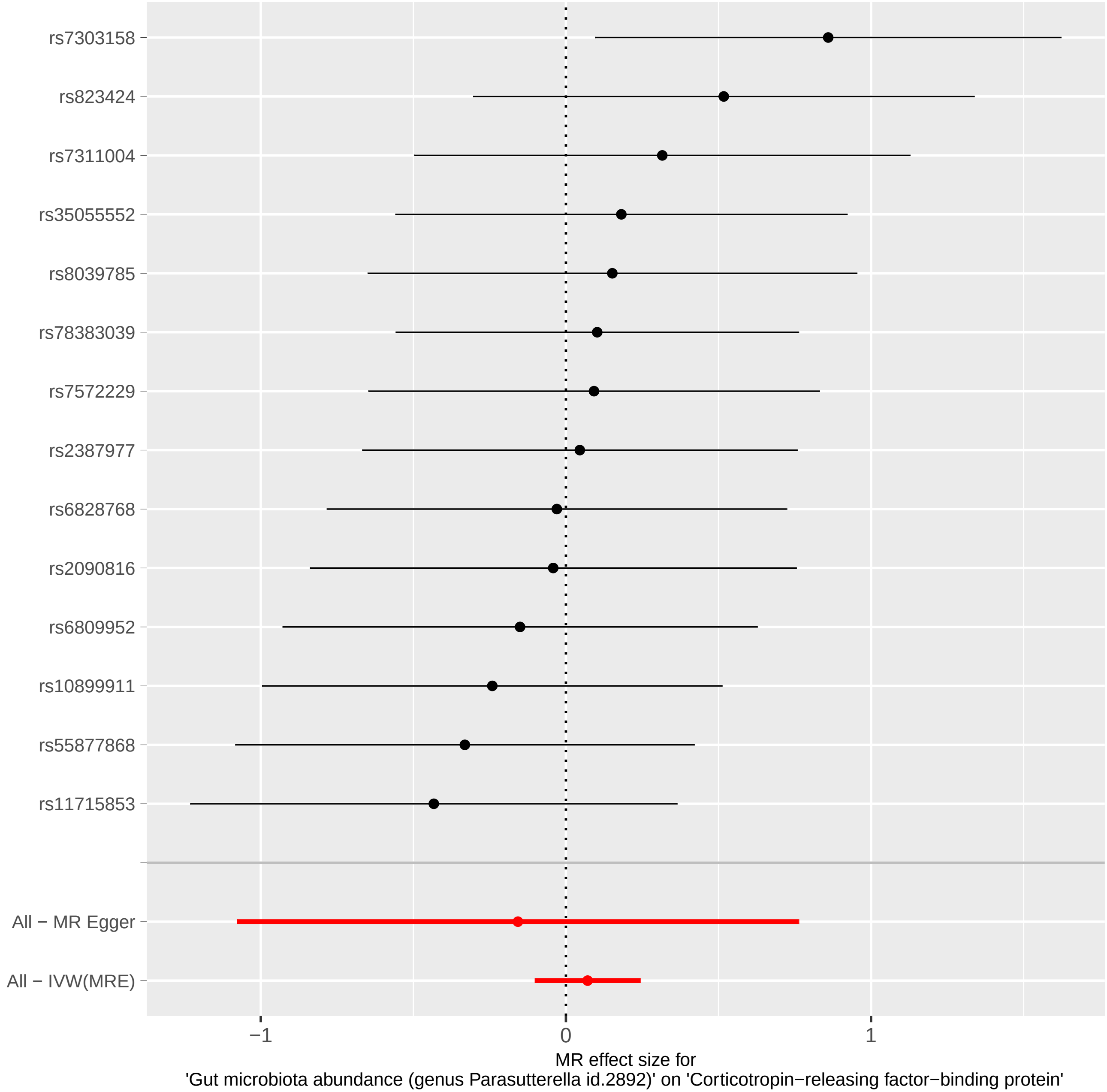

Batch 2−1319 : Gut microbiota abundance (genus Catenibacterium id.2153) on Cysteine−rich motor neuron 1 protein
with a threshold of 1e−05

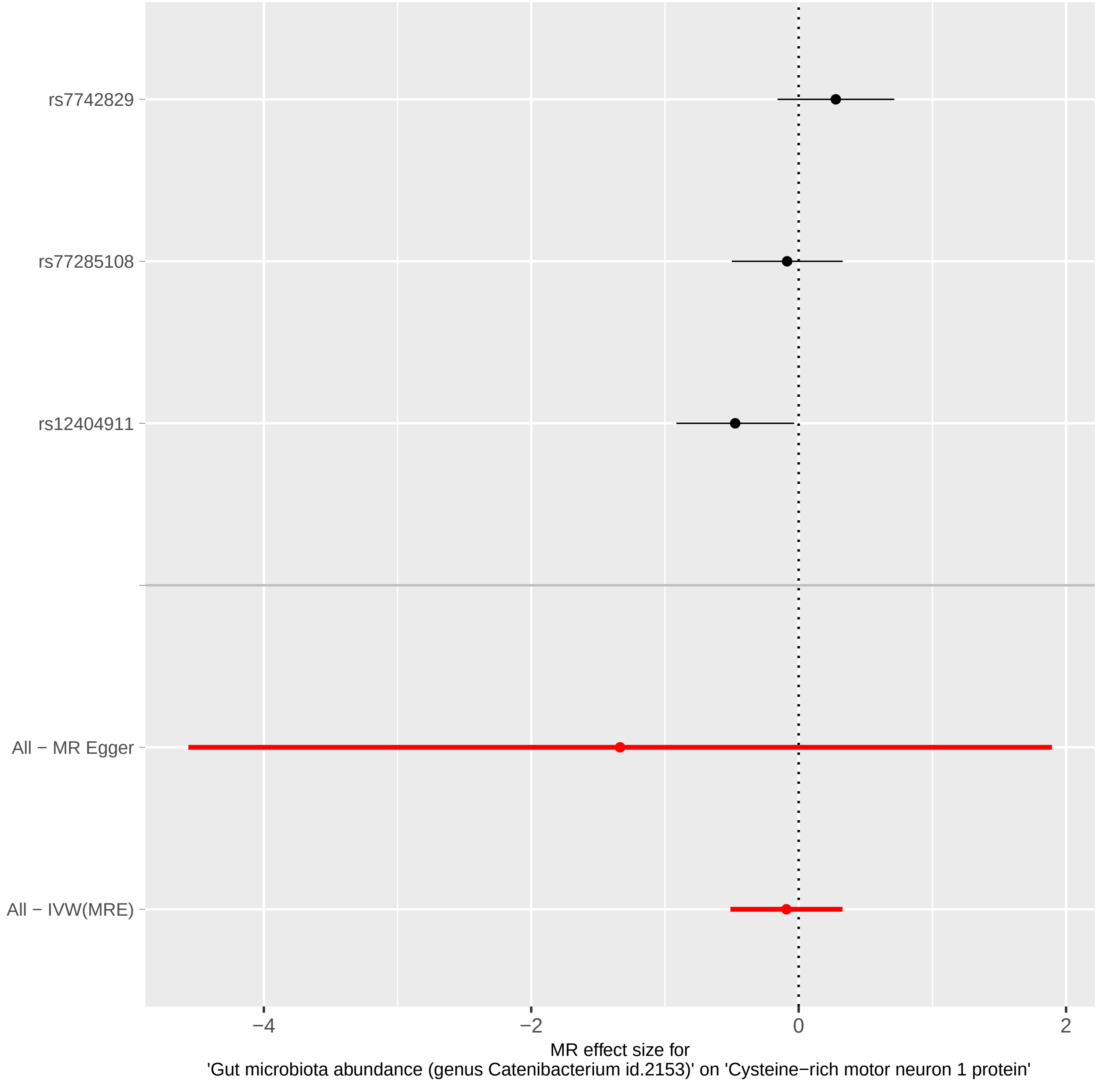

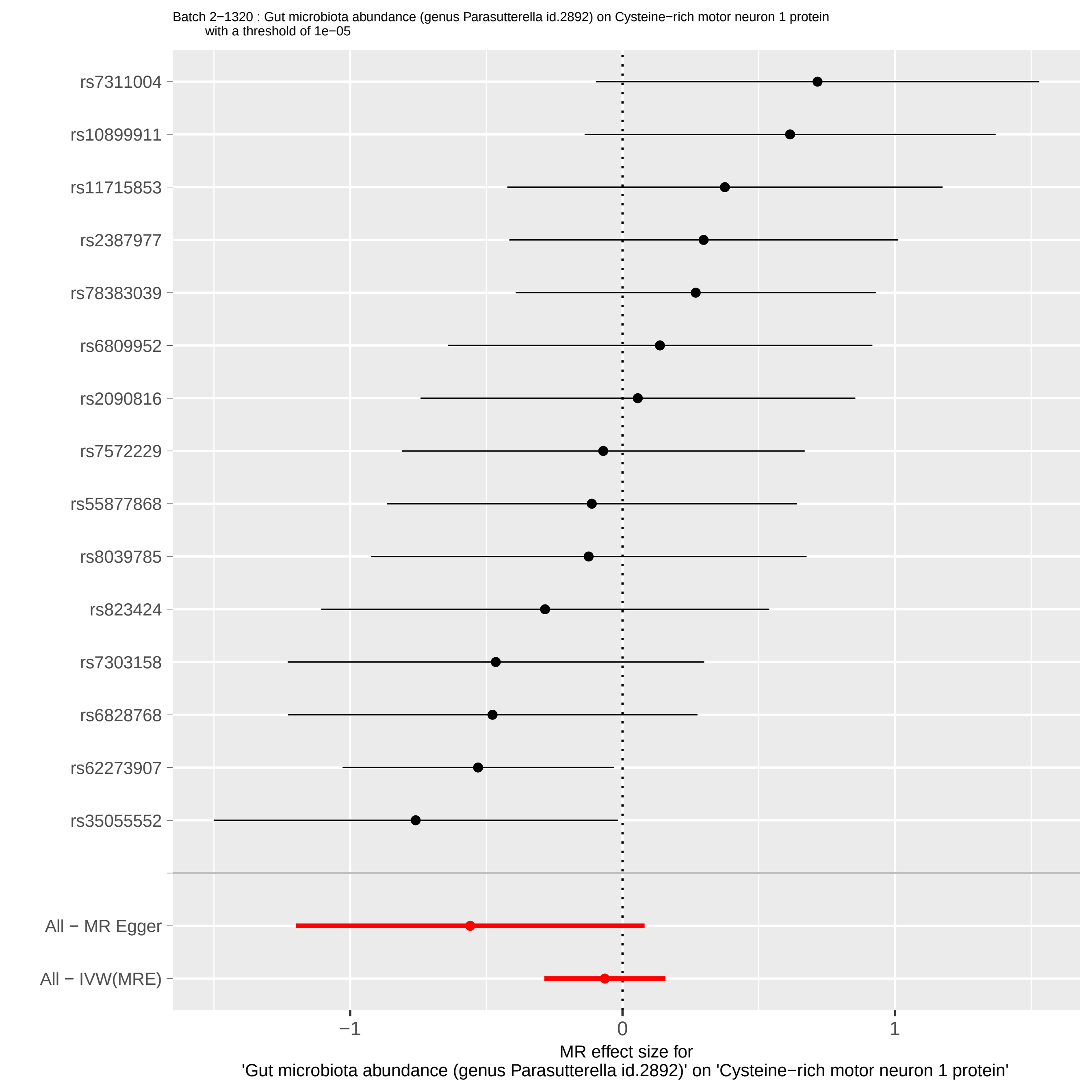

Batch 2−1320 : Gut microbiota abundance (genus Parasutterella id.2892) on Cysteine−rich motor neuron 1 protein
with a threshold of 1e−05
rs7311004
rs10899911
rs11715853
rs2387977
rs78383039
rs6809952
rs2090816
rs7572229
rs55877868
rs8039785
rs823424
rs7303158
rs6828768
rs62273907
rs35055552
All − MR Egger
All − IVW(MRE)
−1
0
1
MR effect size for
'Gut microbiota abundance (genus Parasutterella id.2892)' on 'Cysteine−rich motor neuron 1 protein'

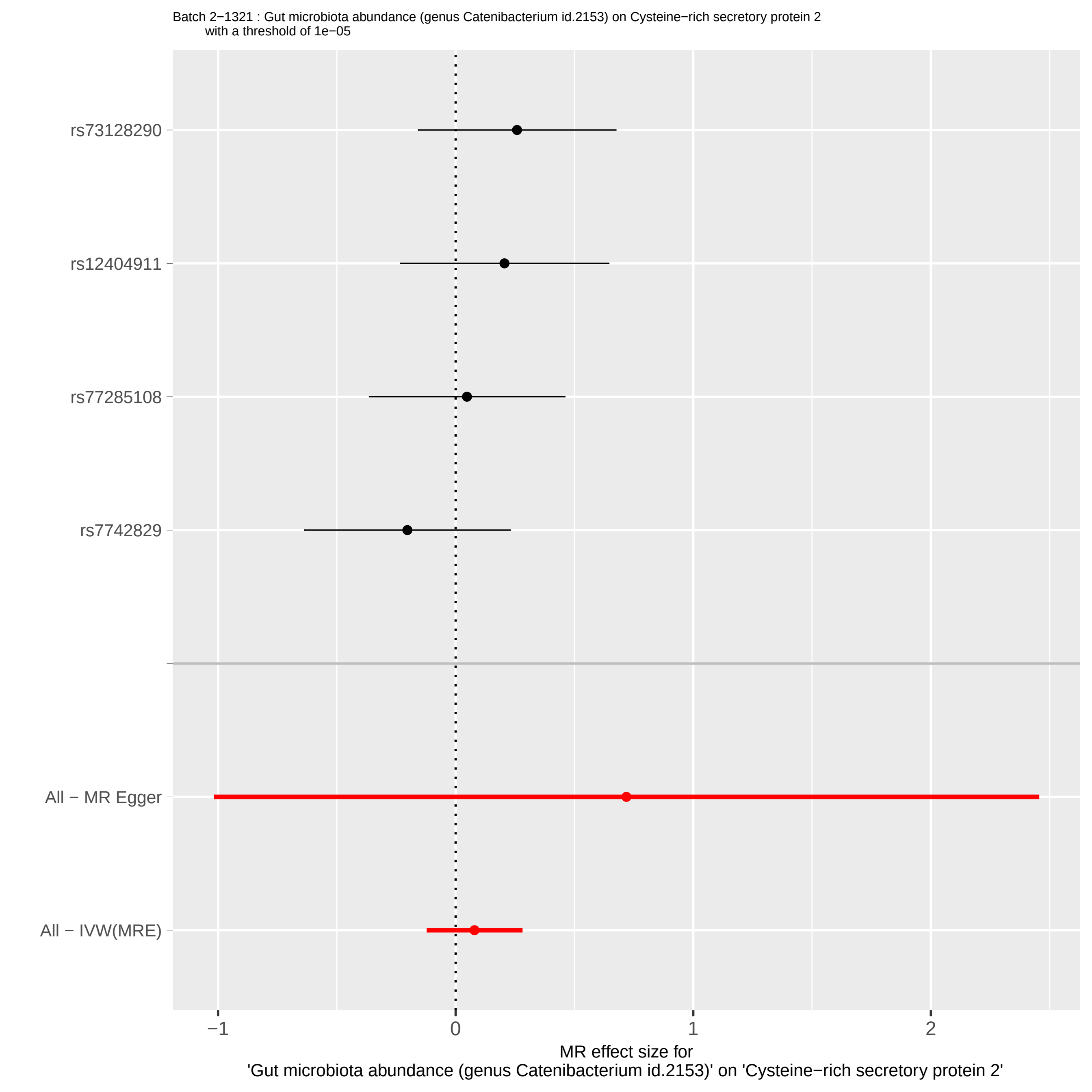

Batch 2−1321 : Gut microbiota abundance (genus Catenibacterium id.2153) on Cysteine−rich secretory protein 2
with a threshold of 1e−05
rs73128290
rs12404911
rs77285108
rs7742829
All − MR Egger
All − IVW(MRE)
−1
0
1
2
MR effect size for
'Gut microbiota abundance (genus Catenibacterium id.2153)' on 'Cysteine−rich secretory protein 2'

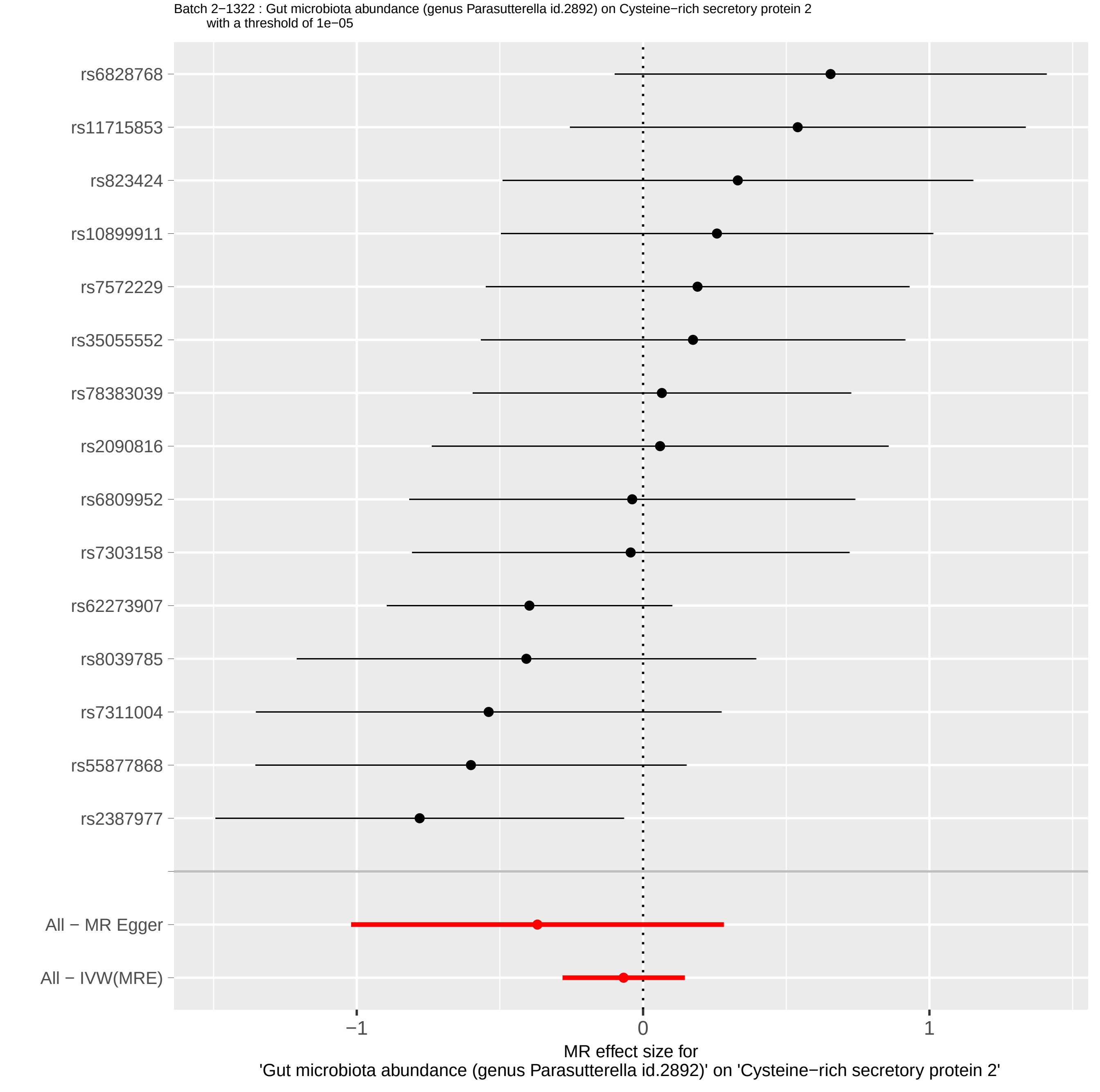
Batch 2−1322 : Gut microbiota abundance (genus Parasutterella id.2892) on Cysteine−rich secretory protein 2
with a threshold of 1e−05
rs6828768
rs11715853
rs823424
rs10899911
rs7572229
rs35055552
rs78383039
rs2090816
rs6809952
rs7303158
rs62273907
rs8039785
rs7311004
rs55877868
rs2387977
All − MR Egger
All − IVW(MRE)
−1
0
1
MR effect size for
'Gut microbiota abundance (genus Parasutterella id.2892)' on 'Cysteine−rich secretory protein 2'

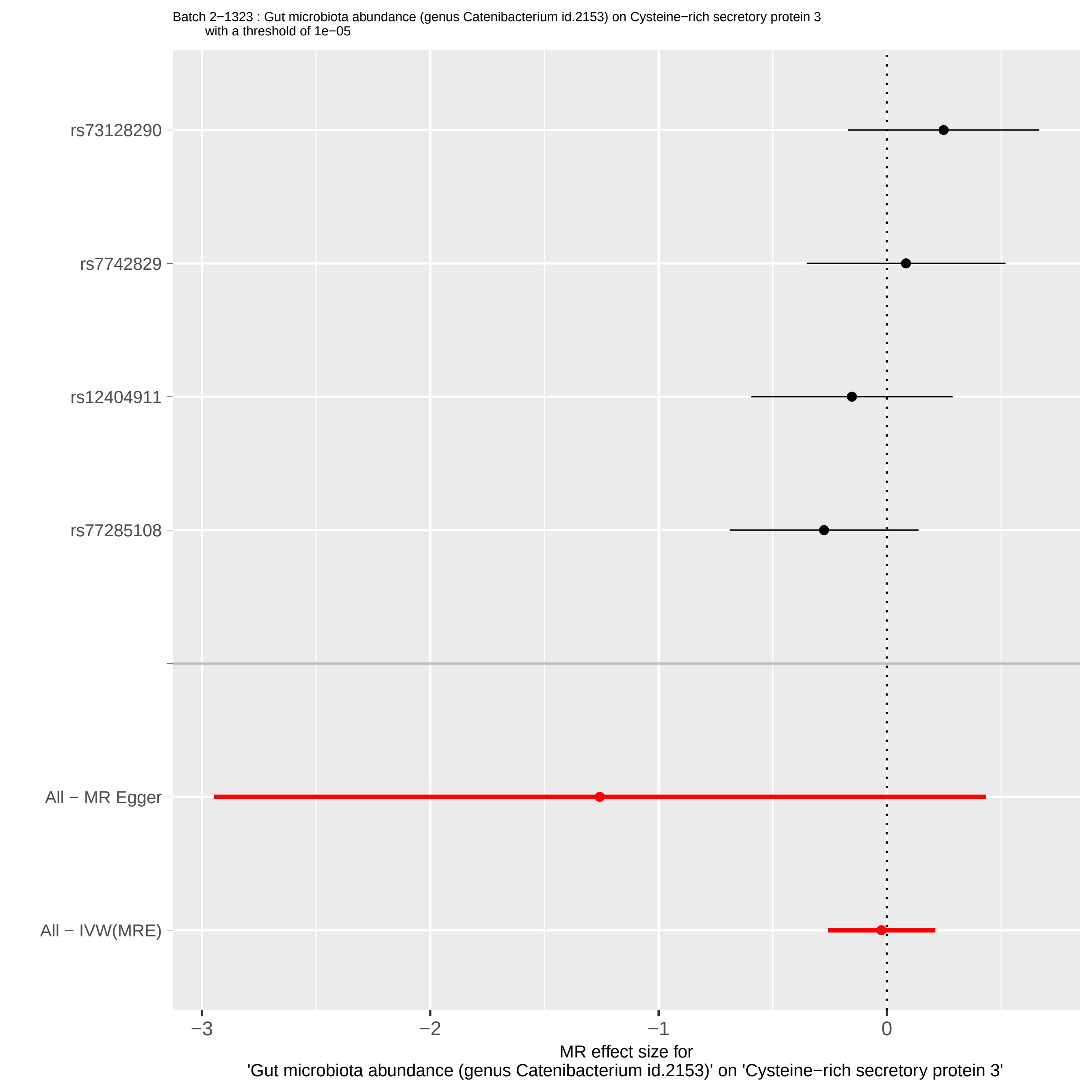
Batch 2−1323 : Gut microbiota abundance (genus Catenibacterium id.2153) on Cysteine−rich secretory protein 3
with a threshold of 1e−05
rs73128290
rs7742829
rs12404911
rs77285108
All − MR Egger
All − IVW(MRE)
−3
−2
−1
0
MR effect size for
'Gut microbiota abundance (genus Catenibacterium id.2153)' on 'Cysteine−rich secretory protein 3'

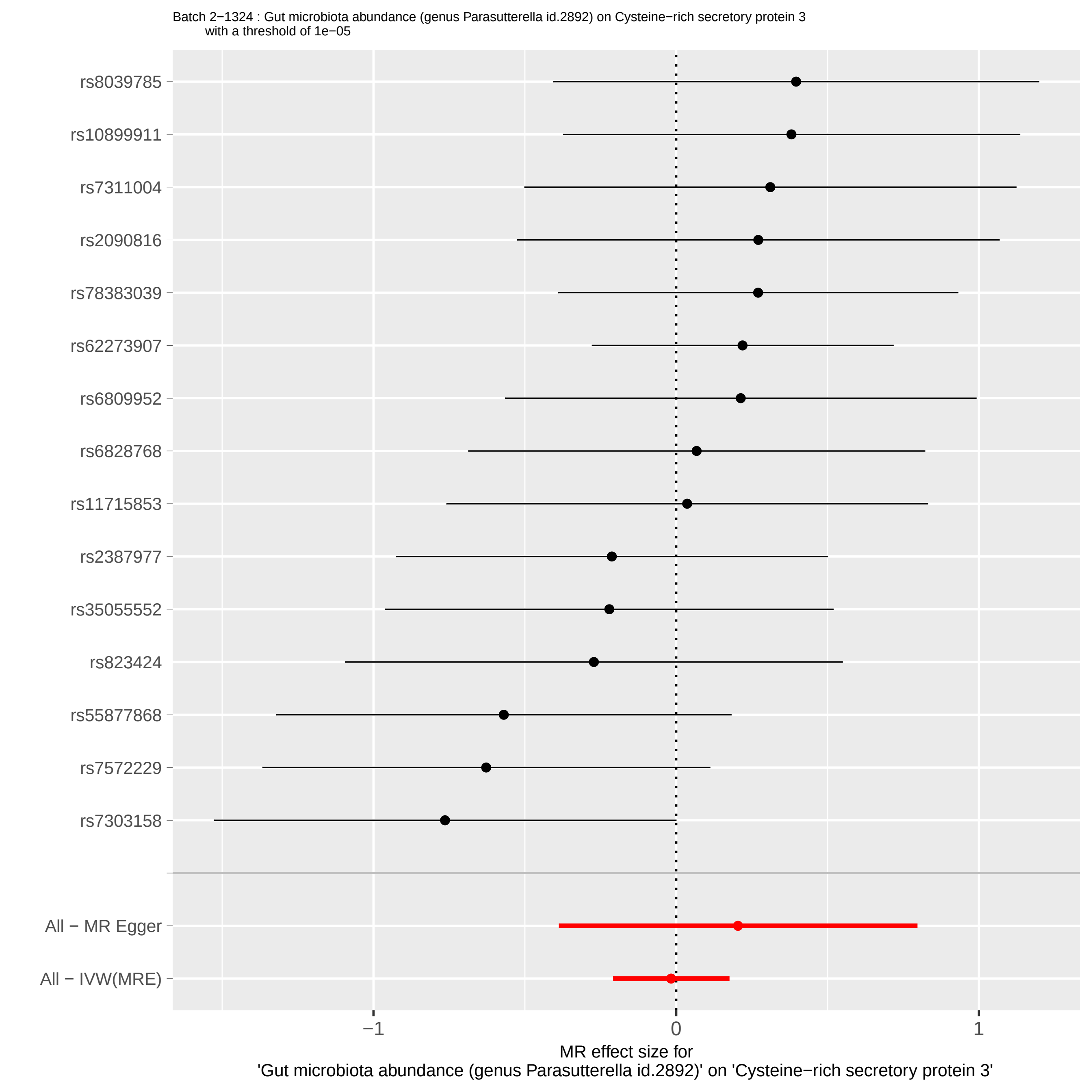

Batch 2−1324 : Gut microbiota abundance (genus Parasutterella id.2892) on Cysteine−rich secretory protein 3
with a threshold of 1e−05
rs8039785
rs10899911
rs7311004
rs2090816
rs78383039
rs62273907
rs6809952
rs6828768
rs11715853
rs2387977
rs35055552
rs823424
rs55877868
rs7572229
rs7303158
All − MR Egger
All − IVW(MRE)
−1
0
1
MR effect size for
'Gut microbiota abundance (genus Parasutterella id.2892)' on 'Cysteine−rich secretory protein 3'

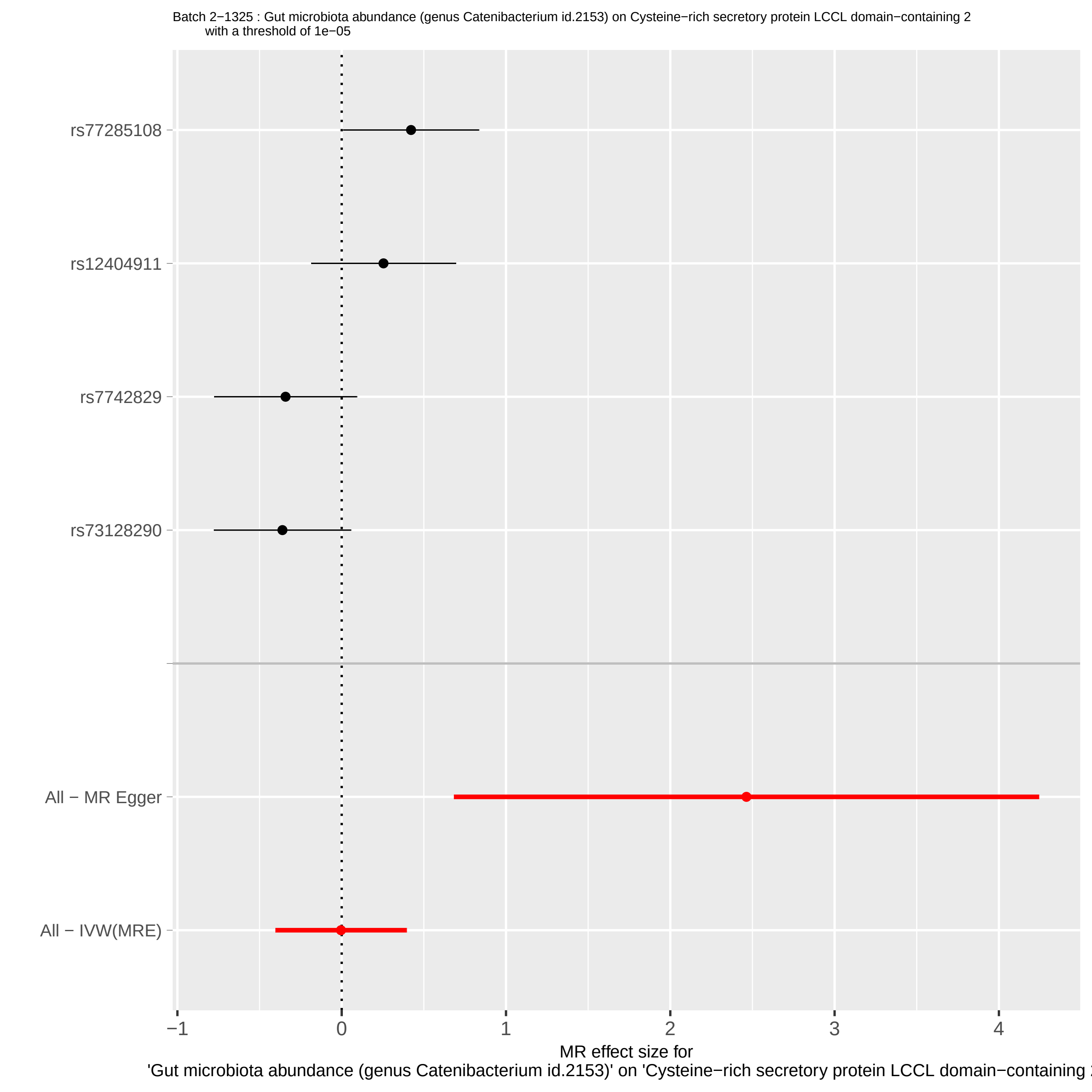
Batch 2−1325 : Gut microbiota abundance (genus Catenibacterium id.2153) on Cysteine−rich secretory protein LCCL domain−containing 2
with a threshold of 1e−05
rs77285108
rs12404911
rs7742829
rs73128290
All − MR Egger
All − IVW(MRE)
−1
0
1
2
3
4
MR effect size for
'Gut microbiota abundance (genus Catenibacterium id.2153)' on 'Cysteine−rich secretory protein LCCL domain−containing

Batch 2−1326 : Gut microbiota abundance (genus Parasutterella id.2892) on Cysteine−rich secretory protein LCCL domain−containing 2 with a threshold of 1e−05

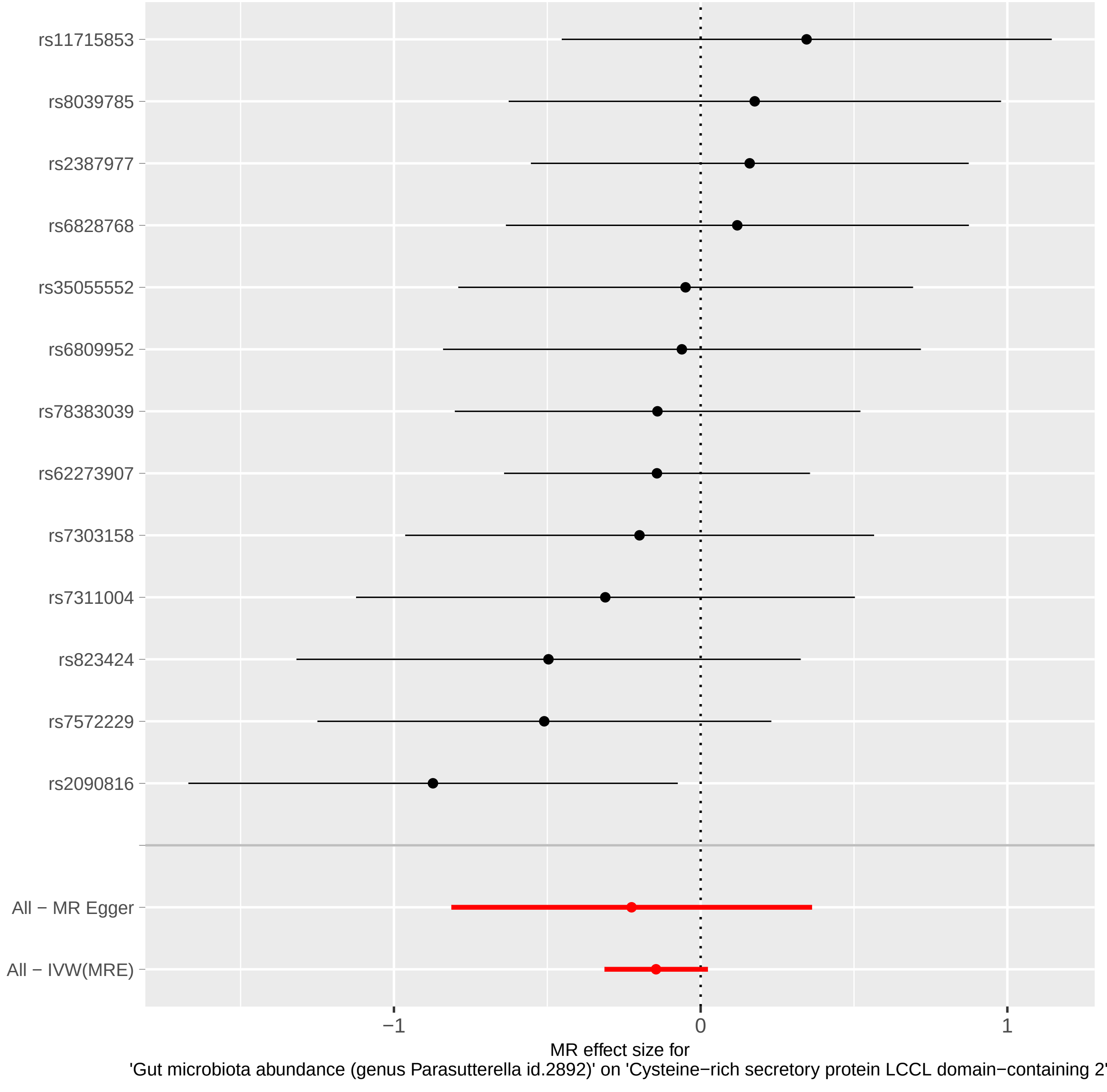

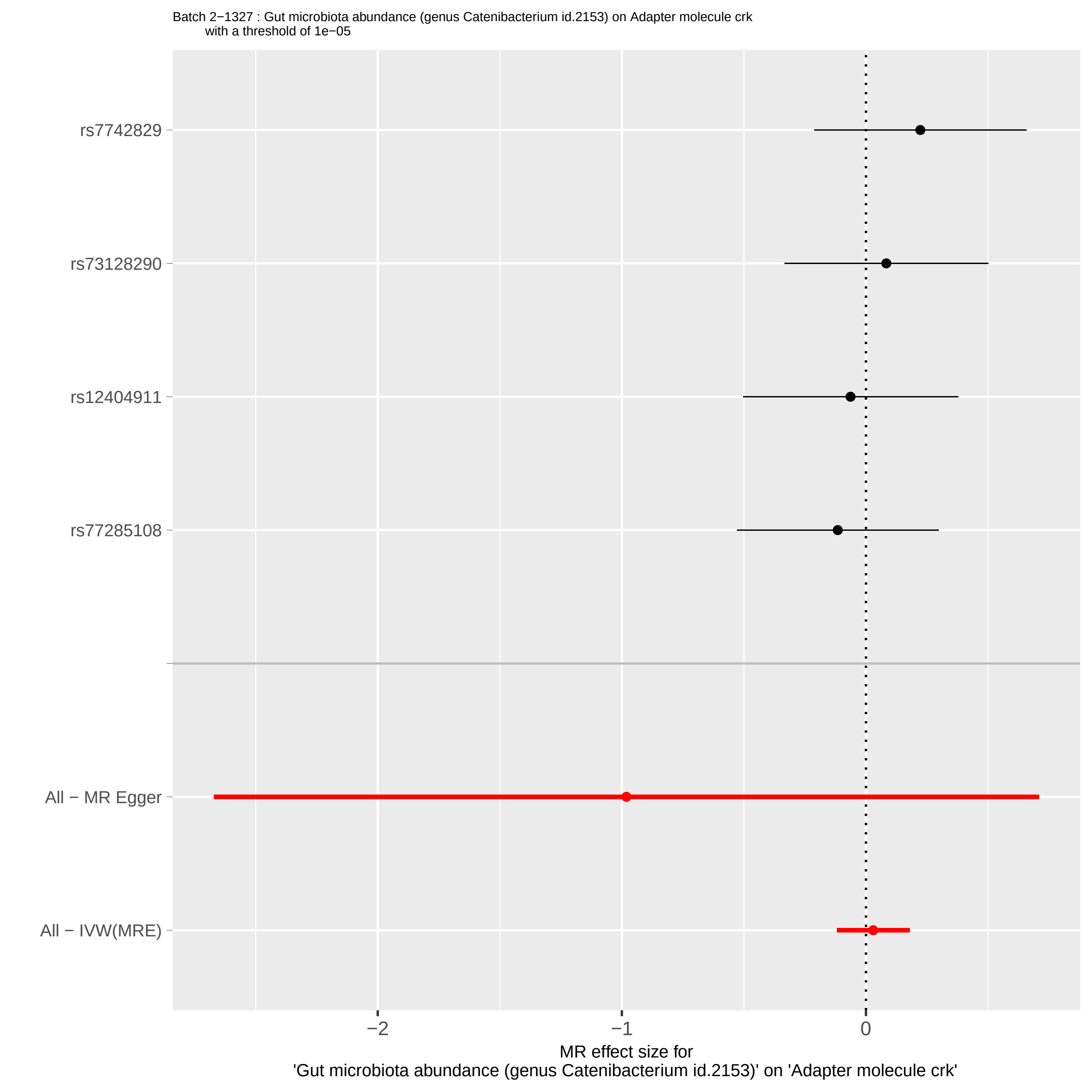
Batch 2−1327 : Gut microbiota abundance (genus Catenibacterium id.2153) on Adapter molecule crk
with a threshold of 1e−05
rs7742829
rs73128290
rs12404911
rs77285108
All − MR Egger
All − IVW(MRE)
−2
−1
0
MR effect size for
'Gut microbiota abundance (genus Catenibacterium id.2153)' on 'Adapter molecule crk'

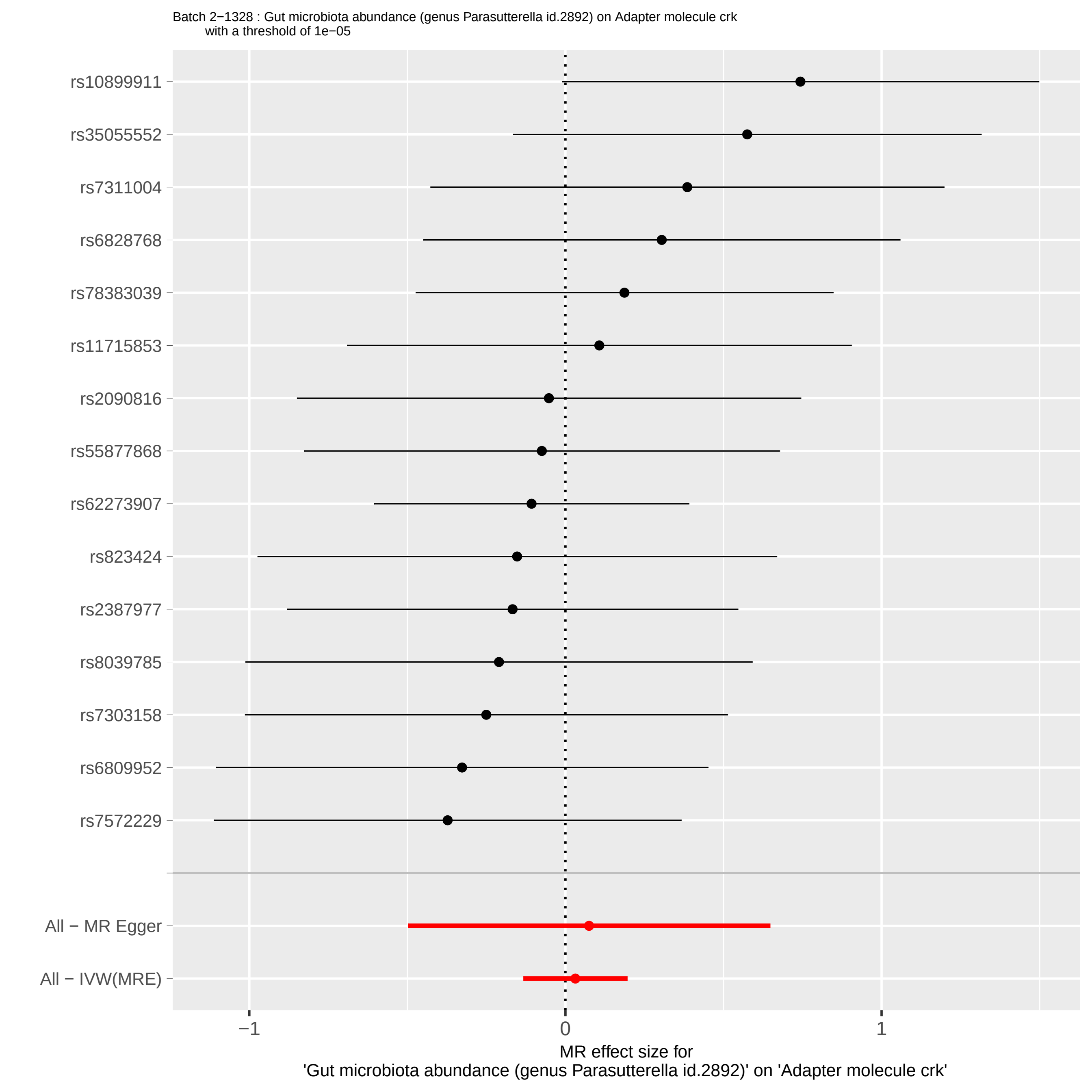
Batch 2−1328 : Gut microbiota abundance (genus Parasutterella id.2892) on Adapter molecule crk
with a threshold of 1e−05
rs10899911
rs35055552
rs7311004
rs6828768
rs78383039
rs11715853
rs2090816
rs55877868
rs62273907
rs823424
rs2387977
rs8039785
rs7303158
rs6809952
rs7572229
All − MR Egger
All − IVW(MRE)
−1
0
1
MR effect size for
'Gut microbiota abundance (genus Parasutterella id.2892)' on 'Adapter molecule crk'

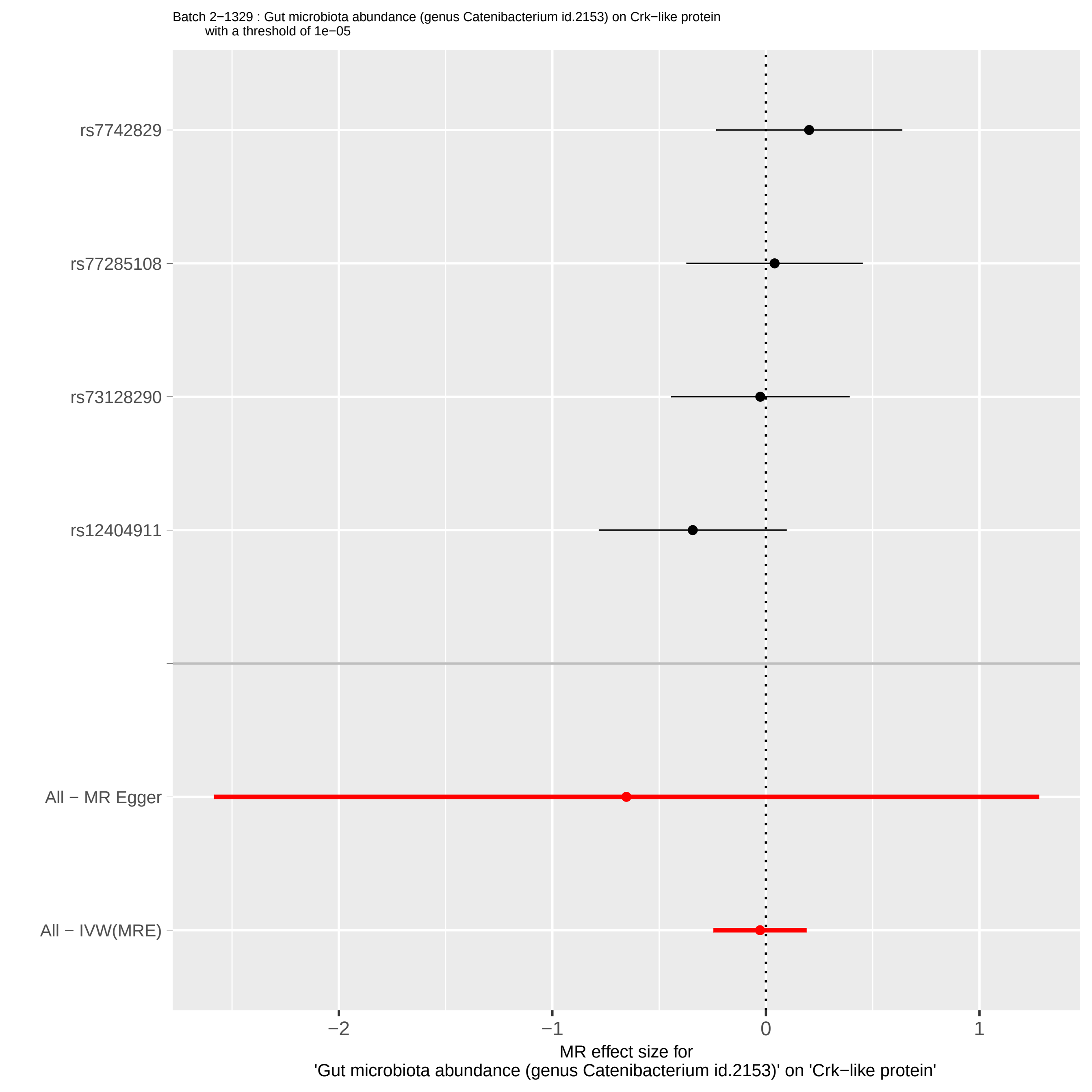

Batch 2−1329 : Gut microbiota abundance (genus Catenibacterium id.2153) on Crk−like protein
with a threshold of 1e−05
rs7742829
rs77285108
rs73128290
rs12404911
All − MR Egger
All − IVW(MRE)
−2
−1
0
1
MR effect size for
'Gut microbiota abundance (genus Catenibacterium id.2153)' on 'Crk−like protein'

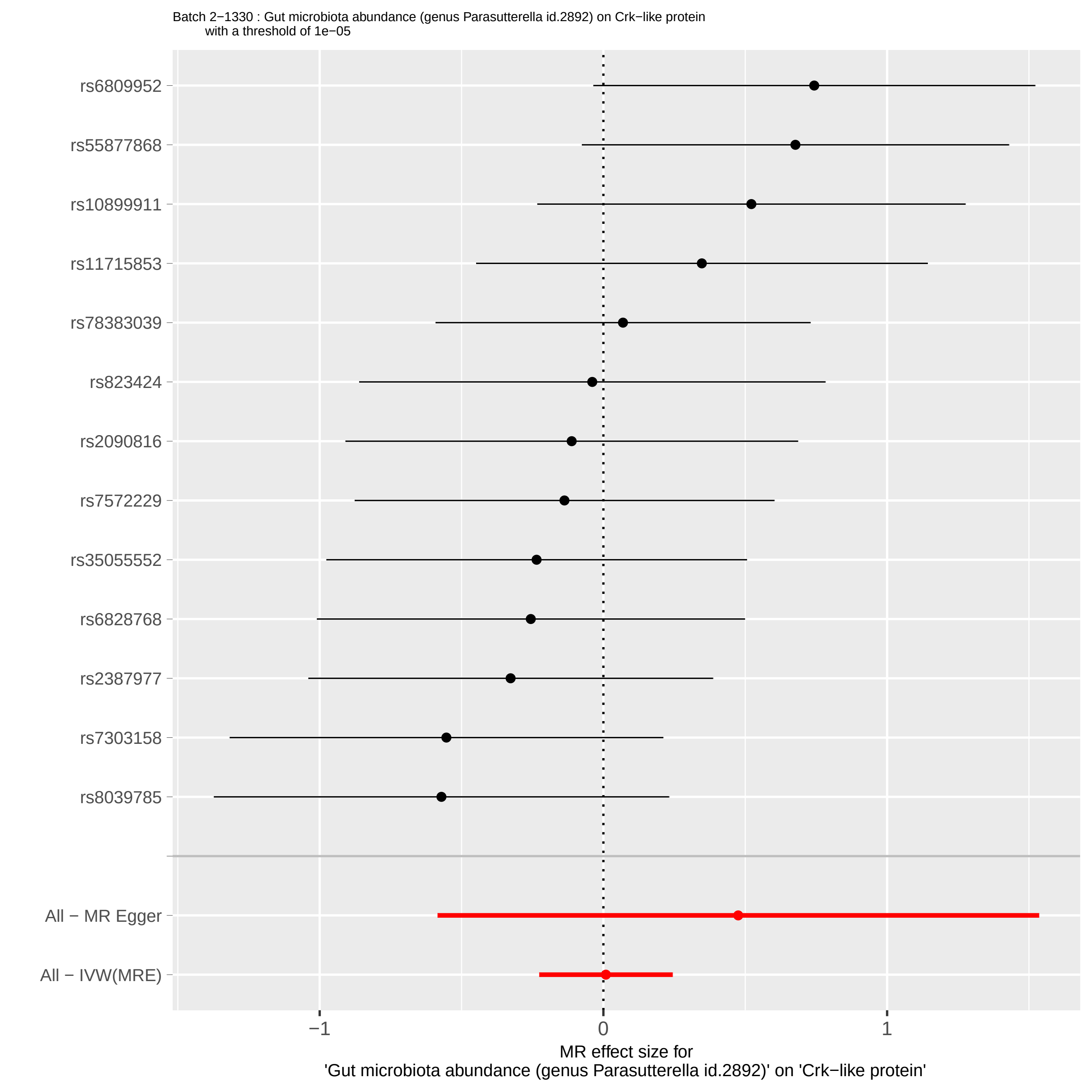

Batch 2−1330 : Gut microbiota abundance (genus Parasutterella id.2892) on Crk−like protein
with a threshold of 1e−05
rs6809952
rs55877868
rs10899911
rs11715853
rs78383039
rs823424
rs2090816
rs7572229
rs35055552
rs6828768
rs2387977
rs7303158
rs8039785
All − MR Egger
All − IVW(MRE)
−1
0
1
MR effect size for
'Gut microbiota abundance (genus Parasutterella id.2892)' on 'Crk−like protein'

Batch 2−1331 : Gut microbiota abundance (genus Catenibacterium id.2153) on Cytokine receptor−like factor 1:Cardiotrophin−like cytokine factor 1 Complex with a threshold of 1e−05

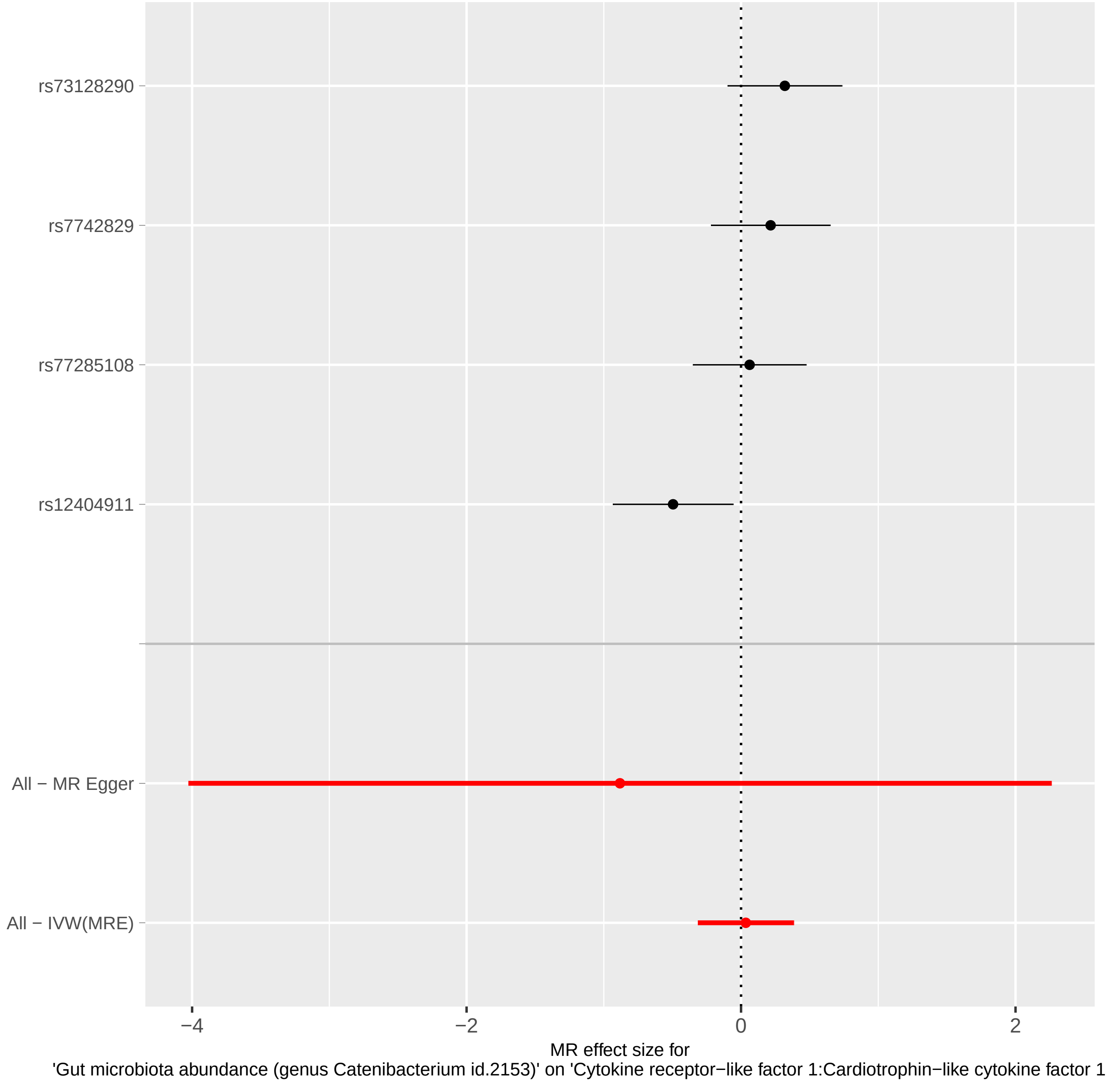

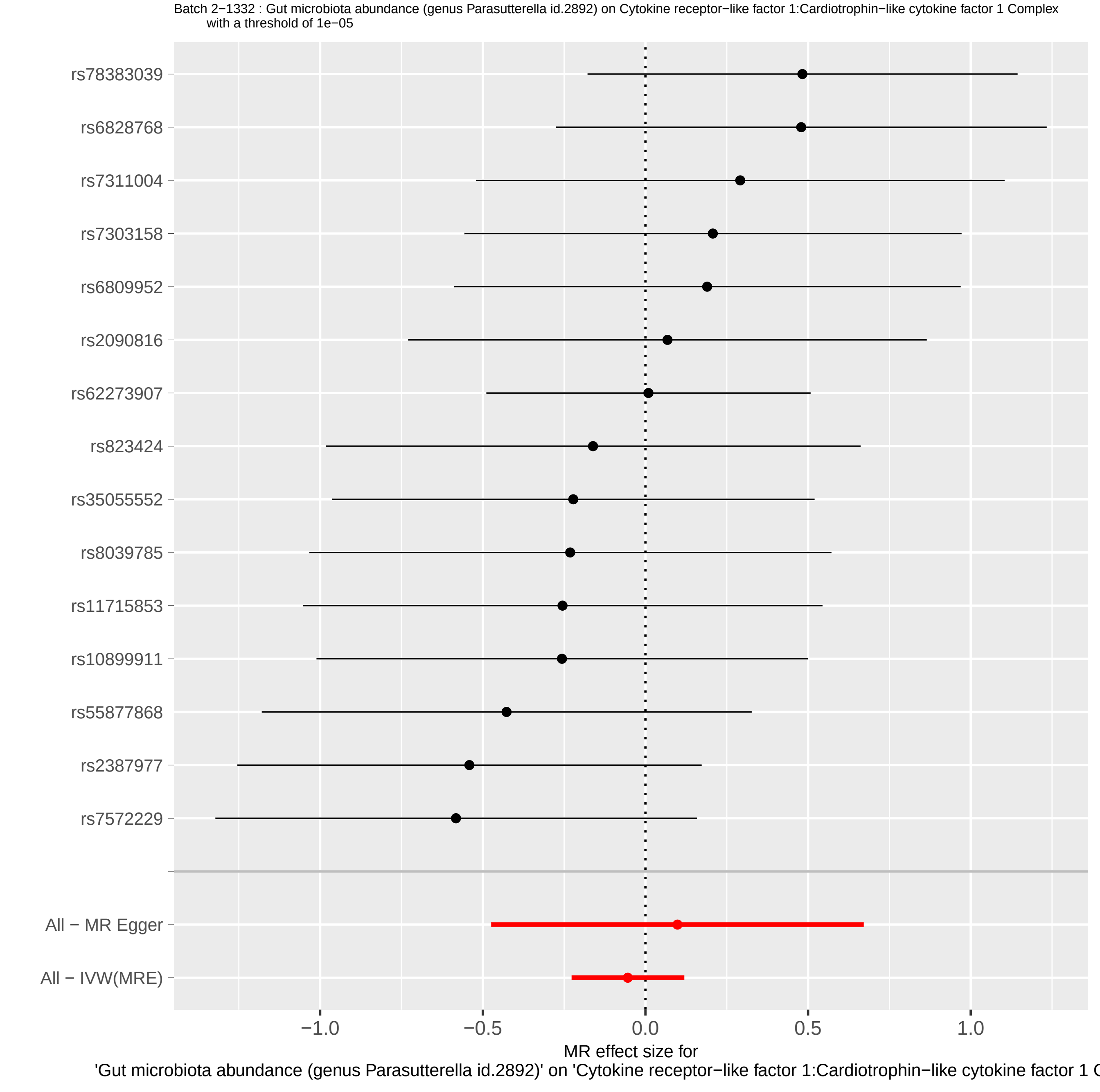

Batch 2−1332 : Gut microbiota abundance (genus Parasutterella id.2892) on Cytokine receptor−like factor 1:Cardiotrophin−like cytokine factor 1 Complex with a threshold of 1e−05
rs78383039
rs6828768
rs7311004
rs7303158
rs6809952
rs2090816
rs62273907
rs823424
rs35055552
rs8039785
rs11715853
rs10899911
rs55877868
rs2387977
rs7572229
All − MR Egger
All − IVW(MRE)
−1.0
−0.5
0.0
0.5
1.0
MR effect size for
'Gut microbiota abundance (genus Parasutterella id.2892)' on 'Cytokine receptor−like factor 1:Cardiotrophin−like cytokine factor 1 C

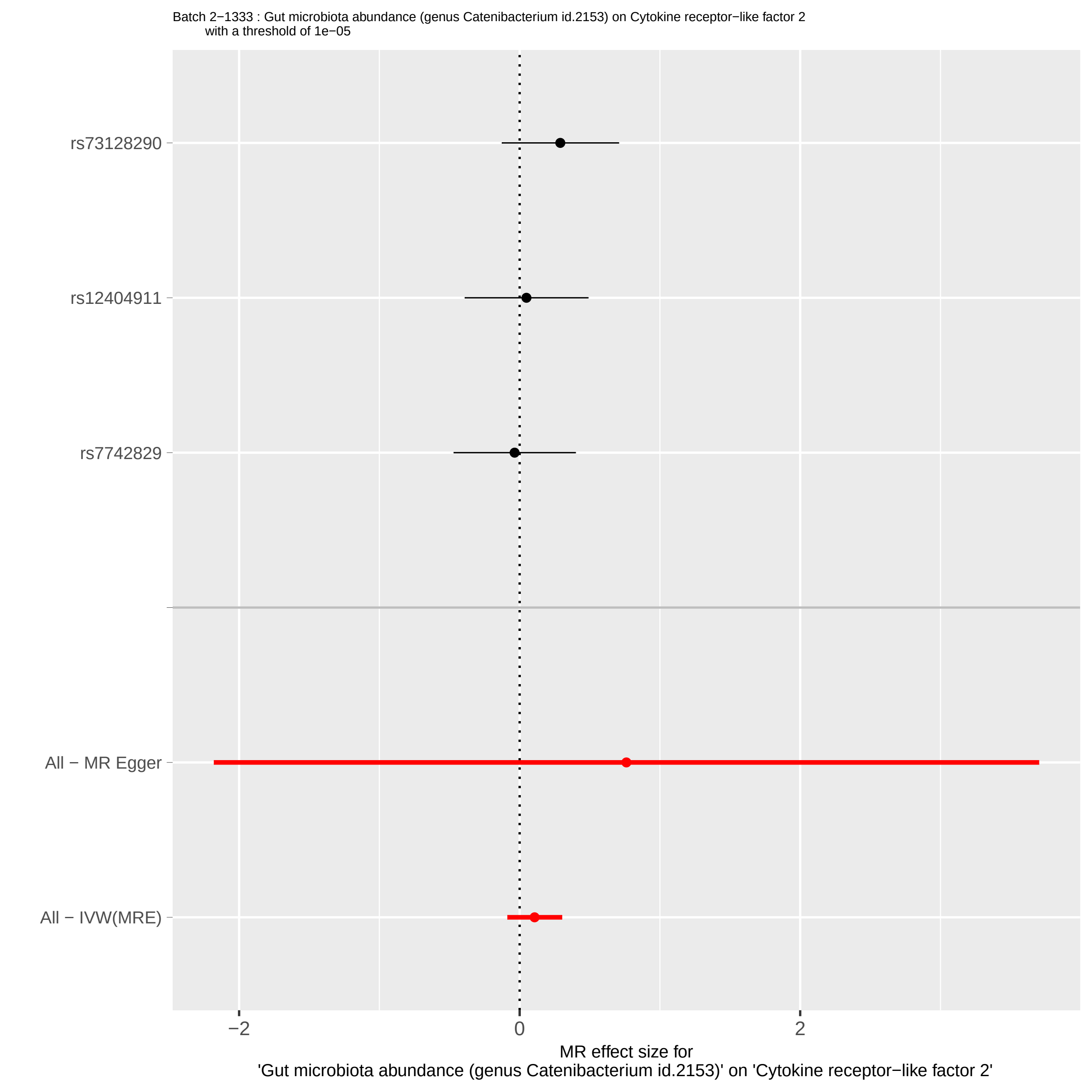
Batch 2−1333 : Gut microbiota abundance (genus Catenibacterium id.2153) on Cytokine receptor−like factor 2
with a threshold of 1e−05
rs73128290
rs12404911
rs7742829
All − MR Egger
All − IVW(MRE)
−2
0
2
MR effect size for
'Gut microbiota abundance (genus Catenibacterium id.2153)' on 'Cytokine receptor−like factor 2'

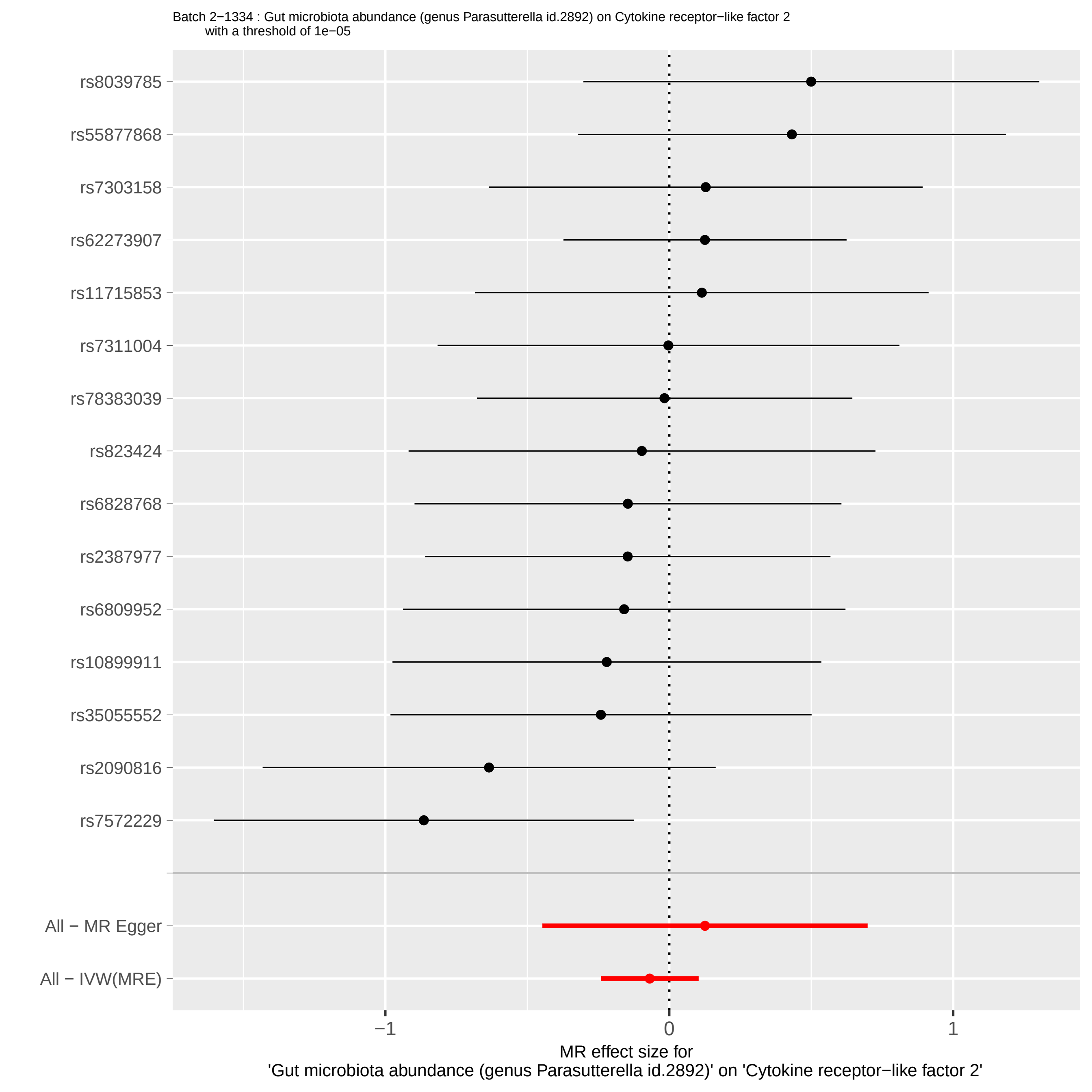
Batch 2−1334 : Gut microbiota abundance (genus Parasutterella id.2892) on Cytokine receptor−like factor 2
with a threshold of 1e−05
rs8039785
rs55877868
rs7303158
rs62273907
rs11715853
rs7311004
rs78383039
rs823424
rs6828768
rs2387977
rs6809952
rs10899911
rs35055552
rs2090816
rs7572229
All − MR Egger
All − IVW(MRE)
−1
0
1
MR effect size for
'Gut microbiota abundance (genus Parasutterella id.2892)' on 'Cytokine receptor−like factor 2'

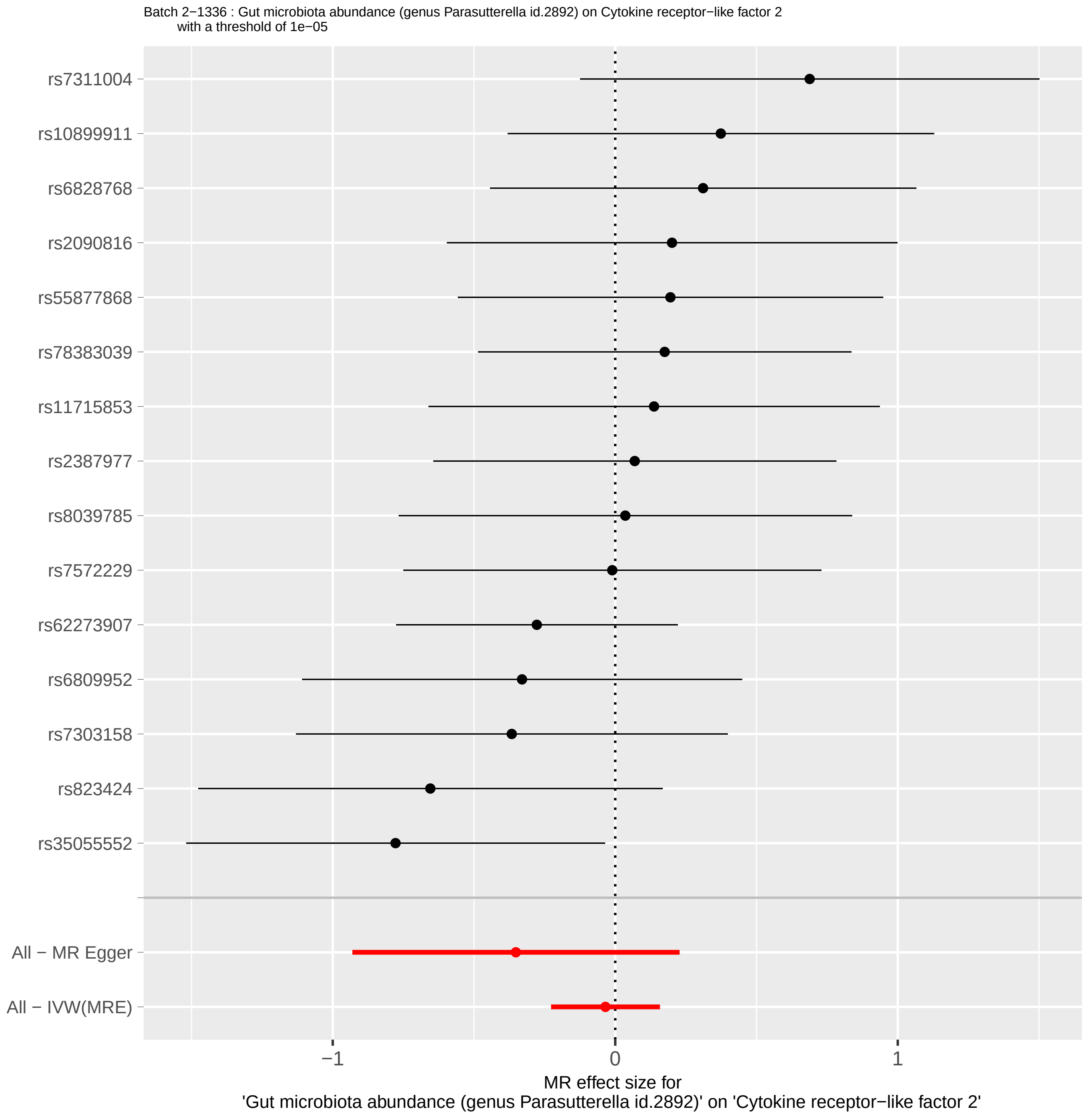
Batch 2−1336 : Gut microbiota abundance (genus Parasutterella id.2892) on Cytokine receptor−like factor 2
with a threshold of 1e−05
rs7311004
rs10899911
rs6828768
rs2090816
rs55877868
rs78383039
rs11715853
rs2387977
rs8039785
rs7572229
rs62273907
rs6809952
rs7303158
rs823424
rs35055552
All − MR Egger
All − IVW(MRE)
−1
0
1
MR effect size for
'Gut microbiota abundance (genus Parasutterella id.2892)' on 'Cytokine receptor−like factor 2'

Batch 2−1337 : Gut microbiota abundance (genus Catenibacterium id.2153) on Peroxisomal carnitine O−octanoyltransferase
with a threshold of 1e−05

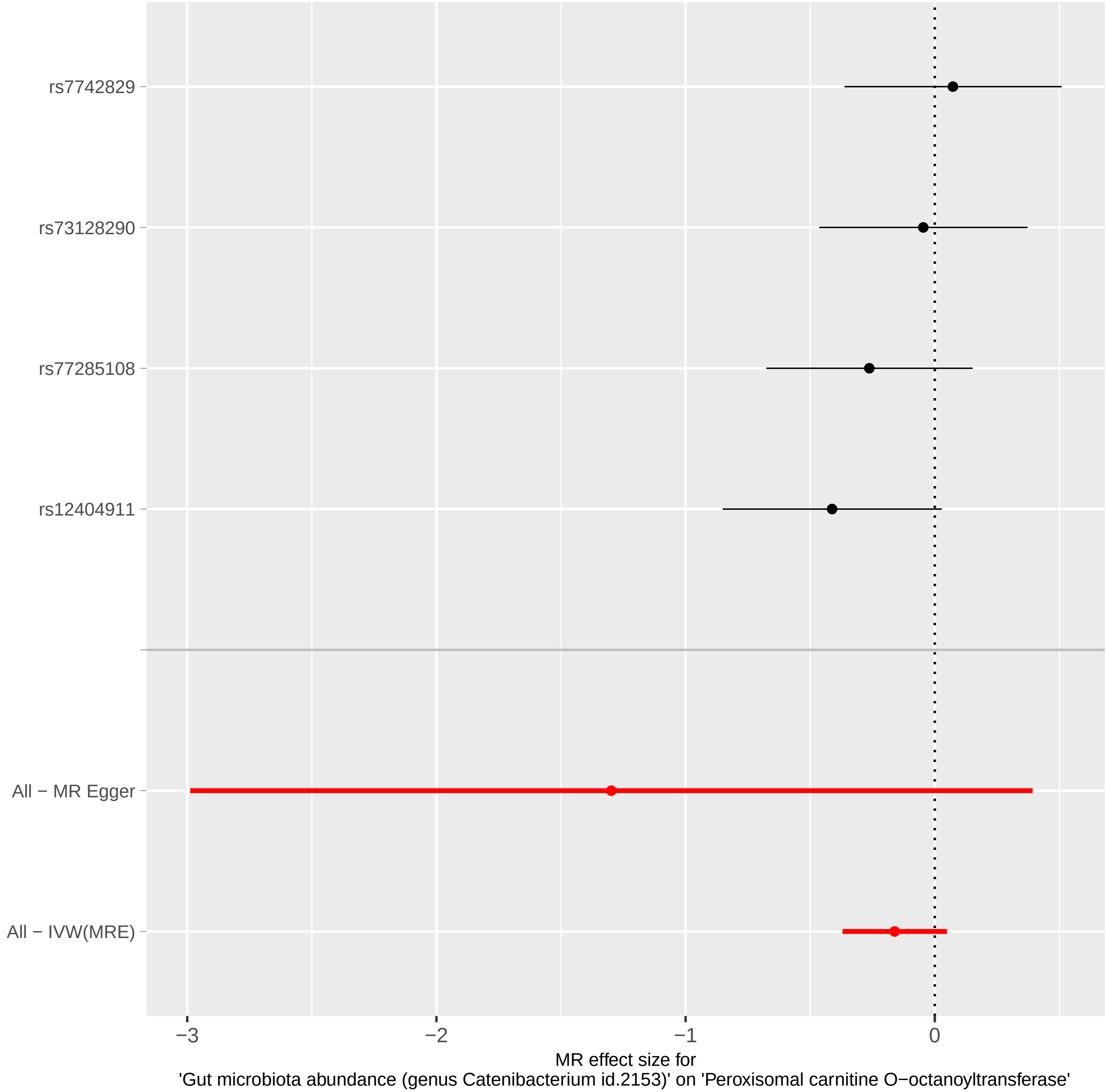

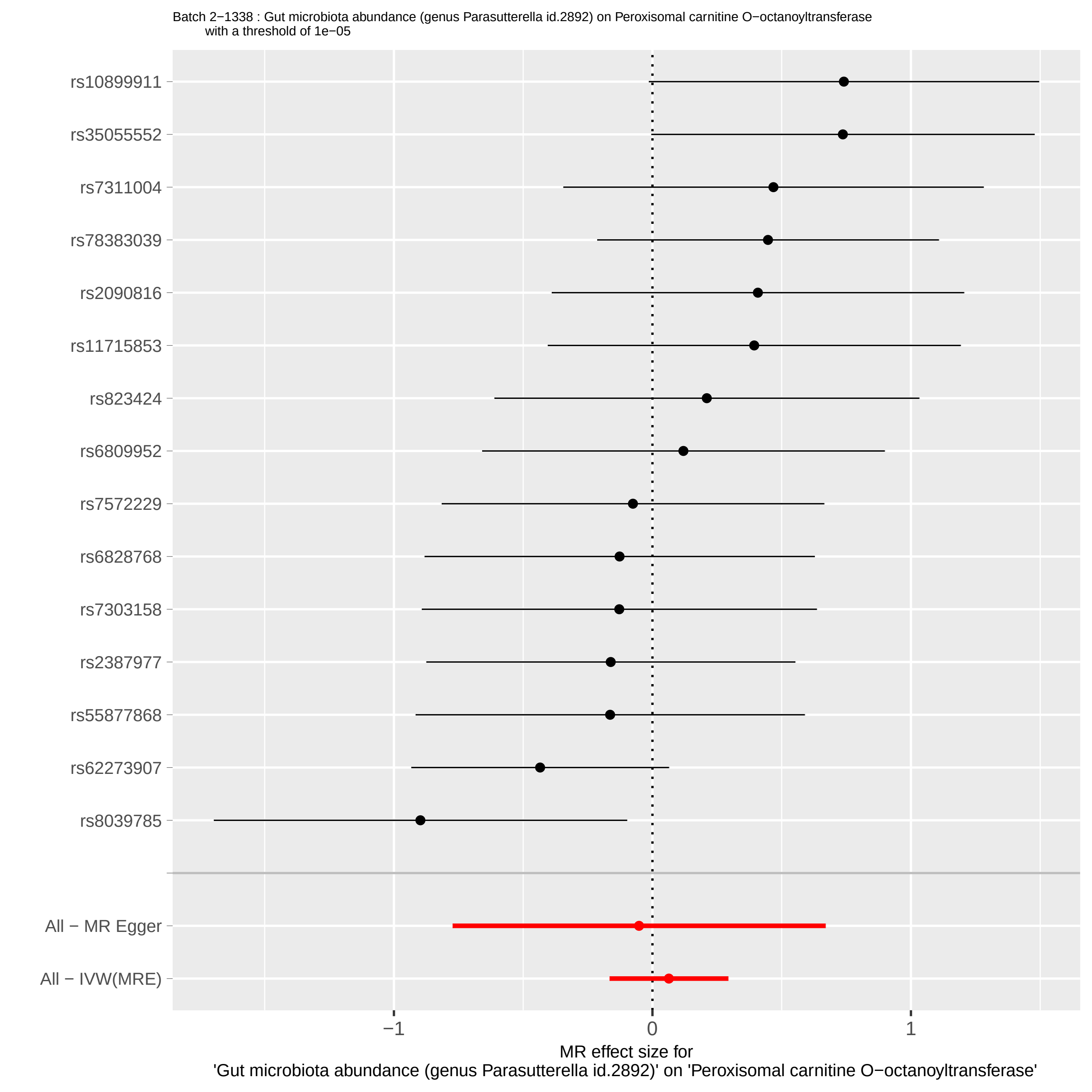

Batch 2−1338 : Gut microbiota abundance (genus Parasutterella id.2892) on Peroxisomal carnitine O−octanoyltransferase
with a threshold of 1e−05
rs10899911
rs35055552
rs7311004
rs78383039
rs2090816
rs11715853
rs823424
rs6809952
rs7572229
rs6828768
rs7303158
rs2387977
rs55877868
rs62273907
rs8039785
All − MR Egger
All − IVW(MRE)
−1
0
1
MR effect size for
'Gut microbiota abundance (genus Parasutterella id.2892)' on 'Peroxisomal carnitine O−octanoyltransferase'

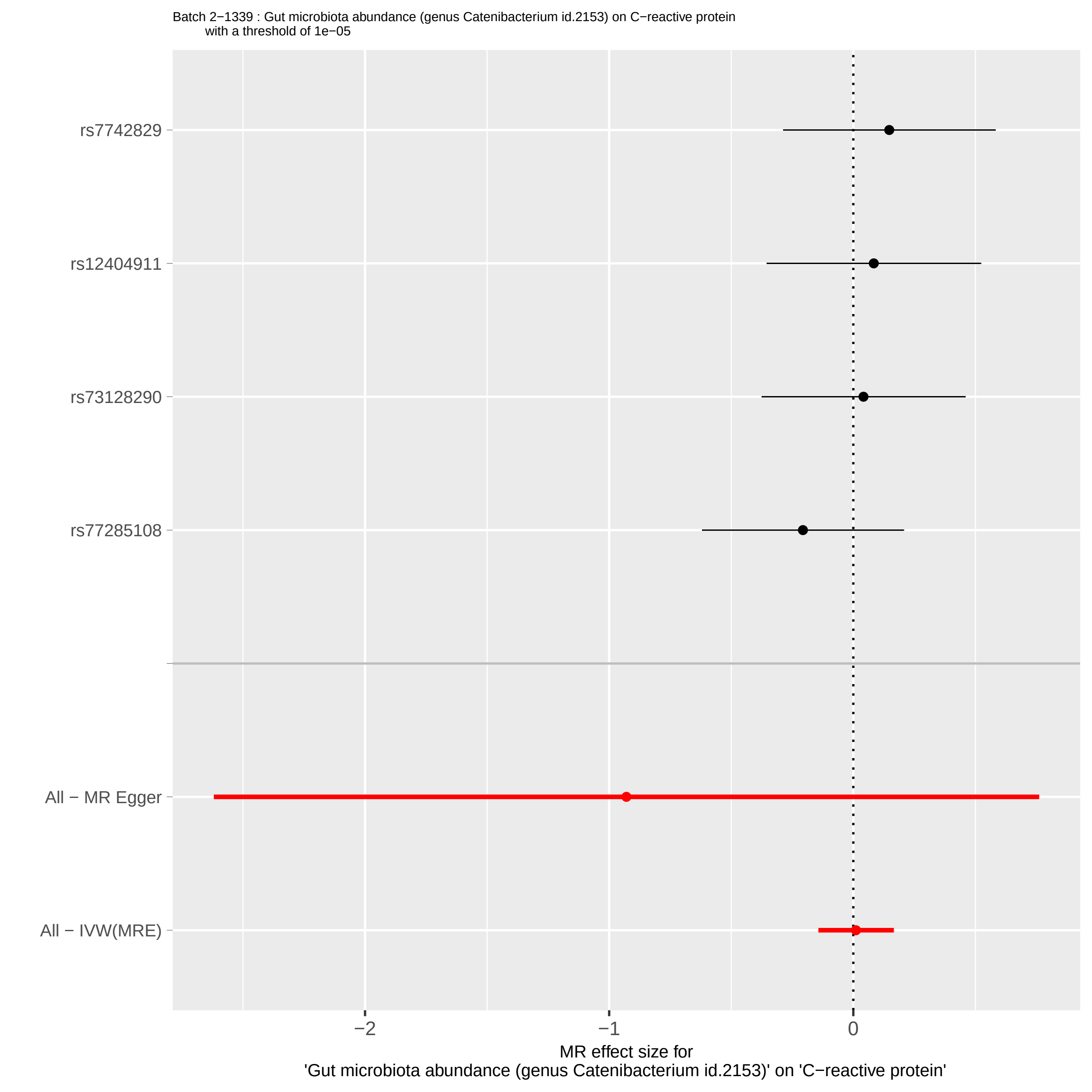
Batch 2−1339 : Gut microbiota abundance (genus Catenibacterium id.2153) on C−reactive protein
with a threshold of 1e−05
rs7742829
rs12404911
rs73128290
rs77285108
All − MR Egger
All − IVW(MRE)
−2
−1
0
MR effect size for
'Gut microbiota abundance (genus Catenibacterium id.2153)' on 'C−reactive protein'

Batch 2−1340 : Gut microbiota abundance (genus Parasutterella id.2892) on C−reactive protein with a threshold of 1e−05

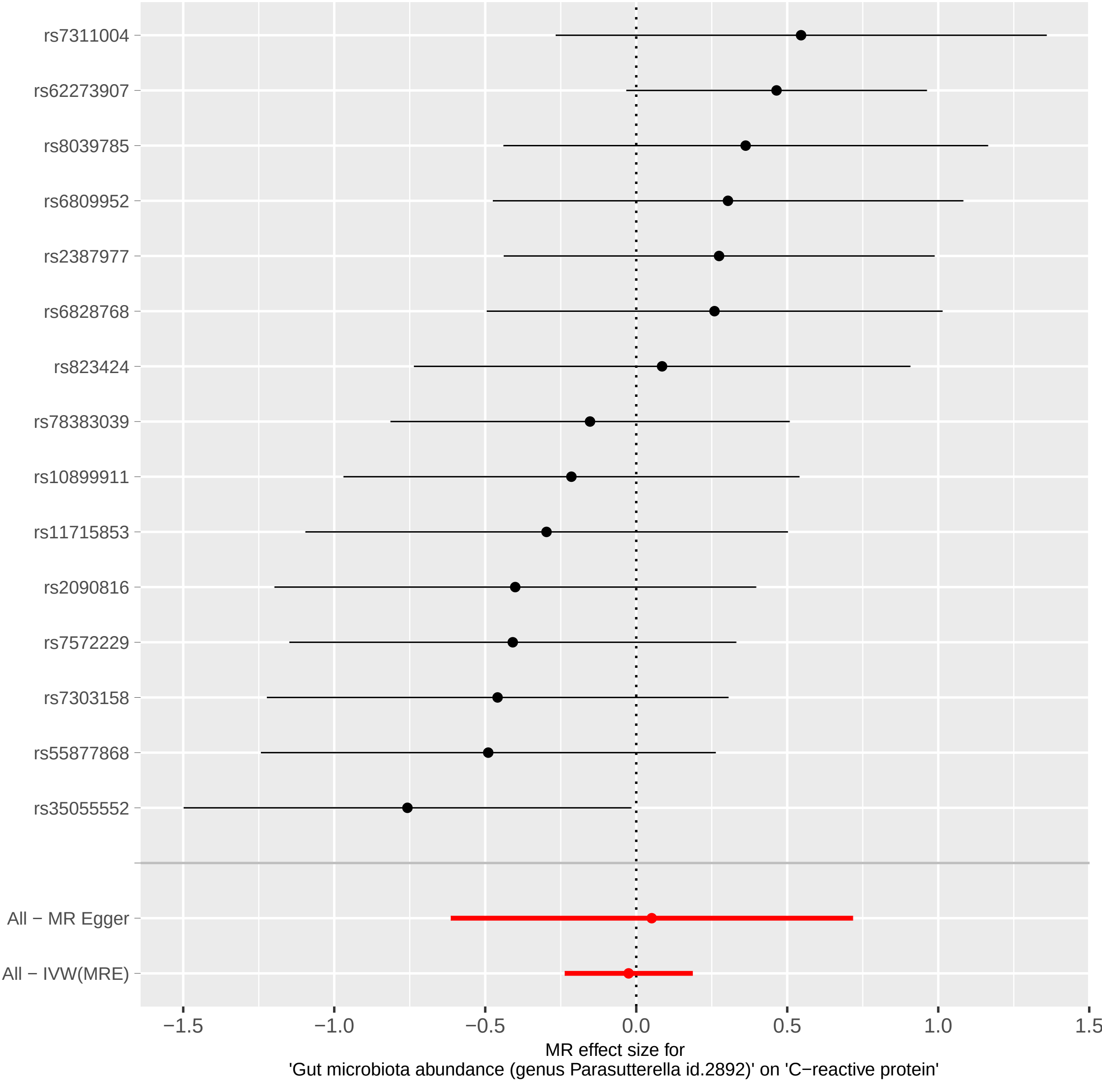

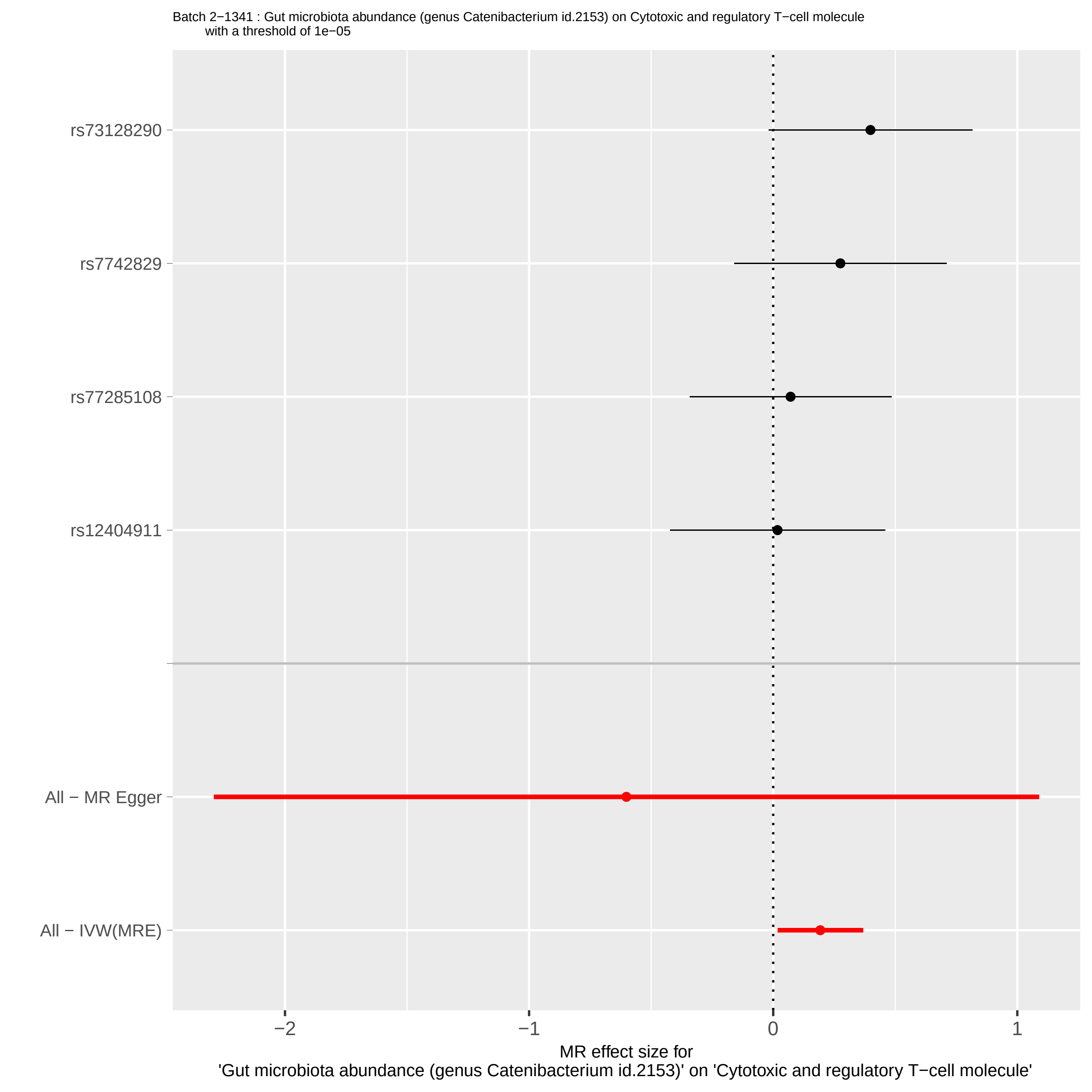
Batch 2−1341 : Gut microbiota abundance (genus Catenibacterium id.2153) on Cytotoxic and regulatory T−cell molecule
with a threshold of 1e−05
rs73128290
rs7742829
rs77285108
rs12404911
All − MR Egger
All − IVW(MRE)
−2
−1
0
1
MR effect size for
'Gut microbiota abundance (genus Catenibacterium id.2153)' on 'Cytotoxic and regulatory T−cell molecule'

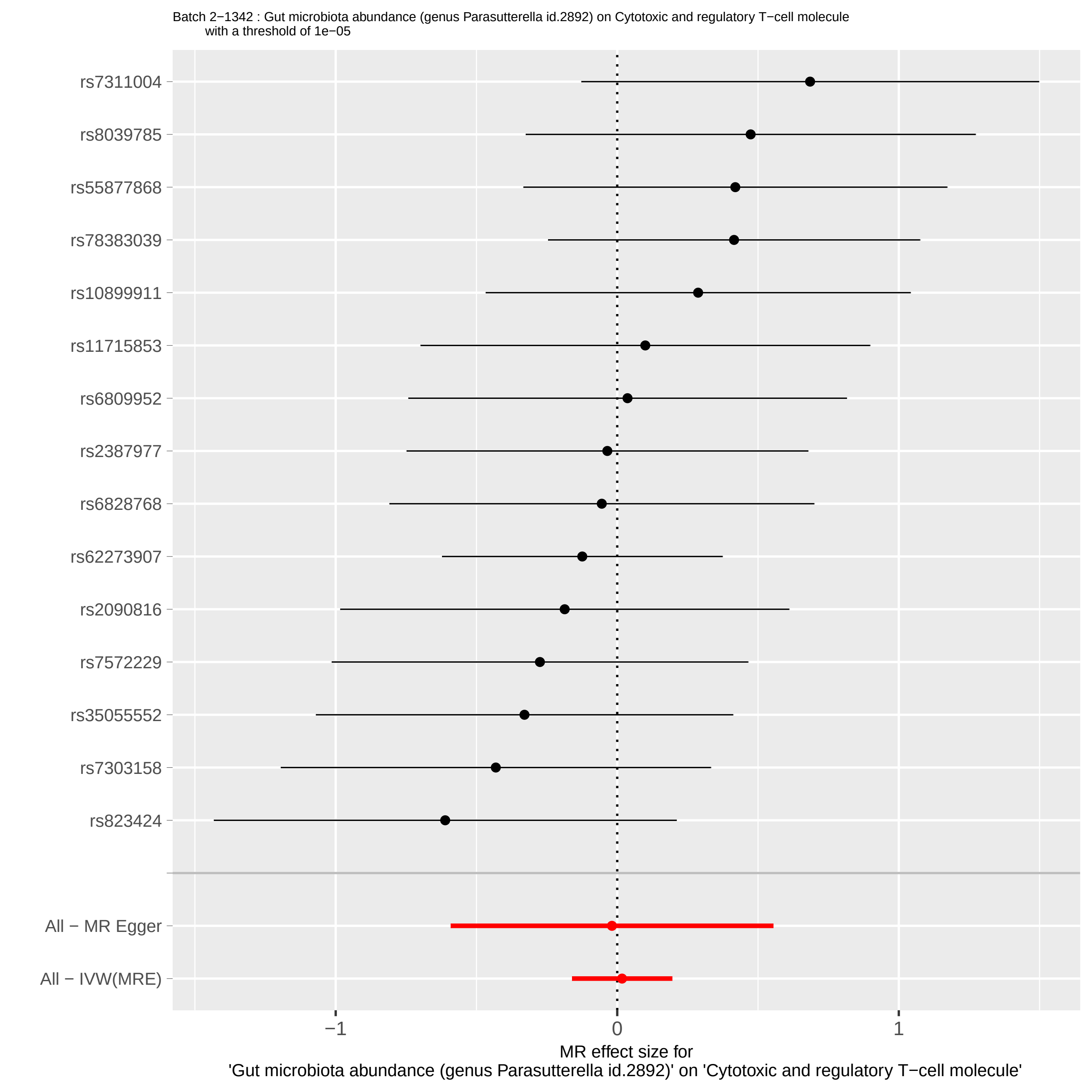

Batch 2−1342 : Gut microbiota abundance (genus Parasutterella id.2892) on Cytotoxic and regulatory T−cell molecule
with a threshold of 1e−05
rs7311004
rs8039785
rs55877868
rs78383039
rs10899911
rs11715853
rs6809952
rs2387977
rs6828768
rs62273907
rs2090816
rs7572229
rs35055552
rs7303158
rs823424
All − MR Egger
All − IVW(MRE)
−1
0
1
MR effect size for
'Gut microbiota abundance (genus Parasutterella id.2892)' on 'Cytotoxic and regulatory T−cell molecule'

Batch 2−1343 : Gut microbiota abundance (genus Catenibacterium id.2153) on Cytotoxic and regulatory T−cell molecule
with a threshold of 1e−05

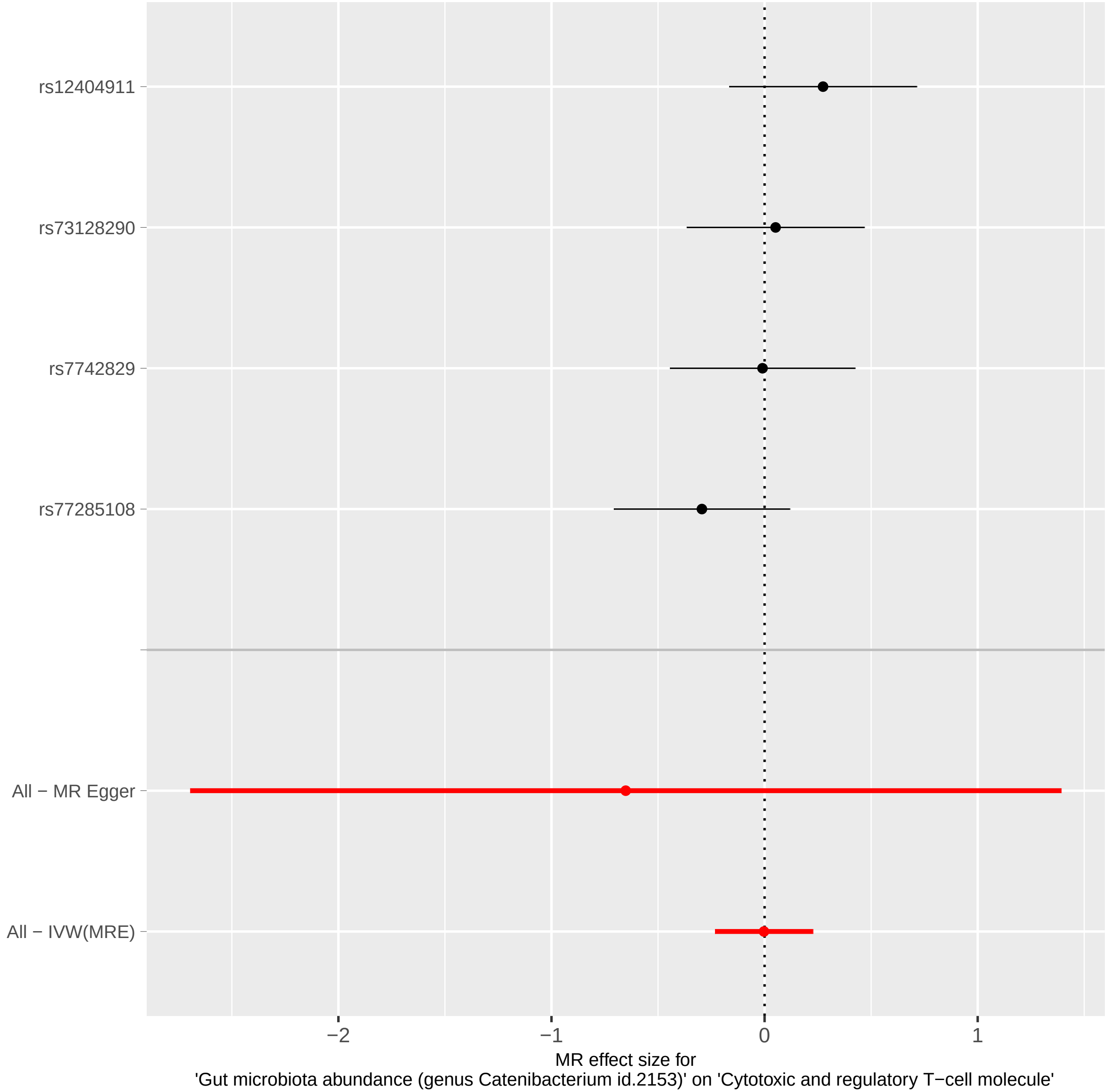

Batch 2−1344 : Gut microbiota abundance (genus Parasutterella id.2892) on Cytotoxic and regulatory T−cell molecule
with a threshold of 1e−05

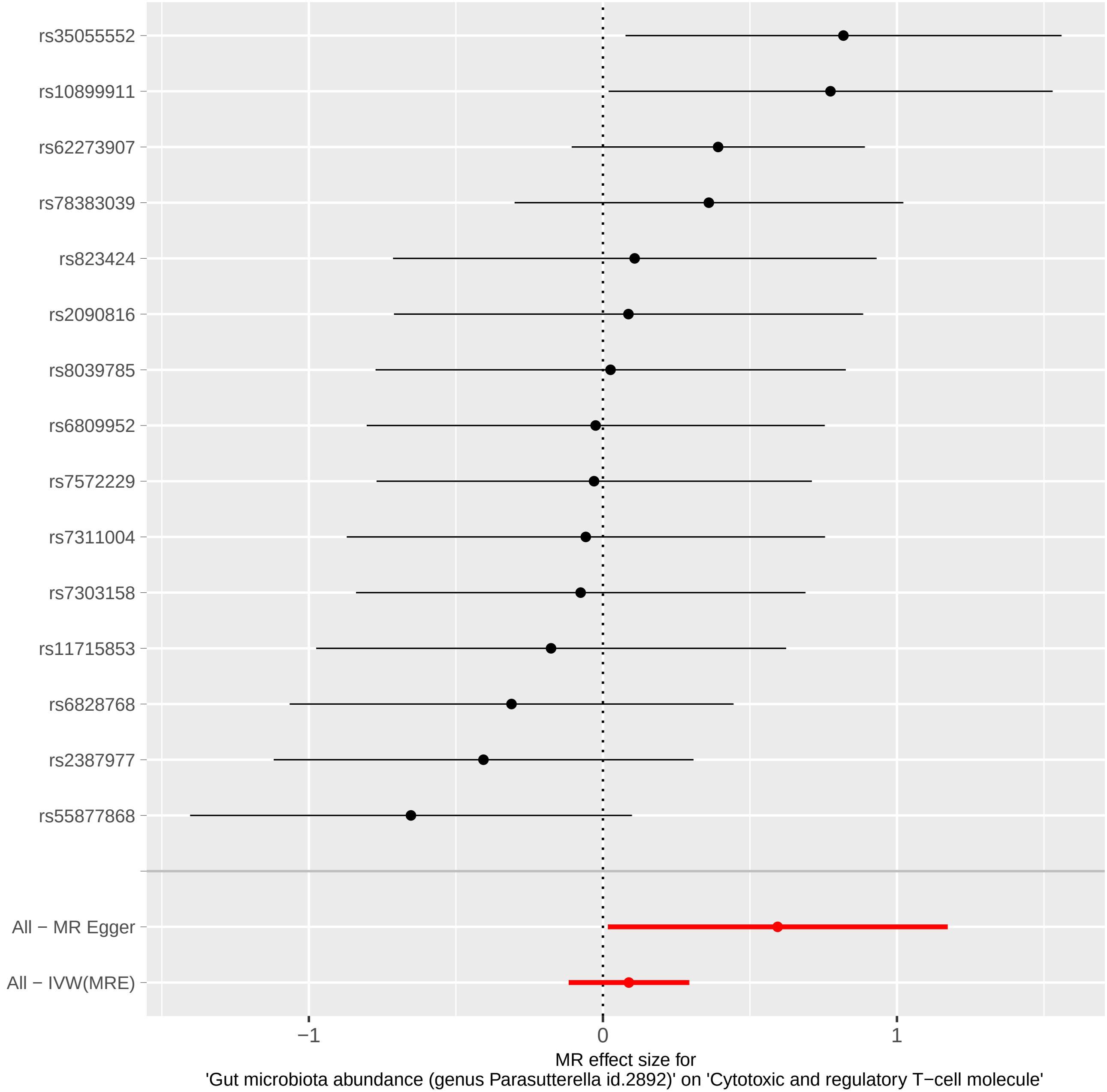

Batch 2−1345 : Gut microbiota abundance (genus Catenibacterium id.2153) on Alpha−crystallin A chain
with a threshold of 1e−05

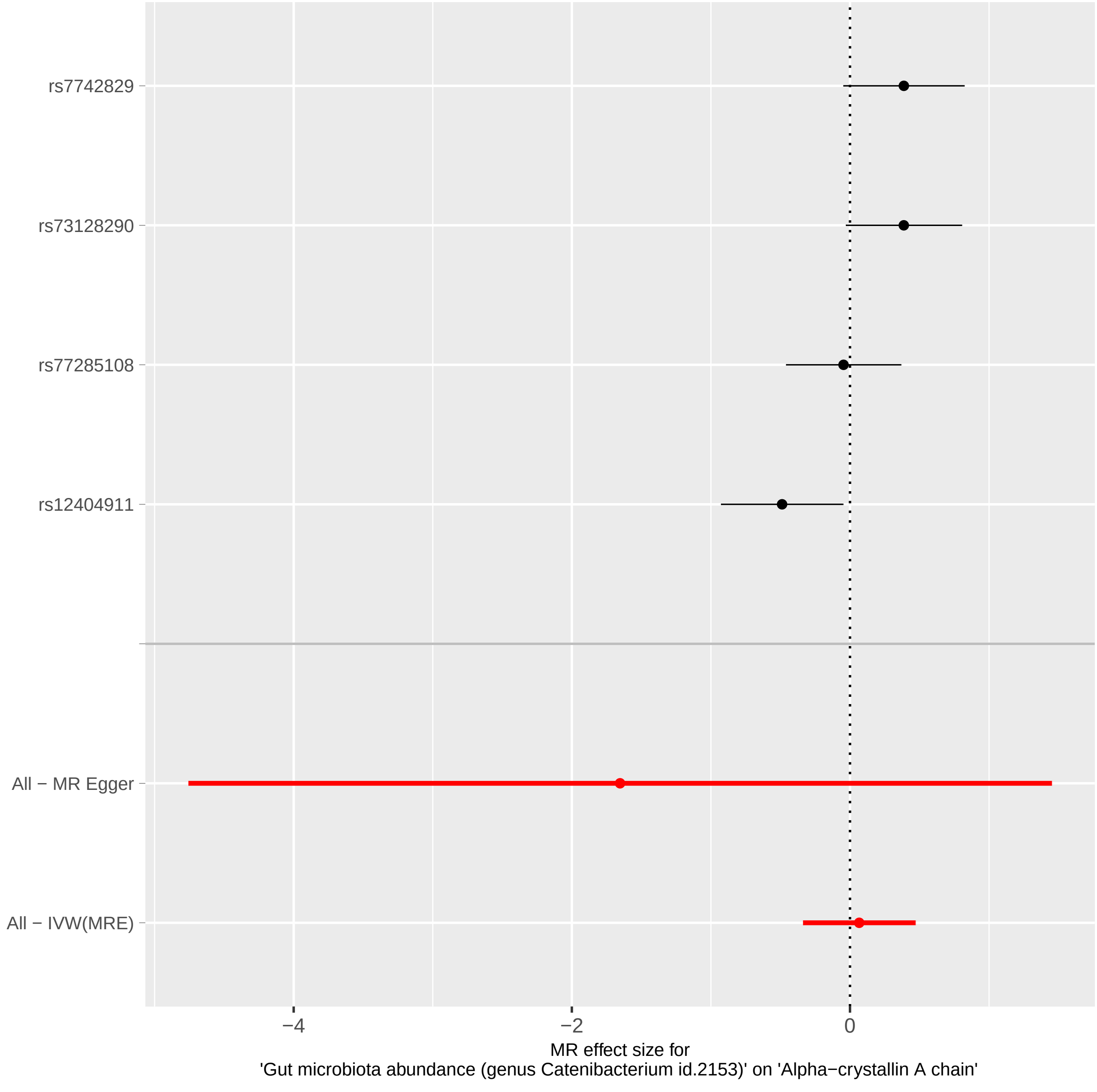

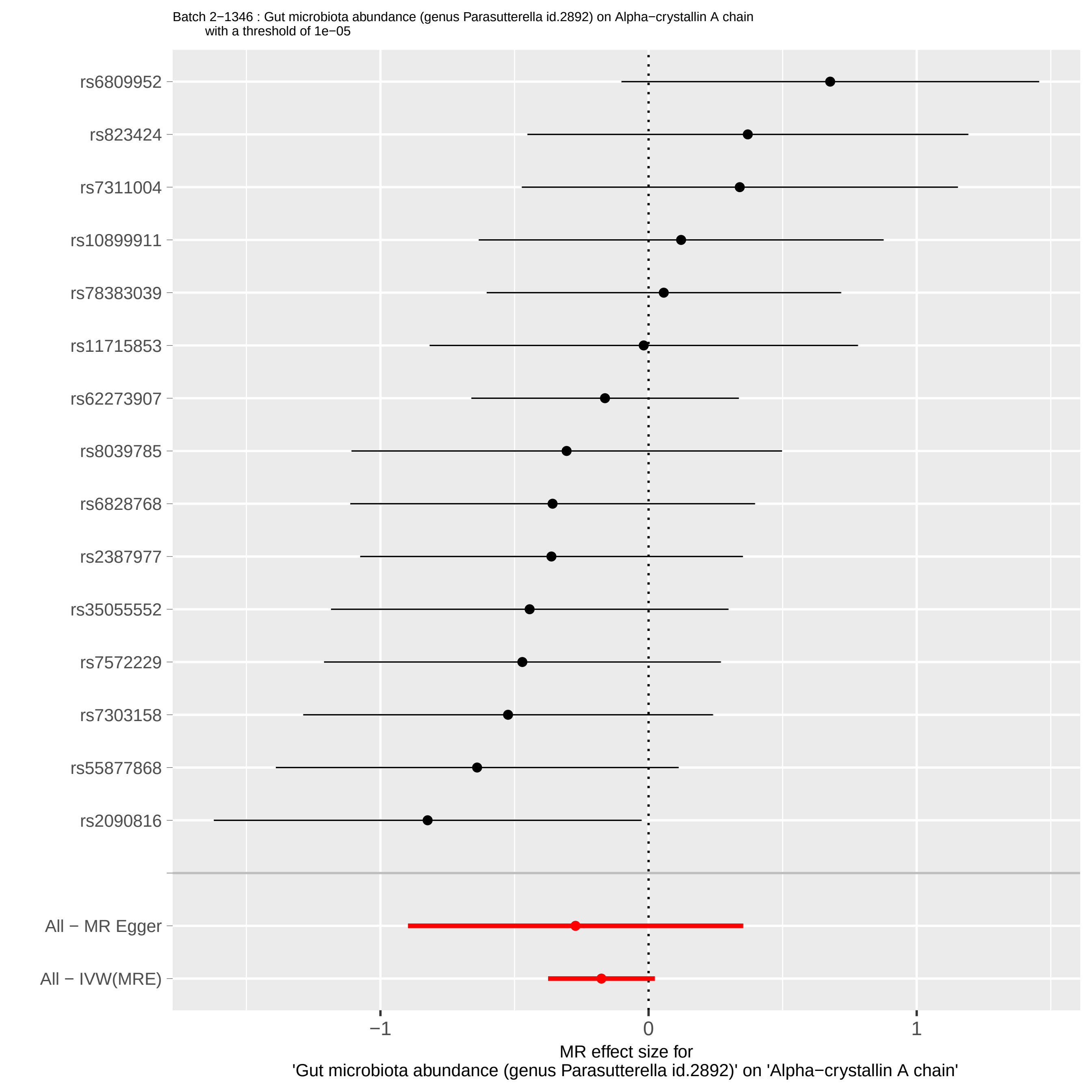
Batch 2−1346 : Gut microbiota abundance (genus Parasutterella id.2892) on Alpha−crystallin A chain
with a threshold of 1e−05
rs6809952
rs823424
rs7311004
rs10899911
rs78383039
rs11715853
rs62273907
rs8039785
rs6828768
rs2387977
rs35055552
rs7572229
rs7303158
rs55877868
rs2090816
All − MR Egger
All − IVW(MRE)
−1
0
1
MR effect size for
'Gut microbiota abundance (genus Parasutterella id.2892)' on 'Alpha−crystallin A chain'

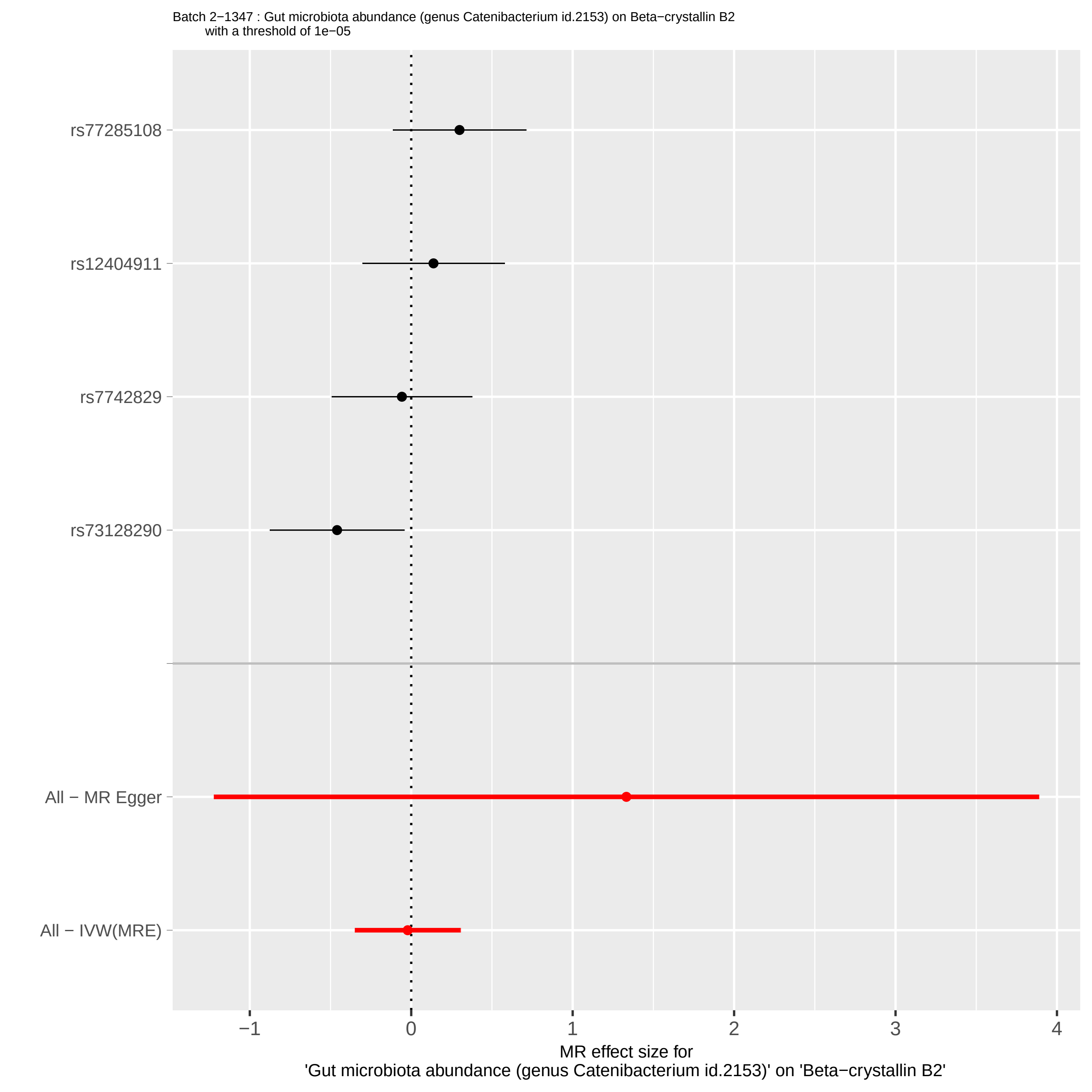
Batch 2−1347 : Gut microbiota abundance (genus Catenibacterium id.2153) on Beta−crystallin B2
with a threshold of 1e−05
rs77285108
rs12404911
rs7742829
rs73128290
All − MR Egger
All − IVW(MRE)
−1
0
1
2
3
4
MR effect size for
'Gut microbiota abundance (genus Catenibacterium id.2153)' on 'Beta−crystallin B2'

Batch 2−1348 : Gut microbiota abundance (genus Parasutterella id.2892) on Beta−crystallin B2
with a threshold of 1e−05

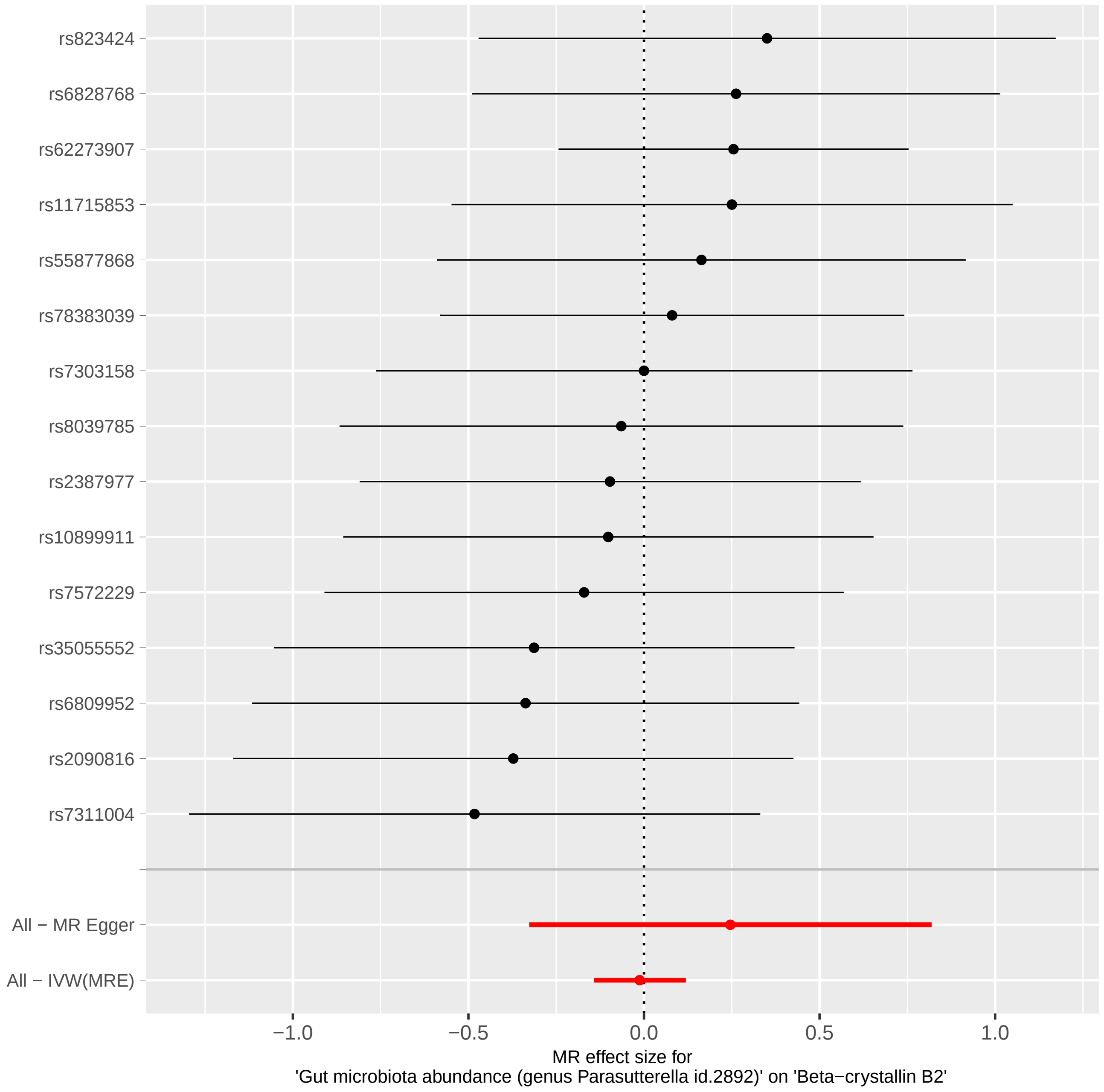

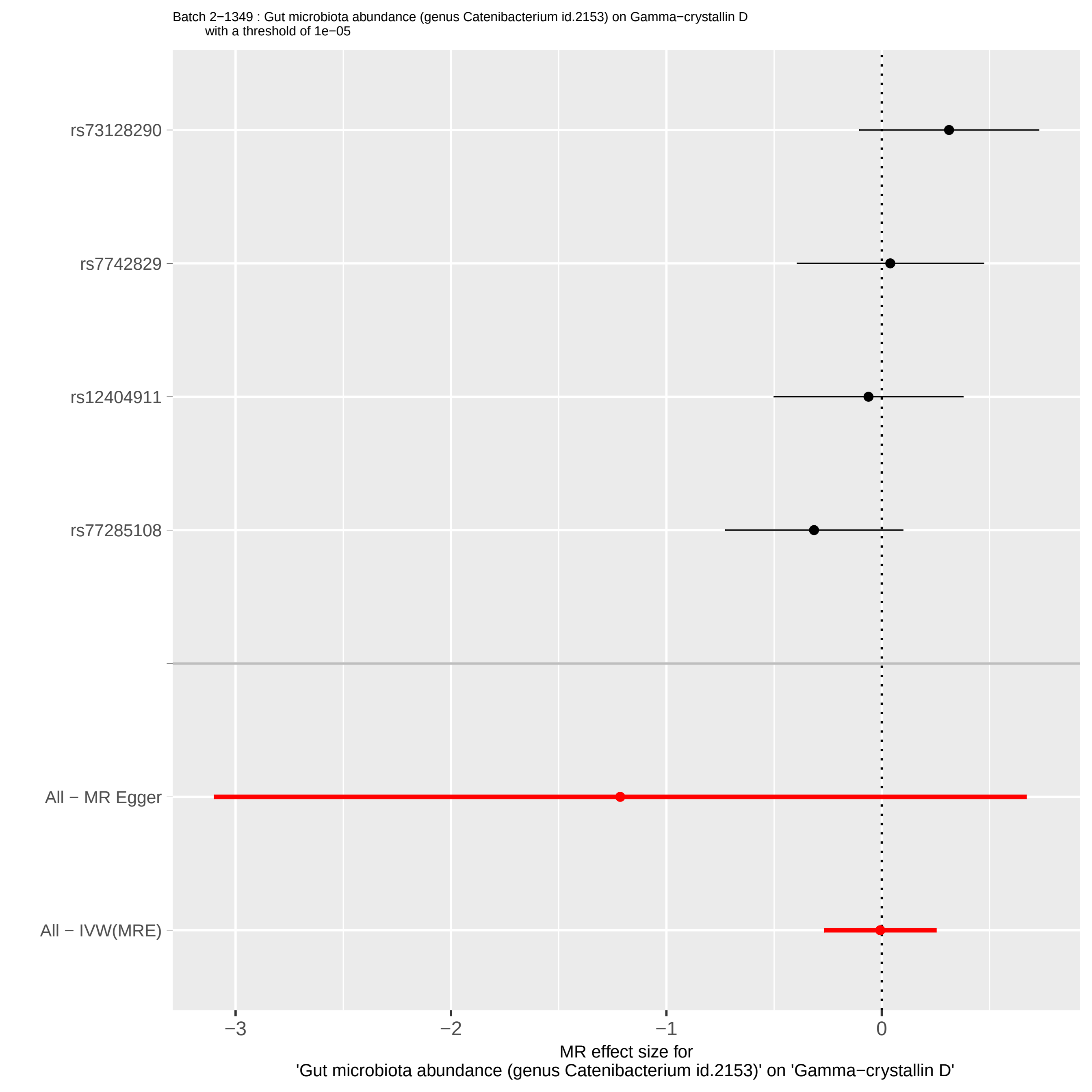

Batch 2−1349 : Gut microbiota abundance (genus Catenibacterium id.2153) on Gamma−crystallin D
with a threshold of 1e−05
rs73128290
rs7742829
rs12404911
rs77285108
All − MR Egger
All − IVW(MRE)
−3
−2
−1
0
MR effect size for
'Gut microbiota abundance (genus Catenibacterium id.2153)' on 'Gamma−crystallin D'

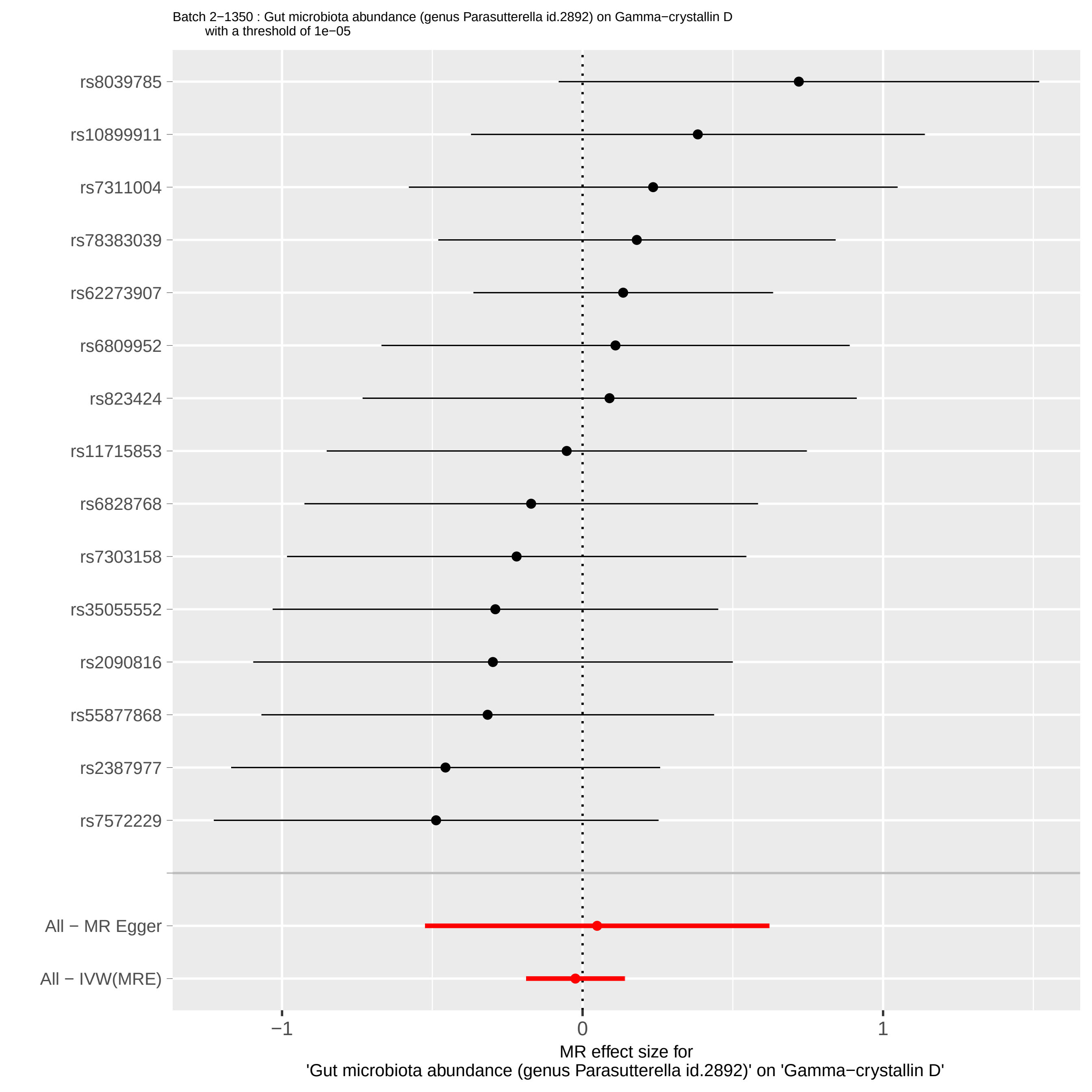

Batch 2−1350 : Gut microbiota abundance (genus Parasutterella id.2892) on Gamma−crystallin D
with a threshold of 1e−05
rs8039785
rs10899911
rs7311004
rs78383039
rs62273907
rs6809952
rs823424
rs11715853
rs6828768
rs7303158
rs35055552
rs2090816
rs55877868
rs2387977
rs7572229
All − MR Egger
All − IVW(MRE)
−1
0
1
MR effect size for
'Gut microbiota abundance (genus Parasutterella id.2892)' on 'Gamma−crystallin D'

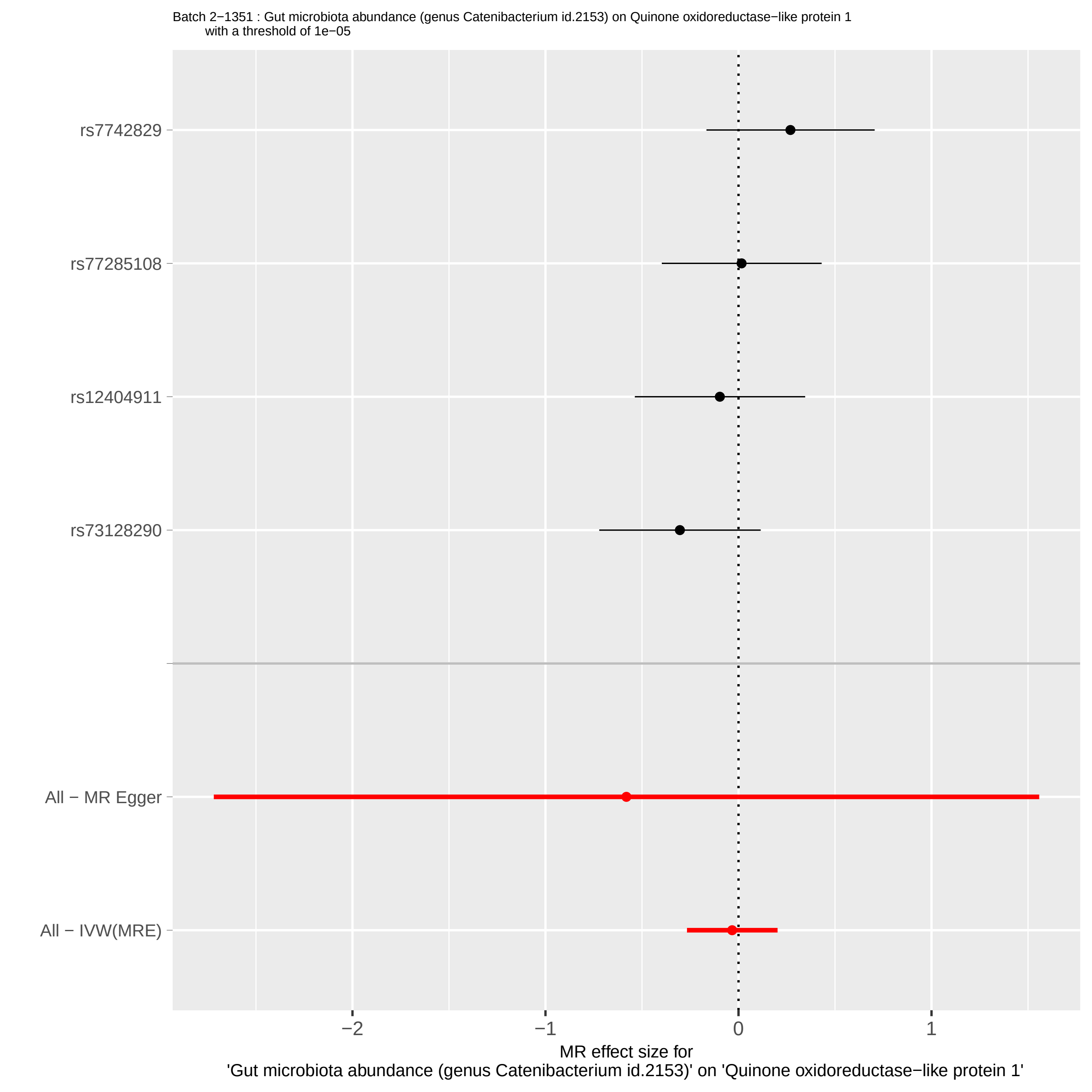

Batch 2−1351 : Gut microbiota abundance (genus Catenibacterium id.2153) on Quinone oxidoreductase−like protein 1
with a threshold of 1e−05
rs7742829
rs77285108
rs12404911
rs73128290
All − MR Egger
All − IVW(MRE)
−2
−1
0
1
MR effect size for
'Gut microbiota abundance (genus Catenibacterium id.2153)' on 'Quinone oxidoreductase−like protein 1'

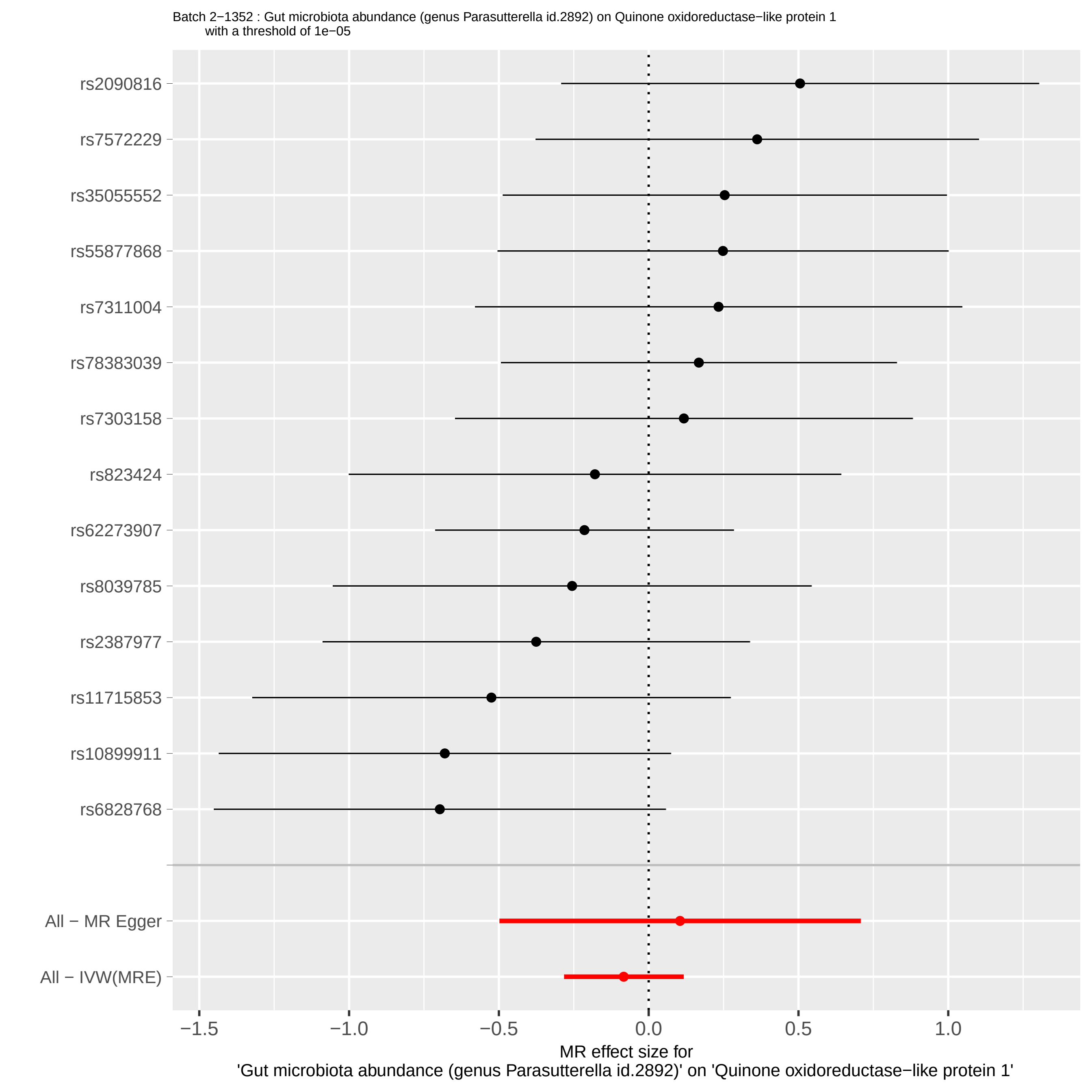

Batch 2−1352 : Gut microbiota abundance (genus Parasutterella id.2892) on Quinone oxidoreductase−like protein 1
with a threshold of 1e−05
rs2090816
rs7572229
rs35055552
rs55877868
rs7311004
rs78383039
rs7303158
rs823424
rs62273907
rs8039785
rs2387977
rs11715853
rs10899911
rs6828768
All − MR Egger
All − IVW(MRE)
−1.5
−1.0
−0.5
0.0
0.5
1.0
MR effect size for
'Gut microbiota abundance (genus Parasutterella id.2892)' on 'Quinone oxidoreductase−like protein 1'

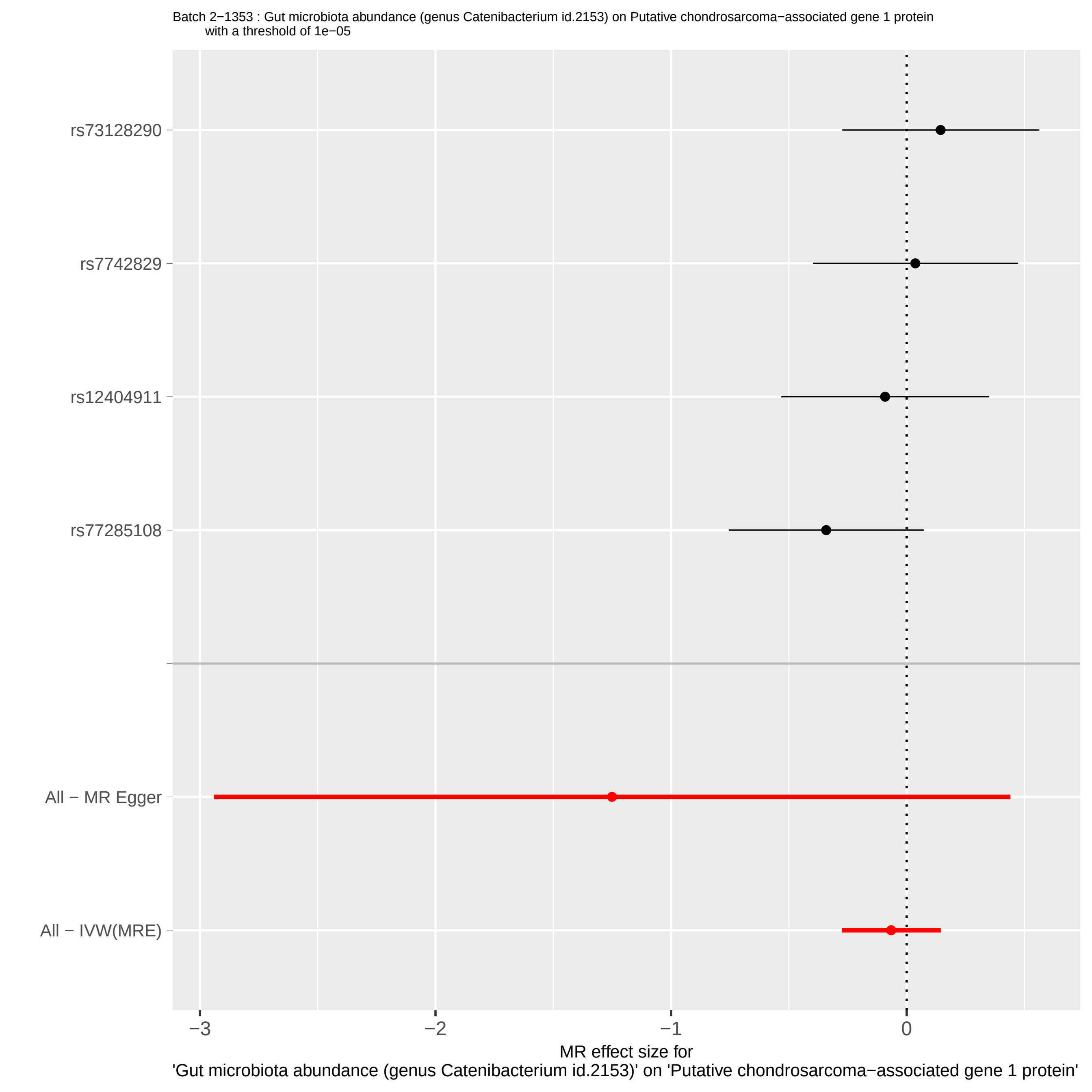

Batch 2−1353 : Gut microbiota abundance (genus Catenibacterium id.2153) on Putative chondrosarcoma−associated gene 1 protein
with a threshold of 1e−05
rs73128290
rs7742829
rs12404911
rs77285108
All − MR Egger
All − IVW(MRE)
−3
−2
−1
0
MR effect size for
'Gut microbiota abundance (genus Catenibacterium id.2153)' on 'Putative chondrosarcoma−associated gene 1 protein'

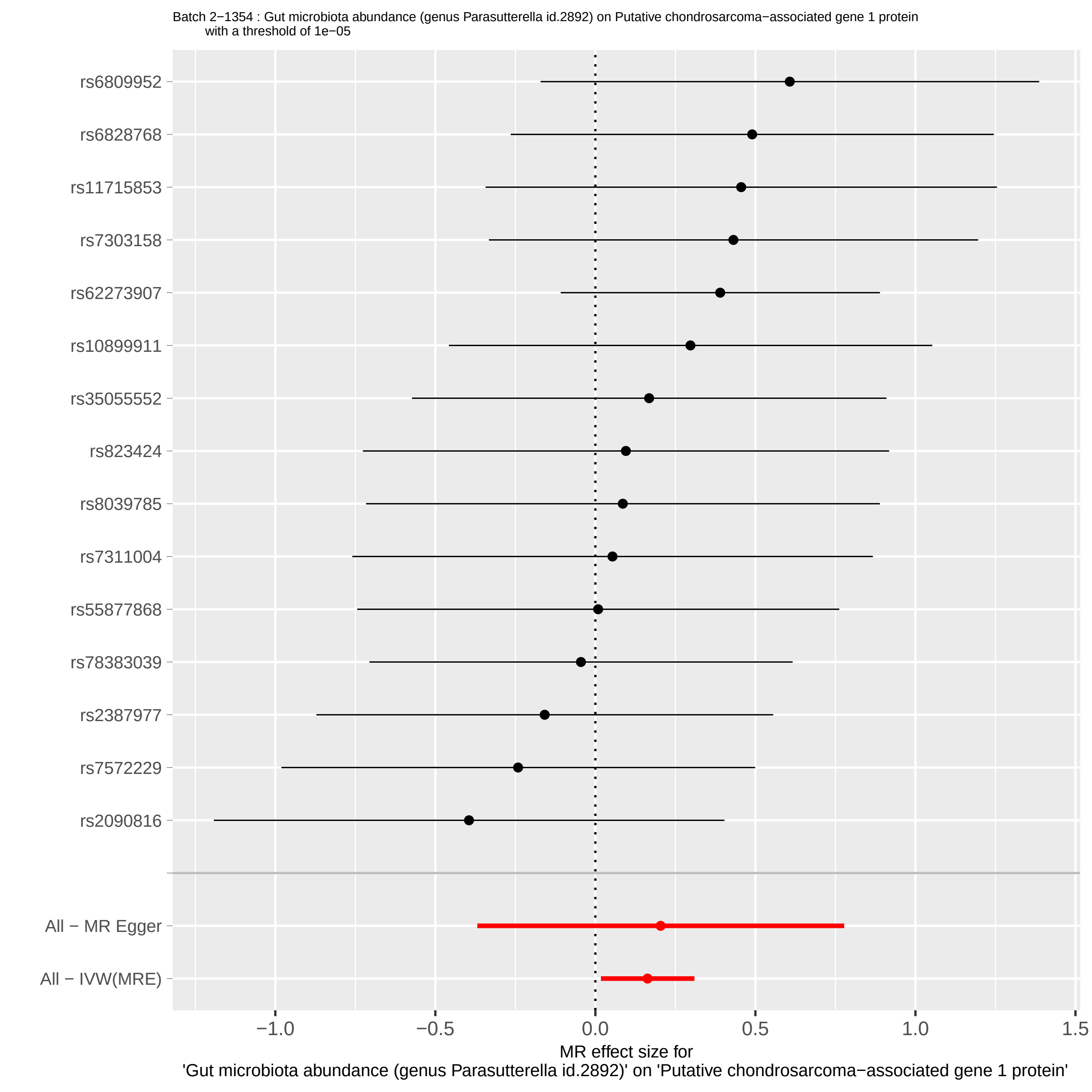

Batch 2−1354 : Gut microbiota abundance (genus Parasutterella id.2892) on Putative chondrosarcoma−associated gene 1 protein
with a threshold of 1e−05
rs6809952
rs6828768
rs11715853
rs7303158
rs62273907
rs10899911
rs35055552
rs823424
rs8039785
rs7311004
rs55877868
rs78383039
rs2387977
rs7572229
rs2090816
All − MR Egger
All − IVW(MRE)
−1.0
−0.5
0.0
0.5
1.0
1.5
MR effect size for
'Gut microbiota abundance (genus Parasutterella id.2892)' on 'Putative chondrosarcoma−associated gene 1 protein'

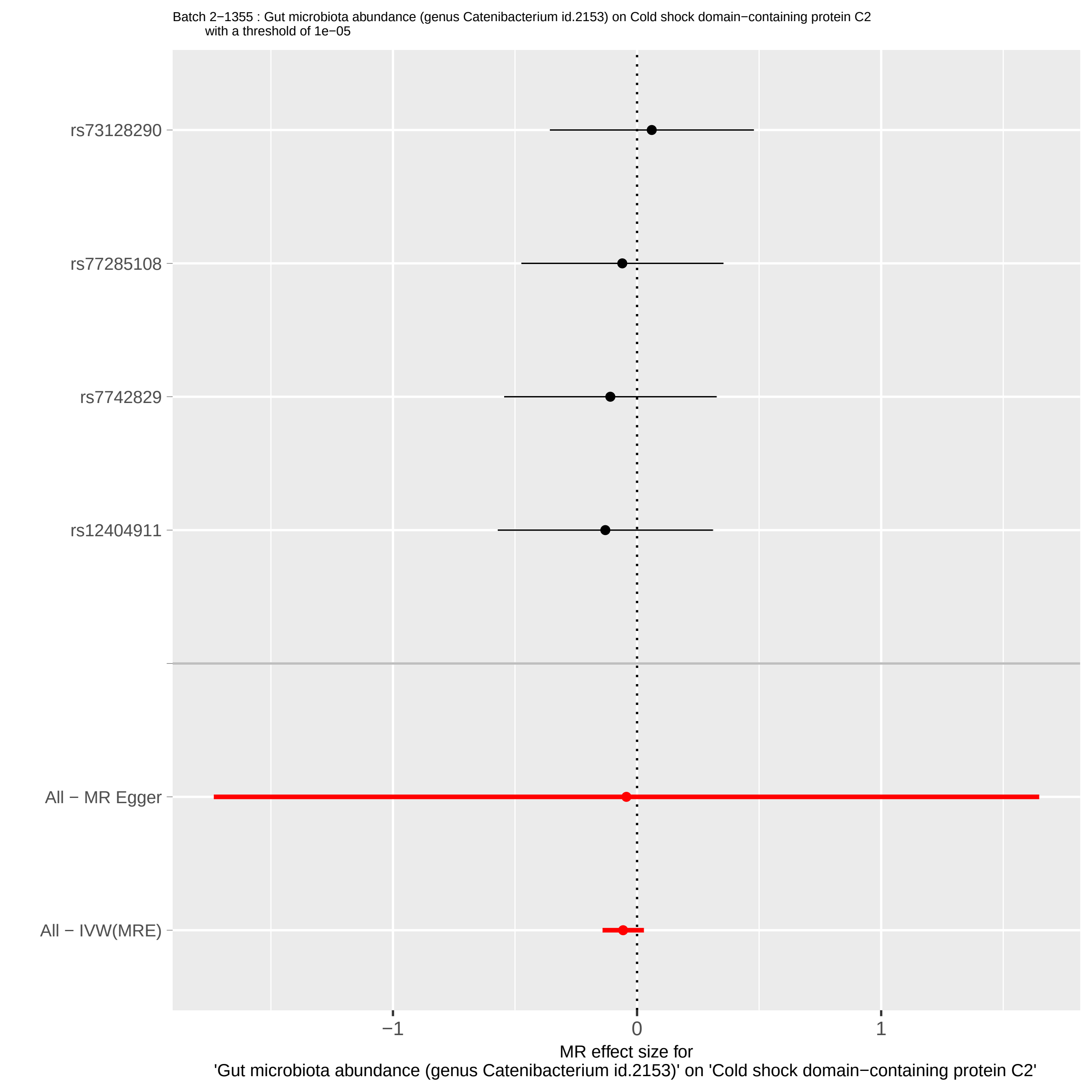

Batch 2−1355 : Gut microbiota abundance (genus Catenibacterium id.2153) on Cold shock domain−containing protein C2
with a threshold of 1e−05
rs73128290
rs77285108
rs7742829
rs12404911
All − MR Egger
All − IVW(MRE)
−1
0
1
MR effect size for
'Gut microbiota abundance (genus Catenibacterium id.2153)' on 'Cold shock domain−containing protein C2'

Batch 2−1356 : Gut microbiota abundance (genus Parasutterella id.2892) on Cold shock domain−containing protein C2
with a threshold of 1e−05

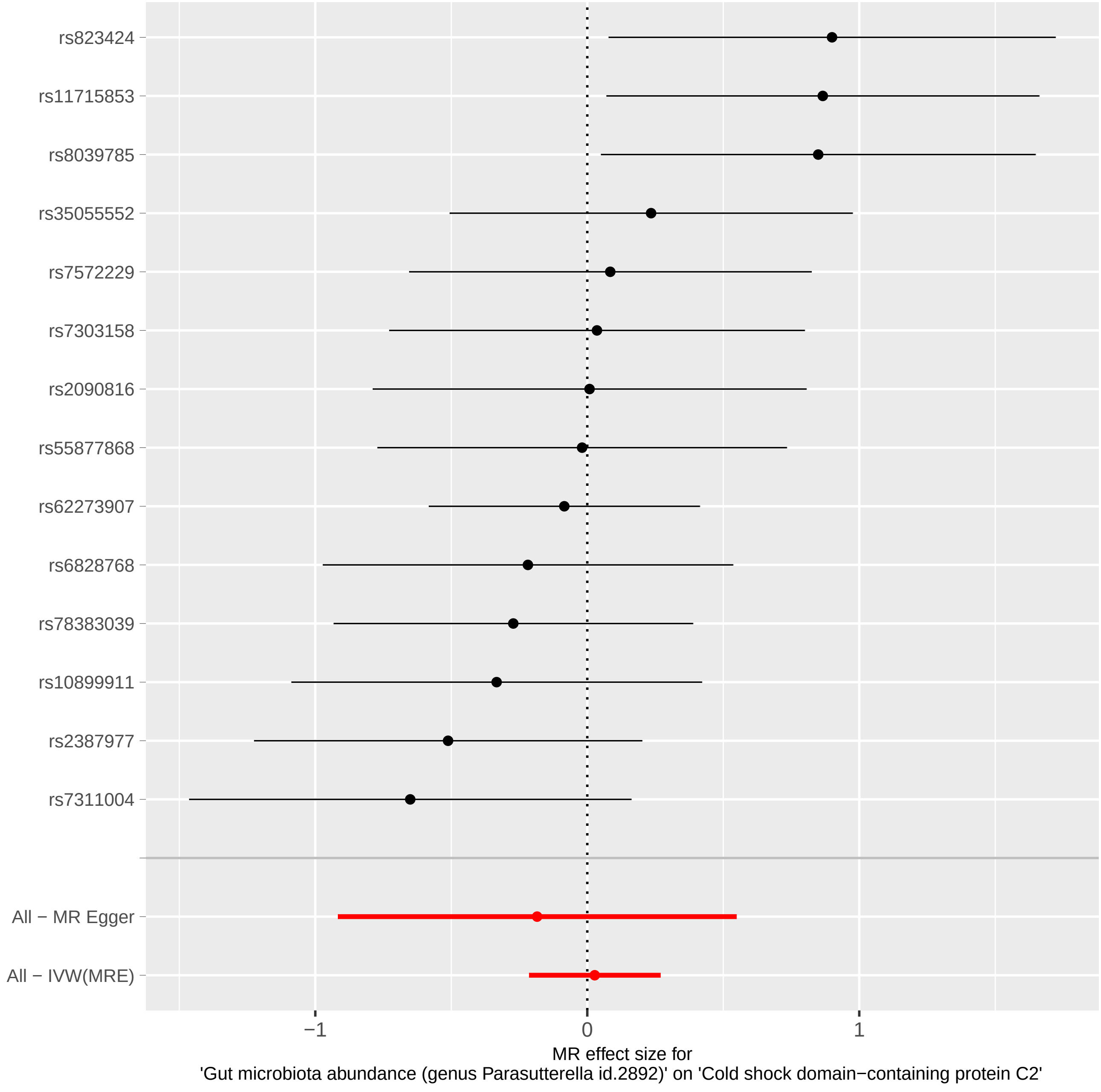

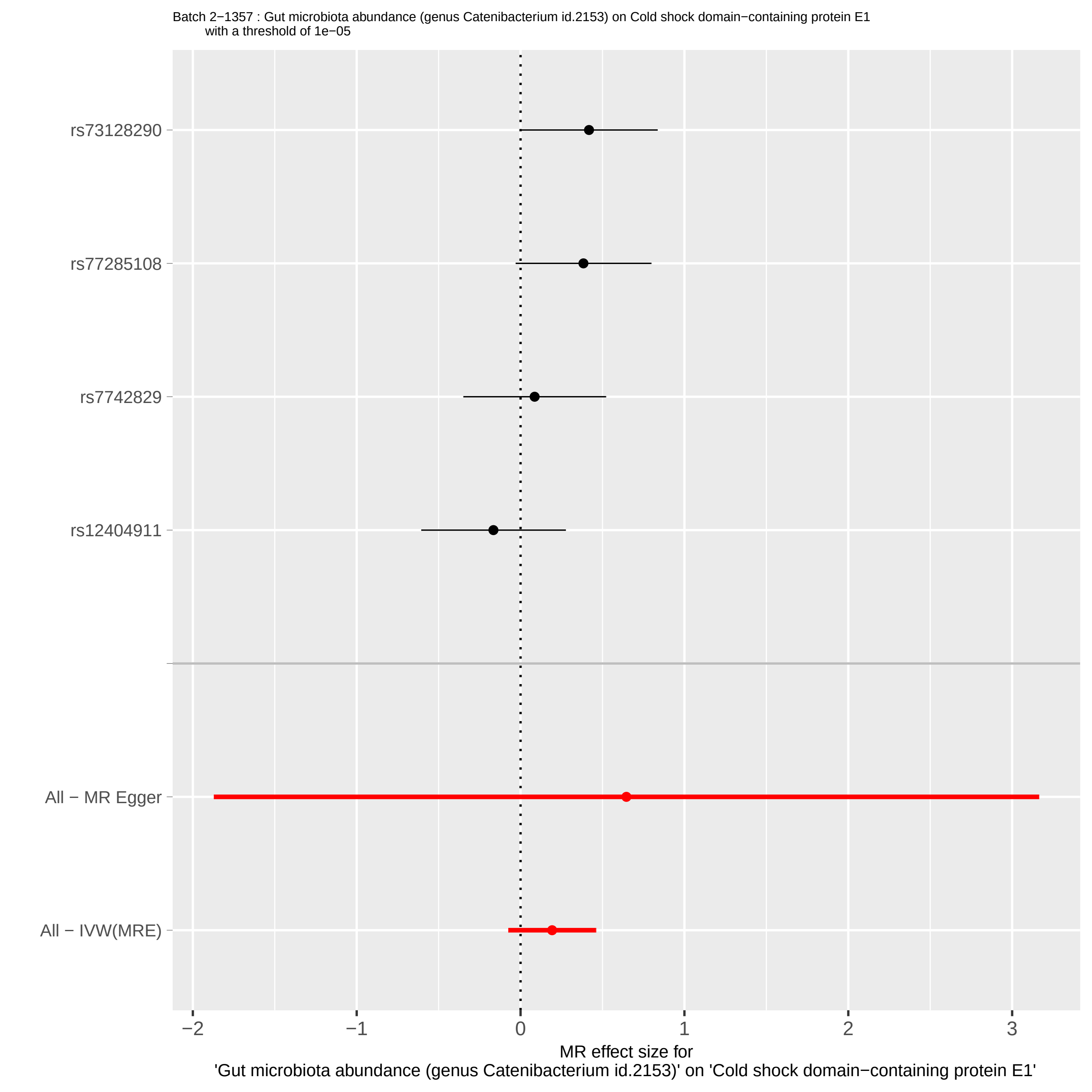

Batch 2−1357 : Gut microbiota abundance (genus Catenibacterium id.2153) on Cold shock domain−containing protein E1
with a threshold of 1e−05
rs73128290
rs77285108
rs7742829
rs12404911
All − MR Egger
All − IVW(MRE)
−2
−1
0
1
2
3
MR effect size for
'Gut microbiota abundance (genus Catenibacterium id.2153)' on 'Cold shock domain−containing protein E1'

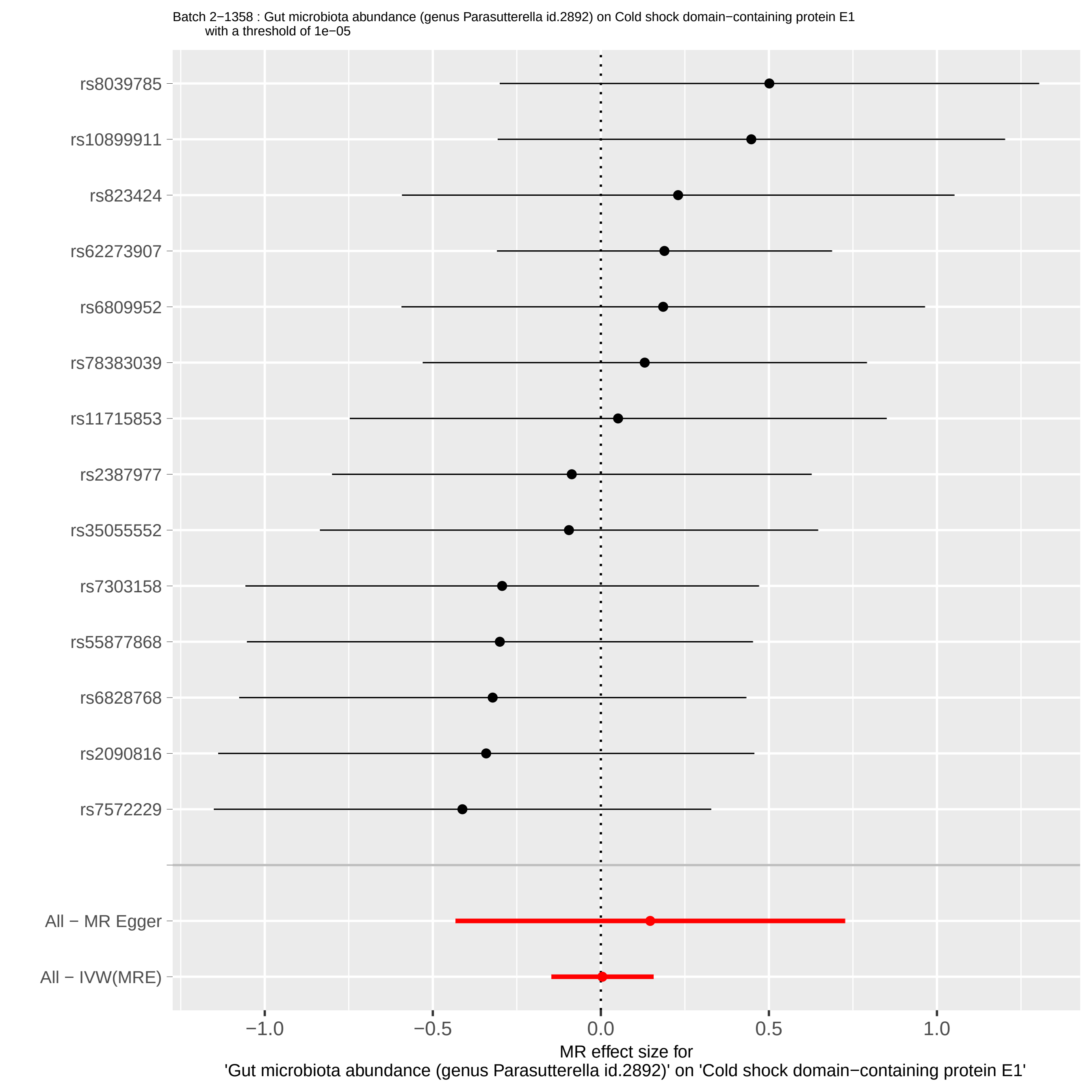
Batch 2−1358 : Gut microbiota abundance (genus Parasutterella id.2892) on Cold shock domain−containing protein E1
with a threshold of 1e−05
rs8039785
rs10899911
rs823424
rs62273907
rs6809952
rs78383039
rs11715853
rs2387977
rs35055552
rs7303158
rs55877868
rs6828768
rs2090816
rs7572229
All − MR Egger
All − IVW(MRE)
−1.0
−0.5
0.0
0.5
1.0
MR effect size for
'Gut microbiota abundance (genus Parasutterella id.2892)' on 'Cold shock domain−containing protein E1'

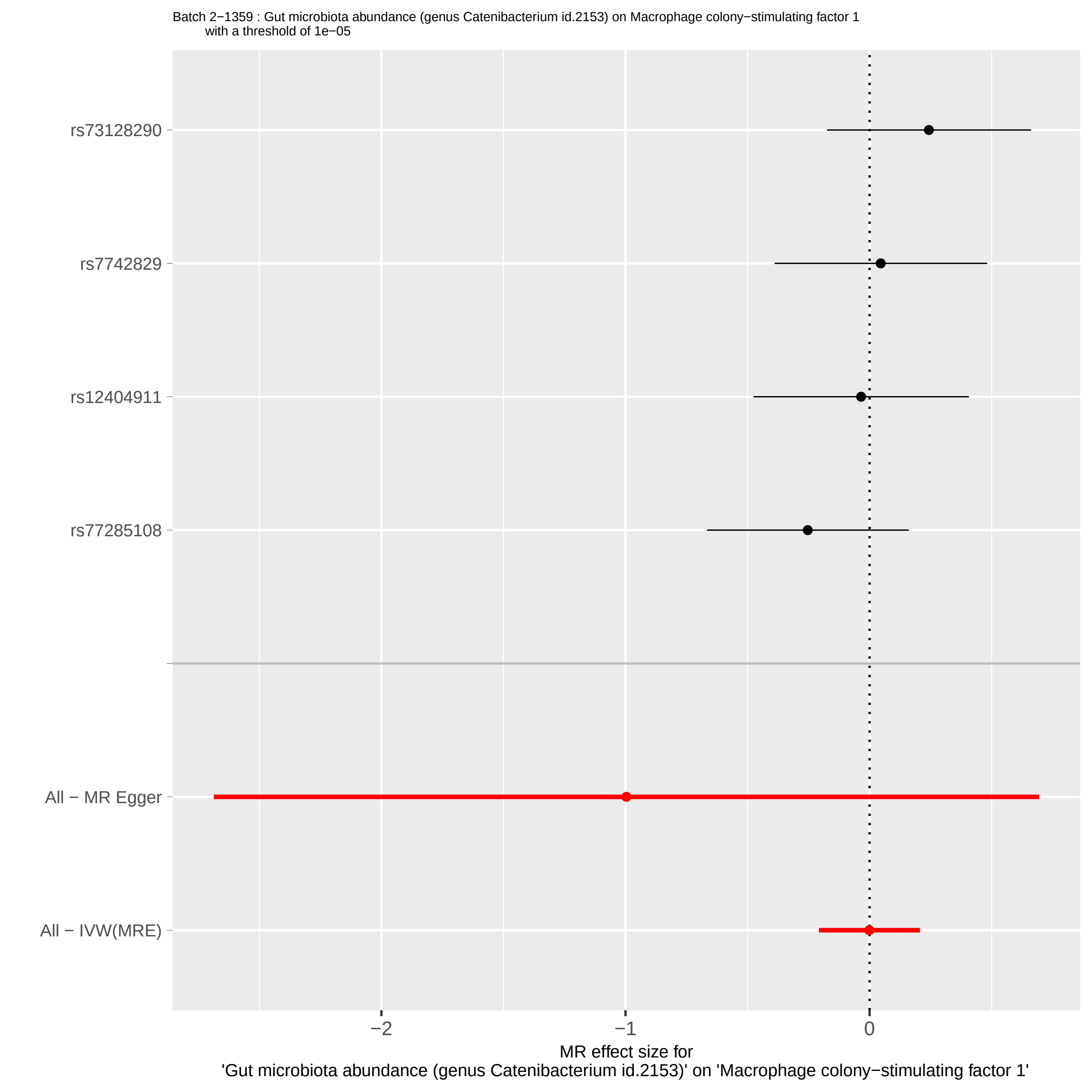

Batch 2−1359 : Gut microbiota abundance (genus Catenibacterium id.2153) on Macrophage colony−stimulating factor 1
with a threshold of 1e−05
rs73128290
rs7742829
rs12404911
rs77285108
All − MR Egger
All − IVW(MRE)
−2
−1
0
MR effect size for
'Gut microbiota abundance (genus Catenibacterium id.2153)' on 'Macrophage colony−stimulating factor 1'

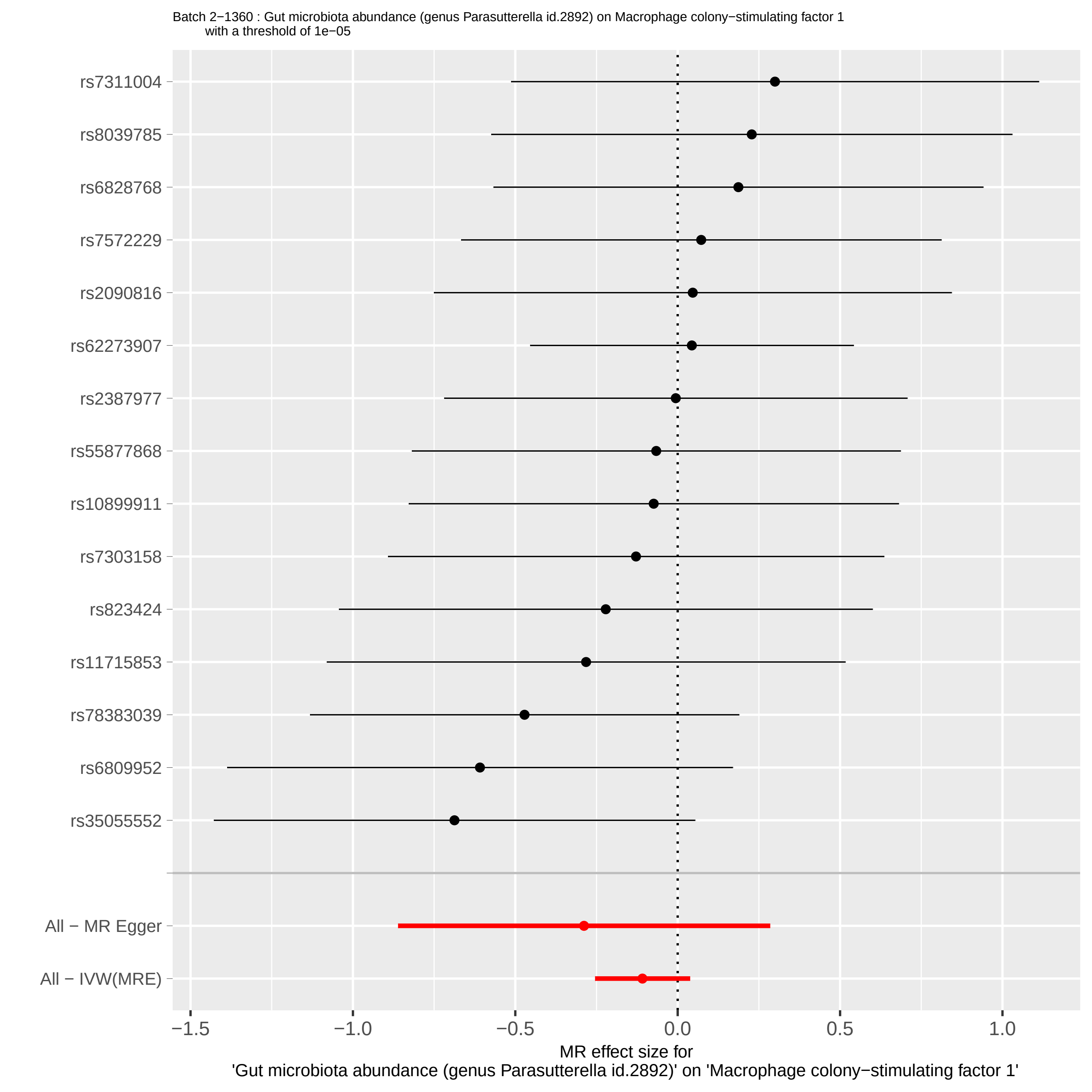

Batch 2−1360 : Gut microbiota abundance (genus Parasutterella id.2892) on Macrophage colony−stimulating factor 1
with a threshold of 1e−05
rs7311004
rs8039785
rs6828768
rs7572229
rs2090816
rs62273907
rs2387977
rs55877868
rs10899911
rs7303158
rs823424
rs11715853
rs78383039
rs6809952
rs35055552
All − MR Egger
All − IVW(MRE)
−1.5
−1.0
−0.5
0.0
0.5
1.0
MR effect size for
'Gut microbiota abundance (genus Parasutterella id.2892)' on 'Macrophage colony−stimulating factor 1'

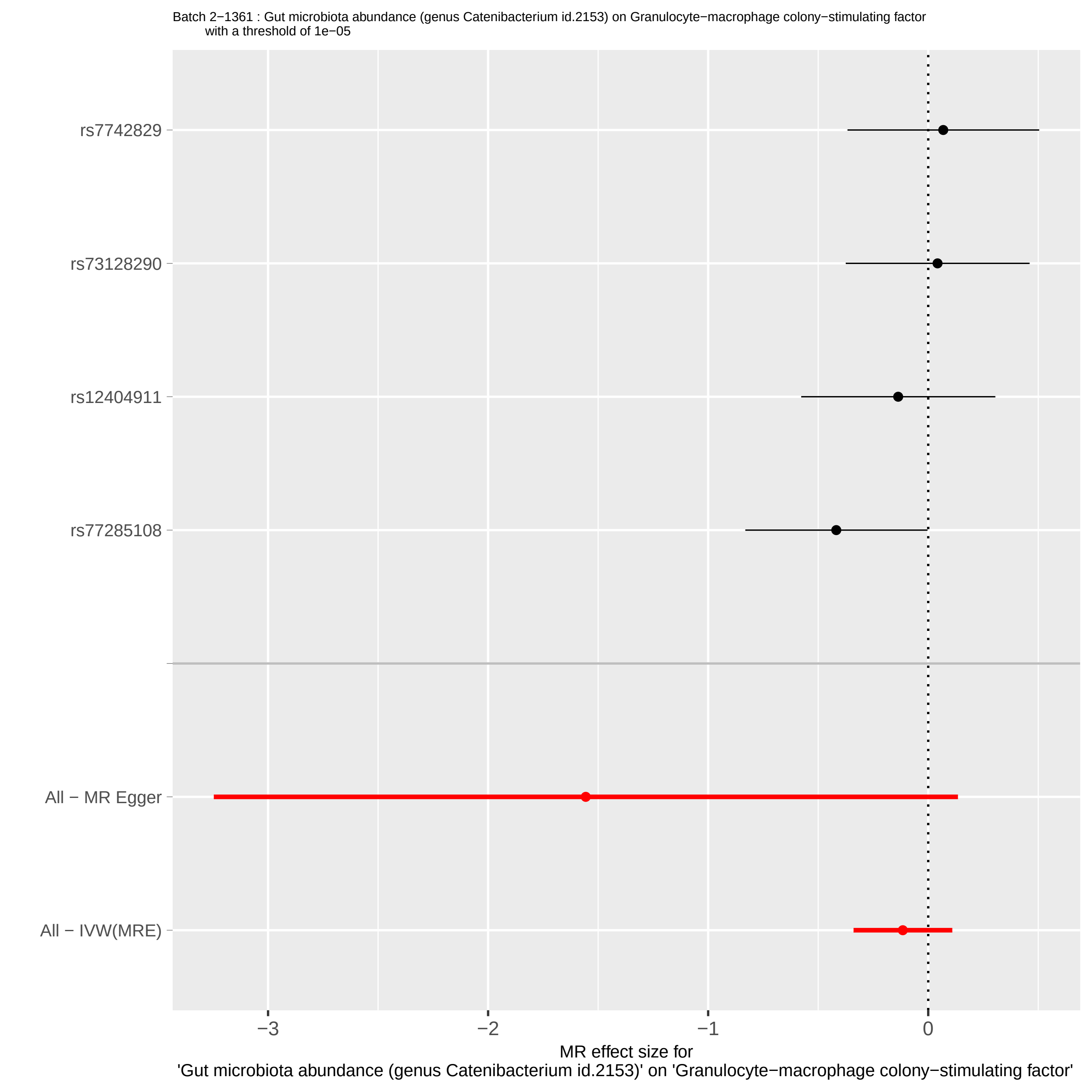
Batch 2−1361 : Gut microbiota abundance (genus Catenibacterium id.2153) on Granulocyte−macrophage colony−stimulating factor
with a threshold of 1e−05
rs7742829
rs73128290
rs12404911
rs77285108
All − MR Egger
All − IVW(MRE)
−3
−2
−1
0
MR effect size for
'Gut microbiota abundance (genus Catenibacterium id.2153)' on 'Granulocyte−macrophage colony−stimulating factor'

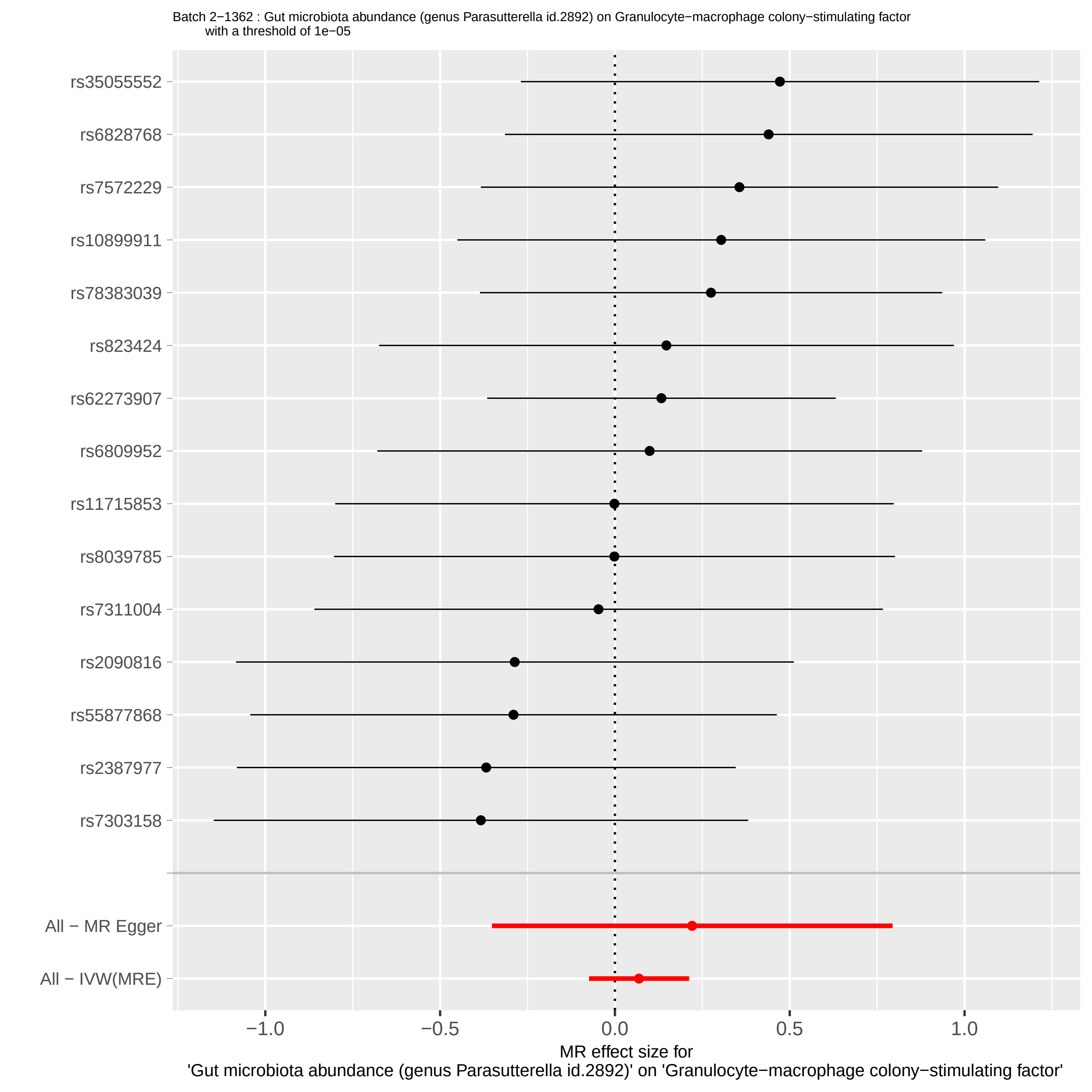
Batch 2−1362 : Gut microbiota abundance (genus Parasutterella id.2892) on Granulocyte−macrophage colony−stimulating factor
with a threshold of 1e−05
rs35055552
rs6828768
rs7572229
rs10899911
rs78383039
rs823424
rs62273907
rs6809952
rs11715853
rs8039785
rs7311004
rs2090816
rs55877868
rs2387977
rs7303158
All − MR Egger
All − IVW(MRE)
−1.0
−0.5
0.0
0.5
1.0
MR effect size for
'Gut microbiota abundance (genus Parasutterella id.2892)' on 'Granulocyte−macrophage colony−stimulating factor'

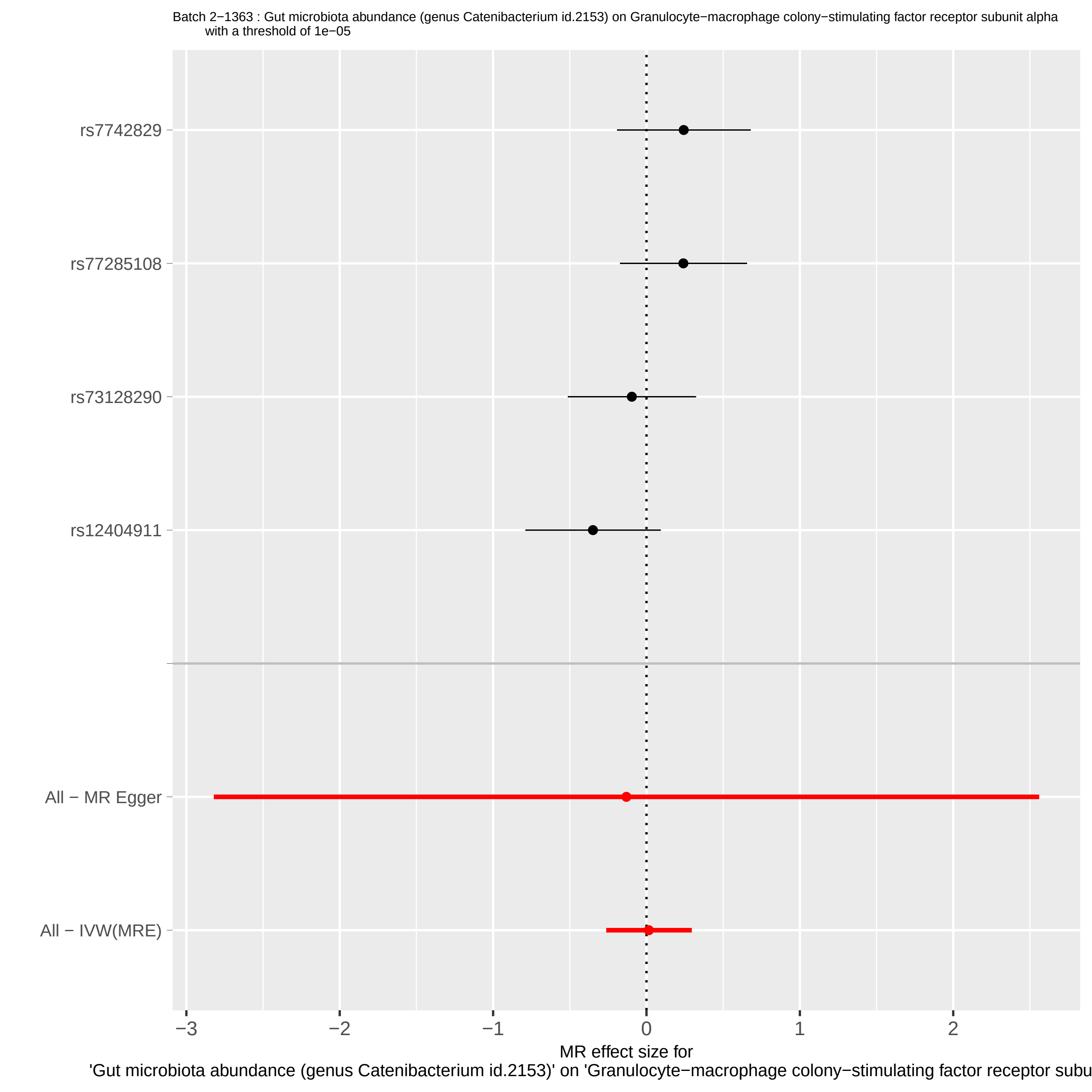
Batch 2−1363 : Gut microbiota abundance (genus Catenibacterium id.2153) on Granulocyte−macrophage colony−stimulating factor receptor subunit alpha
with a threshold of 1e−05
rs7742829
rs77285108
rs73128290
rs12404911
All − MR Egger
All − IVW(MRE)
−3
−2
−1
0
1
2
MR effect size for
'Gut microbiota abundance (genus Catenibacterium id.2153)' on 'Granulocyte−macrophage colony−stimulating factor receptor subu

Batch 2−1364 : Gut microbiota abundance (genus Parasutterella id.2892) on Granulocyte−macrophage colony−stimulating factor receptor subunit alpha with a threshold of 1e−05

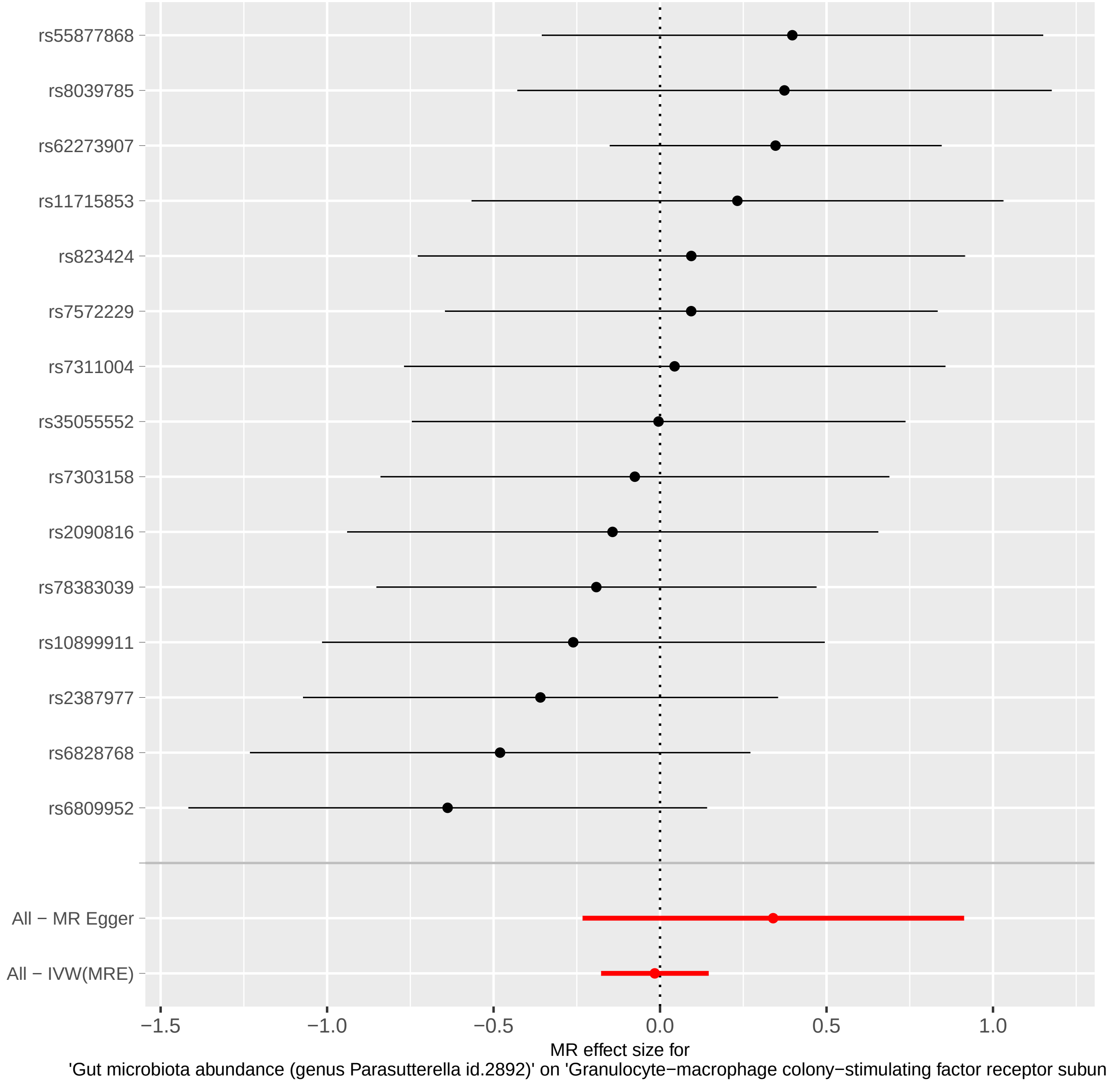

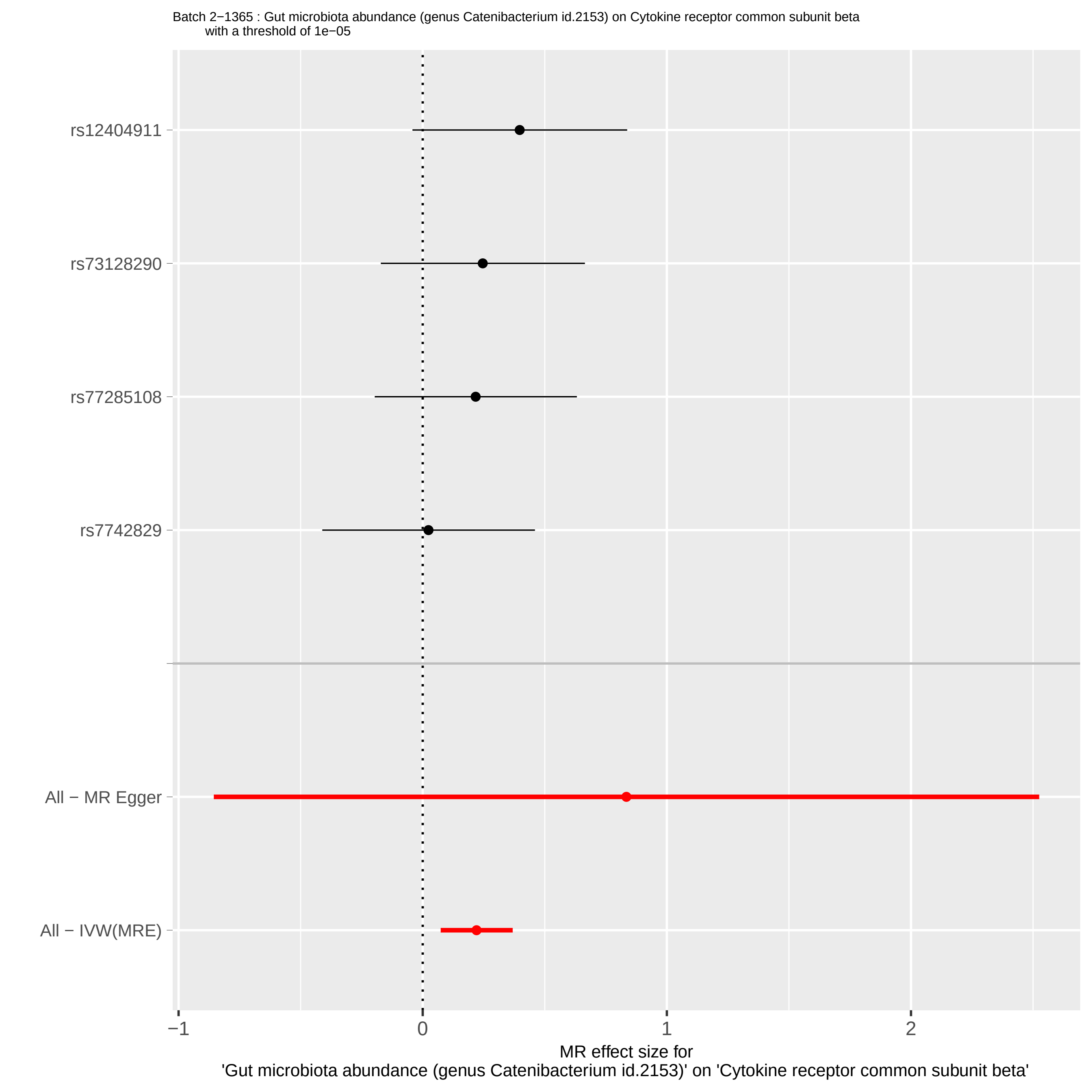

Batch 2−1365 : Gut microbiota abundance (genus Catenibacterium id.2153) on Cytokine receptor common subunit beta
with a threshold of 1e−05
rs12404911
rs73128290
rs77285108
rs7742829
All − MR Egger
All − IVW(MRE)
−1
0
1
2
MR effect size for
'Gut microbiota abundance (genus Catenibacterium id.2153)' on 'Cytokine receptor common subunit beta'

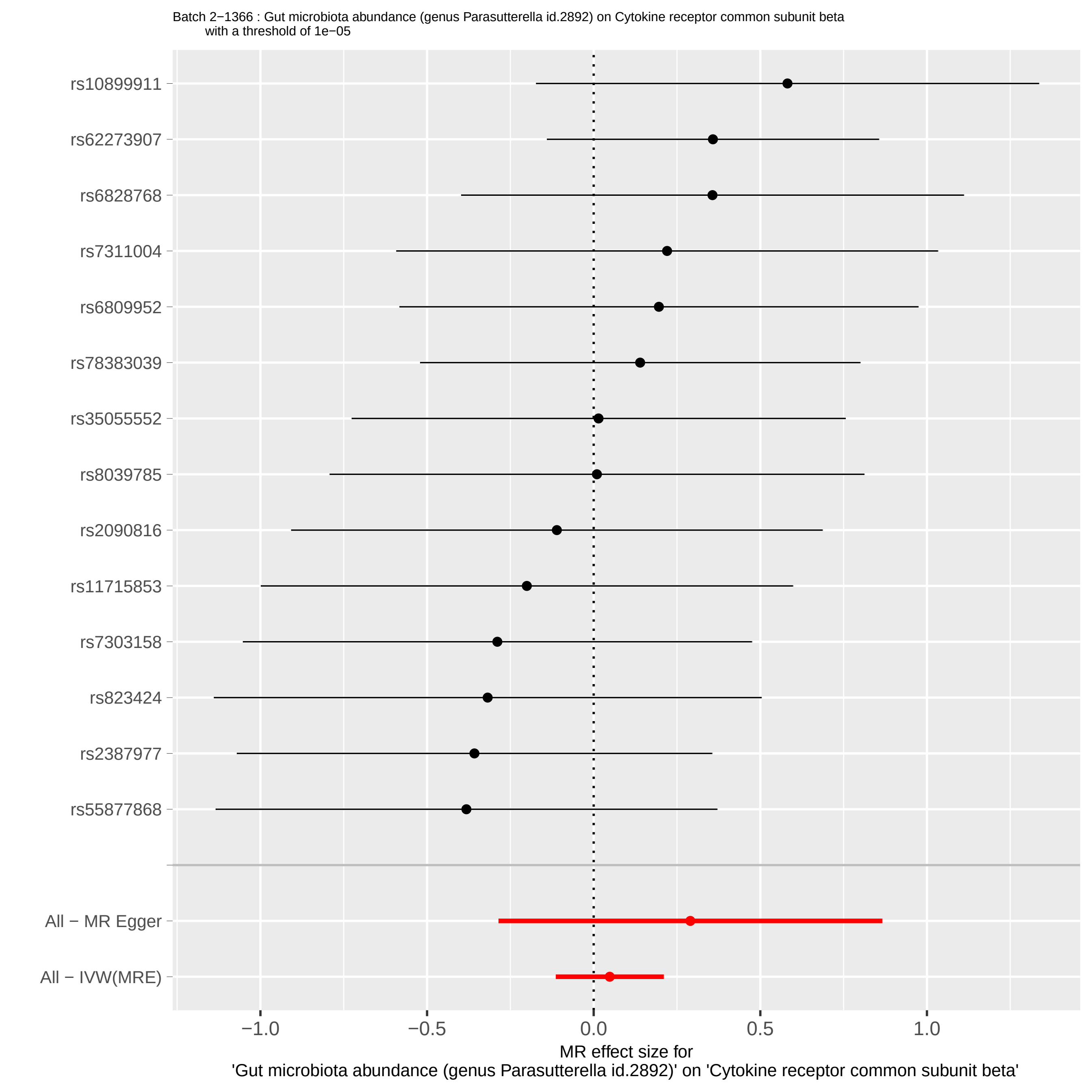
Batch 2−1366 : Gut microbiota abundance (genus Parasutterella id.2892) on Cytokine receptor common subunit beta
with a threshold of 1e−05
rs10899911
rs62273907
rs6828768
rs7311004
rs6809952
rs78383039
rs35055552
rs8039785
rs2090816
rs11715853
rs7303158
rs823424
rs2387977
rs55877868
All − MR Egger
All − IVW(MRE)
−1.0
−0.5
0.0
0.5
1.0
MR effect size for
'Gut microbiota abundance (genus Parasutterella id.2892)' on 'Cytokine receptor common subunit beta'

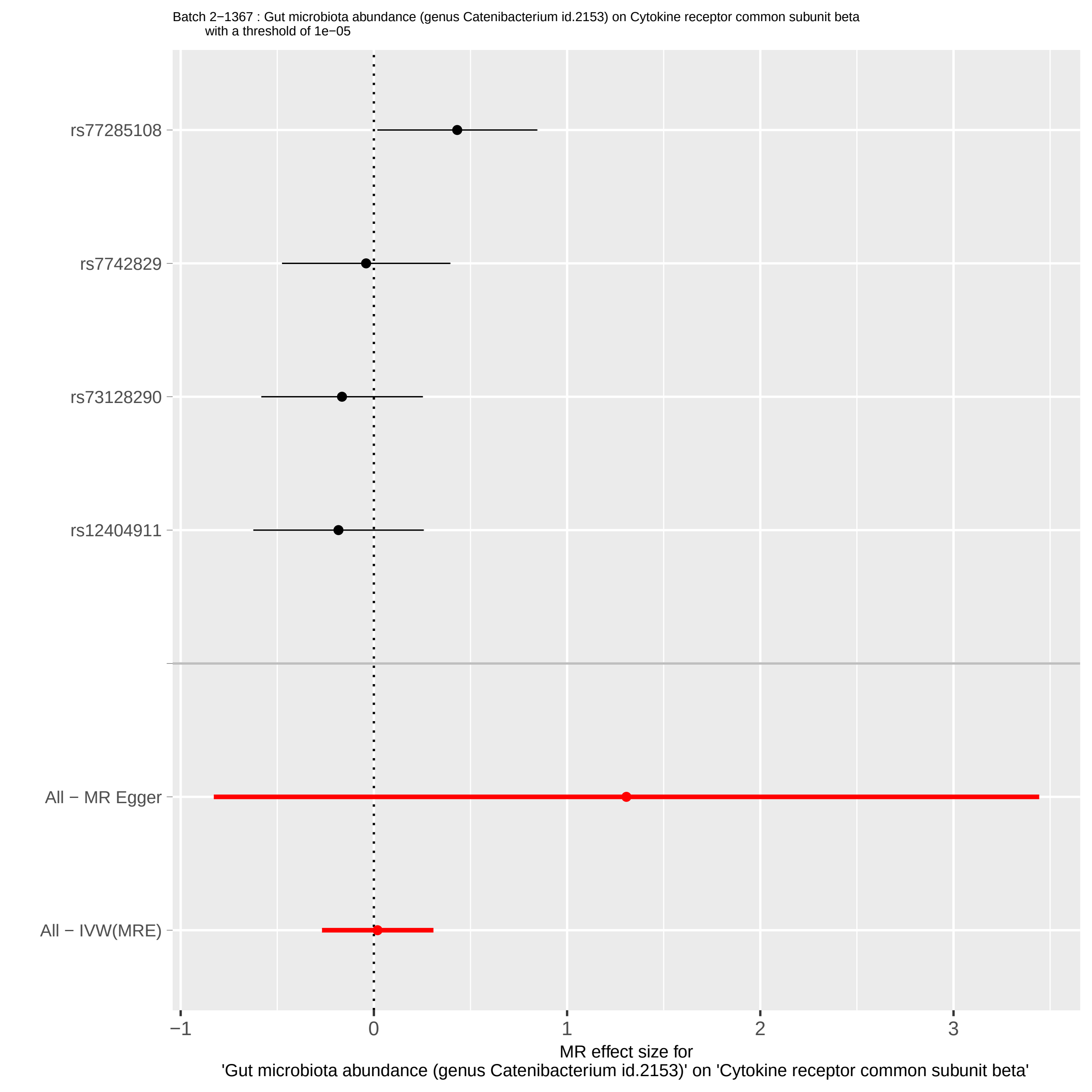

Batch 2−1367 : Gut microbiota abundance (genus Catenibacterium id.2153) on Cytokine receptor common subunit beta
with a threshold of 1e−05
rs77285108
rs7742829
rs73128290
rs12404911
All − MR Egger
All − IVW(MRE)
−1
0
1
2
3
MR effect size for
'Gut microbiota abundance (genus Catenibacterium id.2153)' on 'Cytokine receptor common subunit beta'

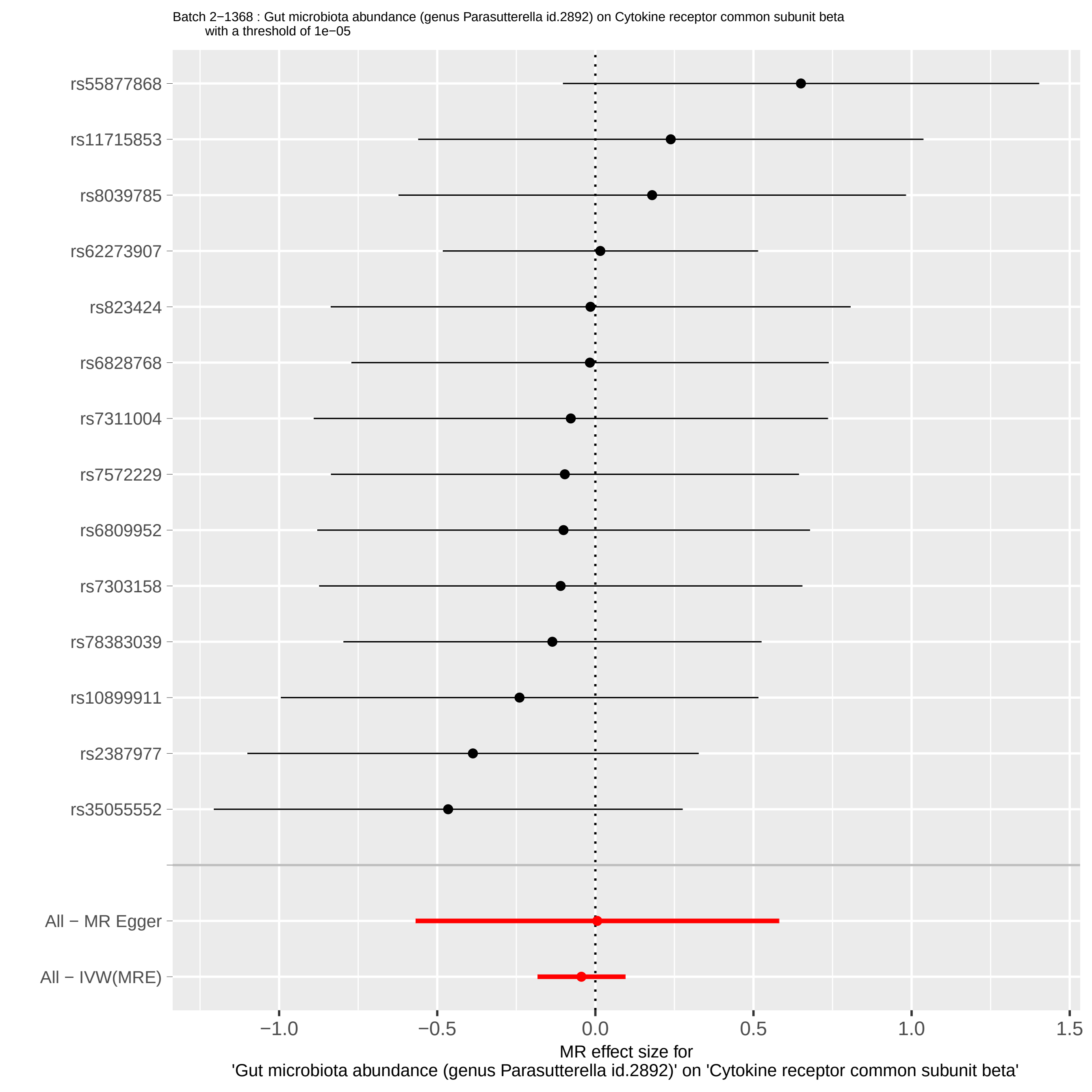

Batch 2−1368 : Gut microbiota abundance (genus Parasutterella id.2892) on Cytokine receptor common subunit beta
with a threshold of 1e−05
rs55877868
rs11715853
rs8039785
rs62273907
rs823424
rs6828768
rs7311004
rs7572229
rs6809952
rs7303158
rs78383039
rs10899911
rs2387977
rs35055552
All − MR Egger
All − IVW(MRE)
−1.0
−0.5
0.0
0.5
1.0
1.5
MR effect size for
'Gut microbiota abundance (genus Parasutterella id.2892)' on 'Cytokine receptor common subunit beta'

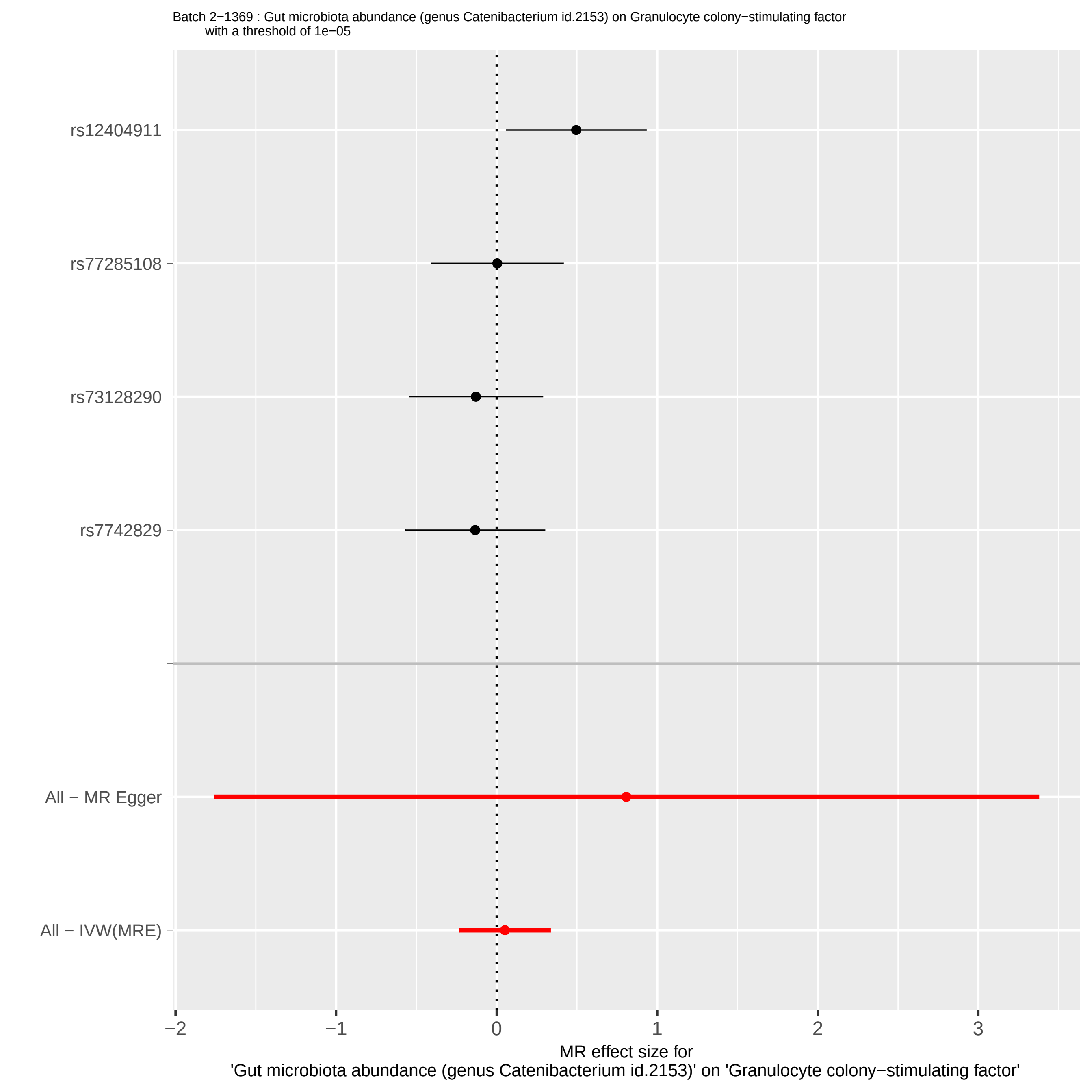

Batch 2−1369 : Gut microbiota abundance (genus Catenibacterium id.2153) on Granulocyte colony−stimulating factor
with a threshold of 1e−05
rs12404911
rs77285108
rs73128290
rs7742829
All − MR Egger
All − IVW(MRE)
−2
−1
0
1
2
3
MR effect size for
'Gut microbiota abundance (genus Catenibacterium id.2153)' on 'Granulocyte colony−stimulating factor'

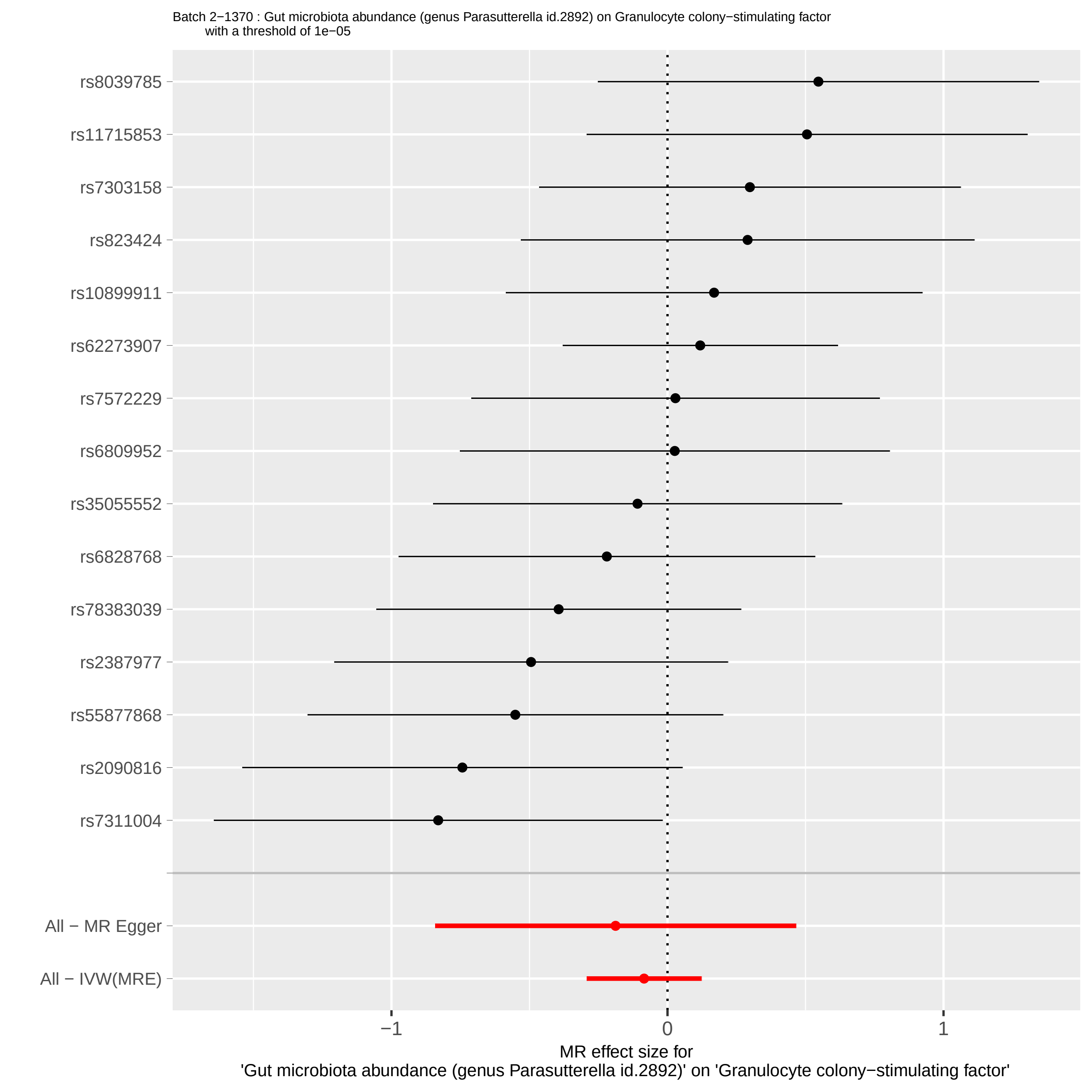
Batch 2−1370 : Gut microbiota abundance (genus Parasutterella id.2892) on Granulocyte colony−stimulating factor
with a threshold of 1e−05
rs8039785
rs11715853
rs7303158
rs823424
rs10899911
rs62273907
rs7572229
rs6809952
rs35055552
rs6828768
rs78383039
rs2387977
rs55877868
rs2090816
rs7311004
All − MR Egger
All − IVW(MRE)
−1
0
1
MR effect size for
'Gut microbiota abundance (genus Parasutterella id.2892)' on 'Granulocyte colony−stimulating factor'

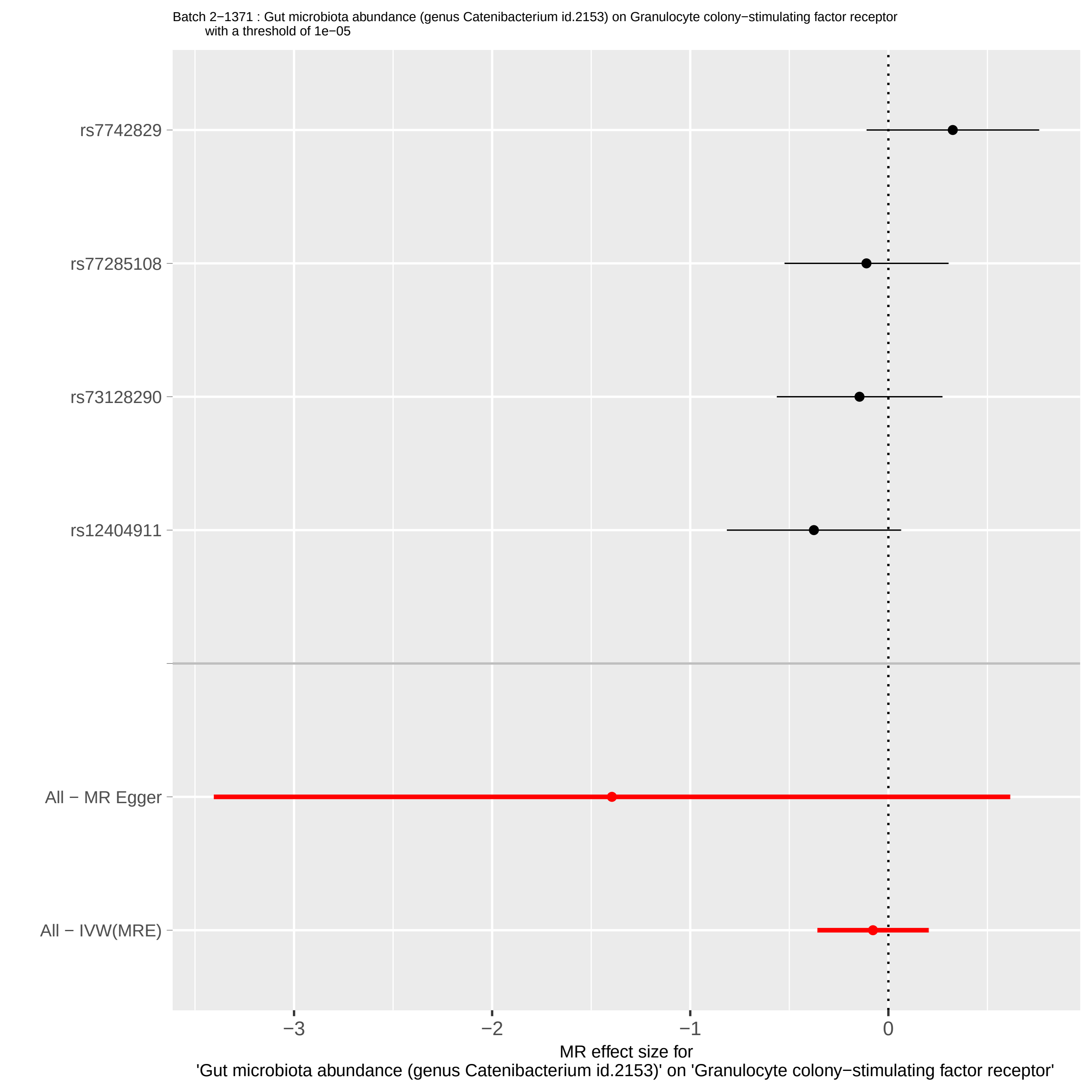

Batch 2−1371 : Gut microbiota abundance (genus Catenibacterium id.2153) on Granulocyte colony−stimulating factor receptor
with a threshold of 1e−05
rs7742829
rs77285108
rs73128290
rs12404911
All − MR Egger
All − IVW(MRE)
−3
−2
−1
0
MR effect size for
'Gut microbiota abundance (genus Catenibacterium id.2153)' on 'Granulocyte colony−stimulating factor receptor'

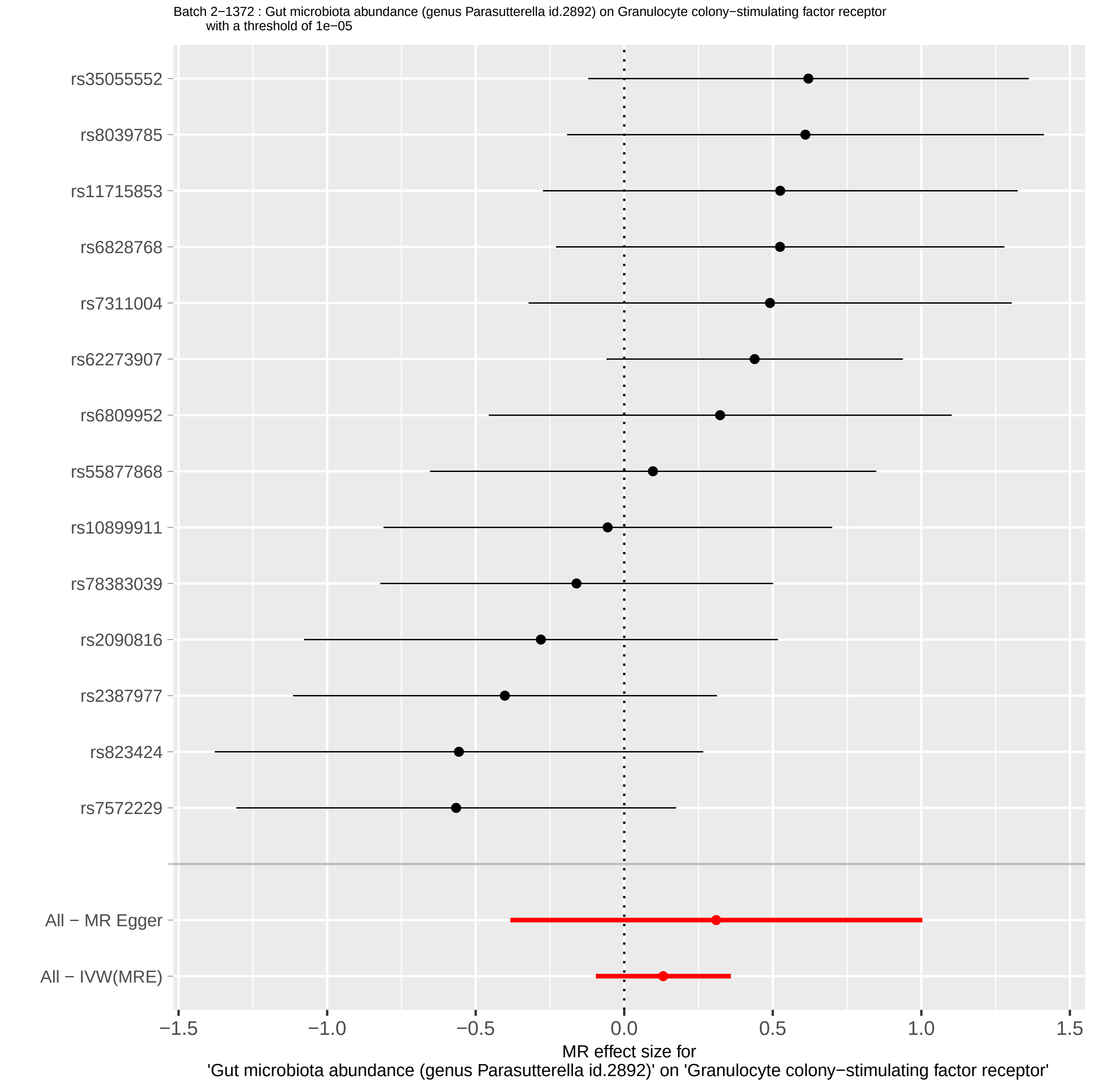

Batch 2−1372 : Gut microbiota abundance (genus Parasutterella id.2892) on Granulocyte colony−stimulating factor receptor
with a threshold of 1e−05
rs35055552
rs8039785
rs11715853
rs6828768
rs7311004
rs62273907
rs6809952
rs55877868
rs10899911
rs78383039
rs2090816
rs2387977
rs823424
rs7572229
All − MR Egger
All − IVW(MRE)
−1.5
−1.0
−0.5
0.0
0.5
1.0
1.5
MR effect size for
'Gut microbiota abundance (genus Parasutterella id.2892)' on 'Granulocyte colony−stimulating factor receptor'

Batch 2−1373 : Gut microbiota abundance (genus Catenibacterium id.2153) on Chondroitin sulfate N−acetylgalactosaminyltransferase 2
with a threshold of 1e−05

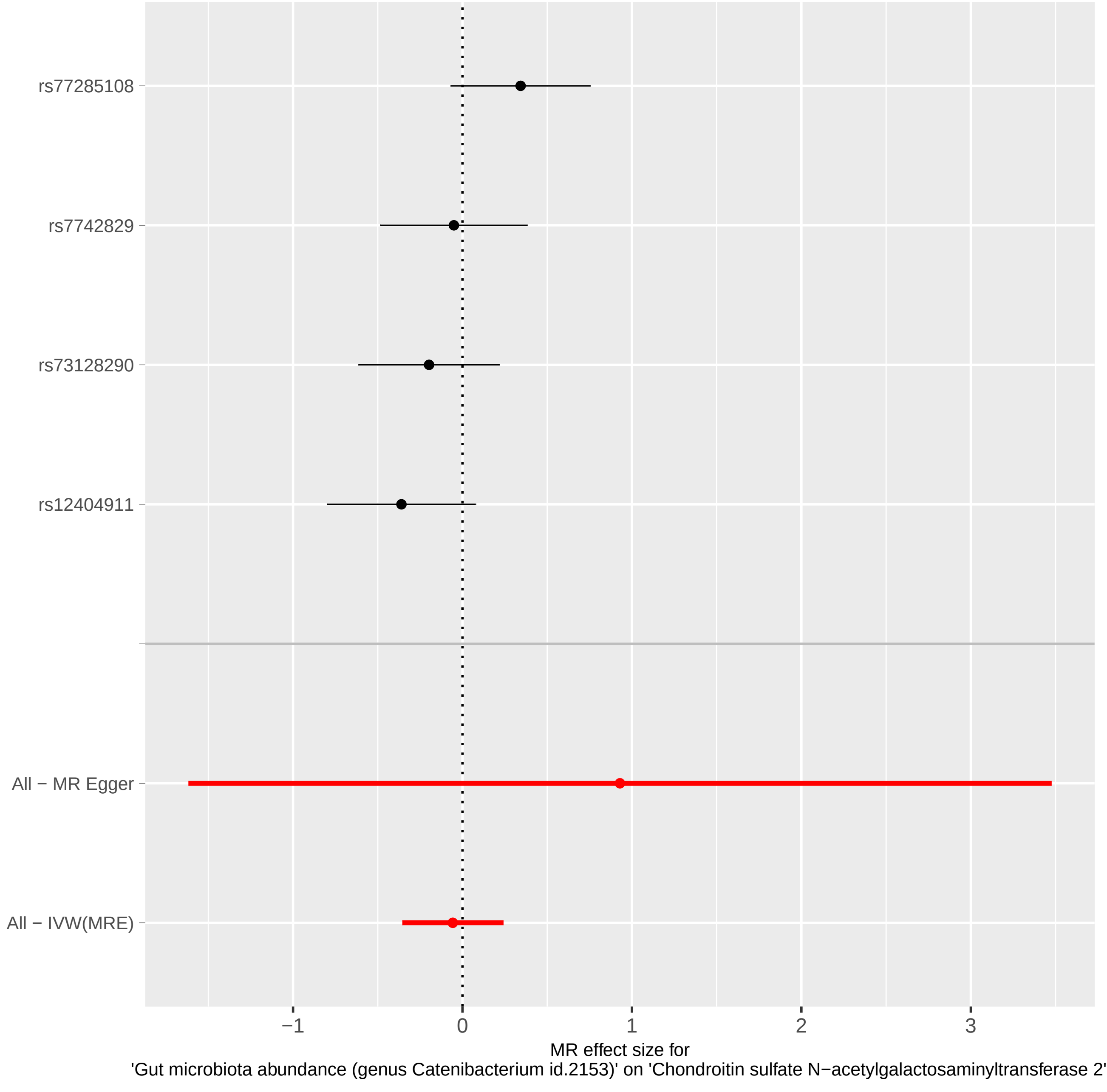

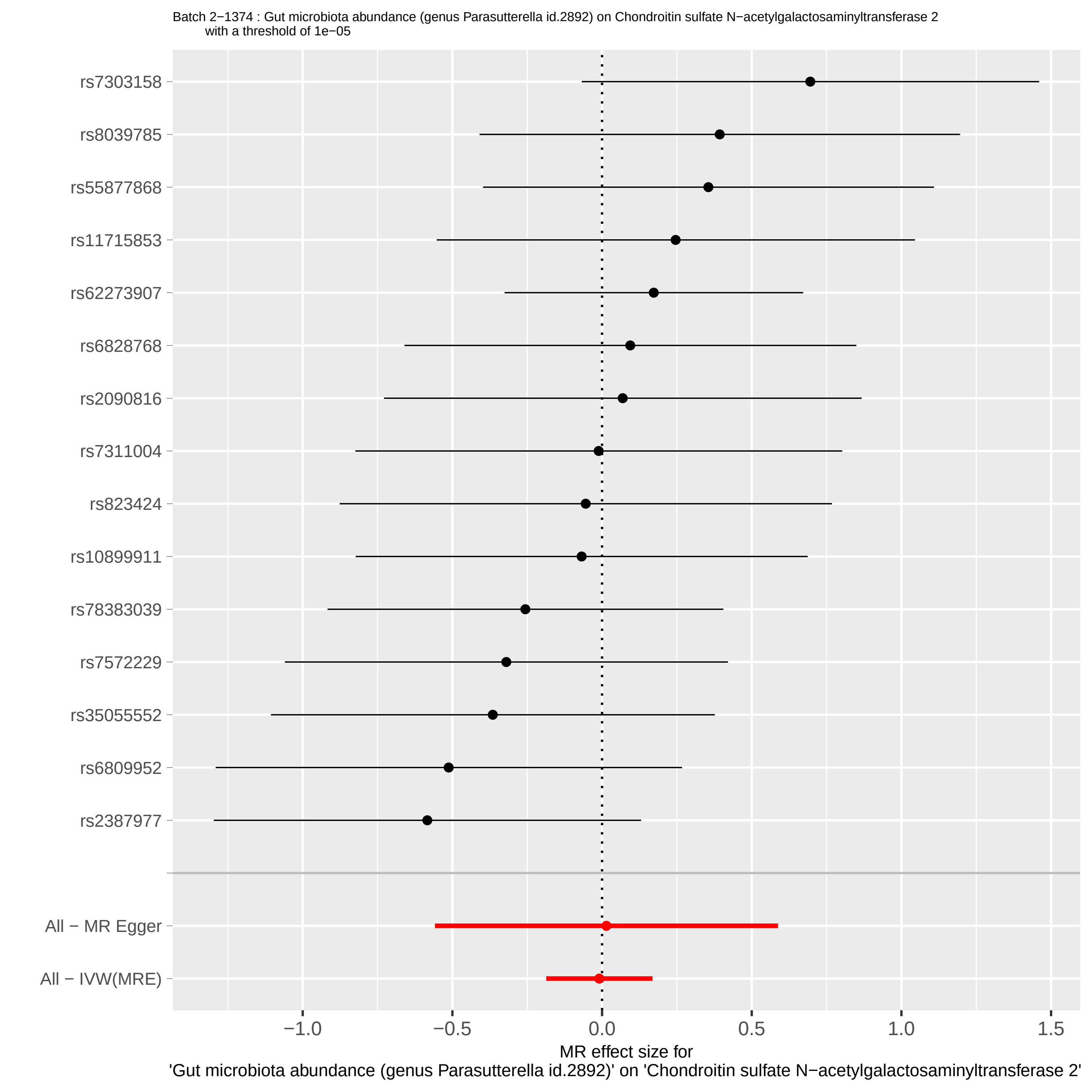
Batch 2−1374 : Gut microbiota abundance (genus Parasutterella id.2892) on Chondroitin sulfate N−acetylgalactosaminyltransferase 2
with a threshold of 1e−05
rs7303158
rs8039785
rs55877868
rs11715853
rs62273907
rs6828768
rs2090816
rs7311004
rs823424
rs10899911
rs78383039
rs7572229
rs35055552
rs6809952
rs2387977
All − MR Egger
All − IVW(MRE)
−1.0
−0.5
0.0
0.5
1.0
1.5
MR effect size for
'Gut microbiota abundance (genus Parasutterella id.2892)' on 'Chondroitin sulfate N−acetylgalactosaminyltransferase 2'

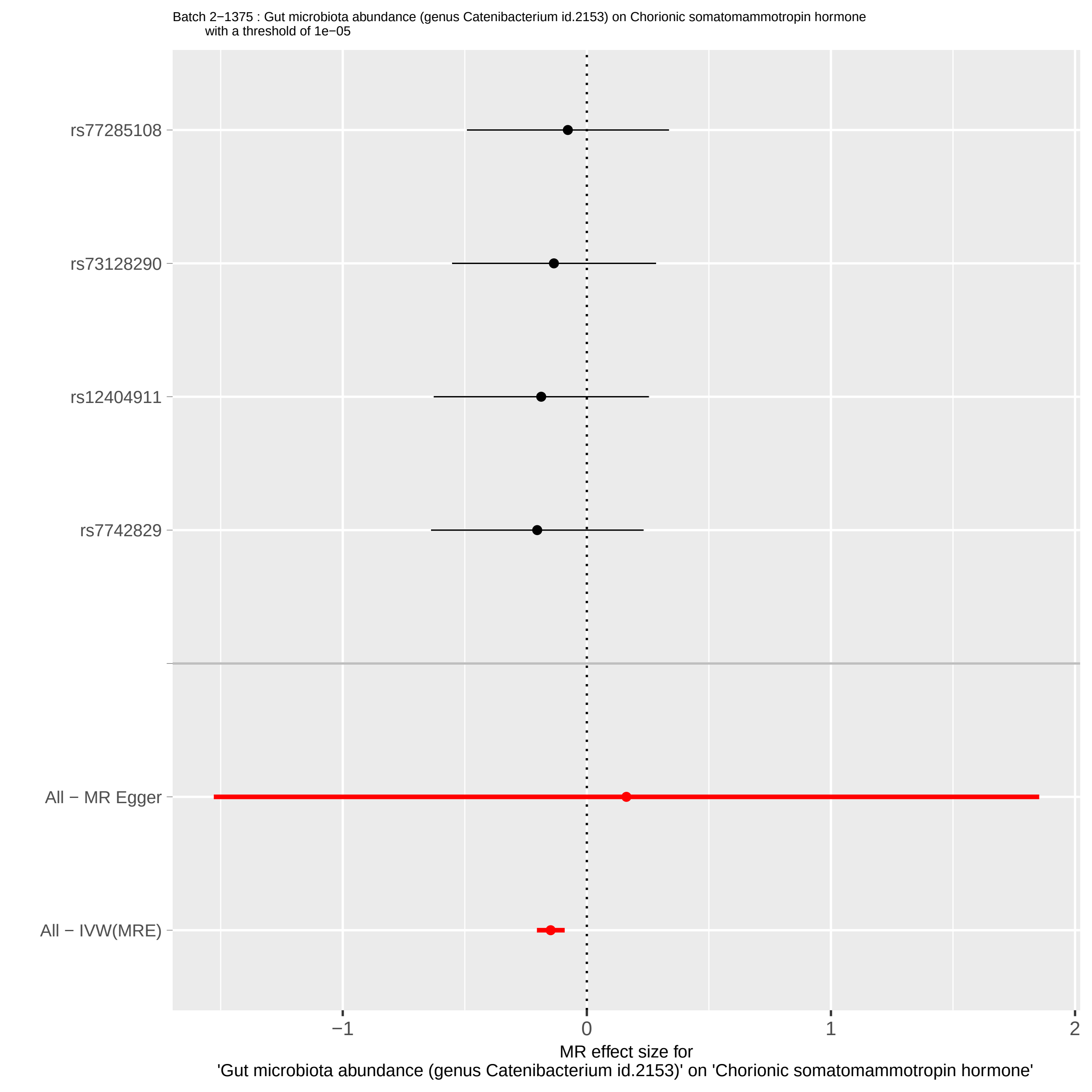
Batch 2−1375 : Gut microbiota abundance (genus Catenibacterium id.2153) on Chorionic somatomammotropin hormone
with a threshold of 1e−05
rs77285108
rs73128290
rs12404911
rs7742829
All − MR Egger
All − IVW(MRE)
−1
0
1
2
MR effect size for
'Gut microbiota abundance (genus Catenibacterium id.2153)' on 'Chorionic somatomammotropin hormone'

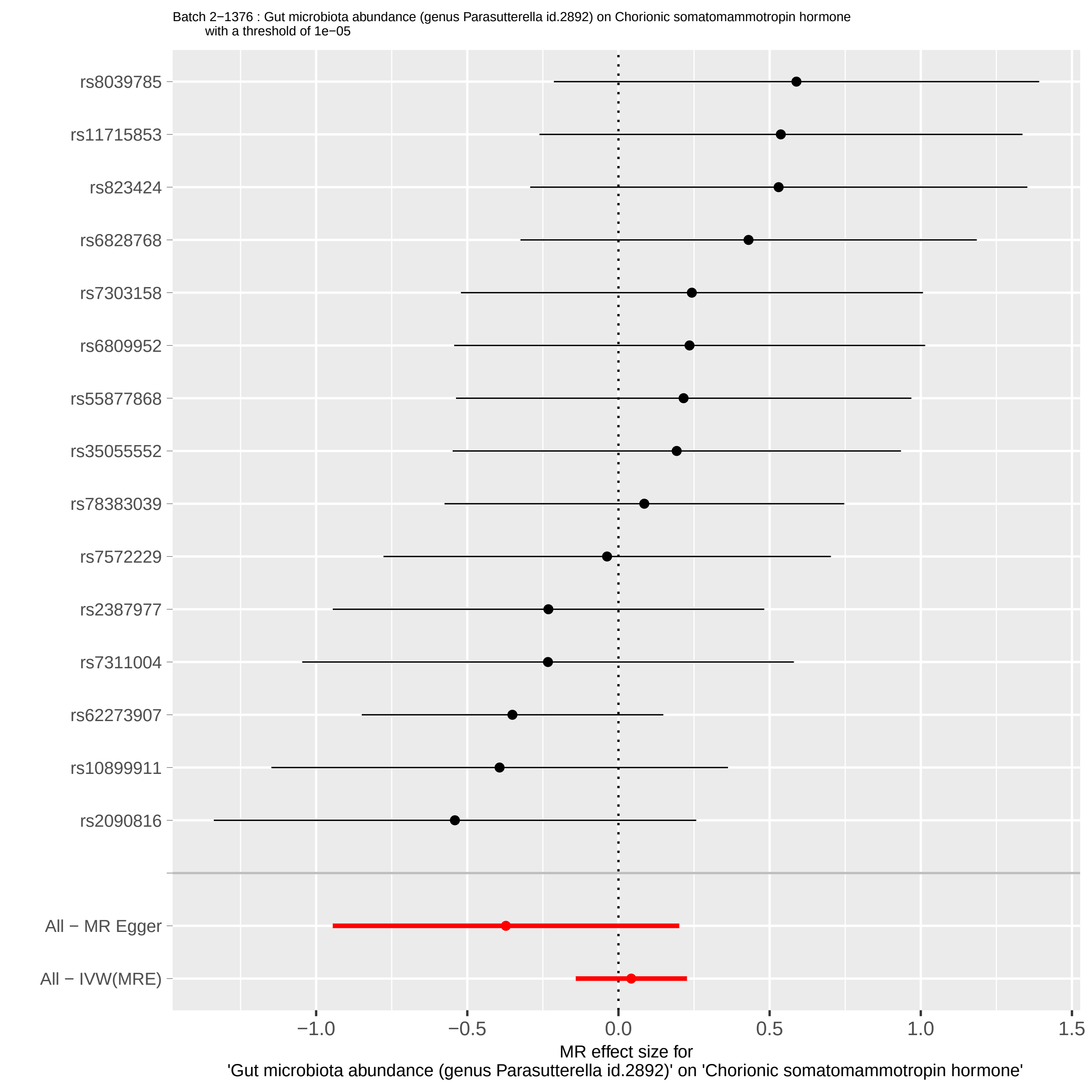

Batch 2−1376 : Gut microbiota abundance (genus Parasutterella id.2892) on Chorionic somatomammotropin hormone
with a threshold of 1e−05
rs8039785
rs11715853
rs823424
rs6828768
rs7303158
rs6809952
rs55877868
rs35055552
rs78383039
rs7572229
rs2387977
rs7311004
rs62273907
rs10899911
rs2090816
All − MR Egger
All − IVW(MRE)
−1.0
−0.5
0.0
0.5
1.0
1.5
MR effect size for
'Gut microbiota abundance (genus Parasutterella id.2892)' on 'Chorionic somatomammotropin hormone'

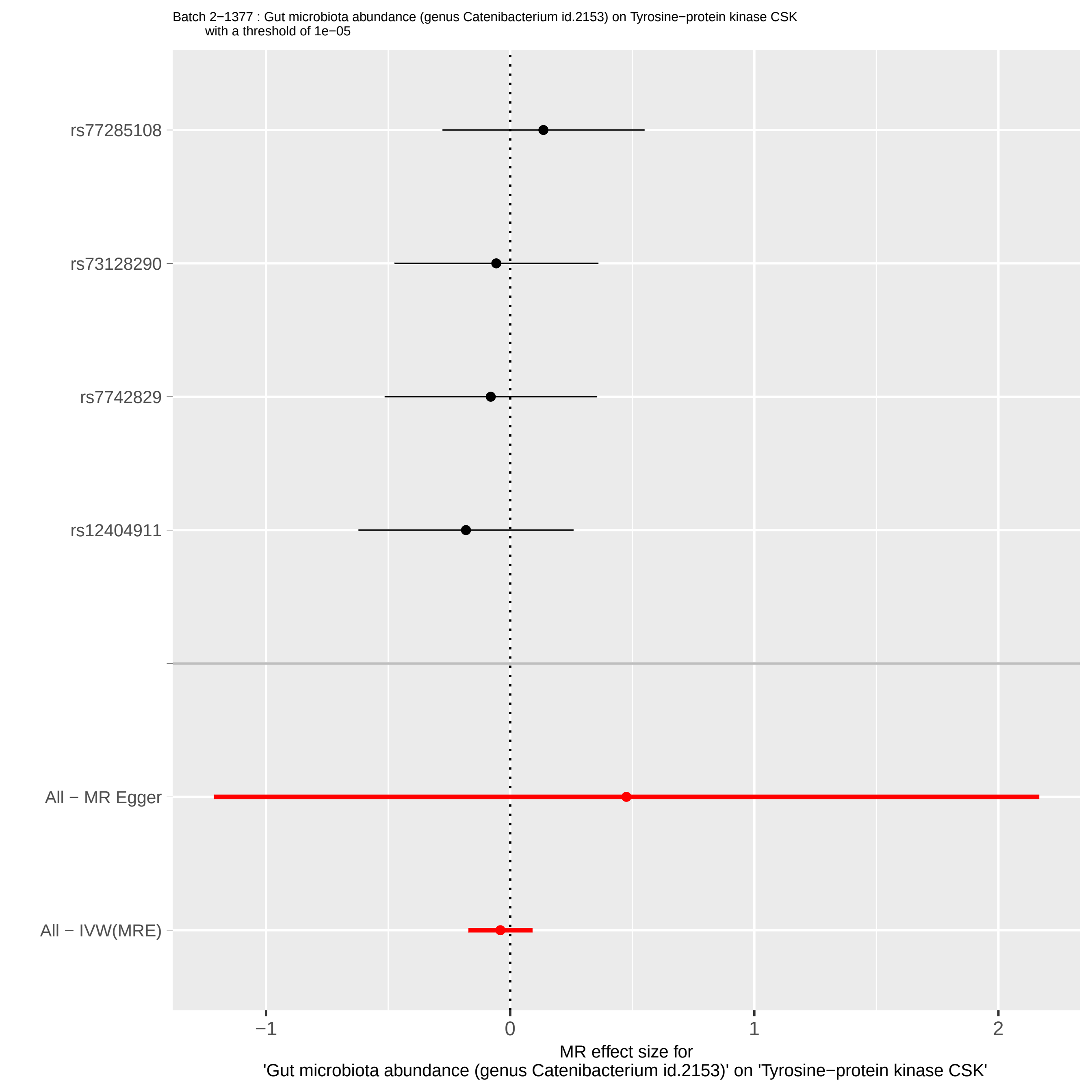
Batch 2−1377 : Gut microbiota abundance (genus Catenibacterium id.2153) on Tyrosine−protein kinase CSK
with a threshold of 1e−05
rs77285108
rs73128290
rs7742829
rs12404911
All − MR Egger
All − IVW(MRE)
−1
0
1
2
MR effect size for
'Gut microbiota abundance (genus Catenibacterium id.2153)' on 'Tyrosine−protein kinase CSK'

Batch 2−1378 : Gut microbiota abundance (genus Parasutterella id.2892) on Tyrosine−protein kinase CSK
with a threshold of 1e−05

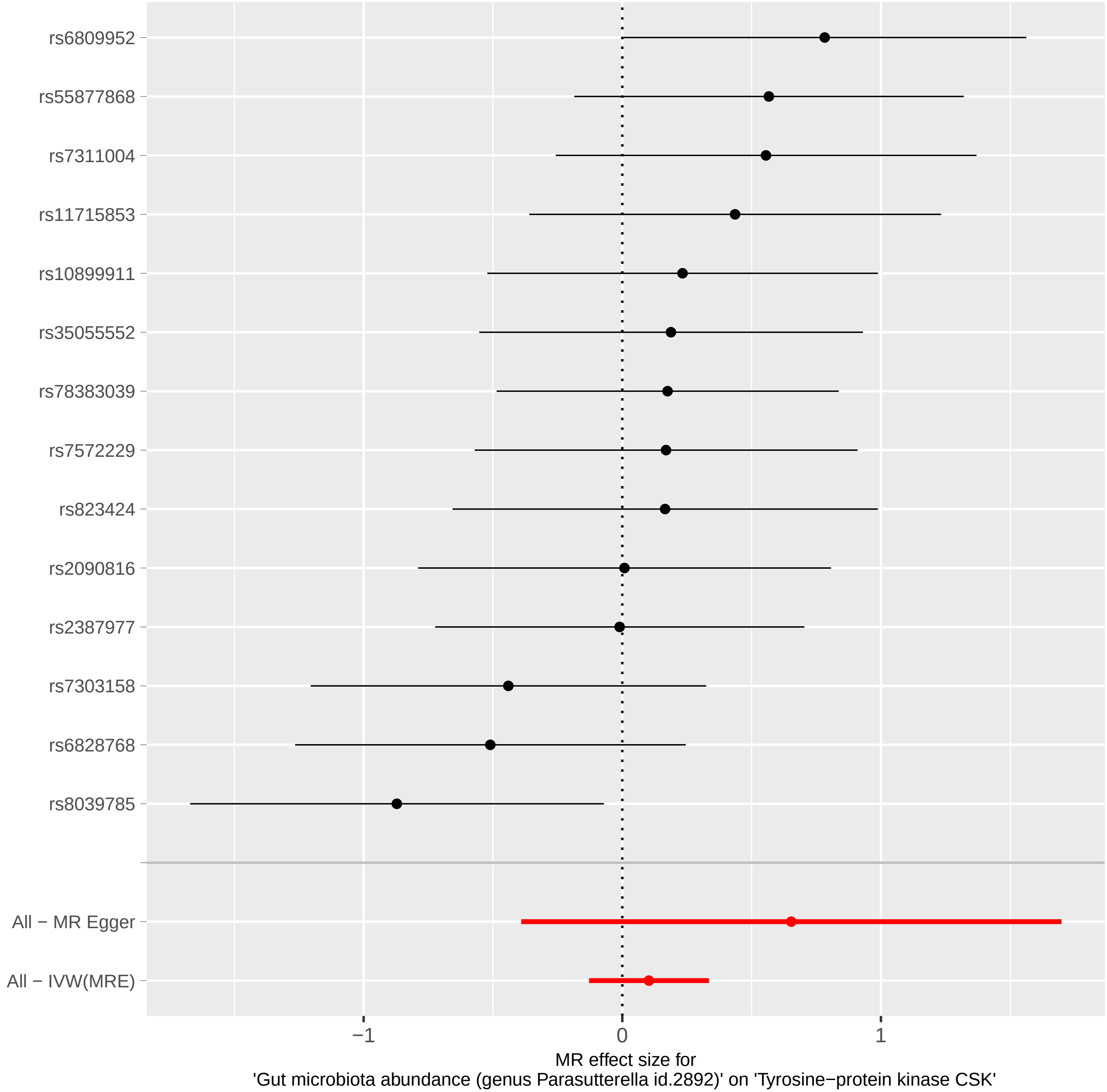

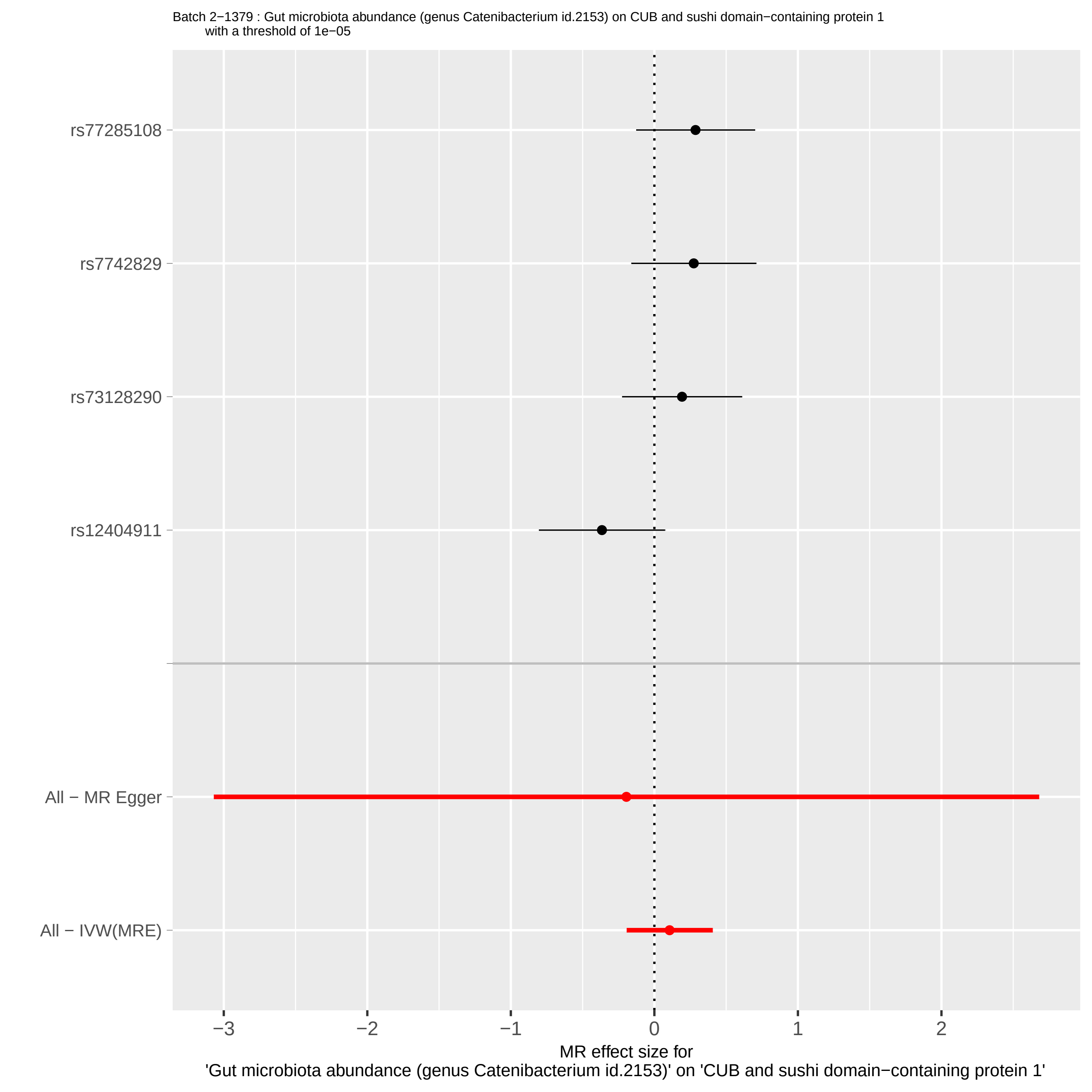

Batch 2−1379 : Gut microbiota abundance (genus Catenibacterium id.2153) on CUB and sushi domain−containing protein 1
with a threshold of 1e−05
rs77285108
rs7742829
rs73128290
rs12404911
All − MR Egger
All − IVW(MRE)
−3
−2
−1
0
1
2
MR effect size for
'Gut microbiota abundance (genus Catenibacterium id.2153)' on 'CUB and sushi domain−containing protein 1'

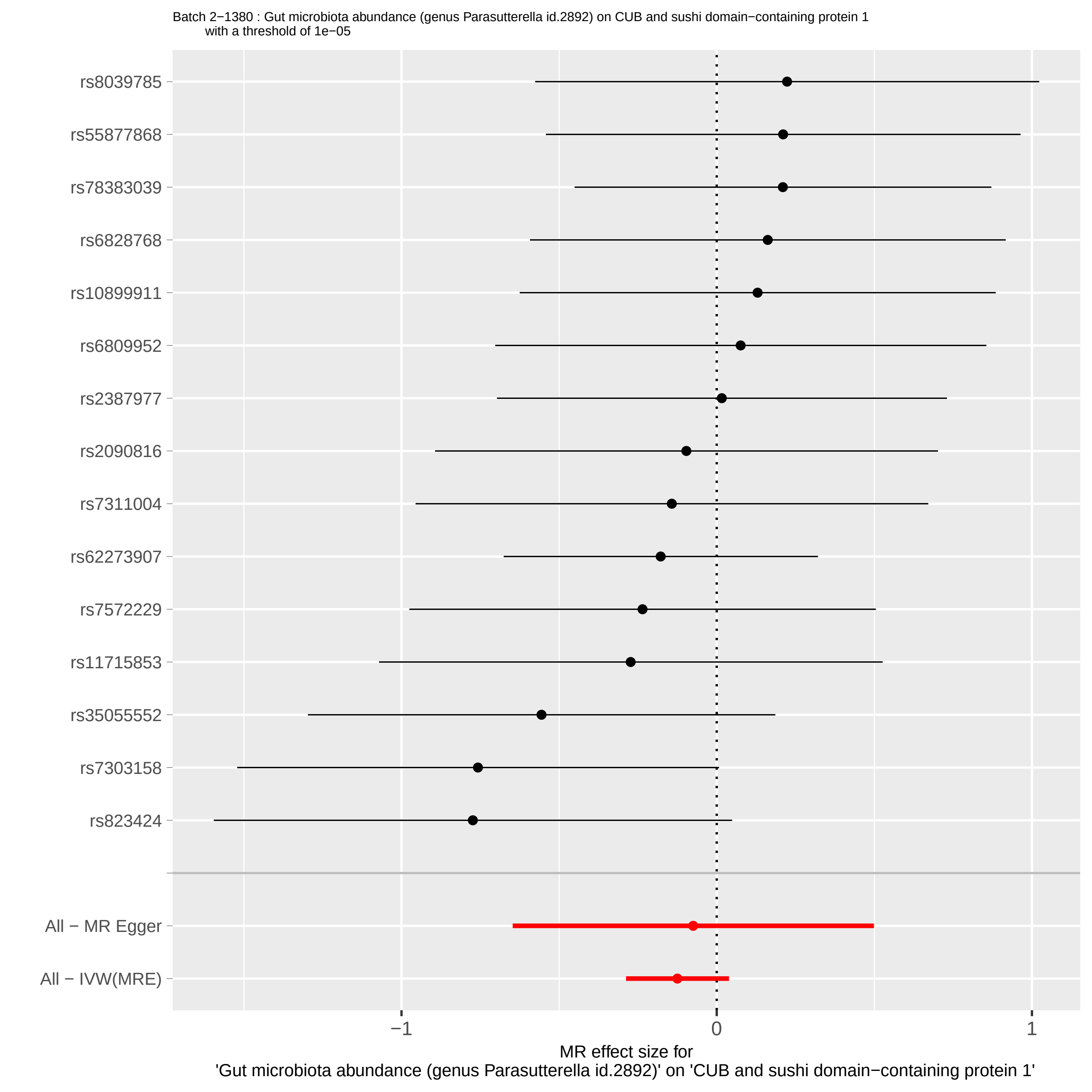

Batch 2−1380 : Gut microbiota abundance (genus Parasutterella id.2892) on CUB and sushi domain−containing protein 1
with a threshold of 1e−05
rs8039785
rs55877868
rs78383039
rs6828768
rs10899911
rs6809952
rs2387977
rs2090816
rs7311004
rs62273907
rs7572229
rs11715853
rs35055552
rs7303158
rs823424
All − MR Egger
All − IVW(MRE)
−1
0
1
MR effect size for
'Gut microbiota abundance (genus Parasutterella id.2892)' on 'CUB and sushi domain−containing protein 1'

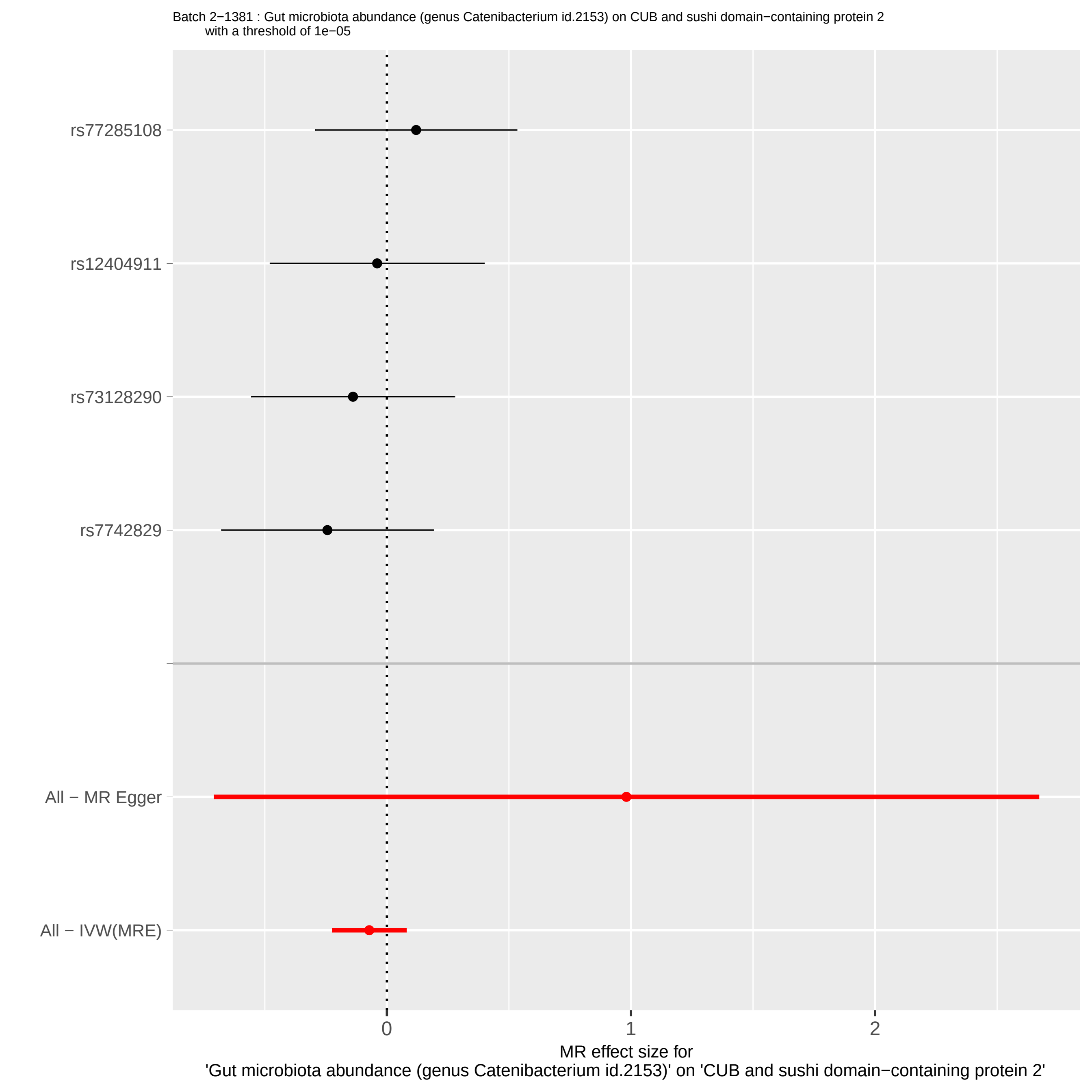
Batch 2−1381 : Gut microbiota abundance (genus Catenibacterium id.2153) on CUB and sushi domain−containing protein 2
with a threshold of 1e−05
rs77285108
rs12404911
rs73128290
rs7742829
All − MR Egger
All − IVW(MRE)
0
1
2
MR effect size for
'Gut microbiota abundance (genus Catenibacterium id.2153)' on 'CUB and sushi domain−containing protein 2'

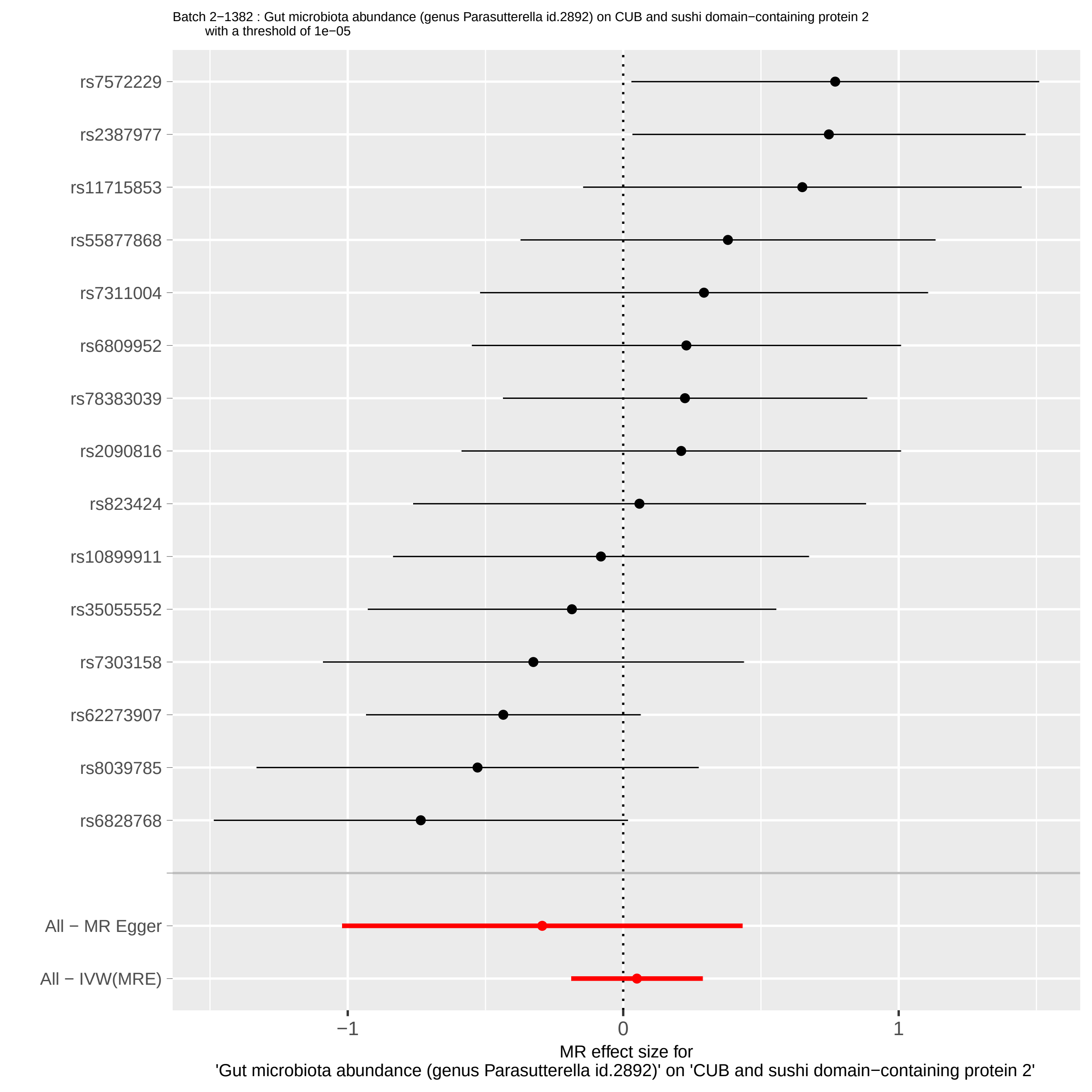

Batch 2−1382 : Gut microbiota abundance (genus Parasutterella id.2892) on CUB and sushi domain−containing protein 2
with a threshold of 1e−05
rs7572229
rs2387977
rs11715853
rs55877868
rs7311004
rs6809952
rs78383039
rs2090816
rs823424
rs10899911
rs35055552
rs7303158
rs62273907
rs8039785
rs6828768
All − MR Egger
All − IVW(MRE)
−1
0
1
MR effect size for
'Gut microbiota abundance (genus Parasutterella id.2892)' on 'CUB and sushi domain−containing protein 2'

Batch 2−1383 : Gut microbiota abundance (genus Catenibacterium id.2153) on alpha−S1−casein
with a threshold of 1e−05

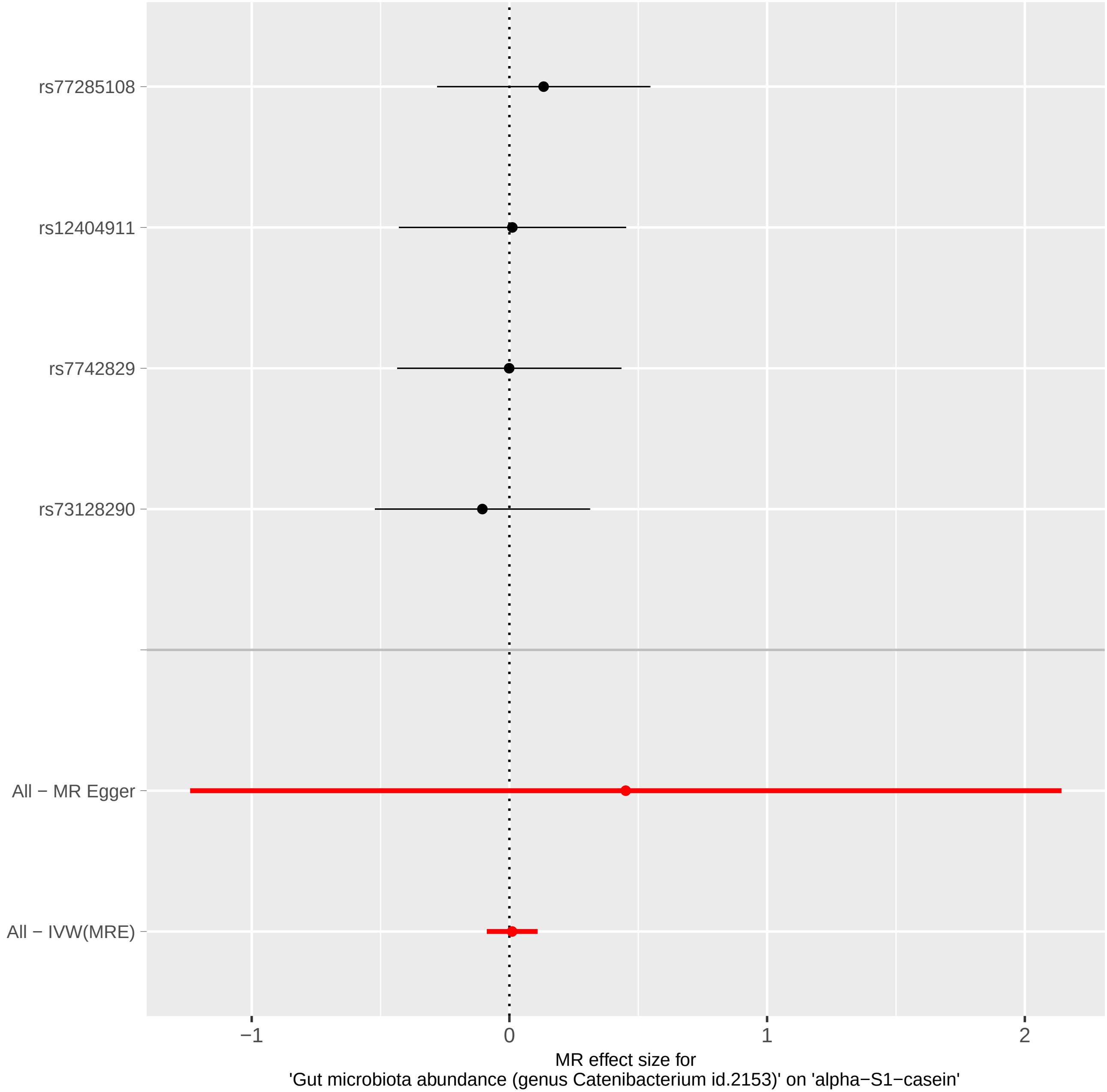

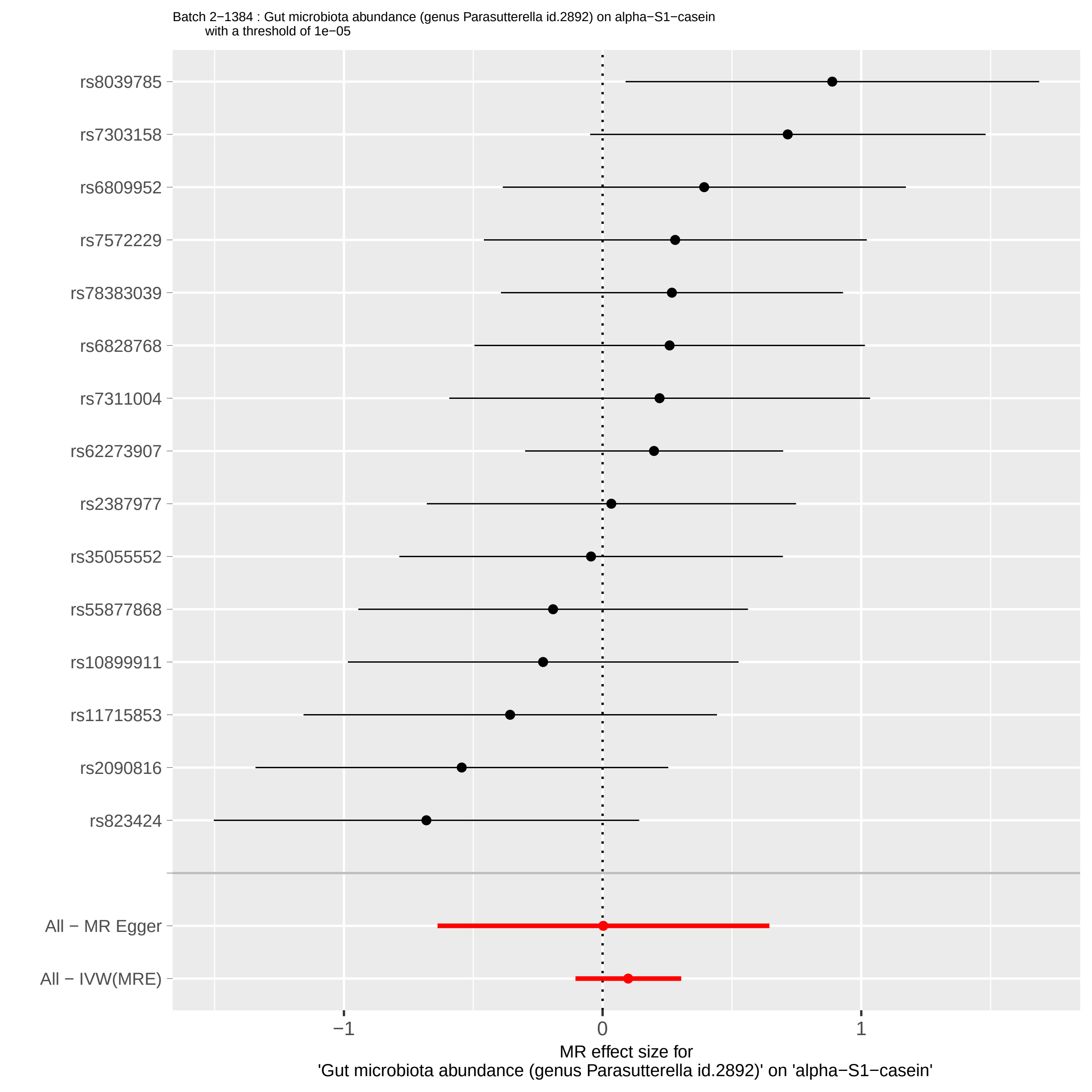

Batch 2−1384 : Gut microbiota abundance (genus Parasutterella id.2892) on alpha−S1−casein
with a threshold of 1e−05
rs8039785
rs7303158
rs6809952
rs7572229
rs78383039
rs6828768
rs7311004
rs62273907
rs2387977
rs35055552
rs55877868
rs10899911
rs11715853
rs2090816
rs823424
All − MR Egger
All − IVW(MRE)
−1
0
1
MR effect size for
'Gut microbiota abundance (genus Parasutterella id.2892)' on 'alpha−S1−casein'

Batch 2−1385 : Gut microbiota abundance (genus Catenibacterium id.2153) on Casein kinase I isoform delta
with a threshold of 1e−05

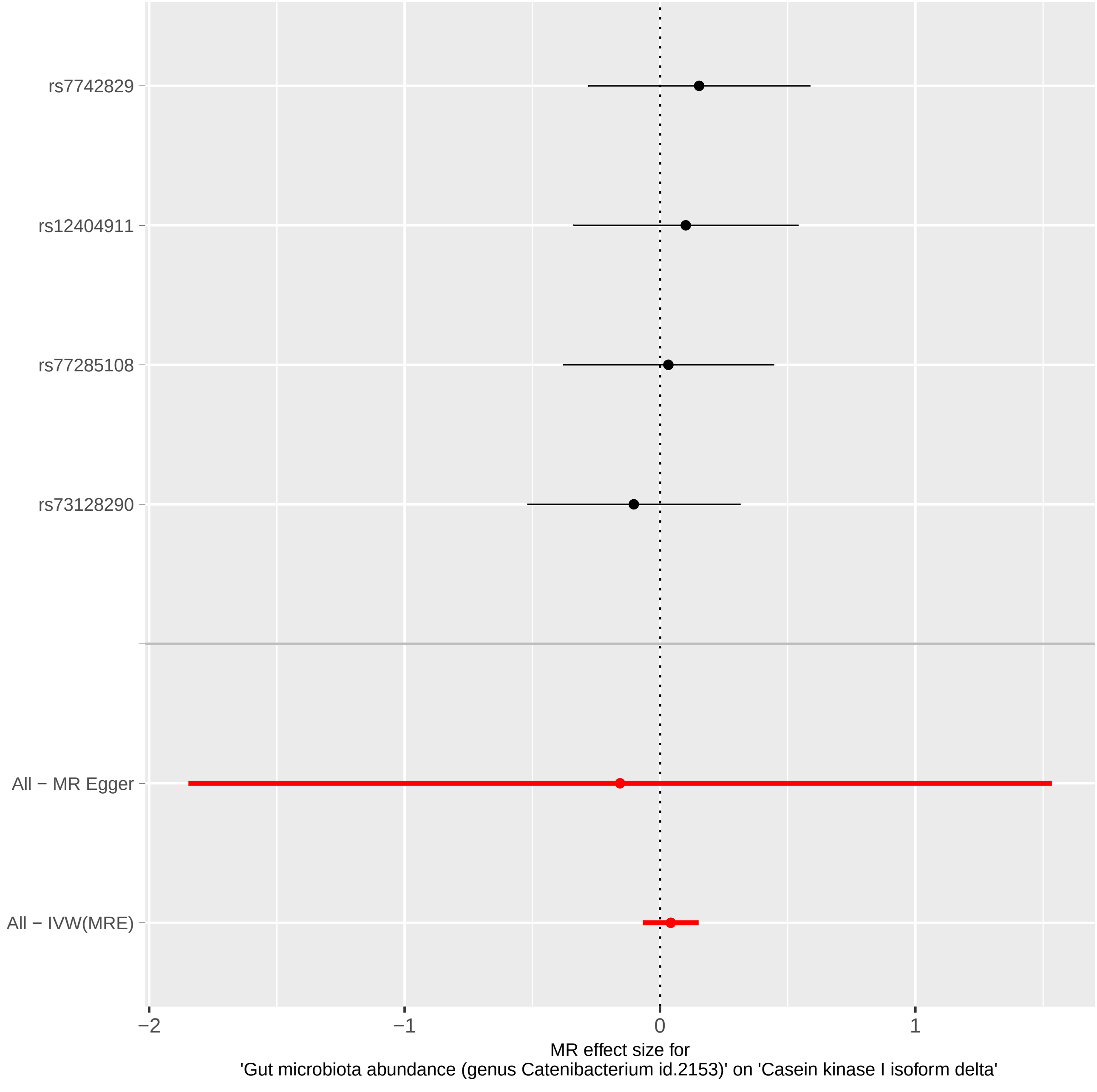

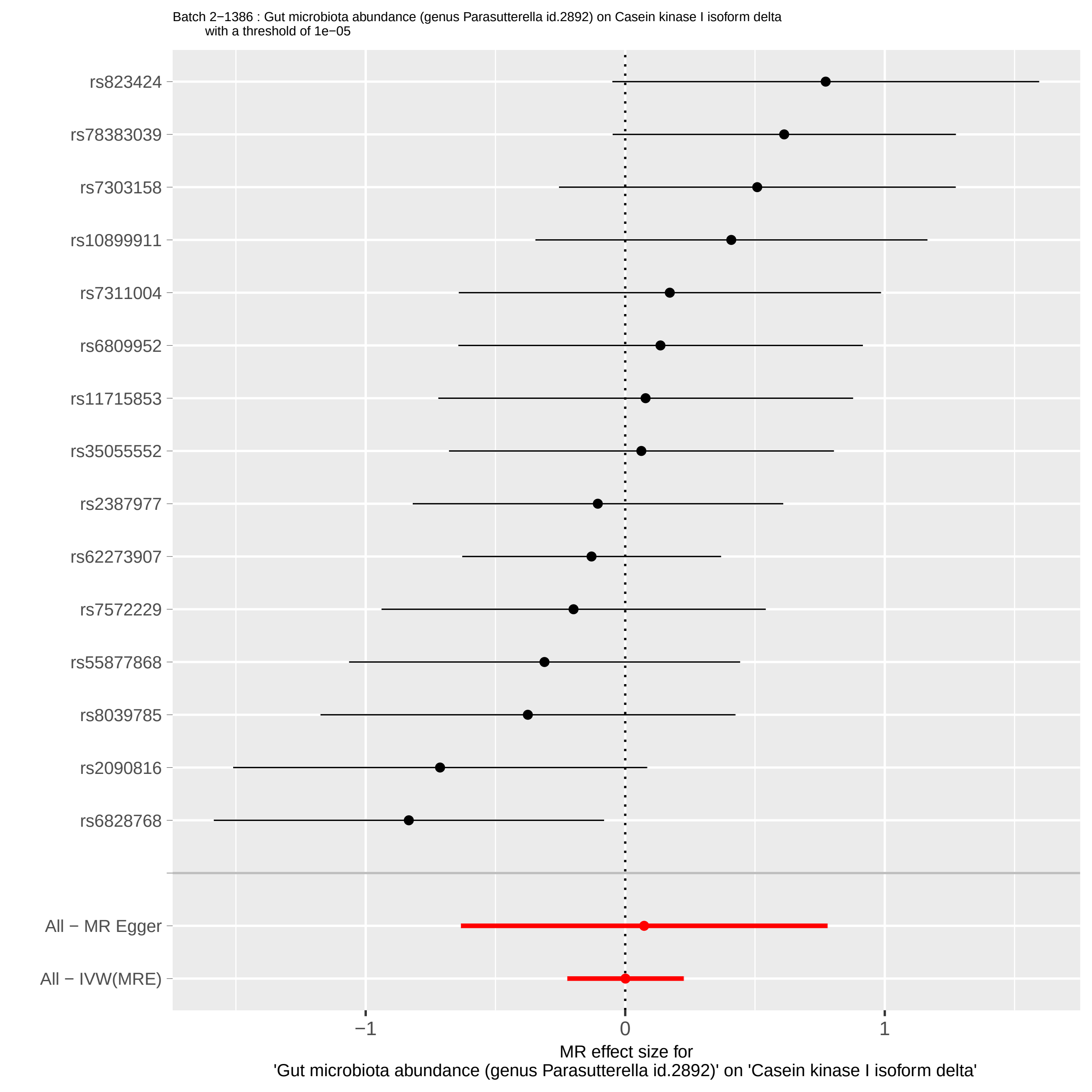
Batch 2−1386 : Gut microbiota abundance (genus Parasutterella id.2892) on Casein kinase I isoform delta
with a threshold of 1e−05
rs823424
rs78383039
rs7303158
rs10899911
rs7311004
rs6809952
rs11715853
rs35055552
rs2387977
rs62273907
rs7572229
rs55877868
rs8039785
rs2090816
rs6828768
All − MR Egger
All − IVW(MRE)
−1
0
1
MR effect size for
'Gut microbiota abundance (genus Parasutterella id.2892)' on 'Casein kinase I isoform delta'

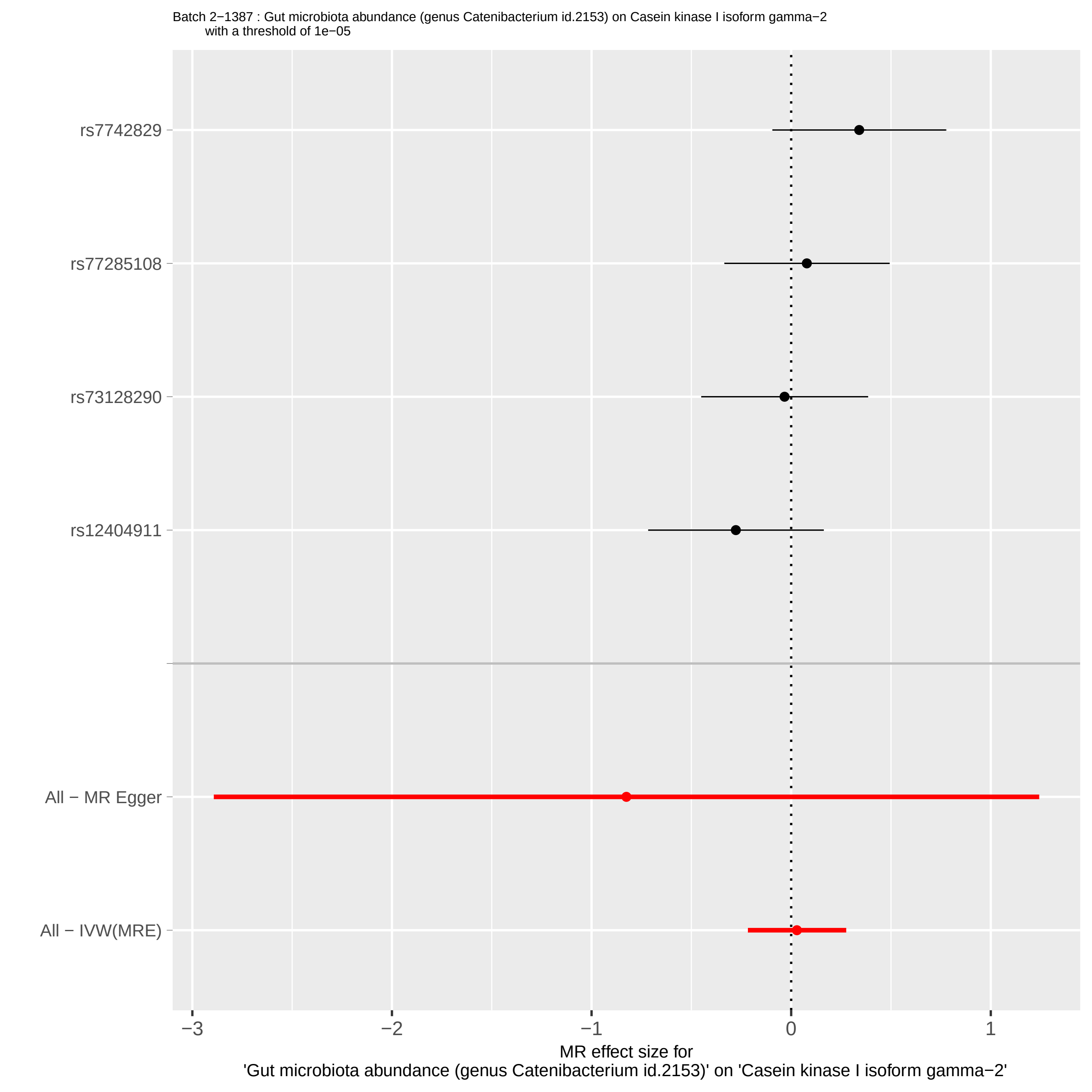
Batch 2−1387 : Gut microbiota abundance (genus Catenibacterium id.2153) on Casein kinase I isoform gamma−2
with a threshold of 1e−05
rs7742829
rs77285108
rs73128290
rs12404911
All − MR Egger
All − IVW(MRE)
−3
−2
−1
0
1
MR effect size for
'Gut microbiota abundance (genus Catenibacterium id.2153)' on 'Casein kinase I isoform gamma−2'

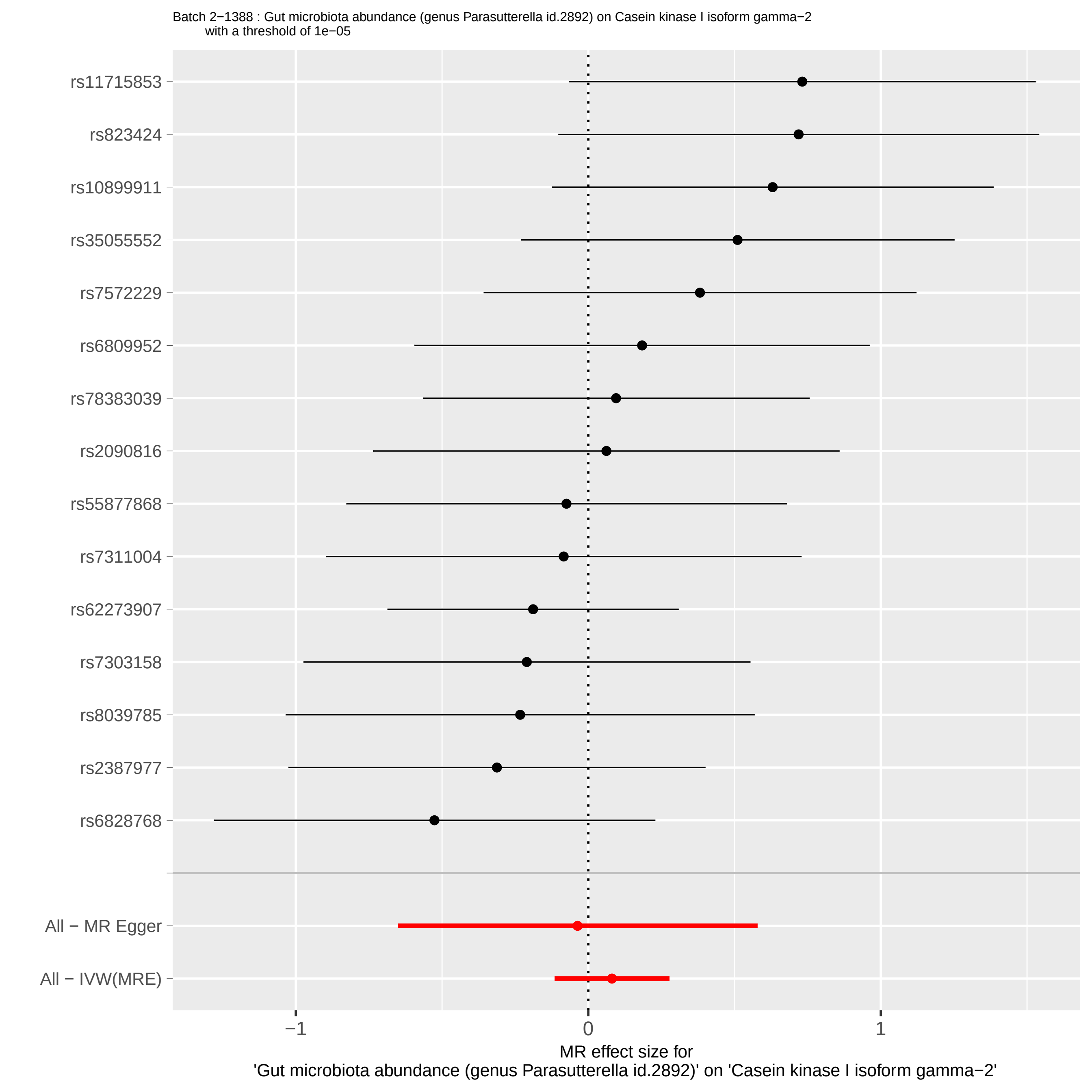

Batch 2−1388 : Gut microbiota abundance (genus Parasutterella id.2892) on Casein kinase I isoform gamma−2
with a threshold of 1e−05
rs11715853
rs823424
rs10899911
rs35055552
rs7572229
rs6809952
rs78383039
rs2090816
rs55877868
rs7311004
rs62273907
rs7303158
rs8039785
rs2387977
rs6828768
All − MR Egger
All − IVW(MRE)
−1
0
1
MR effect size for
'Gut microbiota abundance (genus Parasutterella id.2892)' on 'Casein kinase I isoform gamma−2'

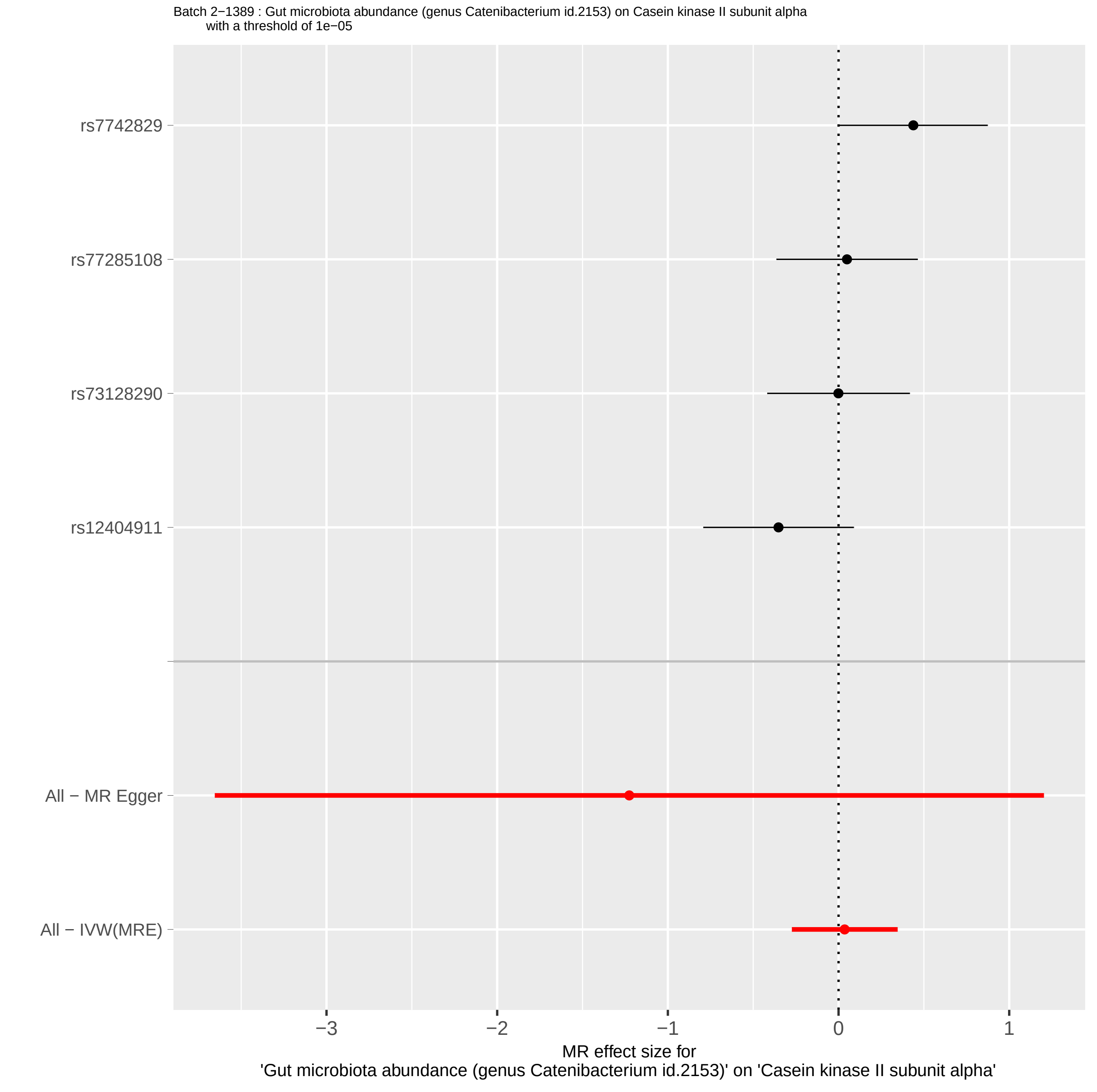
Batch 2−1389 : Gut microbiota abundance (genus Catenibacterium id.2153) on Casein kinase II subunit alpha
with a threshold of 1e−05
rs7742829
rs77285108
rs73128290
rs12404911
All − MR Egger
All − IVW(MRE)
−3
−2
−1
0
1
MR effect size for
'Gut microbiota abundance (genus Catenibacterium id.2153)' on 'Casein kinase II subunit alpha'

Batch 2−1390 : Gut microbiota abundance (genus Parasutterella id.2892) on Casein kinase II subunit alpha
with a threshold of 1e−05

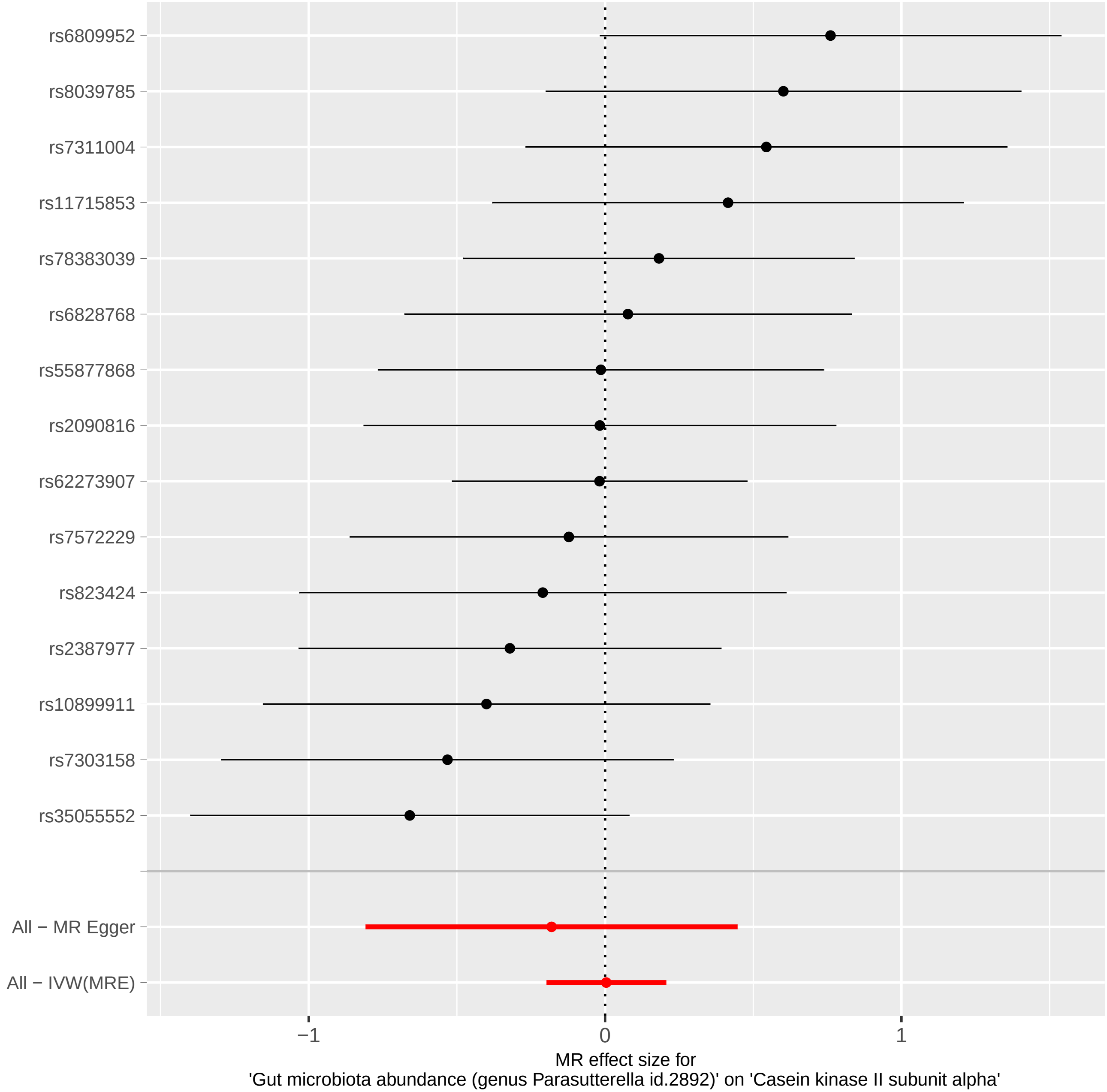

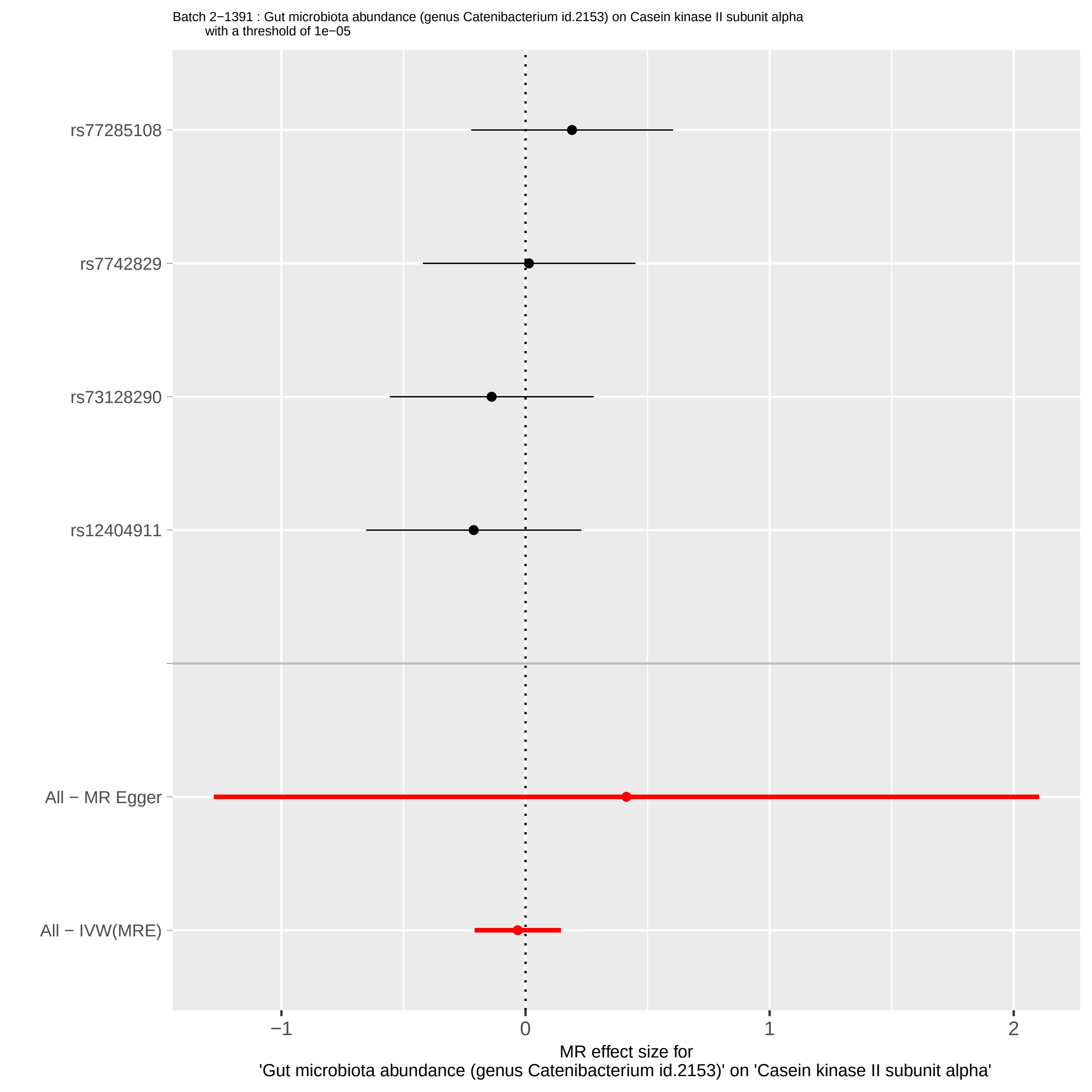

Batch 2−1391 : Gut microbiota abundance (genus Catenibacterium id.2153) on Casein kinase II subunit alpha
with a threshold of 1e−05
rs77285108
rs7742829
rs73128290
rs12404911
All − MR Egger
All − IVW(MRE)
−1
0
1
2
MR effect size for
'Gut microbiota abundance (genus Catenibacterium id.2153)' on 'Casein kinase II subunit alpha'

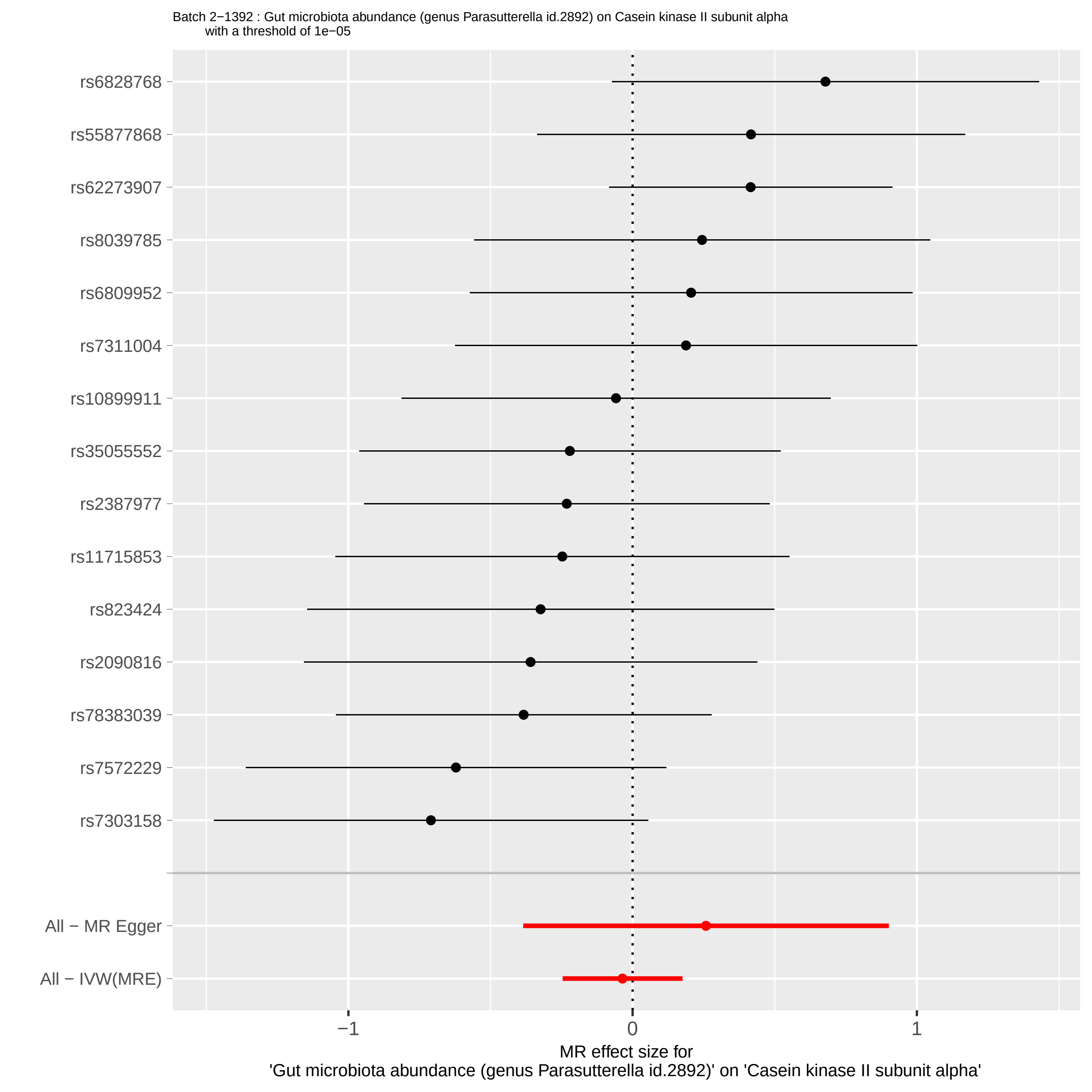

Batch 2−1392 : Gut microbiota abundance (genus Parasutterella id.2892) on Casein kinase II subunit alpha
with a threshold of 1e−05
rs6828768
rs55877868
rs62273907
rs8039785
rs6809952
rs7311004
rs10899911
rs35055552
rs2387977
rs11715853
rs823424
rs2090816
rs78383039
rs7572229
rs7303158
All − MR Egger
All − IVW(MRE)
−1
0
1
MR effect size for
'Gut microbiota abundance (genus Parasutterella id.2892)' on 'Casein kinase II subunit alpha'

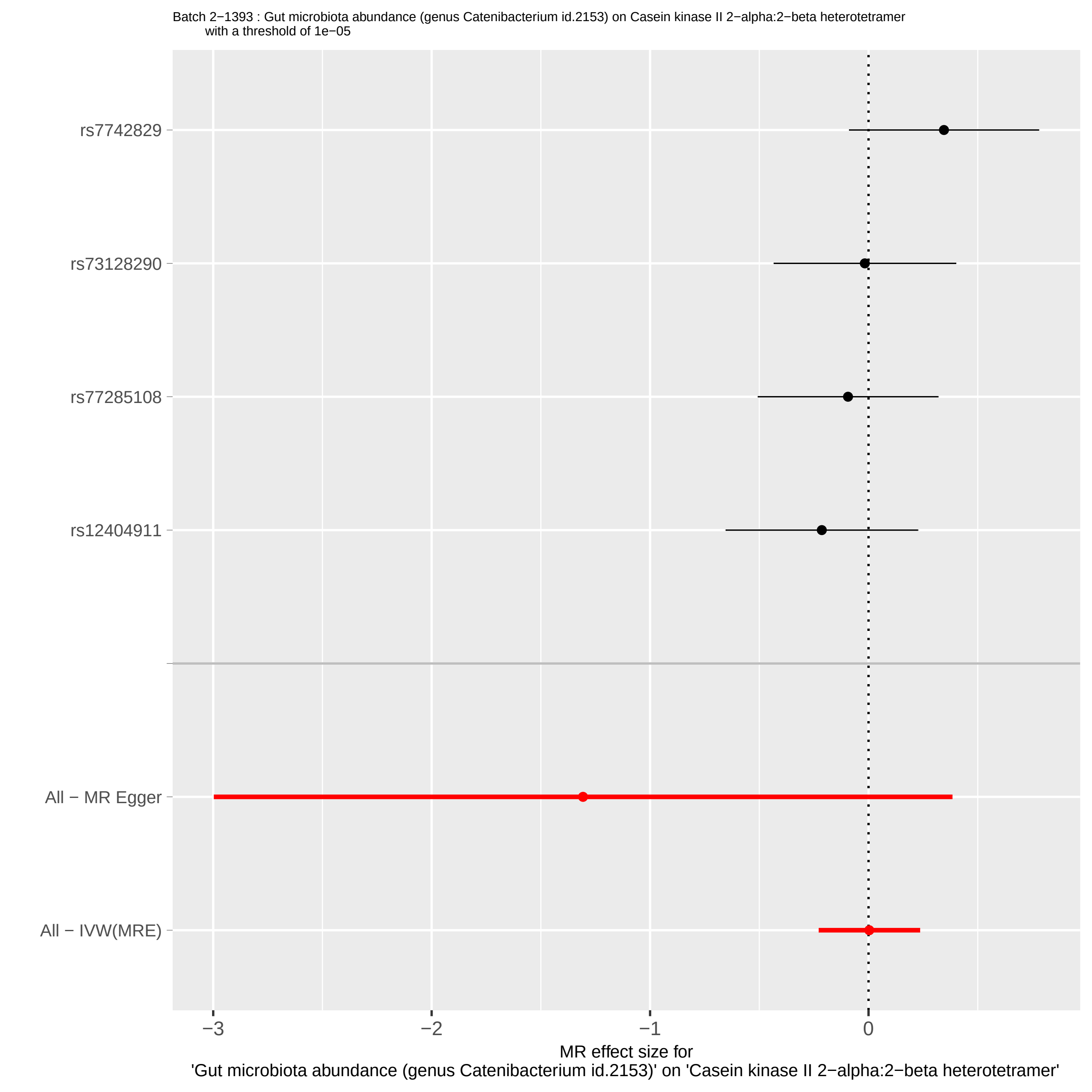

Batch 2−1393 : Gut microbiota abundance (genus Catenibacterium id.2153) on Casein kinase II 2−alpha:2−beta heterotetramer
with a threshold of 1e−05
rs7742829
rs73128290
rs77285108
rs12404911
All − MR Egger
All − IVW(MRE)
−3
−2
−1
0
MR effect size for
'Gut microbiota abundance (genus Catenibacterium id.2153)' on 'Casein kinase II 2−alpha:2−beta heterotetramer'

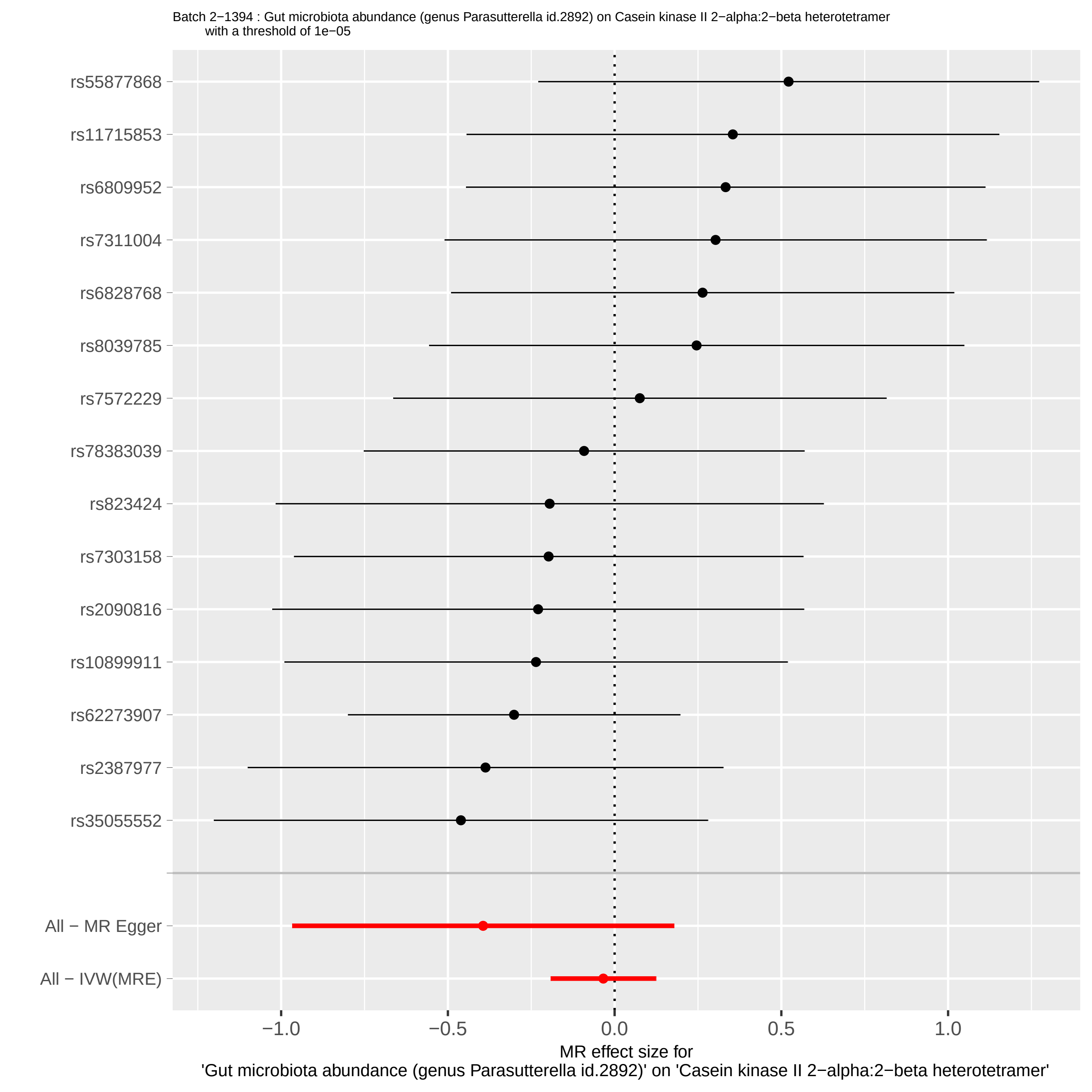

Batch 2−1394 : Gut microbiota abundance (genus Parasutterella id.2892) on Casein kinase II 2−alpha:2−beta heterotetramer
with a threshold of 1e−05
rs55877868
rs11715853
rs6809952
rs7311004
rs6828768
rs8039785
rs7572229
rs78383039
rs823424
rs7303158
rs2090816
rs10899911
rs62273907
rs2387977
rs35055552
All − MR Egger
All − IVW(MRE)
−1.0
−0.5
0.0
0.5
1.0
MR effect size for
'Gut microbiota abundance (genus Parasutterella id.2892)' on 'Casein kinase II 2−alpha:2−beta heterotetramer'

Batch 2−1395 : Gut microbiota abundance (genus Catenibacterium id.2153) on Casein kinase II 2−alpha':2−beta heterotetramer
with a threshold of 1e−05

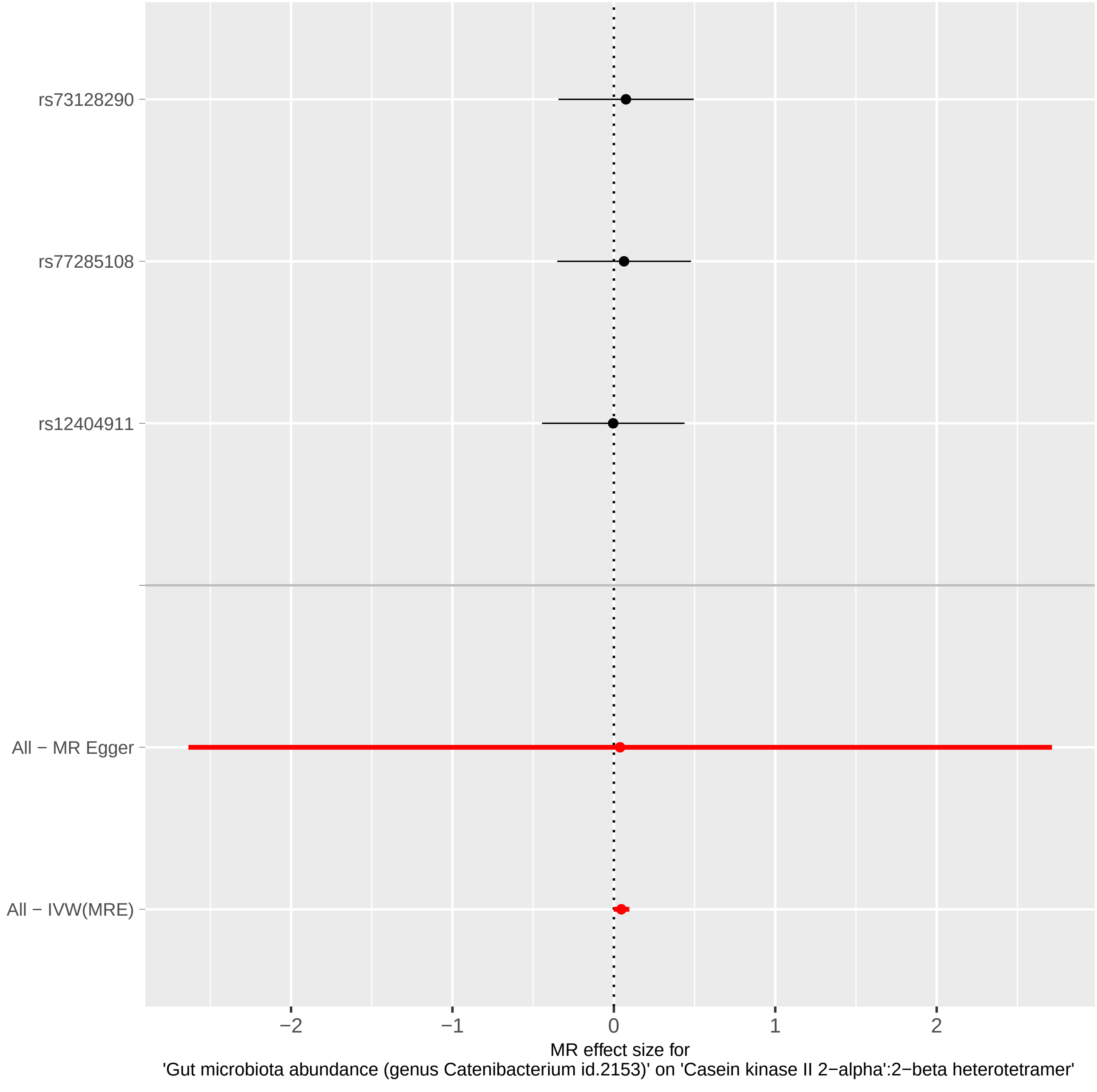

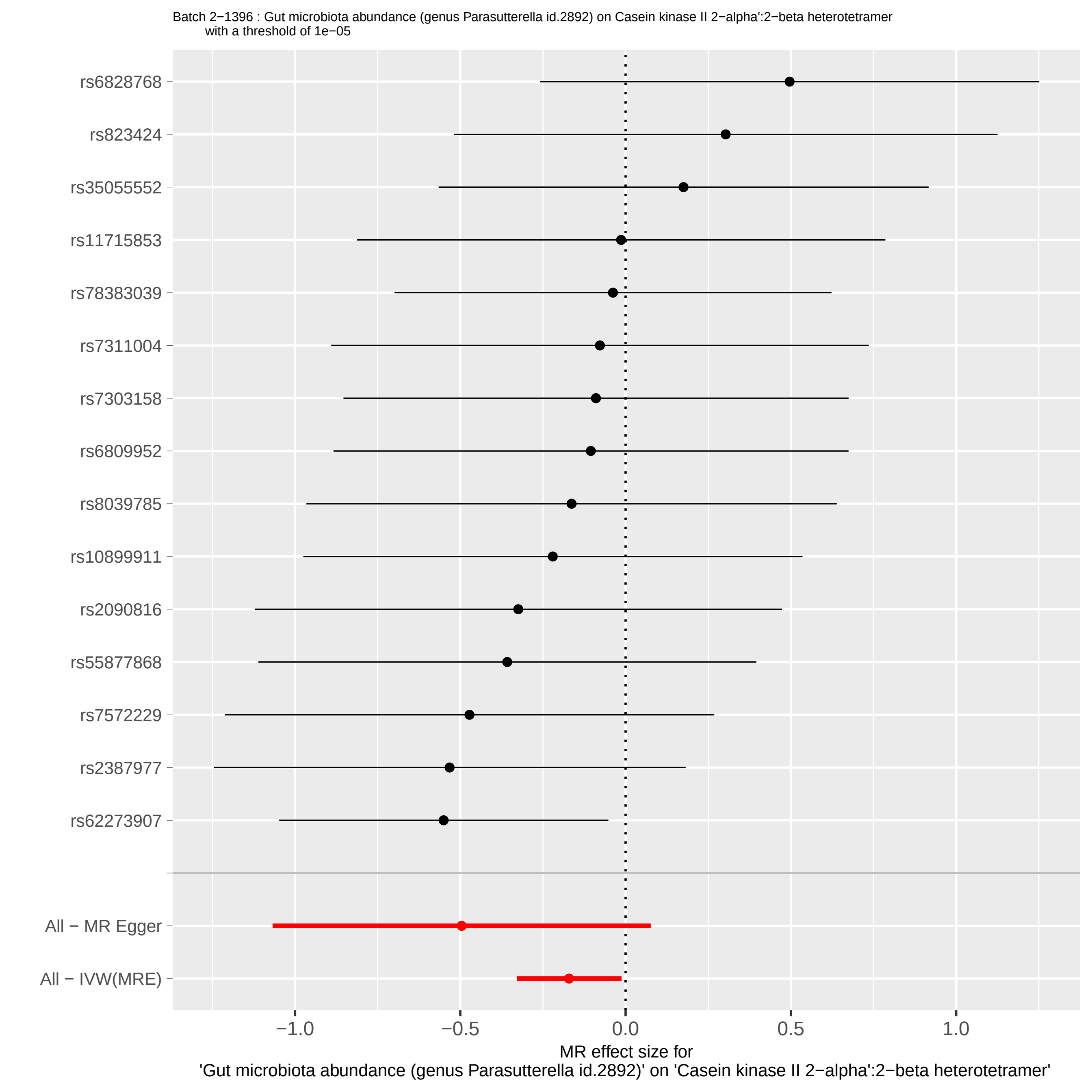
Batch 2−1396 : Gut microbiota abundance (genus Parasutterella id.2892) on Casein kinase II 2−alpha':2−beta heterotetramer
with a threshold of 1e−05
rs6828768
rs823424
rs35055552
rs11715853
rs78383039
rs7311004
rs7303158
rs6809952
rs8039785
rs10899911
rs2090816
rs55877868
rs7572229
rs2387977
rs62273907
All − MR Egger
All − IVW(MRE)
−1.0
−0.5
0.0
0.5
1.0
MR effect size for
'Gut microbiota abundance (genus Parasutterella id.2892)' on 'Casein kinase II 2−alpha':2−beta heterotetramer'

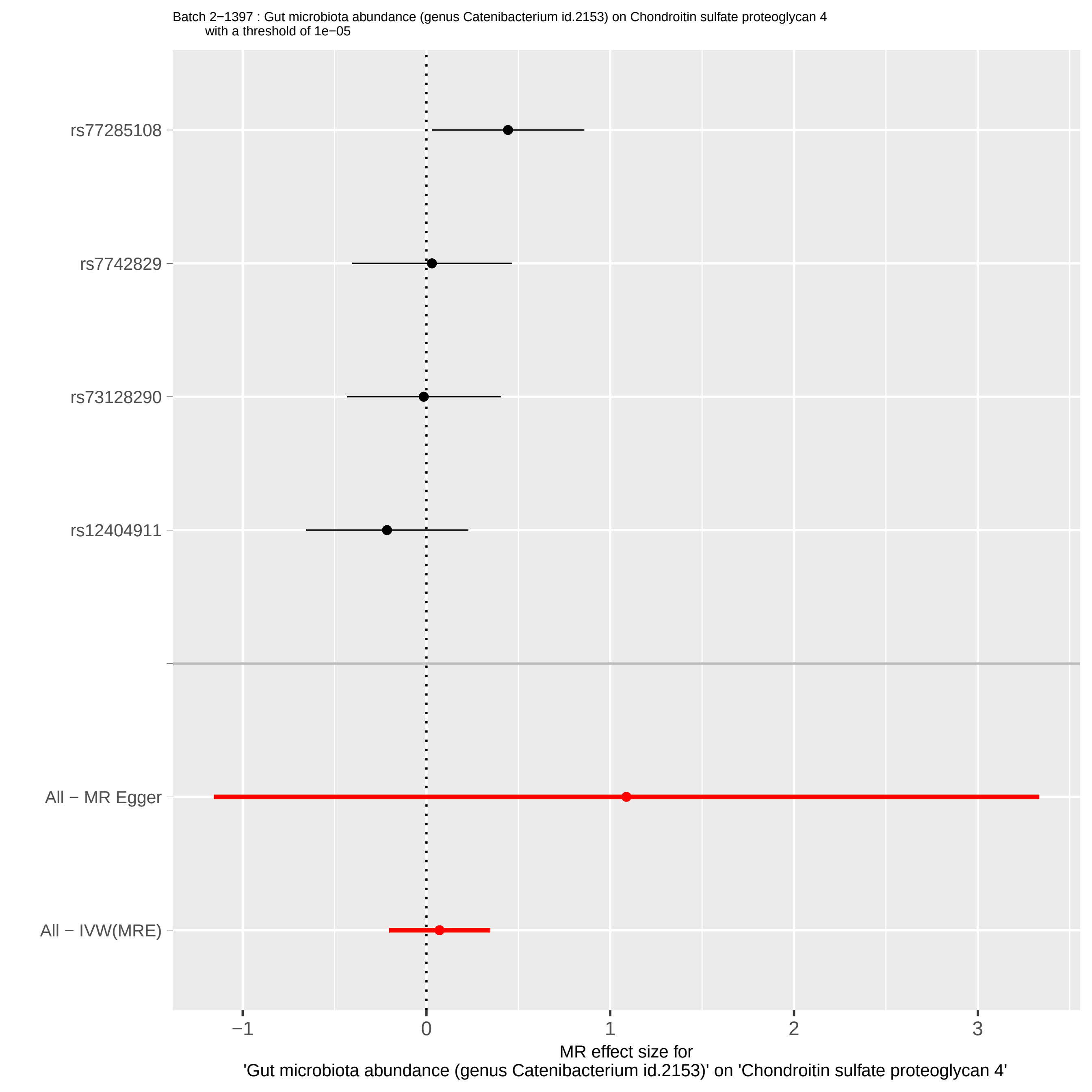

Batch 2−1397 : Gut microbiota abundance (genus Catenibacterium id.2153) on Chondroitin sulfate proteoglycan 4
with a threshold of 1e−05
rs77285108
rs7742829
rs73128290
rs12404911
All − MR Egger
All − IVW(MRE)
−1
0
1
2
3
MR effect size for
'Gut microbiota abundance (genus Catenibacterium id.2153)' on 'Chondroitin sulfate proteoglycan 4'

Batch 2−1398 : Gut microbiota abundance (genus Parasutterella id.2892) on Chondroitin sulfate proteoglycan 4
with a threshold of 1e−05

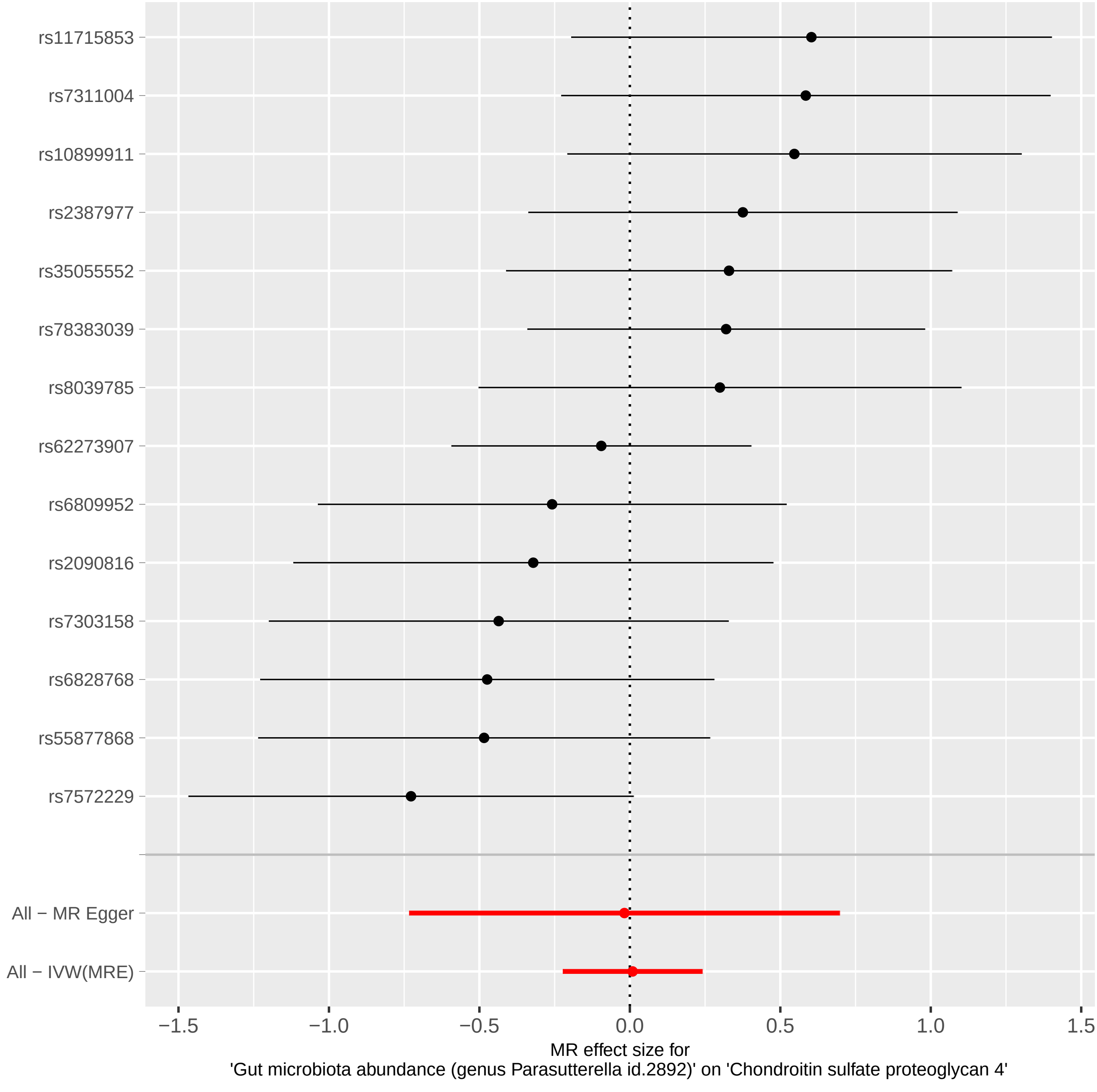

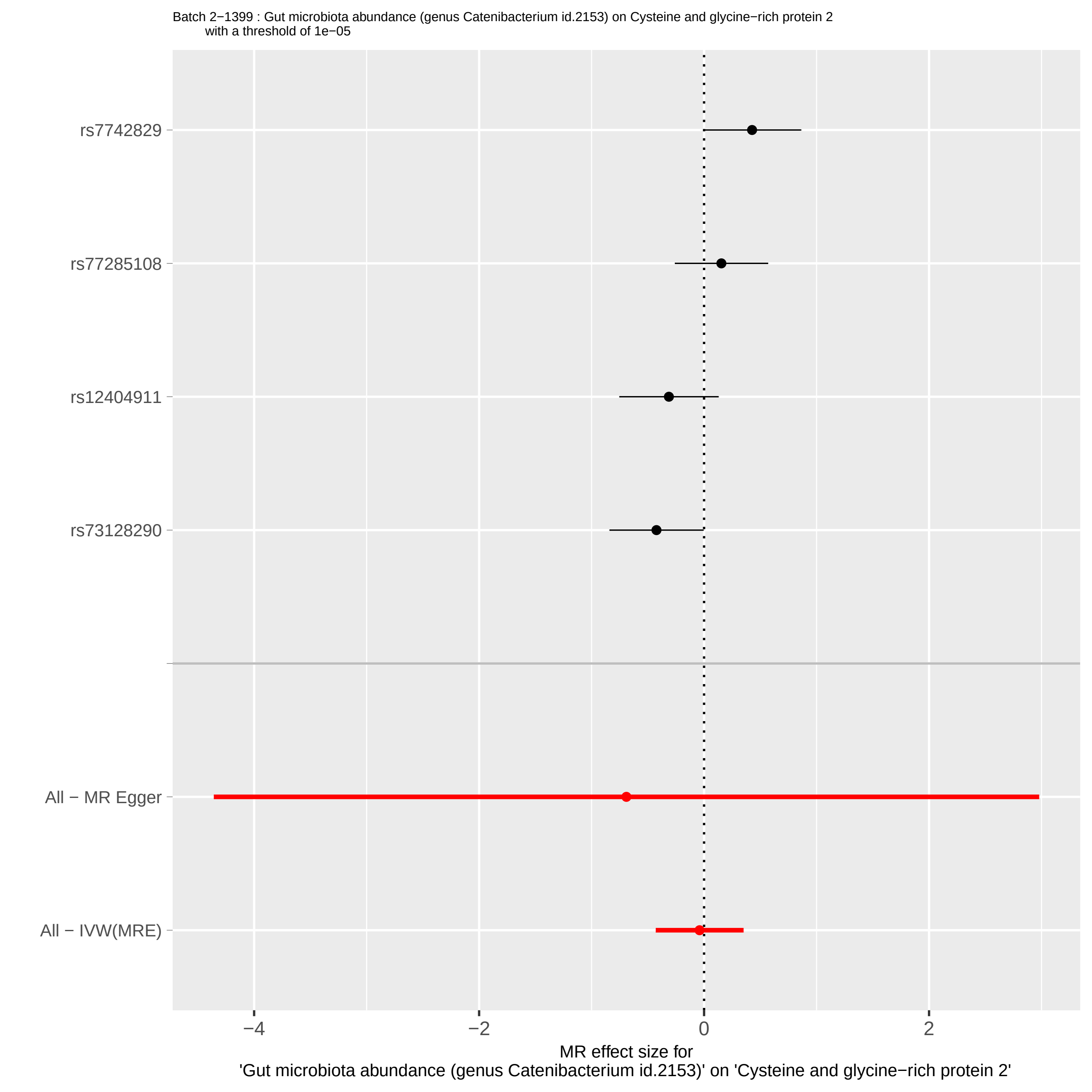
Batch 2−1399 : Gut microbiota abundance (genus Catenibacterium id.2153) on Cysteine and glycine−rich protein 2
with a threshold of 1e−05
rs7742829
rs77285108
rs12404911
rs73128290
All − MR Egger
All − IVW(MRE)
−4
−2
0
2
MR effect size for
'Gut microbiota abundance (genus Catenibacterium id.2153)' on 'Cysteine and glycine−rich protein 2'

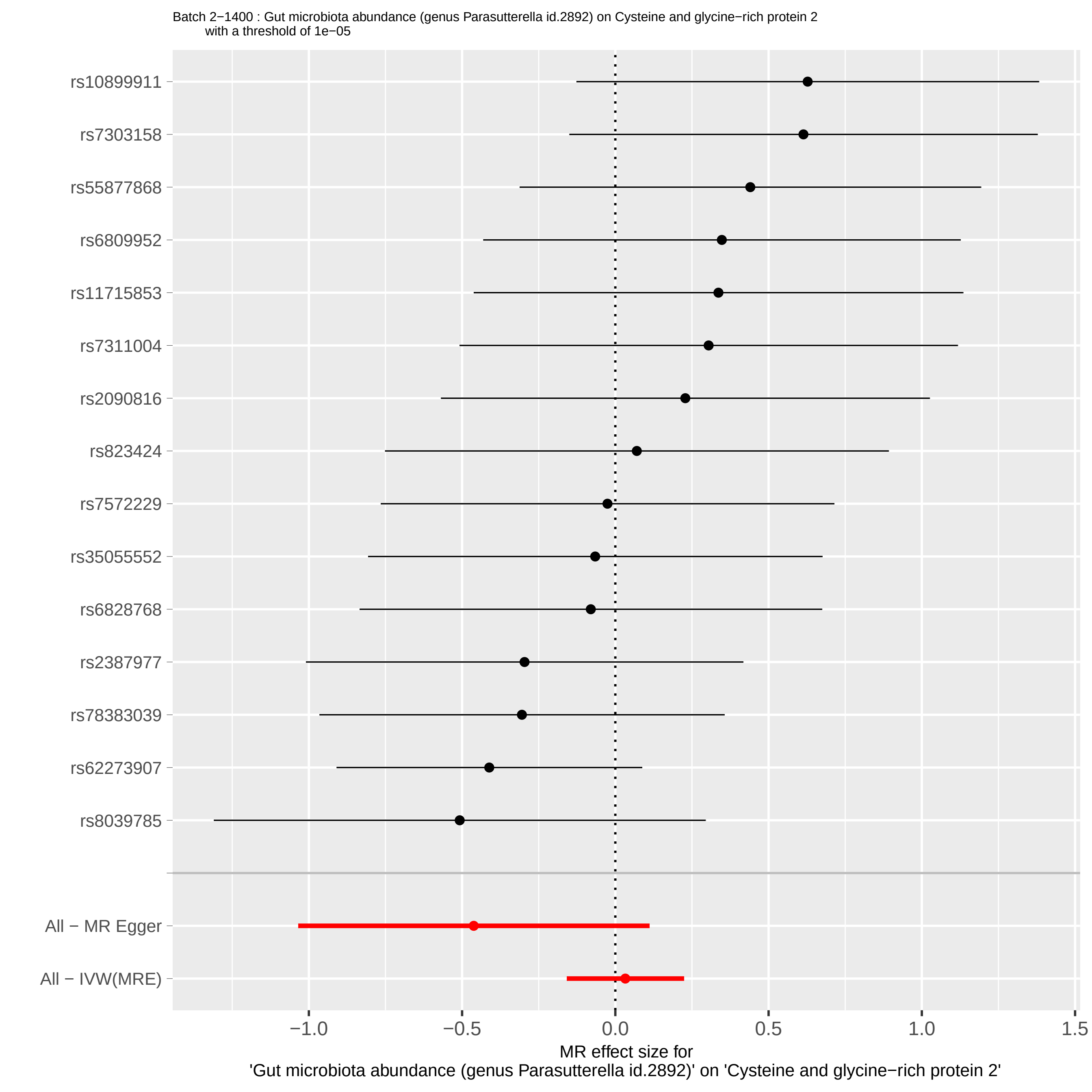
Batch 2−1400 : Gut microbiota abundance (genus Parasutterella id.2892) on Cysteine and glycine−rich protein 2
with a threshold of 1e−05
rs10899911
rs7303158
rs55877868
rs6809952
rs11715853
rs7311004
rs2090816
rs823424
rs7572229
rs35055552
rs6828768
rs2387977
rs78383039
rs62273907
rs8039785
All − MR Egger
All − IVW(MRE)
−1.0
−0.5
0.0
0.5
1.0
1.5
MR effect size for
'Gut microbiota abundance (genus Parasutterella id.2892)' on 'Cysteine and glycine−rich protein 2'

Batch 2−1401 : Gut microbiota abundance (genus Catenibacterium id.2153) on Cysteine and glycine−rich protein 3
with a threshold of 1e−05

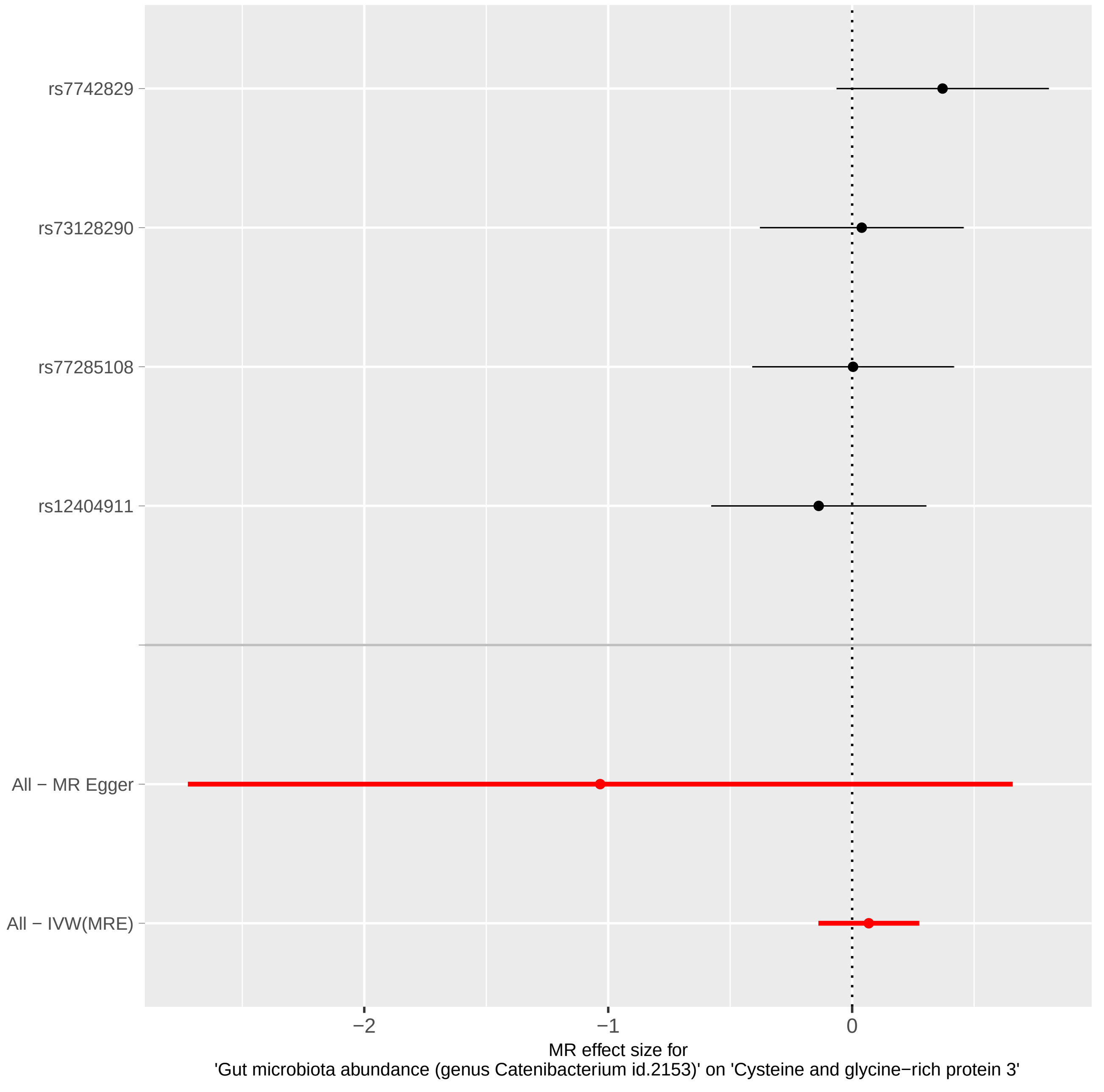

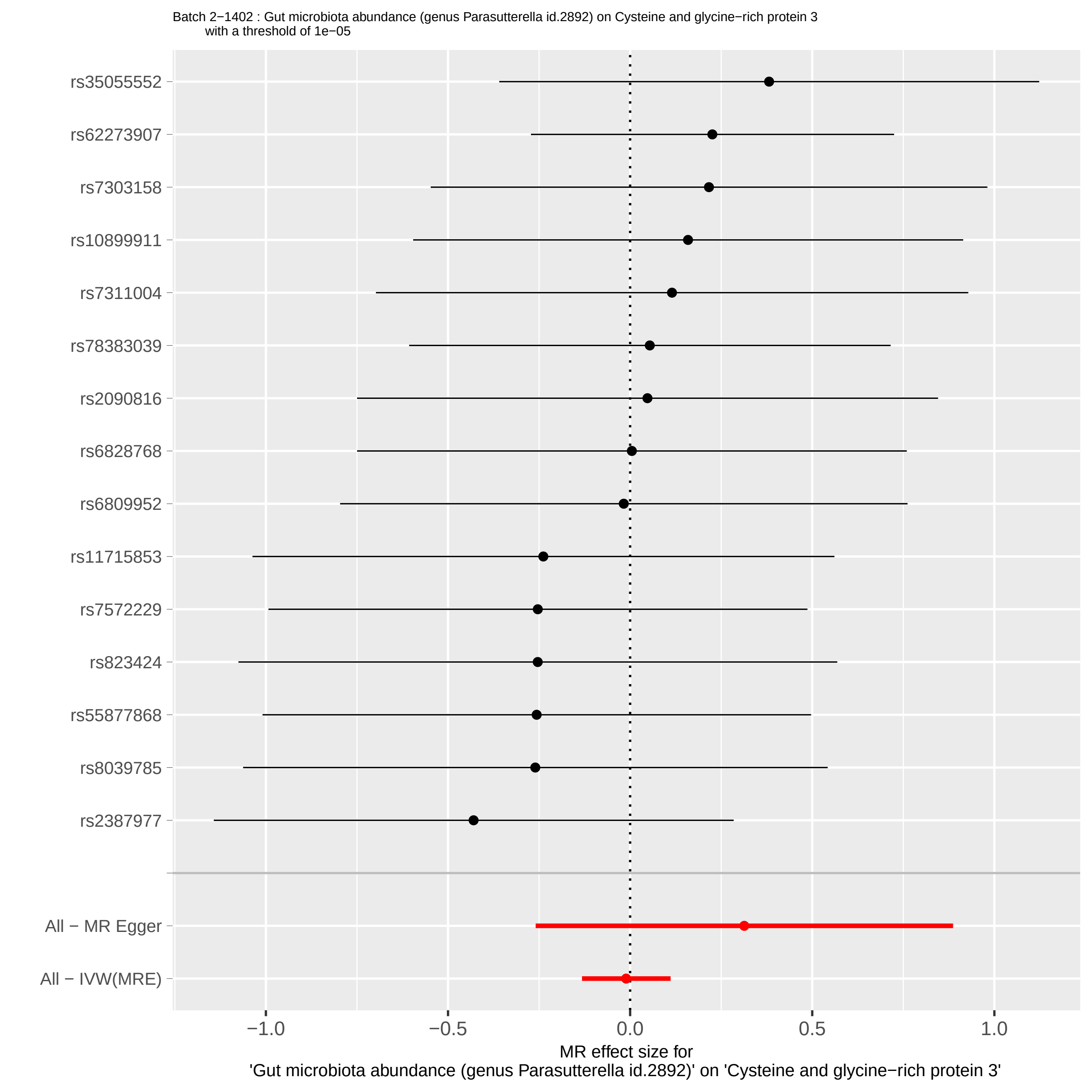

Batch 2−1402 : Gut microbiota abundance (genus Parasutterella id.2892) on Cysteine and glycine−rich protein 3
with a threshold of 1e−05
rs35055552
rs62273907
rs7303158
rs10899911
rs7311004
rs78383039
rs2090816
rs6828768
rs6809952
rs11715853
rs7572229
rs823424
rs55877868
rs8039785
rs2387977
All − MR Egger
All − IVW(MRE)
−1.0
−0.5
0.0
0.5
1.0
MR effect size for
'Gut microbiota abundance (genus Parasutterella id.2892)' on 'Cysteine and glycine−rich protein 3'

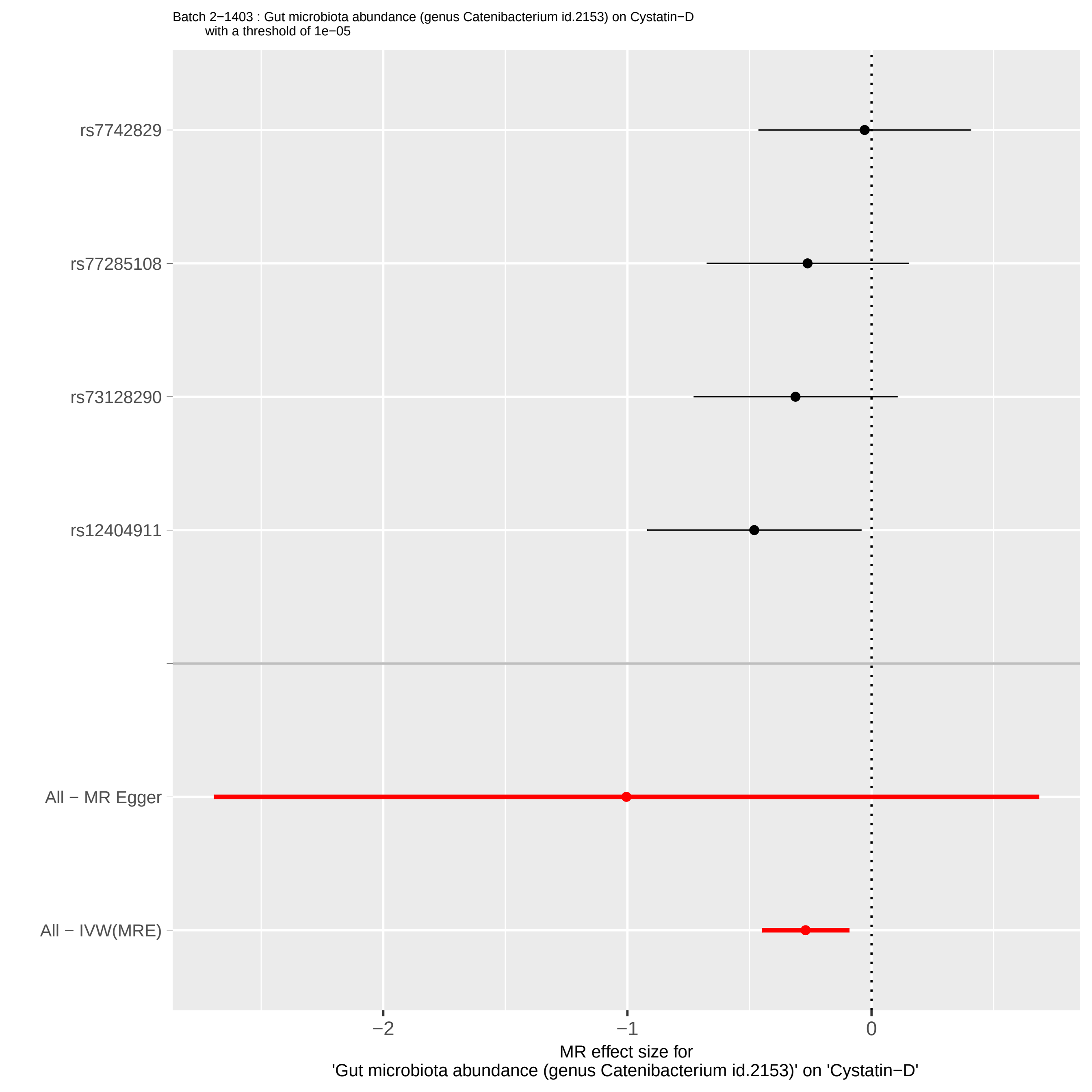

Batch 2−1403 : Gut microbiota abundance (genus Catenibacterium id.2153) on Cystatin−D
with a threshold of 1e−05
rs7742829
rs77285108
rs73128290
rs12404911
All − MR Egger
All − IVW(MRE)
−2
−1
0
MR effect size for
'Gut microbiota abundance (genus Catenibacterium id.2153)' on 'Cystatin−D'

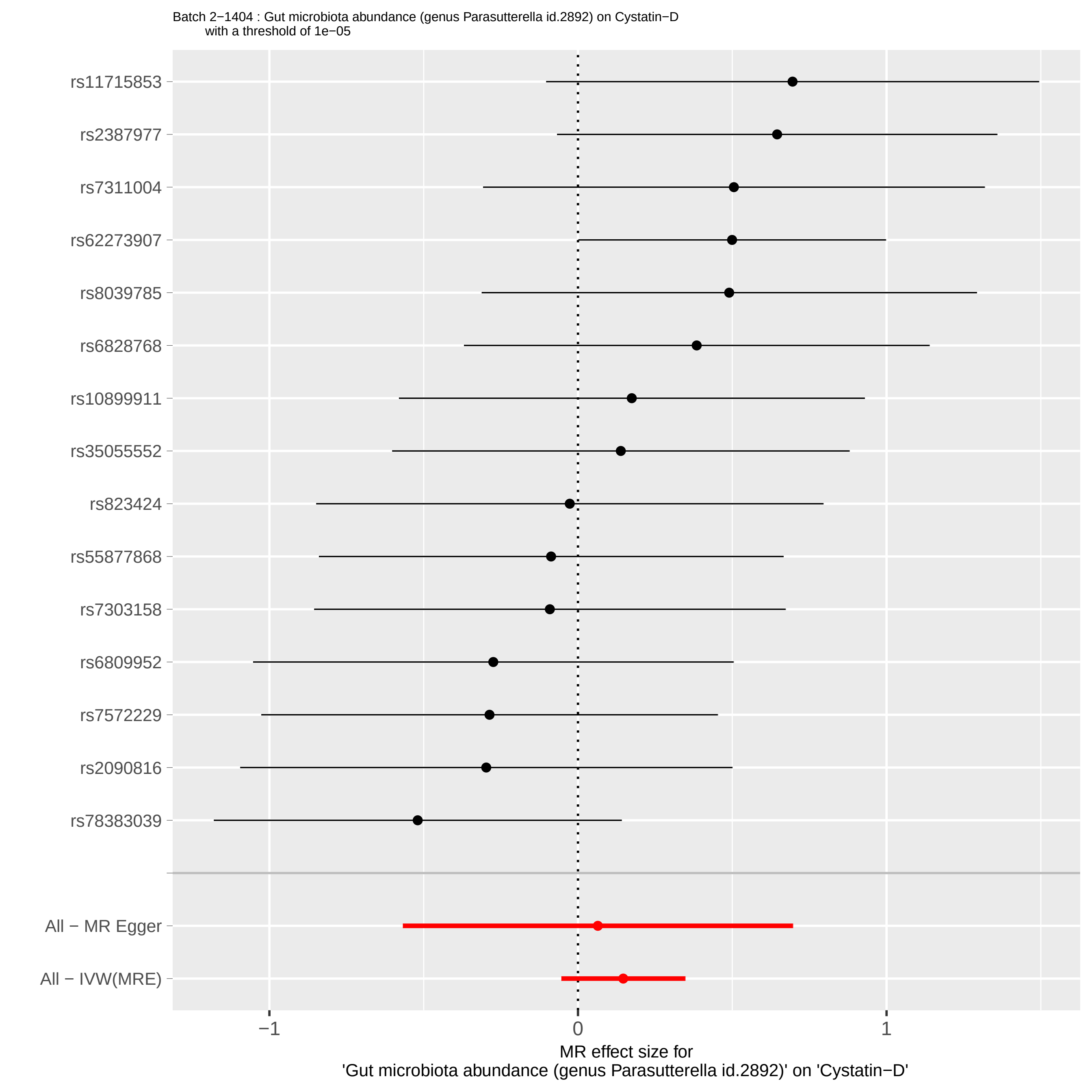

Batch 2−1404 : Gut microbiota abundance (genus Parasutterella id.2892) on Cystatin−D
with a threshold of 1e−05
rs11715853
rs2387977
rs7311004
rs62273907
rs8039785
rs6828768
rs10899911
rs35055552
rs823424
rs55877868
rs7303158
rs6809952
rs7572229
rs2090816
rs78383039
All − MR Egger
All − IVW(MRE)
−1
0
1
MR effect size for
'Gut microbiota abundance (genus Parasutterella id.2892)' on 'Cystatin−D'

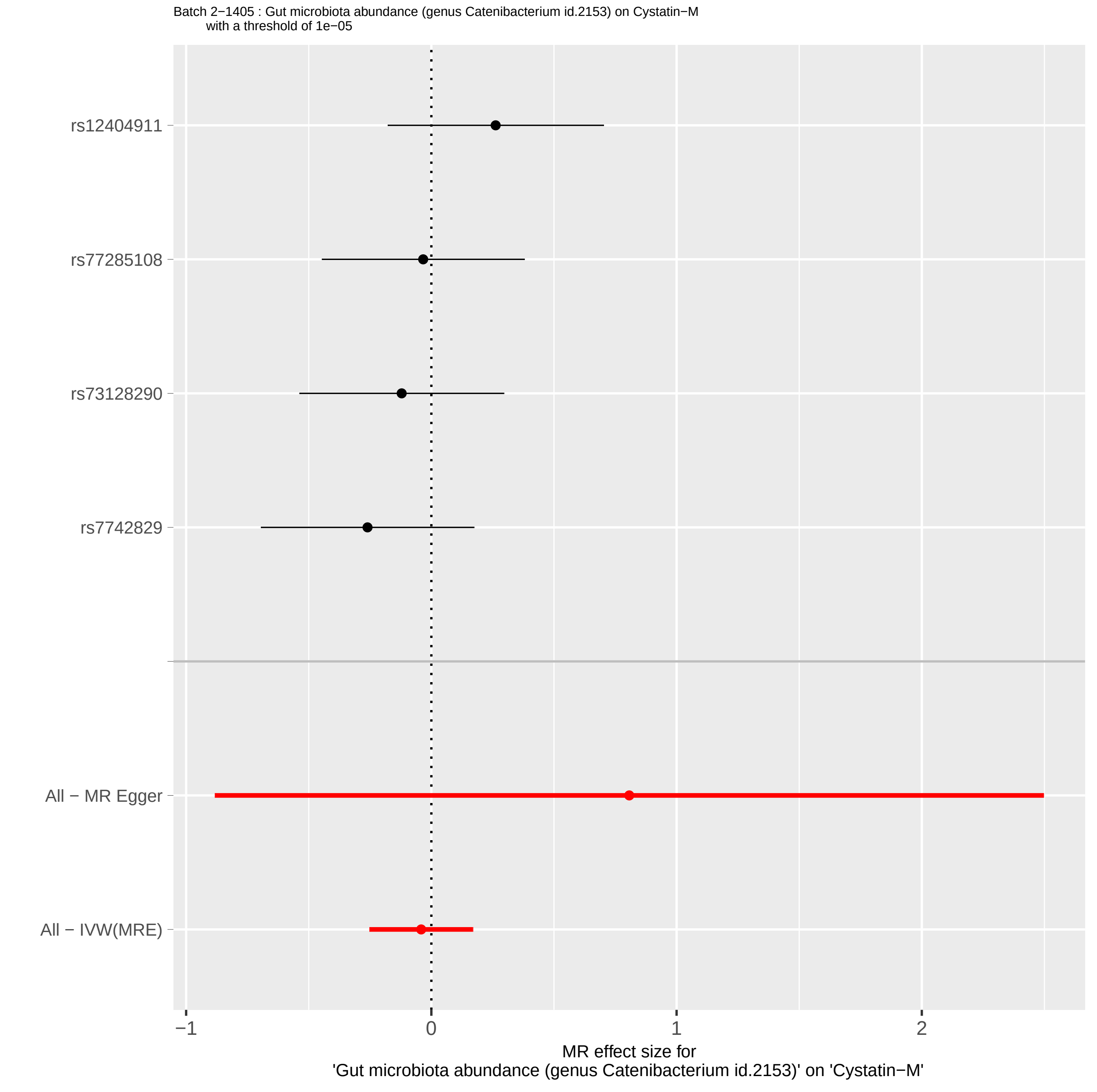
Batch 2−1405 : Gut microbiota abundance (genus Catenibacterium id.2153) on Cystatin−M
with a threshold of 1e−05
rs12404911
rs77285108
rs73128290
rs7742829
All − MR Egger
All − IVW(MRE)
−1
0
1
2
MR effect size for
'Gut microbiota abundance (genus Catenibacterium id.2153)' on 'Cystatin−M'

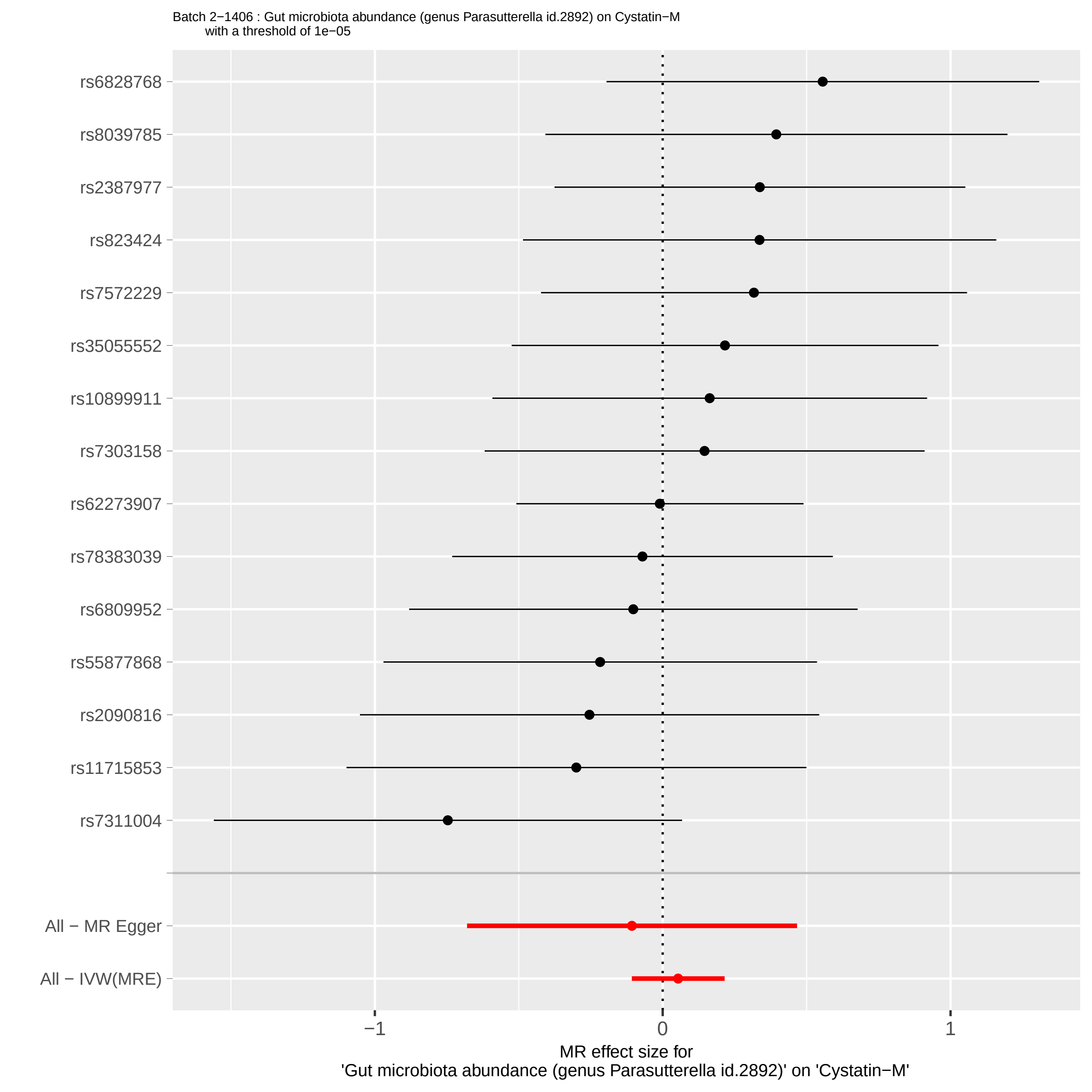

Batch 2−1406 : Gut microbiota abundance (genus Parasutterella id.2892) on Cystatin−M
with a threshold of 1e−05
rs6828768
rs8039785
rs2387977
rs823424
rs7572229
rs35055552
rs10899911
rs7303158
rs62273907
rs78383039
rs6809952
rs55877868
rs2090816
rs11715853
rs7311004
All − MR Egger
All − IVW(MRE)
−1
0
1
MR effect size for
'Gut microbiota abundance (genus Parasutterella id.2892)' on 'Cystatin−M'

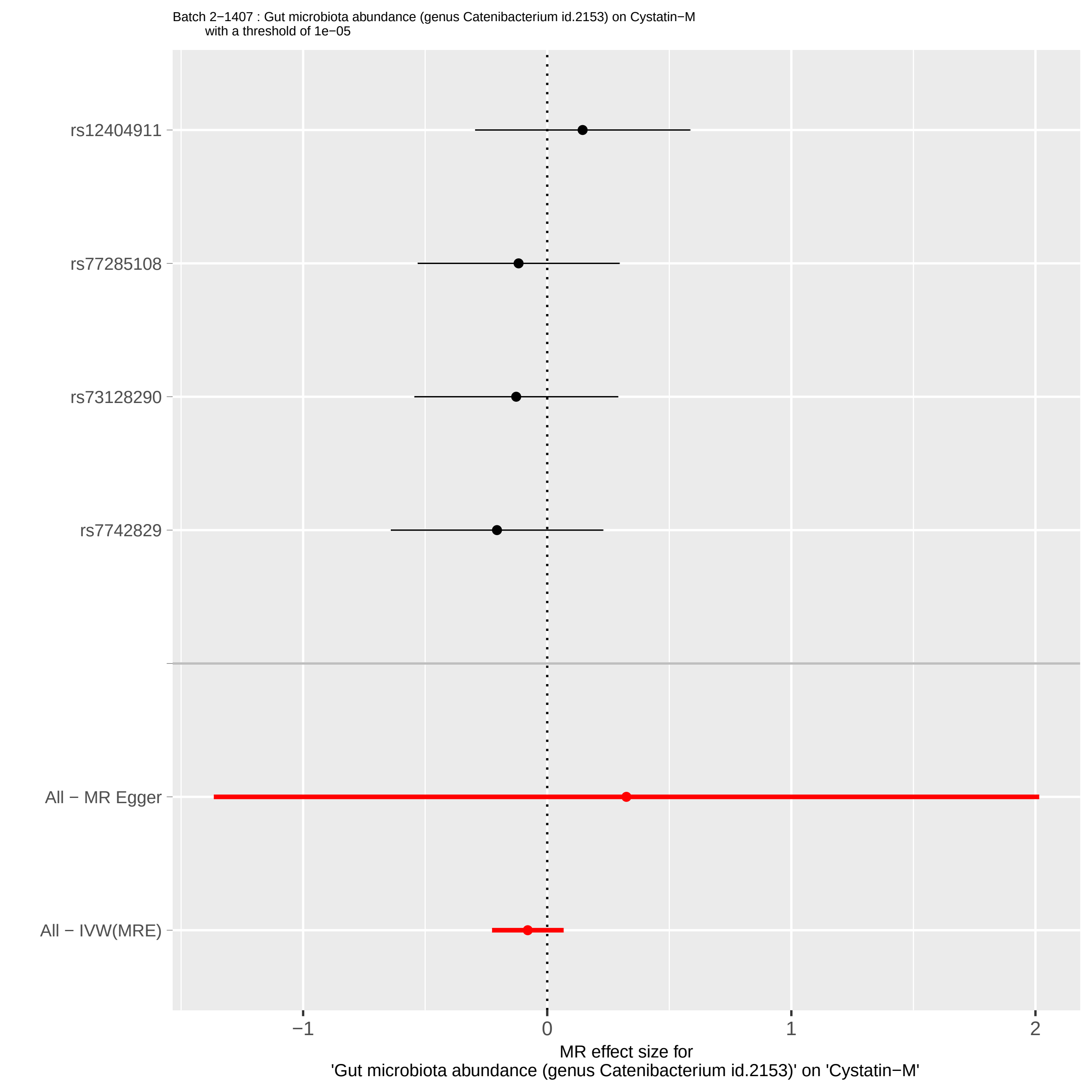
Batch 2−1407 : Gut microbiota abundance (genus Catenibacterium id.2153) on Cystatin−M
with a threshold of 1e−05
rs12404911
rs77285108
rs73128290
rs7742829
All − MR Egger
All − IVW(MRE)
−1
0
1
2
MR effect size for
'Gut microbiota abundance (genus Catenibacterium id.2153)' on 'Cystatin−M'

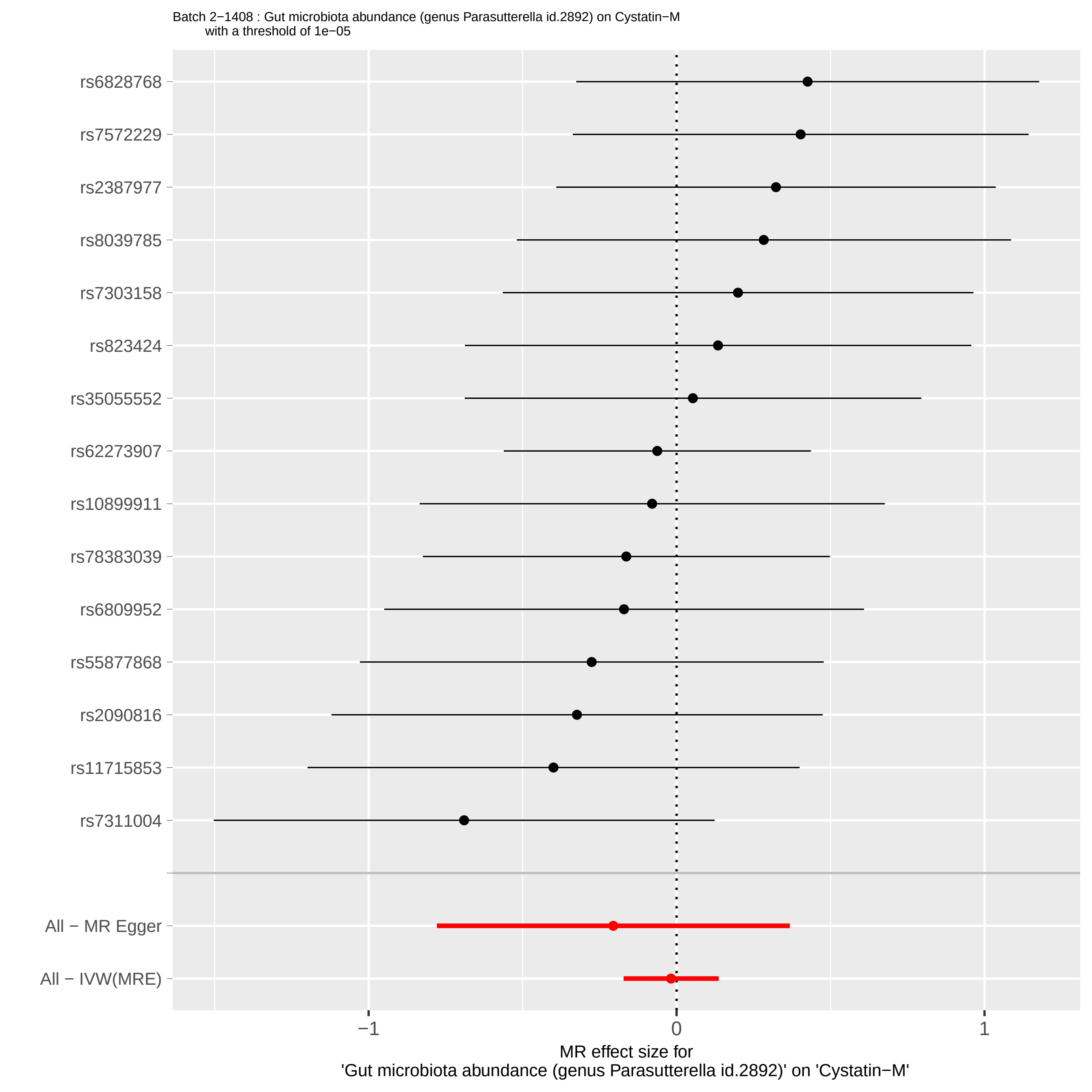

Batch 2−1408 : Gut microbiota abundance (genus Parasutterella id.2892) on Cystatin−M
with a threshold of 1e−05
rs6828768
rs7572229
rs2387977
rs8039785
rs7303158
rs823424
rs35055552
rs62273907
rs10899911
rs78383039
rs6809952
rs55877868
rs2090816
rs11715853
rs7311004
All − MR Egger
All − IVW(MRE)
−1
0
1
MR effect size for
'Gut microbiota abundance (genus Parasutterella id.2892)' on 'Cystatin−M'

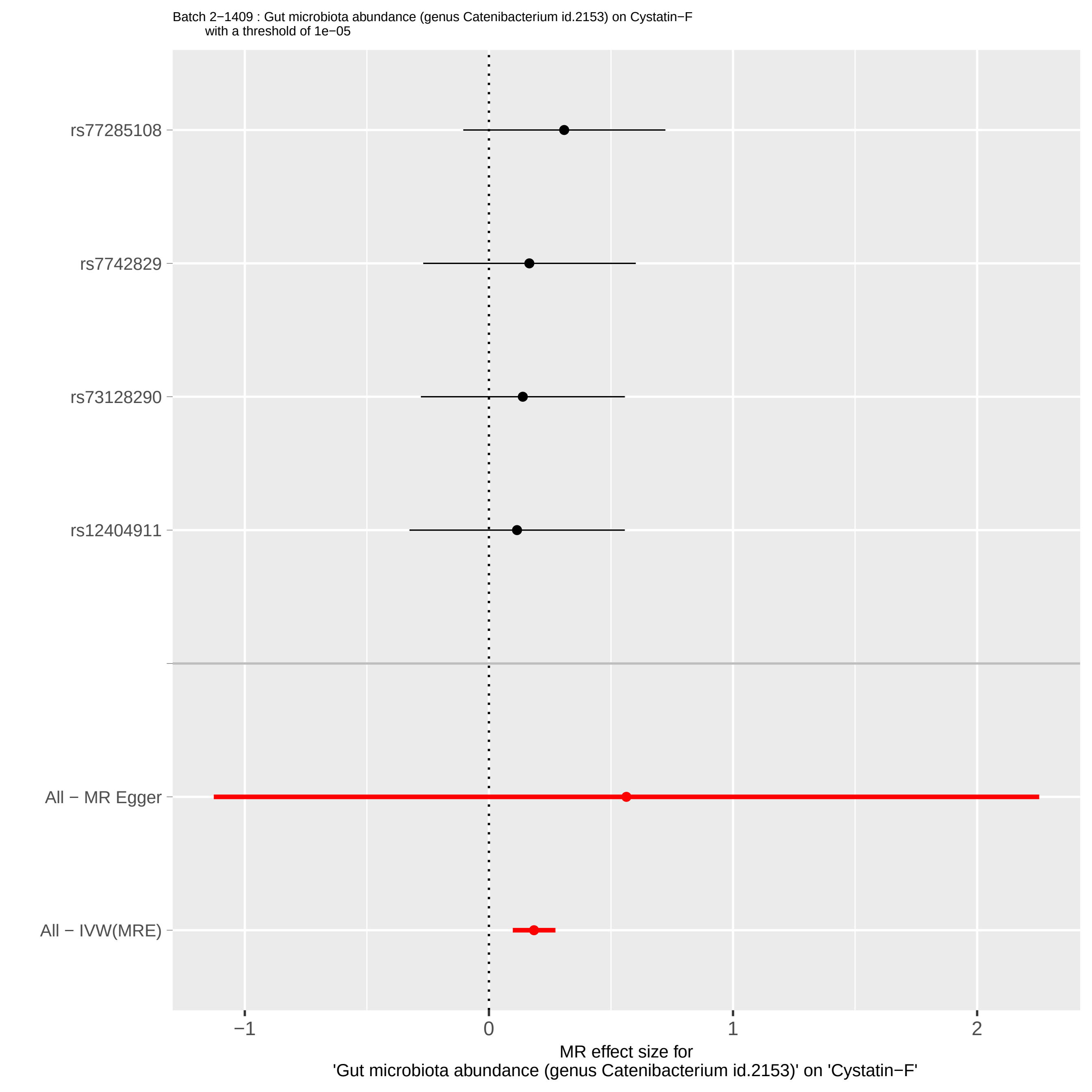
Batch 2−1409 : Gut microbiota abundance (genus Catenibacterium id.2153) on Cystatin−F
with a threshold of 1e−05
rs77285108
rs7742829
rs73128290
rs12404911
All − MR Egger
All − IVW(MRE)
−1
0
1
2
MR effect size for
'Gut microbiota abundance (genus Catenibacterium id.2153)' on 'Cystatin−F'

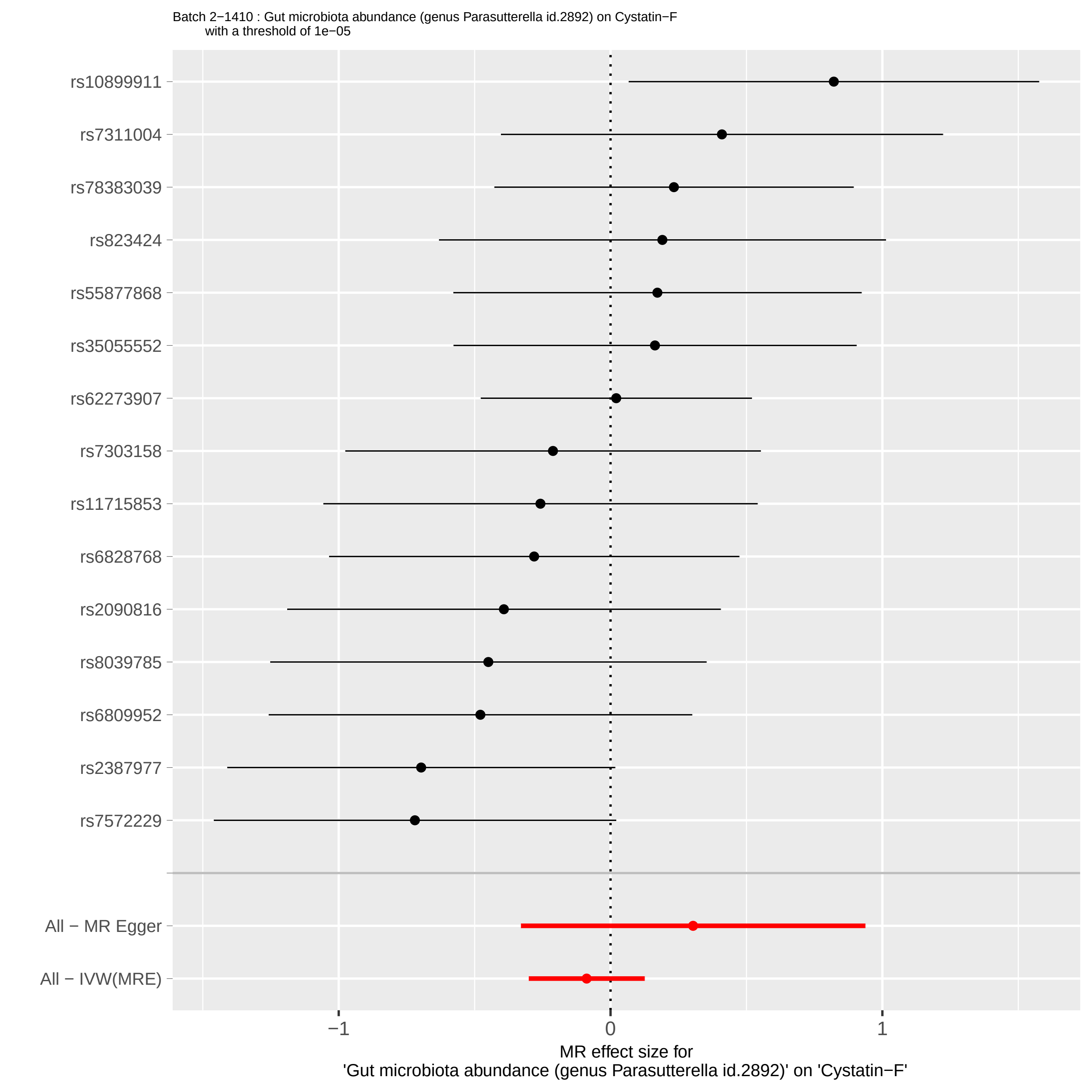

Batch 2−1410 : Gut microbiota abundance (genus Parasutterella id.2892) on Cystatin−F
with a threshold of 1e−05
rs10899911
rs7311004
rs78383039
rs823424
rs55877868
rs35055552
rs62273907
rs7303158
rs11715853
rs6828768
rs2090816
rs8039785
rs6809952
rs2387977
rs7572229
All − MR Egger
All − IVW(MRE)
−1
0
1
MR effect size for
'Gut microbiota abundance (genus Parasutterella id.2892)' on 'Cystatin−F'

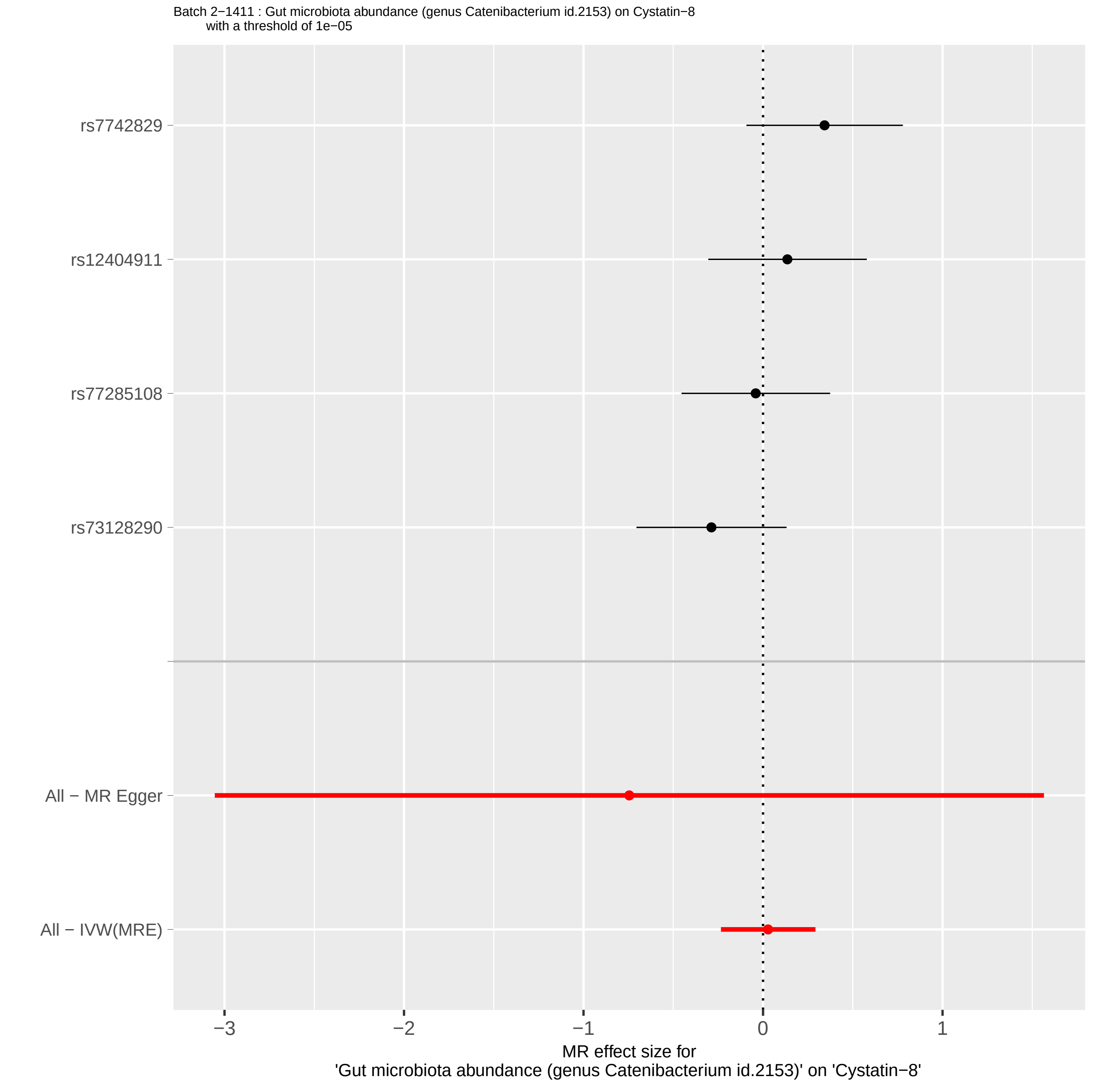
Batch 2−1411 : Gut microbiota abundance (genus Catenibacterium id.2153) on Cystatin−8
with a threshold of 1e−05
rs7742829
rs12404911
rs77285108
rs73128290
All − MR Egger
All − IVW(MRE)
−3
−2
−1
0
1
MR effect size for
'Gut microbiota abundance (genus Catenibacterium id.2153)' on 'Cystatin−8'

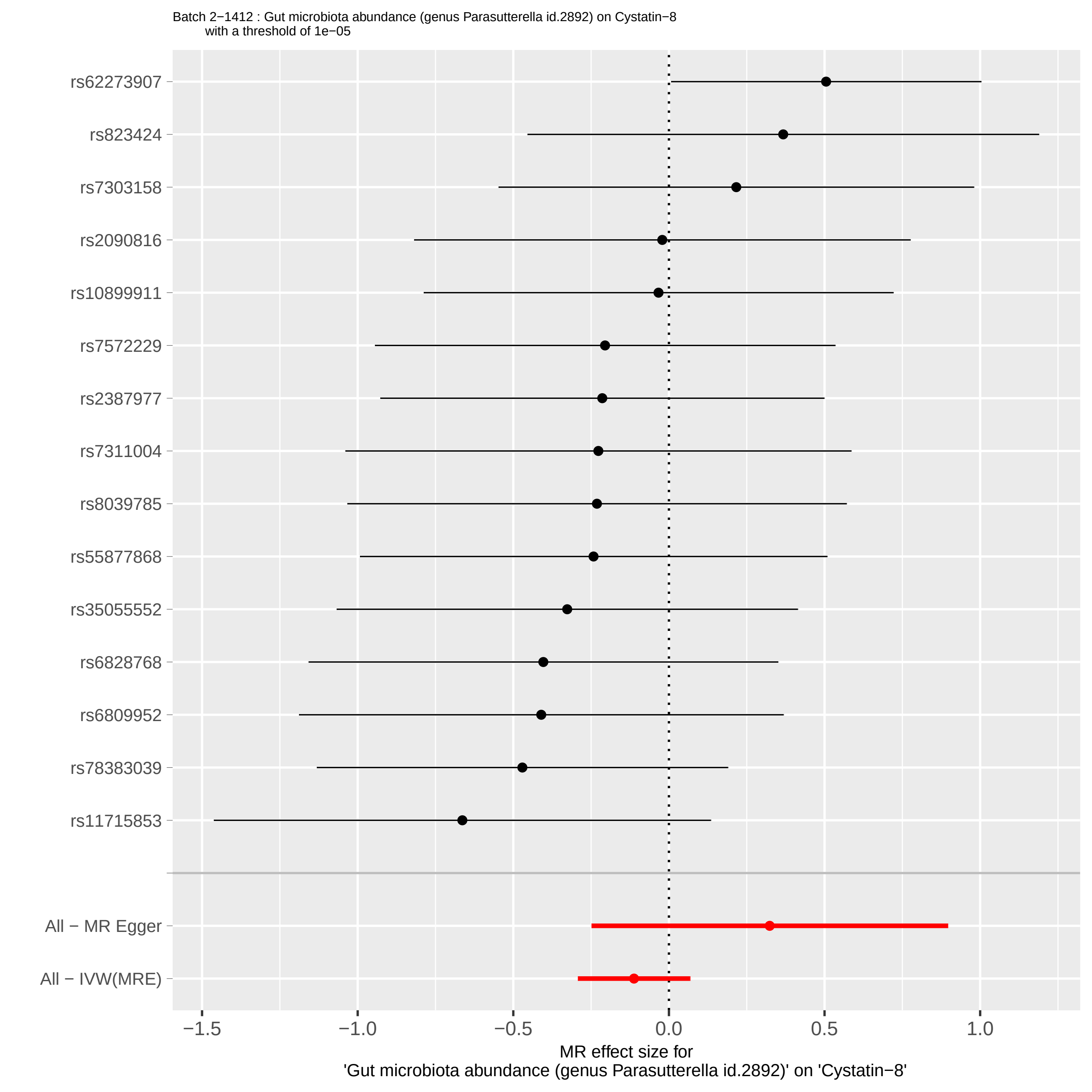

Batch 2−1412 : Gut microbiota abundance (genus Parasutterella id.2892) on Cystatin−8
with a threshold of 1e−05
rs62273907
rs823424
rs7303158
rs2090816
rs10899911
rs7572229
rs2387977
rs7311004
rs8039785
rs55877868
rs35055552
rs6828768
rs6809952
rs78383039
rs11715853
All − MR Egger
All − IVW(MRE)
−1.5
−1.0
−0.5
0.0
0.5
1.0
MR effect size for
'Gut microbiota abundance (genus Parasutterella id.2892)' on 'Cystatin−8'

Batch 2−1413 : Gut microbiota abundance (genus Catenibacterium id.2153) on Cancer/testis antigen 55
with a threshold of 1e−05

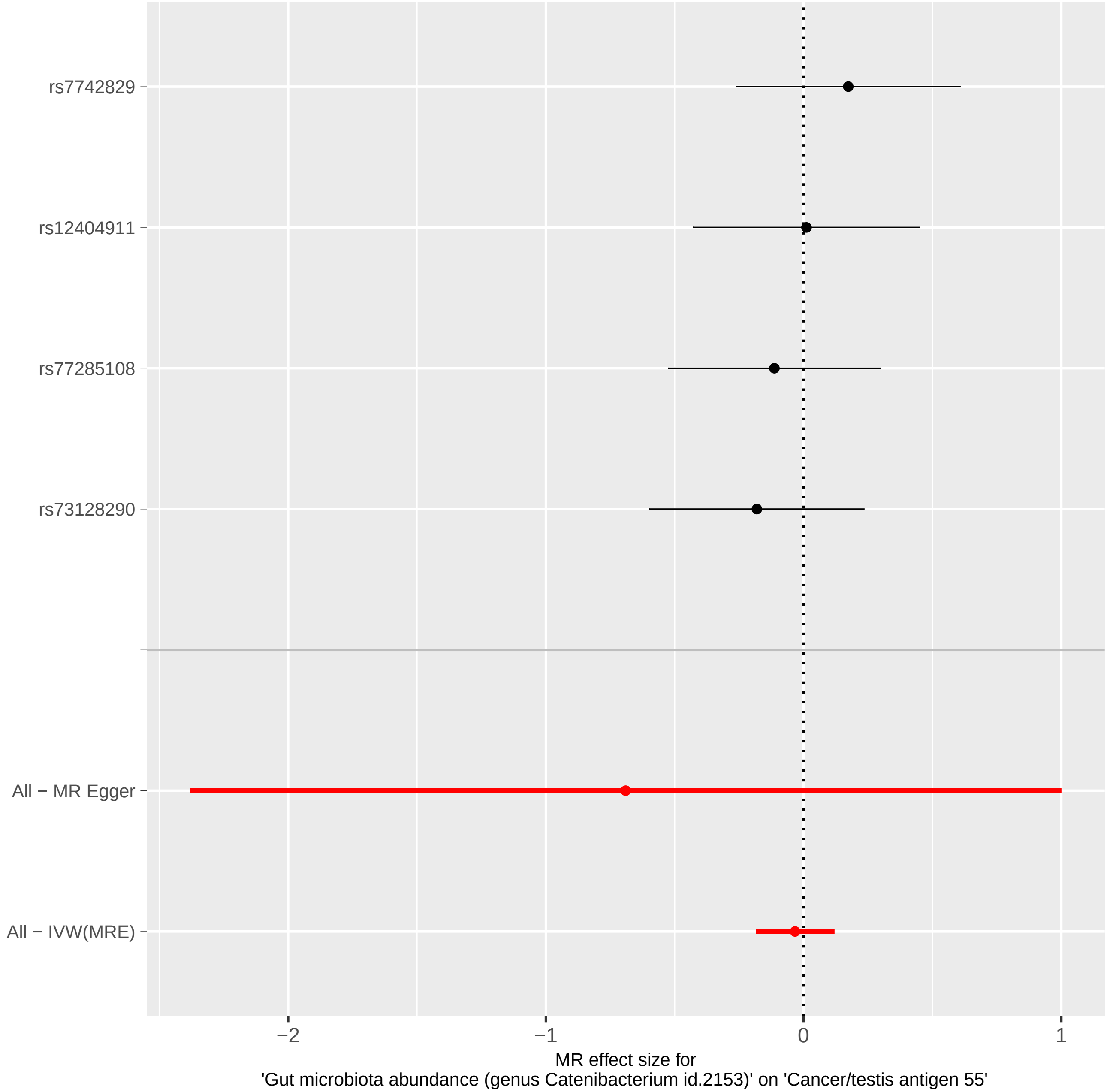

Batch 2−1414 : Gut microbiota abundance (genus Parasutterella id.2892) on Cancer/testis antigen 55
with a threshold of 1e−05

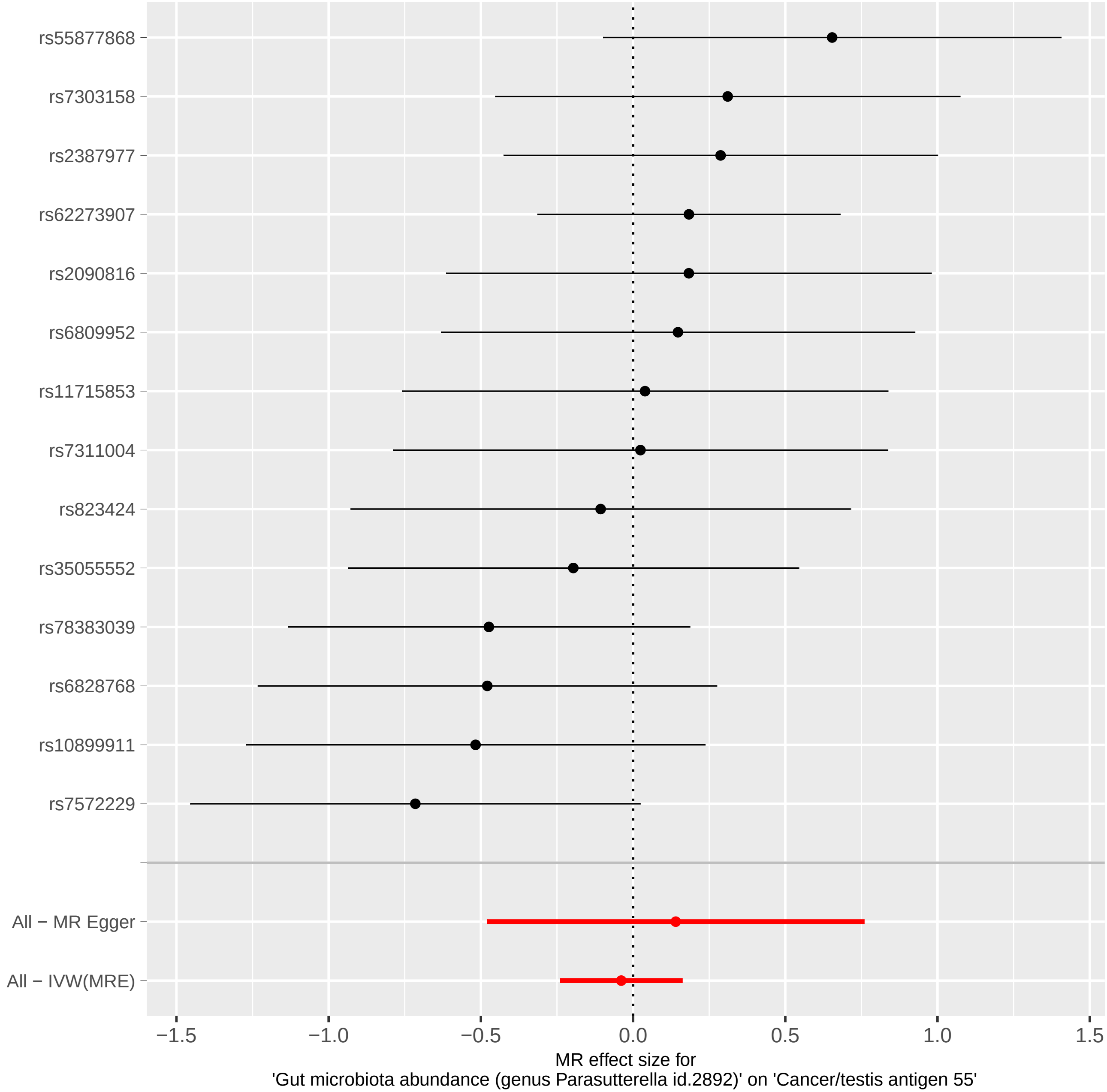

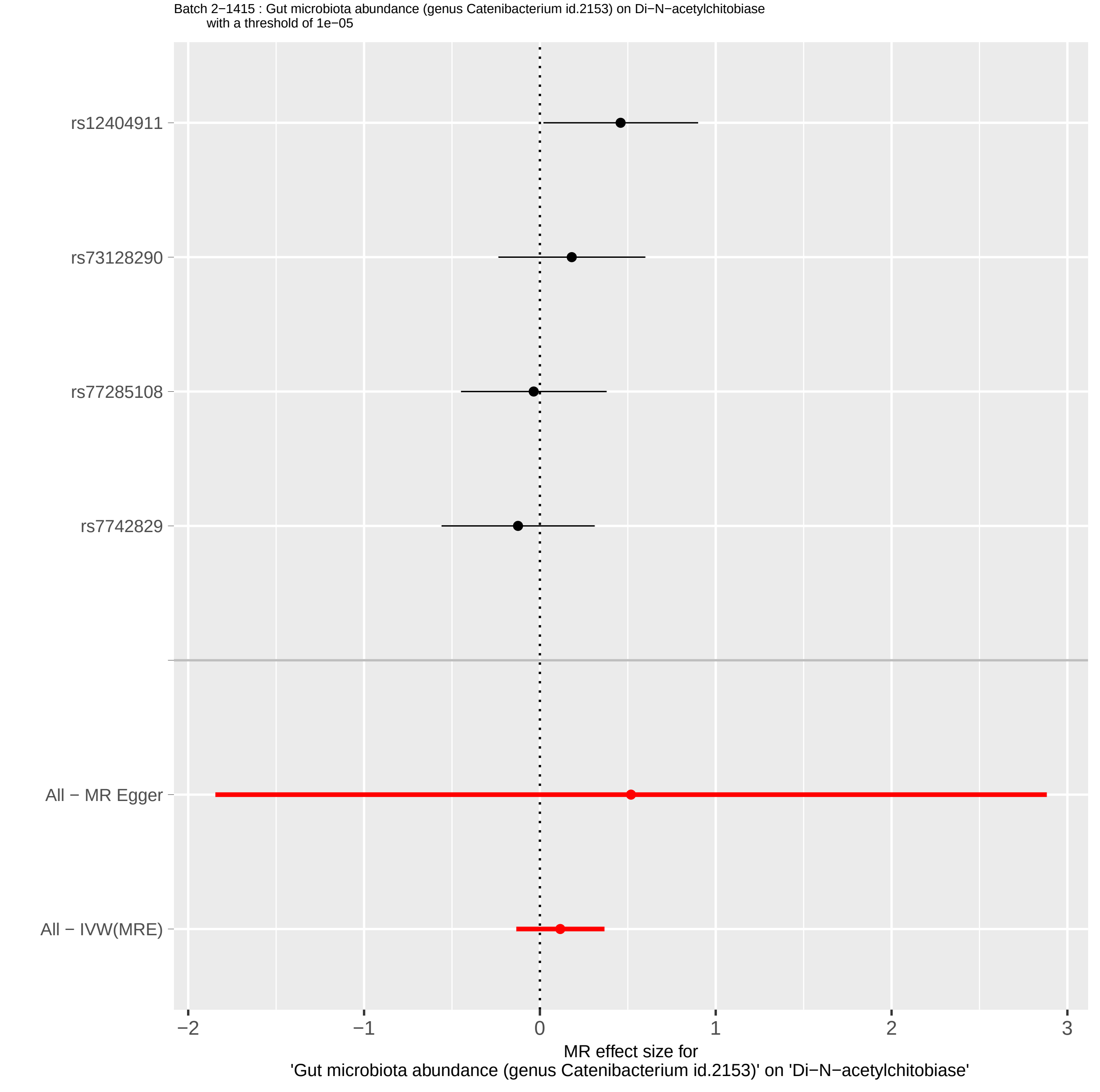

Batch 2−1415 : Gut microbiota abundance (genus Catenibacterium id.2153) on Di−N−acetylchitobiase
with a threshold of 1e−05
rs12404911
rs73128290
rs77285108
rs7742829
All − MR Egger
All − IVW(MRE)
−2
−1
0
1
2
3
MR effect size for
'Gut microbiota abundance (genus Catenibacterium id.2153)' on 'Di−N−acetylchitobiase'

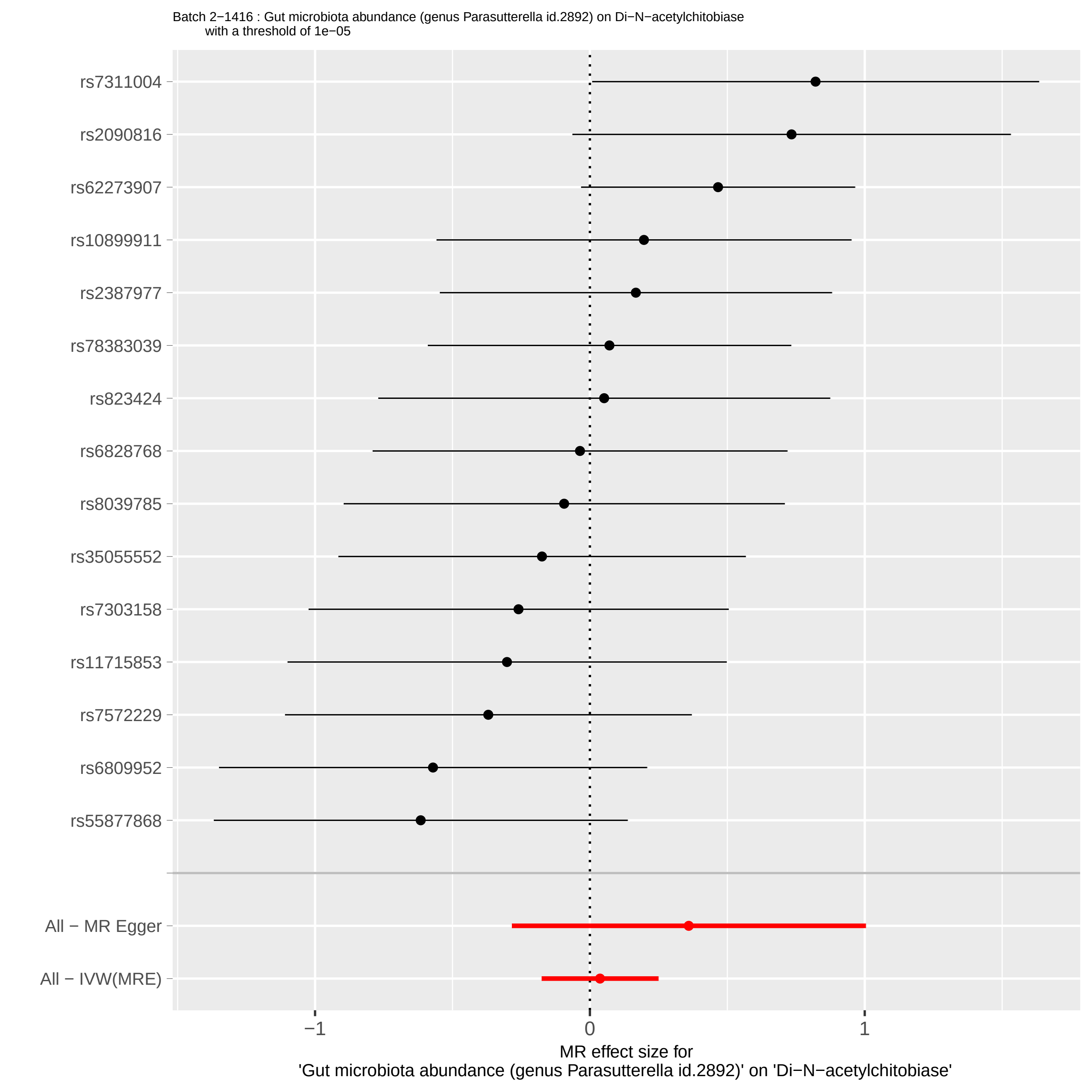
Batch 2−1416 : Gut microbiota abundance (genus Parasutterella id.2892) on Di−N−acetylchitobiase
with a threshold of 1e−05
rs7311004
rs2090816
rs62273907
rs10899911
rs2387977
rs78383039
rs823424
rs6828768
rs8039785
rs35055552
rs7303158
rs11715853
rs7572229
rs6809952
rs55877868
All − MR Egger
All − IVW(MRE)
−1
0
1
MR effect size for
'Gut microbiota abundance (genus Parasutterella id.2892)' on 'Di−N−acetylchitobiase'

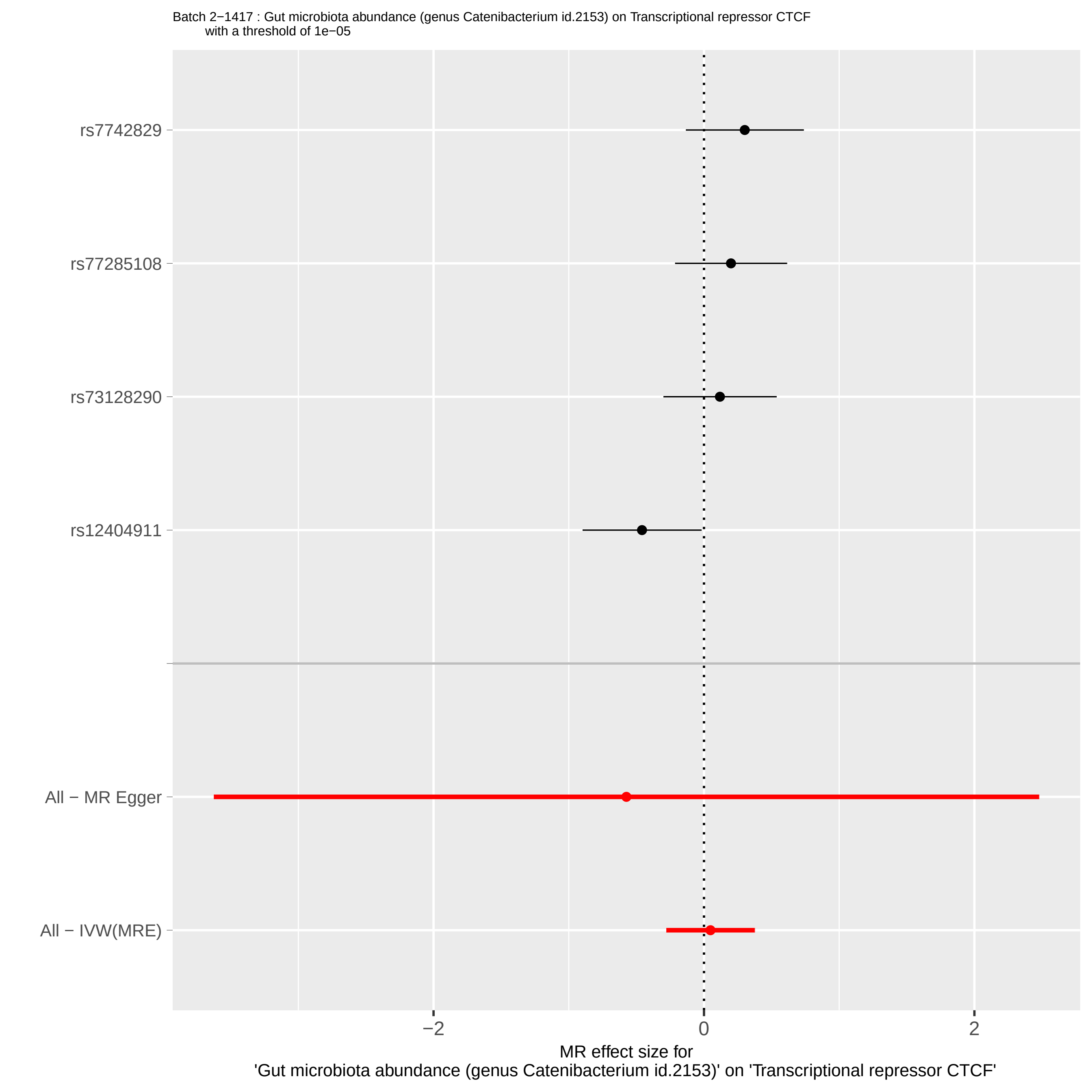
Batch 2−1417 : Gut microbiota abundance (genus Catenibacterium id.2153) on Transcriptional repressor CTCF
with a threshold of 1e−05
rs7742829
rs77285108
rs73128290
rs12404911
All − MR Egger
All − IVW(MRE)
−2
0
2
MR effect size for
'Gut microbiota abundance (genus Catenibacterium id.2153)' on 'Transcriptional repressor CTCF'

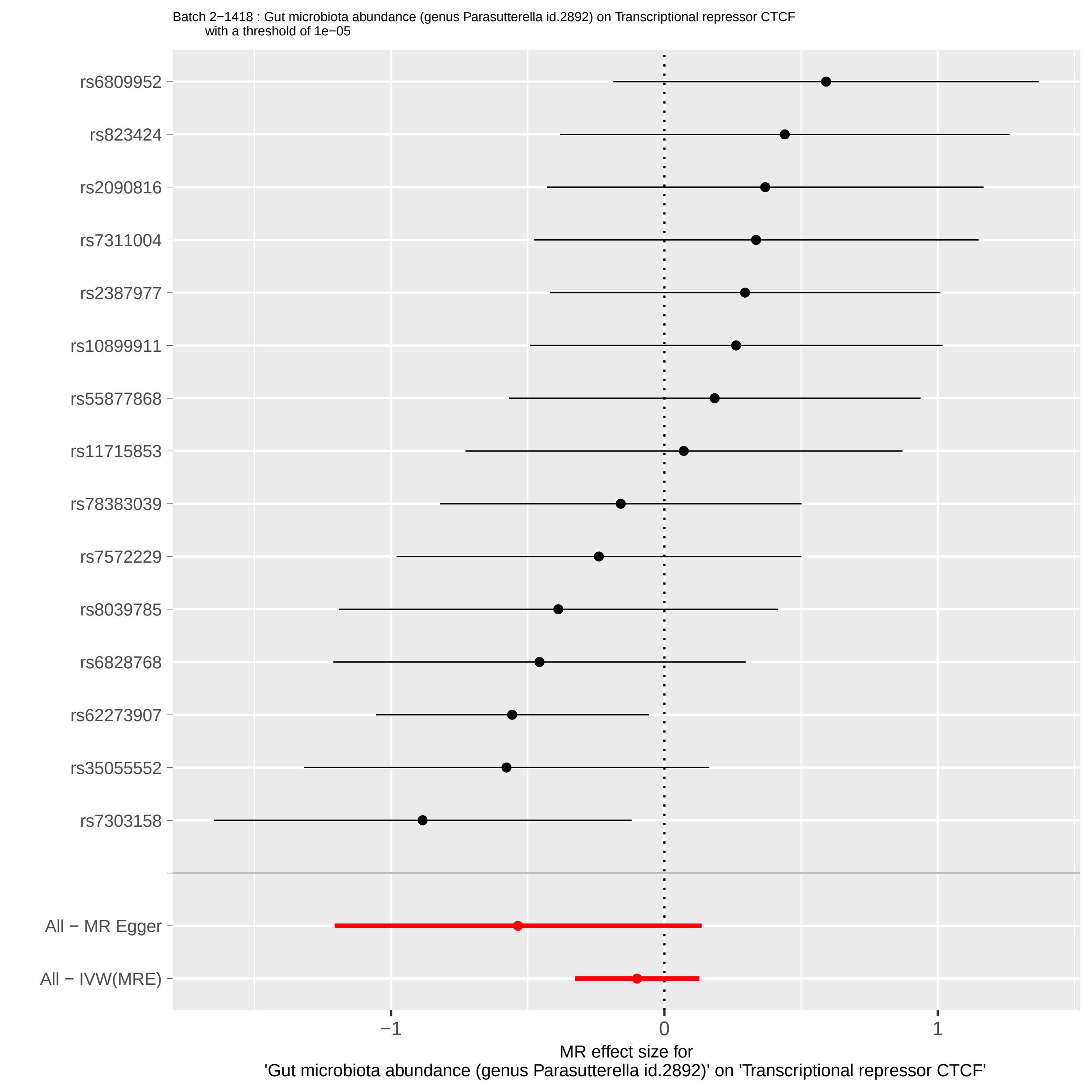

Batch 2−1418 : Gut microbiota abundance (genus Parasutterella id.2892) on Transcriptional repressor CTCF
with a threshold of 1e−05
rs6809952
rs823424
rs2090816
rs7311004
rs2387977
rs10899911
rs55877868
rs11715853
rs78383039
rs7572229
rs8039785
rs6828768
rs62273907
rs35055552
rs7303158
All − MR Egger
All − IVW(MRE)
−1
0
1
MR effect size for
'Gut microbiota abundance (genus Parasutterella id.2892)' on 'Transcriptional repressor CTCF'

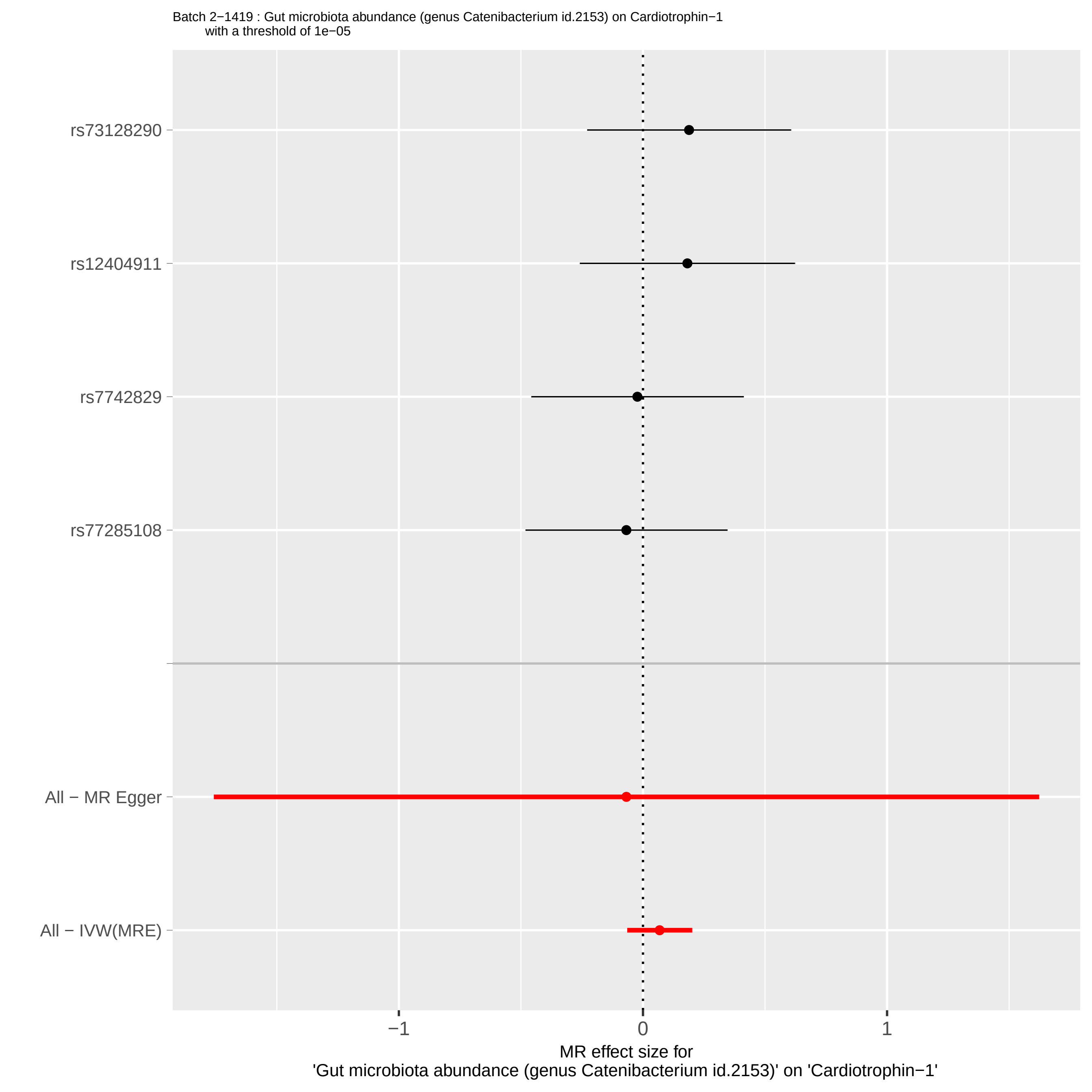

Batch 2−1419 : Gut microbiota abundance (genus Catenibacterium id.2153) on Cardiotrophin−1
with a threshold of 1e−05
rs73128290
rs12404911
rs7742829
rs77285108
All − MR Egger
All − IVW(MRE)
−1
0
1
MR effect size for
'Gut microbiota abundance (genus Catenibacterium id.2153)' on 'Cardiotrophin−1'

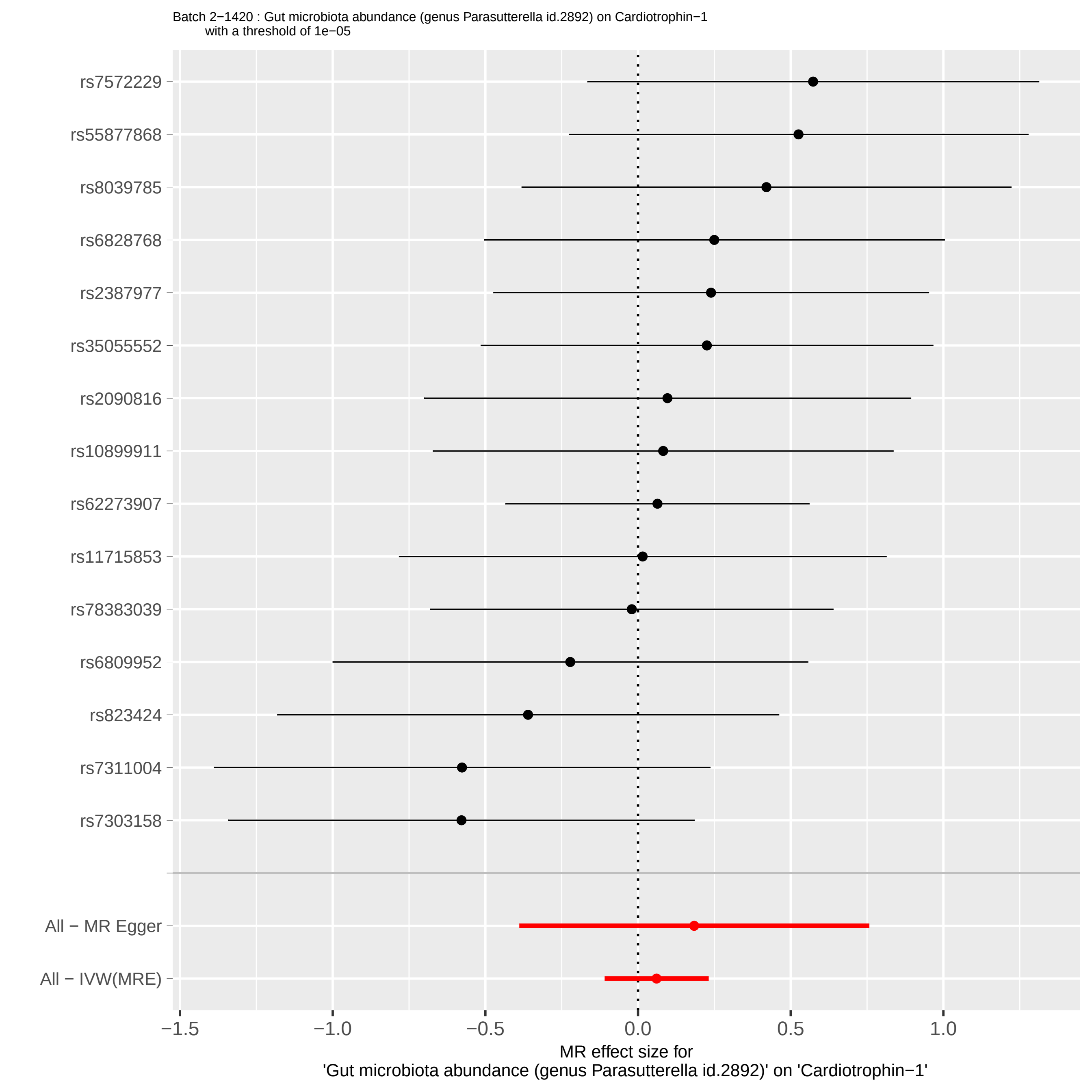

Batch 2−1420 : Gut microbiota abundance (genus Parasutterella id.2892) on Cardiotrophin−1
with a threshold of 1e−05
rs7572229
rs55877868
rs8039785
rs6828768
rs2387977
rs35055552
rs2090816
rs10899911
rs62273907
rs11715853
rs78383039
rs6809952
rs823424
rs7311004
rs7303158
All − MR Egger
All − IVW(MRE)
−1.5
−1.0
−0.5
0.0
0.5
1.0
MR effect size for
'Gut microbiota abundance (genus Parasutterella id.2892)' on 'Cardiotrophin−1'

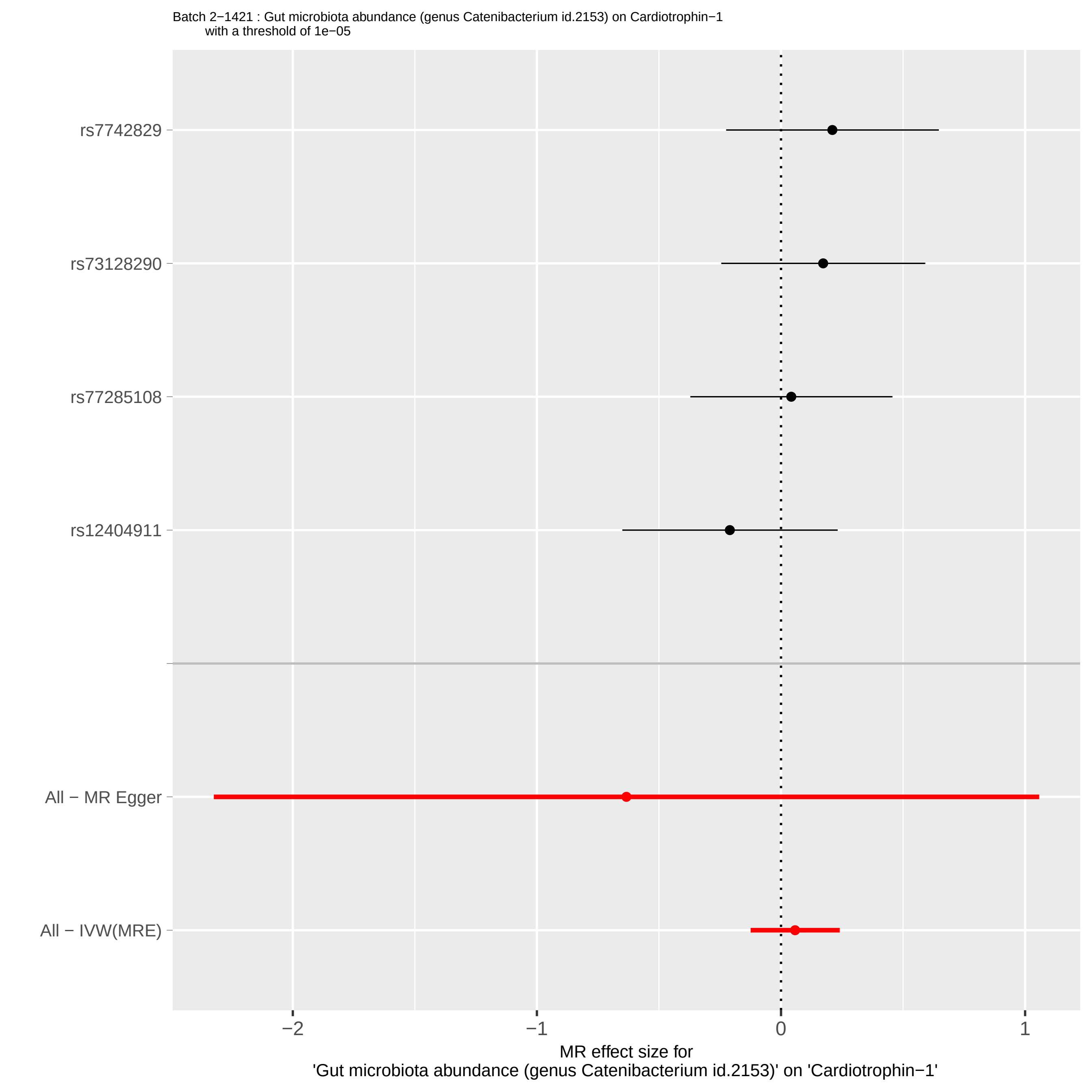

Batch 2−1421 : Gut microbiota abundance (genus Catenibacterium id.2153) on Cardiotrophin−1
with a threshold of 1e−05
rs7742829
rs73128290
rs77285108
rs12404911
All − MR Egger
All − IVW(MRE)
−2
−1
0
1
MR effect size for
'Gut microbiota abundance (genus Catenibacterium id.2153)' on 'Cardiotrophin−1'

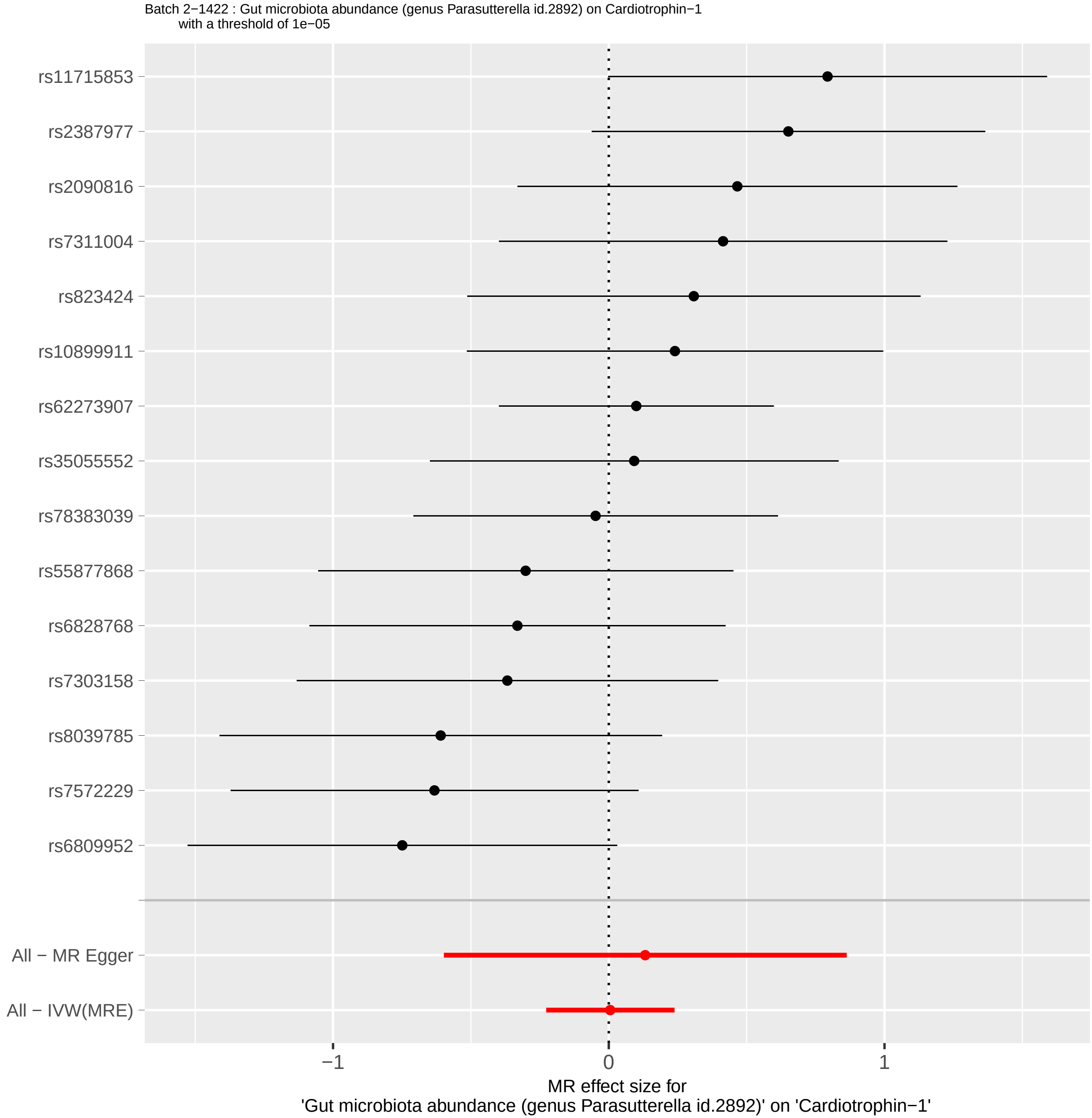

Batch 2−1422 : Gut microbiota abundance (genus Parasutterella id.2892) on Cardiotrophin−1
with a threshold of 1e−05
rs11715853
rs2387977
rs2090816
rs7311004
rs823424
rs10899911
rs62273907
rs35055552
rs78383039
rs55877868
rs6828768
rs7303158
rs8039785
rs7572229
rs6809952
All − MR Egger
All − IVW(MRE)
−1
0
1
MR effect size for
'Gut microbiota abundance (genus Parasutterella id.2892)' on 'Cardiotrophin−1'

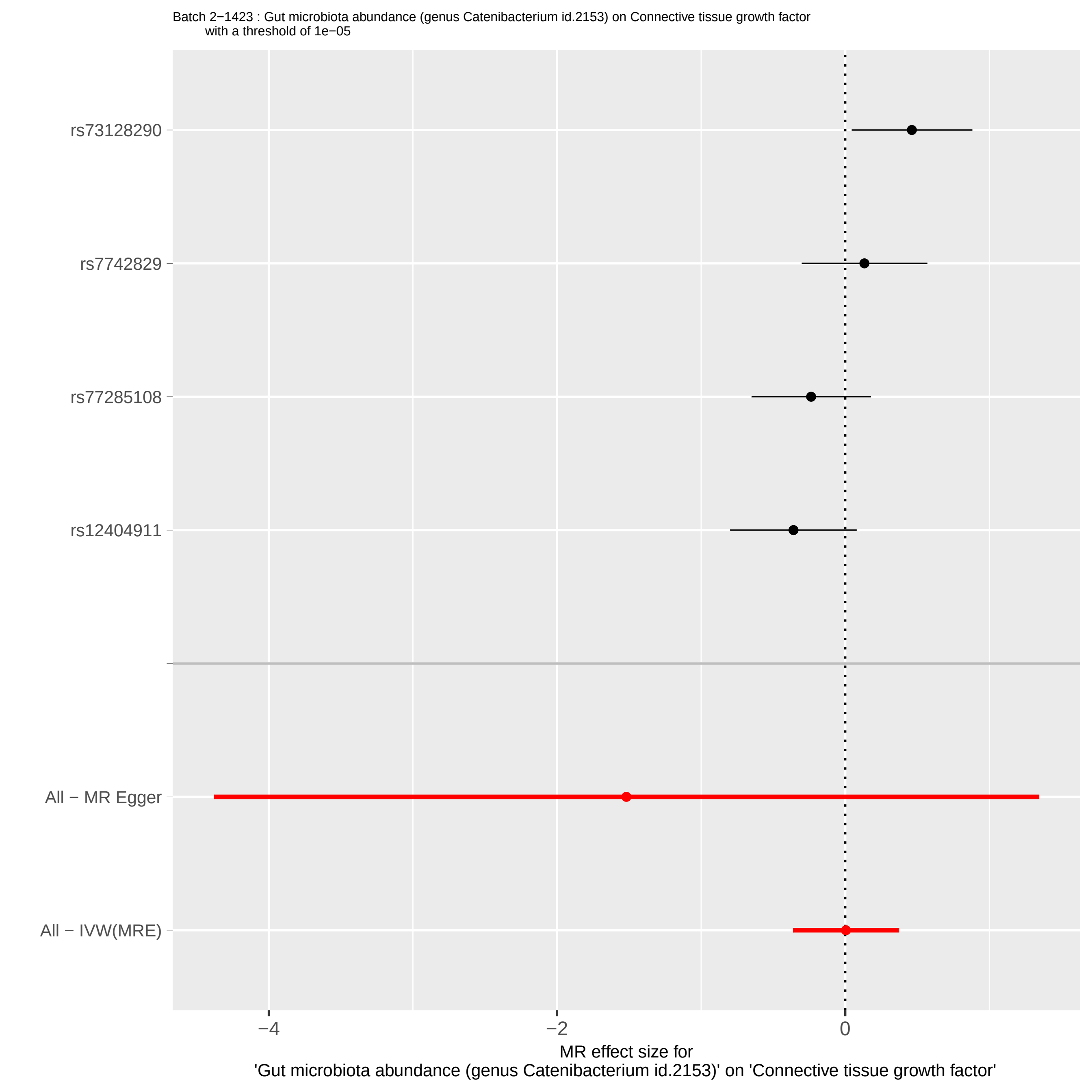
Batch 2−1423 : Gut microbiota abundance (genus Catenibacterium id.2153) on Connective tissue growth factor
with a threshold of 1e−05
rs73128290
rs7742829
rs77285108
rs12404911
All − MR Egger
All − IVW(MRE)
−4
−2
0
MR effect size for
'Gut microbiota abundance (genus Catenibacterium id.2153)' on 'Connective tissue growth factor'

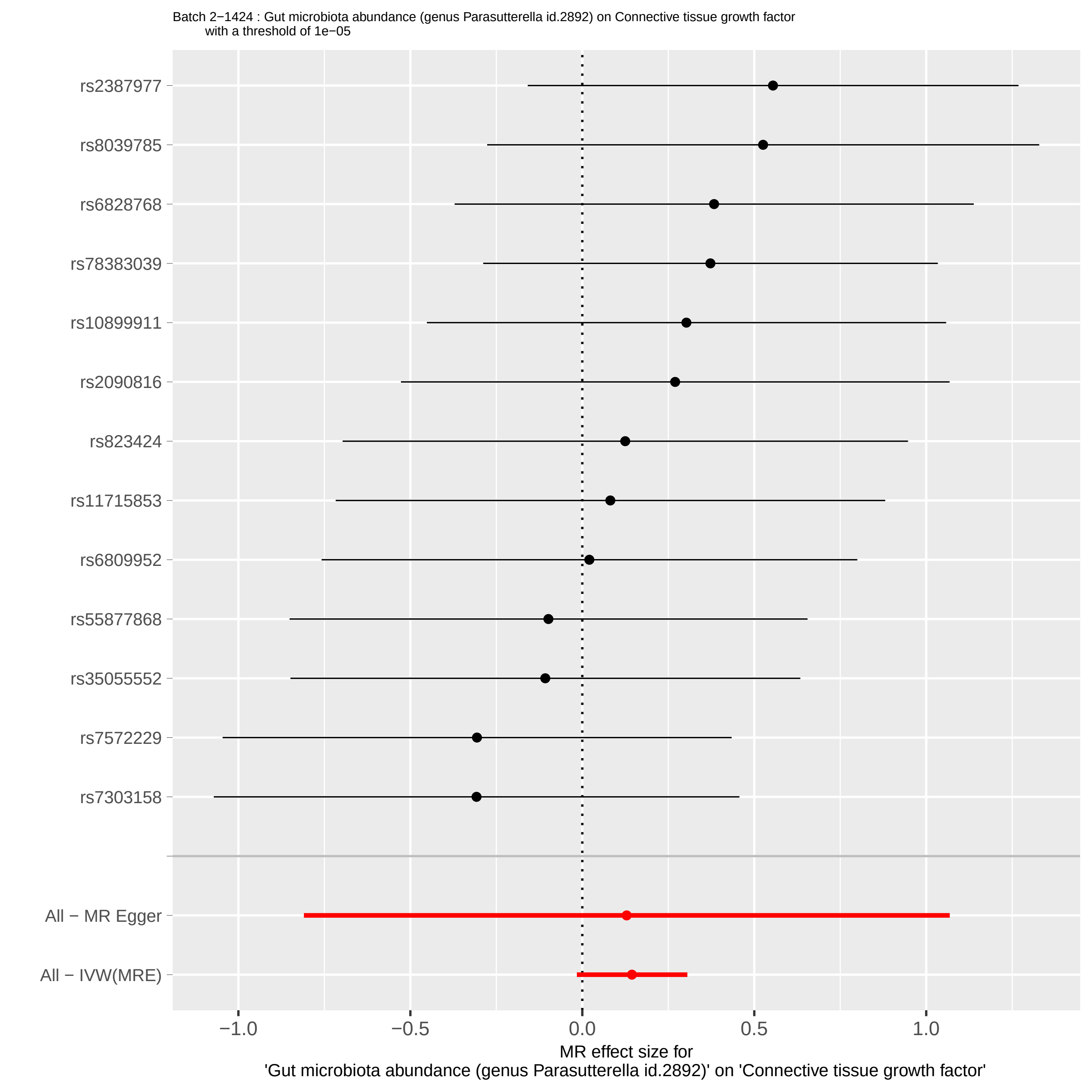
Batch 2−1424 : Gut microbiota abundance (genus Parasutterella id.2892) on Connective tissue growth factor
with a threshold of 1e−05
rs2387977
rs8039785
rs6828768
rs78383039
rs10899911
rs2090816
rs823424
rs11715853
rs6809952
rs55877868
rs35055552
rs7572229
rs7303158
All − MR Egger
All − IVW(MRE)
−1.0
−0.5
0.0
0.5
1.0
MR effect size for
'Gut microbiota abundance (genus Parasutterella id.2892)' on 'Connective tissue growth factor'

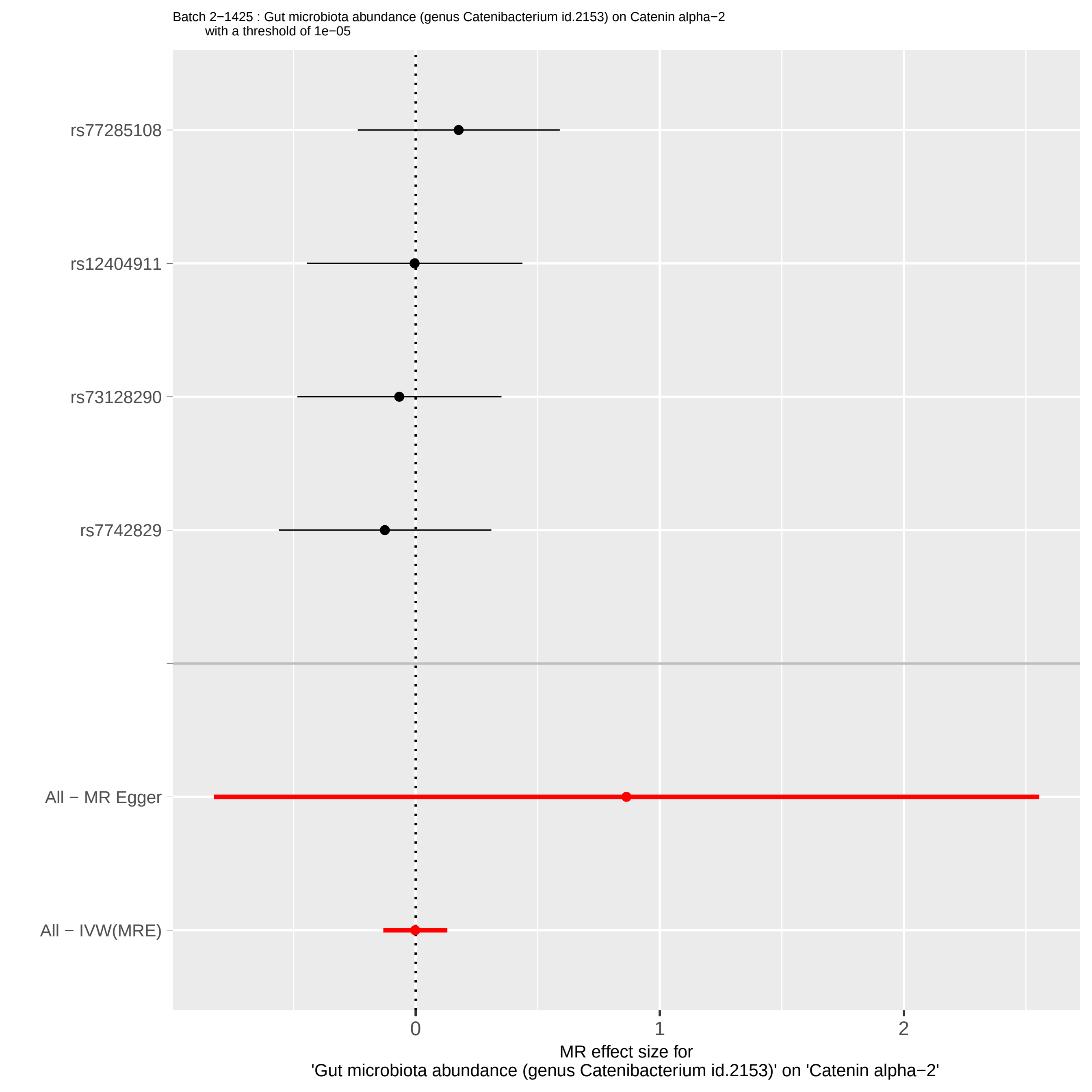
Batch 2−1425 : Gut microbiota abundance (genus Catenibacterium id.2153) on Catenin alpha−2
with a threshold of 1e−05
rs77285108
rs12404911
rs73128290
rs7742829
All − MR Egger
All − IVW(MRE)
0
1
2
MR effect size for
'Gut microbiota abundance (genus Catenibacterium id.2153)' on 'Catenin alpha−2'

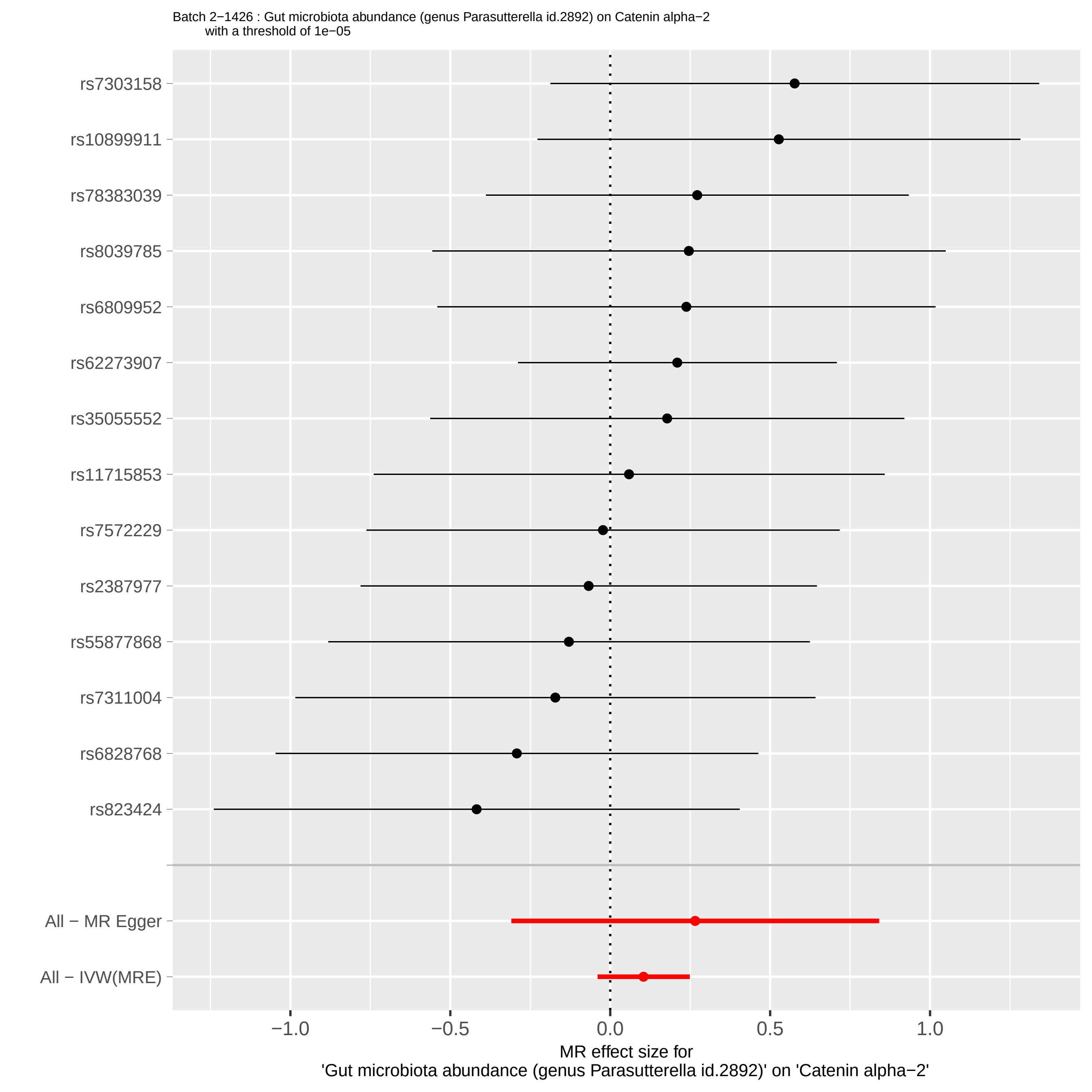
Batch 2−1426 : Gut microbiota abundance (genus Parasutterella id.2892) on Catenin alpha−2
with a threshold of 1e−05
rs7303158
rs10899911
rs78383039
rs8039785
rs6809952
rs62273907
rs35055552
rs11715853
rs7572229
rs2387977
rs55877868
rs7311004
rs6828768
rs823424
All − MR Egger
All − IVW(MRE)
−1.0
−0.5
0.0
0.5
1.0
MR effect size for
'Gut microbiota abundance (genus Parasutterella id.2892)' on 'Catenin alpha−2'

Batch 2−1427 : Gut microbiota abundance (genus Catenibacterium id.2153) on Chymotrypsinogen B
with a threshold of 1e−05

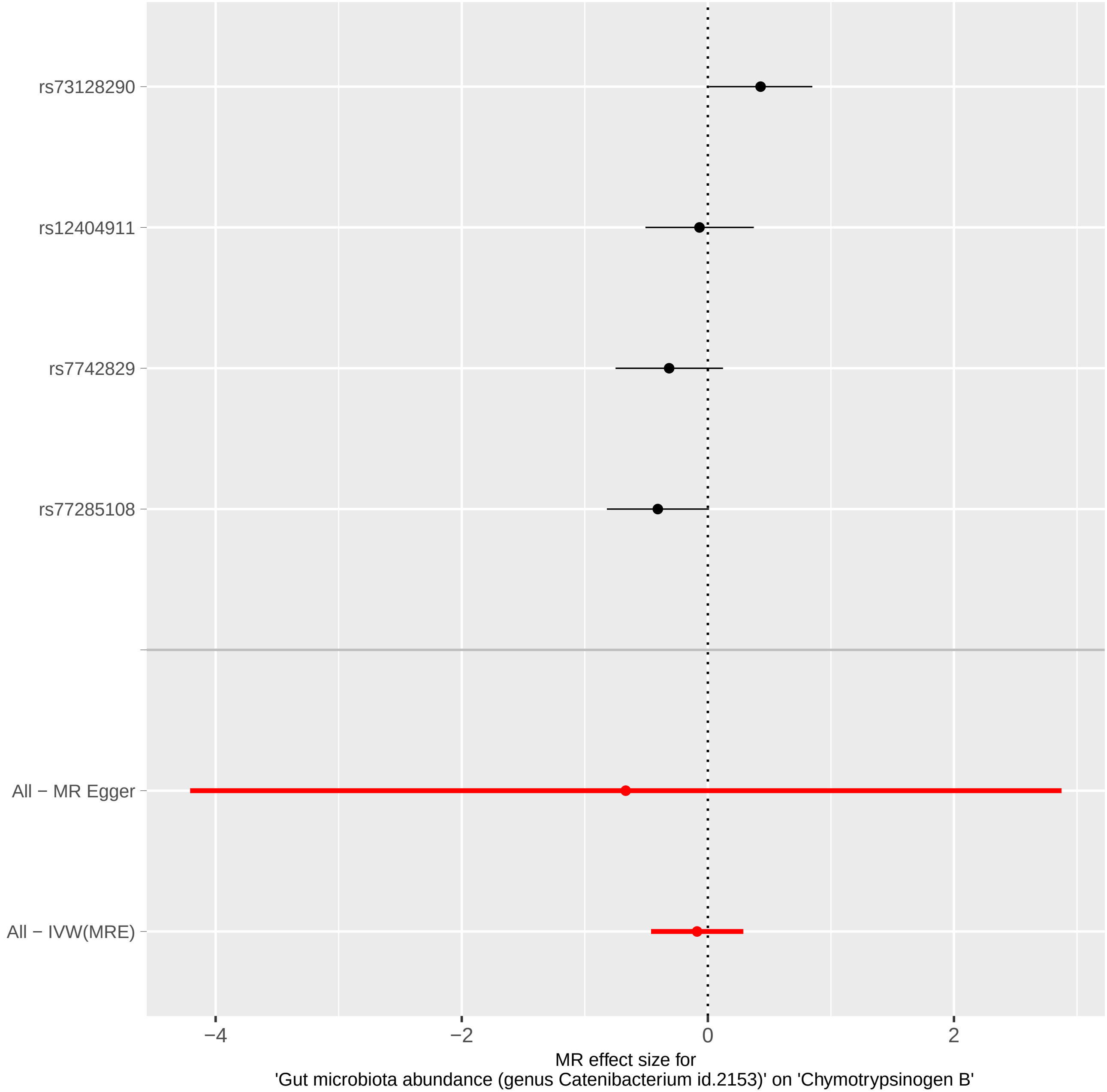

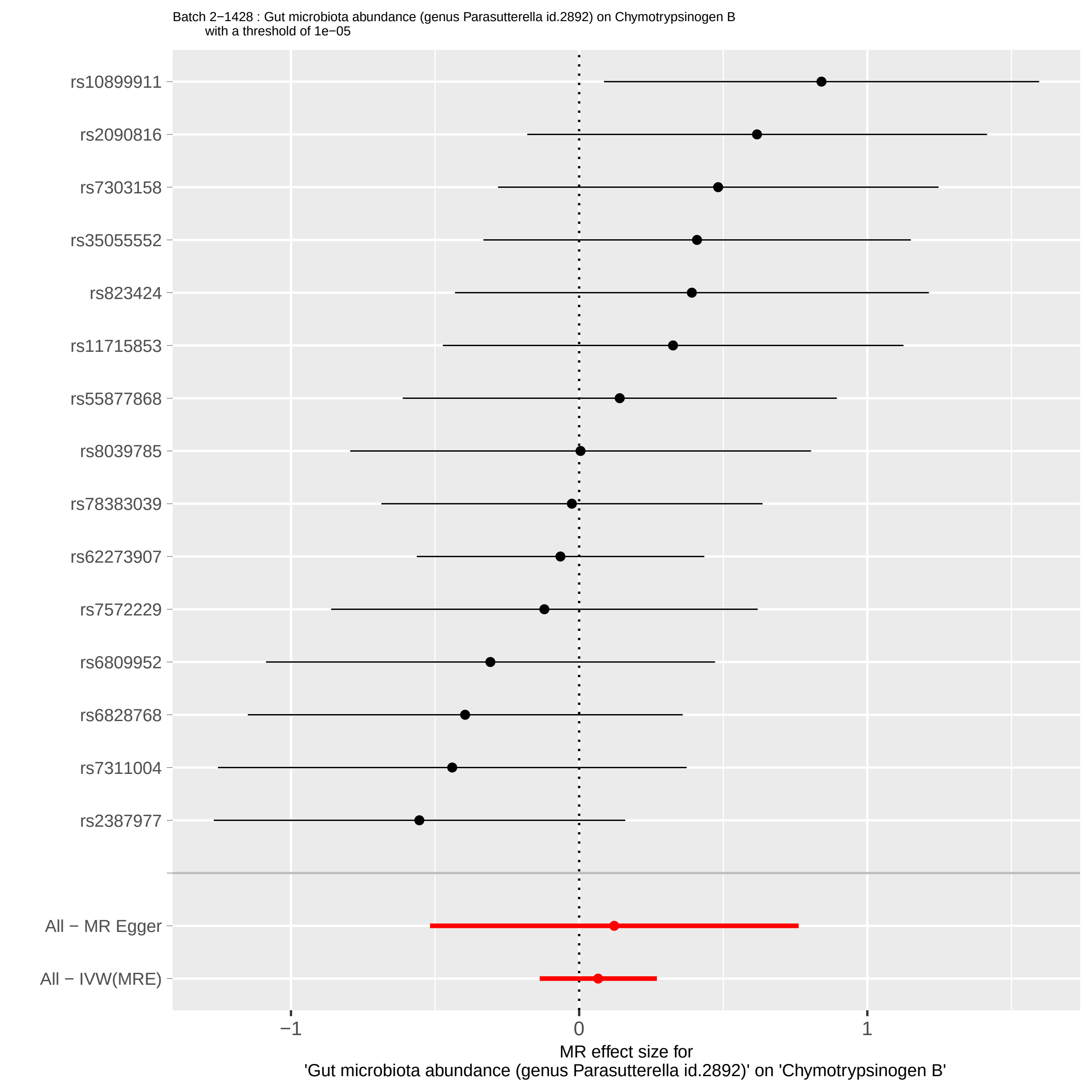

Batch 2−1428 : Gut microbiota abundance (genus Parasutterella id.2892) on Chymotrypsinogen B
with a threshold of 1e−05
rs10899911
rs2090816
rs7303158
rs35055552
rs823424
rs11715853
rs55877868
rs8039785
rs78383039
rs62273907
rs7572229
rs6809952
rs6828768
rs7311004
rs2387977
All − MR Egger
All − IVW(MRE)
−1
0
1
MR effect size for
'Gut microbiota abundance (genus Parasutterella id.2892)' on 'Chymotrypsinogen B'

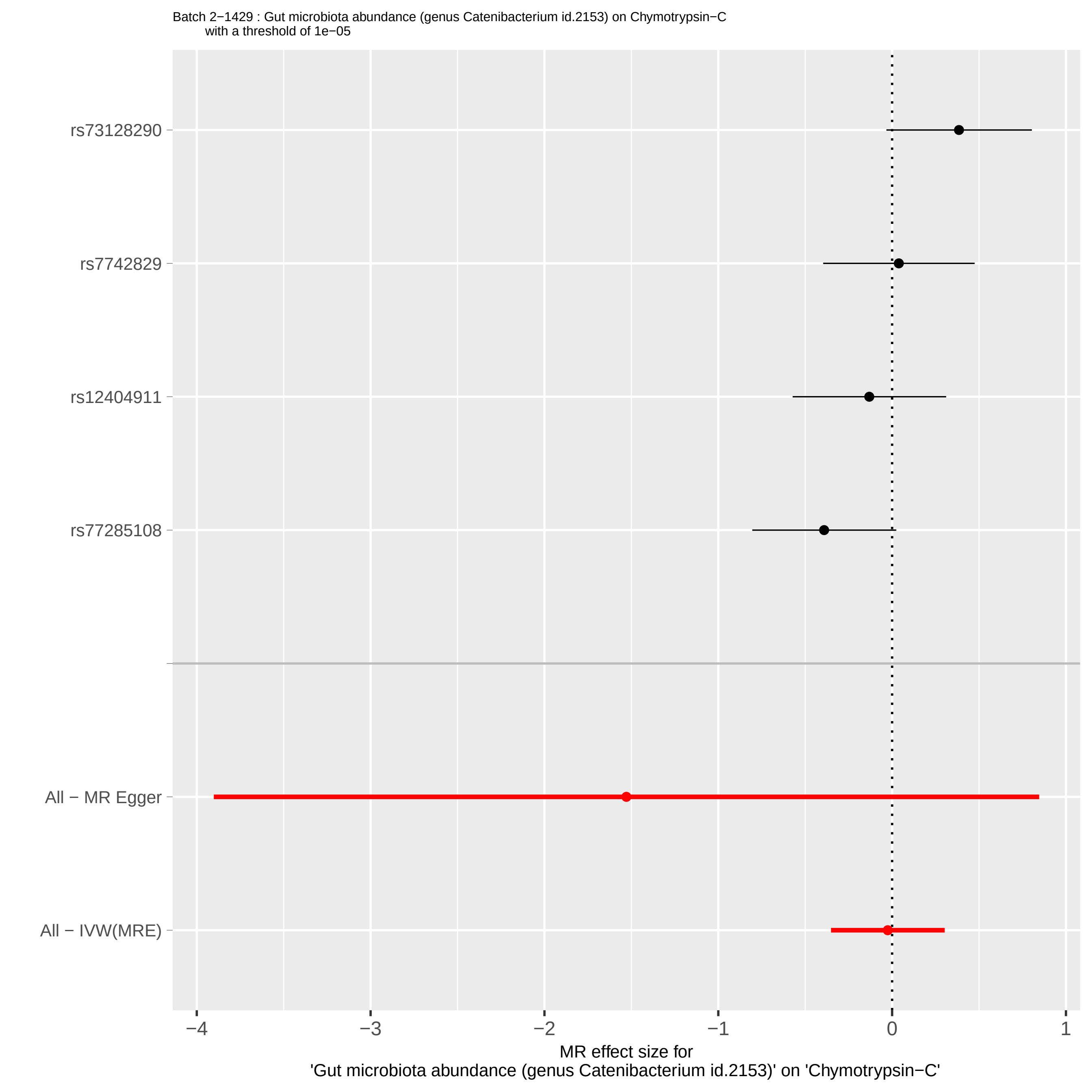

Batch 2−1429 : Gut microbiota abundance (genus Catenibacterium id.2153) on Chymotrypsin−C
with a threshold of 1e−05
rs73128290
rs7742829
rs12404911
rs77285108
All − MR Egger
All − IVW(MRE)
−4
−3
−2
−1
0
1
MR effect size for
'Gut microbiota abundance (genus Catenibacterium id.2153)' on 'Chymotrypsin−C'

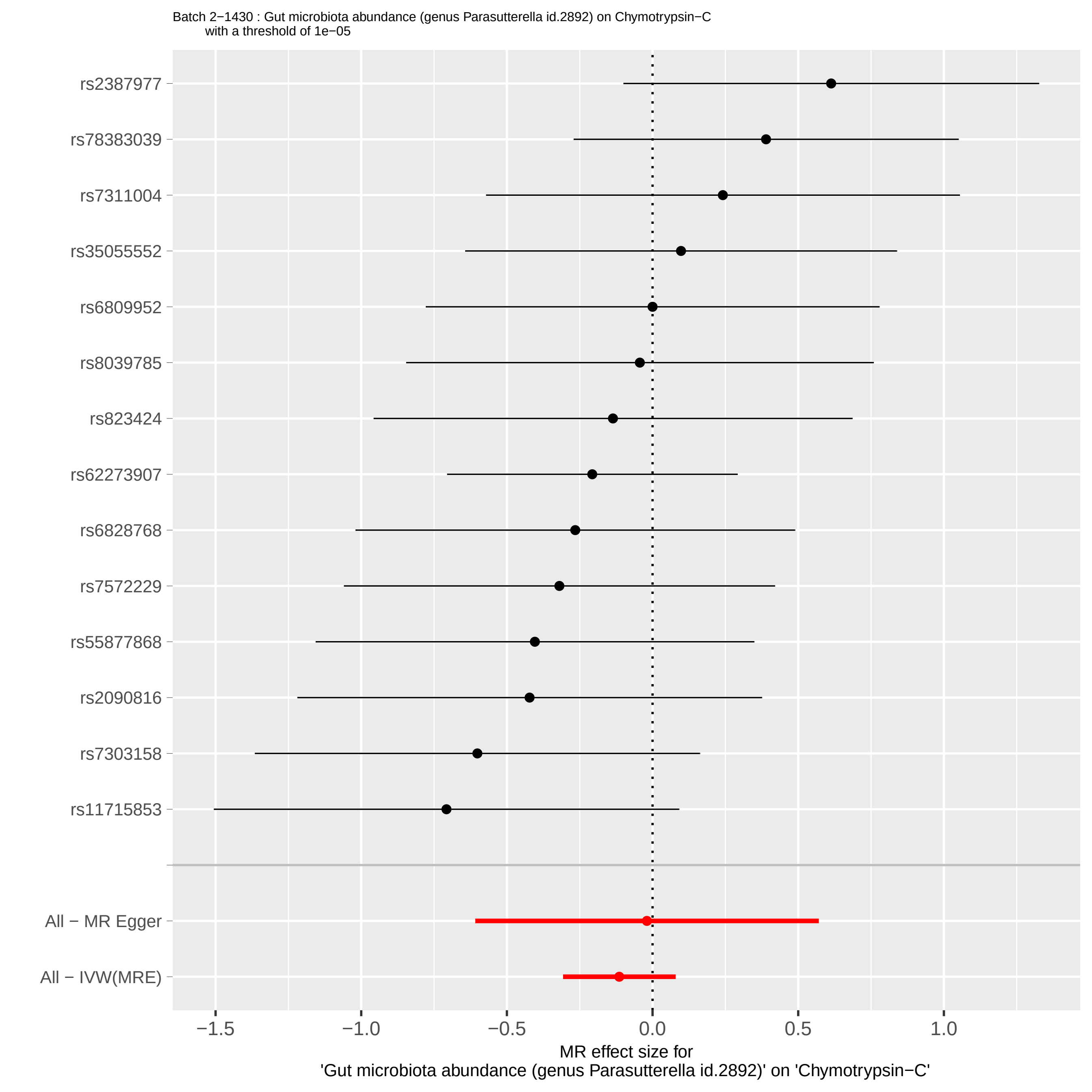

Batch 2−1430 : Gut microbiota abundance (genus Parasutterella id.2892) on Chymotrypsin−C
with a threshold of 1e−05
rs2387977
rs78383039
rs7311004
rs35055552
rs6809952
rs8039785
rs823424
rs62273907
rs6828768
rs7572229
rs55877868
rs2090816
rs7303158
rs11715853
All − MR Egger
All − IVW(MRE)
−1.5
−1.0
−0.5
0.0
0.5
1.0
MR effect size for
'Gut microbiota abundance (genus Parasutterella id.2892)' on 'Chymotrypsin−C'

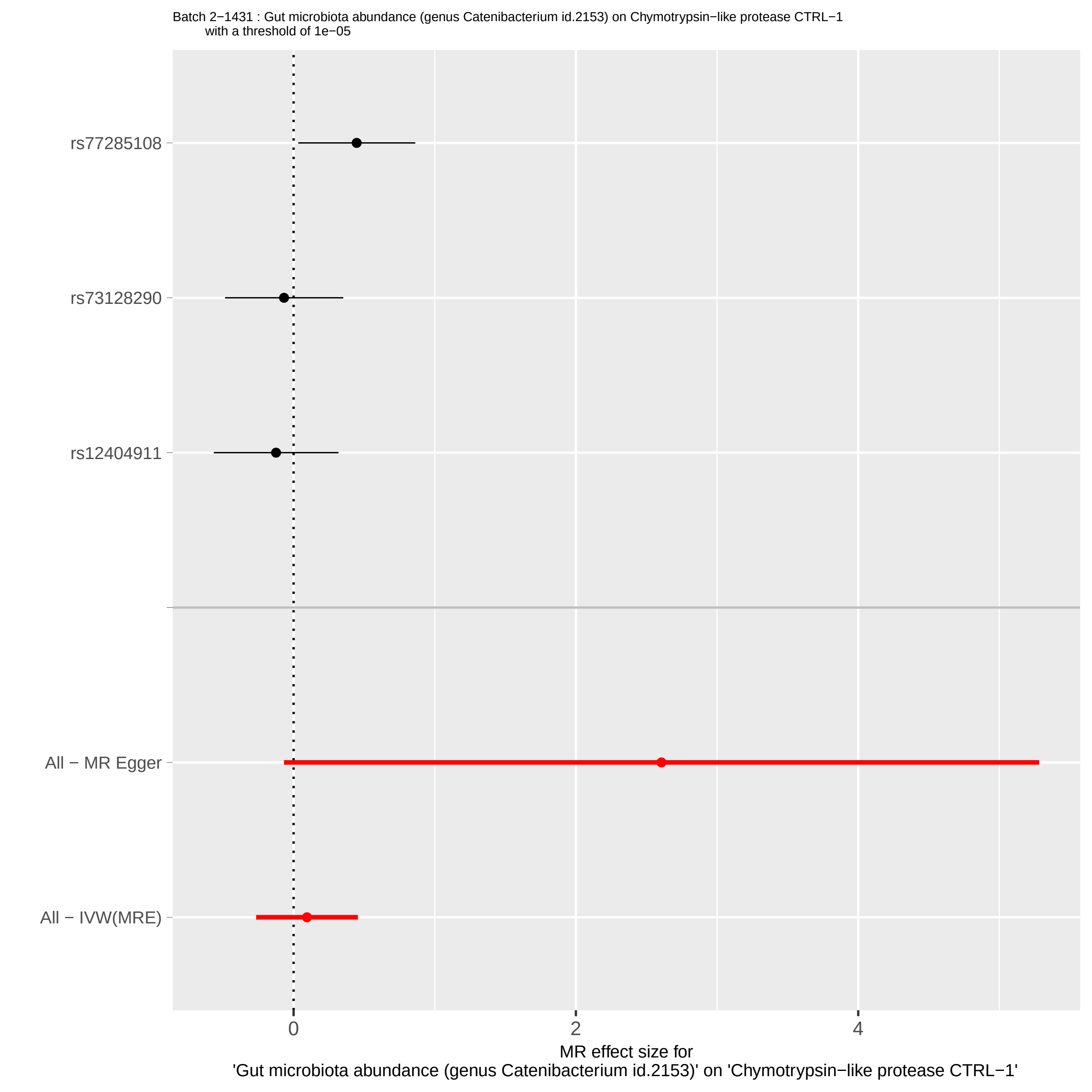

Batch 2−1431 : Gut microbiota abundance (genus Catenibacterium id.2153) on Chymotrypsin−like protease CTRL−1
with a threshold of 1e−05
rs77285108
rs73128290
rs12404911
All − MR Egger
All − IVW(MRE)
0
2
4
MR effect size for
'Gut microbiota abundance (genus Catenibacterium id.2153)' on 'Chymotrypsin−like protease CTRL−1'

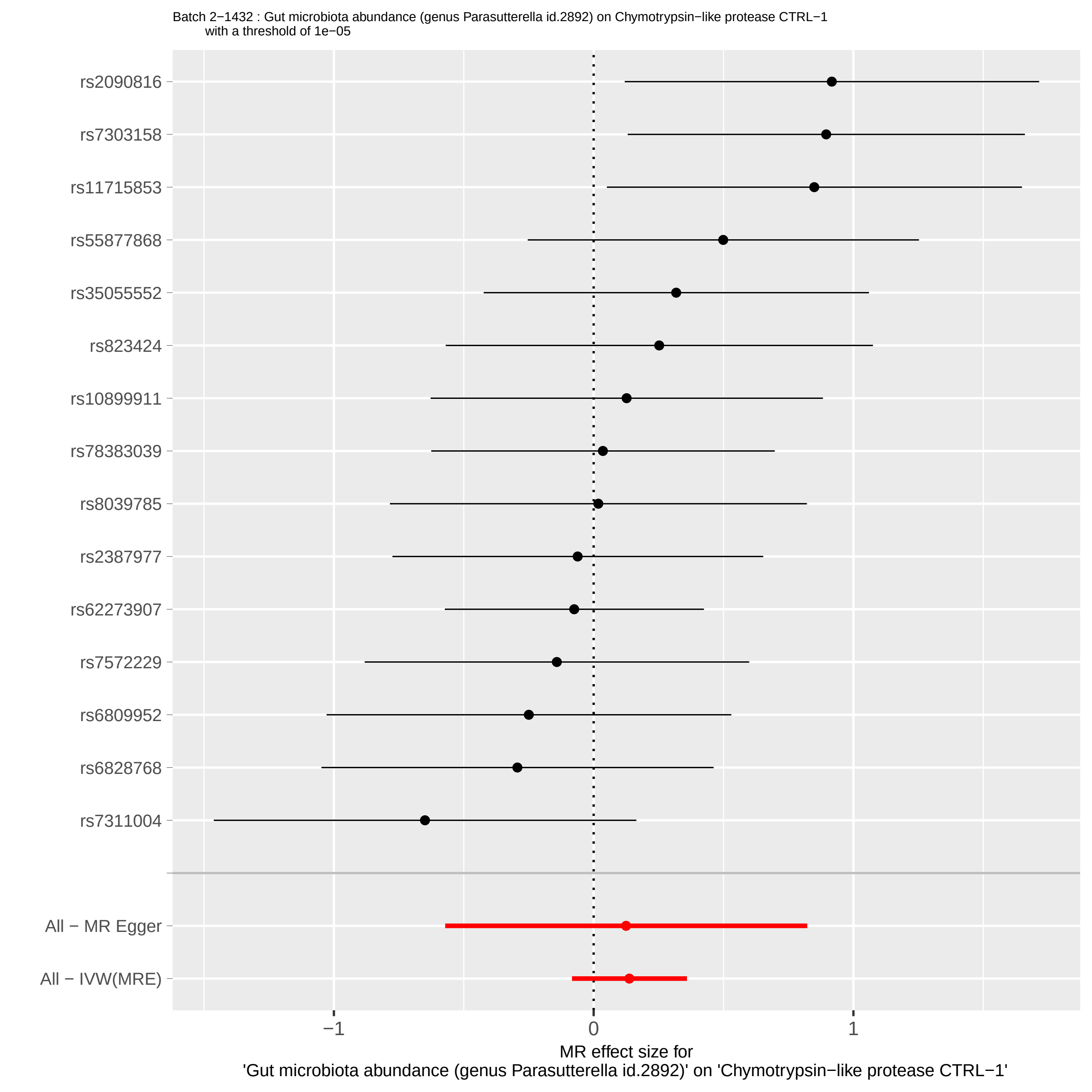

Batch 2−1432 : Gut microbiota abundance (genus Parasutterella id.2892) on Chymotrypsin−like protease CTRL−1
with a threshold of 1e−05
rs2090816
rs7303158
rs11715853
rs55877868
rs35055552
rs823424
rs10899911
rs78383039
rs8039785
rs2387977
rs62273907
rs7572229
rs6809952
rs6828768
rs7311004
All − MR Egger
All − IVW(MRE)
−1
0
1
MR effect size for
'Gut microbiota abundance (genus Parasutterella id.2892)' on 'Chymotrypsin−like protease CTRL−1'

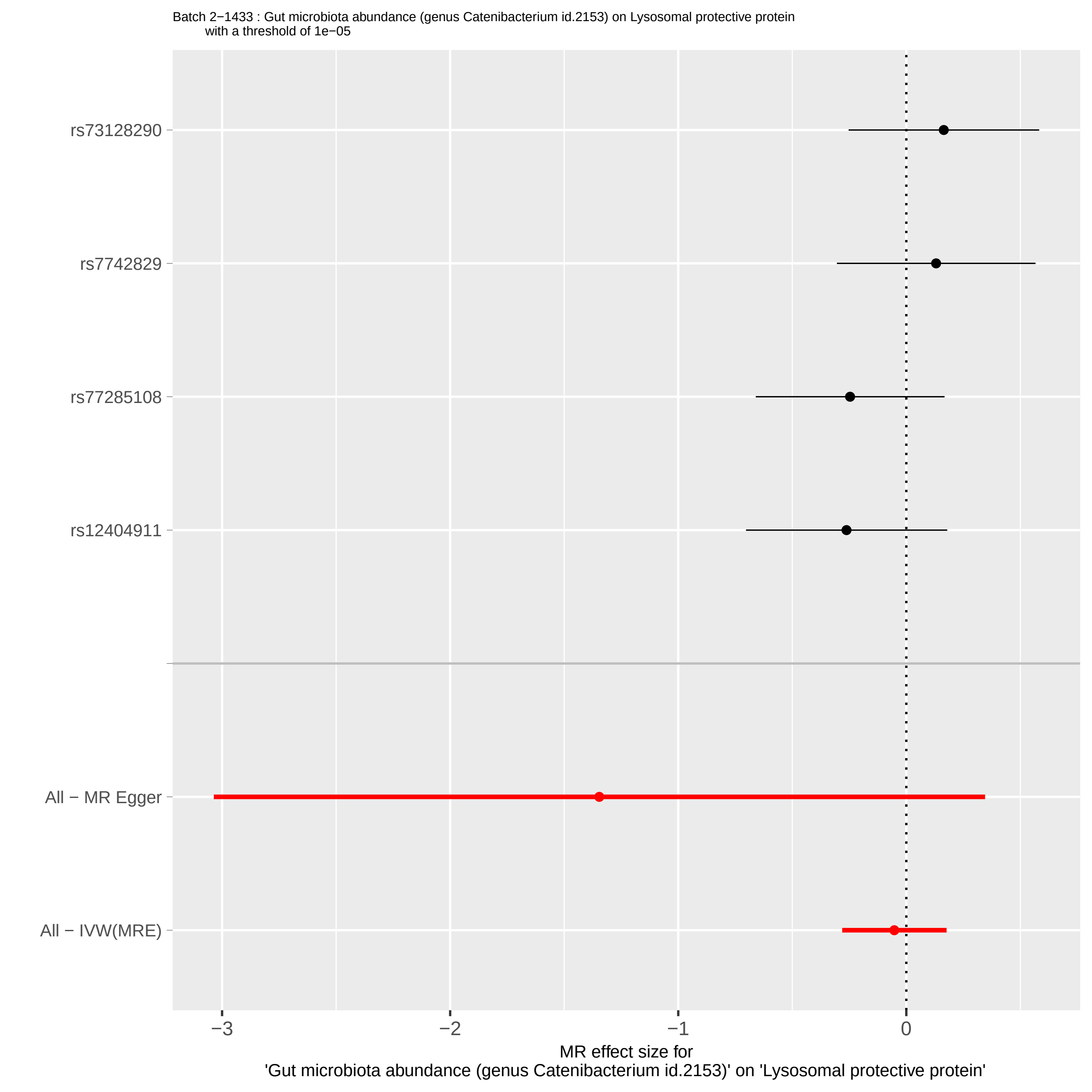
Batch 2−1433 : Gut microbiota abundance (genus Catenibacterium id.2153) on Lysosomal protective protein
with a threshold of 1e−05
rs73128290
rs7742829
rs77285108
rs12404911
All − MR Egger
All − IVW(MRE)
−3
−2
−1
0
MR effect size for
'Gut microbiota abundance (genus Catenibacterium id.2153)' on 'Lysosomal protective protein'

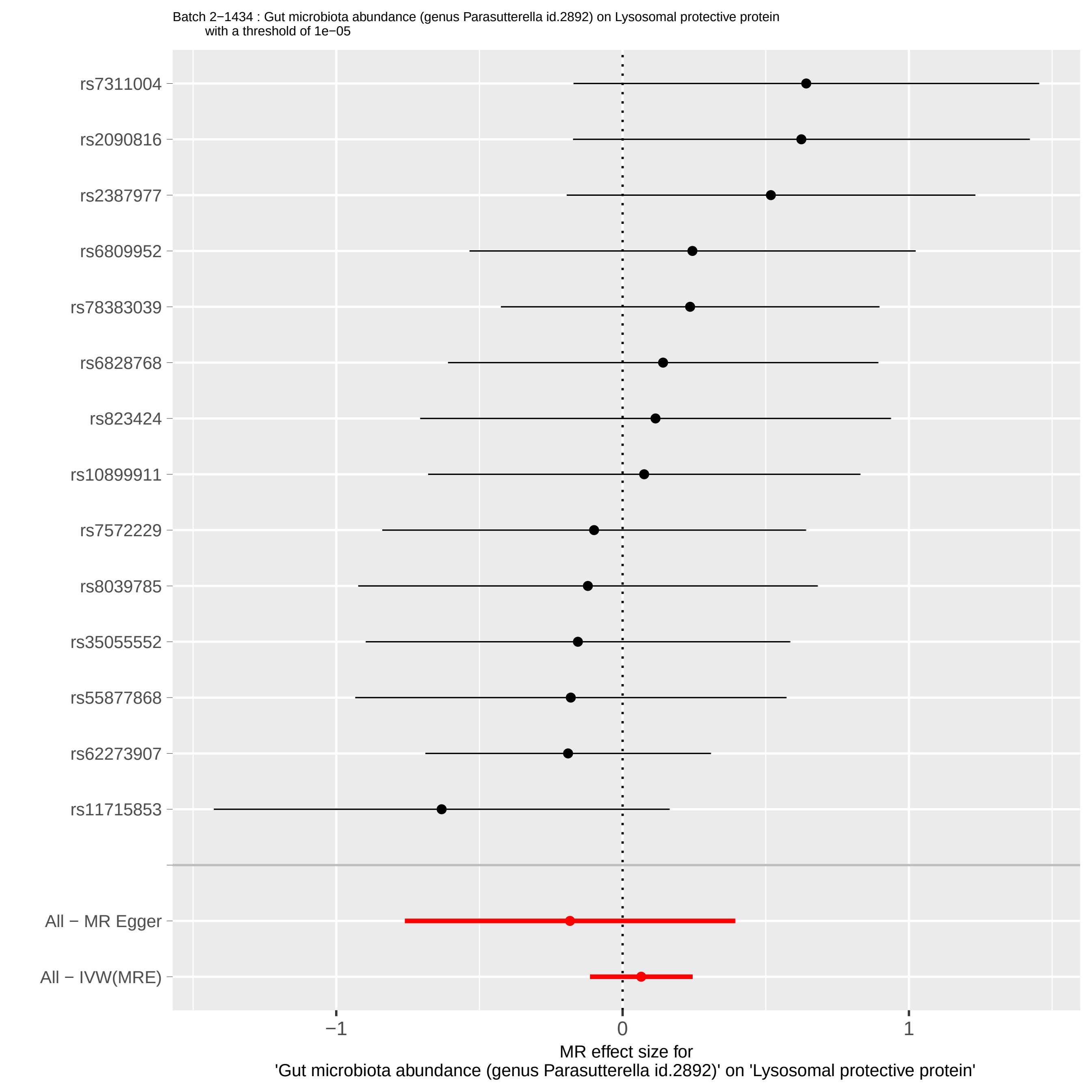
Batch 2−1434 : Gut microbiota abundance (genus Parasutterella id.2892) on Lysosomal protective protein
with a threshold of 1e−05
rs7311004
rs2090816
rs2387977
rs6809952
rs78383039
rs6828768
rs823424
rs10899911
rs7572229
rs8039785
rs35055552
rs55877868
rs62273907
rs11715853
All − MR Egger
All − IVW(MRE)
−1
0
1
MR effect size for
'Gut microbiota abundance (genus Parasutterella id.2892)' on 'Lysosomal protective protein'

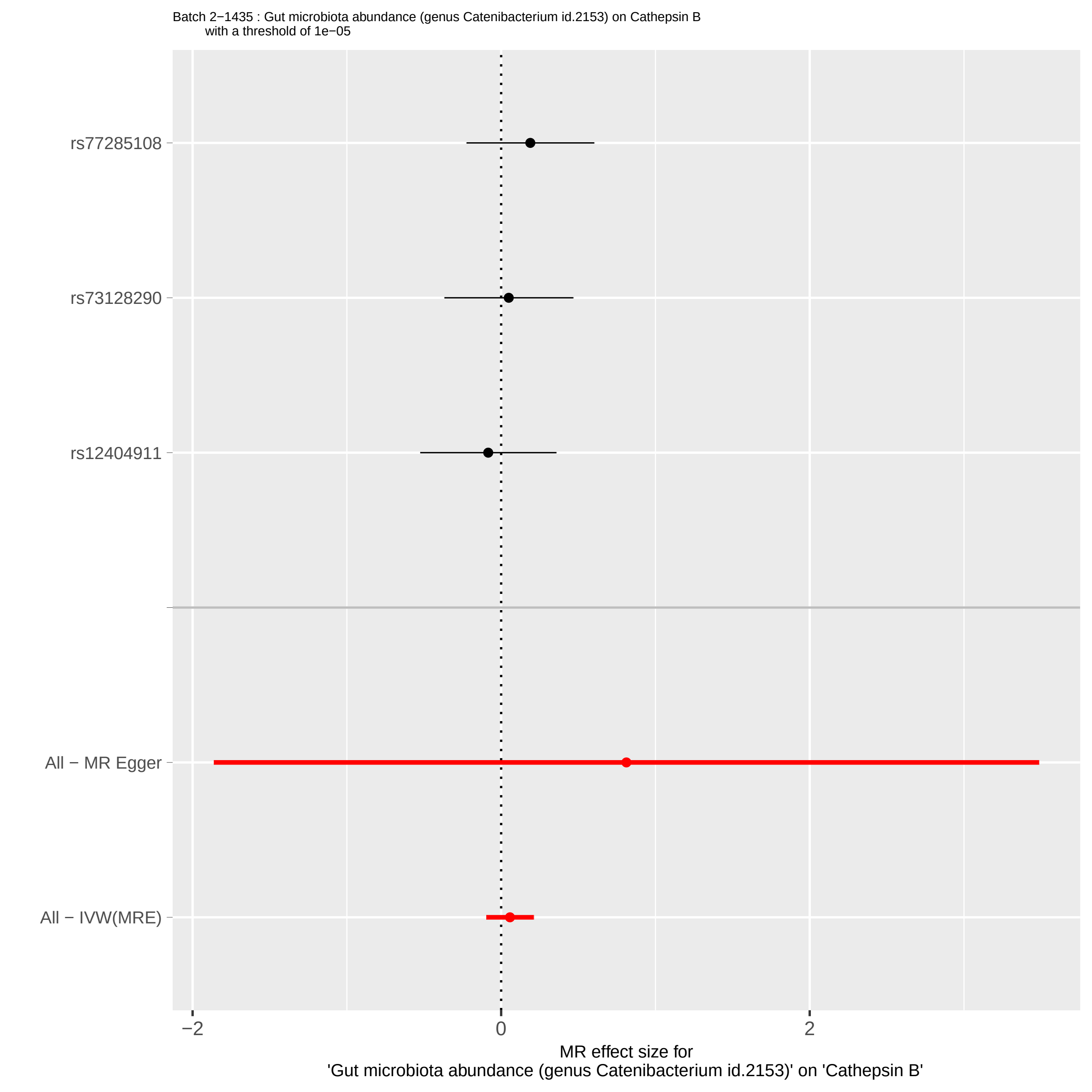

Batch 2−1435 : Gut microbiota abundance (genus Catenibacterium id.2153) on Cathepsin B
with a threshold of 1e−05
rs77285108
rs73128290
rs12404911
All − MR Egger
All − IVW(MRE)
−2
0
2
MR effect size for
'Gut microbiota abundance (genus Catenibacterium id.2153)' on 'Cathepsin B'

Batch 2−1436 : Gut microbiota abundance (genus Parasutterella id.2892) on Cathepsin B
with a threshold of 1e−05

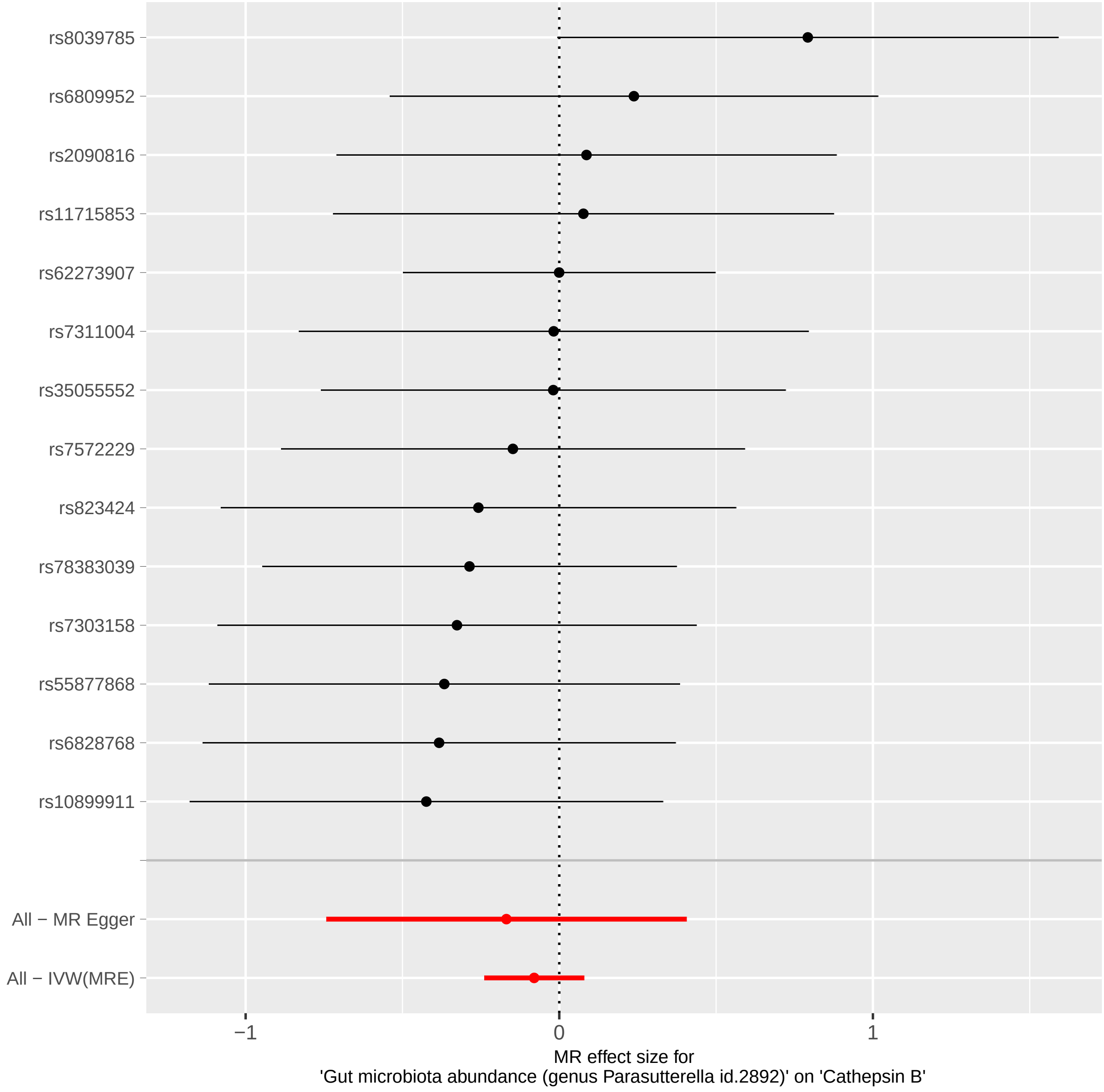

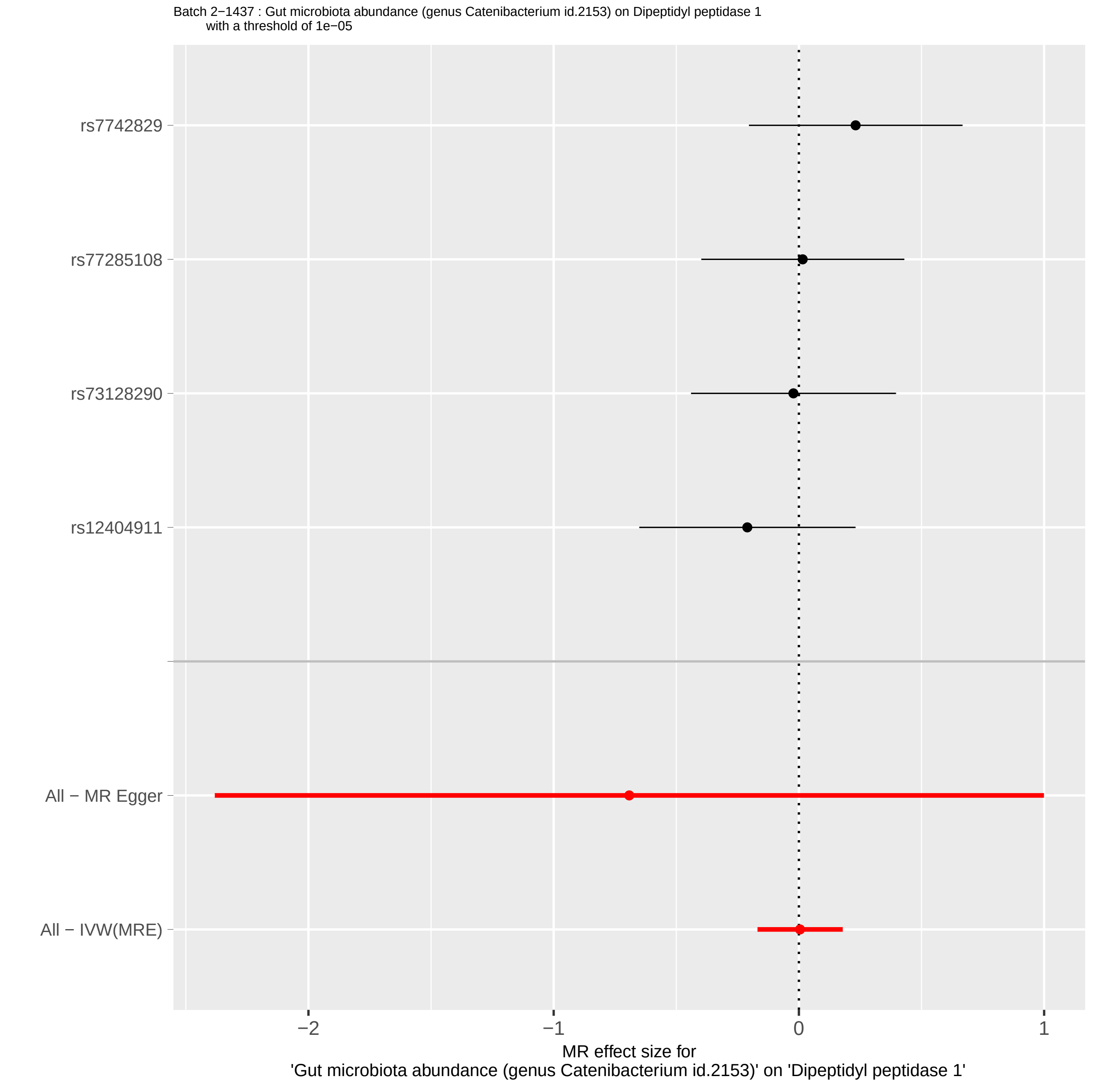
Batch 2−1437 : Gut microbiota abundance (genus Catenibacterium id.2153) on Dipeptidyl peptidase 1
with a threshold of 1e−05
rs7742829
rs77285108
rs73128290
rs12404911
All − MR Egger
All − IVW(MRE)
−2
−1
0
1
MR effect size for
'Gut microbiota abundance (genus Catenibacterium id.2153)' on 'Dipeptidyl peptidase 1'

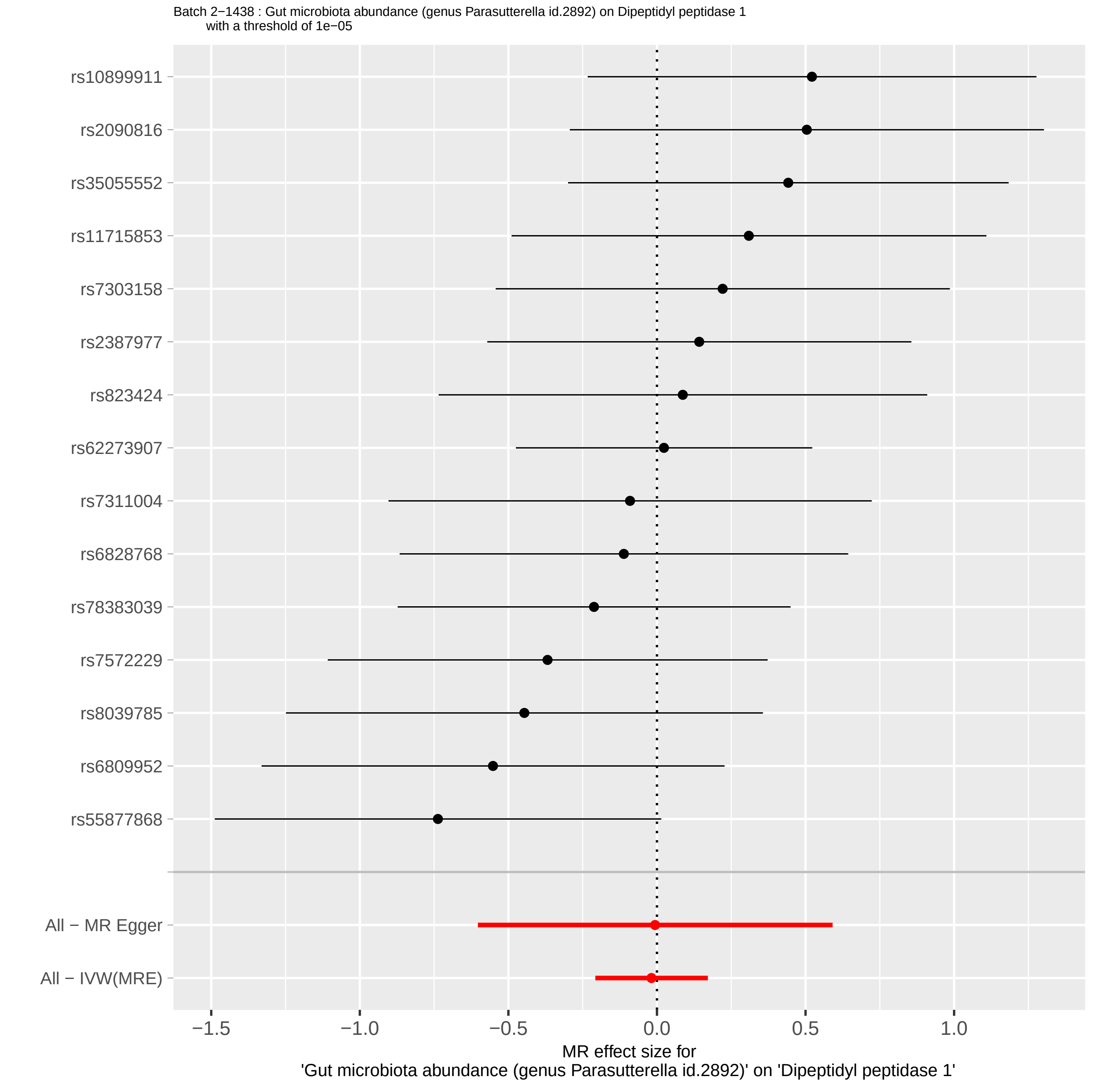
Batch 2−1438 : Gut microbiota abundance (genus Parasutterella id.2892) on Dipeptidyl peptidase 1
with a threshold of 1e−05
rs10899911
rs2090816
rs35055552
rs11715853
rs7303158
rs2387977
rs823424
rs62273907
rs7311004
rs6828768
rs78383039
rs7572229
rs8039785
rs6809952
rs55877868
All − MR Egger
All − IVW(MRE)
−1.5
−1.0
−0.5
0.0
0.5
1.0
MR effect size for
'Gut microbiota abundance (genus Parasutterella id.2892)' on 'Dipeptidyl peptidase 1'

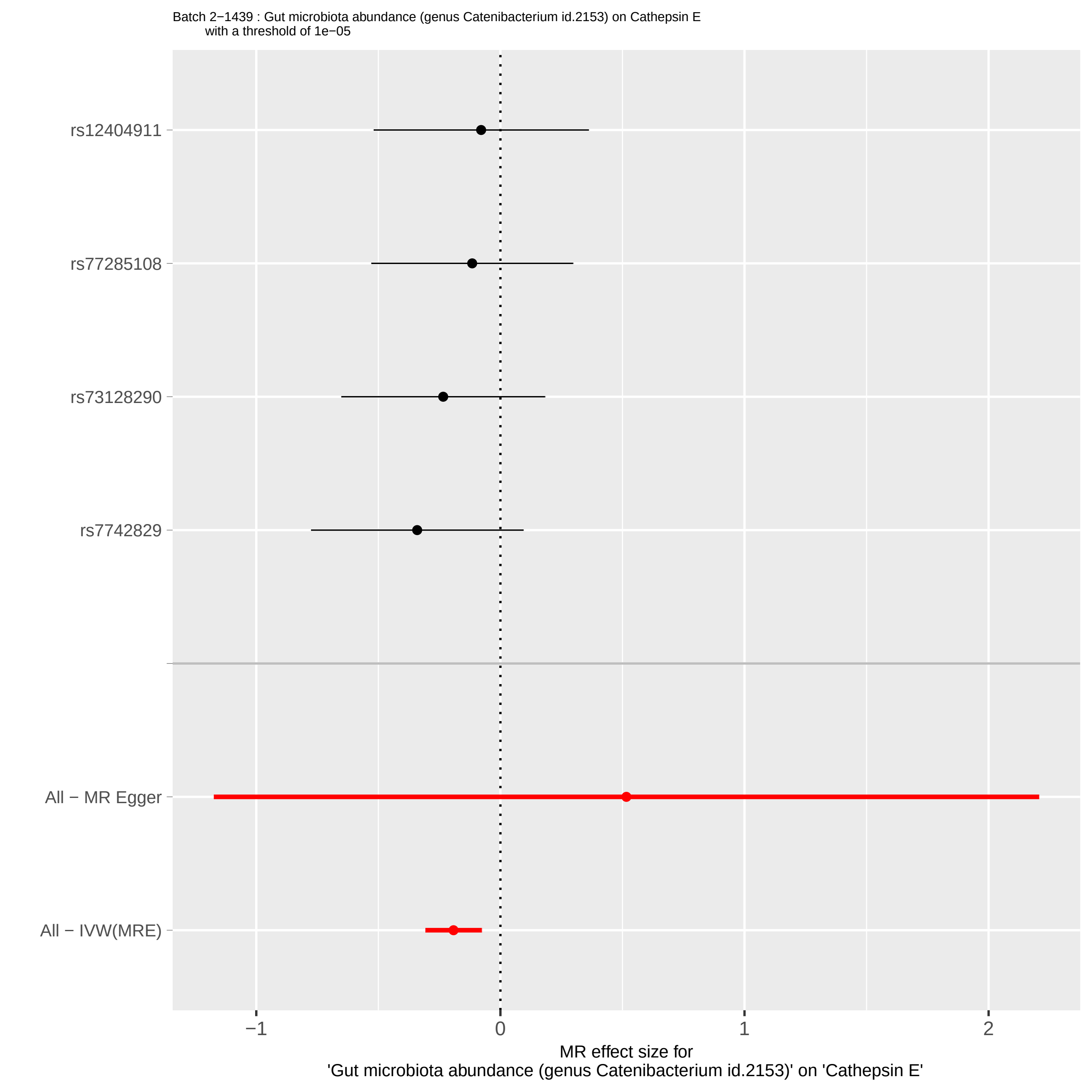

Batch 2−1439 : Gut microbiota abundance (genus Catenibacterium id.2153) on Cathepsin E
with a threshold of 1e−05
rs12404911
rs77285108
rs73128290
rs7742829
All − MR Egger
All − IVW(MRE)
−1
0
1
2
MR effect size for
'Gut microbiota abundance (genus Catenibacterium id.2153)' on 'Cathepsin E'

Batch 2−1440 : Gut microbiota abundance (genus Parasutterella id.2892) on Cathepsin E
with a threshold of 1e−05

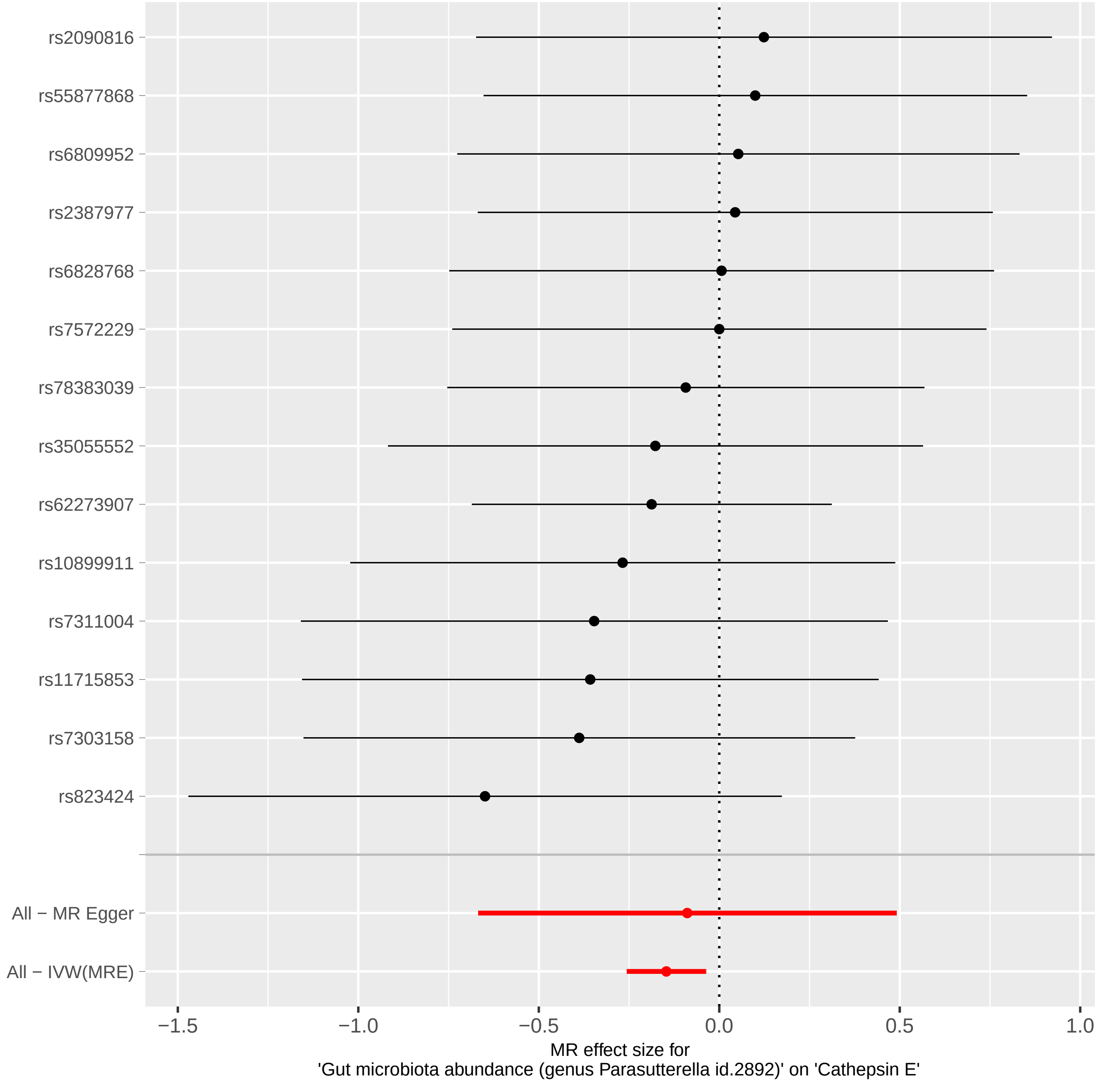

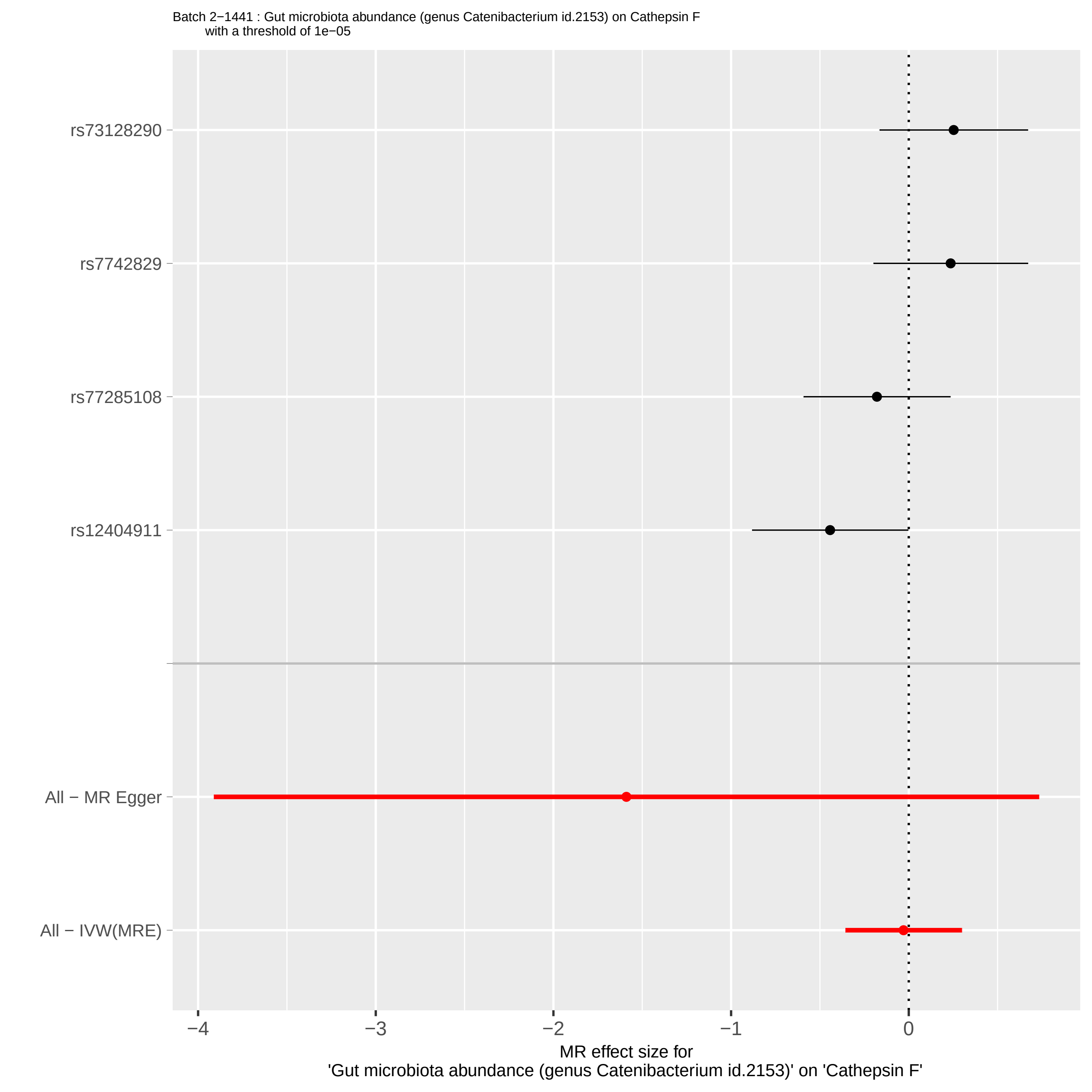
Batch 2−1441 : Gut microbiota abundance (genus Catenibacterium id.2153) on Cathepsin F
with a threshold of 1e−05
rs73128290
rs7742829
rs77285108
rs12404911
All − MR Egger
All − IVW(MRE)
−4
−3
−2
−1
0
MR effect size for
'Gut microbiota abundance (genus Catenibacterium id.2153)' on 'Cathepsin F'

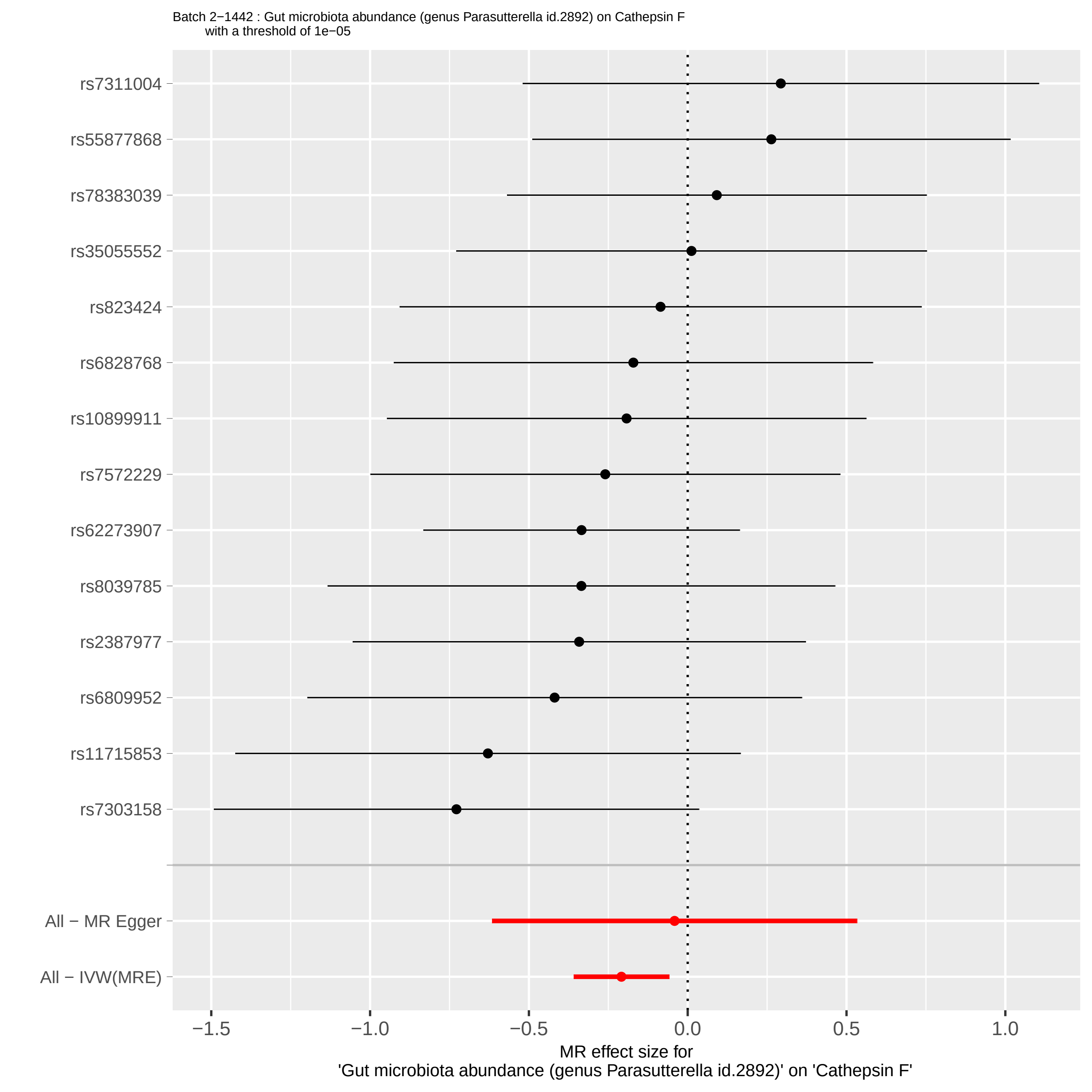

Batch 2−1442 : Gut microbiota abundance (genus Parasutterella id.2892) on Cathepsin F
with a threshold of 1e−05
rs7311004
rs55877868
rs78383039
rs35055552
rs823424
rs6828768
rs10899911
rs7572229
rs62273907
rs8039785
rs2387977
rs6809952
rs11715853
rs7303158
All − MR Egger
All − IVW(MRE)
−1.5
−1.0
−0.5
0.0
0.5
1.0
MR effect size for
'Gut microbiota abundance (genus Parasutterella id.2892)' on 'Cathepsin F'

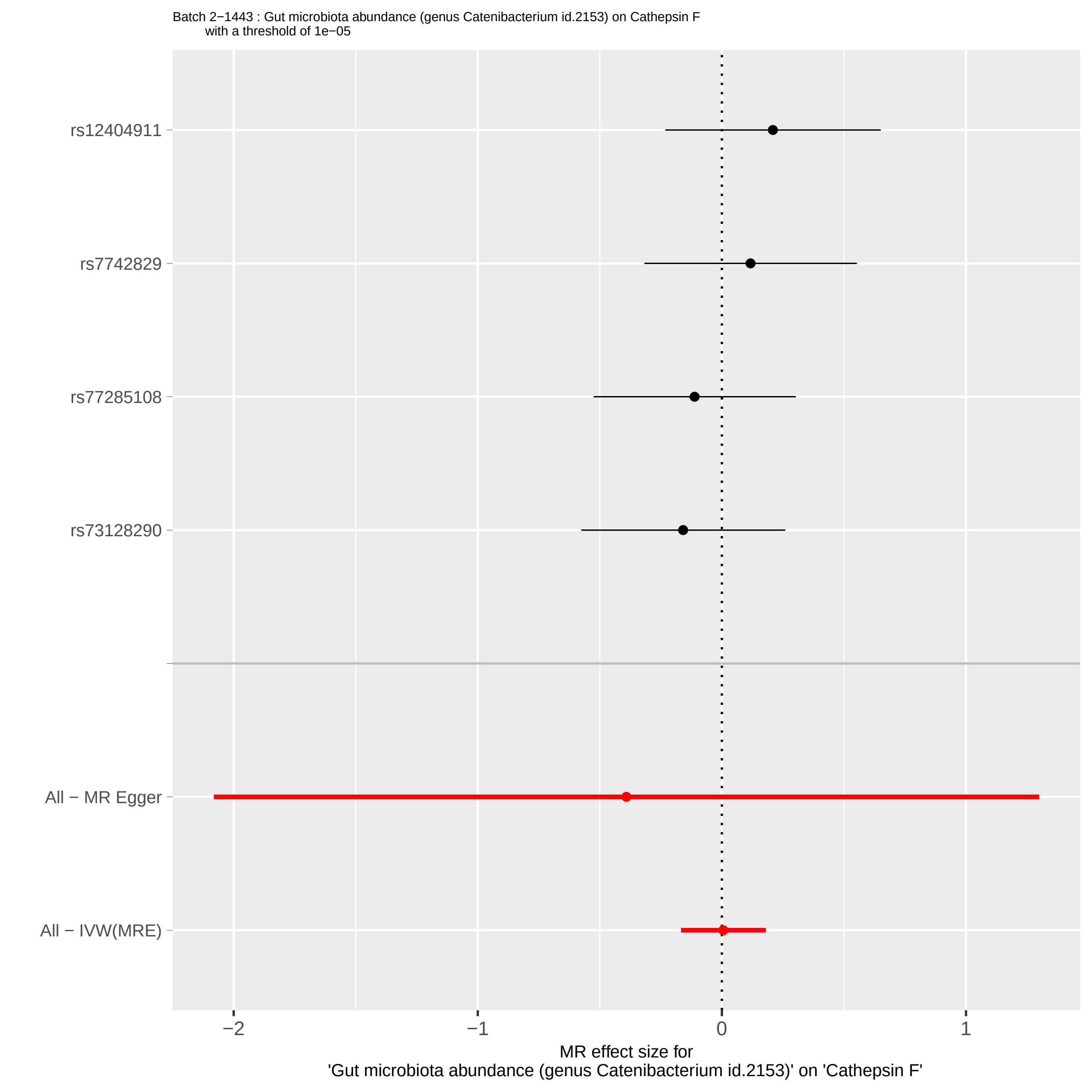
Batch 2−1443 : Gut microbiota abundance (genus Catenibacterium id.2153) on Cathepsin F
with a threshold of 1e−05
rs12404911
rs7742829
rs77285108
rs73128290
All − MR Egger
All − IVW(MRE)
−2
−1
0
1
MR effect size for
'Gut microbiota abundance (genus Catenibacterium id.2153)' on 'Cathepsin F'

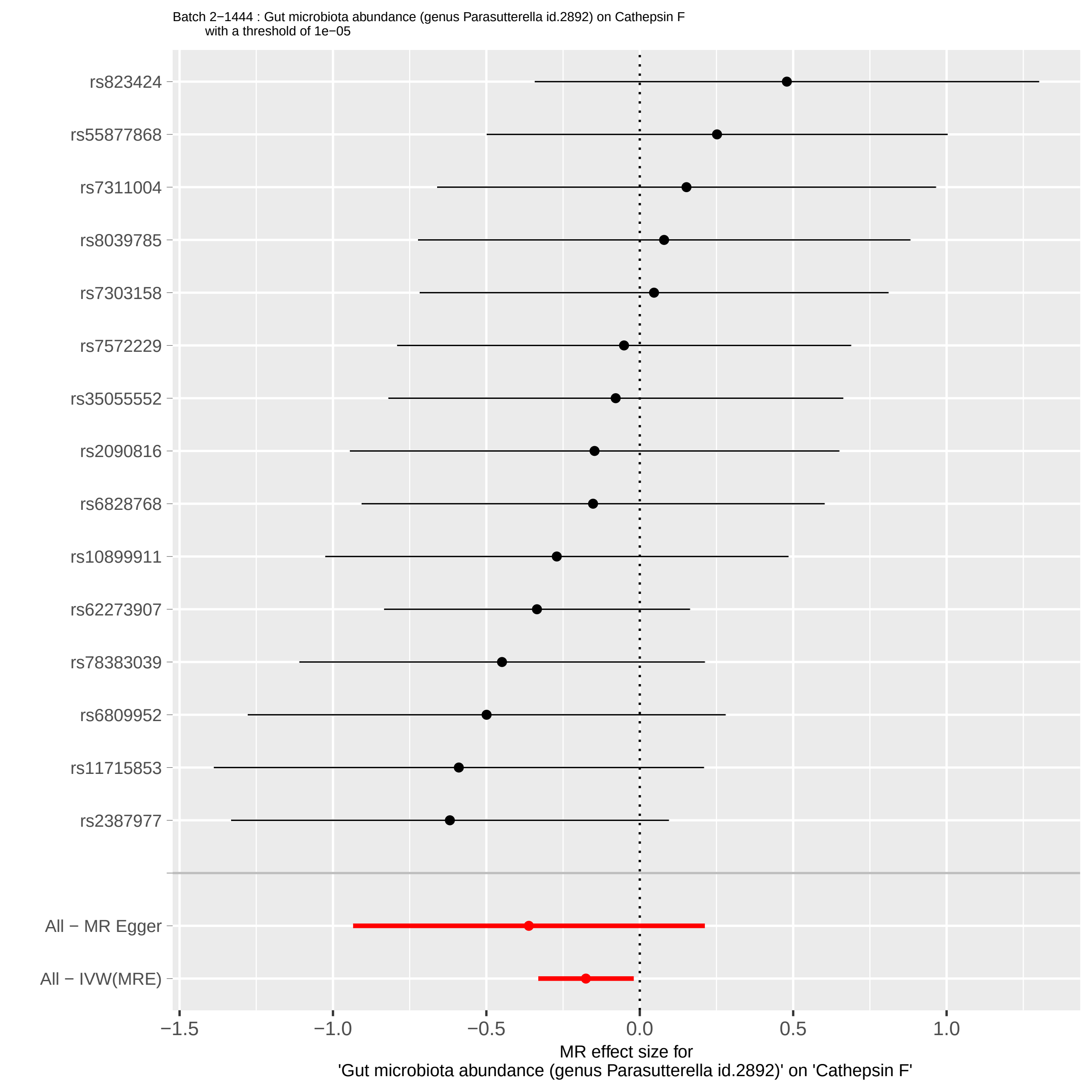

Batch 2−1444 : Gut microbiota abundance (genus Parasutterella id.2892) on Cathepsin F
with a threshold of 1e−05
rs823424
rs55877868
rs7311004
rs8039785
rs7303158
rs7572229
rs35055552
rs2090816
rs6828768
rs10899911
rs62273907
rs78383039
rs6809952
rs11715853
rs2387977
All − MR Egger
All − IVW(MRE)
−1.5
−1.0
−0.5
0.0
0.5
1.0
MR effect size for
'Gut microbiota abundance (genus Parasutterella id.2892)' on 'Cathepsin F'

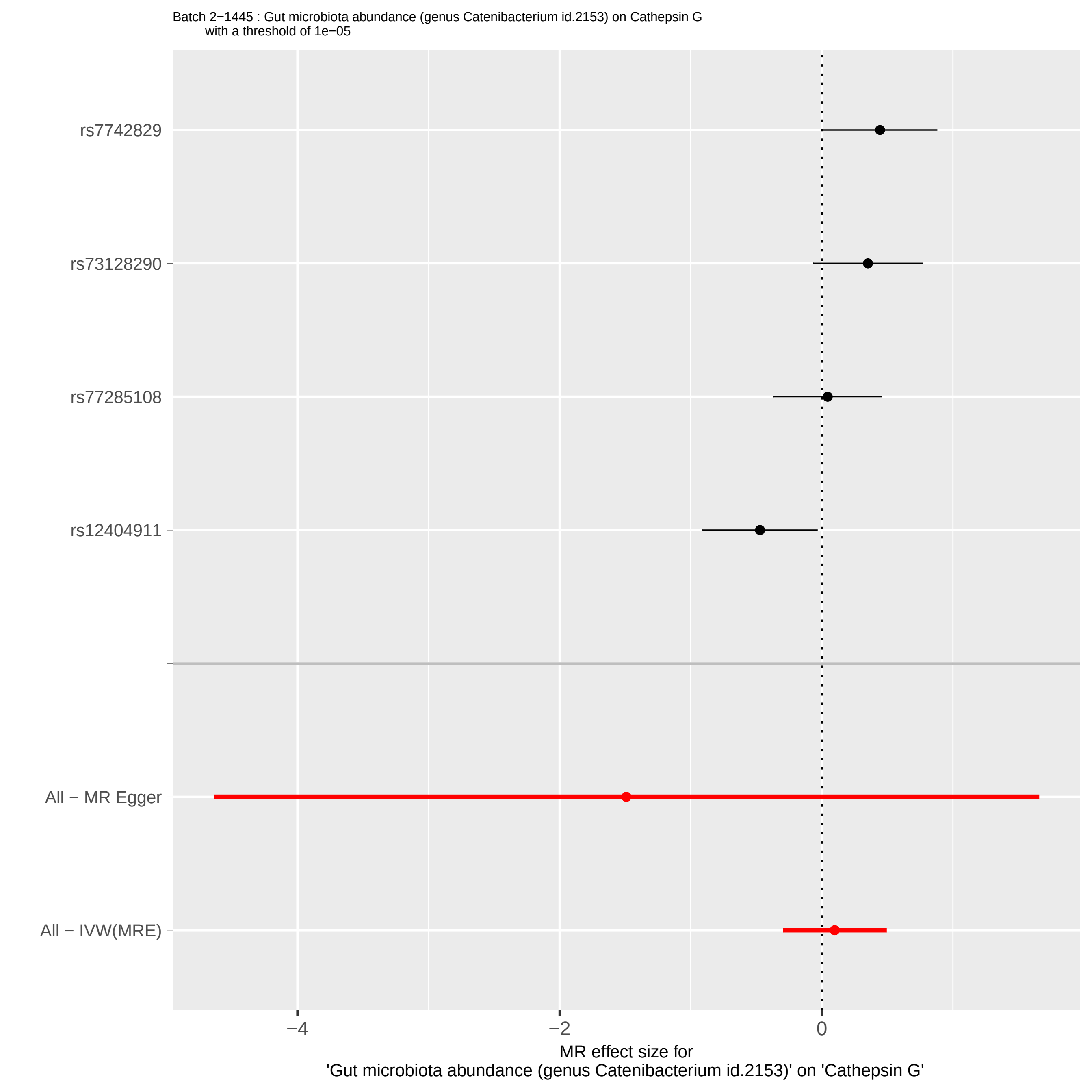

Batch 2−1445 : Gut microbiota abundance (genus Catenibacterium id.2153) on Cathepsin G
with a threshold of 1e−05
rs7742829
rs73128290
rs77285108
rs12404911
All − MR Egger
All − IVW(MRE)
−4
−2
0
MR effect size for
'Gut microbiota abundance (genus Catenibacterium id.2153)' on 'Cathepsin G'

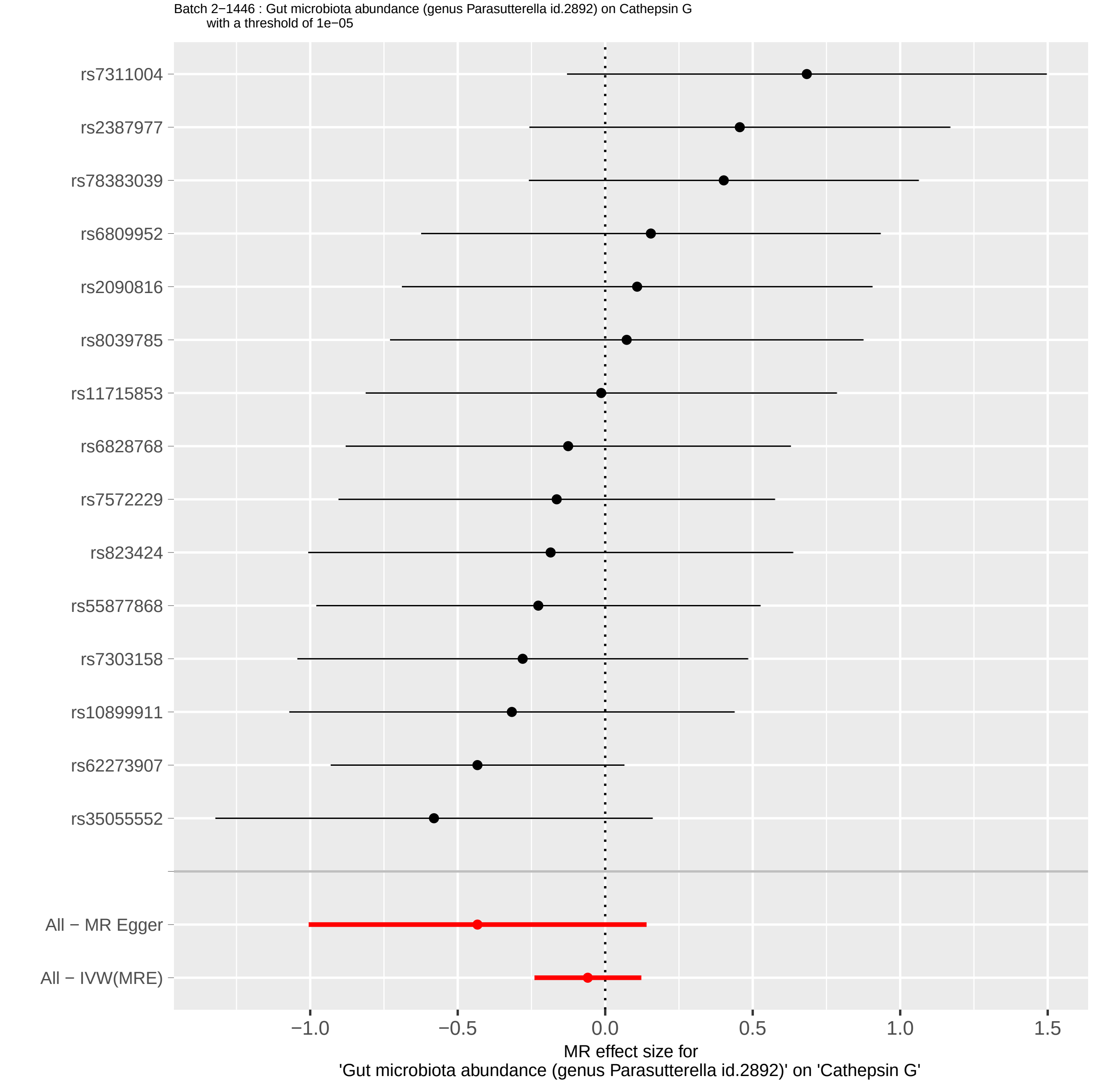
Batch 2−1446 : Gut microbiota abundance (genus Parasutterella id.2892) on Cathepsin G
with a threshold of 1e−05
rs7311004
rs2387977
rs78383039
rs6809952
rs2090816
rs8039785
rs11715853
rs6828768
rs7572229
rs823424
rs55877868
rs7303158
rs10899911
rs62273907
rs35055552
All − MR Egger
All − IVW(MRE)
−1.0
−0.5
0.0
0.5
1.0
1.5
MR effect size for
'Gut microbiota abundance (genus Parasutterella id.2892)' on 'Cathepsin G'

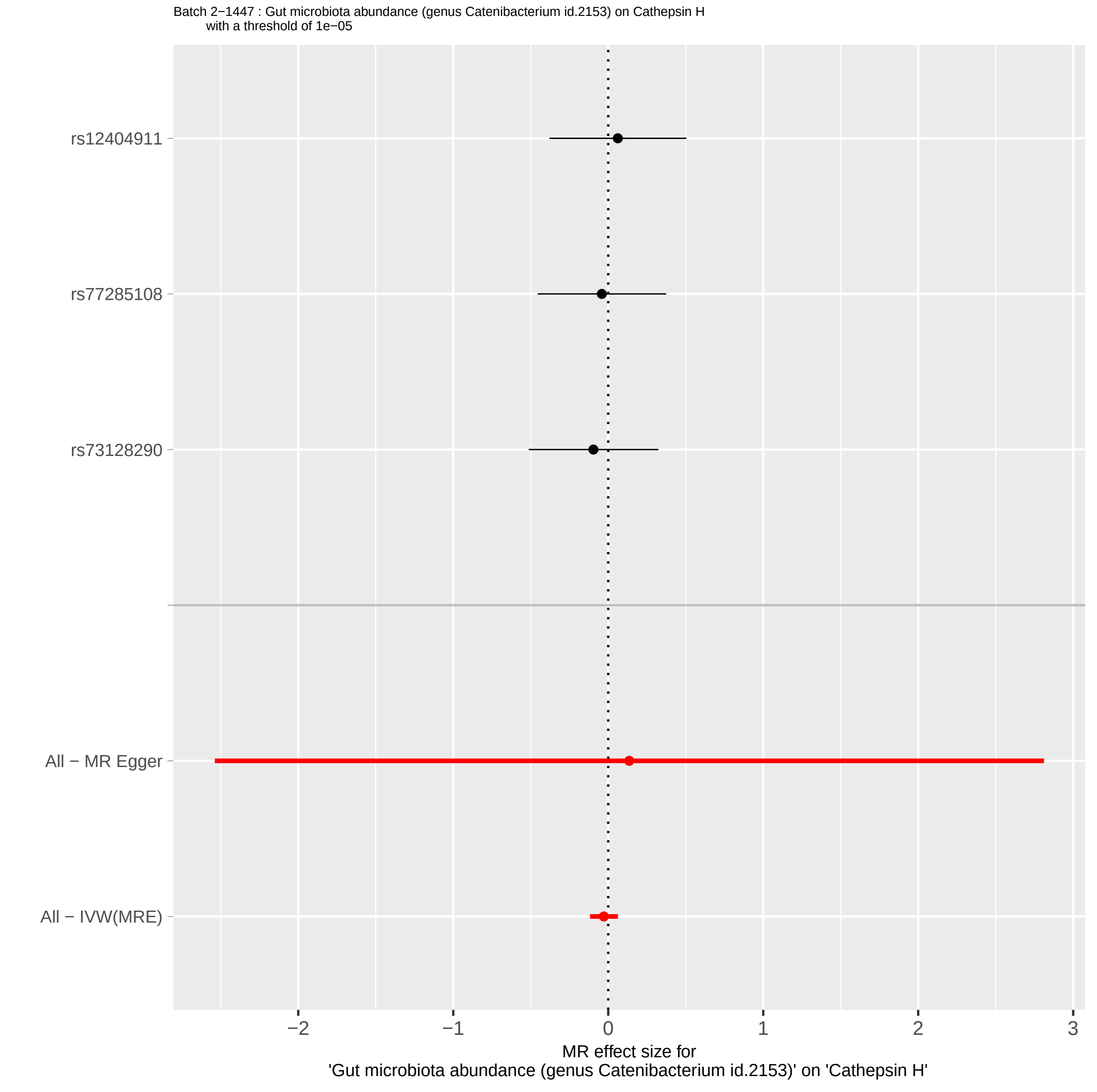
Batch 2−1447 : Gut microbiota abundance (genus Catenibacterium id.2153) on Cathepsin H
with a threshold of 1e−05
rs12404911
rs77285108
rs73128290
All − MR Egger
All − IVW(MRE)
−2
−1
0
1
2
3
MR effect size for
'Gut microbiota abundance (genus Catenibacterium id.2153)' on 'Cathepsin H'

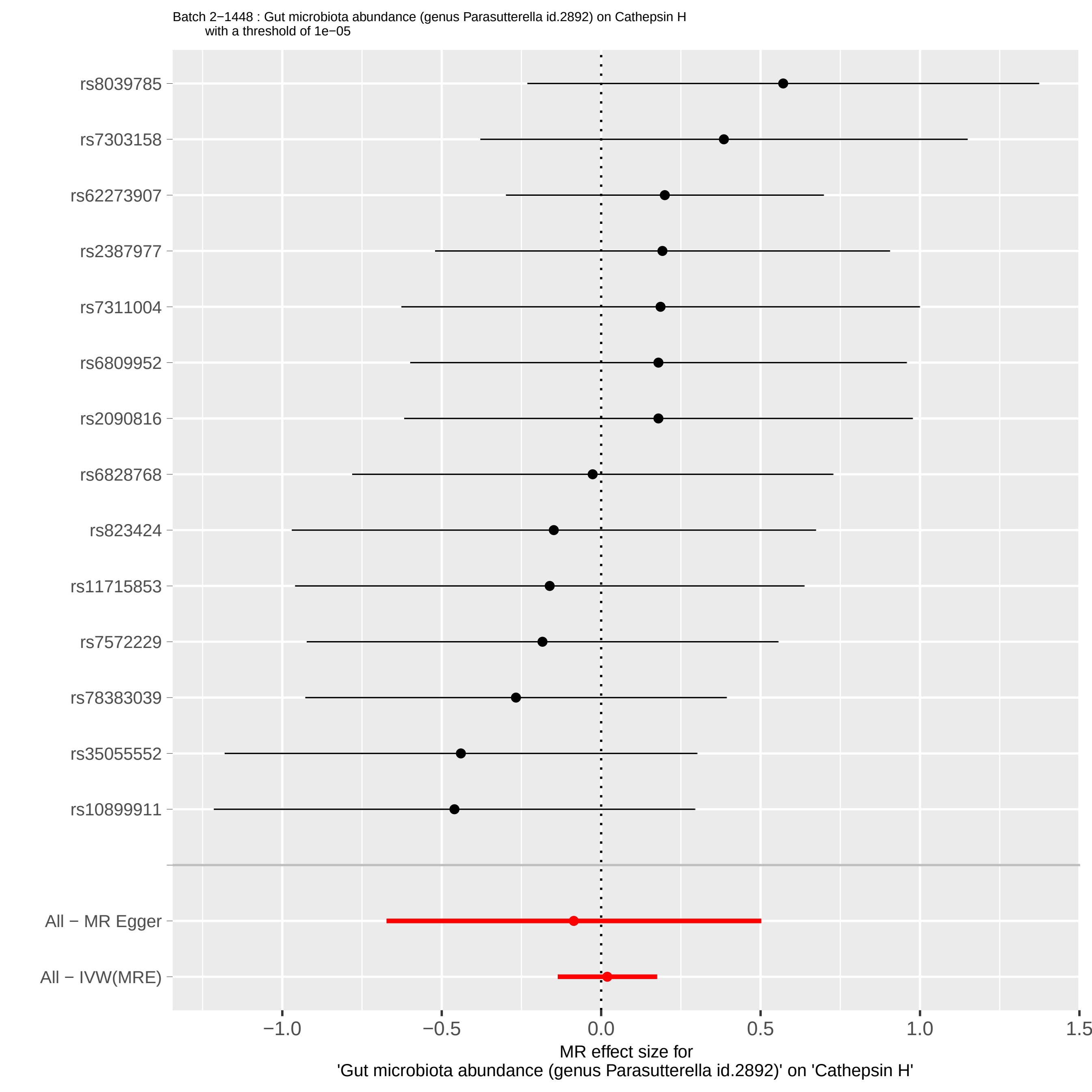

Batch 2−1448 : Gut microbiota abundance (genus Parasutterella id.2892) on Cathepsin H
with a threshold of 1e−05
rs8039785
rs7303158
rs62273907
rs2387977
rs7311004
rs6809952
rs2090816
rs6828768
rs823424
rs11715853
rs7572229
rs78383039
rs35055552
rs10899911
All − MR Egger
All − IVW(MRE)
−1.0
−0.5
0.0
0.5
1.0
1.5
MR effect size for
'Gut microbiota abundance (genus Parasutterella id.2892)' on 'Cathepsin H'

Batch 2−1449 : Gut microbiota abundance (genus Catenibacterium id.2153) on Cathepsin H
with a threshold of 1e−05

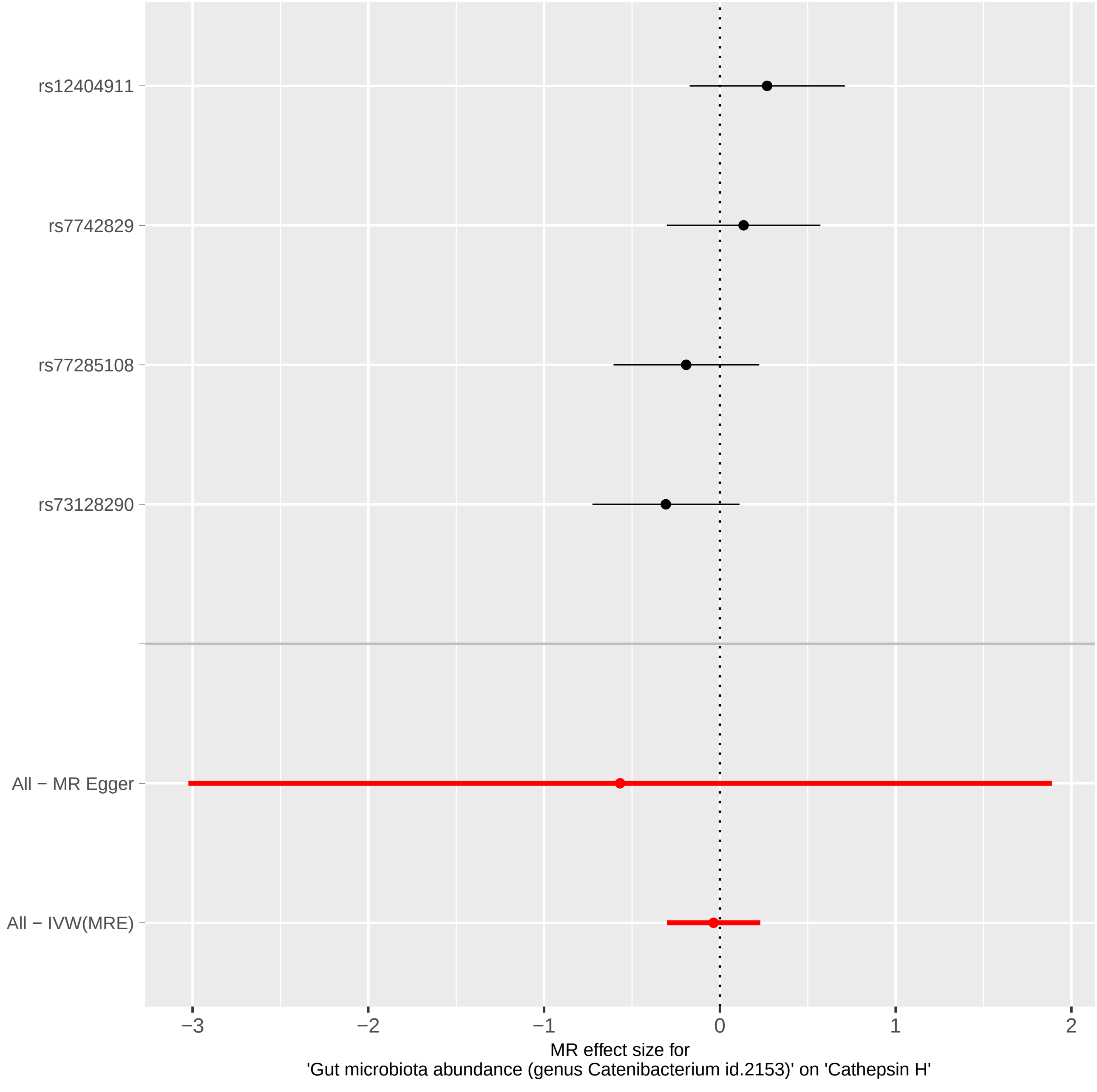

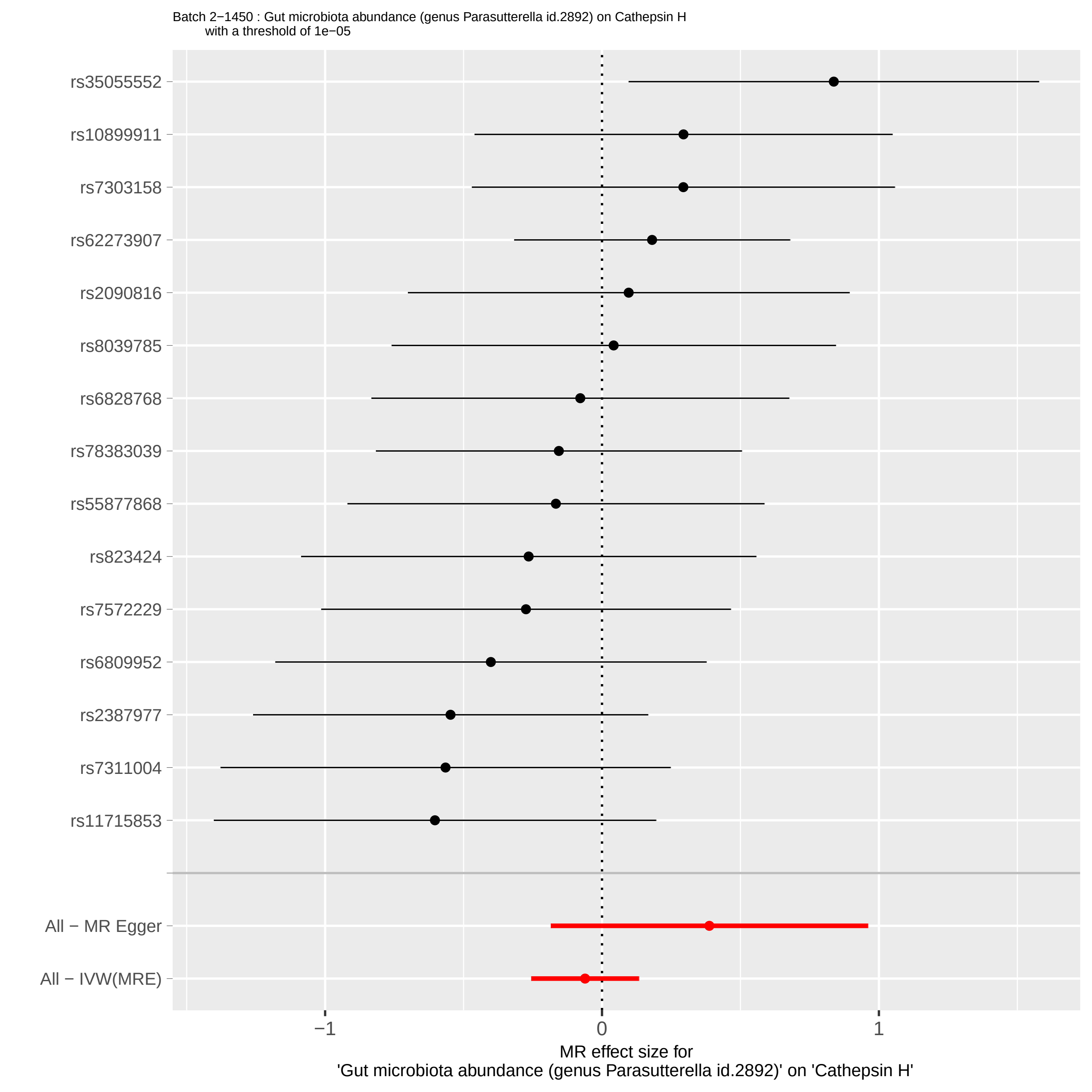

Batch 2−1450 : Gut microbiota abundance (genus Parasutterella id.2892) on Cathepsin H
with a threshold of 1e−05
rs35055552
rs10899911
rs7303158
rs62273907
rs2090816
rs8039785
rs6828768
rs78383039
rs55877868
rs823424
rs7572229
rs6809952
rs2387977
rs7311004
rs11715853
All − MR Egger
All − IVW(MRE)
−1
0
1
MR effect size for
'Gut microbiota abundance (genus Parasutterella id.2892)' on 'Cathepsin H'

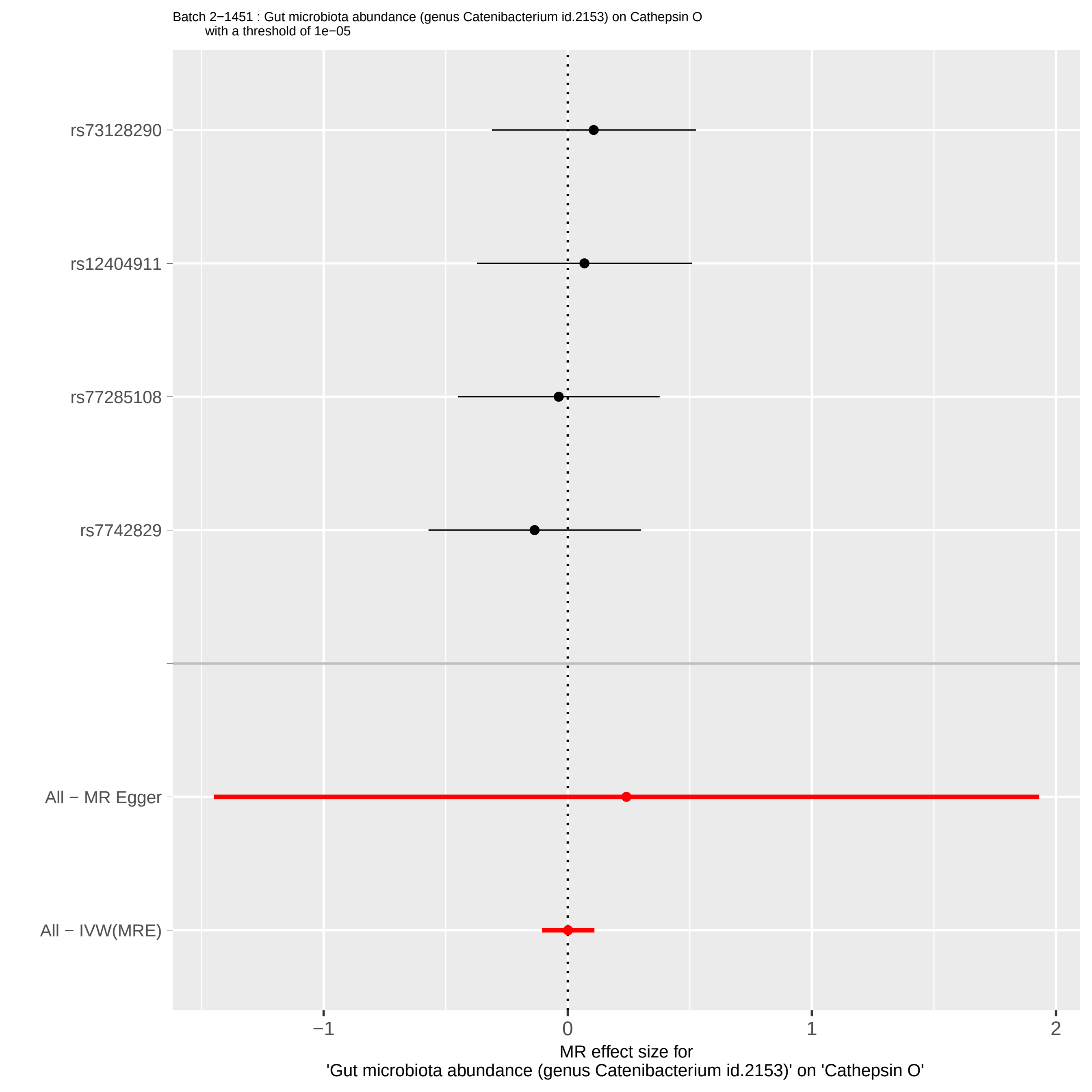

Batch 2−1451 : Gut microbiota abundance (genus Catenibacterium id.2153) on Cathepsin O
with a threshold of 1e−05
rs73128290
rs12404911
rs77285108
rs7742829
All − MR Egger
All − IVW(MRE)
−1
0
1
2
MR effect size for
'Gut microbiota abundance (genus Catenibacterium id.2153)' on 'Cathepsin O'

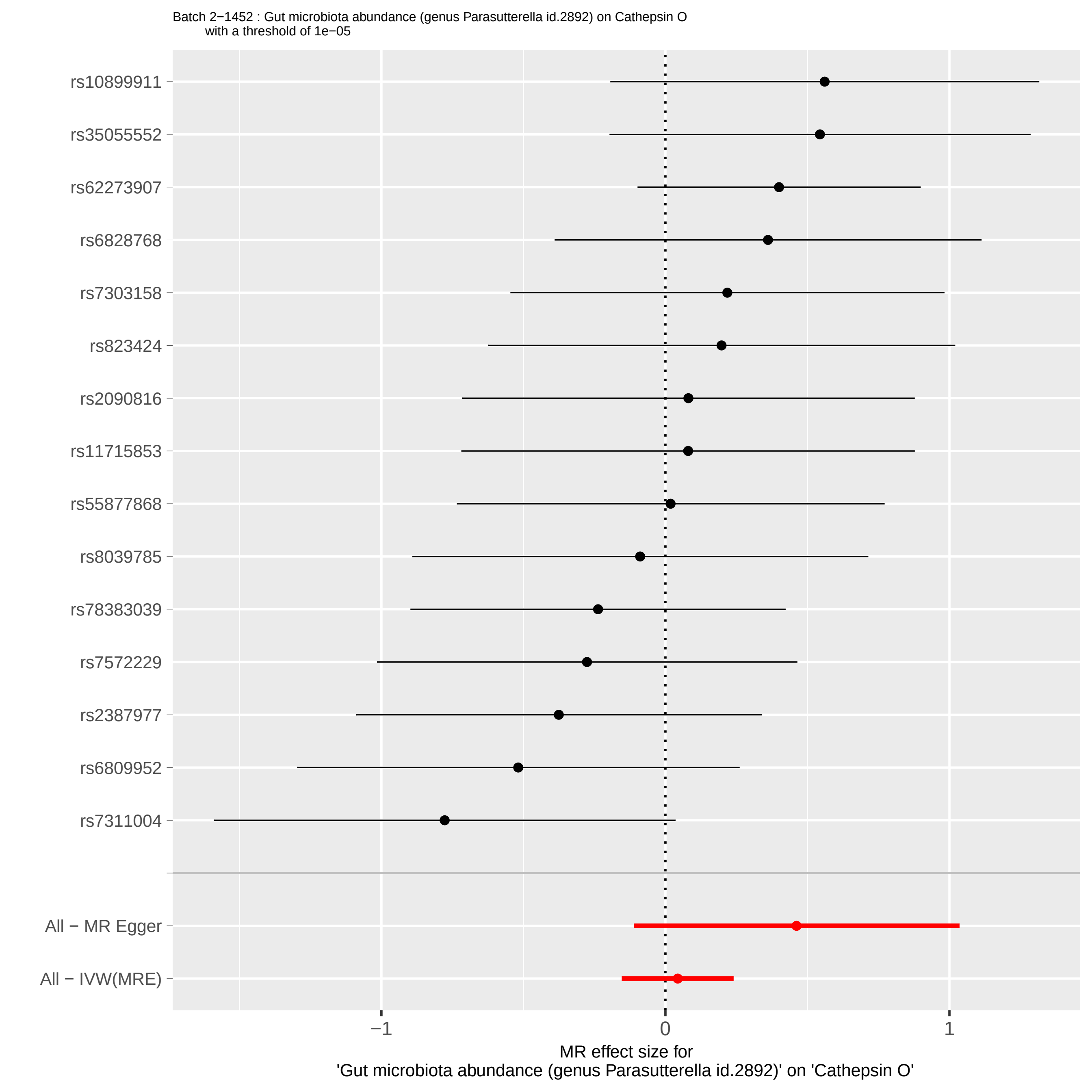

Batch 2−1452 : Gut microbiota abundance (genus Parasutterella id.2892) on Cathepsin O
with a threshold of 1e−05
rs10899911
rs35055552
rs62273907
rs6828768
rs7303158
rs823424
rs2090816
rs11715853
rs55877868
rs8039785
rs78383039
rs7572229
rs2387977
rs6809952
rs7311004
All − MR Egger
All − IVW(MRE)
−1
0
1
MR effect size for
'Gut microbiota abundance (genus Parasutterella id.2892)' on 'Cathepsin O'

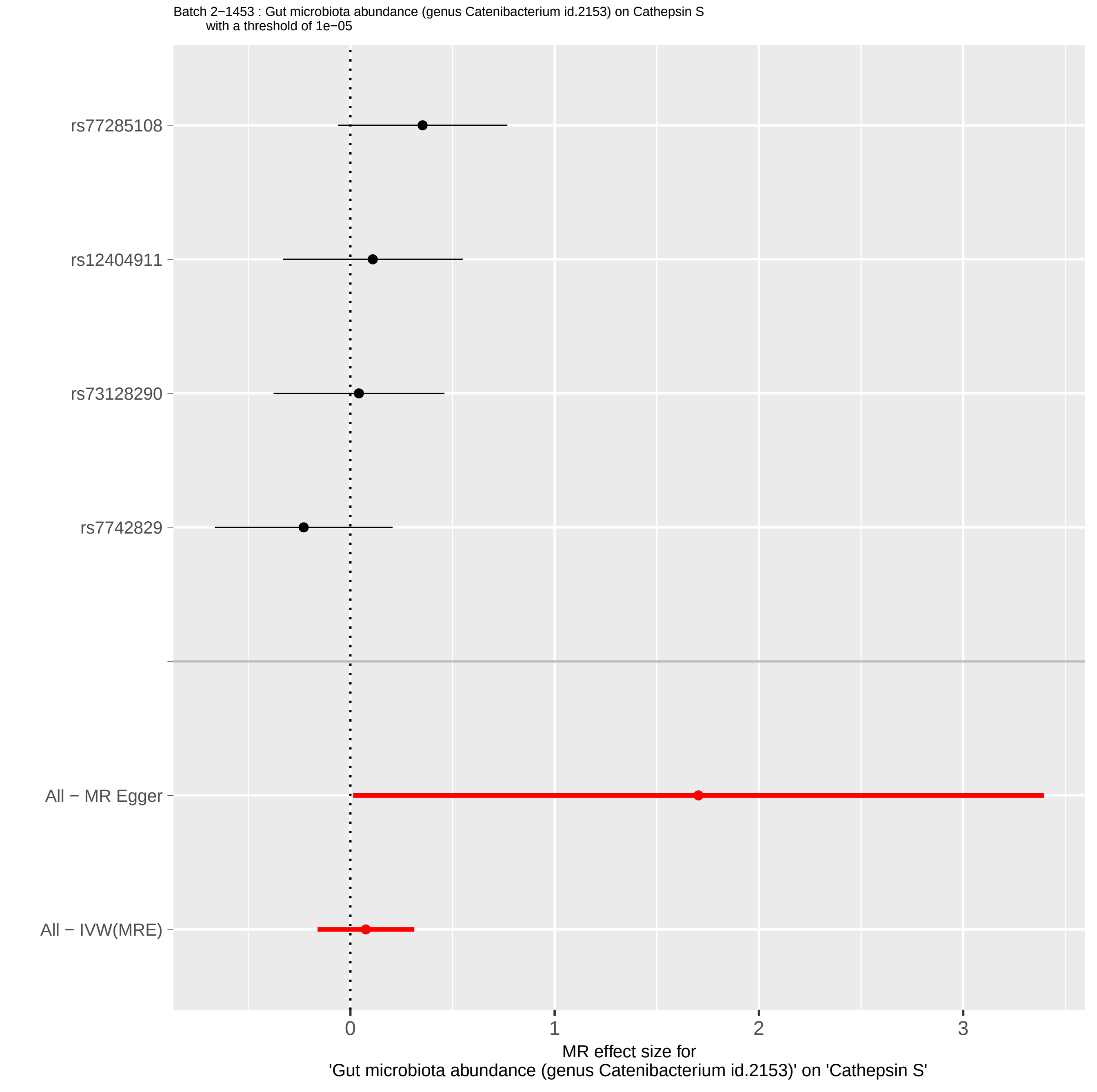

Batch 2−1453 : Gut microbiota abundance (genus Catenibacterium id.2153) on Cathepsin S
with a threshold of 1e−05
rs77285108
rs12404911
rs73128290
rs7742829
All − MR Egger
All − IVW(MRE)
0
1
2
3
MR effect size for
'Gut microbiota abundance (genus Catenibacterium id.2153)' on 'Cathepsin S'

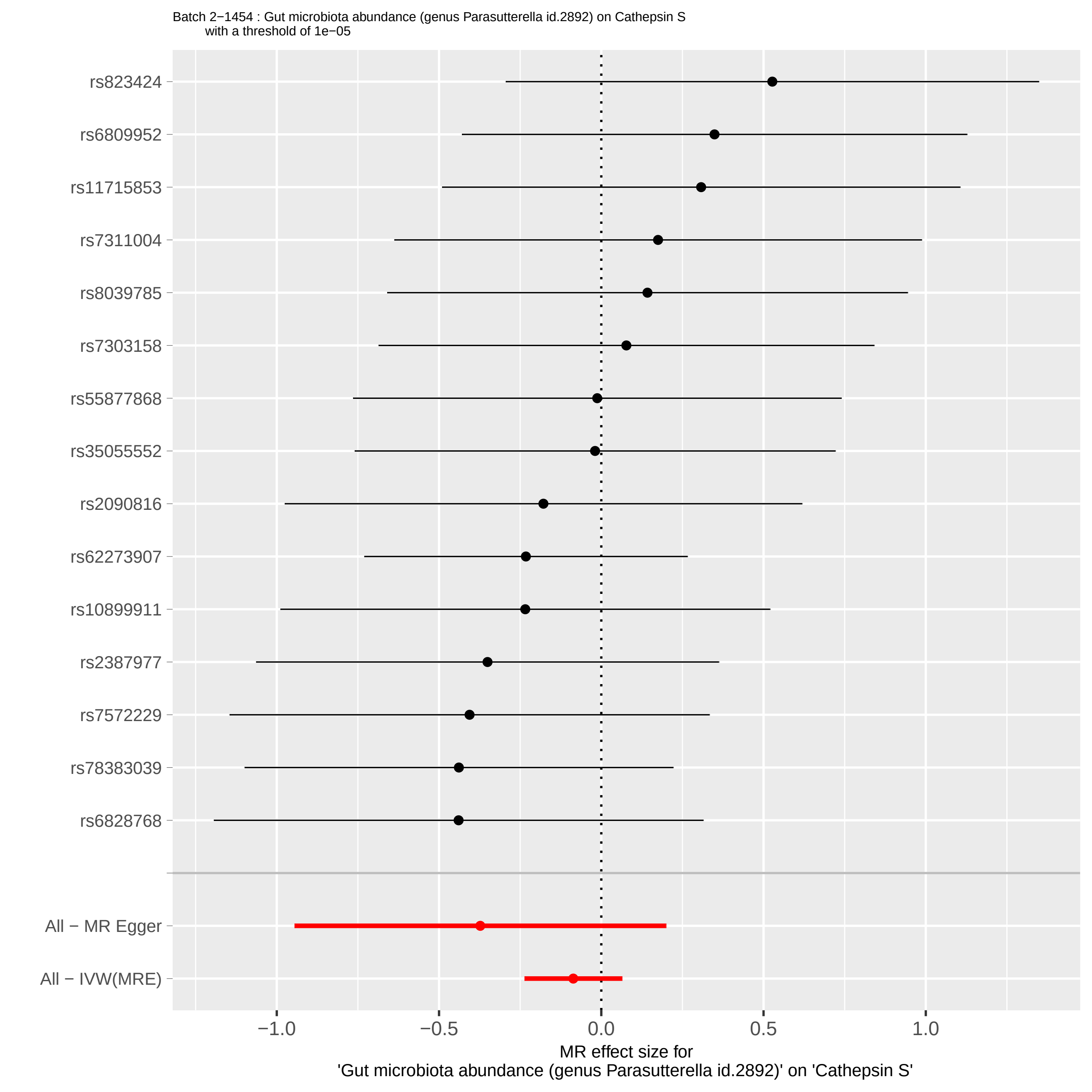
Batch 2−1454 : Gut microbiota abundance (genus Parasutterella id.2892) on Cathepsin S
with a threshold of 1e−05
rs823424
rs6809952
rs11715853
rs7311004
rs8039785
rs7303158
rs55877868
rs35055552
rs2090816
rs62273907
rs10899911
rs2387977
rs7572229
rs78383039
rs6828768
All − MR Egger
All − IVW(MRE)
−1.0
−0.5
0.0
0.5
1.0
MR effect size for
'Gut microbiota abundance (genus Parasutterella id.2892)' on 'Cathepsin S'

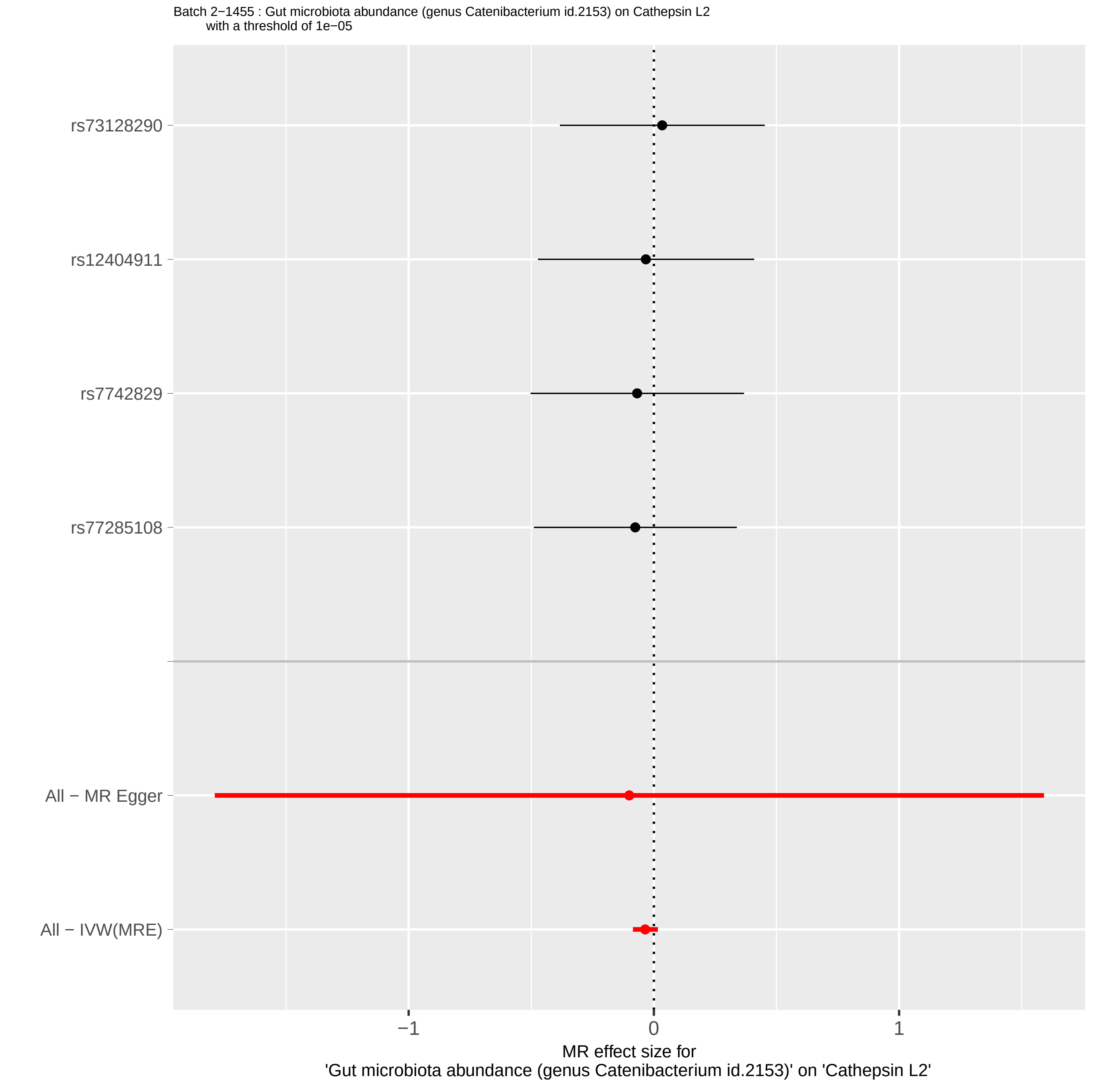
Batch 2−1455 : Gut microbiota abundance (genus Catenibacterium id.2153) on Cathepsin L2
with a threshold of 1e−05
rs73128290
rs12404911
rs7742829
rs77285108
All − MR Egger
All − IVW(MRE)
−1
0
1
MR effect size for
'Gut microbiota abundance (genus Catenibacterium id.2153)' on 'Cathepsin L2'

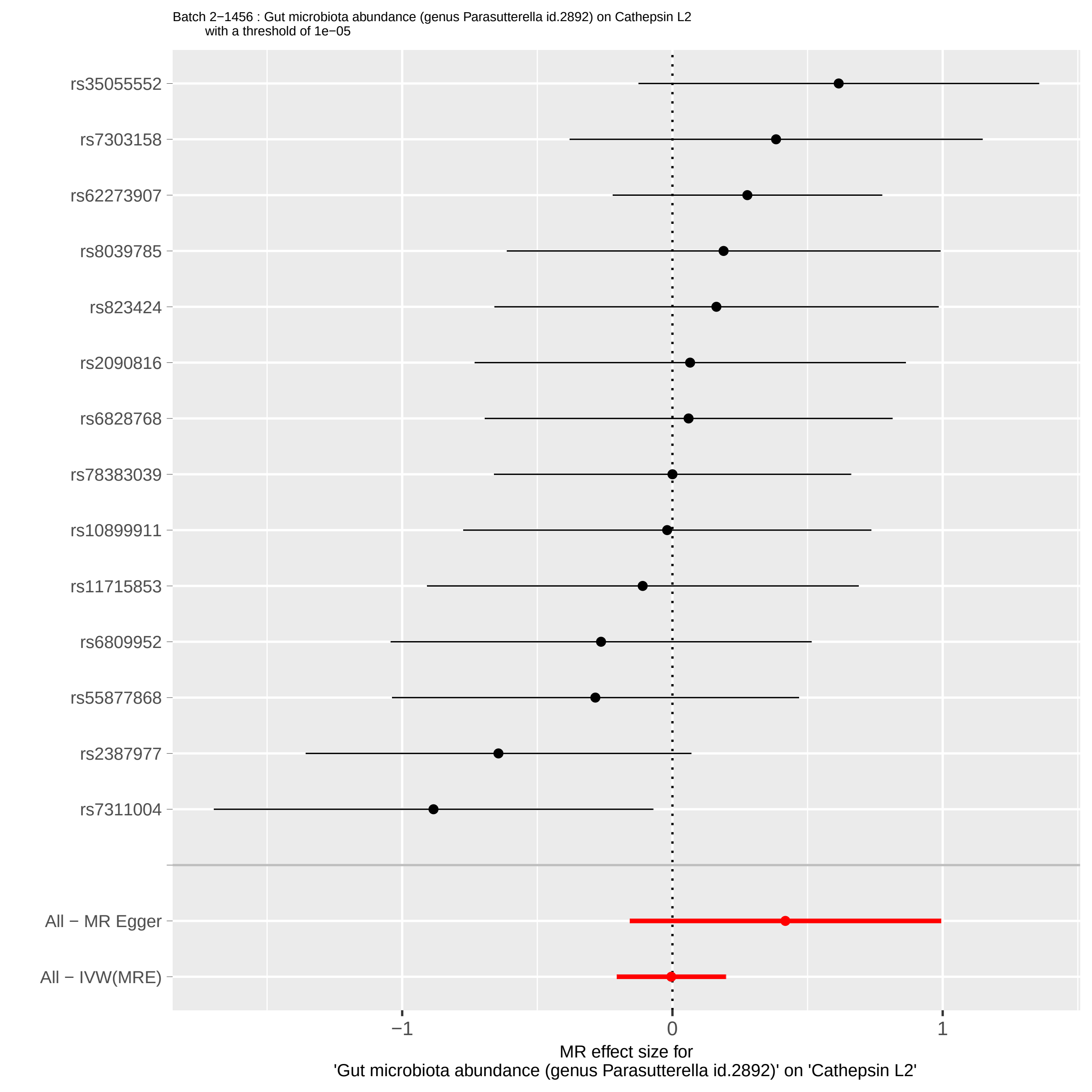

Batch 2−1456 : Gut microbiota abundance (genus Parasutterella id.2892) on Cathepsin L2
with a threshold of 1e−05
rs35055552
rs7303158
rs62273907
rs8039785
rs823424
rs2090816
rs6828768
rs78383039
rs10899911
rs11715853
rs6809952
rs55877868
rs2387977
rs7311004
All − MR Egger
All − IVW(MRE)
−1
0
1
MR effect size for
'Gut microbiota abundance (genus Parasutterella id.2892)' on 'Cathepsin L2'

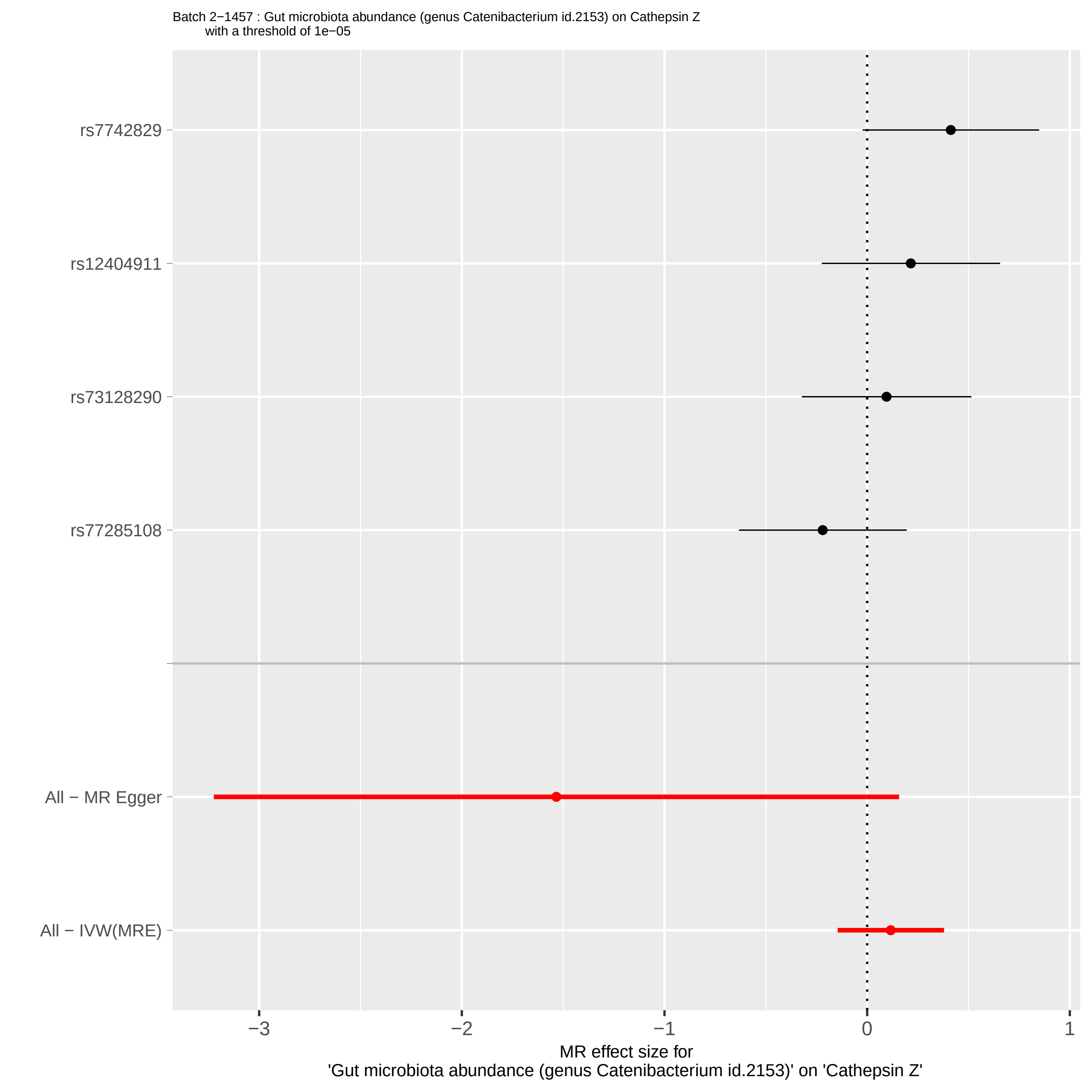

Batch 2−1457 : Gut microbiota abundance (genus Catenibacterium id.2153) on Cathepsin Z
with a threshold of 1e−05
rs7742829
rs12404911
rs73128290
rs77285108
All − MR Egger
All − IVW(MRE)
−3
−2
−1
0
1
MR effect size for
'Gut microbiota abundance (genus Catenibacterium id.2153)' on 'Cathepsin Z'

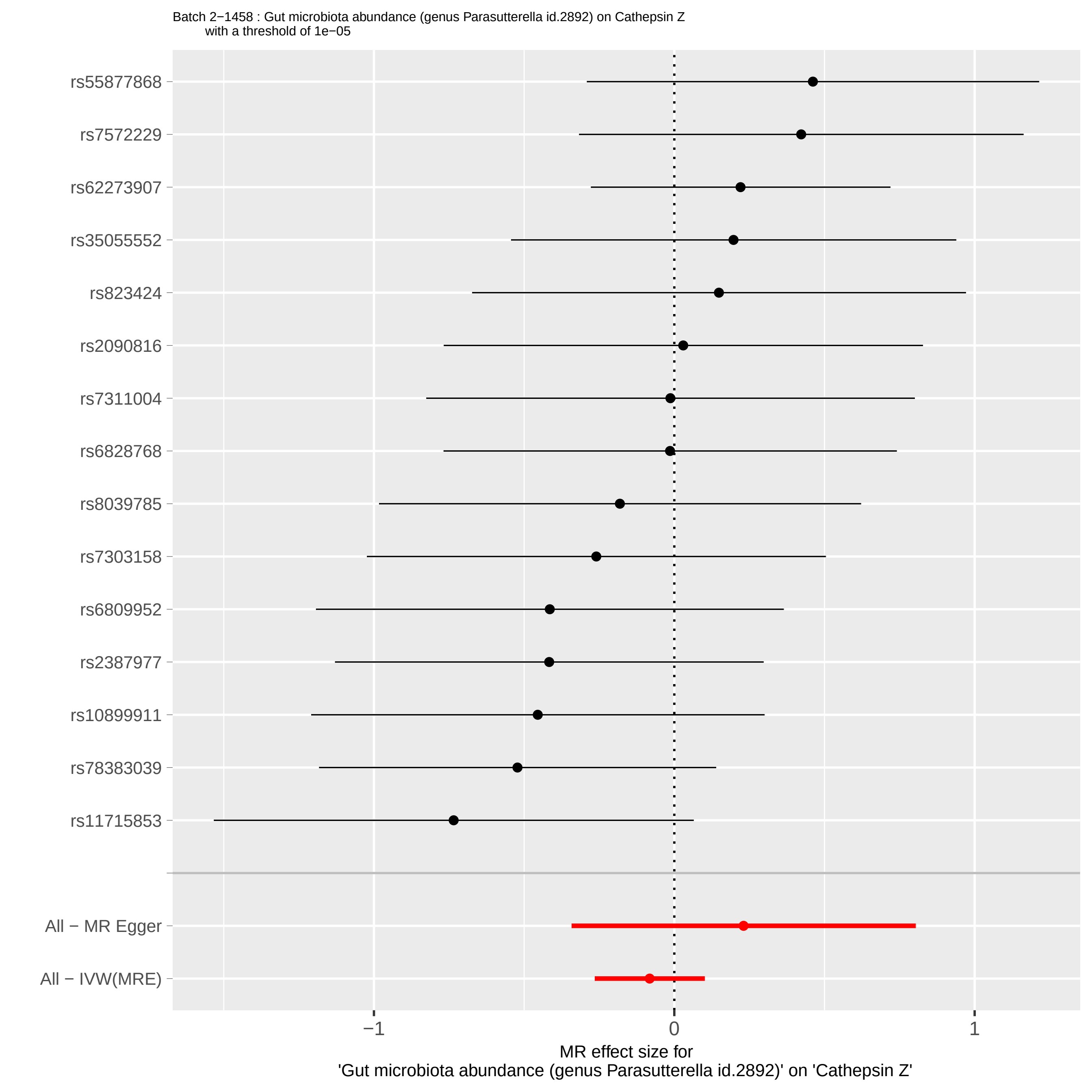
Batch 2−1458 : Gut microbiota abundance (genus Parasutterella id.2892) on Cathepsin Z
with a threshold of 1e−05
rs55877868
rs7572229
rs62273907
rs35055552
rs823424
rs2090816
rs7311004
rs6828768
rs8039785
rs7303158
rs6809952
rs2387977
rs10899911
rs78383039
rs11715853
All − MR Egger
All − IVW(MRE)
−1
0
1
MR effect size for
'Gut microbiota abundance (genus Parasutterella id.2892)' on 'Cathepsin Z'

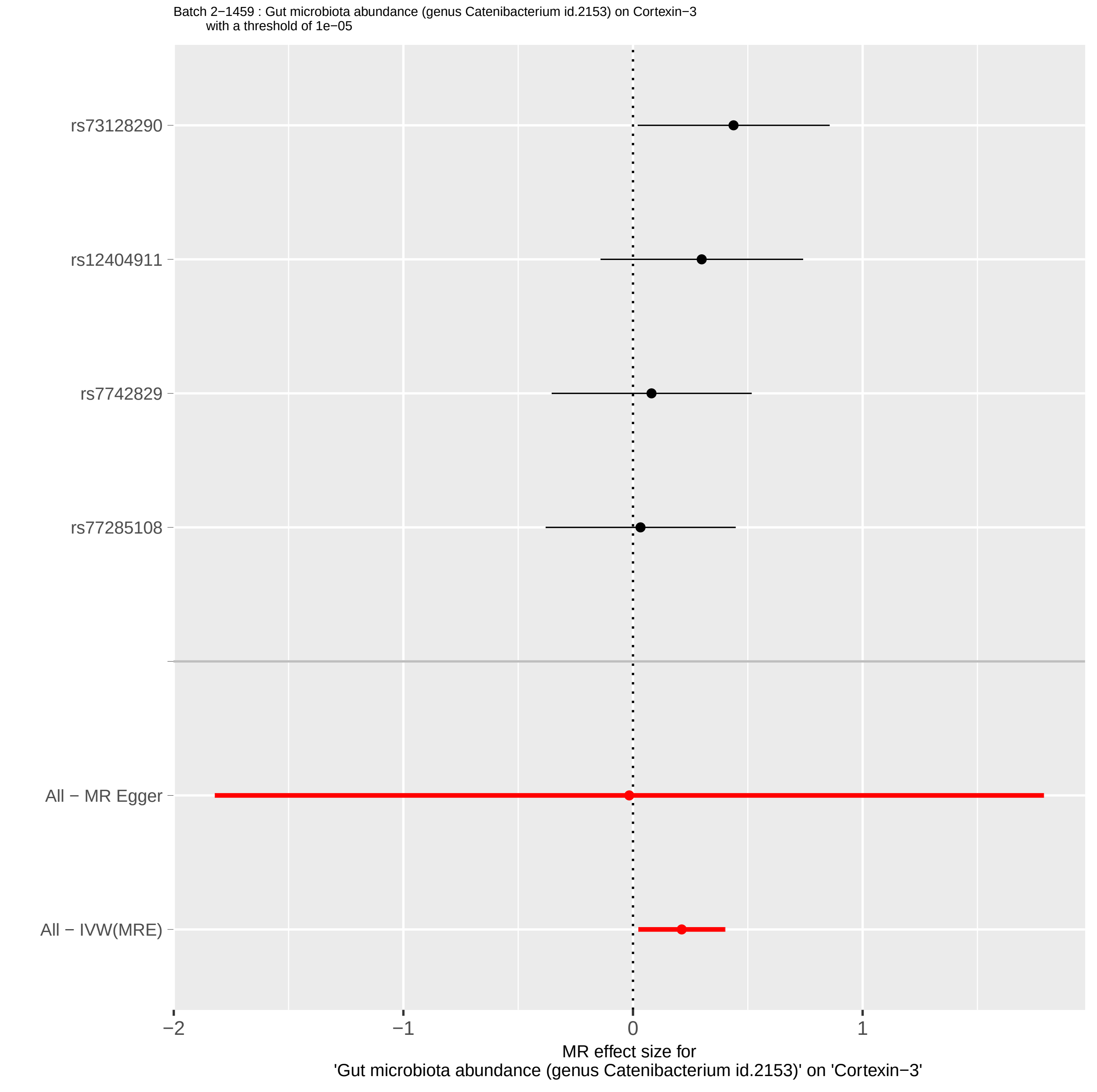

Batch 2−1459 : Gut microbiota abundance (genus Catenibacterium id.2153) on Cortexin−3
with a threshold of 1e−05
rs73128290
rs12404911
rs7742829
rs77285108
All − MR Egger
All − IVW(MRE)
−2
−1
0
1
MR effect size for
'Gut microbiota abundance (genus Catenibacterium id.2153)' on 'Cortexin−3'

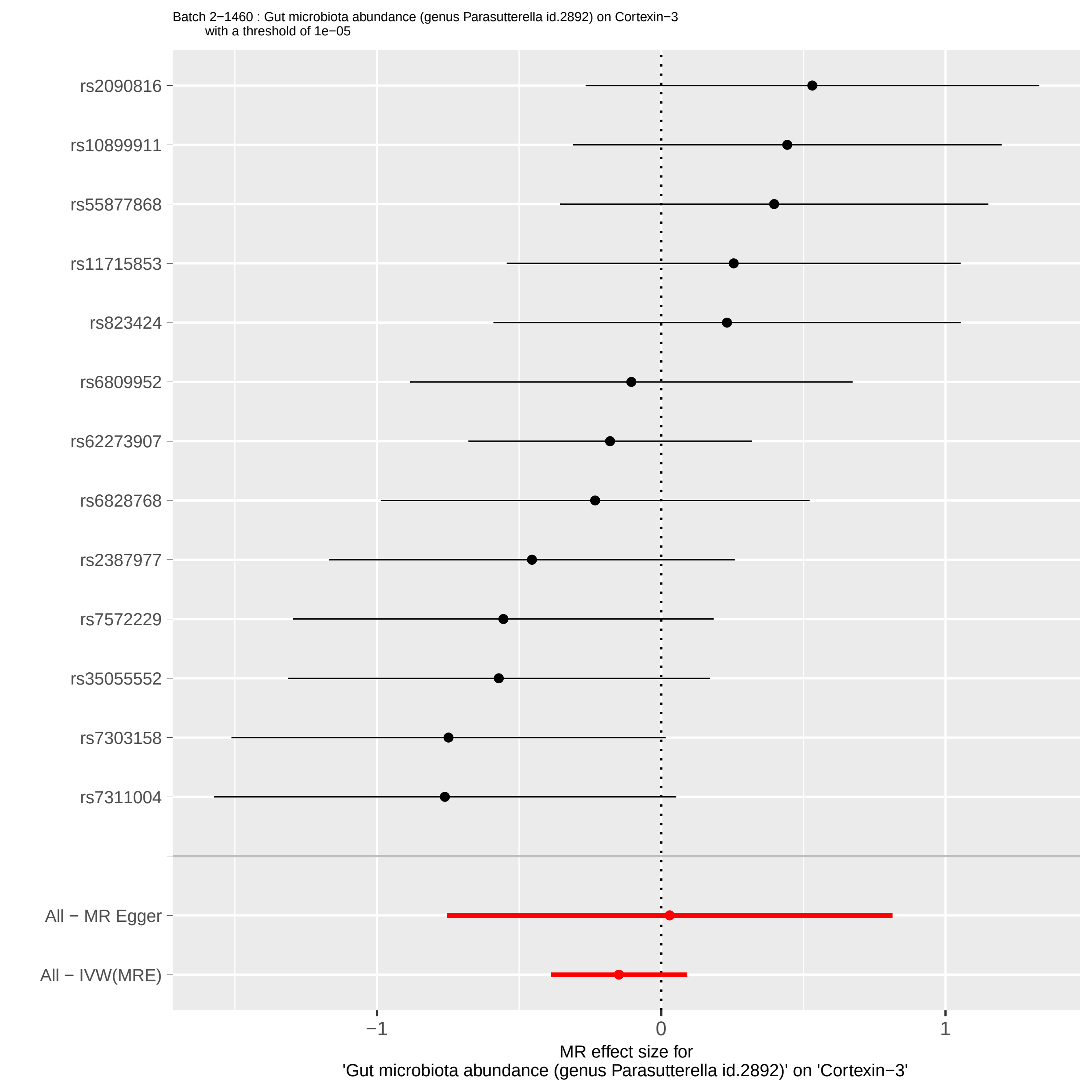
Batch 2−1460 : Gut microbiota abundance (genus Parasutterella id.2892) on Cortexin−3
with a threshold of 1e−05
rs2090816
rs10899911
rs55877868
rs11715853
rs823424
rs6809952
rs62273907
rs6828768
rs2387977
rs7572229
rs35055552
rs7303158
rs7311004
All − MR Egger
All − IVW(MRE)
−1
0
1
MR effect size for
'Gut microbiota abundance (genus Parasutterella id.2892)' on 'Cortexin−3'

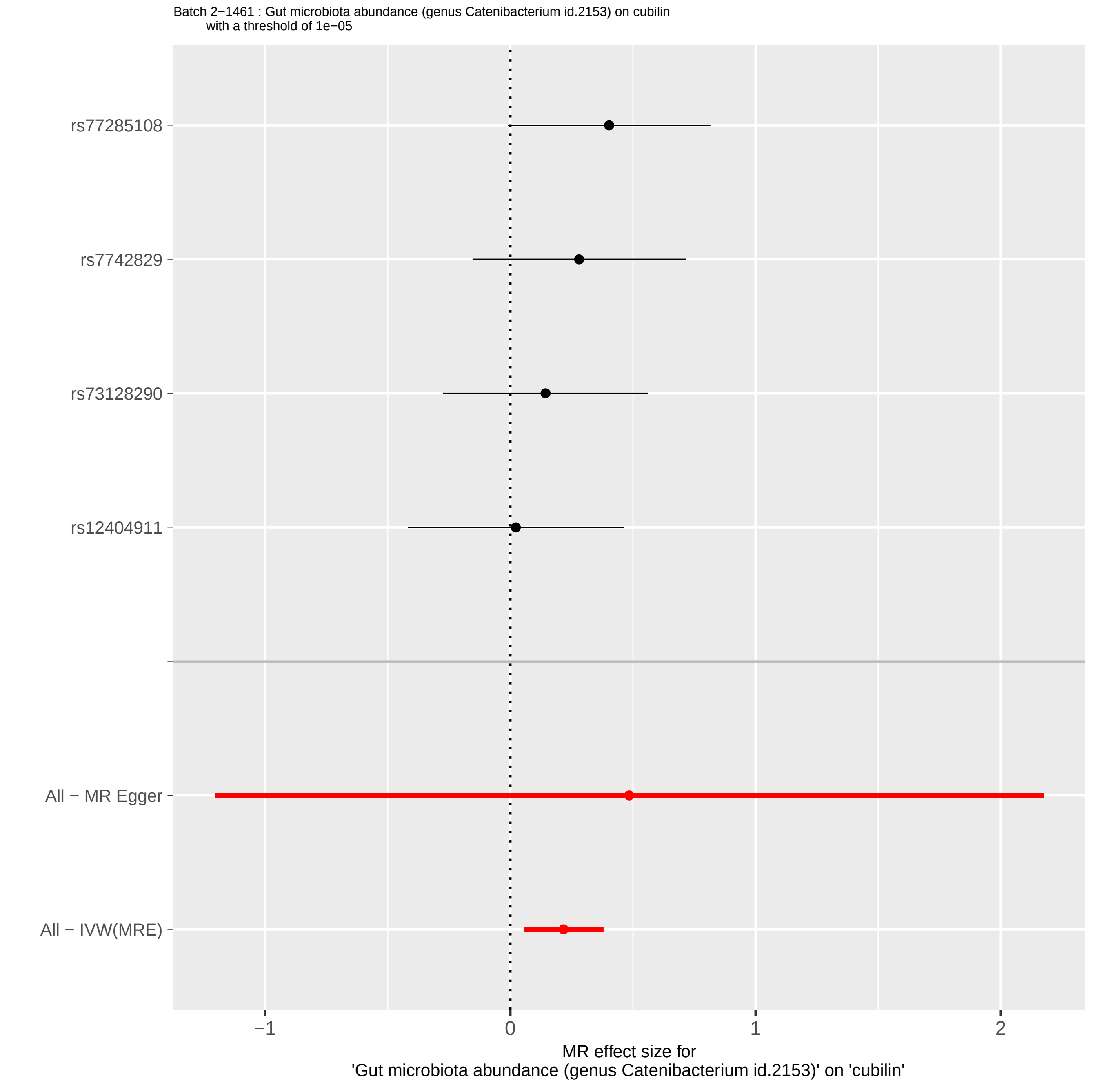
Batch 2−1461 : Gut microbiota abundance (genus Catenibacterium id.2153) on cubilin
with a threshold of 1e−05
rs77285108
rs7742829
rs73128290
rs12404911
All − MR Egger
All − IVW(MRE)
−1
0
1
2
MR effect size for
'Gut microbiota abundance (genus Catenibacterium id.2153)' on 'cubilin'

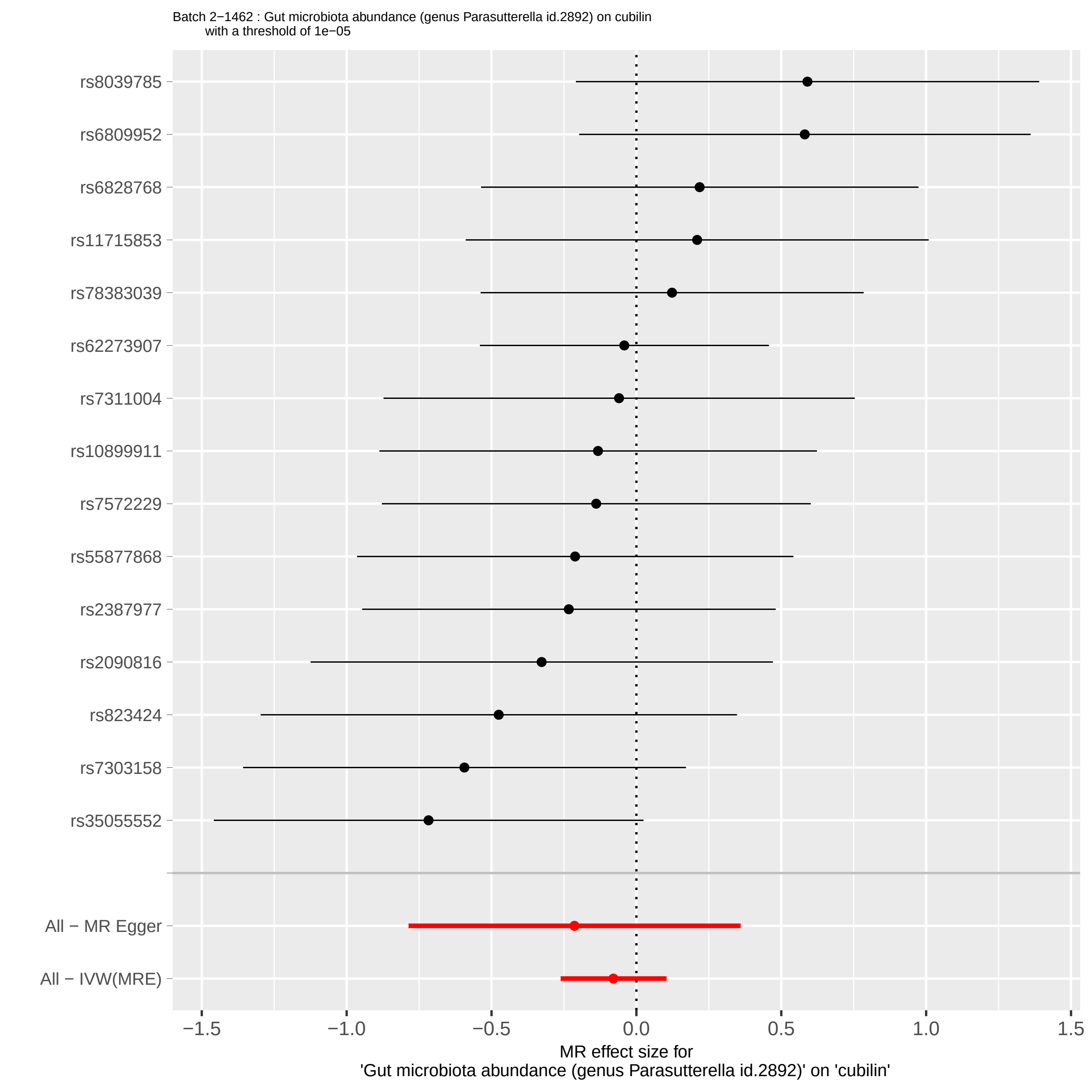
Batch 2−1462 : Gut microbiota abundance (genus Parasutterella id.2892) on cubilin
with a threshold of 1e−05
rs8039785
rs6809952
rs6828768
rs11715853
rs78383039
rs62273907
rs7311004
rs10899911
rs7572229
rs55877868
rs2387977
rs2090816
rs823424
rs7303158
rs35055552
All − MR Egger
All − IVW(MRE)
−1.5
−1.0
−0.5
0.0
0.5
1.0
1.5
MR effect size for
'Gut microbiota abundance (genus Parasutterella id.2892)' on 'cubilin'

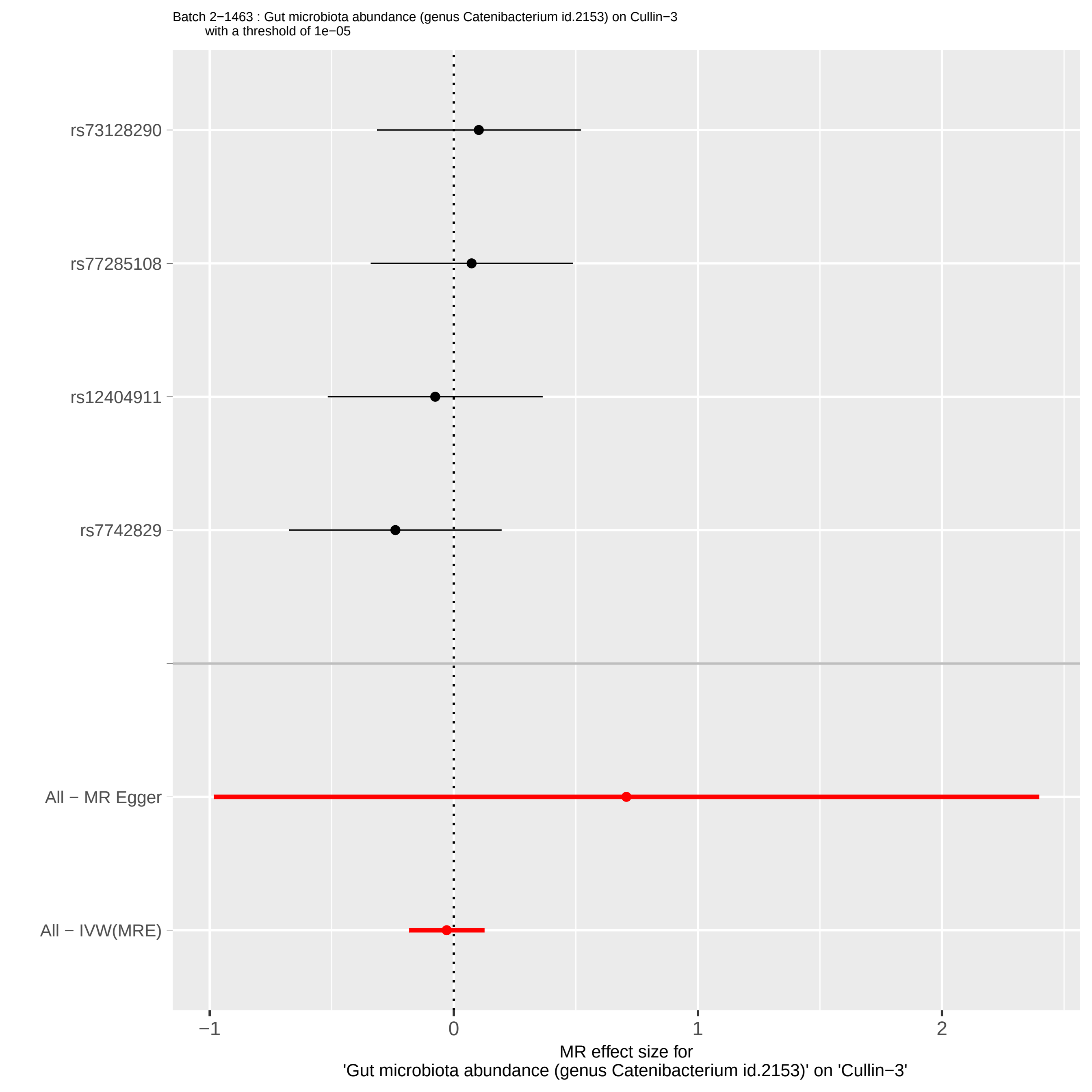

Batch 2−1463 : Gut microbiota abundance (genus Catenibacterium id.2153) on Cullin−3
with a threshold of 1e−05
rs73128290
rs77285108
rs12404911
rs7742829
All − MR Egger
All − IVW(MRE)
−1
0
1
2
MR effect size for
'Gut microbiota abundance (genus Catenibacterium id.2153)' on 'Cullin−3'

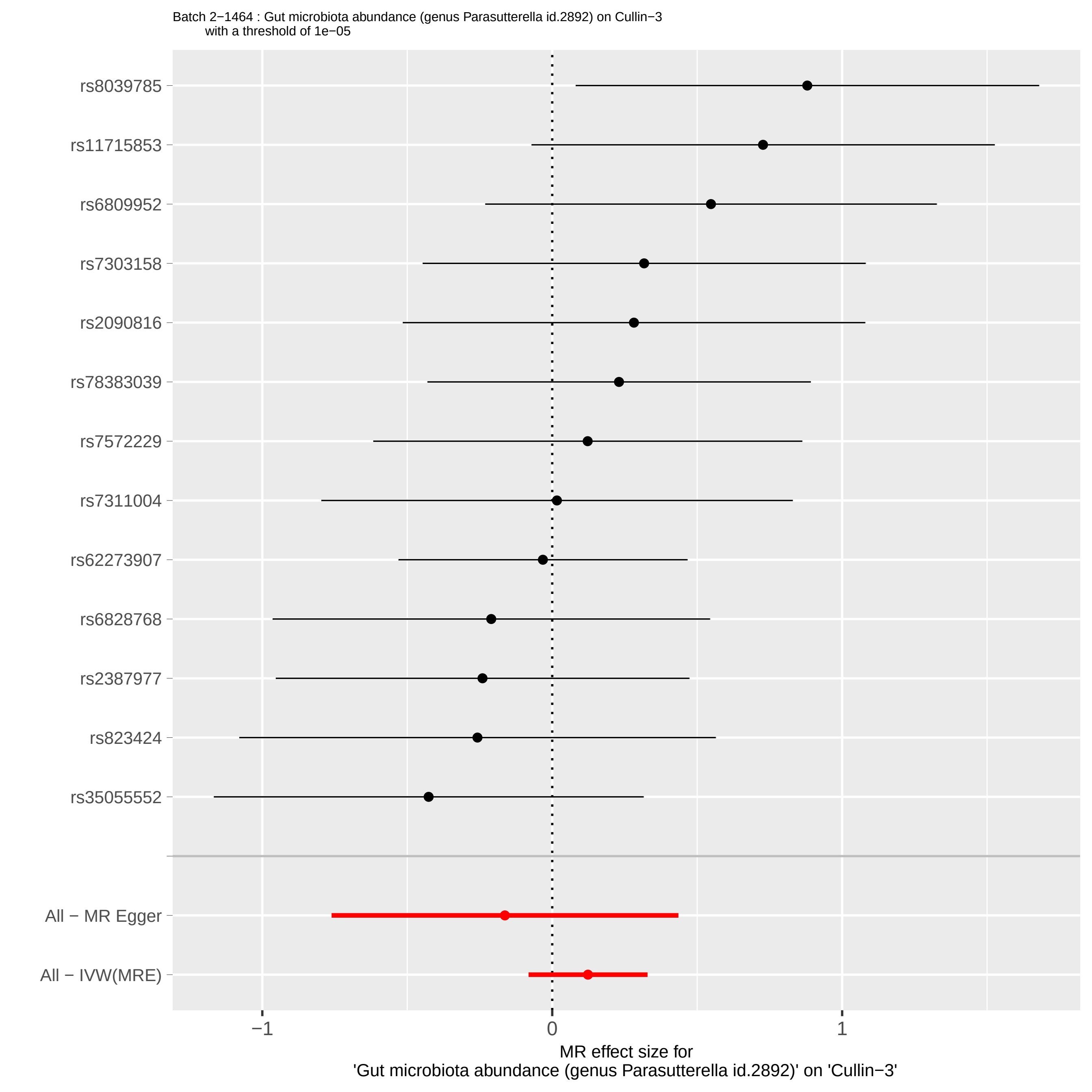
Batch 2−1464 : Gut microbiota abundance (genus Parasutterella id.2892) on Cullin−3
with a threshold of 1e−05
rs8039785
rs11715853
rs6809952
rs7303158
rs2090816
rs78383039
rs7572229
rs7311004
rs62273907
rs6828768
rs2387977
rs823424
rs35055552
All − MR Egger
All − IVW(MRE)
−1
0
1
MR effect size for
'Gut microbiota abundance (genus Parasutterella id.2892)' on 'Cullin−3'

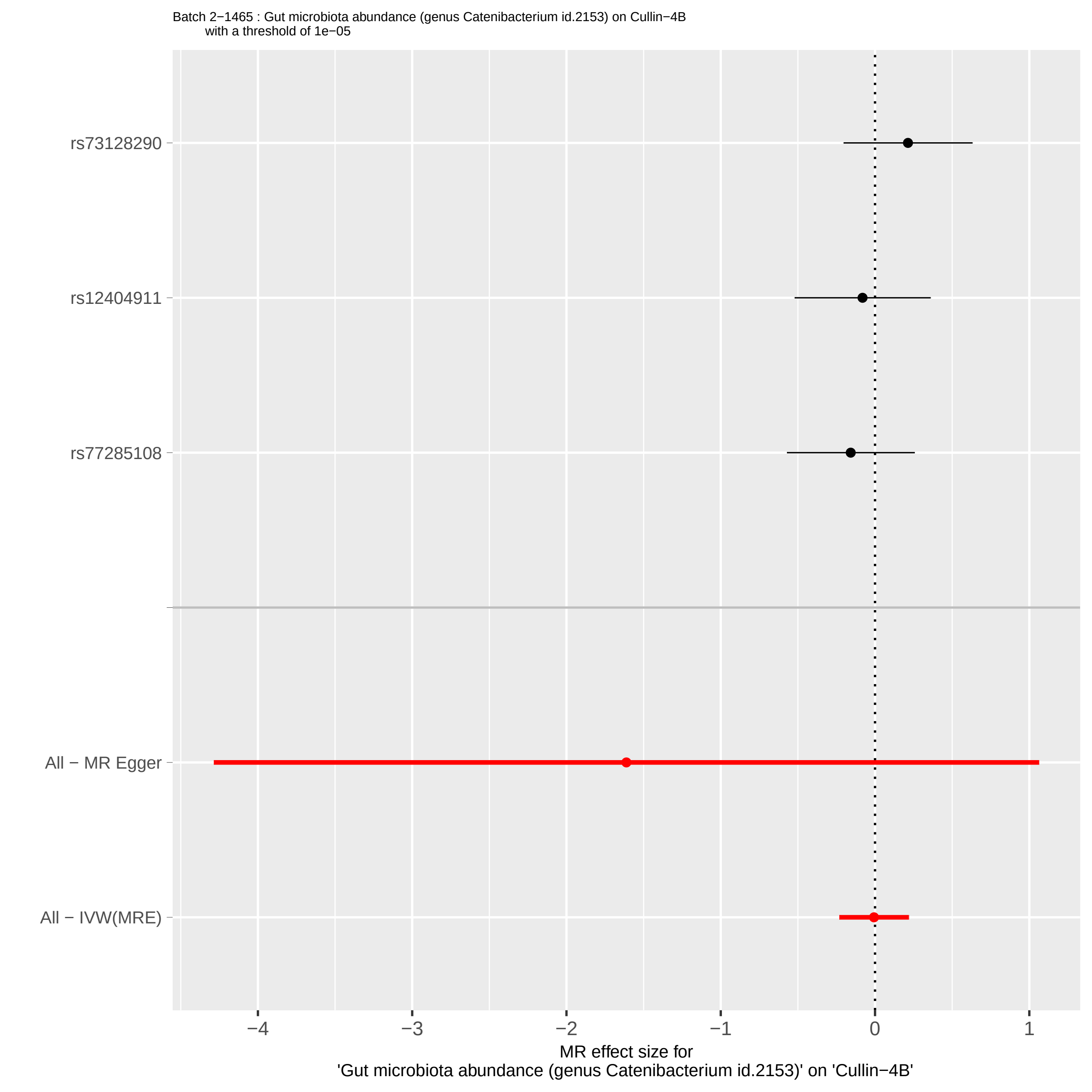

Batch 2−1465 : Gut microbiota abundance (genus Catenibacterium id.2153) on Cullin−4B
with a threshold of 1e−05
rs73128290
rs12404911
rs77285108
All − MR Egger
All − IVW(MRE)
−4
−3
−2
−1
0
1
MR effect size for
'Gut microbiota abundance (genus Catenibacterium id.2153)' on 'Cullin−4B'

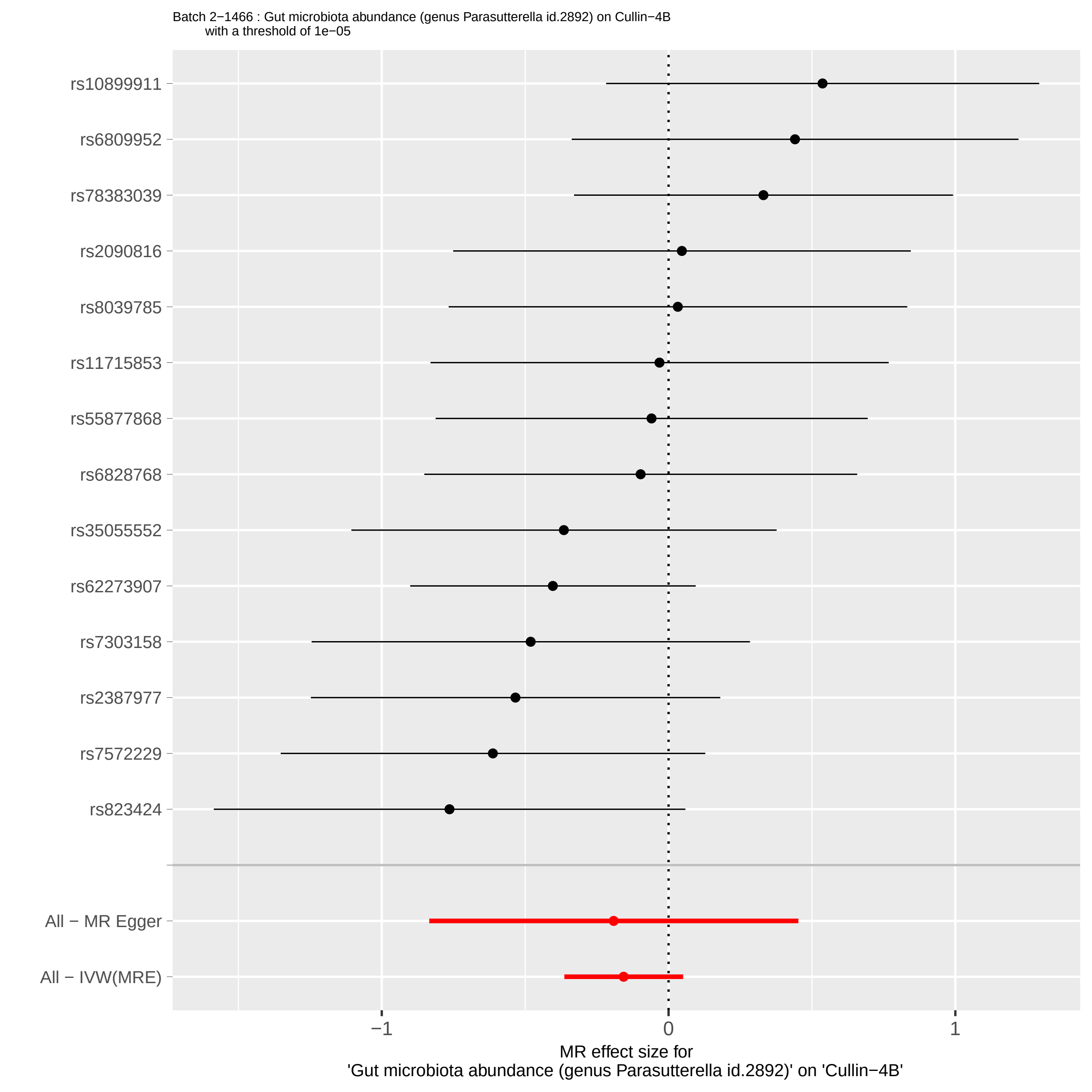

Batch 2−1466 : Gut microbiota abundance (genus Parasutterella id.2892) on Cullin−4B
with a threshold of 1e−05
rs10899911
rs6809952
rs78383039
rs2090816
rs8039785
rs11715853
rs55877868
rs6828768
rs35055552
rs62273907
rs7303158
rs2387977
rs7572229
rs823424
All − MR Egger
All − IVW(MRE)
−1
0
1
MR effect size for
'Gut microbiota abundance (genus Parasutterella id.2892)' on 'Cullin−4B'

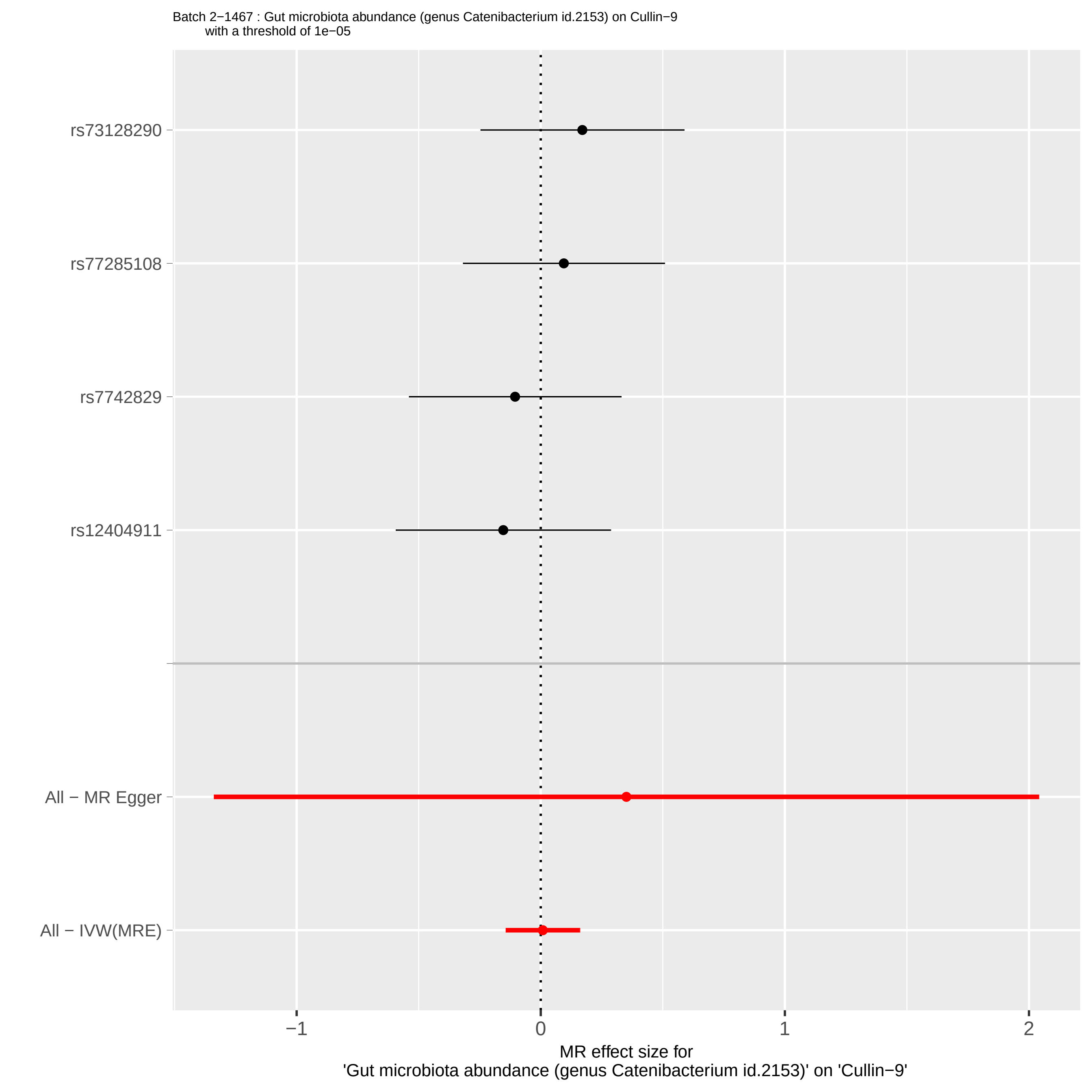
Batch 2−1467 : Gut microbiota abundance (genus Catenibacterium id.2153) on Cullin−9
with a threshold of 1e−05
rs73128290
rs77285108
rs7742829
rs12404911
All − MR Egger
All − IVW(MRE)
−1
0
1
2
MR effect size for
'Gut microbiota abundance (genus Catenibacterium id.2153)' on 'Cullin−9'

Batch 2−1468 : Gut microbiota abundance (genus Parasutterella id.2892) on Cullin−9
with a threshold of 1e−05

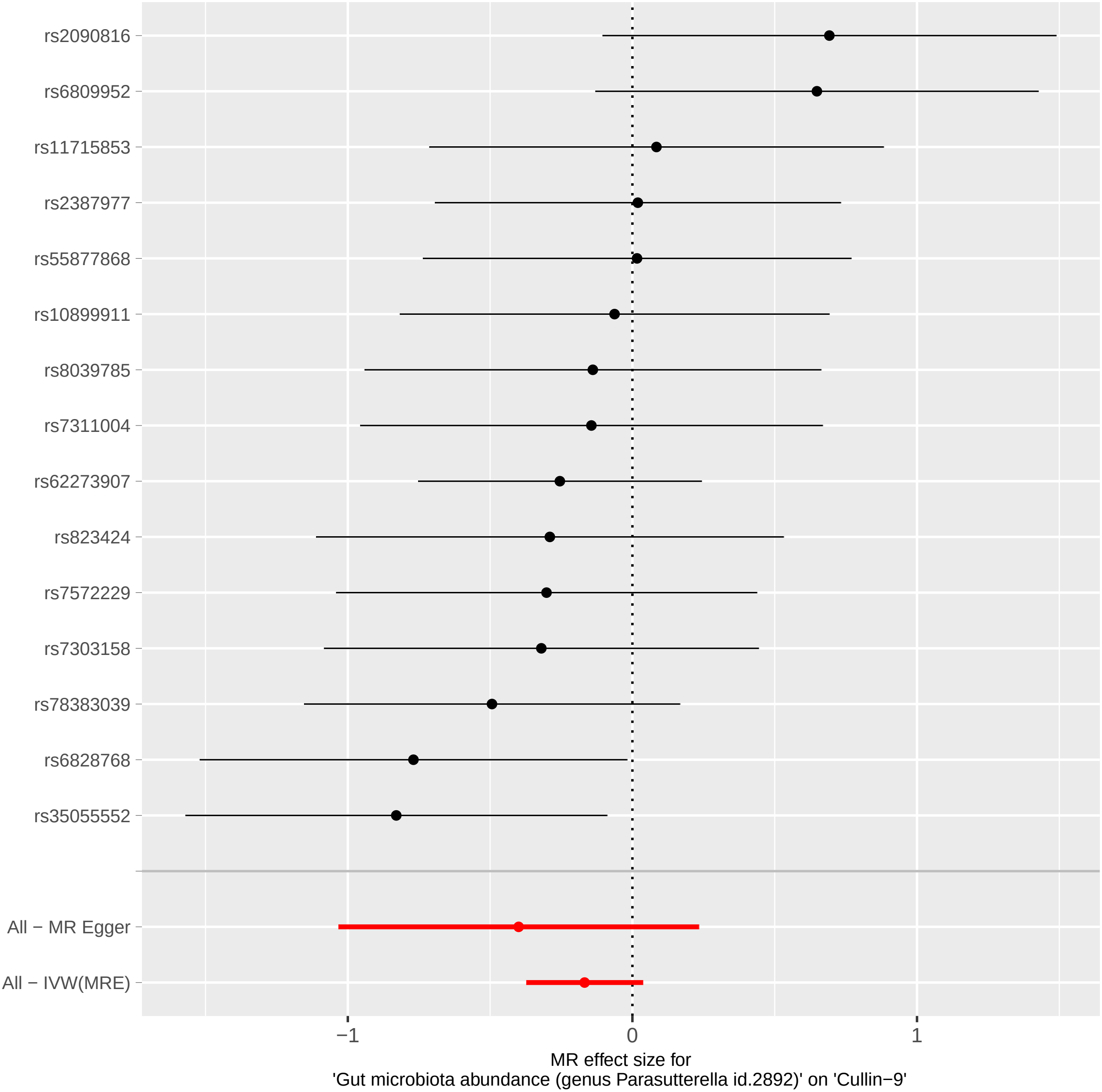

Batch 2−1469 : Gut microbiota abundance (genus Catenibacterium id.2153) on CUB and zona pellucida−like domain−containing protein 1
with a threshold of 1e−05

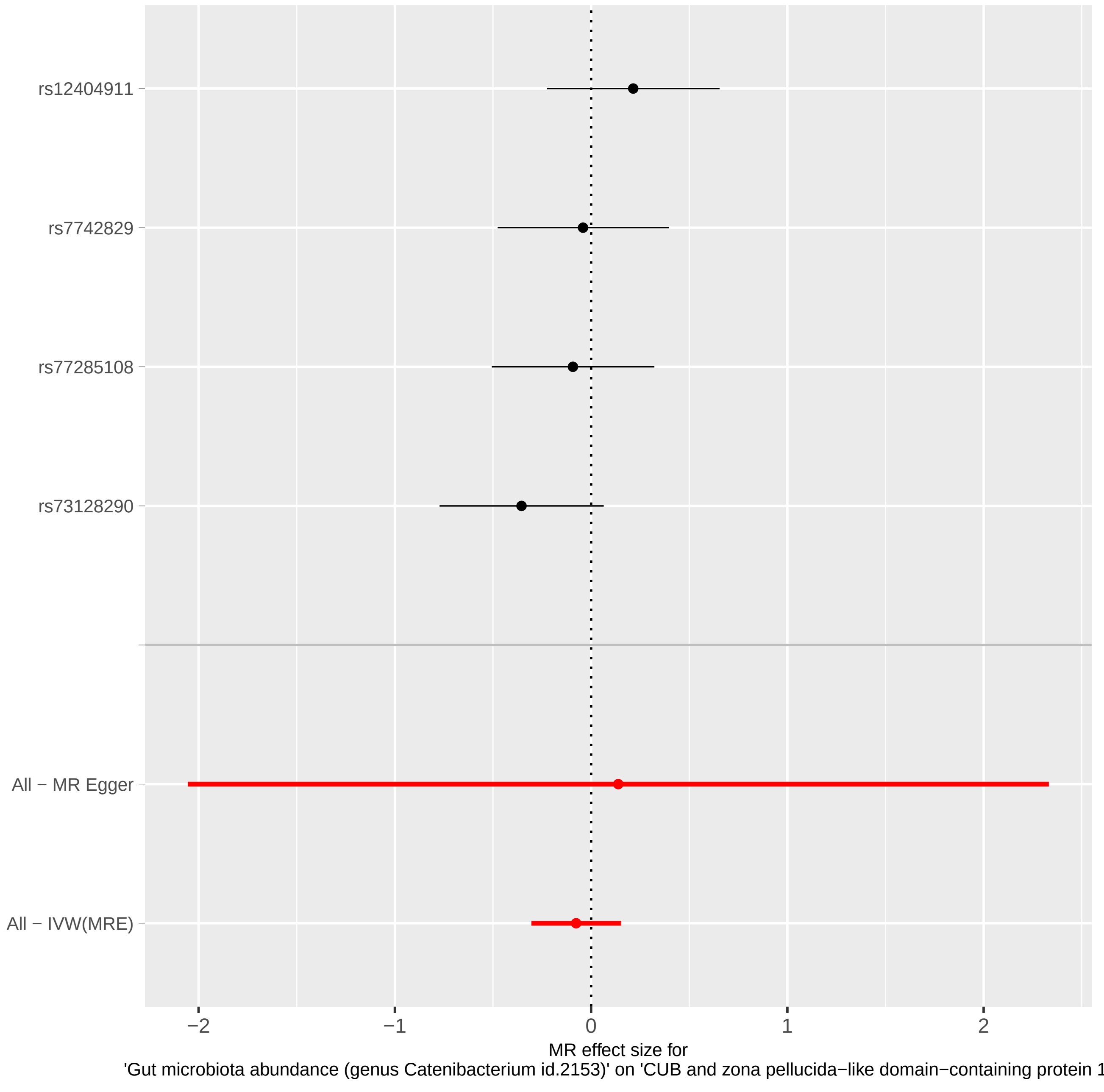

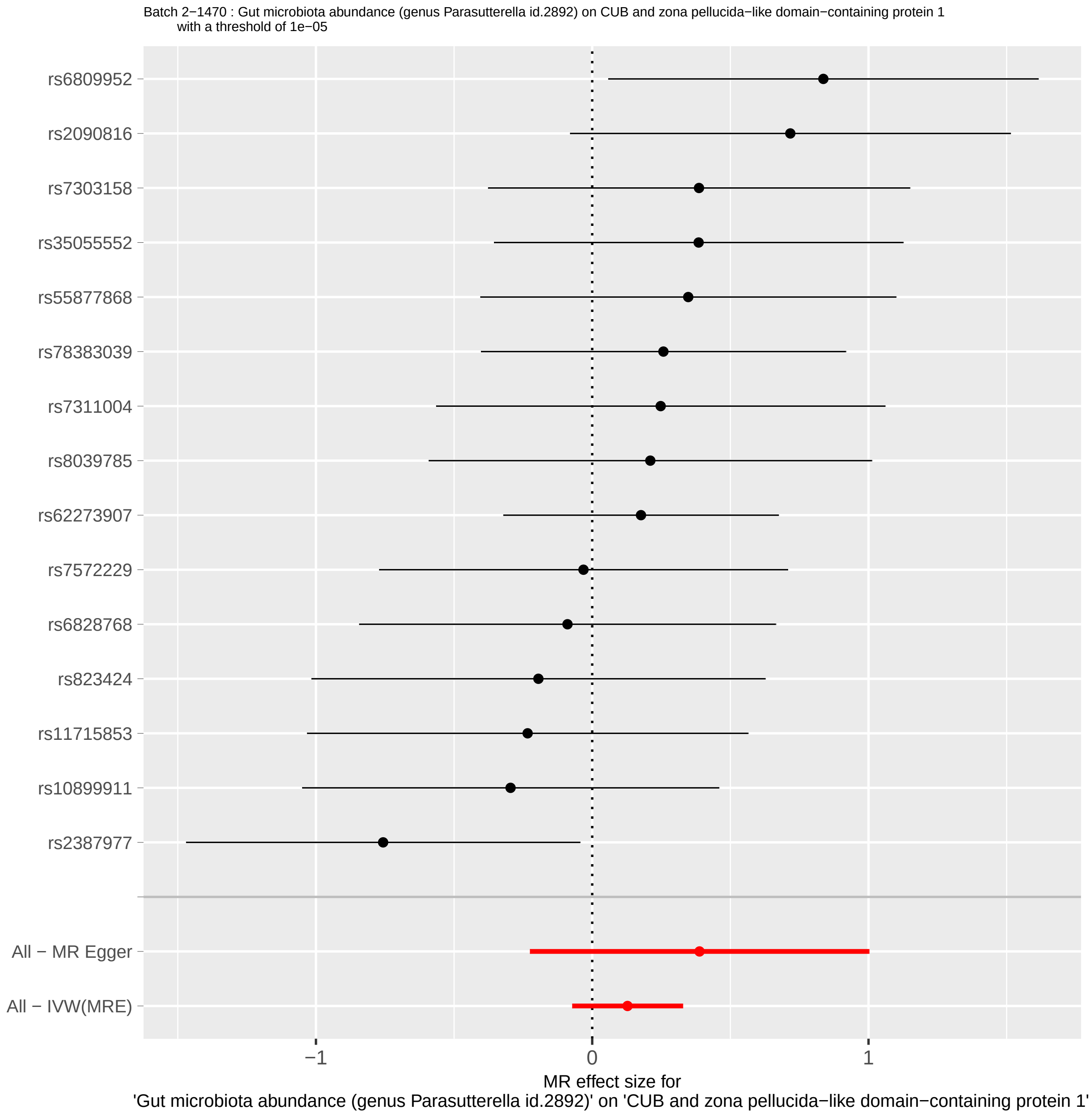

Batch 2−1470 : Gut microbiota abundance (genus Parasutterella id.2892) on CUB and zona pellucida−like domain−containing protein 1
with a threshold of 1e−05
rs6809952
rs2090816
rs7303158
rs35055552
rs55877868
rs78383039
rs7311004
rs8039785
rs62273907
rs7572229
rs6828768
rs823424
rs11715853
rs10899911
rs2387977
All − MR Egger
All − IVW(MRE)
−1
0
1
MR effect size for
'Gut microbiota abundance (genus Parasutterella id.2892)' on 'CUB and zona pellucida−like domain−containing protein 1'

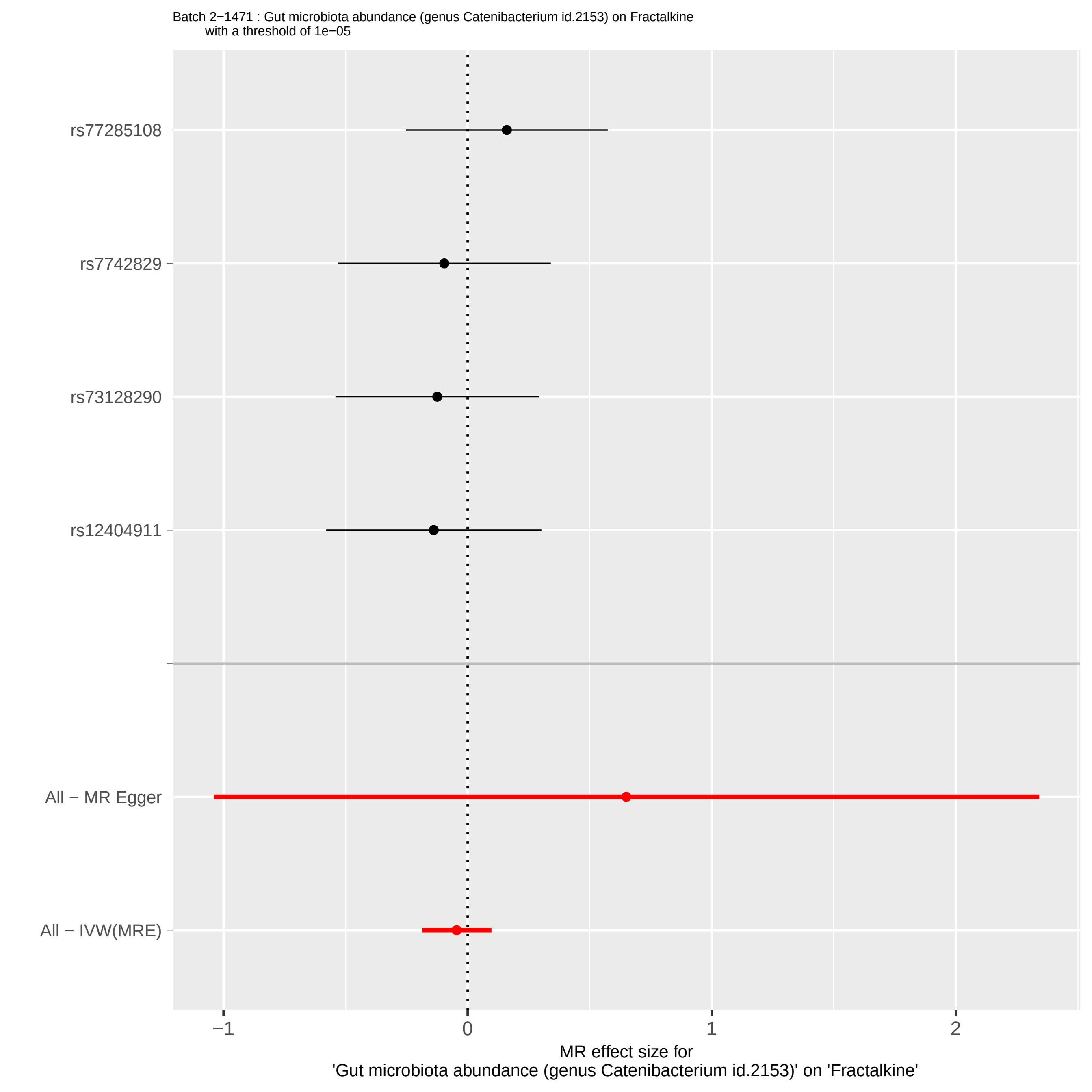
Batch 2−1471 : Gut microbiota abundance (genus Catenibacterium id.2153) on Fractalkine
with a threshold of 1e−05
rs77285108
rs7742829
rs73128290
rs12404911
All − MR Egger
All − IVW(MRE)
−1
0
1
2
MR effect size for
'Gut microbiota abundance (genus Catenibacterium id.2153)' on 'Fractalkine'

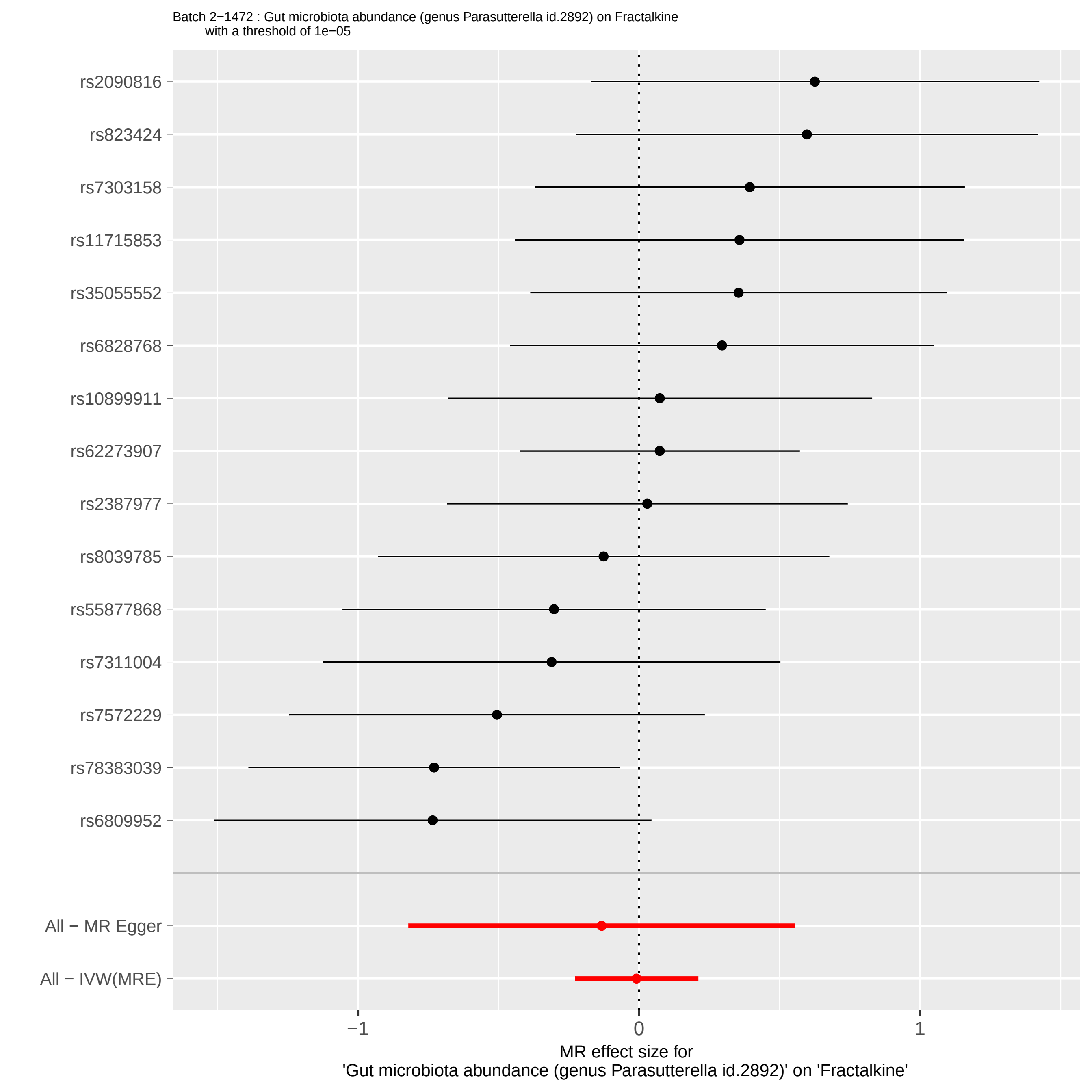

Batch 2−1472 : Gut microbiota abundance (genus Parasutterella id.2892) on Fractalkine
with a threshold of 1e−05
rs2090816
rs823424
rs7303158
rs11715853
rs35055552
rs6828768
rs10899911
rs62273907
rs2387977
rs8039785
rs55877868
rs7311004
rs7572229
rs78383039
rs6809952
All − MR Egger
All − IVW(MRE)
−1
0
1
MR effect size for
'Gut microbiota abundance (genus Parasutterella id.2892)' on 'Fractalkine'

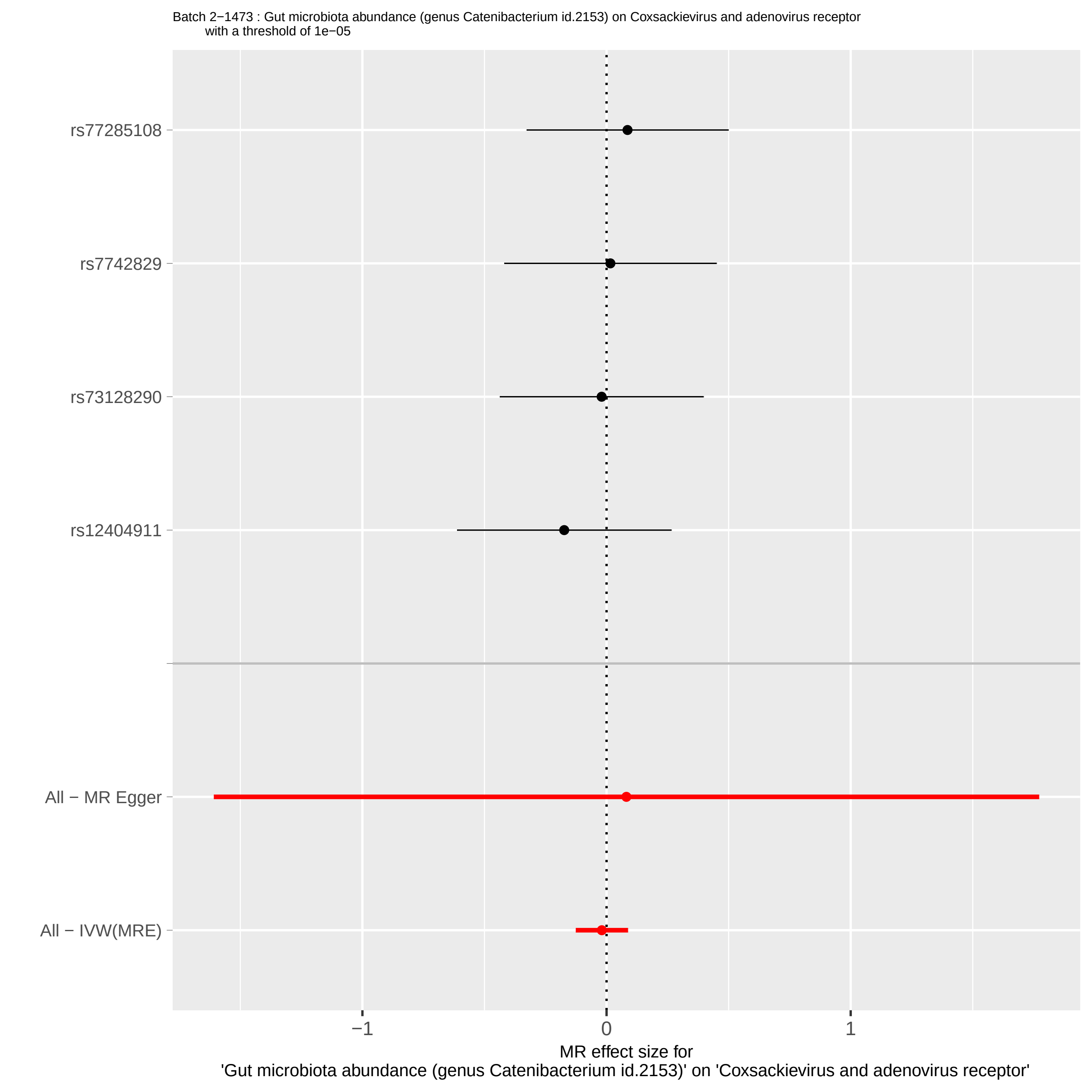

Batch 2−1473 : Gut microbiota abundance (genus Catenibacterium id.2153) on Coxsackievirus and adenovirus receptor
with a threshold of 1e−05
rs77285108
rs7742829
rs73128290
rs12404911
All − MR Egger
All − IVW(MRE)
−1
0
1
MR effect size for
'Gut microbiota abundance (genus Catenibacterium id.2153)' on 'Coxsackievirus and adenovirus receptor'

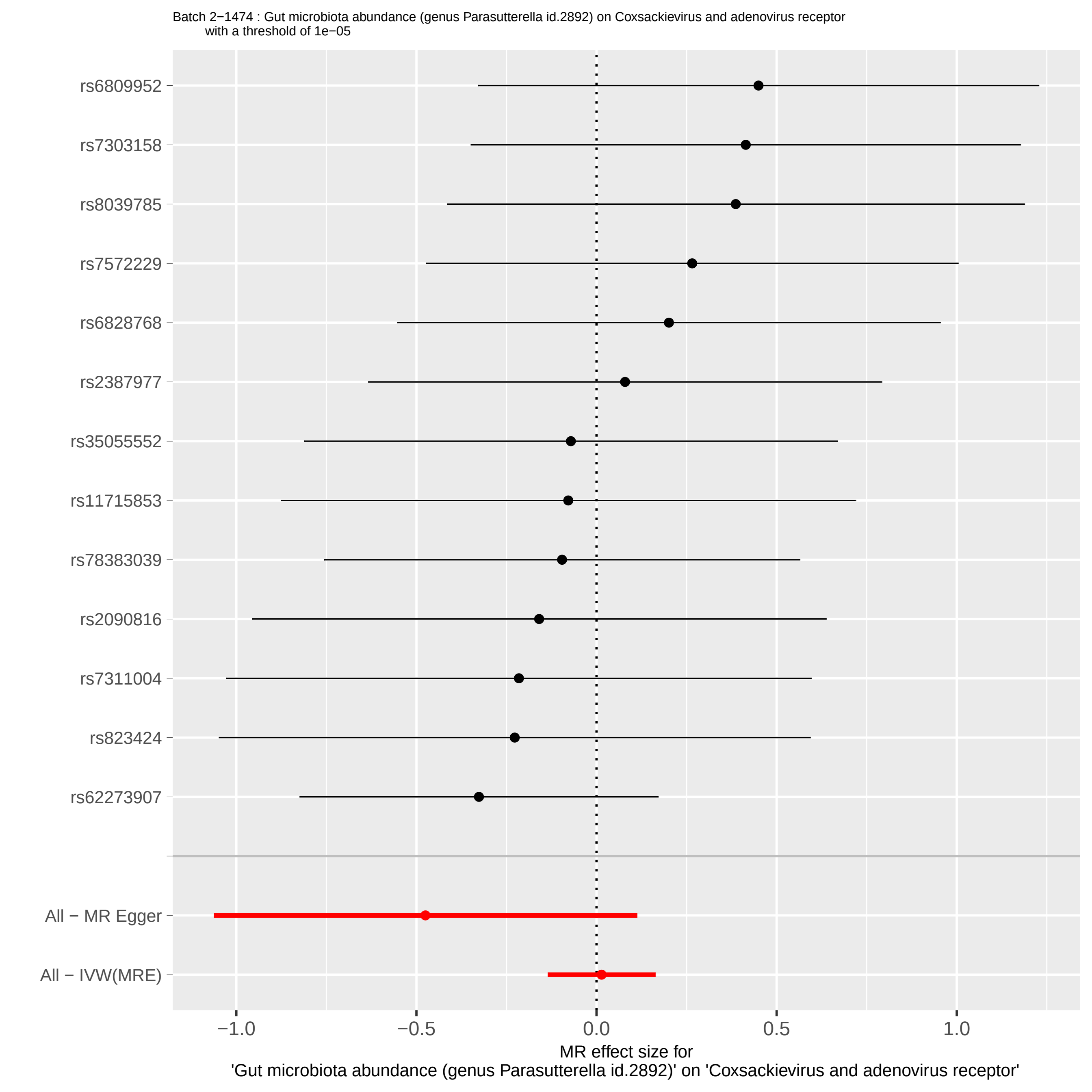
Batch 2−1474 : Gut microbiota abundance (genus Parasutterella id.2892) on Coxsackievirus and adenovirus receptor
with a threshold of 1e−05
rs6809952
rs7303158
rs8039785
rs7572229
rs6828768
rs2387977
rs35055552
rs11715853
rs78383039
rs2090816
rs7311004
rs823424
rs62273907
All − MR Egger
All − IVW(MRE)
−1.0
−0.5
0.0
0.5
1.0
MR effect size for
'Gut microbiota abundance (genus Parasutterella id.2892)' on 'Coxsackievirus and adenovirus receptor'

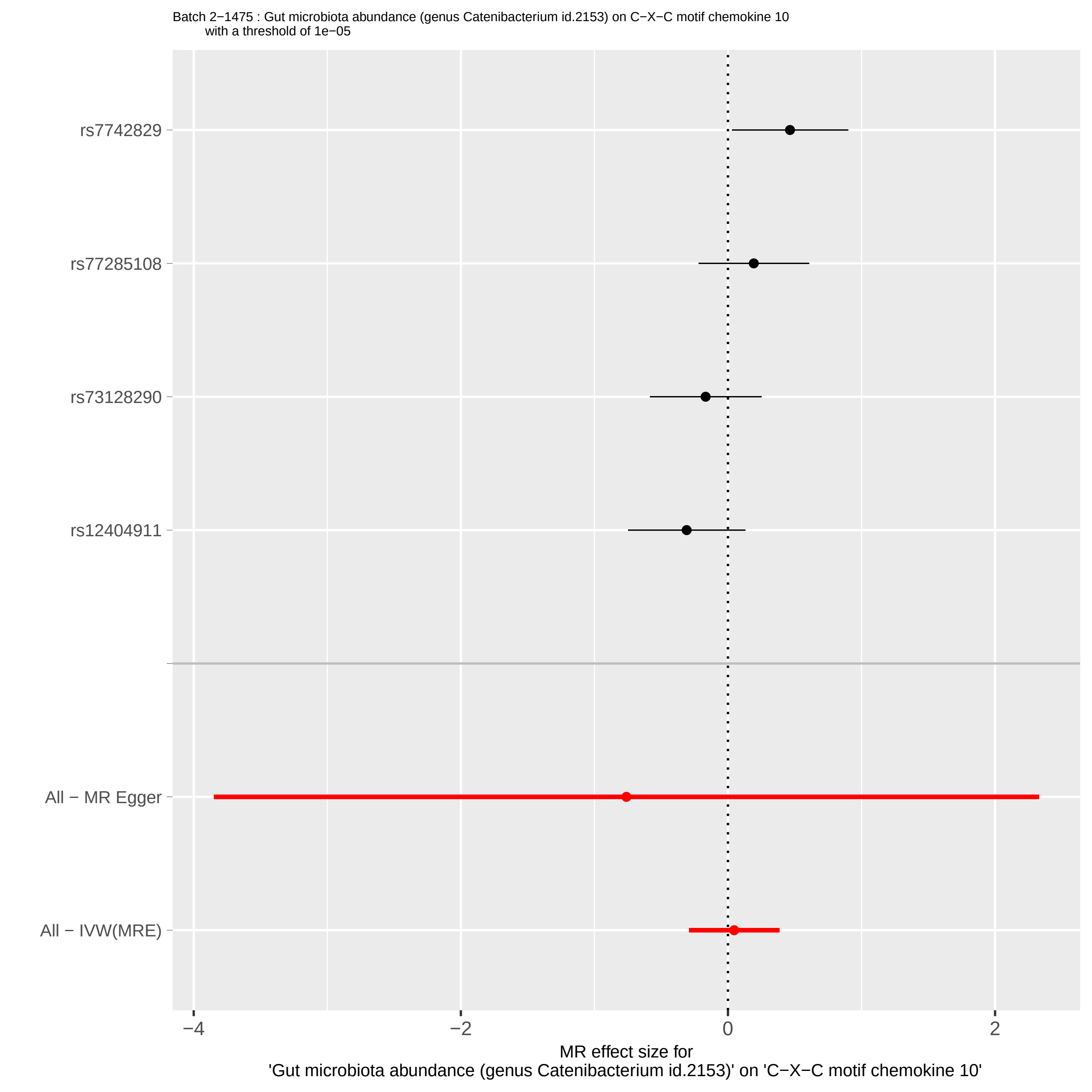
Batch 2−1475 : Gut microbiota abundance (genus Catenibacterium id.2153) on C−X−C motif chemokine 10
with a threshold of 1e−05
rs7742829
rs77285108
rs73128290
rs12404911
All − MR Egger
All − IVW(MRE)
−4
−2
0
2
MR effect size for
'Gut microbiota abundance (genus Catenibacterium id.2153)' on 'C−X−C motif chemokine 10'

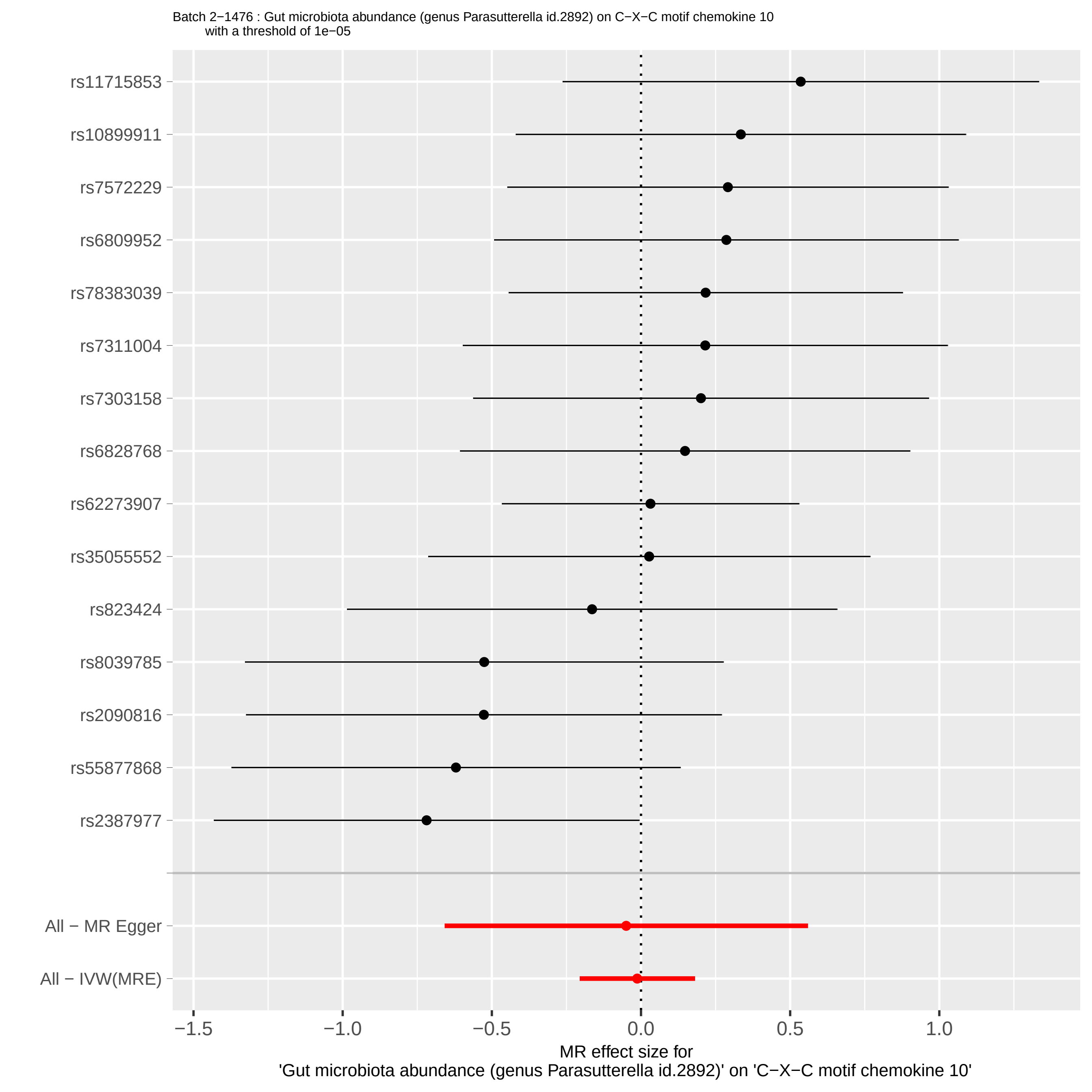

Batch 2−1476 : Gut microbiota abundance (genus Parasutterella id.2892) on C−X−C motif chemokine 10
with a threshold of 1e−05
rs11715853
rs10899911
rs7572229
rs6809952
rs78383039
rs7311004
rs7303158
rs6828768
rs62273907
rs35055552
rs823424
rs8039785
rs2090816
rs55877868
rs2387977
All − MR Egger
All − IVW(MRE)
−1.5
−1.0
−0.5
0.0
0.5
1.0
MR effect size for
'Gut microbiota abundance (genus Parasutterella id.2892)' on 'C−X−C motif chemokine 10'

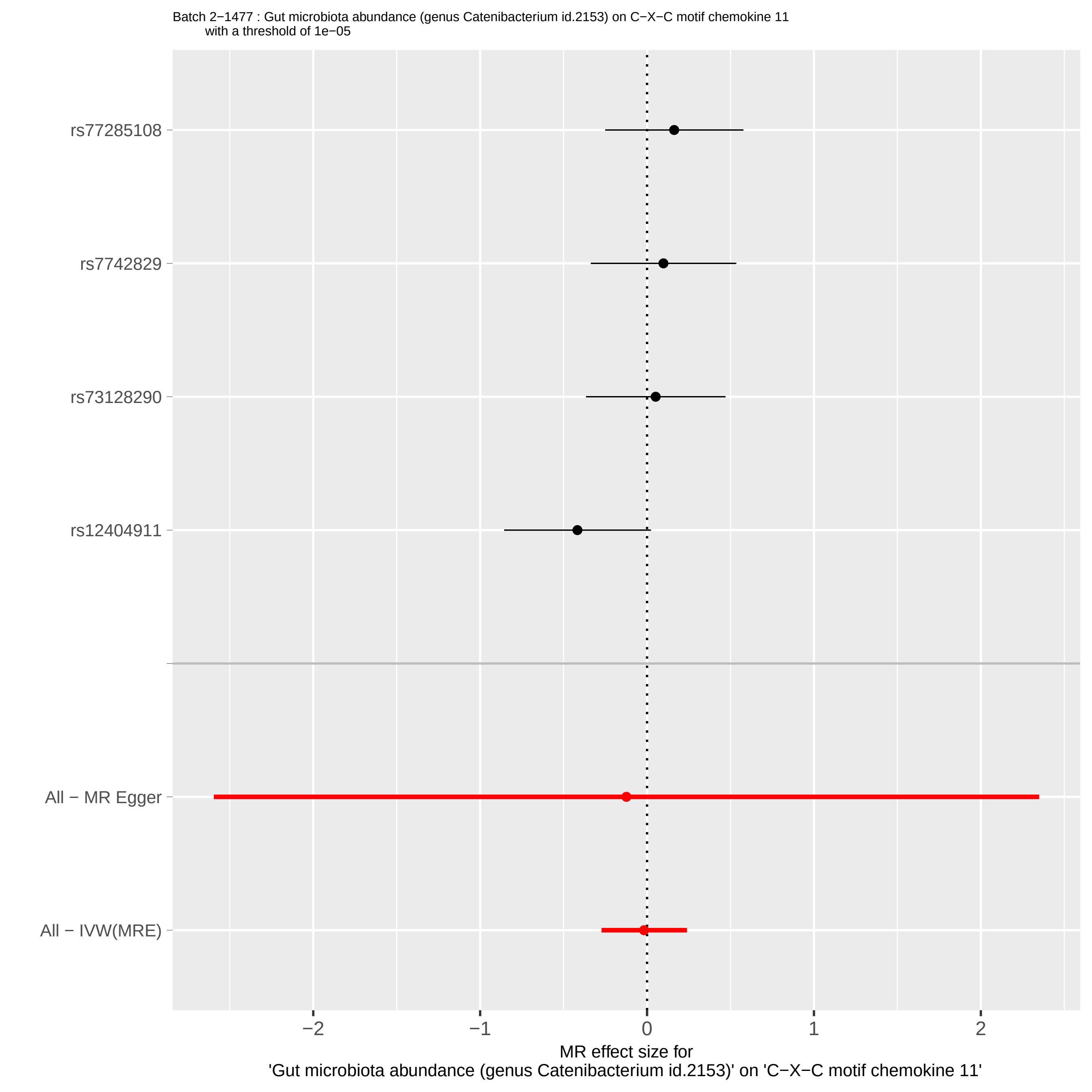
Batch 2−1477 : Gut microbiota abundance (genus Catenibacterium id.2153) on C−X−C motif chemokine 11
with a threshold of 1e−05
rs77285108
rs7742829
rs73128290
rs12404911
All − MR Egger
All − IVW(MRE)
−2
−1
0
1
2
MR effect size for
'Gut microbiota abundance (genus Catenibacterium id.2153)' on 'C−X−C motif chemokine 11'

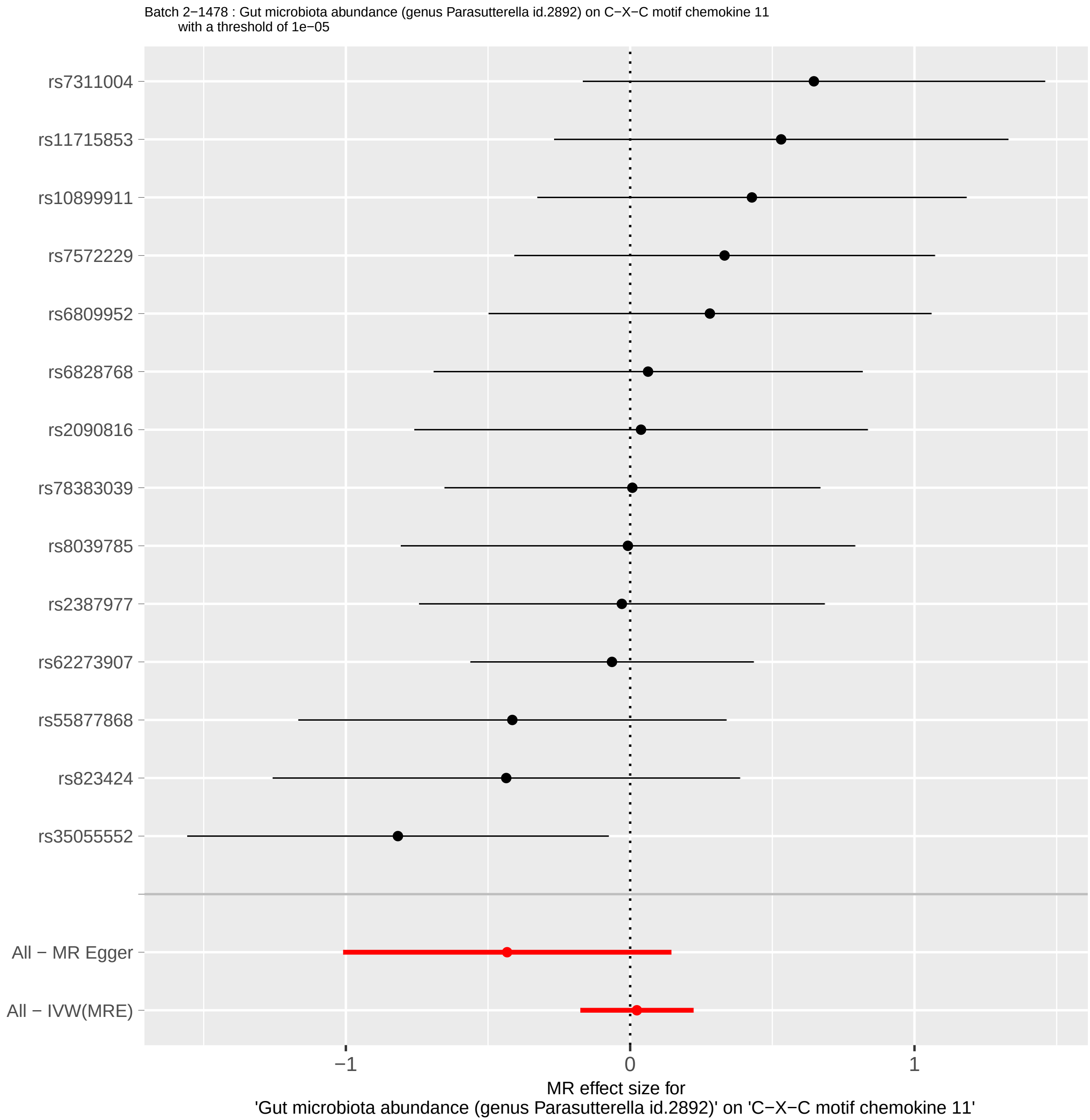

Batch 2−1478 : Gut microbiota abundance (genus Parasutterella id.2892) on C−X−C motif chemokine 11
with a threshold of 1e−05
rs7311004
rs11715853
rs10899911
rs7572229
rs6809952
rs6828768
rs2090816
rs78383039
rs8039785
rs2387977
rs62273907
rs55877868
rs823424
rs35055552
All − MR Egger
All − IVW(MRE)
−1
0
1
MR effect size for
'Gut microbiota abundance (genus Parasutterella id.2892)' on 'C−X−C motif chemokine 11'

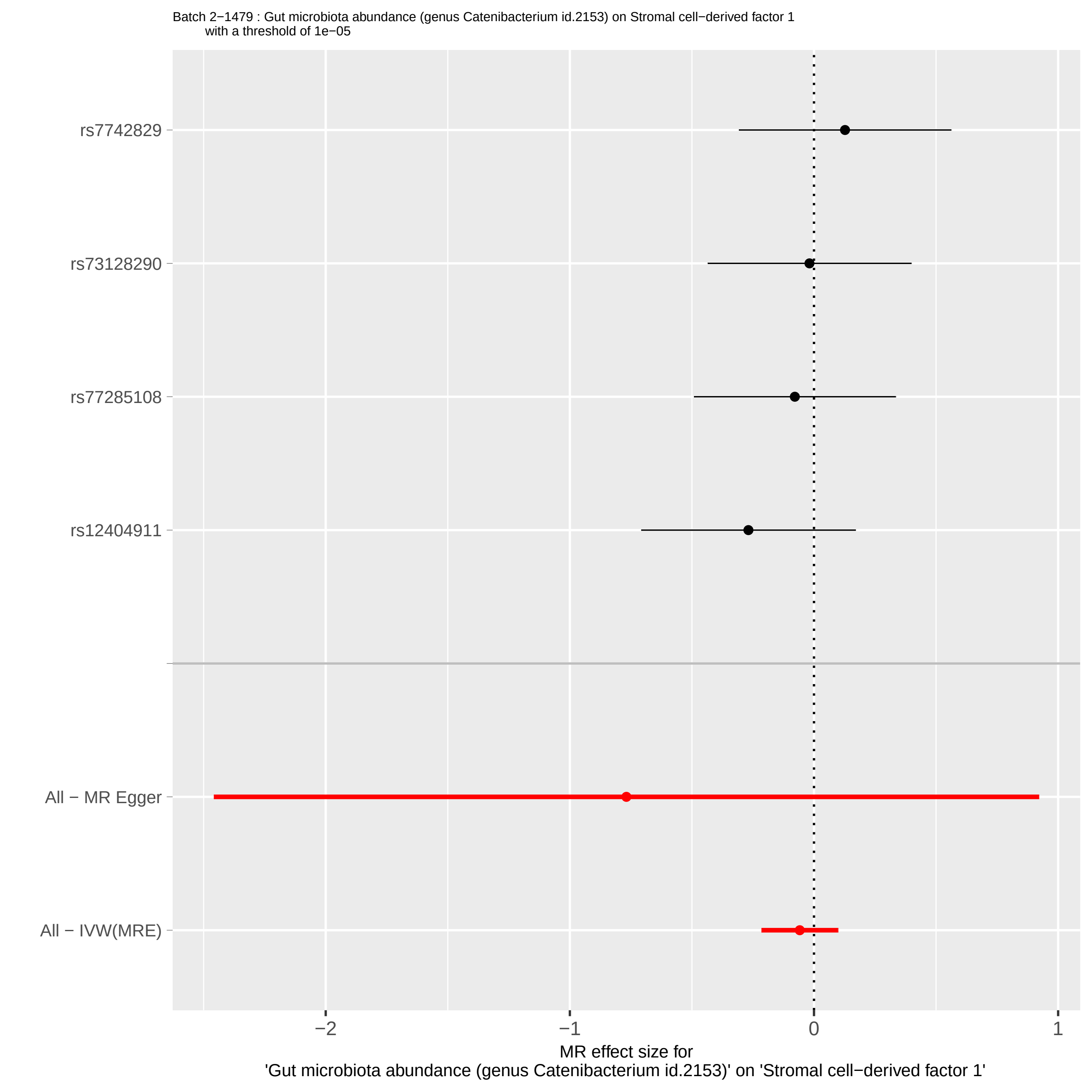

Batch 2−1479 : Gut microbiota abundance (genus Catenibacterium id.2153) on Stromal cell−derived factor 1
with a threshold of 1e−05
rs7742829
rs73128290
rs77285108
rs12404911
All − MR Egger
All − IVW(MRE)
−2
−1
0
1
MR effect size for
'Gut microbiota abundance (genus Catenibacterium id.2153)' on 'Stromal cell−derived factor 1'

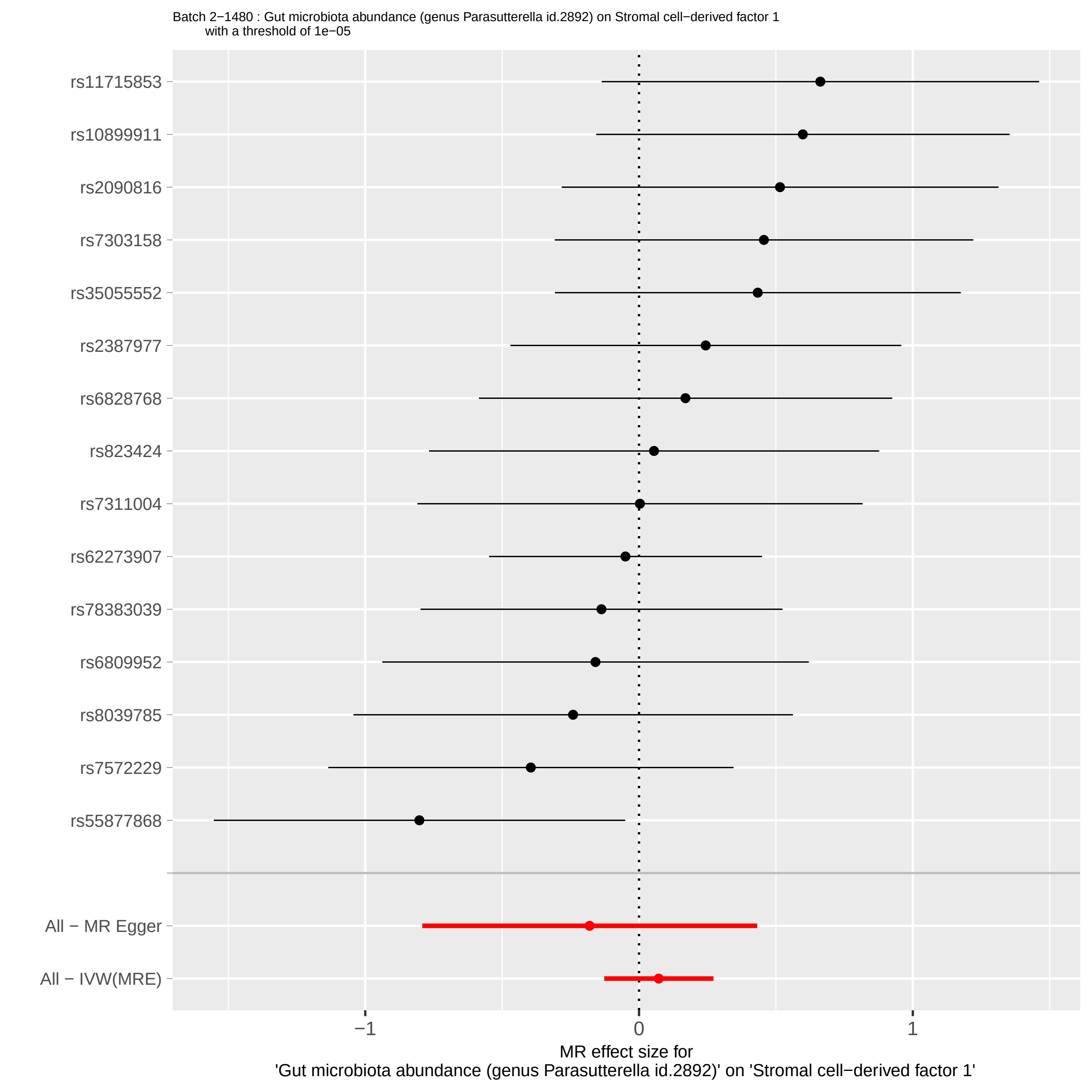

Batch 2−1480 : Gut microbiota abundance (genus Parasutterella id.2892) on Stromal cell−derived factor 1
with a threshold of 1e−05
rs11715853
rs10899911
rs2090816
rs7303158
rs35055552
rs2387977
rs6828768
rs823424
rs7311004
rs62273907
rs78383039
rs6809952
rs8039785
rs7572229
rs55877868
All − MR Egger
All − IVW(MRE)
−1
0
1
MR effect size for
'Gut microbiota abundance (genus Parasutterella id.2892)' on 'Stromal cell−derived factor 1'

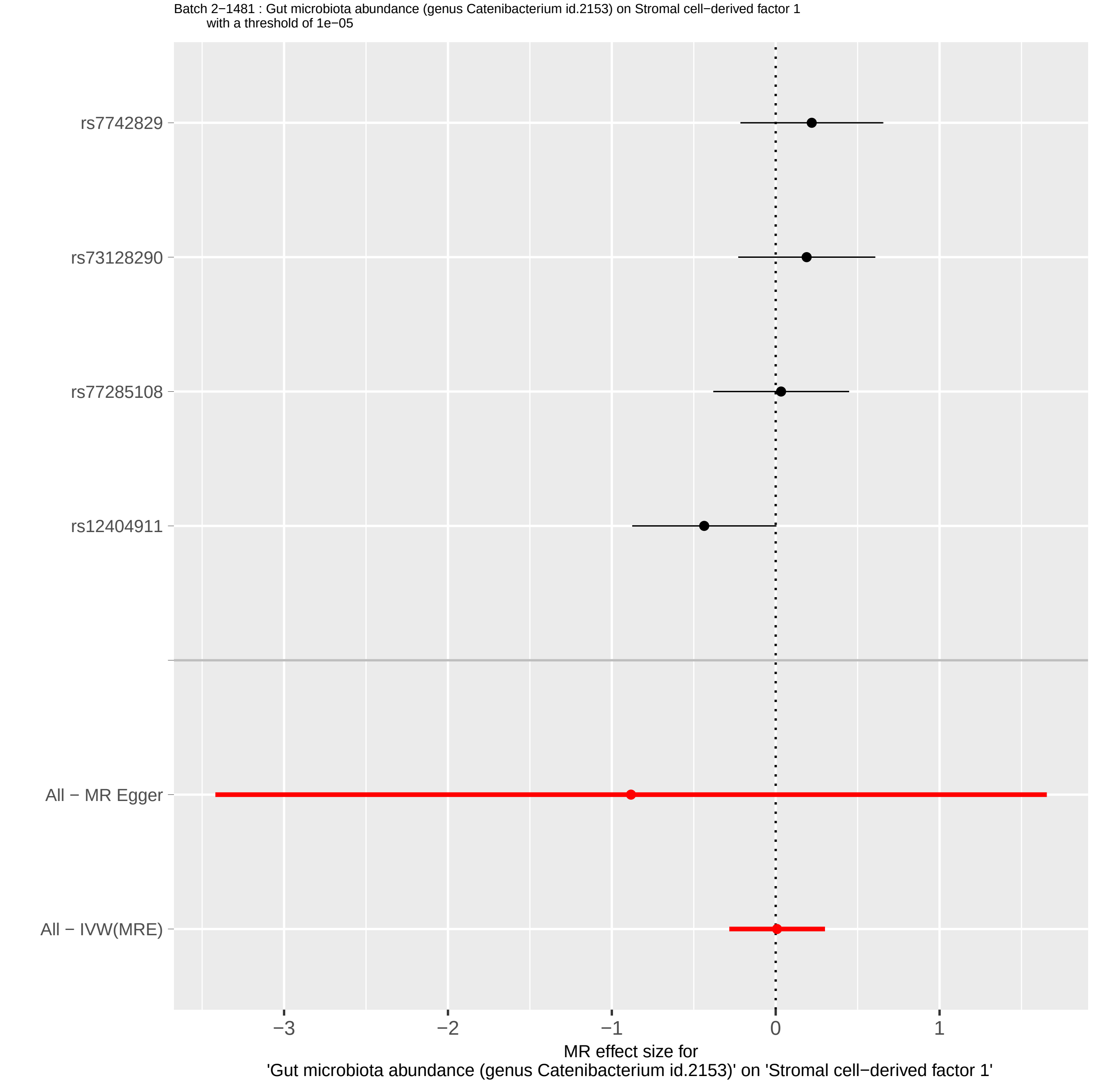

Batch 2−1481 : Gut microbiota abundance (genus Catenibacterium id.2153) on Stromal cell−derived factor 1
with a threshold of 1e−05
rs7742829
rs73128290
rs77285108
rs12404911
All − MR Egger
All − IVW(MRE)
−3
−2
−1
0
1
MR effect size for
'Gut microbiota abundance (genus Catenibacterium id.2153)' on 'Stromal cell−derived factor 1'

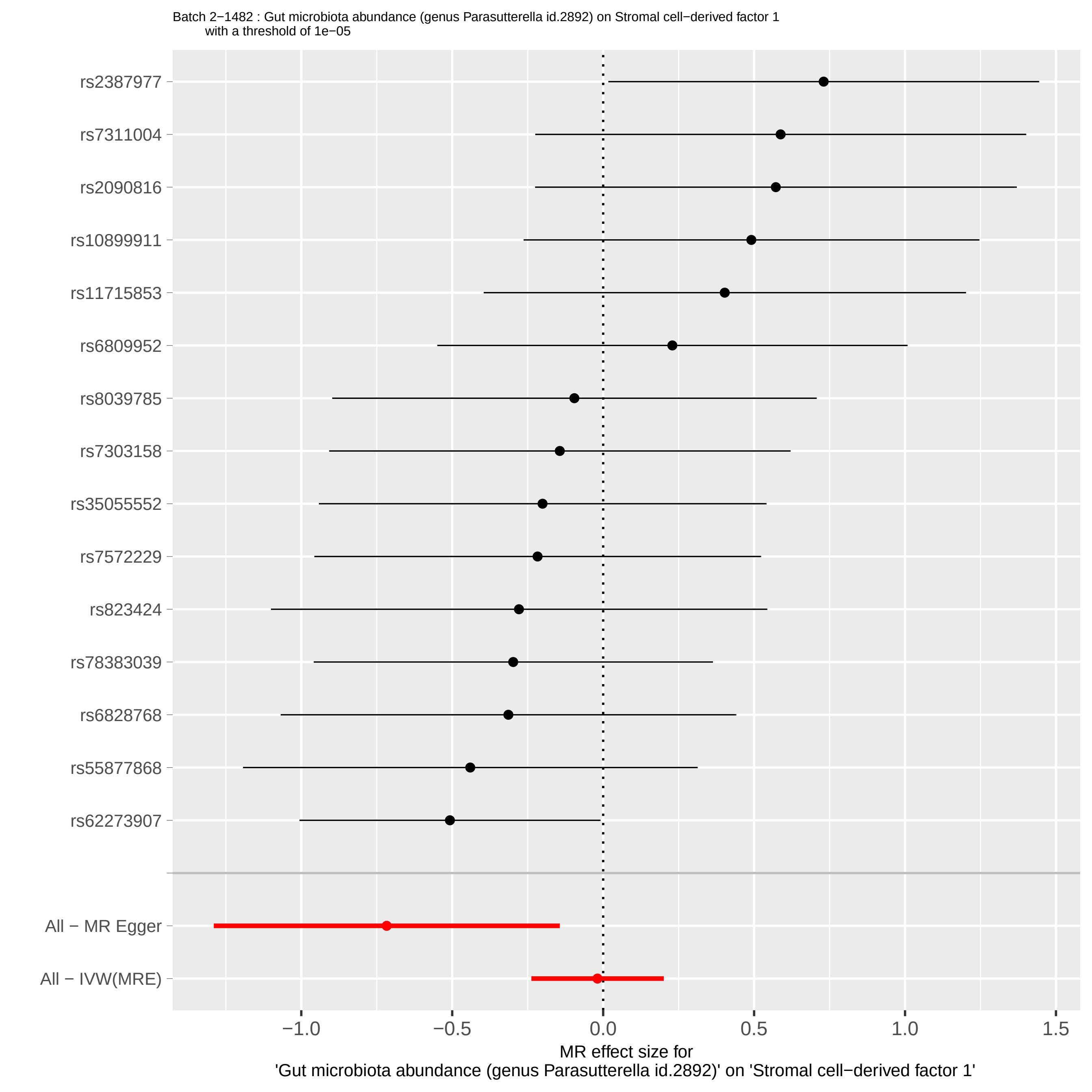

Batch 2−1482 : Gut microbiota abundance (genus Parasutterella id.2892) on Stromal cell−derived factor 1
with a threshold of 1e−05
rs2387977
rs7311004
rs2090816
rs10899911
rs11715853
rs6809952
rs8039785
rs7303158
rs35055552
rs7572229
rs823424
rs78383039
rs6828768
rs55877868
rs62273907
All − MR Egger
All − IVW(MRE)
−1.0
−0.5
0.0
0.5
1.0
1.5
MR effect size for
'Gut microbiota abundance (genus Parasutterella id.2892)' on 'Stromal cell−derived factor 1'

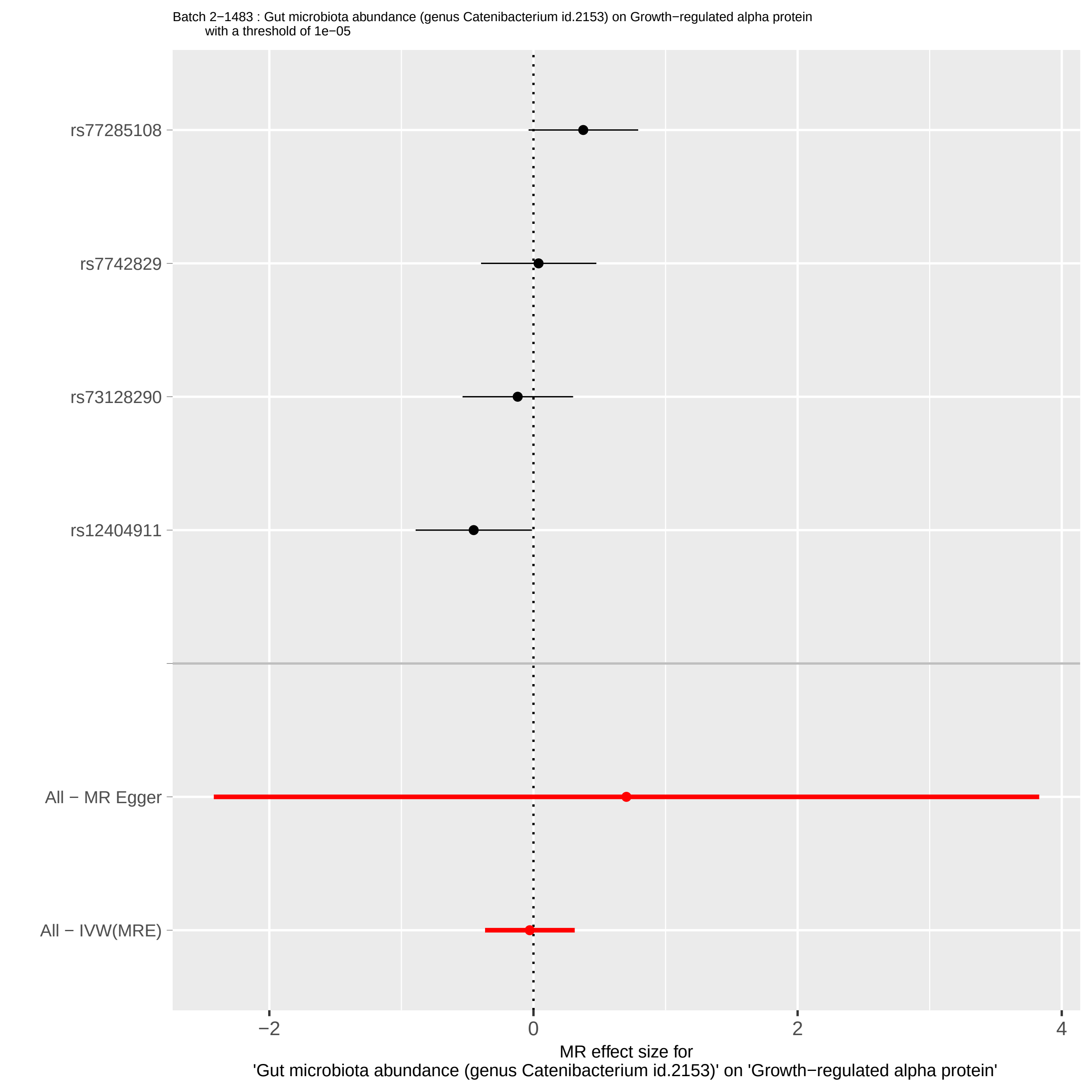

Batch 2−1483 : Gut microbiota abundance (genus Catenibacterium id.2153) on Growth−regulated alpha protein
with a threshold of 1e−05
rs77285108
rs7742829
rs73128290
rs12404911
All − MR Egger
All − IVW(MRE)
−2
0
2
4
MR effect size for
'Gut microbiota abundance (genus Catenibacterium id.2153)' on 'Growth−regulated alpha protein'

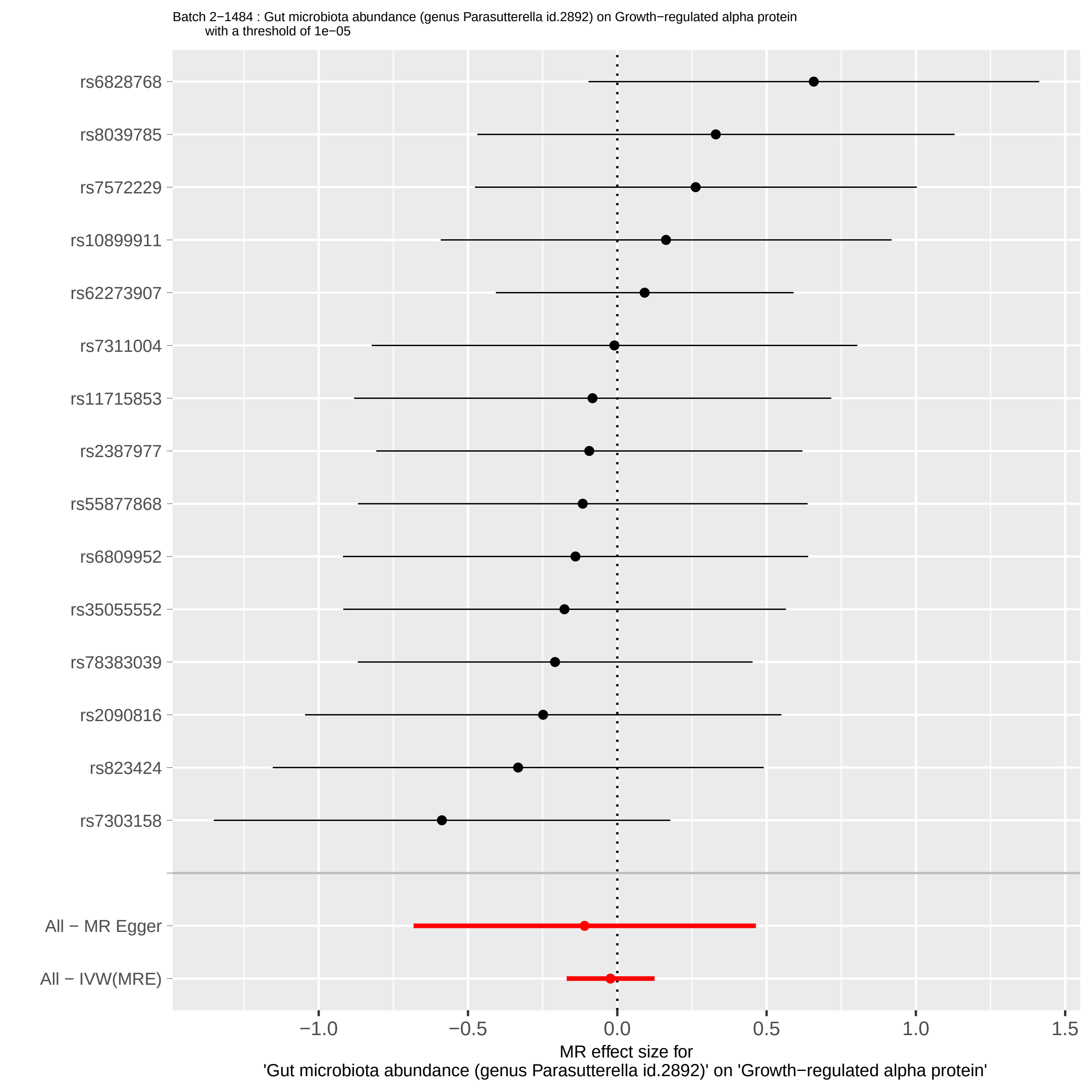

Batch 2−1484 : Gut microbiota abundance (genus Parasutterella id.2892) on Growth−regulated alpha protein
with a threshold of 1e−05
rs6828768
rs8039785
rs7572229
rs10899911
rs62273907
rs7311004
rs11715853
rs2387977
rs55877868
rs6809952
rs35055552
rs78383039
rs2090816
rs823424
rs7303158
All − MR Egger
All − IVW(MRE)
−1.0
−0.5
0.0
0.5
1.0
1.5
MR effect size for
'Gut microbiota abundance (genus Parasutterella id.2892)' on 'Growth−regulated alpha protein'

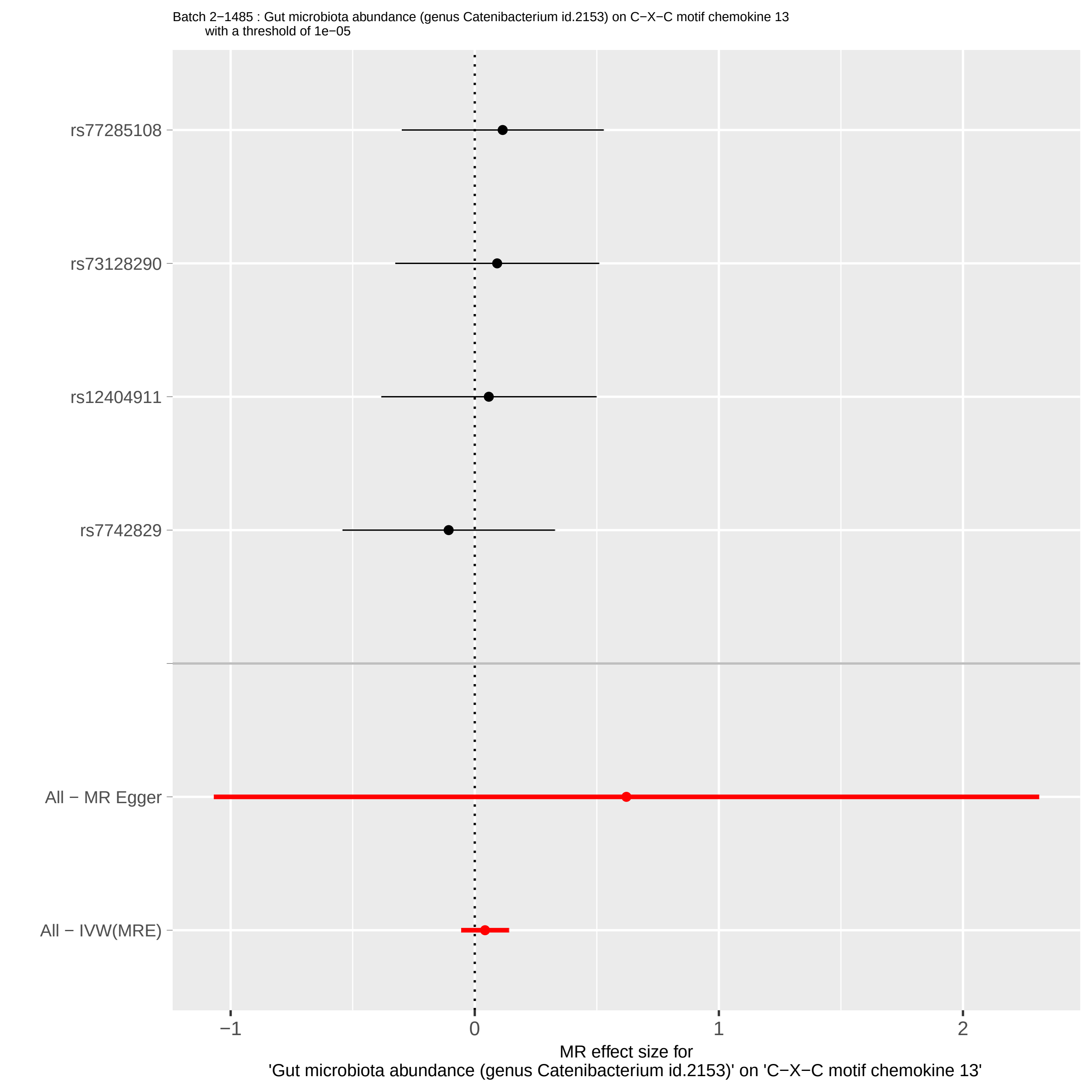
Batch 2−1485 : Gut microbiota abundance (genus Catenibacterium id.2153) on C−X−C motif chemokine 13
with a threshold of 1e−05
rs77285108
rs73128290
rs12404911
rs7742829
All − MR Egger
All − IVW(MRE)
−1
0
1
2
MR effect size for
'Gut microbiota abundance (genus Catenibacterium id.2153)' on 'C−X−C motif chemokine 13'

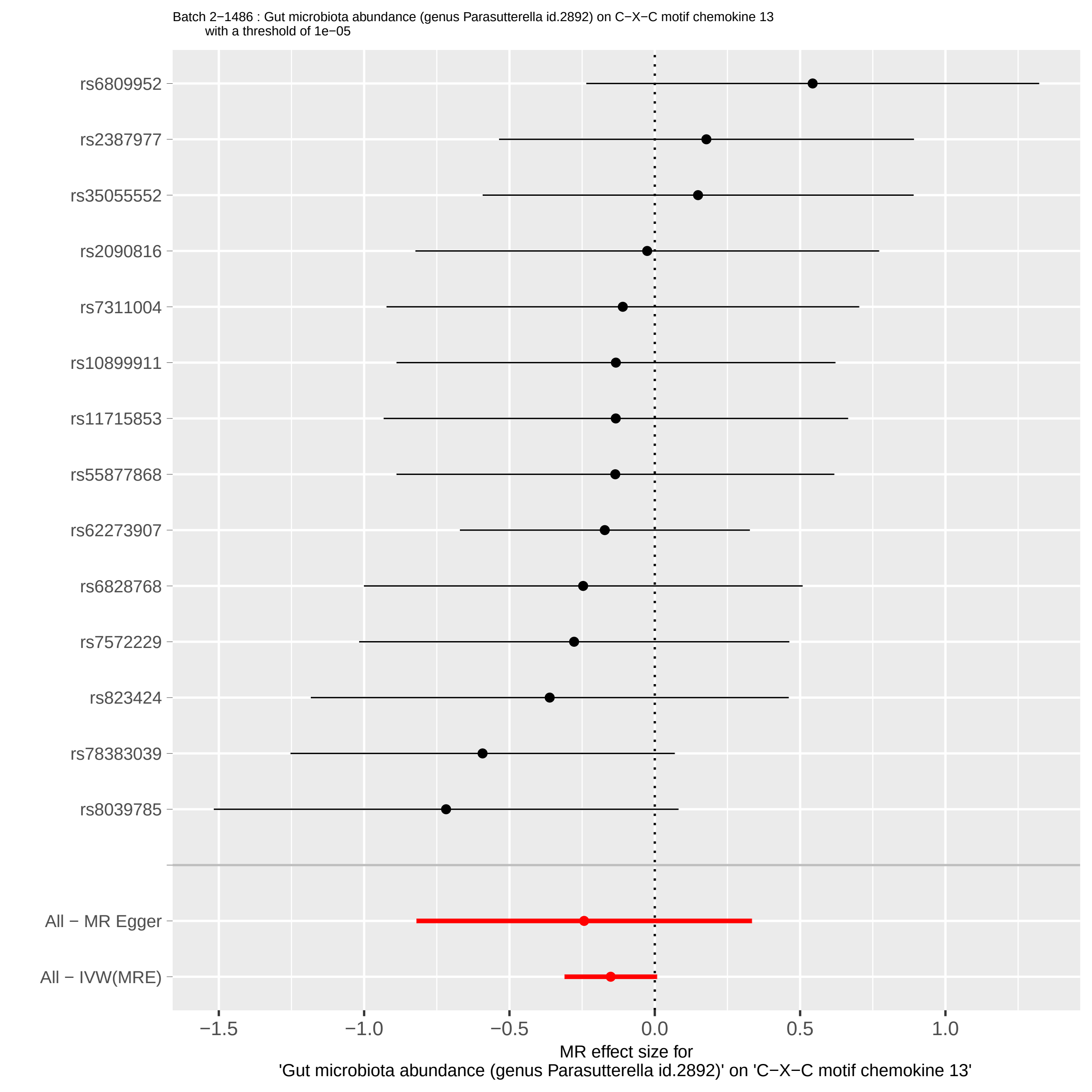

Batch 2−1486 : Gut microbiota abundance (genus Parasutterella id.2892) on C−X−C motif chemokine 13
with a threshold of 1e−05
rs6809952
rs2387977
rs35055552
rs2090816
rs7311004
rs10899911
rs11715853
rs55877868
rs62273907
rs6828768
rs7572229
rs823424
rs78383039
rs8039785
All − MR Egger
All − IVW(MRE)
−1.5
−1.0
−0.5
0.0
0.5
1.0
MR effect size for
'Gut microbiota abundance (genus Parasutterella id.2892)' on 'C−X−C motif chemokine 13'

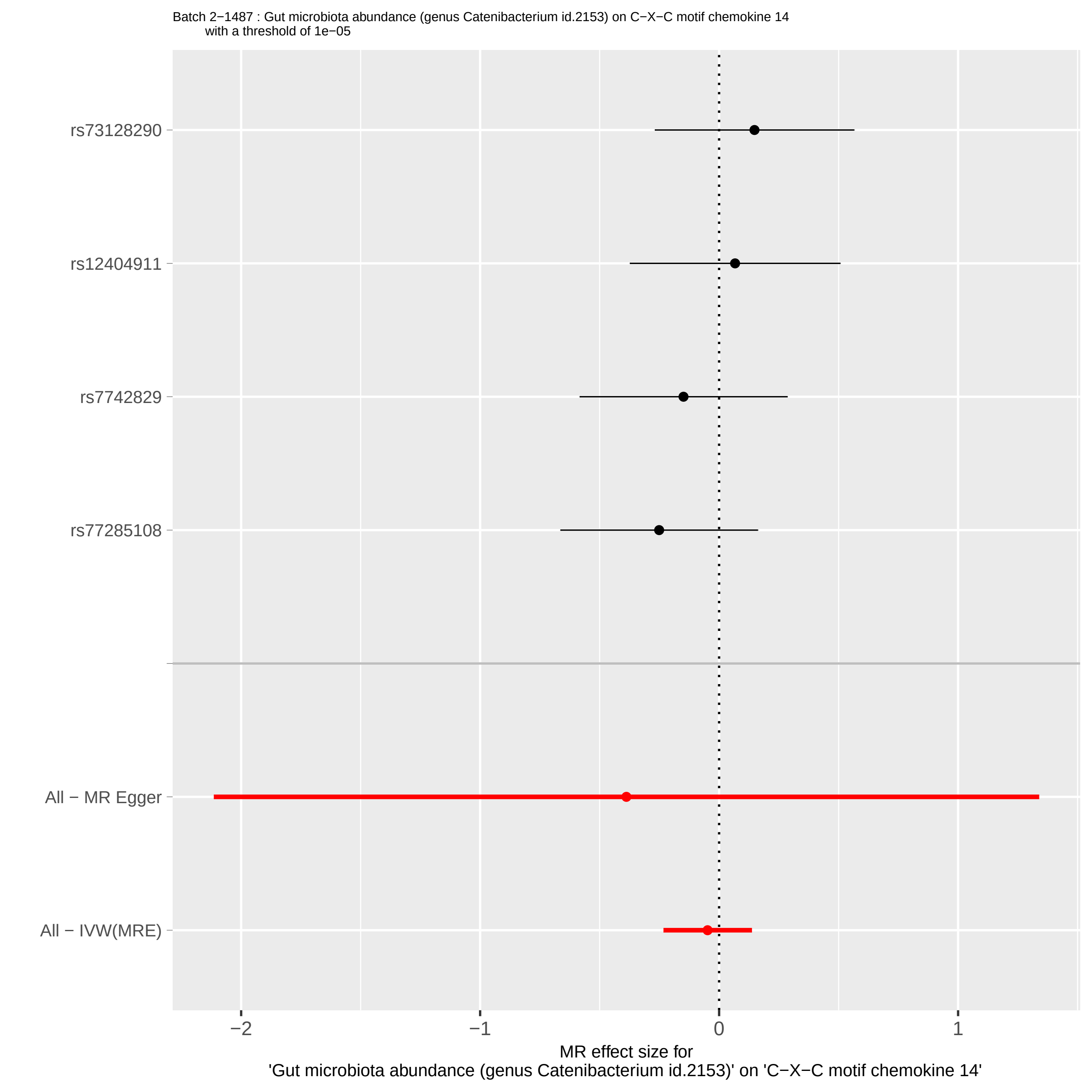

Batch 2−1487 : Gut microbiota abundance (genus Catenibacterium id.2153) on C−X−C motif chemokine 14
with a threshold of 1e−05
rs73128290
rs12404911
rs7742829
rs77285108
All − MR Egger
All − IVW(MRE)
−2
−1
0
1
MR effect size for
'Gut microbiota abundance (genus Catenibacterium id.2153)' on 'C−X−C motif chemokine 14'

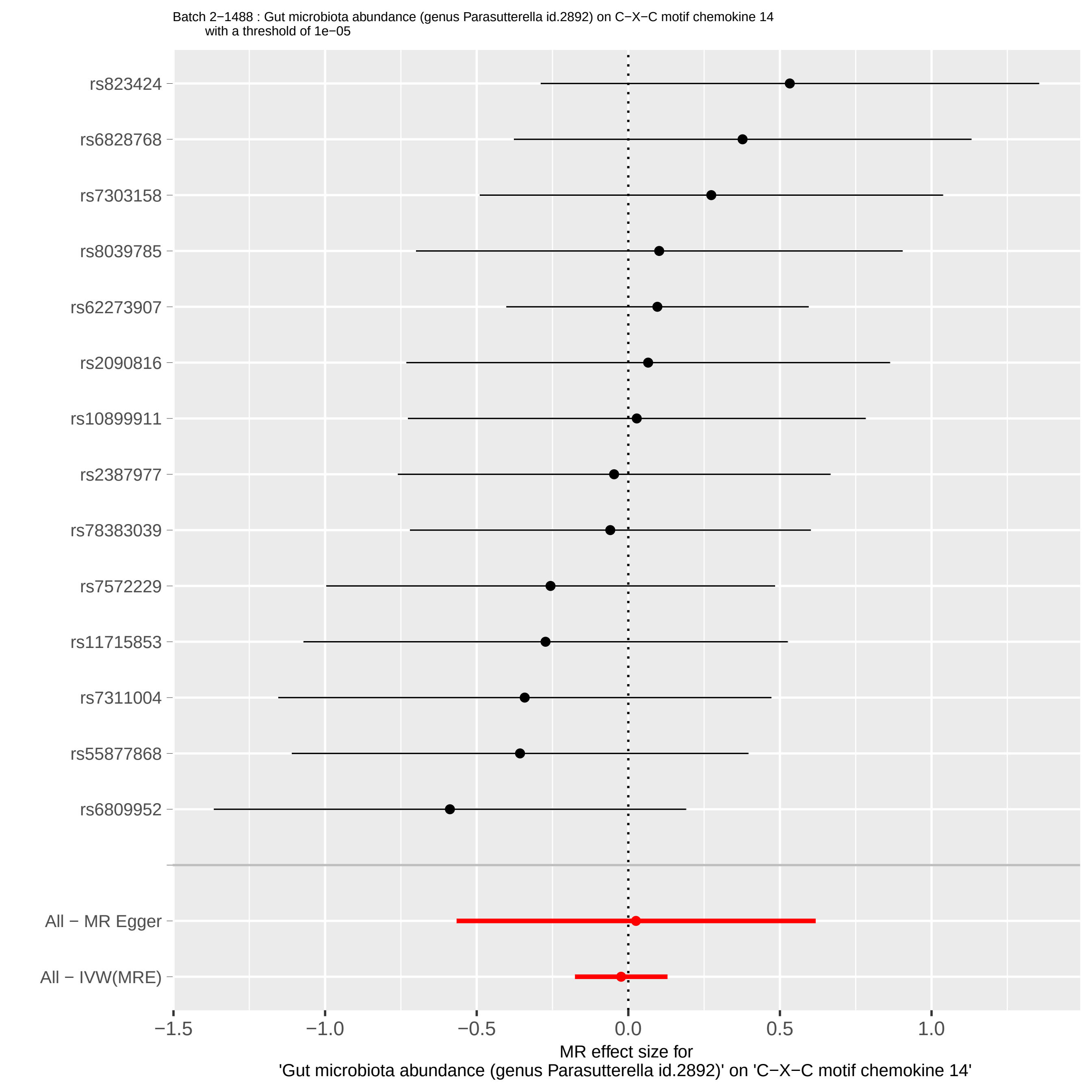

Batch 2−1488 : Gut microbiota abundance (genus Parasutterella id.2892) on C−X−C motif chemokine 14
with a threshold of 1e−05
rs823424
rs6828768
rs7303158
rs8039785
rs62273907
rs2090816
rs10899911
rs2387977
rs78383039
rs7572229
rs11715853
rs7311004
rs55877868
rs6809952
All − MR Egger
All − IVW(MRE)
−1.5
−1.0
−0.5
0.0
0.5
1.0
MR effect size for
'Gut microbiota abundance (genus Parasutterella id.2892)' on 'C−X−C motif chemokine 14'

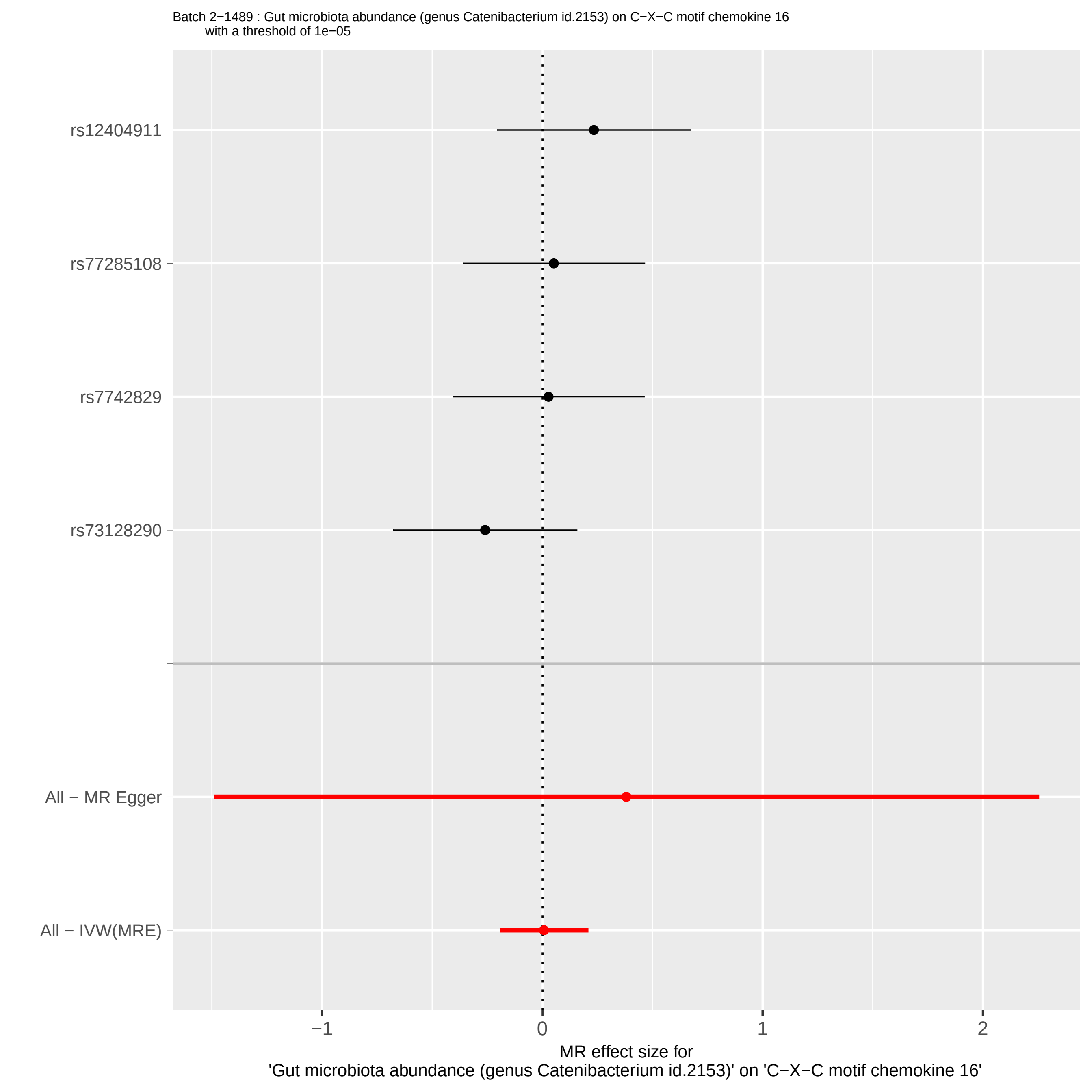
Batch 2−1489 : Gut microbiota abundance (genus Catenibacterium id.2153) on C−X−C motif chemokine 16
with a threshold of 1e−05
rs12404911
rs77285108
rs7742829
rs73128290
All − MR Egger
All − IVW(MRE)
−1
0
1
2
MR effect size for
'Gut microbiota abundance (genus Catenibacterium id.2153)' on 'C−X−C motif chemokine 16'

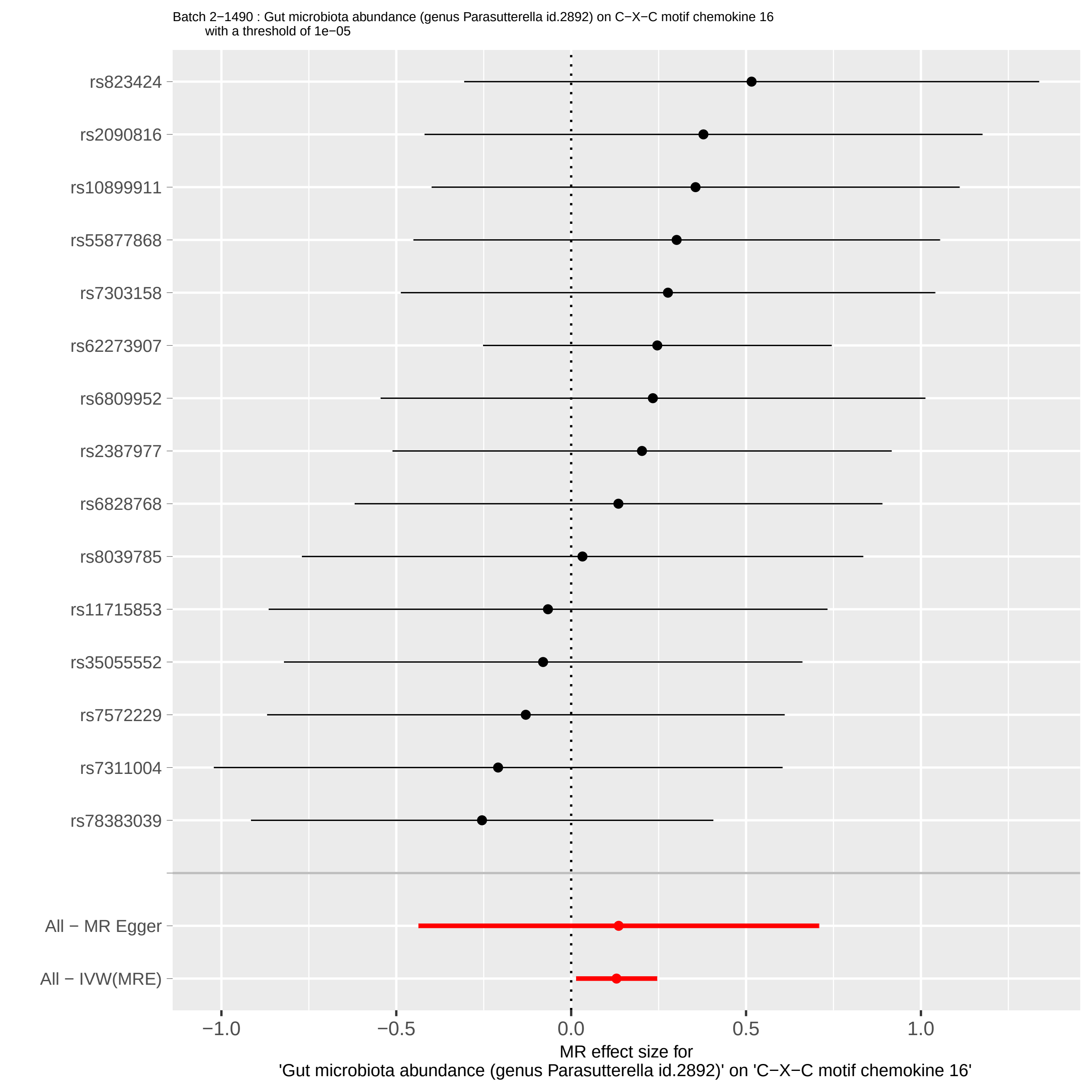

Batch 2−1490 : Gut microbiota abundance (genus Parasutterella id.2892) on C−X−C motif chemokine 16
with a threshold of 1e−05
rs823424
rs2090816
rs10899911
rs55877868
rs7303158
rs62273907
rs6809952
rs2387977
rs6828768
rs8039785
rs11715853
rs35055552
rs7572229
rs7311004
rs78383039
All − MR Egger
All − IVW(MRE)
−1.0
−0.5
0.0
0.5
1.0
MR effect size for
'Gut microbiota abundance (genus Parasutterella id.2892)' on 'C−X−C motif chemokine 16'

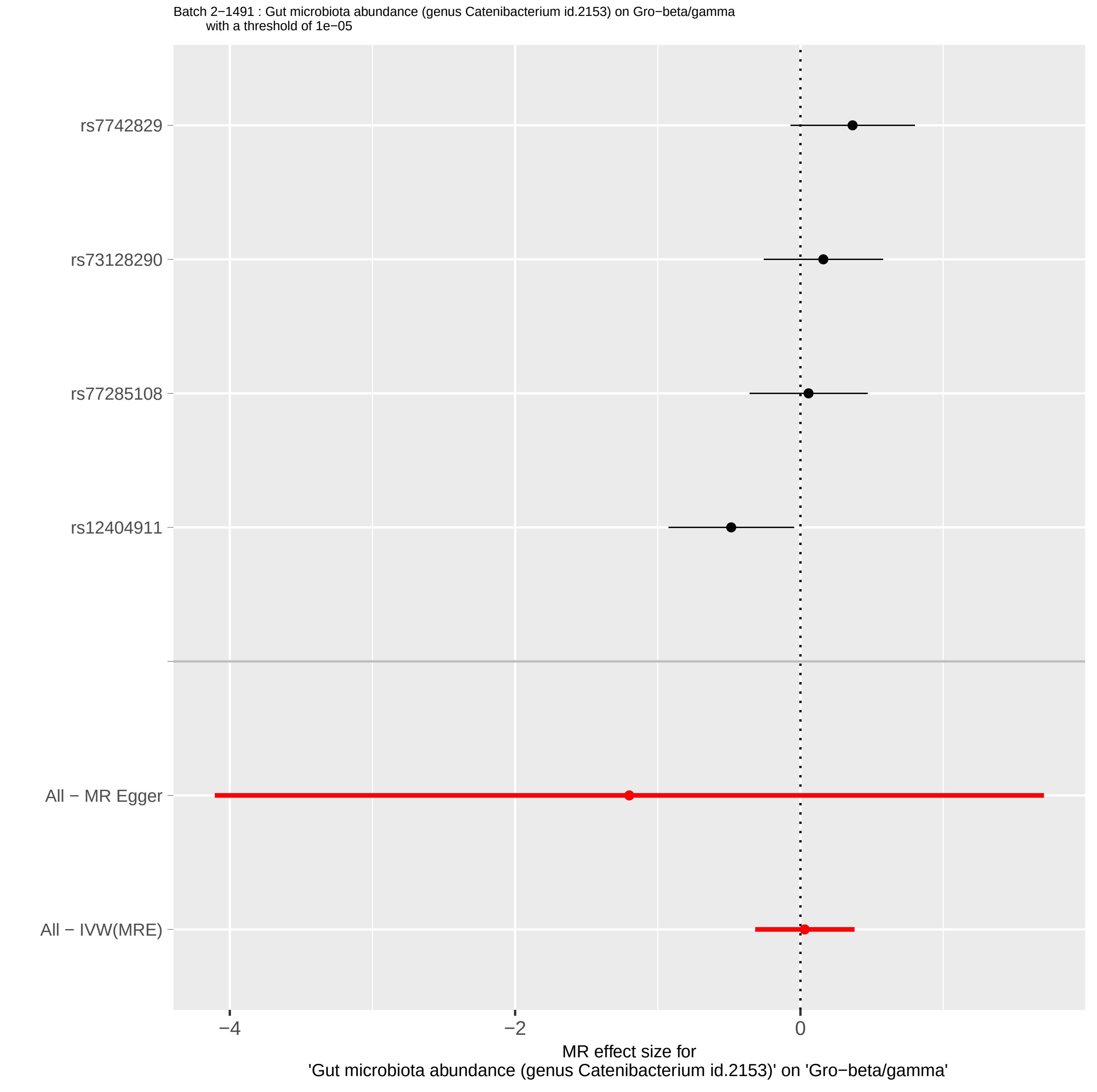

Batch 2−1491 : Gut microbiota abundance (genus Catenibacterium id.2153) on Gro−beta/gamma
with a threshold of 1e−05
rs7742829
rs73128290
rs77285108
rs12404911
All − MR Egger
All − IVW(MRE)
−4
−2
0
MR effect size for
'Gut microbiota abundance (genus Catenibacterium id.2153)' on 'Gro−beta/gamma'

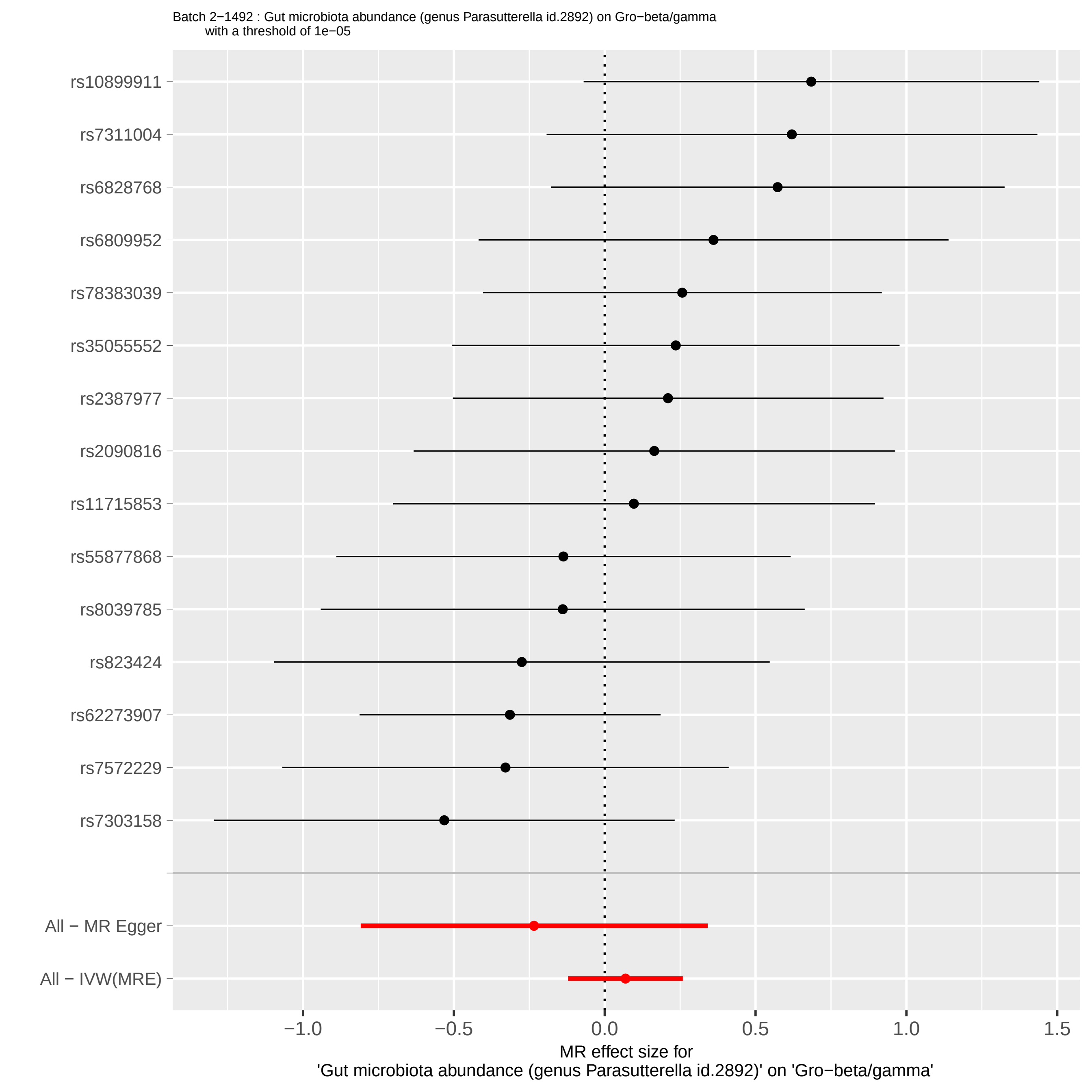
Batch 2−1492 : Gut microbiota abundance (genus Parasutterella id.2892) on Gro−beta/gamma
with a threshold of 1e−05
rs10899911
rs7311004
rs6828768
rs6809952
rs78383039
rs35055552
rs2387977
rs2090816
rs11715853
rs55877868
rs8039785
rs823424
rs62273907
rs7572229
rs7303158
All − MR Egger
All − IVW(MRE)
−1.0
−0.5
0.0
0.5
1.0
1.5
MR effect size for
'Gut microbiota abundance (genus Parasutterella id.2892)' on 'Gro−beta/gamma'

Batch 2−1493 : Gut microbiota abundance (genus Catenibacterium id.2153) on Gro−beta/gamma
with a threshold of 1e−05

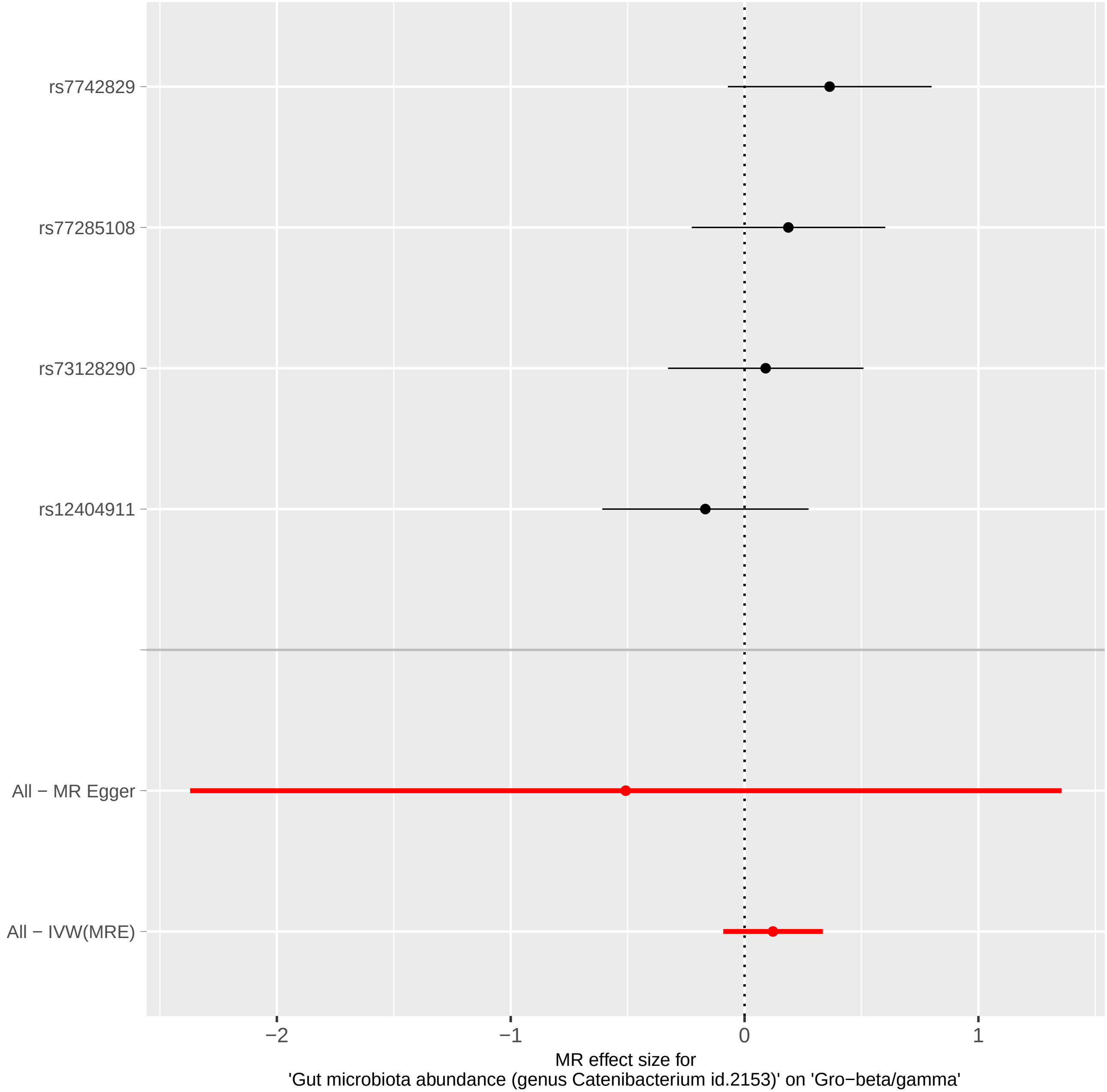

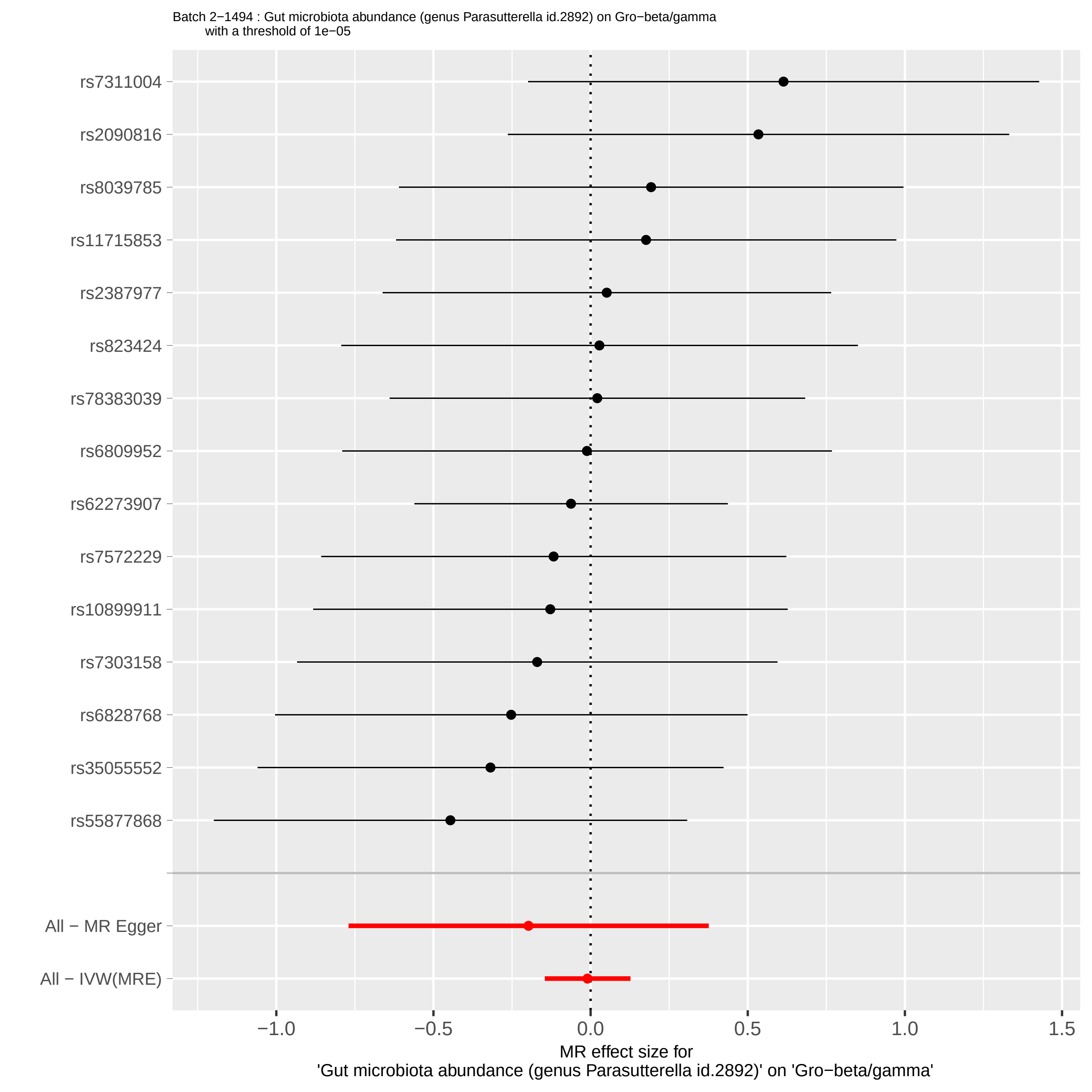
Batch 2−1494 : Gut microbiota abundance (genus Parasutterella id.2892) on Gro−beta/gamma
with a threshold of 1e−05
rs7311004
rs2090816
rs8039785
rs11715853
rs2387977
rs823424
rs78383039
rs6809952
rs62273907
rs7572229
rs10899911
rs7303158
rs6828768
rs35055552
rs55877868
All − MR Egger
All − IVW(MRE)
−1.0
−0.5
0.0
0.5
1.0
1.5
MR effect size for
'Gut microbiota abundance (genus Parasutterella id.2892)' on 'Gro−beta/gamma'

Batch 2−1495 : Gut microbiota abundance (genus Catenibacterium id.2153) on C−X−C motif chemokine 5
with a threshold of 1e−05

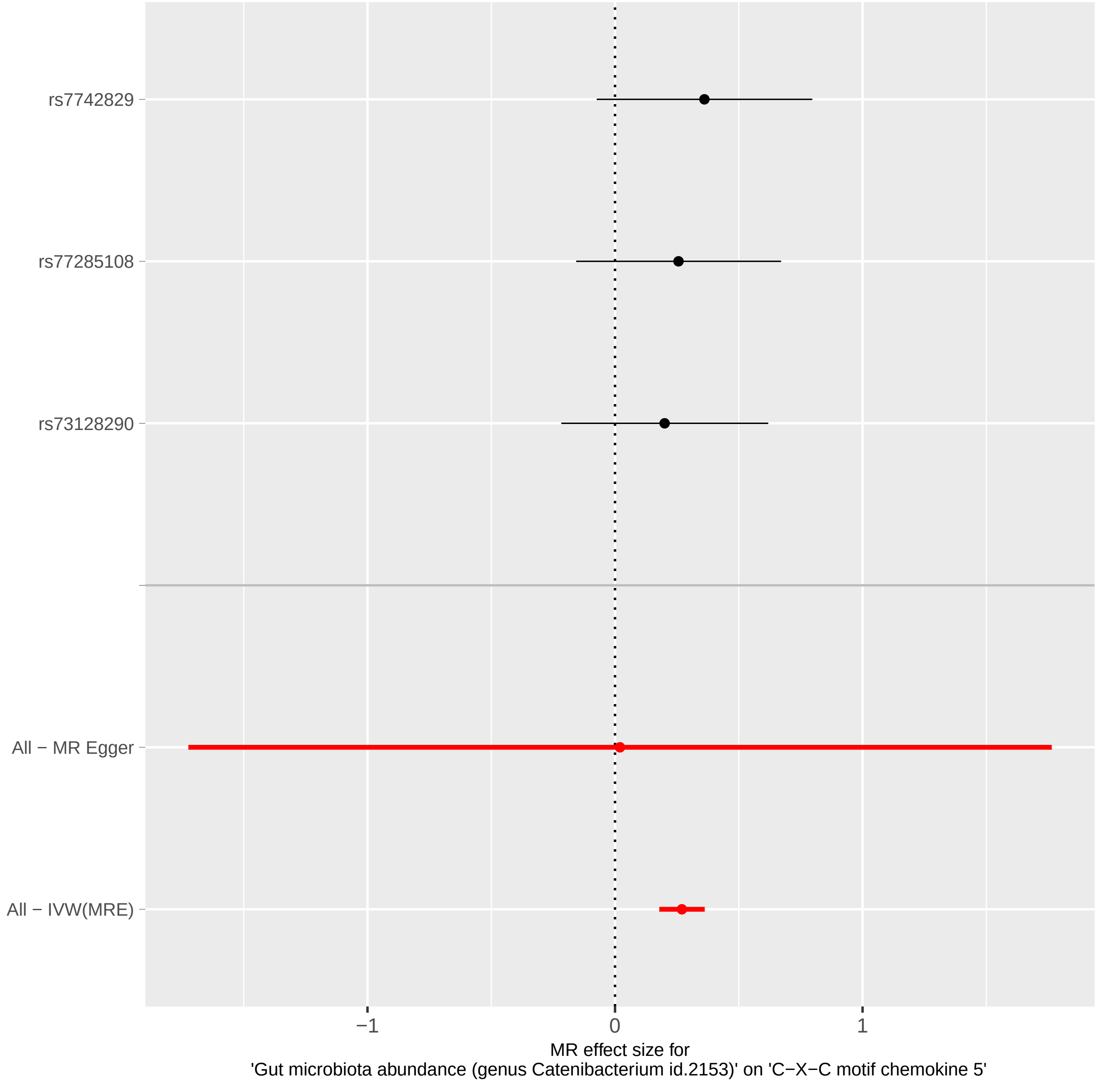

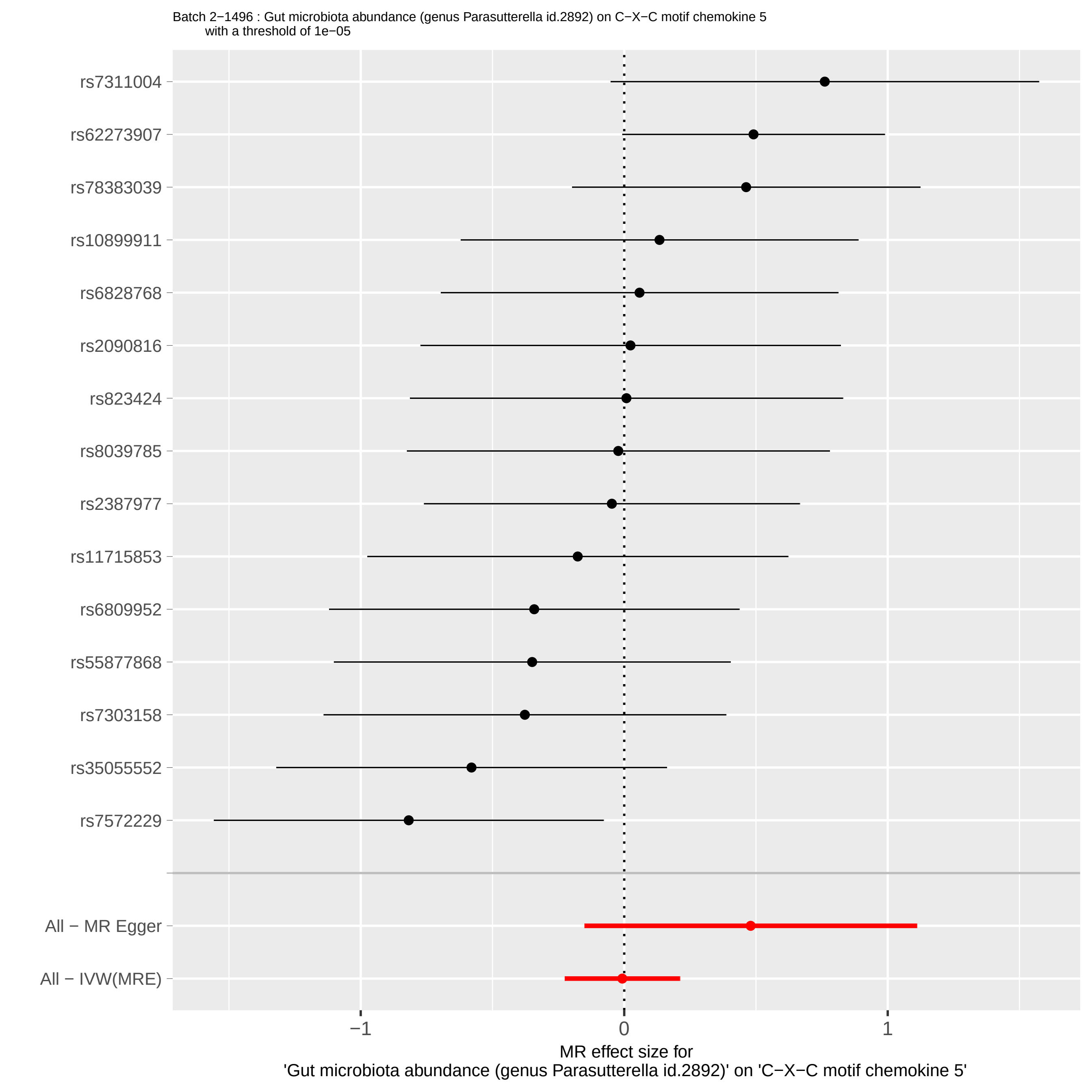

Batch 2−1496 : Gut microbiota abundance (genus Parasutterella id.2892) on C−X−C motif chemokine 5
with a threshold of 1e−05
rs7311004
rs62273907
rs78383039
rs10899911
rs6828768
rs2090816
rs823424
rs8039785
rs2387977
rs11715853
rs6809952
rs55877868
rs7303158
rs35055552
rs7572229
All − MR Egger
All − IVW(MRE)
−1
0
1
MR effect size for
'Gut microbiota abundance (genus Parasutterella id.2892)' on 'C−X−C motif chemokine 5'

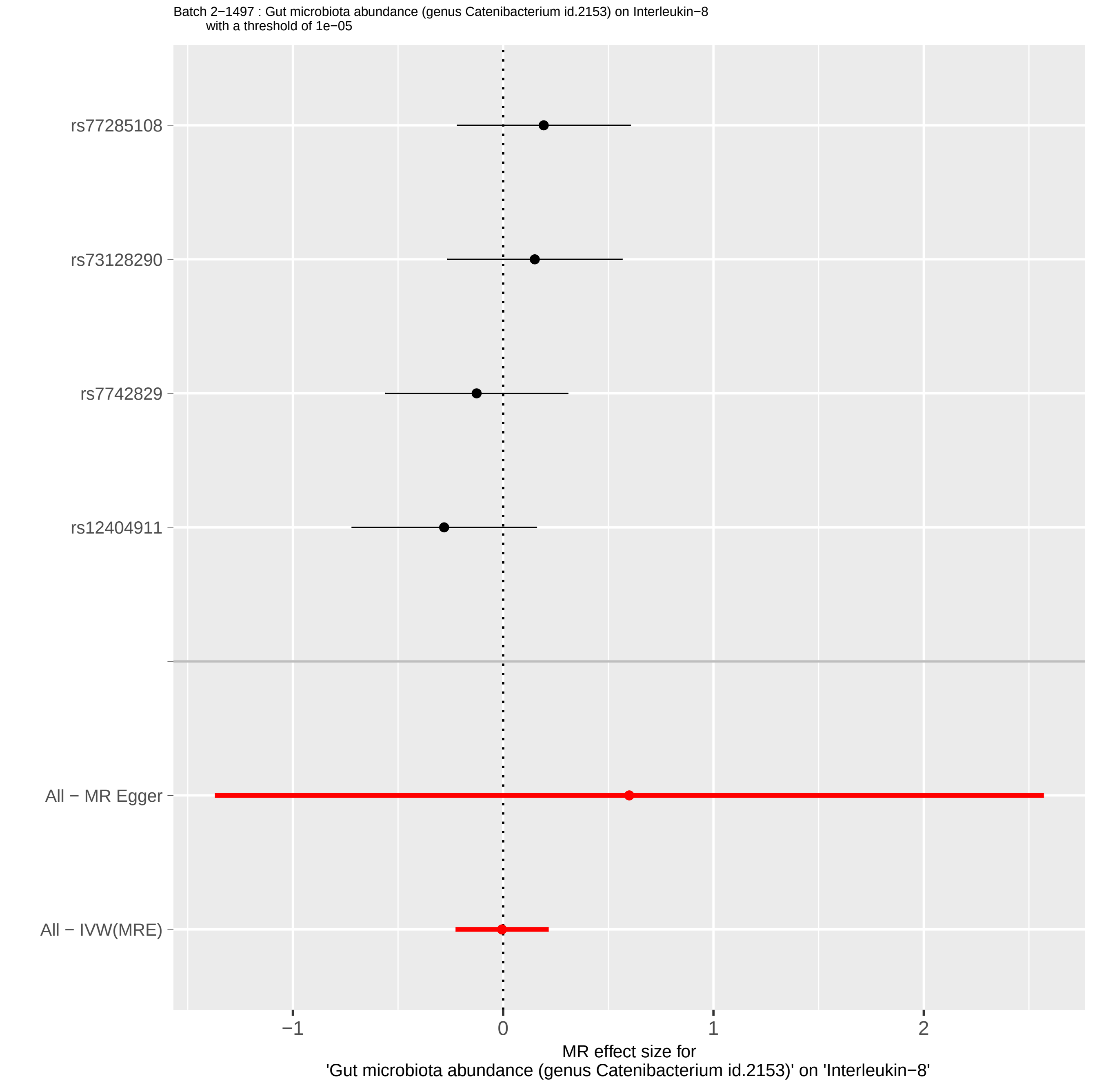

Batch 2−1497 : Gut microbiota abundance (genus Catenibacterium id.2153) on Interleukin−8
with a threshold of 1e−05
rs77285108
rs73128290
rs7742829
rs12404911
All − MR Egger
All − IVW(MRE)
−1
0
1
2
MR effect size for
'Gut microbiota abundance (genus Catenibacterium id.2153)' on 'Interleukin−8'

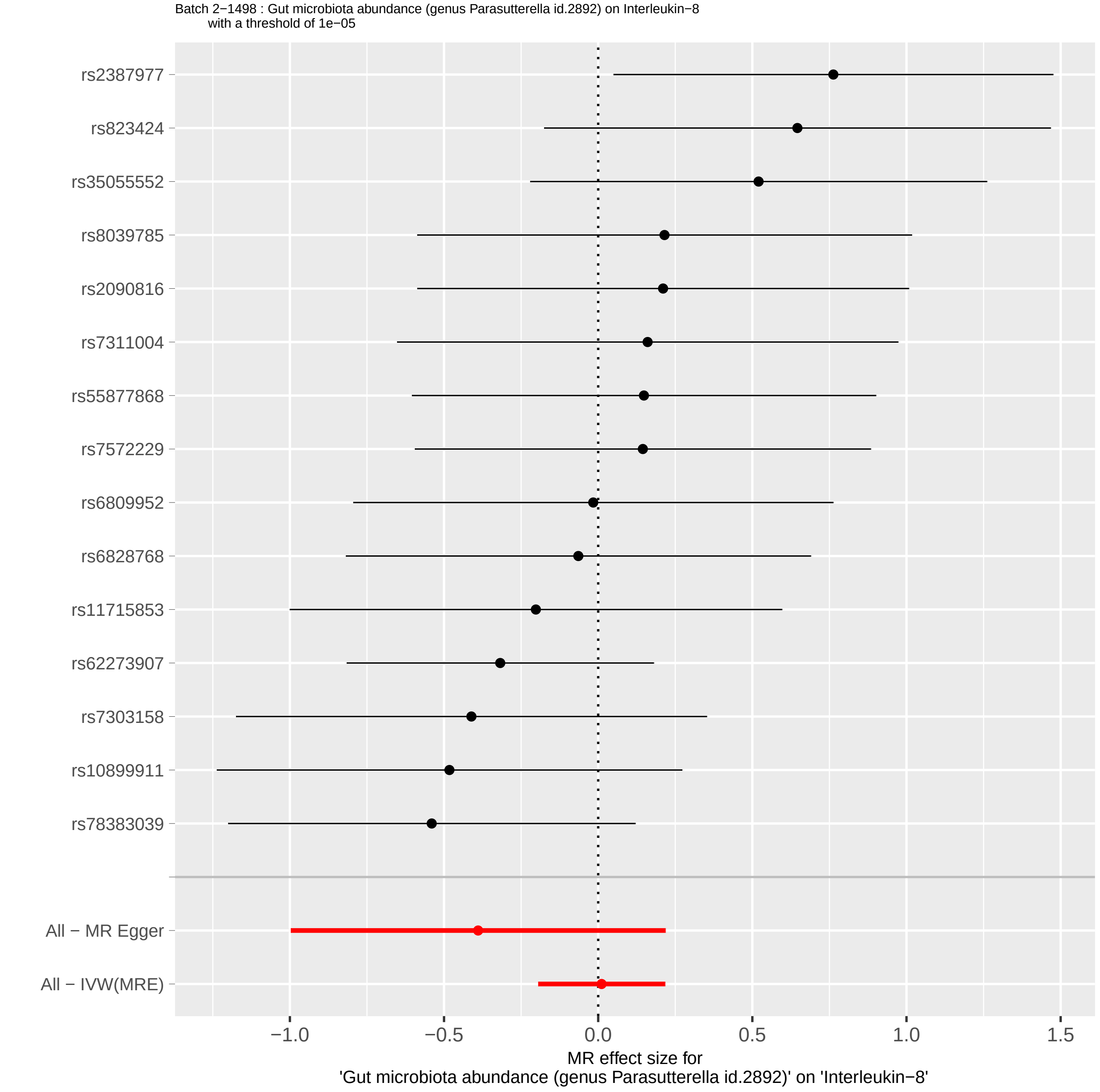

Batch 2−1498 : Gut microbiota abundance (genus Parasutterella id.2892) on Interleukin−8
with a threshold of 1e−05
rs2387977
rs823424
rs35055552
rs8039785
rs2090816
rs7311004
rs55877868
rs7572229
rs6809952
rs6828768
rs11715853
rs62273907
rs7303158
rs10899911
rs78383039
All − MR Egger
All − IVW(MRE)
−1.0
−0.5
0.0
0.5
1.0
1.5
MR effect size for
'Gut microbiota abundance (genus Parasutterella id.2892)' on 'Interleukin−8

Batch 2−1499 : Gut microbiota abundance (genus Catenibacterium id.2153) on C−X−C motif chemokine 9
with a threshold of 1e−05

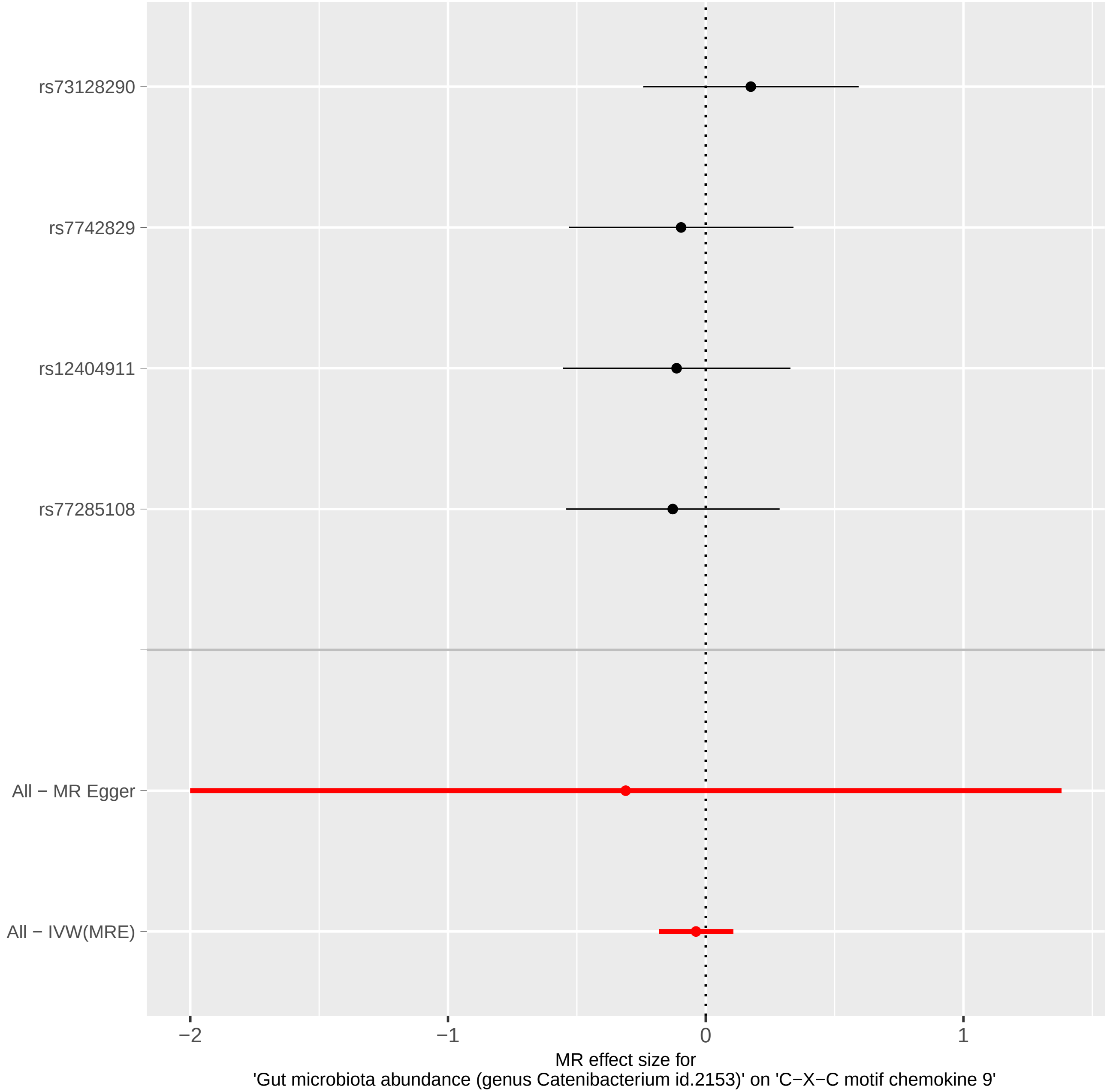

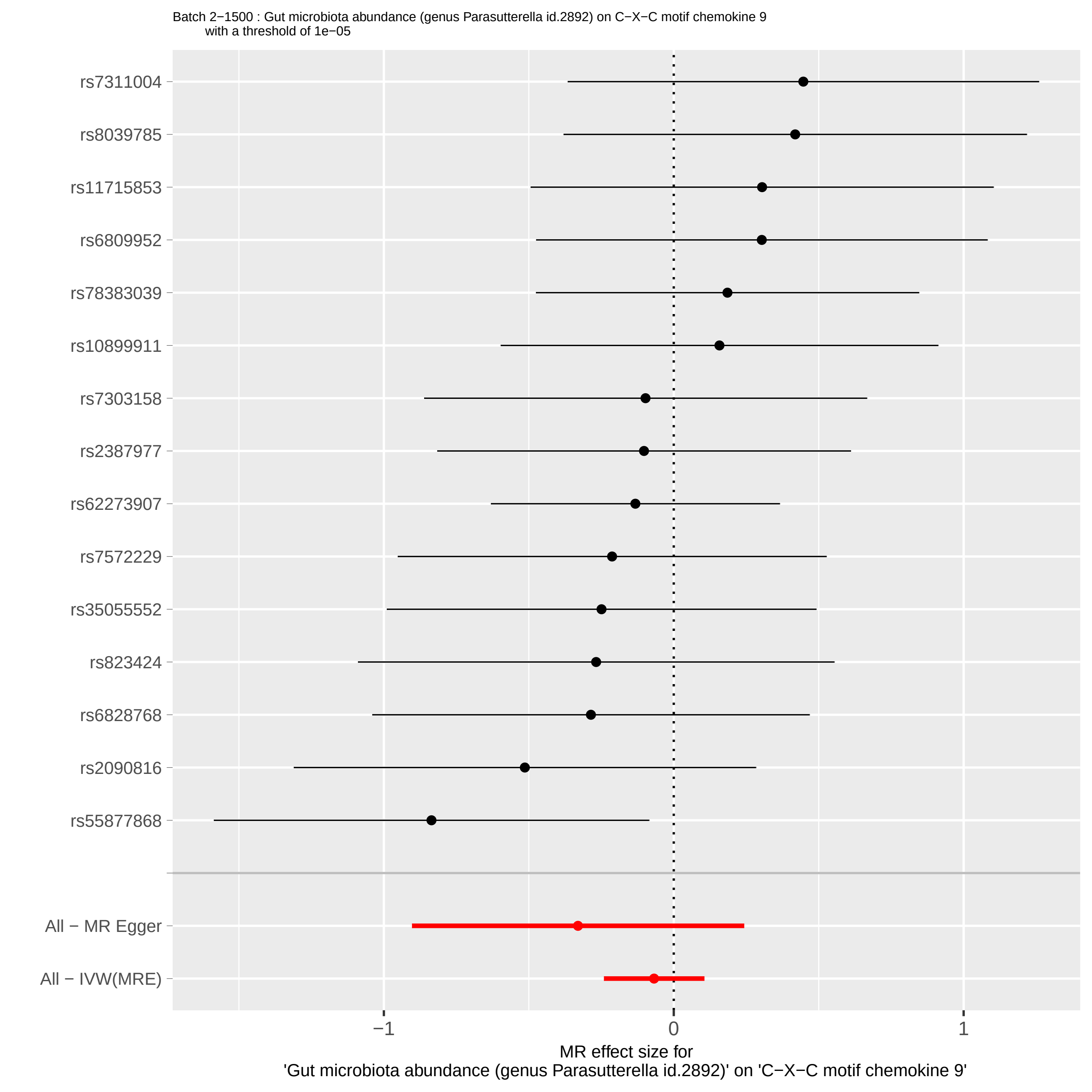

Batch 2−1500 : Gut microbiota abundance (genus Parasutterella id.2892) on C−X−C motif chemokine 9
with a threshold of 1e−05
rs7311004
rs8039785
rs11715853
rs6809952
rs78383039
rs10899911
rs7303158
rs2387977
rs62273907
rs7572229
rs35055552
rs823424
rs6828768
rs2090816
rs55877868
All − MR Egger
All − IVW(MRE)
−1
0
1
MR effect size for
'Gut microbiota abundance (genus Parasutterella id.2892)' on 'C−X−C motif chemokine 9'

Batch 2−1501 : Gut microbiota abundance (genus Catenibacterium id.2153) on C−X−C motif chemokine 9
with a threshold of 1e−05

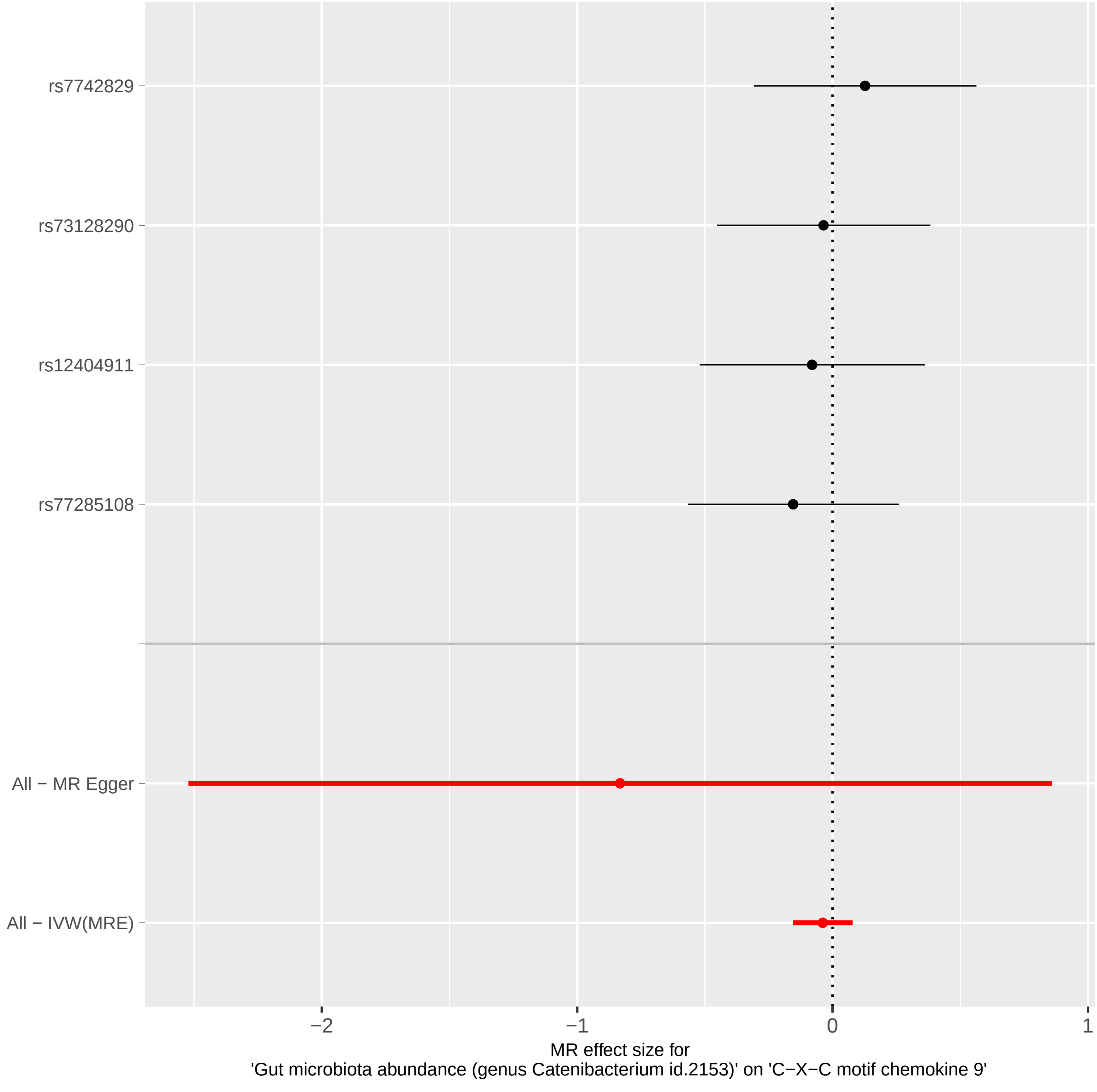

Batch 2−1502 : Gut microbiota abundance (genus Parasutterella id.2892) on C−X−C motif chemokine 9
with a threshold of 1e−05

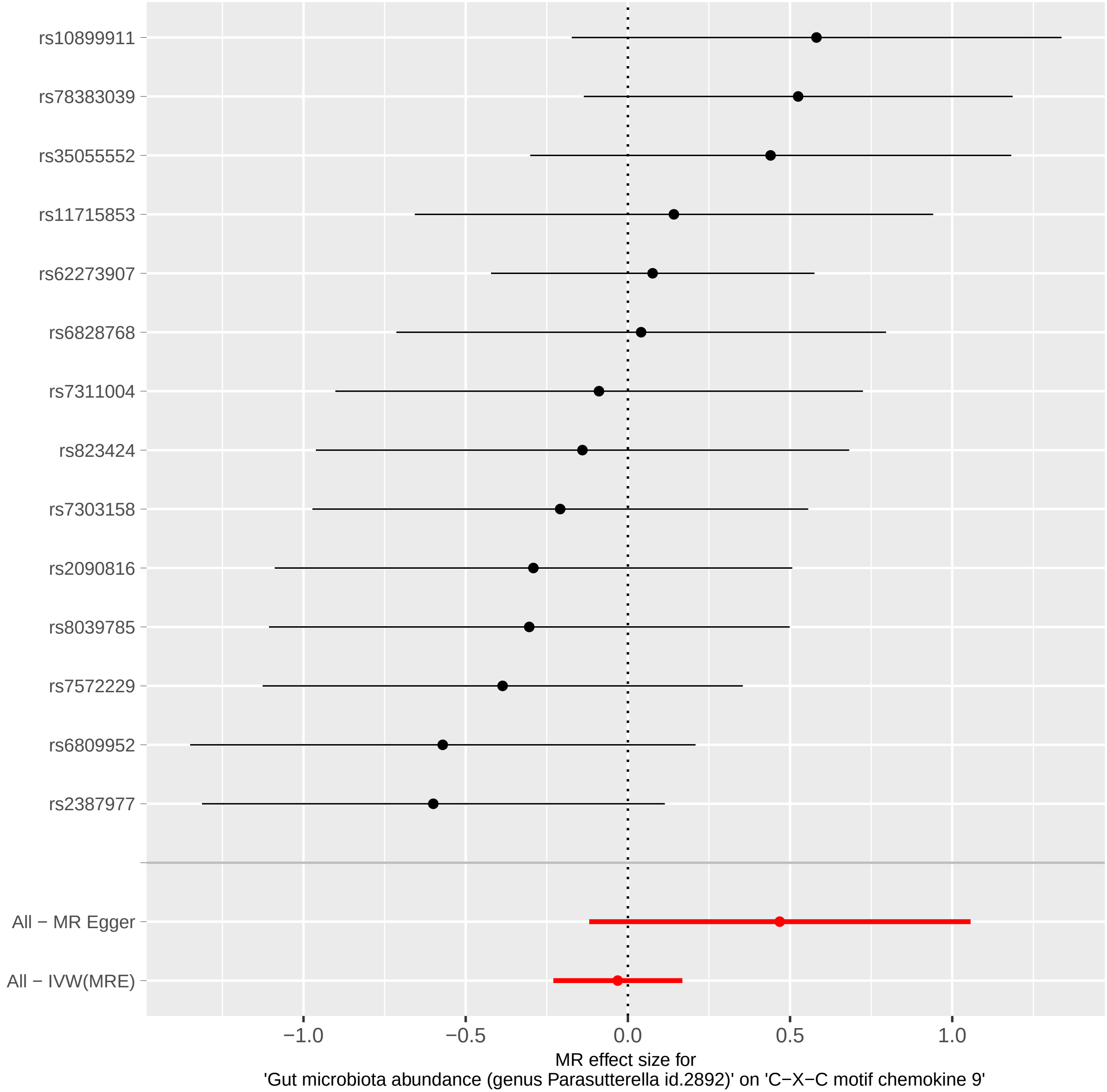

Batch 2−1503 : Gut microbiota abundance (genus Catenibacterium id.2153) on Cytochrome b5
with a threshold of 1e−05

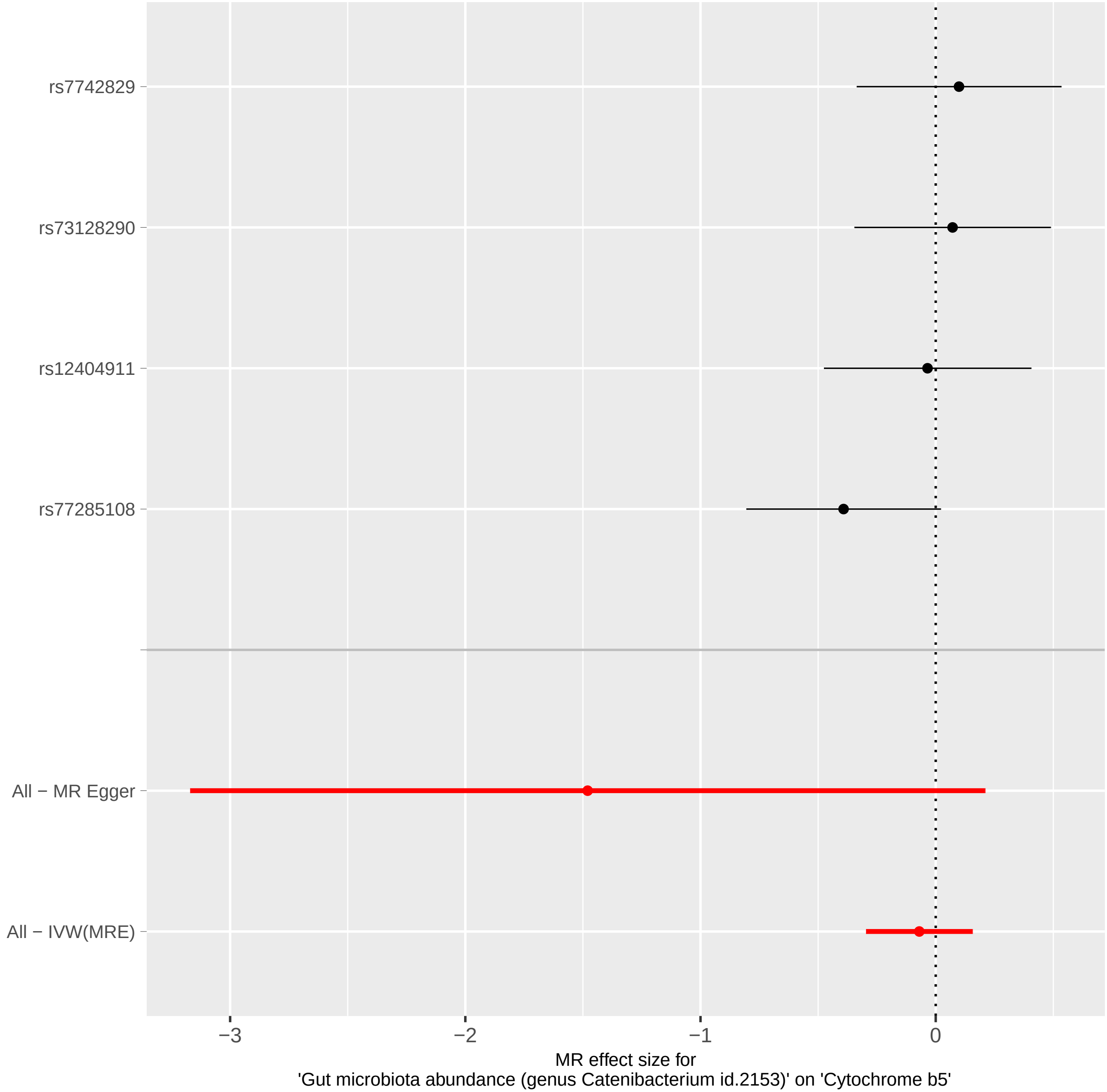

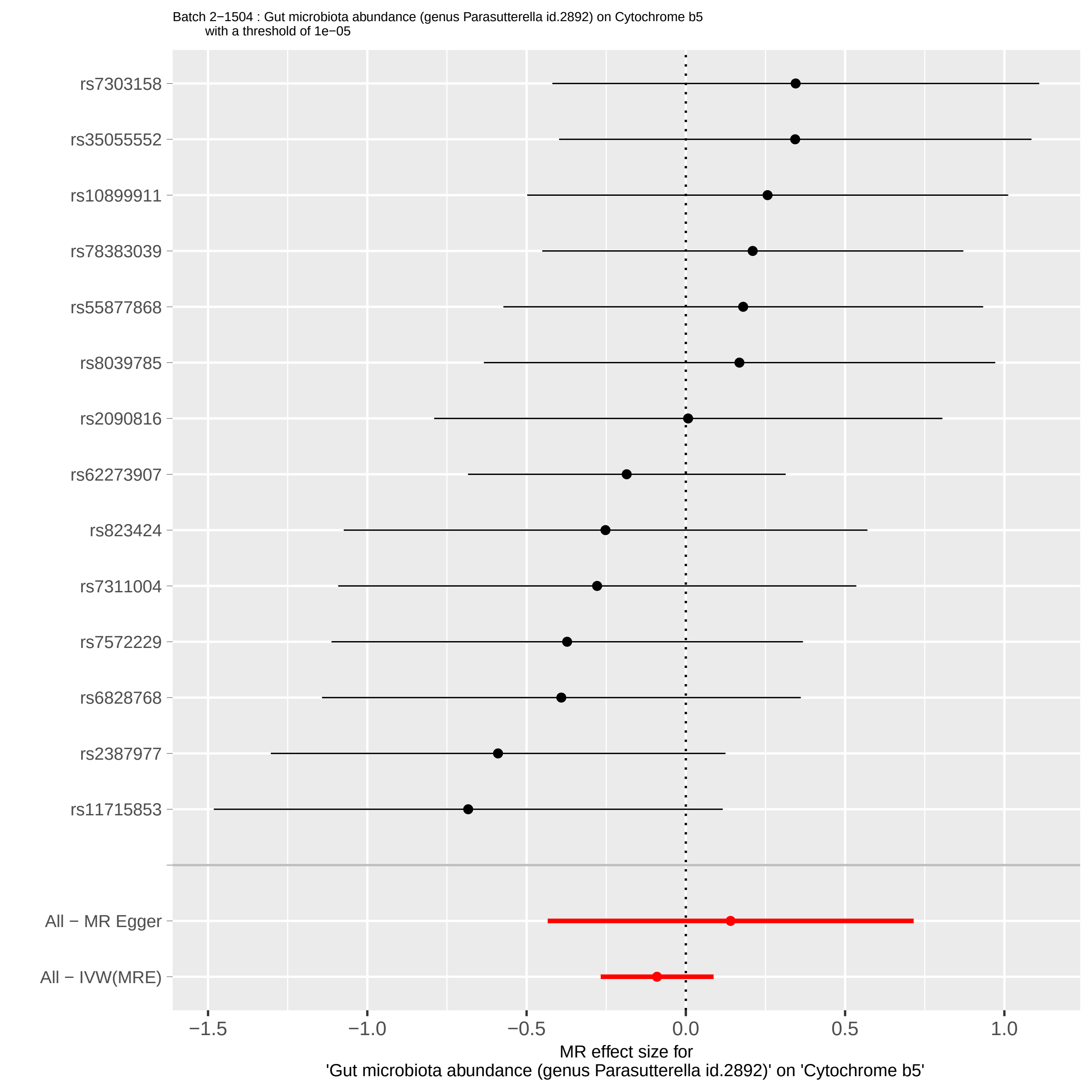
Batch 2−1504 : Gut microbiota abundance (genus Parasutterella id.2892) on Cytochrome b5
with a threshold of 1e−05
rs7303158
rs35055552
rs10899911
rs78383039
rs55877868
rs8039785
rs2090816
rs62273907
rs823424
rs7311004
rs7572229
rs6828768
rs2387977
rs11715853
All − MR Egger
All − IVW(MRE)
−1.5
−1.0
−0.5
0.0
0.5
1.0
MR effect size for
'Gut microbiota abundance (genus Parasutterella id.2892)' on 'Cytochrome b5'

Batch 2−1505 : Gut microbiota abundance (genus Catenibacterium id.2153) on NADH−cytochrome b5 reductase 3
with a threshold of 1e−05

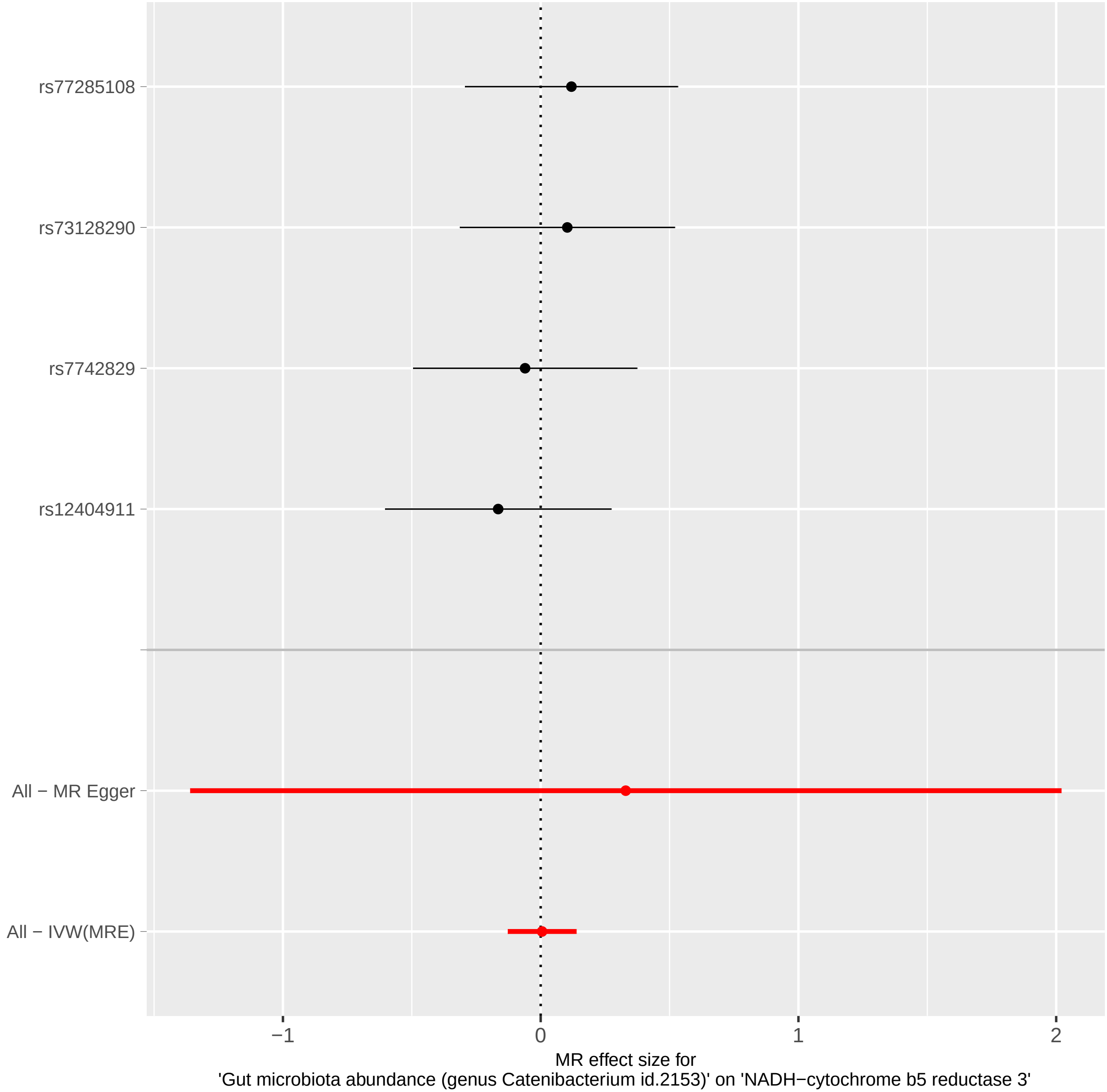

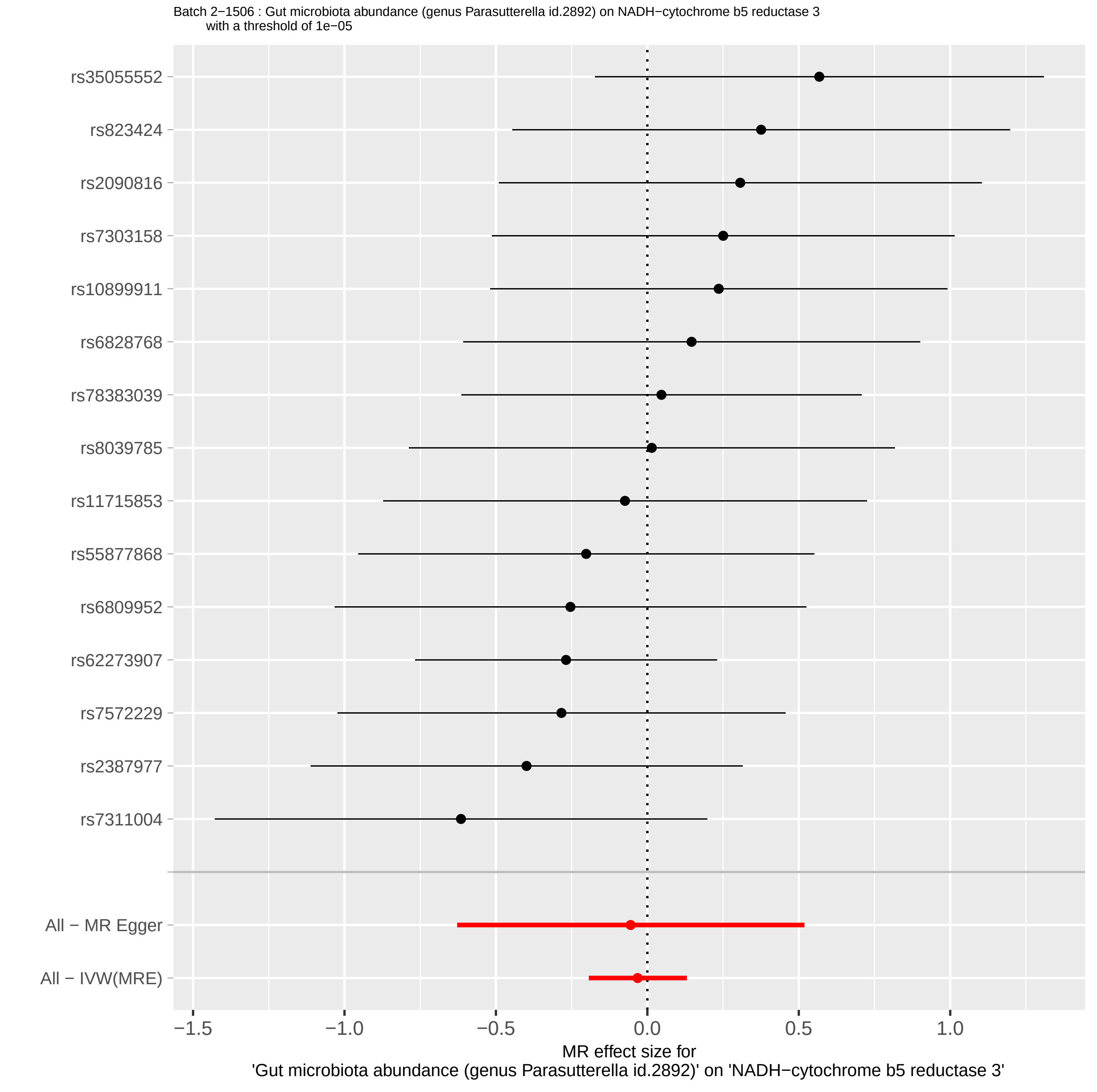

Batch 2−1506 : Gut microbiota abundance (genus Parasutterella id.2892) on NADH−cytochrome b5 reductase 3
with a threshold of 1e−05
rs35055552
rs823424
rs2090816
rs7303158
rs10899911
rs6828768
rs78383039
rs8039785
rs11715853
rs55877868
rs6809952
rs62273907
rs7572229
rs2387977
rs7311004
All − MR Egger
All − IVW(MRE)
−1.5
−1.0
−0.5
0.0
0.5
1.0
MR effect size for
'Gut microbiota abundance (genus Parasutterella id.2892)' on 'NADH−cytochrome b5 reductase 3'

Batch 2−1507 : Gut microbiota abundance (genus Catenibacterium id.2153) on Cytochrome c
with a threshold of 1e−05

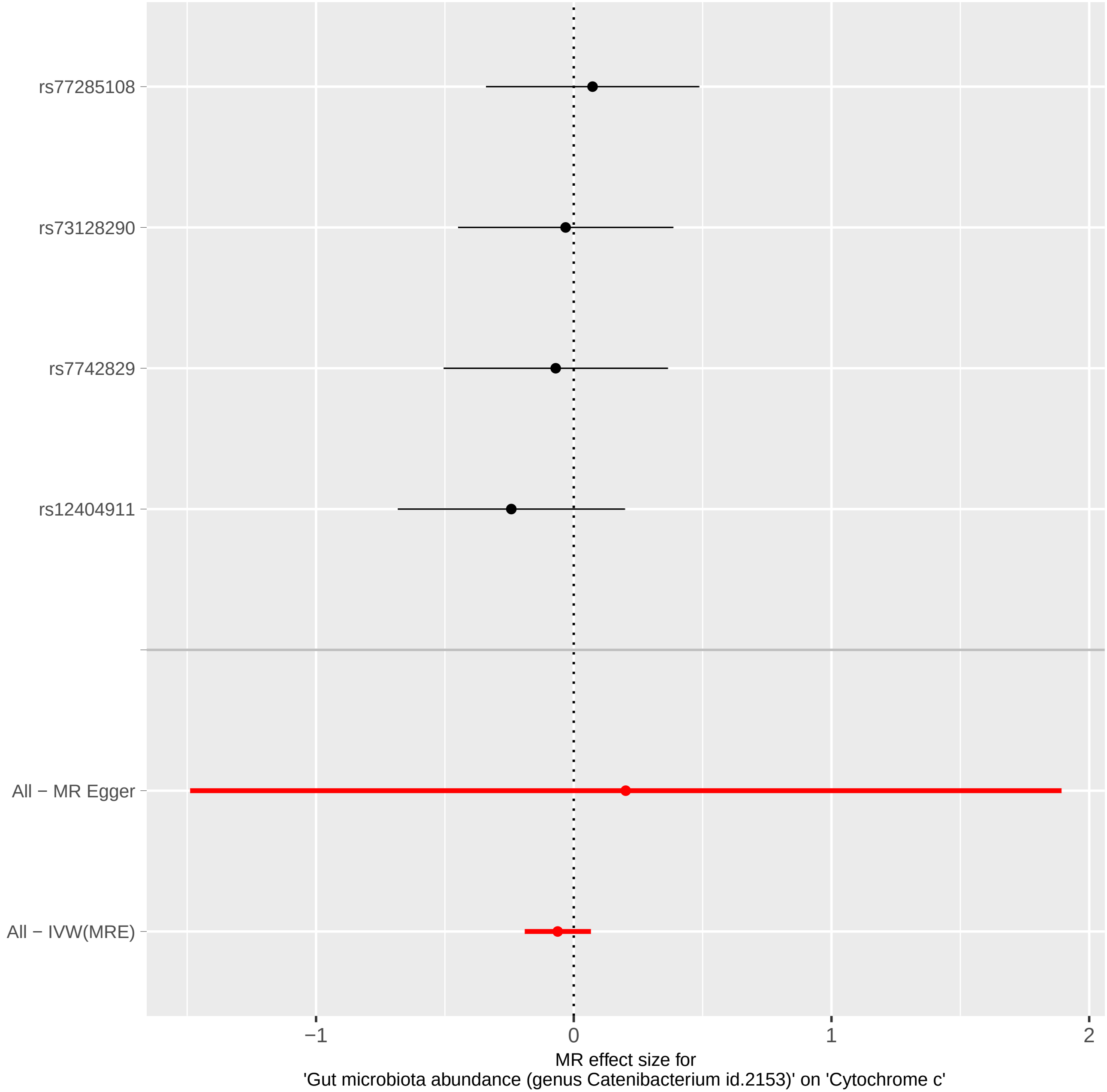

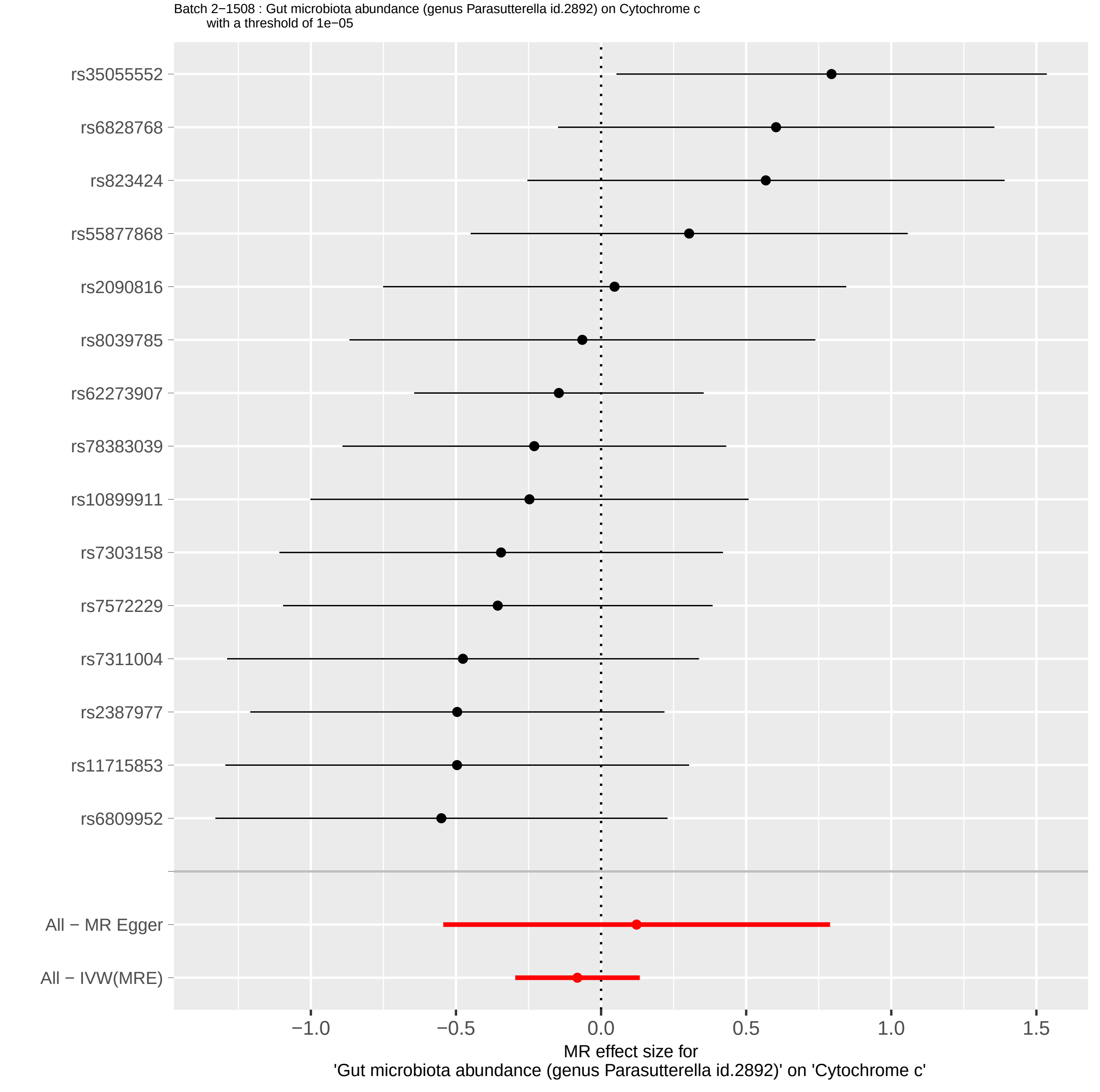

Batch 2−1508 : Gut microbiota abundance (genus Parasutterella id.2892) on Cytochrome c
with a threshold of 1e−05
rs35055552
rs6828768
rs823424
rs55877868
rs2090816
rs8039785
rs62273907
rs78383039
rs10899911
rs7303158
rs7572229
rs7311004
rs2387977
rs11715853
rs6809952
All − MR Egger
All − IVW(MRE)
−1.0
−0.5
0.0
0.5
1.0
1.5
MR effect size for
'Gut microbiota abundance (genus Parasutterella id.2892)' on 'Cytochrome c'

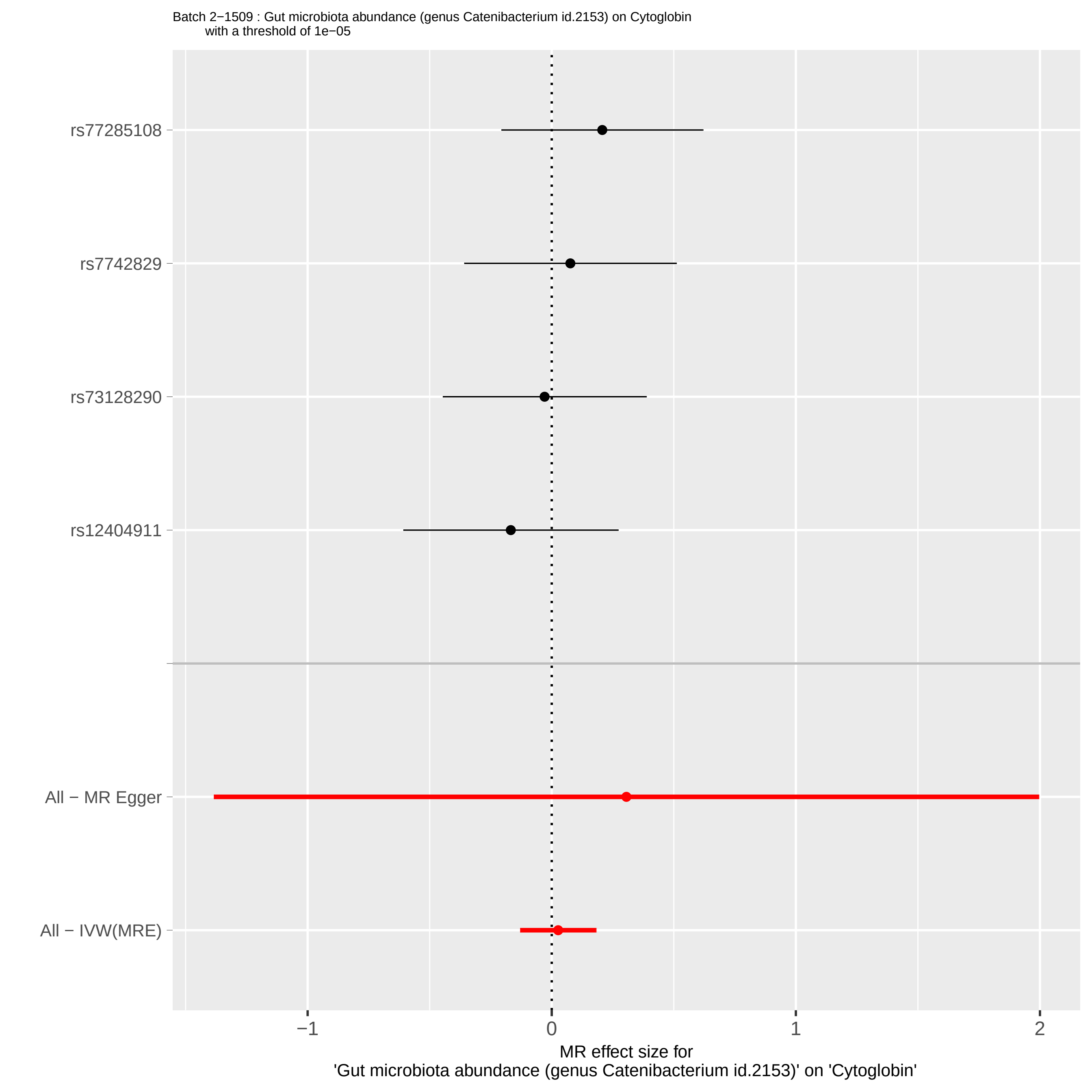
Batch 2−1509 : Gut microbiota abundance (genus Catenibacterium id.2153) on Cytoglobin
with a threshold of 1e−05
rs77285108
rs7742829
rs73128290
rs12404911
All − MR Egger
All − IVW(MRE)
−1
0
1
2
MR effect size for
'Gut microbiota abundance (genus Catenibacterium id.2153)' on 'Cytoglobin'

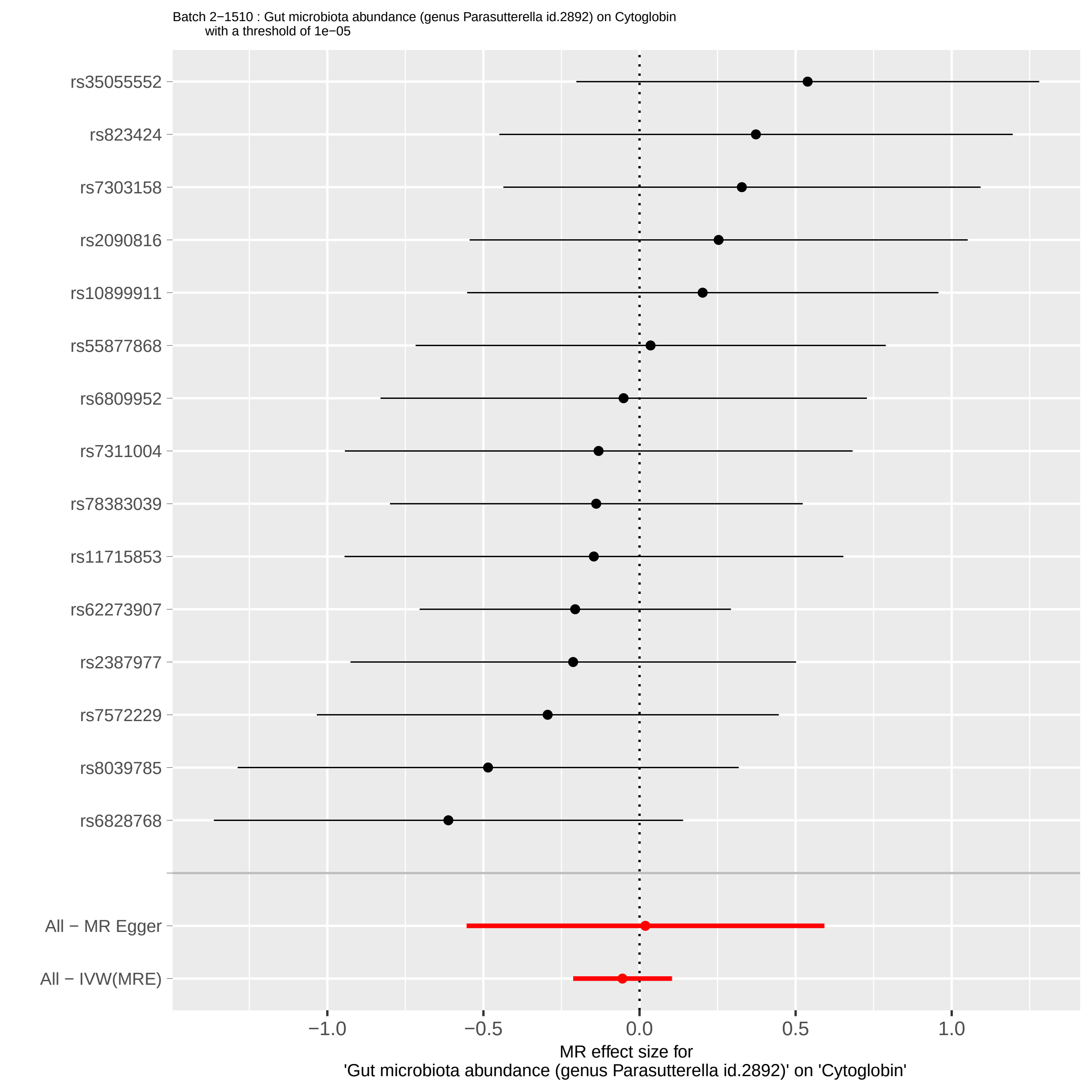

Batch 2−1510 : Gut microbiota abundance (genus Parasutterella id.2892) on Cytoglobin
with a threshold of 1e−05
rs35055552
rs823424
rs7303158
rs2090816
rs10899911
rs55877868
rs6809952
rs7311004
rs78383039
rs11715853
rs62273907
rs2387977
rs7572229
rs8039785
rs6828768
All − MR Egger
All − IVW(MRE)
−1.0
−0.5
0.0
0.5
1.0
MR effect size for
'Gut microbiota abundance (genus Parasutterella id.2892)' on 'Cytoglobin'

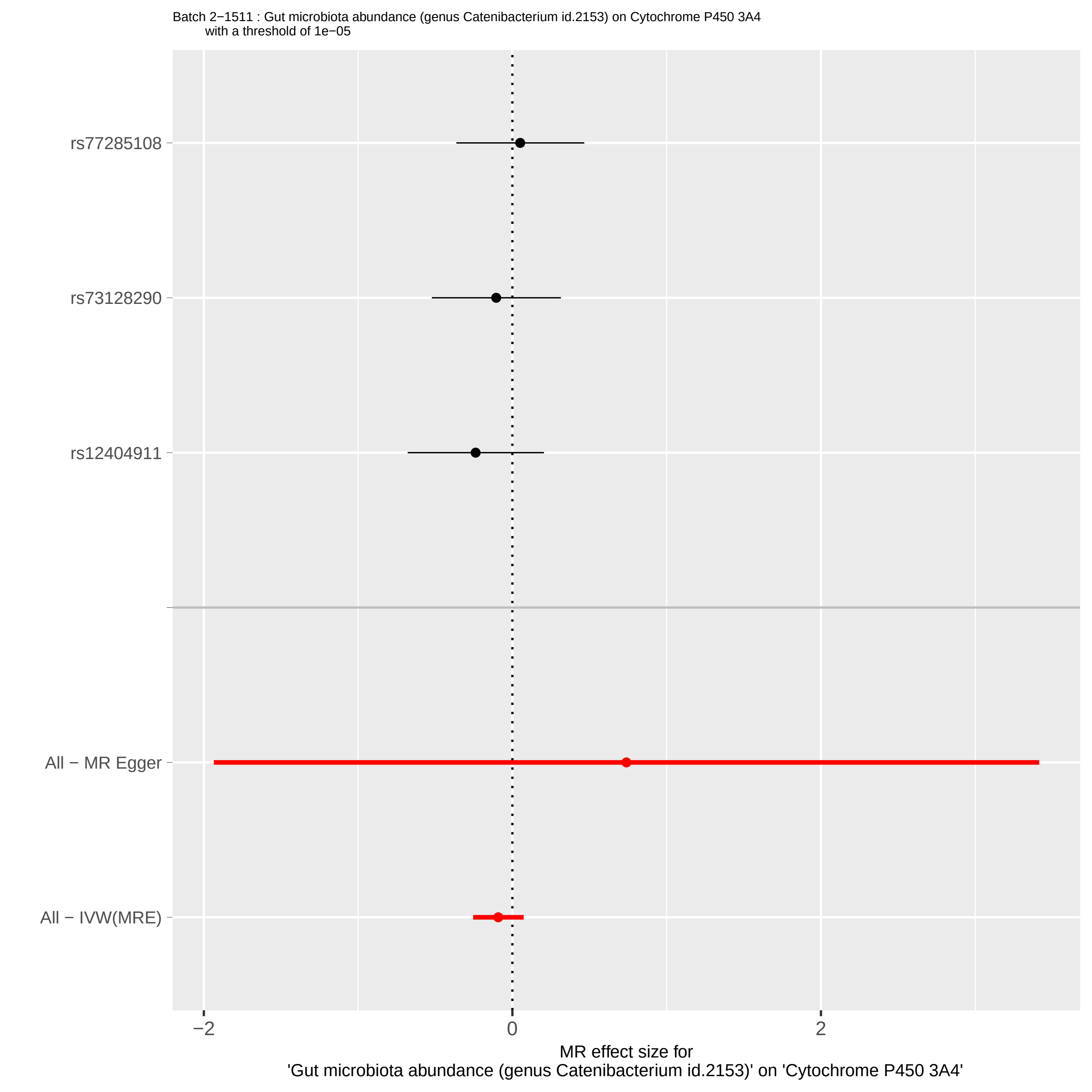
Batch 2−1511 : Gut microbiota abundance (genus Catenibacterium id.2153) on Cytochrome P450 3A4
with a threshold of 1e−05
rs77285108
rs73128290
rs12404911
All − MR Egger
All − IVW(MRE)
−2
0
2
MR effect size for
'Gut microbiota abundance (genus Catenibacterium id.2153)' on 'Cytochrome P450 3A4'

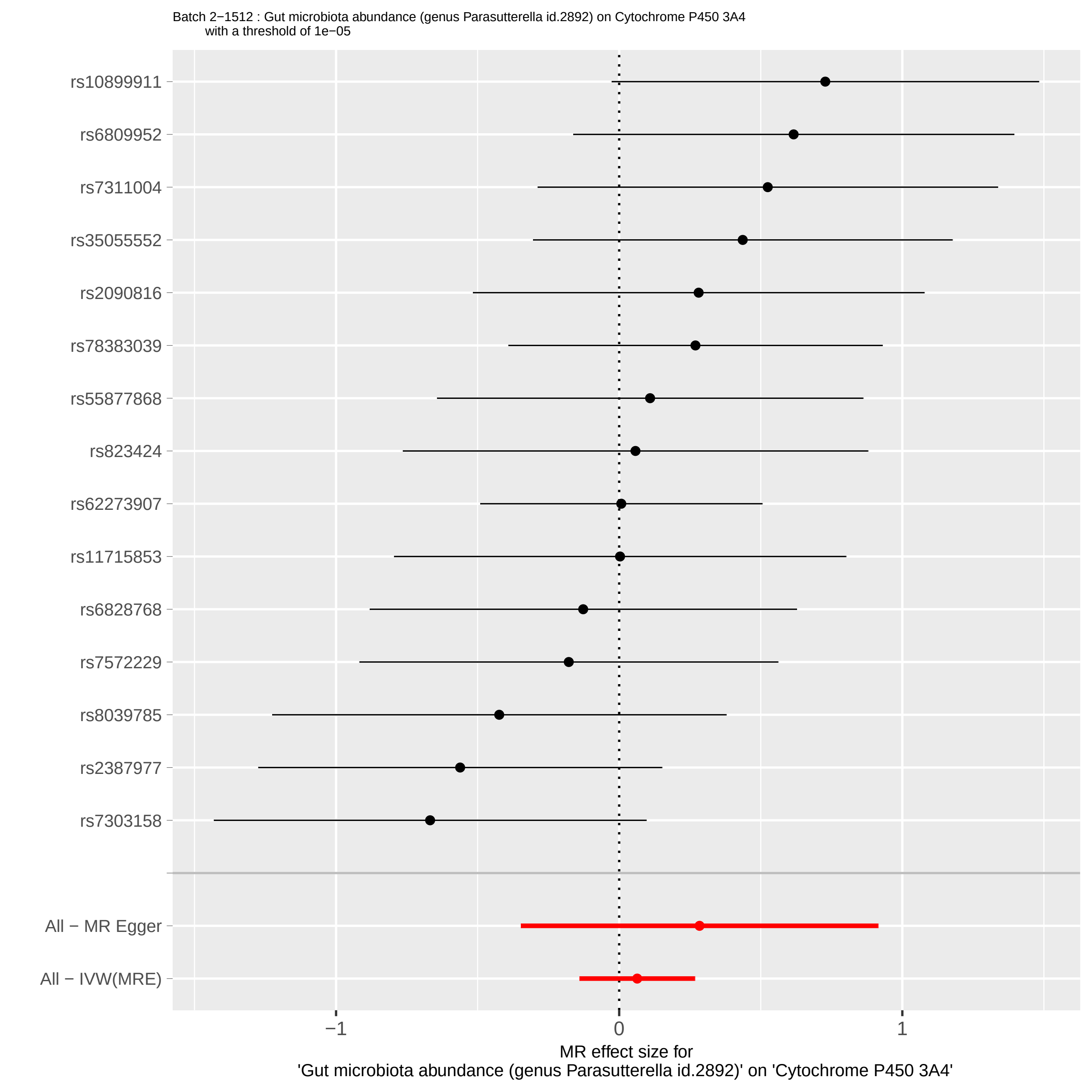

Batch 2−1512 : Gut microbiota abundance (genus Parasutterella id.2892) on Cytochrome P450 3A4
with a threshold of 1e−05
rs10899911
rs6809952
rs7311004
rs35055552
rs2090816
rs78383039
rs55877868
rs823424
rs62273907
rs11715853
rs6828768
rs7572229
rs8039785
rs2387977
rs7303158
All − MR Egger
All − IVW(MRE)
−1
0
1
MR effect size for
'Gut microbiota abundance (genus Parasutterella id.2892)' on 'Cytochrome P450 3A4'

Batch 2−1513 : Gut microbiota abundance (genus Catenibacterium id.2153) on Cytochrome P450 3A4
with a threshold of 1e−05

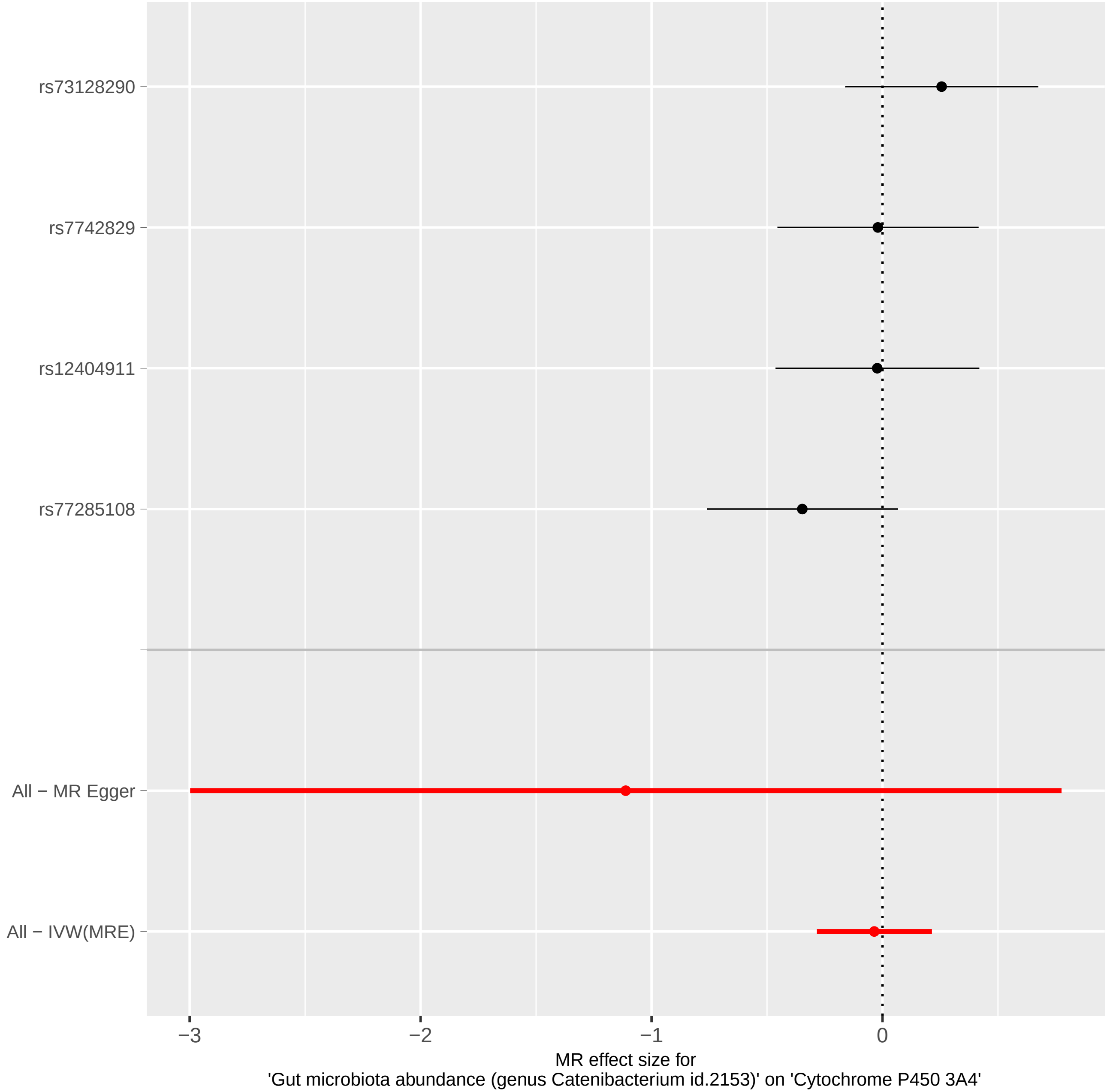

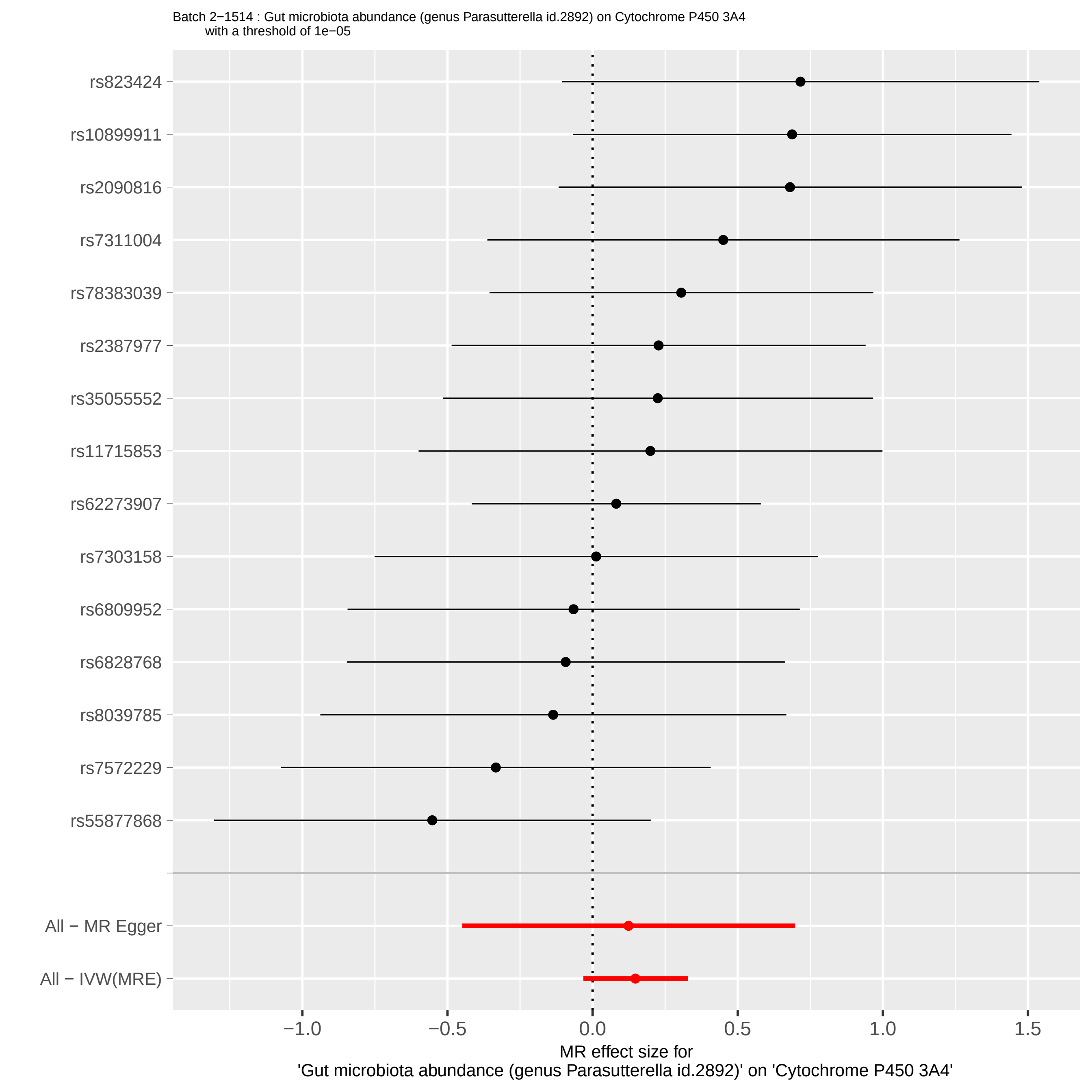

Batch 2−1514 : Gut microbiota abundance (genus Parasutterella id.2892) on Cytochrome P450 3A4
with a threshold of 1e−05
rs823424
rs10899911
rs2090816
rs7311004
rs78383039
rs2387977
rs35055552
rs11715853
rs62273907
rs7303158
rs6809952
rs6828768
rs8039785
rs7572229
rs55877868
All − MR Egger
All − IVW(MRE)
−1.0
−0.5
0.0
0.5
1.0
1.5
MR effect size for
'Gut microbiota abundance (genus Parasutterella id.2892)' on 'Cytochrome P450 3A4'

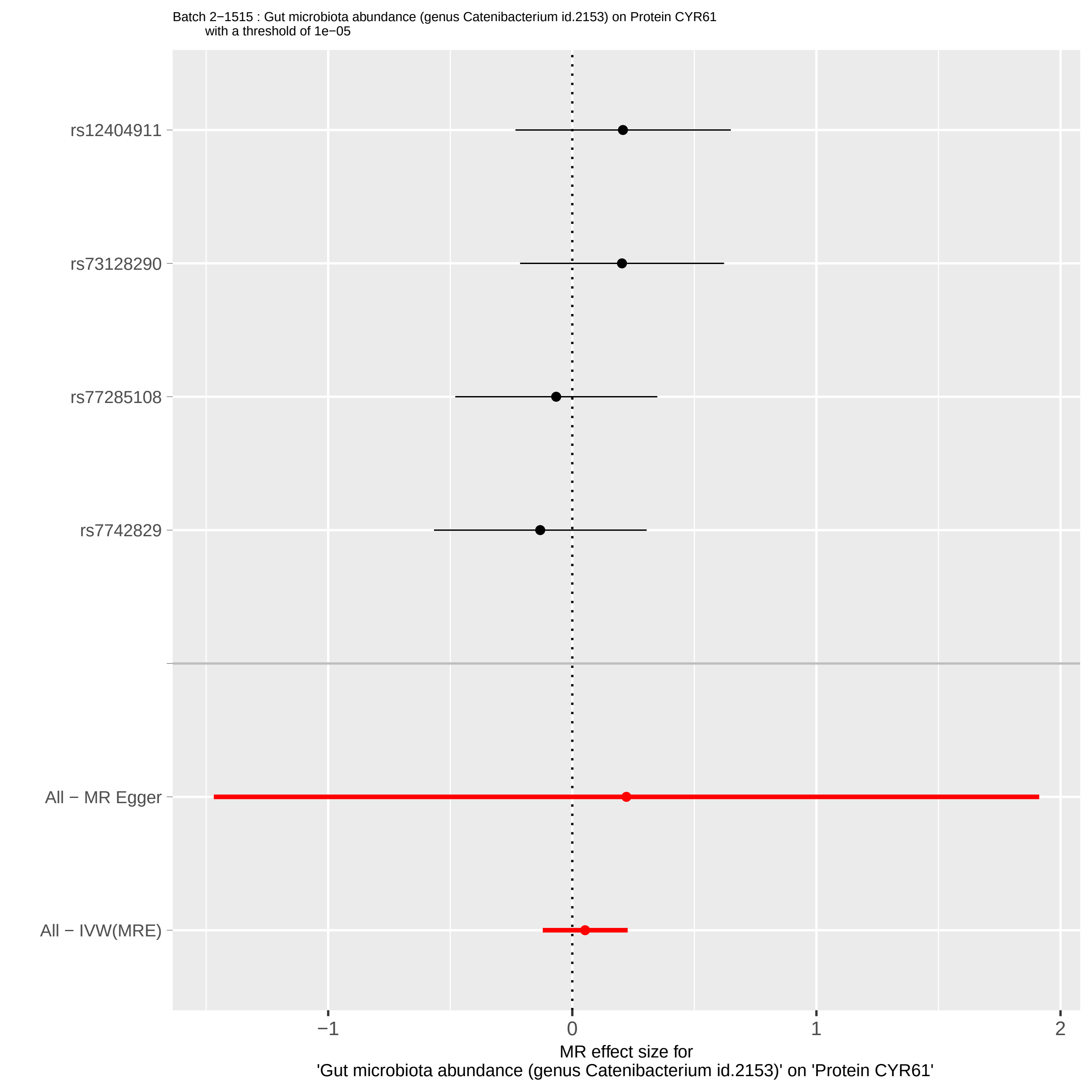
Batch 2−1515 : Gut microbiota abundance (genus Catenibacterium id.2153) on Protein CYR61
with a threshold of 1e−05
rs12404911
rs73128290
rs77285108
rs7742829
All − MR Egger
All − IVW(MRE)
−1
0
1
2
MR effect size for
'Gut microbiota abundance (genus Catenibacterium id.2153)' on 'Protein CYR61'

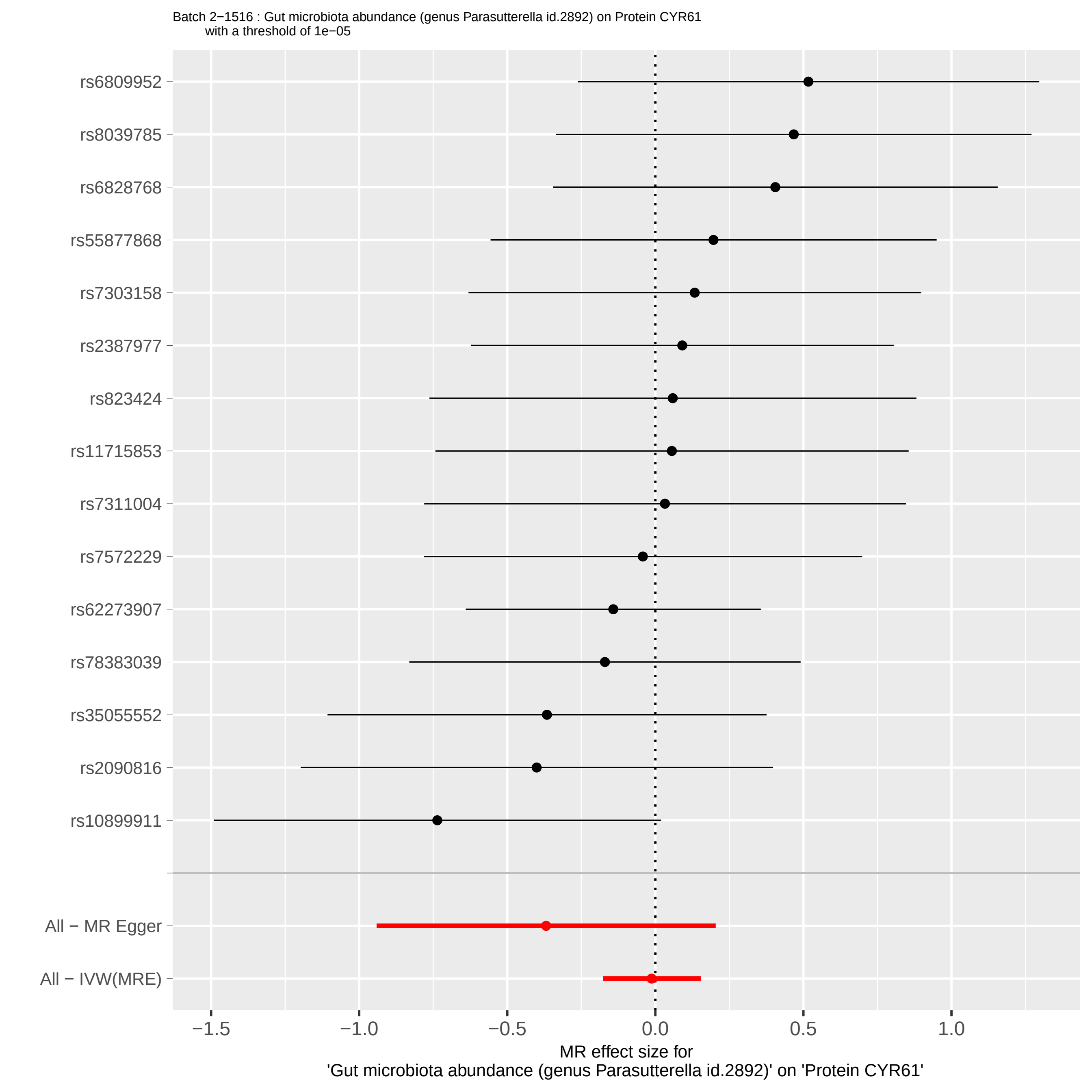

Batch 2−1516 : Gut microbiota abundance (genus Parasutterella id.2892) on Protein CYR61
with a threshold of 1e−05
rs6809952
rs8039785
rs6828768
rs55877868
rs7303158
rs2387977
rs823424
rs11715853
rs7311004
rs7572229
rs62273907
rs78383039
rs35055552
rs2090816
rs10899911
All − MR Egger
All − IVW(MRE)
−1.5
−1.0
−0.5
0.0
0.5
1.0
MR effect size for
'Gut microbiota abundance (genus Parasutterella id.2892)' on 'Protein CYR61'

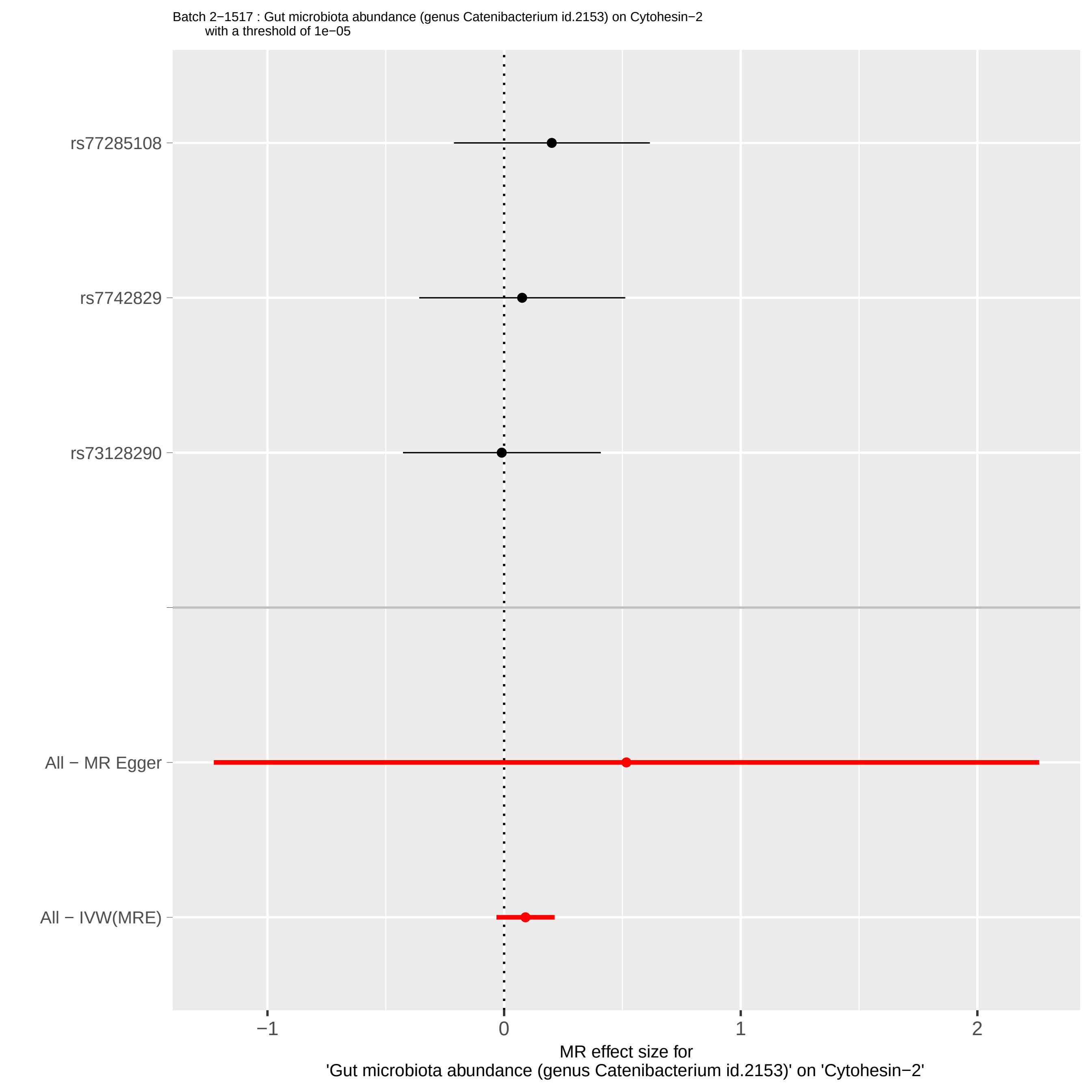
Batch 2−1517 : Gut microbiota abundance (genus Catenibacterium id.2153) on Cytohesin−2
with a threshold of 1e−05
rs77285108
rs7742829
rs73128290
All − MR Egger
All − IVW(MRE)
−1
0
1
2
MR effect size for
'Gut microbiota abundance (genus Catenibacterium id.2153)' on 'Cytohesin−2'

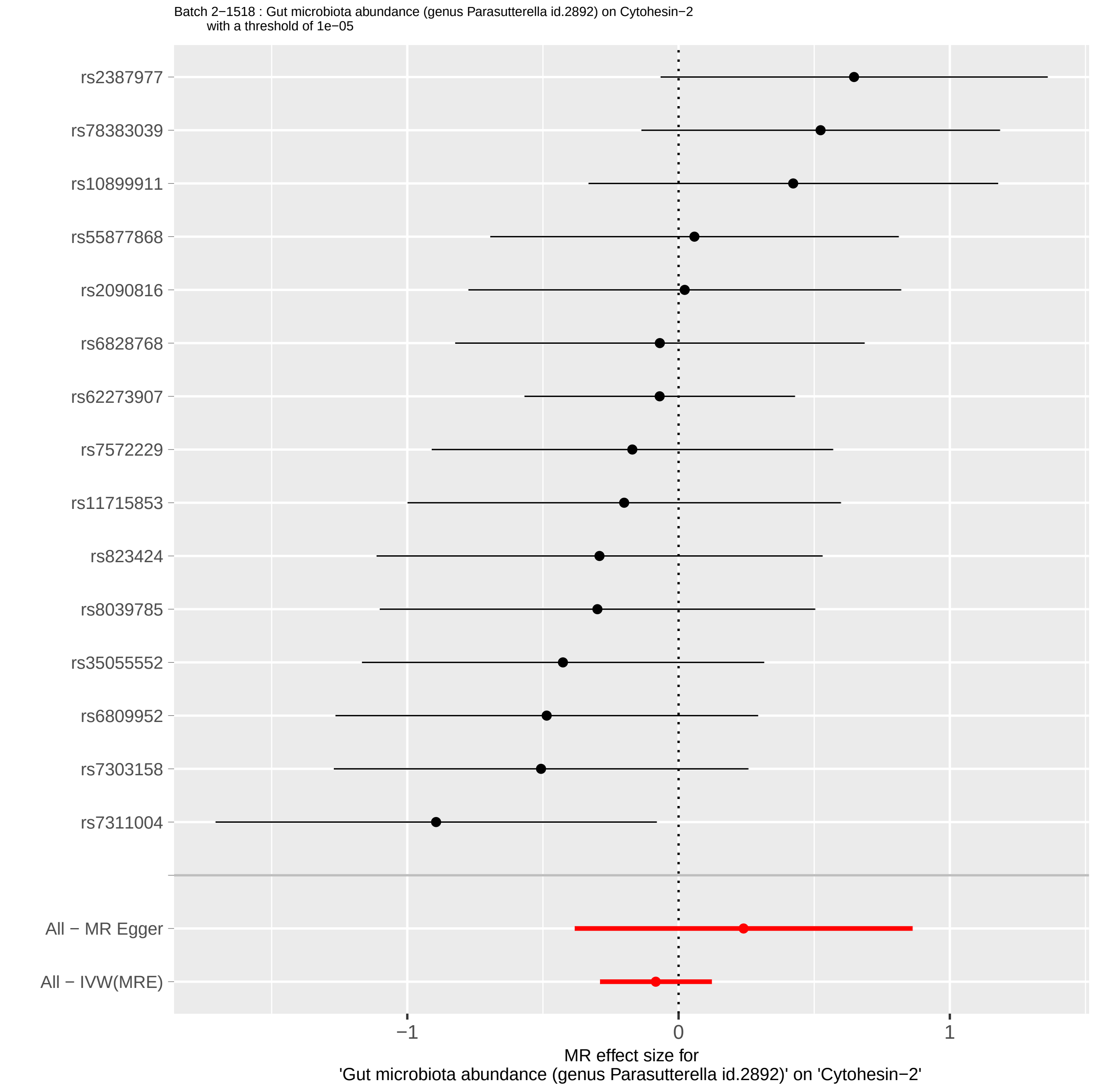

Batch 2−1518 : Gut microbiota abundance (genus Parasutterella id.2892) on Cytohesin−2
with a threshold of 1e−05
rs2387977
rs78383039
rs10899911
rs55877868
rs2090816
rs6828768
rs62273907
rs7572229
rs11715853
rs823424
rs8039785
rs35055552
rs6809952
rs7303158
rs7311004
All − MR Egger
All − IVW(MRE)
−1
0
1
MR effect size for
'Gut microbiota abundance (genus Parasutterella id.2892)' on 'Cytohesin−2'

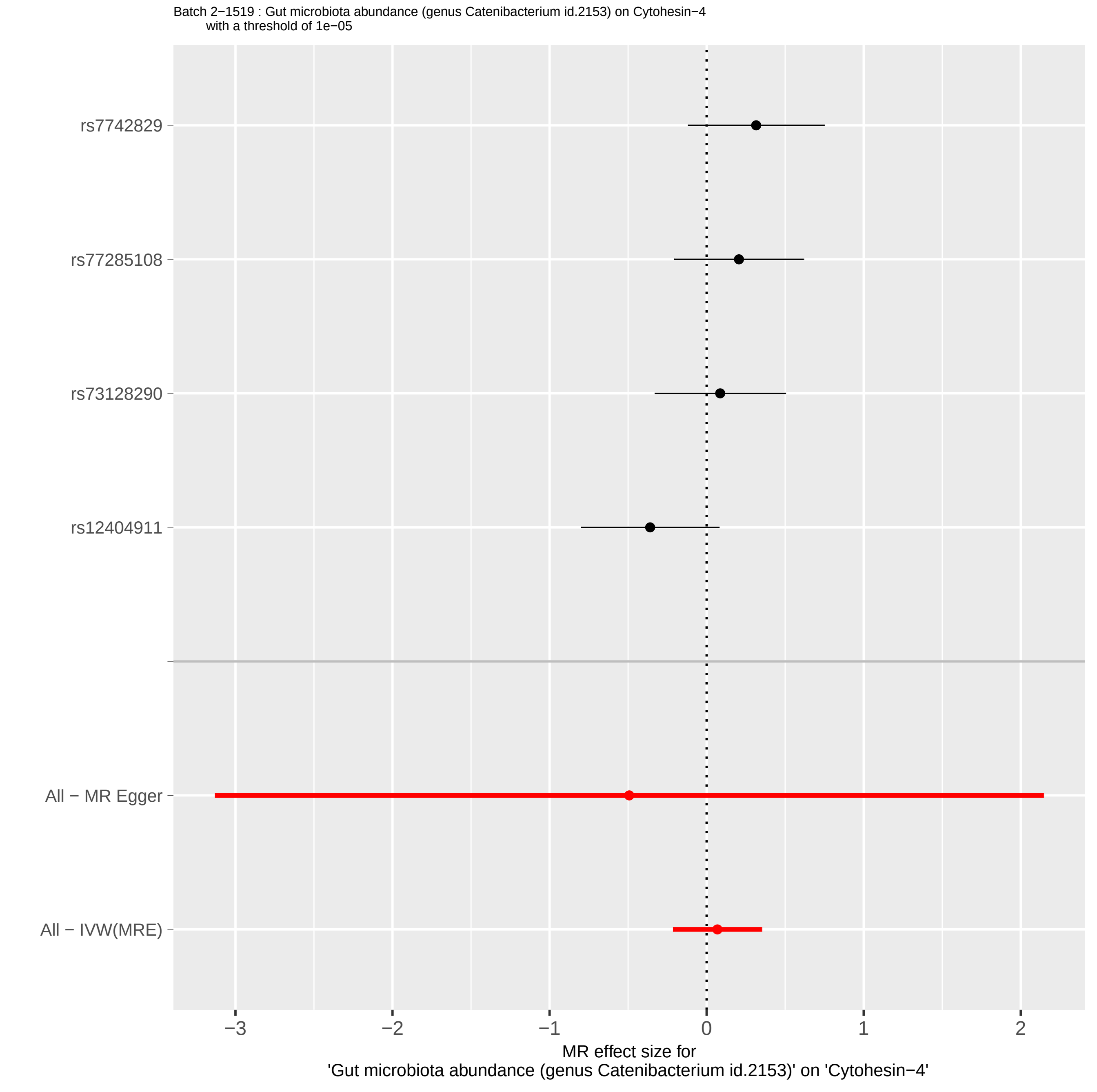

Batch 2−1519 : Gut microbiota abundance (genus Catenibacterium id.2153) on Cytohesin−4
with a threshold of 1e−05
rs7742829
rs77285108
rs73128290
rs12404911
All − MR Egger
All − IVW(MRE)
−3
−2
−1
0
1
2
MR effect size for
'Gut microbiota abundance (genus Catenibacterium id.2153)' on 'Cytohesin−4'

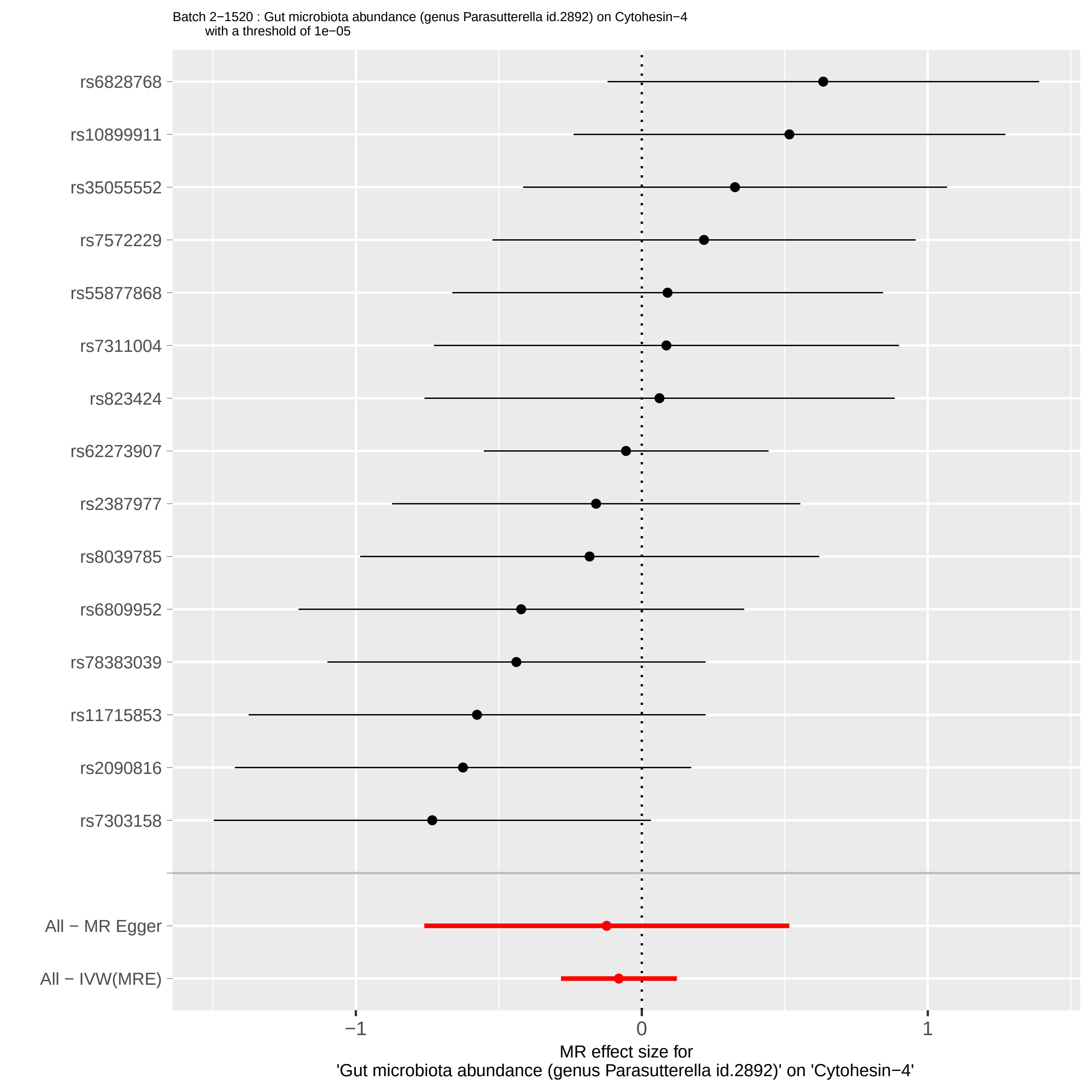
Batch 2−1520 : Gut microbiota abundance (genus Parasutterella id.2892) on Cytohesin−4
with a threshold of 1e−05
rs6828768
rs10899911
rs35055552
rs7572229
rs55877868
rs7311004
rs823424
rs62273907
rs2387977
rs8039785
rs6809952
rs78383039
rs11715853
rs2090816
rs7303158
All − MR Egger
All − IVW(MRE)
−1
0
1
MR effect size for
'Gut microbiota abundance (genus Parasutterella id.2892)' on 'Cytohesin−4'

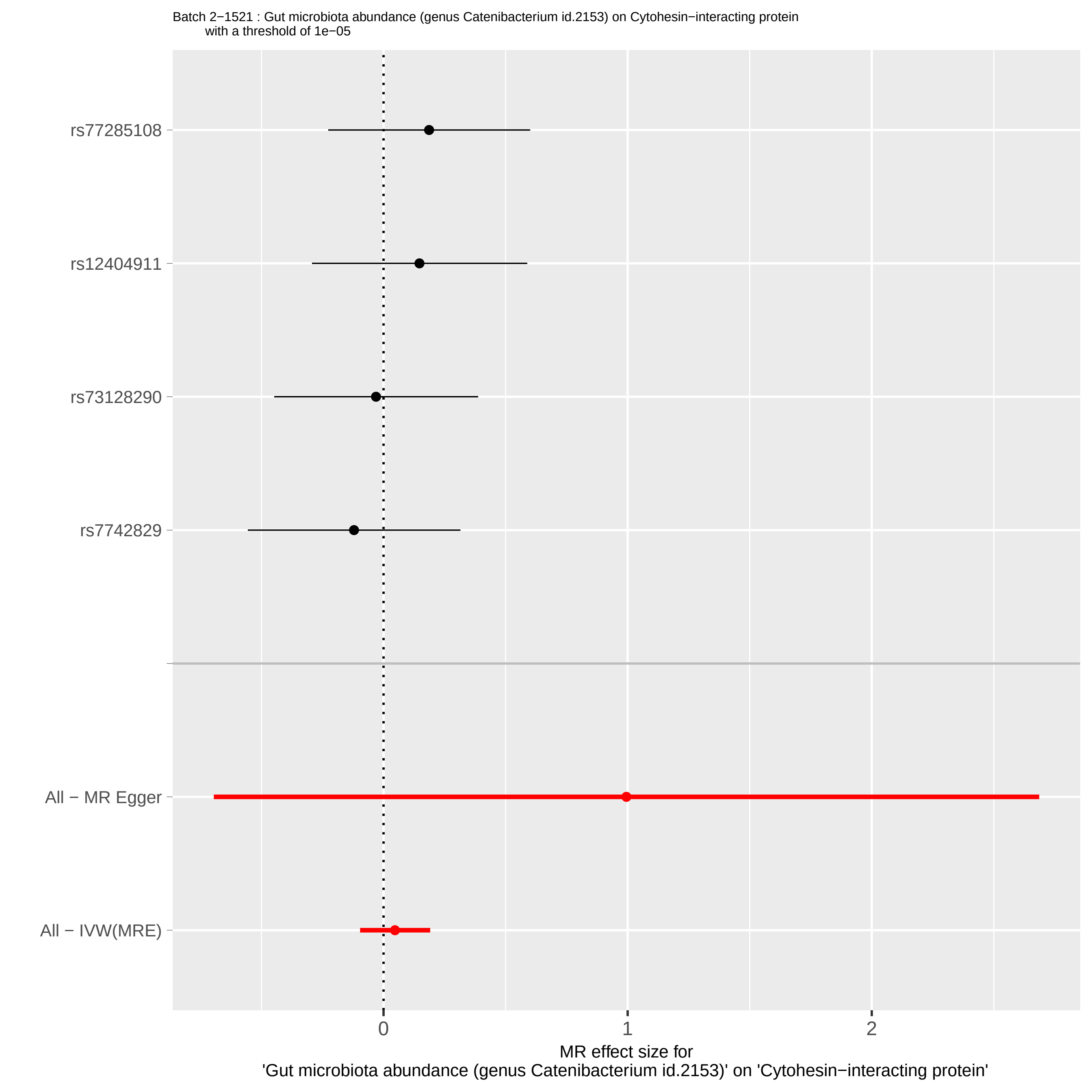
Batch 2−1521 : Gut microbiota abundance (genus Catenibacterium id.2153) on Cytohesin−interacting protein
with a threshold of 1e−05
rs77285108
rs12404911
rs73128290
rs7742829
All − MR Egger
All − IVW(MRE)
0
1
2
MR effect size for
'Gut microbiota abundance (genus Catenibacterium id.2153)' on 'Cytohesin−interacting protein'

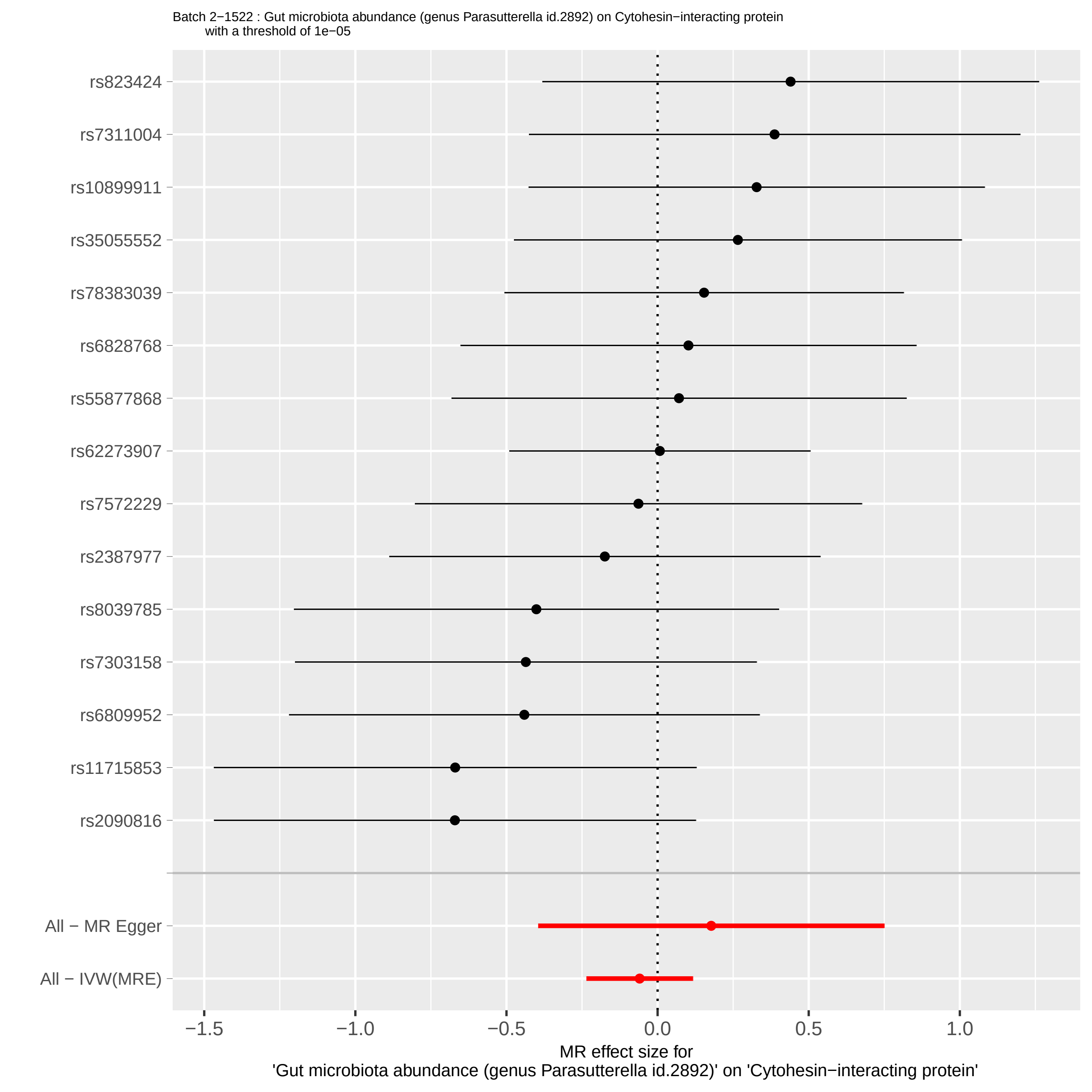

Batch 2−1522 : Gut microbiota abundance (genus Parasutterella id.2892) on Cytohesin−interacting protein
with a threshold of 1e−05
rs823424
rs7311004
rs10899911
rs35055552
rs78383039
rs6828768
rs55877868
rs62273907
rs7572229
rs2387977
rs8039785
rs7303158
rs6809952
rs11715853
rs2090816
All − MR Egger
All − IVW(MRE)
−1.5
−1.0
−0.5
0.0
0.5
1.0
MR effect size for
'Gut microbiota abundance (genus Parasutterella id.2892)' on 'Cytohesin−interacting protein'

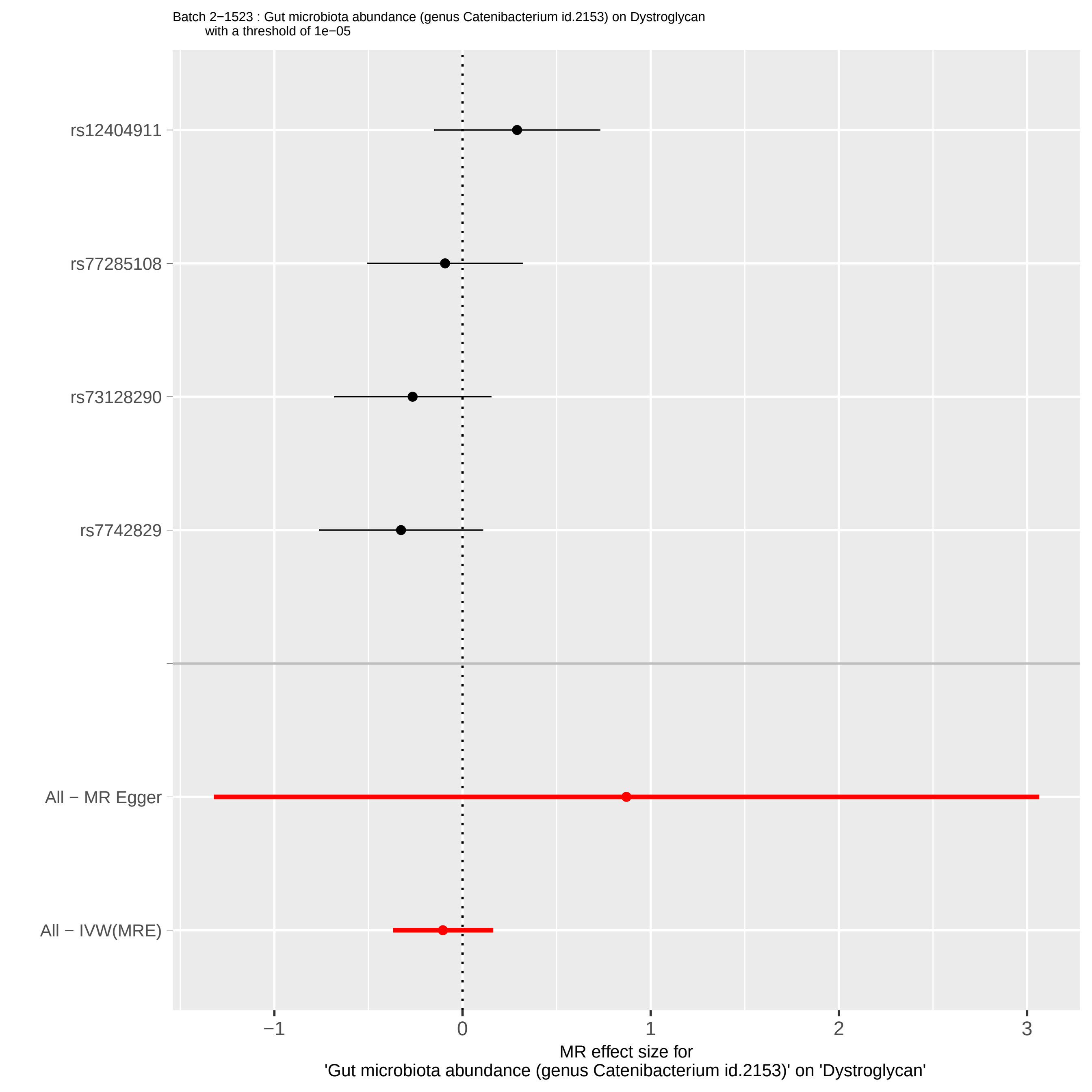
Batch 2−1523 : Gut microbiota abundance (genus Catenibacterium id.2153) on Dystroglycan
with a threshold of 1e−05
rs12404911
rs77285108
rs73128290
rs7742829
All − MR Egger
All − IVW(MRE)
−1
0
1
2
3
MR effect size for
'Gut microbiota abundance (genus Catenibacterium id.2153)' on 'Dystroglycan'

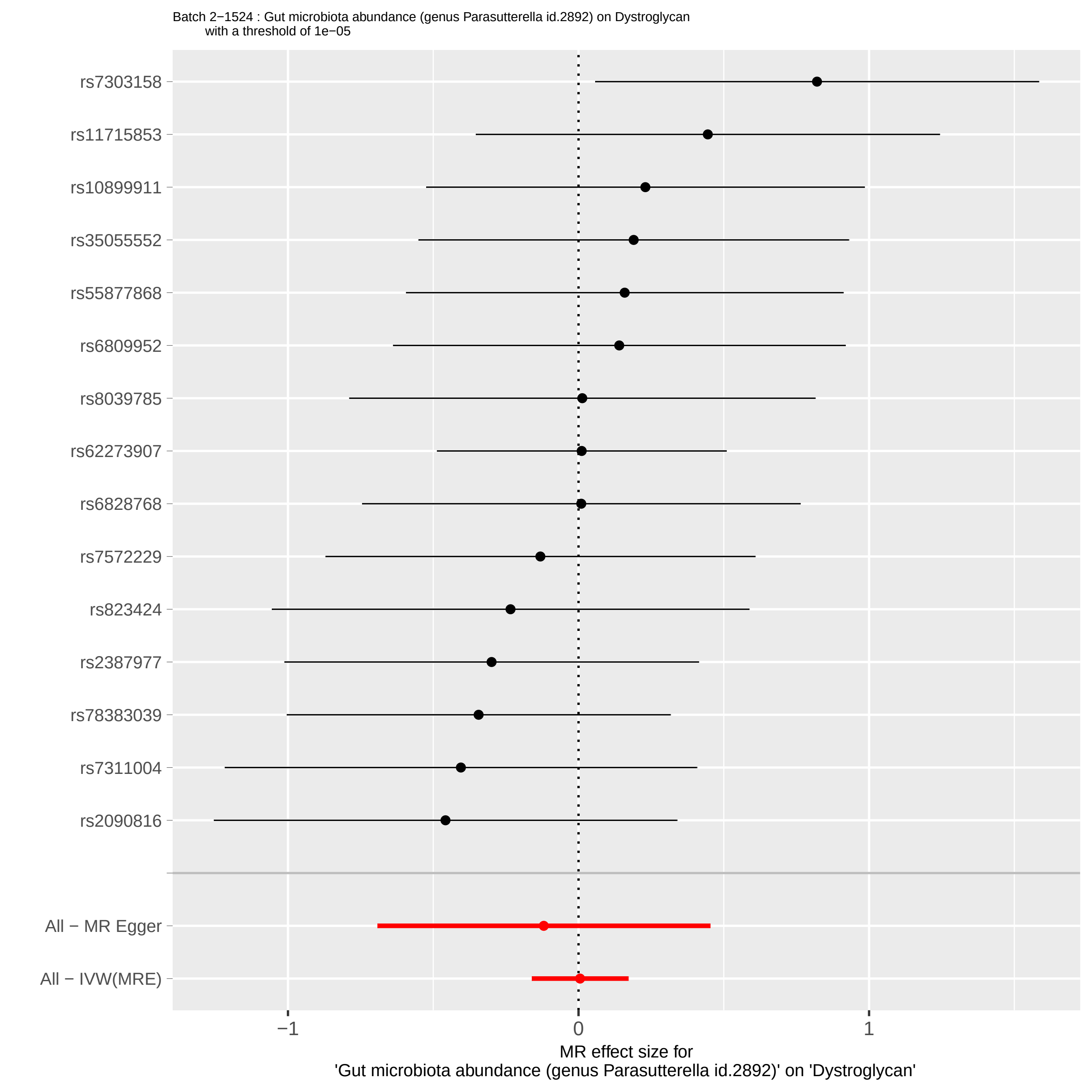
Batch 2−1524 : Gut microbiota abundance (genus Parasutterella id.2892) on Dystroglycan
with a threshold of 1e−05
rs7303158
rs11715853
rs10899911
rs35055552
rs55877868
rs6809952
rs8039785
rs62273907
rs6828768
rs7572229
rs823424
rs2387977
rs78383039
rs7311004
rs2090816
All − MR Egger
All − IVW(MRE)
−1
0
1
MR effect size for
'Gut microbiota abundance (genus Parasutterella id.2892)' on 'Dystroglycan'

Batch 2−1525 : Gut microbiota abundance (genus Catenibacterium id.2153) on Death−associated protein kinase 2
with a threshold of 1e−05

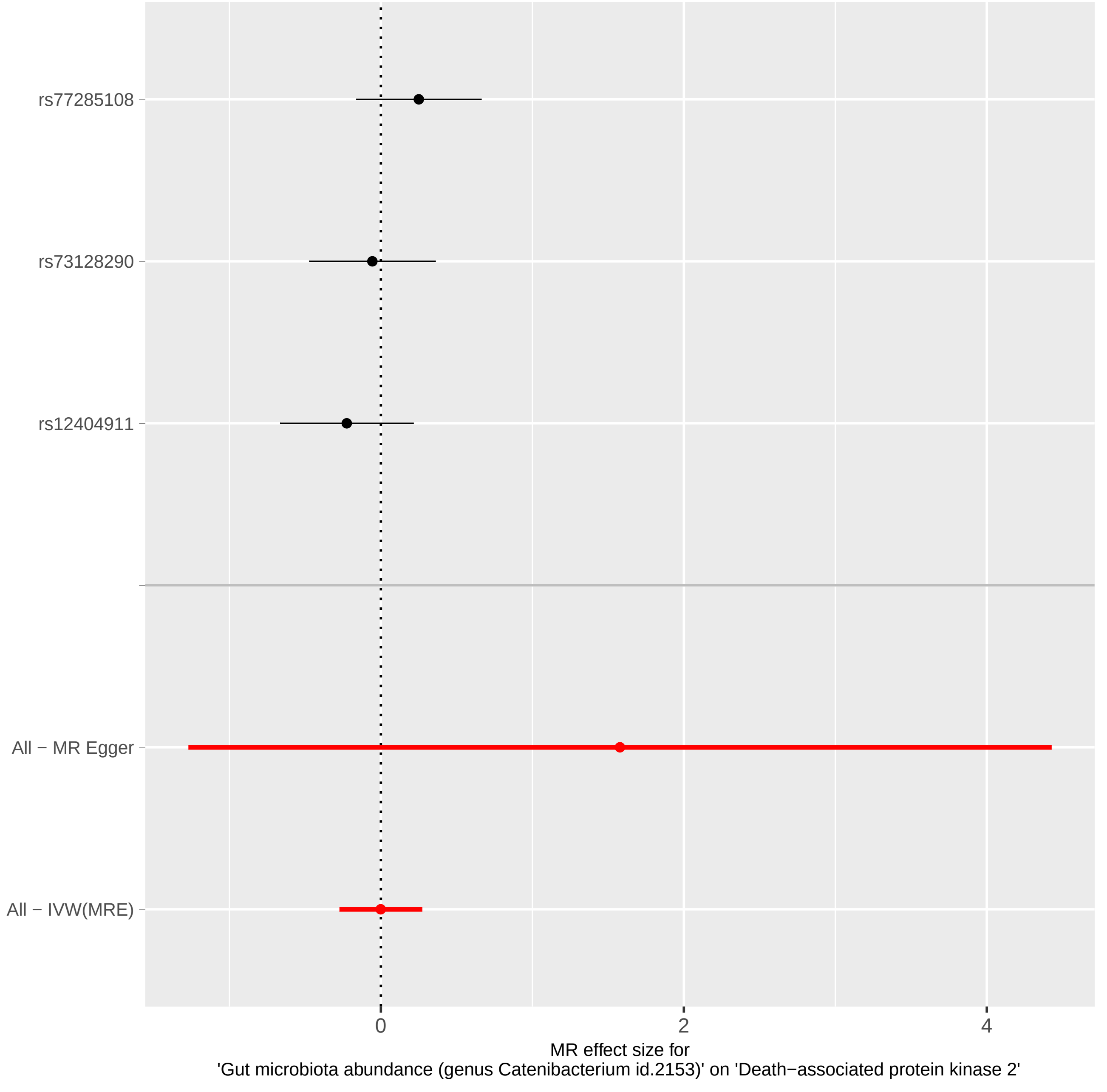

Batch 2−1526 : Gut microbiota abundance (genus Parasutterella id.2892) on Death−associated protein kinase 2
with a threshold of 1e−05

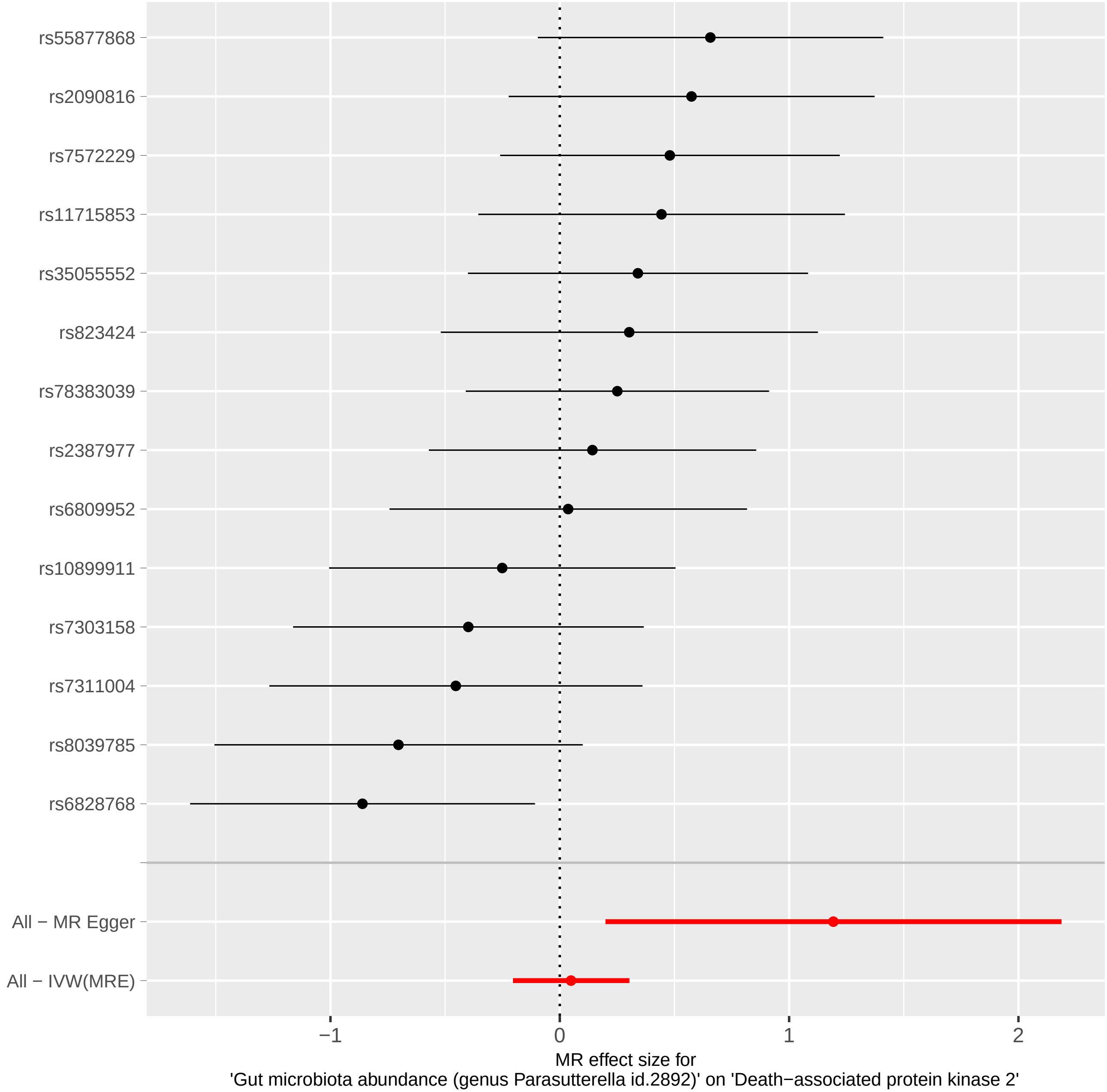

Batch 2−1527 : Gut microbiota abundance (genus Catenibacterium id.2153) on Drebrin−like protein
with a threshold of 1e−05

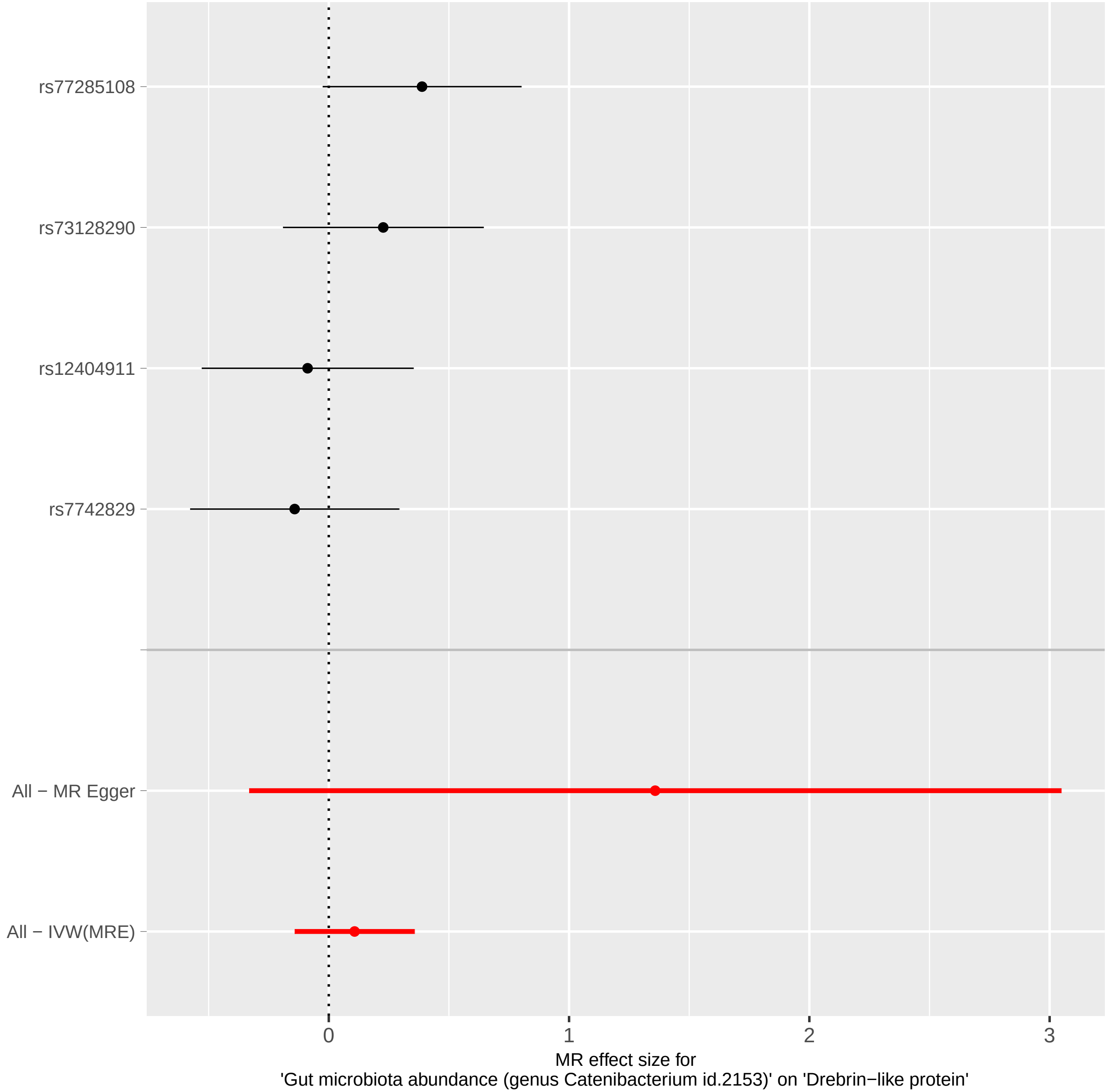

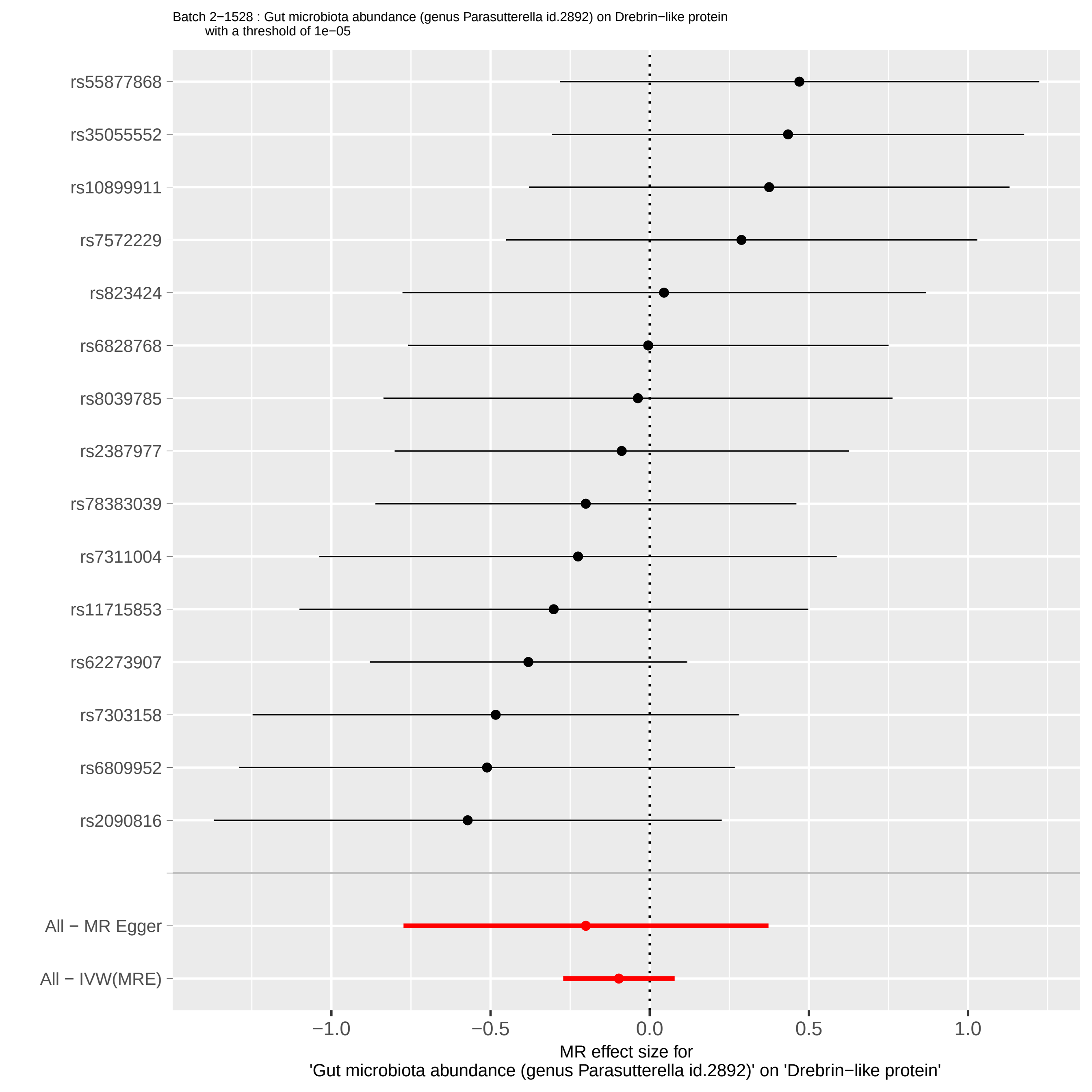

Batch 2−1528 : Gut microbiota abundance (genus Parasutterella id.2892) on Drebrin−like protein
with a threshold of 1e−05
rs55877868
rs35055552
rs10899911
rs7572229
rs823424
rs6828768
rs8039785
rs2387977
rs78383039
rs7311004
rs11715853
rs62273907
rs7303158
rs6809952
rs2090816
All − MR Egger
All − IVW(MRE)
−1.0
−0.5
0.0
0.5
1.0
MR effect size for
'Gut microbiota abundance (genus Parasutterella id.2892)' on 'Drebrin−like protein'

Batch 2−1529 : Gut microbiota abundance (genus Catenibacterium id.2153) on DDB1− and CUL4−associated factor 5
with a threshold of 1e−05

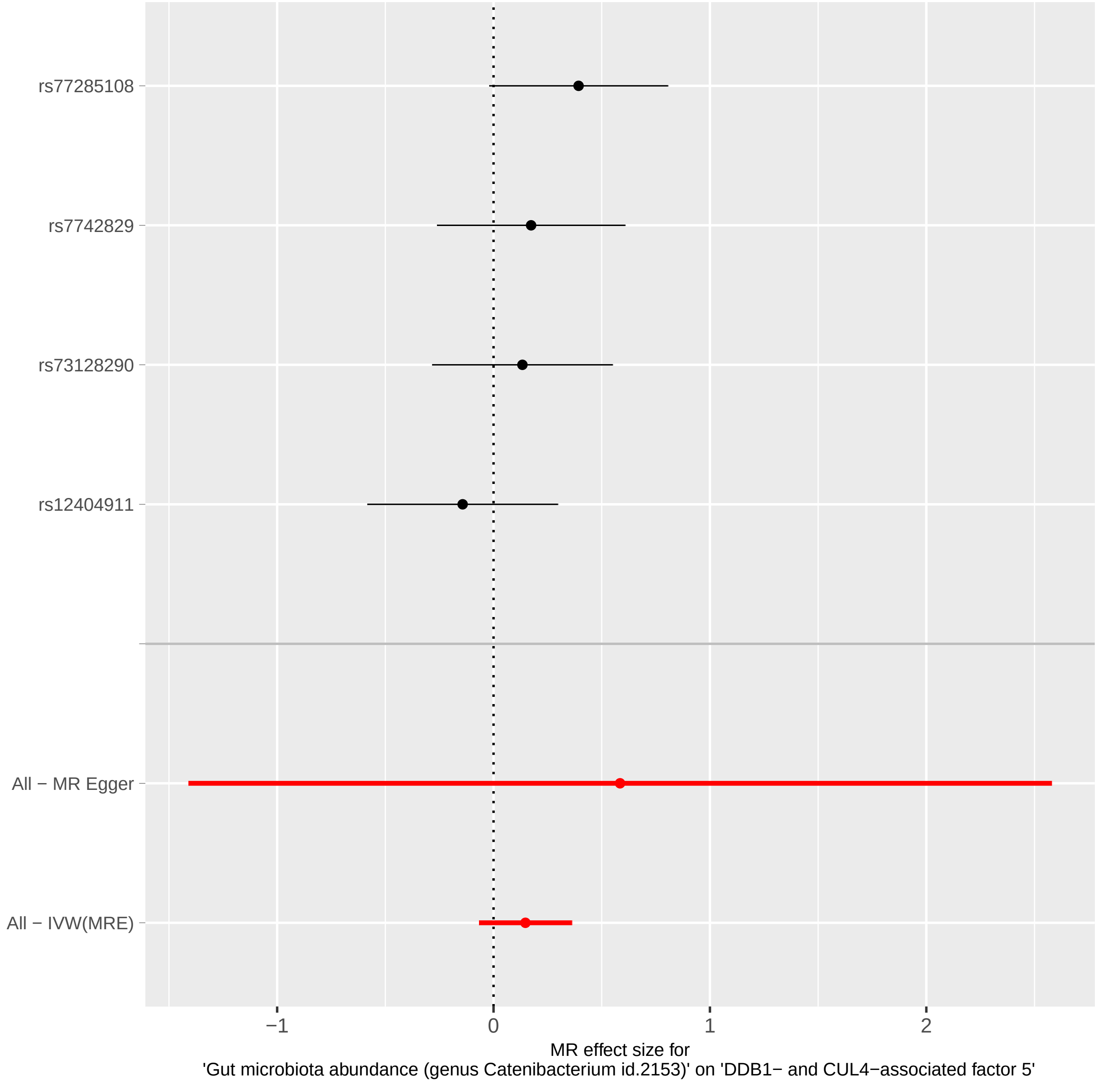

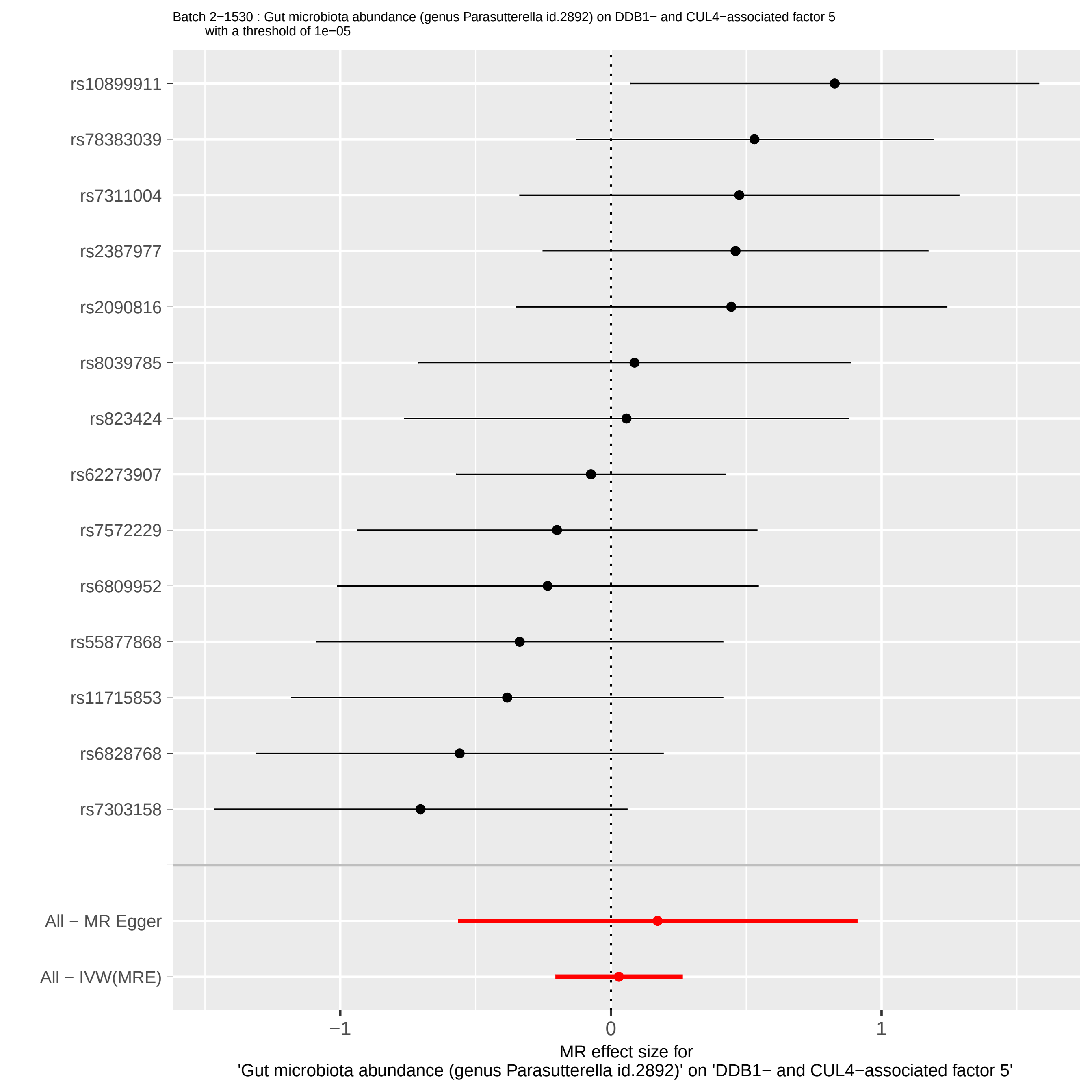
Batch 2−1530 : Gut microbiota abundance (genus Parasutterella id.2892) on DDB1− and CUL4−associated factor 5
with a threshold of 1e−05
rs10899911
rs78383039
rs7311004
rs2387977
rs2090816
rs8039785
rs823424
rs62273907
rs7572229
rs6809952
rs55877868
rs11715853
rs6828768
rs7303158
All − MR Egger
All − IVW(MRE)
−1
0
1
MR effect size for
'Gut microbiota abundance (genus Parasutterella id.2892)' on 'DDB1− and CUL4−associated factor 5'

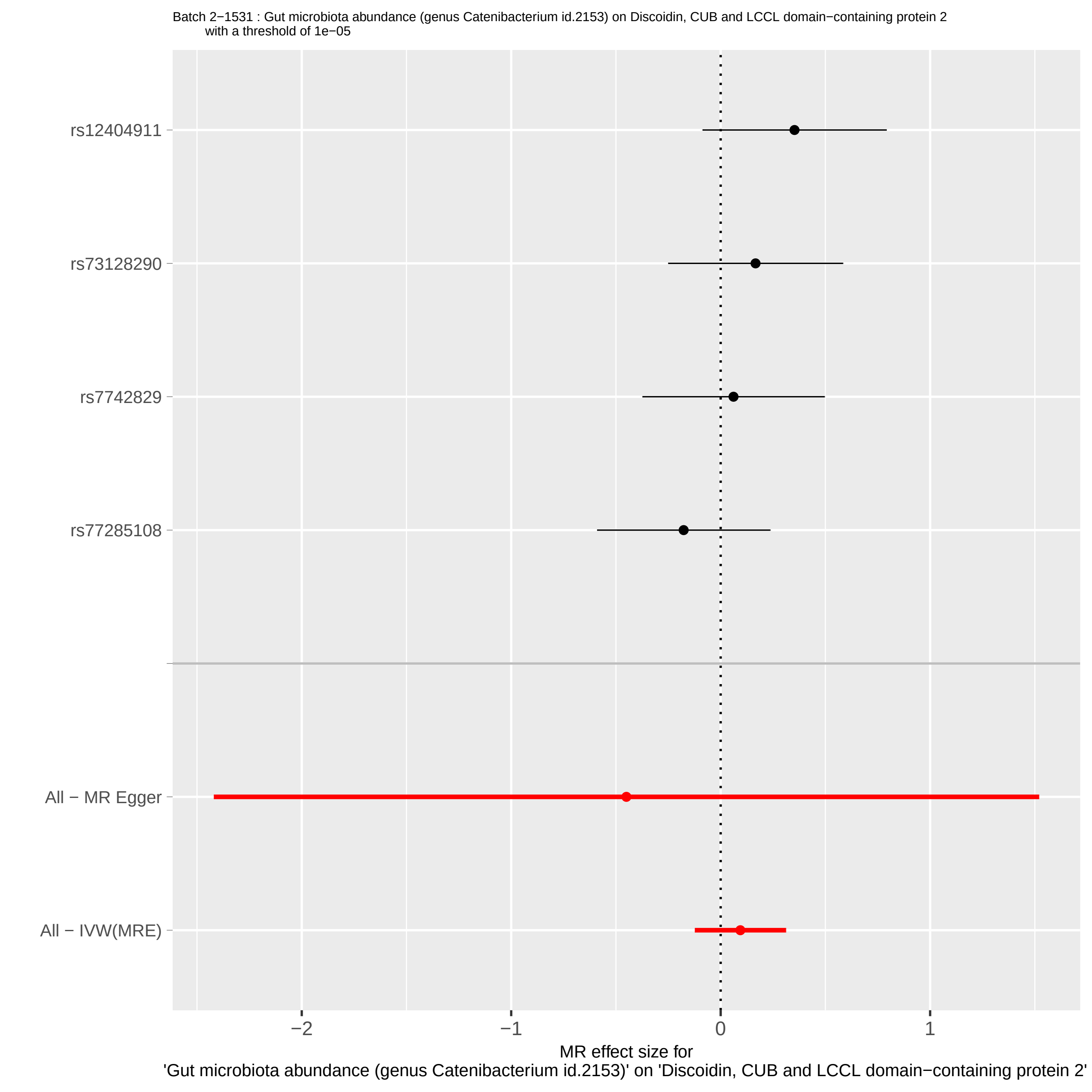

Batch 2−1531 : Gut microbiota abundance (genus Catenibacterium id.2153) on Discoidin, CUB and LCCL domain−containing protein 2
with a threshold of 1e−05
rs12404911
rs73128290
rs7742829
rs77285108
All − MR Egger
All − IVW(MRE)
−2
−1
0
1
MR effect size for
'Gut microbiota abundance (genus Catenibacterium id.2153)' on 'Discoidin, CUB and LCCL domain−containing protein 2'

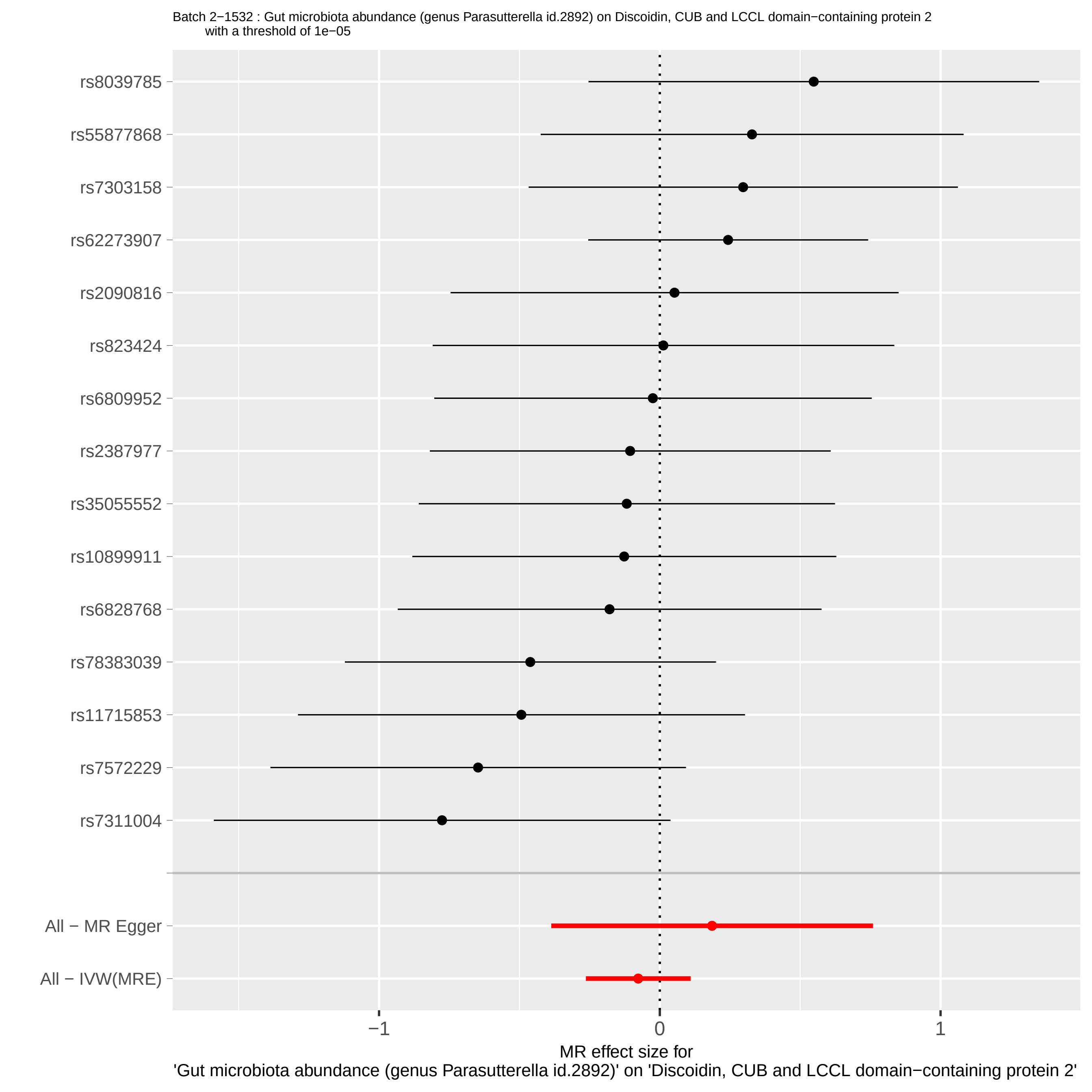

Batch 2−1532 : Gut microbiota abundance (genus Parasutterella id.2892) on Discoidin, CUB and LCCL domain−containing protein 2
with a threshold of 1e−05
rs8039785
rs55877868
rs7303158
rs62273907
rs2090816
rs823424
rs6809952
rs2387977
rs35055552
rs10899911
rs6828768
rs78383039
rs11715853
rs7572229
rs7311004
All − MR Egger
All − IVW(MRE)
−1
0
1
MR effect size for
'Gut microbiota abundance (genus Parasutterella id.2892)' on 'Discoidin, CUB and LCCL domain−containing protein 2'

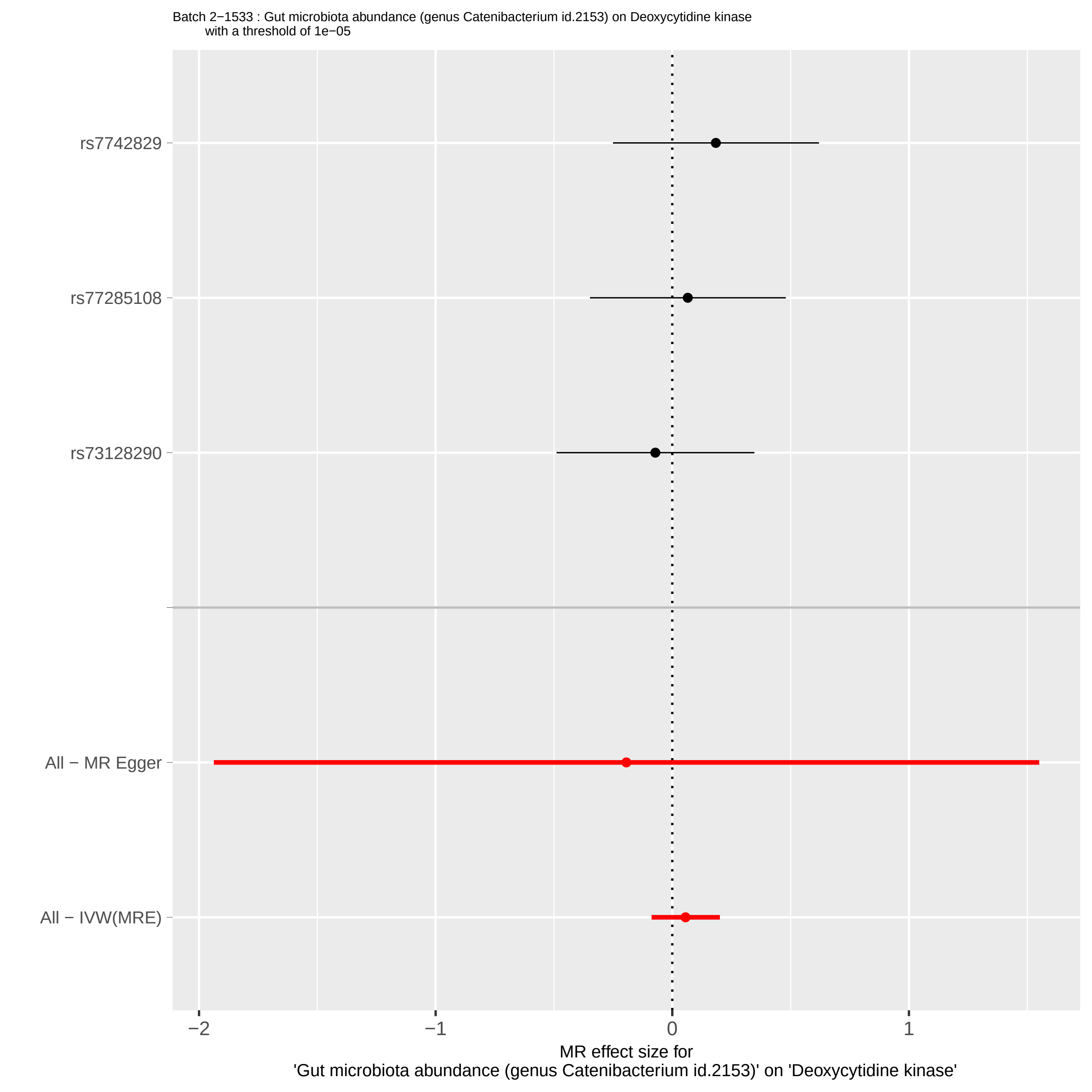
Batch 2−1533 : Gut microbiota abundance (genus Catenibacterium id.2153) on Deoxycytidine kinase
with a threshold of 1e−05
rs7742829
rs77285108
rs73128290
All − MR Egger
All − IVW(MRE)
−2
−1
0
1
MR effect size for
'Gut microbiota abundance (genus Catenibacterium id.2153)' on 'Deoxycytidine kinase'

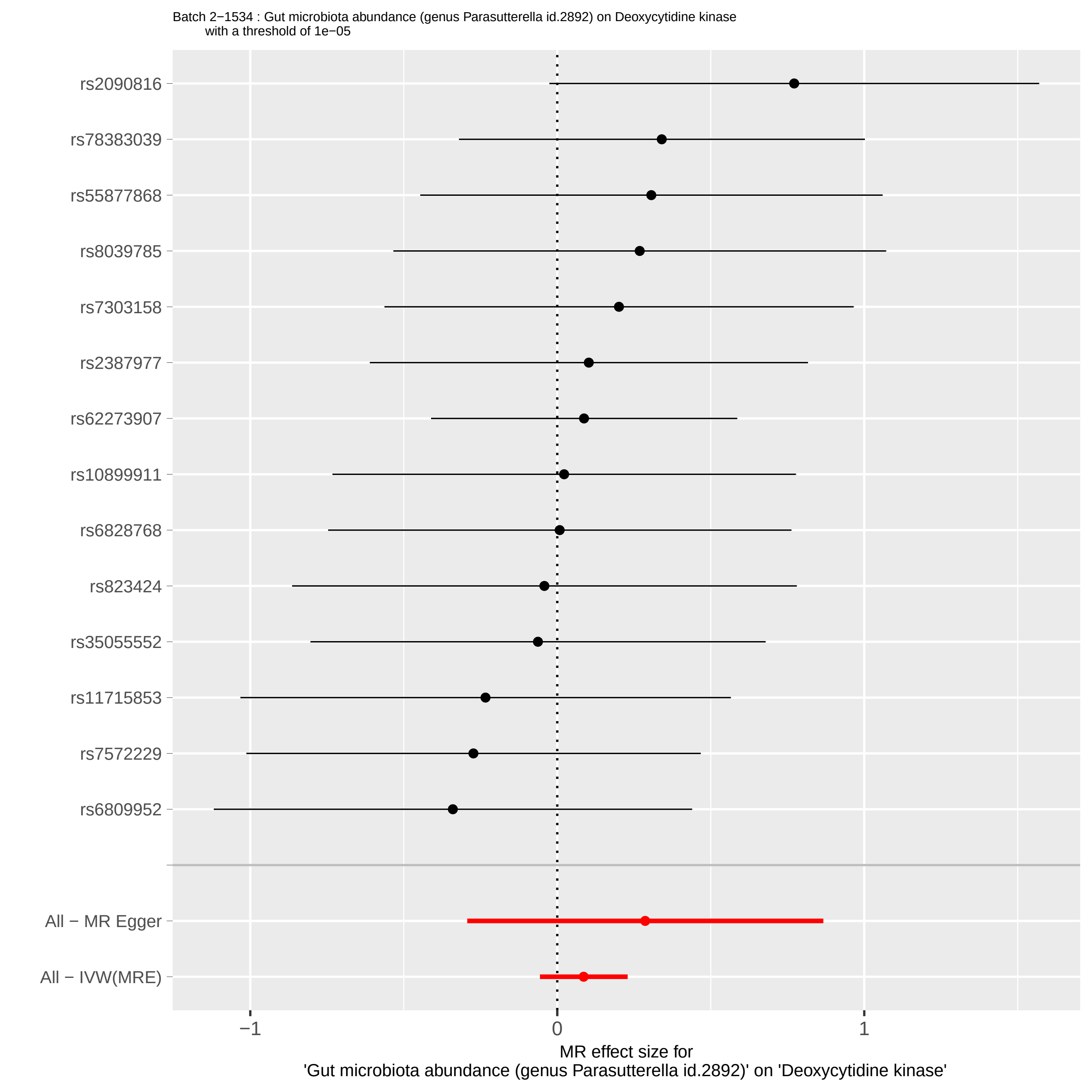
Batch 2−1534 : Gut microbiota abundance (genus Parasutterella id.2892) on Deoxycytidine kinase
with a threshold of 1e−05
rs2090816
rs78383039
rs55877868
rs8039785
rs7303158
rs2387977
rs62273907
rs10899911
rs6828768
rs823424
rs35055552
rs11715853
rs7572229
rs6809952
All − MR Egger
All − IVW(MRE)
−1
0
1
MR effect size for
'Gut microbiota abundance (genus Parasutterella id.2892)' on 'Deoxycytidine kinase'

Batch 2−1535 : Gut microbiota abundance (genus Catenibacterium id.2153) on Serine/threonine−protein kinase DCLK3
with a threshold of 1e−05

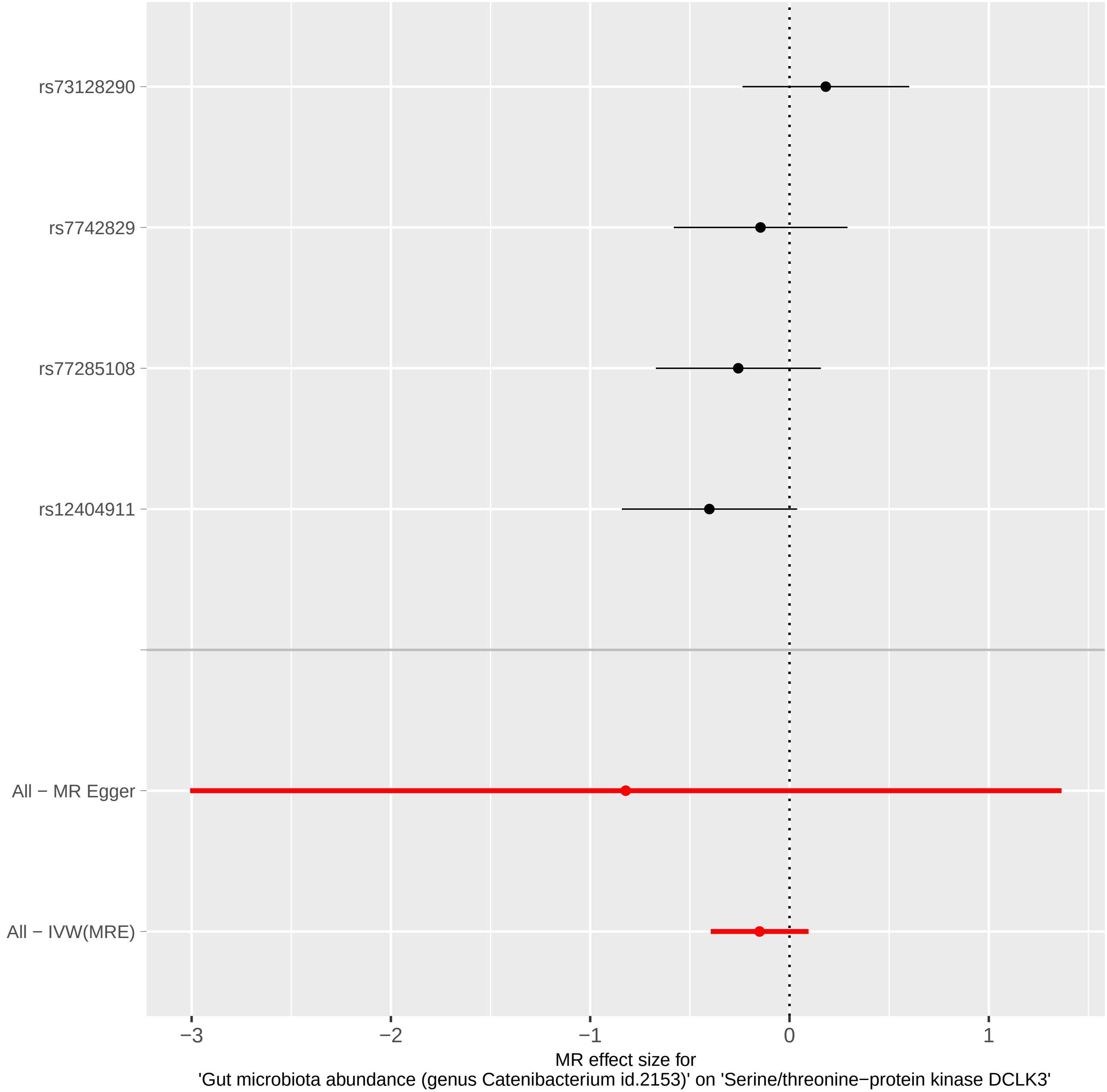

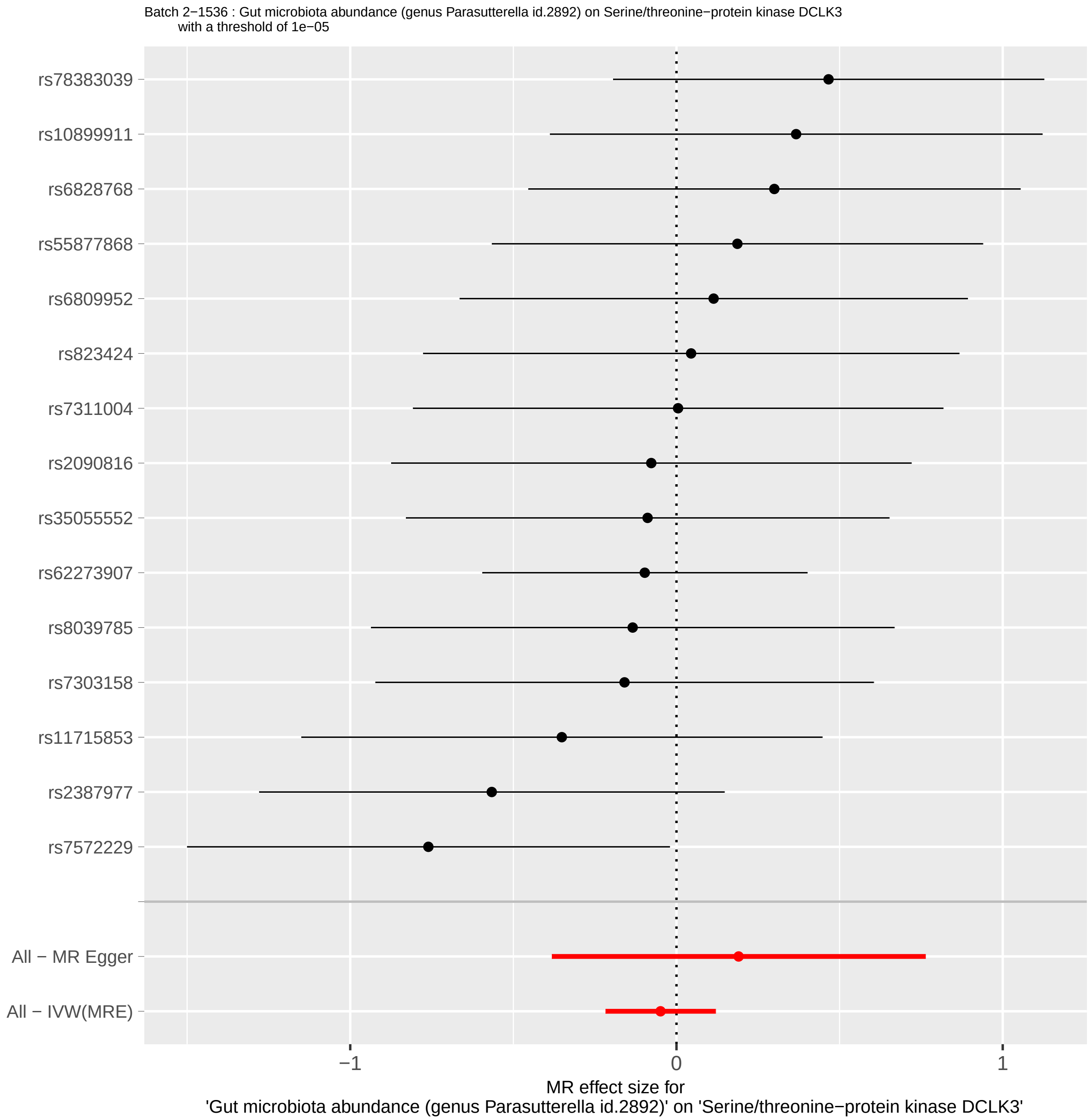

Batch 2−1536 : Gut microbiota abundance (genus Parasutterella id.2892) on Serine/threonine−protein kinase DCLK3
with a threshold of 1e−05
rs78383039
rs10899911
rs6828768
rs55877868
rs6809952
rs823424
rs7311004
rs2090816
rs35055552
rs62273907
rs8039785
rs7303158
rs11715853
rs2387977
rs7572229
All − MR Egger
All − IVW(MRE)
−1
0
1
MR effect size for
'Gut microbiota abundance (genus Parasutterella id.2892)' on 'Serine/threonine−protein kinase DCLK3'

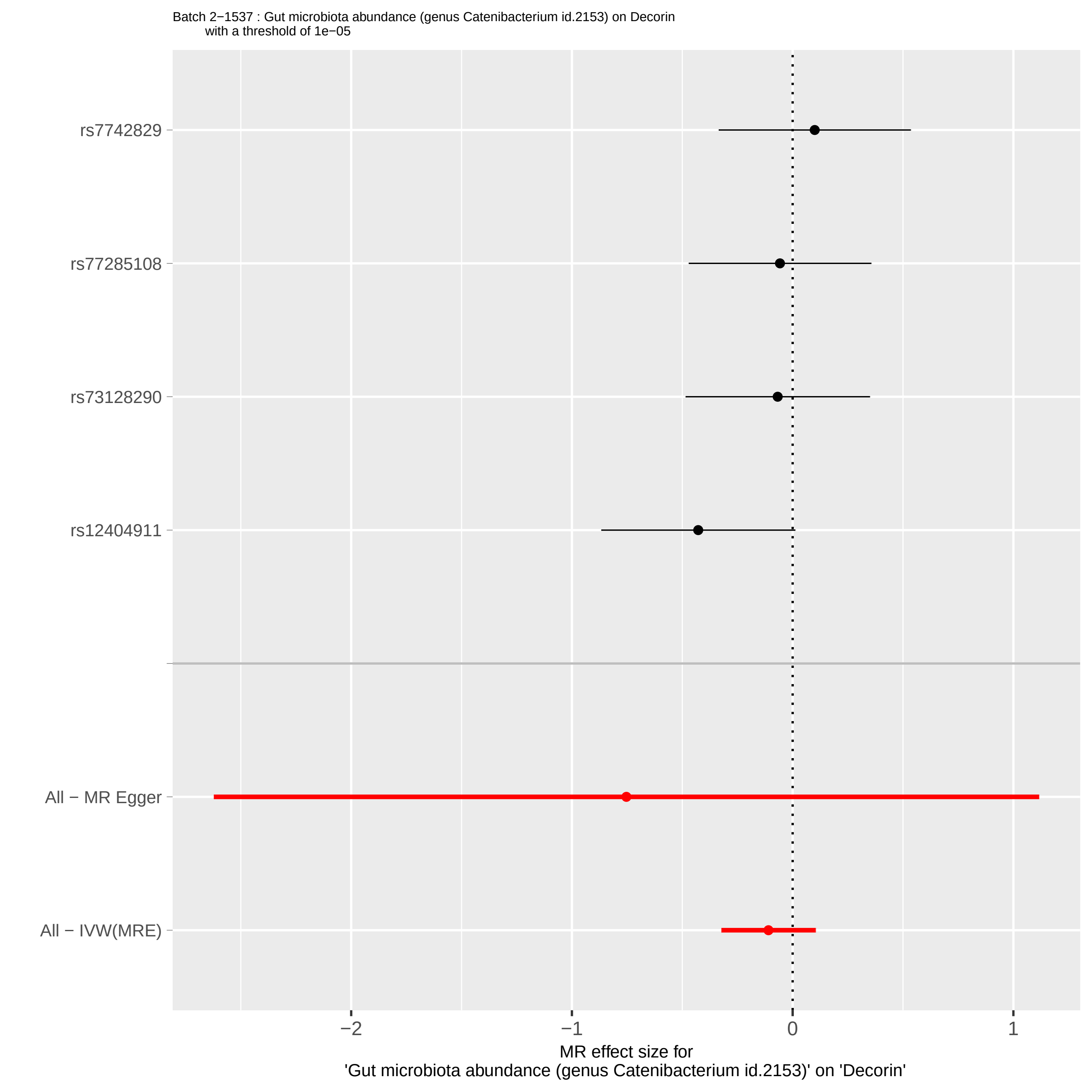
Batch 2−1537 : Gut microbiota abundance (genus Catenibacterium id.2153) on Decorin
with a threshold of 1e−05
rs7742829
rs77285108
rs73128290
rs12404911
All − MR Egger
All − IVW(MRE)
−2
−1
0
1
MR effect size for
'Gut microbiota abundance (genus Catenibacterium id.2153)' on 'Decorin'

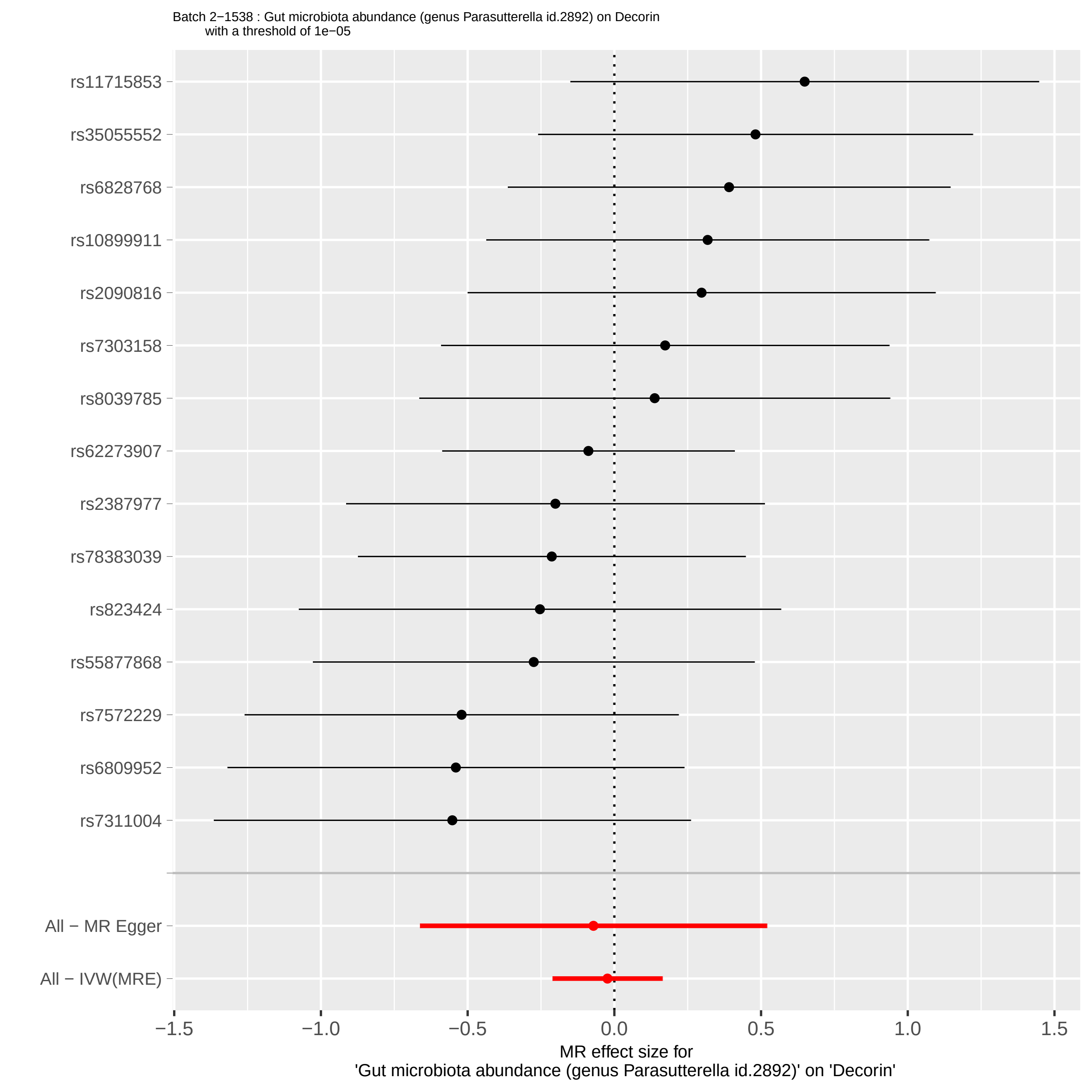

Batch 2−1538 : Gut microbiota abundance (genus Parasutterella id.2892) on Decorin
with a threshold of 1e−05
rs11715853
rs35055552
rs6828768
rs10899911
rs2090816
rs7303158
rs8039785
rs62273907
rs2387977
rs78383039
rs823424
rs55877868
rs7572229
rs6809952
rs7311004
All − MR Egger
All − IVW(MRE)
−1.5
−1.0
−0.5
0.0
0.5
1.0
1.5
MR effect size for
'Gut microbiota abundance (genus Parasutterella id.2892)' on 'Decorin'

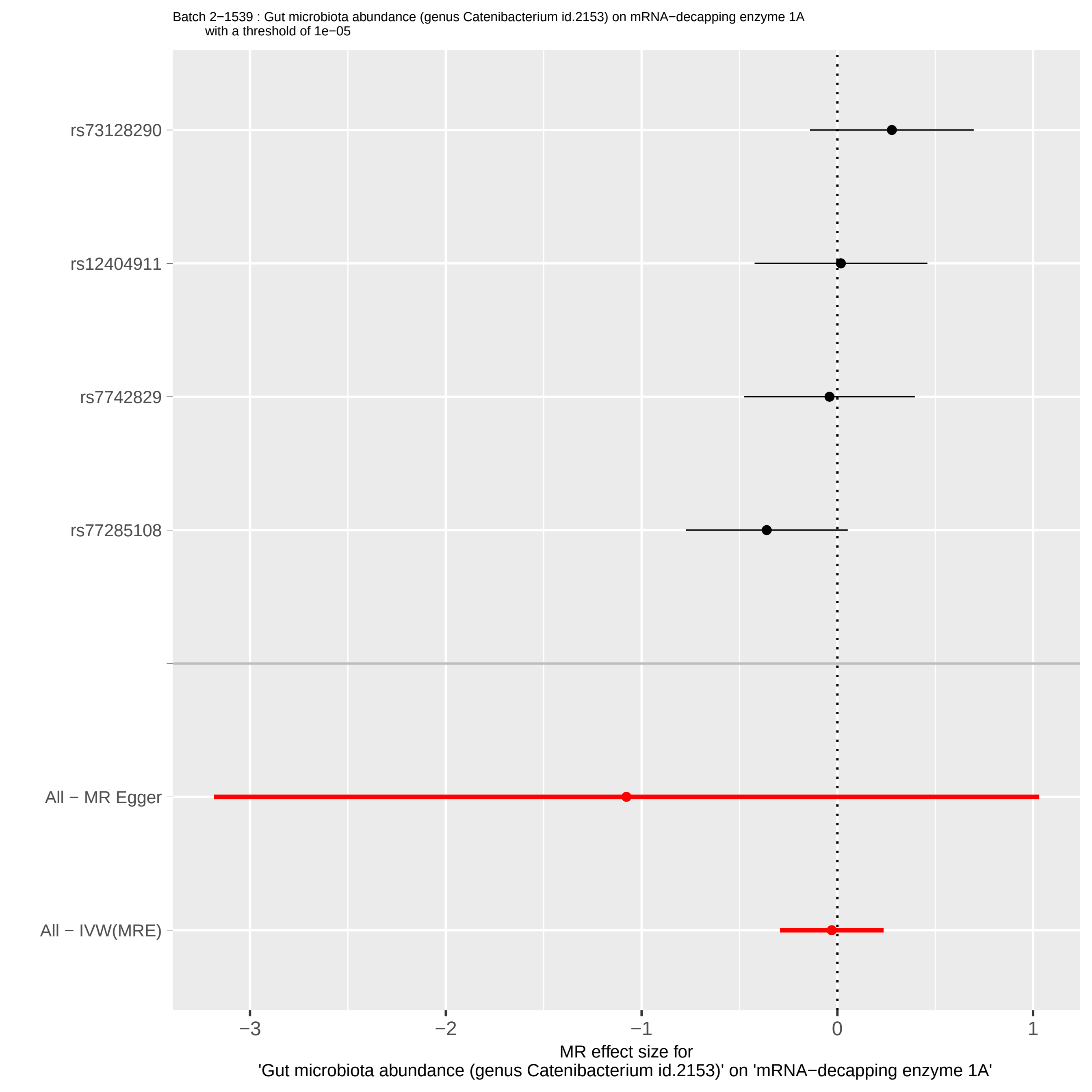

Batch 2−1539 : Gut microbiota abundance (genus Catenibacterium id.2153) on mRNA−decapping enzyme 1A
with a threshold of 1e−05
rs73128290
rs12404911
rs7742829
rs77285108
All − MR Egger
All − IVW(MRE)
−3
−2
−1
0
1
MR effect size for
'Gut microbiota abundance (genus Catenibacterium id.2153)' on 'mRNA−decapping enzyme 1A'

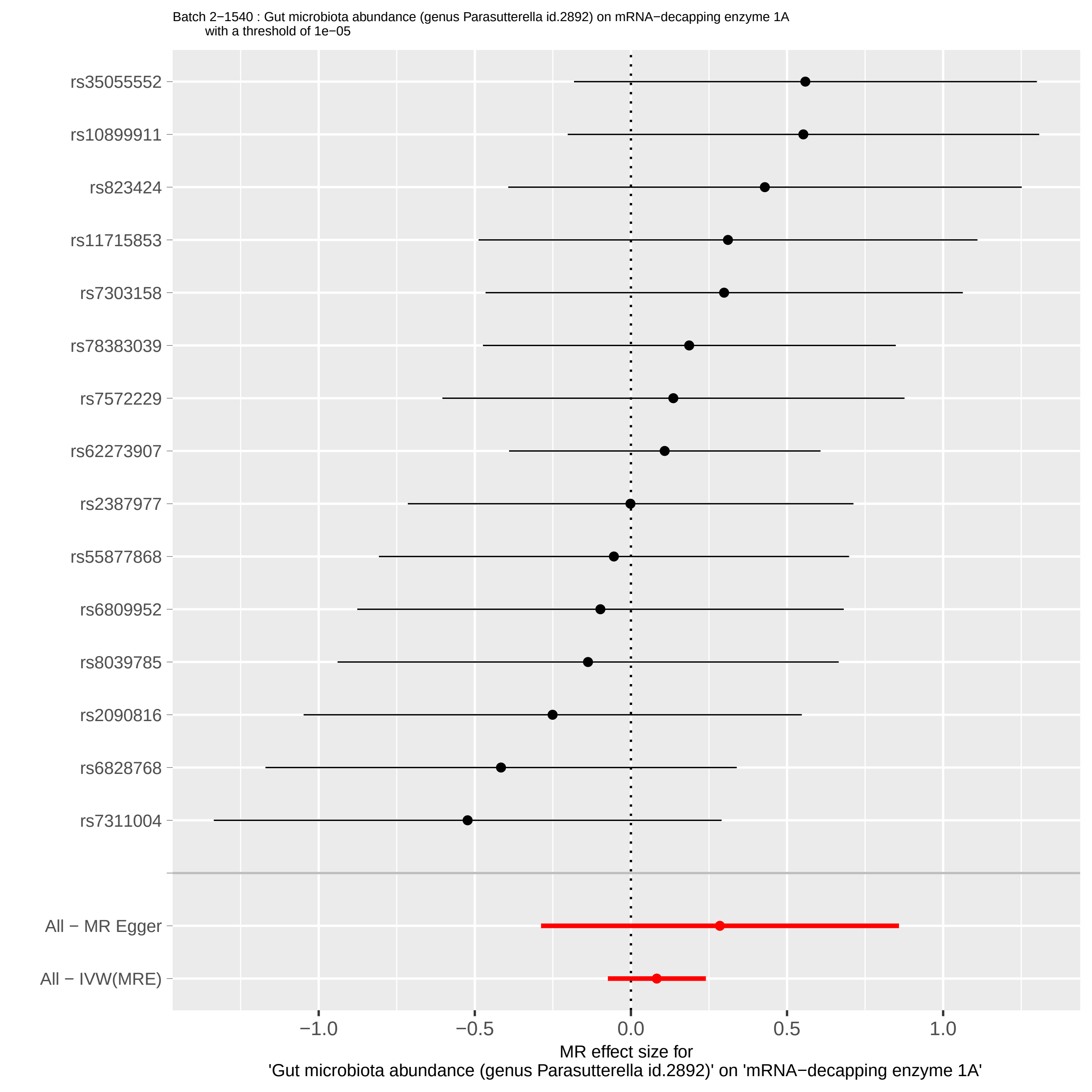
Batch 2−1540 : Gut microbiota abundance (genus Parasutterella id.2892) on mRNA−decapping enzyme 1A
with a threshold of 1e−05
rs35055552
rs10899911
rs823424
rs11715853
rs7303158
rs78383039
rs7572229
rs62273907
rs2387977
rs55877868
rs6809952
rs8039785
rs2090816
rs6828768
rs7311004
All − MR Egger
All − IVW(MRE)
−1.0
−0.5
0.0
0.5
1.0
MR effect size for
'Gut microbiota abundance (genus Parasutterella id.2892)' on 'mRNA−decapping enzyme 1A'

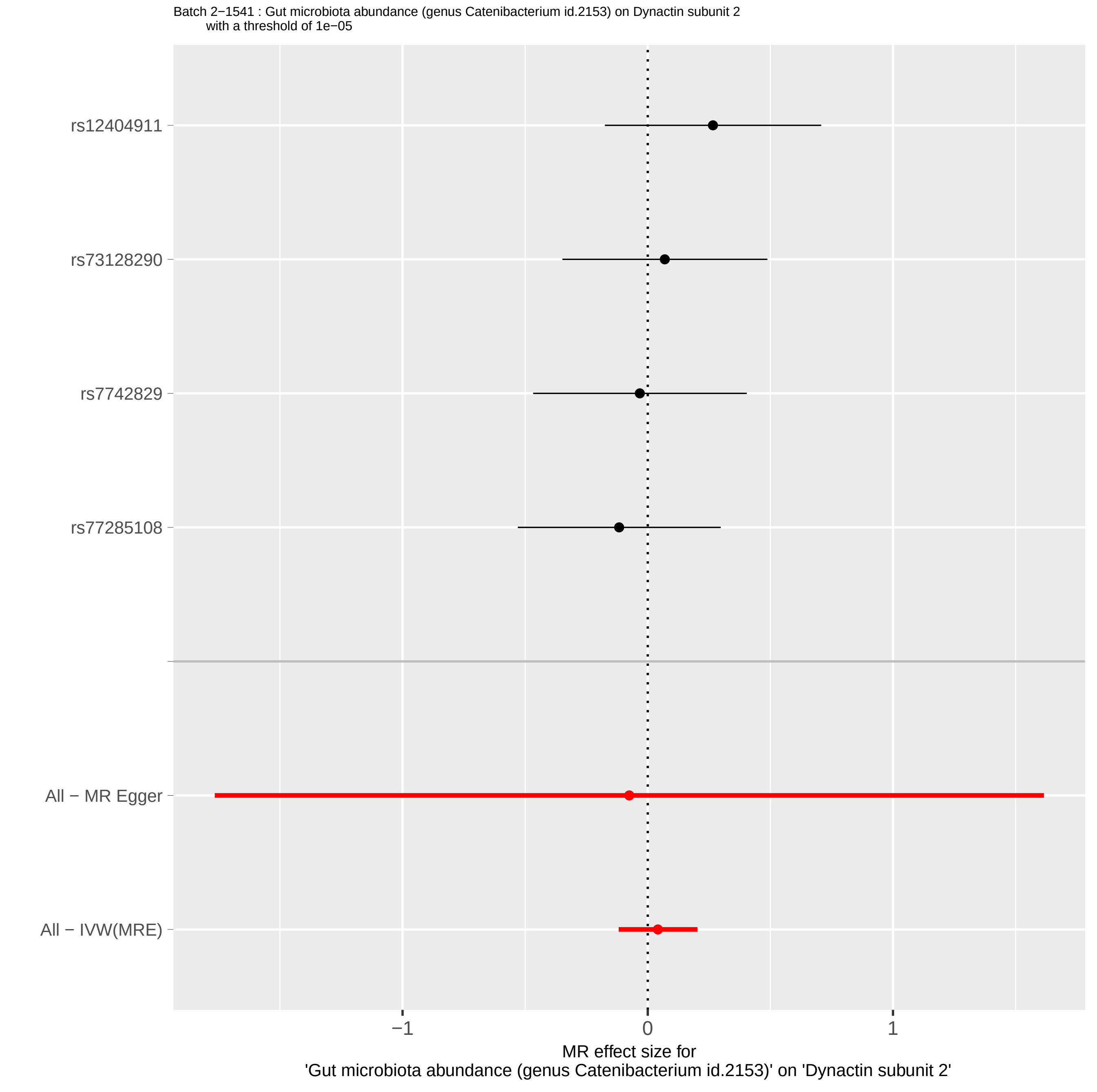

Batch 2−1541 : Gut microbiota abundance (genus Catenibacterium id.2153) on Dynactin subunit 2
with a threshold of 1e−05
rs12404911
rs73128290
rs7742829
rs77285108
All − MR Egger
All − IVW(MRE)
−1
0
1
MR effect size for
'Gut microbiota abundance (genus Catenibacterium id.2153)' on 'Dynactin subunit 2'

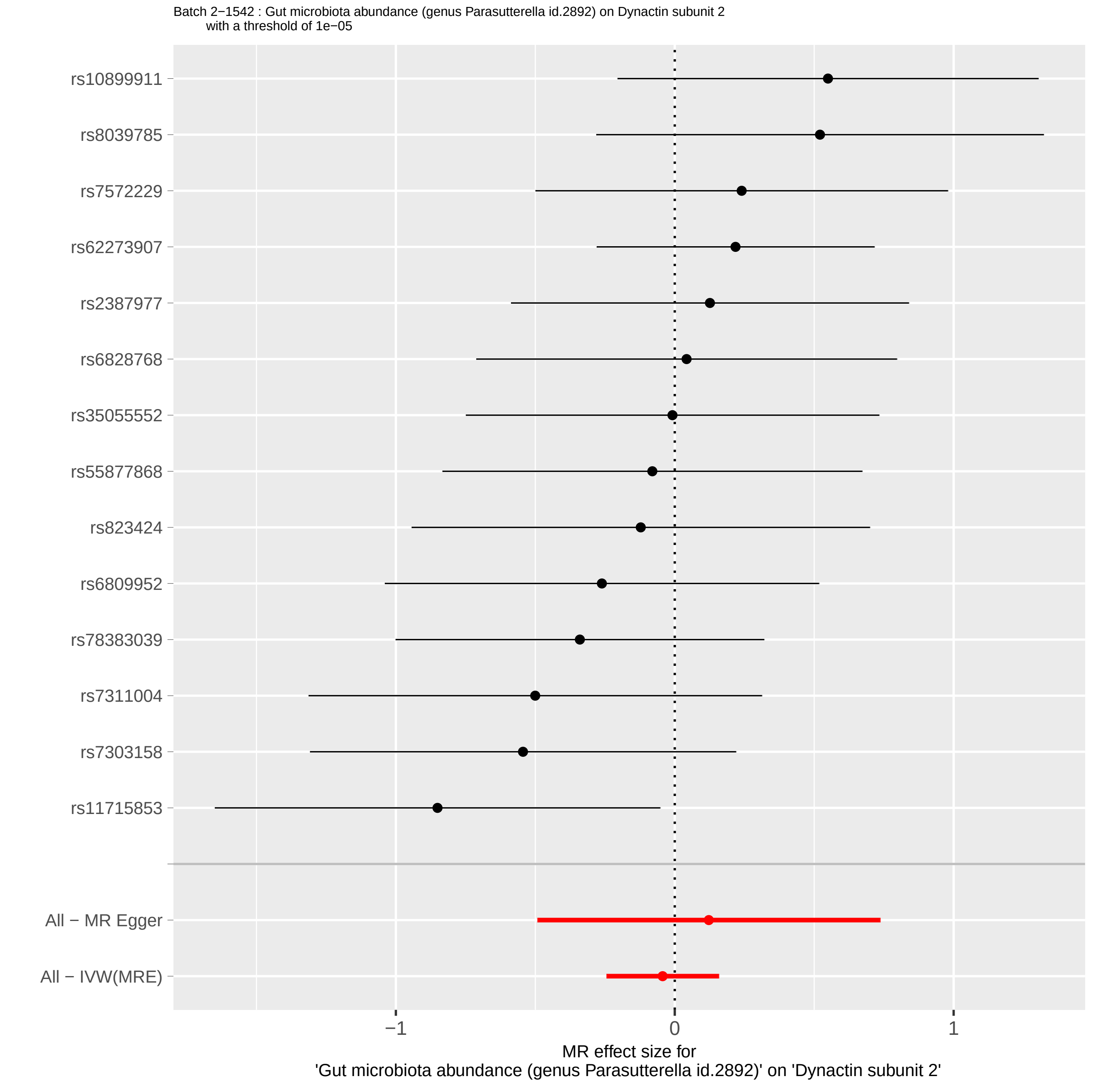

Batch 2−1542 : Gut microbiota abundance (genus Parasutterella id.2892) on Dynactin subunit 2
with a threshold of 1e−05
rs10899911
rs8039785
rs7572229
rs62273907
rs2387977
rs6828768
rs35055552
rs55877868
rs823424
rs6809952
rs78383039
rs7311004
rs7303158
rs11715853
All − MR Egger
All − IVW(MRE)
−1
0
1
MR effect size for
'Gut microbiota abundance (genus Parasutterella id.2892)' on 'Dynactin subunit 2'

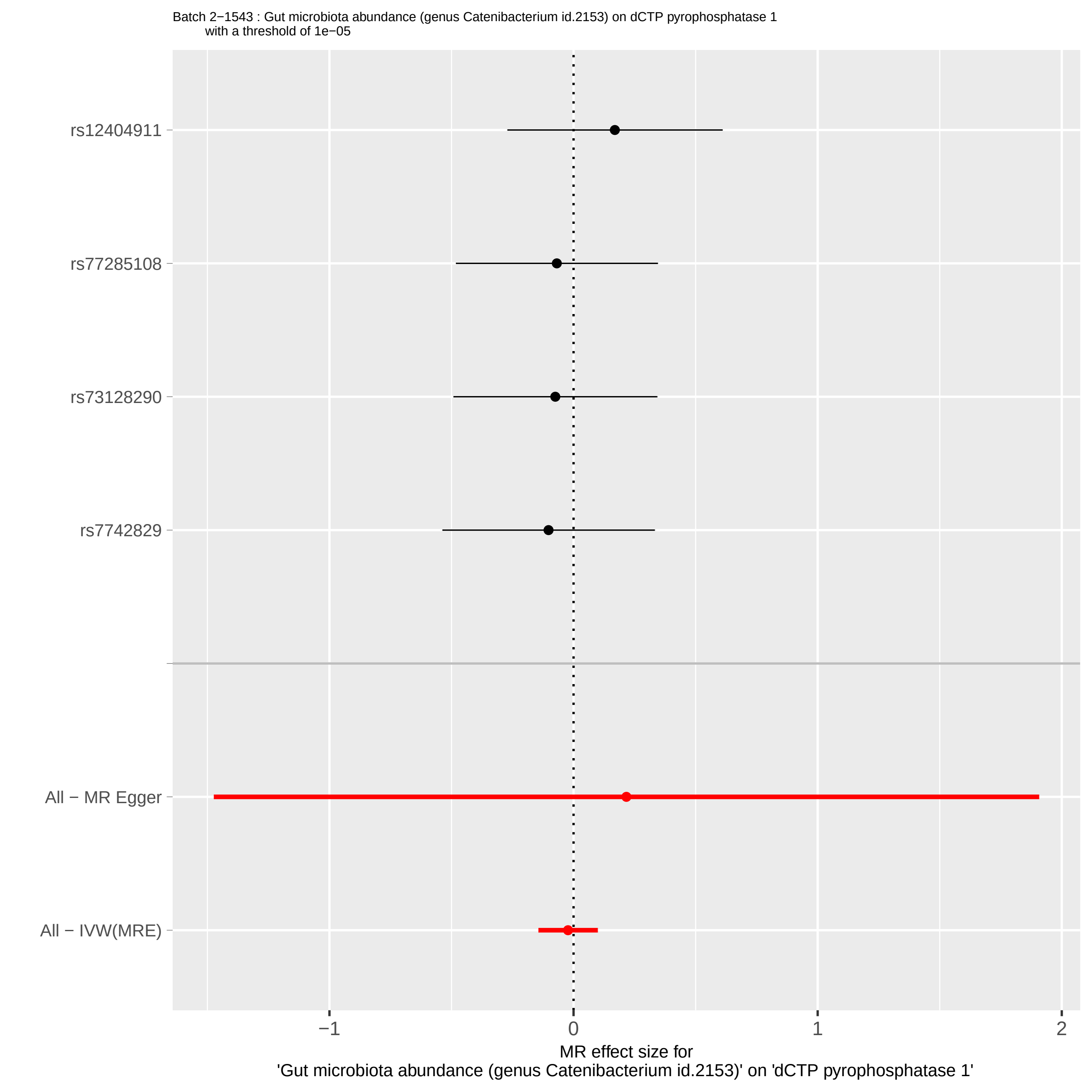
Batch 2−1543 : Gut microbiota abundance (genus Catenibacterium id.2153) on dCTP pyrophosphatase 1
with a threshold of 1e−05
rs12404911
rs77285108
rs73128290
rs7742829
All − MR Egger
All − IVW(MRE)
−1
0
1
2
MR effect size for
'Gut microbiota abundance (genus Catenibacterium id.2153)' on 'dCTP pyrophosphatase 1'

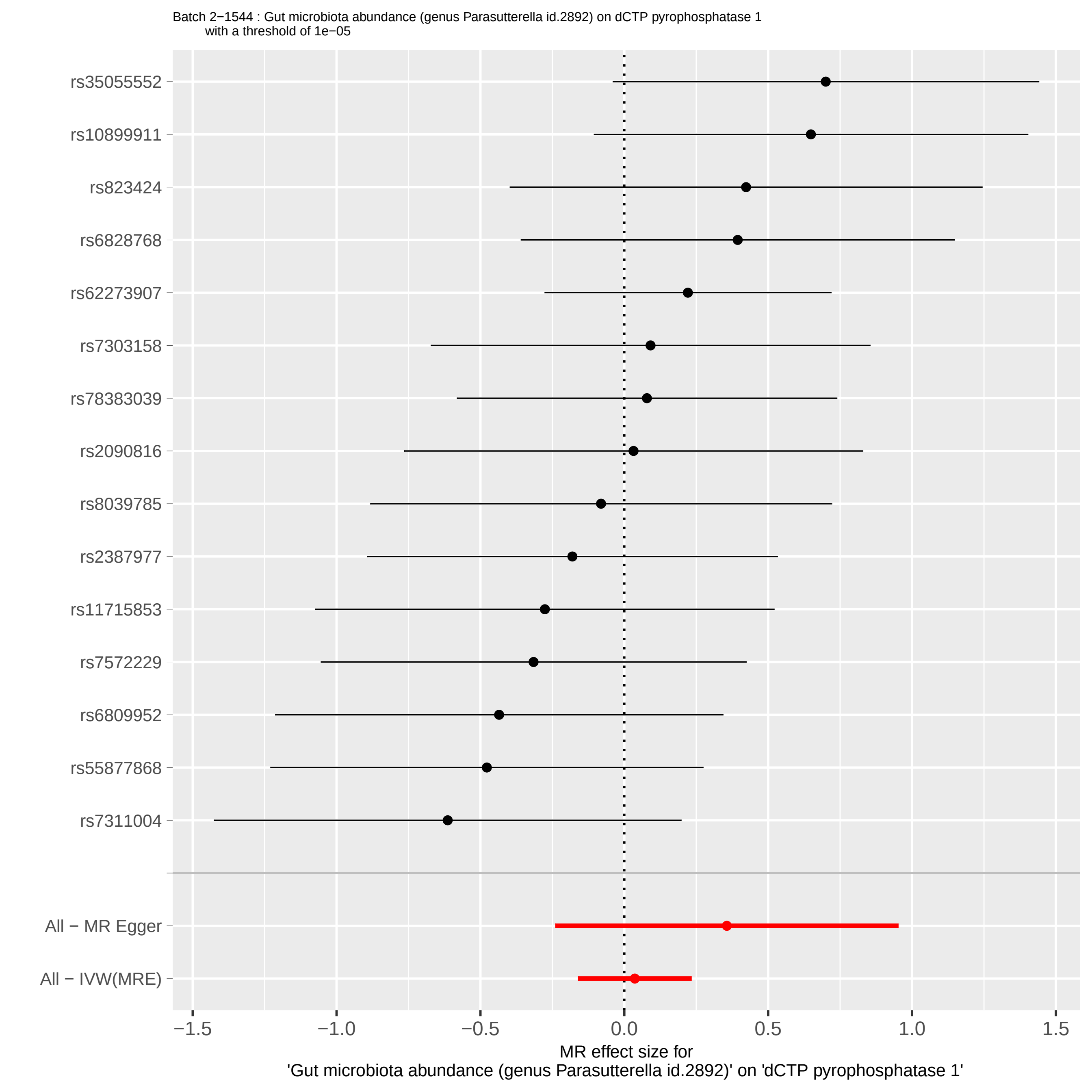

Batch 2−1544 : Gut microbiota abundance (genus Parasutterella id.2892) on dCTP pyrophosphatase 1
with a threshold of 1e−05
rs35055552
rs10899911
rs823424
rs6828768
rs62273907
rs7303158
rs78383039
rs2090816
rs8039785
rs2387977
rs11715853
rs7572229
rs6809952
rs55877868
rs7311004
All − MR Egger
All − IVW(MRE)
−1.5
−1.0
−0.5
0.0
0.5
1.0
1.5
MR effect size for
'Gut microbiota abundance (genus Parasutterella id.2892)' on 'dCTP pyrophosphatase 1'

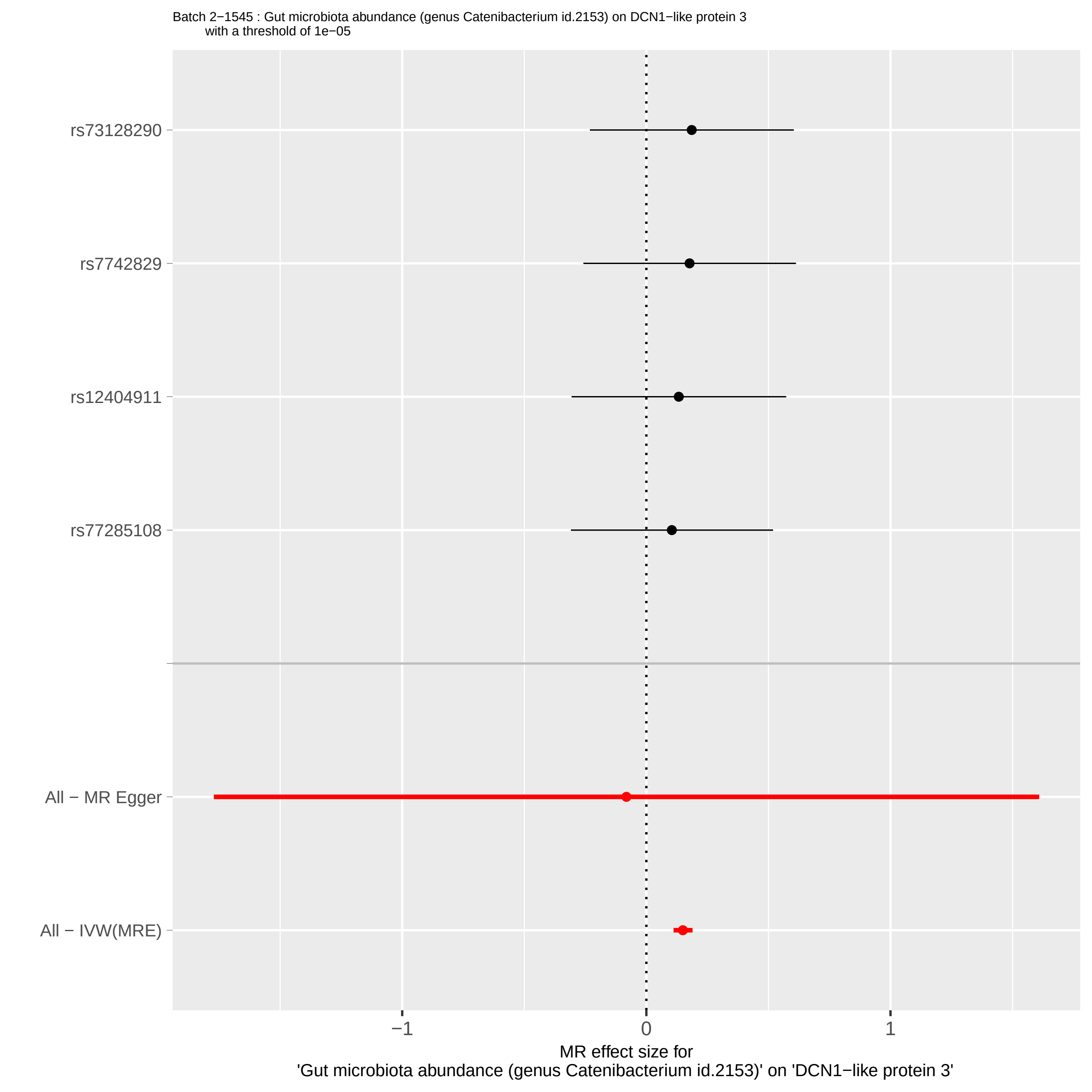
Batch 2−1545 : Gut microbiota abundance (genus Catenibacterium id.2153) on DCN1−like protein 3
with a threshold of 1e−05
rs73128290
rs7742829
rs12404911
rs77285108
All − MR Egger
All − IVW(MRE)
−1
0
1
MR effect size for
'Gut microbiota abundance (genus Catenibacterium id.2153)' on 'DCN1−like protein 3'

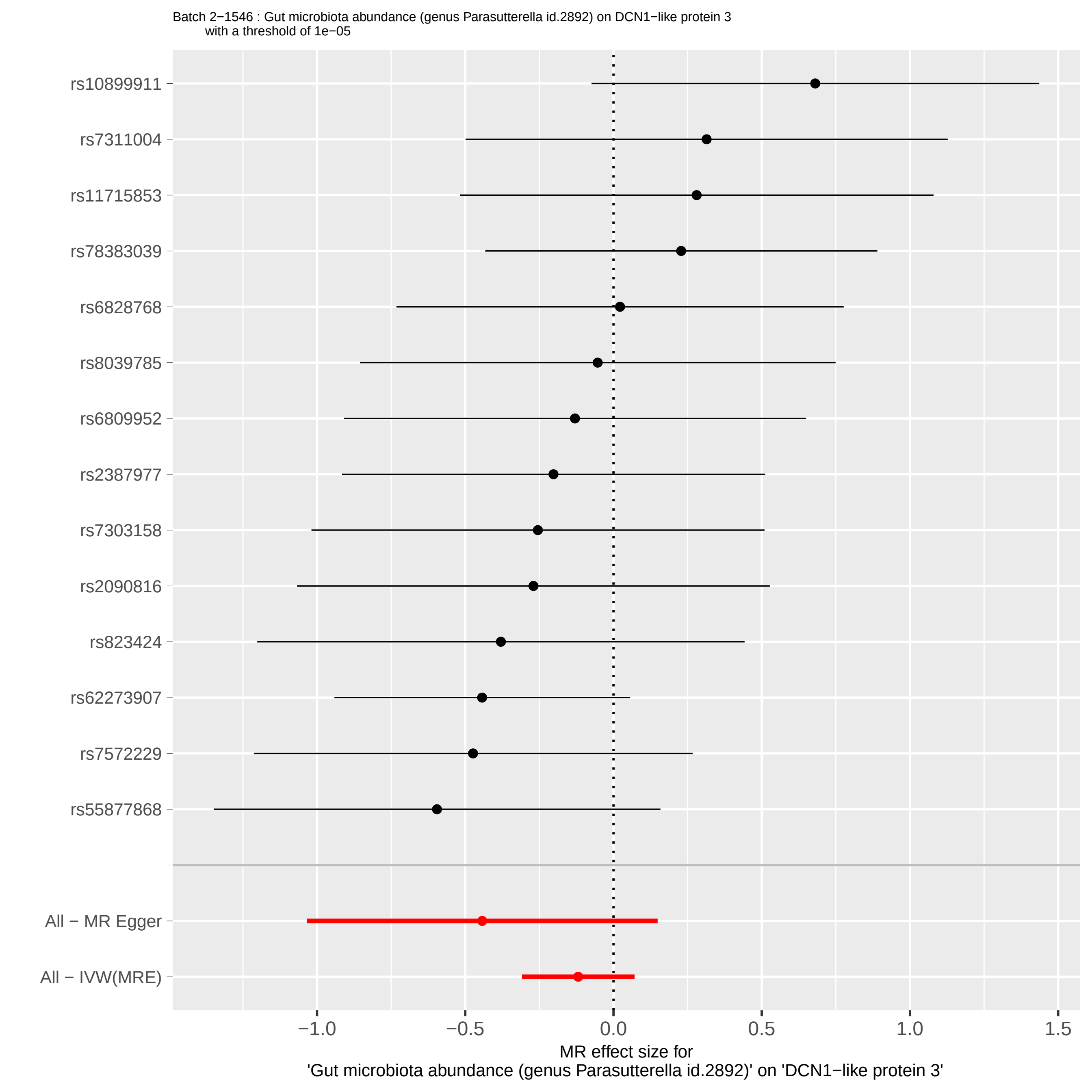

Batch 2−1546 : Gut microbiota abundance (genus Parasutterella id.2892) on DCN1−like protein 3
with a threshold of 1e−05
rs10899911
rs7311004
rs11715853
rs78383039
rs6828768
rs8039785
rs6809952
rs2387977
rs7303158
rs2090816
rs823424
rs62273907
rs7572229
rs55877868
All − MR Egger
All − IVW(MRE)
−1.0
−0.5
0.0
0.5
1.0
1.5
MR effect size for
'Gut microbiota abundance (genus Parasutterella id.2892)' on 'DCN1−like protein 3'

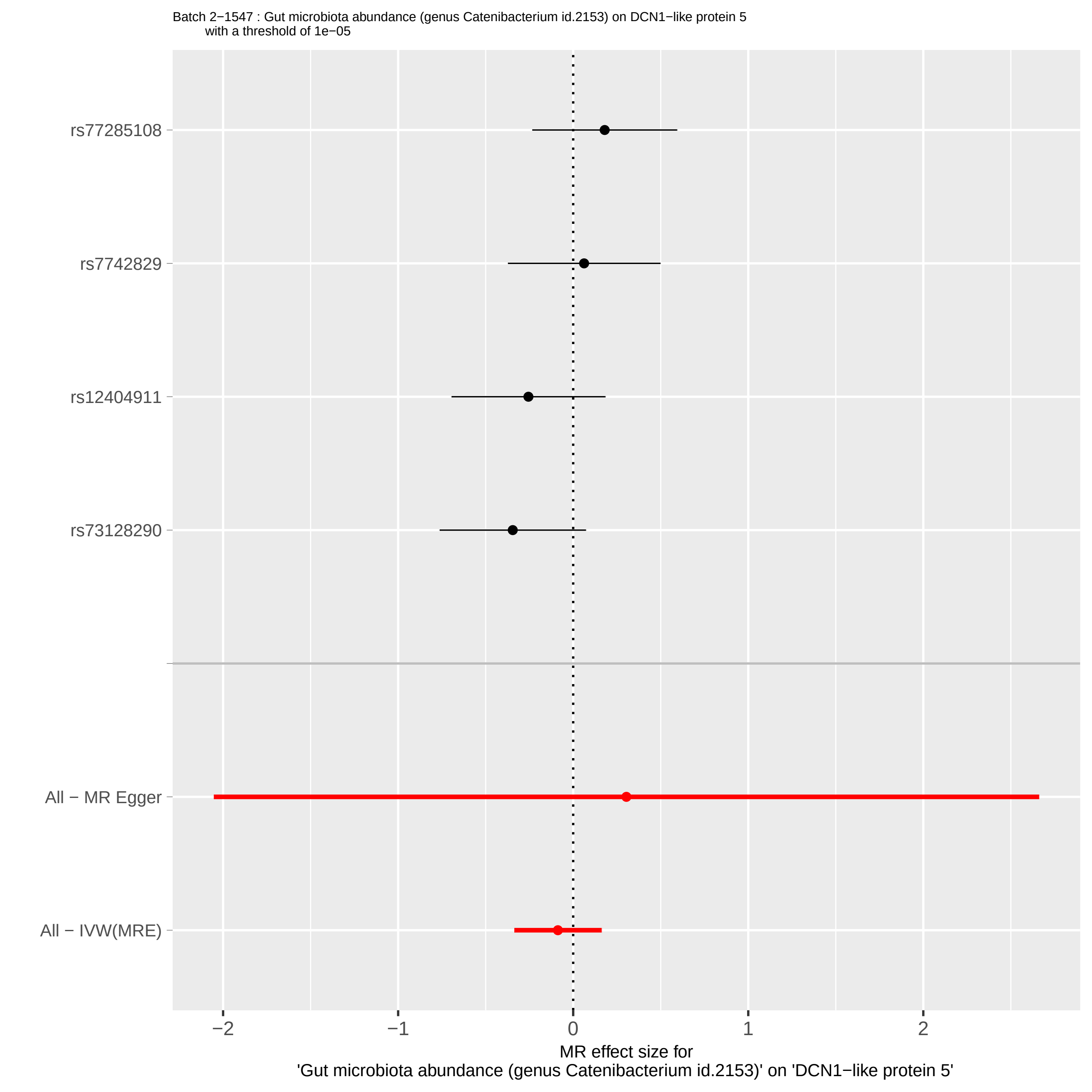

Batch 2−1547 : Gut microbiota abundance (genus Catenibacterium id.2153) on DCN1−like protein 5
with a threshold of 1e−05
rs77285108
rs7742829
rs12404911
rs73128290
All − MR Egger
All − IVW(MRE)
−2
−1
0
1
2
MR effect size for
'Gut microbiota abundance (genus Catenibacterium id.2153)' on 'DCN1−like protein 5'

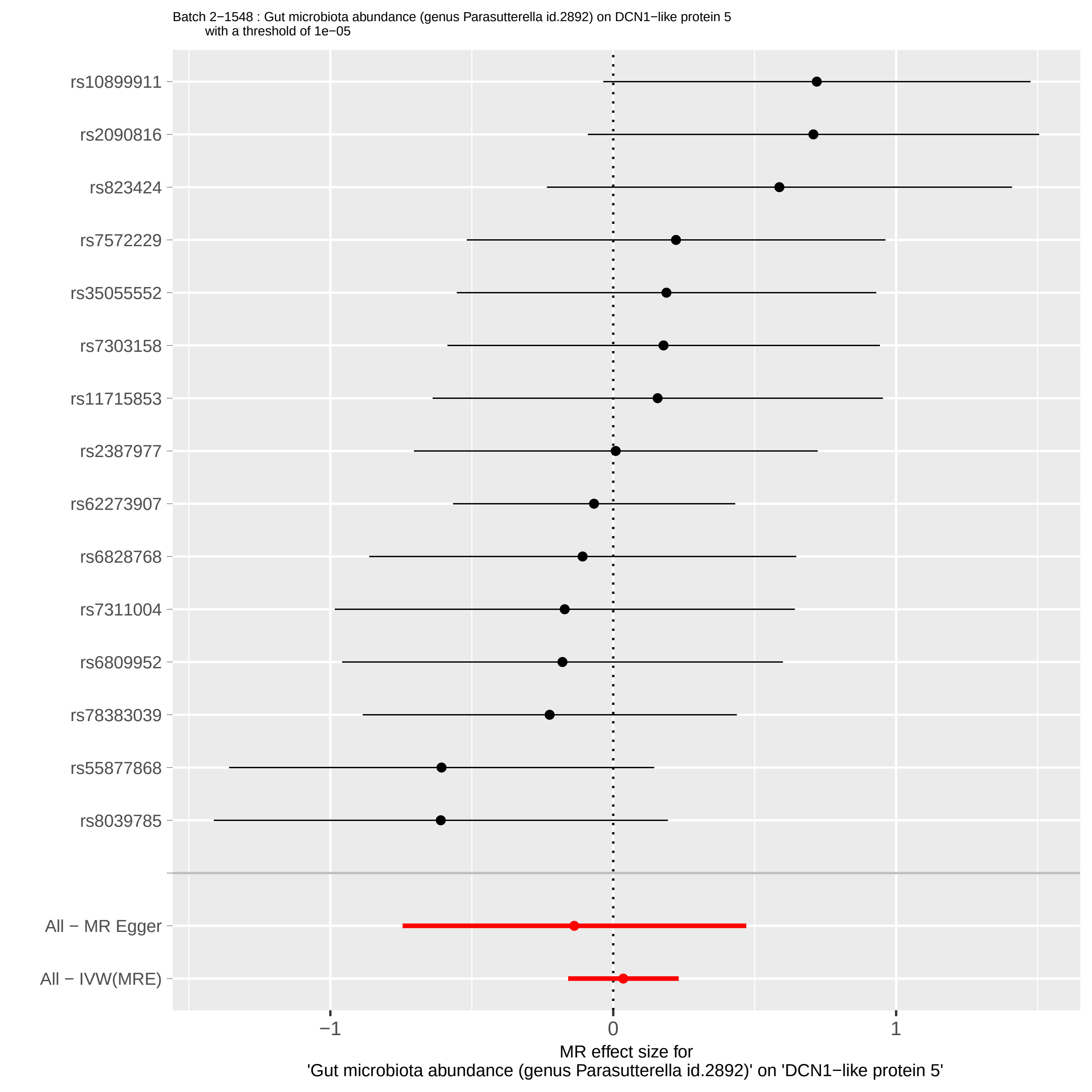

Batch 2−1548 : Gut microbiota abundance (genus Parasutterella id.2892) on DCN1−like protein 5
with a threshold of 1e−05
rs10899911
rs2090816
rs823424
rs7572229
rs35055552
rs7303158
rs11715853
rs2387977
rs62273907
rs6828768
rs7311004
rs6809952
rs78383039
rs55877868
rs8039785
All − MR Egger
All − IVW(MRE)
−1
0
1
MR effect size for
'Gut microbiota abundance (genus Parasutterella id.2892)' on 'DCN1−like protein 5'

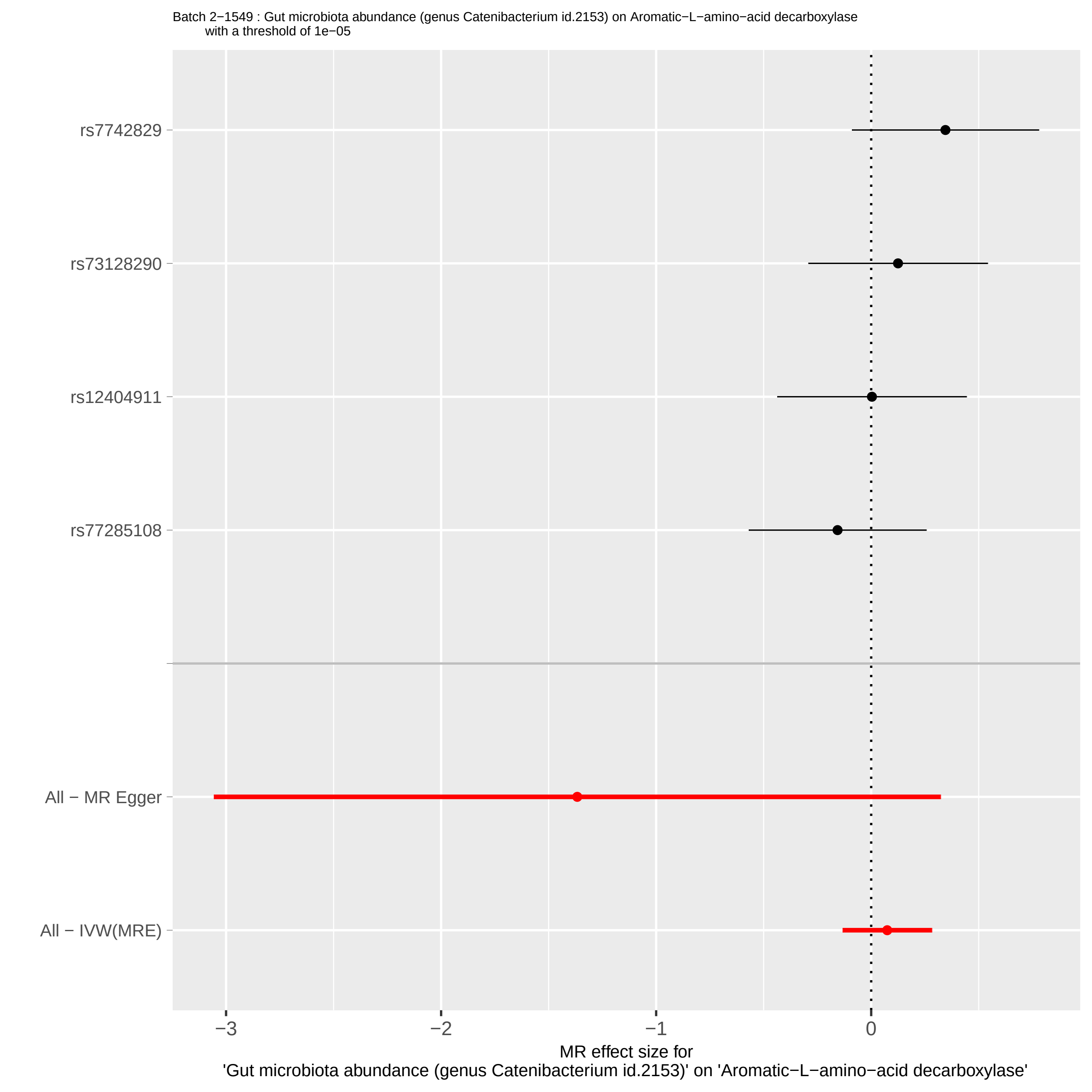

Batch 2−1549 : Gut microbiota abundance (genus Catenibacterium id.2153) on Aromatic−L−amino−acid decarboxylase
with a threshold of 1e−05
rs7742829
rs73128290
rs12404911
rs77285108
All − MR Egger
All − IVW(MRE)
−3
−2
−1
0
MR effect size for
'Gut microbiota abundance (genus Catenibacterium id.2153)' on 'Aromatic−L−amino−acid decarboxylase'

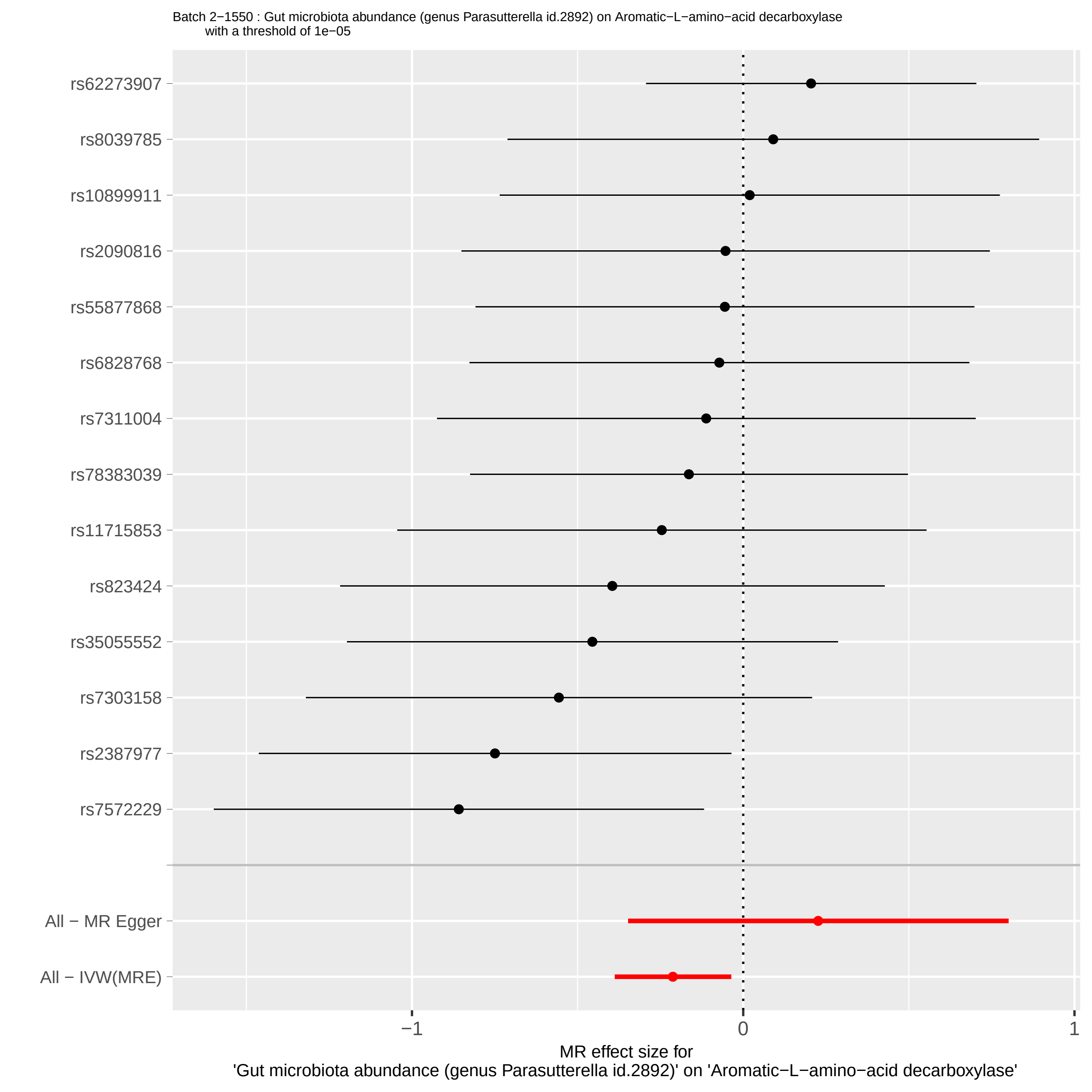

Batch 2−1550 : Gut microbiota abundance (genus Parasutterella id.2892) on Aromatic−L−amino−acid decarboxylase
with a threshold of 1e−05
rs62273907
rs8039785
rs10899911
rs2090816
rs55877868
rs6828768
rs7311004
rs78383039
rs11715853
rs823424
rs35055552
rs7303158
rs2387977
rs7572229
All − MR Egger
All − IVW(MRE)
−1
0
1
MR effect size for
'Gut microbiota abundance (genus Parasutterella id.2892)' on 'Aromatic−L−amino−acid decarboxylase'

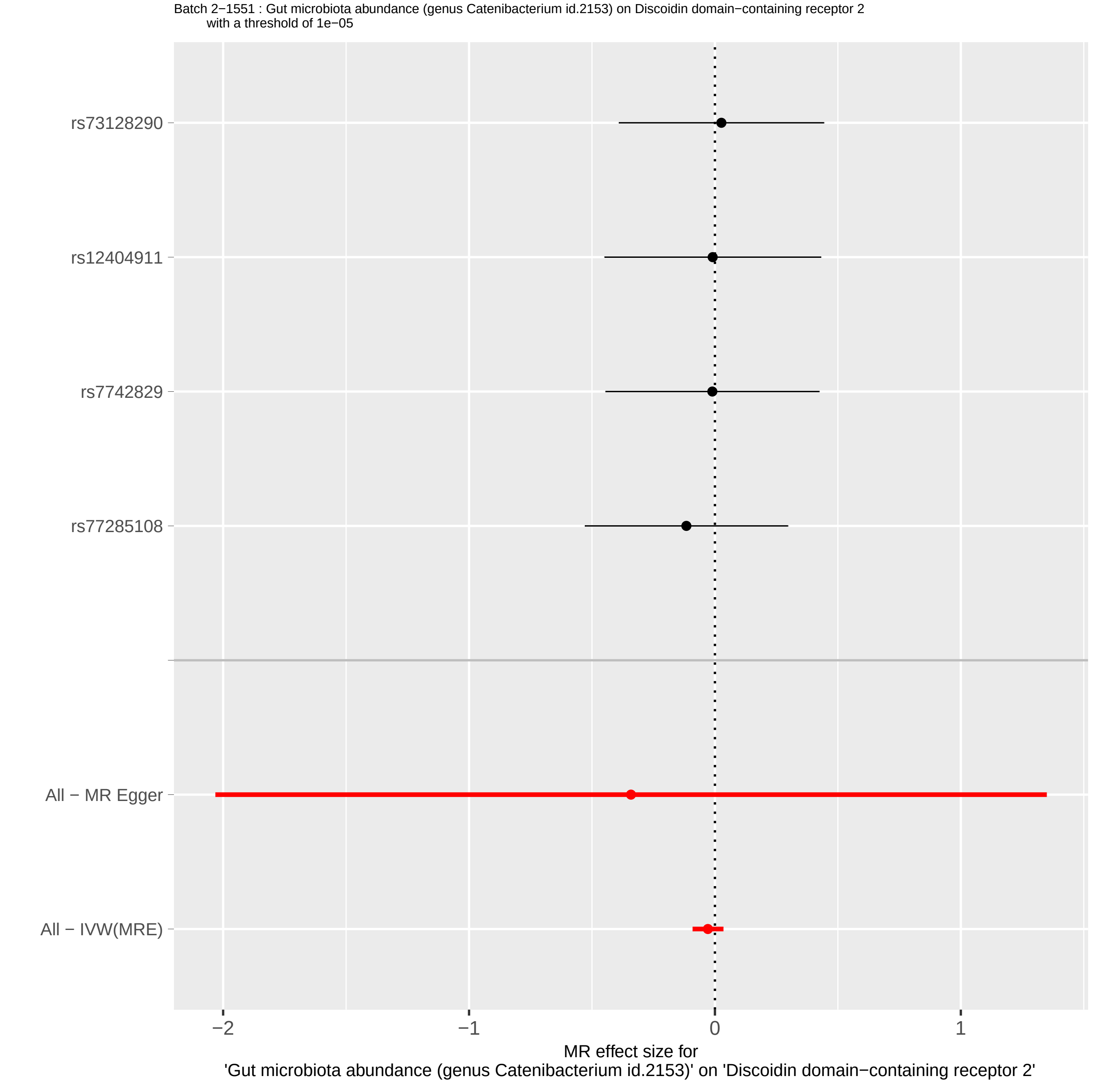

Batch 2−1551 : Gut microbiota abundance (genus Catenibacterium id.2153) on Discoidin domain−containing receptor 2
with a threshold of 1e−05
rs73128290
rs12404911
rs7742829
rs77285108
All − MR Egger
All − IVW(MRE)
−2
−1
0
1
MR effect size for
'Gut microbiota abundance (genus Catenibacterium id.2153)' on 'Discoidin domain−containing receptor 2'

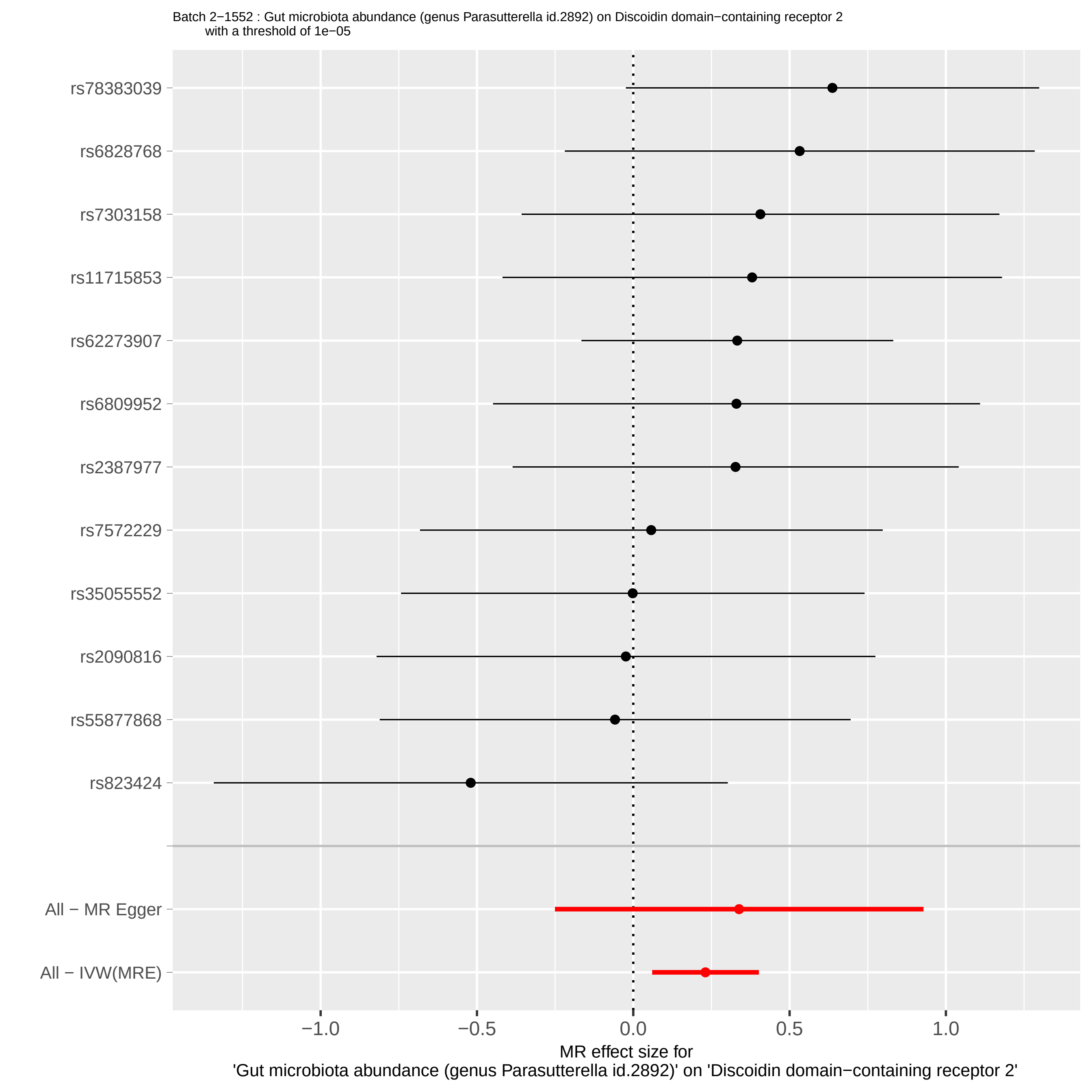

Batch 2−1552 : Gut microbiota abundance (genus Parasutterella id.2892) on Discoidin domain−containing receptor 2 with a threshold of 1e−05
rs78383039
rs6828768
rs7303158
rs11715853
rs62273907
rs6809952
rs2387977
rs7572229
rs35055552
rs2090816
rs55877868
rs823424
All − MR Egger
All − IVW(MRE)
−1.0
−0.5
0.0
0.5
1.0
MR effect size for
'Gut microbiota abundance (genus Parasutterella id.2892)' on 'Discoidin domain−containing receptor 2'

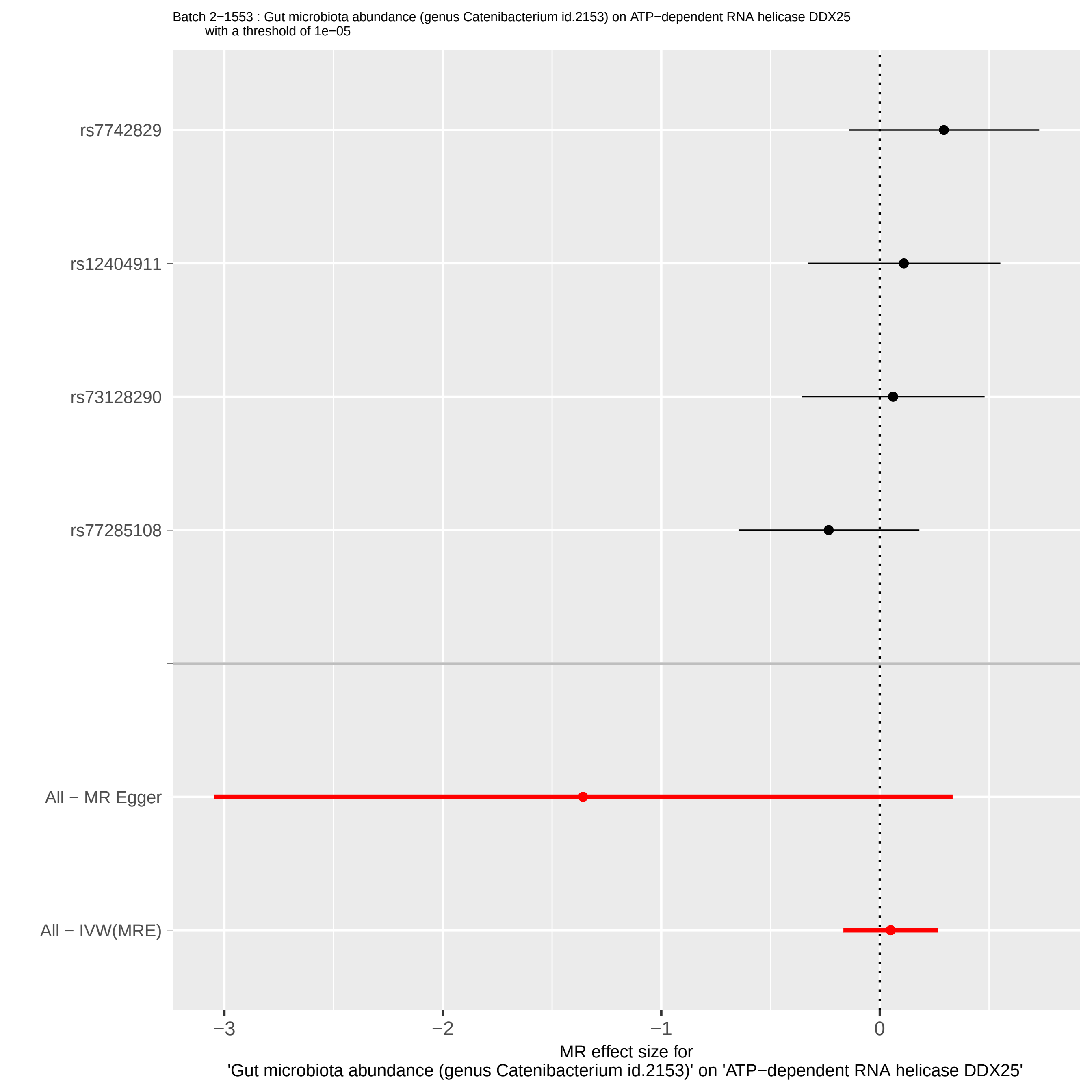

Batch 2−1553 : Gut microbiota abundance (genus Catenibacterium id.2153) on ATP−dependent RNA helicase DDX25
with a threshold of 1e−05
rs7742829
rs12404911
rs73128290
rs77285108
All − MR Egger
All − IVW(MRE)
−3
−2
−1
0
MR effect size for
'Gut microbiota abundance (genus Catenibacterium id.2153)' on 'ATP−dependent RNA helicase DDX25'

Batch 2−1554 : Gut microbiota abundance (genus Parasutterella id.2892) on ATP−dependent RNA helicase DDX25
with a threshold of 1e−05

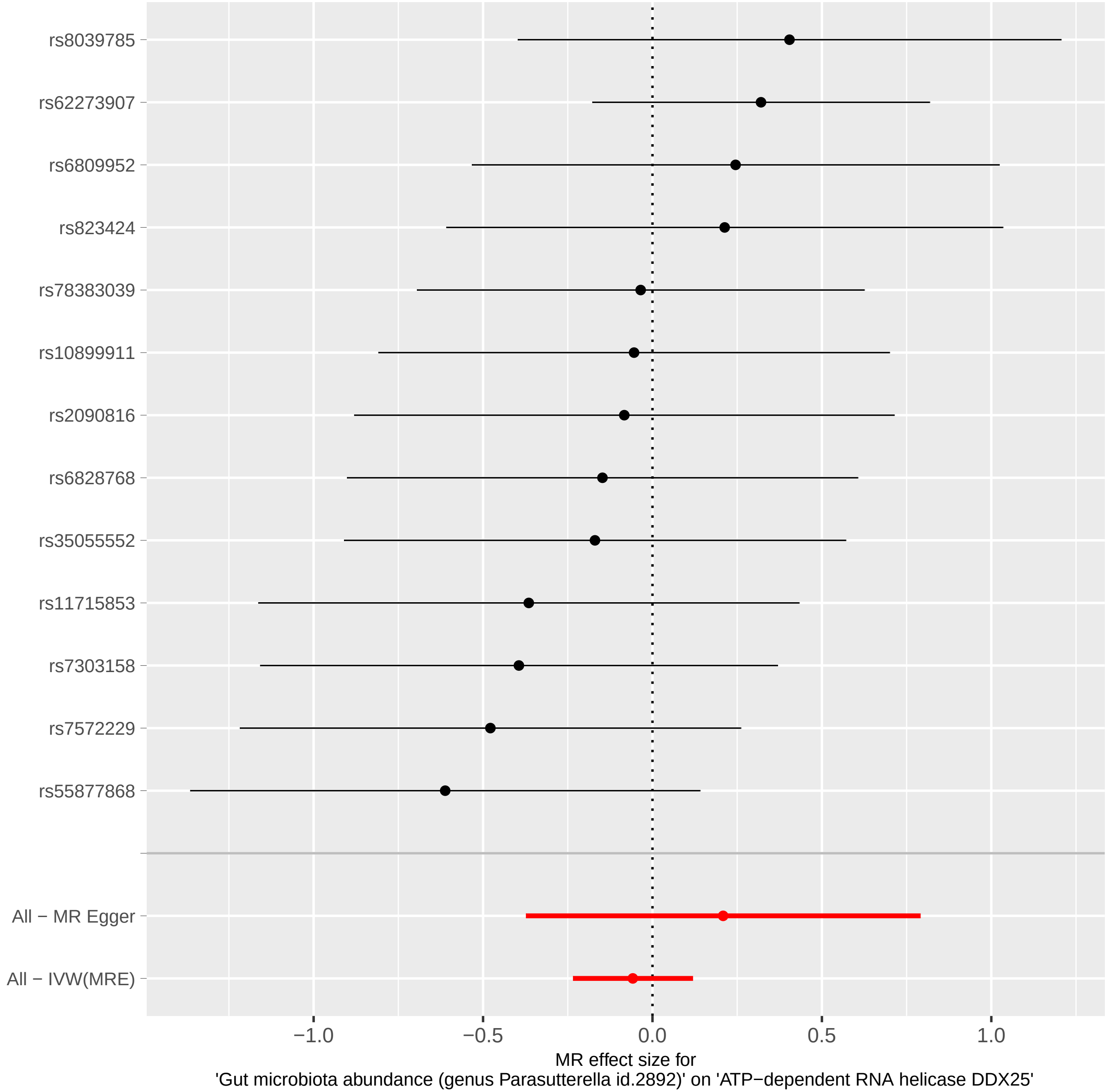

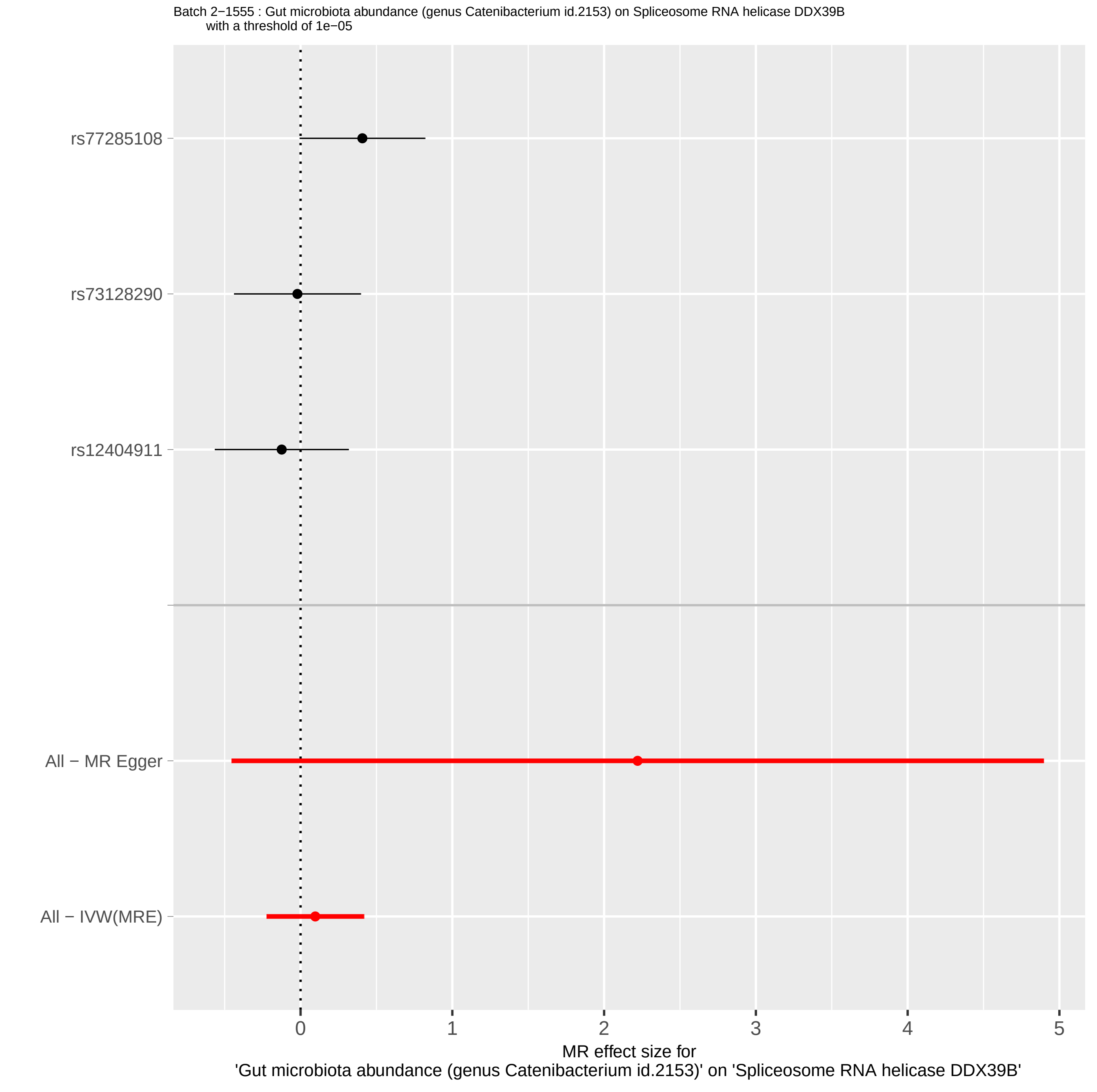
Batch 2−1555 : Gut microbiota abundance (genus Catenibacterium id.2153) on Spliceosome RNA helicase DDX39B
with a threshold of 1e−05
rs77285108
rs73128290
rs12404911
All − MR Egger
All − IVW(MRE)
0
1
2
3
4
5
MR effect size for
'Gut microbiota abundance (genus Catenibacterium id.2153)' on 'Spliceosome RNA helicase DDX39B'

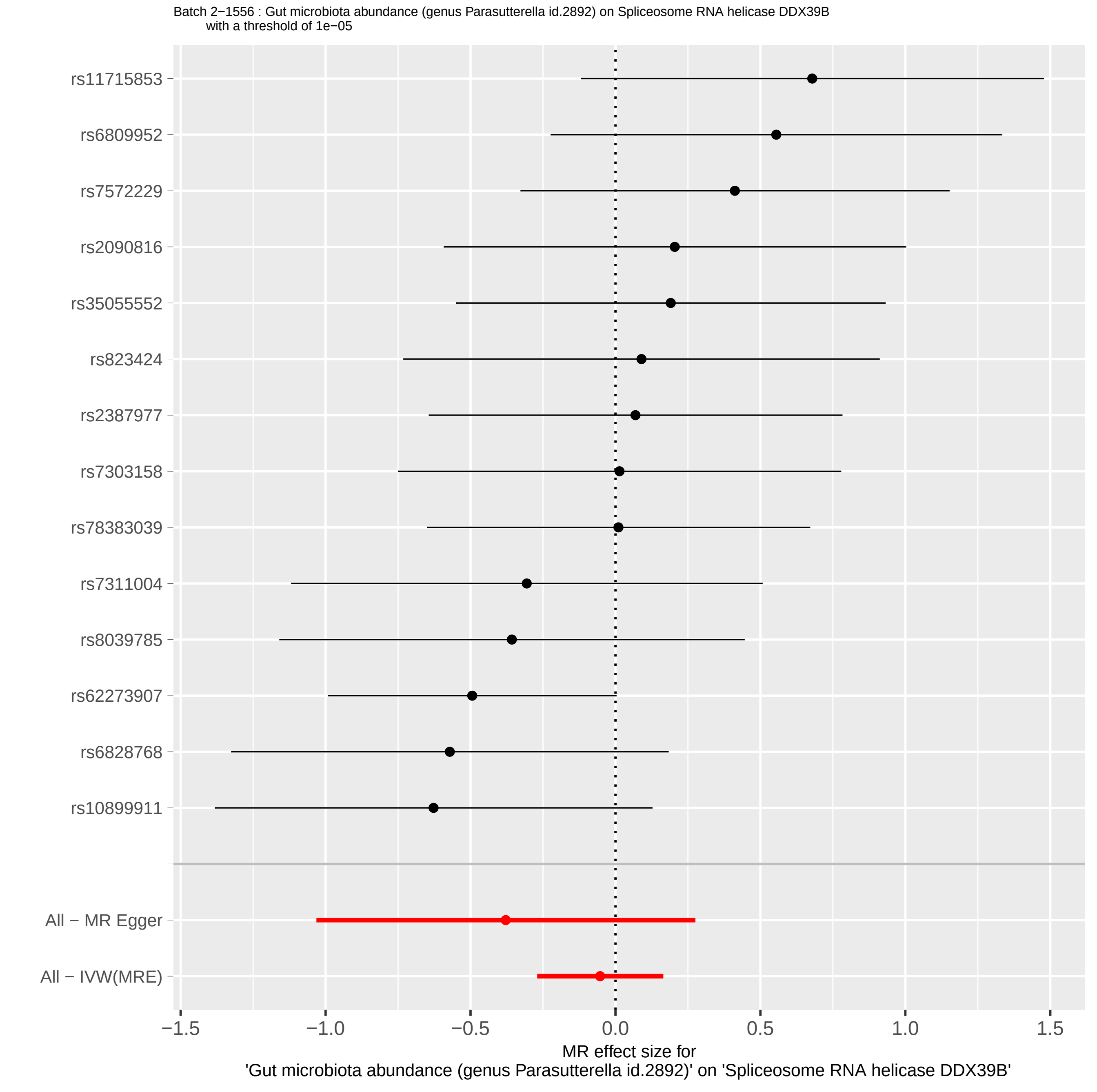
Batch 2−1556 : Gut microbiota abundance (genus Parasutterella id.2892) on Spliceosome RNA helicase DDX39B
with a threshold of 1e−05
rs11715853
rs6809952
rs7572229
rs2090816
rs35055552
rs823424
rs2387977
rs7303158
rs78383039
rs7311004
rs8039785
rs62273907
rs6828768
rs10899911
All − MR Egger
All − IVW(MRE)
−1.5
−1.0
−0.5
0.0
0.5
1.0
1.5
MR effect size for
'Gut microbiota abundance (genus Parasutterella id.2892)' on 'Spliceosome RNA helicase DDX39B'

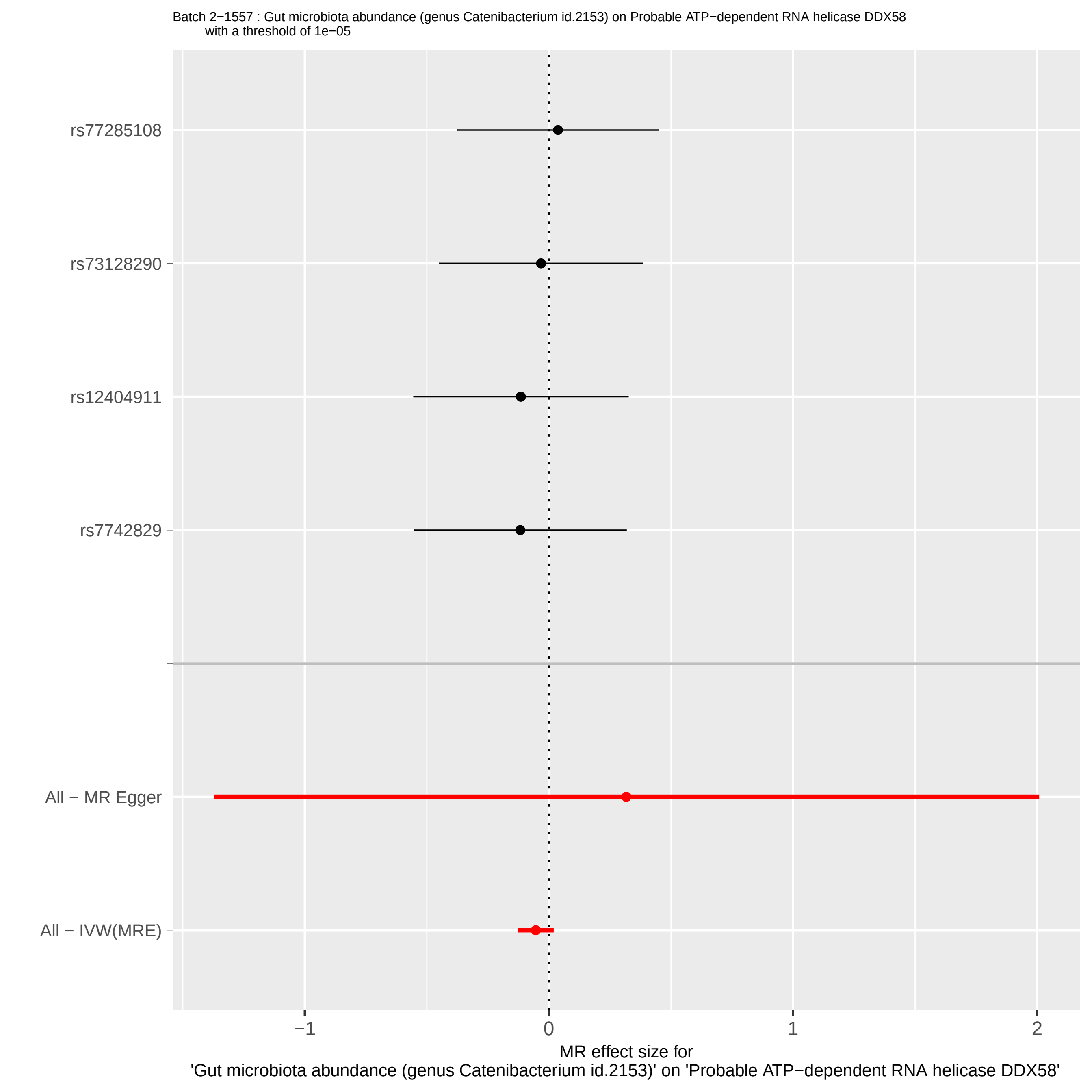

Batch 2−1557 : Gut microbiota abundance (genus Catenibacterium id.2153) on Probable ATP−dependent RNA helicase DDX58
with a threshold of 1e−05
rs77285108
rs73128290
rs12404911
rs7742829
All − MR Egger
All − IVW(MRE)
−1
0
1
2
MR effect size for
'Gut microbiota abundance (genus Catenibacterium id.2153)' on 'Probable ATP−dependent RNA helicase DDX58'

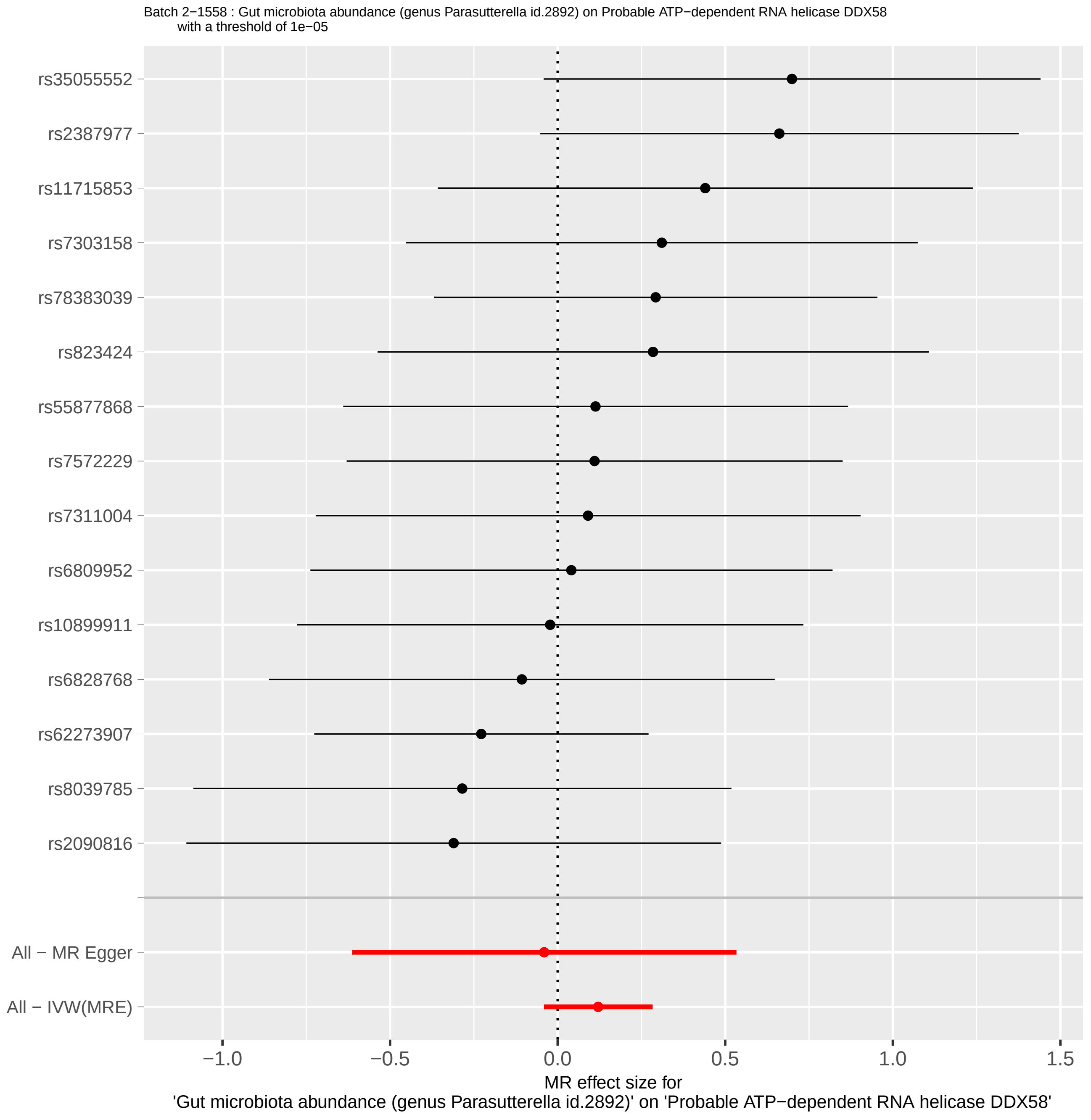
Batch 2−1558 : Gut microbiota abundance (genus Parasutterella id.2892) on Probable ATP−dependent RNA helicase DDX58
with a threshold of 1e−05
rs35055552
rs2387977
rs11715853
rs7303158
rs78383039
rs823424
rs55877868
rs7572229
rs7311004
rs6809952
rs10899911
rs6828768
rs62273907
rs8039785
rs2090816
All − MR Egger
All − IVW(MRE)
−1.0
−0.5
0.0
0.5
1.0
1.5
MR effect size for
'Gut microbiota abundance (genus Parasutterella id.2892)' on 'Probable ATP−dependent RNA helicase DDX58'

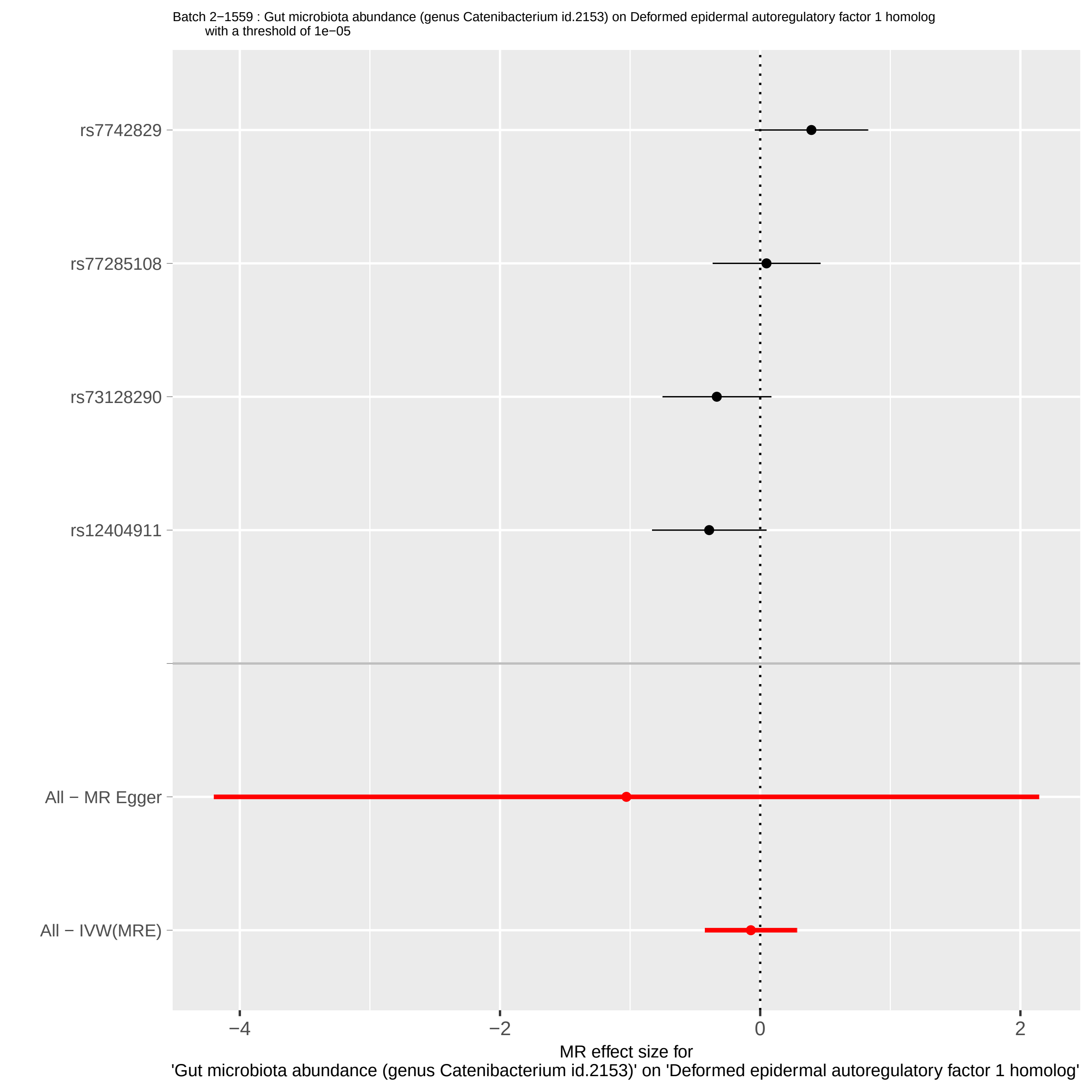

Batch 2−1559 : Gut microbiota abundance (genus Catenibacterium id.2153) on Deformed epidermal autoregulatory factor 1 homolog
with a threshold of 1e−05
rs7742829
rs77285108
rs73128290
rs12404911
All − MR Egger
All − IVW(MRE)
−4
−2
0
2
MR effect size for
'Gut microbiota abundance (genus Catenibacterium id.2153)' on 'Deformed epidermal autoregulatory factor 1 homolog'

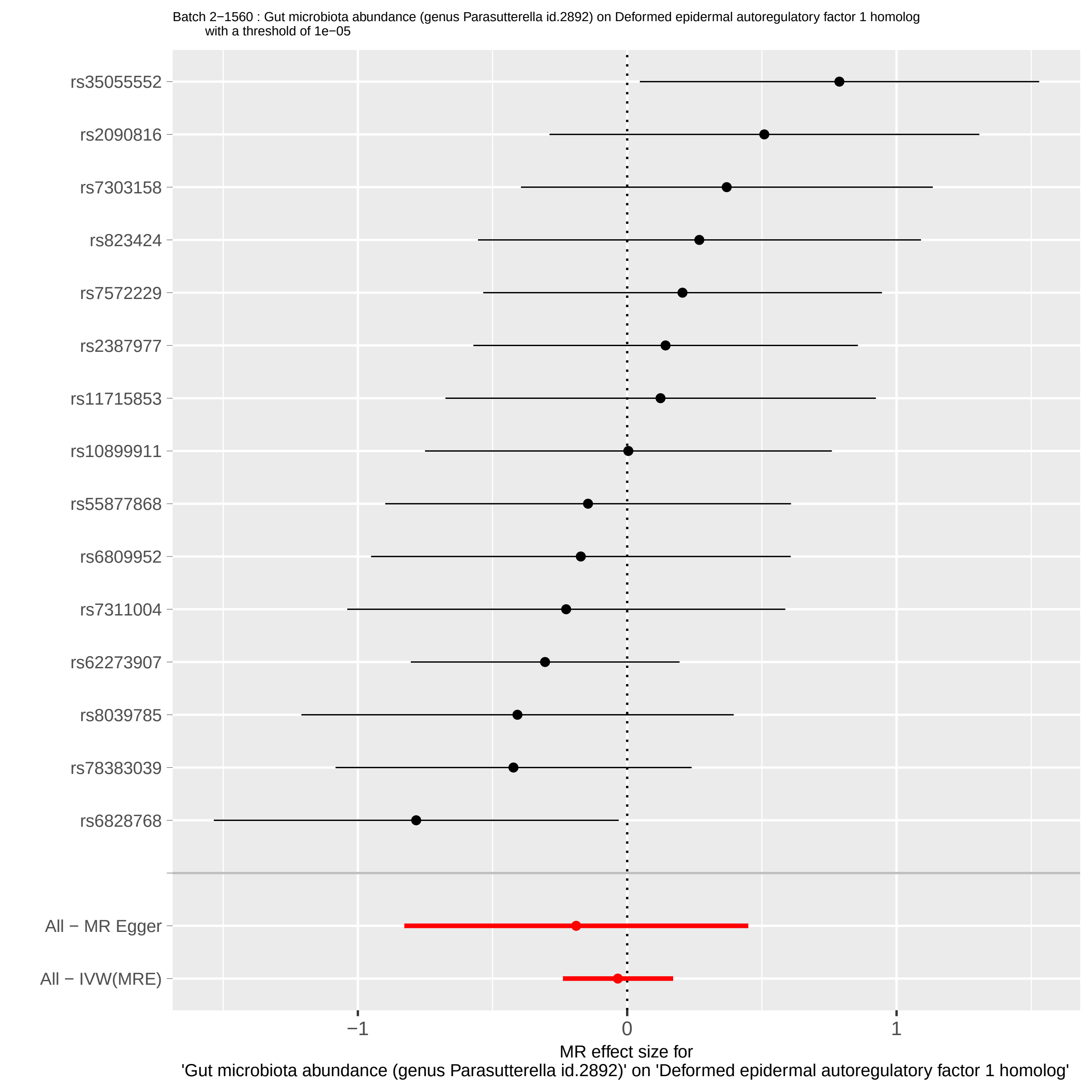

Batch 2−1560 : Gut microbiota abundance (genus Parasutterella id.2892) on Deformed epidermal autoregulatory factor 1 homolog
with a threshold of 1e−05
rs35055552
rs2090816
rs7303158
rs823424
rs7572229
rs2387977
rs11715853
rs10899911
rs55877868
rs6809952
rs7311004
rs62273907
rs8039785
rs78383039
rs6828768
All − MR Egger
All − IVW(MRE)
−1
0
1
MR effect size for
'Gut microbiota abundance (genus Parasutterella id.2892)' on 'Deformed epidermal autoregulatory factor 1 homolog'

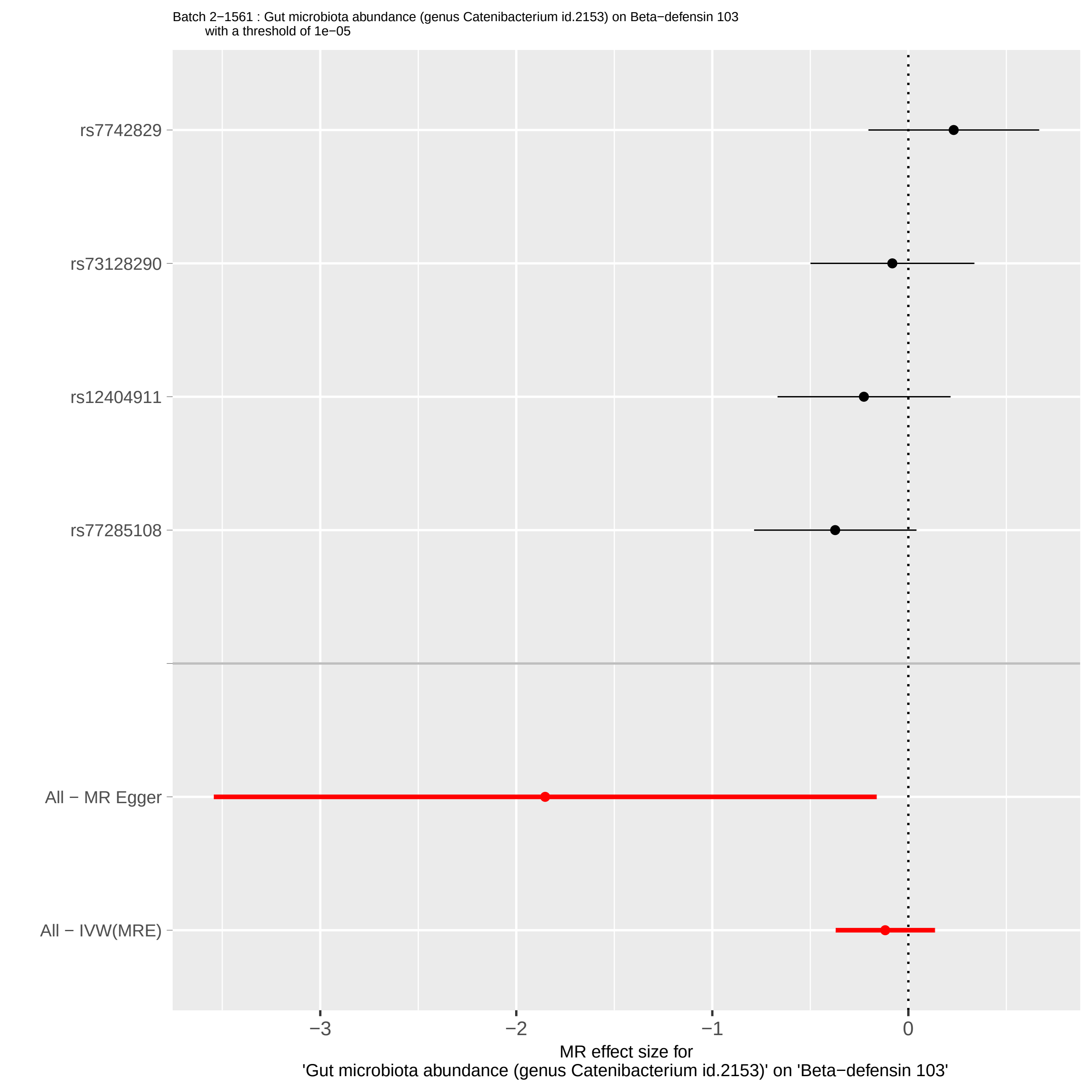
Batch 2−1561 : Gut microbiota abundance (genus Catenibacterium id.2153) on Beta−defensin 103
with a threshold of 1e−05
rs7742829
rs73128290
rs12404911
rs77285108
All − MR Egger
All − IVW(MRE)
−3
−2
−1
0
MR effect size for
'Gut microbiota abundance (genus Catenibacterium id.2153)' on 'Beta−defensin 103'

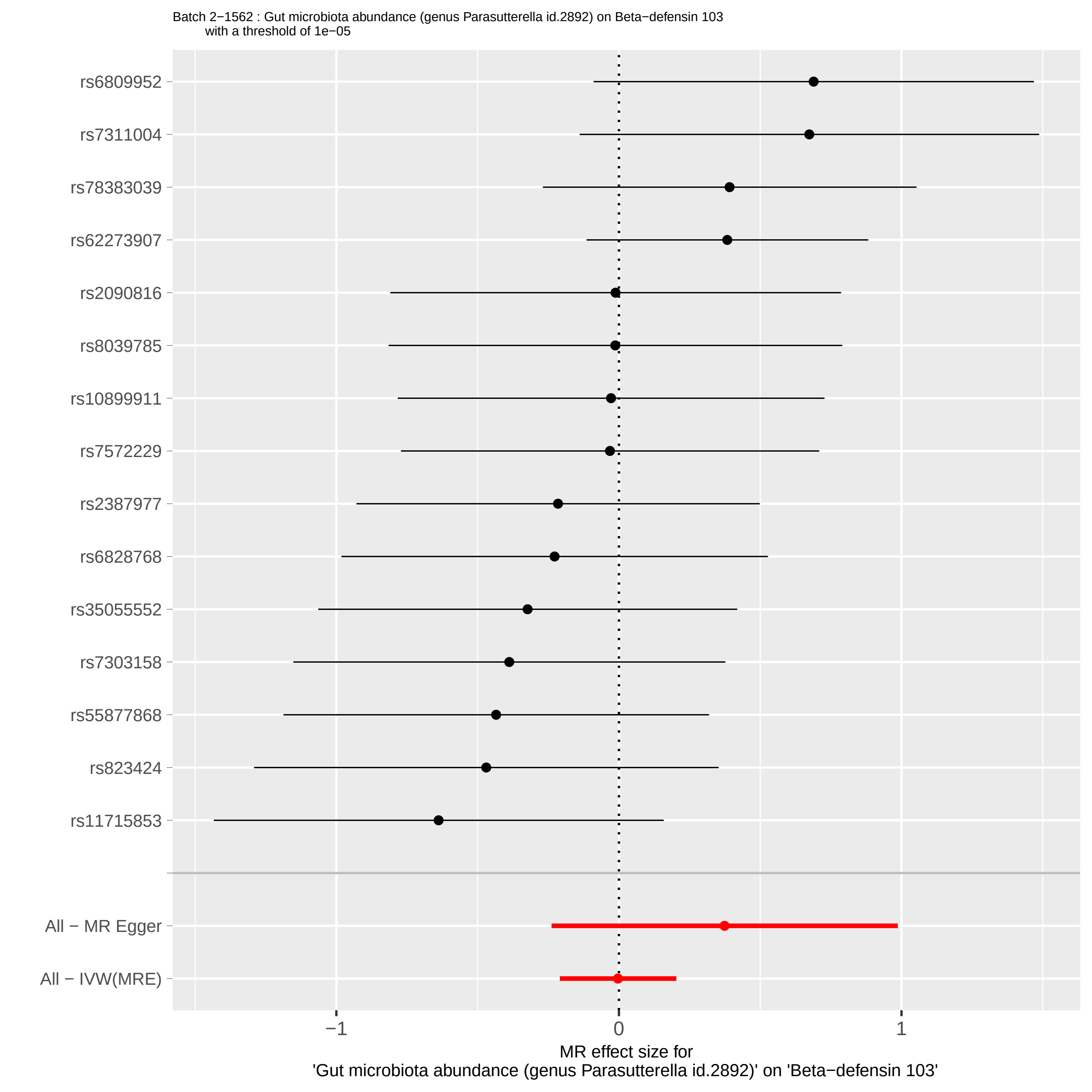

Batch 2−1562 : Gut microbiota abundance (genus Parasutterella id.2892) on Beta−defensin 103
with a threshold of 1e−05
rs6809952
rs7311004
rs78383039
rs62273907
rs2090816
rs8039785
rs10899911
rs7572229
rs2387977
rs6828768
rs35055552
rs7303158
rs55877868
rs823424
rs11715853
All − MR Egger
All − IVW(MRE)
−1
0
1
MR effect size for
'Gut microbiota abundance (genus Parasutterella id.2892)' on 'Beta−defensin 103'

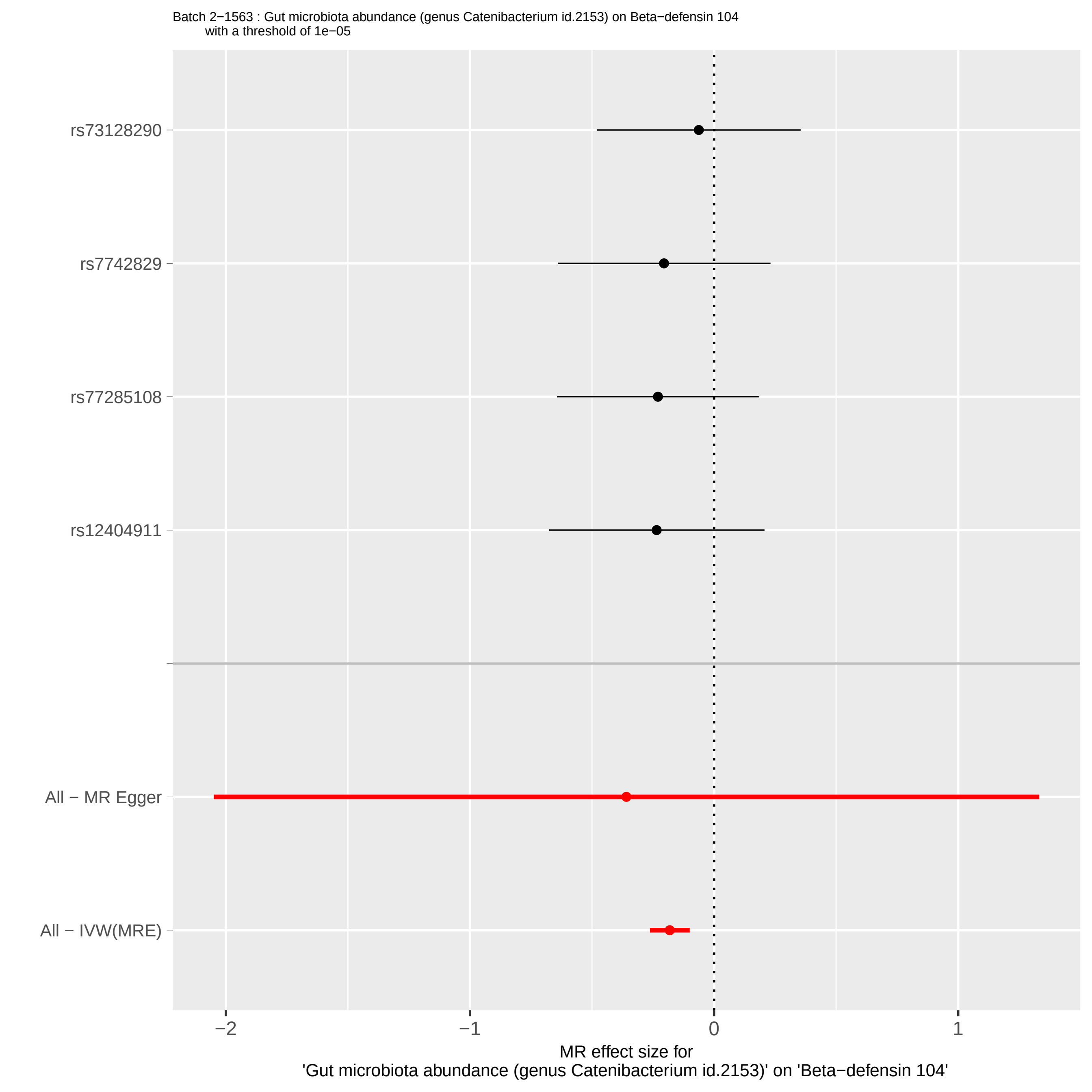

Batch 2−1563 : Gut microbiota abundance (genus Catenibacterium id.2153) on Beta−defensin 104
with a threshold of 1e−05
rs73128290
rs7742829
rs77285108
rs12404911
All − MR Egger
All − IVW(MRE)
−2
−1
0
1
MR effect size for
'Gut microbiota abundance (genus Catenibacterium id.2153)' on 'Beta−defensin 104'

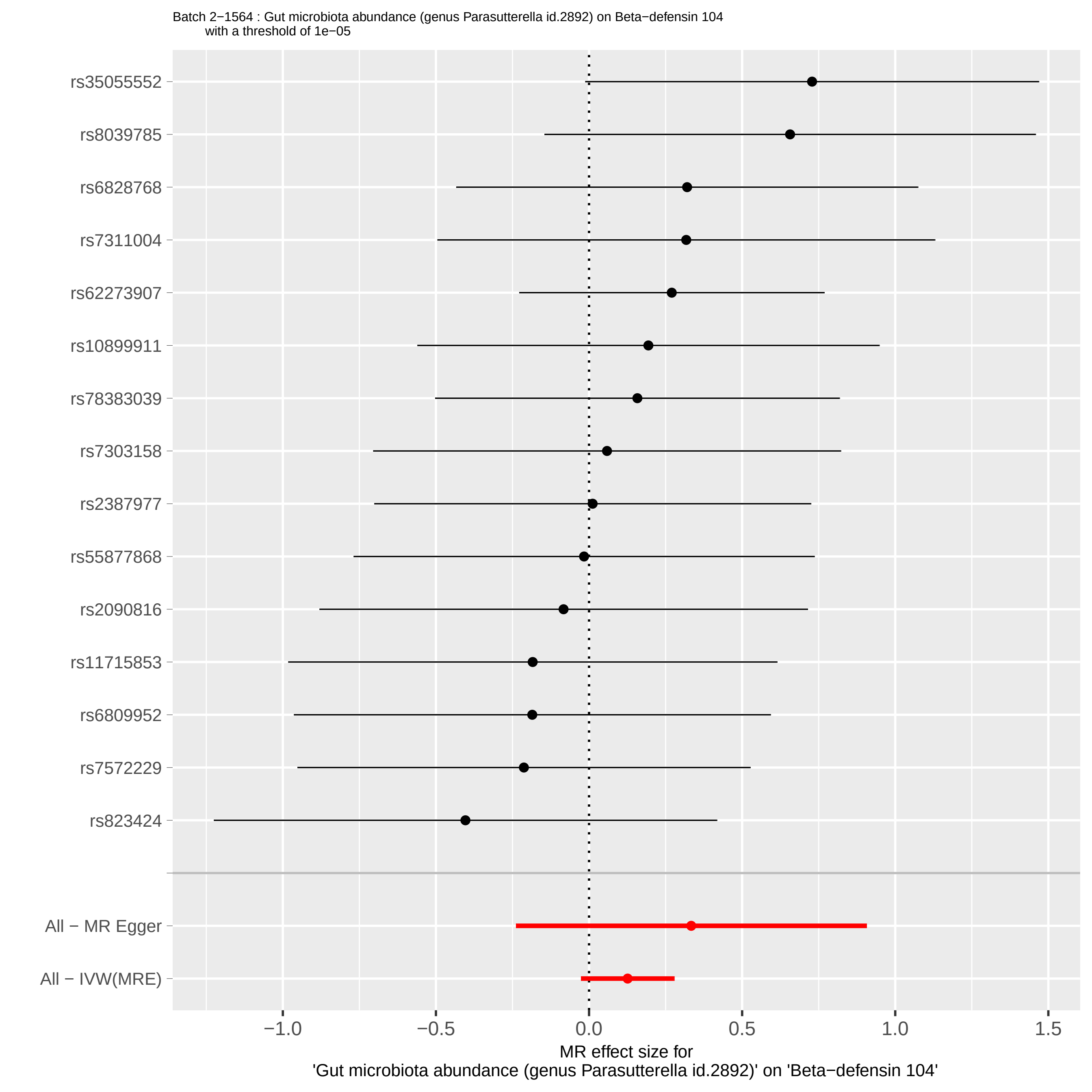

Batch 2−1564 : Gut microbiota abundance (genus Parasutterella id.2892) on Beta−defensin 104
with a threshold of 1e−05
rs35055552
rs8039785
rs6828768
rs7311004
rs62273907
rs10899911
rs78383039
rs7303158
rs2387977
rs55877868
rs2090816
rs11715853
rs6809952
rs7572229
rs823424
All − MR Egger
All − IVW(MRE)
−1.0
−0.5
0.0
0.5
1.0
1.5
MR effect size for
'Gut microbiota abundance (genus Parasutterella id.2892)' on 'Beta−defensin 104'

Batch 2−1565 : Gut microbiota abundance (genus Catenibacterium id.2153) on Beta−defensin 105
with a threshold of 1e−05

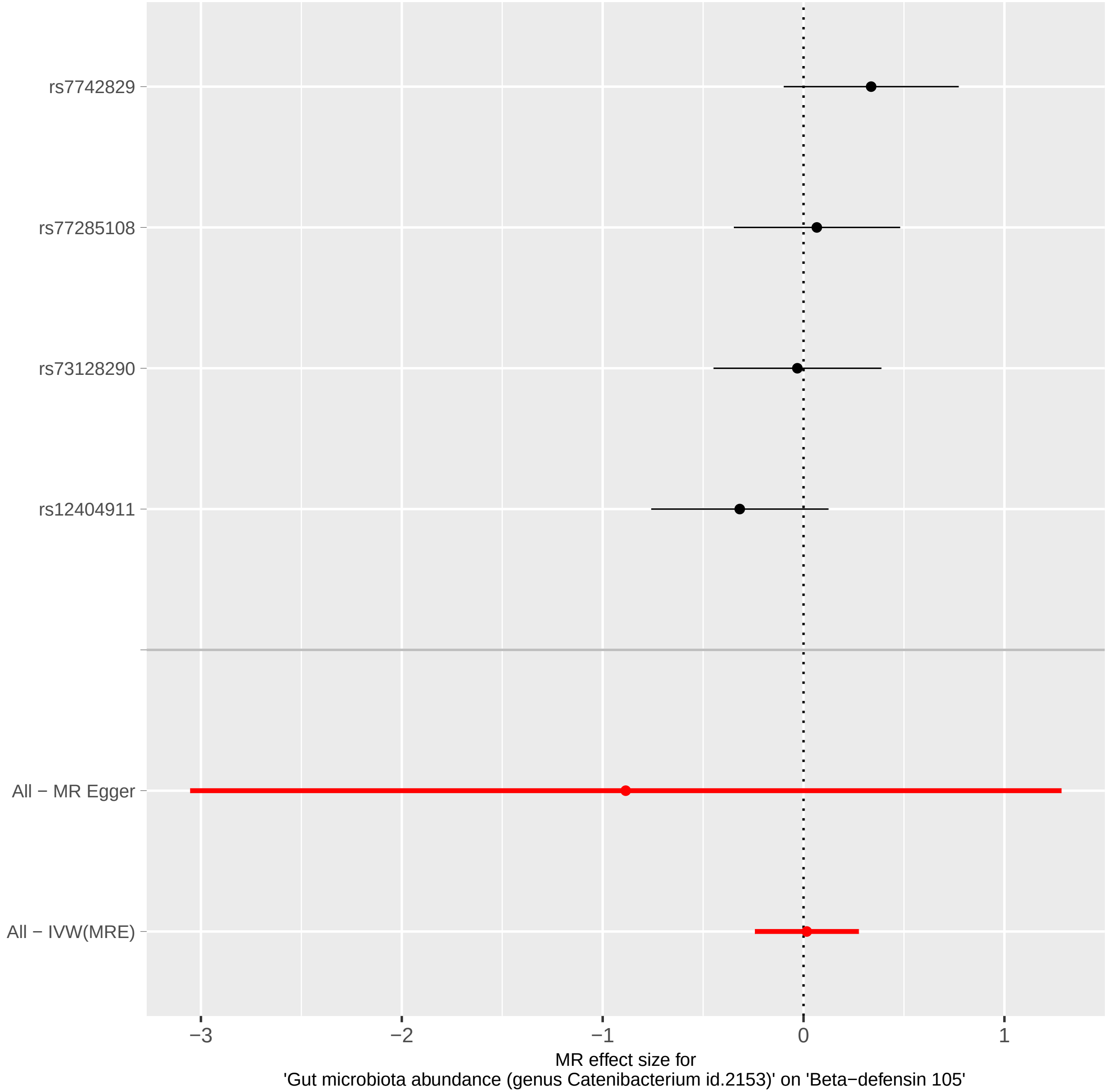

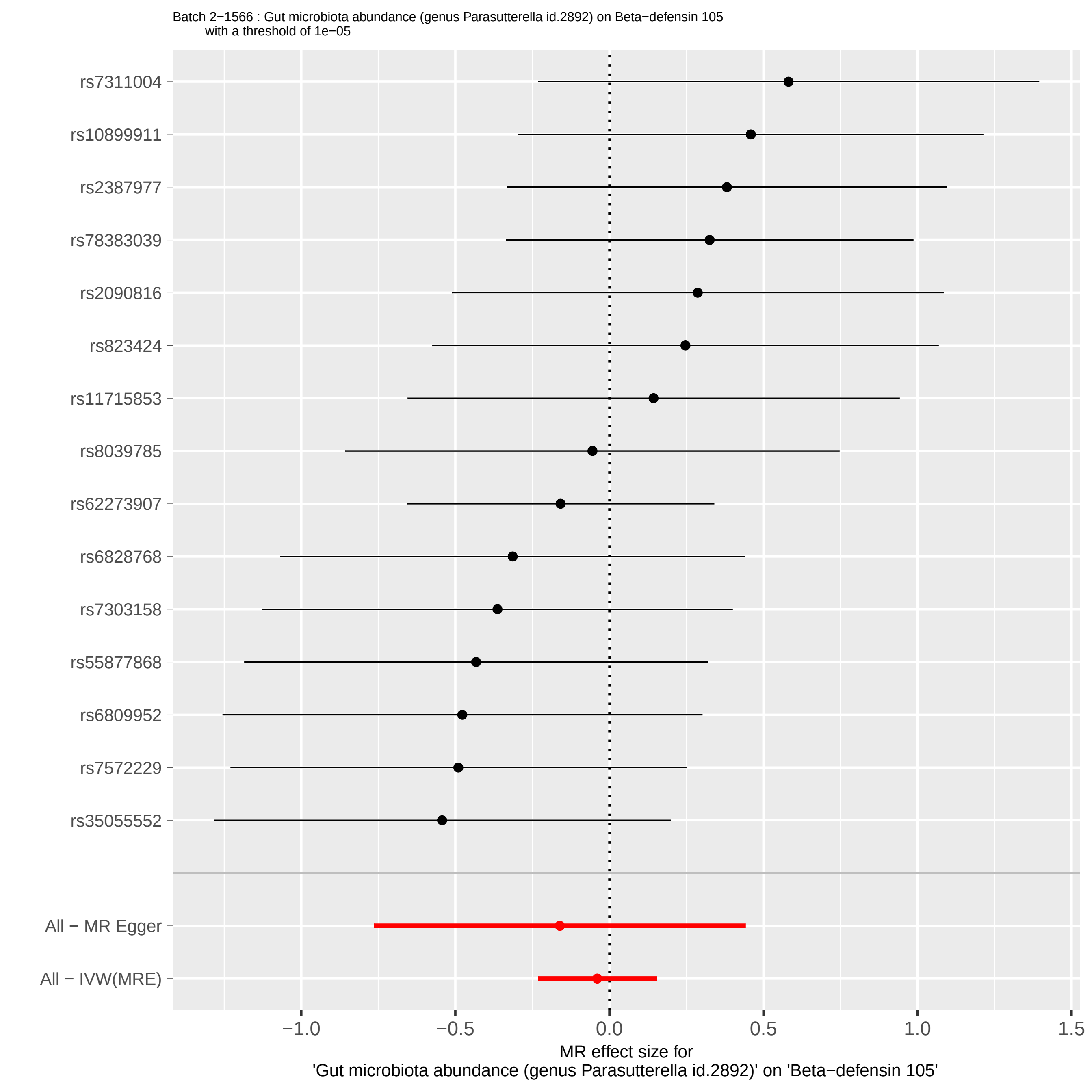

Batch 2−1566 : Gut microbiota abundance (genus Parasutterella id.2892) on Beta−defensin 105
with a threshold of 1e−05
rs7311004
rs10899911
rs2387977
rs78383039
rs2090816
rs823424
rs11715853
rs8039785
rs62273907
rs6828768
rs7303158
rs55877868
rs6809952
rs7572229
rs35055552
All − MR Egger
All − IVW(MRE)
−1.0
−0.5
0.0
0.5
1.0
1.5
MR effect size for
'Gut microbiota abundance (genus Parasutterella id.2892)' on 'Beta−defensin 105'

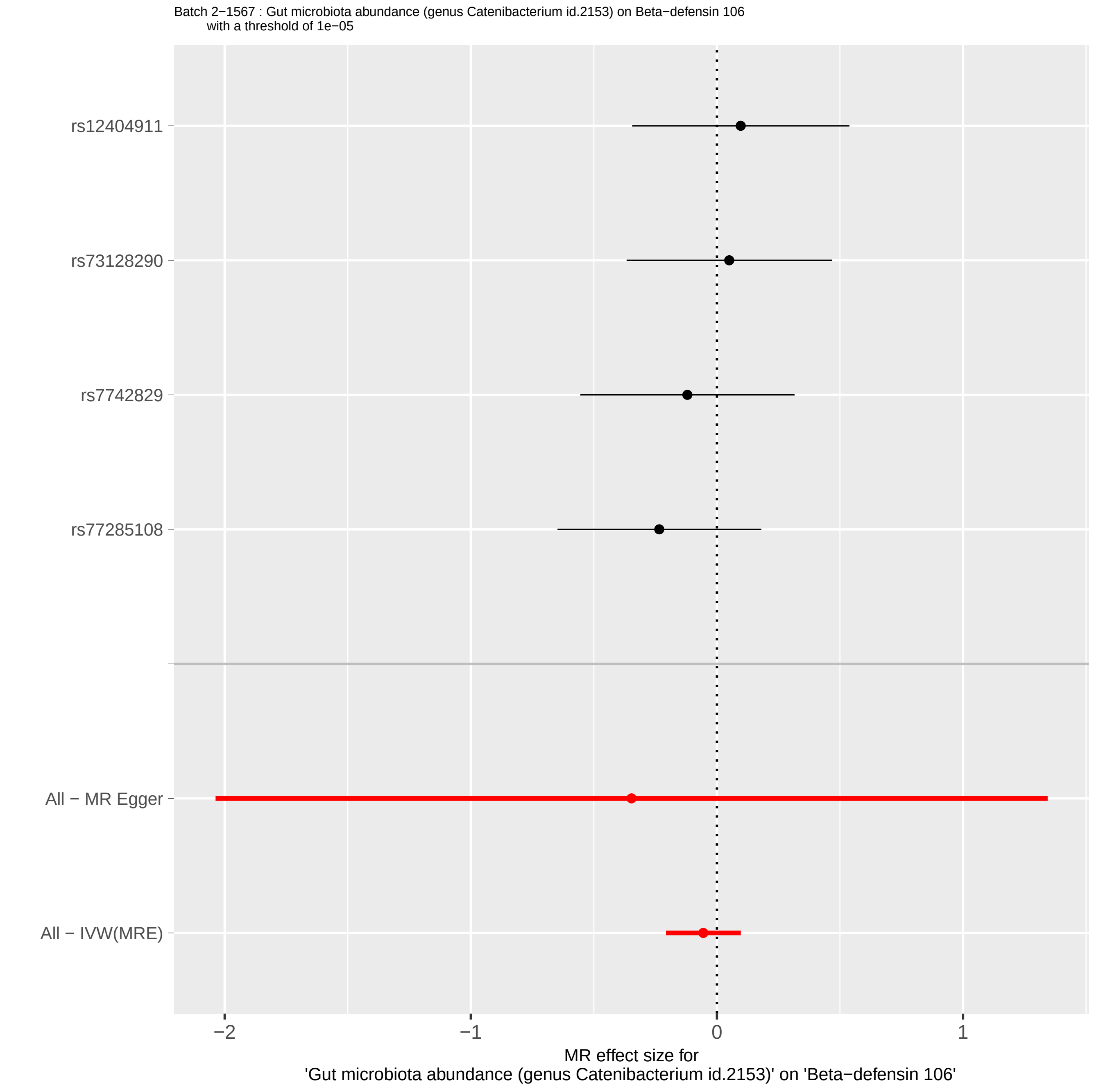

Batch 2−1567 : Gut microbiota abundance (genus Catenibacterium id.2153) on Beta−defensin 106
with a threshold of 1e−05
rs12404911
rs73128290
rs7742829
rs77285108
All − MR Egger
All − IVW(MRE)
−2
−1
0
1
MR effect size for
'Gut microbiota abundance (genus Catenibacterium id.2153)' on 'Beta−defensin 106'

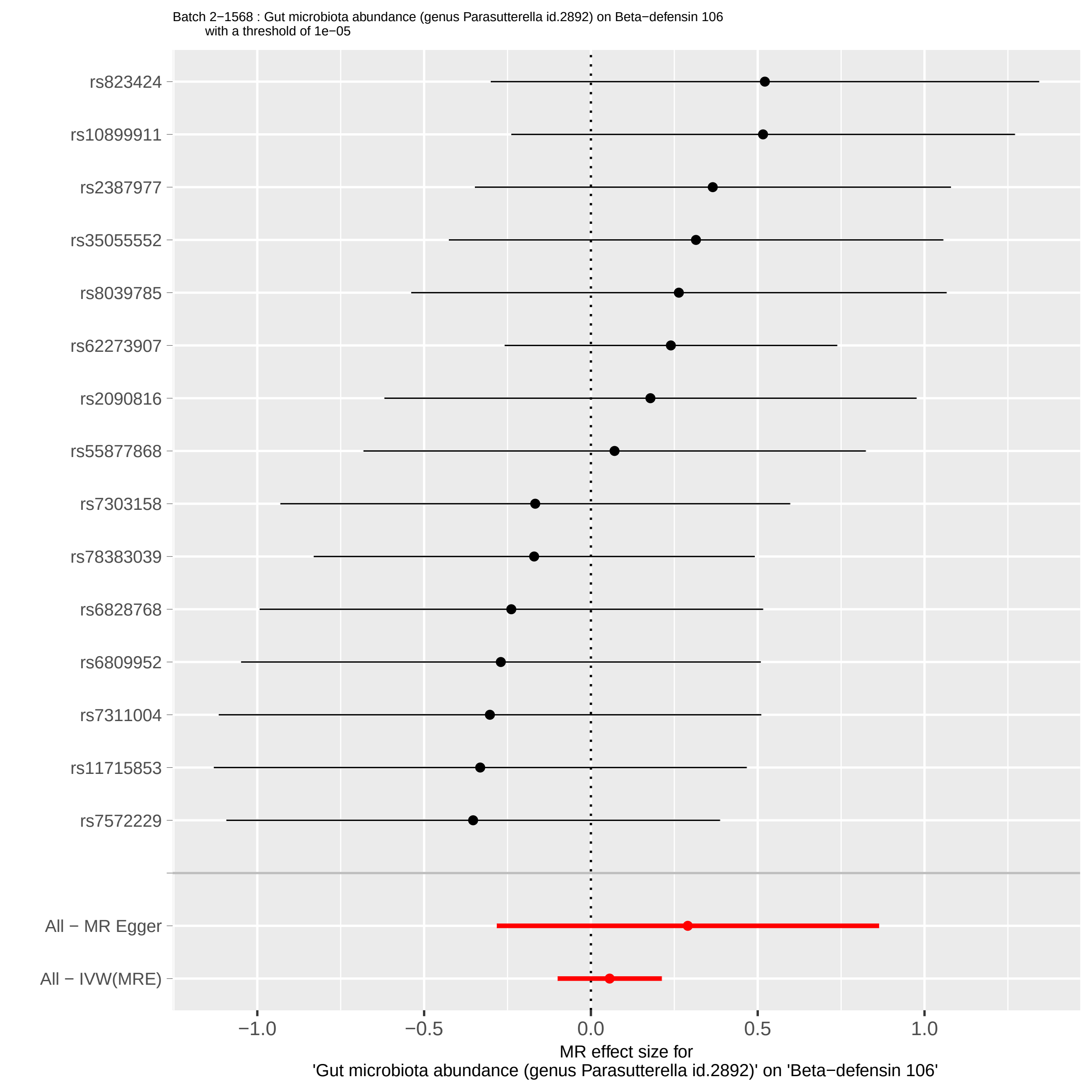

Batch 2−1568 : Gut microbiota abundance (genus Parasutterella id.2892) on Beta−defensin 106
with a threshold of 1e−05
rs823424
rs10899911
rs2387977
rs35055552
rs8039785
rs62273907
rs2090816
rs55877868
rs7303158
rs78383039
rs6828768
rs6809952
rs7311004
rs11715853
rs7572229
All − MR Egger
All − IVW(MRE)
−1.0
−0.5
0.0
0.5
1.0
MR effect size for
'Gut microbiota abundance (genus Parasutterella id.2892)' on 'Beta−defensin 106'

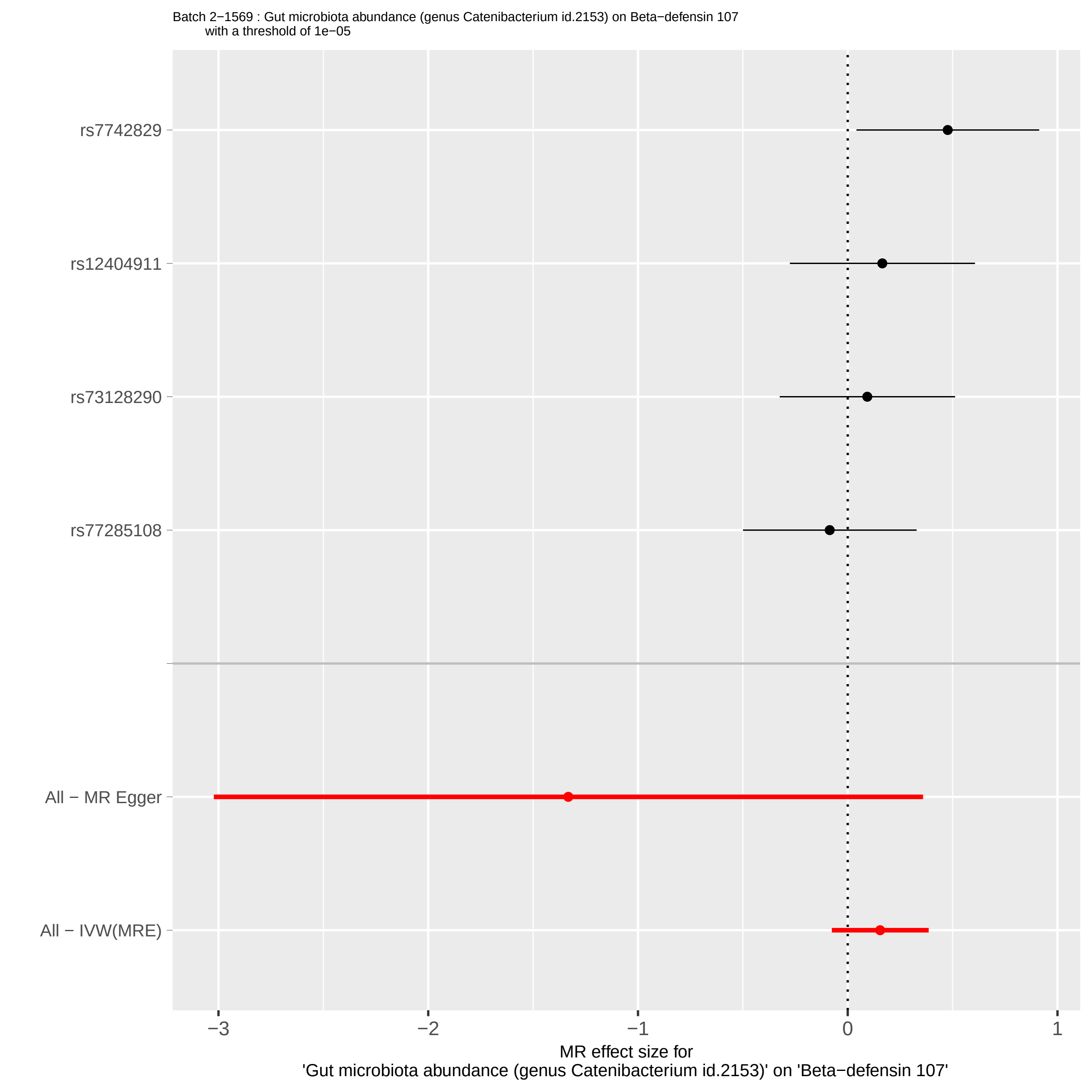

Batch 2−1569 : Gut microbiota abundance (genus Catenibacterium id.2153) on Beta−defensin 107
with a threshold of 1e−05
rs7742829
rs12404911
rs73128290
rs77285108
All − MR Egger
All − IVW(MRE)
−3
−2
−1
0
1
MR effect size for
'Gut microbiota abundance (genus Catenibacterium id.2153)' on 'Beta−defensin 107'

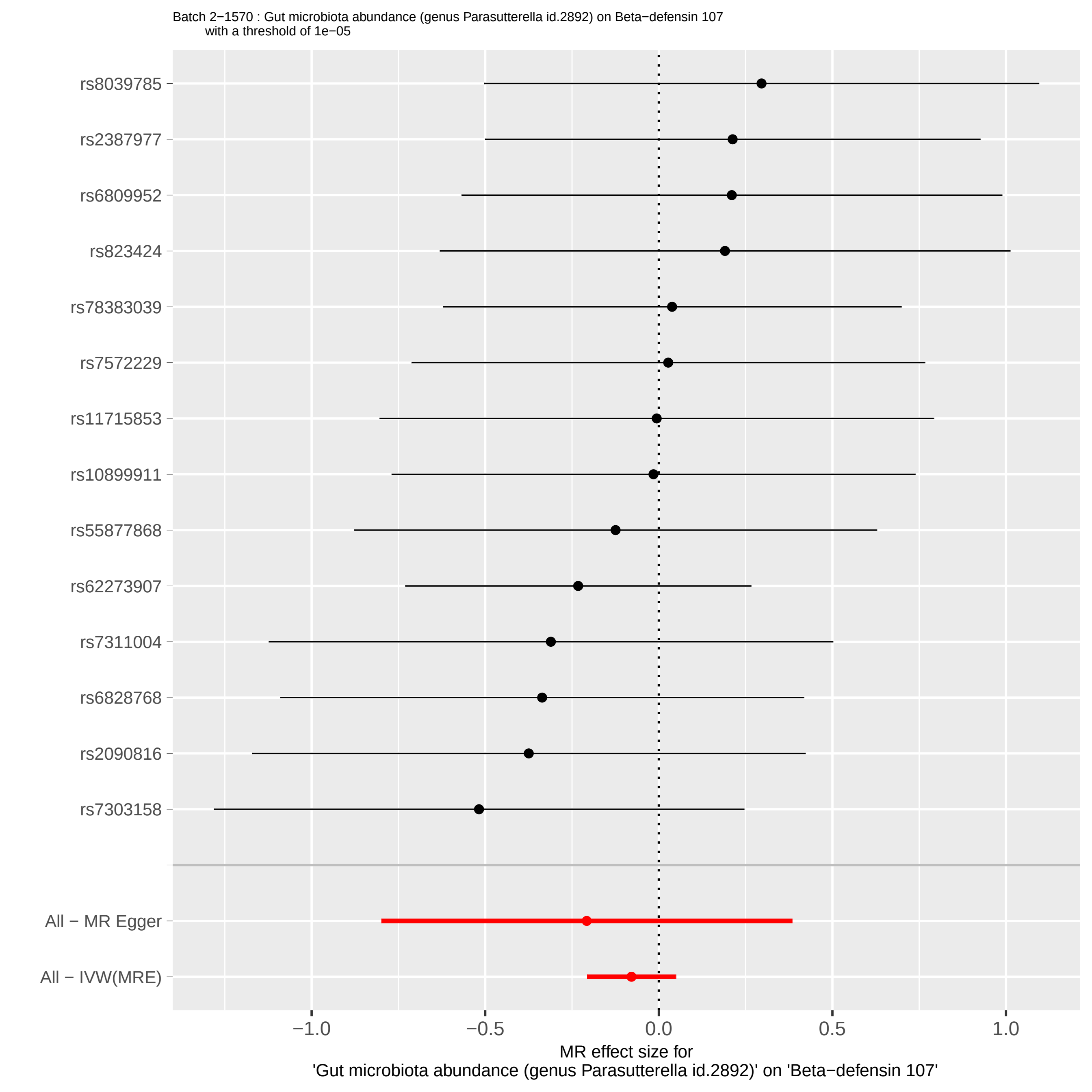

Batch 2−1570 : Gut microbiota abundance (genus Parasutterella id.2892) on Beta−defensin 107
with a threshold of 1e−05
rs8039785
rs2387977
rs6809952
rs823424
rs78383039
rs7572229
rs11715853
rs10899911
rs55877868
rs62273907
rs7311004
rs6828768
rs2090816
rs7303158
All − MR Egger
All − IVW(MRE)
−1.0
−0.5
0.0
0.5
1.0
MR effect size for
'Gut microbiota abundance (genus Parasutterella id.2892)' on 'Beta−defensin 107'

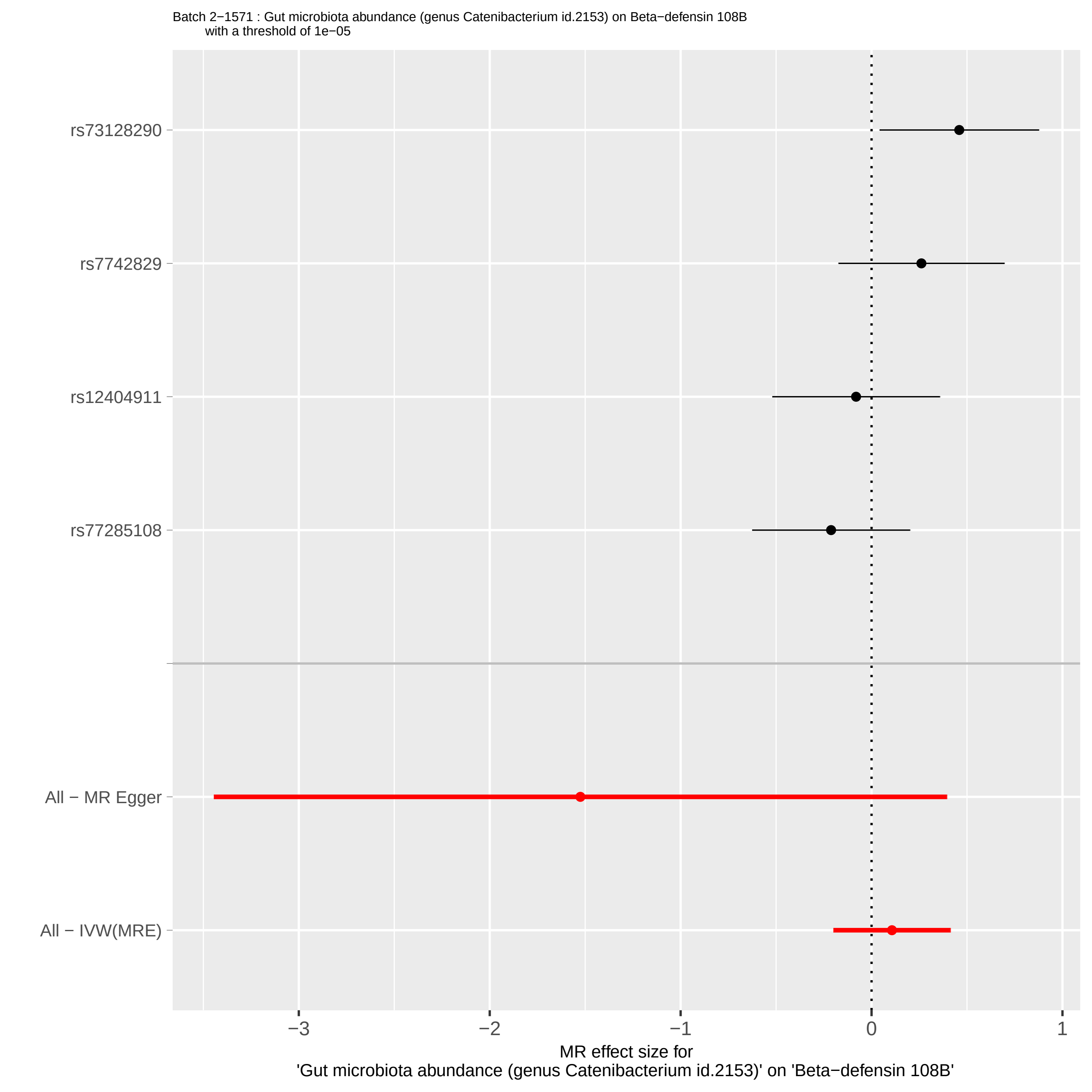

Batch 2−1571 : Gut microbiota abundance (genus Catenibacterium id.2153) on Beta−defensin 108B
with a threshold of 1e−05
rs73128290
rs7742829
rs12404911
rs77285108
All − MR Egger
All − IVW(MRE)
−3
−2
−1
0
1
MR effect size for
'Gut microbiota abundance (genus Catenibacterium id.2153)' on 'Beta−defensin 108B'

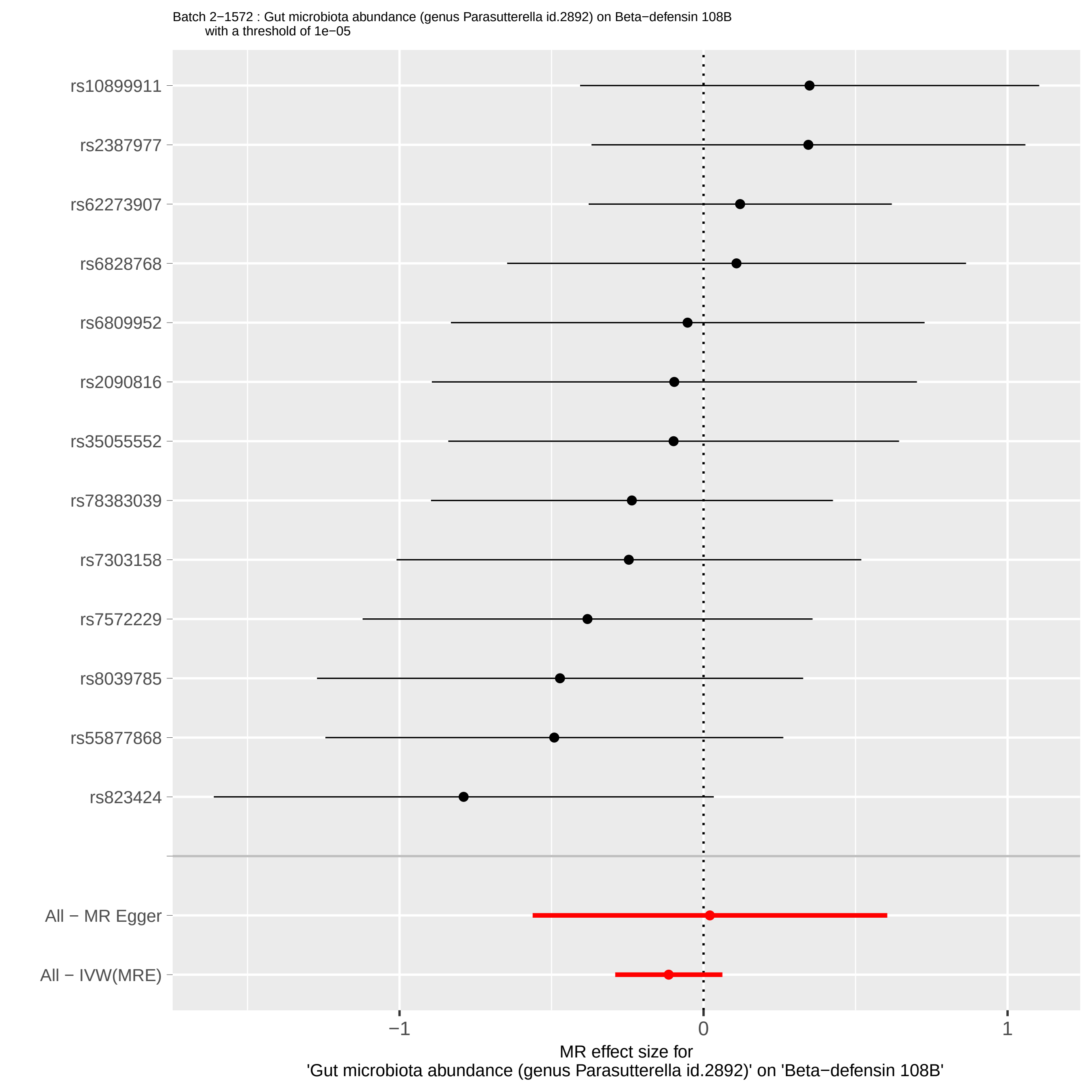

Batch 2−1572 : Gut microbiota abundance (genus Parasutterella id.2892) on Beta−defensin 108B
with a threshold of 1e−05
rs10899911
rs2387977
rs62273907
rs6828768
rs6809952
rs2090816
rs35055552
rs78383039
rs7303158
rs7572229
rs8039785
rs55877868
rs823424
All − MR Egger
All − IVW(MRE)
−1
0
1
MR effect size for
'Gut microbiota abundance (genus Parasutterella id.2892)' on 'Beta−defensin 108B'

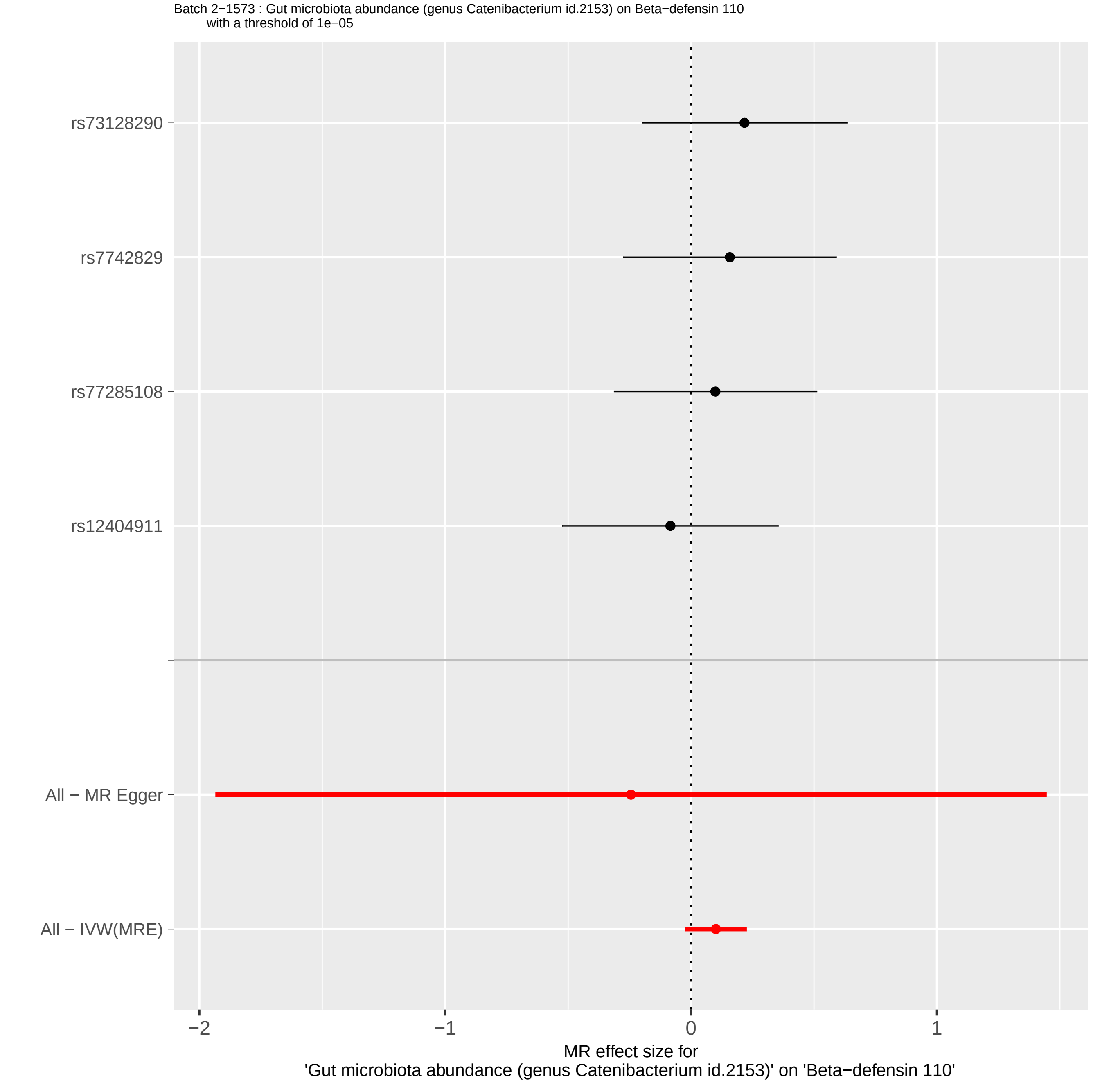

Batch 2−1573 : Gut microbiota abundance (genus Catenibacterium id.2153) on Beta−defensin 110
with a threshold of 1e−05
rs73128290
rs7742829
rs77285108
rs12404911
All − MR Egger
All − IVW(MRE)
−2
−1
0
1
MR effect size for
'Gut microbiota abundance (genus Catenibacterium id.2153)' on 'Beta−defensin 110'

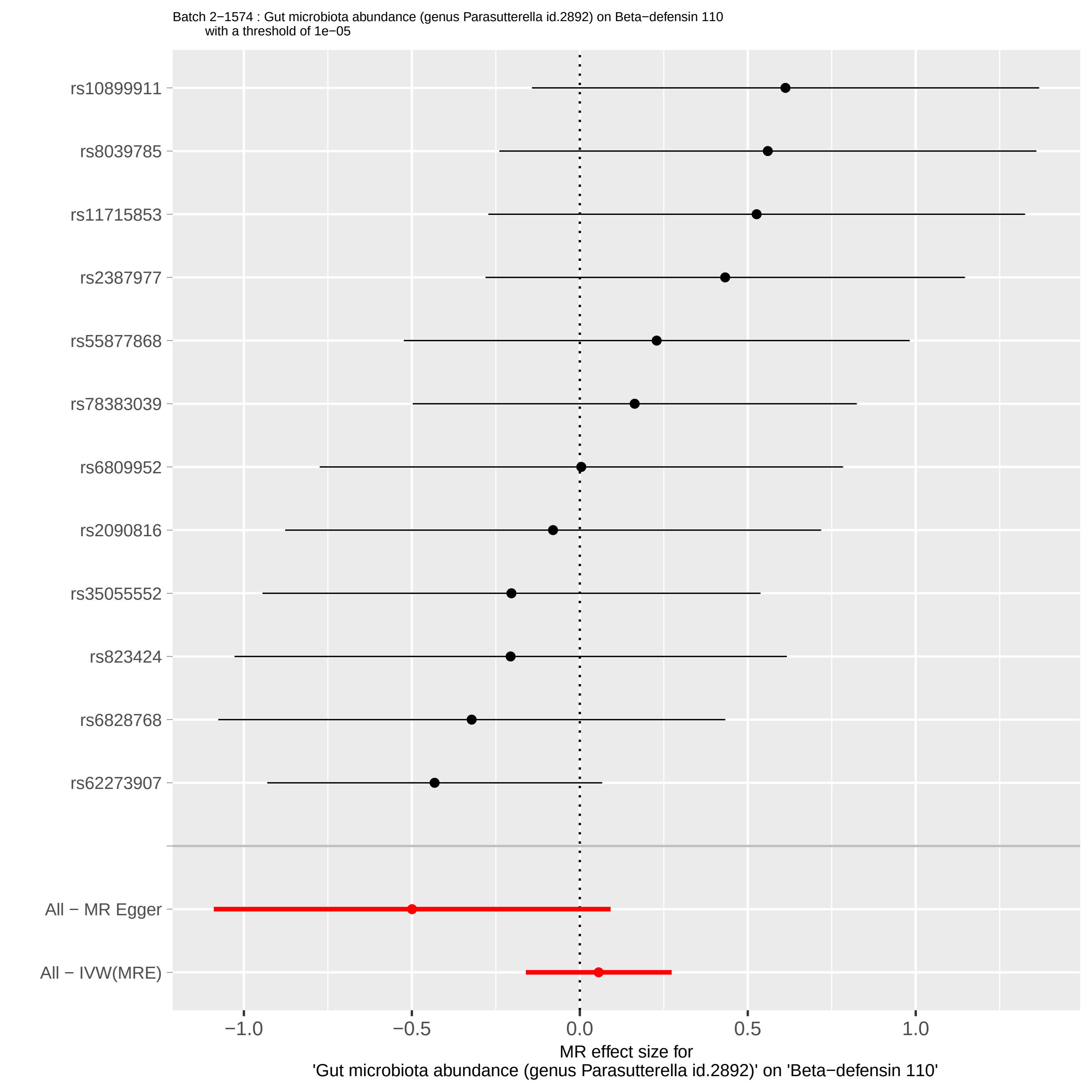

Batch 2−1574 : Gut microbiota abundance (genus Parasutterella id.2892) on Beta−defensin 110
with a threshold of 1e−05
rs10899911
rs8039785
rs11715853
rs2387977
rs55877868
rs78383039
rs6809952
rs2090816
rs35055552
rs823424
rs6828768
rs62273907
All − MR Egger
All − IVW(MRE)
−1.0
−0.5
0.0
0.5
1.0
MR effect size for
'Gut microbiota abundance (genus Parasutterella id.2892)' on 'Beta−defensin 110'

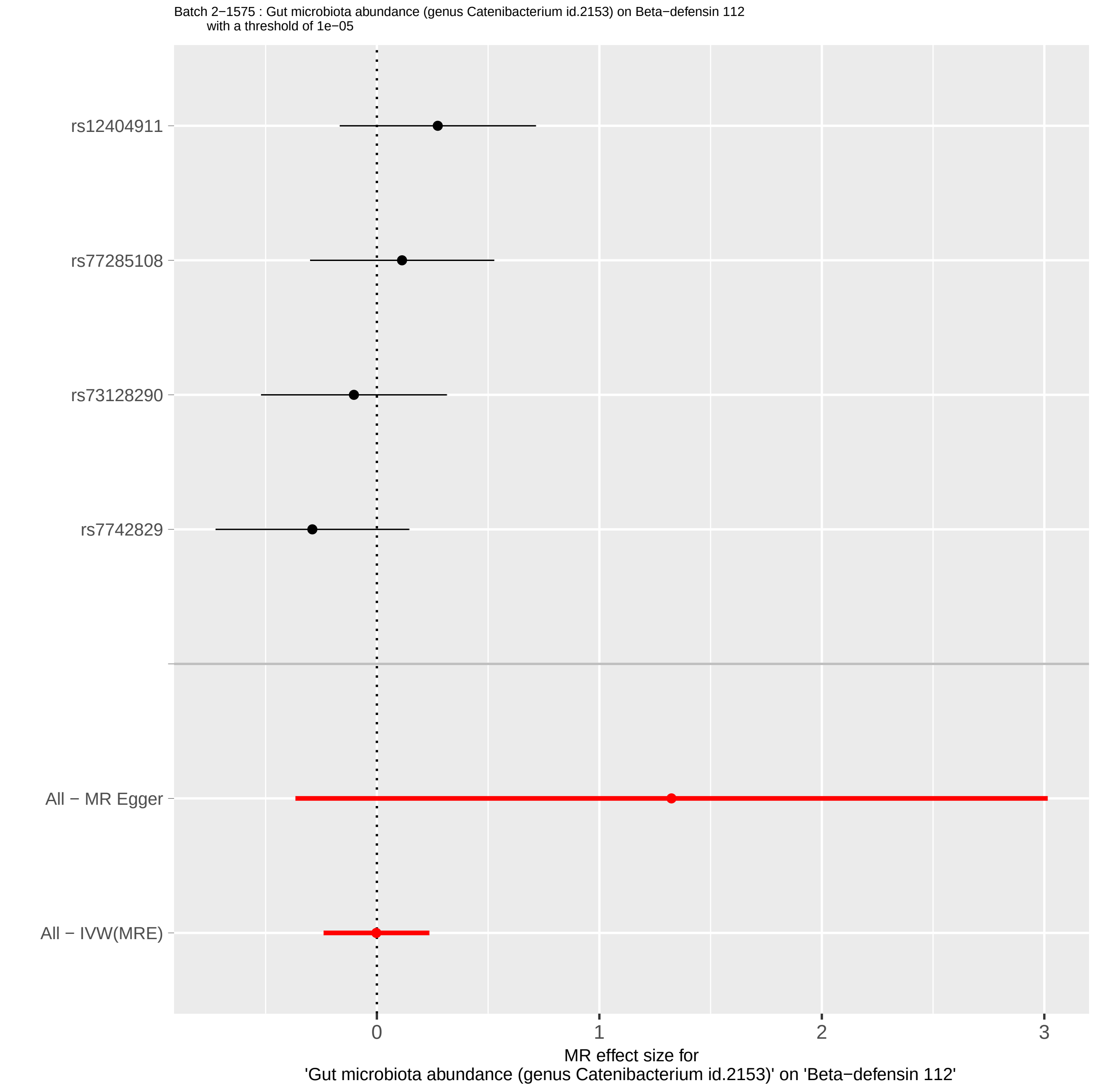
Batch 2−1575 : Gut microbiota abundance (genus Catenibacterium id.2153) on Beta−defensin 112
with a threshold of 1e−05
rs12404911
rs77285108
rs73128290
rs7742829
All − MR Egger
All − IVW(MRE)
0
1
2
3
MR effect size for
'Gut microbiota abundance (genus Catenibacterium id.2153)' on 'Beta−defensin 112'

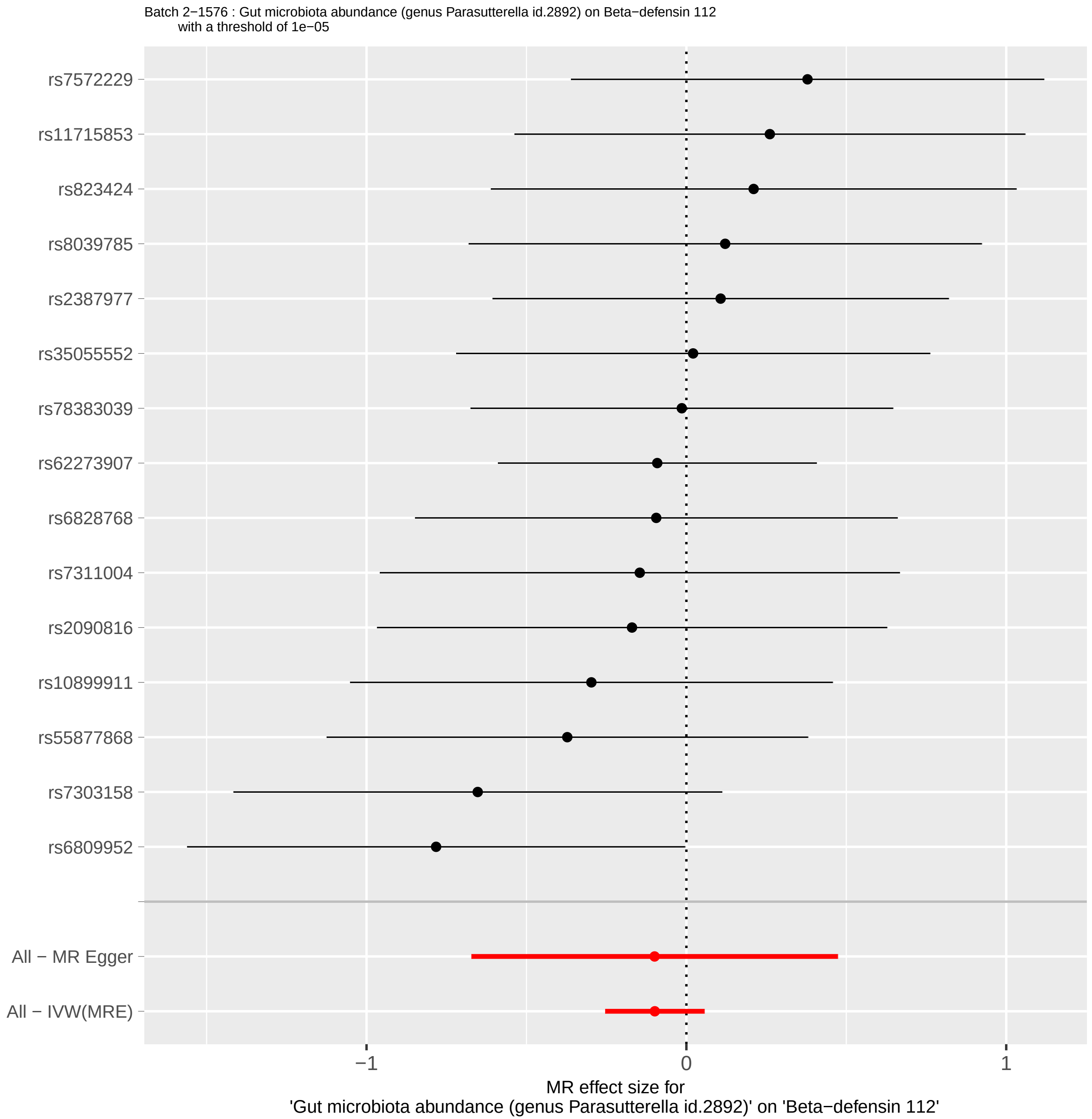
Batch 2−1576 : Gut microbiota abundance (genus Parasutterella id.2892) on Beta−defensin 112
with a threshold of 1e−05
rs7572229
rs11715853
rs823424
rs8039785
rs2387977
rs35055552
rs78383039
rs62273907
rs6828768
rs7311004
rs2090816
rs10899911
rs55877868
rs7303158
rs6809952
All − MR Egger
All − IVW(MRE)
−1
0
1
MR effect size for
'Gut microbiota abundance (genus Parasutterella id.2892)' on 'Beta−defensin 112'

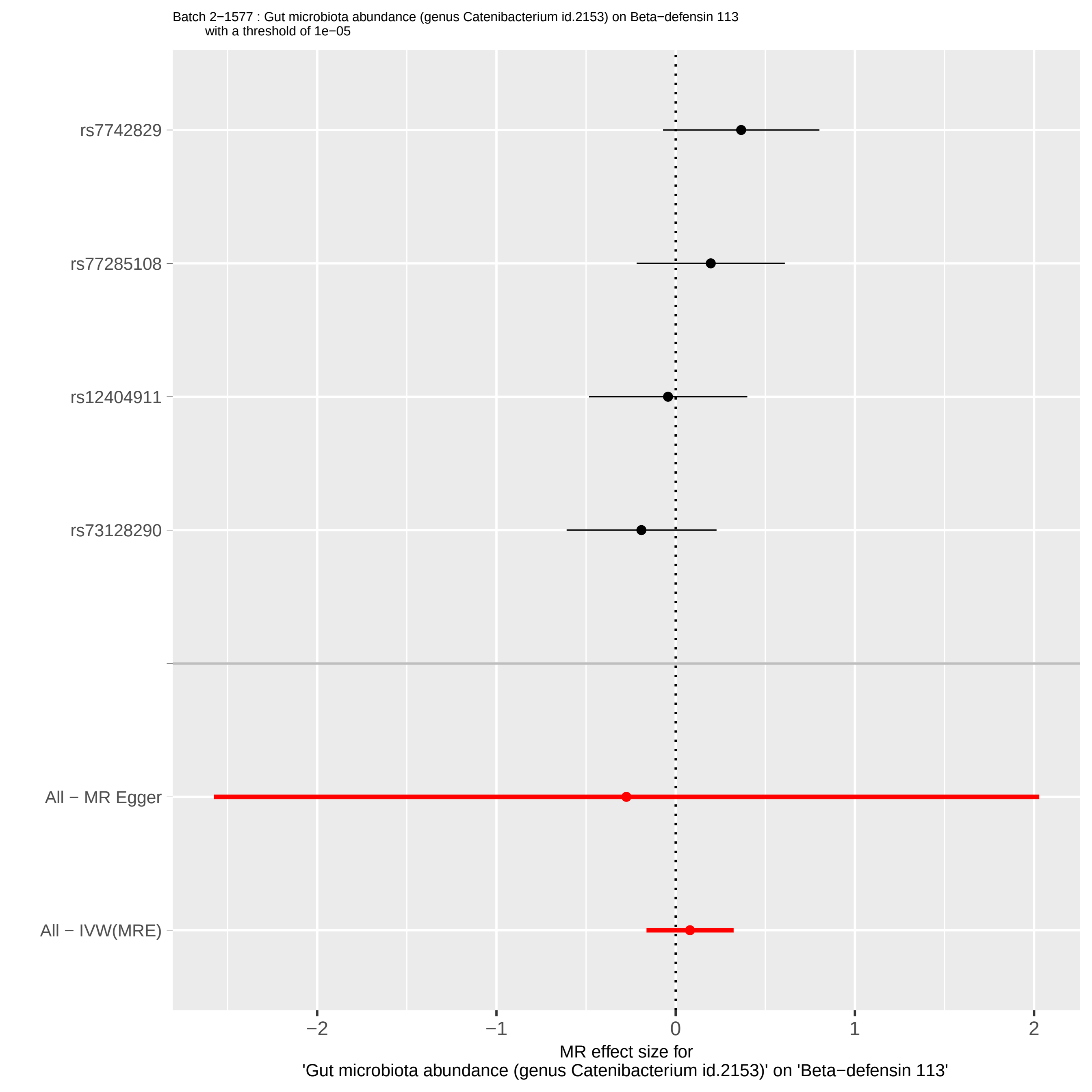
Batch 2−1577 : Gut microbiota abundance (genus Catenibacterium id.2153) on Beta−defensin 113
with a threshold of 1e−05
rs7742829
rs77285108
rs12404911
rs73128290
All − MR Egger
All − IVW(MRE)
−2
−1
0
1
2
MR effect size for
'Gut microbiota abundance (genus Catenibacterium id.2153)' on 'Beta−defensin 113'

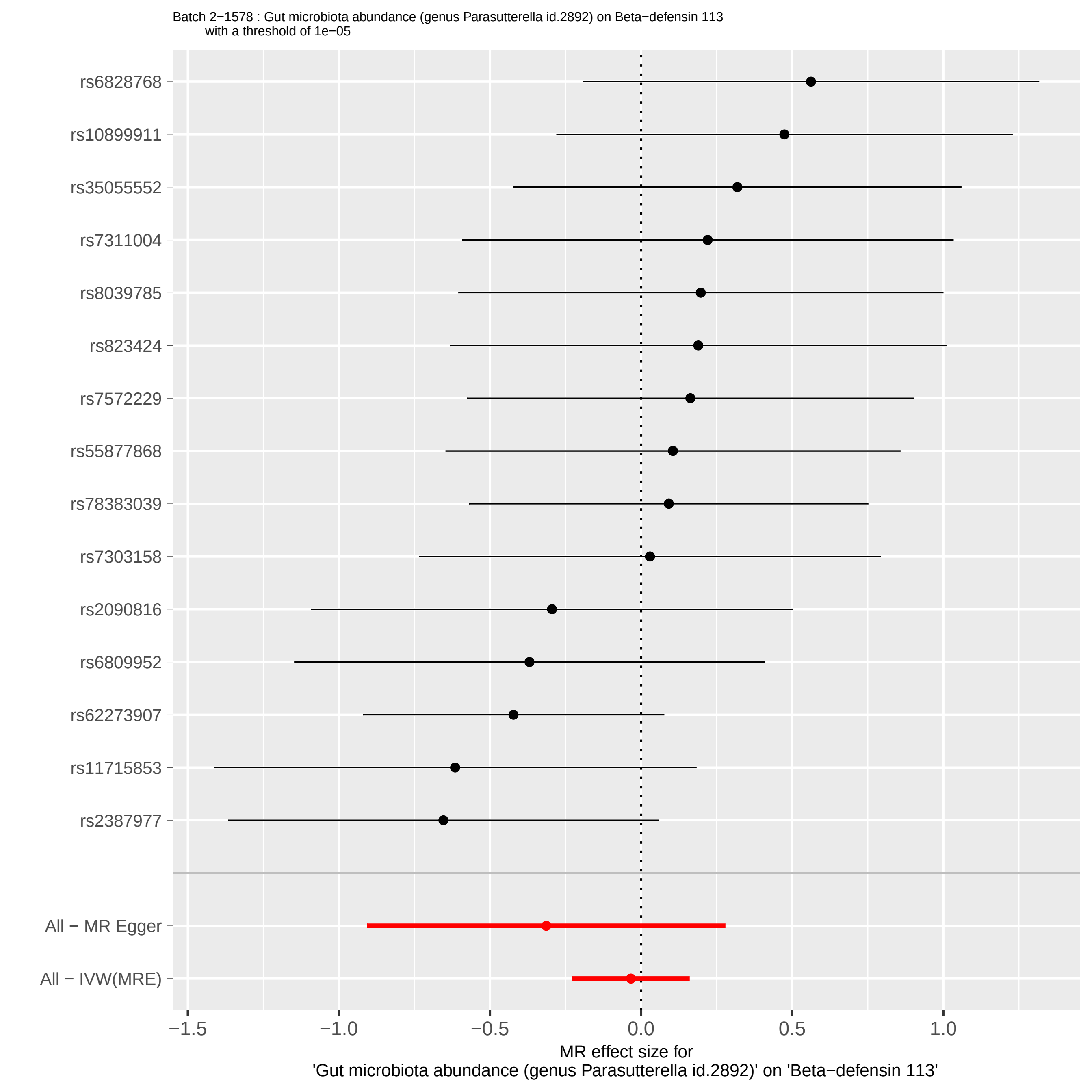

Batch 2−1578 : Gut microbiota abundance (genus Parasutterella id.2892) on Beta−defensin 113
with a threshold of 1e−05
rs6828768
rs10899911
rs35055552
rs7311004
rs8039785
rs823424
rs7572229
rs55877868
rs78383039
rs7303158
rs2090816
rs6809952
rs62273907
rs11715853
rs2387977
All − MR Egger
All − IVW(MRE)
−1.5
−1.0
−0.5
0.0
0.5
1.0
MR effect size for
'Gut microbiota abundance (genus Parasutterella id.2892)' on 'Beta−defensin 113'

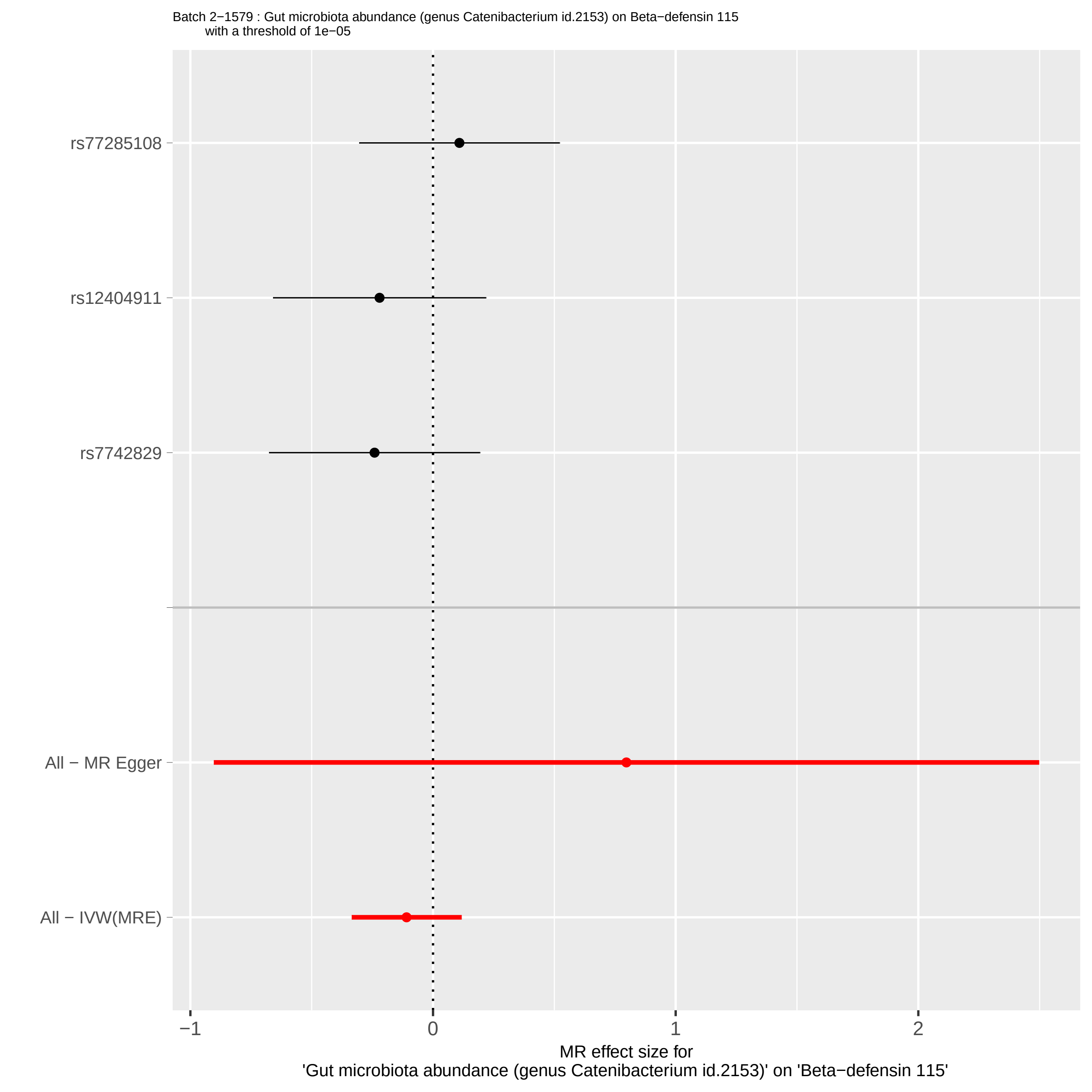
Batch 2−1579 : Gut microbiota abundance (genus Catenibacterium id.2153) on Beta−defensin 115
with a threshold of 1e−05
rs77285108
rs12404911
rs7742829
All − MR Egger
All − IVW(MRE)
−1
0
1
2
MR effect size for
'Gut microbiota abundance (genus Catenibacterium id.2153)' on 'Beta−defensin 115'

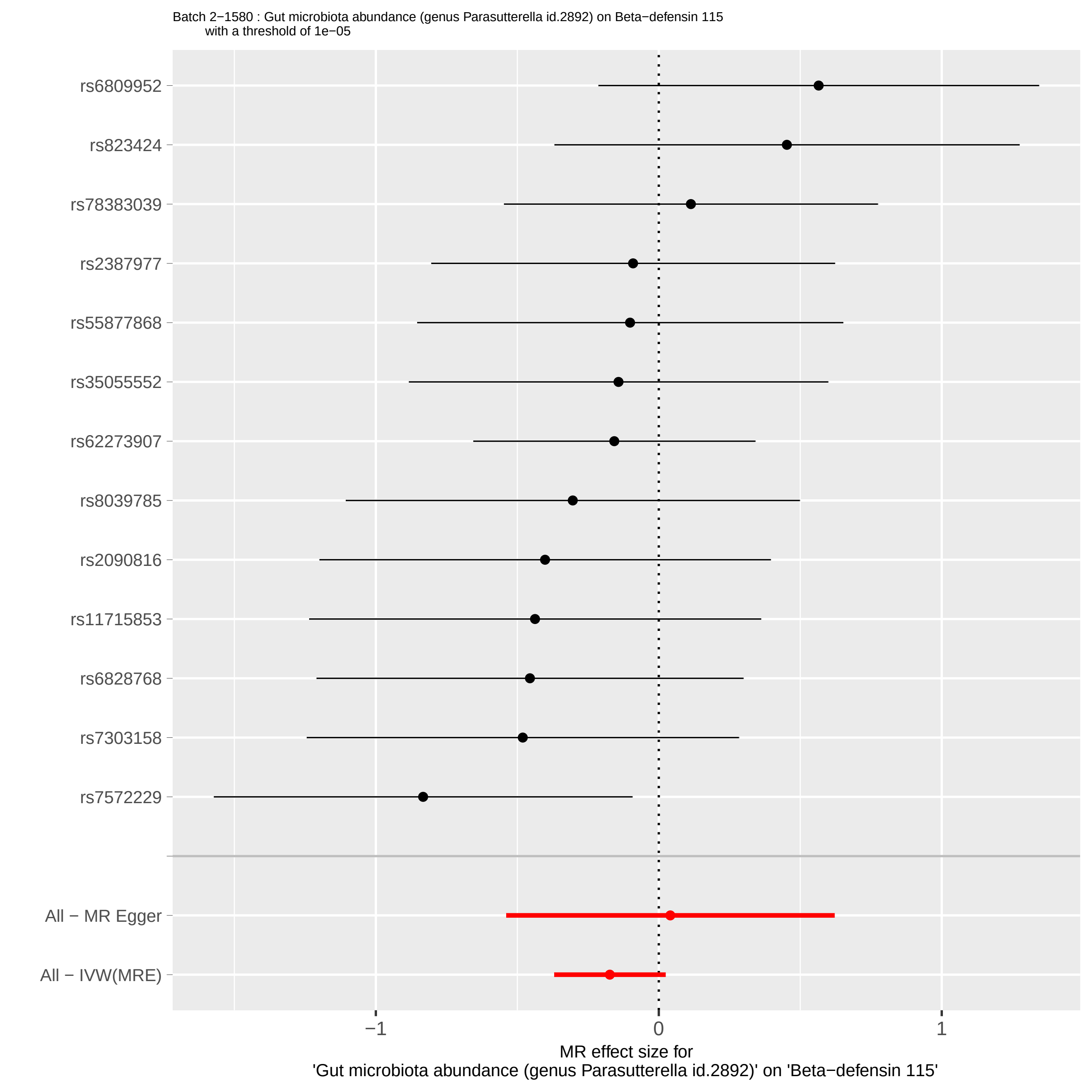
Batch 2−1580 : Gut microbiota abundance (genus Parasutterella id.2892) on Beta−defensin 115
with a threshold of 1e−05
rs6809952
rs823424
rs78383039
rs2387977
rs55877868
rs35055552
rs62273907
rs8039785
rs2090816
rs11715853
rs6828768
rs7303158
rs7572229
All − MR Egger
All − IVW(MRE)
−1
0
1
MR effect size for
'Gut microbiota abundance (genus Parasutterella id.2892)' on 'Beta−defensin 115'

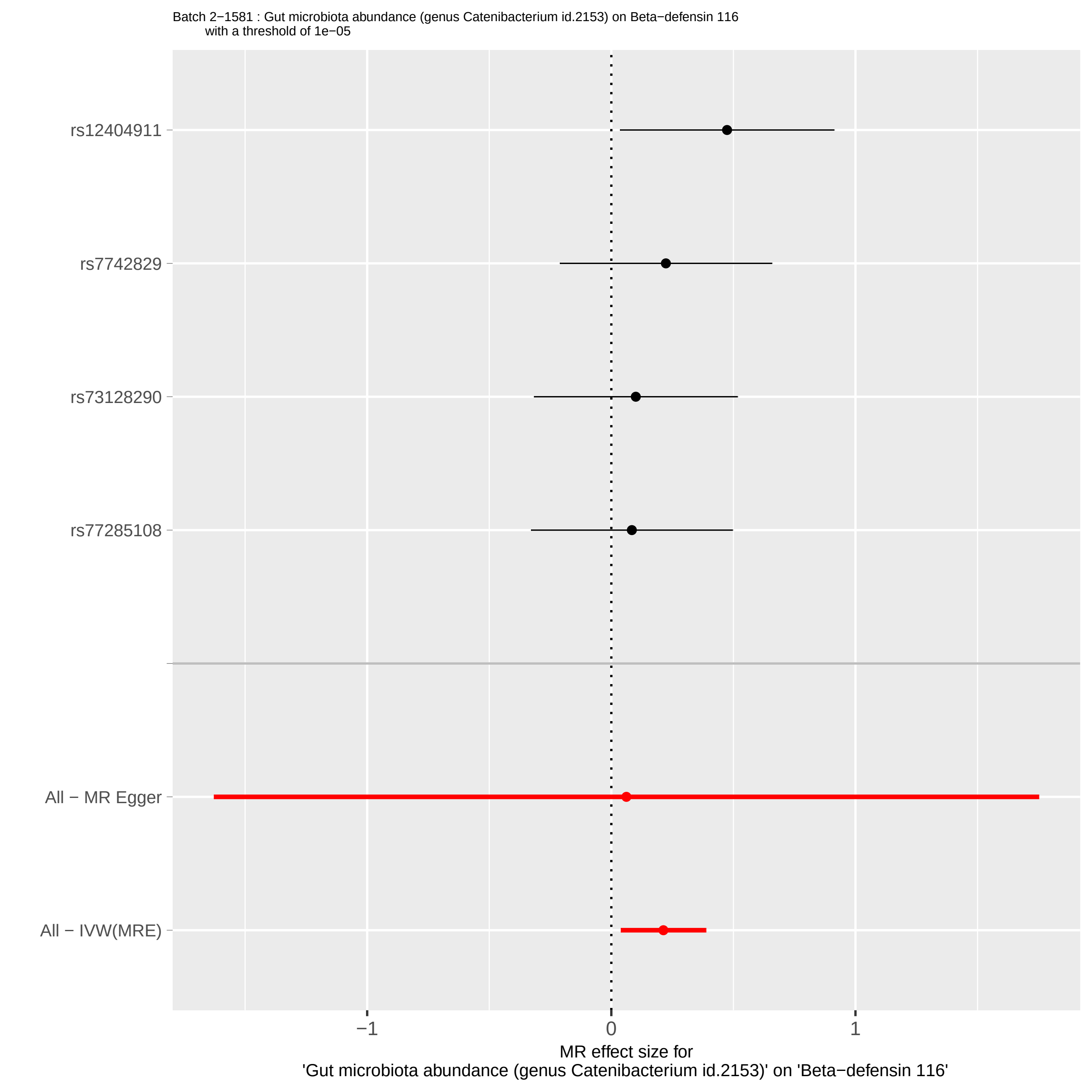

Batch 2−1581 : Gut microbiota abundance (genus Catenibacterium id.2153) on Beta−defensin 116
with a threshold of 1e−05
rs12404911
rs7742829
rs73128290
rs77285108
All − MR Egger
All − IVW(MRE)
−1
0
1
MR effect size for
'Gut microbiota abundance (genus Catenibacterium id.2153)' on 'Beta−defensin 116'

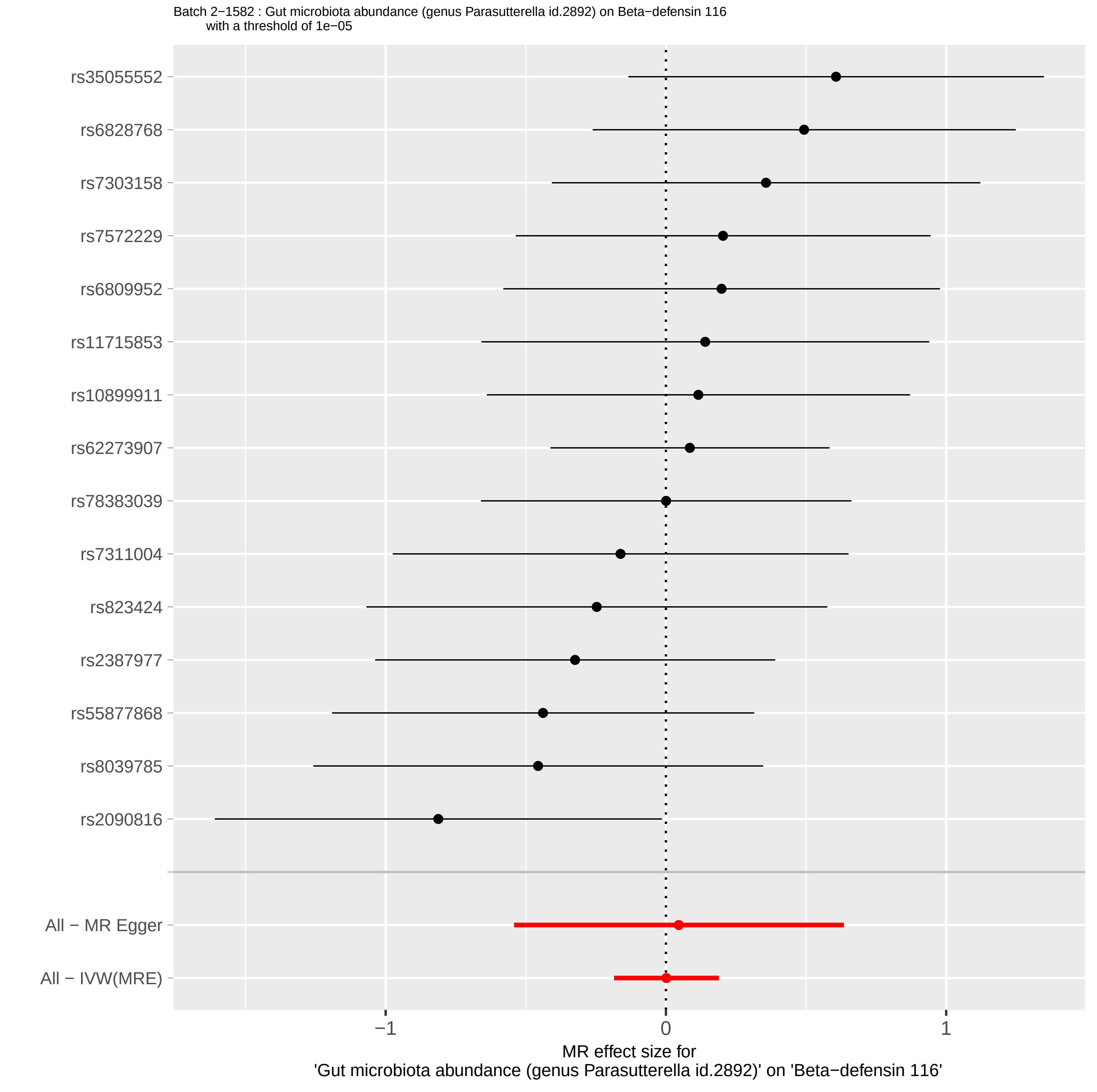

Batch 2−1582 : Gut microbiota abundance (genus Parasutterella id.2892) on Beta−defensin 116
with a threshold of 1e−05
rs35055552
rs6828768
rs7303158
rs7572229
rs6809952
rs11715853
rs10899911
rs62273907
rs78383039
rs7311004
rs823424
rs2387977
rs55877868
rs8039785
rs2090816
All − MR Egger
All − IVW(MRE)
−1
0
1
MR effect size for
'Gut microbiota abundance (genus Parasutterella id.2892)' on 'Beta−defensin 116'

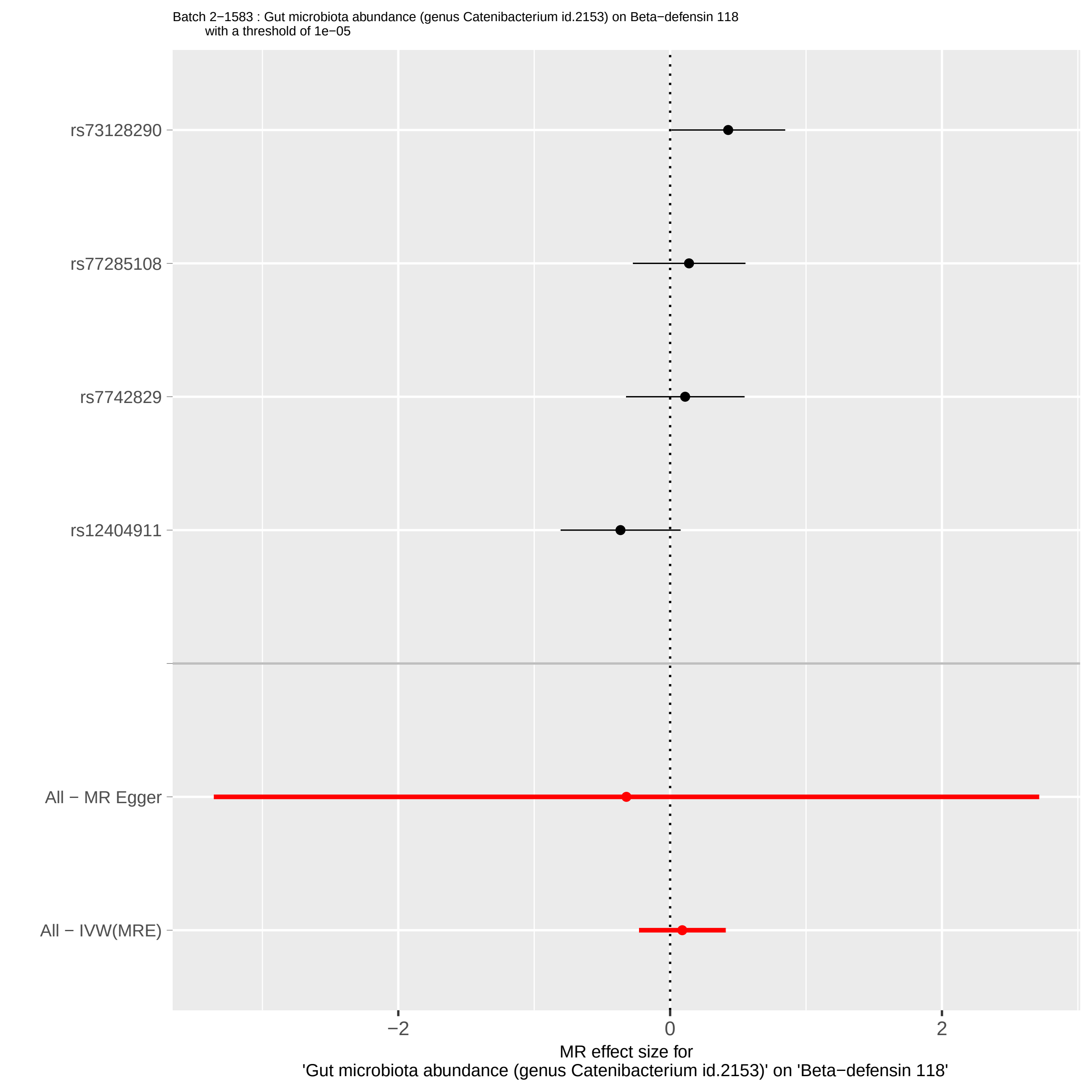

Batch 2−1583 : Gut microbiota abundance (genus Catenibacterium id.2153) on Beta−defensin 118
with a threshold of 1e−05
rs73128290
rs77285108
rs7742829
rs12404911
All − MR Egger
All − IVW(MRE)
−2
0
2
MR effect size for
'Gut microbiota abundance (genus Catenibacterium id.2153)' on 'Beta−defensin 118'

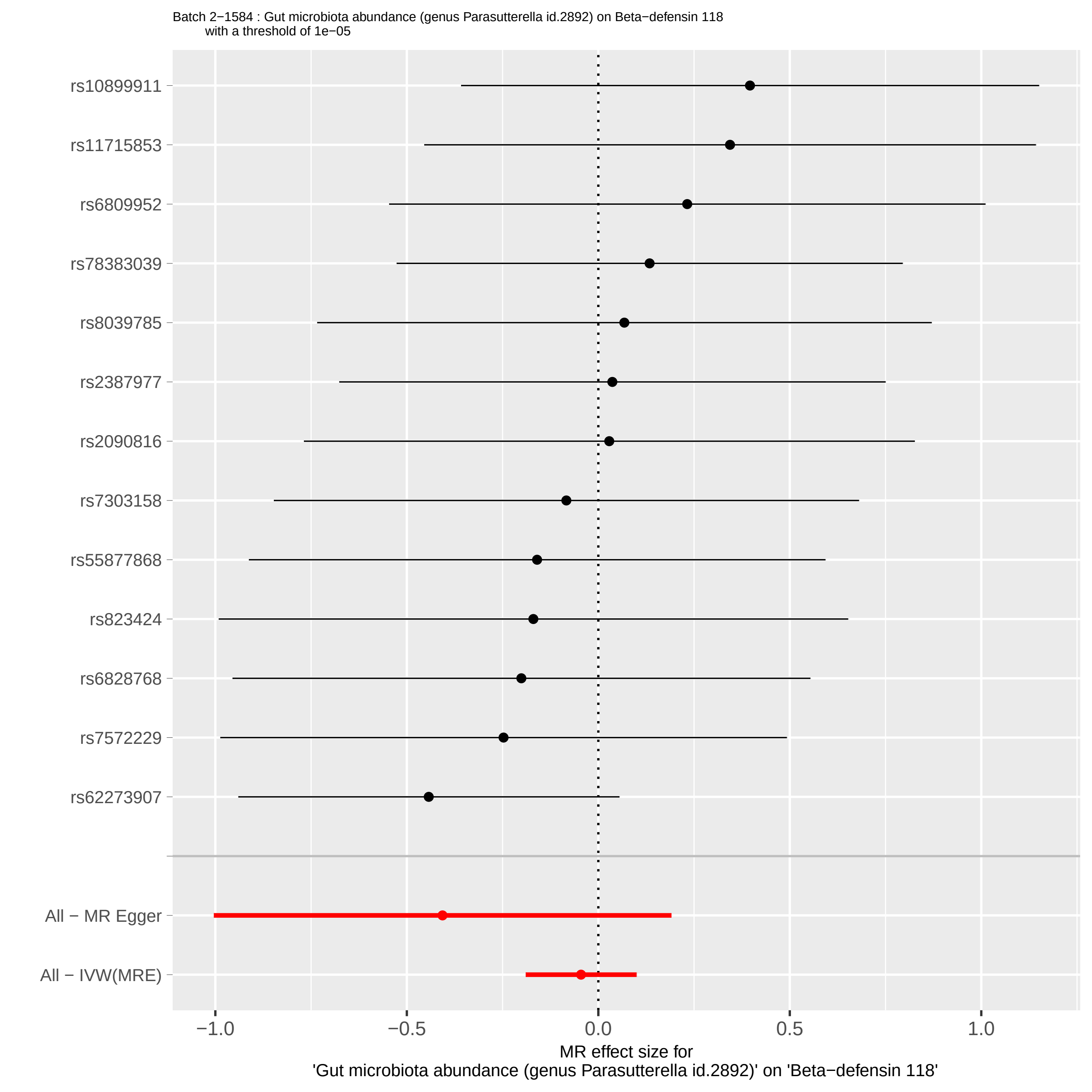

Batch 2−1584 : Gut microbiota abundance (genus Parasutterella id.2892) on Beta−defensin 118
with a threshold of 1e−05
rs10899911
rs11715853
rs6809952
rs78383039
rs8039785
rs2387977
rs2090816
rs7303158
rs55877868
rs823424
rs6828768
rs7572229
rs62273907
All − MR Egger
All − IVW(MRE)
−1.0
−0.5
0.0
0.5
1.0
MR effect size for
'Gut microbiota abundance (genus Parasutterella id.2892)' on 'Beta−defensin 118'

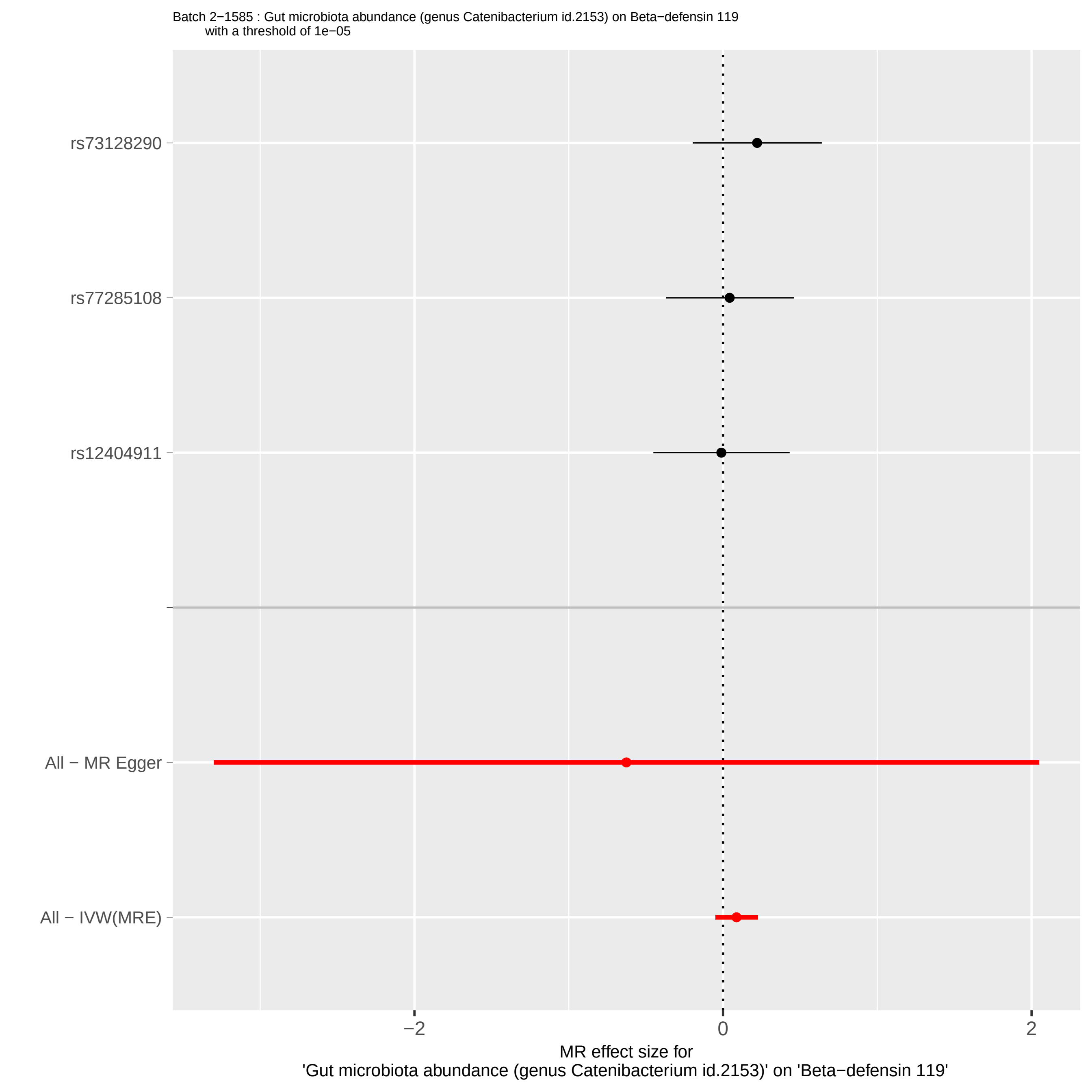

Batch 2−1585 : Gut microbiota abundance (genus Catenibacterium id.2153) on Beta−defensin 119
with a threshold of 1e−05
rs73128290
rs77285108
rs12404911
All − MR Egger
All − IVW(MRE)
−2
0
2
MR effect size for
'Gut microbiota abundance (genus Catenibacterium id.2153)' on 'Beta−defensin 119'

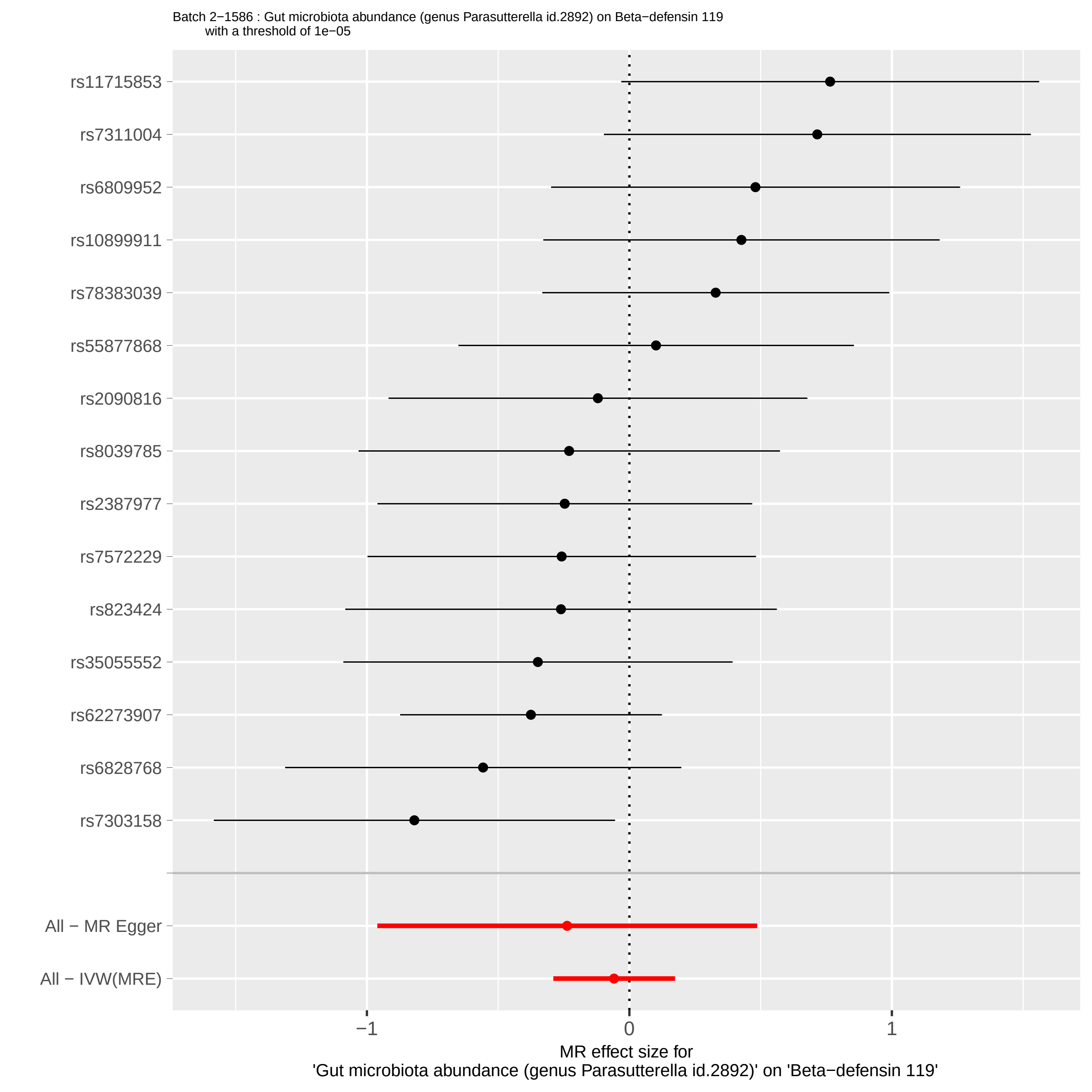

Batch 2−1586 : Gut microbiota abundance (genus Parasutterella id.2892) on Beta−defensin 119
with a threshold of 1e−05
rs11715853
rs7311004
rs6809952
rs10899911
rs78383039
rs55877868
rs2090816
rs8039785
rs2387977
rs7572229
rs823424
rs35055552
rs62273907
rs6828768
rs7303158
All − MR Egger
All − IVW(MRE)
−1
0
1
MR effect size for
'Gut microbiota abundance (genus Parasutterella id.2892)' on 'Beta−defensin 119'

Batch 2−1587 : Gut microbiota abundance (genus Catenibacterium id.2153) on Beta−defensin 119
with a threshold of 1e−05

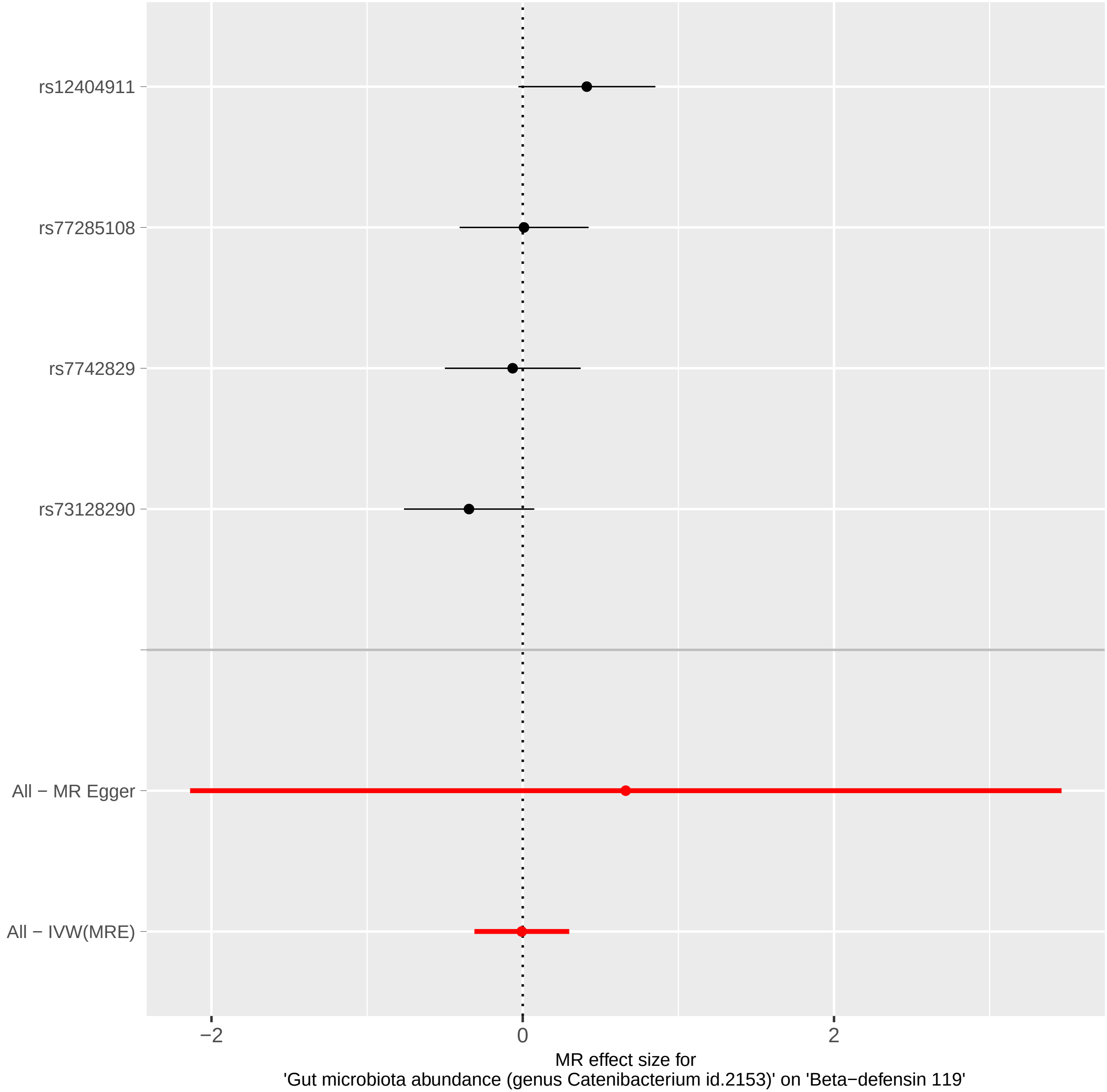

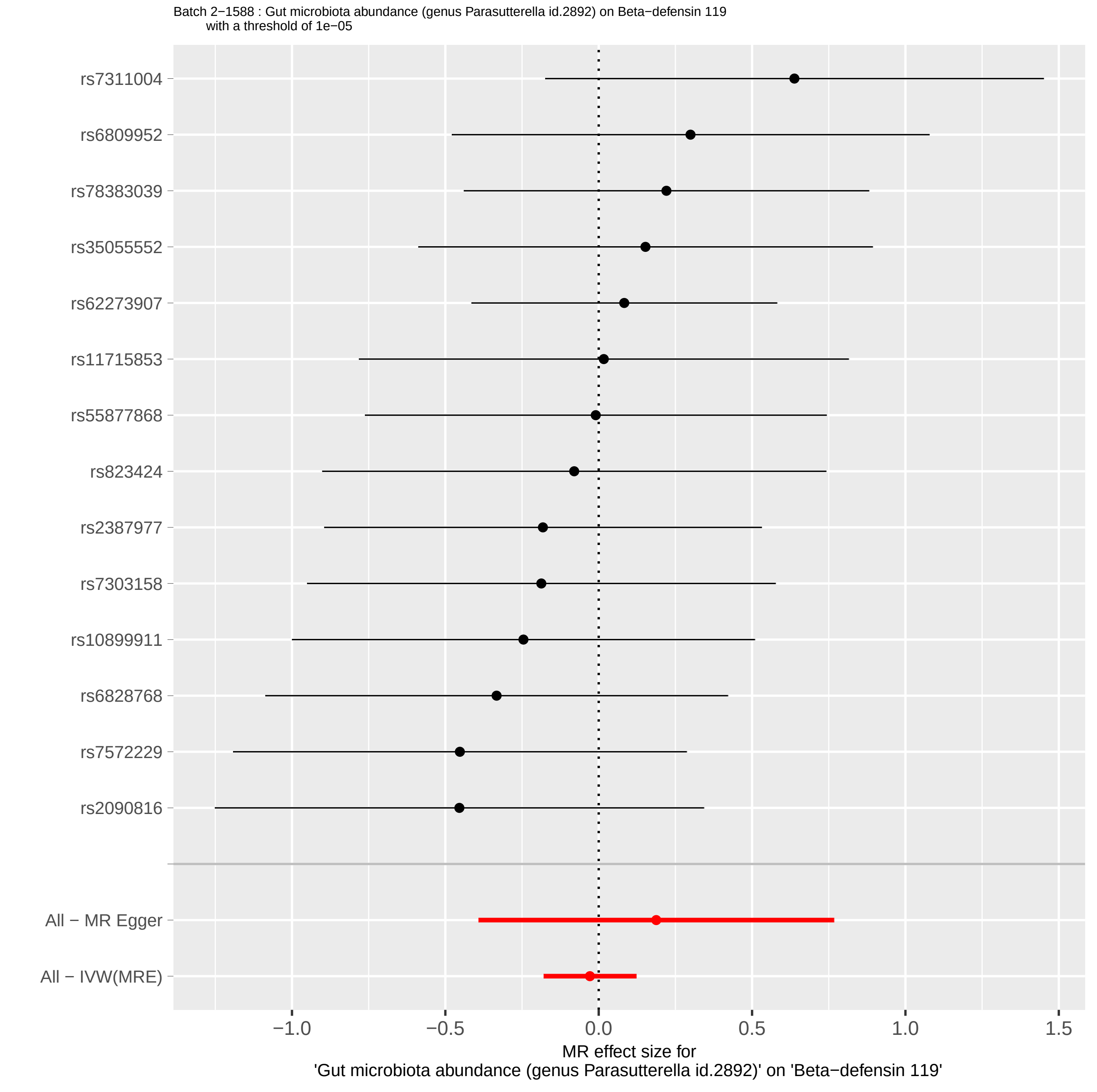

Batch 2−1588 : Gut microbiota abundance (genus Parasutterella id.2892) on Beta−defensin 119
with a threshold of 1e−05
rs7311004
rs6809952
rs78383039
rs35055552
rs62273907
rs11715853
rs55877868
rs823424
rs2387977
rs7303158
rs10899911
rs6828768
rs7572229
rs2090816
All − MR Egger
All − IVW(MRE)
−1.0
−0.5
0.0
0.5
1.0
1.5
MR effect size for
'Gut microbiota abundance (genus Parasutterella id.2892)' on 'Beta−defensin 119'

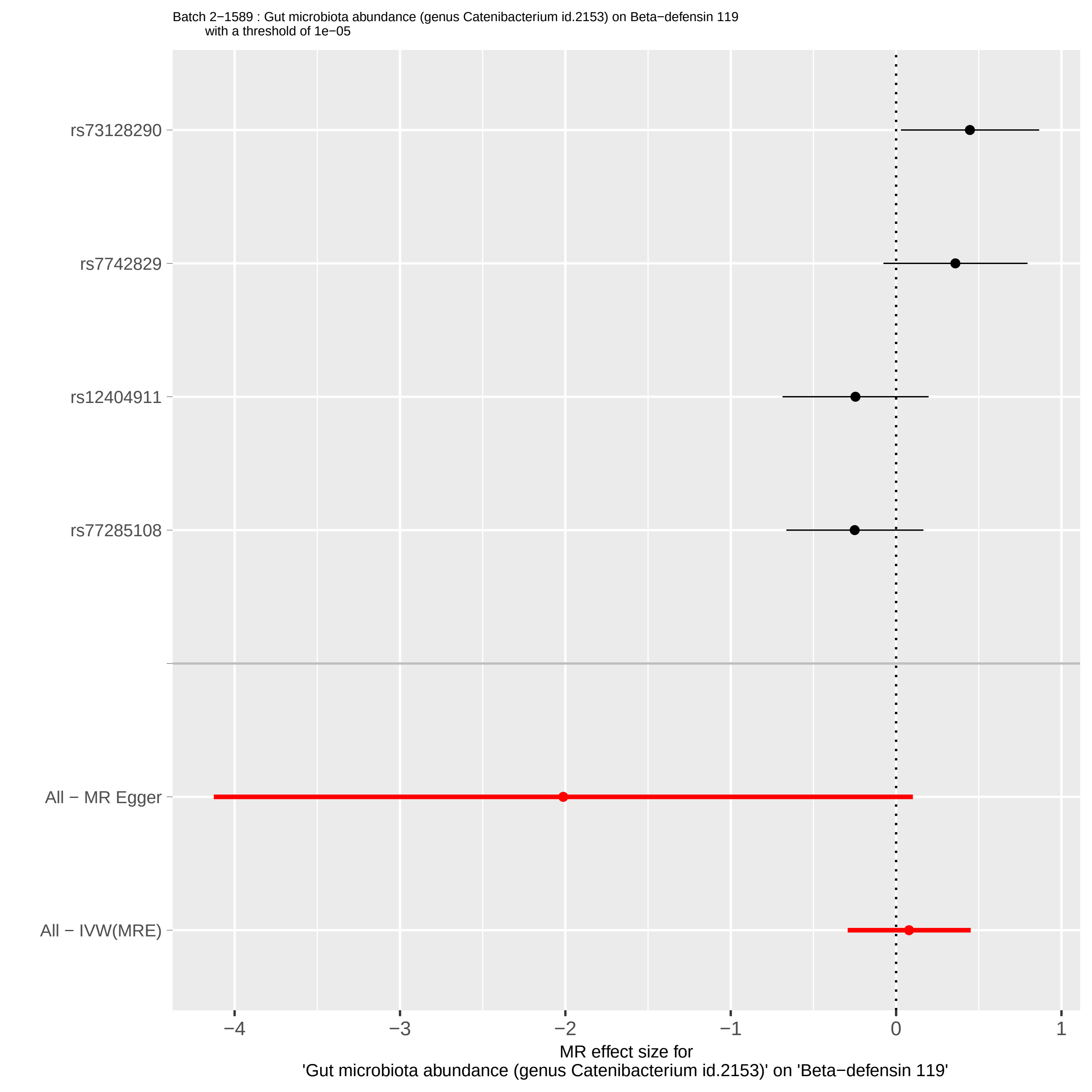
Batch 2−1589 : Gut microbiota abundance (genus Catenibacterium id.2153) on Beta−defensin 119
with a threshold of 1e−05
rs73128290
rs7742829
rs12404911
rs77285108
All − MR Egger
All − IVW(MRE)
−4
−3
−2
−1
0
1
MR effect size for
'Gut microbiota abundance (genus Catenibacterium id.2153)' on 'Beta−defensin 119'

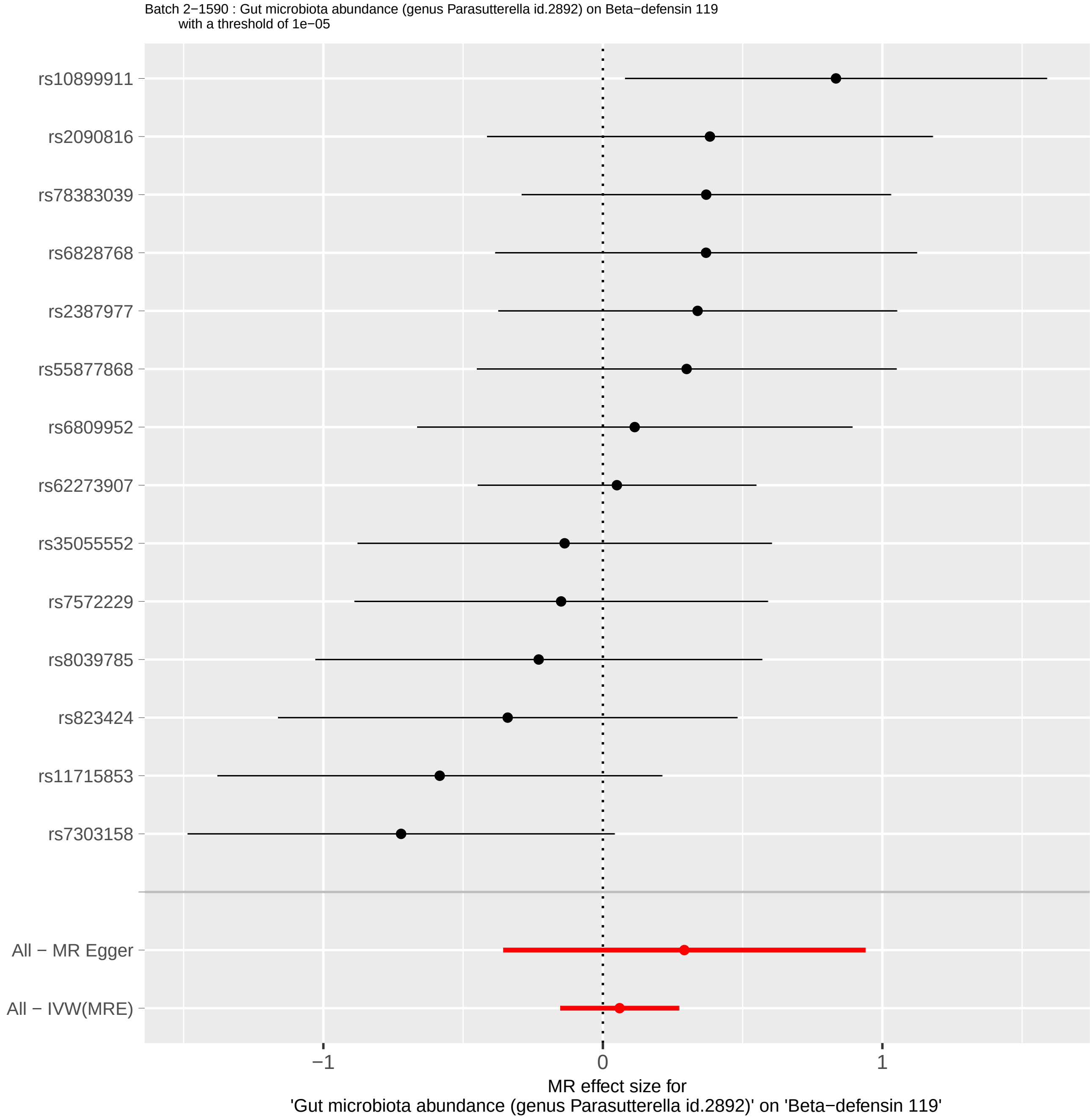

Batch 2−1590 : Gut microbiota abundance (genus Parasutterella id.2892) on Beta−defensin 119
with a threshold of 1e−05
rs10899911
rs2090816
rs78383039
rs6828768
rs2387977
rs55877868
rs6809952
rs62273907
rs35055552
rs7572229
rs8039785
rs823424
rs11715853
rs7303158
All − MR Egger
All − IVW(MRE)
−1
0
1
MR effect size for
'Gut microbiota abundance (genus Parasutterella id.2892)' on 'Beta−defensin 119'

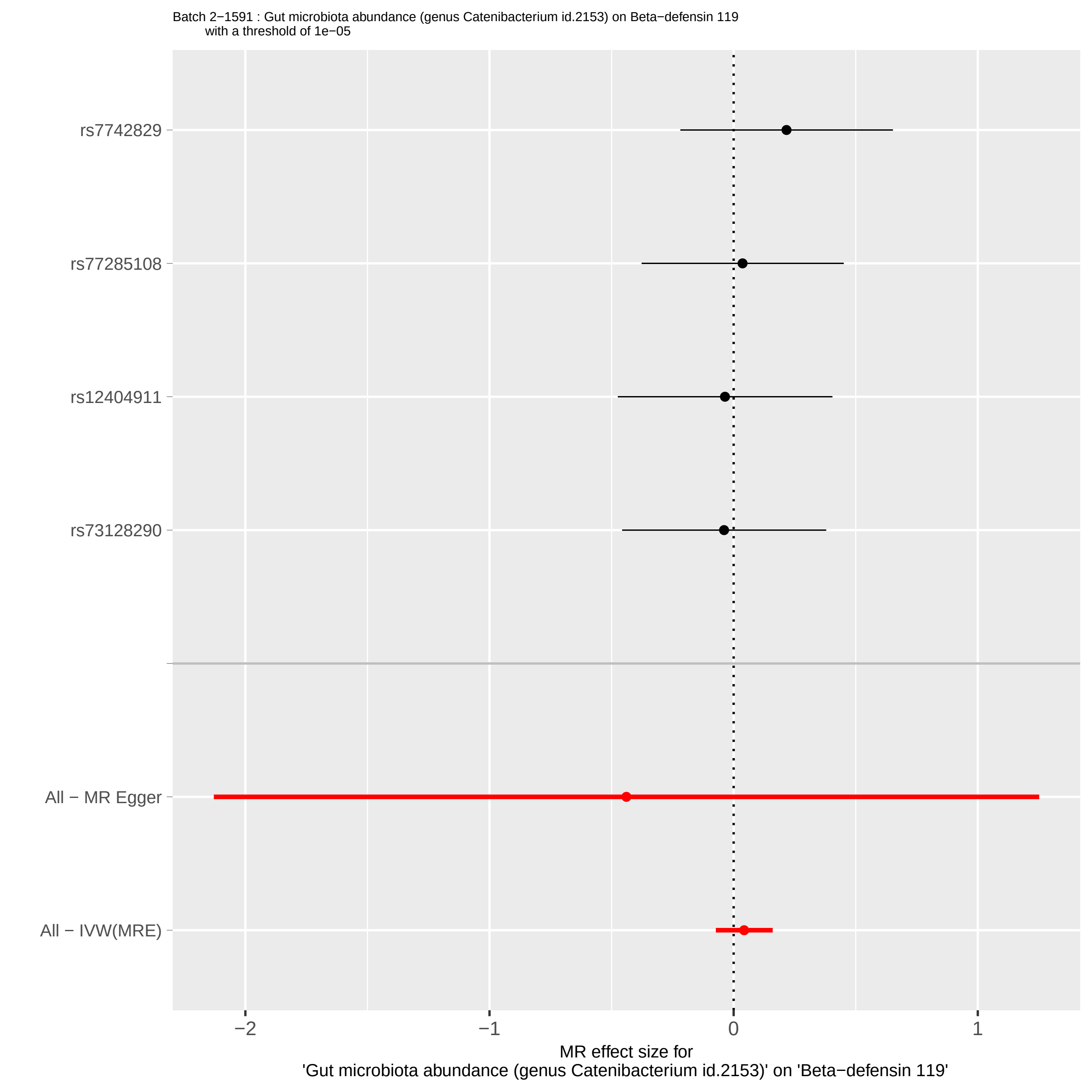

Batch 2−1591 : Gut microbiota abundance (genus Catenibacterium id.2153) on Beta−defensin 119
with a threshold of 1e−05
rs7742829
rs77285108
rs12404911
rs73128290
All − MR Egger
All − IVW(MRE)
−2
−1
0
1
MR effect size for
'Gut microbiota abundance (genus Catenibacterium id.2153)' on 'Beta−defensin 119'

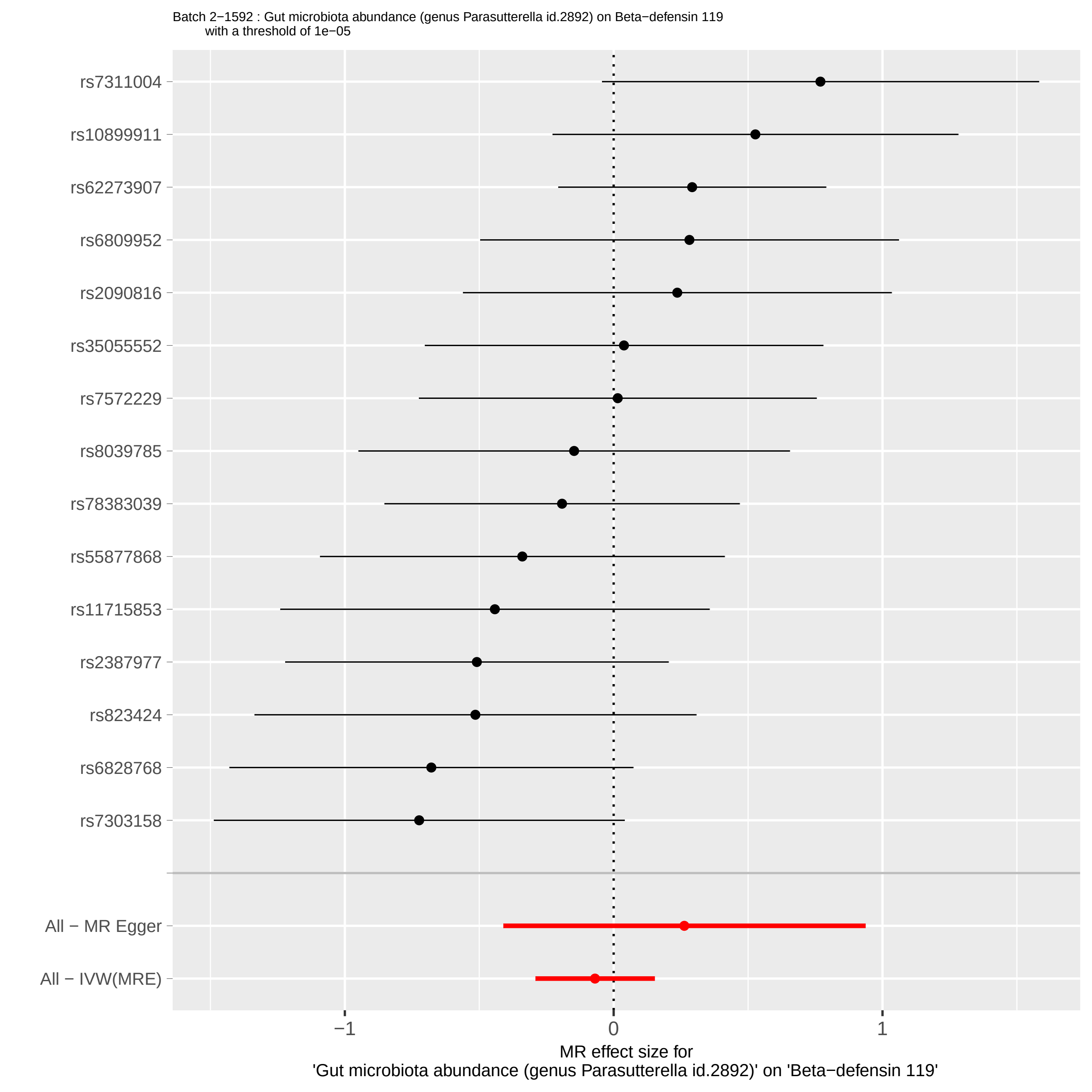

Batch 2−1592 : Gut microbiota abundance (genus Parasutterella id.2892) on Beta−defensin 119
with a threshold of 1e−05
rs7311004
rs10899911
rs62273907
rs6809952
rs2090816
rs35055552
rs7572229
rs8039785
rs78383039
rs55877868
rs11715853
rs2387977
rs823424
rs6828768
rs7303158
All − MR Egger
All − IVW(MRE)
−1
0
1
MR effect size for
'Gut microbiota abundance (genus Parasutterella id.2892)' on 'Beta−defensin 119'

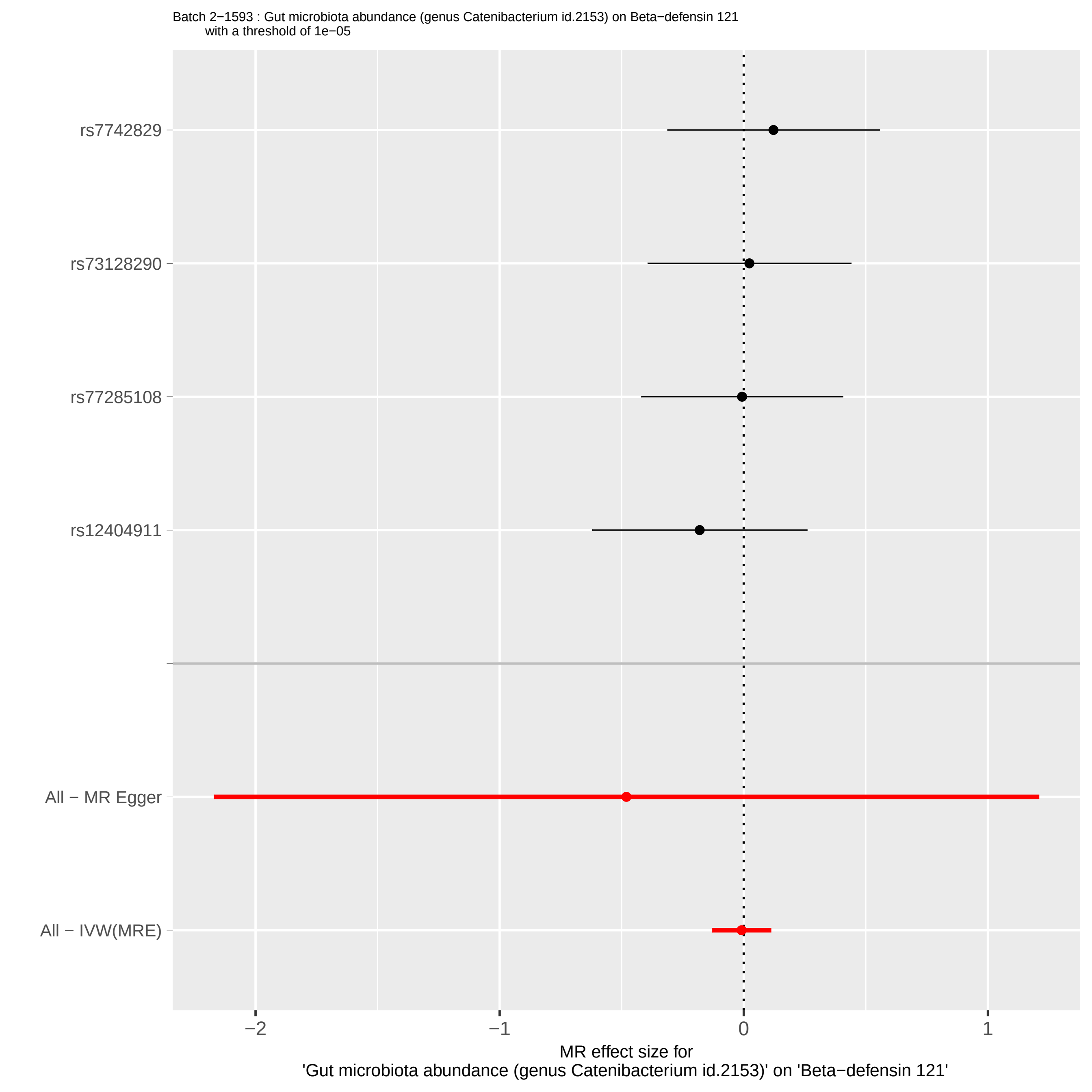

Batch 2−1593 : Gut microbiota abundance (genus Catenibacterium id.2153) on Beta−defensin 121
with a threshold of 1e−05
rs7742829
rs73128290
rs77285108
rs12404911
All − MR Egger
All − IVW(MRE)
−2
−1
0
1
MR effect size for
'Gut microbiota abundance (genus Catenibacterium id.2153)' on 'Beta−defensin 121'

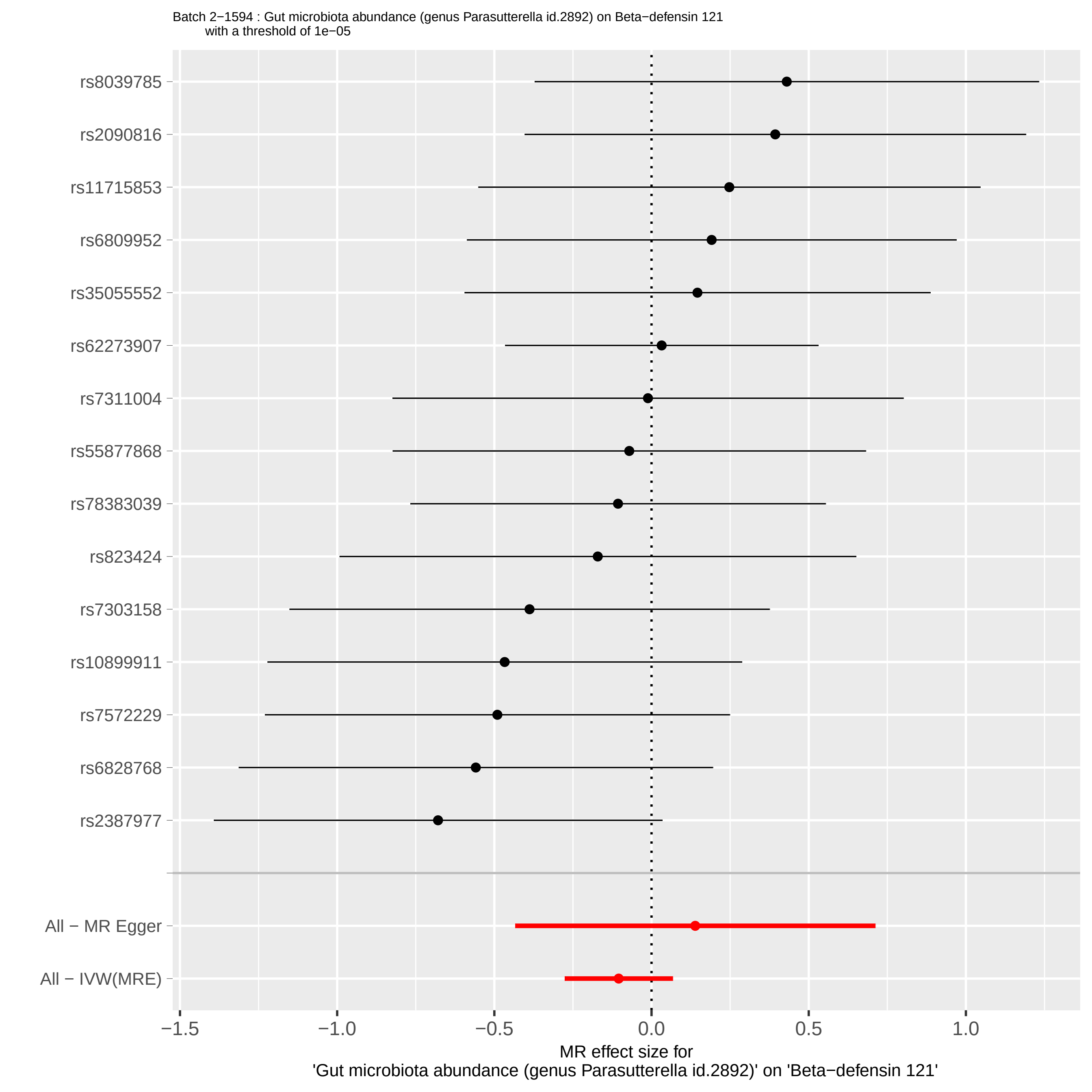

Batch 2−1594 : Gut microbiota abundance (genus Parasutterella id.2892) on Beta−defensin 121
with a threshold of 1e−05
rs8039785
rs2090816
rs11715853
rs6809952
rs35055552
rs62273907
rs7311004
rs55877868
rs78383039
rs823424
rs7303158
rs10899911
rs7572229
rs6828768
rs2387977
All − MR Egger
All − IVW(MRE)
−1.5
−1.0
−0.5
0.0
0.5
1.0
MR effect size for
'Gut microbiota abundance (genus Parasutterella id.2892)' on 'Beta−defensin 121'

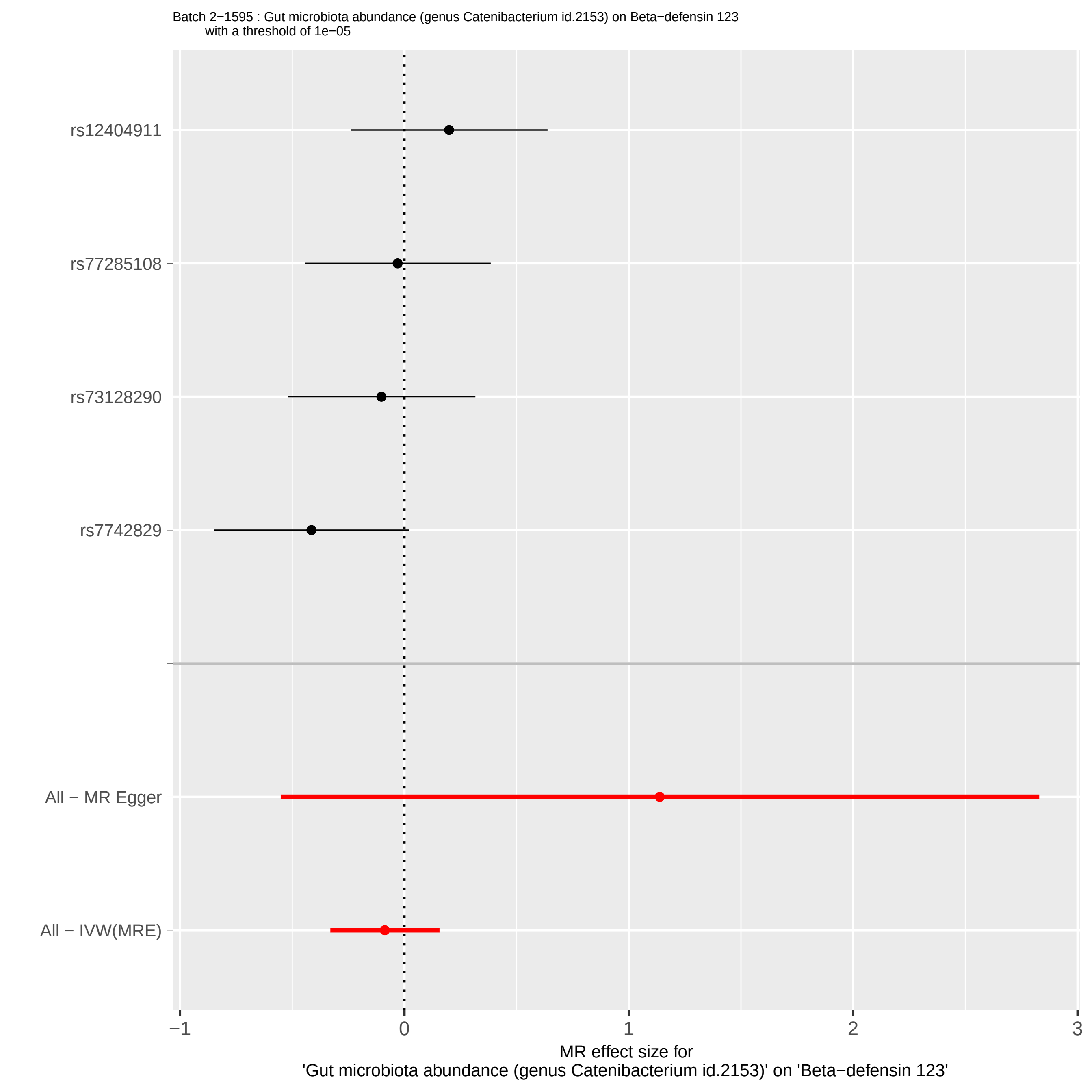
Batch 2−1595 : Gut microbiota abundance (genus Catenibacterium id.2153) on Beta−defensin 123
with a threshold of 1e−05
rs12404911
rs77285108
rs73128290
rs7742829
All − MR Egger
All − IVW(MRE)
−1
0
1
2
3
MR effect size for
'Gut microbiota abundance (genus Catenibacterium id.2153)' on 'Beta−defensin 123'

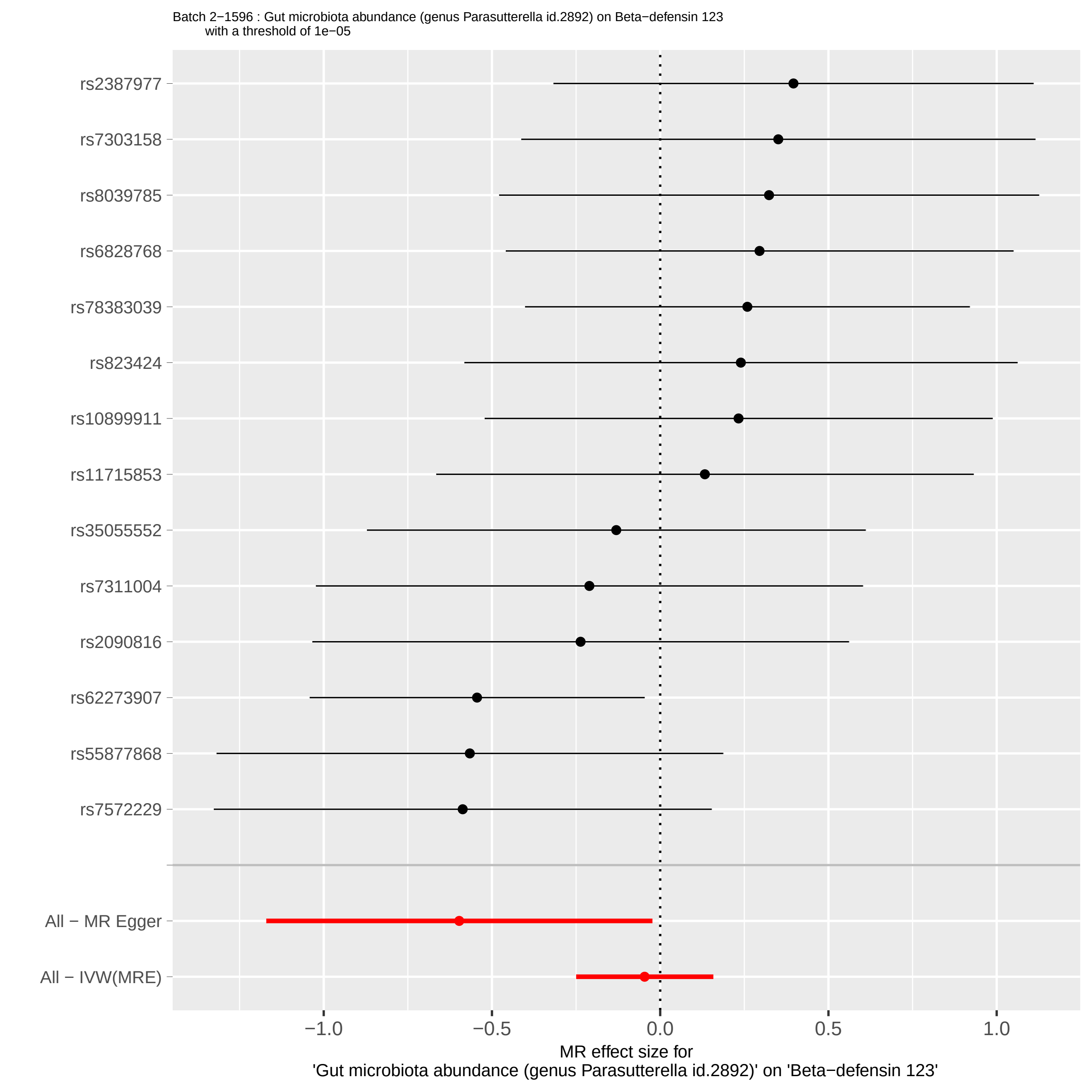
Batch 2−1596 : Gut microbiota abundance (genus Parasutterella id.2892) on Beta−defensin 123
with a threshold of 1e−05
rs2387977
rs7303158
rs8039785
rs6828768
rs78383039
rs823424
rs10899911
rs11715853
rs35055552
rs7311004
rs2090816
rs62273907
rs55877868
rs7572229
All − MR Egger
All − IVW(MRE)
−1.0
−0.5
0.0
0.5
1.0
MR effect size for
'Gut microbiota abundance (genus Parasutterella id.2892)' on 'Beta−defensin 123'

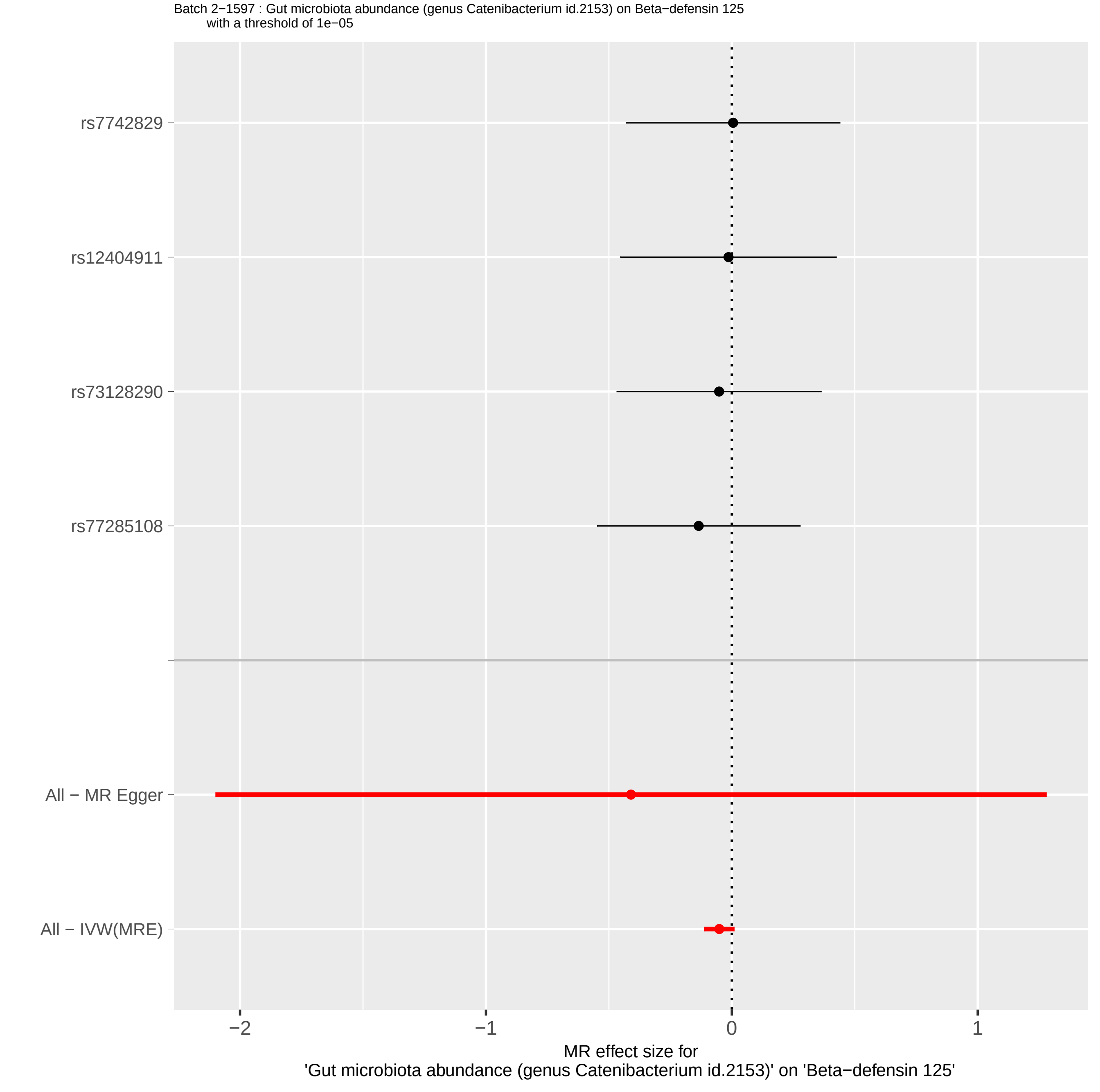
Batch 2−1597 : Gut microbiota abundance (genus Catenibacterium id.2153) on Beta−defensin 125
with a threshold of 1e−05
rs7742829
rs12404911
rs73128290
rs77285108
All − MR Egger
All − IVW(MRE)
−2
−1
0
1
MR effect size for
'Gut microbiota abundance (genus Catenibacterium id.2153)' on 'Beta−defensin 125'

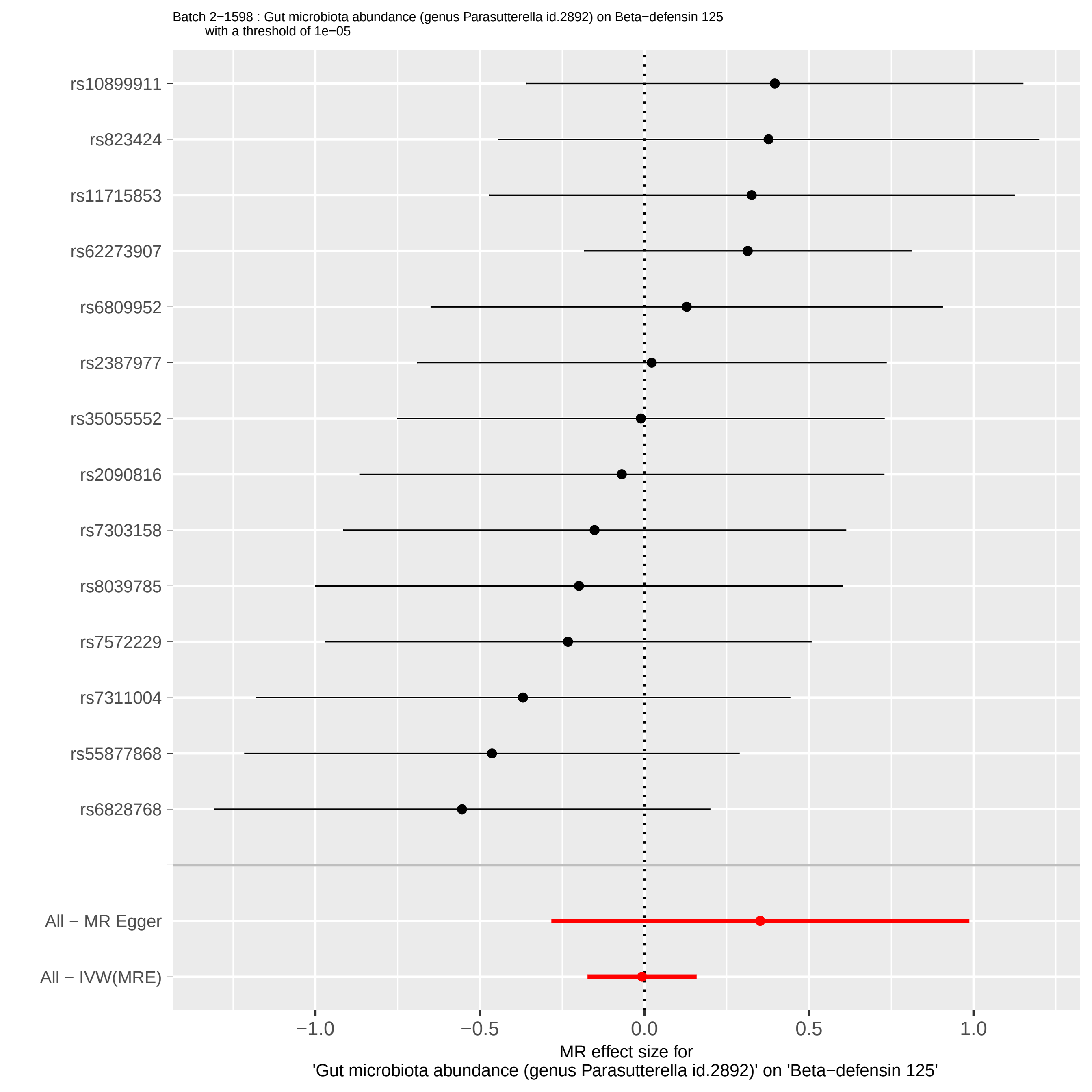

Batch 2−1598 : Gut microbiota abundance (genus Parasutterella id.2892) on Beta−defensin 125
with a threshold of 1e−05
rs10899911
rs823424
rs11715853
rs62273907
rs6809952
rs2387977
rs35055552
rs2090816
rs7303158
rs8039785
rs7572229
rs7311004
rs55877868
rs6828768
All − MR Egger
All − IVW(MRE)
−1.0
−0.5
0.0
0.5
1.0
MR effect size for
'Gut microbiota abundance (genus Parasutterella id.2892)' on 'Beta−defensin 125'

Batch 2−1599 : Gut microbiota abundance (genus Catenibacterium id.2153) on Beta−defensin 128
with a threshold of 1e−05

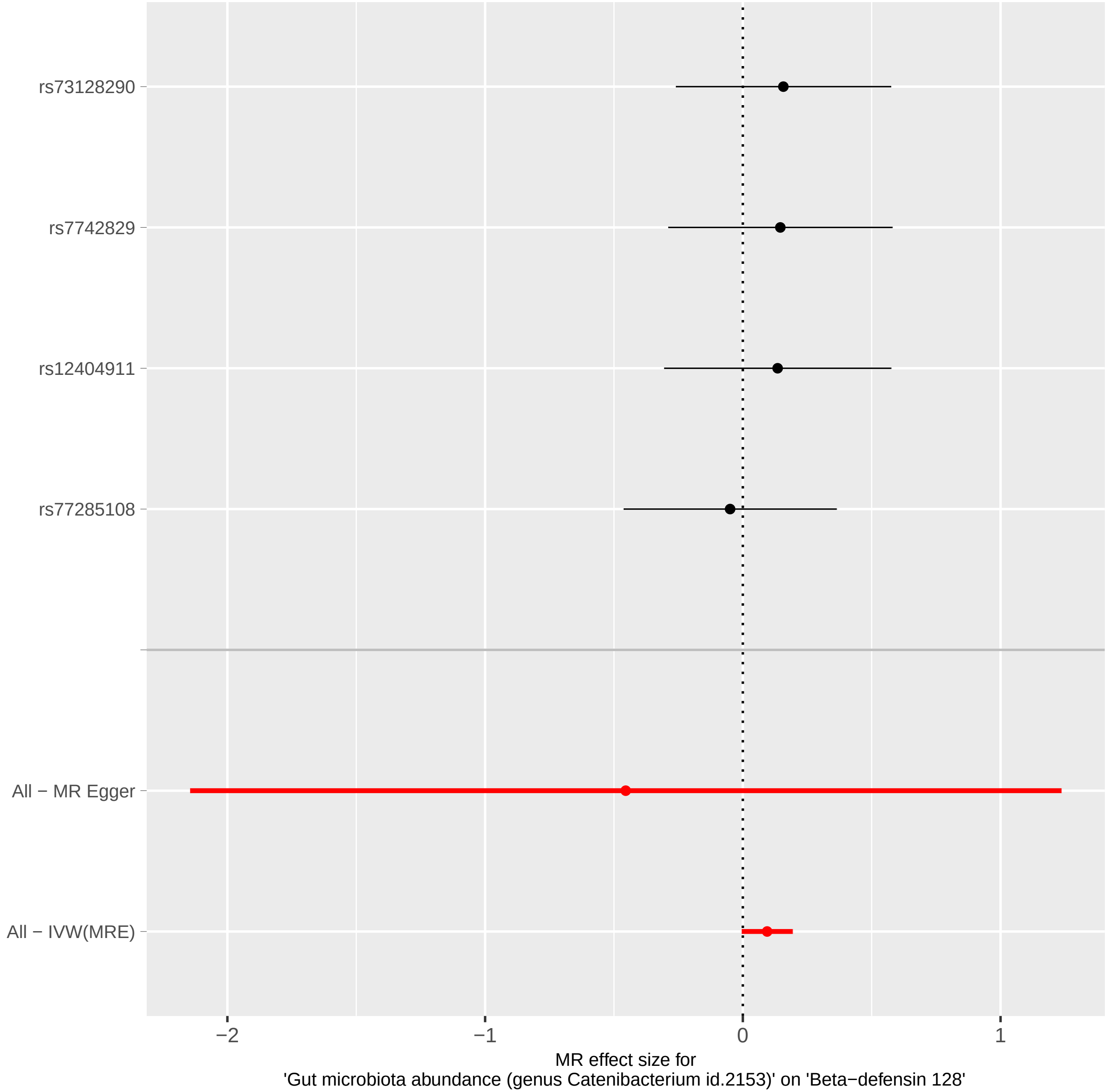

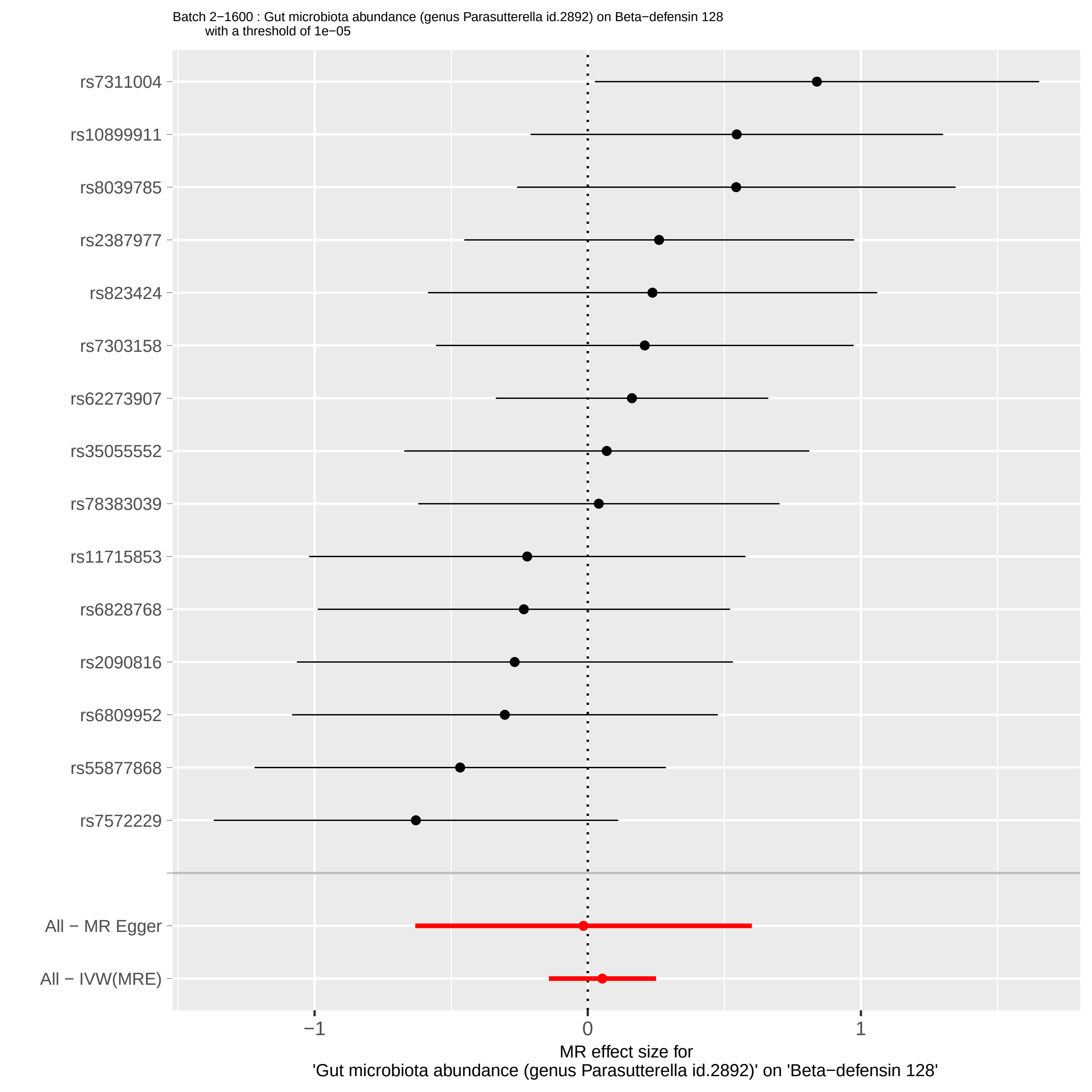

Batch 2−1600 : Gut microbiota abundance (genus Parasutterella id.2892) on Beta−defensin 128
with a threshold of 1e−05
rs7311004
rs10899911
rs8039785
rs2387977
rs823424
rs7303158
rs62273907
rs35055552
rs78383039
rs11715853
rs6828768
rs2090816
rs6809952
rs55877868
rs7572229
All − MR Egger
All − IVW(MRE)
−1
0
1
MR effect size for
'Gut microbiota abundance (genus Parasutterella id.2892)' on 'Beta−defensin 128'

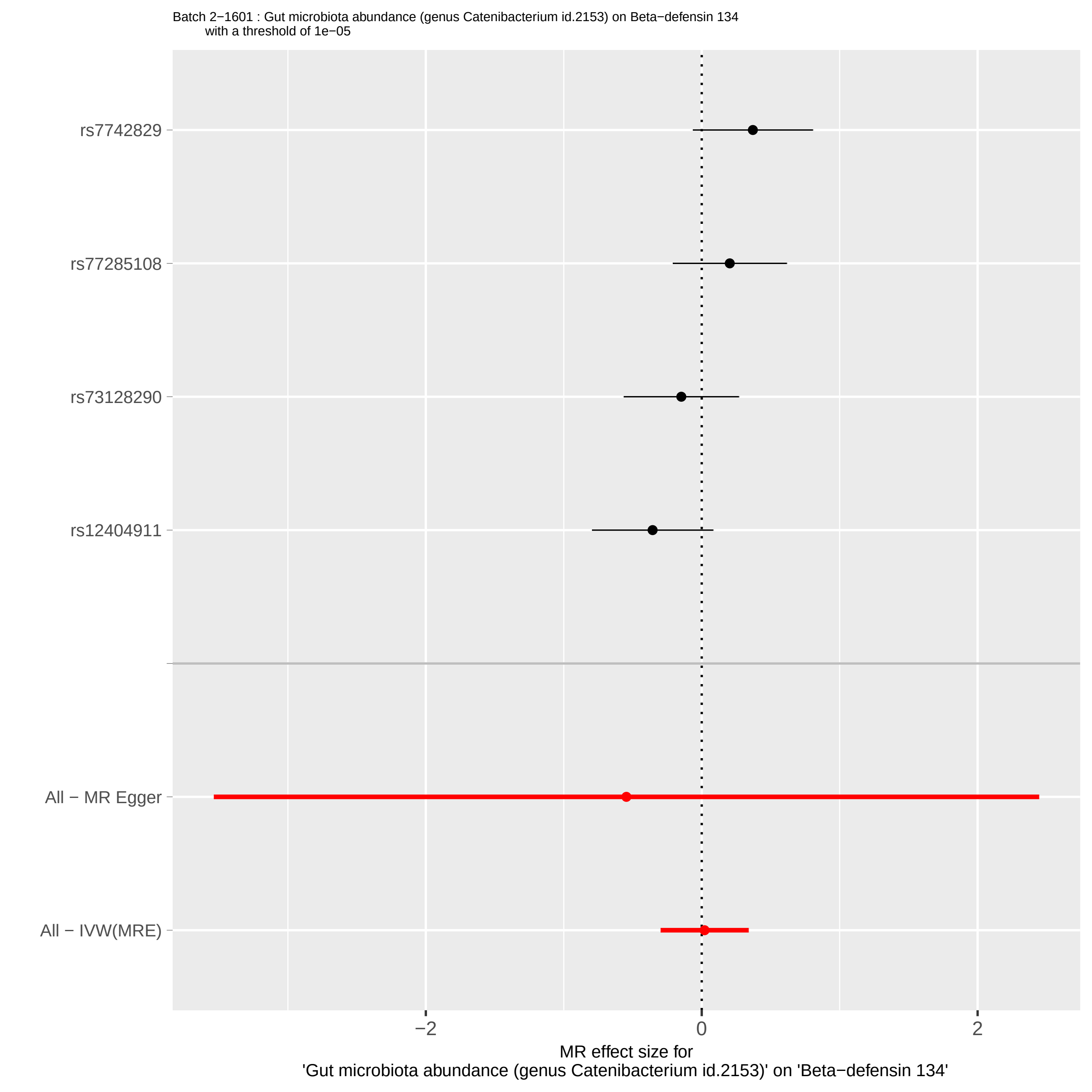

Batch 2−1601 : Gut microbiota abundance (genus Catenibacterium id.2153) on Beta−defensin 134
with a threshold of 1e−05
rs7742829
rs77285108
rs73128290
rs12404911
All − MR Egger
All − IVW(MRE)
−2
0
2
MR effect size for
'Gut microbiota abundance (genus Catenibacterium id.2153)' on 'Beta−defensin 134'

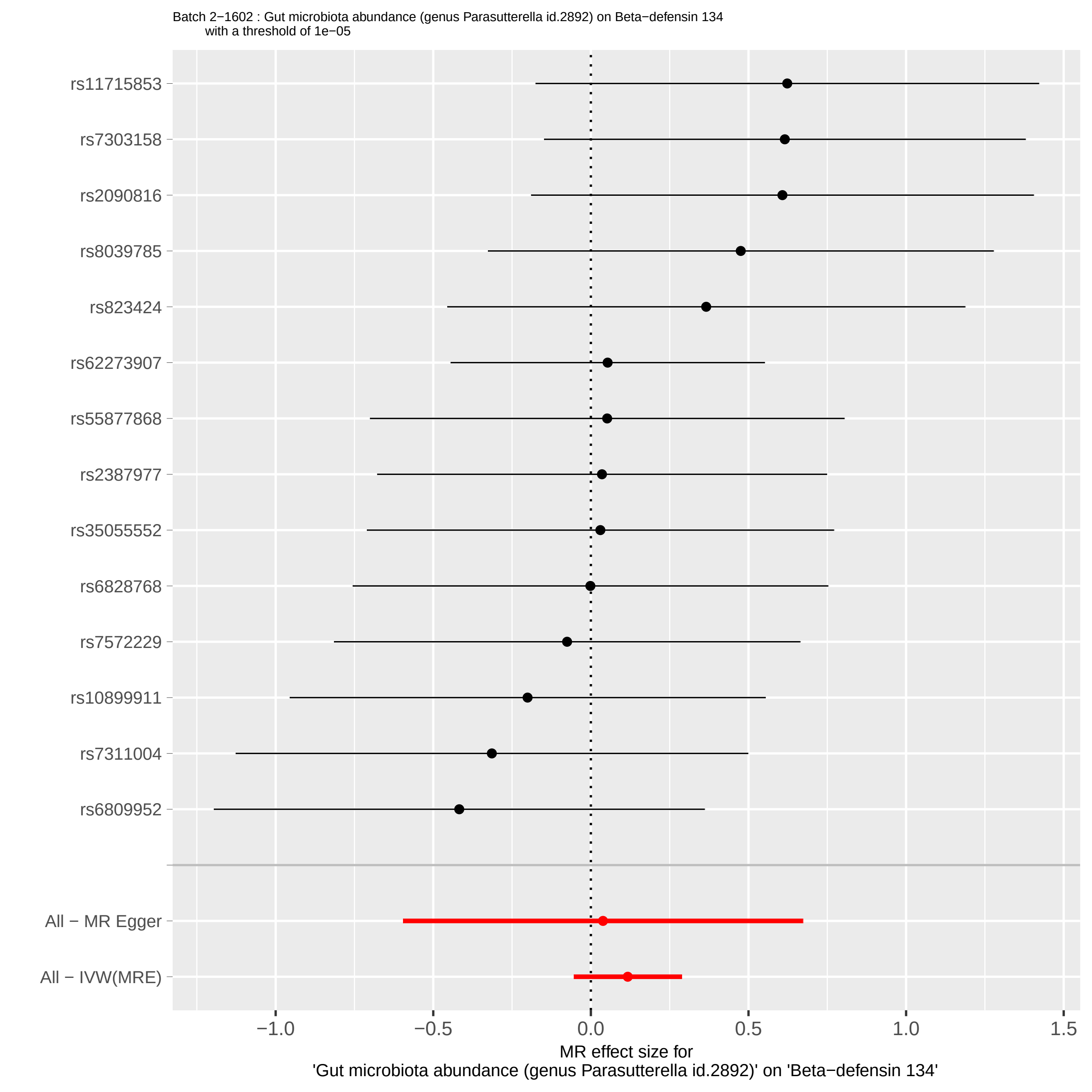

Batch 2−1602 : Gut microbiota abundance (genus Parasutterella id.2892) on Beta−defensin 134
with a threshold of 1e−05
rs11715853
rs7303158
rs2090816
rs8039785
rs823424
rs62273907
rs55877868
rs2387977
rs35055552
rs6828768
rs7572229
rs10899911
rs7311004
rs6809952
All − MR Egger
All − IVW(MRE)
−1.0
−0.5
0.0
0.5
1.0
1.5
MR effect size for
'Gut microbiota abundance (genus Parasutterella id.2892)' on 'Beta−defensin 134'

Batch 2−1603 : Gut microbiota abundance (genus Catenibacterium id.2153) on Beta−defensin 135
with a threshold of 1e−05

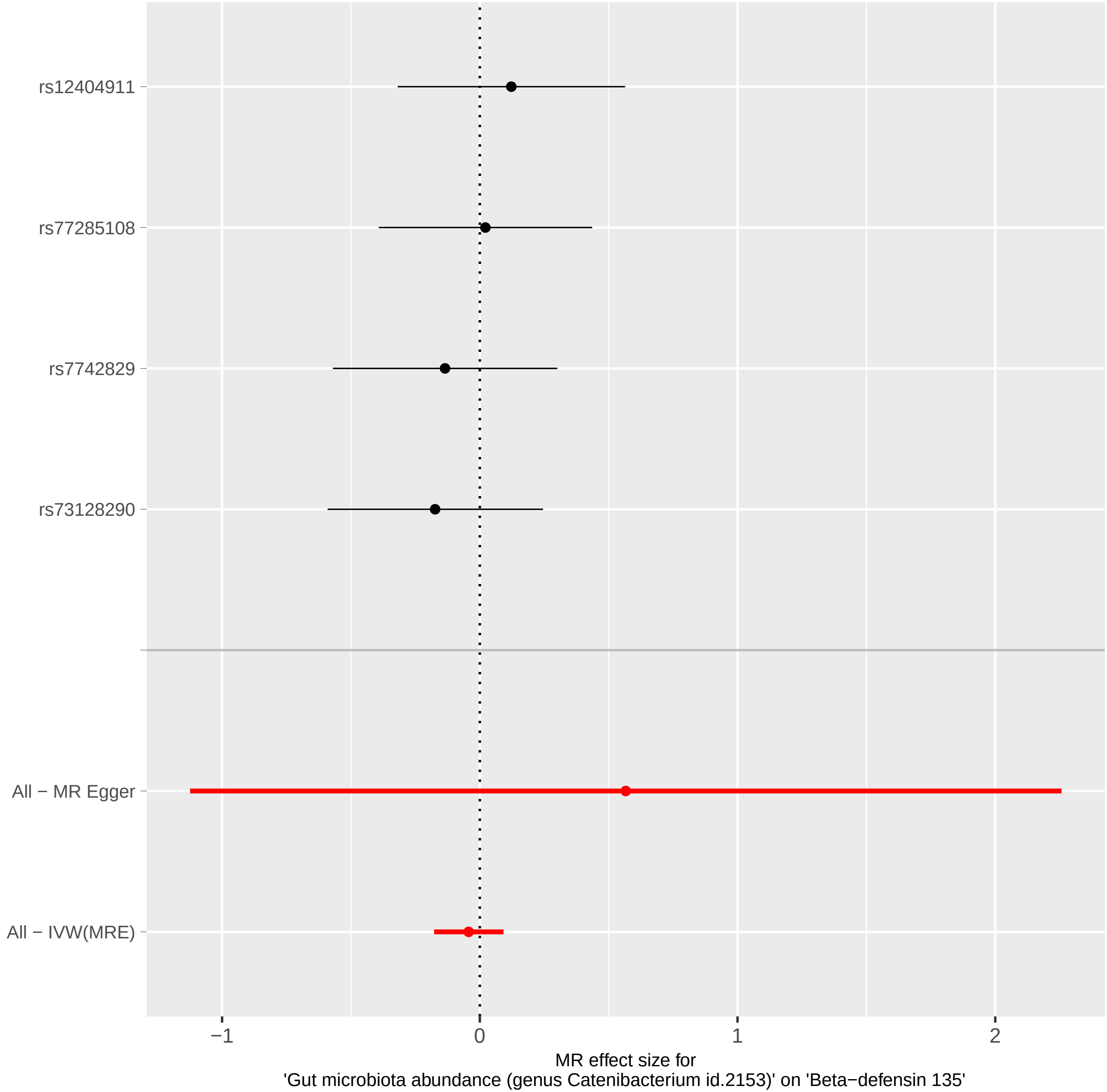

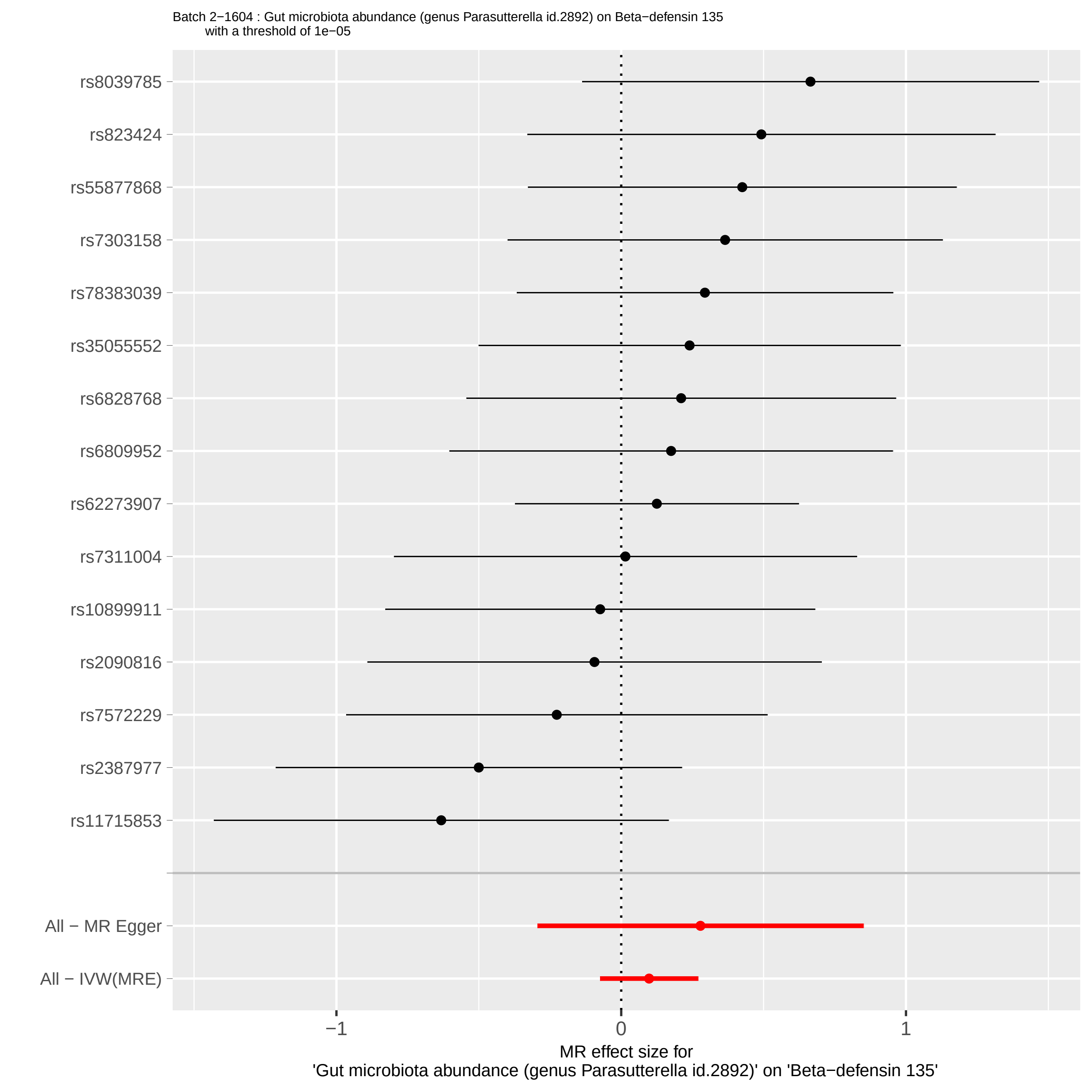

Batch 2−1604 : Gut microbiota abundance (genus Parasutterella id.2892) on Beta−defensin 135
with a threshold of 1e−05
rs8039785
rs823424
rs55877868
rs7303158
rs78383039
rs35055552
rs6828768
rs6809952
rs62273907
rs7311004
rs10899911
rs2090816
rs7572229
rs2387977
rs11715853
All − MR Egger
All − IVW(MRE)
−1
0
1
MR effect size for
'Gut microbiota abundance (genus Parasutterella id.2892)' on 'Beta−defensin 135'

Batch 2−1605 : Gut microbiota abundance (genus Catenibacterium id.2153) on Beta−defensin 136
with a threshold of 1e−05

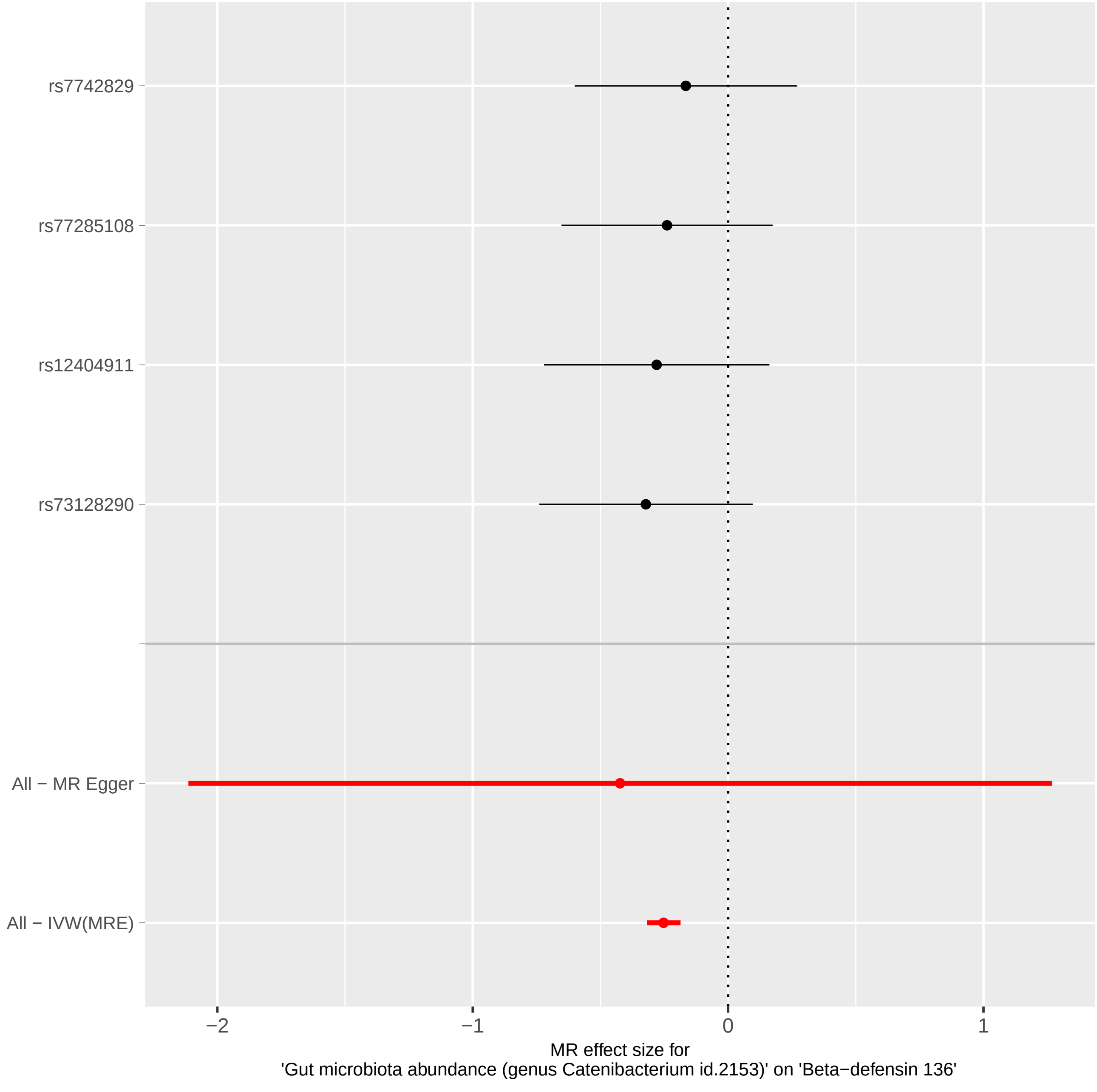

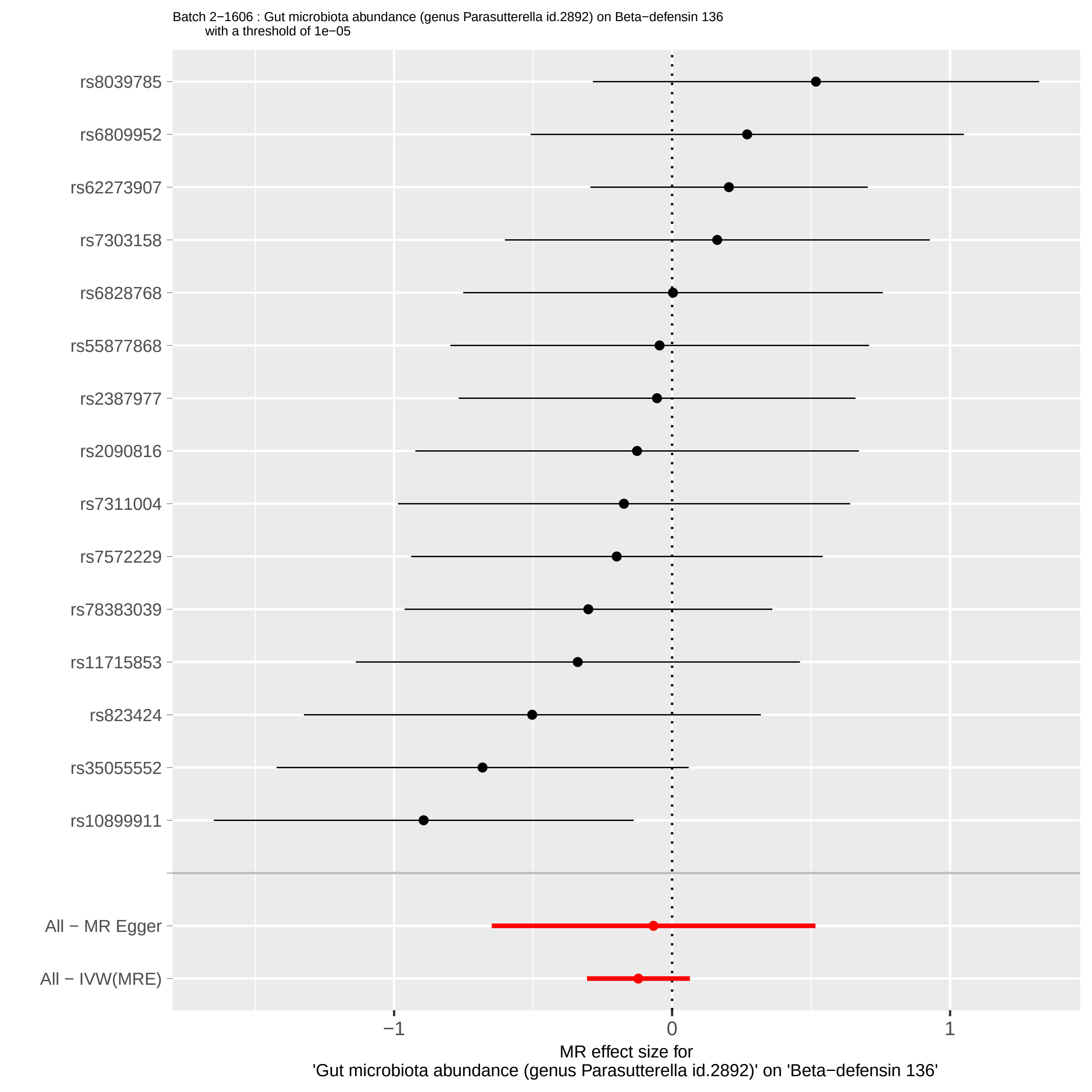

Batch 2−1606 : Gut microbiota abundance (genus Parasutterella id.2892) on Beta−defensin 136
with a threshold of 1e−05
rs8039785
rs6809952
rs62273907
rs7303158
rs6828768
rs55877868
rs2387977
rs2090816
rs7311004
rs7572229
rs78383039
rs11715853
rs823424
rs35055552
rs10899911
All − MR Egger
All − IVW(MRE)
−1
0
1
MR effect size for
'Gut microbiota abundance (genus Parasutterella id.2892)' on 'Beta−defensin 136'

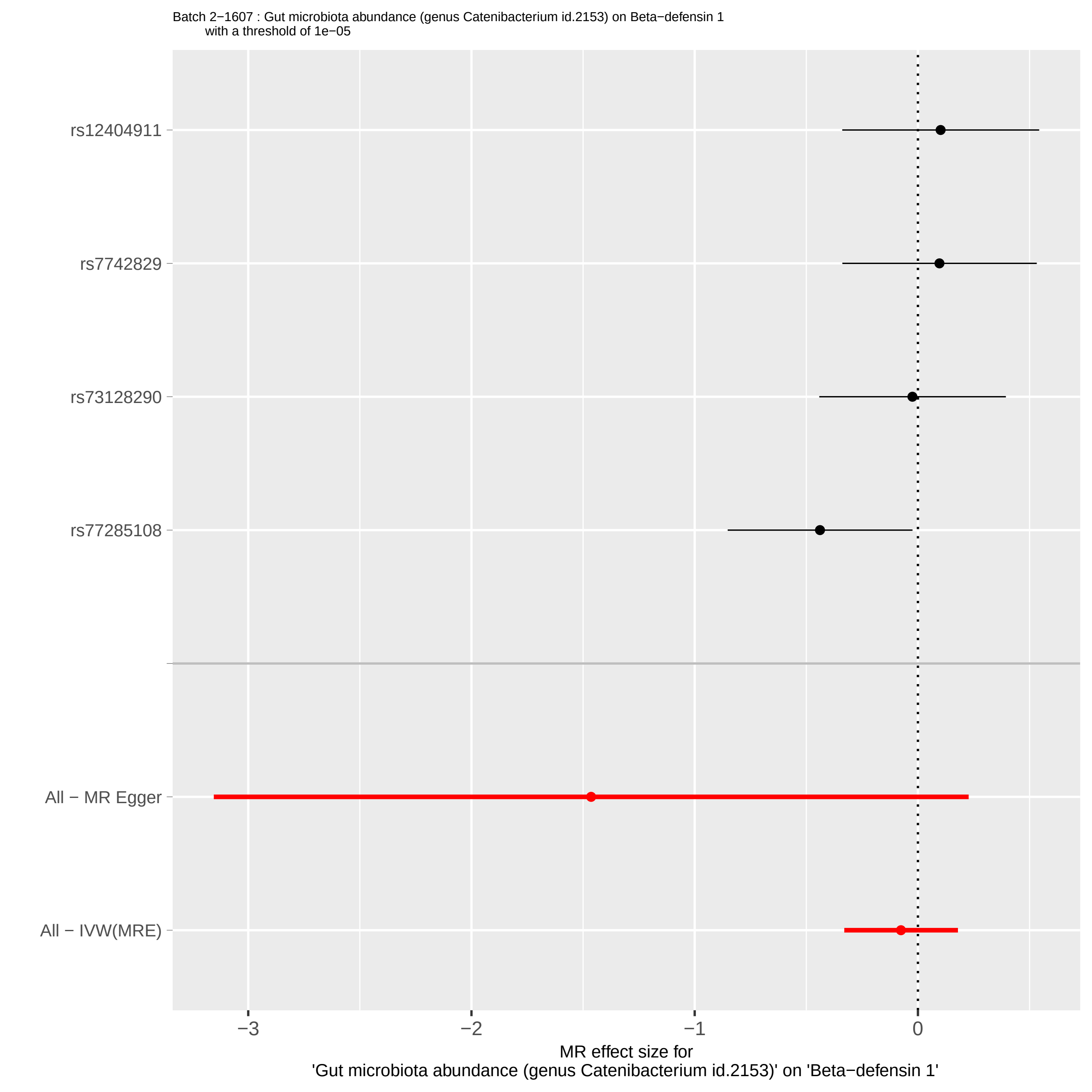
Batch 2−1607 : Gut microbiota abundance (genus Catenibacterium id.2153) on Beta−defensin 1
with a threshold of 1e−05
rs12404911
rs7742829
rs73128290
rs77285108
All − MR Egger
All − IVW(MRE)
−3
−2
−1
0
MR effect size for
'Gut microbiota abundance (genus Catenibacterium id.2153)' on 'Beta−defensin 1'

Batch 2−1608 : Gut microbiota abundance (genus Parasutterella id.2892) on Beta−defensin 1
with a threshold of 1e−05

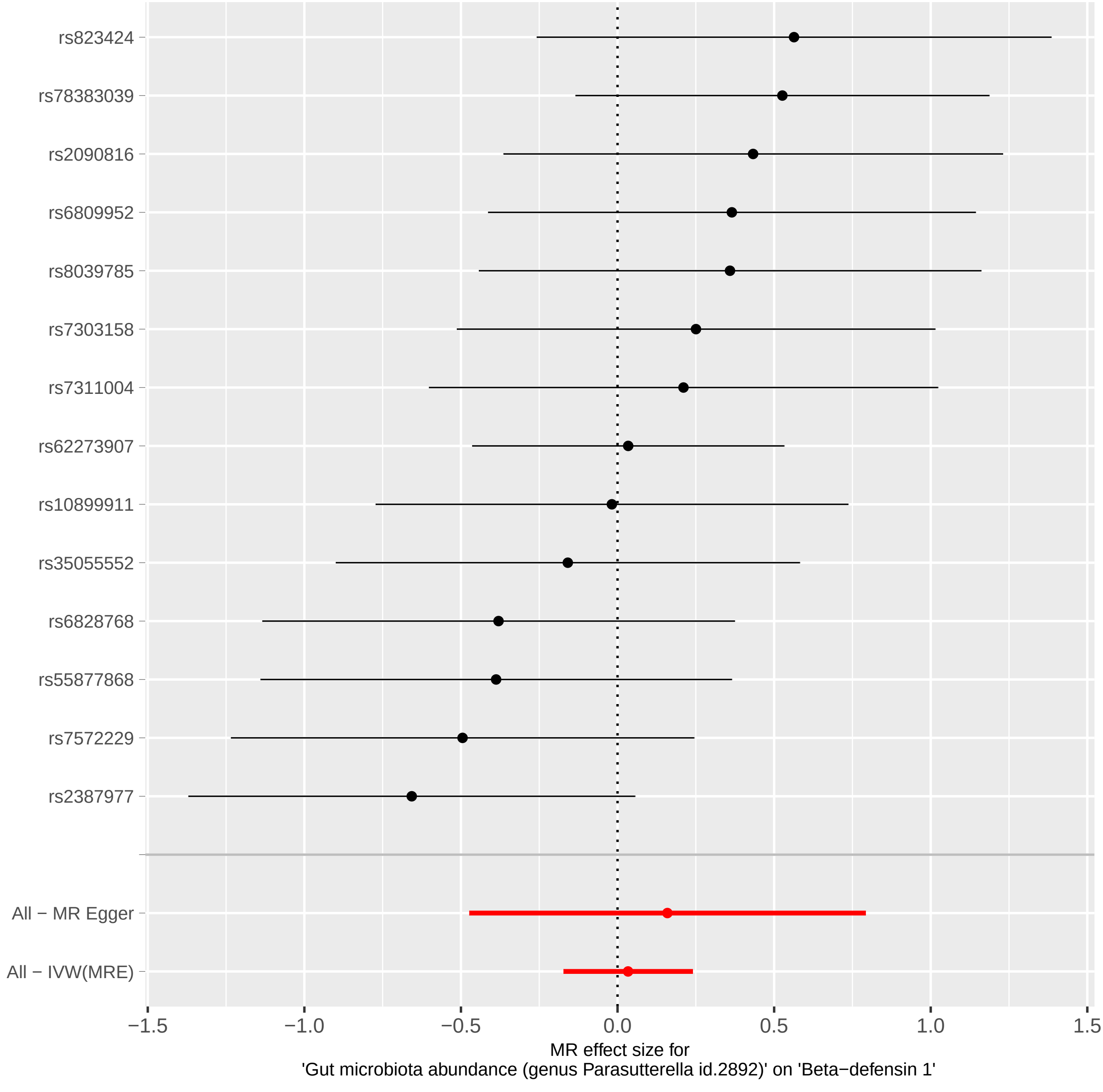

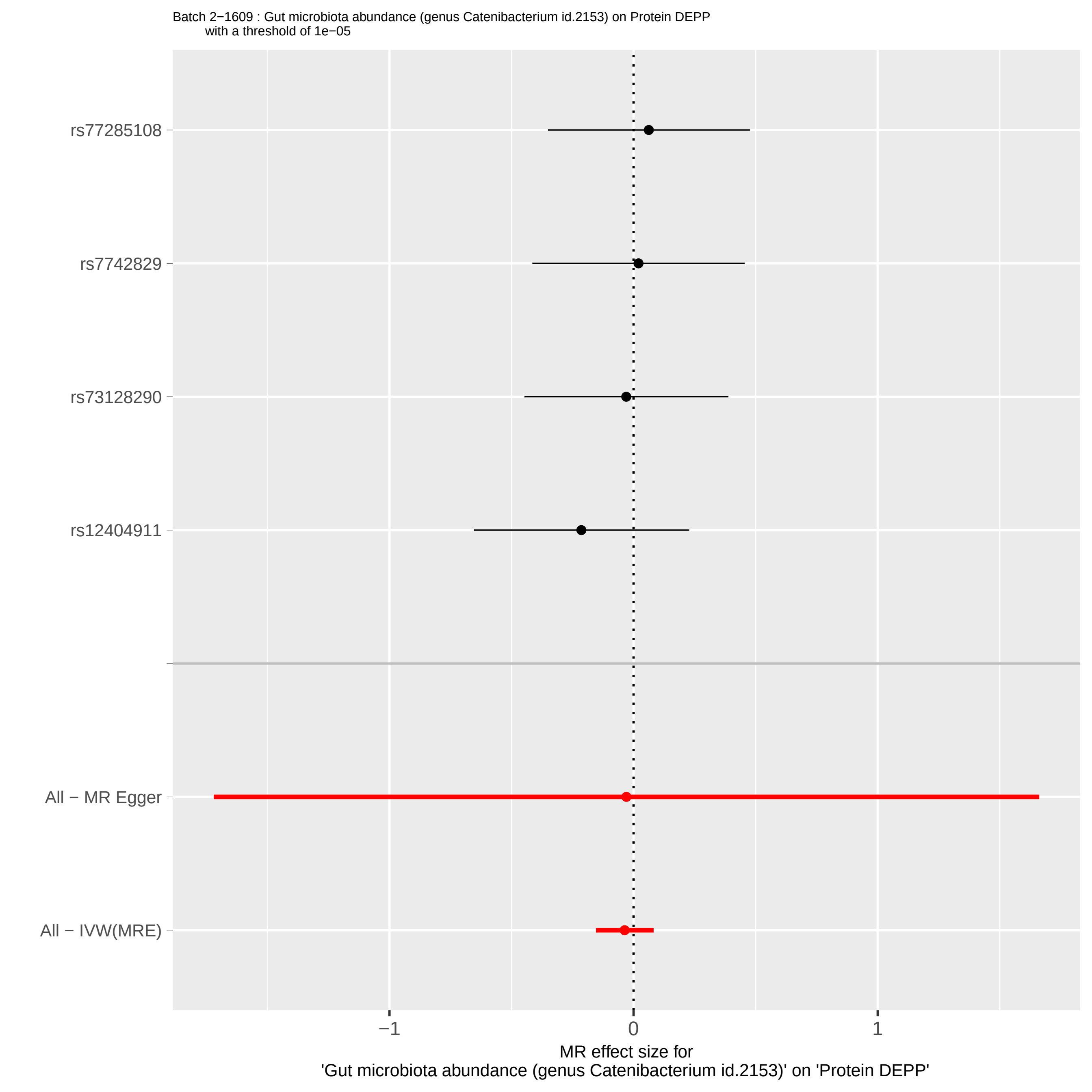

Batch 2−1609 : Gut microbiota abundance (genus Catenibacterium id.2153) on Protein DEPP
with a threshold of 1e−05
rs77285108
rs7742829
rs73128290
rs12404911
All − MR Egger
All − IVW(MRE)
−1
0
1
MR effect size for
'Gut microbiota abundance (genus Catenibacterium id.2153)' on 'Protein DEPP'

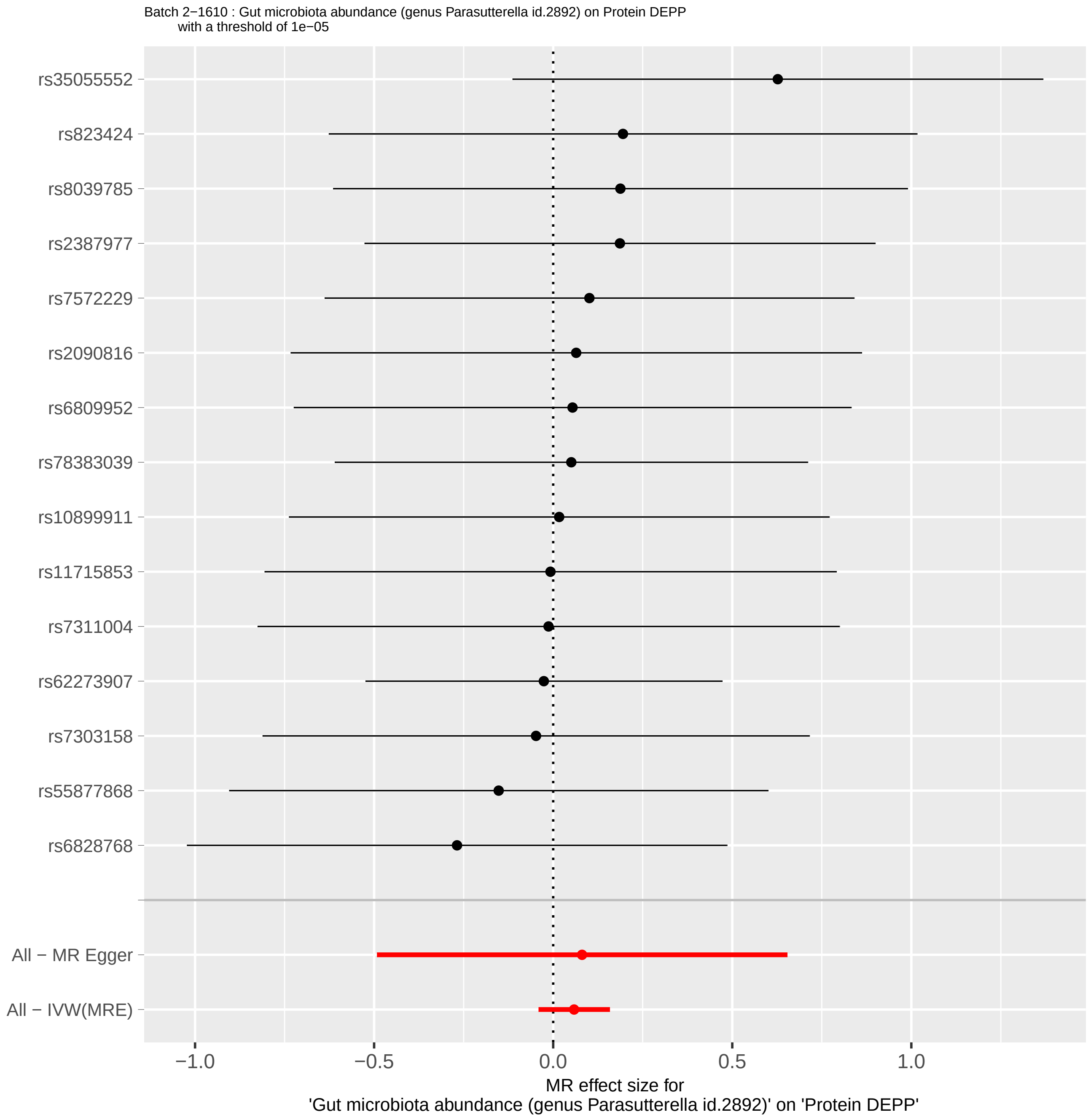

Batch 2−1610 : Gut microbiota abundance (genus Parasutterella id.2892) on Protein DEPP
with a threshold of 1e−05
rs35055552
rs823424
rs8039785
rs2387977
rs7572229
rs2090816
rs6809952
rs78383039
rs10899911
rs11715853
rs7311004
rs62273907
rs7303158
rs55877868
rs6828768
All − MR Egger
All − IVW(MRE)
−1.0
−0.5
0.0
0.5
1.0
MR effect size for
'Gut microbiota abundance (genus Parasutterella id.2892)' on 'Protein DEPP'

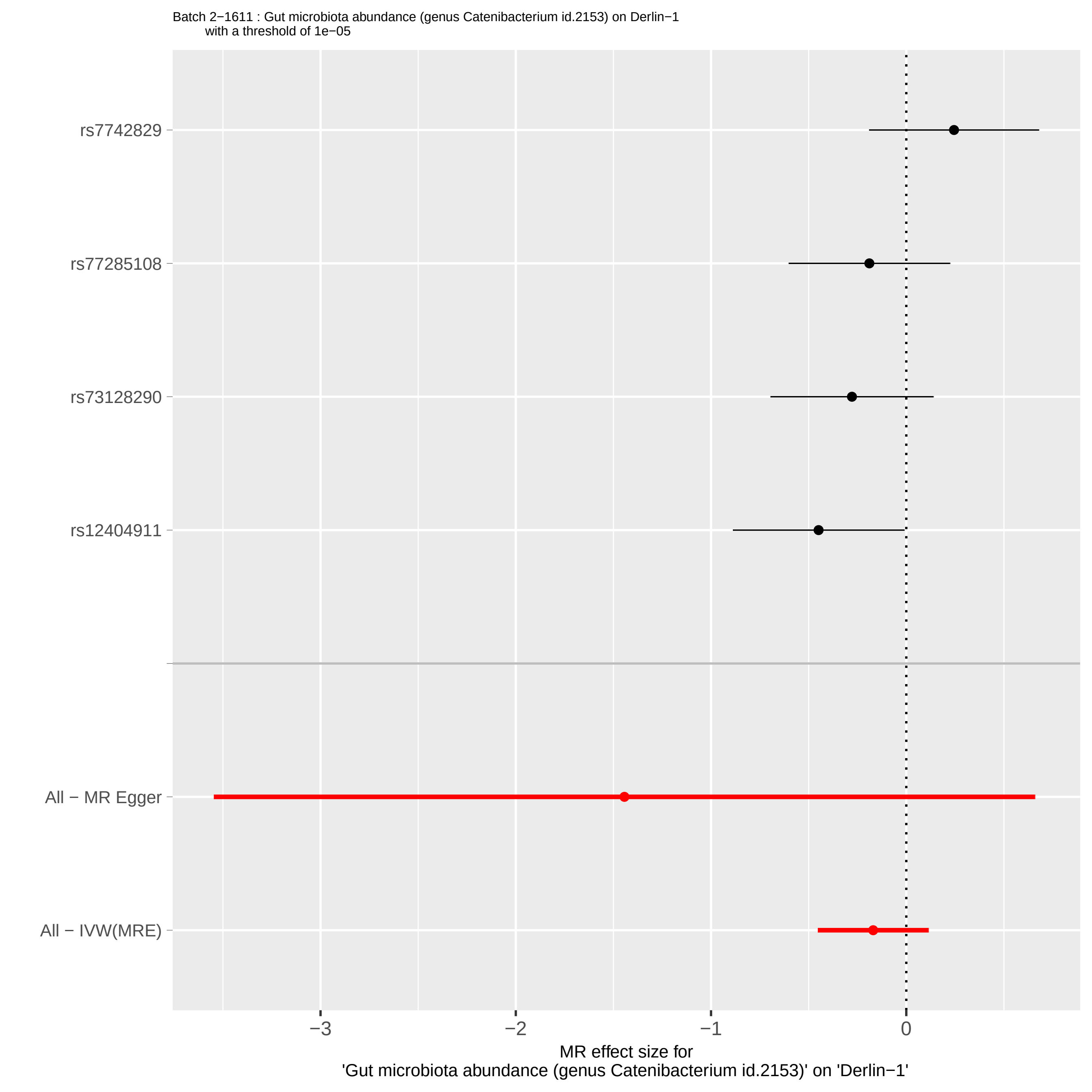

Batch 2−1611 : Gut microbiota abundance (genus Catenibacterium id.2153) on Derlin−1
with a threshold of 1e−05
rs7742829
rs77285108
rs73128290
rs12404911
All − MR Egger
All − IVW(MRE)
−3
−2
−1
0
MR effect size for
'Gut microbiota abundance (genus Catenibacterium id.2153)' on 'Derlin−1'

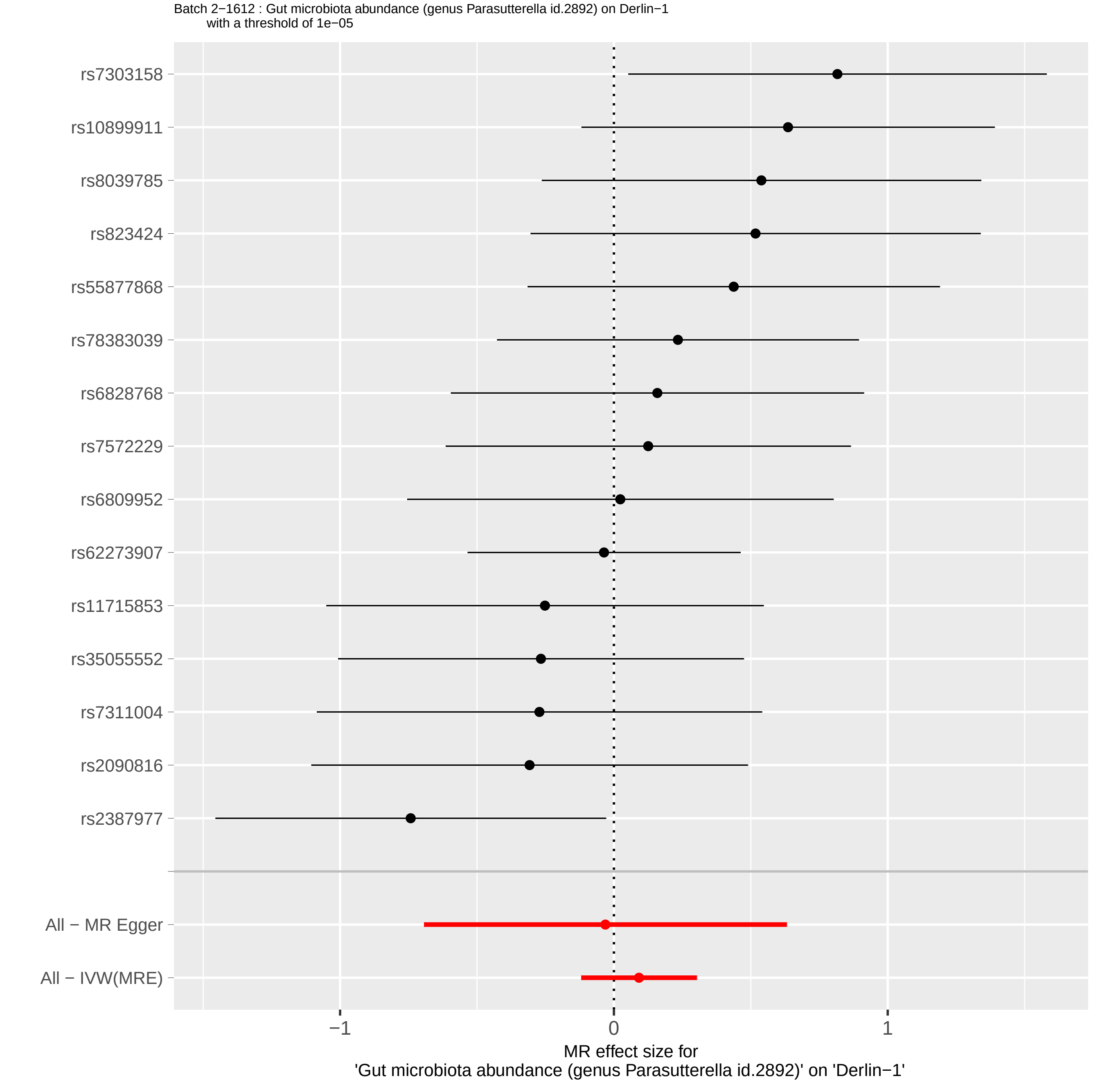
Batch 2−1612 : Gut microbiota abundance (genus Parasutterella id.2892) on Derlin−1
with a threshold of 1e−05
rs7303158
rs10899911
rs8039785
rs823424
rs55877868
rs78383039
rs6828768
rs7572229
rs6809952
rs62273907
rs11715853
rs35055552
rs7311004
rs2090816
rs2387977
All − MR Egger
All − IVW(MRE)
−1
0
1
MR effect size for
'Gut microbiota abundance (genus Parasutterella id.2892)' on 'Derlin−1'

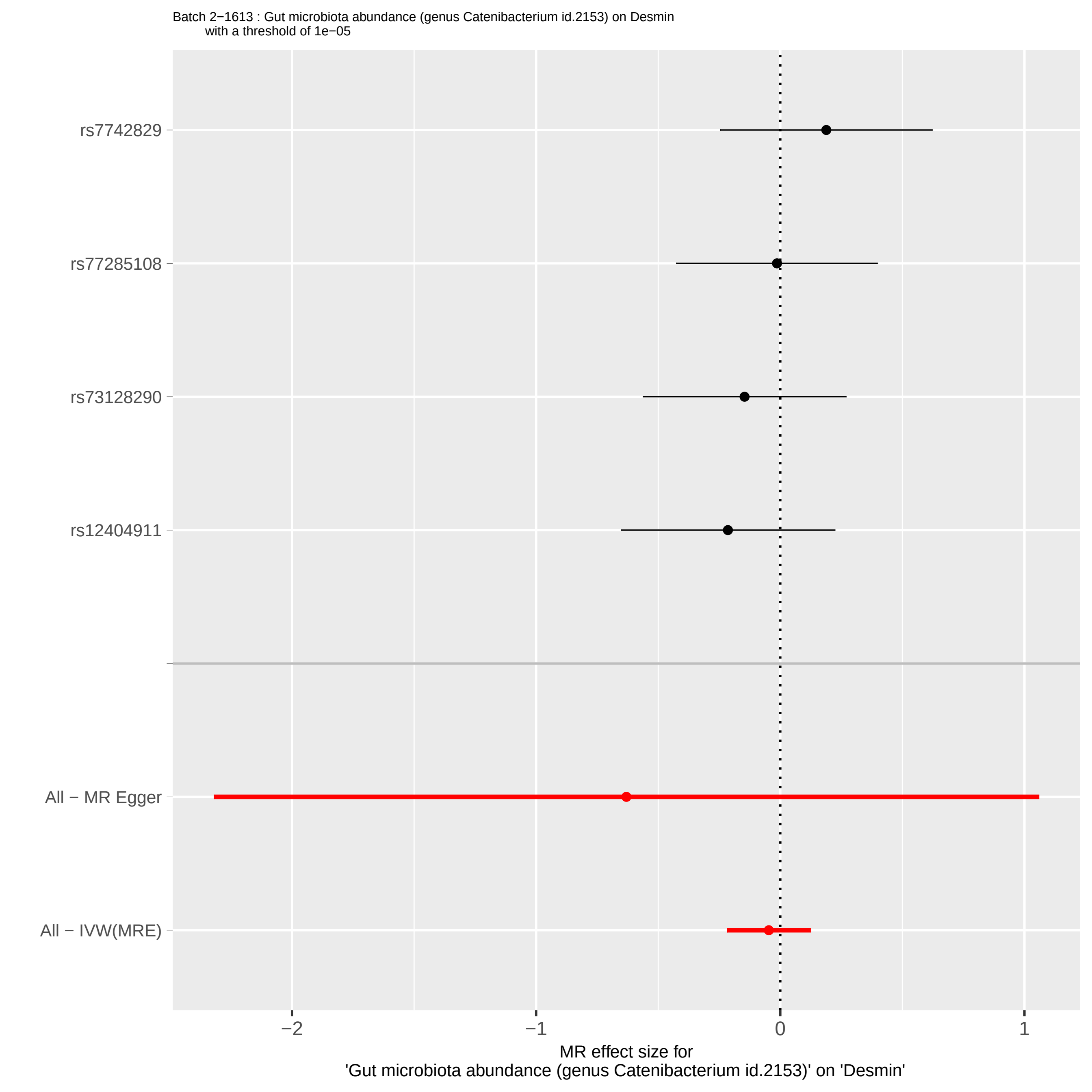

Batch 2−1613 : Gut microbiota abundance (genus Catenibacterium id.2153) on Desmin
with a threshold of 1e−05
rs7742829
rs77285108
rs73128290
rs12404911
All − MR Egger
All − IVW(MRE)
−2
−1
0
1
MR effect size for
'Gut microbiota abundance (genus Catenibacterium id.2153)' on 'Desmin'

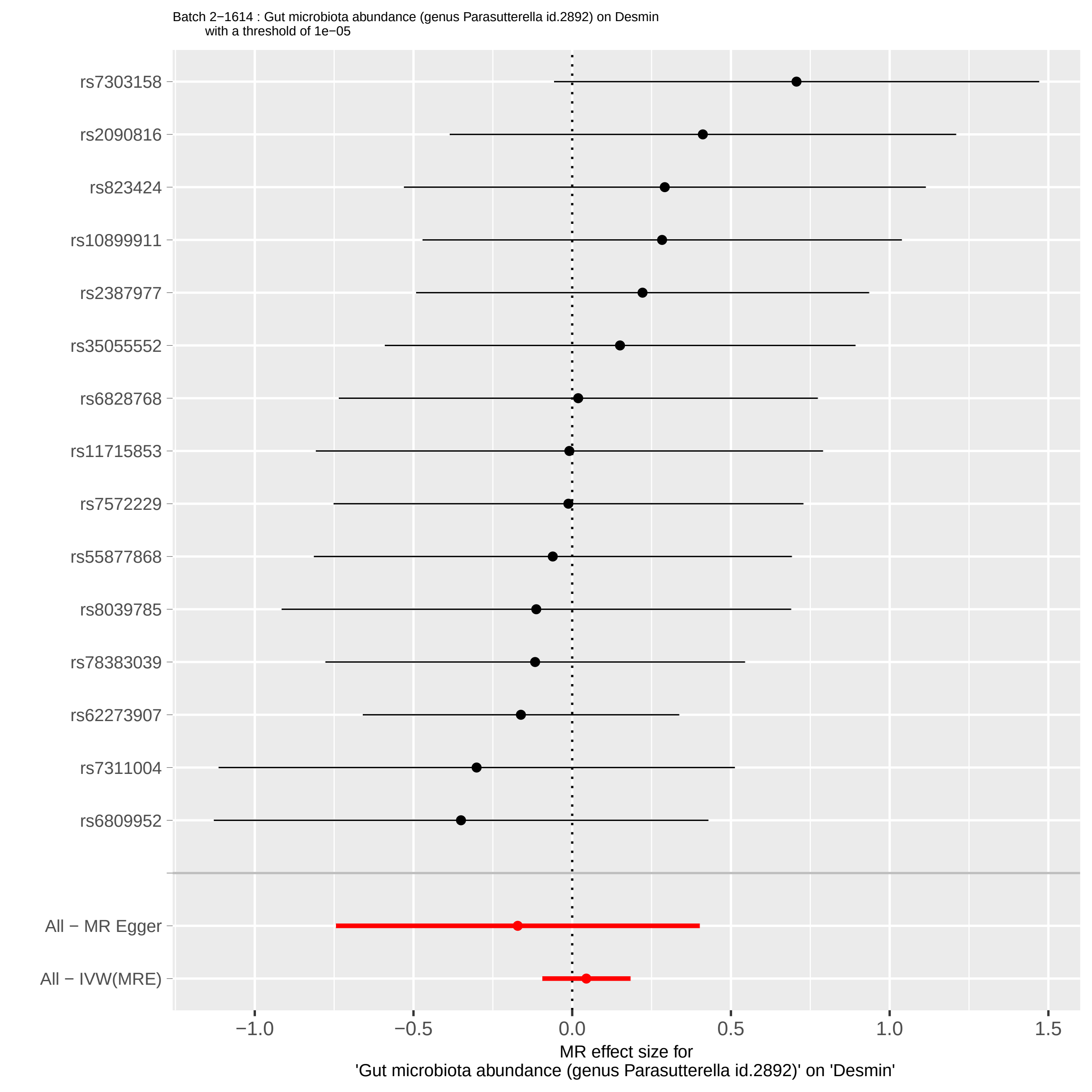

Batch 2−1614 : Gut microbiota abundance (genus Parasutterella id.2892) on Desmin
with a threshold of 1e−05
rs7303158
rs2090816
rs823424
rs10899911
rs2387977
rs35055552
rs6828768
rs11715853
rs7572229
rs55877868
rs8039785
rs78383039
rs62273907
rs7311004
rs6809952
All − MR Egger
All − IVW(MRE)
−1.0
−0.5
0.0
0.5
1.0
1.5
MR effect size for
'Gut microbiota abundance (genus Parasutterella id.2892)' on 'Desmin'

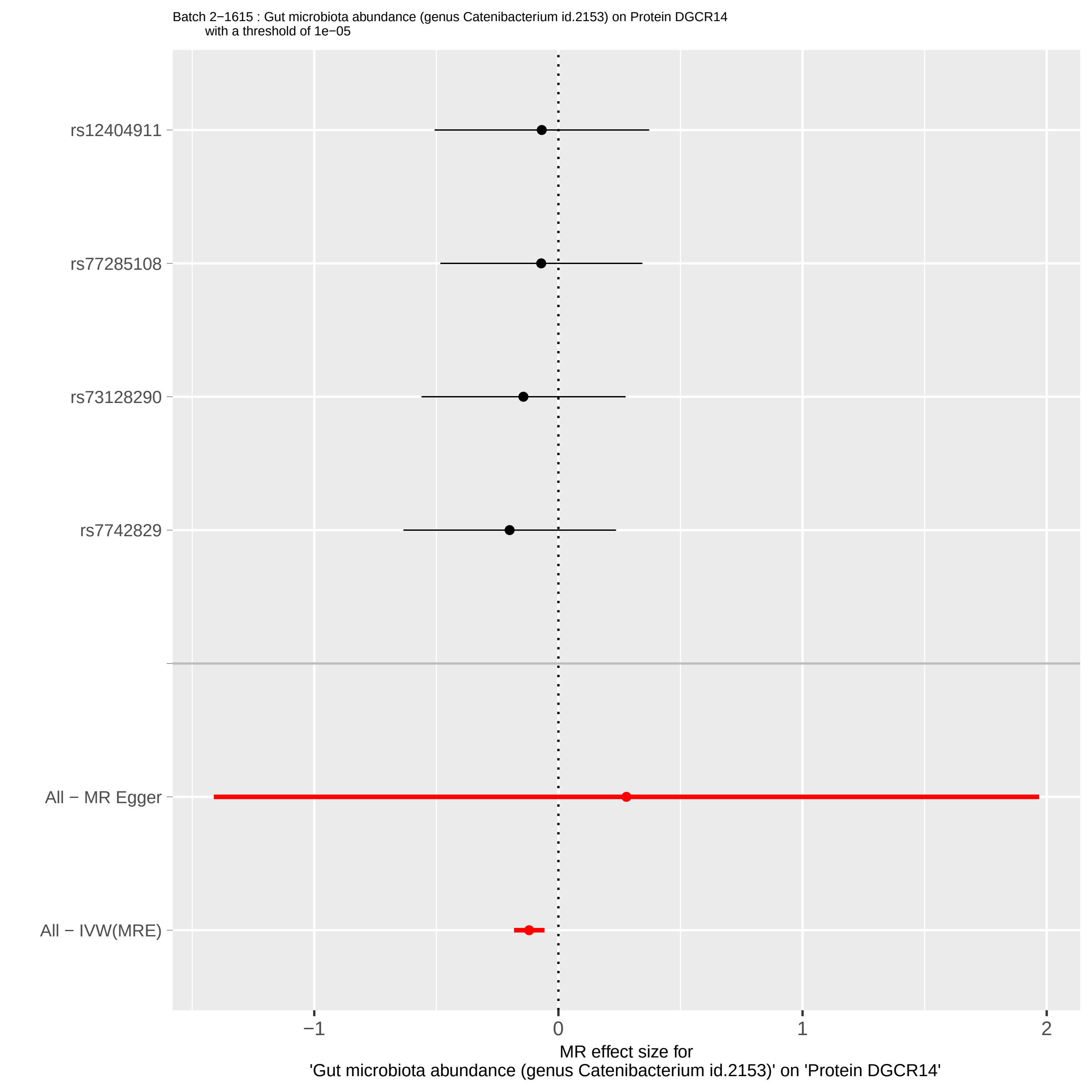
Batch 2−1615 : Gut microbiota abundance (genus Catenibacterium id.2153) on Protein DGCR14
with a threshold of 1e−05
rs12404911
rs77285108
rs73128290
rs7742829
All − MR Egger
All − IVW(MRE)
−1
0
1
2
MR effect size for
'Gut microbiota abundance (genus Catenibacterium id.2153)' on 'Protein DGCR14'

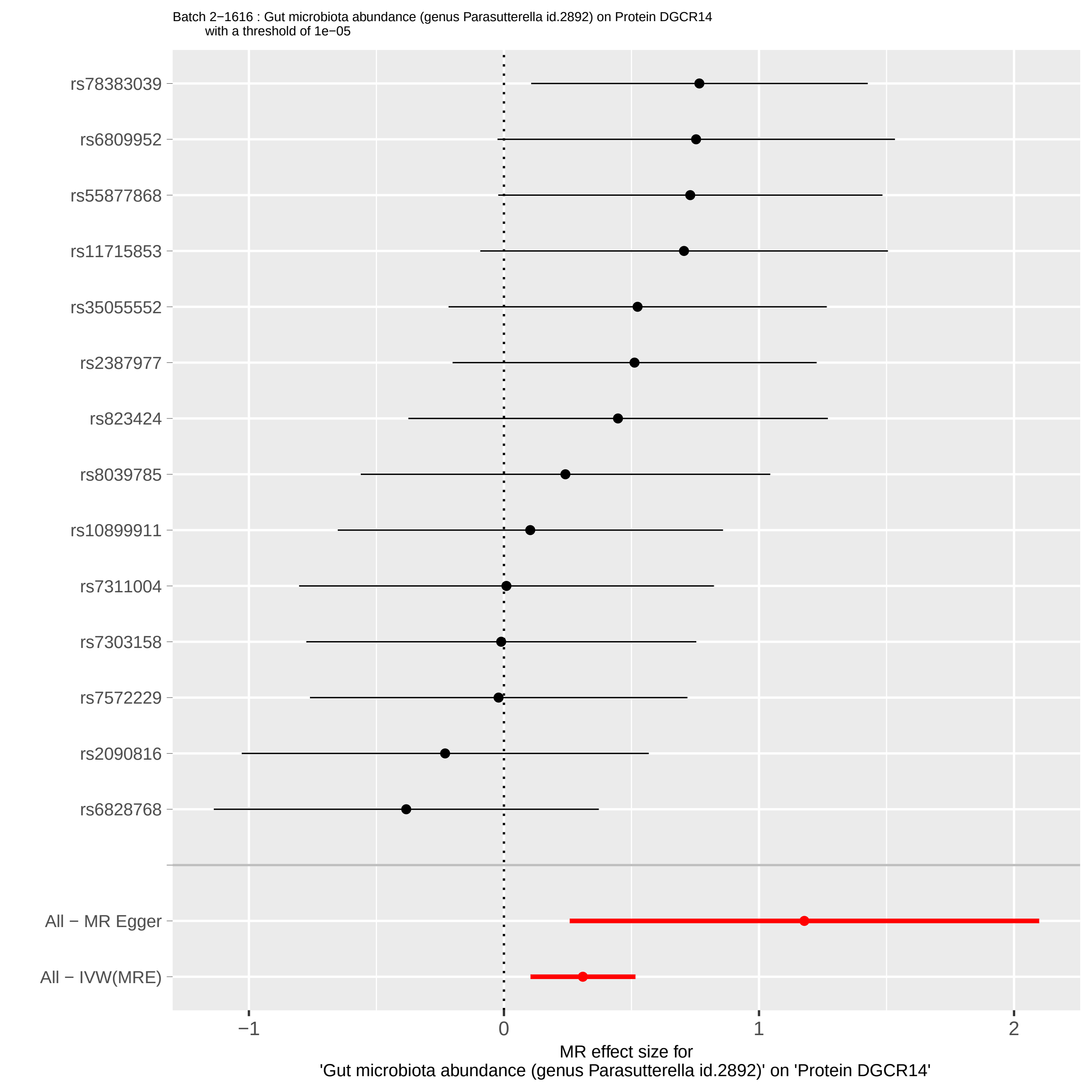

Batch 2−1616 : Gut microbiota abundance (genus Parasutterella id.2892) on Protein DGCR14
with a threshold of 1e−05
rs78383039
rs6809952
rs55877868
rs11715853
rs35055552
rs2387977
rs823424
rs8039785
rs10899911
rs7311004
rs7303158
rs7572229
rs2090816
rs6828768
All − MR Egger
All − IVW(MRE)
−1
0
1
2
MR effect size for
'Gut microbiota abundance (genus Parasutterella id.2892)' on 'Protein DGCR14'

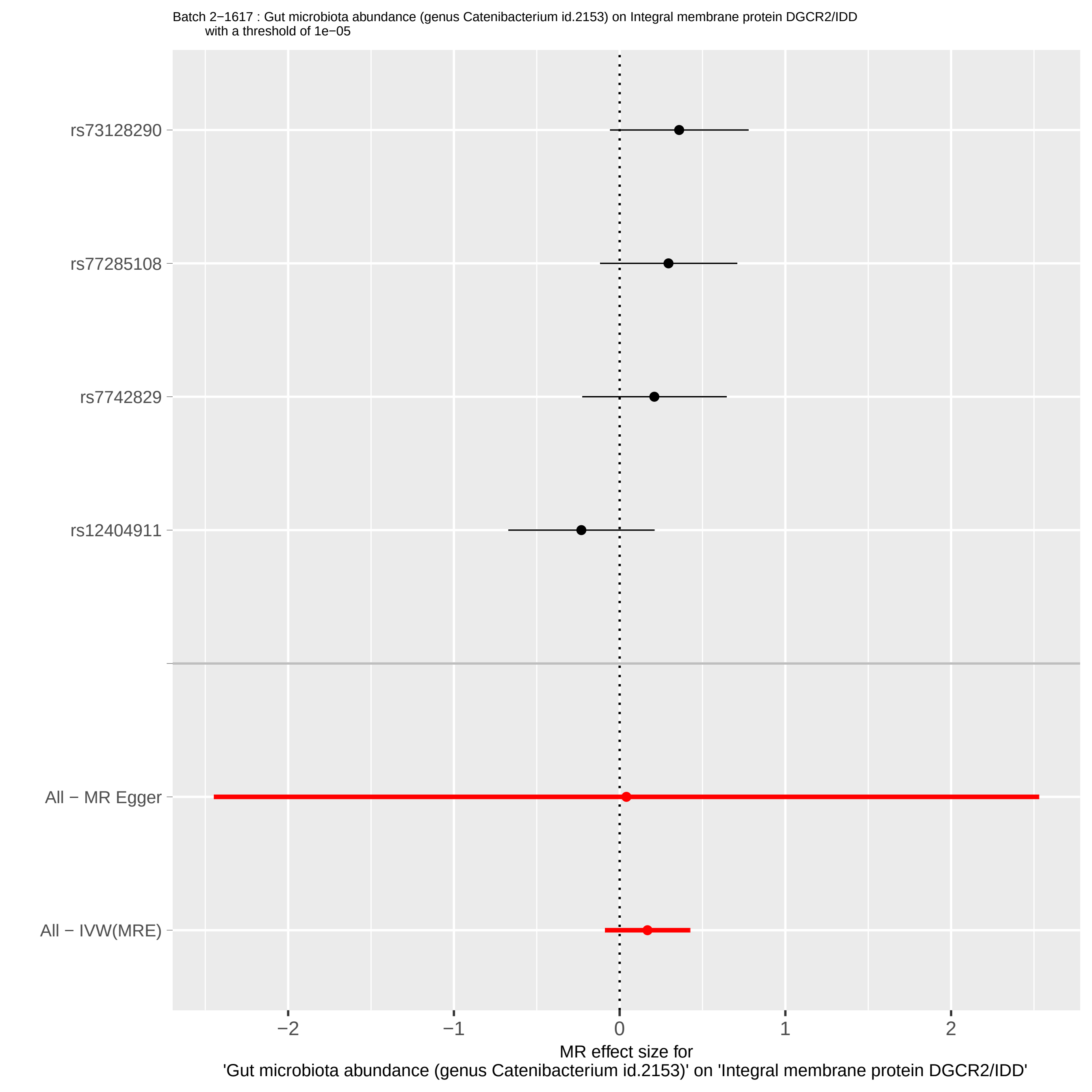

Batch 2−1617 : Gut microbiota abundance (genus Catenibacterium id.2153) on Integral membrane protein DGCR2/IDD
with a threshold of 1e−05
rs73128290
rs77285108
rs7742829
rs12404911
All − MR Egger
All − IVW(MRE)
−2
−1
0
1
2
MR effect size for
'Gut microbiota abundance (genus Catenibacterium id.2153)' on 'Integral membrane protein DGCR2/IDD'

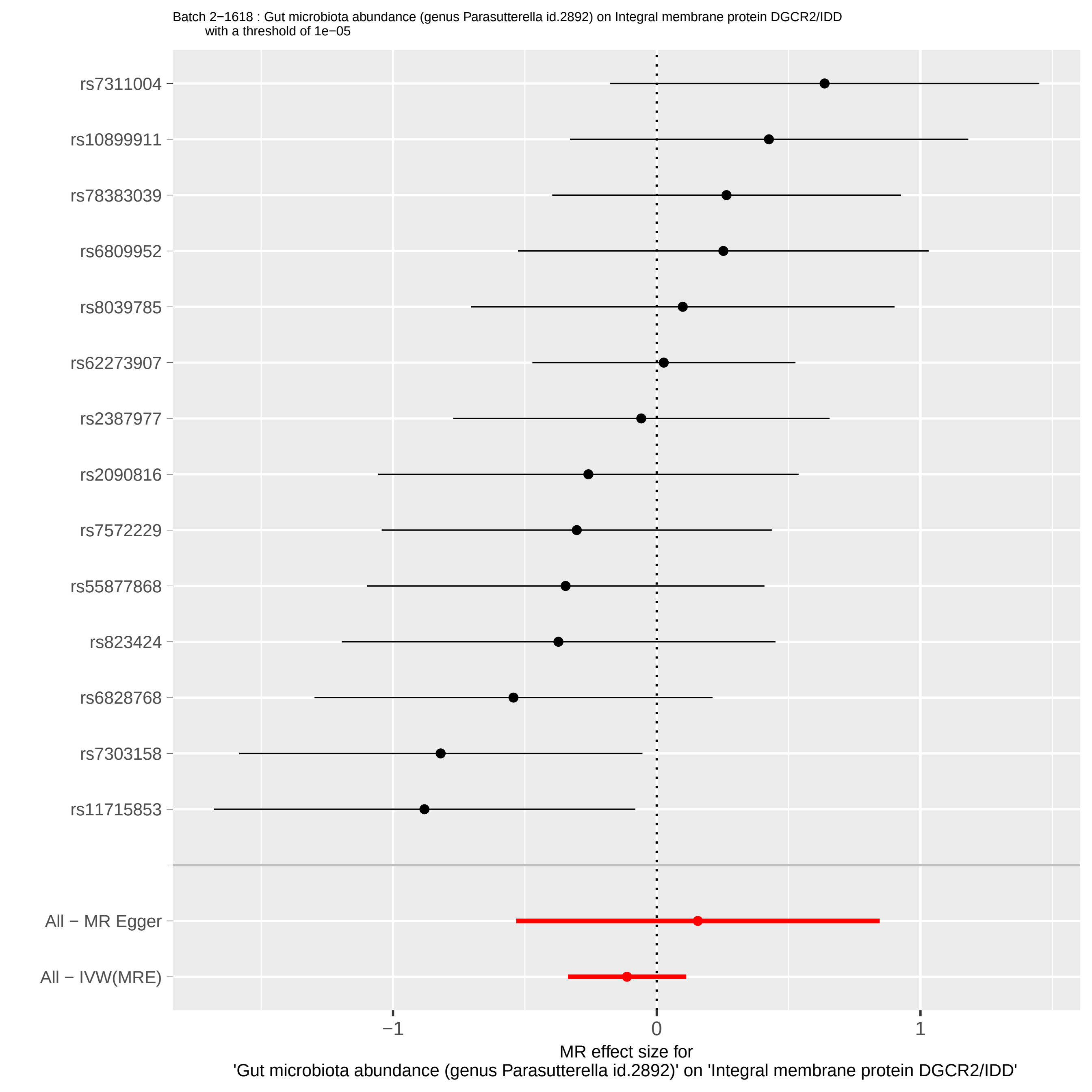
Batch 2−1618 : Gut microbiota abundance (genus Parasutterella id.2892) on Integral membrane protein DGCR2/IDD
with a threshold of 1e−05
rs7311004
rs10899911
rs78383039
rs6809952
rs8039785
rs62273907
rs2387977
rs2090816
rs7572229
rs55877868
rs823424
rs6828768
rs7303158
rs11715853
All − MR Egger
All − IVW(MRE)
−1
0
1
MR effect size for
'Gut microbiota abundance (genus Parasutterella id.2892)' on 'Integral membrane protein DGCR2/IDD'

Batch 2−1619 : Gut microbiota abundance (genus Catenibacterium id.2153) on Protein DGCR6
with a threshold of 1e−05

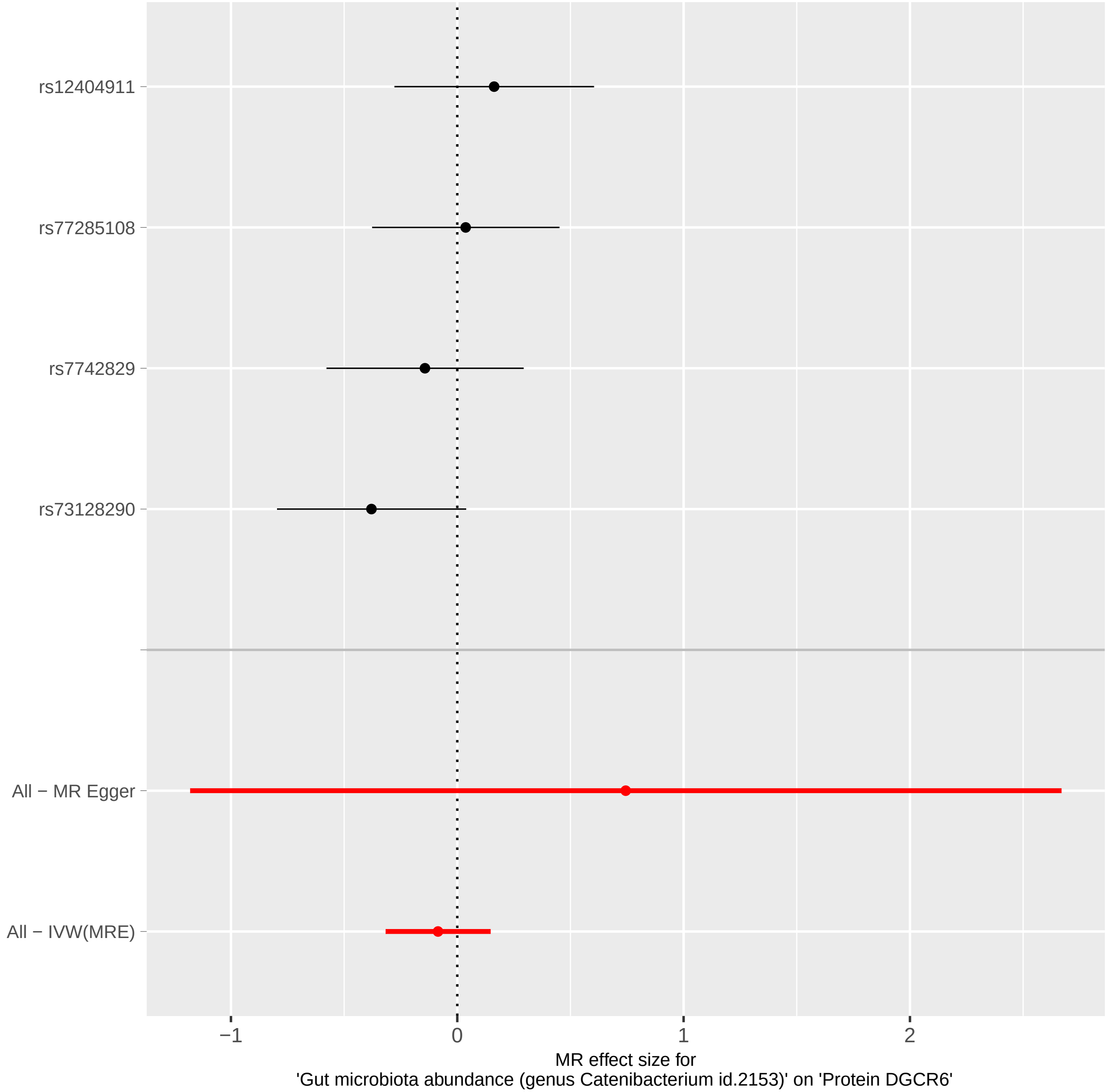

Batch 2−1620 : Gut microbiota abundance (genus Parasutterella id.2892) on Protein DGCR6
with a threshold of 1e−05

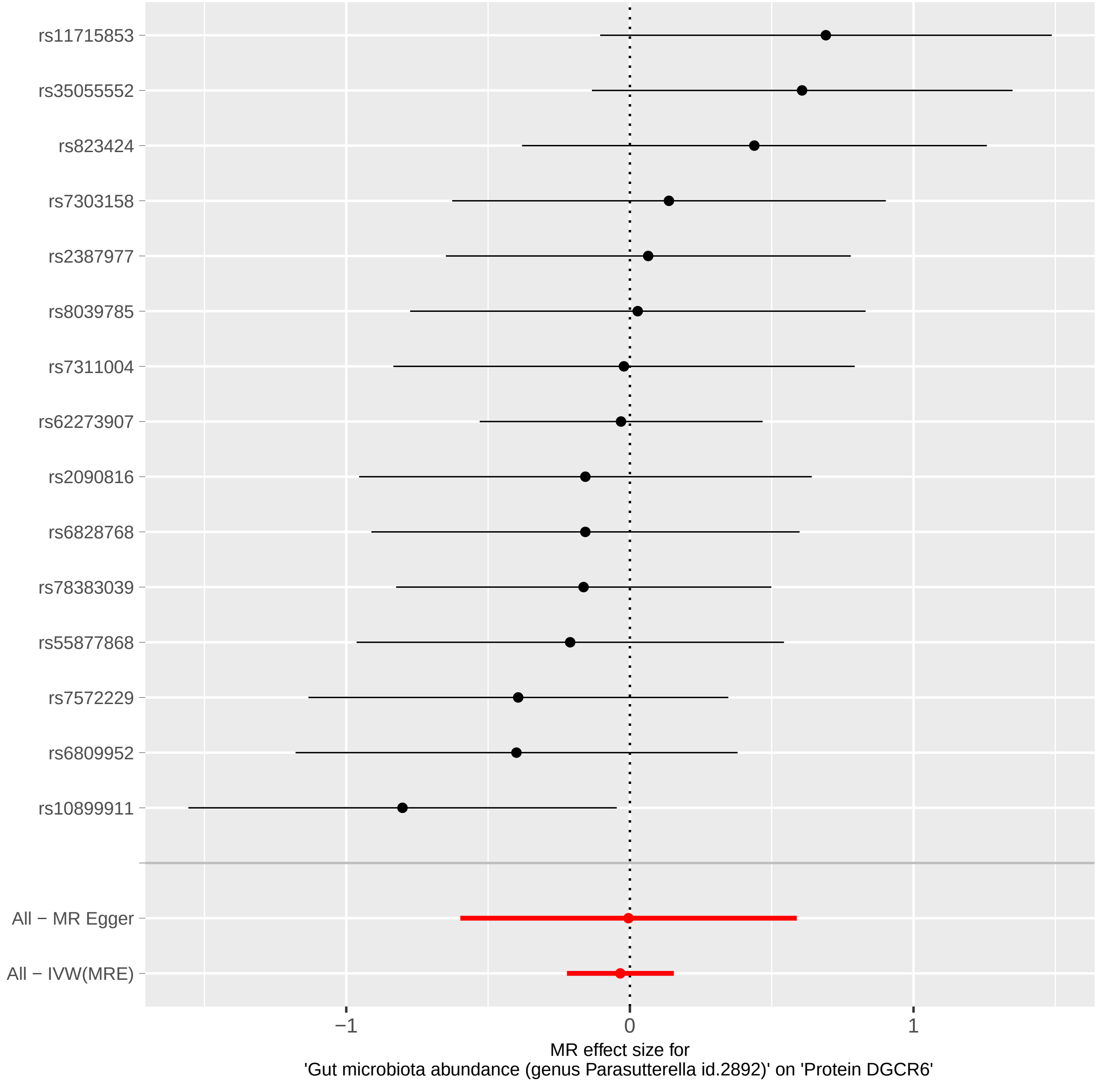

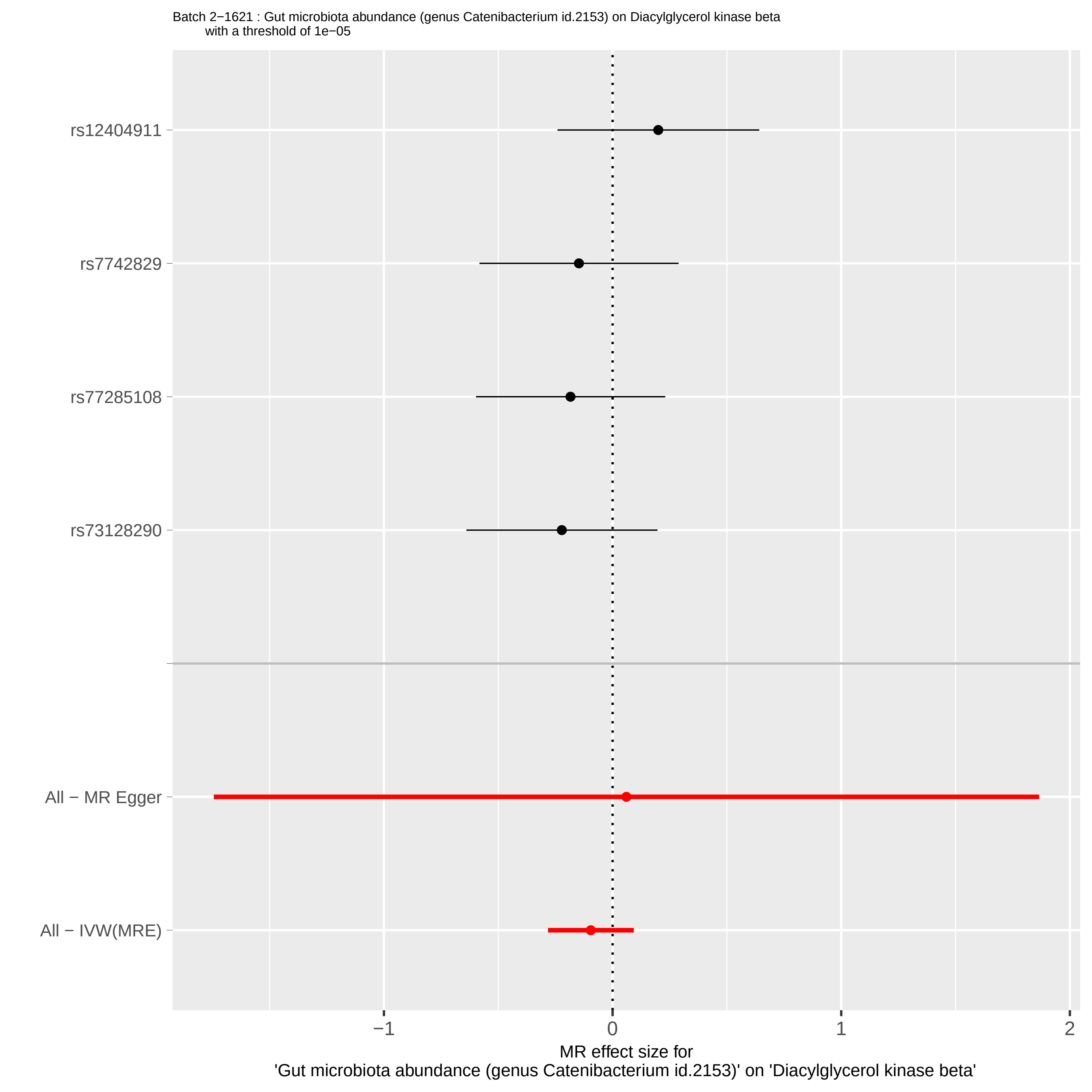
Batch 2−1621 : Gut microbiota abundance (genus Catenibacterium id.2153) on Diacylglycerol kinase beta
with a threshold of 1e−05
rs12404911
rs7742829
rs77285108
rs73128290
All − MR Egger
All − IVW(MRE)
−1
0
1
2
MR effect size for
'Gut microbiota abundance (genus Catenibacterium id.2153)' on 'Diacylglycerol kinase beta'

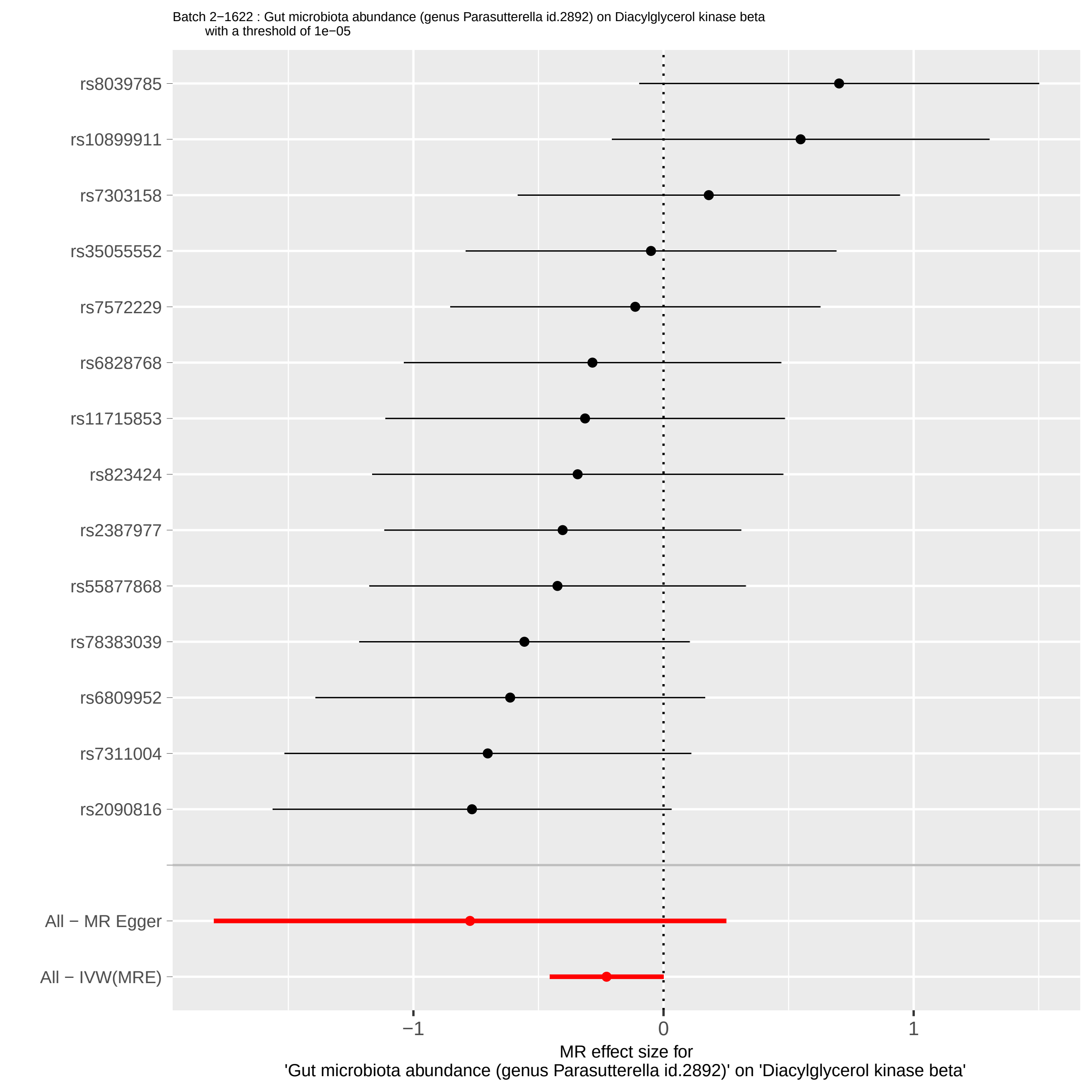

Batch 2−1622 : Gut microbiota abundance (genus Parasutterella id.2892) on Diacylglycerol kinase beta
with a threshold of 1e−05
rs8039785
rs10899911
rs7303158
rs35055552
rs7572229
rs6828768
rs11715853
rs823424
rs2387977
rs55877868
rs78383039
rs6809952
rs7311004
rs2090816
All − MR Egger
All − IVW(MRE)
−1
0
1
MR effect size for
'Gut microbiota abundance (genus Parasutterella id.2892)' on 'Diacylglycerol kinase beta'

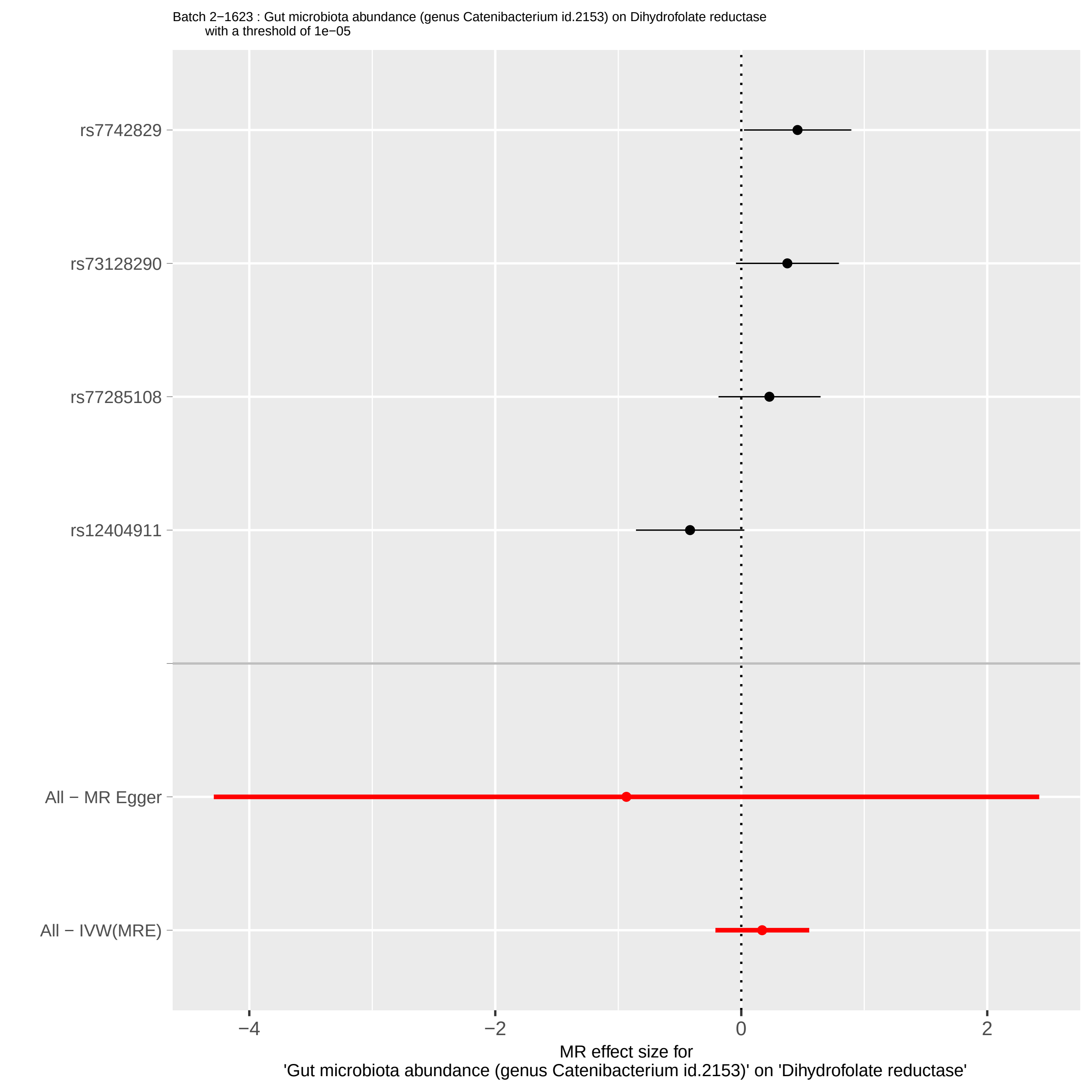

Batch 2−1623 : Gut microbiota abundance (genus Catenibacterium id.2153) on Dihydrofolate reductase
with a threshold of 1e−05
rs7742829
rs73128290
rs77285108
rs12404911
All − MR Egger
All − IVW(MRE)
−4
−2
0
2
MR effect size for
'Gut microbiota abundance (genus Catenibacterium id.2153)' on 'Dihydrofolate reductase'

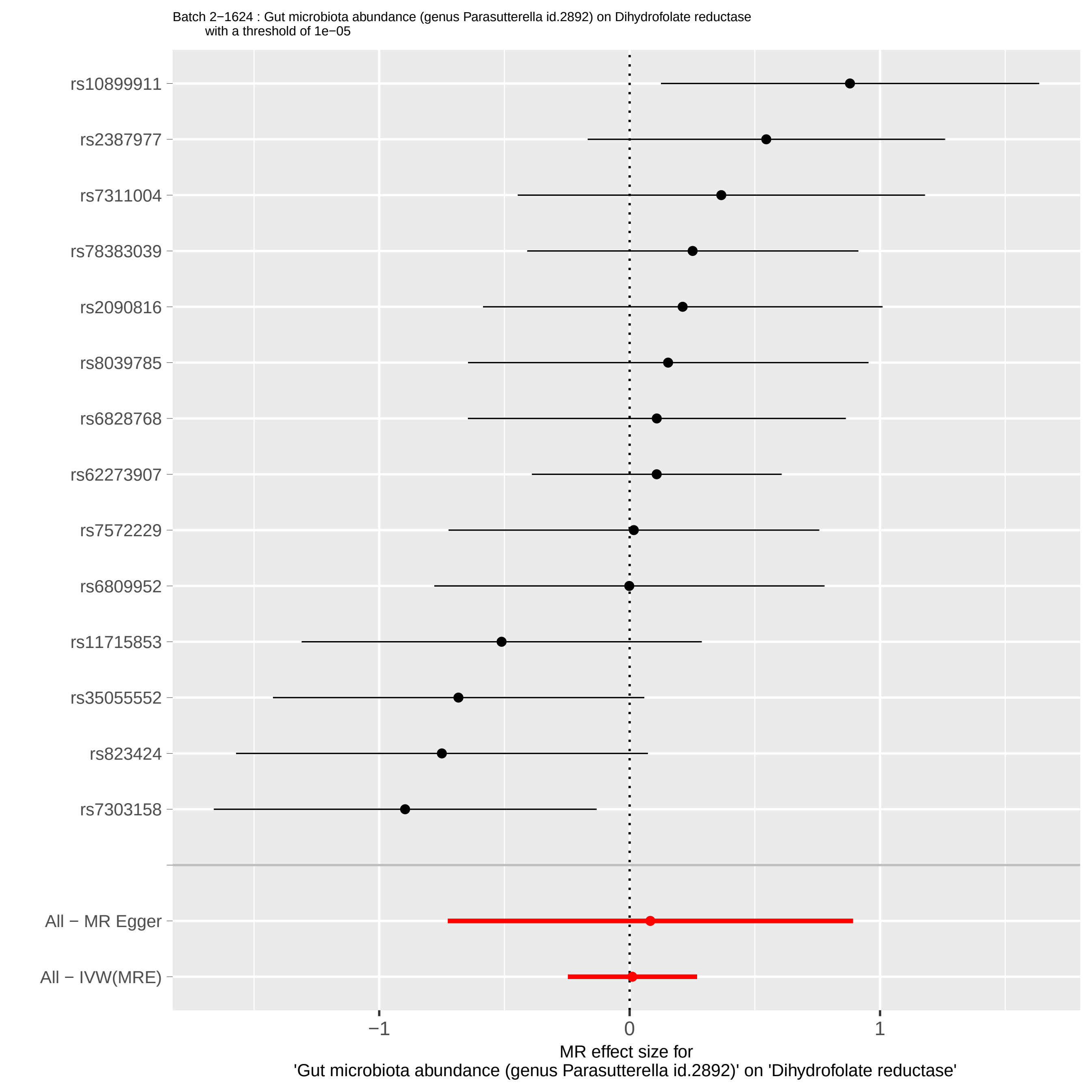
Batch 2−1624 : Gut microbiota abundance (genus Parasutterella id.2892) on Dihydrofolate reductase
with a threshold of 1e−05
rs10899911
rs2387977
rs7311004
rs78383039
rs2090816
rs8039785
rs6828768
rs62273907
rs7572229
rs6809952
rs11715853
rs35055552
rs823424
rs7303158
All − MR Egger
All − IVW(MRE)
−1
0
1
MR effect size for
'Gut microbiota abundance (genus Parasutterella id.2892)' on 'Dihydrofolate reductase'

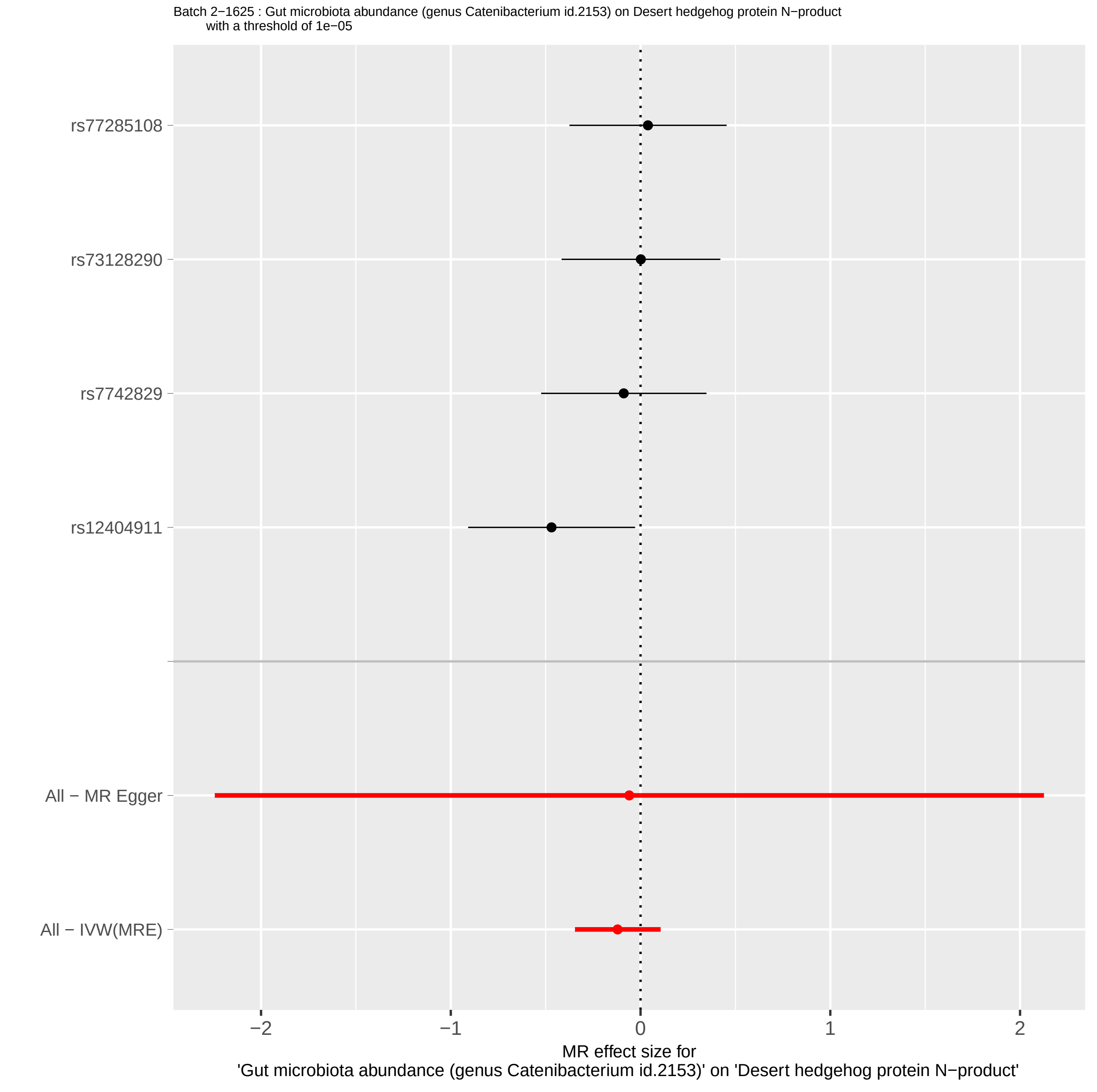

Batch 2−1625 : Gut microbiota abundance (genus Catenibacterium id.2153) on Desert hedgehog protein N−product
with a threshold of 1e−05
rs77285108
rs73128290
rs7742829
rs12404911
All − MR Egger
All − IVW(MRE)
−2
−1
0
1
2
MR effect size for
'Gut microbiota abundance (genus Catenibacterium id.2153)' on 'Desert hedgehog protein N−product'

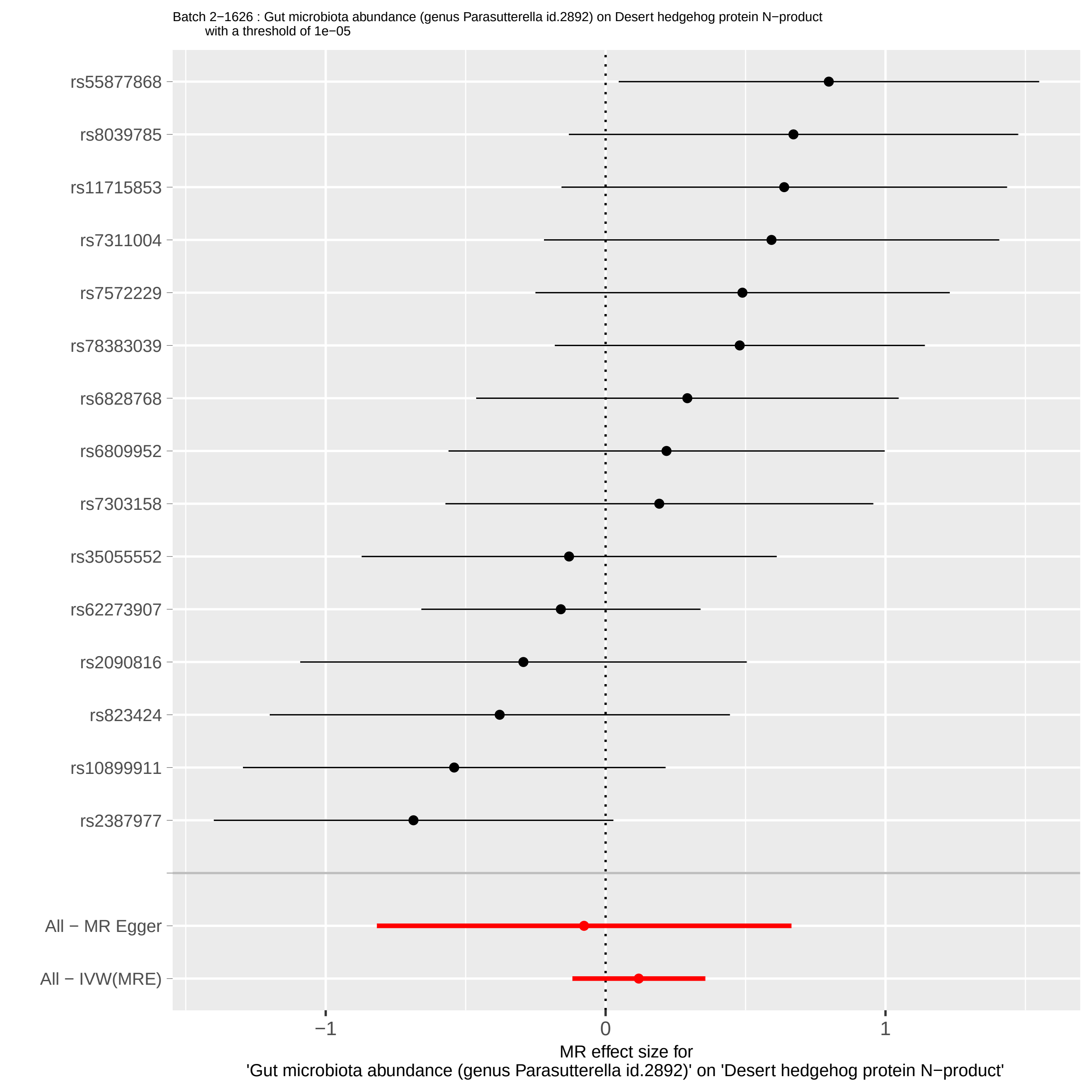
Batch 2−1626 : Gut microbiota abundance (genus Parasutterella id.2892) on Desert hedgehog protein N−product
with a threshold of 1e−05
rs55877868
rs8039785
rs11715853
rs7311004
rs7572229
rs78383039
rs6828768
rs6809952
rs7303158
rs35055552
rs62273907
rs2090816
rs823424
rs10899911
rs2387977
All − MR Egger
All − IVW(MRE)
−1
0
1
MR effect size for
'Gut microbiota abundance (genus Parasutterella id.2892)' on 'Desert hedgehog protein N−product'

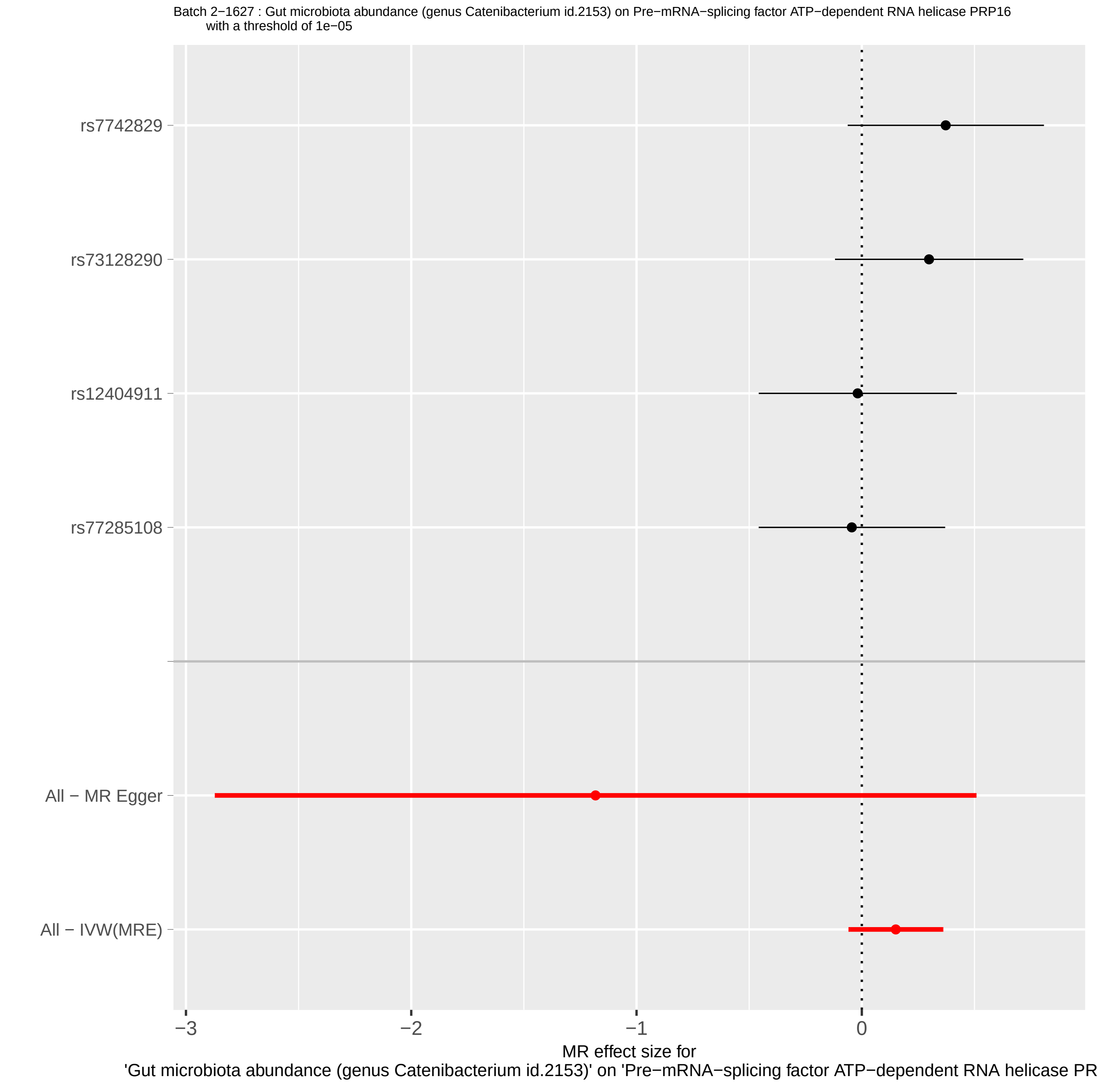

Batch 2−1627 : Gut microbiota abundance (genus Catenibacterium id.2153) on Pre−mRNA−splicing factor ATP−dependent RNA helicase PRP16
with a threshold of 1e−05
rs7742829
rs73128290
rs12404911
rs77285108
All − MR Egger
All − IVW(MRE)
−3
−2
−1
0
MR effect size for
'Gut microbiota abundance (genus Catenibacterium id.2153)' on 'Pre−mRNA−splicing factor ATP−dependent RNA helicase PR

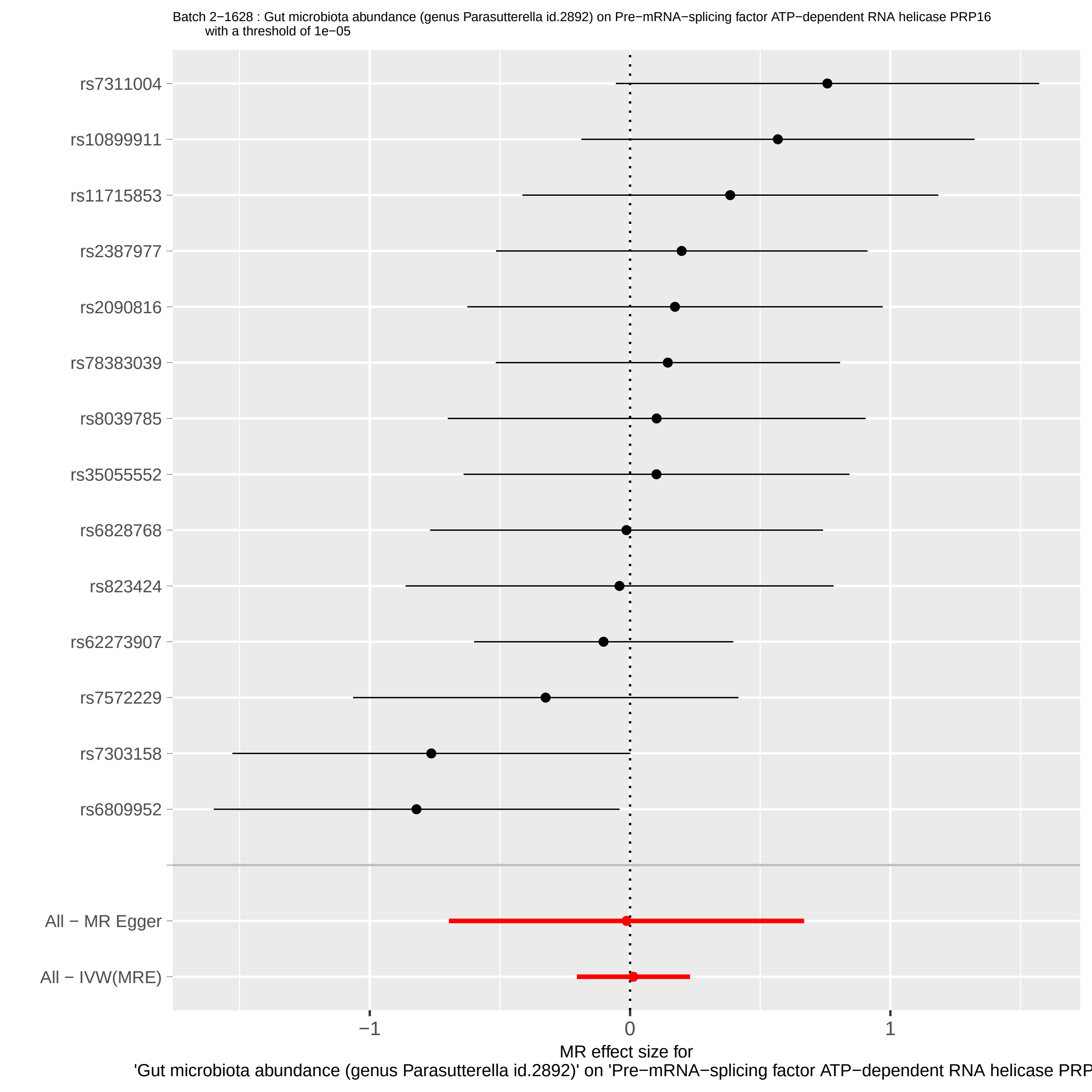
Batch 2−1628 : Gut microbiota abundance (genus Parasutterella id.2892) on Pre−mRNA−splicing factor ATP−dependent RNA helicase PRP16
with a threshold of 1e−05
rs7311004
rs10899911
rs11715853
rs2387977
rs2090816
rs78383039
rs8039785
rs35055552
rs6828768
rs823424
rs62273907
rs7572229
rs7303158
rs6809952
All − MR Egger
All − IVW(MRE)
−1
0
1
MR effect size for
'Gut microbiota abundance (genus Parasutterella id.2892)' on 'Pre−mRNA−splicing factor ATP−dependent RNA helicase PRP

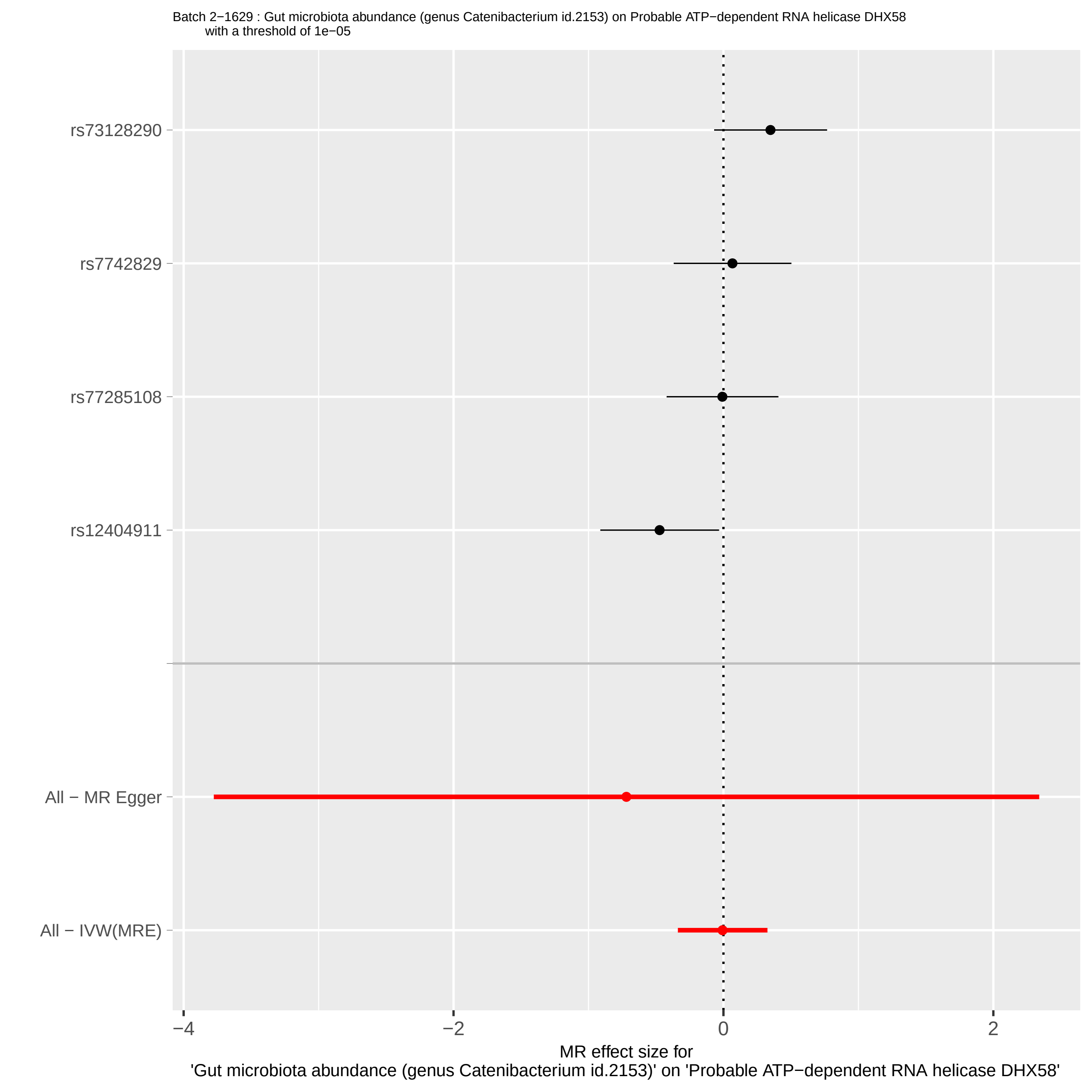

Batch 2−1629 : Gut microbiota abundance (genus Catenibacterium id.2153) on Probable ATP−dependent RNA helicase DHX58
with a threshold of 1e−05
rs73128290
rs7742829
rs77285108
rs12404911
All − MR Egger
All − IVW(MRE)
−4
−2
0
2
MR effect size for
'Gut microbiota abundance (genus Catenibacterium id.2153)' on 'Probable ATP−dependent RNA helicase DHX58'

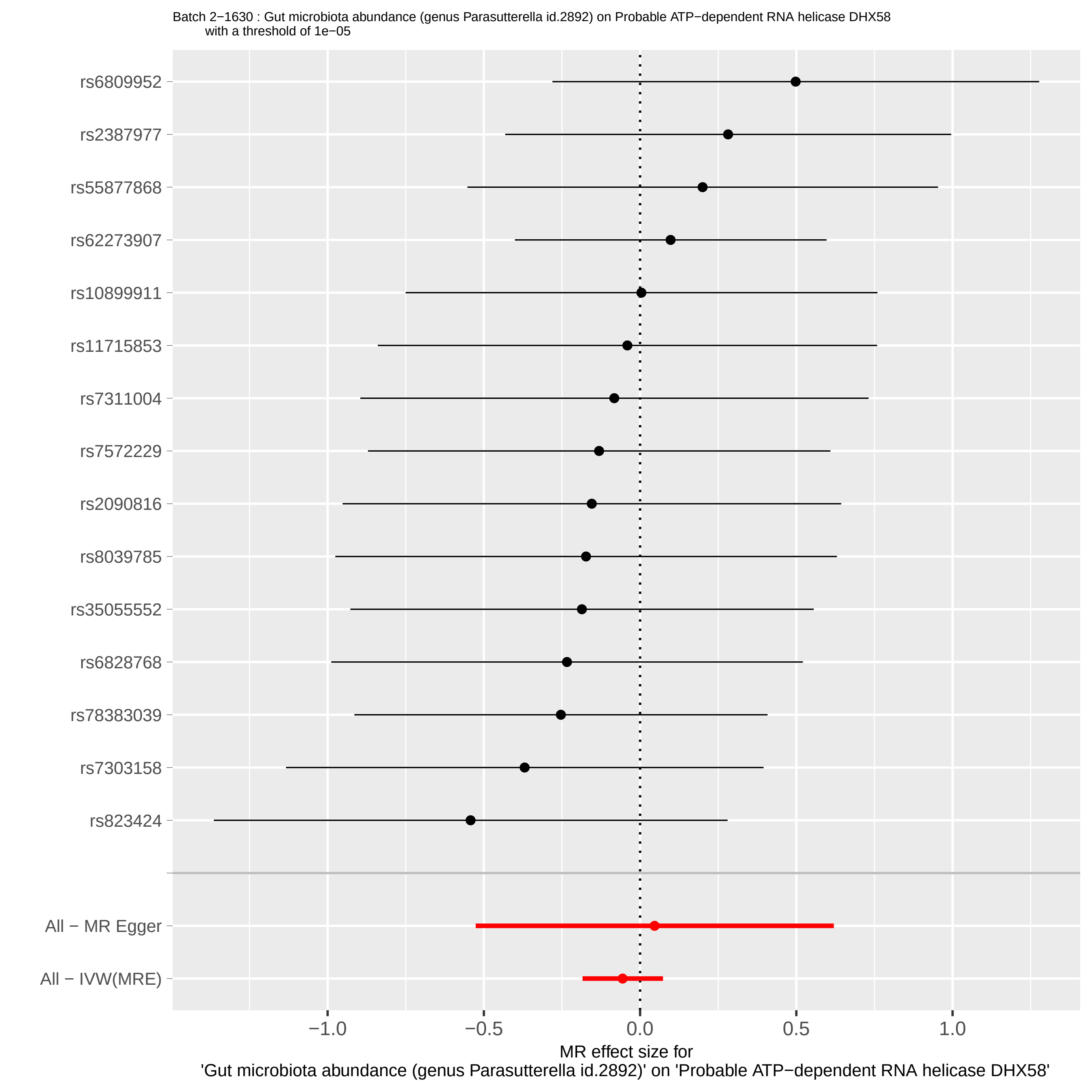
Batch 2−1630 : Gut microbiota abundance (genus Parasutterella id.2892) on Probable ATP−dependent RNA helicase DHX58
with a threshold of 1e−05
rs6809952
rs2387977
rs55877868
rs62273907
rs10899911
rs11715853
rs7311004
rs7572229
rs2090816
rs8039785
rs35055552
rs6828768
rs78383039
rs7303158
rs823424
All − MR Egger
All − IVW(MRE)
−1.0
−0.5
0.0
0.5
1.0
MR effect size for
'Gut microbiota abundance (genus Parasutterella id.2892)' on 'Probable ATP−dependent RNA helicase DHX58'

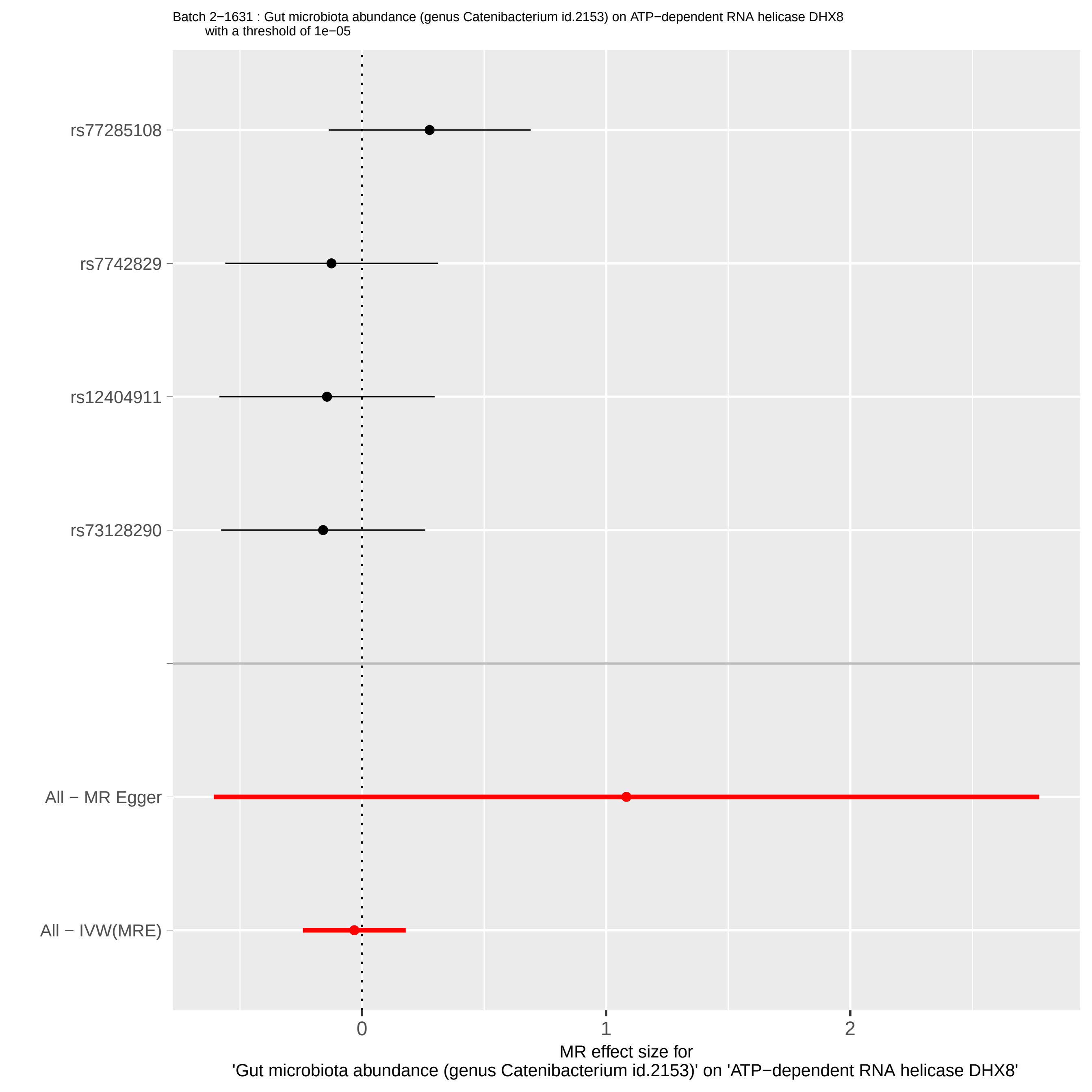
Batch 2−1631 : Gut microbiota abundance (genus Catenibacterium id.2153) on ATP−dependent RNA helicase DHX8
with a threshold of 1e−05
rs77285108
rs7742829
rs12404911
rs73128290
All − MR Egger
All − IVW(MRE)
0
1
2
MR effect size for
'Gut microbiota abundance (genus Catenibacterium id.2153)' on 'ATP−dependent RNA helicase DHX8'

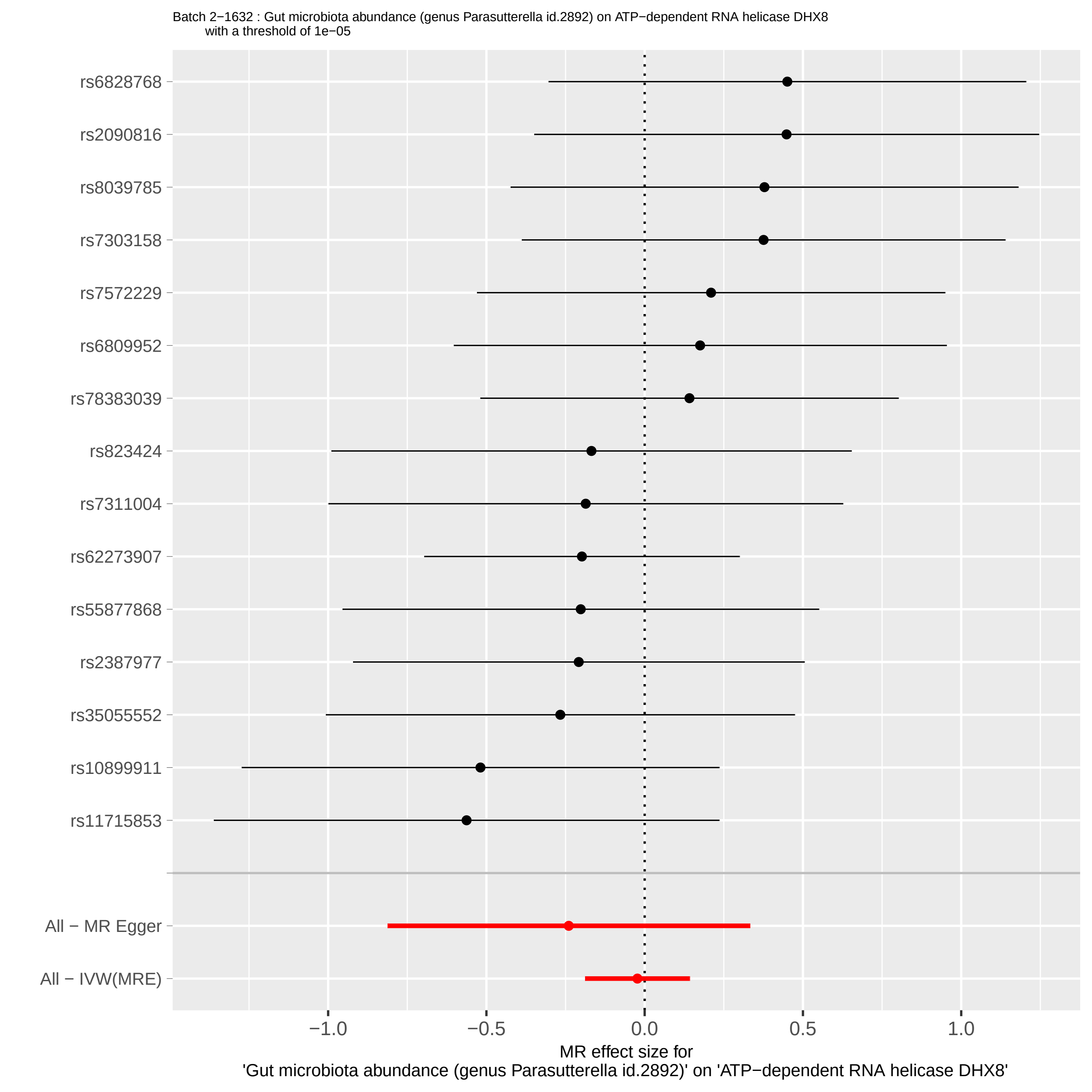

Batch 2−1632 : Gut microbiota abundance (genus Parasutterella id.2892) on ATP−dependent RNA helicase DHX8
with a threshold of 1e−05
rs6828768
rs2090816
rs8039785
rs7303158
rs7572229
rs6809952
rs78383039
rs823424
rs7311004
rs62273907
rs55877868
rs2387977
rs35055552
rs10899911
rs11715853
All − MR Egger
All − IVW(MRE)
−1.0
−0.5
0.0
0.5
1.0
MR effect size for
'Gut microbiota abundance (genus Parasutterella id.2892)' on 'ATP−dependent RNA helicase DHX8'

Batch 2−1633 : Gut microbiota abundance (genus Catenibacterium id.2153) on ATP−dependent RNA helicase A
with a threshold of 1e−05

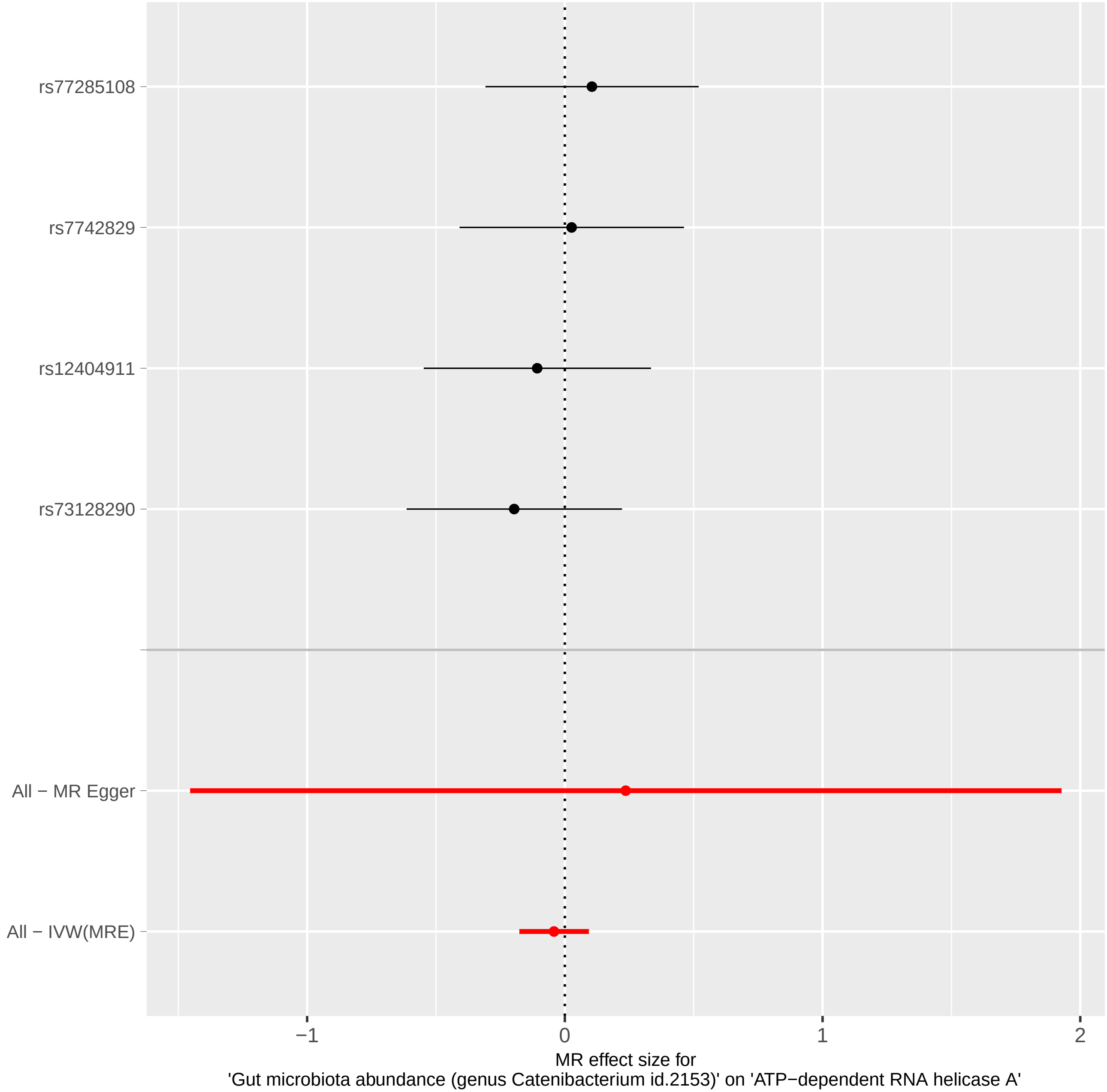

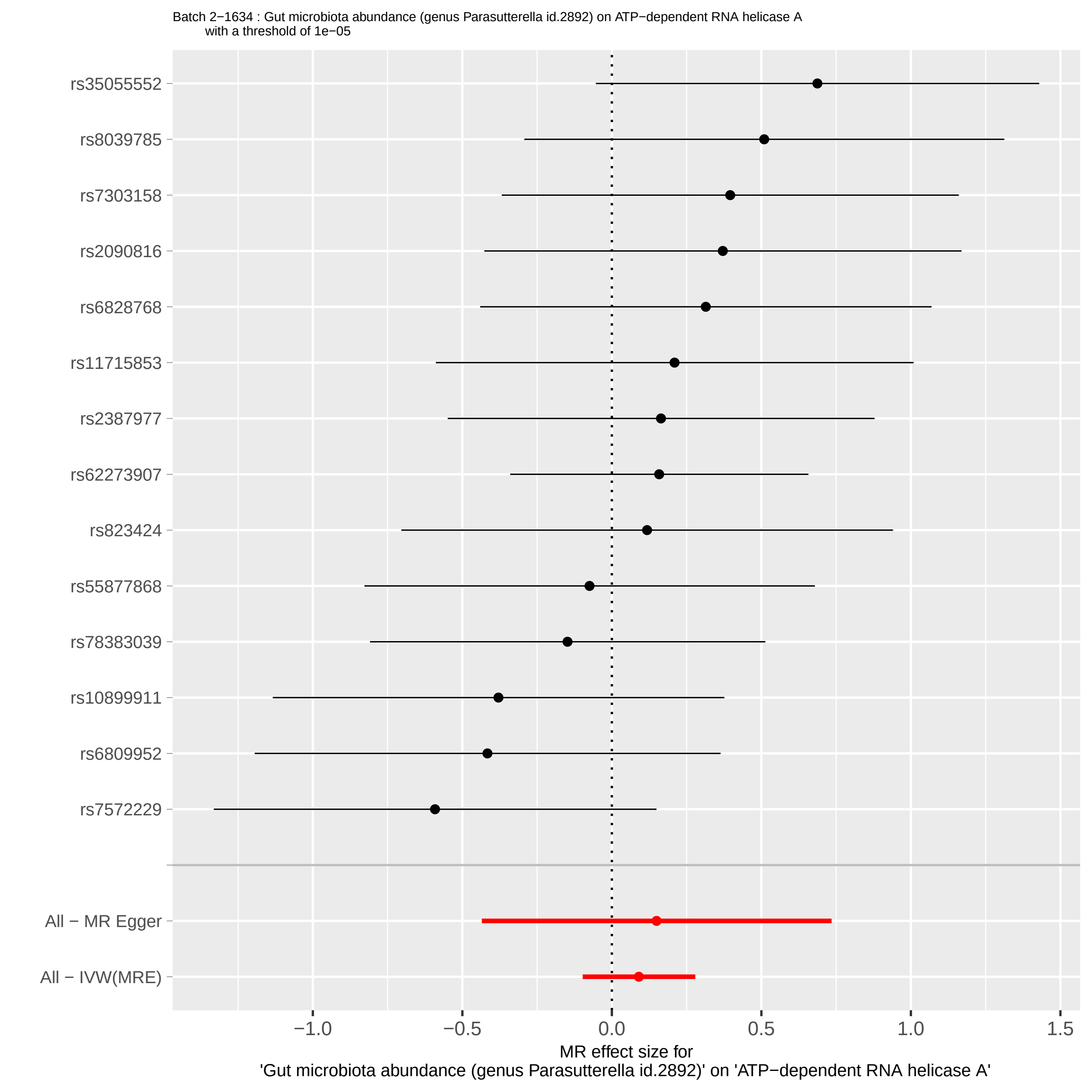

Batch 2−1634 : Gut microbiota abundance (genus Parasutterella id.2892) on ATP−dependent RNA helicase A
with a threshold of 1e−05
rs35055552
rs8039785
rs7303158
rs2090816
rs6828768
rs11715853
rs2387977
rs62273907
rs823424
rs55877868
rs78383039
rs10899911
rs6809952
rs7572229
All − MR Egger
All − IVW(MRE)
−1.0
−0.5
0.0
0.5
1.0
1.5
MR effect size for
'Gut microbiota abundance (genus Parasutterella id.2892)' on 'ATP−dependent RNA helicase A'

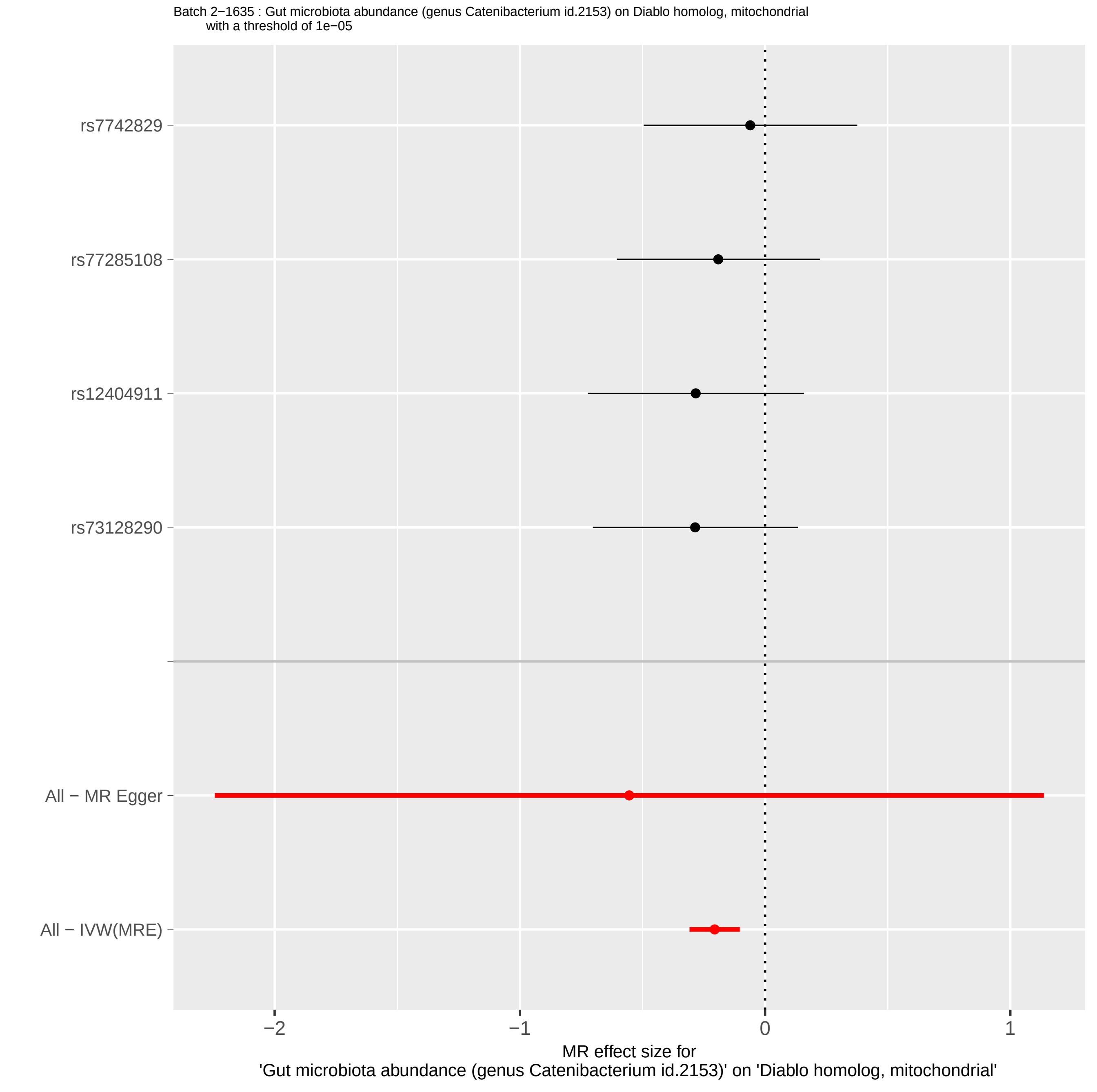

Batch 2−1635 : Gut microbiota abundance (genus Catenibacterium id.2153) on Diablo homolog, mitochondrial
with a threshold of 1e−05
rs7742829
rs77285108
rs12404911
rs73128290
All − MR Egger
All − IVW(MRE)
−2
−1
0
1
MR effect size for
'Gut microbiota abundance (genus Catenibacterium id.2153)' on 'Diablo homolog, mitochondrial'

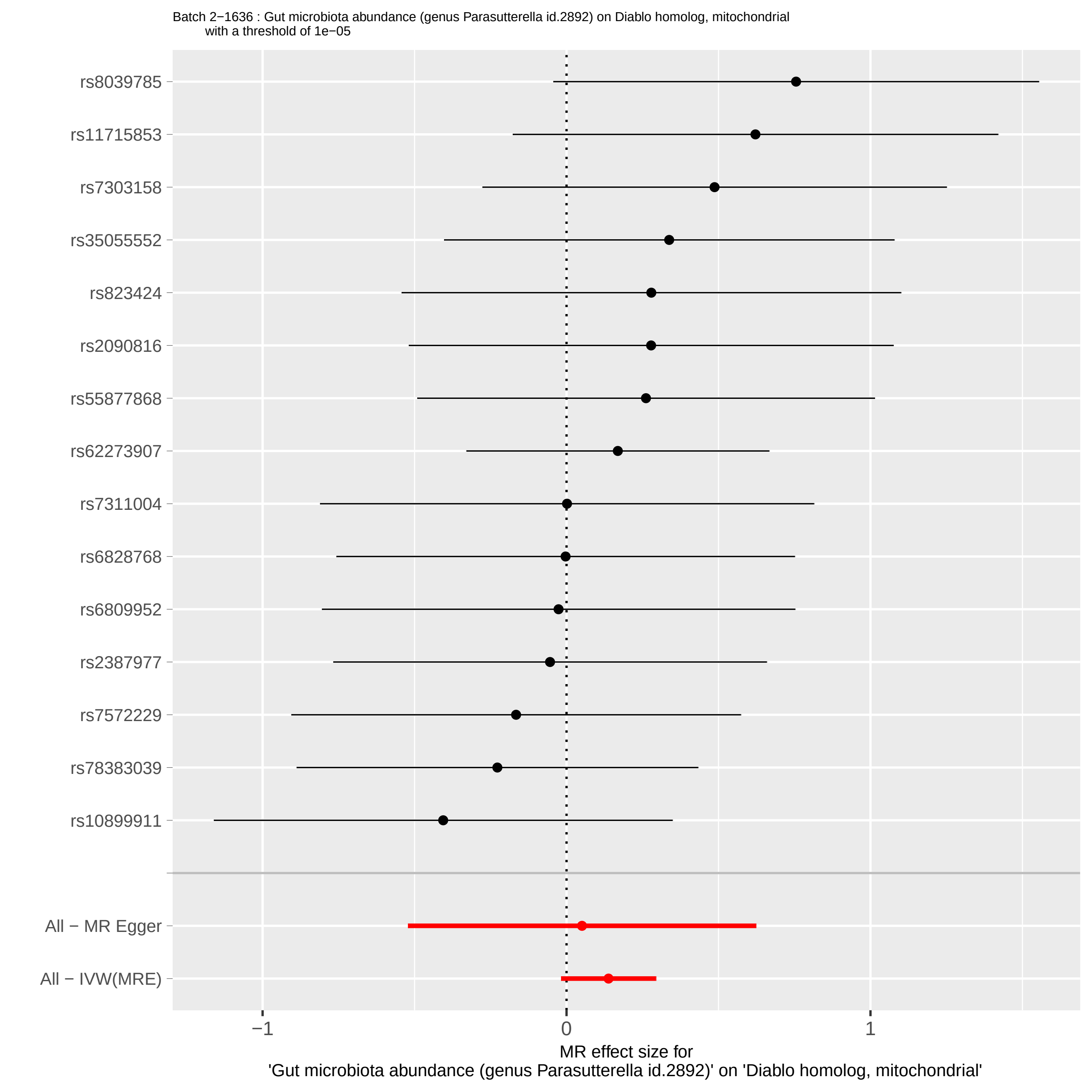

Batch 2−1636 : Gut microbiota abundance (genus Parasutterella id.2892) on Diablo homolog, mitochondrial
with a threshold of 1e−05
rs8039785
rs11715853
rs7303158
rs35055552
rs823424
rs2090816
rs55877868
rs62273907
rs7311004
rs6828768
rs6809952
rs2387977
rs7572229
rs78383039
rs10899911
All − MR Egger
All − IVW(MRE)
−1
0
1
MR effect size for
'Gut microbiota abundance (genus Parasutterella id.2892)' on 'Diablo homolog, mitochondrial'

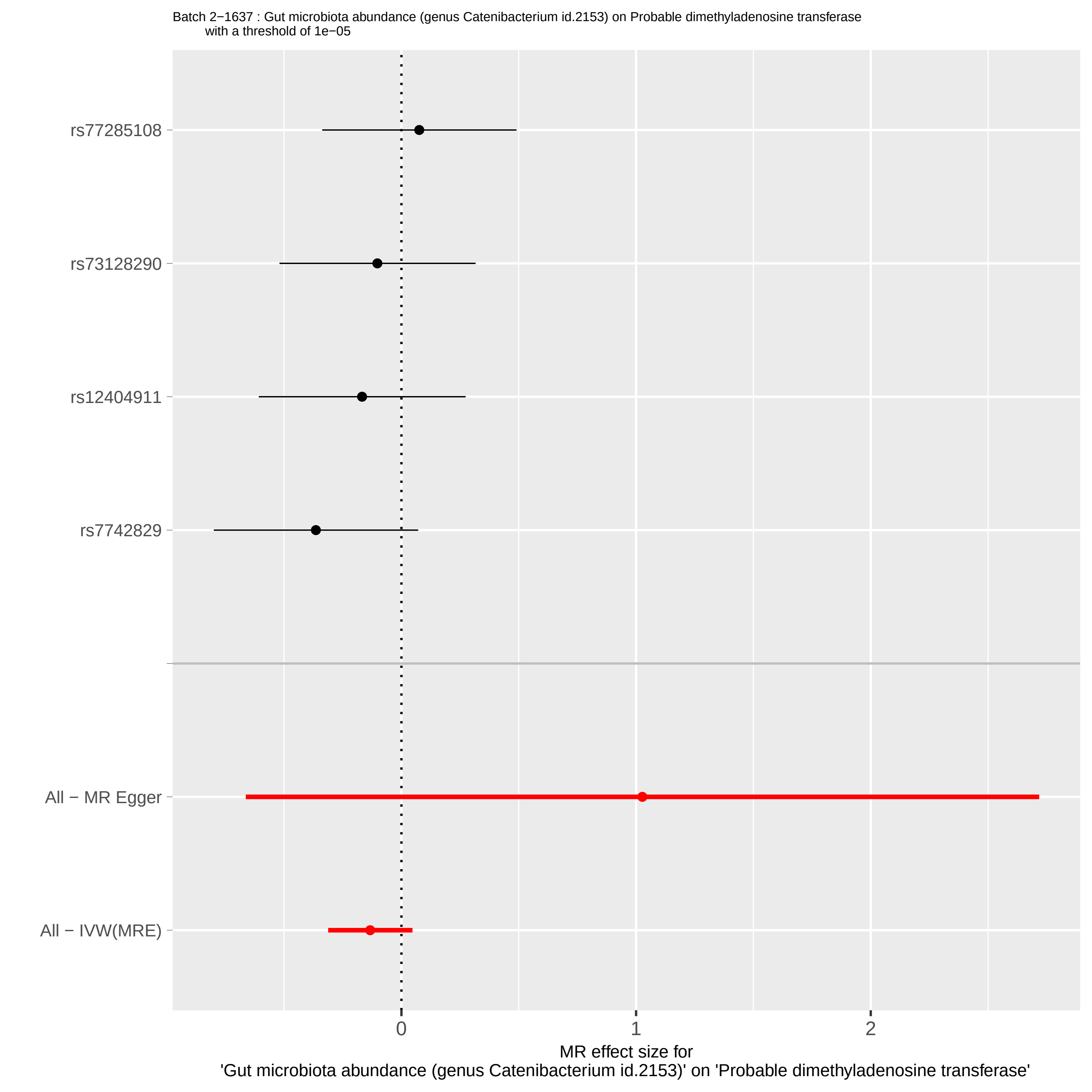

Batch 2−1637 : Gut microbiota abundance (genus Catenibacterium id.2153) on Probable dimethyladenosine transferase
with a threshold of 1e−05
rs77285108
rs73128290
rs12404911
rs7742829
All − MR Egger
All − IVW(MRE)
0
1
2
MR effect size for
'Gut microbiota abundance (genus Catenibacterium id.2153)' on 'Probable dimethyladenosine transferase'

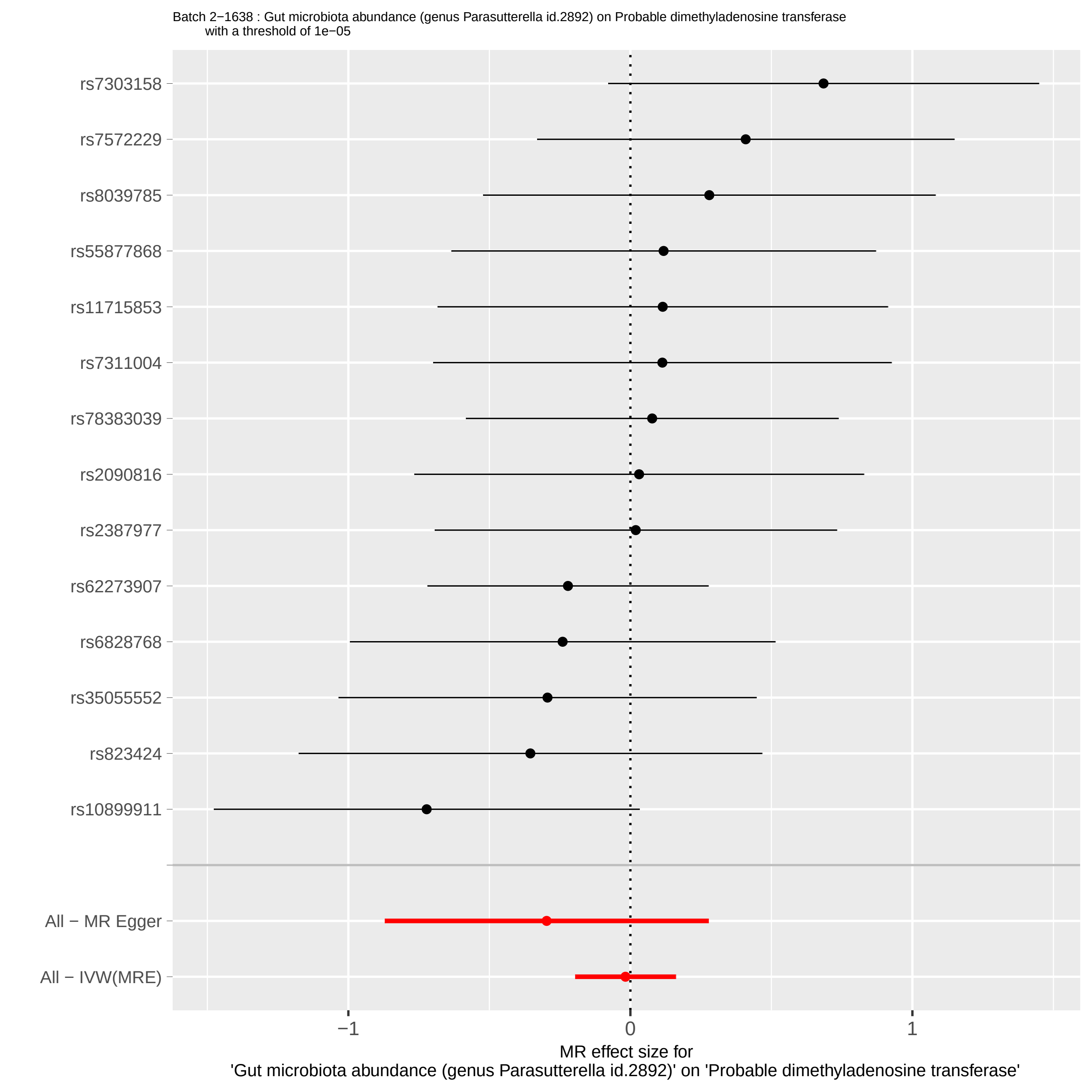
Batch 2−1638 : Gut microbiota abundance (genus Parasutterella id.2892) on Probable dimethyladenosine transferase
with a threshold of 1e−05
rs7303158
rs7572229
rs8039785
rs55877868
rs11715853
rs7311004
rs78383039
rs2090816
rs2387977
rs62273907
rs6828768
rs35055552
rs823424
rs10899911
All − MR Egger
All − IVW(MRE)
−1
0
1
MR effect size for
'Gut microbiota abundance (genus Parasutterella id.2892)' on 'Probable dimethyladenosine transferase'

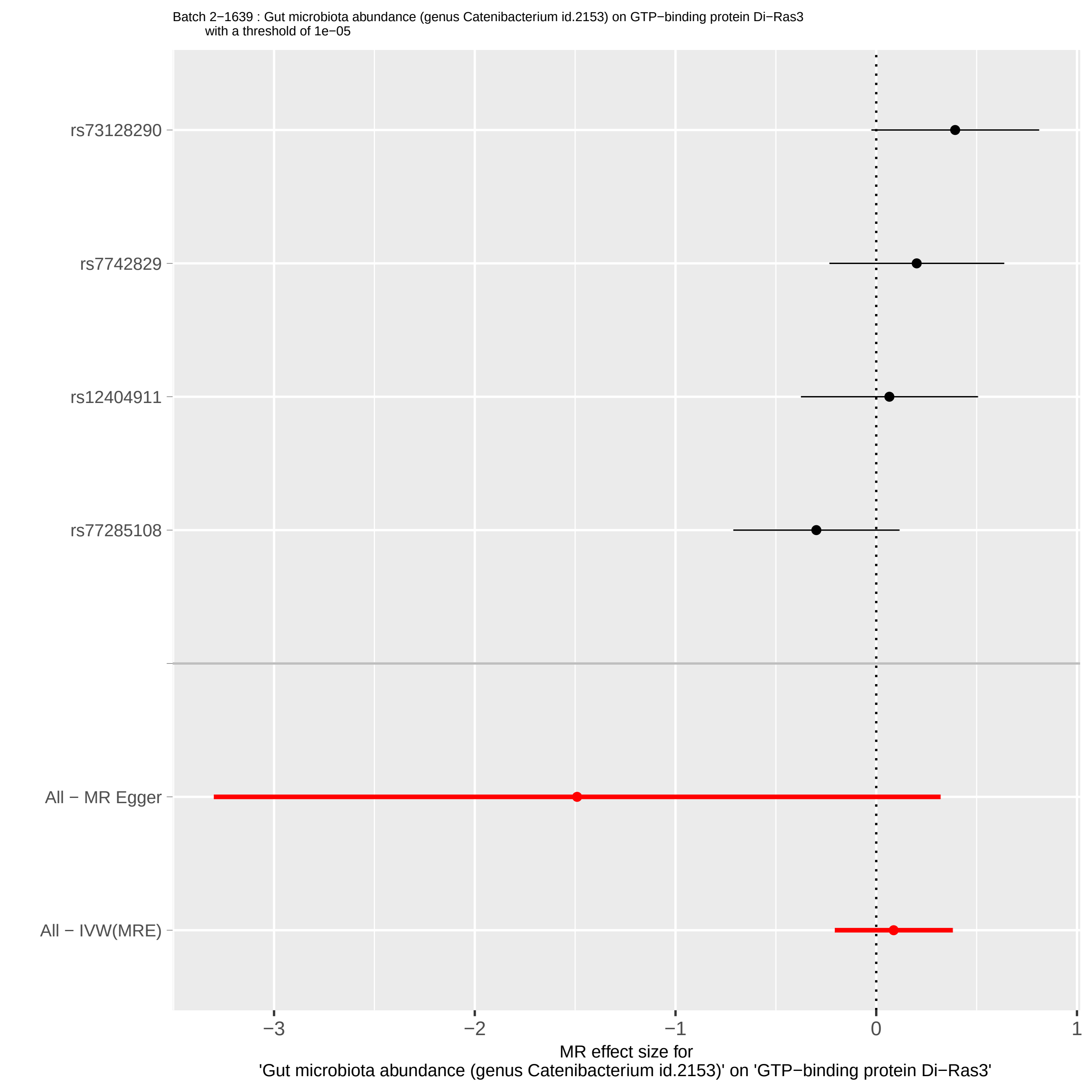

Batch 2−1639 : Gut microbiota abundance (genus Catenibacterium id.2153) on GTP−binding protein Di−Ras3
with a threshold of 1e−05
rs73128290
rs7742829
rs12404911
rs77285108
All − MR Egger
All − IVW(MRE)
−3
−2
−1
0
1
MR effect size for
'Gut microbiota abundance (genus Catenibacterium id.2153)' on 'GTP−binding protein Di−Ras3'

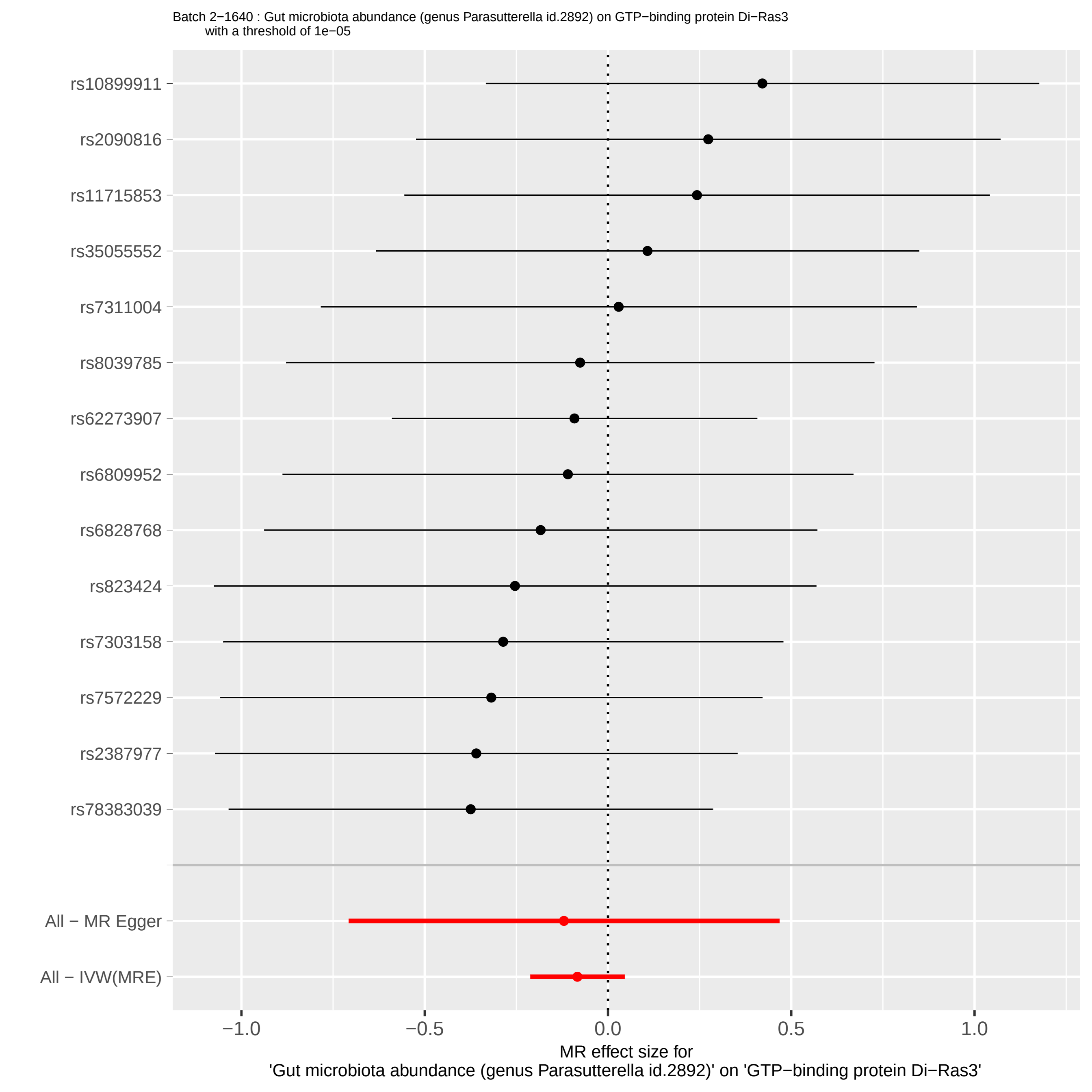

Batch 2−1640 : Gut microbiota abundance (genus Parasutterella id.2892) on GTP−binding protein Di−Ras3
with a threshold of 1e−05
rs10899911
rs2090816
rs11715853
rs35055552
rs7311004
rs8039785
rs62273907
rs6809952
rs6828768
rs823424
rs7303158
rs7572229
rs2387977
rs78383039
All − MR Egger
All − IVW(MRE)
−1.0
−0.5
0.0
0.5
1.0
MR effect size for
'Gut microbiota abundance (genus Parasutterella id.2892)' on 'GTP−binding protein Di−Ras3'

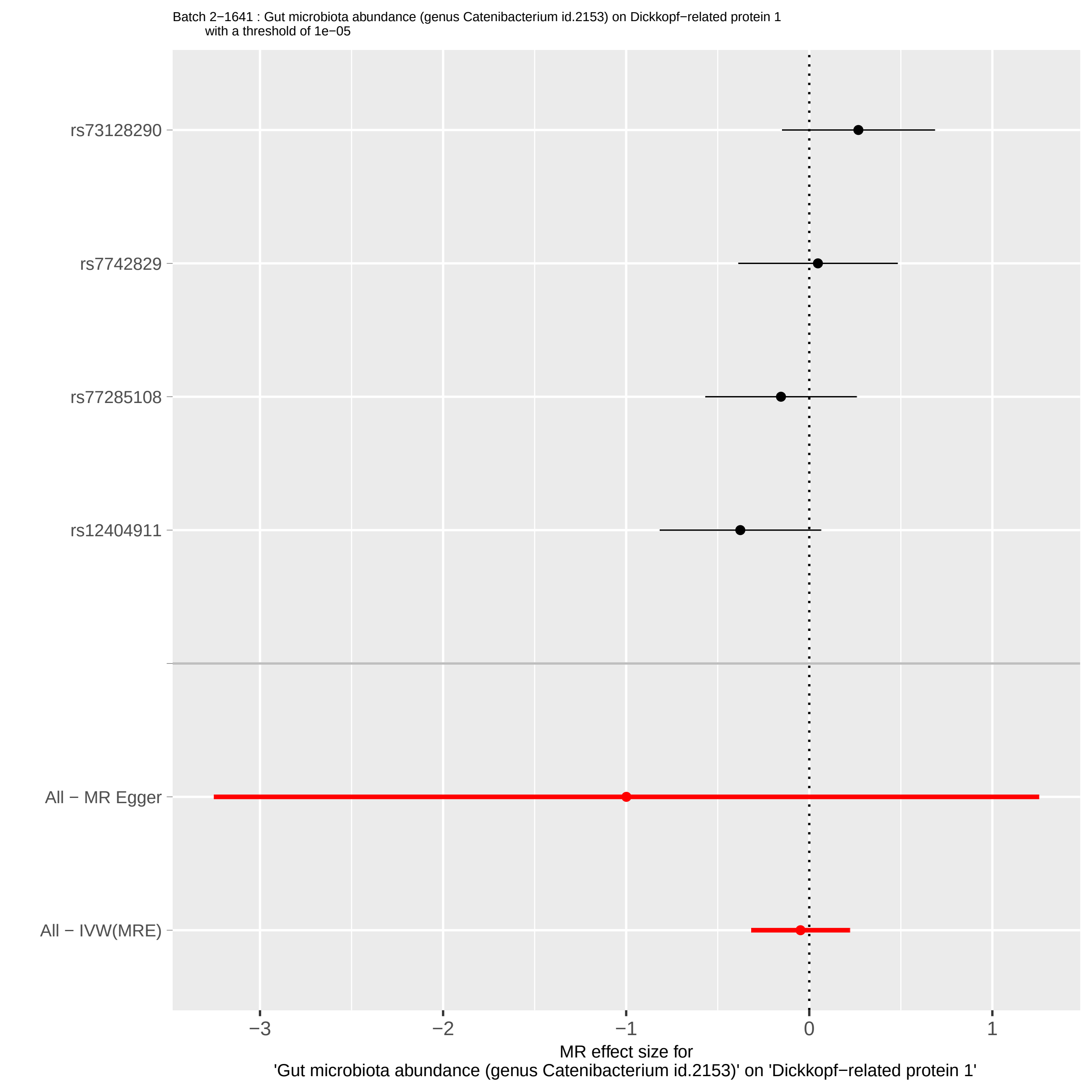

Batch 2−1641 : Gut microbiota abundance (genus Catenibacterium id.2153) on Dickkopf−related protein 1
with a threshold of 1e−05
rs73128290
rs7742829
rs77285108
rs12404911
All − MR Egger
All − IVW(MRE)
−3
−2
−1
0
1
MR effect size for
'Gut microbiota abundance (genus Catenibacterium id.2153)' on 'Dickkopf−related protein 1'

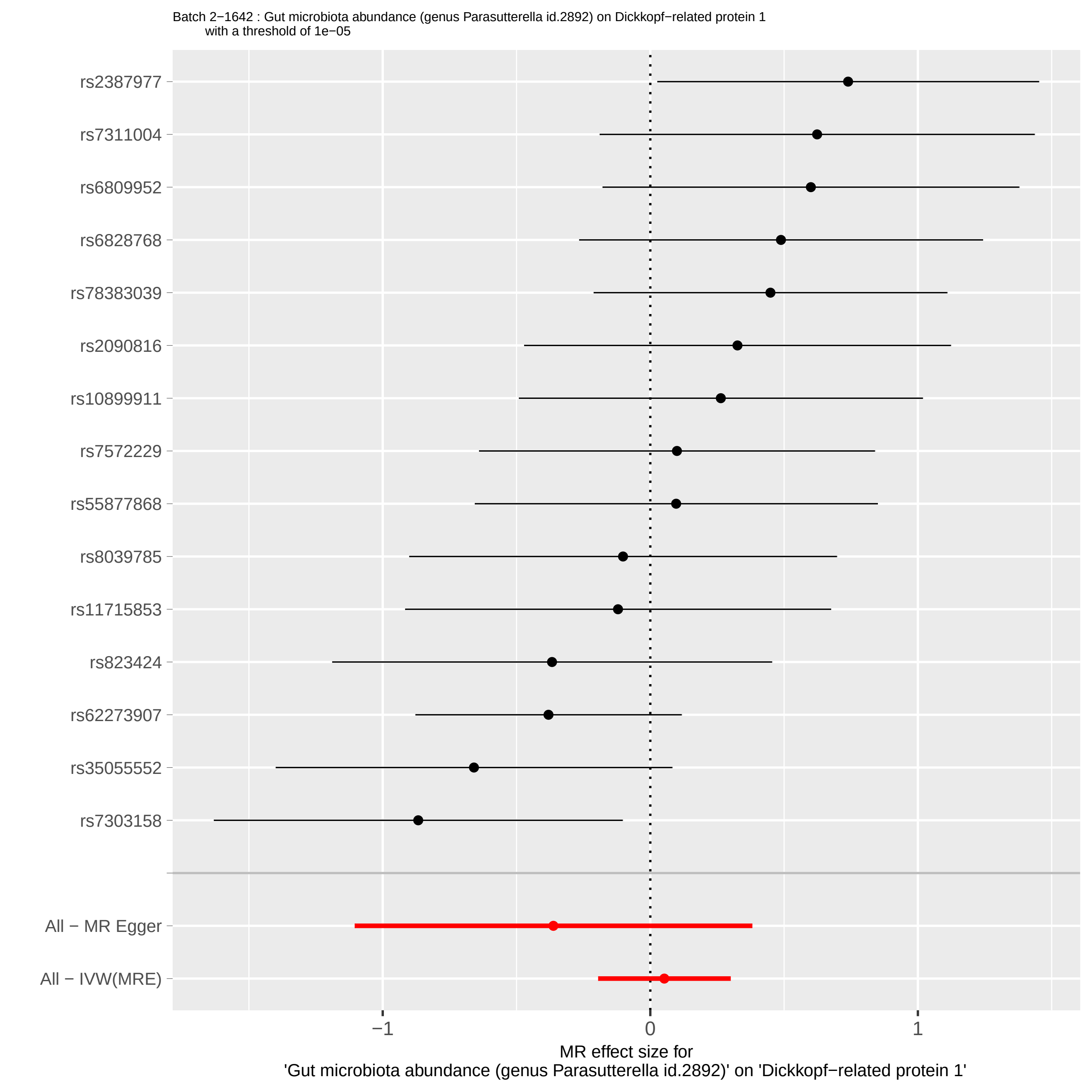
Batch 2−1642 : Gut microbiota abundance (genus Parasutterella id.2892) on Dickkopf−related protein 1
with a threshold of 1e−05
rs2387977
rs7311004
rs6809952
rs6828768
rs78383039
rs2090816
rs10899911
rs7572229
rs55877868
rs8039785
rs11715853
rs823424
rs62273907
rs35055552
rs7303158
All − MR Egger
All − IVW(MRE)
−1
0
1
MR effect size for
'Gut microbiota abundance (genus Parasutterella id.2892)' on 'Dickkopf−related protein 1'

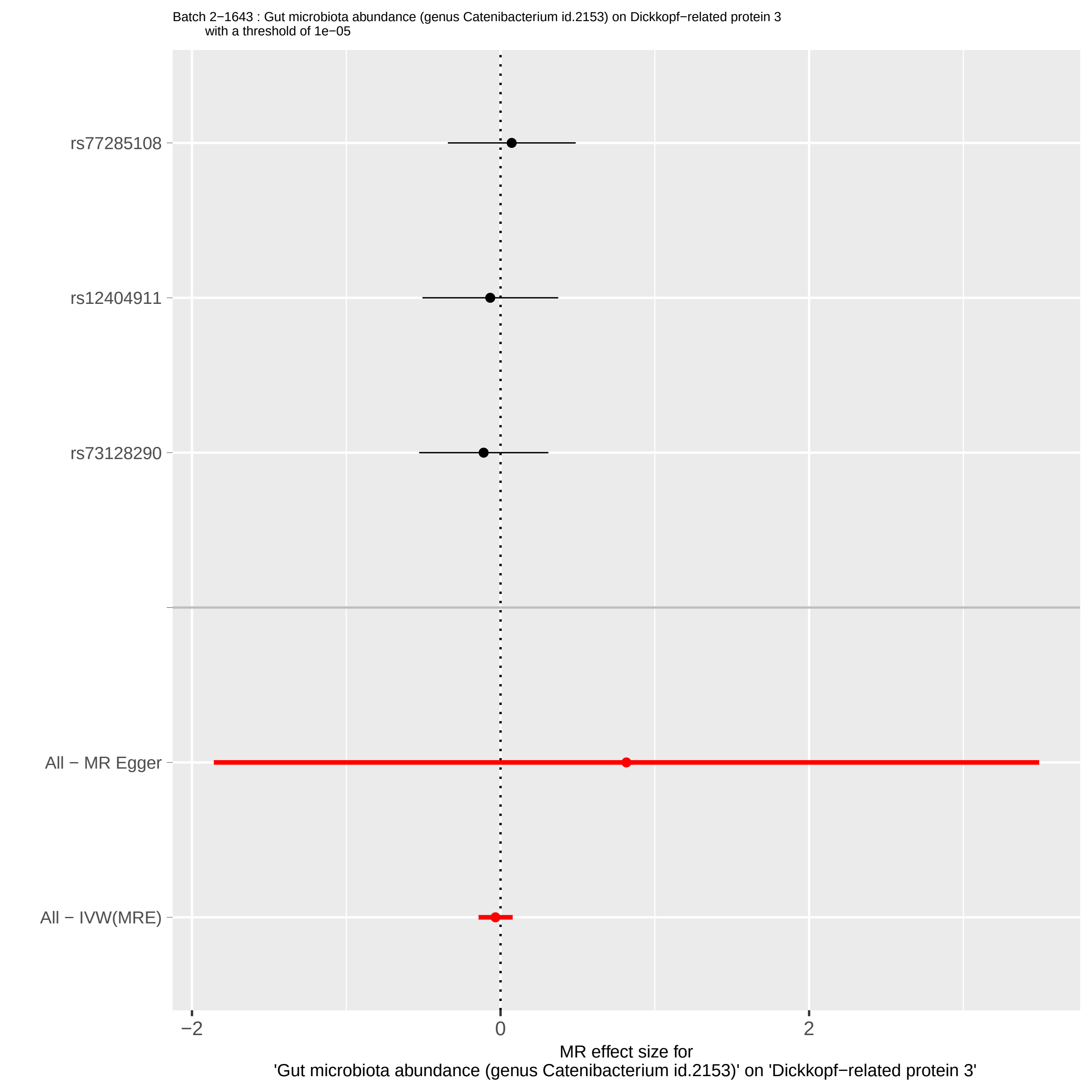
Batch 2−1643 : Gut microbiota abundance (genus Catenibacterium id.2153) on Dickkopf−related protein 3
with a threshold of 1e−05
rs77285108
rs12404911
rs73128290
All − MR Egger
All − IVW(MRE)
−2
0
2
MR effect size for
'Gut microbiota abundance (genus Catenibacterium id.2153)' on 'Dickkopf−related protein 3'

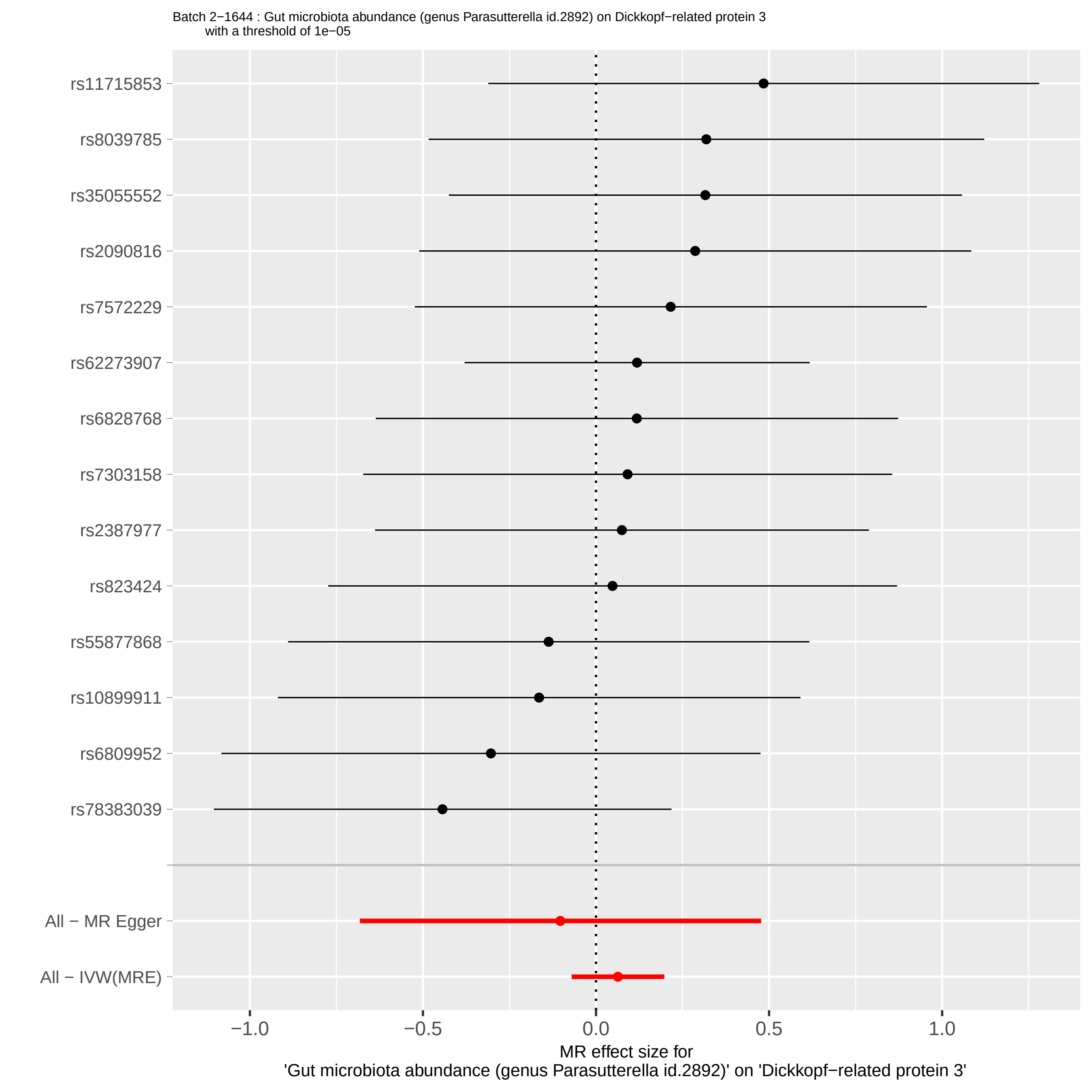
Batch 2−1644 : Gut microbiota abundance (genus Parasutterella id.2892) on Dickkopf−related protein 3
with a threshold of 1e−05
rs11715853
rs8039785
rs35055552
rs2090816
rs7572229
rs62273907
rs6828768
rs7303158
rs2387977
rs823424
rs55877868
rs10899911
rs6809952
rs78383039
All − MR Egger
All − IVW(MRE)
−1.0
−0.5
0.0
0.5
1.0
MR effect size for
'Gut microbiota abundance (genus Parasutterella id.2892)' on 'Dickkopf−related protein 3'

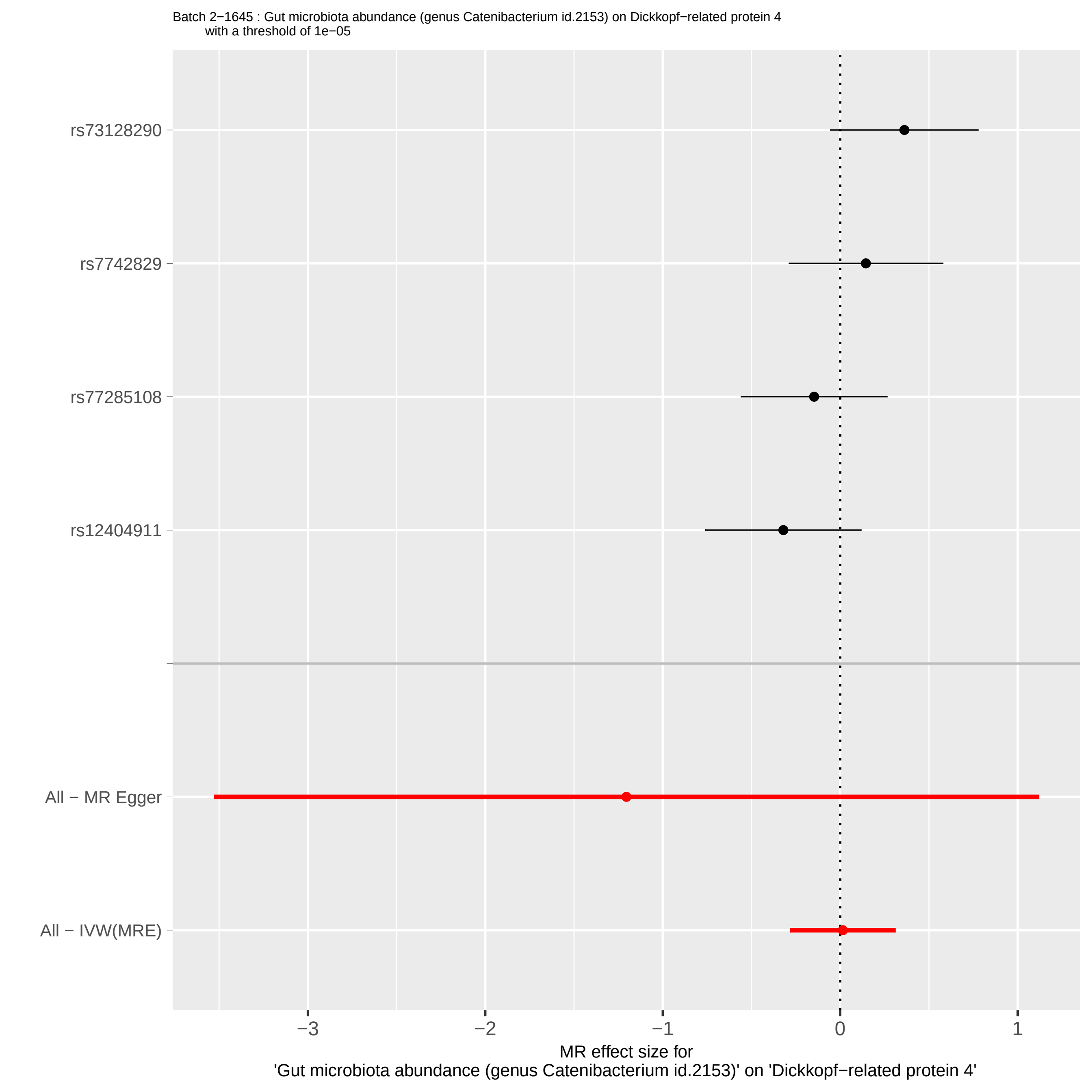

Batch 2−1645 : Gut microbiota abundance (genus Catenibacterium id.2153) on Dickkopf−related protein 4
with a threshold of 1e−05
rs73128290
rs7742829
rs77285108
rs12404911
All − MR Egger
All − IVW(MRE)
−3
−2
−1
0
1
MR effect size for
'Gut microbiota abundance (genus Catenibacterium id.2153)' on 'Dickkopf−related protein 4'

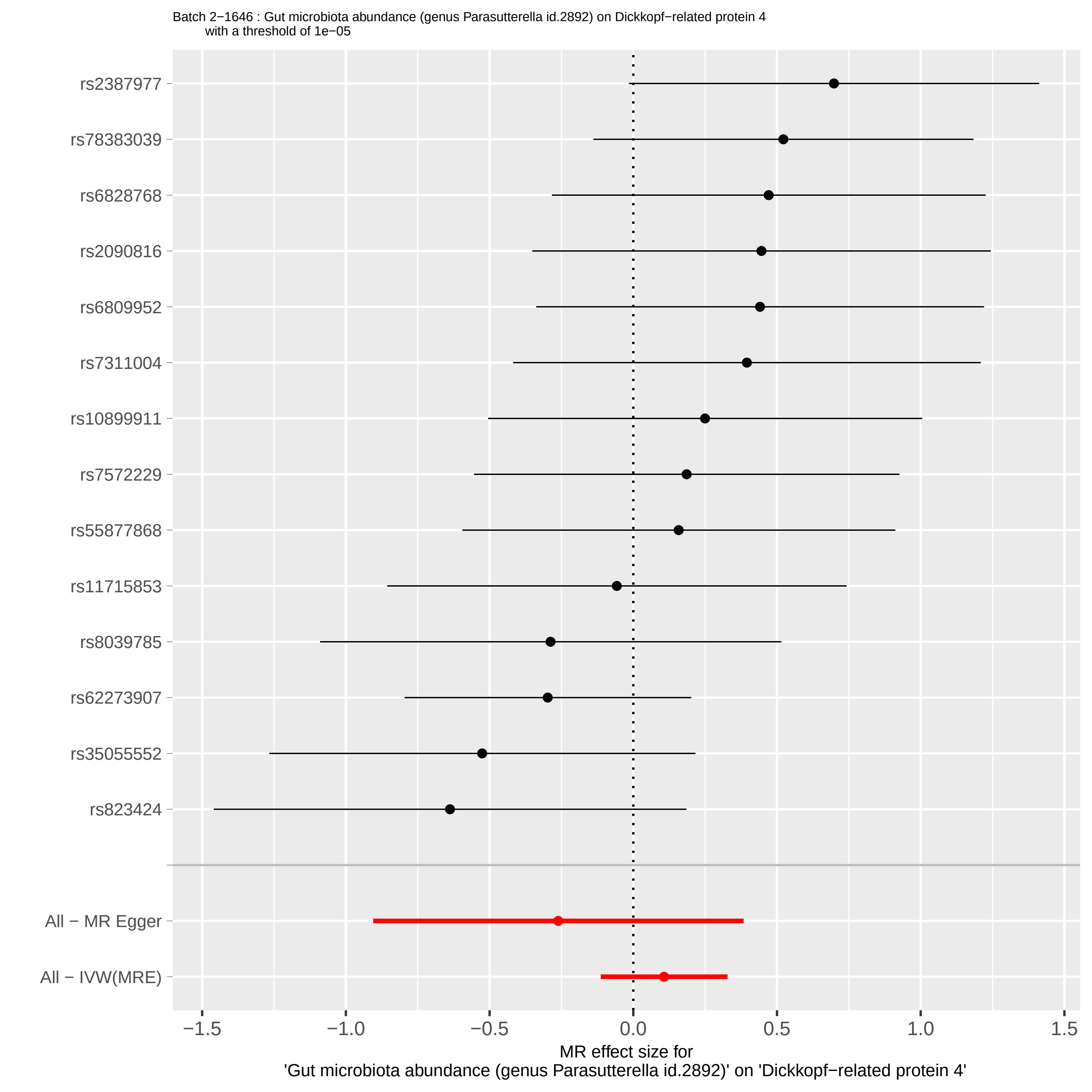

Batch 2−1646 : Gut microbiota abundance (genus Parasutterella id.2892) on Dickkopf−related protein 4
with a threshold of 1e−05
rs2387977
rs78383039
rs6828768
rs2090816
rs6809952
rs7311004
rs10899911
rs7572229
rs55877868
rs11715853
rs8039785
rs62273907
rs35055552
rs823424
All − MR Egger
All − IVW(MRE)
−1.5
−1.0
−0.5
0.0
0.5
1.0
1.5
MR effect size for
'Gut microbiota abundance (genus Parasutterella id.2892)' on 'Dickkopf−related protein 4'

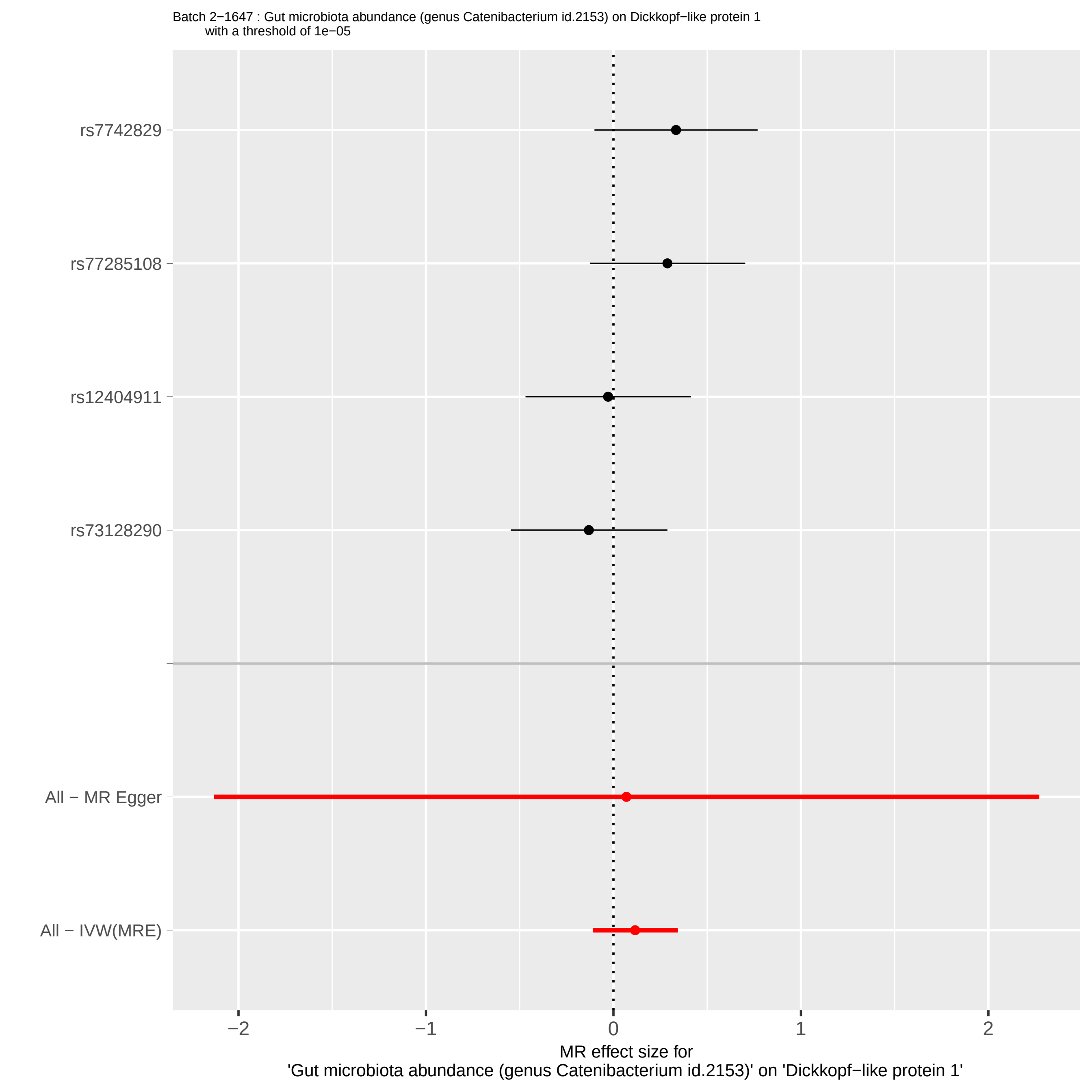
Batch 2−1647 : Gut microbiota abundance (genus Catenibacterium id.2153) on Dickkopf−like protein 1
with a threshold of 1e−05
rs7742829
rs77285108
rs12404911
rs73128290
All − MR Egger
All − IVW(MRE)
−2
−1
0
1
2
MR effect size for
'Gut microbiota abundance (genus Catenibacterium id.2153)' on 'Dickkopf−like protein 1'

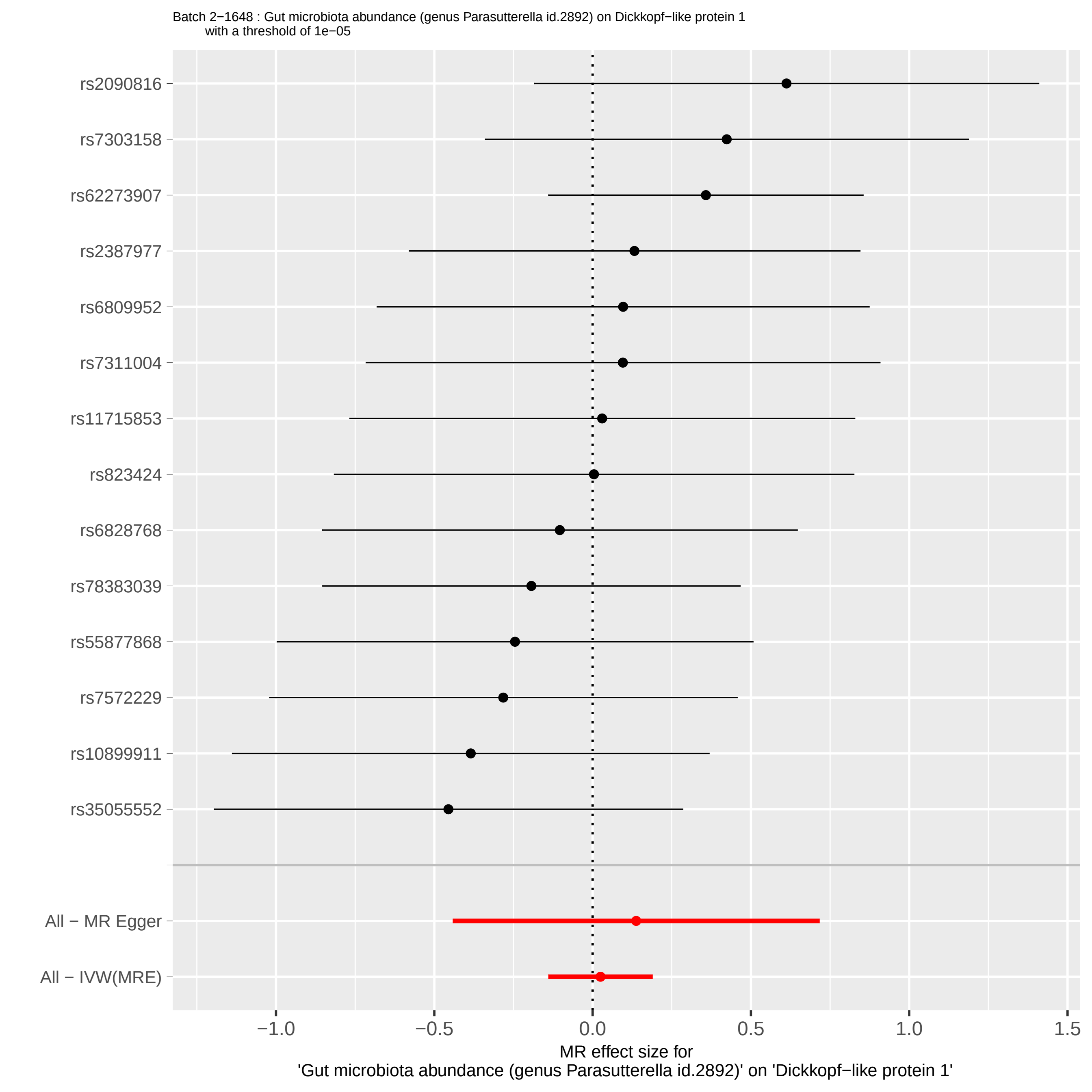
Batch 2−1648 : Gut microbiota abundance (genus Parasutterella id.2892) on Dickkopf−like protein 1
with a threshold of 1e−05
rs2090816
rs7303158
rs62273907
rs2387977
rs6809952
rs7311004
rs11715853
rs823424
rs6828768
rs78383039
rs55877868
rs7572229
rs10899911
rs35055552
All − MR Egger
All − IVW(MRE)
−1.0
−0.5
0.0
0.5
1.0
1.5
MR effect size for
'Gut microbiota abundance (genus Parasutterella id.2892)' on 'Dickkopf−like protein 1'

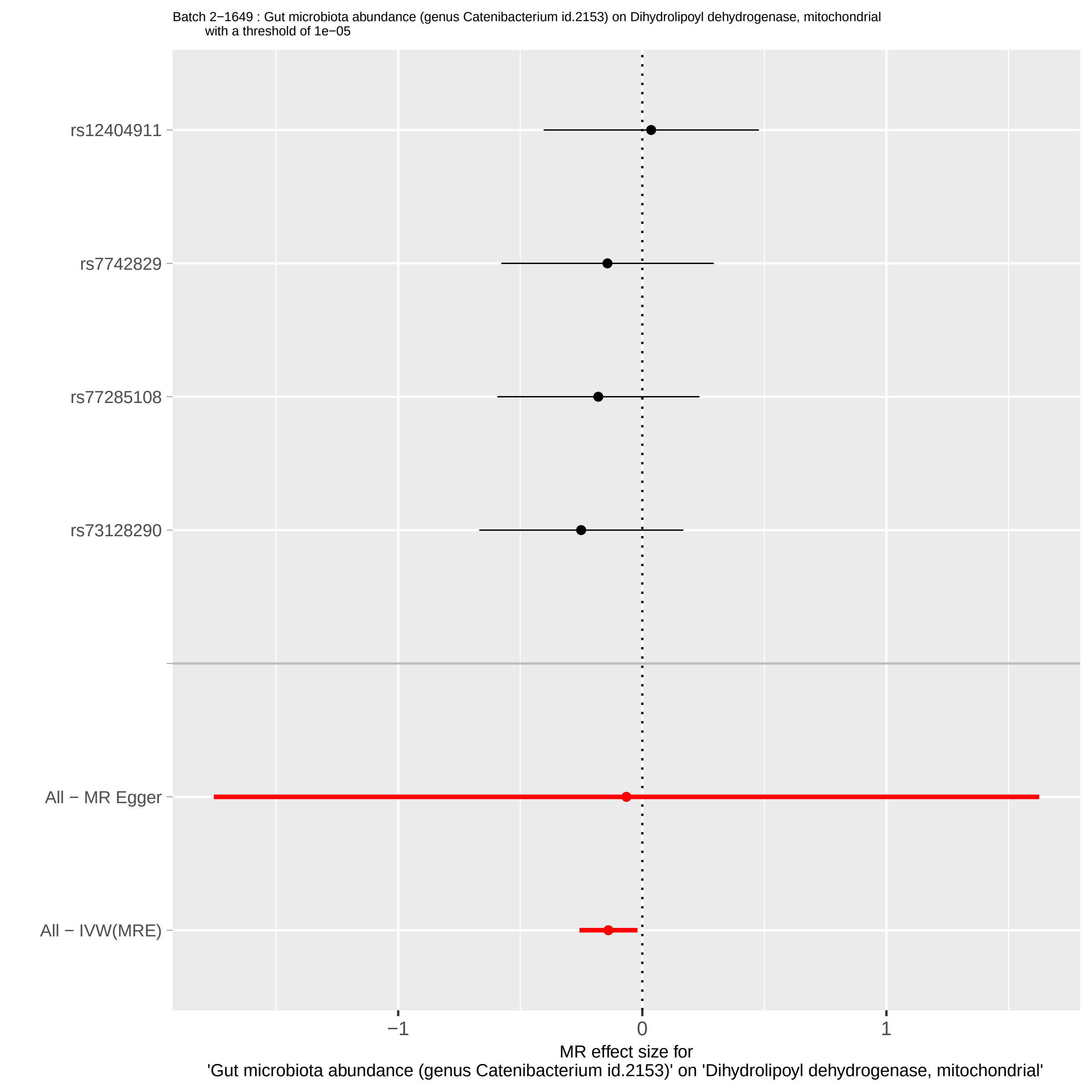
Batch 2−1649 : Gut microbiota abundance (genus Catenibacterium id.2153) on Dihydrolipoyl dehydrogenase, mitochondrial
with a threshold of 1e−05
rs12404911
rs7742829
rs77285108
rs73128290
All − MR Egger
All − IVW(MRE)
−1
0
1
MR effect size for
'Gut microbiota abundance (genus Catenibacterium id.2153)' on 'Dihydrolipoyl dehydrogenase, mitochondrial'

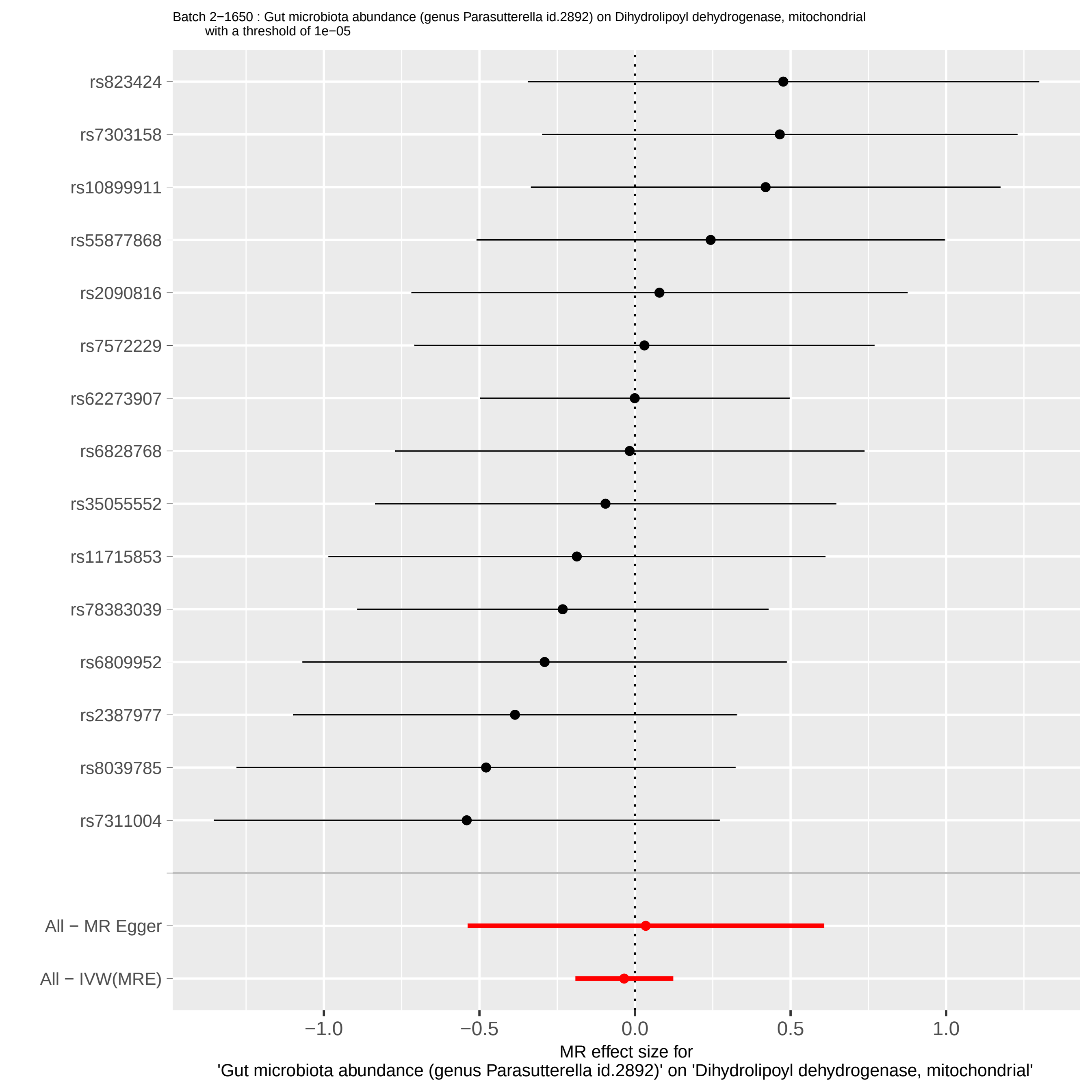

Batch 2−1650 : Gut microbiota abundance (genus Parasutterella id.2892) on Dihydrolipoyl dehydrogenase, mitochondrial
with a threshold of 1e−05
rs823424
rs7303158
rs10899911
rs55877868
rs2090816
rs7572229
rs62273907
rs6828768
rs35055552
rs11715853
rs78383039
rs6809952
rs2387977
rs8039785
rs7311004
All − MR Egger
All − IVW(MRE)
−1.0
−0.5
0.0
0.5
1.0
MR effect size for
'Gut microbiota abundance (genus Parasutterella id.2892)' on 'Dihydrolipoyl dehydrogenase, mitochondrial'

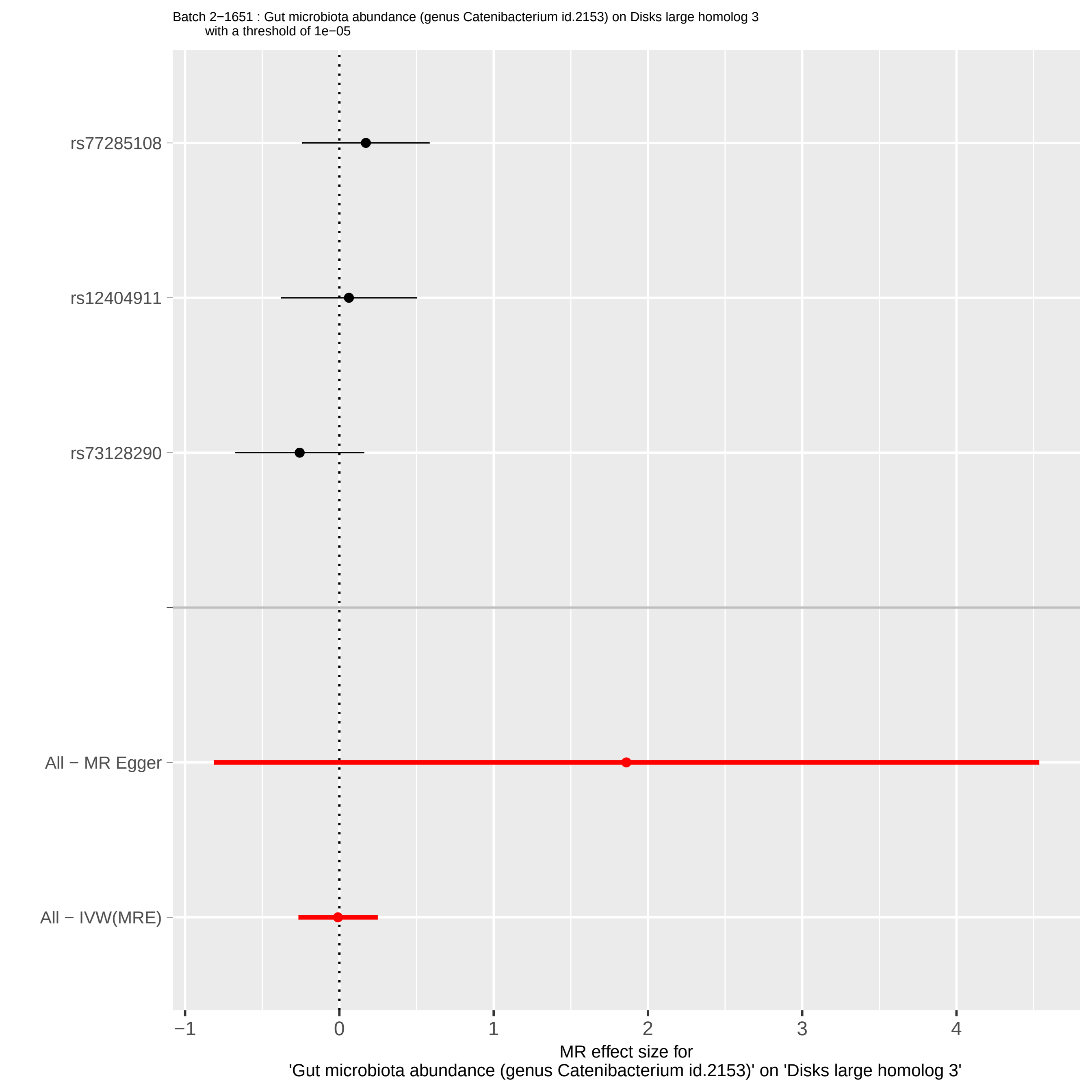

Batch 2−1651 : Gut microbiota abundance (genus Catenibacterium id.2153) on Disks large homolog 3
with a threshold of 1e−05
rs77285108
rs12404911
rs73128290
All − MR Egger
All − IVW(MRE)
−1
0
1
2
3
4
MR effect size for
'Gut microbiota abundance (genus Catenibacterium id.2153)' on 'Disks large homolog 3'

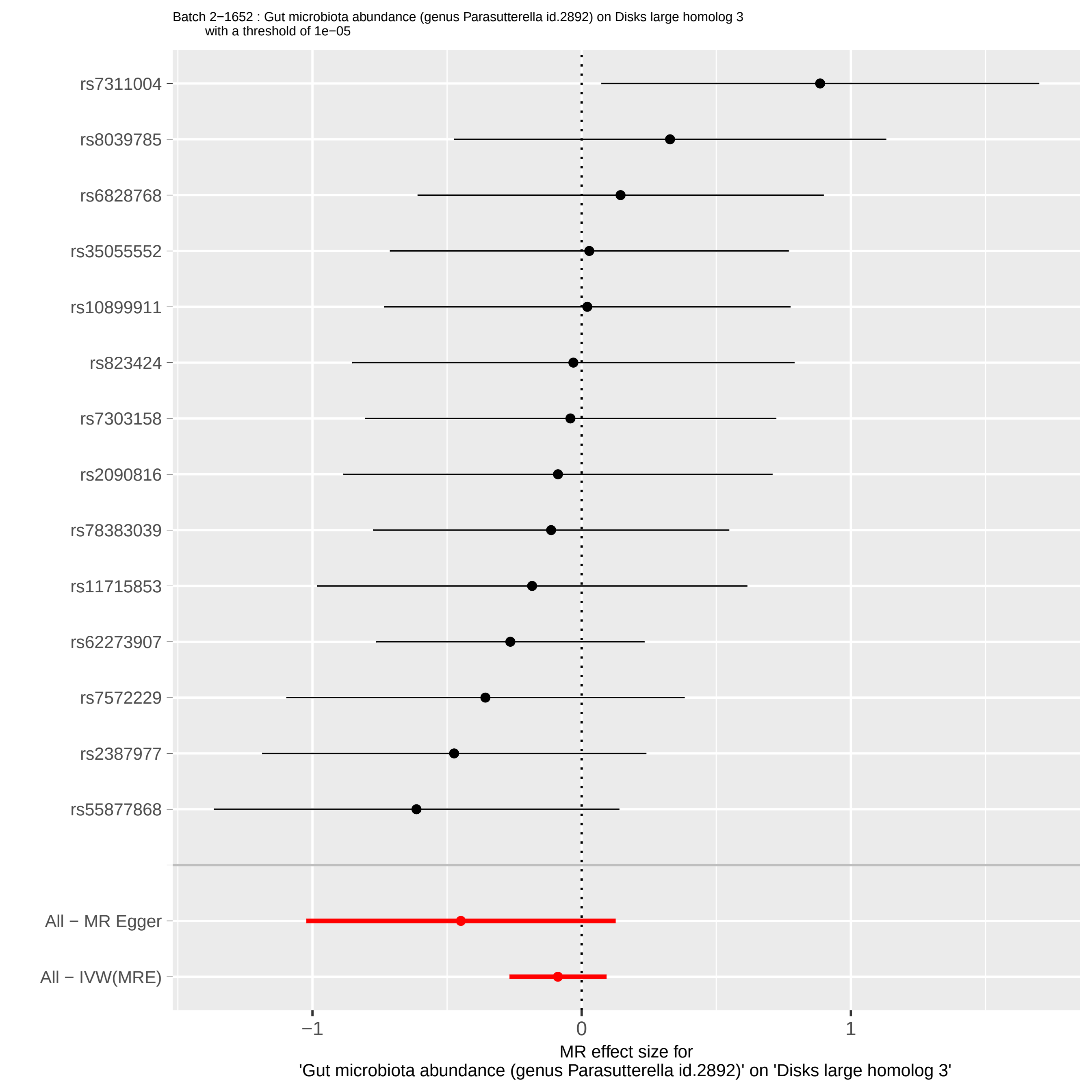

Batch 2−1652 : Gut microbiota abundance (genus Parasutterella id.2892) on Disks large homolog 3
with a threshold of 1e−05
rs7311004
rs8039785
rs6828768
rs35055552
rs10899911
rs823424
rs7303158
rs2090816
rs78383039
rs11715853
rs62273907
rs7572229
rs2387977
rs55877868
All − MR Egger
All − IVW(MRE)
−1
0
1
MR effect size for
'Gut microbiota abundance (genus Parasutterella id.2892)' on 'Disks large homolog 3'

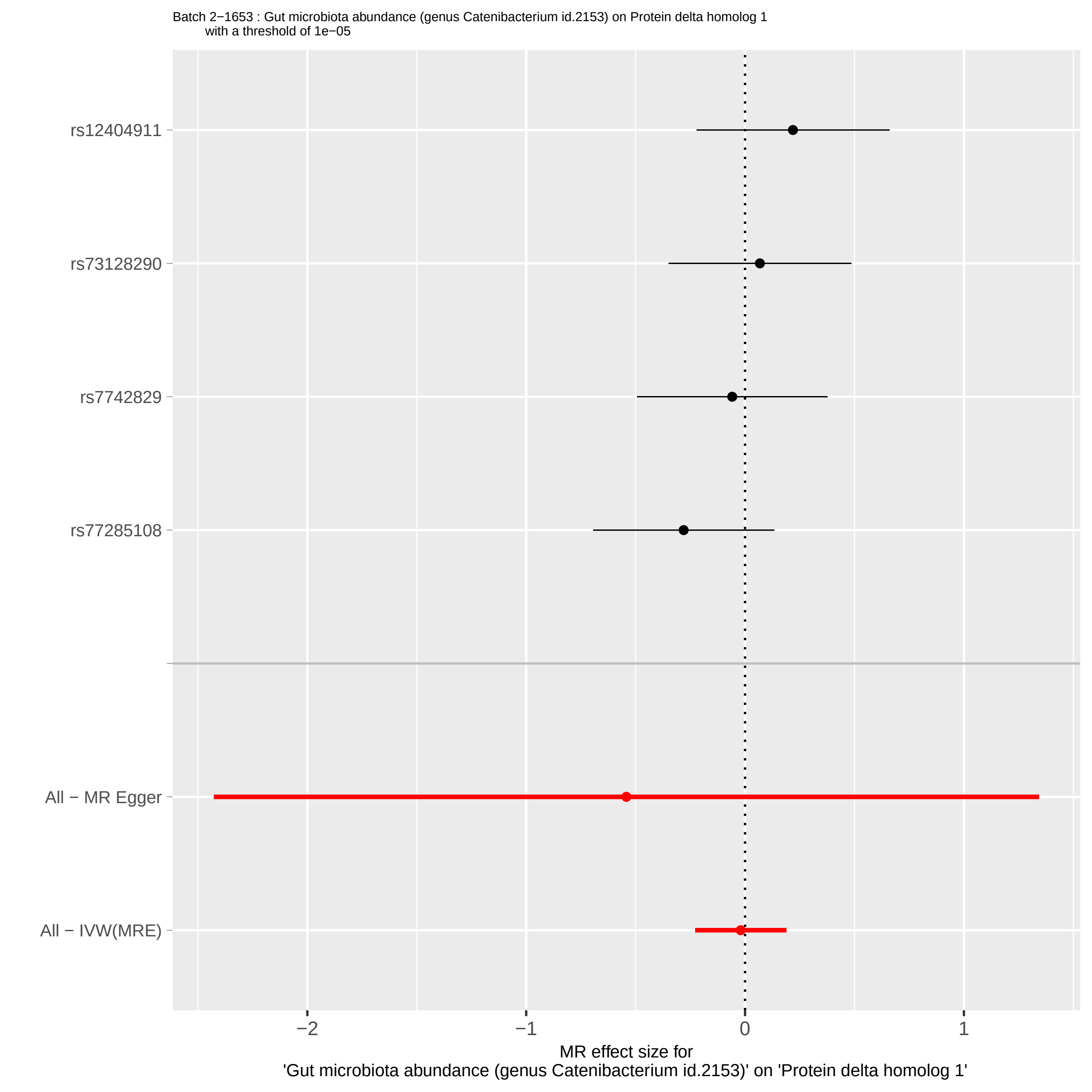
Batch 2−1653 : Gut microbiota abundance (genus Catenibacterium id.2153) on Protein delta homolog 1
with a threshold of 1e−05
rs12404911
rs73128290
rs7742829
rs77285108
All − MR Egger
All − IVW(MRE)
−2
−1
0
1
MR effect size for
'Gut microbiota abundance (genus Catenibacterium id.2153)' on 'Protein delta homolog 1'

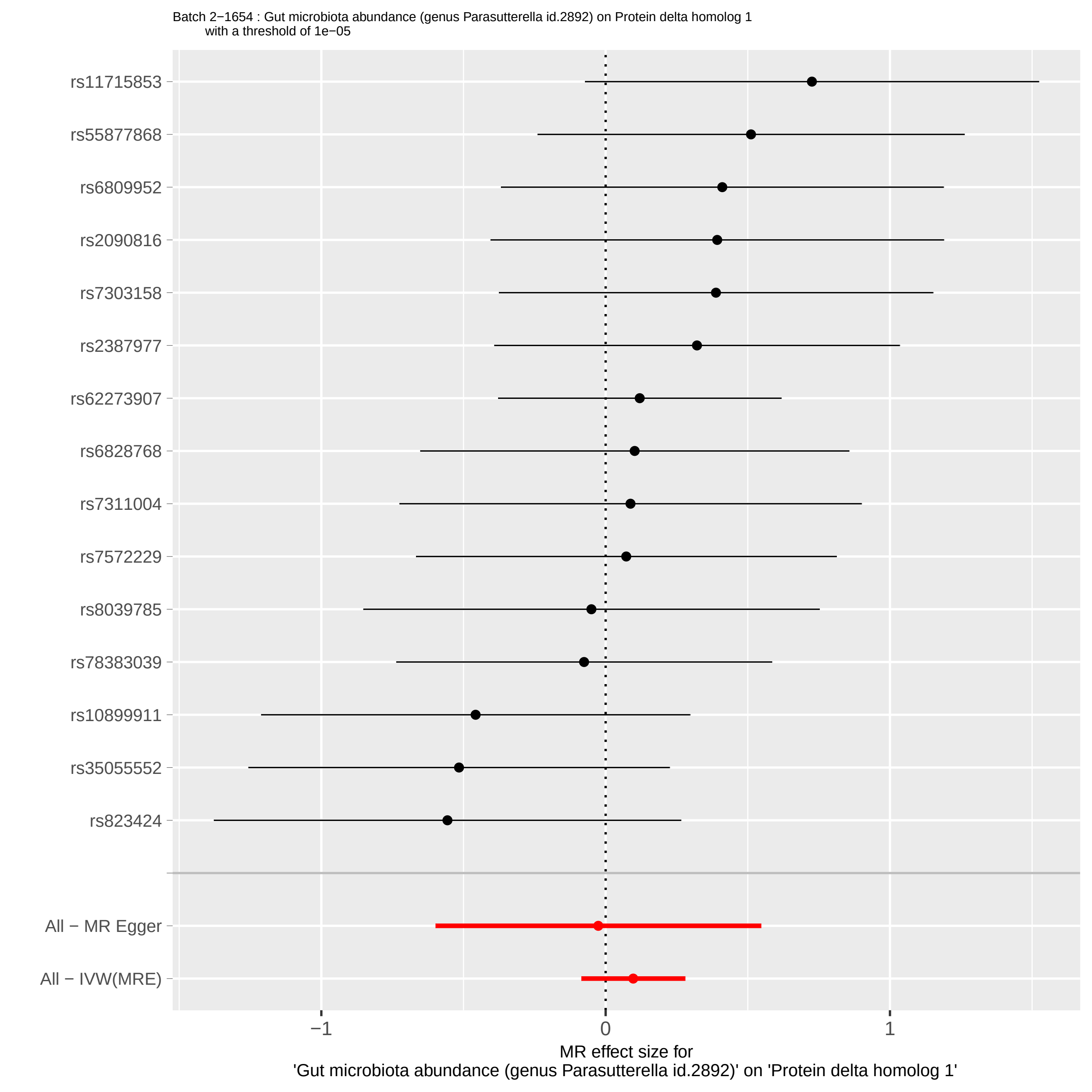
Batch 2−1654 : Gut microbiota abundance (genus Parasutterella id.2892) on Protein delta homolog 1
with a threshold of 1e−05
rs11715853
rs55877868
rs6809952
rs2090816
rs7303158
rs2387977
rs62273907
rs6828768
rs7311004
rs7572229
rs8039785
rs78383039
rs10899911
rs35055552
rs823424
All − MR Egger
All − IVW(MRE)
−1
0
1
MR effect size for
'Gut microbiota abundance (genus Parasutterella id.2892)' on 'Protein delta homolog 1'

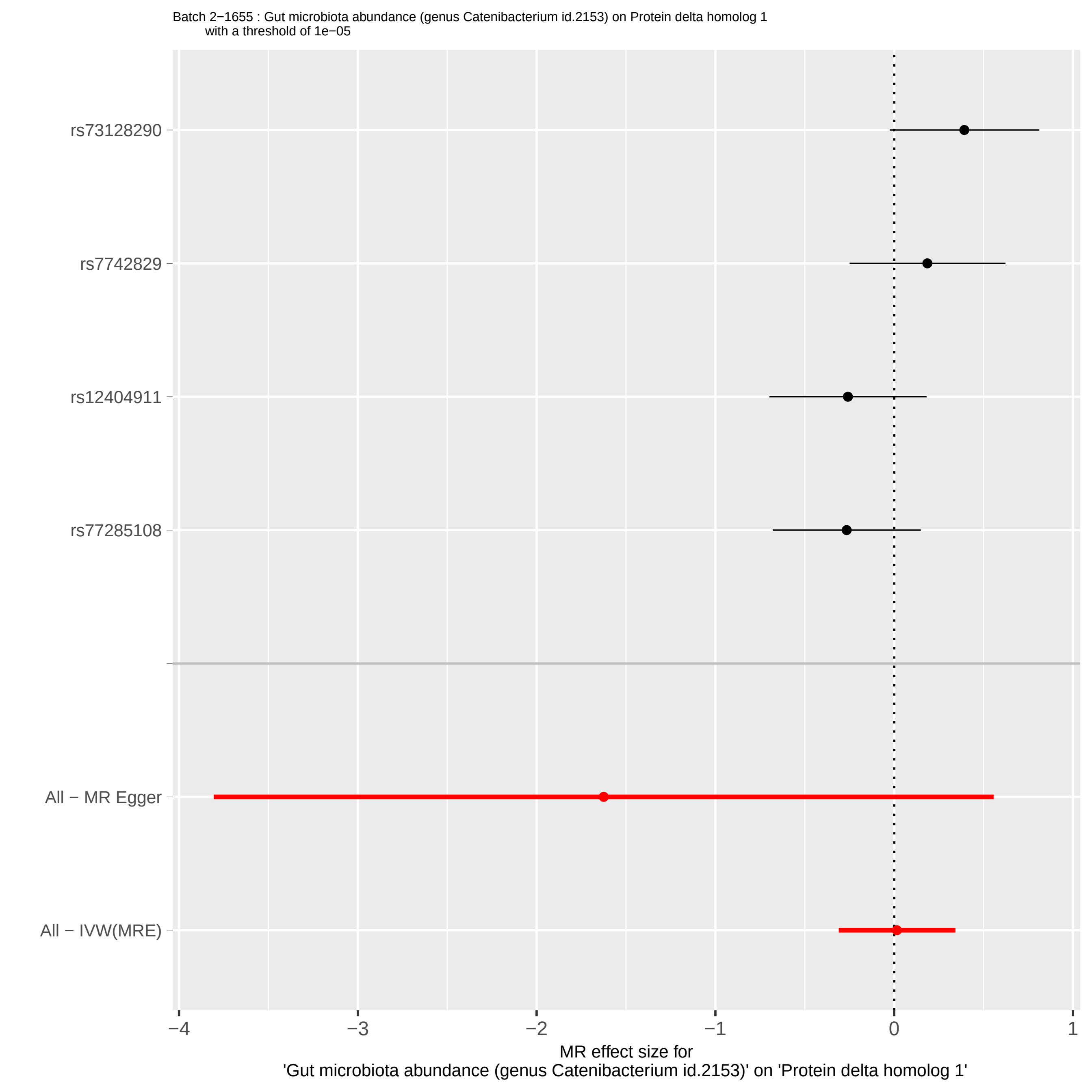

Batch 2−1655 : Gut microbiota abundance (genus Catenibacterium id.2153) on Protein delta homolog 1
with a threshold of 1e−05
rs73128290
rs7742829
rs12404911
rs77285108
All − MR Egger
All − IVW(MRE)
−4
−3
−2
−1
0
1
MR effect size for
'Gut microbiota abundance (genus Catenibacterium id.2153)' on 'Protein delta homolog 1'

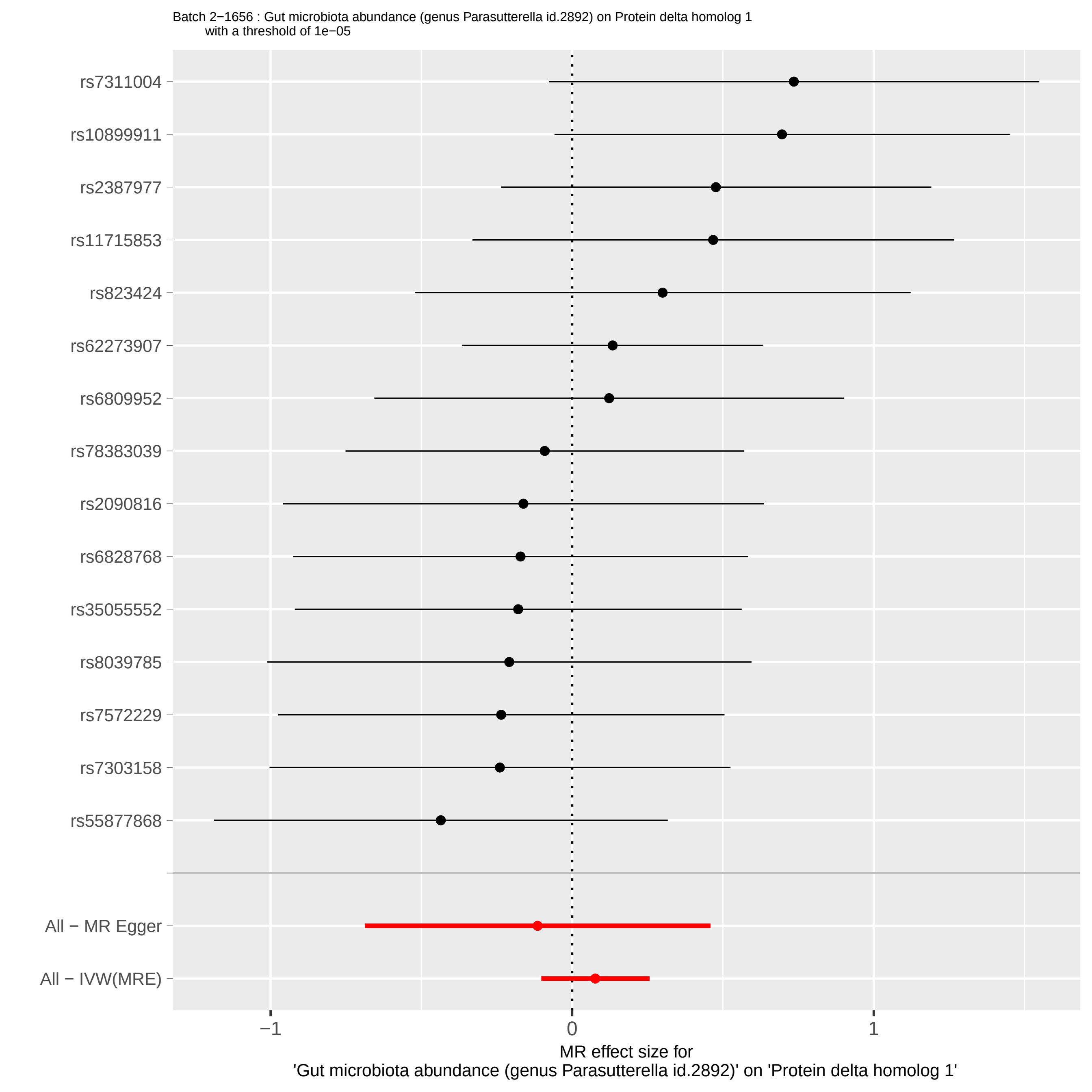

Batch 2−1656 : Gut microbiota abundance (genus Parasutterella id.2892) on Protein delta homolog 1
with a threshold of 1e−05
rs7311004
rs10899911
rs2387977
rs11715853
rs823424
rs62273907
rs6809952
rs78383039
rs2090816
rs6828768
rs35055552
rs8039785
rs7572229
rs7303158
rs55877868
All − MR Egger
All − IVW(MRE)
−1
0
1
MR effect size for
'Gut microbiota abundance (genus Parasutterella id.2892)' on 'Protein delta homolog 1'

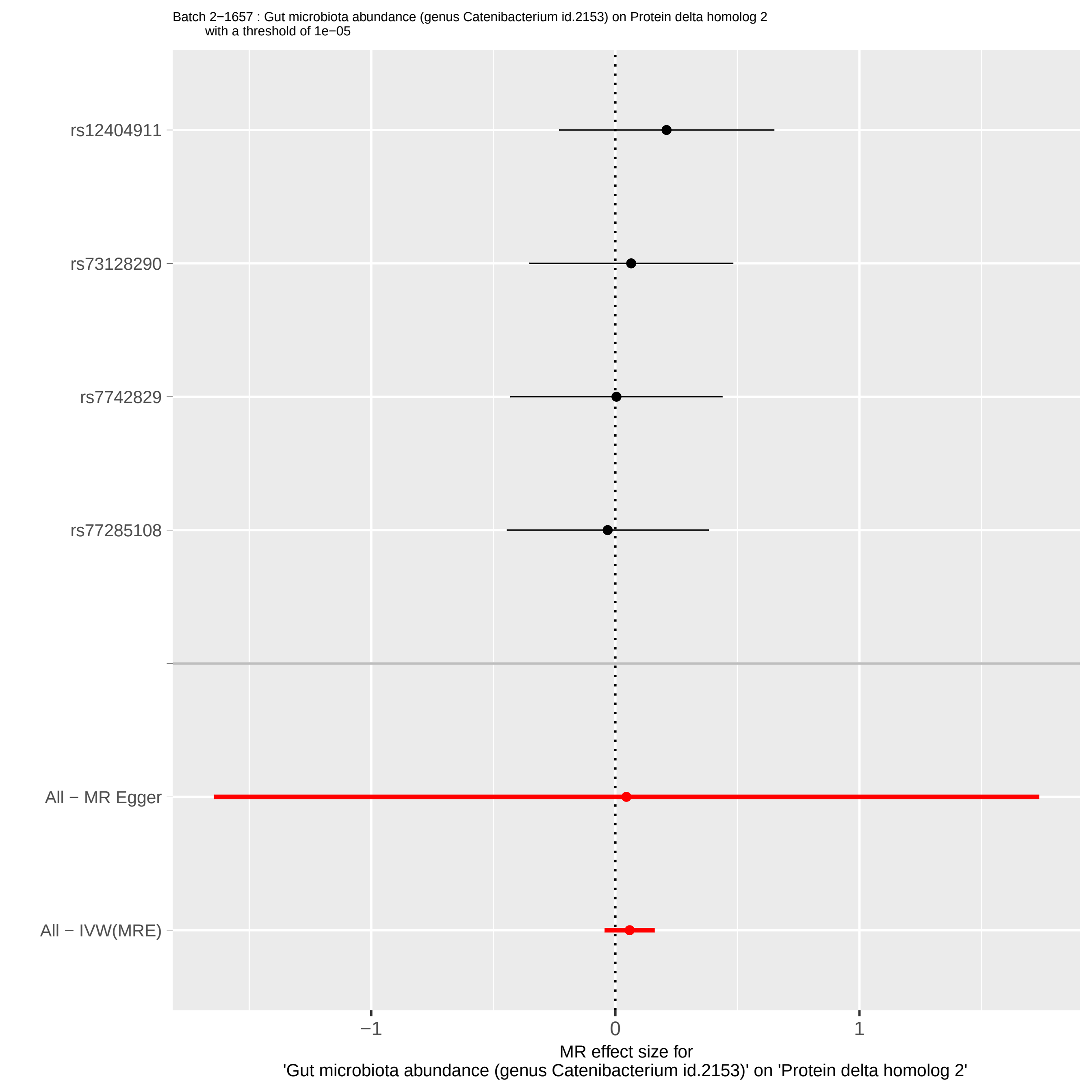
Batch 2−1657 : Gut microbiota abundance (genus Catenibacterium id.2153) on Protein delta homolog 2
with a threshold of 1e−05
rs12404911
rs73128290
rs7742829
rs77285108
All − MR Egger
All − IVW(MRE)
−1
0
1
MR effect size for
'Gut microbiota abundance (genus Catenibacterium id.2153)' on 'Protein delta homolog 2'

Batch 2−1658 : Gut microbiota abundance (genus Parasutterella id.2892) on Protein delta homolog 2
with a threshold of 1e−05

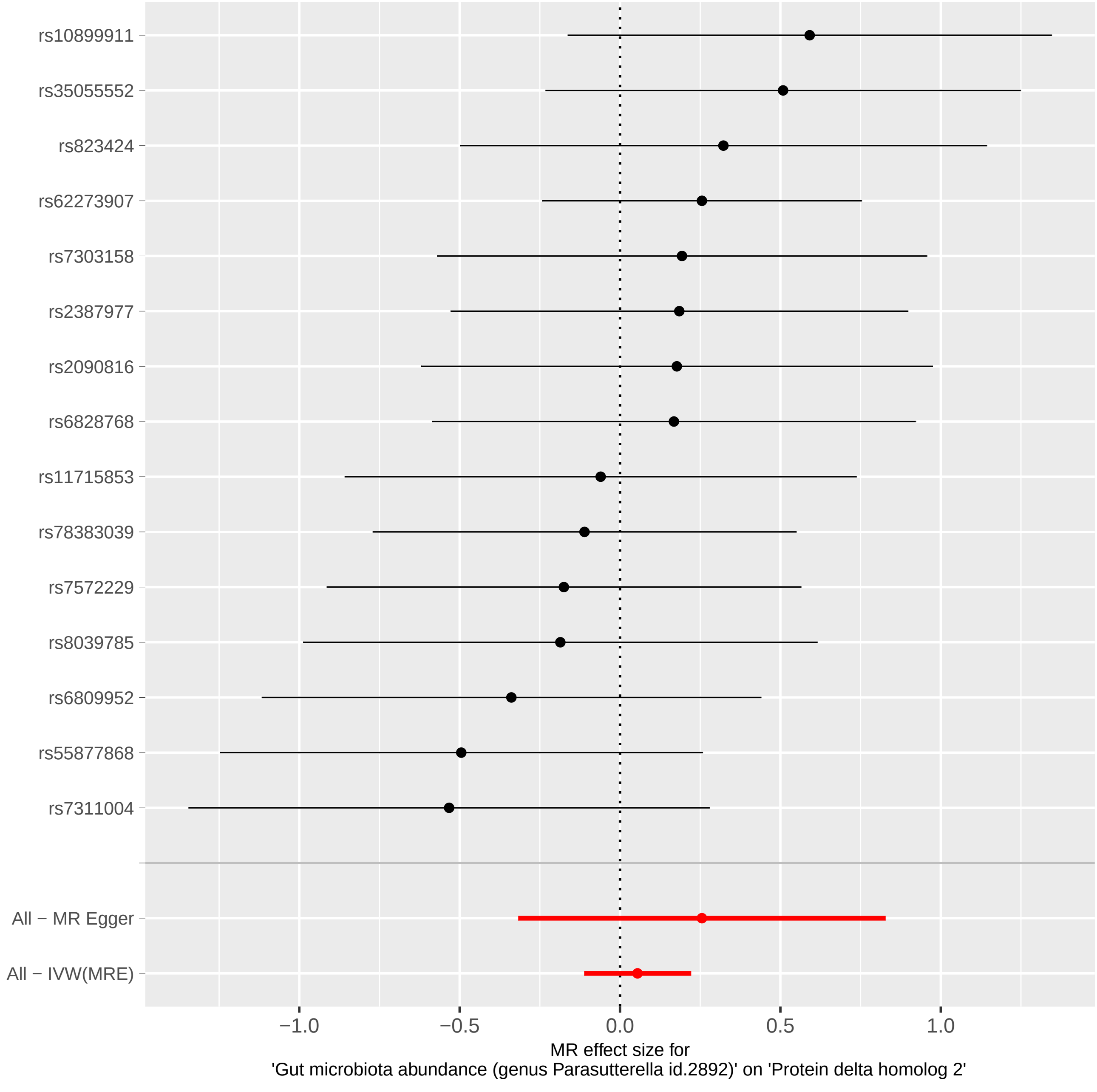

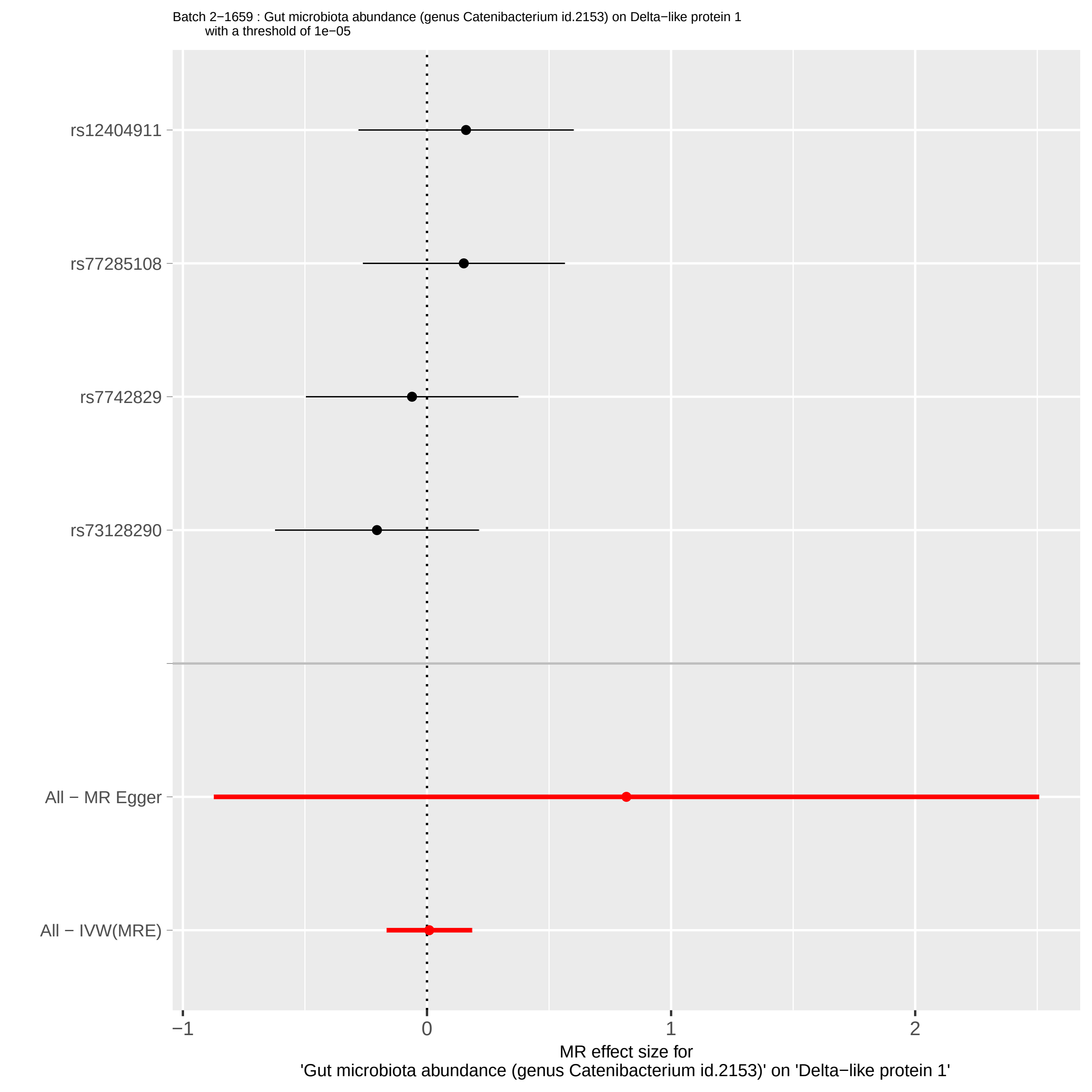

Batch 2−1659 : Gut microbiota abundance (genus Catenibacterium id.2153) on Delta−like protein 1
with a threshold of 1e−05
rs12404911
rs77285108
rs7742829
rs73128290
All − MR Egger
All − IVW(MRE)
−1
0
1
2
MR effect size for
'Gut microbiota abundance (genus Catenibacterium id.2153)' on 'Delta−like protein 1'

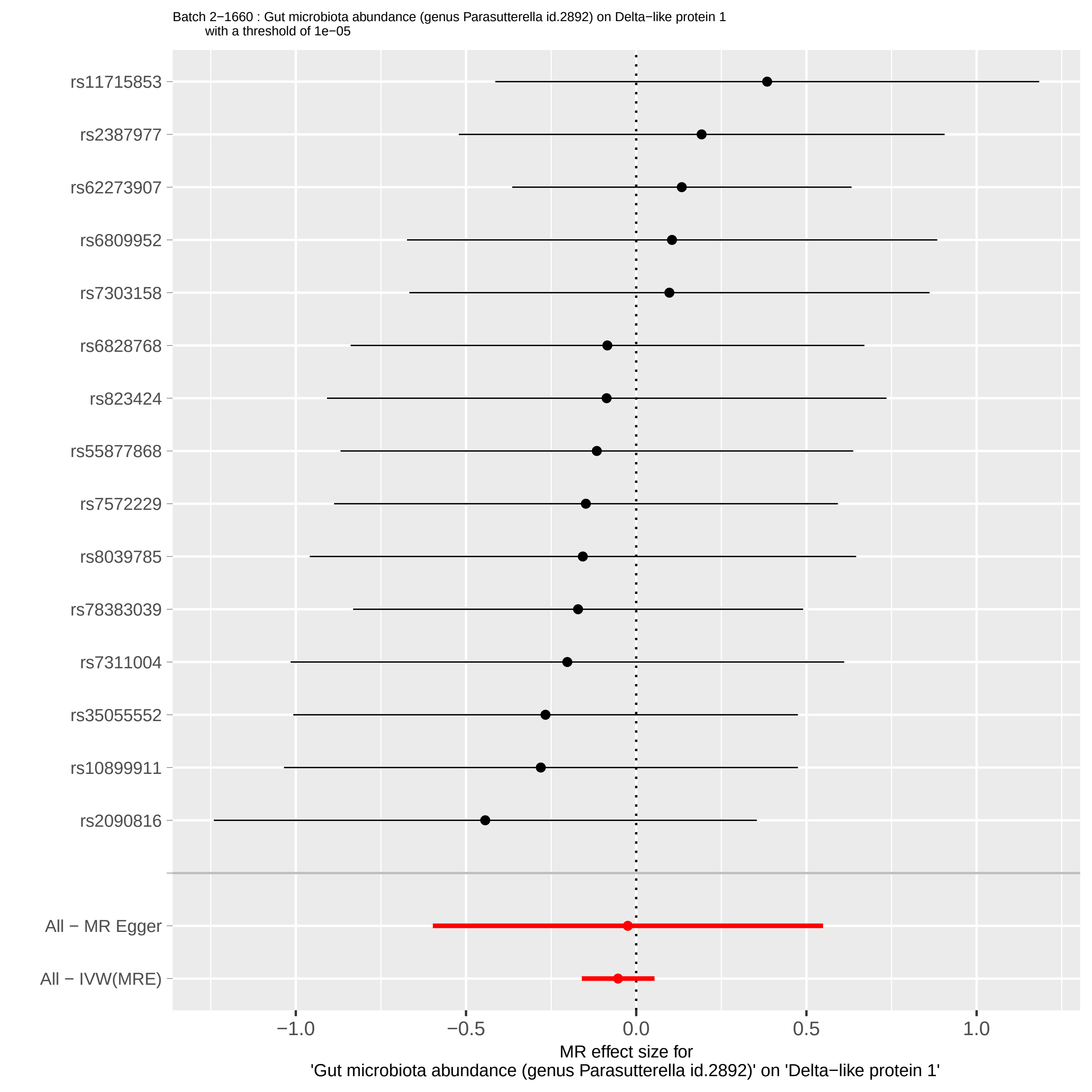

Batch 2−1660 : Gut microbiota abundance (genus Parasutterella id.2892) on Delta−like protein 1
with a threshold of 1e−05
rs11715853
rs2387977
rs62273907
rs6809952
rs7303158
rs6828768
rs823424
rs55877868
rs7572229
rs8039785
rs78383039
rs7311004
rs35055552
rs10899911
rs2090816
All − MR Egger
All − IVW(MRE)
−1.0
−0.5
0.0
0.5
1.0
MR effect size for
'Gut microbiota abundance (genus Parasutterella id.2892)' on 'Delta−like protein 1'

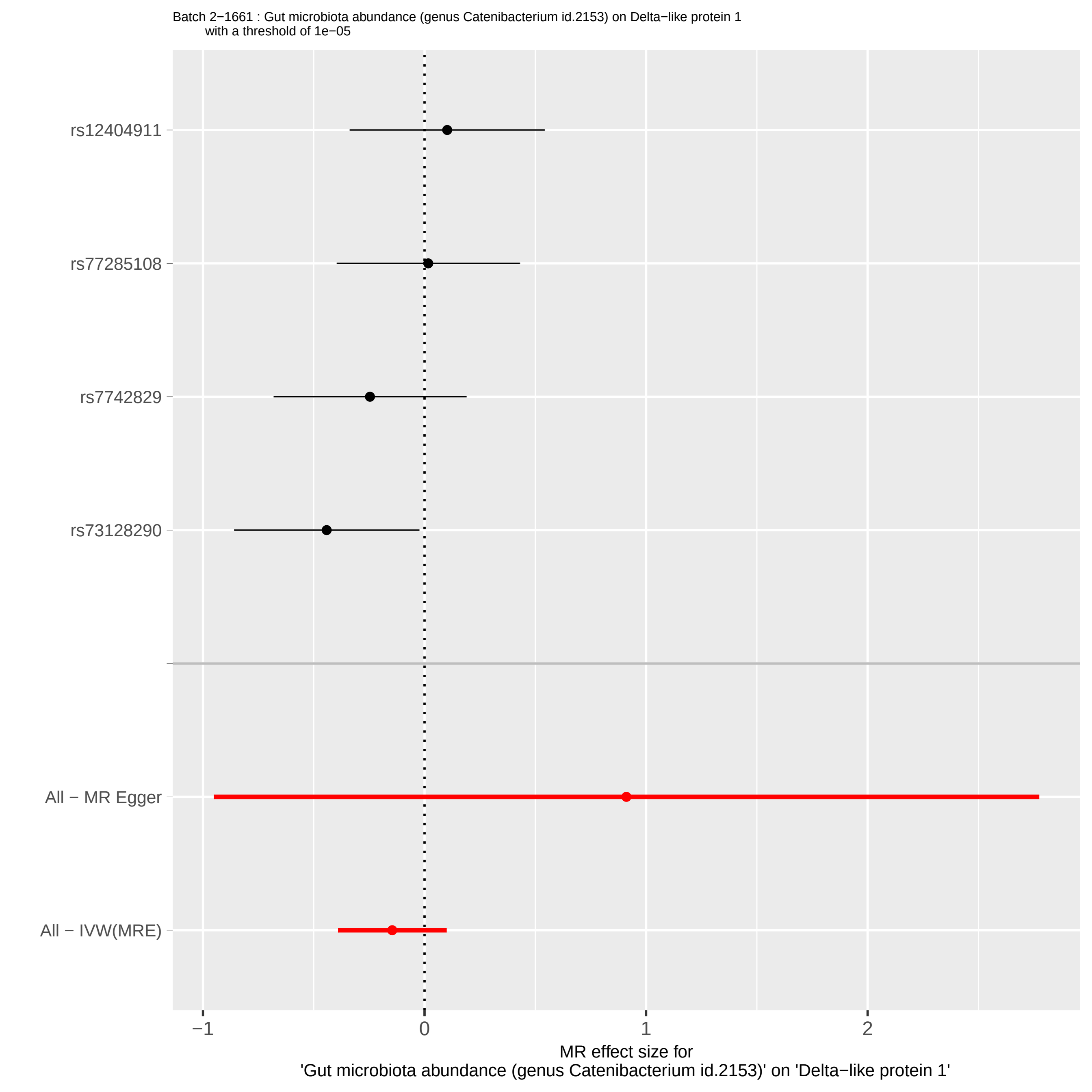
Batch 2−1661 : Gut microbiota abundance (genus Catenibacterium id.2153) on Delta−like protein 1
with a threshold of 1e−05
rs12404911
rs77285108
rs7742829
rs73128290
All − MR Egger
All − IVW(MRE)
−1
0
1
2
MR effect size for
'Gut microbiota abundance (genus Catenibacterium id.2153)' on 'Delta−like protein 1'

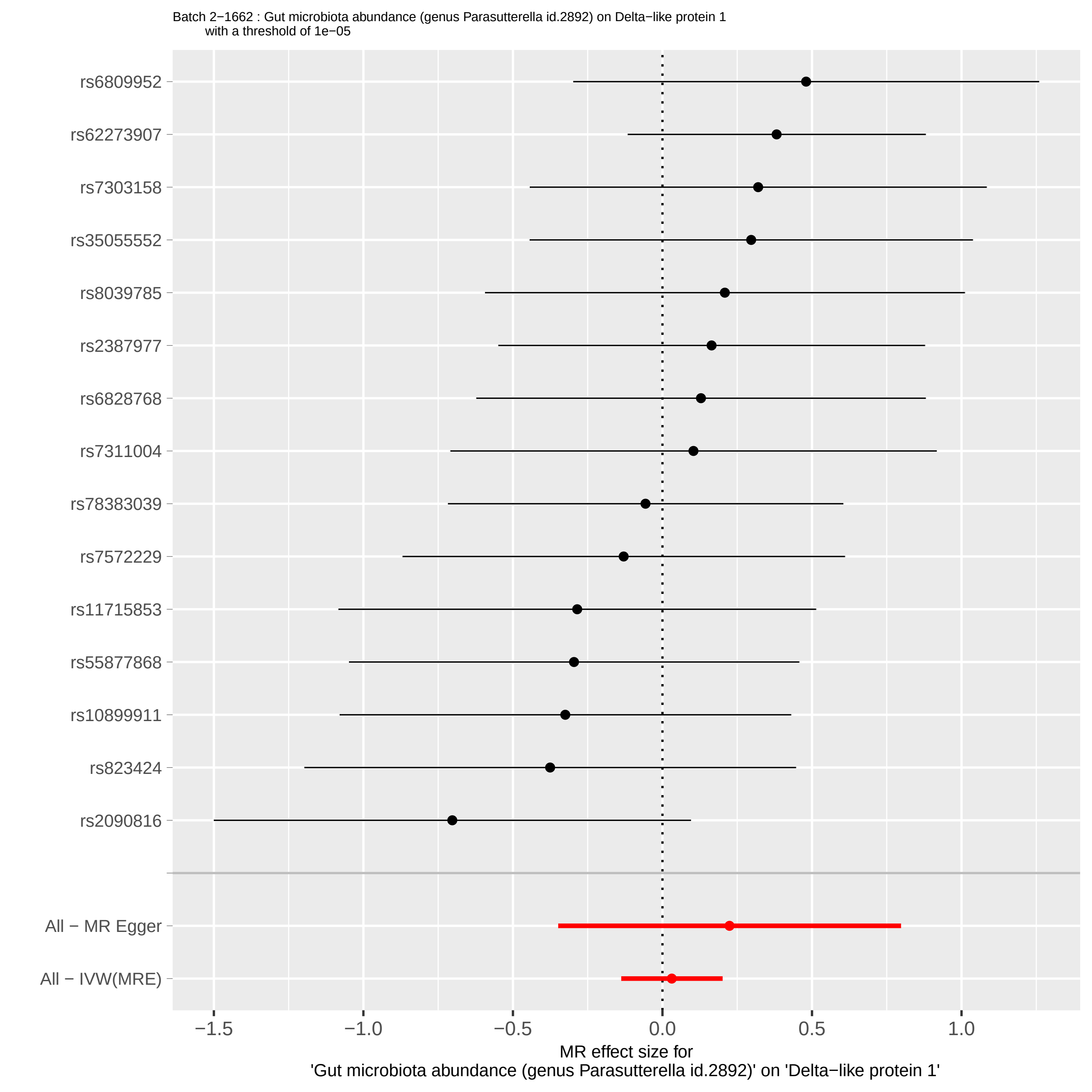

Batch 2−1662 : Gut microbiota abundance (genus Parasutterella id.2892) on Delta−like protein 1
with a threshold of 1e−05
rs6809952
rs62273907
rs7303158
rs35055552
rs8039785
rs2387977
rs6828768
rs7311004
rs78383039
rs7572229
rs11715853
rs55877868
rs10899911
rs823424
rs2090816
All − MR Egger
All − IVW(MRE)
−1.5
−1.0
−0.5
0.0
0.5
1.0
MR effect size for
'Gut microbiota abundance (genus Parasutterella id.2892)' on 'Delta−like protein 1'

Batch 2−1663 : Gut microbiota abundance (genus Catenibacterium id.2153) on Delta−like protein 4
with a threshold of 1e−05

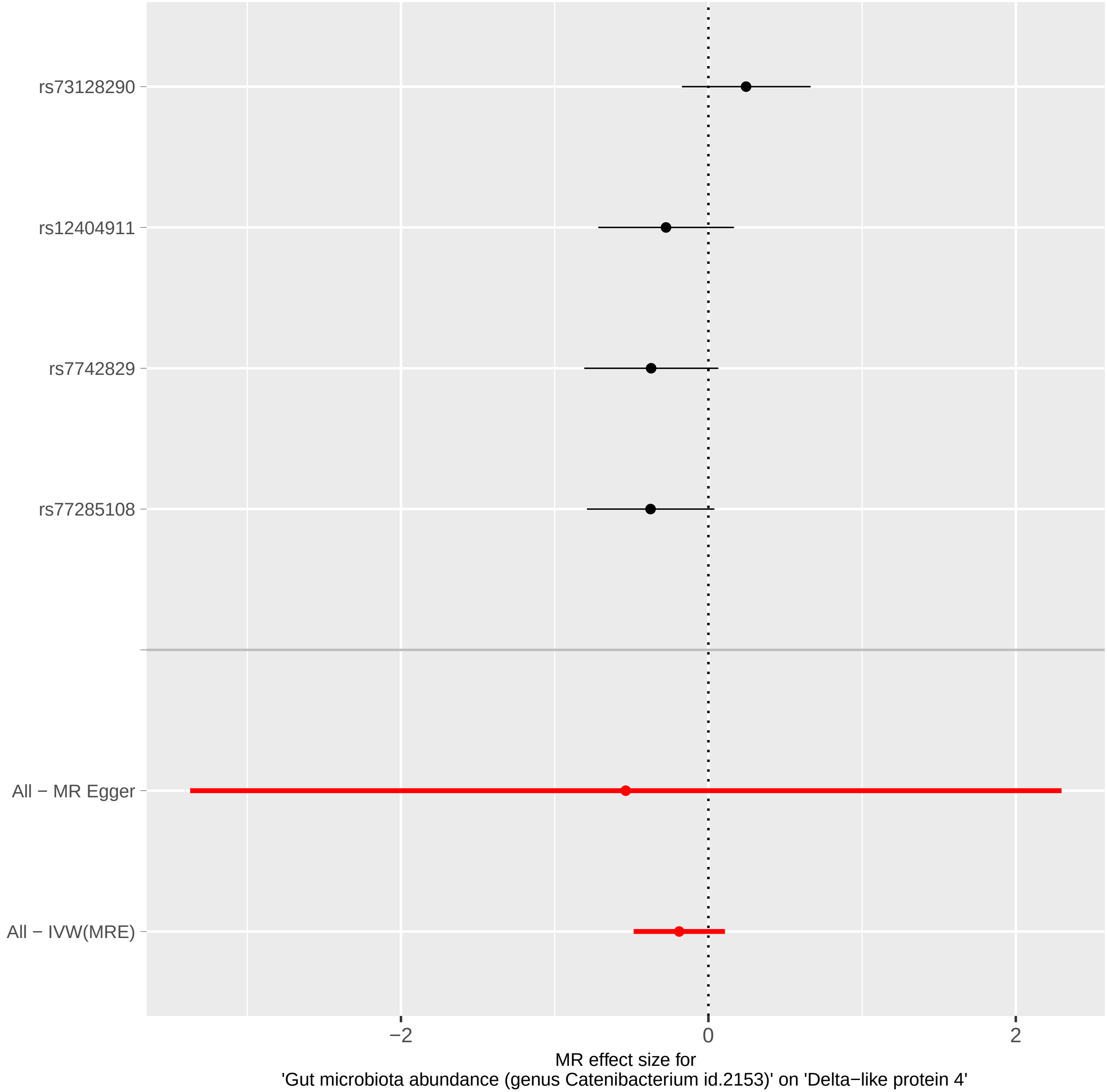

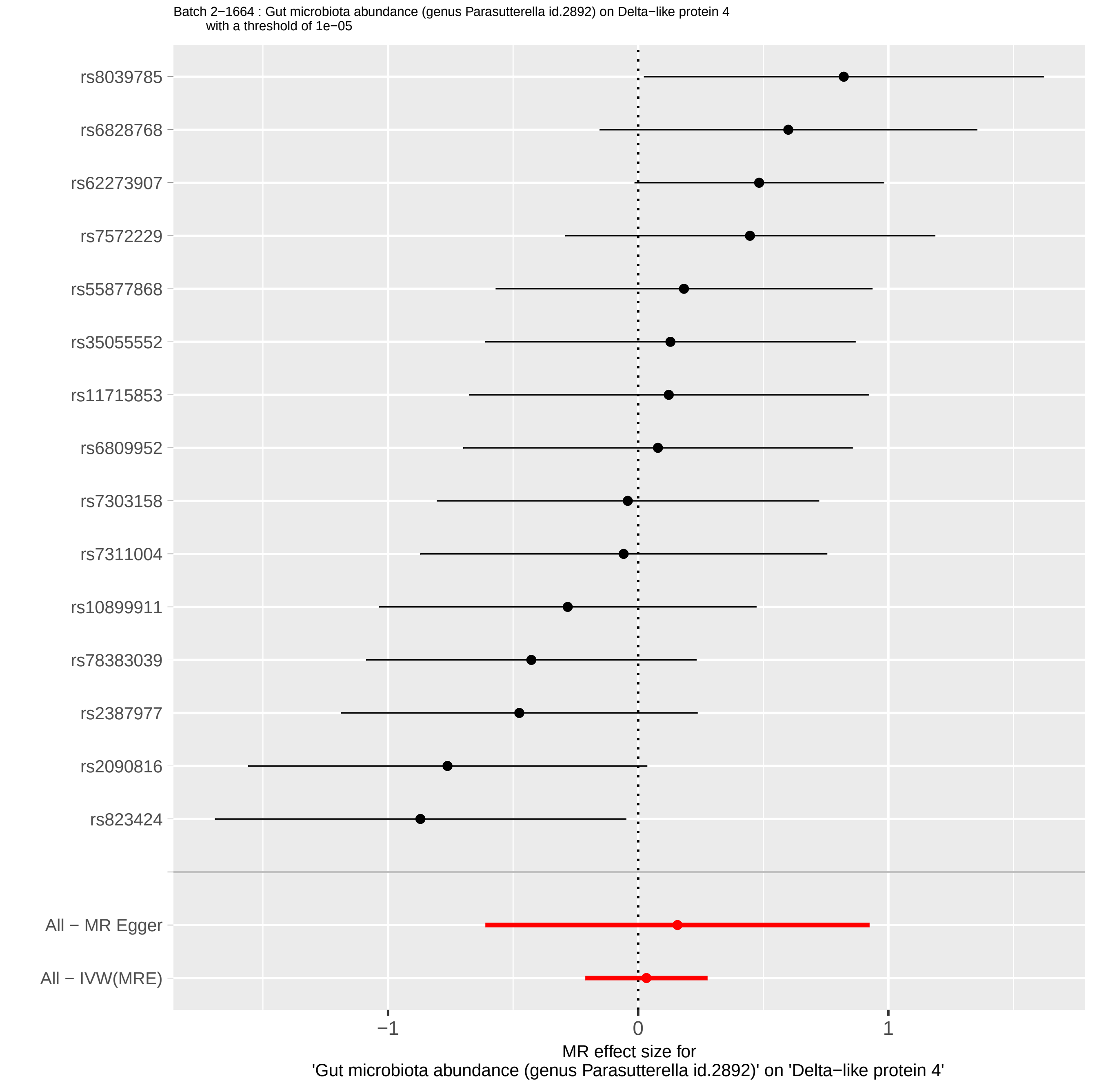

Batch 2−1664 : Gut microbiota abundance (genus Parasutterella id.2892) on Delta−like protein 4
with a threshold of 1e−05
rs8039785
rs6828768
rs62273907
rs7572229
rs55877868
rs35055552
rs11715853
rs6809952
rs7303158
rs7311004
rs10899911
rs78383039
rs2387977
rs2090816
rs823424
All − MR Egger
All − IVW(MRE)
−1
0
1
MR effect size for
'Gut microbiota abundance (genus Parasutterella id.2892)' on 'Delta−like protein 4'

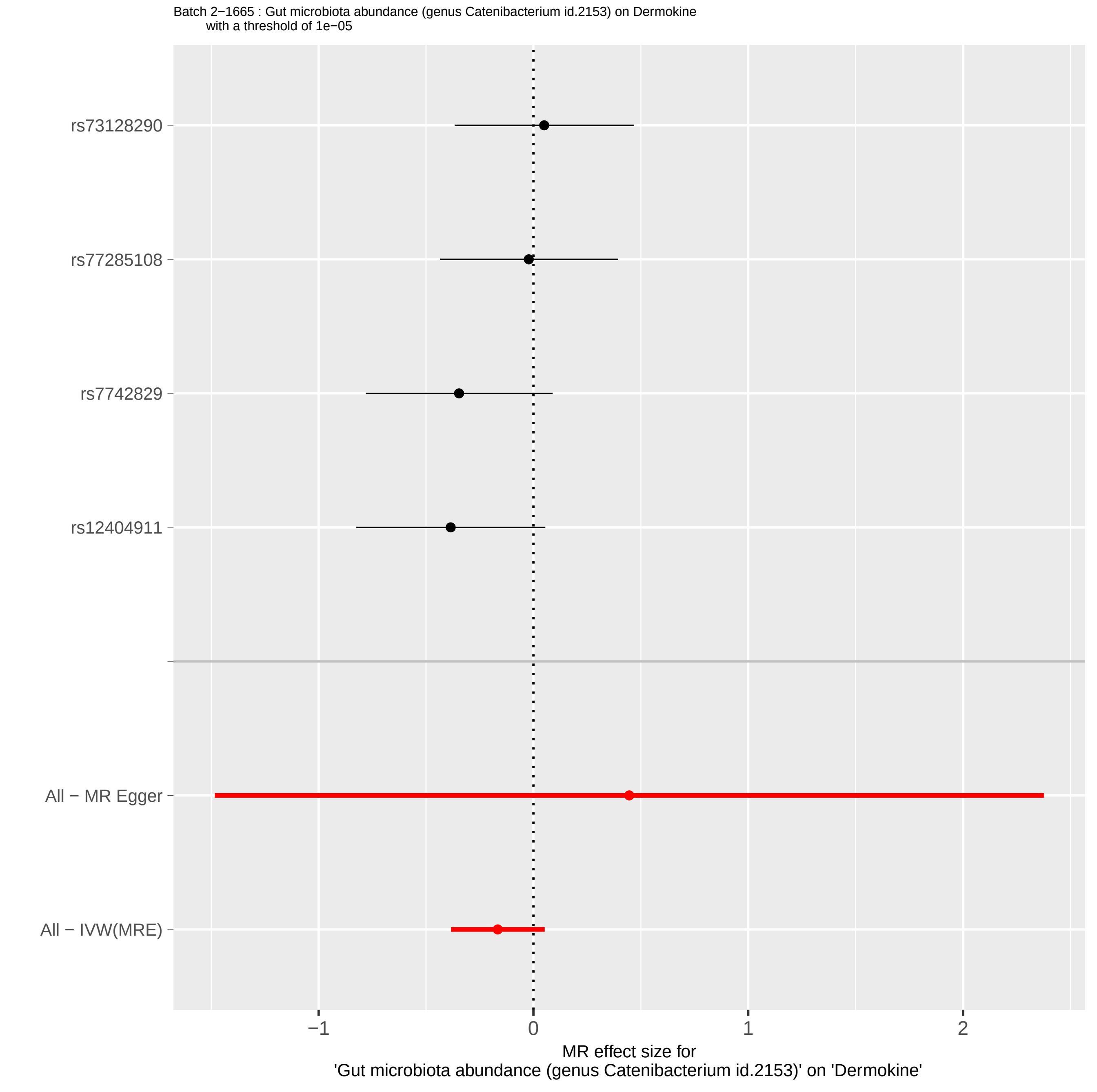

Batch 2−1665 : Gut microbiota abundance (genus Catenibacterium id.2153) on Dermokine
with a threshold of 1e−05
rs73128290
rs77285108
rs7742829
rs12404911
All − MR Egger
All − IVW(MRE)
−1
0
1
2
MR effect size for
'Gut microbiota abundance (genus Catenibacterium id.2153)' on 'Dermokine'

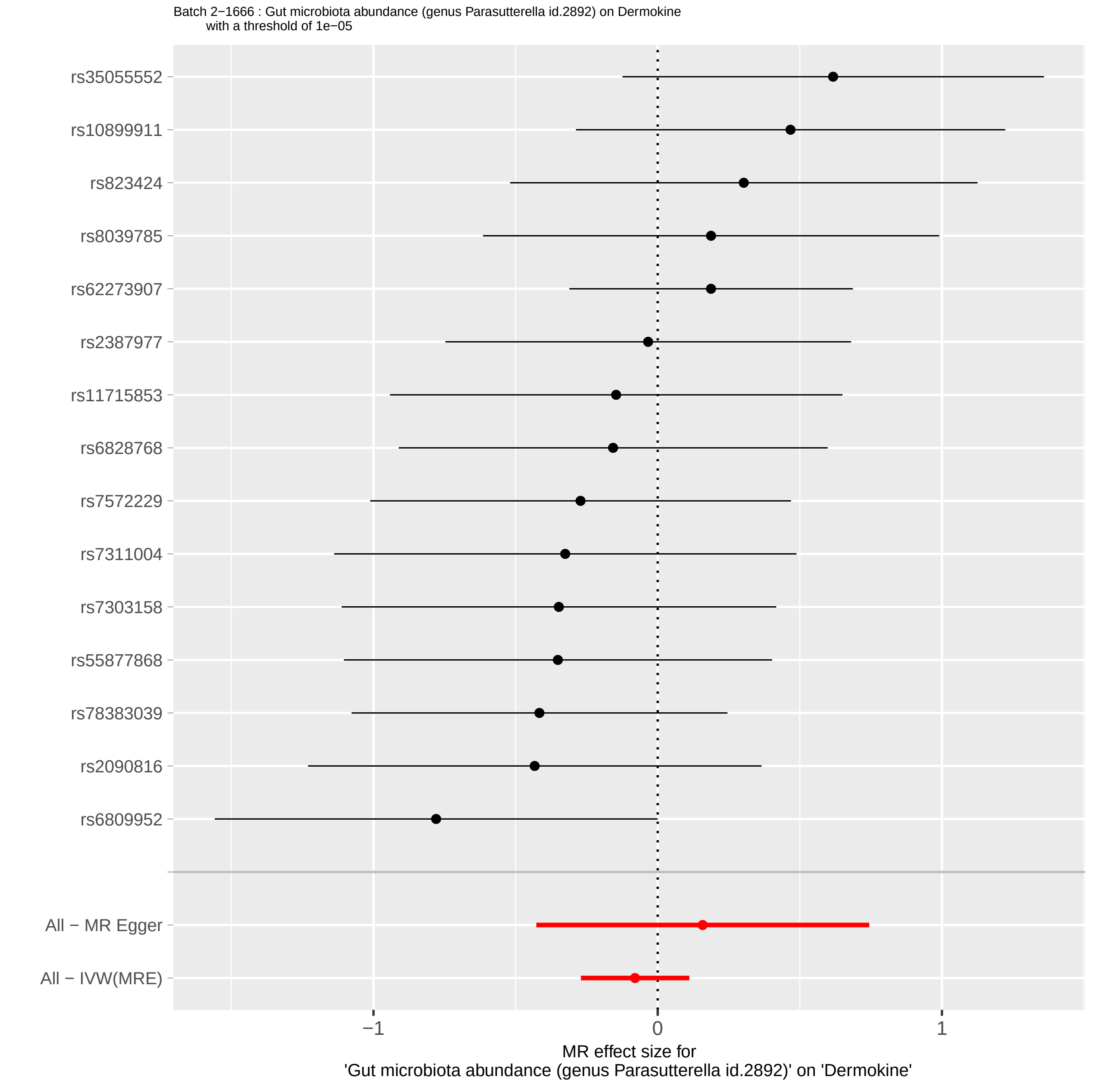
Batch 2−1666 : Gut microbiota abundance (genus Parasutterella id.2892) on Dermokine
with a threshold of 1e−05
rs35055552
rs10899911
rs823424
rs8039785
rs62273907
rs2387977
rs11715853
rs6828768
rs7572229
rs7311004
rs7303158
rs55877868
rs78383039
rs2090816
rs6809952
All − MR Egger
All − IVW(MRE)
−1
0
1
MR effect size for
'Gut microbiota abundance (genus Parasutterella id.2892)' on 'Dermokine'

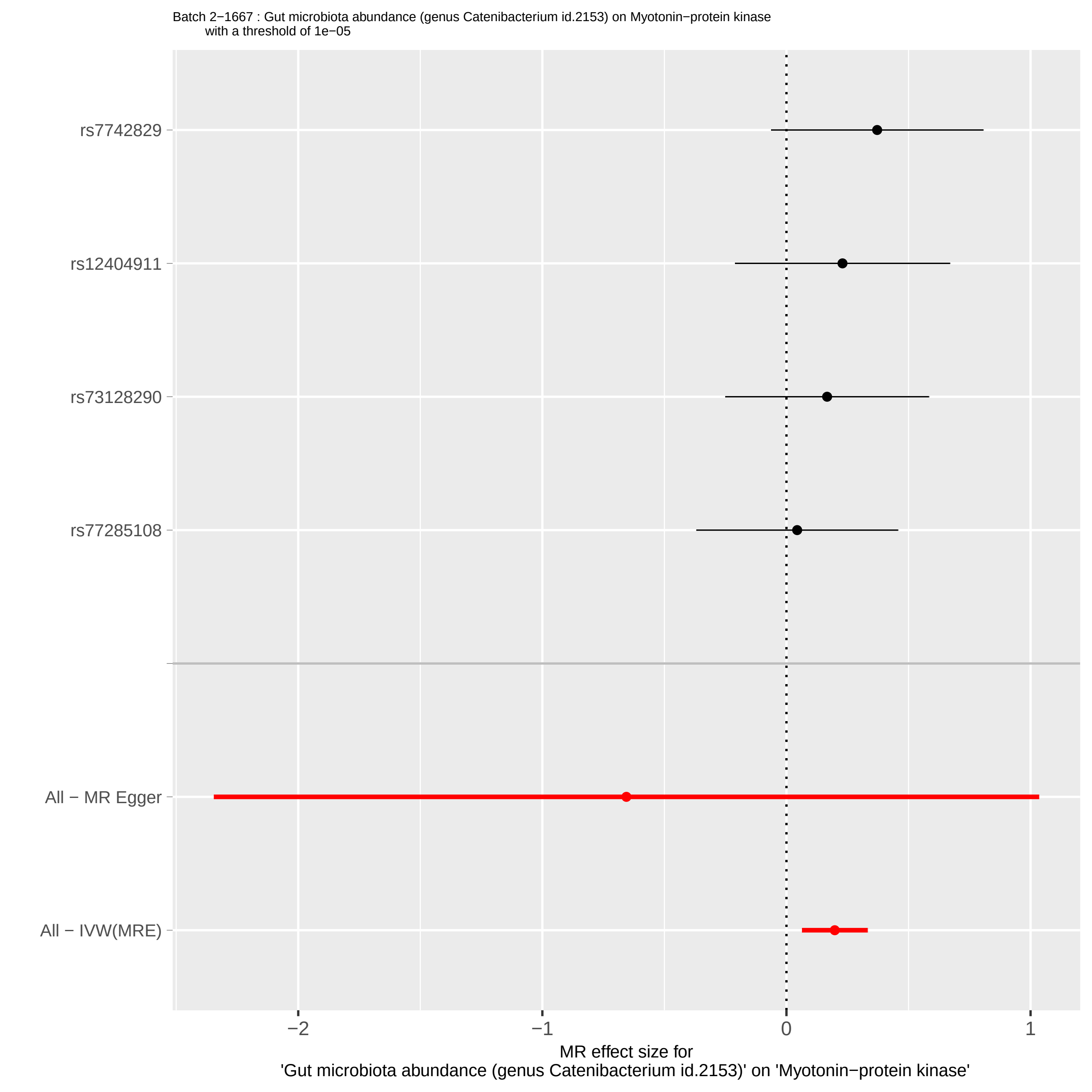

Batch 2−1667 : Gut microbiota abundance (genus Catenibacterium id.2153) on Myotonin−protein kinase
with a threshold of 1e−05
rs7742829
rs12404911
rs73128290
rs77285108
All − MR Egger
All − IVW(MRE)
−2
−1
0
1
MR effect size for
'Gut microbiota abundance (genus Catenibacterium id.2153)' on 'Myotonin−protein kinase'

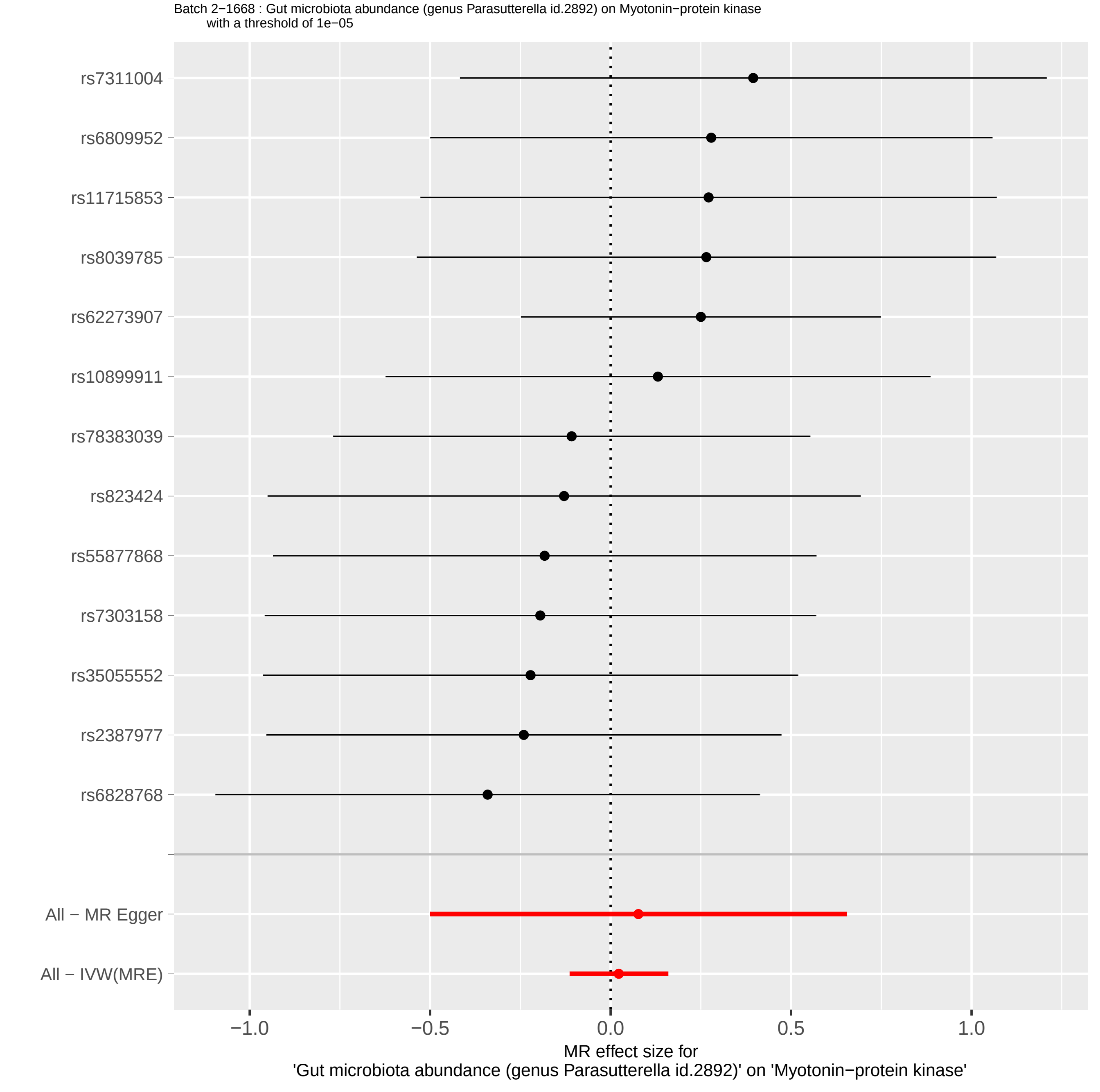
Batch 2−1668 : Gut microbiota abundance (genus Parasutterella id.2892) on Myotonin−protein kinase
with a threshold of 1e−05
rs7311004
rs6809952
rs11715853
rs8039785
rs62273907
rs10899911
rs78383039
rs823424
rs55877868
rs7303158
rs35055552
rs2387977
rs6828768
All − MR Egger
All − IVW(MRE)
−1.0
−0.5
0.0
0.5
1.0
MR effect size for
'Gut microbiota abundance (genus Parasutterella id.2892)' on 'Myotonin−protein kinase'

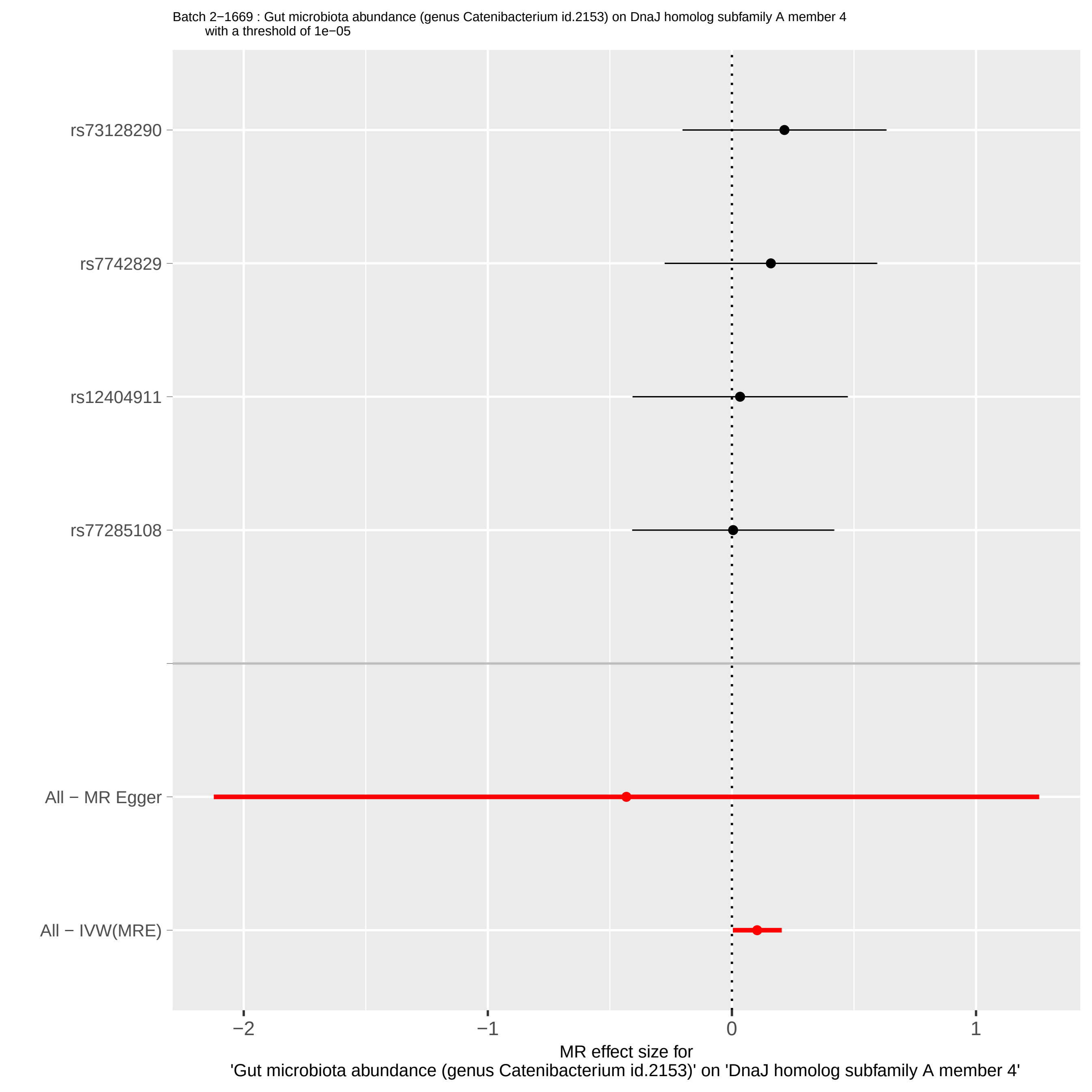

Batch 2−1669 : Gut microbiota abundance (genus Catenibacterium id.2153) on DnaJ homolog subfamily A member 4
with a threshold of 1e−05
rs73128290
rs7742829
rs12404911
rs77285108
All − MR Egger
All − IVW(MRE)
−2
−1
0
1
MR effect size for
'Gut microbiota abundance (genus Catenibacterium id.2153)' on 'DnaJ homolog subfamily A member 4'

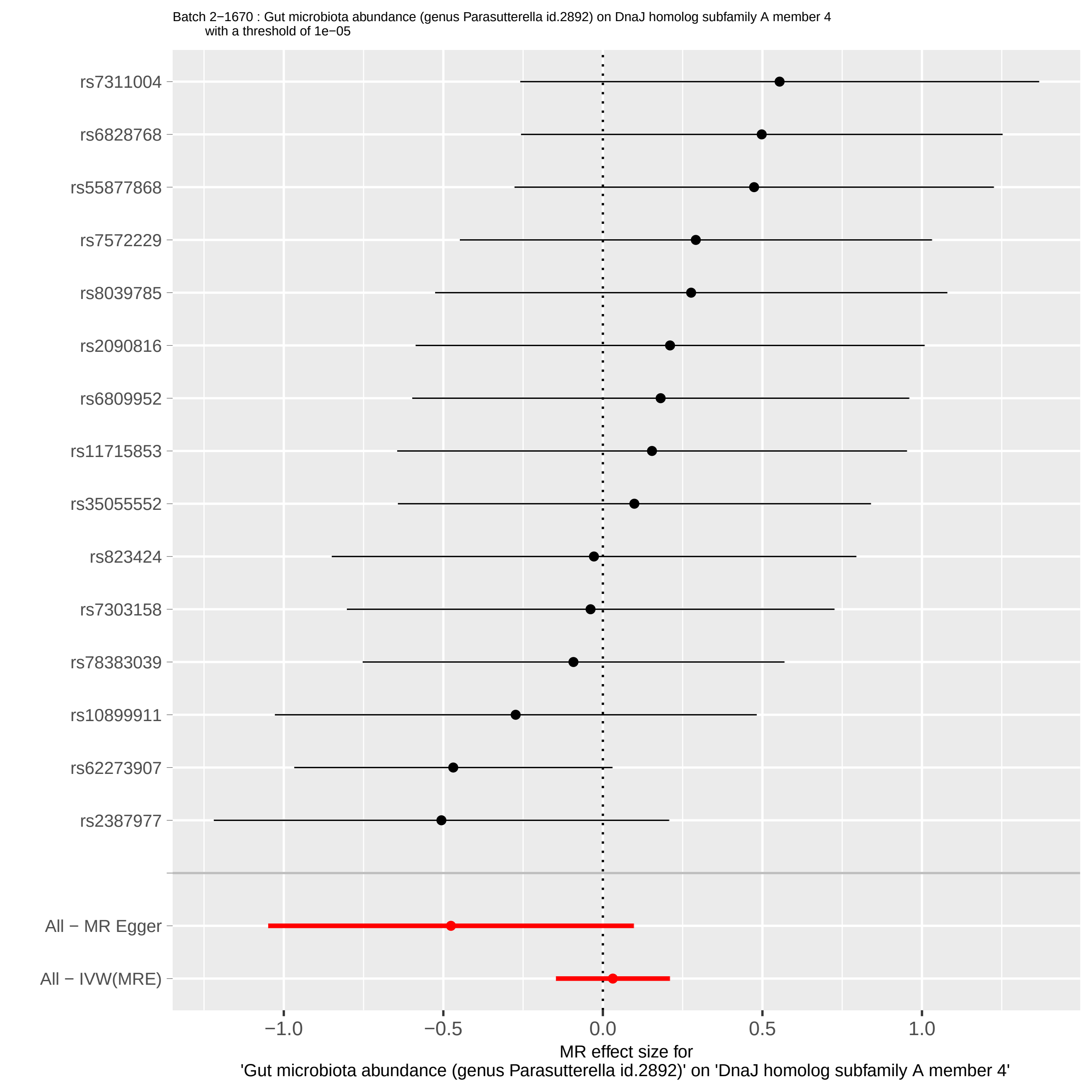
Batch 2−1670 : Gut microbiota abundance (genus Parasutterella id.2892) on DnaJ homolog subfamily A member 4
with a threshold of 1e−05
rs7311004
rs6828768
rs55877868
rs7572229
rs8039785
rs2090816
rs6809952
rs11715853
rs35055552
rs823424
rs7303158
rs78383039
rs10899911
rs62273907
rs2387977
All − MR Egger
All − IVW(MRE)
−1.0
−0.5
0.0
0.5
1.0
MR effect size for
'Gut microbiota abundance (genus Parasutterella id.2892)' on 'DnaJ homolog subfamily A member 4'

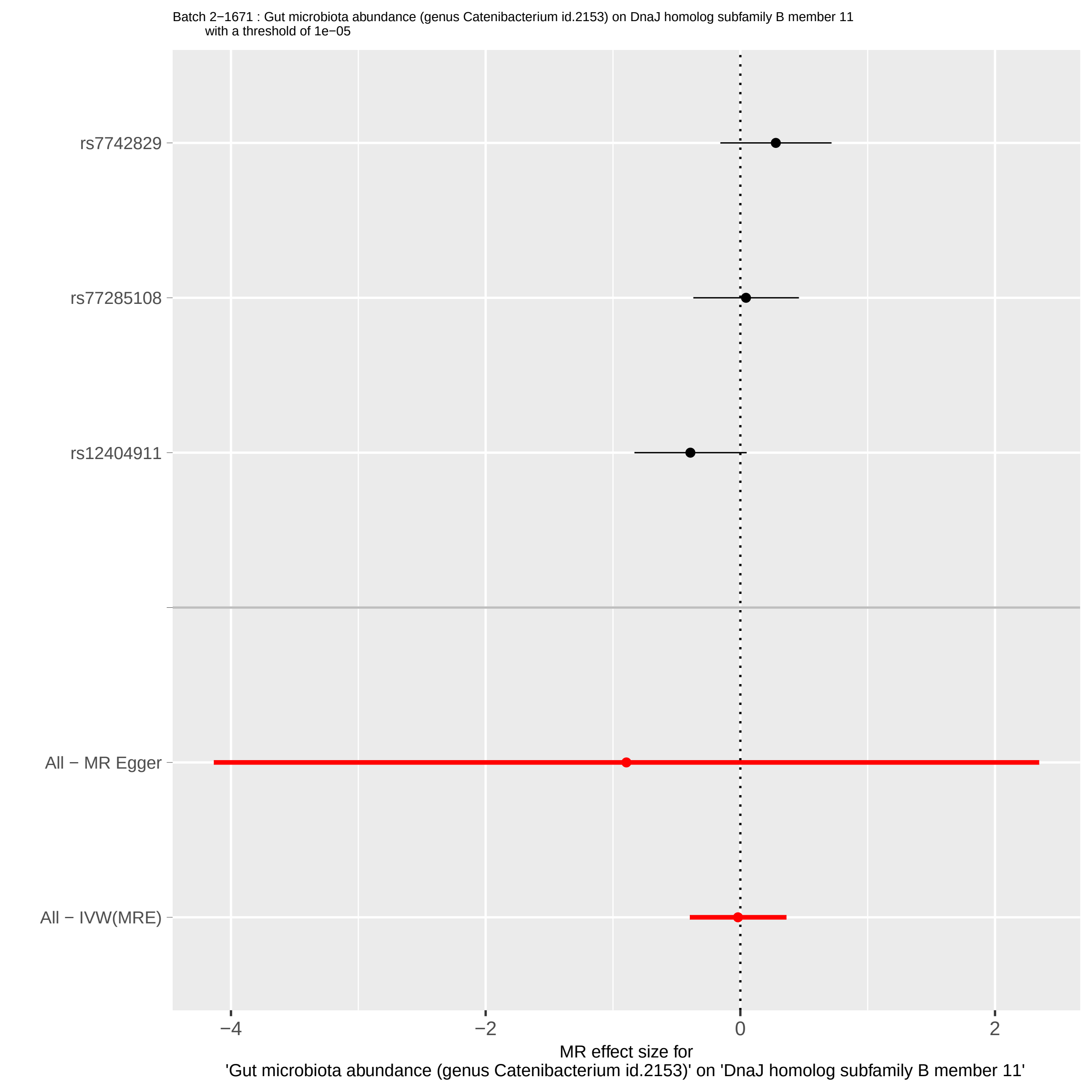

Batch 2−1671 : Gut microbiota abundance (genus Catenibacterium id.2153) on DnaJ homolog subfamily B member 11
with a threshold of 1e−05
rs7742829
rs77285108
rs12404911
All − MR Egger
All − IVW(MRE)
−4
−2
0
2
MR effect size for
'Gut microbiota abundance (genus Catenibacterium id.2153)' on 'DnaJ homolog subfamily B member 11'

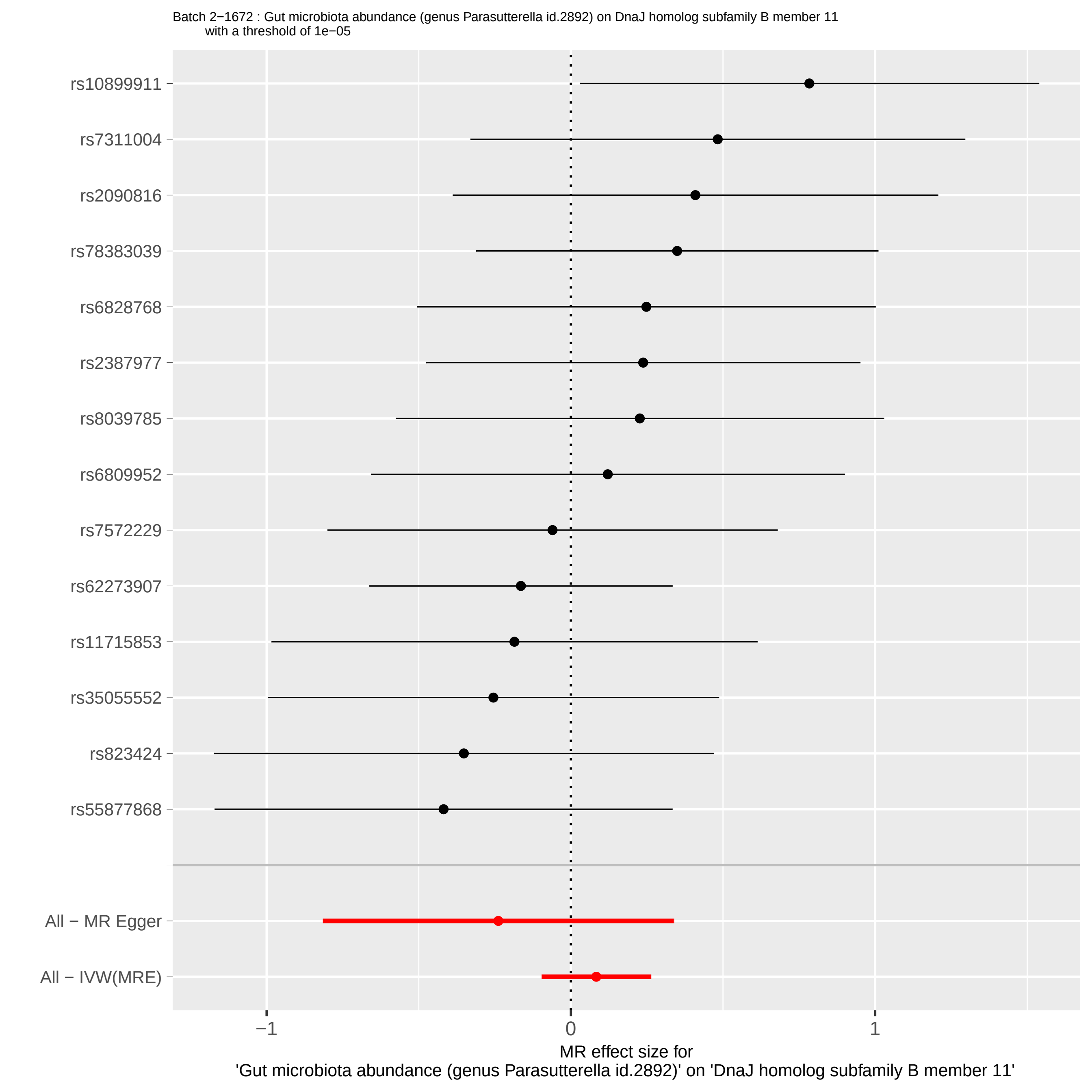
Batch 2−1672 : Gut microbiota abundance (genus Parasutterella id.2892) on DnaJ homolog subfamily B member 11
with a threshold of 1e−05
rs10899911
rs7311004
rs2090816
rs78383039
rs6828768
rs2387977
rs8039785
rs6809952
rs7572229
rs62273907
rs11715853
rs35055552
rs823424
rs55877868
All − MR Egger
All − IVW(MRE)
−1
0
1
MR effect size for
'Gut microbiota abundance (genus Parasutterella id.2892)' on 'DnaJ homolog subfamily B member 11'

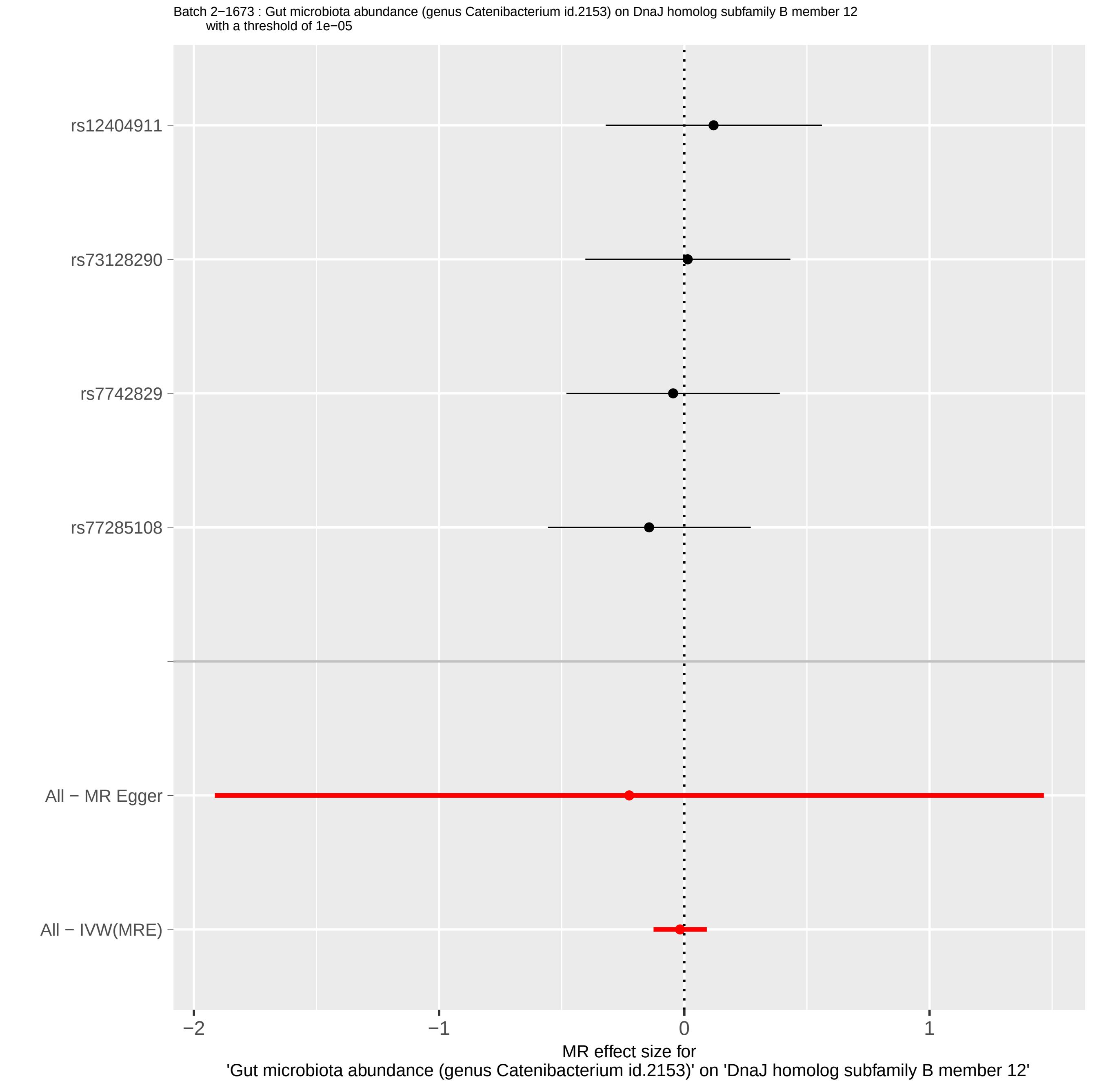
Batch 2−1673 : Gut microbiota abundance (genus Catenibacterium id.2153) on DnaJ homolog subfamily B member 12
with a threshold of 1e−05
rs12404911
rs73128290
rs7742829
rs77285108
All − MR Egger
All − IVW(MRE)
−2
−1
0
1
MR effect size for
'Gut microbiota abundance (genus Catenibacterium id.2153)' on 'DnaJ homolog subfamily B member 12'

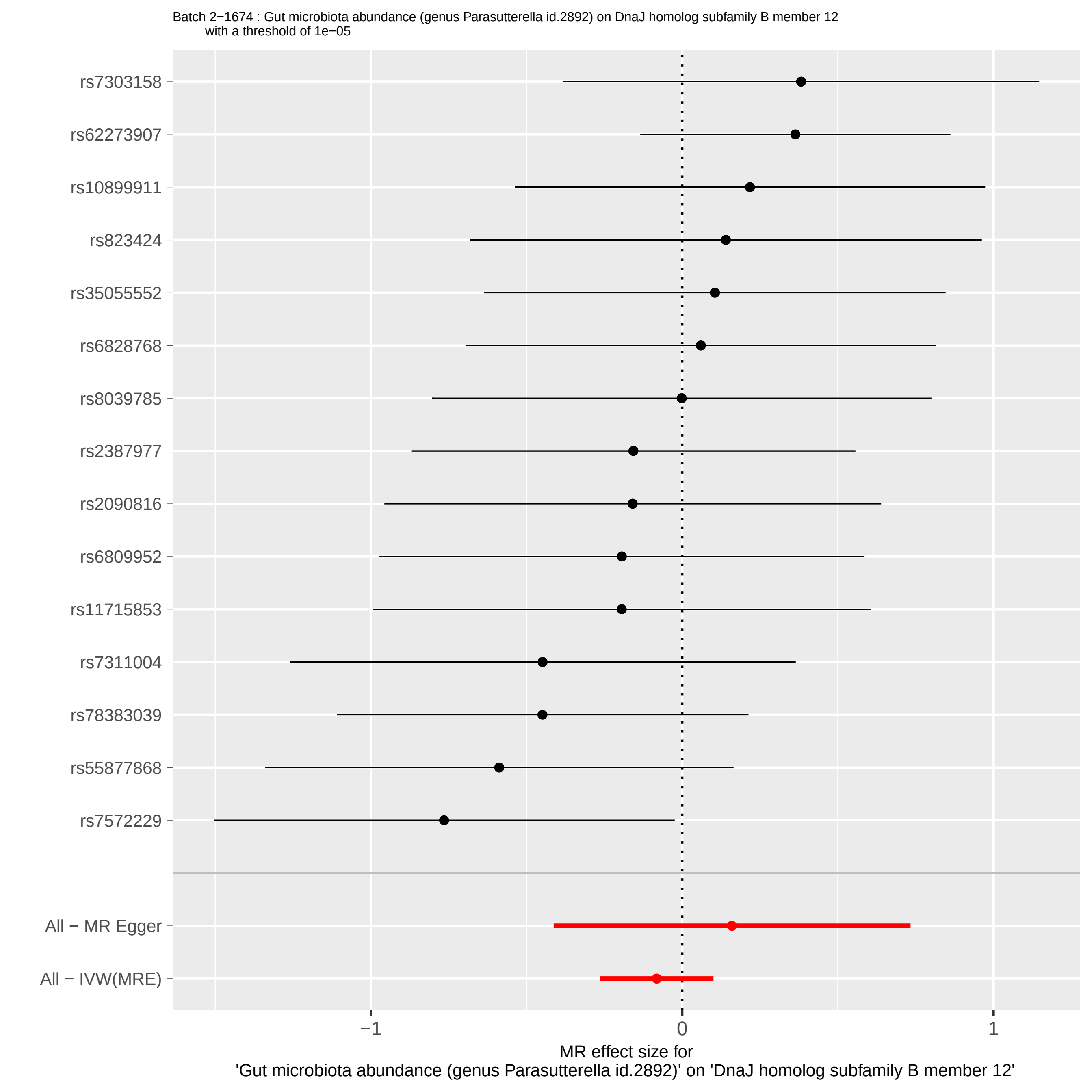
Batch 2−1674 : Gut microbiota abundance (genus Parasutterella id.2892) on DnaJ homolog subfamily B member 12
with a threshold of 1e−05
rs7303158
rs62273907
rs10899911
rs823424
rs35055552
rs6828768
rs8039785
rs2387977
rs2090816
rs6809952
rs11715853
rs7311004
rs78383039
rs55877868
rs7572229
All − MR Egger
All − IVW(MRE)
−1
0
1
MR effect size for
'Gut microbiota abundance (genus Parasutterella id.2892)' on 'DnaJ homolog subfamily B member 12'

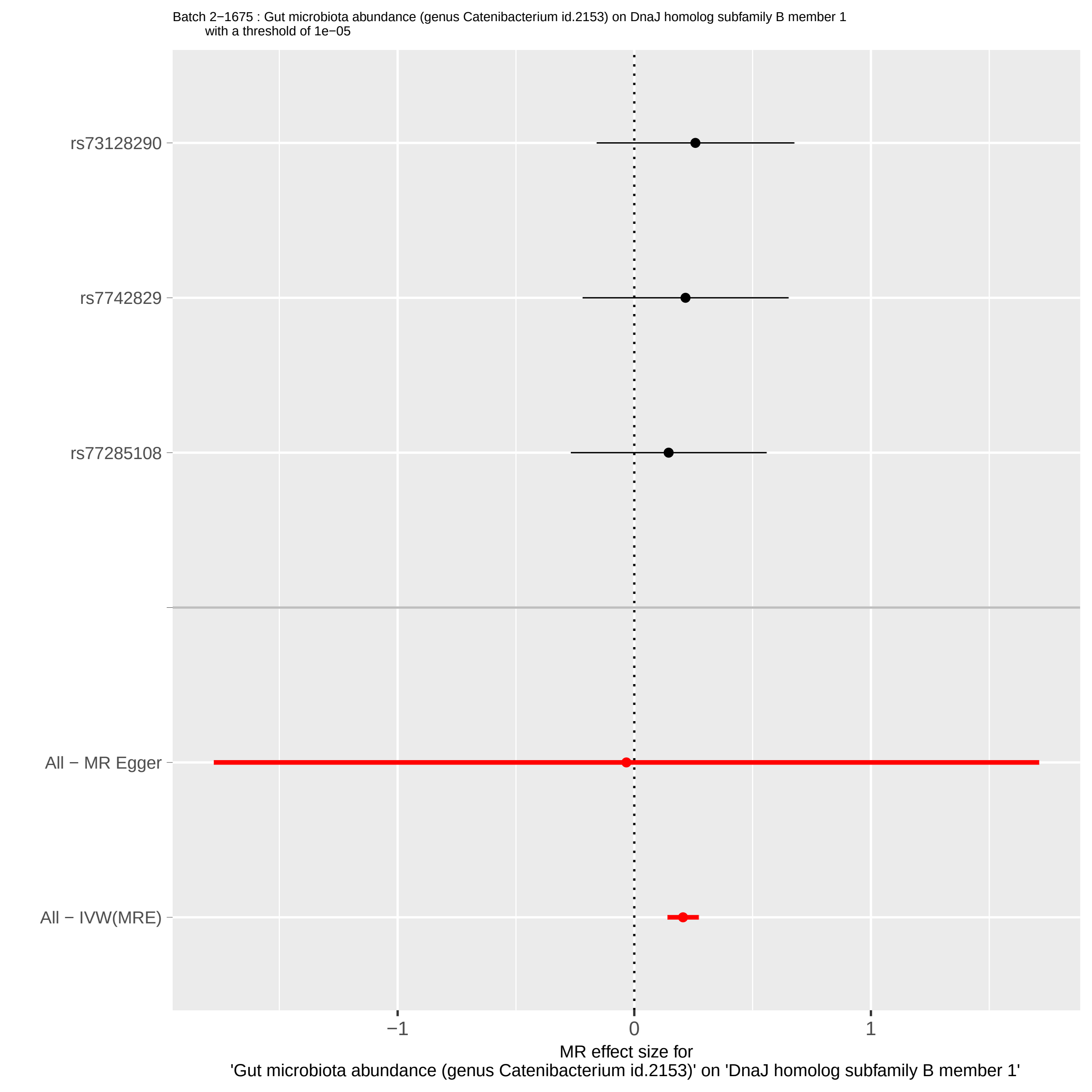

Batch 2−1675 : Gut microbiota abundance (genus Catenibacterium id.2153) on DnaJ homolog subfamily B member 1
with a threshold of 1e−05
rs73128290
rs7742829
rs77285108
All − MR Egger
All − IVW(MRE)
−1
0
1
MR effect size for
'Gut microbiota abundance (genus Catenibacterium id.2153)' on 'DnaJ homolog subfamily B member 1'

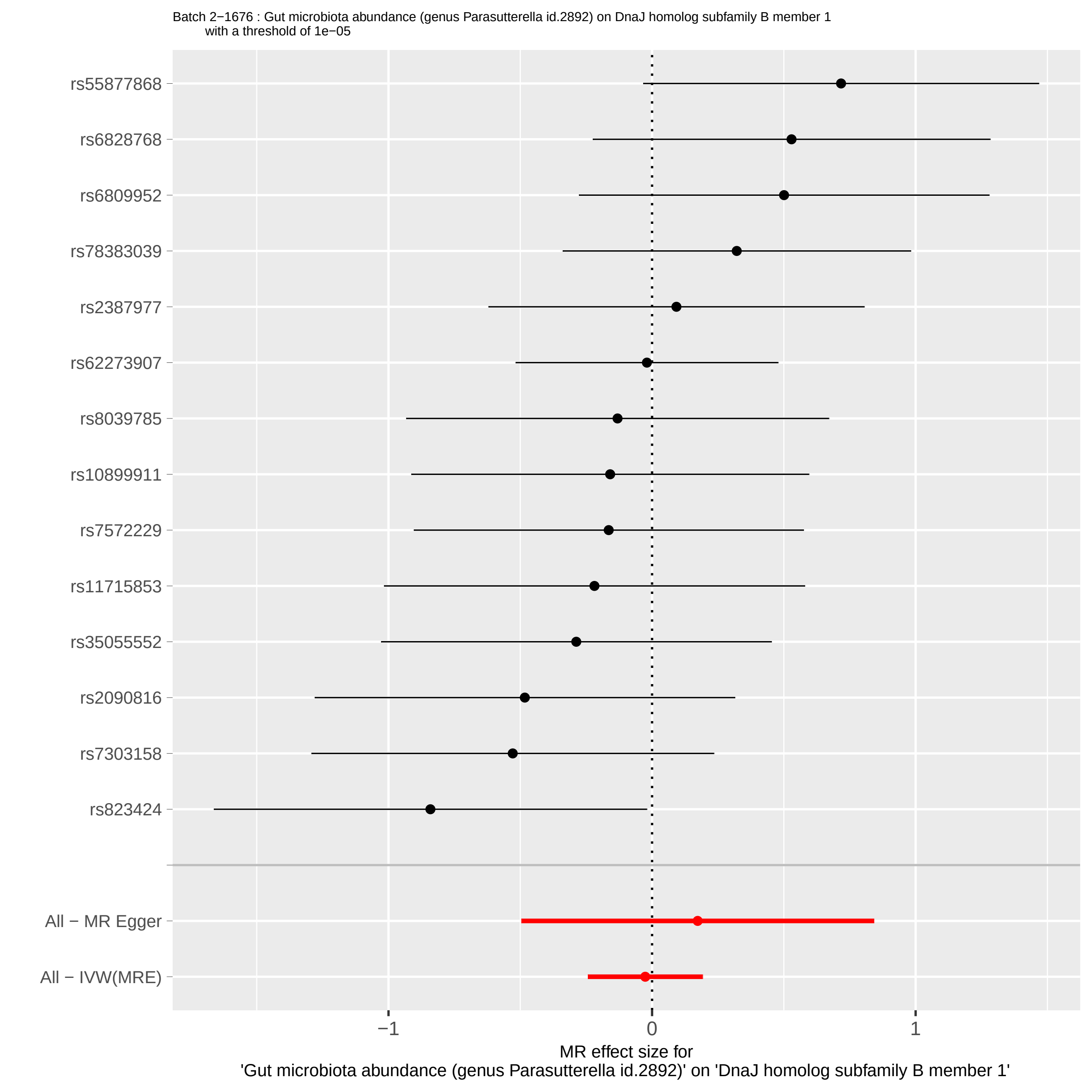
Batch 2−1676 : Gut microbiota abundance (genus Parasutterella id.2892) on DnaJ homolog subfamily B member 1
with a threshold of 1e−05
rs55877868
rs6828768
rs6809952
rs78383039
rs2387977
rs62273907
rs8039785
rs10899911
rs7572229
rs11715853
rs35055552
rs2090816
rs7303158
rs823424
All − MR Egger
All − IVW(MRE)
−1
0
1
MR effect size for
'Gut microbiota abundance (genus Parasutterella id.2892)' on 'DnaJ homolog subfamily B member 1'

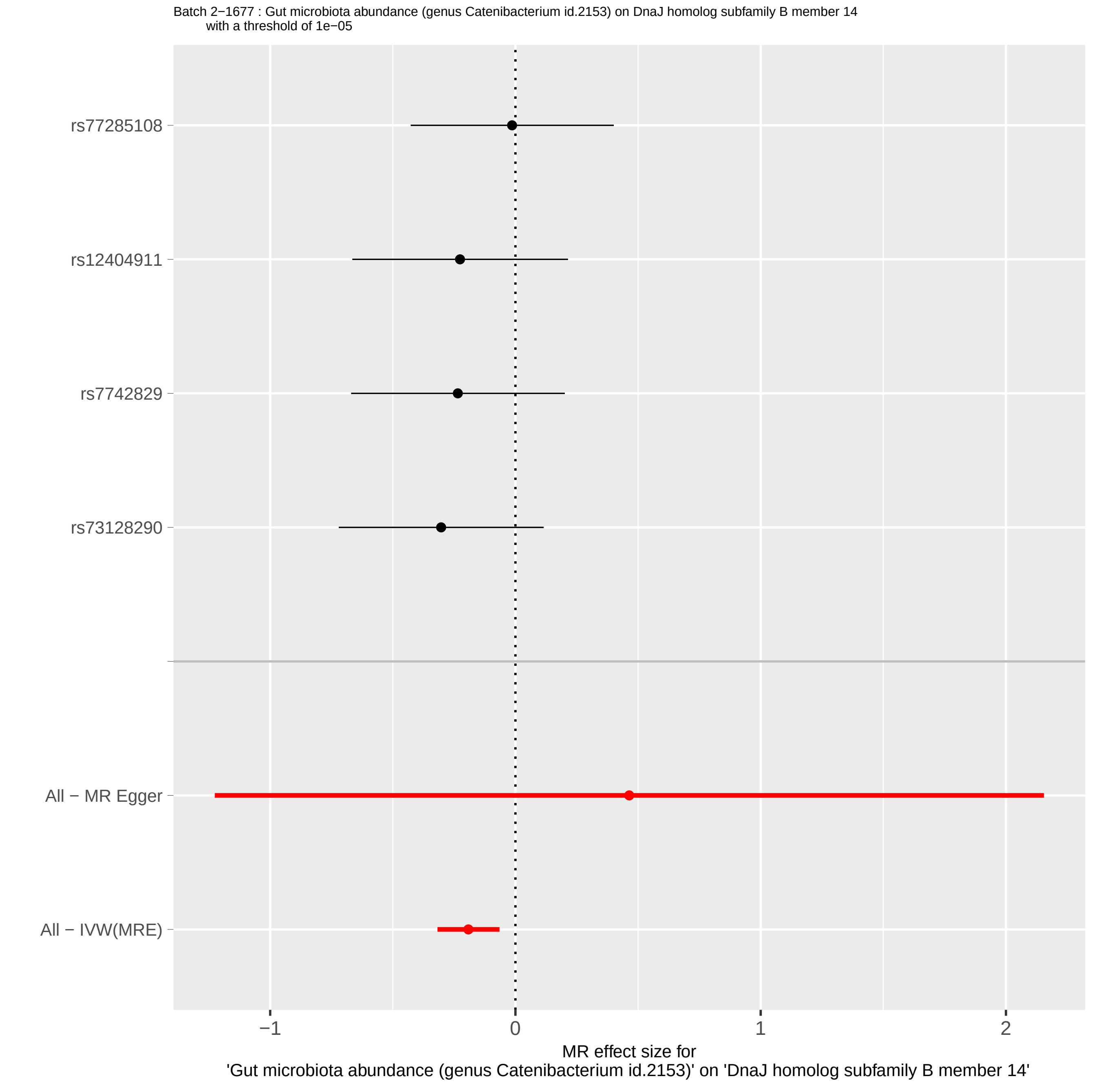

Batch 2−1677 : Gut microbiota abundance (genus Catenibacterium id.2153) on DnaJ homolog subfamily B member 14 with a threshold of 1e−05
rs77285108
rs12404911
rs7742829
rs73128290
All − MR Egger
All − IVW(MRE)
−1
0
1
2
MR effect size for
'Gut microbiota abundance (genus Catenibacterium id.2153)' on 'DnaJ homolog subfamily B member 14'

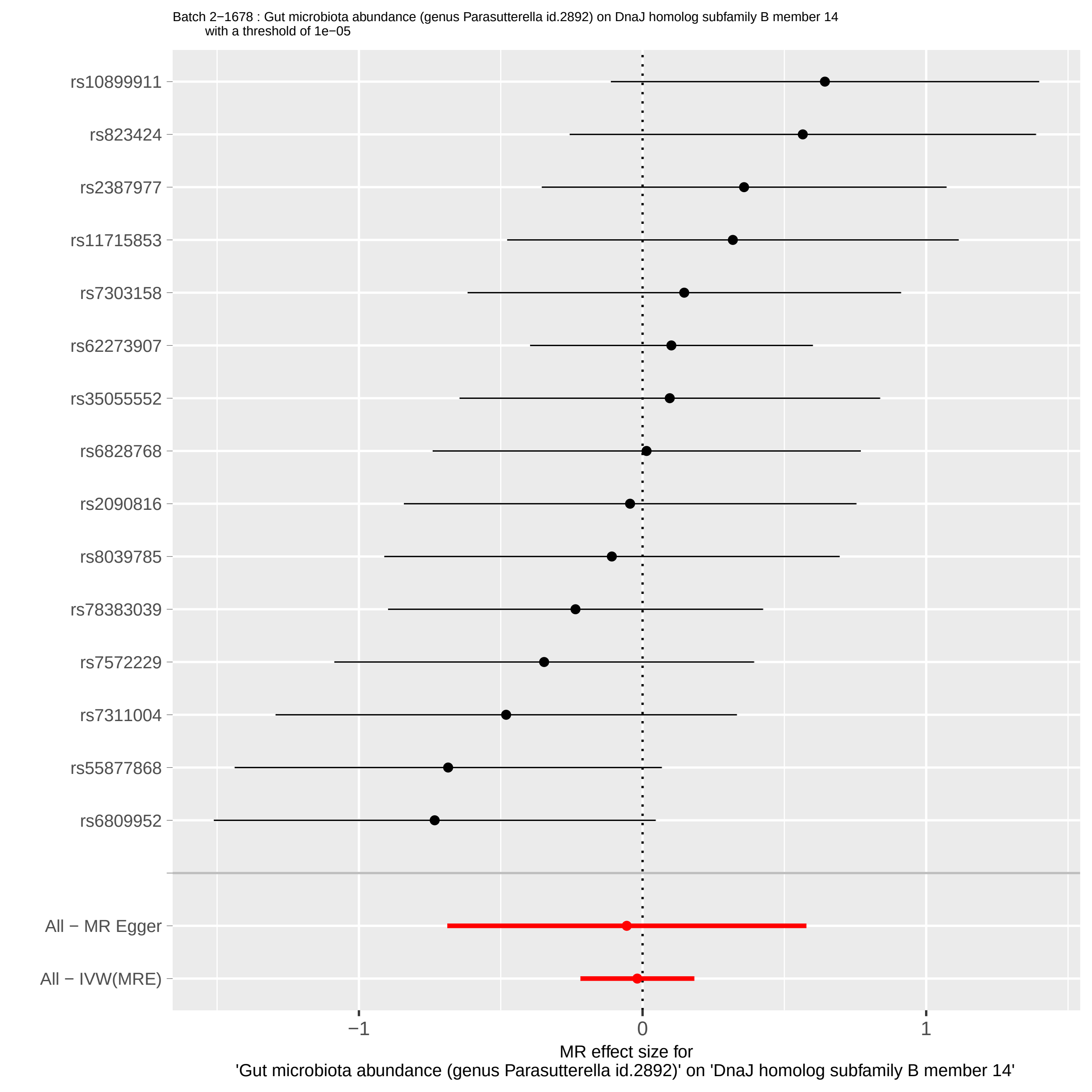
Batch 2−1678 : Gut microbiota abundance (genus Parasutterella id.2892) on DnaJ homolog subfamily B member 14
with a threshold of 1e−05
rs10899911
rs823424
rs2387977
rs11715853
rs7303158
rs62273907
rs35055552
rs6828768
rs2090816
rs8039785
rs78383039
rs7572229
rs7311004
rs55877868
rs6809952
All − MR Egger
All − IVW(MRE)
−1
0
1
MR effect size for
'Gut microbiota abundance (genus Parasutterella id.2892)' on 'DnaJ homolog subfamily B member 14'

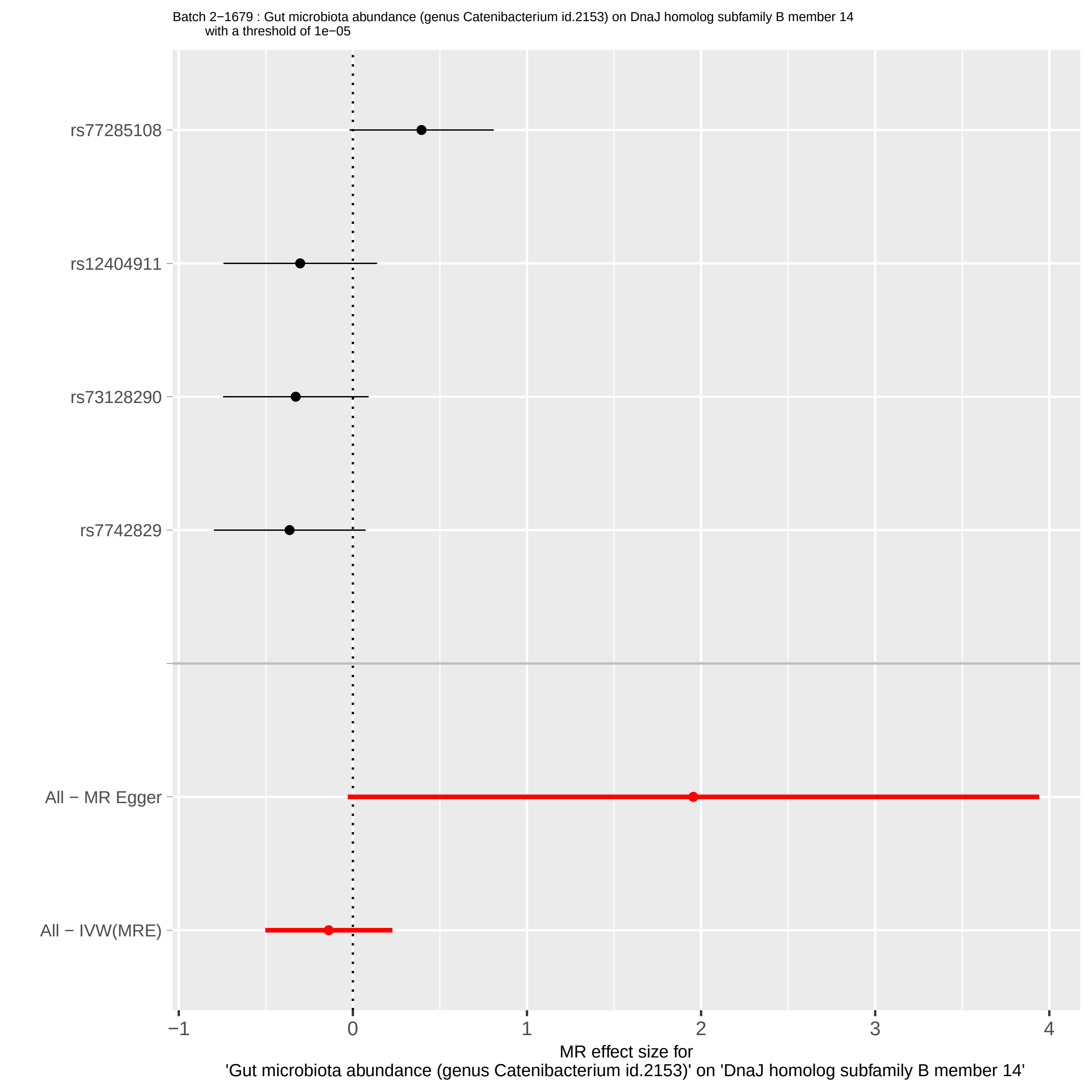
Batch 2−1679 : Gut microbiota abundance (genus Catenibacterium id.2153) on DnaJ homolog subfamily B member 14
with a threshold of 1e−05
rs77285108
rs12404911
rs73128290
rs7742829
All − MR Egger
All − IVW(MRE)
−1
0
1
2
3
4
MR effect size for
'Gut microbiota abundance (genus Catenibacterium id.2153)' on 'DnaJ homolog subfamily B member 14'

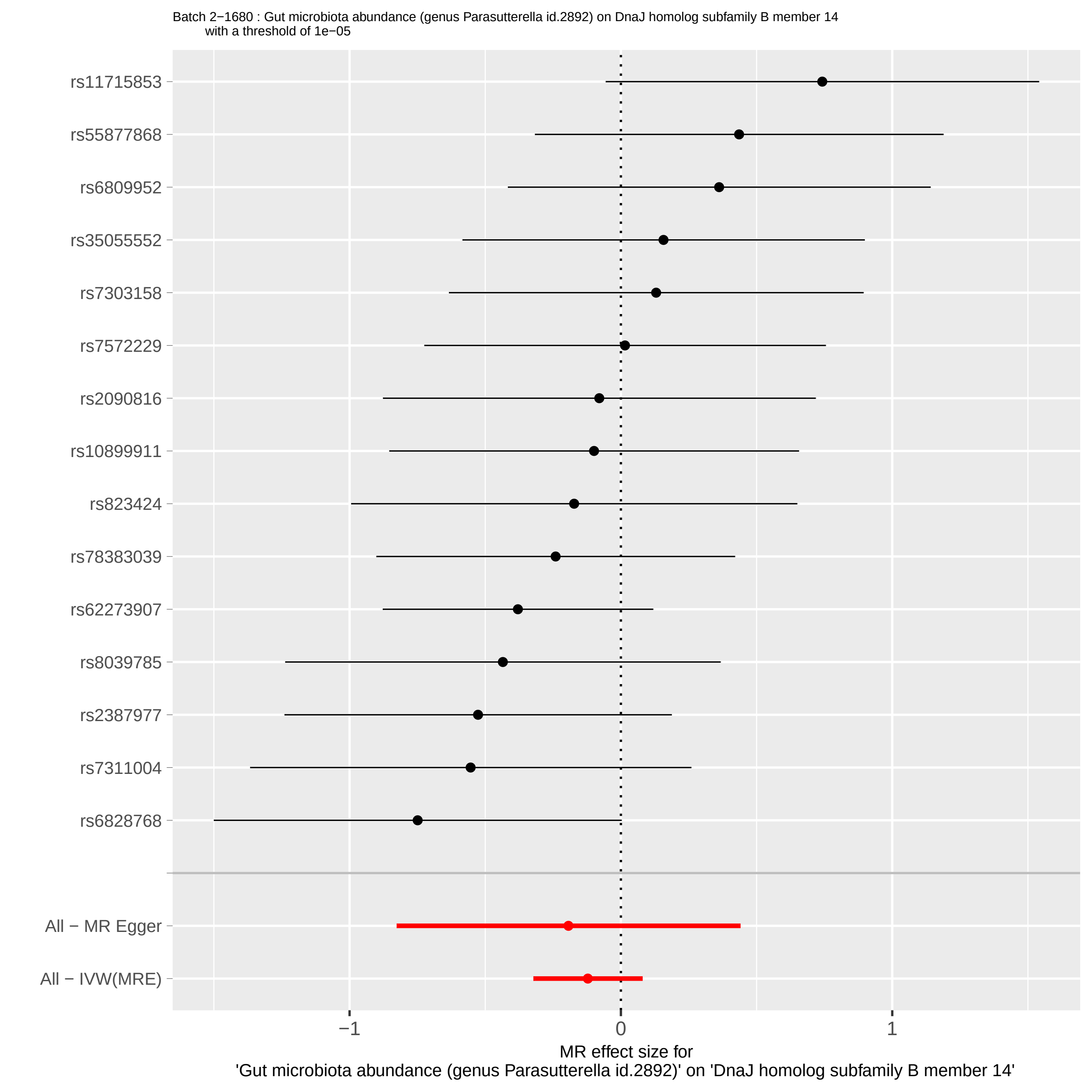
Batch 2−1680 : Gut microbiota abundance (genus Parasutterella id.2892) on DnaJ homolog subfamily B member 14
with a threshold of 1e−05
rs11715853
rs55877868
rs6809952
rs35055552
rs7303158
rs7572229
rs2090816
rs10899911
rs823424
rs78383039
rs62273907
rs8039785
rs2387977
rs7311004
rs6828768
All − MR Egger
All − IVW(MRE)
−1
0
1
MR effect size for
'Gut microbiota abundance (genus Parasutterella id.2892)' on 'DnaJ homolog subfamily B member 14'

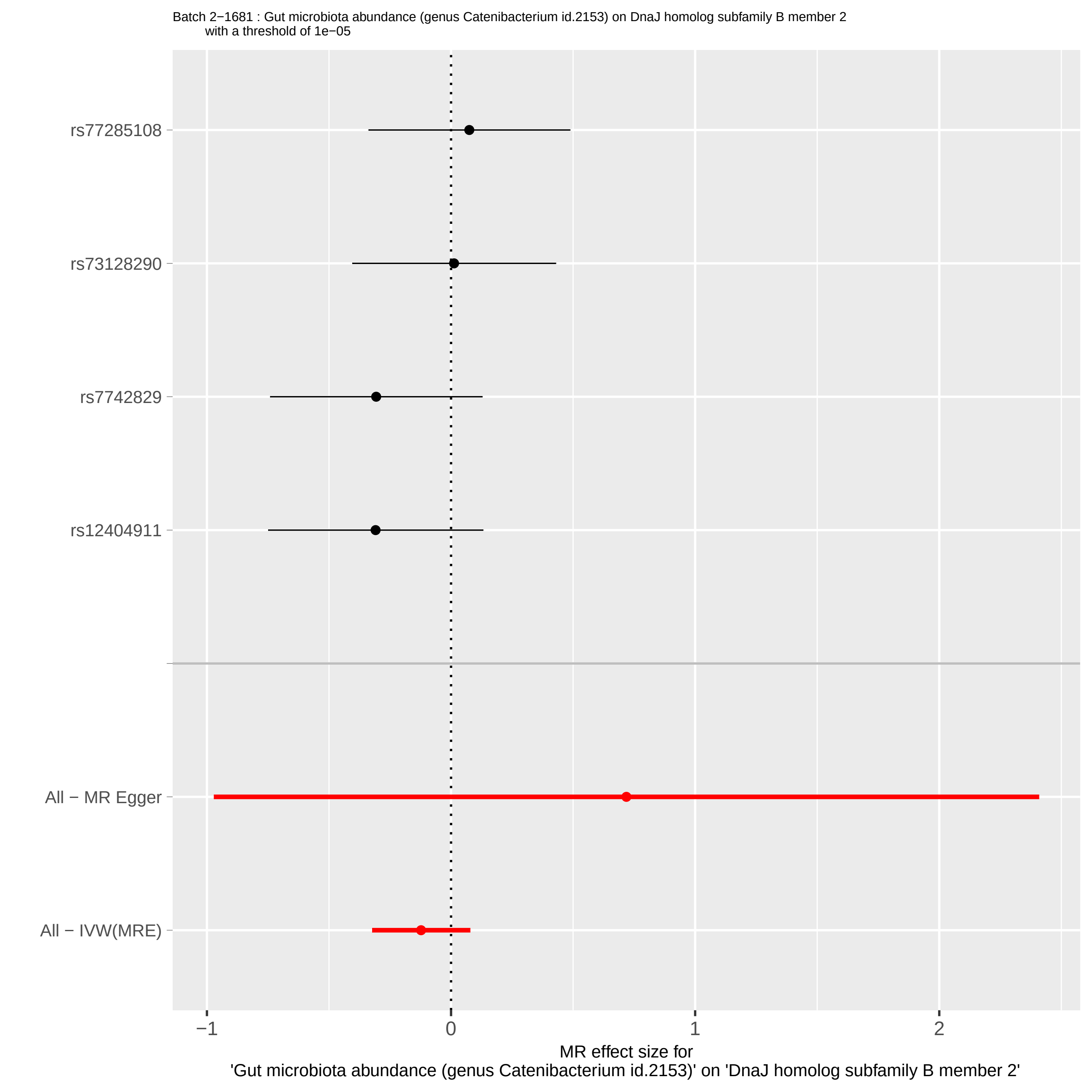

Batch 2−1681 : Gut microbiota abundance (genus Catenibacterium id.2153) on DnaJ homolog subfamily B member 2
with a threshold of 1e−05
rs77285108
rs73128290
rs7742829
rs12404911
All − MR Egger
All − IVW(MRE)
−1
0
1
2
MR effect size for
'Gut microbiota abundance (genus Catenibacterium id.2153)' on 'DnaJ homolog subfamily B member 2'

Batch 2−1682 : Gut microbiota abundance (genus Parasutterella id.2892) on DnaJ homolog subfamily B member 2 with a threshold of 1e−05

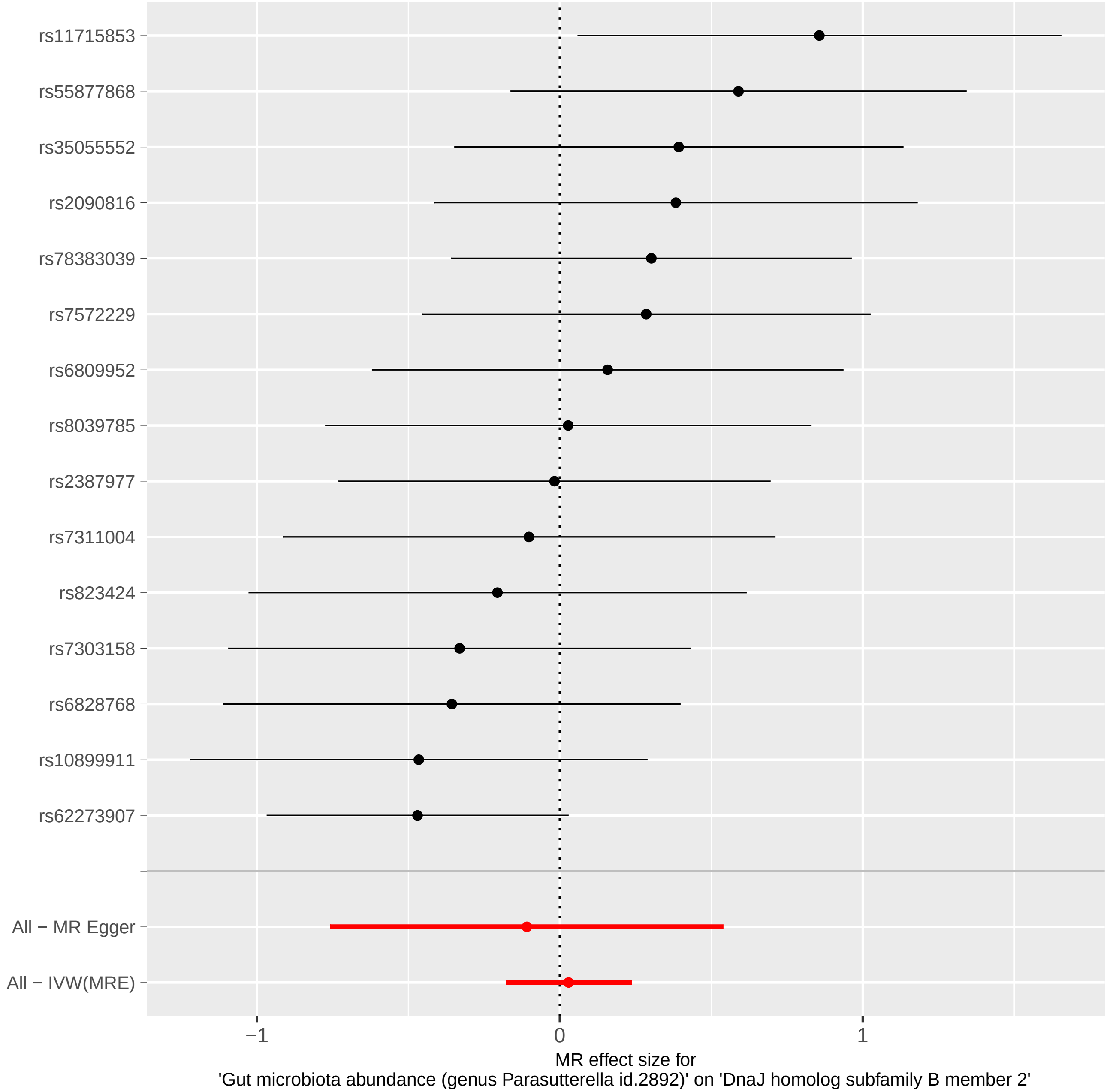

Batch 2−1683 : Gut microbiota abundance (genus Catenibacterium id.2153) on DnaJ homolog subfamily B member 9
with a threshold of 1e−05

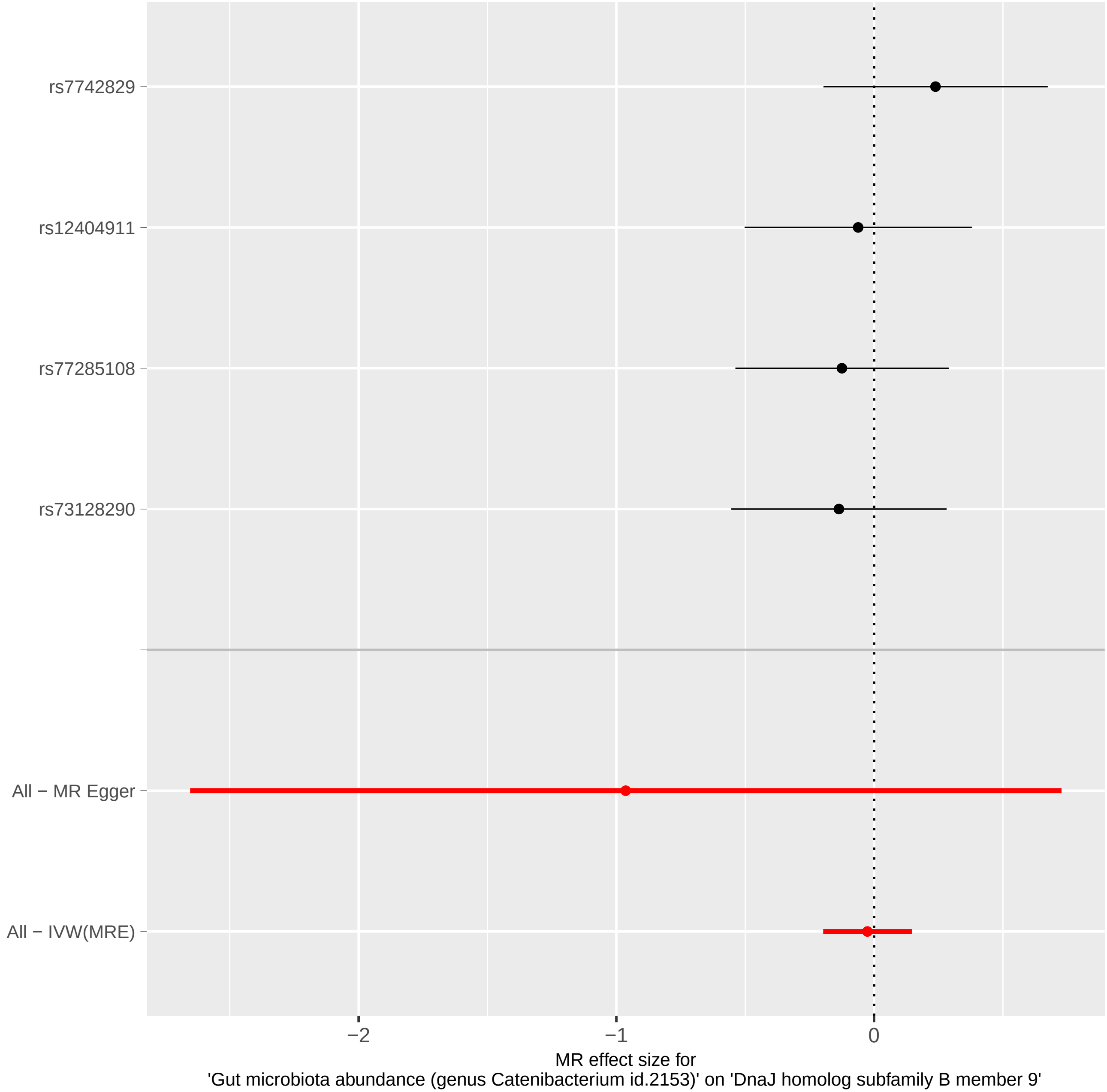

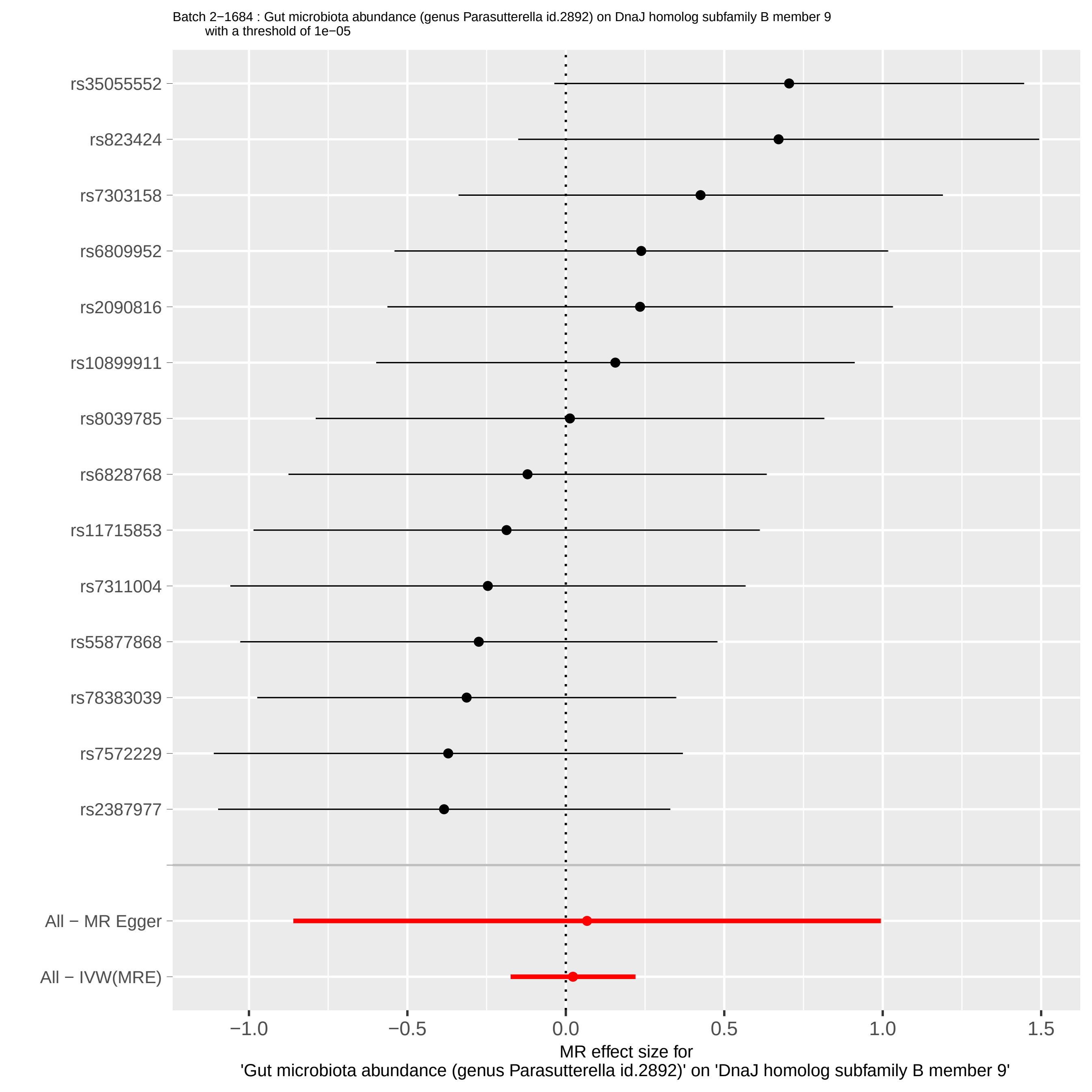
Batch 2−1684 : Gut microbiota abundance (genus Parasutterella id.2892) on DnaJ homolog subfamily B member 9
with a threshold of 1e−05
rs35055552
rs823424
rs7303158
rs6809952
rs2090816
rs10899911
rs8039785
rs6828768
rs11715853
rs7311004
rs55877868
rs78383039
rs7572229
rs2387977
All − MR Egger
All − IVW(MRE)
−1.0
−0.5
0.0
0.5
1.0
1.5
MR effect size for
'Gut microbiota abundance (genus Parasutterella id.2892)' on 'DnaJ homolog subfamily B member 9'

Batch 2−1686 : Gut microbiota abundance (genus Parasutterella id.2892) on DnaJ homolog subfamily C member 10
with a threshold of 1e−05

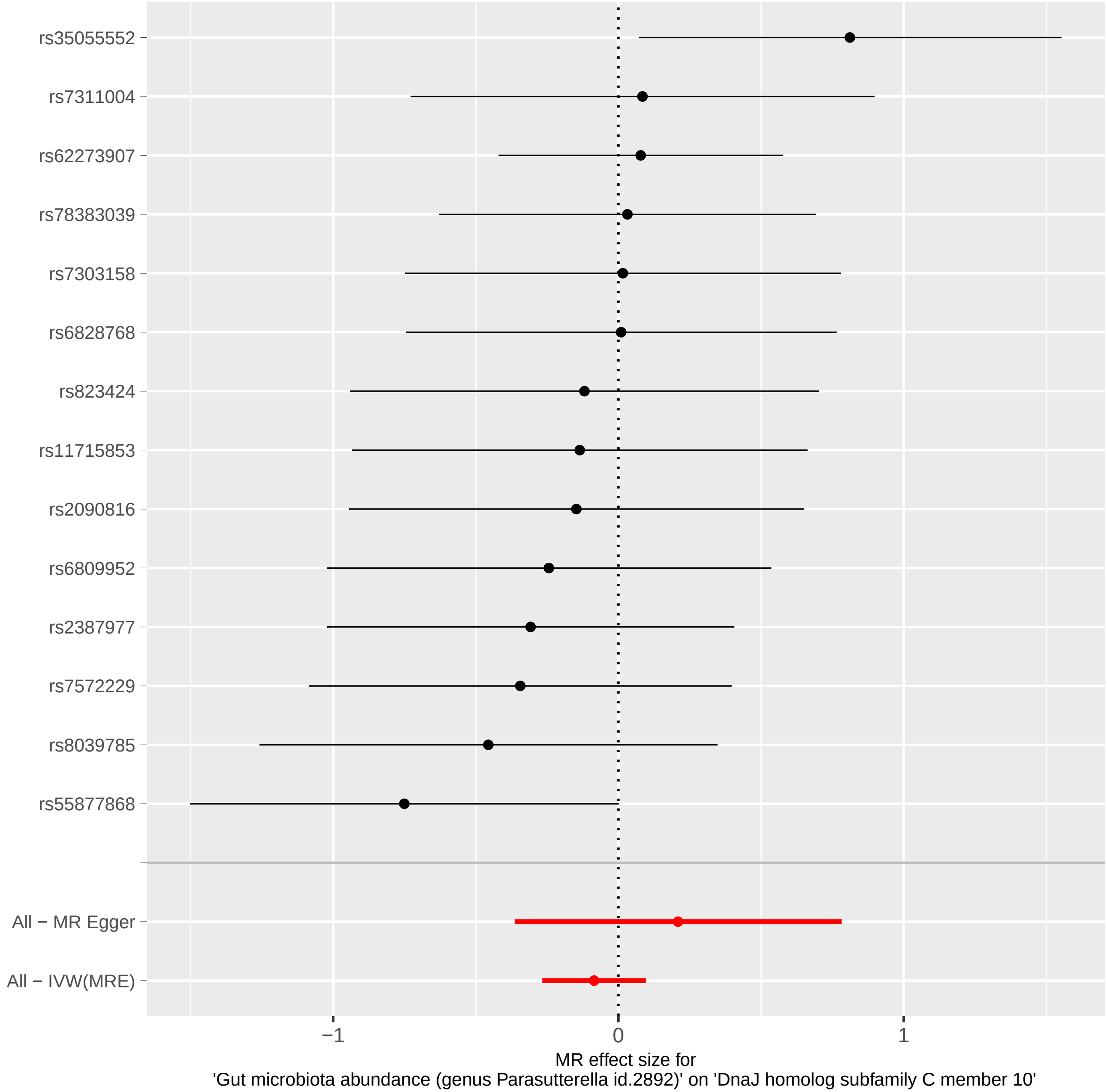

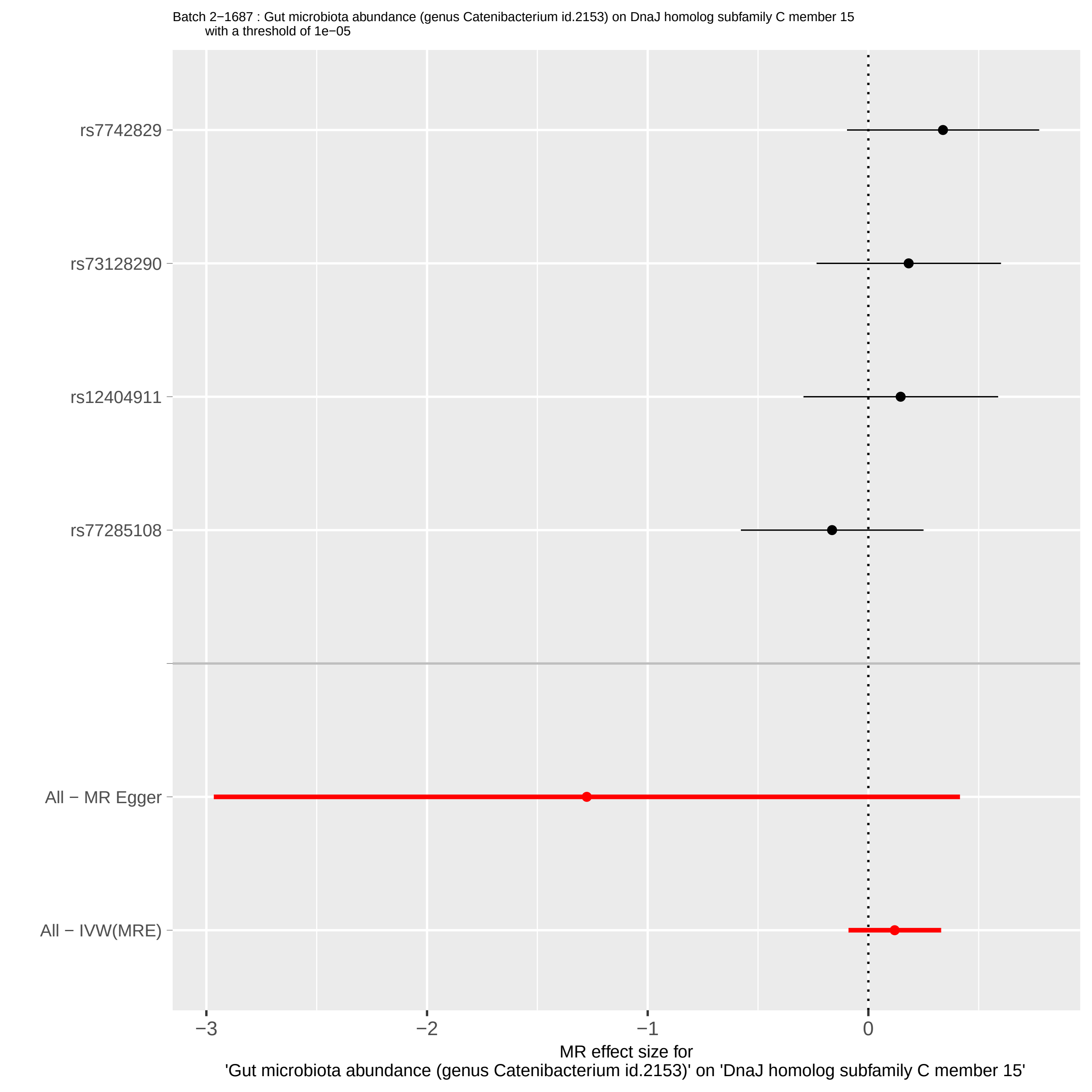
Batch 2−1687 : Gut microbiota abundance (genus Catenibacterium id.2153) on DnaJ homolog subfamily C member 15
with a threshold of 1e−05
rs7742829
rs73128290
rs12404911
rs77285108
All − MR Egger
All − IVW(MRE)
−3
−2
−1
0
MR effect size for
'Gut microbiota abundance (genus Catenibacterium id.2153)' on 'DnaJ homolog subfamily C member 15'

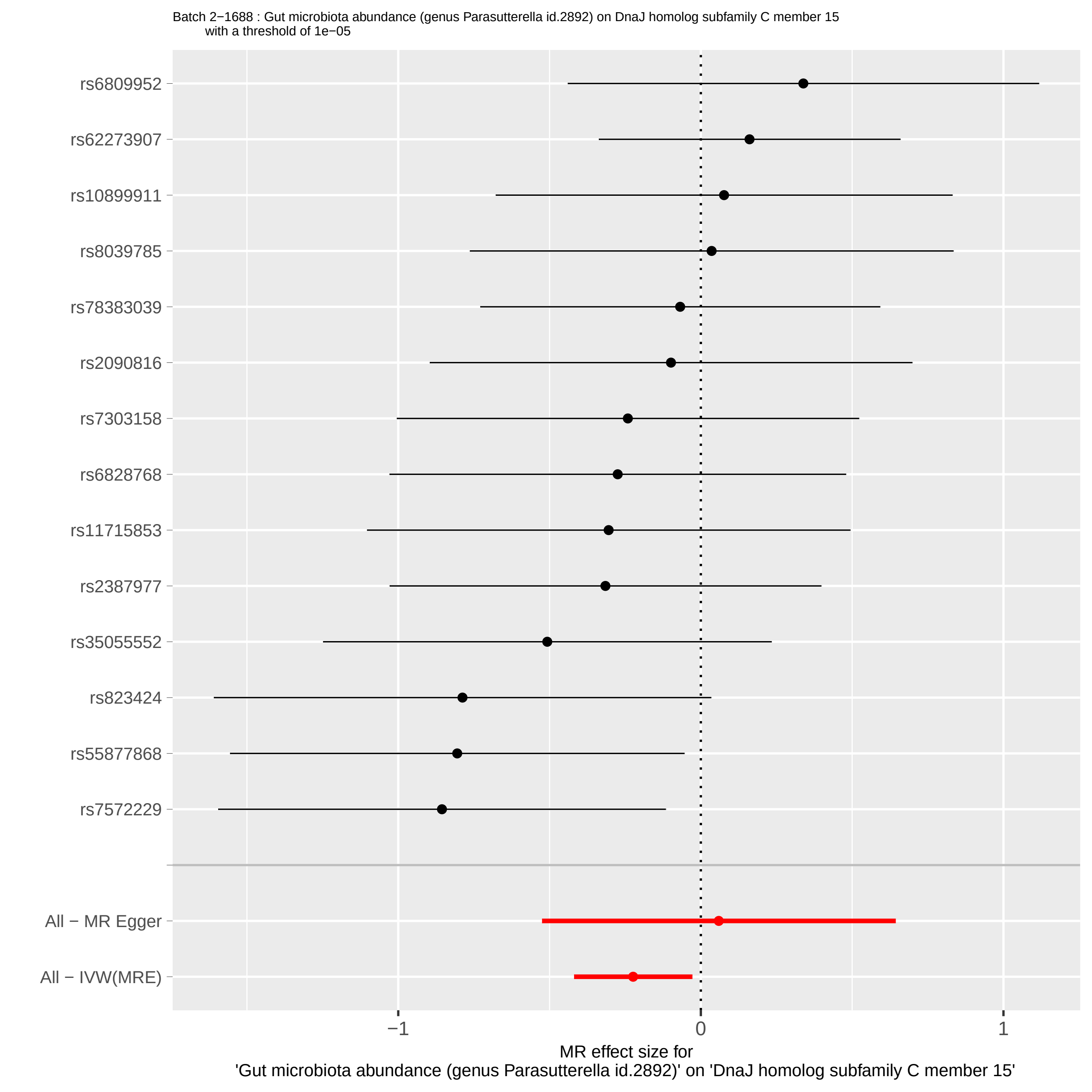

Batch 2−1688 : Gut microbiota abundance (genus Parasutterella id.2892) on DnaJ homolog subfamily C member 15
with a threshold of 1e−05
rs6809952
rs62273907
rs10899911
rs8039785
rs78383039
rs2090816
rs7303158
rs6828768
rs11715853
rs2387977
rs35055552
rs823424
rs55877868
rs7572229
All − MR Egger
All − IVW(MRE)
−1
0
1
MR effect size for
'Gut microbiota abundance (genus Parasutterella id.2892)' on 'DnaJ homolog subfamily C member 15'

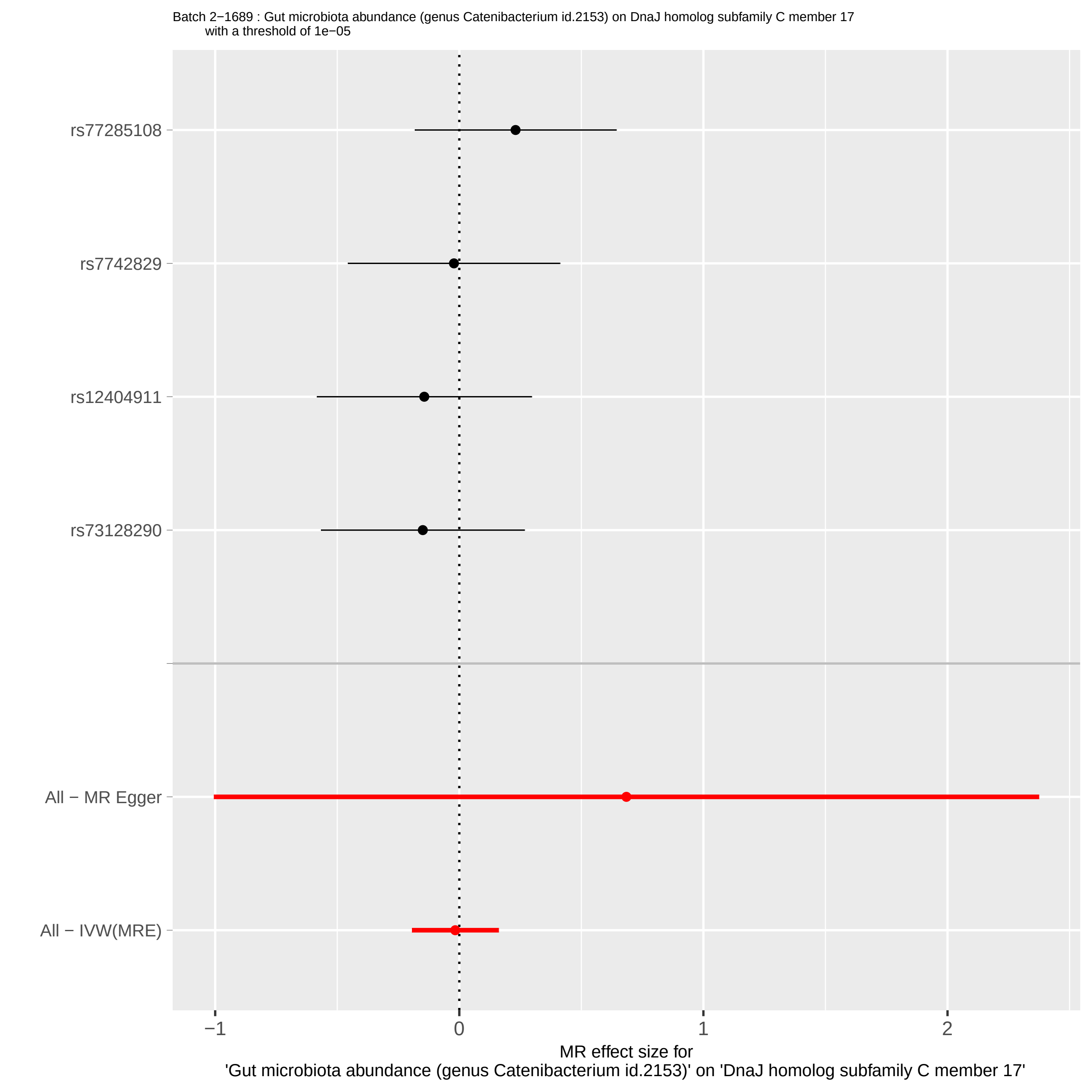

Batch 2−1689 : Gut microbiota abundance (genus Catenibacterium id.2153) on DnaJ homolog subfamily C member 17
with a threshold of 1e−05
rs77285108
rs7742829
rs12404911
rs73128290
All − MR Egger
All − IVW(MRE)
−1
0
1
2
MR effect size for
'Gut microbiota abundance (genus Catenibacterium id.2153)' on 'DnaJ homolog subfamily C member 17'

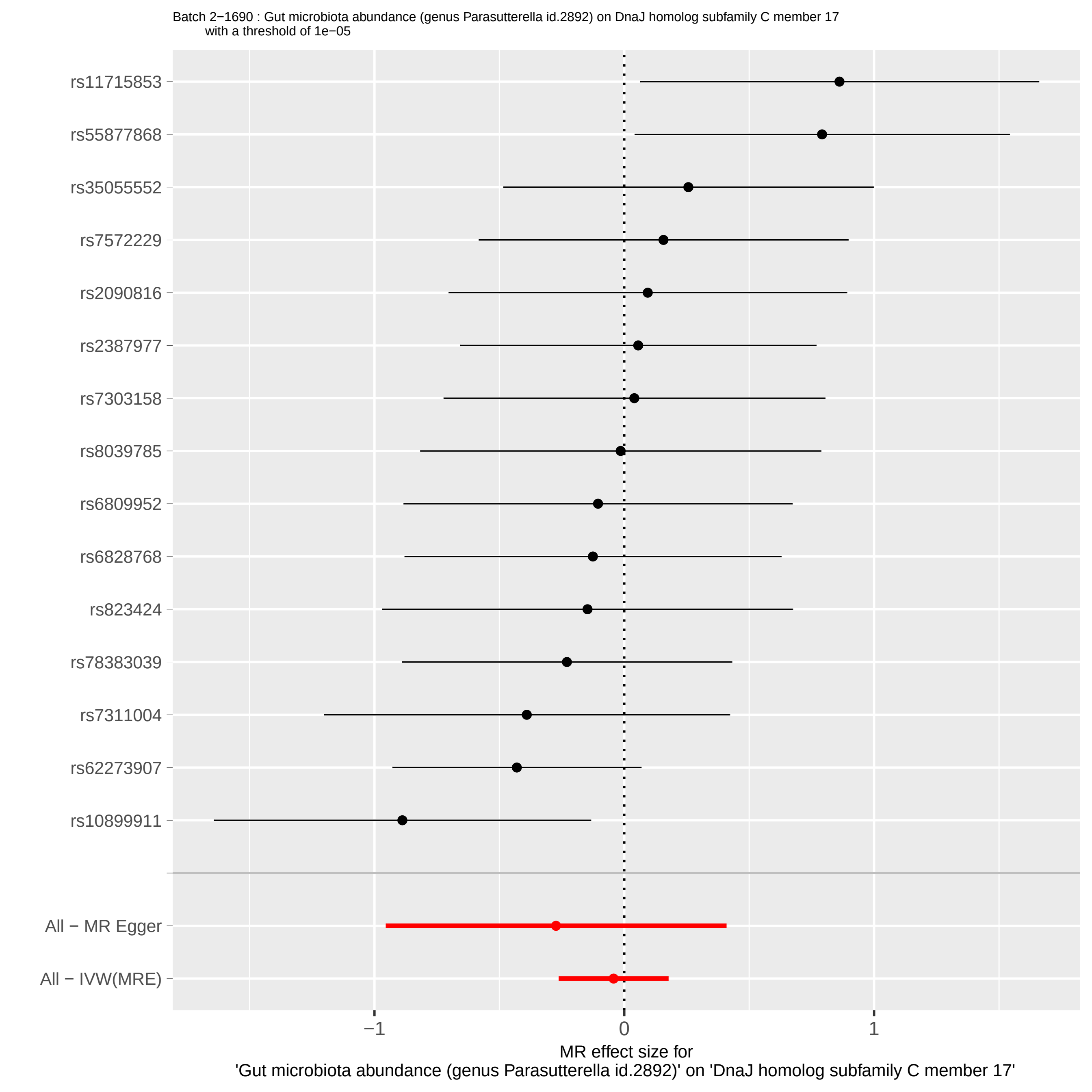

Batch 2−1690 : Gut microbiota abundance (genus Parasutterella id.2892) on DnaJ homolog subfamily C member 17
with a threshold of 1e−05
rs11715853
rs55877868
rs35055552
rs7572229
rs2090816
rs2387977
rs7303158
rs8039785
rs6809952
rs6828768
rs823424
rs78383039
rs7311004
rs62273907
rs10899911
All − MR Egger
All − IVW(MRE)
−1
0
1
MR effect size for
'Gut microbiota abundance (genus Parasutterella id.2892)' on 'DnaJ homolog subfamily C member 17'

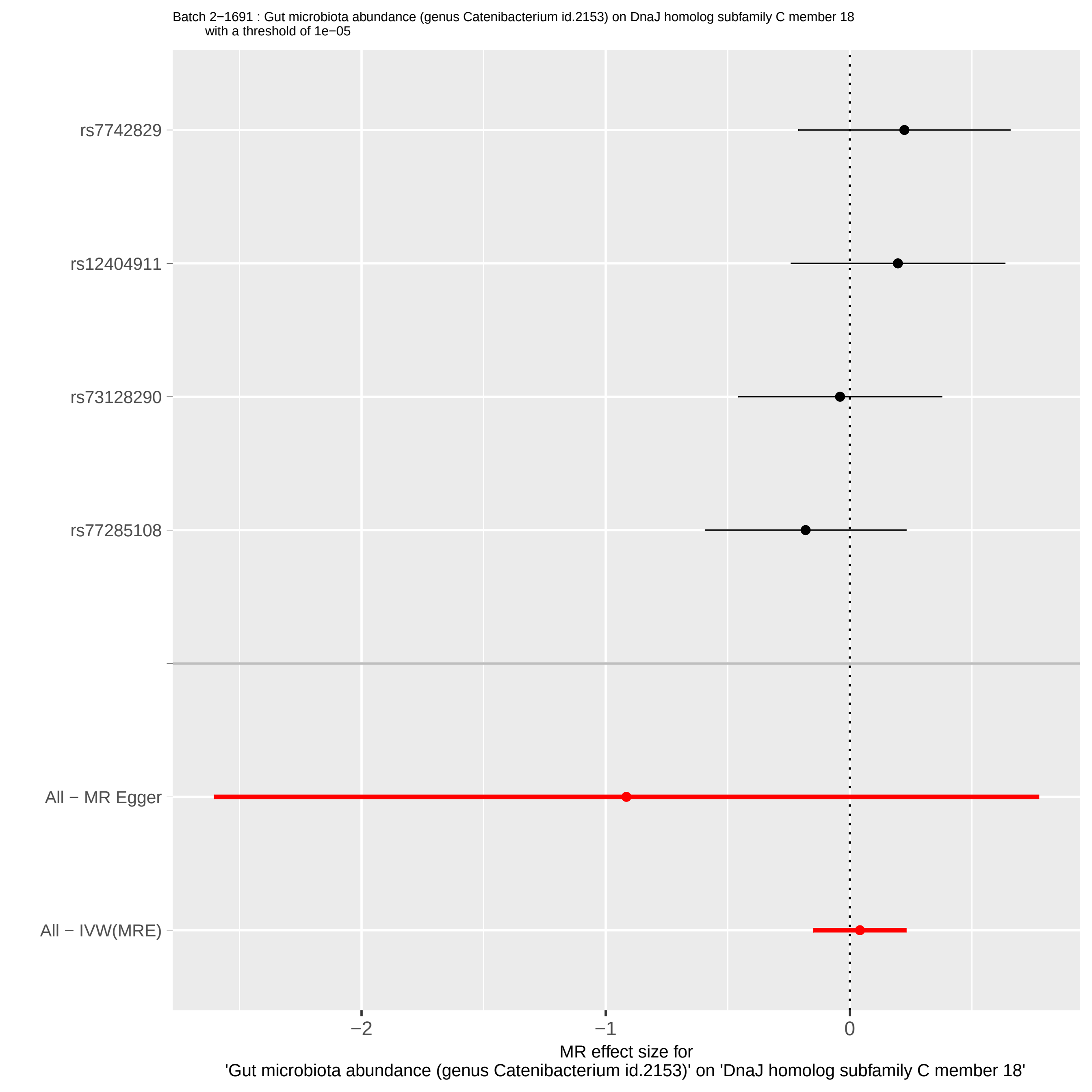

Batch 2−1691 : Gut microbiota abundance (genus Catenibacterium id.2153) on DnaJ homolog subfamily C member 18
with a threshold of 1e−05
rs7742829
rs12404911
rs73128290
rs77285108
All − MR Egger
All − IVW(MRE)
−2
−1
0
MR effect size for
'Gut microbiota abundance (genus Catenibacterium id.2153)' on 'DnaJ homolog subfamily C member 18'

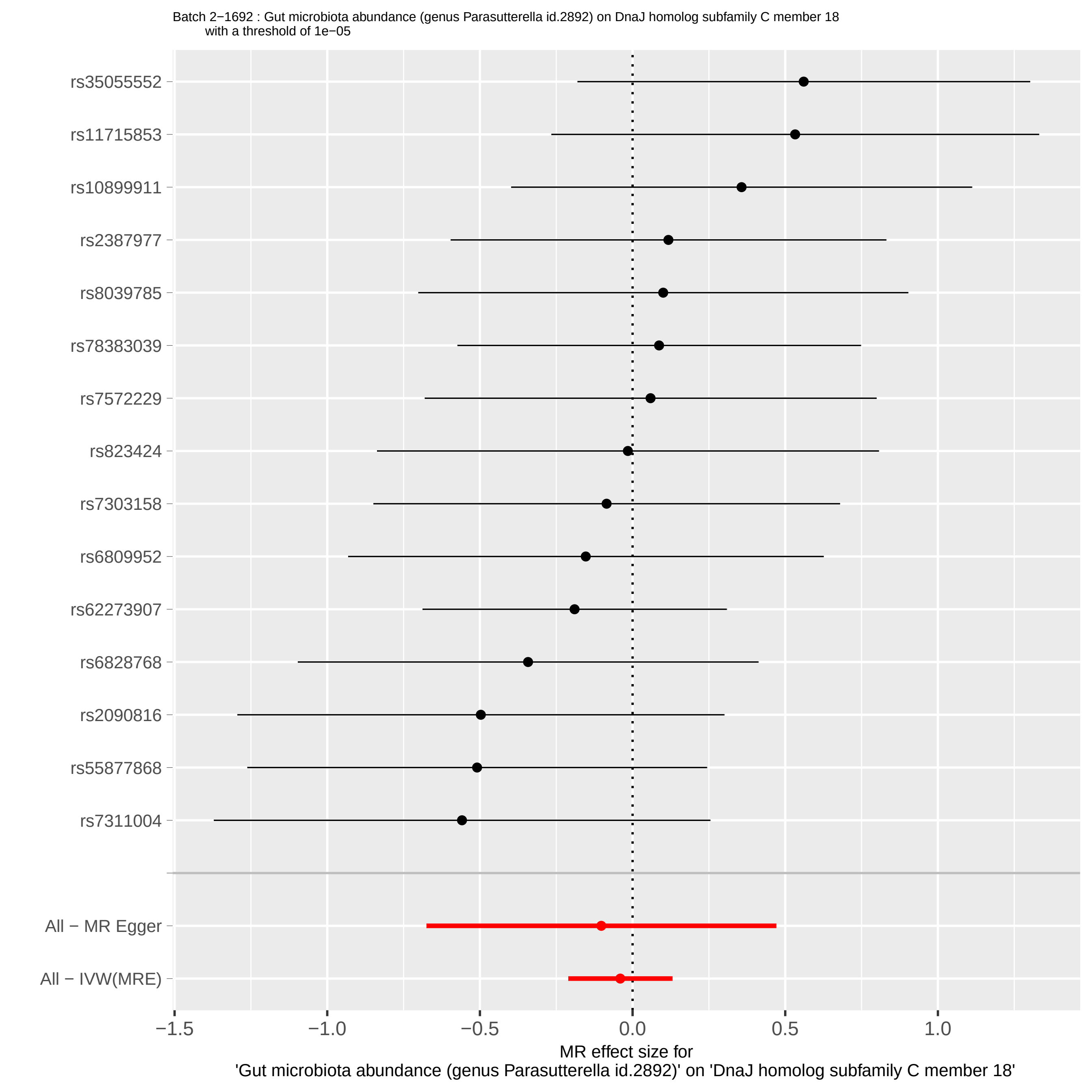

Batch 2−1692 : Gut microbiota abundance (genus Parasutterella id.2892) on DnaJ homolog subfamily C member 18
with a threshold of 1e−05
rs35055552
rs11715853
rs10899911
rs2387977
rs8039785
rs78383039
rs7572229
rs823424
rs7303158
rs6809952
rs62273907
rs6828768
rs2090816
rs55877868
rs7311004
All − MR Egger
All − IVW(MRE)
−1.5
−1.0
−0.5
0.0
0.5
1.0
MR effect size for
'Gut microbiota abundance (genus Parasutterella id.2892)' on 'DnaJ homolog subfamily C member 18'

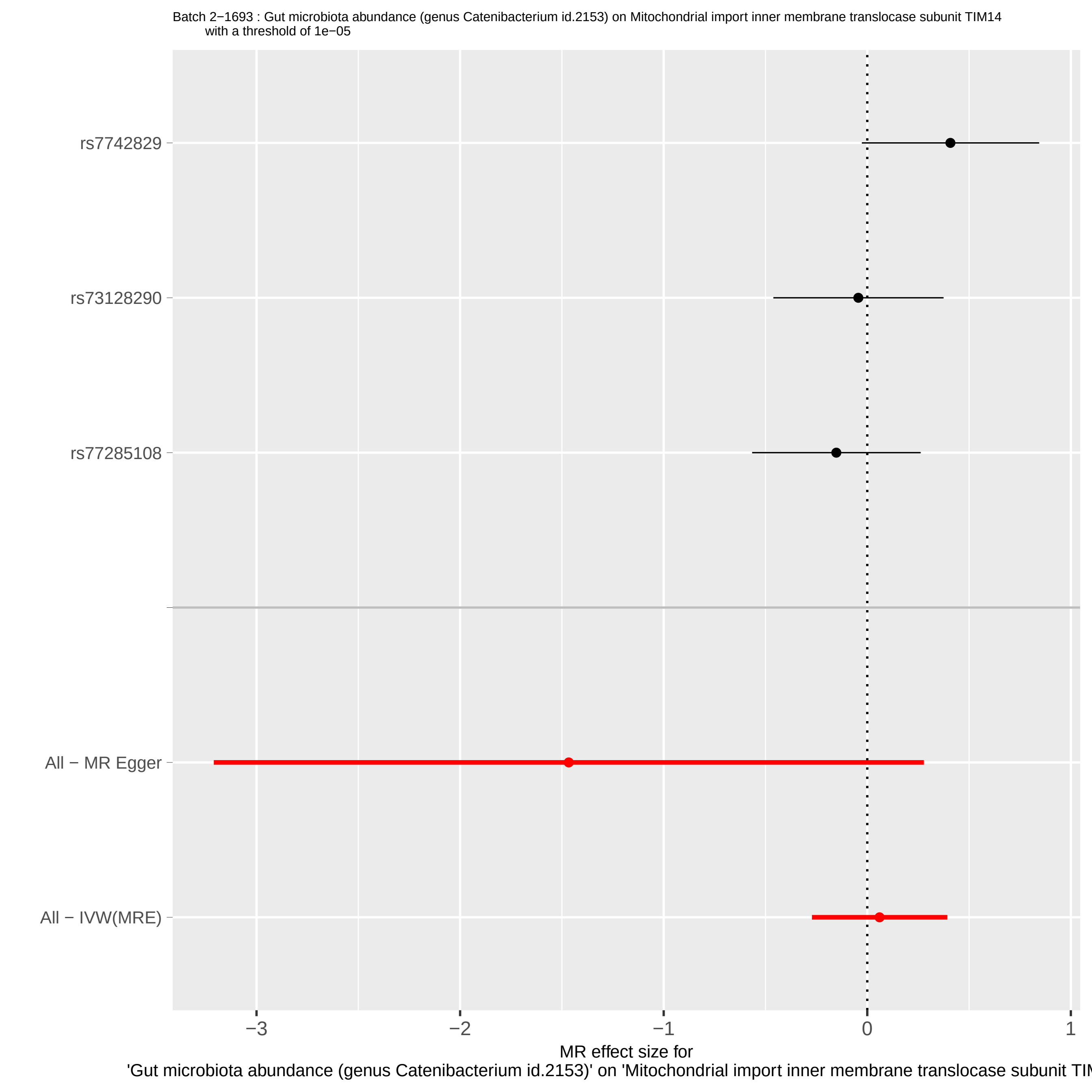
Batch 2−1693 : Gut microbiota abundance (genus Catenibacterium id.2153) on Mitochondrial import inner membrane translocase subunit TIM14
with a threshold of 1e−05
rs7742829
rs73128290
rs77285108
All − MR Egger
All − IVW(MRE)
−3
−2
−1
0
1
MR effect size for
'Gut microbiota abundance (genus Catenibacterium id.2153)' on 'Mitochondrial import inner membrane translocase subunit TIM

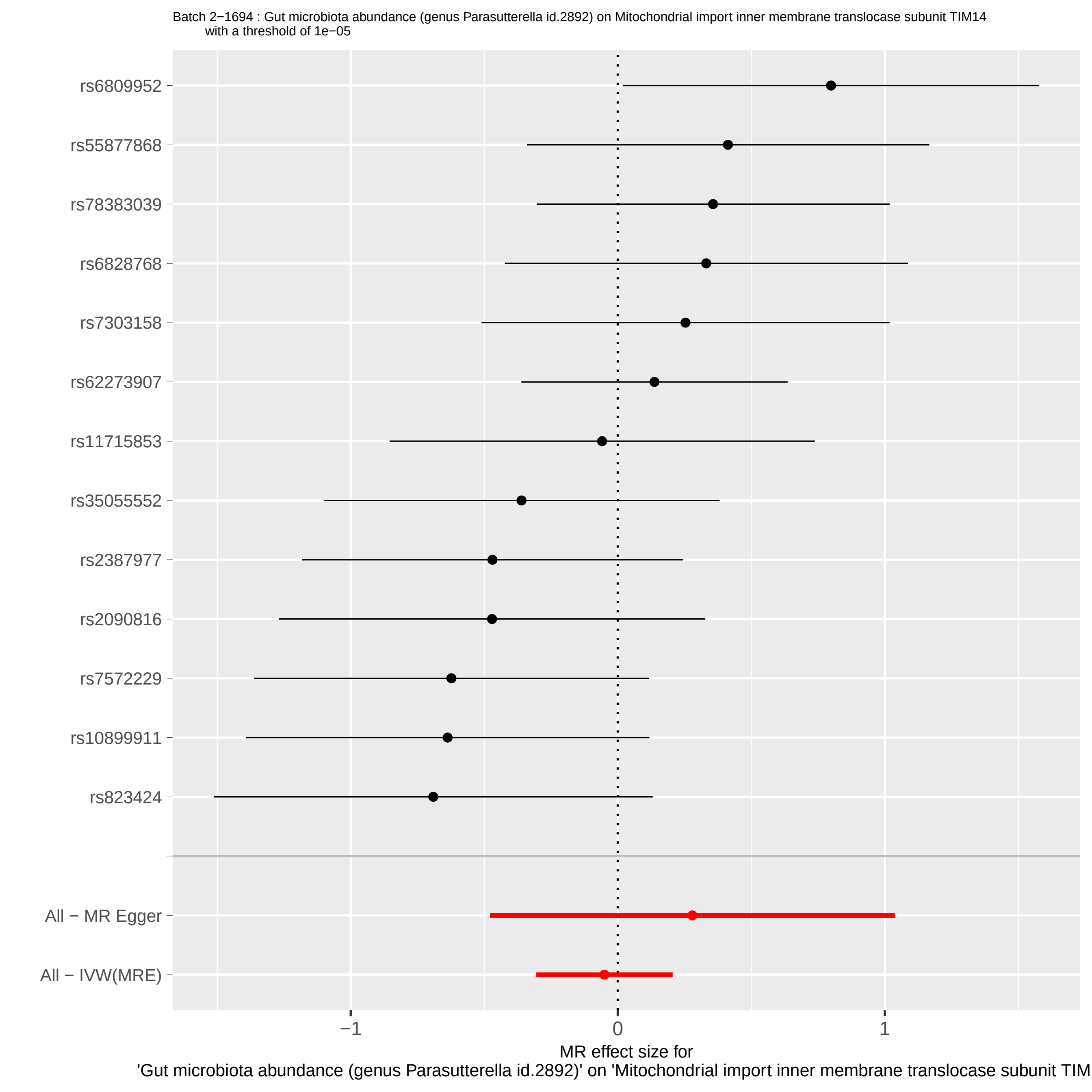

Batch 2−1694 : Gut microbiota abundance (genus Parasutterella id.2892) on Mitochondrial import inner membrane translocase subunit TIM14
with a threshold of 1e−05
rs6809952
rs55877868
rs78383039
rs6828768
rs7303158
rs62273907
rs11715853
rs35055552
rs2387977
rs2090816
rs7572229
rs10899911
rs823424
All − MR Egger
All − IVW(MRE)
−1
0
1
MR effect size for
'Gut microbiota abundance (genus Parasutterella id.2892)' on 'Mitochondrial import inner membrane translocase subunit TIM

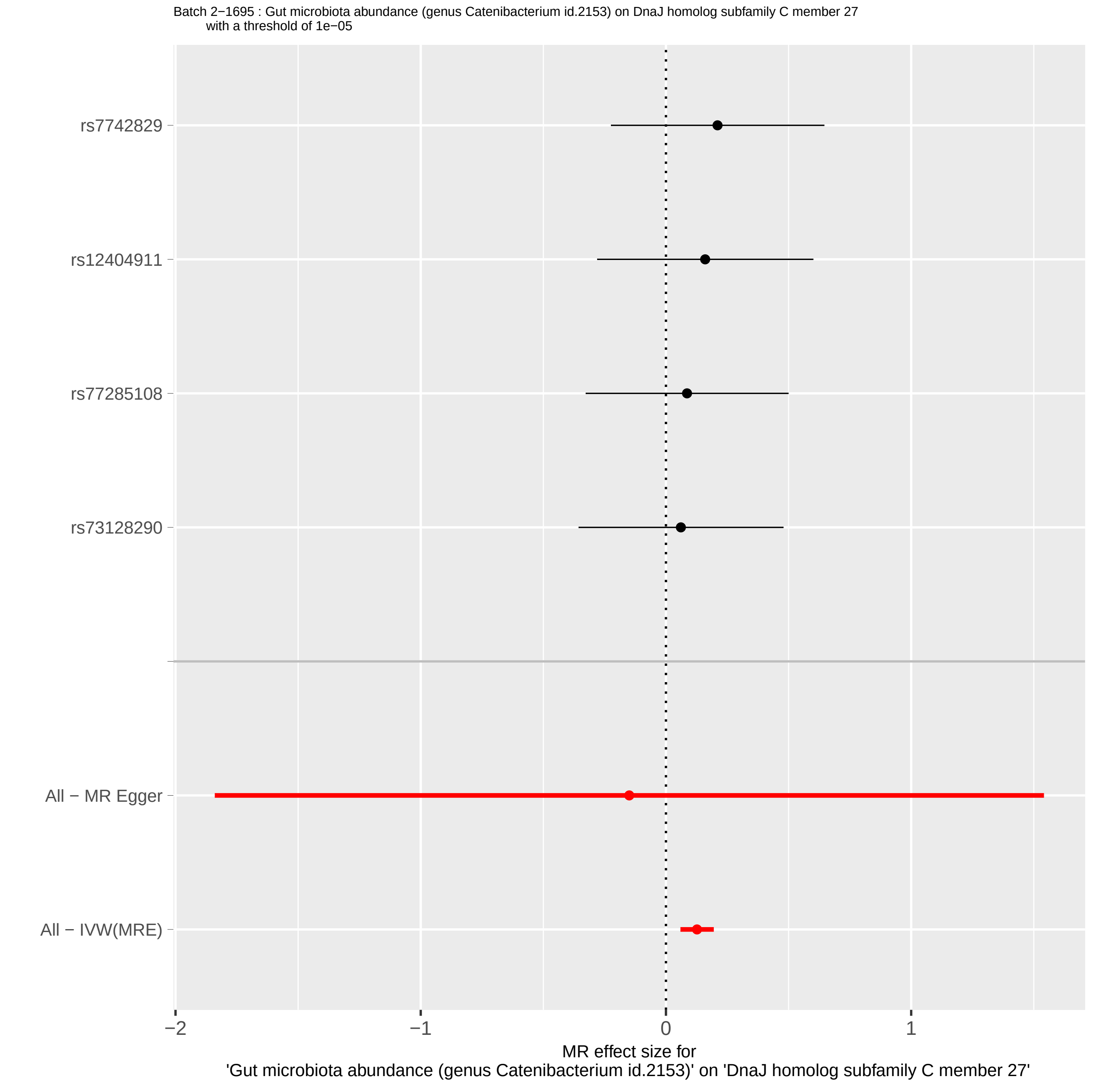

Batch 2−1695 : Gut microbiota abundance (genus Catenibacterium id.2153) on DnaJ homolog subfamily C member 27
with a threshold of 1e−05
rs7742829
rs12404911
rs77285108
rs73128290
All − MR Egger
All − IVW(MRE)
−2
−1
0
1
MR effect size for
'Gut microbiota abundance (genus Catenibacterium id.2153)' on 'DnaJ homolog subfamily C member 27'

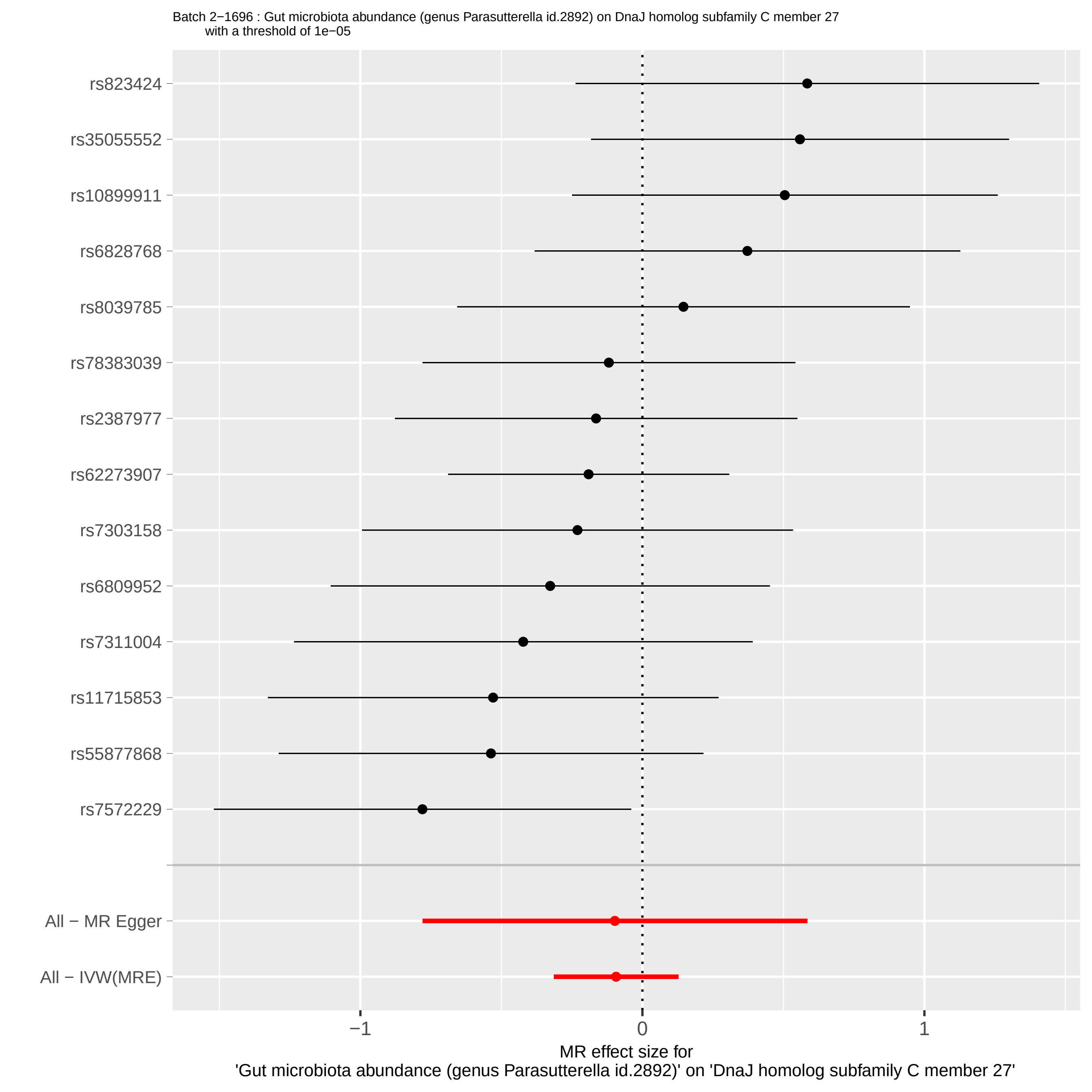

Batch 2−1696 : Gut microbiota abundance (genus Parasutterella id.2892) on DnaJ homolog subfamily C member 27
with a threshold of 1e−05
rs823424
rs35055552
rs10899911
rs6828768
rs8039785
rs78383039
rs2387977
rs62273907
rs7303158
rs6809952
rs7311004
rs11715853
rs55877868
rs7572229
All − MR Egger
All − IVW(MRE)
−1
0
1
MR effect size for
'Gut microbiota abundance (genus Parasutterella id.2892)' on 'DnaJ homolog subfamily C member 27'

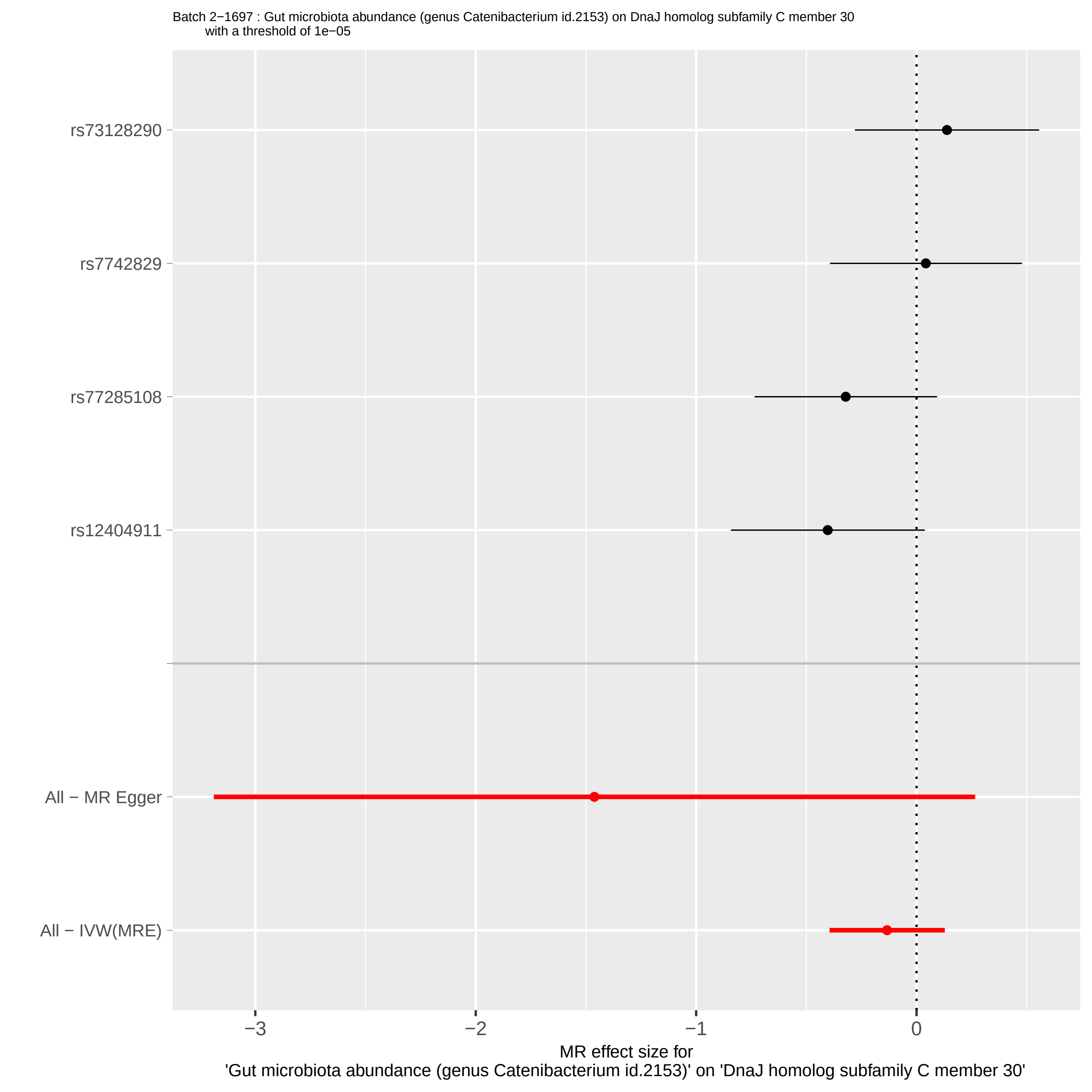

Batch 2−1697 : Gut microbiota abundance (genus Catenibacterium id.2153) on DnaJ homolog subfamily C member 30
with a threshold of 1e−05
rs73128290
rs7742829
rs77285108
rs12404911
All − MR Egger
All − IVW(MRE)
−3
−2
−1
0
MR effect size for
'Gut microbiota abundance (genus Catenibacterium id.2153)' on 'DnaJ homolog subfamily C member 30'

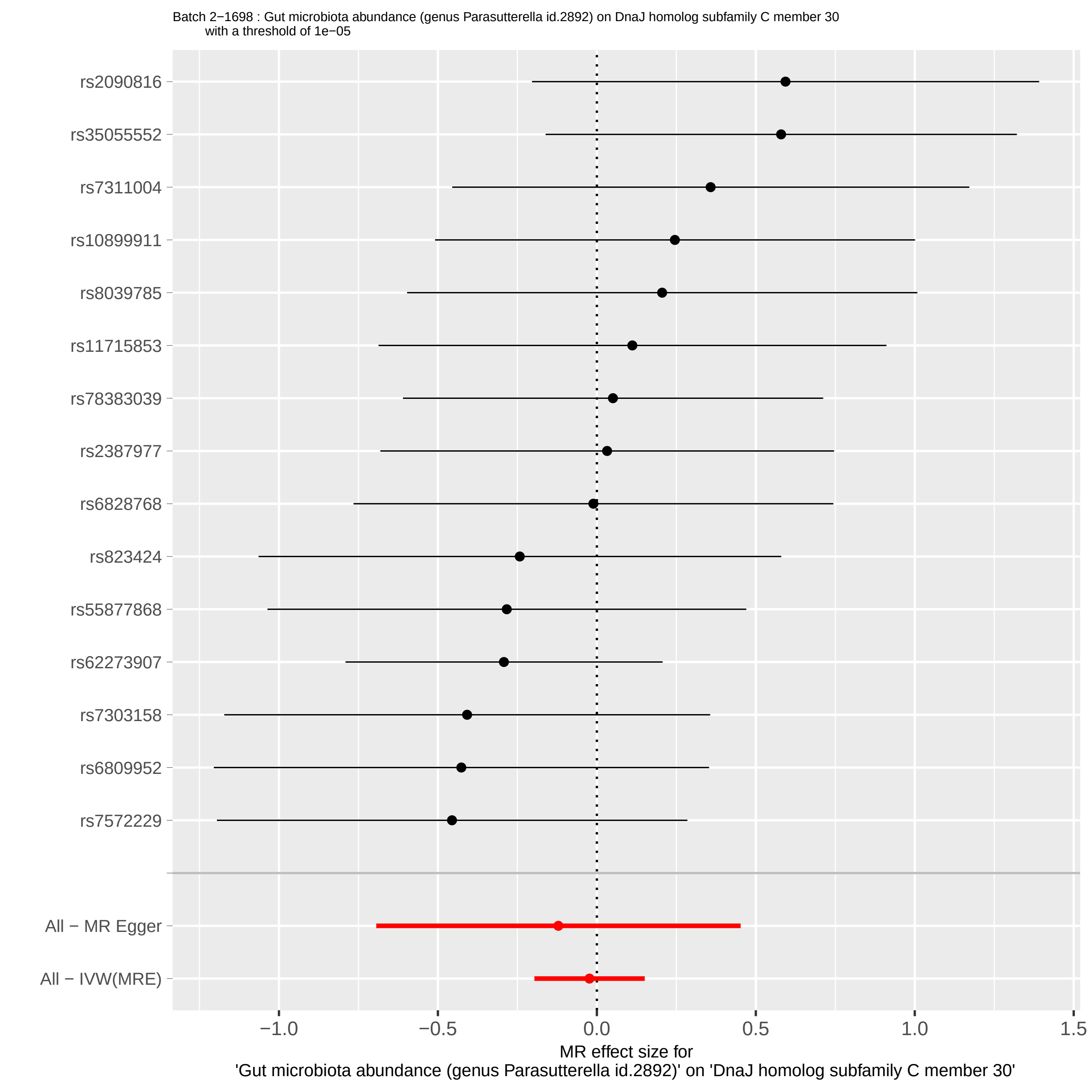

Batch 2−1698 : Gut microbiota abundance (genus Parasutterella id.2892) on DnaJ homolog subfamily C member 30
with a threshold of 1e−05
rs2090816
rs35055552
rs7311004
rs10899911
rs8039785
rs11715853
rs78383039
rs2387977
rs6828768
rs823424
rs55877868
rs62273907
rs7303158
rs6809952
rs7572229
All − MR Egger
All − IVW(MRE)
−1.0
−0.5
0.0
0.5
1.0
1.5
MR effect size for
'Gut microbiota abundance (genus Parasutterella id.2892)' on 'DnaJ homolog subfamily C member 30'

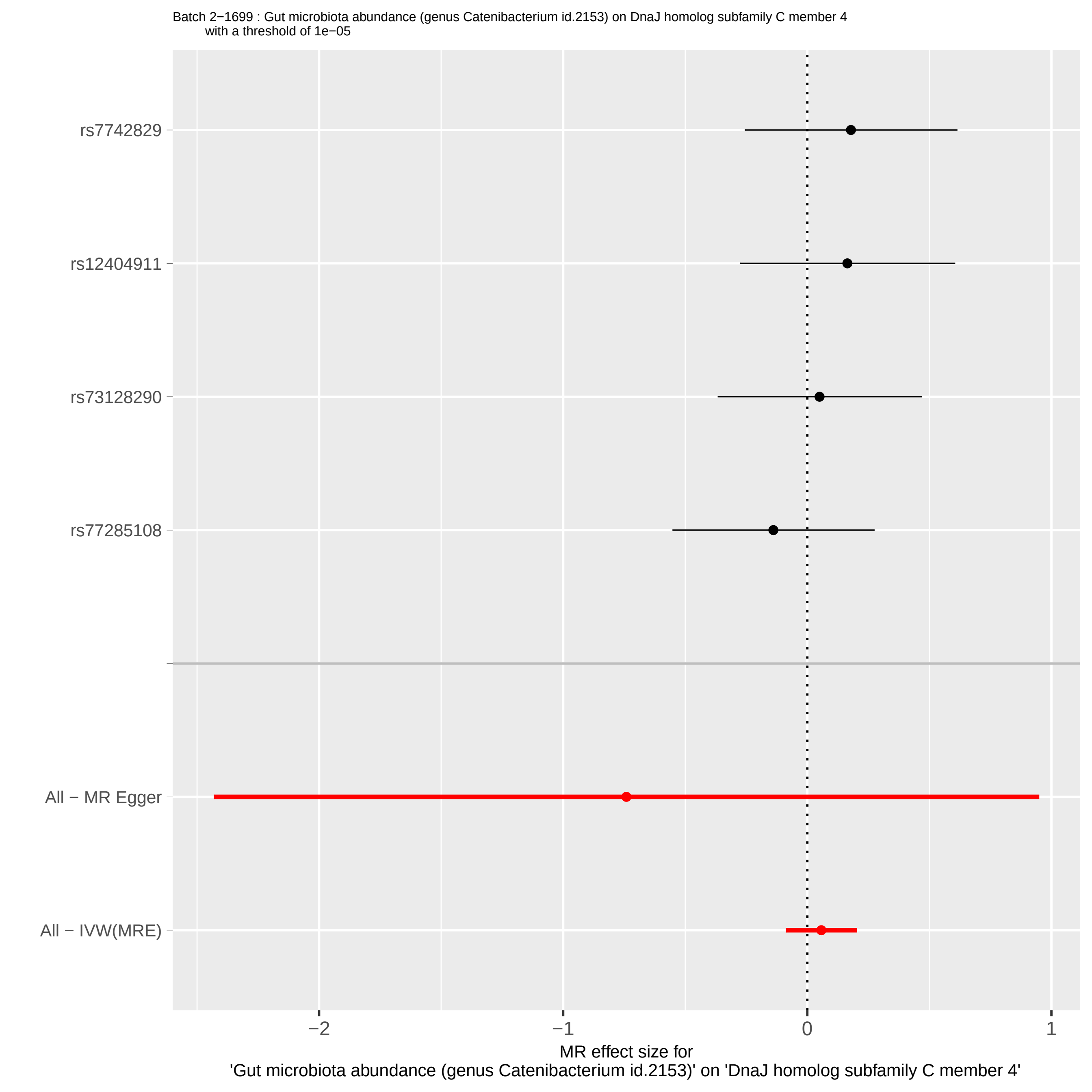
Batch 2−1699 : Gut microbiota abundance (genus Catenibacterium id.2153) on DnaJ homolog subfamily C member 4
with a threshold of 1e−05
rs7742829
rs12404911
rs73128290
rs77285108
All − MR Egger
All − IVW(MRE)
−2
−1
0
1
MR effect size for
'Gut microbiota abundance (genus Catenibacterium id.2153)' on 'DnaJ homolog subfamily C member 4'

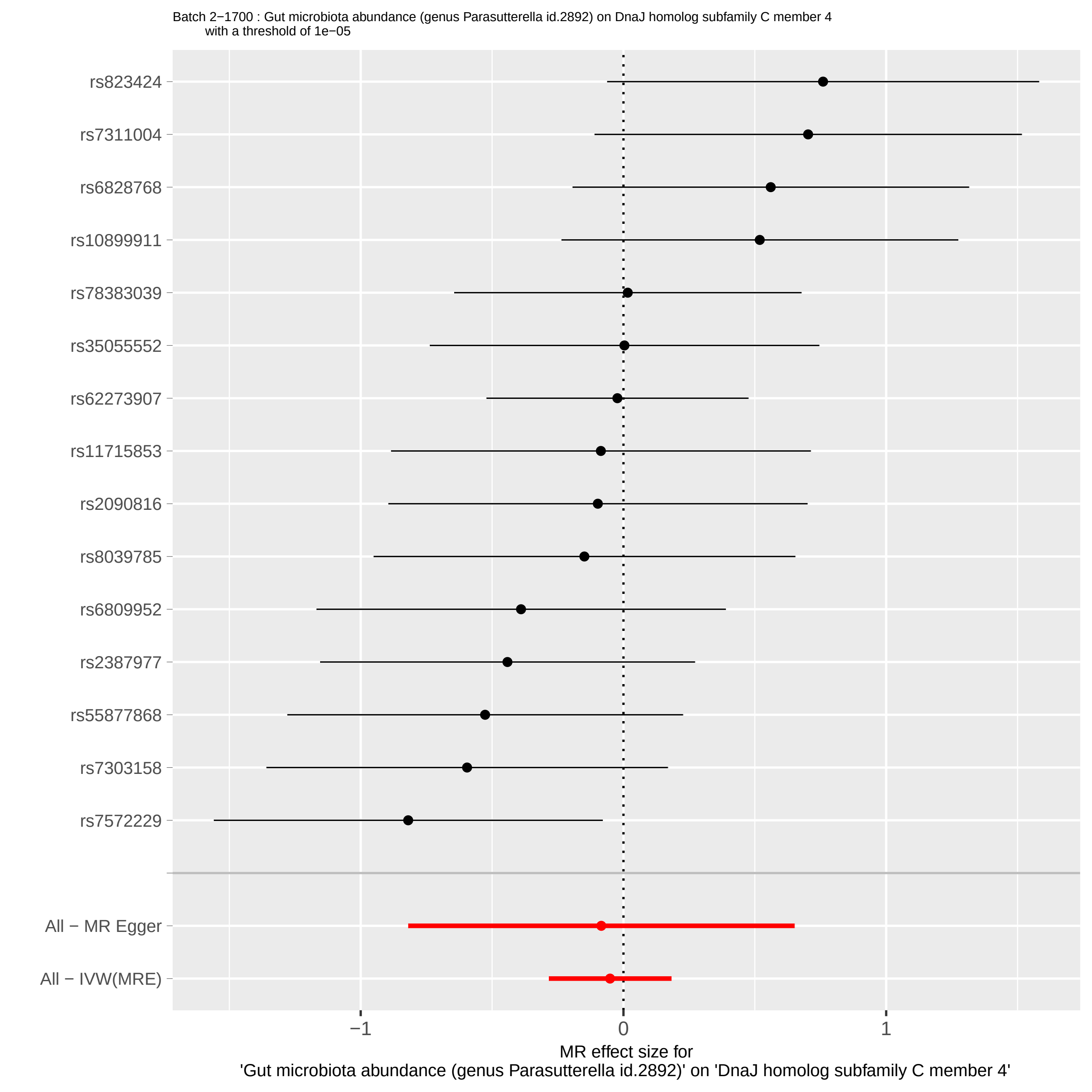

Batch 2−1700 : Gut microbiota abundance (genus Parasutterella id.2892) on DnaJ homolog subfamily C member 4
with a threshold of 1e−05
rs823424
rs7311004
rs6828768
rs10899911
rs78383039
rs35055552
rs62273907
rs11715853
rs2090816
rs8039785
rs6809952
rs2387977
rs55877868
rs7303158
rs7572229
All − MR Egger
All − IVW(MRE)
−1
0
1
MR effect size for
'Gut microbiota abundance (genus Parasutterella id.2892)' on 'DnaJ homolog subfamily C member 4'

Batch 2−1701 : Gut microbiota abundance (genus Catenibacterium id.2153) on Deoxyribonuclease−2−beta
with a threshold of 1e−05

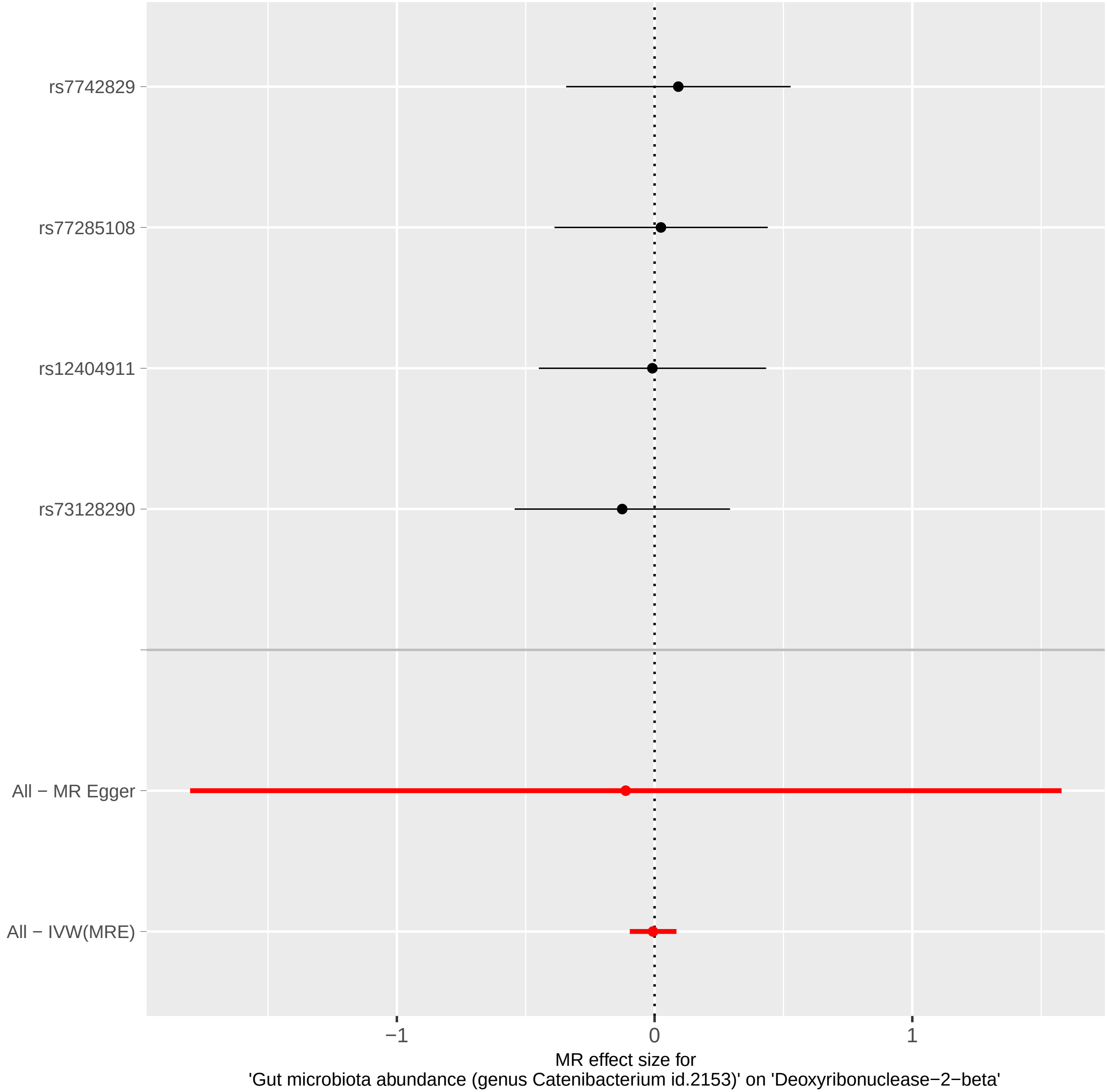

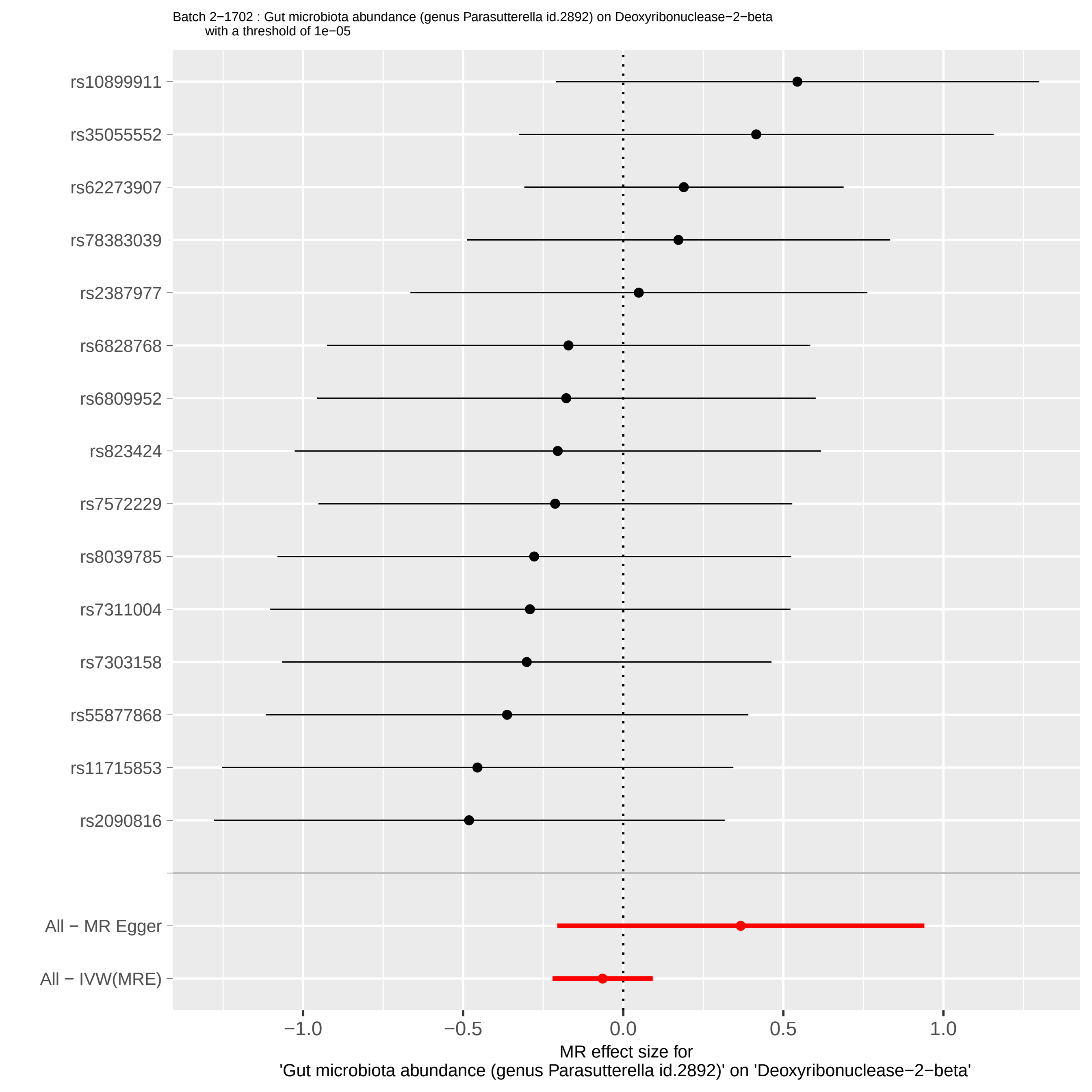
Batch 2−1702 : Gut microbiota abundance (genus Parasutterella id.2892) on Deoxyribonuclease−2−beta
with a threshold of 1e−05
rs10899911
rs35055552
rs62273907
rs78383039
rs2387977
rs6828768
rs6809952
rs823424
rs7572229
rs8039785
rs7311004
rs7303158
rs55877868
rs11715853
rs2090816
All − MR Egger
All − IVW(MRE)
−1.0
−0.5
0.0
0.5
1.0
MR effect size for
'Gut microbiota abundance (genus Parasutterella id.2892)' on 'Deoxyribonuclease−2−beta'

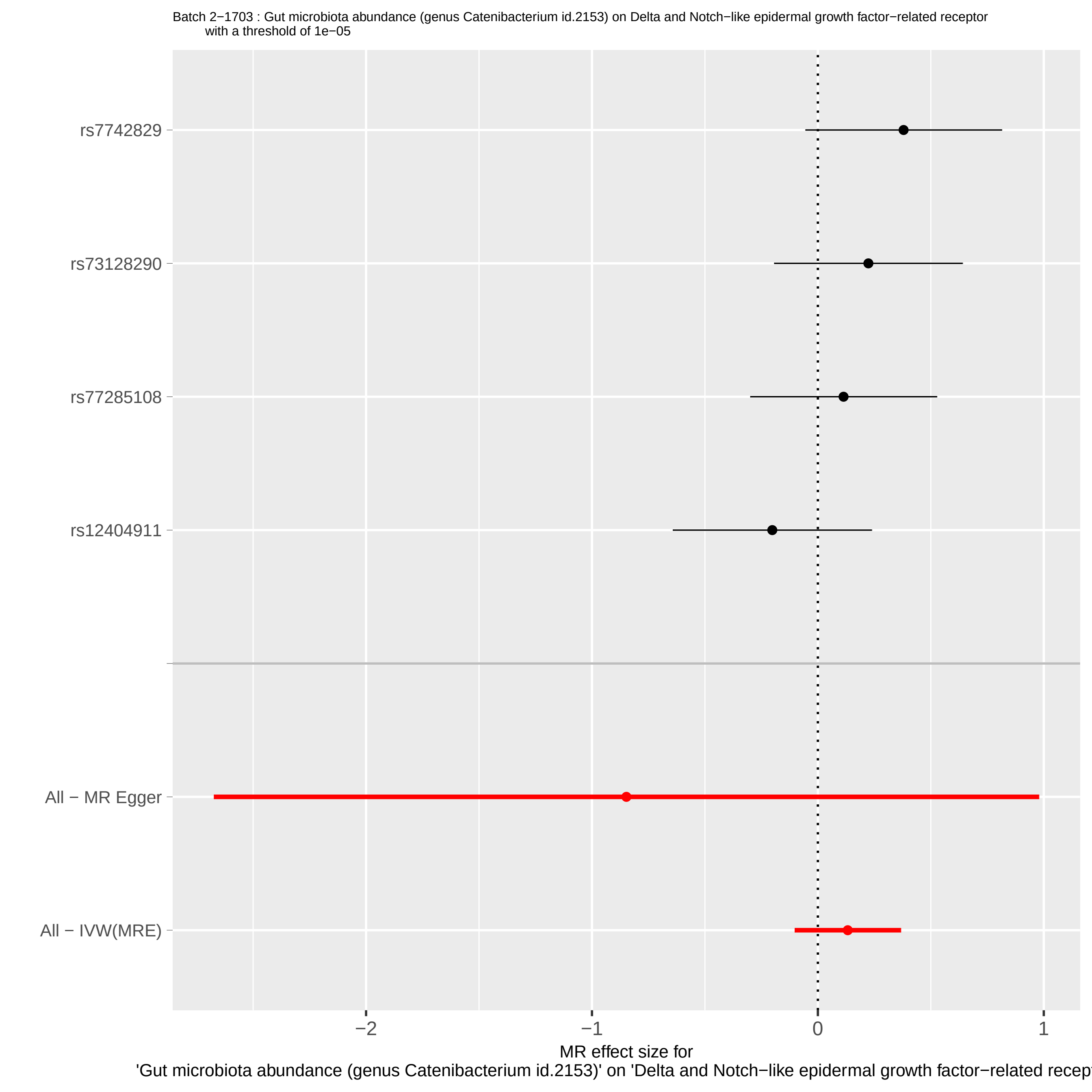
Batch 2−1703 : Gut microbiota abundance (genus Catenibacterium id.2153) on Delta and Notch−like epidermal growth factor−related receptor
with a threshold of 1e−05
rs7742829
rs73128290
rs77285108
rs12404911
All − MR Egger
All − IVW(MRE)
−2
−1
0
1
MR effect size for
'Gut microbiota abundance (genus Catenibacterium id.2153)' on 'Delta and Notch−like epidermal growth factor−related recep

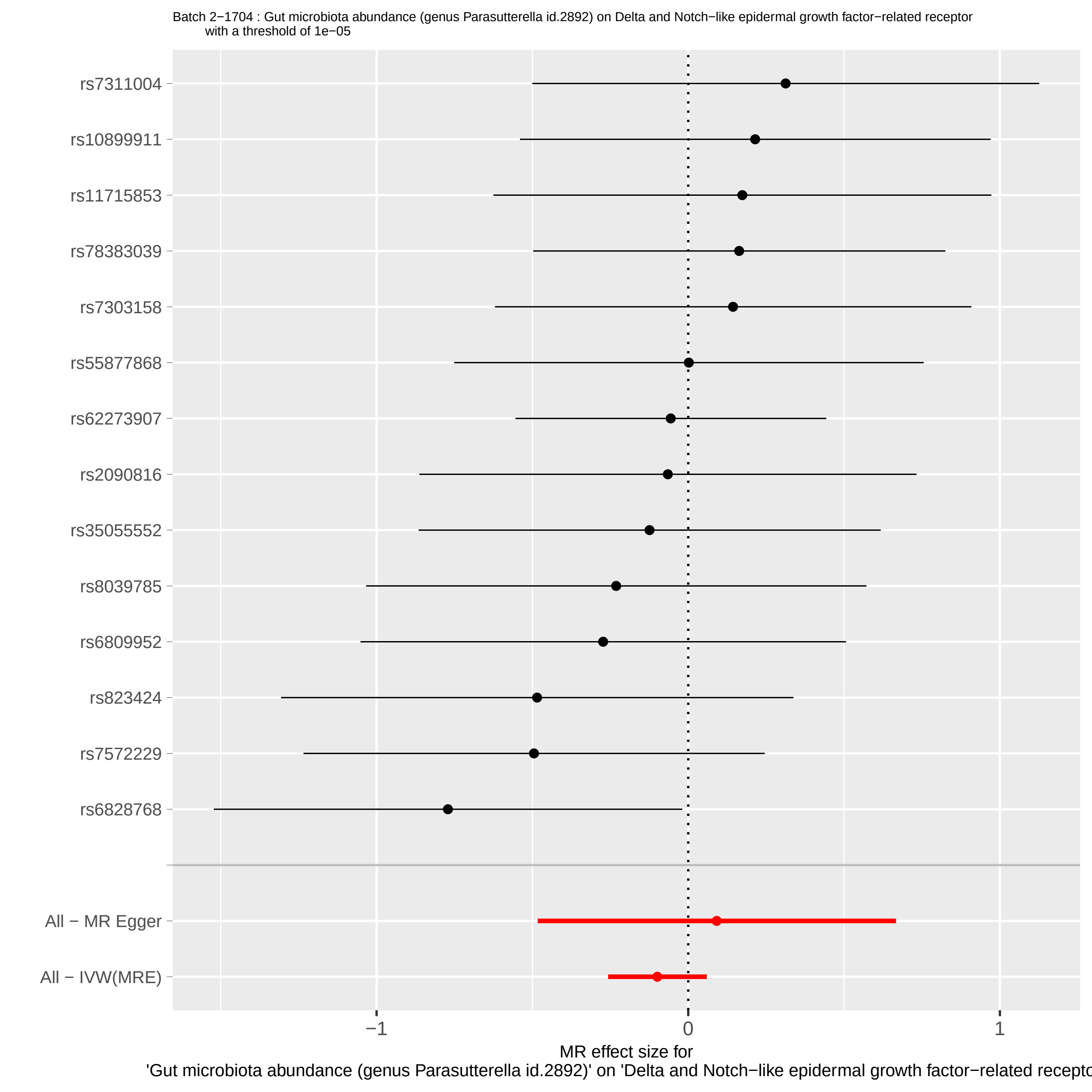

Batch 2−1704 : Gut microbiota abundance (genus Parasutterella id.2892) on Delta and Notch−like epidermal growth factor−related receptor
with a threshold of 1e−05
rs7311004
rs10899911
rs11715853
rs78383039
rs7303158
rs55877868
rs62273907
rs2090816
rs35055552
rs8039785
rs6809952
rs823424
rs7572229
rs6828768
All − MR Egger
All − IVW(MRE)
−1
0
1
MR effect size for
'Gut microbiota abundance (genus Parasutterella id.2892)' on 'Delta and Notch−like epidermal growth factor−related recepto

Batch 2−1705 : Gut microbiota abundance (genus Catenibacterium id.2153) on Delta and Notch−like epidermal growth factor−related receptor
with a threshold of 1e−05

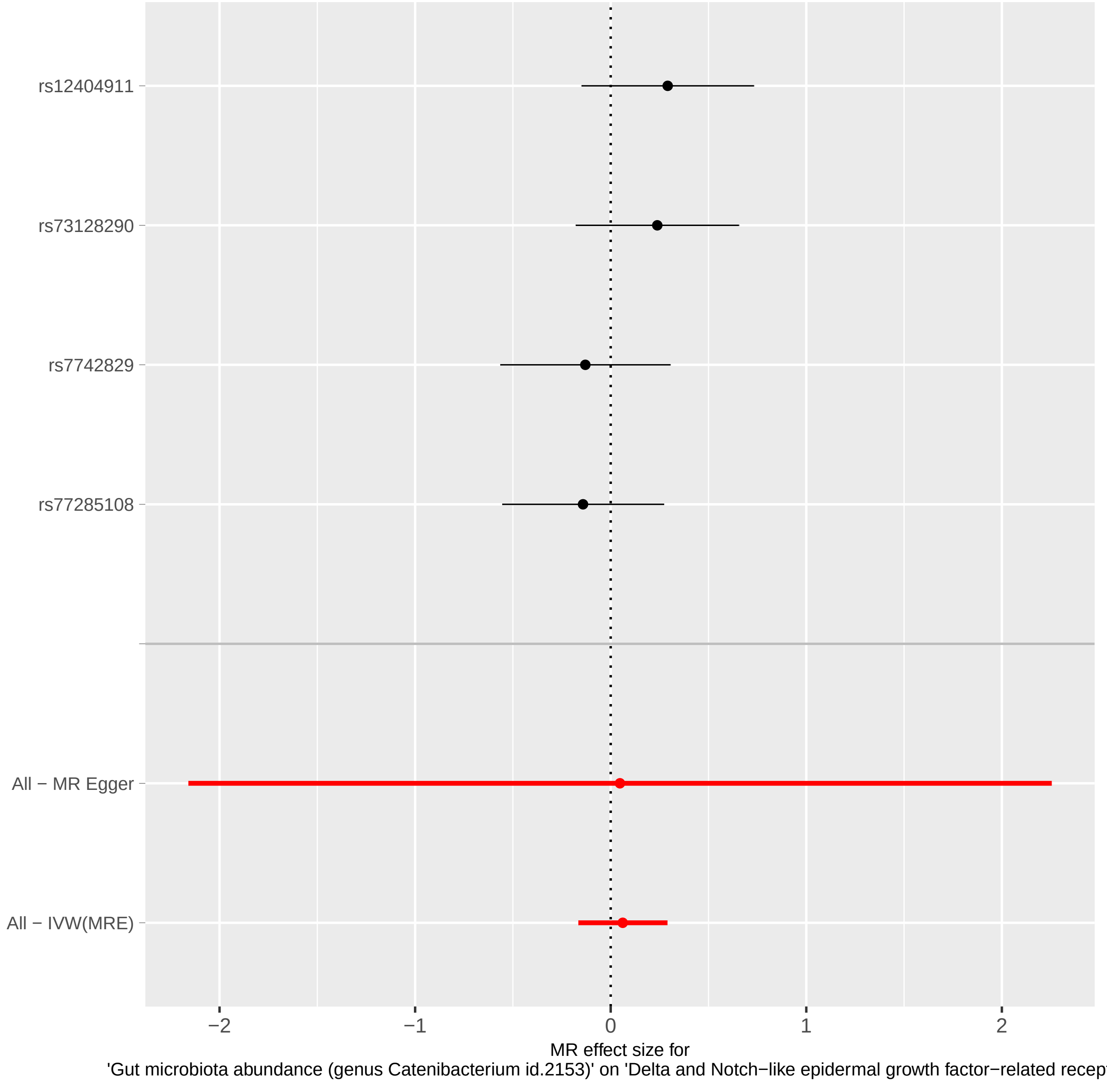

Batch 2−1706 : Gut microbiota abundance (genus Parasutterella id.2892) on Delta and Notch−like epidermal growth factor−related receptor with a threshold of 1e−05

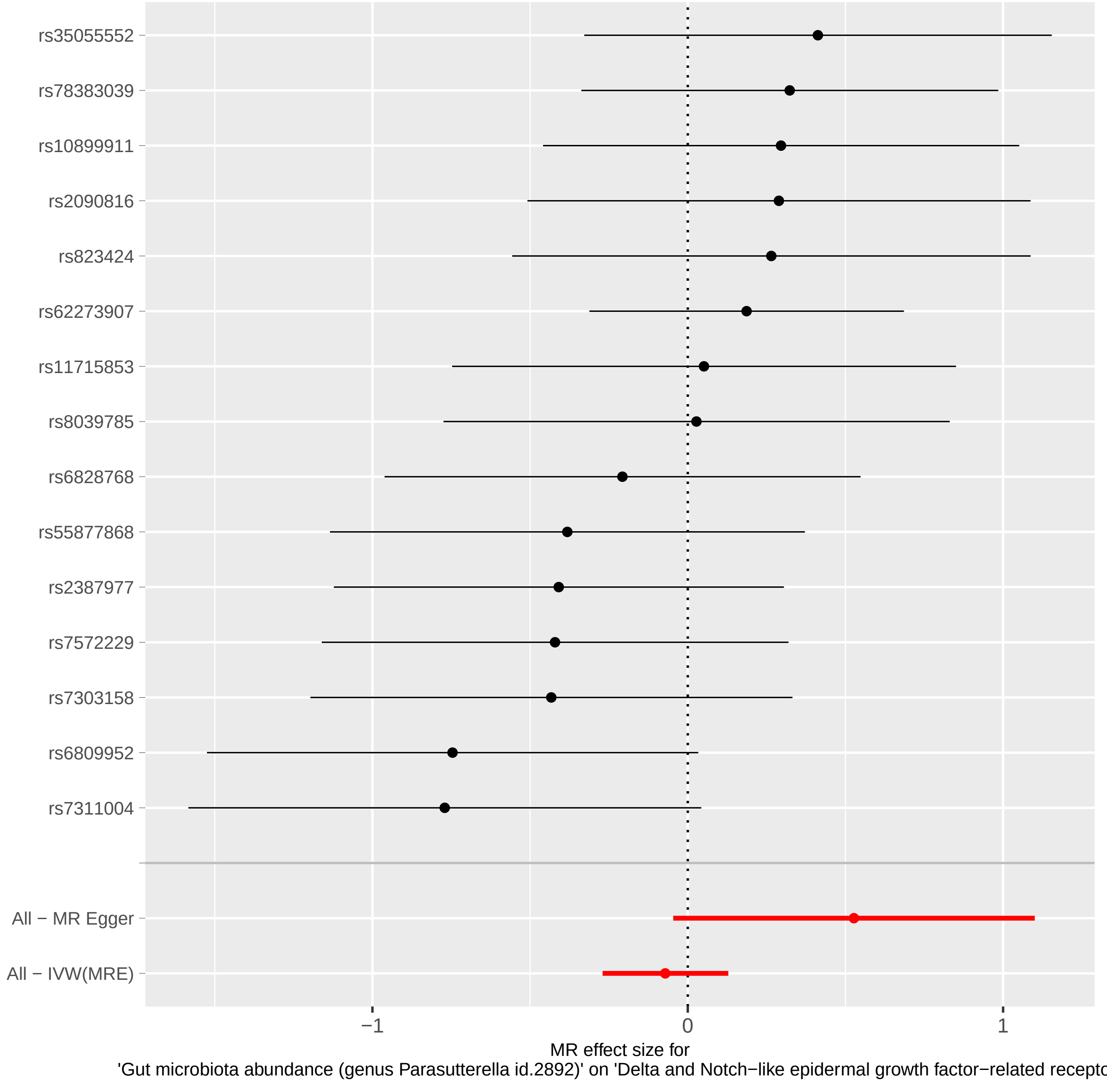

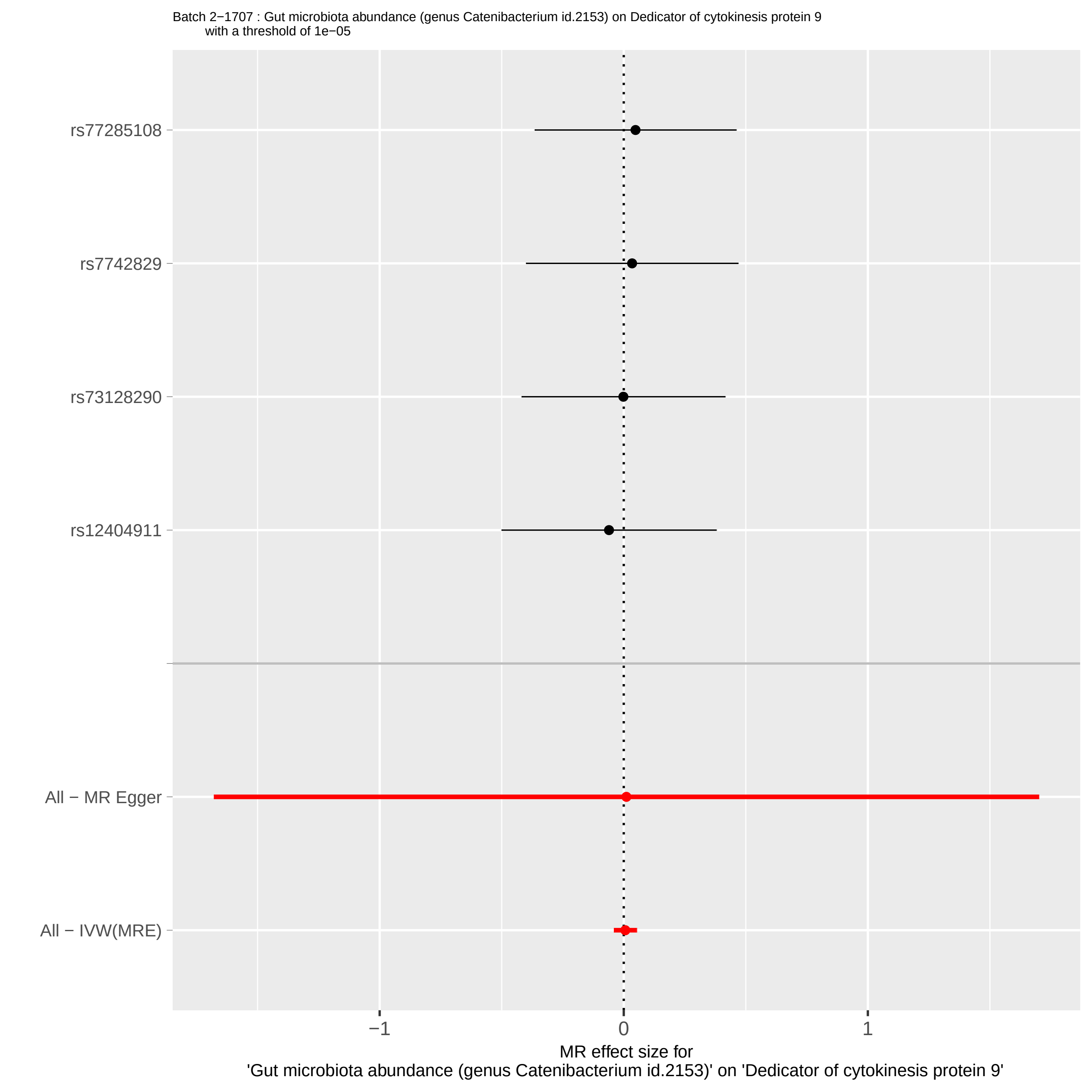

Batch 2−1707 : Gut microbiota abundance (genus Catenibacterium id.2153) on Dedicator of cytokinesis protein 9
with a threshold of 1e−05
rs77285108
rs7742829
rs73128290
rs12404911
All − MR Egger
All − IVW(MRE)
−1
0
1
MR effect size for
'Gut microbiota abundance (genus Catenibacterium id.2153)' on 'Dedicator of cytokinesis protein 9'

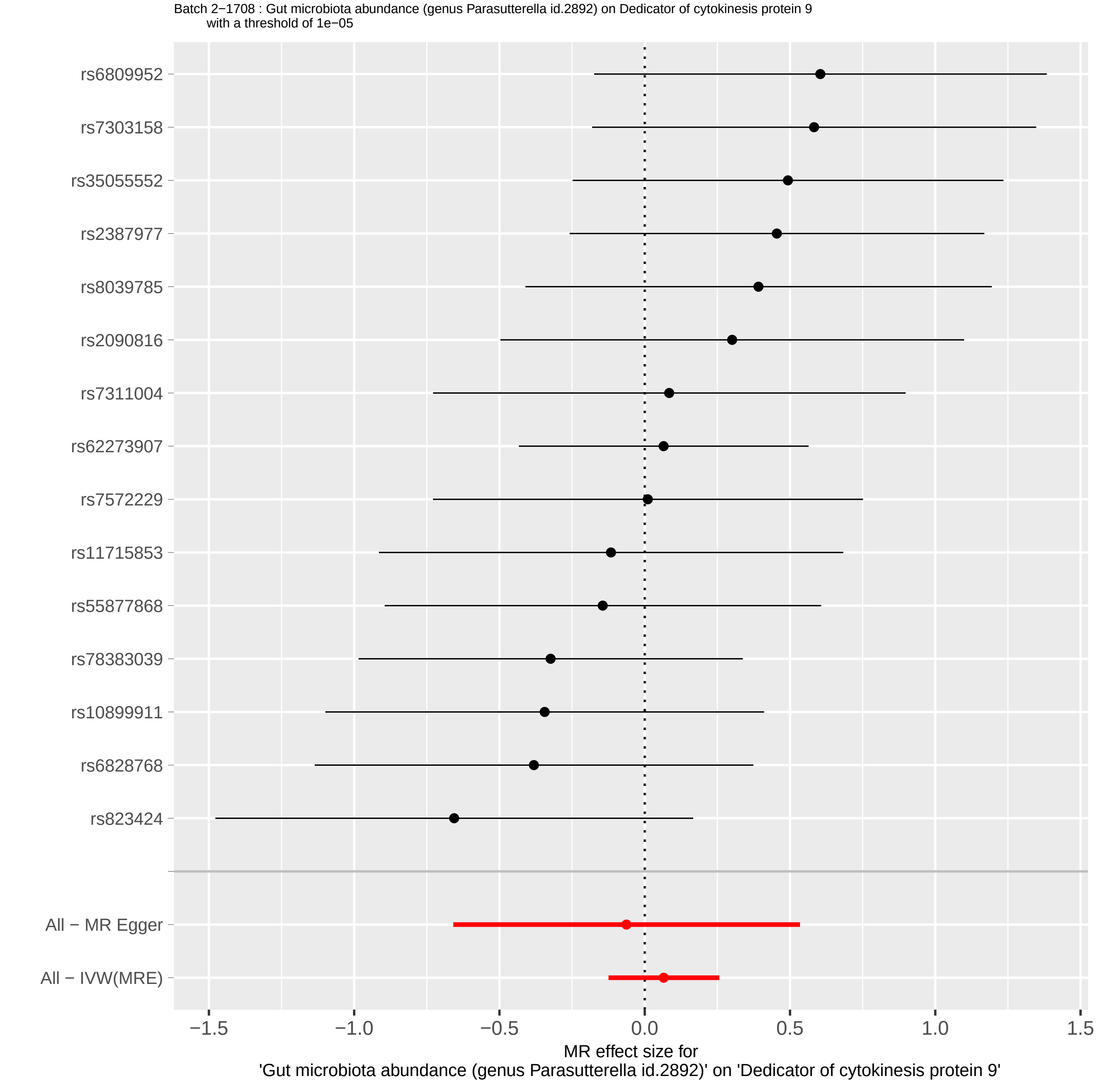
Batch 2−1708 : Gut microbiota abundance (genus Parasutterella id.2892) on Dedicator of cytokinesis protein 9
with a threshold of 1e−05
rs6809952
rs7303158
rs35055552
rs2387977
rs8039785
rs2090816
rs7311004
rs62273907
rs7572229
rs11715853
rs55877868
rs78383039
rs10899911
rs6828768
rs823424
All − MR Egger
All − IVW(MRE)
−1.5
−1.0
−0.5
0.0
0.5
1.0
1.5
MR effect size for
'Gut microbiota abundance (genus Parasutterella id.2892)' on 'Dedicator of cytokinesis protein 9'

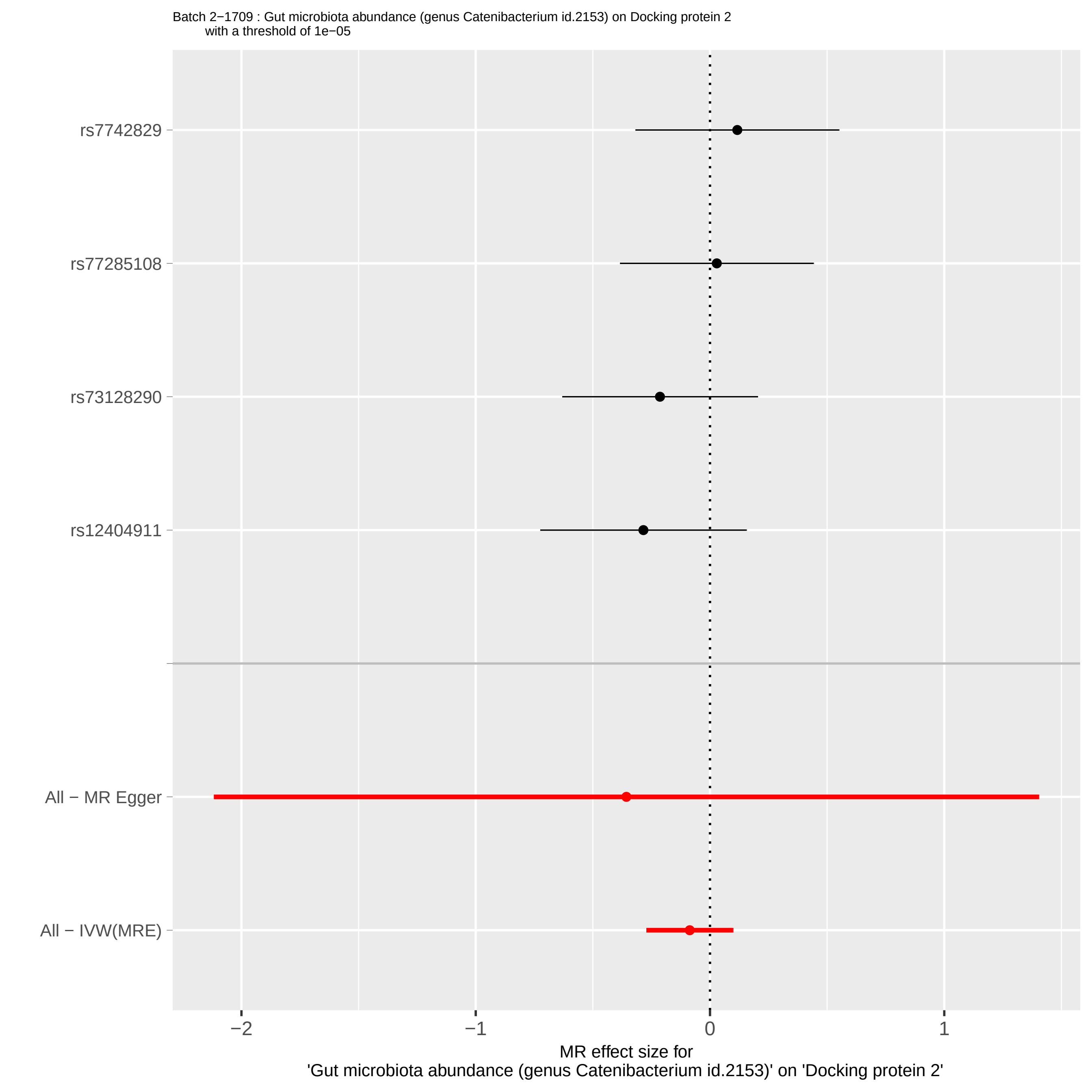
Batch 2−1709 : Gut microbiota abundance (genus Catenibacterium id.2153) on Docking protein 2
with a threshold of 1e−05
rs7742829
rs77285108
rs73128290
rs12404911
All − MR Egger
All − IVW(MRE)
−2
−1
0
1
MR effect size for
'Gut microbiota abundance (genus Catenibacterium id.2153)' on 'Docking protein 2'

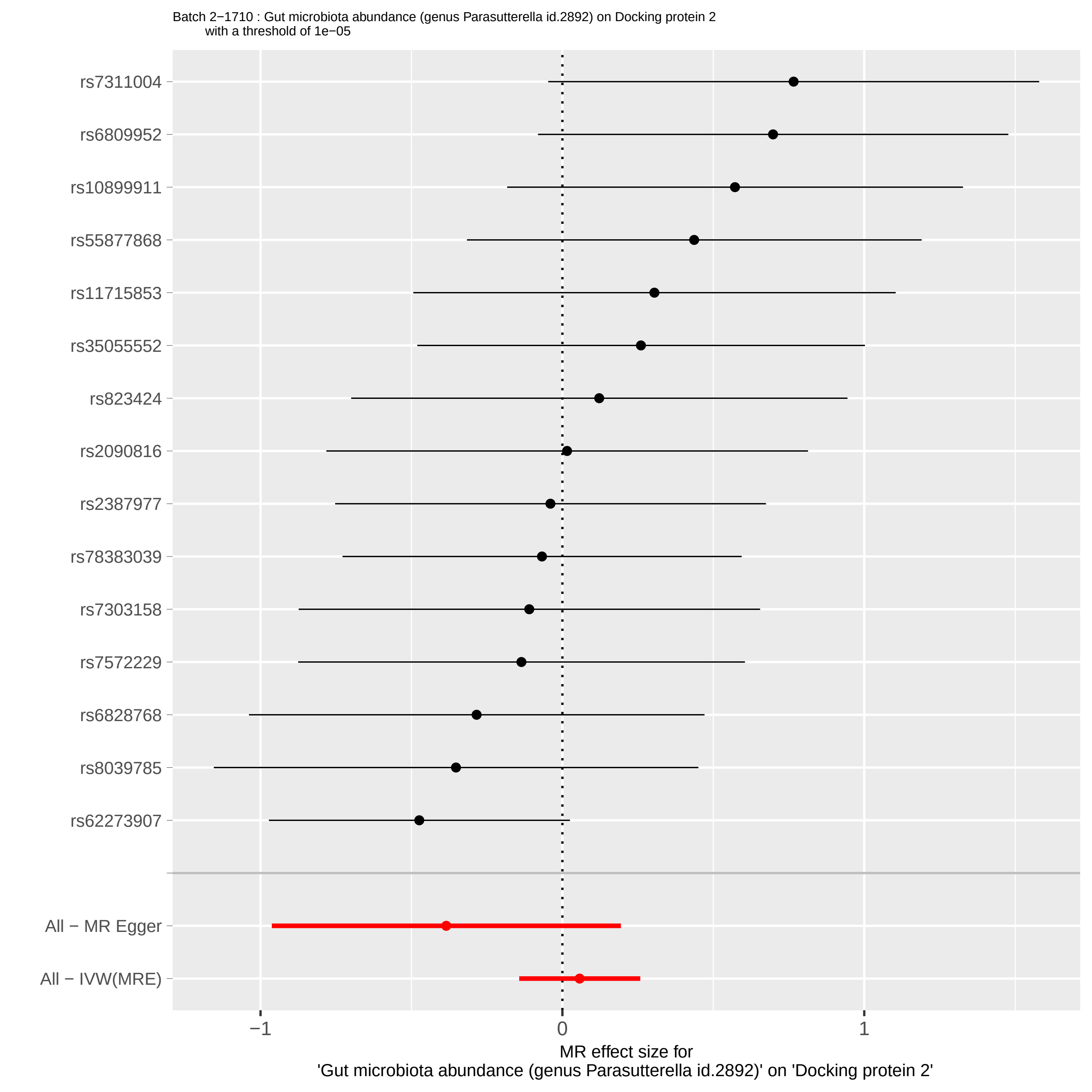
Batch 2−1710 : Gut microbiota abundance (genus Parasutterella id.2892) on Docking protein 2
with a threshold of 1e−05
rs7311004
rs6809952
rs10899911
rs55877868
rs11715853
rs35055552
rs823424
rs2090816
rs2387977
rs78383039
rs7303158
rs7572229
rs6828768
rs8039785
rs62273907
All − MR Egger
All − IVW(MRE)
−1
0
1
MR effect size for
'Gut microbiota abundance (genus Parasutterella id.2892)' on 'Docking protein 2'

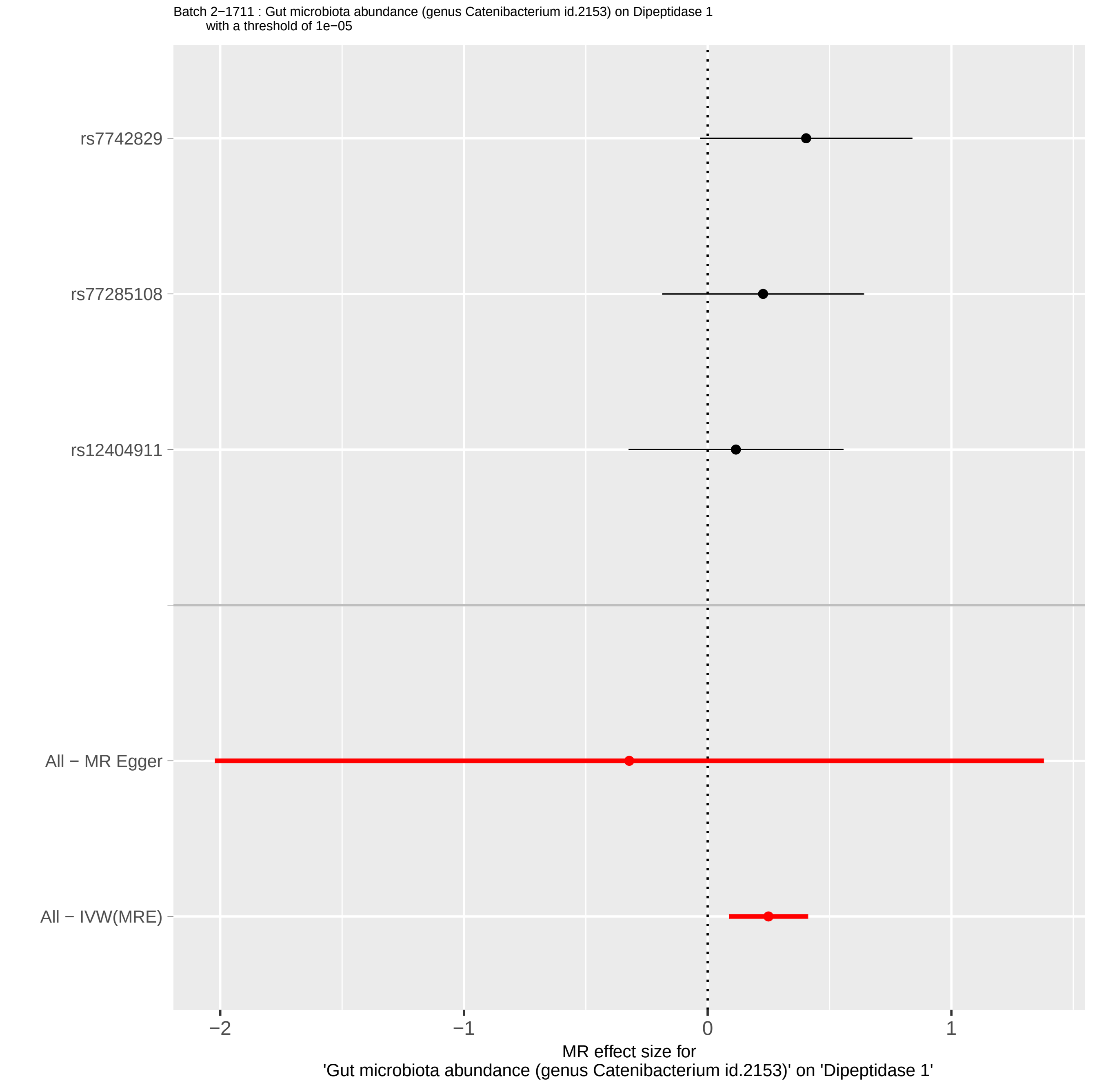
Batch 2−1711 : Gut microbiota abundance (genus Catenibacterium id.2153) on Dipeptidase 1
with a threshold of 1e−05
rs7742829
rs77285108
rs12404911
All − MR Egger
All − IVW(MRE)
−2
−1
0
1
MR effect size for
'Gut microbiota abundance (genus Catenibacterium id.2153)' on 'Dipeptidase 1'

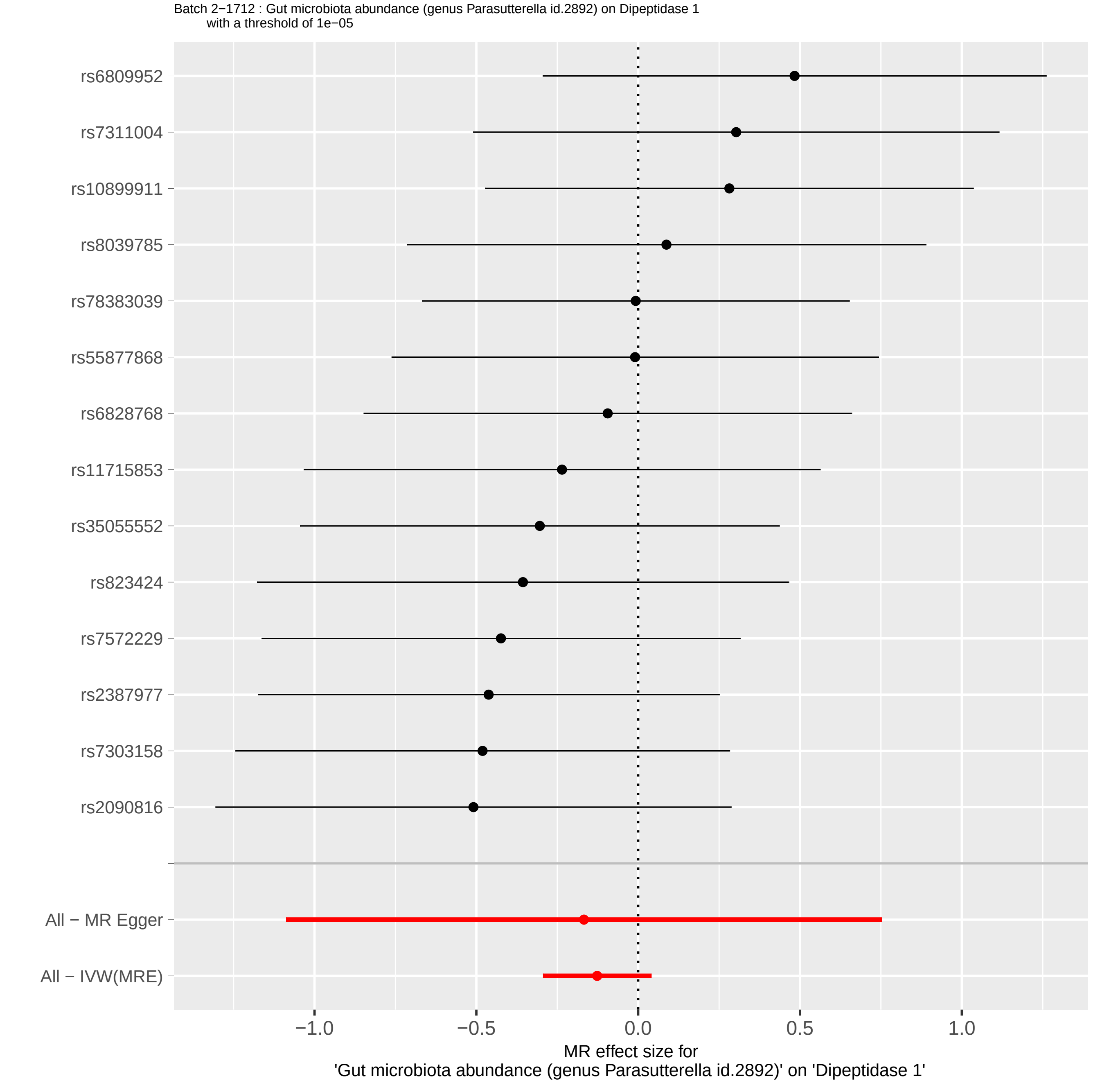
Batch 2−1712 : Gut microbiota abundance (genus Parasutterella id.2892) on Dipeptidase 1
with a threshold of 1e−05
rs6809952
rs7311004
rs10899911
rs8039785
rs78383039
rs55877868
rs6828768
rs11715853
rs35055552
rs823424
rs7572229
rs2387977
rs7303158
rs2090816
All − MR Egger
All − IVW(MRE)
−1.0
−0.5
0.0
0.5
1.0
MR effect size for
'Gut microbiota abundance (genus Parasutterella id.2892)' on 'Dipeptidase 1'

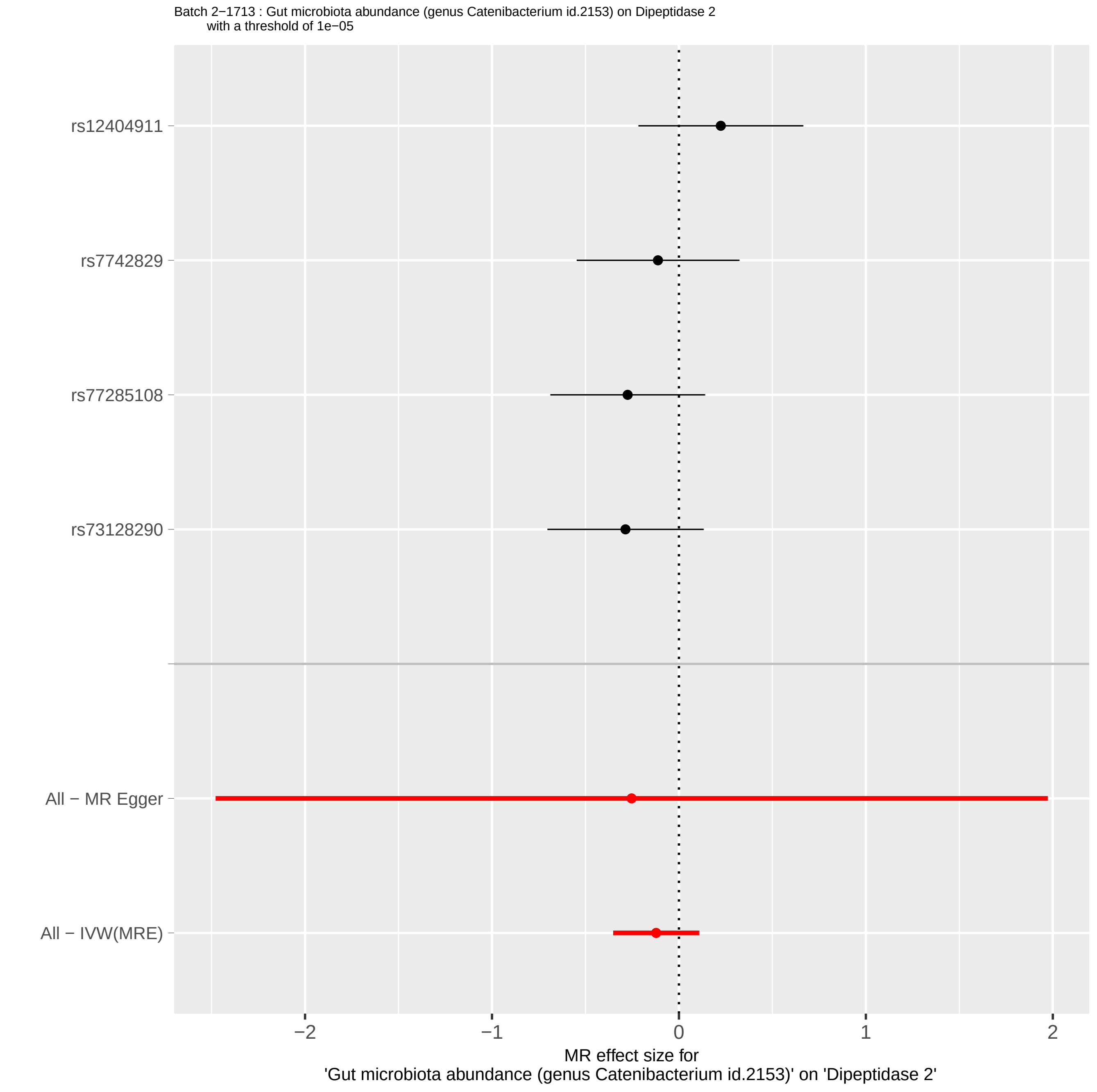
Batch 2−1713 : Gut microbiota abundance (genus Catenibacterium id.2153) on Dipeptidase 2
with a threshold of 1e−05
rs12404911
rs7742829
rs77285108
rs73128290
All − MR Egger
All − IVW(MRE)
−2
−1
0
1
2
MR effect size for
'Gut microbiota abundance (genus Catenibacterium id.2153)' on 'Dipeptidase 2'

Batch 2−1714 : Gut microbiota abundance (genus Parasutterella id.2892) on Dipeptidase 2
with a threshold of 1e−05

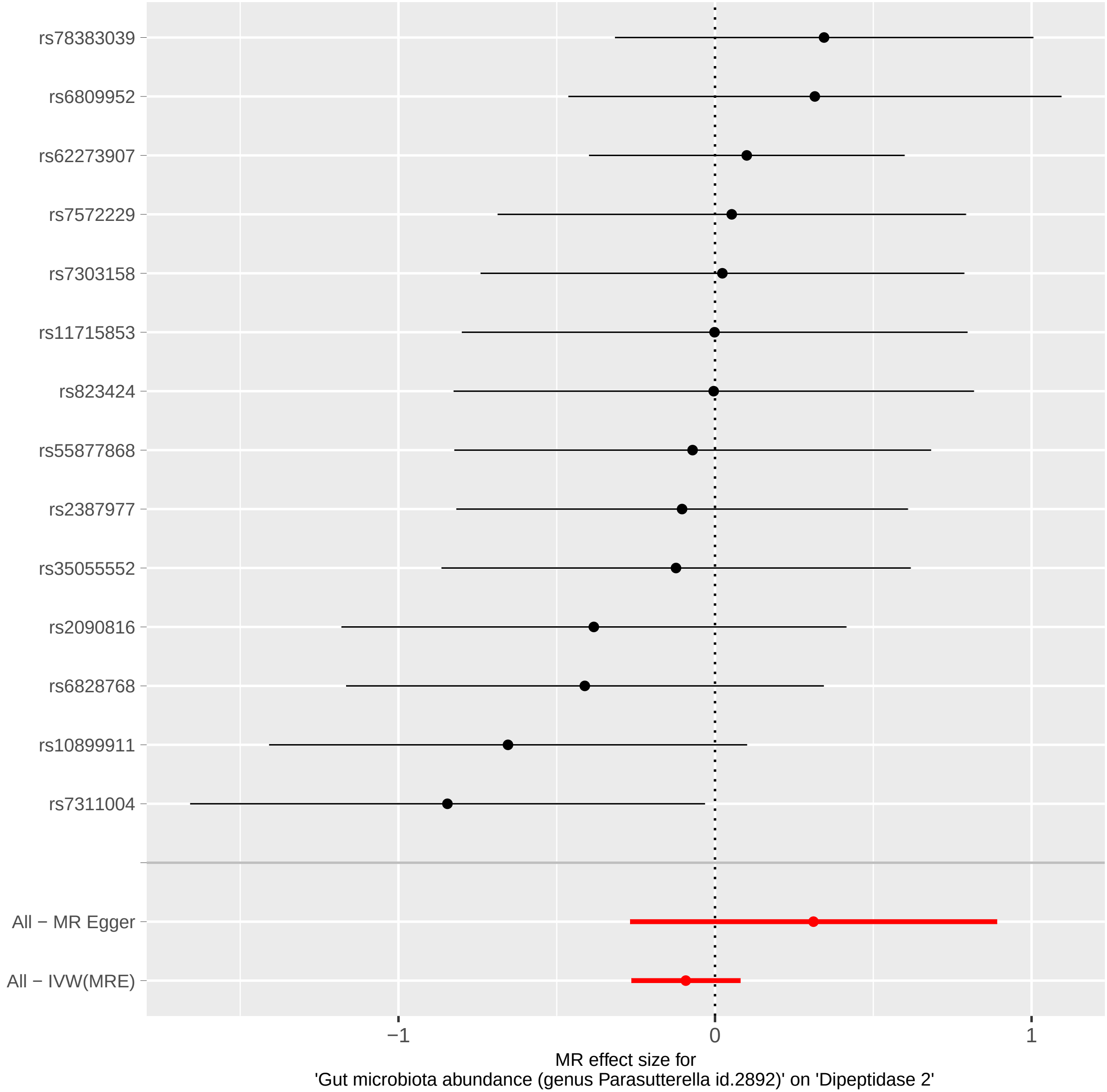

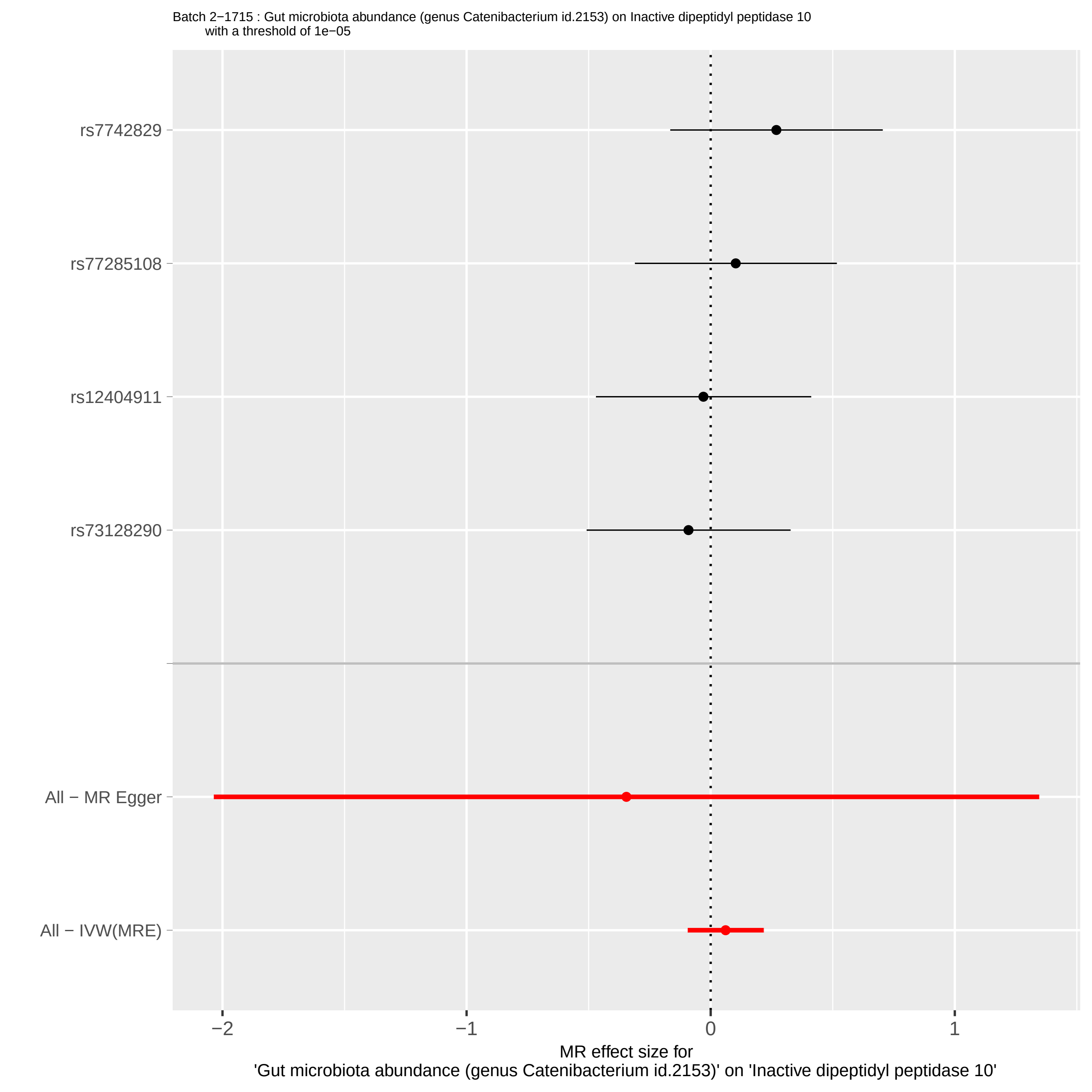

Batch 2−1715 : Gut microbiota abundance (genus Catenibacterium id.2153) on Inactive dipeptidyl peptidase 10
with a threshold of 1e−05
rs7742829
rs77285108
rs12404911
rs73128290
All − MR Egger
All − IVW(MRE)
−2
−1
0
1
MR effect size for
'Gut microbiota abundance (genus Catenibacterium id.2153)' on 'Inactive dipeptidyl peptidase 10'

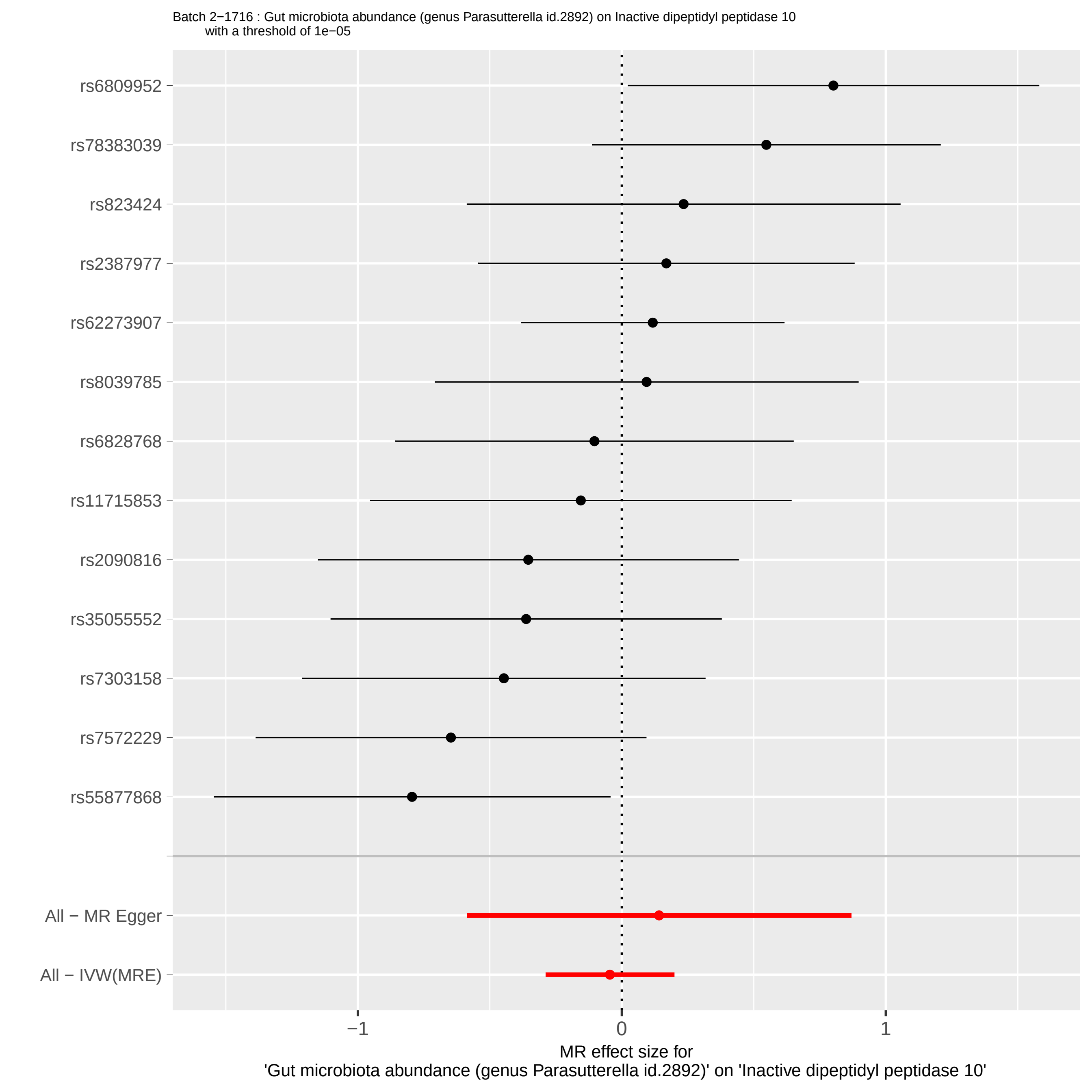

Batch 2−1716 : Gut microbiota abundance (genus Parasutterella id.2892) on Inactive dipeptidyl peptidase 10
with a threshold of 1e−05
rs6809952
rs78383039
rs823424
rs2387977
rs62273907
rs8039785
rs6828768
rs11715853
rs2090816
rs35055552
rs7303158
rs7572229
rs55877868
All − MR Egger
All − IVW(MRE)
−1
0
1
MR effect size for
'Gut microbiota abundance (genus Parasutterella id.2892)' on 'Inactive dipeptidyl peptidase 10'

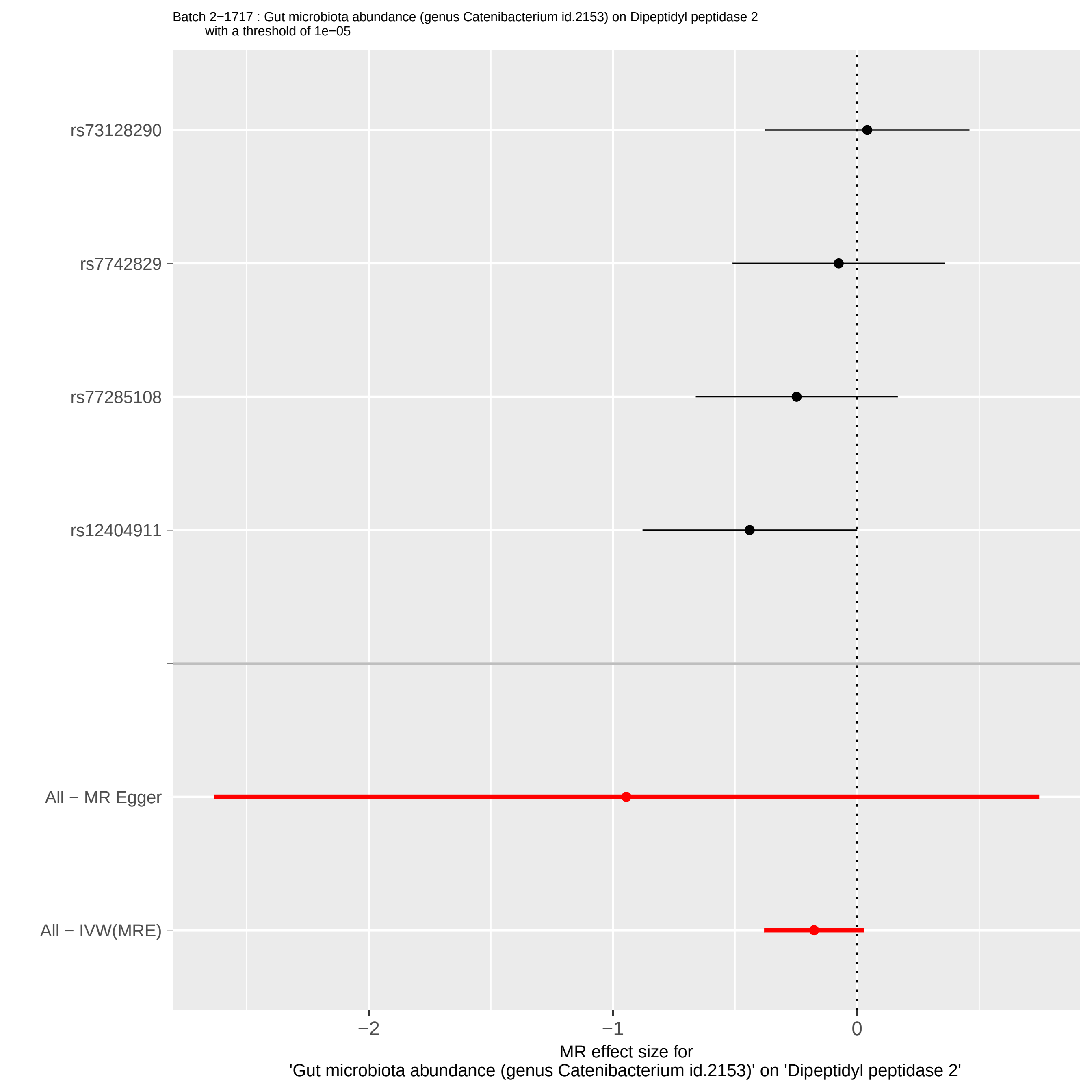

Batch 2−1717 : Gut microbiota abundance (genus Catenibacterium id.2153) on Dipeptidyl peptidase 2
with a threshold of 1e−05
rs73128290
rs7742829
rs77285108
rs12404911
All − MR Egger
All − IVW(MRE)
−2
−1
0
MR effect size for
'Gut microbiota abundance (genus Catenibacterium id.2153)' on 'Dipeptidyl peptidase 2'

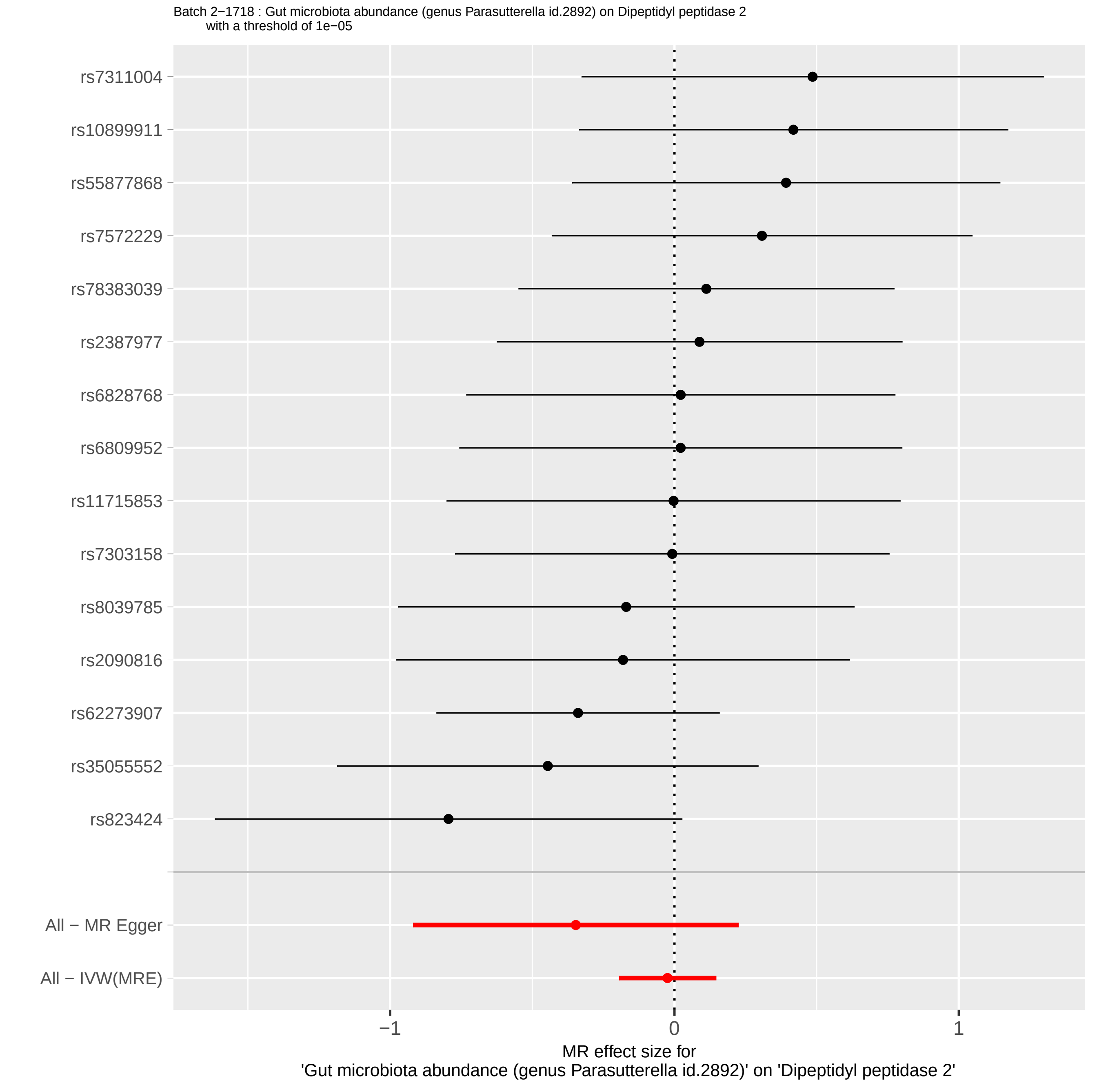

Batch 2−1718 : Gut microbiota abundance (genus Parasutterella id.2892) on Dipeptidyl peptidase 2
with a threshold of 1e−05
rs7311004
rs10899911
rs55877868
rs7572229
rs78383039
rs2387977
rs6828768
rs6809952
rs11715853
rs7303158
rs8039785
rs2090816
rs62273907
rs35055552
rs823424
All − MR Egger
All − IVW(MRE)
−1
0
1
MR effect size for
'Gut microbiota abundance (genus Parasutterella id.2892)' on 'Dipeptidyl peptidase 2'

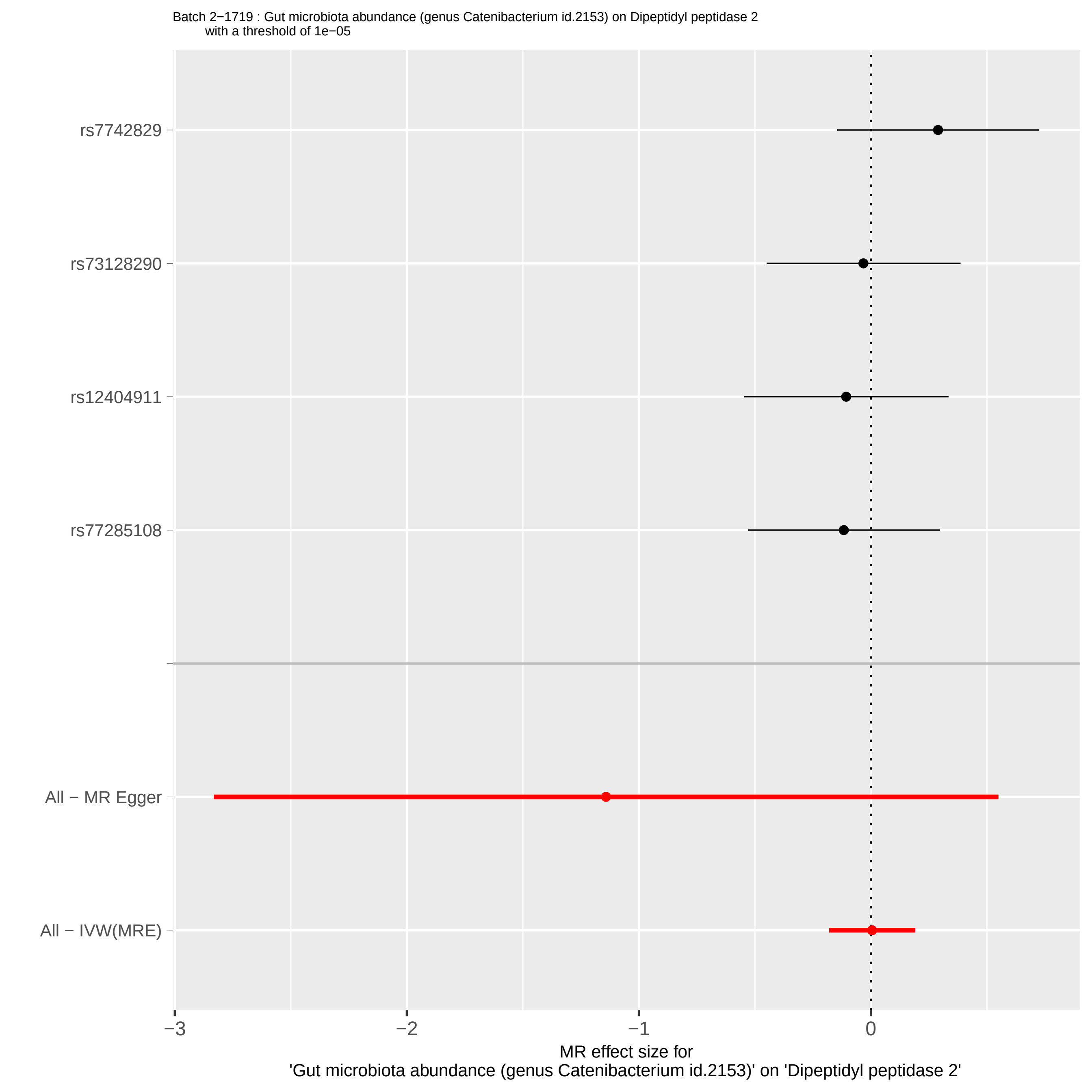

Batch 2−1719 : Gut microbiota abundance (genus Catenibacterium id.2153) on Dipeptidyl peptidase 2
with a threshold of 1e−05
rs7742829
rs73128290
rs12404911
rs77285108
All − MR Egger
All − IVW(MRE)
−3
−2
−1
0
MR effect size for
'Gut microbiota abundance (genus Catenibacterium id.2153)' on 'Dipeptidyl peptidase 2'

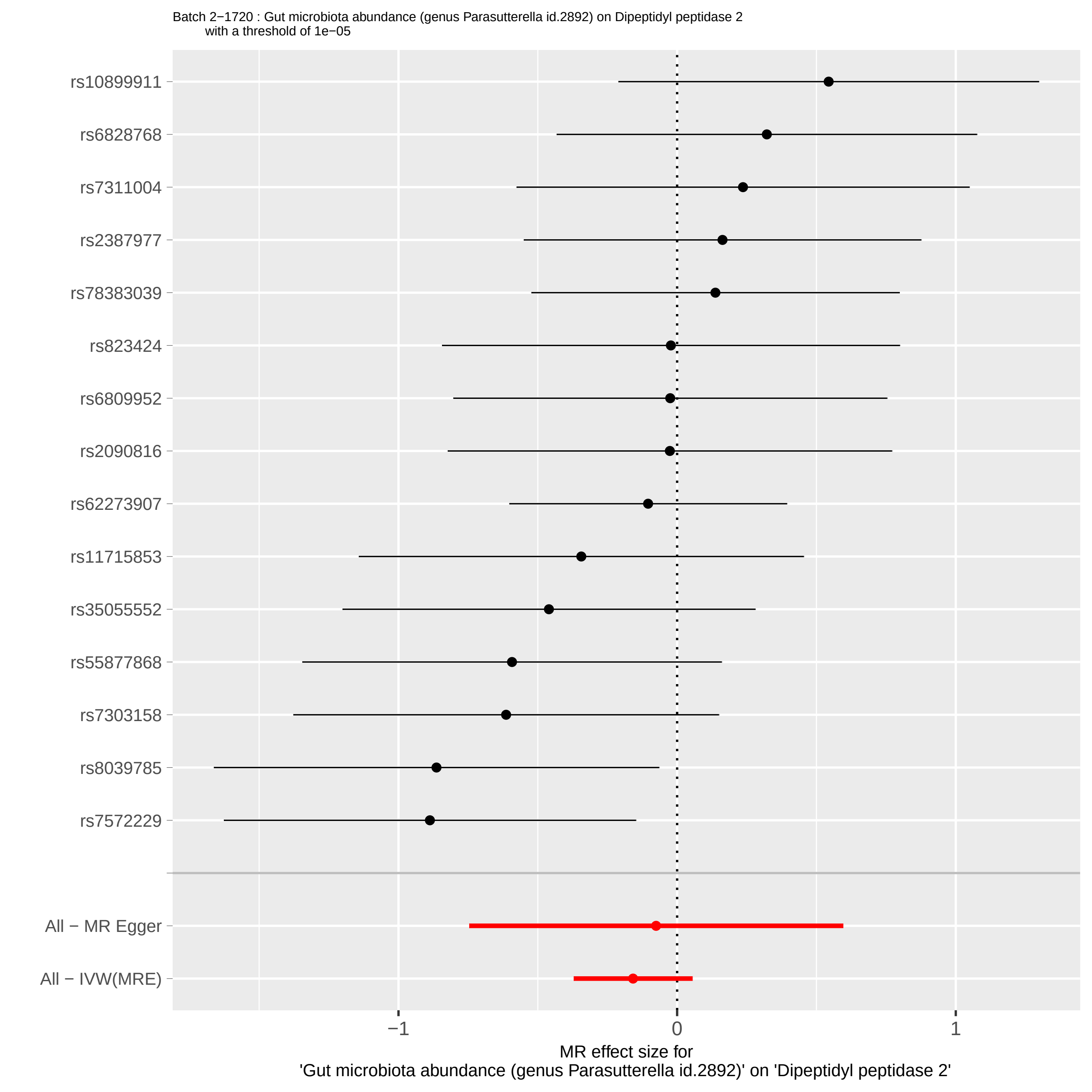

Batch 2−1720 : Gut microbiota abundance (genus Parasutterella id.2892) on Dipeptidyl peptidase 2
with a threshold of 1e−05
rs10899911
rs6828768
rs7311004
rs2387977
rs78383039
rs823424
rs6809952
rs2090816
rs62273907
rs11715853
rs35055552
rs55877868
rs7303158
rs8039785
rs7572229
All − MR Egger
All − IVW(MRE)
−1
0
1
MR effect size for
'Gut microbiota abundance (genus Parasutterella id.2892)' on 'Dipeptidyl peptidase 2'

Batch 2−1721 : Gut microbiota abundance (genus Catenibacterium id.2153) on Dermatopontin
with a threshold of 1e−05

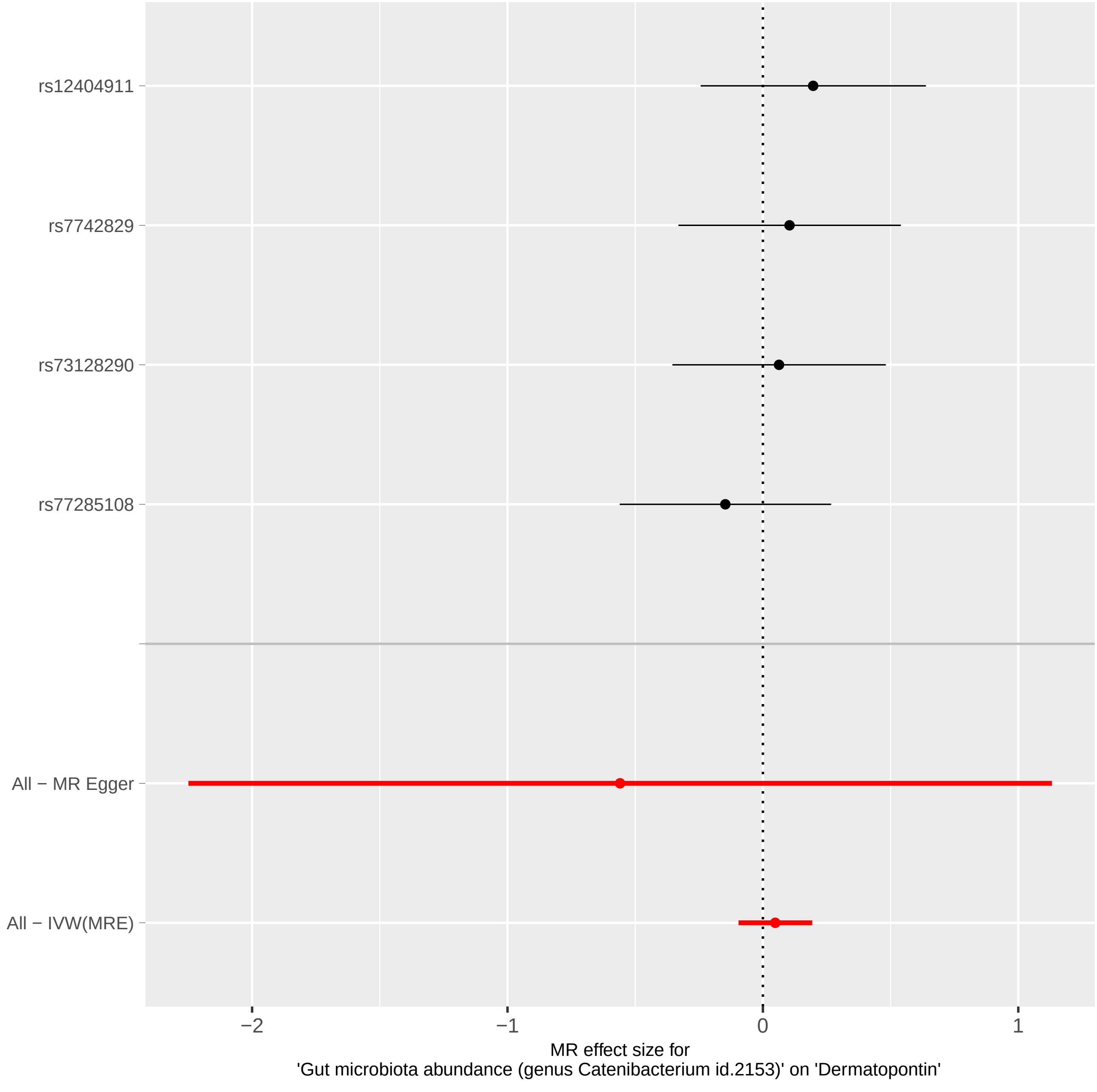

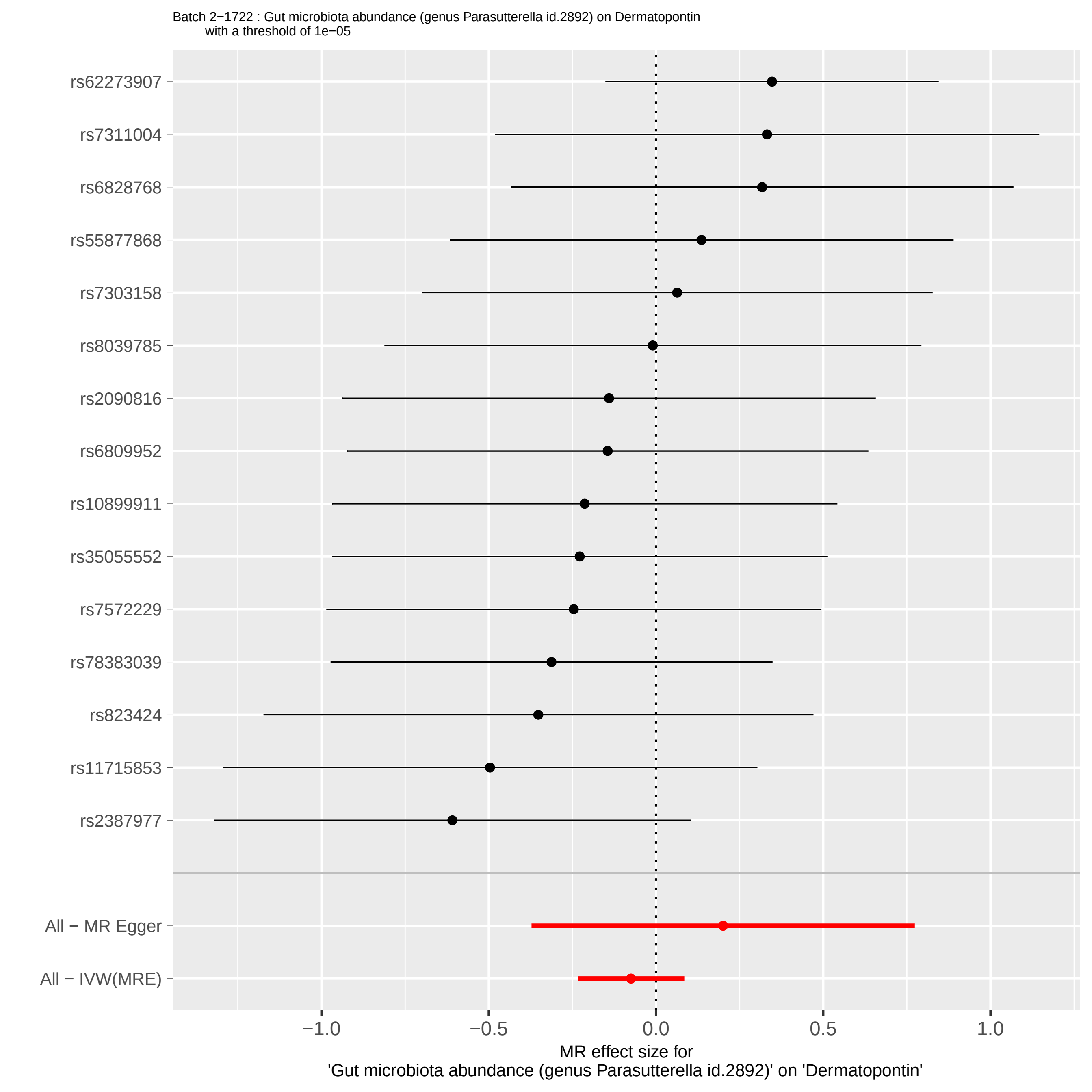

Batch 2−1722 : Gut microbiota abundance (genus Parasutterella id.2892) on Dermatopontin
with a threshold of 1e−05
rs62273907
rs7311004
rs6828768
rs55877868
rs7303158
rs8039785
rs2090816
rs6809952
rs10899911
rs35055552
rs7572229
rs78383039
rs823424
rs11715853
rs2387977
All − MR Egger
All − IVW(MRE)
−1.0
−0.5
0.0
0.5
1.0
MR effect size for
'Gut microbiota abundance (genus Parasutterella id.2892)' on 'Dermatopontin'

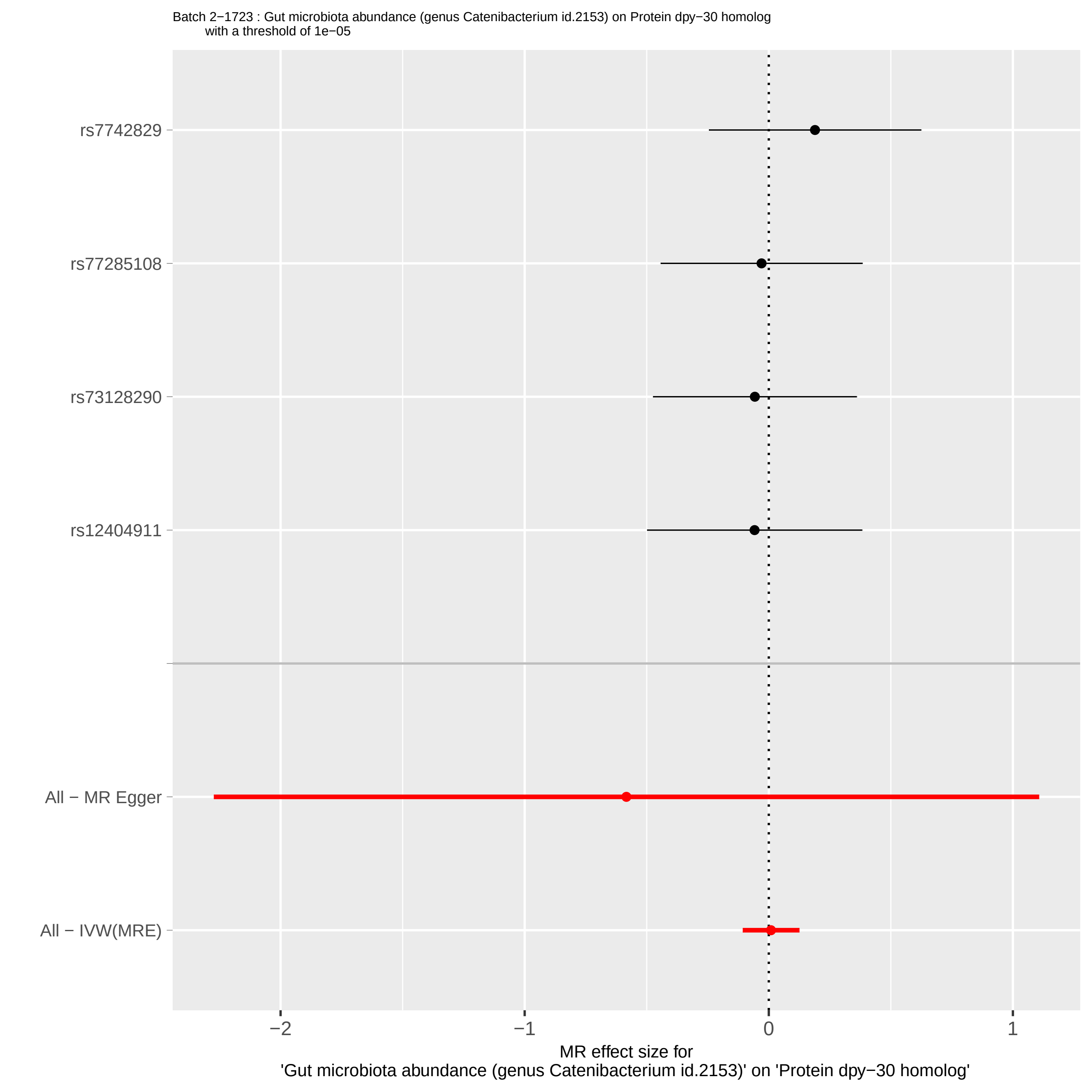

Batch 2−1723 : Gut microbiota abundance (genus Catenibacterium id.2153) on Protein dpy−30 homolog
with a threshold of 1e−05
rs7742829
rs77285108
rs73128290
rs12404911
All − MR Egger
All − IVW(MRE)
−2
−1
0
1
MR effect size for
'Gut microbiota abundance (genus Catenibacterium id.2153)' on 'Protein dpy−30 homolog'

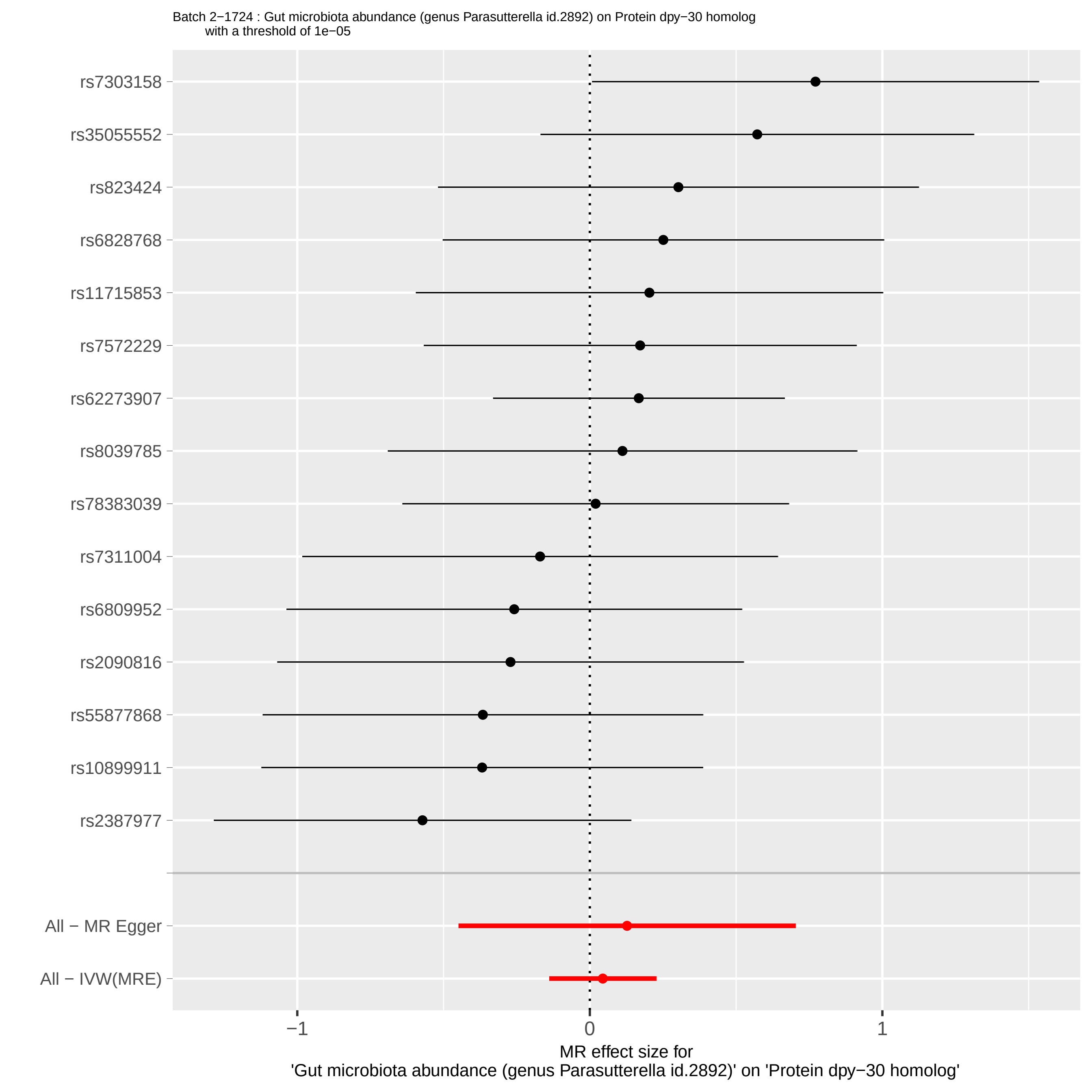
Batch 2−1724 : Gut microbiota abundance (genus Parasutterella id.2892) on Protein dpy−30 homolog
with a threshold of 1e−05
rs7303158
rs35055552
rs823424
rs6828768
rs11715853
rs7572229
rs62273907
rs8039785
rs78383039
rs7311004
rs6809952
rs2090816
rs55877868
rs10899911
rs2387977
All − MR Egger
All − IVW(MRE)
−1
0
1
MR effect size for
'Gut microbiota abundance (genus Parasutterella id.2892)' on 'Protein dpy−30 homolog'

Batch 2−1725 : Gut microbiota abundance (genus Catenibacterium id.2153) on Dihydropyrimidinase−related protein 3
with a threshold of 1e−05

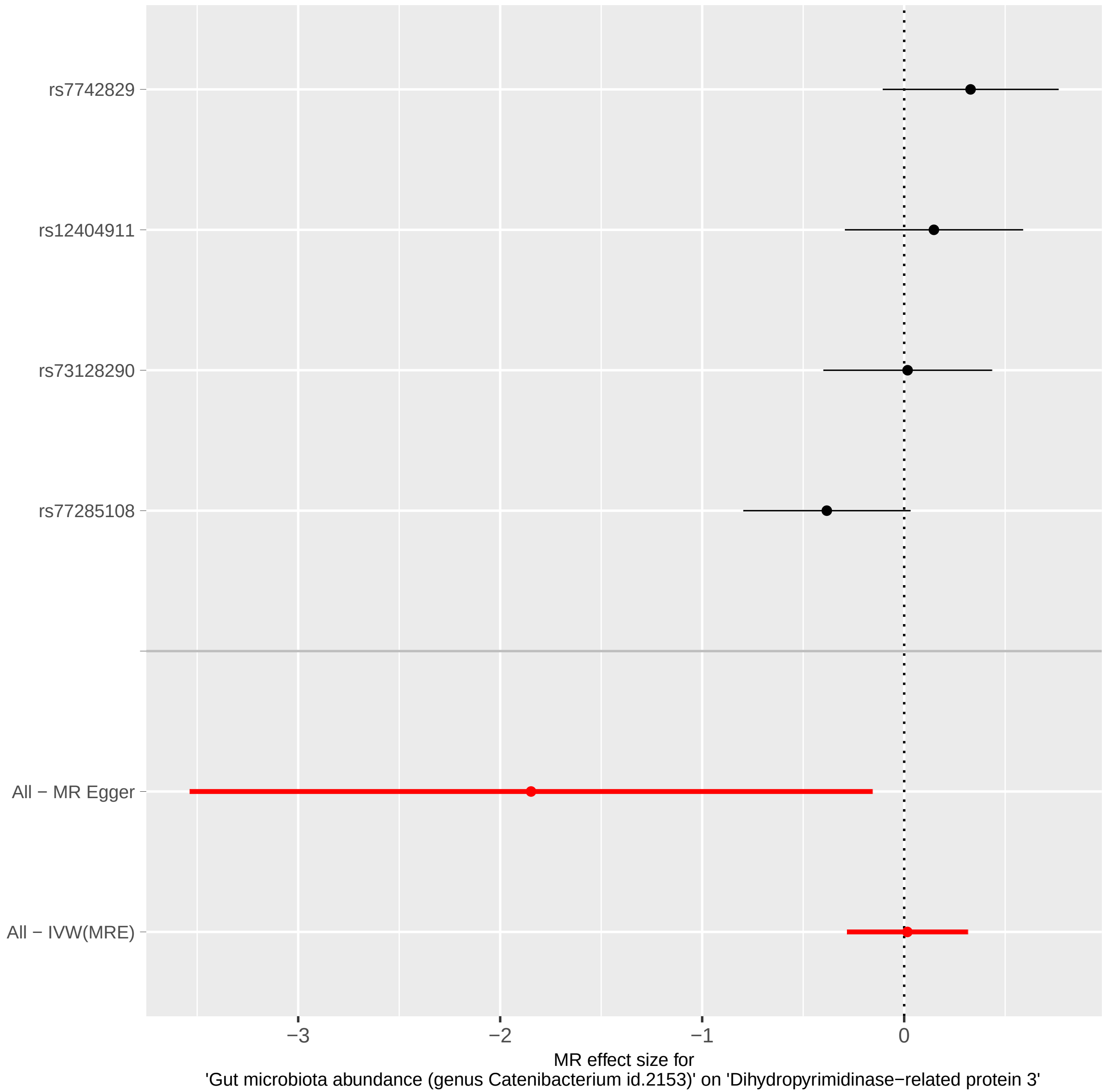

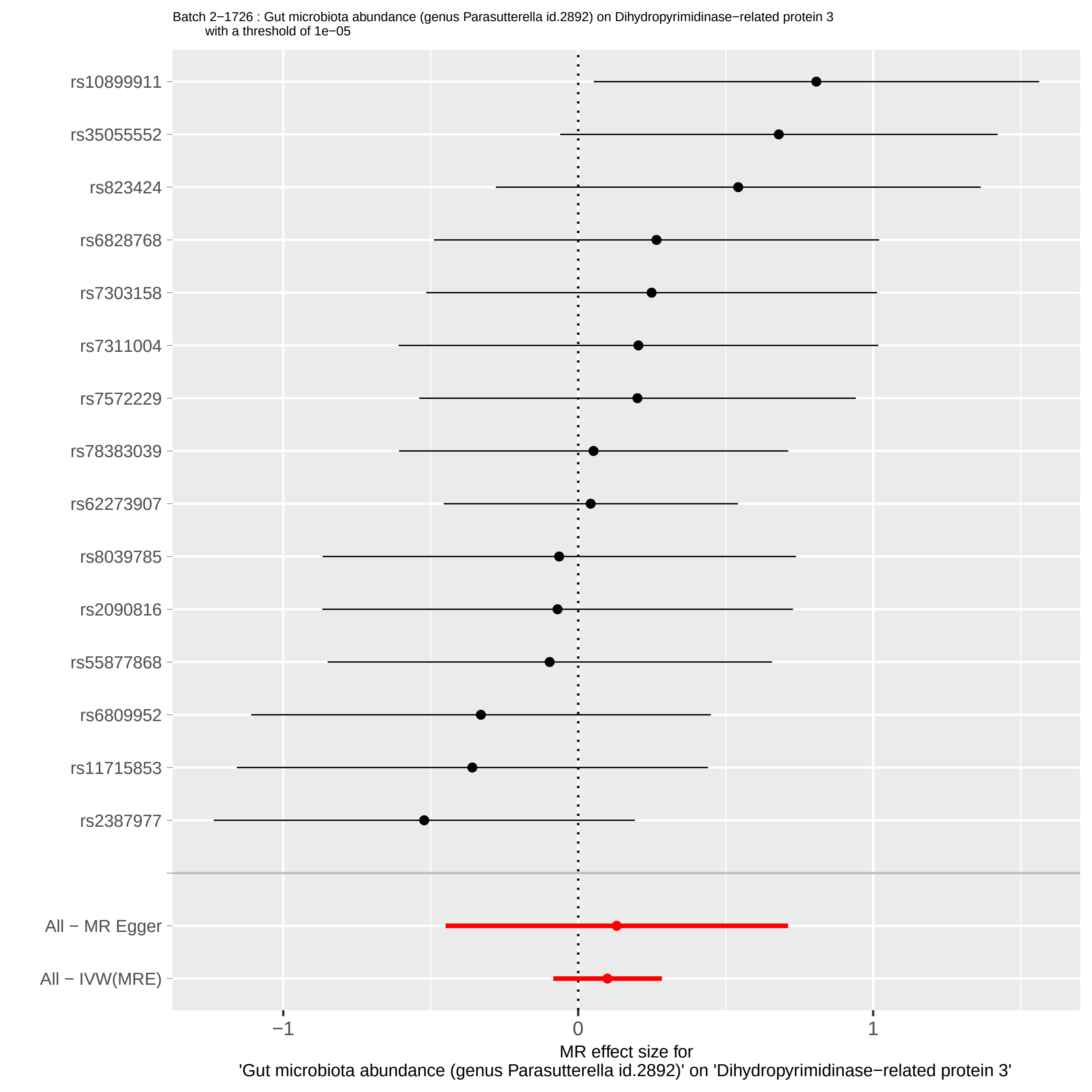
Batch 2−1726 : Gut microbiota abundance (genus Parasutterella id.2892) on Dihydropyrimidinase−related protein 3
with a threshold of 1e−05
rs10899911
rs35055552
rs823424
rs6828768
rs7303158
rs7311004
rs7572229
rs78383039
rs62273907
rs8039785
rs2090816
rs55877868
rs6809952
rs11715853
rs2387977
All − MR Egger
All − IVW(MRE)
−1
0
1
MR effect size for
'Gut microbiota abundance (genus Parasutterella id.2892)' on 'Dihydropyrimidinase−related protein 3'

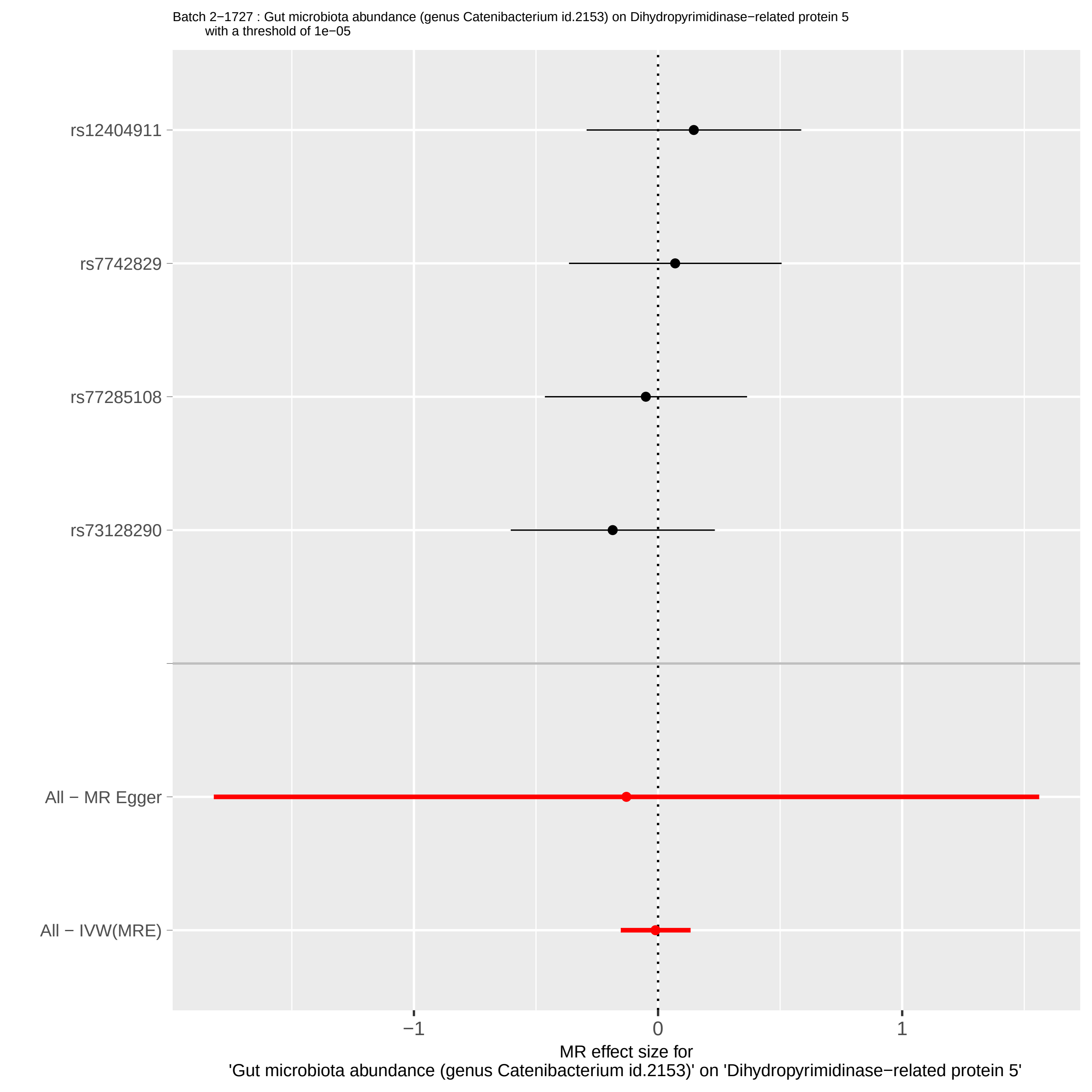
Batch 2−1727 : Gut microbiota abundance (genus Catenibacterium id.2153) on Dihydropyrimidinase−related protein 5
with a threshold of 1e−05
rs12404911
rs7742829
rs77285108
rs73128290
All − MR Egger
All − IVW(MRE)
−1
0
1
MR effect size for
'Gut microbiota abundance (genus Catenibacterium id.2153)' on 'Dihydropyrimidinase−related protein 5'

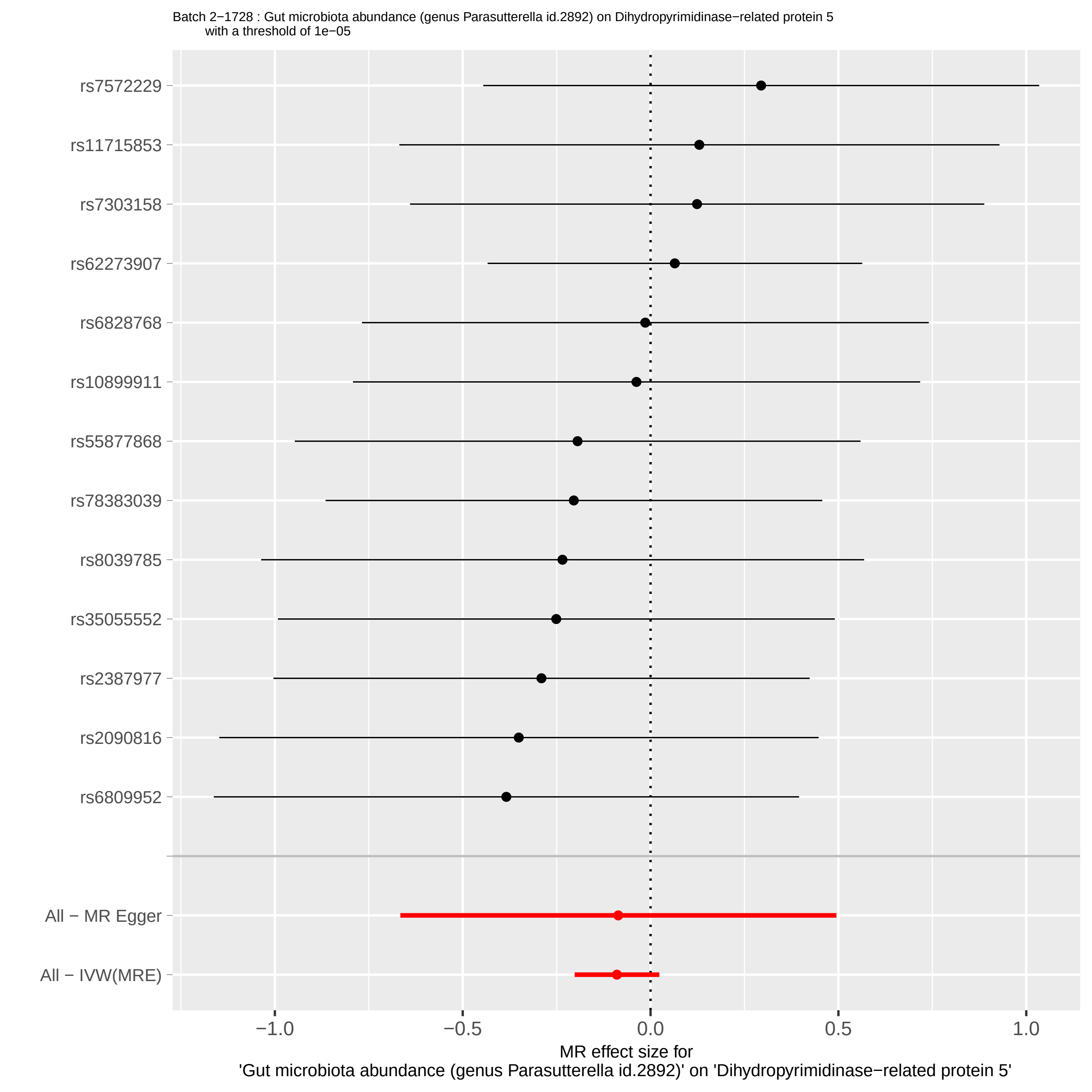

Batch 2−1728 : Gut microbiota abundance (genus Parasutterella id.2892) on Dihydropyrimidinase−related protein 5
with a threshold of 1e−05
rs7572229
rs11715853
rs7303158
rs62273907
rs6828768
rs10899911
rs55877868
rs78383039
rs8039785
rs35055552
rs2387977
rs2090816
rs6809952
All − MR Egger
All − IVW(MRE)
−1.0
−0.5
0.0
0.5
1.0
MR effect size for
'Gut microbiota abundance (genus Parasutterella id.2892)' on 'Dihydropyrimidinase−related protein 5'

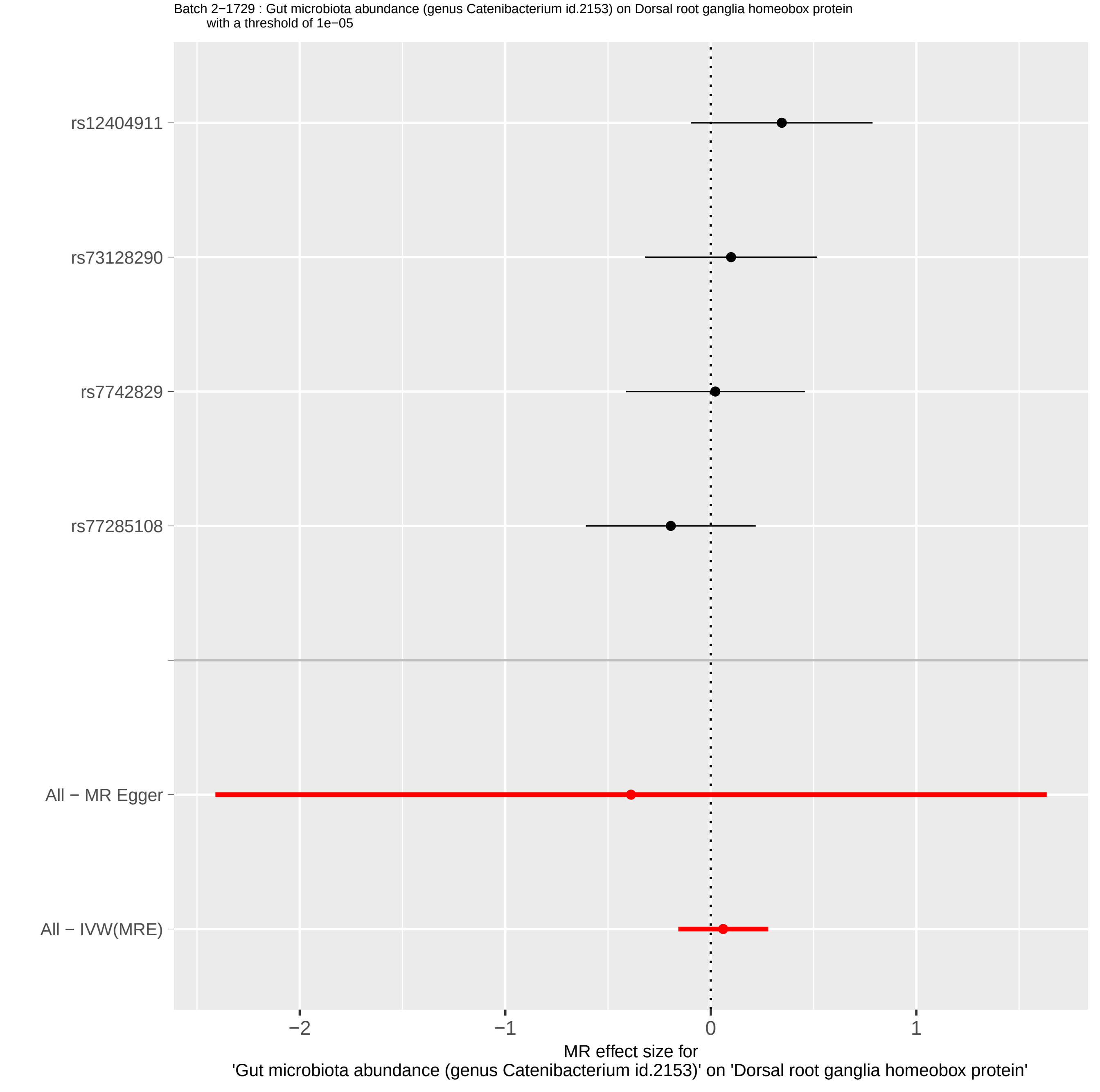

Batch 2−1729 : Gut microbiota abundance (genus Catenibacterium id.2153) on Dorsal root ganglia homeobox protein
with a threshold of 1e−05
rs12404911
rs73128290
rs7742829
rs77285108
All − MR Egger
All − IVW(MRE)
−2
−1
0
1
MR effect size for
'Gut microbiota abundance (genus Catenibacterium id.2153)' on 'Dorsal root ganglia homeobox protein'

Batch 2−1730 : Gut microbiota abundance (genus Parasutterella id.2892) on Dorsal root ganglia homeobox protein
with a threshold of 1e−05

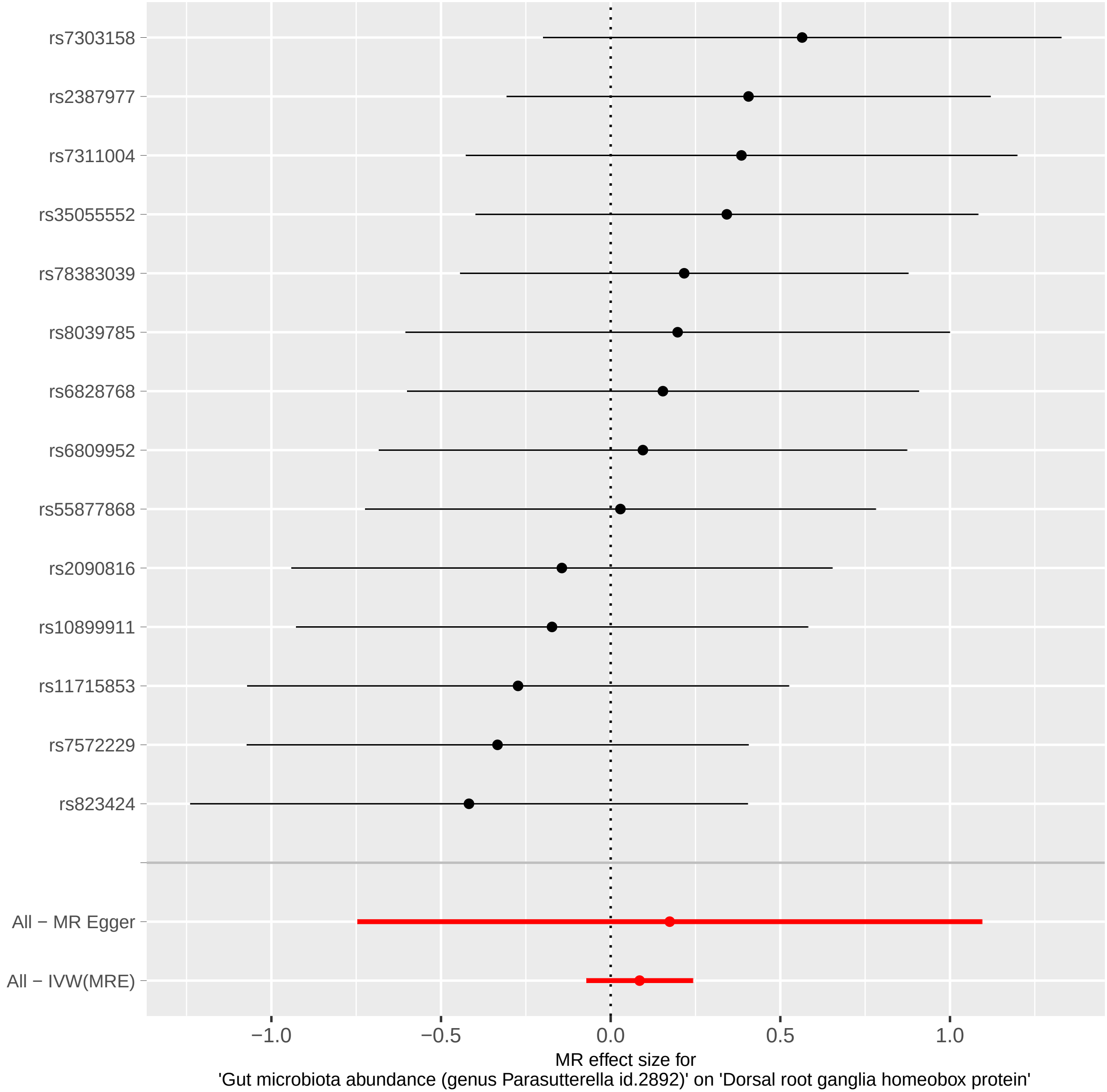

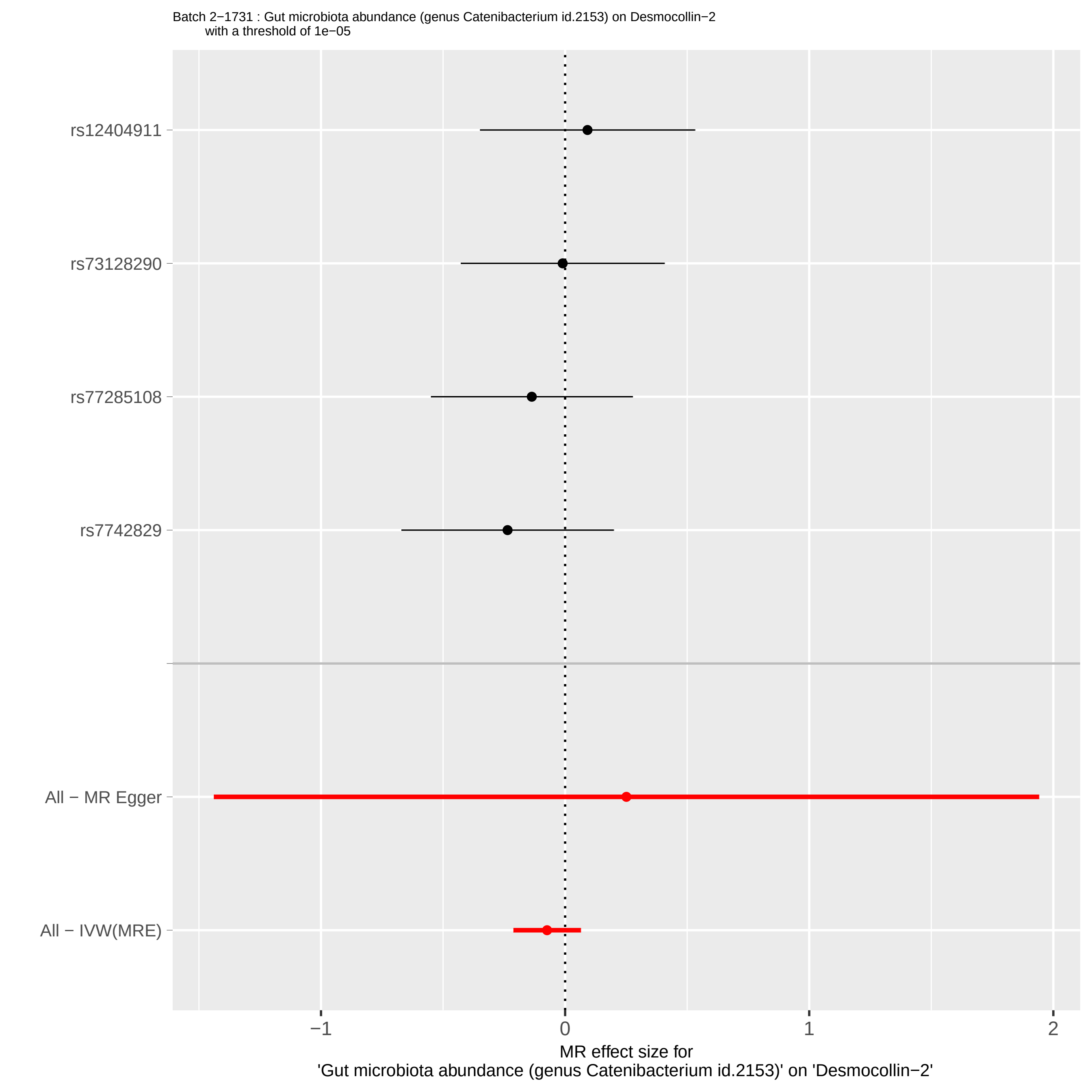
Batch 2−1731 : Gut microbiota abundance (genus Catenibacterium id.2153) on Desmocollin−2
with a threshold of 1e−05
rs12404911
rs73128290
rs77285108
rs7742829
All − MR Egger
All − IVW(MRE)
−1
0
1
2
MR effect size for
'Gut microbiota abundance (genus Catenibacterium id.2153)' on 'Desmocollin−2'

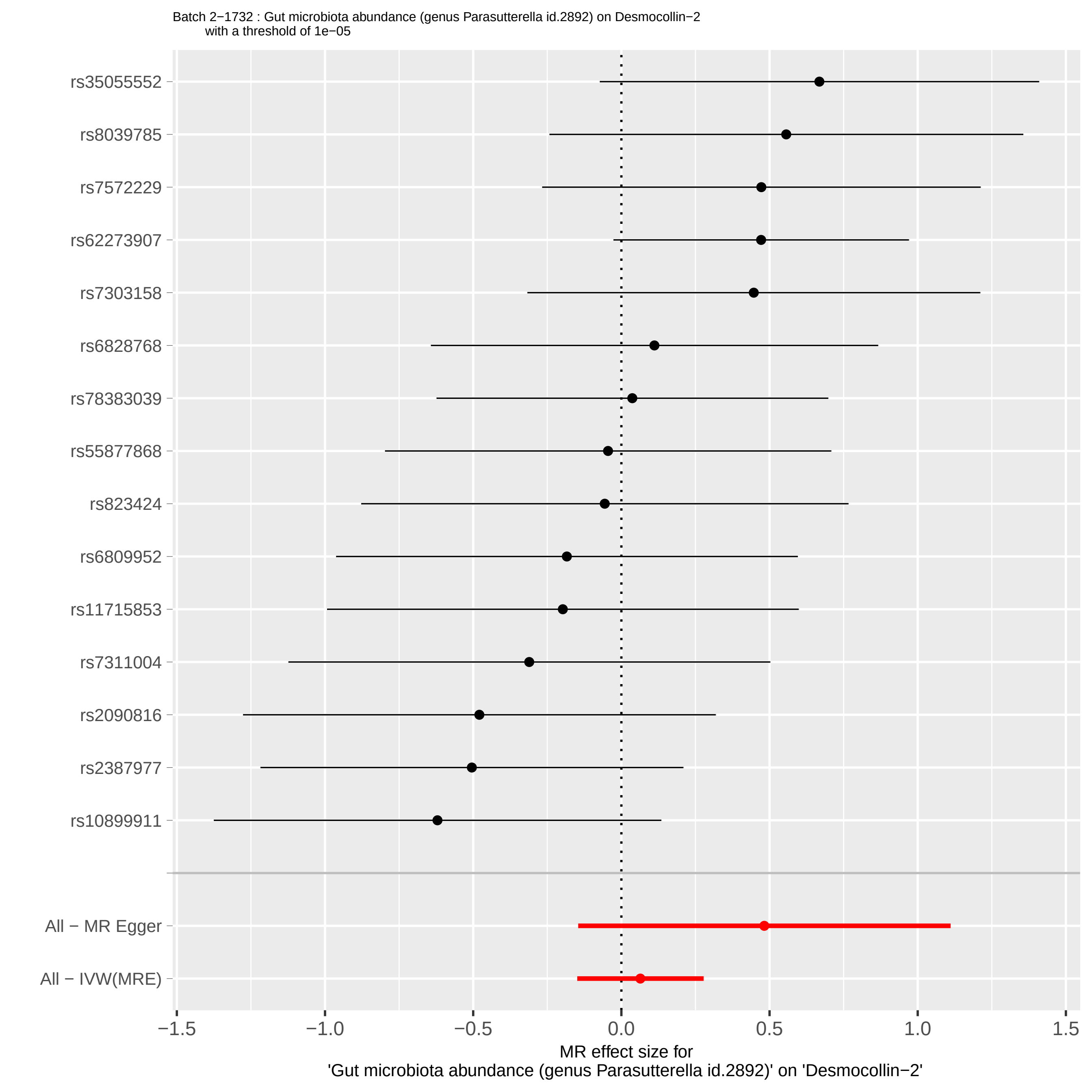

Batch 2−1732 : Gut microbiota abundance (genus Parasutterella id.2892) on Desmocollin−2
with a threshold of 1e−05
rs35055552
rs8039785
rs7572229
rs62273907
rs7303158
rs6828768
rs78383039
rs55877868
rs823424
rs6809952
rs11715853
rs7311004
rs2090816
rs2387977
rs10899911
All − MR Egger
All − IVW(MRE)
−1.5
−1.0
−0.5
0.0
0.5
1.0
1.5
MR effect size for
'Gut microbiota abundance (genus Parasutterella id.2892)' on 'Desmocollin−2'

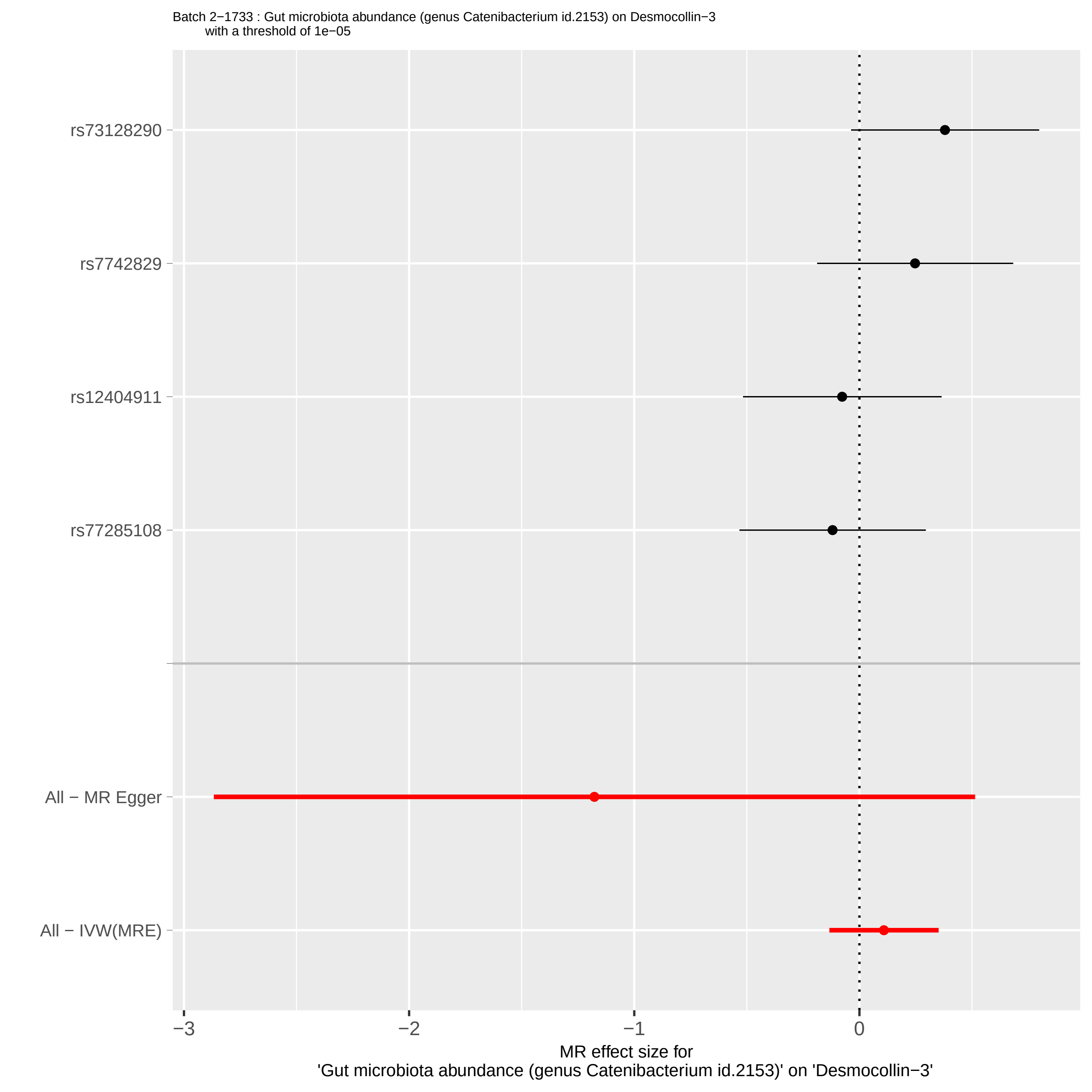
Batch 2−1733 : Gut microbiota abundance (genus Catenibacterium id.2153) on Desmocollin−3
with a threshold of 1e−05
rs73128290
rs7742829
rs12404911
rs77285108
All − MR Egger
All − IVW(MRE)
−3
−2
−1
0
MR effect size for
'Gut microbiota abundance (genus Catenibacterium id.2153)' on 'Desmocollin−3'

Batch 2−1734 : Gut microbiota abundance (genus Parasutterella id.2892) on Desmocollin−3
with a threshold of 1e−05

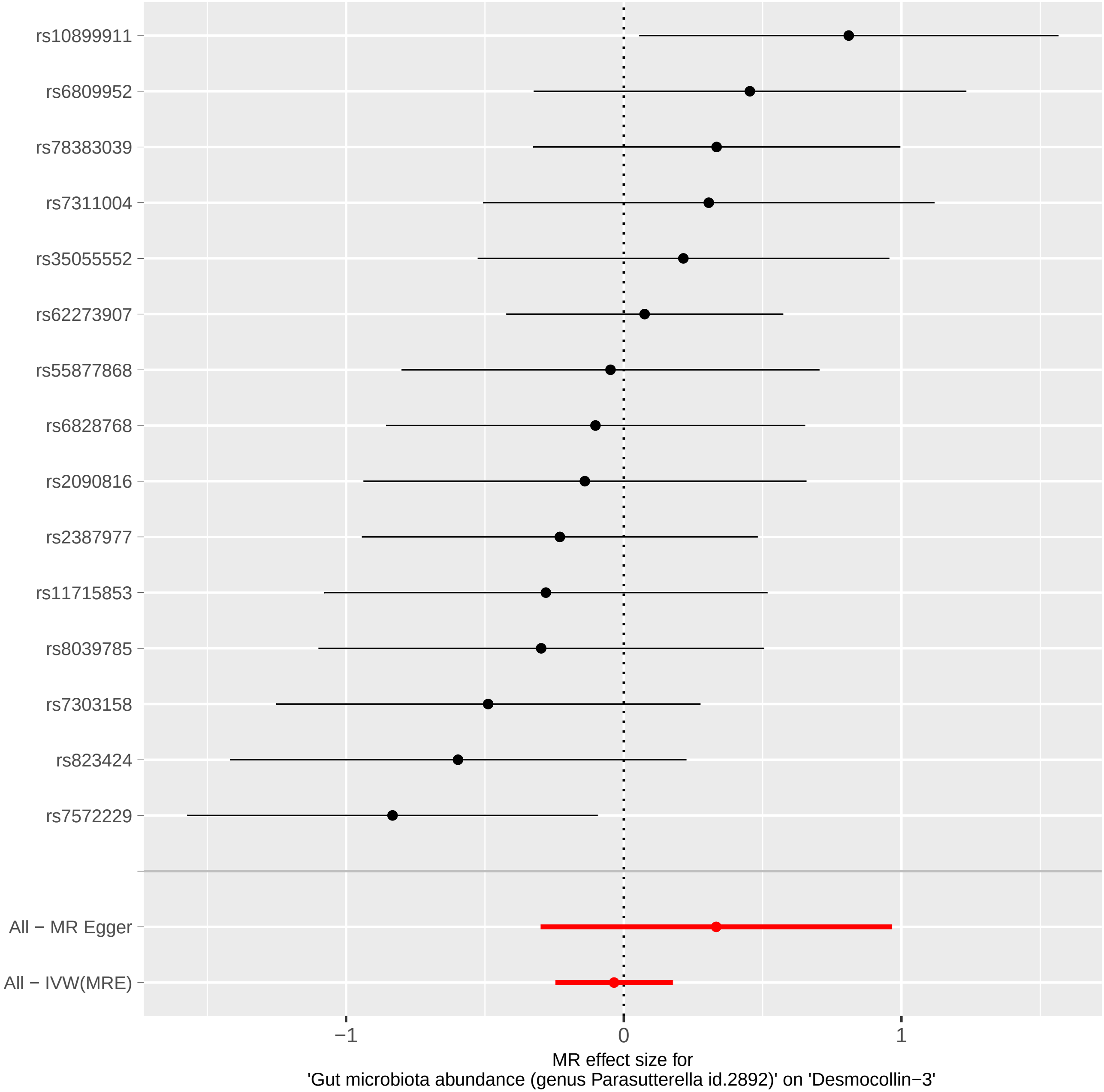

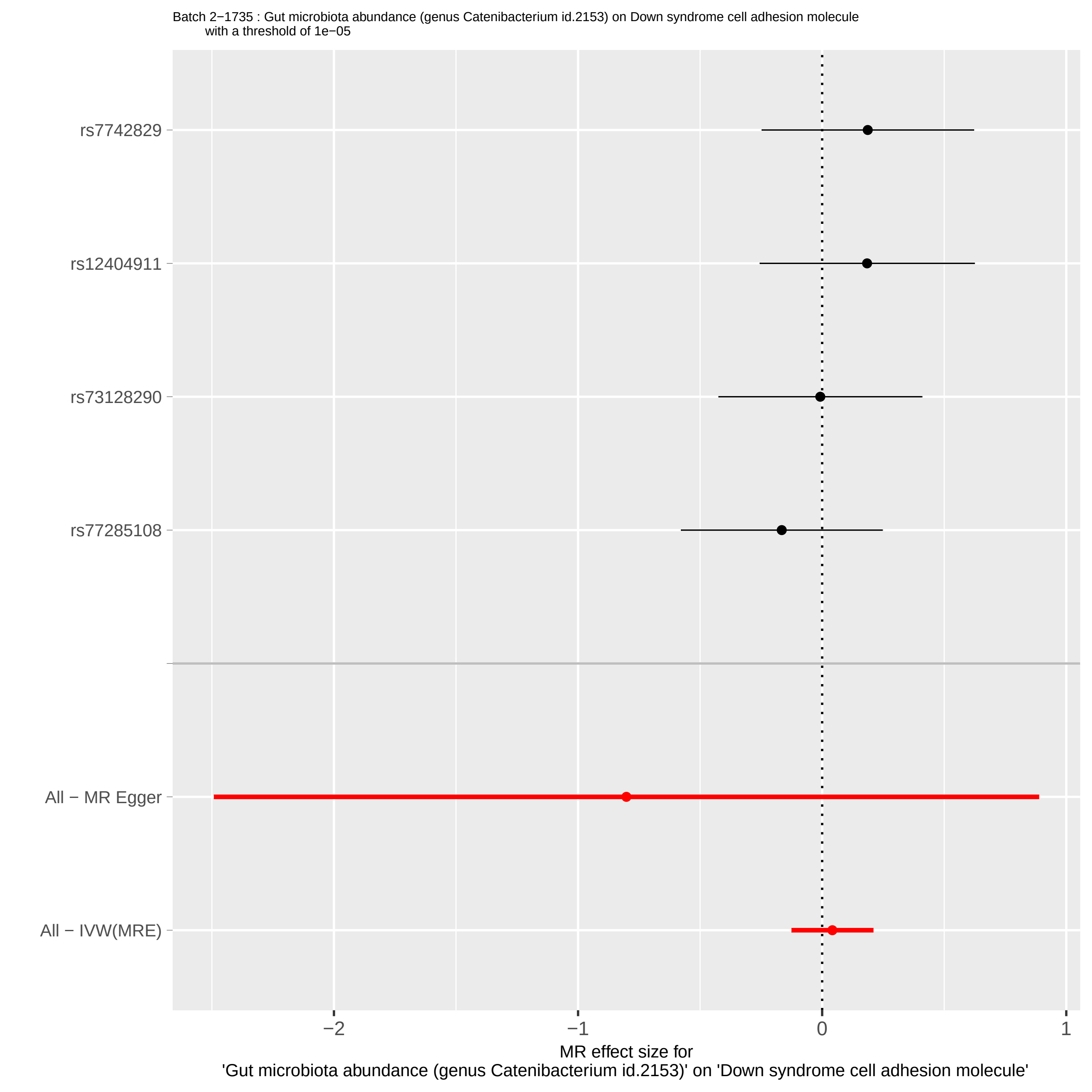

Batch 2−1735 : Gut microbiota abundance (genus Catenibacterium id.2153) on Down syndrome cell adhesion molecule
with a threshold of 1e−05
rs7742829
rs12404911
rs73128290
rs77285108
All − MR Egger
All − IVW(MRE)
−2
−1
0
1
MR effect size for
'Gut microbiota abundance (genus Catenibacterium id.2153)' on 'Down syndrome cell adhesion molecule'

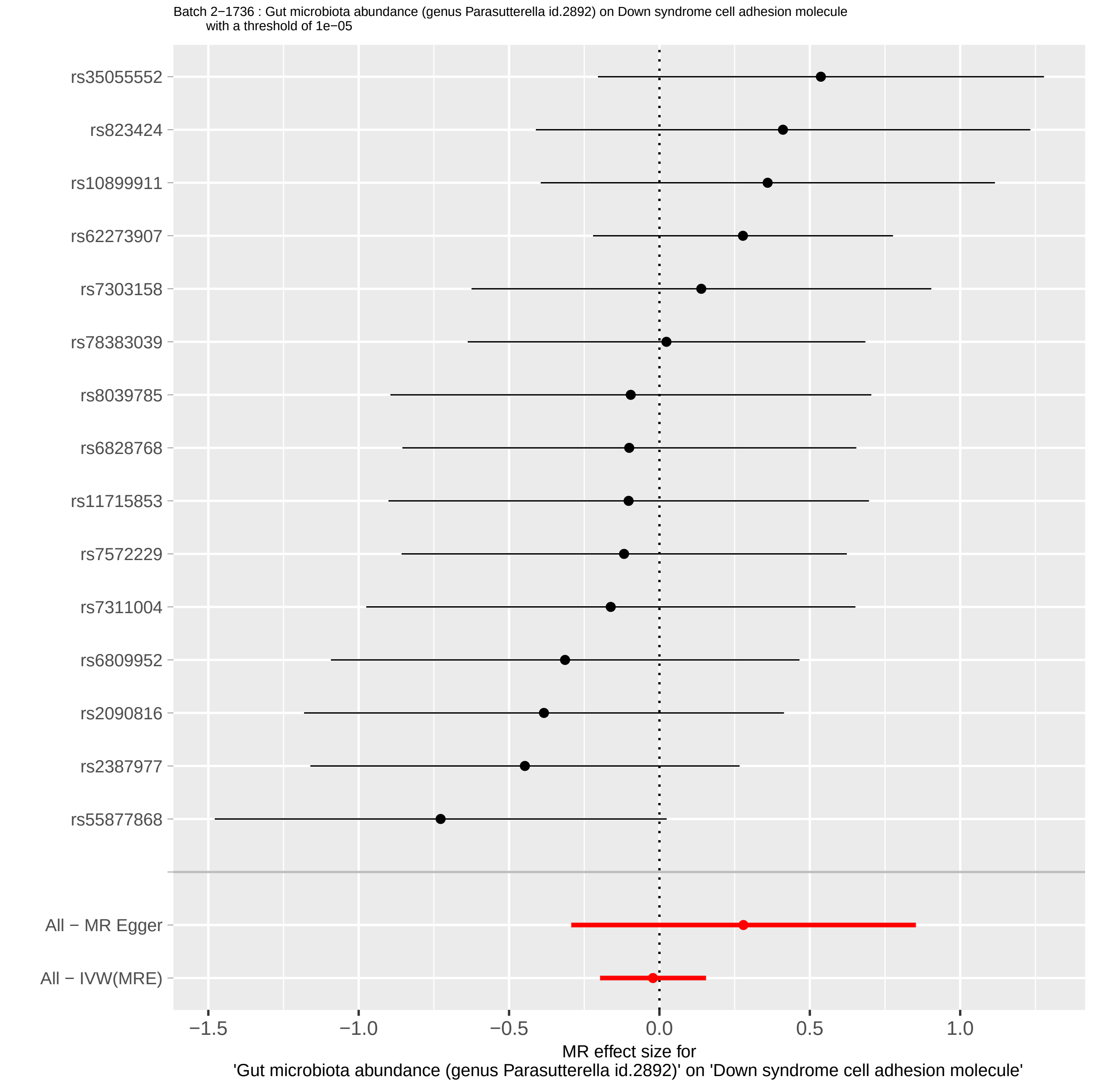

Batch 2−1736 : Gut microbiota abundance (genus Parasutterella id.2892) on Down syndrome cell adhesion molecule
with a threshold of 1e−05
rs35055552
rs823424
rs10899911
rs62273907
rs7303158
rs78383039
rs8039785
rs6828768
rs11715853
rs7572229
rs7311004
rs6809952
rs2090816
rs2387977
rs55877868
All − MR Egger
All − IVW(MRE)
−1.5
−1.0
−0.5
0.0
0.5
1.0
MR effect size for
'Gut microbiota abundance (genus Parasutterella id.2892)' on 'Down syndrome cell adhesion molecule'

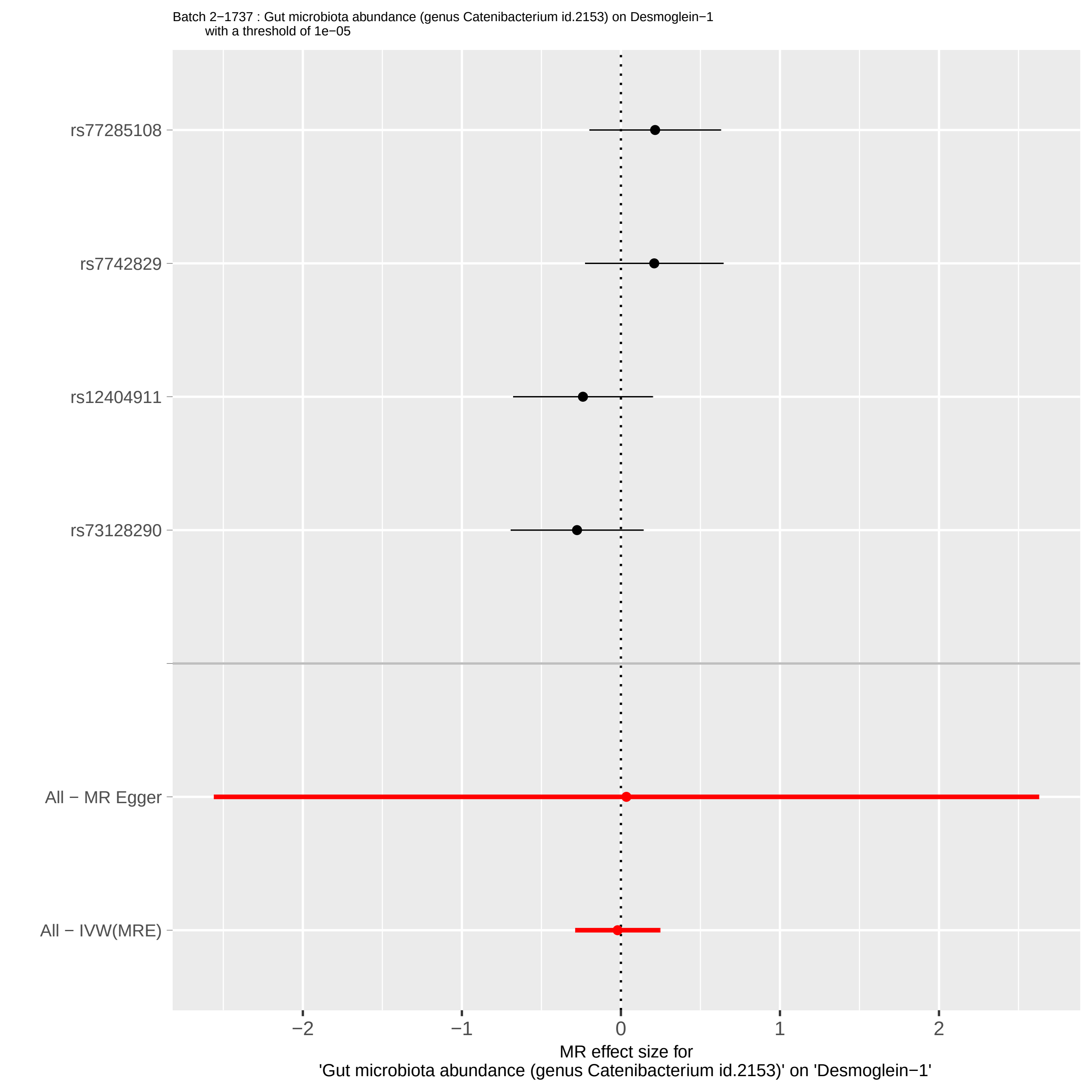

Batch 2−1737 : Gut microbiota abundance (genus Catenibacterium id.2153) on Desmoglein−1
with a threshold of 1e−05
rs77285108
rs7742829
rs12404911
rs73128290
All − MR Egger
All − IVW(MRE)
−2
−1
0
1
2
MR effect size for
'Gut microbiota abundance (genus Catenibacterium id.2153)' on 'Desmoglein−1'

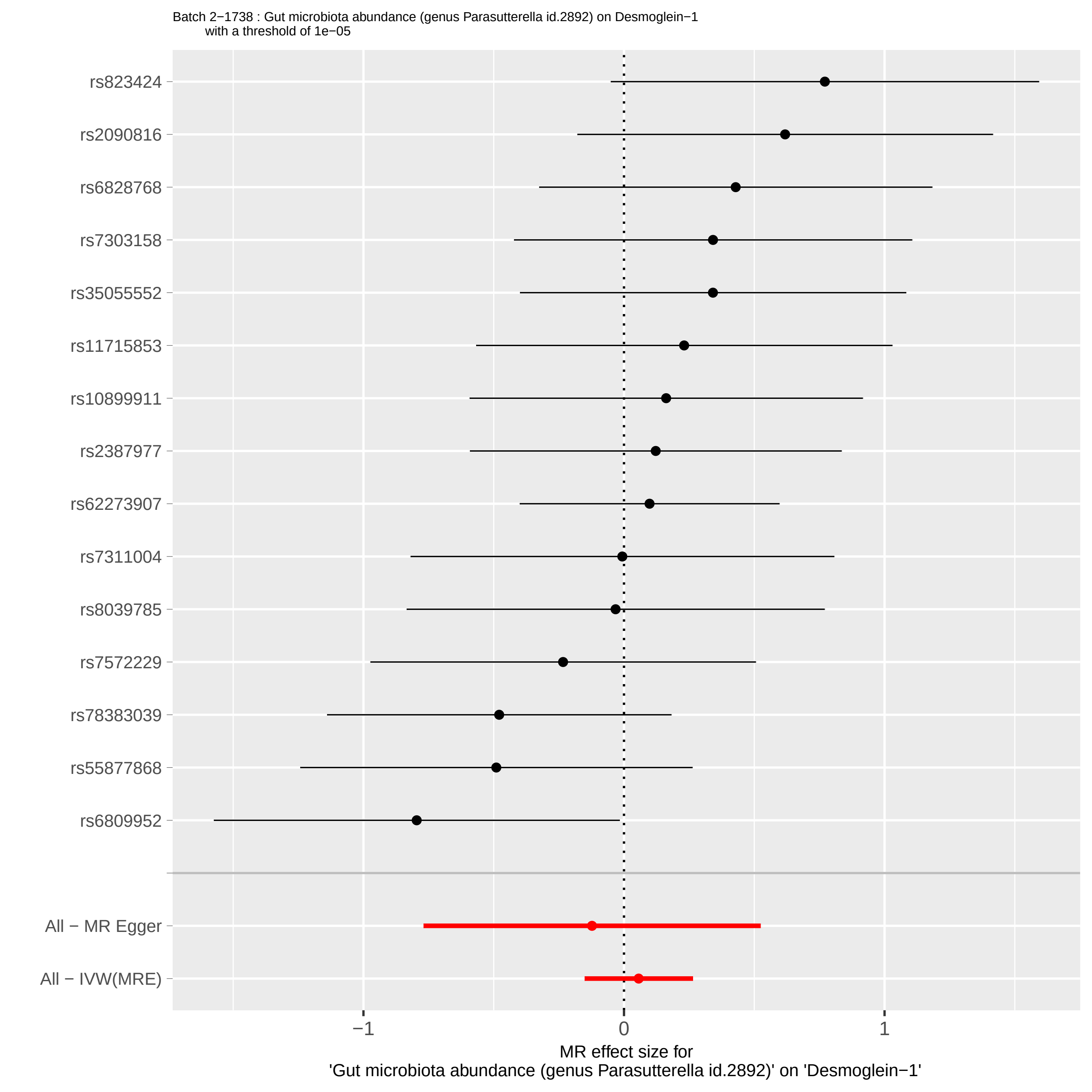

Batch 2−1738 : Gut microbiota abundance (genus Parasutterella id.2892) on Desmoglein−1
with a threshold of 1e−05
rs823424
rs2090816
rs6828768
rs7303158
rs35055552
rs11715853
rs10899911
rs2387977
rs62273907
rs7311004
rs8039785
rs7572229
rs78383039
rs55877868
rs6809952
All − MR Egger
All − IVW(MRE)
−1
0
1
MR effect size for
'Gut microbiota abundance (genus Parasutterella id.2892)' on 'Desmoglein−1'

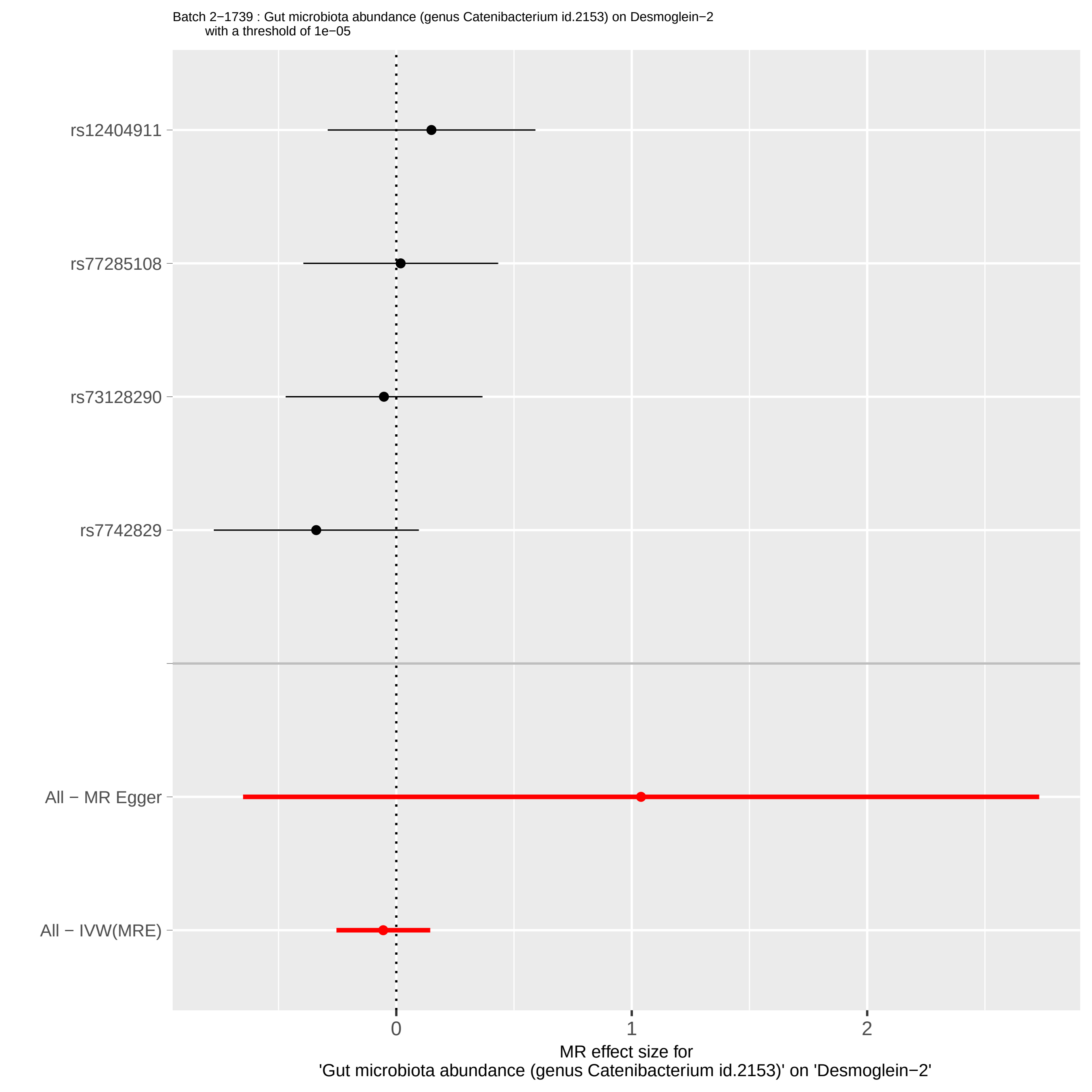

Batch 2−1739 : Gut microbiota abundance (genus Catenibacterium id.2153) on Desmoglein−2
with a threshold of 1e−05
rs12404911
rs77285108
rs73128290
rs7742829
All − MR Egger
All − IVW(MRE)
0
1
2
MR effect size for
'Gut microbiota abundance (genus Catenibacterium id.2153)' on 'Desmoglein−2'

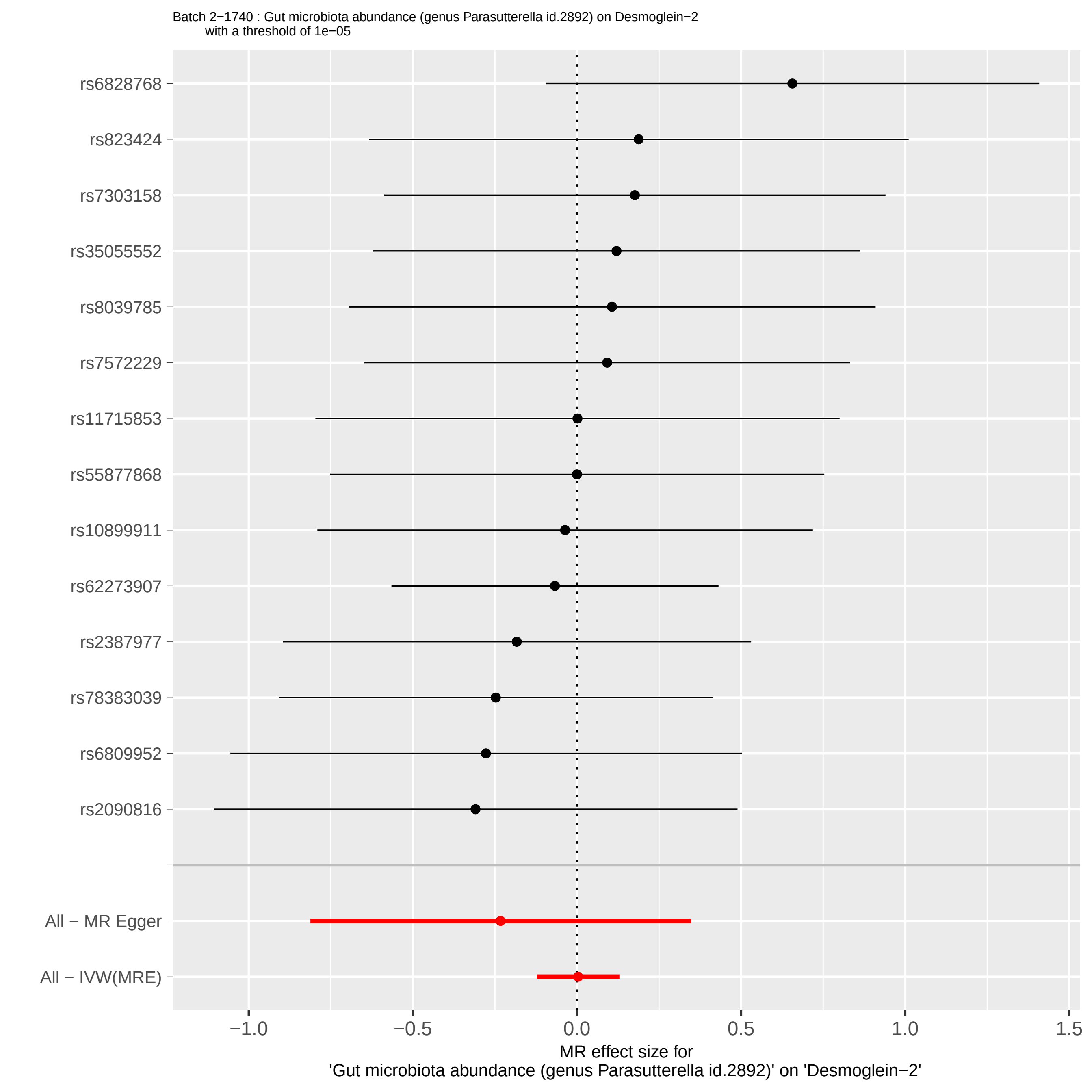

Batch 2−1740 : Gut microbiota abundance (genus Parasutterella id.2892) on Desmoglein−2
with a threshold of 1e−05
rs6828768
rs823424
rs7303158
rs35055552
rs8039785
rs7572229
rs11715853
rs55877868
rs10899911
rs62273907
rs2387977
rs78383039
rs6809952
rs2090816
All − MR Egger
All − IVW(MRE)
−1.0
−0.5
0.0
0.5
1.0
1.5
MR effect size for
'Gut microbiota abundance (genus Parasutterella id.2892)' on 'Desmoglein−2'

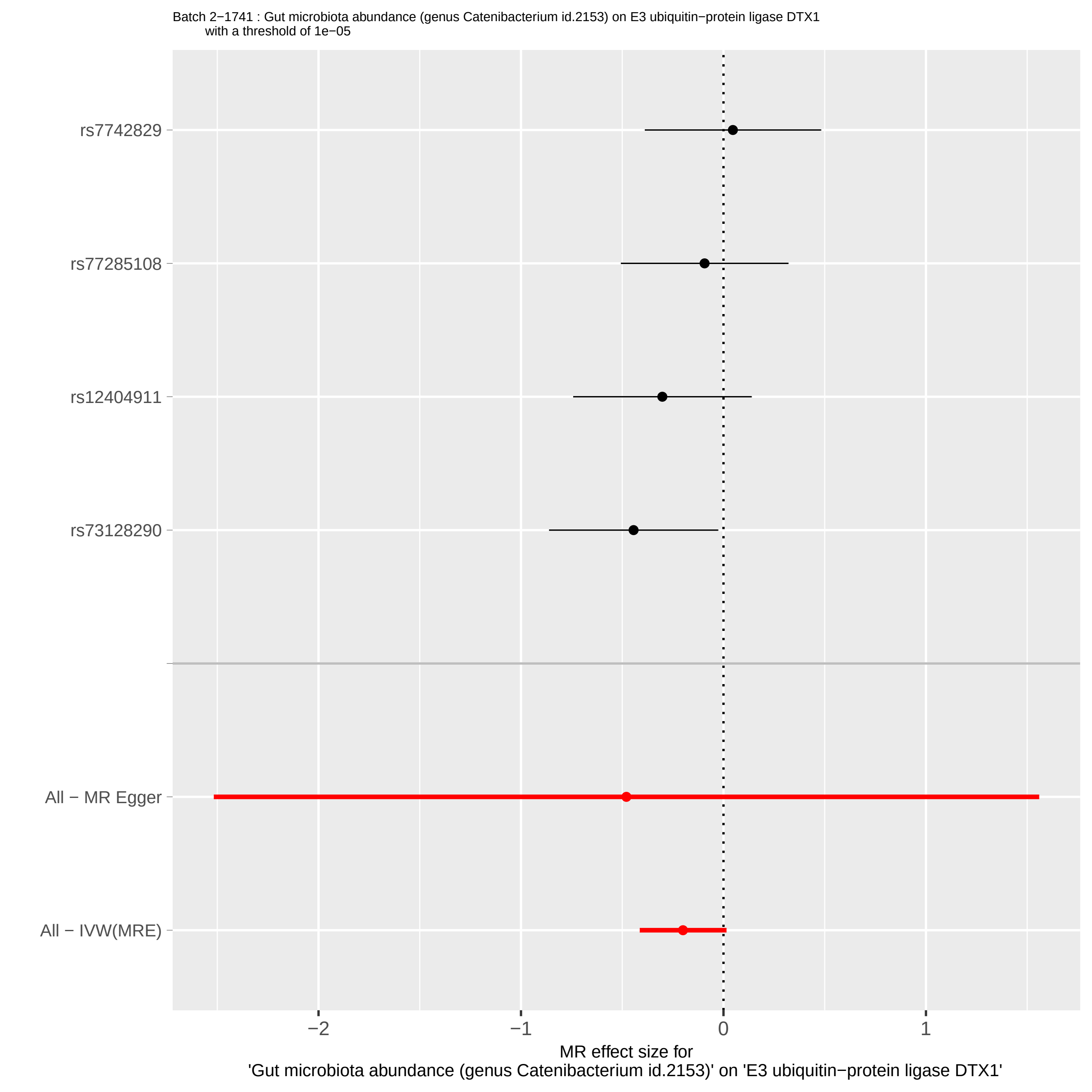
Batch 2−1741 : Gut microbiota abundance (genus Catenibacterium id.2153) on E3 ubiquitin−protein ligase DTX1
with a threshold of 1e−05
rs7742829
rs77285108
rs12404911
rs73128290
All − MR Egger
All − IVW(MRE)
−2
−1
0
1
MR effect size for
'Gut microbiota abundance (genus Catenibacterium id.2153)' on 'E3 ubiquitin−protein ligase DTX1'

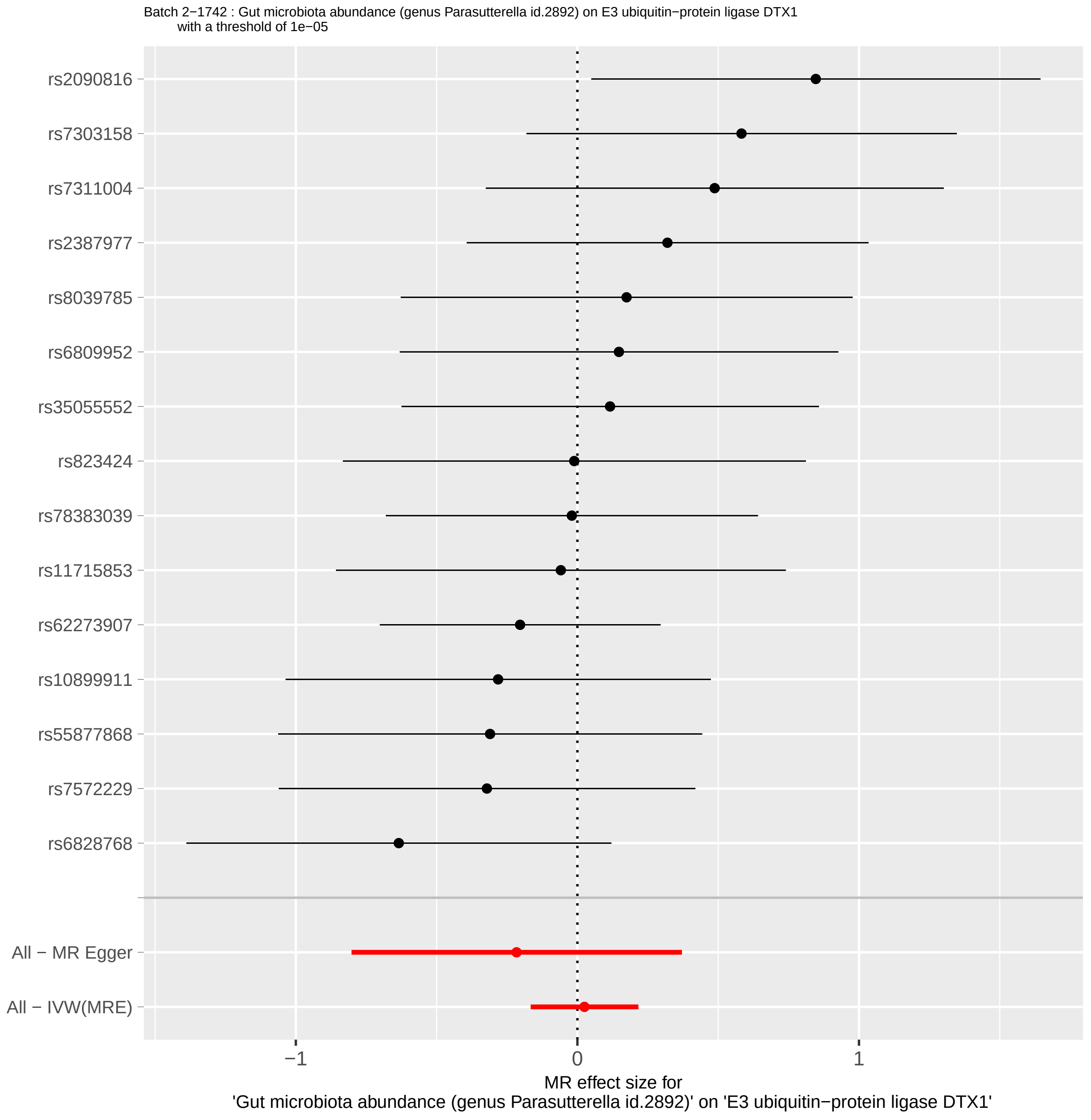
Batch 2−1742 : Gut microbiota abundance (genus Parasutterella id.2892) on E3 ubiquitin−protein ligase DTX1
with a threshold of 1e−05
rs2090816
rs7303158
rs7311004
rs2387977
rs8039785
rs6809952
rs35055552
rs823424
rs78383039
rs11715853
rs62273907
rs10899911
rs55877868
rs7572229
rs6828768
All − MR Egger
All − IVW(MRE)
−1
0
1
MR effect size for
'Gut microbiota abundance (genus Parasutterella id.2892)' on 'E3 ubiquitin−protein ligase DTX1'

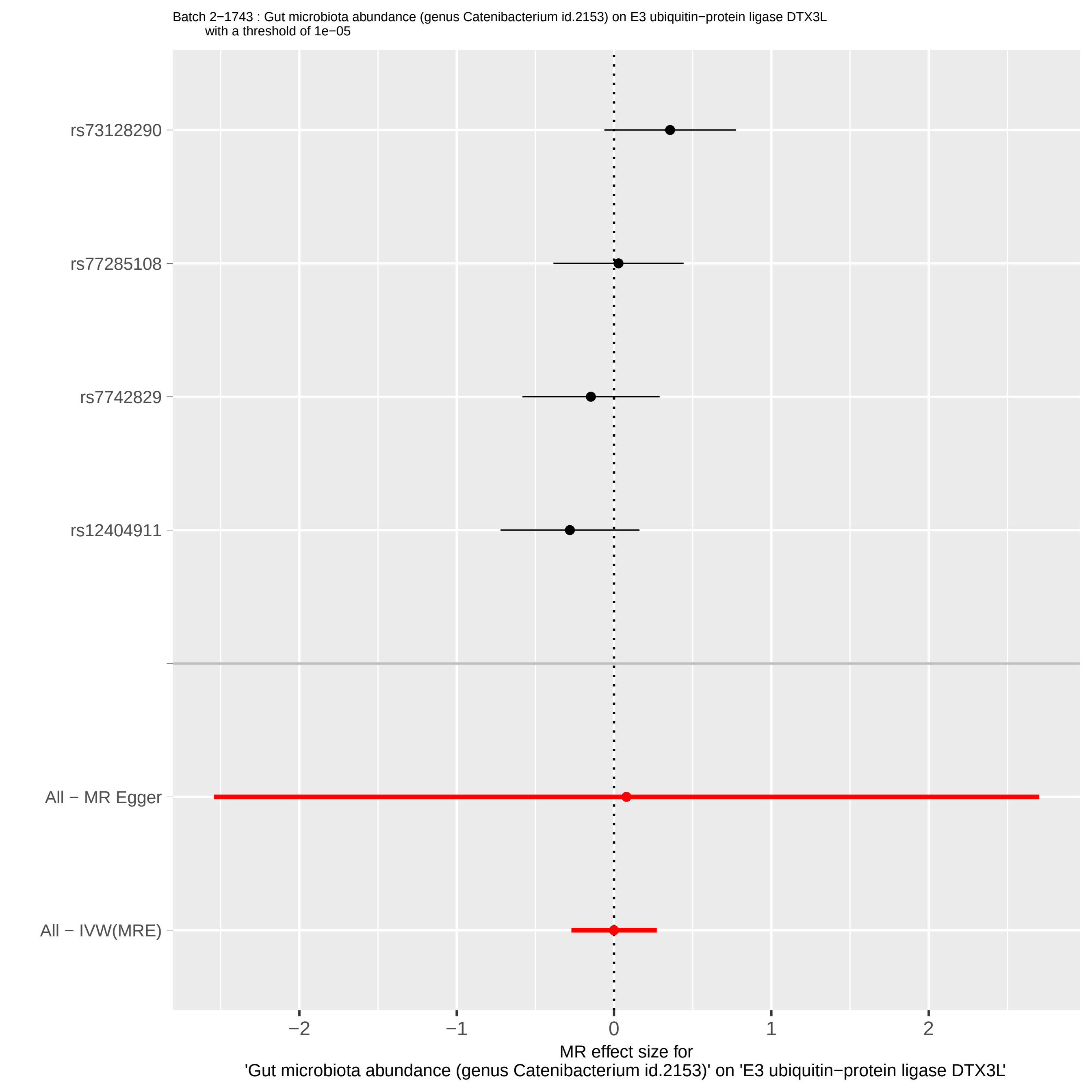
Batch 2−1743 : Gut microbiota abundance (genus Catenibacterium id.2153) on E3 ubiquitin−protein ligase DTX3L
with a threshold of 1e−05
rs73128290
rs77285108
rs7742829
rs12404911
All − MR Egger
All − IVW(MRE)
−2
−1
0
1
2
MR effect size for
'Gut microbiota abundance (genus Catenibacterium id.2153)' on 'E3 ubiquitin−protein ligase DTX3L'

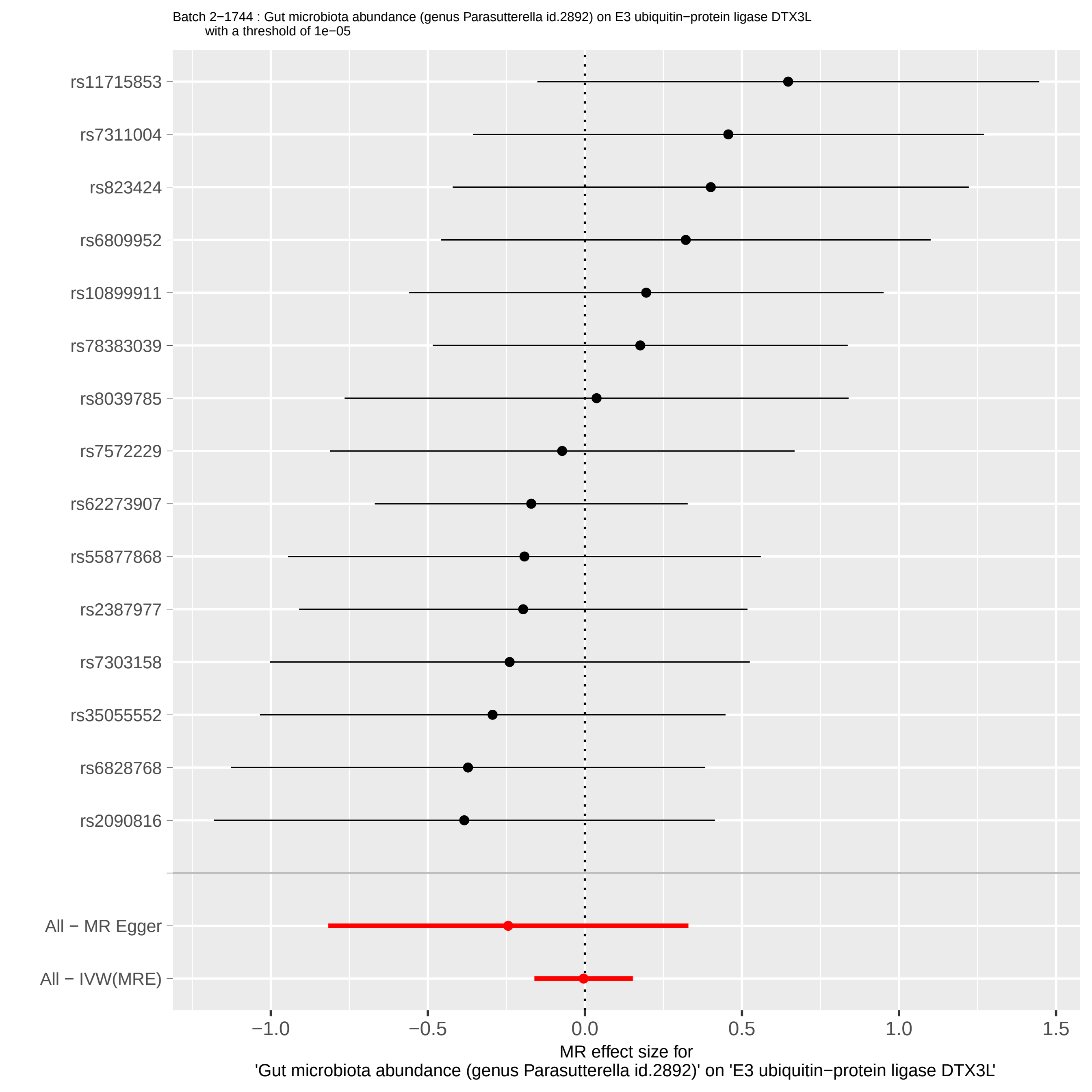
Batch 2−1744 : Gut microbiota abundance (genus Parasutterella id.2892) on E3 ubiquitin−protein ligase DTX3L
with a threshold of 1e−05
rs11715853
rs7311004
rs823424
rs6809952
rs10899911
rs78383039
rs8039785
rs7572229
rs62273907
rs55877868
rs2387977
rs7303158
rs35055552
rs6828768
rs2090816
All − MR Egger
All − IVW(MRE)
−1.0
−0.5
0.0
0.5
1.0
1.5
MR effect size for
'Gut microbiota abundance (genus Parasutterella id.2892)' on 'E3 ubiquitin−protein ligase DTX3L'

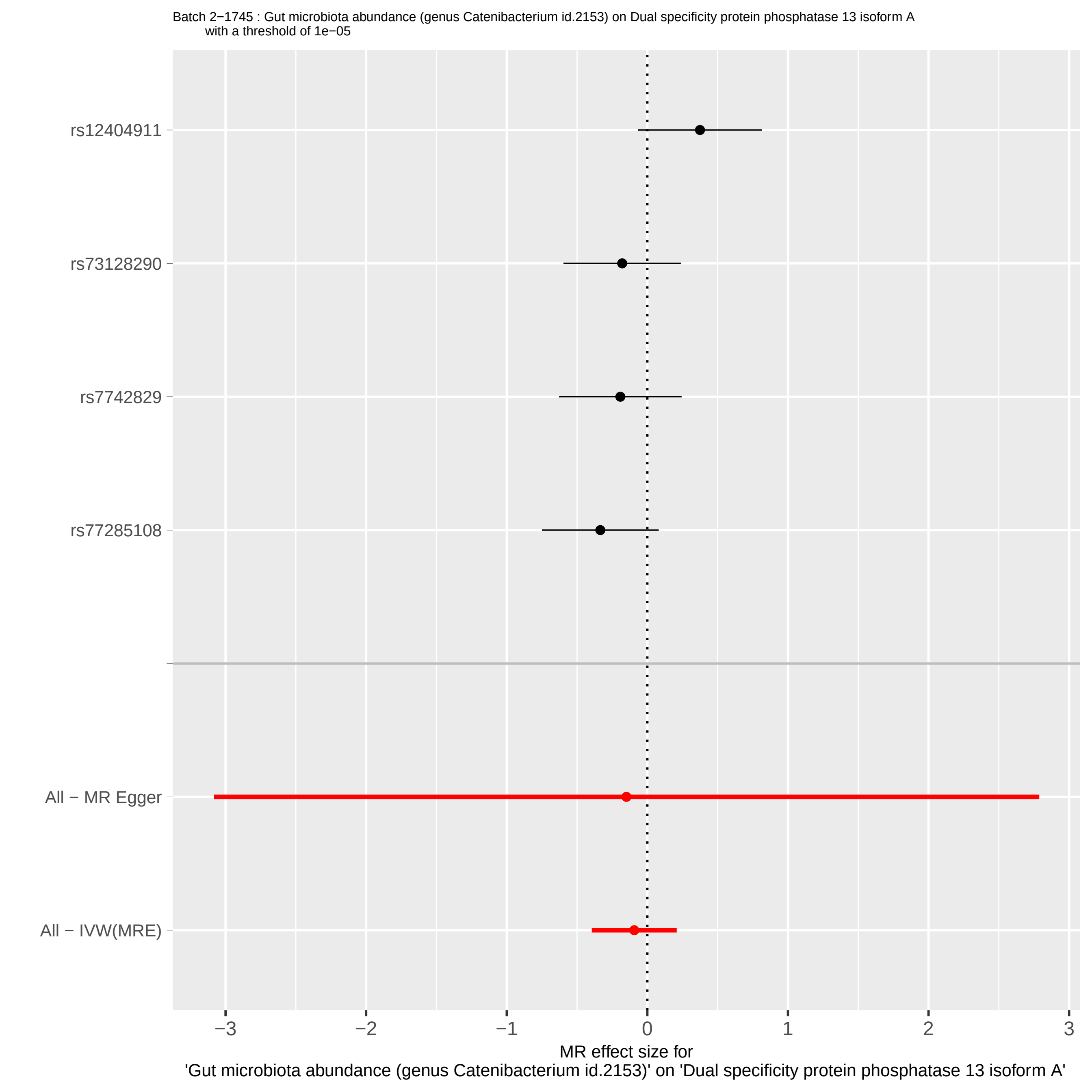
Batch 2−1745 : Gut microbiota abundance (genus Catenibacterium id.2153) on Dual specificity protein phosphatase 13 isoform A with a threshold of 1e−05
rs12404911
rs73128290
rs7742829
rs77285108
All − MR Egger
All − IVW(MRE)
−3
−2
−1
0
1
2
3
MR effect size for
'Gut microbiota abundance (genus Catenibacterium id.2153)' on 'Dual specificity protein phosphatase 13 isoform A'

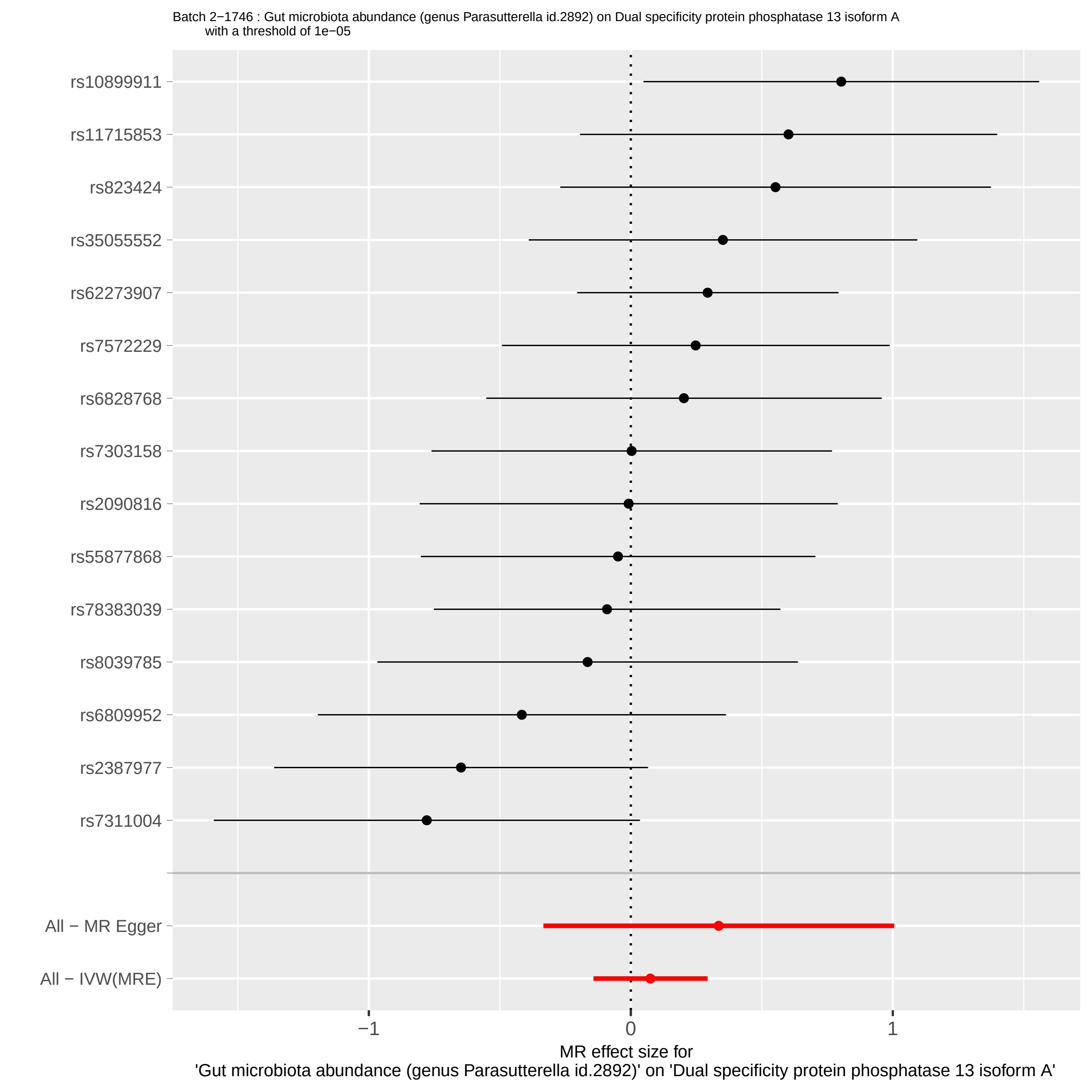
Batch 2−1746 : Gut microbiota abundance (genus Parasutterella id.2892) on Dual specificity protein phosphatase 13 isoform A
with a threshold of 1e−05
rs10899911
rs11715853
rs823424
rs35055552
rs62273907
rs7572229
rs6828768
rs7303158
rs2090816
rs55877868
rs78383039
rs8039785
rs6809952
rs2387977
rs7311004
All − MR Egger
All − IVW(MRE)
−1
0
1
MR effect size for
'Gut microbiota abundance (genus Parasutterella id.2892)' on 'Dual specificity protein phosphatase 13 isoform A'

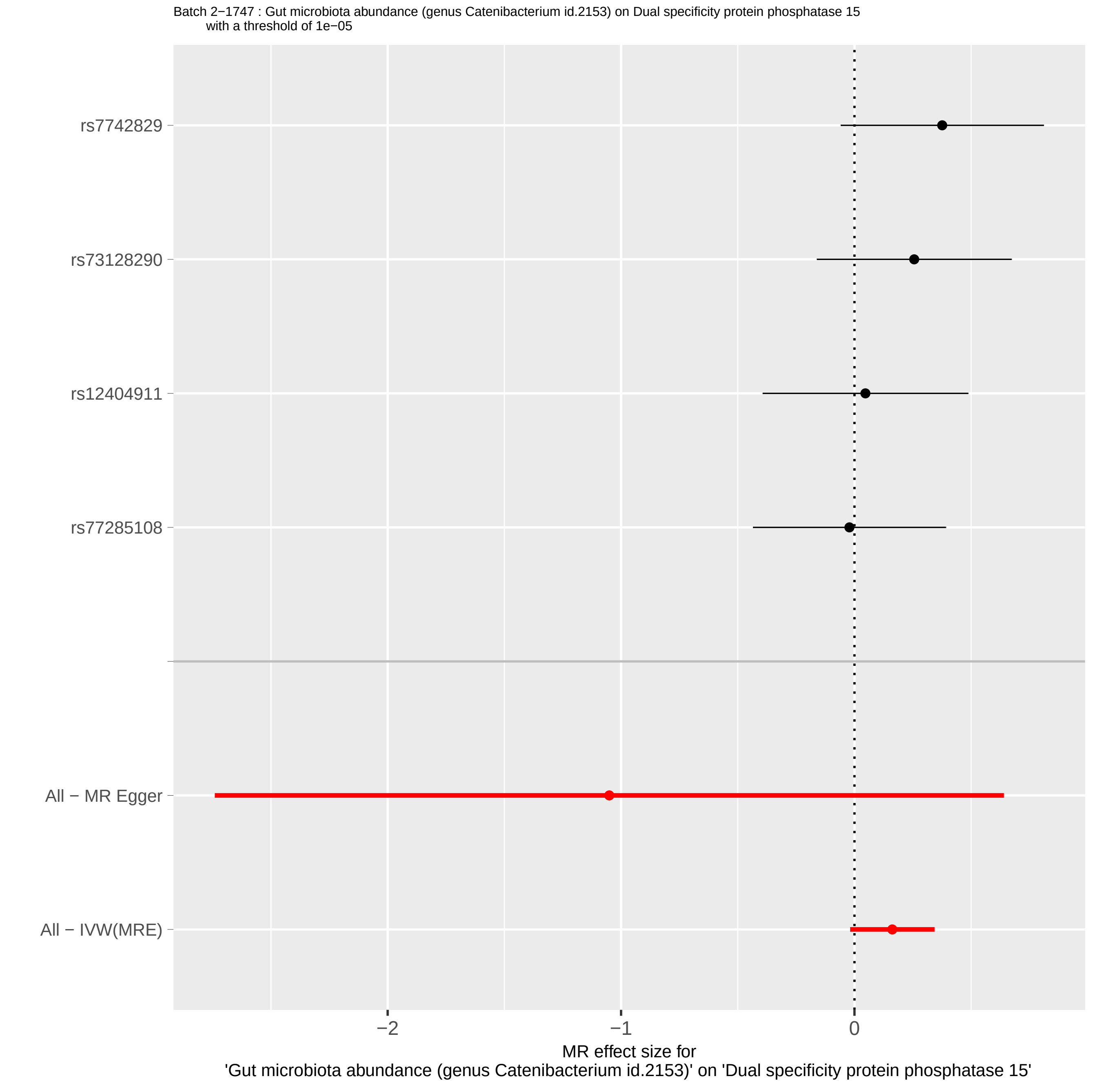

Batch 2−1747 : Gut microbiota abundance (genus Catenibacterium id.2153) on Dual specificity protein phosphatase 15
with a threshold of 1e−05
rs7742829
rs73128290
rs12404911
rs77285108
All − MR Egger
All − IVW(MRE)
−2
−1
0
MR effect size for
'Gut microbiota abundance (genus Catenibacterium id.2153)' on 'Dual specificity protein phosphatase 15'

Batch 2−1748 : Gut microbiota abundance (genus Parasutterella id.2892) on Dual specificity protein phosphatase 15
with a threshold of 1e−05

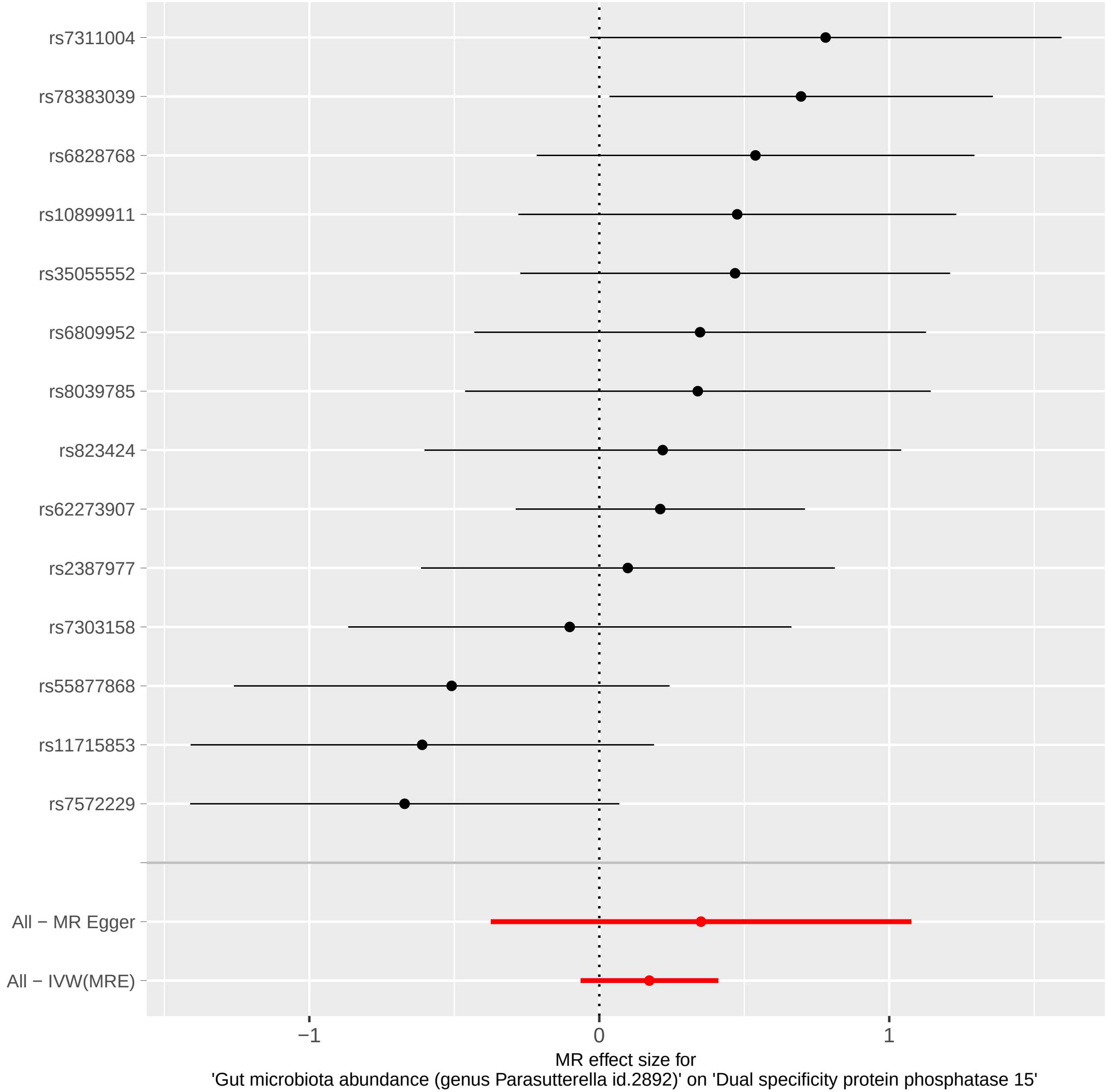

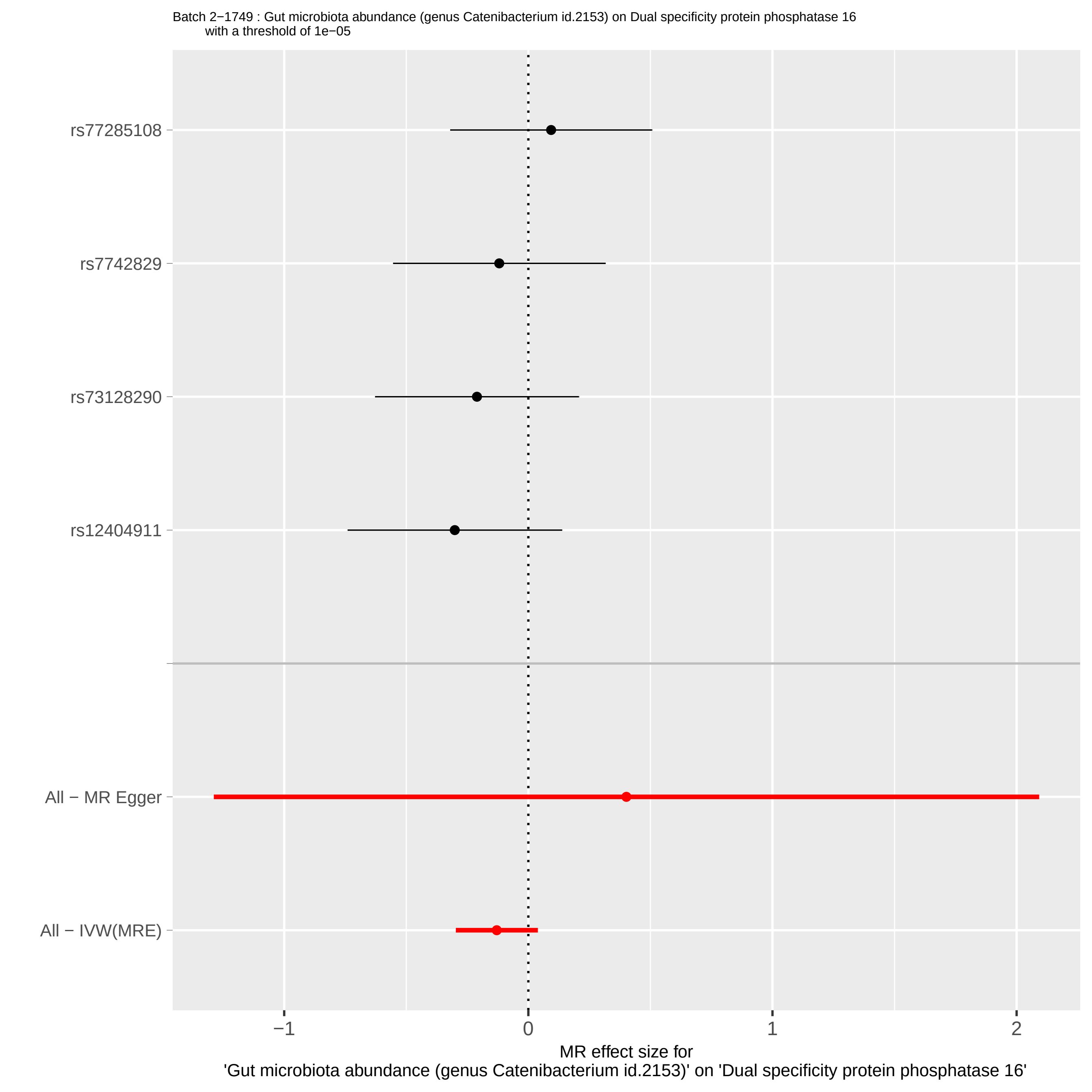
Batch 2−1749 : Gut microbiota abundance (genus Catenibacterium id.2153) on Dual specificity protein phosphatase 16
with a threshold of 1e−05
rs77285108
rs7742829
rs73128290
rs12404911
All − MR Egger
All − IVW(MRE)
−1
0
1
2
MR effect size for
'Gut microbiota abundance (genus Catenibacterium id.2153)' on 'Dual specificity protein phosphatase 16'

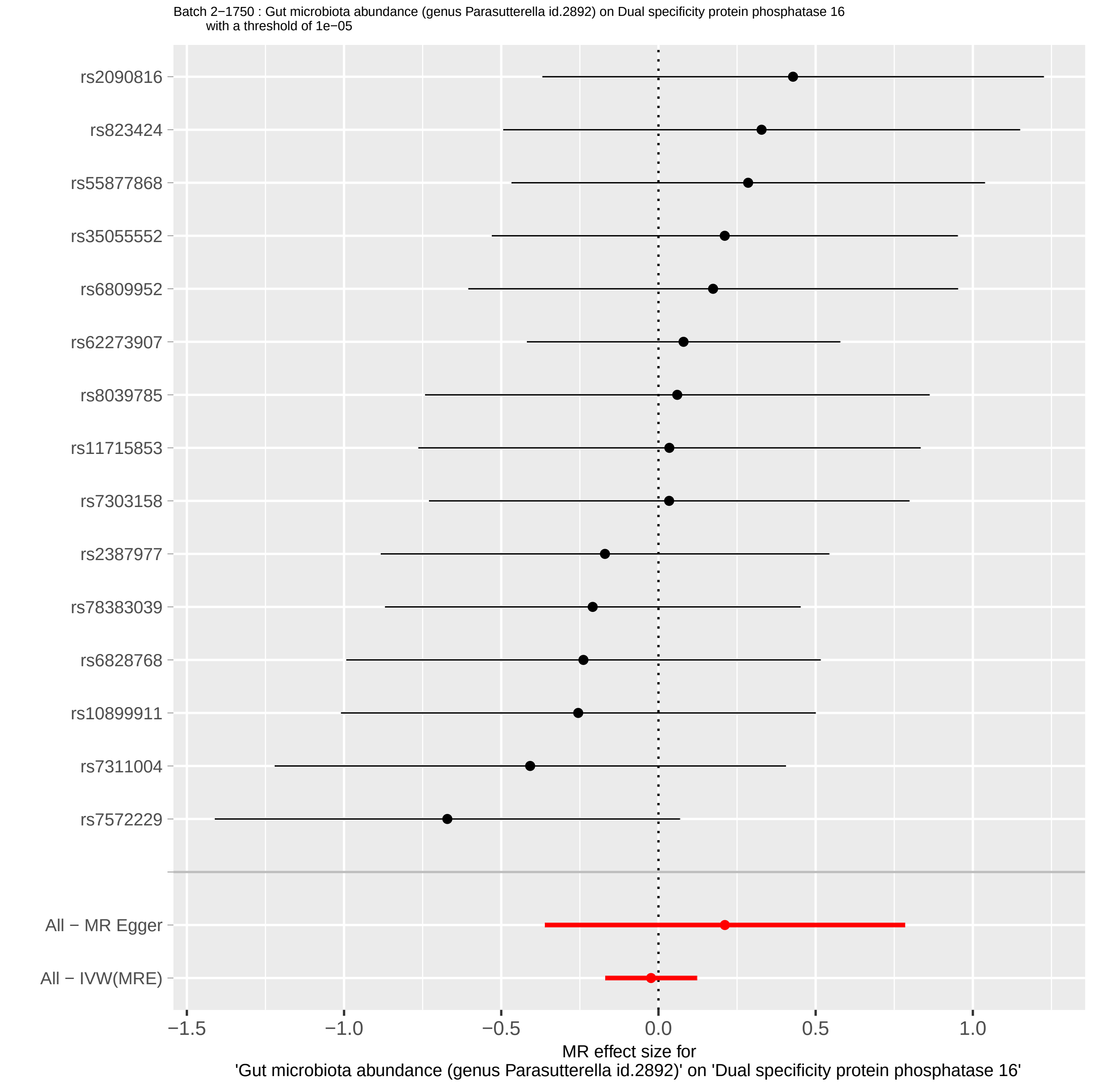

Batch 2−1750 : Gut microbiota abundance (genus Parasutterella id.2892) on Dual specificity protein phosphatase 16
with a threshold of 1e−05
rs2090816
rs823424
rs55877868
rs35055552
rs6809952
rs62273907
rs8039785
rs11715853
rs7303158
rs2387977
rs78383039
rs6828768
rs10899911
rs7311004
rs7572229
All − MR Egger
All − IVW(MRE)
−1.5
−1.0
−0.5
0.0
0.5
1.0
MR effect size for
'Gut microbiota abundance (genus Parasutterella id.2892)' on 'Dual specificity protein phosphatase 16'

Batch 2−1751 : Gut microbiota abundance (genus Catenibacterium id.2153) on Dual specificity protein phosphatase 4
with a threshold of 1e−05

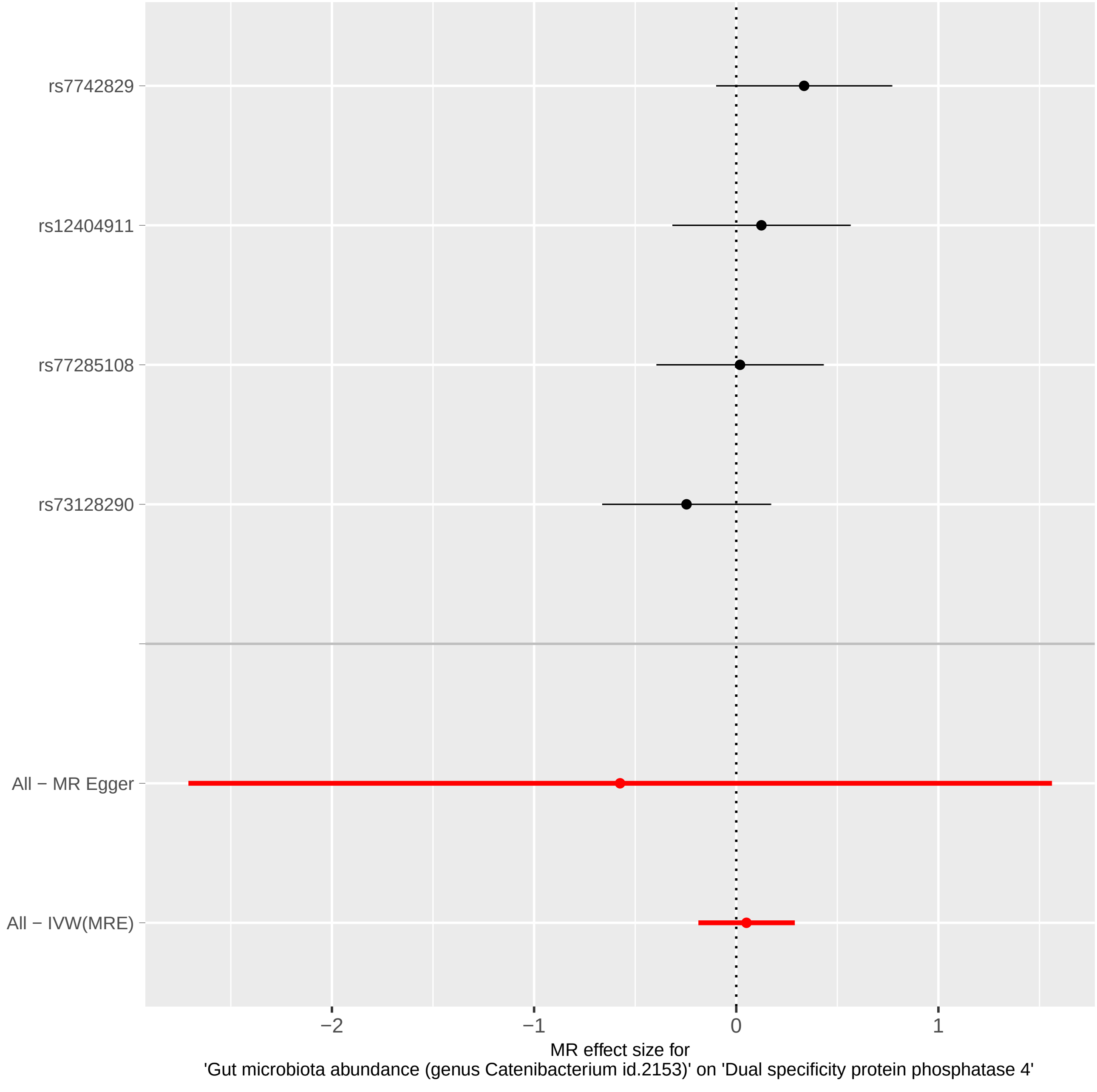

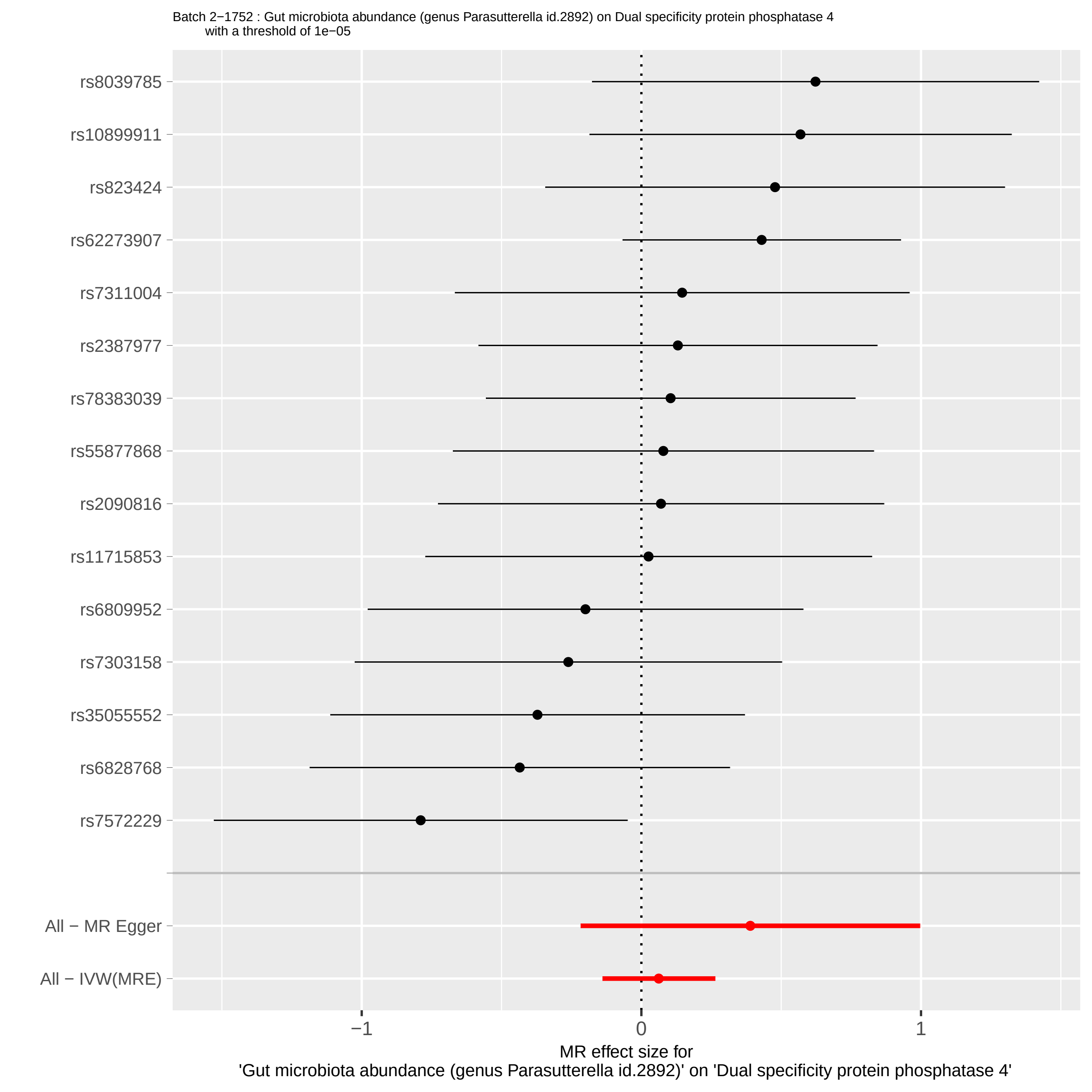

Batch 2−1752 : Gut microbiota abundance (genus Parasutterella id.2892) on Dual specificity protein phosphatase 4
with a threshold of 1e−05
rs8039785
rs10899911
rs823424
rs62273907
rs7311004
rs2387977
rs78383039
rs55877868
rs2090816
rs11715853
rs6809952
rs7303158
rs35055552
rs6828768
rs7572229
All − MR Egger
All − IVW(MRE)
−1
0
1
MR effect size for
'Gut microbiota abundance (genus Parasutterella id.2892)' on 'Dual specificity protein phosphatase 4'

Batch 2−1753 : Gut microbiota abundance (genus Catenibacterium id.2153) on Dual specificity protein phosphatase 6
with a threshold of 1e−05

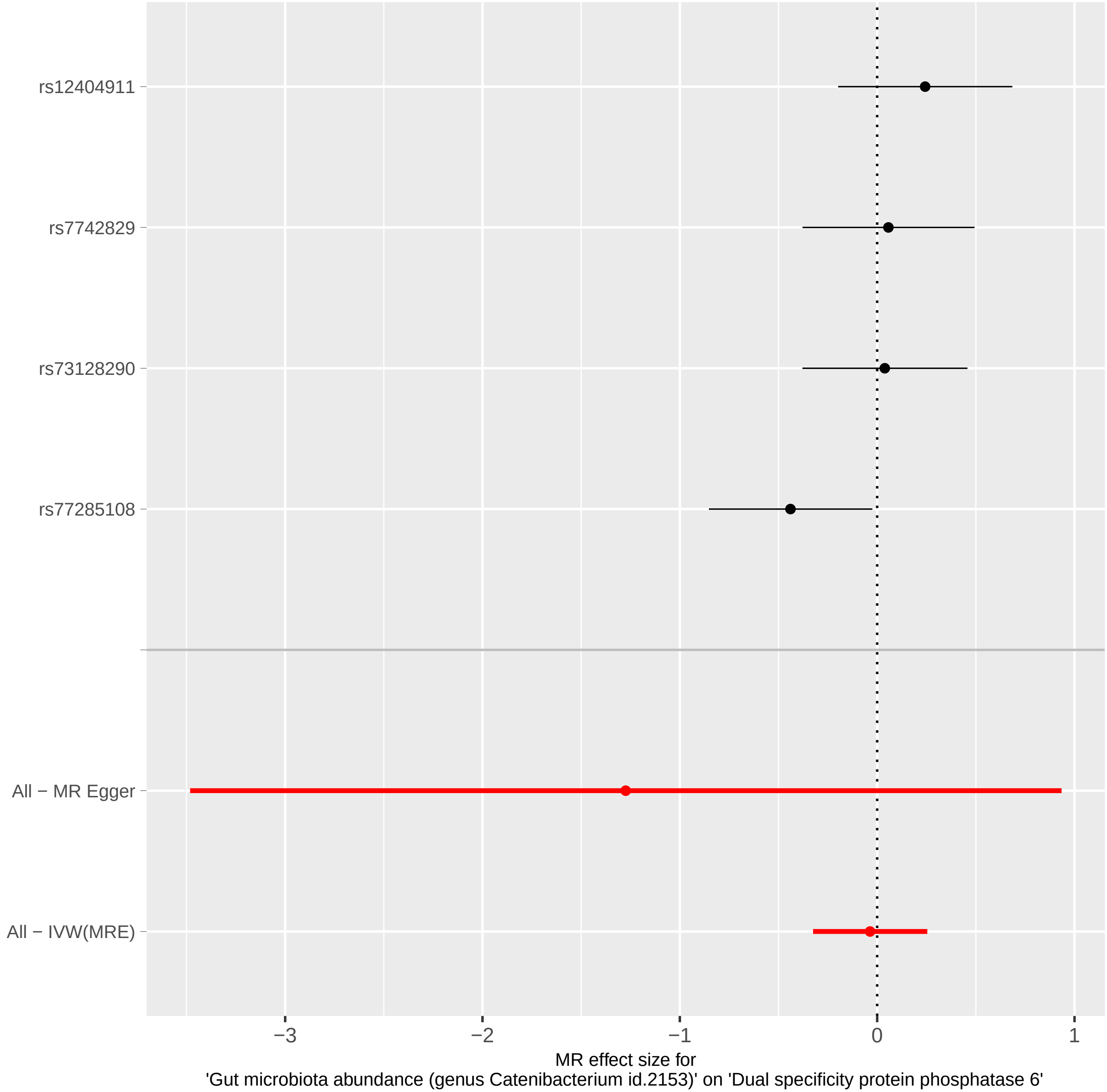

Batch 2−1754 : Gut microbiota abundance (genus Parasutterella id.2892) on Dual specificity protein phosphatase 6
with a threshold of 1e−05

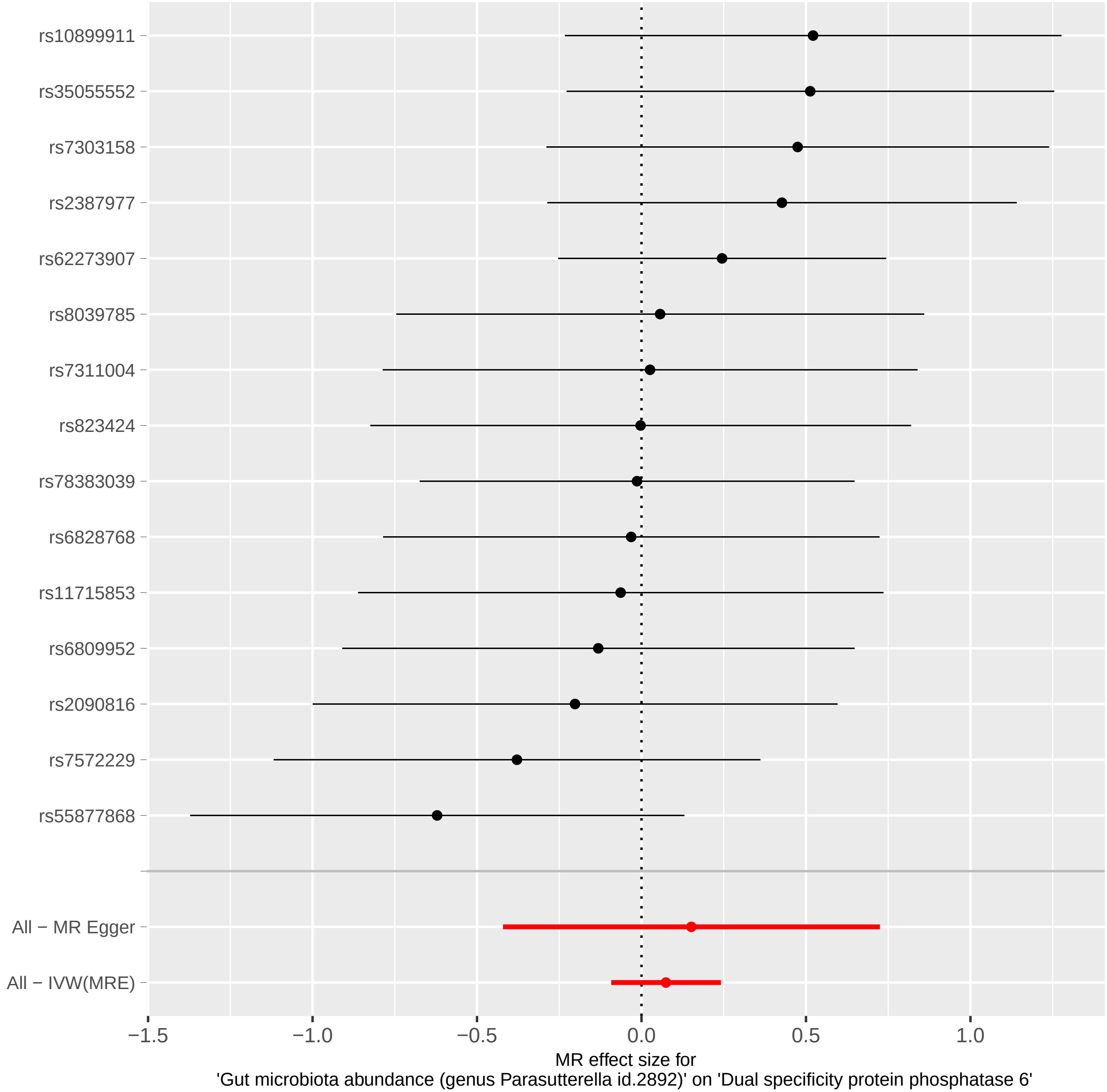

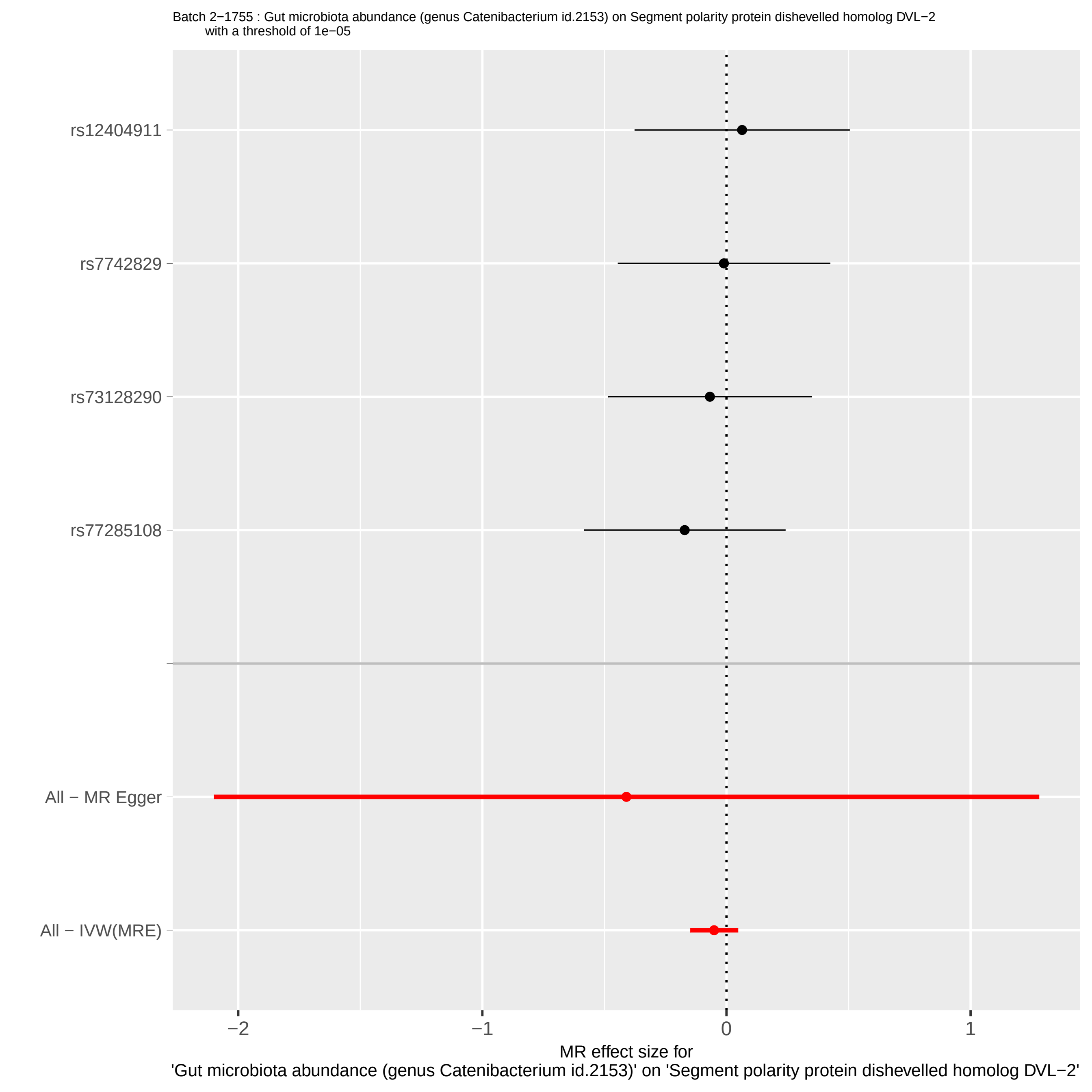
Batch 2−1755 : Gut microbiota abundance (genus Catenibacterium id.2153) on Segment polarity protein dishevelled homolog DVL−2
with a threshold of 1e−05
rs12404911
rs7742829
rs73128290
rs77285108
All − MR Egger
All − IVW(MRE)
−2
−1
0
1
MR effect size for
'Gut microbiota abundance (genus Catenibacterium id.2153)' on 'Segment polarity protein dishevelled homolog DVL−2'

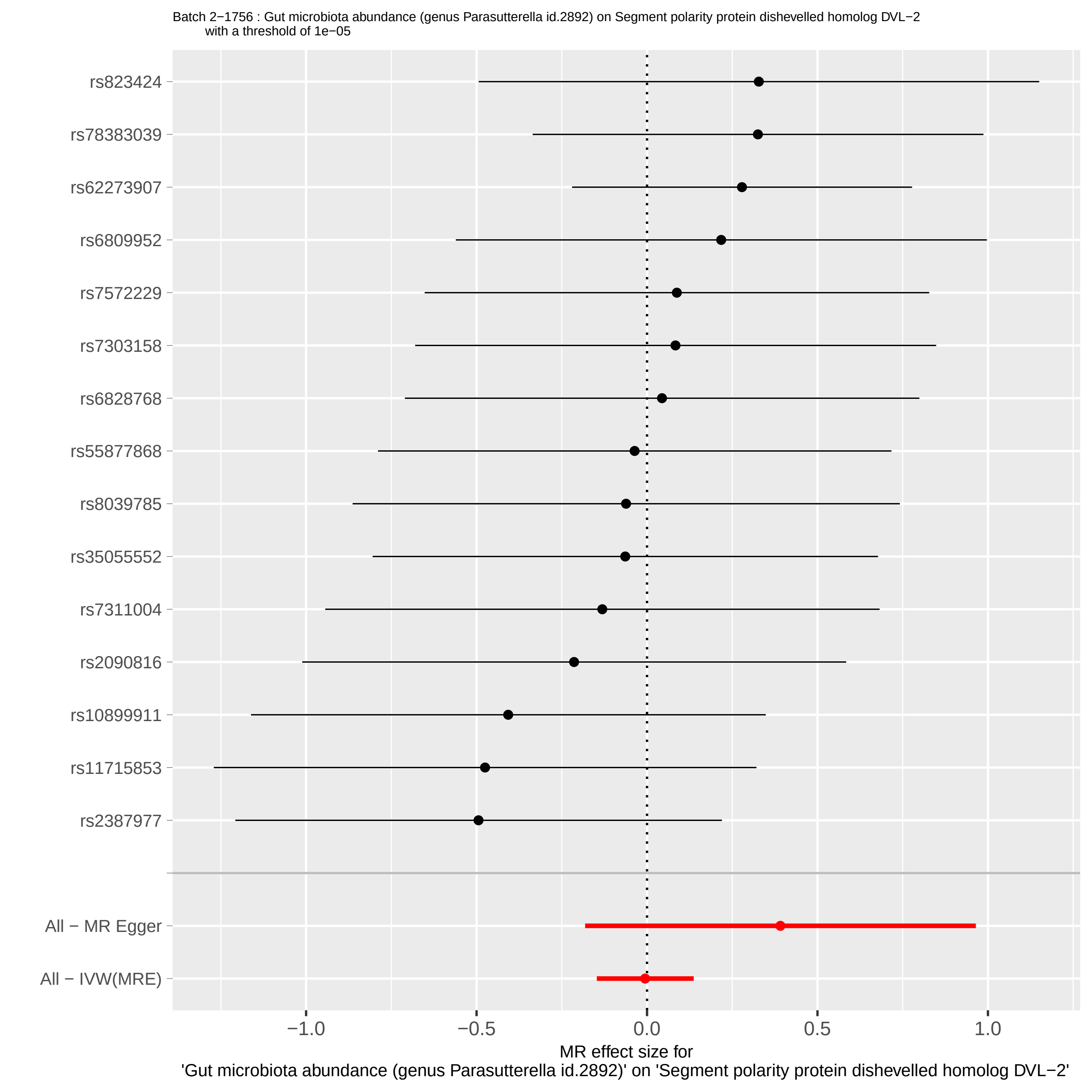
Batch 2−1756 : Gut microbiota abundance (genus Parasutterella id.2892) on Segment polarity protein dishevelled homolog DVL−2
with a threshold of 1e−05
rs823424
rs78383039
rs62273907
rs6809952
rs7572229
rs7303158
rs6828768
rs55877868
rs8039785
rs35055552
rs7311004
rs2090816
rs10899911
rs11715853
rs2387977
All − MR Egger
All − IVW(MRE)
−1.0
−0.5
0.0
0.5
1.0
MR effect size for
'Gut microbiota abundance (genus Parasutterella id.2892)' on 'Segment polarity protein dishevelled homolog DVL−2'

Batch 2−1757 : Gut microbiota abundance (genus Catenibacterium id.2153) on Dynactin−associated protein
with a threshold of 1e−05

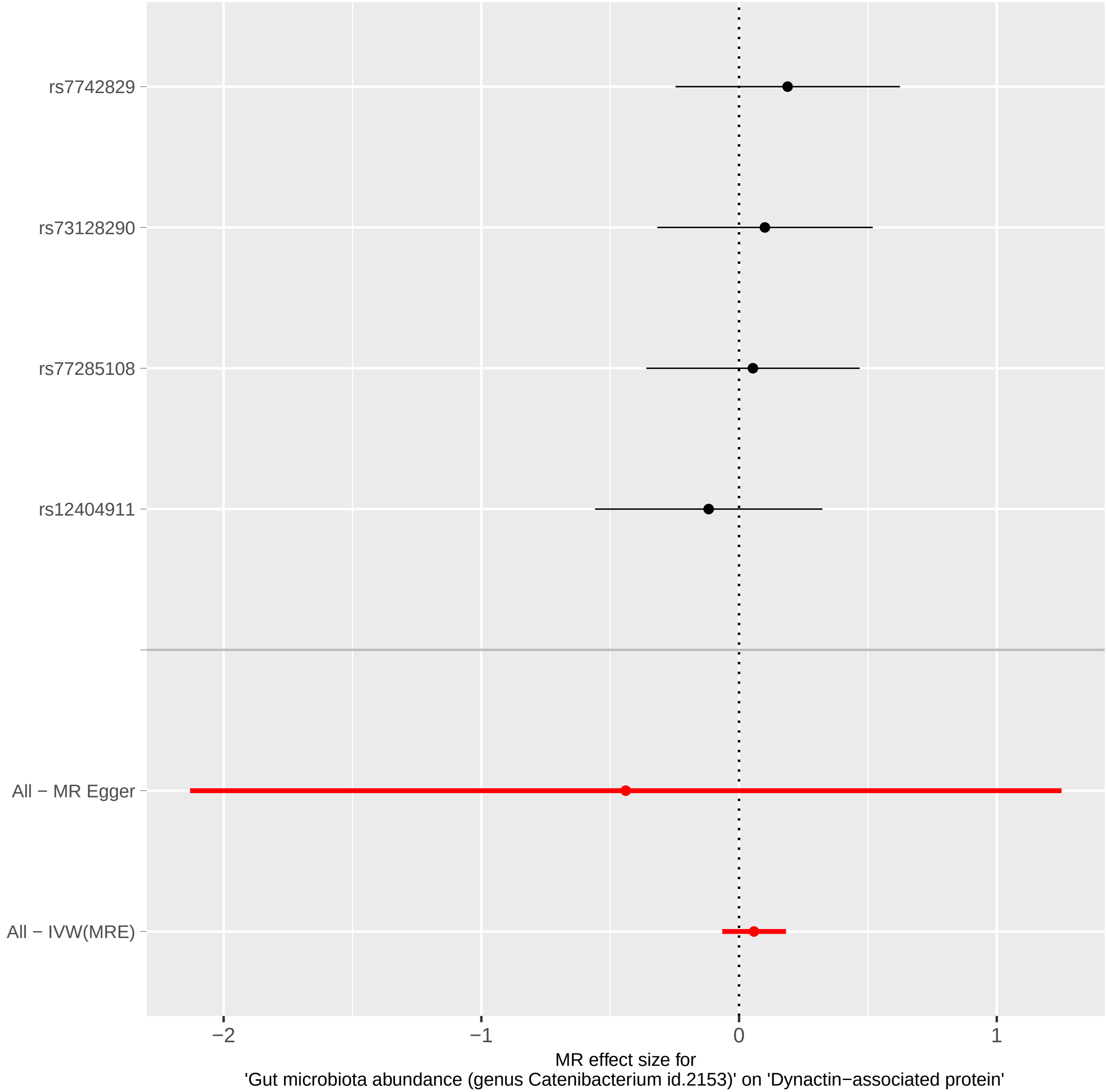

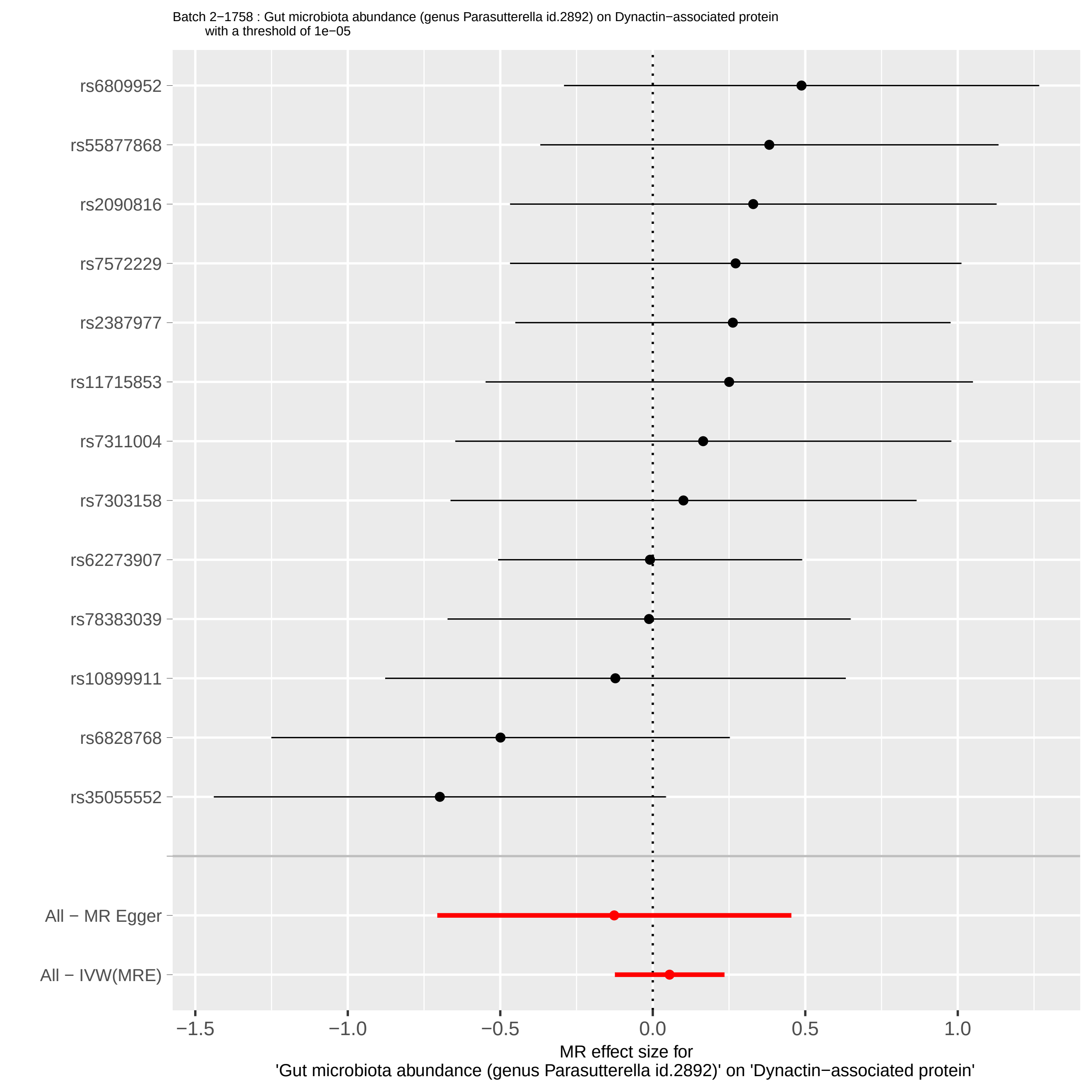
Batch 2−1758 : Gut microbiota abundance (genus Parasutterella id.2892) on Dynactin−associated protein
with a threshold of 1e−05
rs6809952
rs55877868
rs2090816
rs7572229
rs2387977
rs11715853
rs7311004
rs7303158
rs62273907
rs78383039
rs10899911
rs6828768
rs35055552
All − MR Egger
All − IVW(MRE)
−1.5
−1.0
−0.5
0.0
0.5
1.0
MR effect size for
'Gut microbiota abundance (genus Parasutterella id.2892)' on 'Dynactin−associated protein

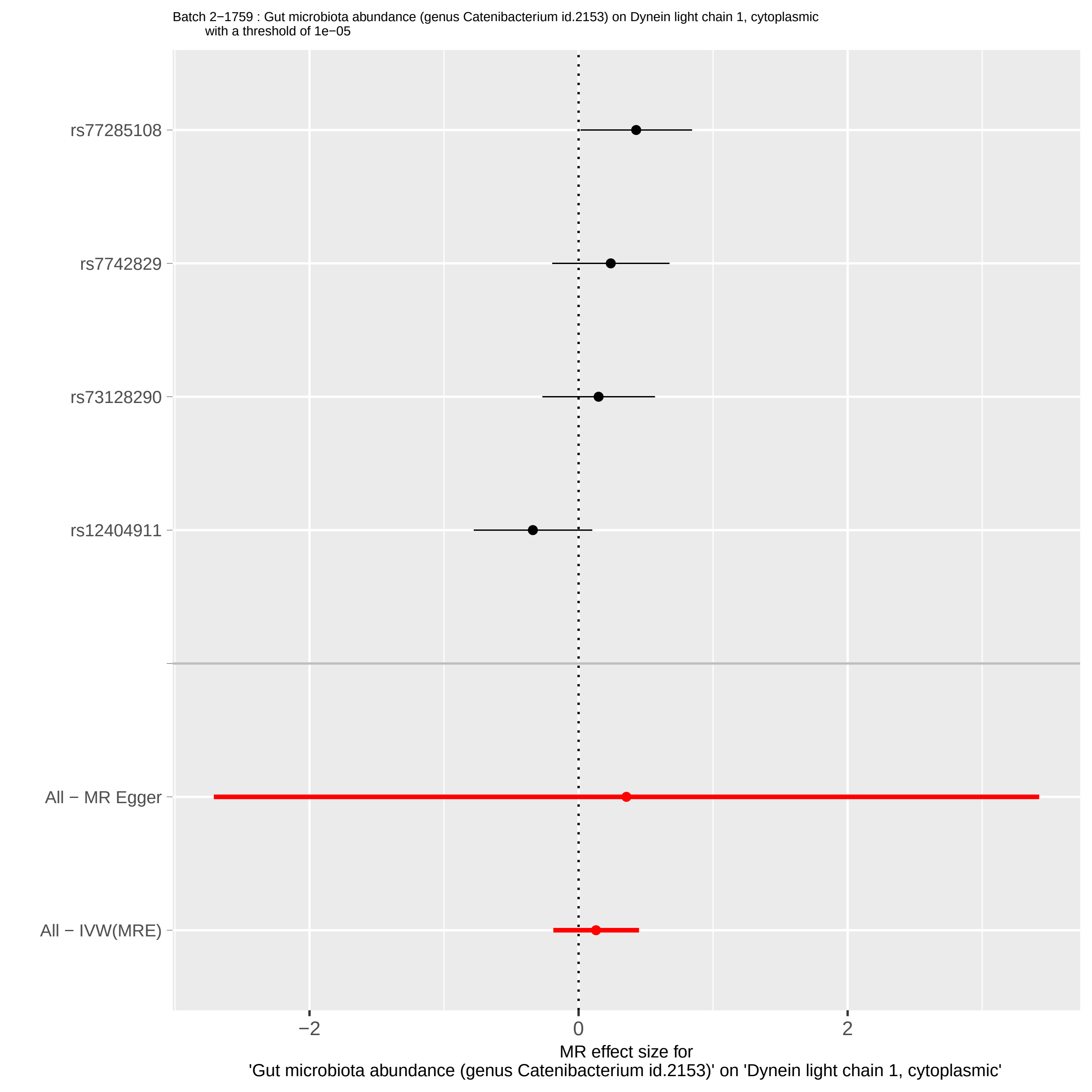

Batch 2−1759 : Gut microbiota abundance (genus Catenibacterium id.2153) on Dynein light chain 1, cytoplasmic
with a threshold of 1e−05
rs77285108
rs7742829
rs73128290
rs12404911
All − MR Egger
All − IVW(MRE)
−2
0
2
MR effect size for
'Gut microbiota abundance (genus Catenibacterium id.2153)' on 'Dynein light chain 1, cytoplasmic'

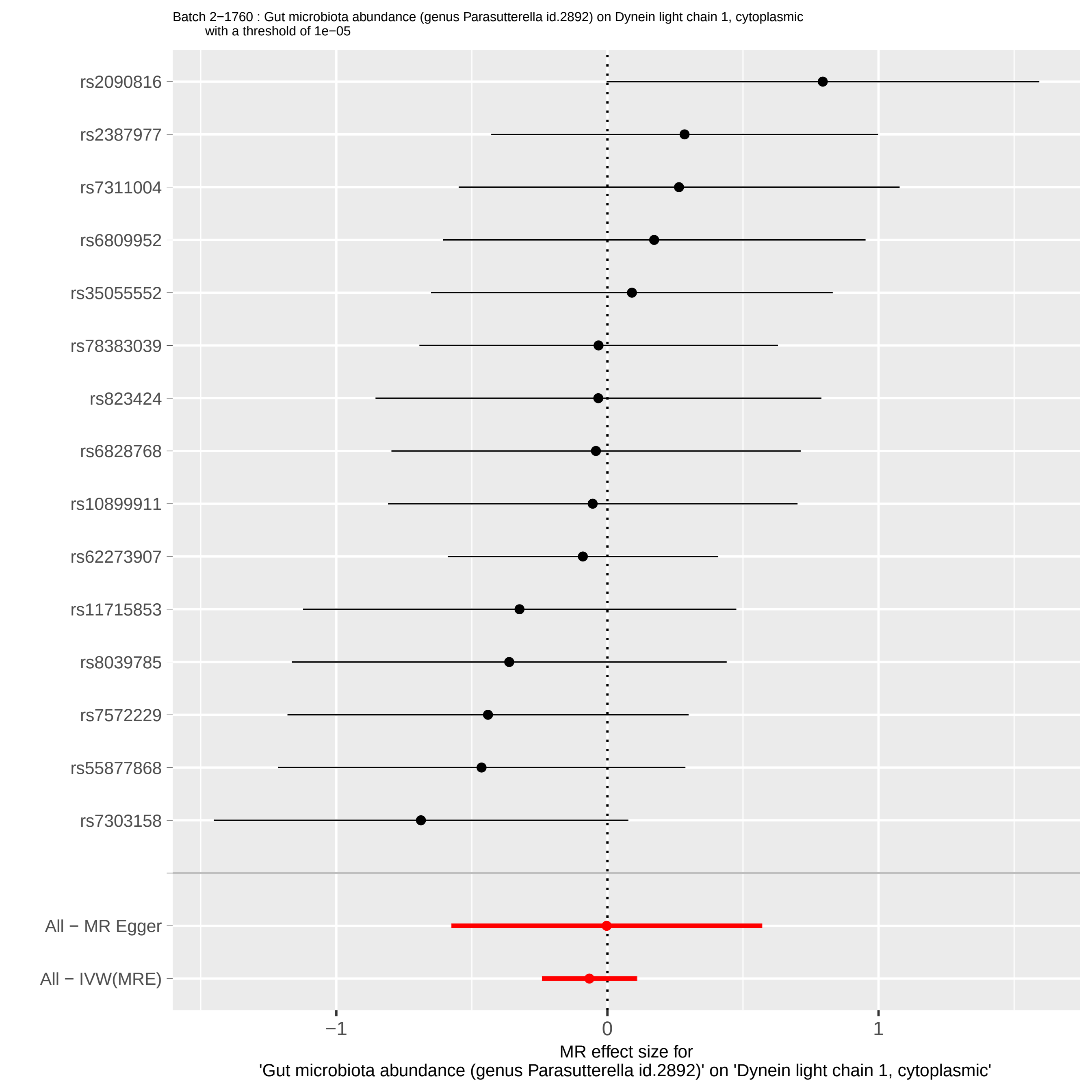
Batch 2−1760 : Gut microbiota abundance (genus Parasutterella id.2892) on Dynein light chain 1, cytoplasmic
with a threshold of 1e−05
rs2090816
rs2387977
rs7311004
rs6809952
rs35055552
rs78383039
rs823424
rs6828768
rs10899911
rs62273907
rs11715853
rs8039785
rs7572229
rs55877868
rs7303158
All − MR Egger
All − IVW(MRE)
−1
0
1
MR effect size for
'Gut microbiota abundance (genus Parasutterella id.2892)' on 'Dynein light chain 1, cytoplasmic'

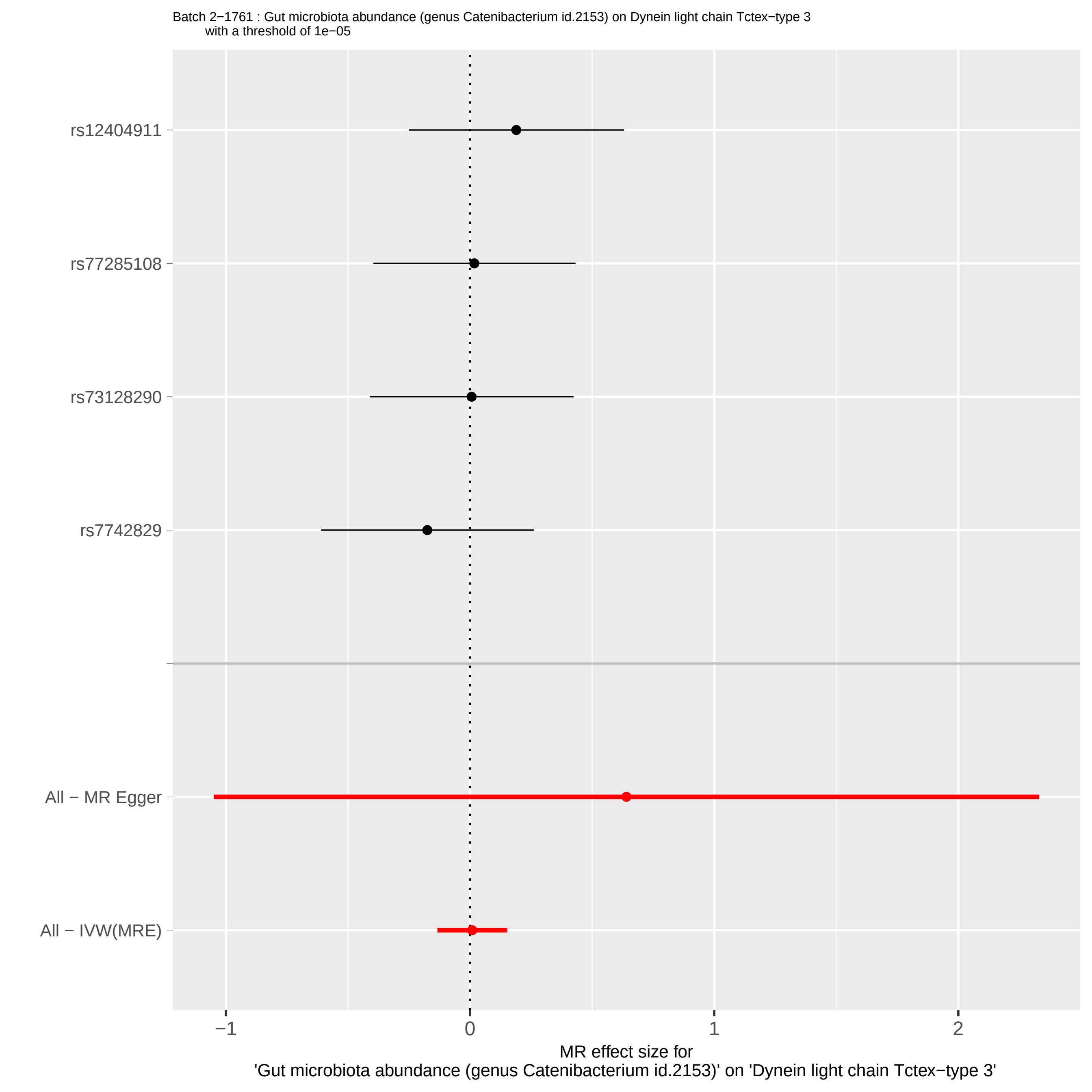

Batch 2−1761 : Gut microbiota abundance (genus Catenibacterium id.2153) on Dynein light chain Tctex−type 3
with a threshold of 1e−05
rs12404911
rs77285108
rs73128290
rs7742829
All − MR Egger
All − IVW(MRE)
−1
0
1
2
MR effect size for
'Gut microbiota abundance (genus Catenibacterium id.2153)' on 'Dynein light chain Tctex−type 3'

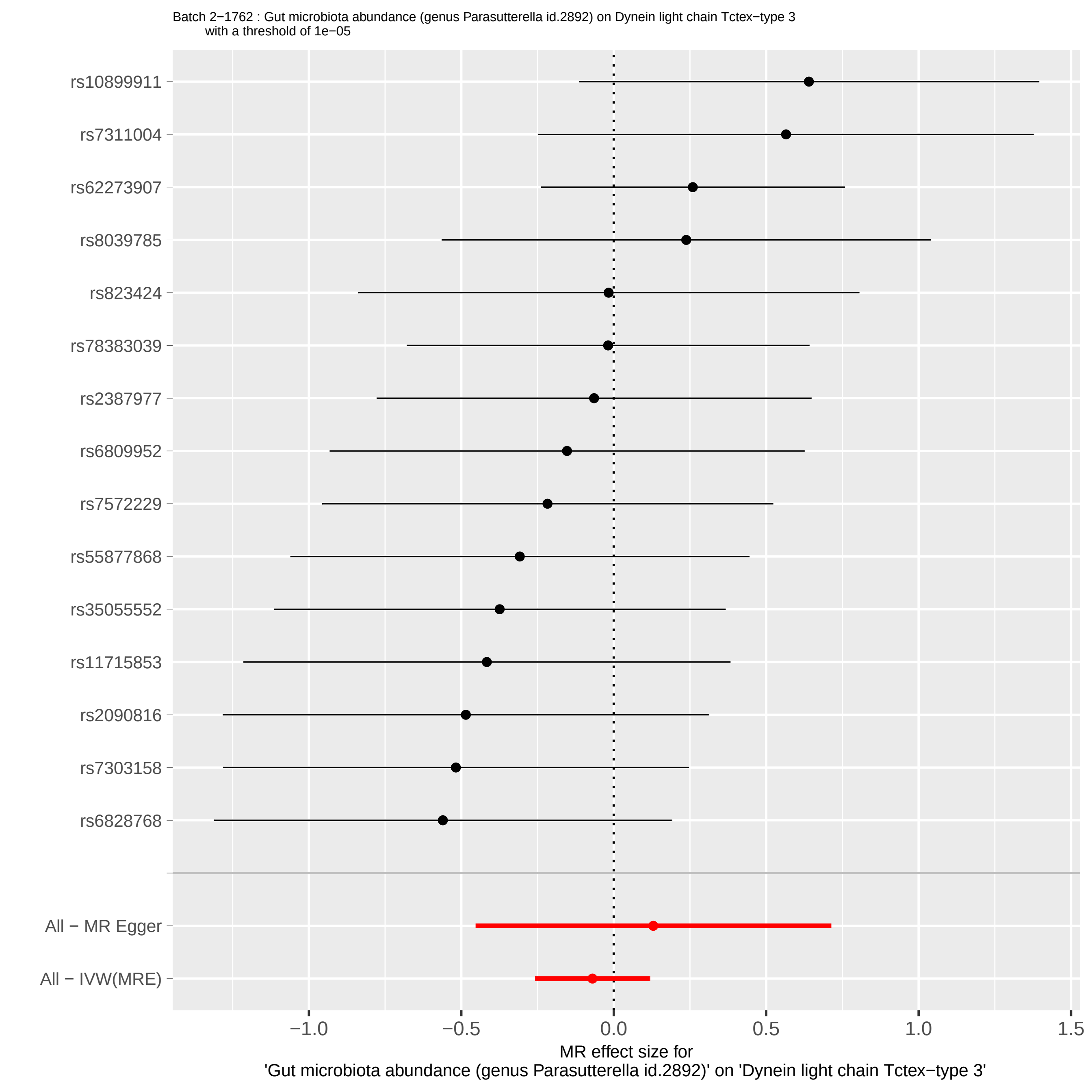

Batch 2−1762 : Gut microbiota abundance (genus Parasutterella id.2892) on Dynein light chain Tctex−type 3
with a threshold of 1e−05
rs10899911
rs7311004
rs62273907
rs8039785
rs823424
rs78383039
rs2387977
rs6809952
rs7572229
rs55877868
rs35055552
rs11715853
rs2090816
rs7303158
rs6828768
All − MR Egger
All − IVW(MRE)
−1.0
−0.5
0.0
0.5
1.0
1.5
MR effect size for
'Gut microbiota abundance (genus Parasutterella id.2892)' on 'Dynein light chain Tctex−type 3'

Batch 2−1763 : Gut microbiota abundance (genus Catenibacterium id.2153) on Dual specificity tyrosine−phosphorylation−regulated kinase 3
with a threshold of 1e−05

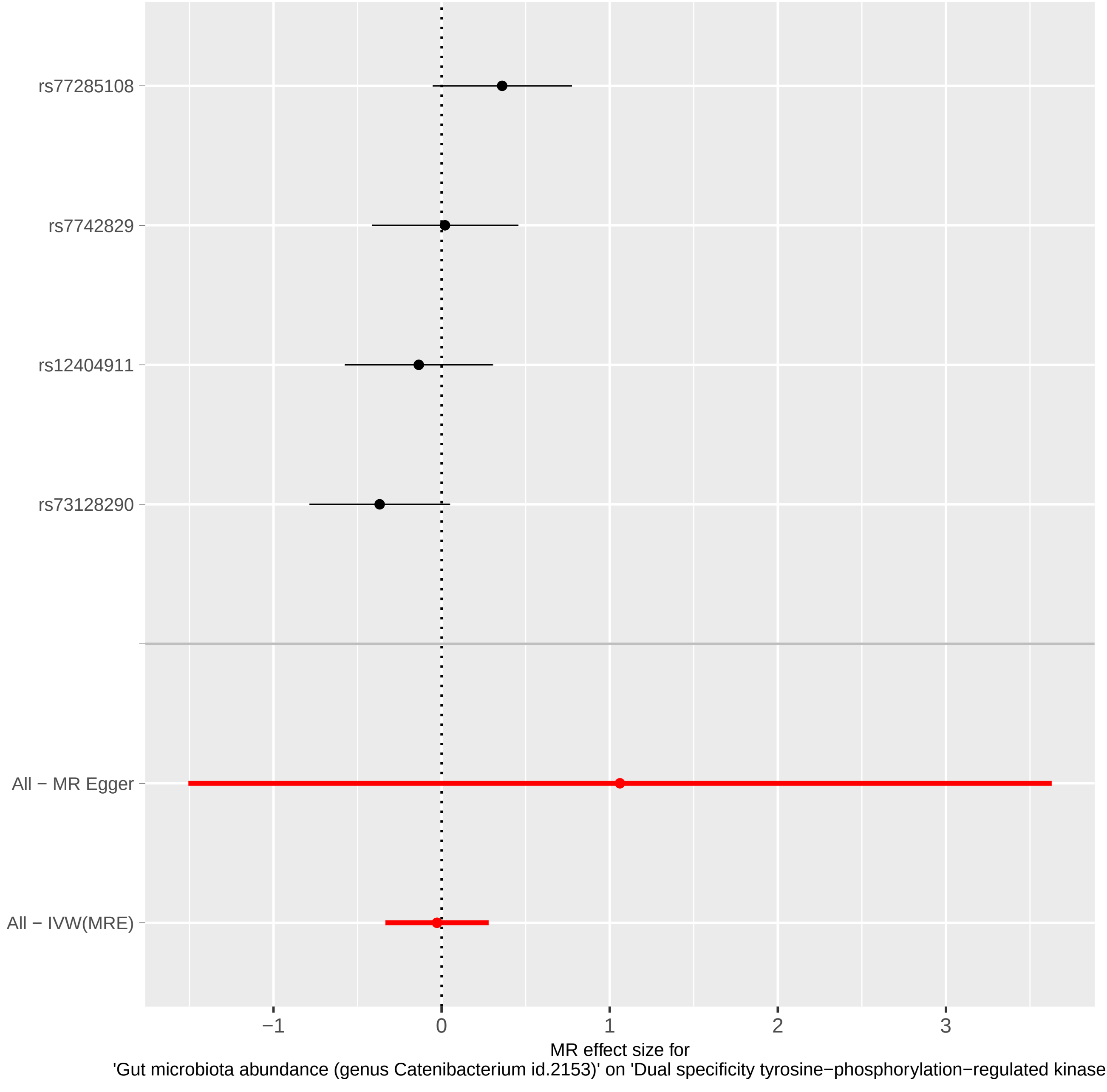

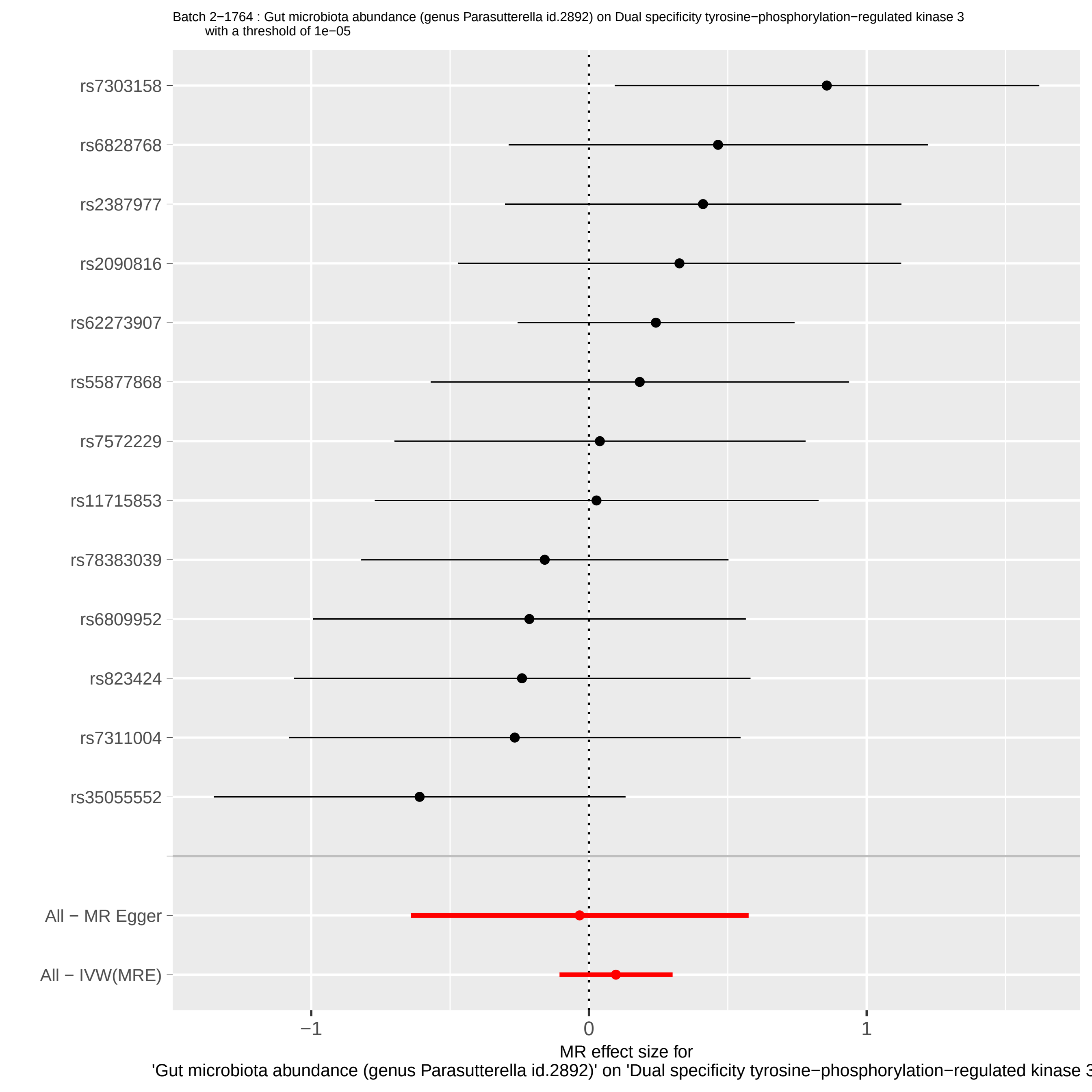

Batch 2−1764 : Gut microbiota abundance (genus Parasutterella id.2892) on Dual specificity tyrosine−phosphorylation−regulated kinase 3
with a threshold of 1e−05
rs7303158
rs6828768
rs2387977
rs2090816
rs62273907
rs55877868
rs7572229
rs11715853
rs78383039
rs6809952
rs823424
rs7311004
rs35055552
All − MR Egger
All − IVW(MRE)
−1
0
1
MR effect size for
'Gut microbiota abundance (genus Parasutterella id.2892)' on 'Dual specificity tyrosine−phosphorylation−regulated kinase 3

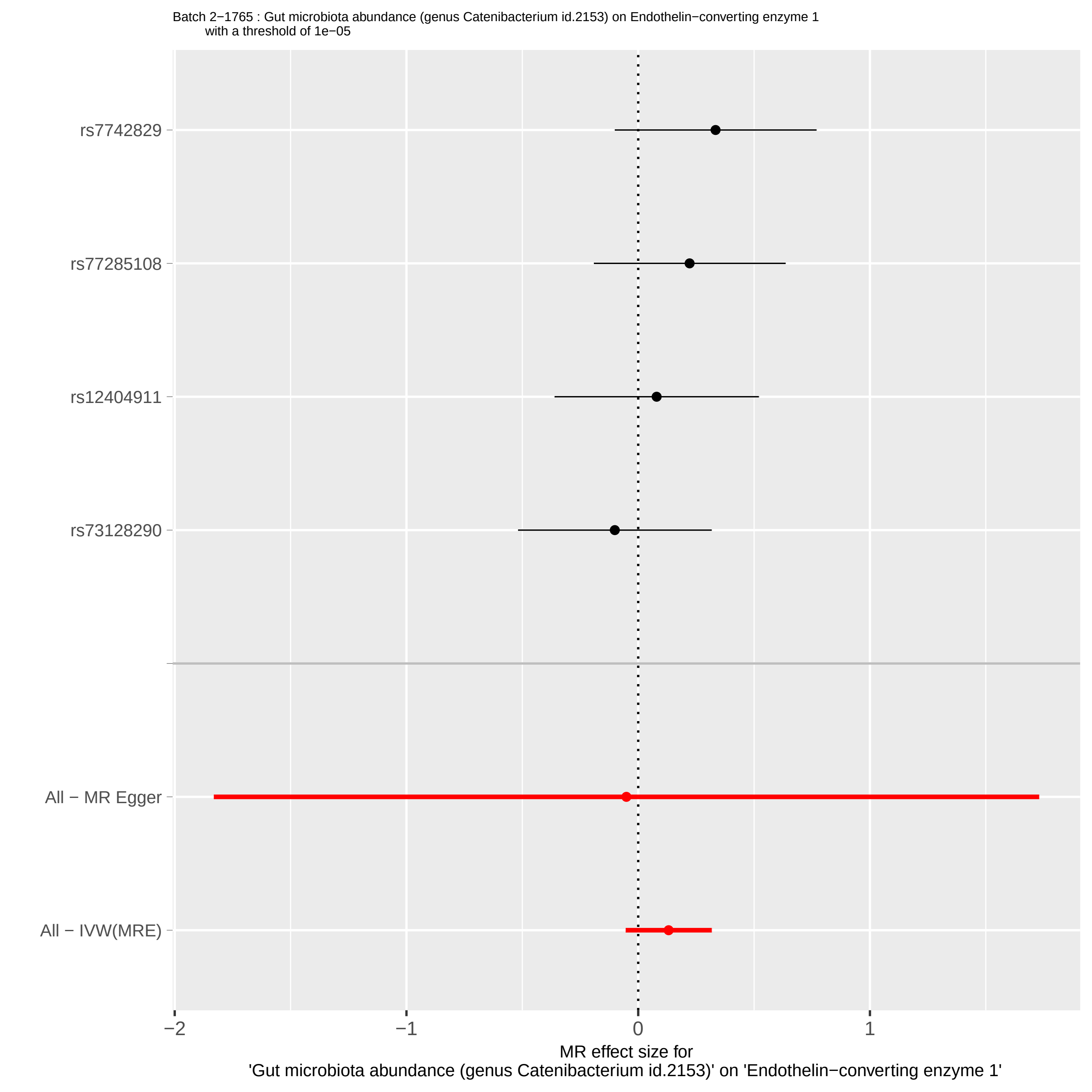

Batch 2−1765 : Gut microbiota abundance (genus Catenibacterium id.2153) on Endothelin−converting enzyme 1
with a threshold of 1e−05
rs7742829
rs77285108
rs12404911
rs73128290
All − MR Egger
All − IVW(MRE)
−2
−1
0
1
MR effect size for
'Gut microbiota abundance (genus Catenibacterium id.2153)' on 'Endothelin−converting enzyme 1'

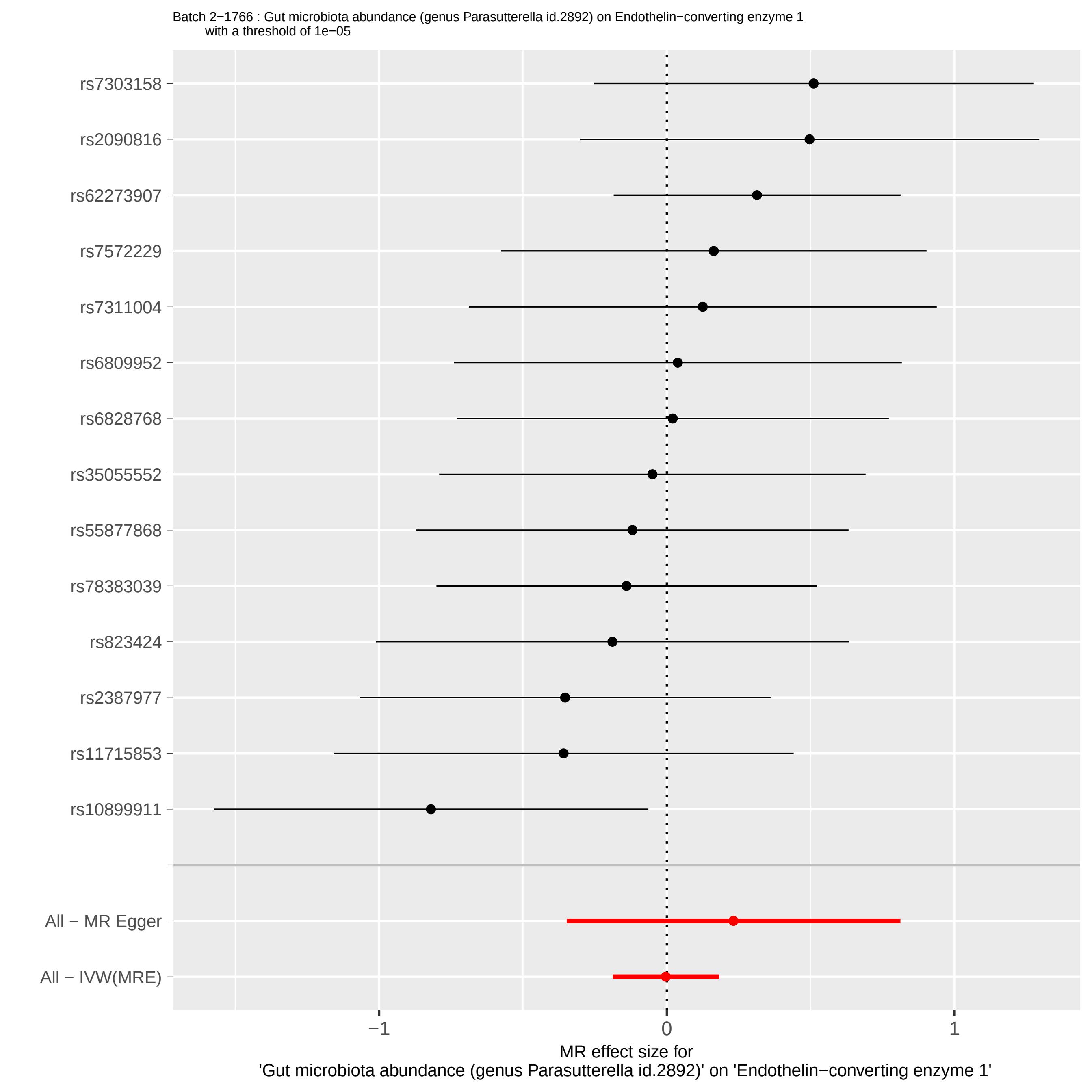
Batch 2−1766 : Gut microbiota abundance (genus Parasutterella id.2892) on Endothelin−converting enzyme 1
with a threshold of 1e−05
rs7303158
rs2090816
rs62273907
rs7572229
rs7311004
rs6809952
rs6828768
rs35055552
rs55877868
rs78383039
rs823424
rs2387977
rs11715853
rs10899911
All − MR Egger
All − IVW(MRE)
−1
0
1
MR effect size for
'Gut microbiota abundance (genus Parasutterella id.2892)' on 'Endothelin−converting enzyme 1'

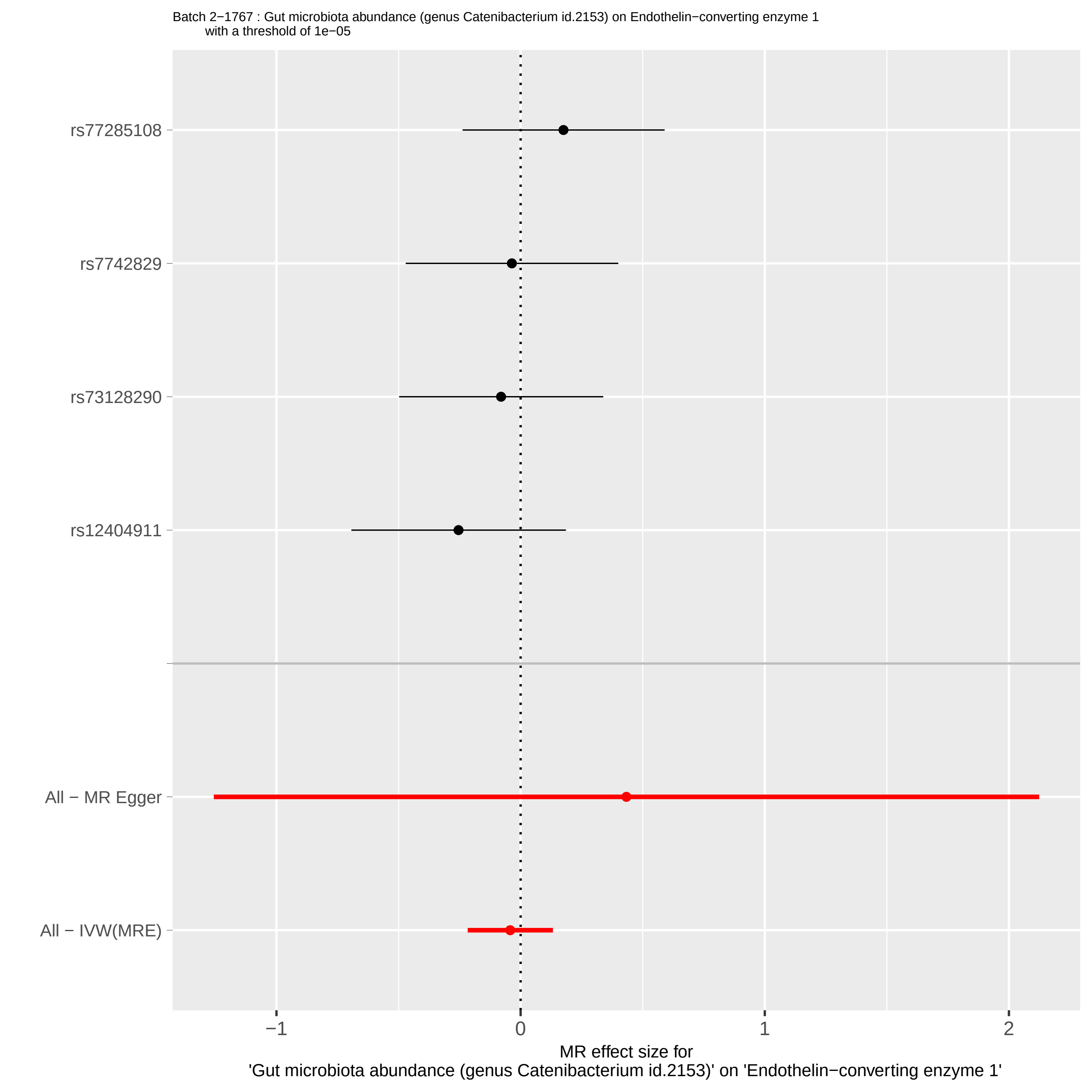
Batch 2−1767 : Gut microbiota abundance (genus Catenibacterium id.2153) on Endothelin−converting enzyme 1
with a threshold of 1e−05
rs77285108
rs7742829
rs73128290
rs12404911
All − MR Egger
All − IVW(MRE)
−1
0
1
2
MR effect size for
'Gut microbiota abundance (genus Catenibacterium id.2153)' on 'Endothelin−converting enzyme 1'

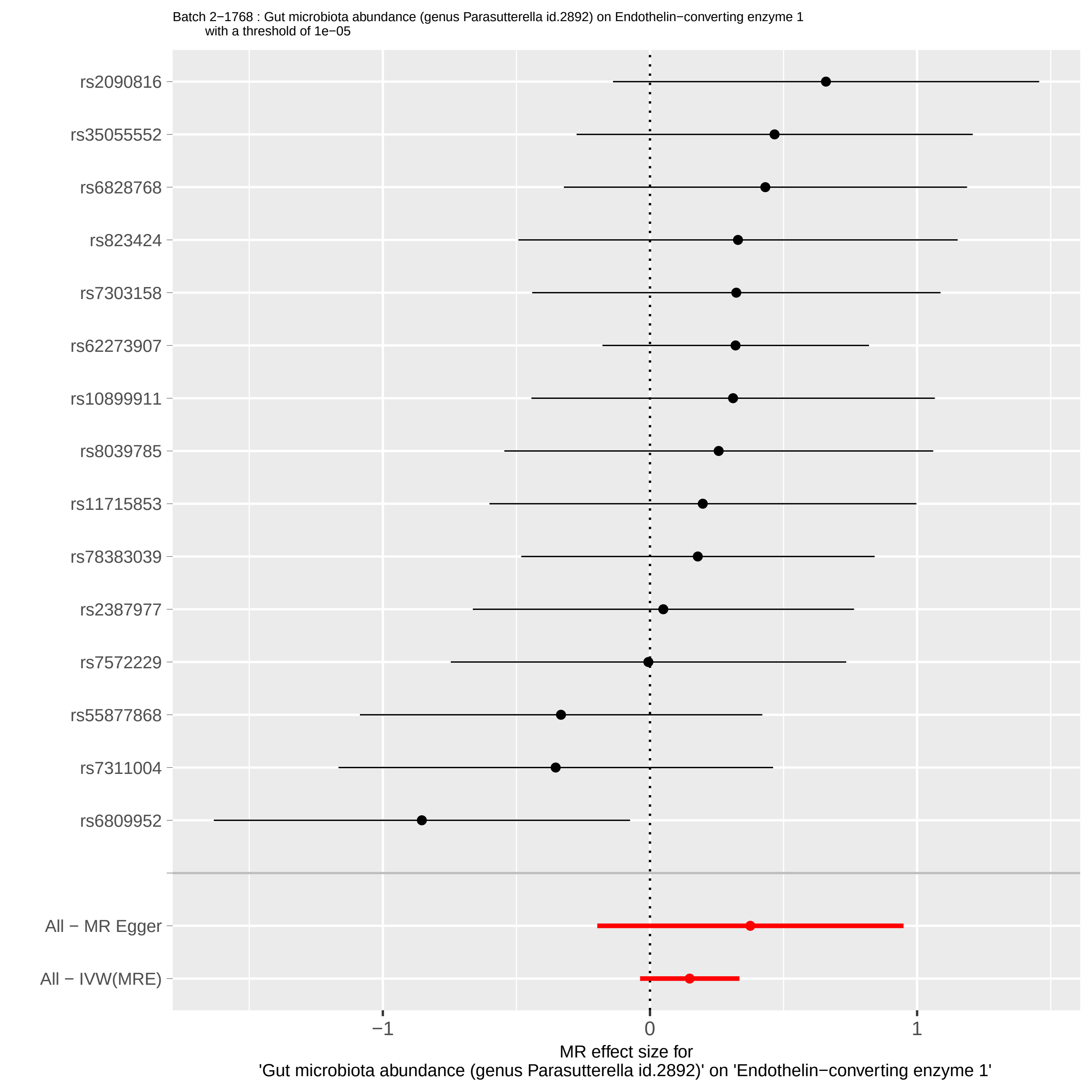

Batch 2−1768 : Gut microbiota abundance (genus Parasutterella id.2892) on Endothelin−converting enzyme 1
with a threshold of 1e−05
rs2090816
rs35055552
rs6828768
rs823424
rs7303158
rs62273907
rs10899911
rs8039785
rs11715853
rs78383039
rs2387977
rs7572229
rs55877868
rs7311004
rs6809952
All − MR Egger
All − IVW(MRE)
−1
0
1
MR effect size for
'Gut microbiota abundance (genus Parasutterella id.2892)' on 'Endothelin−converting enzyme 1'

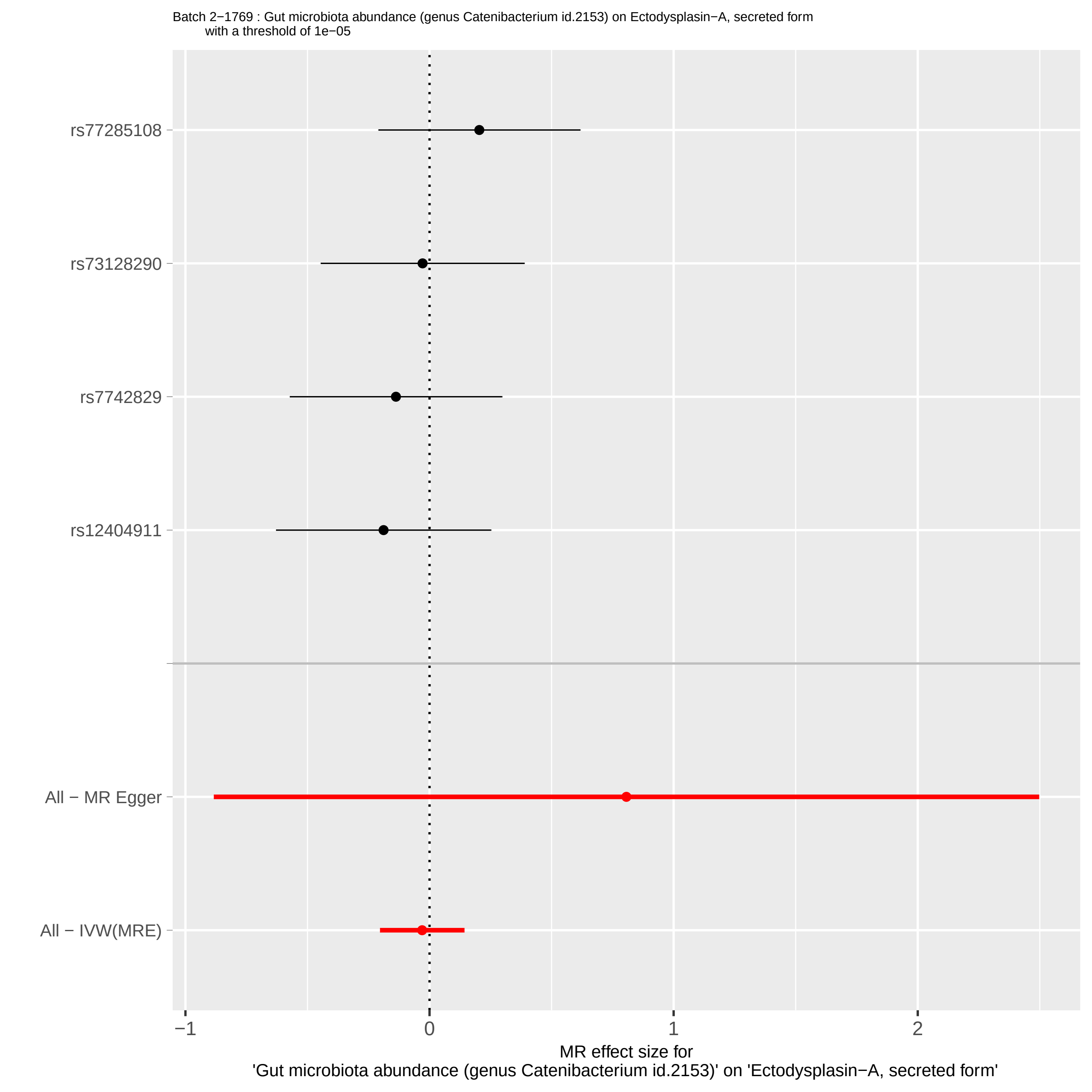
Batch 2−1769 : Gut microbiota abundance (genus Catenibacterium id.2153) on Ectodysplasin−A, secreted form
with a threshold of 1e−05
rs77285108
rs73128290
rs7742829
rs12404911
All − MR Egger
All − IVW(MRE)
−1
0
1
2
MR effect size for
'Gut microbiota abundance (genus Catenibacterium id.2153)' on 'Ectodysplasin−A, secreted form'

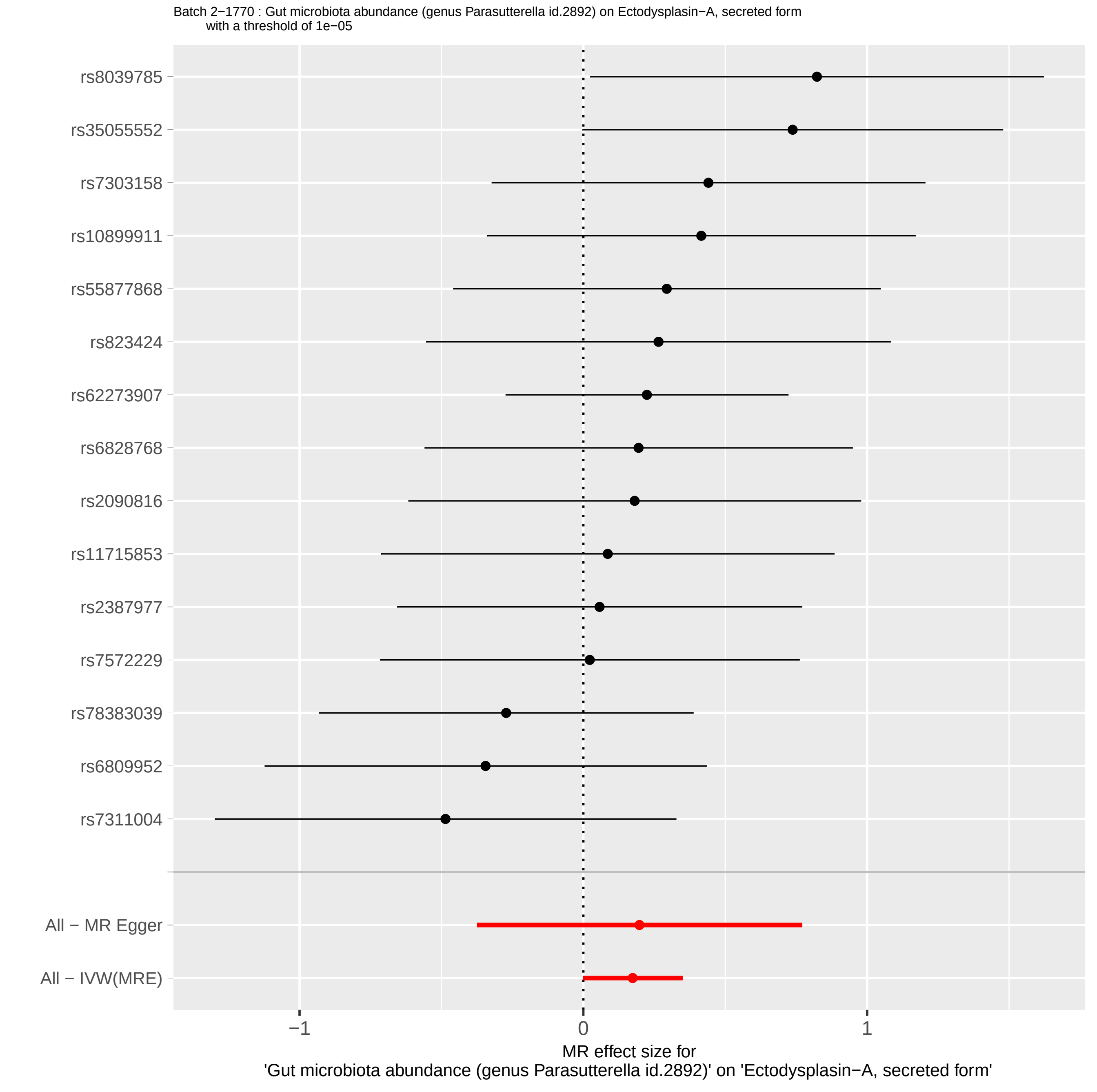

Batch 2−1770 : Gut microbiota abundance (genus Parasutterella id.2892) on Ectodysplasin−A, secreted form
with a threshold of 1e−05
rs8039785
rs35055552
rs7303158
rs10899911
rs55877868
rs823424
rs62273907
rs6828768
rs2090816
rs11715853
rs2387977
rs7572229
rs78383039
rs6809952
rs7311004
All − MR Egger
All − IVW(MRE)
−1
0
1
MR effect size for
'Gut microbiota abundance (genus Parasutterella id.2892)' on 'Ectodysplasin−A, secreted form'

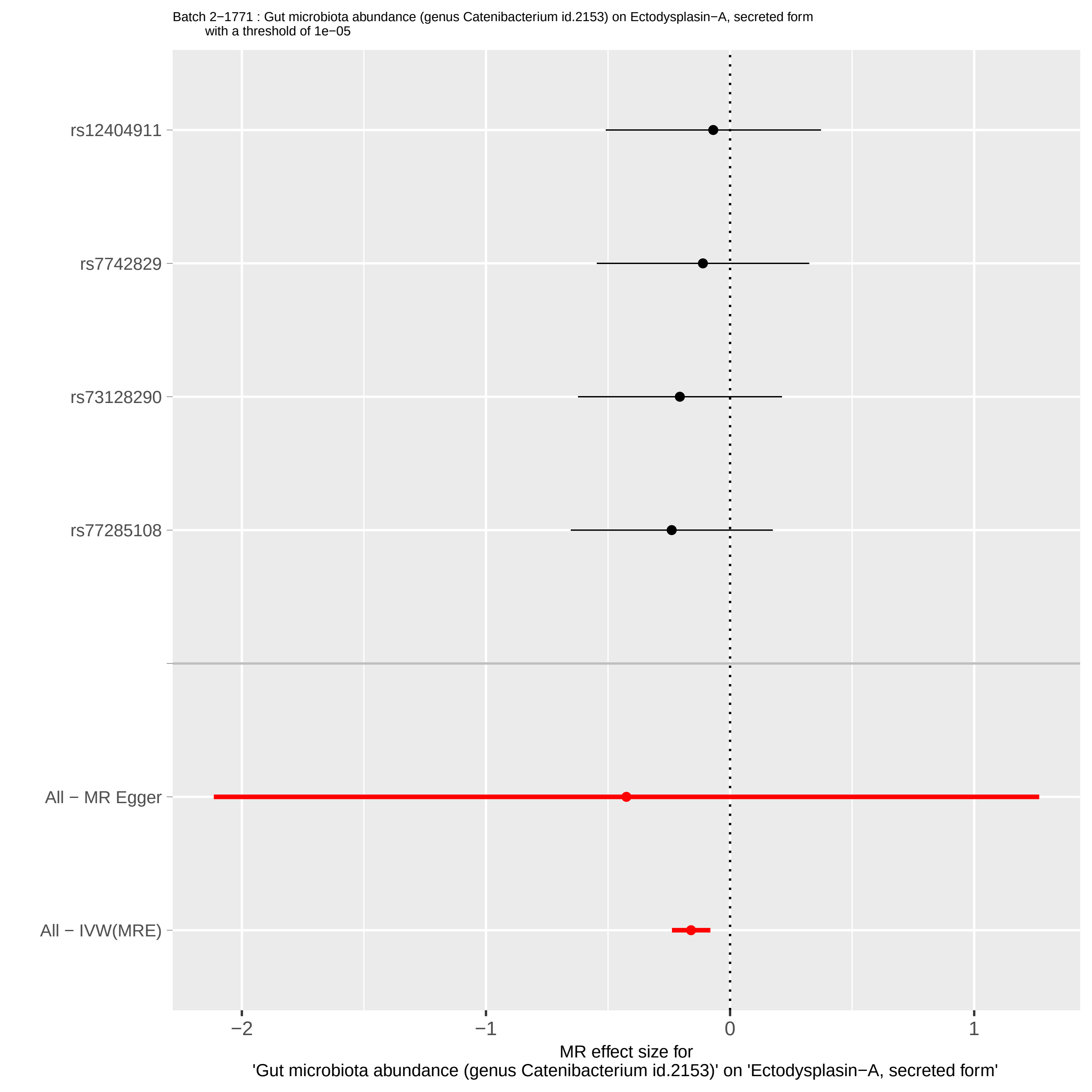
Batch 2−1771 : Gut microbiota abundance (genus Catenibacterium id.2153) on Ectodysplasin−A, secreted form
with a threshold of 1e−05
rs12404911
rs7742829
rs73128290
rs77285108
All − MR Egger
All − IVW(MRE)
−2
−1
0
1
MR effect size for
'Gut microbiota abundance (genus Catenibacterium id.2153)' on 'Ectodysplasin−A, secreted form'

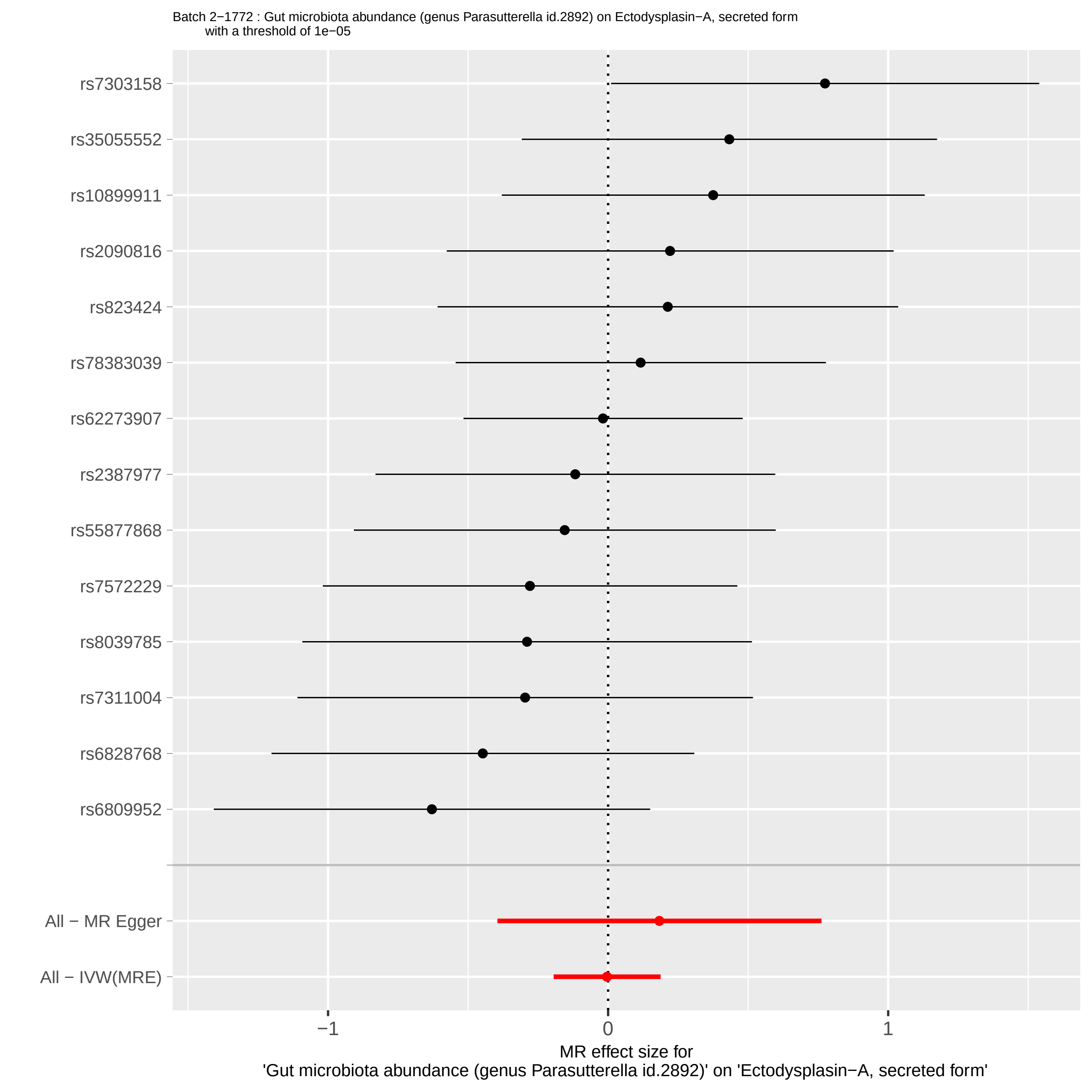

Batch 2−1772 : Gut microbiota abundance (genus Parasutterella id.2892) on Ectodysplasin−A, secreted form
with a threshold of 1e−05
rs7303158
rs35055552
rs10899911
rs2090816
rs823424
rs78383039
rs62273907
rs2387977
rs55877868
rs7572229
rs8039785
rs7311004
rs6828768
rs6809952
All − MR Egger
All − IVW(MRE)
−1
0
1
MR effect size for
'Gut microbiota abundance (genus Parasutterella id.2892)' on 'Ectodysplasin−A, secreted form'

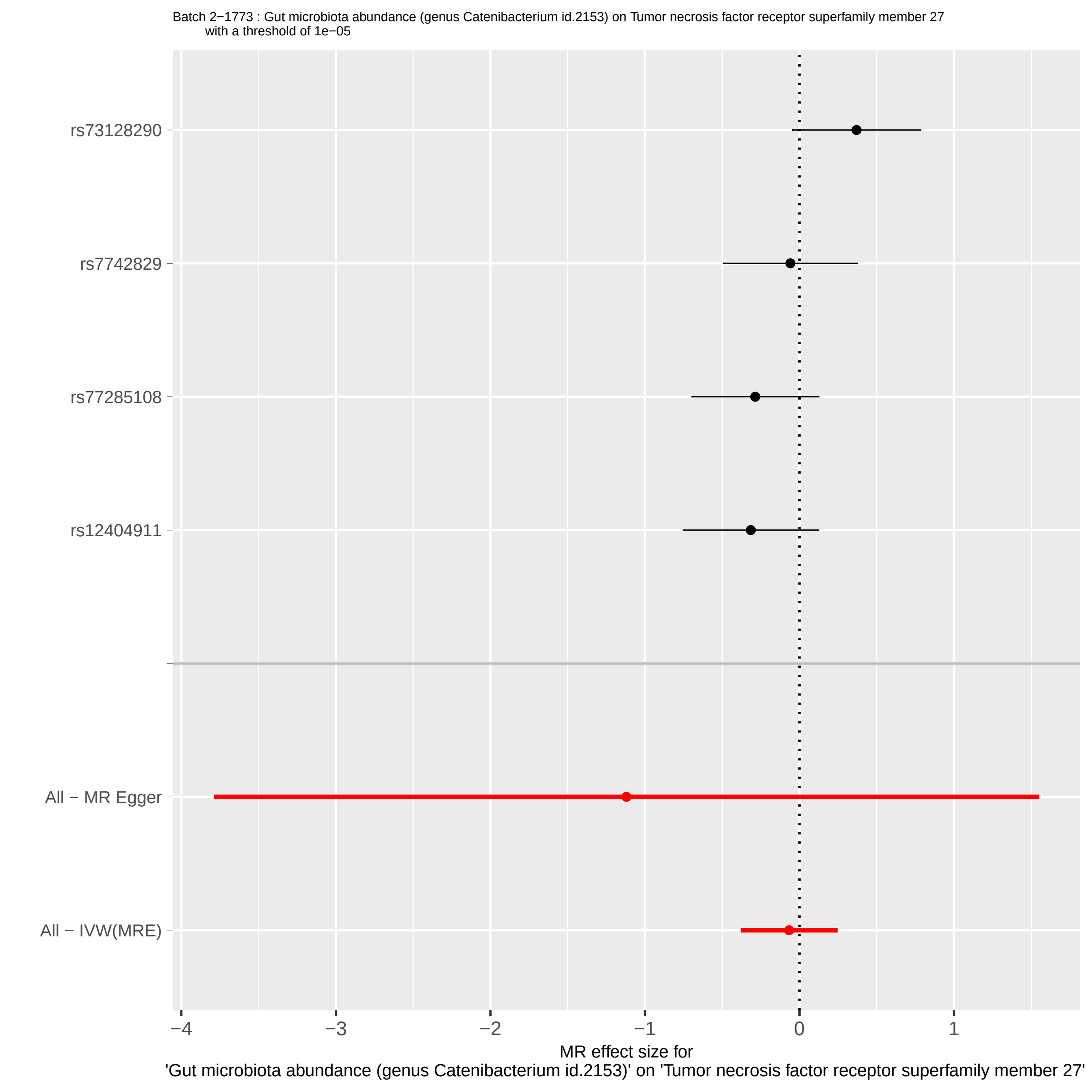

Batch 2−1773 : Gut microbiota abundance (genus Catenibacterium id.2153) on Tumor necrosis factor receptor superfamily member 27
with a threshold of 1e−05
rs73128290
rs7742829
rs77285108
rs12404911
All − MR Egger
All − IVW(MRE)
−4
−3
−2
−1
0
1
MR effect size for
'Gut microbiota abundance (genus Catenibacterium id.2153)' on 'Tumor necrosis factor receptor superfamily member 27'

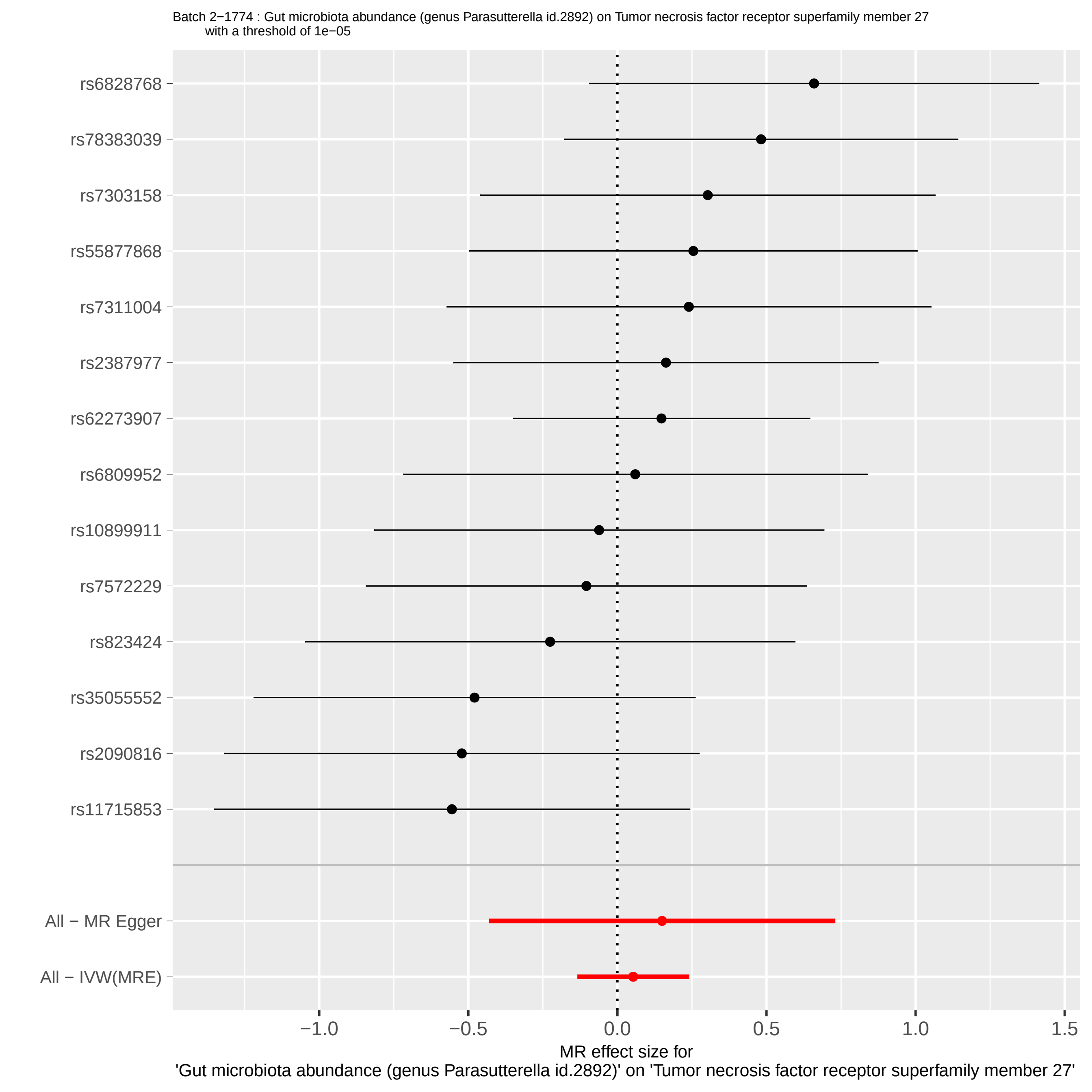
Batch 2−1774 : Gut microbiota abundance (genus Parasutterella id.2892) on Tumor necrosis factor receptor superfamily member 27
with a threshold of 1e−05
rs6828768
rs78383039
rs7303158
rs55877868
rs7311004
rs2387977
rs62273907
rs6809952
rs10899911
rs7572229
rs823424
rs35055552
rs2090816
rs11715853
All − MR Egger
All − IVW(MRE)
−1.0
−0.5
0.0
0.5
1.0
1.5
MR effect size for
'Gut microbiota abundance (genus Parasutterella id.2892)' on 'Tumor necrosis factor receptor superfamily member 27'

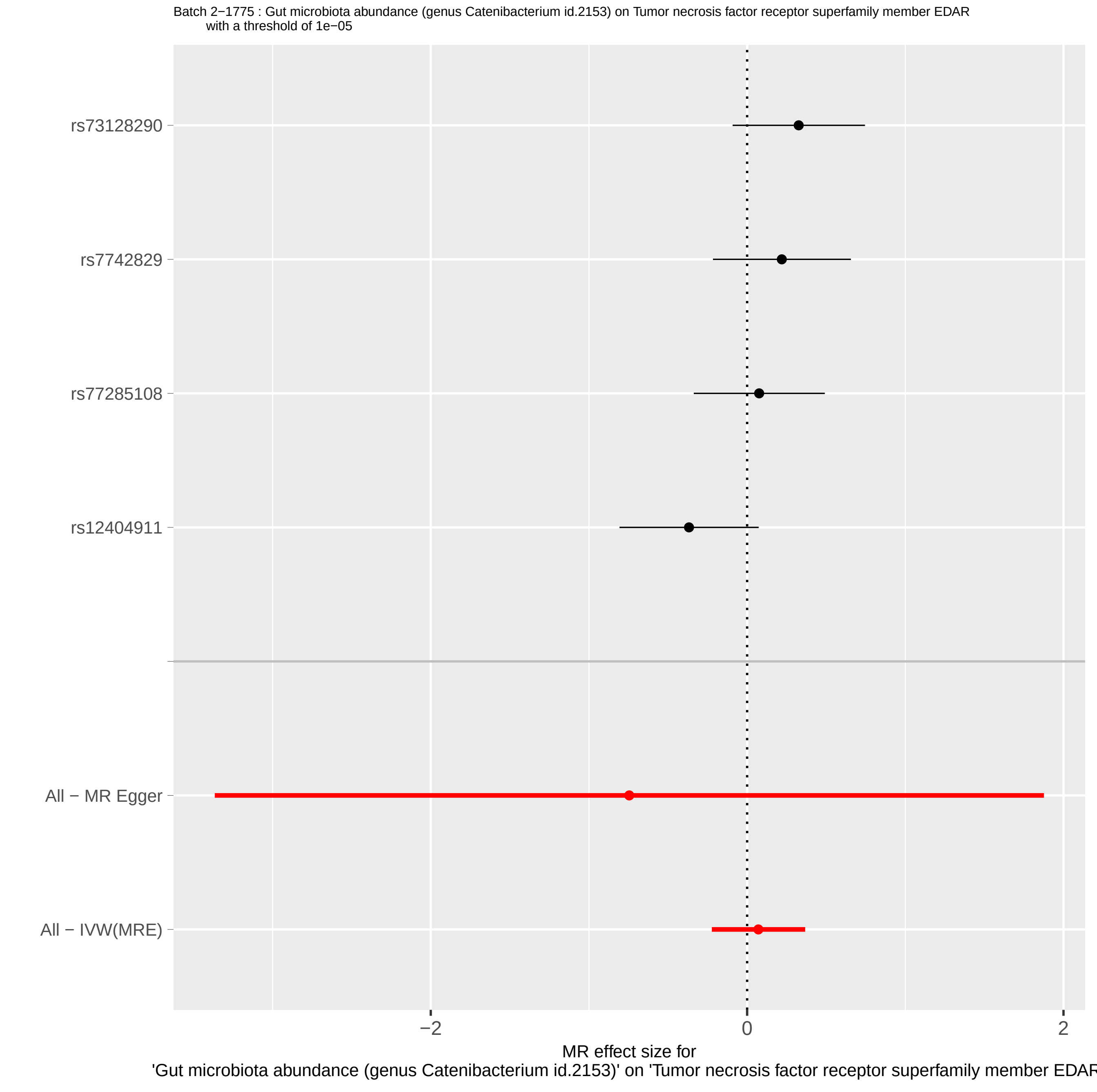
Batch 2−1775 : Gut microbiota abundance (genus Catenibacterium id.2153) on Tumor necrosis factor receptor superfamily member EDAR
with a threshold of 1e−05
rs73128290
rs7742829
rs77285108
rs12404911
All − MR Egger
All − IVW(MRE)
−2
0
2
MR effect size for
'Gut microbiota abundance (genus Catenibacterium id.2153)' on 'Tumor necrosis factor receptor superfamily member EDAR

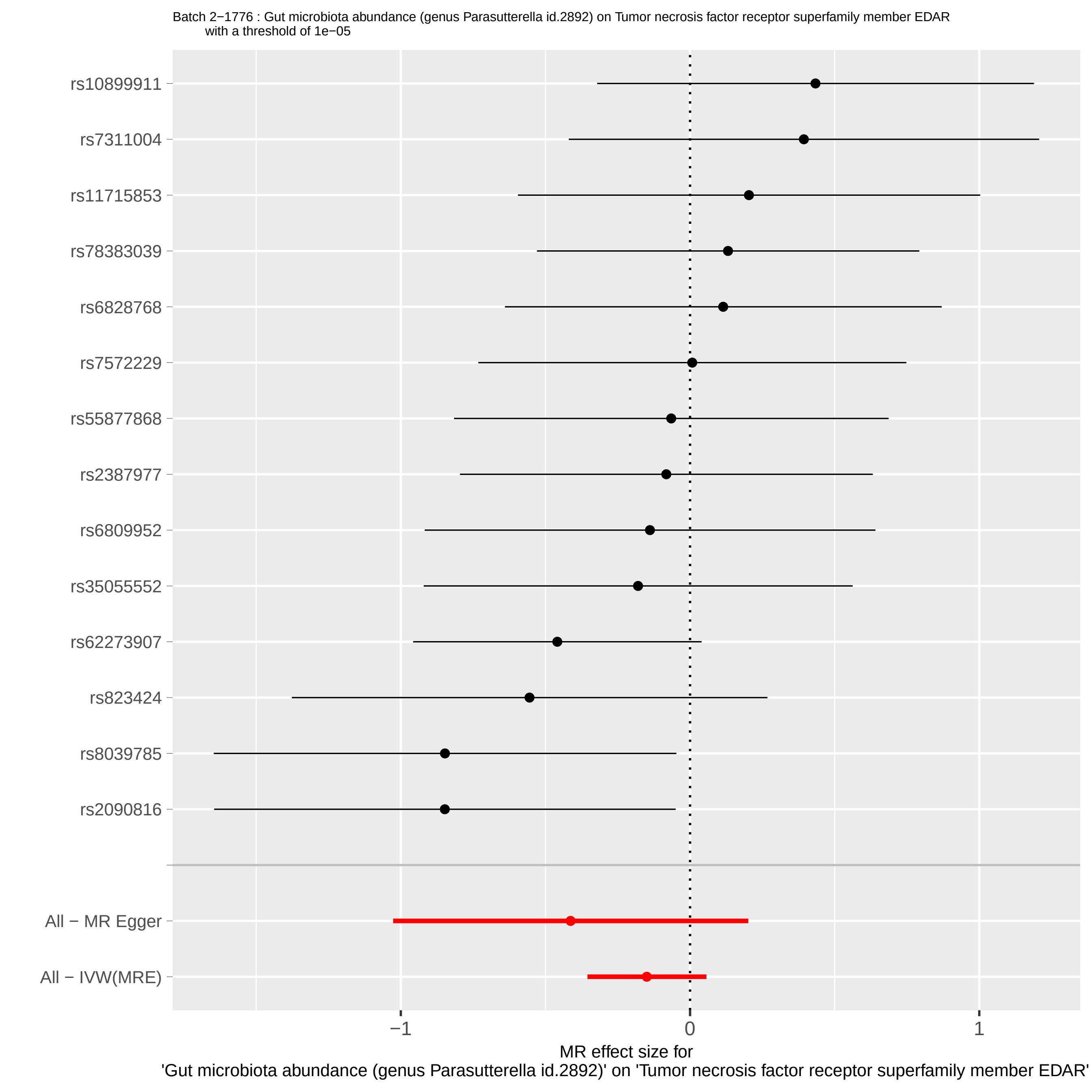
Batch 2−1776 : Gut microbiota abundance (genus Parasutterella id.2892) on Tumor necrosis factor receptor superfamily member EDAR
with a threshold of 1e−05
rs10899911
rs7311004
rs11715853
rs78383039
rs6828768
rs7572229
rs55877868
rs2387977
rs6809952
rs35055552
rs62273907
rs823424
rs8039785
rs2090816
All − MR Egger
All − IVW(MRE)
−1
0
1
MR effect size for
'Gut microbiota abundance (genus Parasutterella id.2892)' on 'Tumor necrosis factor receptor superfamily member EDAR'

Batch 2−1777 : Gut microbiota abundance (genus Catenibacterium id.2153) on Enhancer of mRNA−decapping protein 4 with a threshold of 1e−05

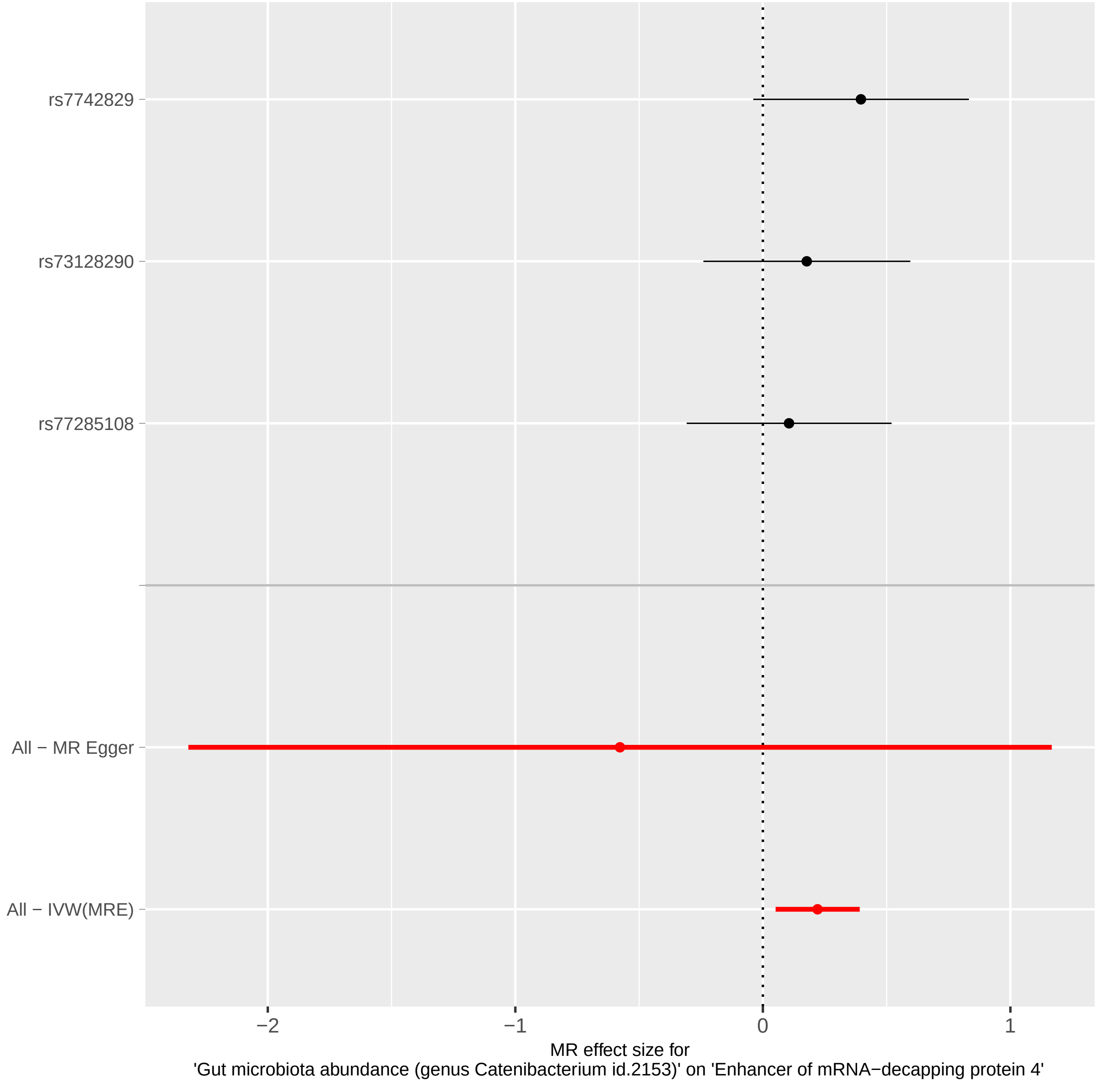

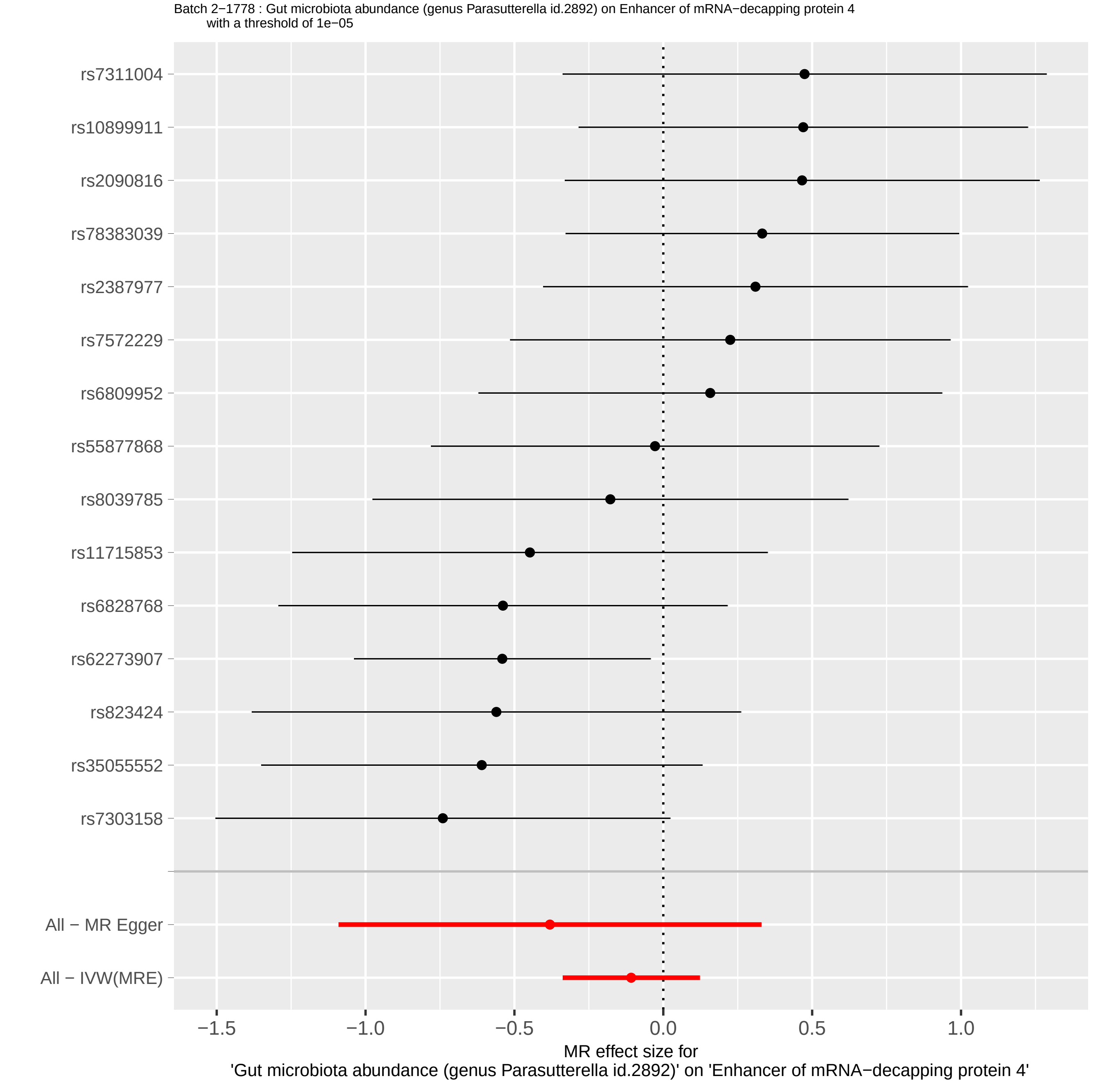

Batch 2−1778 : Gut microbiota abundance (genus Parasutterella id.2892) on Enhancer of mRNA−decapping protein 4
with a threshold of 1e−05
rs7311004
rs10899911
rs2090816
rs78383039
rs2387977
rs7572229
rs6809952
rs55877868
rs8039785
rs11715853
rs6828768
rs62273907
rs823424
rs35055552
rs7303158
All − MR Egger
All − IVW(MRE)
−1.5
−1.0
−0.5
0.0
0.5
1.0
MR effect size for
'Gut microbiota abundance (genus Parasutterella id.2892)' on 'Enhancer of mRNA−decapping protein 4'

Batch 2−1779 : Gut microbiota abundance (genus Catenibacterium id.2153) on Epididymal secretory protein E3−beta
with a threshold of 1e−05

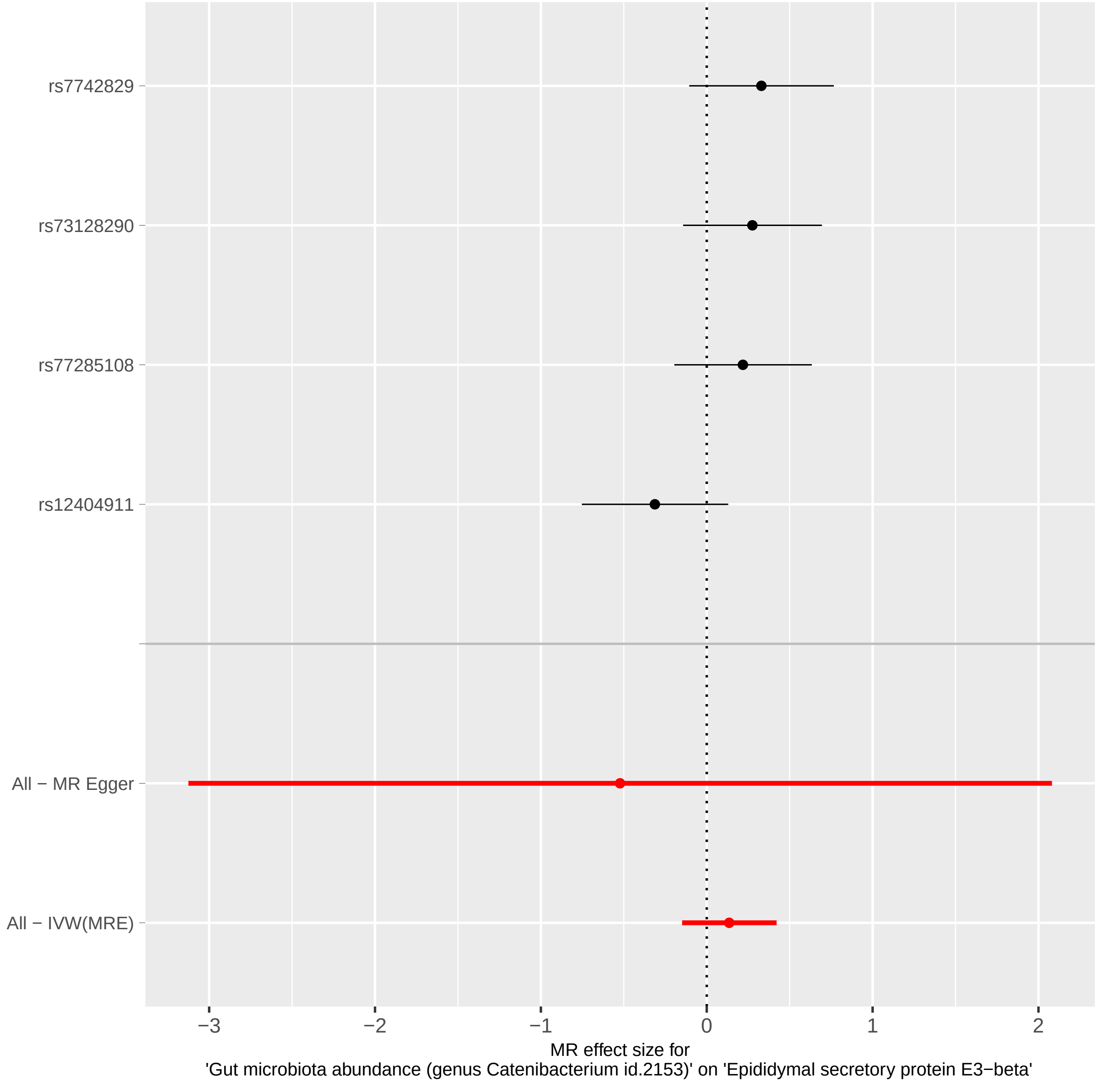

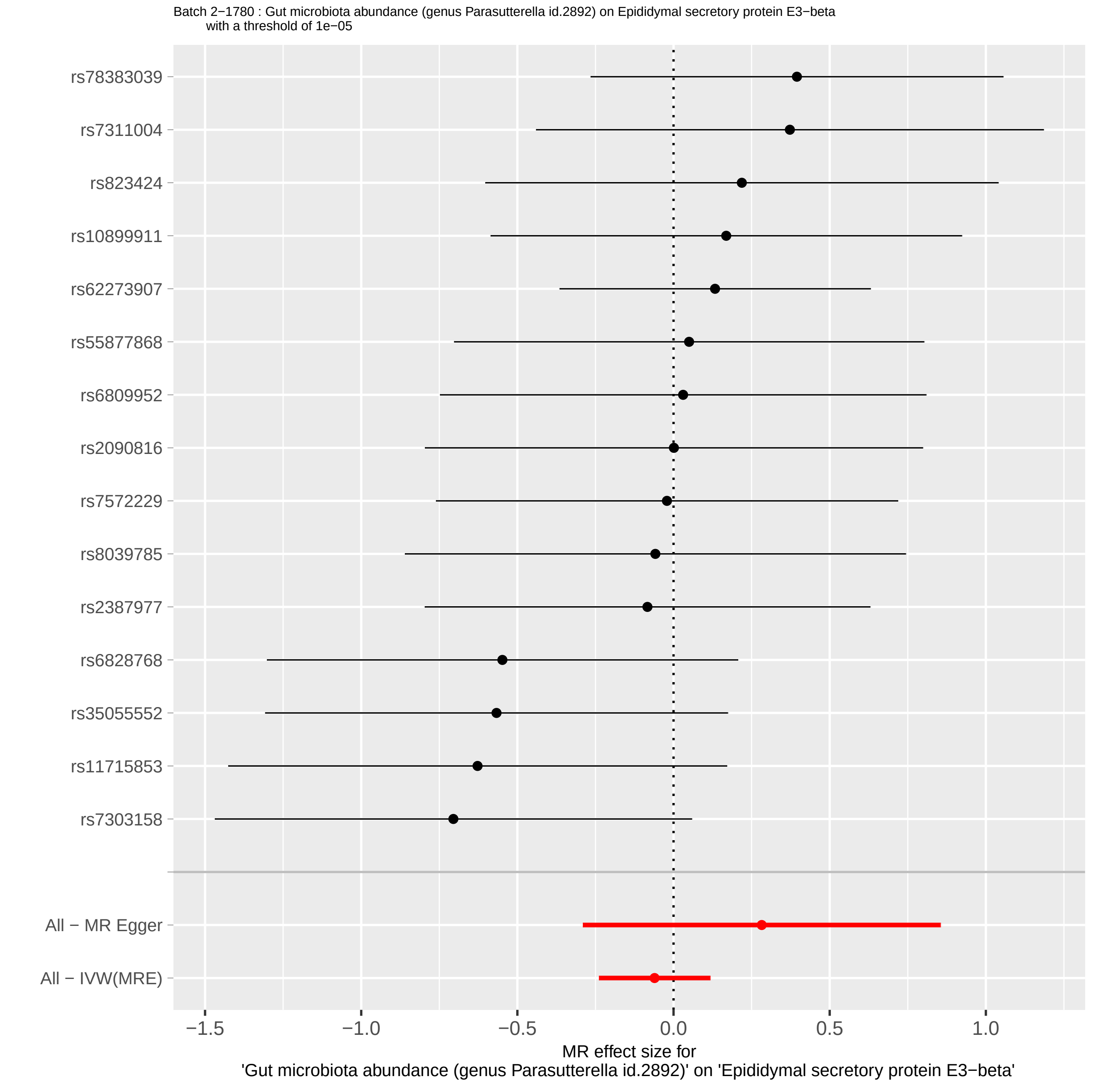

Batch 2−1780 : Gut microbiota abundance (genus Parasutterella id.2892) on Epididymal secretory protein E3−beta
with a threshold of 1e−05
rs78383039
rs7311004
rs823424
rs10899911
rs62273907
rs55877868
rs6809952
rs2090816
rs7572229
rs8039785
rs2387977
rs6828768
rs35055552
rs11715853
rs7303158
All − MR Egger
All − IVW(MRE)
−1.5
−1.0
−0.5
0.0
0.5
1.0
MR effect size for
'Gut microbiota abundance (genus Parasutterella id.2892)' on 'Epididymal secretory protein E3−beta'

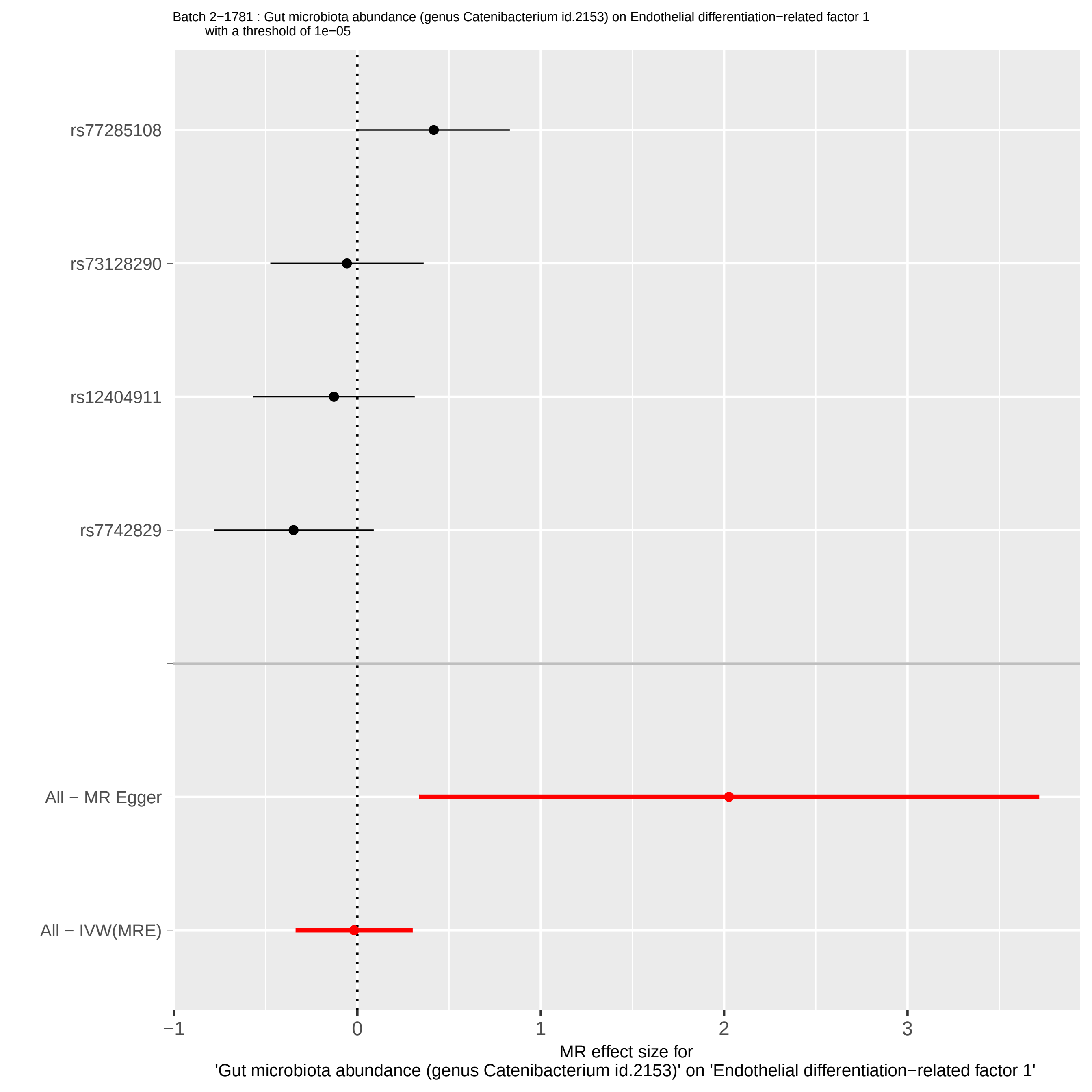

Batch 2−1781 : Gut microbiota abundance (genus Catenibacterium id.2153) on Endothelial differentiation−related factor 1
with a threshold of 1e−05
rs77285108
rs73128290
rs12404911
rs7742829
All − MR Egger
All − IVW(MRE)
−1
0
1
2
3
MR effect size for
'Gut microbiota abundance (genus Catenibacterium id.2153)' on 'Endothelial differentiation−related factor 1'

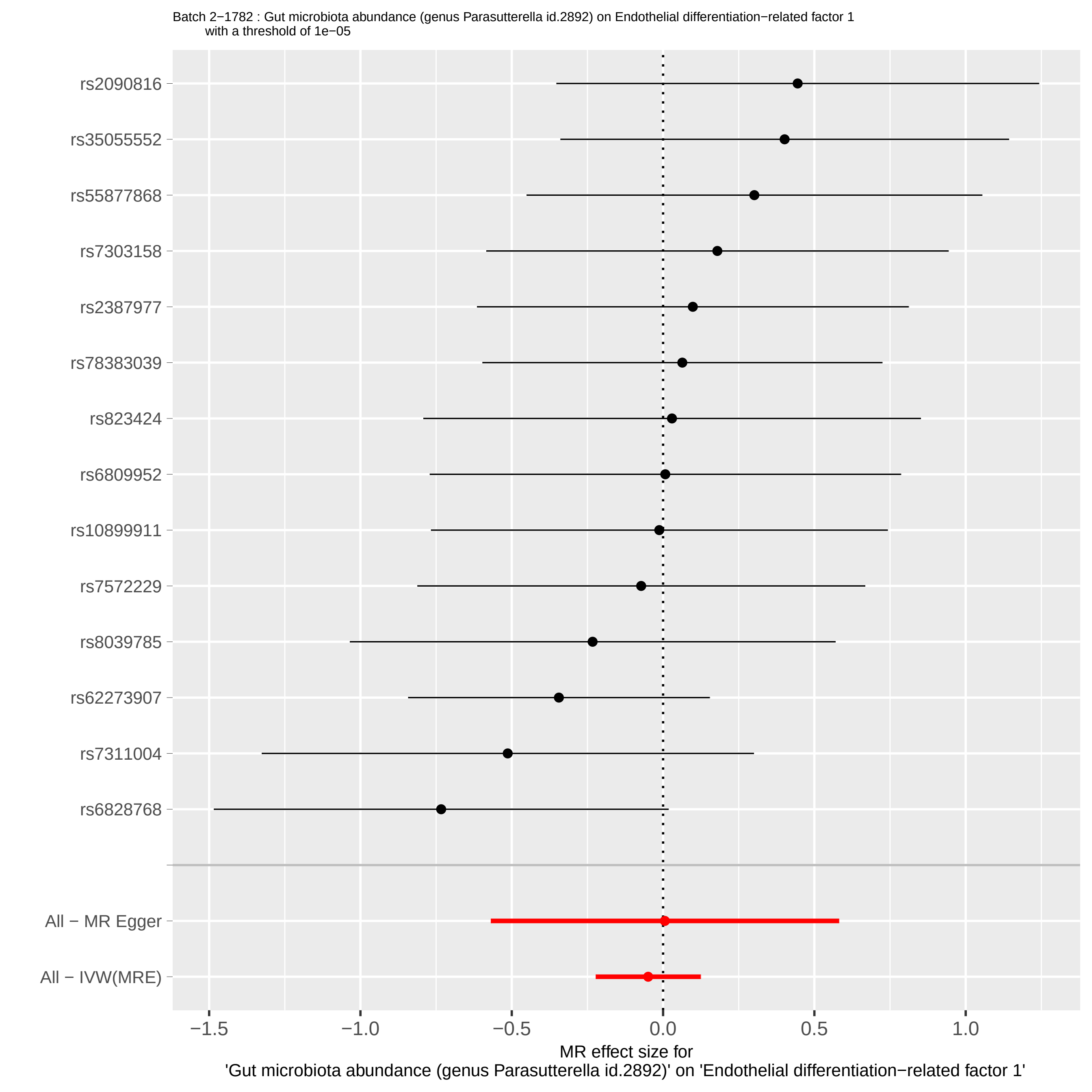

Batch 2−1782 : Gut microbiota abundance (genus Parasutterella id.2892) on Endothelial differentiation−related factor 1
with a threshold of 1e−05
rs2090816
rs35055552
rs55877868
rs7303158
rs2387977
rs78383039
rs823424
rs6809952
rs10899911
rs7572229
rs8039785
rs62273907
rs7311004
rs6828768
All − MR Egger
All − IVW(MRE)
−1.5
−1.0
−0.5
0.0
0.5
1.0
MR effect size for
'Gut microbiota abundance (genus Parasutterella id.2892)' on 'Endothelial differentiation−related factor 1'

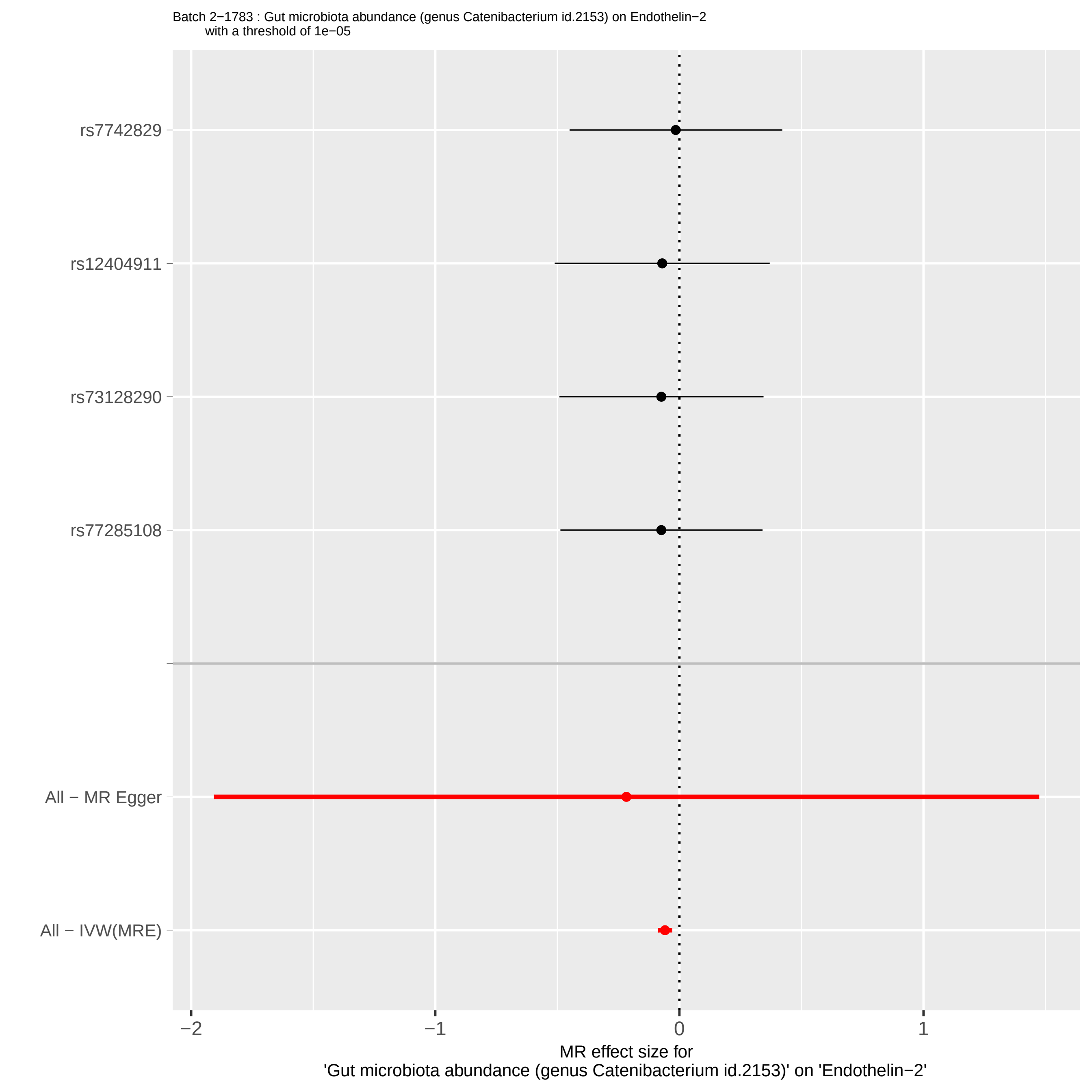

Batch 2−1783 : Gut microbiota abundance (genus Catenibacterium id.2153) on Endothelin−2
with a threshold of 1e−05
rs7742829
rs12404911
rs73128290
rs77285108
All − MR Egger
All − IVW(MRE)
−2
−1
0
1
MR effect size for
'Gut microbiota abundance (genus Catenibacterium id.2153)' on 'Endothelin−2'

Batch 2−1784 : Gut microbiota abundance (genus Parasutterella id.2892) on Endothelin−2
with a threshold of 1e−05

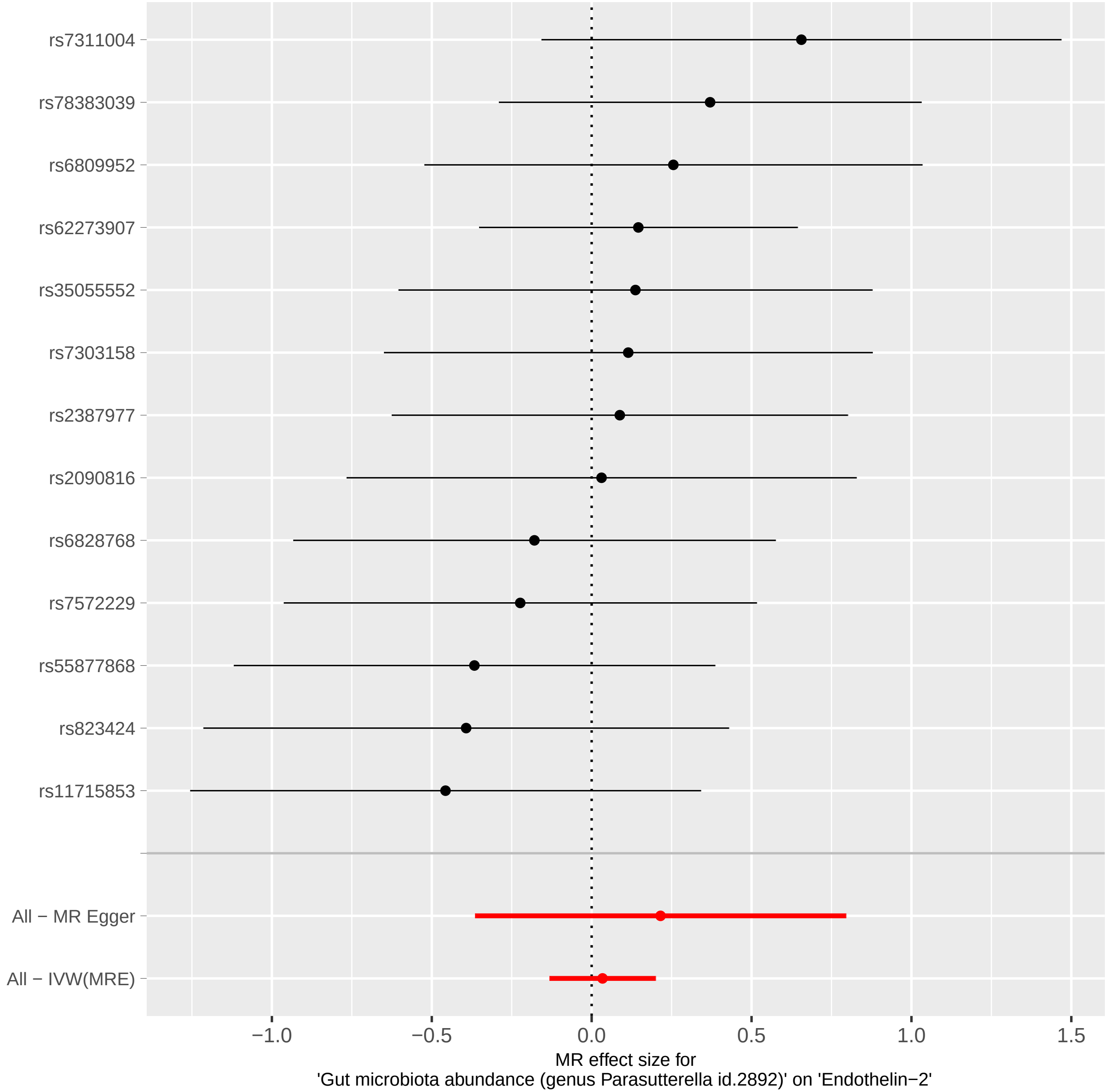

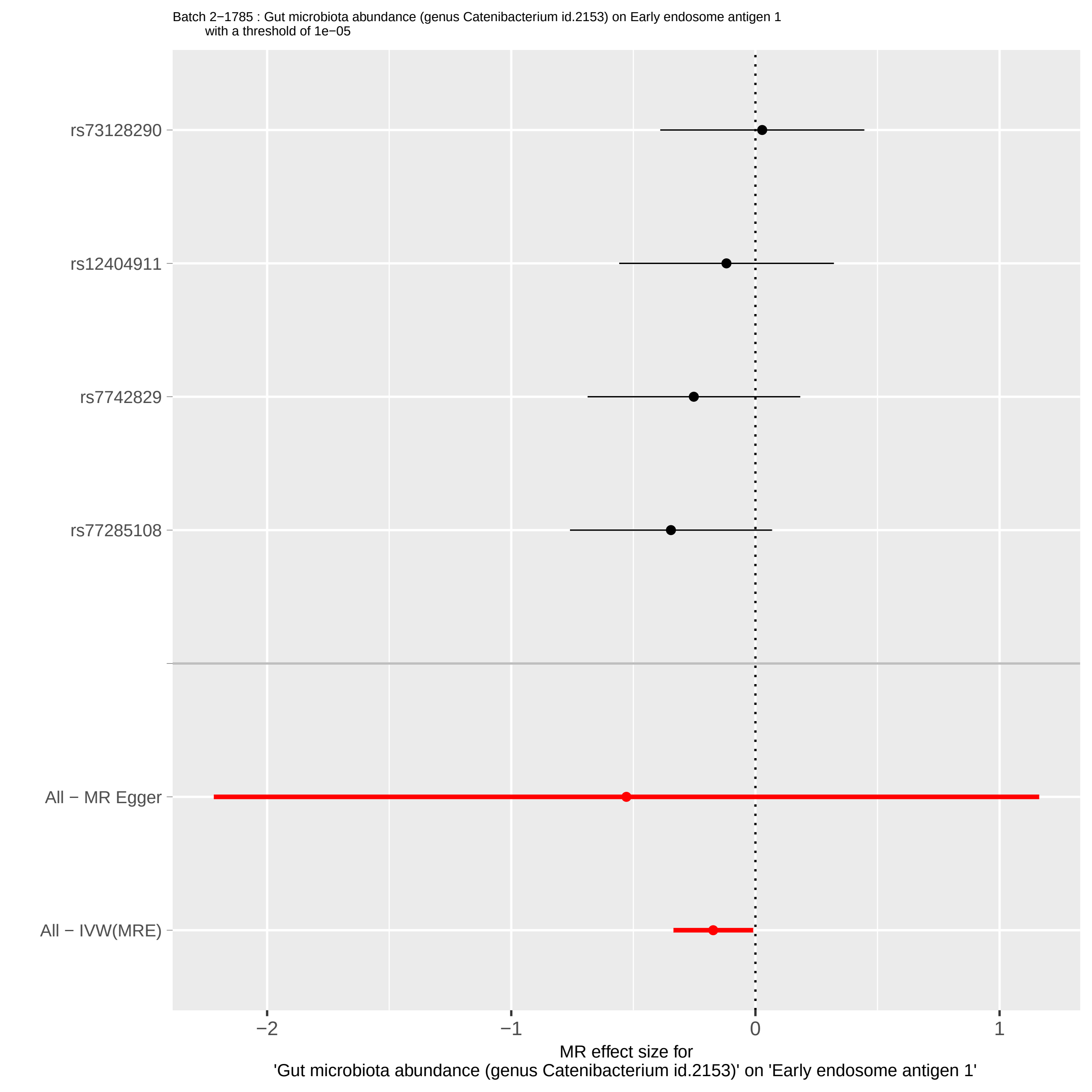

Batch 2−1785 : Gut microbiota abundance (genus Catenibacterium id.2153) on Early endosome antigen 1
with a threshold of 1e−05
rs73128290
rs12404911
rs7742829
rs77285108
All − MR Egger
All − IVW(MRE)
−2
−1
0
1
MR effect size for
'Gut microbiota abundance (genus Catenibacterium id.2153)' on 'Early endosome antigen 1'

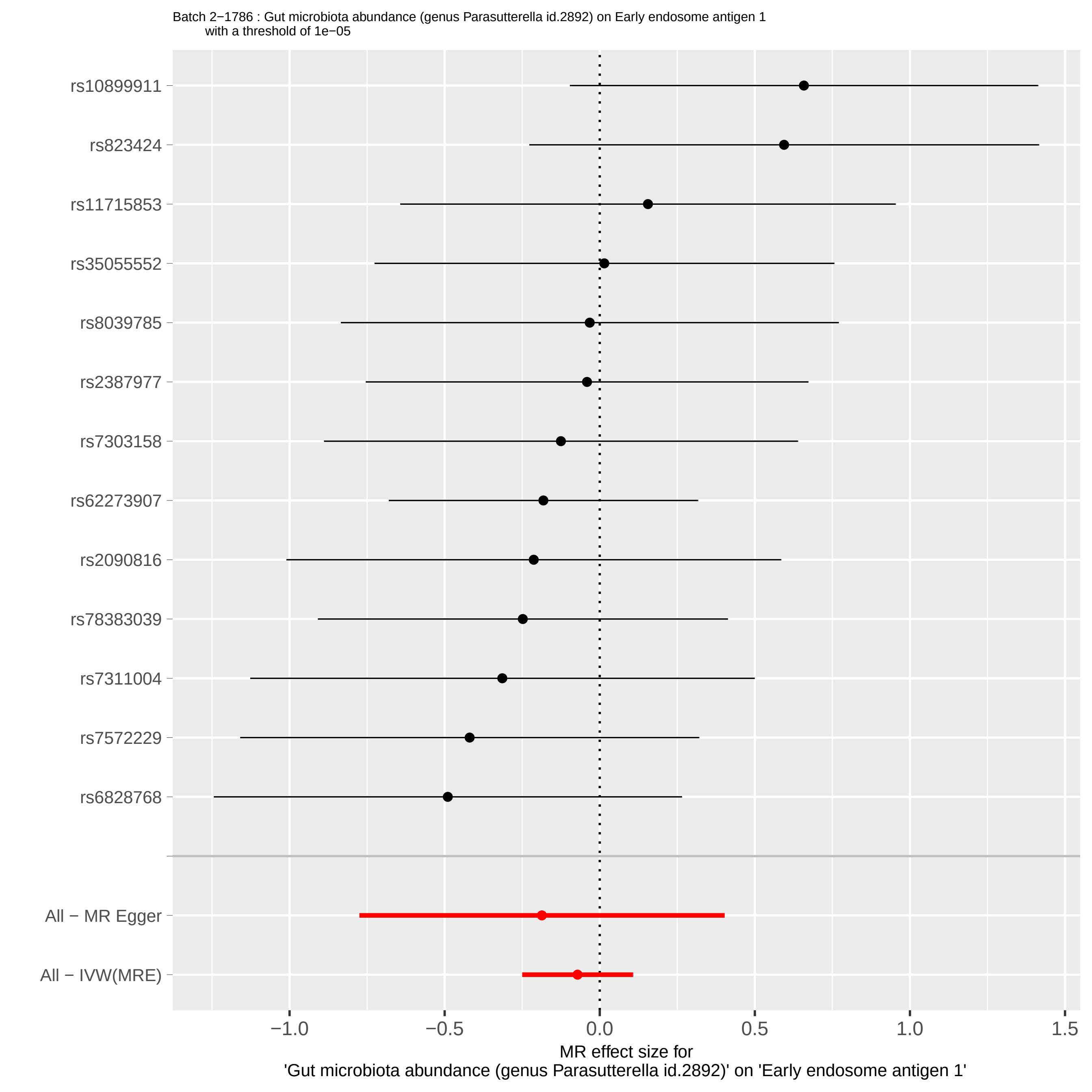

Batch 2−1786 : Gut microbiota abundance (genus Parasutterella id.2892) on Early endosome antigen 1
with a threshold of 1e−05
rs10899911
rs823424
rs11715853
rs35055552
rs8039785
rs2387977
rs7303158
rs62273907
rs2090816
rs78383039
rs7311004
rs7572229
rs6828768
All − MR Egger
All − IVW(MRE)
−1.0
−0.5
0.0
0.5
1.0
1.5
MR effect size for
'Gut microbiota abundance (genus Parasutterella id.2892)' on 'Early endosome antigen 1'

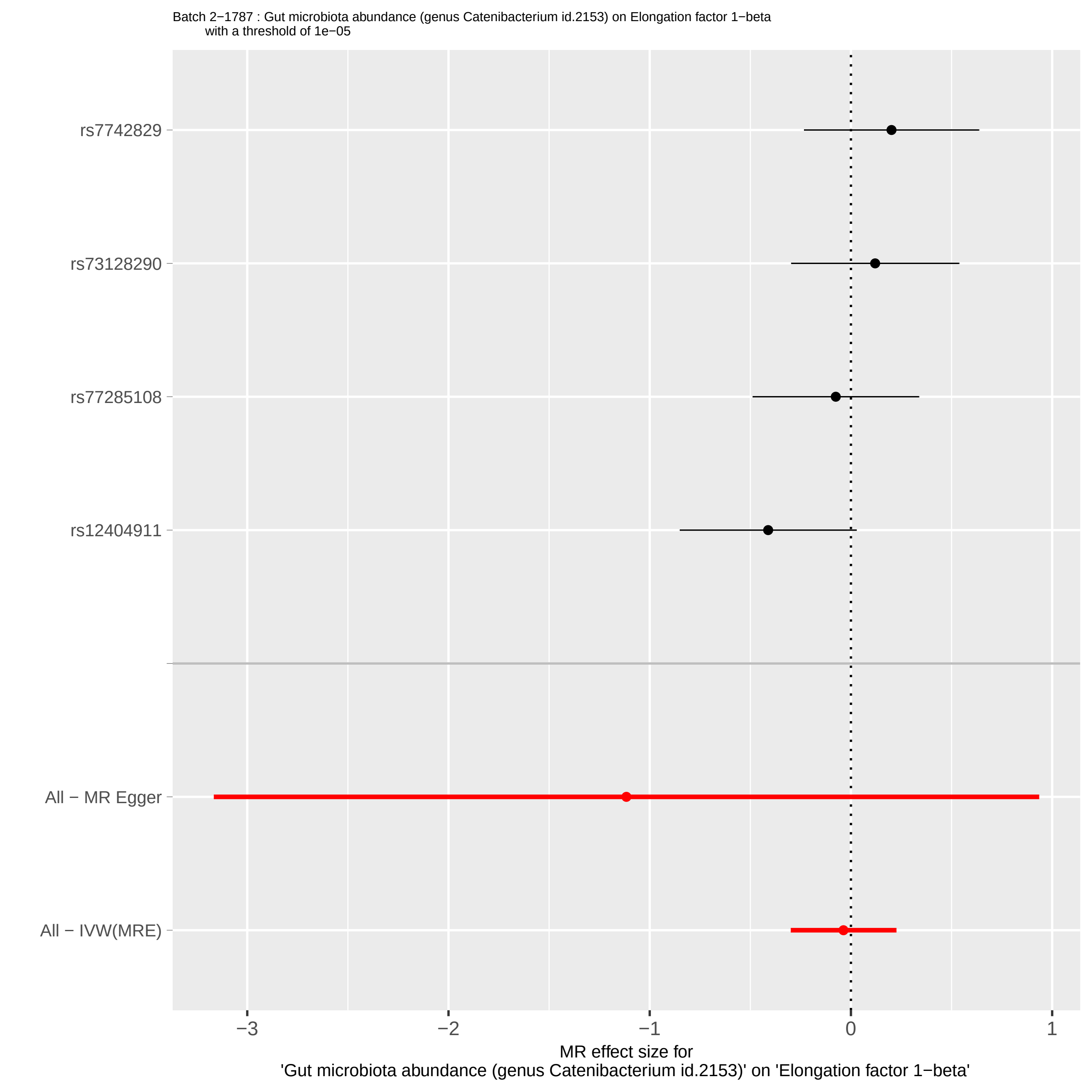
Batch 2−1787 : Gut microbiota abundance (genus Catenibacterium id.2153) on Elongation factor 1−beta
with a threshold of 1e−05
rs7742829
rs73128290
rs77285108
rs12404911
All − MR Egger
All − IVW(MRE)
−3
−2
−1
0
1
MR effect size for
'Gut microbiota abundance (genus Catenibacterium id.2153)' on 'Elongation factor 1−beta'

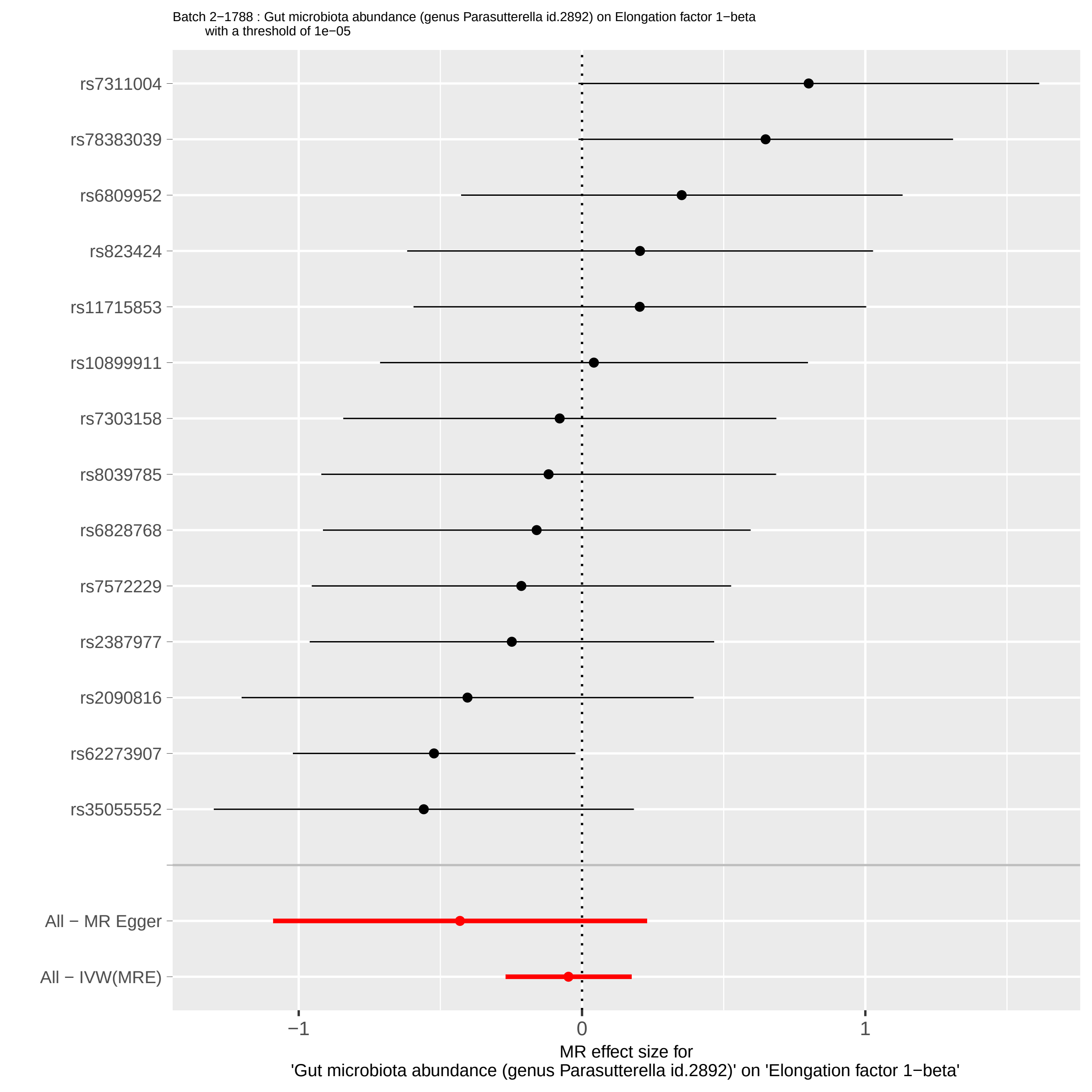
Batch 2−1788 : Gut microbiota abundance (genus Parasutterella id.2892) on Elongation factor 1−beta
with a threshold of 1e−05
rs7311004
rs78383039
rs6809952
rs823424
rs11715853
rs10899911
rs7303158
rs8039785
rs6828768
rs7572229
rs2387977
rs2090816
rs62273907
rs35055552
All − MR Egger
All − IVW(MRE)
−1
0
1
MR effect size for
'Gut microbiota abundance (genus Parasutterella id.2892)' on 'Elongation factor 1−beta'

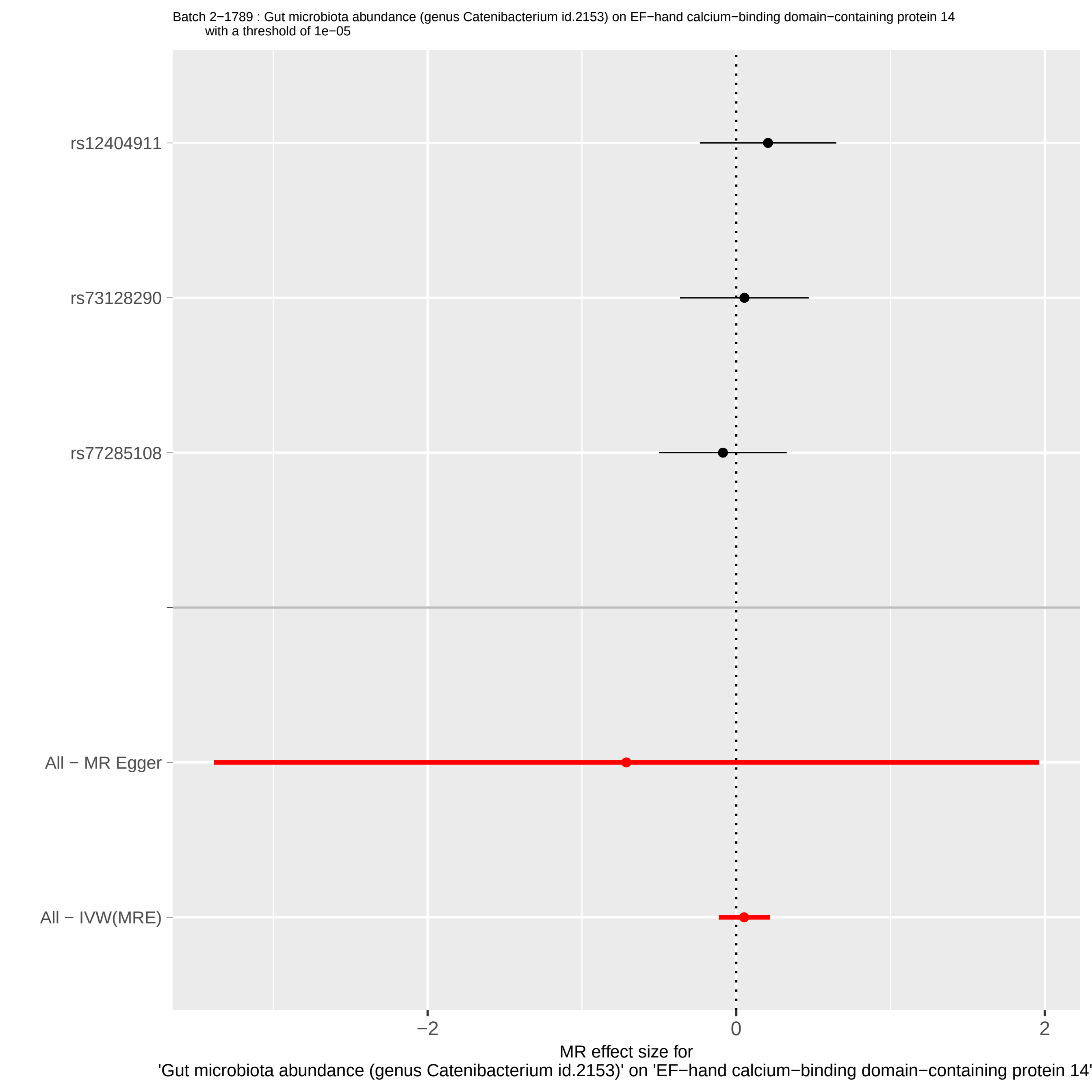

Batch 2−1789 : Gut microbiota abundance (genus Catenibacterium id.2153) on EF−hand calcium−binding domain−containing protein 14
with a threshold of 1e−05
rs12404911
rs73128290
rs77285108
All − MR Egger
All − IVW(MRE)
−2
0
2
MR effect size for
'Gut microbiota abundance (genus Catenibacterium id.2153)' on 'EF−hand calcium−binding domain−containing protein 14'

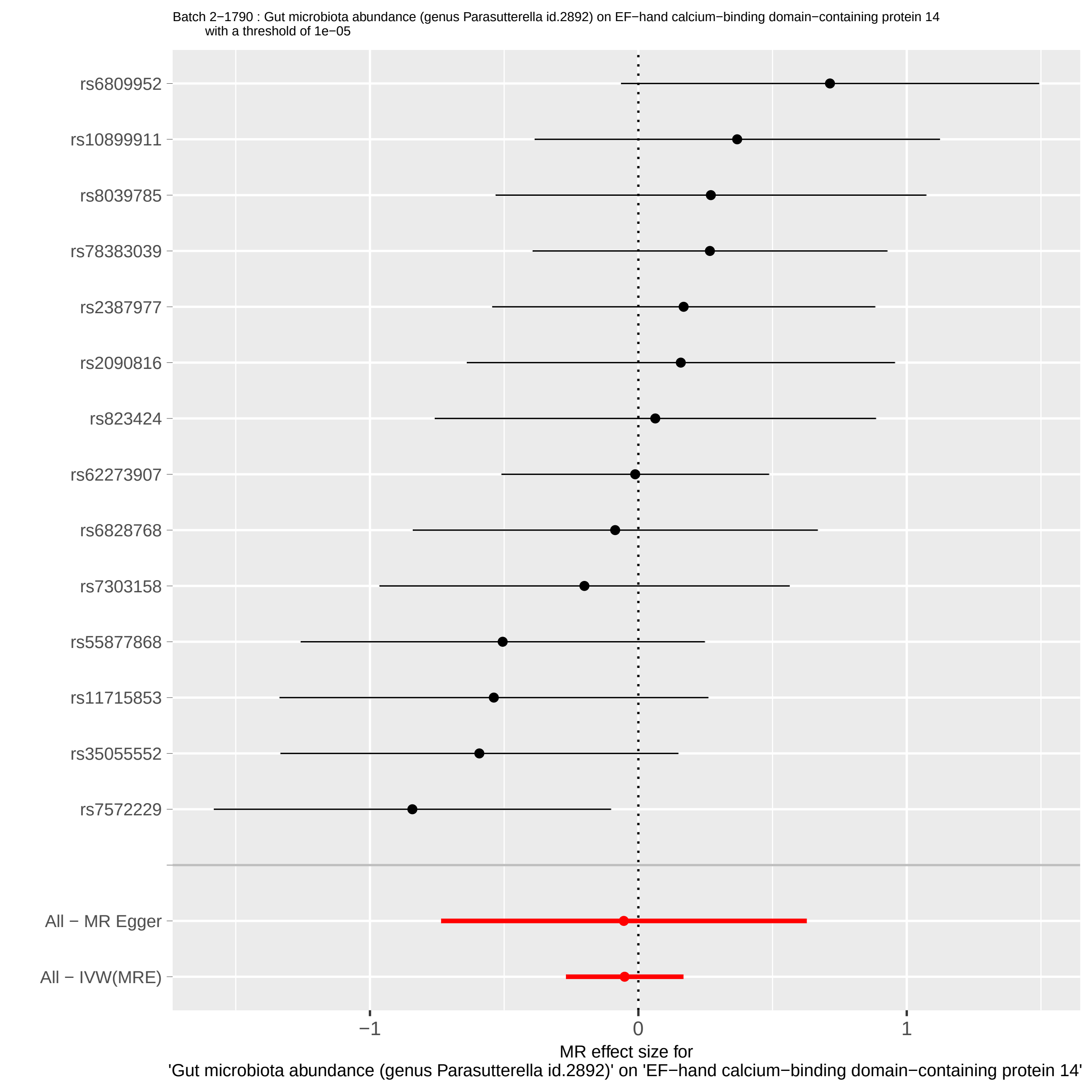
Batch 2−1790 : Gut microbiota abundance (genus Parasutterella id.2892) on EF−hand calcium−binding domain−containing protein 14
with a threshold of 1e−05
rs6809952
rs10899911
rs8039785
rs78383039
rs2387977
rs2090816
rs823424
rs62273907
rs6828768
rs7303158
rs55877868
rs11715853
rs35055552
rs7572229
All − MR Egger
All − IVW(MRE)
−1
0
1
MR effect size for
'Gut microbiota abundance (genus Parasutterella id.2892)' on 'EF−hand calcium−binding domain−containing protein 14'

Batch 2−1791 : Gut microbiota abundance (genus Catenibacterium id.2153) on Calcium uptake protein 3, mitochondrial
with a threshold of 1e−05

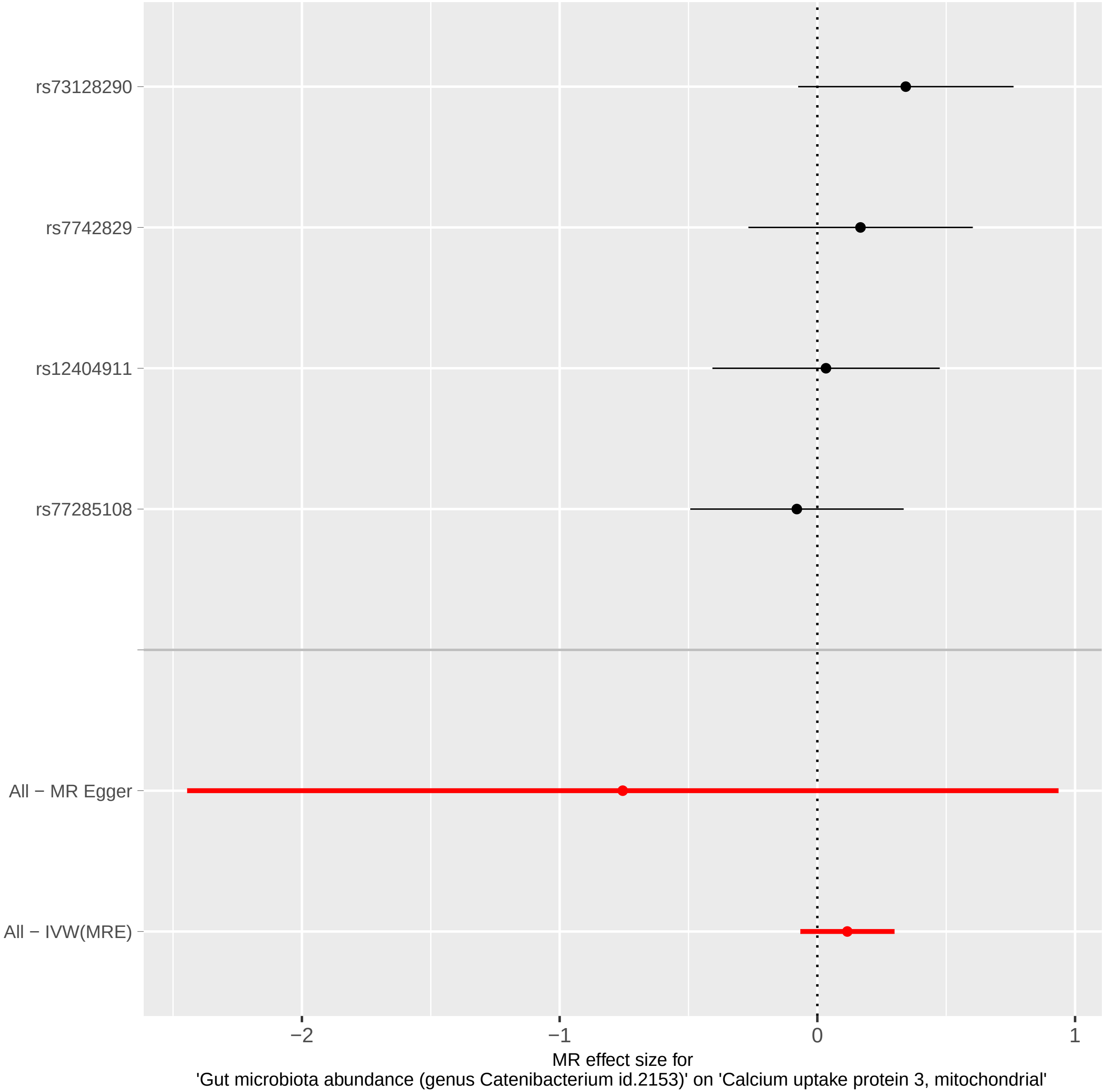

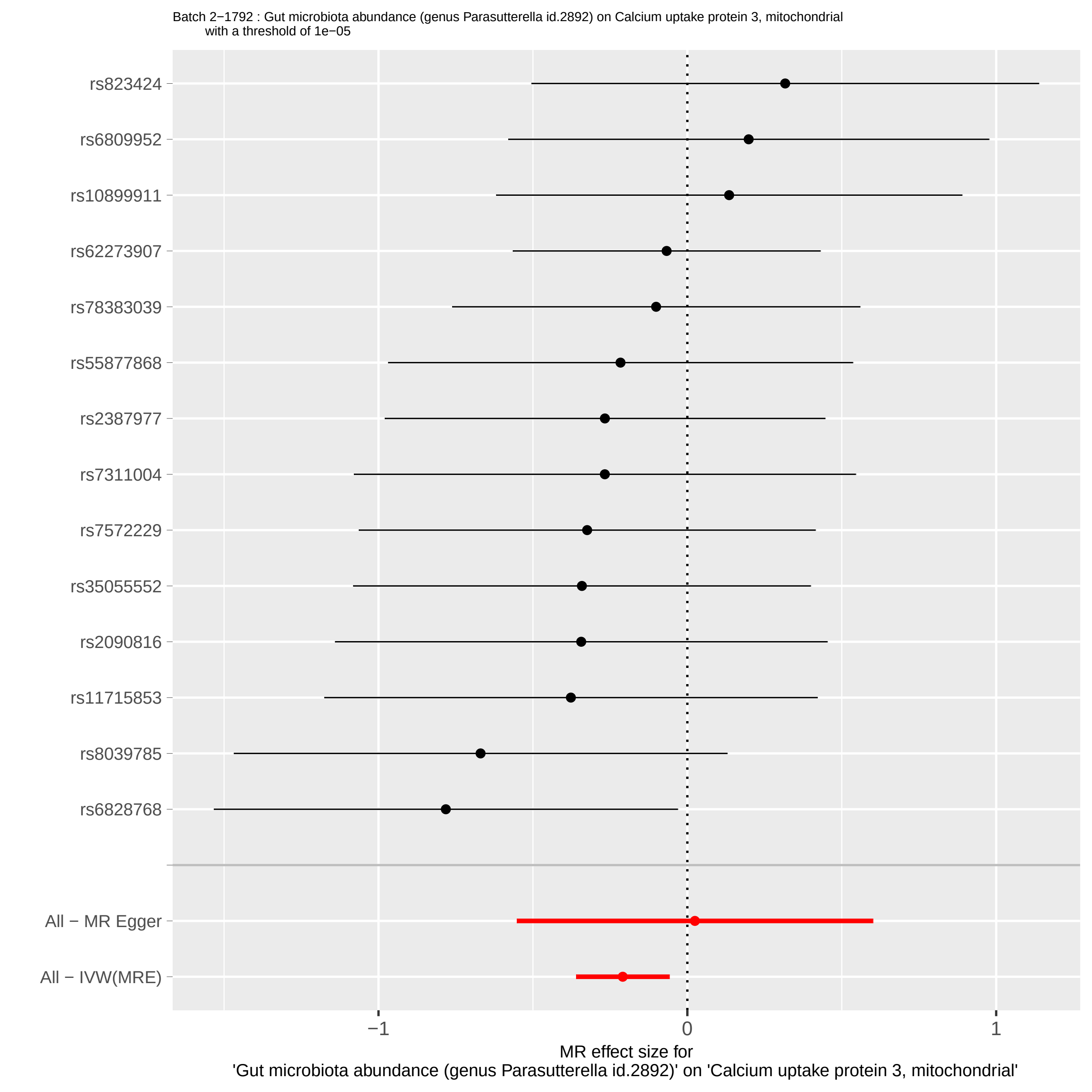
Batch 2−1792 : Gut microbiota abundance (genus Parasutterella id.2892) on Calcium uptake protein 3, mitochondrial
with a threshold of 1e−05
rs823424
rs6809952
rs10899911
rs62273907
rs78383039
rs55877868
rs2387977
rs7311004
rs7572229
rs35055552
rs2090816
rs11715853
rs8039785
rs6828768
All − MR Egger
All − IVW(MRE)
−1
0
1
MR effect size for
'Gut microbiota abundance (genus Parasutterella id.2892)' on 'Calcium uptake protein 3, mitochondrial'

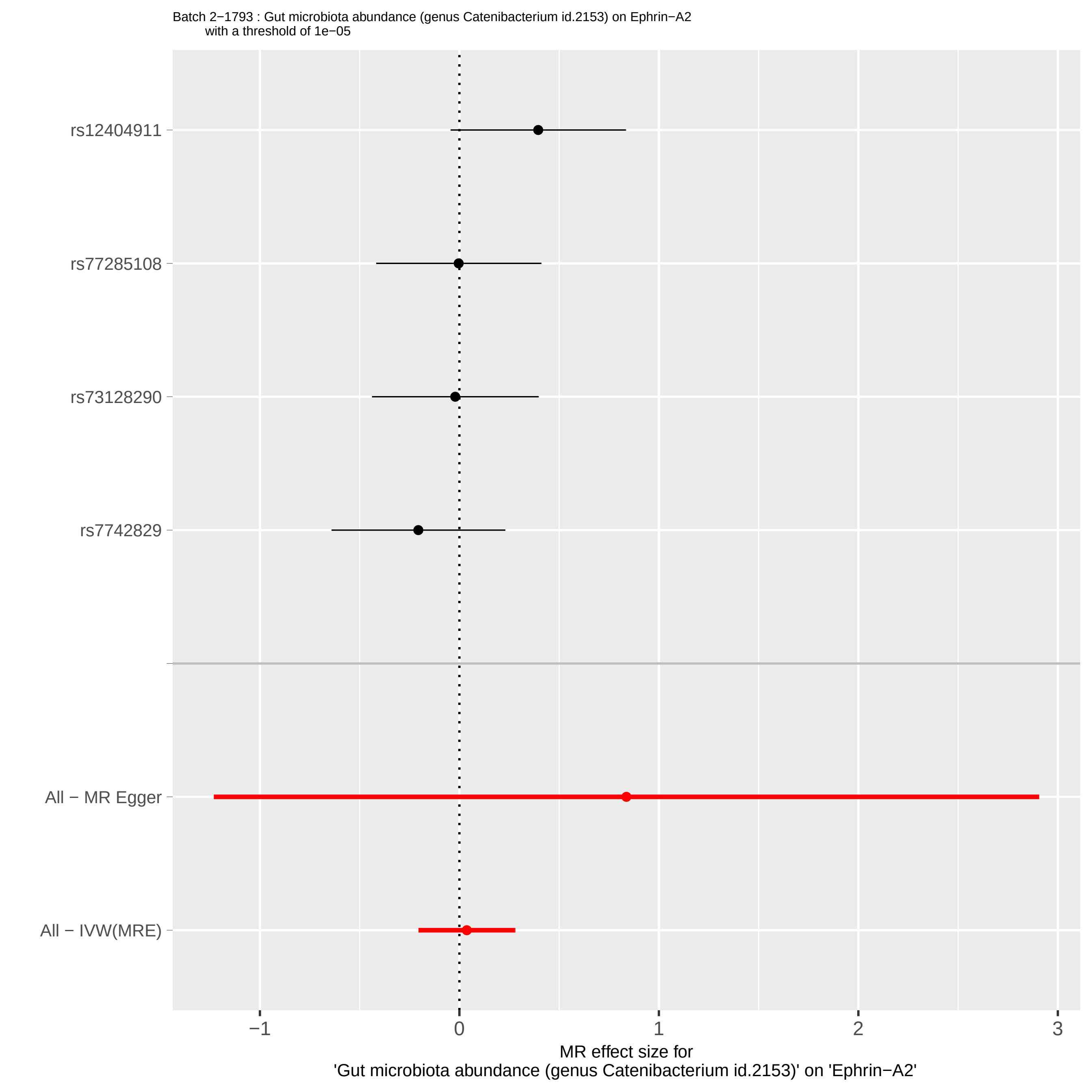
Batch 2−1793 : Gut microbiota abundance (genus Catenibacterium id.2153) on Ephrin−A2
with a threshold of 1e−05
rs12404911
rs77285108
rs73128290
rs7742829
All − MR Egger
All − IVW(MRE)
−1
0
1
2
3
MR effect size for
'Gut microbiota abundance (genus Catenibacterium id.2153)' on 'Ephrin−A2'

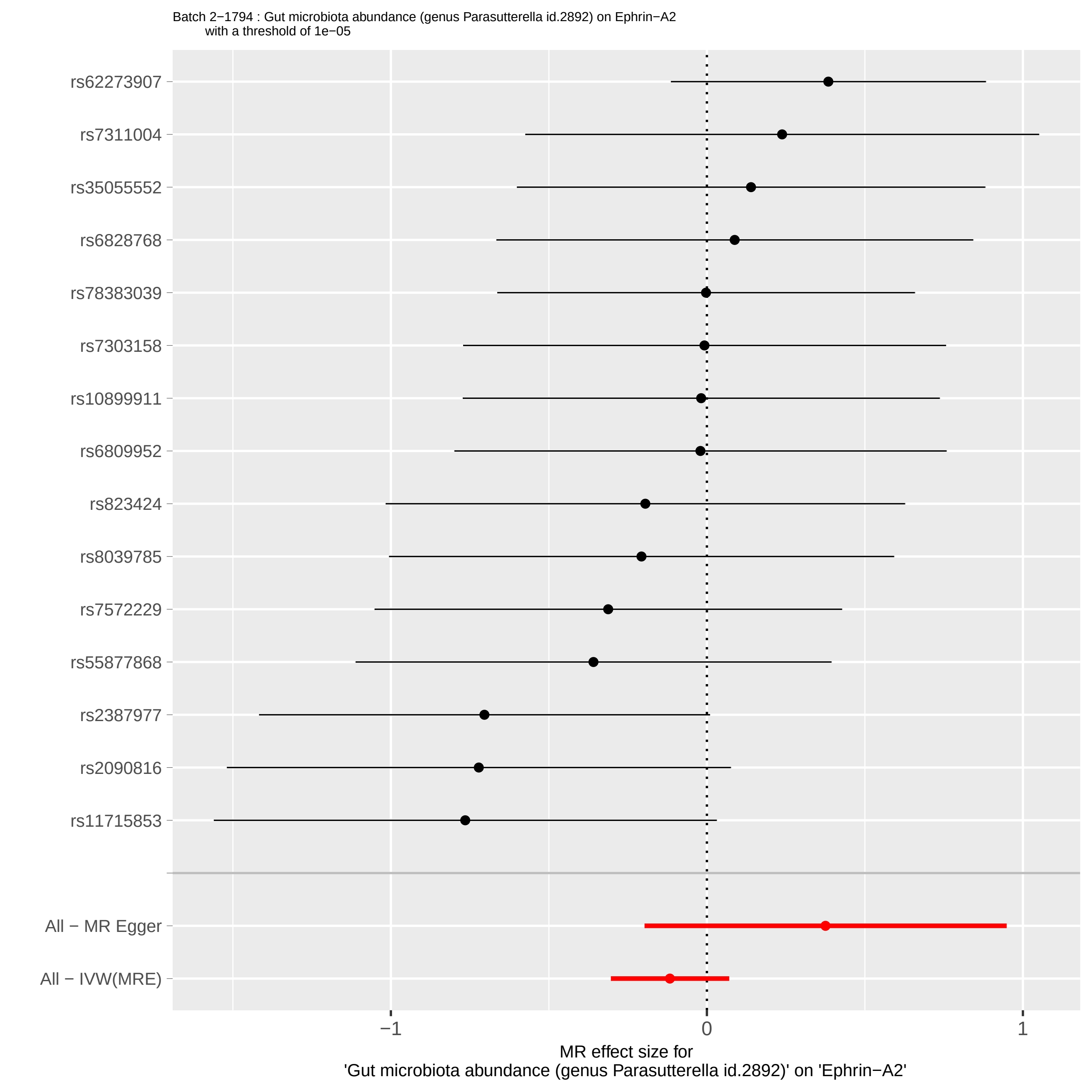
Batch 2−1794 : Gut microbiota abundance (genus Parasutterella id.2892) on Ephrin−A2
with a threshold of 1e−05
rs62273907
rs7311004
rs35055552
rs6828768
rs78383039
rs7303158
rs10899911
rs6809952
rs823424
rs8039785
rs7572229
rs55877868
rs2387977
rs2090816
rs11715853
All − MR Egger
All − IVW(MRE)
−1
0
1
MR effect size for
'Gut microbiota abundance (genus Parasutterella id.2892)' on 'Ephrin−A2'

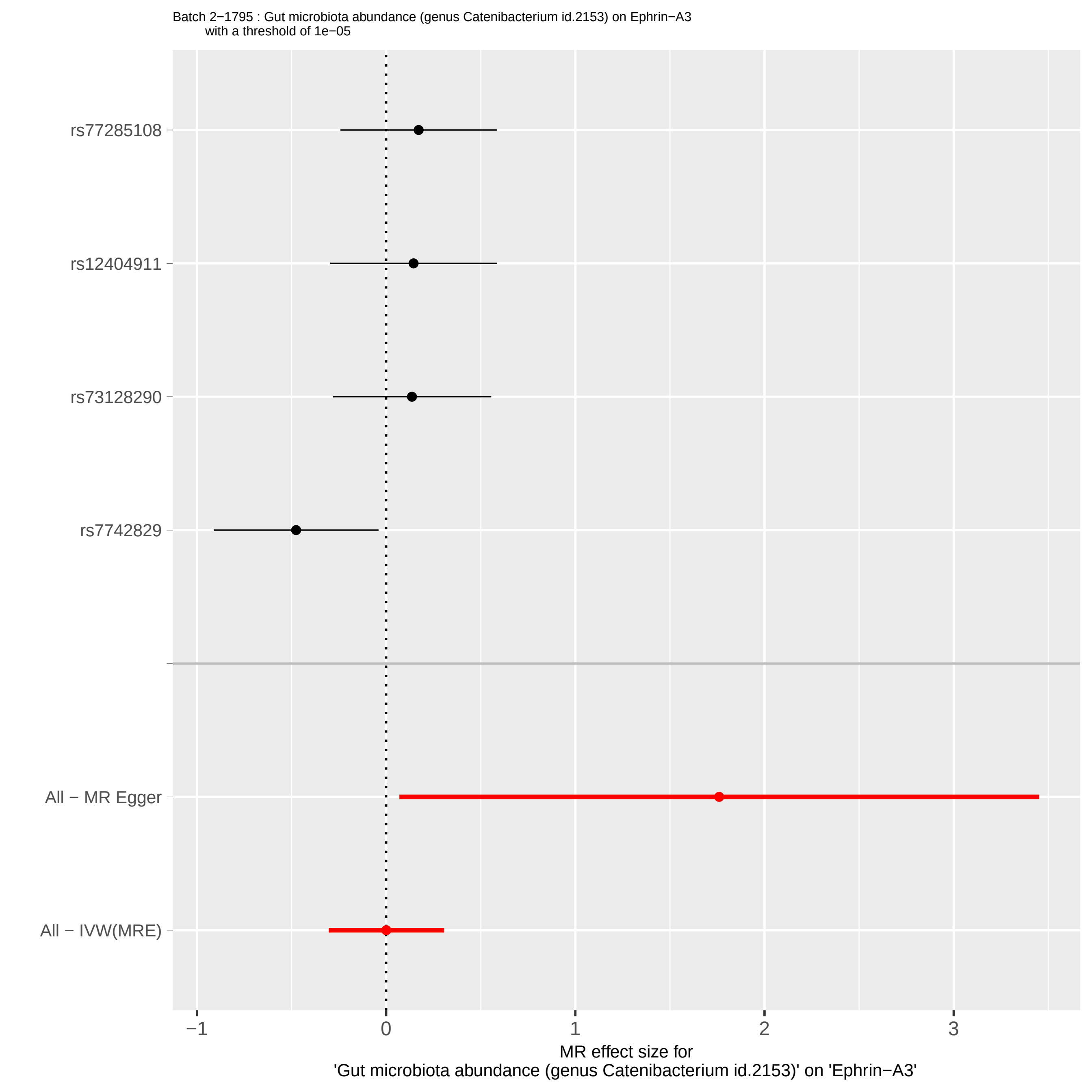
Batch 2−1795 : Gut microbiota abundance (genus Catenibacterium id.2153) on Ephrin−A3
with a threshold of 1e−05
rs77285108
rs12404911
rs73128290
rs7742829
All − MR Egger
All − IVW(MRE)
−1
0
1
2
3
MR effect size for
'Gut microbiota abundance (genus Catenibacterium id.2153)' on 'Ephrin−A3'

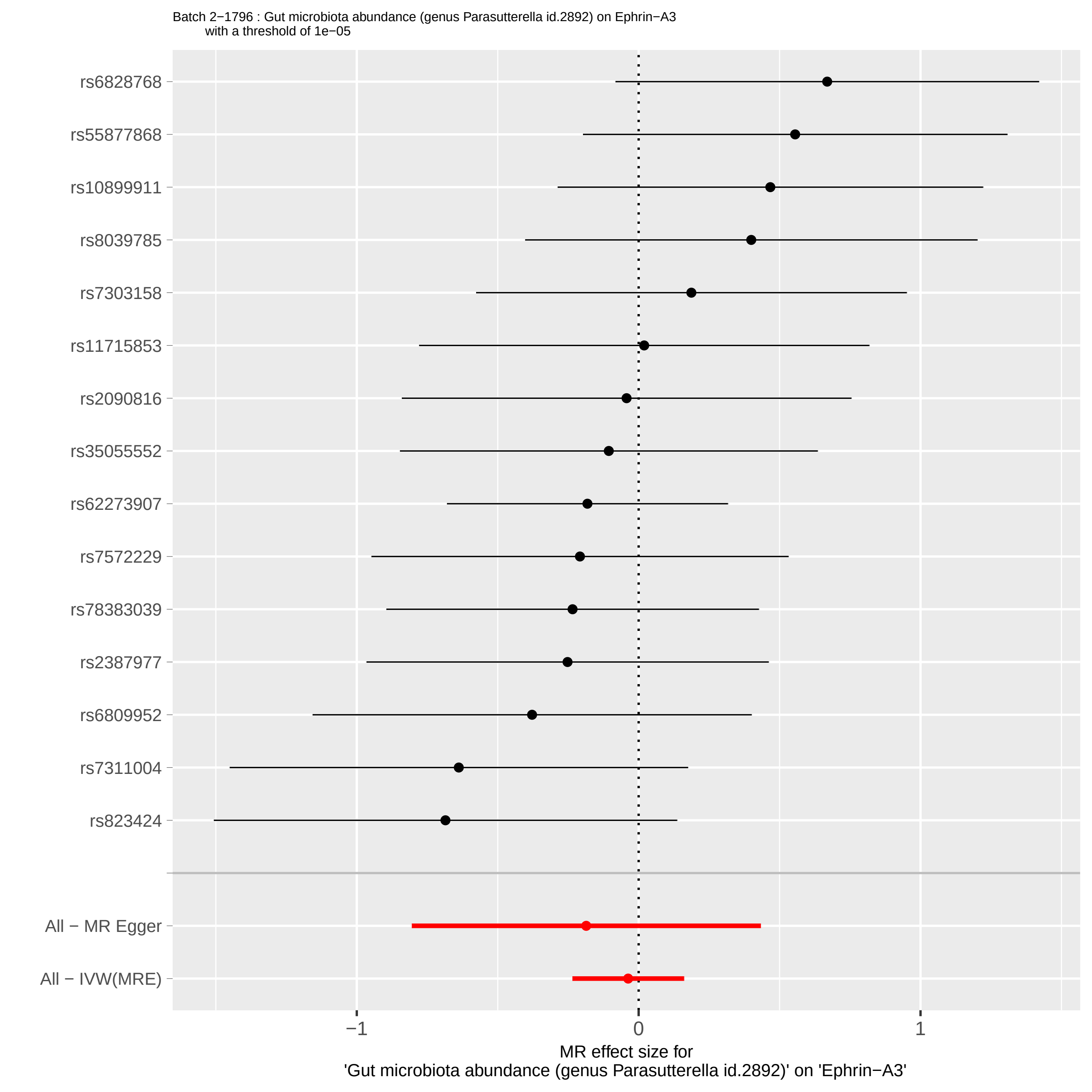
Batch 2−1796 : Gut microbiota abundance (genus Parasutterella id.2892) on Ephrin−A3
with a threshold of 1e−05
rs6828768
rs55877868
rs10899911
rs8039785
rs7303158
rs11715853
rs2090816
rs35055552
rs62273907
rs7572229
rs78383039
rs2387977
rs6809952
rs7311004
rs823424
All − MR Egger
All − IVW(MRE)
−1
0
1
MR effect size for
'Gut microbiota abundance (genus Parasutterella id.2892)' on 'Ephrin−A3'

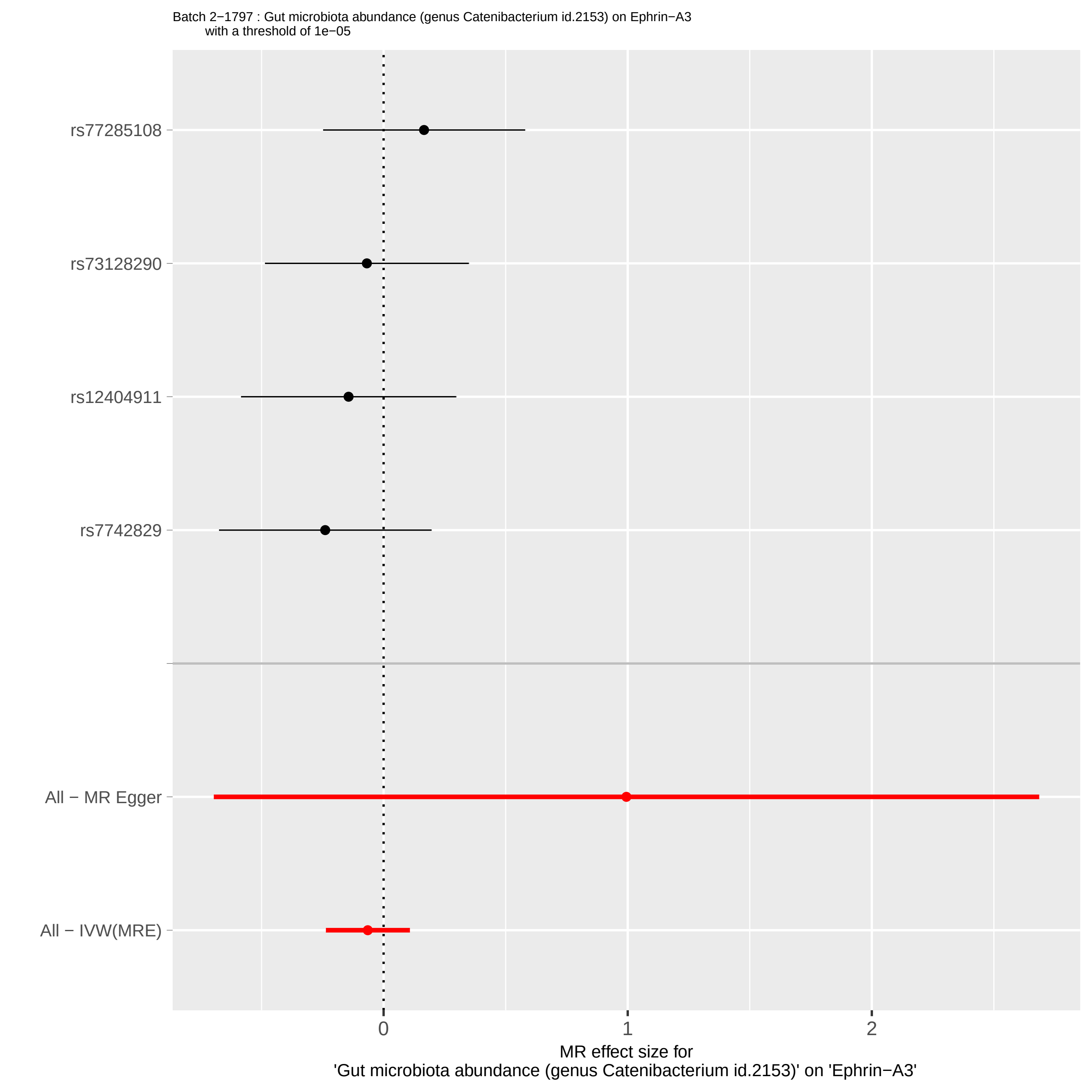

Batch 2−1797 : Gut microbiota abundance (genus Catenibacterium id.2153) on Ephrin−A3
with a threshold of 1e−05
rs77285108
rs73128290
rs12404911
rs7742829
All − MR Egger
All − IVW(MRE)
0
1
2
MR effect size for
'Gut microbiota abundance (genus Catenibacterium id.2153)' on 'Ephrin−A3'

Batch 2−1798 : Gut microbiota abundance (genus Parasutterella id.2892) on Ephrin−A3
with a threshold of 1e−05

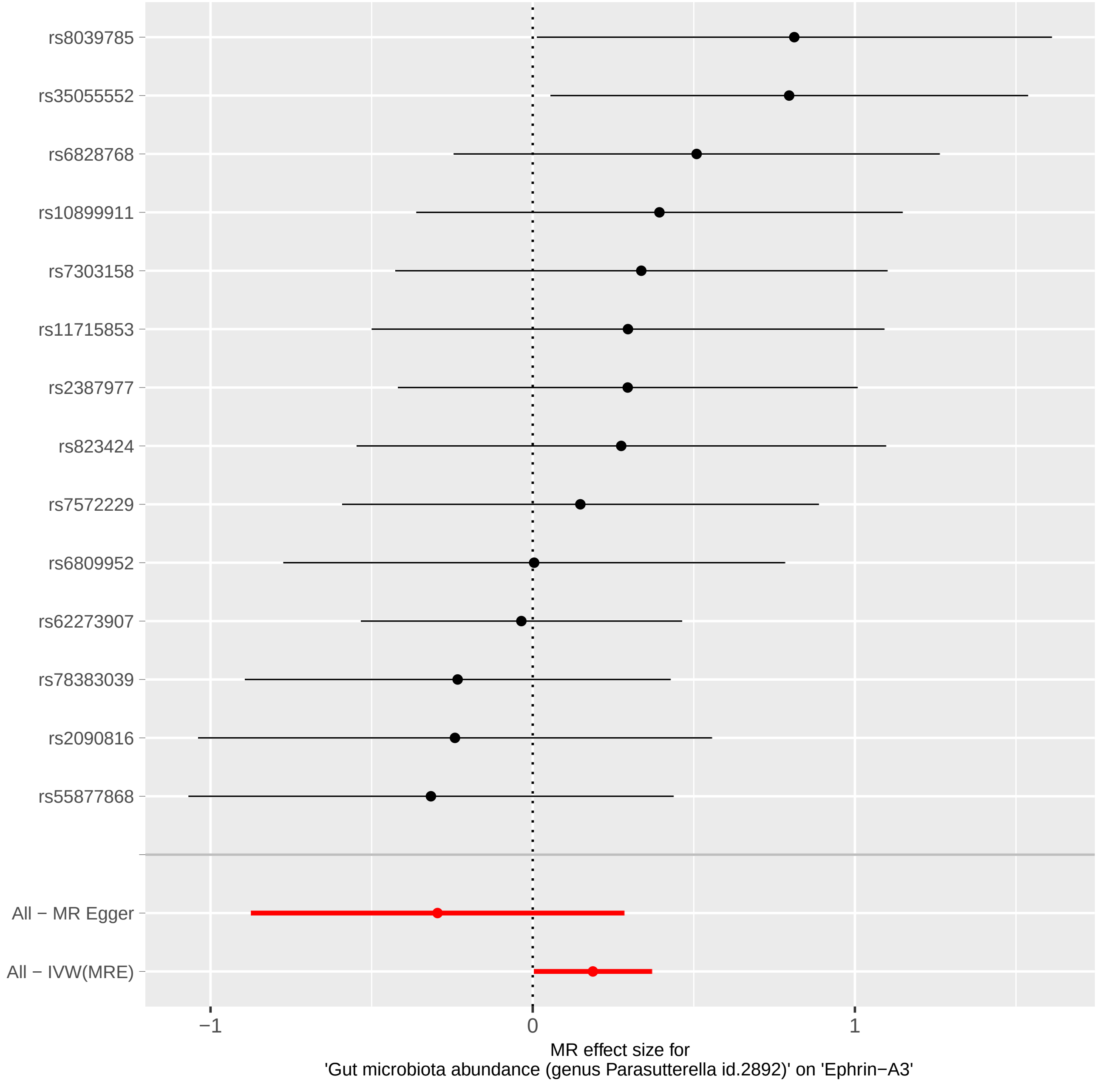

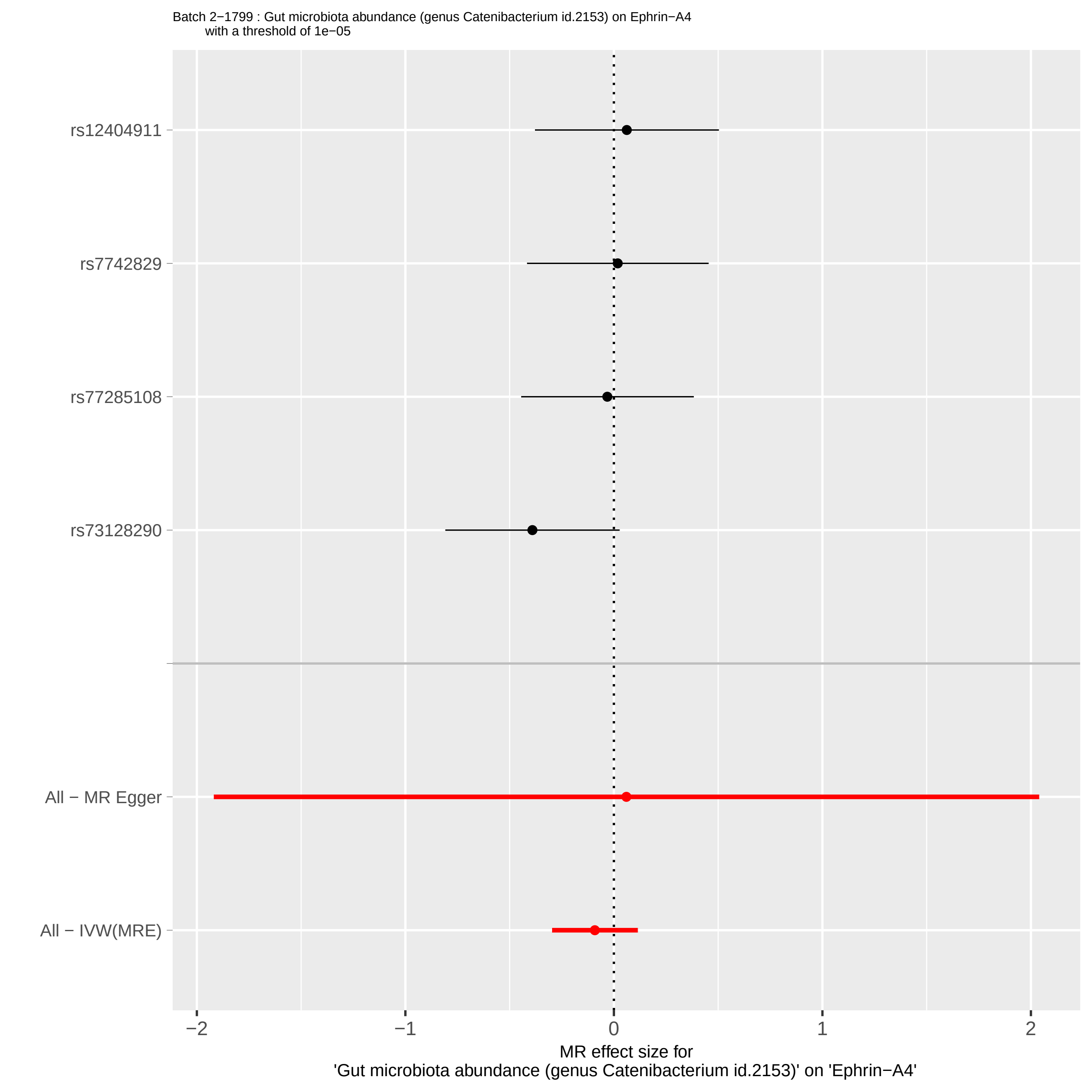

Batch 2−1799 : Gut microbiota abundance (genus Catenibacterium id.2153) on Ephrin−A4
with a threshold of 1e−05
rs12404911
rs7742829
rs77285108
rs73128290
All − MR Egger
All − IVW(MRE)
−2
−1
0
1
2
MR effect size for
'Gut microbiota abundance (genus Catenibacterium id.2153)' on 'Ephrin−A4'

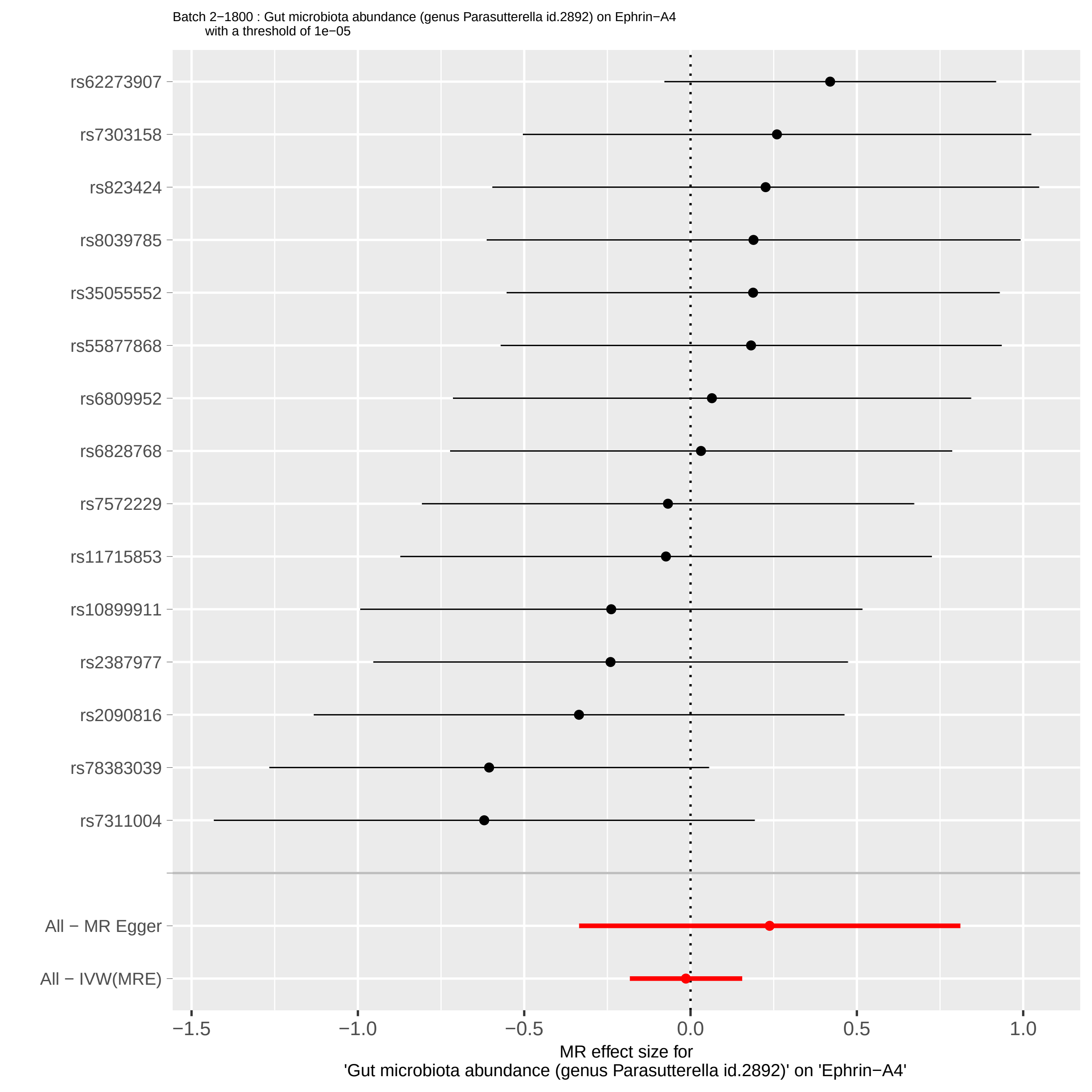

Batch 2−1800 : Gut microbiota abundance (genus Parasutterella id.2892) on Ephrin−A4
with a threshold of 1e−05
rs62273907
rs7303158
rs823424
rs8039785
rs35055552
rs55877868
rs6809952
rs6828768
rs7572229
rs11715853
rs10899911
rs2387977
rs2090816
rs78383039
rs7311004
All − MR Egger
All − IVW(MRE)
−1.5
−1.0
−0.5
0.0
0.5
1.0
MR effect size for
'Gut microbiota abundance (genus Parasutterella id.2892)' on 'Ephrin−A4'

Batch 2−1801 : Gut microbiota abundance (genus Catenibacterium id.2153) on Ephrin−A5
with a threshold of 1e−05

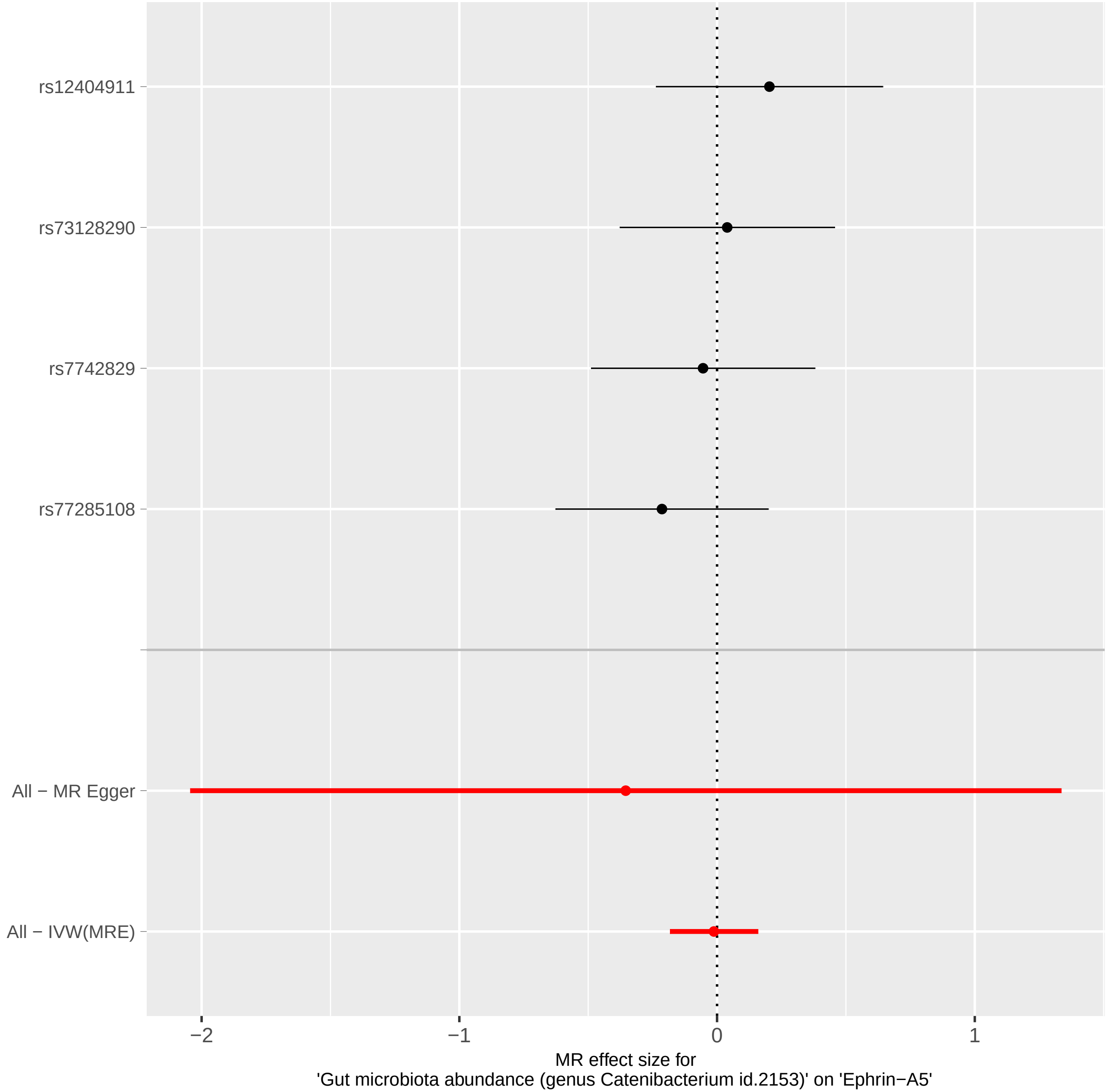

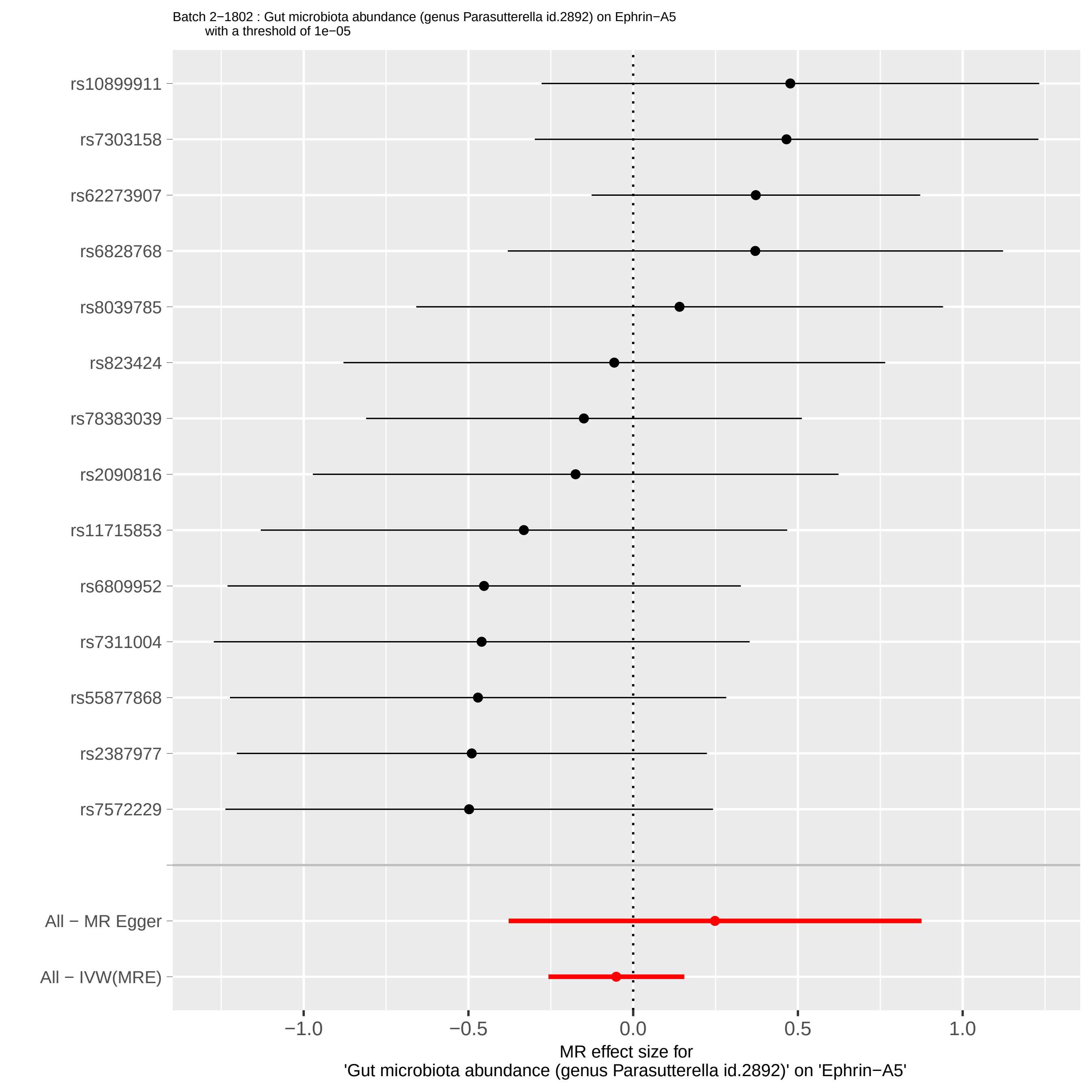

Batch 2−1802 : Gut microbiota abundance (genus Parasutterella id.2892) on Ephrin−A5
with a threshold of 1e−05
rs10899911
rs7303158
rs62273907
rs6828768
rs8039785
rs823424
rs78383039
rs2090816
rs11715853
rs6809952
rs7311004
rs55877868
rs2387977
rs7572229
All − MR Egger
All − IVW(MRE)
−1.0
−0.5
0.0
0.5
1.0
MR effect size for
'Gut microbiota abundance (genus Parasutterella id.2892)' on 'Ephrin−A5'

Batch 2−1803 : Gut microbiota abundance (genus Catenibacterium id.2153) on Ephrin−B1
with a threshold of 1e−05

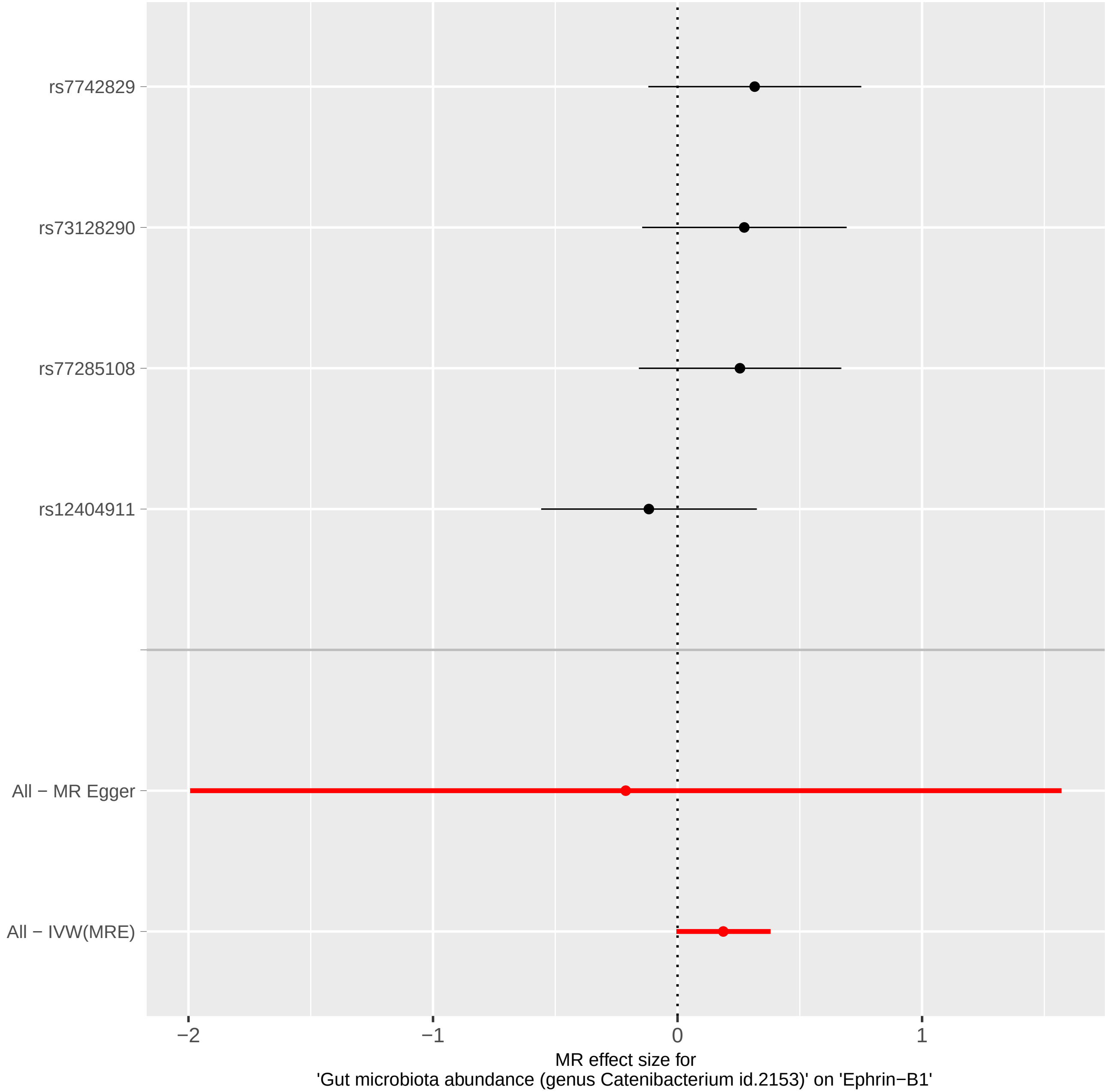

Batch 2−1804 : Gut microbiota abundance (genus Parasutterella id.2892) on Ephrin−B1
with a threshold of 1e−05

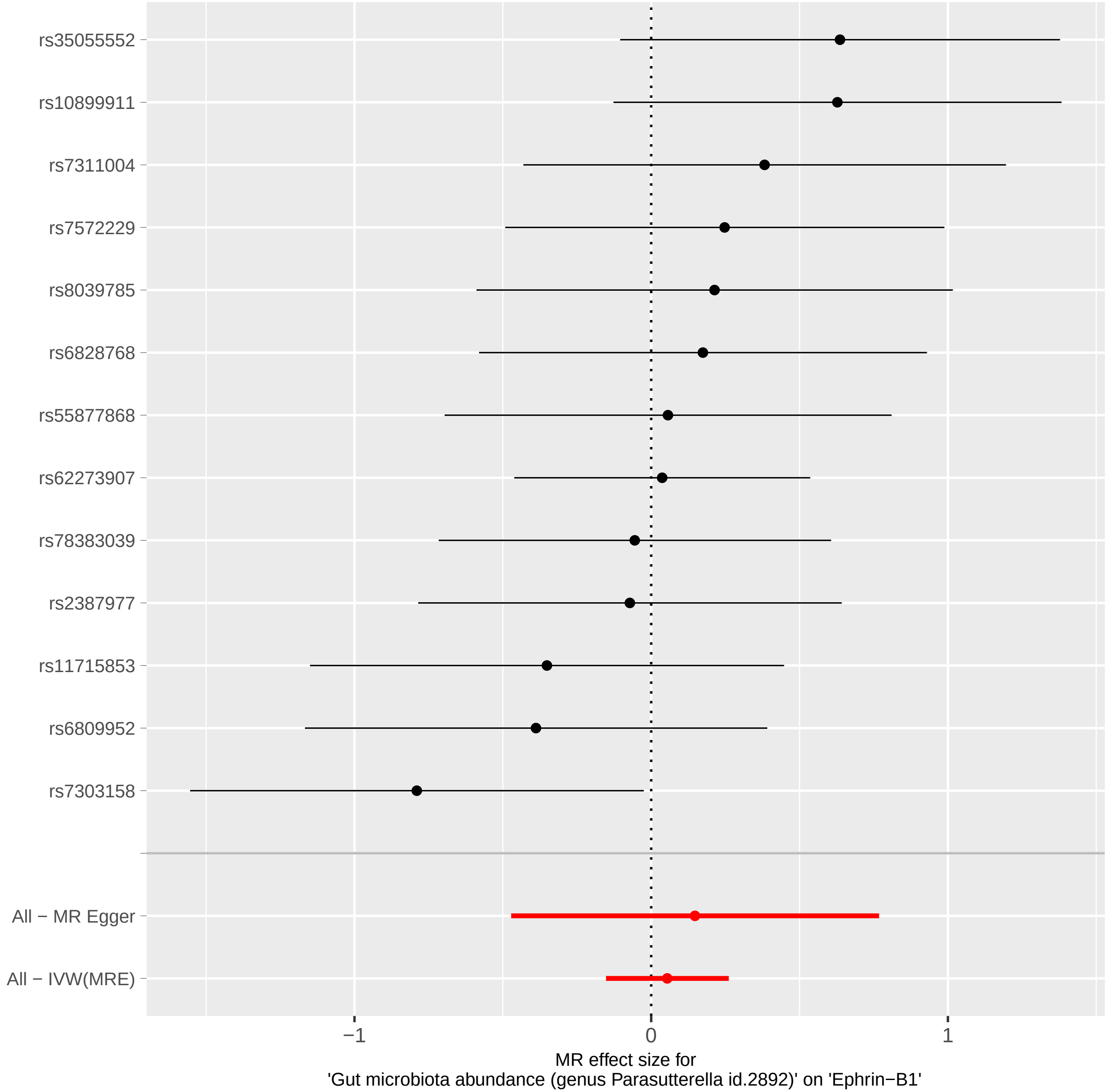

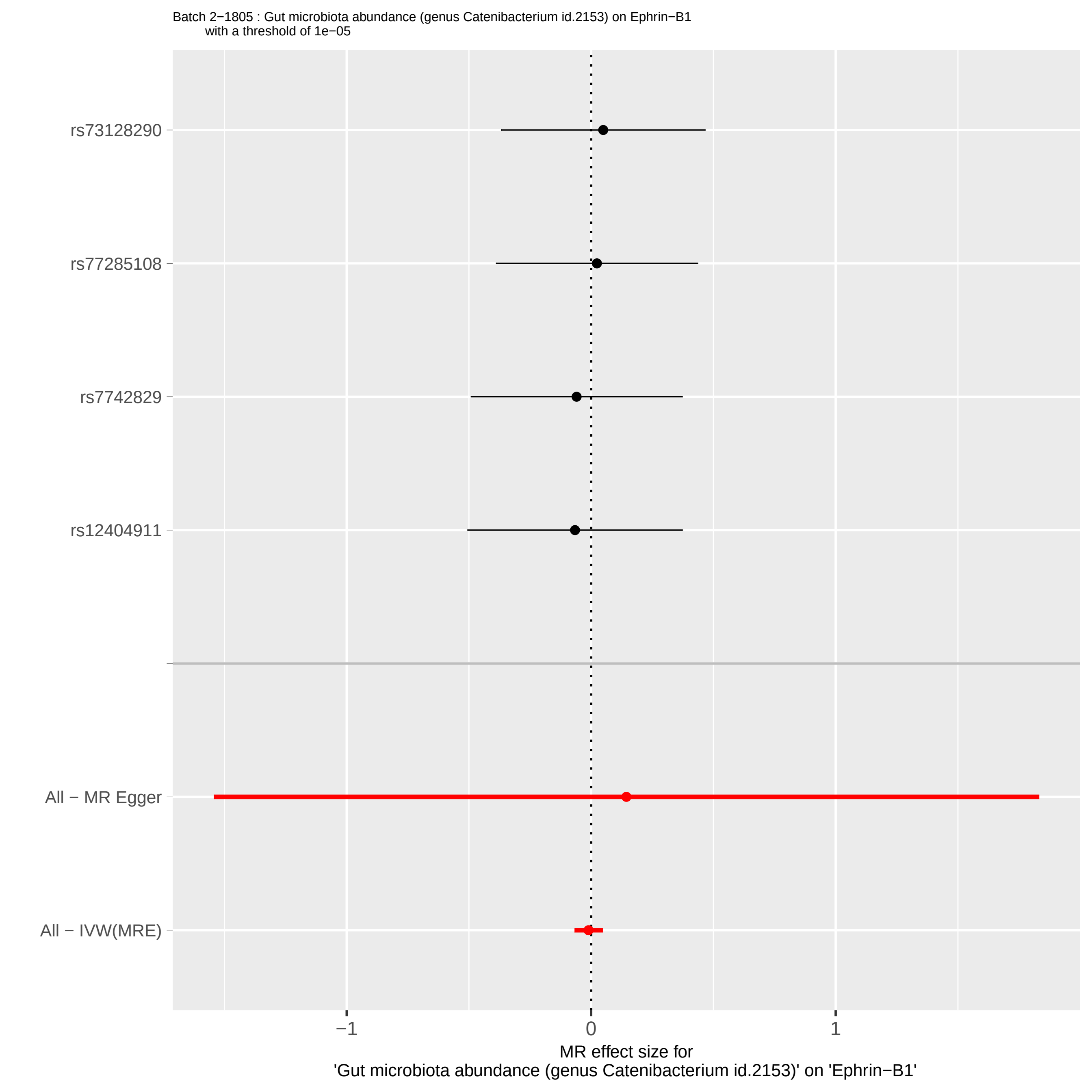
Batch 2−1805 : Gut microbiota abundance (genus Catenibacterium id.2153) on Ephrin−B1
with a threshold of 1e−05
rs73128290
rs77285108
rs7742829
rs12404911
All − MR Egger
All − IVW(MRE)
−1
0
1
MR effect size for
'Gut microbiota abundance (genus Catenibacterium id.2153)' on 'Ephrin−B1'

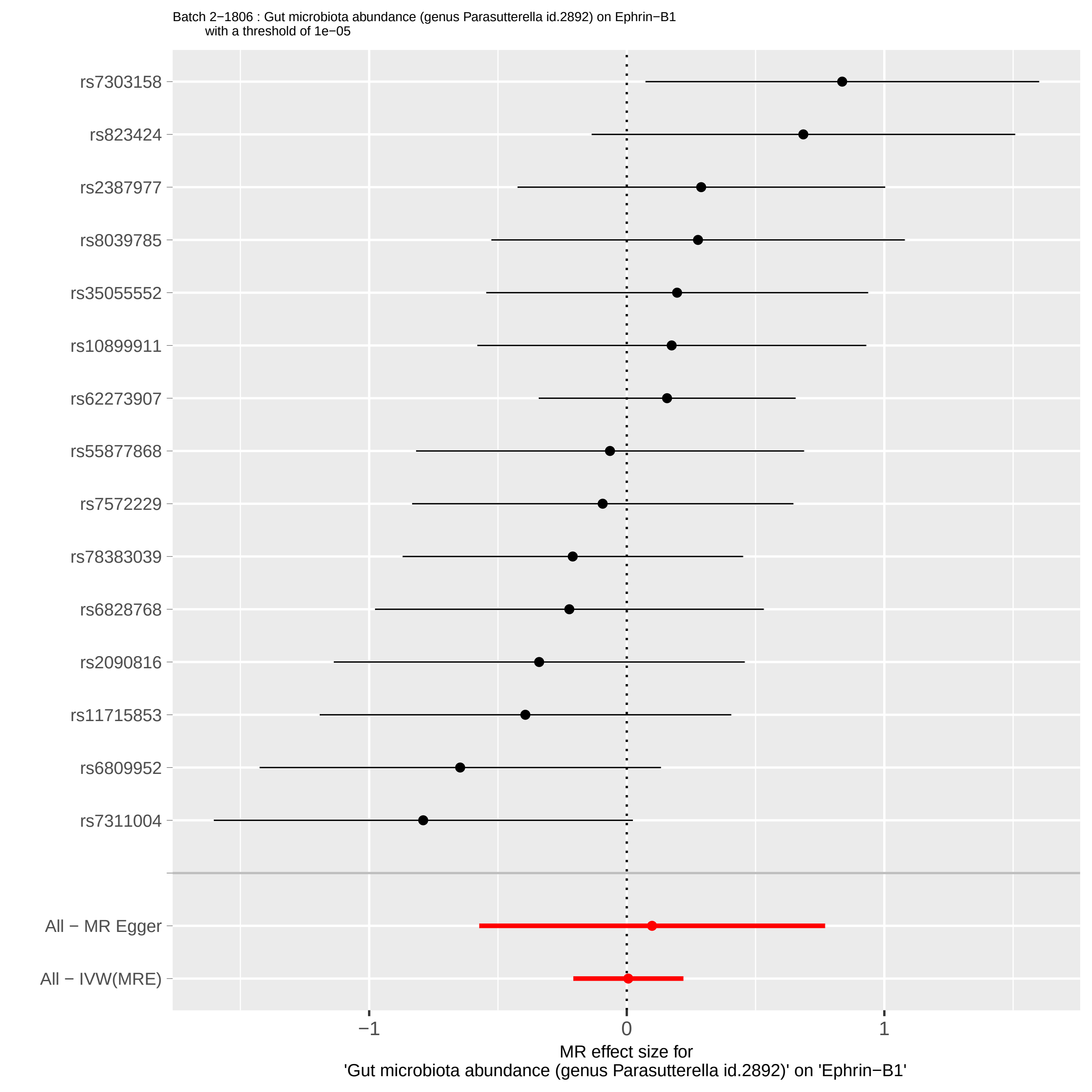
Batch 2−1806 : Gut microbiota abundance (genus Parasutterella id.2892) on Ephrin−B1
with a threshold of 1e−05
rs7303158
rs823424
rs2387977
rs8039785
rs35055552
rs10899911
rs62273907
rs55877868
rs7572229
rs78383039
rs6828768
rs2090816
rs11715853
rs6809952
rs7311004
All − MR Egger
All − IVW(MRE)
−1
0
1
MR effect size for
'Gut microbiota abundance (genus Parasutterella id.2892)' on 'Ephrin−B1'

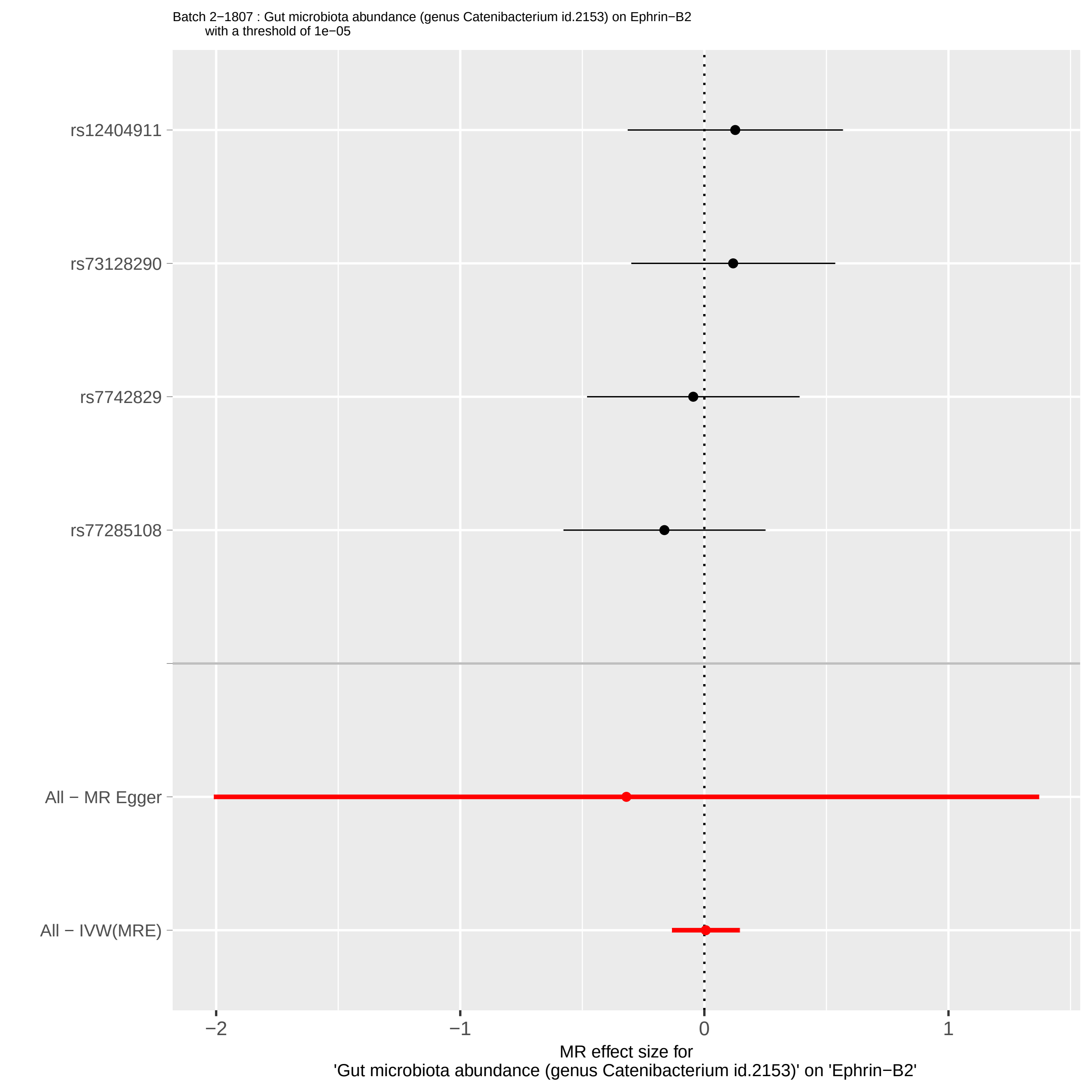

Batch 2−1807 : Gut microbiota abundance (genus Catenibacterium id.2153) on Ephrin−B2
with a threshold of 1e−05
rs12404911
rs73128290
rs7742829
rs77285108
All − MR Egger
All − IVW(MRE)
−2
−1
0
1
MR effect size for
'Gut microbiota abundance (genus Catenibacterium id.2153)' on 'Ephrin−B2'

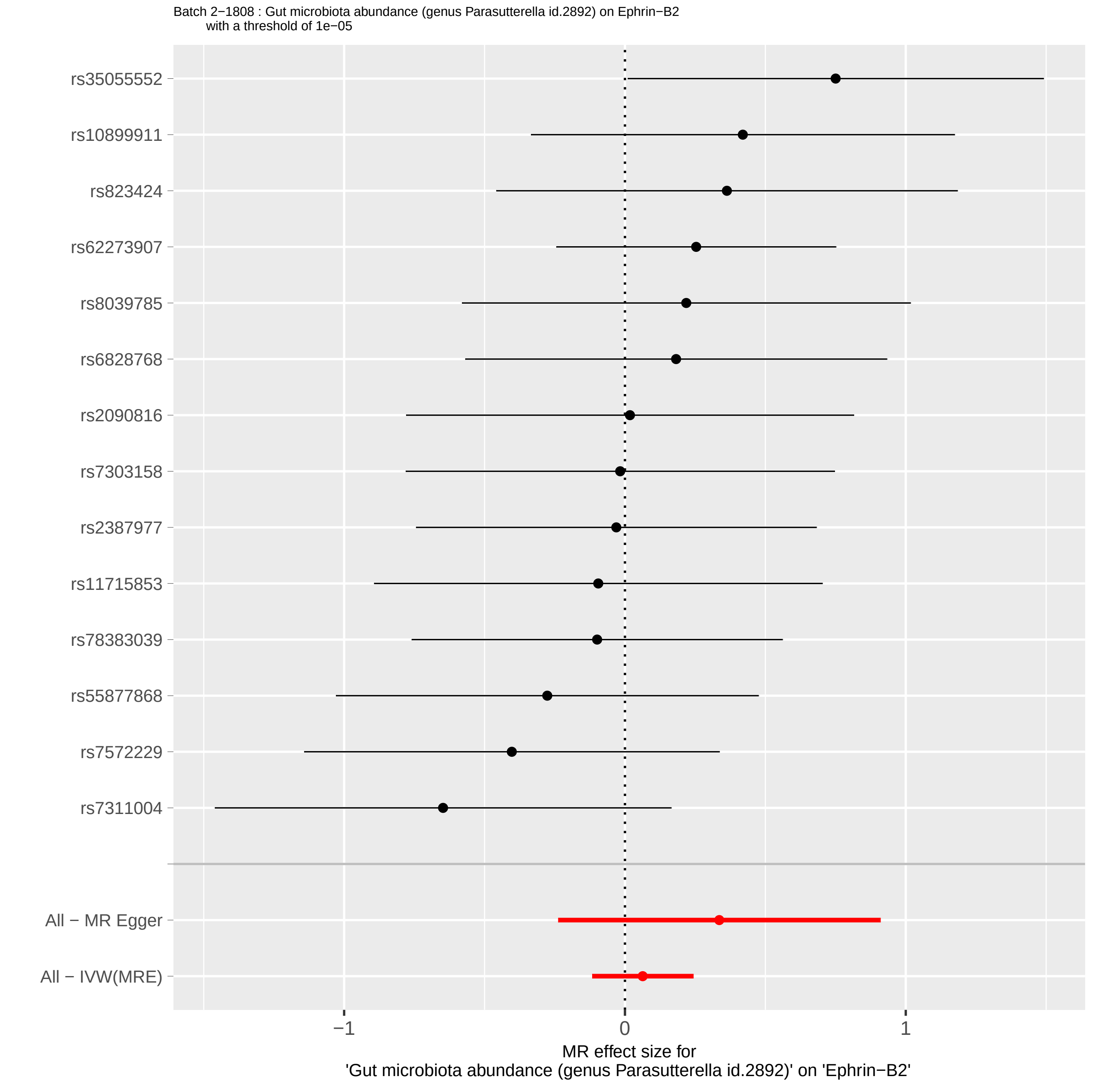
Batch 2−1808 : Gut microbiota abundance (genus Parasutterella id.2892) on Ephrin−B2
with a threshold of 1e−05
rs35055552
rs10899911
rs823424
rs62273907
rs8039785
rs6828768
rs2090816
rs7303158
rs2387977
rs11715853
rs78383039
rs55877868
rs7572229
rs7311004
All − MR Egger
All − IVW(MRE)
−1
0
1
MR effect size for
'Gut microbiota abundance (genus Parasutterella id.2892)' on 'Ephrin−B2'

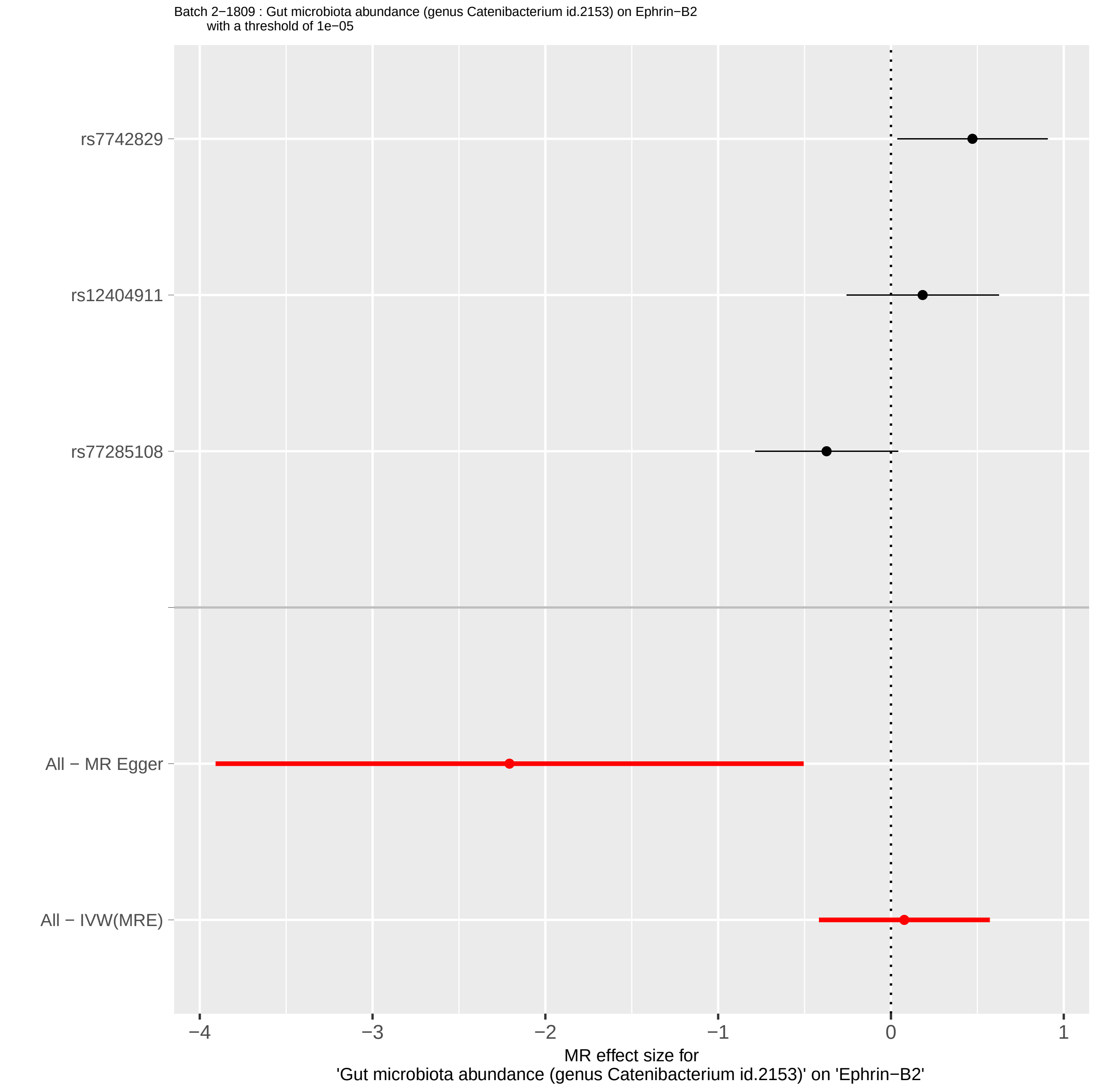

Batch 2−1809 : Gut microbiota abundance (genus Catenibacterium id.2153) on Ephrin−B2
with a threshold of 1e−05
rs7742829
rs12404911
rs77285108
All − MR Egger
All − IVW(MRE)
−4
−3
−2
−1
0
1
MR effect size for
'Gut microbiota abundance (genus Catenibacterium id.2153)' on 'Ephrin−B2'

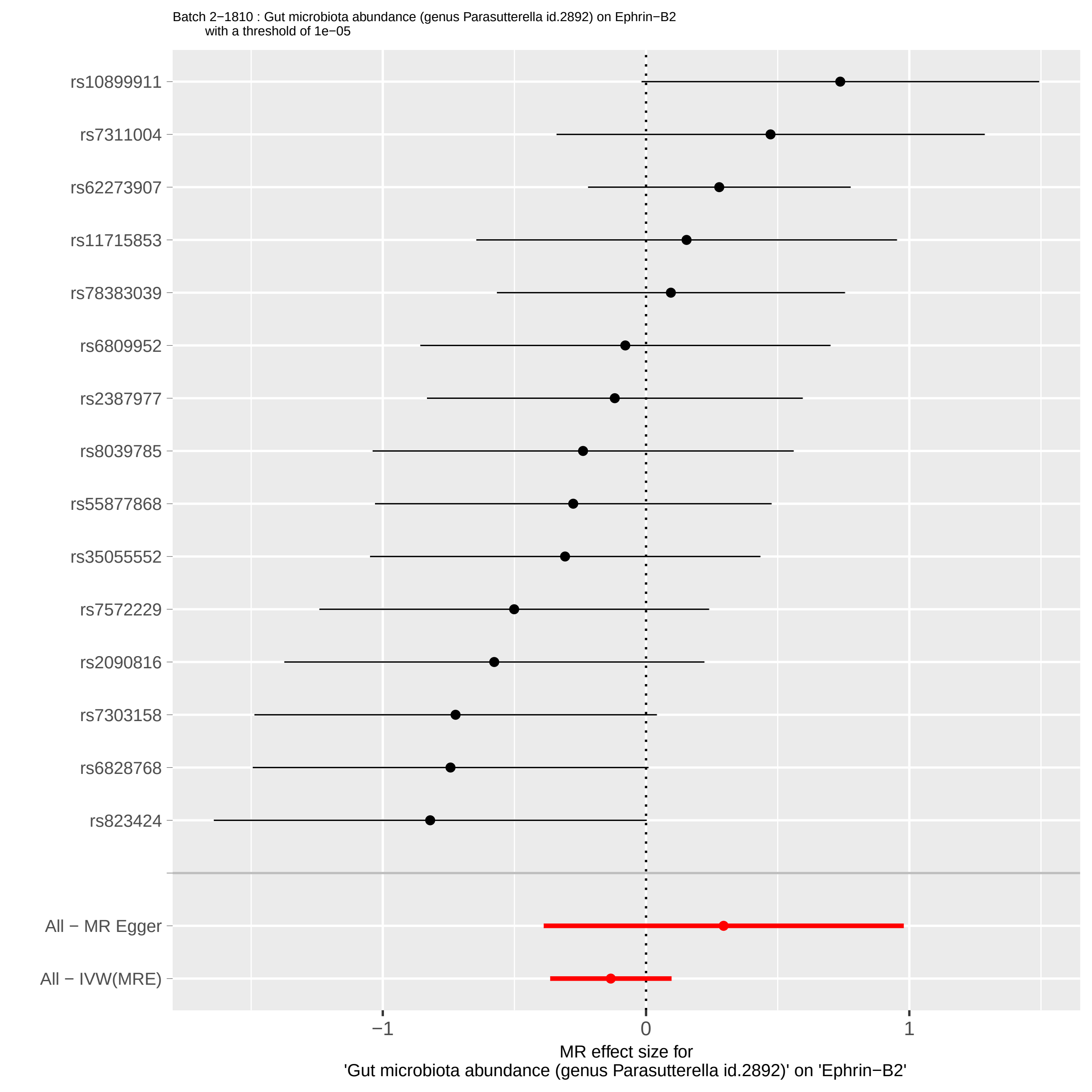
Batch 2−1810 : Gut microbiota abundance (genus Parasutterella id.2892) on Ephrin−B2
with a threshold of 1e−05
rs10899911
rs7311004
rs62273907
rs11715853
rs78383039
rs6809952
rs2387977
rs8039785
rs55877868
rs35055552
rs7572229
rs2090816
rs7303158
rs6828768
rs823424
All − MR Egger
All − IVW(MRE)
−1
0
1
MR effect size for
'Gut microbiota abundance (genus Parasutterella id.2892)' on 'Ephrin−B2'

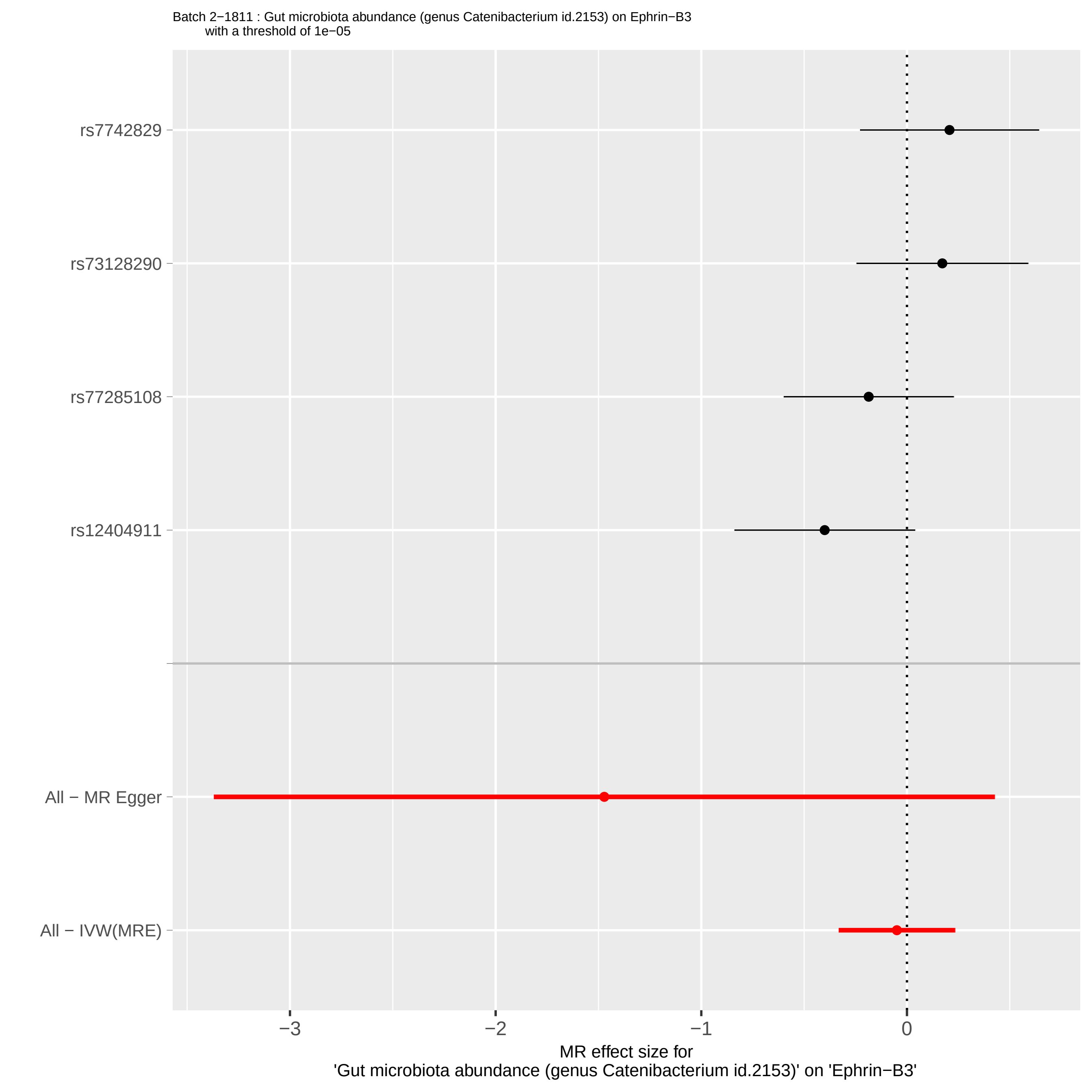

Batch 2−1811 : Gut microbiota abundance (genus Catenibacterium id.2153) on Ephrin−B3
with a threshold of 1e−05
rs7742829
rs73128290
rs77285108
rs12404911
All − MR Egger
All − IVW(MRE)
−3
−2
−1
0
MR effect size for
'Gut microbiota abundance (genus Catenibacterium id.2153)' on 'Ephrin−B3'

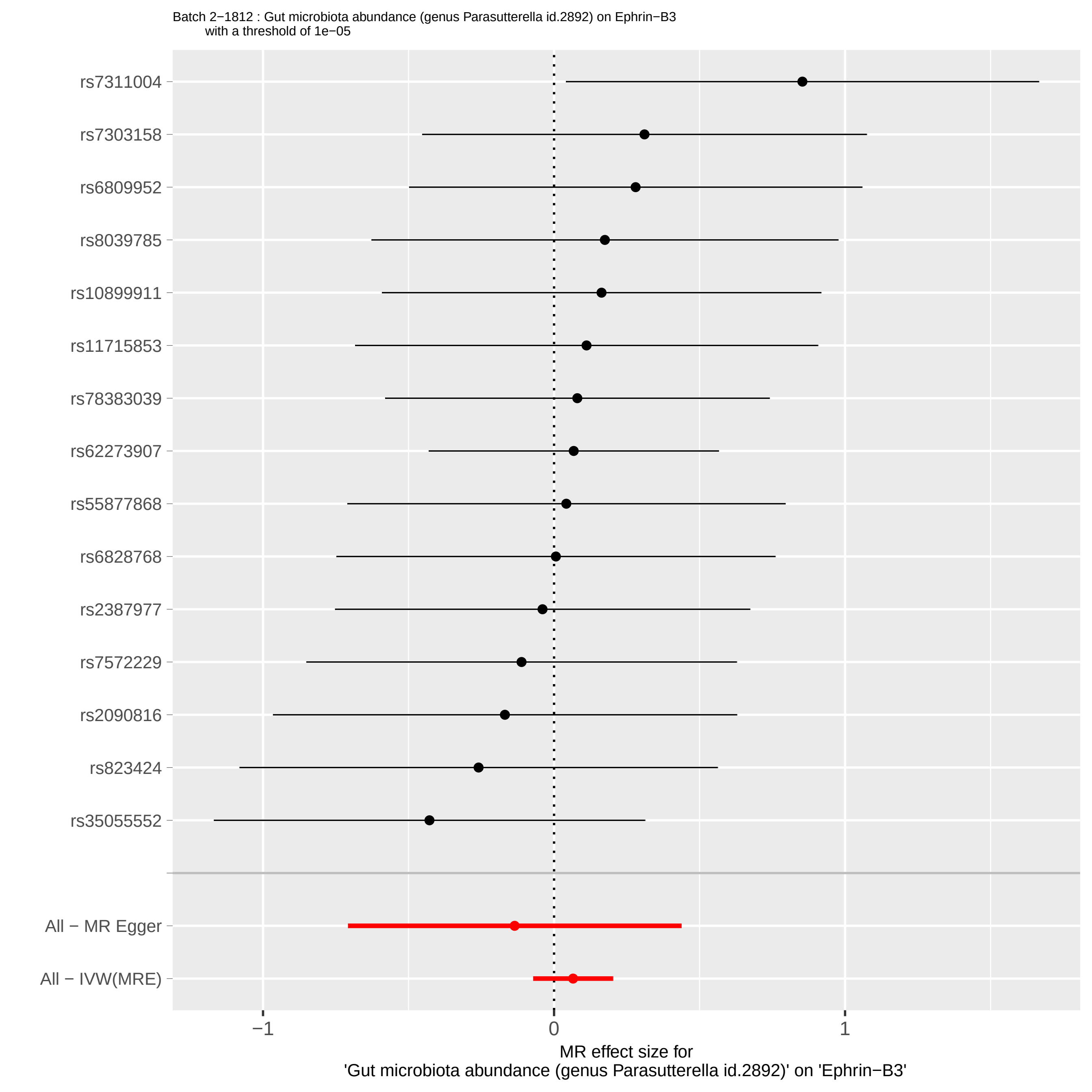
Batch 2−1812 : Gut microbiota abundance (genus Parasutterella id.2892) on Ephrin−B3
with a threshold of 1e−05
rs7311004
rs7303158
rs6809952
rs8039785
rs10899911
rs11715853
rs78383039
rs62273907
rs55877868
rs6828768
rs2387977
rs7572229
rs2090816
rs823424
rs35055552
All − MR Egger
All − IVW(MRE)
−1
0
1
MR effect size for
'Gut microbiota abundance (genus Parasutterella id.2892)' on 'Ephrin−B3'

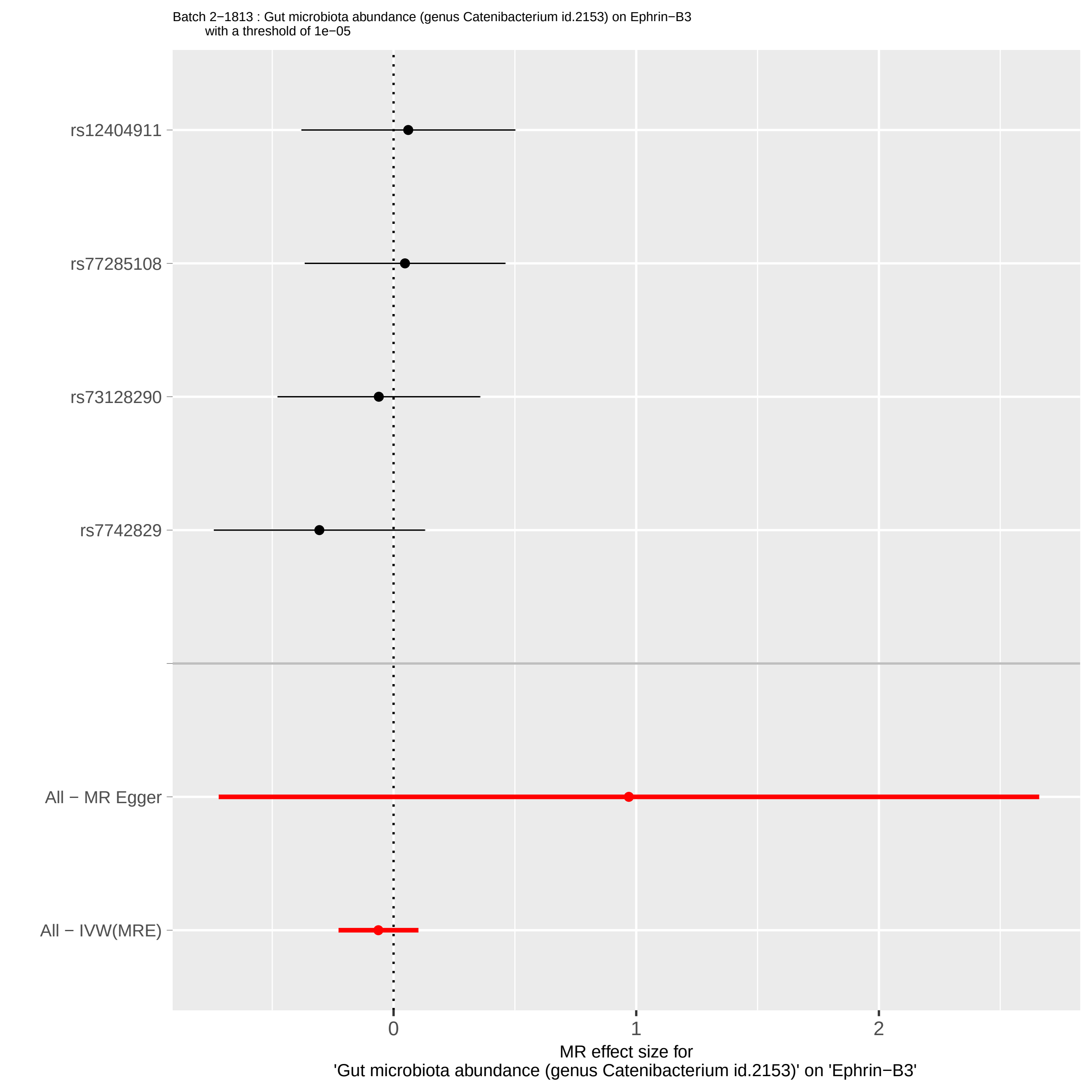

Batch 2−1813 : Gut microbiota abundance (genus Catenibacterium id.2153) on Ephrin−B3
with a threshold of 1e−05
rs12404911
rs77285108
rs73128290
rs7742829
All − MR Egger
All − IVW(MRE)
0
1
2
MR effect size for
'Gut microbiota abundance (genus Catenibacterium id.2153)' on 'Ephrin−B3'

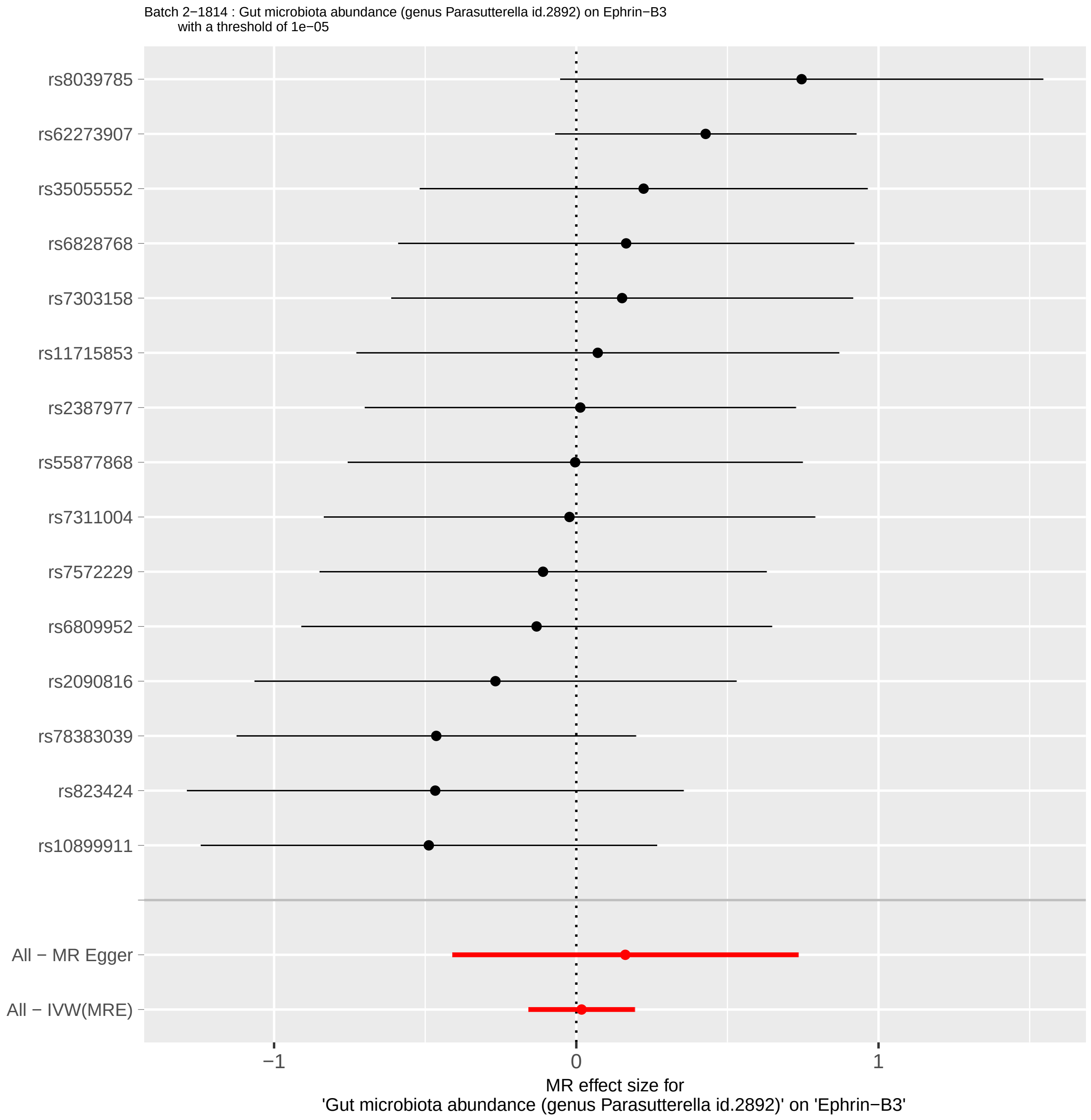

Batch 2−1814 : Gut microbiota abundance (genus Parasutterella id.2892) on Ephrin−B3
with a threshold of 1e−05
rs8039785
rs62273907
rs35055552
rs6828768
rs7303158
rs11715853
rs2387977
rs55877868
rs7311004
rs7572229
rs6809952
rs2090816
rs78383039
rs823424
rs10899911
All − MR Egger
All − IVW(MRE)
−1
0
1
MR effect size for
'Gut microbiota abundance (genus Parasutterella id.2892)' on 'Ephrin−B3'

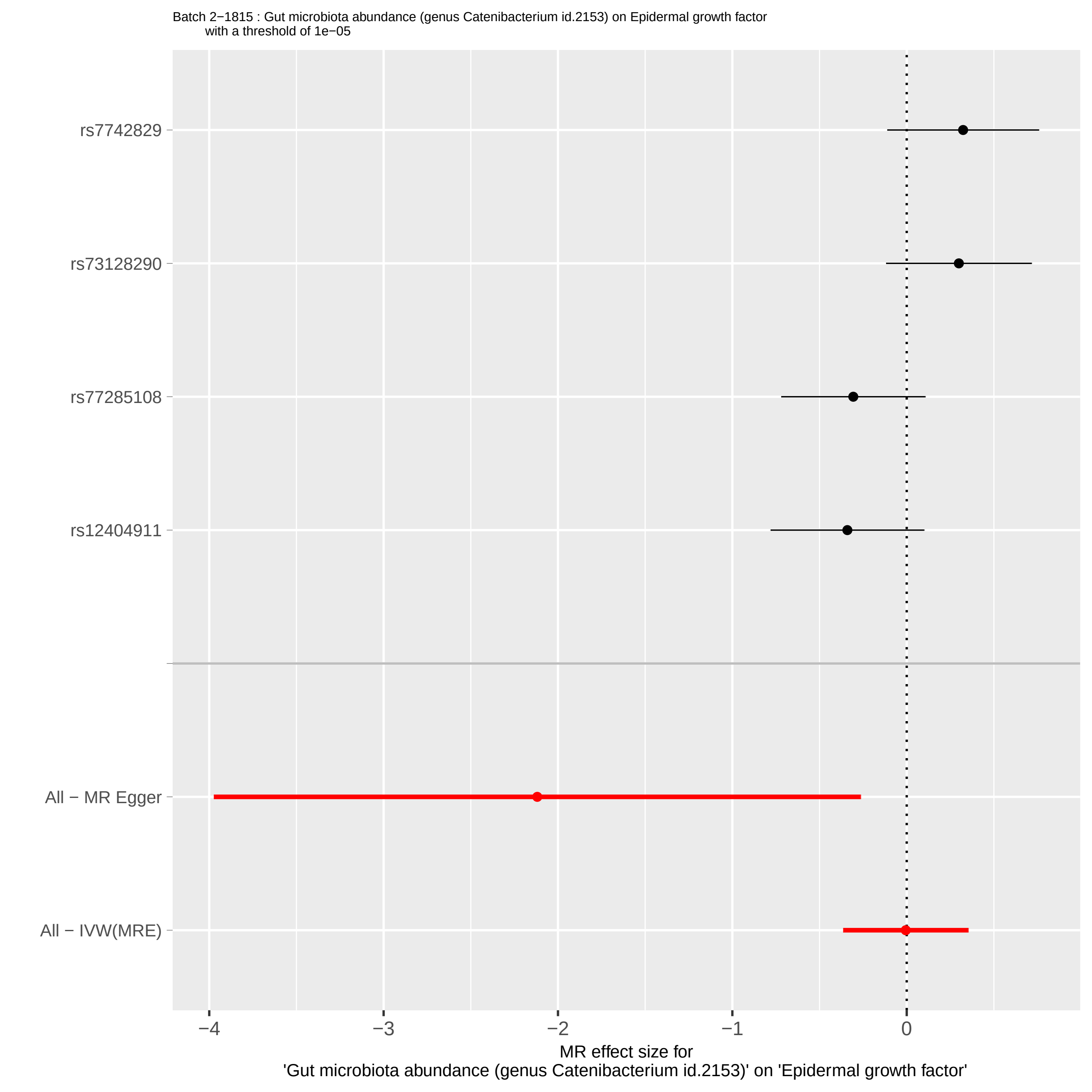
Batch 2−1815 : Gut microbiota abundance (genus Catenibacterium id.2153) on Epidermal growth factor
with a threshold of 1e−05
rs7742829
rs73128290
rs77285108
rs12404911
All − MR Egger
All − IVW(MRE)
−4
−3
−2
−1
0
MR effect size for
'Gut microbiota abundance (genus Catenibacterium id.2153)' on 'Epidermal growth factor'

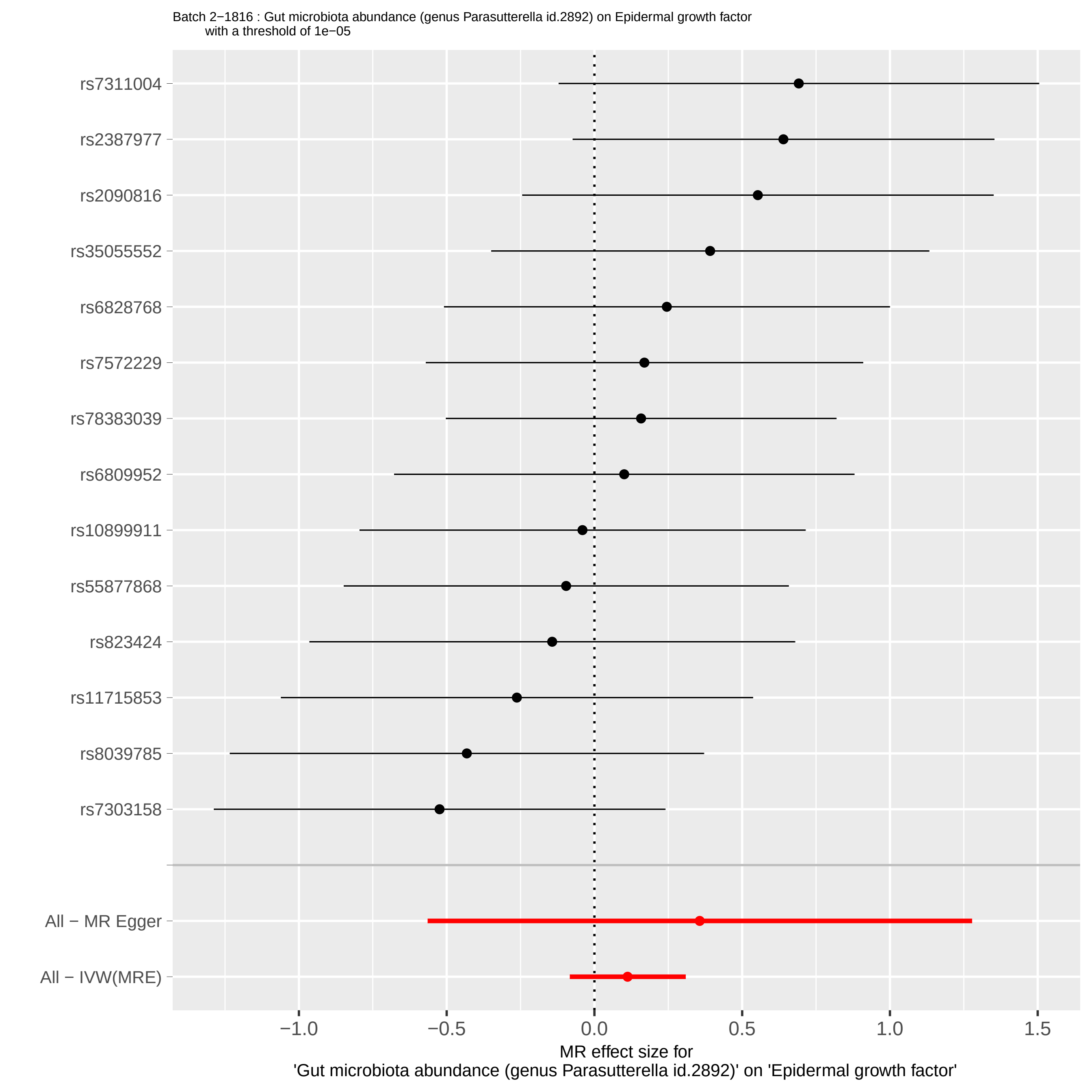
Batch 2−1816 : Gut microbiota abundance (genus Parasutterella id.2892) on Epidermal growth factor
with a threshold of 1e−05
rs7311004
rs2387977
rs2090816
rs35055552
rs6828768
rs7572229
rs78383039
rs6809952
rs10899911
rs55877868
rs823424
rs11715853
rs8039785
rs7303158
All − MR Egger
All − IVW(MRE)
−1.0
−0.5
0.0
0.5
1.0
1.5
MR effect size for
'Gut microbiota abundance (genus Parasutterella id.2892)' on 'Epidermal growth factor'

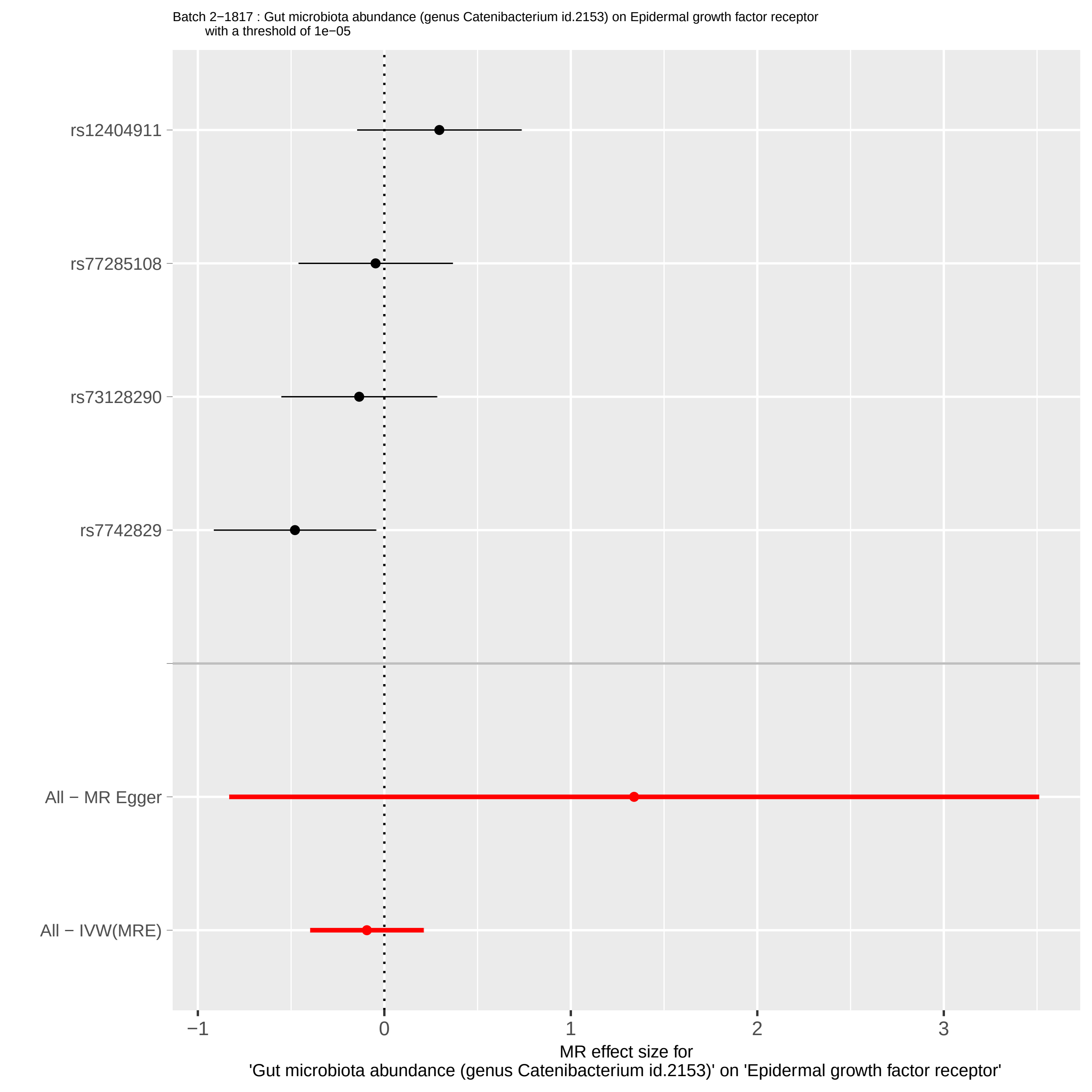

Batch 2−1817 : Gut microbiota abundance (genus Catenibacterium id.2153) on Epidermal growth factor receptor
with a threshold of 1e−05
rs12404911
rs77285108
rs73128290
rs7742829
All − MR Egger
All − IVW(MRE)
−1
0
1
2
3
MR effect size for
'Gut microbiota abundance (genus Catenibacterium id.2153)' on 'Epidermal growth factor receptor'

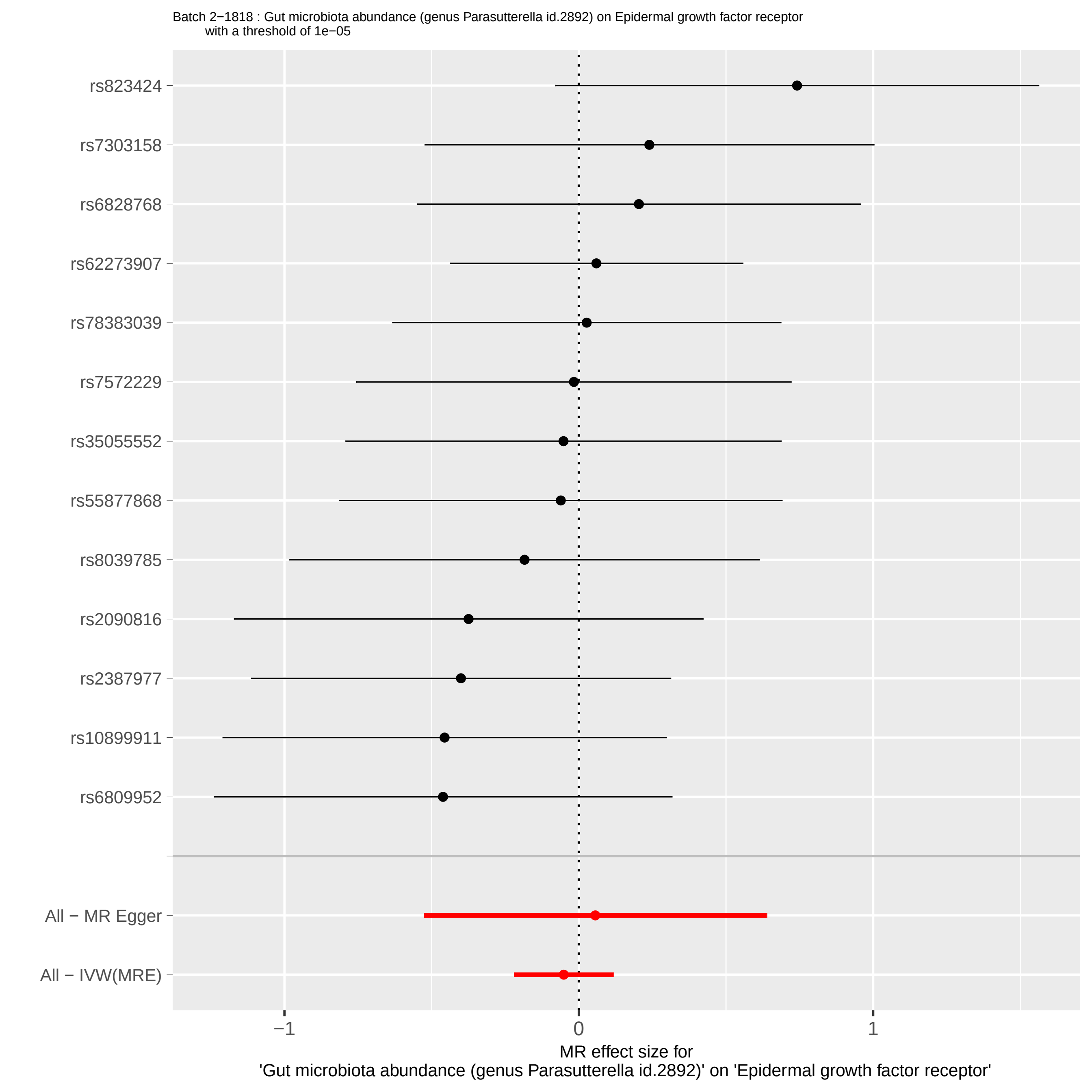

Batch 2−1818 : Gut microbiota abundance (genus Parasutterella id.2892) on Epidermal growth factor receptor
with a threshold of 1e−05
rs823424
rs7303158
rs6828768
rs62273907
rs78383039
rs7572229
rs35055552
rs55877868
rs8039785
rs2090816
rs2387977
rs10899911
rs6809952
All − MR Egger
All − IVW(MRE)
−1
0
1
MR effect size for
'Gut microbiota abundance (genus Parasutterella id.2892)' on 'Epidermal growth factor receptor'

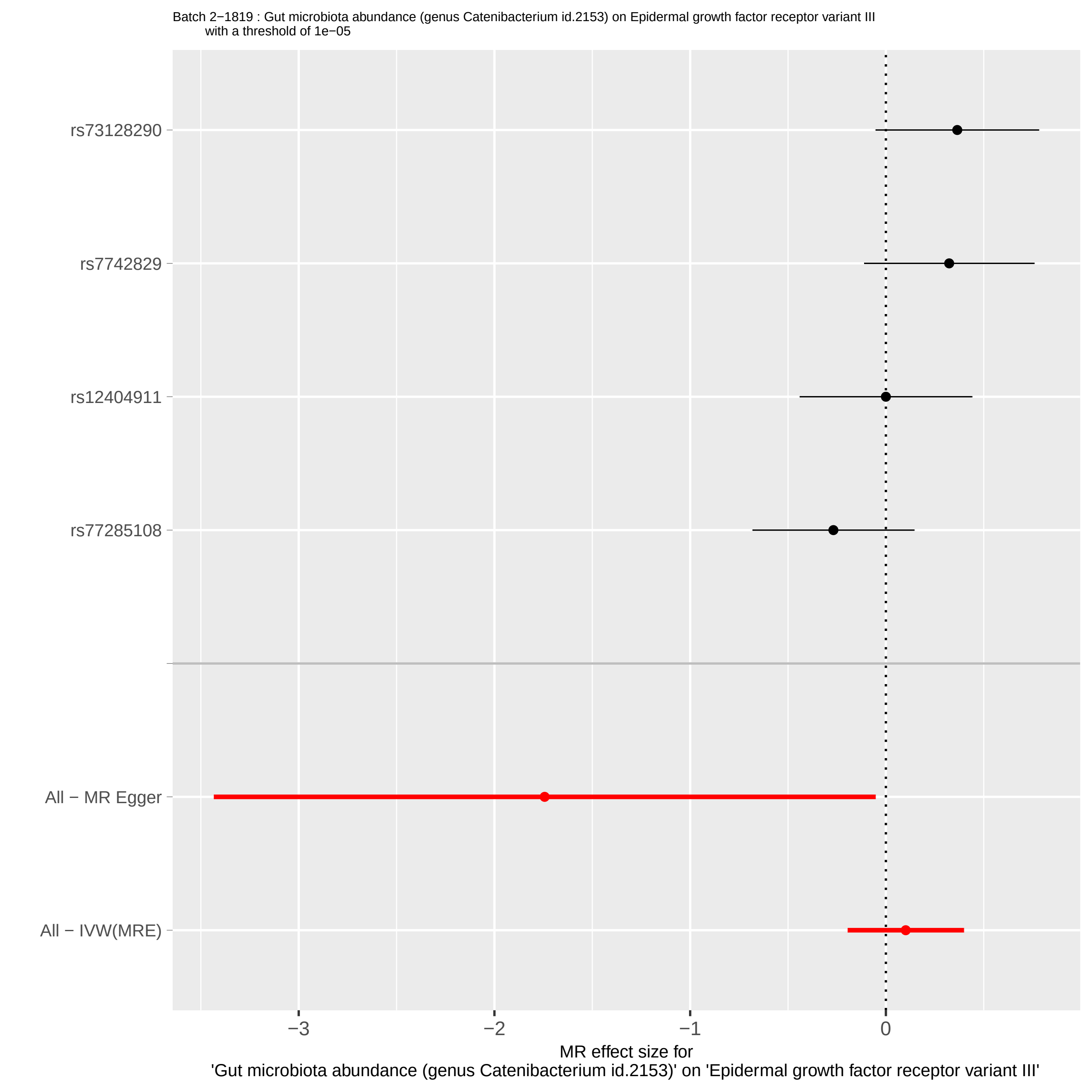

Batch 2−1819 : Gut microbiota abundance (genus Catenibacterium id.2153) on Epidermal growth factor receptor variant III
with a threshold of 1e−05
rs73128290
rs7742829
rs12404911
rs77285108
All − MR Egger
All − IVW(MRE)
−3
−2
−1
0
MR effect size for
'Gut microbiota abundance (genus Catenibacterium id.2153)' on 'Epidermal growth factor receptor variant III'

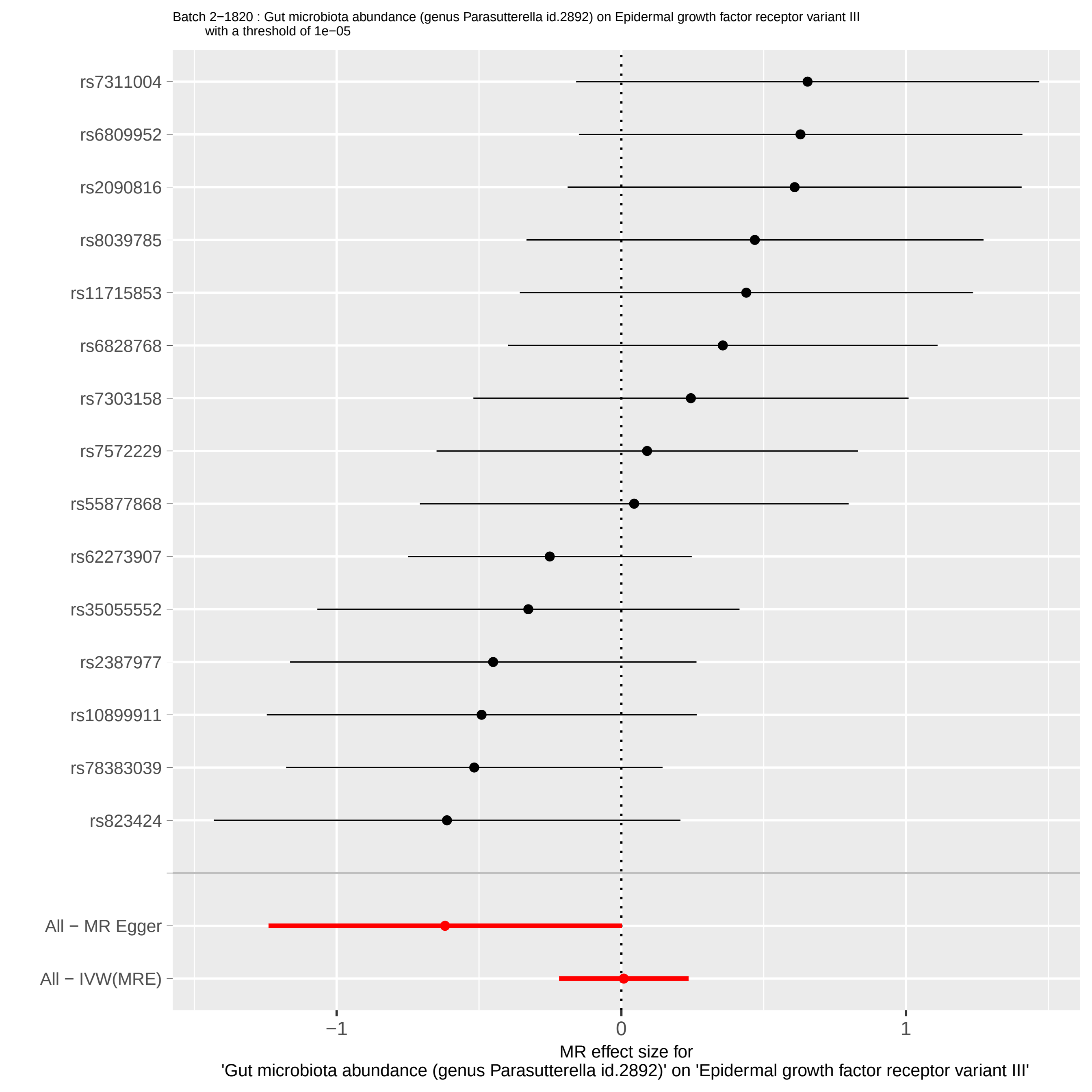

Batch 2−1820 : Gut microbiota abundance (genus Parasutterella id.2892) on Epidermal growth factor receptor variant III
with a threshold of 1e−05
rs7311004
rs6809952
rs2090816
rs8039785
rs11715853
rs6828768
rs7303158
rs7572229
rs55877868
rs62273907
rs35055552
rs2387977
rs10899911
rs78383039
rs823424
All − MR Egger
All − IVW(MRE)
−1
0
1
MR effect size for
'Gut microbiota abundance (genus Parasutterella id.2892)' on 'Epidermal growth factor receptor variant III'

Batch 2−1821 : Gut microbiota abundance (genus Catenibacterium id.2153) on EH domain−binding protein 1
with a threshold of 1e−05

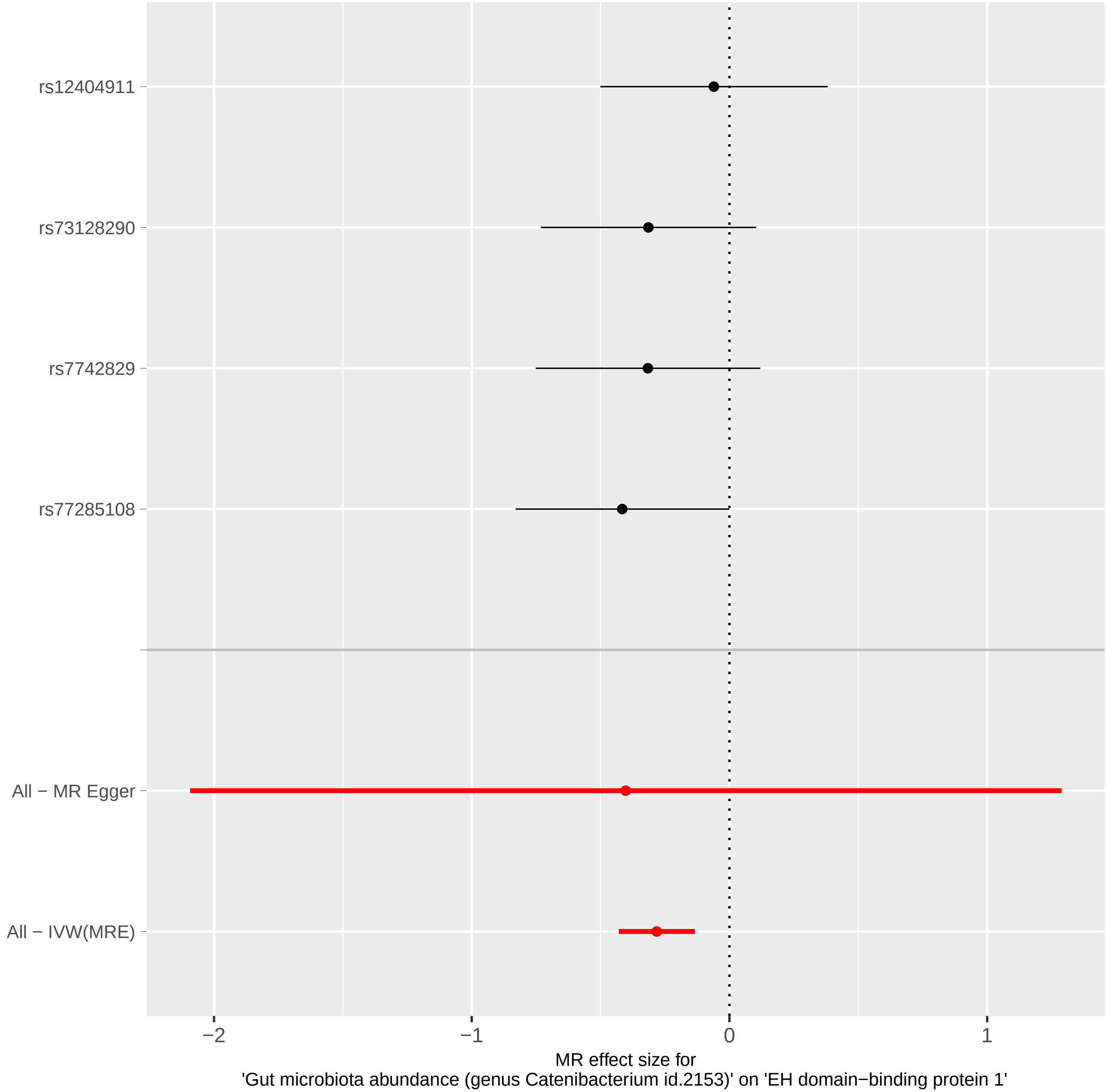

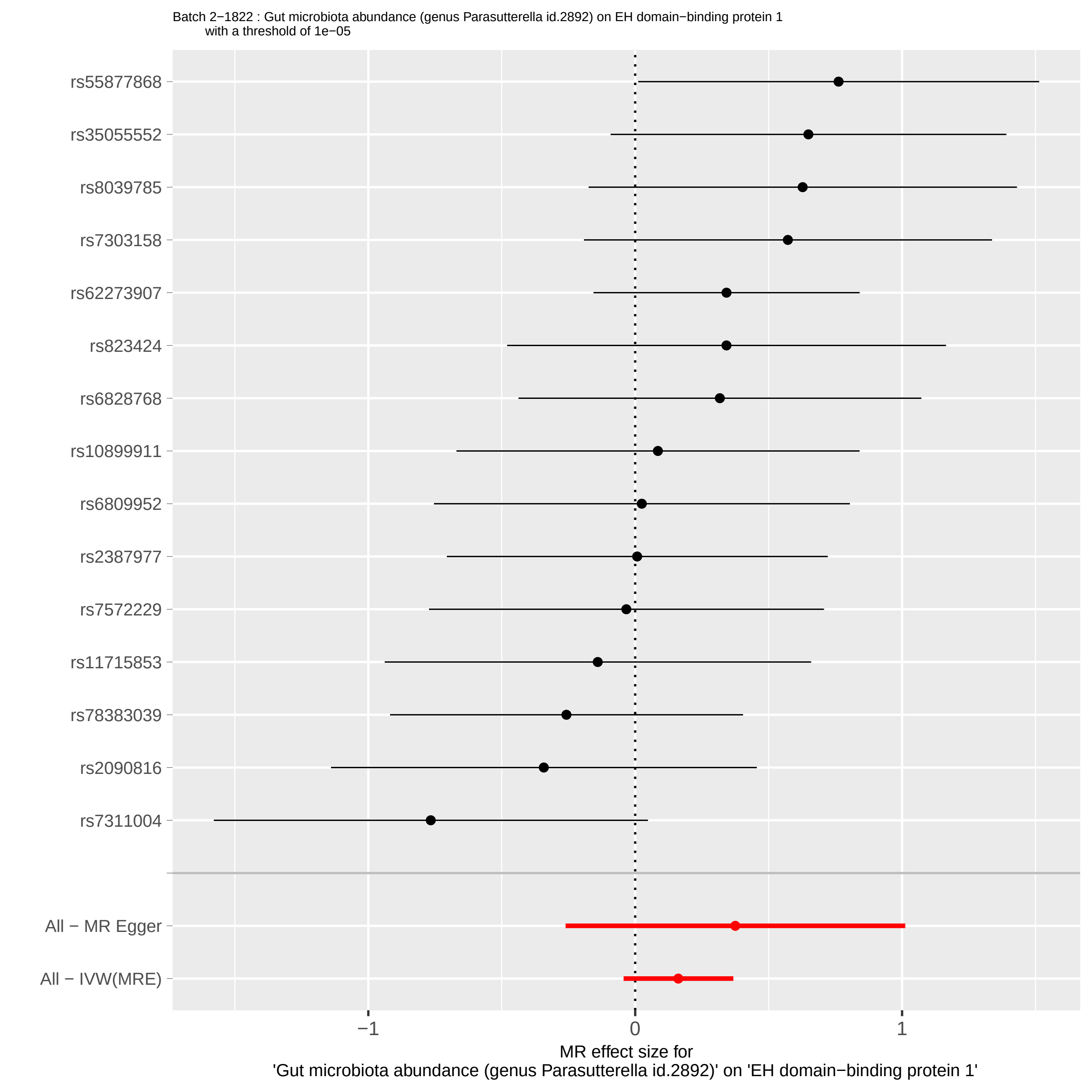
Batch 2−1822 : Gut microbiota abundance (genus Parasutterella id.2892) on EH domain−binding protein 1
with a threshold of 1e−05
rs55877868
rs35055552
rs8039785
rs7303158
rs62273907
rs823424
rs6828768
rs10899911
rs6809952
rs2387977
rs7572229
rs11715853
rs78383039
rs2090816
rs7311004
All − MR Egger
All − IVW(MRE)
−1
0
1
MR effect size for
'Gut microbiota abundance (genus Parasutterella id.2892)' on 'EH domain−binding protein 1'

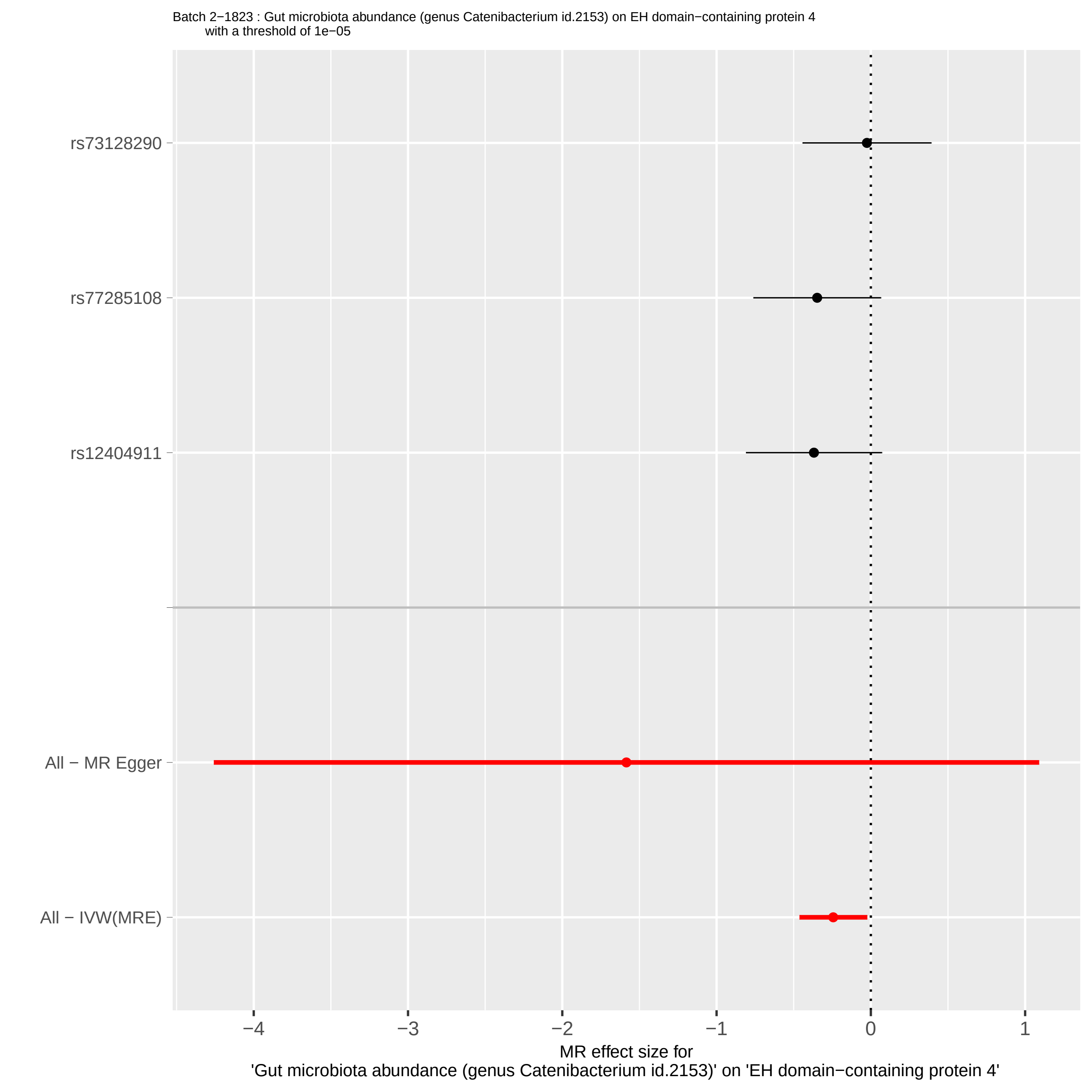

Batch 2−1823 : Gut microbiota abundance (genus Catenibacterium id.2153) on EH domain−containing protein 4
with a threshold of 1e−05
rs73128290
rs77285108
rs12404911
All − MR Egger
All − IVW(MRE)
−4
−3
−2
−1
0
1
MR effect size for
'Gut microbiota abundance (genus Catenibacterium id.2153)' on 'EH domain−containing protein 4'

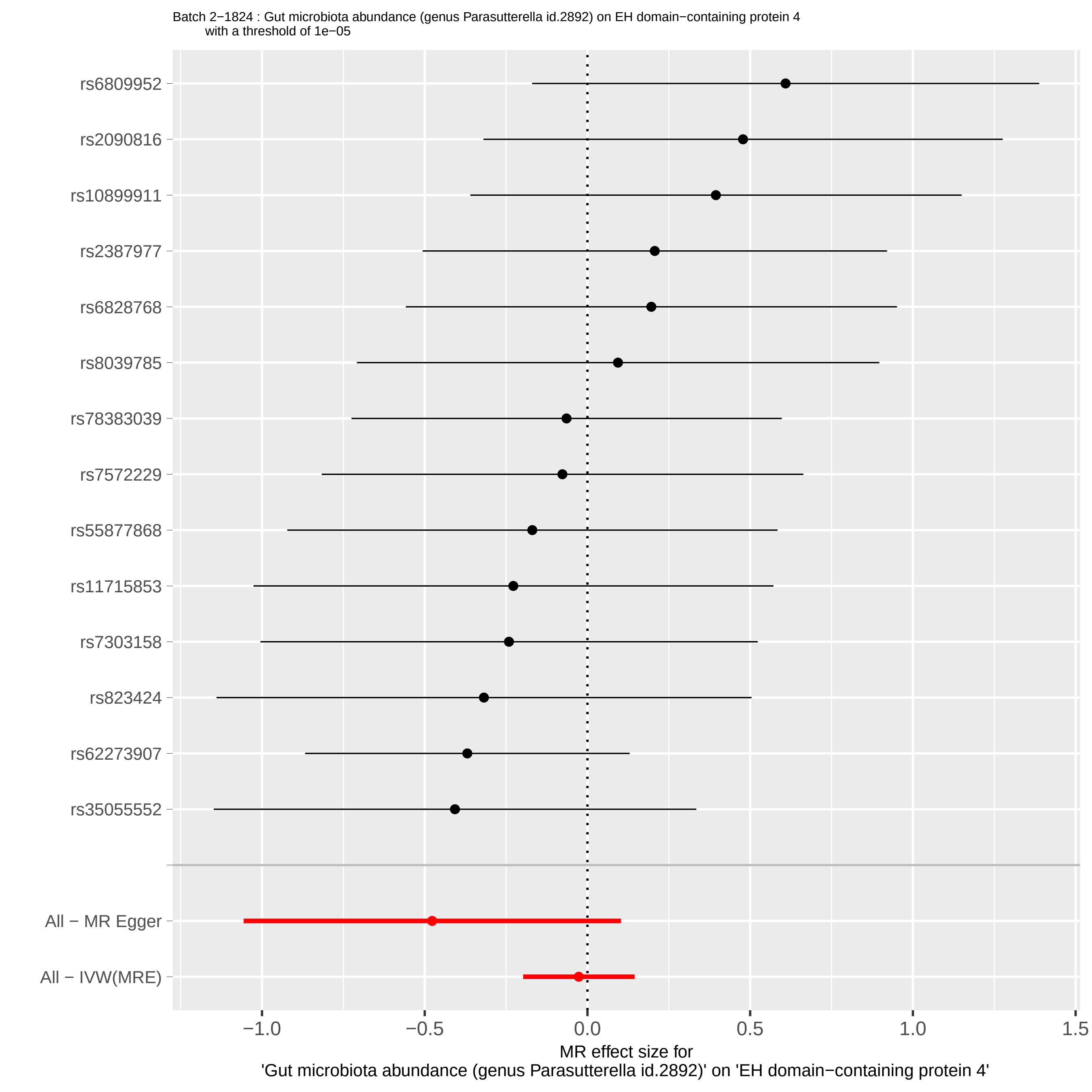

Batch 2−1824 : Gut microbiota abundance (genus Parasutterella id.2892) on EH domain−containing protein 4
with a threshold of 1e−05
rs6809952
rs2090816
rs10899911
rs2387977
rs6828768
rs8039785
rs78383039
rs7572229
rs55877868
rs11715853
rs7303158
rs823424
rs62273907
rs35055552
All − MR Egger
All − IVW(MRE)
−1.0
−0.5
0.0
0.5
1.0
1.5
MR effect size for
'Gut microbiota abundance (genus Parasutterella id.2892)' on 'EH domain−containing protein 4'

Batch 2−1825 : Gut microbiota abundance (genus Catenibacterium id.2153) on ETS homologous factor with a threshold of 1e−05

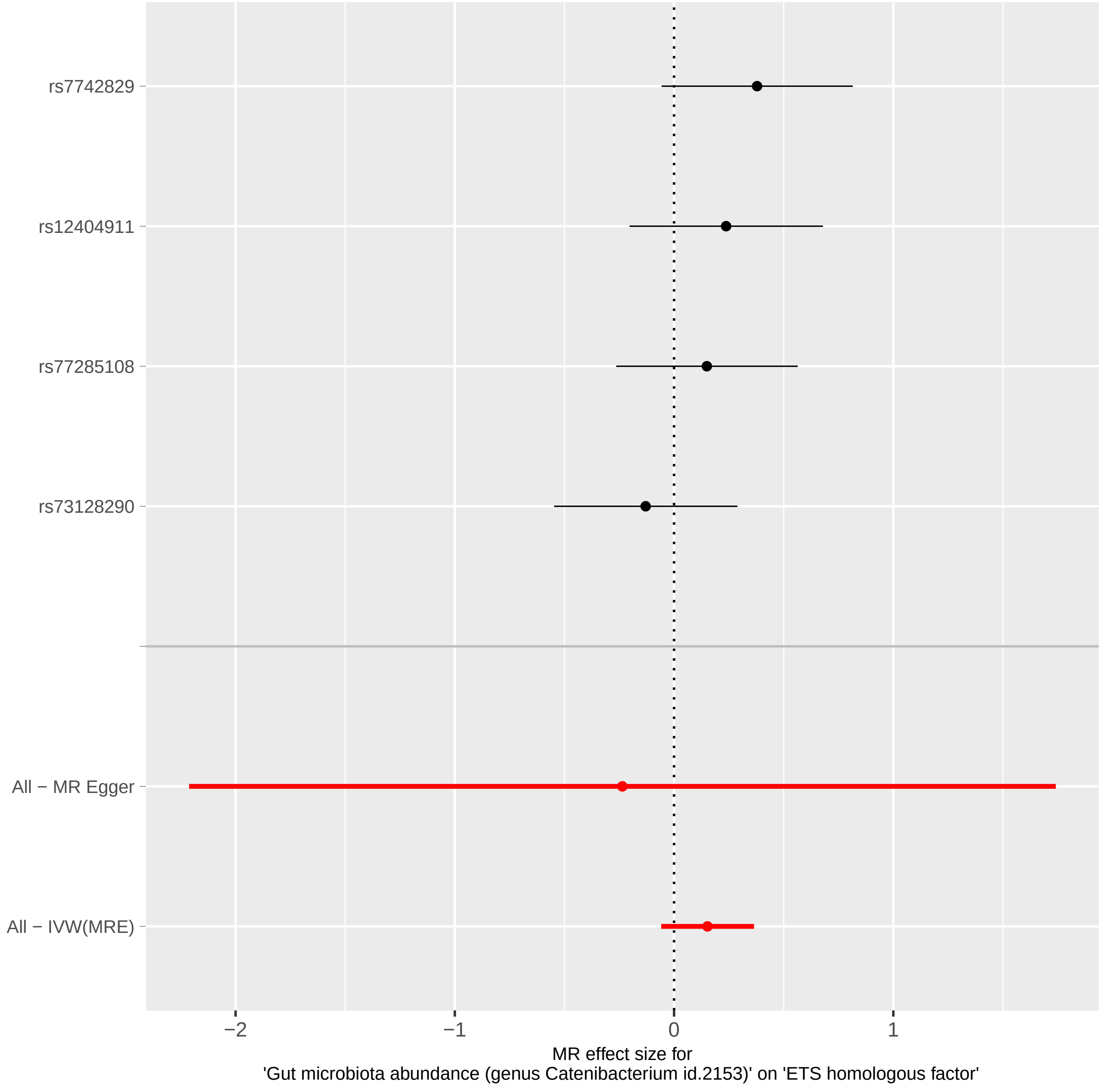

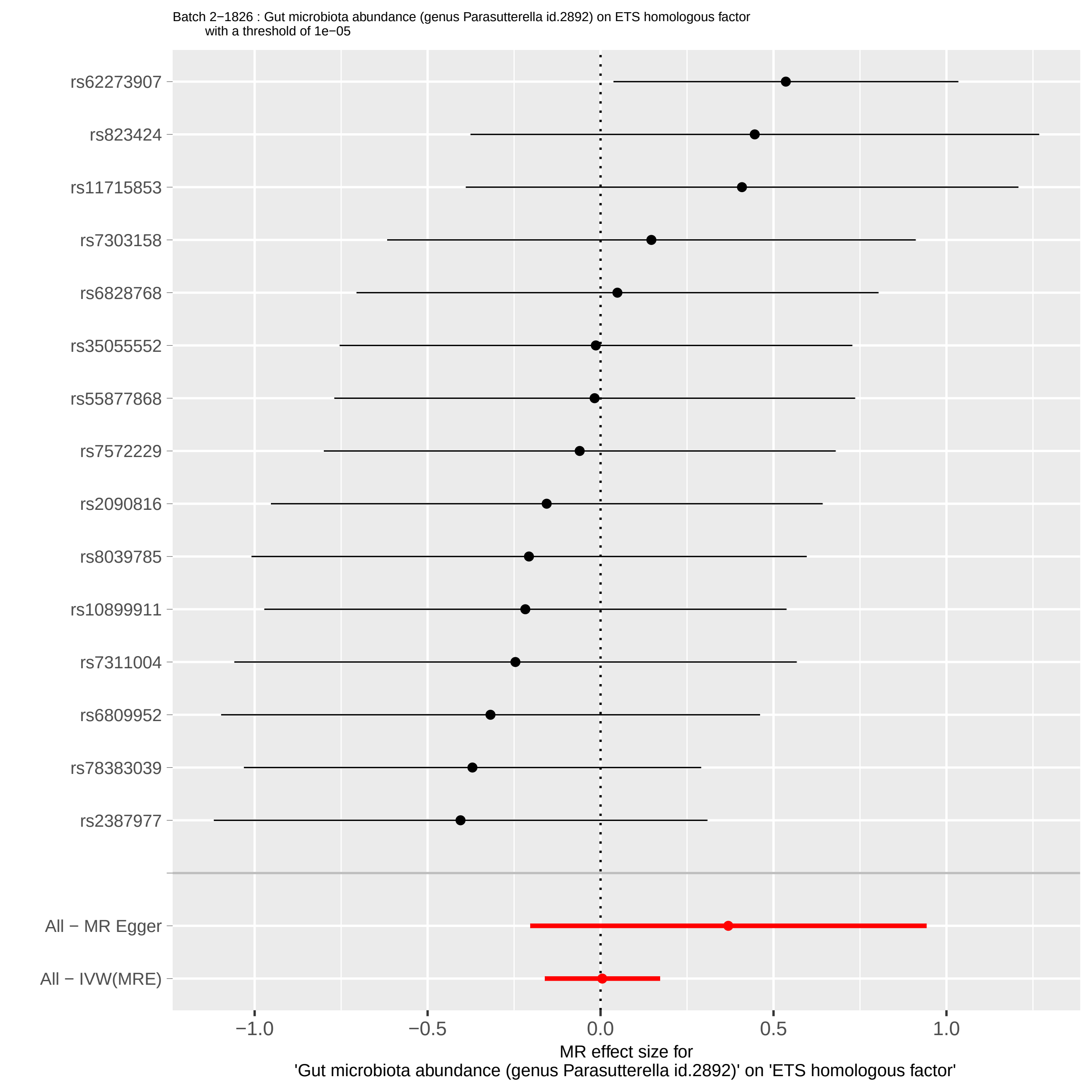
Batch 2−1826 : Gut microbiota abundance (genus Parasutterella id.2892) on ETS homologous factor
with a threshold of 1e−05
rs62273907
rs823424
rs11715853
rs7303158
rs6828768
rs35055552
rs55877868
rs7572229
rs2090816
rs8039785
rs10899911
rs7311004
rs6809952
rs78383039
rs2387977
All − MR Egger
All − IVW(MRE)
−1.0
−0.5
0.0
0.5
1.0
MR effect size for
'Gut microbiota abundance (genus Parasutterella id.2892)' on 'ETS homologous factor'

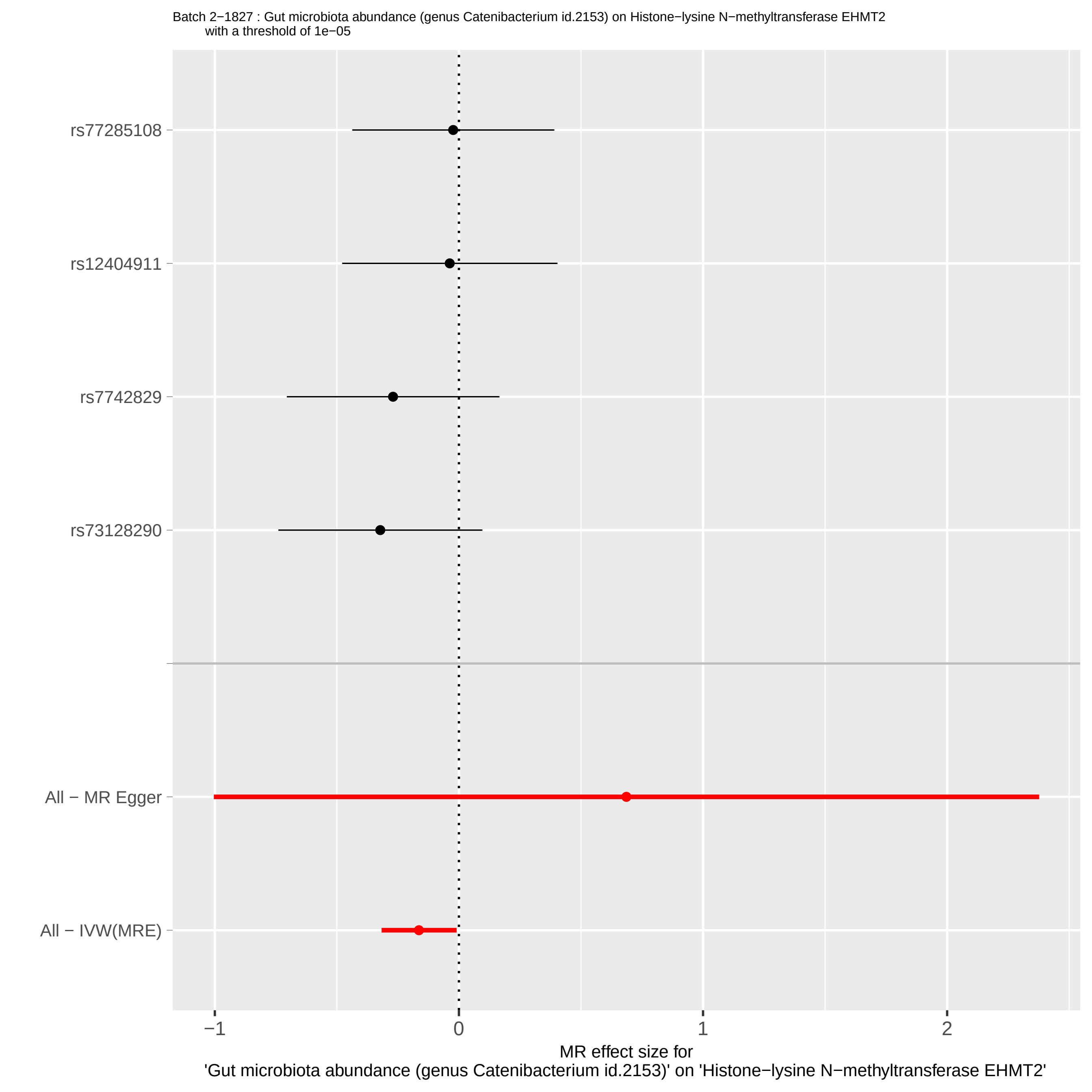
Batch 2−1827 : Gut microbiota abundance (genus Catenibacterium id.2153) on Histone−lysine N−methyltransferase EHMT2
with a threshold of 1e−05
rs77285108
rs12404911
rs7742829
rs73128290
All − MR Egger
All − IVW(MRE)
−1
0
1
2
MR effect size for
'Gut microbiota abundance (genus Catenibacterium id.2153)' on 'Histone−lysine N−methyltransferase EHMT2'

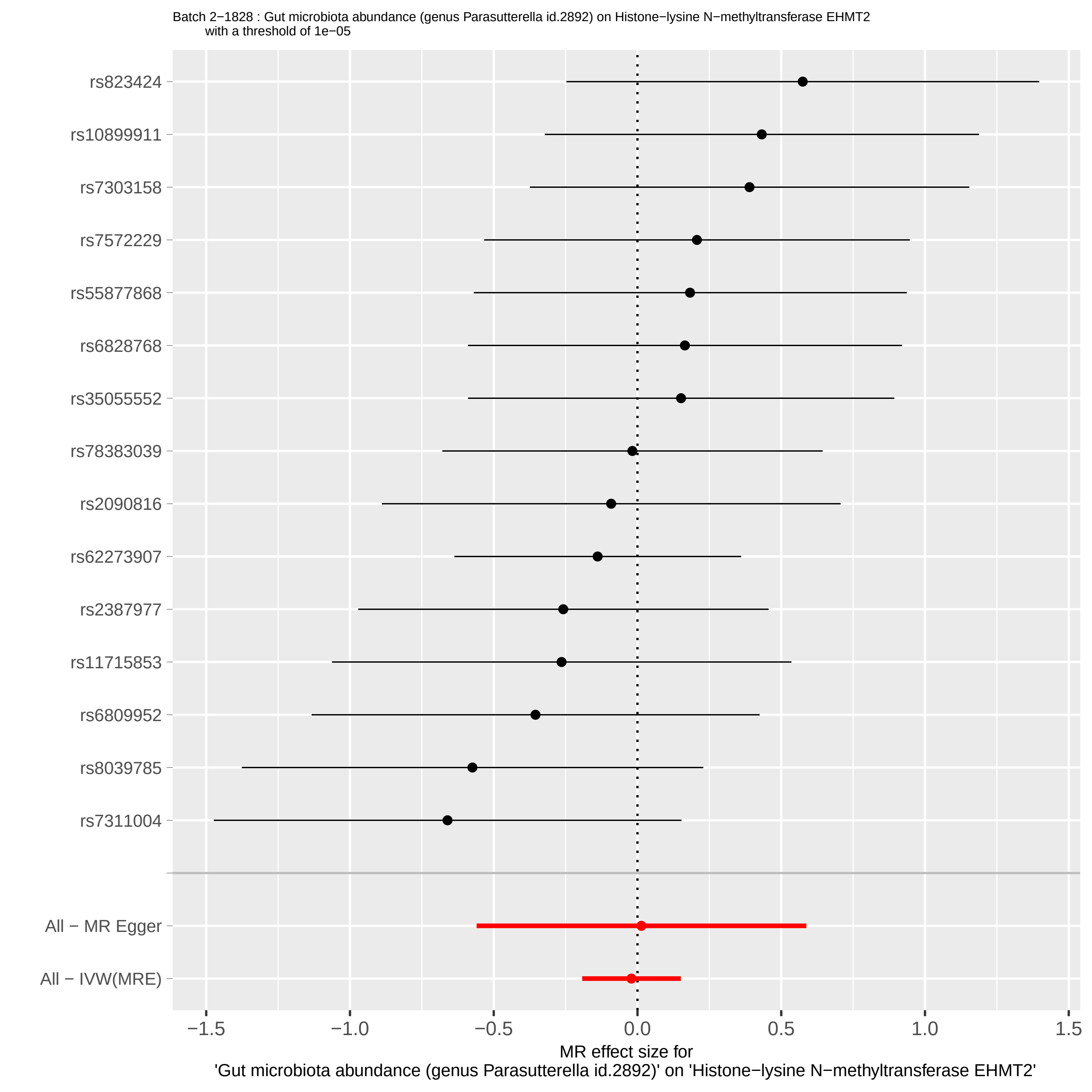

Batch 2−1828 : Gut microbiota abundance (genus Parasutterella id.2892) on Histone−lysine N−methyltransferase EHMT2
with a threshold of 1e−05
rs823424
rs10899911
rs7303158
rs7572229
rs55877868
rs6828768
rs35055552
rs78383039
rs2090816
rs62273907
rs2387977
rs11715853
rs6809952
rs8039785
rs7311004
All − MR Egger
All − IVW(MRE)
−1.5
−1.0
−0.5
0.0
0.5
1.0
1.5
MR effect size for
'Gut microbiota abundance (genus Parasutterella id.2892)' on 'Histone−lysine N−methyltransferase EHMT2'

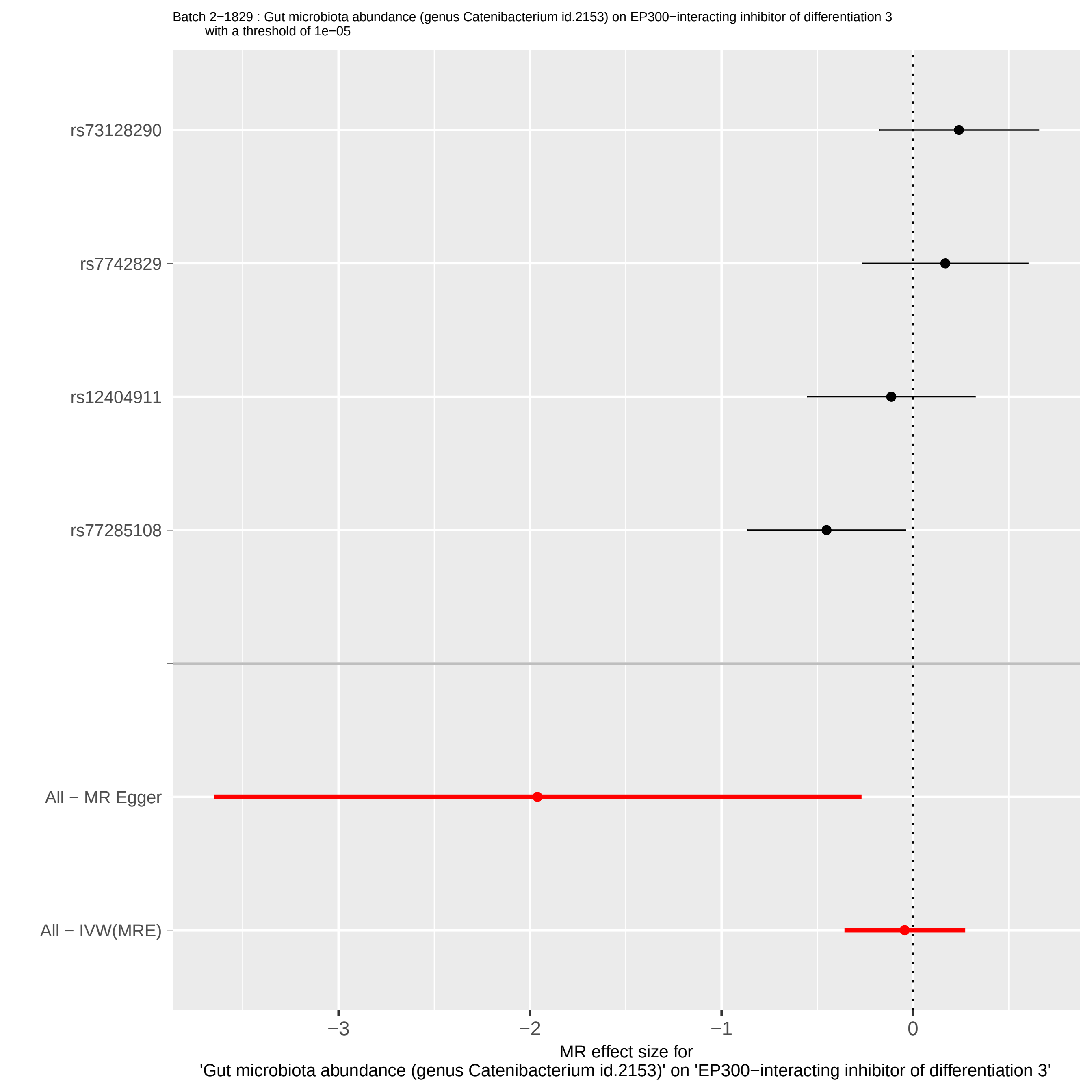
Batch 2−1829 : Gut microbiota abundance (genus Catenibacterium id.2153) on EP300−interacting inhibitor of differentiation 3
with a threshold of 1e−05
rs73128290
rs7742829
rs12404911
rs77285108
All − MR Egger
All − IVW(MRE)
−3
−2
−1
0
MR effect size for
'Gut microbiota abundance (genus Catenibacterium id.2153)' on 'EP300−interacting inhibitor of differentiation 3'

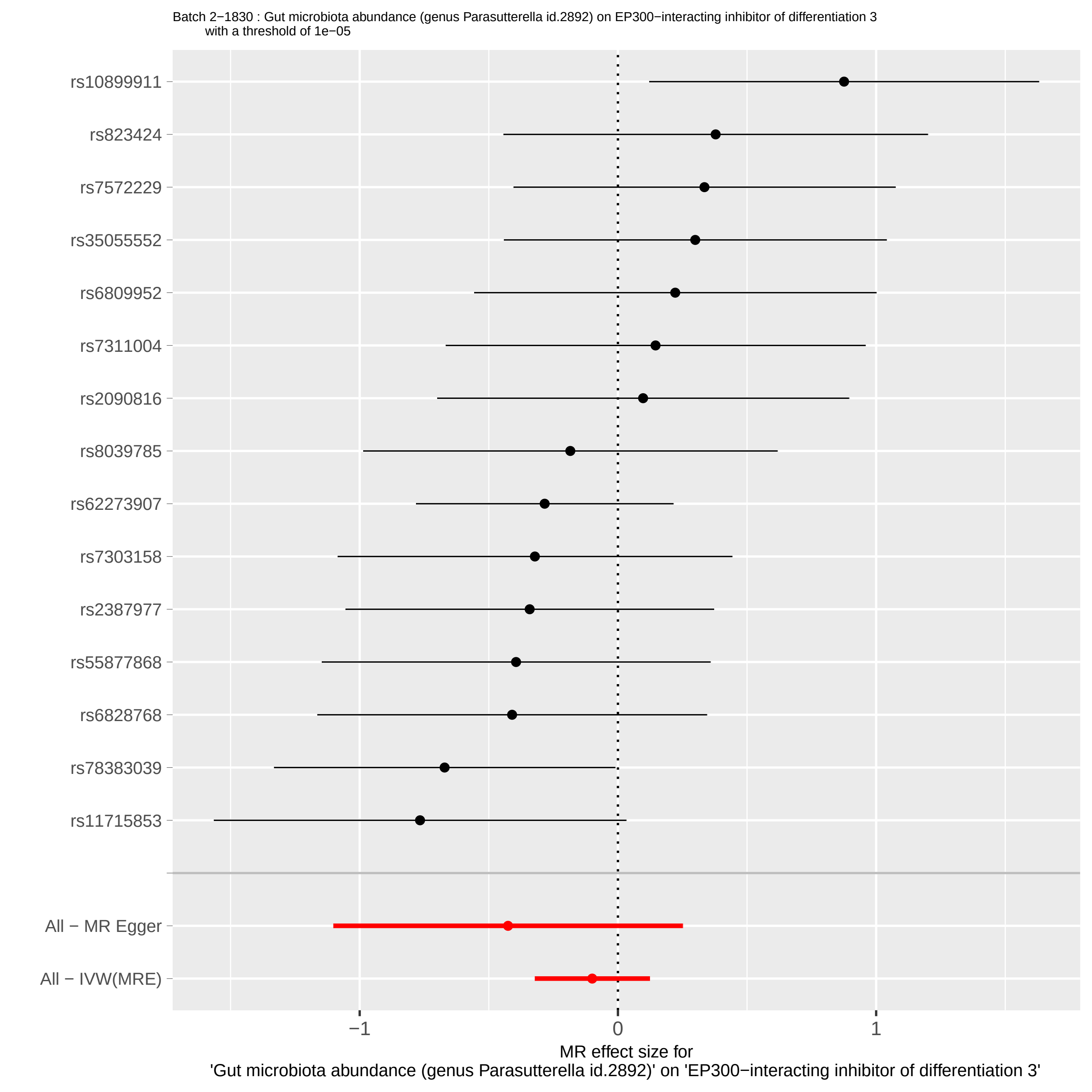
Batch 2−1830 : Gut microbiota abundance (genus Parasutterella id.2892) on EP300−interacting inhibitor of differentiation 3
with a threshold of 1e−05
rs10899911
rs823424
rs7572229
rs35055552
rs6809952
rs7311004
rs2090816
rs8039785
rs62273907
rs7303158
rs2387977
rs55877868
rs6828768
rs78383039
rs11715853
All − MR Egger
All − IVW(MRE)
−1
0
1
MR effect size for
'Gut microbiota abundance (genus Parasutterella id.2892)' on 'EP300−interacting inhibitor of differentiation 3'

Batch 2−1831 : Gut microbiota abundance (genus Catenibacterium id.2153) on Probable RNA−binding protein EIF1AD
with a threshold of 1e−05

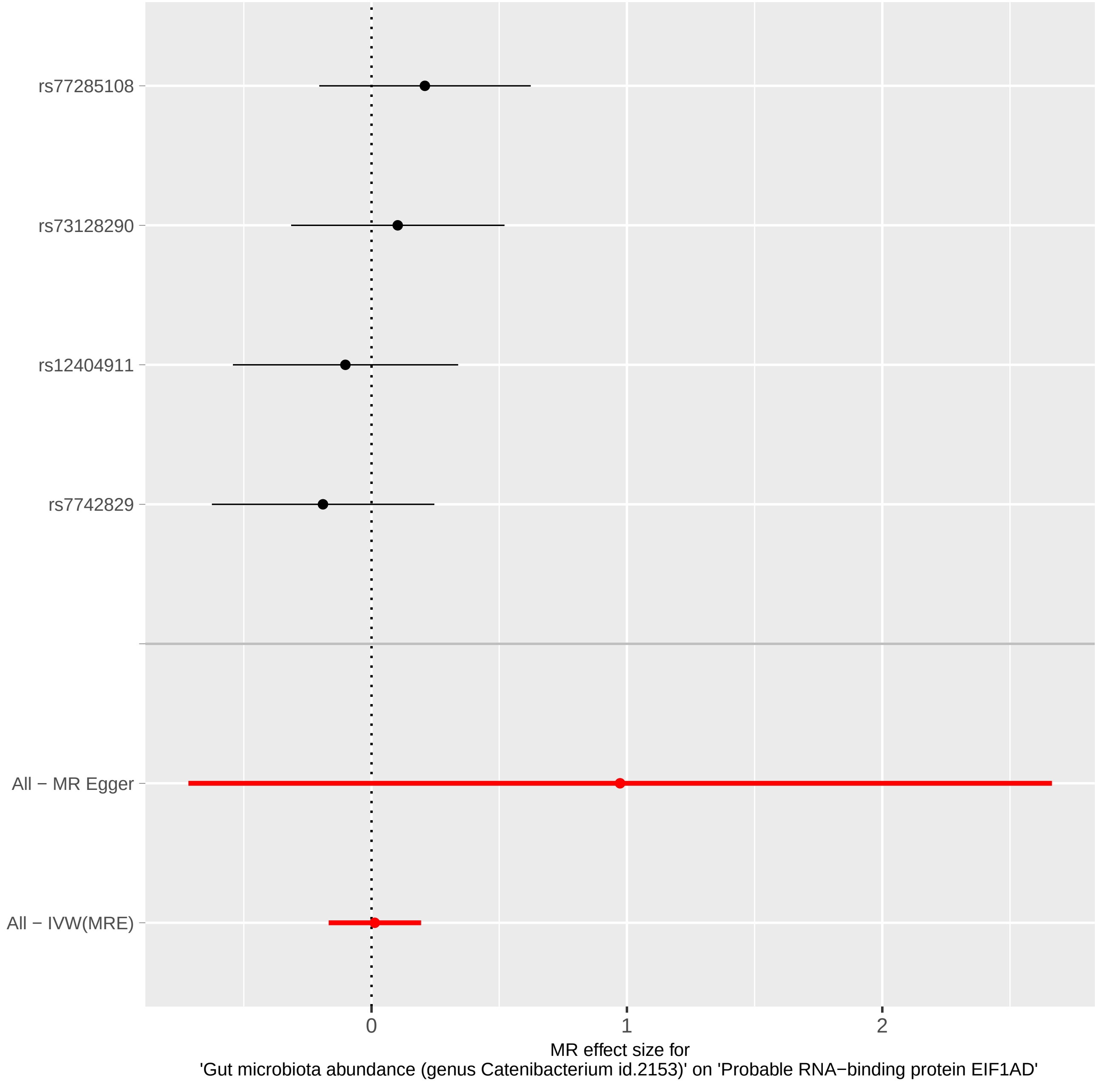

Batch 2−1832 : Gut microbiota abundance (genus Parasutterella id.2892) on Probable RNA−binding protein EIF1AD
with a threshold of 1e−05

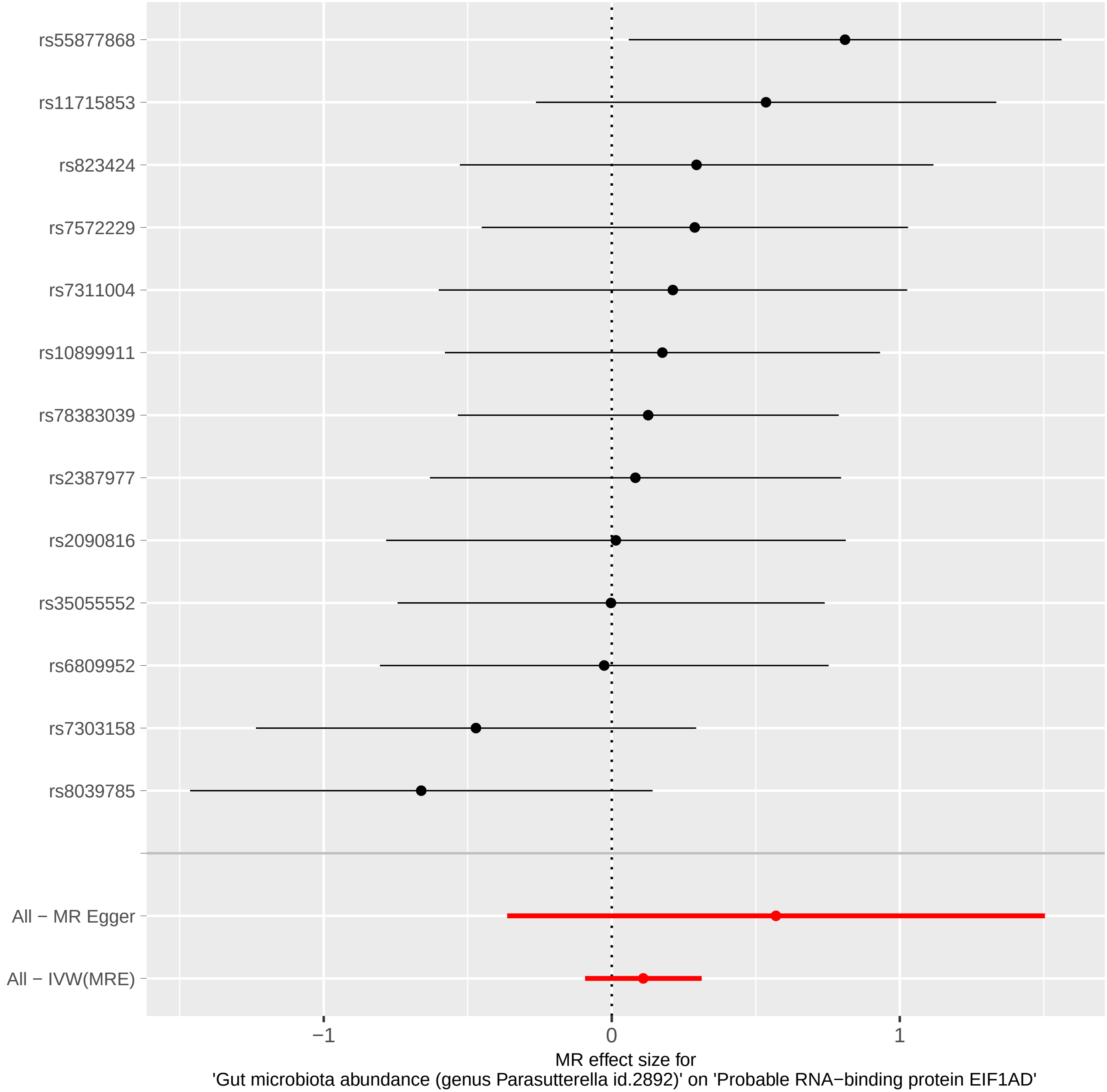

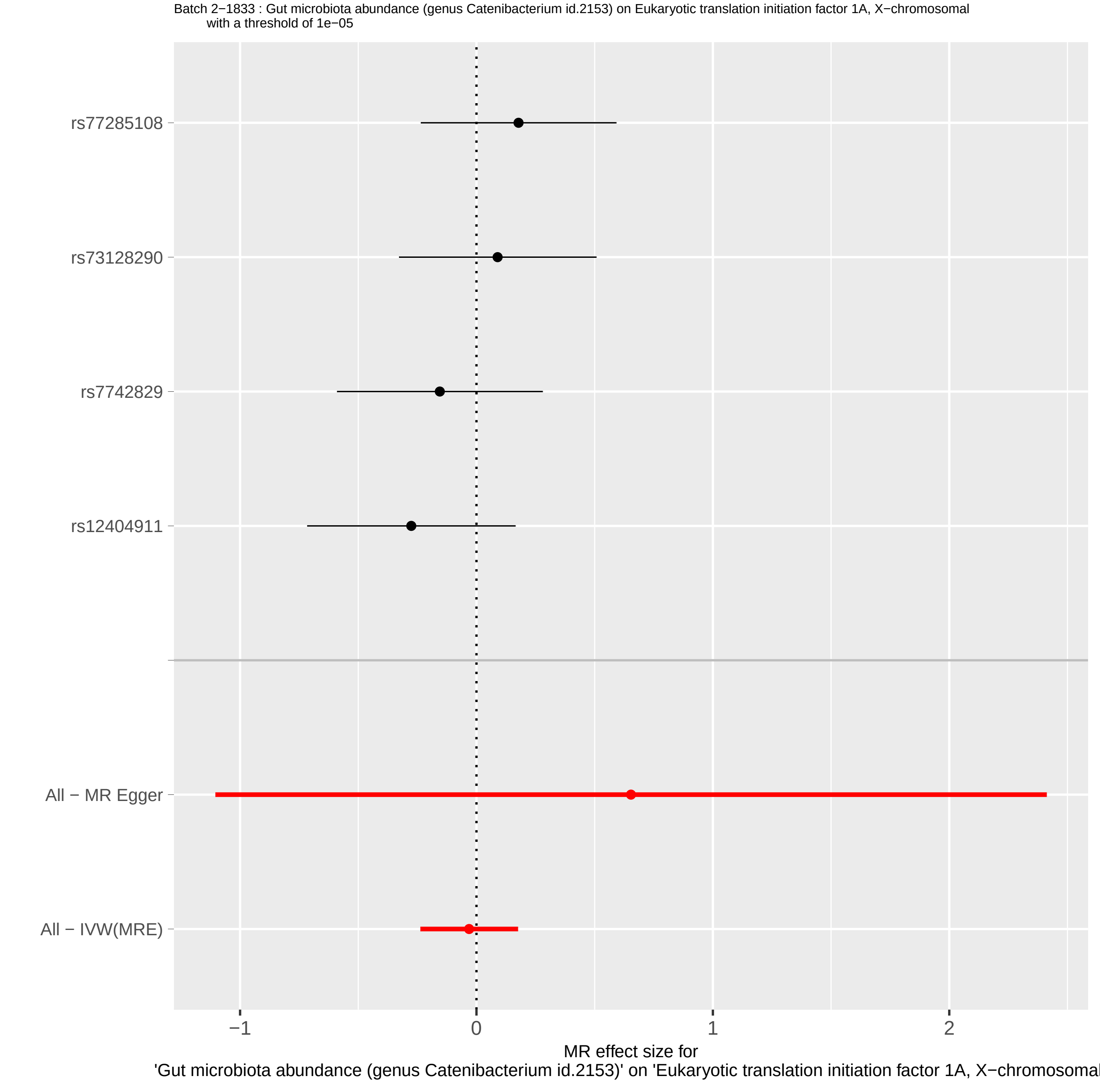

Batch 2−1833 : Gut microbiota abundance (genus Catenibacterium id.2153) on Eukaryotic translation initiation factor 1A, X−chromosomal
with a threshold of 1e−05
rs77285108
rs73128290
rs7742829
rs12404911
All − MR Egger
All − IVW(MRE)
−1
0
1
2
MR effect size for
'Gut microbiota abundance (genus Catenibacterium id.2153)' on 'Eukaryotic translation initiation factor 1A, X−chromosomal

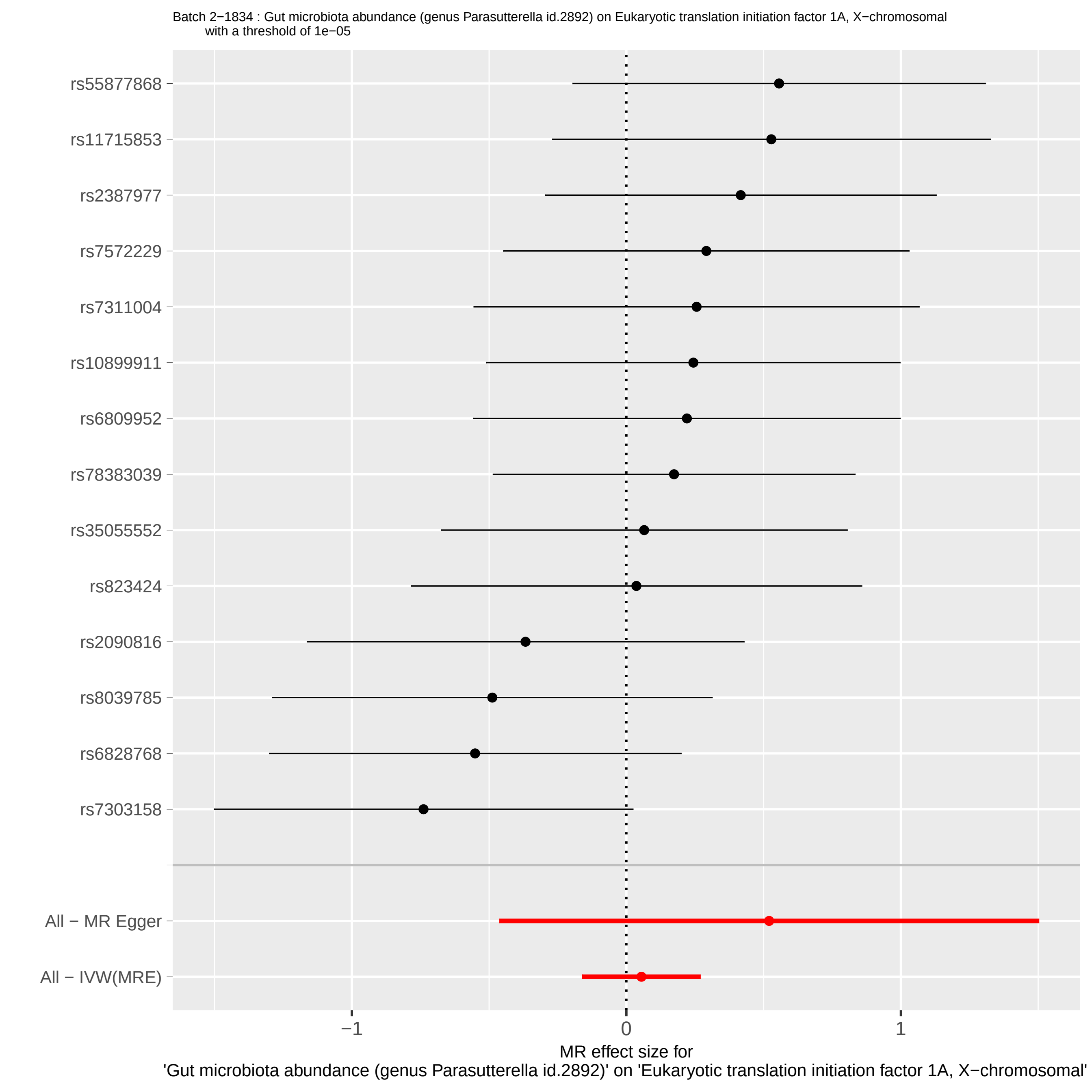

Batch 2−1834 : Gut microbiota abundance (genus Parasutterella id.2892) on Eukaryotic translation initiation factor 1A, X−chromosomal
with a threshold of 1e−05
rs55877868
rs11715853
rs2387977
rs7572229
rs7311004
rs10899911
rs6809952
rs78383039
rs35055552
rs823424
rs2090816
rs8039785
rs6828768
rs7303158
All − MR Egger
All − IVW(MRE)
−1
0
1
MR effect size for
'Gut microbiota abundance (genus Parasutterella id.2892)' on 'Eukaryotic translation initiation factor 1A, X−chromosomal'

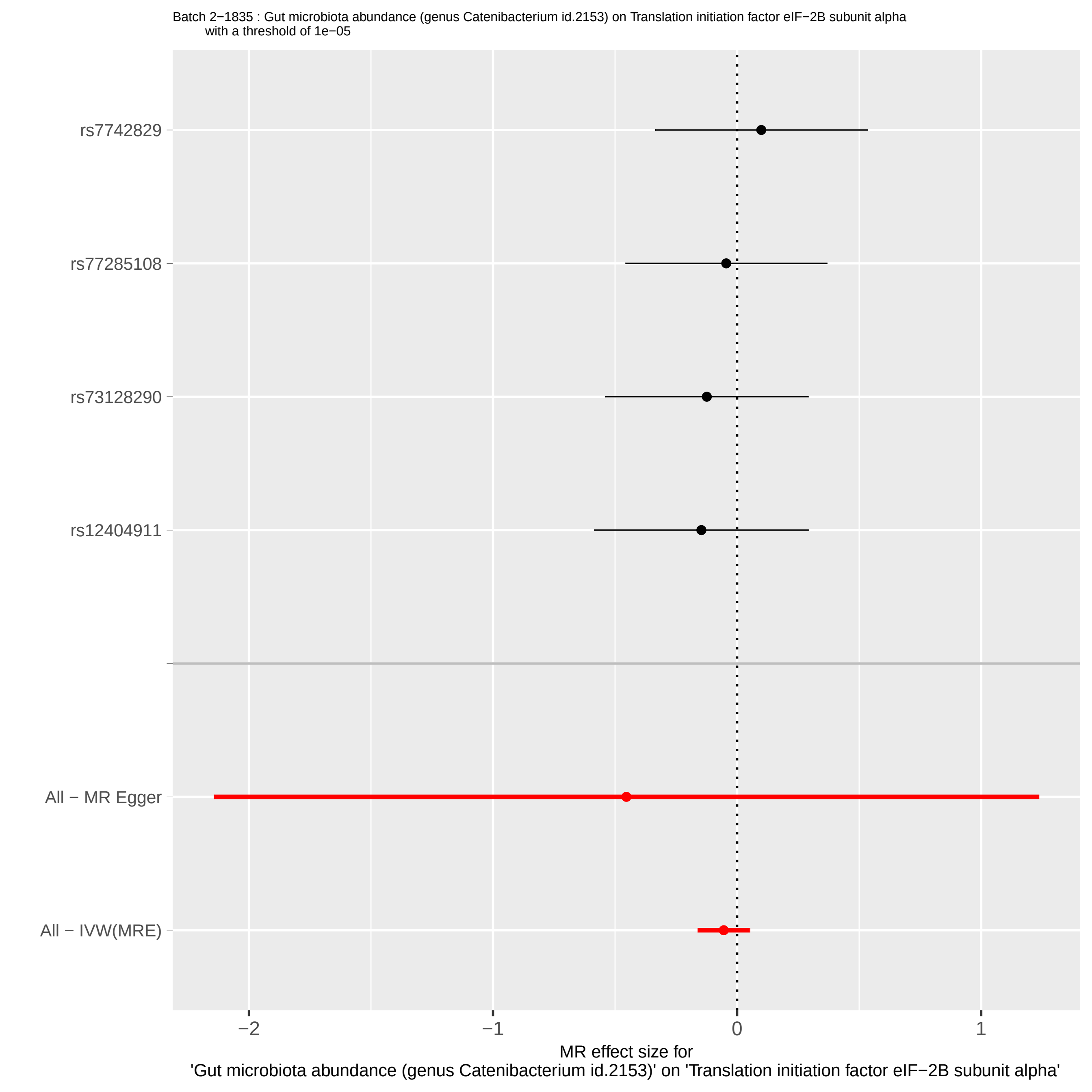
Batch 2−1835 : Gut microbiota abundance (genus Catenibacterium id.2153) on Translation initiation factor eIF−2B subunit alpha
with a threshold of 1e−05
rs7742829
rs77285108
rs73128290
rs12404911
All − MR Egger
All − IVW(MRE)
−2
−1
0
1
MR effect size for
'Gut microbiota abundance (genus Catenibacterium id.2153)' on 'Translation initiation factor eIF−2B subunit alpha'

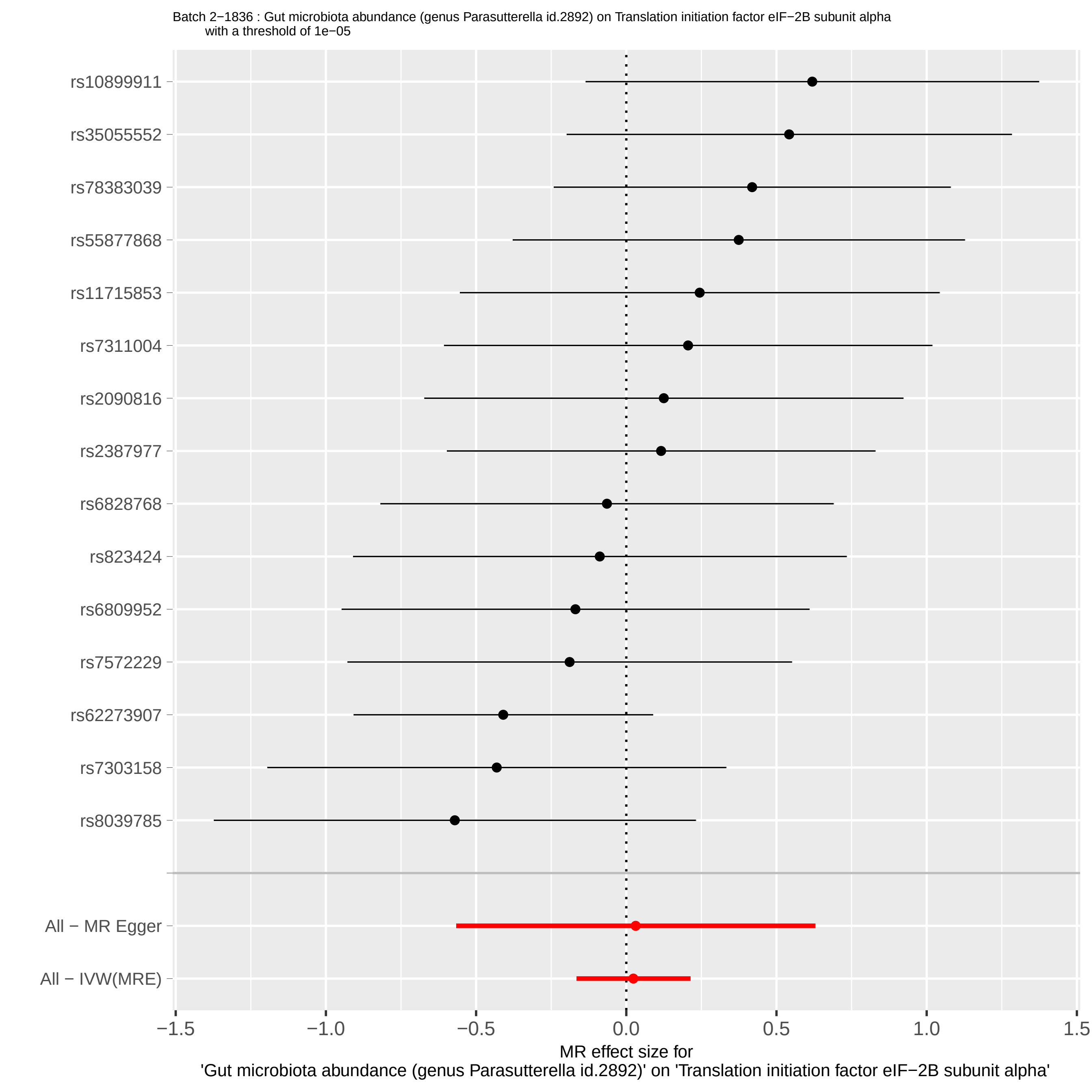

Batch 2−1836 : Gut microbiota abundance (genus Parasutterella id.2892) on Translation initiation factor eIF−2B subunit alpha
with a threshold of 1e−05
rs10899911
rs35055552
rs78383039
rs55877868
rs11715853
rs7311004
rs2090816
rs2387977
rs6828768
rs823424
rs6809952
rs7572229
rs62273907
rs7303158
rs8039785
All − MR Egger
All − IVW(MRE)
−1.5
−1.0
−0.5
0.0
0.5
1.0
1.5
MR effect size for
'Gut microbiota abundance (genus Parasutterella id.2892)' on 'Translation initiation factor eIF−2B subunit alpha'

Batch 2−1837 : Gut microbiota abundance (genus Catenibacterium id.2153) on Eukaryotic translation initiation factor 3 subunit G
with a threshold of 1e−05

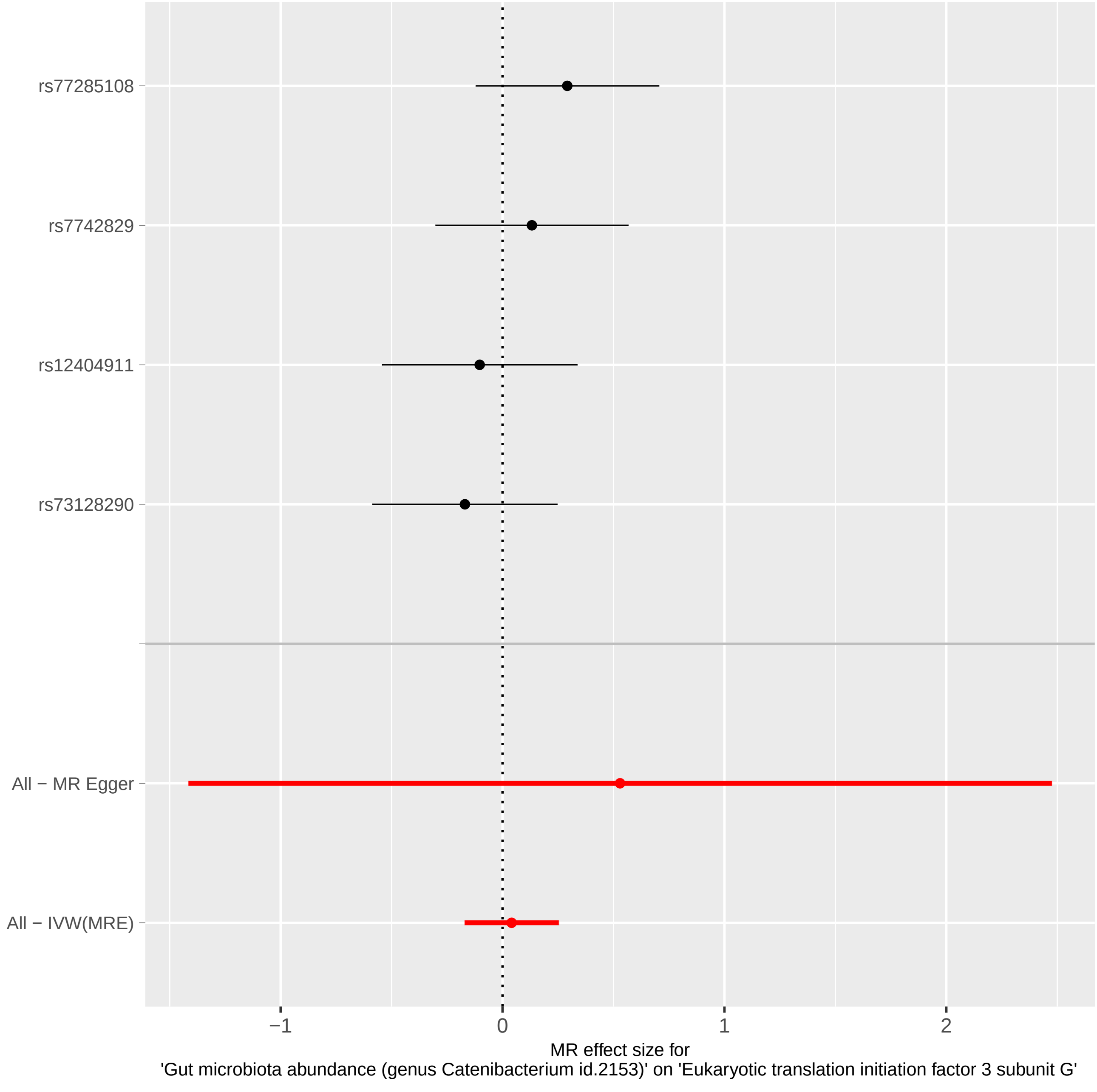

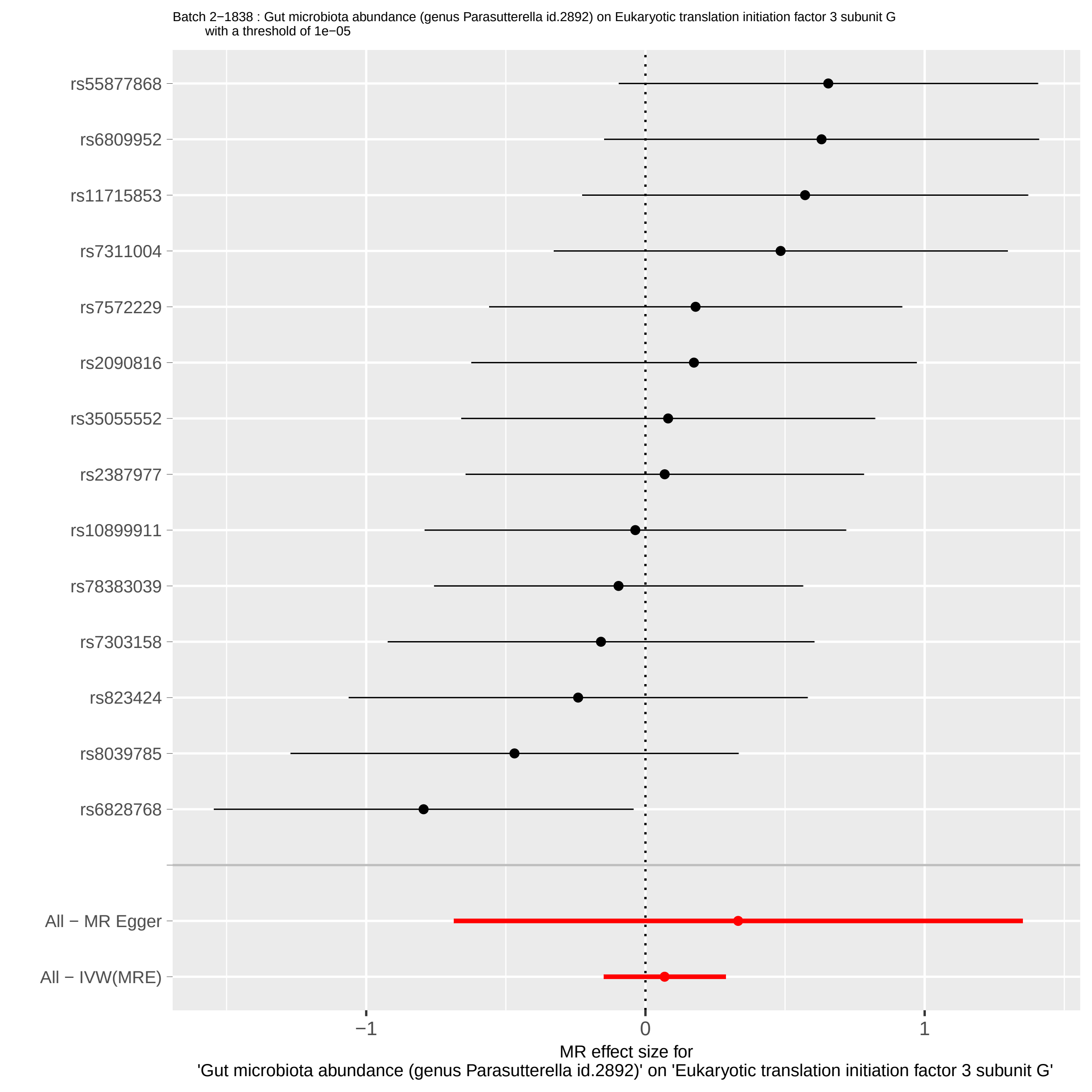

Batch 2−1838 : Gut microbiota abundance (genus Parasutterella id.2892) on Eukaryotic translation initiation factor 3 subunit G
with a threshold of 1e−05
rs55877868
rs6809952
rs11715853
rs7311004
rs7572229
rs2090816
rs35055552
rs2387977
rs10899911
rs78383039
rs7303158
rs823424
rs8039785
rs6828768
All − MR Egger
All − IVW(MRE)
−1
0
1
MR effect size for
'Gut microbiota abundance (genus Parasutterella id.2892)' on 'Eukaryotic translation initiation factor 3 subunit G'

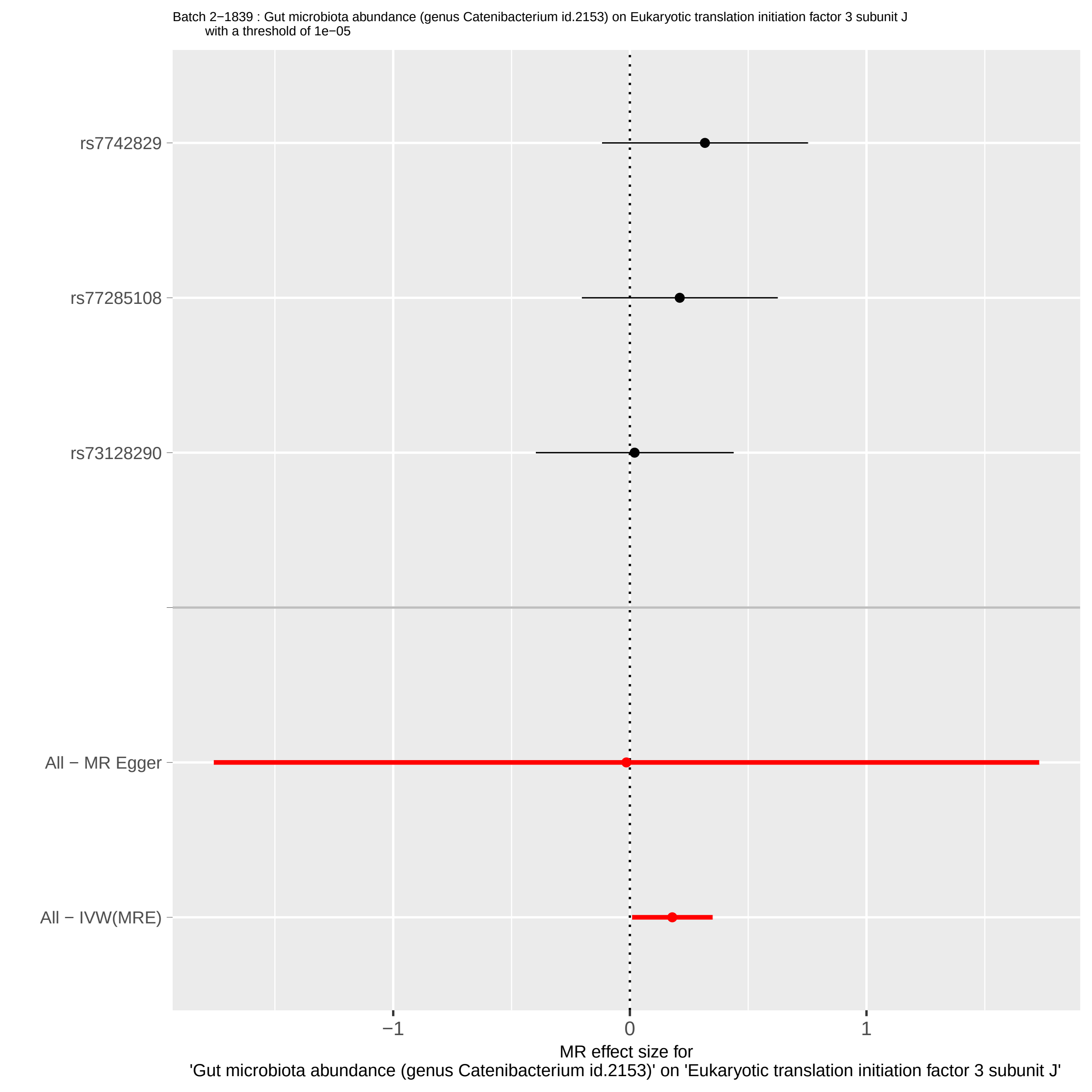
Batch 2−1839 : Gut microbiota abundance (genus Catenibacterium id.2153) on Eukaryotic translation initiation factor 3 subunit J
with a threshold of 1e−05
rs7742829
rs77285108
rs73128290
All − MR Egger
All − IVW(MRE)
−1
0
1
MR effect size for
'Gut microbiota abundance (genus Catenibacterium id.2153)' on 'Eukaryotic translation initiation factor 3 subunit J'

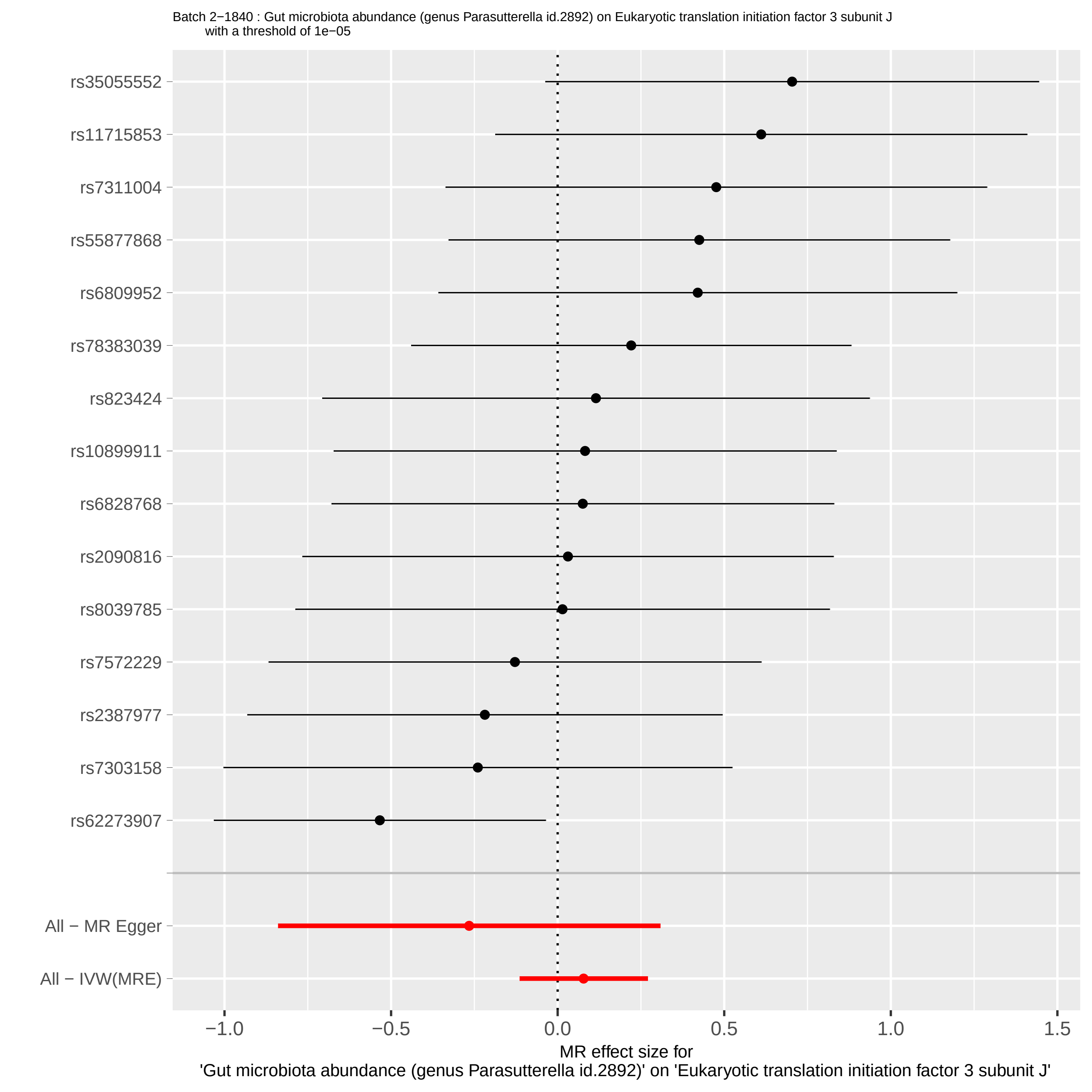

Batch 2−1840 : Gut microbiota abundance (genus Parasutterella id.2892) on Eukaryotic translation initiation factor 3 subunit J
with a threshold of 1e−05
rs35055552
rs11715853
rs7311004
rs55877868
rs6809952
rs78383039
rs823424
rs10899911
rs6828768
rs2090816
rs8039785
rs7572229
rs2387977
rs7303158
rs62273907
All − MR Egger
All − IVW(MRE)
−1.0
−0.5
0.0
0.5
1.0
1.5
MR effect size for
'Gut microbiota abundance (genus Parasutterella id.2892)' on 'Eukaryotic translation initiation factor 3 subunit J'

Batch 2−1841 : Gut microbiota abundance (genus Catenibacterium id.2153) on Eukaryotic initiation factor 4A−III
with a threshold of 1e−05

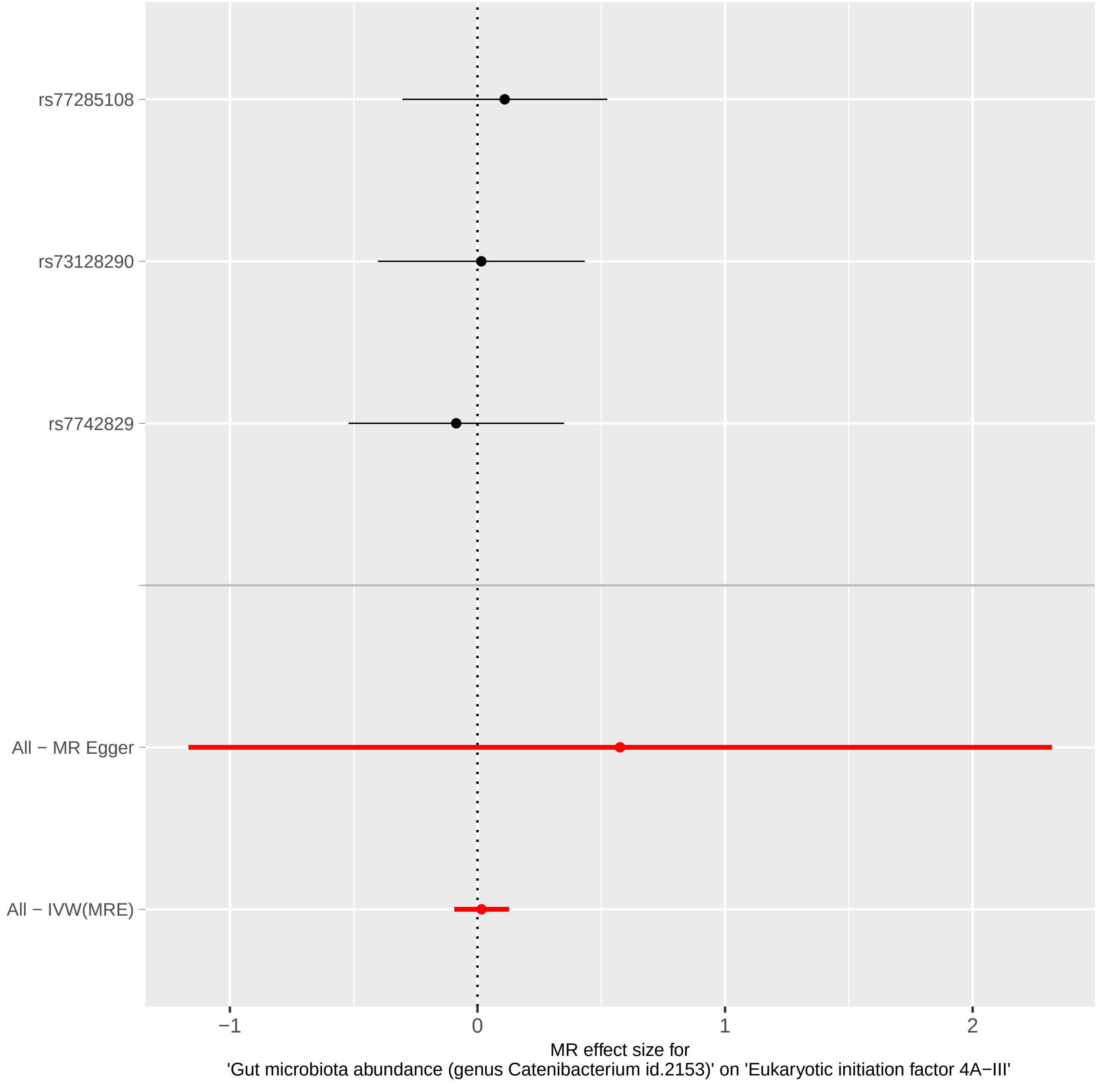

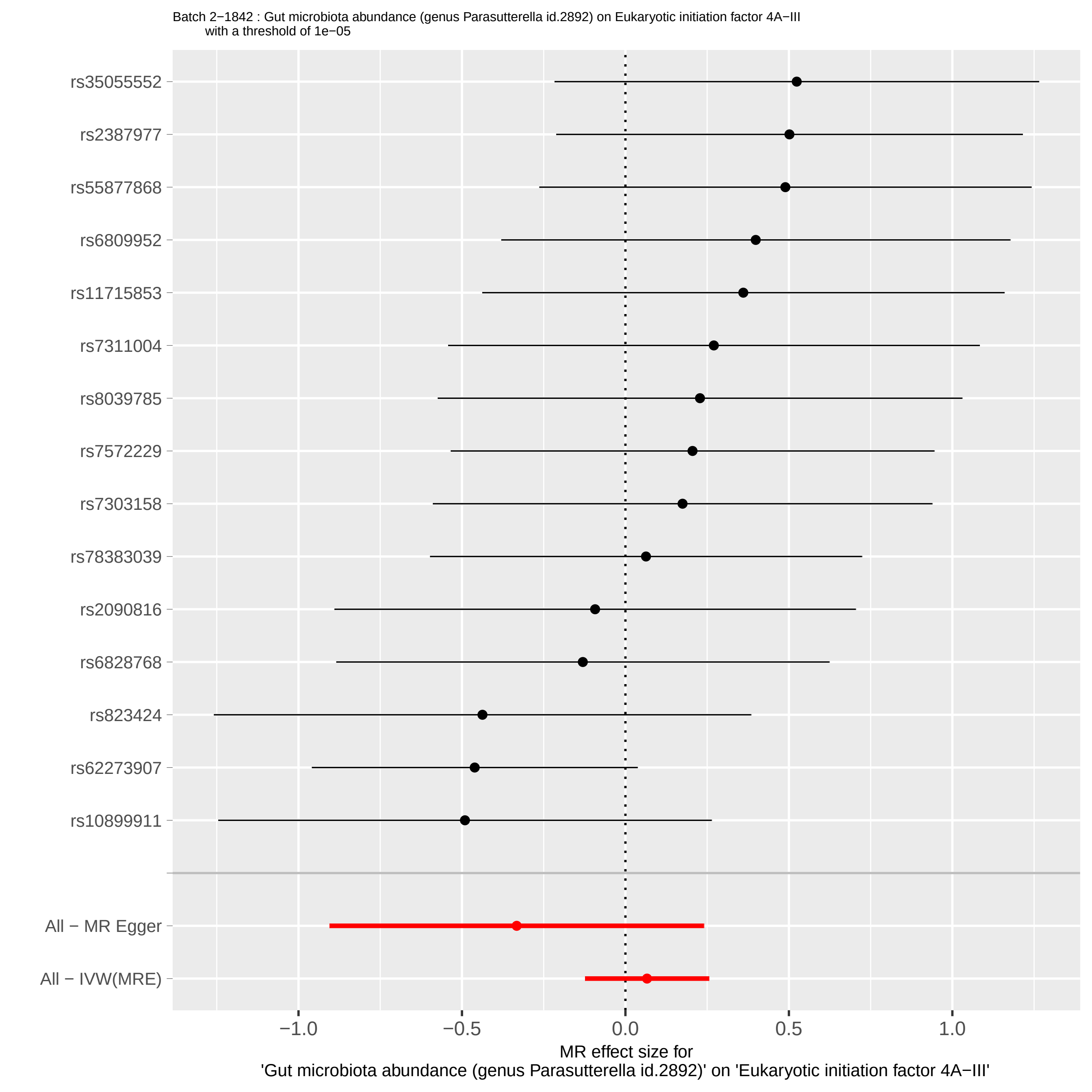
Batch 2−1842 : Gut microbiota abundance (genus Parasutterella id.2892) on Eukaryotic initiation factor 4A−III
with a threshold of 1e−05
rs35055552
rs2387977
rs55877868
rs6809952
rs11715853
rs7311004
rs8039785
rs7572229
rs7303158
rs78383039
rs2090816
rs6828768
rs823424
rs62273907
rs10899911
All − MR Egger
All − IVW(MRE)
−1.0
−0.5
0.0
0.5
1.0
MR effect size for
'Gut microbiota abundance (genus Parasutterella id.2892)' on 'Eukaryotic initiation factor 4A−III'

Batch 2−1843 : Gut microbiota abundance (genus Catenibacterium id.2153) on Eukaryotic translation initiation factor 4B
with a threshold of 1e−05

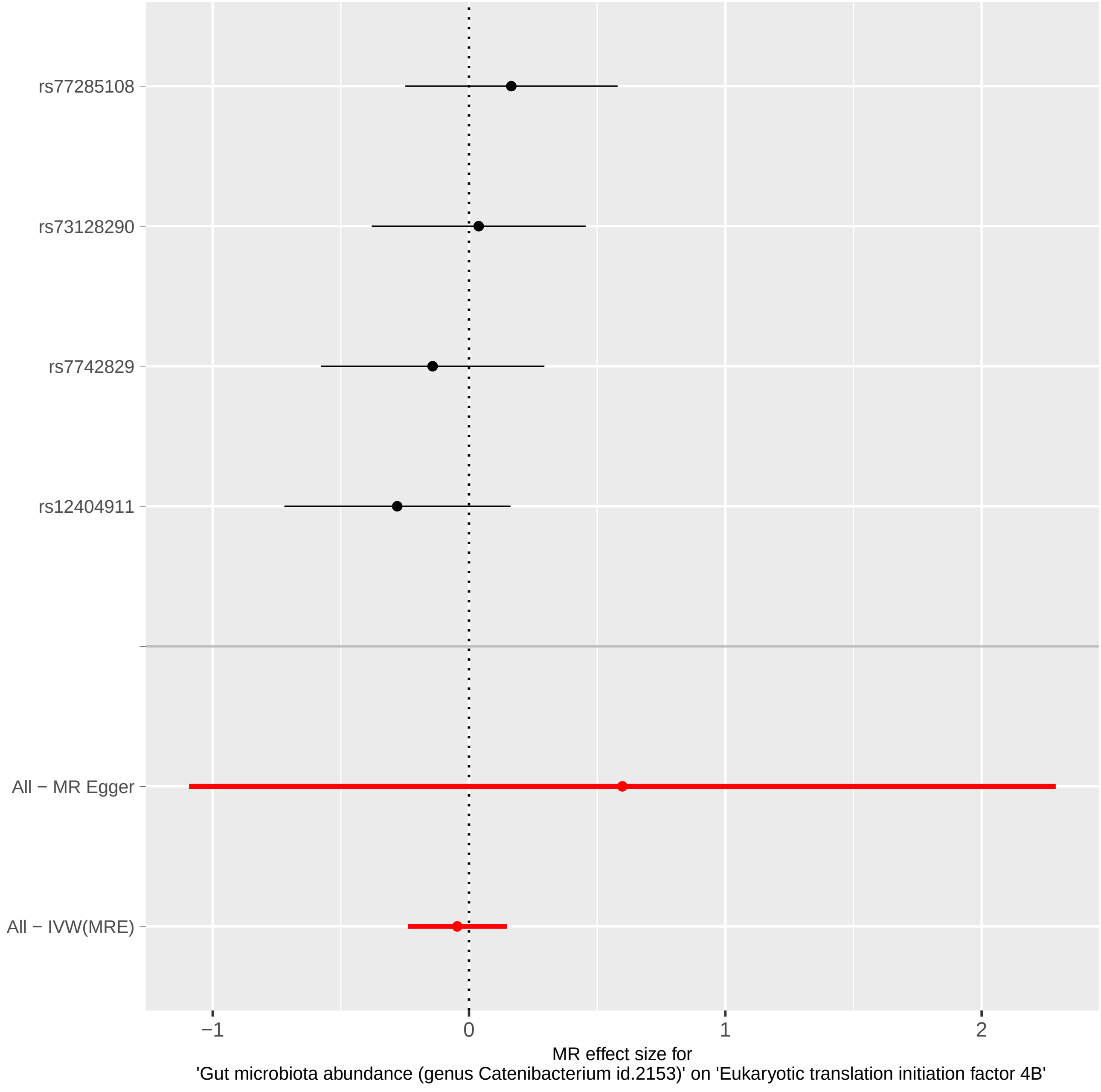

Batch 2−1844 : Gut microbiota abundance (genus Parasutterella id.2892) on Eukaryotic translation initiation factor 4B
with a threshold of 1e−05

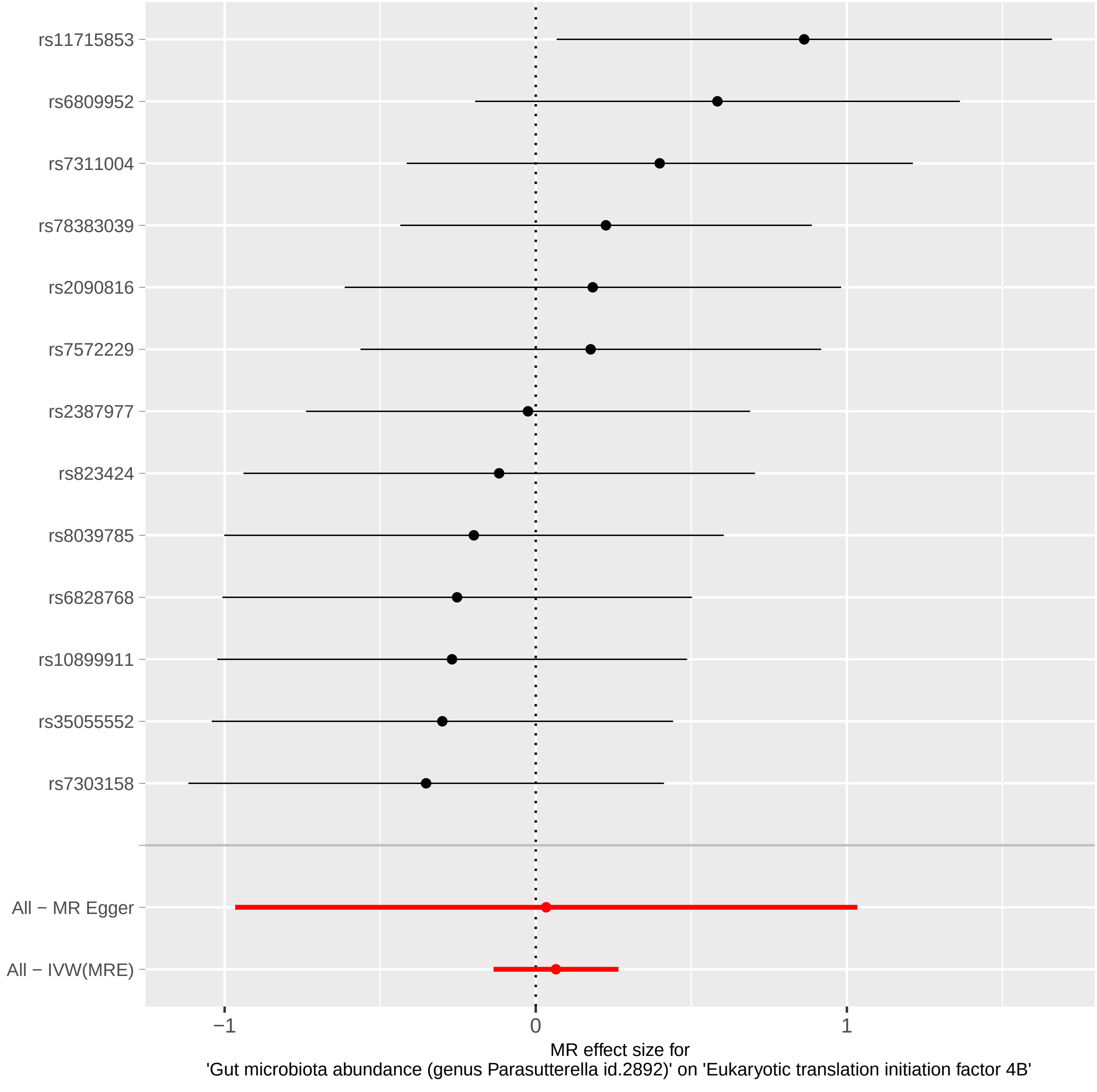

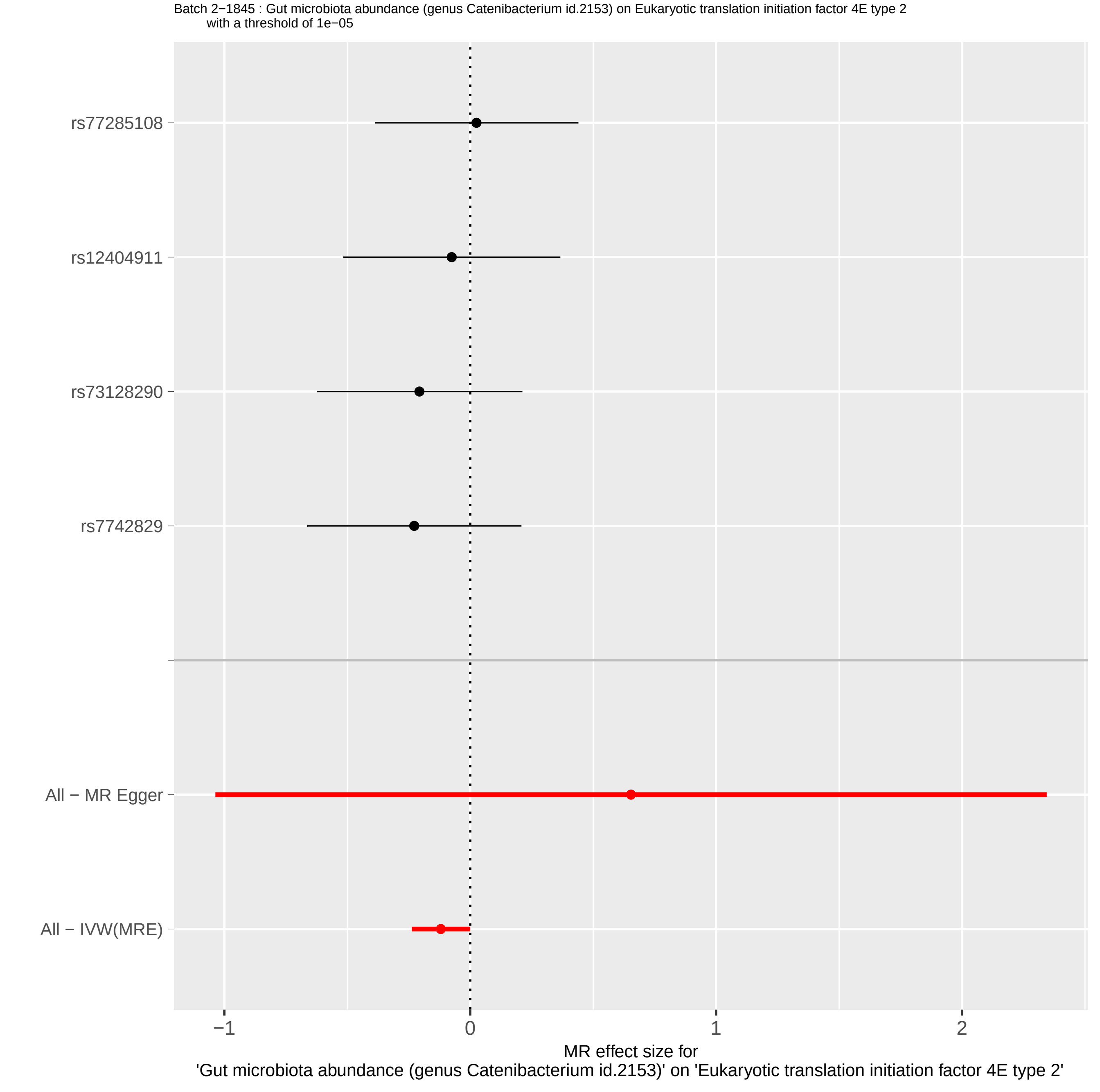

Batch 2−1845 : Gut microbiota abundance (genus Catenibacterium id.2153) on Eukaryotic translation initiation factor 4E type 2
with a threshold of 1e−05
rs77285108
rs12404911
rs73128290
rs7742829
All − MR Egger
All − IVW(MRE)
−1
0
1
2
MR effect size for
'Gut microbiota abundance (genus Catenibacterium id.2153)' on 'Eukaryotic translation initiation factor 4E type 2'

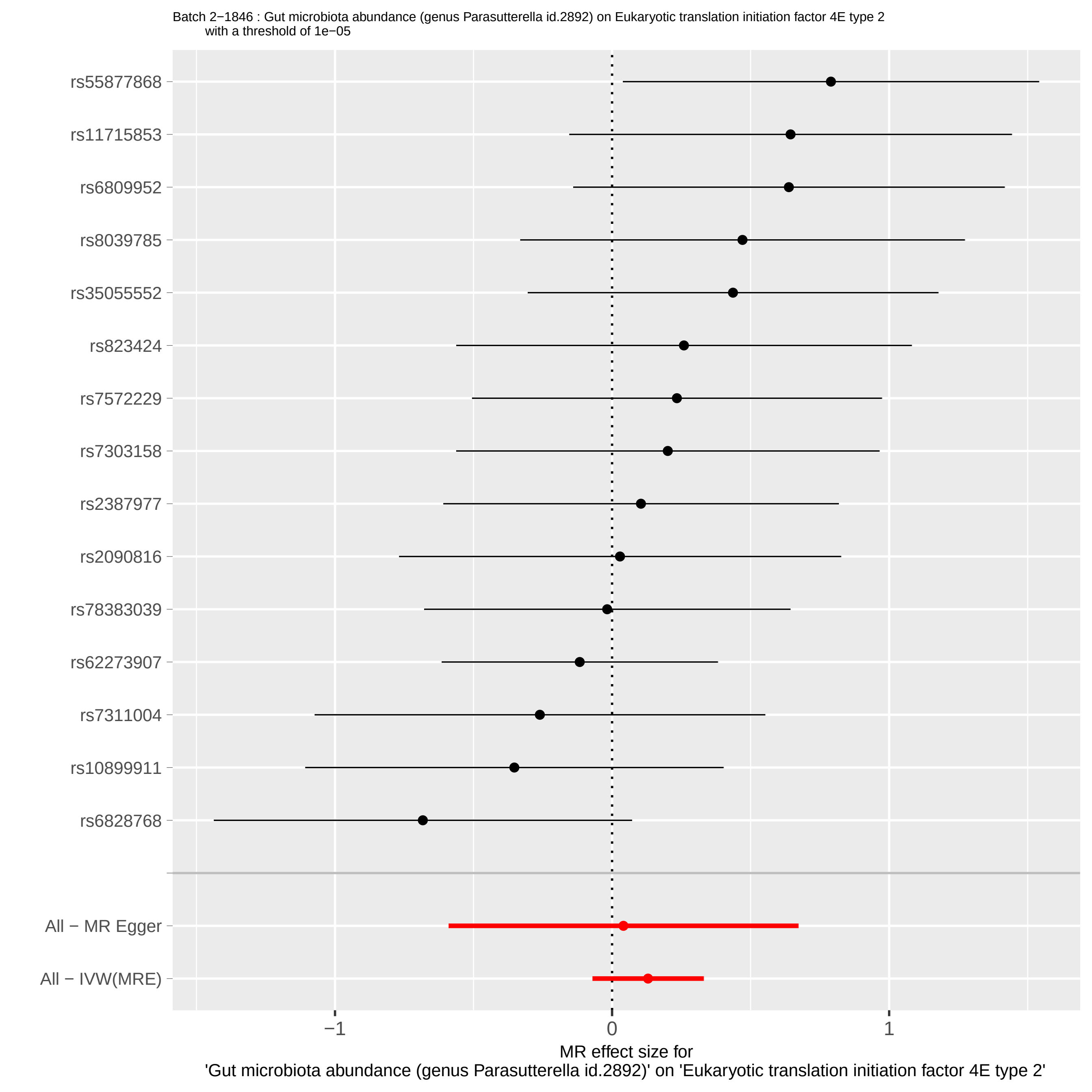

Batch 2−1846 : Gut microbiota abundance (genus Parasutterella id.2892) on Eukaryotic translation initiation factor 4E type 2
with a threshold of 1e−05
rs55877868
rs11715853
rs6809952
rs8039785
rs35055552
rs823424
rs7572229
rs7303158
rs2387977
rs2090816
rs78383039
rs62273907
rs7311004
rs10899911
rs6828768
All − MR Egger
All − IVW(MRE)
−1
0
1
MR effect size for
'Gut microbiota abundance (genus Parasutterella id.2892)' on 'Eukaryotic translation initiation factor 4E type 2'

Batch 2−1847 : Gut microbiota abundance (genus Catenibacterium id.2153) on Eukaryotic translation initiation factor 4E−binding protein 2
with a threshold of 1e−05

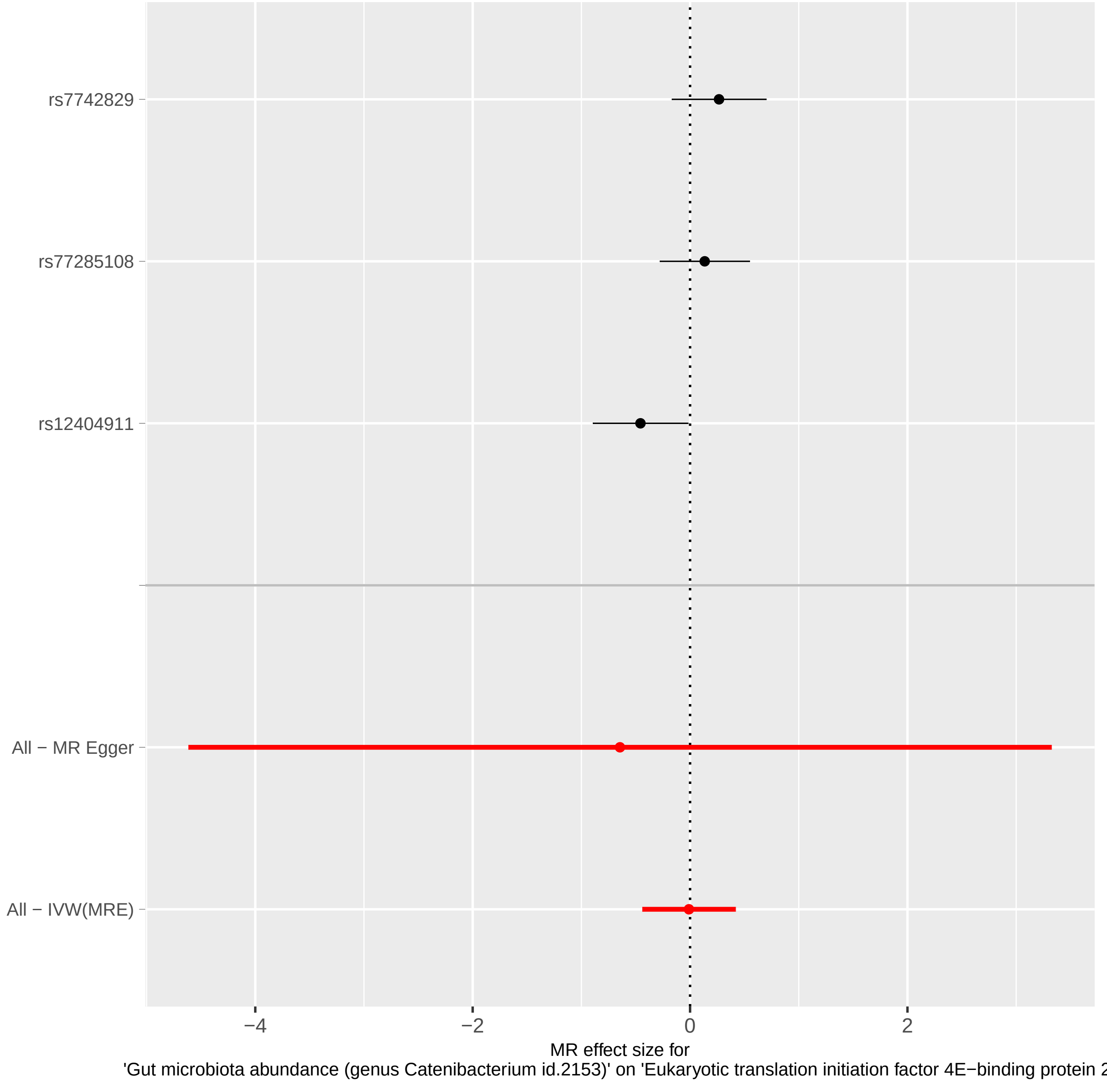

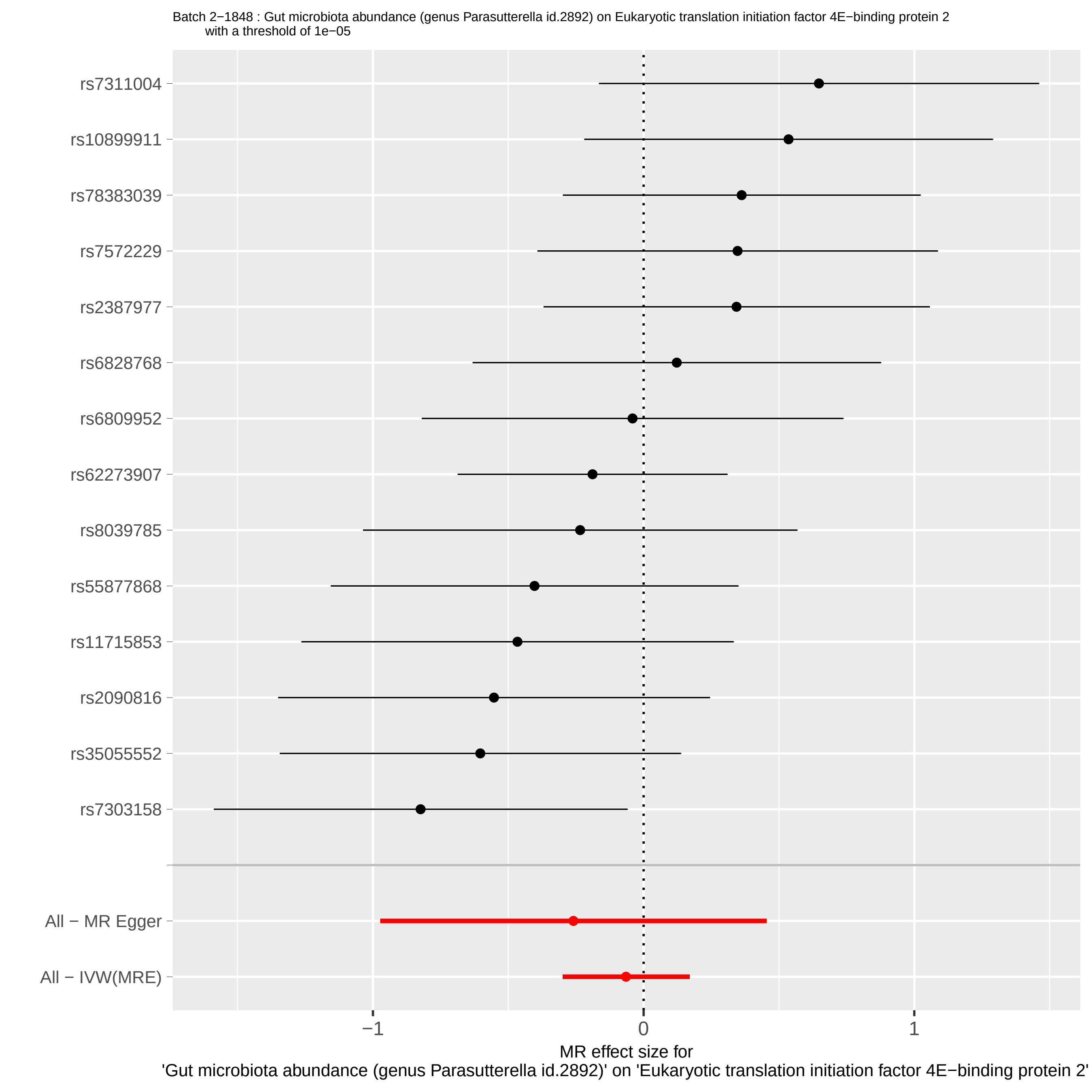

Batch 2−1848 : Gut microbiota abundance (genus Parasutterella id.2892) on Eukaryotic translation initiation factor 4E−binding protein 2
with a threshold of 1e−05
rs7311004
rs10899911
rs78383039
rs7572229
rs2387977
rs6828768
rs6809952
rs62273907
rs8039785
rs55877868
rs11715853
rs2090816
rs35055552
rs7303158
All − MR Egger
All − IVW(MRE)
−1
0
1
MR effect size for
'Gut microbiota abundance (genus Parasutterella id.2892)' on 'Eukaryotic translation initiation factor 4E−binding protein 2'

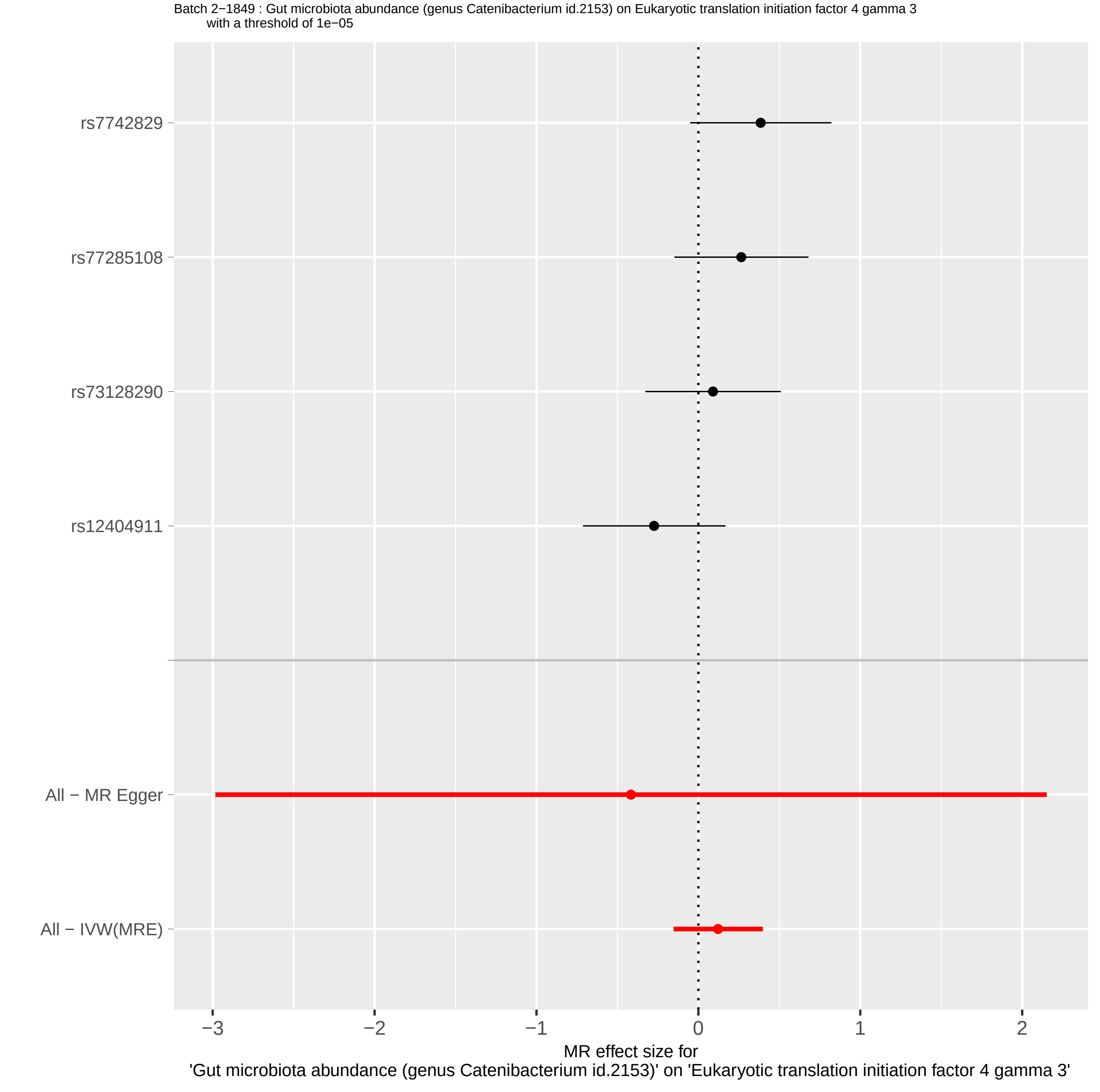

Batch 2−1849 : Gut microbiota abundance (genus Catenibacterium id.2153) on Eukaryotic translation initiation factor 4 gamma 3
with a threshold of 1e−05
rs7742829
rs77285108
rs73128290
rs12404911
All − MR Egger
All − IVW(MRE)
−3
−2
−1
0
1
2
MR effect size for
'Gut microbiota abundance (genus Catenibacterium id.2153)' on 'Eukaryotic translation initiation factor 4 gamma 3'

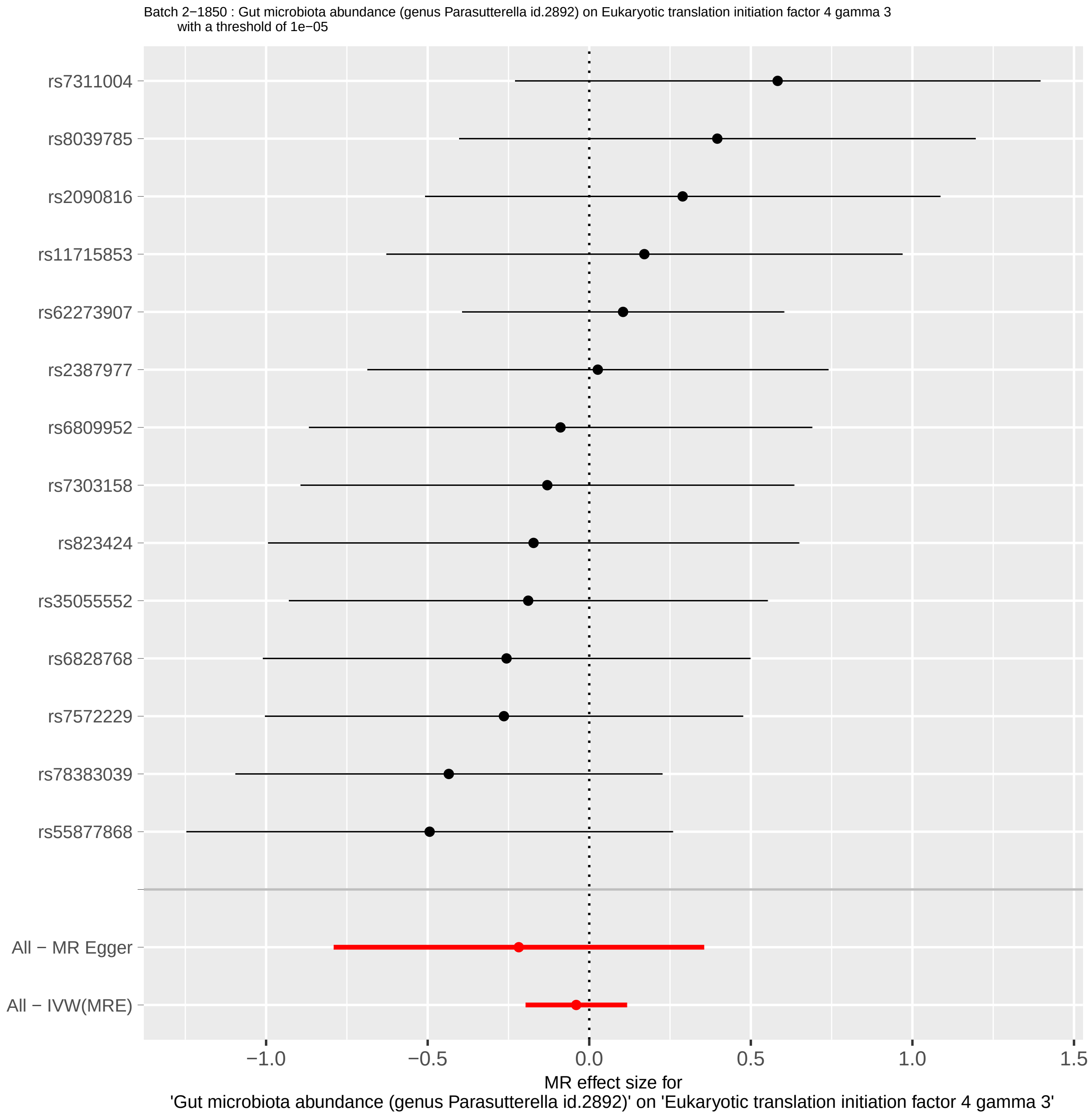

Batch 2−1850 : Gut microbiota abundance (genus Parasutterella id.2892) on Eukaryotic translation initiation factor 4 gamma 3
with a threshold of 1e−05
rs7311004
rs8039785
rs2090816
rs11715853
rs62273907
rs2387977
rs6809952
rs7303158
rs823424
rs35055552
rs6828768
rs7572229
rs78383039
rs55877868
All − MR Egger
All − IVW(MRE)
−1.0
−0.5
0.0
0.5
1.0
1.5
MR effect size for
'Gut microbiota abundance (genus Parasutterella id.2892)' on 'Eukaryotic translation initiation factor 4 gamma 3'

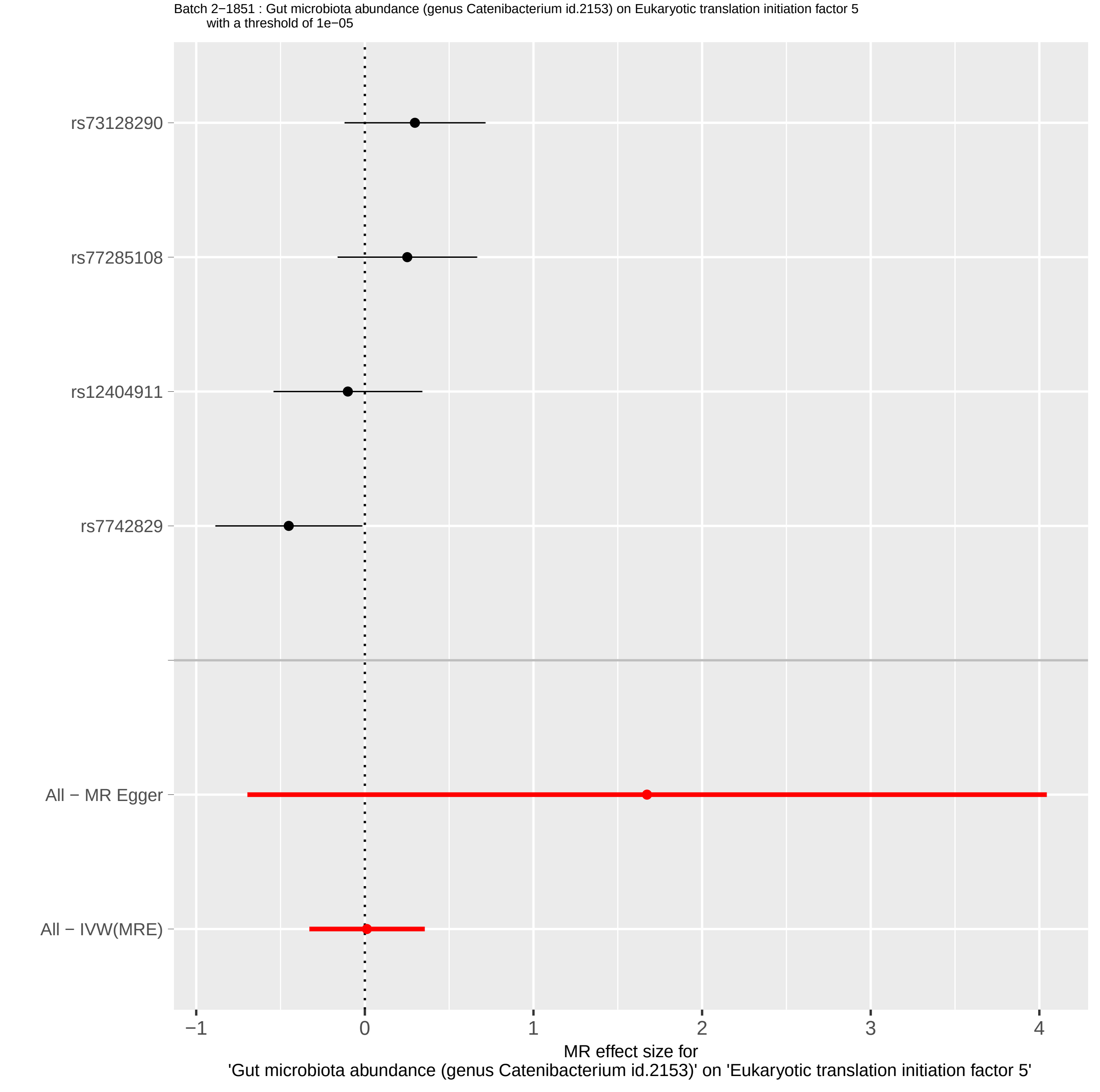
Batch 2−1851 : Gut microbiota abundance (genus Catenibacterium id.2153) on Eukaryotic translation initiation factor 5
with a threshold of 1e−05
rs73128290
rs77285108
rs12404911
rs7742829
All − MR Egger
All − IVW(MRE)
−1
0
1
2
3
4
MR effect size for
'Gut microbiota abundance (genus Catenibacterium id.2153)' on 'Eukaryotic translation initiation factor 5'

Batch 2−1852 : Gut microbiota abundance (genus Parasutterella id.2892) on Eukaryotic translation initiation factor 5
with a threshold of 1e−05

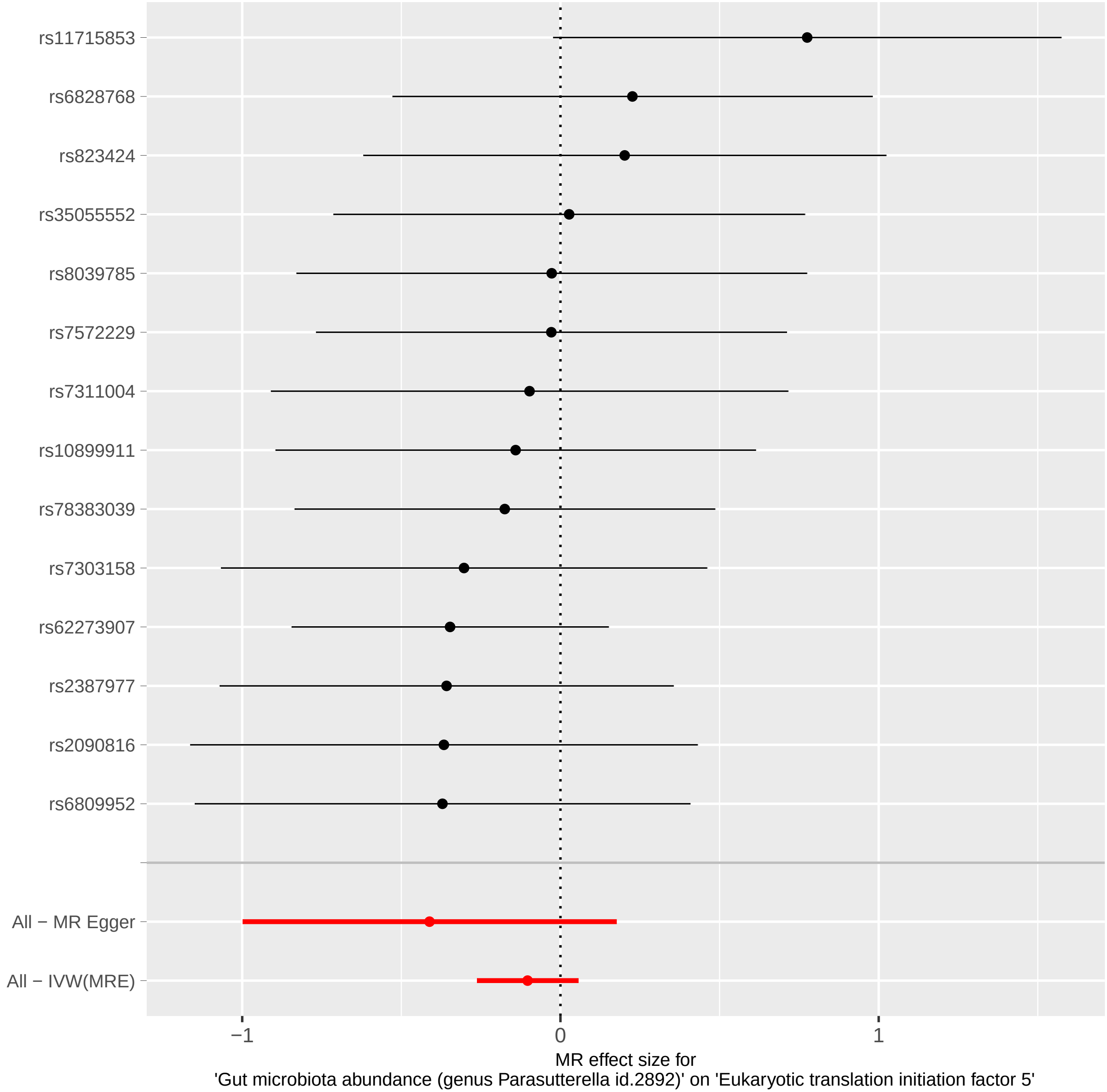

Batch 2−1853 : Gut microbiota abundance (genus Catenibacterium id.2153) on Eukaryotic translation initiation factor 5A−1
with a threshold of 1e−05

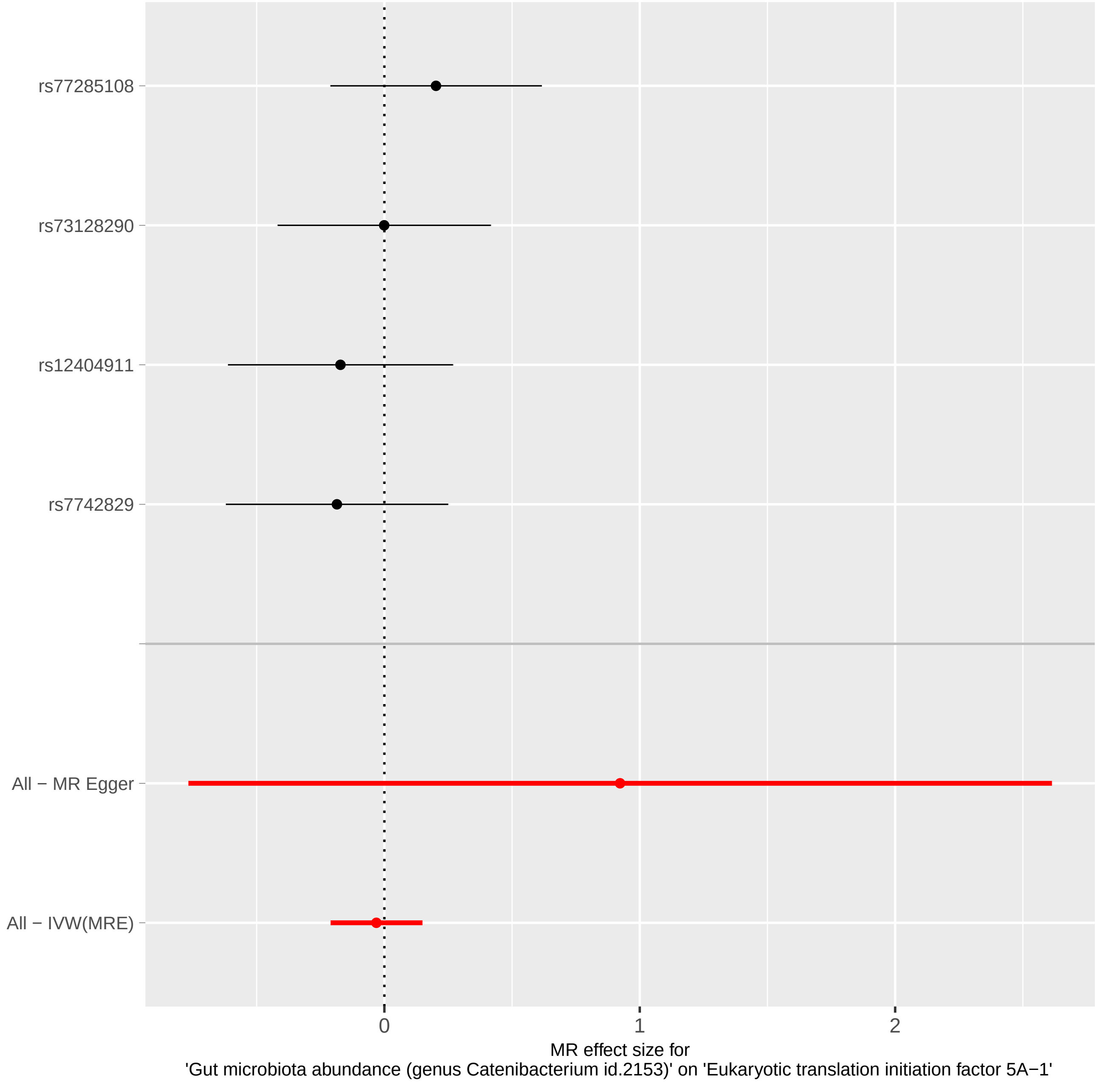

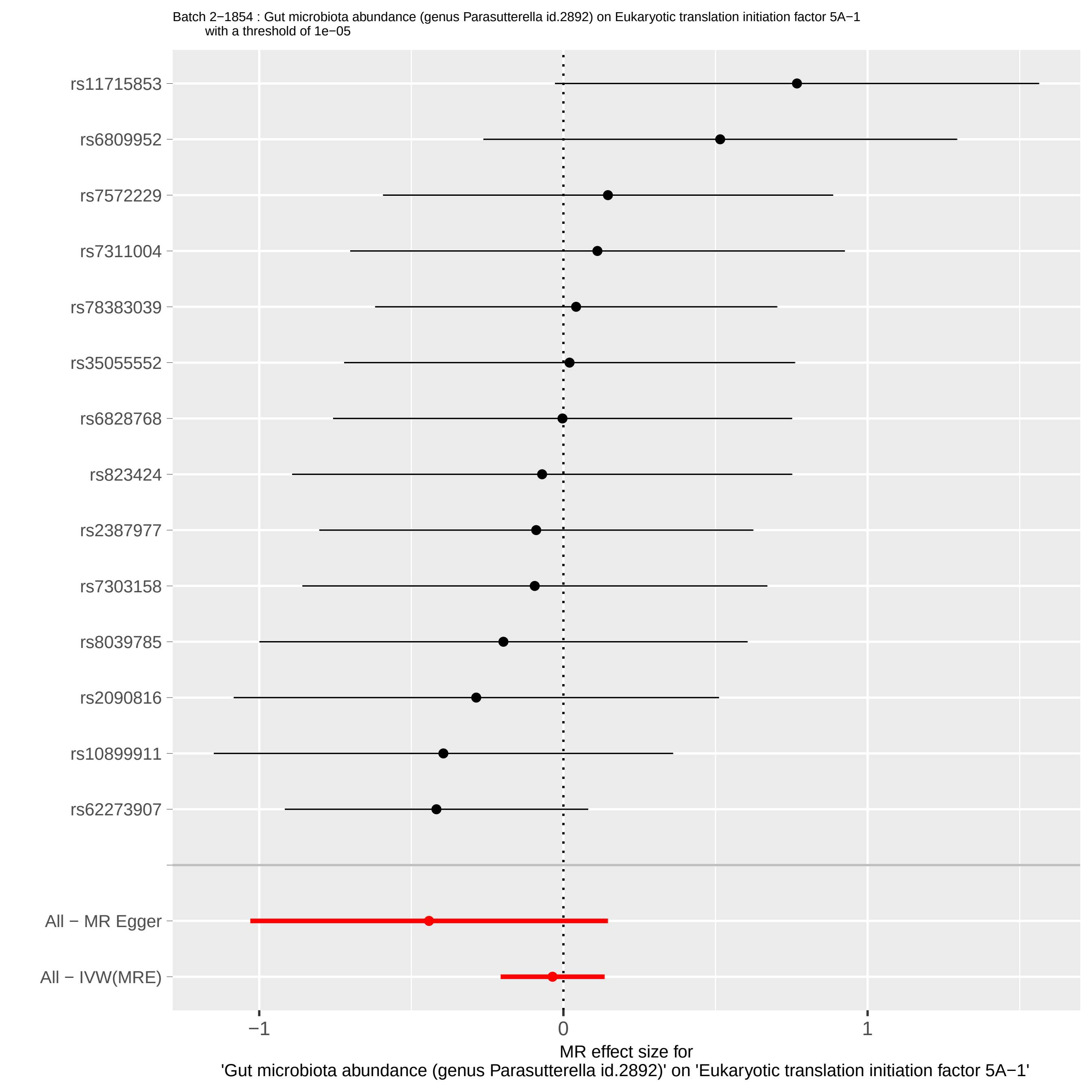

Batch 2−1854 : Gut microbiota abundance (genus Parasutterella id.2892) on Eukaryotic translation initiation factor 5A−1
with a threshold of 1e−05
rs11715853
rs6809952
rs7572229
rs7311004
rs78383039
rs35055552
rs6828768
rs823424
rs2387977
rs7303158
rs8039785
rs2090816
rs10899911
rs62273907
All − MR Egger
All − IVW(MRE)
−1
0
1
MR effect size for
'Gut microbiota abundance (genus Parasutterella id.2892)' on 'Eukaryotic translation initiation factor 5A−1'

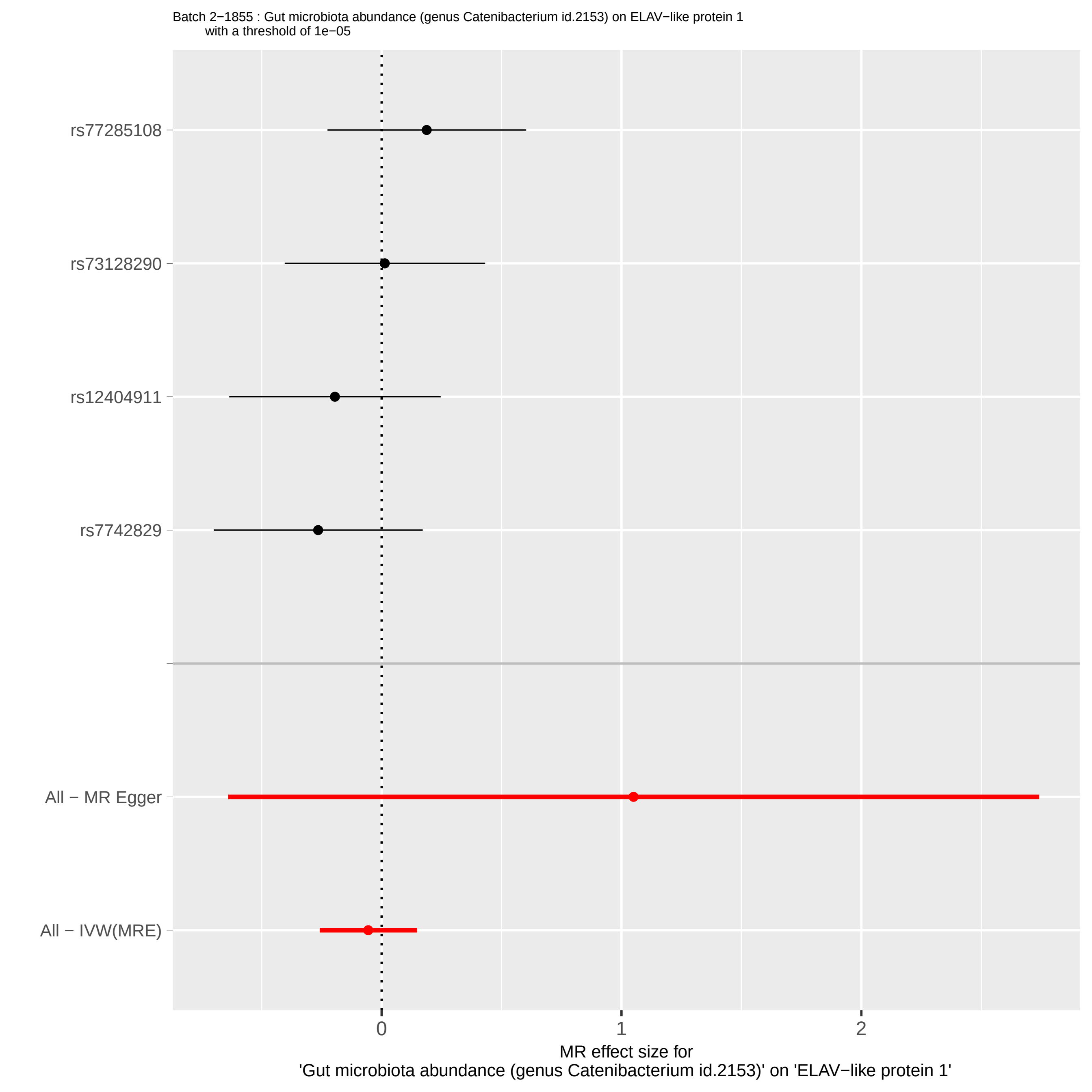

Batch 2−1855 : Gut microbiota abundance (genus Catenibacterium id.2153) on ELAV−like protein 1
with a threshold of 1e−05
rs77285108
rs73128290
rs12404911
rs7742829
All − MR Egger
All − IVW(MRE)
0
1
2
MR effect size for
'Gut microbiota abundance (genus Catenibacterium id.2153)' on 'ELAV−like protein 1'

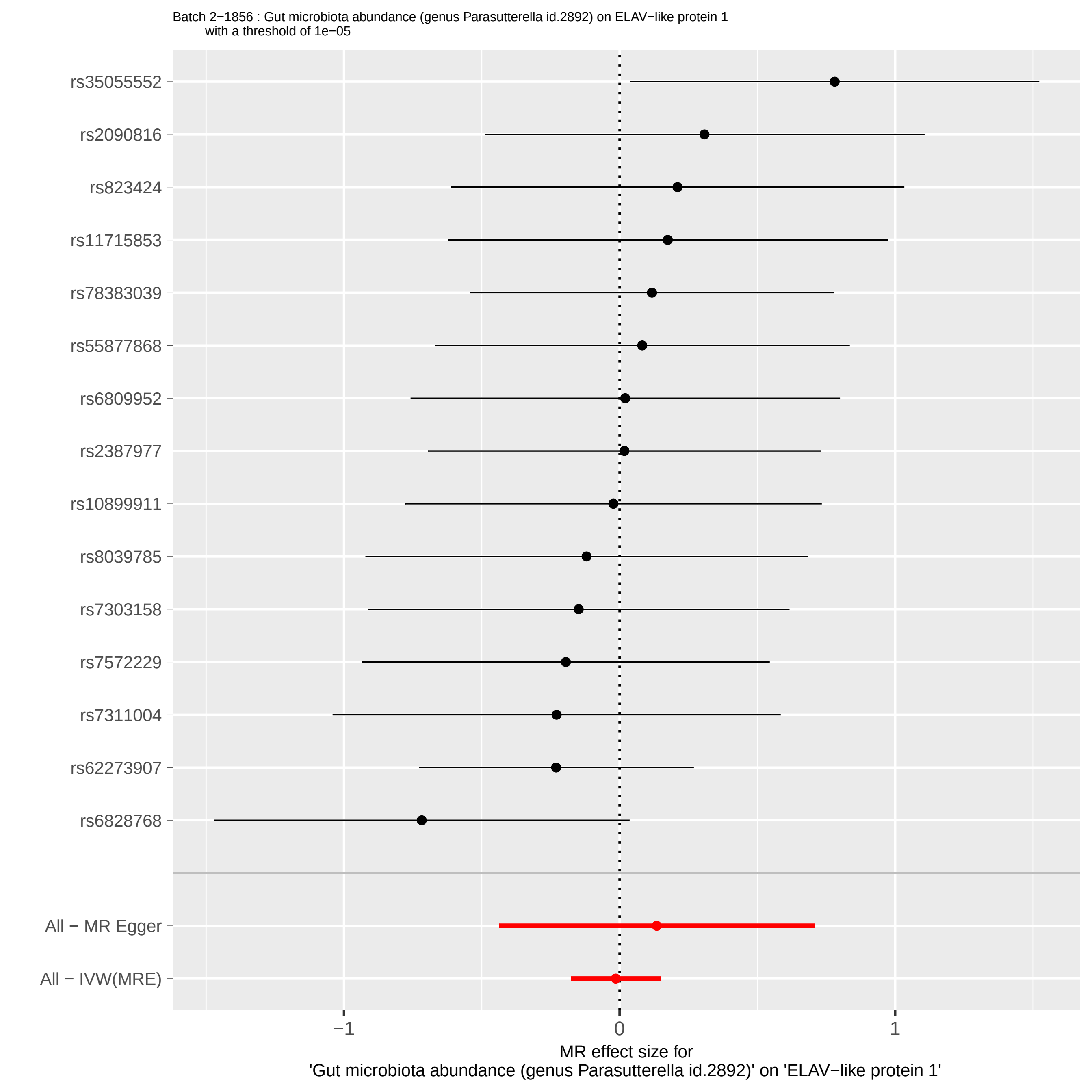

Batch 2−1856 : Gut microbiota abundance (genus Parasutterella id.2892) on ELAV−like protein 1
with a threshold of 1e−05
rs35055552
rs2090816
rs823424
rs11715853
rs78383039
rs55877868
rs6809952
rs2387977
rs10899911
rs8039785
rs7303158
rs7572229
rs7311004
rs62273907
rs6828768
All − MR Egger
All − IVW(MRE)
−1
0
1
MR effect size for
'Gut microbiota abundance (genus Parasutterella id.2892)' on 'ELAV−like protein 1'

Batch 2−1857 : Gut microbiota abundance (genus Catenibacterium id.2153) on ETS−related transcription factor Elf−5
with a threshold of 1e−05

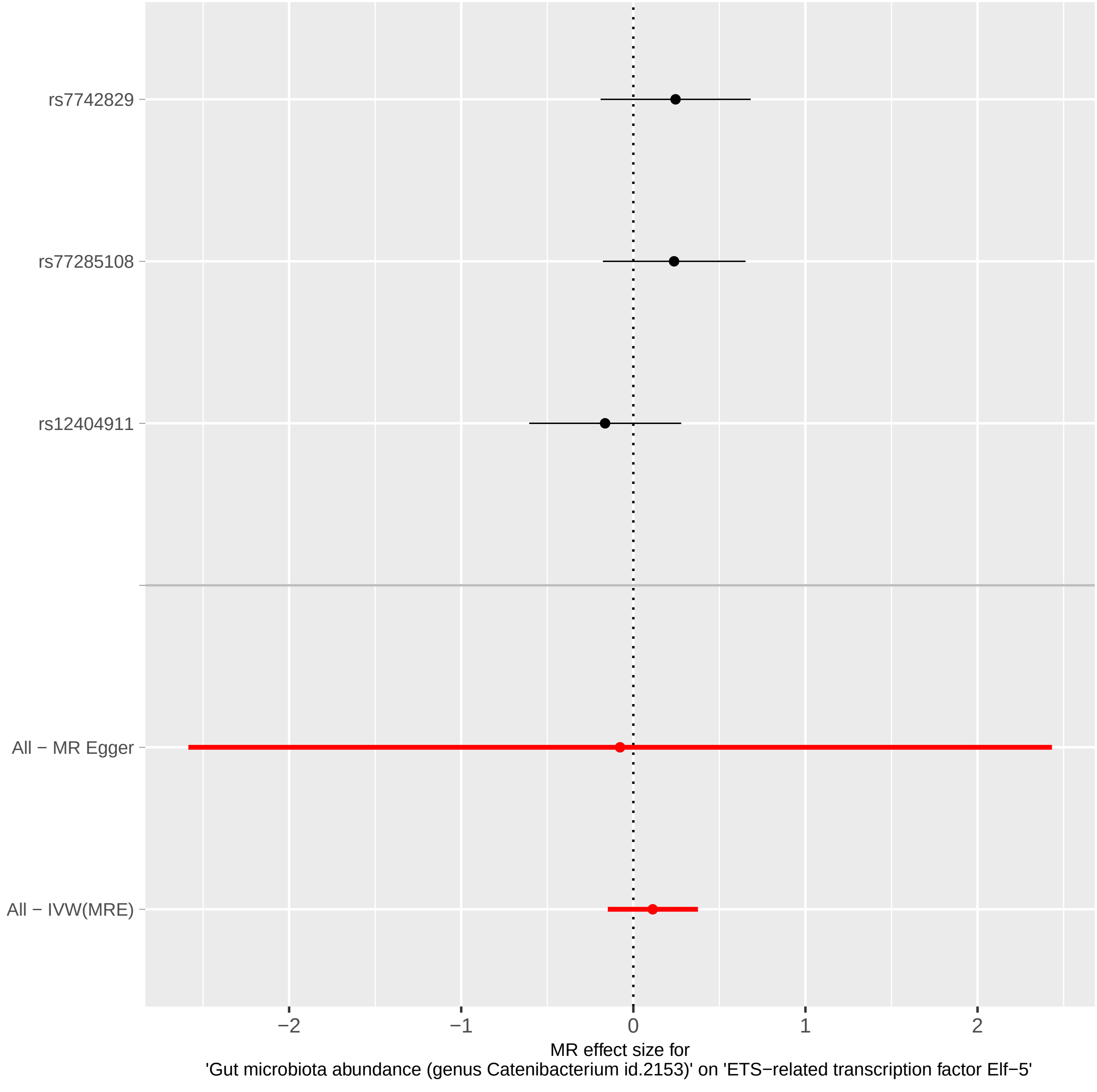

Batch 2−1858 : Gut microbiota abundance (genus Parasutterella id.2892) on ETS−related transcription factor Elf−5
with a threshold of 1e−05

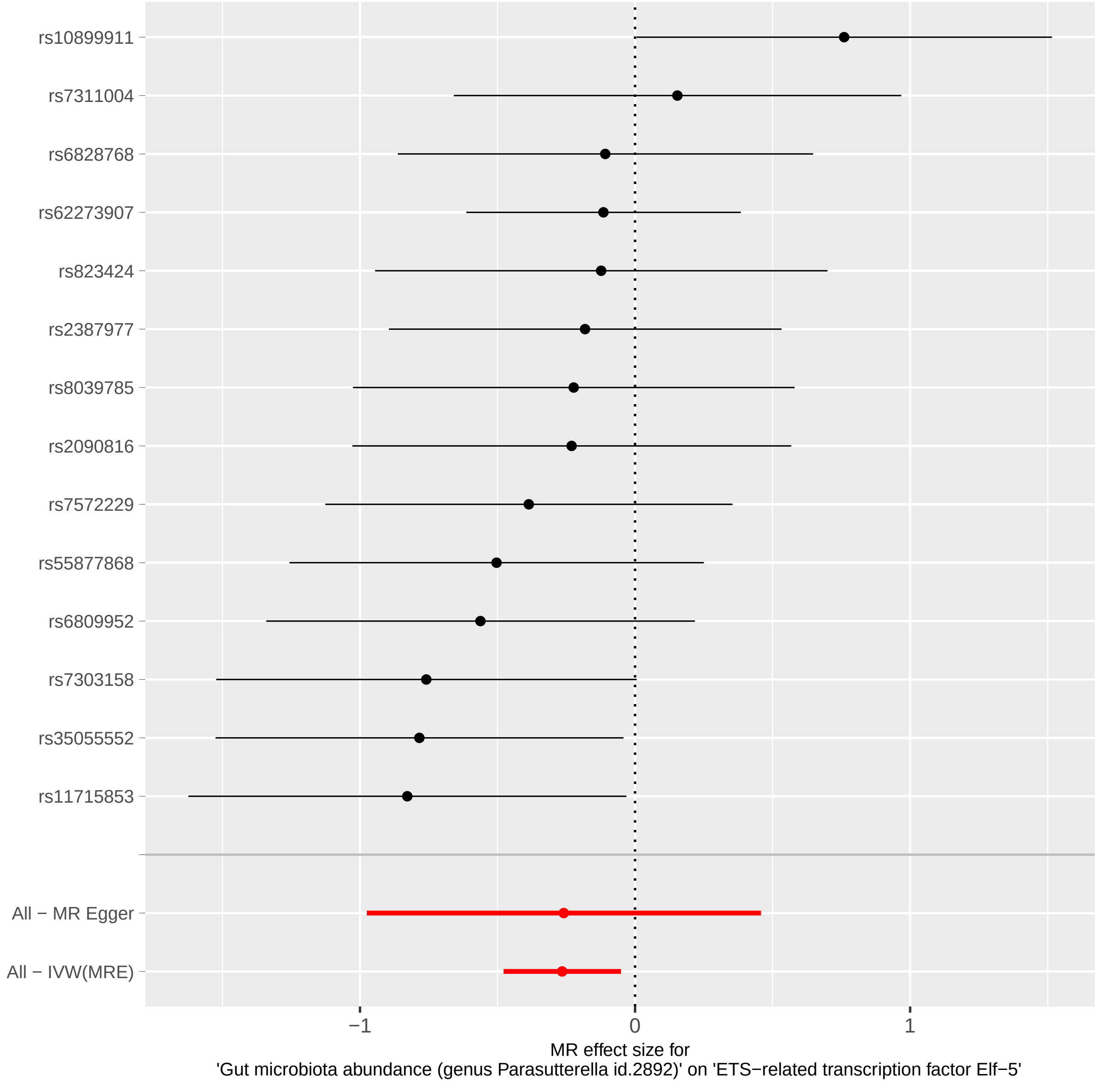

Batch 2−1860 : Gut microbiota abundance (genus Parasutterella id.2892) on ETS domain−containing protein Elk−1
with a threshold of 1e−05

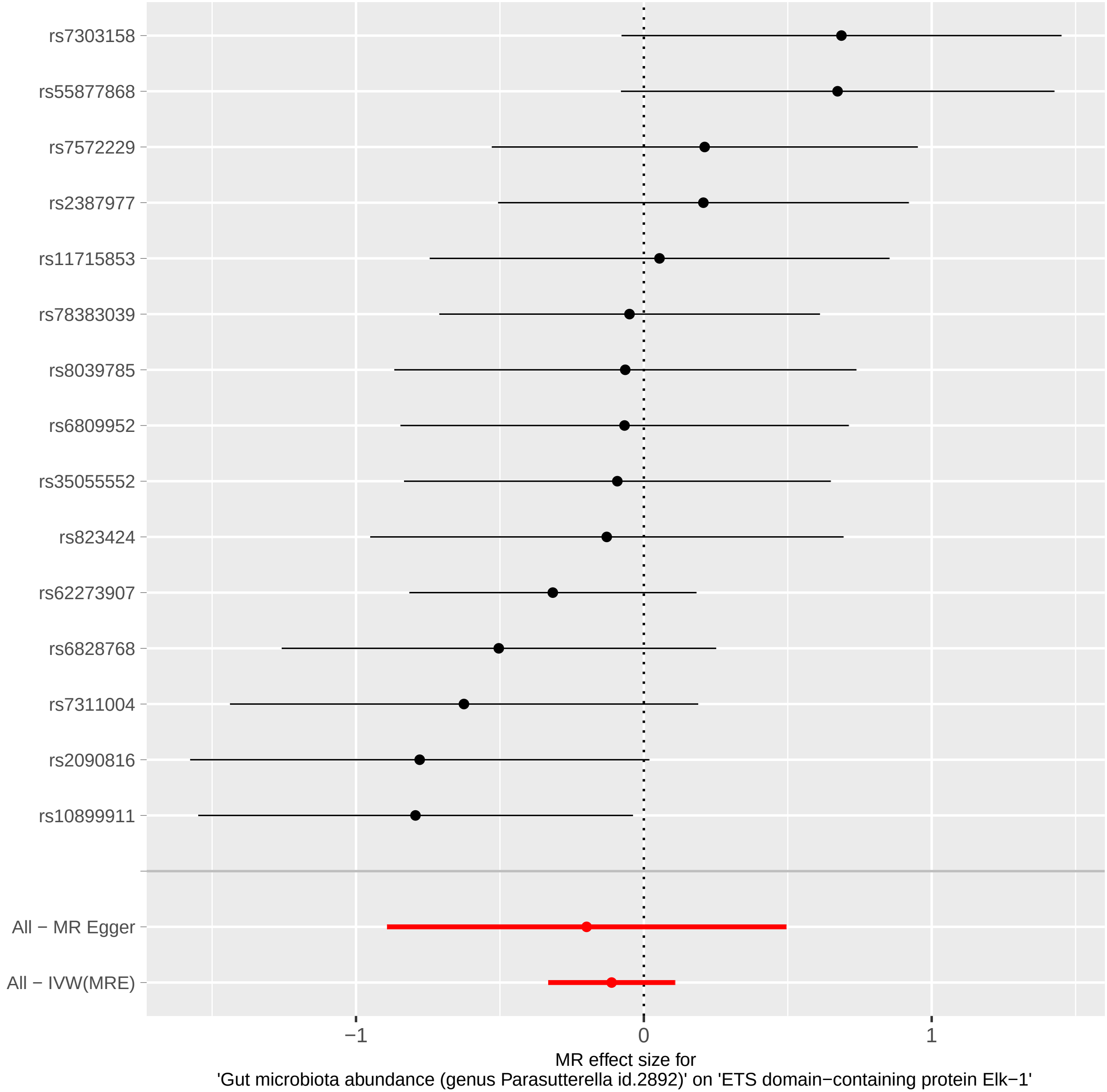

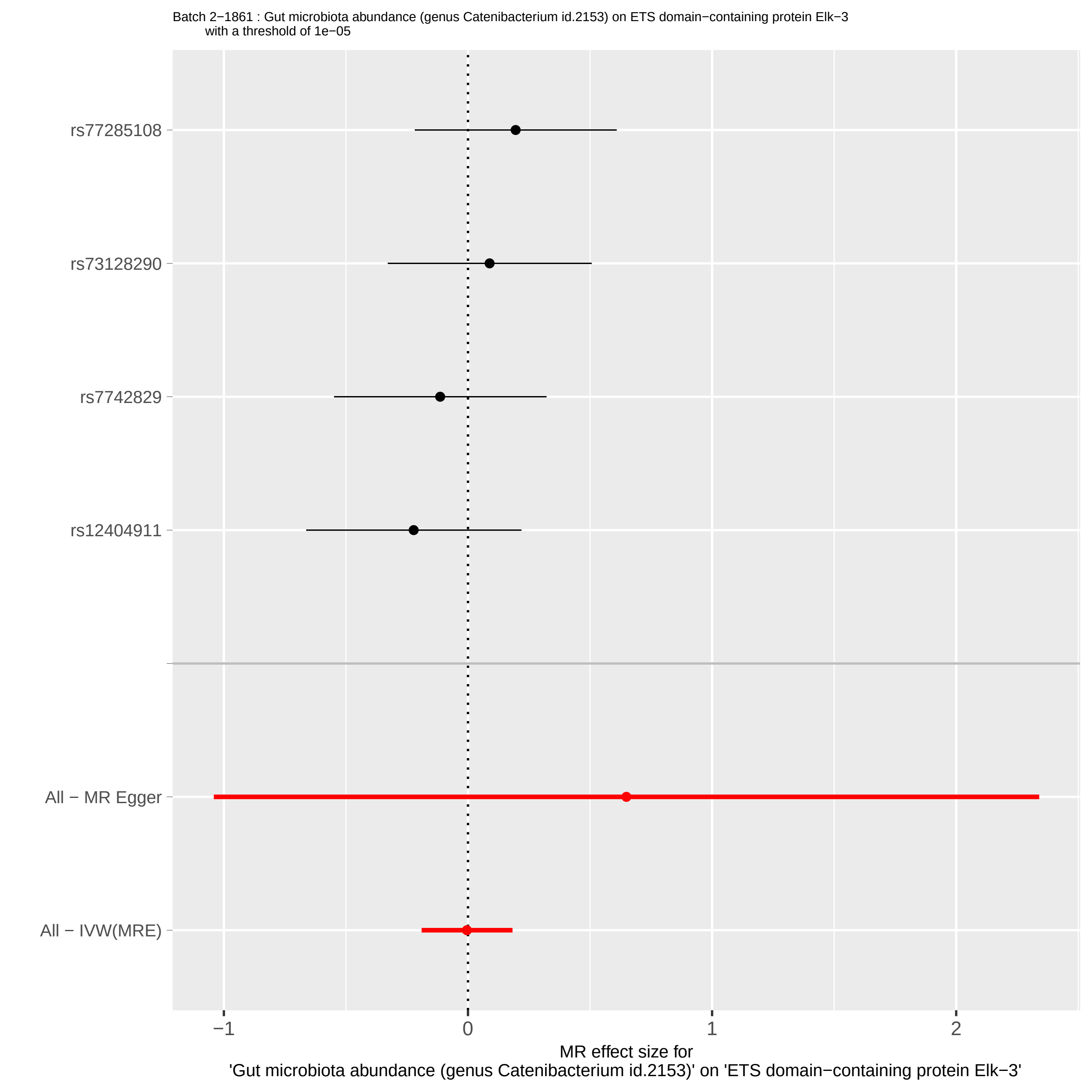

Batch 2−1861 : Gut microbiota abundance (genus Catenibacterium id.2153) on ETS domain−containing protein Elk−3
with a threshold of 1e−05
rs77285108
rs73128290
rs7742829
rs12404911
All − MR Egger
All − IVW(MRE)
−1
0
1
2
MR effect size for
'Gut microbiota abundance (genus Catenibacterium id.2153)' on 'ETS domain−containing protein Elk−3'

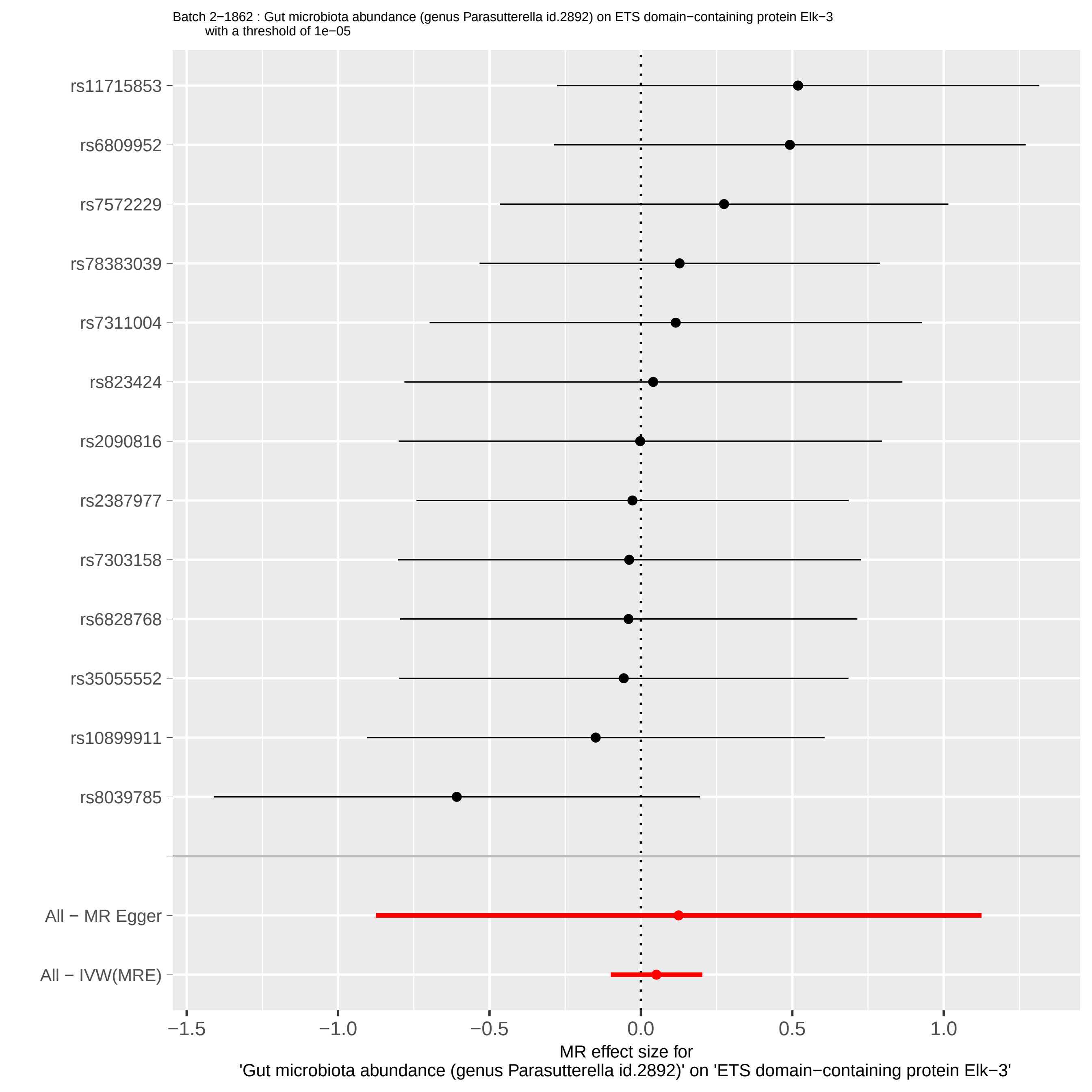

Batch 2−1862 : Gut microbiota abundance (genus Parasutterella id.2892) on ETS domain−containing protein Elk−3
with a threshold of 1e−05
rs11715853
rs6809952
rs7572229
rs78383039
rs7311004
rs823424
rs2090816
rs2387977
rs7303158
rs6828768
rs35055552
rs10899911
rs8039785
All − MR Egger
All − IVW(MRE)
−1.5
−1.0
−0.5
0.0
0.5
1.0
MR effect size for
'Gut microbiota abundance (genus Parasutterella id.2892)' on 'ETS domain−containing protein Elk−3'

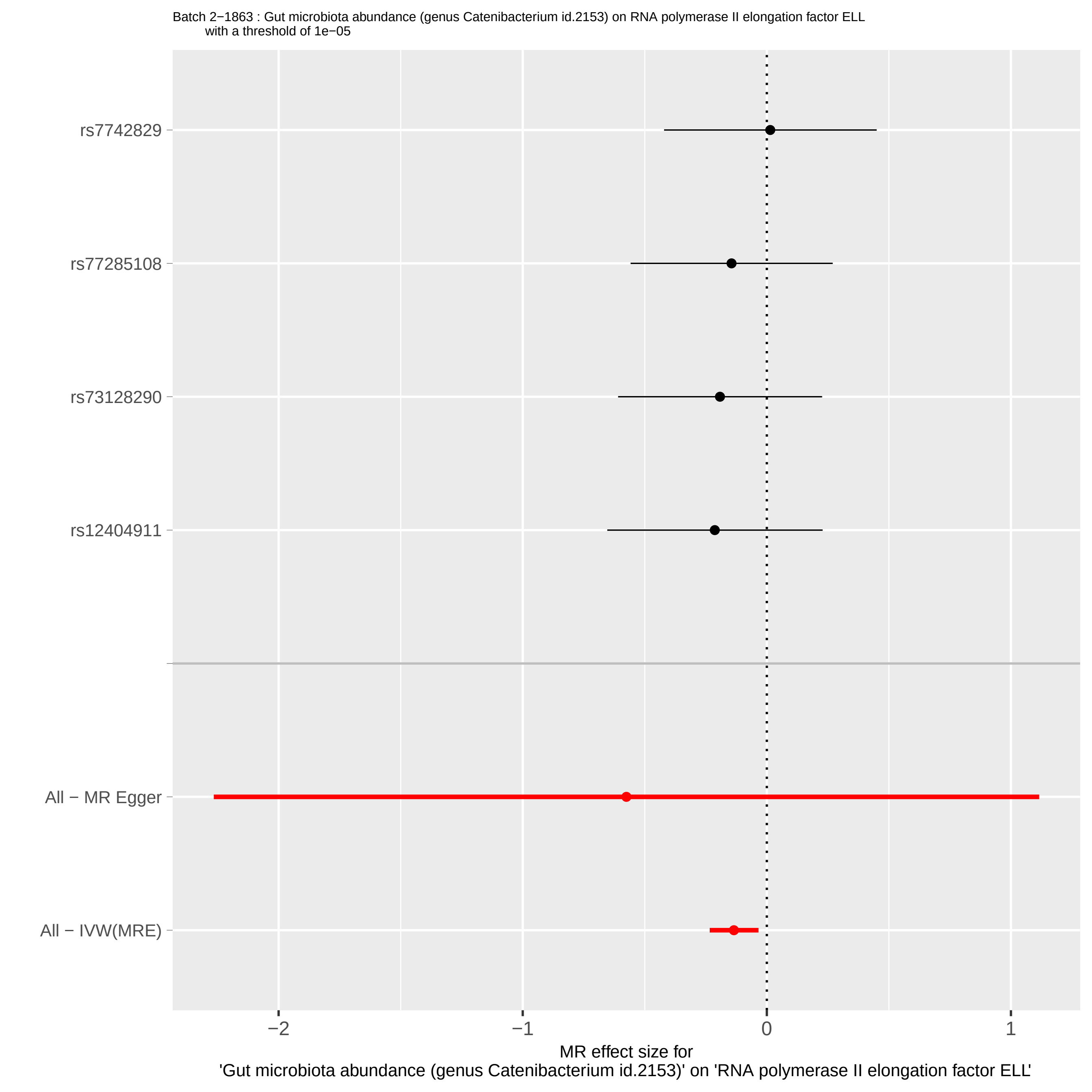

Batch 2−1863 : Gut microbiota abundance (genus Catenibacterium id.2153) on RNA polymerase II elongation factor ELL
with a threshold of 1e−05
rs7742829
rs77285108
rs73128290
rs12404911
All − MR Egger
All − IVW(MRE)
−2
−1
0
1
MR effect size for
'Gut microbiota abundance (genus Catenibacterium id.2153)' on 'RNA polymerase II elongation factor ELL'

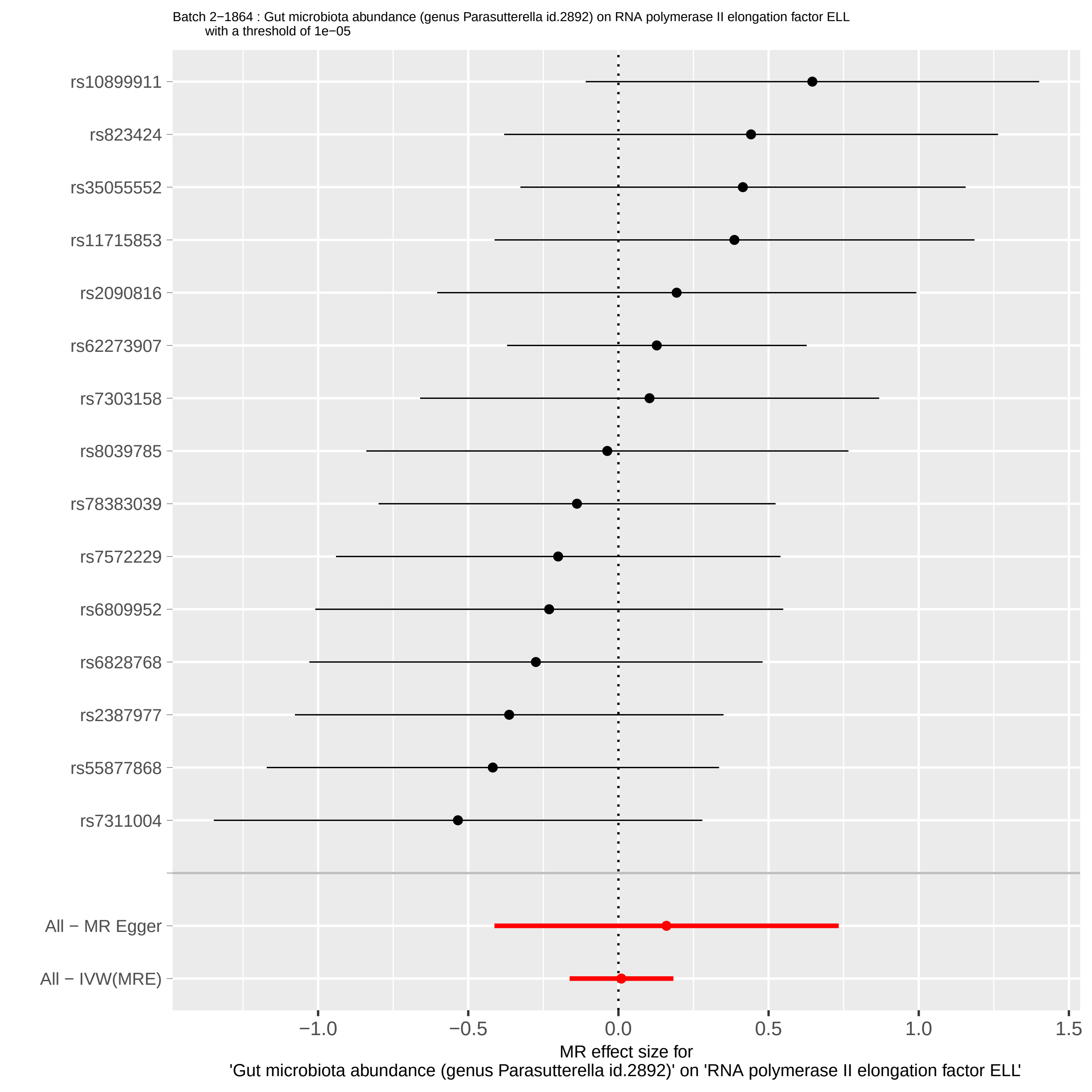
Batch 2−1864 : Gut microbiota abundance (genus Parasutterella id.2892) on RNA polymerase II elongation factor ELL
with a threshold of 1e−05
rs10899911
rs823424
rs35055552
rs11715853
rs2090816
rs62273907
rs7303158
rs8039785
rs78383039
rs7572229
rs6809952
rs6828768
rs2387977
rs55877868
rs7311004
All − MR Egger
All − IVW(MRE)
−1.0
−0.5
0.0
0.5
1.0
1.5
MR effect size for
'Gut microbiota abundance (genus Parasutterella id.2892)' on 'RNA polymerase II elongation factor ELL'

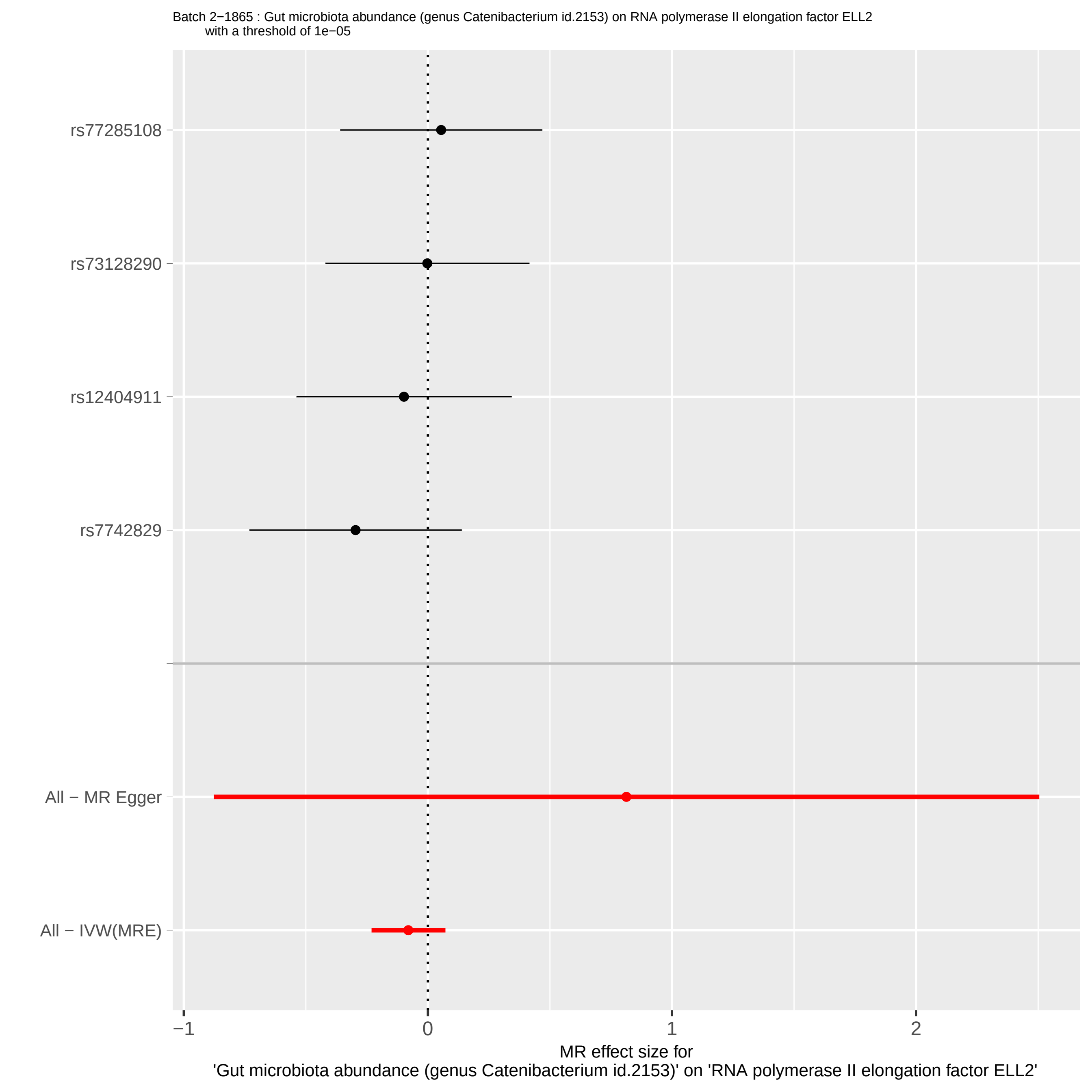

Batch 2−1865 : Gut microbiota abundance (genus Catenibacterium id.2153) on RNA polymerase II elongation factor ELL2
with a threshold of 1e−05
rs77285108
rs73128290
rs12404911
rs7742829
All − MR Egger
All − IVW(MRE)
−1
0
1
2
MR effect size for
'Gut microbiota abundance (genus Catenibacterium id.2153)' on 'RNA polymerase II elongation factor ELL2'

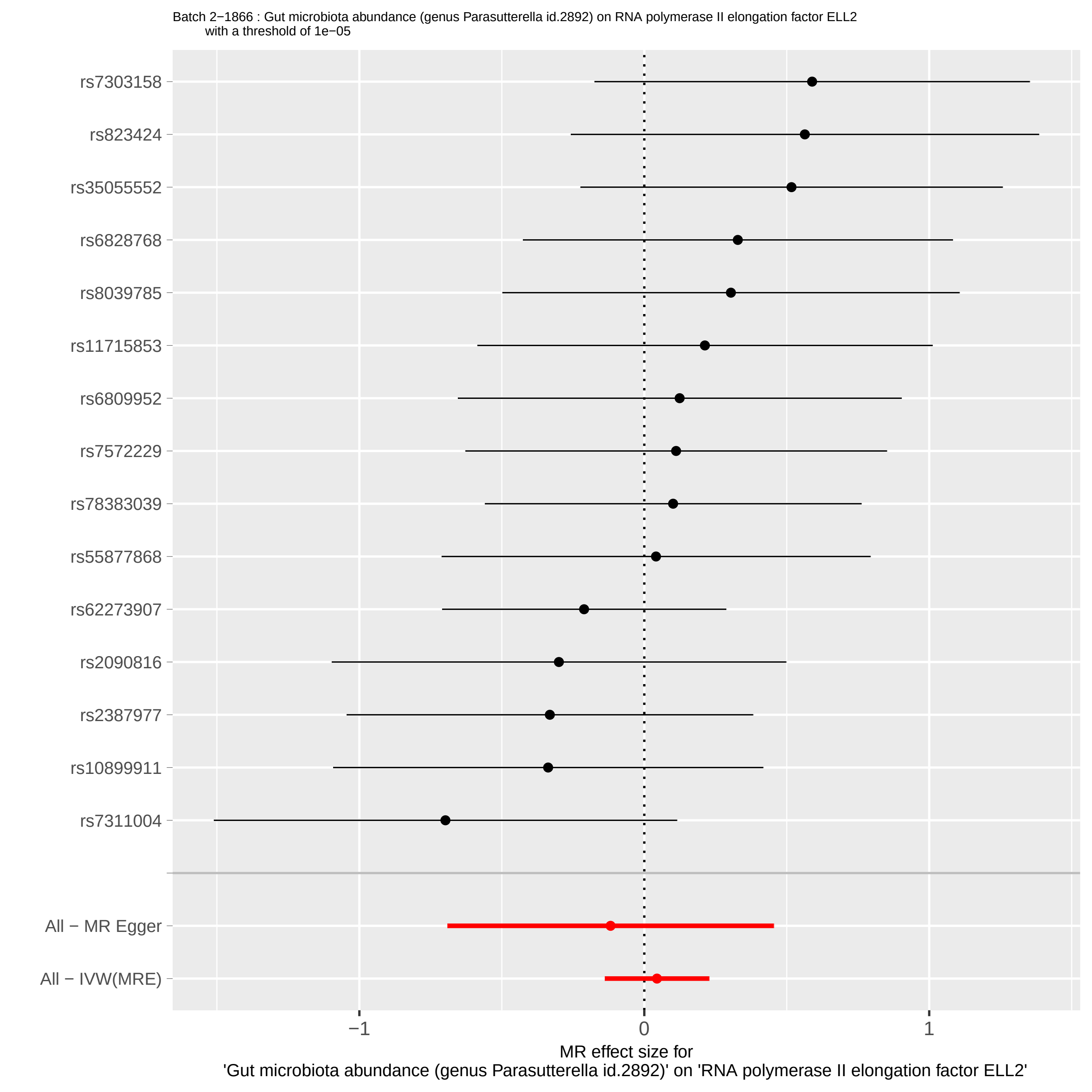
Batch 2−1866 : Gut microbiota abundance (genus Parasutterella id.2892) on RNA polymerase II elongation factor ELL2
with a threshold of 1e−05
rs7303158
rs823424
rs35055552
rs6828768
rs8039785
rs11715853
rs6809952
rs7572229
rs78383039
rs55877868
rs62273907
rs2090816
rs2387977
rs10899911
rs7311004
All − MR Egger
All − IVW(MRE)
−1
0
1
MR effect size for
'Gut microbiota abundance (genus Parasutterella id.2892)' on 'RNA polymerase II elongation factor ELL2'

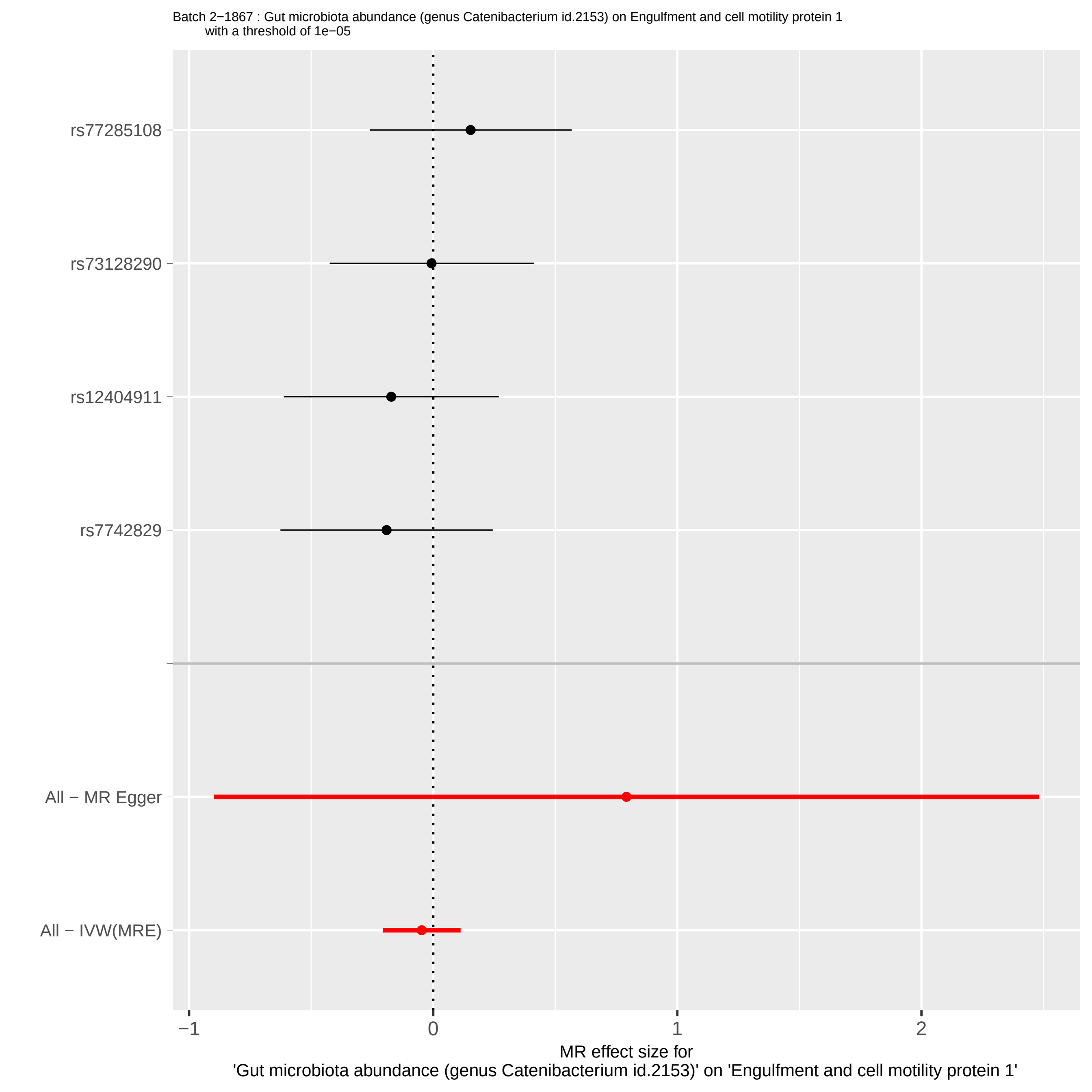

Batch 2−1867 : Gut microbiota abundance (genus Catenibacterium id.2153) on Engulfment and cell motility protein 1
with a threshold of 1e−05
rs77285108
rs73128290
rs12404911
rs7742829
All − MR Egger
All − IVW(MRE)
−1
0
1
2
MR effect size for
'Gut microbiota abundance (genus Catenibacterium id.2153)' on 'Engulfment and cell motility protein 1'

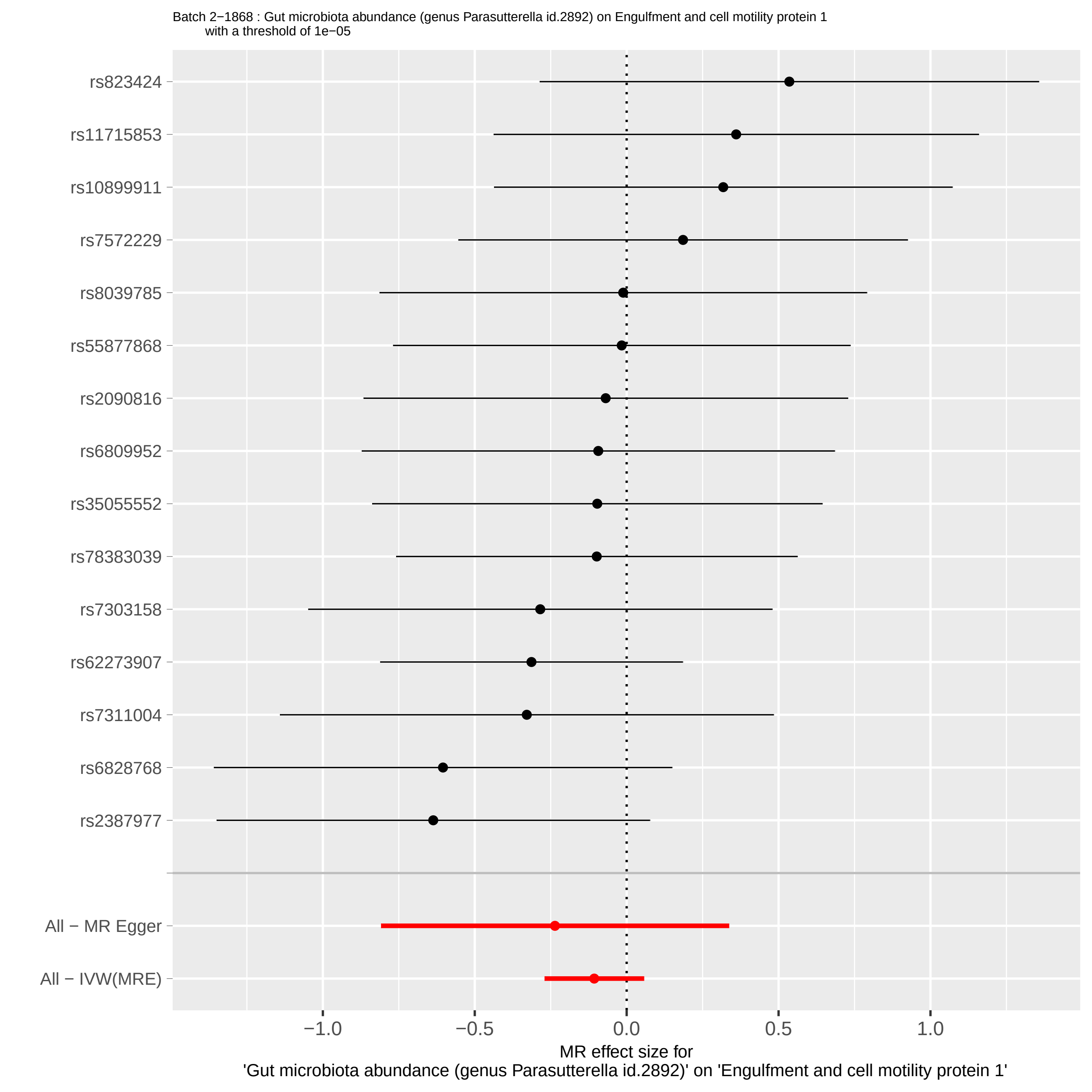
Batch 2−1868 : Gut microbiota abundance (genus Parasutterella id.2892) on Engulfment and cell motility protein 1
with a threshold of 1e−05
rs823424
rs11715853
rs10899911
rs7572229
rs8039785
rs55877868
rs2090816
rs6809952
rs35055552
rs78383039
rs7303158
rs62273907
rs7311004
rs6828768
rs2387977
All − MR Egger
All − IVW(MRE)
−1.0
−0.5
0.0
0.5
1.0
MR effect size for
'Gut microbiota abundance (genus Parasutterella id.2892)' on 'Engulfment and cell motility protein 1'

Batch 2−1869 : Gut microbiota abundance (genus Catenibacterium id.2153) on ER membrane protein complex subunit 1
with a threshold of 1e−05

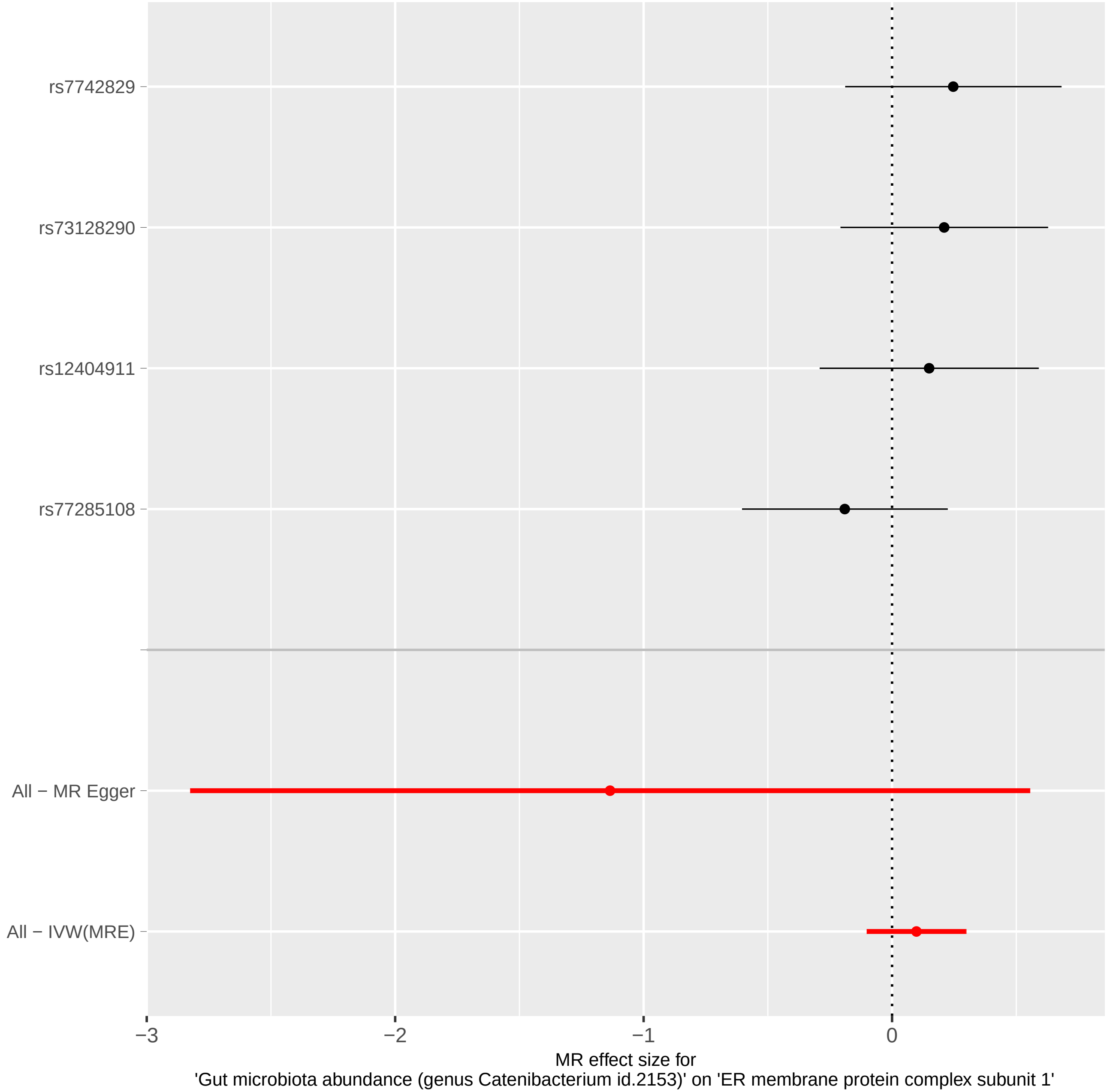

Batch 2−1870 : Gut microbiota abundance (genus Parasutterella id.2892) on ER membrane protein complex subunit 1
with a threshold of 1e−05

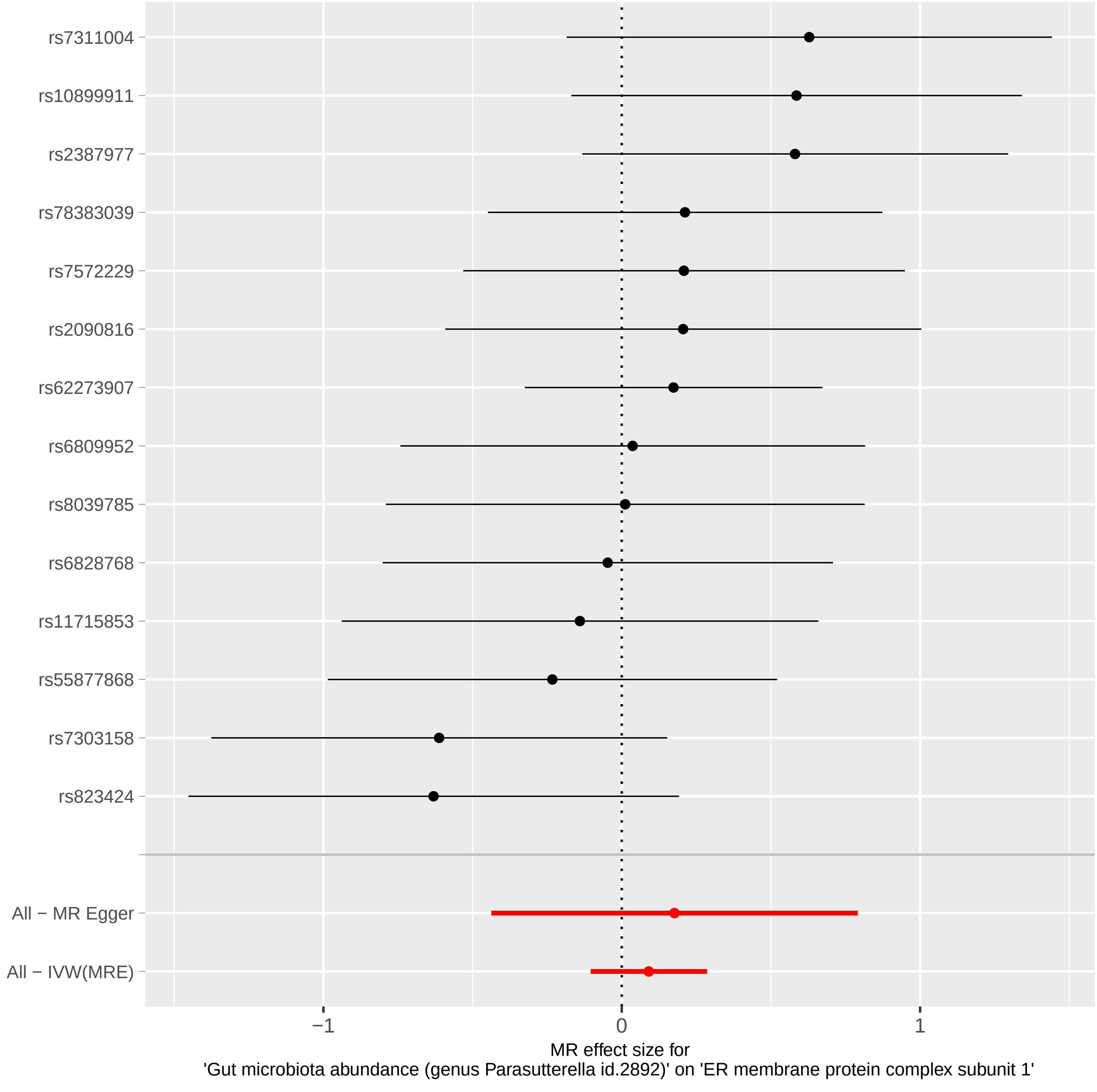

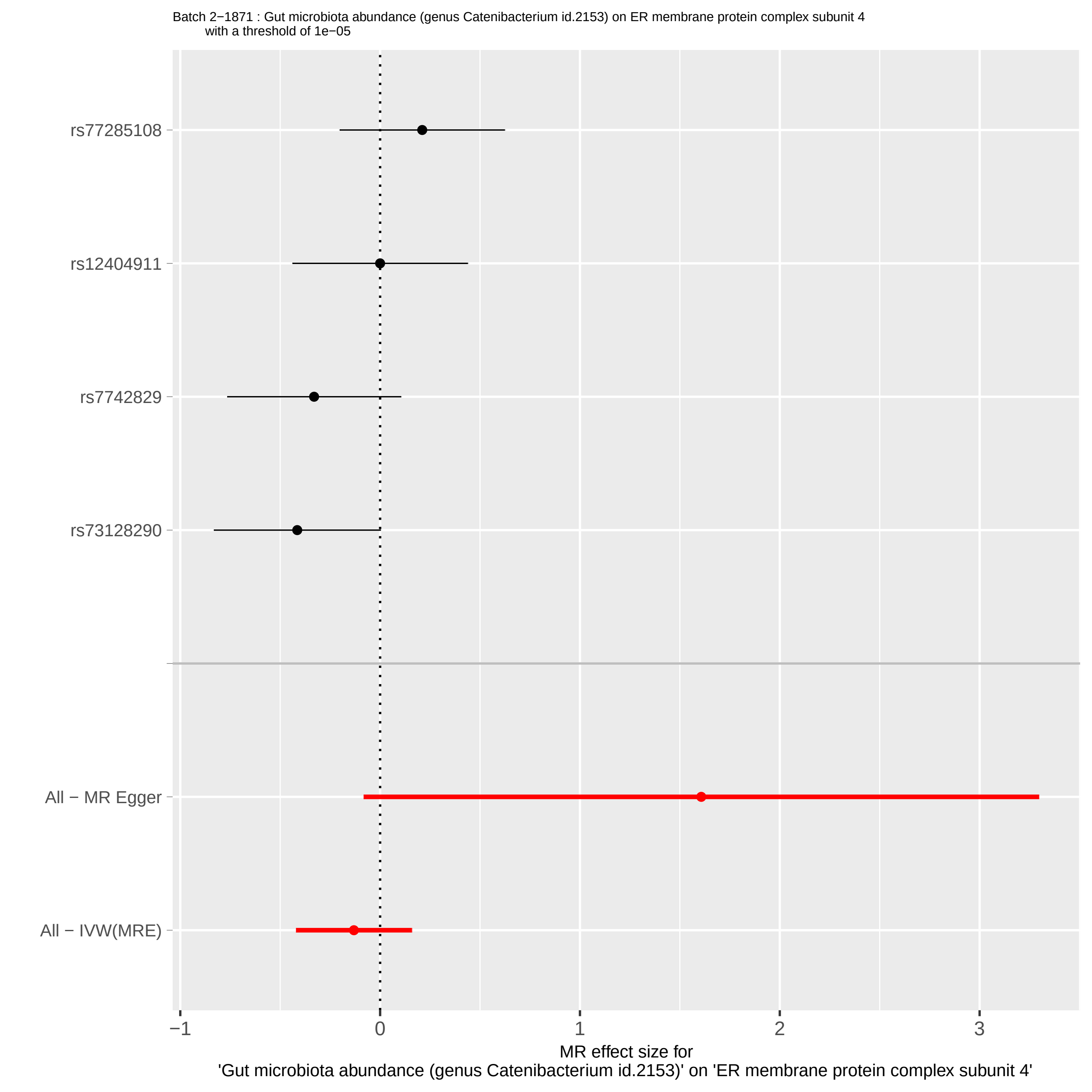

Batch 2−1871 : Gut microbiota abundance (genus Catenibacterium id.2153) on ER membrane protein complex subunit 4
with a threshold of 1e−05
rs77285108
rs12404911
rs7742829
rs73128290
All − MR Egger
All − IVW(MRE)
−1
0
1
2
3
MR effect size for
'Gut microbiota abundance (genus Catenibacterium id.2153)' on 'ER membrane protein complex subunit 4'

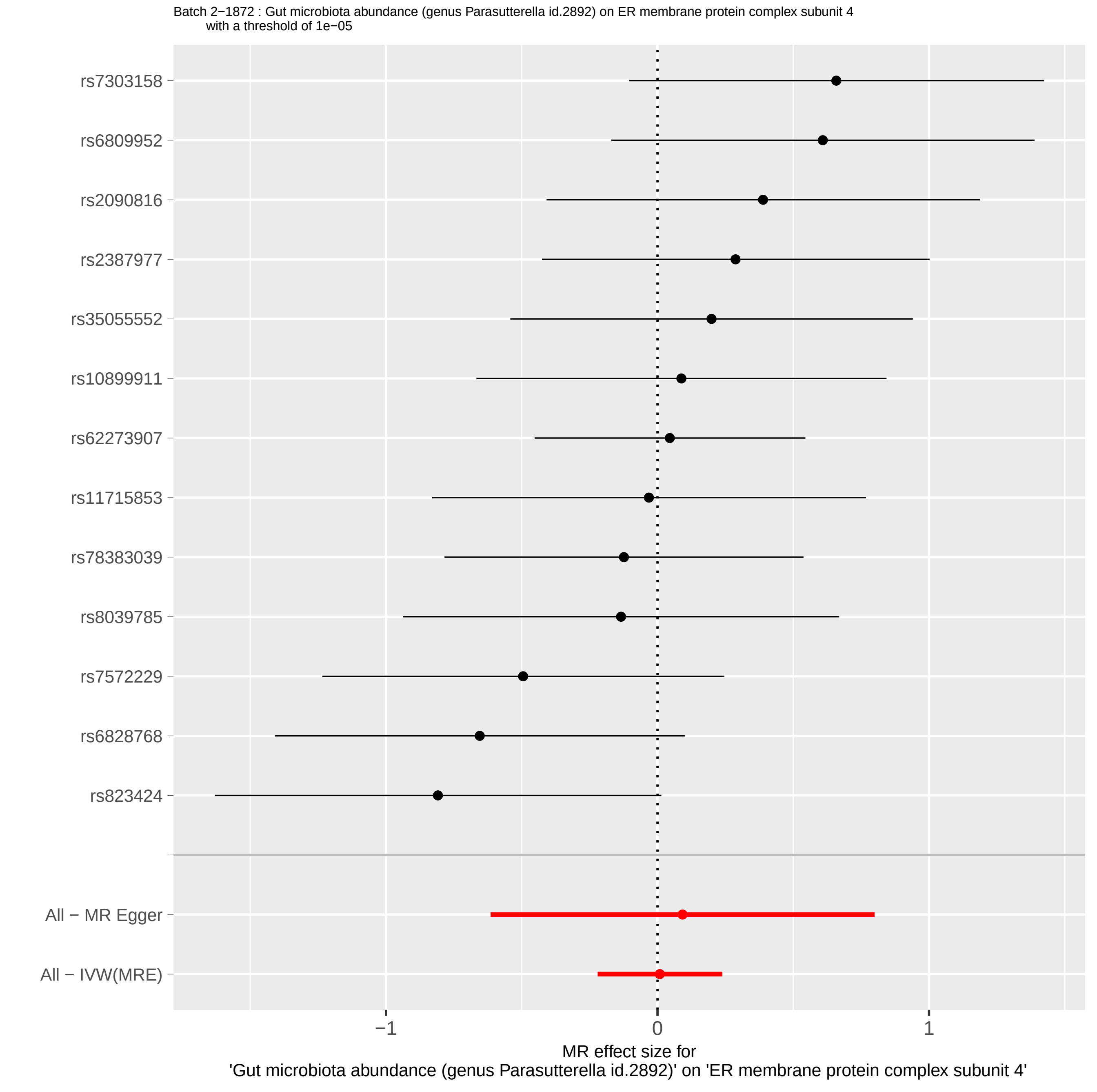

Batch 2−1872 : Gut microbiota abundance (genus Parasutterella id.2892) on ER membrane protein complex subunit 4
with a threshold of 1e−05
rs7303158
rs6809952
rs2090816
rs2387977
rs35055552
rs10899911
rs62273907
rs11715853
rs78383039
rs8039785
rs7572229
rs6828768
rs823424
All − MR Egger
All − IVW(MRE)
−1
0
1
MR effect size for
'Gut microbiota abundance (genus Parasutterella id.2892)' on 'ER membrane protein complex subunit 4'

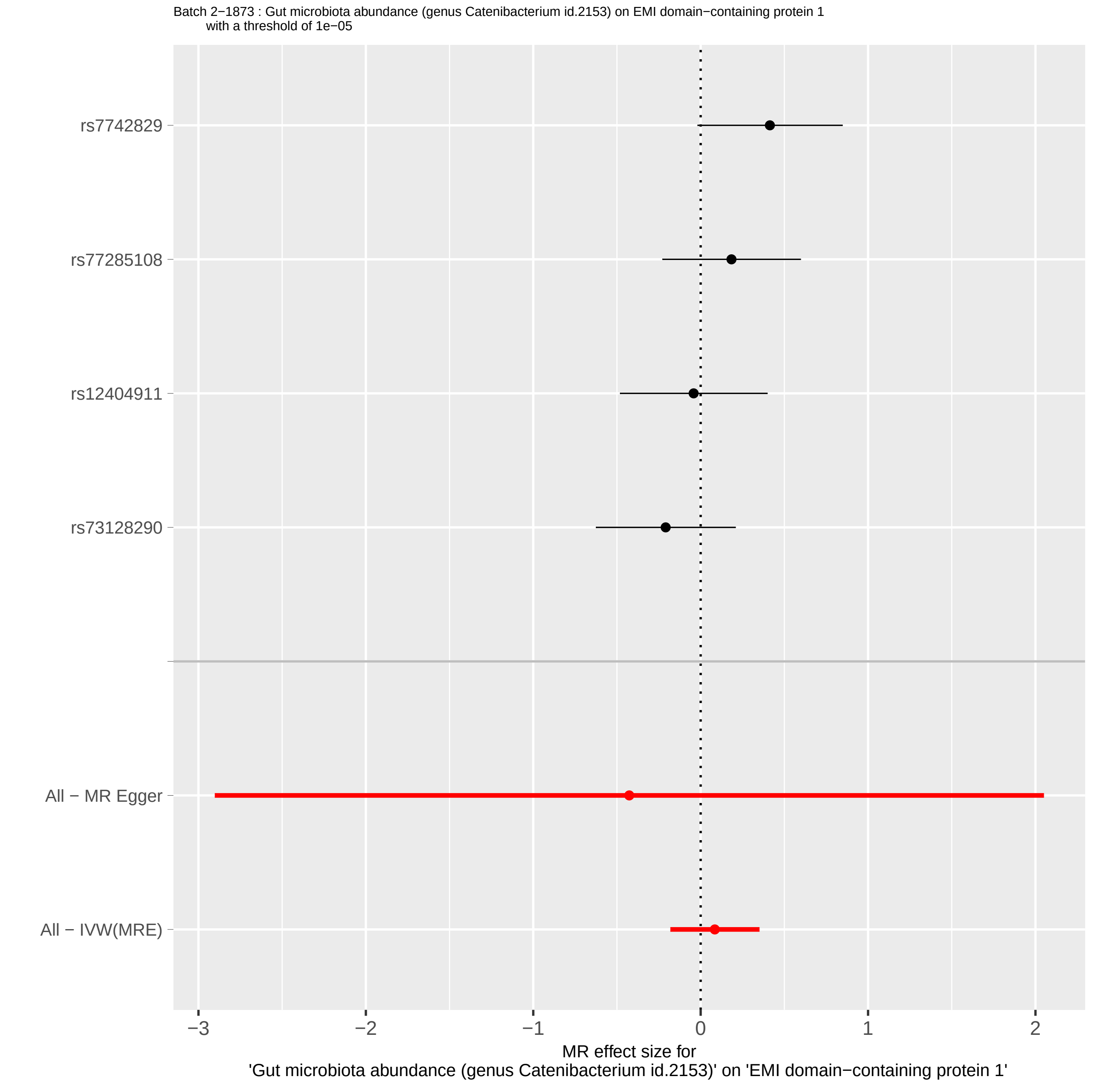
Batch 2−1873 : Gut microbiota abundance (genus Catenibacterium id.2153) on EMI domain−containing protein 1
with a threshold of 1e−05
rs7742829
rs77285108
rs12404911
rs73128290
All − MR Egger
All − IVW(MRE)
−3
−2
−1
0
1
2
MR effect size for
'Gut microbiota abundance (genus Catenibacterium id.2153)' on 'EMI domain−containing protein 1'

Batch 2−1874 : Gut microbiota abundance (genus Parasutterella id.2892) on EMI domain−containing protein 1
with a threshold of 1e−05

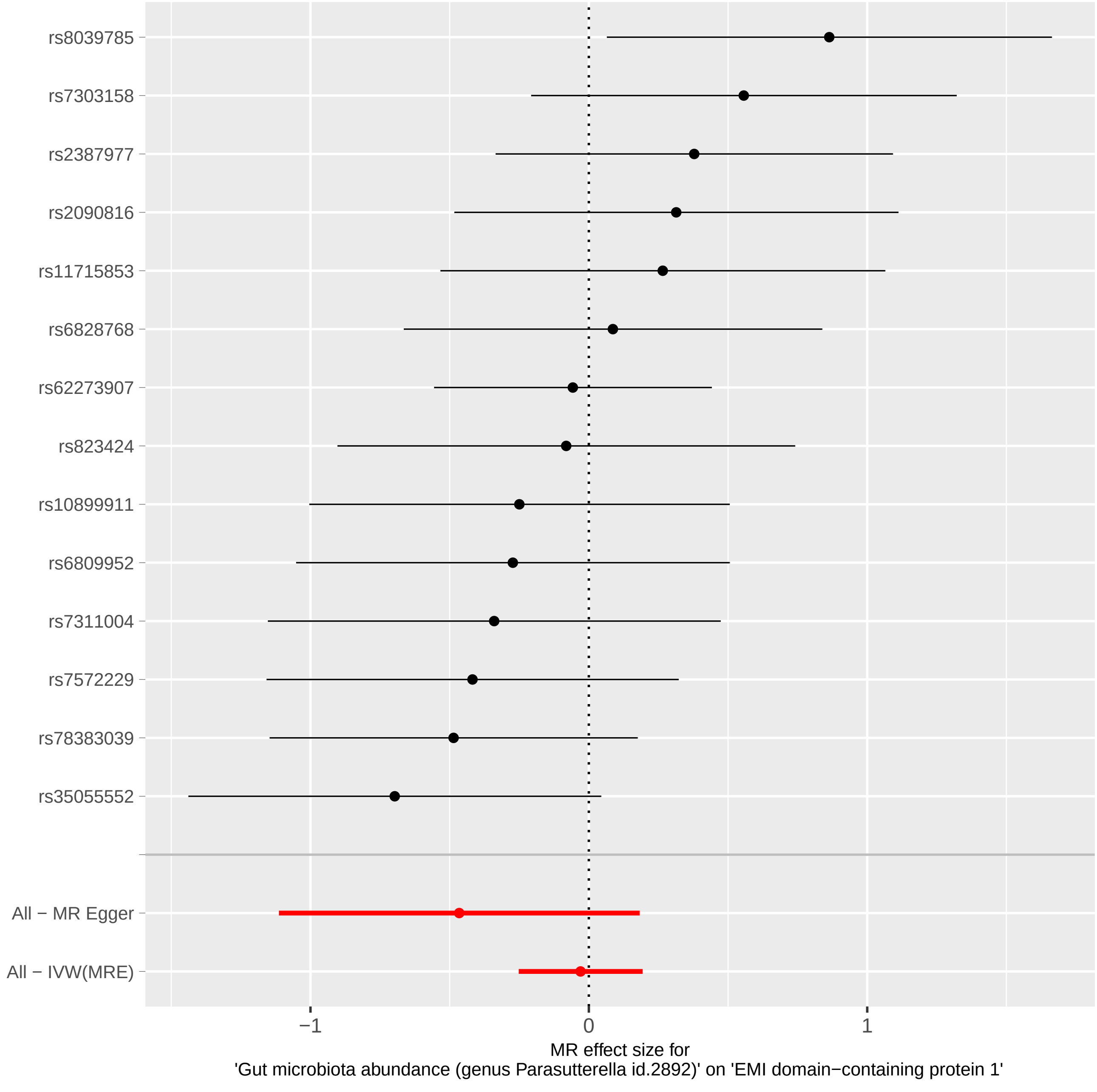

Batch 2−1875 : Gut microbiota abundance (genus Catenibacterium id.2153) on EMILIN−3
with a threshold of 1e−05

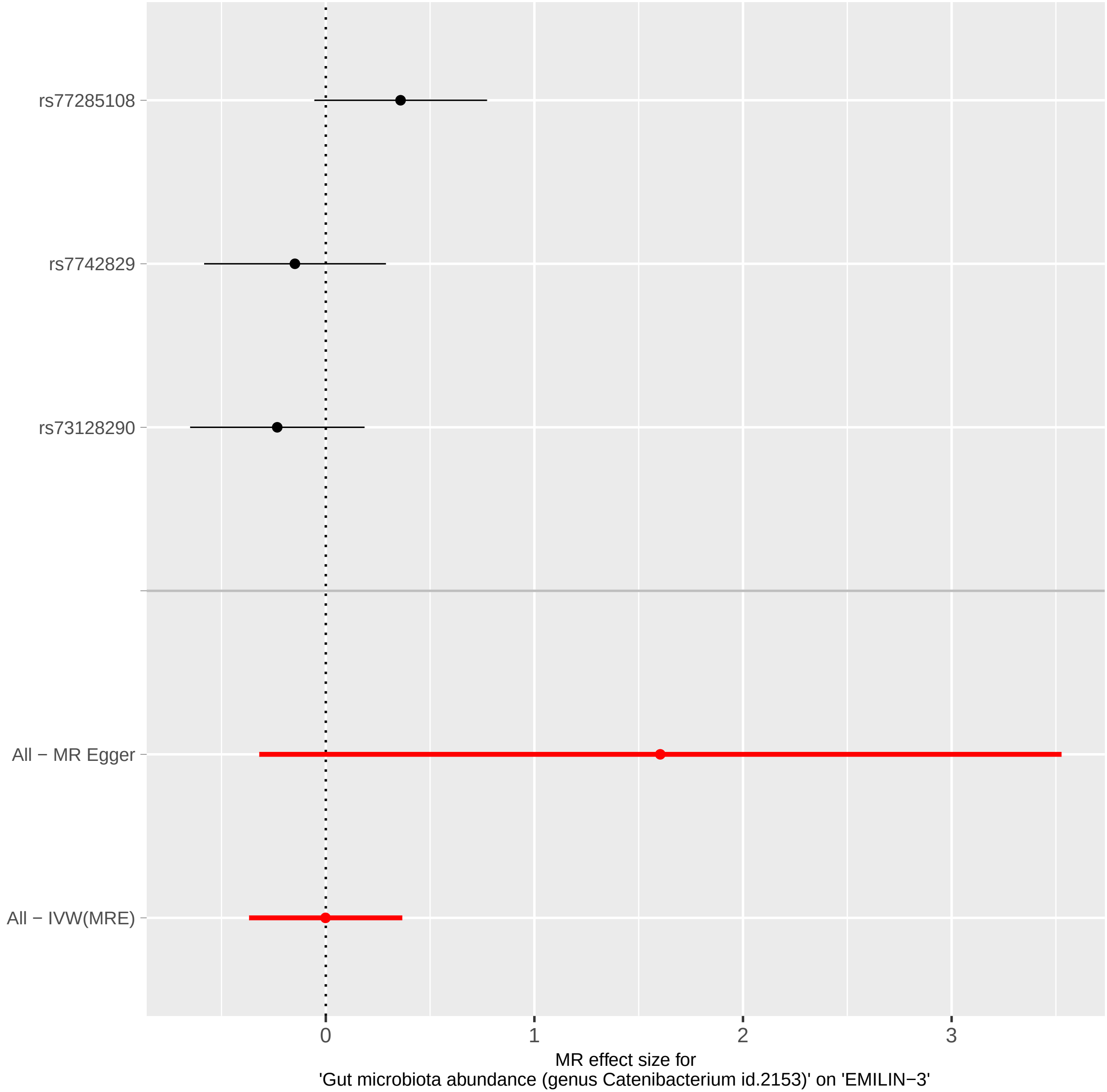

Batch 2−1876 : Gut microbiota abundance (genus Parasutterella id.2892) on EMILIN−3
with a threshold of 1e−05

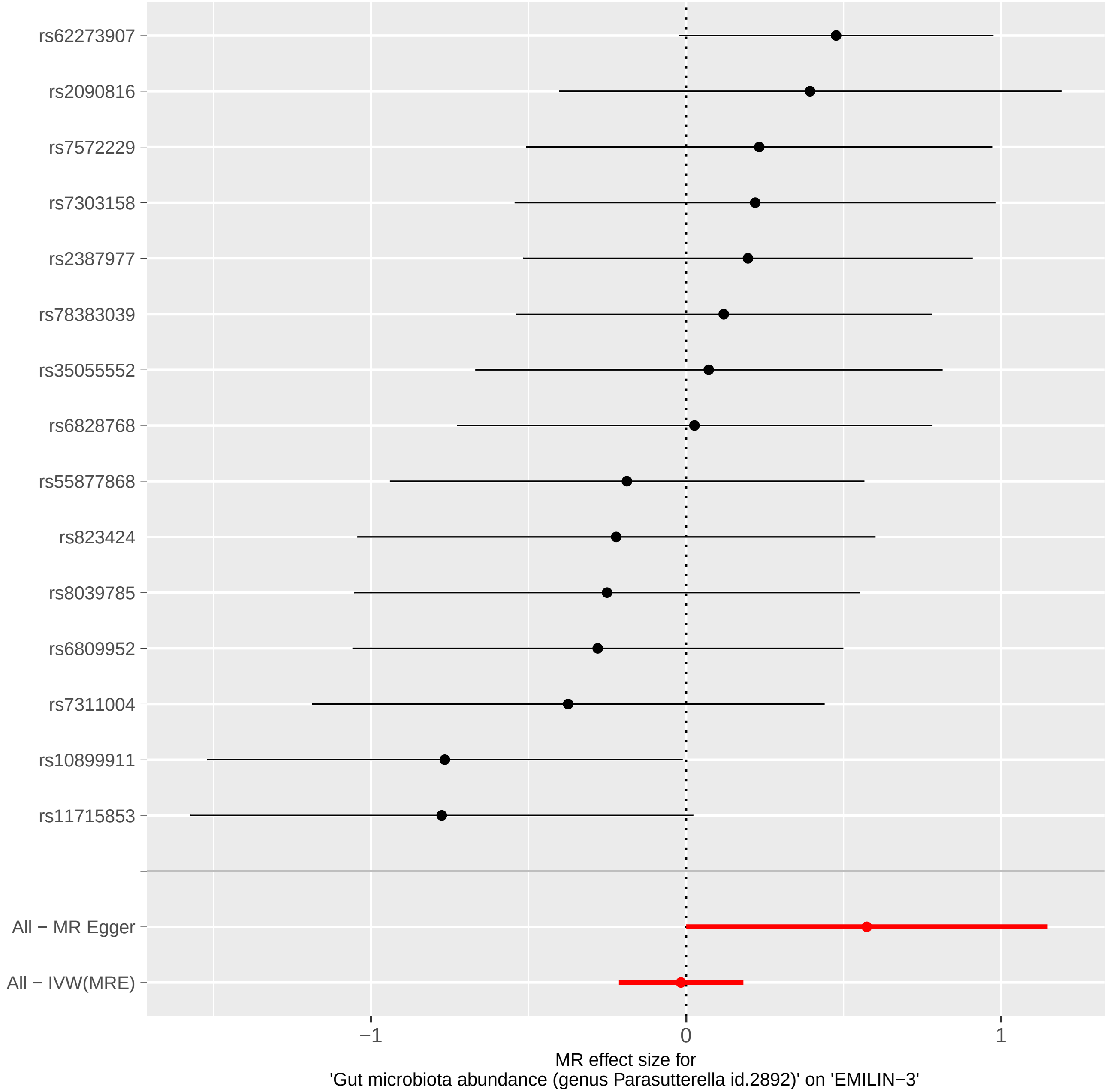

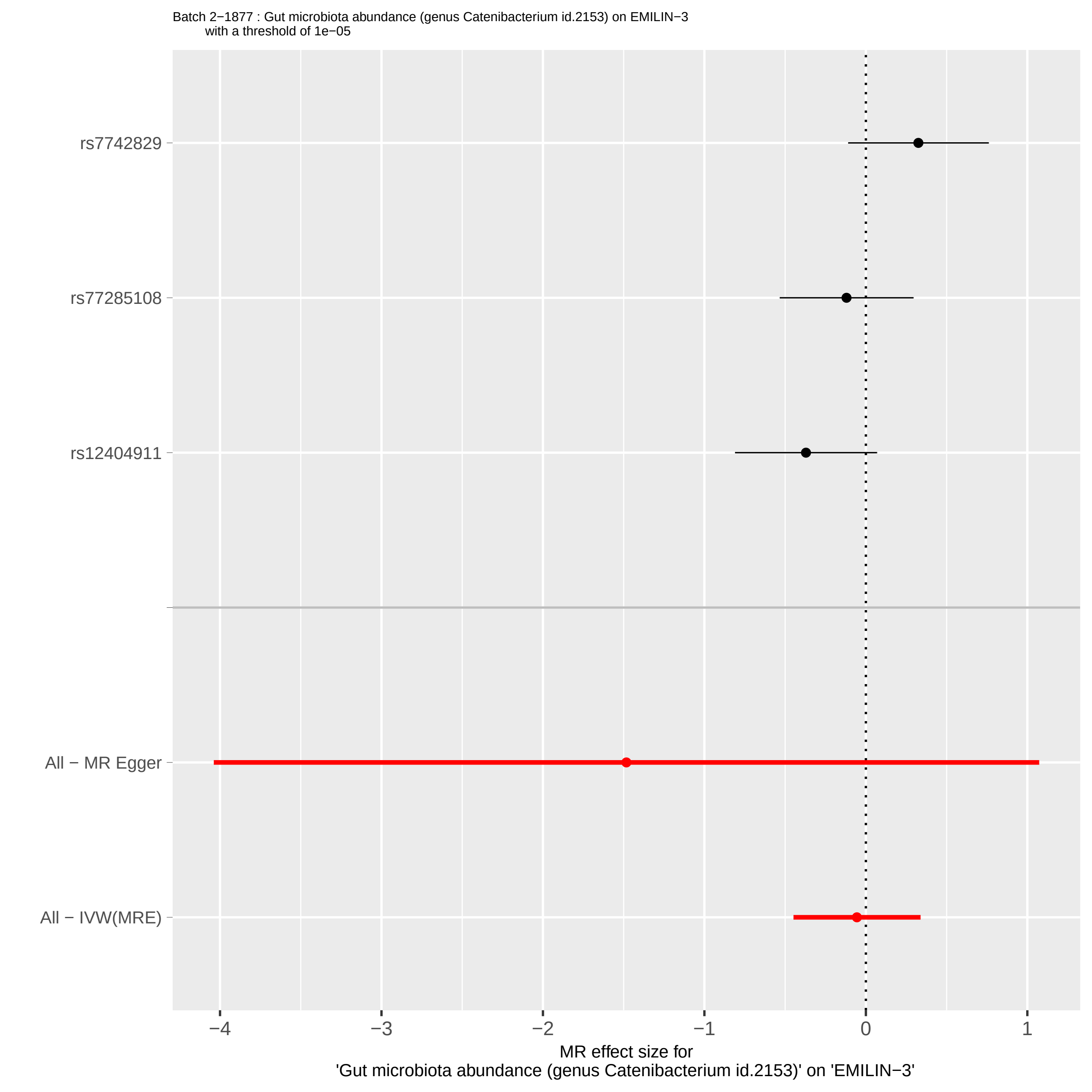

Batch 2−1877 : Gut microbiota abundance (genus Catenibacterium id.2153) on EMILIN−3
with a threshold of 1e−05
rs7742829
rs77285108
rs12404911
All − MR Egger
All − IVW(MRE)
−4
−3
−2
−1
0
1
MR effect size for
'Gut microbiota abundance (genus Catenibacterium id.2153)' on 'EMILIN−3'

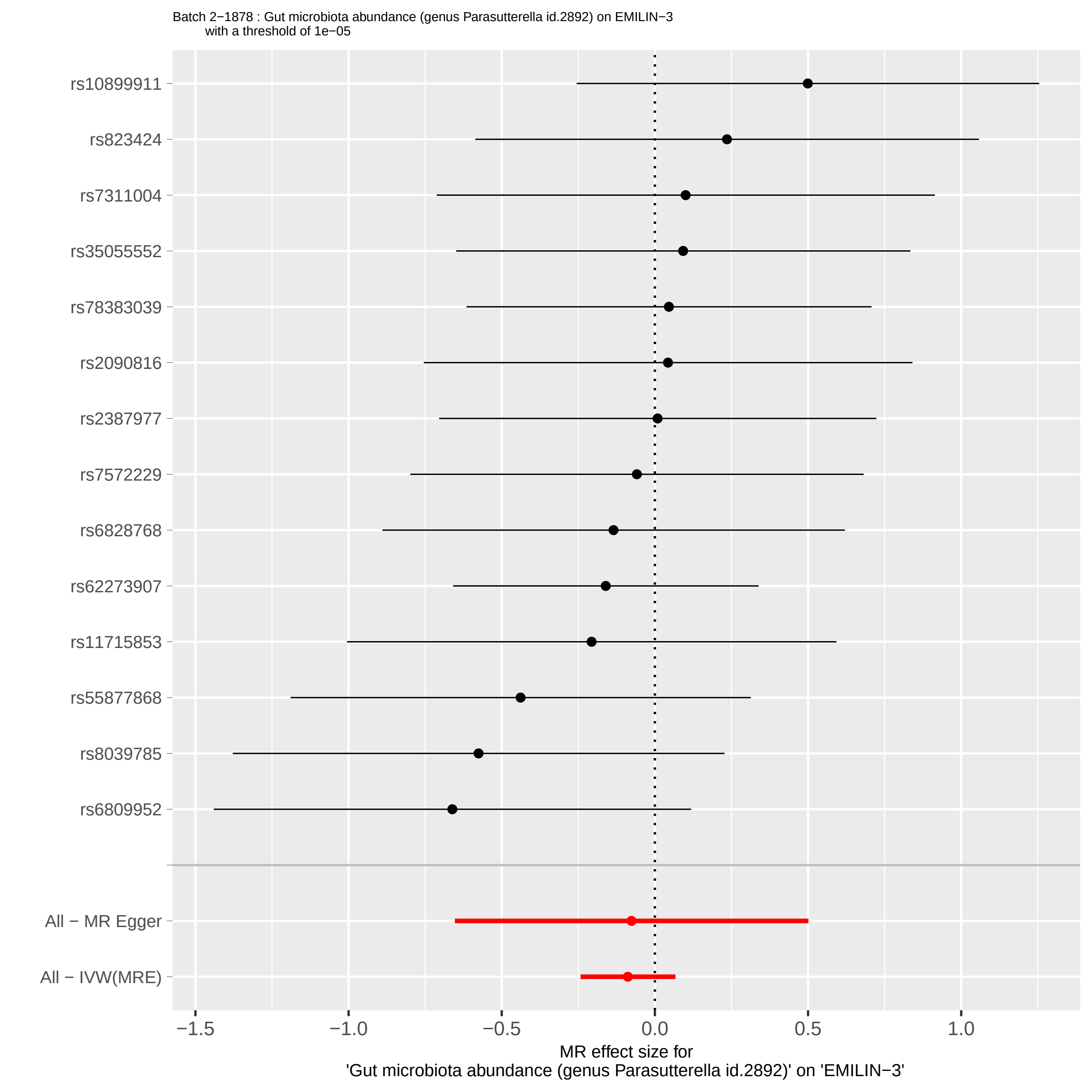

Batch 2−1878 : Gut microbiota abundance (genus Parasutterella id.2892) on EMILIN−3
with a threshold of 1e−05
rs10899911
rs823424
rs7311004
rs35055552
rs78383039
rs2090816
rs2387977
rs7572229
rs6828768
rs62273907
rs11715853
rs55877868
rs8039785
rs6809952
All − MR Egger
All − IVW(MRE)
−1.5
−1.0
−0.5
0.0
0.5
1.0
MR effect size for
'Gut microbiota abundance (genus Parasutterella id.2892)' on 'EMILIN−3'

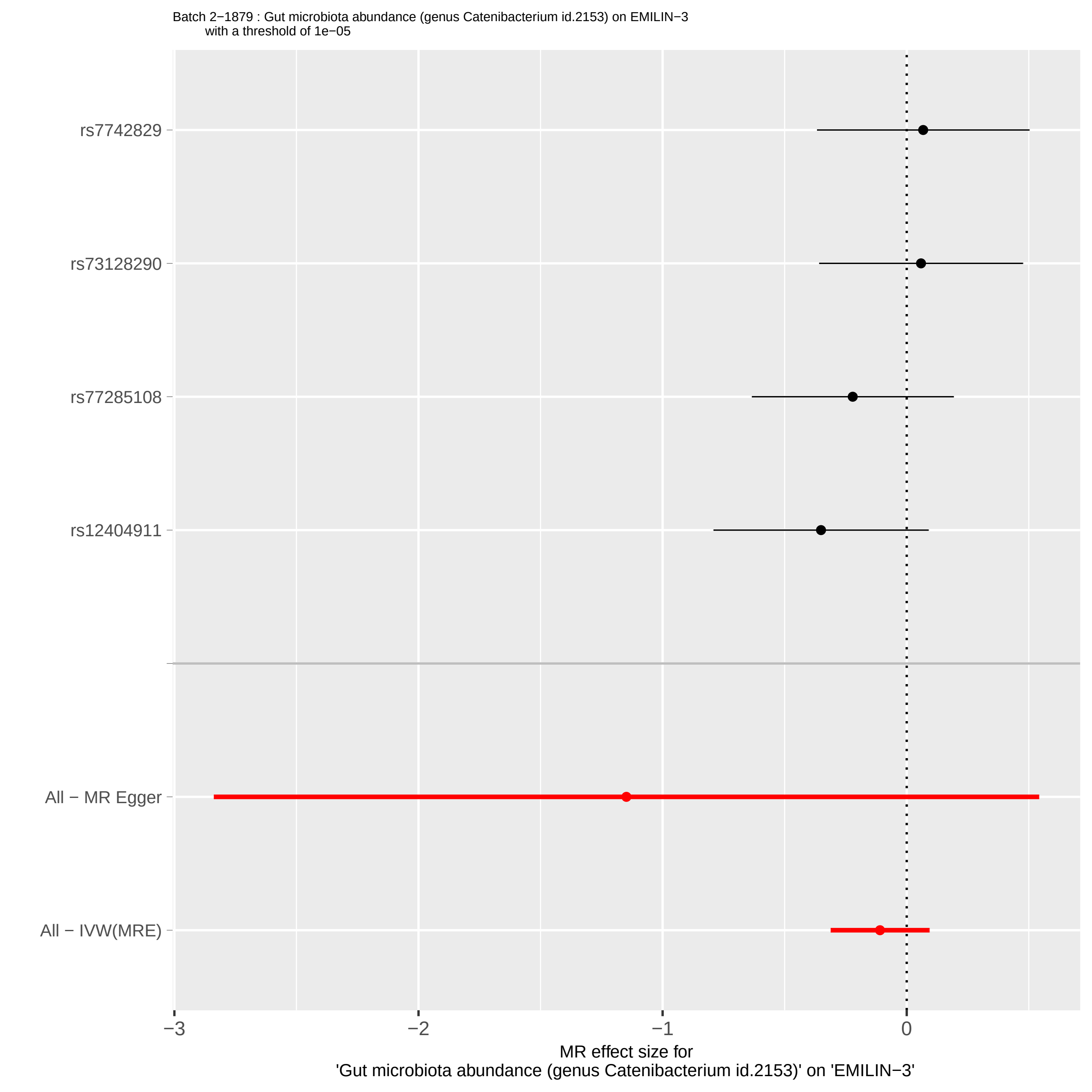

Batch 2−1879 : Gut microbiota abundance (genus Catenibacterium id.2153) on EMILIN−3
with a threshold of 1e−05
rs7742829
rs73128290
rs77285108
rs12404911
All − MR Egger
All − IVW(MRE)
−3
−2
−1
0
MR effect size for
'Gut microbiota abundance (genus Catenibacterium id.2153)' on 'EMILIN−3'

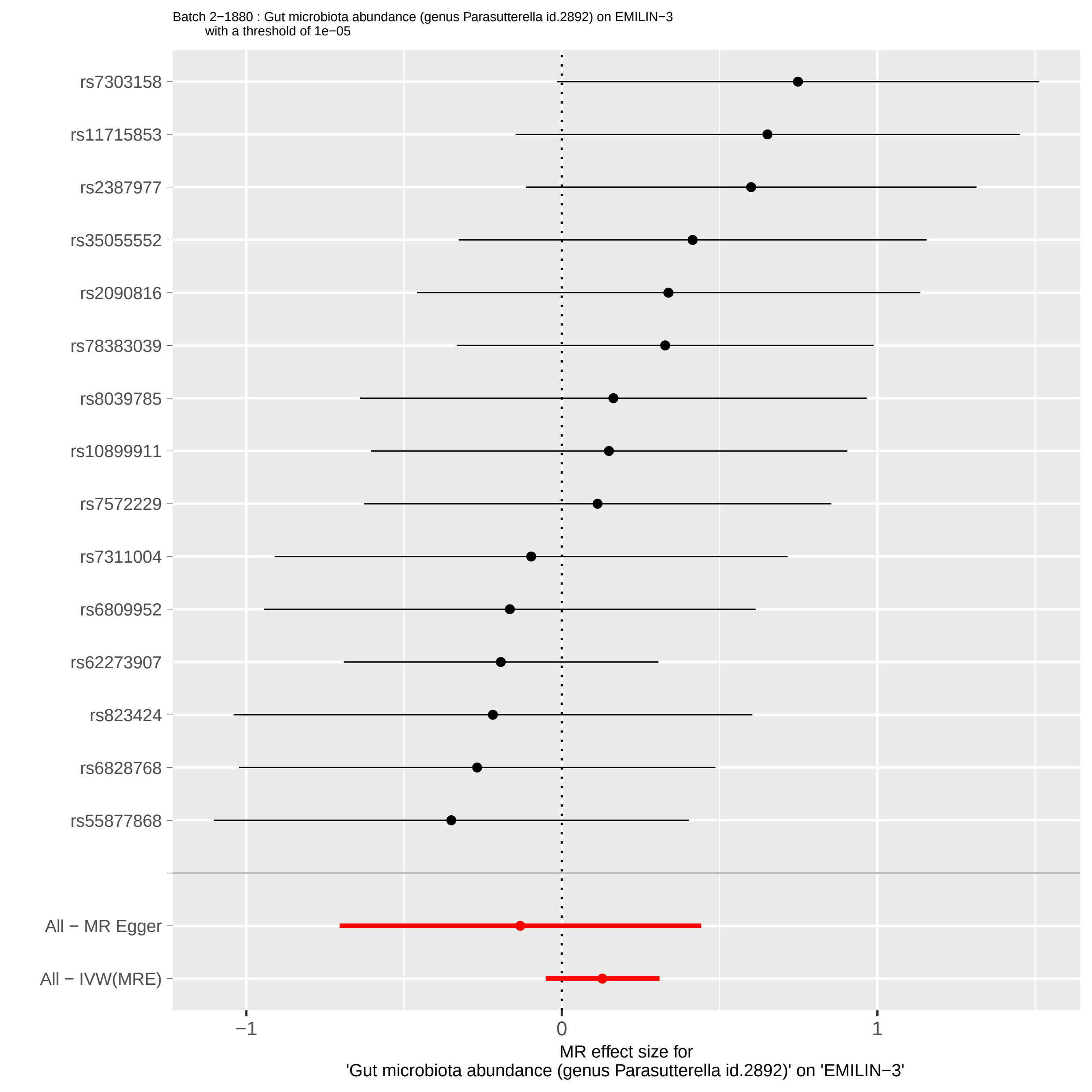

Batch 2−1880 : Gut microbiota abundance (genus Parasutterella id.2892) on EMILIN−3
with a threshold of 1e−05
rs7303158
rs11715853
rs2387977
rs35055552
rs2090816
rs78383039
rs8039785
rs10899911
rs7572229
rs7311004
rs6809952
rs62273907
rs823424
rs6828768
rs55877868
All − MR Egger
All − IVW(MRE)
−1
0
1
MR effect size for
'Gut microbiota abundance (genus Parasutterella id.2892)' on 'EMILIN−3'

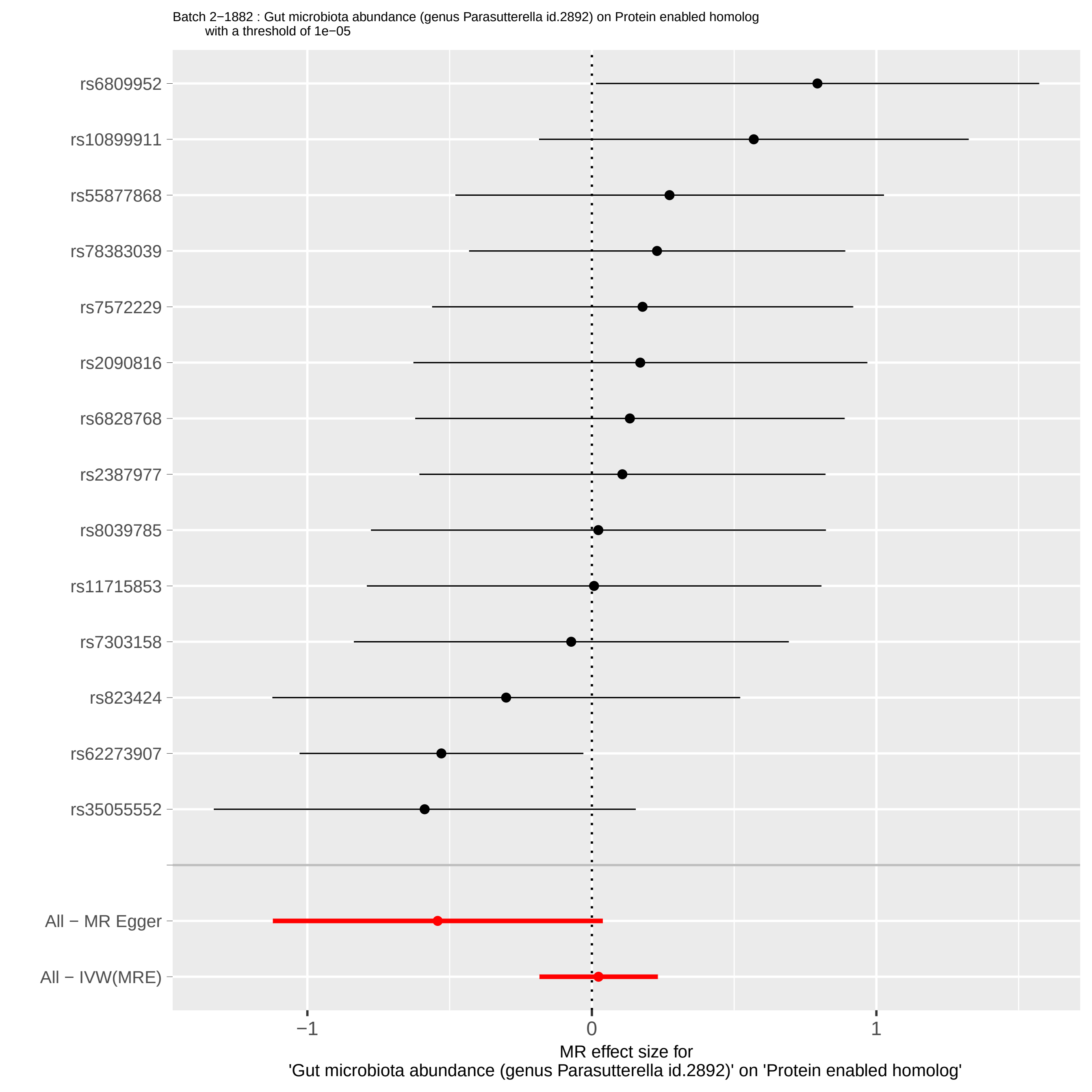

Batch 2−1882 : Gut microbiota abundance (genus Parasutterella id.2892) on Protein enabled homolog
with a threshold of 1e−05
rs6809952
rs10899911
rs55877868
rs78383039
rs7572229
rs2090816
rs6828768
rs2387977
rs8039785
rs11715853
rs7303158
rs823424
rs62273907
rs35055552
All − MR Egger
All − IVW(MRE)
−1
0
1
MR effect size for
'Gut microbiota abundance (genus Parasutterella id.2892)' on 'Protein enabled homolog'

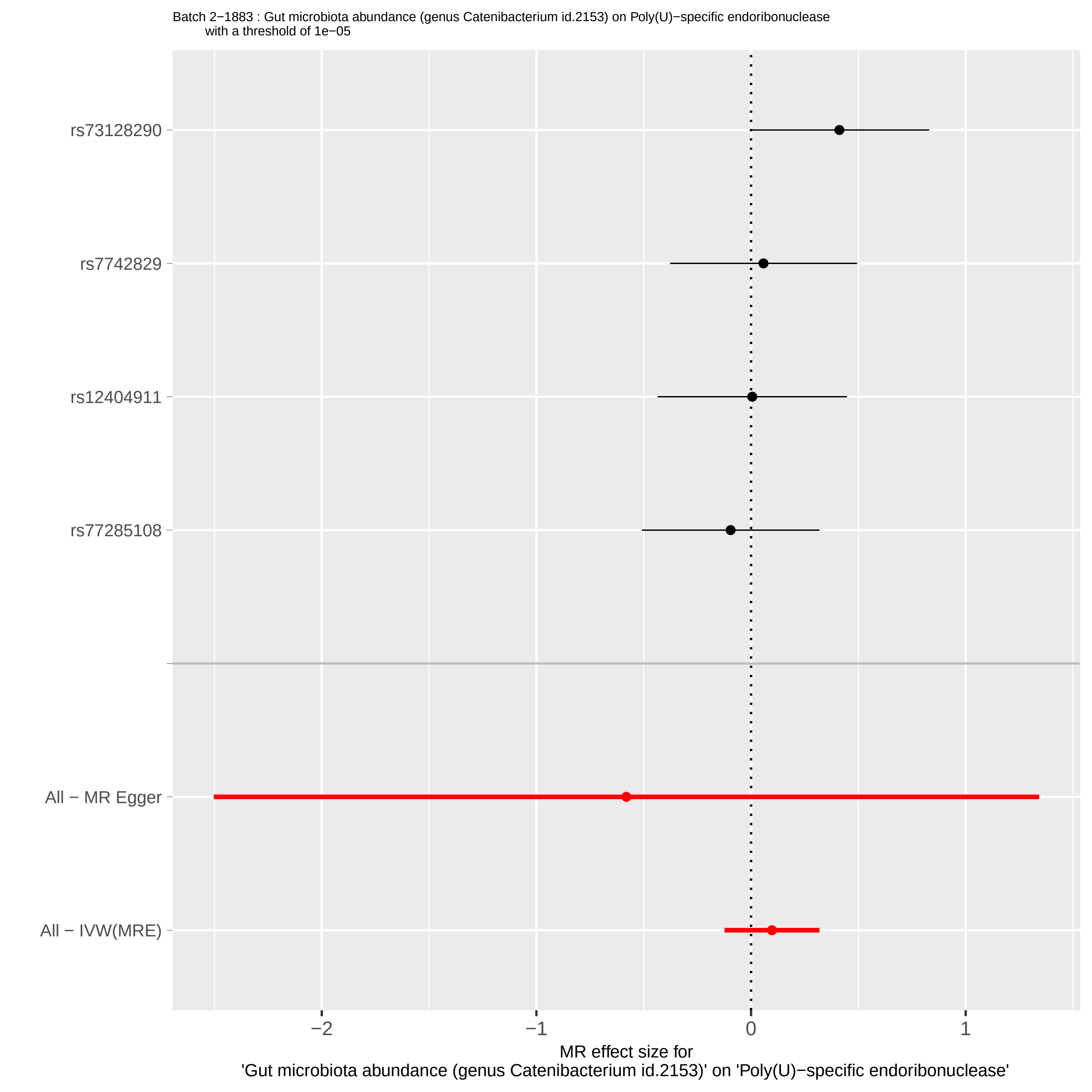
Batch 2−1883 : Gut microbiota abundance (genus Catenibacterium id.2153) on Poly(U)−specific endoribonuclease
with a threshold of 1e−05
rs73128290
rs7742829
rs12404911
rs77285108
All − MR Egger
All − IVW(MRE)
−2
−1
0
1
MR effect size for
'Gut microbiota abundance (genus Catenibacterium id.2153)' on 'Poly(U)−specific endoribonuclease'

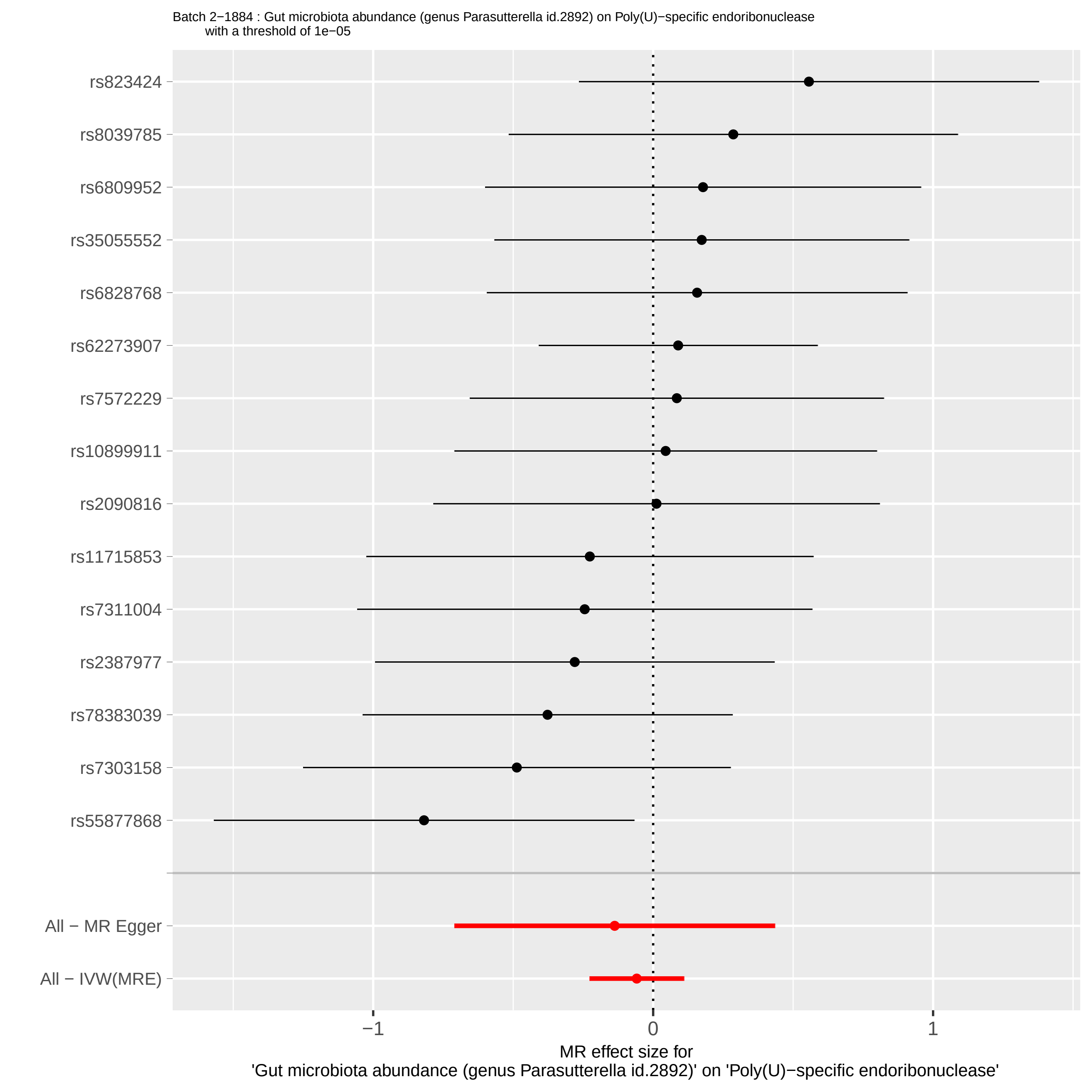
Batch 2−1884 : Gut microbiota abundance (genus Parasutterella id.2892) on Poly(U)−specific endoribonuclease
with a threshold of 1e−05
rs823424
rs8039785
rs6809952
rs35055552
rs6828768
rs62273907
rs7572229
rs10899911
rs2090816
rs11715853
rs7311004
rs2387977
rs78383039
rs7303158
rs55877868
All − MR Egger
All − IVW(MRE)
−1
0
1
MR effect size for
'Gut microbiota abundance (genus Parasutterella id.2892)' on 'Poly(U)−specific endoribonuclease'

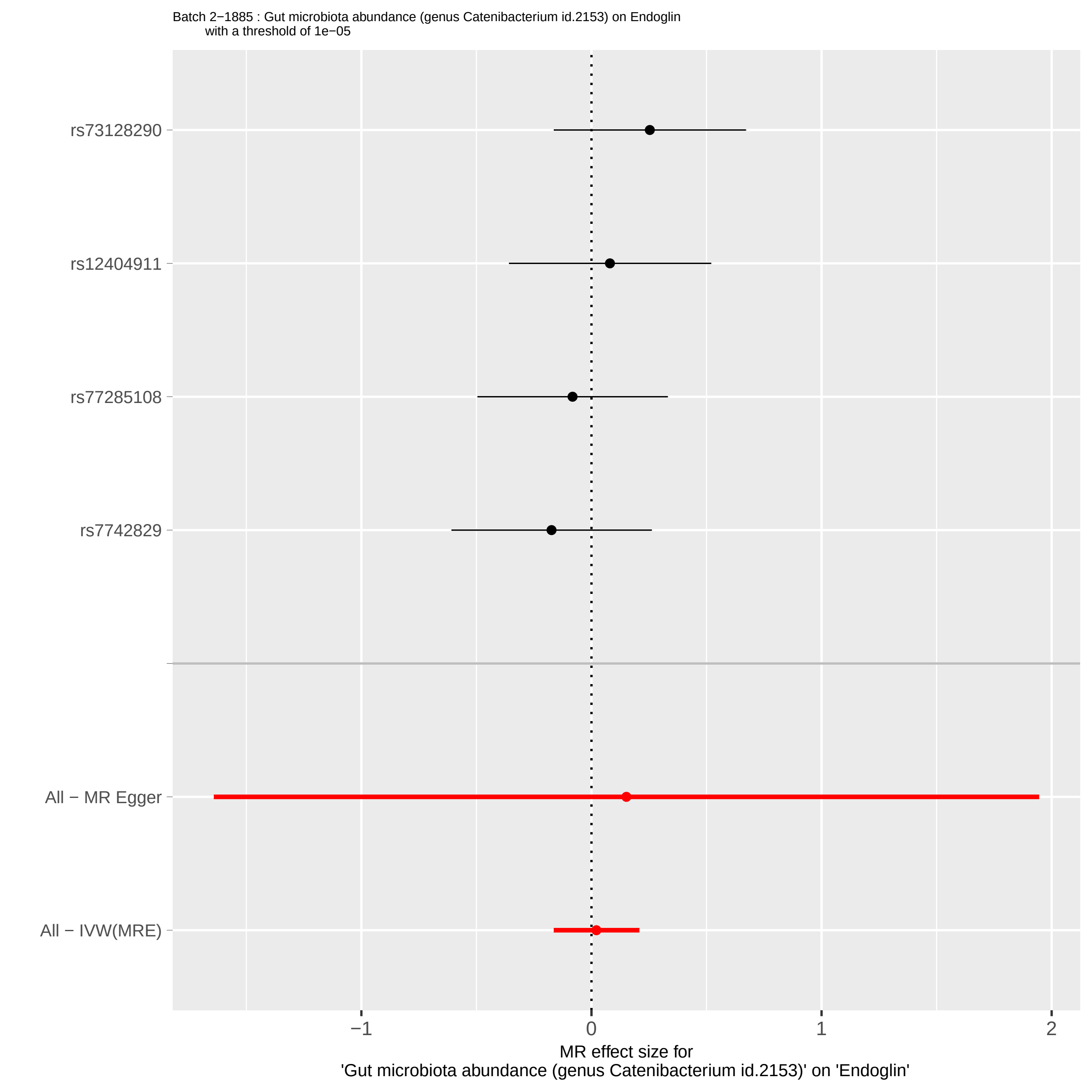

Batch 2−1885 : Gut microbiota abundance (genus Catenibacterium id.2153) on Endoglin
with a threshold of 1e−05
rs73128290
rs12404911
rs77285108
rs7742829
All − MR Egger
All − IVW(MRE)
−1
0
1
2
MR effect size for
'Gut microbiota abundance (genus Catenibacterium id.2153)' on 'Endoglin'

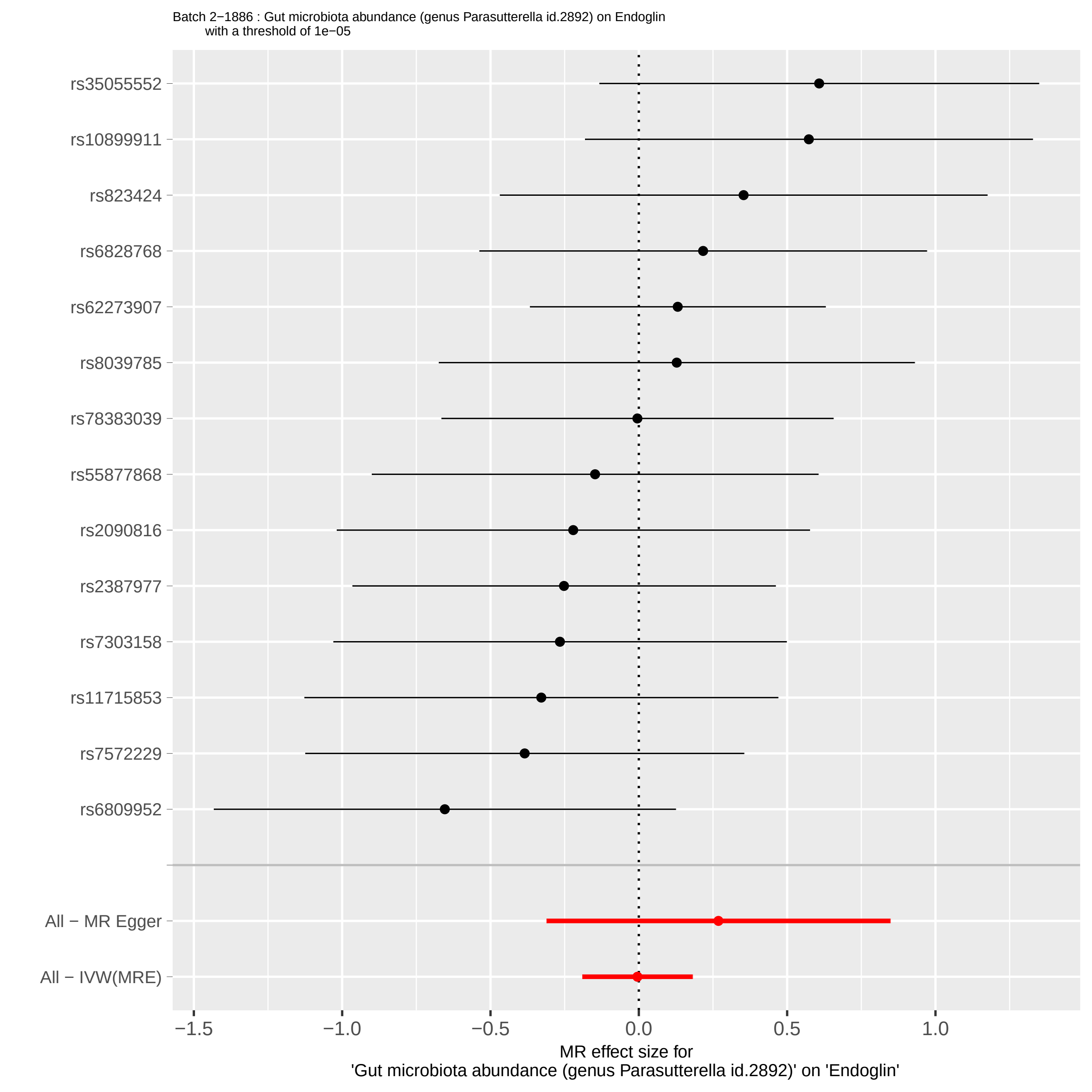

Batch 2−1886 : Gut microbiota abundance (genus Parasutterella id.2892) on Endoglin
with a threshold of 1e−05
rs35055552
rs10899911
rs823424
rs6828768
rs62273907
rs8039785
rs78383039
rs55877868
rs2090816
rs2387977
rs7303158
rs11715853
rs7572229
rs6809952
All − MR Egger
All − IVW(MRE)
−1.5
−1.0
−0.5
0.0
0.5
1.0
MR effect size for
'Gut microbiota abundance (genus Parasutterella id.2892)' on 'Endoglin'

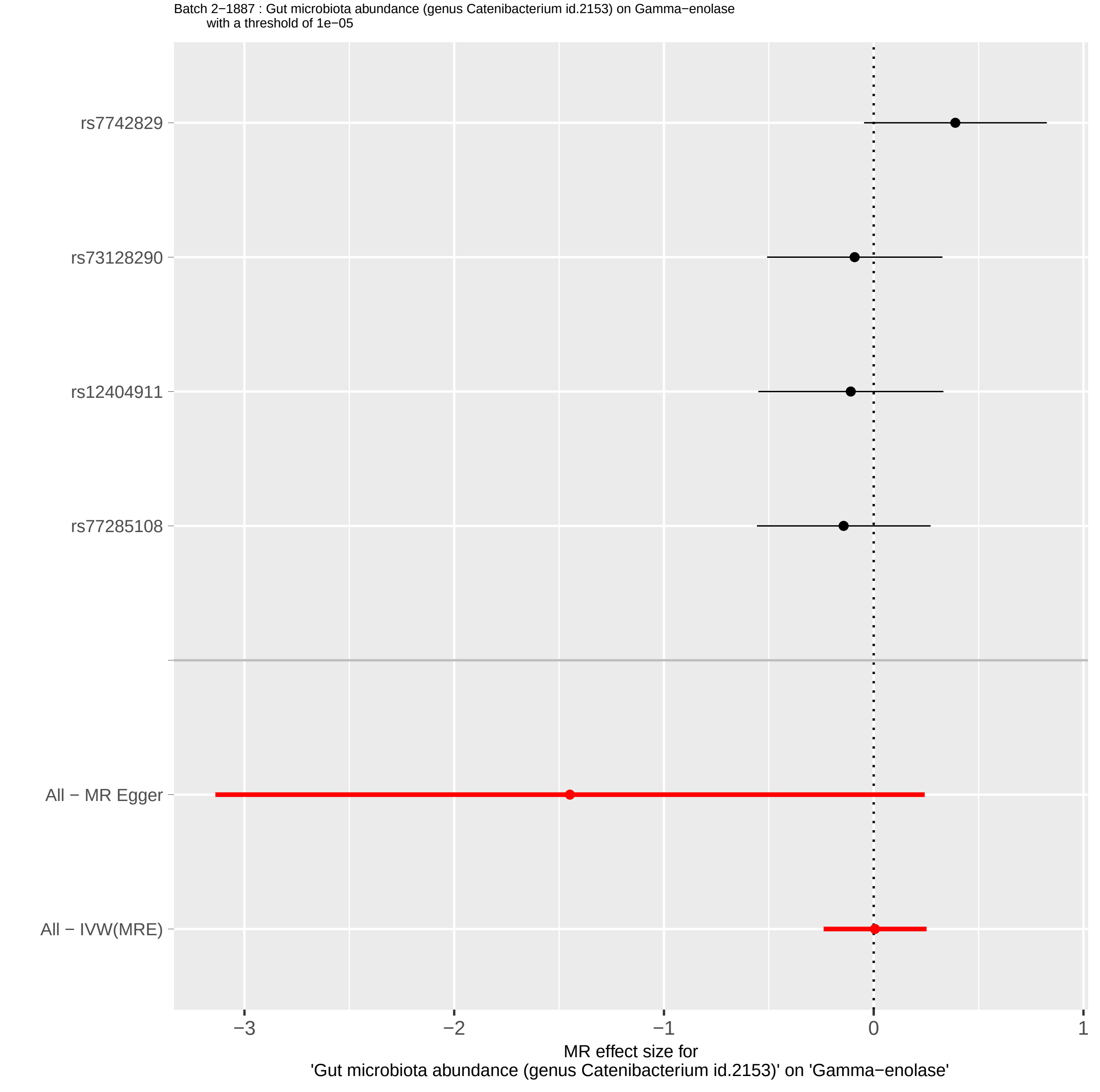

Batch 2−1887 : Gut microbiota abundance (genus Catenibacterium id.2153) on Gamma−enolase
with a threshold of 1e−05
rs7742829
rs73128290
rs12404911
rs77285108
All − MR Egger
All − IVW(MRE)
−3
−2
−1
0
1
MR effect size for
'Gut microbiota abundance (genus Catenibacterium id.2153)' on 'Gamma−enolase'

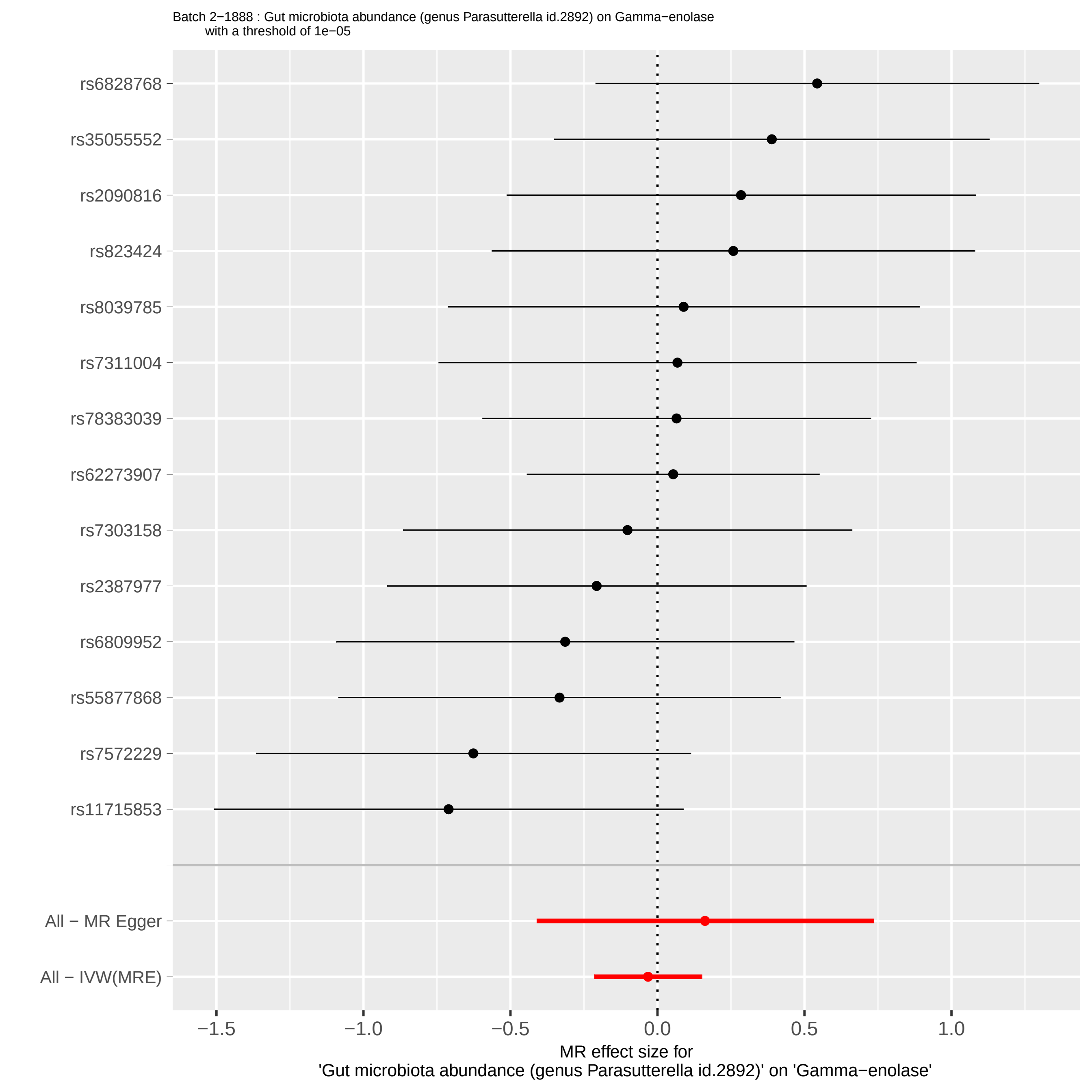
Batch 2−1888 : Gut microbiota abundance (genus Parasutterella id.2892) on Gamma−enolase
with a threshold of 1e−05
rs6828768
rs35055552
rs2090816
rs823424
rs8039785
rs7311004
rs78383039
rs62273907
rs7303158
rs2387977
rs6809952
rs55877868
rs7572229
rs11715853
All − MR Egger
All − IVW(MRE)
−1.5
−1.0
−0.5
0.0
0.5
1.0
MR effect size for
'Gut microbiota abundance (genus Parasutterella id.2892)' on 'Gamma−enolase'

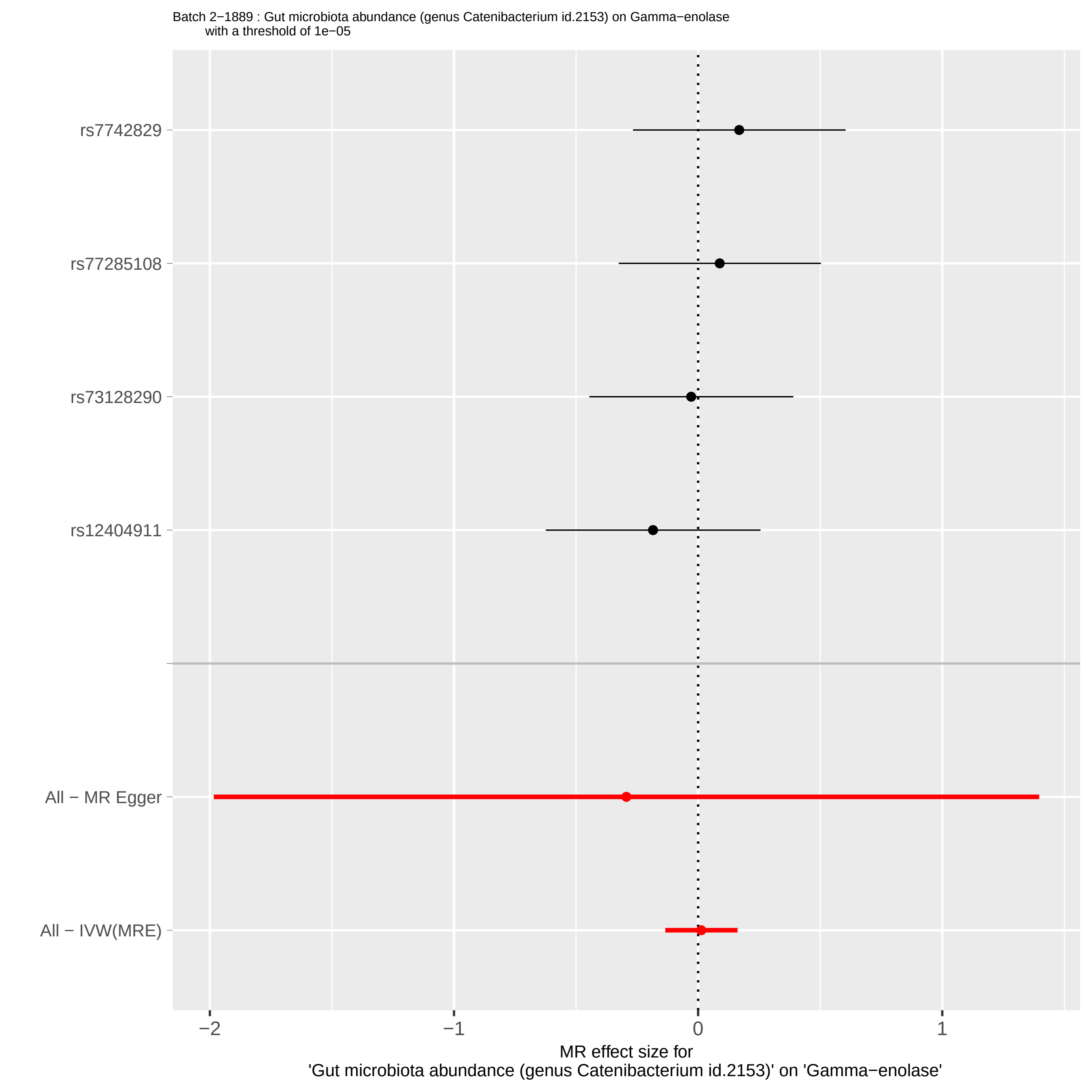
Batch 2−1889 : Gut microbiota abundance (genus Catenibacterium id.2153) on Gamma−enolase
with a threshold of 1e−05
rs7742829
rs77285108
rs73128290
rs12404911
All − MR Egger
All − IVW(MRE)
−2
−1
0
1
MR effect size for
'Gut microbiota abundance (genus Catenibacterium id.2153)' on 'Gamma−enolase'

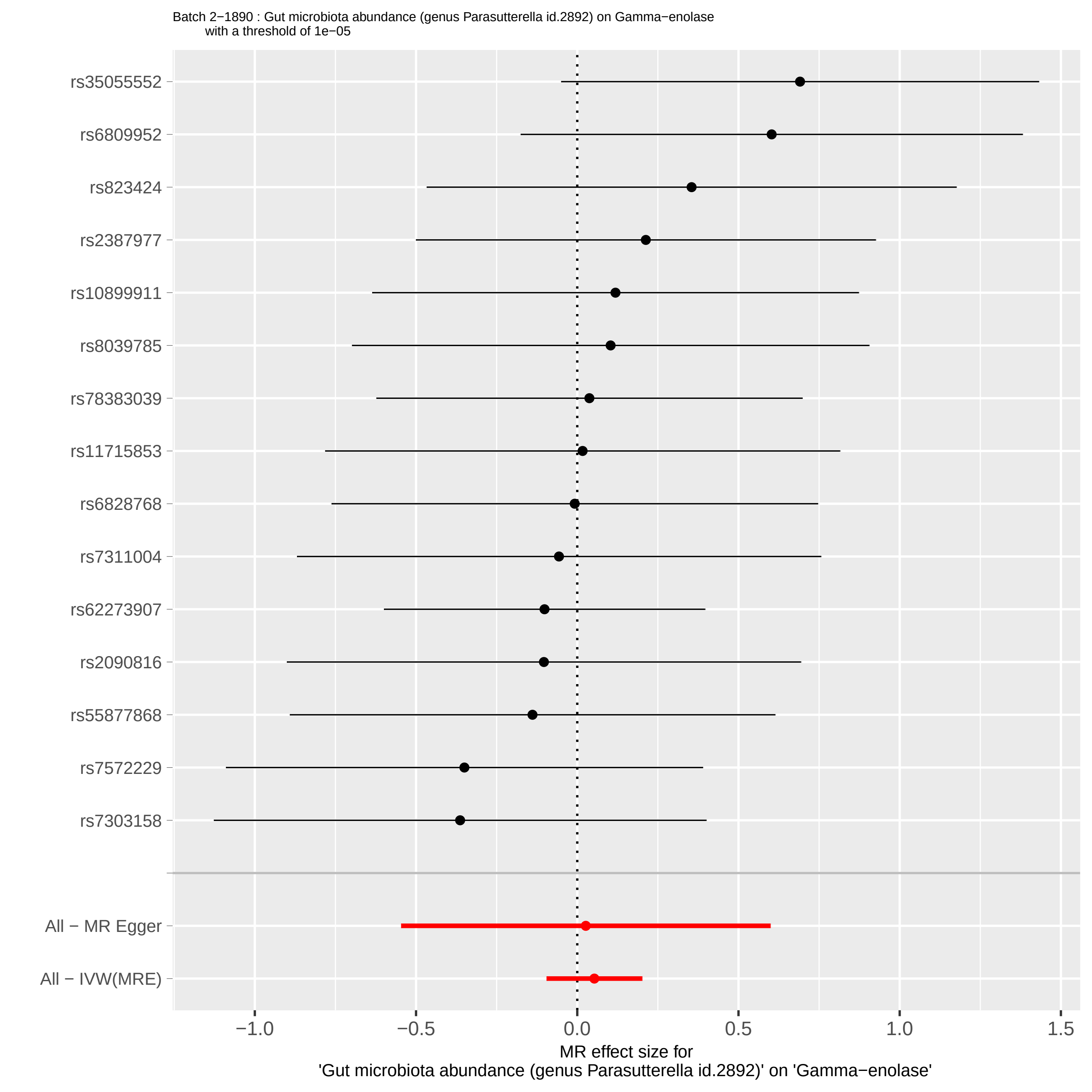

Batch 2−1890 : Gut microbiota abundance (genus Parasutterella id.2892) on Gamma−enolase
with a threshold of 1e−05
rs35055552
rs6809952
rs823424
rs2387977
rs10899911
rs8039785
rs78383039
rs11715853
rs6828768
rs7311004
rs62273907
rs2090816
rs55877868
rs7572229
rs7303158
All − MR Egger
All − IVW(MRE)
−1.0
−0.5
0.0
0.5
1.0
1.5
MR effect size for
'Gut microbiota abundance (genus Parasutterella id.2892)' on 'Gamma−enolase'

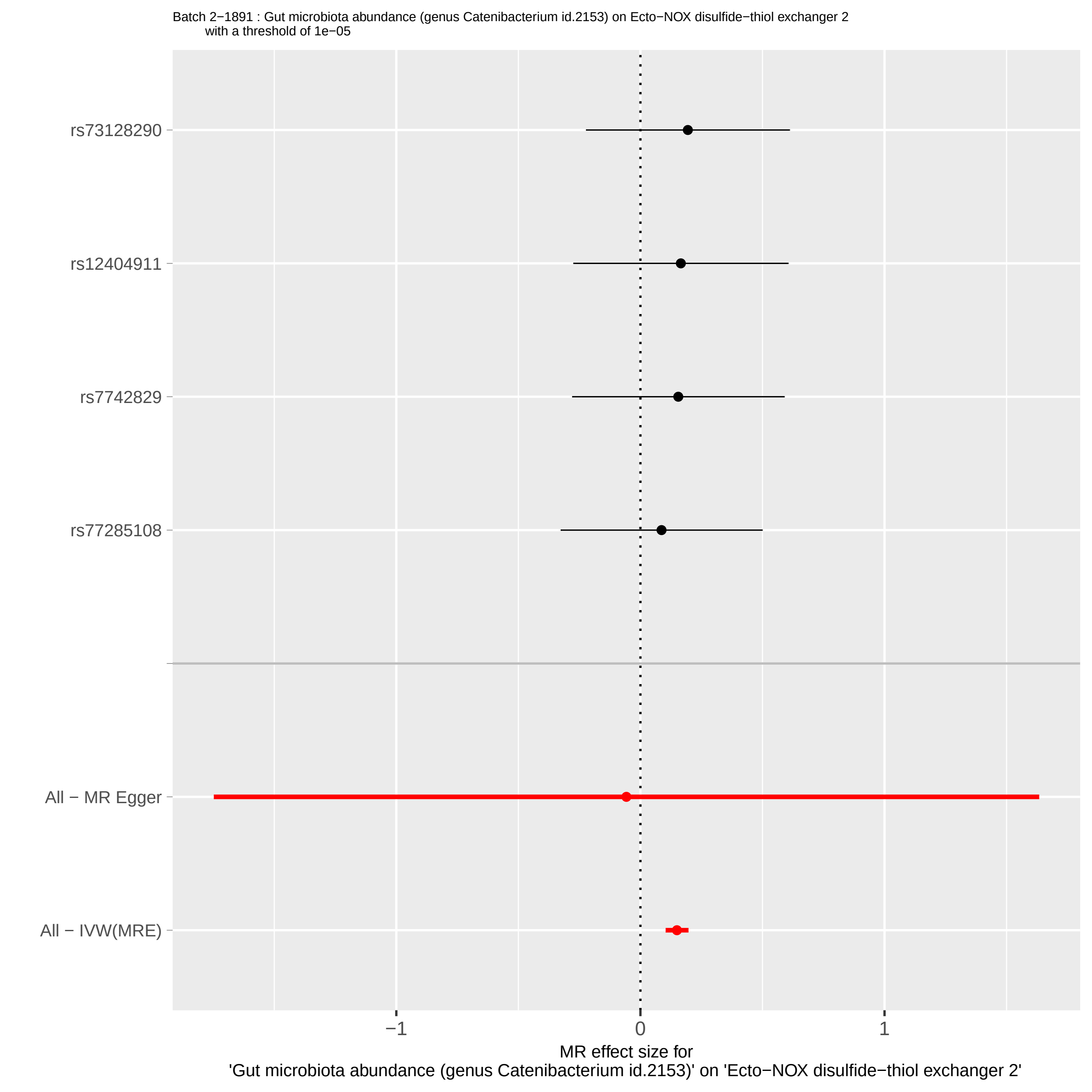
Batch 2−1891 : Gut microbiota abundance (genus Catenibacterium id.2153) on Ecto−NOX disulfide−thiol exchanger 2
with a threshold of 1e−05
rs73128290
rs12404911
rs7742829
rs77285108
All − MR Egger
All − IVW(MRE)
−1
0
1
MR effect size for
'Gut microbiota abundance (genus Catenibacterium id.2153)' on 'Ecto−NOX disulfide−thiol exchanger 2'

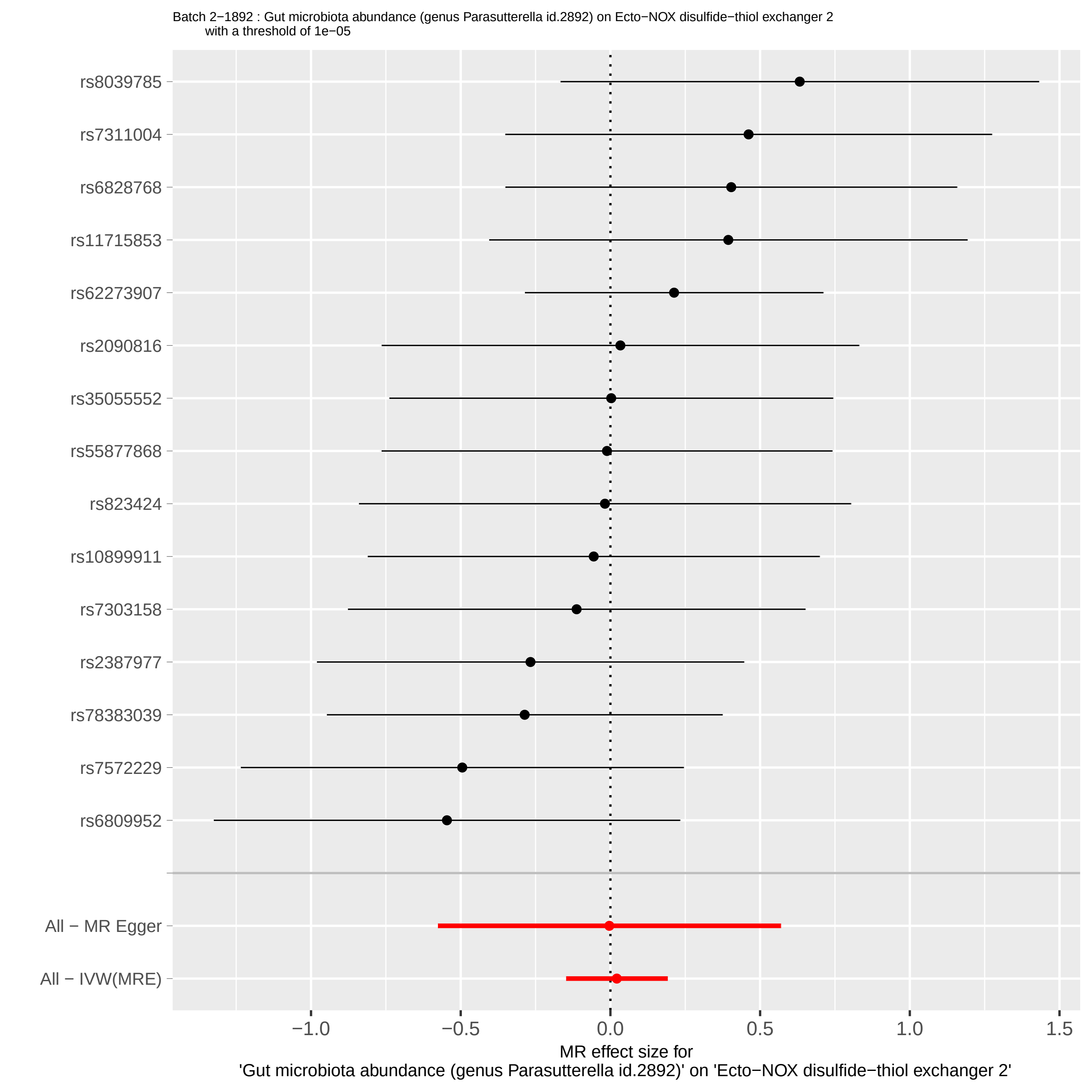

Batch 2−1892 : Gut microbiota abundance (genus Parasutterella id.2892) on Ecto−NOX disulfide−thiol exchanger 2
with a threshold of 1e−05
rs8039785
rs7311004
rs6828768
rs11715853
rs62273907
rs2090816
rs35055552
rs55877868
rs823424
rs10899911
rs7303158
rs2387977
rs78383039
rs7572229
rs6809952
All − MR Egger
All − IVW(MRE)
−1.0
−0.5
0.0
0.5
1.0
1.5
MR effect size for
'Gut microbiota abundance (genus Parasutterella id.2892)' on 'Ecto−NOX disulfide−thiol exchanger 2'

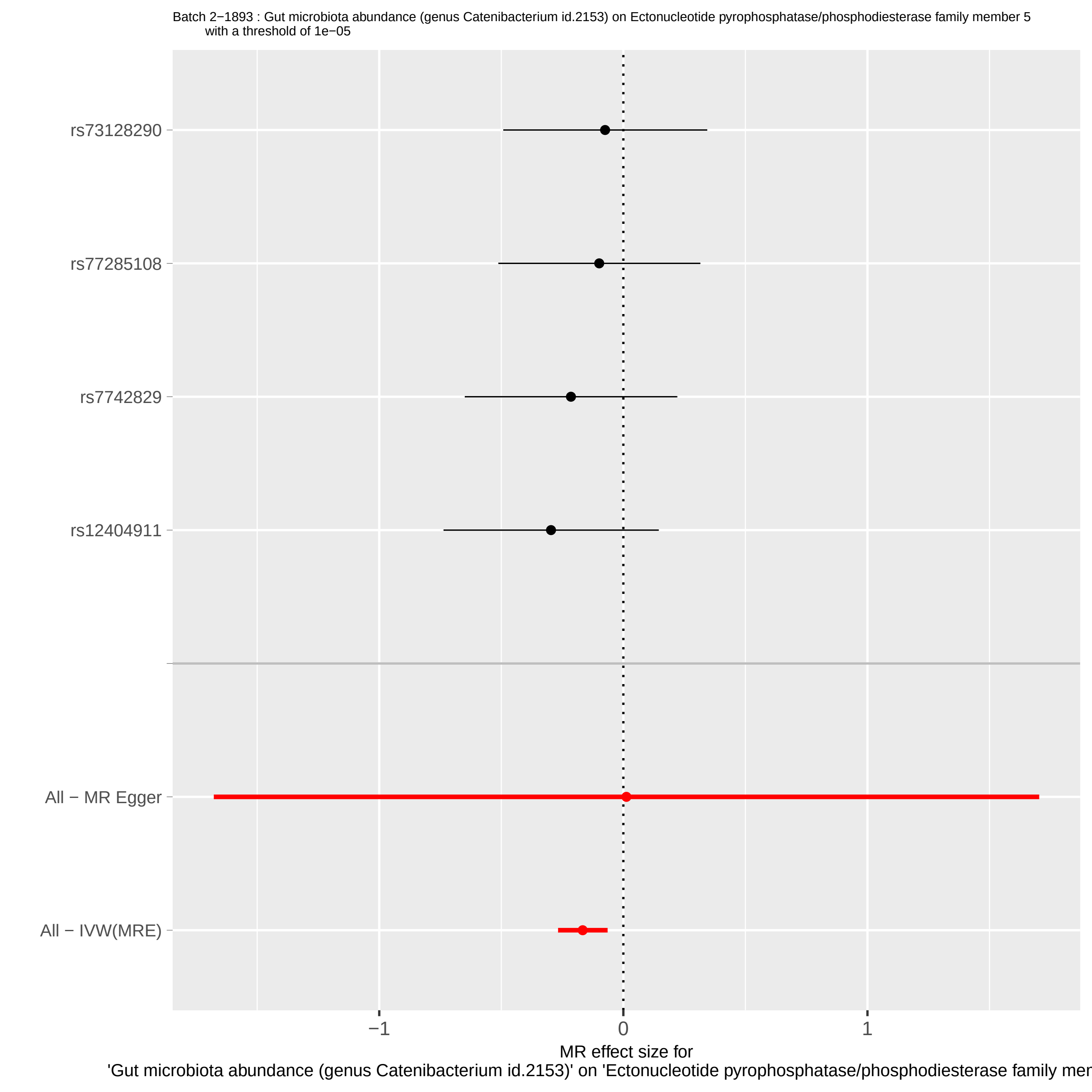

Batch 2−1893 : Gut microbiota abundance (genus Catenibacterium id.2153) on Ectonucleotide pyrophosphatase/phosphodiesterase family member 5
with a threshold of 1e−05
rs73128290
rs77285108
rs7742829
rs12404911
All − MR Egger
All − IVW(MRE)
−1
0
1
MR effect size for
'Gut microbiota abundance (genus Catenibacterium id.2153)' on 'Ectonucleotide pyrophosphatase/phosphodiesterase family mem

Batch 2−1894 : Gut microbiota abundance (genus Parasutterella id.2892) on Ectonucleotide pyrophosphatase/phosphodiesterase family member 5 with a threshold of 1e−05

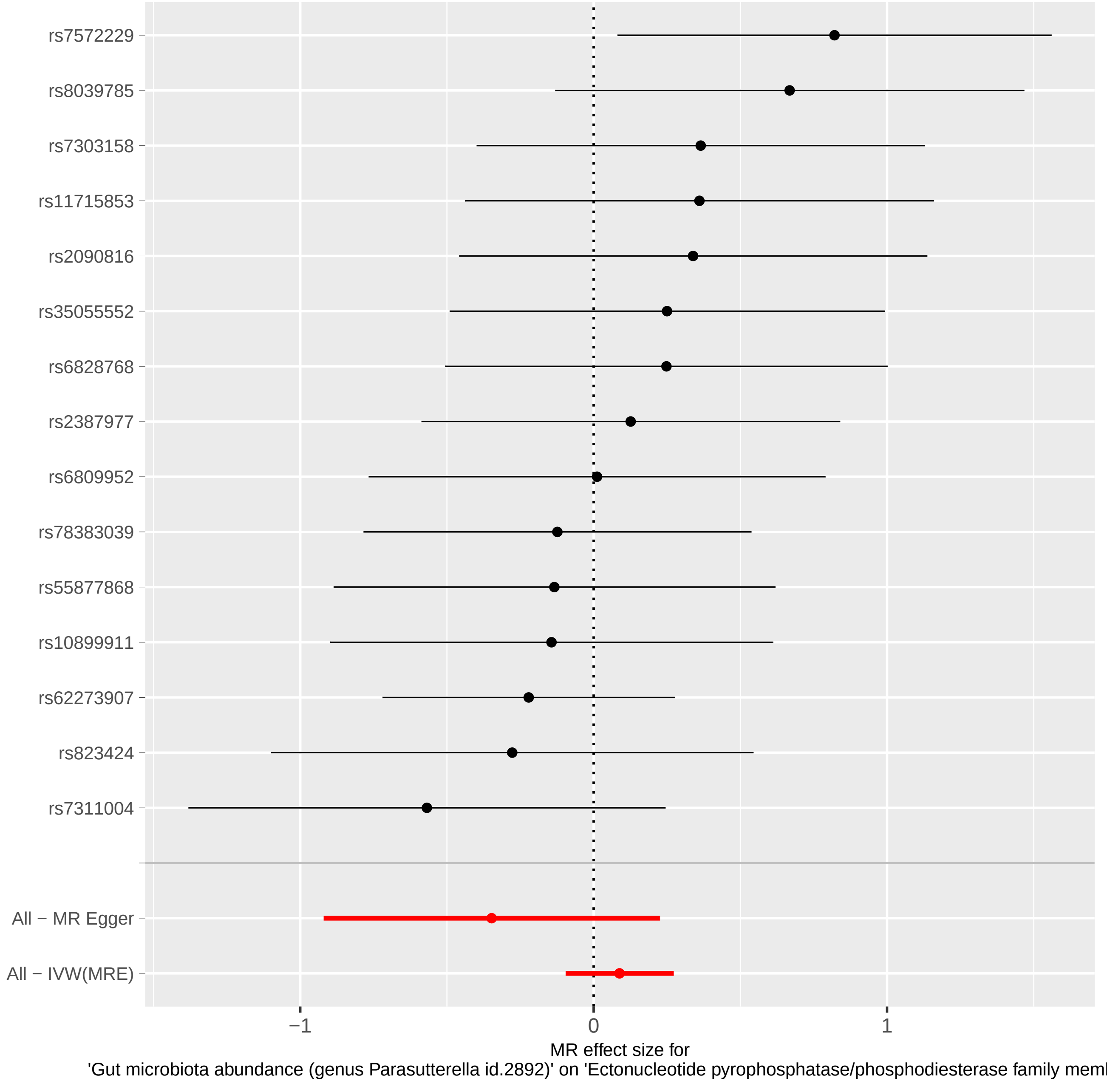

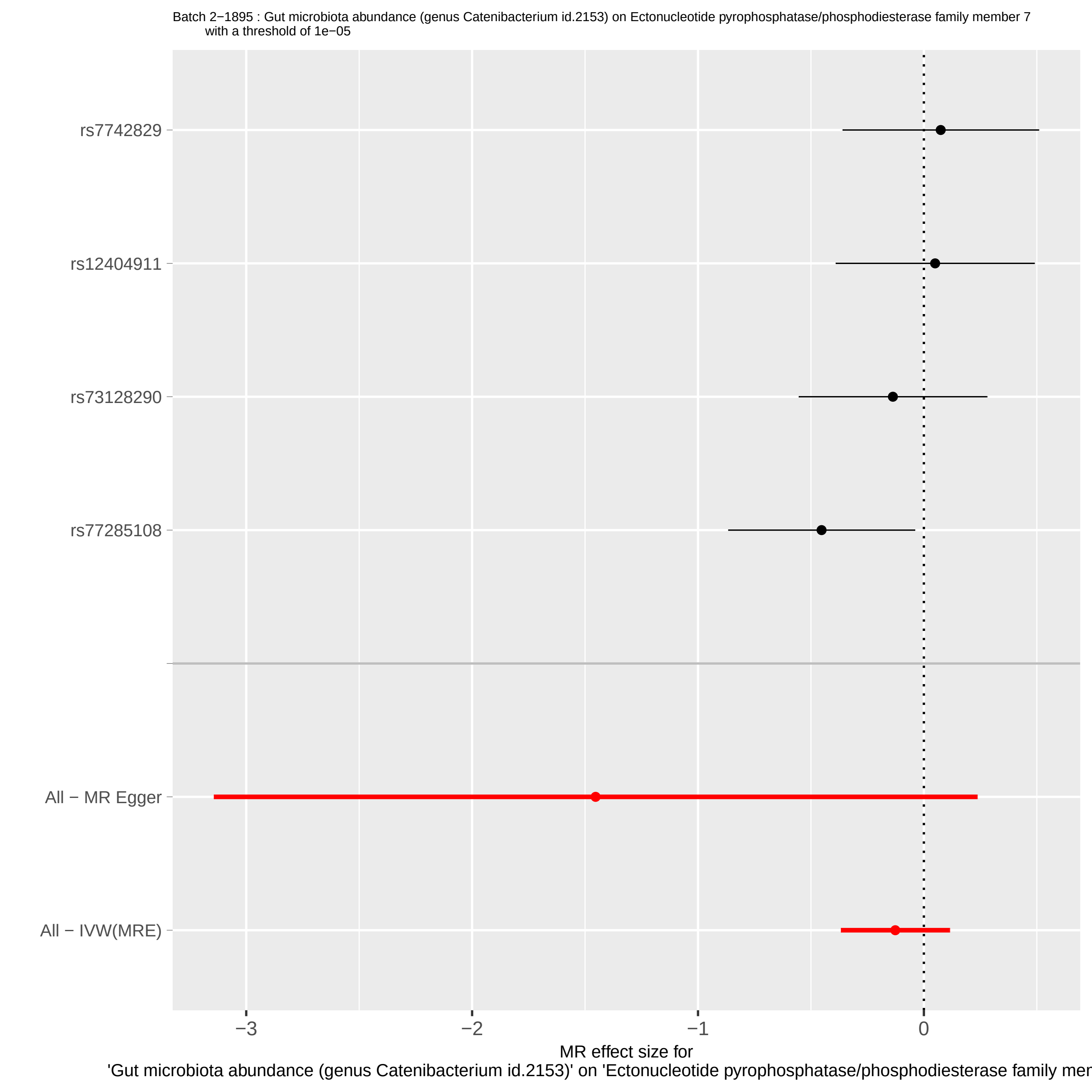

Batch 2−1895 : Gut microbiota abundance (genus Catenibacterium id.2153) on Ectonucleotide pyrophosphatase/phosphodiesterase family member 7
with a threshold of 1e−05
rs7742829
rs12404911
rs73128290
rs77285108
All − MR Egger
All − IVW(MRE)
−3
−2
−1
0
MR effect size for
'Gut microbiota abundance (genus Catenibacterium id.2153)' on 'Ectonucleotide pyrophosphatase/phosphodiesterase family mem

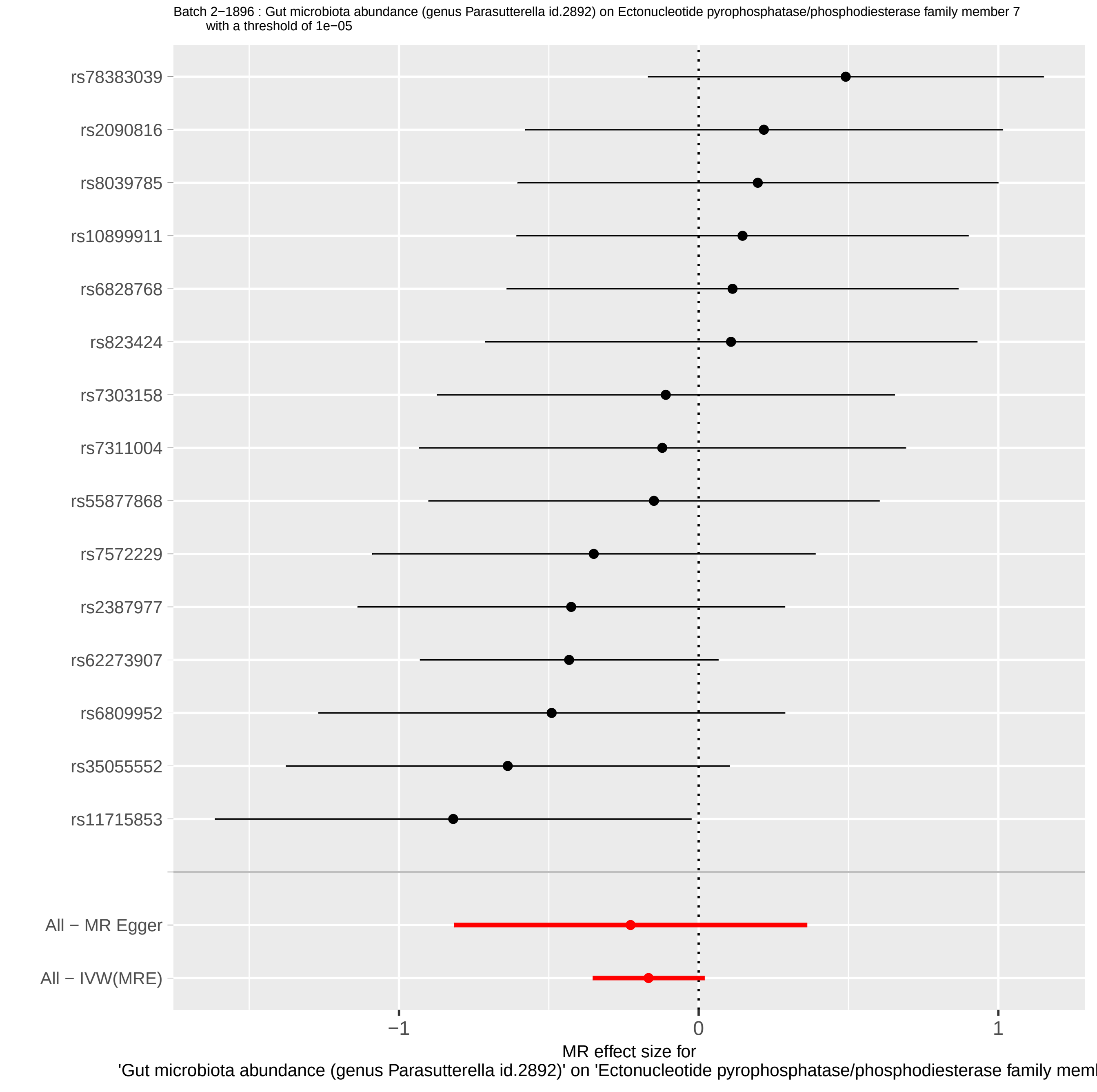

Batch 2−1896 : Gut microbiota abundance (genus Parasutterella id.2892) on Ectonucleotide pyrophosphatase/phosphodiesterase family member 7
with a threshold of 1e−05
rs78383039
rs2090816
rs8039785
rs10899911
rs6828768
rs823424
rs7303158
rs7311004
rs55877868
rs7572229
rs2387977
rs62273907
rs6809952
rs35055552
rs11715853
All − MR Egger
All − IVW(MRE)
−1
0
1
MR effect size for
'Gut microbiota abundance (genus Parasutterella id.2892)' on 'Ectonucleotide pyrophosphatase/phosphodiesterase family memb

Batch 2−1897 : Gut microbiota abundance (genus Catenibacterium id.2153) on AP−4 complex accessory subunit tepsin
with a threshold of 1e−05

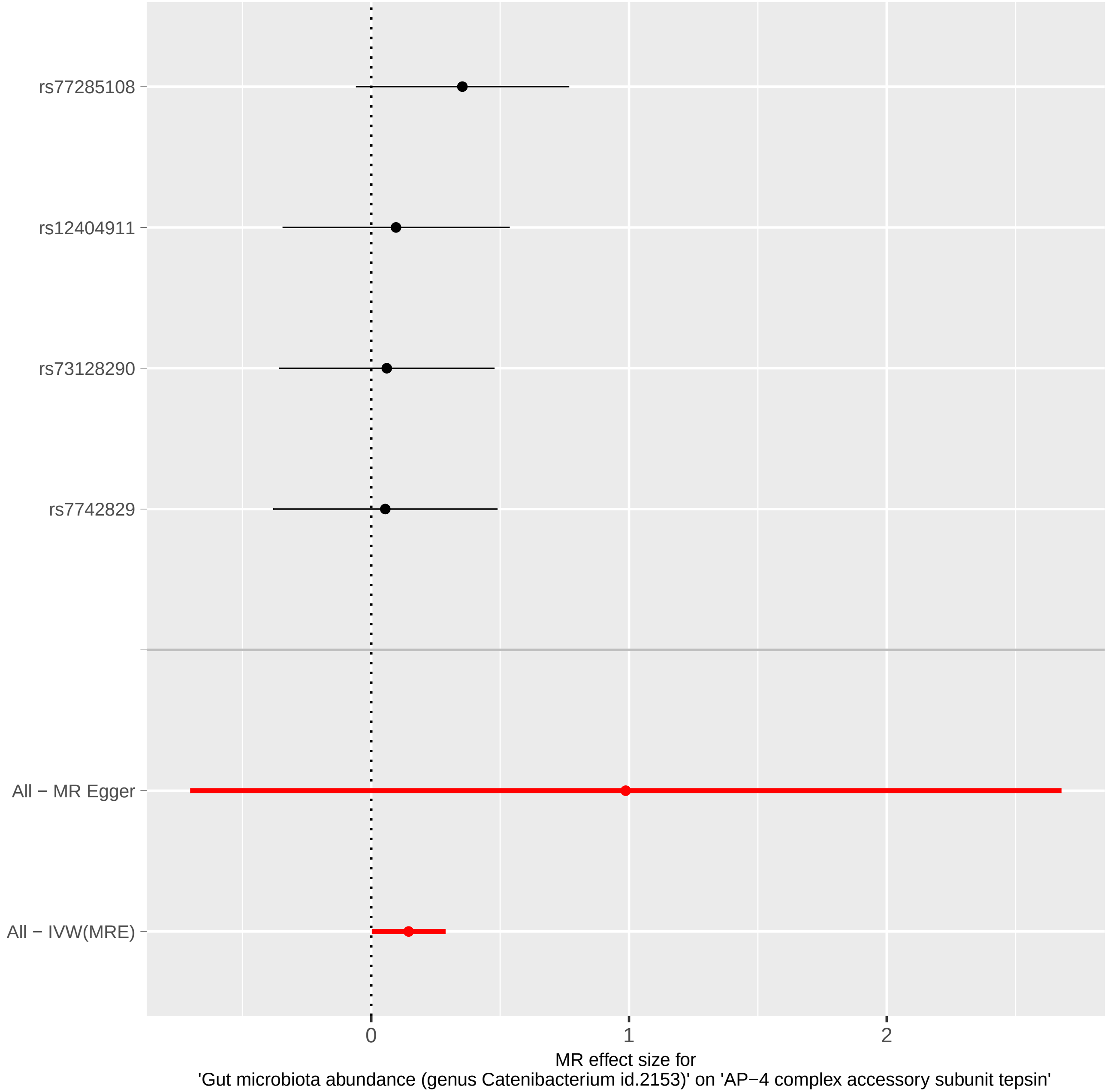

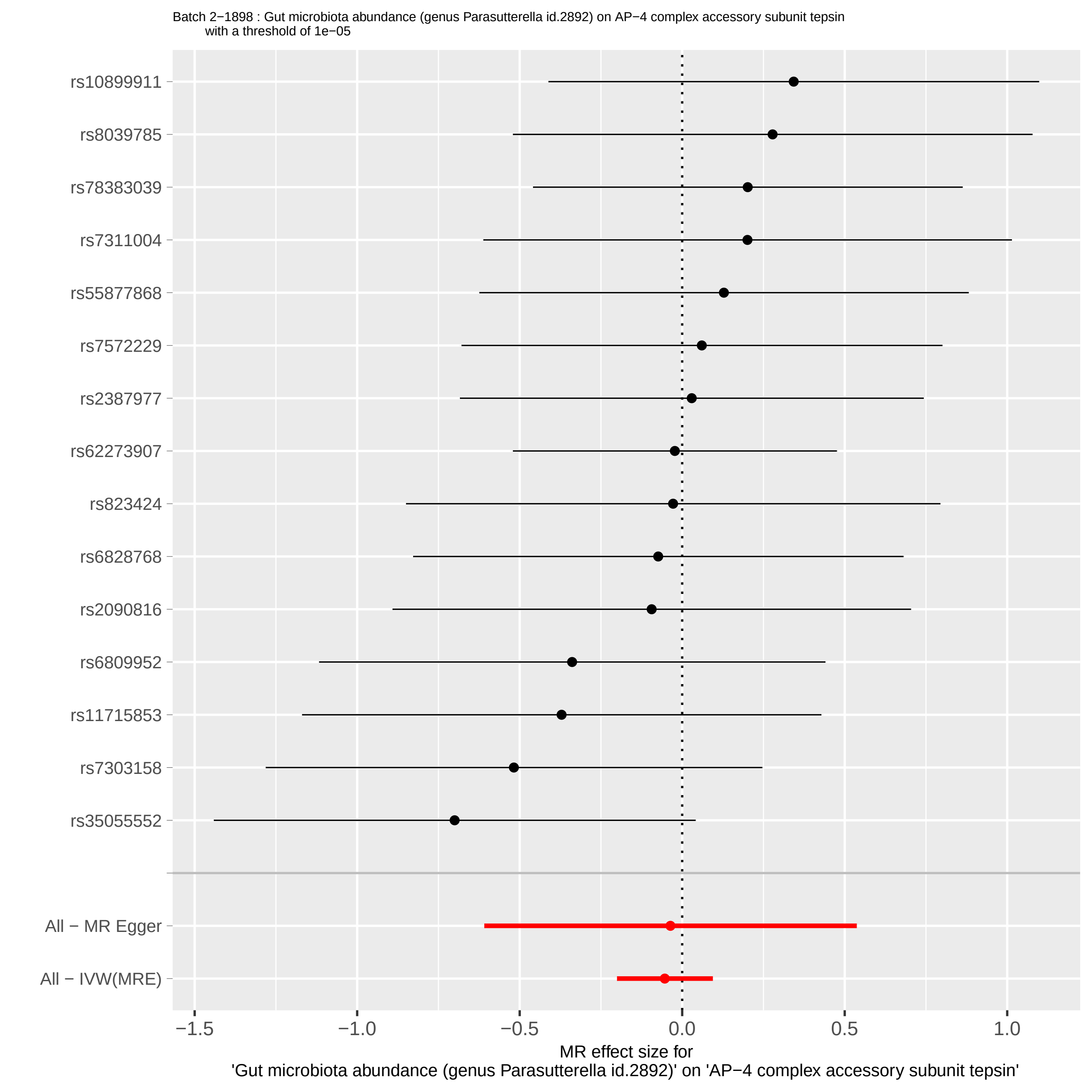

Batch 2−1898 : Gut microbiota abundance (genus Parasutterella id.2892) on AP−4 complex accessory subunit tepsin
with a threshold of 1e−05
rs10899911
rs8039785
rs78383039
rs7311004
rs55877868
rs7572229
rs2387977
rs62273907
rs823424
rs6828768
rs2090816
rs6809952
rs11715853
rs7303158
rs35055552
All − MR Egger
All − IVW(MRE)
−1.5
−1.0
−0.5
0.0
0.5
1.0
MR effect size for
'Gut microbiota abundance (genus Parasutterella id.2892)' on 'AP−4 complex accessory subunit tepsin'

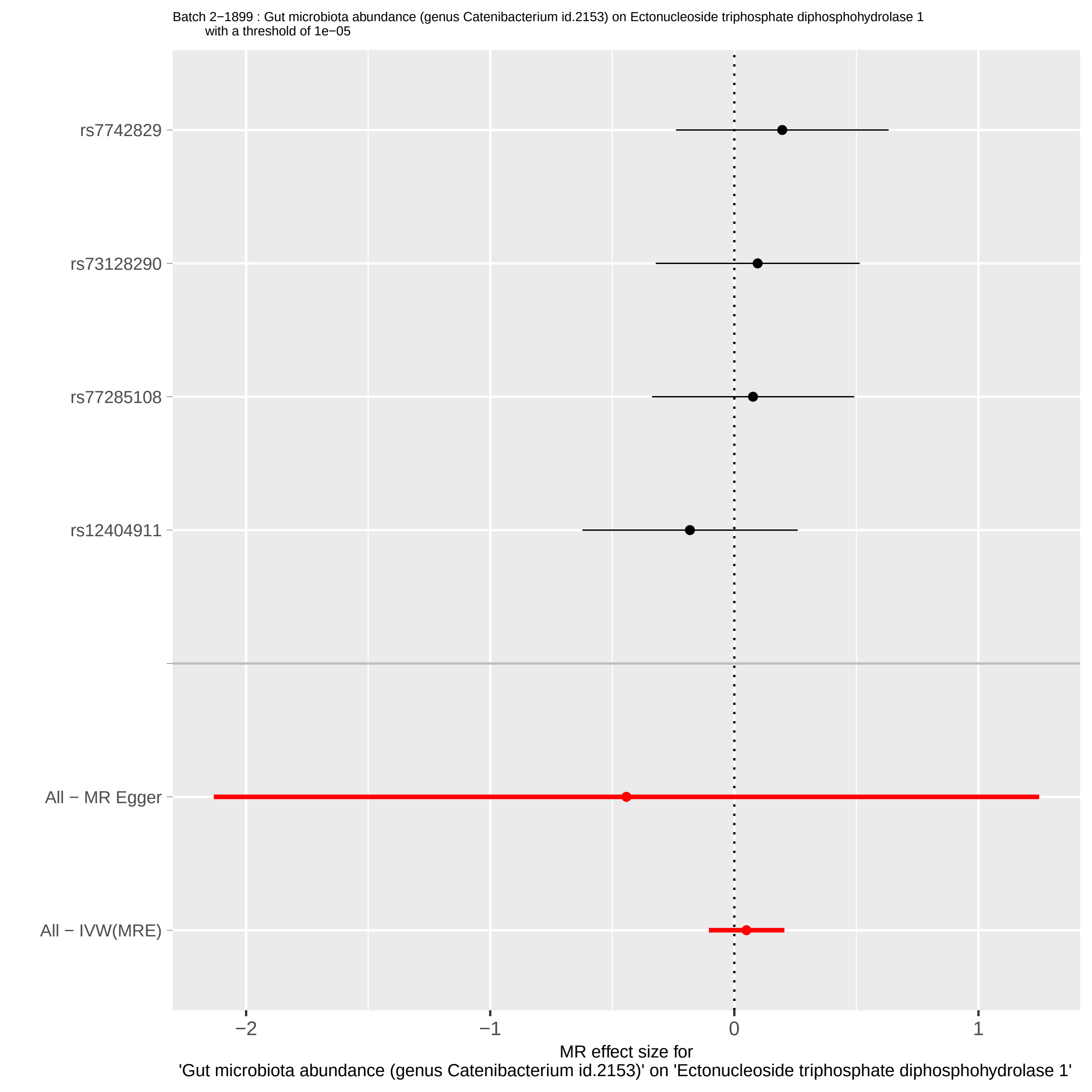

Batch 2−1899 : Gut microbiota abundance (genus Catenibacterium id.2153) on Ectonucleoside triphosphate diphosphohydrolase 1
with a threshold of 1e−05
rs7742829
rs73128290
rs77285108
rs12404911
All − MR Egger
All − IVW(MRE)
−2
−1
0
1
MR effect size for
'Gut microbiota abundance (genus Catenibacterium id.2153)' on 'Ectonucleoside triphosphate diphosphohydrolase 1'

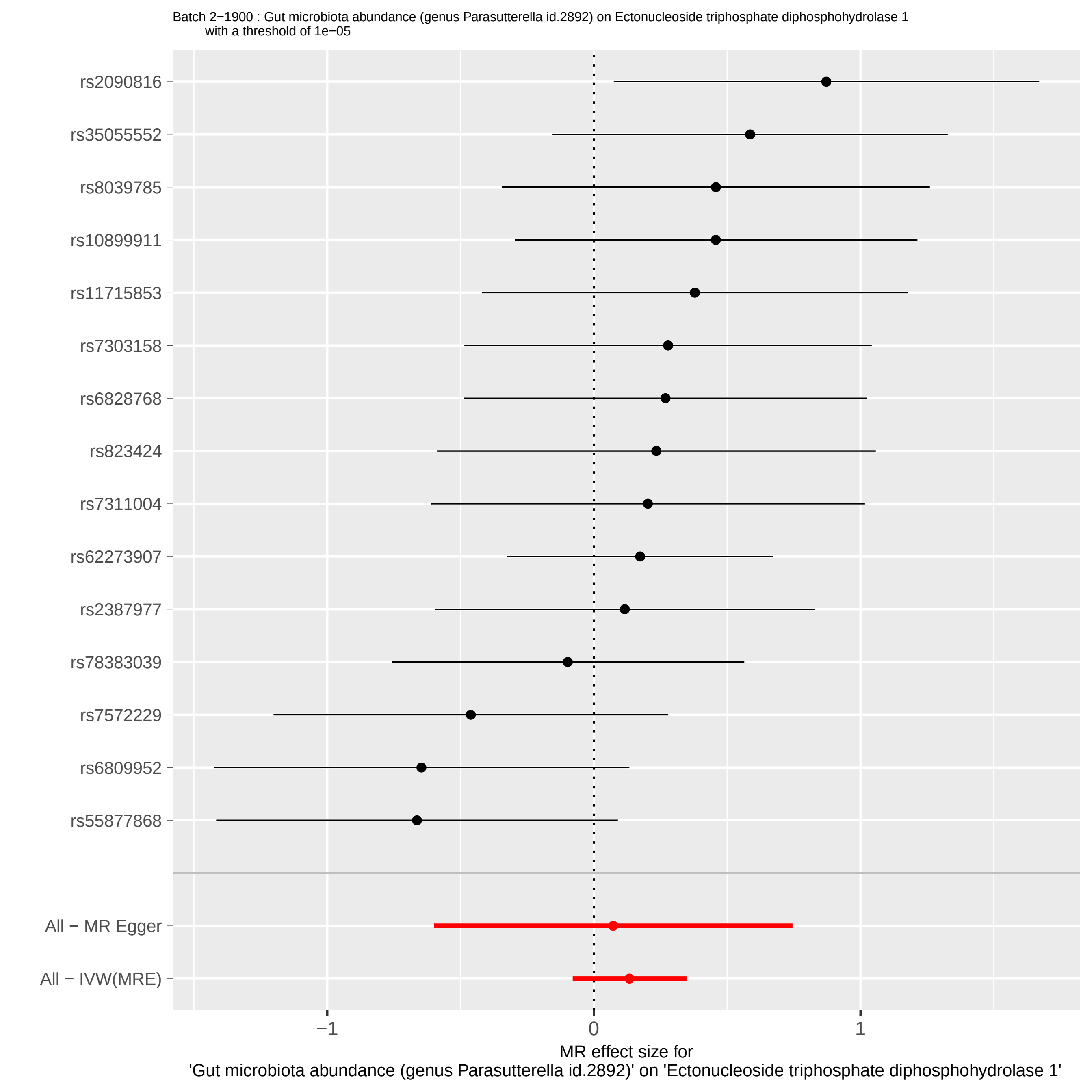
Batch 2−1900 : Gut microbiota abundance (genus Parasutterella id.2892) on Ectonucleoside triphosphate diphosphohydrolase 1
with a threshold of 1e−05
rs2090816
rs35055552
rs8039785
rs10899911
rs11715853
rs7303158
rs6828768
rs823424
rs7311004
rs62273907
rs2387977
rs78383039
rs7572229
rs6809952
rs55877868
All − MR Egger
All − IVW(MRE)
−1
0
1
MR effect size for
'Gut microbiota abundance (genus Parasutterella id.2892)' on 'Ectonucleoside triphosphate diphosphohydrolase 1'

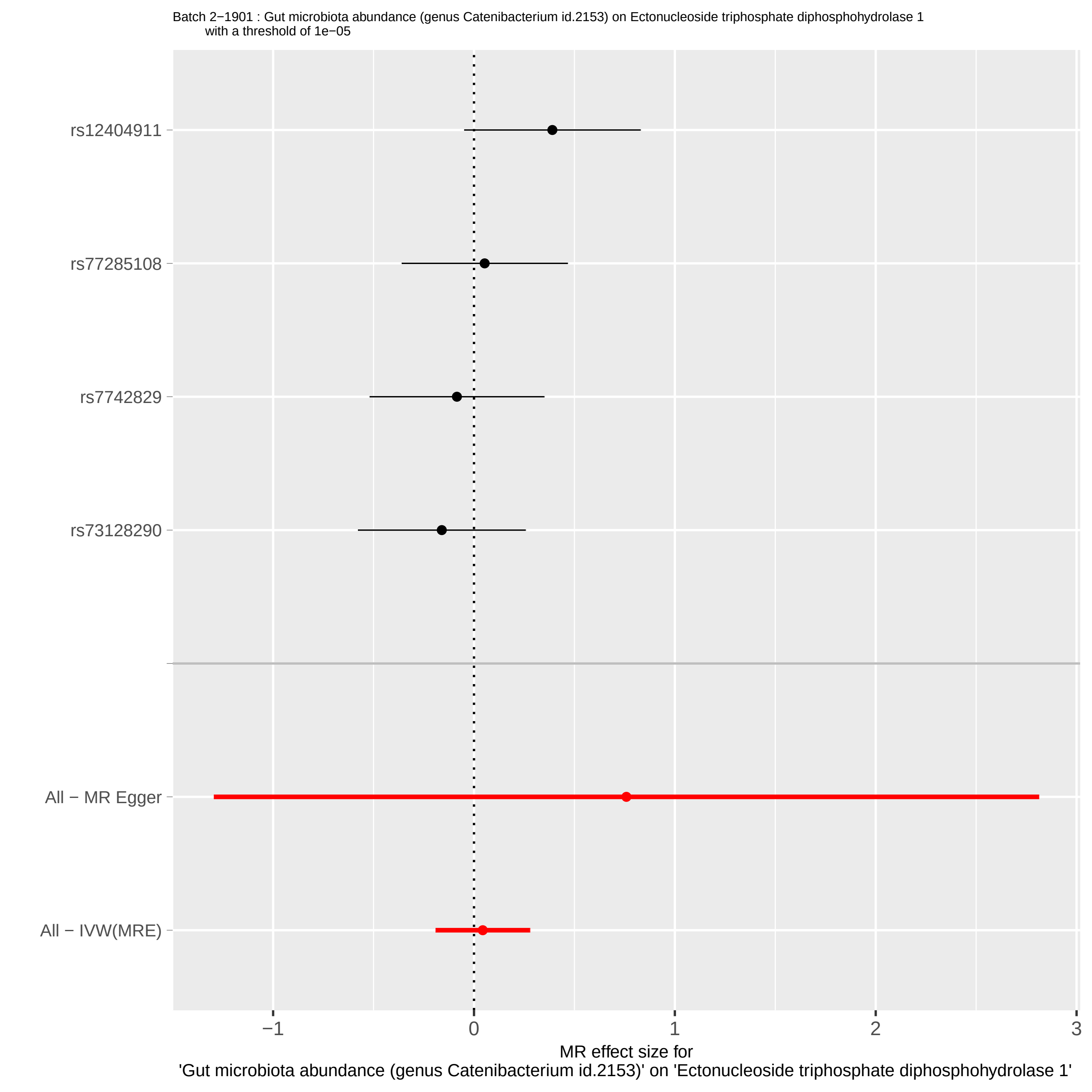
Batch 2−1901 : Gut microbiota abundance (genus Catenibacterium id.2153) on Ectonucleoside triphosphate diphosphohydrolase 1
with a threshold of 1e−05
rs12404911
rs77285108
rs7742829
rs73128290
All − MR Egger
All − IVW(MRE)
−1
0
1
2
3
MR effect size for
'Gut microbiota abundance (genus Catenibacterium id.2153)' on 'Ectonucleoside triphosphate diphosphohydrolase 1'

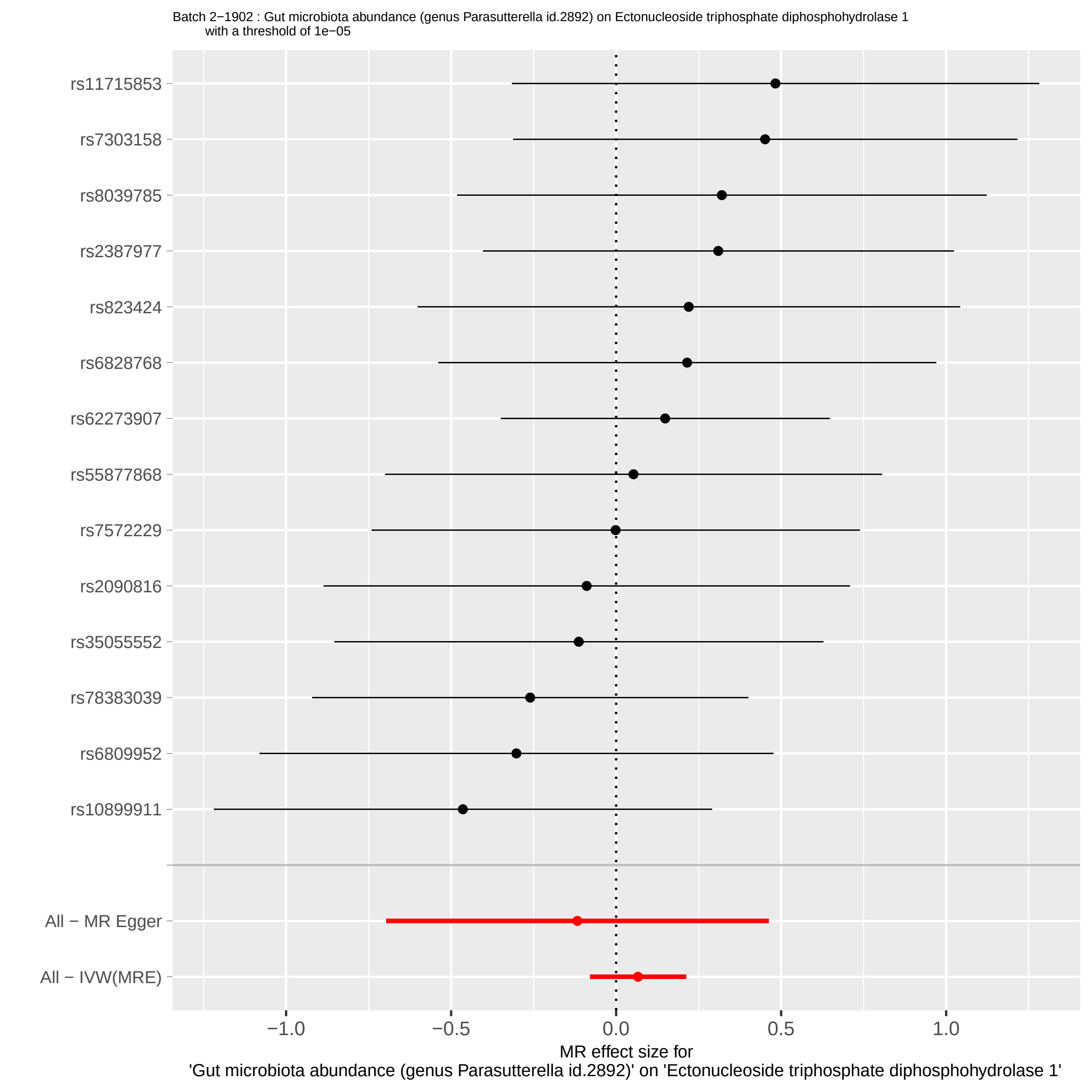

Batch 2−1902 : Gut microbiota abundance (genus Parasutterella id.2892) on Ectonucleoside triphosphate diphosphohydrolase 1
with a threshold of 1e−05
rs11715853
rs7303158
rs8039785
rs2387977
rs823424
rs6828768
rs62273907
rs55877868
rs7572229
rs2090816
rs35055552
rs78383039
rs6809952
rs10899911
All − MR Egger
All − IVW(MRE)
−1.0
−0.5
0.0
0.5
1.0
MR effect size for
'Gut microbiota abundance (genus Parasutterella id.2892)' on 'Ectonucleoside triphosphate diphosphohydrolase 1'

Batch 2−1903 : Gut microbiota abundance (genus Catenibacterium id.2153) on Ectonucleoside triphosphate diphosphohydrolase 3 with a threshold of 1e−05

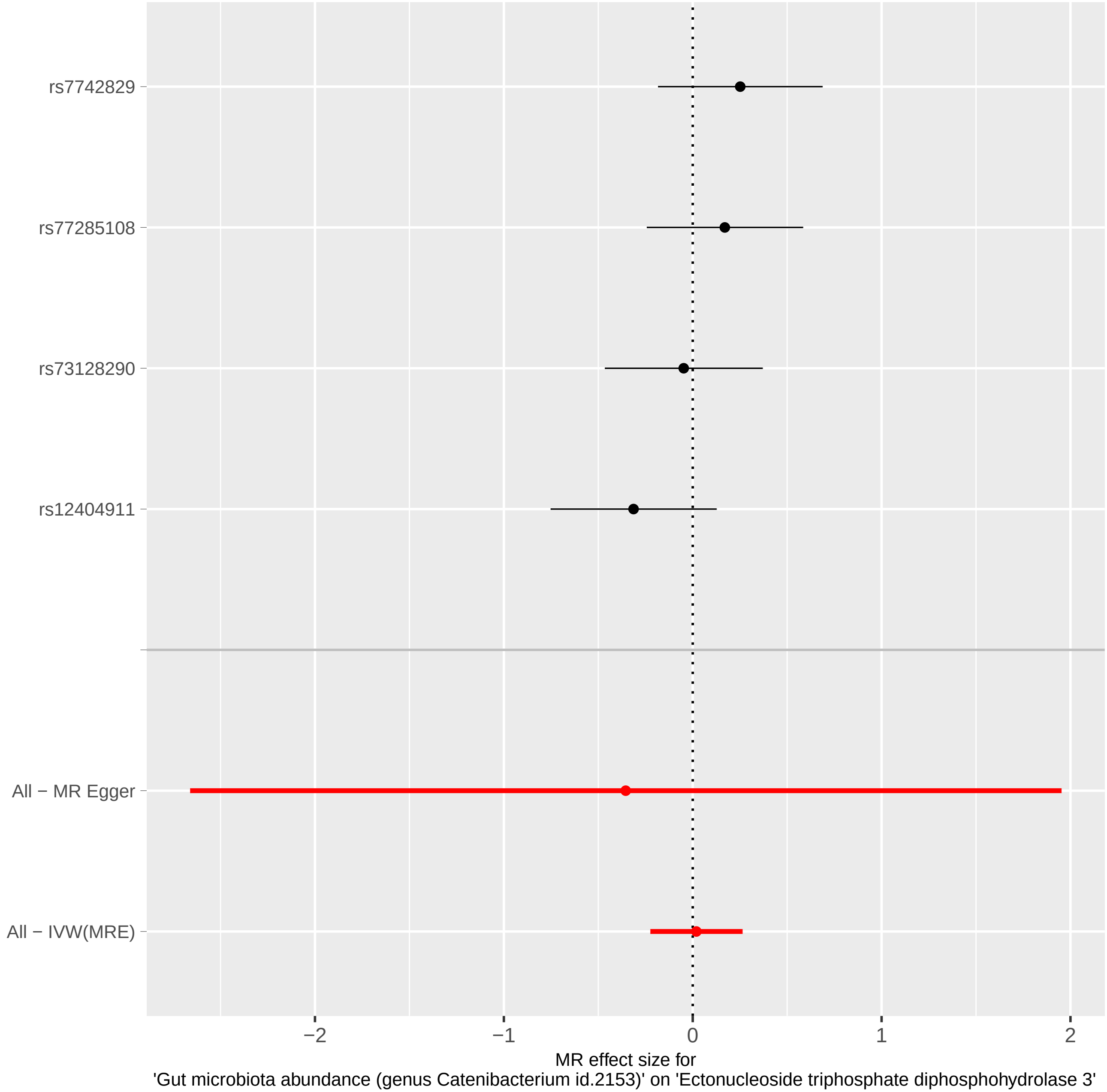

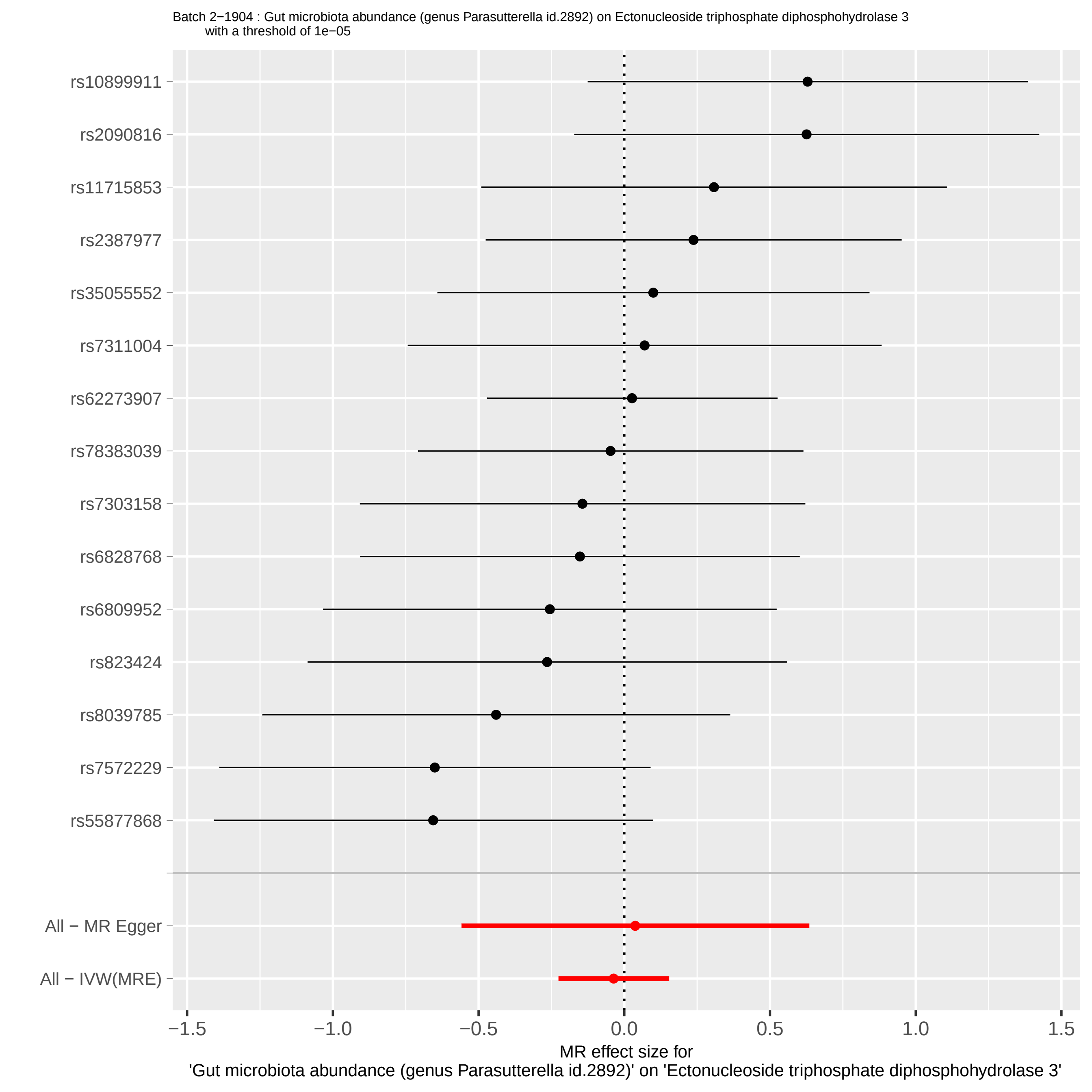

Batch 2−1904 : Gut microbiota abundance (genus Parasutterella id.2892) on Ectonucleoside triphosphate diphosphohydrolase 3
with a threshold of 1e−05
rs10899911
rs2090816
rs11715853
rs2387977
rs35055552
rs7311004
rs62273907
rs78383039
rs7303158
rs6828768
rs6809952
rs823424
rs8039785
rs7572229
rs55877868
All − MR Egger
All − IVW(MRE)
−1.5
−1.0
−0.5
0.0
0.5
1.0
1.5
MR effect size for
'Gut microbiota abundance (genus Parasutterella id.2892)' on 'Ectonucleoside triphosphate diphosphohydrolase 3'

Batch 2−1905 : Gut microbiota abundance (genus Catenibacterium id.2153) on Ectonucleoside triphosphate diphosphohydrolase 5
with a threshold of 1e−05

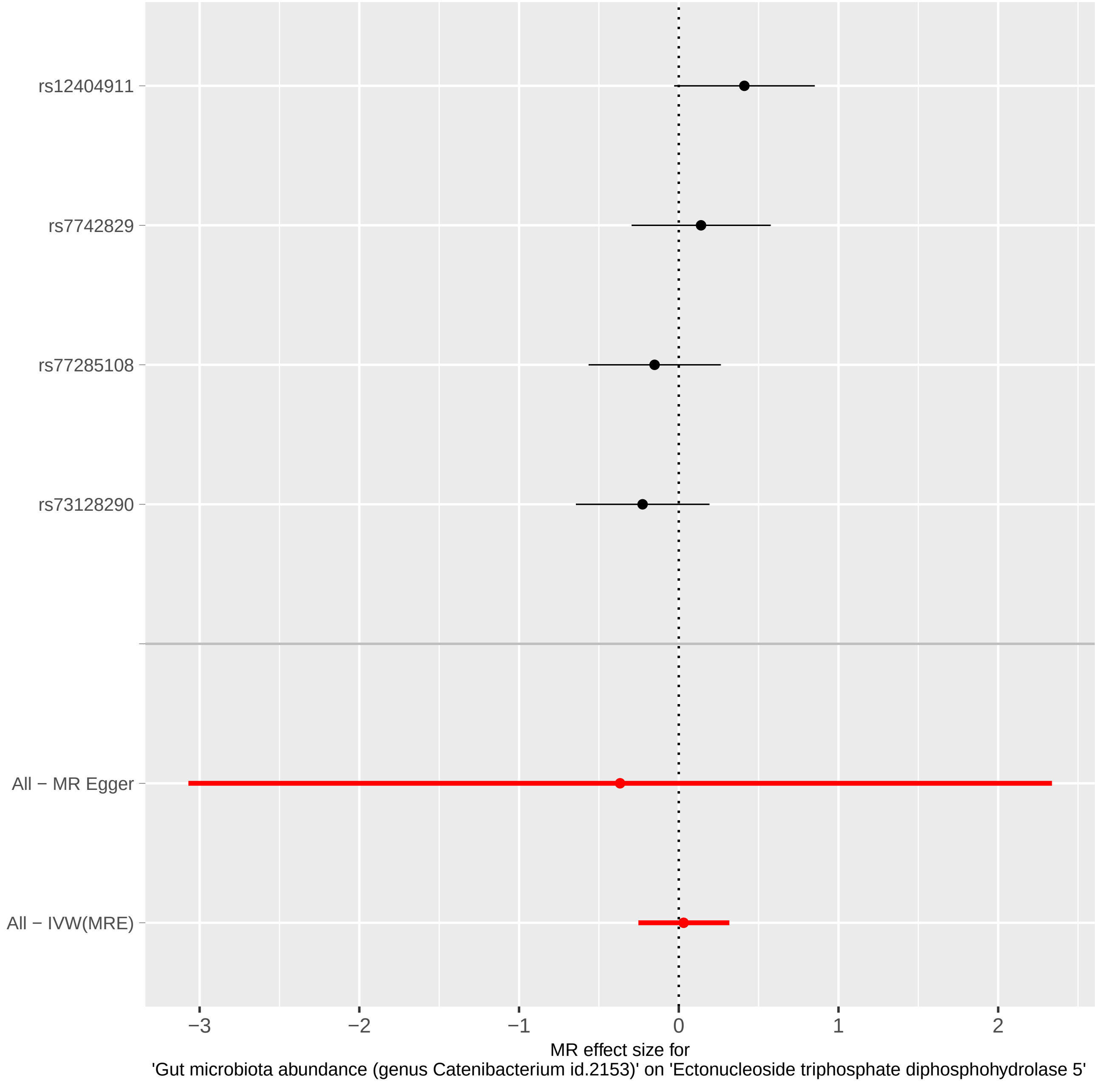

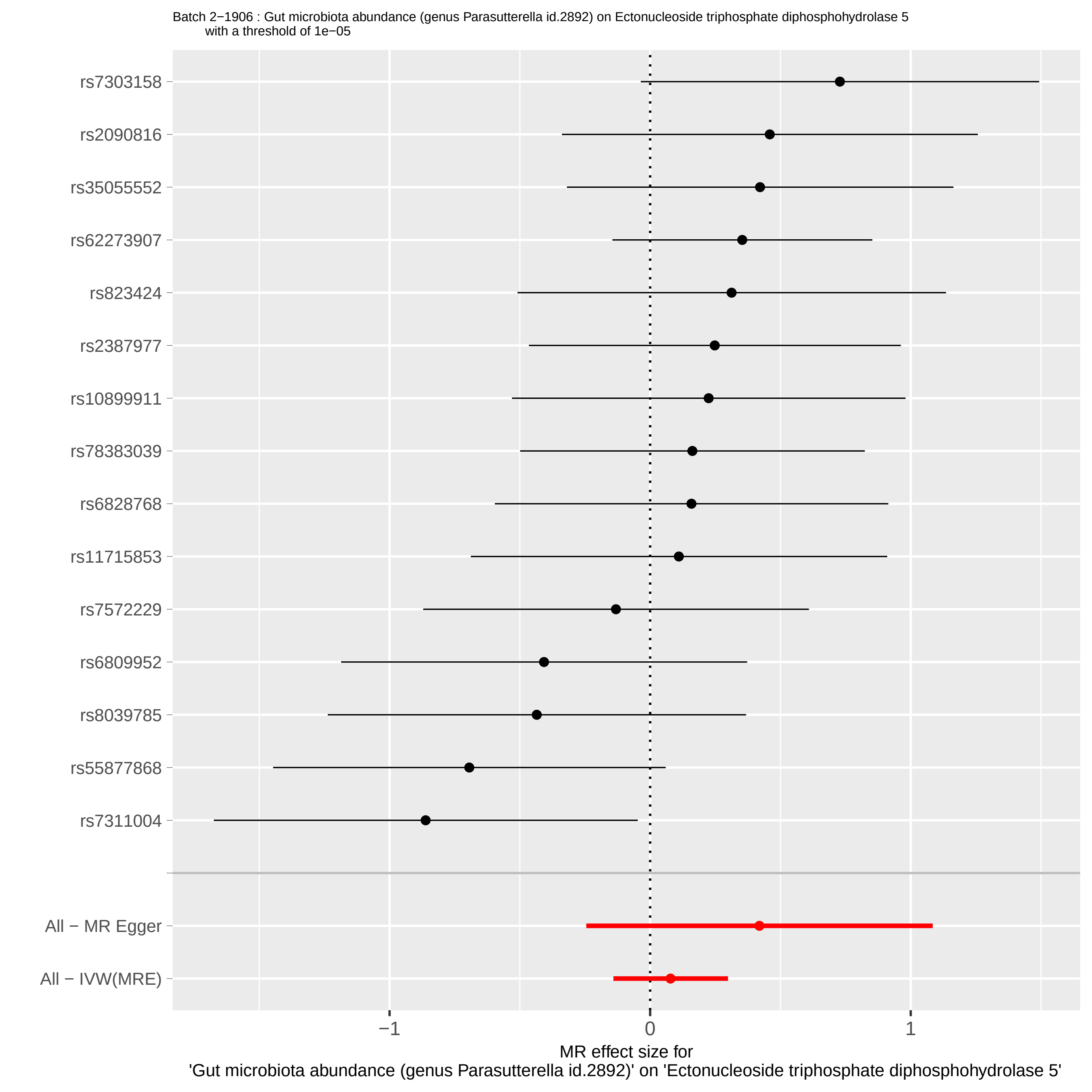

Batch 2−1906 : Gut microbiota abundance (genus Parasutterella id.2892) on Ectonucleoside triphosphate diphosphohydrolase 5
with a threshold of 1e−05
rs7303158
rs2090816
rs35055552
rs62273907
rs823424
rs2387977
rs10899911
rs78383039
rs6828768
rs11715853
rs7572229
rs6809952
rs8039785
rs55877868
rs7311004
All − MR Egger
All − IVW(MRE)
−1
0
1
MR effect size for
'Gut microbiota abundance (genus Parasutterella id.2892)' on 'Ectonucleoside triphosphate diphosphohydrolase 5'

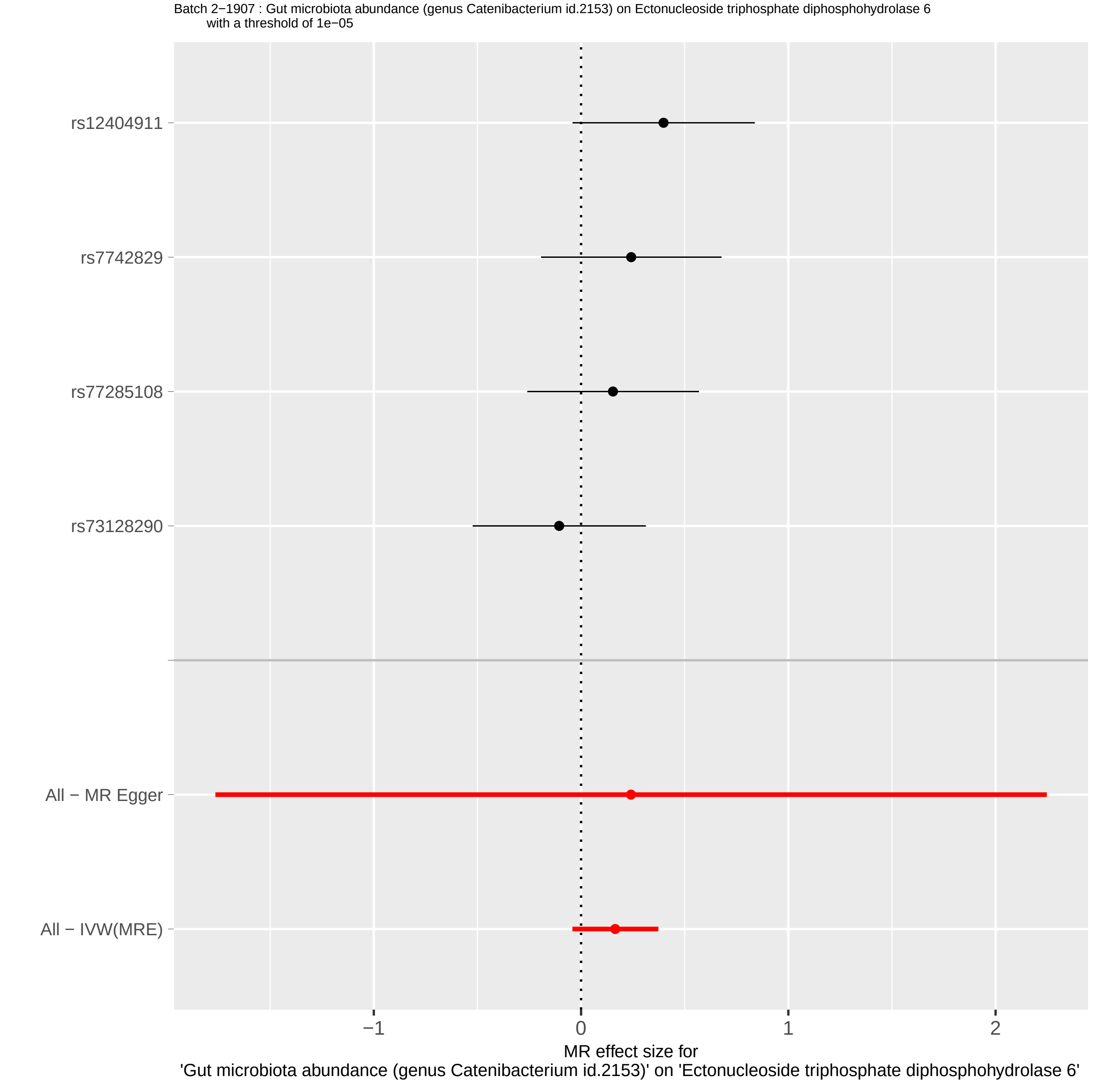

Batch 2−1907 : Gut microbiota abundance (genus Catenibacterium id.2153) on Ectonucleoside triphosphate diphosphohydrolase 6
with a threshold of 1e−05
rs12404911
rs7742829
rs77285108
rs73128290
All − MR Egger
All − IVW(MRE)
−1
0
1
2
MR effect size for
'Gut microbiota abundance (genus Catenibacterium id.2153)' on 'Ectonucleoside triphosphate diphosphohydrolase 6'

Batch 2−1908 : Gut microbiota abundance (genus Parasutterella id.2892) on Ectonucleoside triphosphate diphosphohydrolase 6 with a threshold of 1e−05

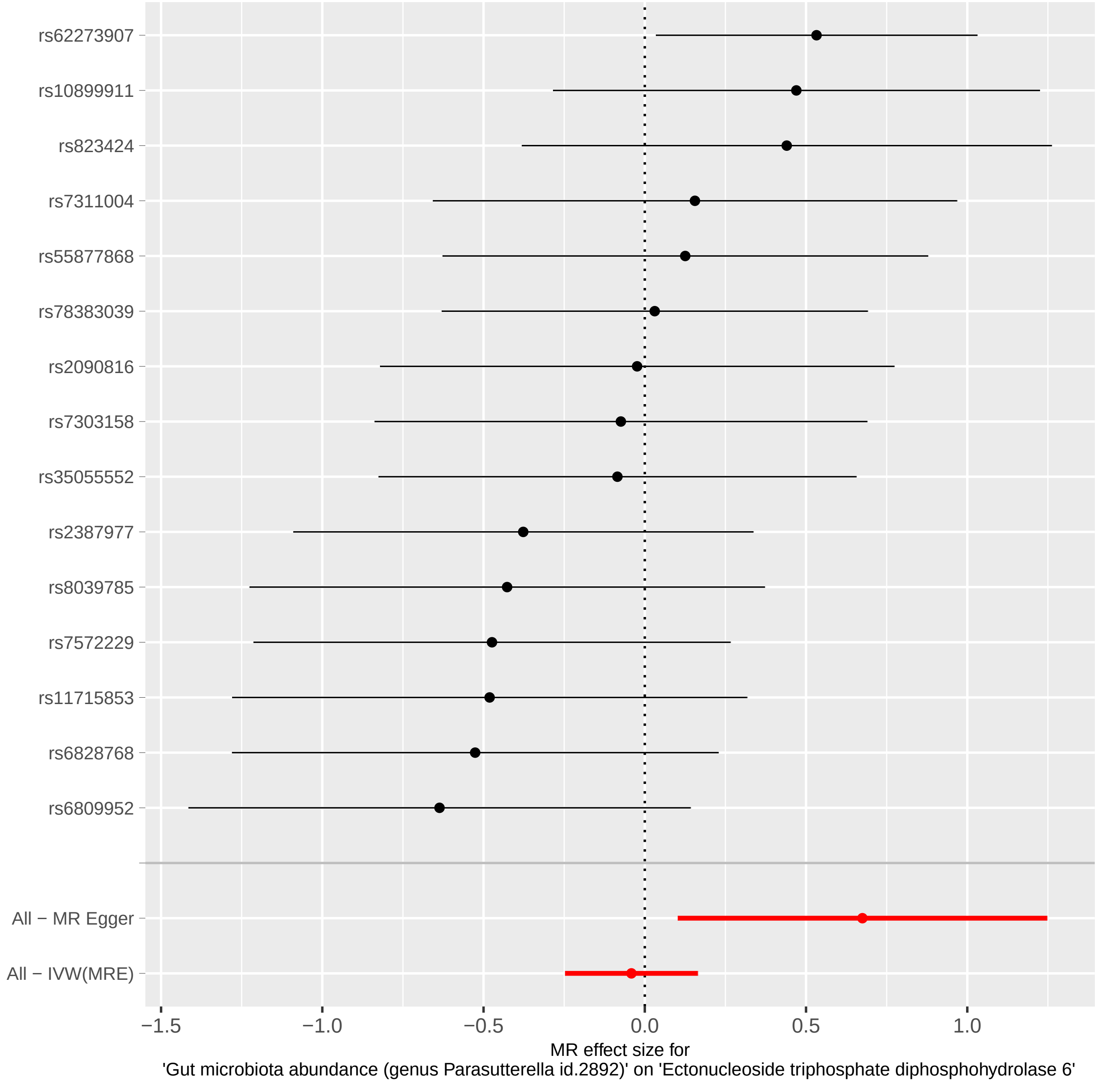

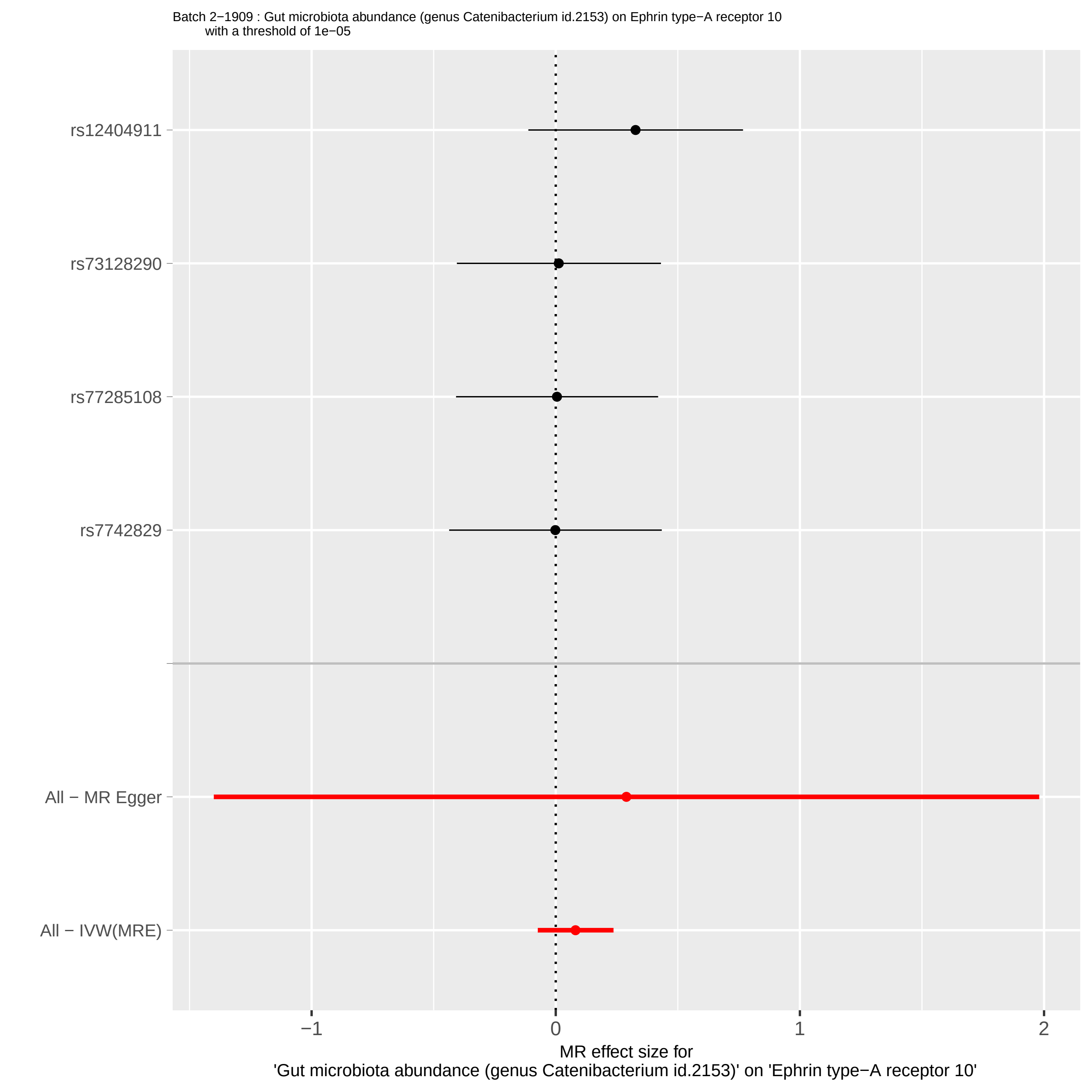

Batch 2−1909 : Gut microbiota abundance (genus Catenibacterium id.2153) on Ephrin type−A receptor 10
with a threshold of 1e−05
rs12404911
rs73128290
rs77285108
rs7742829
All − MR Egger
All − IVW(MRE)
−1
0
1
2
MR effect size for
'Gut microbiota abundance (genus Catenibacterium id.2153)' on 'Ephrin type−A receptor 10'

Batch 2−1910 : Gut microbiota abundance (genus Parasutterella id.2892) on Ephrin type−A receptor 10
with a threshold of 1e−05

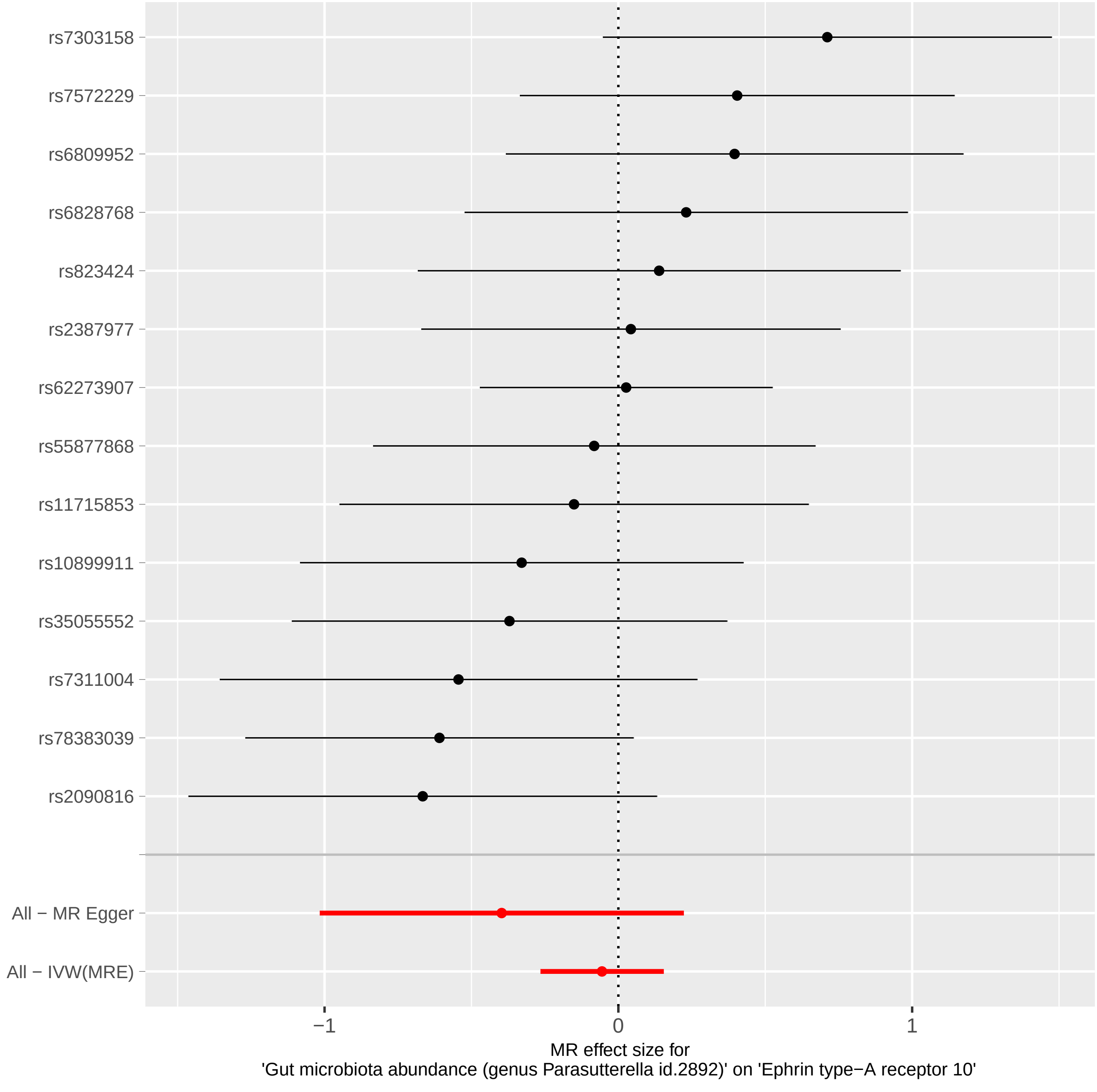

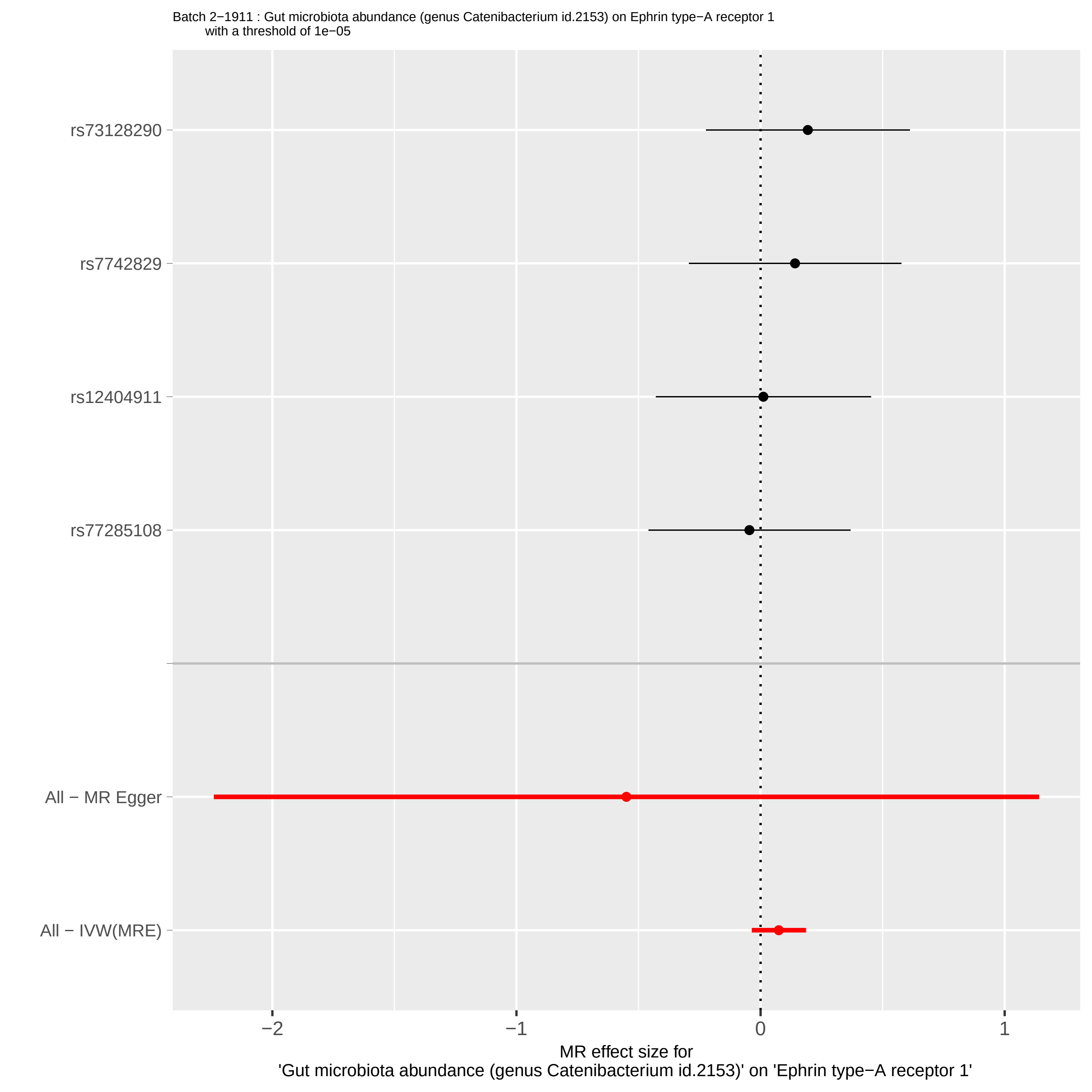
Batch 2−1911 : Gut microbiota abundance (genus Catenibacterium id.2153) on Ephrin type−A receptor 1
with a threshold of 1e−05
rs73128290
rs7742829
rs12404911
rs77285108
All − MR Egger
All − IVW(MRE)
−2
−1
0
1
MR effect size for
'Gut microbiota abundance (genus Catenibacterium id.2153)' on 'Ephrin type−A receptor 1'

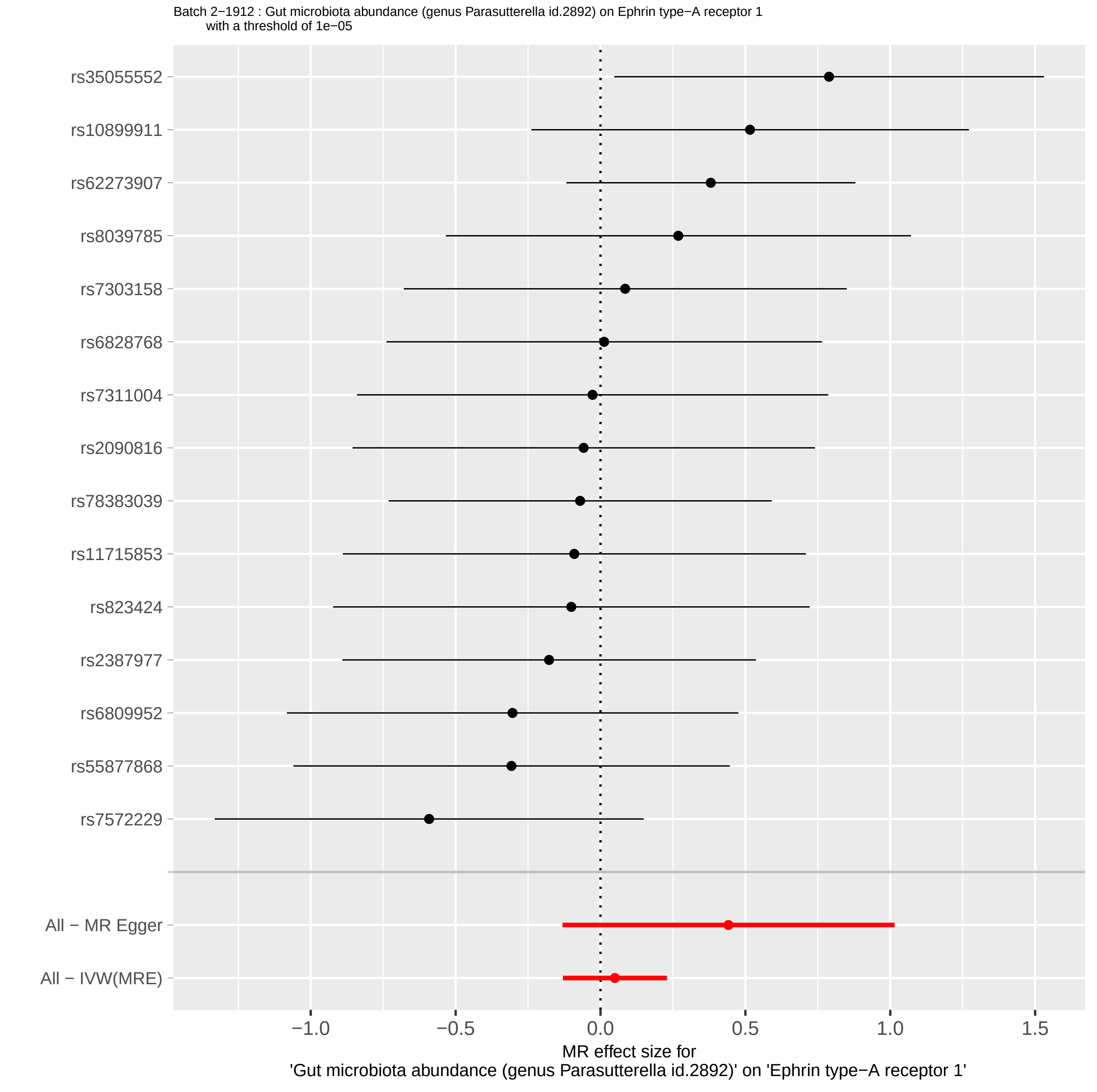
Batch 2−1912 : Gut microbiota abundance (genus Parasutterella id.2892) on Ephrin type−A receptor 1
with a threshold of 1e−05
rs35055552
rs10899911
rs62273907
rs8039785
rs7303158
rs6828768
rs7311004
rs2090816
rs78383039
rs11715853
rs823424
rs2387977
rs6809952
rs55877868
rs7572229
All − MR Egger
All − IVW(MRE)
−1.0
−0.5
0.0
0.5
1.0
1.5
MR effect size for
'Gut microbiota abundance (genus Parasutterella id.2892)' on 'Ephrin type−A receptor 1'

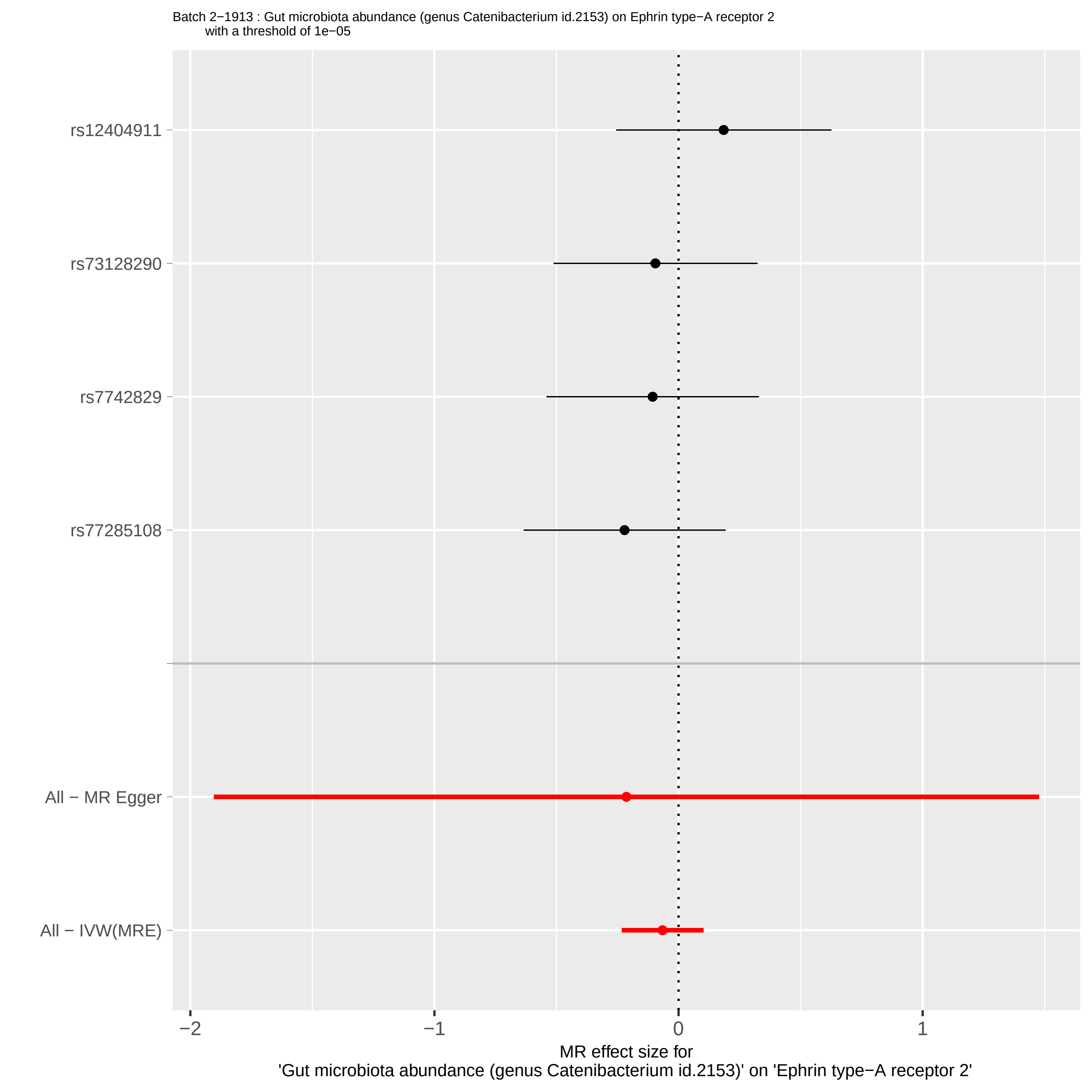

Batch 2−1913 : Gut microbiota abundance (genus Catenibacterium id.2153) on Ephrin type−A receptor 2
with a threshold of 1e−05
rs12404911
rs73128290
rs7742829
rs77285108
All − MR Egger
All − IVW(MRE)
−2
−1
0
1
MR effect size for
'Gut microbiota abundance (genus Catenibacterium id.2153)' on 'Ephrin type−A receptor 2'

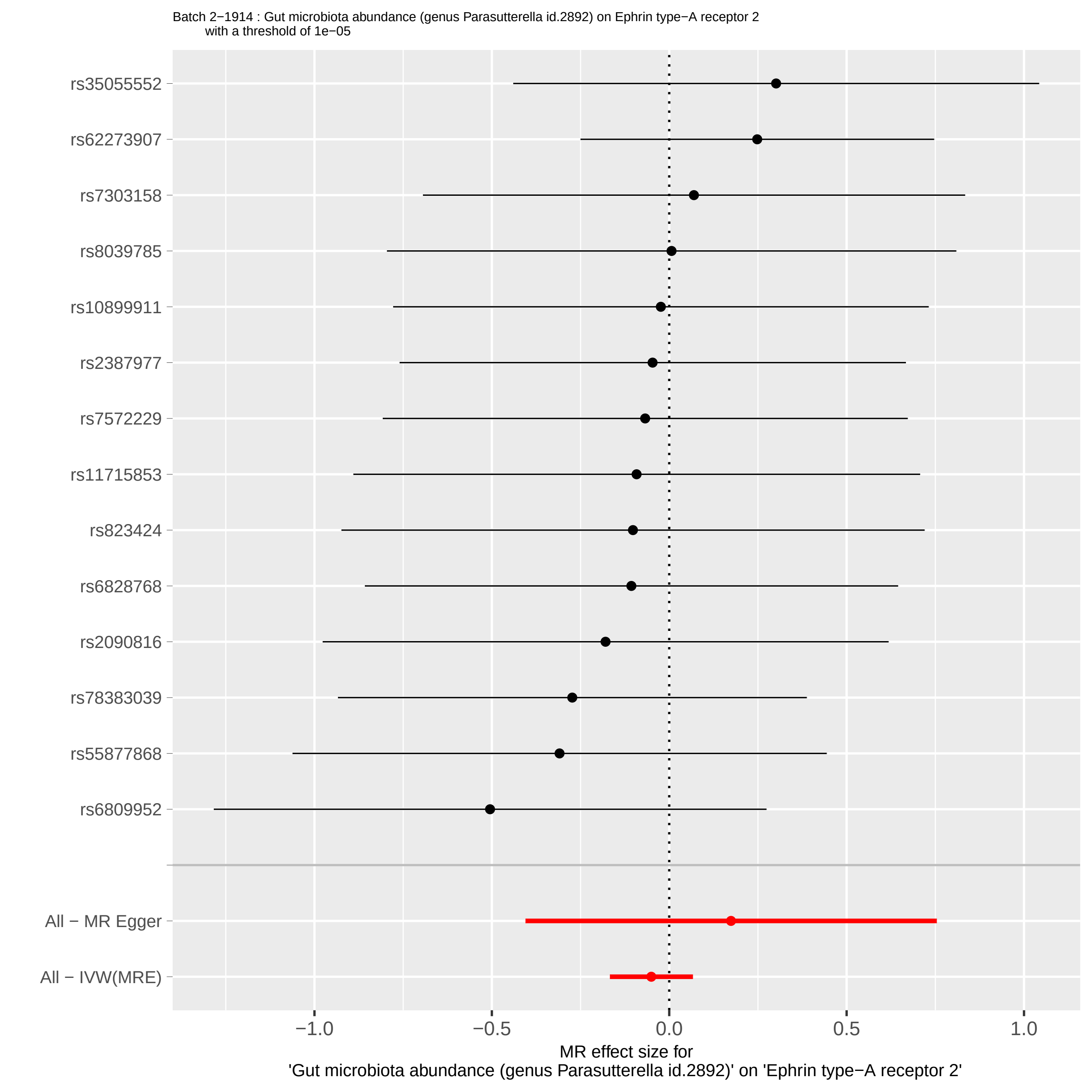

Batch 2−1914 : Gut microbiota abundance (genus Parasutterella id.2892) on Ephrin type−A receptor 2
with a threshold of 1e−05
rs35055552
rs62273907
rs7303158
rs8039785
rs10899911
rs2387977
rs7572229
rs11715853
rs823424
rs6828768
rs2090816
rs78383039
rs55877868
rs6809952
All − MR Egger
All − IVW(MRE)
−1.0
−0.5
0.0
0.5
1.0
MR effect size for
'Gut microbiota abundance (genus Parasutterella id.2892)' on 'Ephrin type−A receptor 2'

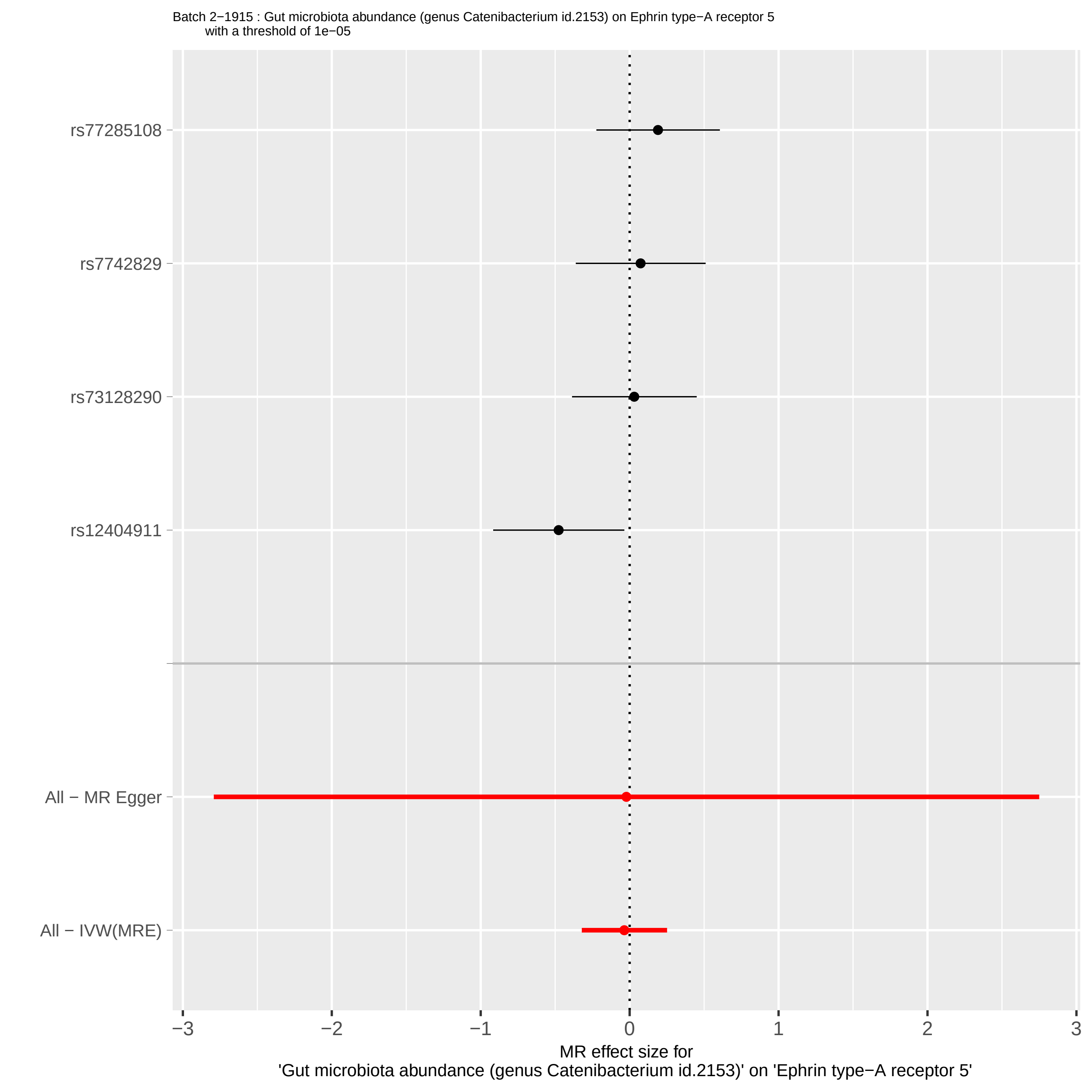

Batch 2−1915 : Gut microbiota abundance (genus Catenibacterium id.2153) on Ephrin type−A receptor 5
with a threshold of 1e−05
rs77285108
rs7742829
rs73128290
rs12404911
All − MR Egger
All − IVW(MRE)
−3
−2
−1
0
1
2
3
MR effect size for
'Gut microbiota abundance (genus Catenibacterium id.2153)' on 'Ephrin type−A receptor 5'

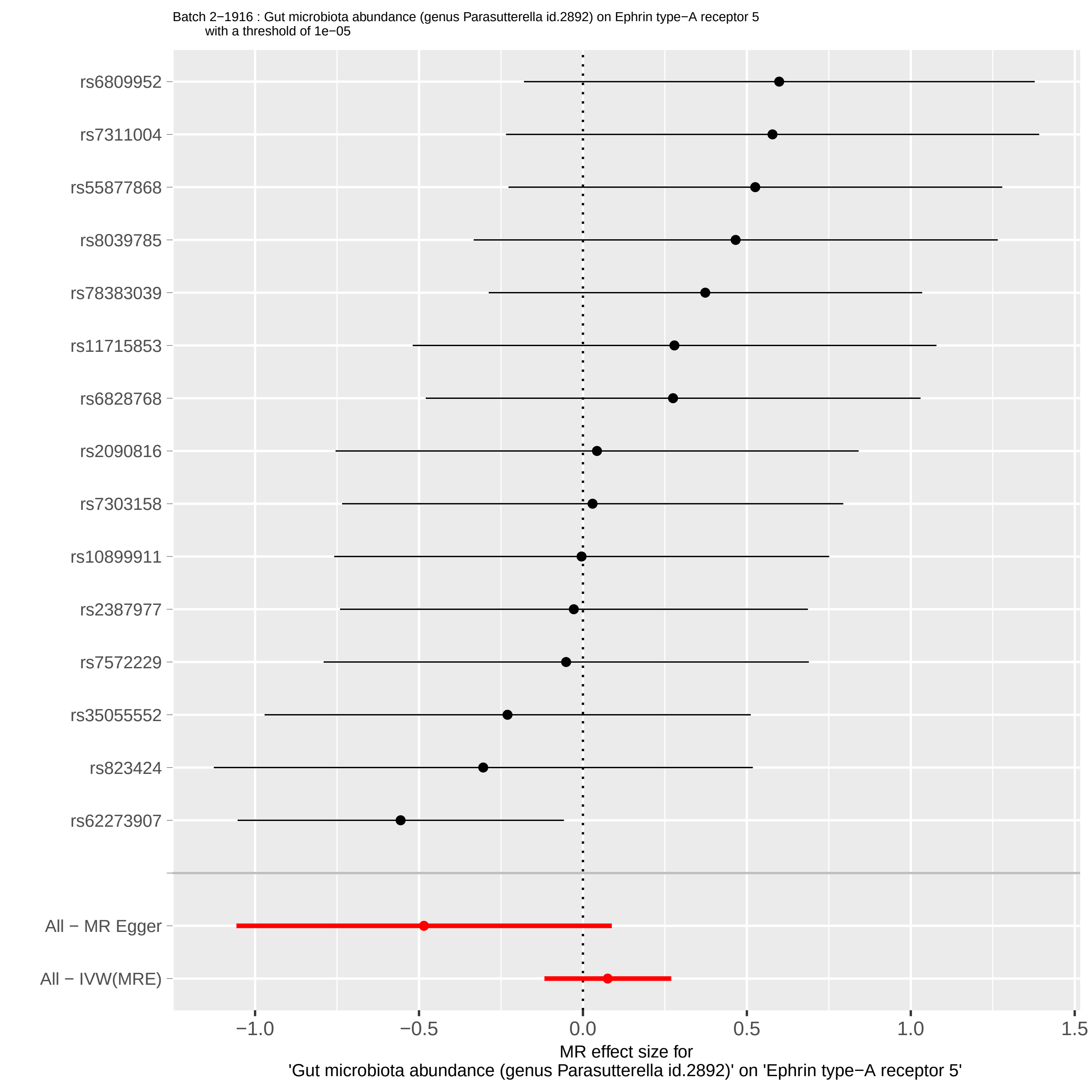
Batch 2−1916 : Gut microbiota abundance (genus Parasutterella id.2892) on Ephrin type−A receptor 5
with a threshold of 1e−05
rs6809952
rs7311004
rs55877868
rs8039785
rs78383039
rs11715853
rs6828768
rs2090816
rs7303158
rs10899911
rs2387977
rs7572229
rs35055552
rs823424
rs62273907
All − MR Egger
All − IVW(MRE)
−1.0
−0.5
0.0
0.5
1.0
1.5
MR effect size for
'Gut microbiota abundance (genus Parasutterella id.2892)' on 'Ephrin type−A receptor 5'

Batch 2−1917 : Gut microbiota abundance (genus Catenibacterium id.2153) on Ephrin type−A receptor 5
with a threshold of 1e−05

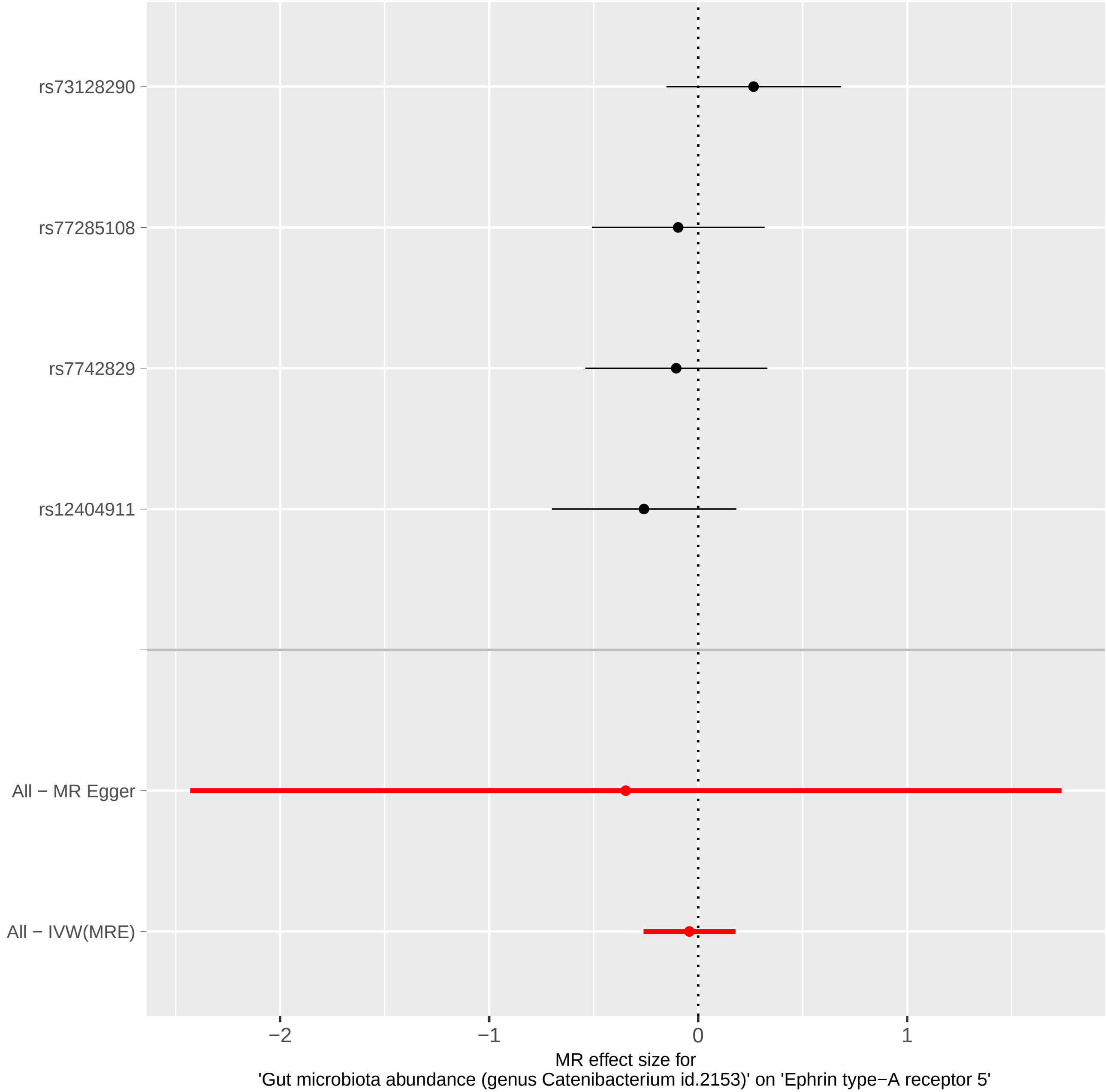

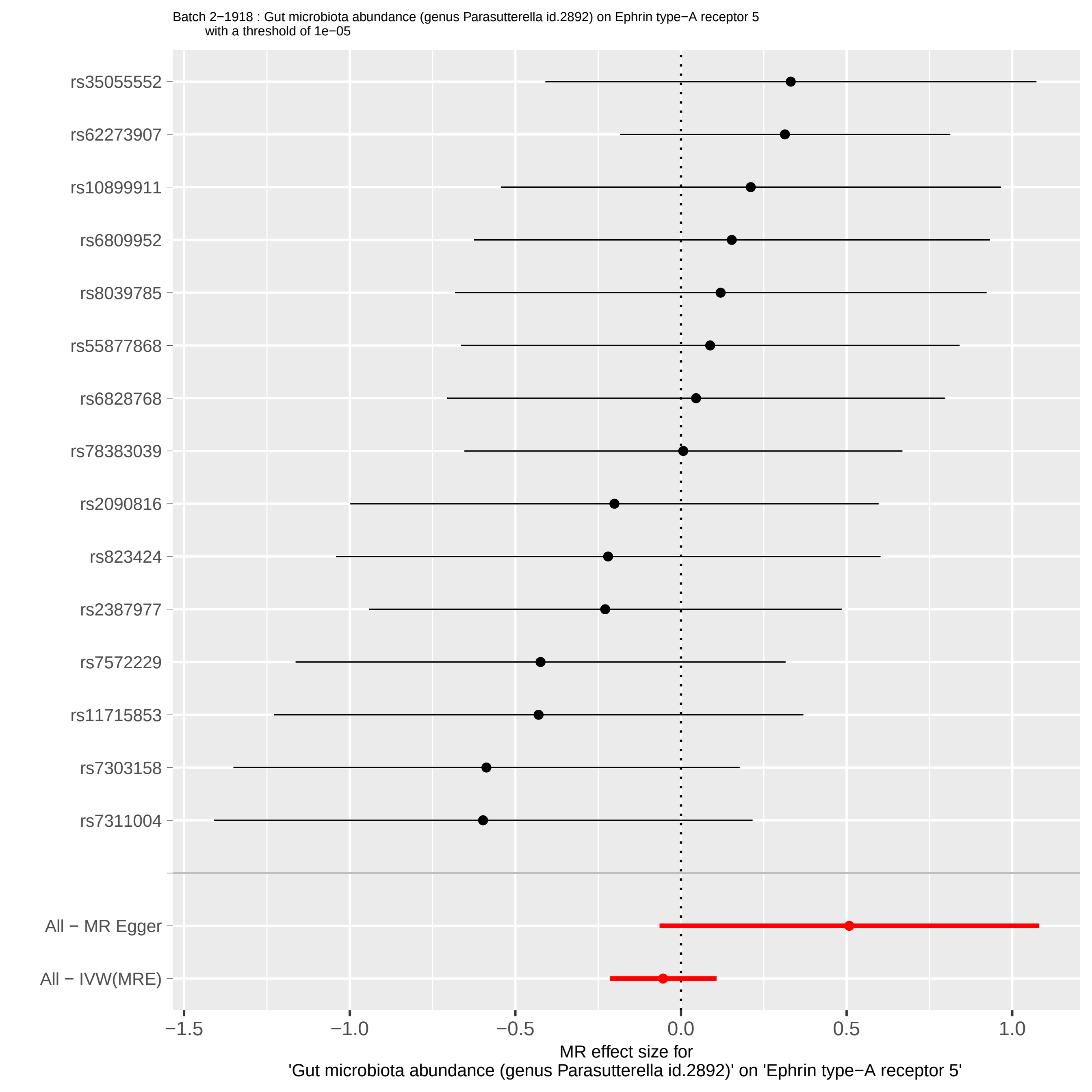

Batch 2−1918 : Gut microbiota abundance (genus Parasutterella id.2892) on Ephrin type−A receptor 5
with a threshold of 1e−05
rs35055552
rs62273907
rs10899911
rs6809952
rs8039785
rs55877868
rs6828768
rs78383039
rs2090816
rs823424
rs2387977
rs7572229
rs11715853
rs7303158
rs7311004
All − MR Egger
All − IVW(MRE)
−1.5
−1.0
−0.5
0.0
0.5
1.0
MR effect size for
'Gut microbiota abundance (genus Parasutterella id.2892)' on 'Ephrin type−A receptor 5'

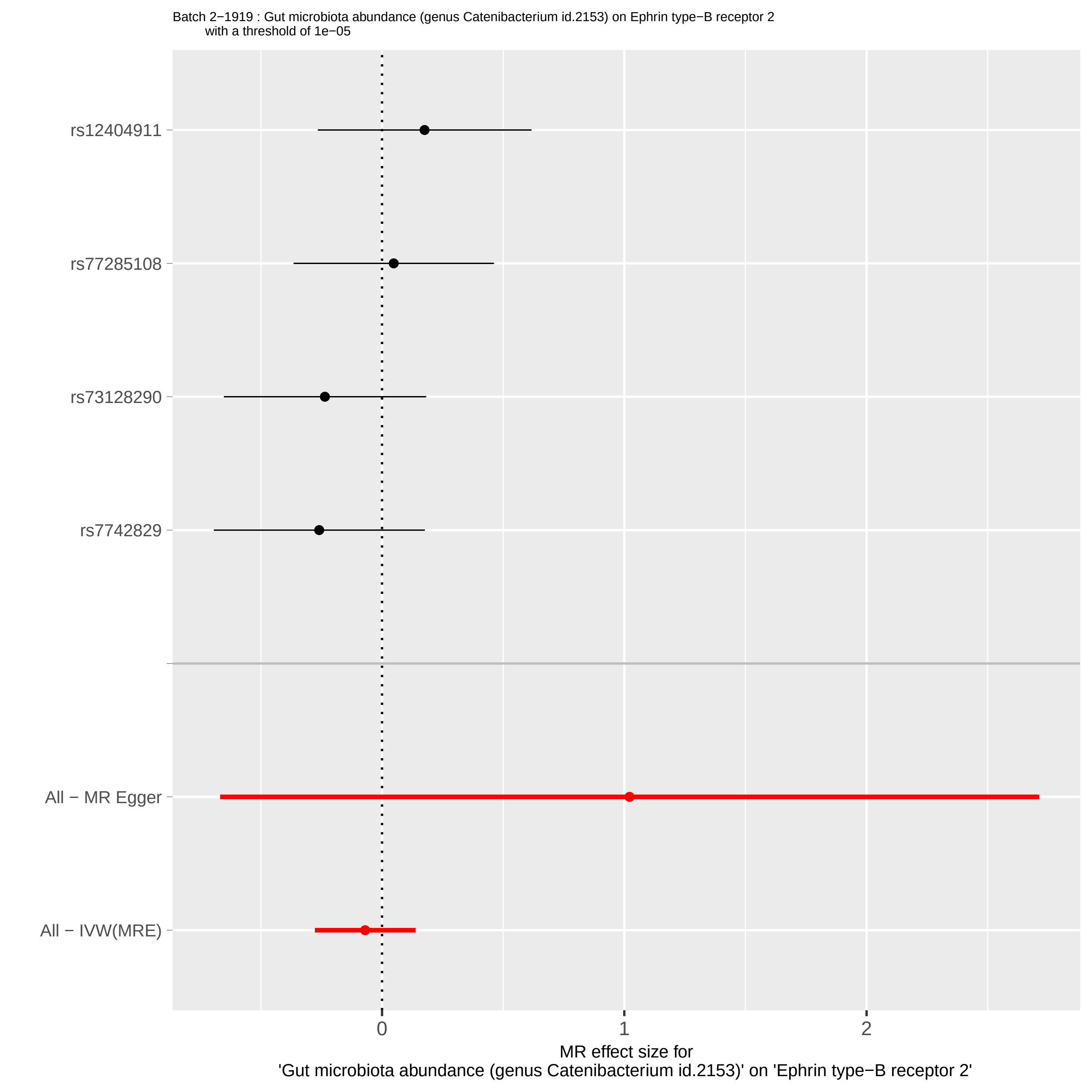

Batch 2−1919 : Gut microbiota abundance (genus Catenibacterium id.2153) on Ephrin type−B receptor 2
with a threshold of 1e−05
rs12404911
rs77285108
rs73128290
rs7742829
All − MR Egger
All − IVW(MRE)
0
1
2
MR effect size for
'Gut microbiota abundance (genus Catenibacterium id.2153)' on 'Ephrin type−B receptor 2'

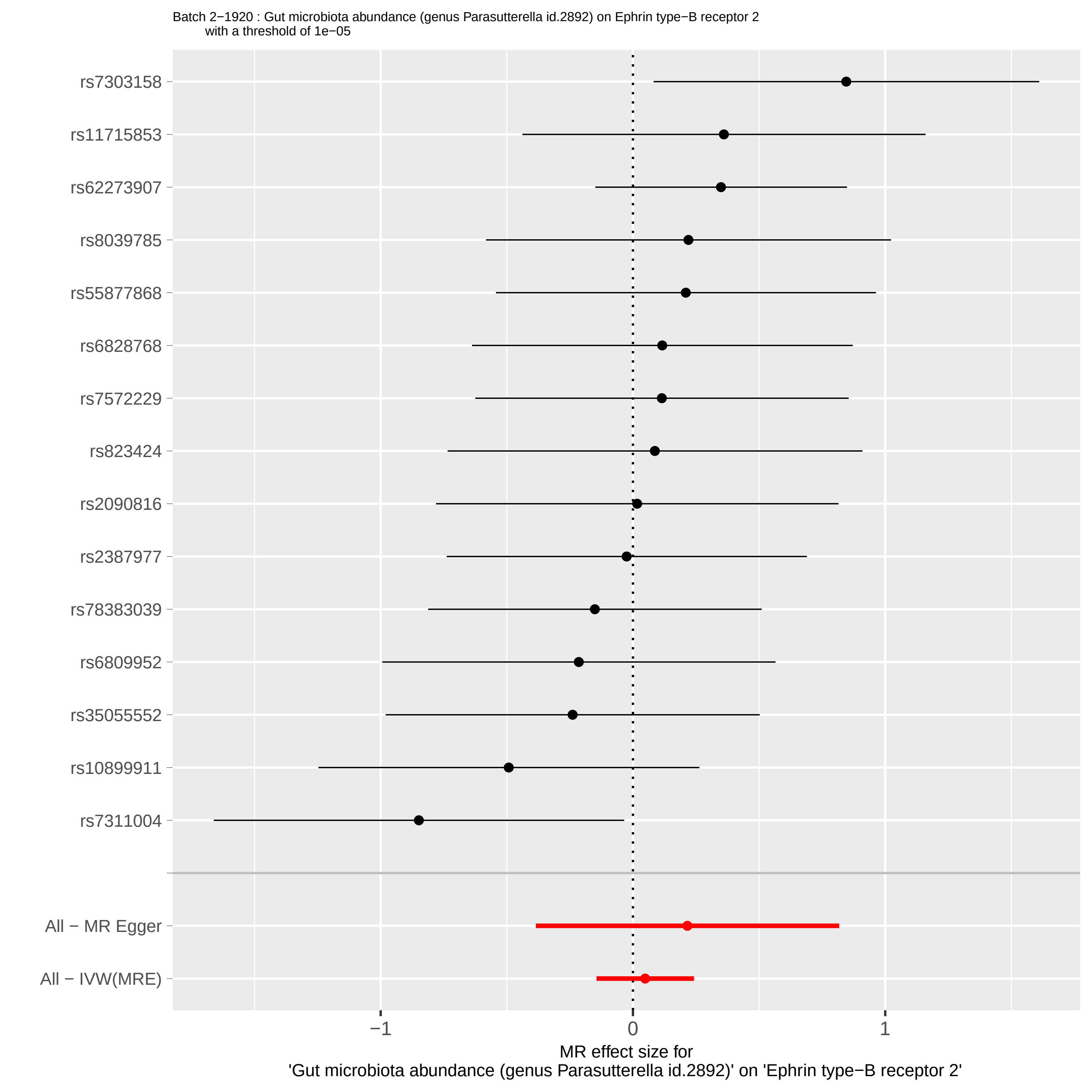

Batch 2−1920 : Gut microbiota abundance (genus Parasutterella id.2892) on Ephrin type−B receptor 2
with a threshold of 1e−05
rs7303158
rs11715853
rs62273907
rs8039785
rs55877868
rs6828768
rs7572229
rs823424
rs2090816
rs2387977
rs78383039
rs6809952
rs35055552
rs10899911
rs7311004
All − MR Egger
All − IVW(MRE)
−1
0
1
MR effect size for
'Gut microbiota abundance (genus Parasutterella id.2892)' on 'Ephrin type−B receptor 2'

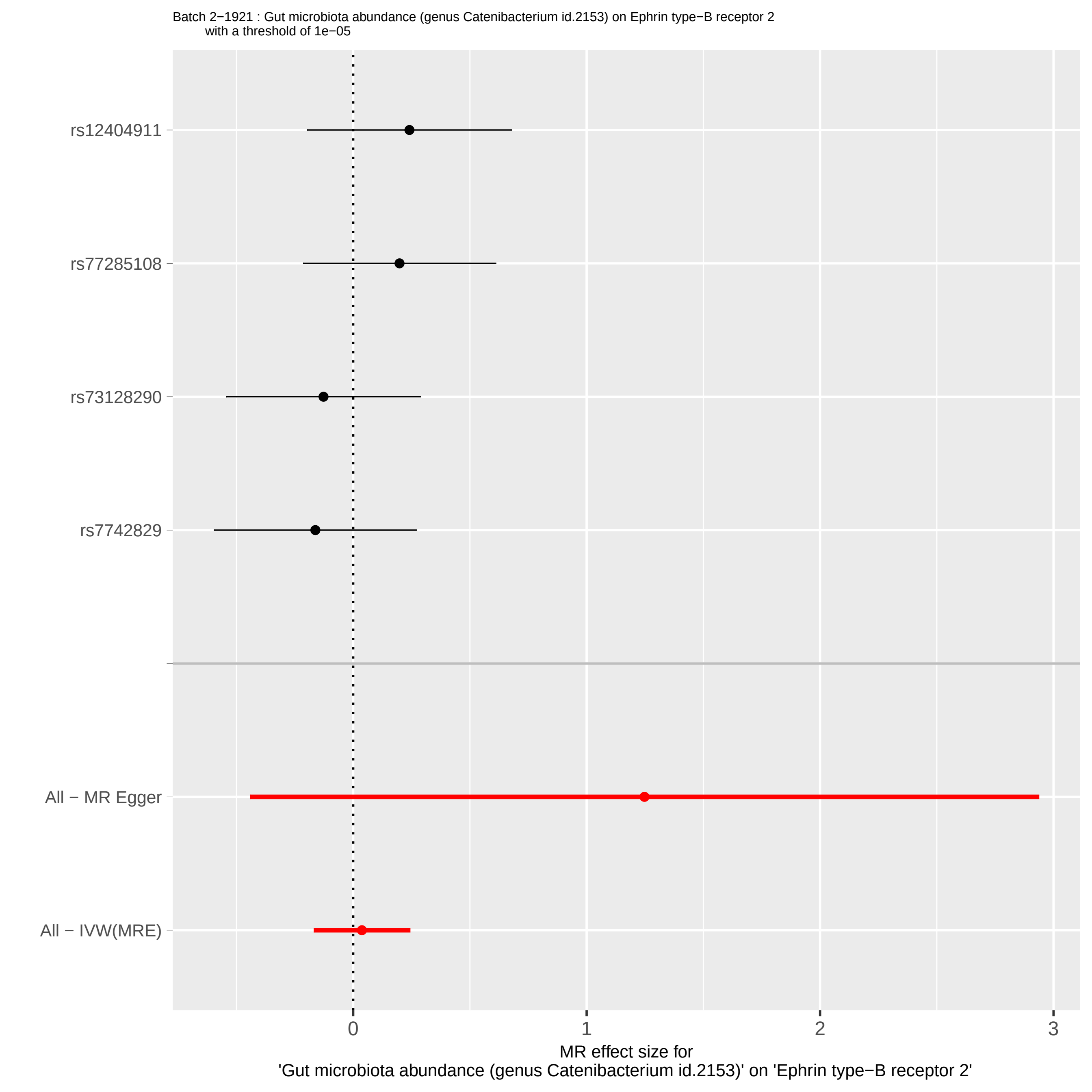
Batch 2−1921 : Gut microbiota abundance (genus Catenibacterium id.2153) on Ephrin type−B receptor 2
with a threshold of 1e−05
rs12404911
rs77285108
rs73128290
rs7742829
All − MR Egger
All − IVW(MRE)
0
1
2
3
MR effect size for
'Gut microbiota abundance (genus Catenibacterium id.2153)' on 'Ephrin type−B receptor 2'

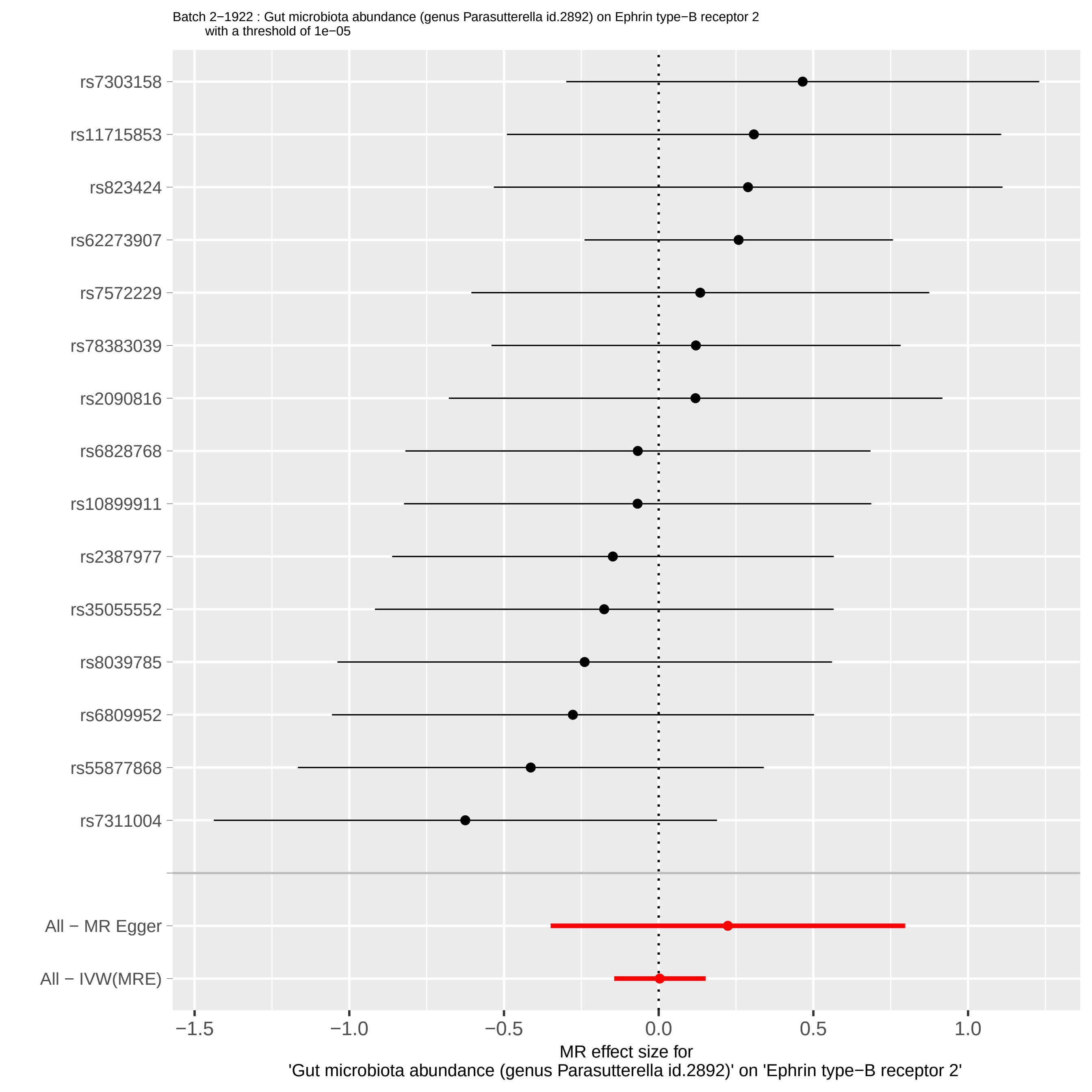

Batch 2−1922 : Gut microbiota abundance (genus Parasutterella id.2892) on Ephrin type−B receptor 2
with a threshold of 1e−05
rs7303158
rs11715853
rs823424
rs62273907
rs7572229
rs78383039
rs2090816
rs6828768
rs10899911
rs2387977
rs35055552
rs8039785
rs6809952
rs55877868
rs7311004
All − MR Egger
All − IVW(MRE)
−1.5
−1.0
−0.5
0.0
0.5
1.0
MR effect size for
'Gut microbiota abundance (genus Parasutterella id.2892)' on 'Ephrin type−B receptor 2'

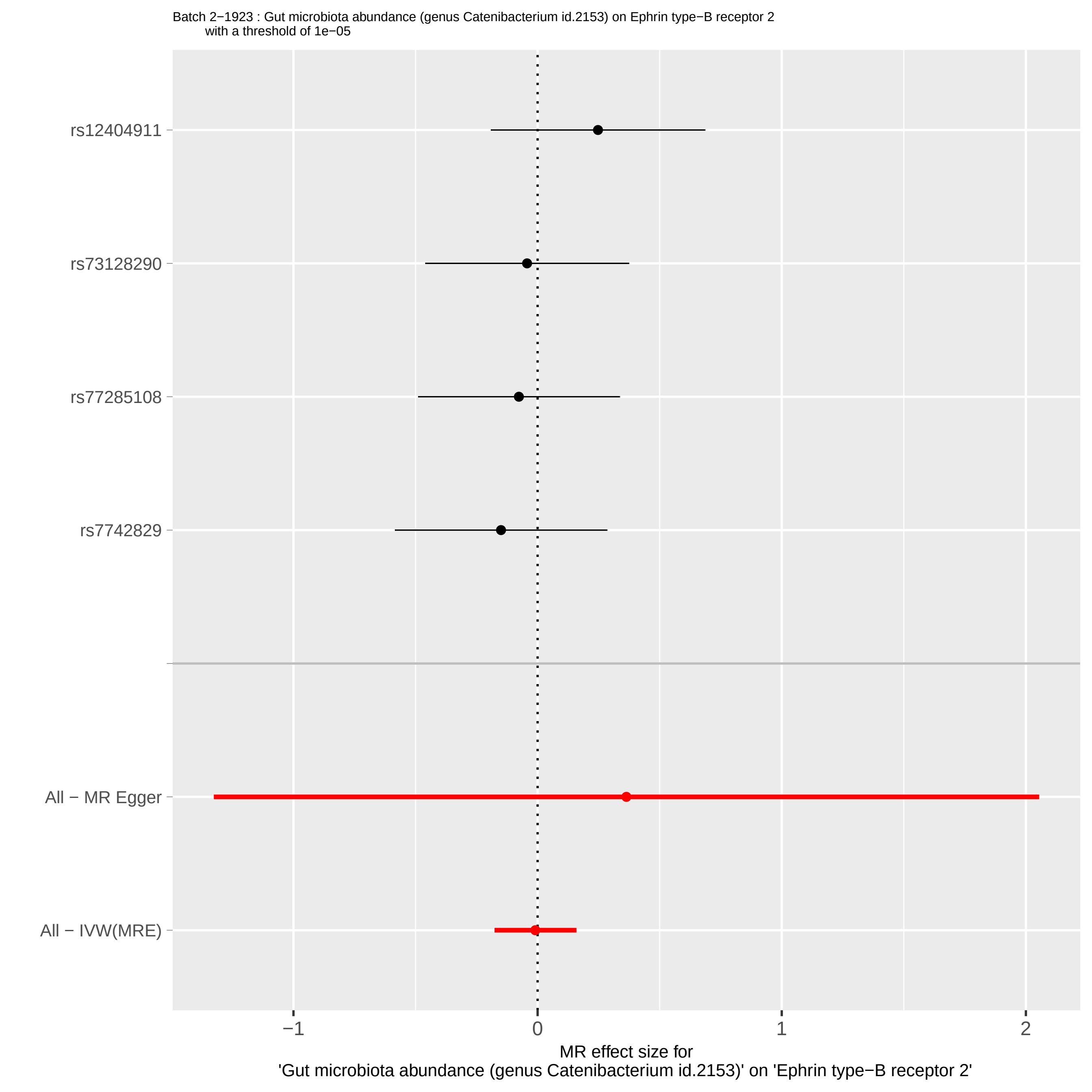
Batch 2−1923 : Gut microbiota abundance (genus Catenibacterium id.2153) on Ephrin type−B receptor 2
with a threshold of 1e−05
rs12404911
rs73128290
rs77285108
rs7742829
All − MR Egger
All − IVW(MRE)
−1
0
1
2
MR effect size for
'Gut microbiota abundance (genus Catenibacterium id.2153)' on 'Ephrin type−B receptor 2'

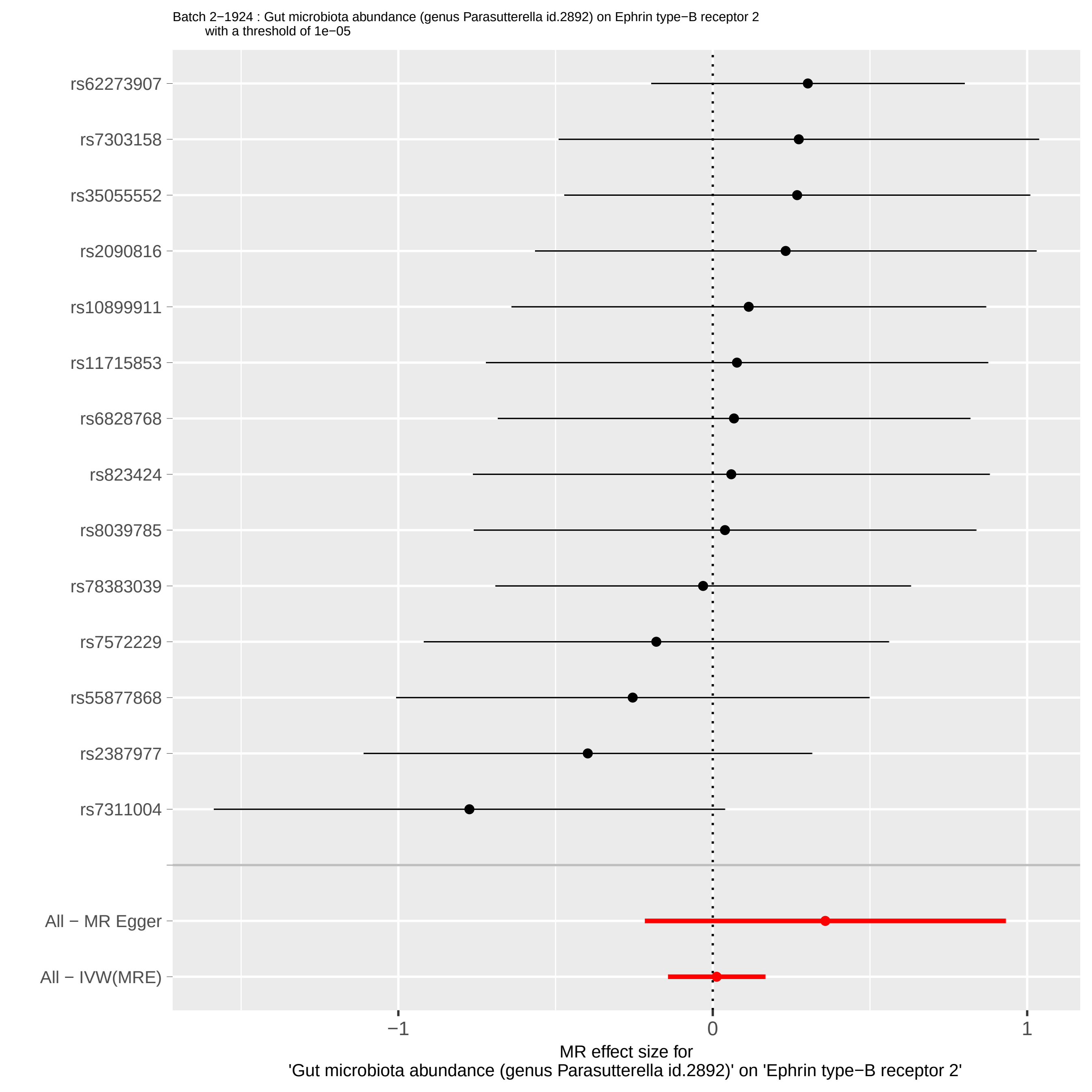
Batch 2−1924 : Gut microbiota abundance (genus Parasutterella id.2892) on Ephrin type−B receptor 2
with a threshold of 1e−05
rs62273907
rs7303158
rs35055552
rs2090816
rs10899911
rs11715853
rs6828768
rs823424
rs8039785
rs78383039
rs7572229
rs55877868
rs2387977
rs7311004
All − MR Egger
All − IVW(MRE)
−1
0
1
MR effect size for
'Gut microbiota abundance (genus Parasutterella id.2892)' on 'Ephrin type−B receptor 2'

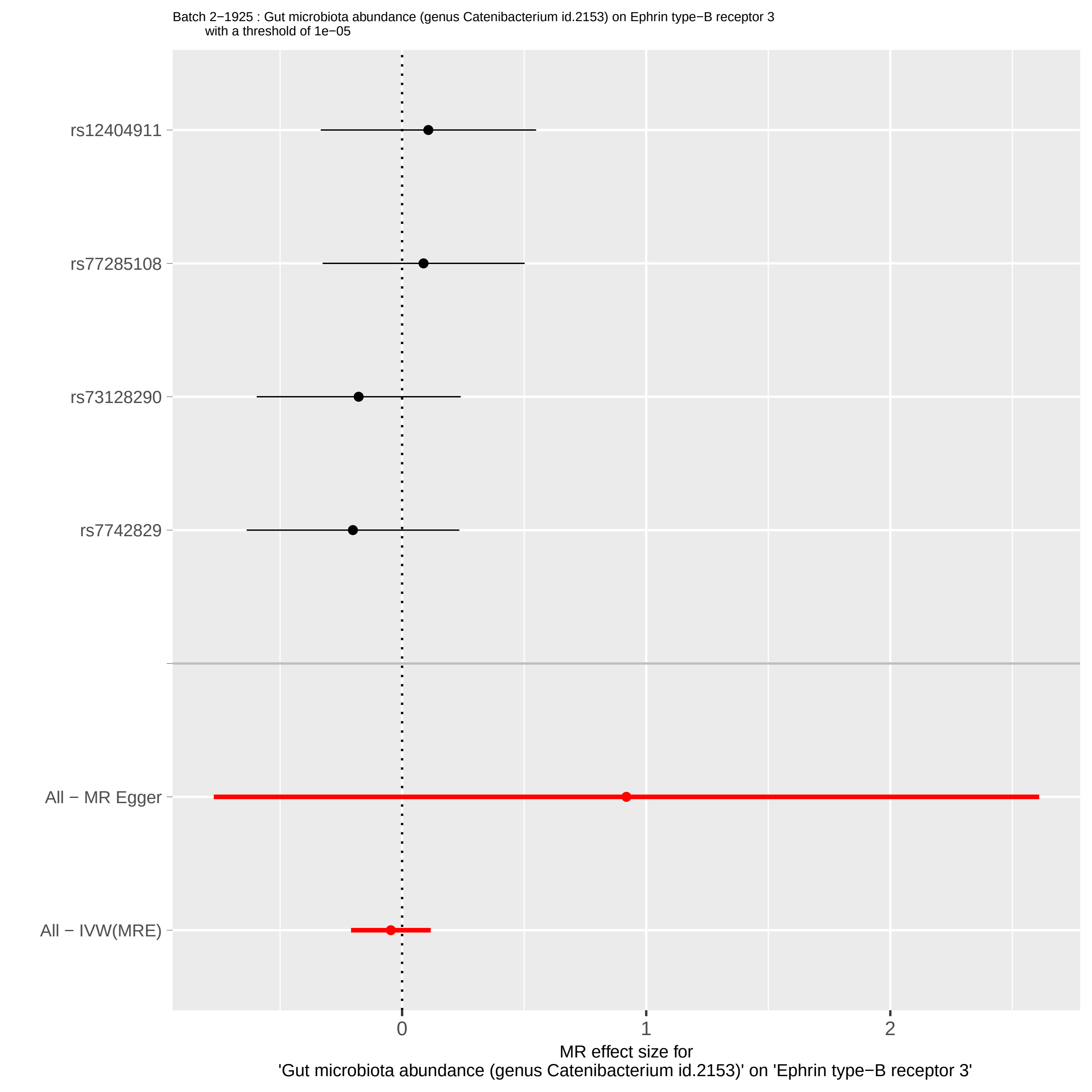
Batch 2−1925 : Gut microbiota abundance (genus Catenibacterium id.2153) on Ephrin type−B receptor 3
with a threshold of 1e−05
rs12404911
rs77285108
rs73128290
rs7742829
All − MR Egger
All − IVW(MRE)
0
1
2
MR effect size for
'Gut microbiota abundance (genus Catenibacterium id.2153)' on 'Ephrin type−B receptor 3'

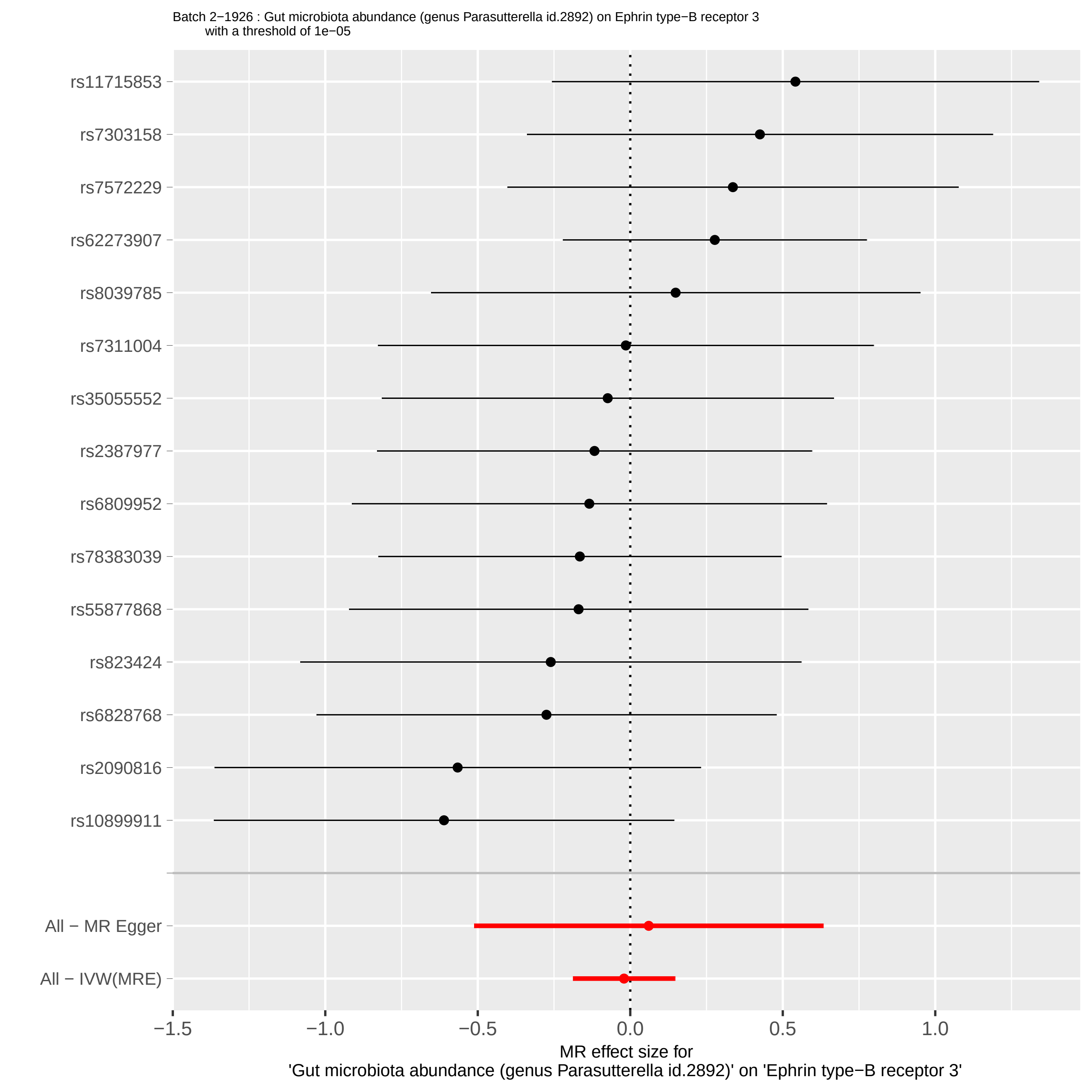

Batch 2−1926 : Gut microbiota abundance (genus Parasutterella id.2892) on Ephrin type−B receptor 3
with a threshold of 1e−05
rs11715853
rs7303158
rs7572229
rs62273907
rs8039785
rs7311004
rs35055552
rs2387977
rs6809952
rs78383039
rs55877868
rs823424
rs6828768
rs2090816
rs10899911
All − MR Egger
All − IVW(MRE)
−1.5
−1.0
−0.5
0.0
0.5
1.0
MR effect size for
'Gut microbiota abundance (genus Parasutterella id.2892)' on 'Ephrin type−B receptor 3'

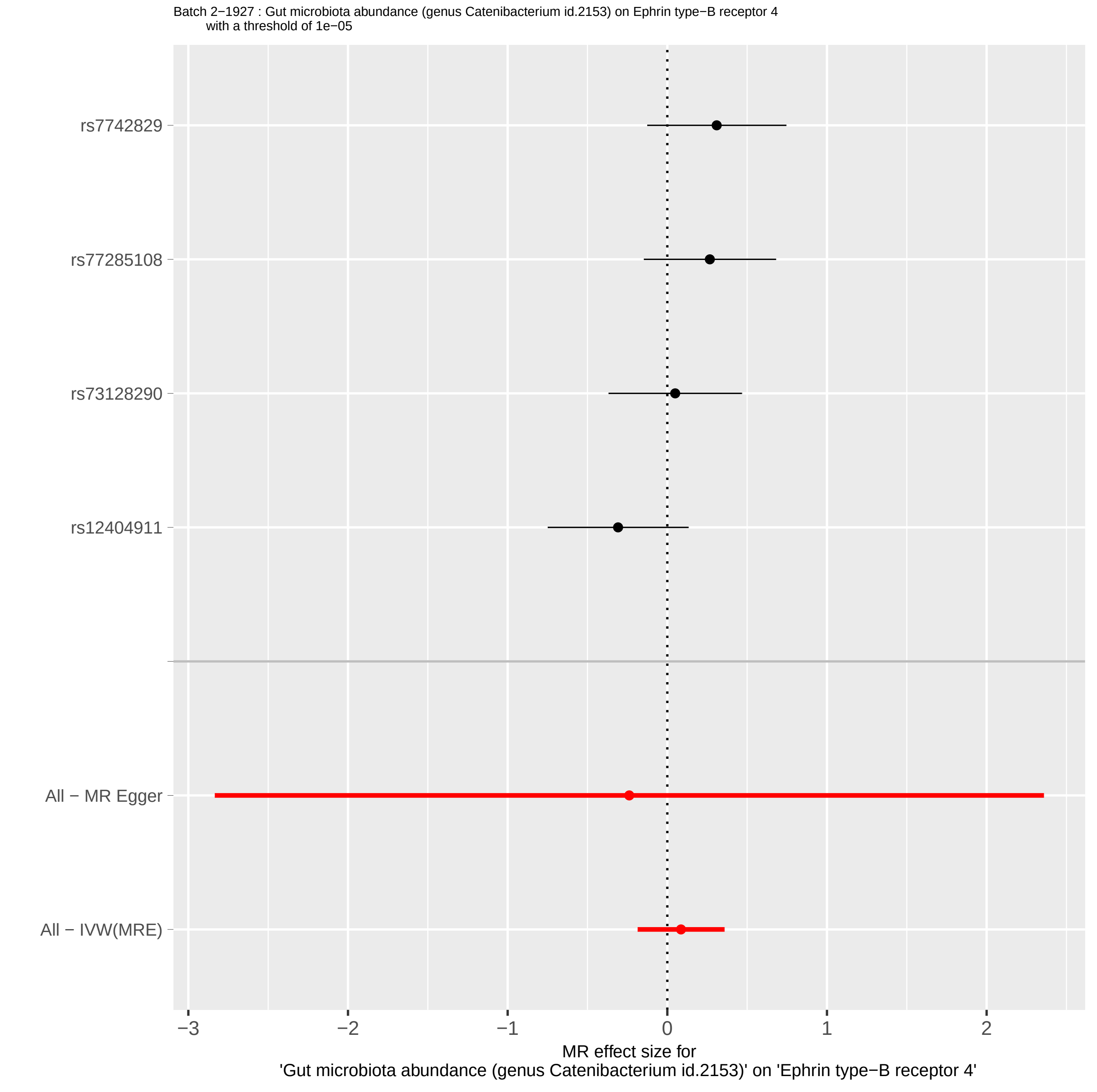

Batch 2−1927 : Gut microbiota abundance (genus Catenibacterium id.2153) on Ephrin type−B receptor 4
with a threshold of 1e−05
rs7742829
rs77285108
rs73128290
rs12404911
All − MR Egger
All − IVW(MRE)
−3
−2
−1
0
1
2
MR effect size for
'Gut microbiota abundance (genus Catenibacterium id.2153)' on 'Ephrin type−B receptor 4'

Batch 2−1928 : Gut microbiota abundance (genus Parasutterella id.2892) on Ephrin type−B receptor 4
with a threshold of 1e−05

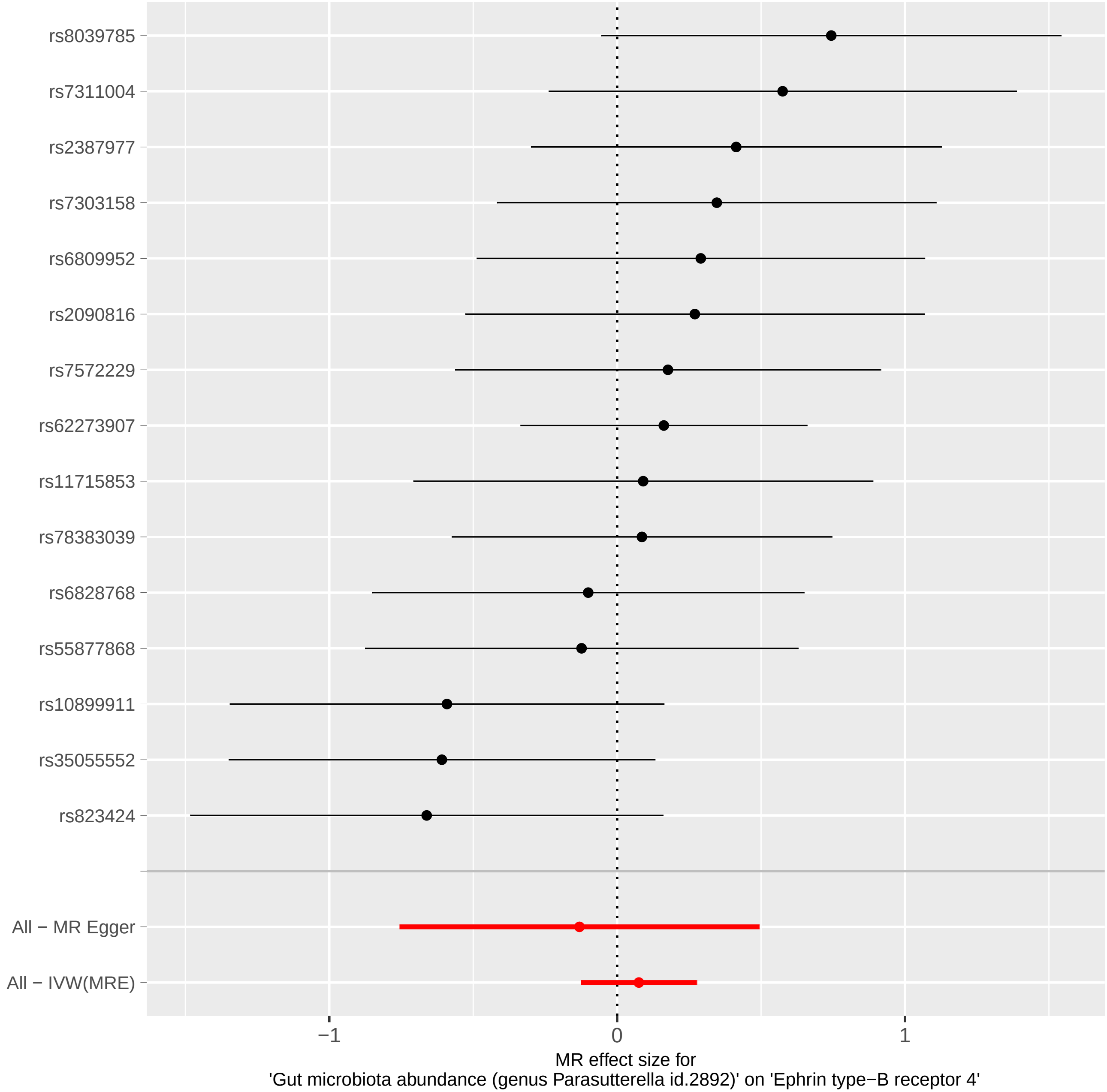

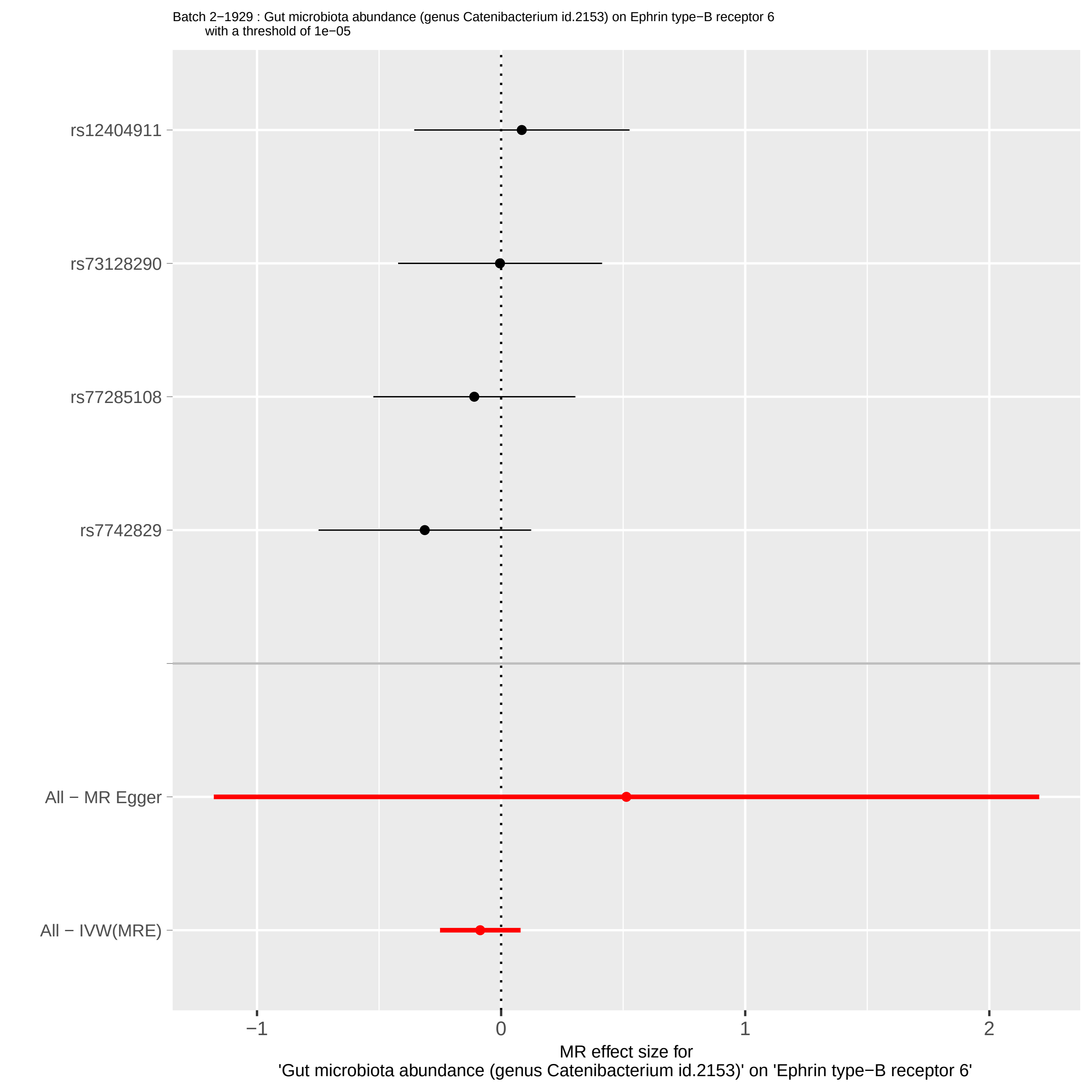

Batch 2−1929 : Gut microbiota abundance (genus Catenibacterium id.2153) on Ephrin type−B receptor 6
with a threshold of 1e−05
rs12404911
rs73128290
rs77285108
rs7742829
All − MR Egger
All − IVW(MRE)
−1
0
1
2
MR effect size for
'Gut microbiota abundance (genus Catenibacterium id.2153)' on 'Ephrin type−B receptor 6'

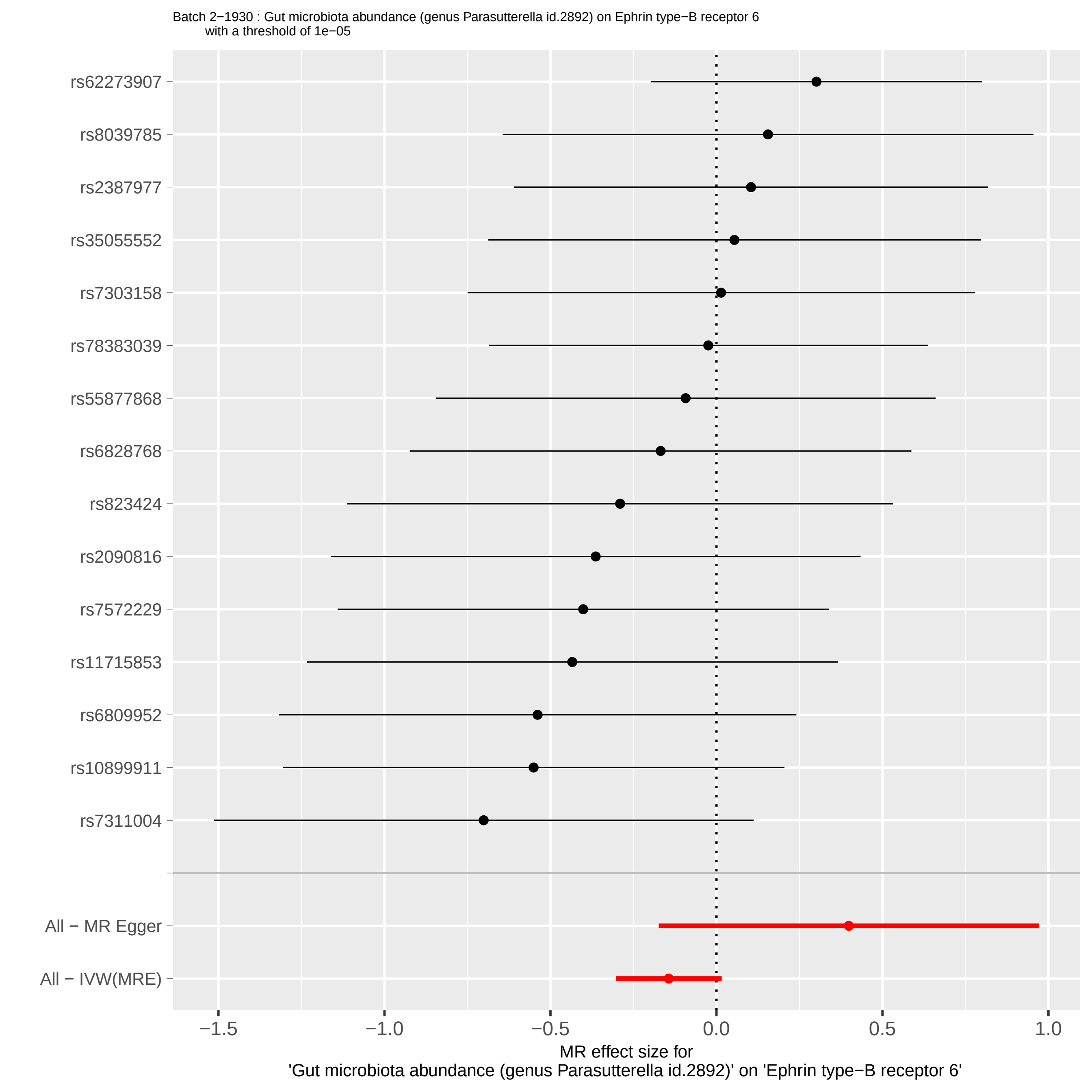
Batch 2−1930 : Gut microbiota abundance (genus Parasutterella id.2892) on Ephrin type−B receptor 6
with a threshold of 1e−05
rs62273907
rs8039785
rs2387977
rs35055552
rs7303158
rs78383039
rs55877868
rs6828768
rs823424
rs2090816
rs7572229
rs11715853
rs6809952
rs10899911
rs7311004
All − MR Egger
All − IVW(MRE)
−1.5
−1.0
−0.5
0.0
0.5
1.0
MR effect size for
'Gut microbiota abundance (genus Parasutterella id.2892)' on 'Ephrin type−B receptor 6'

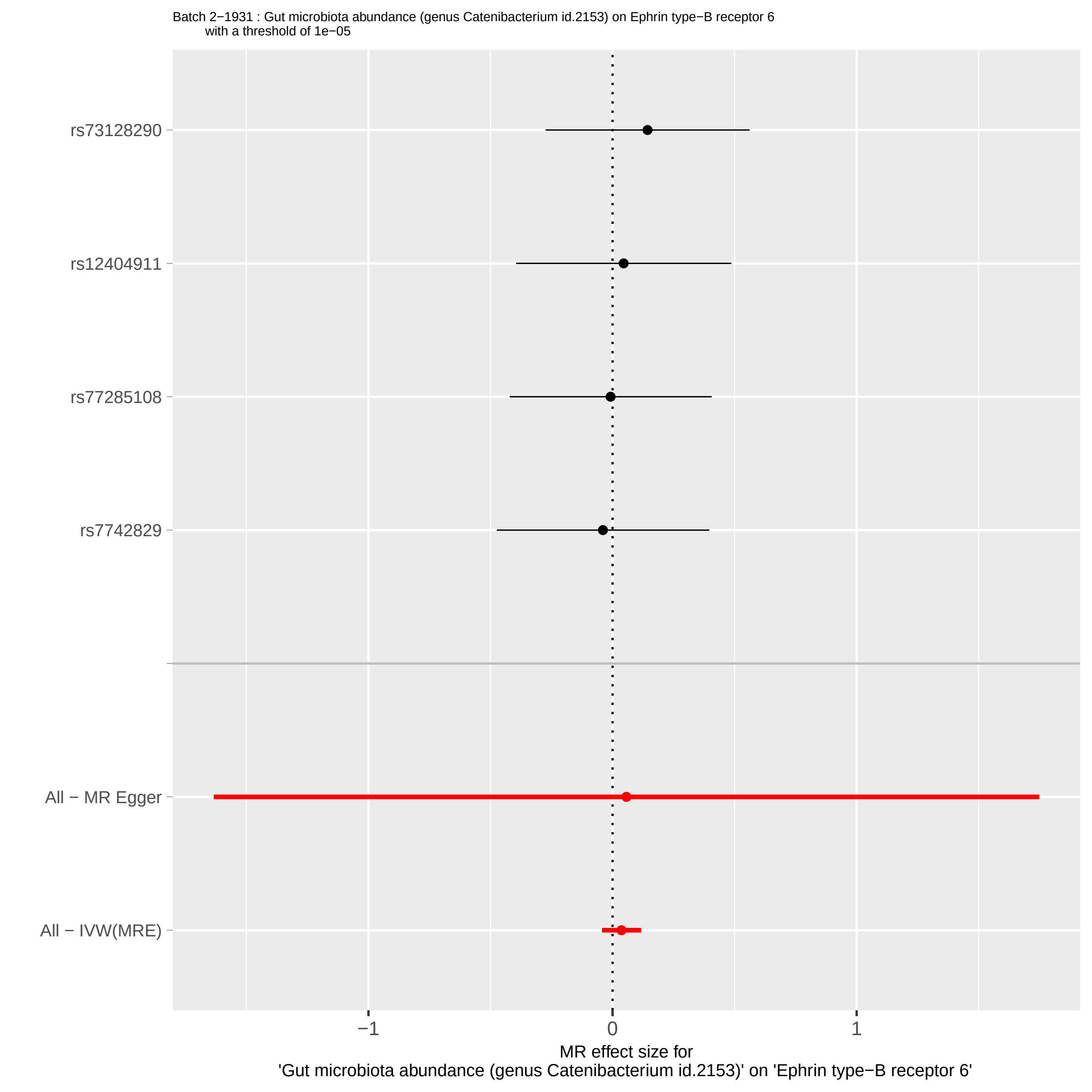
Batch 2−1931 : Gut microbiota abundance (genus Catenibacterium id.2153) on Ephrin type−B receptor 6
with a threshold of 1e−05
rs73128290
rs12404911
rs77285108
rs7742829
All − MR Egger
All − IVW(MRE)
−1
0
1
MR effect size for
'Gut microbiota abundance (genus Catenibacterium id.2153)' on 'Ephrin type−B receptor 6'

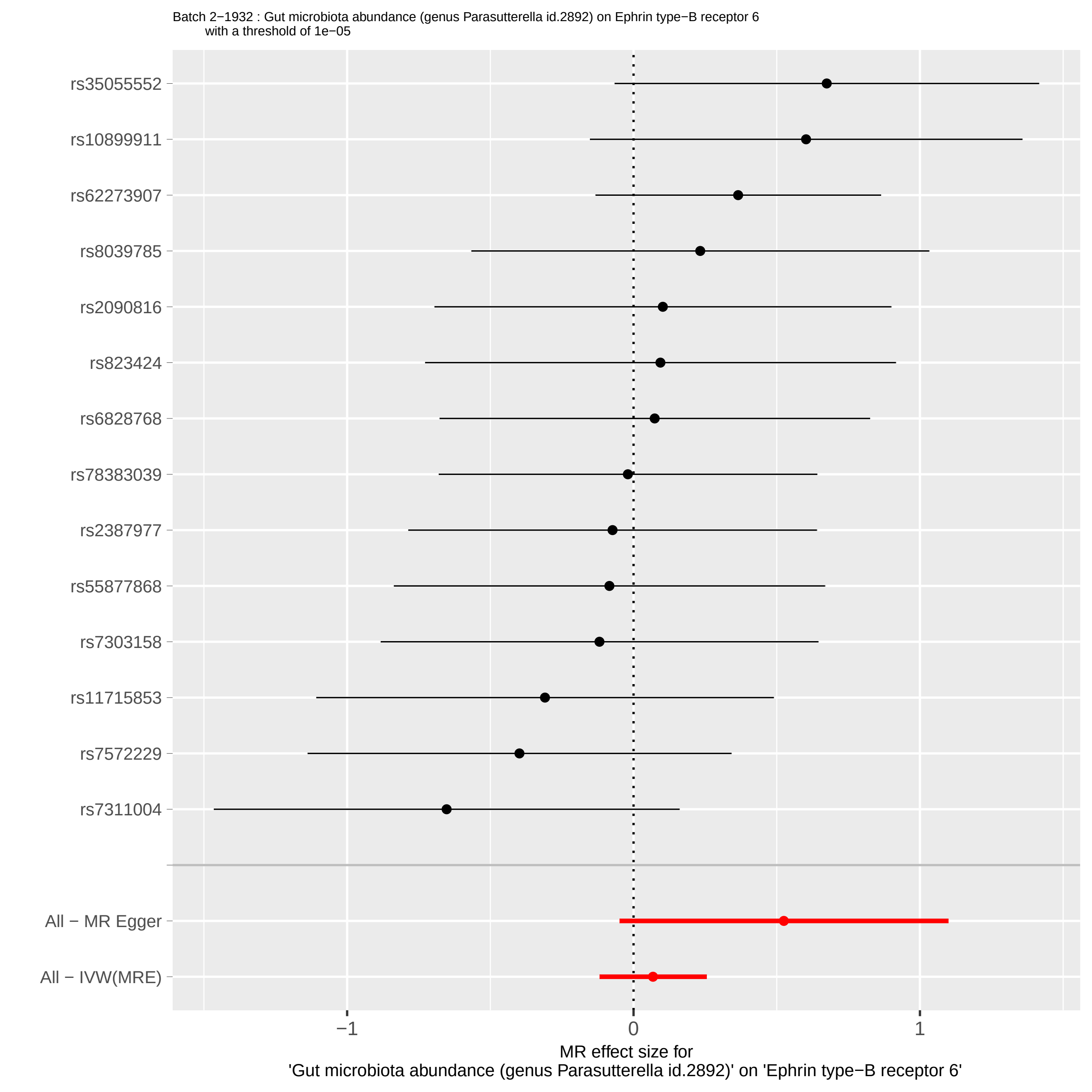
Batch 2−1932 : Gut microbiota abundance (genus Parasutterella id.2892) on Ephrin type−B receptor 6
with a threshold of 1e−05
rs35055552
rs10899911
rs62273907
rs8039785
rs2090816
rs823424
rs6828768
rs78383039
rs2387977
rs55877868
rs7303158
rs11715853
rs7572229
rs7311004
All − MR Egger
All − IVW(MRE)
−1
0
1
MR effect size for
'Gut microbiota abundance (genus Parasutterella id.2892)' on 'Ephrin type−B receptor 6'

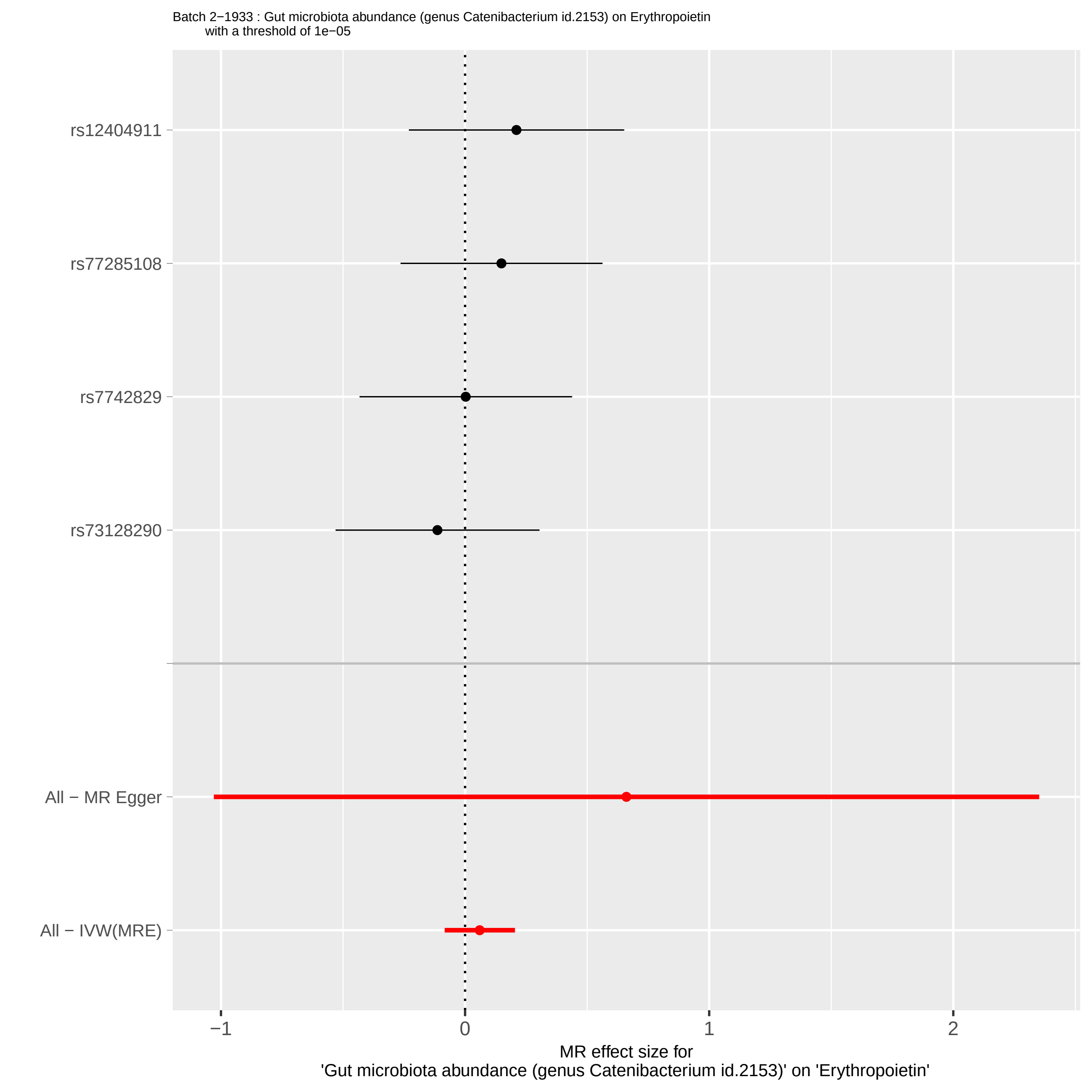

Batch 2−1933 : Gut microbiota abundance (genus Catenibacterium id.2153) on Erythropoietin
with a threshold of 1e−05
rs12404911
rs77285108
rs7742829
rs73128290
All − MR Egger
All − IVW(MRE)
−1
0
1
2
MR effect size for
'Gut microbiota abundance (genus Catenibacterium id.2153)' on 'Erythropoietin'

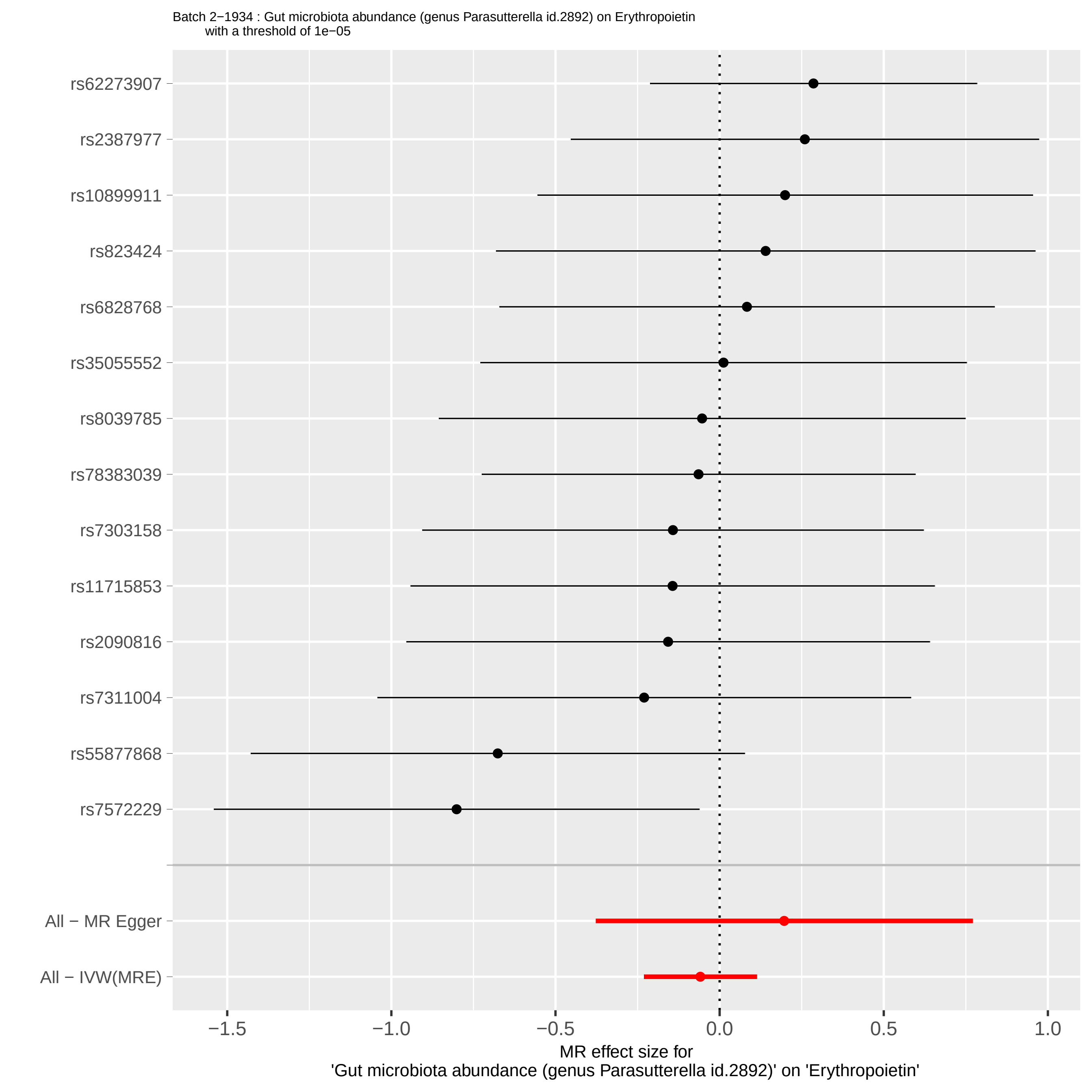
Batch 2−1934 : Gut microbiota abundance (genus Parasutterella id.2892) on Erythropoietin
with a threshold of 1e−05
rs62273907
rs2387977
rs10899911
rs823424
rs6828768
rs35055552
rs8039785
rs78383039
rs7303158
rs11715853
rs2090816
rs7311004
rs55877868
rs7572229
All − MR Egger
All − IVW(MRE)
−1.5
−1.0
−0.5
0.0
0.5
1.0
MR effect size for
'Gut microbiota abundance (genus Parasutterella id.2892)' on 'Erythropoietin'

Batch 2−1935 : Gut microbiota abundance (genus Catenibacterium id.2153) on Erythropoietin receptor
with a threshold of 1e−05

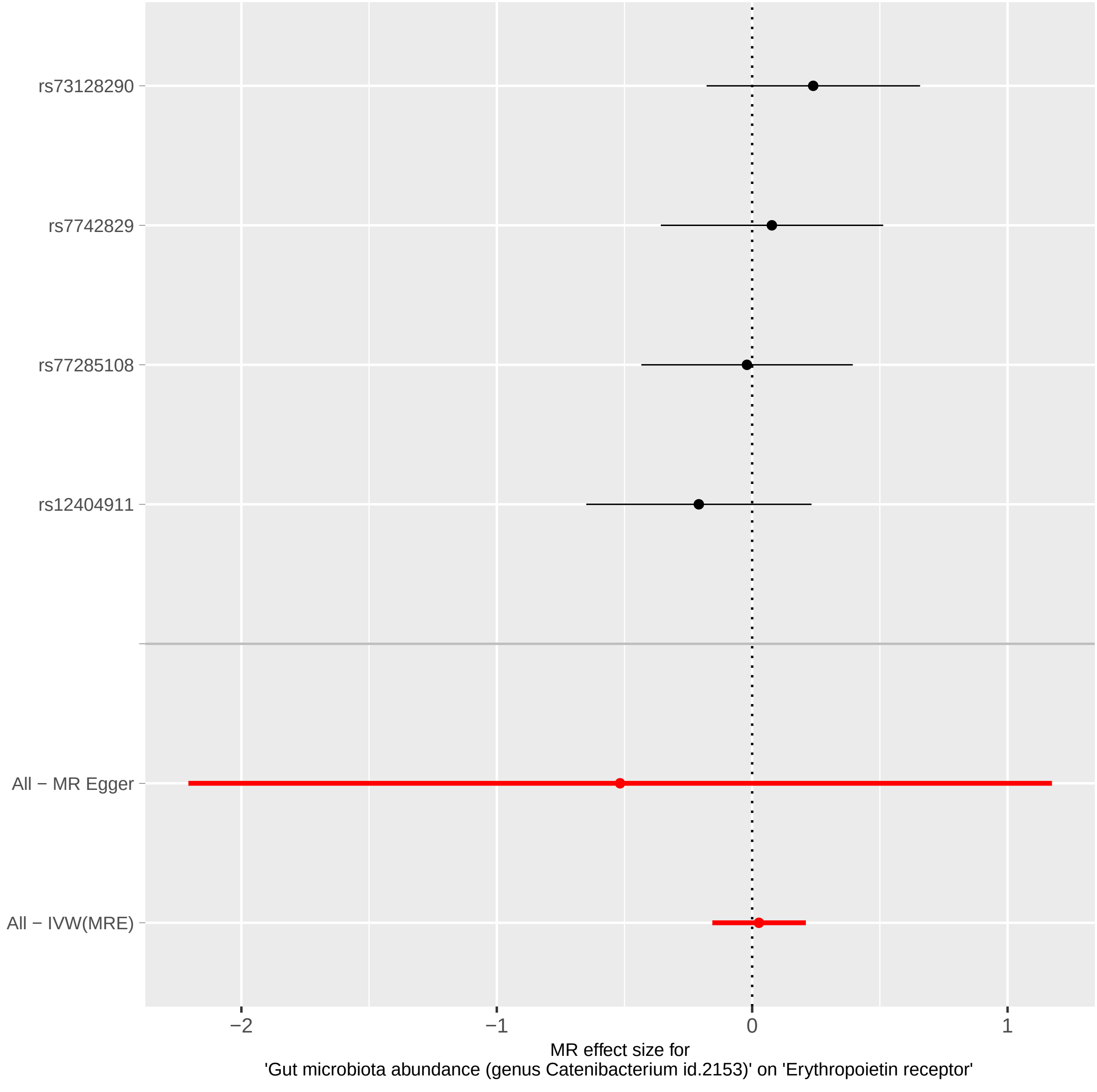

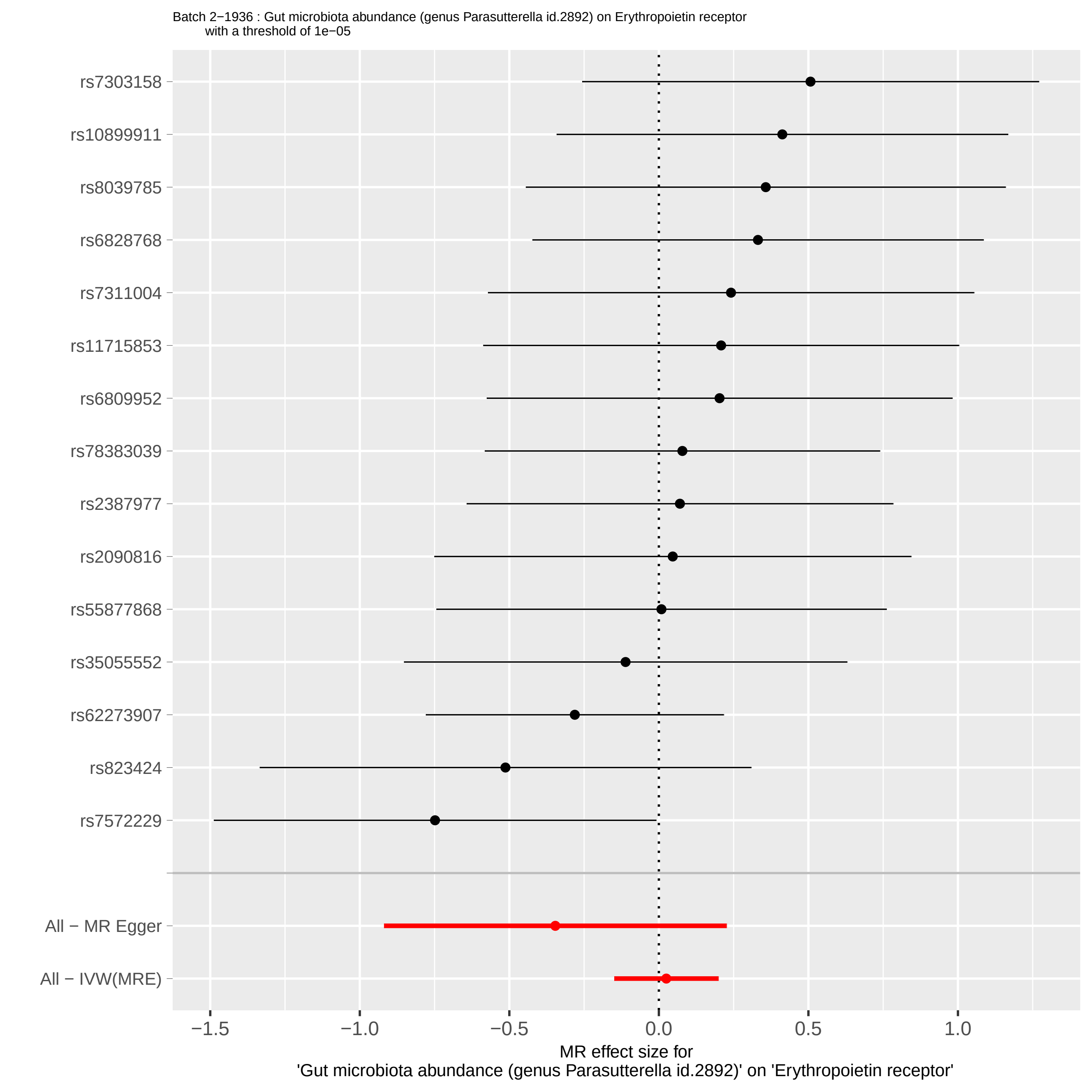

Batch 2−1936 : Gut microbiota abundance (genus Parasutterella id.2892) on Erythropoietin receptor
with a threshold of 1e−05
rs7303158
rs10899911
rs8039785
rs6828768
rs7311004
rs11715853
rs6809952
rs78383039
rs2387977
rs2090816
rs55877868
rs35055552
rs62273907
rs823424
rs7572229
All − MR Egger
All − IVW(MRE)
−1.5
−1.0
−0.5
0.0
0.5
1.0
MR effect size for
'Gut microbiota abundance (genus Parasutterella id.2892)' on 'Erythropoietin receptor'

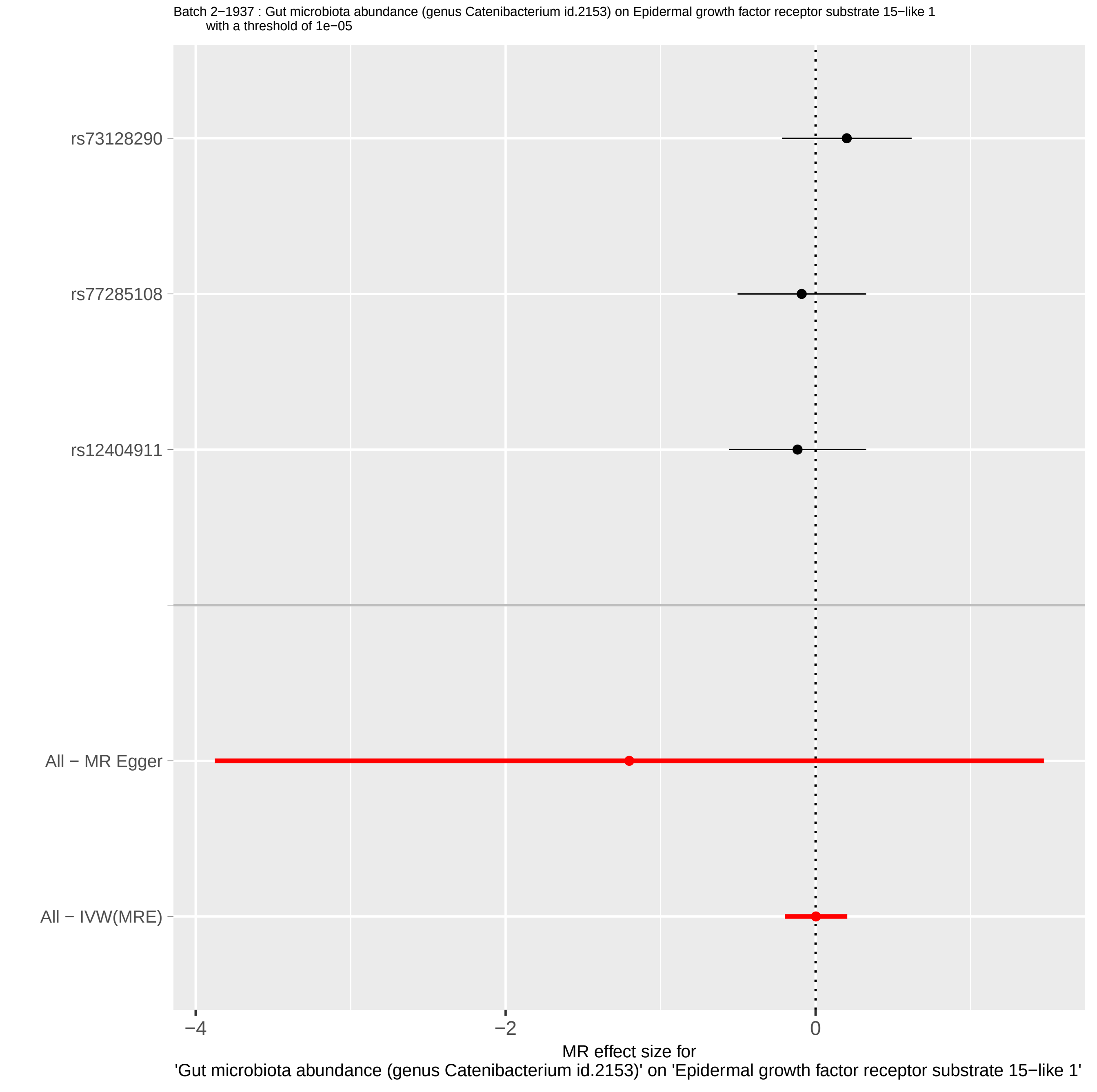

Batch 2−1937 : Gut microbiota abundance (genus Catenibacterium id.2153) on Epidermal growth factor receptor substrate 15−like 1
with a threshold of 1e−05
rs73128290
rs77285108
rs12404911
All − MR Egger
All − IVW(MRE)
−4
−2
0
MR effect size for
'Gut microbiota abundance (genus Catenibacterium id.2153)' on 'Epidermal growth factor receptor substrate 15−like 1'

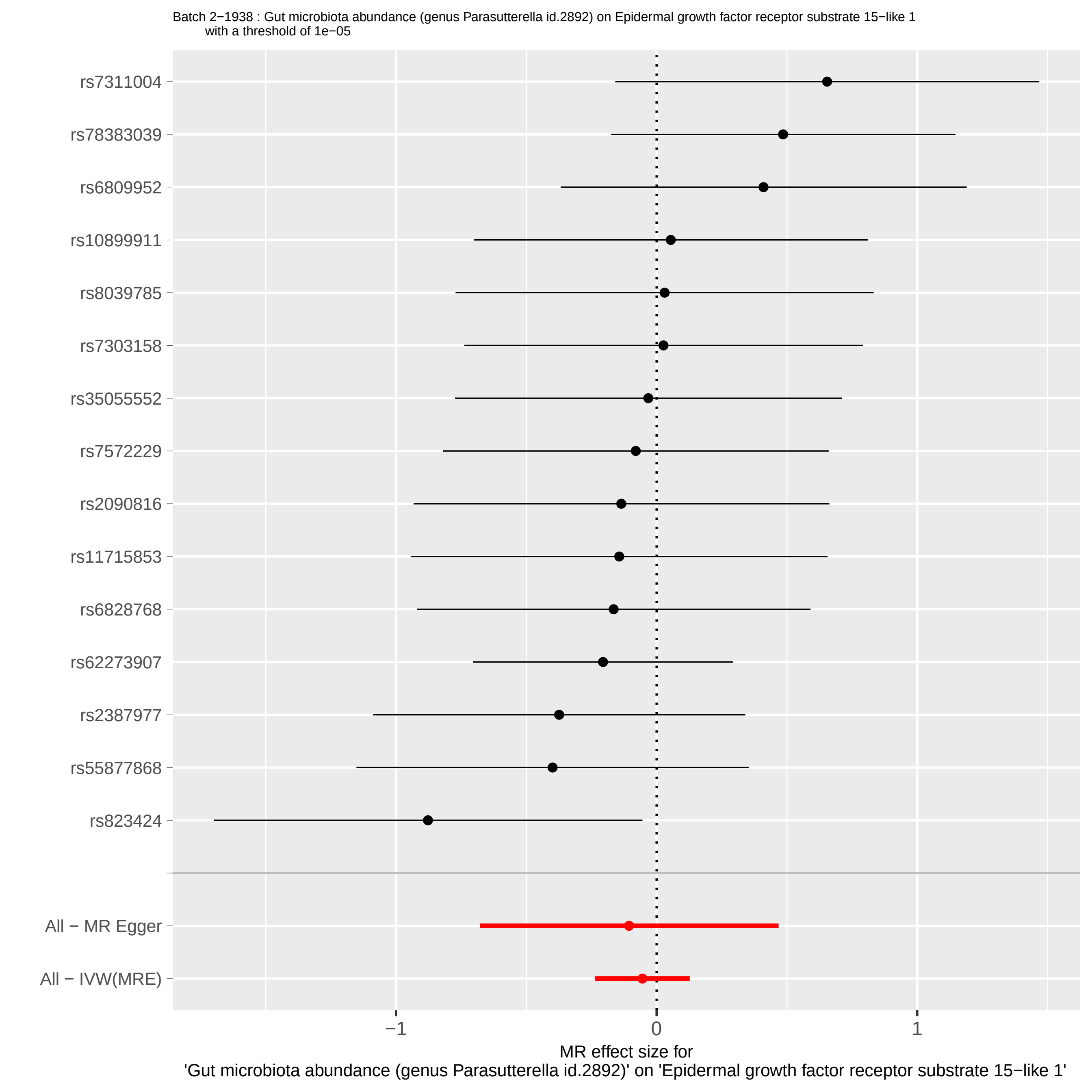
Batch 2−1938 : Gut microbiota abundance (genus Parasutterella id.2892) on Epidermal growth factor receptor substrate 15−like 1
with a threshold of 1e−05
rs7311004
rs78383039
rs6809952
rs10899911
rs8039785
rs7303158
rs35055552
rs7572229
rs2090816
rs11715853
rs6828768
rs62273907
rs2387977
rs55877868
rs823424
All − MR Egger
All − IVW(MRE)
−1
0
1
MR effect size for
'Gut microbiota abundance (genus Parasutterella id.2892)' on 'Epidermal growth factor receptor substrate 15−like 1'

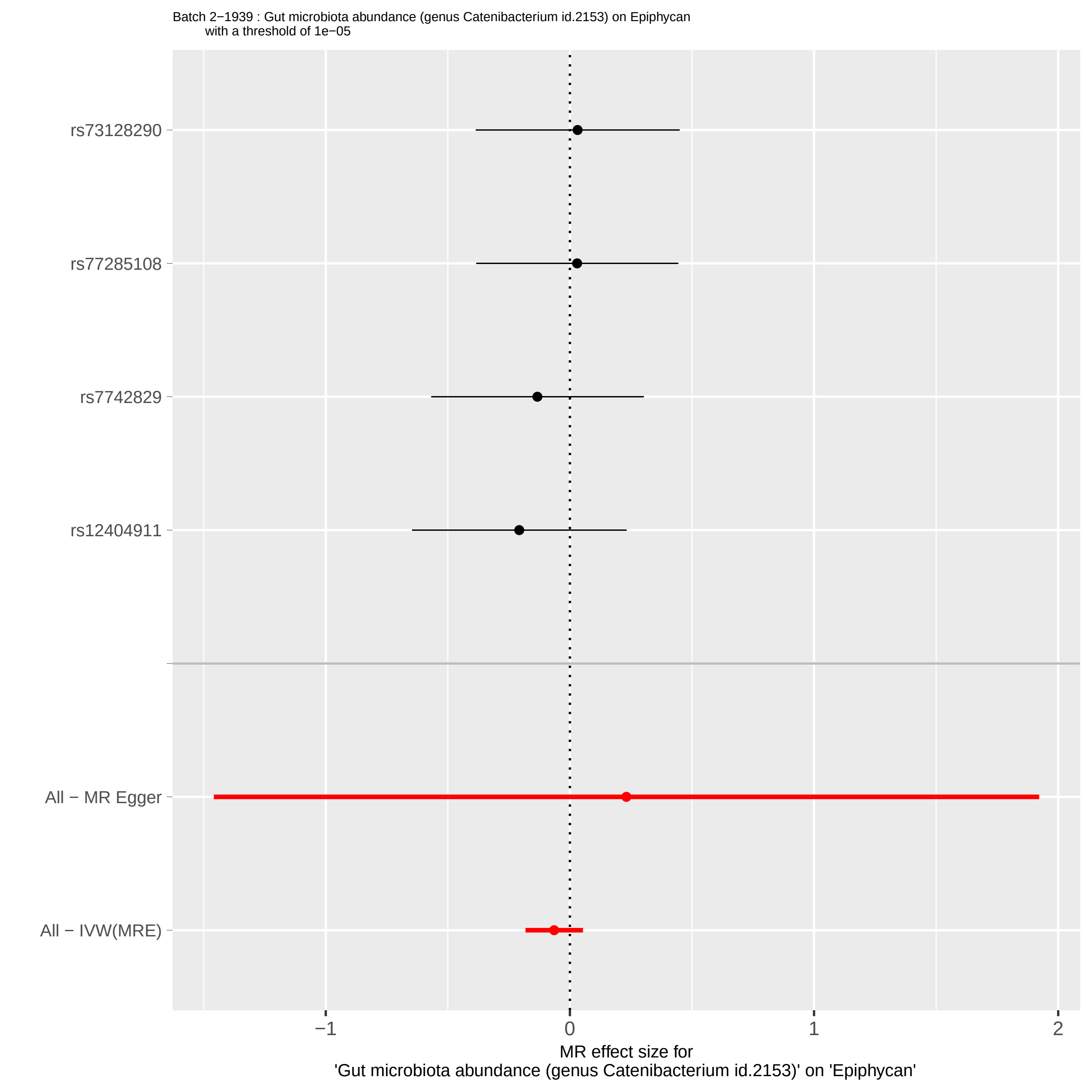

Batch 2−1939 : Gut microbiota abundance (genus Catenibacterium id.2153) on Epiphycan
with a threshold of 1e−05
rs73128290
rs77285108
rs7742829
rs12404911
All − MR Egger
All − IVW(MRE)
−1
0
1
2
MR effect size for
'Gut microbiota abundance (genus Catenibacterium id.2153)' on 'Epiphycan'

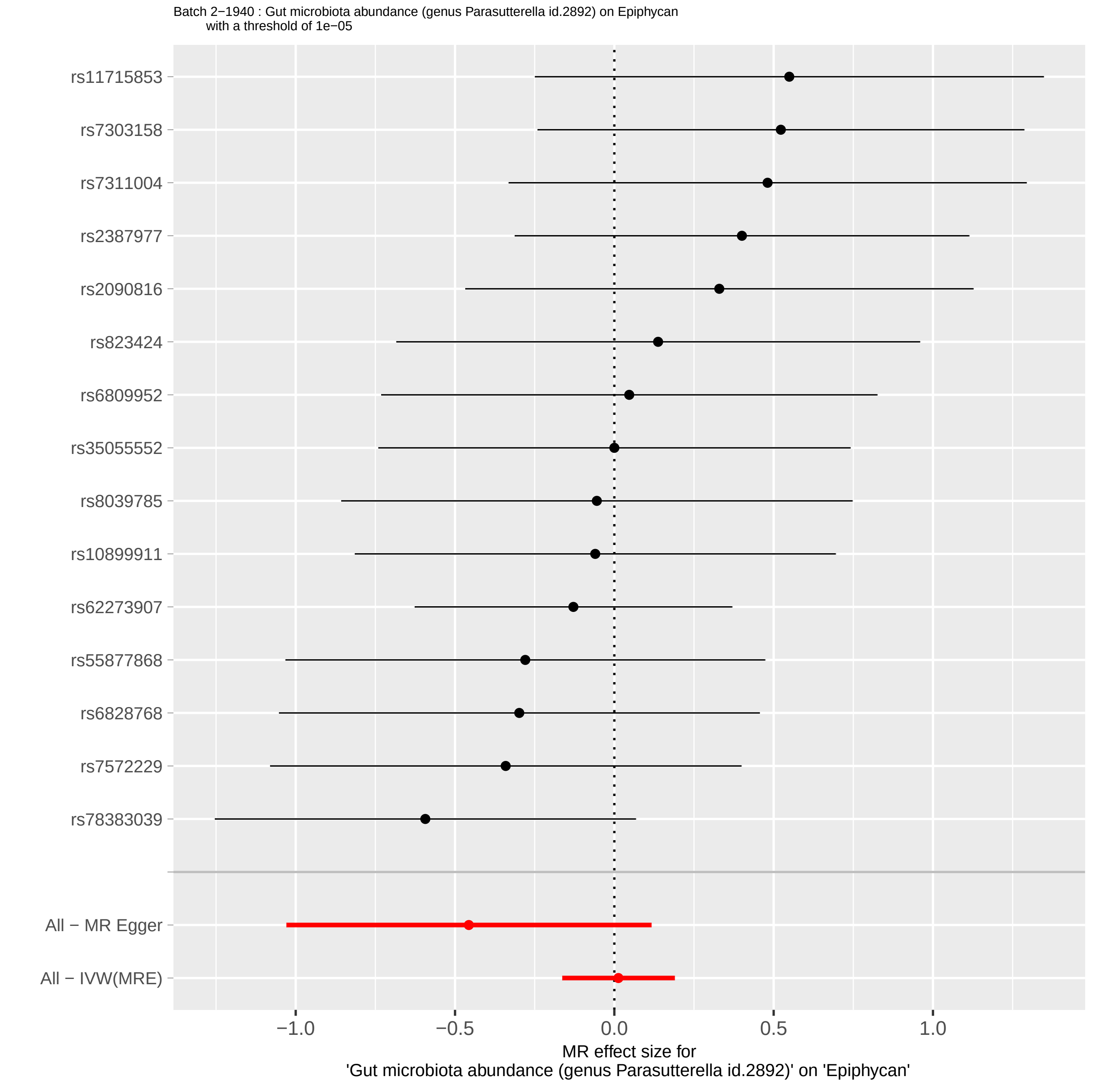
Batch 2−1940 : Gut microbiota abundance (genus Parasutterella id.2892) on Epiphycan
with a threshold of 1e−05
rs11715853
rs7303158
rs7311004
rs2387977
rs2090816
rs823424
rs6809952
rs35055552
rs8039785
rs10899911
rs62273907
rs55877868
rs6828768
rs7572229
rs78383039
All − MR Egger
All − IVW(MRE)
−1.0
−0.5
0.0
0.5
1.0
MR effect size for
'Gut microbiota abundance (genus Parasutterella id.2892)' on 'Epiphycan'

Batch 2−1941 : Gut microbiota abundance (genus Catenibacterium id.2153) on Endoplasmic reticulum aminopeptidase 1
with a threshold of 1e−05

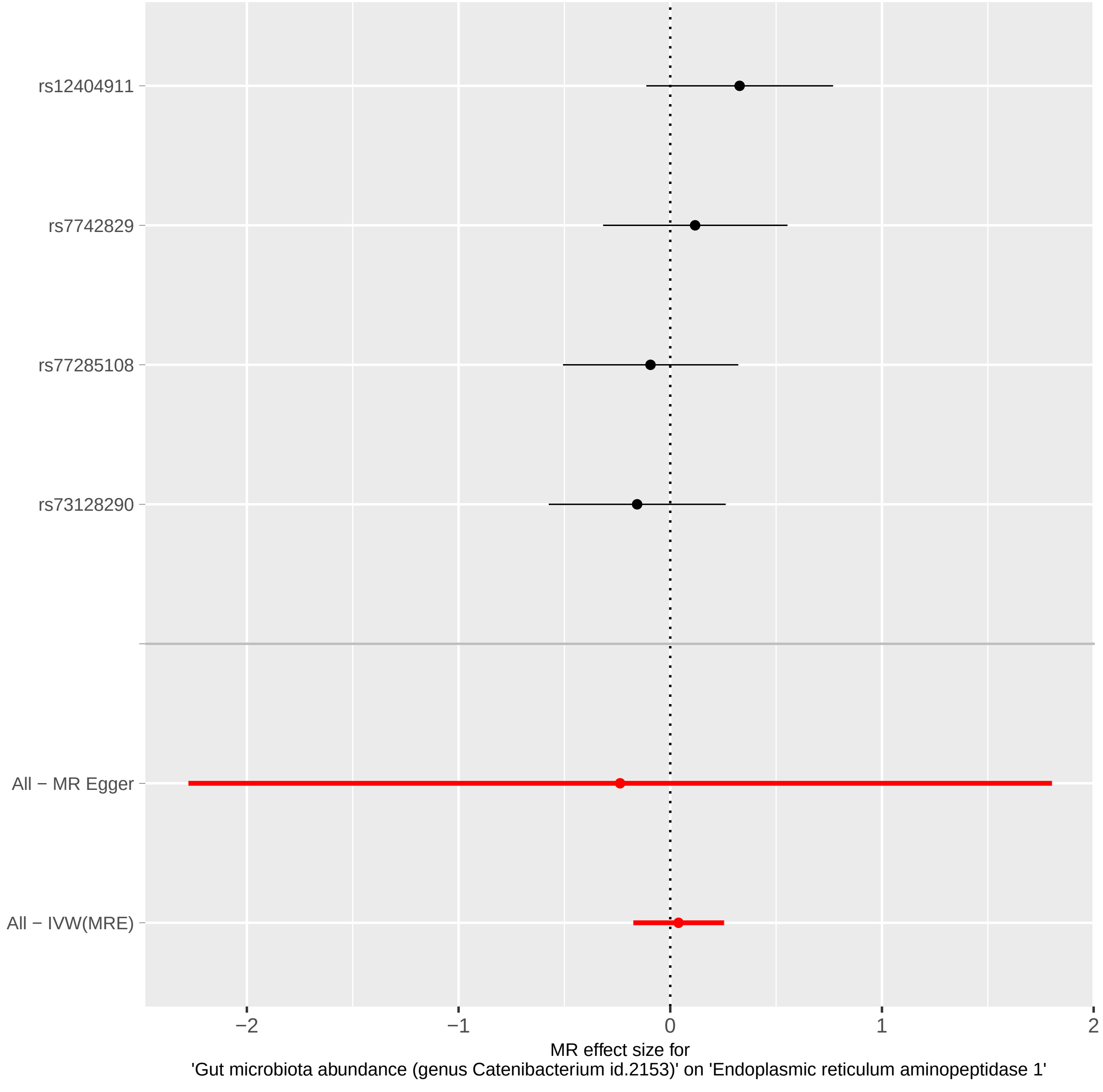

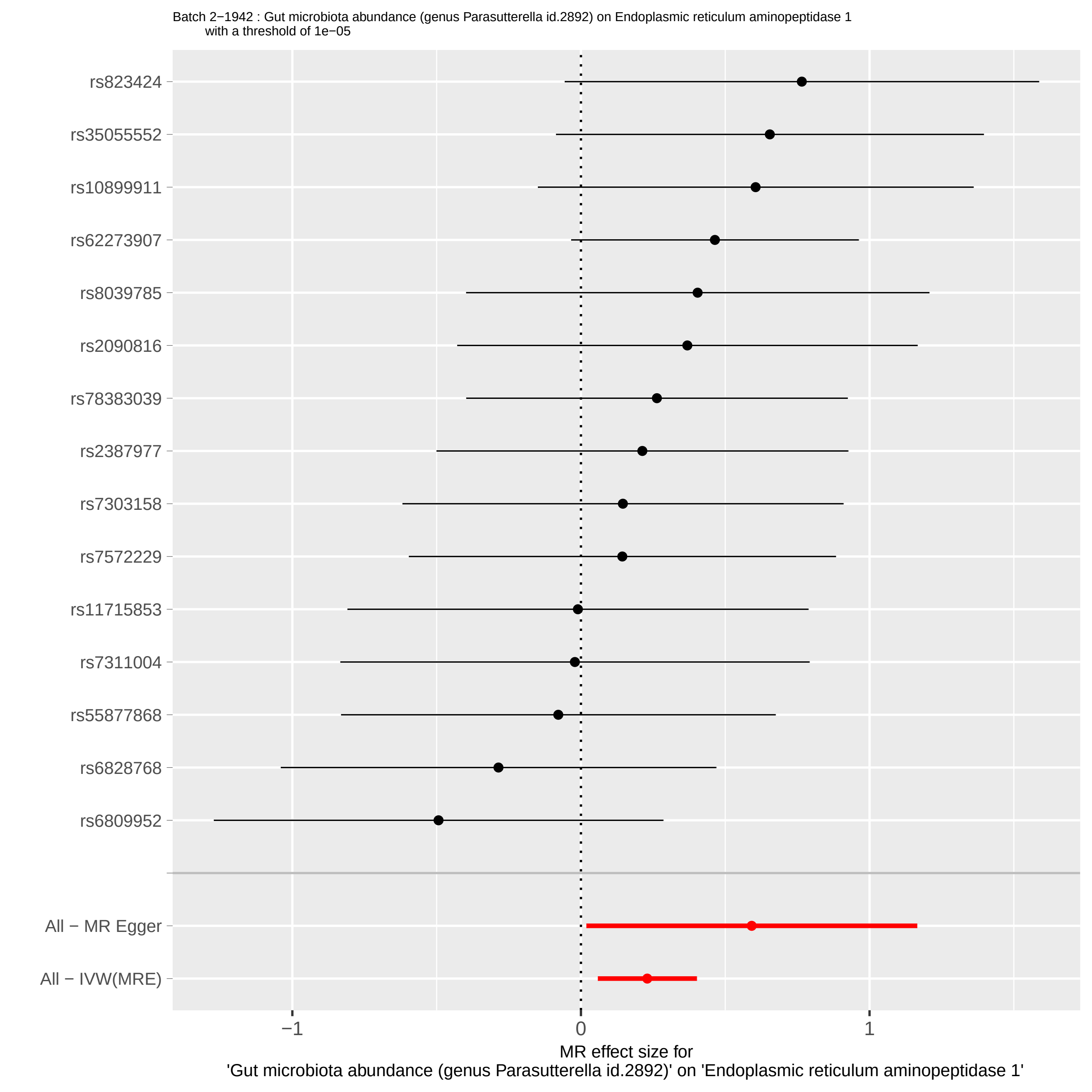
Batch 2−1942 : Gut microbiota abundance (genus Parasutterella id.2892) on Endoplasmic reticulum aminopeptidase 1
with a threshold of 1e−05
rs823424
rs35055552
rs10899911
rs62273907
rs8039785
rs2090816
rs78383039
rs2387977
rs7303158
rs7572229
rs11715853
rs7311004
rs55877868
rs6828768
rs6809952
All − MR Egger
All − IVW(MRE)
−1
0
1
MR effect size for
'Gut microbiota abundance (genus Parasutterella id.2892)' on 'Endoplasmic reticulum aminopeptidase 1'

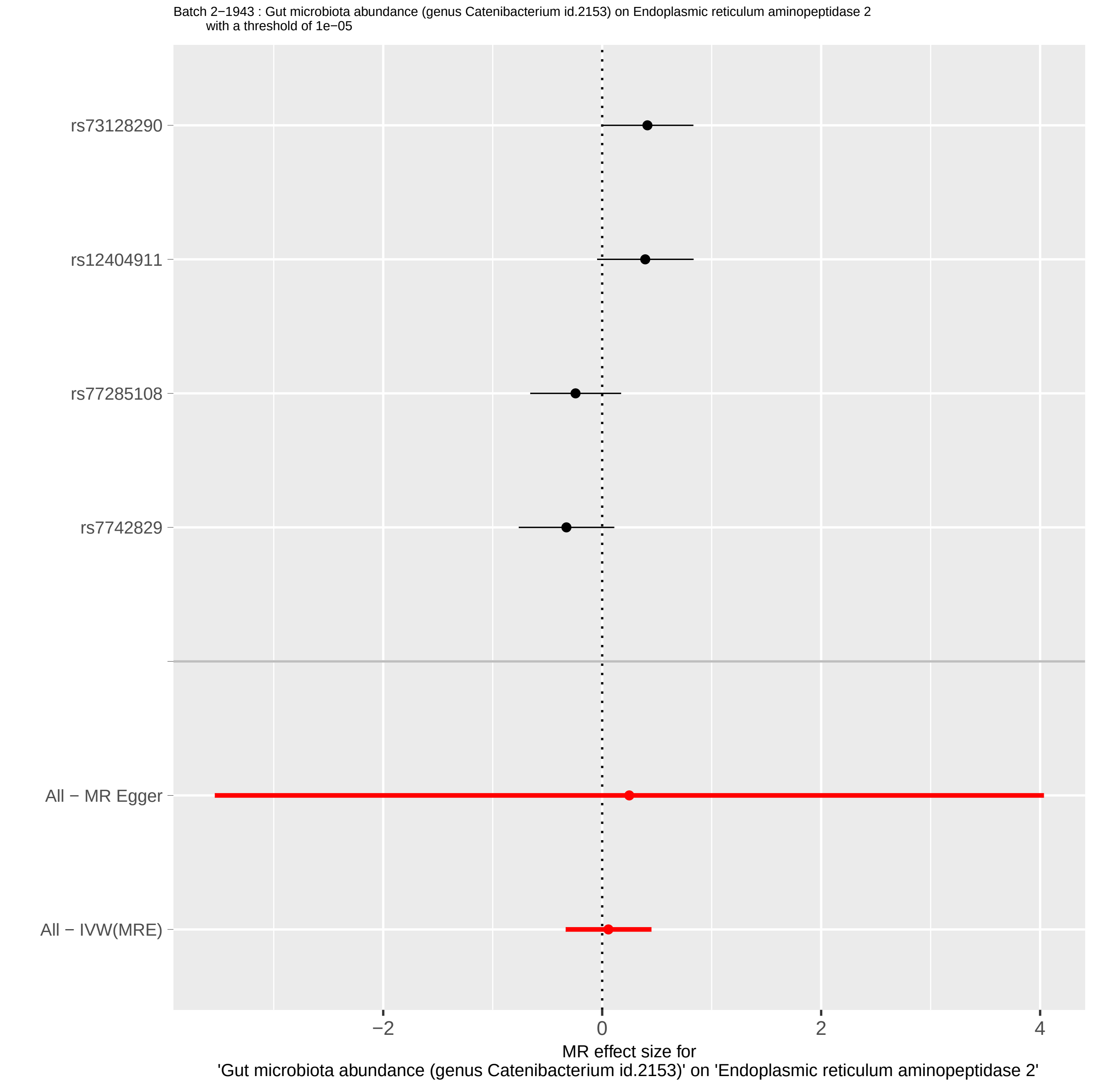

Batch 2−1943 : Gut microbiota abundance (genus Catenibacterium id.2153) on Endoplasmic reticulum aminopeptidase 2
with a threshold of 1e−05
rs73128290
rs12404911
rs77285108
rs7742829
All − MR Egger
All − IVW(MRE)
−2
0
2
4
MR effect size for
'Gut microbiota abundance (genus Catenibacterium id.2153)' on 'Endoplasmic reticulum aminopeptidase 2'

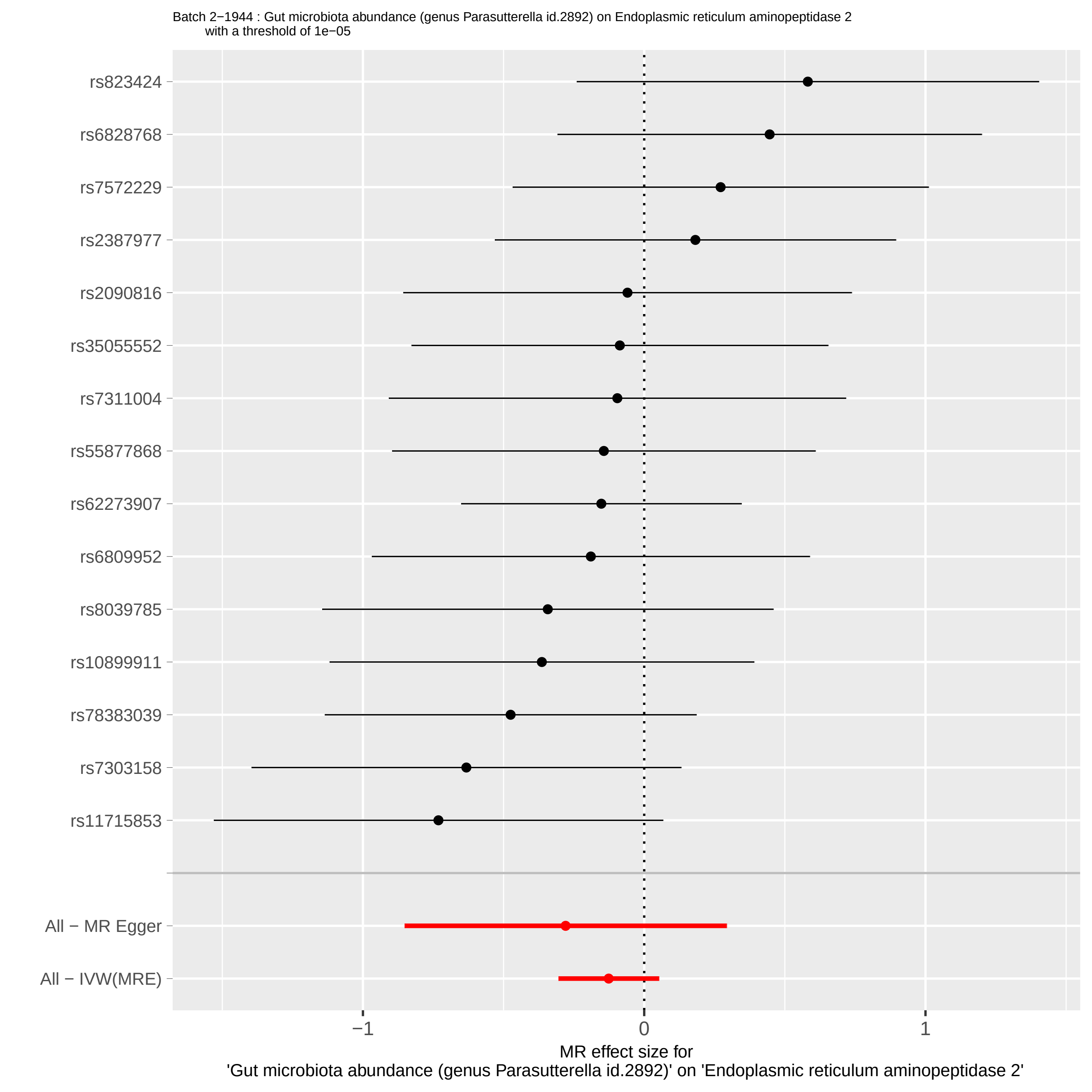
Batch 2−1944 : Gut microbiota abundance (genus Parasutterella id.2892) on Endoplasmic reticulum aminopeptidase 2
with a threshold of 1e−05
rs823424
rs6828768
rs7572229
rs2387977
rs2090816
rs35055552
rs7311004
rs55877868
rs62273907
rs6809952
rs8039785
rs10899911
rs78383039
rs7303158
rs11715853
All − MR Egger
All − IVW(MRE)
−1
0
1
MR effect size for
'Gut microbiota abundance (genus Parasutterella id.2892)' on 'Endoplasmic reticulum aminopeptidase 2'

Batch 2−1945 : Gut microbiota abundance (genus Catenibacterium id.2153) on Receptor tyrosine−protein kinase erbB−2
with a threshold of 1e−05

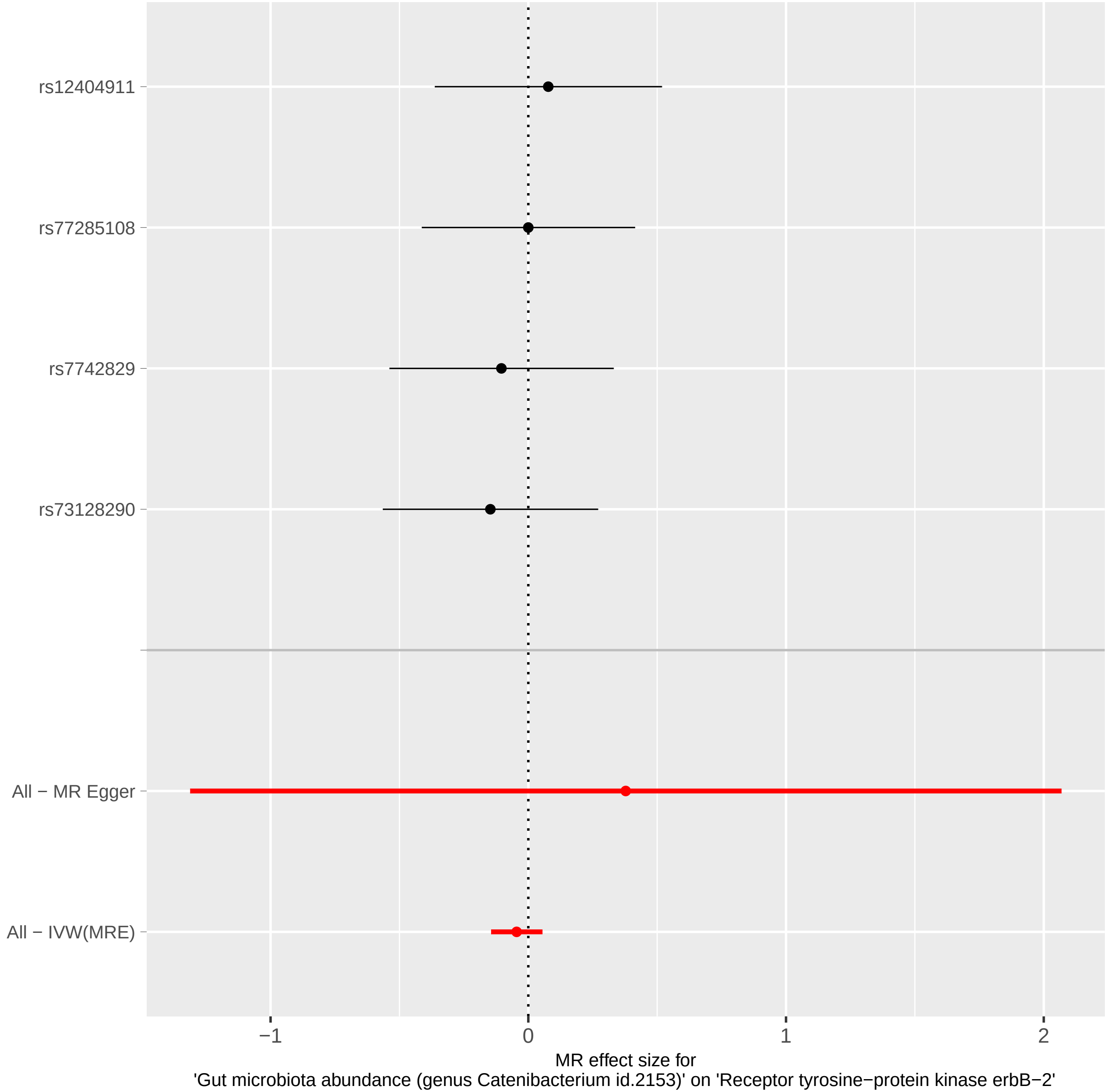

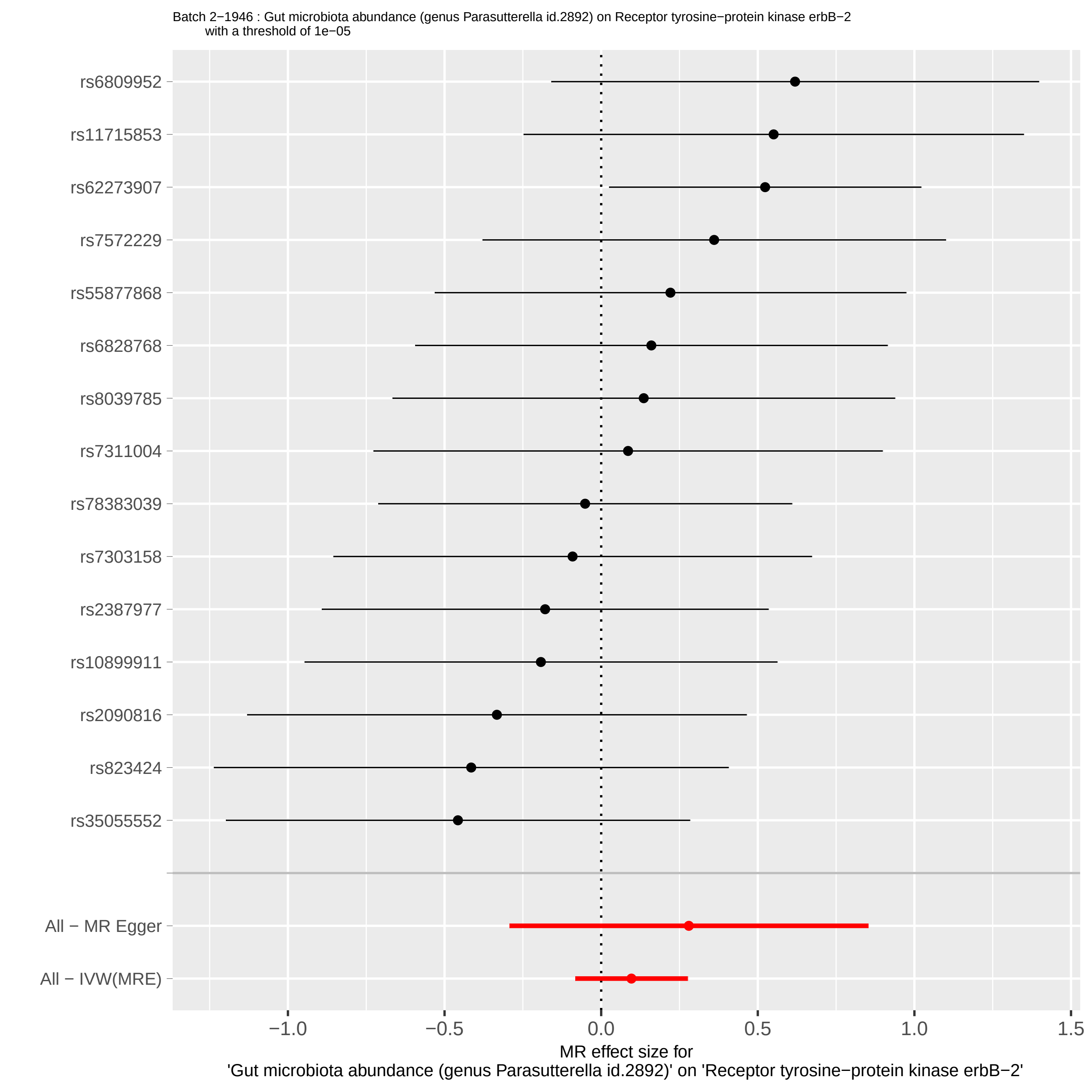
Batch 2−1946 : Gut microbiota abundance (genus Parasutterella id.2892) on Receptor tyrosine−protein kinase erbB−2
with a threshold of 1e−05
rs6809952
rs11715853
rs62273907
rs7572229
rs55877868
rs6828768
rs8039785
rs7311004
rs78383039
rs7303158
rs2387977
rs10899911
rs2090816
rs823424
rs35055552
All − MR Egger
All − IVW(MRE)
−1.0
−0.5
0.0
0.5
1.0
1.5
MR effect size for
'Gut microbiota abundance (genus Parasutterella id.2892)' on 'Receptor tyrosine−protein kinase erbB−2'

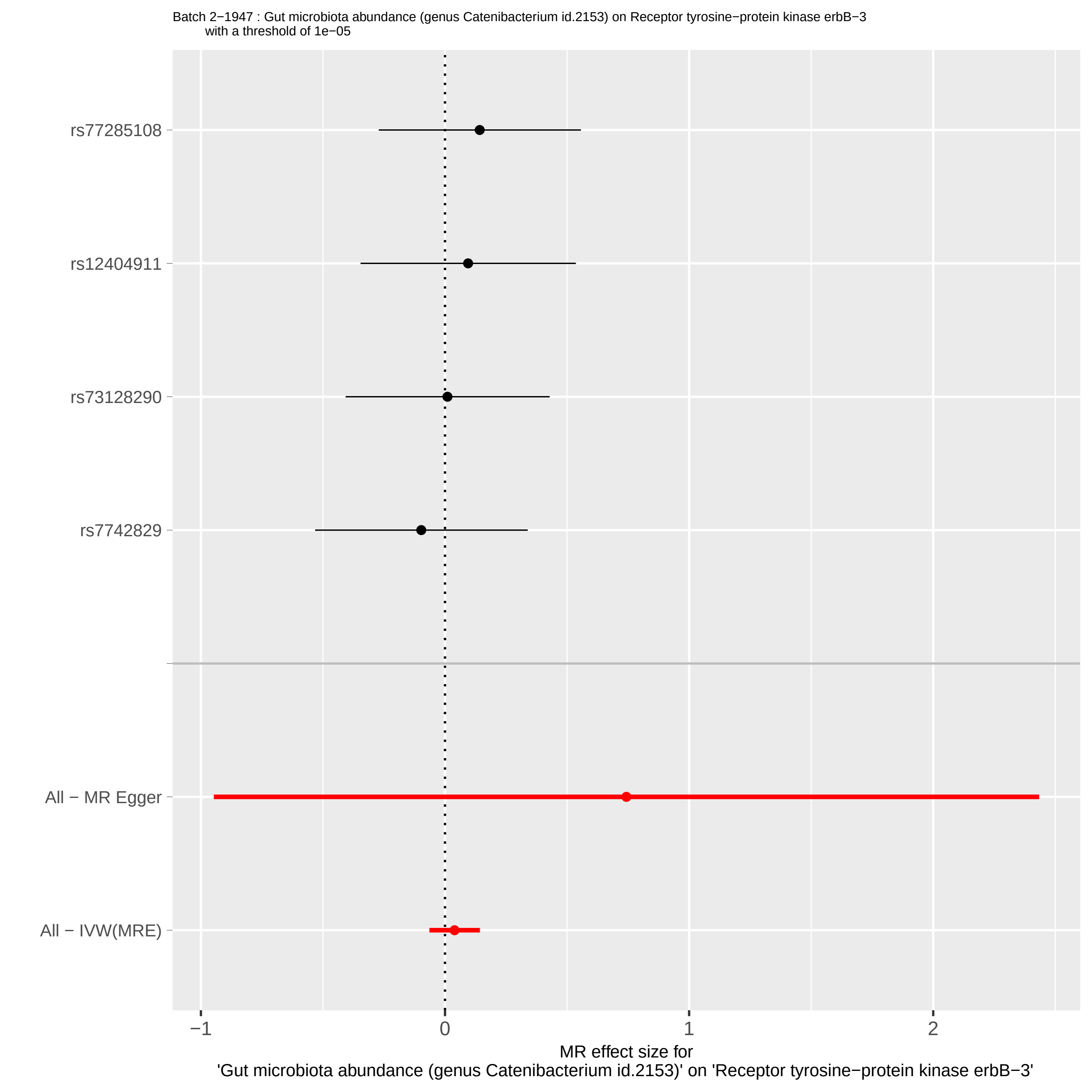
Batch 2−1947 : Gut microbiota abundance (genus Catenibacterium id.2153) on Receptor tyrosine−protein kinase erbB−3
with a threshold of 1e−05
rs77285108
rs12404911
rs73128290
rs7742829
All − MR Egger
All − IVW(MRE)
−1
0
1
2
MR effect size for
'Gut microbiota abundance (genus Catenibacterium id.2153)' on 'Receptor tyrosine−protein kinase erbB−3'

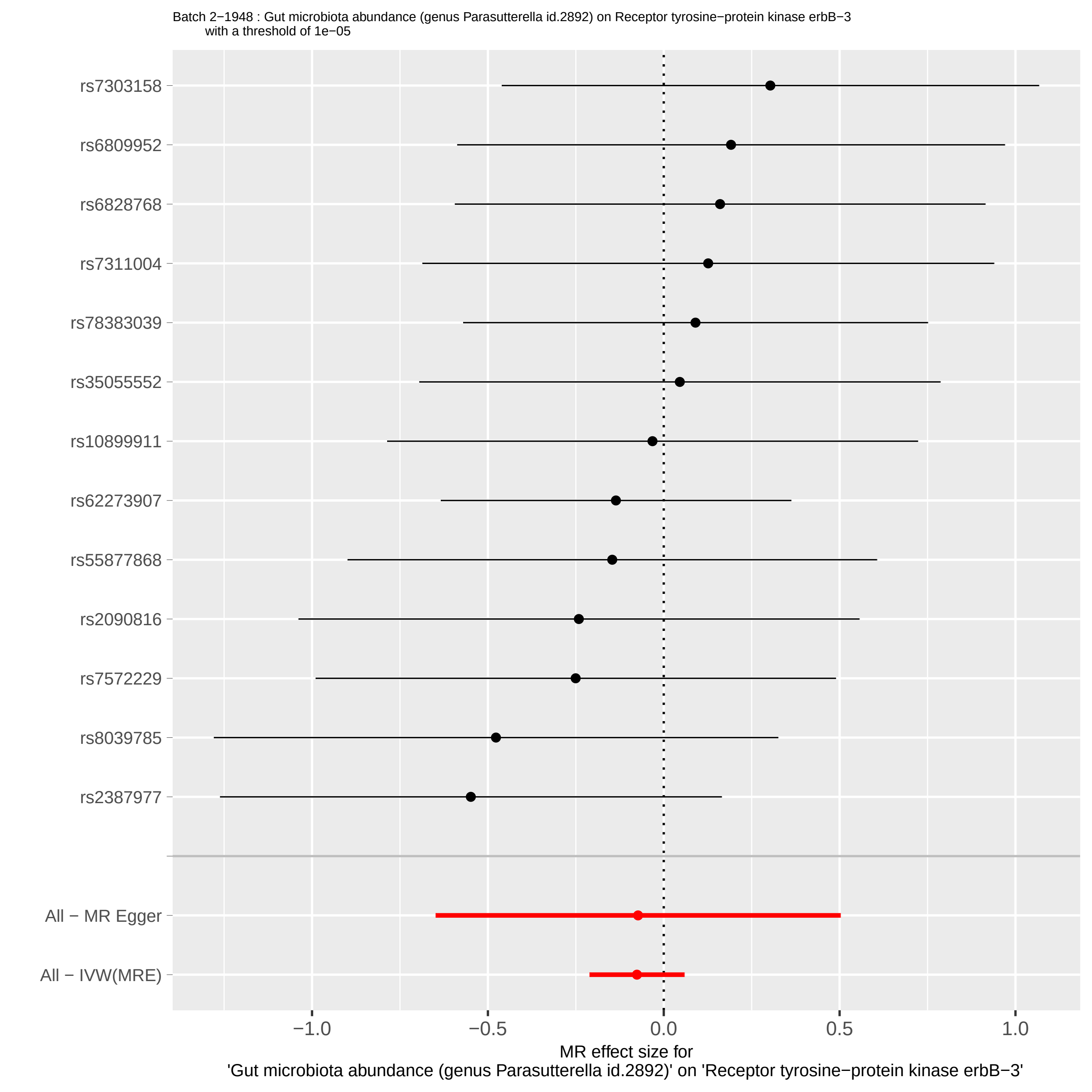

Batch 2−1948 : Gut microbiota abundance (genus Parasutterella id.2892) on Receptor tyrosine−protein kinase erbB−3
with a threshold of 1e−05
rs7303158
rs6809952
rs6828768
rs7311004
rs78383039
rs35055552
rs10899911
rs62273907
rs55877868
rs2090816
rs7572229
rs8039785
rs2387977
All − MR Egger
All − IVW(MRE)
−1.0
−0.5
0.0
0.5
1.0
MR effect size for
'Gut microbiota abundance (genus Parasutterella id.2892)' on 'Receptor tyrosine−protein kinase erbB−3'

Batch 2−1949 : Gut microbiota abundance (genus Catenibacterium id.2153) on DNA excision repair protein ERCC−1
with a threshold of 1e−05

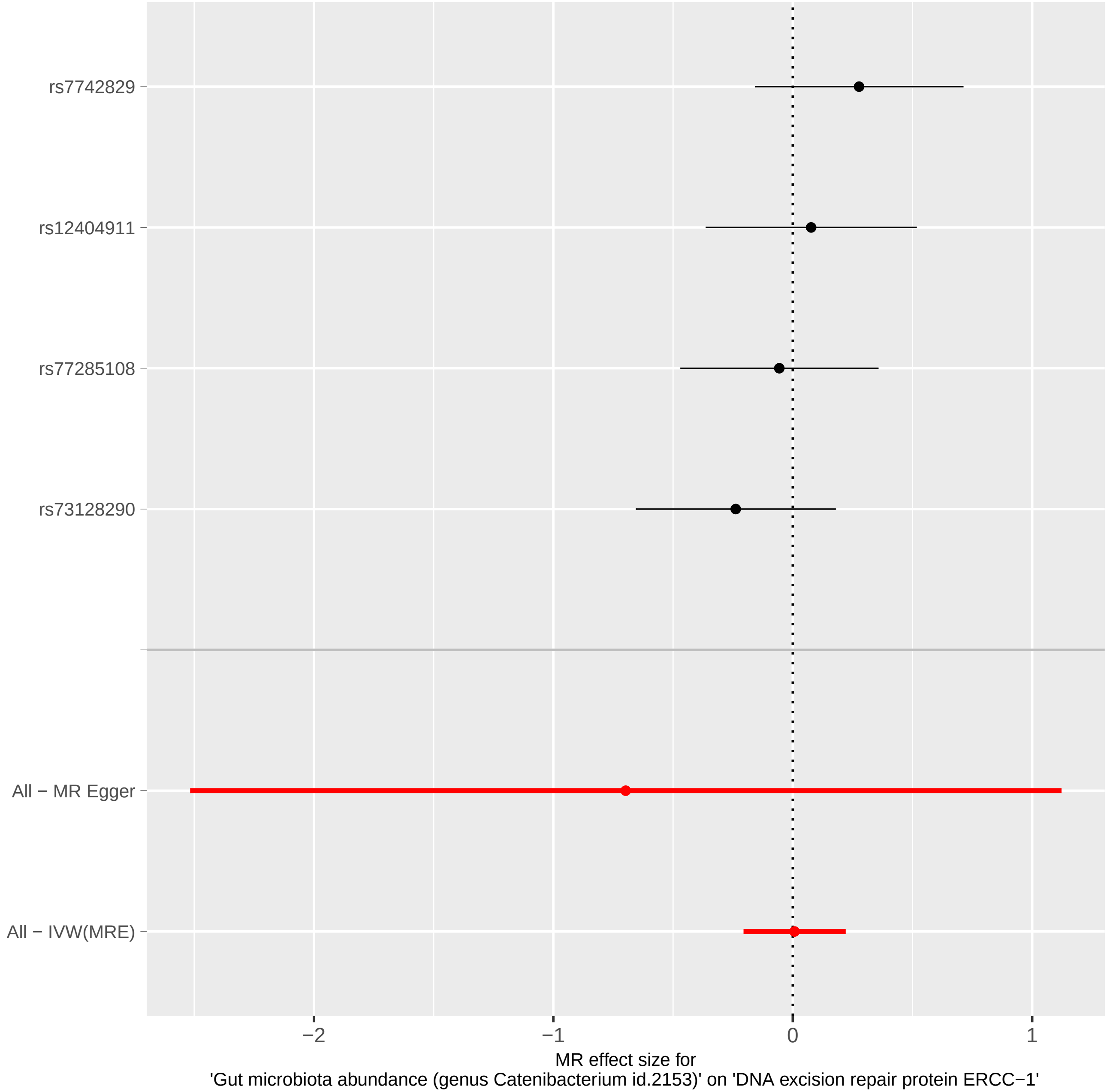

Batch 2−1950 : Gut microbiota abundance (genus Parasutterella id.2892) on DNA excision repair protein ERCC−1
with a threshold of 1e−05

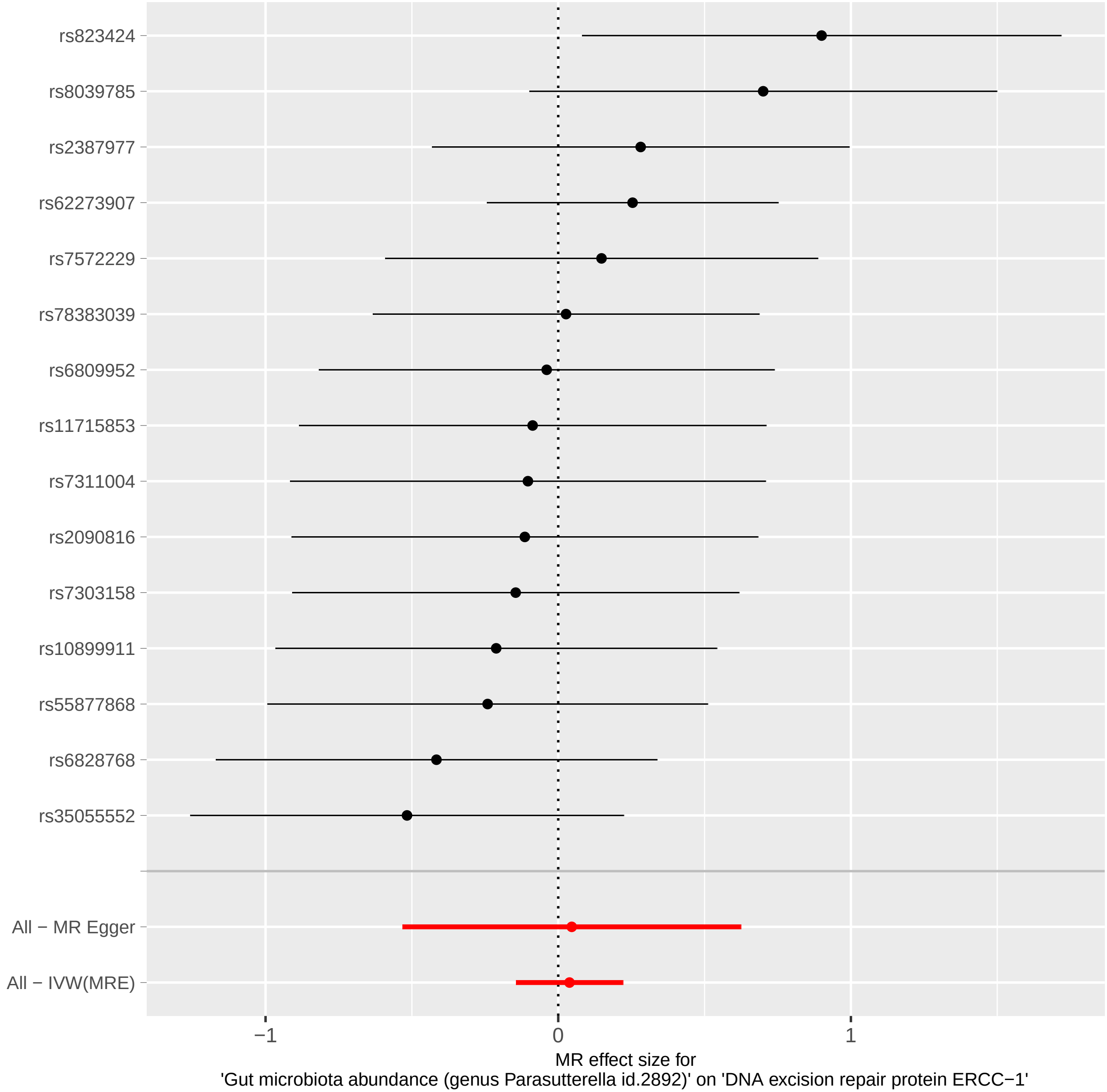

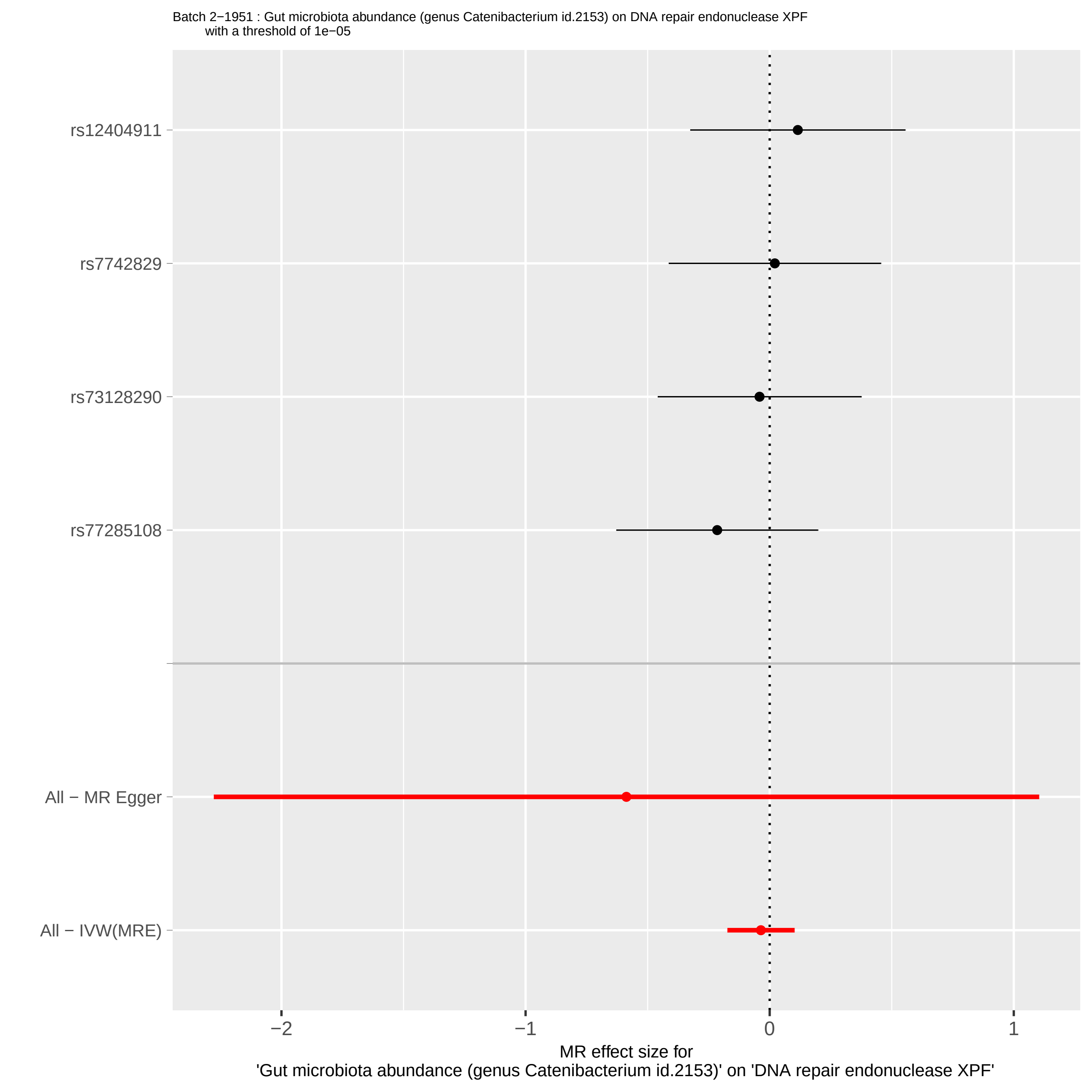
Batch 2−1951 : Gut microbiota abundance (genus Catenibacterium id.2153) on DNA repair endonuclease XPF
with a threshold of 1e−05
rs12404911
rs7742829
rs73128290
rs77285108
All − MR Egger
All − IVW(MRE)
−2
−1
0
1
MR effect size for
'Gut microbiota abundance (genus Catenibacterium id.2153)' on 'DNA repair endonuclease XPF'

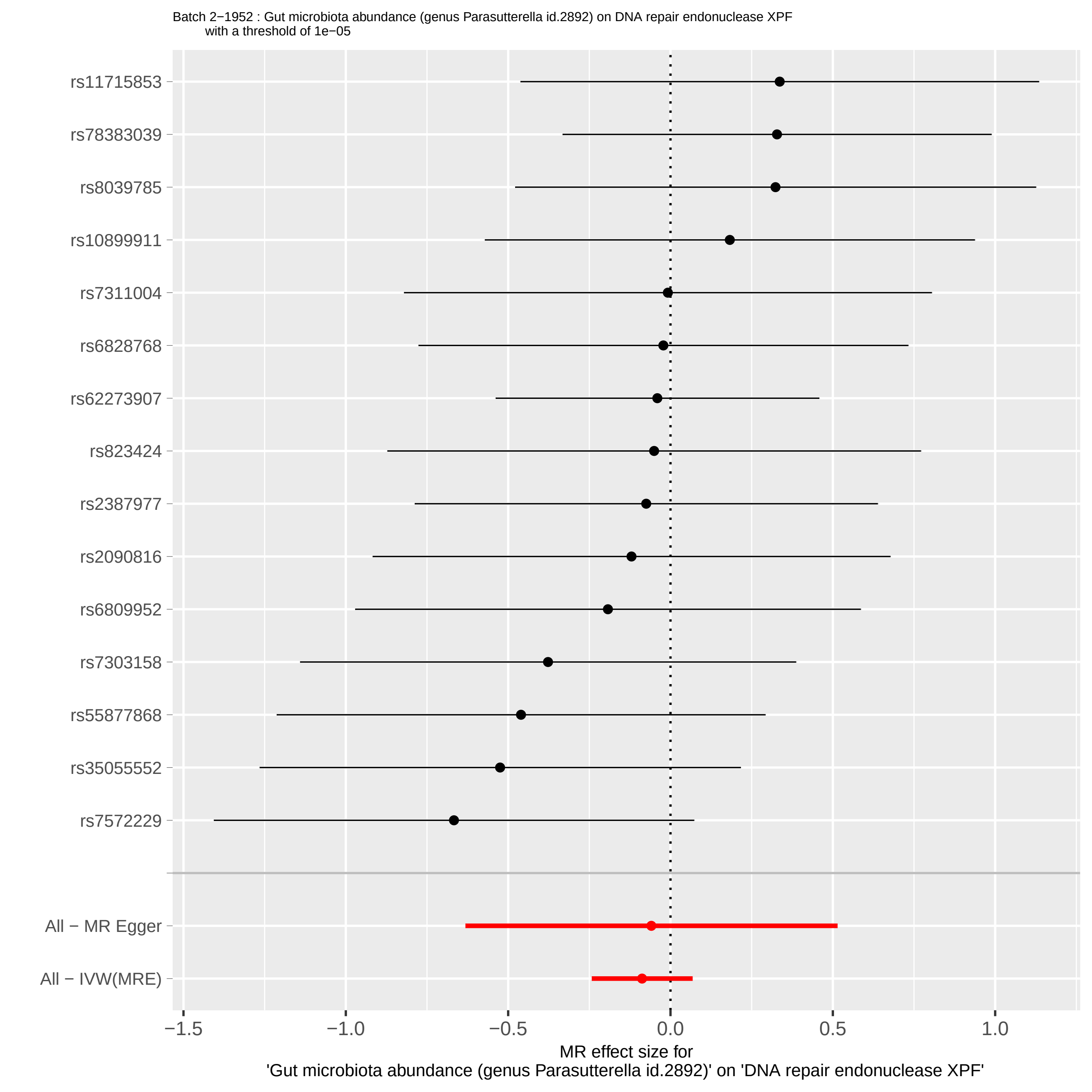

Batch 2−1952 : Gut microbiota abundance (genus Parasutterella id.2892) on DNA repair endonuclease XPF
with a threshold of 1e−05
rs11715853
rs78383039
rs8039785
rs10899911
rs7311004
rs6828768
rs62273907
rs823424
rs2387977
rs2090816
rs6809952
rs7303158
rs55877868
rs35055552
rs7572229
All − MR Egger
All − IVW(MRE)
−1.5
−1.0
−0.5
0.0
0.5
1.0
MR effect size for
'Gut microbiota abundance (genus Parasutterella id.2892)' on 'DNA repair endonuclease XPF'

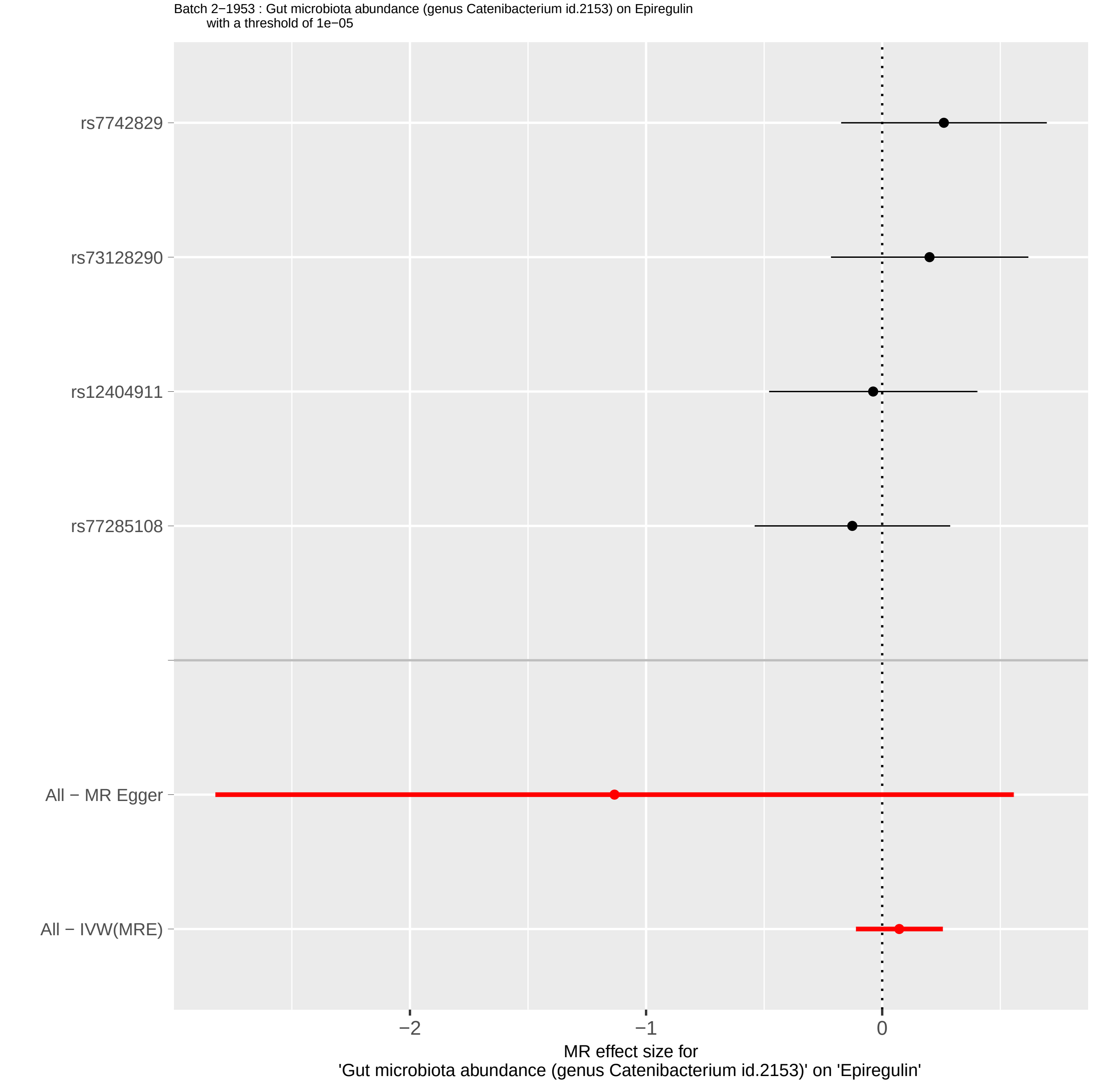

Batch 2−1953 : Gut microbiota abundance (genus Catenibacterium id.2153) on Epiregulin
with a threshold of 1e−05
rs7742829
rs73128290
rs12404911
rs77285108
All − MR Egger
All − IVW(MRE)
−2
−1
0
MR effect size for
'Gut microbiota abundance (genus Catenibacterium id.2153)' on 'Epiregulin'

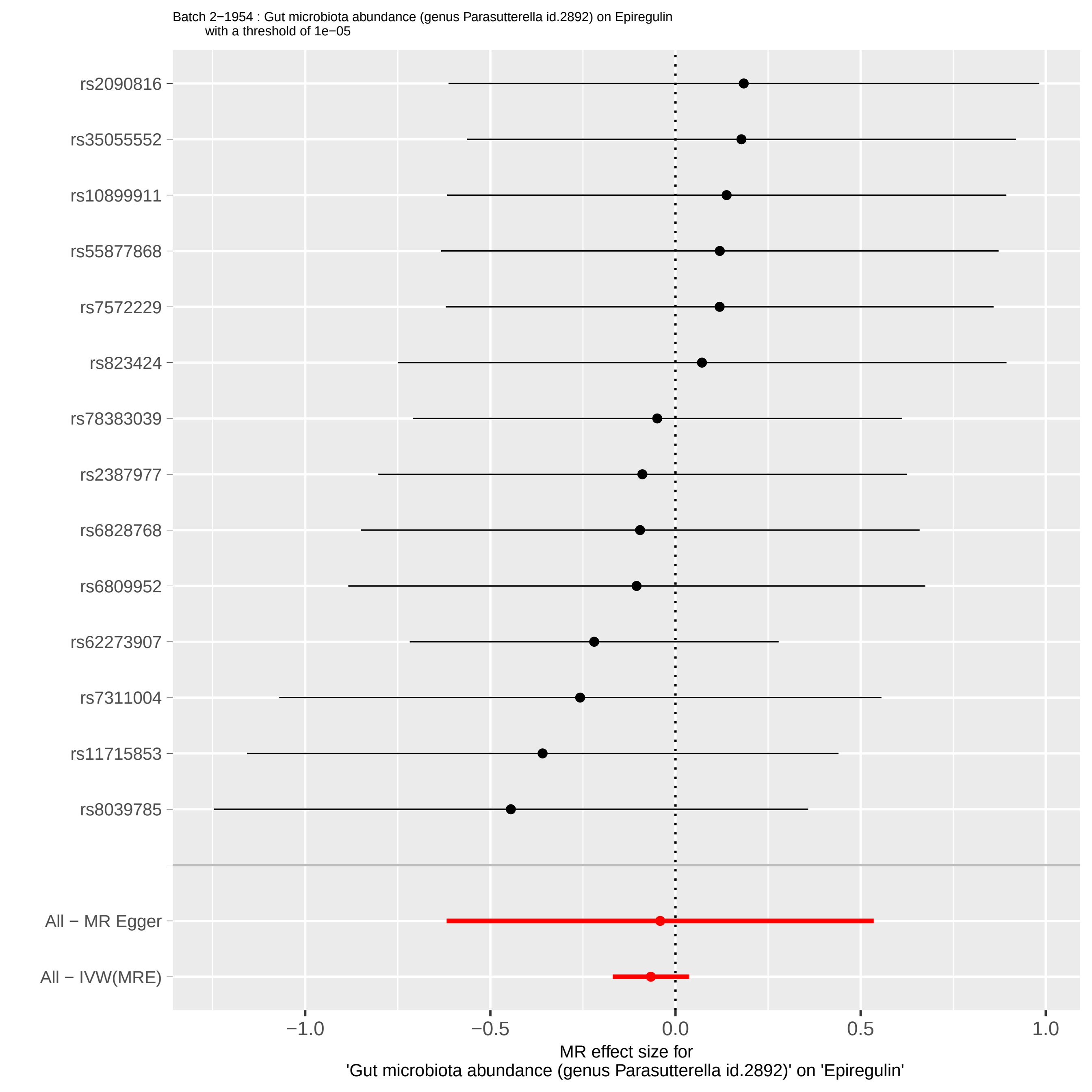

Batch 2−1954 : Gut microbiota abundance (genus Parasutterella id.2892) on Epiregulin
with a threshold of 1e−05
rs2090816
rs35055552
rs10899911
rs55877868
rs7572229
rs823424
rs78383039
rs2387977
rs6828768
rs6809952
rs62273907
rs7311004
rs11715853
rs8039785
All − MR Egger
All − IVW(MRE)
−1.0
−0.5
0.0
0.5
1.0
MR effect size for
'Gut microbiota abundance (genus Parasutterella id.2892)' on 'Epiregulin'

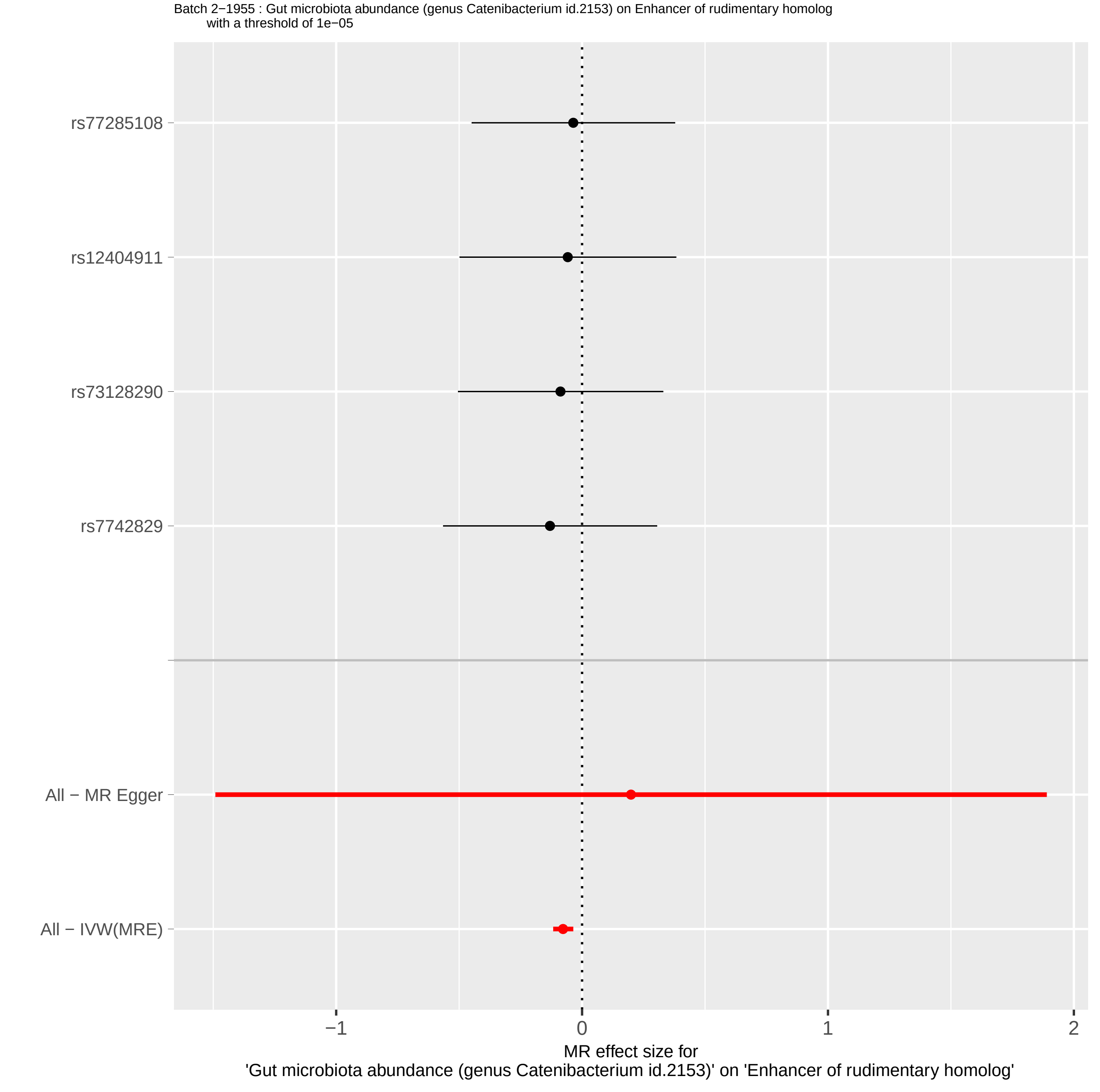

Batch 2−1955 : Gut microbiota abundance (genus Catenibacterium id.2153) on Enhancer of rudimentary homolog
with a threshold of 1e−05
rs77285108
rs12404911
rs73128290
rs7742829
All − MR Egger
All − IVW(MRE)
−1
0
1
2
MR effect size for
'Gut microbiota abundance (genus Catenibacterium id.2153)' on 'Enhancer of rudimentary homolog'

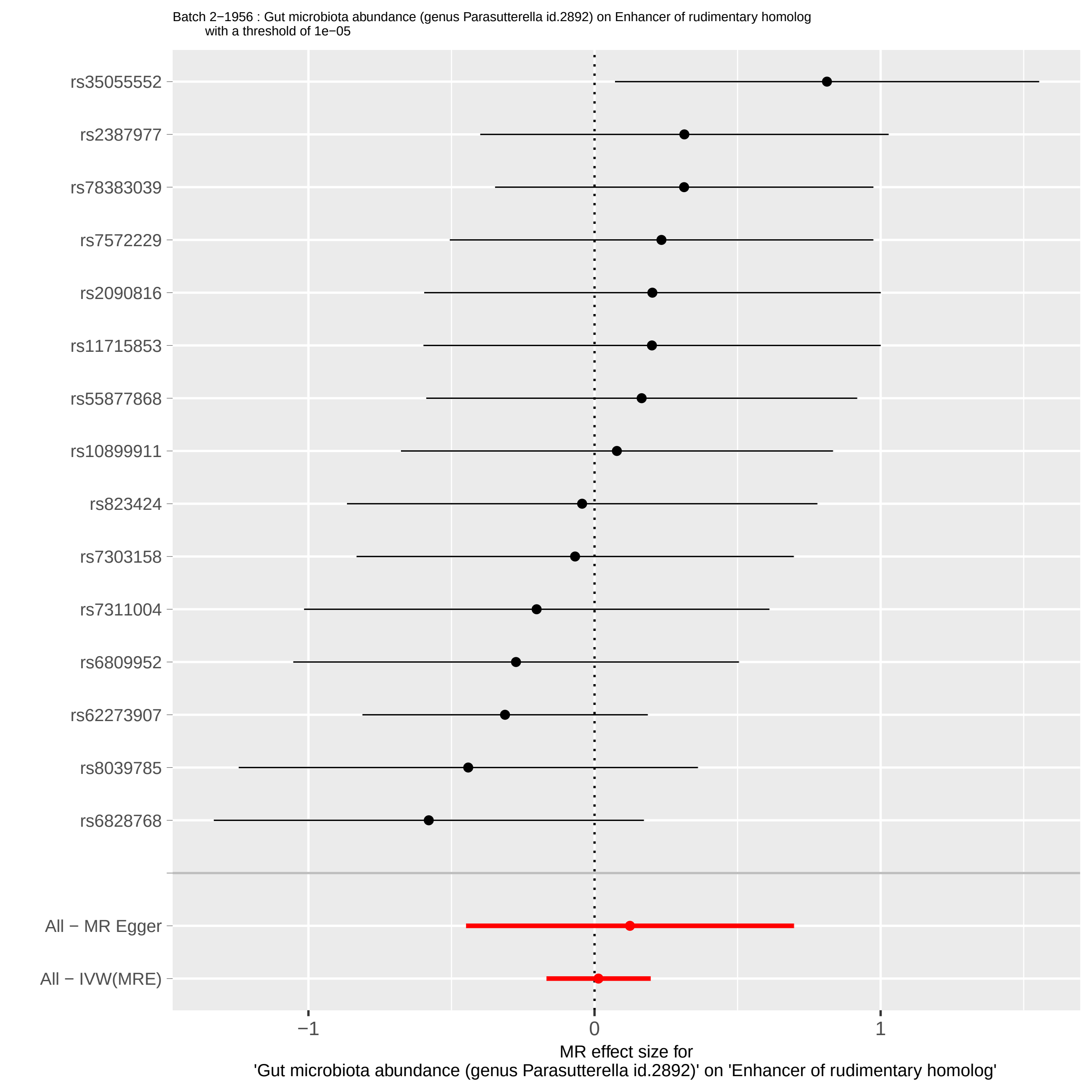
Batch 2−1956 : Gut microbiota abundance (genus Parasutterella id.2892) on Enhancer of rudimentary homolog
with a threshold of 1e−05
rs35055552
rs2387977
rs78383039
rs7572229
rs2090816
rs11715853
rs55877868
rs10899911
rs823424
rs7303158
rs7311004
rs6809952
rs62273907
rs8039785
rs6828768
All − MR Egger
All − IVW(MRE)
−1
0
1
MR effect size for
'Gut microbiota abundance (genus Parasutterella id.2892)' on 'Enhancer of rudimentary homolog'

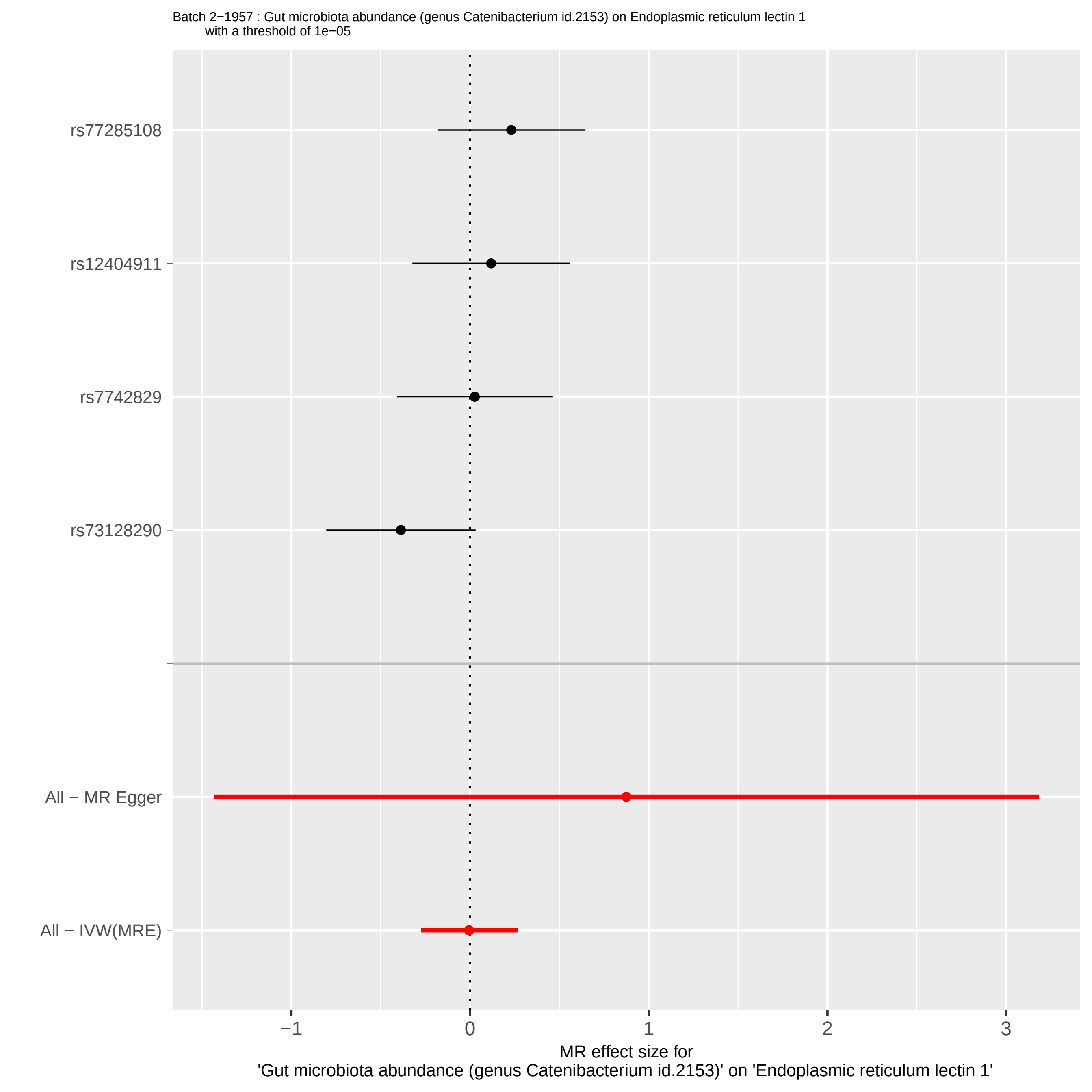
Batch 2−1957 : Gut microbiota abundance (genus Catenibacterium id.2153) on Endoplasmic reticulum lectin 1
with a threshold of 1e−05
rs77285108
rs12404911
rs7742829
rs73128290
All − MR Egger
All − IVW(MRE)
−1
0
1
2
3
MR effect size for
'Gut microbiota abundance (genus Catenibacterium id.2153)' on 'Endoplasmic reticulum lectin 1'

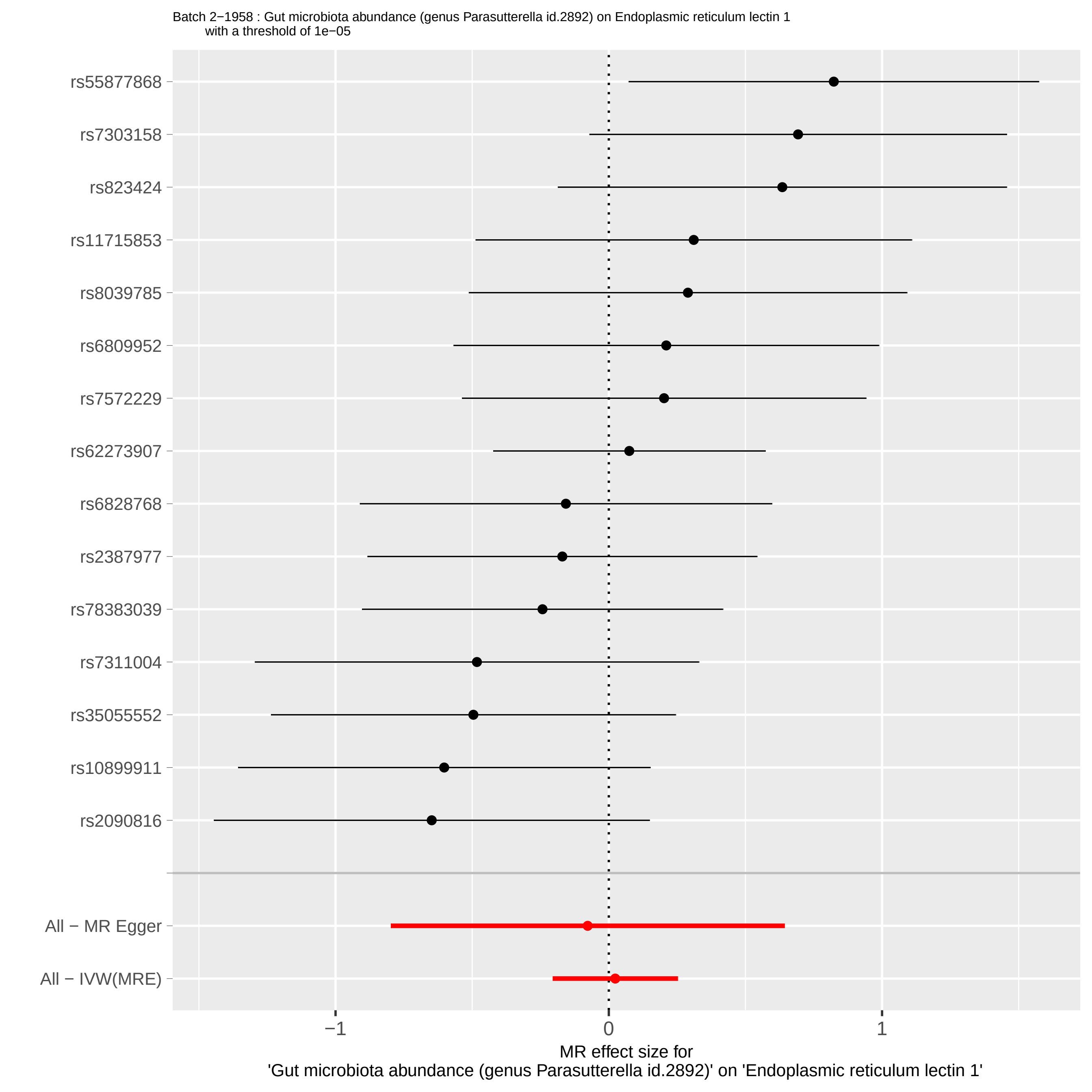
Batch 2−1958 : Gut microbiota abundance (genus Parasutterella id.2892) on Endoplasmic reticulum lectin 1
with a threshold of 1e−05
rs55877868
rs7303158
rs823424
rs11715853
rs8039785
rs6809952
rs7572229
rs62273907
rs6828768
rs2387977
rs78383039
rs7311004
rs35055552
rs10899911
rs2090816
All − MR Egger
All − IVW(MRE)
−1
0
1
MR effect size for
'Gut microbiota abundance (genus Parasutterella id.2892)' on 'Endoplasmic reticulum lectin 1'

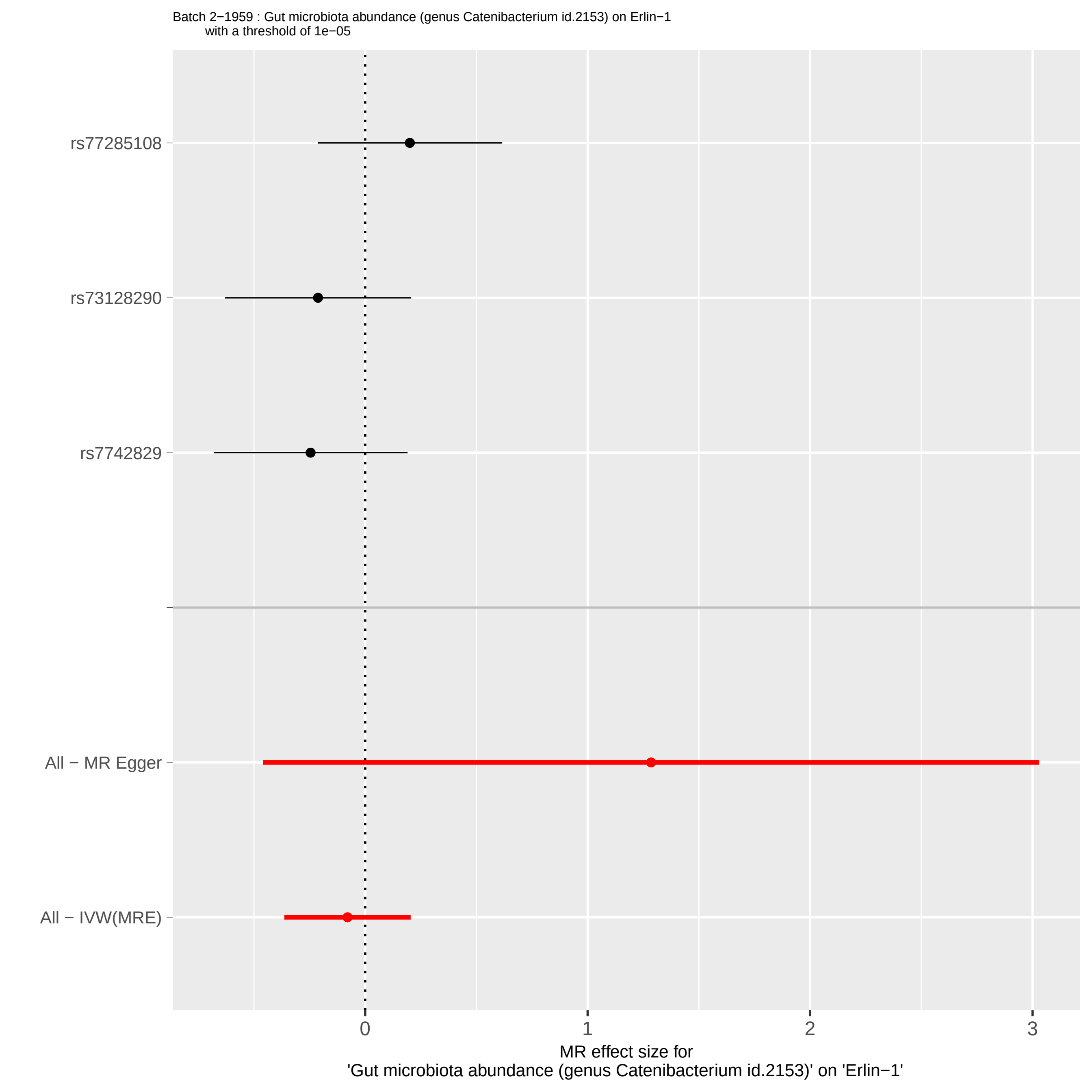

Batch 2−1959 : Gut microbiota abundance (genus Catenibacterium id.2153) on Erlin−1
with a threshold of 1e−05
rs77285108
rs73128290
rs7742829
All − MR Egger
All − IVW(MRE)
0
1
2
3
MR effect size for
'Gut microbiota abundance (genus Catenibacterium id.2153)' on 'Erlin−1'

Batch 2−1960 : Gut microbiota abundance (genus Parasutterella id.2892) on Erlin−1
with a threshold of 1e−05

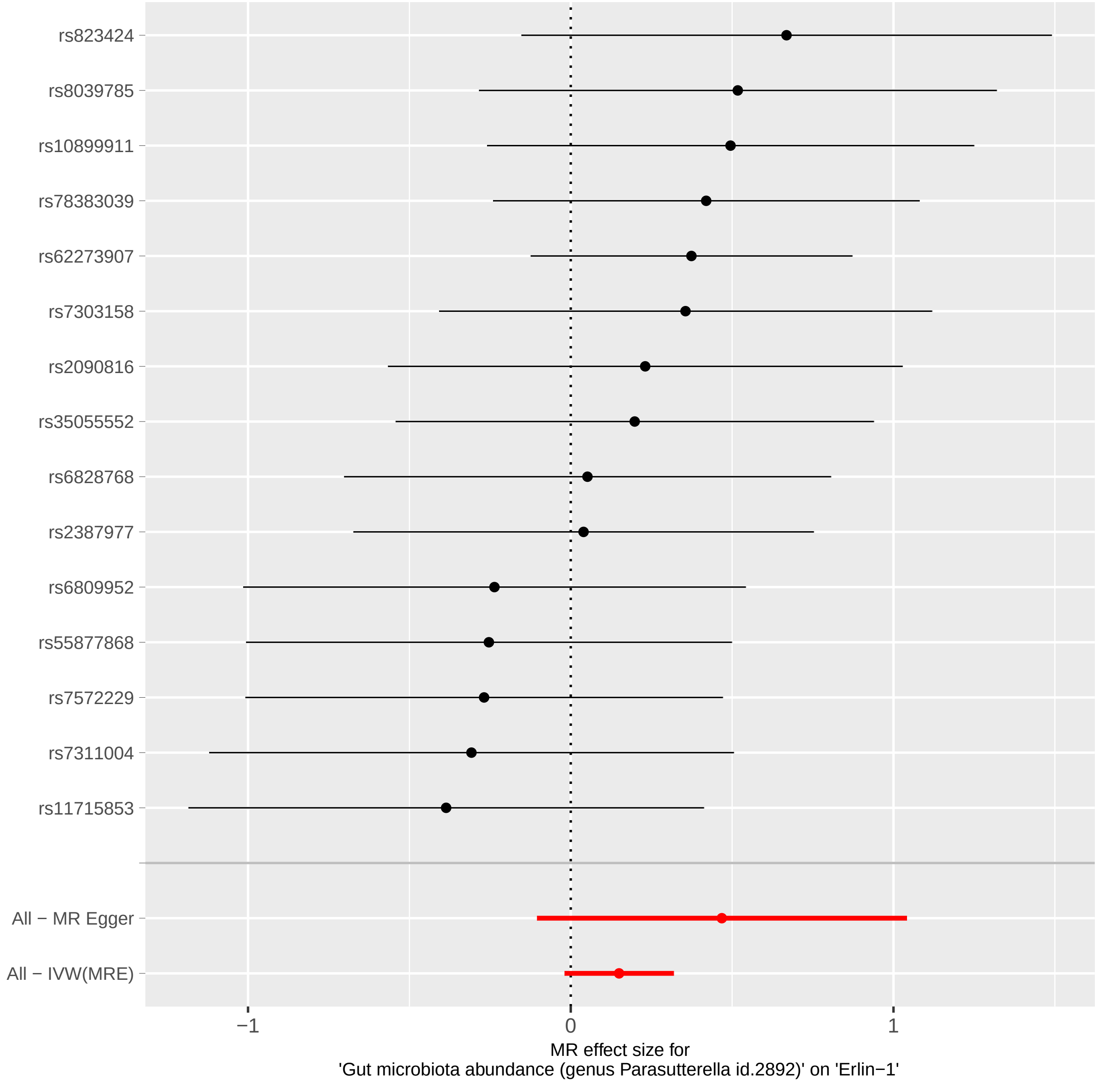

Batch 2−1961 : Gut microbiota abundance (genus Catenibacterium id.2153) on Erythroid membrane−associated protein with a threshold of 1e−05

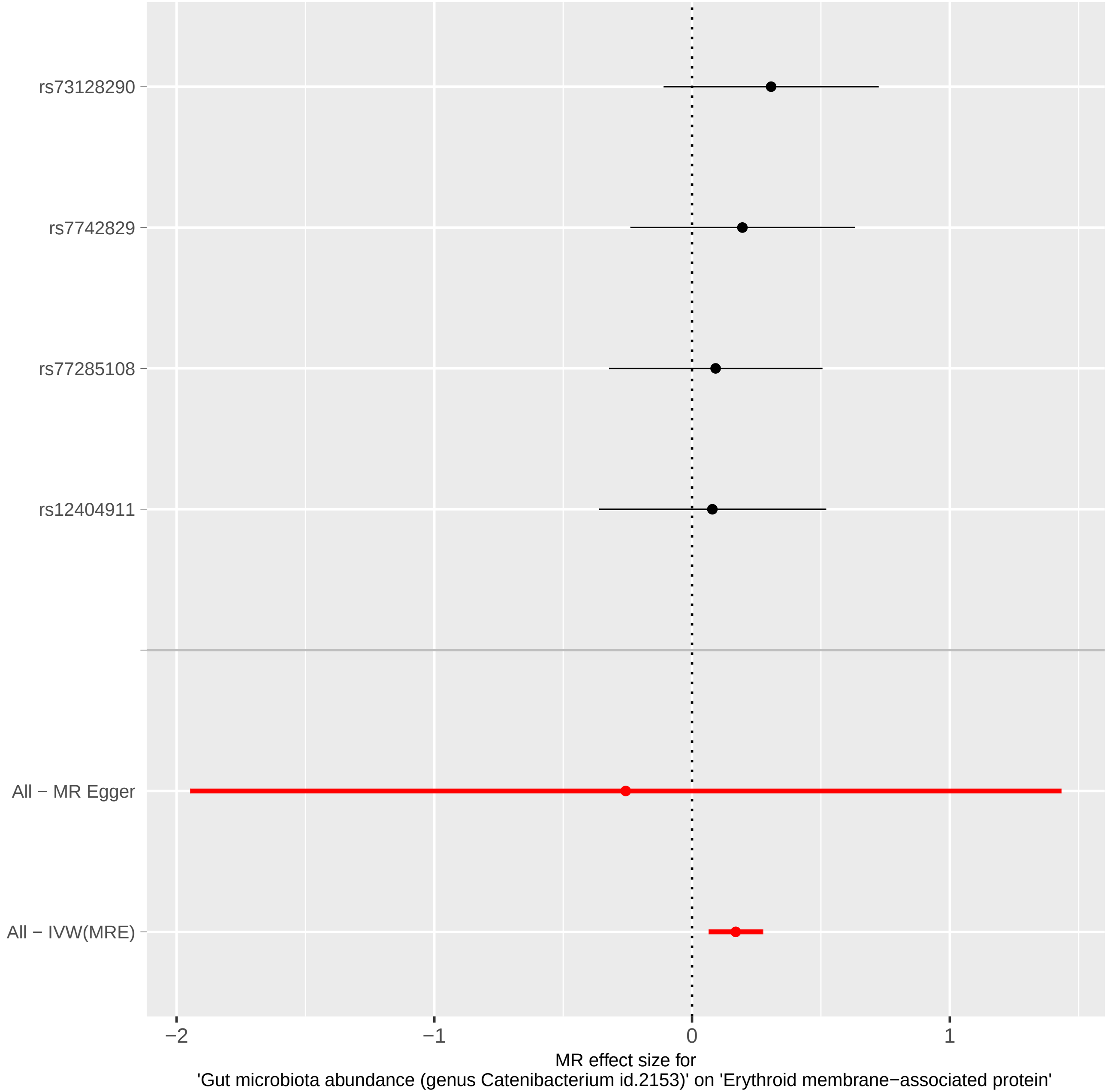

Batch 2−1962 : Gut microbiota abundance (genus Parasutterella id.2892) on Erythroid membrane−associated protein with a threshold of 1e−05

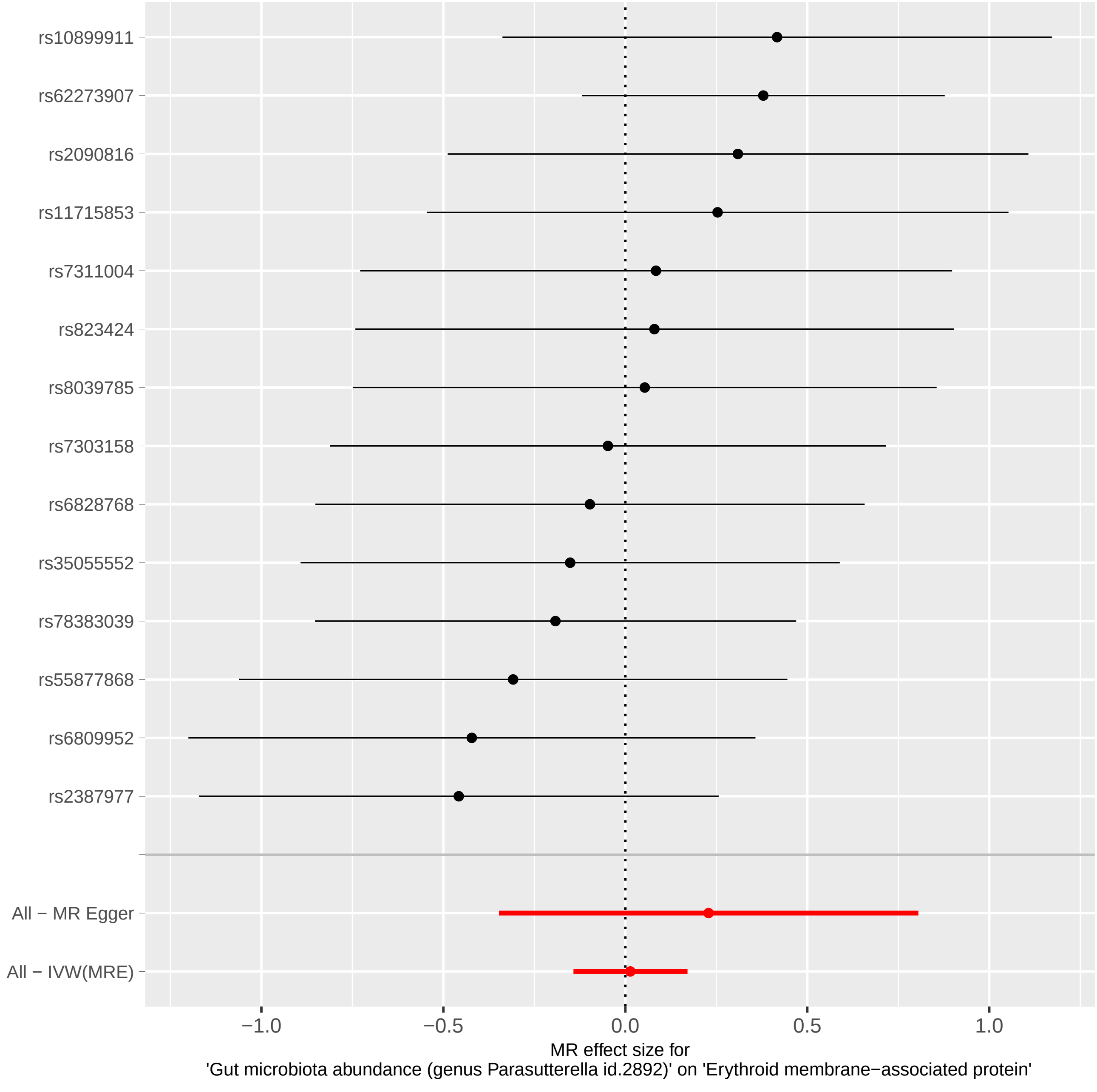

Batch 2−1963 : Gut microbiota abundance (genus Catenibacterium id.2153) on ERO1−like protein alpha
with a threshold of 1e−05

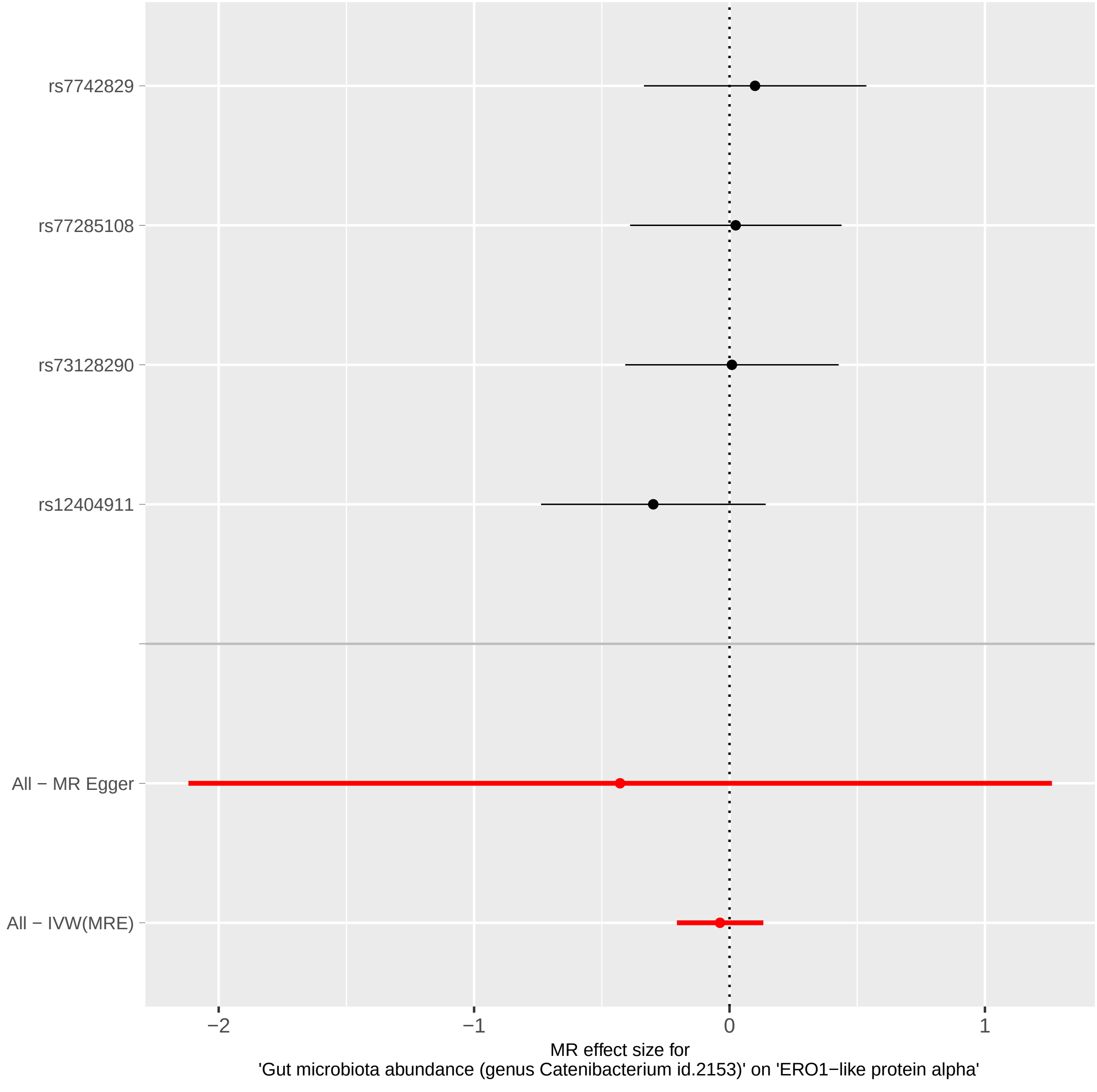

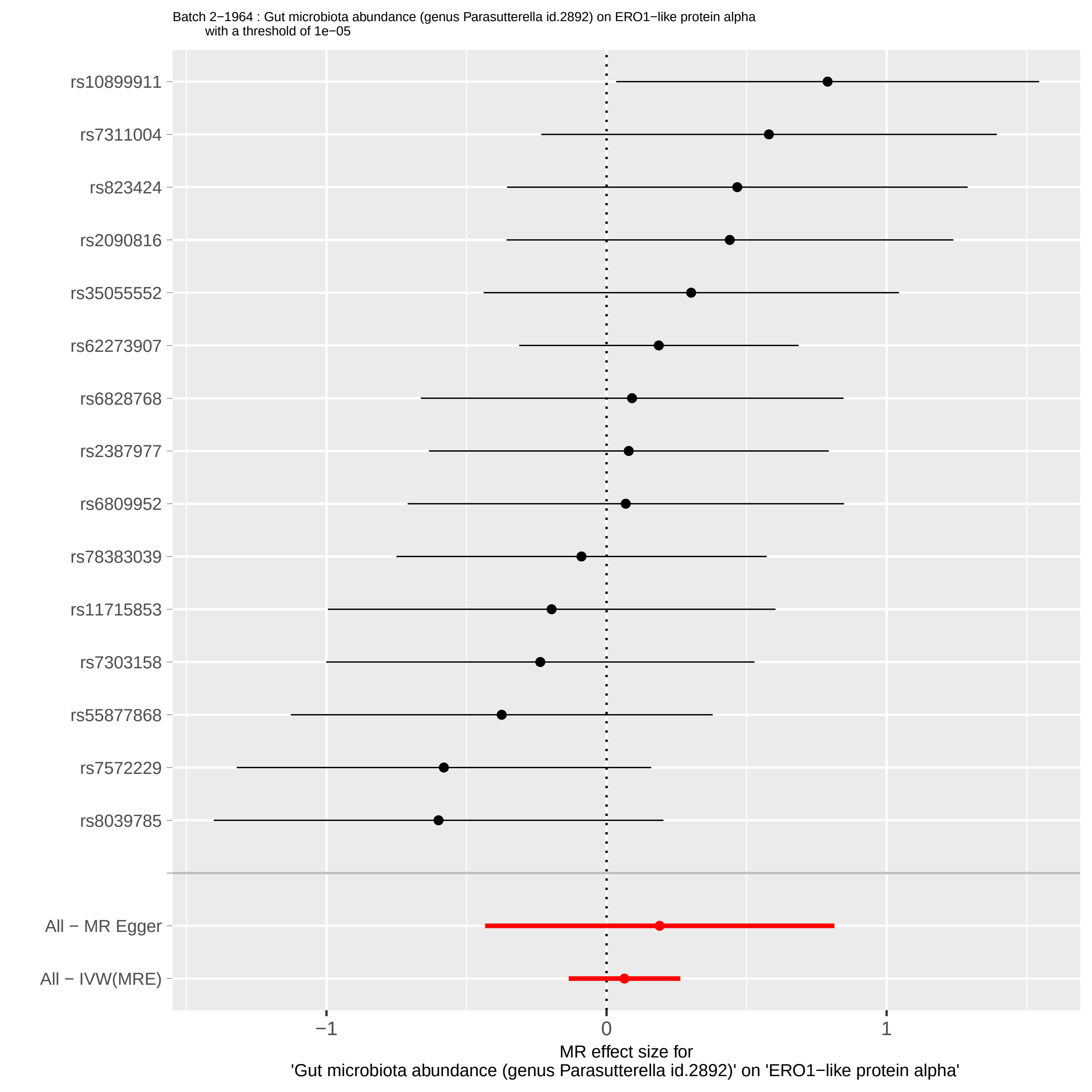

Batch 2−1964 : Gut microbiota abundance (genus Parasutterella id.2892) on ERO1−like protein alpha
with a threshold of 1e−05
rs10899911
rs7311004
rs823424
rs2090816
rs35055552
rs62273907
rs6828768
rs2387977
rs6809952
rs78383039
rs11715853
rs7303158
rs55877868
rs7572229
rs8039785
All − MR Egger
All − IVW(MRE)
−1
0
1
MR effect size for
'Gut microbiota abundance (genus Parasutterella id.2892)' on 'ERO1−like protein alpha'

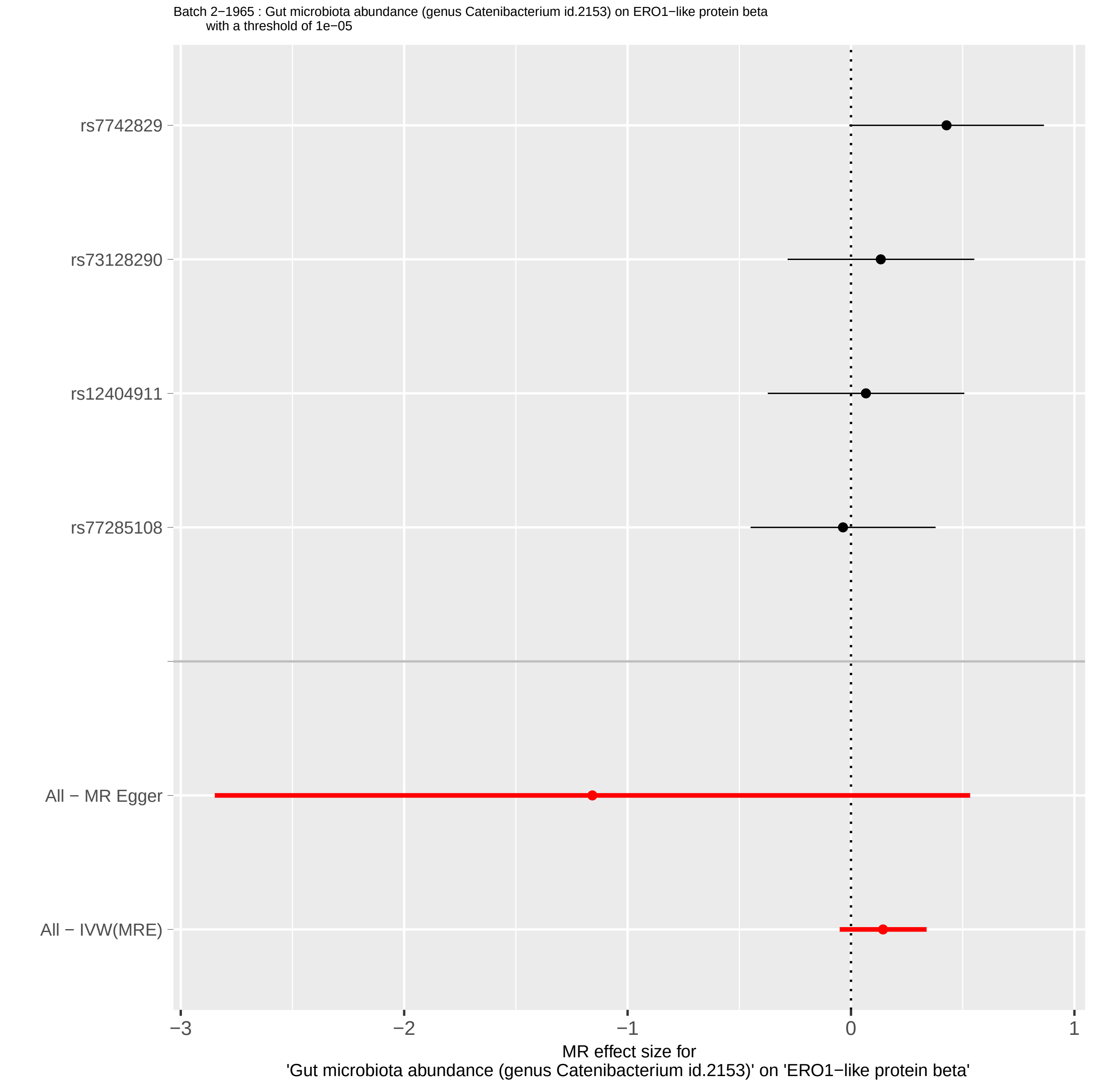

Batch 2−1965 : Gut microbiota abundance (genus Catenibacterium id.2153) on ERO1−like protein beta
with a threshold of 1e−05
rs7742829
rs73128290
rs12404911
rs77285108
All − MR Egger
All − IVW(MRE)
−3
−2
−1
0
1
MR effect size for
'Gut microbiota abundance (genus Catenibacterium id.2153)' on 'ERO1−like protein beta'

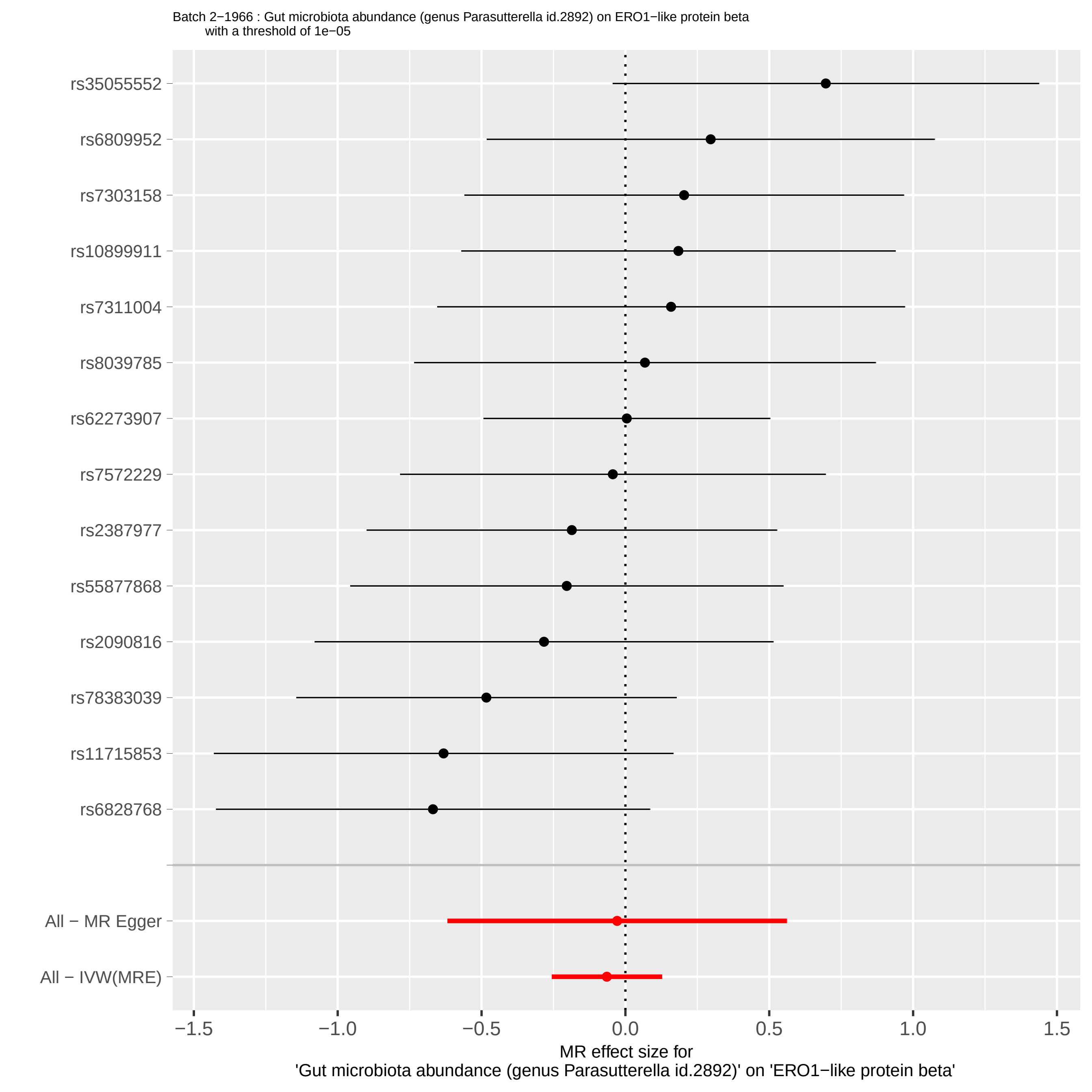

Batch 2−1966 : Gut microbiota abundance (genus Parasutterella id.2892) on ERO1−like protein beta
with a threshold of 1e−05
rs35055552
rs6809952
rs7303158
rs10899911
rs7311004
rs8039785
rs62273907
rs7572229
rs2387977
rs55877868
rs2090816
rs78383039
rs11715853
rs6828768
All − MR Egger
All − IVW(MRE)
−1.5
−1.0
−0.5
0.0
0.5
1.0
1.5
MR effect size for
'Gut microbiota abundance (genus Parasutterella id.2892)' on 'ERO1−like protein beta'

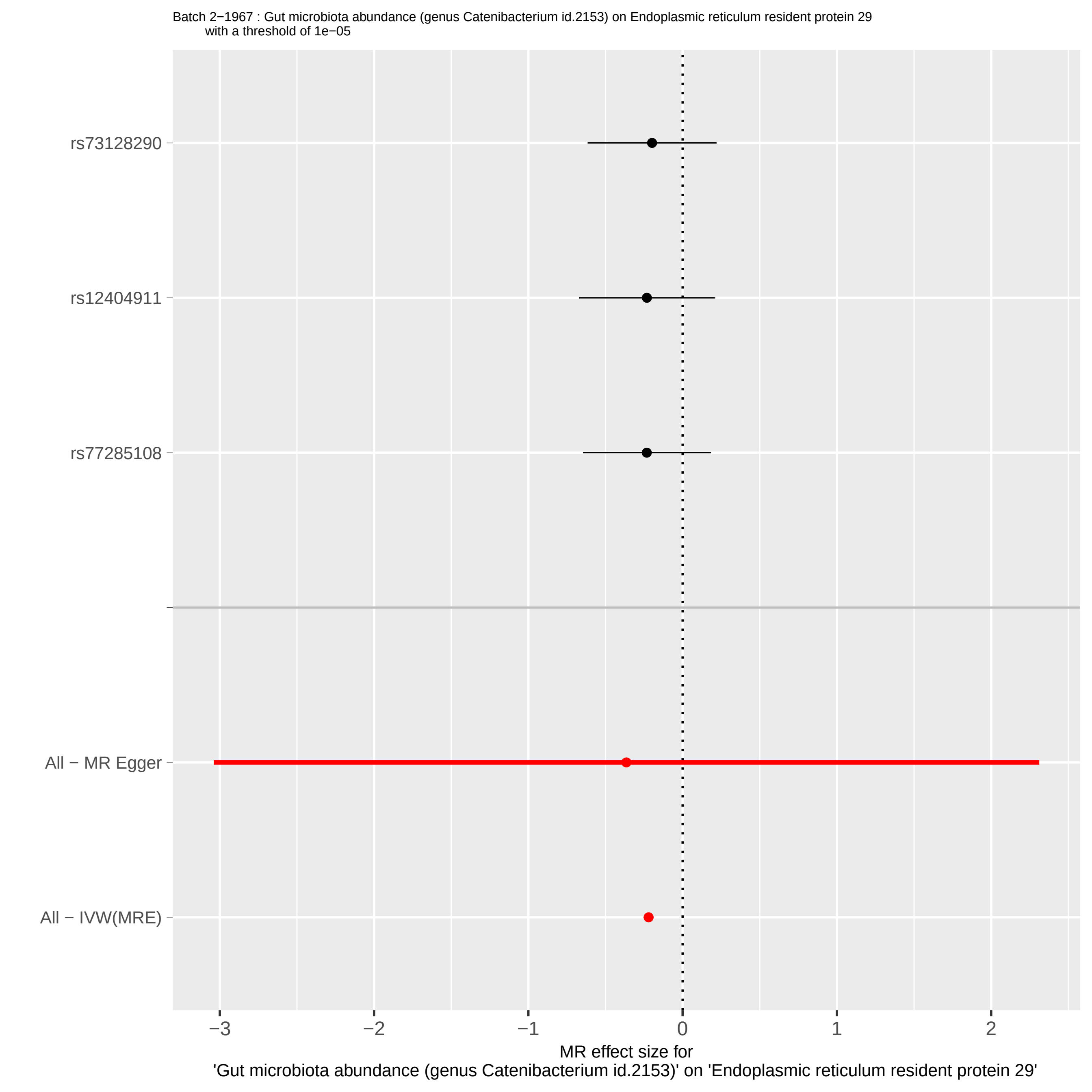

Batch 2−1967 : Gut microbiota abundance (genus Catenibacterium id.2153) on Endoplasmic reticulum resident protein 29
with a threshold of 1e−05
rs73128290
rs12404911
rs77285108
All − MR Egger
All − IVW(MRE)
−3
−2
−1
0
1
2
MR effect size for
'Gut microbiota abundance (genus Catenibacterium id.2153)' on 'Endoplasmic reticulum resident protein 29'

Batch 2−1968 : Gut microbiota abundance (genus Parasutterella id.2892) on Endoplasmic reticulum resident protein 29
with a threshold of 1e−05

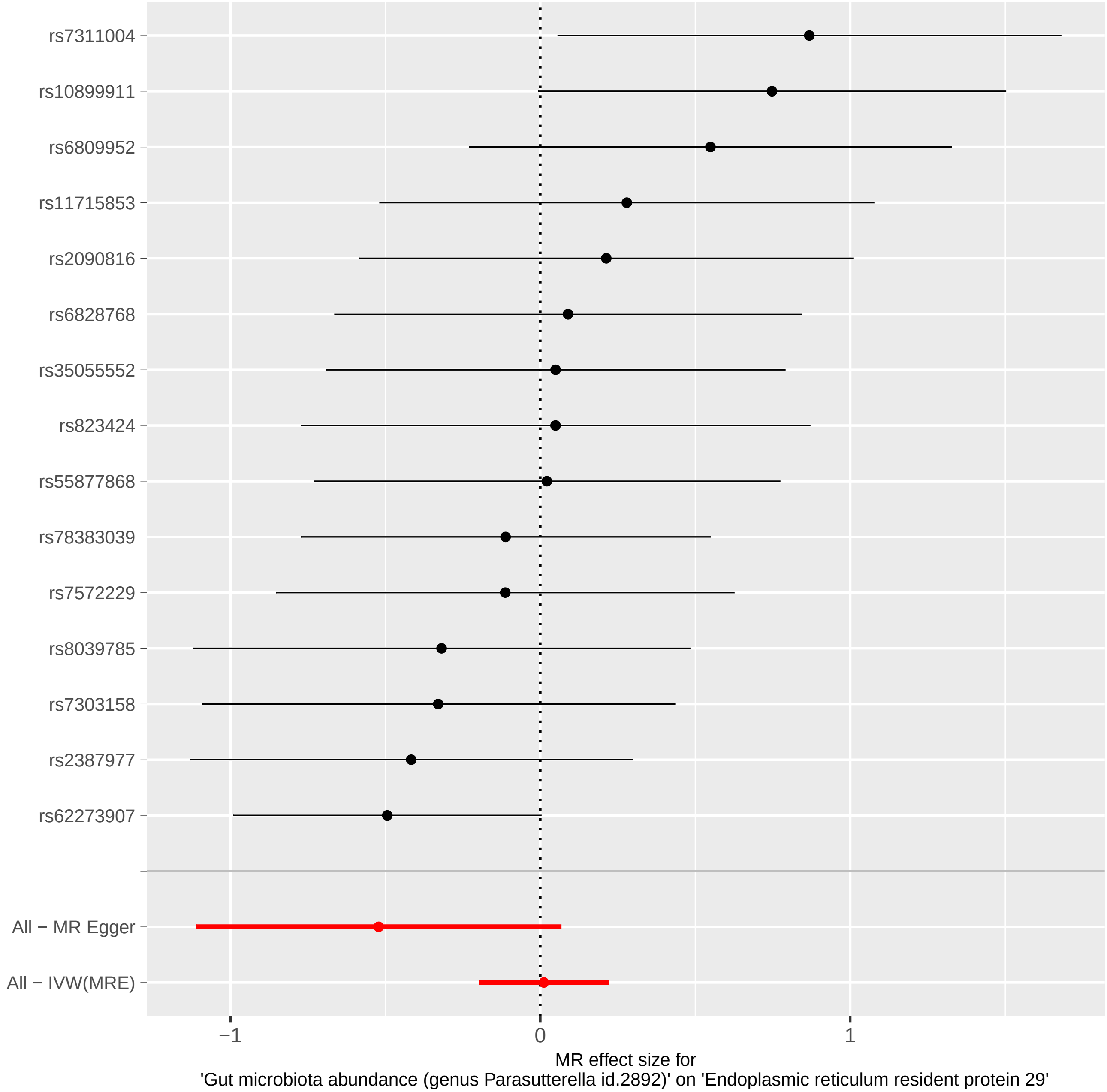

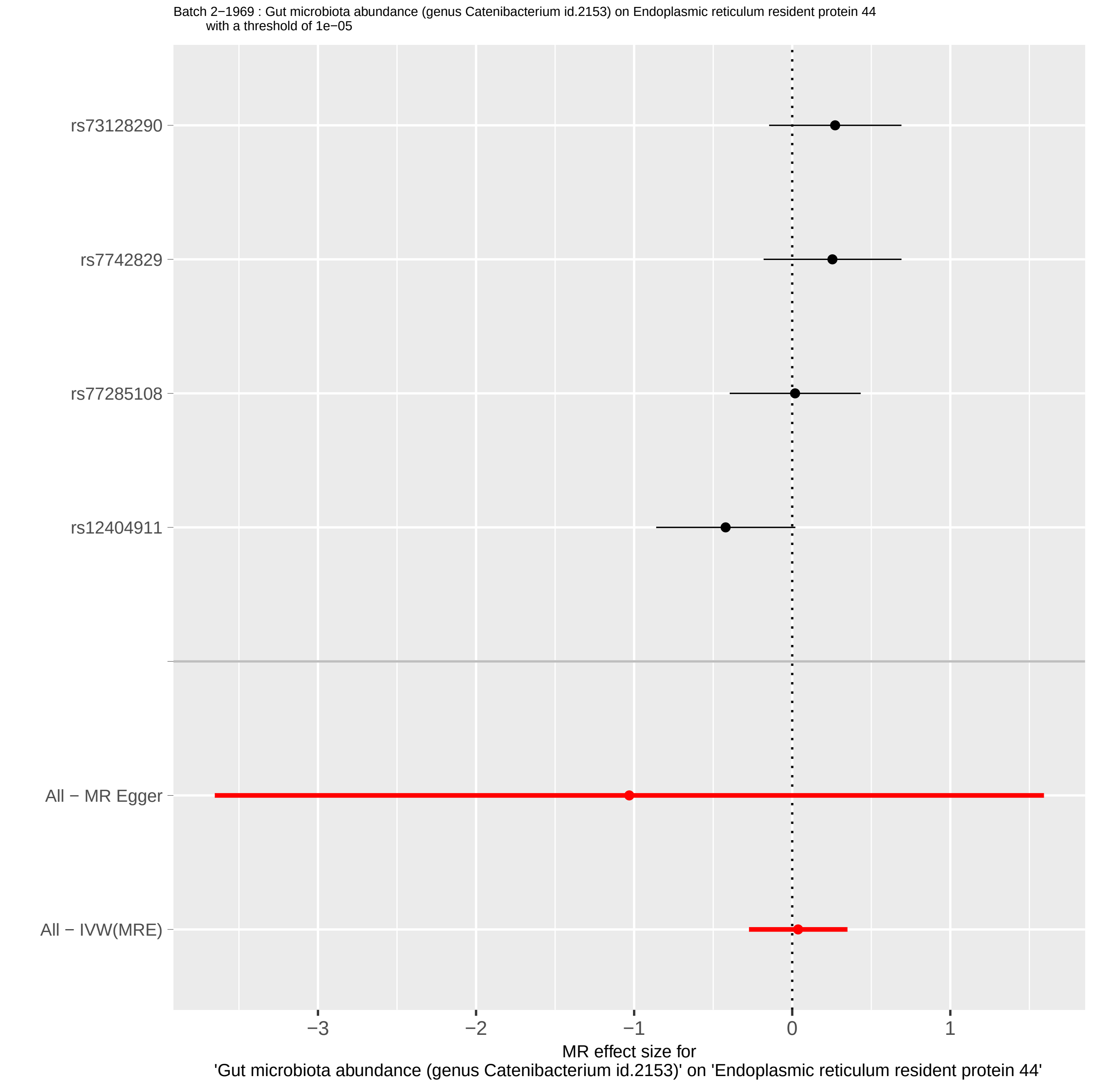

Batch 2−1969 : Gut microbiota abundance (genus Catenibacterium id.2153) on Endoplasmic reticulum resident protein 44
with a threshold of 1e−05
rs73128290
rs7742829
rs77285108
rs12404911
All − MR Egger
All − IVW(MRE)
−3
−2
−1
0
1
MR effect size for
'Gut microbiota abundance (genus Catenibacterium id.2153)' on 'Endoplasmic reticulum resident protein 44'

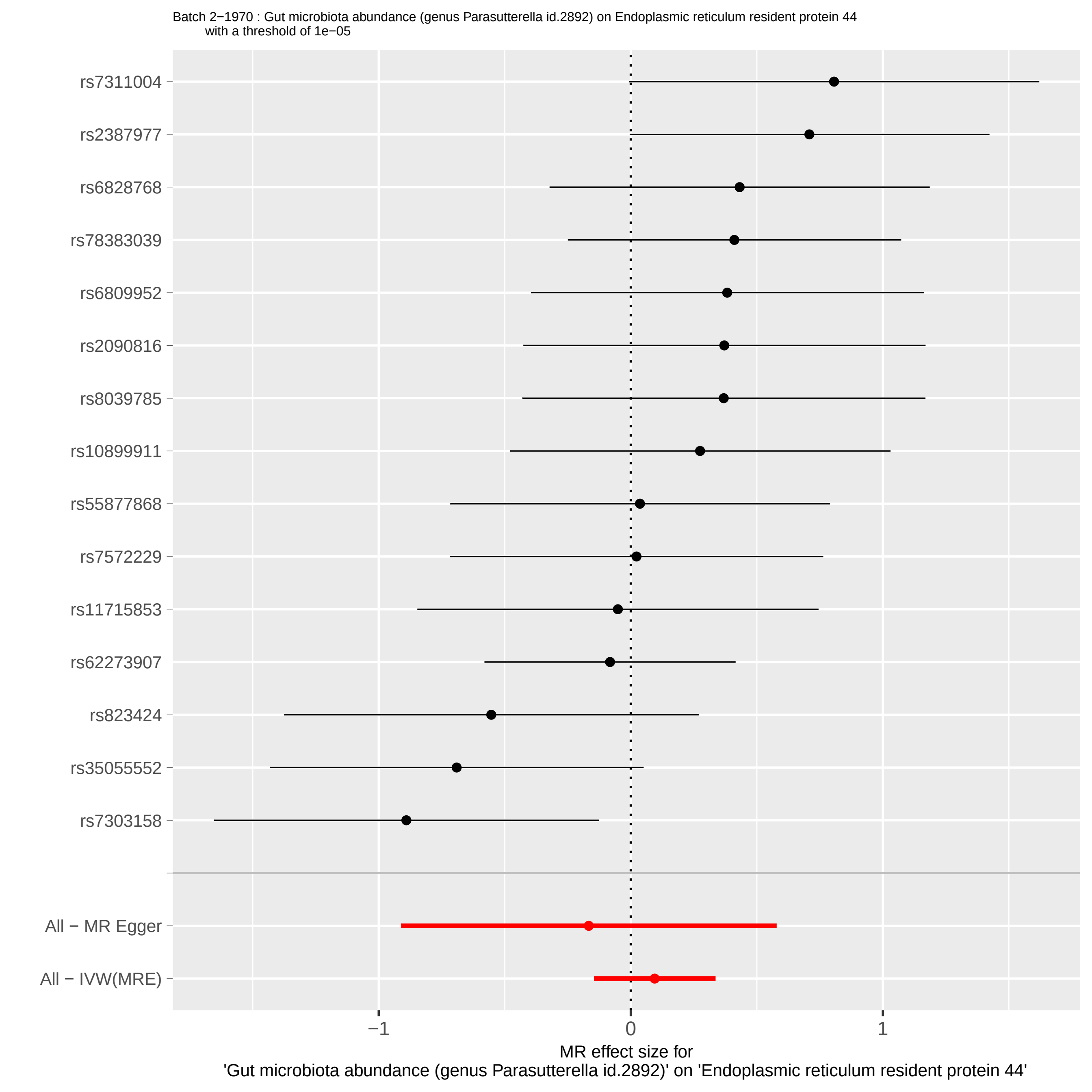

Batch 2−1970 : Gut microbiota abundance (genus Parasutterella id.2892) on Endoplasmic reticulum resident protein 44
with a threshold of 1e−05
rs7311004
rs2387977
rs6828768
rs78383039
rs6809952
rs2090816
rs8039785
rs10899911
rs55877868
rs7572229
rs11715853
rs62273907
rs823424
rs35055552
rs7303158
All − MR Egger
All − IVW(MRE)
−1
0
1
MR effect size for
'Gut microbiota abundance (genus Parasutterella id.2892)' on 'Endoplasmic reticulum resident protein 44'

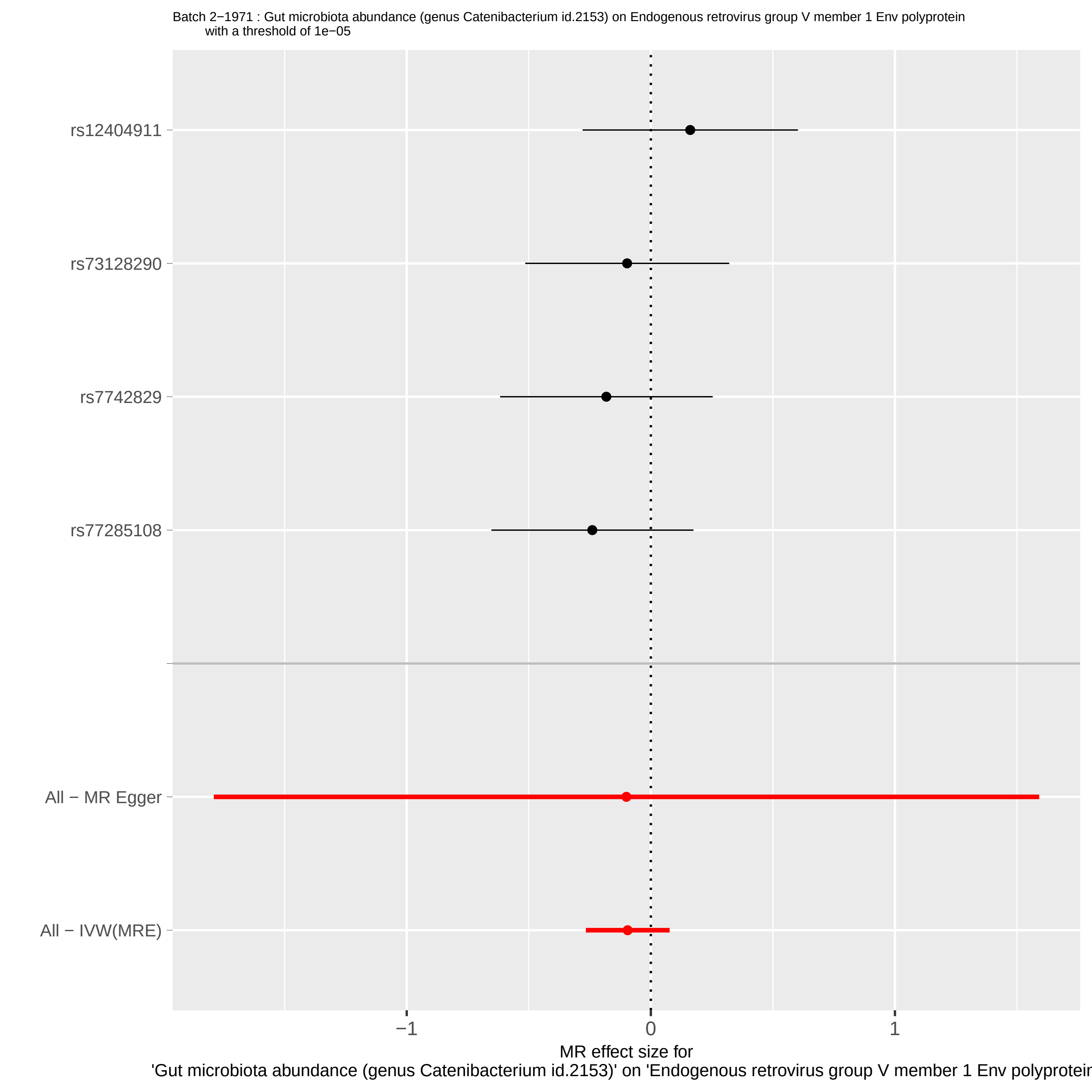

Batch 2−1971 : Gut microbiota abundance (genus Catenibacterium id.2153) on Endogenous retrovirus group V member 1 Env polyprotein
with a threshold of 1e−05
rs12404911
rs73128290
rs7742829
rs77285108
All − MR Egger
All − IVW(MRE)
−1
0
1
MR effect size for
'Gut microbiota abundance (genus Catenibacterium id.2153)' on 'Endogenous retrovirus group V member 1 Env polyprotein

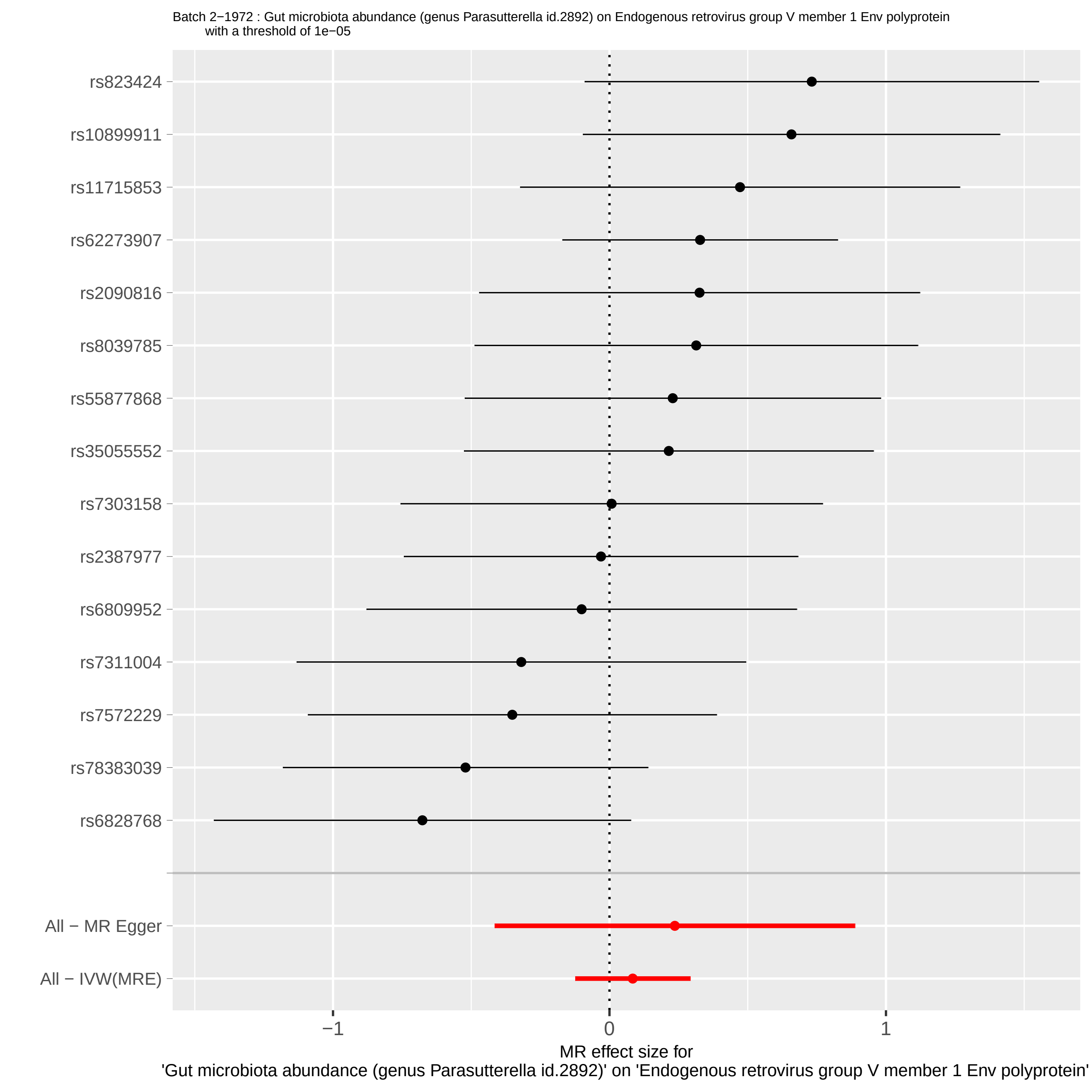

Batch 2−1972 : Gut microbiota abundance (genus Parasutterella id.2892) on Endogenous retrovirus group V member 1 Env polyprotein with a threshold of 1e−05
rs823424
rs10899911
rs11715853
rs62273907
rs2090816
rs8039785
rs55877868
rs35055552
rs7303158
rs2387977
rs6809952
rs7311004
rs7572229
rs78383039
rs6828768
All − MR Egger
All − IVW(MRE)
−1
0
1
MR effect size for
'Gut microbiota abundance (genus Parasutterella id.2892)' on 'Endogenous retrovirus group V member 1 Env polyprotein'

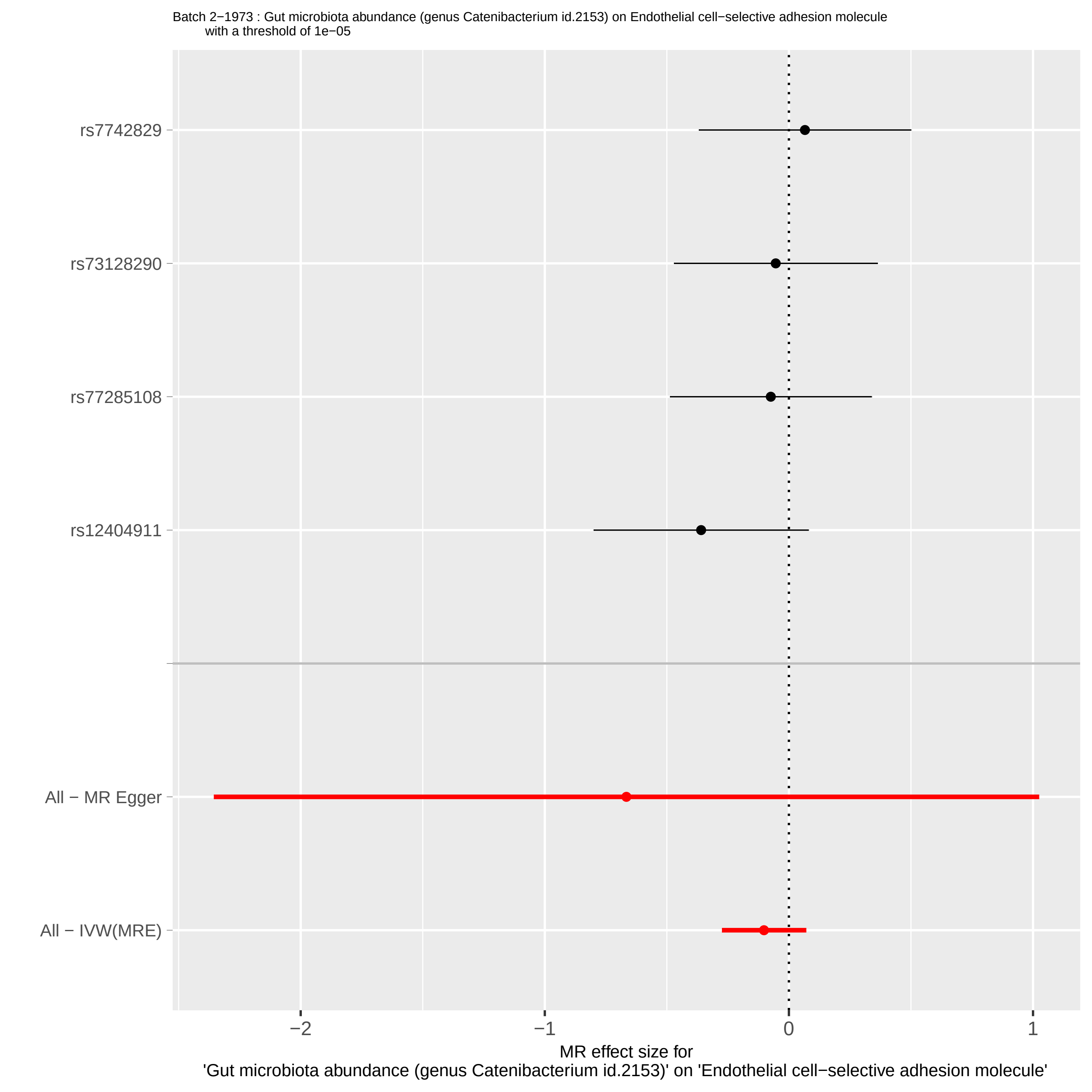

Batch 2−1973 : Gut microbiota abundance (genus Catenibacterium id.2153) on Endothelial cell−selective adhesion molecule
with a threshold of 1e−05
rs7742829
rs73128290
rs77285108
rs12404911
All − MR Egger
All − IVW(MRE)
−2
−1
0
1
MR effect size for
'Gut microbiota abundance (genus Catenibacterium id.2153)' on 'Endothelial cell−selective adhesion molecule'

Batch 2−1974 : Gut microbiota abundance (genus Parasutterella id.2892) on Endothelial cell−selective adhesion molecule with a threshold of 1e−05

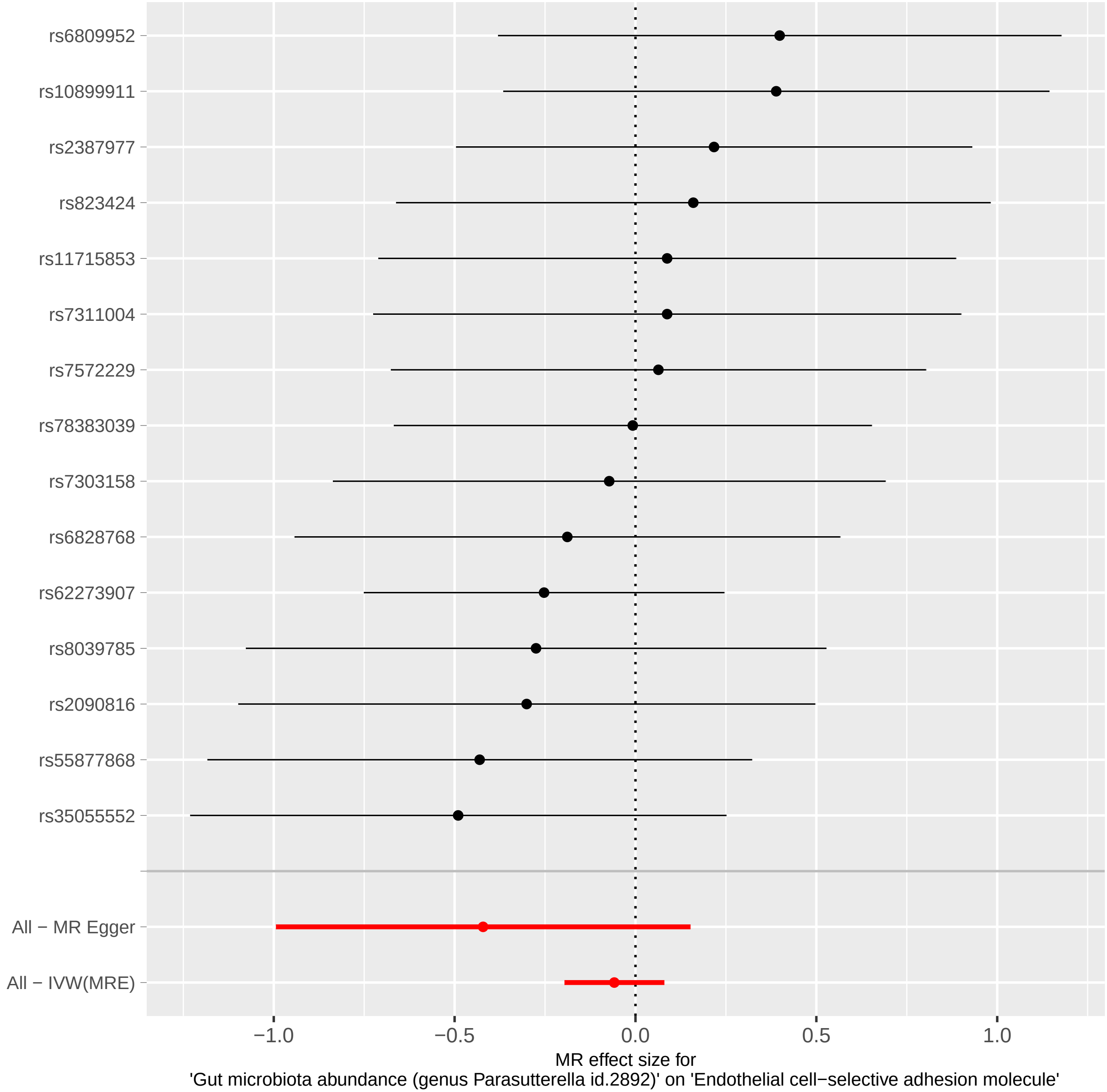

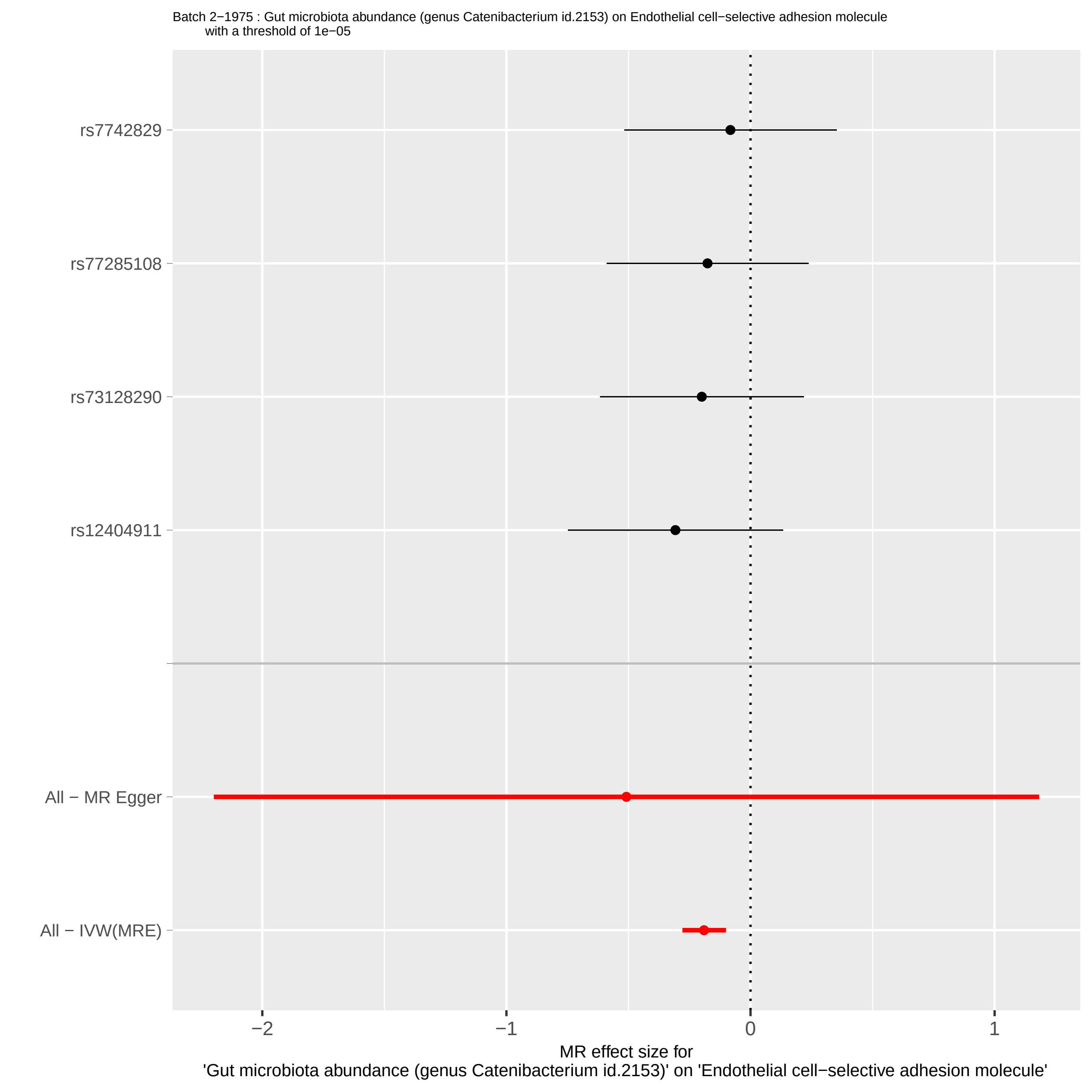

Batch 2−1975 : Gut microbiota abundance (genus Catenibacterium id.2153) on Endothelial cell−selective adhesion molecule with a threshold of 1e−05
rs7742829
rs77285108
rs73128290
rs12404911
All − MR Egger
All − IVW(MRE)
−2
−1
0
1
MR effect size for
'Gut microbiota abundance (genus Catenibacterium id.2153)' on 'Endothelial cell−selective adhesion molecule'

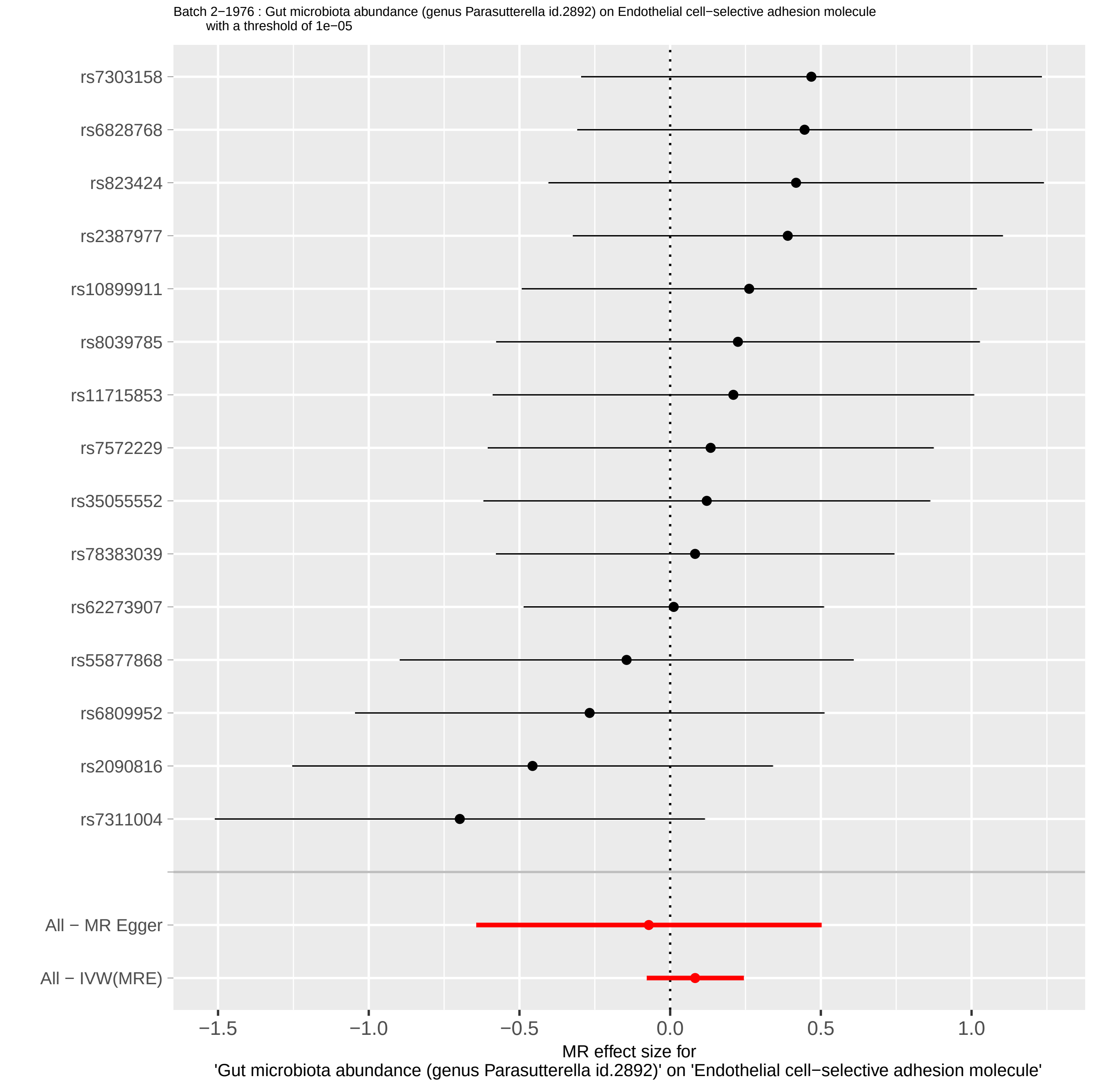
Batch 2−1976 : Gut microbiota abundance (genus Parasutterella id.2892) on Endothelial cell−selective adhesion molecule
with a threshold of 1e−05
rs7303158
rs6828768
rs823424
rs2387977
rs10899911
rs8039785
rs11715853
rs7572229
rs35055552
rs78383039
rs62273907
rs55877868
rs6809952
rs2090816
rs7311004
All − MR Egger
All − IVW(MRE)
−1.5
−1.0
−0.5
0.0
0.5
1.0
MR effect size for
'Gut microbiota abundance (genus Parasutterella id.2892)' on 'Endothelial cell−selective adhesion molecule'

Batch 2−1977 : Gut microbiota abundance (genus Catenibacterium id.2153) on S−formylglutathione hydrolase with a threshold of 1e−05

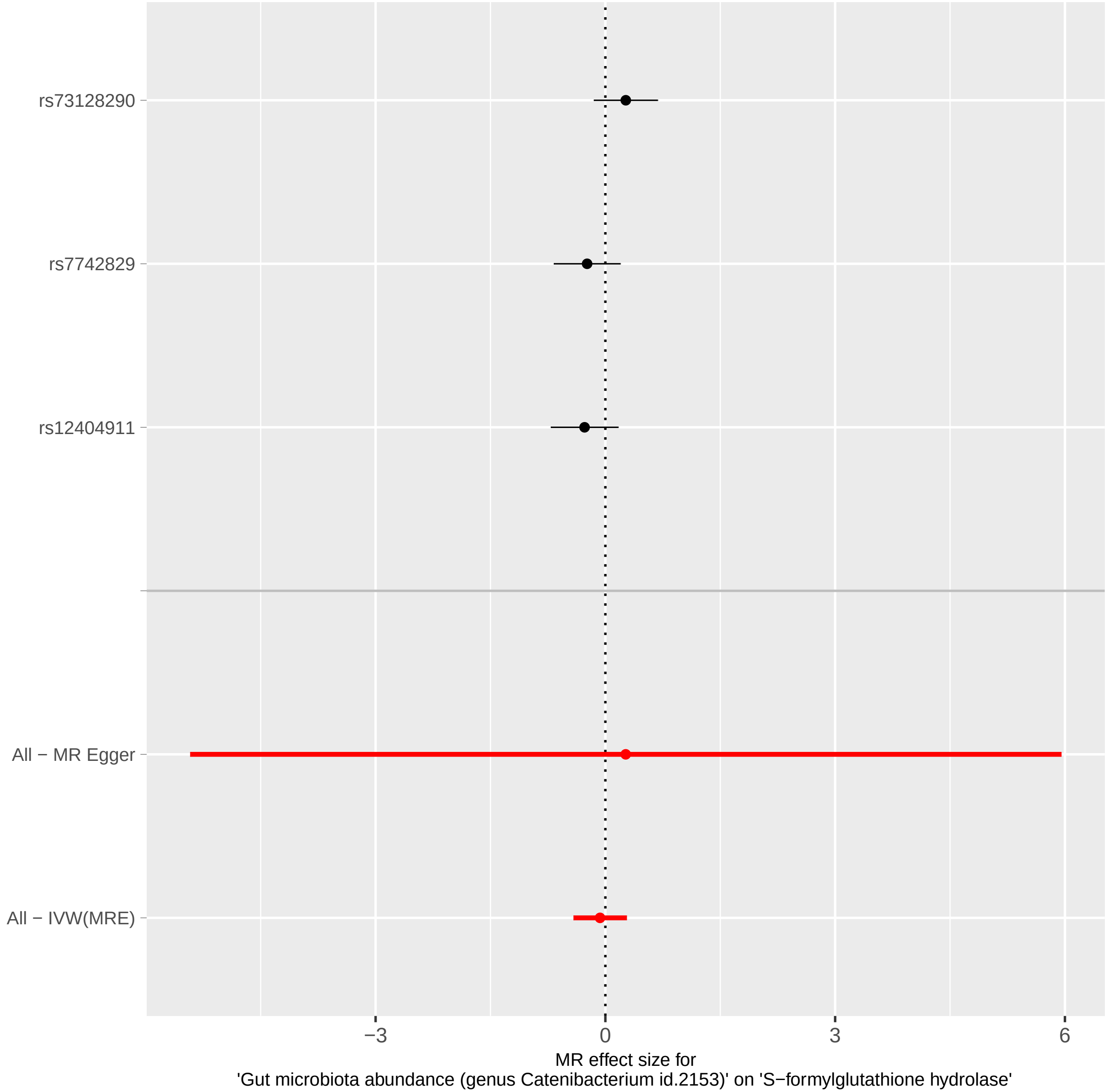

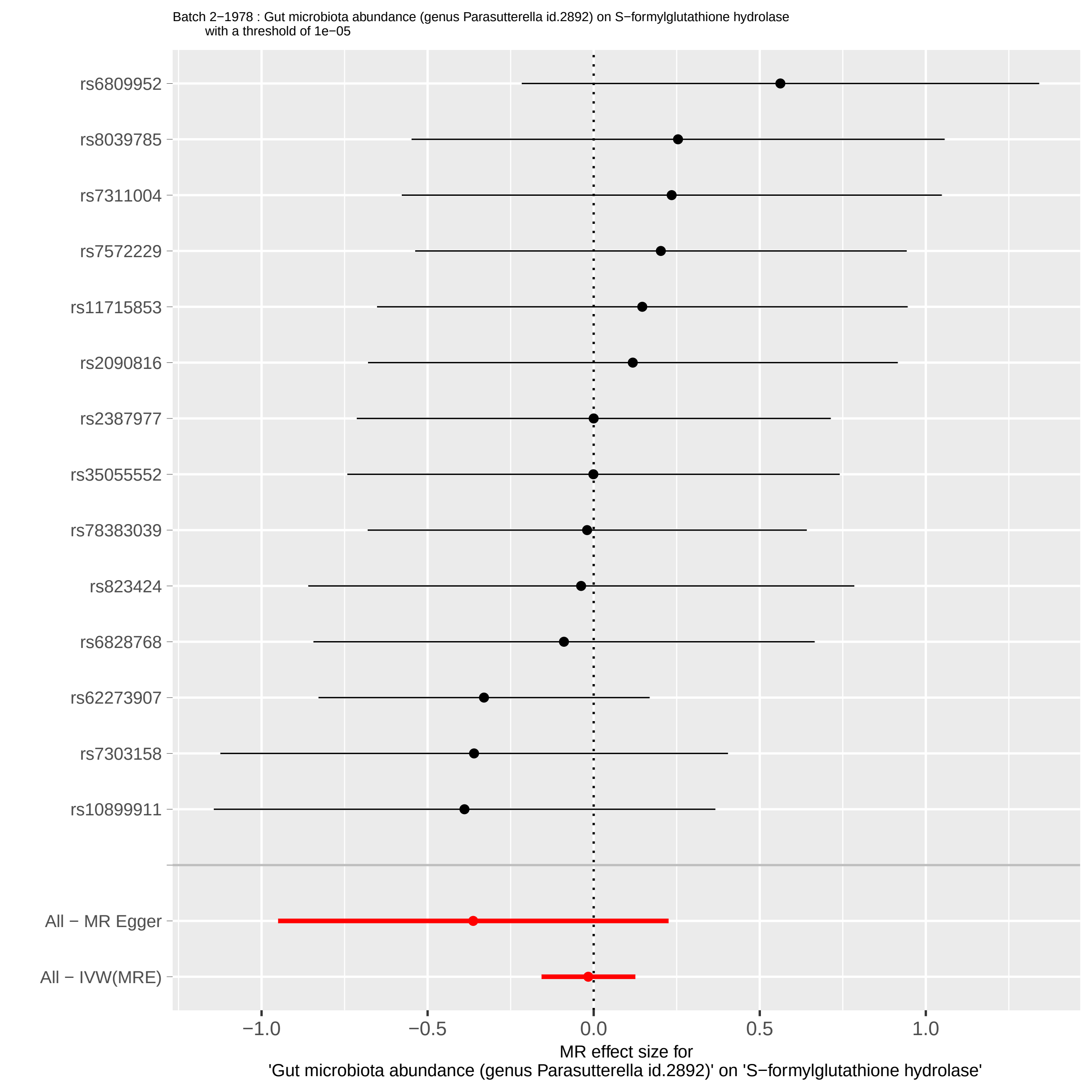
Batch 2−1978 : Gut microbiota abundance (genus Parasutterella id.2892) on S−formylglutathione hydrolase
with a threshold of 1e−05
rs6809952
rs8039785
rs7311004
rs7572229
rs11715853
rs2090816
rs2387977
rs35055552
rs78383039
rs823424
rs6828768
rs62273907
rs7303158
rs10899911
All − MR Egger
All − IVW(MRE)
−1.0
−0.5
0.0
0.5
1.0
MR effect size for
'Gut microbiota abundance (genus Parasutterella id.2892)' on 'S−formylglutathione hydrolase'

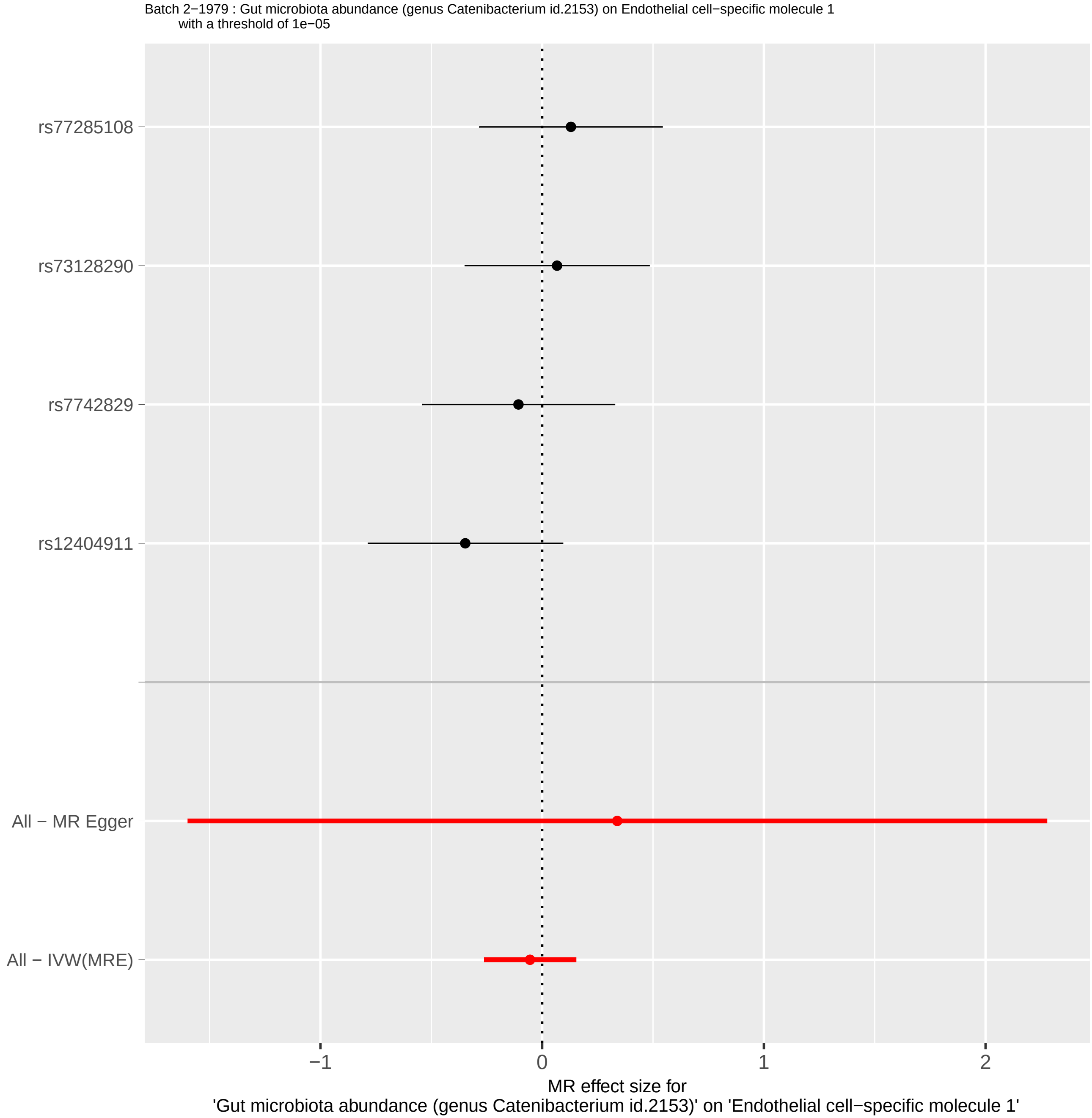

Batch 2−1979 : Gut microbiota abundance (genus Catenibacterium id.2153) on Endothelial cell−specific molecule 1
with a threshold of 1e−05
rs77285108
rs73128290
rs7742829
rs12404911
All − MR Egger
All − IVW(MRE)
−1
0
1
2
MR effect size for
'Gut microbiota abundance (genus Catenibacterium id.2153)' on 'Endothelial cell−specific molecule 1'

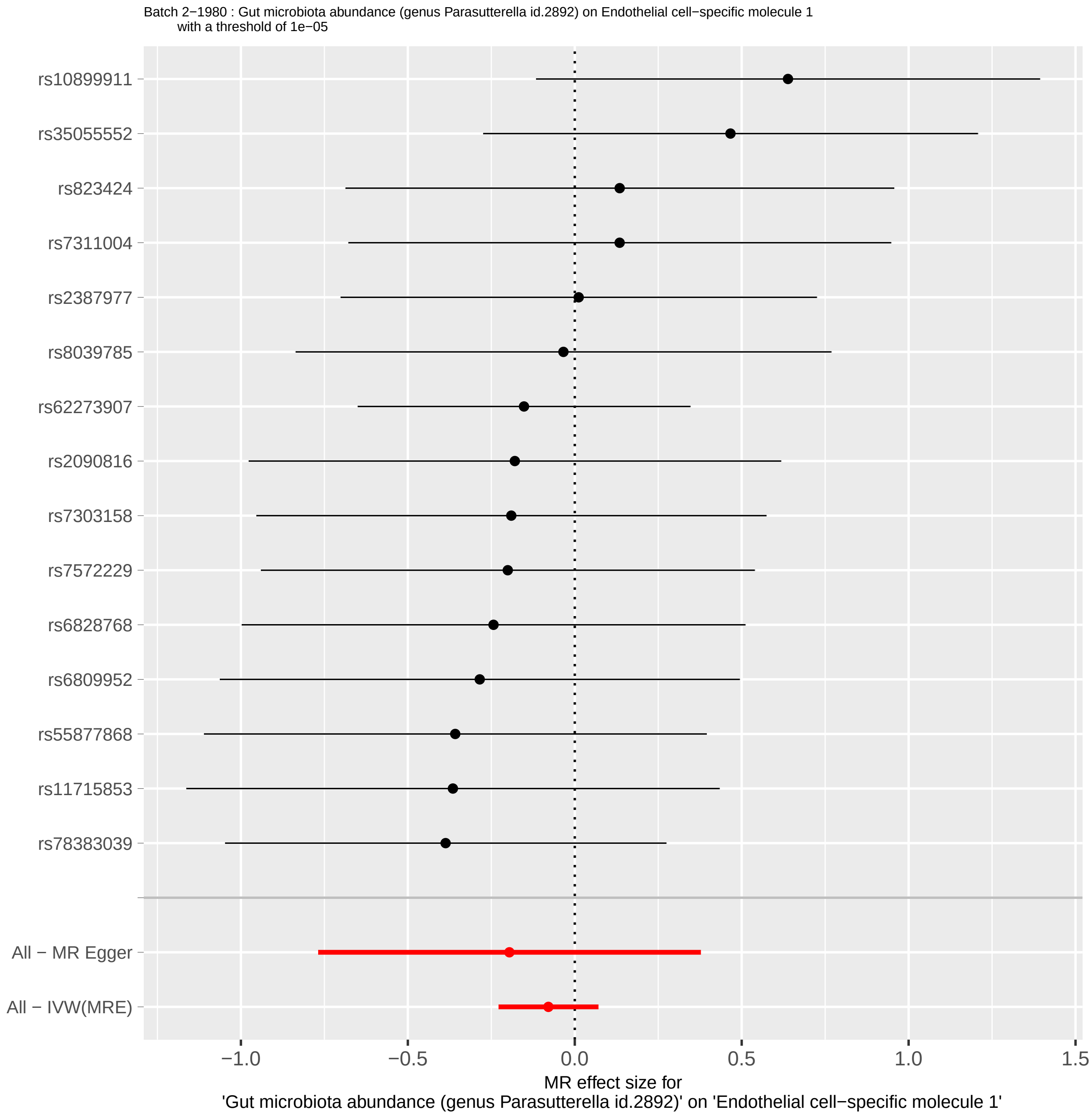

Batch 2−1980 : Gut microbiota abundance (genus Parasutterella id.2892) on Endothelial cell−specific molecule 1
with a threshold of 1e−05
rs10899911
rs35055552
rs823424
rs7311004
rs2387977
rs8039785
rs62273907
rs2090816
rs7303158
rs7572229
rs6828768
rs6809952
rs55877868
rs11715853
rs78383039
All − MR Egger
All − IVW(MRE)
−1.0
−0.5
0.0
0.5
1.0
1.5
MR effect size for
'Gut microbiota abundance (genus Parasutterella id.2892)' on 'Endothelial cell−specific molecule 1'

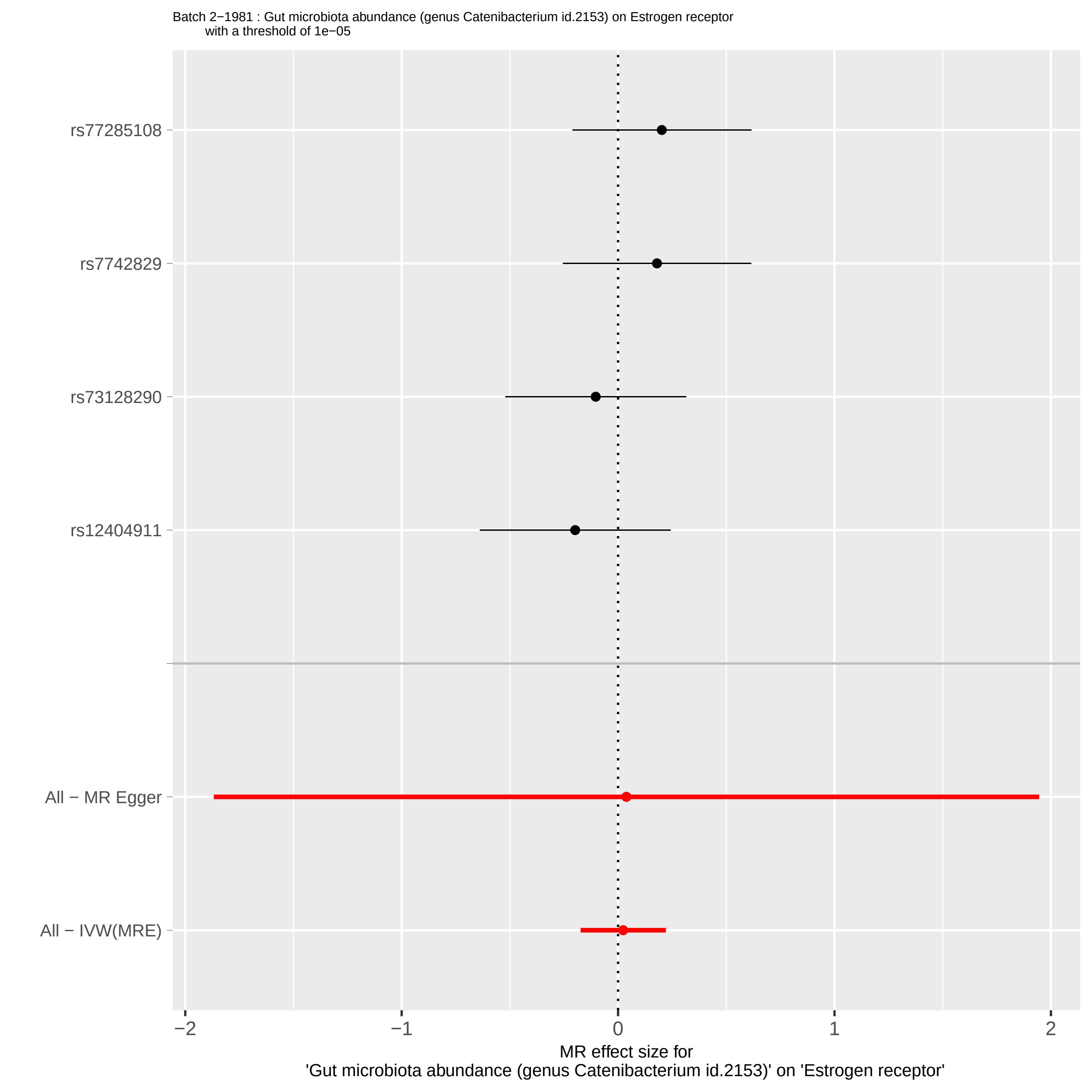

Batch 2−1981 : Gut microbiota abundance (genus Catenibacterium id.2153) on Estrogen receptor
with a threshold of 1e−05
rs77285108
rs7742829
rs73128290
rs12404911
All − MR Egger
All − IVW(MRE)
−2
−1
0
1
2
MR effect size for
'Gut microbiota abundance (genus Catenibacterium id.2153)' on 'Estrogen receptor'

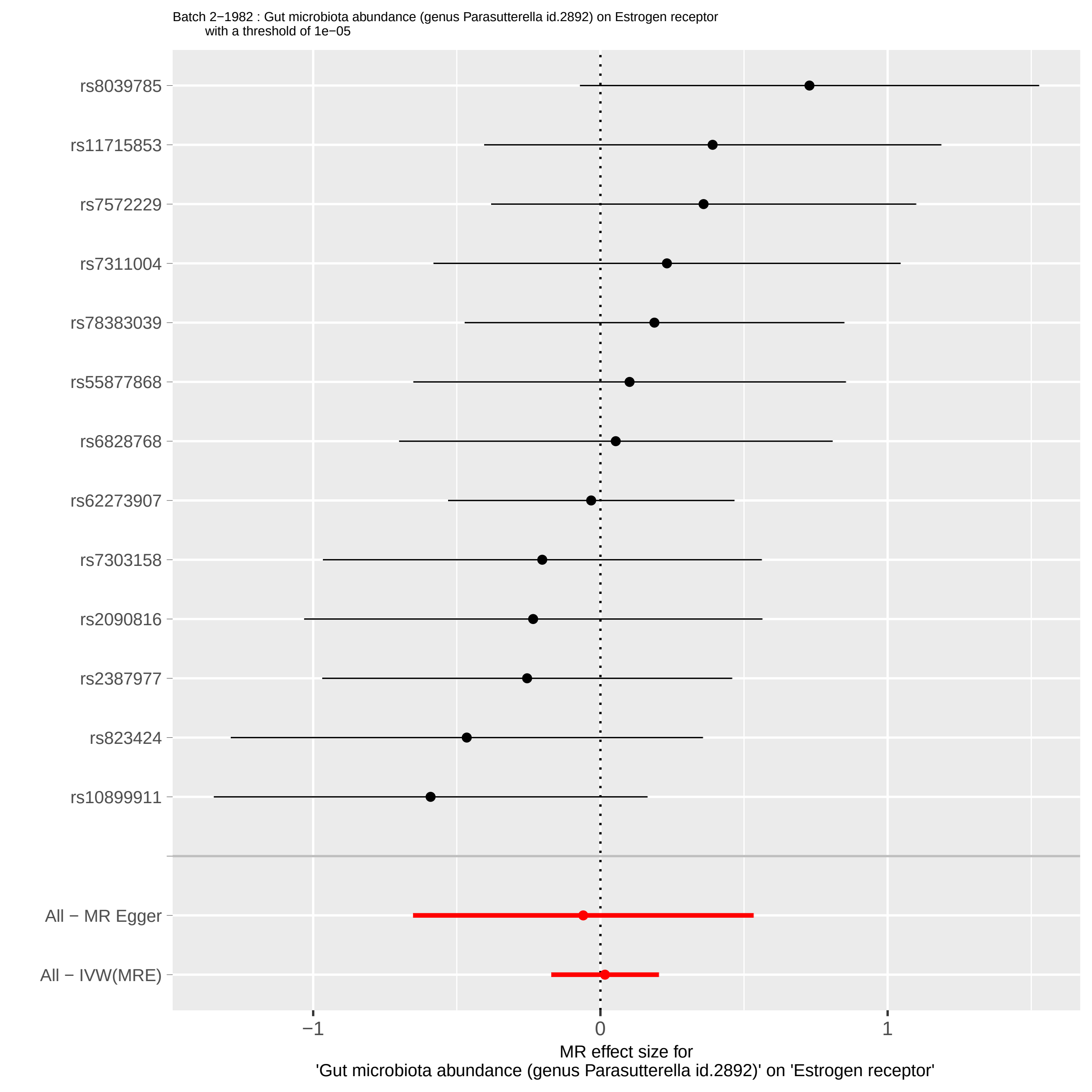
Batch 2−1982 : Gut microbiota abundance (genus Parasutterella id.2892) on Estrogen receptor
with a threshold of 1e−05
rs8039785
rs11715853
rs7572229
rs7311004
rs78383039
rs55877868
rs6828768
rs62273907
rs7303158
rs2090816
rs2387977
rs823424
rs10899911
All − MR Egger
All − IVW(MRE)
−1
0
1
MR effect size for
'Gut microbiota abundance (genus Parasutterella id.2892)' on 'Estrogen receptor'

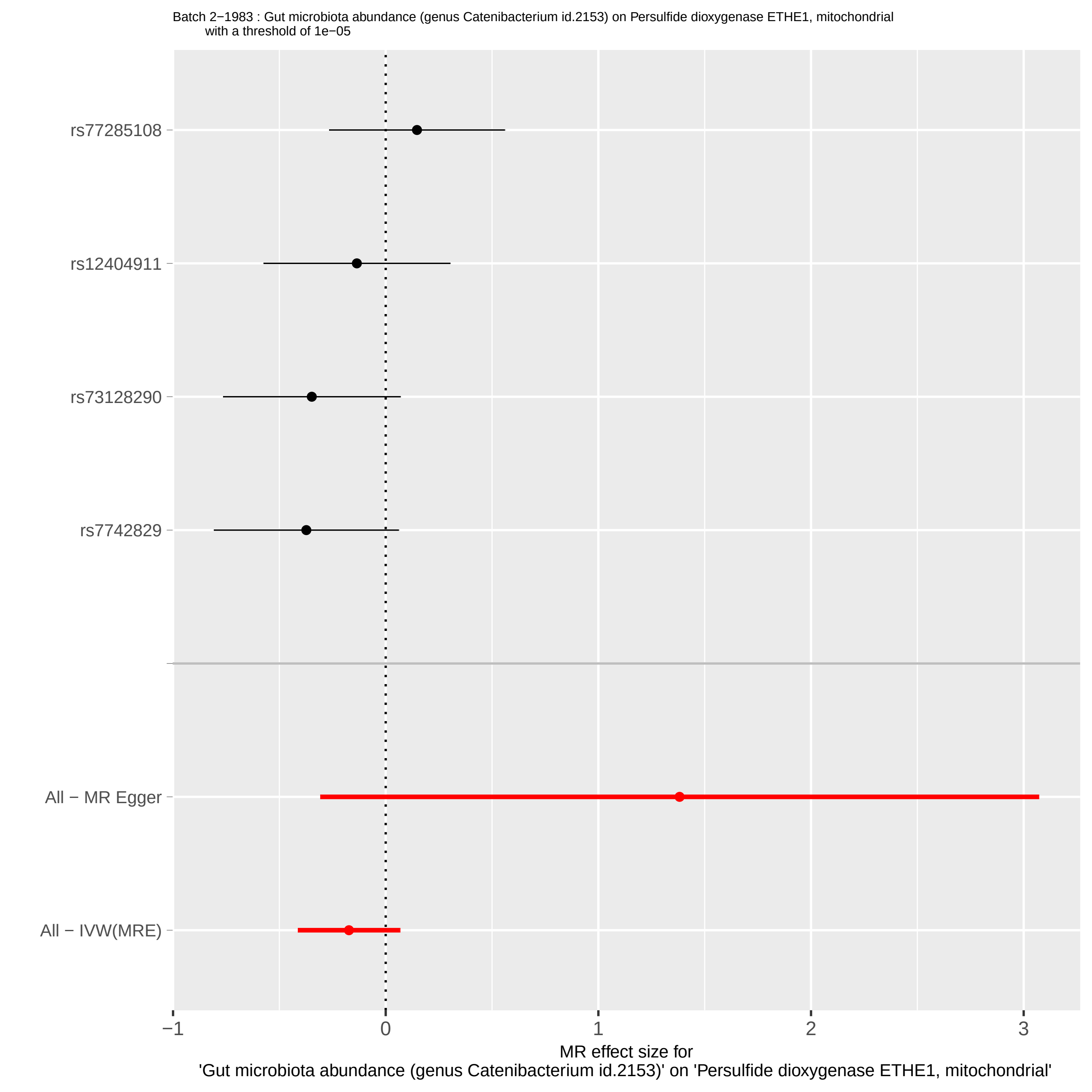
Batch 2−1983 : Gut microbiota abundance (genus Catenibacterium id.2153) on Persulfide dioxygenase ETHE1, mitochondrial
with a threshold of 1e−05
rs77285108
rs12404911
rs73128290
rs7742829
All − MR Egger
All − IVW(MRE)
−1
0
1
2
3
MR effect size for
'Gut microbiota abundance (genus Catenibacterium id.2153)' on 'Persulfide dioxygenase ETHE1, mitochondrial'

Batch 2−1984 : Gut microbiota abundance (genus Parasutterella id.2892) on Persulfide dioxygenase ETHE1, mitochondrial
with a threshold of 1e−05

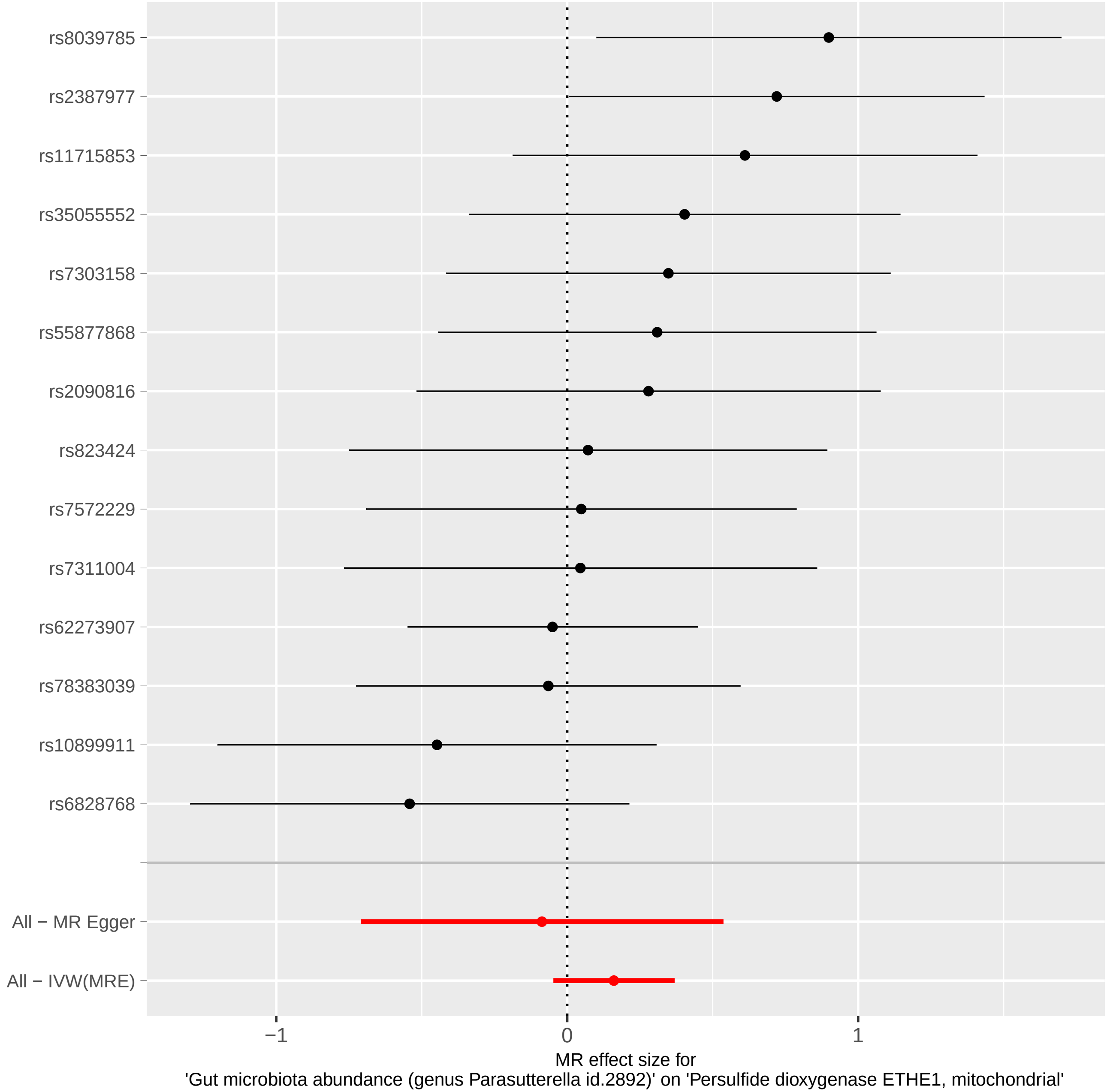

Batch 2−1985 : Gut microbiota abundance (genus Catenibacterium id.2153) on Ethanolamine kinase 1
with a threshold of 1e−05

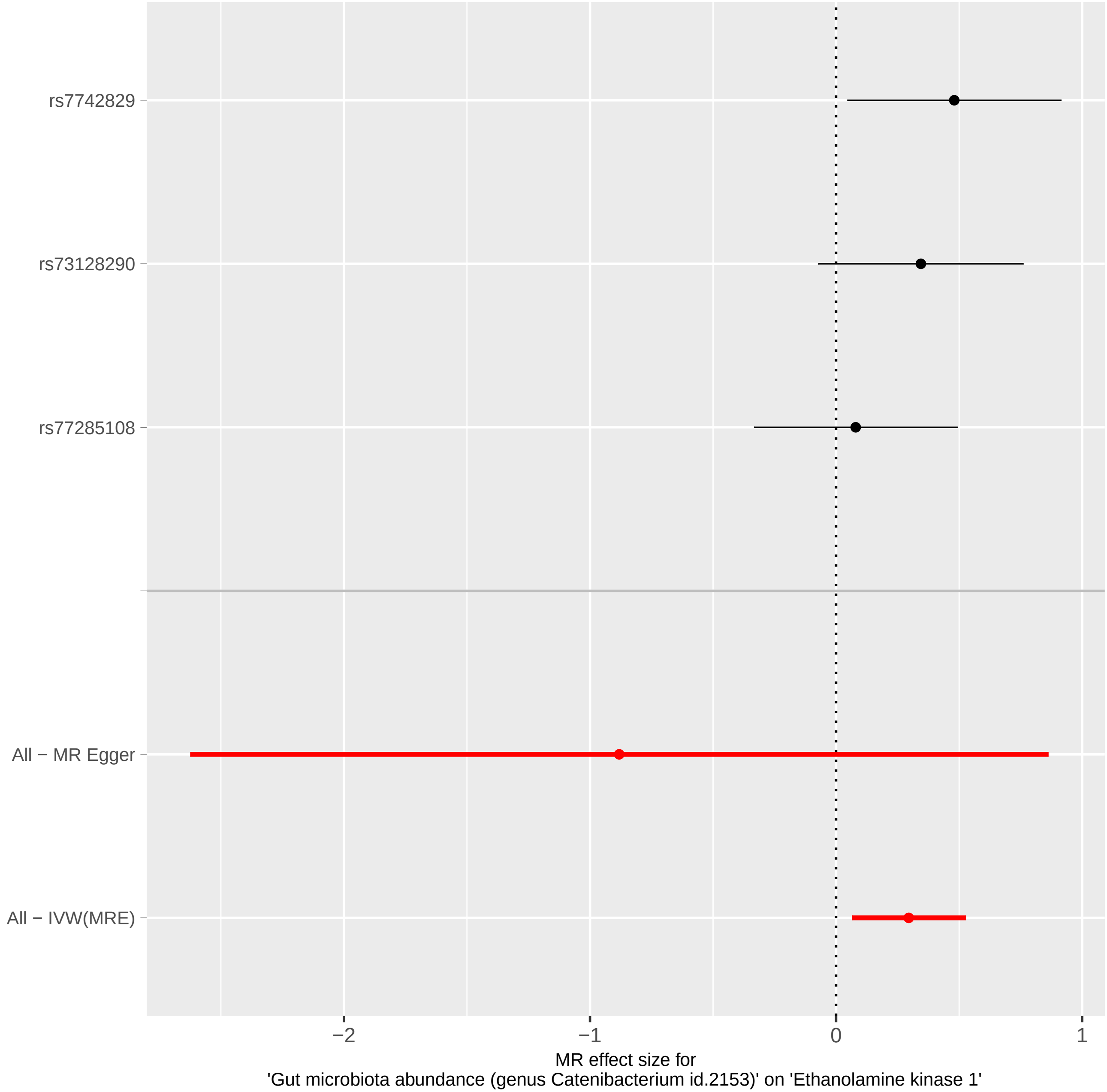

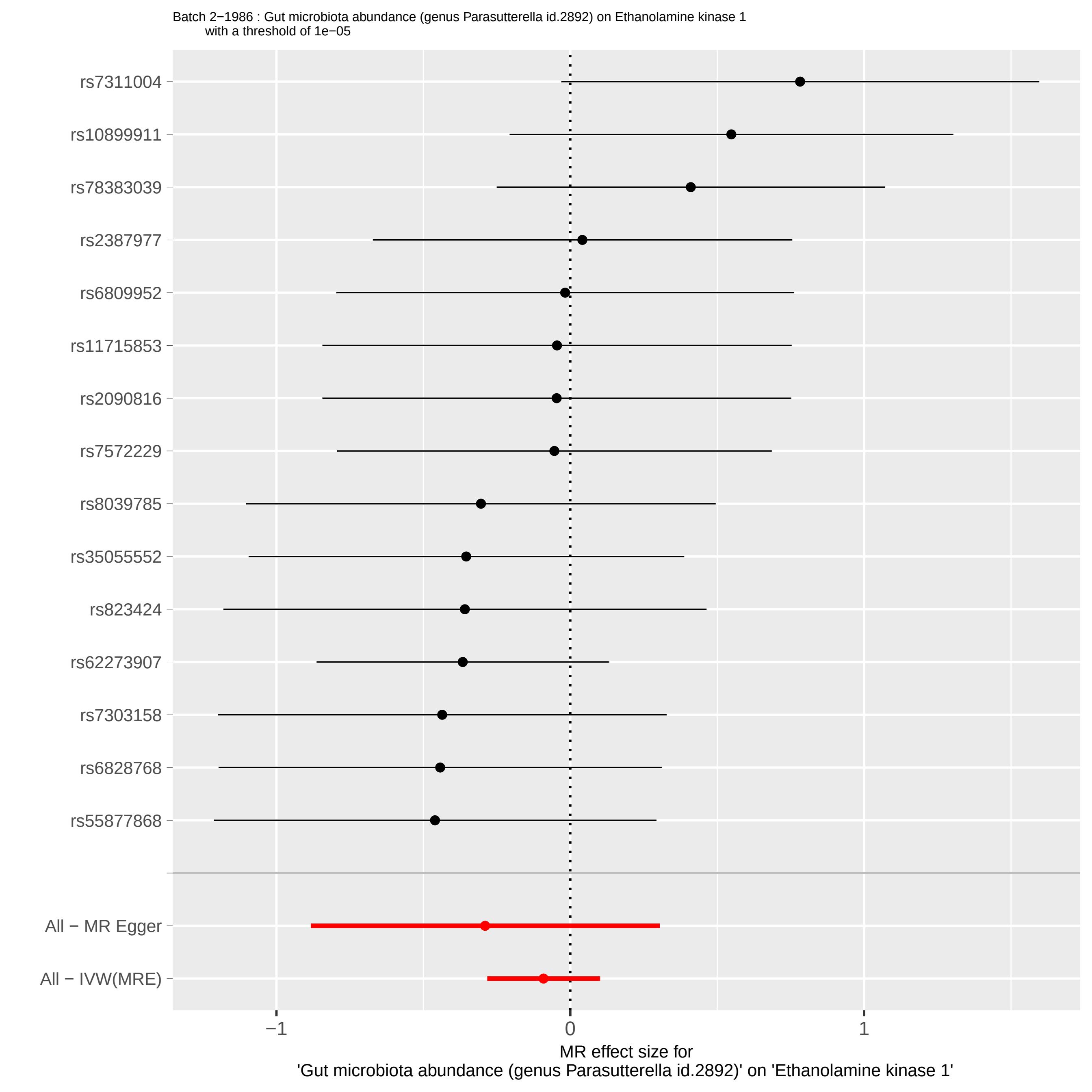

Batch 2−1986 : Gut microbiota abundance (genus Parasutterella id.2892) on Ethanolamine kinase 1
with a threshold of 1e−05
rs7311004
rs10899911
rs78383039
rs2387977
rs6809952
rs11715853
rs2090816
rs7572229
rs8039785
rs35055552
rs823424
rs62273907
rs7303158
rs6828768
rs55877868
All − MR Egger
All − IVW(MRE)
−1
0
1
MR effect size for
'Gut microbiota abundance (genus Parasutterella id.2892)' on 'Ethanolamine kinase 1'

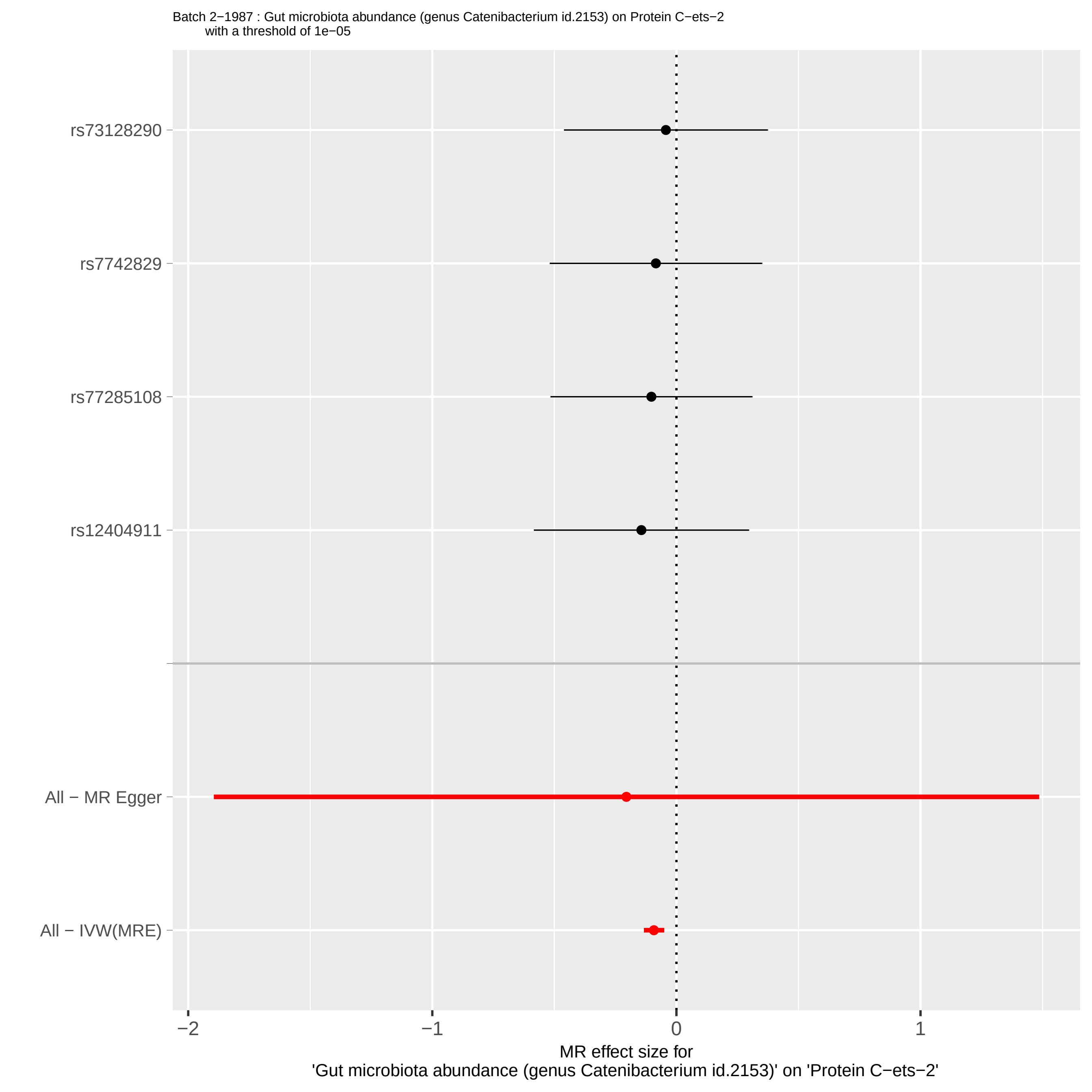

Batch 2−1987 : Gut microbiota abundance (genus Catenibacterium id.2153) on Protein C−ets−2
with a threshold of 1e−05
rs73128290
rs7742829
rs77285108
rs12404911
All − MR Egger
All − IVW(MRE)
−2
−1
0
1
MR effect size for
'Gut microbiota abundance (genus Catenibacterium id.2153)' on 'Protein C−ets−2'

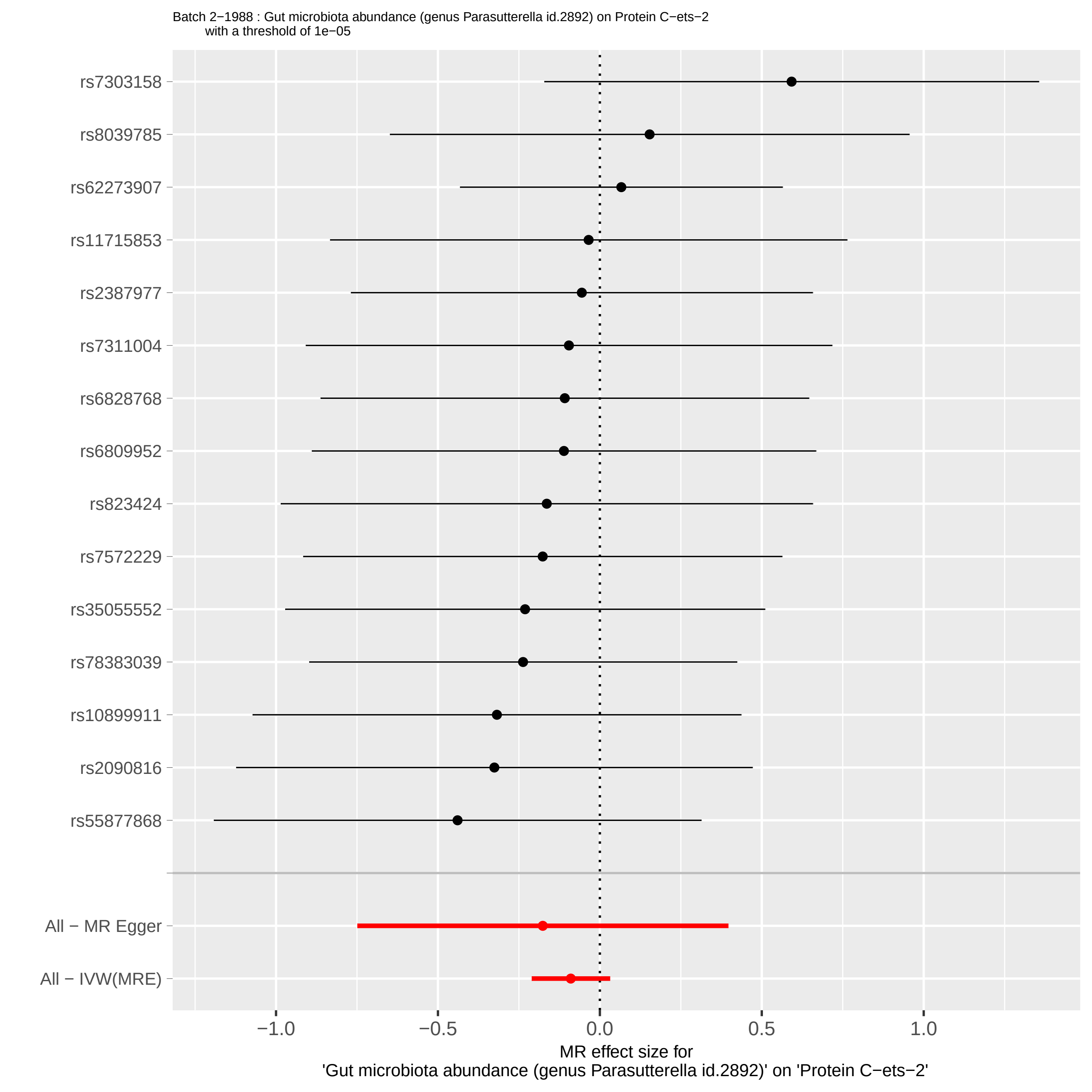

Batch 2−1988 : Gut microbiota abundance (genus Parasutterella id.2892) on Protein C−ets−2
with a threshold of 1e−05
rs7303158
rs8039785
rs62273907
rs11715853
rs2387977
rs7311004
rs6828768
rs6809952
rs823424
rs7572229
rs35055552
rs78383039
rs10899911
rs2090816
rs55877868
All − MR Egger
All − IVW(MRE)
−1.0
−0.5
0.0
0.5
1.0
MR effect size for
'Gut microbiota abundance (genus Parasutterella id.2892)' on 'Protein C−ets−2'

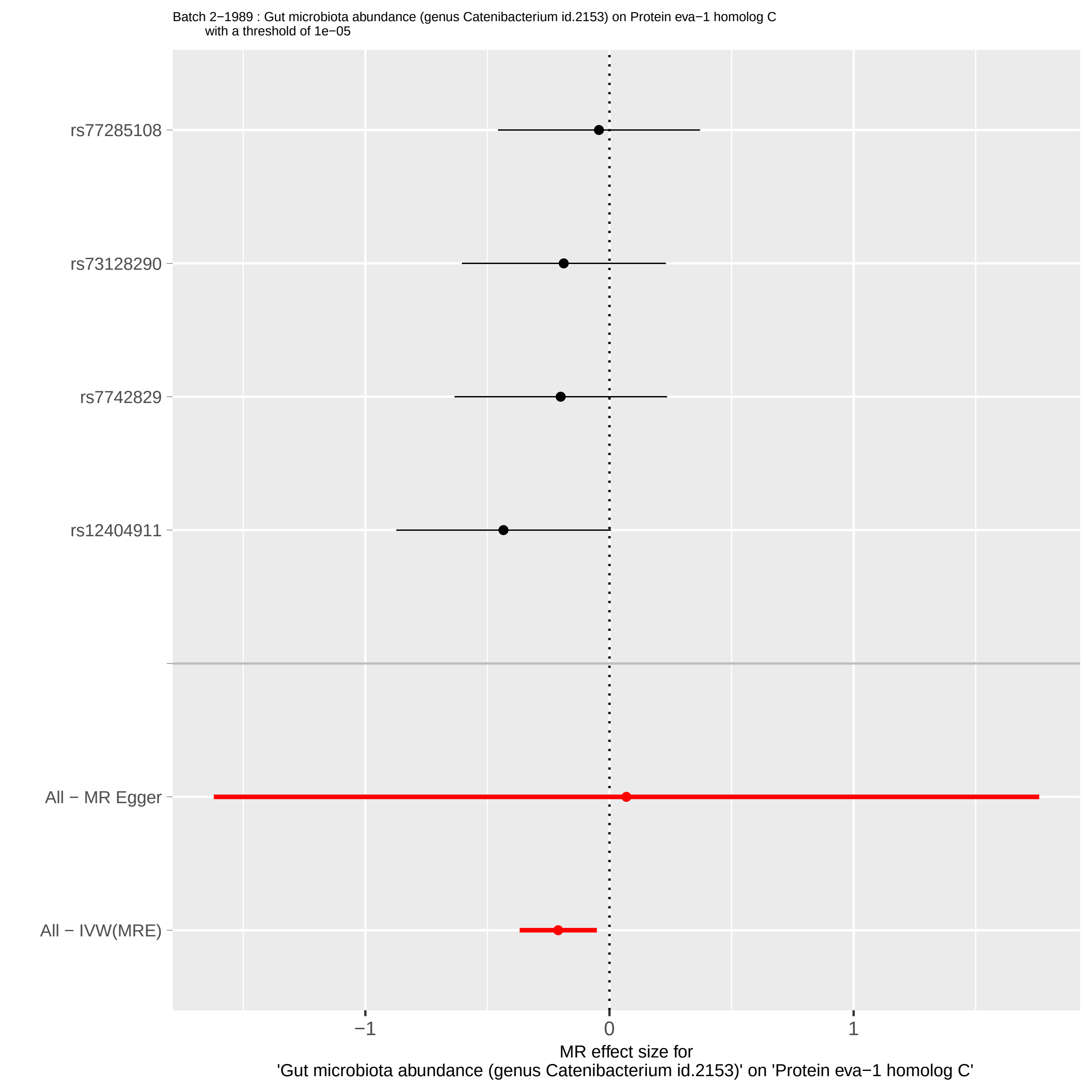
Batch 2−1989 : Gut microbiota abundance (genus Catenibacterium id.2153) on Protein eva−1 homolog C
with a threshold of 1e−05
rs77285108
rs73128290
rs7742829
rs12404911
All − MR Egger
All − IVW(MRE)
−1
0
1
MR effect size for
'Gut microbiota abundance (genus Catenibacterium id.2153)' on 'Protein eva−1 homolog C'

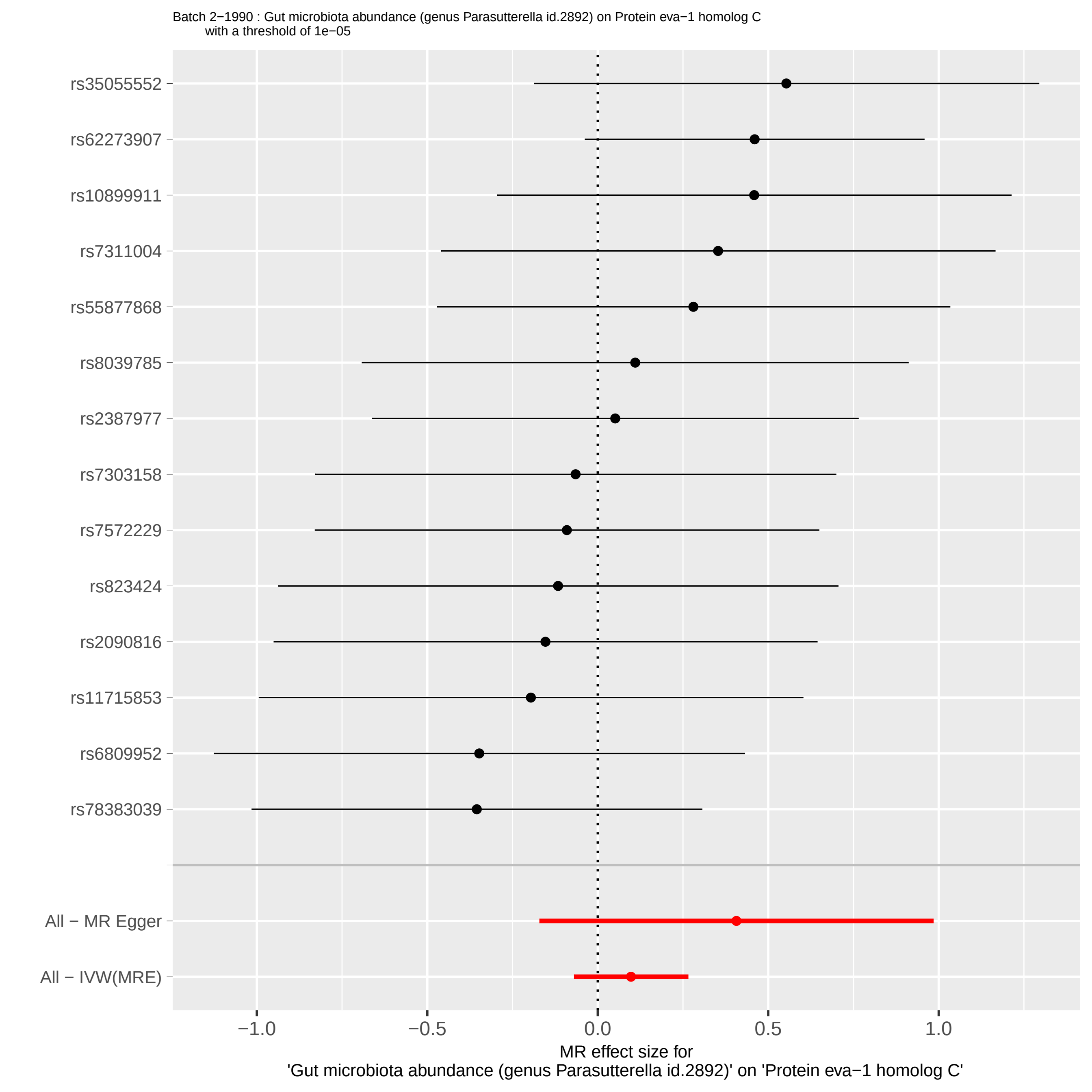

Batch 2−1990 : Gut microbiota abundance (genus Parasutterella id.2892) on Protein eva−1 homolog C
with a threshold of 1e−05
rs35055552
rs62273907
rs10899911
rs7311004
rs55877868
rs8039785
rs2387977
rs7303158
rs7572229
rs823424
rs2090816
rs11715853
rs6809952
rs78383039
All − MR Egger
All − IVW(MRE)
−1.0
−0.5
0.0
0.5
1.0
MR effect size for
'Gut microbiota abundance (genus Parasutterella id.2892)' on 'Protein eva−1 homolog C'

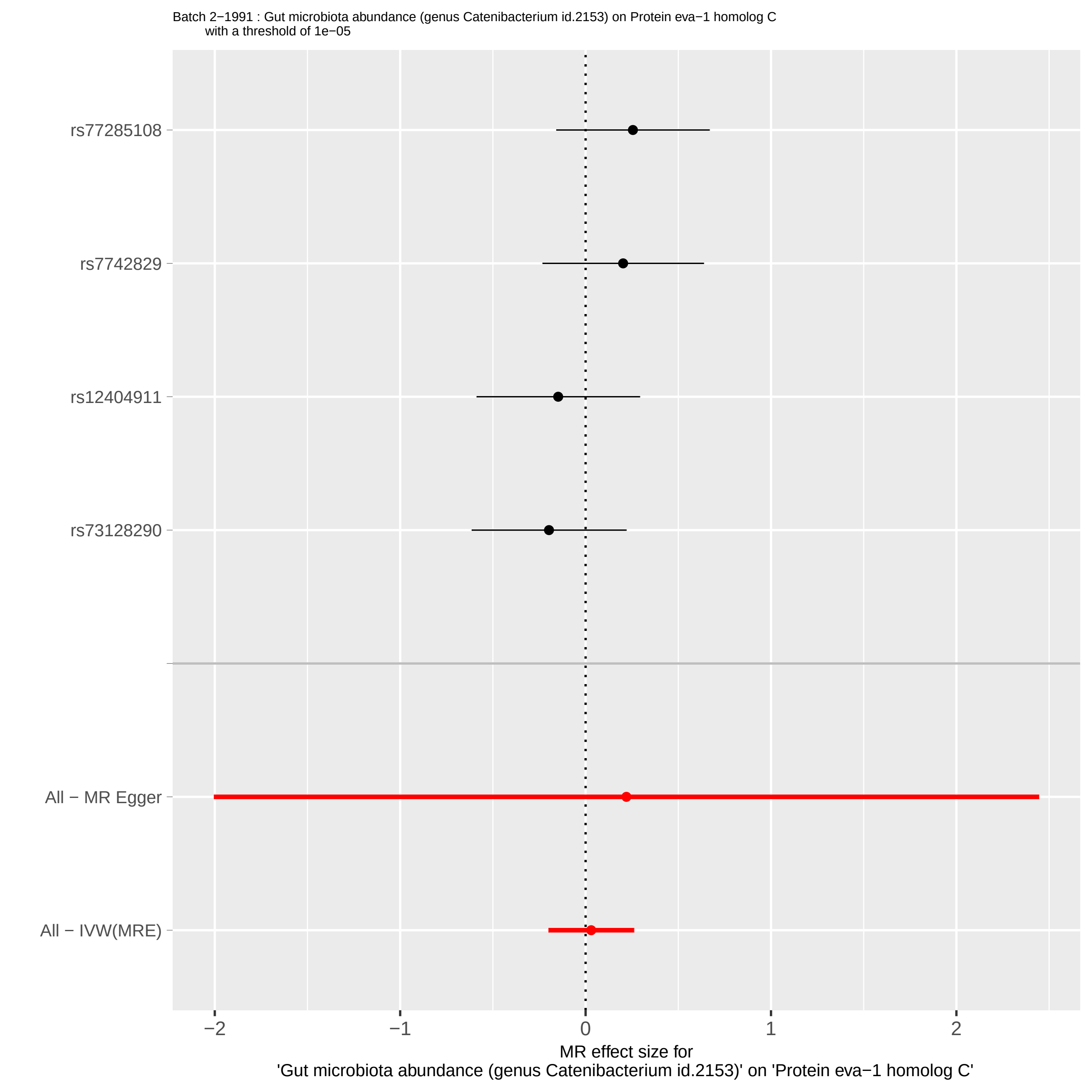

Batch 2−1991 : Gut microbiota abundance (genus Catenibacterium id.2153) on Protein eva−1 homolog C
with a threshold of 1e−05
rs77285108
rs7742829
rs12404911
rs73128290
All − MR Egger
All − IVW(MRE)
−2
−1
0
1
2
MR effect size for
'Gut microbiota abundance (genus Catenibacterium id.2153)' on 'Protein eva−1 homolog C'

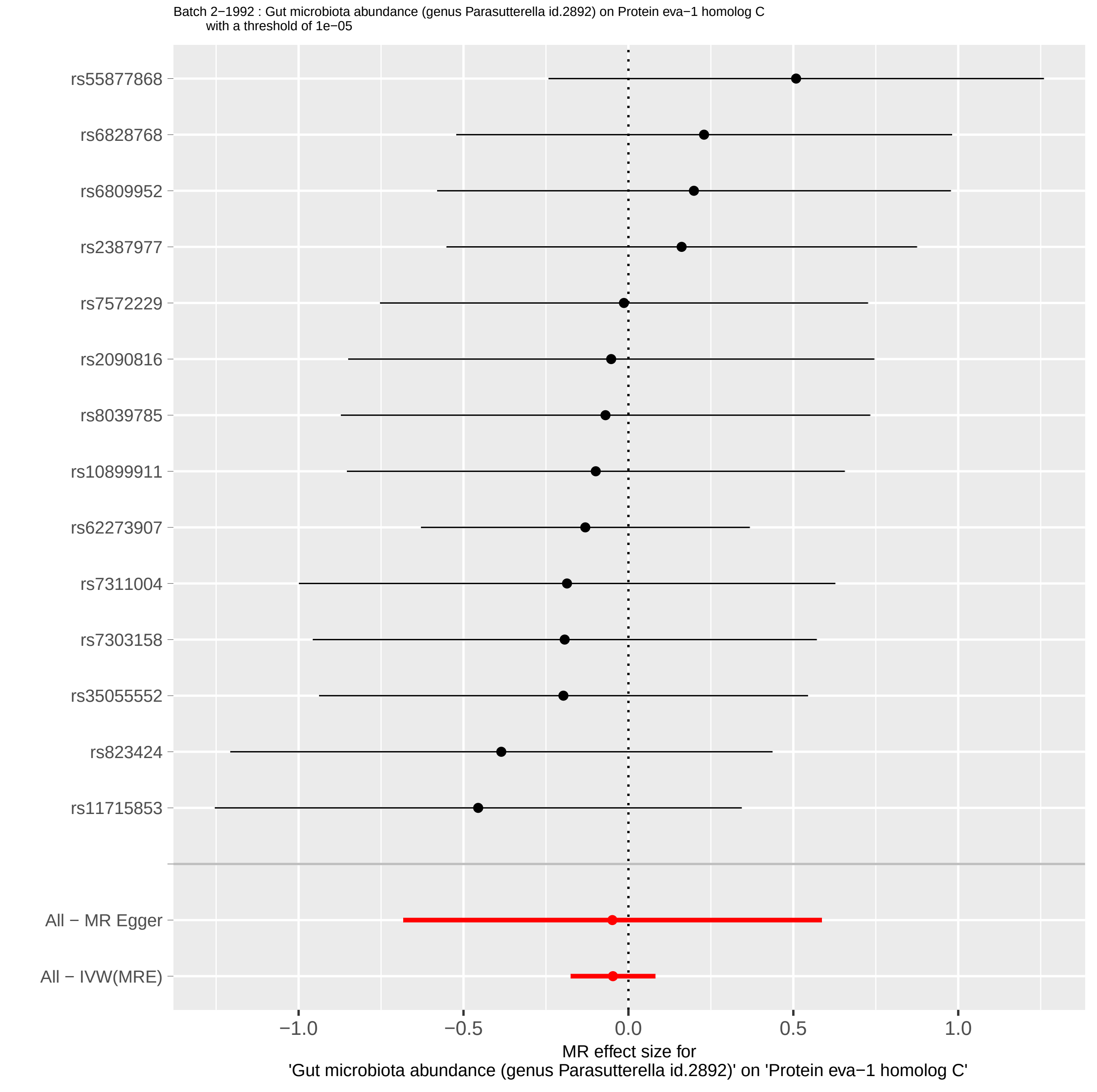

Batch 2−1992 : Gut microbiota abundance (genus Parasutterella id.2892) on Protein eva−1 homolog C
with a threshold of 1e−05
rs55877868
rs6828768
rs6809952
rs2387977
rs7572229
rs2090816
rs8039785
rs10899911
rs62273907
rs7311004
rs7303158
rs35055552
rs823424
rs11715853
All − MR Egger
All − IVW(MRE)
−1.0
−0.5
0.0
0.5
1.0
MR effect size for
'Gut microbiota abundance (genus Parasutterella id.2892)' on 'Protein eva−1 homolog C'

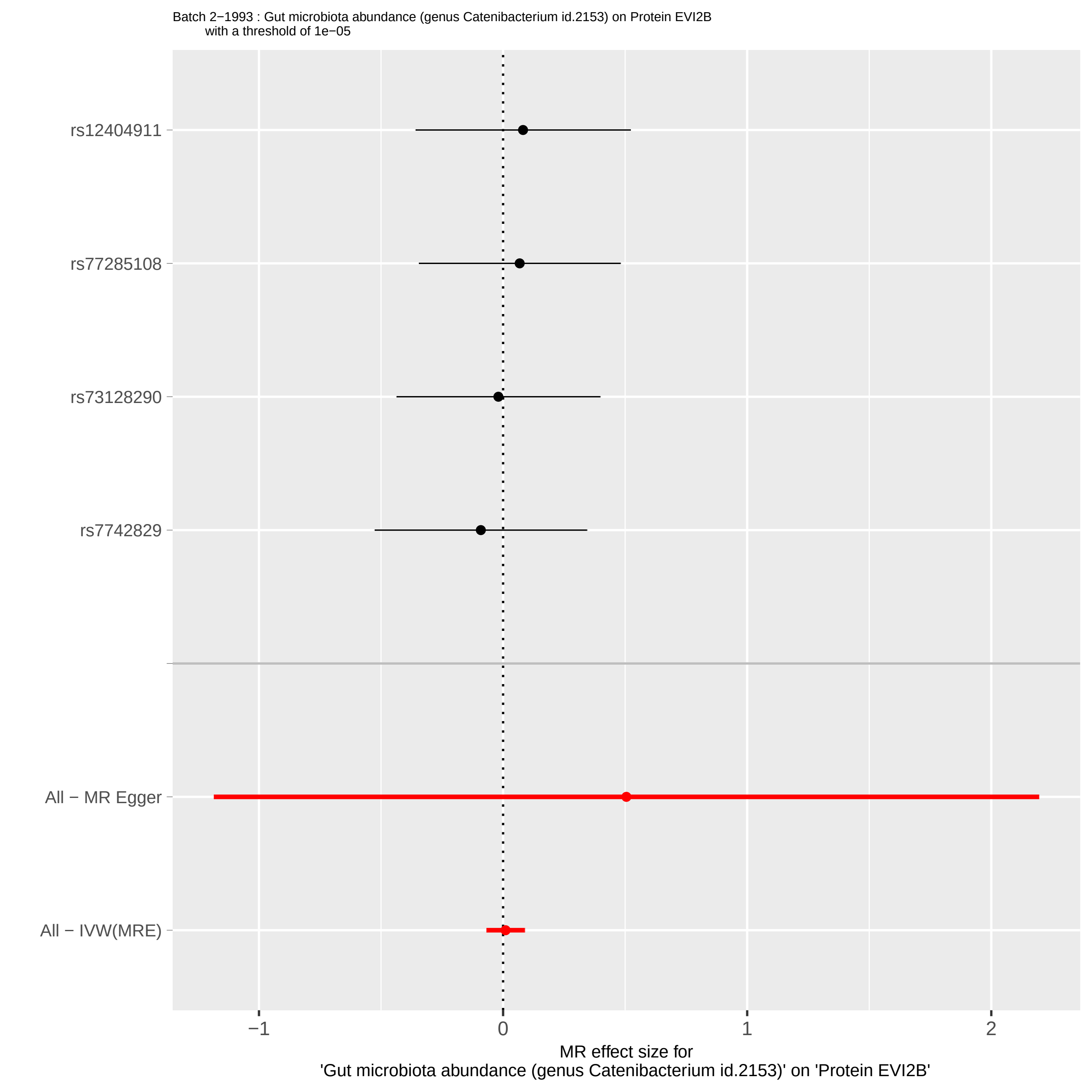
Batch 2−1993 : Gut microbiota abundance (genus Catenibacterium id.2153) on Protein EVI2B
with a threshold of 1e−05
rs12404911
rs77285108
rs73128290
rs7742829
All − MR Egger
All − IVW(MRE)
−1
0
1
2
MR effect size for
'Gut microbiota abundance (genus Catenibacterium id.2153)' on 'Protein EVI2B'

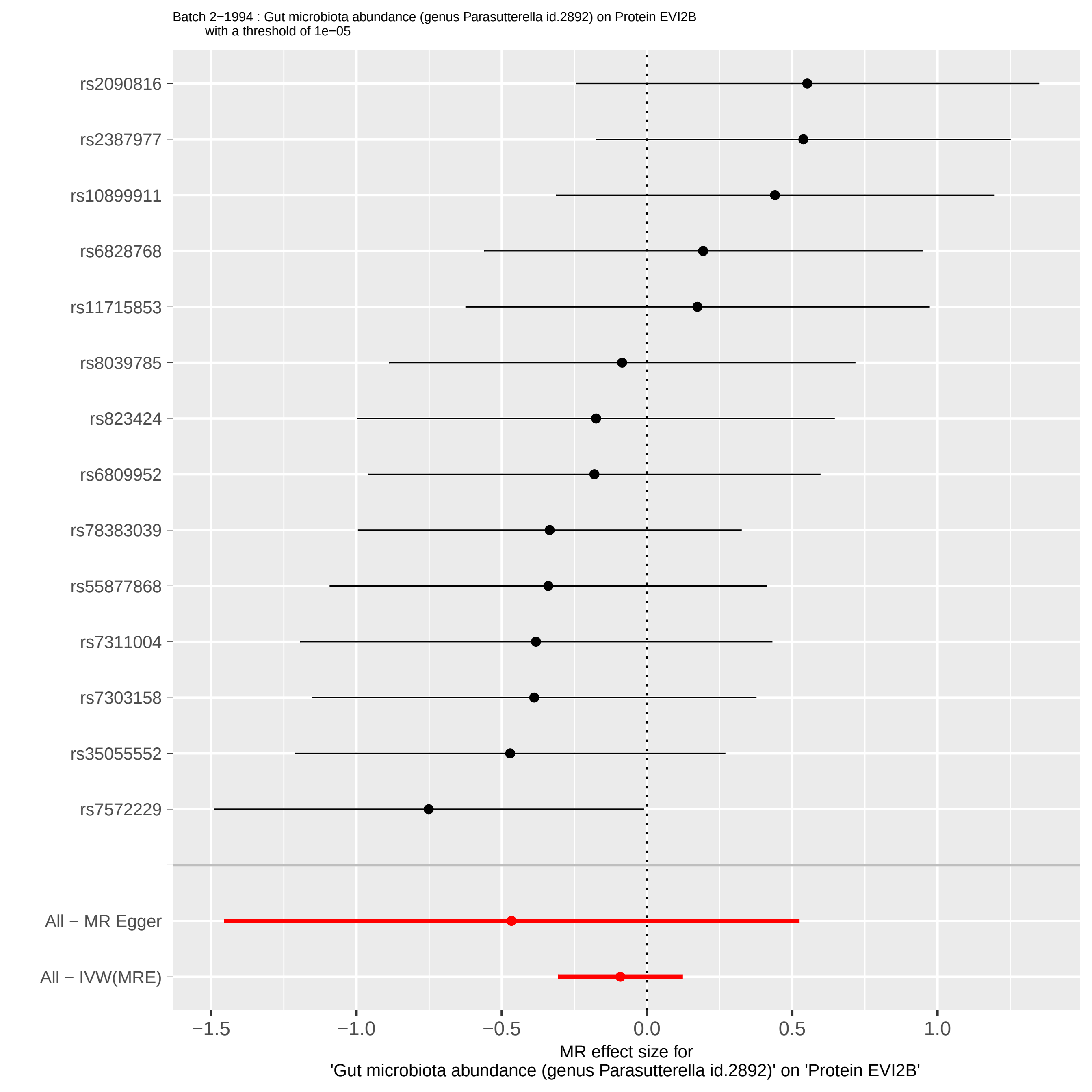

Batch 2−1994 : Gut microbiota abundance (genus Parasutterella id.2892) on Protein EVI2B
with a threshold of 1e−05
rs2090816
rs2387977
rs10899911
rs6828768
rs11715853
rs8039785
rs823424
rs6809952
rs78383039
rs55877868
rs7311004
rs7303158
rs35055552
rs7572229
All − MR Egger
All − IVW(MRE)
−1.5
−1.0
−0.5
0.0
0.5
1.0
MR effect size for
'Gut microbiota abundance (genus Parasutterella id.2892)' on 'Protein EVI2B'

Batch 2−1995 : Gut microbiota abundance (genus Catenibacterium id.2153) on Ena/VASP−like protein
with a threshold of 1e−05

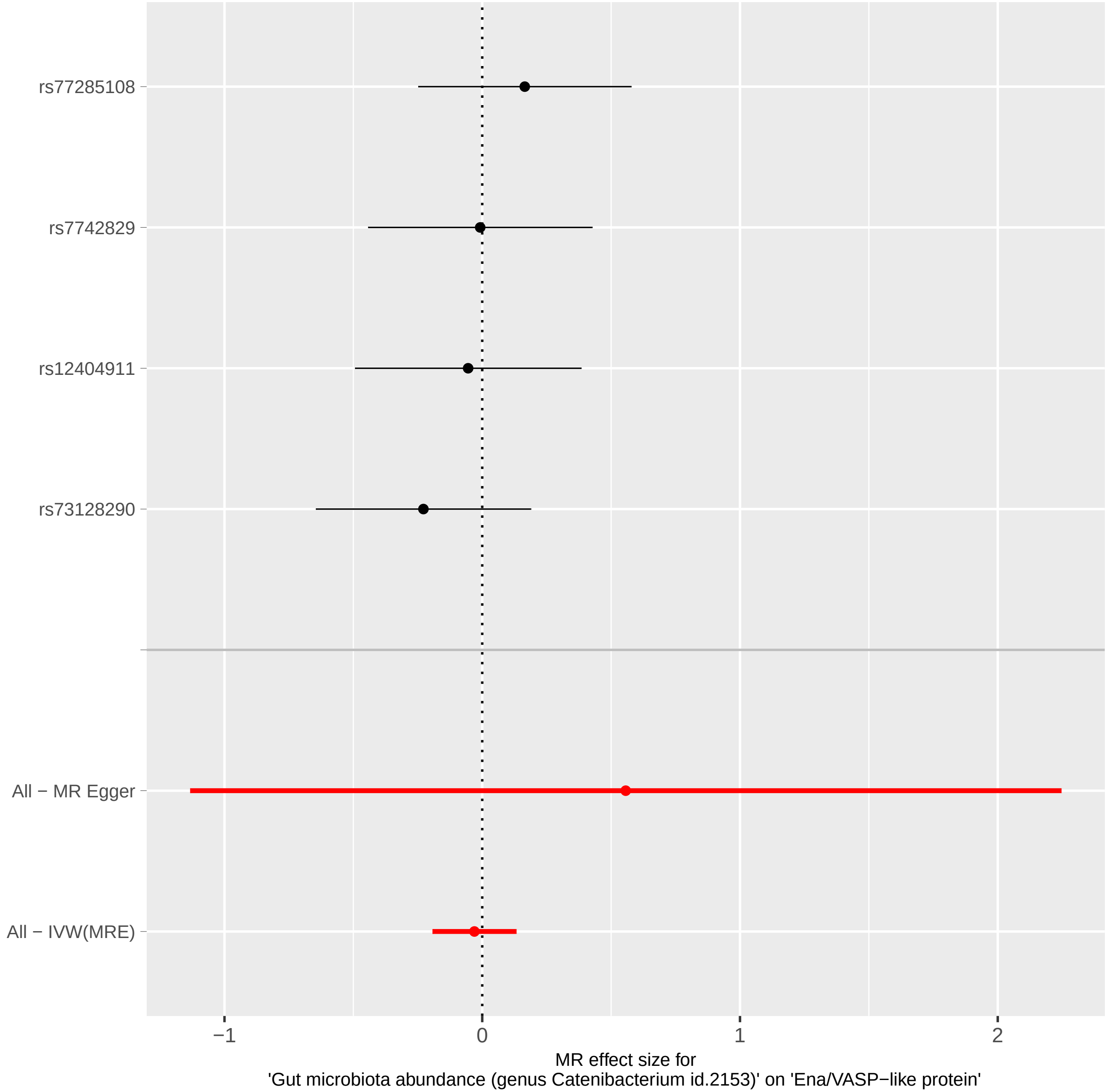

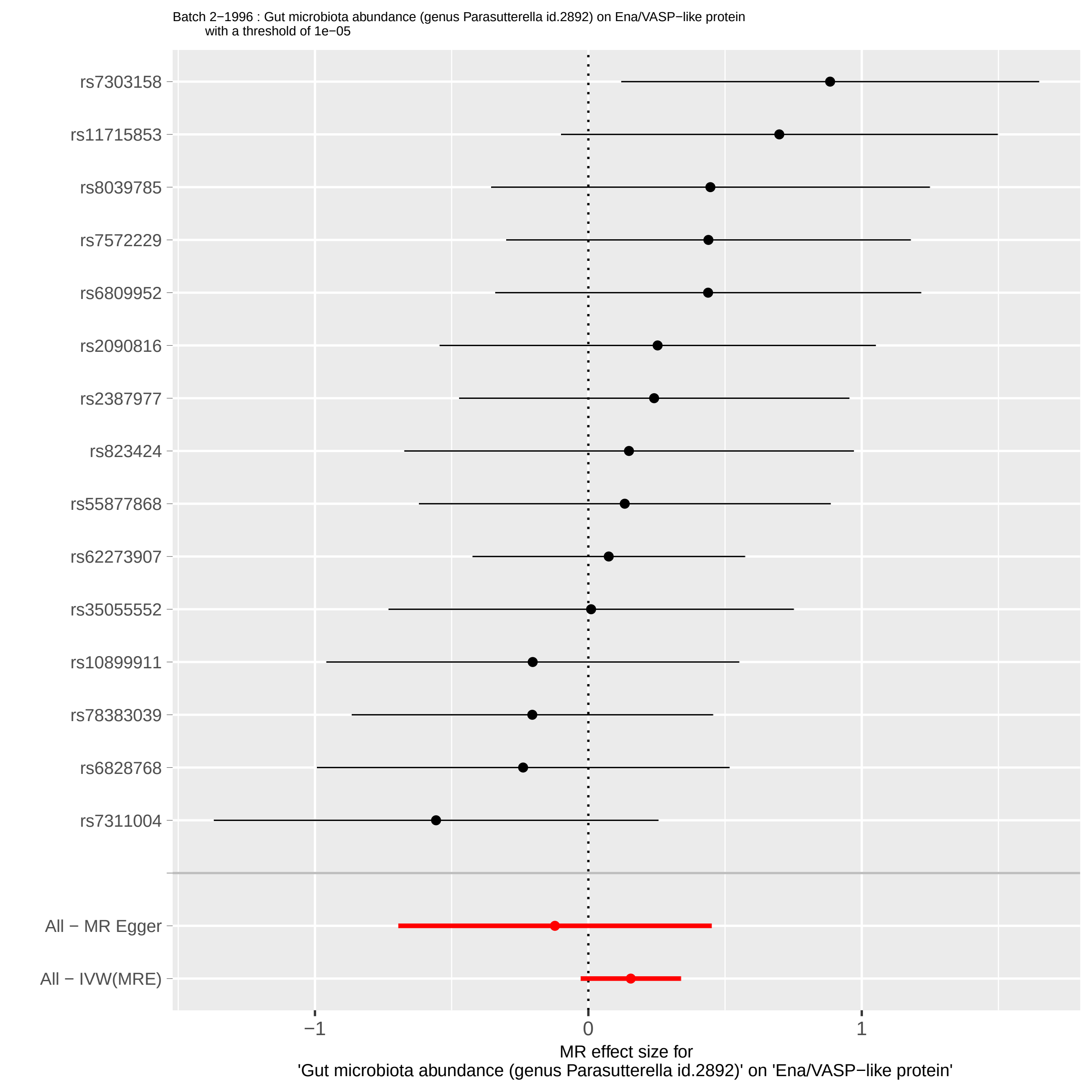

Batch 2−1996 : Gut microbiota abundance (genus Parasutterella id.2892) on Ena/VASP−like protein
with a threshold of 1e−05
rs7303158
rs11715853
rs8039785
rs7572229
rs6809952
rs2090816
rs2387977
rs823424
rs55877868
rs62273907
rs35055552
rs10899911
rs78383039
rs6828768
rs7311004
All − MR Egger
All − IVW(MRE)
−1
0
1
MR effect size for
'Gut microbiota abundance (genus Parasutterella id.2892)' on 'Ena/VASP−like protein'

Batch 2−1997 : Gut microbiota abundance (genus Catenibacterium id.2153) on Envoplakin
with a threshold of 1e−05

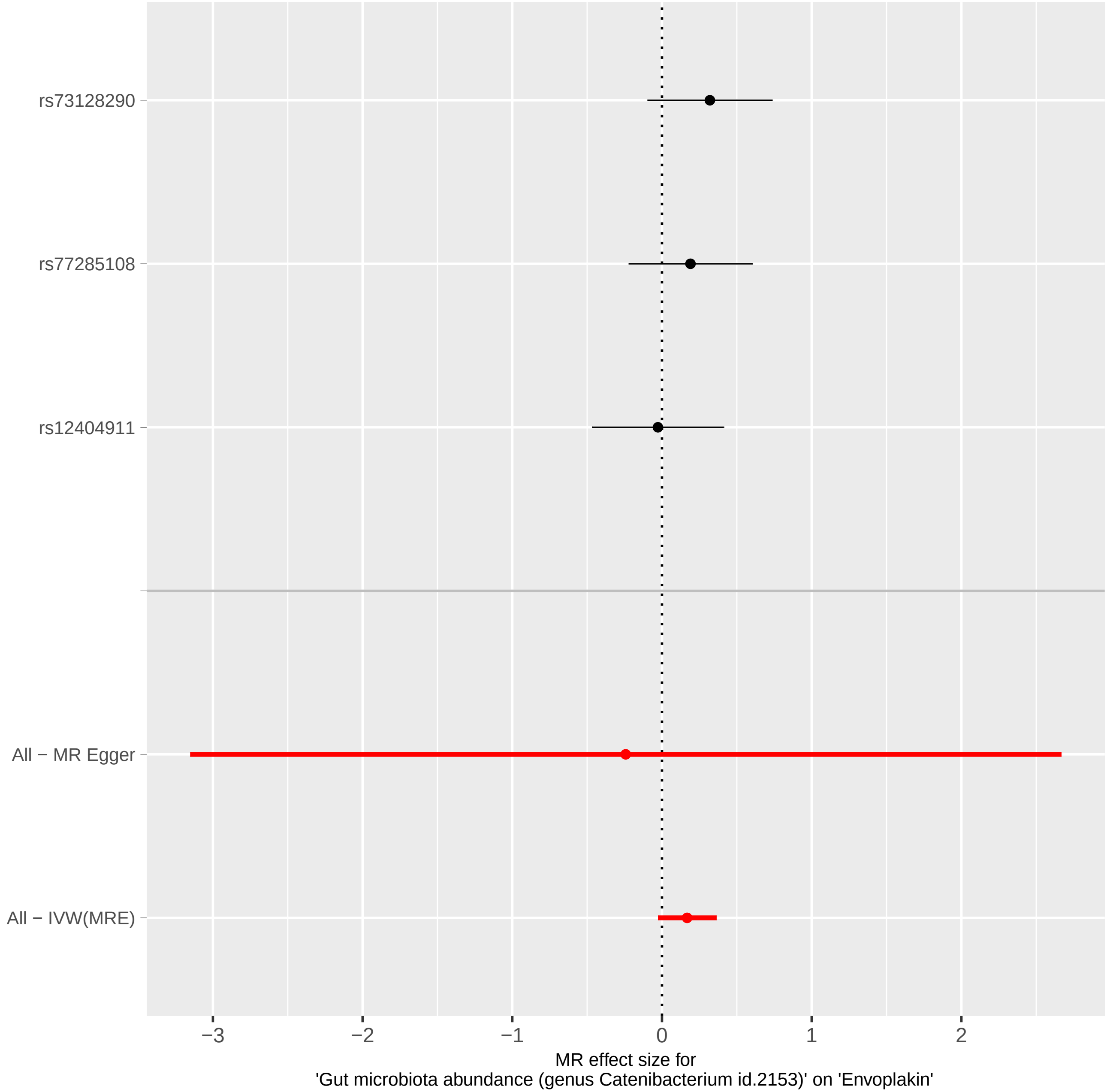

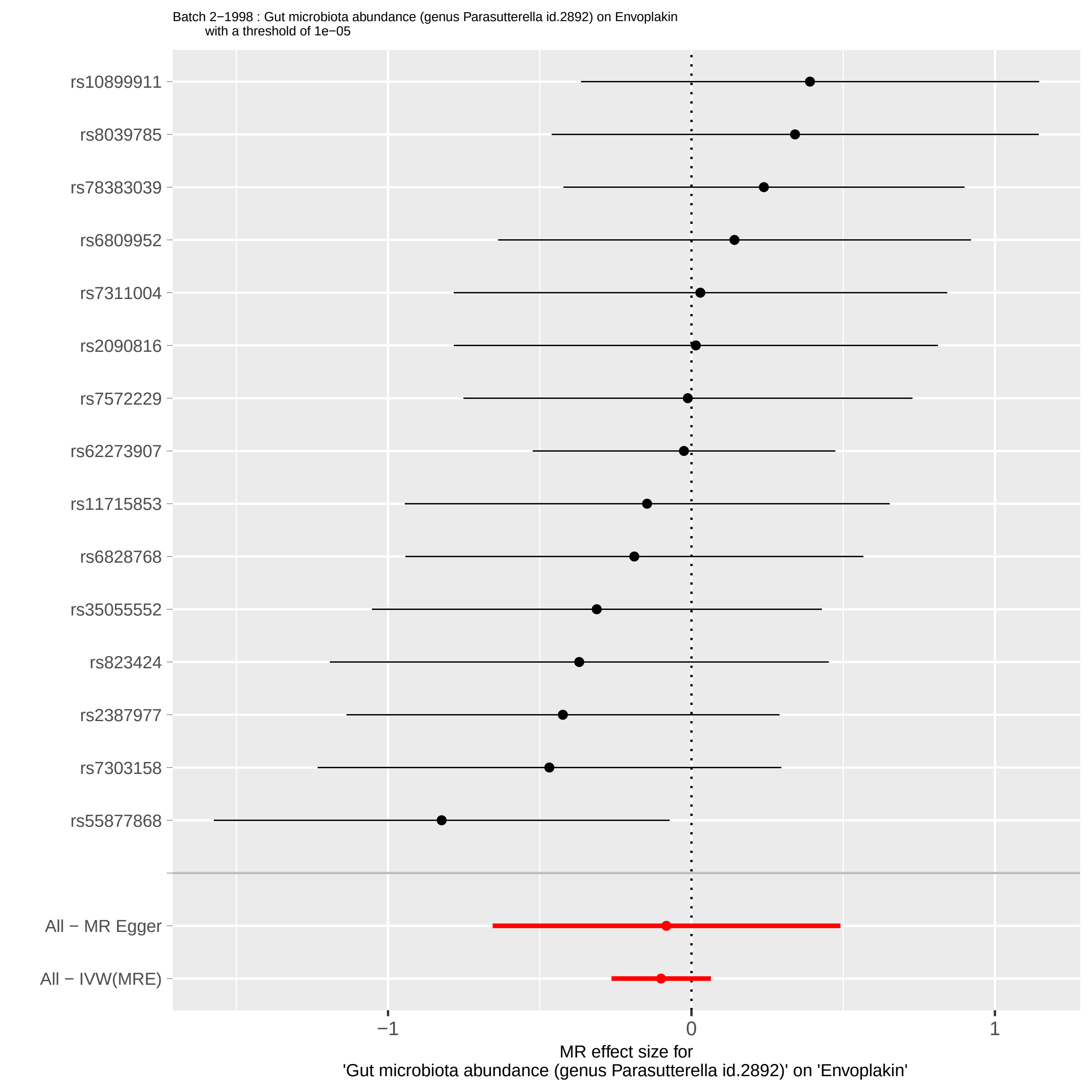

Batch 2−1998 : Gut microbiota abundance (genus Parasutterella id.2892) on Envoplakin
with a threshold of 1e−05
rs10899911
rs8039785
rs78383039
rs6809952
rs7311004
rs2090816
rs7572229
rs62273907
rs11715853
rs6828768
rs35055552
rs823424
rs2387977
rs7303158
rs55877868
All − MR Egger
All − IVW(MRE)
−1
0
1
MR effect size for
'Gut microbiota abundance (genus Parasutterella id.2892)' on 'Envoplakin'

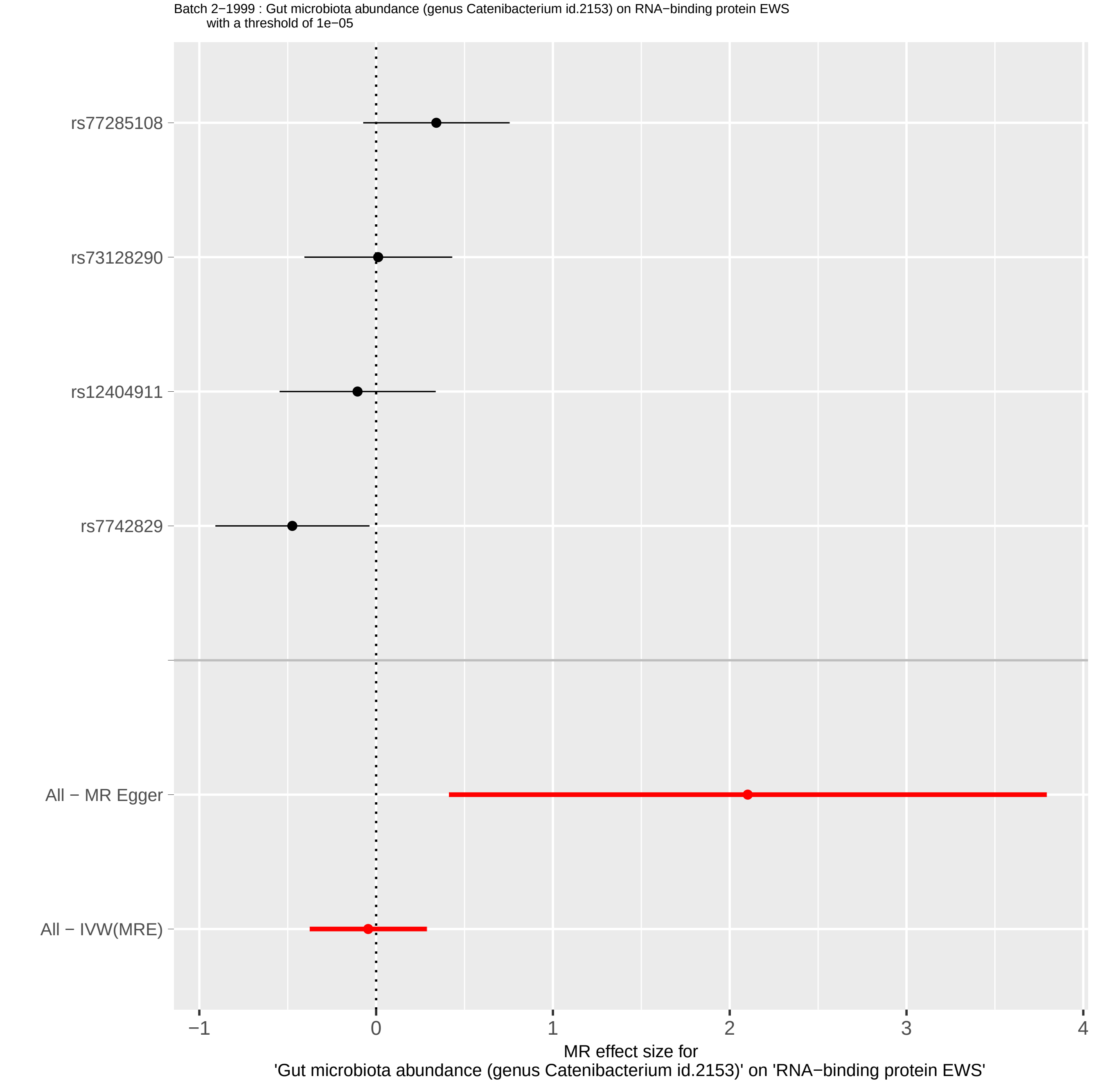
Batch 2−1999 : Gut microbiota abundance (genus Catenibacterium id.2153) on RNA−binding protein EWS
with a threshold of 1e−05
rs77285108
rs73128290
rs12404911
rs7742829
All − MR Egger
All − IVW(MRE)
−1
0
1
2
3
4
MR effect size for
'Gut microbiota abundance (genus Catenibacterium id.2153)' on 'RNA−binding protein EWS'

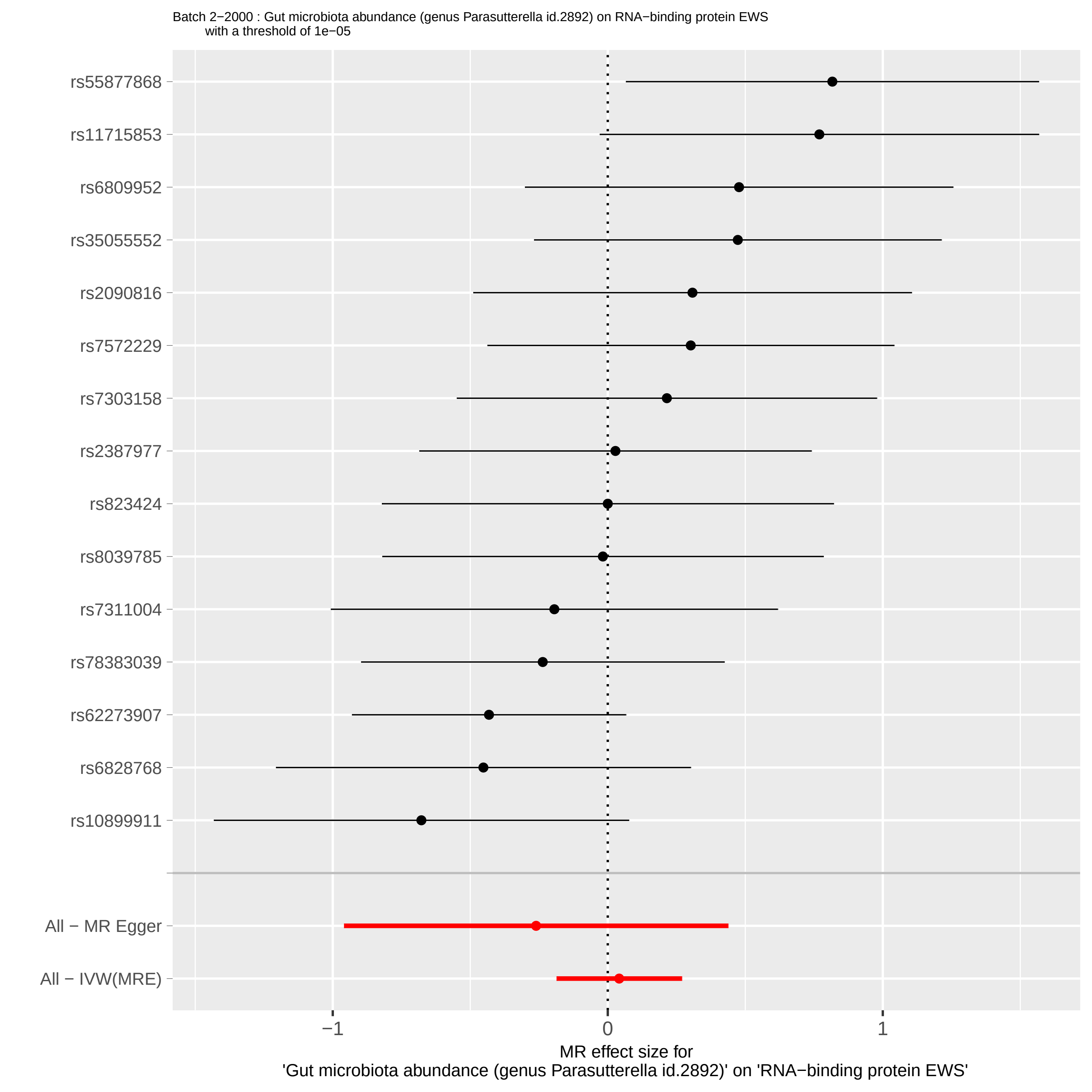

Batch 2−2000 : Gut microbiota abundance (genus Parasutterella id.2892) on RNA−binding protein EWS
with a threshold of 1e−05
rs55877868
rs11715853
rs6809952
rs35055552
rs2090816
rs7572229
rs7303158
rs2387977
rs823424
rs8039785
rs7311004
rs78383039
rs62273907
rs6828768
rs10899911
All − MR Egger
All − IVW(MRE)
−1
0
1
MR effect size for
'Gut microbiota abundance (genus Parasutterella id.2892)' on 'RNA−binding protein EWS'

Batch 2−2001 : Gut microbiota abundance (genus Catenibacterium id.2153) on Exosome complex component CSL4
with a threshold of 1e−05

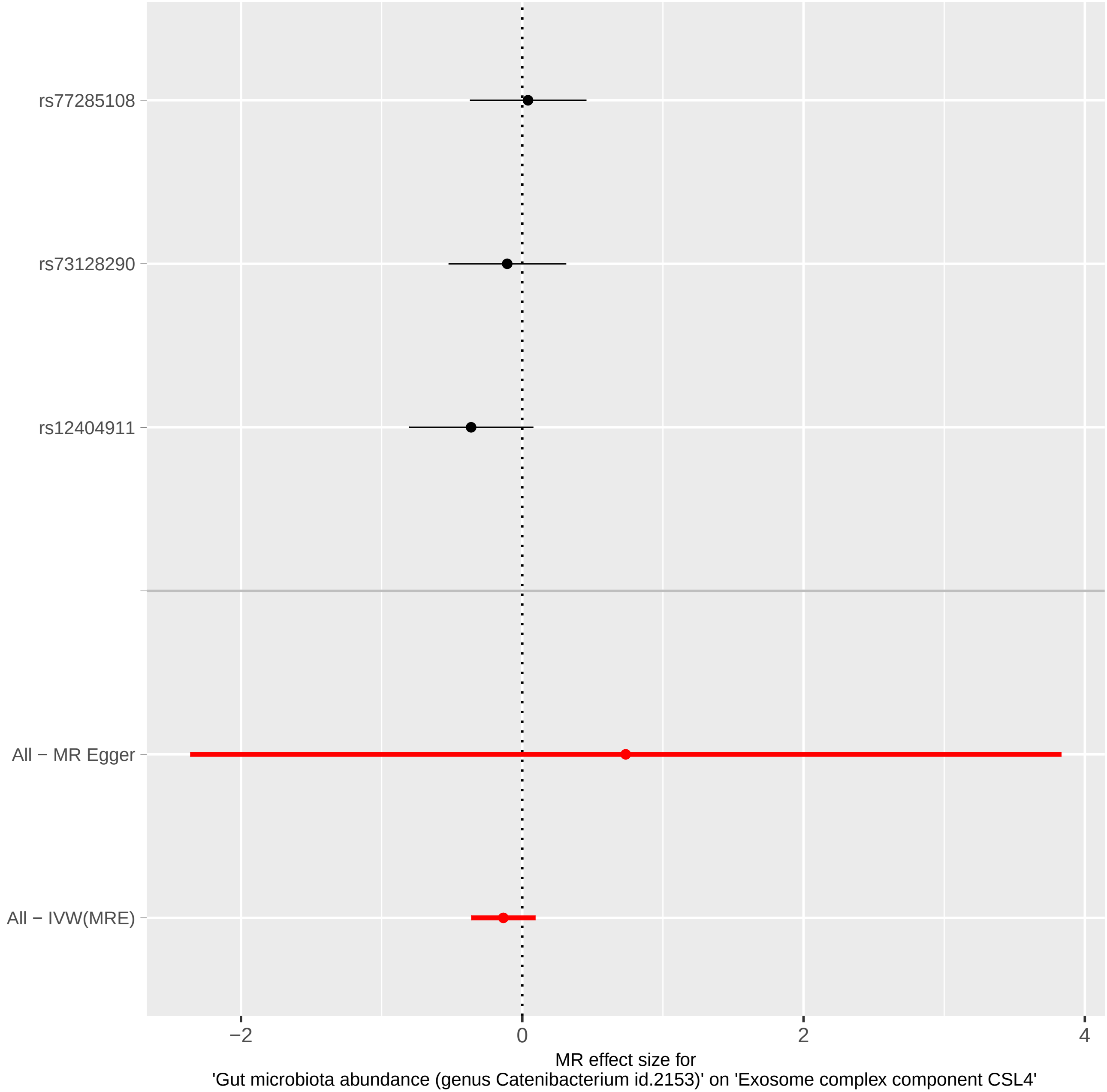

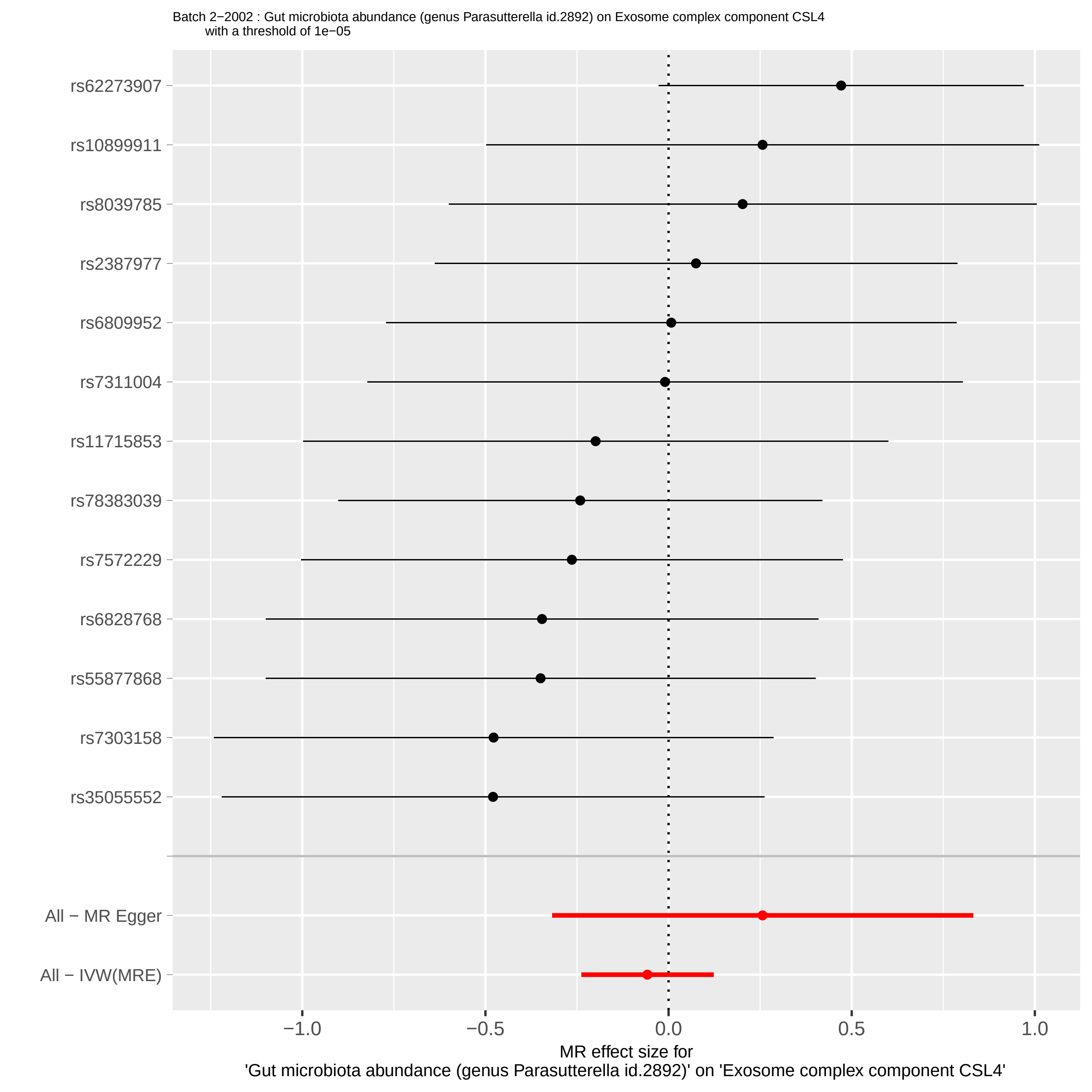
Batch 2−2002 : Gut microbiota abundance (genus Parasutterella id.2892) on Exosome complex component CSL4
with a threshold of 1e−05
rs62273907
rs10899911
rs8039785
rs2387977
rs6809952
rs7311004
rs11715853
rs78383039
rs7572229
rs6828768
rs55877868
rs7303158
rs35055552
All − MR Egger
All − IVW(MRE)
−1.0
−0.5
0.0
0.5
1.0
MR effect size for
'Gut microbiota abundance (genus Parasutterella id.2892)' on 'Exosome complex component CSL4'

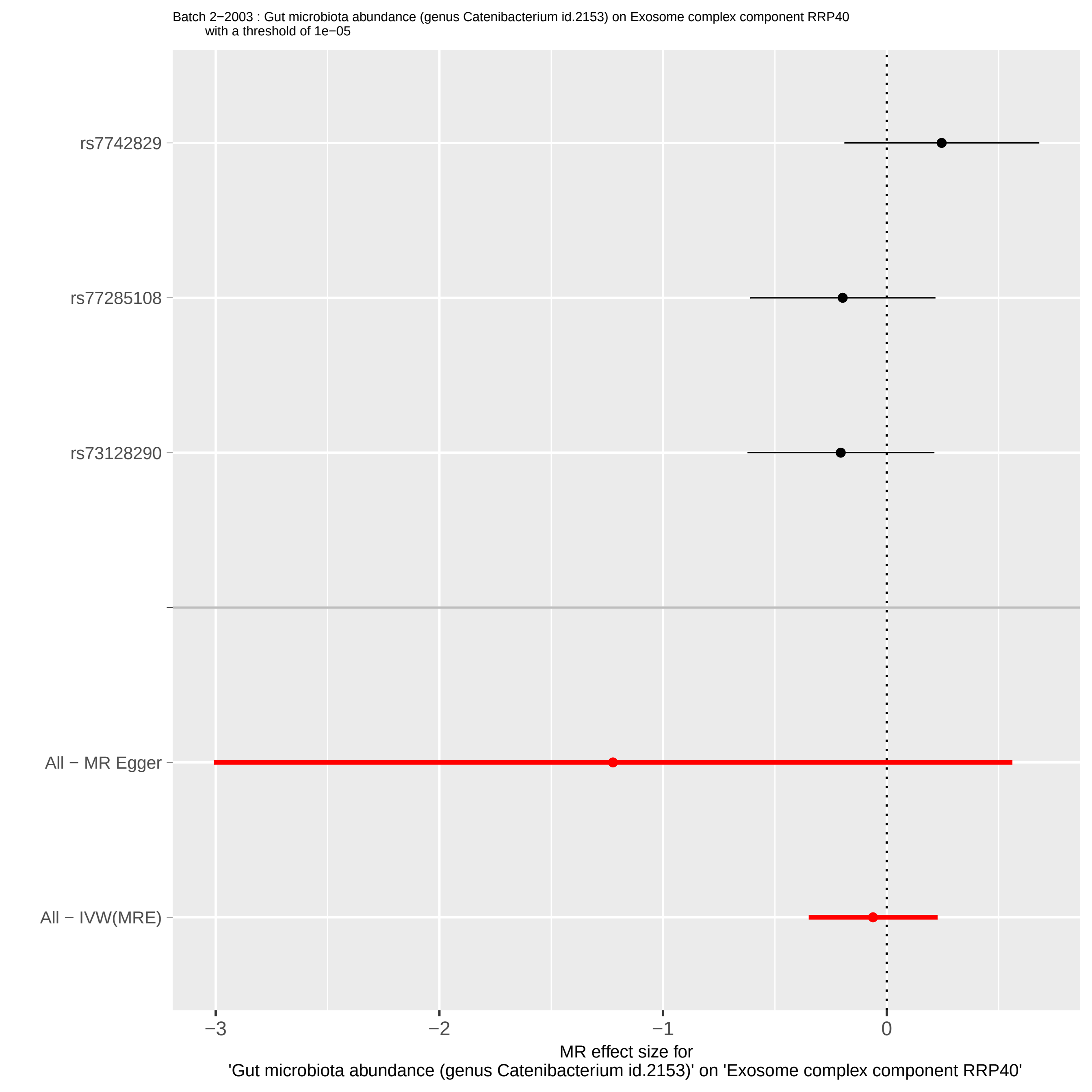

Batch 2−2003 : Gut microbiota abundance (genus Catenibacterium id.2153) on Exosome complex component RRP40
with a threshold of 1e−05
rs7742829
rs77285108
rs73128290
All − MR Egger
All − IVW(MRE)
−3
−2
−1
0
MR effect size for
'Gut microbiota abundance (genus Catenibacterium id.2153)' on 'Exosome complex component RRP40'

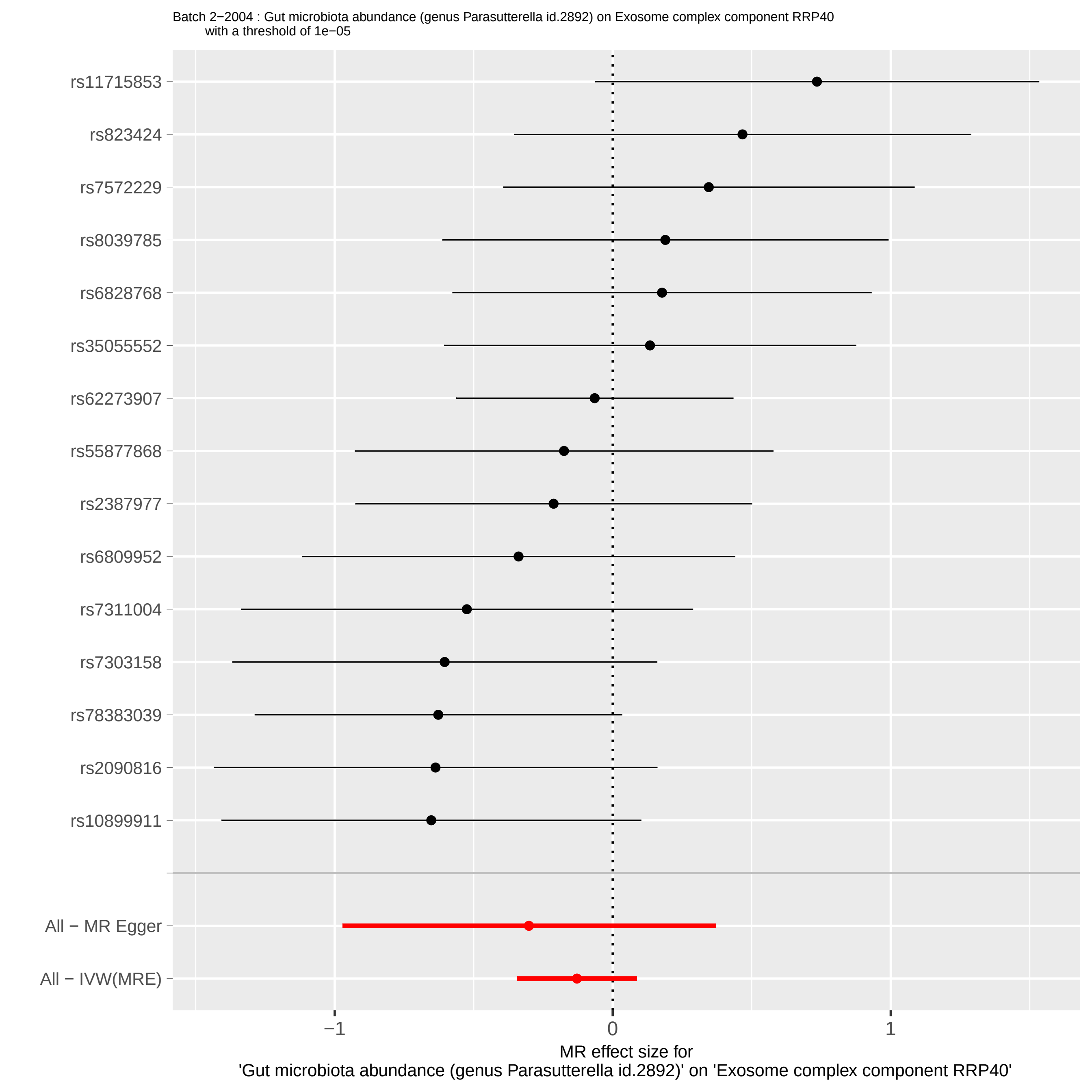
Batch 2−2004 : Gut microbiota abundance (genus Parasutterella id.2892) on Exosome complex component RRP40
with a threshold of 1e−05
rs11715853
rs823424
rs7572229
rs8039785
rs6828768
rs35055552
rs62273907
rs55877868
rs2387977
rs6809952
rs7311004
rs7303158
rs78383039
rs2090816
rs10899911
All − MR Egger
All − IVW(MRE)
−1
0
1
MR effect size for
'Gut microbiota abundance (genus Parasutterella id.2892)' on 'Exosome complex component RRP40'

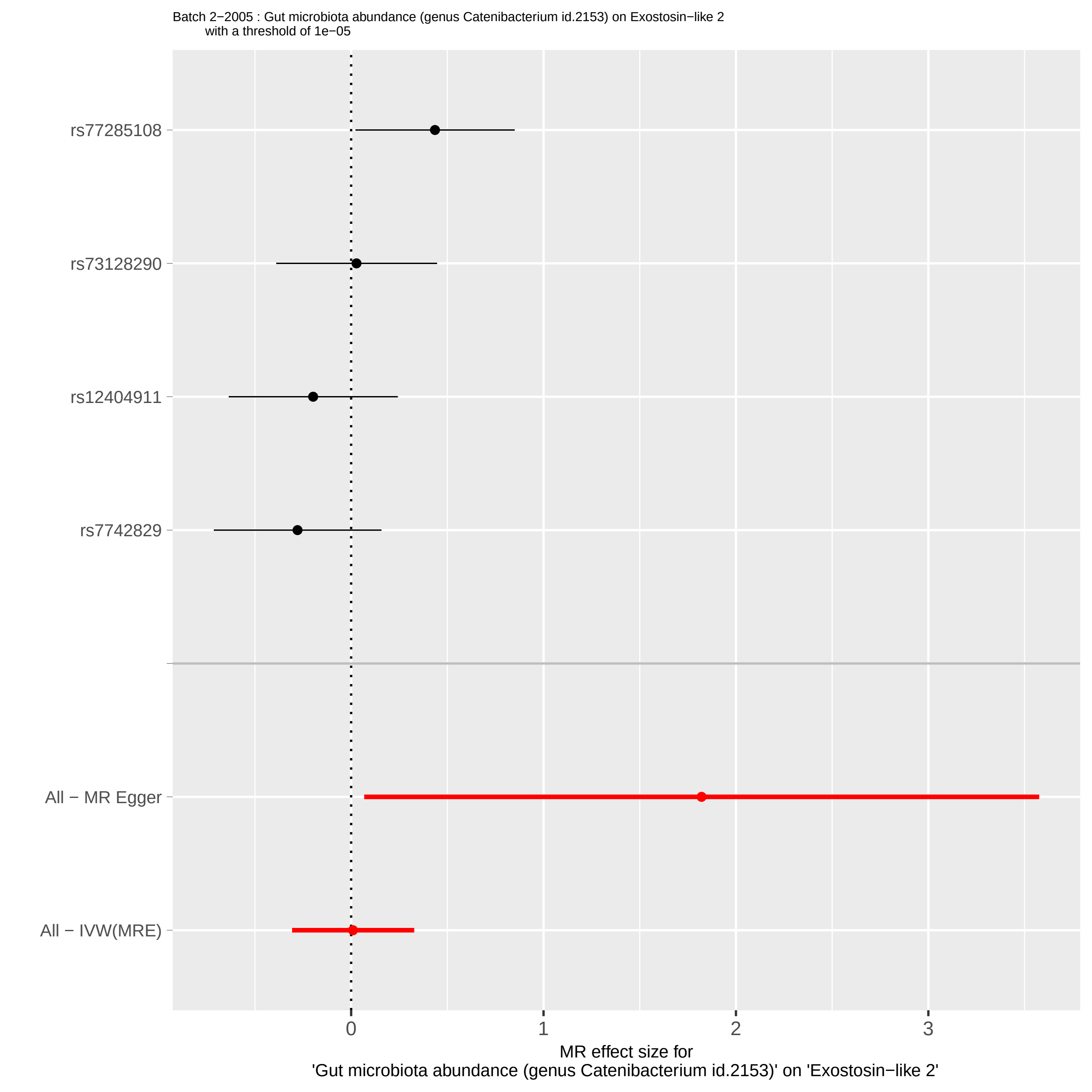

Batch 2−2005 : Gut microbiota abundance (genus Catenibacterium id.2153) on Exostosin−like 2
with a threshold of 1e−05
rs77285108
rs73128290
rs12404911
rs7742829
All − MR Egger
All − IVW(MRE)
0
1
2
3
MR effect size for
'Gut microbiota abundance (genus Catenibacterium id.2153)' on 'Exostosin−like 2'

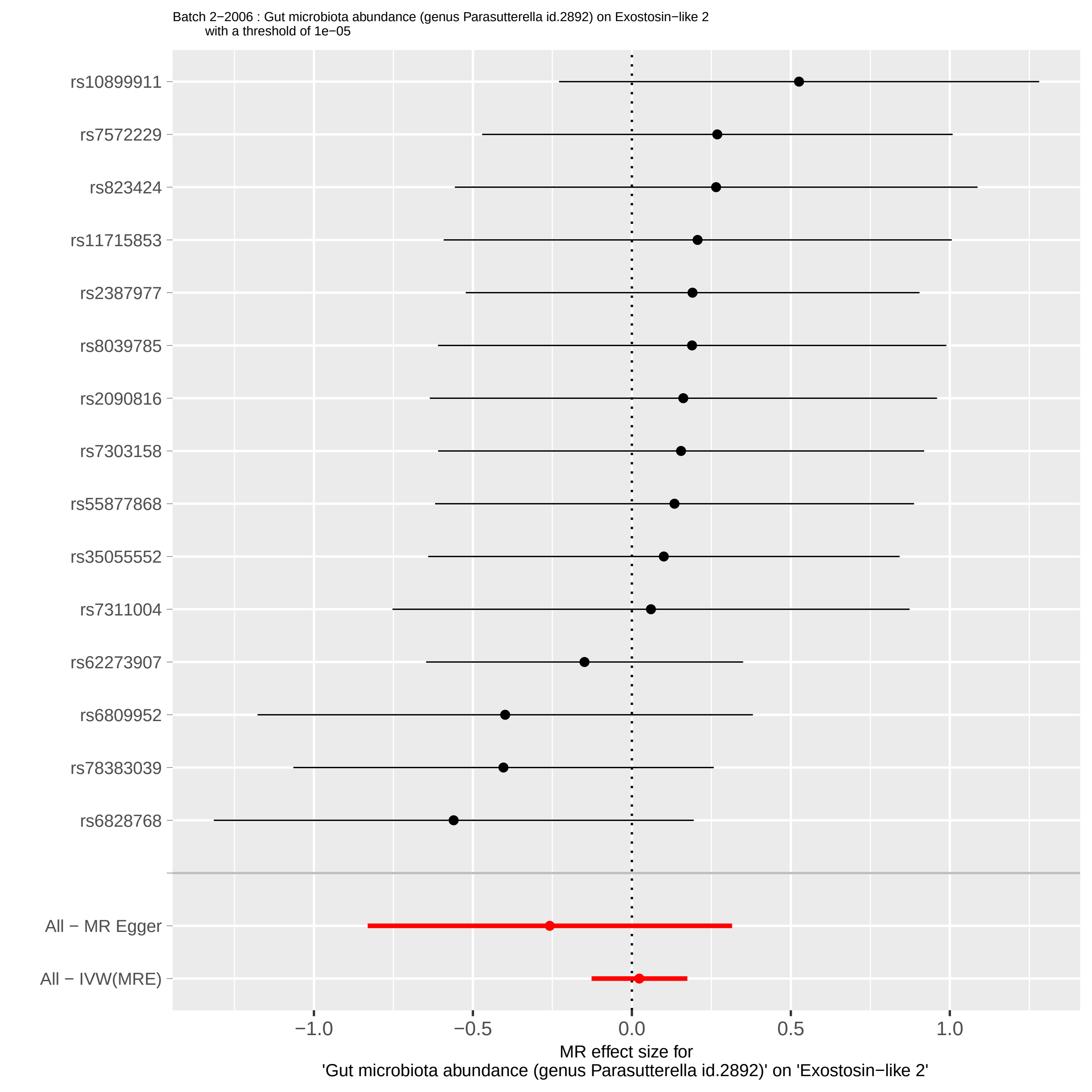

Batch 2−2006 : Gut microbiota abundance (genus Parasutterella id.2892) on Exostosin−like 2
with a threshold of 1e−05
rs10899911
rs7572229
rs823424
rs11715853
rs2387977
rs8039785
rs2090816
rs7303158
rs55877868
rs35055552
rs7311004
rs62273907
rs6809952
rs78383039
rs6828768
All − MR Egger
All − IVW(MRE)
−1.0
−0.5
0.0
0.5
1.0
MR effect size for
'Gut microbiota abundance (genus Parasutterella id.2892)' on 'Exostosin−like 2'

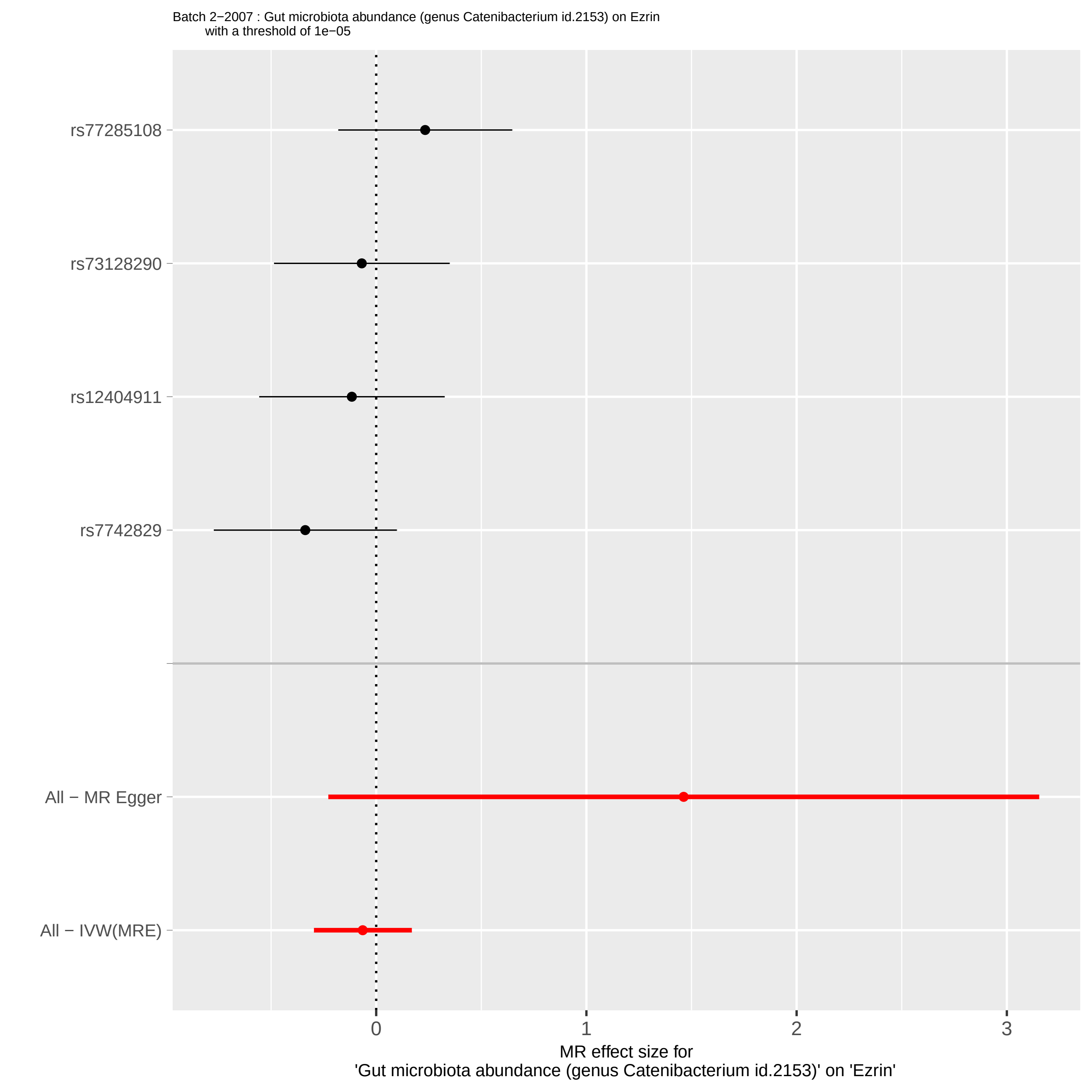

Batch 2−2007 : Gut microbiota abundance (genus Catenibacterium id.2153) on Ezrin
with a threshold of 1e−05
rs77285108
rs73128290
rs12404911
rs7742829
All − MR Egger
All − IVW(MRE)
0
1
2
3
MR effect size for
'Gut microbiota abundance (genus Catenibacterium id.2153)' on 'Ezrin'

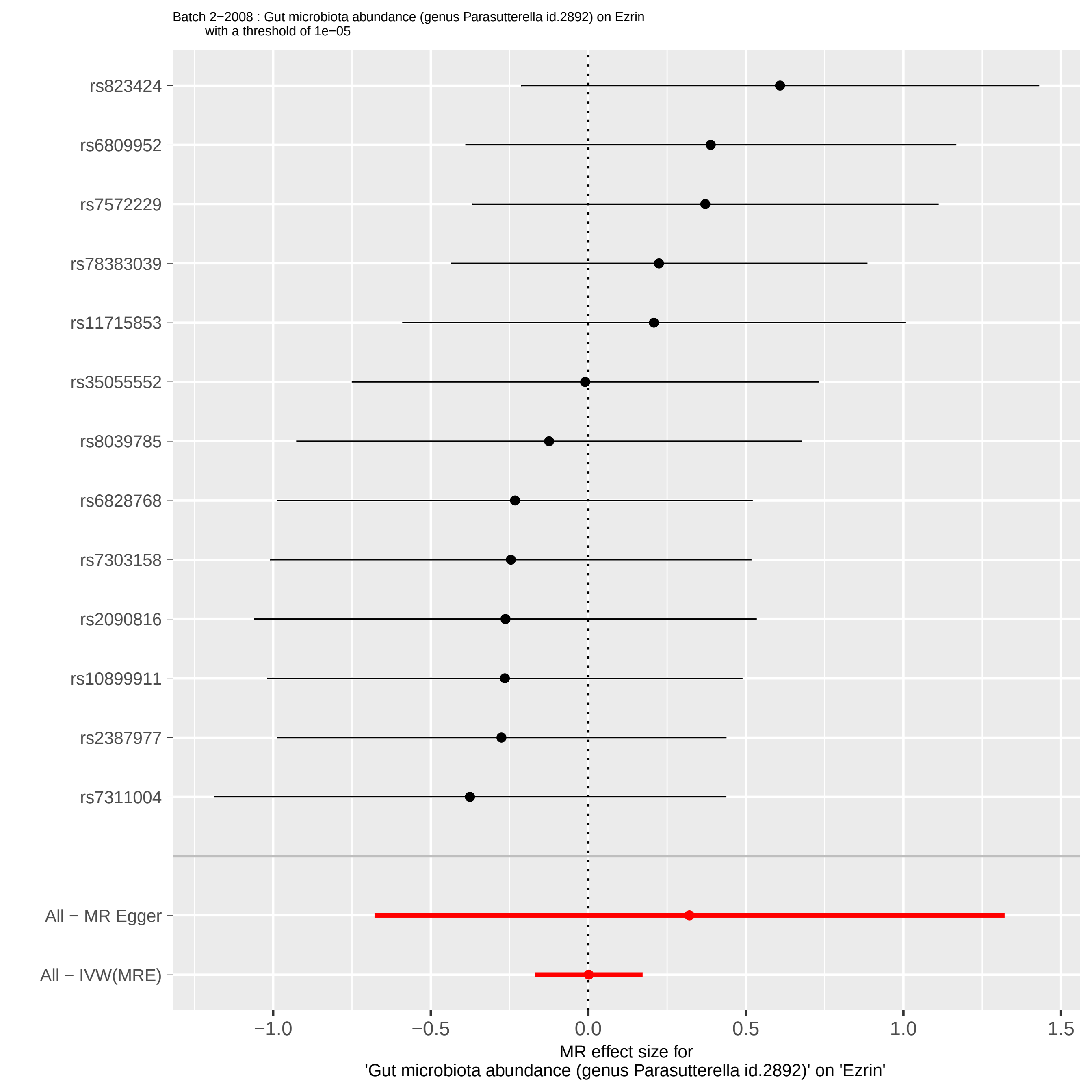

Batch 2−2008 : Gut microbiota abundance (genus Parasutterella id.2892) on Ezrin
with a threshold of 1e−05
rs823424
rs6809952
rs7572229
rs78383039
rs11715853
rs35055552
rs8039785
rs6828768
rs7303158
rs2090816
rs10899911
rs2387977
rs7311004
All − MR Egger
All − IVW(MRE)
−1.0
−0.5
0.0
0.5
1.0
1.5
MR effect size for
'Gut microbiota abundance (genus Parasutterella id.2892)' on 'Ezrin'

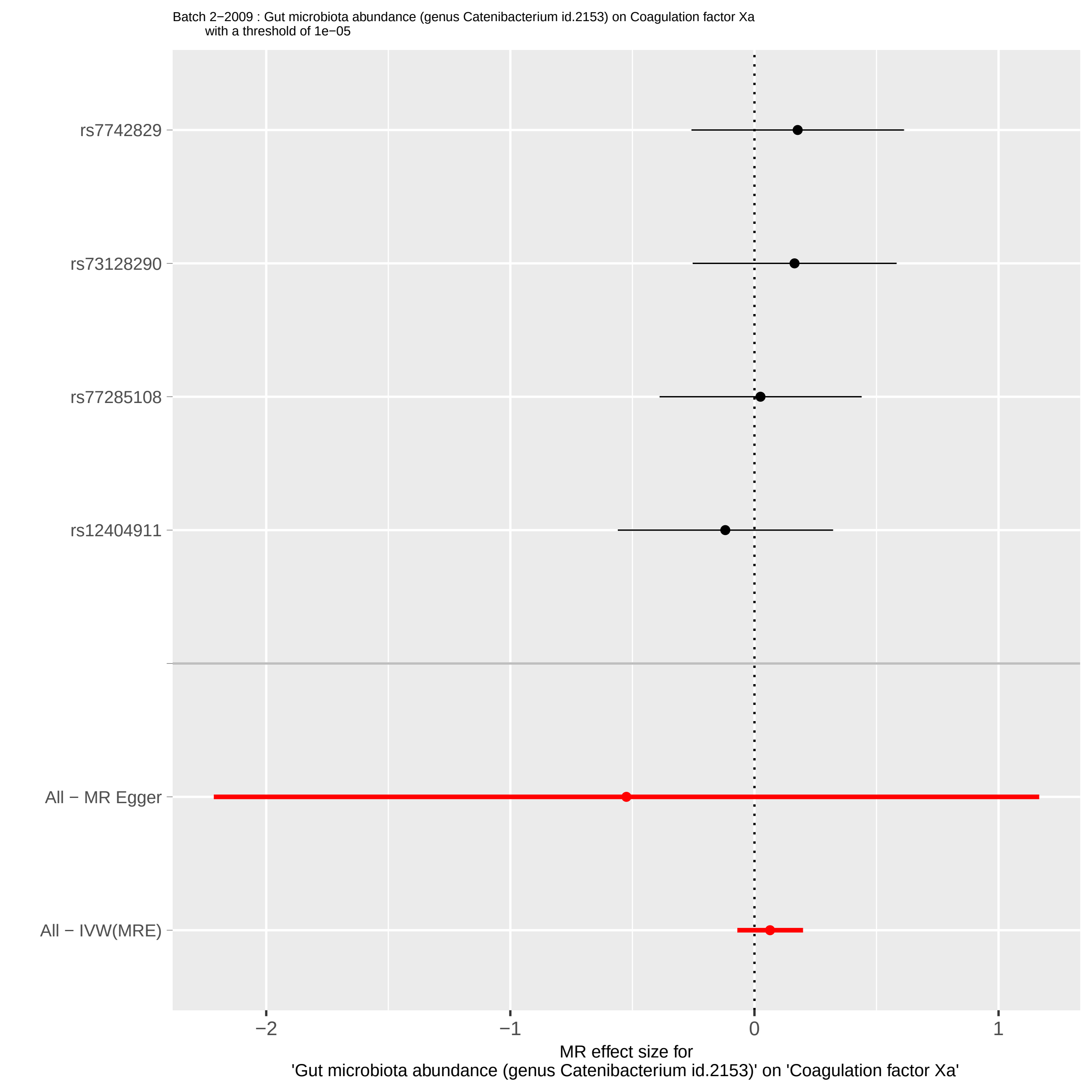
Batch 2−2009 : Gut microbiota abundance (genus Catenibacterium id.2153) on Coagulation factor Xa
with a threshold of 1e−05
rs7742829
rs73128290
rs77285108
rs12404911
All − MR Egger
All − IVW(MRE)
−2
−1
0
1
MR effect size for
'Gut microbiota abundance (genus Catenibacterium id.2153)' on 'Coagulation factor Xa'

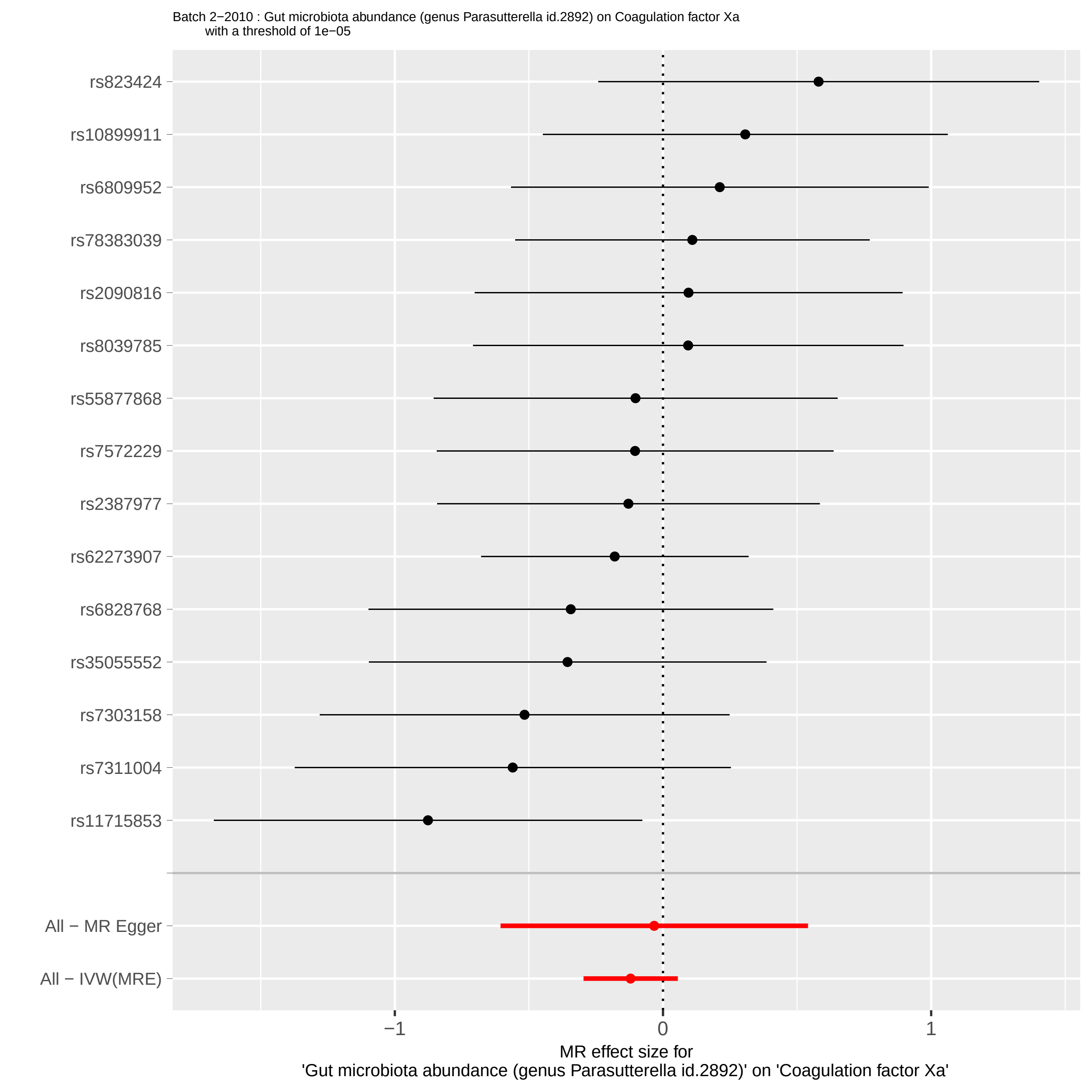

Batch 2−2010 : Gut microbiota abundance (genus Parasutterella id.2892) on Coagulation factor Xa
with a threshold of 1e−05
rs823424
rs10899911
rs6809952
rs78383039
rs2090816
rs8039785
rs55877868
rs7572229
rs2387977
rs62273907
rs6828768
rs35055552
rs7303158
rs7311004
rs11715853
All − MR Egger
All − IVW(MRE)
−1
0
1
MR effect size for
'Gut microbiota abundance (genus Parasutterella id.2892)' on 'Coagulation factor Xa'

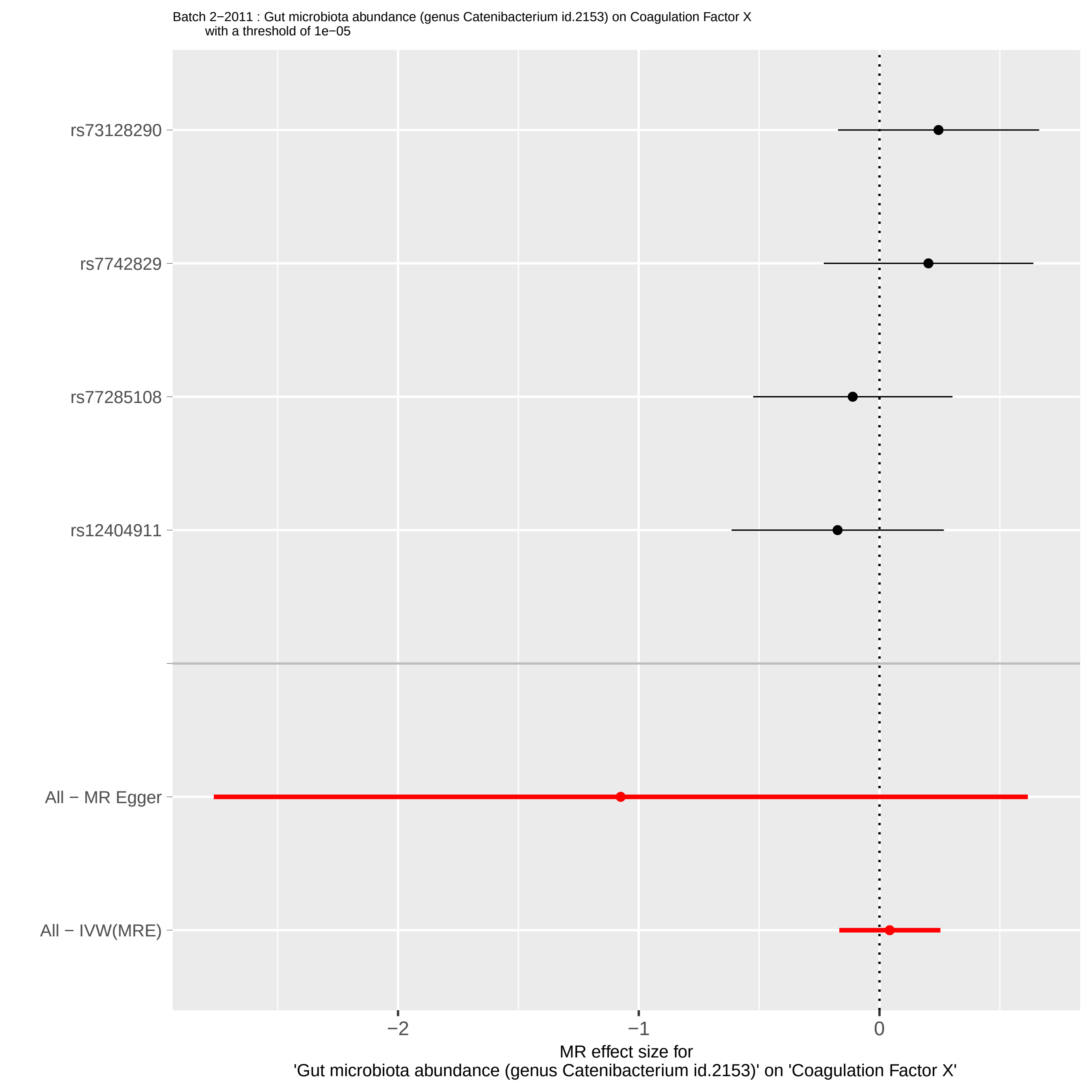

Batch 2−2011 : Gut microbiota abundance (genus Catenibacterium id.2153) on Coagulation Factor X
with a threshold of 1e−05
rs73128290
rs7742829
rs77285108
rs12404911
All − MR Egger
All − IVW(MRE)
−2
−1
0
MR effect size for
'Gut microbiota abundance (genus Catenibacterium id.2153)' on 'Coagulation Factor X'

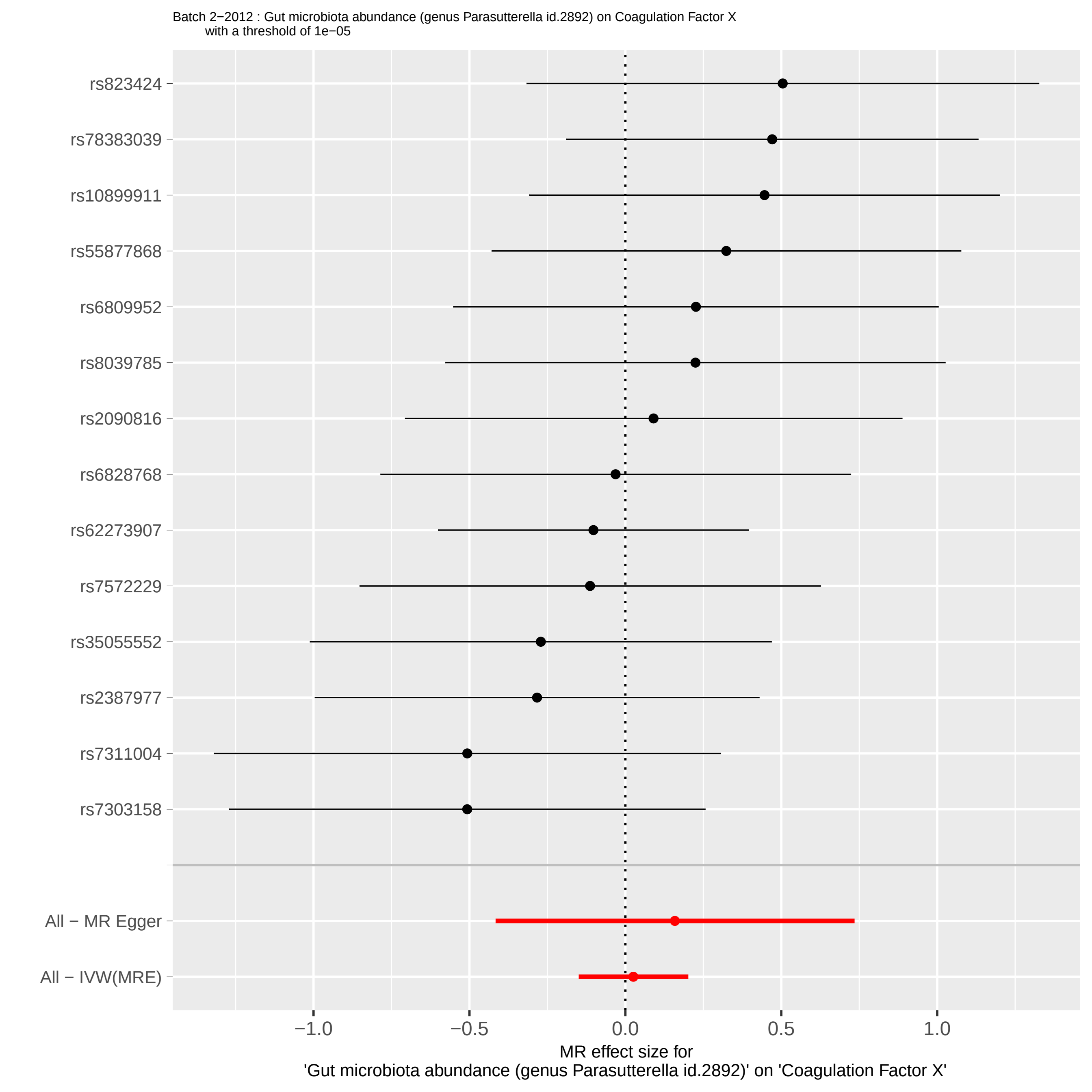
Batch 2−2012 : Gut microbiota abundance (genus Parasutterella id.2892) on Coagulation Factor X
with a threshold of 1e−05
rs823424
rs78383039
rs10899911
rs55877868
rs6809952
rs8039785
rs2090816
rs6828768
rs62273907
rs7572229
rs35055552
rs2387977
rs7311004
rs7303158
All − MR Egger
All − IVW(MRE)
−1.0
−0.5
0.0
0.5
1.0
MR effect size for
'Gut microbiota abundance (genus Parasutterella id.2892)' on 'Coagulation Factor X'

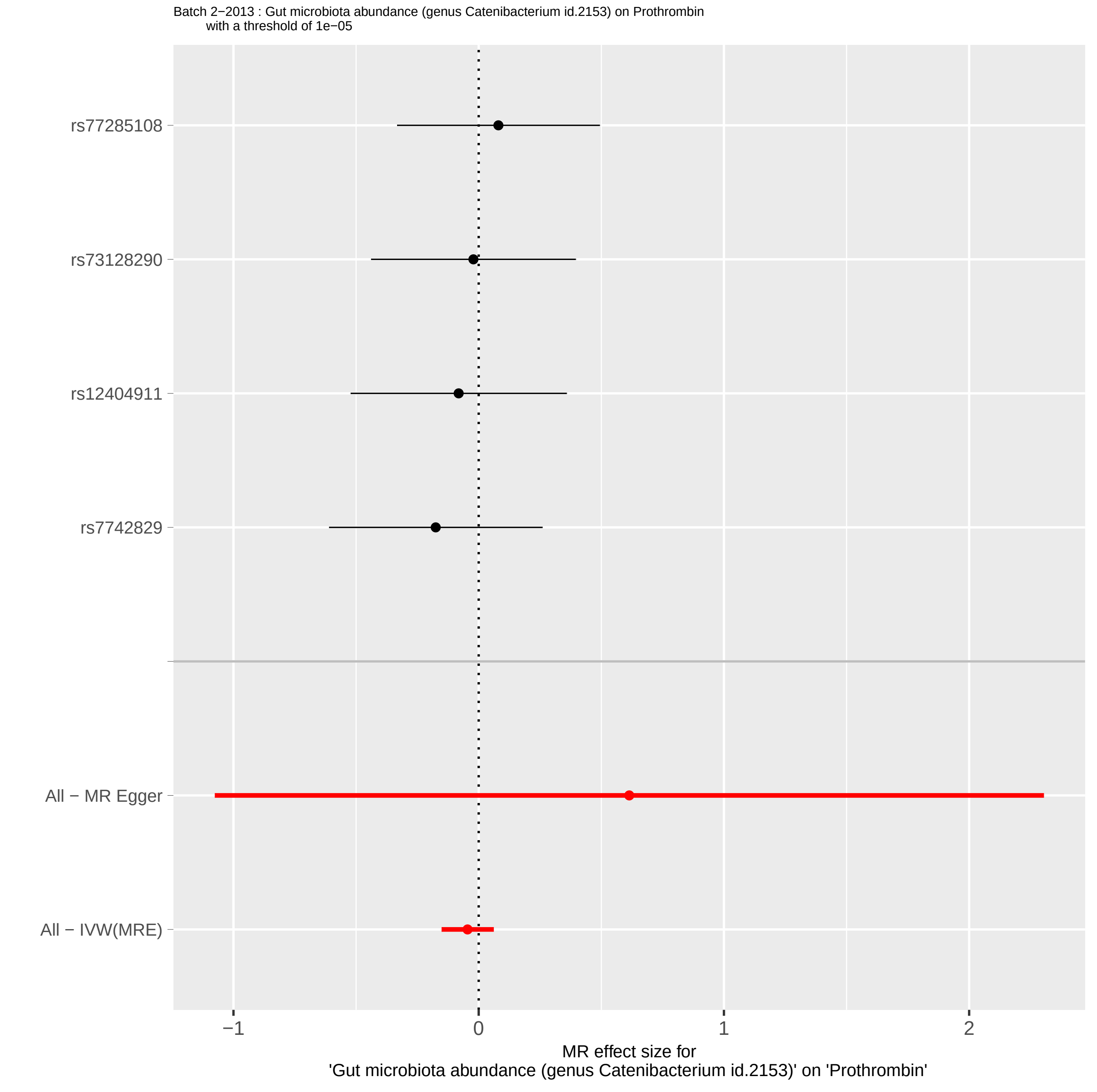

Batch 2−2013 : Gut microbiota abundance (genus Catenibacterium id.2153) on Prothrombin
with a threshold of 1e−05
rs77285108
rs73128290
rs12404911
rs7742829
All − MR Egger
All − IVW(MRE)
−1
0
1
2
MR effect size for
'Gut microbiota abundance (genus Catenibacterium id.2153)' on 'Prothrombin'

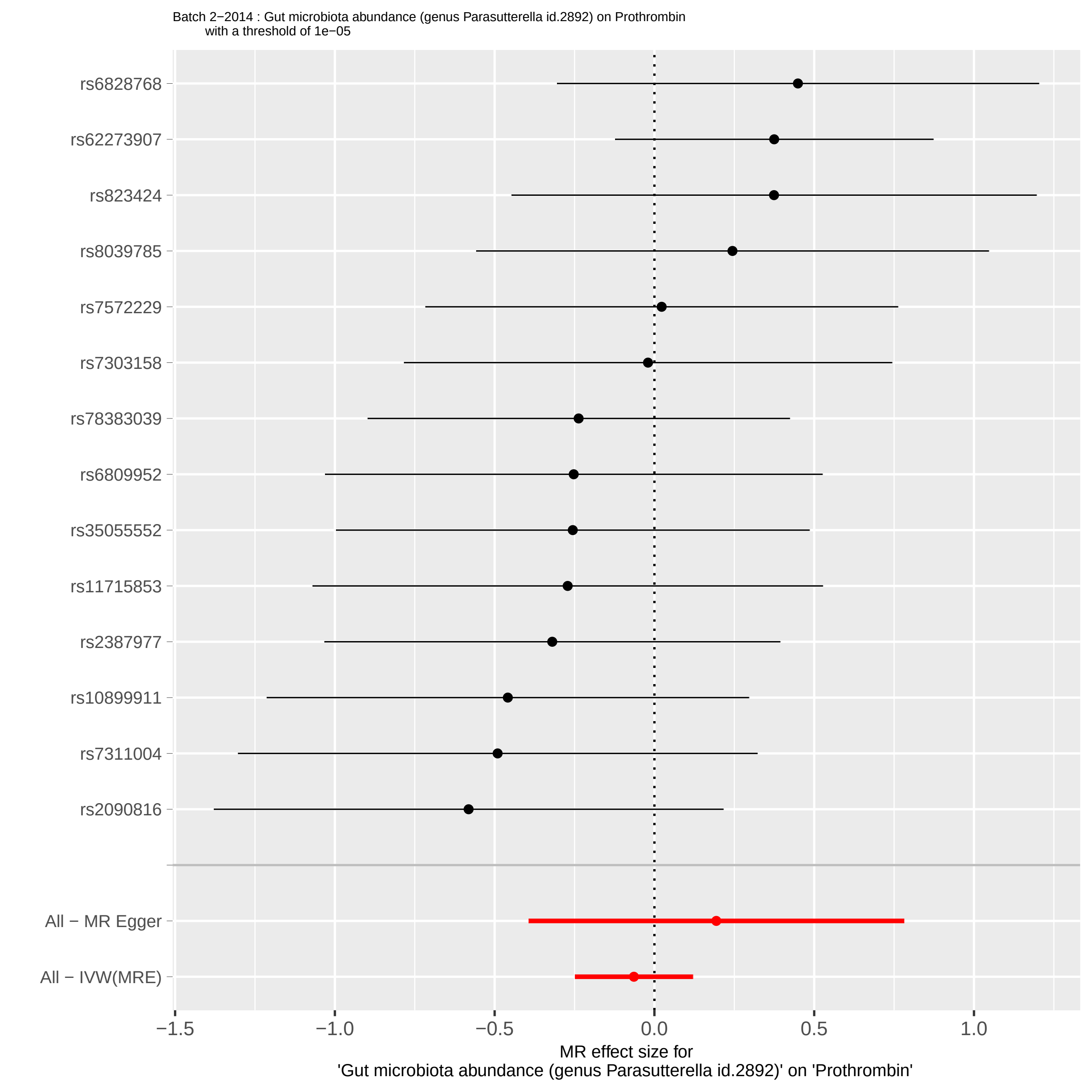
Batch 2−2014 : Gut microbiota abundance (genus Parasutterella id.2892) on Prothrombin
with a threshold of 1e−05
rs6828768
rs62273907
rs823424
rs8039785
rs7572229
rs7303158
rs78383039
rs6809952
rs35055552
rs11715853
rs2387977
rs10899911
rs7311004
rs2090816
All − MR Egger
All − IVW(MRE)
−1.5
−1.0
−0.5
0.0
0.5
1.0
MR effect size for
'Gut microbiota abundance (genus Parasutterella id.2892)' on 'Prothrombin'

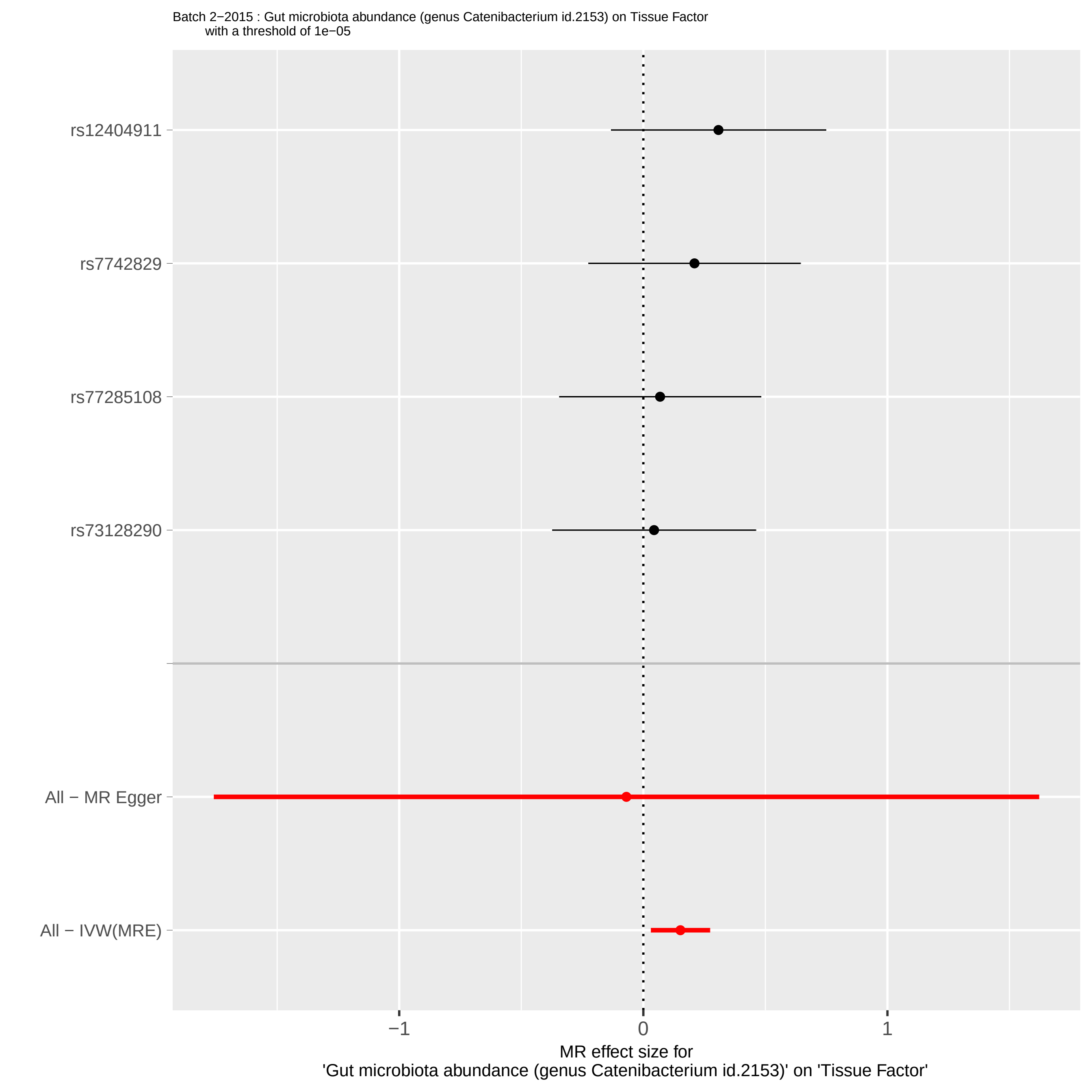

Batch 2−2015 : Gut microbiota abundance (genus Catenibacterium id.2153) on Tissue Factor
with a threshold of 1e−05
rs12404911
rs7742829
rs77285108
rs73128290
All − MR Egger
All − IVW(MRE)
−1
0
1
MR effect size for
'Gut microbiota abundance (genus Catenibacterium id.2153)' on 'Tissue Factor'

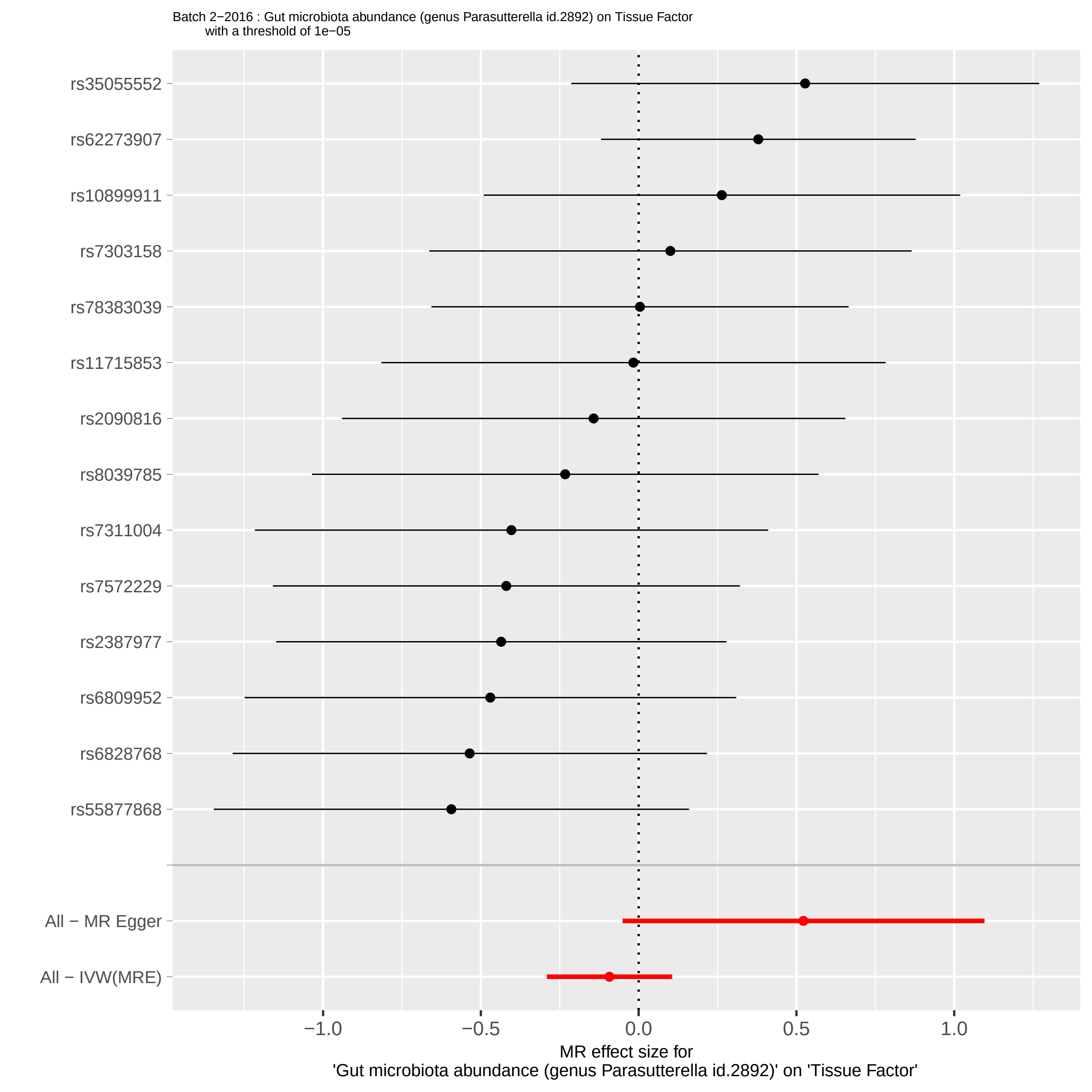

Batch 2−2016 : Gut microbiota abundance (genus Parasutterella id.2892) on Tissue Factor
with a threshold of 1e−05
rs35055552
rs62273907
rs10899911
rs7303158
rs78383039
rs11715853
rs2090816
rs8039785
rs7311004
rs7572229
rs2387977
rs6809952
rs6828768
rs55877868
All − MR Egger
All − IVW(MRE)
−1.0
−0.5
0.0
0.5
1.0
MR effect size for
'Gut microbiota abundance (genus Parasutterella id.2892)' on 'Tissue Factor'

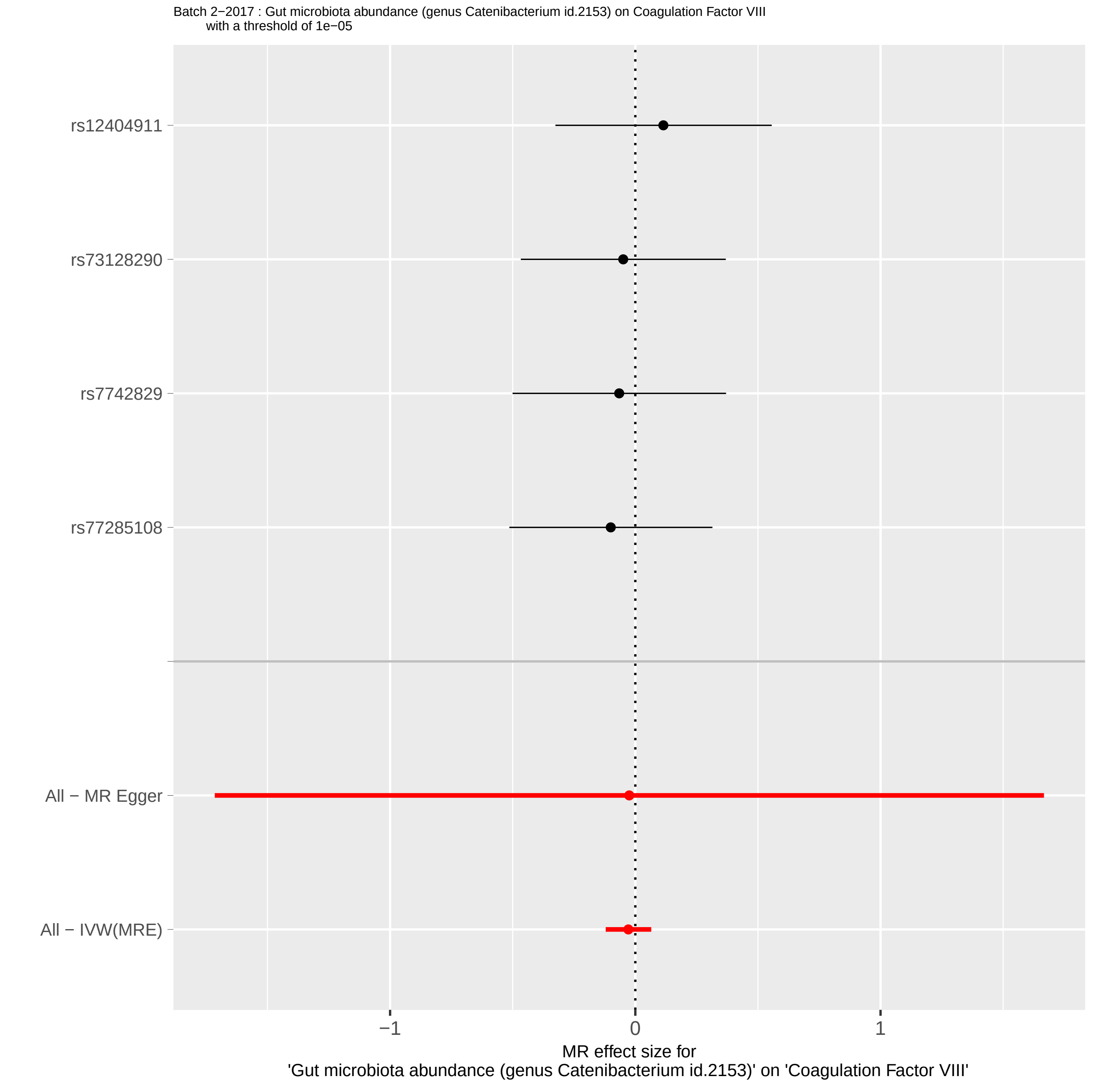
Batch 2−2017 : Gut microbiota abundance (genus Catenibacterium id.2153) on Coagulation Factor VIII
with a threshold of 1e−05
rs12404911
rs73128290
rs7742829
rs77285108
All − MR Egger
All − IVW(MRE)
−1
0
1
MR effect size for
'Gut microbiota abundance (genus Catenibacterium id.2153)' on 'Coagulation Factor VIII'

Batch 2−2018 : Gut microbiota abundance (genus Parasutterella id.2892) on Coagulation Factor VIII
with a threshold of 1e−05

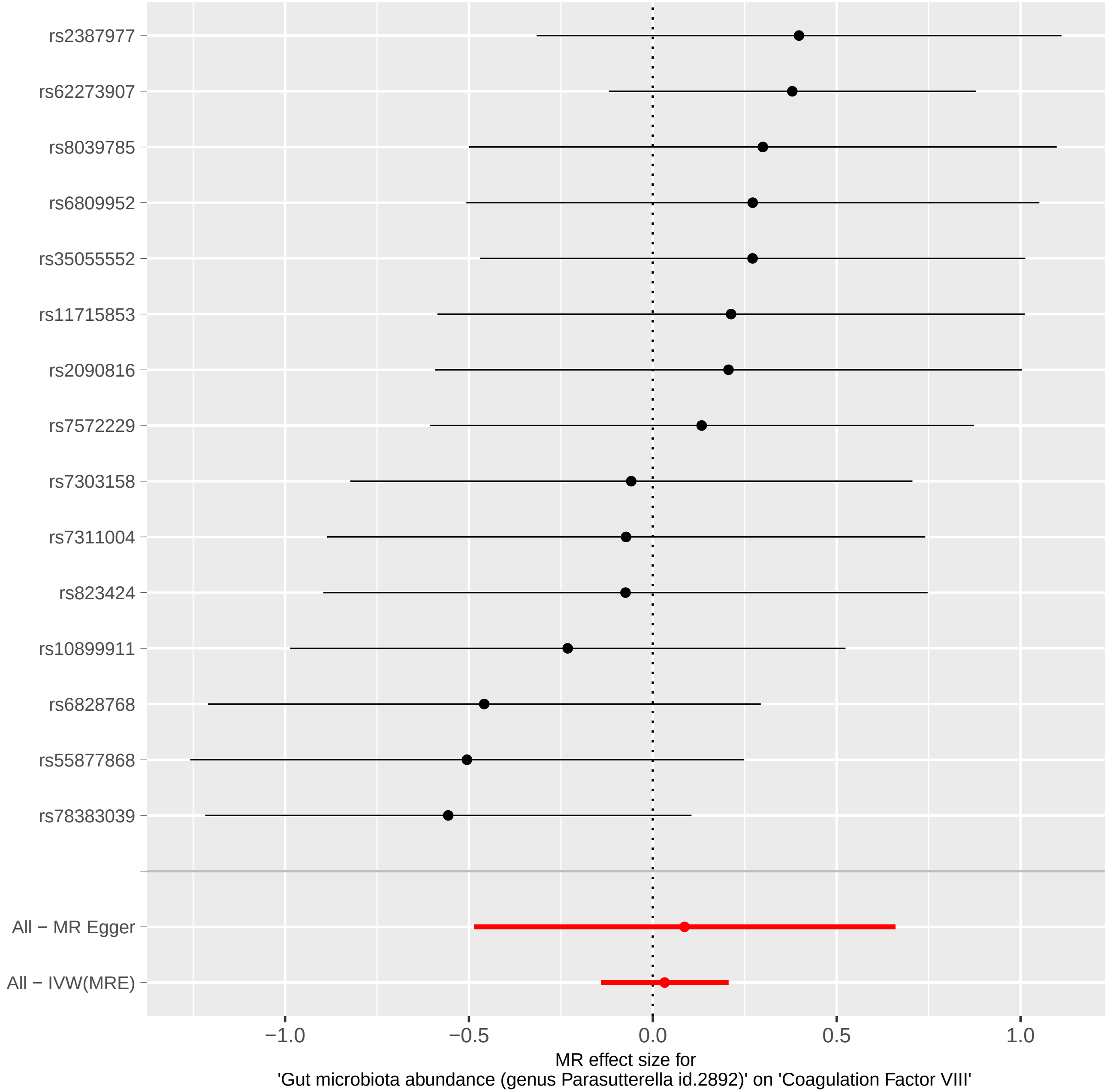

Batch 2−2019 : Gut microbiota abundance (genus Catenibacterium id.2153) on Fatty−acid amide hydrolase 2
with a threshold of 1e−05

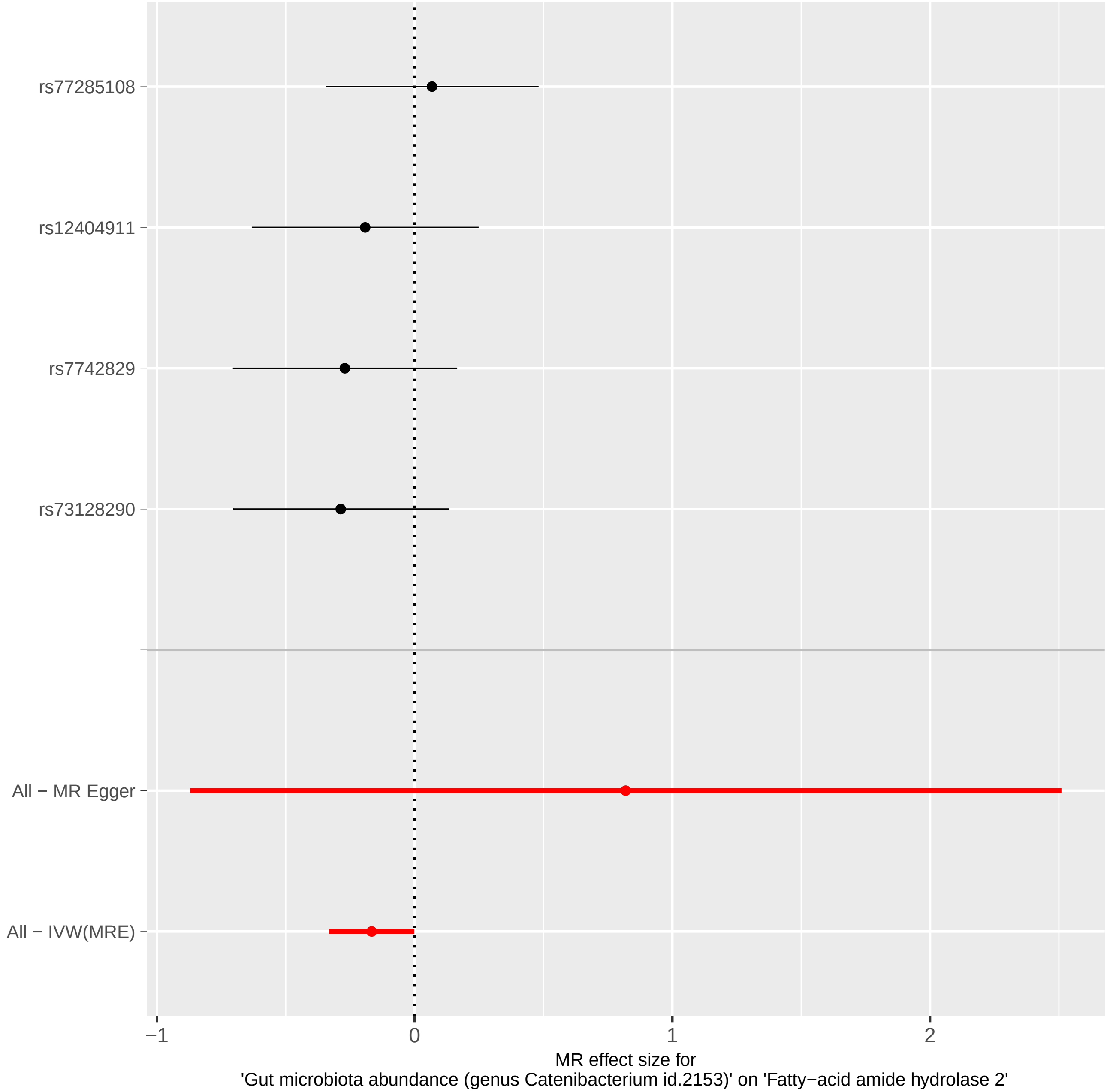

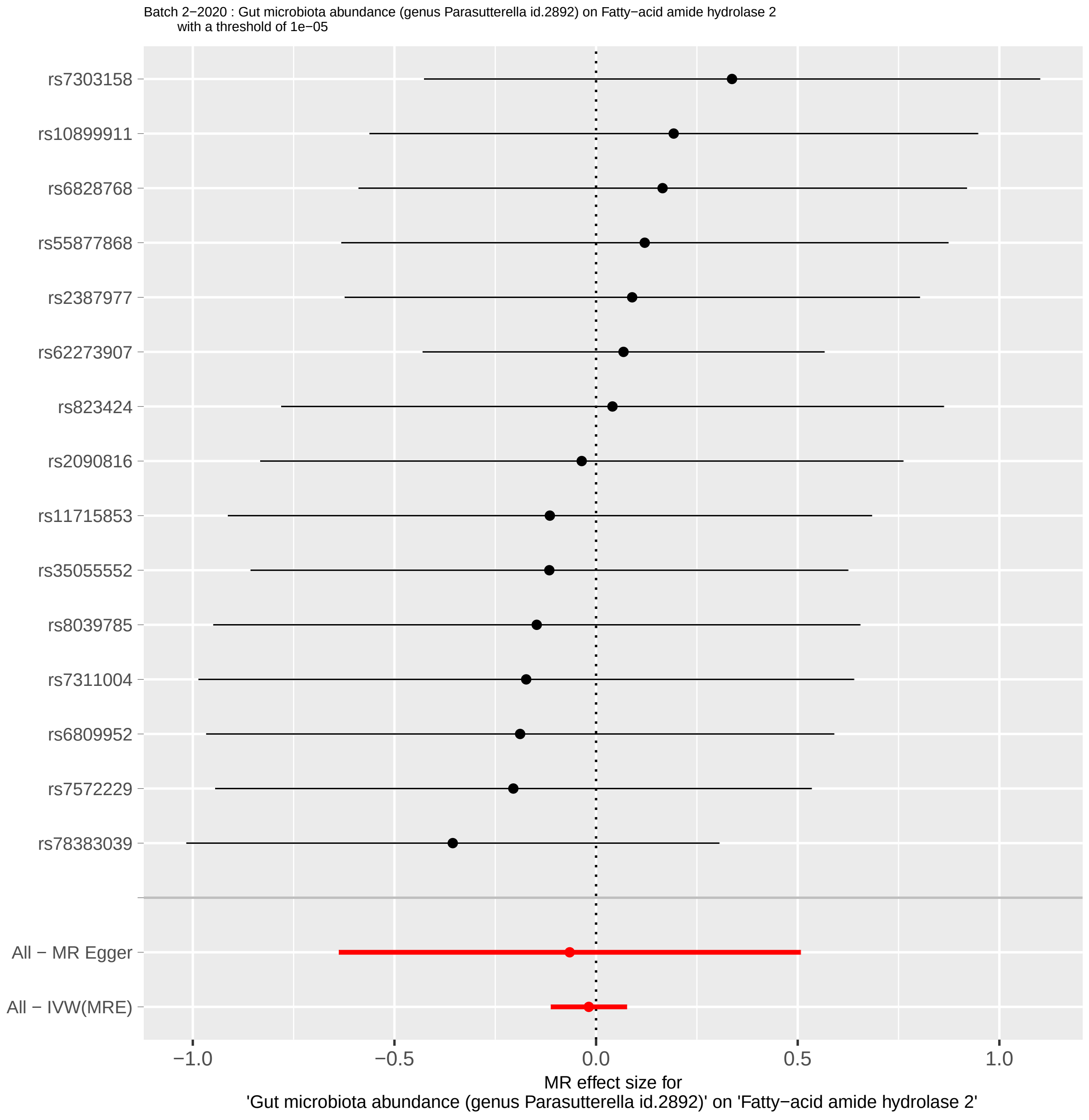

Batch 2−2020 : Gut microbiota abundance (genus Parasutterella id.2892) on Fatty−acid amide hydrolase 2
with a threshold of 1e−05
rs7303158
rs10899911
rs6828768
rs55877868
rs2387977
rs62273907
rs823424
rs2090816
rs11715853
rs35055552
rs8039785
rs7311004
rs6809952
rs7572229
rs78383039
All − MR Egger
All − IVW(MRE)
−1.0
−0.5
0.0
0.5
1.0
MR effect size for
'Gut microbiota abundance (genus Parasutterella id.2892)' on 'Fatty−acid amide hydrolase 2'

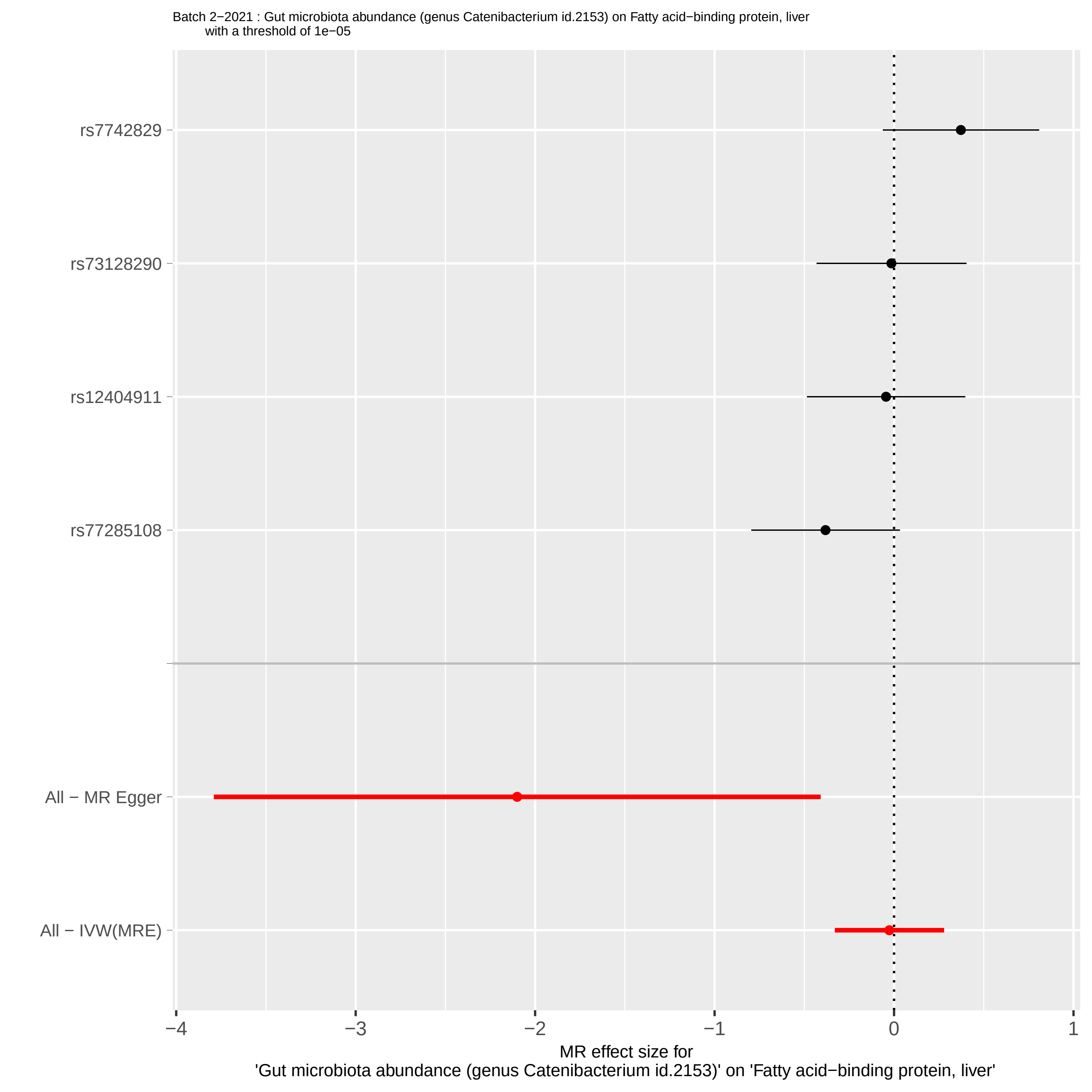
Batch 2−2021 : Gut microbiota abundance (genus Catenibacterium id.2153) on Fatty acid−binding protein, liver
with a threshold of 1e−05
rs7742829
rs73128290
rs12404911
rs77285108
All − MR Egger
All − IVW(MRE)
−4
−3
−2
−1
0
1
MR effect size for
'Gut microbiota abundance (genus Catenibacterium id.2153)' on 'Fatty acid−binding protein, liver'

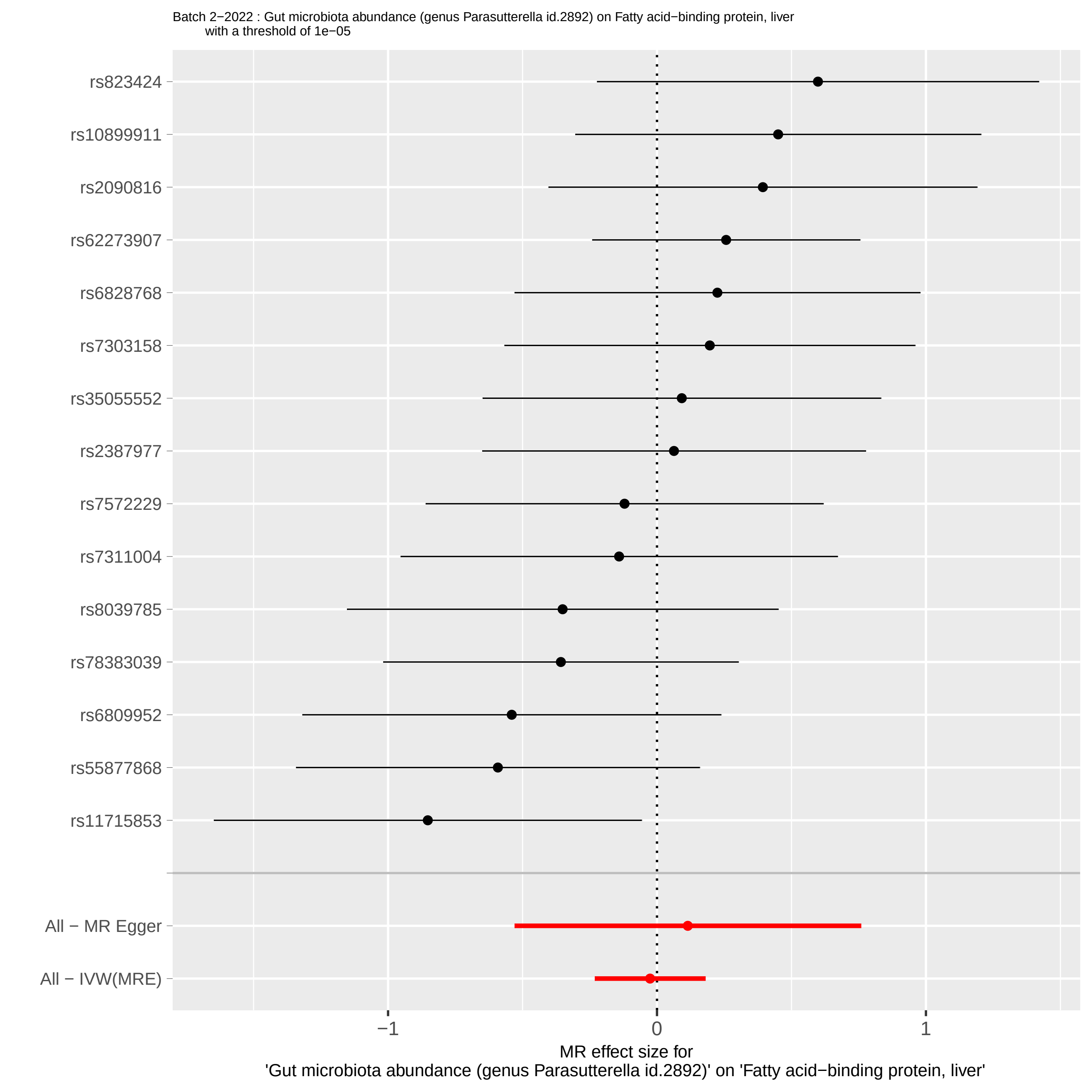
Batch 2−2022 : Gut microbiota abundance (genus Parasutterella id.2892) on Fatty acid−binding protein, liver
with a threshold of 1e−05
rs823424
rs10899911
rs2090816
rs62273907
rs6828768
rs7303158
rs35055552
rs2387977
rs7572229
rs7311004
rs8039785
rs78383039
rs6809952
rs55877868
rs11715853
All − MR Egger
All − IVW(MRE)
−1
0
1
MR effect size for
'Gut microbiota abundance (genus Parasutterella id.2892)' on 'Fatty acid−binding protein, liver'

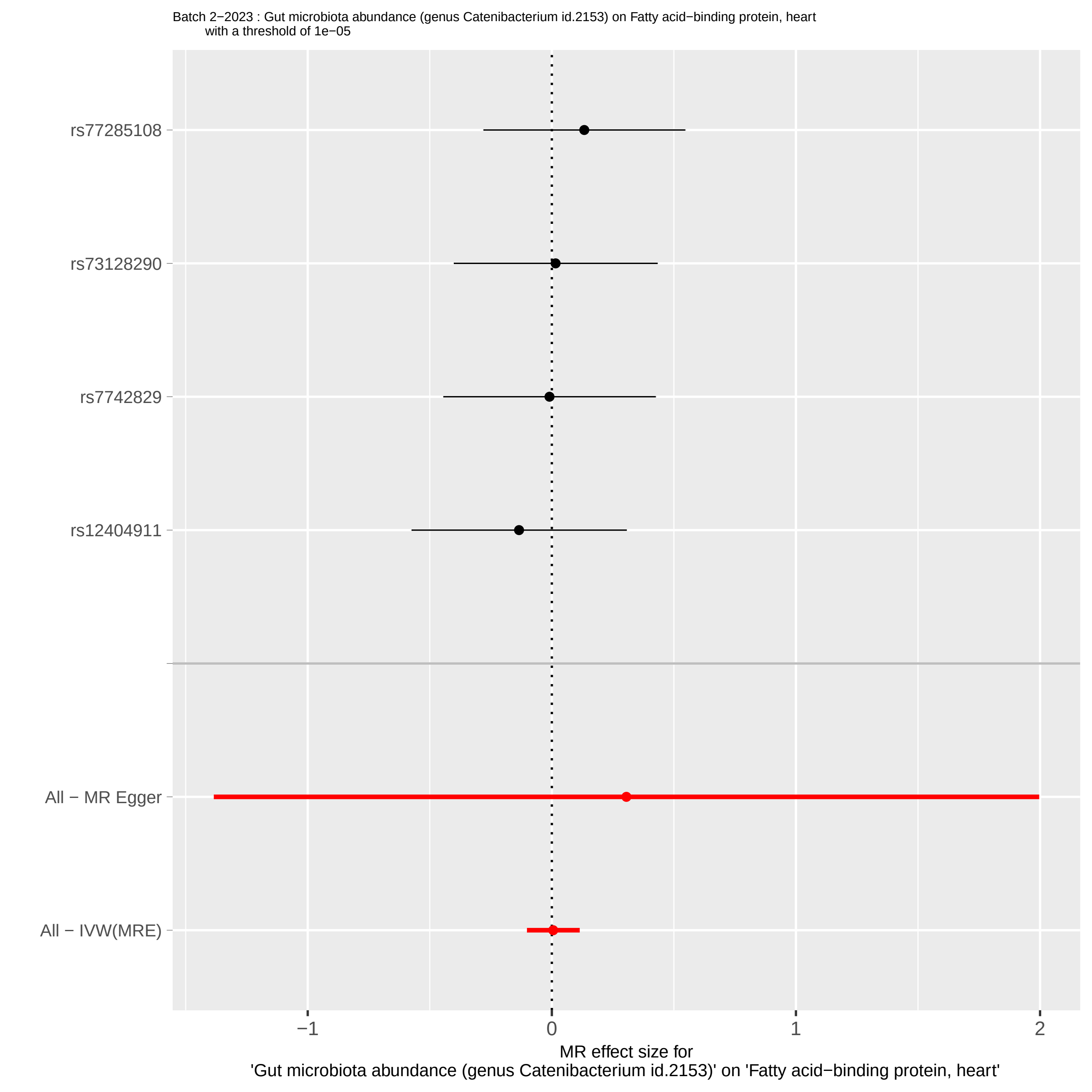

Batch 2−2023 : Gut microbiota abundance (genus Catenibacterium id.2153) on Fatty acid−binding protein, heart
with a threshold of 1e−05
rs77285108
rs73128290
rs7742829
rs12404911
All − MR Egger
All − IVW(MRE)
−1
0
1
2
MR effect size for
'Gut microbiota abundance (genus Catenibacterium id.2153)' on 'Fatty acid−binding protein, heart'

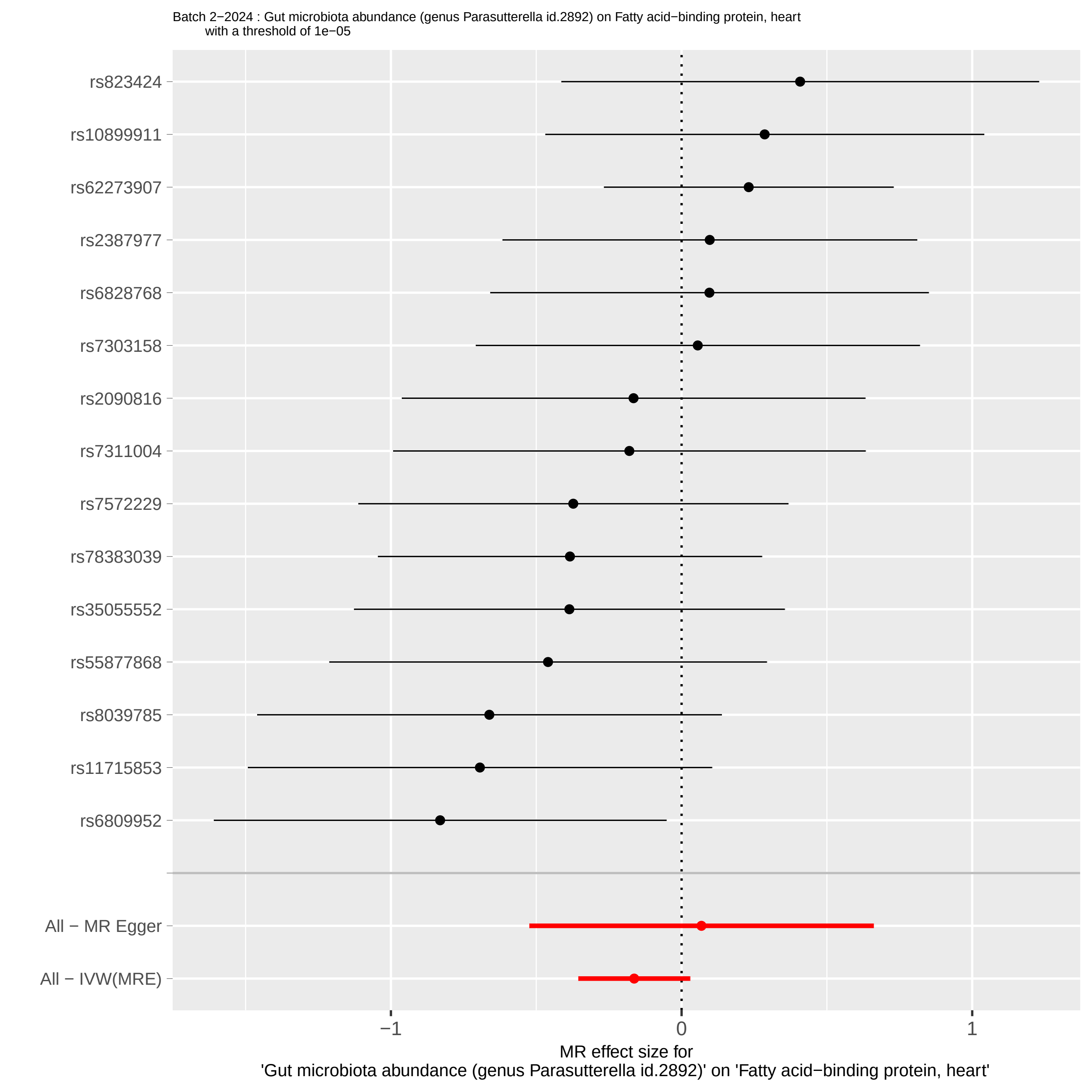
Batch 2−2024 : Gut microbiota abundance (genus Parasutterella id.2892) on Fatty acid−binding protein, heart
with a threshold of 1e−05
rs823424
rs10899911
rs62273907
rs2387977
rs6828768
rs7303158
rs2090816
rs7311004
rs7572229
rs78383039
rs35055552
rs55877868
rs8039785
rs11715853
rs6809952
All − MR Egger
All − IVW(MRE)
−1
0
1
MR effect size for
'Gut microbiota abundance (genus Parasutterella id.2892)' on 'Fatty acid−binding protein, heart'

Batch 2−2025 : Gut microbiota abundance (genus Catenibacterium id.2153) on Fatty acid−binding protein, adipocyte
with a threshold of 1e−05

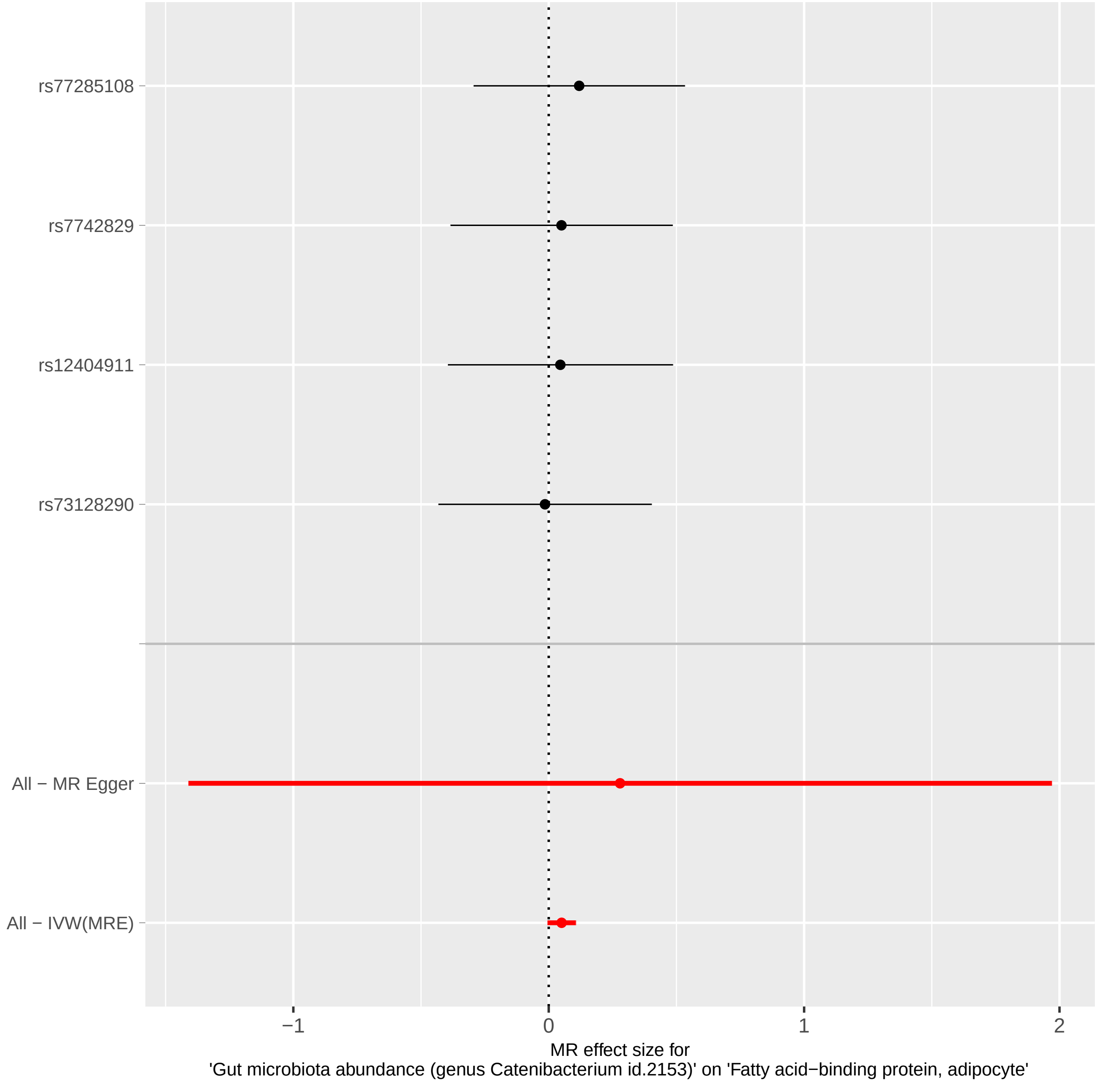

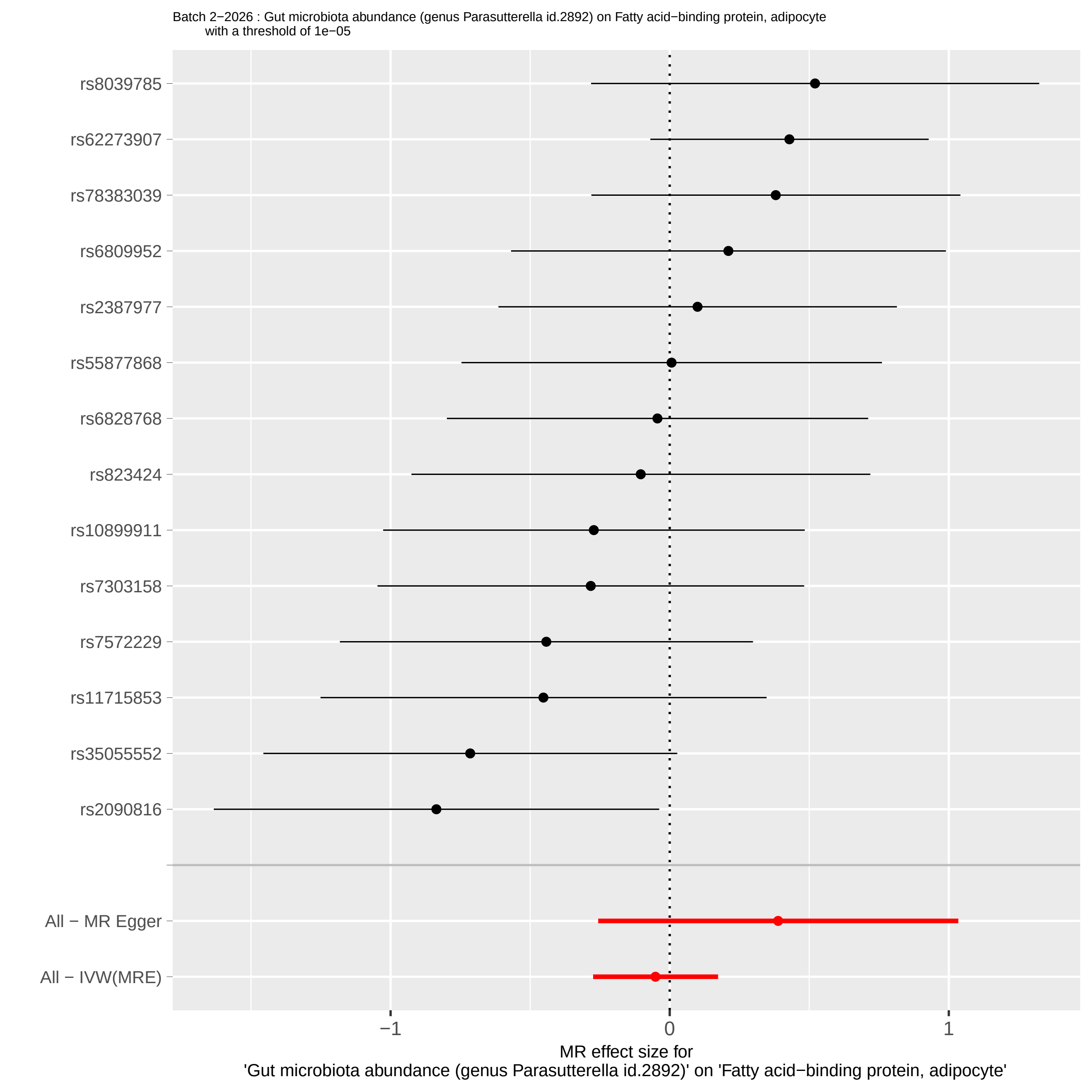

Batch 2−2026 : Gut microbiota abundance (genus Parasutterella id.2892) on Fatty acid−binding protein, adipocyte
with a threshold of 1e−05
rs8039785
rs62273907
rs78383039
rs6809952
rs2387977
rs55877868
rs6828768
rs823424
rs10899911
rs7303158
rs7572229
rs11715853
rs35055552
rs2090816
All − MR Egger
All − IVW(MRE)
−1
0
1
MR effect size for
'Gut microbiota abundance (genus Parasutterella id.2892)' on 'Fatty acid−binding protein, adipocyte'

Batch 2−2027 : Gut microbiota abundance (genus Catenibacterium id.2153) on Fatty acid−binding protein, epidermal
with a threshold of 1e−05

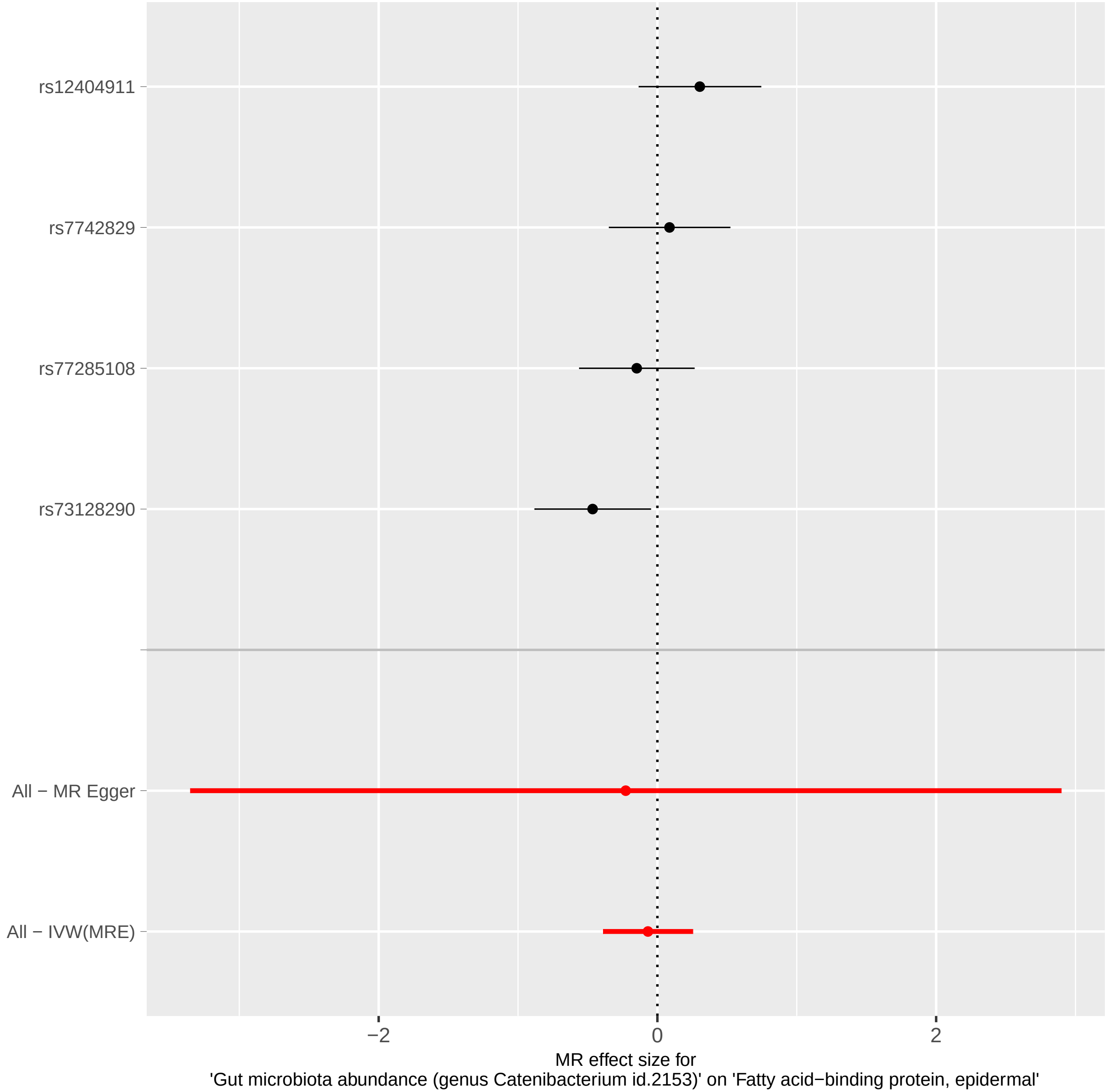

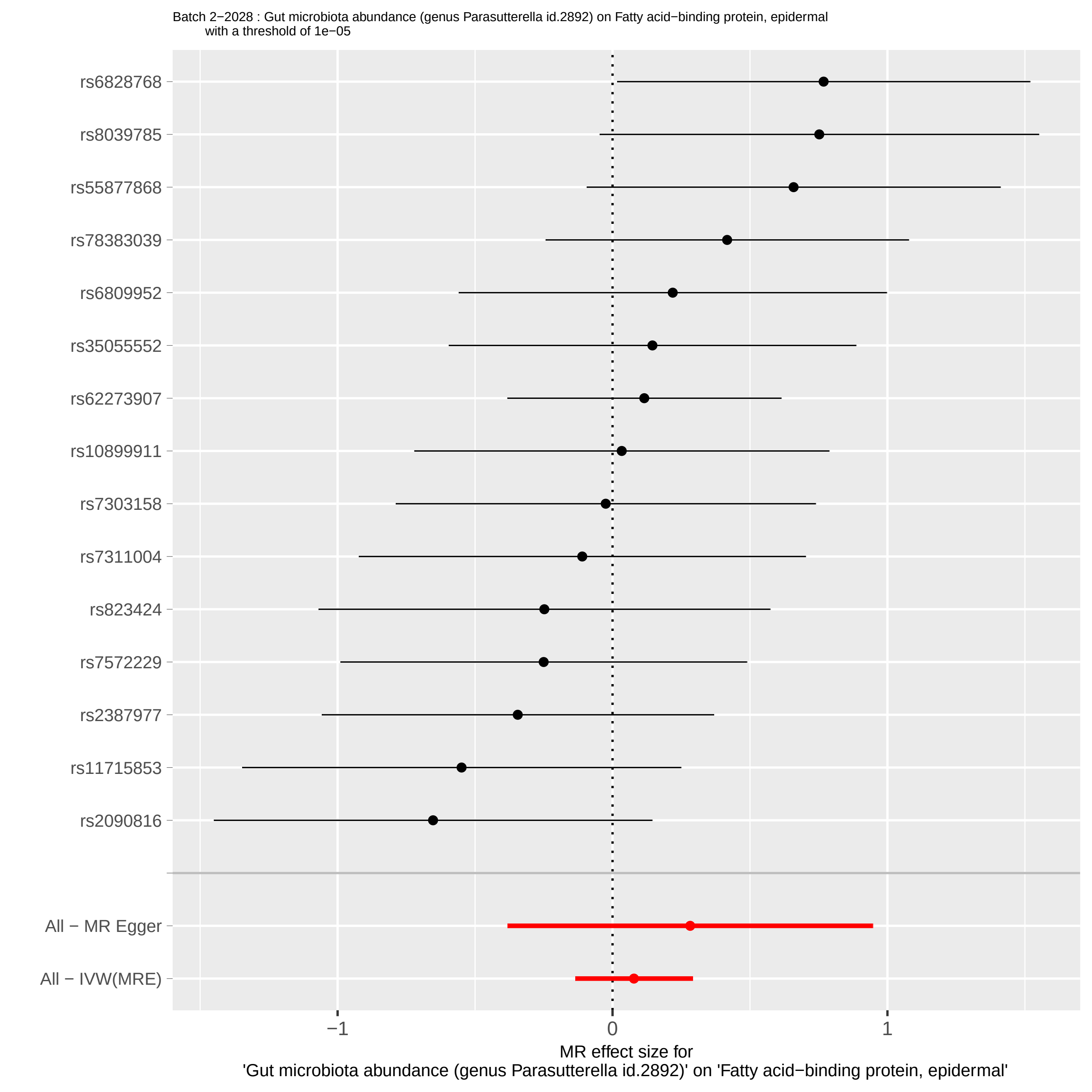
Batch 2−2028 : Gut microbiota abundance (genus Parasutterella id.2892) on Fatty acid−binding protein, epidermal
with a threshold of 1e−05
rs6828768
rs8039785
rs55877868
rs78383039
rs6809952
rs35055552
rs62273907
rs10899911
rs7303158
rs7311004
rs823424
rs7572229
rs2387977
rs11715853
rs2090816
All − MR Egger
All − IVW(MRE)
−1
0
1
MR effect size for
'Gut microbiota abundance (genus Parasutterella id.2892)' on 'Fatty acid−binding protein, epidermal'

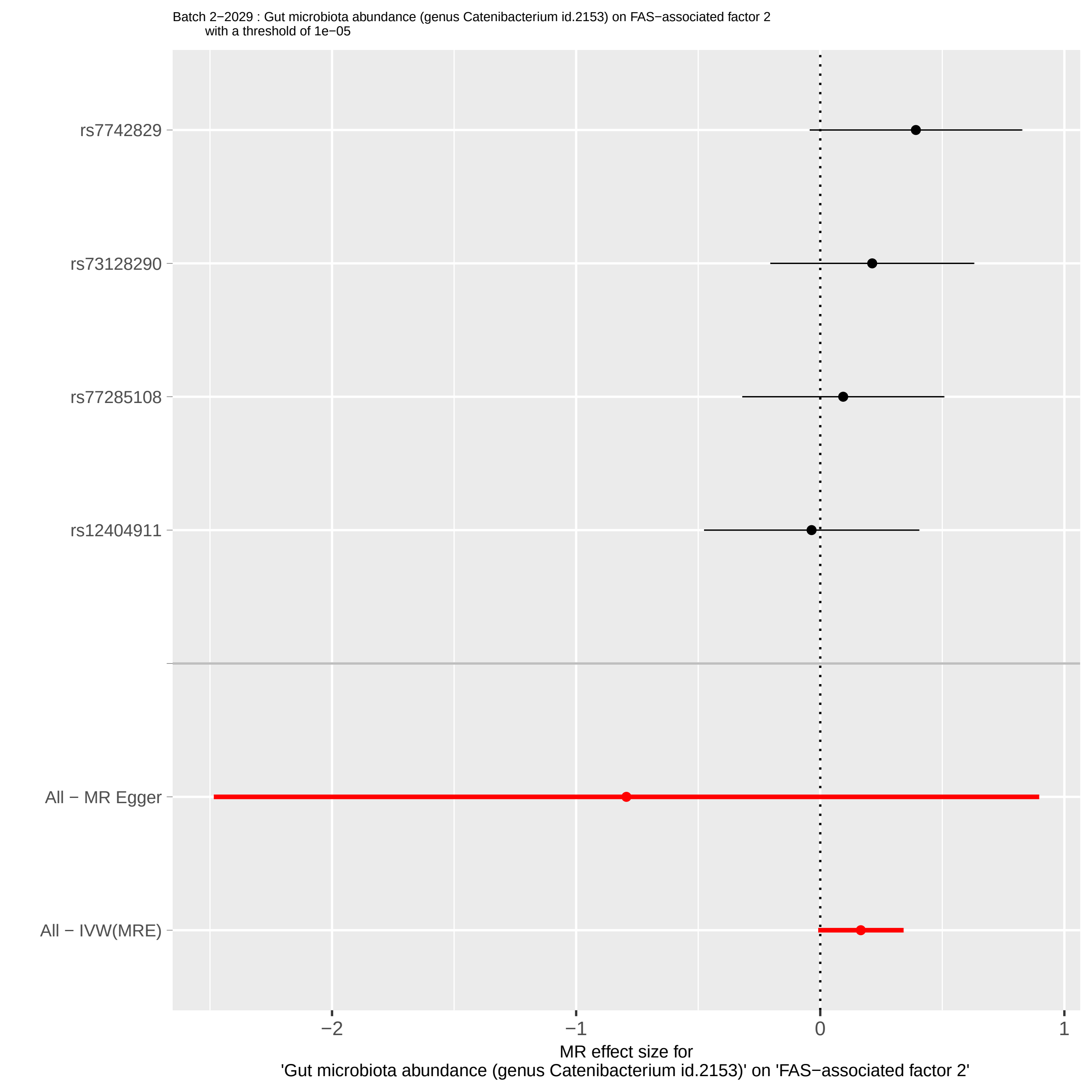
Batch 2−2029 : Gut microbiota abundance (genus Catenibacterium id.2153) on FAS−associated factor 2
with a threshold of 1e−05
rs7742829
rs73128290
rs77285108
rs12404911
All − MR Egger
All − IVW(MRE)
−2
−1
0
1
MR effect size for
'Gut microbiota abundance (genus Catenibacterium id.2153)' on 'FAS−associated factor 2'

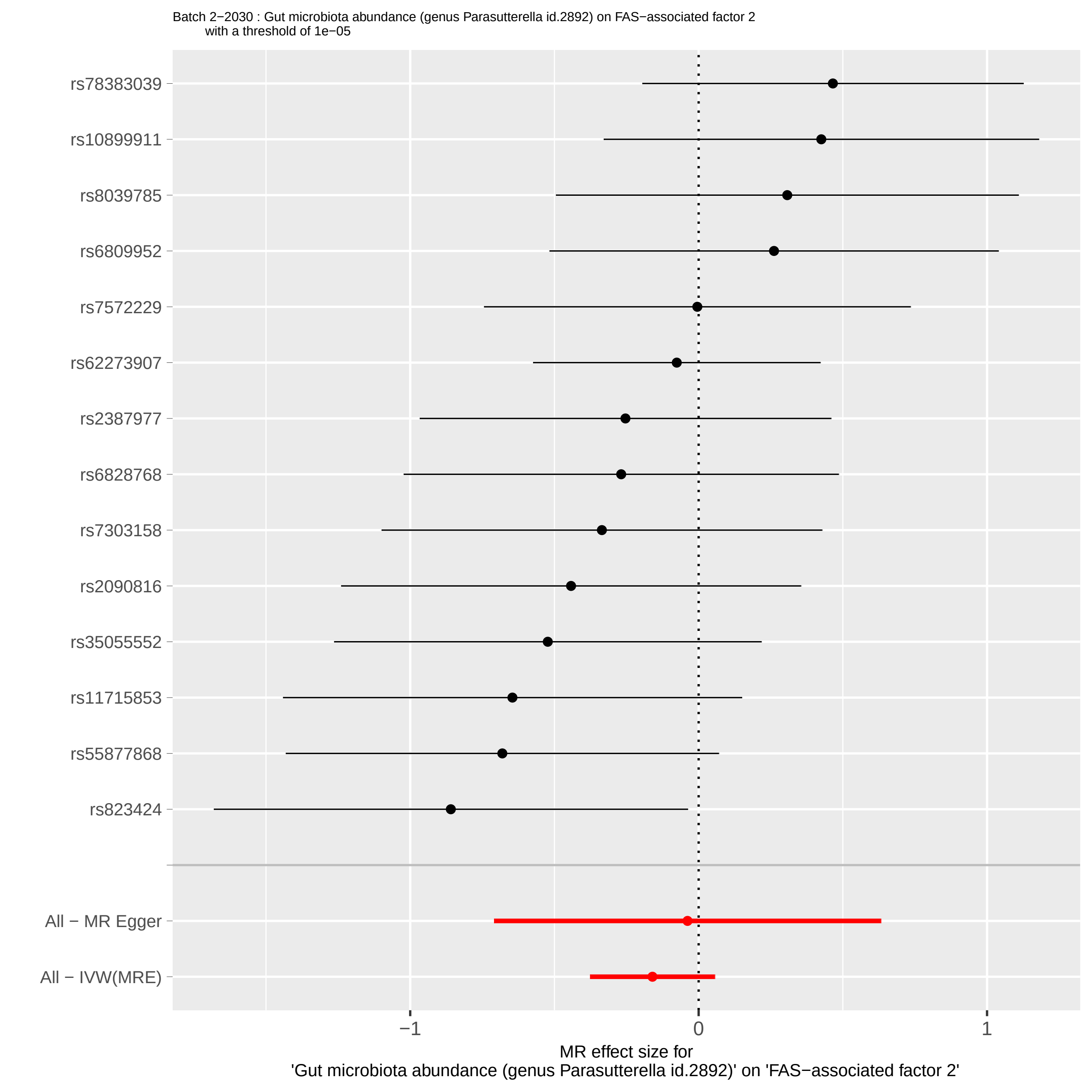

Batch 2−2030 : Gut microbiota abundance (genus Parasutterella id.2892) on FAS−associated factor 2
with a threshold of 1e−05
rs78383039
rs10899911
rs8039785
rs6809952
rs7572229
rs62273907
rs2387977
rs6828768
rs7303158
rs2090816
rs35055552
rs11715853
rs55877868
rs823424
All − MR Egger
All − IVW(MRE)
−1
0
1
MR effect size for
'Gut microbiota abundance (genus Parasutterella id.2892)' on 'FAS−associated factor 2'

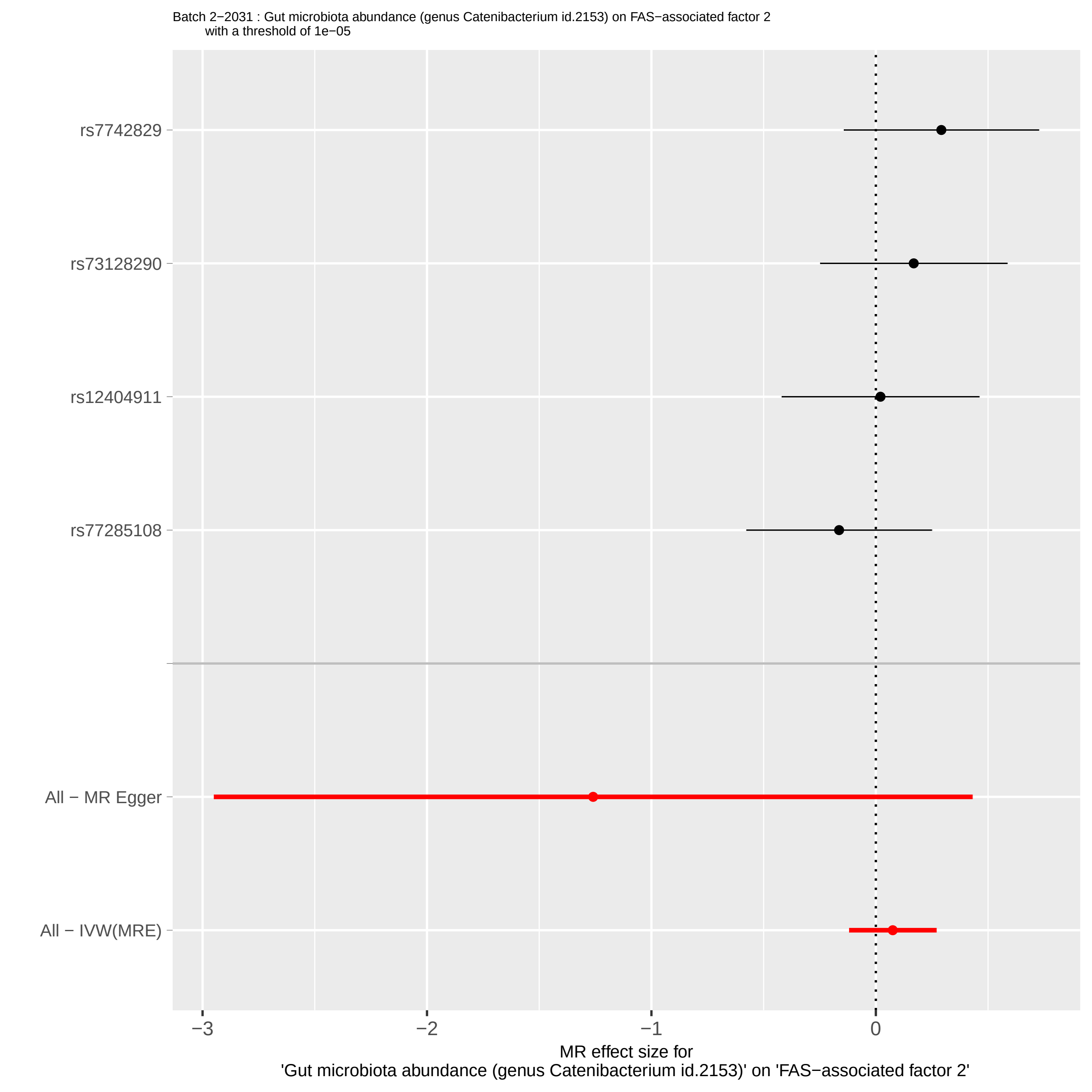
Batch 2−2031 : Gut microbiota abundance (genus Catenibacterium id.2153) on FAS−associated factor 2
with a threshold of 1e−05
rs7742829
rs73128290
rs12404911
rs77285108
All − MR Egger
All − IVW(MRE)
−3
−2
−1
0
MR effect size for
'Gut microbiota abundance (genus Catenibacterium id.2153)' on 'FAS−associated factor 2'

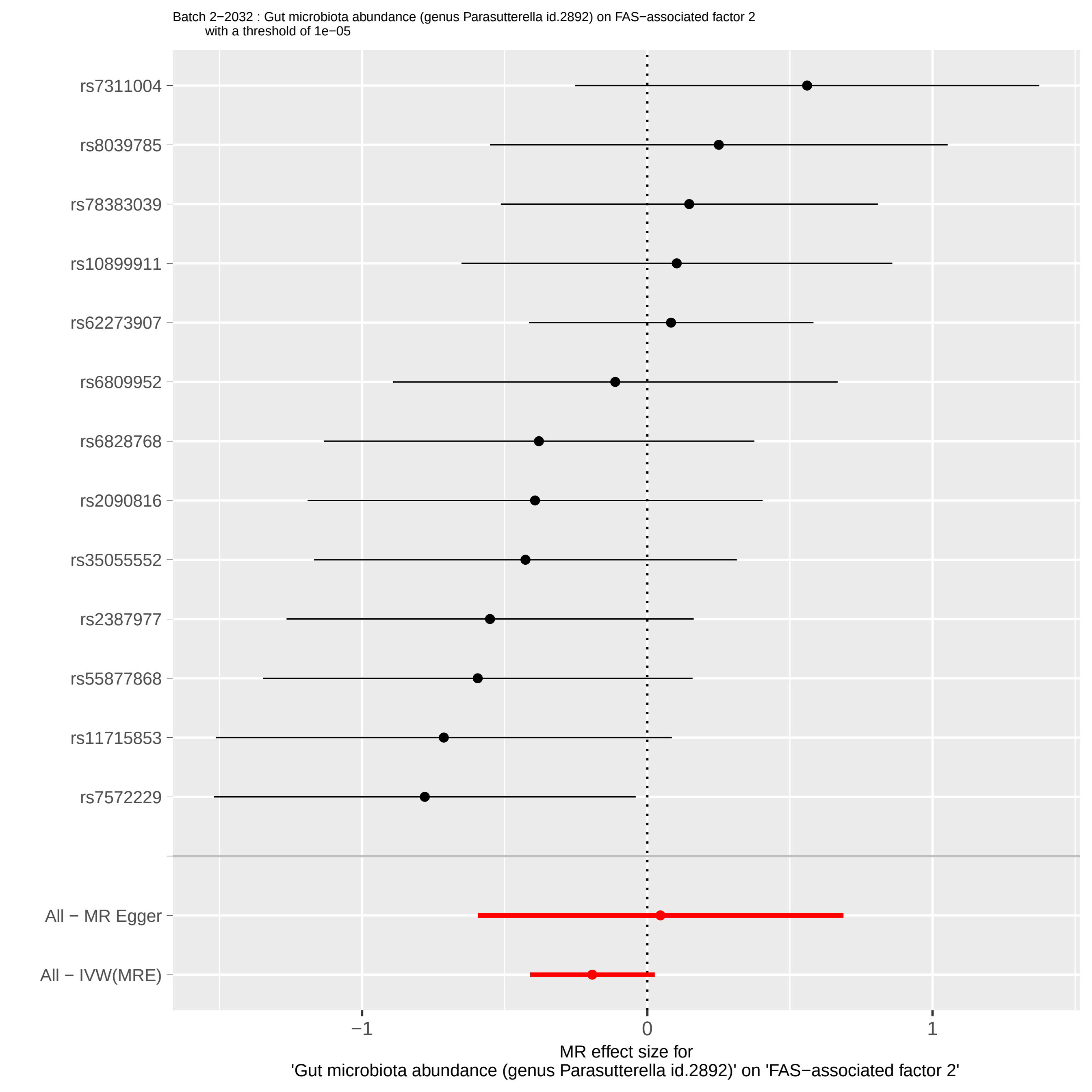

Batch 2−2032 : Gut microbiota abundance (genus Parasutterella id.2892) on FAS−associated factor 2
with a threshold of 1e−05
rs7311004
rs8039785
rs78383039
rs10899911
rs62273907
rs6809952
rs6828768
rs2090816
rs35055552
rs2387977
rs55877868
rs11715853
rs7572229
All − MR Egger
All − IVW(MRE)
−1
0
1
MR effect size for
'Gut microbiota abundance (genus Parasutterella id.2892)' on 'FAS−associated factor 2'

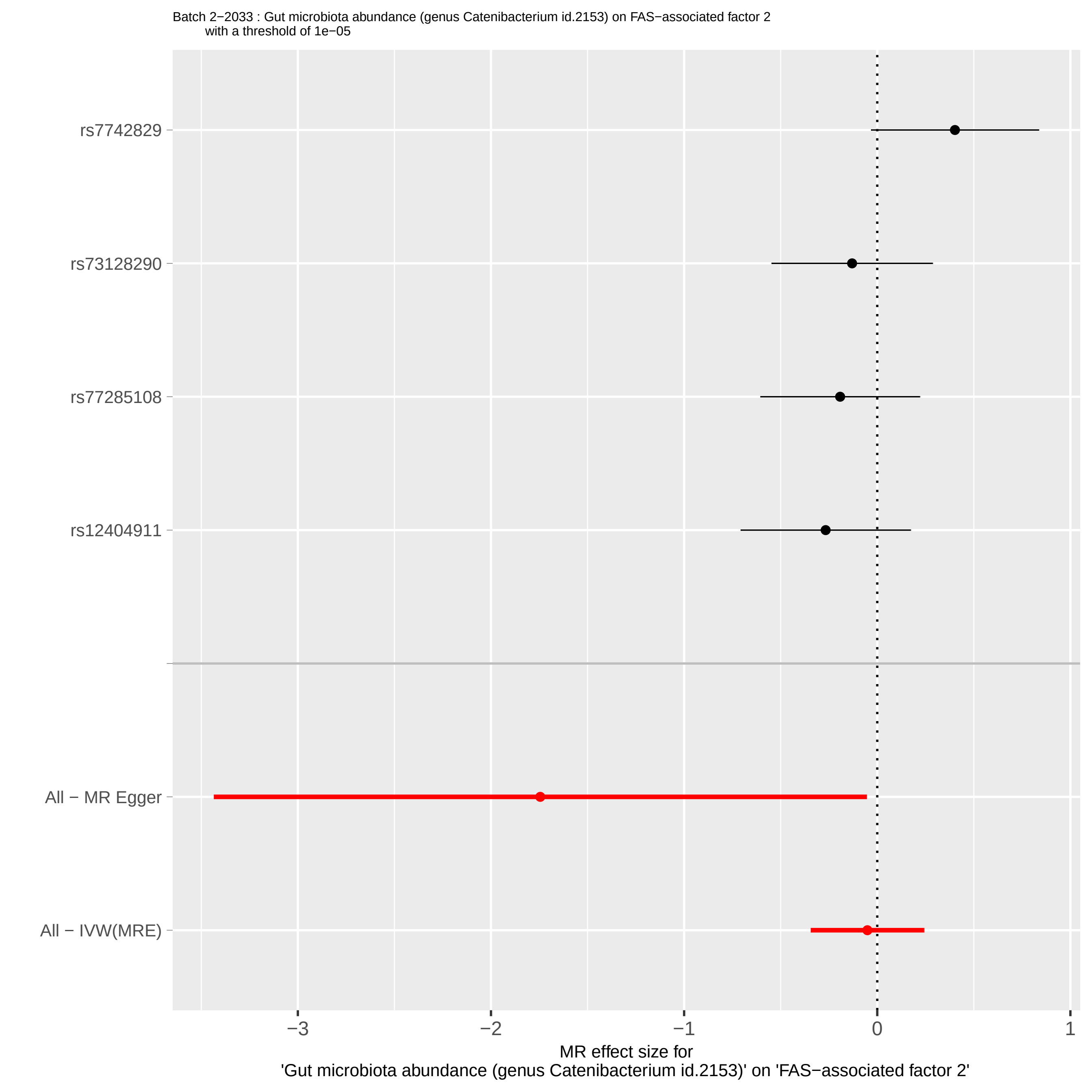

Batch 2−2033 : Gut microbiota abundance (genus Catenibacterium id.2153) on FAS−associated factor 2
with a threshold of 1e−05
rs7742829
rs73128290
rs77285108
rs12404911
All − MR Egger
All − IVW(MRE)
−3
−2
−1
0
1
MR effect size for
'Gut microbiota abundance (genus Catenibacterium id.2153)' on 'FAS−associated factor 2'

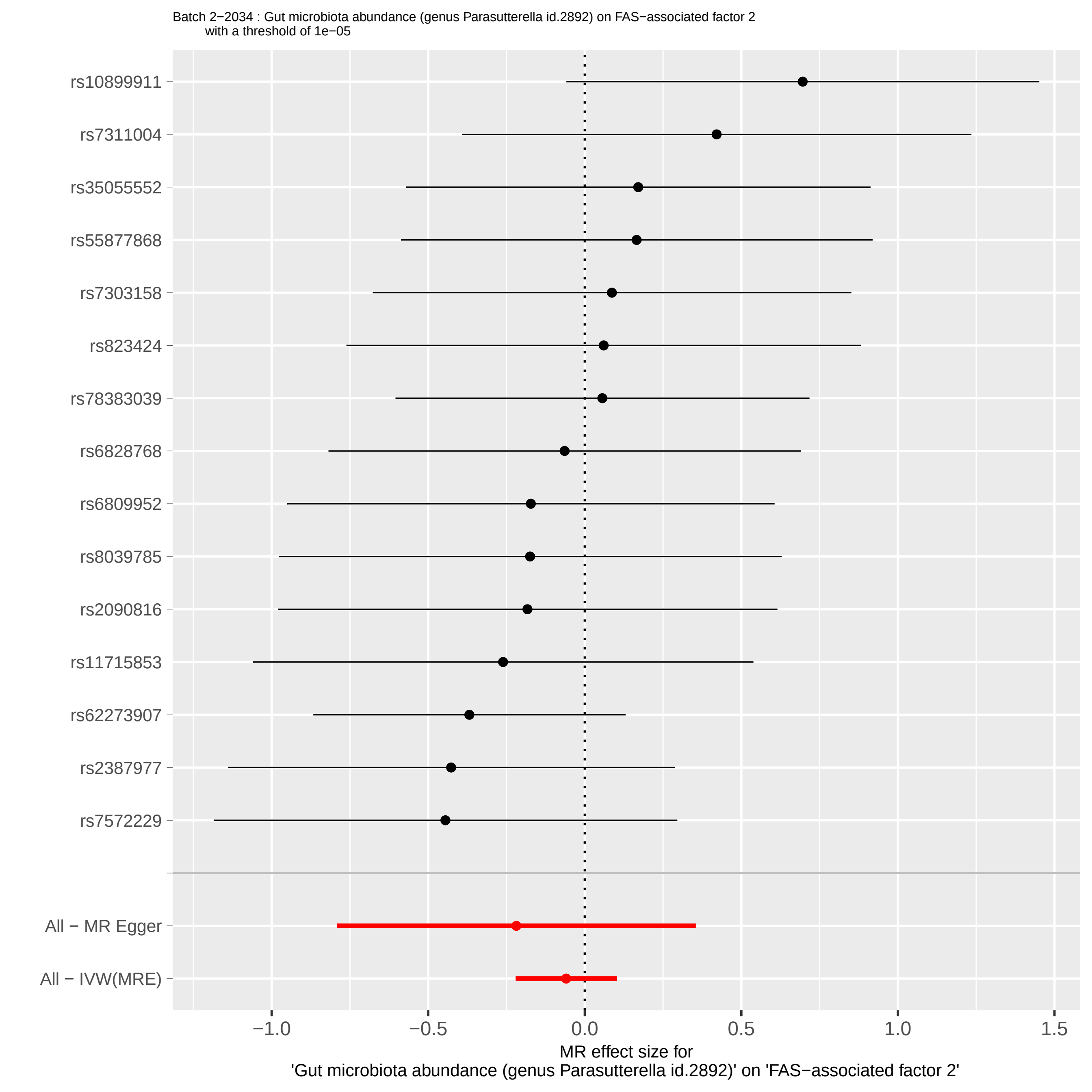
Batch 2−2034 : Gut microbiota abundance (genus Parasutterella id.2892) on FAS−associated factor 2
with a threshold of 1e−05
rs10899911
rs7311004
rs35055552
rs55877868
rs7303158
rs823424
rs78383039
rs6828768
rs6809952
rs8039785
rs2090816
rs11715853
rs62273907
rs2387977
rs7572229
All − MR Egger
All − IVW(MRE)
−1.0
−0.5
0.0
0.5
1.0
1.5
MR effect size for
'Gut microbiota abundance (genus Parasutterella id.2892)' on 'FAS−associated factor 2'

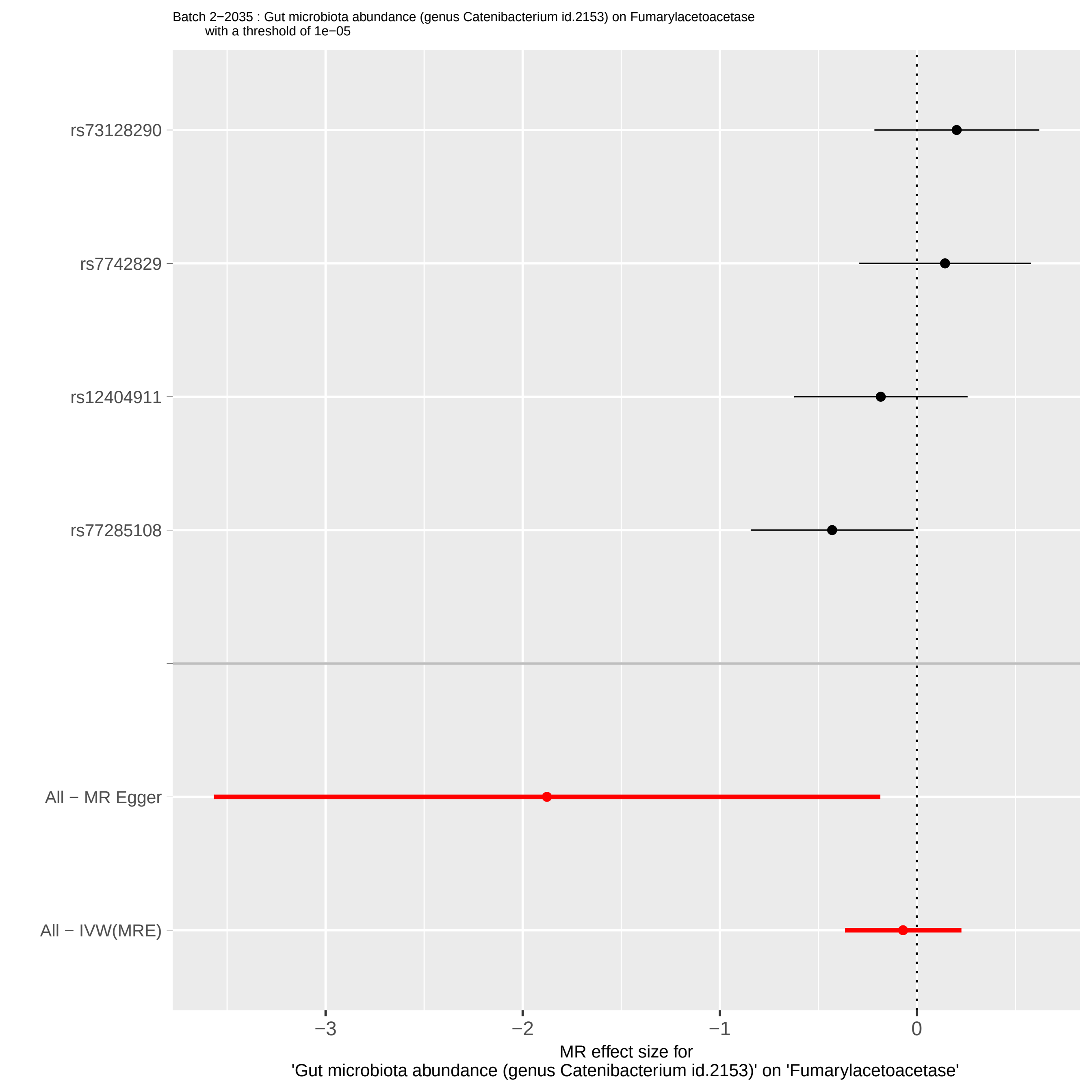

Batch 2−2035 : Gut microbiota abundance (genus Catenibacterium id.2153) on Fumarylacetoacetase
with a threshold of 1e−05
rs73128290
rs7742829
rs12404911
rs77285108
All − MR Egger
All − IVW(MRE)
−3
−2
−1
0
MR effect size for
'Gut microbiota abundance (genus Catenibacterium id.2153)' on 'Fumarylacetoacetase'

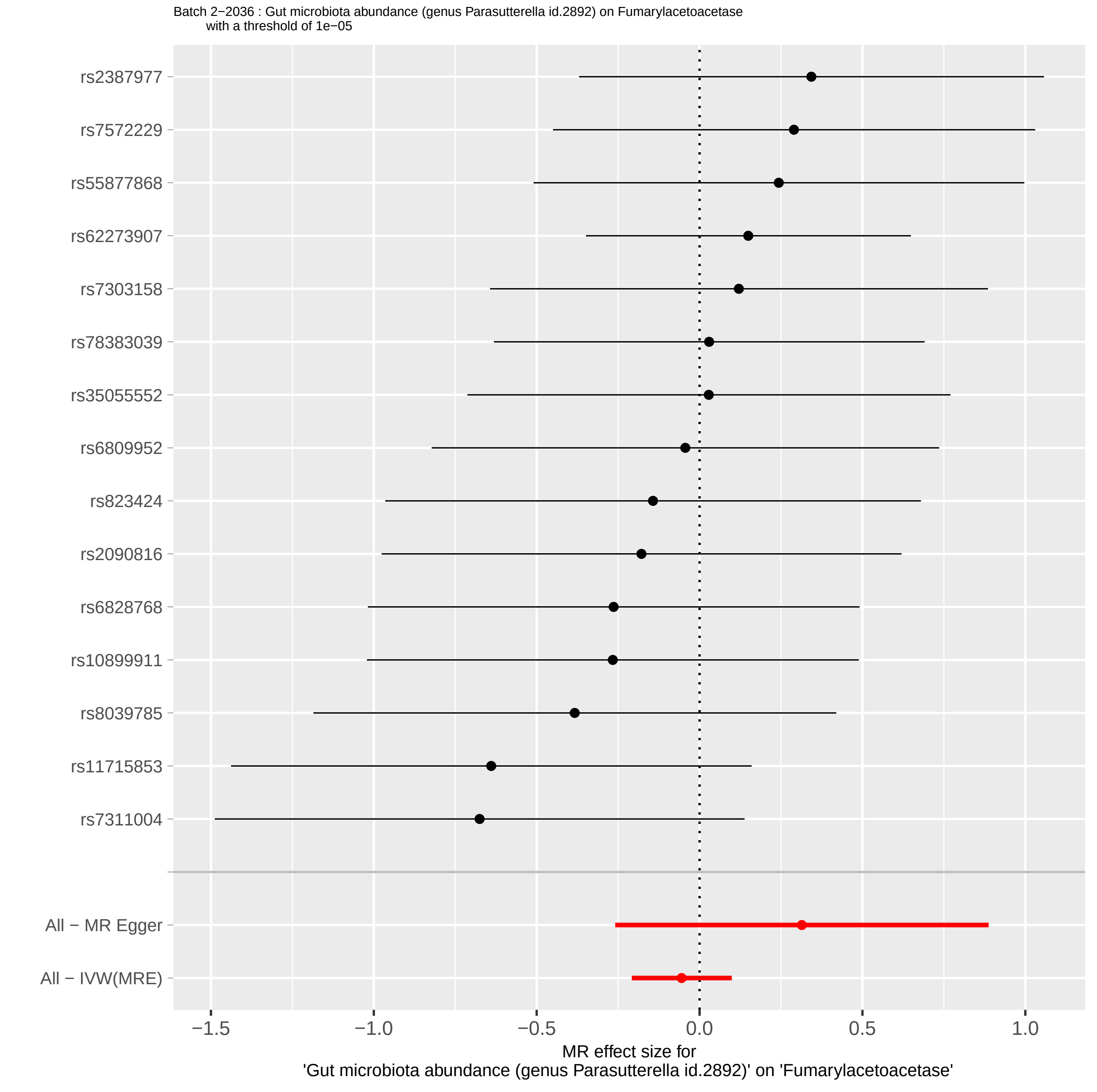

Batch 2−2036 : Gut microbiota abundance (genus Parasutterella id.2892) on Fumarylacetoacetase
with a threshold of 1e−05
rs2387977
rs7572229
rs55877868
rs62273907
rs7303158
rs78383039
rs35055552
rs6809952
rs823424
rs2090816
rs6828768
rs10899911
rs8039785
rs11715853
rs7311004
All − MR Egger
All − IVW(MRE)
−1.5
−1.0
−0.5
0.0
0.5
1.0
MR effect size for
'Gut microbiota abundance (genus Parasutterella id.2892)' on 'Fumarylacetoacetase'

Batch 2−2037 : Gut microbiota abundance (genus Catenibacterium id.2153) on Fas apoptotic inhibitory molecule 3
with a threshold of 1e−05

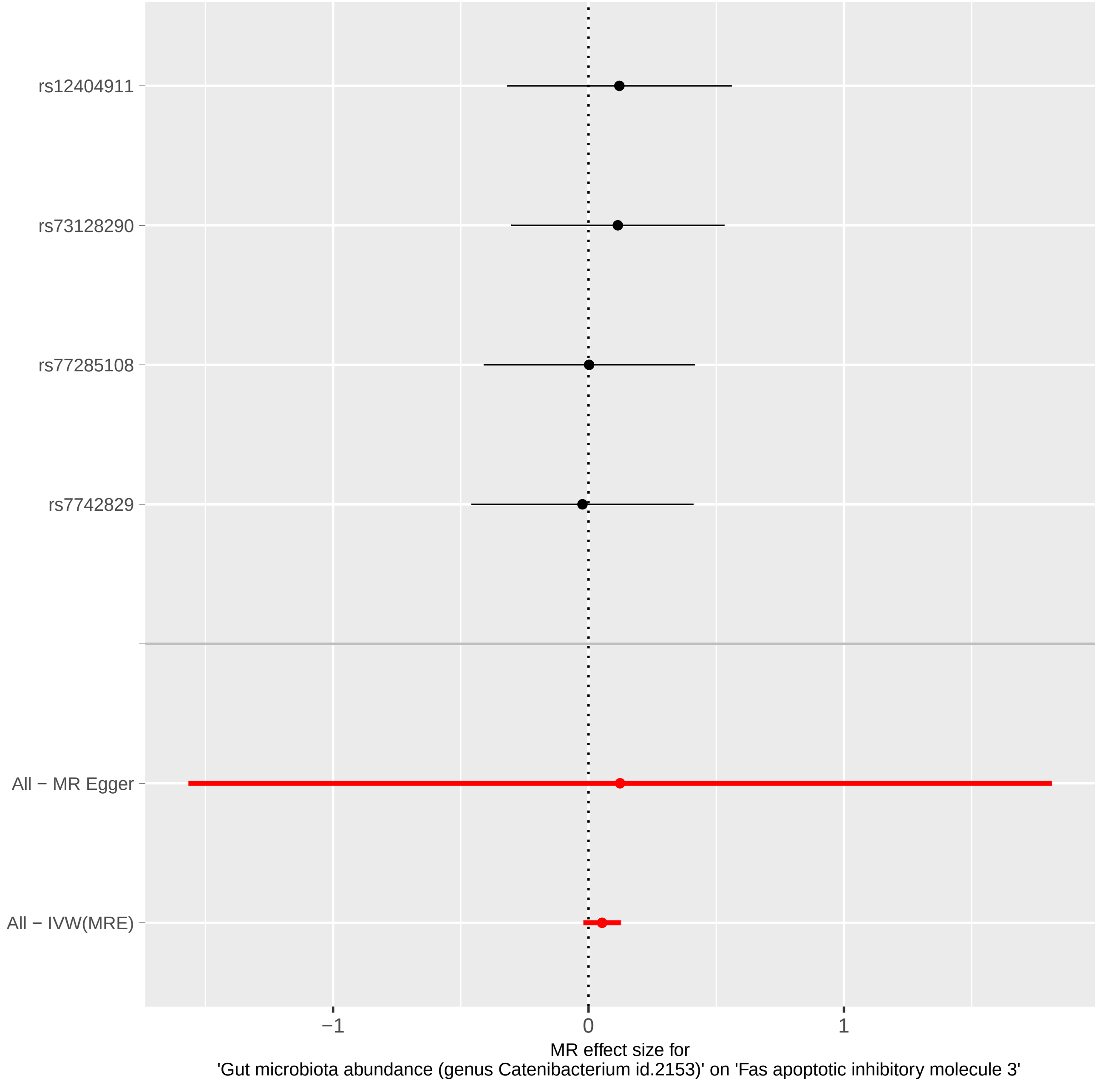

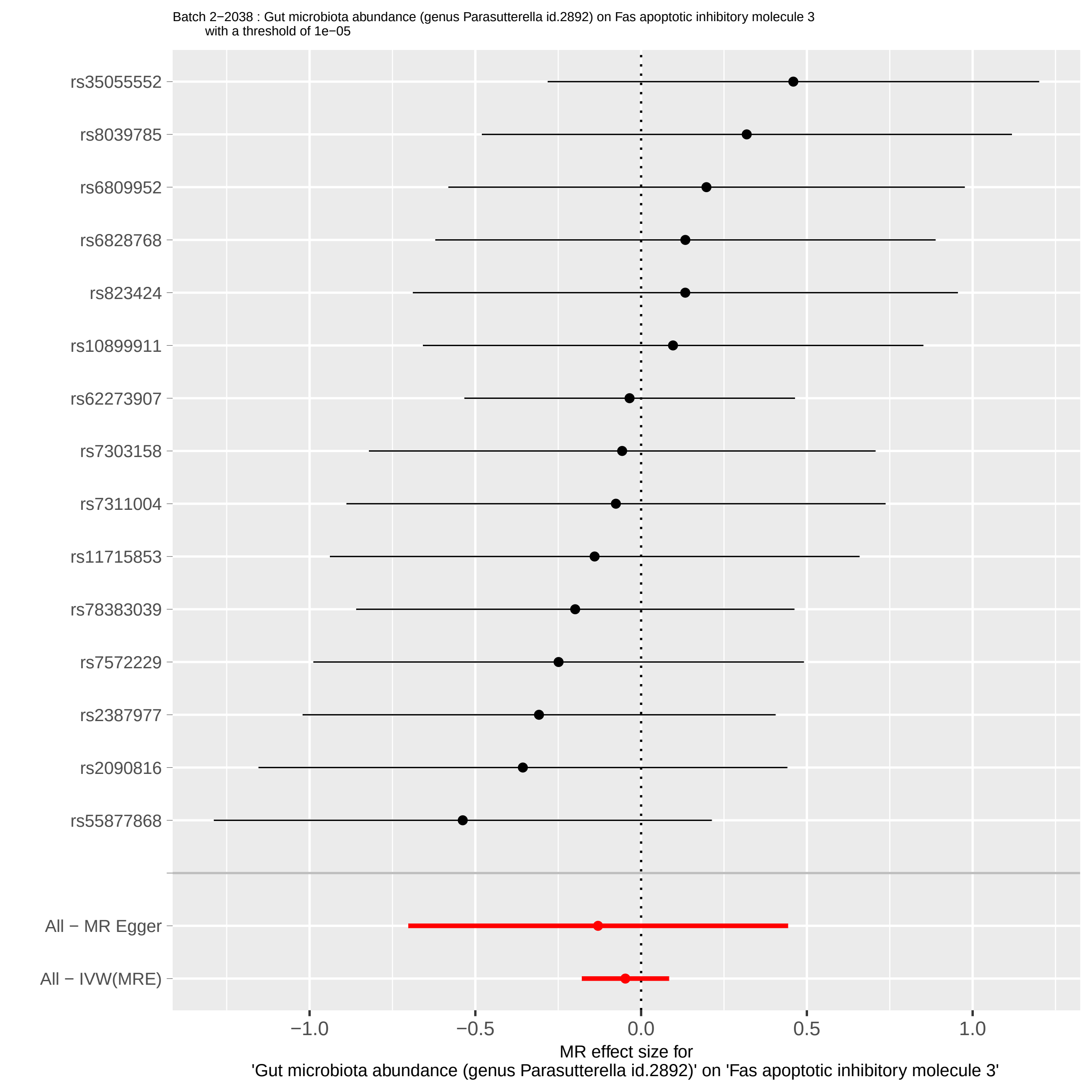
Batch 2−2038 : Gut microbiota abundance (genus Parasutterella id.2892) on Fas apoptotic inhibitory molecule 3
with a threshold of 1e−05
rs35055552
rs8039785
rs6809952
rs6828768
rs823424
rs10899911
rs62273907
rs7303158
rs7311004
rs11715853
rs78383039
rs7572229
rs2387977
rs2090816
rs55877868
All − MR Egger
All − IVW(MRE)
−1.0
−0.5
0.0
0.5
1.0
MR effect size for
'Gut microbiota abundance (genus Parasutterella id.2892)' on 'Fas apoptotic inhibitory molecule 3'

Batch 2−2039 : Gut microbiota abundance (genus Catenibacterium id.2153) on Protein FAM107A
with a threshold of 1e−05

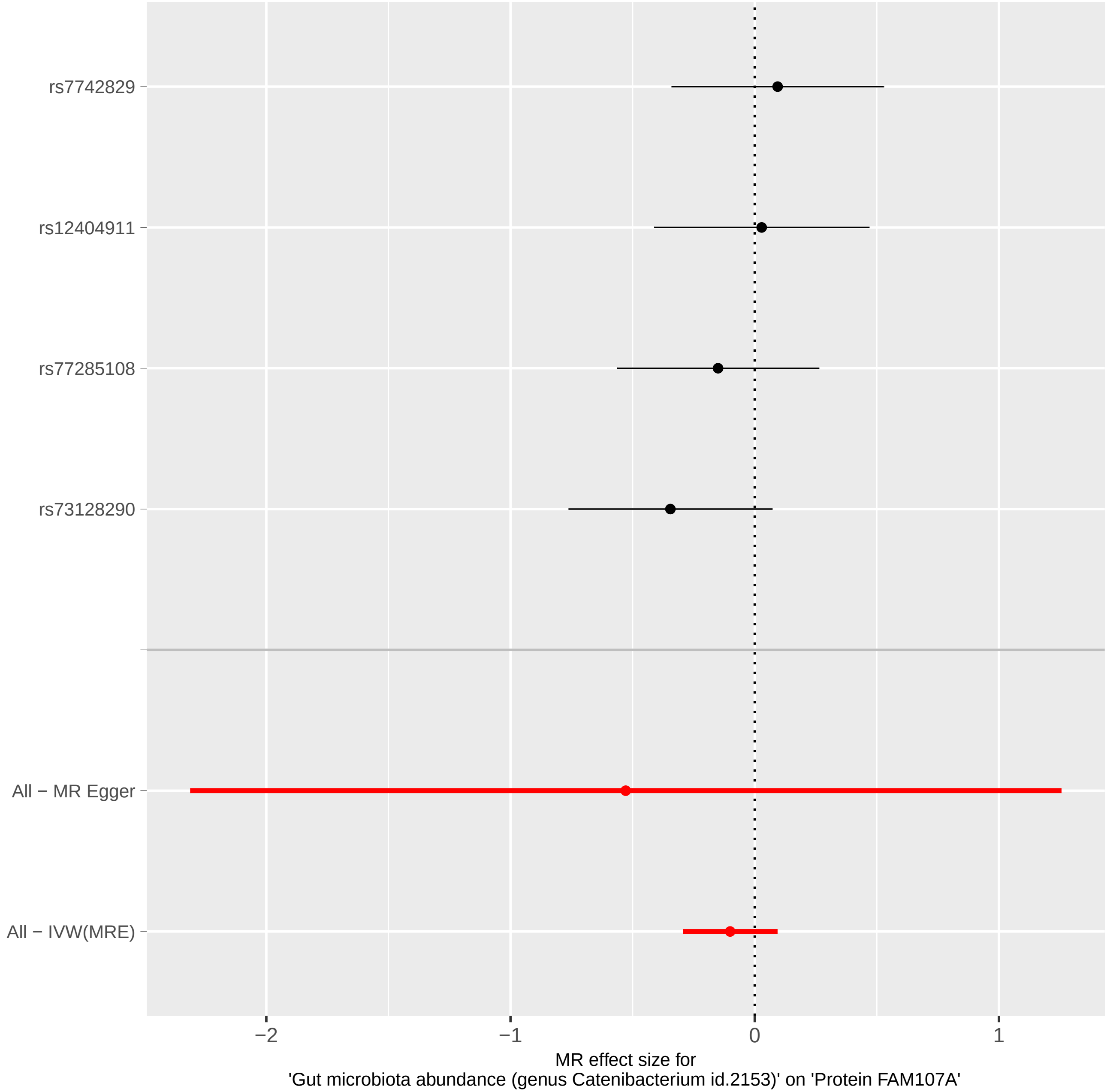

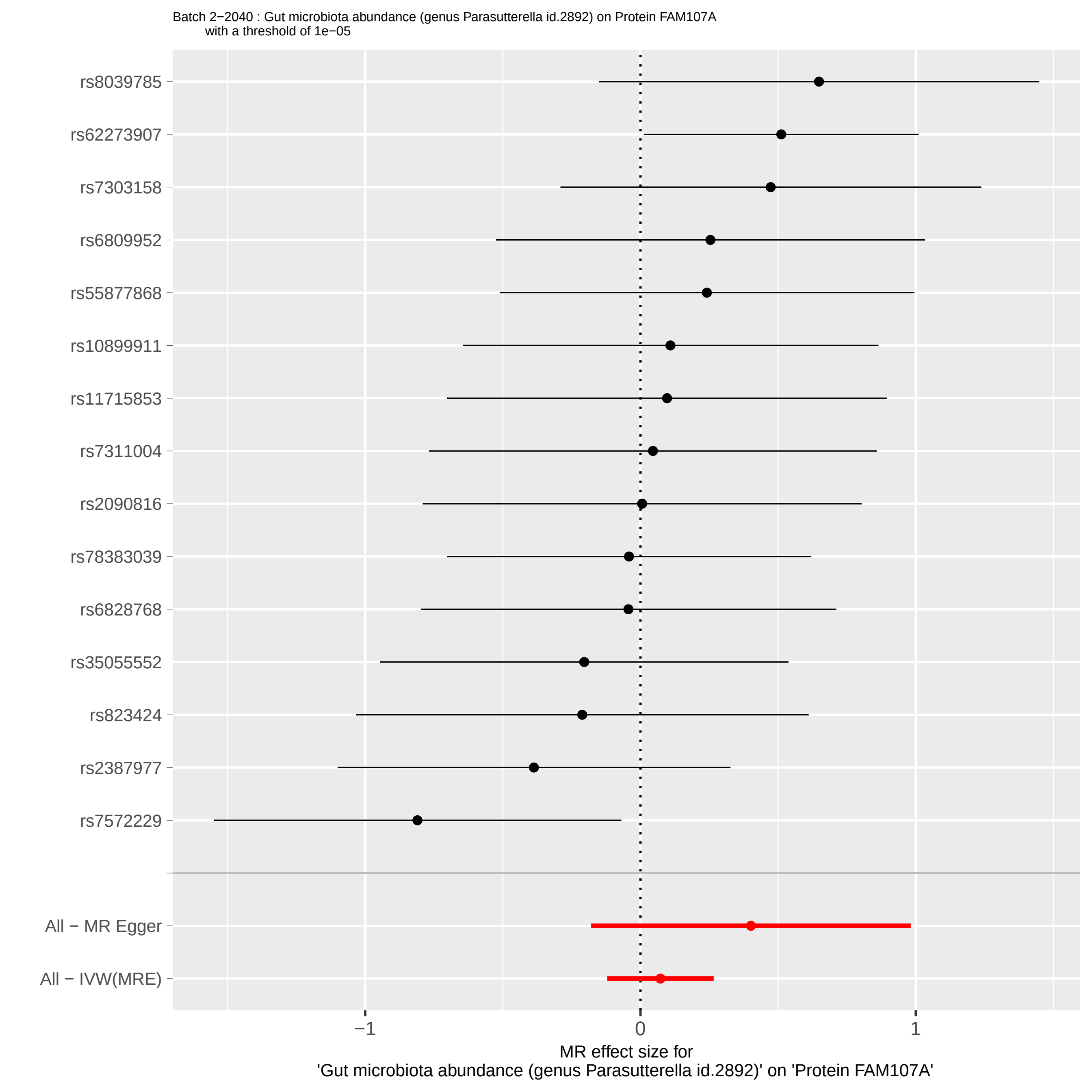

Batch 2−2040 : Gut microbiota abundance (genus Parasutterella id.2892) on Protein FAM107A
with a threshold of 1e−05
rs8039785
rs62273907
rs7303158
rs6809952
rs55877868
rs10899911
rs11715853
rs7311004
rs2090816
rs78383039
rs6828768
rs35055552
rs823424
rs2387977
rs7572229
All − MR Egger
All − IVW(MRE)
−1
0
1
MR effect size for
'Gut microbiota abundance (genus Parasutterella id.2892)' on 'Protein FAM107A'

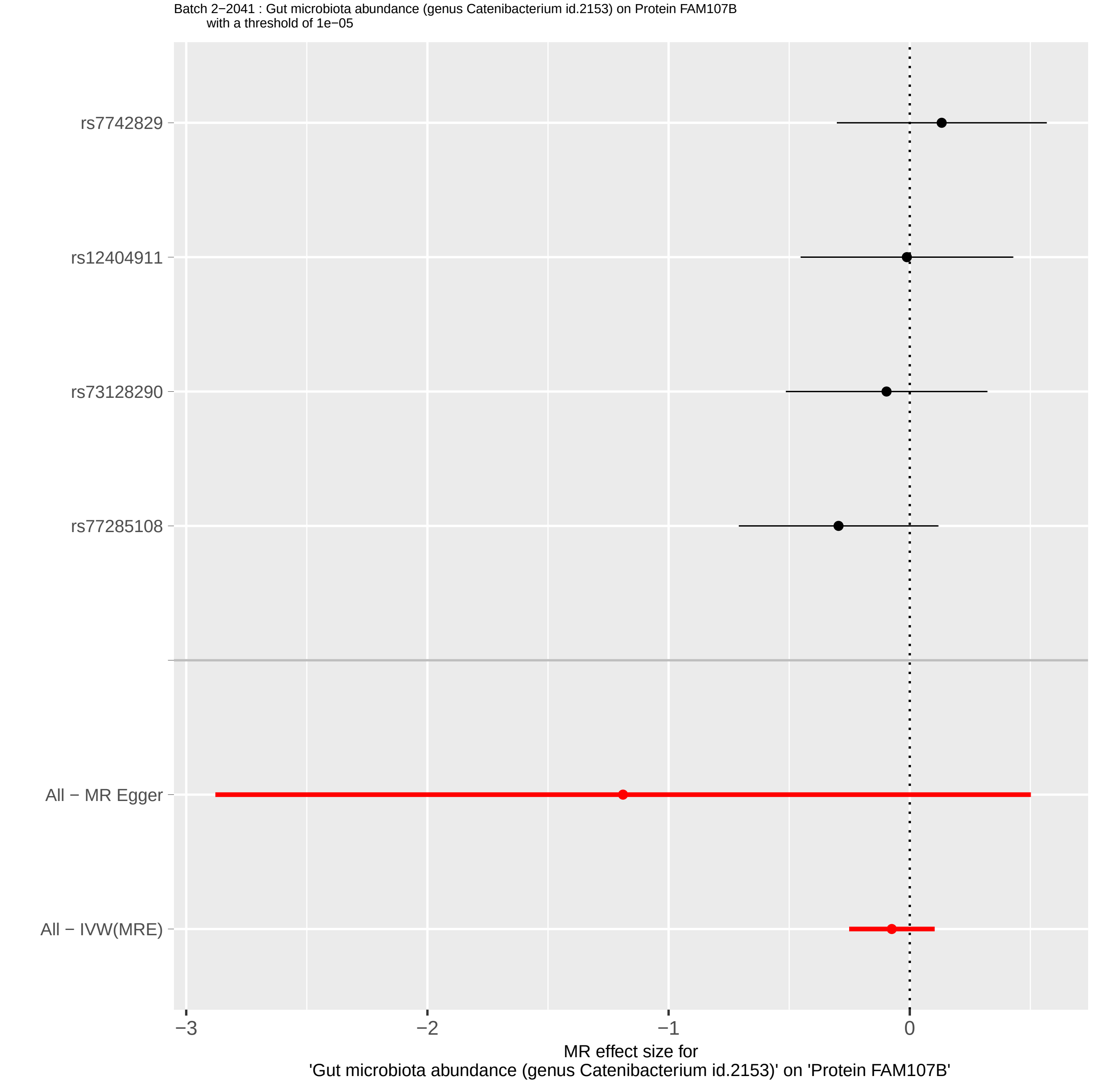

Batch 2−2041 : Gut microbiota abundance (genus Catenibacterium id.2153) on Protein FAM107B
with a threshold of 1e−05
rs7742829
rs12404911
rs73128290
rs77285108
All − MR Egger
All − IVW(MRE)
−3
−2
−1
0
MR effect size for
'Gut microbiota abundance (genus Catenibacterium id.2153)' on 'Protein FAM107B'

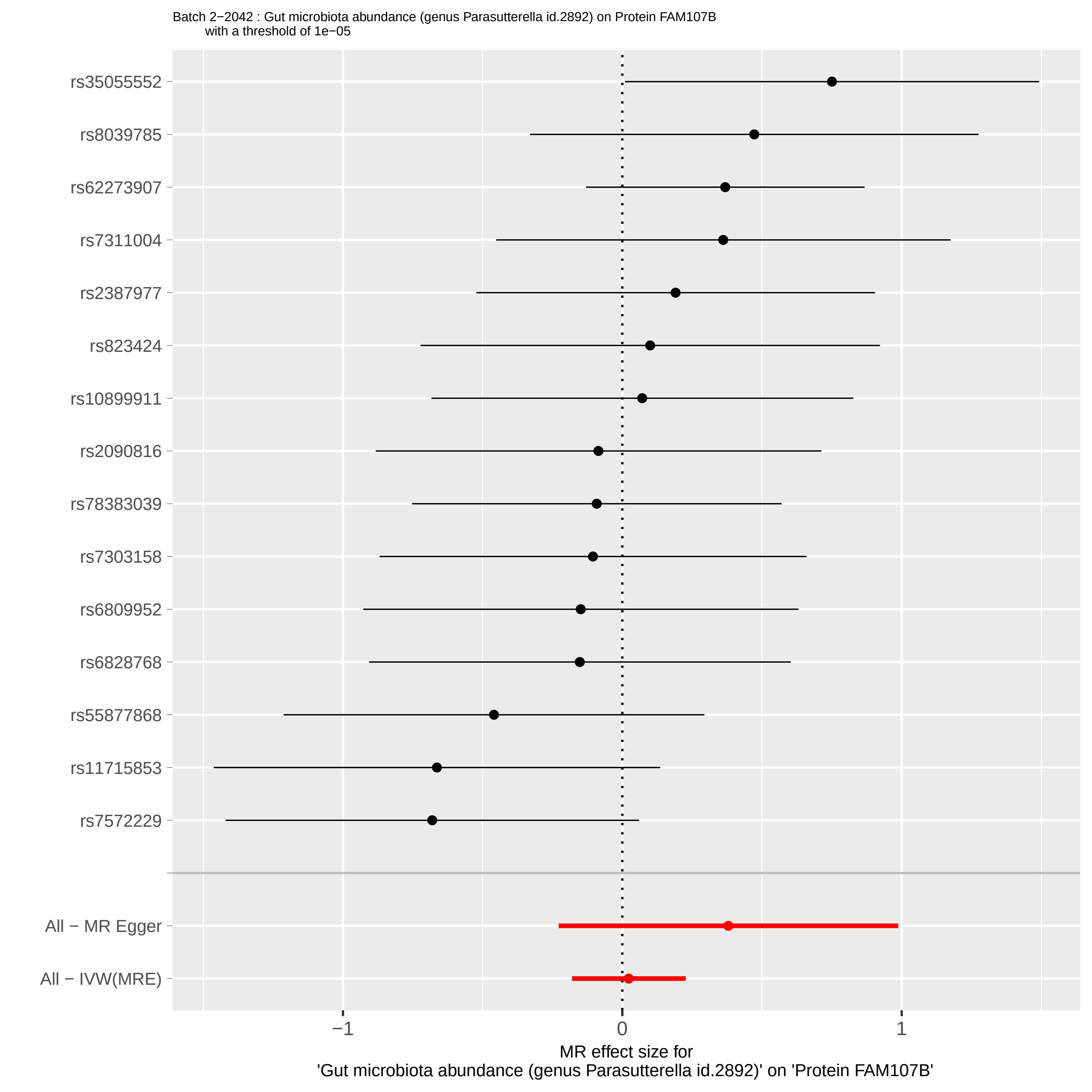

Batch 2−2042 : Gut microbiota abundance (genus Parasutterella id.2892) on Protein FAM107B
with a threshold of 1e−05
rs35055552
rs8039785
rs62273907
rs7311004
rs2387977
rs823424
rs10899911
rs2090816
rs78383039
rs7303158
rs6809952
rs6828768
rs55877868
rs11715853
rs7572229
All − MR Egger
All − IVW(MRE)
−1
0
1
MR effect size for
'Gut microbiota abundance (genus Parasutterella id.2892)' on 'Protein FAM107B'

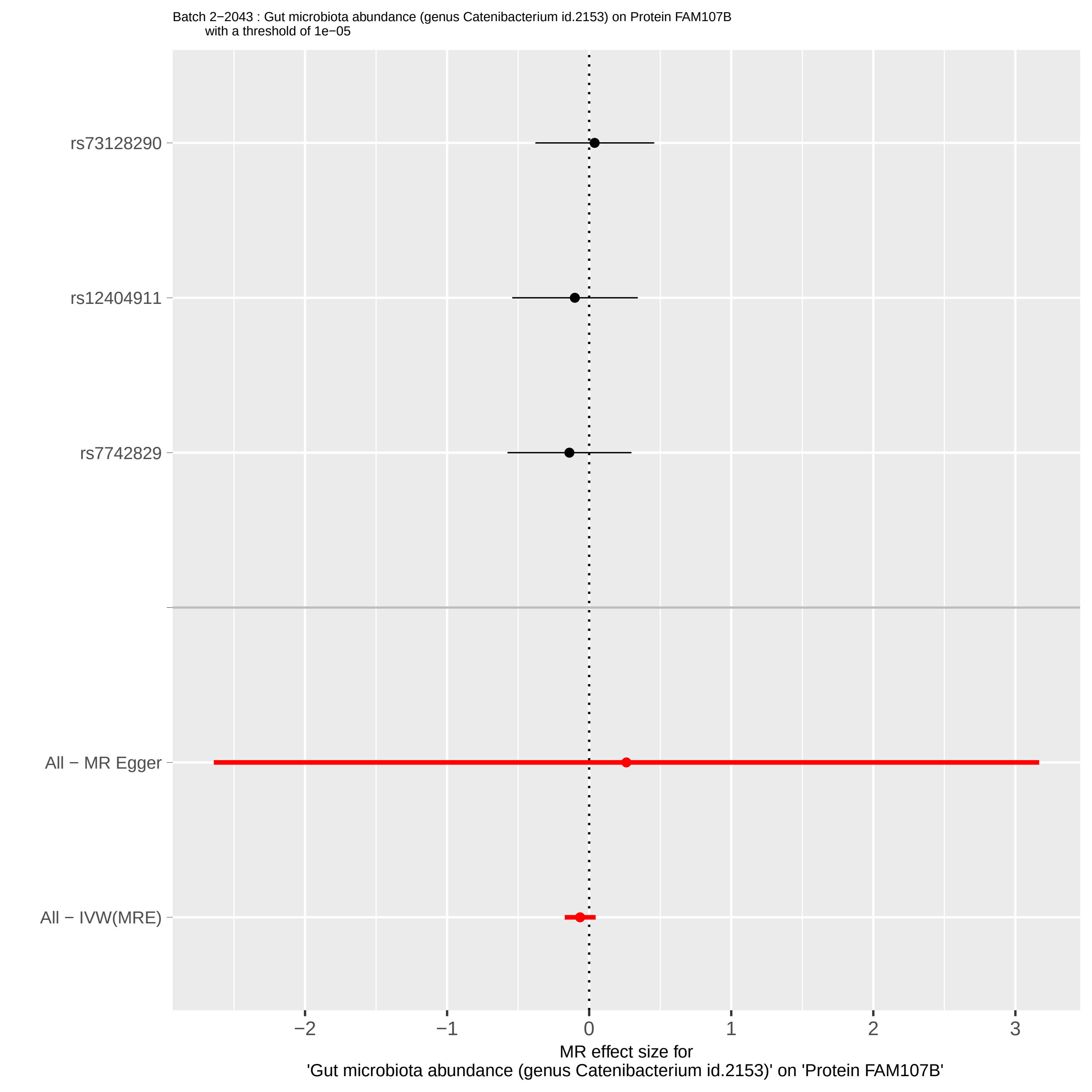

Batch 2−2043 : Gut microbiota abundance (genus Catenibacterium id.2153) on Protein FAM107B
with a threshold of 1e−05
rs73128290
rs12404911
rs7742829
All − MR Egger
All − IVW(MRE)
−2
−1
0
1
2
3
MR effect size for
'Gut microbiota abundance (genus Catenibacterium id.2153)' on 'Protein FAM107B'

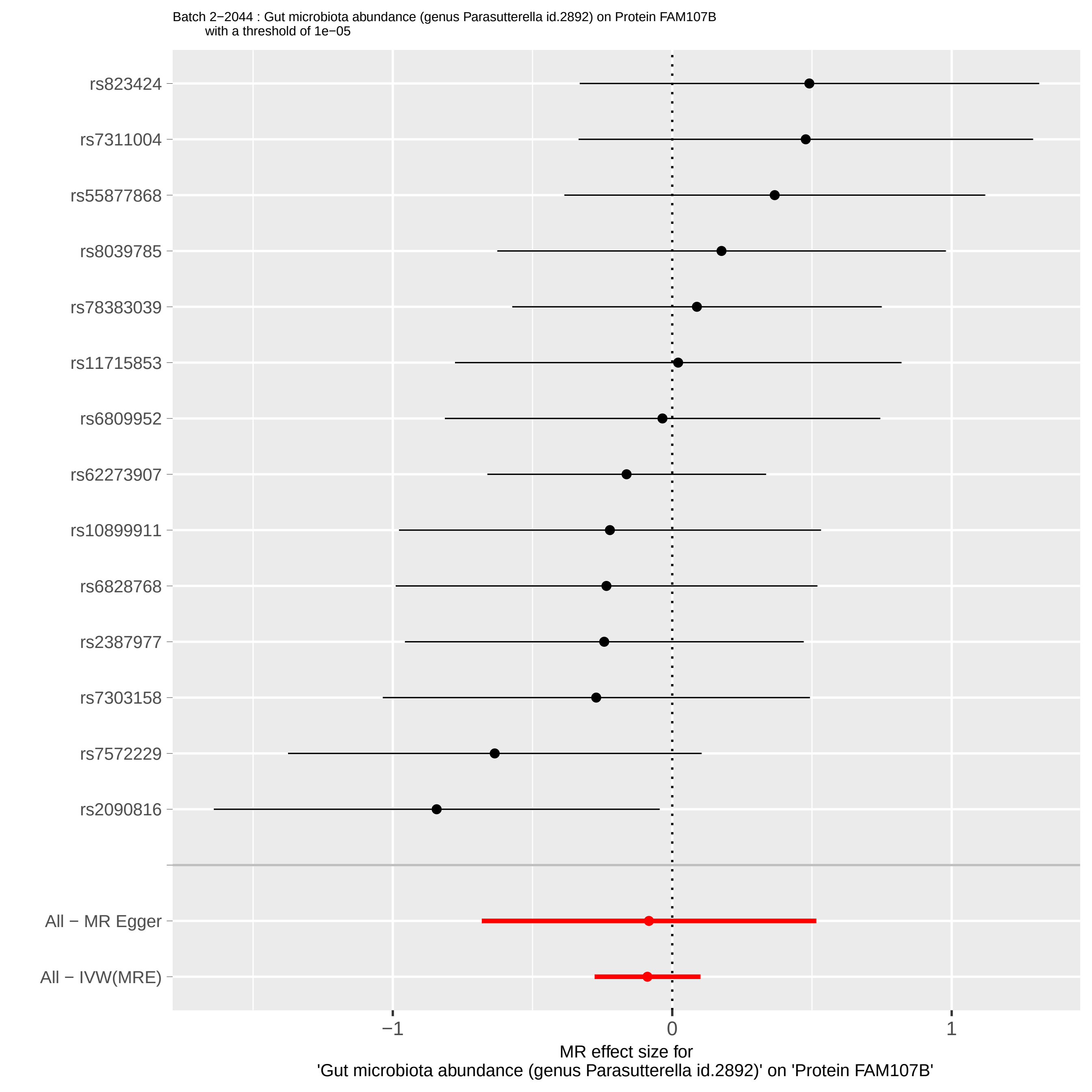

Batch 2−2044 : Gut microbiota abundance (genus Parasutterella id.2892) on Protein FAM107B
with a threshold of 1e−05
rs823424
rs7311004
rs55877868
rs8039785
rs78383039
rs11715853
rs6809952
rs62273907
rs10899911
rs6828768
rs2387977
rs7303158
rs7572229
rs2090816
All − MR Egger
All − IVW(MRE)
−1
0
1
MR effect size for
'Gut microbiota abundance (genus Parasutterella id.2892)' on 'Protein FAM107B'

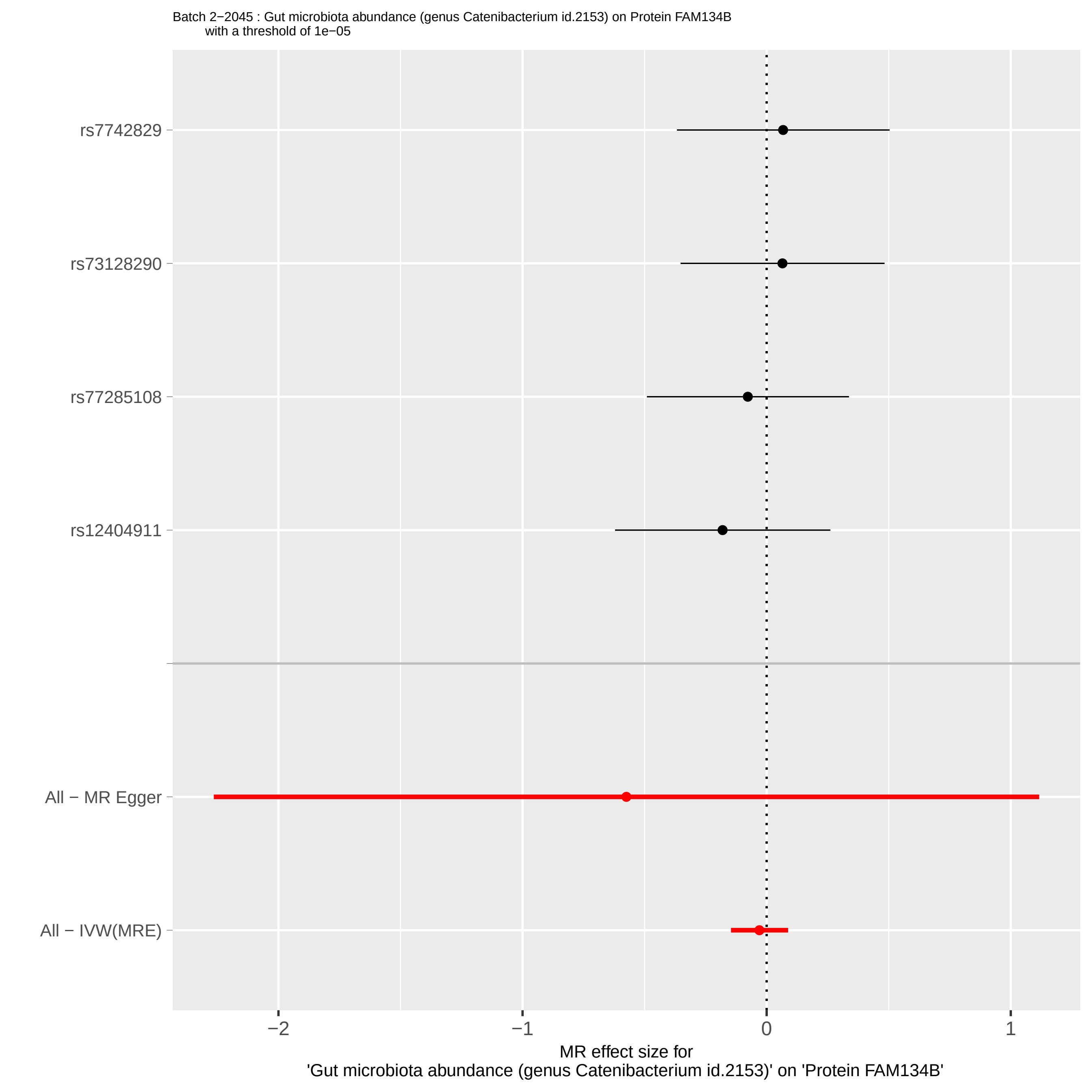

Batch 2−2045 : Gut microbiota abundance (genus Catenibacterium id.2153) on Protein FAM134B
with a threshold of 1e−05
rs7742829
rs73128290
rs77285108
rs12404911
All − MR Egger
All − IVW(MRE)
−2
−1
0
1
MR effect size for
'Gut microbiota abundance (genus Catenibacterium id.2153)' on 'Protein FAM134B'

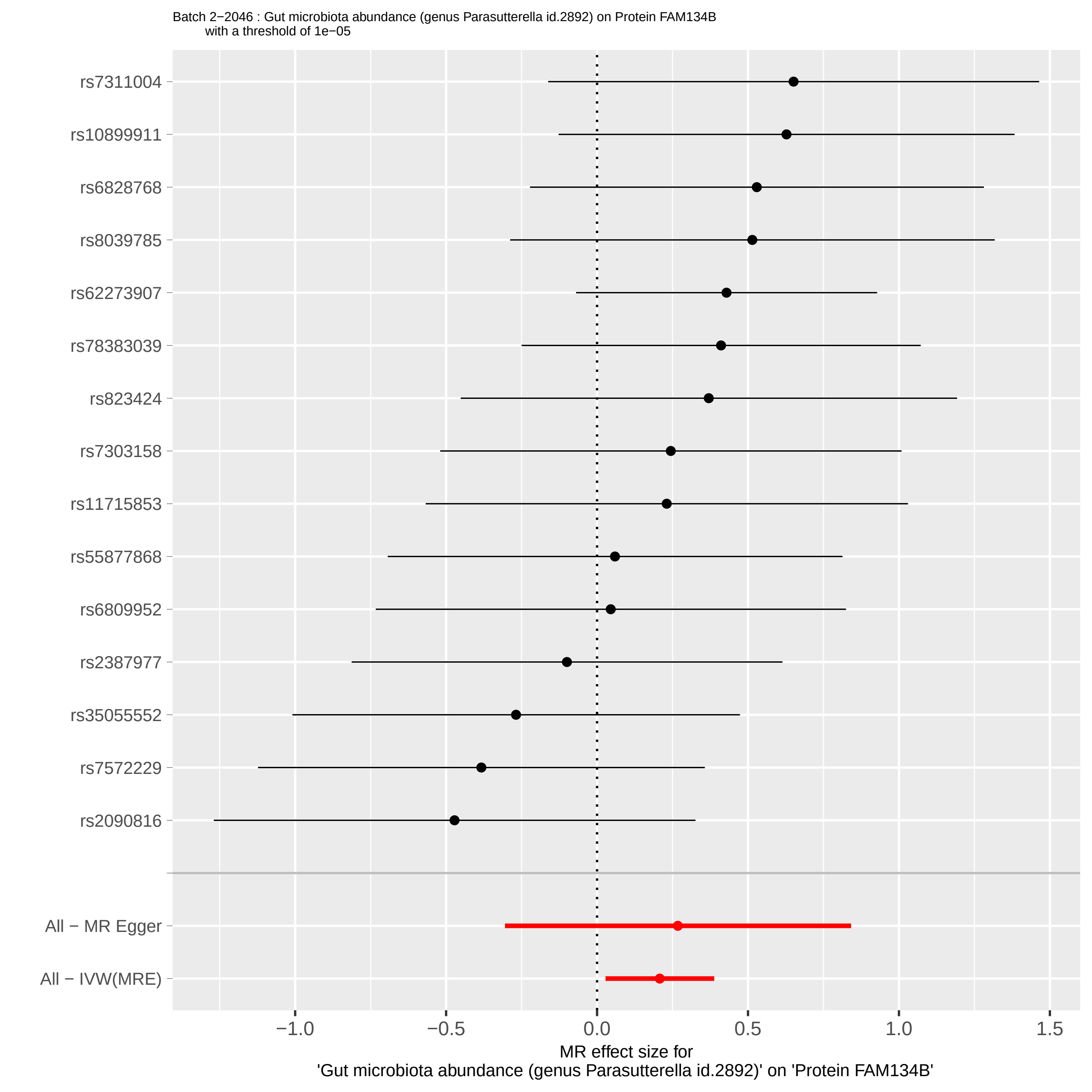

Batch 2−2046 : Gut microbiota abundance (genus Parasutterella id.2892) on Protein FAM134B
with a threshold of 1e−05
rs7311004
rs10899911
rs6828768
rs8039785
rs62273907
rs78383039
rs823424
rs7303158
rs11715853
rs55877868
rs6809952
rs2387977
rs35055552
rs7572229
rs2090816
All − MR Egger
All − IVW(MRE)
−1.0
−0.5
0.0
0.5
1.0
1.5
MR effect size for
'Gut microbiota abundance (genus Parasutterella id.2892)' on 'Protein FAM134B'

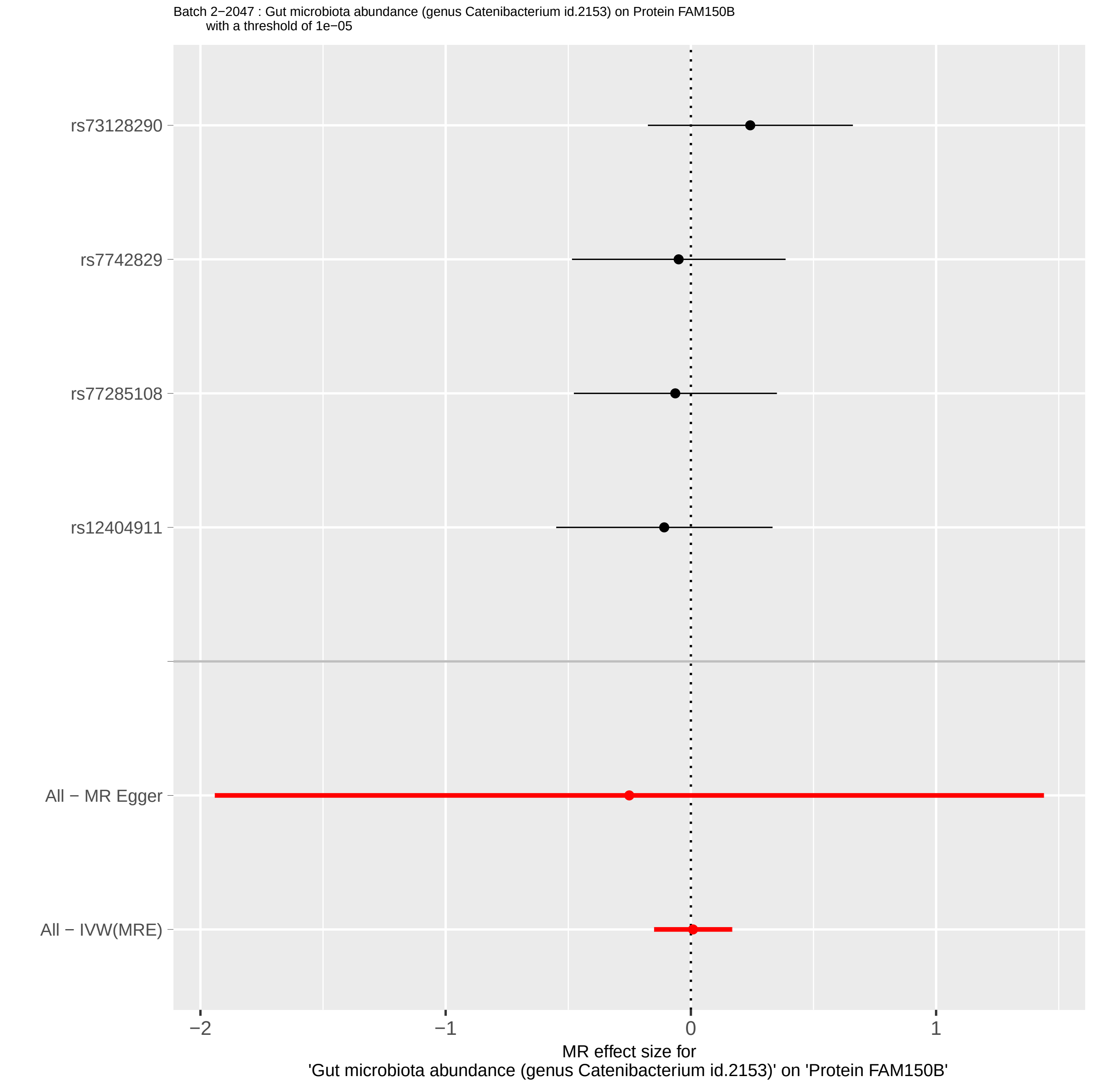

Batch 2−2047 : Gut microbiota abundance (genus Catenibacterium id.2153) on Protein FAM150B
with a threshold of 1e−05
rs73128290
rs7742829
rs77285108
rs12404911
All − MR Egger
All − IVW(MRE)
−2
−1
0
1
MR effect size for
'Gut microbiota abundance (genus Catenibacterium id.2153)' on 'Protein FAM150B'

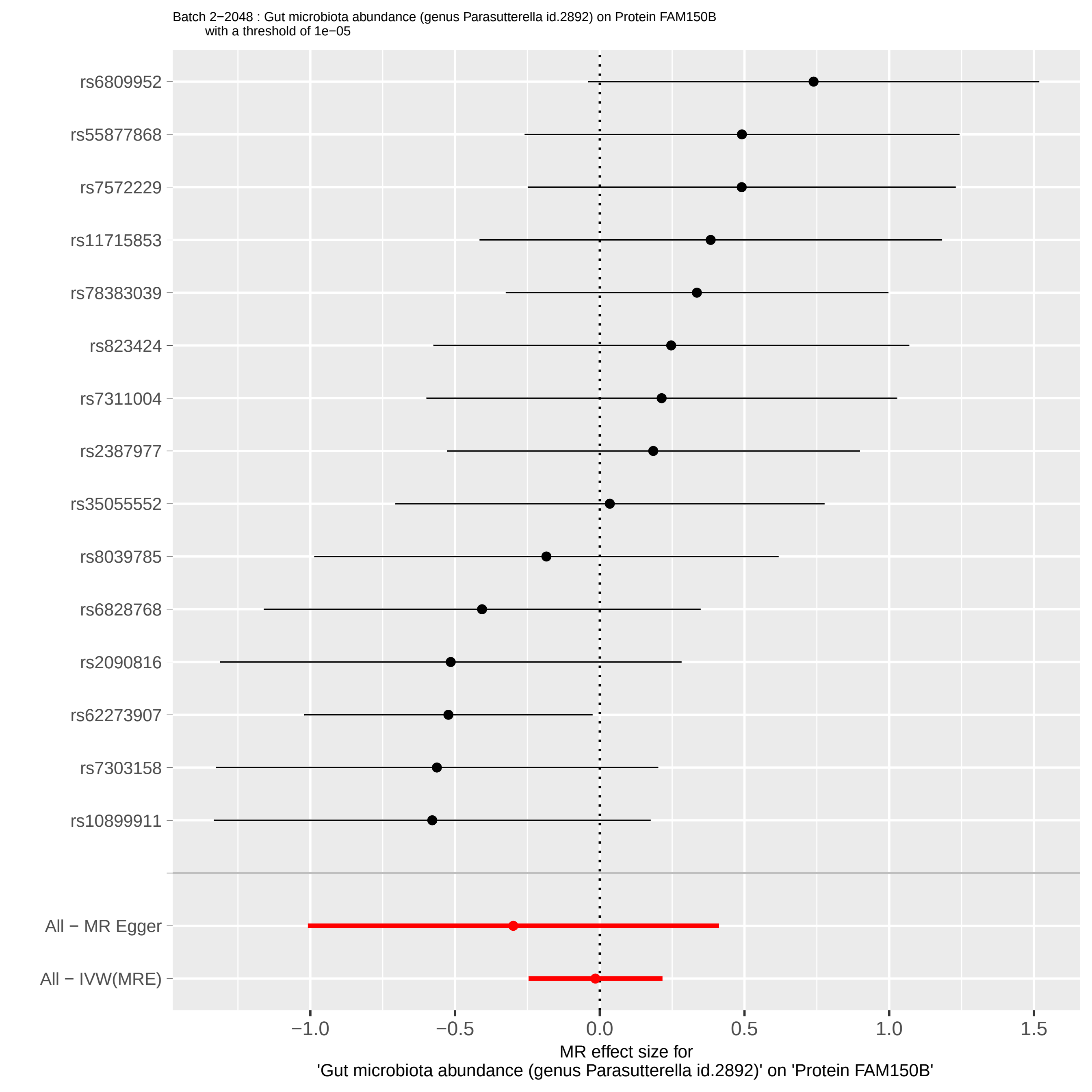

Batch 2−2048 : Gut microbiota abundance (genus Parasutterella id.2892) on Protein FAM150B
with a threshold of 1e−05
rs6809952
rs55877868
rs7572229
rs11715853
rs78383039
rs823424
rs7311004
rs2387977
rs35055552
rs8039785
rs6828768
rs2090816
rs62273907
rs7303158
rs10899911
All − MR Egger
All − IVW(MRE)
−1.0
−0.5
0.0
0.5
1.0
1.5
MR effect size for
'Gut microbiota abundance (genus Parasutterella id.2892)' on 'Protein FAM150B'

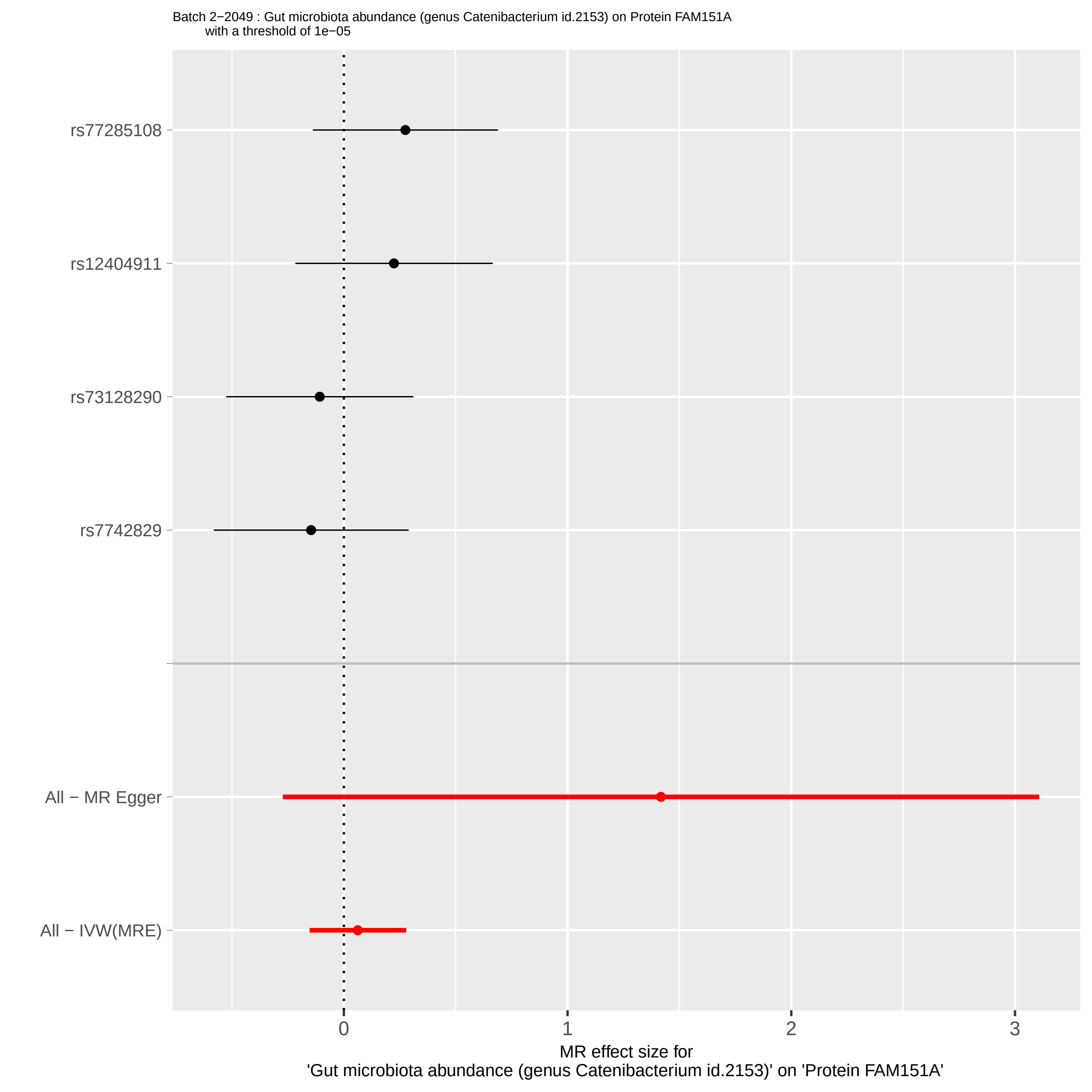
Batch 2−2049 : Gut microbiota abundance (genus Catenibacterium id.2153) on Protein FAM151A
with a threshold of 1e−05
rs77285108
rs12404911
rs73128290
rs7742829
All − MR Egger
All − IVW(MRE)
0
1
2
3
MR effect size for
'Gut microbiota abundance (genus Catenibacterium id.2153)' on 'Protein FAM151A'

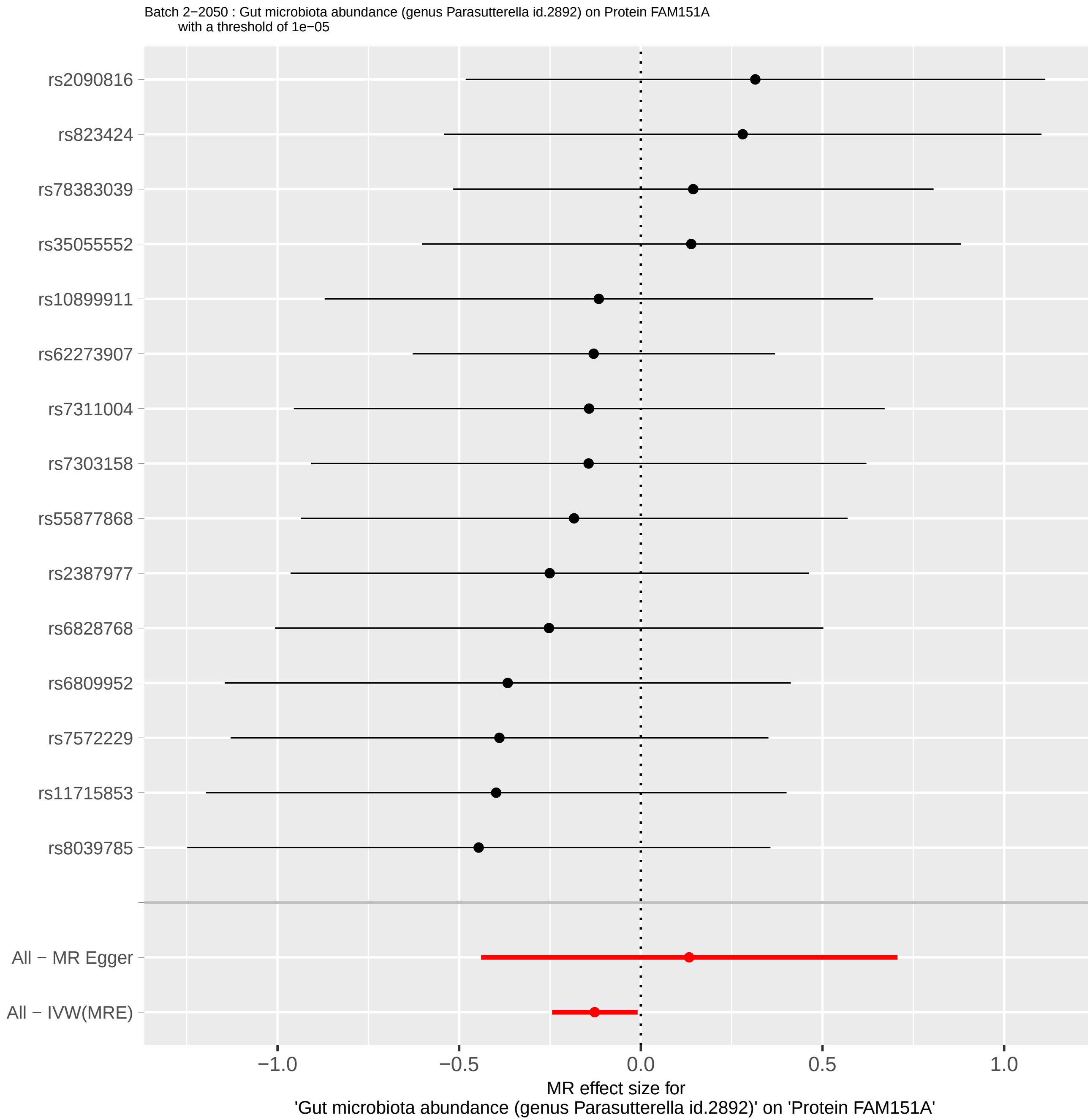

Batch 2−2050 : Gut microbiota abundance (genus Parasutterella id.2892) on Protein FAM151A
with a threshold of 1e−05
rs2090816
rs823424
rs78383039
rs35055552
rs10899911
rs62273907
rs7311004
rs7303158
rs55877868
rs2387977
rs6828768
rs6809952
rs7572229
rs11715853
rs8039785
All − MR Egger
All − IVW(MRE)
−1.0
−0.5
0.0
0.5
1.0
MR effect size for
'Gut microbiota abundance (genus Parasutterella id.2892)' on 'Protein FAM151A'

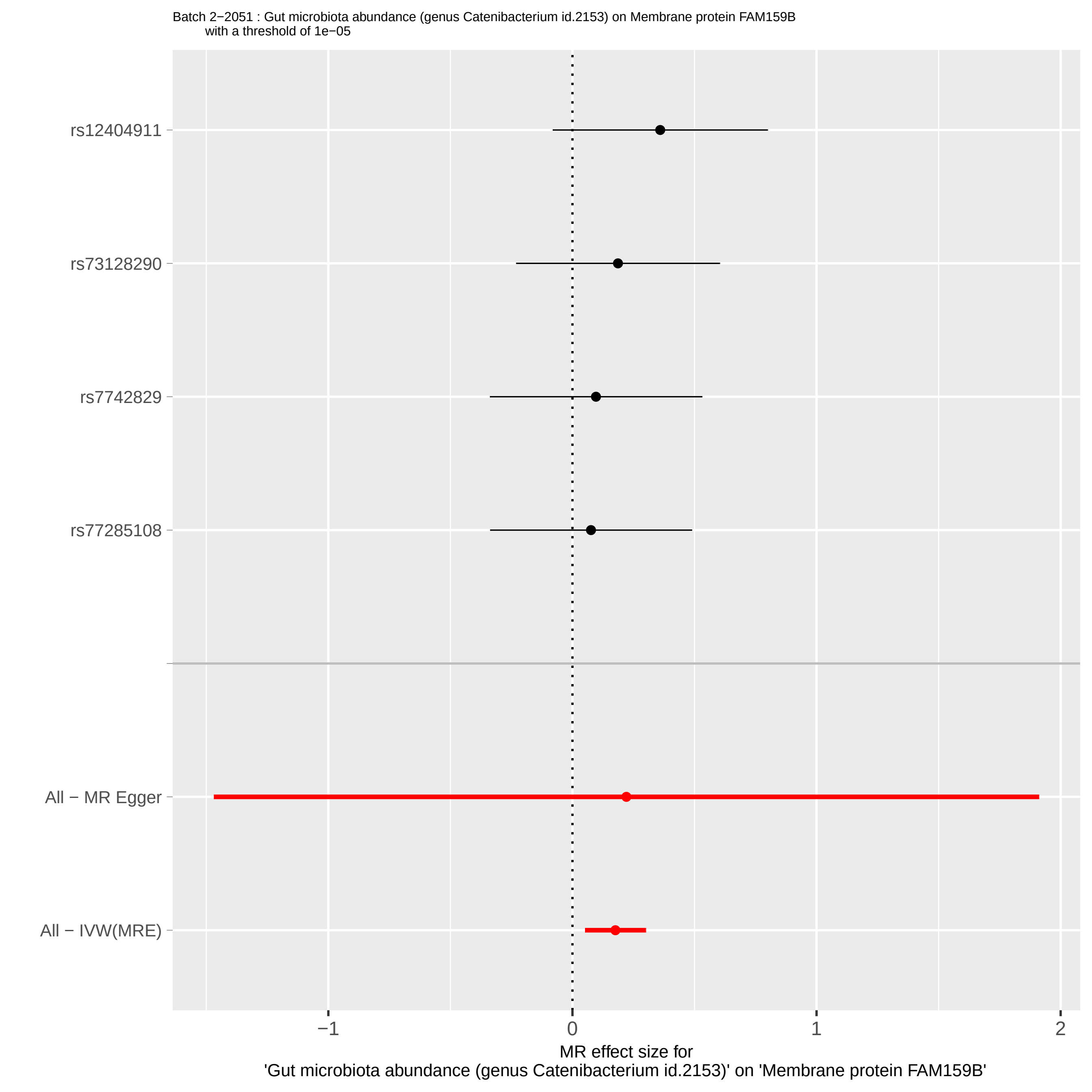
Batch 2−2051 : Gut microbiota abundance (genus Catenibacterium id.2153) on Membrane protein FAM159B
with a threshold of 1e−05
rs12404911
rs73128290
rs7742829
rs77285108
All − MR Egger
All − IVW(MRE)
−1
0
1
2
MR effect size for
'Gut microbiota abundance (genus Catenibacterium id.2153)' on 'Membrane protein FAM159B'

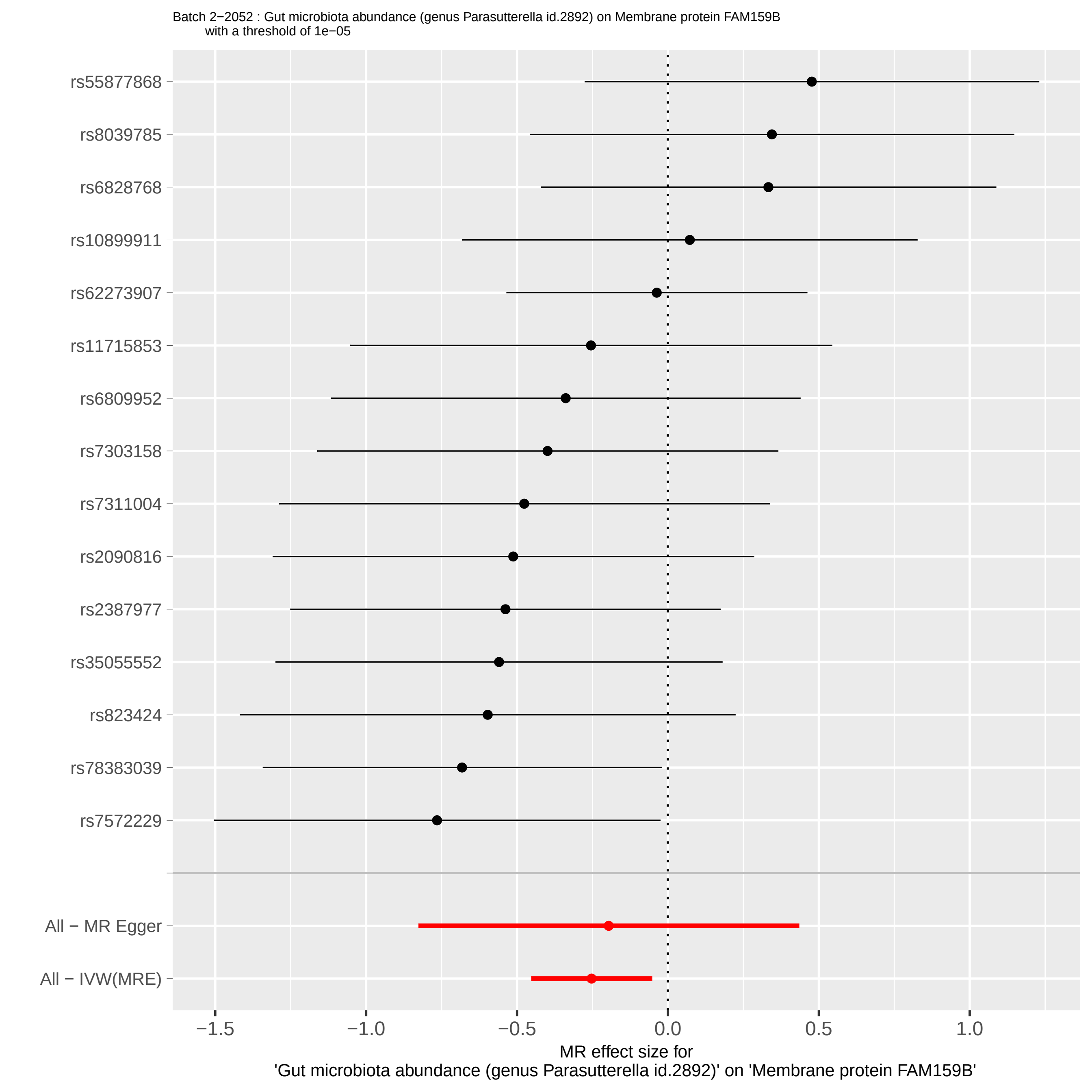
Batch 2−2052 : Gut microbiota abundance (genus Parasutterella id.2892) on Membrane protein FAM159B
with a threshold of 1e−05
rs55877868
rs8039785
rs6828768
rs10899911
rs62273907
rs11715853
rs6809952
rs7303158
rs7311004
rs2090816
rs2387977
rs35055552
rs823424
rs78383039
rs7572229
All − MR Egger
All − IVW(MRE)
−1.5
−1.0
−0.5
0.0
0.5
1.0
MR effect size for
'Gut microbiota abundance (genus Parasutterella id.2892)' on 'Membrane protein FAM159B'

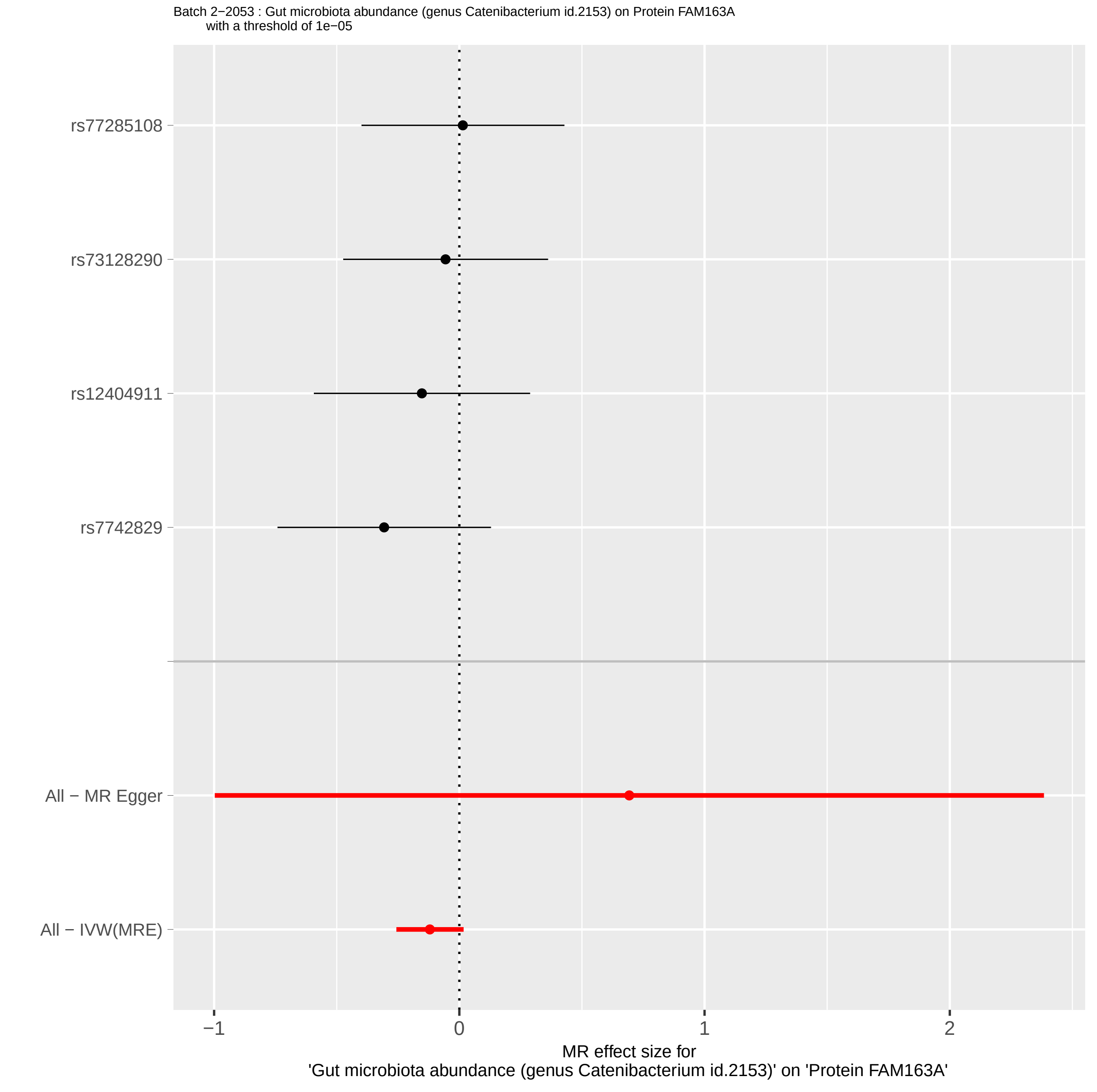

Batch 2−2053 : Gut microbiota abundance (genus Catenibacterium id.2153) on Protein FAM163A
with a threshold of 1e−05
rs77285108
rs73128290
rs12404911
rs7742829
All − MR Egger
All − IVW(MRE)
−1
0
1
2
MR effect size for
'Gut microbiota abundance (genus Catenibacterium id.2153)' on 'Protein FAM163A'

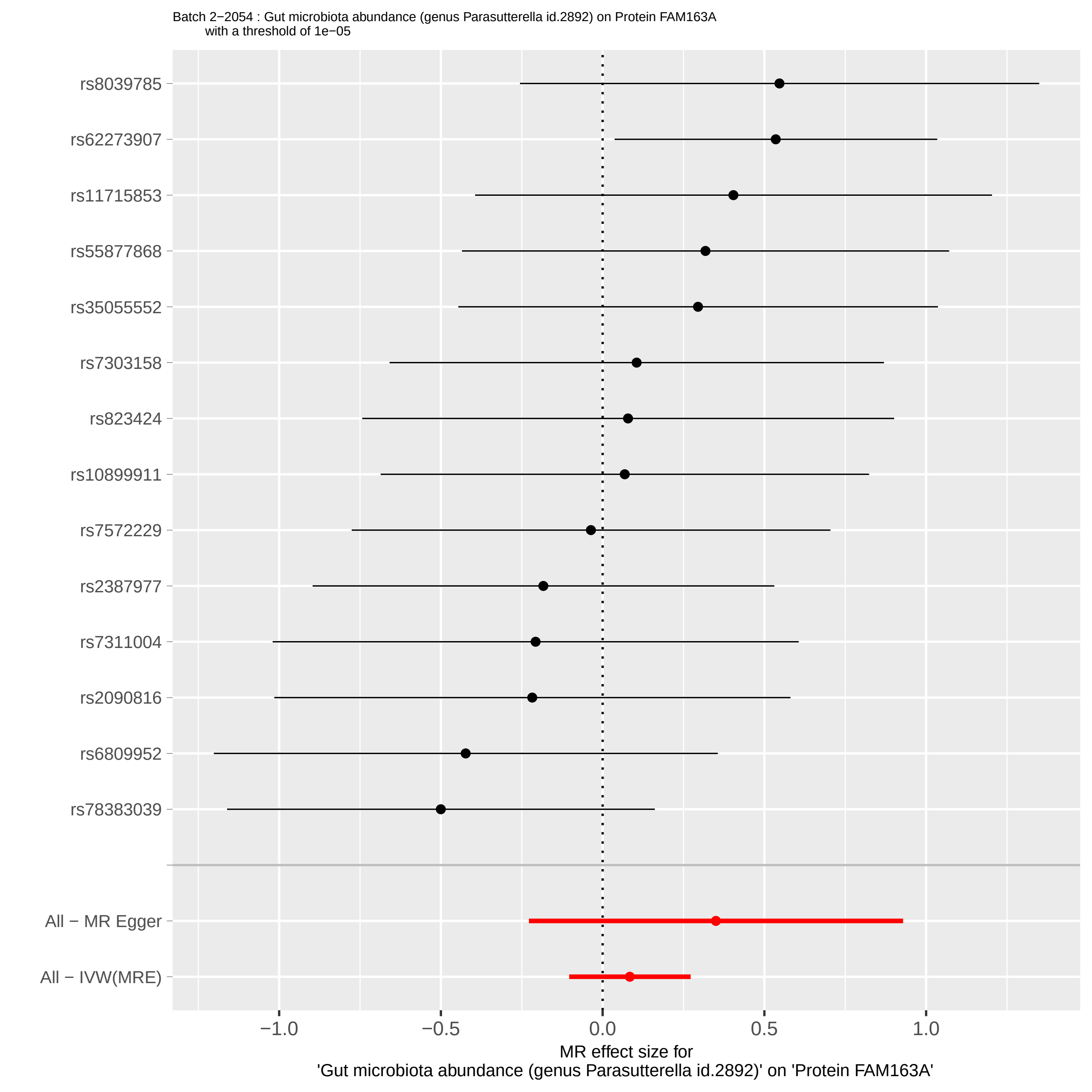
Batch 2−2054 : Gut microbiota abundance (genus Parasutterella id.2892) on Protein FAM163A
with a threshold of 1e−05
rs8039785
rs62273907
rs11715853
rs55877868
rs35055552
rs7303158
rs823424
rs10899911
rs7572229
rs2387977
rs7311004
rs2090816
rs6809952
rs78383039
All − MR Egger
All − IVW(MRE)
−1.0
−0.5
0.0
0.5
1.0
MR effect size for
'Gut microbiota abundance (genus Parasutterella id.2892)' on 'Protein FAM163A'

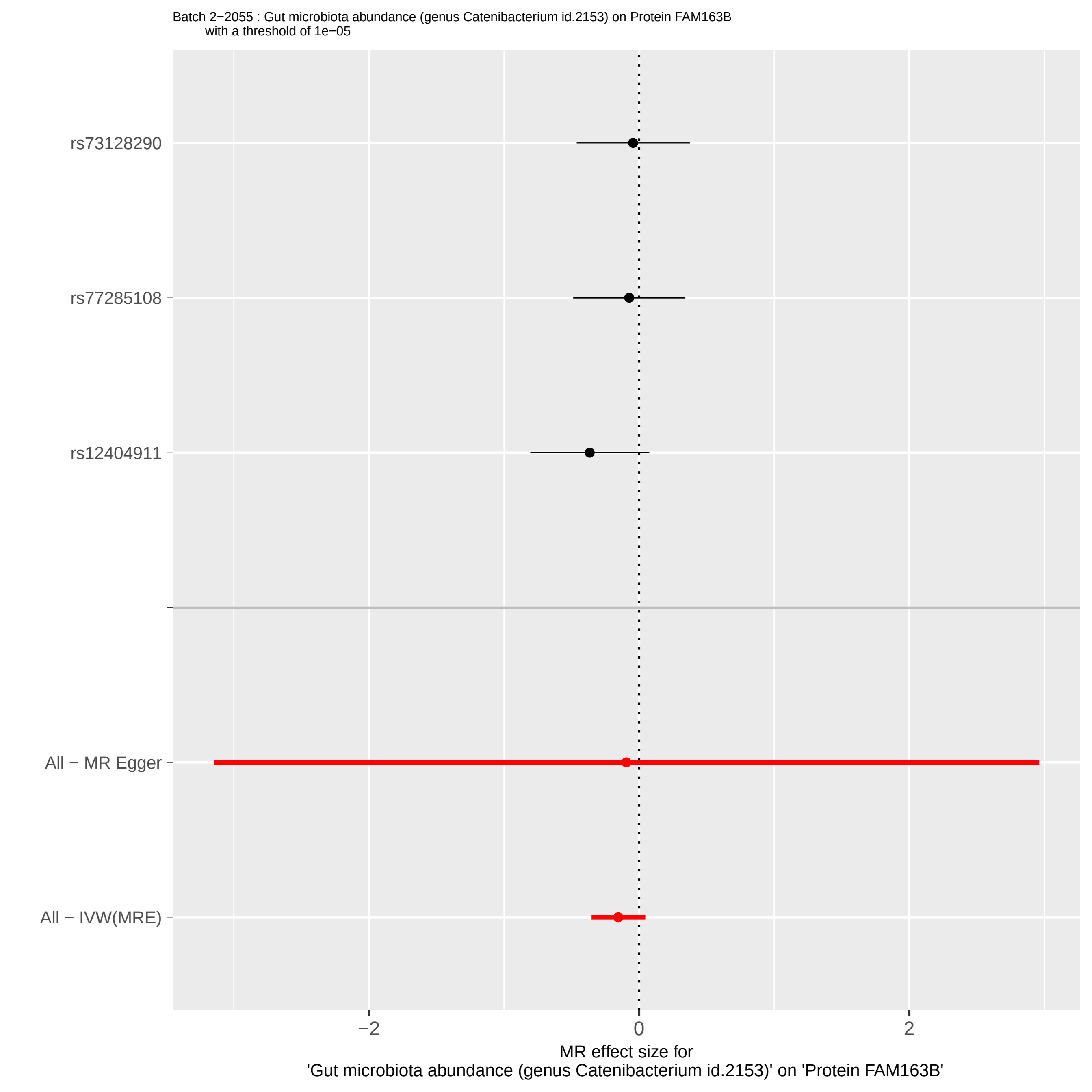

Batch 2−2055 : Gut microbiota abundance (genus Catenibacterium id.2153) on Protein FAM163B
with a threshold of 1e−05
rs73128290
rs77285108
rs12404911
All − MR Egger
All − IVW(MRE)
−2
0
2
MR effect size for
'Gut microbiota abundance (genus Catenibacterium id.2153)' on 'Protein FAM163B'

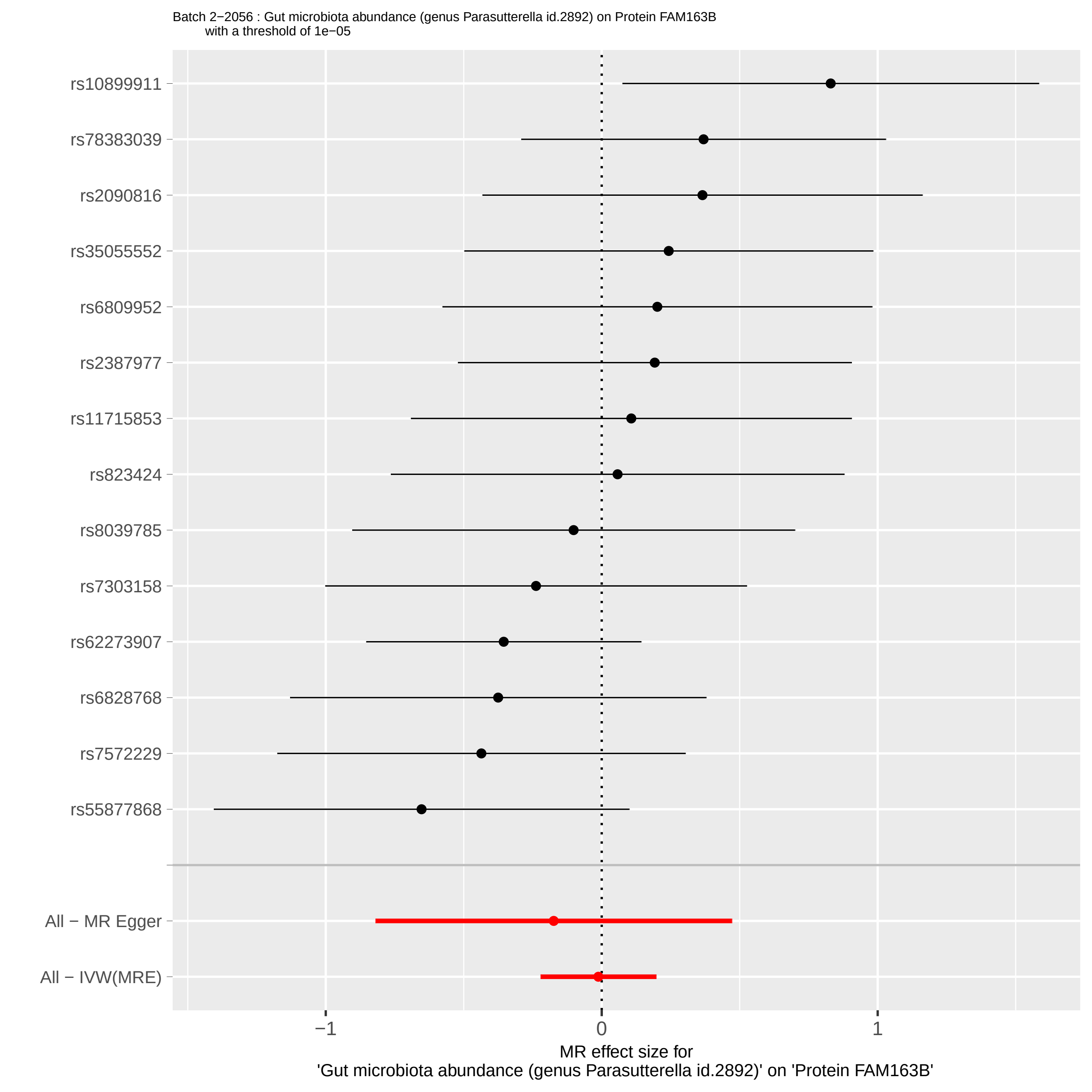
Batch 2−2056 : Gut microbiota abundance (genus Parasutterella id.2892) on Protein FAM163B
with a threshold of 1e−05
rs10899911
rs78383039
rs2090816
rs35055552
rs6809952
rs2387977
rs11715853
rs823424
rs8039785
rs7303158
rs62273907
rs6828768
rs7572229
rs55877868
All − MR Egger
All − IVW(MRE)
−1
0
1
MR effect size for
'Gut microbiota abundance (genus Parasutterella id.2892)' on 'Protein FAM163B'

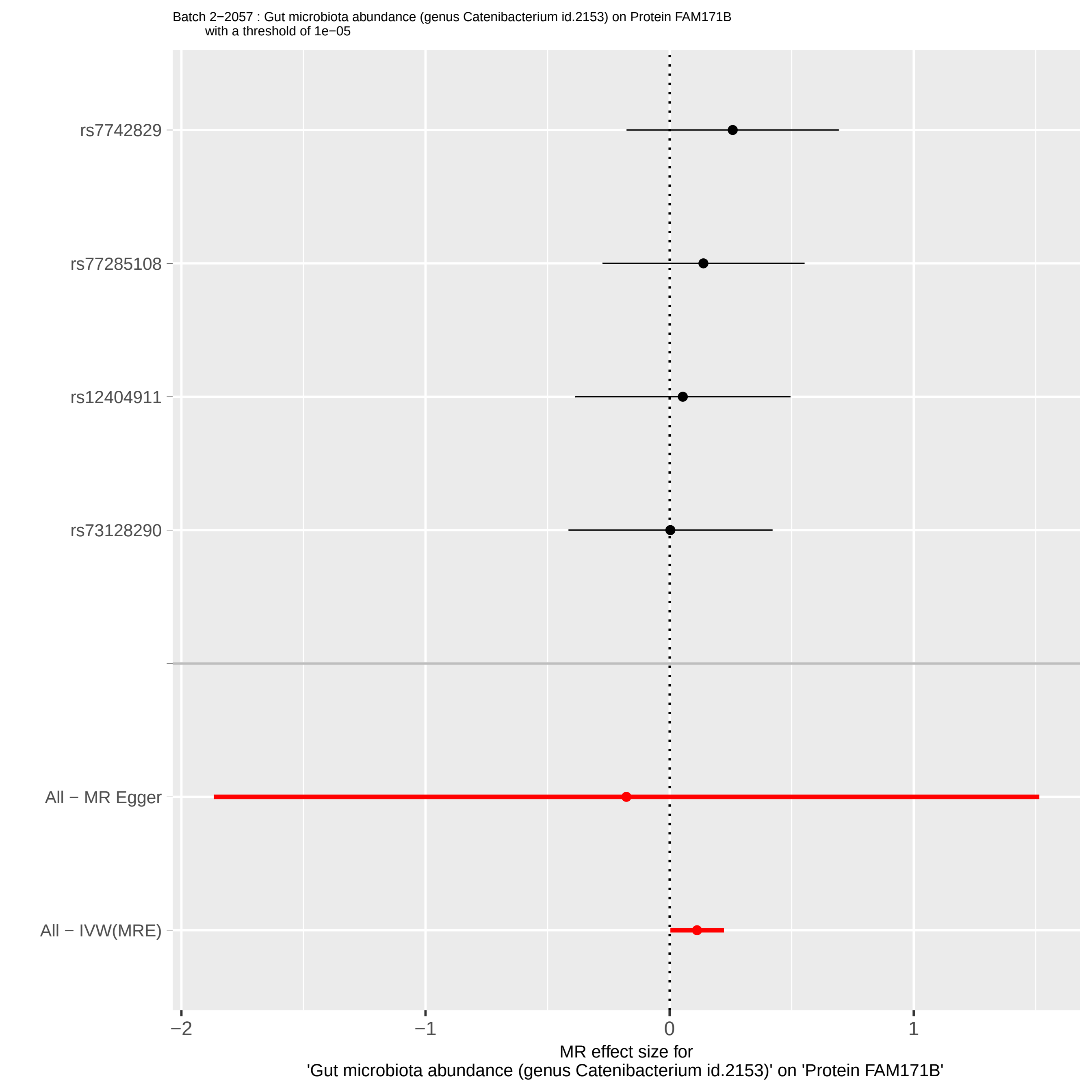

Batch 2−2057 : Gut microbiota abundance (genus Catenibacterium id.2153) on Protein FAM171B
with a threshold of 1e−05
rs7742829
rs77285108
rs12404911
rs73128290
All − MR Egger
All − IVW(MRE)
−2
−1
0
1
MR effect size for
'Gut microbiota abundance (genus Catenibacterium id.2153)' on 'Protein FAM171B'

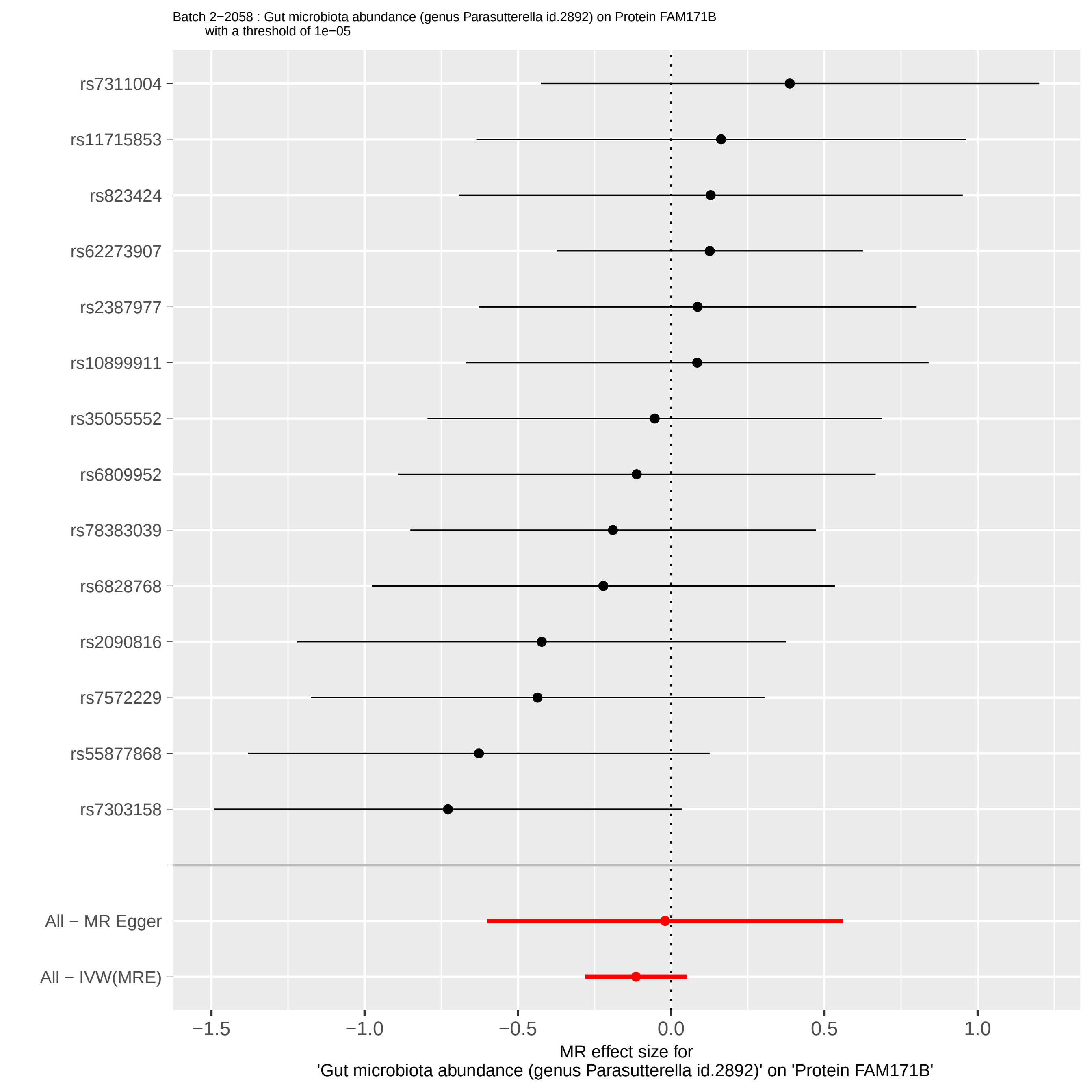

Batch 2−2058 : Gut microbiota abundance (genus Parasutterella id.2892) on Protein FAM171B
with a threshold of 1e−05
rs7311004
rs11715853
rs823424
rs62273907
rs2387977
rs10899911
rs35055552
rs6809952
rs78383039
rs6828768
rs2090816
rs7572229
rs55877868
rs7303158
All − MR Egger
All − IVW(MRE)
−1.5
−1.0
−0.5
0.0
0.5
1.0
MR effect size for
'Gut microbiota abundance (genus Parasutterella id.2892)' on 'Protein FAM171B'

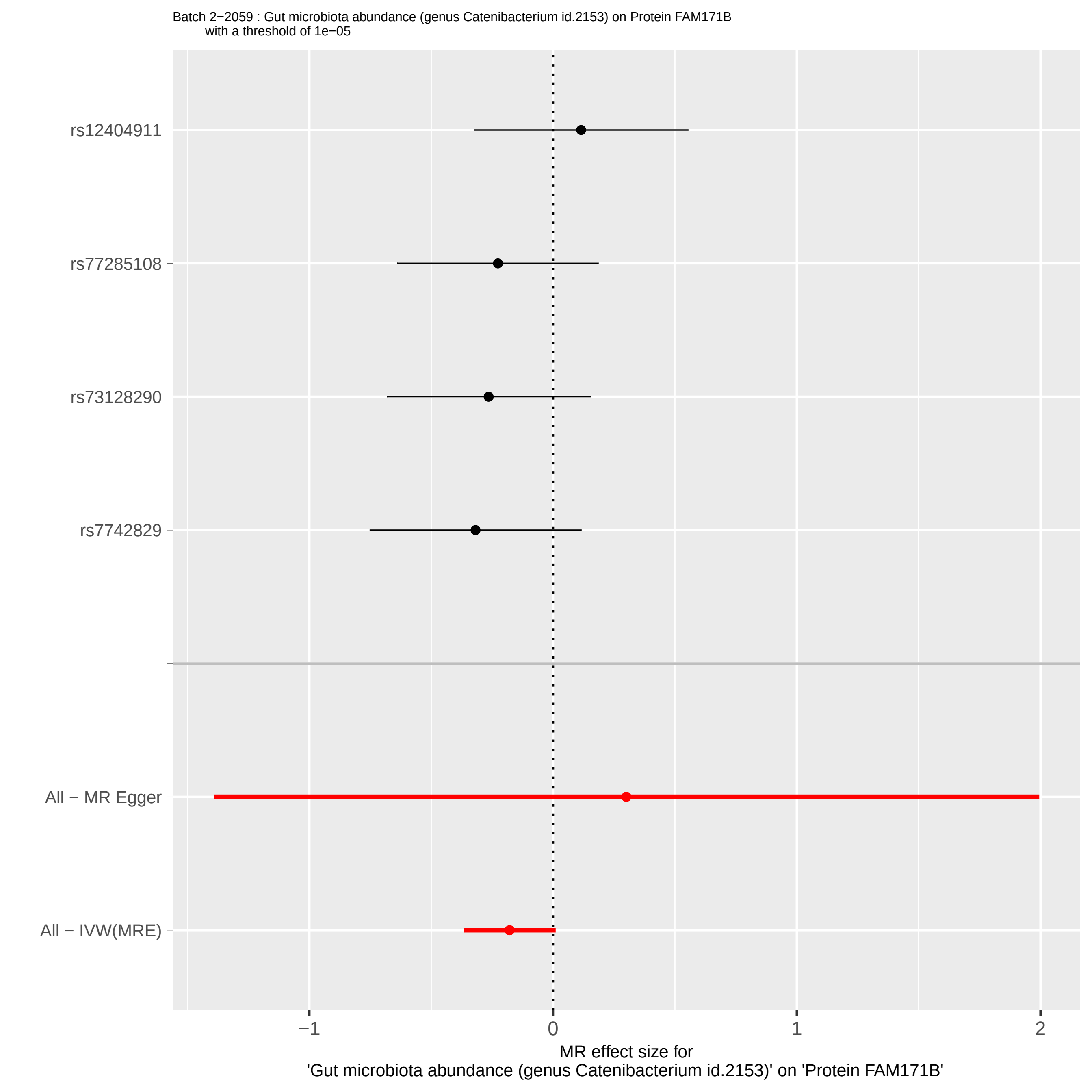
Batch 2−2059 : Gut microbiota abundance (genus Catenibacterium id.2153) on Protein FAM171B
with a threshold of 1e−05
rs12404911
rs77285108
rs73128290
rs7742829
All − MR Egger
All − IVW(MRE)
−1
0
1
2
MR effect size for
'Gut microbiota abundance (genus Catenibacterium id.2153)' on 'Protein FAM171B'

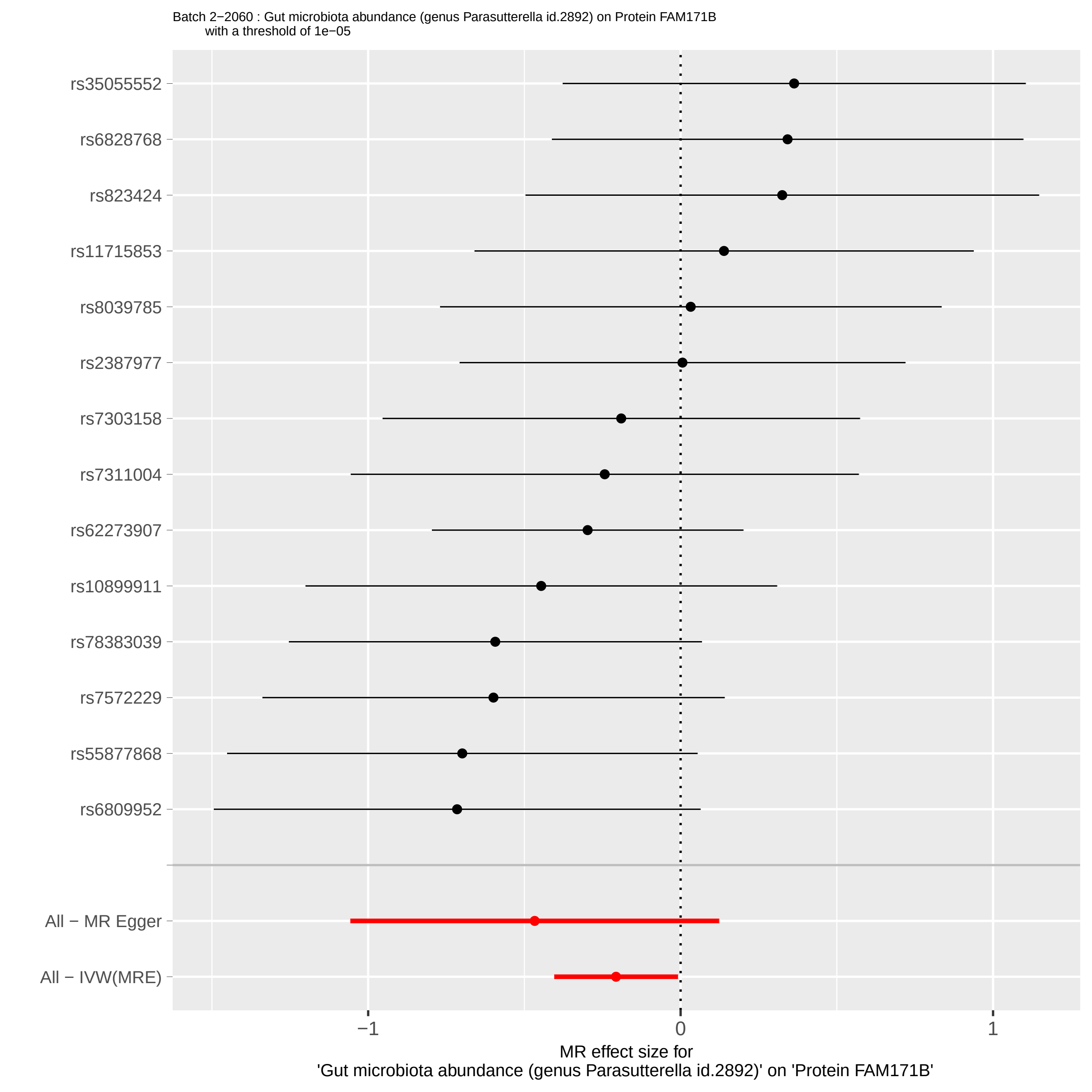
Batch 2−2060 : Gut microbiota abundance (genus Parasutterella id.2892) on Protein FAM171B
with a threshold of 1e−05
rs35055552
rs6828768
rs823424
rs11715853
rs8039785
rs2387977
rs7303158
rs7311004
rs62273907
rs10899911
rs78383039
rs7572229
rs55877868
rs6809952
All − MR Egger
All − IVW(MRE)
−1
0
1
MR effect size for
'Gut microbiota abundance (genus Parasutterella id.2892)' on 'Protein FAM171B'

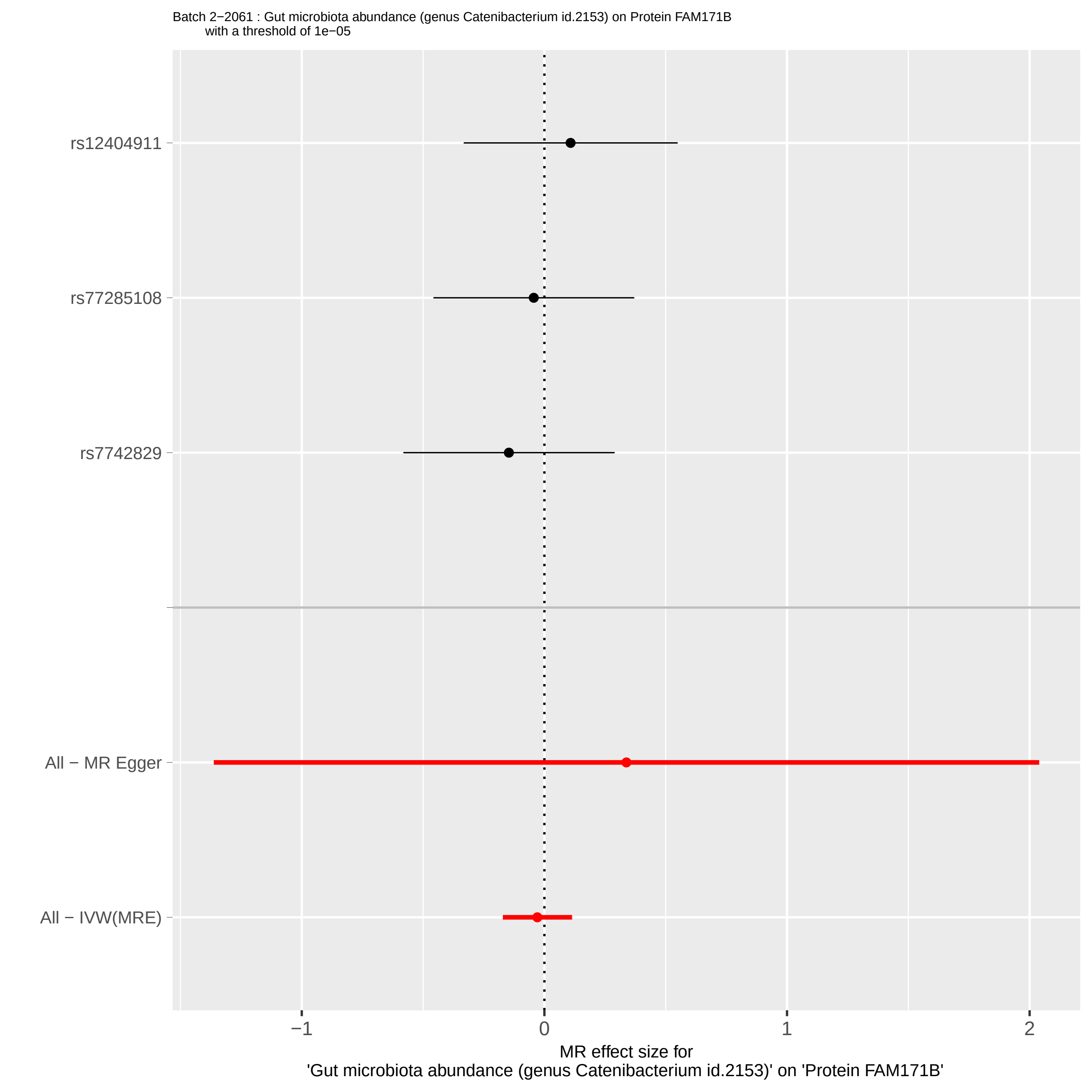
Batch 2−2061 : Gut microbiota abundance (genus Catenibacterium id.2153) on Protein FAM171B
with a threshold of 1e−05
rs12404911
rs77285108
rs7742829
All − MR Egger
All − IVW(MRE)
−1
0
1
2
MR effect size for
'Gut microbiota abundance (genus Catenibacterium id.2153)' on 'Protein FAM171B'

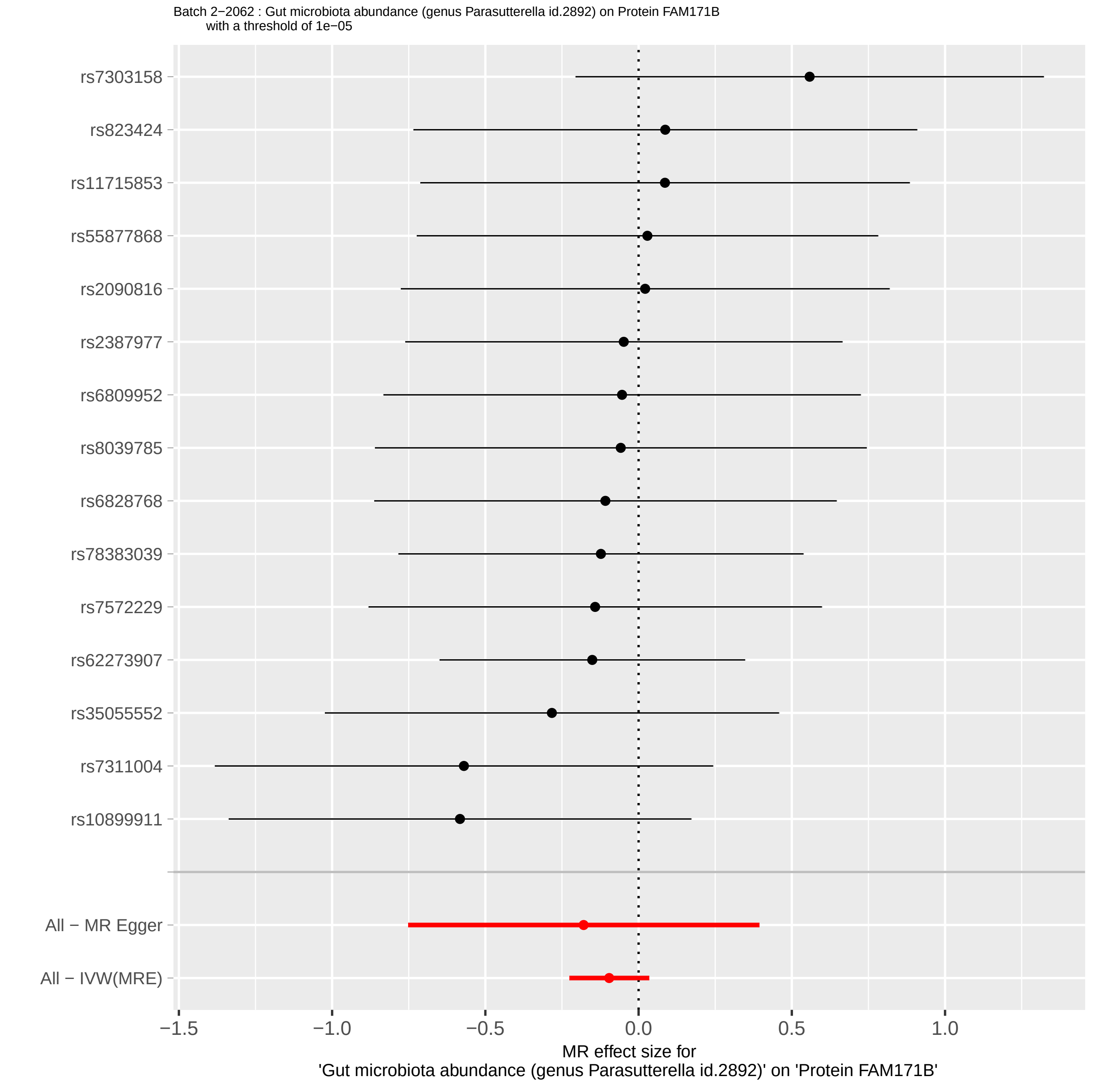

Batch 2−2062 : Gut microbiota abundance (genus Parasutterella id.2892) on Protein FAM171B
with a threshold of 1e−05
rs7303158
rs823424
rs11715853
rs55877868
rs2090816
rs2387977
rs6809952
rs8039785
rs6828768
rs78383039
rs7572229
rs62273907
rs35055552
rs7311004
rs10899911
All − MR Egger
All − IVW(MRE)
−1.5
−1.0
−0.5
0.0
0.5
1.0
MR effect size for
'Gut microbiota abundance (genus Parasutterella id.2892)' on 'Protein FAM171B'

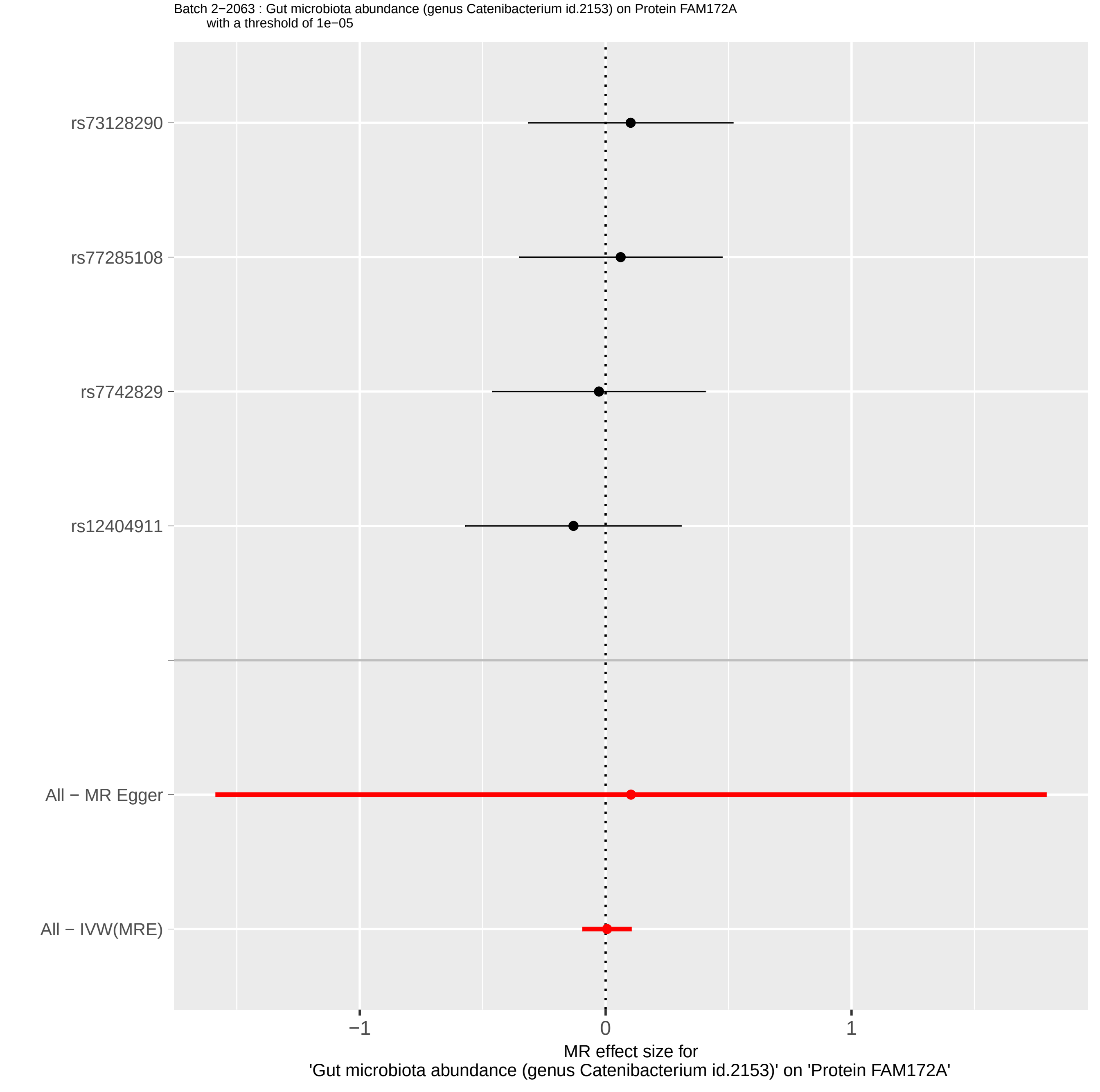

Batch 2−2063 : Gut microbiota abundance (genus Catenibacterium id.2153) on Protein FAM172A
with a threshold of 1e−05
rs73128290
rs77285108
rs7742829
rs12404911
All − MR Egger
All − IVW(MRE)
−1
0
1
MR effect size for
'Gut microbiota abundance (genus Catenibacterium id.2153)' on 'Protein FAM172A'

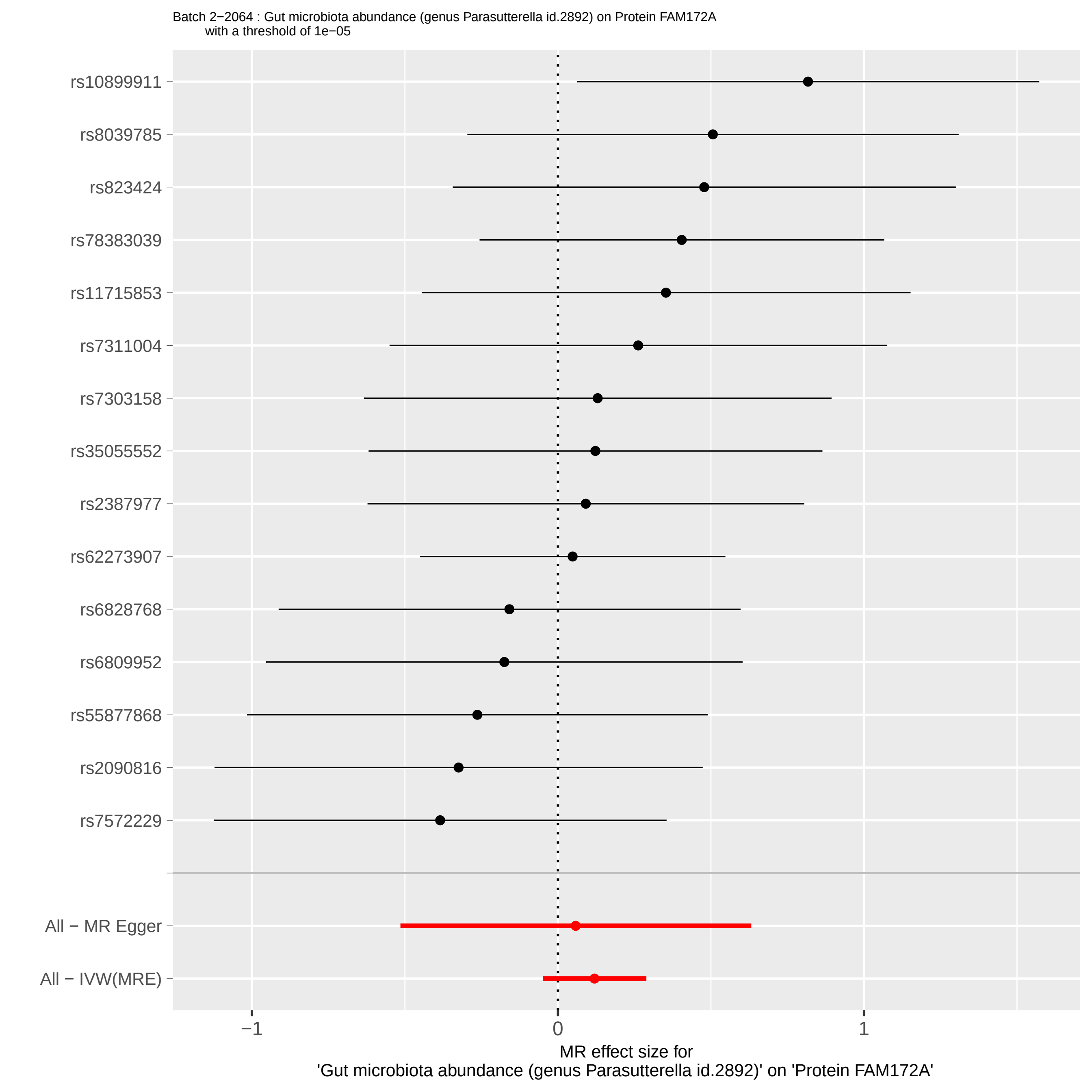
Batch 2−2064 : Gut microbiota abundance (genus Parasutterella id.2892) on Protein FAM172A
with a threshold of 1e−05
rs10899911
rs8039785
rs823424
rs78383039
rs11715853
rs7311004
rs7303158
rs35055552
rs2387977
rs62273907
rs6828768
rs6809952
rs55877868
rs2090816
rs7572229
All − MR Egger
All − IVW(MRE)
−1
0
1
MR effect size for
'Gut microbiota abundance (genus Parasutterella id.2892)' on 'Protein FAM172A'

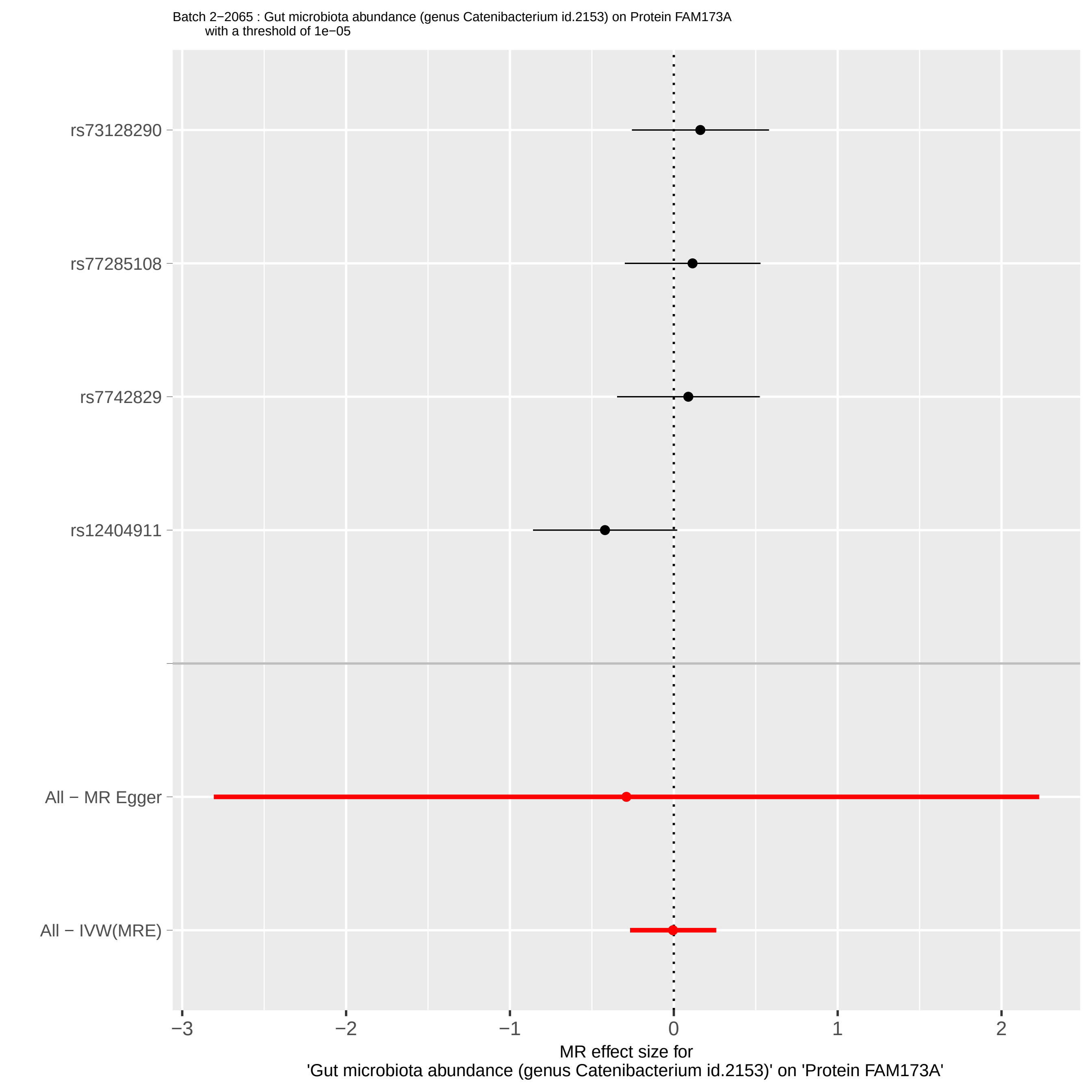
Batch 2−2065 : Gut microbiota abundance (genus Catenibacterium id.2153) on Protein FAM173A
with a threshold of 1e−05
rs73128290
rs77285108
rs7742829
rs12404911
All − MR Egger
All − IVW(MRE)
−3
−2
−1
0
1
2
MR effect size for
'Gut microbiota abundance (genus Catenibacterium id.2153)' on 'Protein FAM173A'

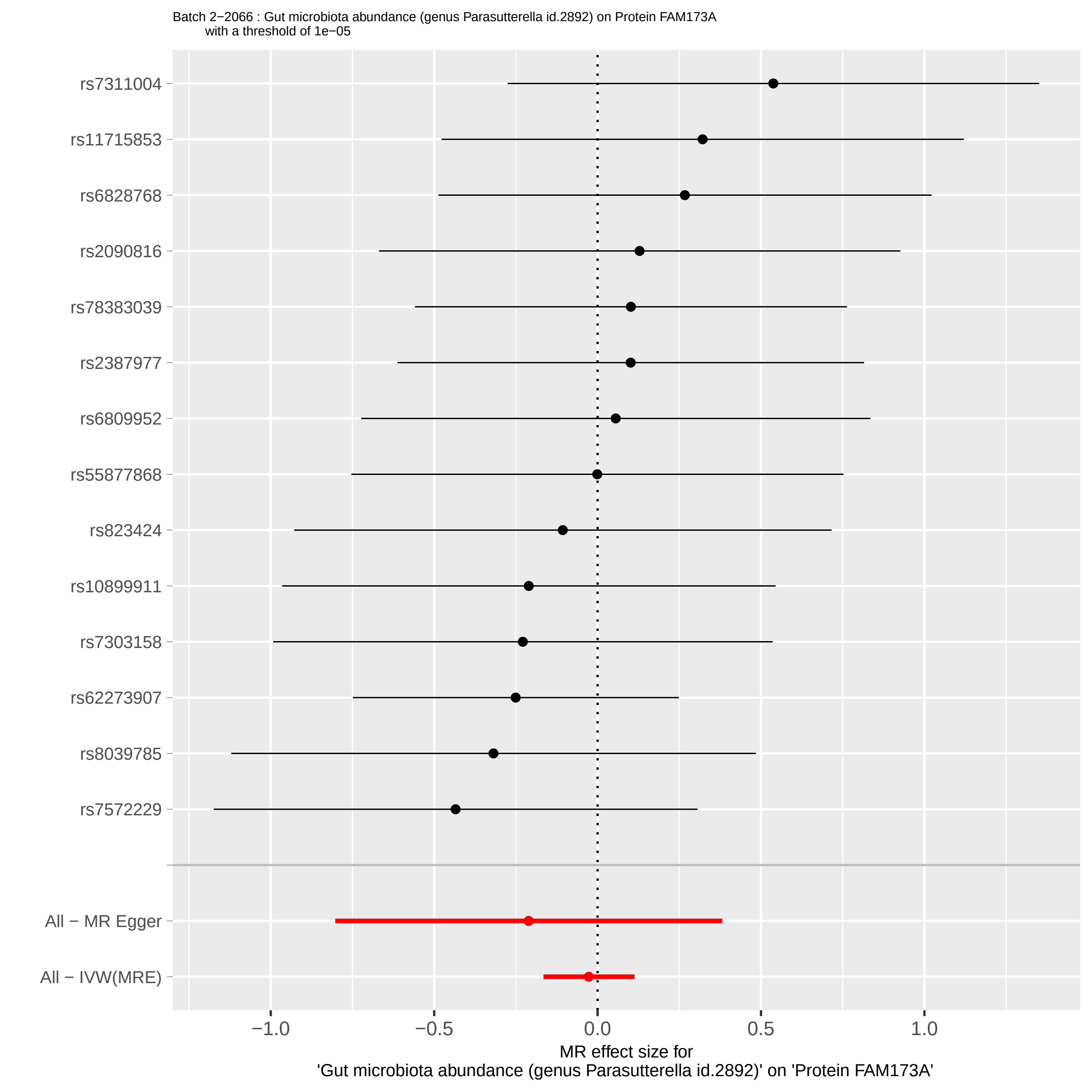

Batch 2−2066 : Gut microbiota abundance (genus Parasutterella id.2892) on Protein FAM173A
with a threshold of 1e−05
rs7311004
rs11715853
rs6828768
rs2090816
rs78383039
rs2387977
rs6809952
rs55877868
rs823424
rs10899911
rs7303158
rs62273907
rs8039785
rs7572229
All − MR Egger
All − IVW(MRE)
−1.0
−0.5
0.0
0.5
1.0
MR effect size for
'Gut microbiota abundance (genus Parasutterella id.2892)' on 'Protein FAM173A'

Batch 2−2067 : Gut microbiota abundance (genus Catenibacterium id.2153) on Membrane protein FAM174A
with a threshold of 1e−05

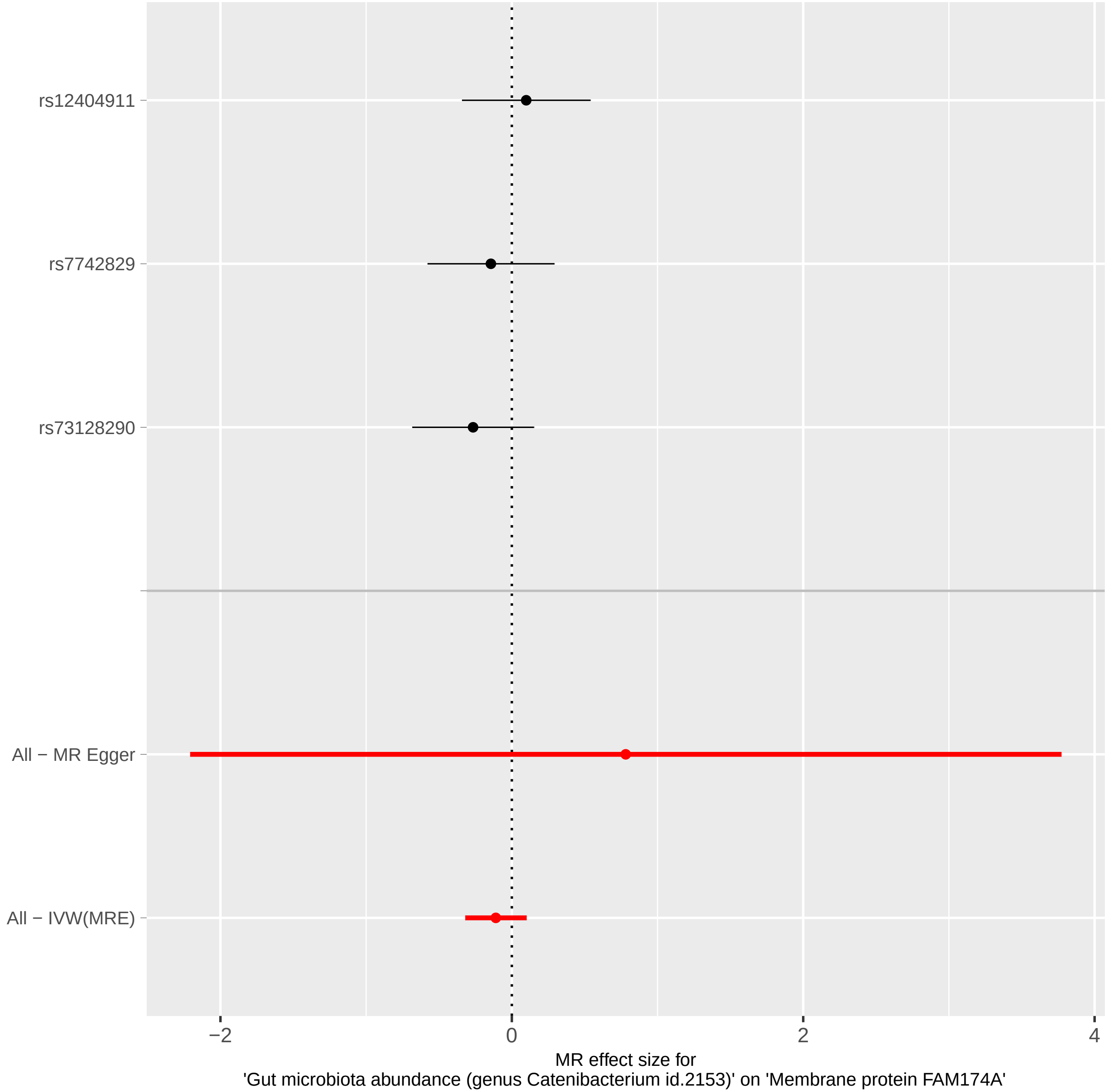

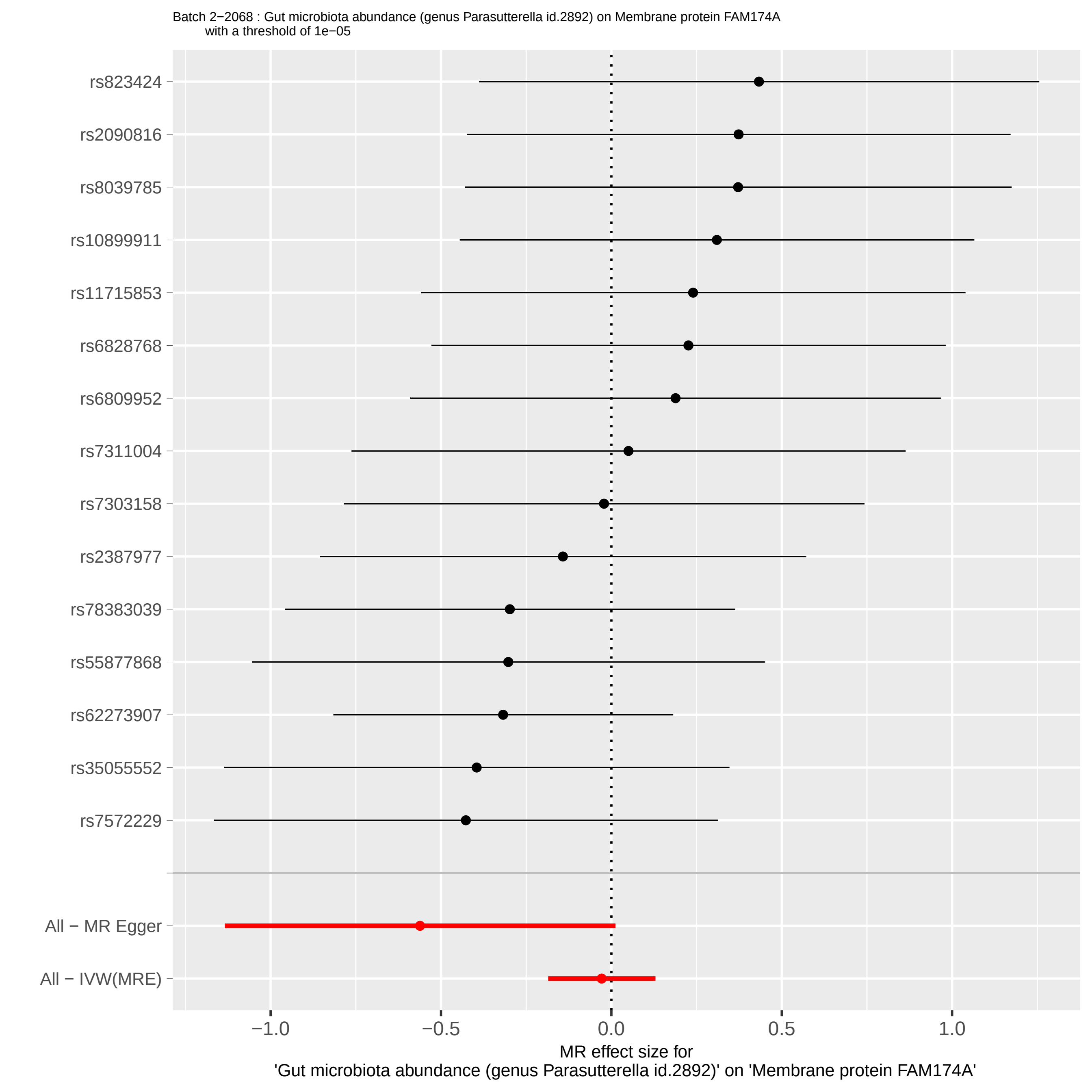

Batch 2−2068 : Gut microbiota abundance (genus Parasutterella id.2892) on Membrane protein FAM174A
with a threshold of 1e−05
rs823424
rs2090816
rs8039785
rs10899911
rs11715853
rs6828768
rs6809952
rs7311004
rs7303158
rs2387977
rs78383039
rs55877868
rs62273907
rs35055552
rs7572229
All − MR Egger
All − IVW(MRE)
−1.0
−0.5
0.0
0.5
1.0
MR effect size for
'Gut microbiota abundance (genus Parasutterella id.2892)' on 'Membrane protein FAM174A'

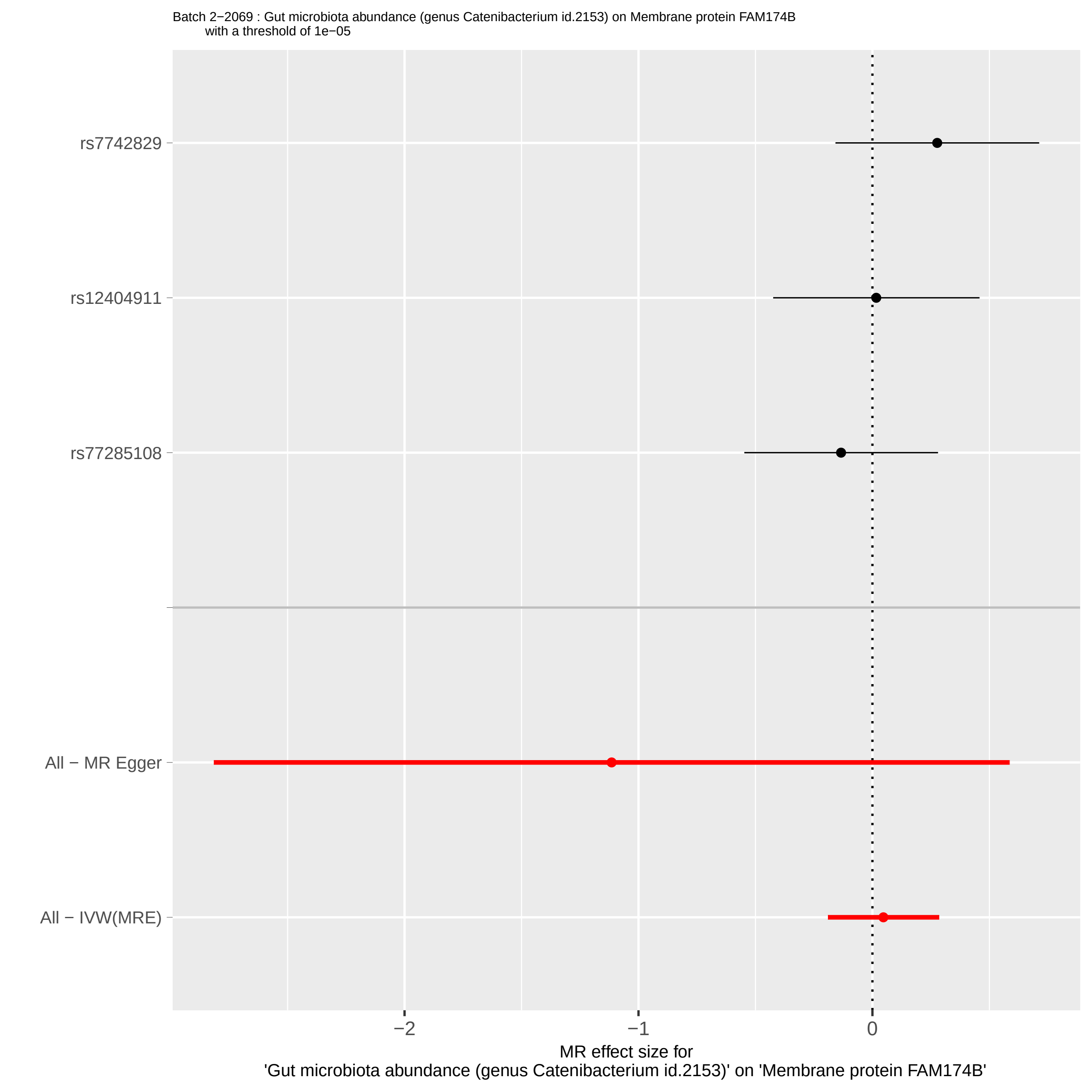

Batch 2−2069 : Gut microbiota abundance (genus Catenibacterium id.2153) on Membrane protein FAM174B
with a threshold of 1e−05
rs7742829
rs12404911
rs77285108
All − MR Egger
All − IVW(MRE)
−2
−1
0
MR effect size for
'Gut microbiota abundance (genus Catenibacterium id.2153)' on 'Membrane protein FAM174B'

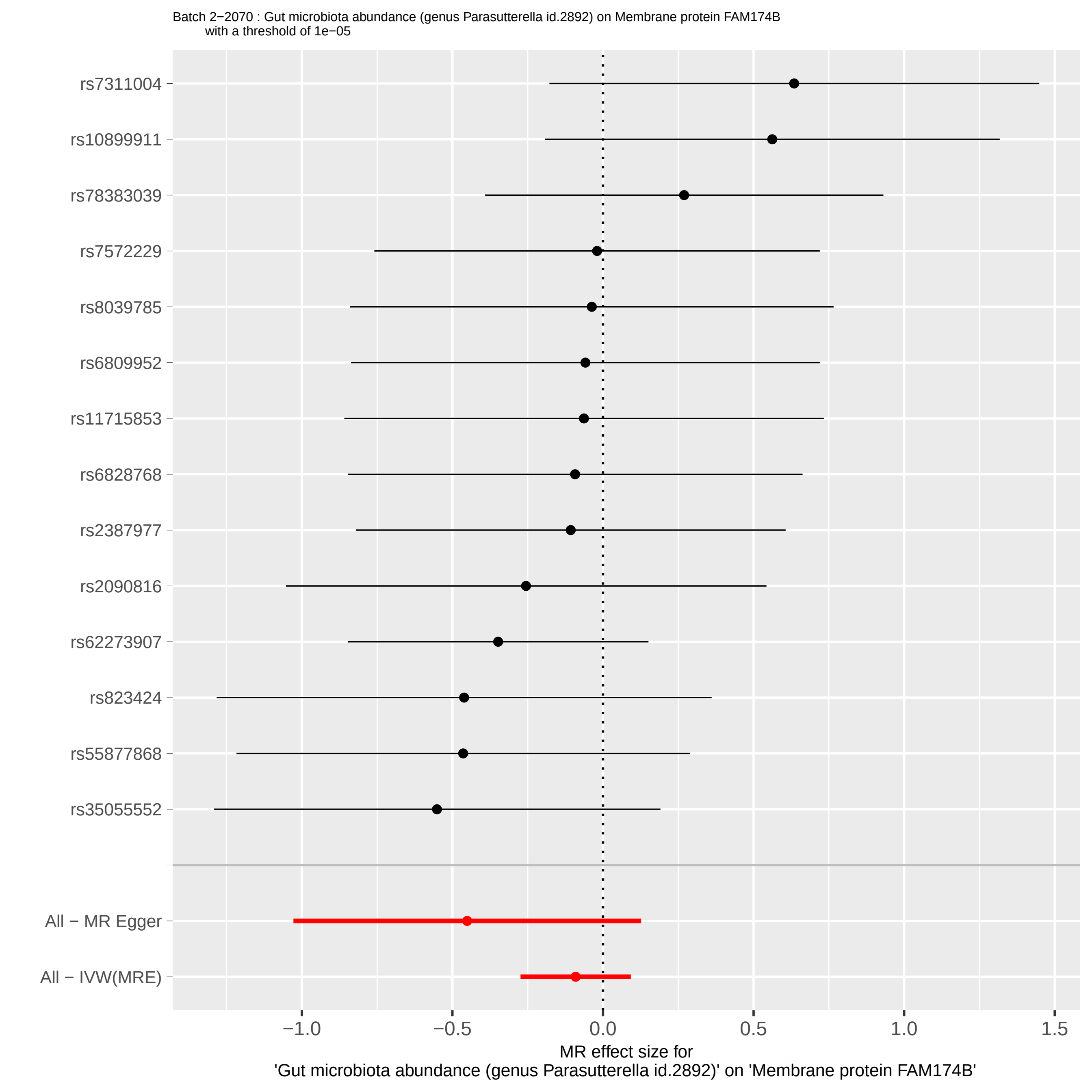
Batch 2−2070 : Gut microbiota abundance (genus Parasutterella id.2892) on Membrane protein FAM174B
with a threshold of 1e−05
rs7311004
rs10899911
rs78383039
rs7572229
rs8039785
rs6809952
rs11715853
rs6828768
rs2387977
rs2090816
rs62273907
rs823424
rs55877868
rs35055552
All − MR Egger
All − IVW(MRE)
−1.0
−0.5
0.0
0.5
1.0
1.5
MR effect size for
'Gut microbiota abundance (genus Parasutterella id.2892)' on 'Membrane protein FAM174B'

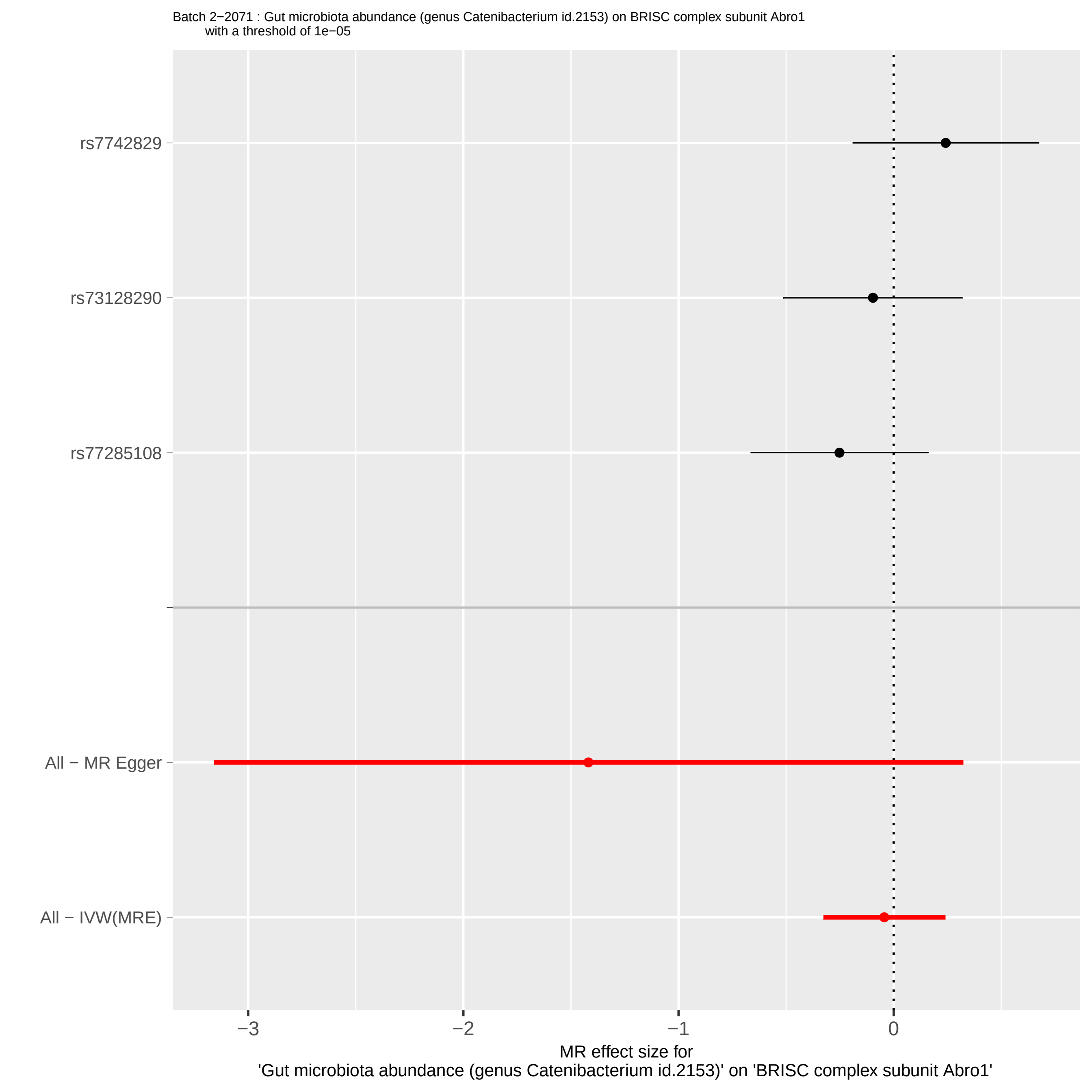
Batch 2−2071 : Gut microbiota abundance (genus Catenibacterium id.2153) on BRISC complex subunit Abro1
with a threshold of 1e−05
rs7742829
rs73128290
rs77285108
All − MR Egger
All − IVW(MRE)
−3
−2
−1
0
MR effect size for
'Gut microbiota abundance (genus Catenibacterium id.2153)' on 'BRISC complex subunit Abro1'

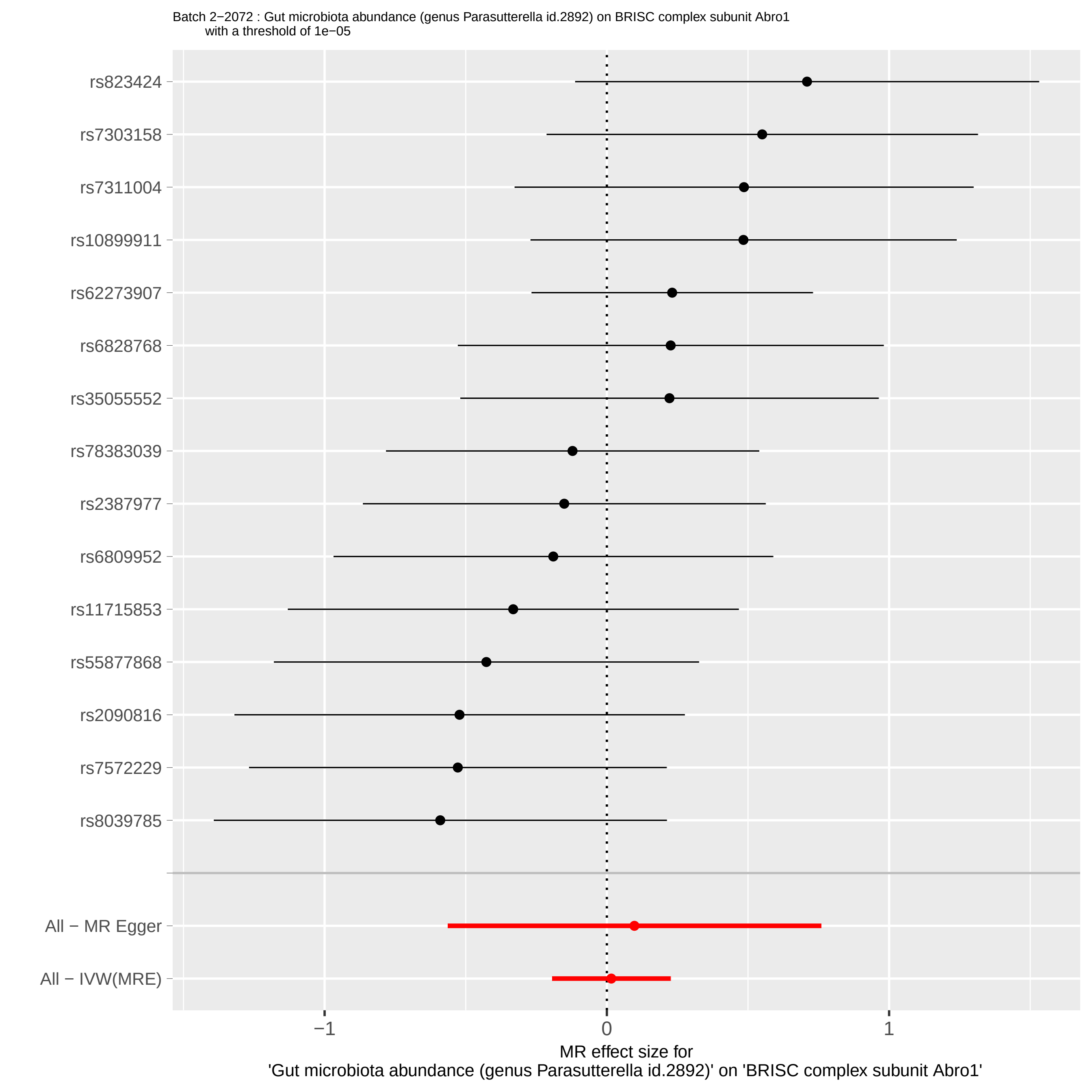

Batch 2−2072 : Gut microbiota abundance (genus Parasutterella id.2892) on BRISC complex subunit Abro1
with a threshold of 1e−05
rs823424
rs7303158
rs7311004
rs10899911
rs62273907
rs6828768
rs35055552
rs78383039
rs2387977
rs6809952
rs11715853
rs55877868
rs2090816
rs7572229
rs8039785
All − MR Egger
All − IVW(MRE)
−1
0
1
MR effect size for
'Gut microbiota abundance (genus Parasutterella id.2892)' on 'BRISC complex subunit Abro1'

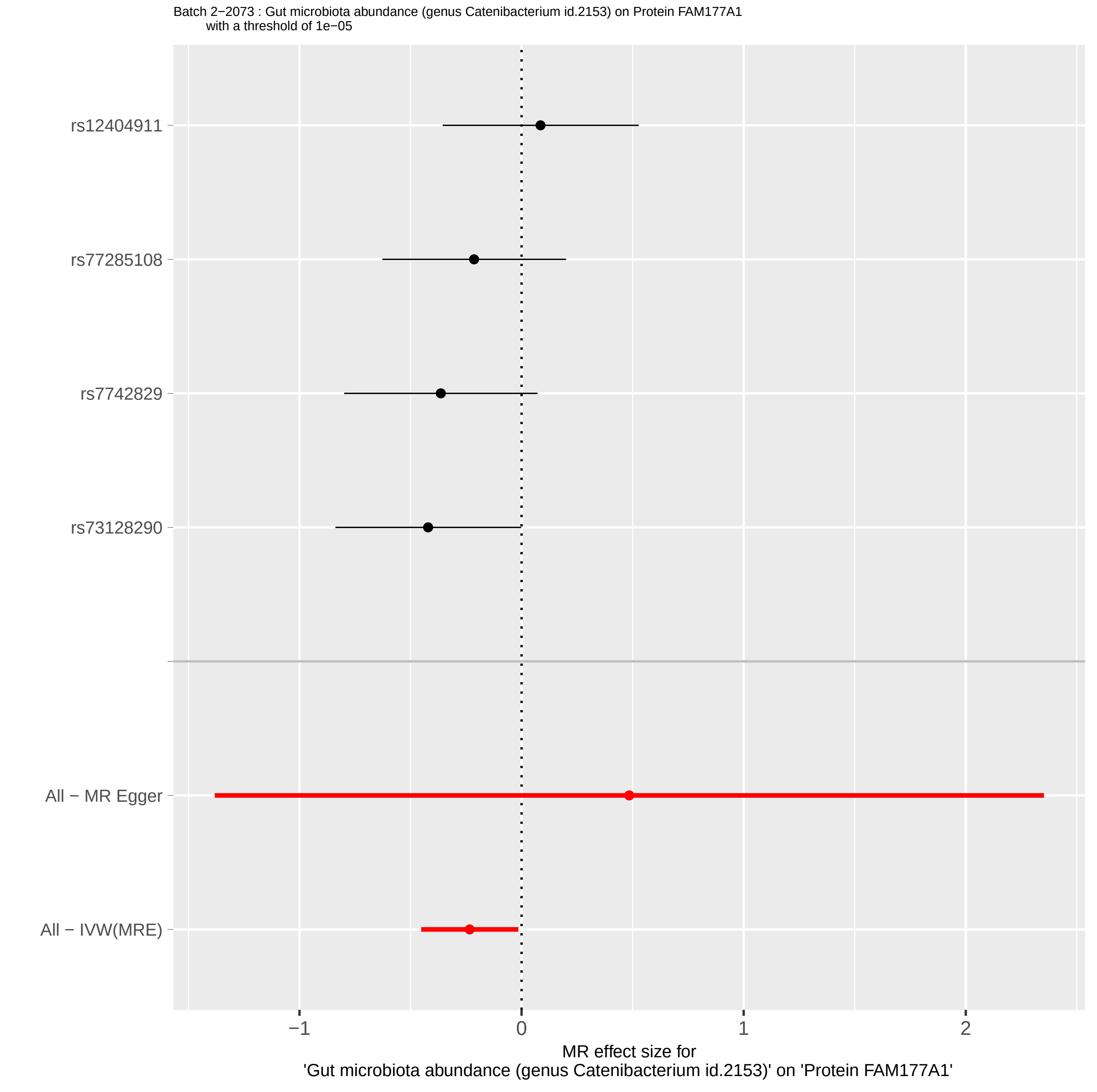

Batch 2−2073 : Gut microbiota abundance (genus Catenibacterium id.2153) on Protein FAM177A1
with a threshold of 1e−05
rs12404911
rs77285108
rs7742829
rs73128290
All − MR Egger
All − IVW(MRE)
−1
0
1
2
MR effect size for
'Gut microbiota abundance (genus Catenibacterium id.2153)' on 'Protein FAM177A1'

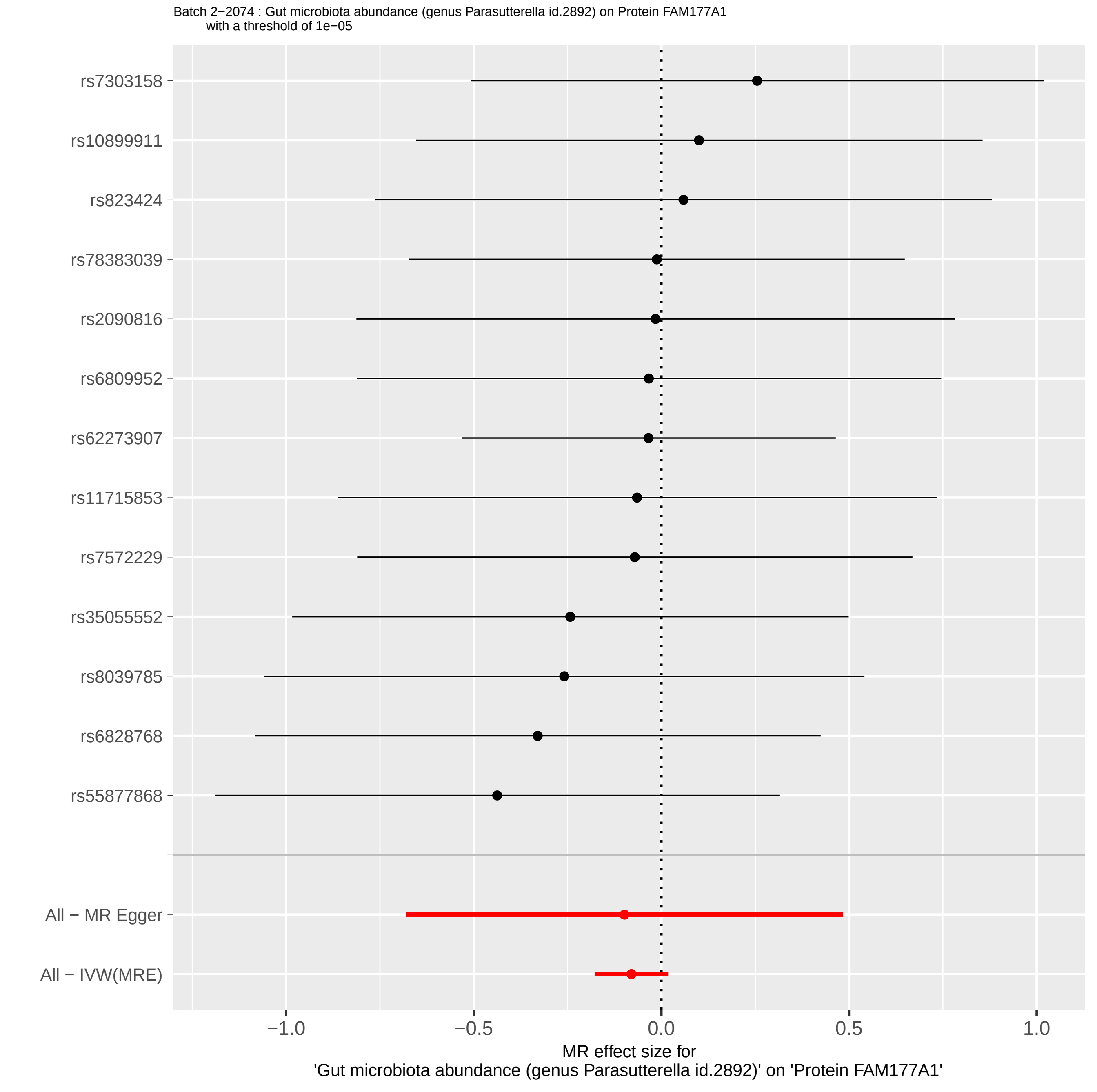
Batch 2−2074 : Gut microbiota abundance (genus Parasutterella id.2892) on Protein FAM177A1
with a threshold of 1e−05
rs7303158
rs10899911
rs823424
rs78383039
rs2090816
rs6809952
rs62273907
rs11715853
rs7572229
rs35055552
rs8039785
rs6828768
rs55877868
All − MR Egger
All − IVW(MRE)
−1.0
−0.5
0.0
0.5
1.0
MR effect size for
'Gut microbiota abundance (genus Parasutterella id.2892)' on 'Protein FAM177A1'

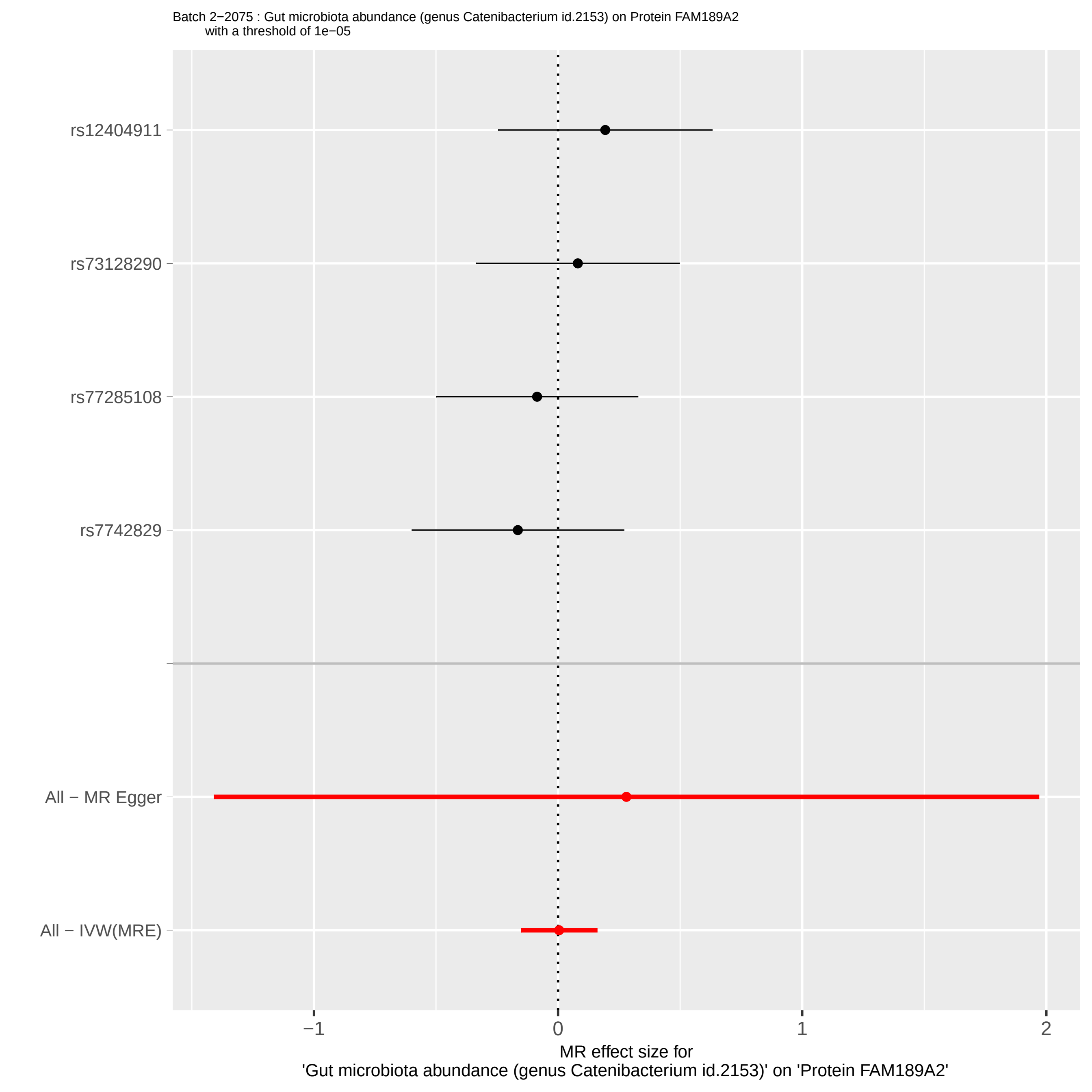
Batch 2−2075 : Gut microbiota abundance (genus Catenibacterium id.2153) on Protein FAM189A2
with a threshold of 1e−05
rs12404911
rs73128290
rs77285108
rs7742829
All − MR Egger
All − IVW(MRE)
−1
0
1
2
MR effect size for
'Gut microbiota abundance (genus Catenibacterium id.2153)' on 'Protein FAM189A2'

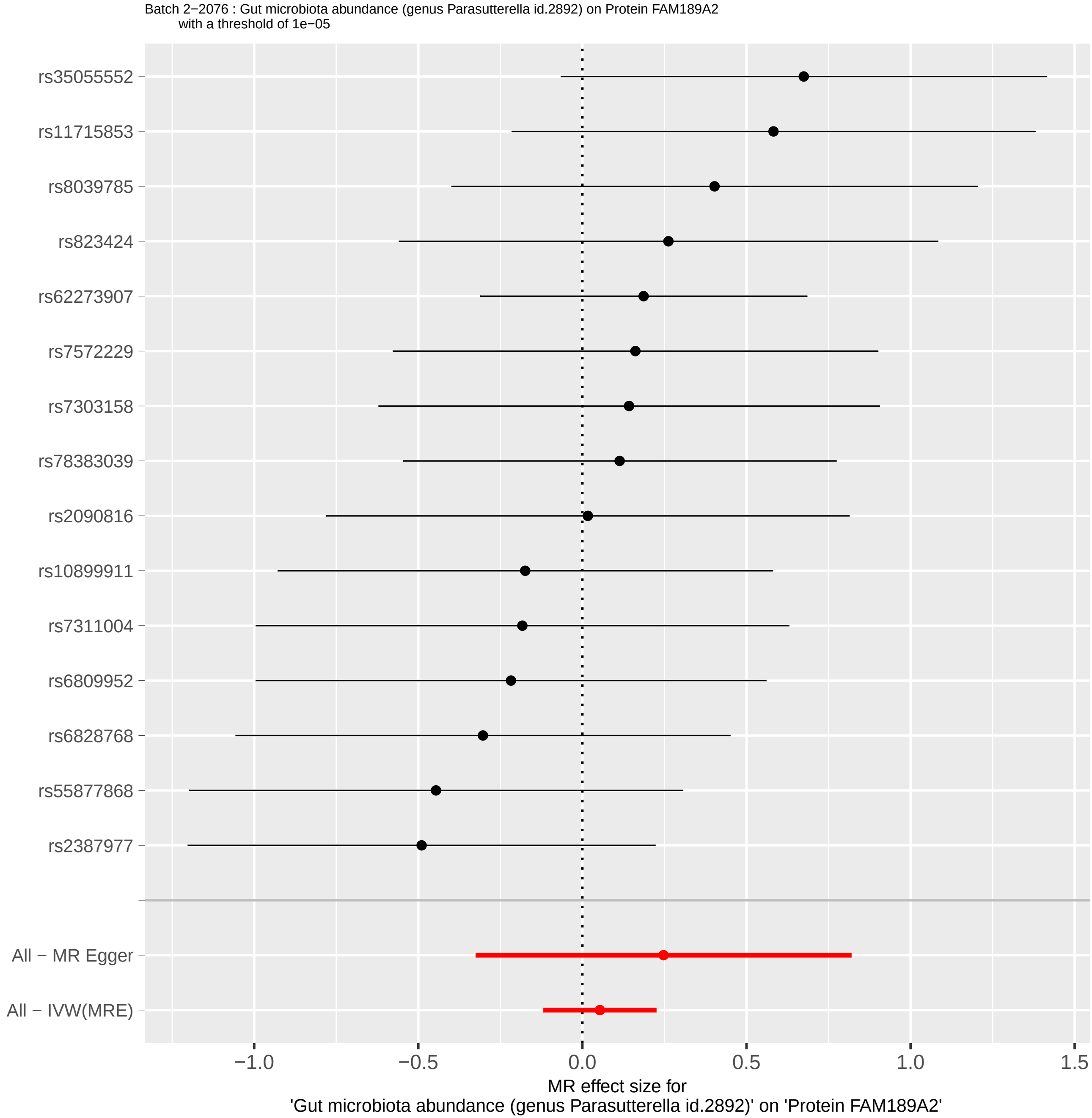

Batch 2−2076 : Gut microbiota abundance (genus Parasutterella id.2892) on Protein FAM189A2
with a threshold of 1e−05
rs35055552
rs11715853
rs8039785
rs823424
rs62273907
rs7572229
rs7303158
rs78383039
rs2090816
rs10899911
rs7311004
rs6809952
rs6828768
rs55877868
rs2387977
All − MR Egger
All − IVW(MRE)
−1.0
−0.5
0.0
0.5
1.0
1.5
MR effect size for
'Gut microbiota abundance (genus Parasutterella id.2892)' on 'Protein FAM189A2'

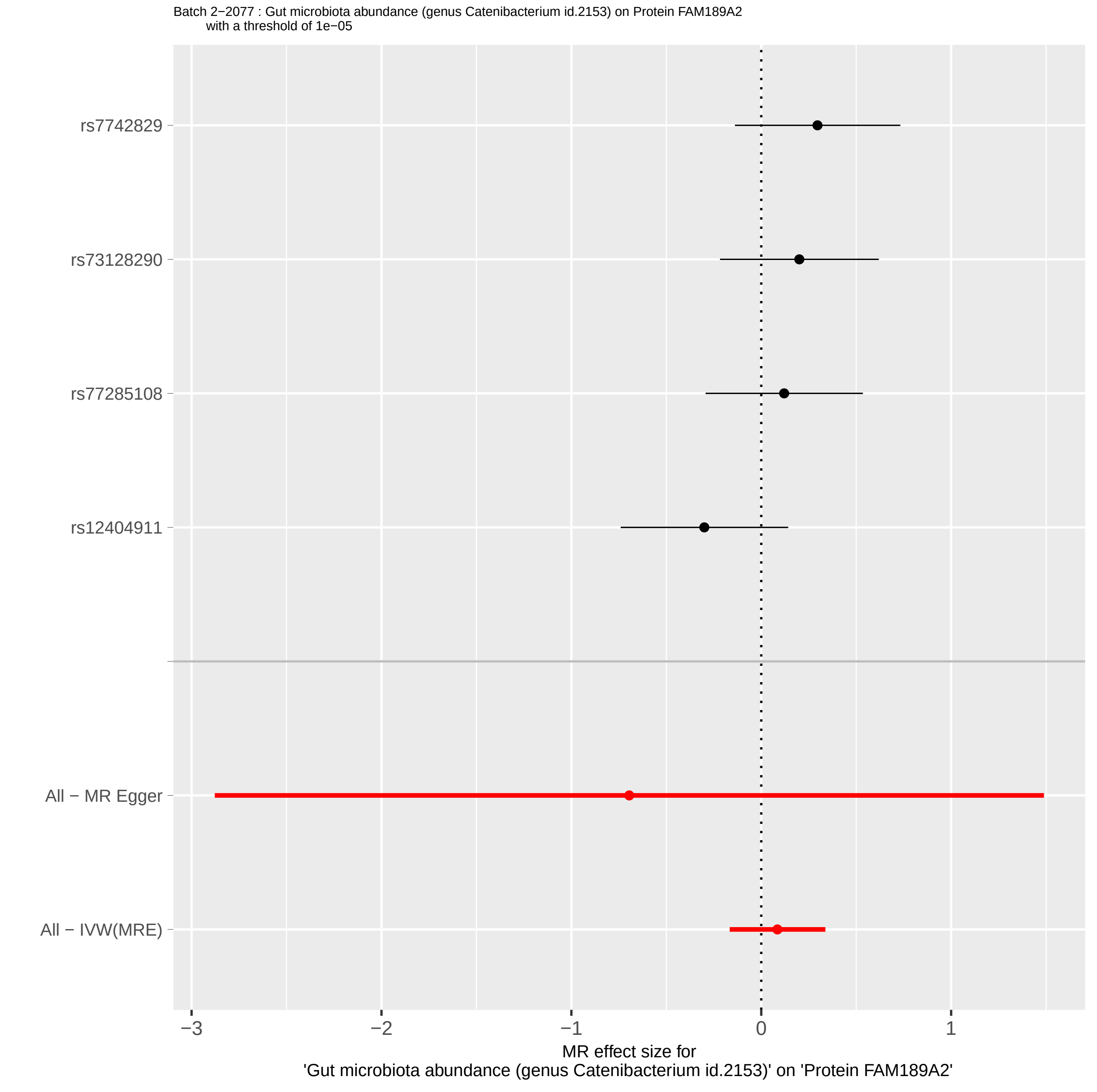
Batch 2−2077 : Gut microbiota abundance (genus Catenibacterium id.2153) on Protein FAM189A2
with a threshold of 1e−05
rs7742829
rs73128290
rs77285108
rs12404911
All − MR Egger
All − IVW(MRE)
−3
−2
−1
0
1
MR effect size for
'Gut microbiota abundance (genus Catenibacterium id.2153)' on 'Protein FAM189A2'

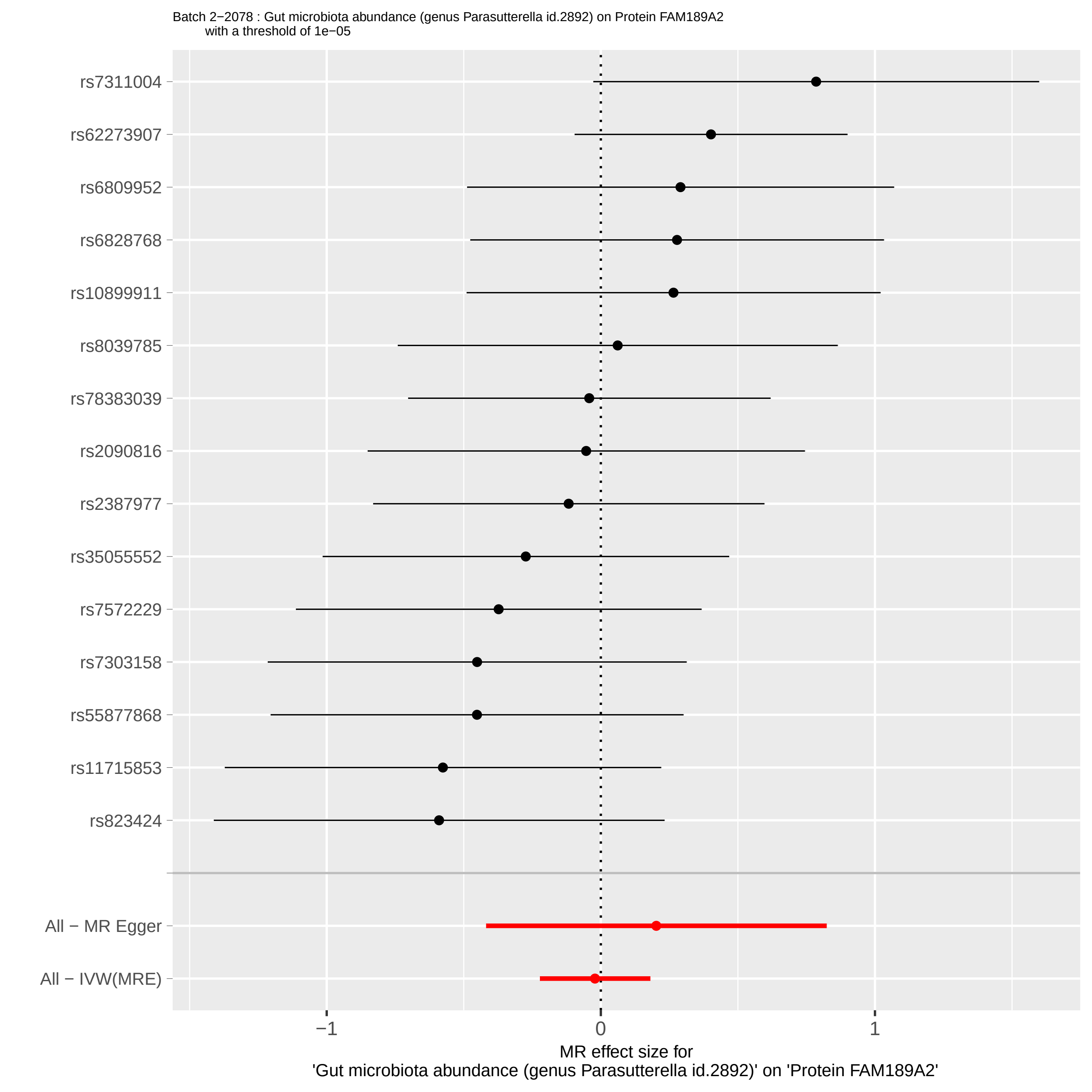

Batch 2−2078 : Gut microbiota abundance (genus Parasutterella id.2892) on Protein FAM189A2
with a threshold of 1e−05
rs7311004
rs62273907
rs6809952
rs6828768
rs10899911
rs8039785
rs78383039
rs2090816
rs2387977
rs35055552
rs7572229
rs7303158
rs55877868
rs11715853
rs823424
All − MR Egger
All − IVW(MRE)
−1
0
1
MR effect size for
'Gut microbiota abundance (genus Parasutterella id.2892)' on 'Protein FAM189A2'

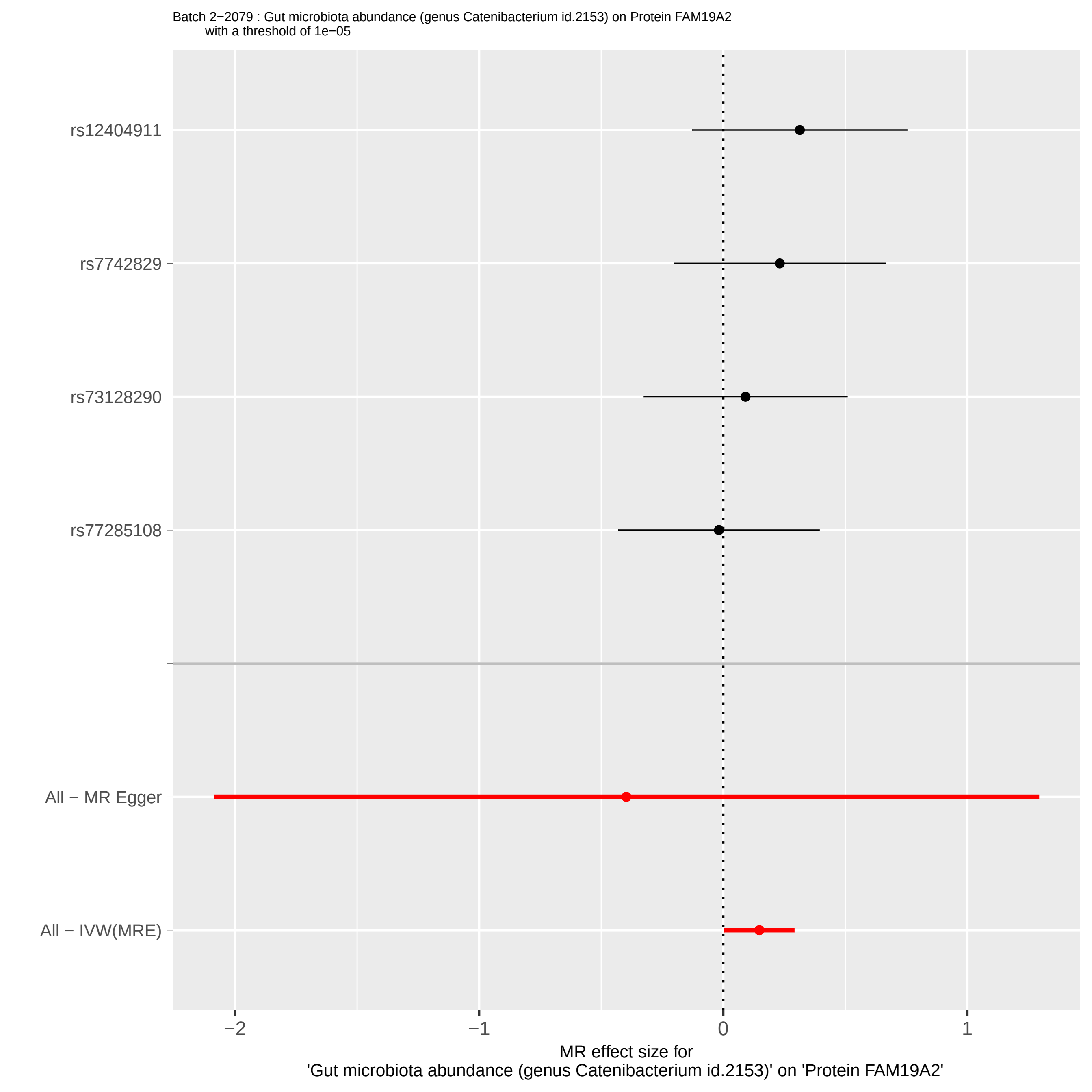
Batch 2−2079 : Gut microbiota abundance (genus Catenibacterium id.2153) on Protein FAM19A2
with a threshold of 1e−05
rs12404911
rs7742829
rs73128290
rs77285108
All − MR Egger
All − IVW(MRE)
−2
−1
0
1
MR effect size for
'Gut microbiota abundance (genus Catenibacterium id.2153)' on 'Protein FAM19A2'

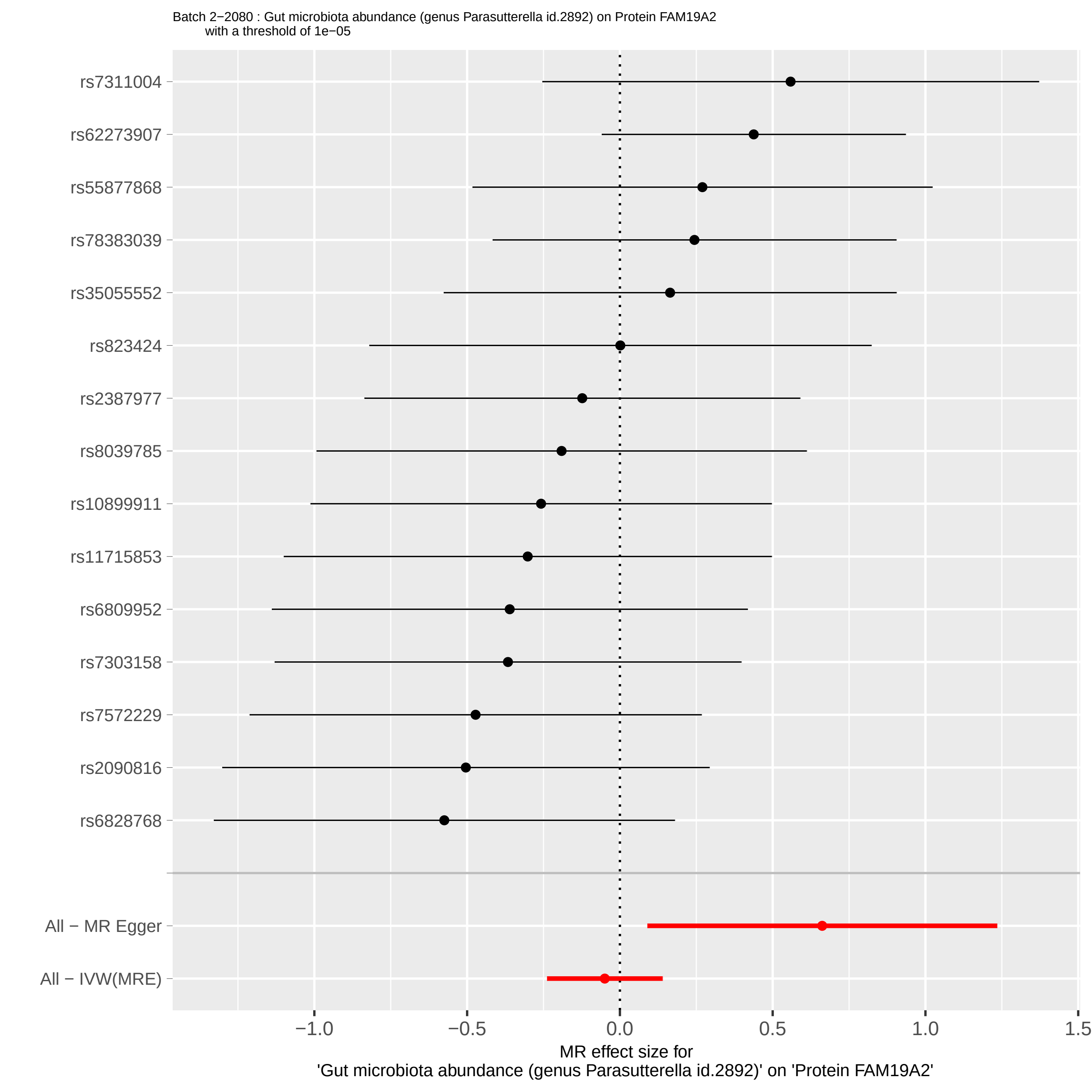
Batch 2−2080 : Gut microbiota abundance (genus Parasutterella id.2892) on Protein FAM19A2
with a threshold of 1e−05
rs7311004
rs62273907
rs55877868
rs78383039
rs35055552
rs823424
rs2387977
rs8039785
rs10899911
rs11715853
rs6809952
rs7303158
rs7572229
rs2090816
rs6828768
All − MR Egger
All − IVW(MRE)
−1.0
−0.5
0.0
0.5
1.0
1.5
MR effect size for
'Gut microbiota abundance (genus Parasutterella id.2892)' on 'Protein FAM19A2'

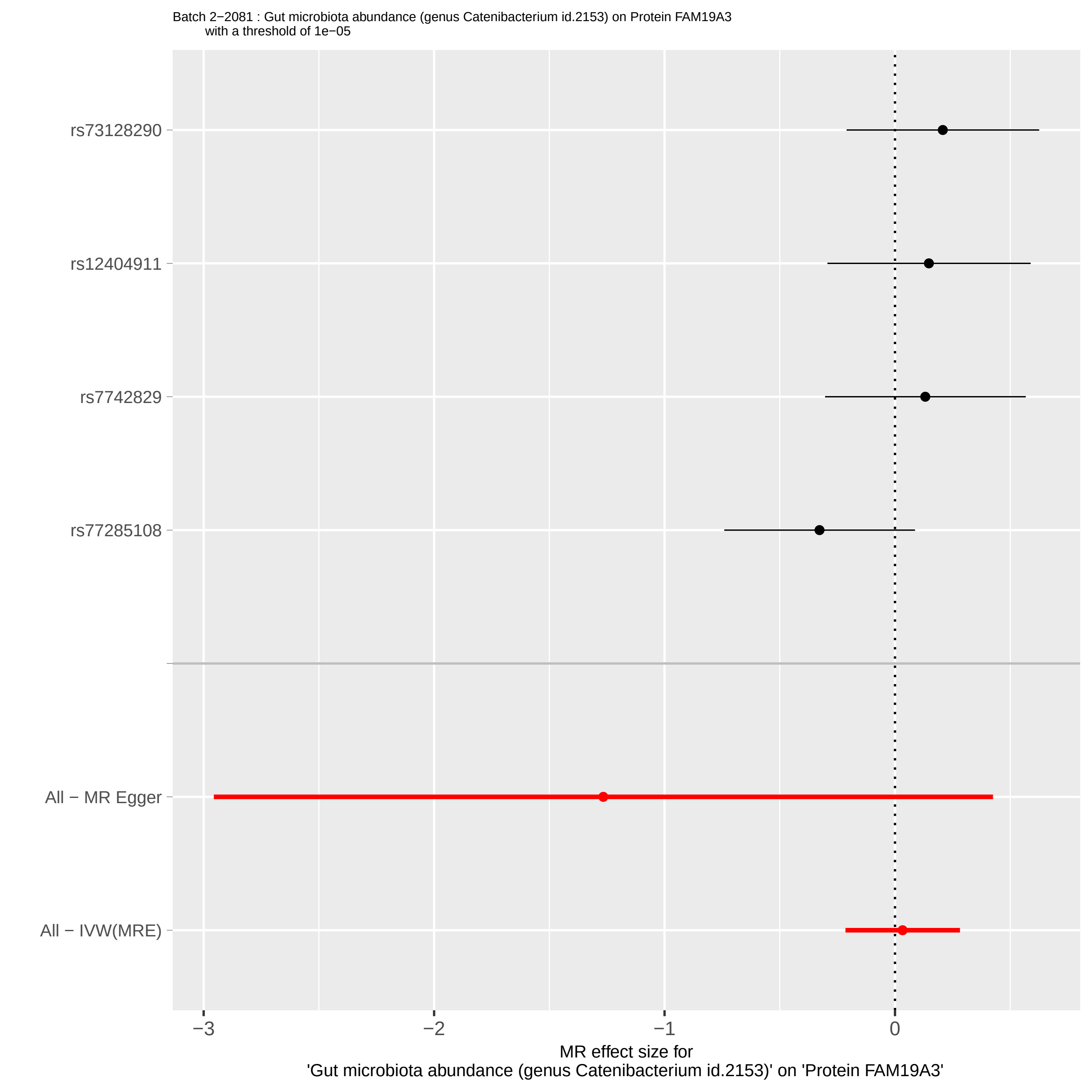

Batch 2−2081 : Gut microbiota abundance (genus Catenibacterium id.2153) on Protein FAM19A3
with a threshold of 1e−05
rs73128290
rs12404911
rs7742829
rs77285108
All − MR Egger
All − IVW(MRE)
−3
−2
−1
0
MR effect size for
'Gut microbiota abundance (genus Catenibacterium id.2153)' on 'Protein FAM19A3'

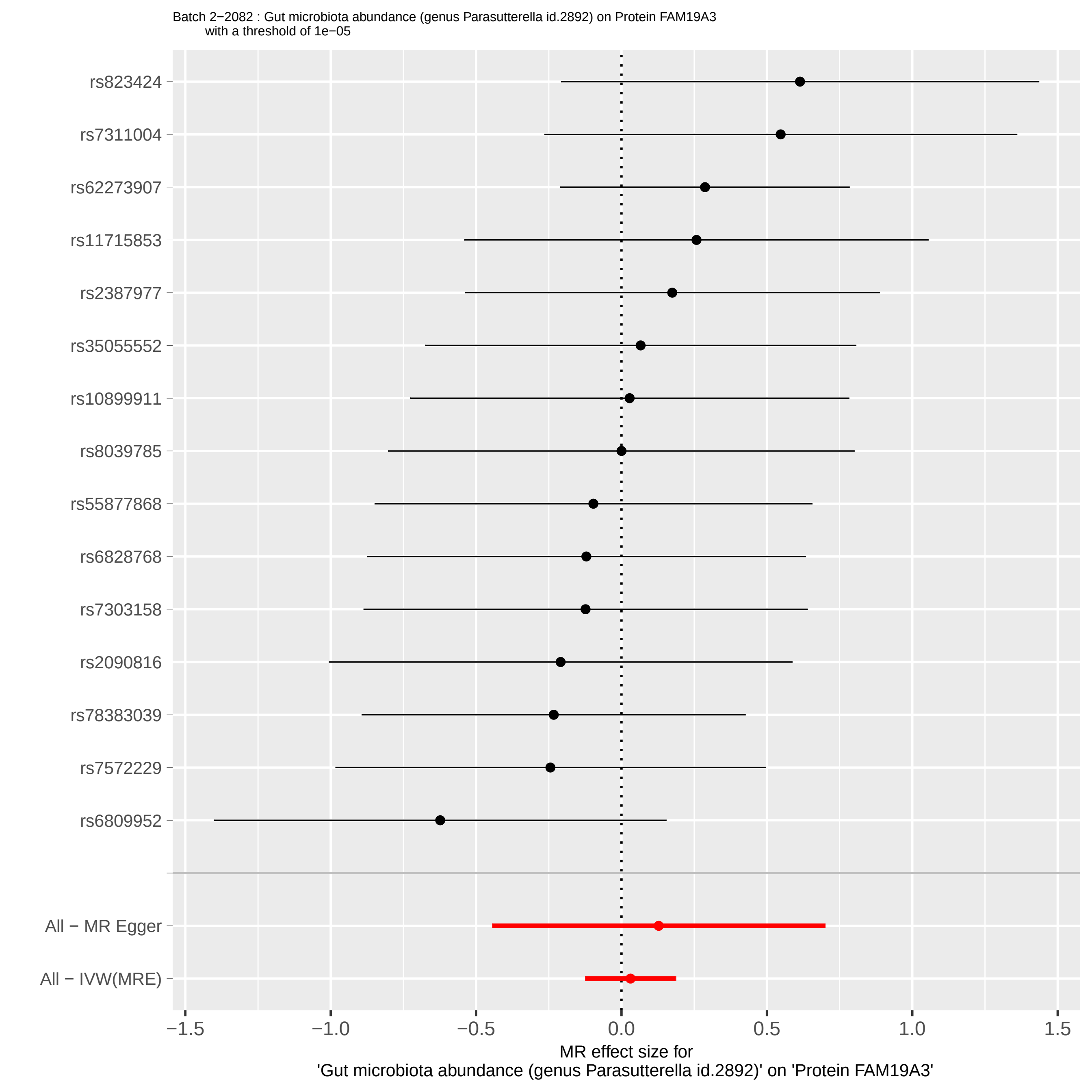
Batch 2−2082 : Gut microbiota abundance (genus Parasutterella id.2892) on Protein FAM19A3
with a threshold of 1e−05
rs823424
rs7311004
rs62273907
rs11715853
rs2387977
rs35055552
rs10899911
rs8039785
rs55877868
rs6828768
rs7303158
rs2090816
rs78383039
rs7572229
rs6809952
All − MR Egger
All − IVW(MRE)
−1.5
−1.0
−0.5
0.0
0.5
1.0
1.5
MR effect size for
'Gut microbiota abundance (genus Parasutterella id.2892)' on 'Protein FAM19A3'

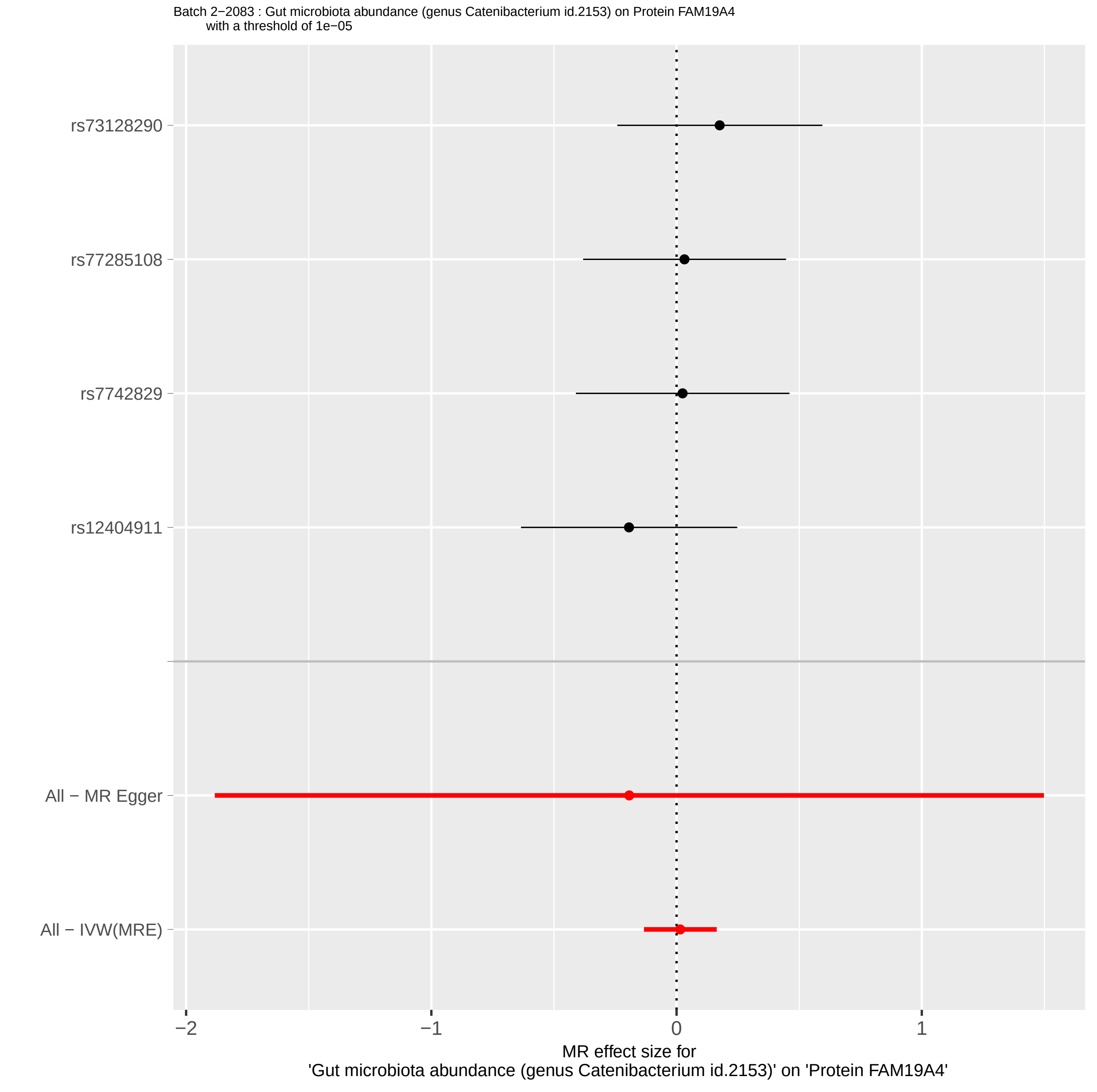
Batch 2−2083 : Gut microbiota abundance (genus Catenibacterium id.2153) on Protein FAM19A4
with a threshold of 1e−05
rs73128290
rs77285108
rs7742829
rs12404911
All − MR Egger
All − IVW(MRE)
−2
−1
0
1
MR effect size for
'Gut microbiota abundance (genus Catenibacterium id.2153)' on 'Protein FAM19A4'

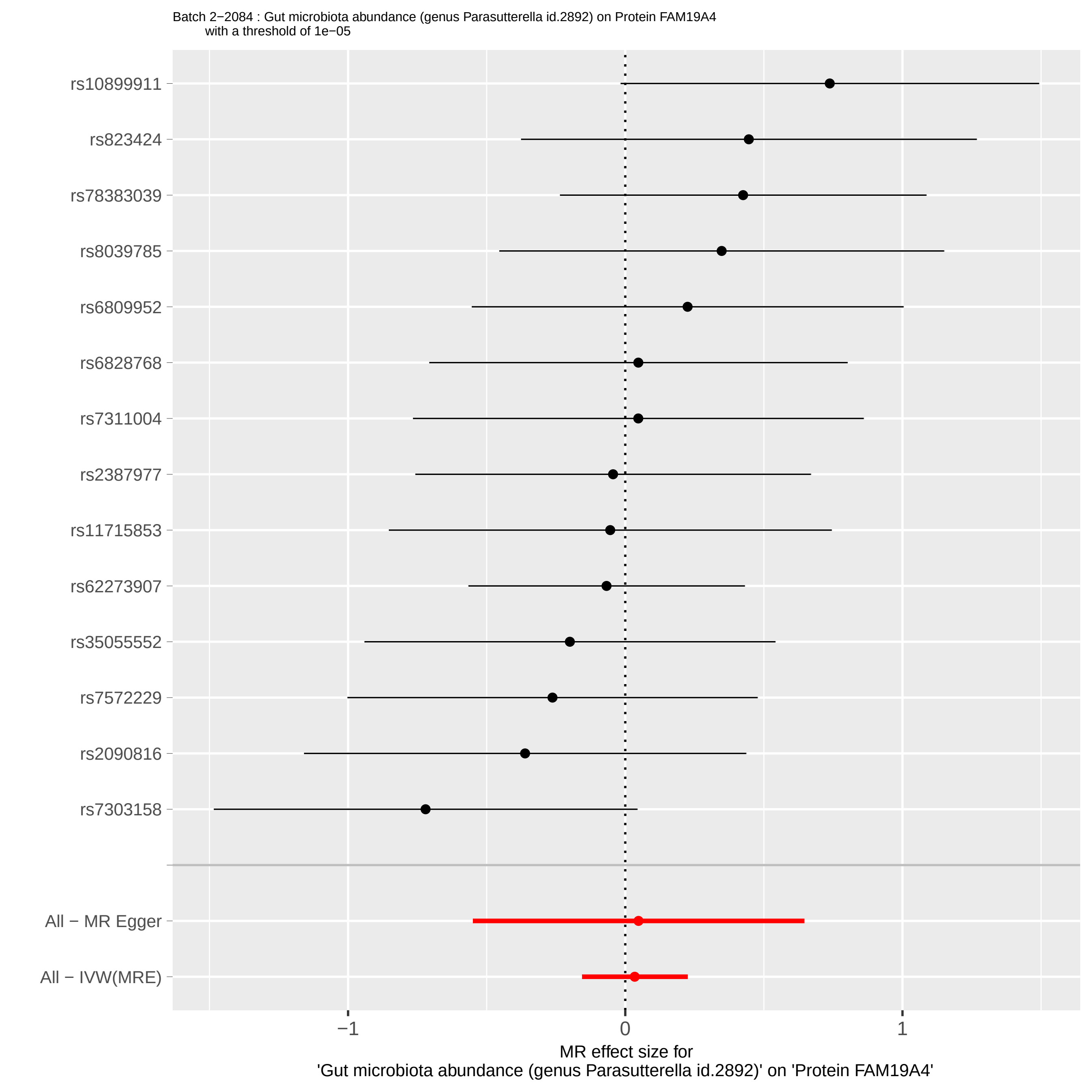
Batch 2−2084 : Gut microbiota abundance (genus Parasutterella id.2892) on Protein FAM19A4
with a threshold of 1e−05
rs10899911
rs823424
rs78383039
rs8039785
rs6809952
rs6828768
rs7311004
rs2387977
rs11715853
rs62273907
rs35055552
rs7572229
rs2090816
rs7303158
All − MR Egger
All − IVW(MRE)
−1
0
1
MR effect size for
'Gut microbiota abundance (genus Parasutterella id.2892)' on 'Protein FAM19A4'

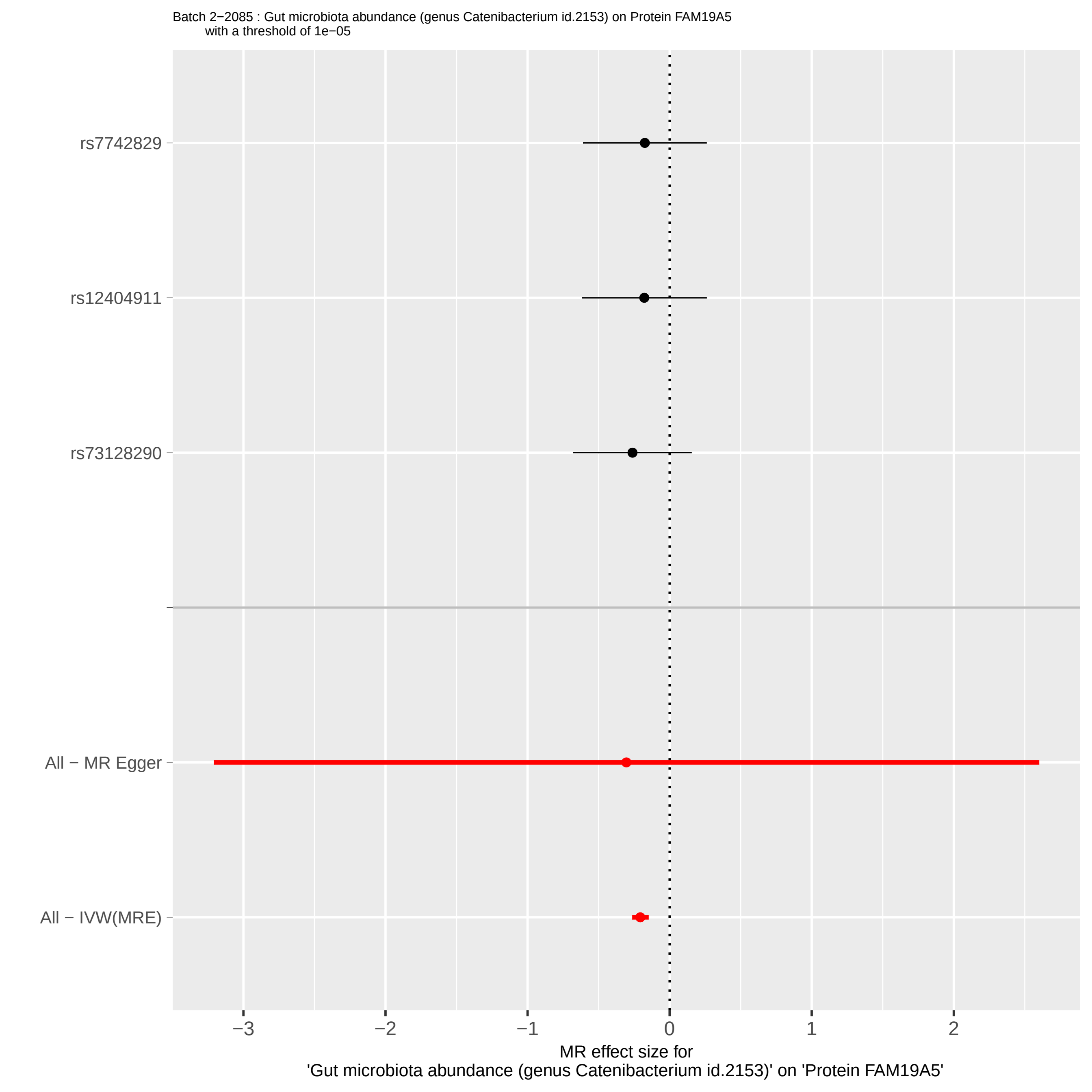

Batch 2−2085 : Gut microbiota abundance (genus Catenibacterium id.2153) on Protein FAM19A5
with a threshold of 1e−05
rs7742829
rs12404911
rs73128290
All − MR Egger
All − IVW(MRE)
−3
−2
−1
0
1
2
MR effect size for
'Gut microbiota abundance (genus Catenibacterium id.2153)' on 'Protein FAM19A5'

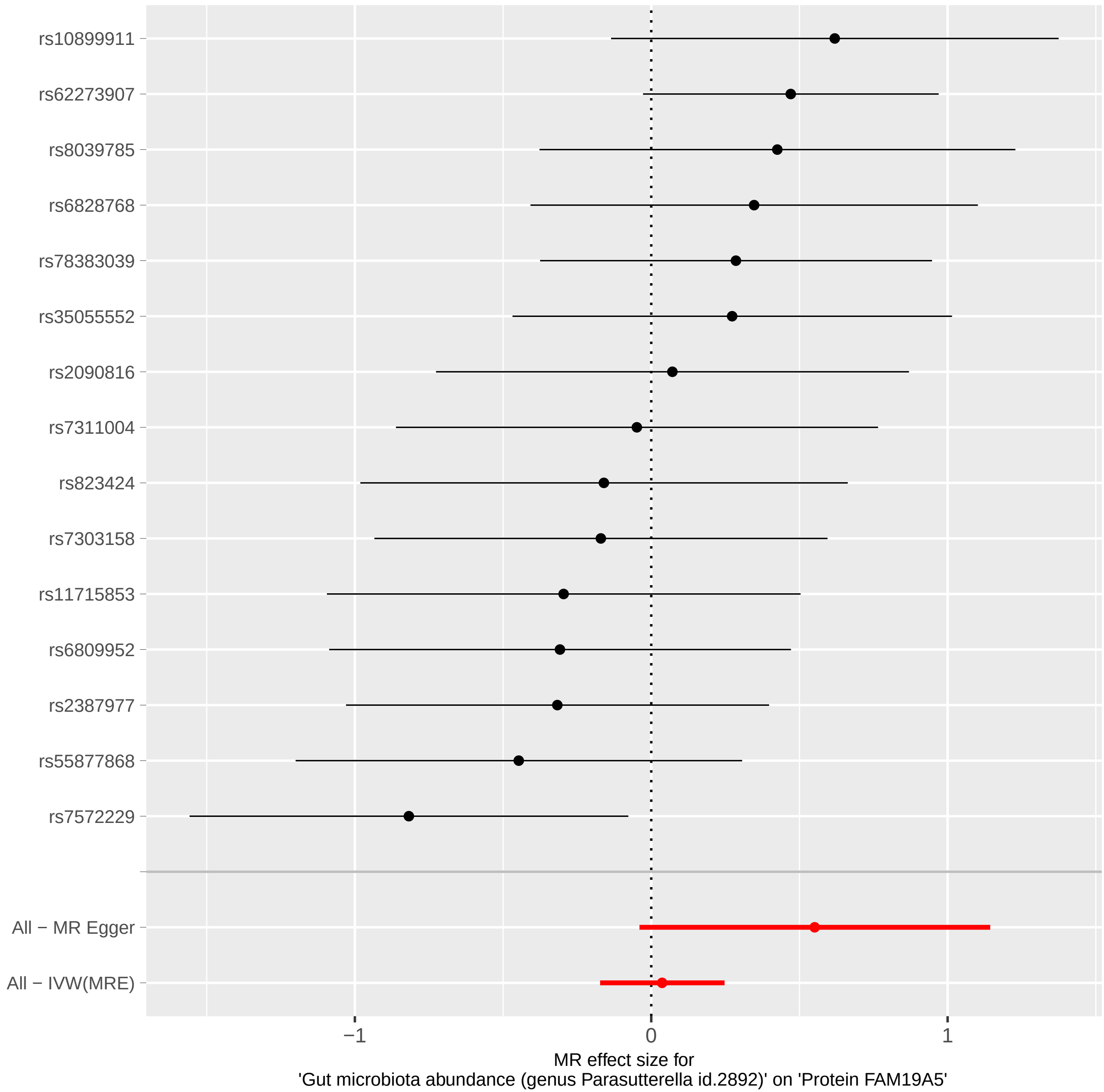

Batch 2−2086 : Gut microbiota abundance (genus Parasutterella id.2892) on Protein FAM19A5
with a threshold of 1e−05
rs10899911
rs62273907
rs8039785
rs6828768
rs78383039
rs35055552
rs2090816
rs7311004
rs823424
rs7303158
rs11715853
rs6809952
rs2387977
rs55877868
rs7572229
All − MR Egger
All − IVW(MRE)
−1
0
1
MR effect size for
'Gut microbiota abundance (genus Parasutterella id.2892)' on 'Protein FAM19A5'

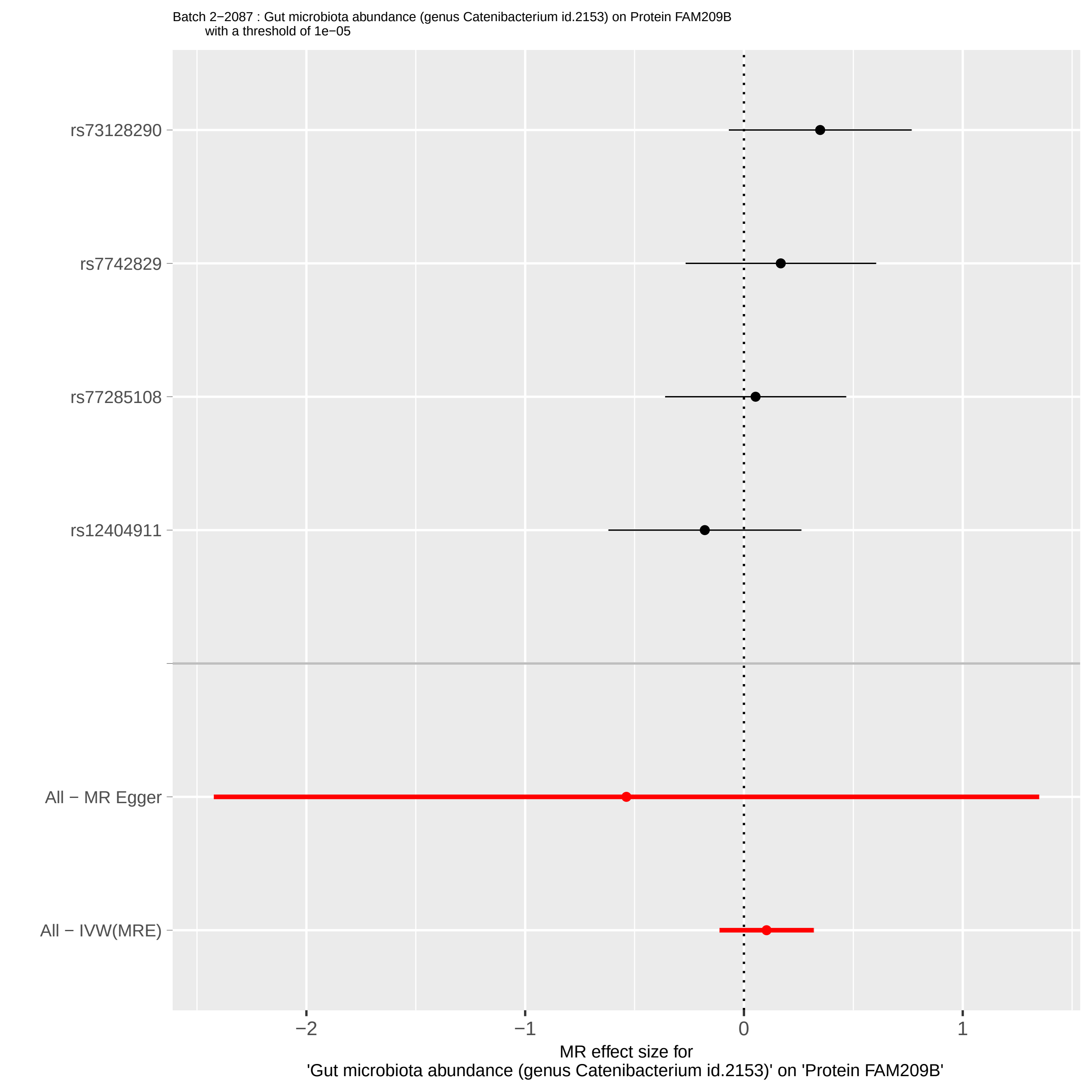

Batch 2−2087 : Gut microbiota abundance (genus Catenibacterium id.2153) on Protein FAM209B
with a threshold of 1e−05
rs73128290
rs7742829
rs77285108
rs12404911
All − MR Egger
All − IVW(MRE)
−2
−1
0
1
MR effect size for
'Gut microbiota abundance (genus Catenibacterium id.2153)' on 'Protein FAM209B'

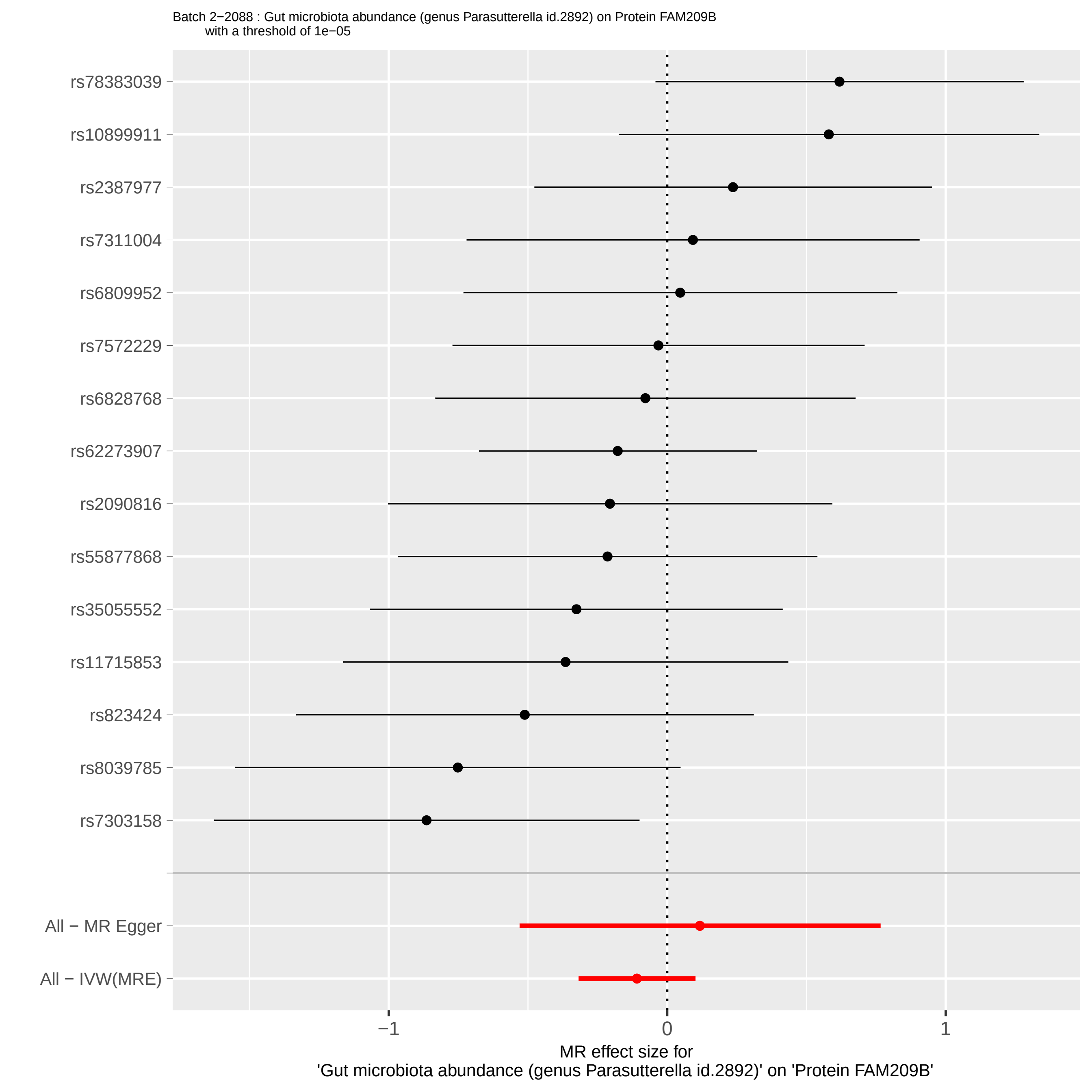

Batch 2−2088 : Gut microbiota abundance (genus Parasutterella id.2892) on Protein FAM209B
with a threshold of 1e−05
rs78383039
rs10899911
rs2387977
rs7311004
rs6809952
rs7572229
rs6828768
rs62273907
rs2090816
rs55877868
rs35055552
rs11715853
rs823424
rs8039785
rs7303158
All − MR Egger
All − IVW(MRE)
−1
0
1
MR effect size for
'Gut microbiota abundance (genus Parasutterella id.2892)' on 'Protein FAM209B'

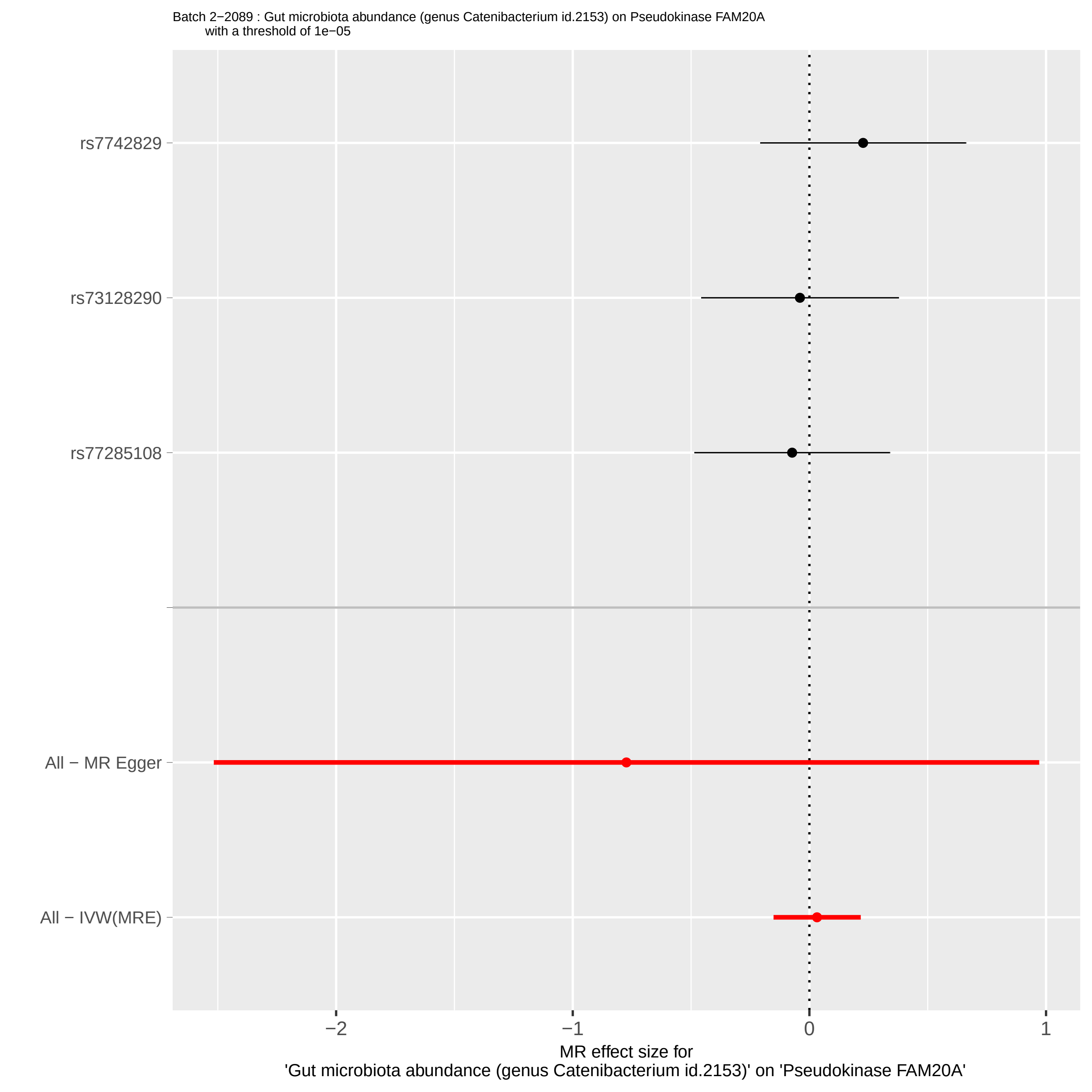
Batch 2−2089 : Gut microbiota abundance (genus Catenibacterium id.2153) on Pseudokinase FAM20A
with a threshold of 1e−05
rs7742829
rs73128290
rs77285108
All − MR Egger
All − IVW(MRE)
−2
−1
0
1
MR effect size for
'Gut microbiota abundance (genus Catenibacterium id.2153)' on 'Pseudokinase FAM20A'

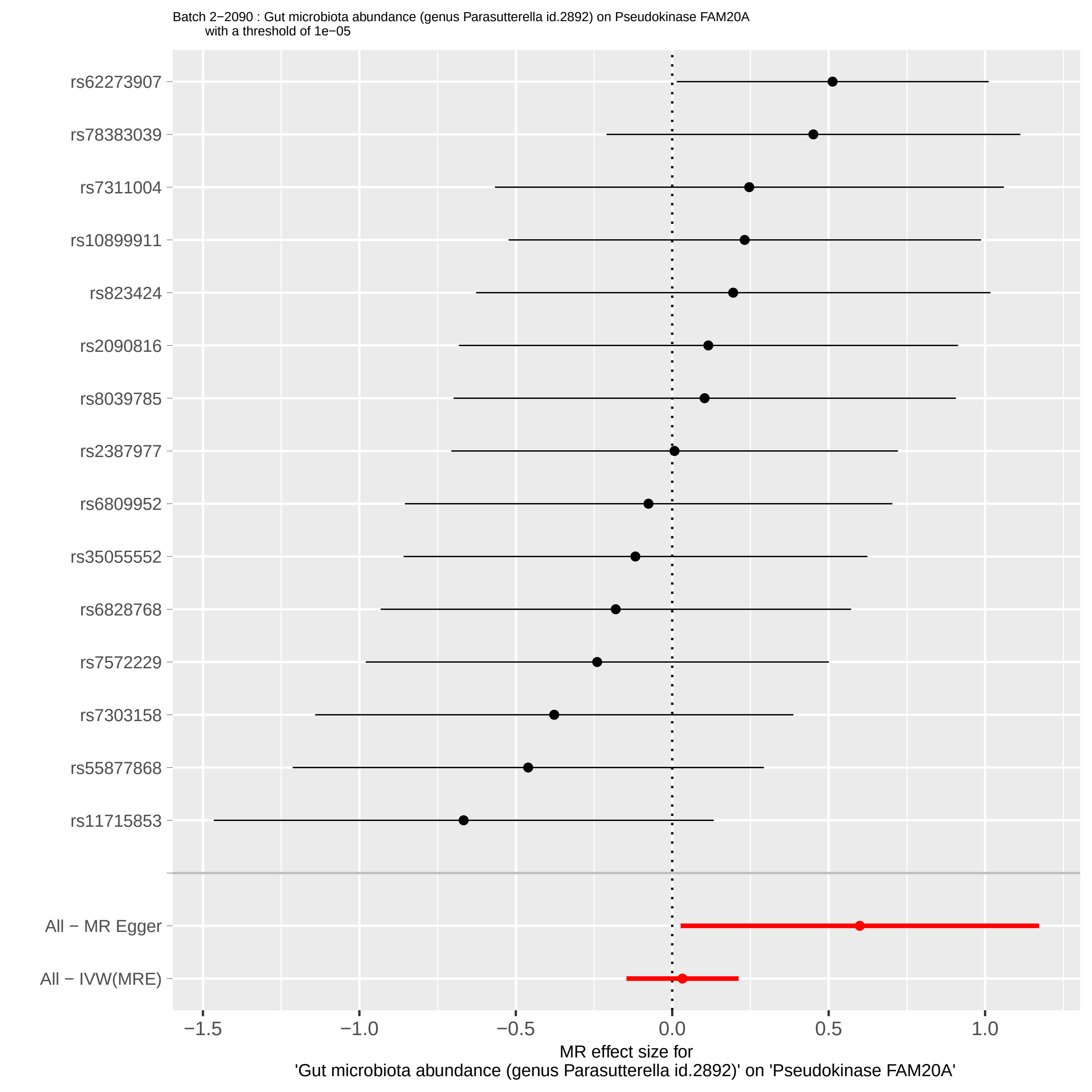
Batch 2−2090 : Gut microbiota abundance (genus Parasutterella id.2892) on Pseudokinase FAM20A
with a threshold of 1e−05
rs62273907
rs78383039
rs7311004
rs10899911
rs823424
rs2090816
rs8039785
rs2387977
rs6809952
rs35055552
rs6828768
rs7572229
rs7303158
rs55877868
rs11715853
All − MR Egger
All − IVW(MRE)
−1.5
−1.0
−0.5
0.0
0.5
1.0
MR effect size for
'Gut microbiota abundance (genus Parasutterella id.2892)' on 'Pseudokinase FAM20A'

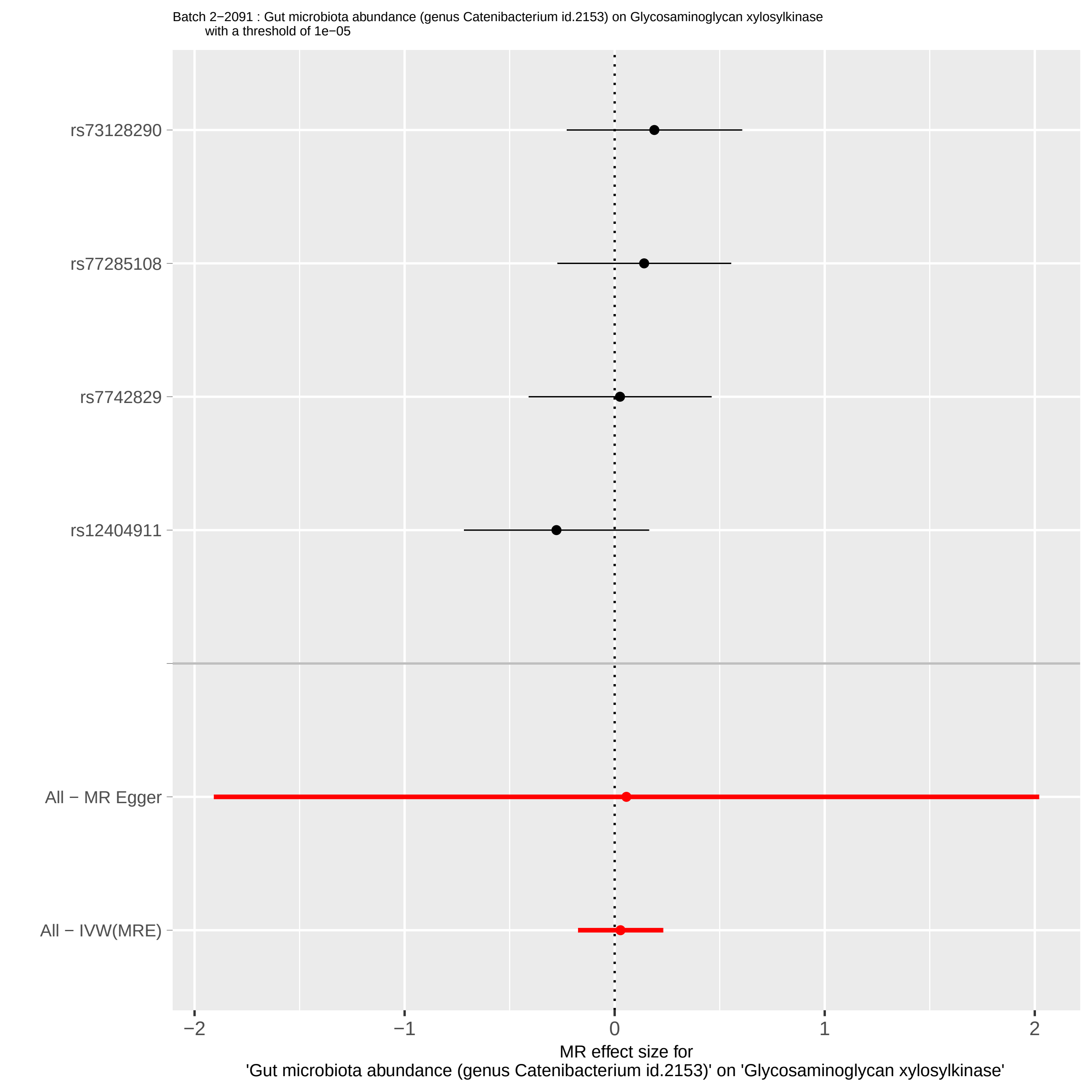

Batch 2−2091 : Gut microbiota abundance (genus Catenibacterium id.2153) on Glycosaminoglycan xylosylkinase
with a threshold of 1e−05
rs73128290
rs77285108
rs7742829
rs12404911
All − MR Egger
All − IVW(MRE)
−2
−1
0
1
2
MR effect size for
'Gut microbiota abundance (genus Catenibacterium id.2153)' on 'Glycosaminoglycan xylosylkinase'

Batch 2−2092 : Gut microbiota abundance (genus Parasutterella id.2892) on Glycosaminoglycan xylosylkinase
with a threshold of 1e−05

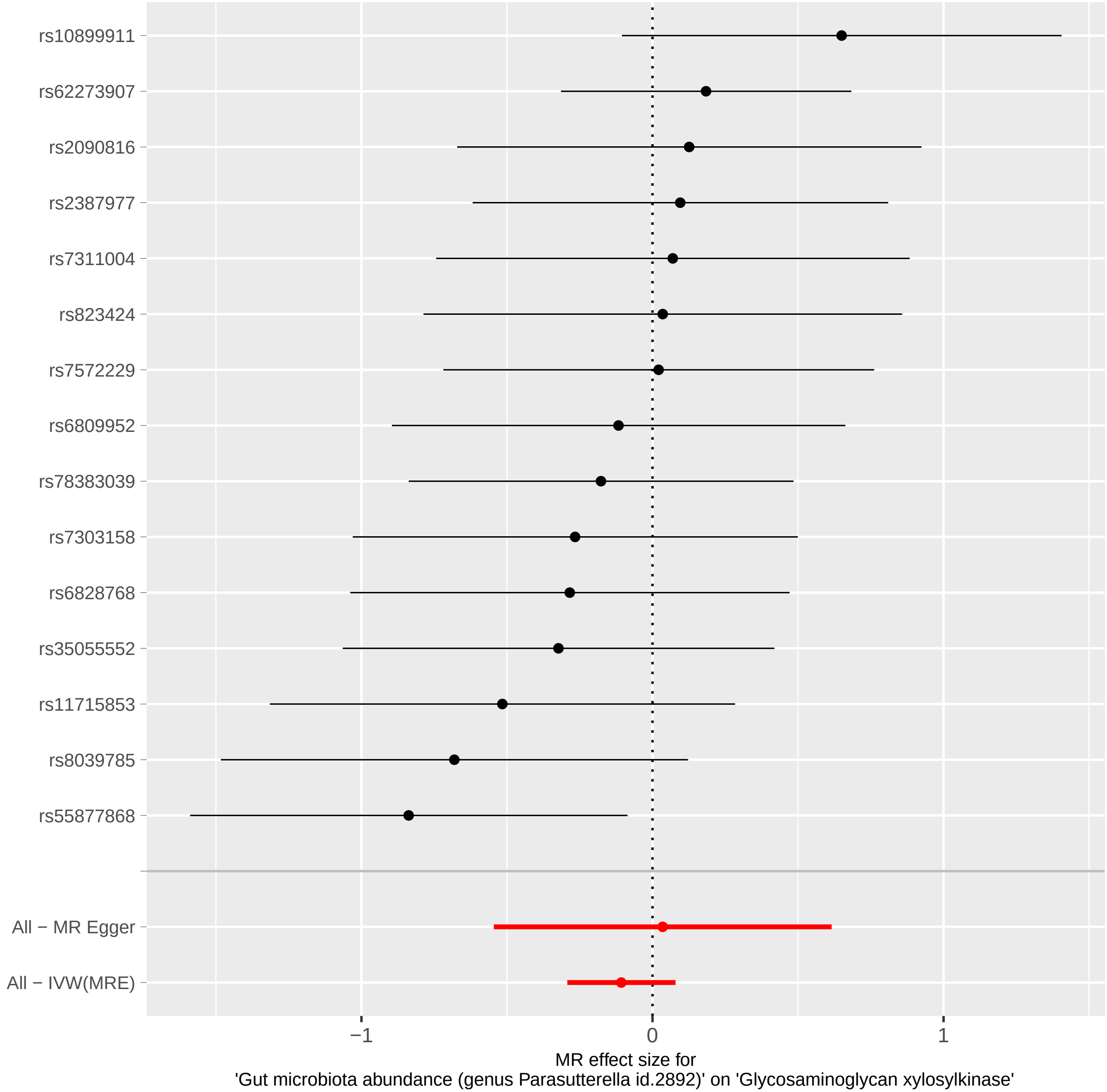

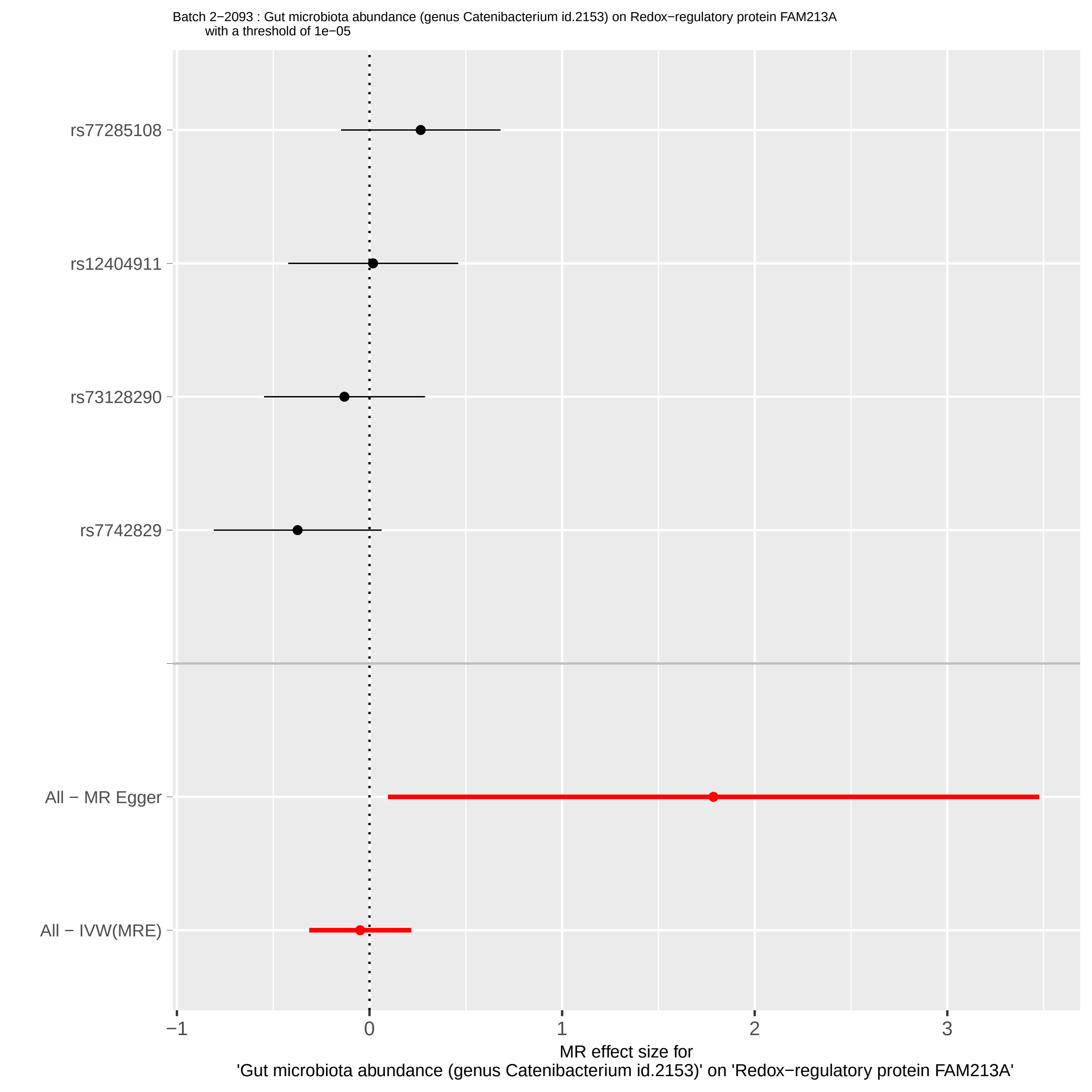

Batch 2−2093 : Gut microbiota abundance (genus Catenibacterium id.2153) on Redox−regulatory protein FAM213A
with a threshold of 1e−05
rs77285108
rs12404911
rs73128290
rs7742829
All − MR Egger
All − IVW(MRE)
−1
0
1
2
3
MR effect size for
'Gut microbiota abundance (genus Catenibacterium id.2153)' on 'Redox−regulatory protein FAM213A'

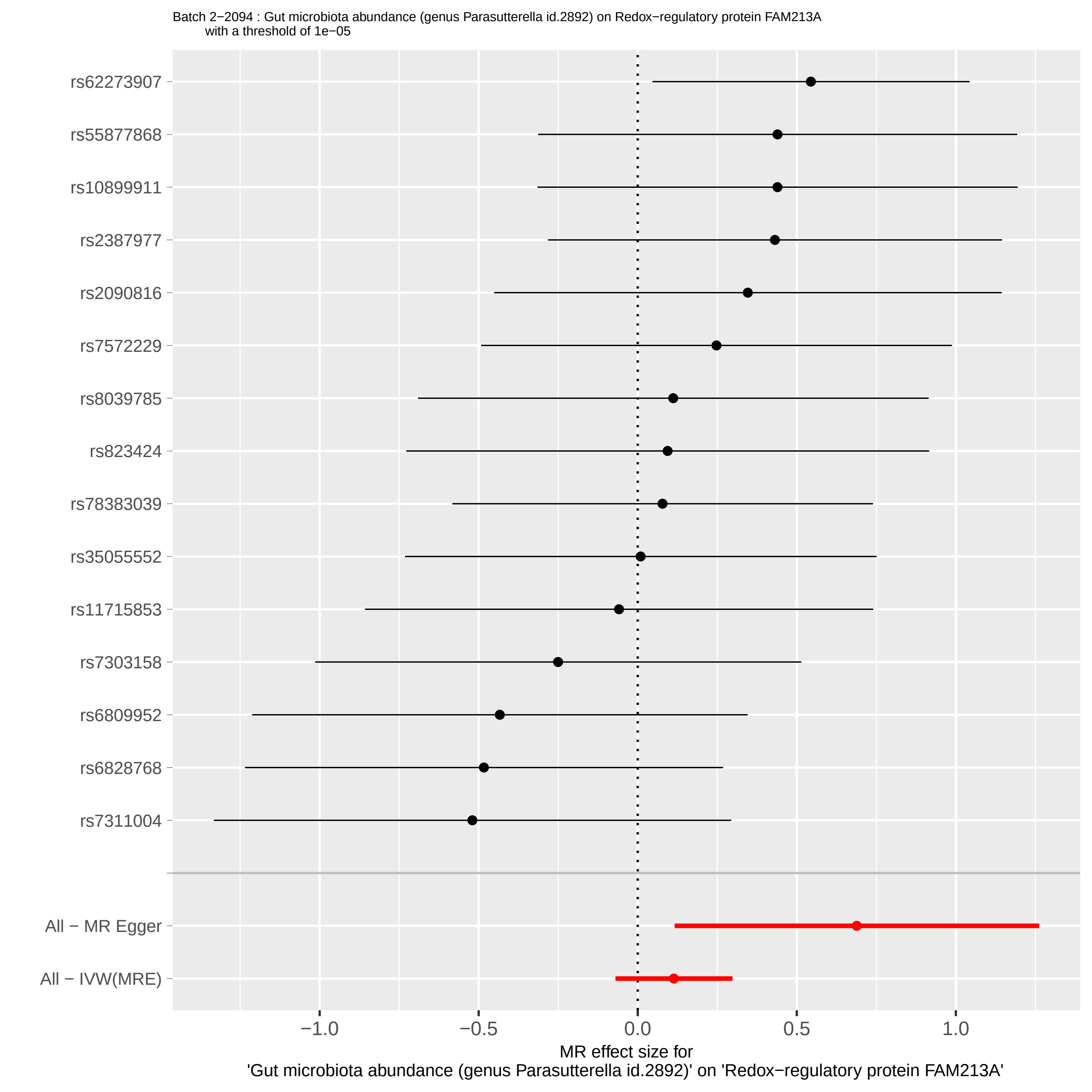

Batch 2−2094 : Gut microbiota abundance (genus Parasutterella id.2892) on Redox−regulatory protein FAM213A
with a threshold of 1e−05
rs62273907
rs55877868
rs10899911
rs2387977
rs2090816
rs7572229
rs8039785
rs823424
rs78383039
rs35055552
rs11715853
rs7303158
rs6809952
rs6828768
rs7311004
All − MR Egger
All − IVW(MRE)
−1.0
−0.5
0.0
0.5
1.0
MR effect size for
'Gut microbiota abundance (genus Parasutterella id.2892)' on 'Redox−regulatory protein FAM213A'

Batch 2−2095 : Gut microbiota abundance (genus Catenibacterium id.2153) on Protein FAM24B
with a threshold of 1e−05

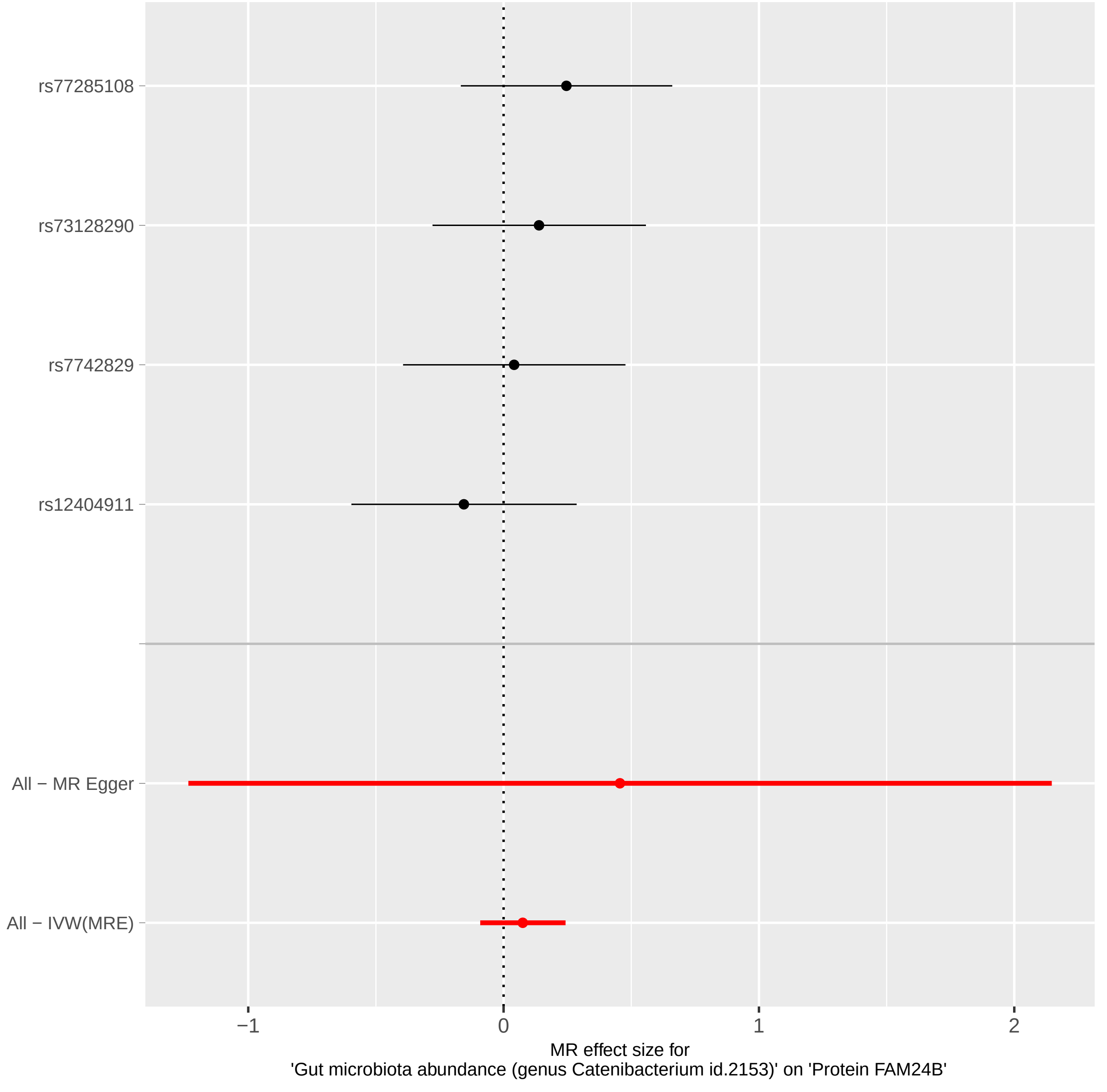

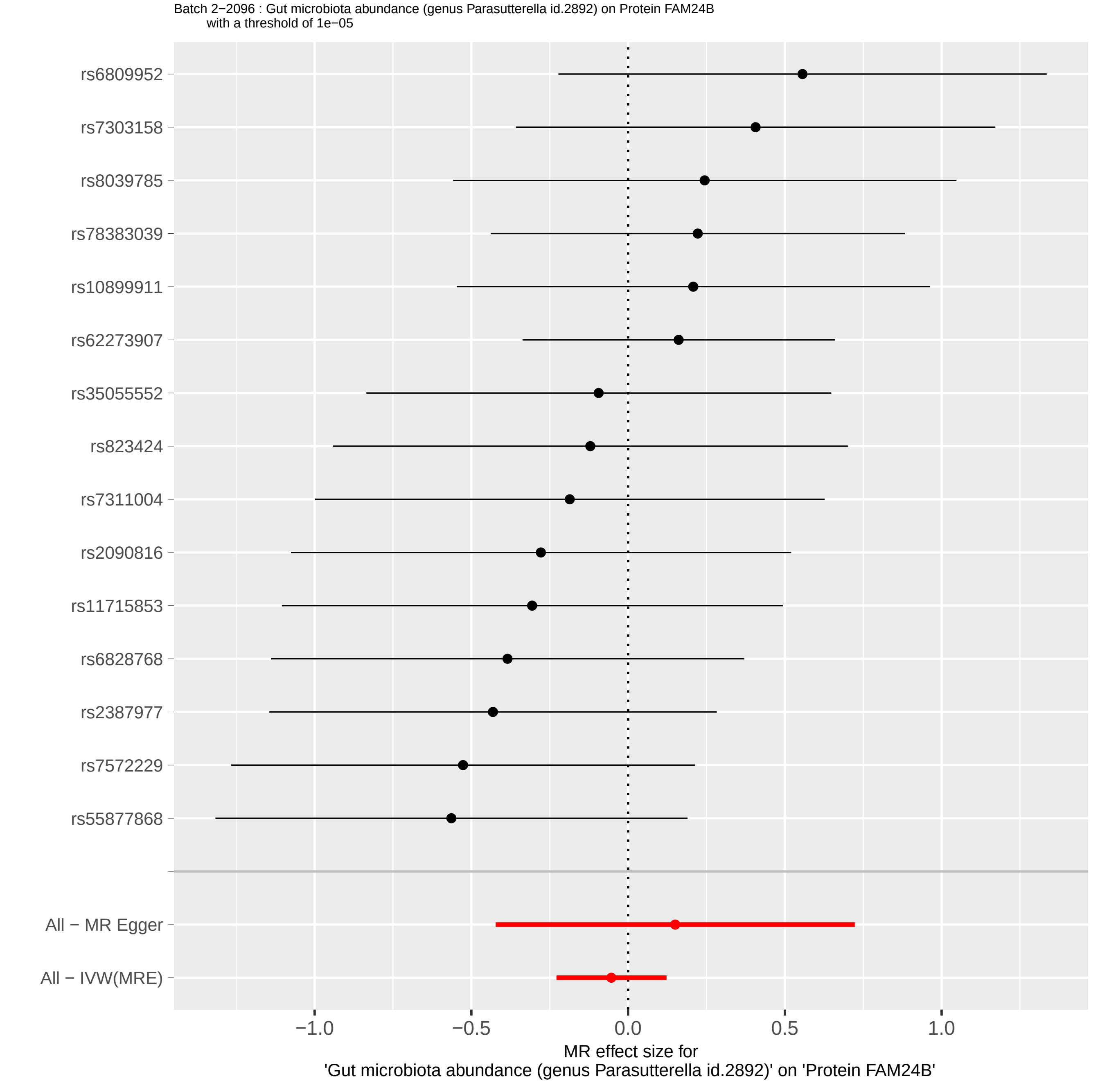

Batch 2−2096 : Gut microbiota abundance (genus Parasutterella id.2892) on Protein FAM24B
with a threshold of 1e−05
rs6809952
rs7303158
rs8039785
rs78383039
rs10899911
rs62273907
rs35055552
rs823424
rs7311004
rs2090816
rs11715853
rs6828768
rs2387977
rs7572229
rs55877868
All − MR Egger
All − IVW(MRE)
−1.0
−0.5
0.0
0.5
1.0
MR effect size for
'Gut microbiota abundance (genus Parasutterella id.2892)' on 'Protein FAM24B'

Batch 2−2097 : Gut microbiota abundance (genus Catenibacterium id.2153) on Protein FAM3B
with a threshold of 1e−05

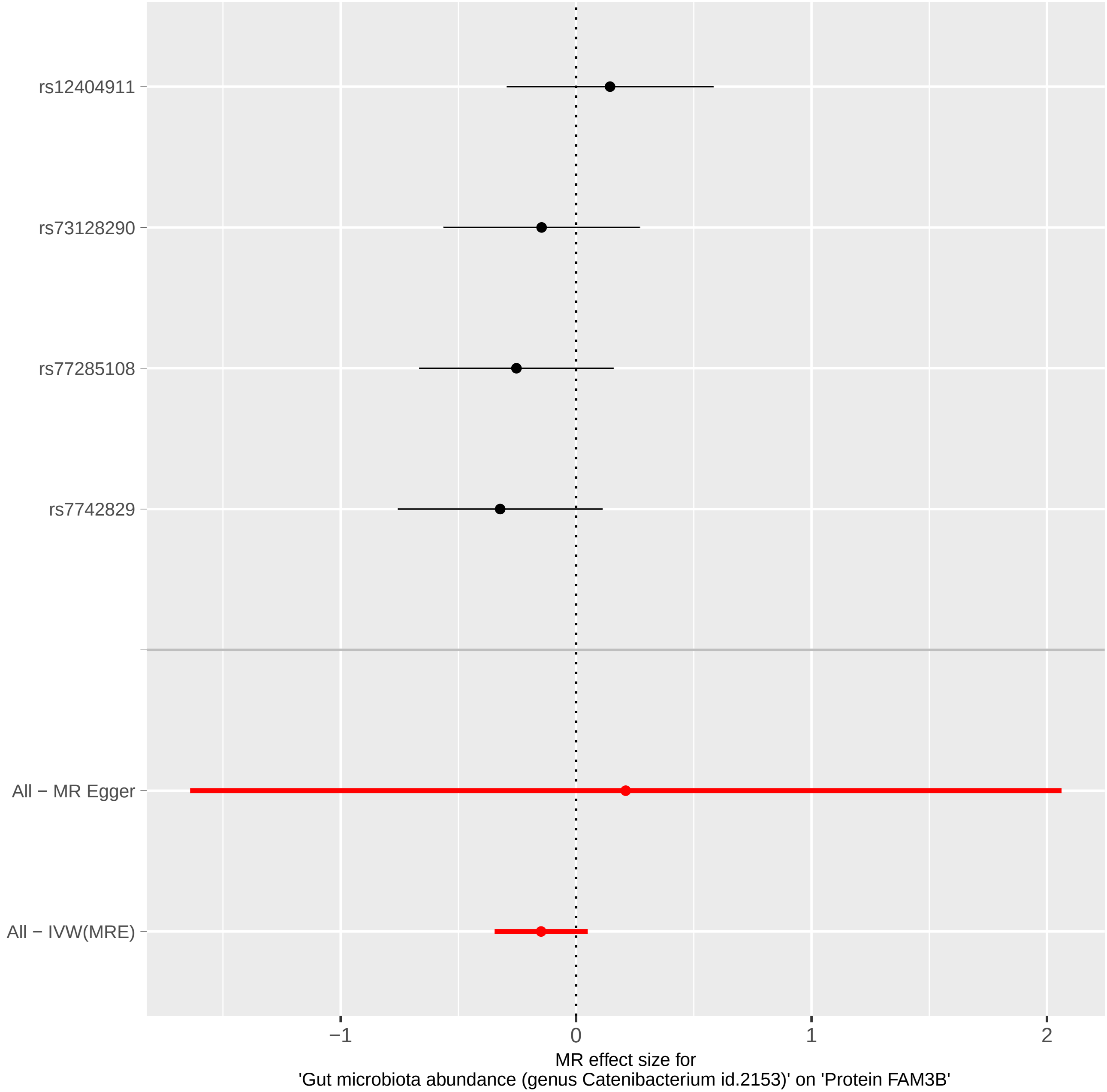

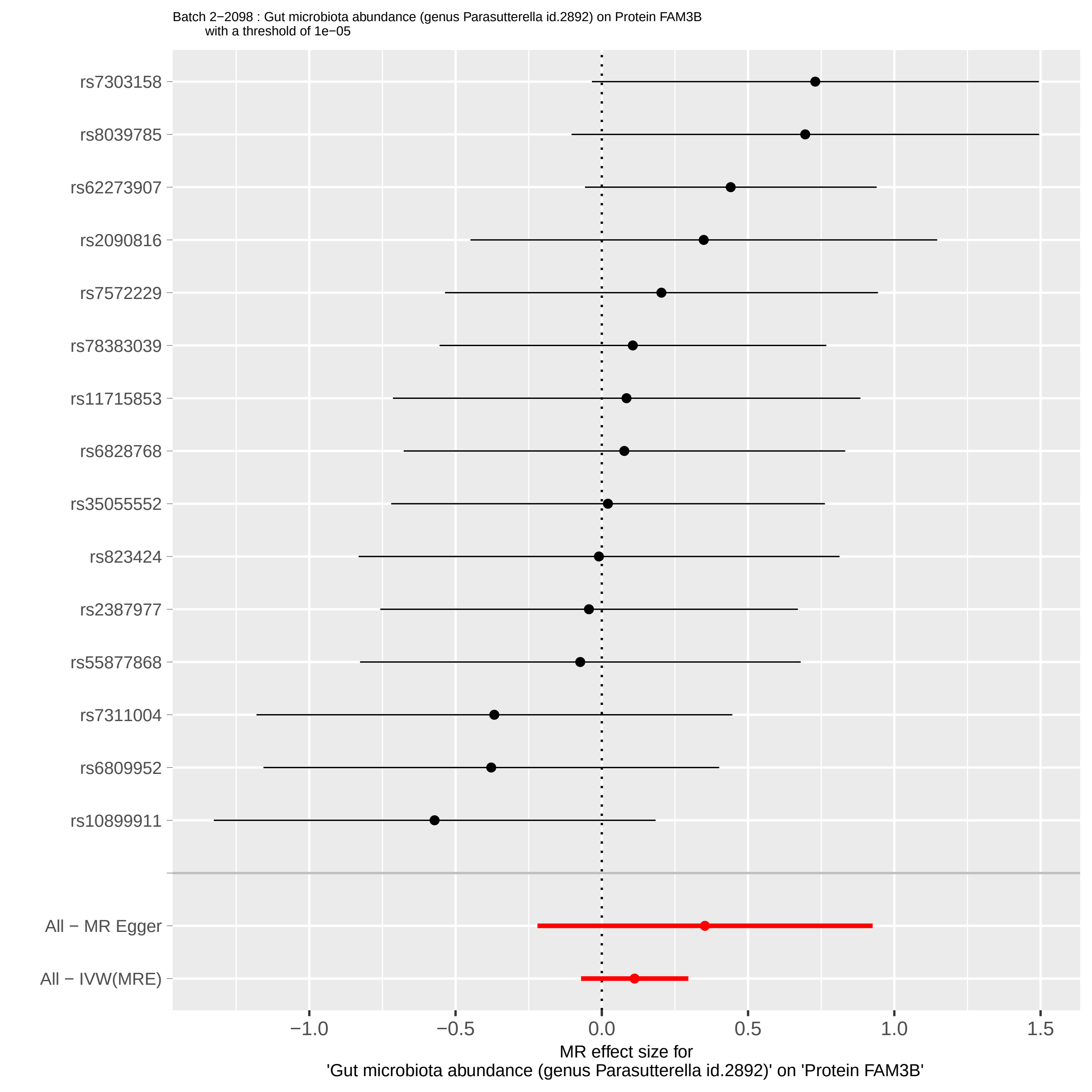

Batch 2−2098 : Gut microbiota abundance (genus Parasutterella id.2892) on Protein FAM3B
with a threshold of 1e−05
rs7303158
rs8039785
rs62273907
rs2090816
rs7572229
rs78383039
rs11715853
rs6828768
rs35055552
rs823424
rs2387977
rs55877868
rs7311004
rs6809952
rs10899911
All − MR Egger
All − IVW(MRE)
−1.0
−0.5
0.0
0.5
1.0
1.5
MR effect size for
'Gut microbiota abundance (genus Parasutterella id.2892)' on 'Protein FAM3B'

Batch 2−2099 : Gut microbiota abundance (genus Catenibacterium id.2153) on Protein FAM3B
with a threshold of 1e−05

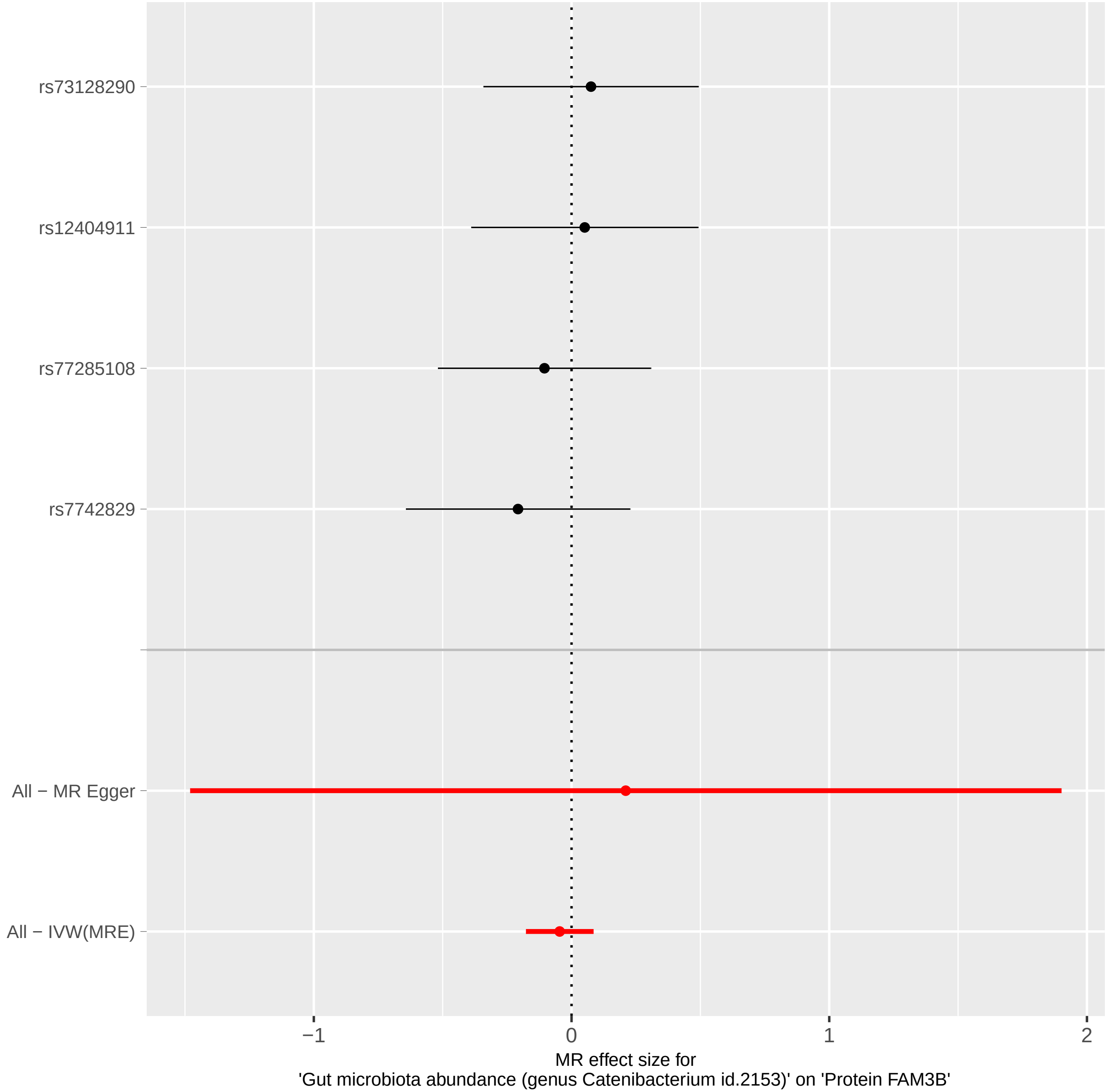

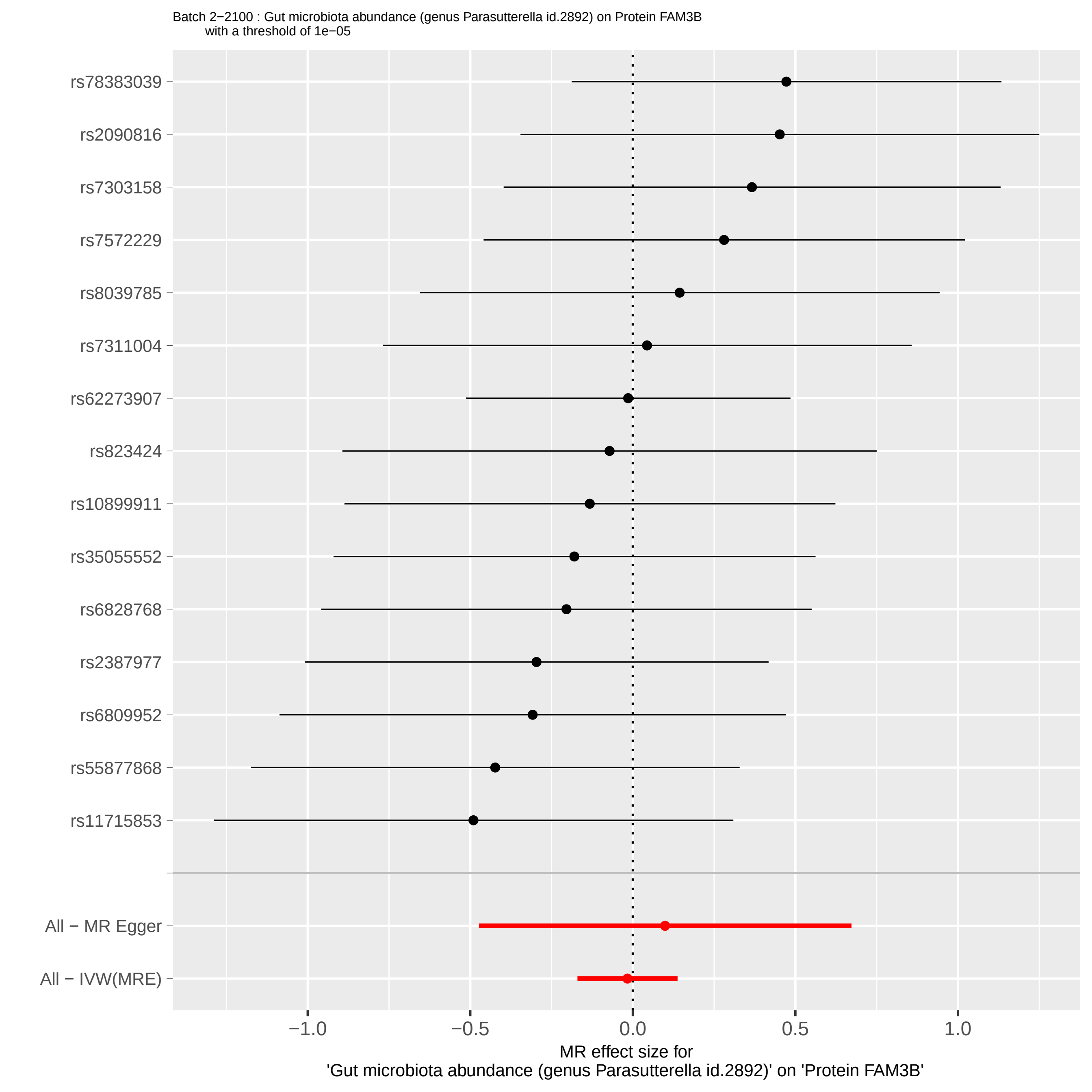
Batch 2−2100 : Gut microbiota abundance (genus Parasutterella id.2892) on Protein FAM3B
with a threshold of 1e−05
rs78383039
rs2090816
rs7303158
rs7572229
rs8039785
rs7311004
rs62273907
rs823424
rs10899911
rs35055552
rs6828768
rs2387977
rs6809952
rs55877868
rs11715853
All − MR Egger
All − IVW(MRE)
−1.0
−0.5
0.0
0.5
1.0
MR effect size for
'Gut microbiota abundance (genus Parasutterella id.2892)' on 'Protein FAM3B'

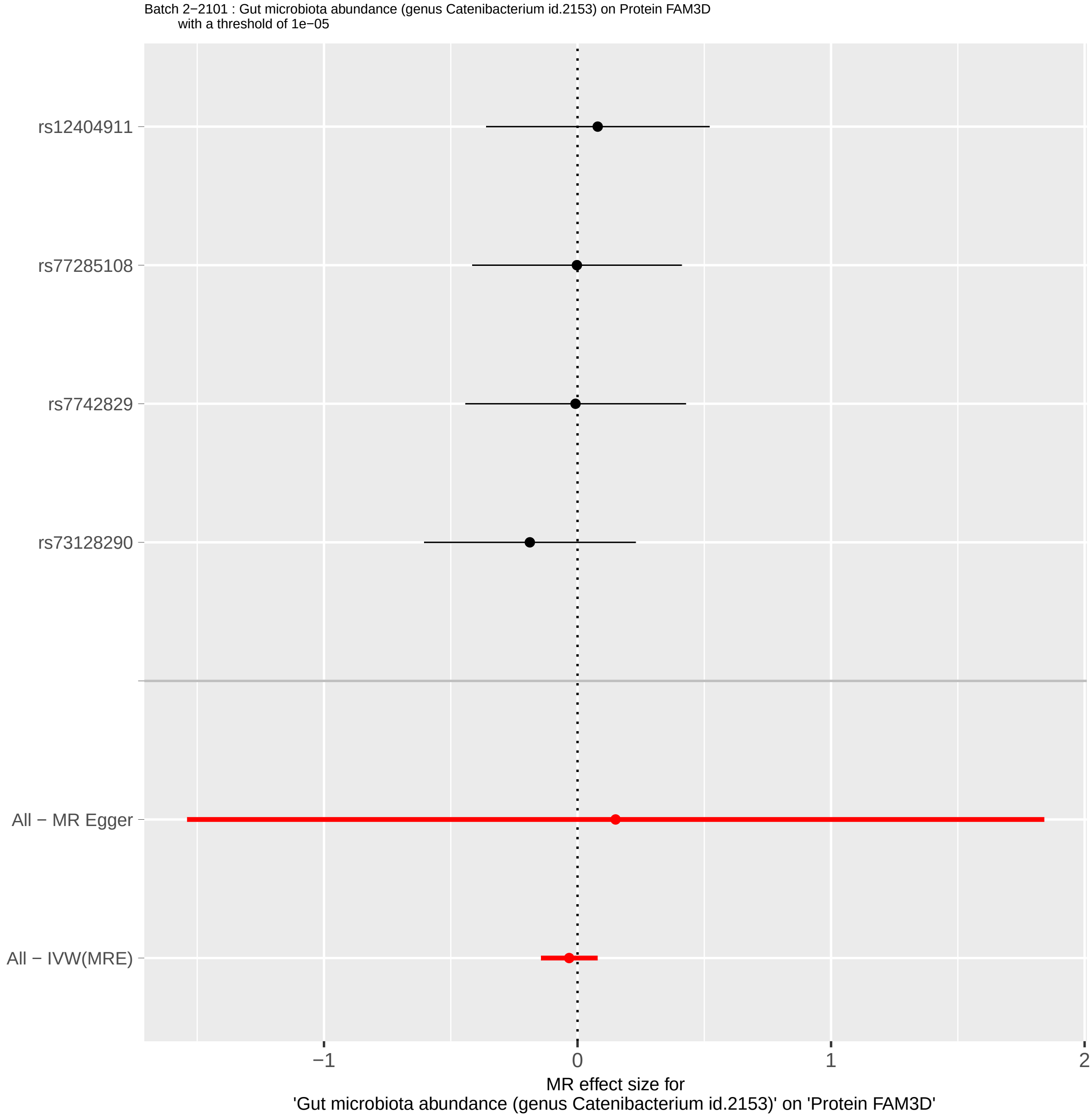

Batch 2−2101 : Gut microbiota abundance (genus Catenibacterium id.2153) on Protein FAM3D
with a threshold of 1e−05
rs12404911
rs77285108
rs7742829
rs73128290
All − MR Egger
All − IVW(MRE)
−1
0
1
2
MR effect size for
'Gut microbiota abundance (genus Catenibacterium id.2153)' on 'Protein FAM3D'

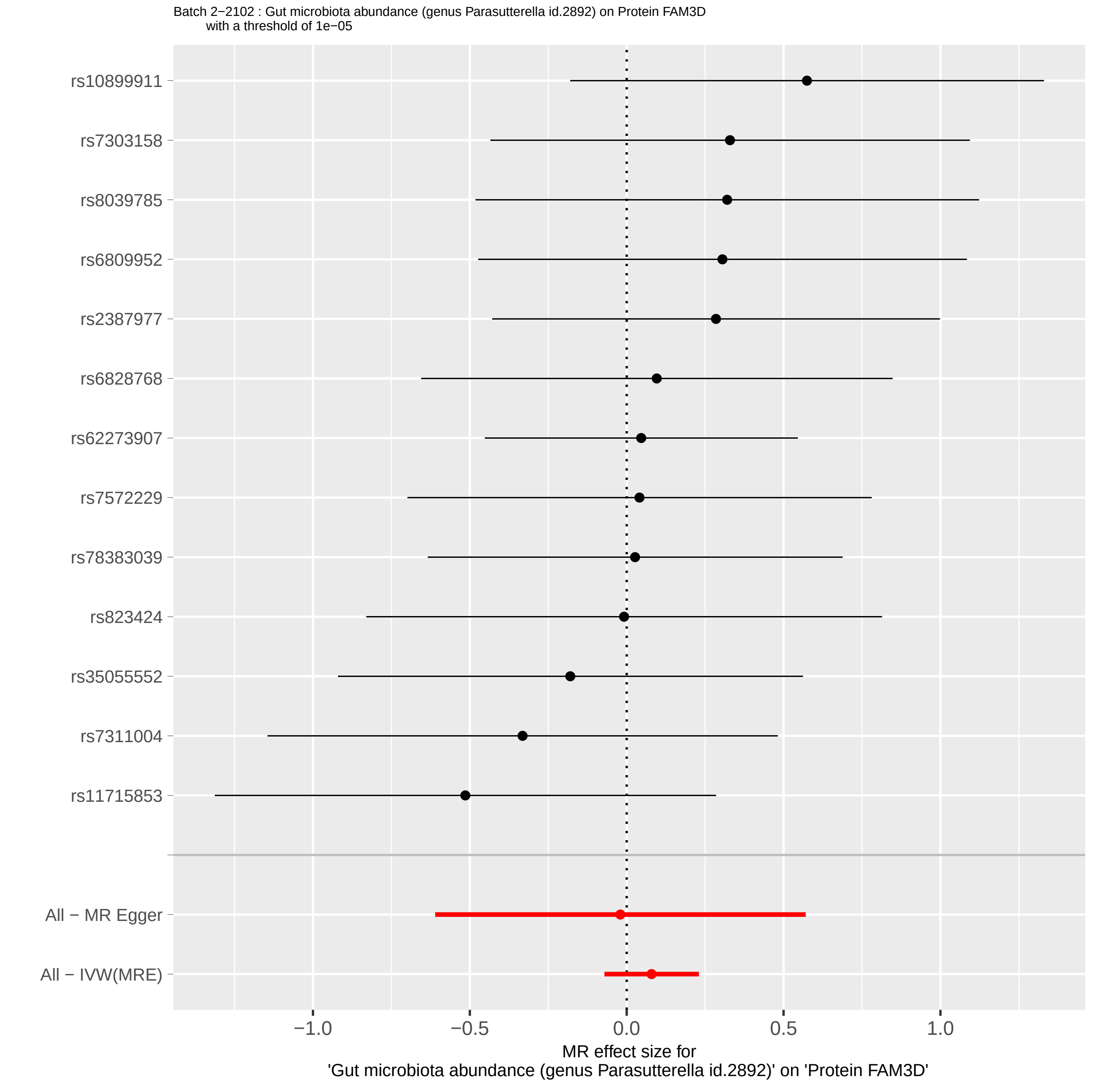

Batch 2−2102 : Gut microbiota abundance (genus Parasutterella id.2892) on Protein FAM3D
with a threshold of 1e−05
rs10899911
rs7303158
rs8039785
rs6809952
rs2387977
rs6828768
rs62273907
rs7572229
rs78383039
rs823424
rs35055552
rs7311004
rs11715853
All − MR Egger
All − IVW(MRE)
−1.0
−0.5
0.0
0.5
1.0
MR effect size for
'Gut microbiota abundance (genus Parasutterella id.2892)' on 'Protein FAM3D'

Batch 2−2103 : Gut microbiota abundance (genus Catenibacterium id.2153) on MIP18 family protein FAM96A
with a threshold of 1e−05

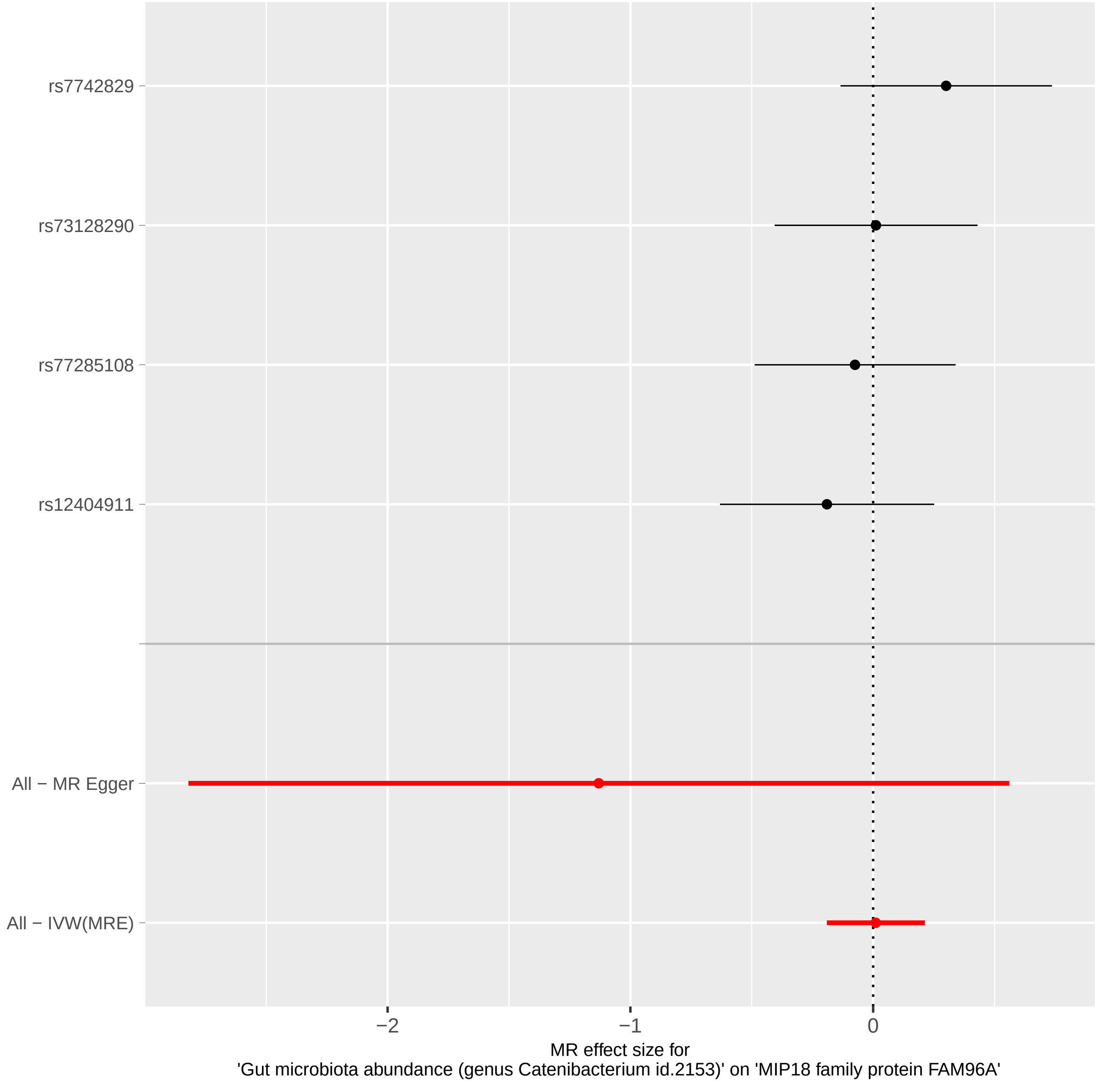

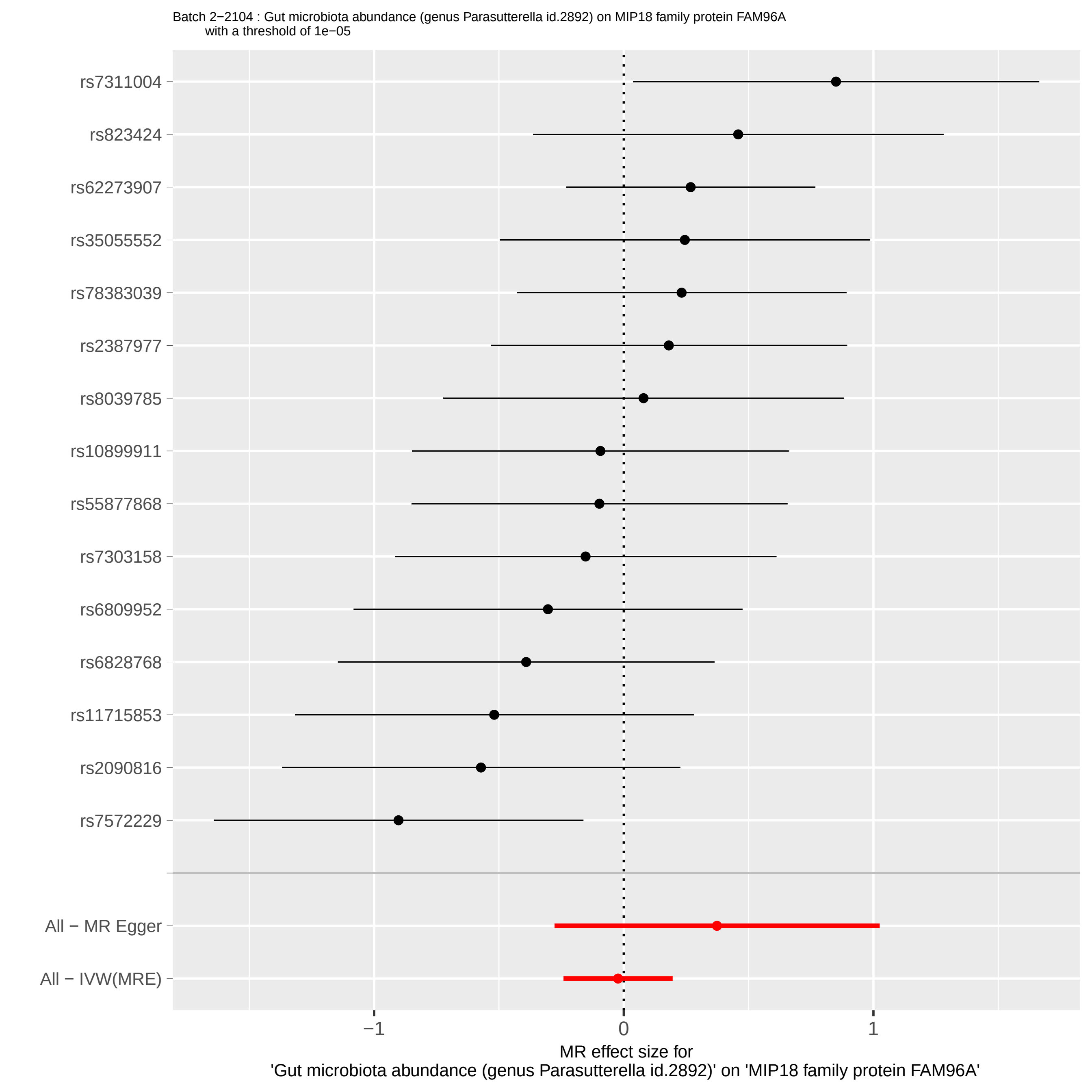

Batch 2−2104 : Gut microbiota abundance (genus Parasutterella id.2892) on MIP18 family protein FAM96A
with a threshold of 1e−05
rs7311004
rs823424
rs62273907
rs35055552
rs78383039
rs2387977
rs8039785
rs10899911
rs55877868
rs7303158
rs6809952
rs6828768
rs11715853
rs2090816
rs7572229
All − MR Egger
All − IVW(MRE)
−1
0
1
MR effect size for
'Gut microbiota abundance (genus Parasutterella id.2892)' on 'MIP18 family protein FAM96A'

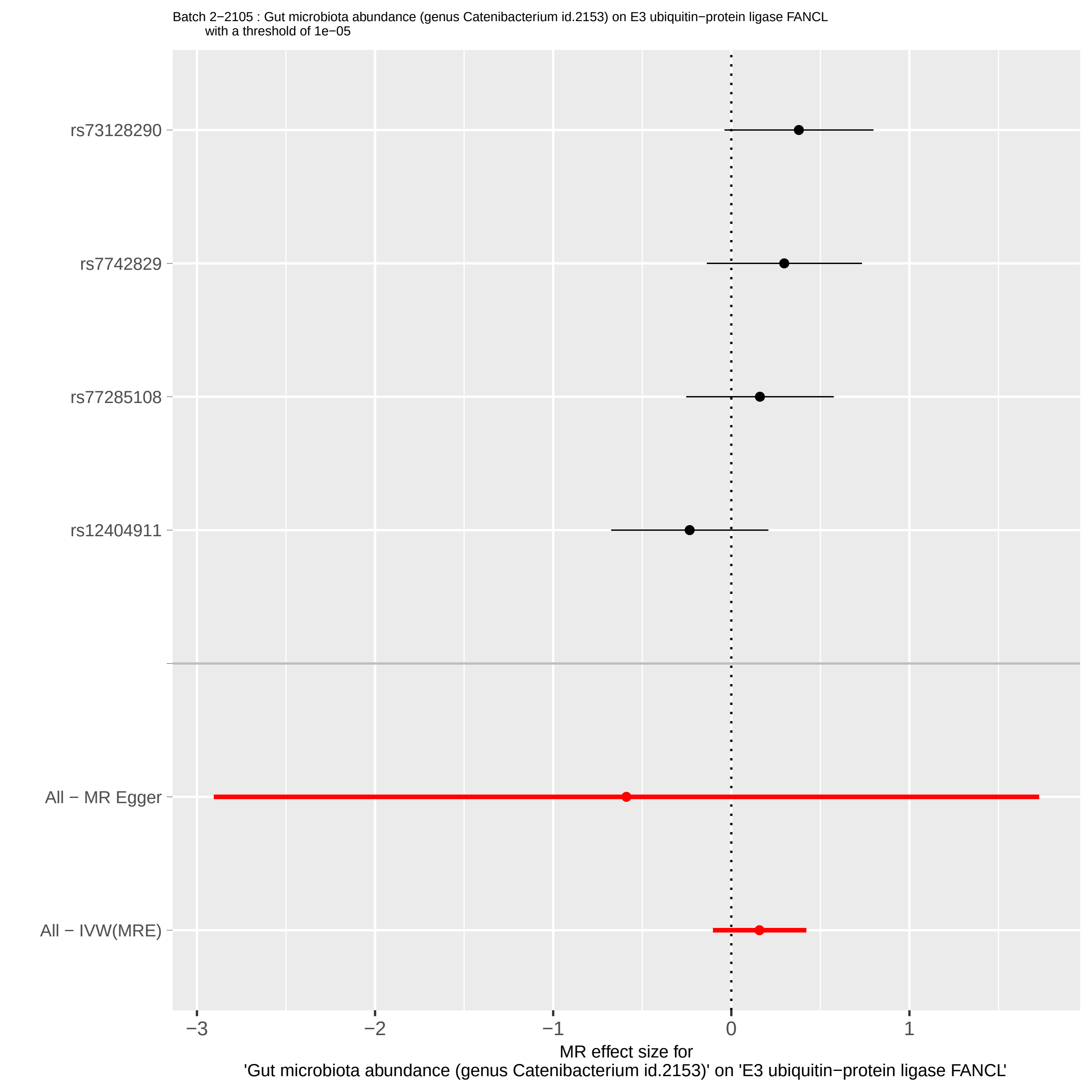

Batch 2−2105 : Gut microbiota abundance (genus Catenibacterium id.2153) on E3 ubiquitin−protein ligase FANCL
with a threshold of 1e−05
rs73128290
rs7742829
rs77285108
rs12404911
All − MR Egger
All − IVW(MRE)
−3
−2
−1
0
1
MR effect size for
'Gut microbiota abundance (genus Catenibacterium id.2153)' on 'E3 ubiquitin−protein ligase FANCL'

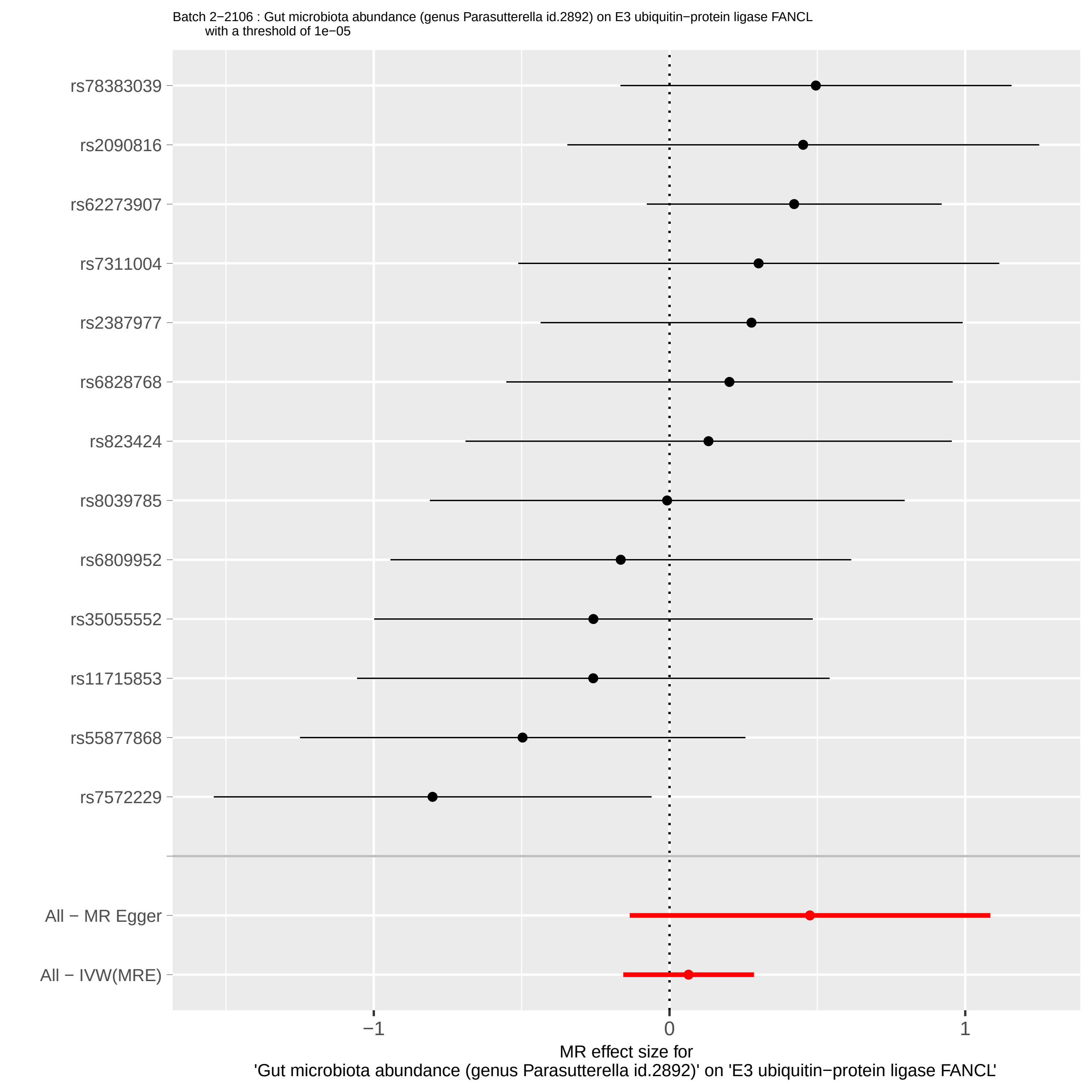

Batch 2−2106 : Gut microbiota abundance (genus Parasutterella id.2892) on E3 ubiquitin−protein ligase FANCL
with a threshold of 1e−05
rs78383039
rs2090816
rs62273907
rs7311004
rs2387977
rs6828768
rs823424
rs8039785
rs6809952
rs35055552
rs11715853
rs55877868
rs7572229
All − MR Egger
All − IVW(MRE)
−1
0
1
MR effect size for
'Gut microbiota abundance (genus Parasutterella id.2892)' on 'E3 ubiquitin−protein ligase FANCL'

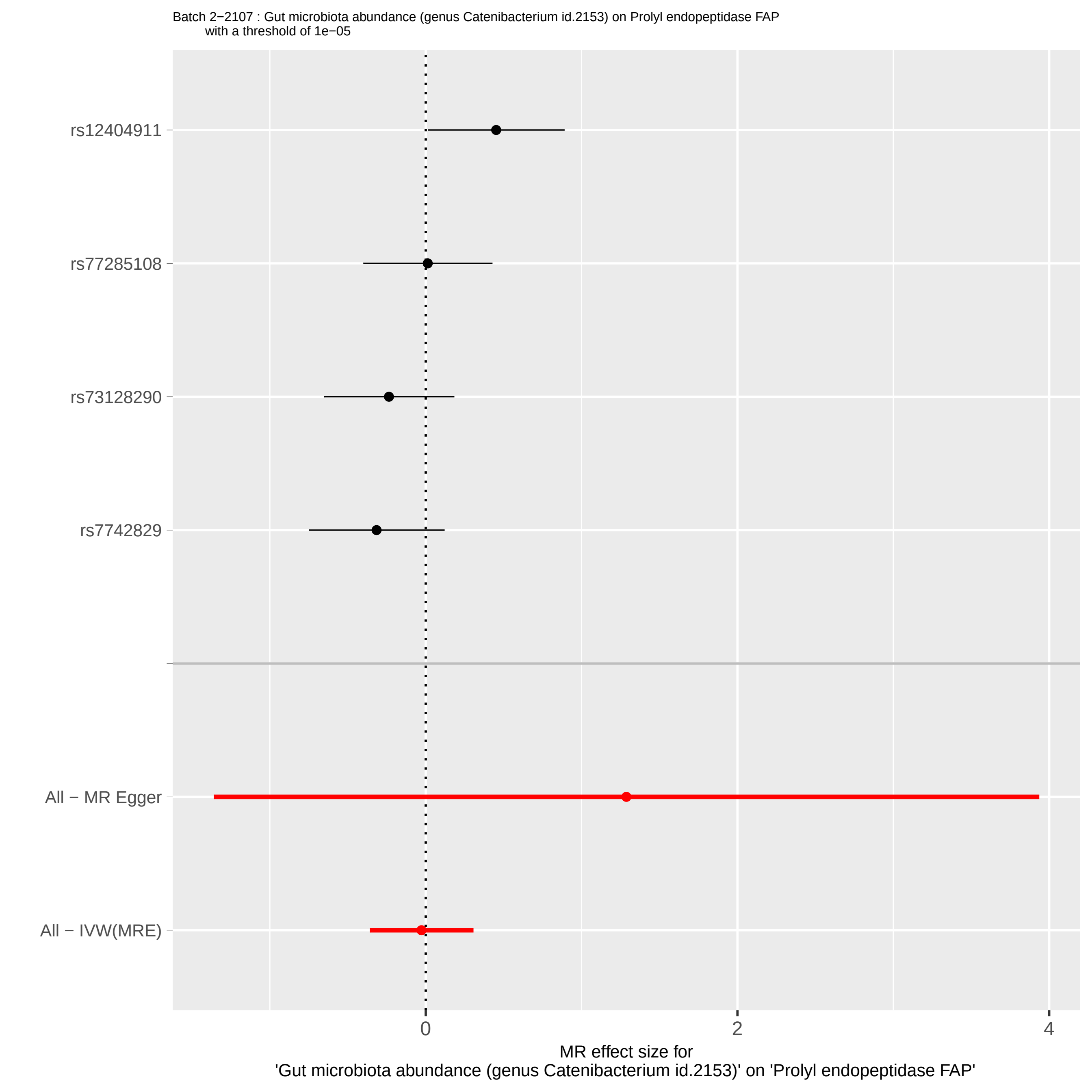

Batch 2−2107 : Gut microbiota abundance (genus Catenibacterium id.2153) on Prolyl endopeptidase FAP
with a threshold of 1e−05
rs12404911
rs77285108
rs73128290
rs7742829
All − MR Egger
All − IVW(MRE)
0
2
4
MR effect size for
'Gut microbiota abundance (genus Catenibacterium id.2153)' on 'Prolyl endopeptidase FAP'

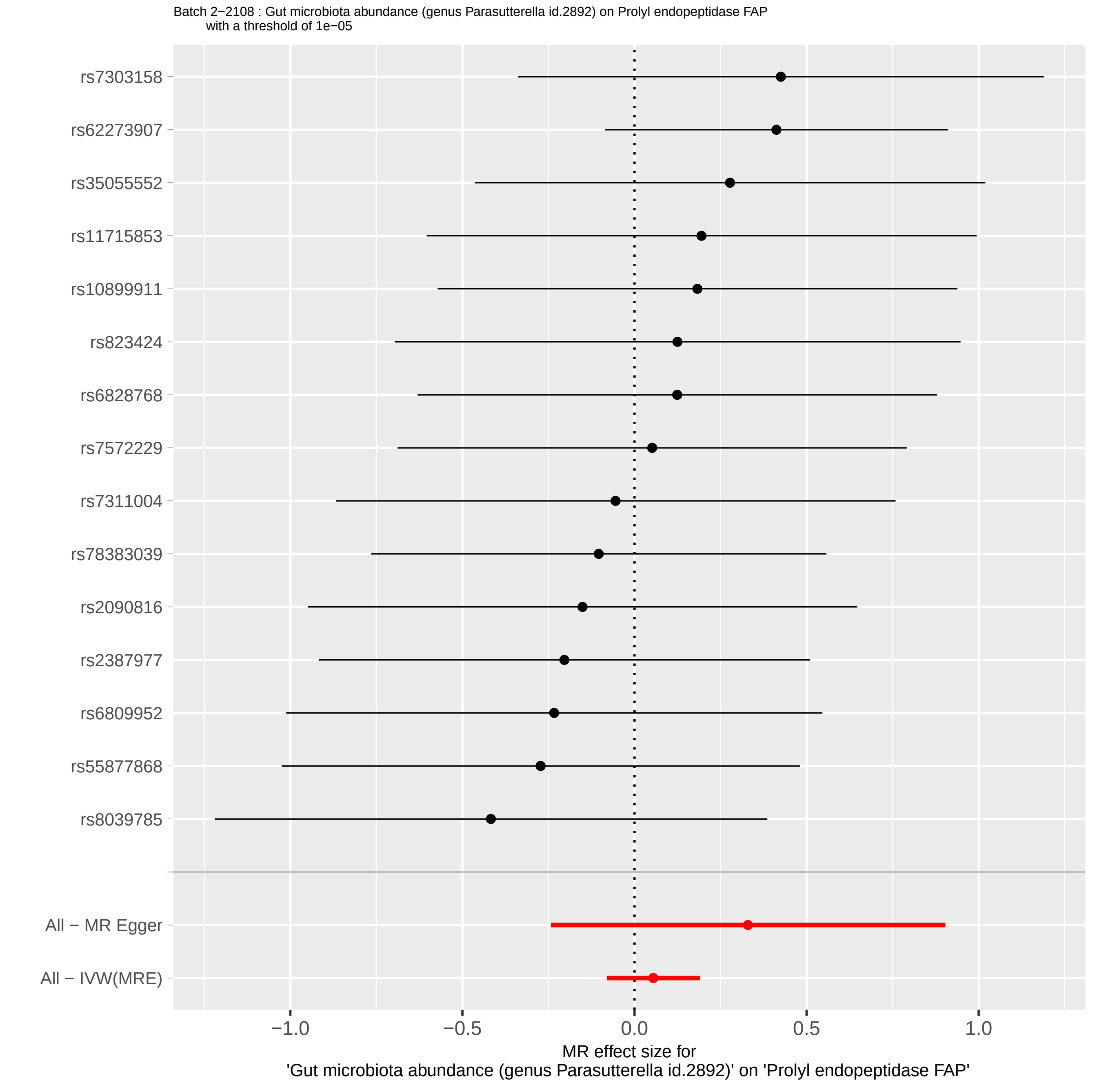
Batch 2−2108 : Gut microbiota abundance (genus Parasutterella id.2892) on Prolyl endopeptidase FAP
with a threshold of 1e−05
rs7303158
rs62273907
rs35055552
rs11715853
rs10899911
rs823424
rs6828768
rs7572229
rs7311004
rs78383039
rs2090816
rs2387977
rs6809952
rs55877868
rs8039785
All − MR Egger
All − IVW(MRE)
−1.0
−0.5
0.0
0.5
1.0
MR effect size for
'Gut microbiota abundance (genus Parasutterella id.2892)' on 'Prolyl endopeptidase FAP'

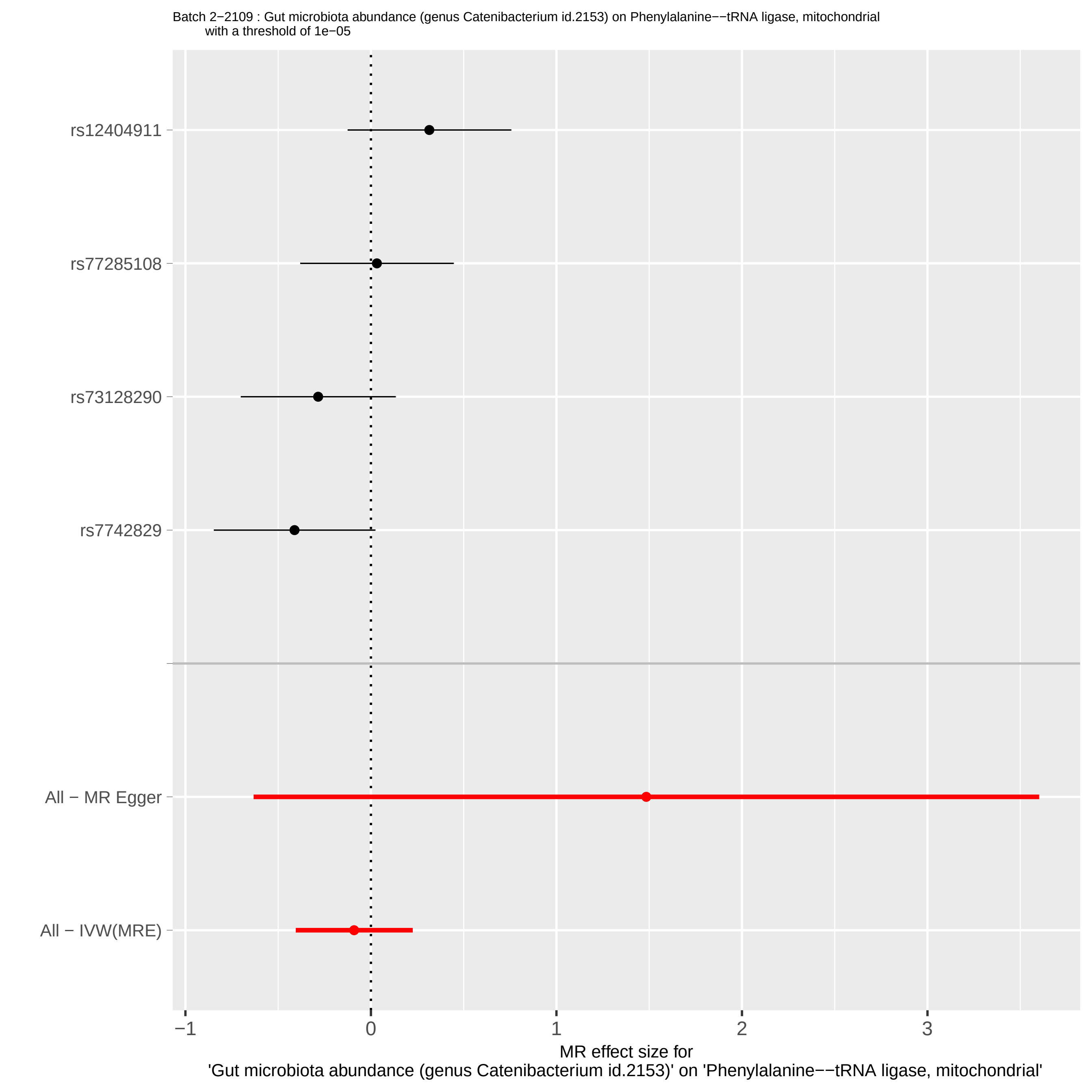

Batch 2−2109 : Gut microbiota abundance (genus Catenibacterium id.2153) on Phenylalanine−−tRNA ligase, mitochondrial
with a threshold of 1e−05
rs12404911
rs77285108
rs73128290
rs7742829
All − MR Egger
All − IVW(MRE)
−1
0
1
2
3
MR effect size for
'Gut microbiota abundance (genus Catenibacterium id.2153)' on 'Phenylalanine−−tRNA ligase, mitochondrial'

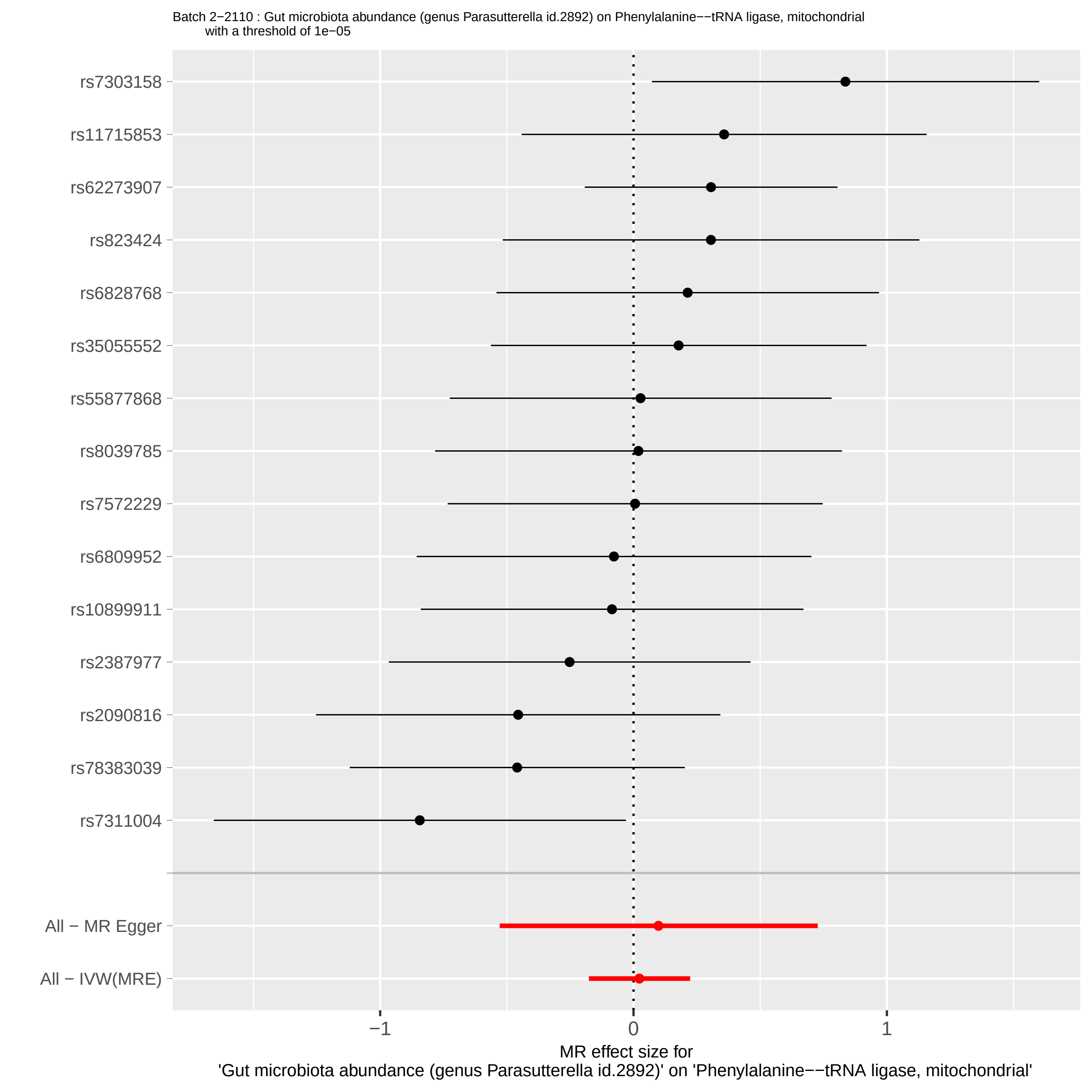

Batch 2−2110 : Gut microbiota abundance (genus Parasutterella id.2892) on Phenylalanine−−tRNA ligase, mitochondrial
with a threshold of 1e−05
rs7303158
rs11715853
rs62273907
rs823424
rs6828768
rs35055552
rs55877868
rs8039785
rs7572229
rs6809952
rs10899911
rs2387977
rs2090816
rs78383039
rs7311004
All − MR Egger
All − IVW(MRE)
−1
0
1
MR effect size for
'Gut microbiota abundance (genus Parasutterella id.2892)' on 'Phenylalanine−−tRNA ligase, mitochondrial'

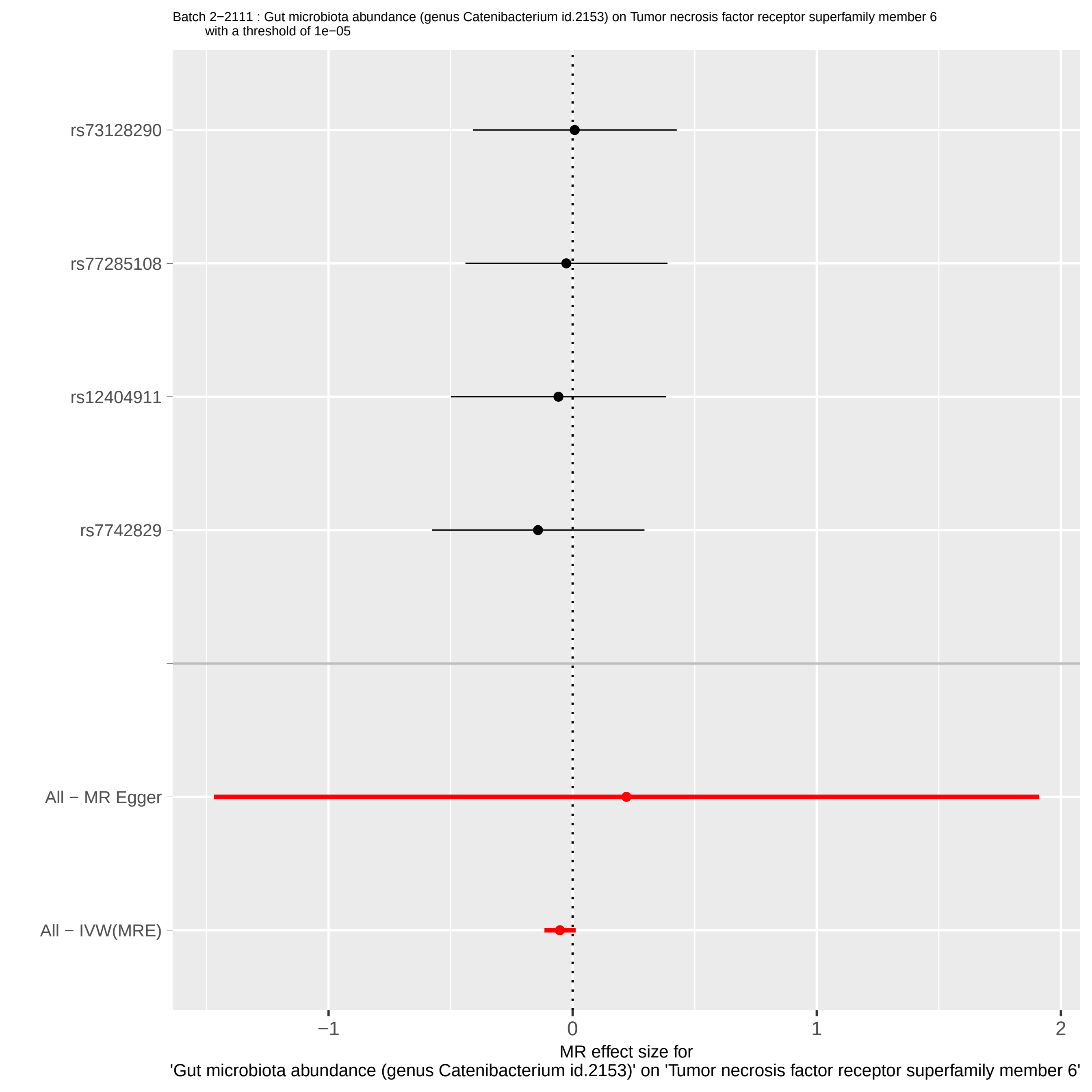

Batch 2−2111 : Gut microbiota abundance (genus Catenibacterium id.2153) on Tumor necrosis factor receptor superfamily member 6
with a threshold of 1e−05
rs73128290
rs77285108
rs12404911
rs7742829
All − MR Egger
All − IVW(MRE)
−1
0
1
2
MR effect size for
'Gut microbiota abundance (genus Catenibacterium id.2153)' on 'Tumor necrosis factor receptor superfamily member 6'

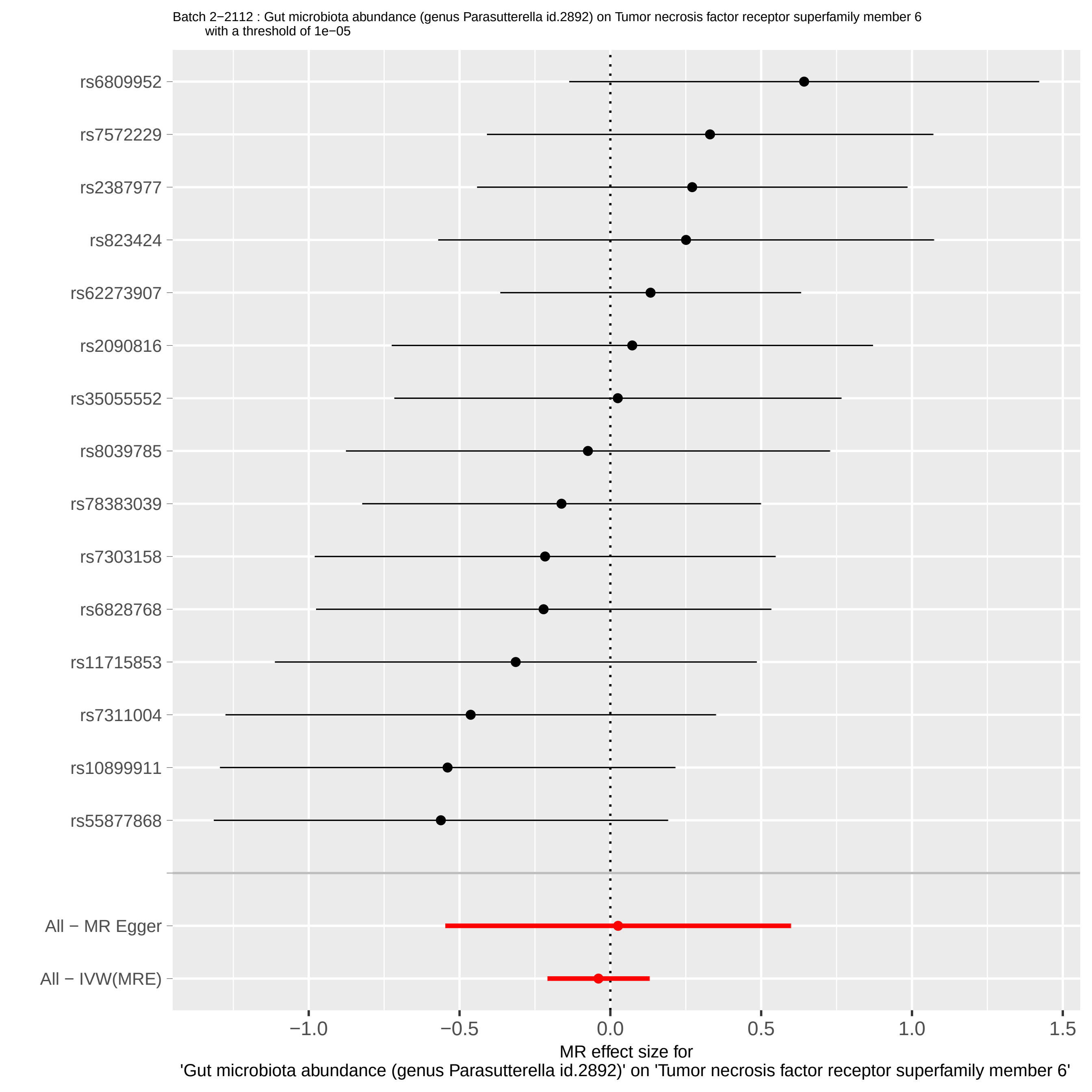

Batch 2−2112 : Gut microbiota abundance (genus Parasutterella id.2892) on Tumor necrosis factor receptor superfamily member 6
with a threshold of 1e−05
rs6809952
rs7572229
rs2387977
rs823424
rs62273907
rs2090816
rs35055552
rs8039785
rs78383039
rs7303158
rs6828768
rs11715853
rs7311004
rs10899911
rs55877868
All − MR Egger
All − IVW(MRE)
−1.0
−0.5
0.0
0.5
1.0
1.5
MR effect size for
'Gut microbiota abundance (genus Parasutterella id.2892)' on 'Tumor necrosis factor receptor superfamily member 6'

Batch 2−2113 : Gut microbiota abundance (genus Catenibacterium id.2153) on Tumor necrosis factor receptor superfamily member 6
with a threshold of 1e−05

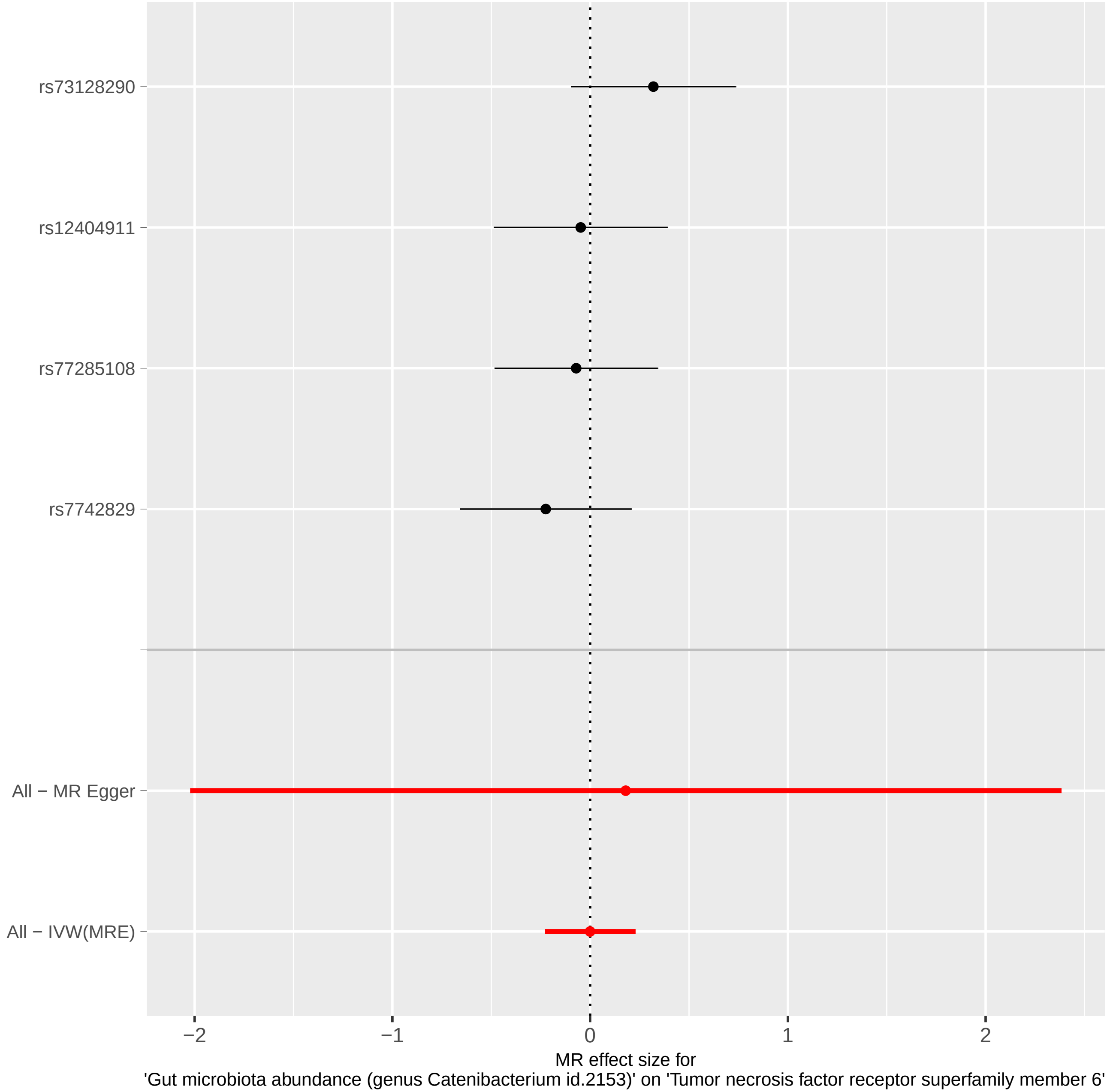

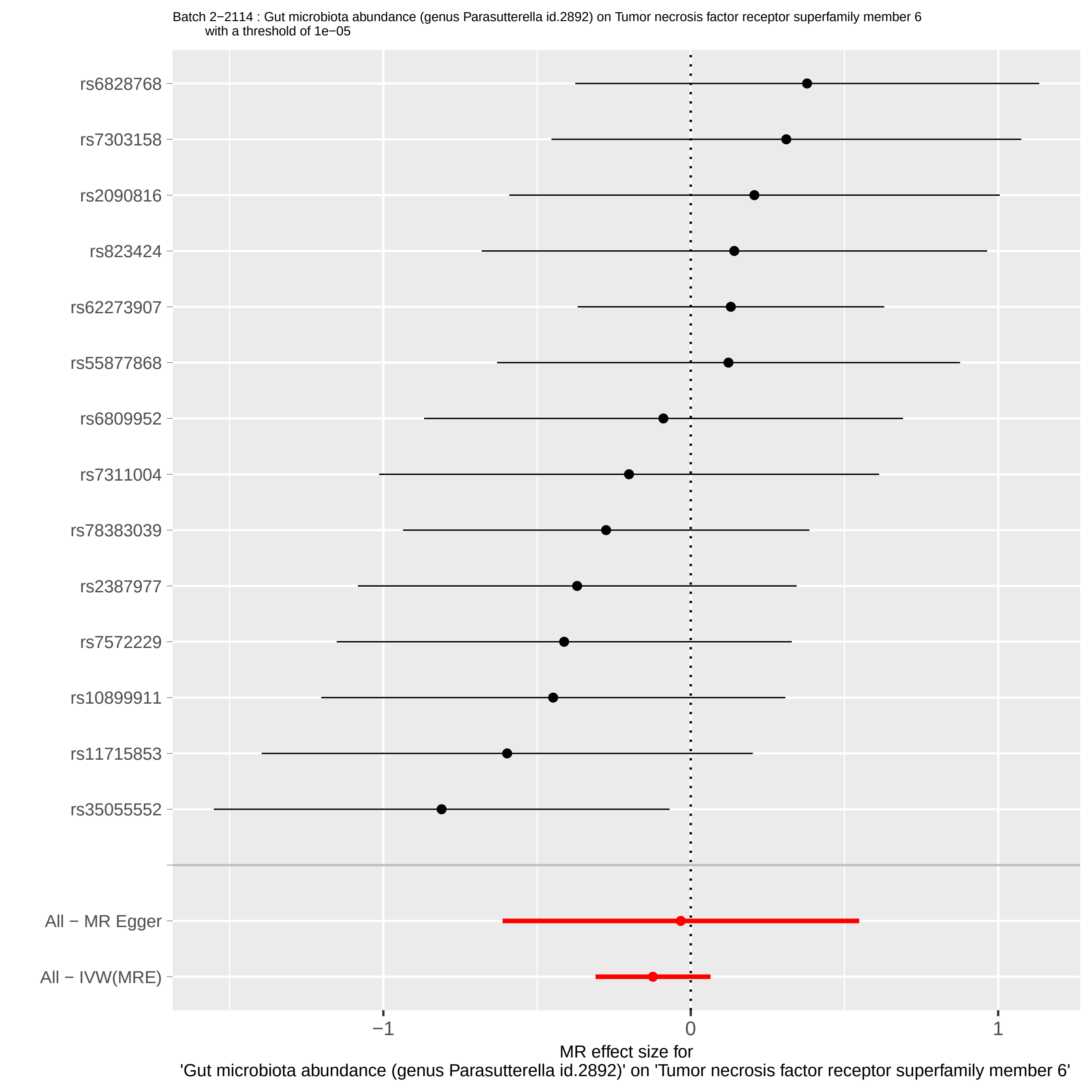

Batch 2−2114 : Gut microbiota abundance (genus Parasutterella id.2892) on Tumor necrosis factor receptor superfamily member 6
with a threshold of 1e−05
rs6828768
rs7303158
rs2090816
rs823424
rs62273907
rs55877868
rs6809952
rs7311004
rs78383039
rs2387977
rs7572229
rs10899911
rs11715853
rs35055552
All − MR Egger
All − IVW(MRE)
−1
0
1
MR effect size for
'Gut microbiota abundance (genus Parasutterella id.2892)' on 'Tumor necrosis factor receptor superfamily member 6'

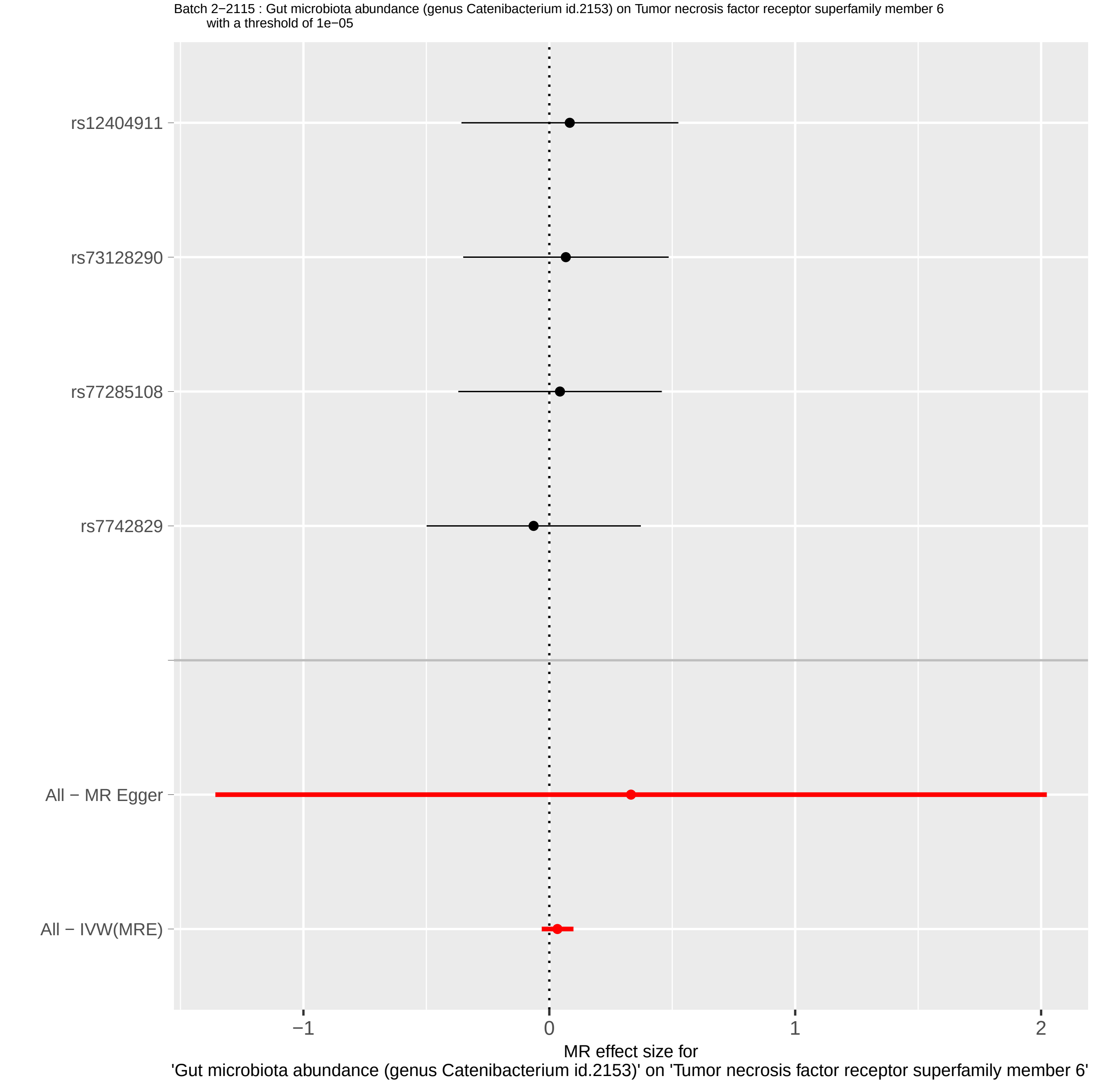

Batch 2−2115 : Gut microbiota abundance (genus Catenibacterium id.2153) on Tumor necrosis factor receptor superfamily member 6
with a threshold of 1e−05
rs12404911
rs73128290
rs77285108
rs7742829
All − MR Egger
All − IVW(MRE)
−1
0
1
2
MR effect size for
'Gut microbiota abundance (genus Catenibacterium id.2153)' on 'Tumor necrosis factor receptor superfamily member 6'

Batch 2−2116 : Gut microbiota abundance (genus Parasutterella id.2892) on Tumor necrosis factor receptor superfamily member 6
with a threshold of 1e−05

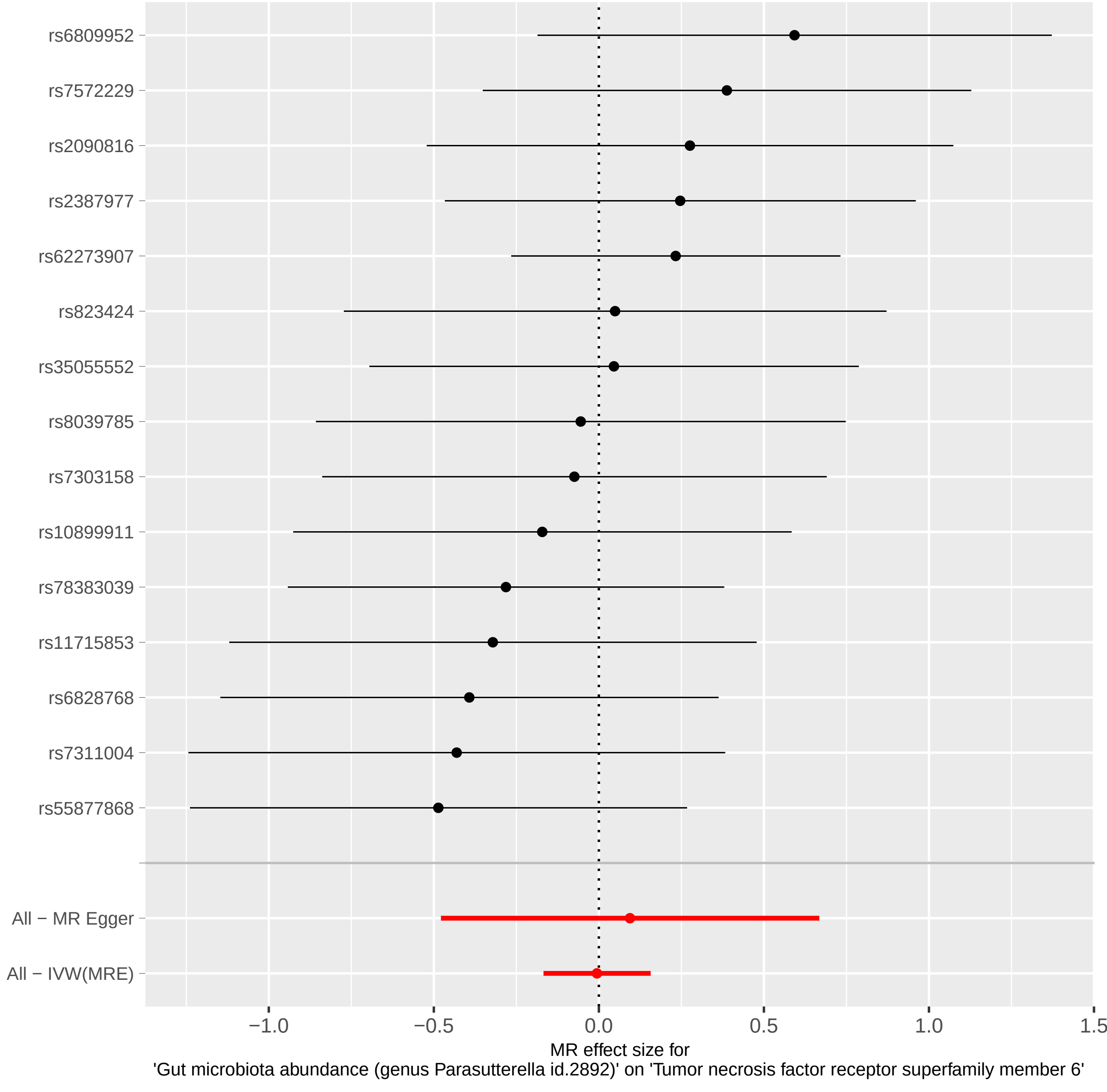

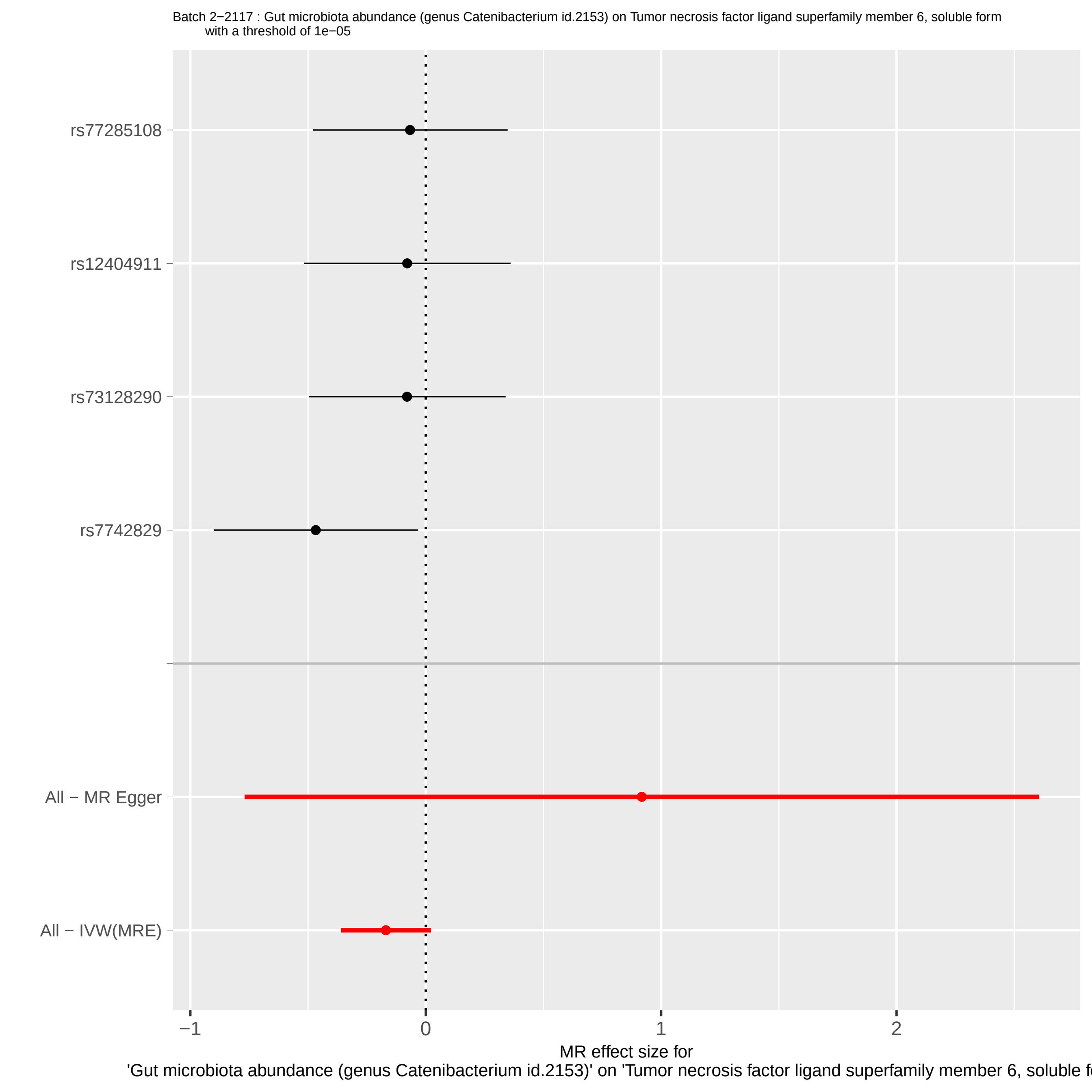

Batch 2−2117 : Gut microbiota abundance (genus Catenibacterium id.2153) on Tumor necrosis factor ligand superfamily member 6, soluble form
with a threshold of 1e−05
rs77285108
rs12404911
rs73128290
rs7742829
All − MR Egger
All − IVW(MRE)
−1
0
1
2
MR effect size for
'Gut microbiota abundance (genus Catenibacterium id.2153)' on 'Tumor necrosis factor ligand superfamily member 6, soluble fo

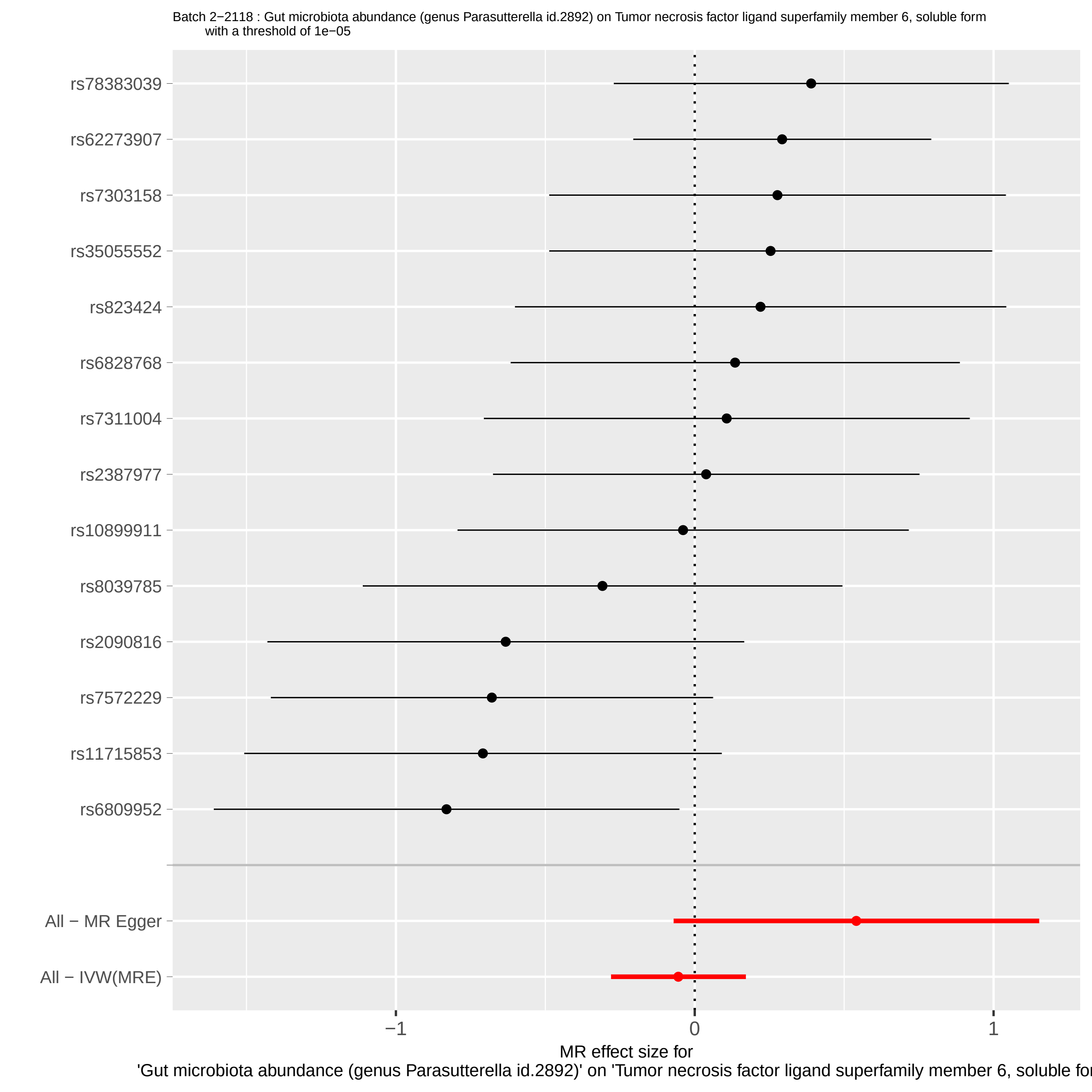

Batch 2−2118 : Gut microbiota abundance (genus Parasutterella id.2892) on Tumor necrosis factor ligand superfamily member 6, soluble form with a threshold of 1e−05
rs78383039
rs62273907
rs7303158
rs35055552
rs823424
rs6828768
rs7311004
rs2387977
rs10899911
rs8039785
rs2090816
rs7572229
rs11715853
rs6809952
All − MR Egger
All − IVW(MRE)
−1
0
1
MR effect size for
'Gut microbiota abundance (genus Parasutterella id.2892)' on 'Tumor necrosis factor ligand superfamily member 6, soluble for

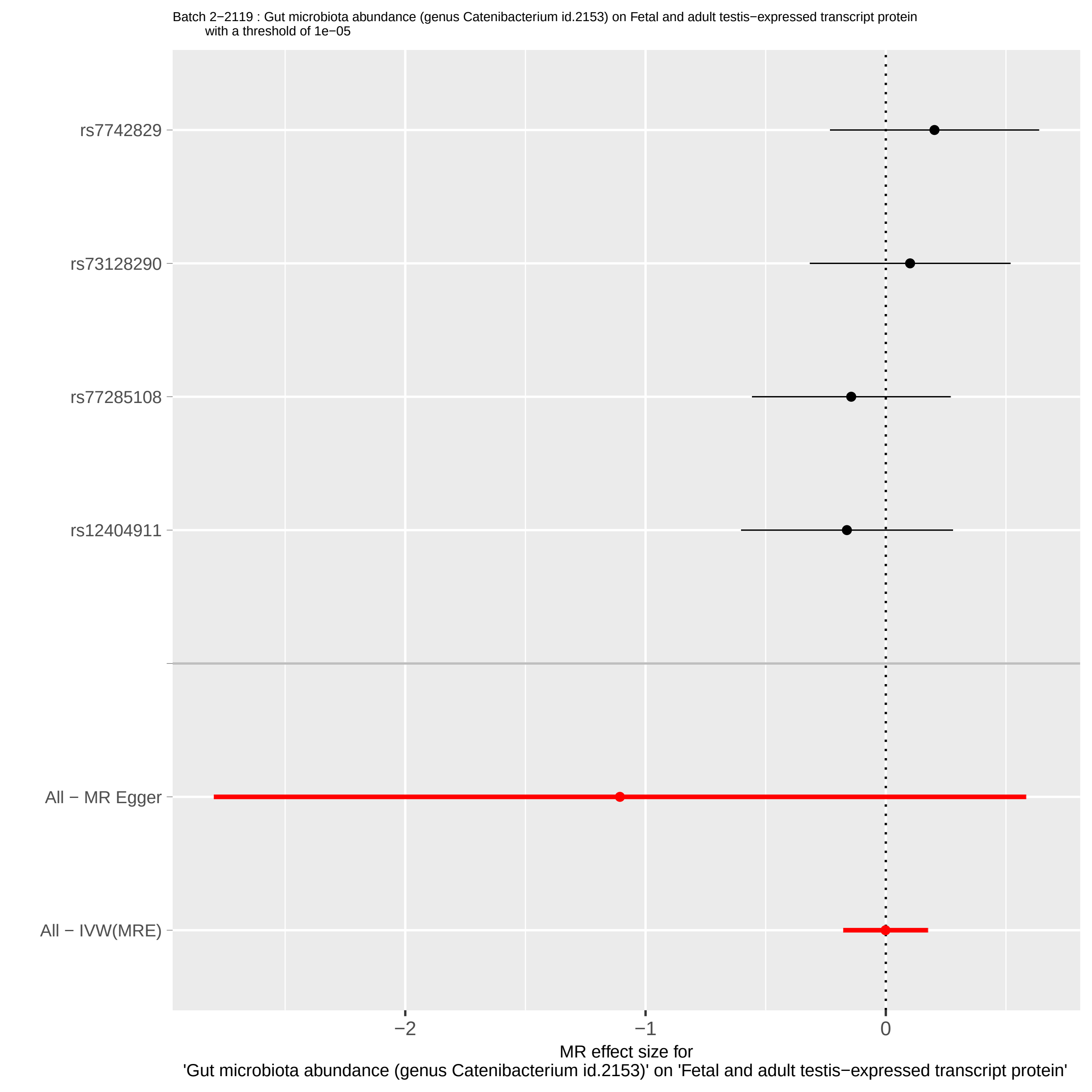

Batch 2−2119 : Gut microbiota abundance (genus Catenibacterium id.2153) on Fetal and adult testis−expressed transcript protein
with a threshold of 1e−05
rs7742829
rs73128290
rs77285108
rs12404911
All − MR Egger
All − IVW(MRE)
−2
−1
0
MR effect size for
'Gut microbiota abundance (genus Catenibacterium id.2153)' on 'Fetal and adult testis−expressed transcript protein'

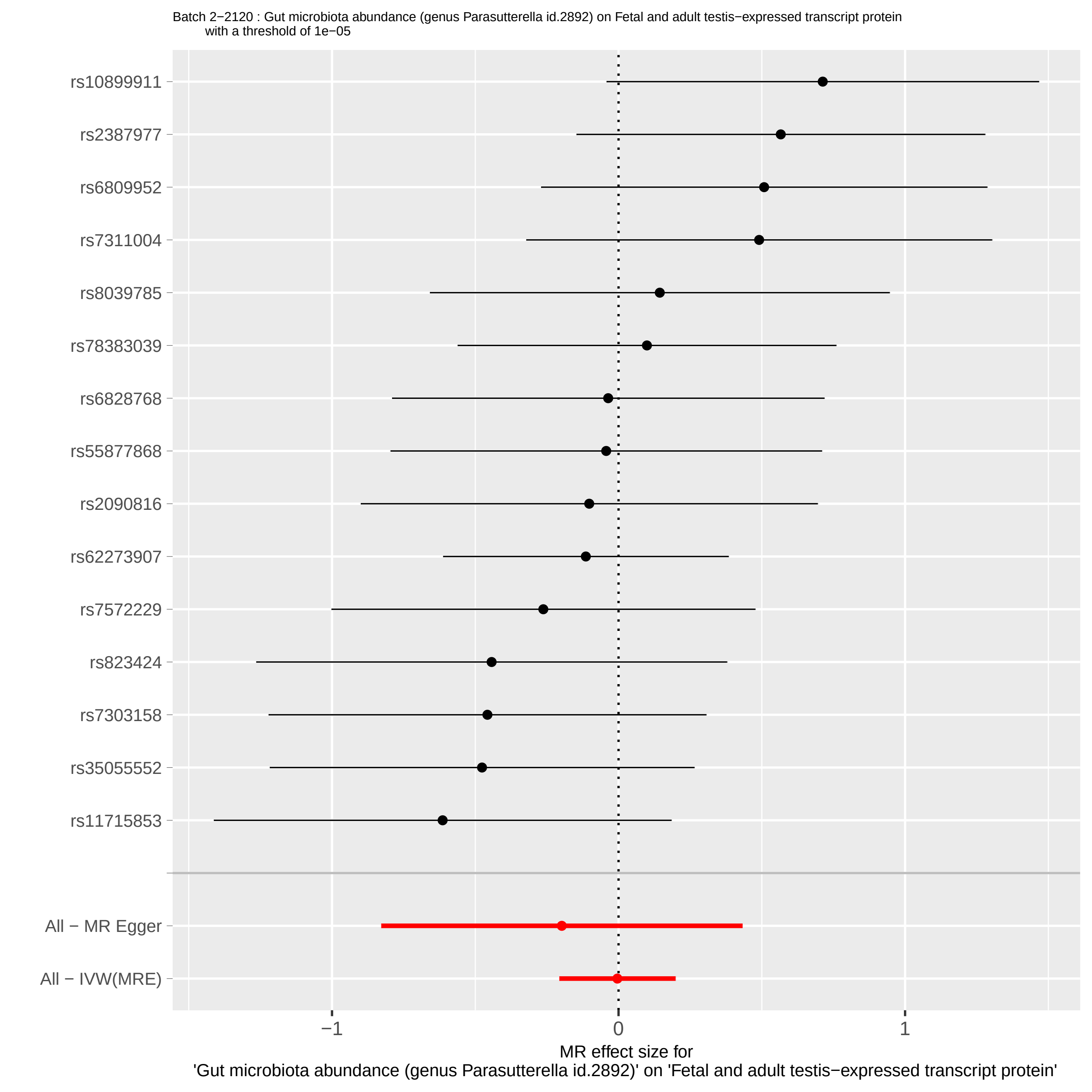
Batch 2−2120 : Gut microbiota abundance (genus Parasutterella id.2892) on Fetal and adult testis−expressed transcript protein
with a threshold of 1e−05
rs10899911
rs2387977
rs6809952
rs7311004
rs8039785
rs78383039
rs6828768
rs55877868
rs2090816
rs62273907
rs7572229
rs823424
rs7303158
rs35055552
rs11715853
All − MR Egger
All − IVW(MRE)
−1
0
1
MR effect size for
'Gut microbiota abundance (genus Parasutterella id.2892)' on 'Fetal and adult testis−expressed transcript protein'

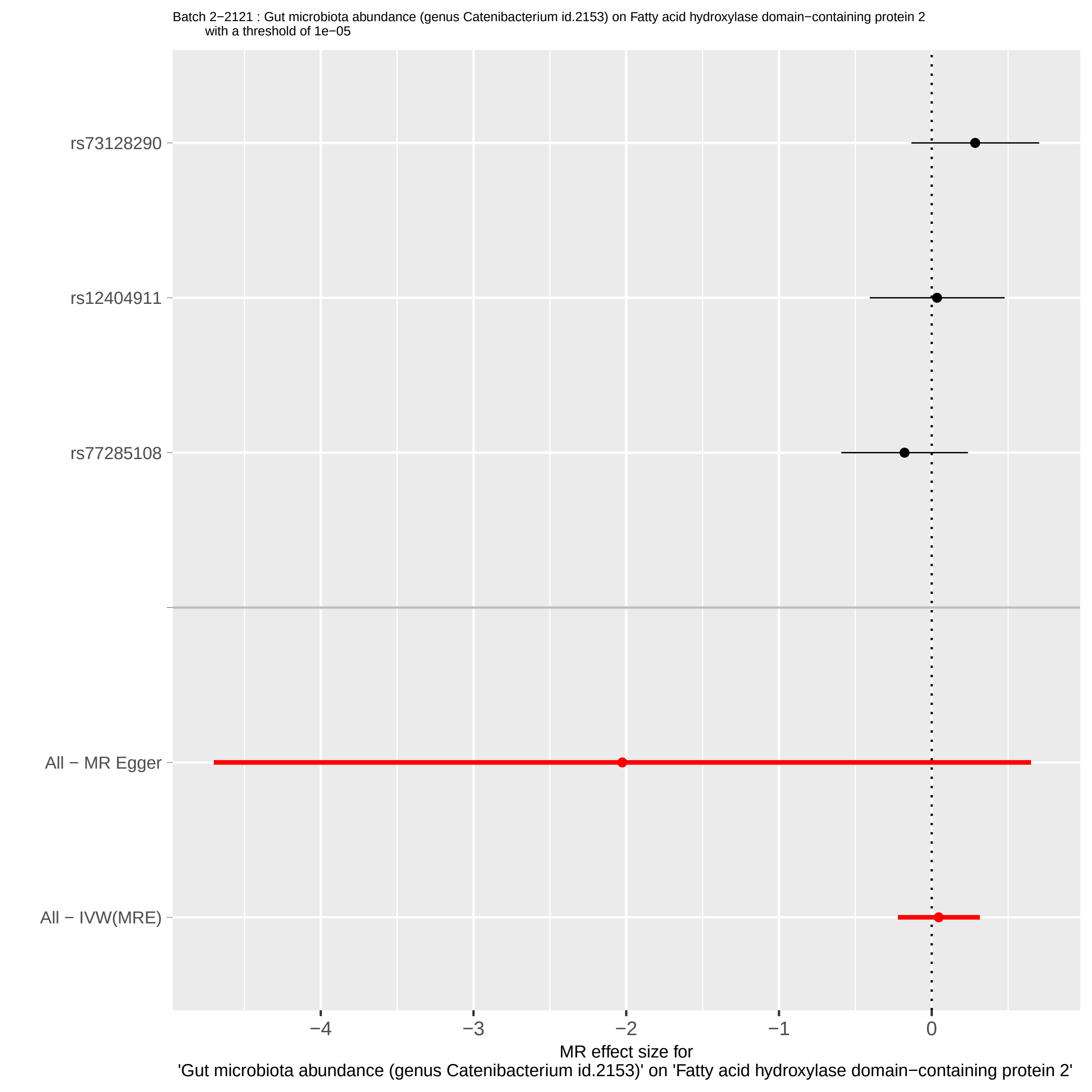

Batch 2−2121 : Gut microbiota abundance (genus Catenibacterium id.2153) on Fatty acid hydroxylase domain−containing protein 2
with a threshold of 1e−05
rs73128290
rs12404911
rs77285108
All − MR Egger
All − IVW(MRE)
−4
−3
−2
−1
0
MR effect size for
'Gut microbiota abundance (genus Catenibacterium id.2153)' on 'Fatty acid hydroxylase domain−containing protein 2'

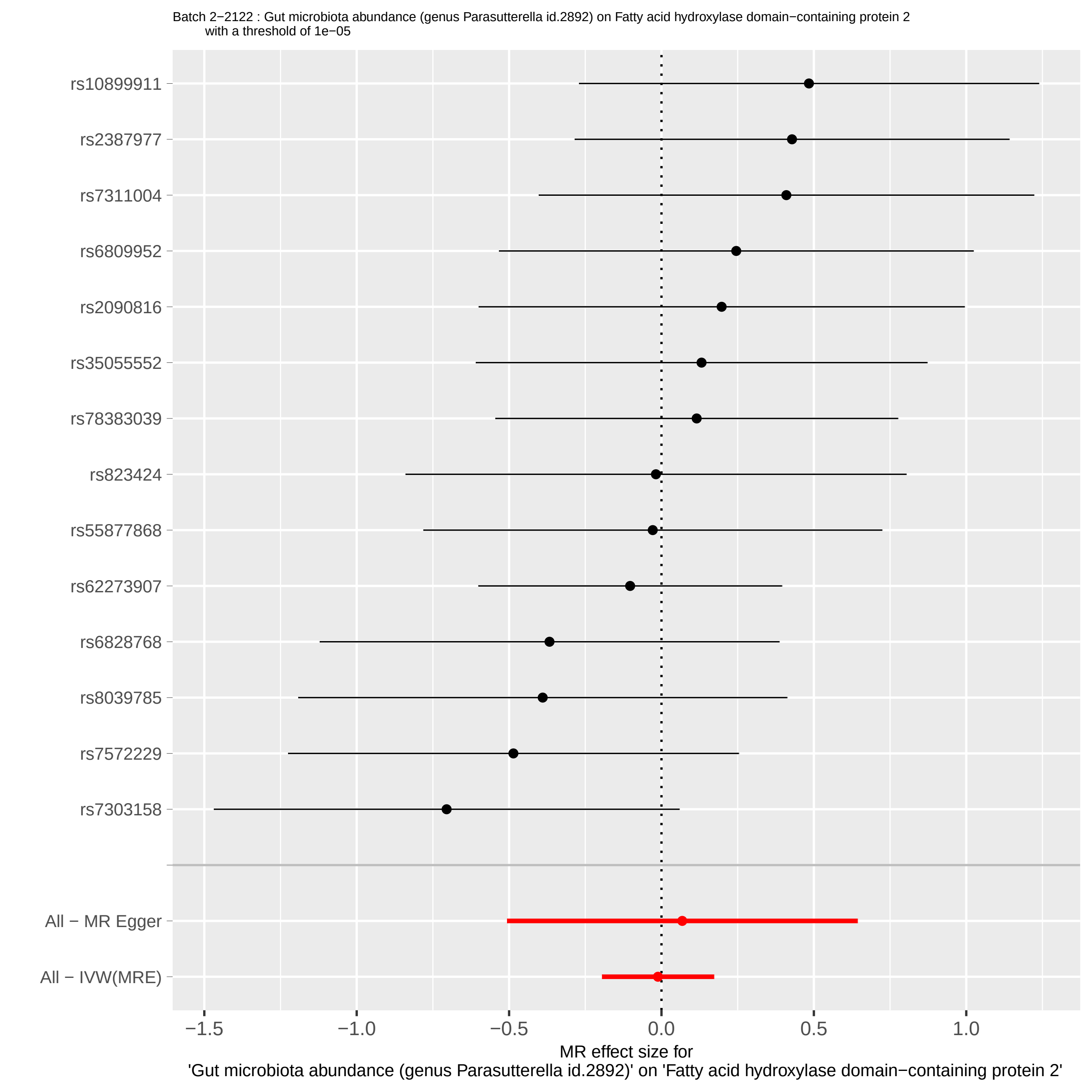

Batch 2−2122 : Gut microbiota abundance (genus Parasutterella id.2892) on Fatty acid hydroxylase domain−containing protein 2
with a threshold of 1e−05
rs10899911
rs2387977
rs7311004
rs6809952
rs2090816
rs35055552
rs78383039
rs823424
rs55877868
rs62273907
rs6828768
rs8039785
rs7572229
rs7303158
All − MR Egger
All − IVW(MRE)
−1.5
−1.0
−0.5
0.0
0.5
1.0
MR effect size for
'Gut microbiota abundance (genus Parasutterella id.2892)' on 'Fatty acid hydroxylase domain−containing protein 2'

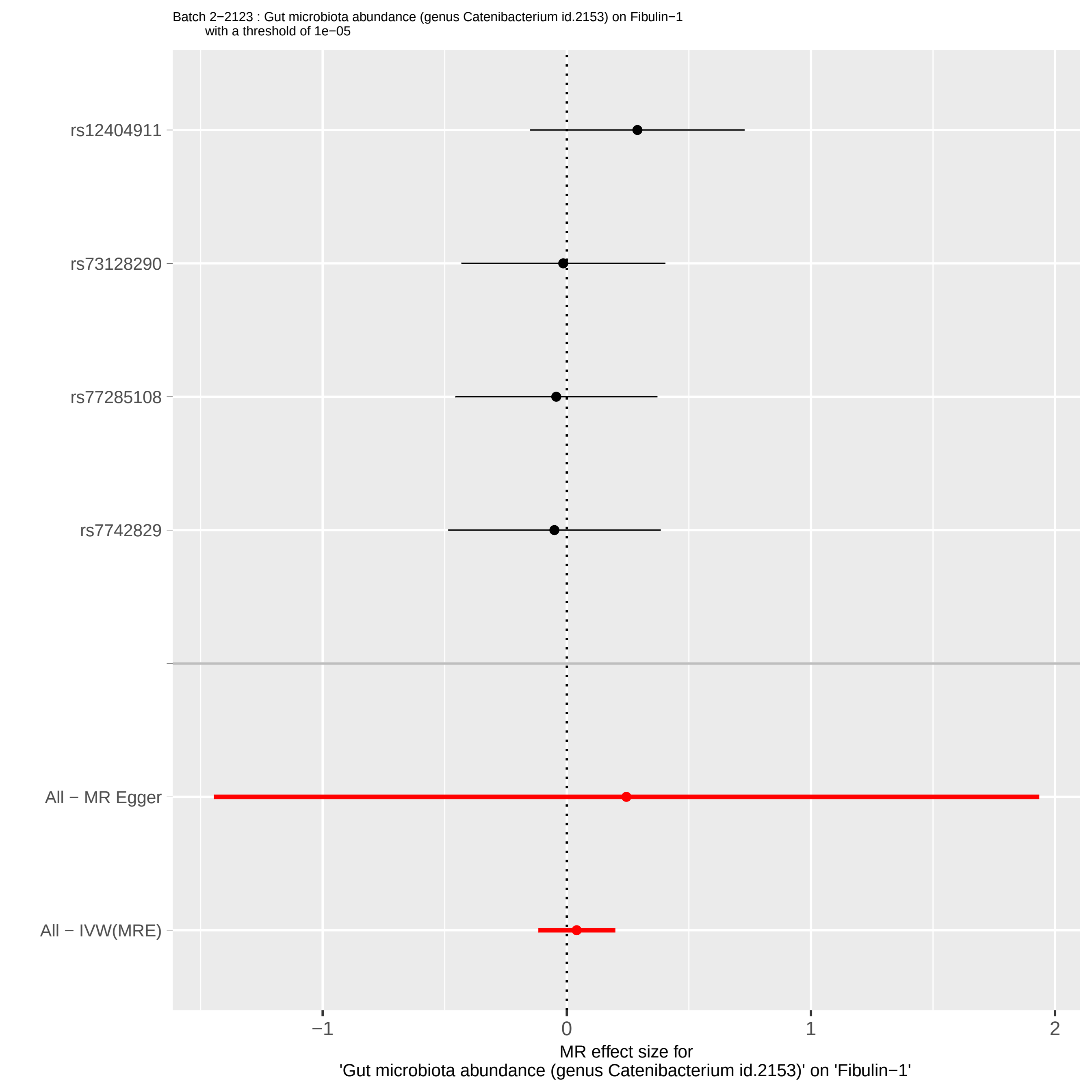

Batch 2−2123 : Gut microbiota abundance (genus Catenibacterium id.2153) on Fibulin−1
with a threshold of 1e−05
rs12404911
rs73128290
rs77285108
rs7742829
All − MR Egger
All − IVW(MRE)
−1
0
1
2
MR effect size for
'Gut microbiota abundance (genus Catenibacterium id.2153)' on 'Fibulin−1'

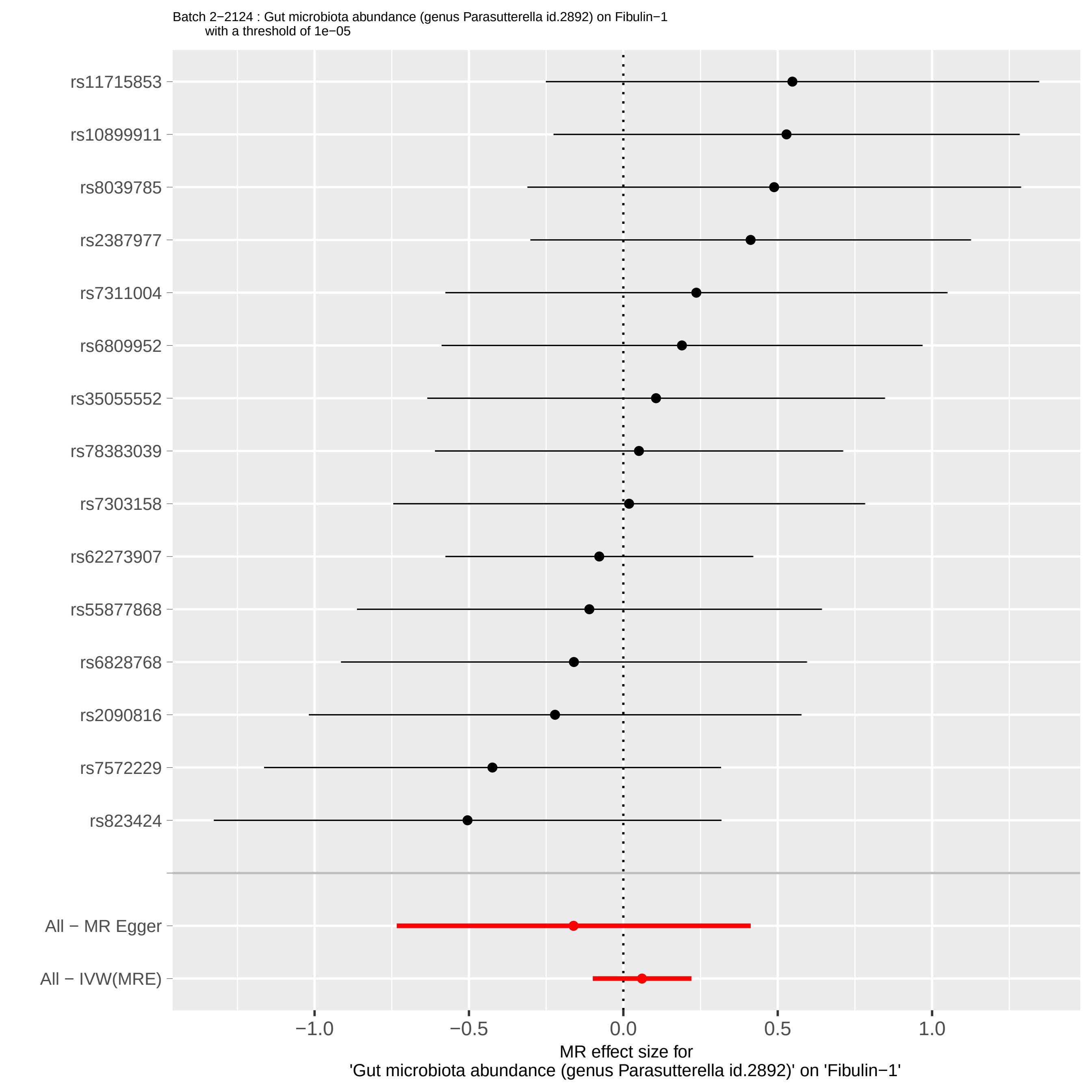
Batch 2−2124 : Gut microbiota abundance (genus Parasutterella id.2892) on Fibulin−1
with a threshold of 1e−05
rs11715853
rs10899911
rs8039785
rs2387977
rs7311004
rs6809952
rs35055552
rs78383039
rs7303158
rs62273907
rs55877868
rs6828768
rs2090816
rs7572229
rs823424
All − MR Egger
All − IVW(MRE)
−1.0
−0.5
0.0
0.5
1.0
MR effect size for
'Gut microbiota abundance (genus Parasutterella id.2892)' on 'Fibulin−1'

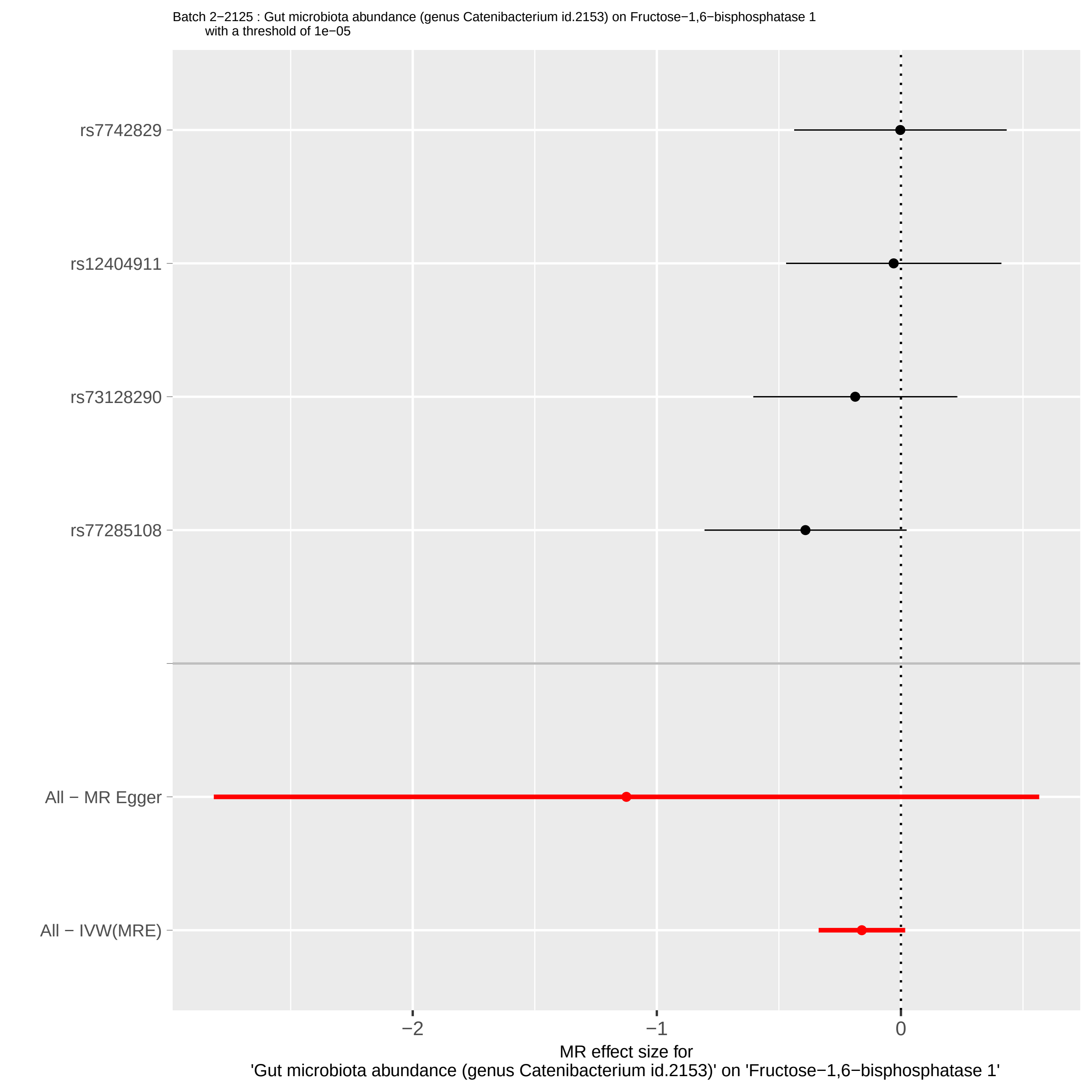

Batch 2−2125 : Gut microbiota abundance (genus Catenibacterium id.2153) on Fructose−1,6−bisphosphatase 1
with a threshold of 1e−05
rs7742829
rs12404911
rs73128290
rs77285108
All − MR Egger
All − IVW(MRE)
−2
−1
0
MR effect size for
'Gut microbiota abundance (genus Catenibacterium id.2153)' on 'Fructose−1,6−bisphosphatase 1'

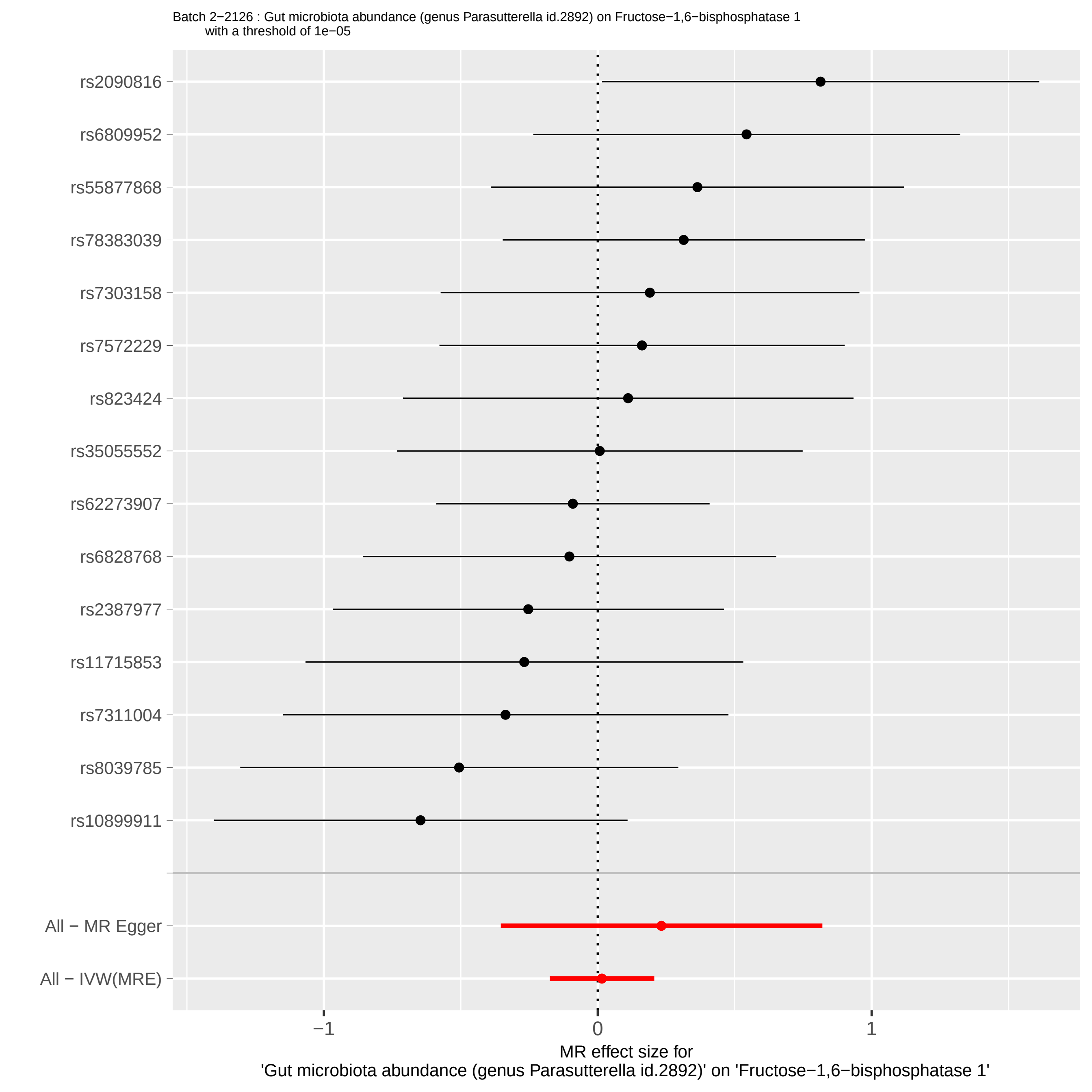

Batch 2−2126 : Gut microbiota abundance (genus Parasutterella id.2892) on Fructose−1,6−bisphosphatase 1
with a threshold of 1e−05
rs2090816
rs6809952
rs55877868
rs78383039
rs7303158
rs7572229
rs823424
rs35055552
rs62273907
rs6828768
rs2387977
rs11715853
rs7311004
rs8039785
rs10899911
All − MR Egger
All − IVW(MRE)
−1
0
1
MR effect size for
'Gut microbiota abundance (genus Parasutterella id.2892)' on 'Fructose−1,6−bisphosphatase 1'

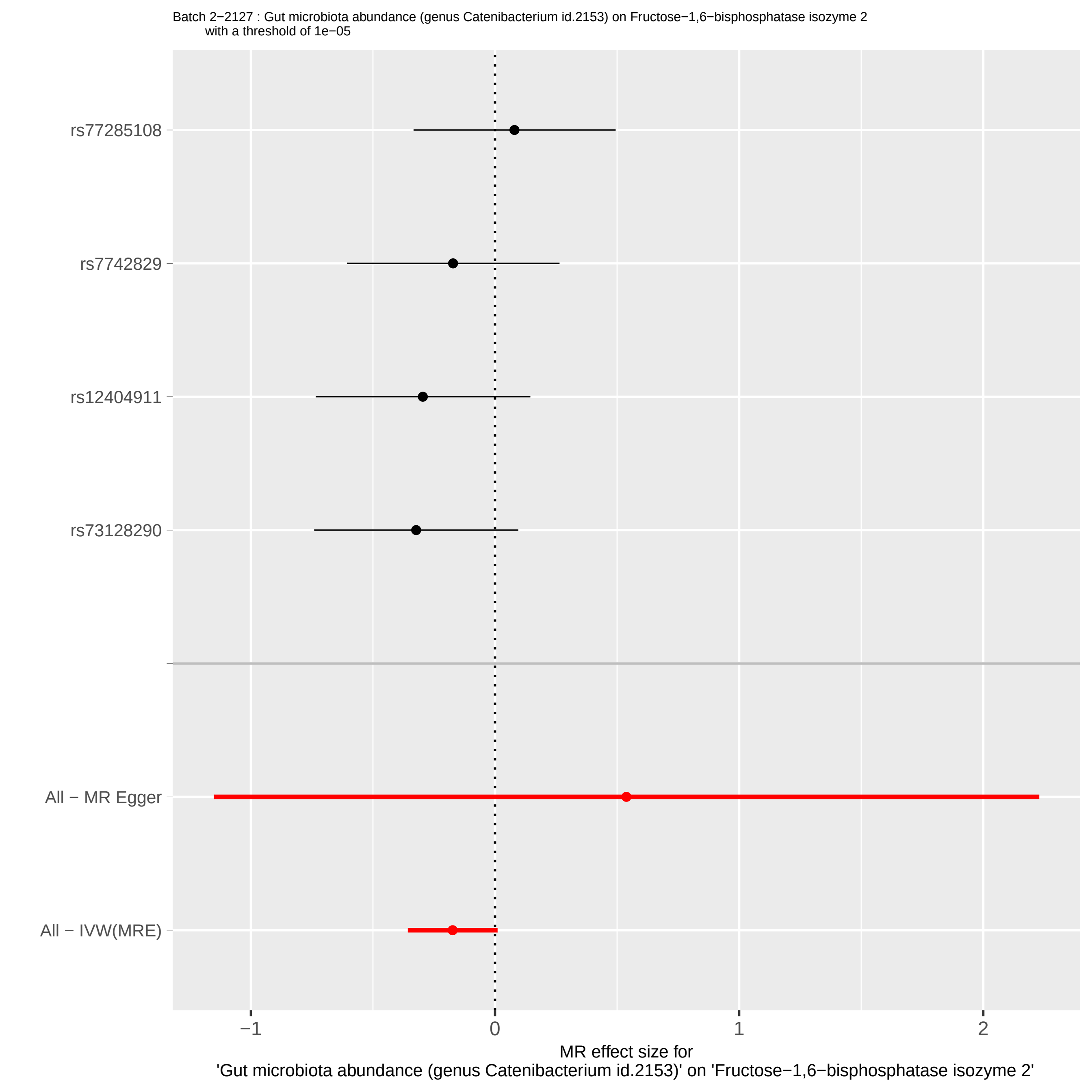

Batch 2−2127 : Gut microbiota abundance (genus Catenibacterium id.2153) on Fructose−1,6−bisphosphatase isozyme 2
with a threshold of 1e−05
rs77285108
rs7742829
rs12404911
rs73128290
All − MR Egger
All − IVW(MRE)
−1
0
1
2
MR effect size for
'Gut microbiota abundance (genus Catenibacterium id.2153)' on 'Fructose−1,6−bisphosphatase isozyme 2'

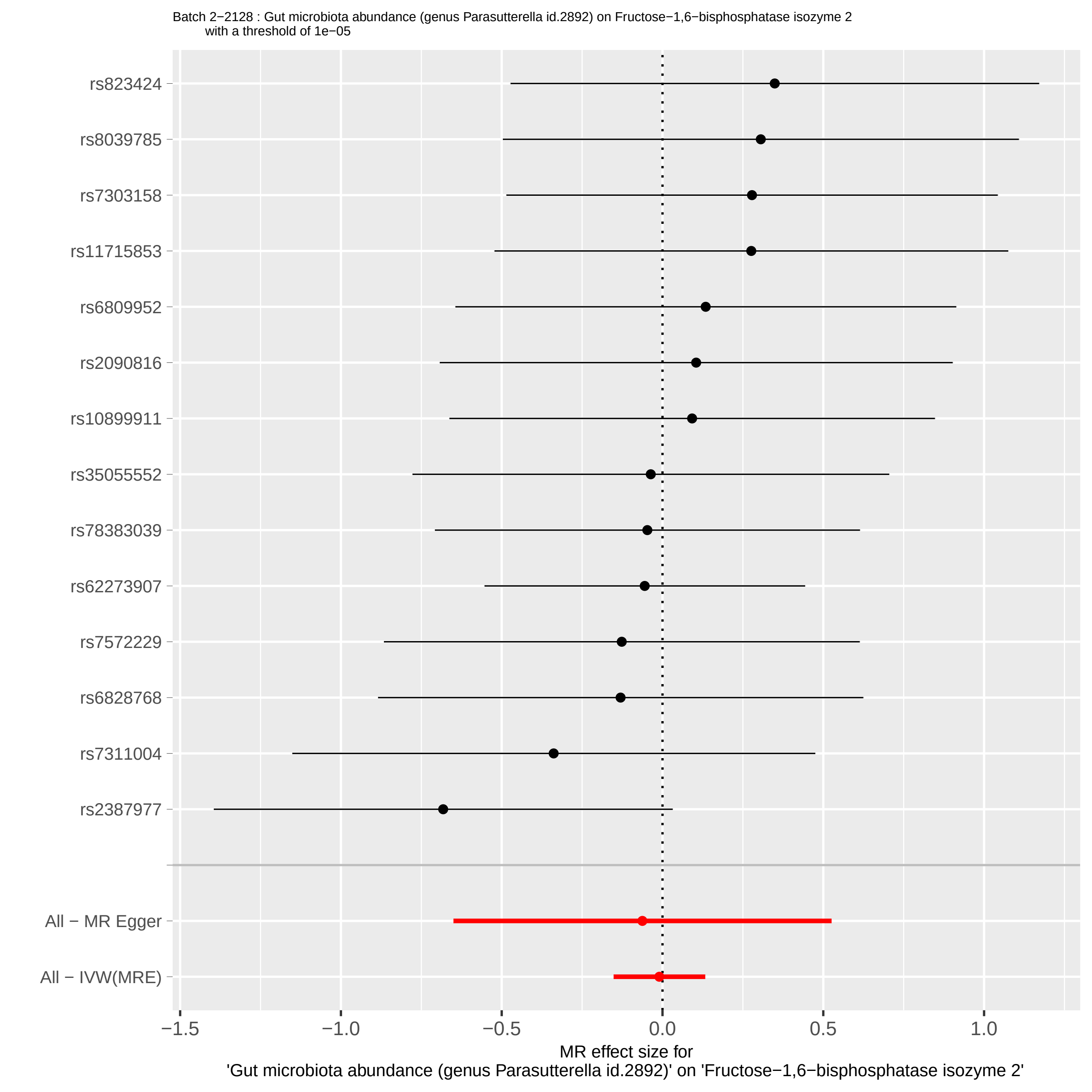

Batch 2−2128 : Gut microbiota abundance (genus Parasutterella id.2892) on Fructose−1,6−bisphosphatase isozyme 2
with a threshold of 1e−05
rs823424
rs8039785
rs7303158
rs11715853
rs6809952
rs2090816
rs10899911
rs35055552
rs78383039
rs62273907
rs7572229
rs6828768
rs7311004
rs2387977
All − MR Egger
All − IVW(MRE)
−1.5
−1.0
−0.5
0.0
0.5
1.0
MR effect size for
'Gut microbiota abundance (genus Parasutterella id.2892)' on 'Fructose−1,6−bisphosphatase isozyme 2'

Batch 2−2129 : Gut microbiota abundance (genus Catenibacterium id.2153) on F−box/LRR−repeat protein 4
with a threshold of 1e−05

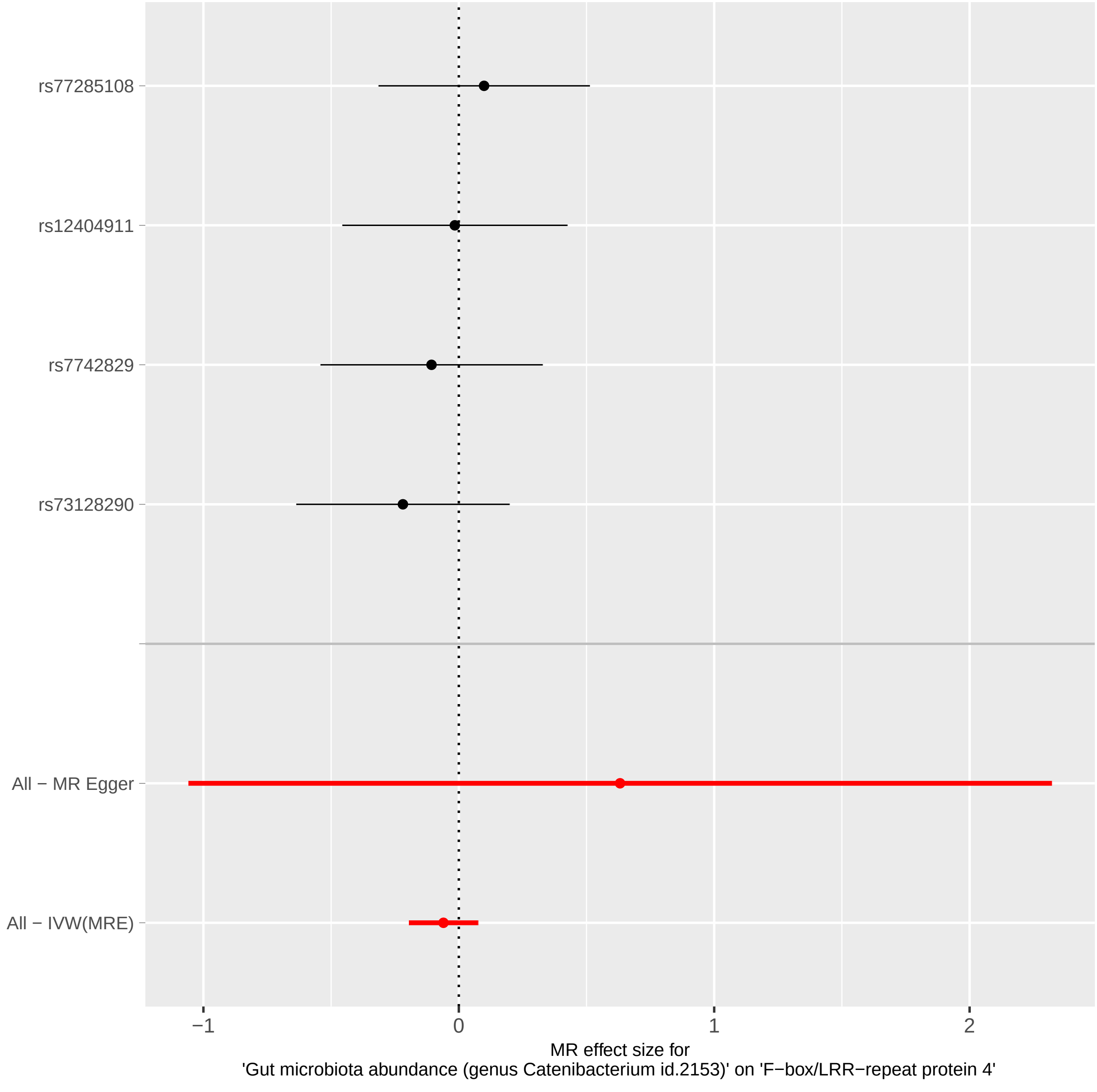

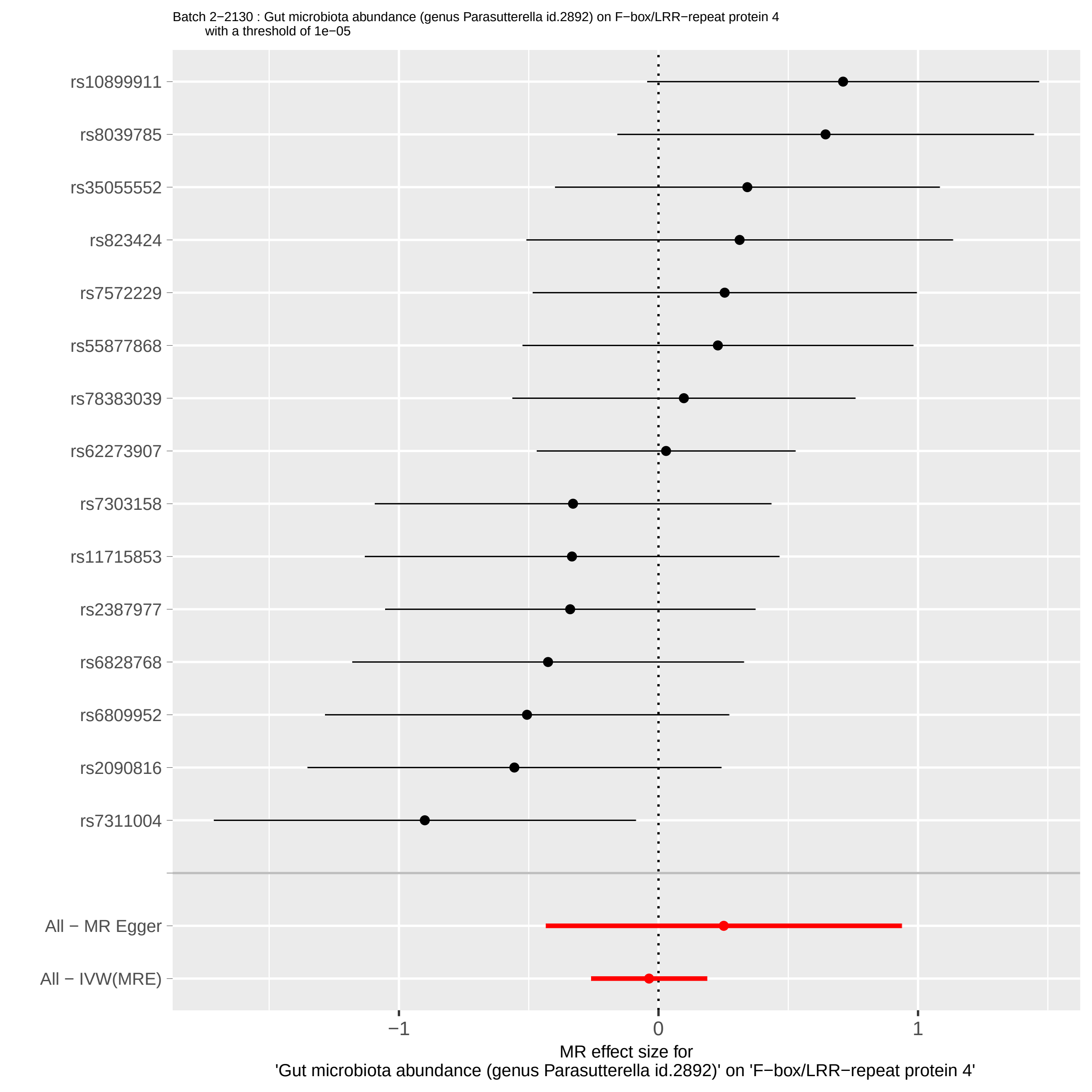

Batch 2−2130 : Gut microbiota abundance (genus Parasutterella id.2892) on F−box/LRR−repeat protein 4
with a threshold of 1e−05
rs10899911
rs8039785
rs35055552
rs823424
rs7572229
rs55877868
rs78383039
rs62273907
rs7303158
rs11715853
rs2387977
rs6828768
rs6809952
rs2090816
rs7311004
All − MR Egger
All − IVW(MRE)
−1
0
1
MR effect size for
'Gut microbiota abundance (genus Parasutterella id.2892)' on 'F−box/LRR−repeat protein 4'

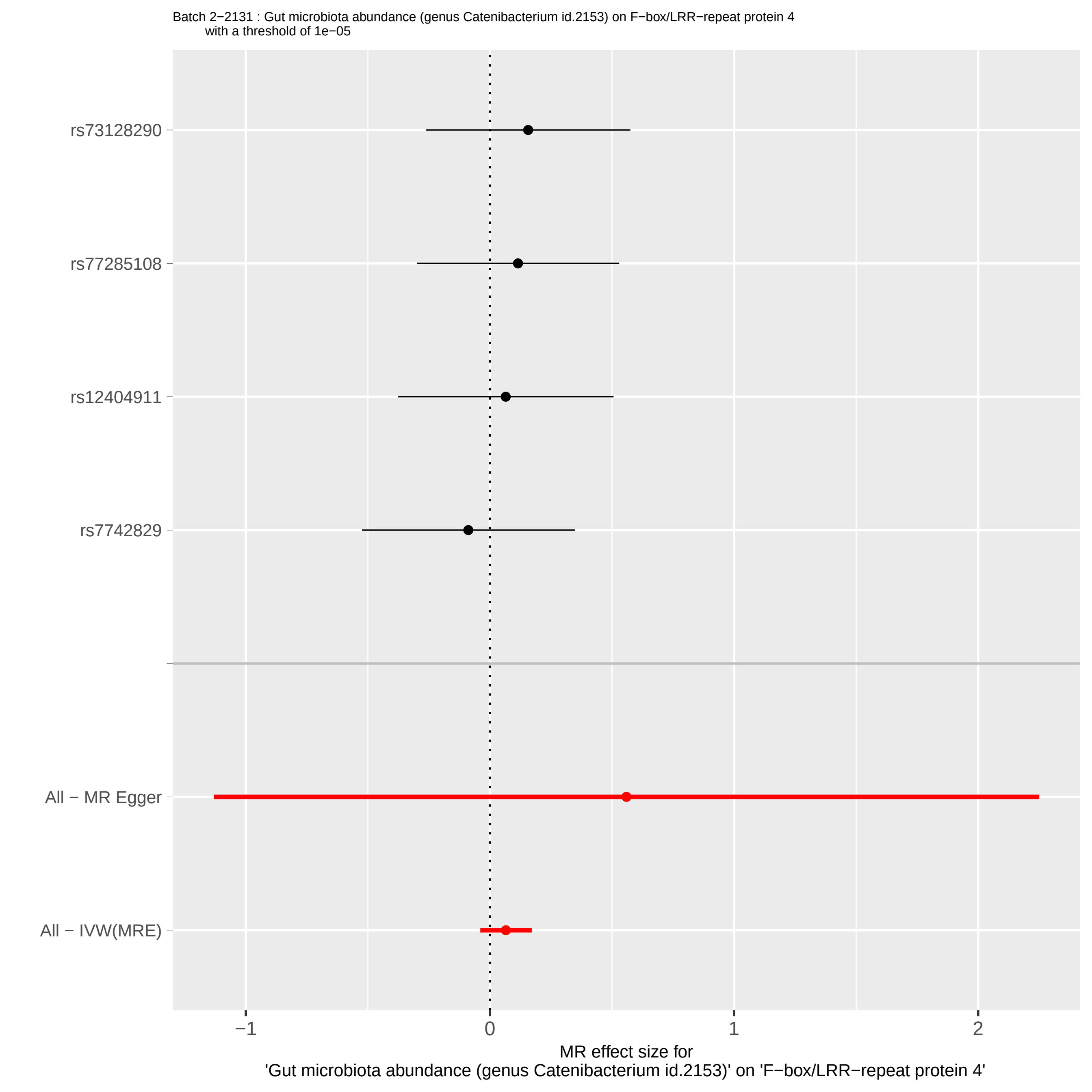

Batch 2−2131 : Gut microbiota abundance (genus Catenibacterium id.2153) on F−box/LRR−repeat protein 4
with a threshold of 1e−05
rs73128290
rs77285108
rs12404911
rs7742829
All − MR Egger
All − IVW(MRE)
−1
0
1
2
MR effect size for
'Gut microbiota abundance (genus Catenibacterium id.2153)' on 'F−box/LRR−repeat protein 4'

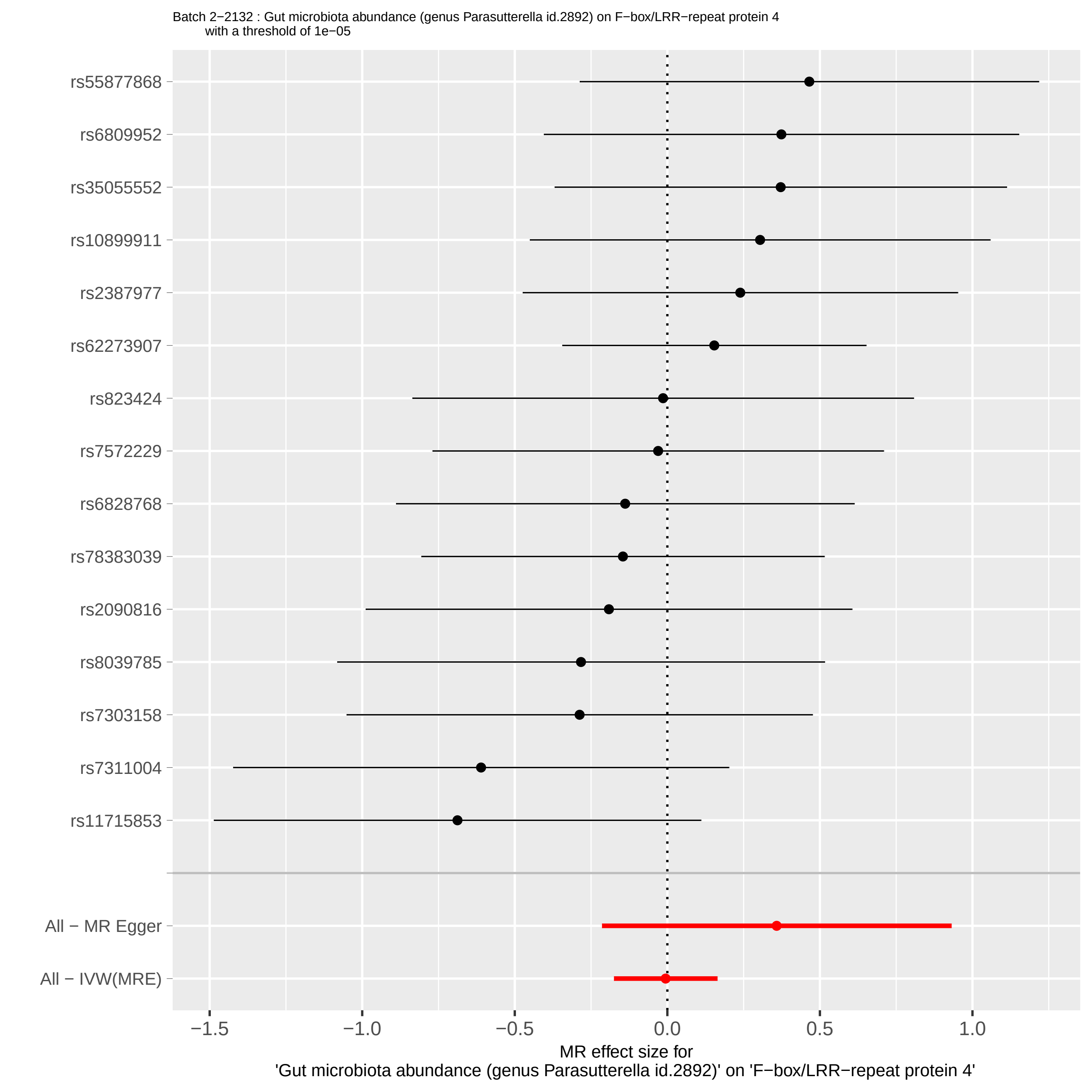

Batch 2−2132 : Gut microbiota abundance (genus Parasutterella id.2892) on F−box/LRR−repeat protein 4
with a threshold of 1e−05
rs55877868
rs6809952
rs35055552
rs10899911
rs2387977
rs62273907
rs823424
rs7572229
rs6828768
rs78383039
rs2090816
rs8039785
rs7303158
rs7311004
rs11715853
All − MR Egger
All − IVW(MRE)
−1.5
−1.0
−0.5
0.0
0.5
1.0
MR effect size for
'Gut microbiota abundance (genus Parasutterella id.2892)' on 'F−box/LRR−repeat protein 4'

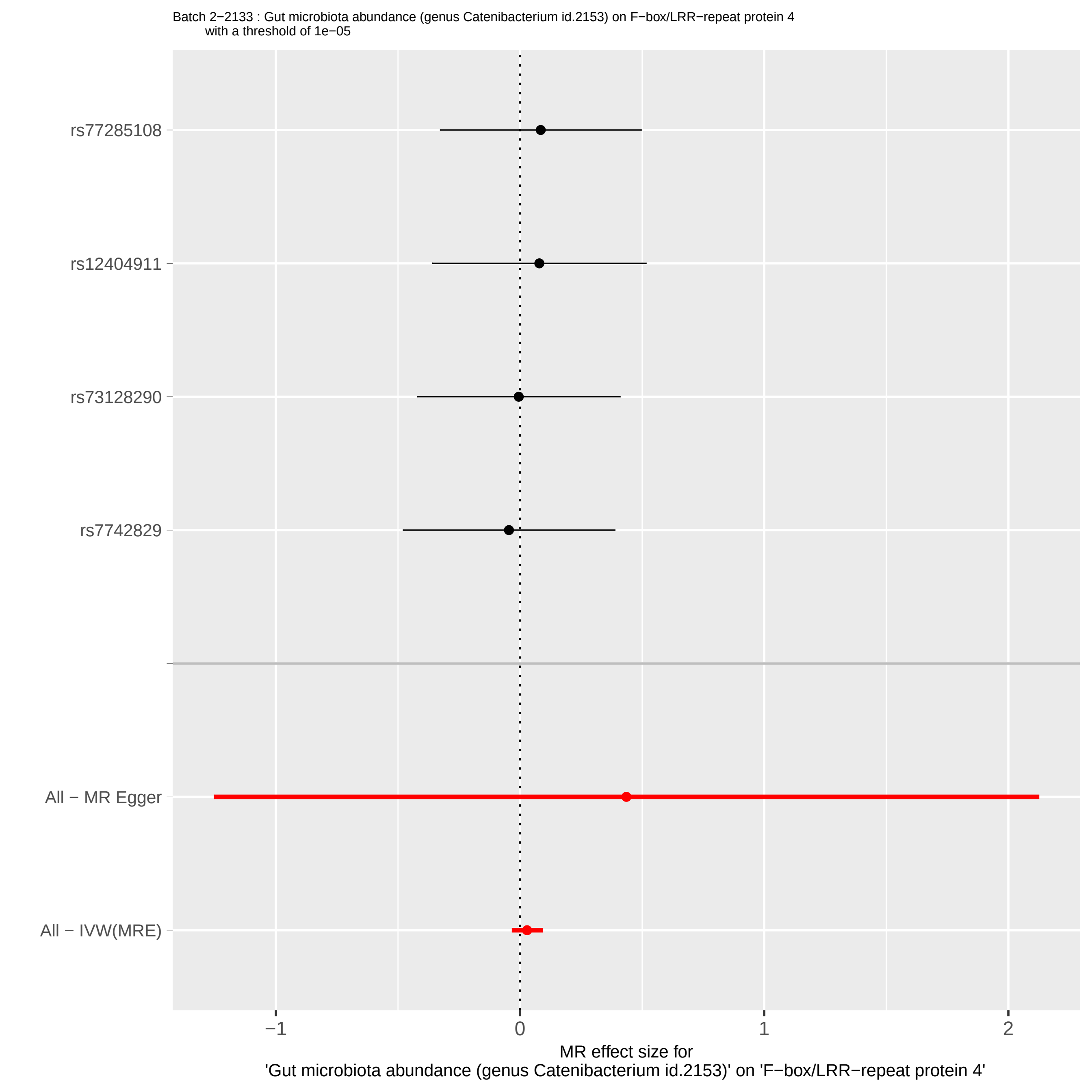
Batch 2−2133 : Gut microbiota abundance (genus Catenibacterium id.2153) on F−box/LRR−repeat protein 4
with a threshold of 1e−05
rs77285108
rs12404911
rs73128290
rs7742829
All − MR Egger
All − IVW(MRE)
−1
0
1
2
MR effect size for
'Gut microbiota abundance (genus Catenibacterium id.2153)' on 'F−box/LRR−repeat protein 4'

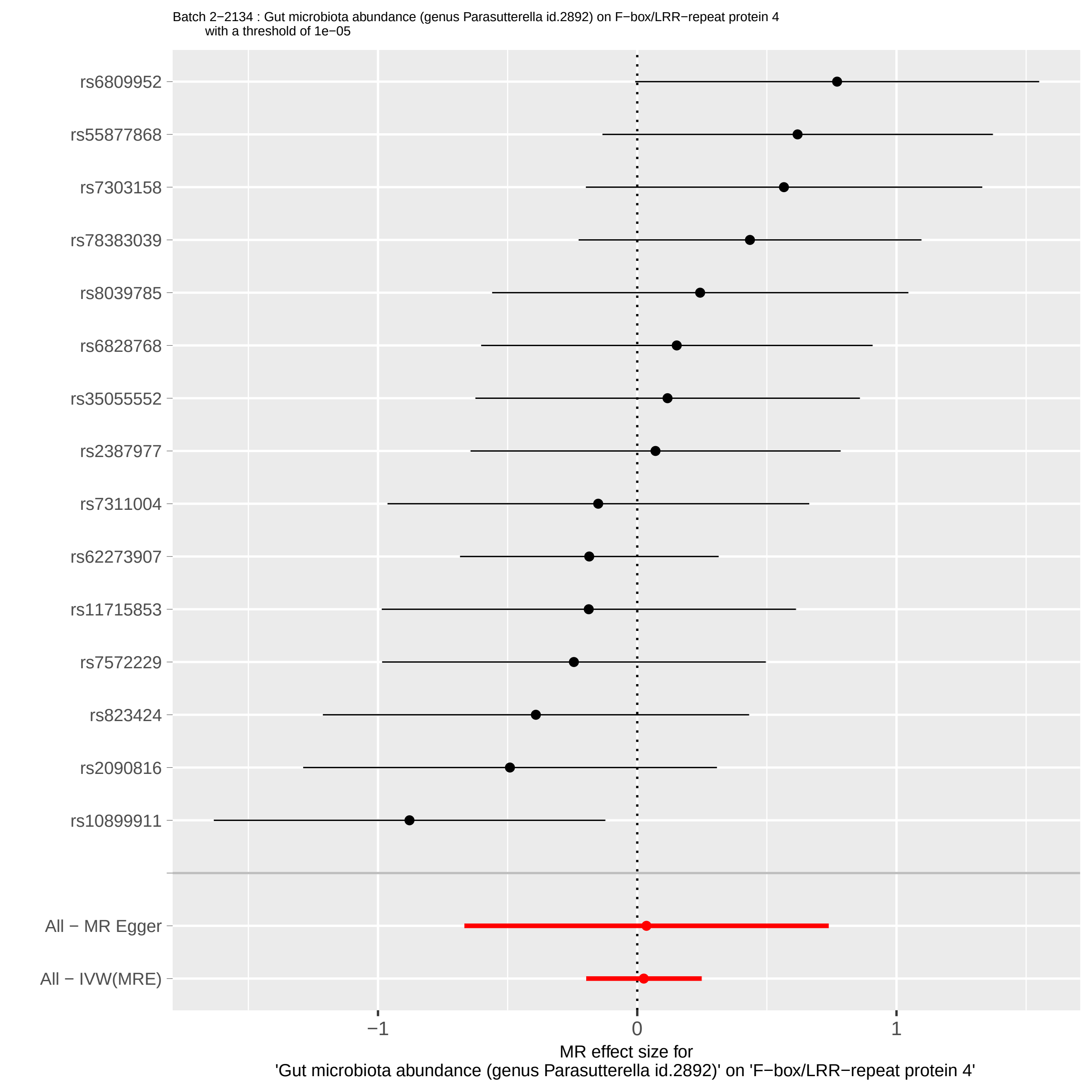

Batch 2−2134 : Gut microbiota abundance (genus Parasutterella id.2892) on F−box/LRR−repeat protein 4
with a threshold of 1e−05
rs6809952
rs55877868
rs7303158
rs78383039
rs8039785
rs6828768
rs35055552
rs2387977
rs7311004
rs62273907
rs11715853
rs7572229
rs823424
rs2090816
rs10899911
All − MR Egger
All − IVW(MRE)
−1
0
1
MR effect size for
'Gut microbiota abundance (genus Parasutterella id.2892)' on 'F−box/LRR−repeat protein 4'

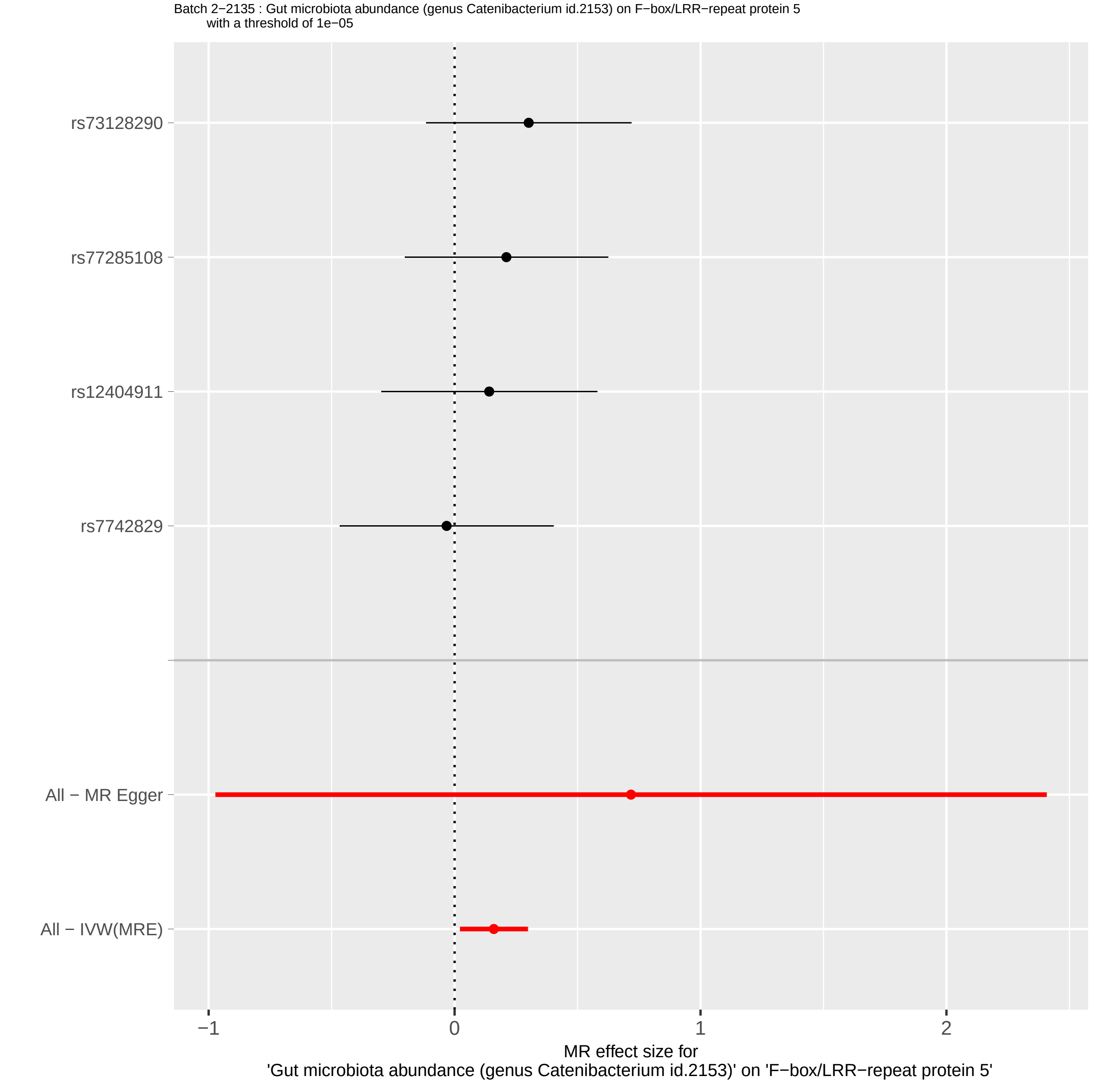

Batch 2−2135 : Gut microbiota abundance (genus Catenibacterium id.2153) on F−box/LRR−repeat protein 5
with a threshold of 1e−05
rs73128290
rs77285108
rs12404911
rs7742829
All − MR Egger
All − IVW(MRE)
−1
0
1
2
MR effect size for
'Gut microbiota abundance (genus Catenibacterium id.2153)' on 'F−box/LRR−repeat protein 5'

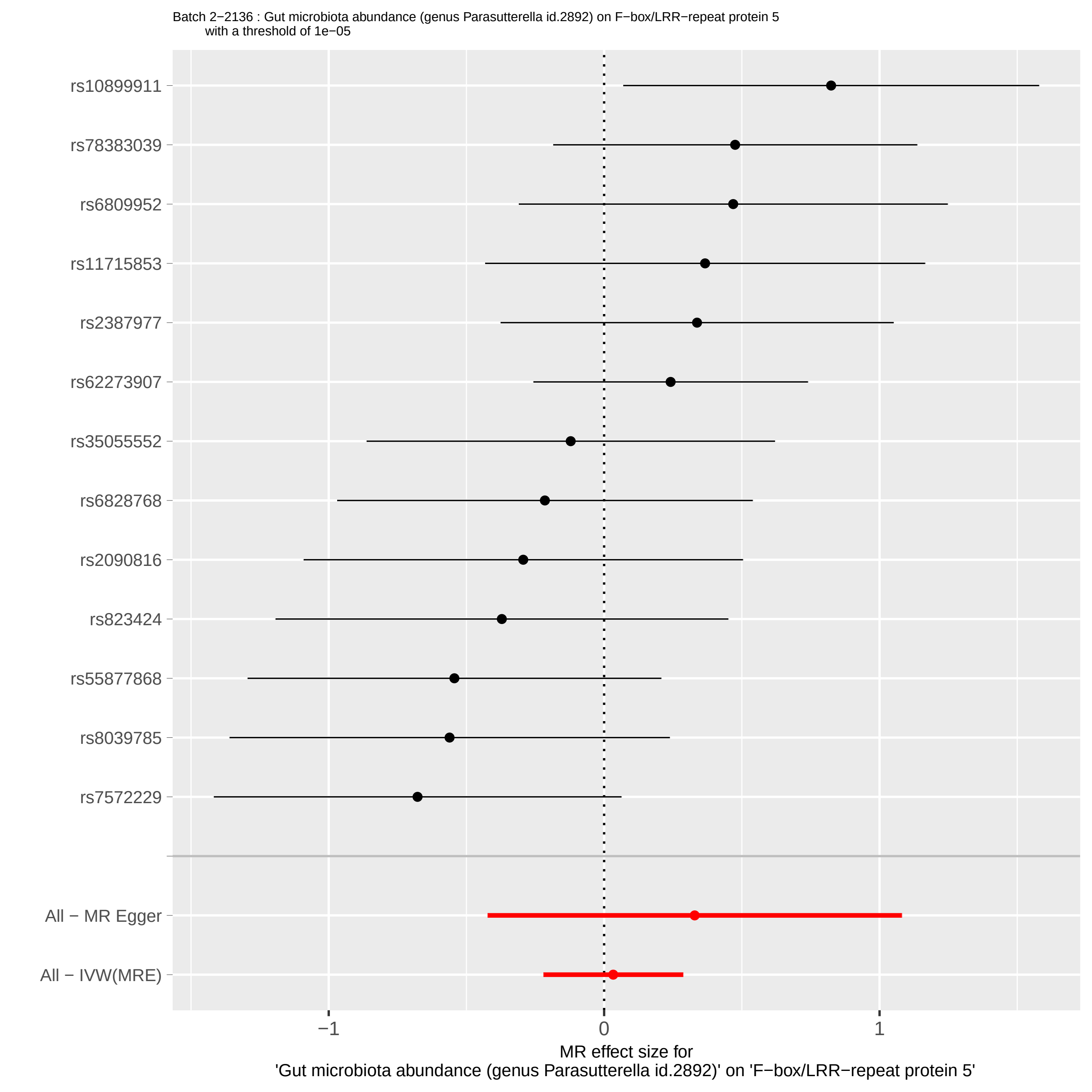

Batch 2−2136 : Gut microbiota abundance (genus Parasutterella id.2892) on F−box/LRR−repeat protein 5
with a threshold of 1e−05
rs10899911
rs78383039
rs6809952
rs11715853
rs2387977
rs62273907
rs35055552
rs6828768
rs2090816
rs823424
rs55877868
rs8039785
rs7572229
All − MR Egger
All − IVW(MRE)
−1
0
1
MR effect size for
'Gut microbiota abundance (genus Parasutterella id.2892)' on 'F−box/LRR−repeat protein 5'

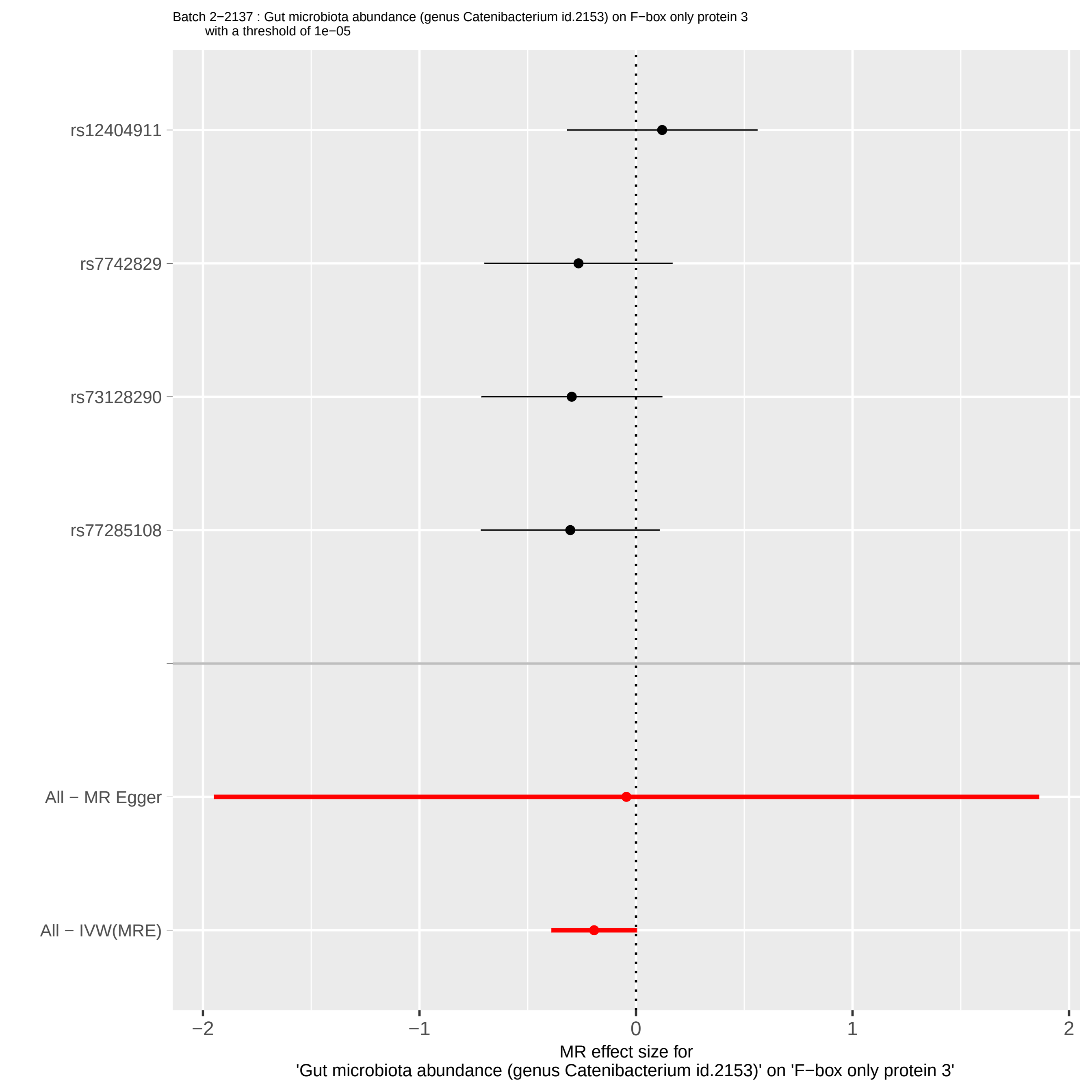

Batch 2−2137 : Gut microbiota abundance (genus Catenibacterium id.2153) on F−box only protein 3
with a threshold of 1e−05
rs12404911
rs7742829
rs73128290
rs77285108
All − MR Egger
All − IVW(MRE)
−2
−1
0
1
2
MR effect size for
'Gut microbiota abundance (genus Catenibacterium id.2153)' on 'F−box only protein 3'

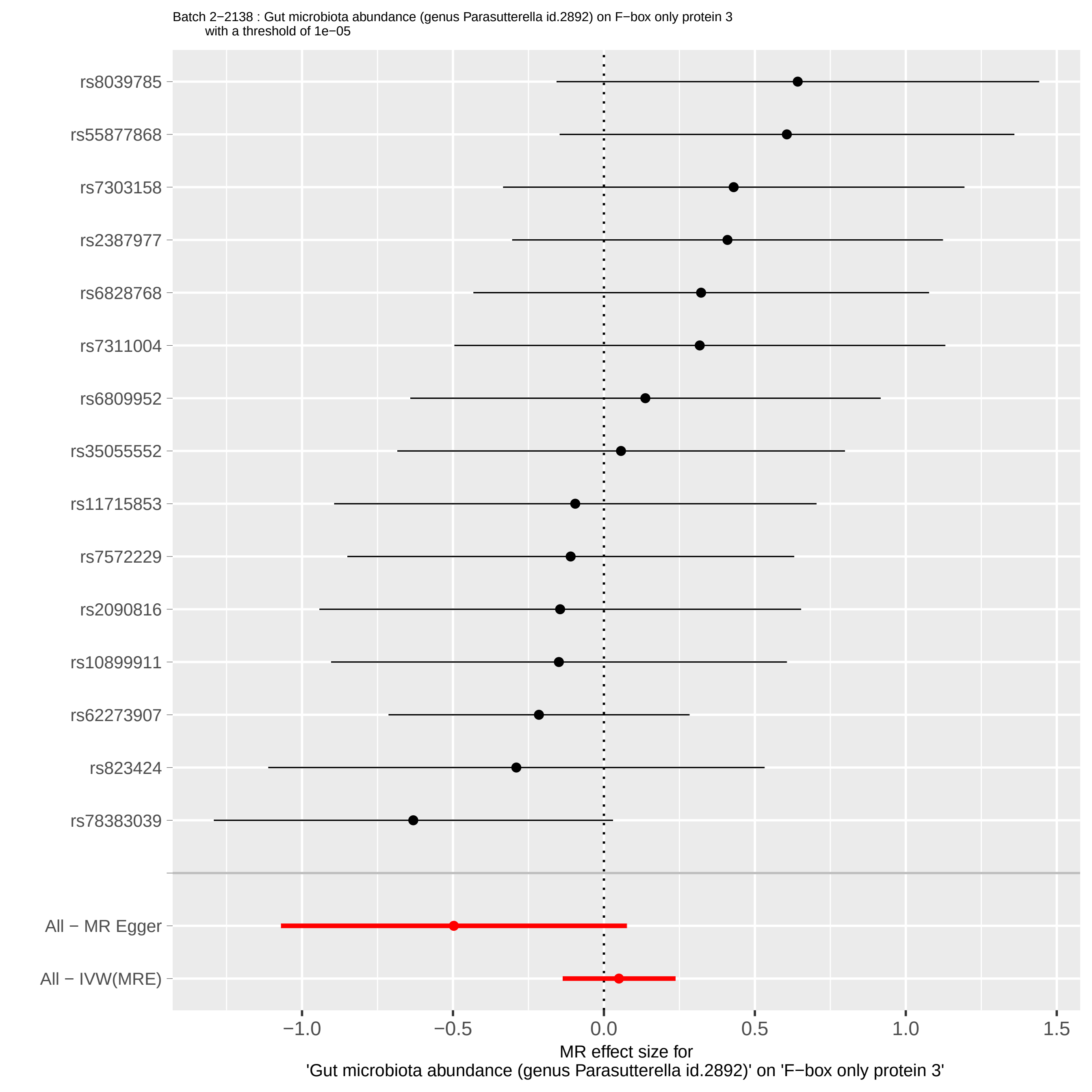

Batch 2−2138 : Gut microbiota abundance (genus Parasutterella id.2892) on F−box only protein 3
with a threshold of 1e−05
rs8039785
rs55877868
rs7303158
rs2387977
rs6828768
rs7311004
rs6809952
rs35055552
rs11715853
rs7572229
rs2090816
rs10899911
rs62273907
rs823424
rs78383039
All − MR Egger
All − IVW(MRE)
−1.0
−0.5
0.0
0.5
1.0
1.5
MR effect size for
'Gut microbiota abundance (genus Parasutterella id.2892)' on 'F−box only protein 3'

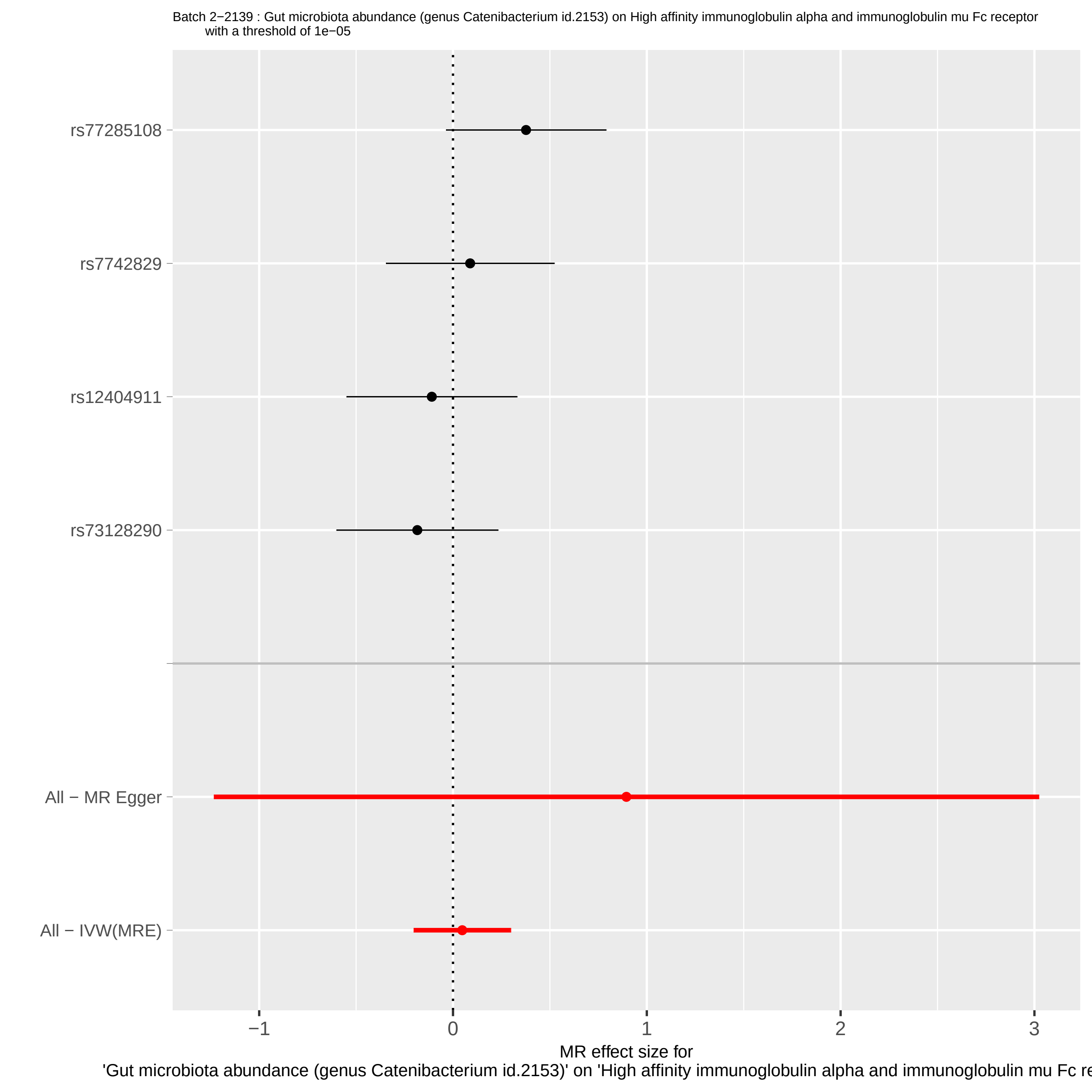
Batch 2−2139 : Gut microbiota abundance (genus Catenibacterium id.2153) on High affinity immunoglobulin alpha and immunoglobulin mu Fc receptor
with a threshold of 1e−05
rs77285108
rs7742829
rs12404911
rs73128290
All − MR Egger
All − IVW(MRE)
−1
0
1
2
3
MR effect size for
'Gut microbiota abundance (genus Catenibacterium id.2153)' on 'High affinity immunoglobulin alpha and immunoglobulin mu Fc re

Batch 2−2140 : Gut microbiota abundance (genus Parasutterella id.2892) on High affinity immunoglobulin alpha and immunoglobulin mu Fc receptor with a threshold of 1e−05

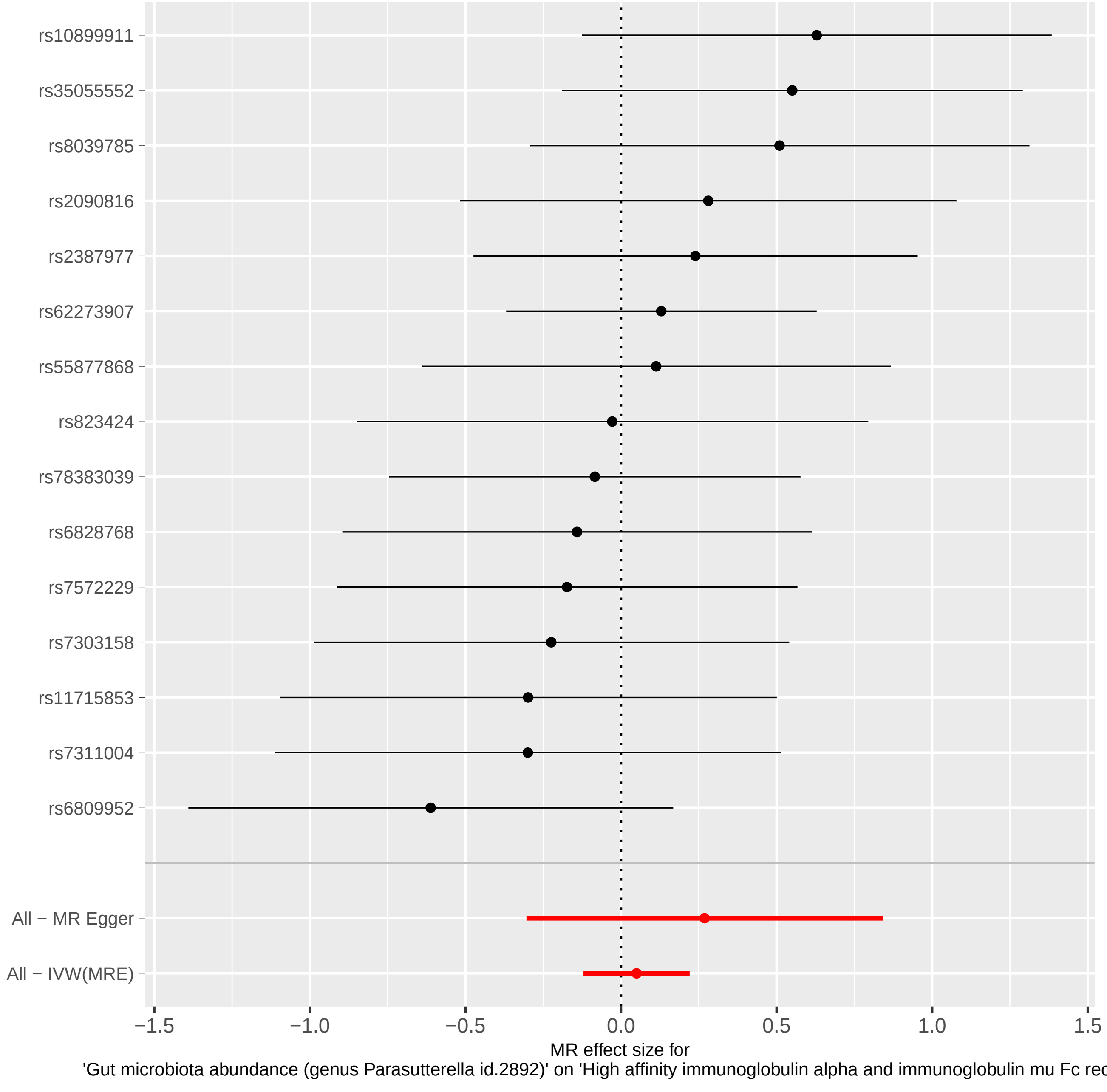

Batch 2−2141 : Gut microbiota abundance (genus Catenibacterium id.2153) on Immunoglobulin alpha Fc receptor
with a threshold of 1e−05

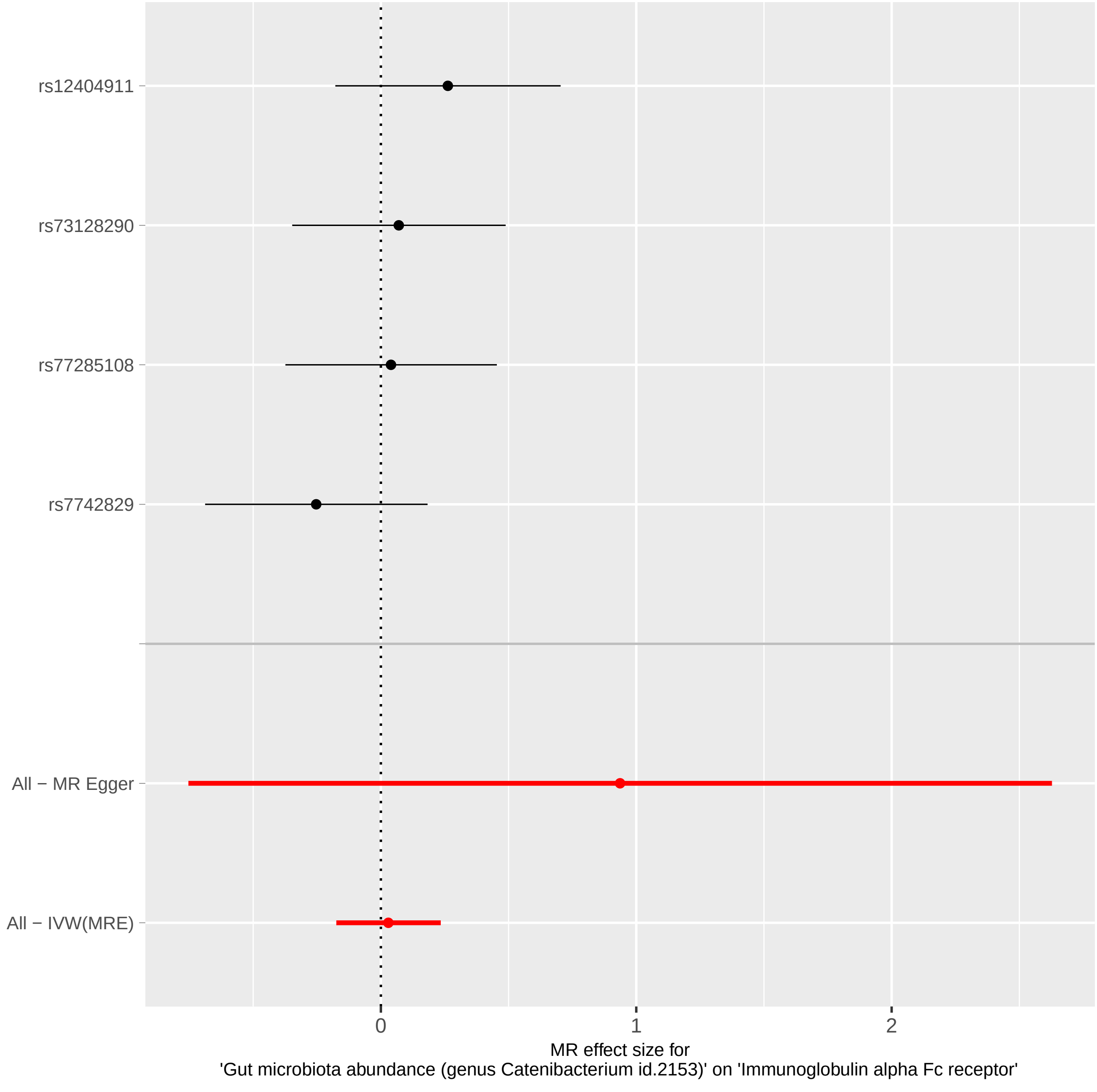

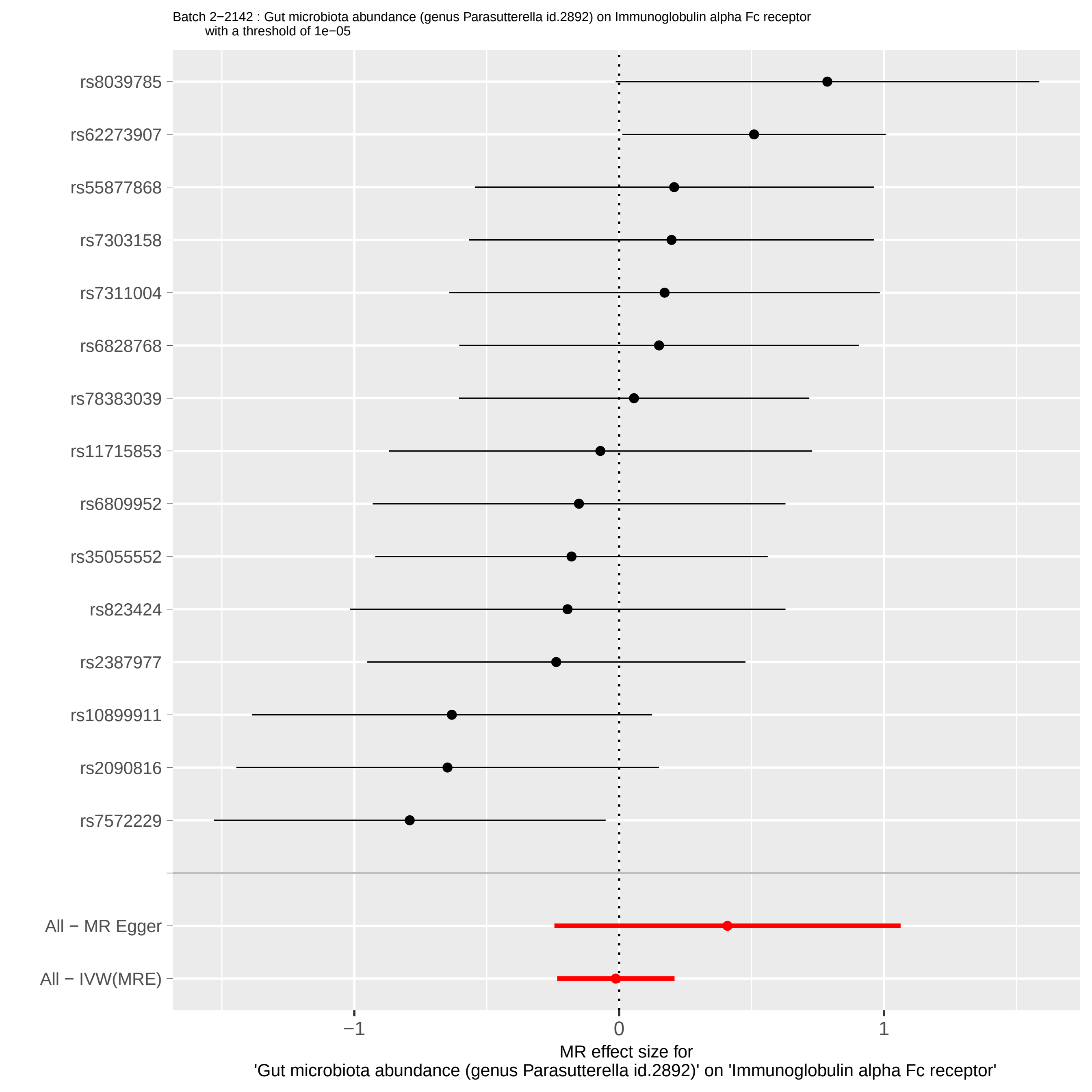
Batch 2−2142 : Gut microbiota abundance (genus Parasutterella id.2892) on Immunoglobulin alpha Fc receptor
with a threshold of 1e−05
rs8039785
rs62273907
rs55877868
rs7303158
rs7311004
rs6828768
rs78383039
rs11715853
rs6809952
rs35055552
rs823424
rs2387977
rs10899911
rs2090816
rs7572229
All − MR Egger
All − IVW(MRE)
−1
0
1
MR effect size for
'Gut microbiota abundance (genus Parasutterella id.2892)' on 'Immunoglobulin alpha Fc receptor'

Batch 2−2143 : Gut microbiota abundance (genus Catenibacterium id.2153) on Low affinity immunoglobulin epsilon Fc receptor
with a threshold of 1e−05

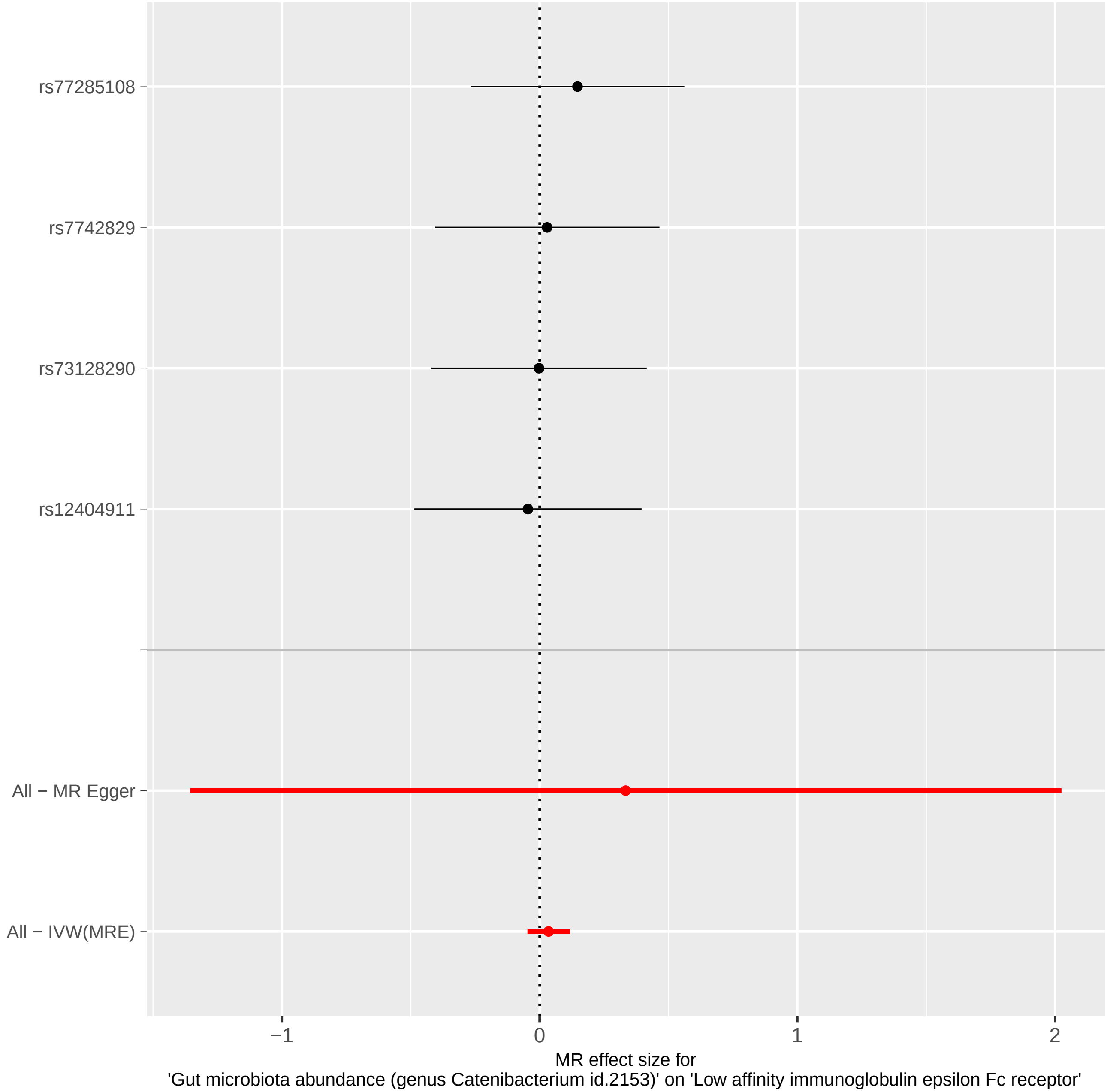

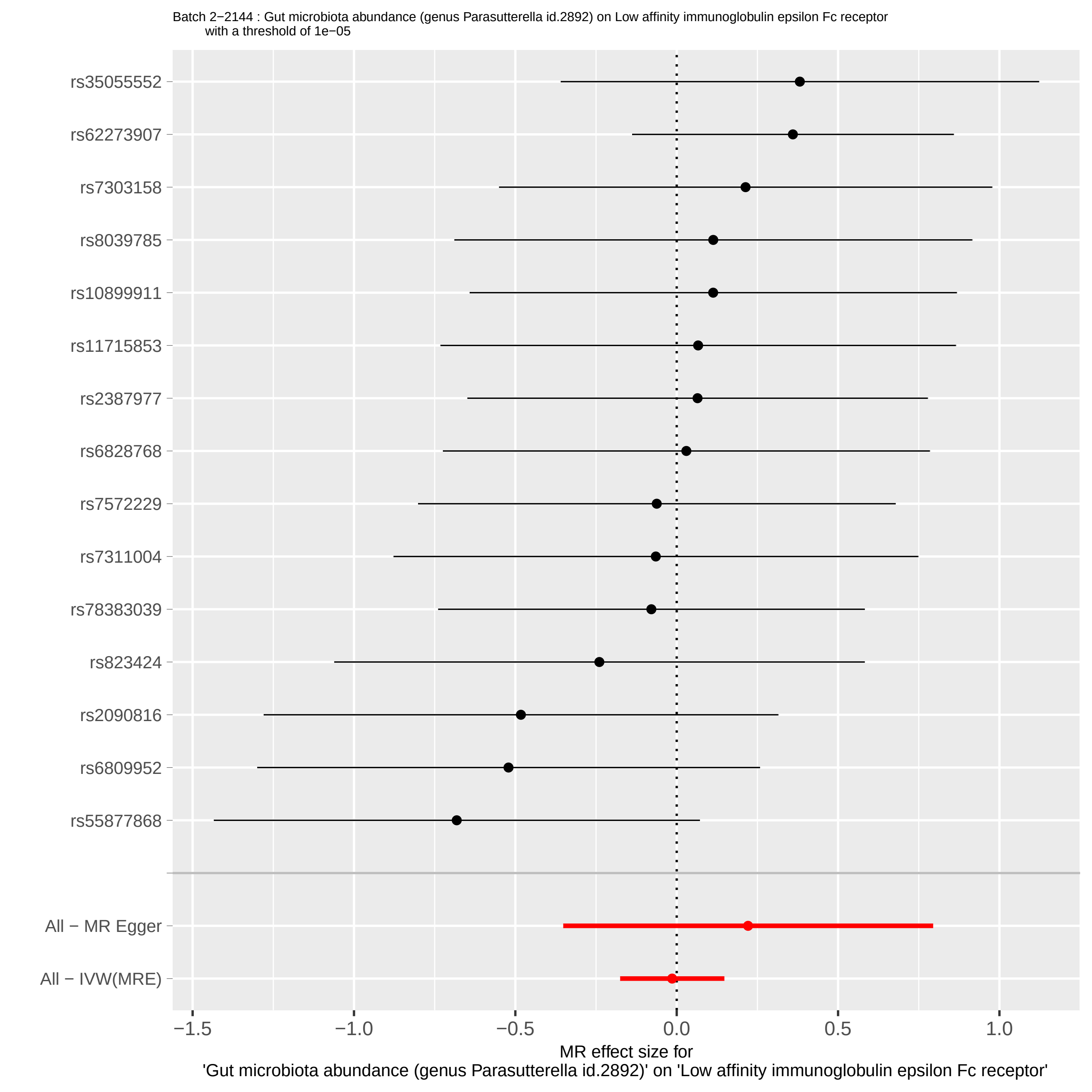
Batch 2−2144 : Gut microbiota abundance (genus Parasutterella id.2892) on Low affinity immunoglobulin epsilon Fc receptor
with a threshold of 1e−05
rs35055552
rs62273907
rs7303158
rs8039785
rs10899911
rs11715853
rs2387977
rs6828768
rs7572229
rs7311004
rs78383039
rs823424
rs2090816
rs6809952
rs55877868
All − MR Egger
All − IVW(MRE)
−1.5
−1.0
−0.5
0.0
0.5
1.0
MR effect size for
'Gut microbiota abundance (genus Parasutterella id.2892)' on 'Low affinity immunoglobulin epsilon Fc receptor'

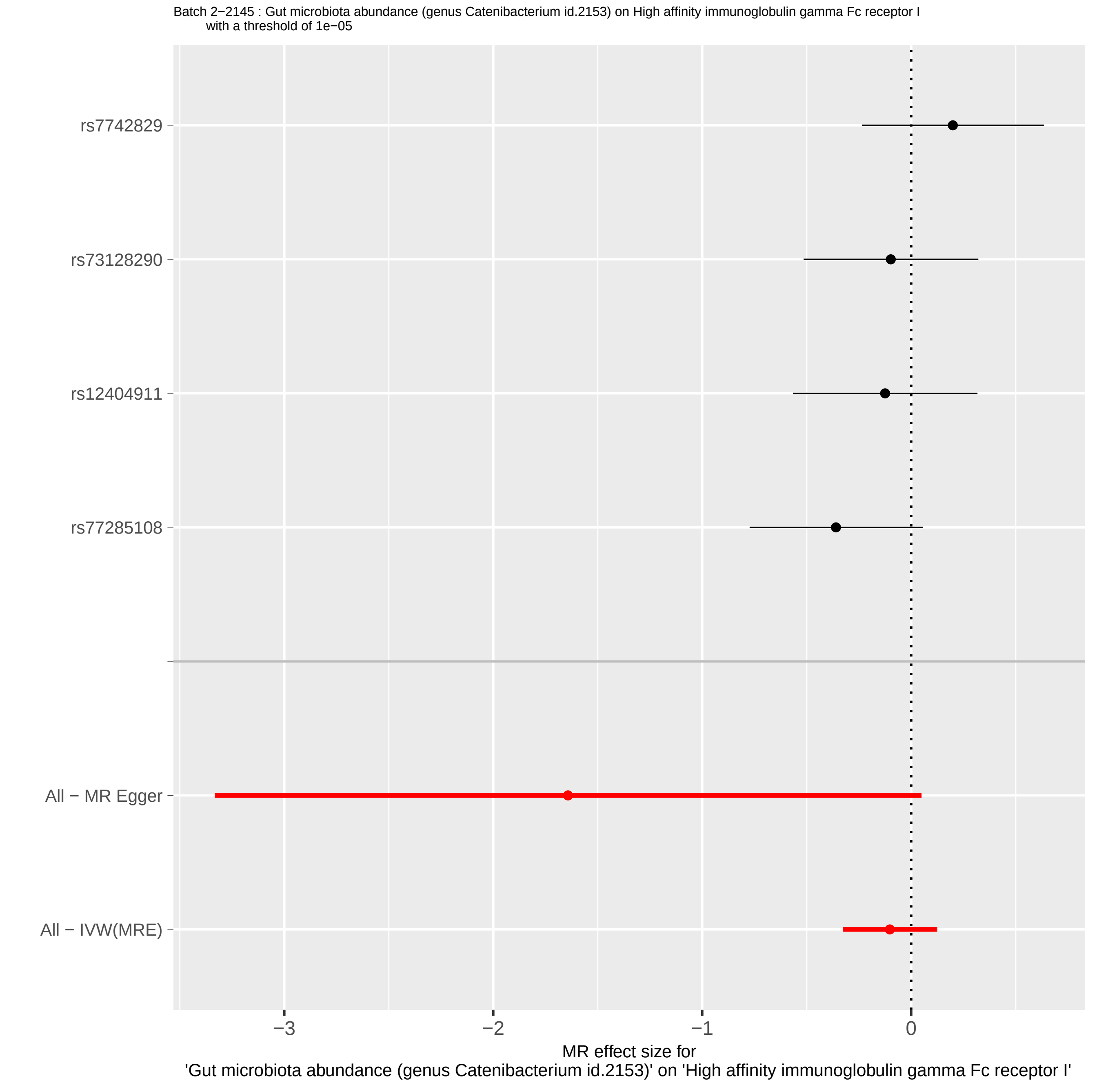
Batch 2−2145 : Gut microbiota abundance (genus Catenibacterium id.2153) on High affinity immunoglobulin gamma Fc receptor I
with a threshold of 1e−05
rs7742829
rs73128290
rs12404911
rs77285108
All − MR Egger
All − IVW(MRE)
−3
−2
−1
0
MR effect size for
'Gut microbiota abundance (genus Catenibacterium id.2153)' on 'High affinity immunoglobulin gamma Fc receptor I'

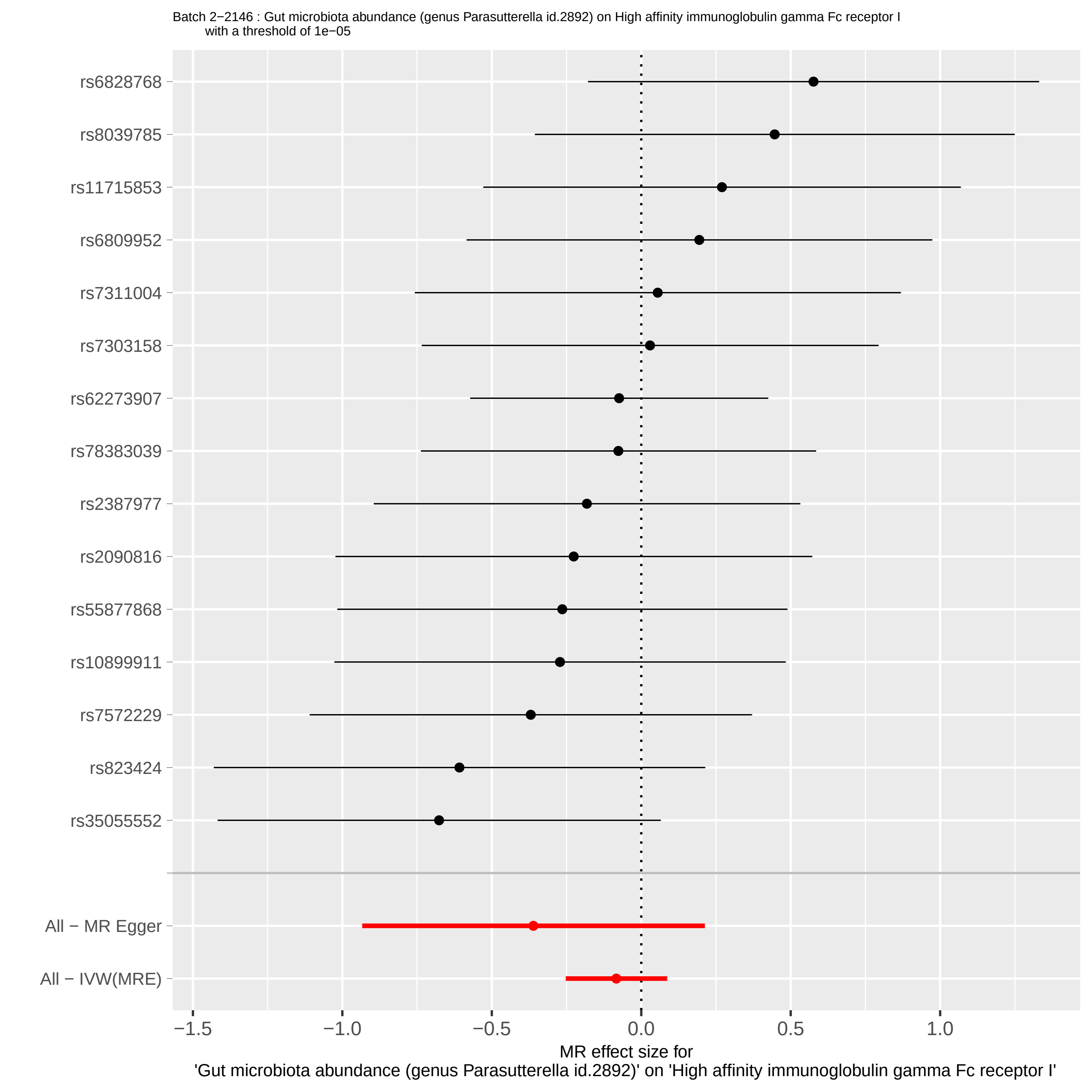
Batch 2−2146 : Gut microbiota abundance (genus Parasutterella id.2892) on High affinity immunoglobulin gamma Fc receptor I
with a threshold of 1e−05
rs6828768
rs8039785
rs11715853
rs6809952
rs7311004
rs7303158
rs62273907
rs78383039
rs2387977
rs2090816
rs55877868
rs10899911
rs7572229
rs823424
rs35055552
All − MR Egger
All − IVW(MRE)
−1.5
−1.0
−0.5
0.0
0.5
1.0
MR effect size for
'Gut microbiota abundance (genus Parasutterella id.2892)' on 'High affinity immunoglobulin gamma Fc receptor I'

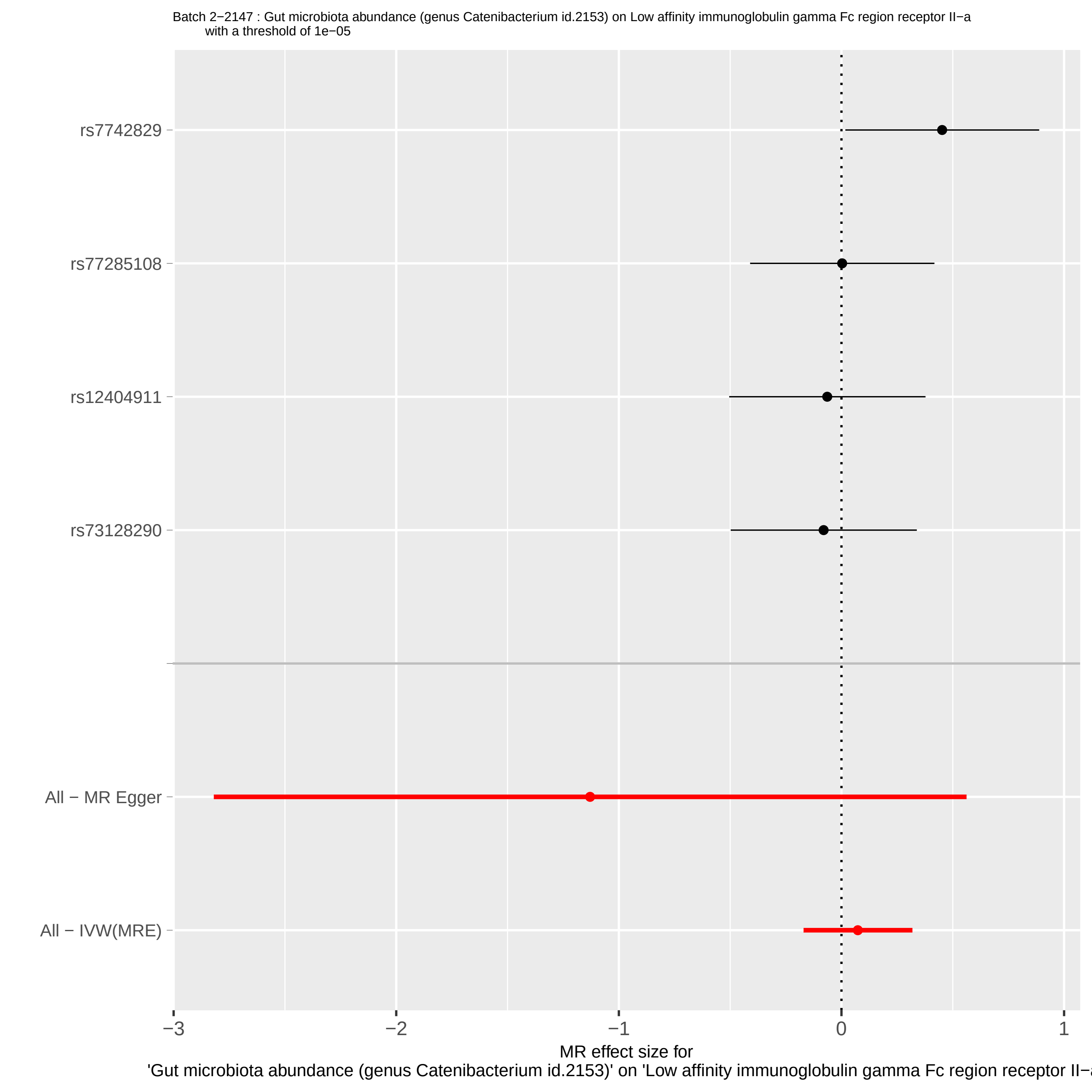

Batch 2−2147 : Gut microbiota abundance (genus Catenibacterium id.2153) on Low affinity immunoglobulin gamma Fc region receptor II−a
with a threshold of 1e−05
rs7742829
rs77285108
rs12404911
rs73128290
All − MR Egger
All − IVW(MRE)
−3
−2
−1
0
1
MR effect size for
'Gut microbiota abundance (genus Catenibacterium id.2153)' on 'Low affinity immunoglobulin gamma Fc region receptor II−a

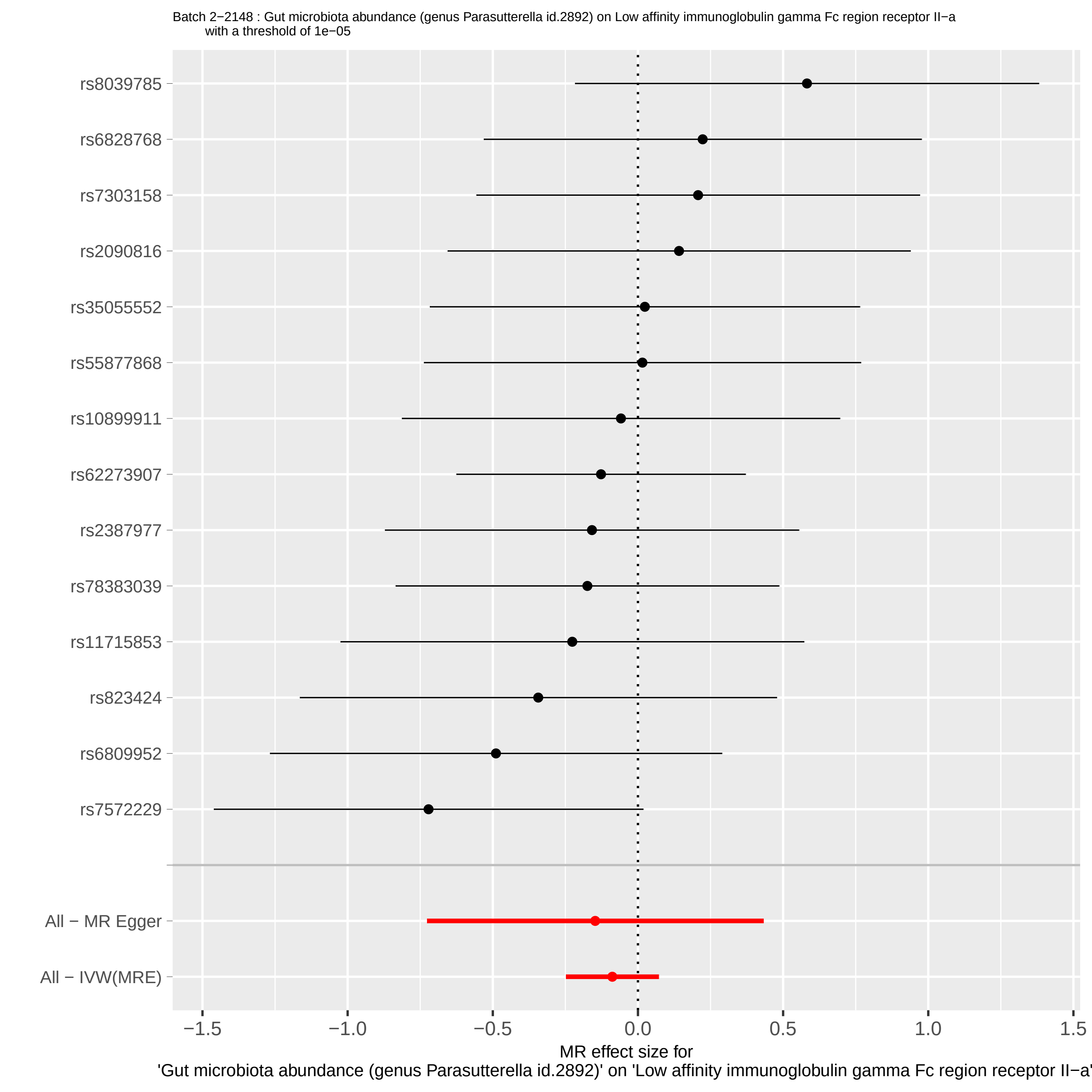
Batch 2−2148 : Gut microbiota abundance (genus Parasutterella id.2892) on Low affinity immunoglobulin gamma Fc region receptor II−a with a threshold of 1e−05
rs8039785
rs6828768
rs7303158
rs2090816
rs35055552
rs55877868
rs10899911
rs62273907
rs2387977
rs78383039
rs11715853
rs823424
rs6809952
rs7572229
All − MR Egger
All − IVW(MRE)
−1.5
−1.0
−0.5
0.0
0.5
1.0
1.5
MR effect size for
'Gut microbiota abundance (genus Parasutterella id.2892)' on 'Low affinity immunoglobulin gamma Fc region receptor II−a'

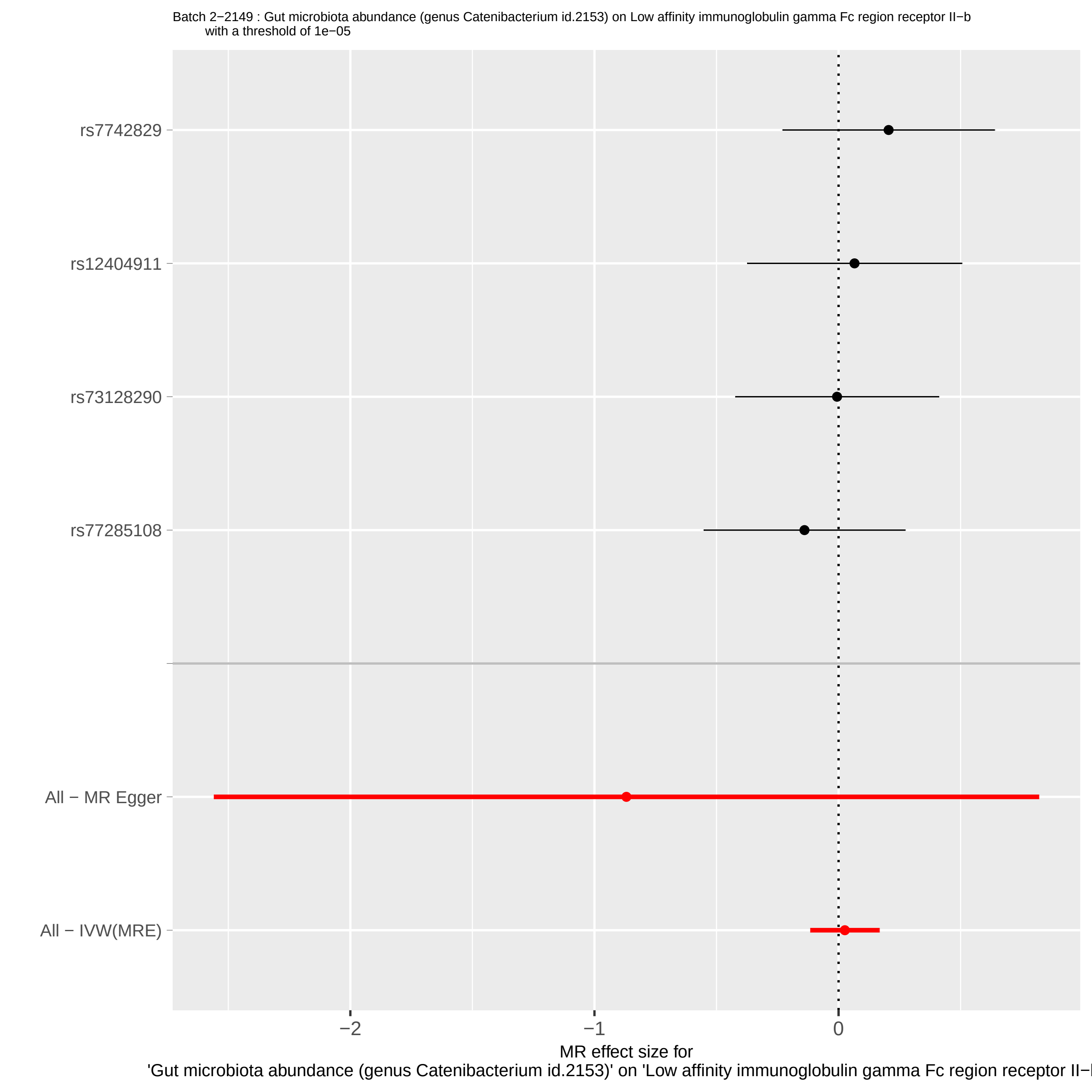

Batch 2−2149 : Gut microbiota abundance (genus Catenibacterium id.2153) on Low affinity immunoglobulin gamma Fc region receptor II−b
with a threshold of 1e−05
rs7742829
rs12404911
rs73128290
rs77285108
All − MR Egger
All − IVW(MRE)
−2
−1
0
MR effect size for
'Gut microbiota abundance (genus Catenibacterium id.2153)' on 'Low affinity immunoglobulin gamma Fc region receptor II−

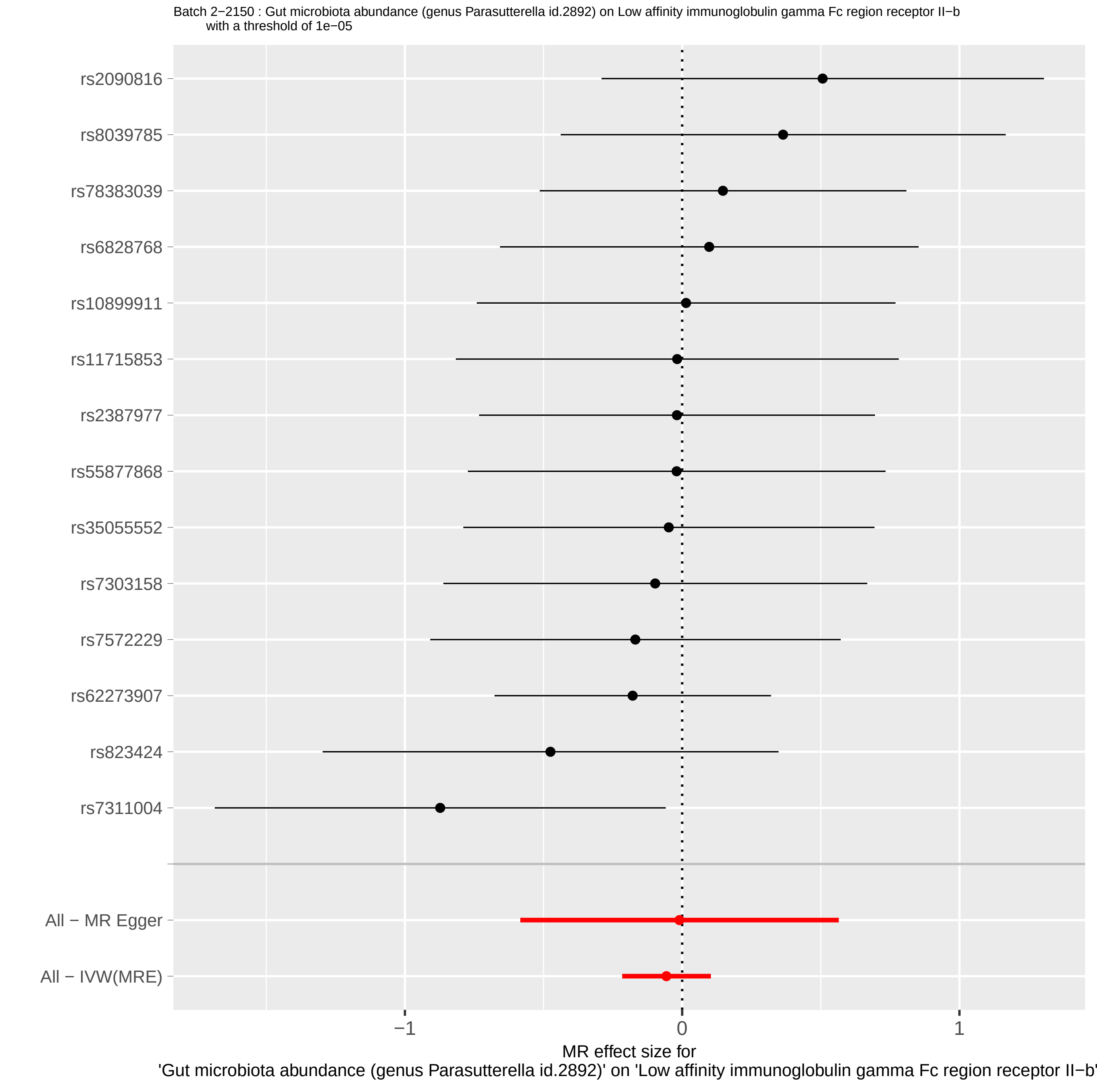

Batch 2−2150 : Gut microbiota abundance (genus Parasutterella id.2892) on Low affinity immunoglobulin gamma Fc region receptor II−b
with a threshold of 1e−05
rs2090816
rs8039785
rs78383039
rs6828768
rs10899911
rs11715853
rs2387977
rs55877868
rs35055552
rs7303158
rs7572229
rs62273907
rs823424
rs7311004
All − MR Egger
All − IVW(MRE)
−1
0
1
MR effect size for
'Gut microbiota abundance (genus Parasutterella id.2892)' on 'Low affinity immunoglobulin gamma Fc region receptor II−b'

Batch 2−2151 : Gut microbiota abundance (genus Catenibacterium id.2153) on Low affinity immunoglobulin gamma Fc region receptor III−B
with a threshold of 1e−05

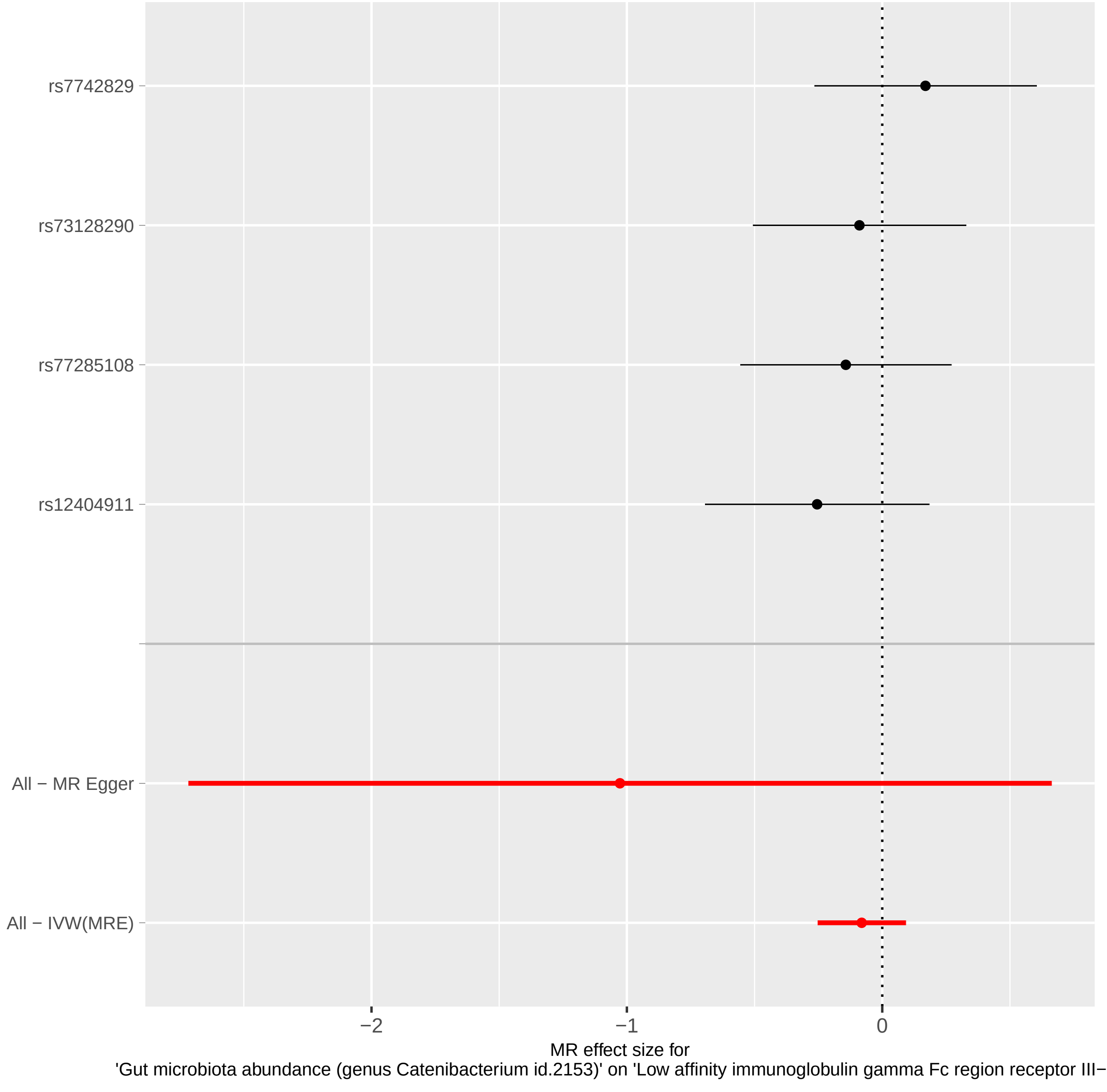

Batch 2−2152 : Gut microbiota abundance (genus Parasutterella id.2892) on Low affinity immunoglobulin gamma Fc region receptor III−B with a threshold of 1e−05

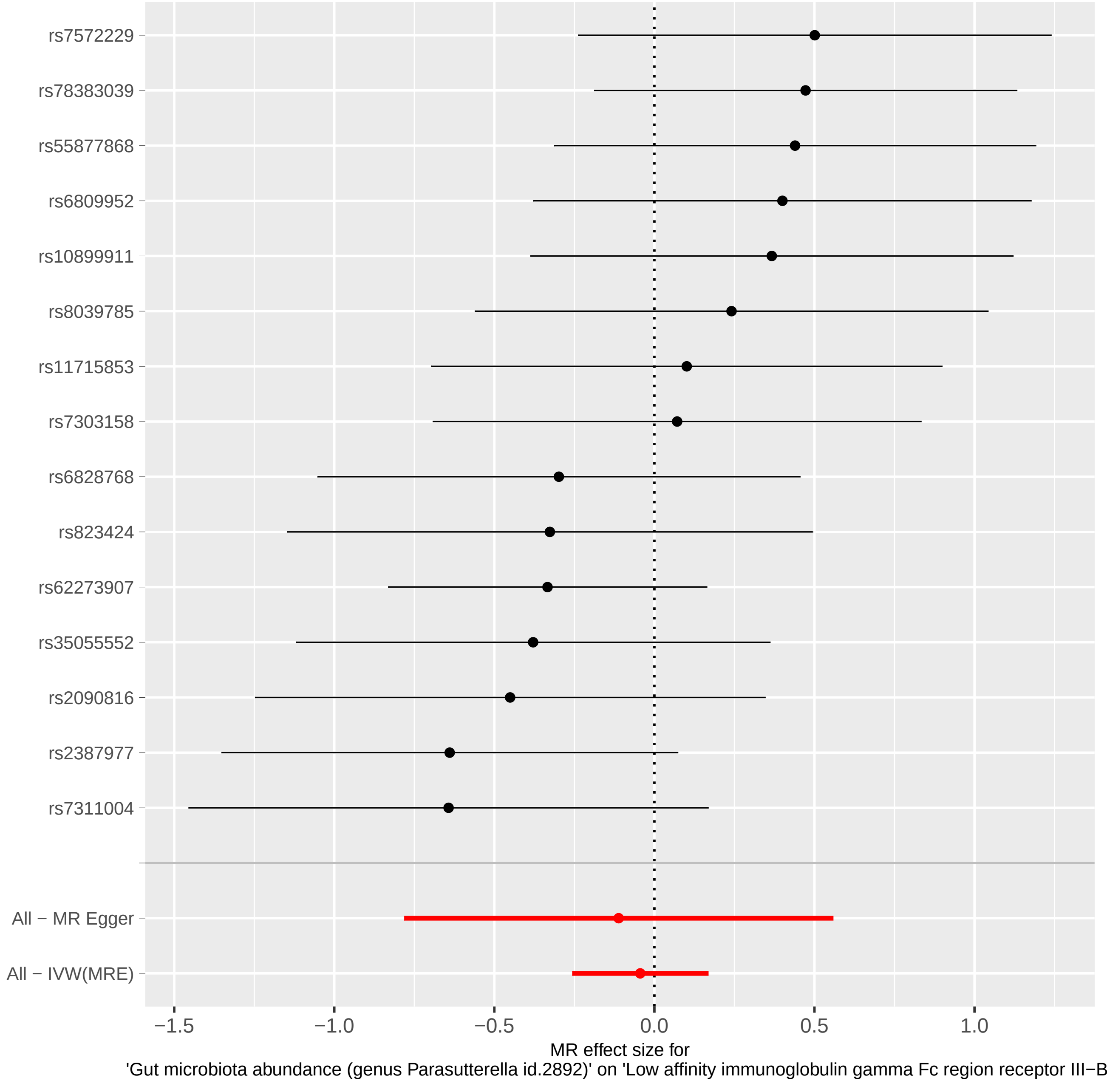

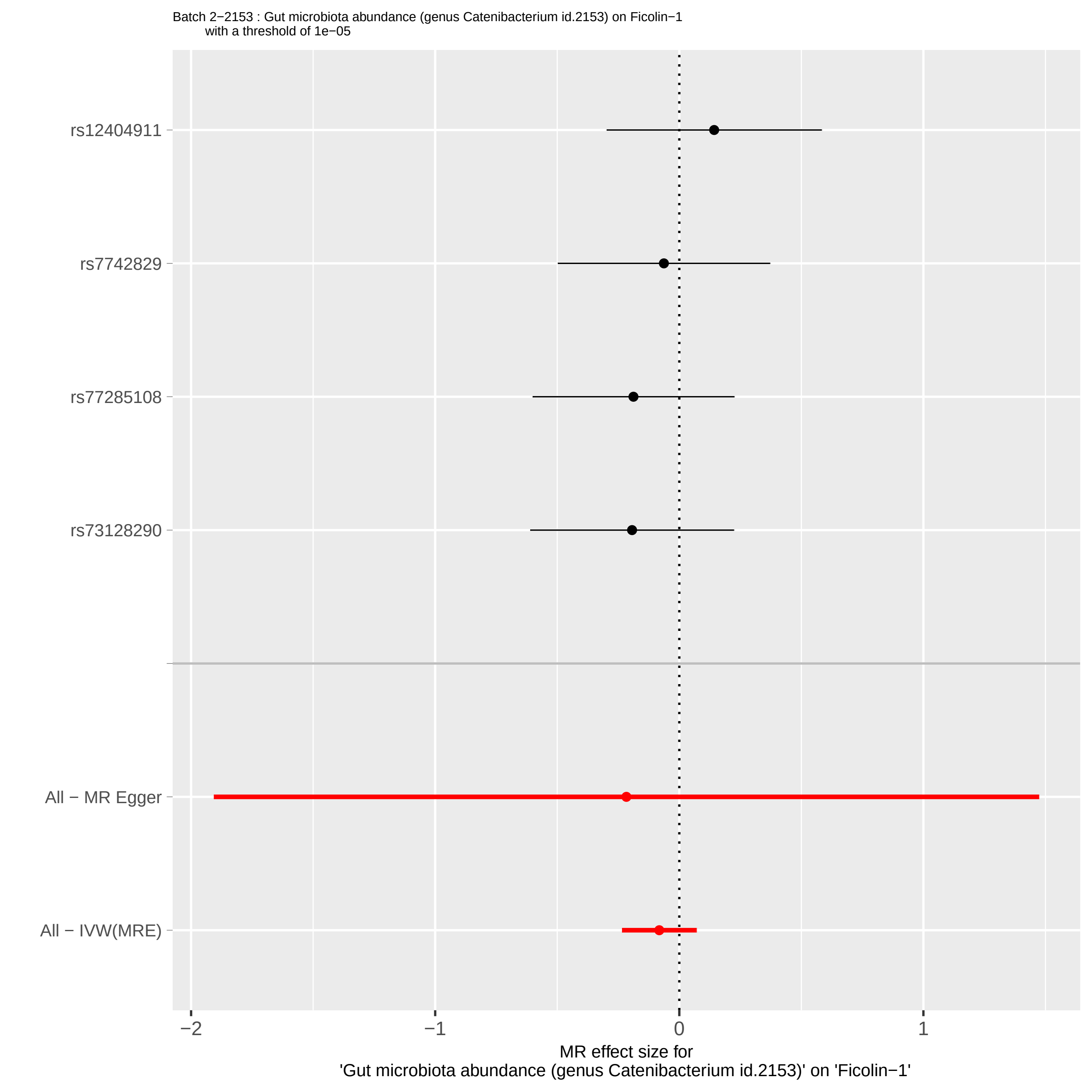
Batch 2−2153 : Gut microbiota abundance (genus Catenibacterium id.2153) on Ficolin−1
with a threshold of 1e−05
rs12404911
rs7742829
rs77285108
rs73128290
All − MR Egger
All − IVW(MRE)
−2
−1
0
1
MR effect size for
'Gut microbiota abundance (genus Catenibacterium id.2153)' on 'Ficolin−1'

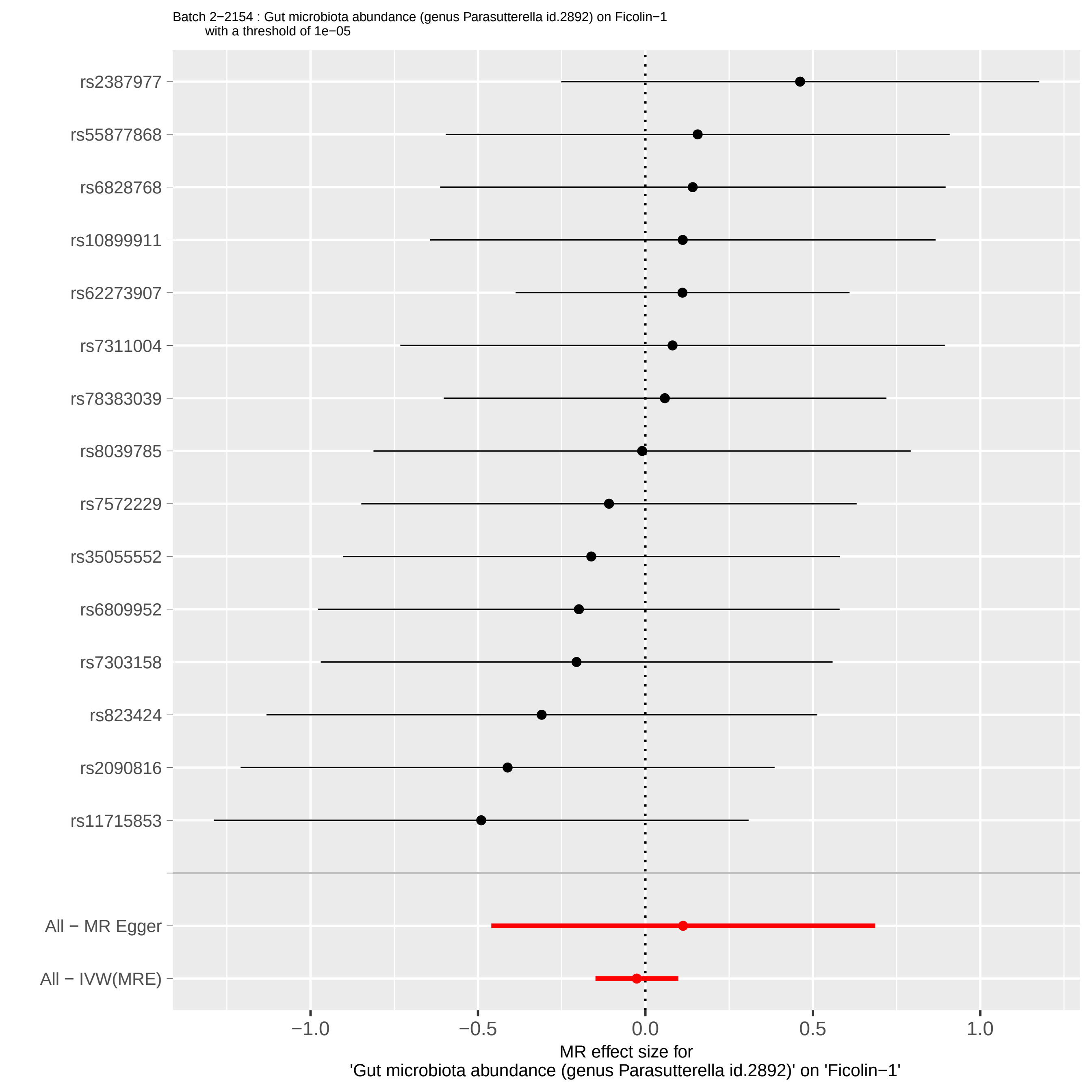

Batch 2−2154 : Gut microbiota abundance (genus Parasutterella id.2892) on Ficolin−1
with a threshold of 1e−05
rs2387977
rs55877868
rs6828768
rs10899911
rs62273907
rs7311004
rs78383039
rs8039785
rs7572229
rs35055552
rs6809952
rs7303158
rs823424
rs2090816
rs11715853
All − MR Egger
All − IVW(MRE)
−1.0
−0.5
0.0
0.5
1.0
MR effect size for
'Gut microbiota abundance (genus Parasutterella id.2892)' on 'Ficolin−1'

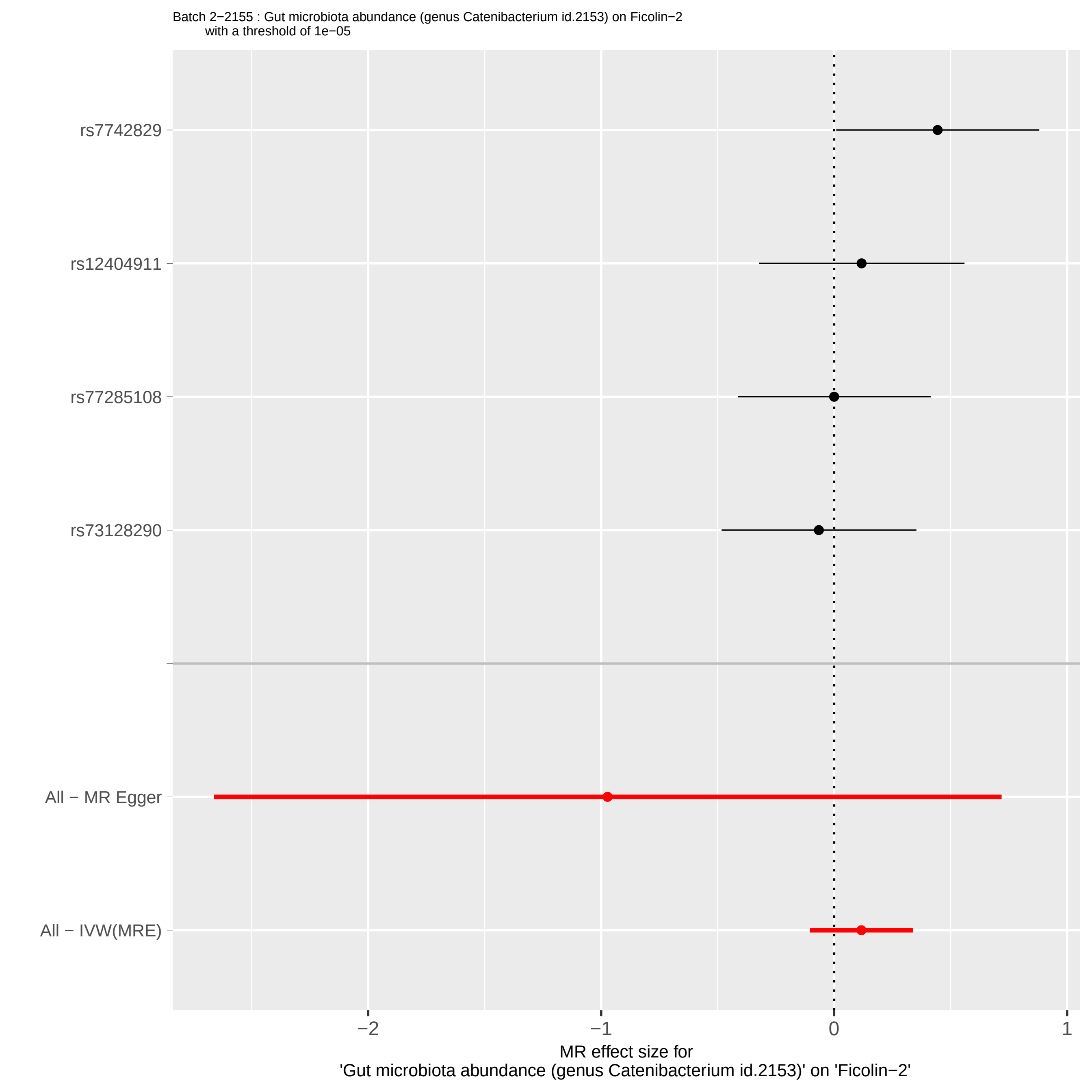

Batch 2−2155 : Gut microbiota abundance (genus Catenibacterium id.2153) on Ficolin−2
with a threshold of 1e−05
rs7742829
rs12404911
rs77285108
rs73128290
All − MR Egger
All − IVW(MRE)
−2
−1
0
1
MR effect size for
'Gut microbiota abundance (genus Catenibacterium id.2153)' on 'Ficolin−2'

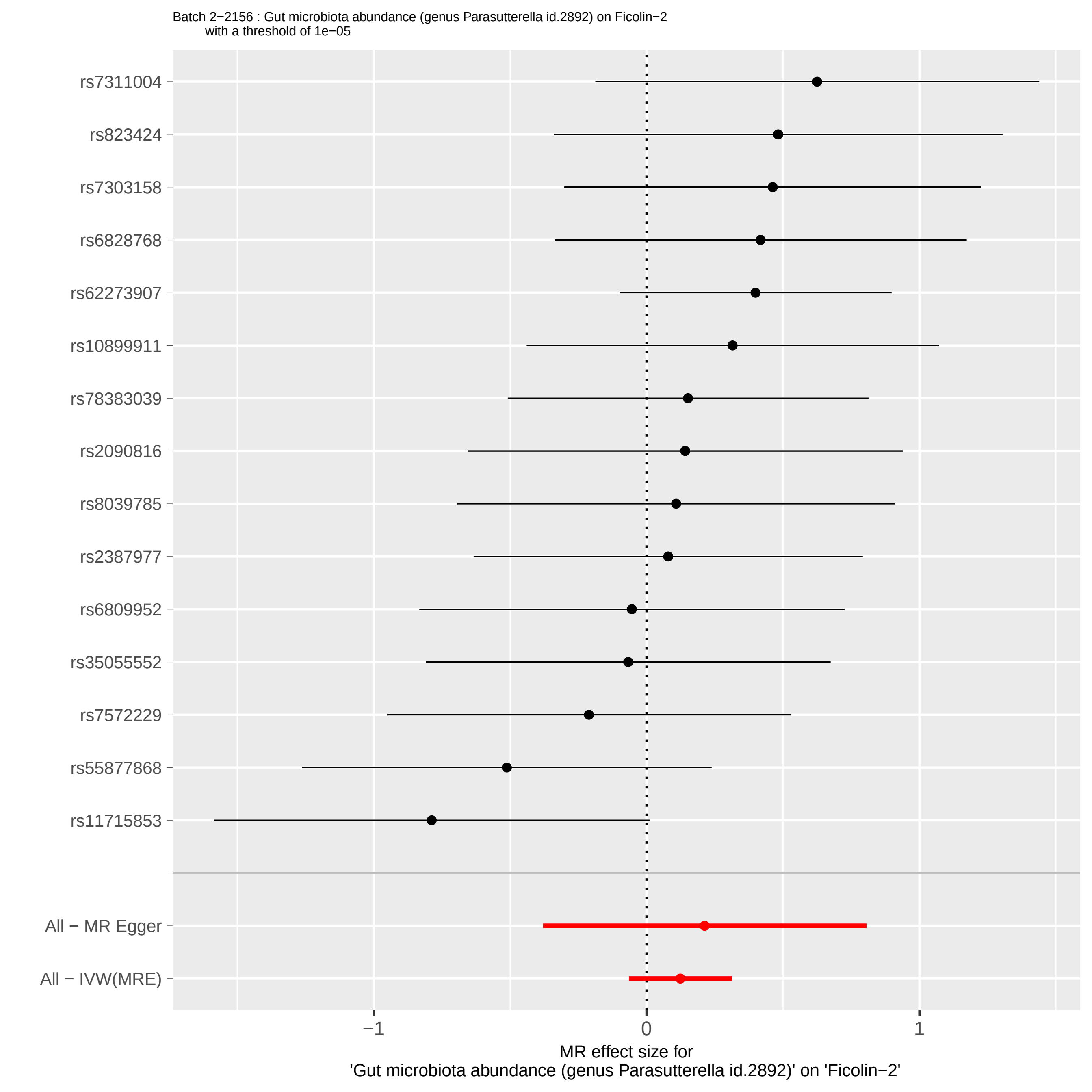

Batch 2−2156 : Gut microbiota abundance (genus Parasutterella id.2892) on Ficolin−2
with a threshold of 1e−05
rs7311004
rs823424
rs7303158
rs6828768
rs62273907
rs10899911
rs78383039
rs2090816
rs8039785
rs2387977
rs6809952
rs35055552
rs7572229
rs55877868
rs11715853
All − MR Egger
All − IVW(MRE)
−1
0
1
MR effect size for
'Gut microbiota abundance (genus Parasutterella id.2892)' on 'Ficolin−2'

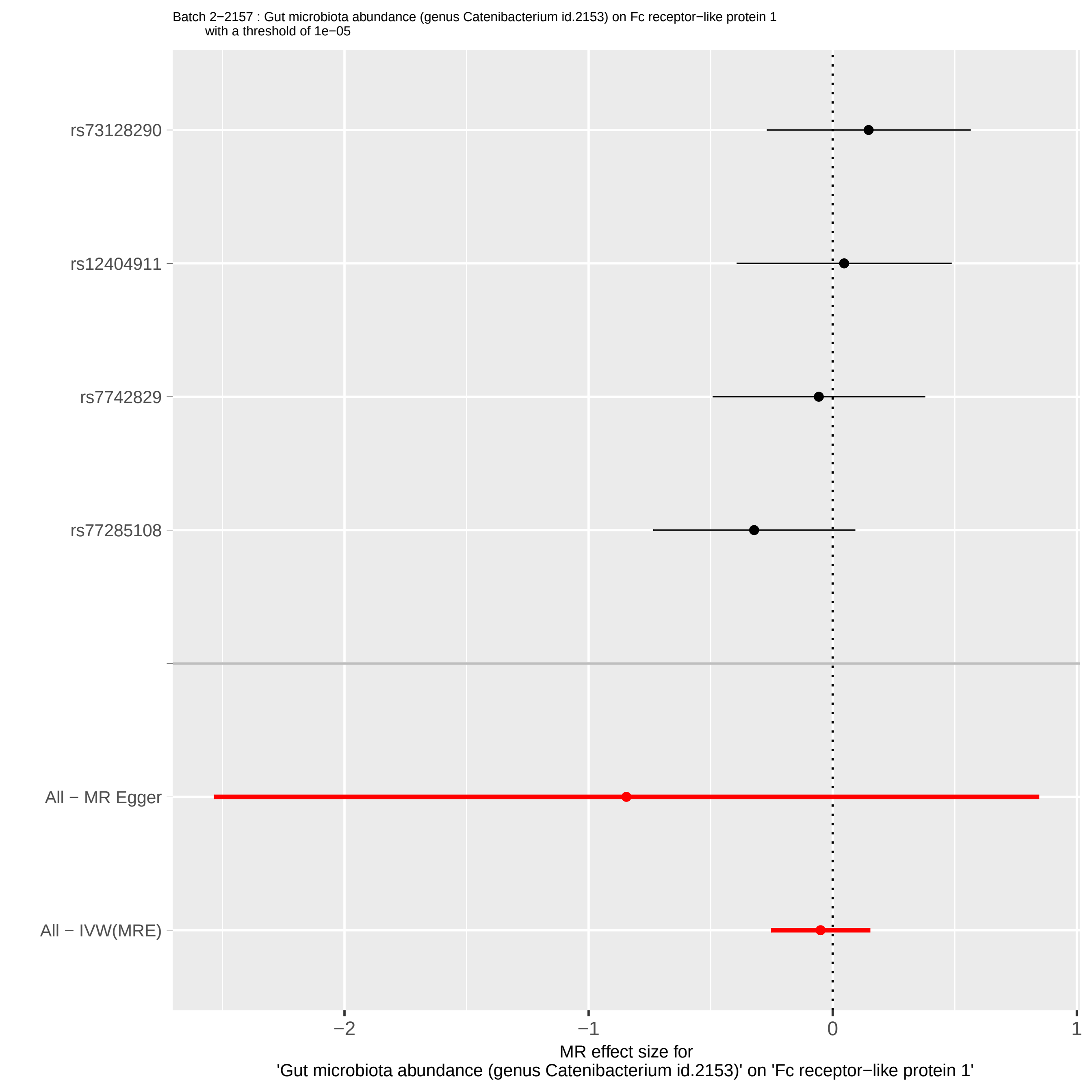
Batch 2−2157 : Gut microbiota abundance (genus Catenibacterium id.2153) on Fc receptor−like protein 1
with a threshold of 1e−05
rs73128290
rs12404911
rs7742829
rs77285108
All − MR Egger
All − IVW(MRE)
−2
−1
0
1
MR effect size for
'Gut microbiota abundance (genus Catenibacterium id.2153)' on 'Fc receptor−like protein 1'

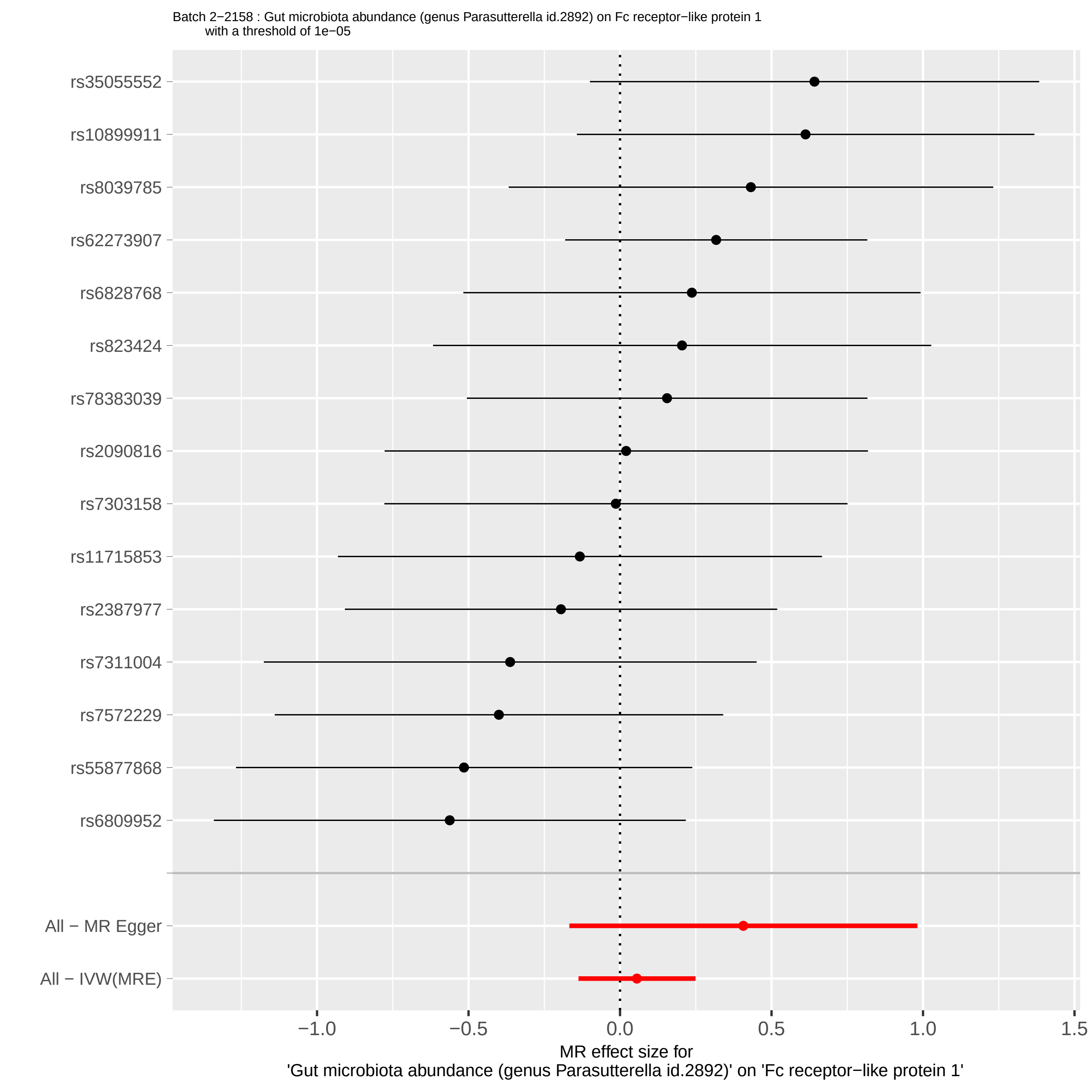
Batch 2−2158 : Gut microbiota abundance (genus Parasutterella id.2892) on Fc receptor−like protein 1
with a threshold of 1e−05
rs35055552
rs10899911
rs8039785
rs62273907
rs6828768
rs823424
rs78383039
rs2090816
rs7303158
rs11715853
rs2387977
rs7311004
rs7572229
rs55877868
rs6809952
All − MR Egger
All − IVW(MRE)
−1.0
−0.5
0.0
0.5
1.0
1.5
MR effect size for
'Gut microbiota abundance (genus Parasutterella id.2892)' on 'Fc receptor−like protein 1'

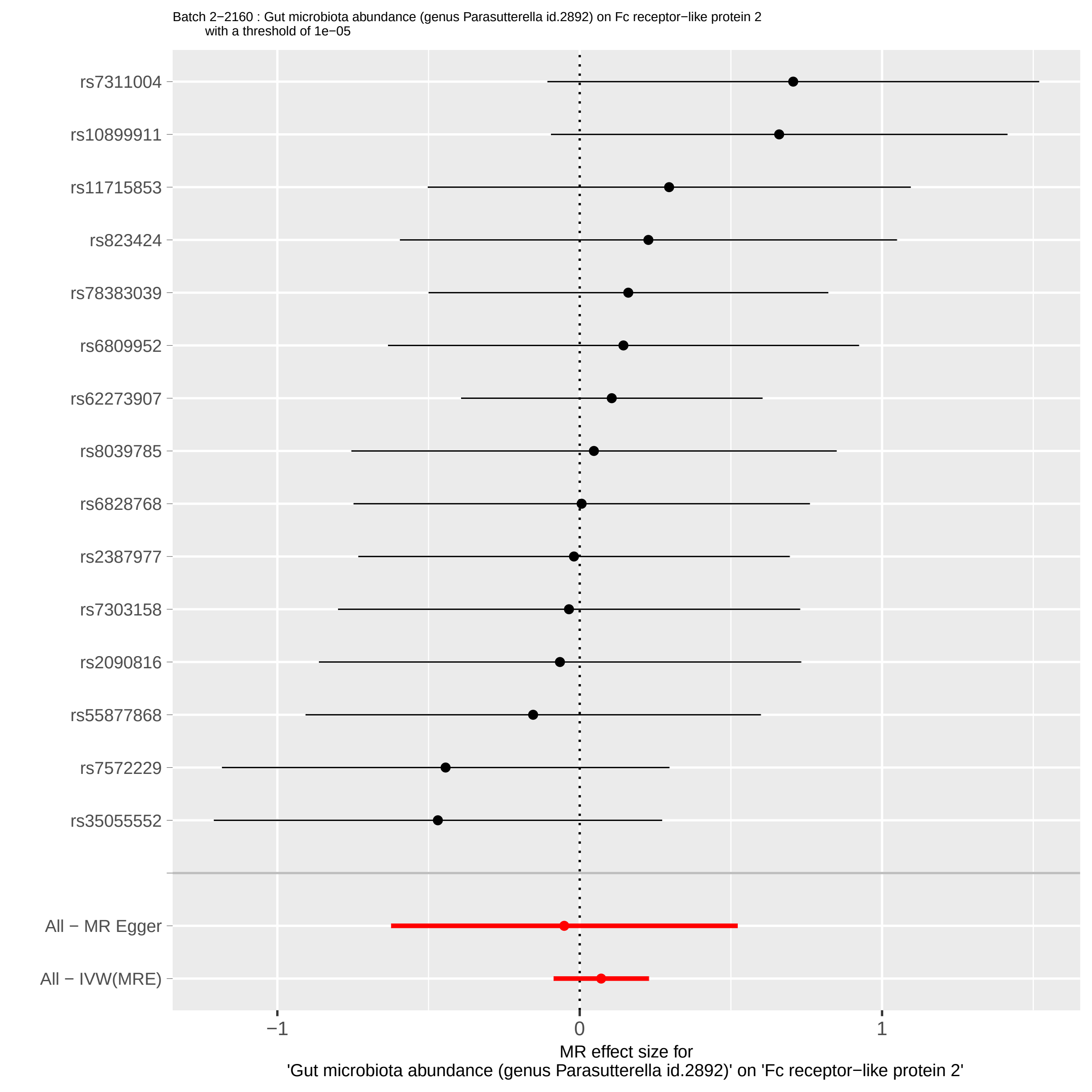

Batch 2−2160 : Gut microbiota abundance (genus Parasutterella id.2892) on Fc receptor−like protein 2
with a threshold of 1e−05
rs7311004
rs10899911
rs11715853
rs823424
rs78383039
rs6809952
rs62273907
rs8039785
rs6828768
rs2387977
rs7303158
rs2090816
rs55877868
rs7572229
rs35055552
All − MR Egger
All − IVW(MRE)
−1
0
1
MR effect size for
'Gut microbiota abundance (genus Parasutterella id.2892)' on 'Fc receptor−like protein 2'

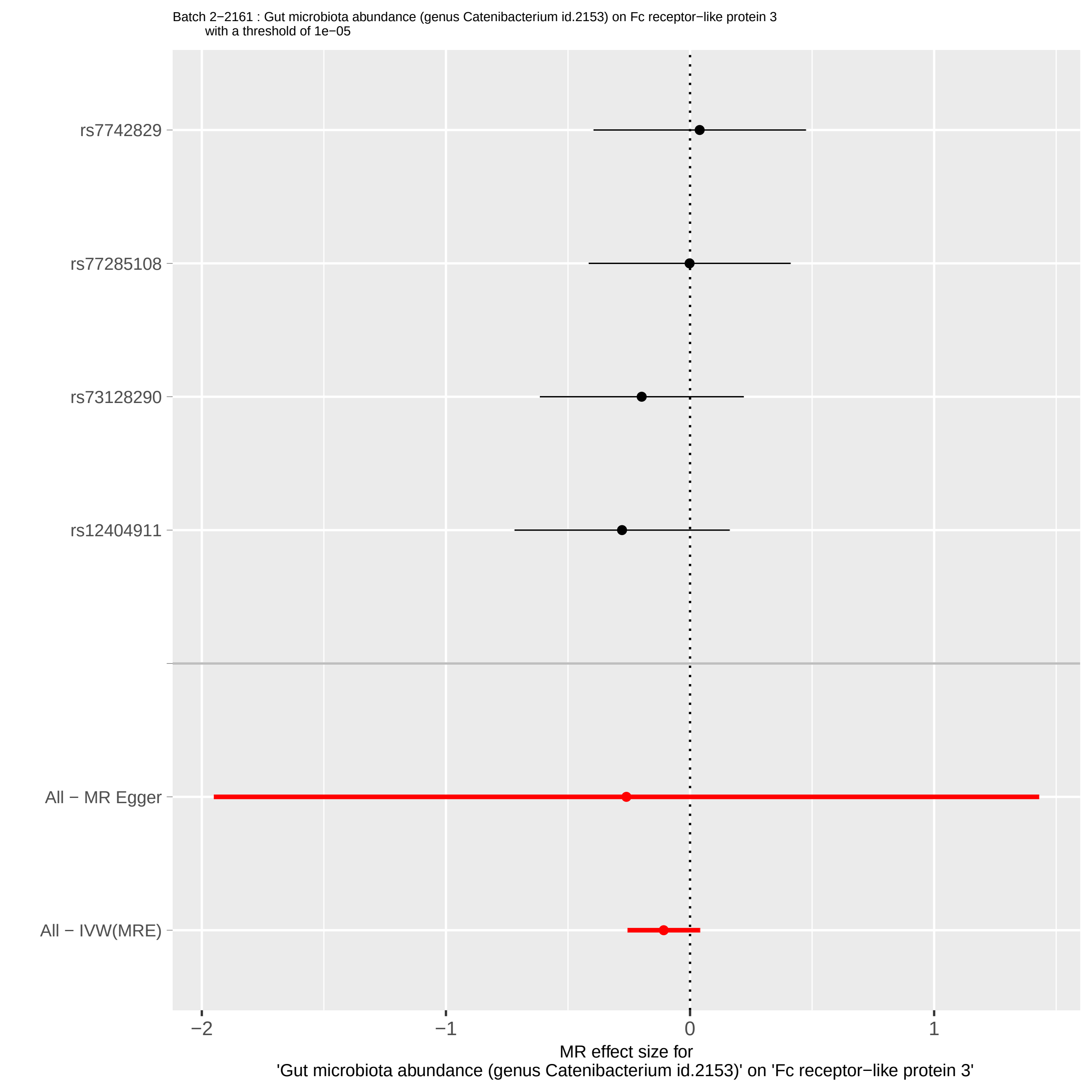

Batch 2−2161 : Gut microbiota abundance (genus Catenibacterium id.2153) on Fc receptor−like protein 3
with a threshold of 1e−05
rs7742829
rs77285108
rs73128290
rs12404911
All − MR Egger
All − IVW(MRE)
−2
−1
0
1
MR effect size for
'Gut microbiota abundance (genus Catenibacterium id.2153)' on 'Fc receptor−like protein 3'

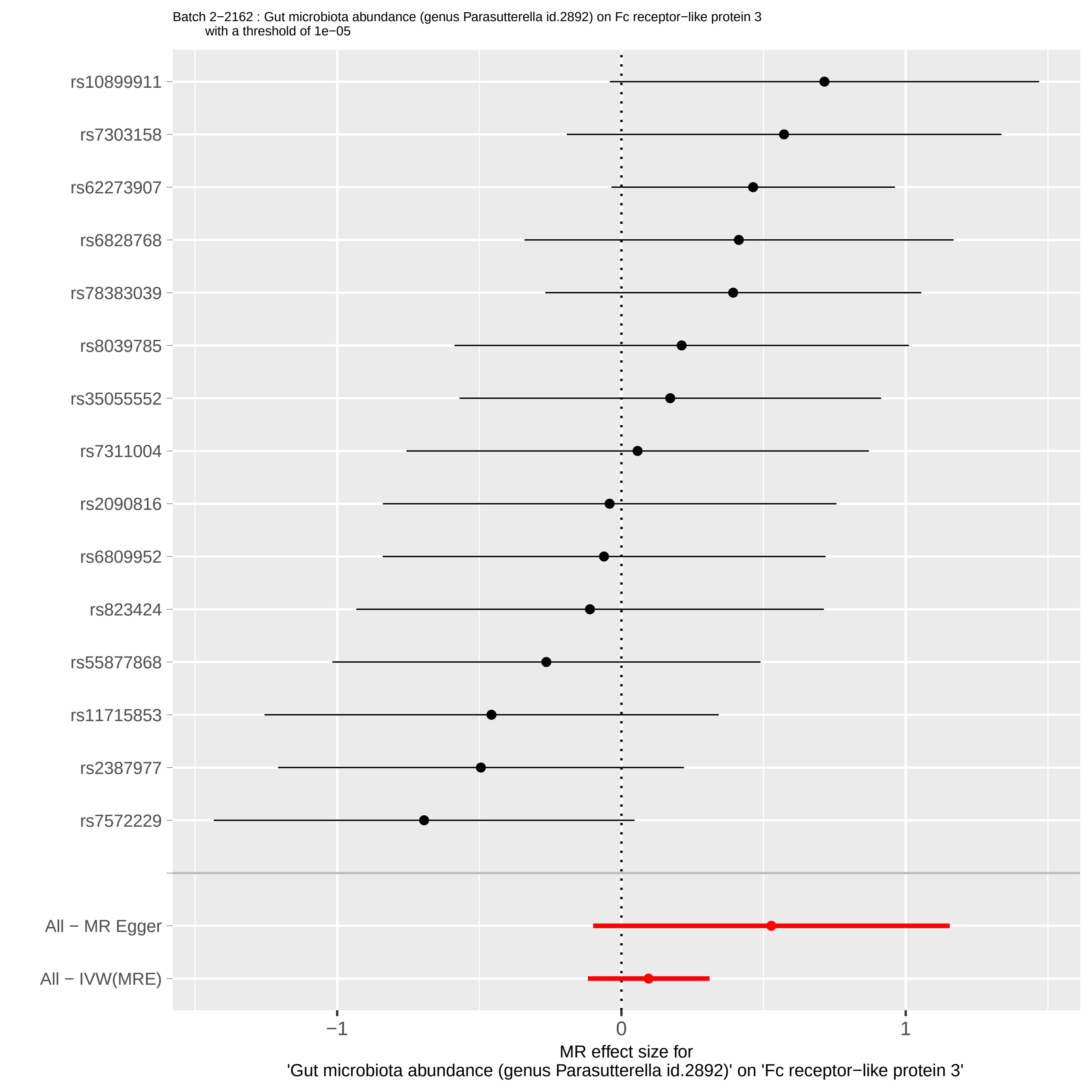
Batch 2−2162 : Gut microbiota abundance (genus Parasutterella id.2892) on Fc receptor−like protein 3
with a threshold of 1e−05
rs10899911
rs7303158
rs62273907
rs6828768
rs78383039
rs8039785
rs35055552
rs7311004
rs2090816
rs6809952
rs823424
rs55877868
rs11715853
rs2387977
rs7572229
All − MR Egger
All − IVW(MRE)
−1
0
1
MR effect size for
'Gut microbiota abundance (genus Parasutterella id.2892)' on 'Fc receptor−like protein 3'

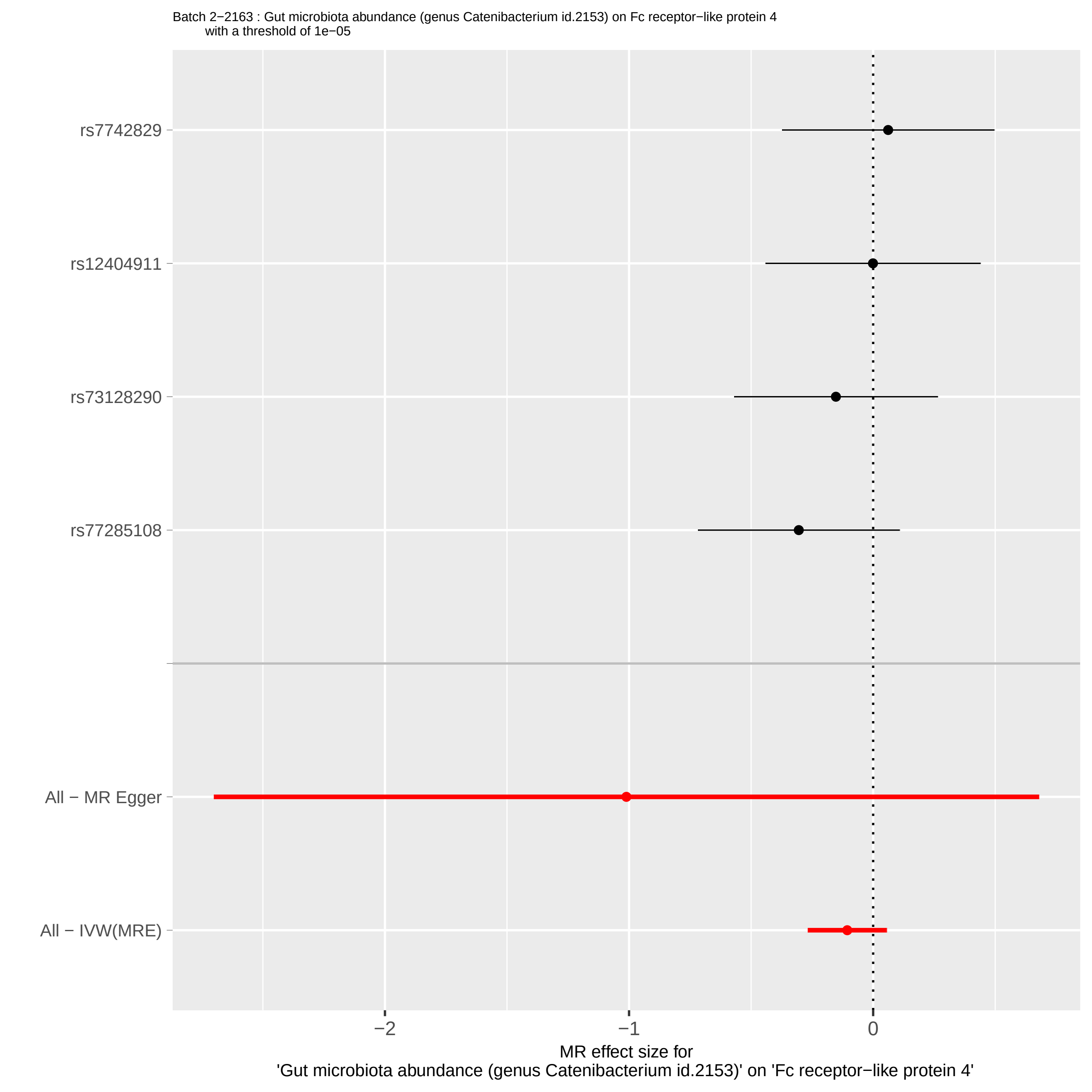

Batch 2−2163 : Gut microbiota abundance (genus Catenibacterium id.2153) on Fc receptor−like protein 4
with a threshold of 1e−05
rs7742829
rs12404911
rs73128290
rs77285108
All − MR Egger
All − IVW(MRE)
−2
−1
0
MR effect size for
'Gut microbiota abundance (genus Catenibacterium id.2153)' on 'Fc receptor−like protein 4'

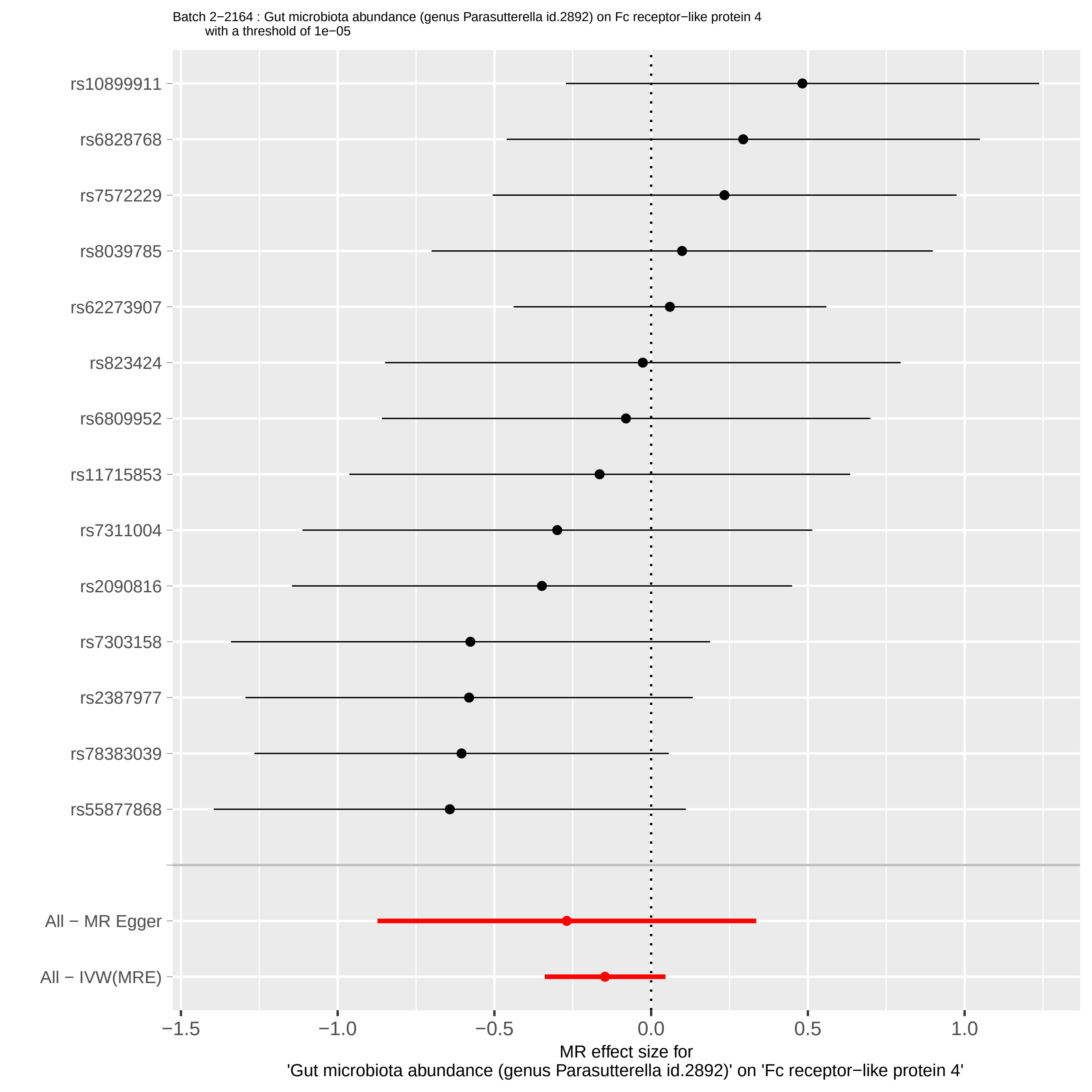

Batch 2−2164 : Gut microbiota abundance (genus Parasutterella id.2892) on Fc receptor−like protein 4
with a threshold of 1e−05
rs10899911
rs6828768
rs7572229
rs8039785
rs62273907
rs823424
rs6809952
rs11715853
rs7311004
rs2090816
rs7303158
rs2387977
rs78383039
rs55877868
All − MR Egger
All − IVW(MRE)
−1.5
−1.0
−0.5
0.0
0.5
1.0
MR effect size for
'Gut microbiota abundance (genus Parasutterella id.2892)' on 'Fc receptor−like protein 4'

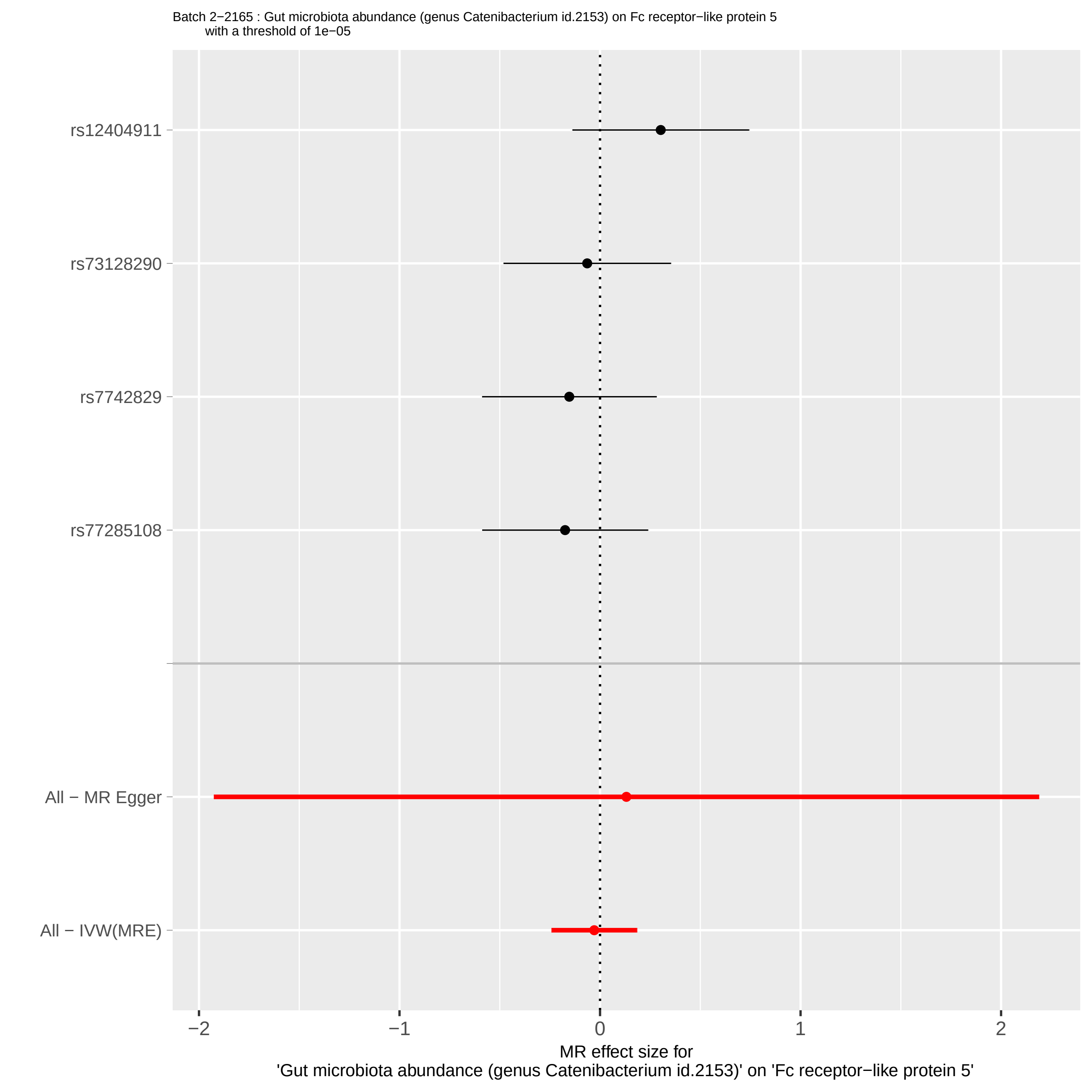

Batch 2−2165 : Gut microbiota abundance (genus Catenibacterium id.2153) on Fc receptor−like protein 5
with a threshold of 1e−05
rs12404911
rs73128290
rs7742829
rs77285108
All − MR Egger
All − IVW(MRE)
−2
−1
0
1
2
MR effect size for
'Gut microbiota abundance (genus Catenibacterium id.2153)' on 'Fc receptor−like protein 5'

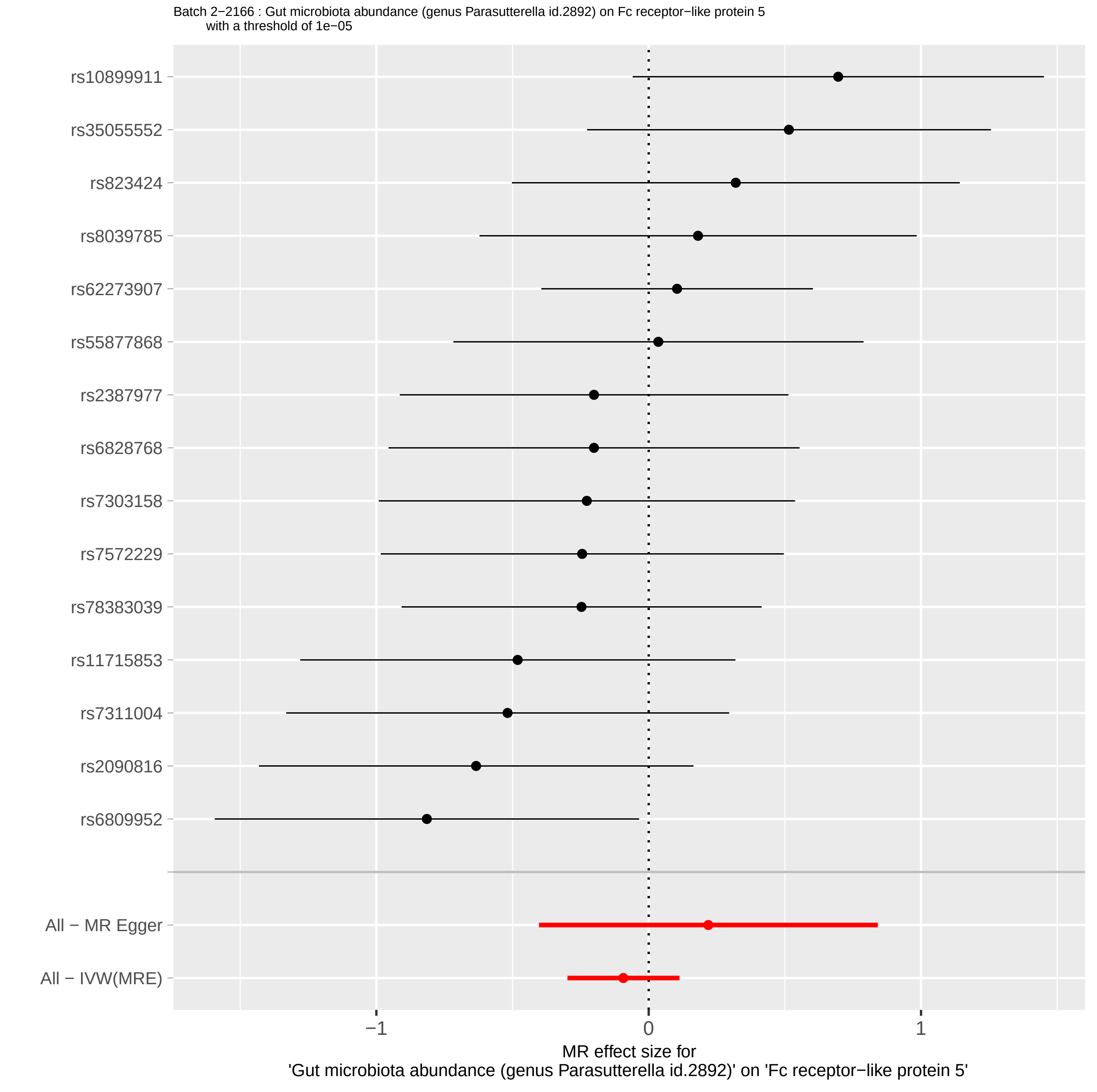

Batch 2−2166 : Gut microbiota abundance (genus Parasutterella id.2892) on Fc receptor−like protein 5
with a threshold of 1e−05
rs10899911
rs35055552
rs823424
rs8039785
rs62273907
rs55877868
rs2387977
rs6828768
rs7303158
rs7572229
rs78383039
rs11715853
rs7311004
rs2090816
rs6809952
All − MR Egger
All − IVW(MRE)
−1
0
1
MR effect size for
'Gut microbiota abundance (genus Parasutterella id.2892)' on 'Fc receptor−like protein 5'

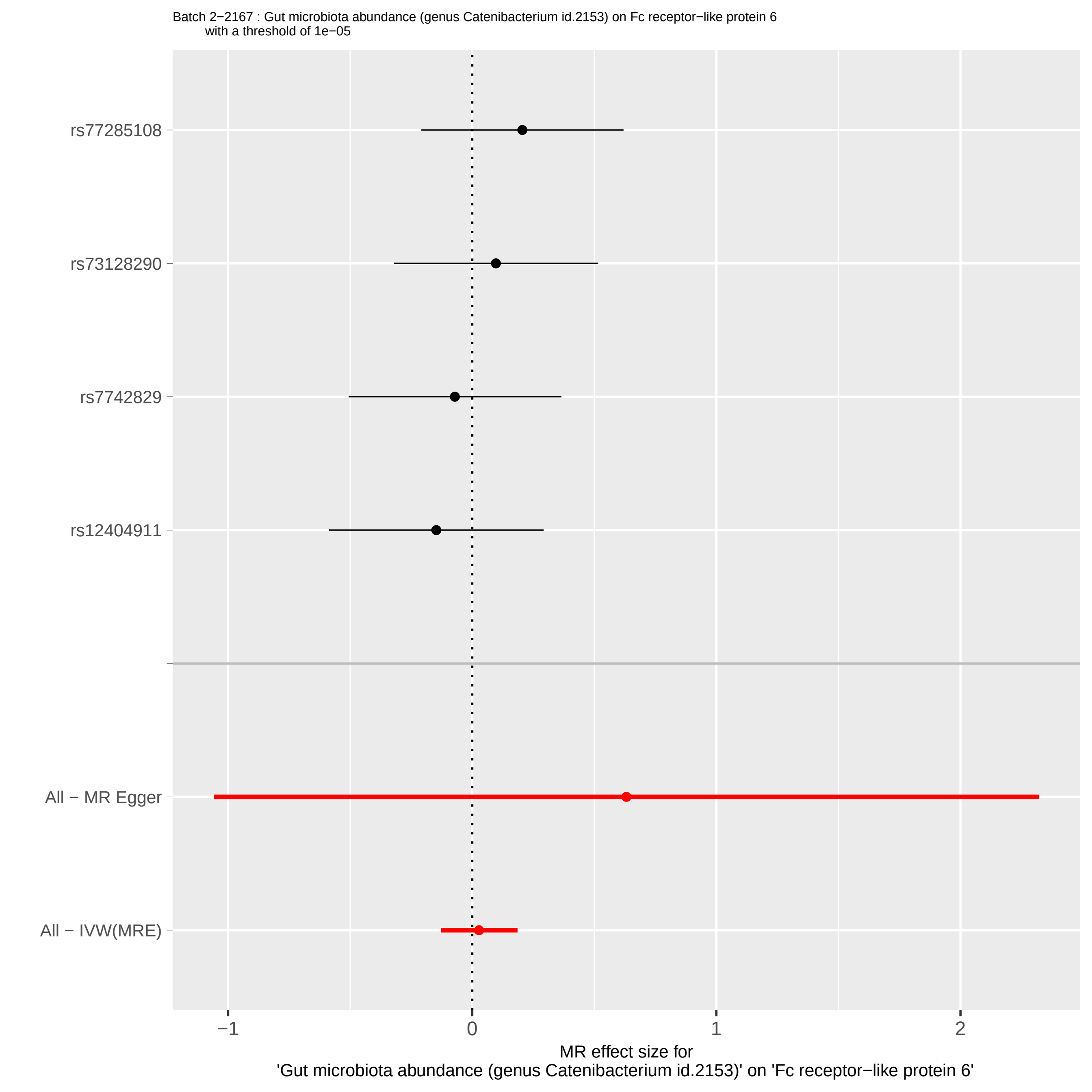

Batch 2−2167 : Gut microbiota abundance (genus Catenibacterium id.2153) on Fc receptor−like protein 6
with a threshold of 1e−05
rs77285108
rs73128290
rs7742829
rs12404911
All − MR Egger
All − IVW(MRE)
−1
0
1
2
MR effect size for
'Gut microbiota abundance (genus Catenibacterium id.2153)' on 'Fc receptor−like protein 6'

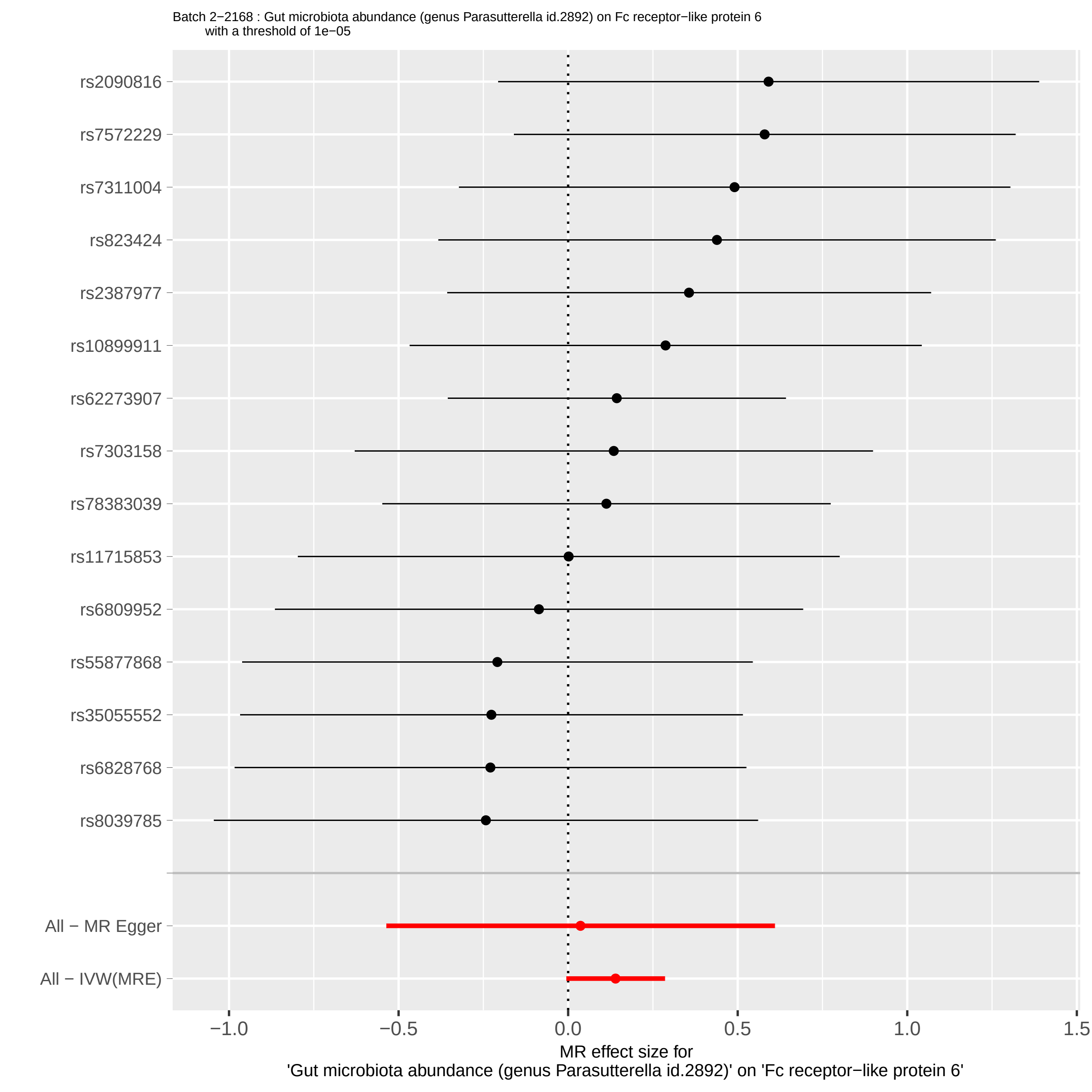

Batch 2−2168 : Gut microbiota abundance (genus Parasutterella id.2892) on Fc receptor−like protein 6
with a threshold of 1e−05
rs2090816
rs7572229
rs7311004
rs823424
rs2387977
rs10899911
rs62273907
rs7303158
rs78383039
rs11715853
rs6809952
rs55877868
rs35055552
rs6828768
rs8039785
All − MR Egger
All − IVW(MRE)
−1.0
−0.5
0.0
0.5
1.0
1.5
MR effect size for
'Gut microbiota abundance (genus Parasutterella id.2892)' on 'Fc receptor−like protein 6'

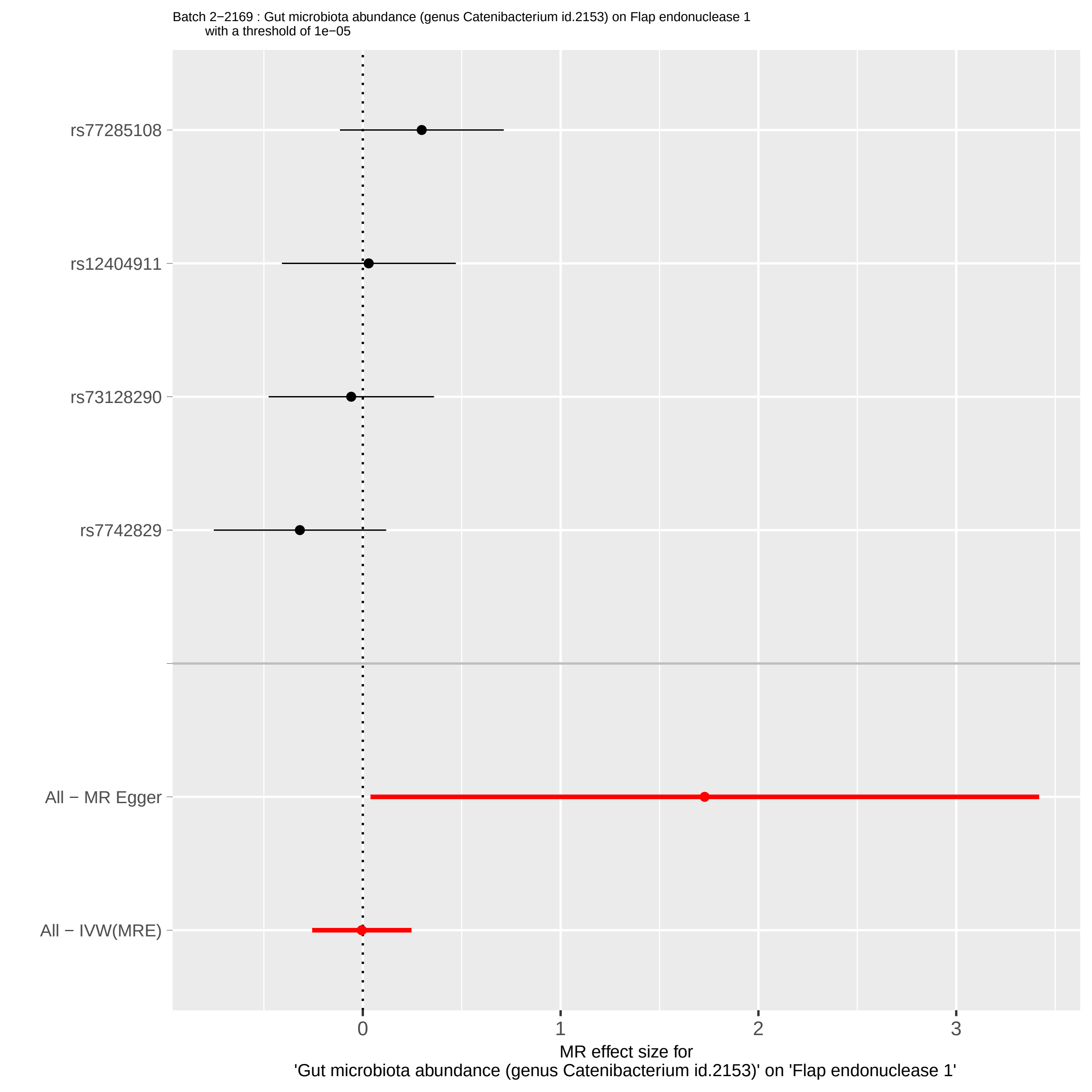
Batch 2−2169 : Gut microbiota abundance (genus Catenibacterium id.2153) on Flap endonuclease 1
with a threshold of 1e−05
rs77285108
rs12404911
rs73128290
rs7742829
All − MR Egger
All − IVW(MRE)
0
1
2
3
MR effect size for
'Gut microbiota abundance (genus Catenibacterium id.2153)' on 'Flap endonuclease 1'

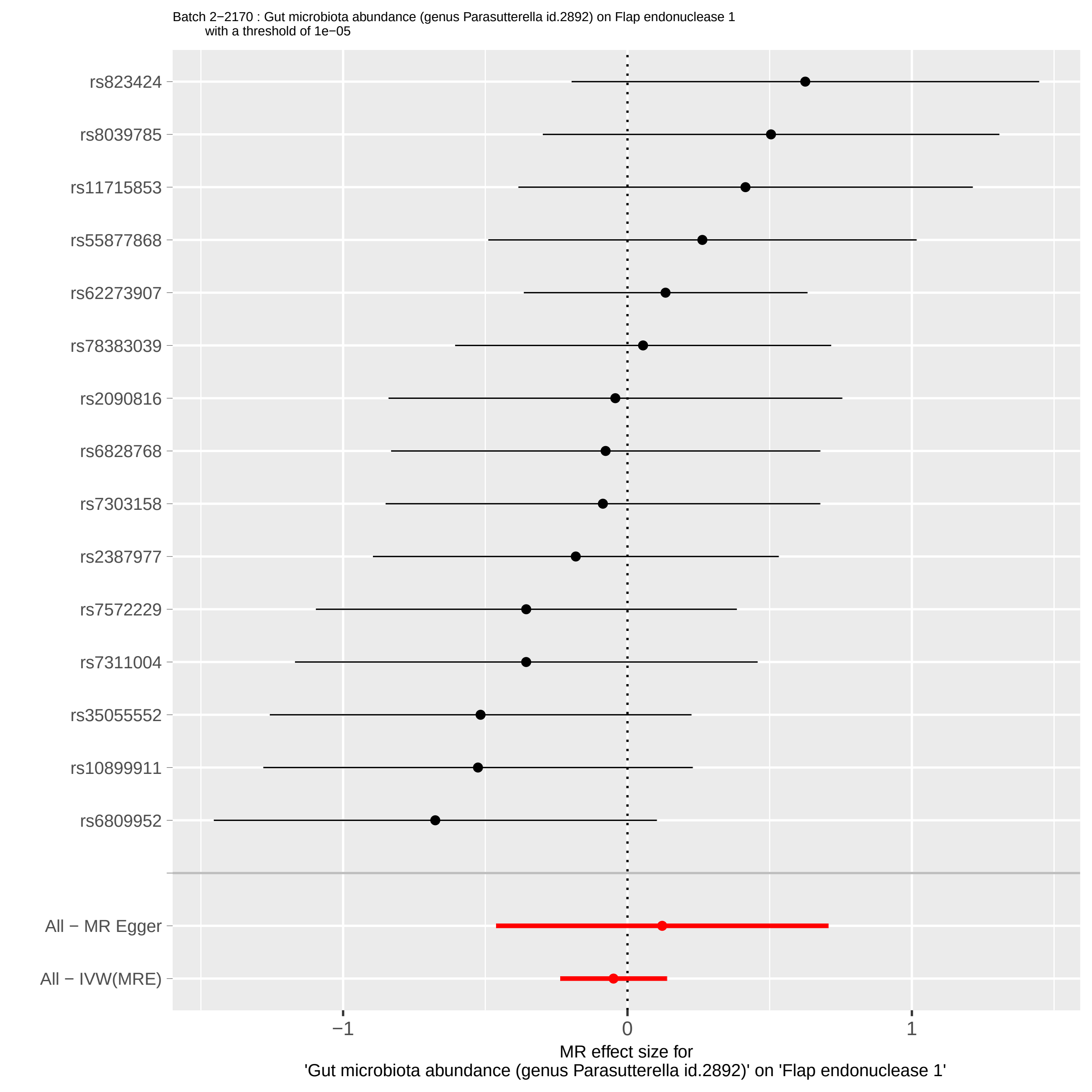

Batch 2−2170 : Gut microbiota abundance (genus Parasutterella id.2892) on Flap endonuclease 1
with a threshold of 1e−05
rs823424
rs8039785
rs11715853
rs55877868
rs62273907
rs78383039
rs2090816
rs6828768
rs7303158
rs2387977
rs7572229
rs7311004
rs35055552
rs10899911
rs6809952
All − MR Egger
All − IVW(MRE)
−1
0
1
MR effect size for
'Gut microbiota abundance (genus Parasutterella id.2892)' on 'Flap endonuclease 1'

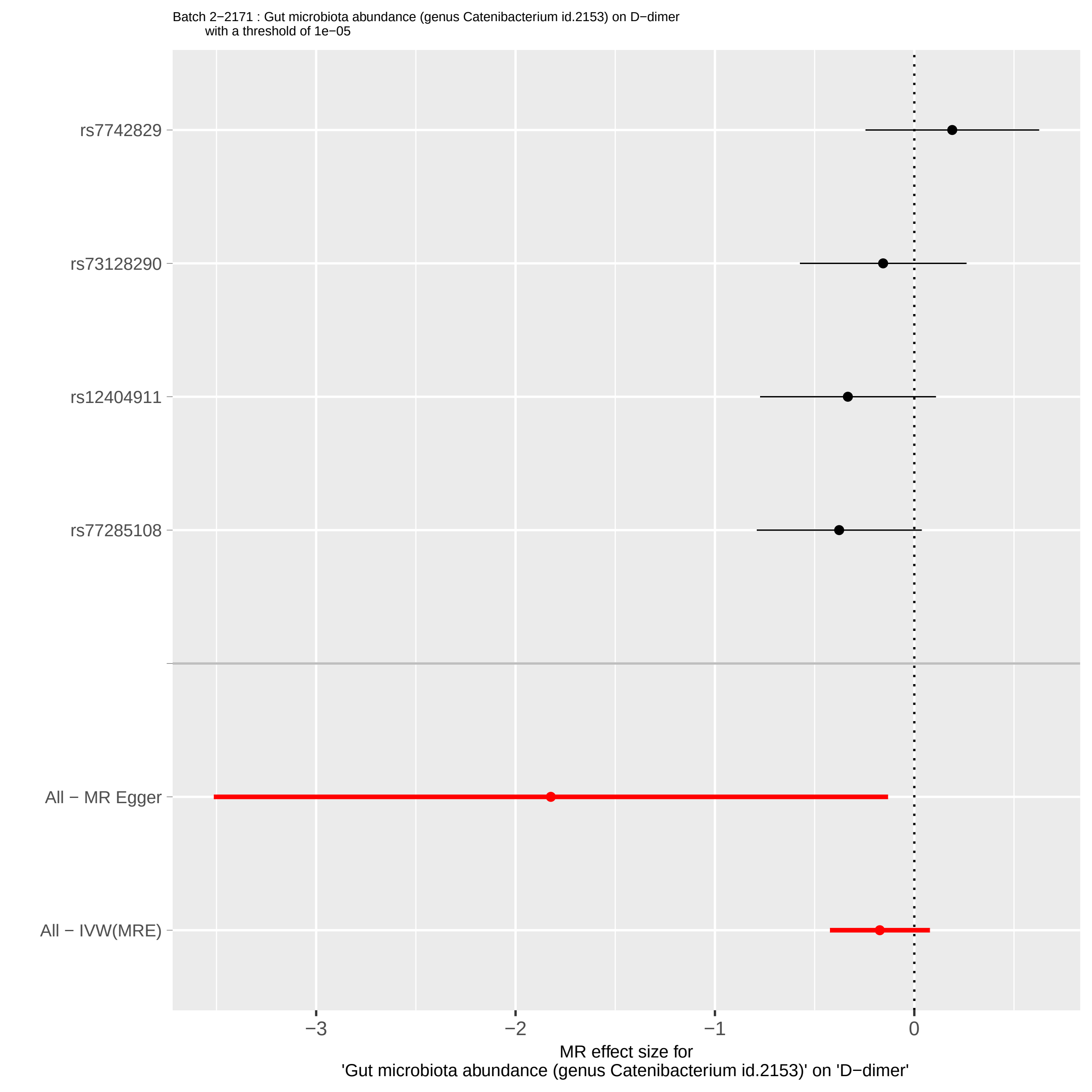

Batch 2−2171 : Gut microbiota abundance (genus Catenibacterium id.2153) on D−dimer
with a threshold of 1e−05
rs7742829
rs73128290
rs12404911
rs77285108
All − MR Egger
All − IVW(MRE)
−3
−2
−1
0
MR effect size for
'Gut microbiota abundance (genus Catenibacterium id.2153)' on 'D−dimer'

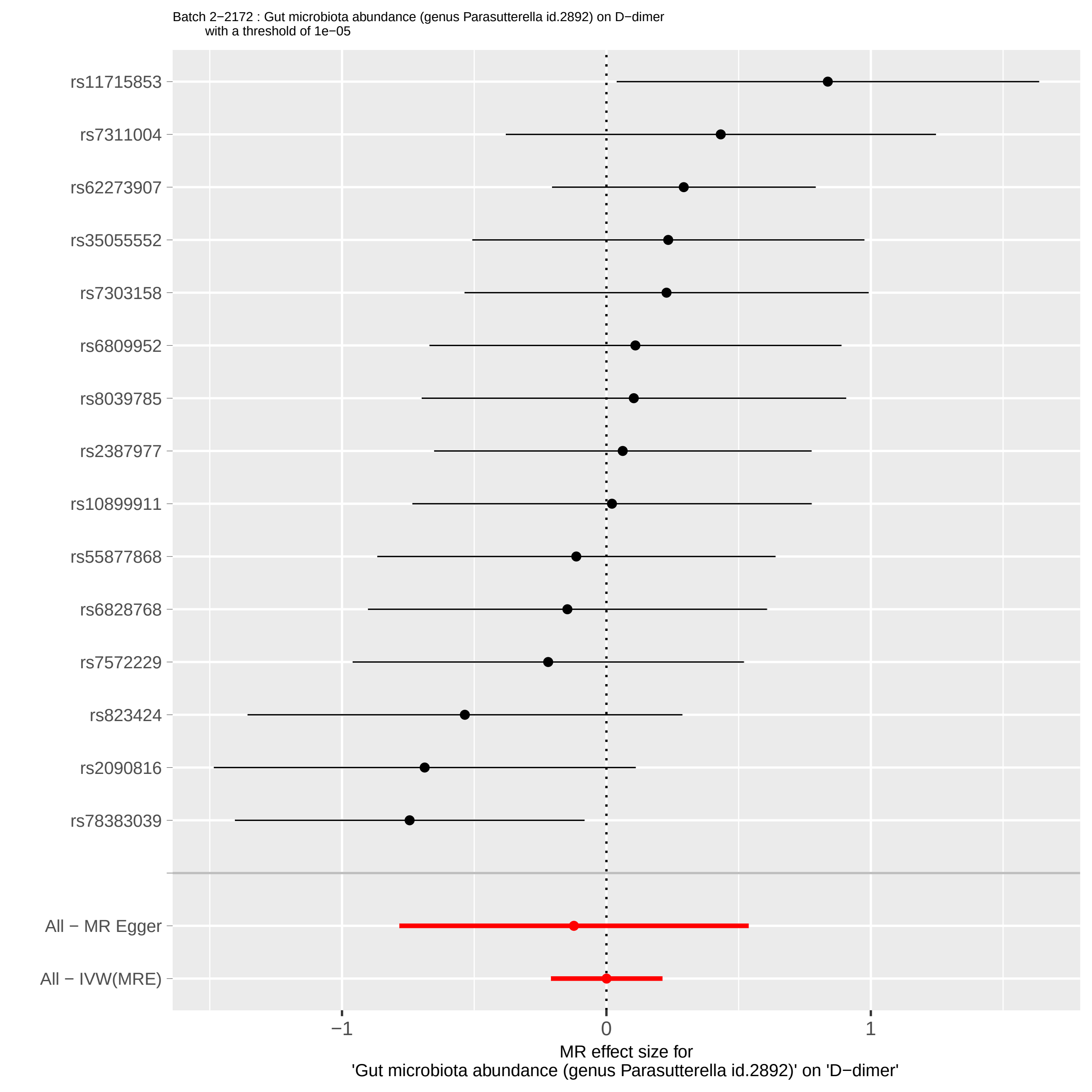

Batch 2−2172 : Gut microbiota abundance (genus Parasutterella id.2892) on D−dimer
with a threshold of 1e−05
rs11715853
rs7311004
rs62273907
rs35055552
rs7303158
rs6809952
rs8039785
rs2387977
rs10899911
rs55877868
rs6828768
rs7572229
rs823424
rs2090816
rs78383039
All − MR Egger
All − IVW(MRE)
−1
0
1
MR effect size for
'Gut microbiota abundance (genus Parasutterella id.2892)' on 'D−dimer'

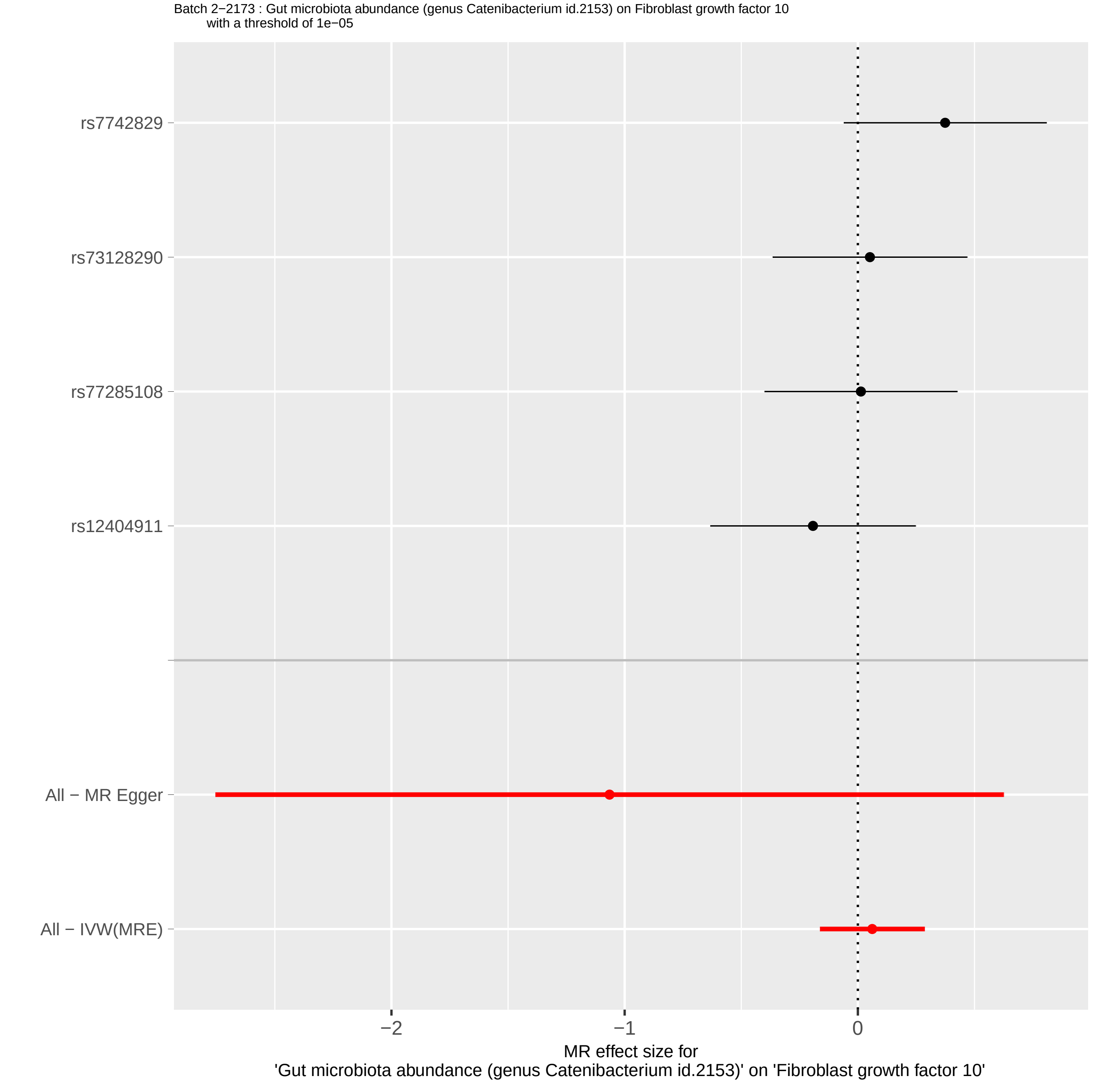

Batch 2−2173 : Gut microbiota abundance (genus Catenibacterium id.2153) on Fibroblast growth factor 10
with a threshold of 1e−05
rs7742829
rs73128290
rs77285108
rs12404911
All − MR Egger
All − IVW(MRE)
−2
−1
0
MR effect size for
'Gut microbiota abundance (genus Catenibacterium id.2153)' on 'Fibroblast growth factor 10'

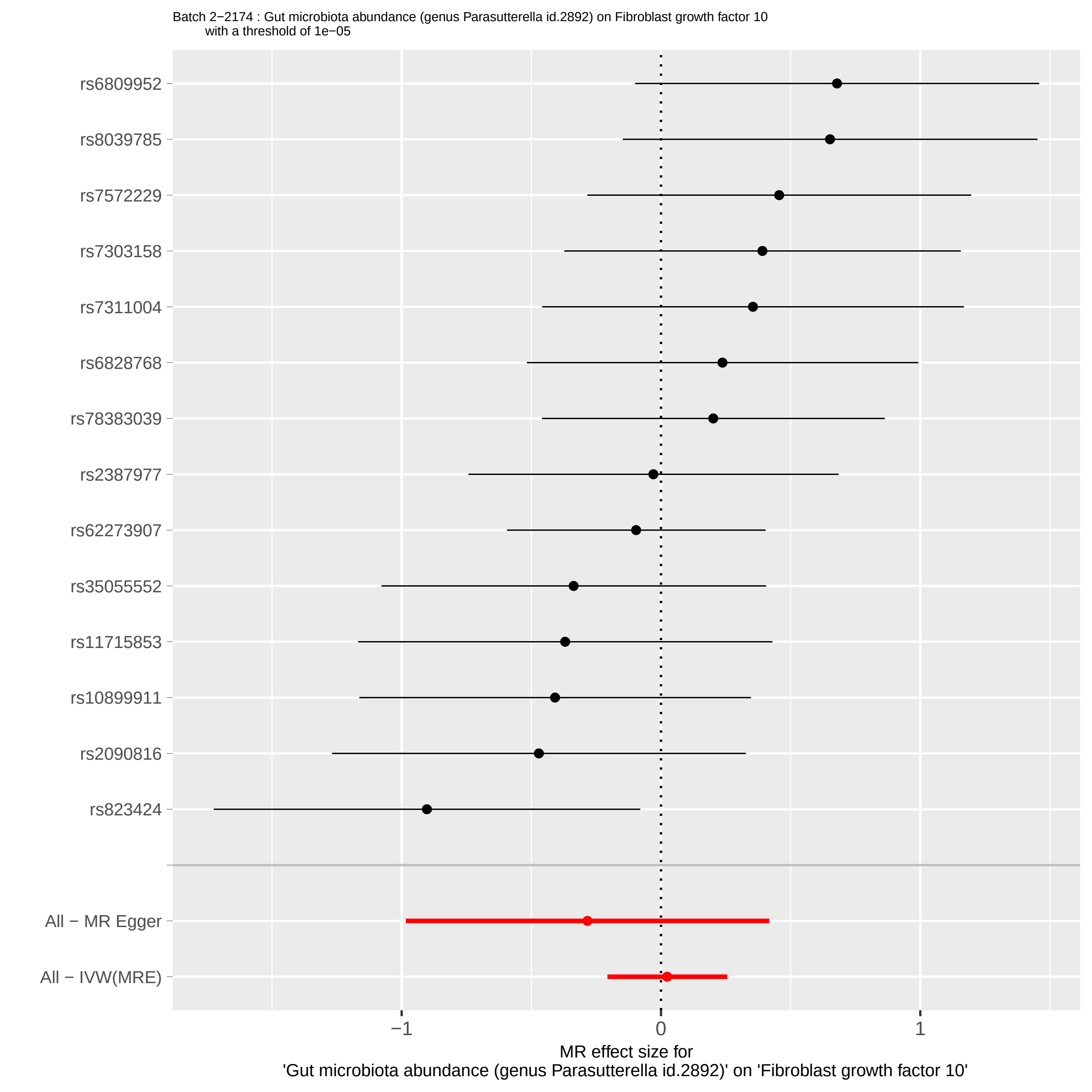
Batch 2−2174 : Gut microbiota abundance (genus Parasutterella id.2892) on Fibroblast growth factor 10
with a threshold of 1e−05
rs6809952
rs8039785
rs7572229
rs7303158
rs7311004
rs6828768
rs78383039
rs2387977
rs62273907
rs35055552
rs11715853
rs10899911
rs2090816
rs823424
All − MR Egger
All − IVW(MRE)
−1
0
1
MR effect size for
'Gut microbiota abundance (genus Parasutterella id.2892)' on 'Fibroblast growth factor 10'

Batch 2−2175 : Gut microbiota abundance (genus Catenibacterium id.2153) on Fibroblast growth factor 1
with a threshold of 1e−05

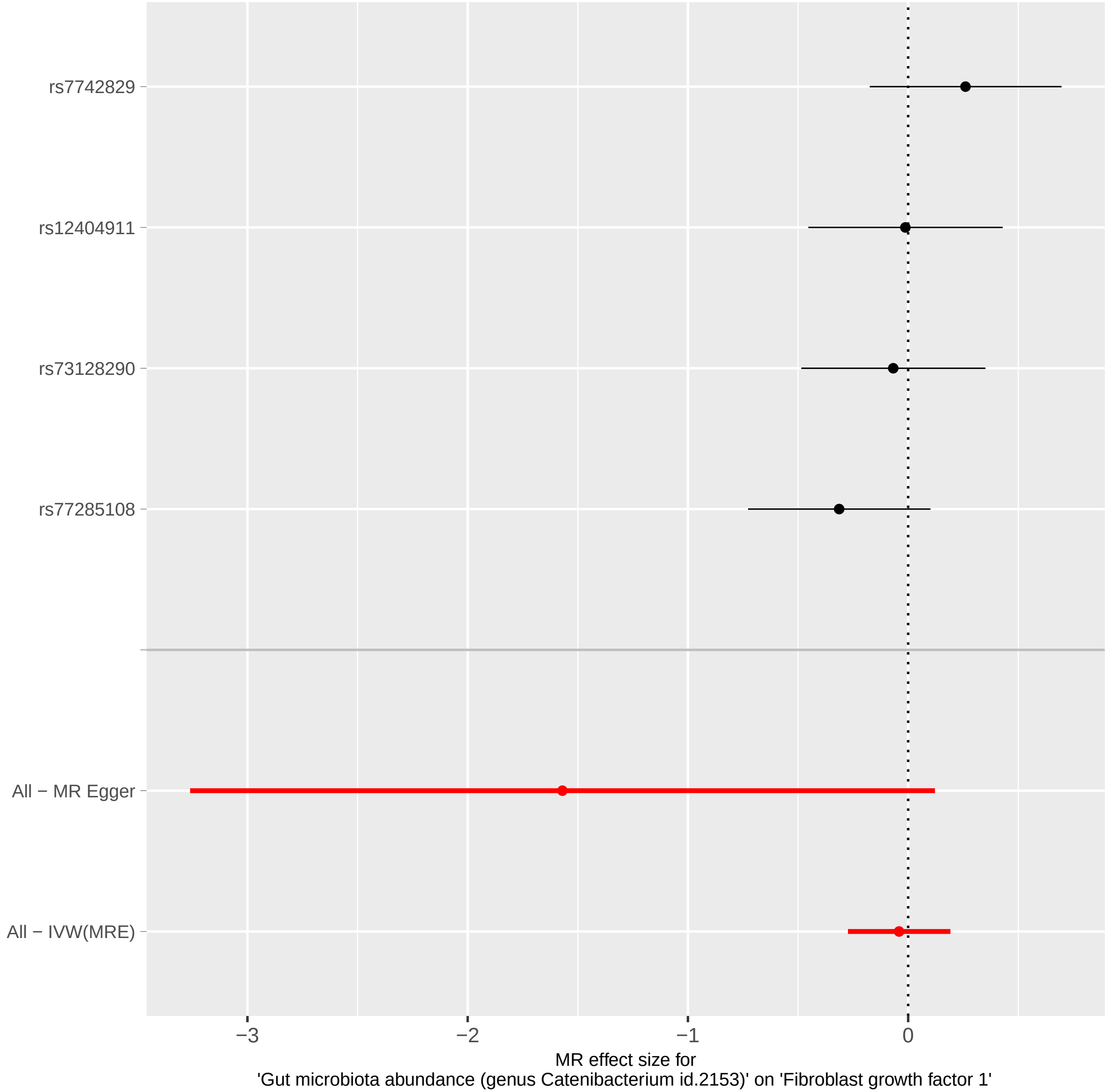

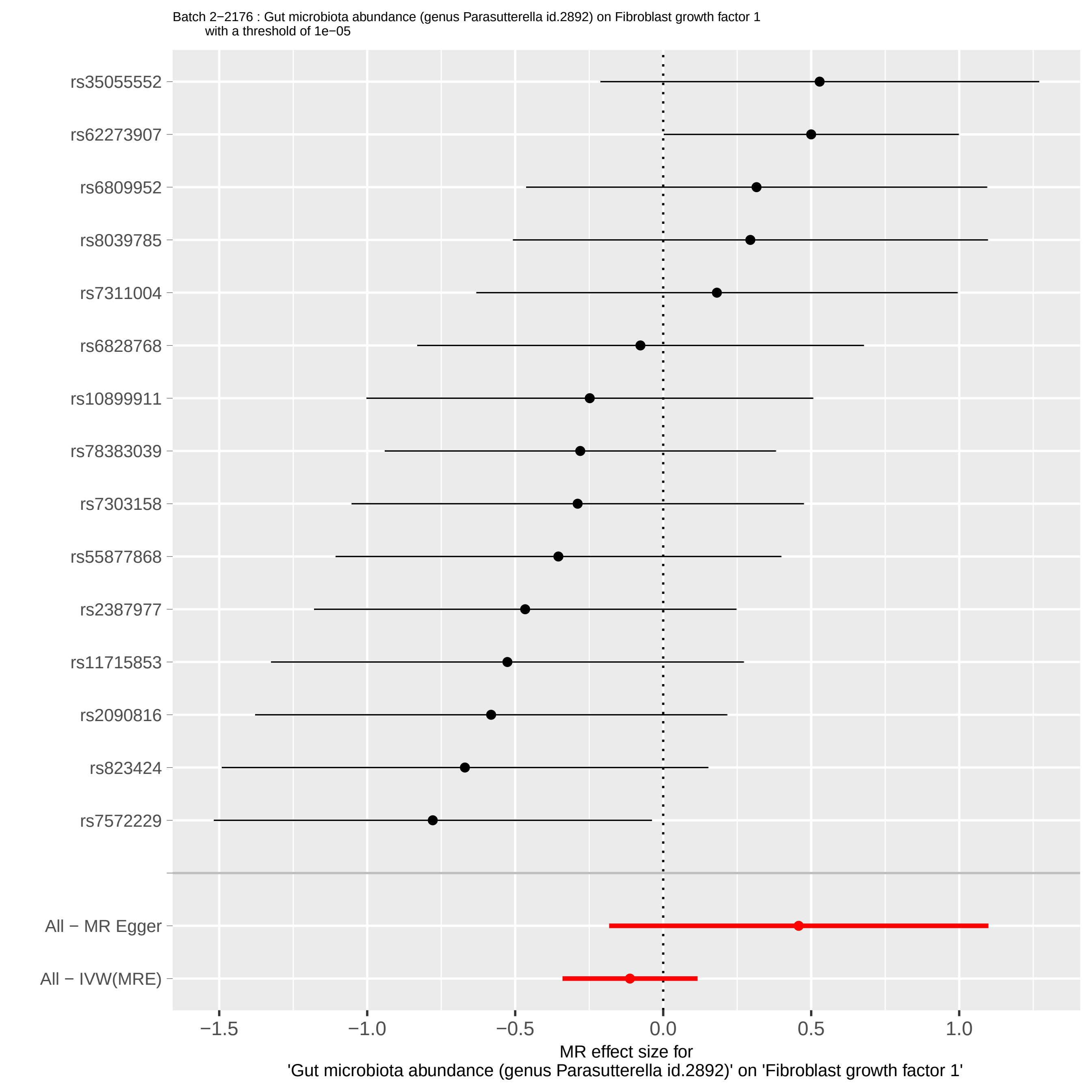

Batch 2−2176 : Gut microbiota abundance (genus Parasutterella id.2892) on Fibroblast growth factor 1
with a threshold of 1e−05
rs35055552
rs62273907
rs6809952
rs8039785
rs7311004
rs6828768
rs10899911
rs78383039
rs7303158
rs55877868
rs2387977
rs11715853
rs2090816
rs823424
rs7572229
All − MR Egger
All − IVW(MRE)
−1.5
−1.0
−0.5
0.0
0.5
1.0
MR effect size for
'Gut microbiota abundance (genus Parasutterella id.2892)' on 'Fibroblast growth factor 1'

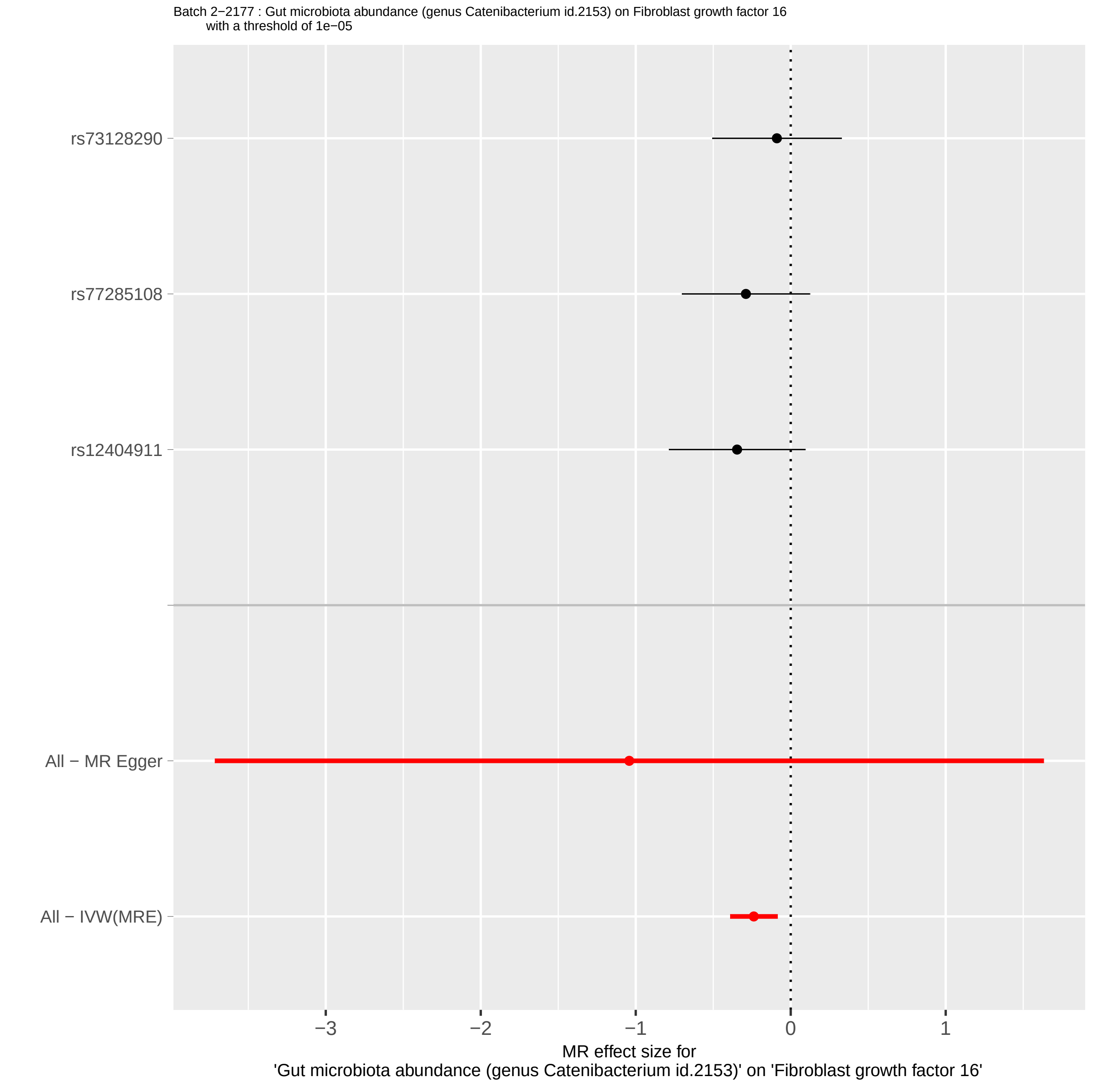

Batch 2−2177 : Gut microbiota abundance (genus Catenibacterium id.2153) on Fibroblast growth factor 16
with a threshold of 1e−05
rs73128290
rs77285108
rs12404911
All − MR Egger
All − IVW(MRE)
−3
−2
−1
0
1
MR effect size for
'Gut microbiota abundance (genus Catenibacterium id.2153)' on 'Fibroblast growth factor 16'

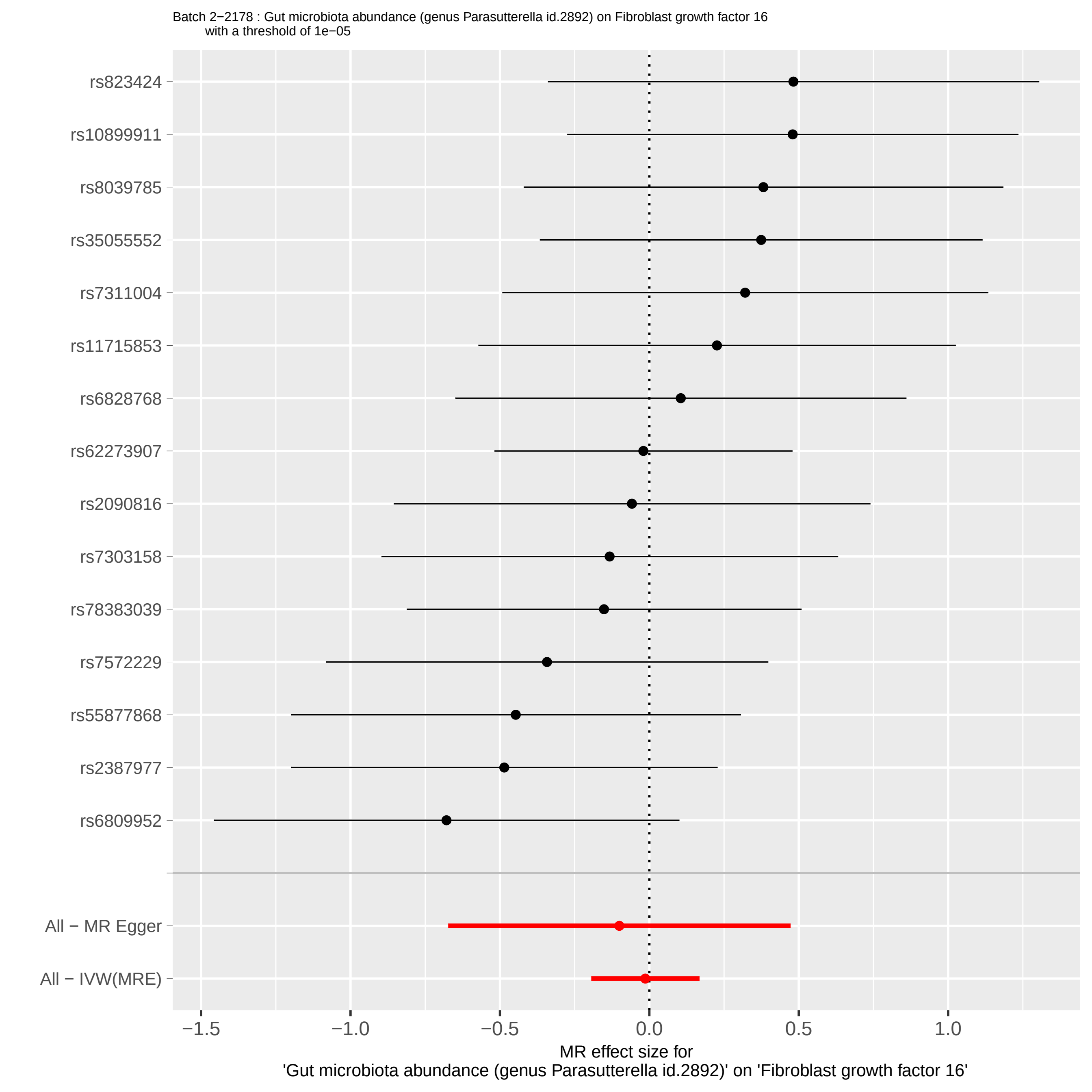

Batch 2−2178 : Gut microbiota abundance (genus Parasutterella id.2892) on Fibroblast growth factor 16
with a threshold of 1e−05
rs823424
rs10899911
rs8039785
rs35055552
rs7311004
rs11715853
rs6828768
rs62273907
rs2090816
rs7303158
rs78383039
rs7572229
rs55877868
rs2387977
rs6809952
All − MR Egger
All − IVW(MRE)
−1.5
−1.0
−0.5
0.0
0.5
1.0
MR effect size for
'Gut microbiota abundance (genus Parasutterella id.2892)' on 'Fibroblast growth factor 16'

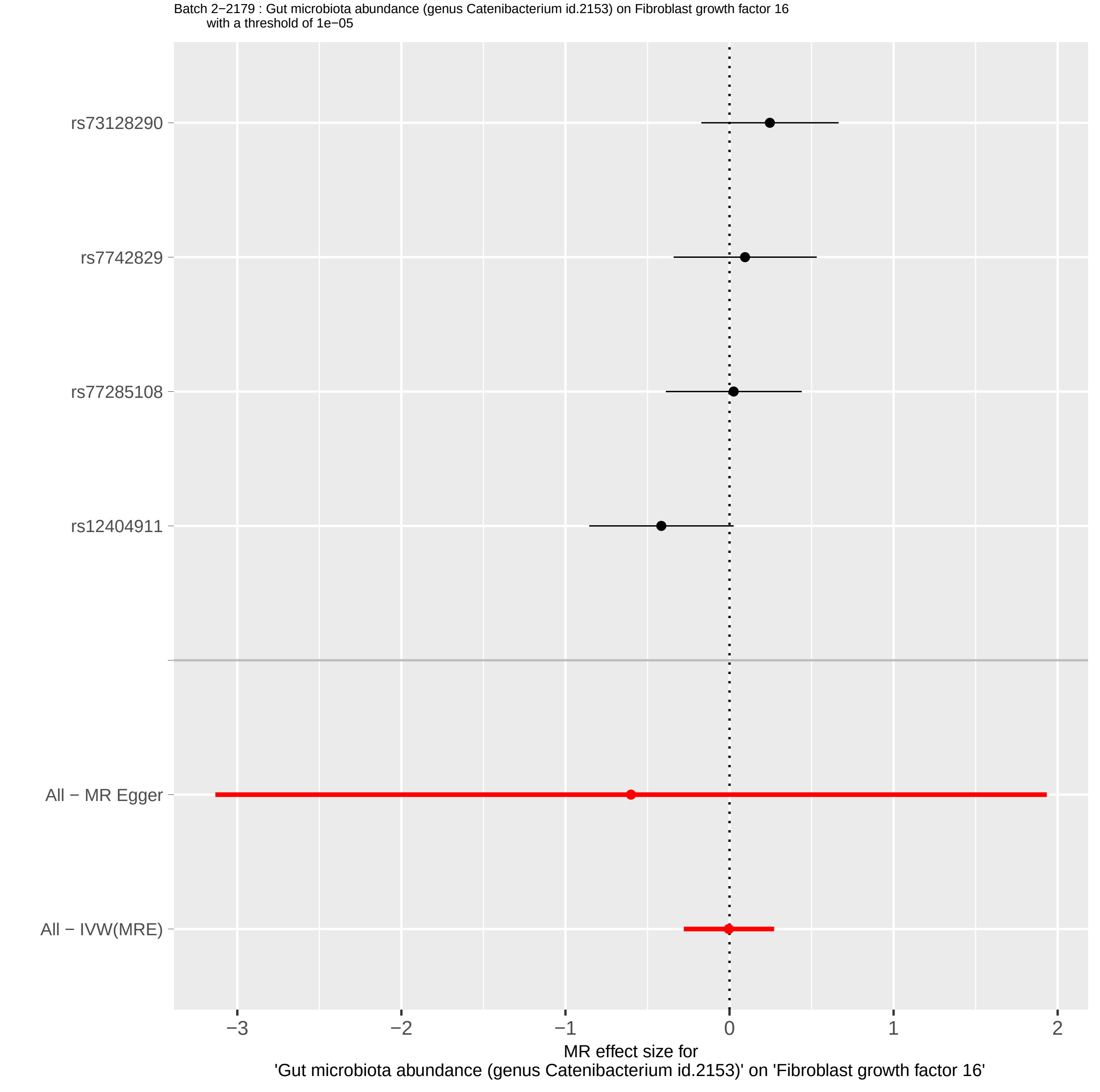
Batch 2−2179 : Gut microbiota abundance (genus Catenibacterium id.2153) on Fibroblast growth factor 16
with a threshold of 1e−05
rs73128290
rs7742829
rs77285108
rs12404911
All − MR Egger
All − IVW(MRE)
−3
−2
−1
0
1
2
MR effect size for
'Gut microbiota abundance (genus Catenibacterium id.2153)' on 'Fibroblast growth factor 16'

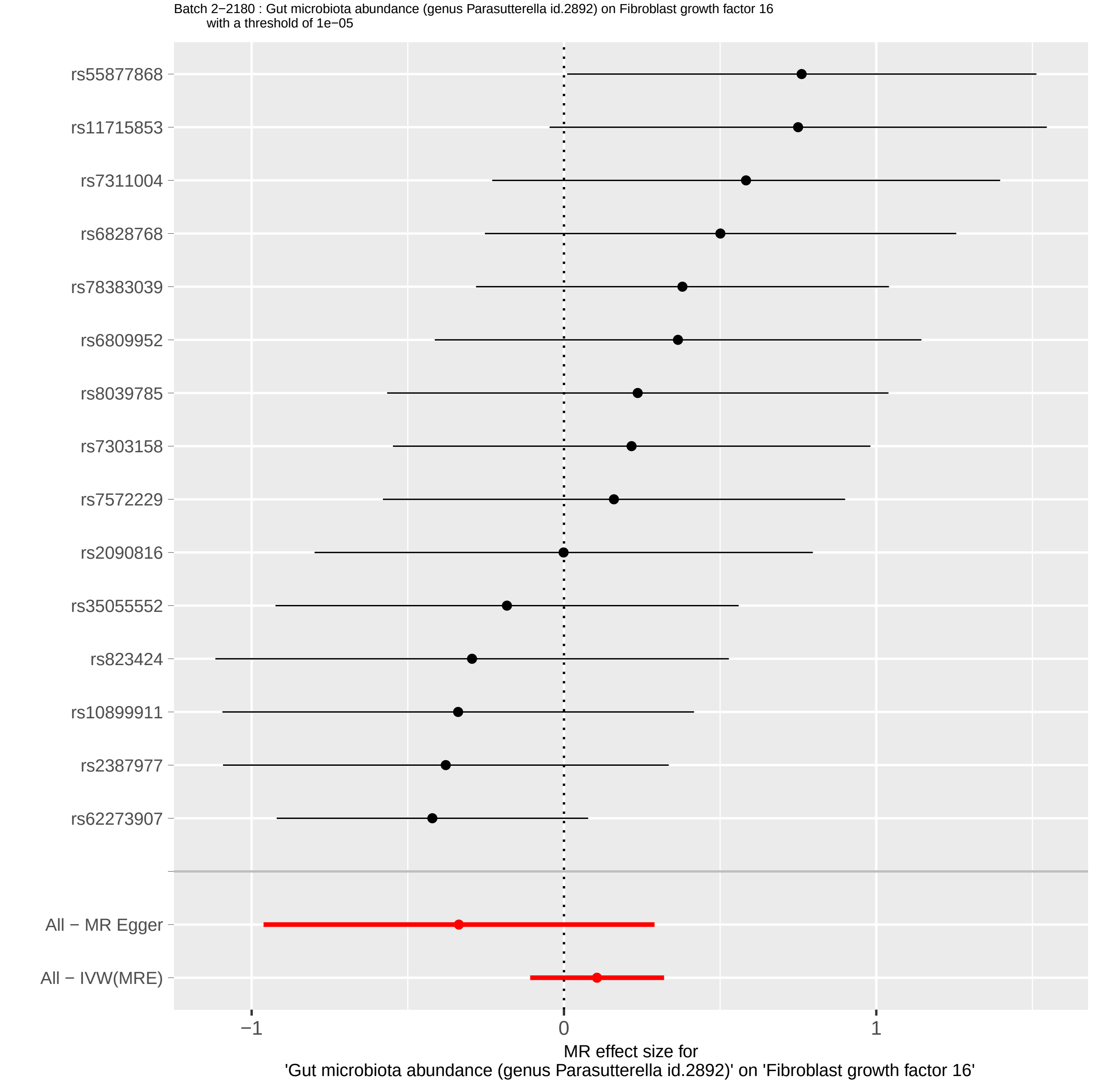
Batch 2−2180 : Gut microbiota abundance (genus Parasutterella id.2892) on Fibroblast growth factor 16
with a threshold of 1e−05
rs55877868
rs11715853
rs7311004
rs6828768
rs78383039
rs6809952
rs8039785
rs7303158
rs7572229
rs2090816
rs35055552
rs823424
rs10899911
rs2387977
rs62273907
All − MR Egger
All − IVW(MRE)
−1
0
1
MR effect size for
'Gut microbiota abundance (genus Parasutterella id.2892)' on 'Fibroblast growth factor 16'

Batch 2−2181 : Gut microbiota abundance (genus Catenibacterium id.2153) on Fibroblast growth factor 19
with a threshold of 1e−05

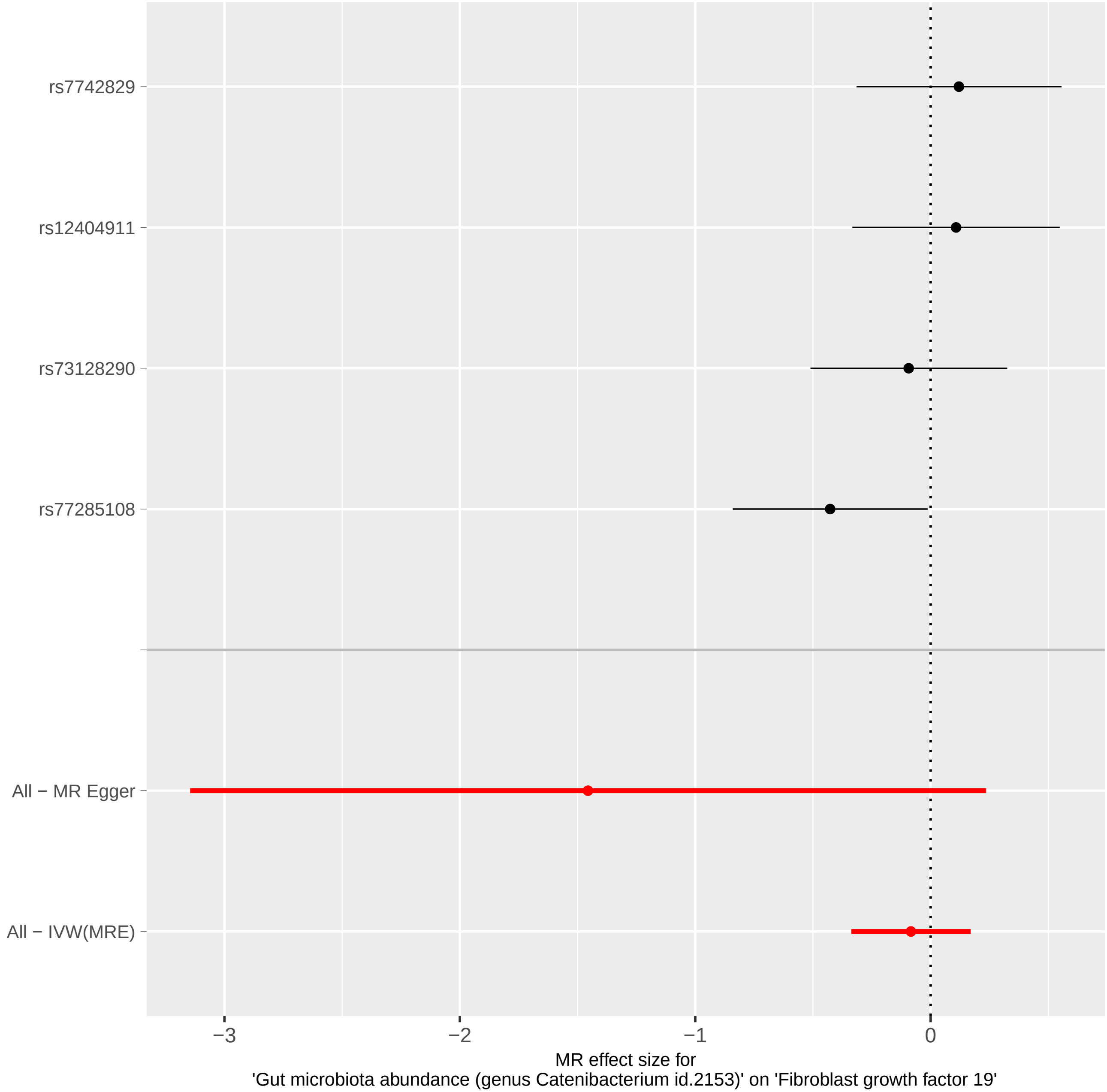

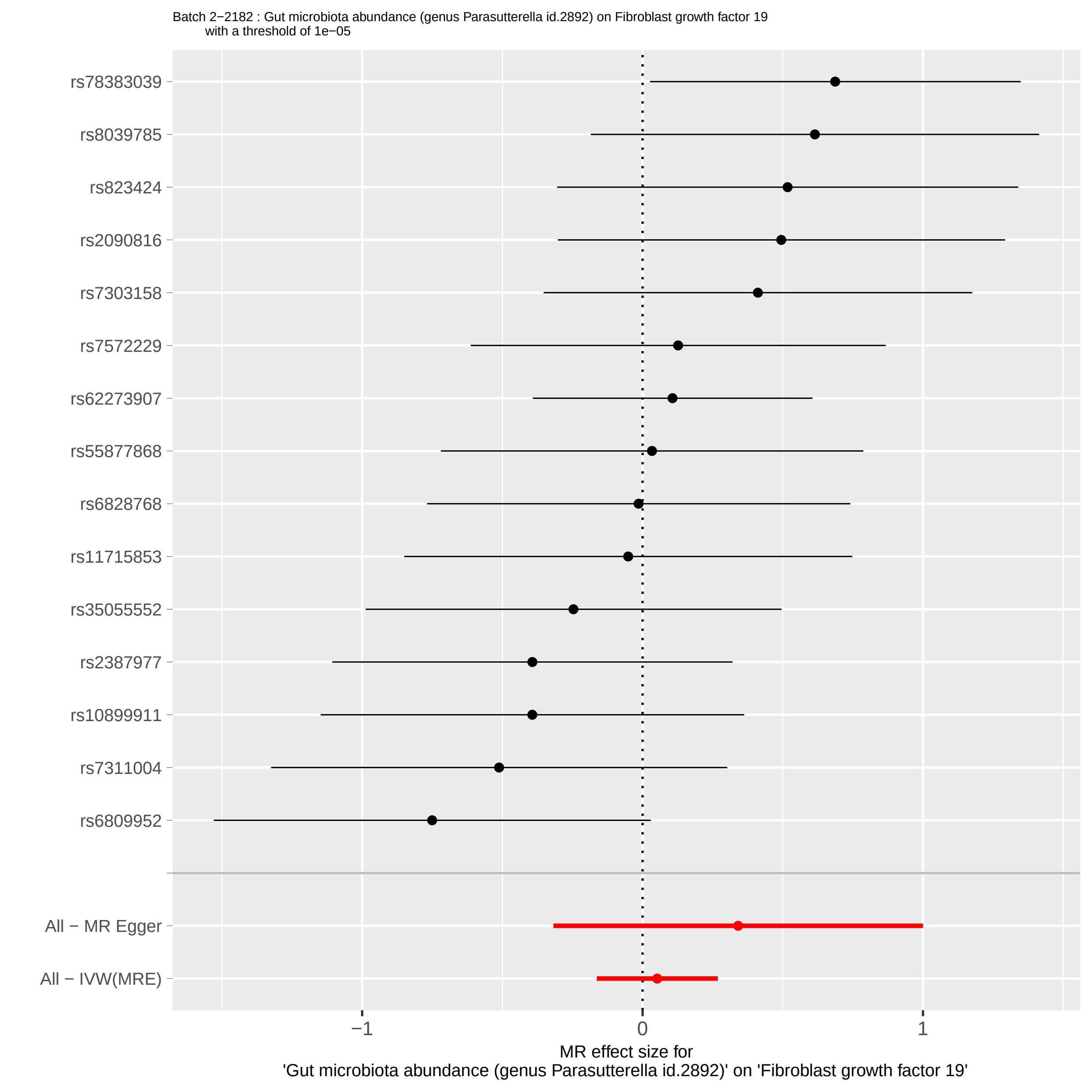

Batch 2−2182 : Gut microbiota abundance (genus Parasutterella id.2892) on Fibroblast growth factor 19
with a threshold of 1e−05
rs78383039
rs8039785
rs823424
rs2090816
rs7303158
rs7572229
rs62273907
rs55877868
rs6828768
rs11715853
rs35055552
rs2387977
rs10899911
rs7311004
rs6809952
All − MR Egger
All − IVW(MRE)
−1
0
1
MR effect size for
'Gut microbiota abundance (genus Parasutterella id.2892)' on 'Fibroblast growth factor 19'

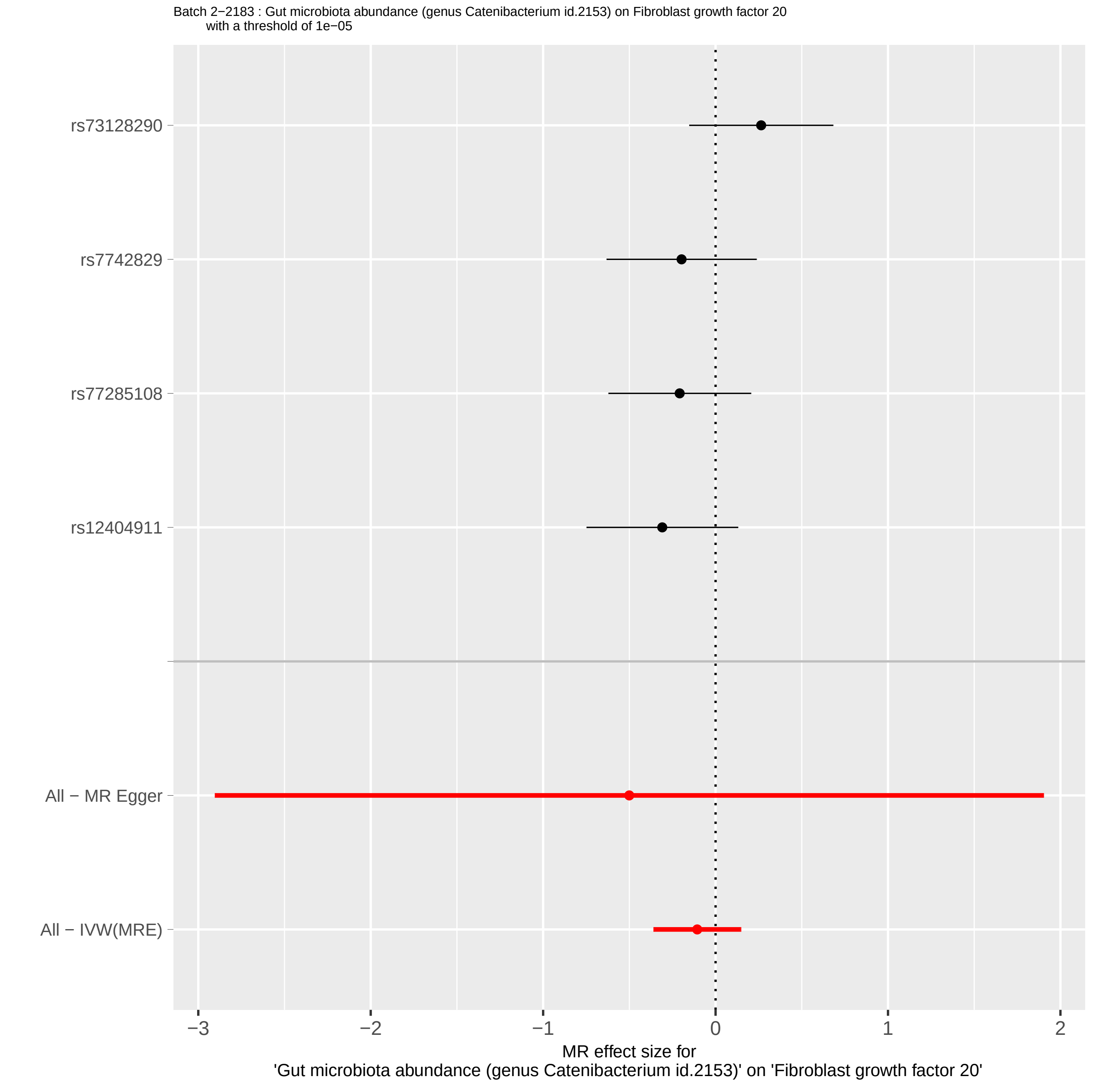

Batch 2−2183 : Gut microbiota abundance (genus Catenibacterium id.2153) on Fibroblast growth factor 20
with a threshold of 1e−05
rs73128290
rs7742829
rs77285108
rs12404911
All − MR Egger
All − IVW(MRE)
−3
−2
−1
0
1
2
MR effect size for
'Gut microbiota abundance (genus Catenibacterium id.2153)' on 'Fibroblast growth factor 20'

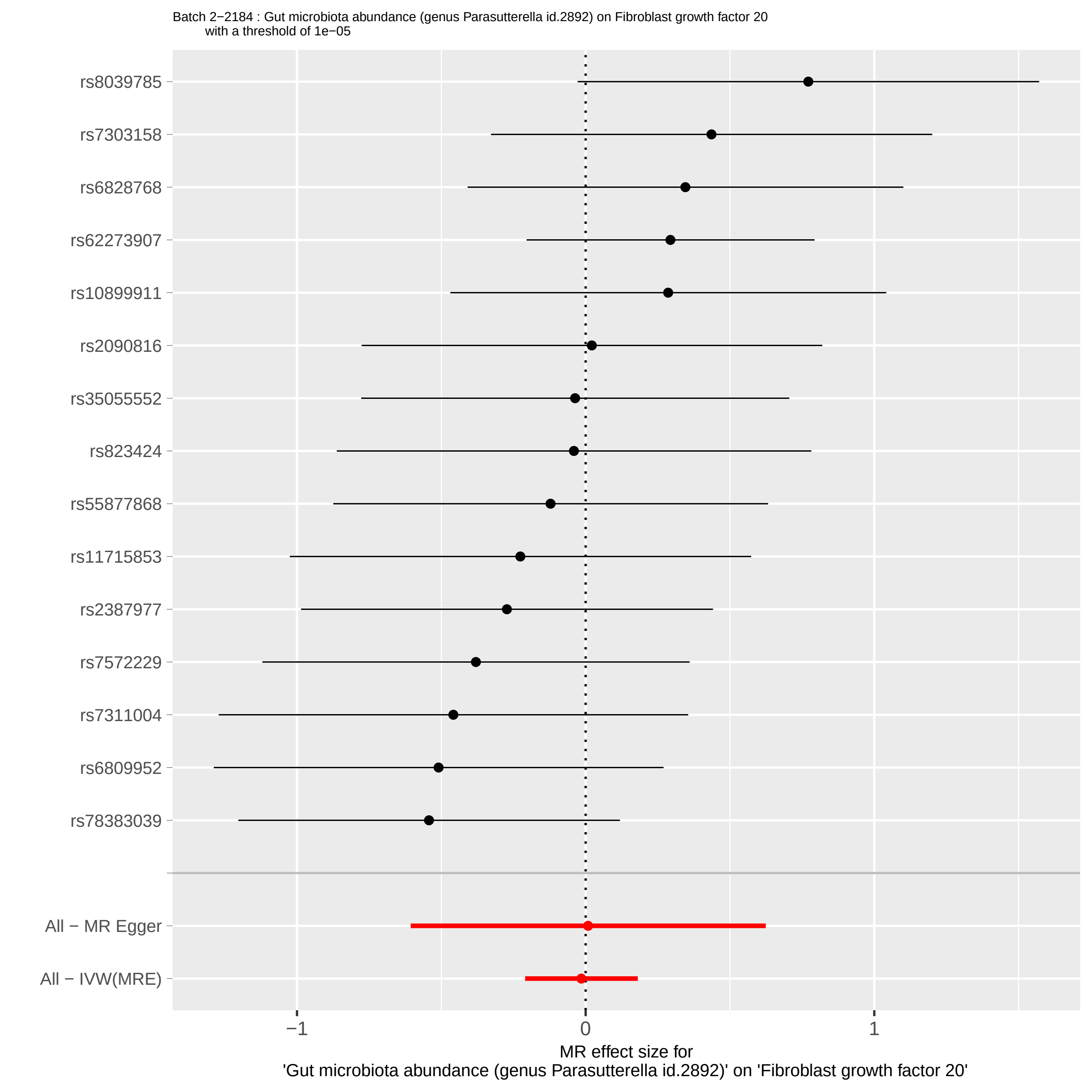

Batch 2−2184 : Gut microbiota abundance (genus Parasutterella id.2892) on Fibroblast growth factor 20
with a threshold of 1e−05
rs8039785
rs7303158
rs6828768
rs62273907
rs10899911
rs2090816
rs35055552
rs823424
rs55877868
rs11715853
rs2387977
rs7572229
rs7311004
rs6809952
rs78383039
All − MR Egger
All − IVW(MRE)
−1
0
1
MR effect size for
'Gut microbiota abundance (genus Parasutterella id.2892)' on 'Fibroblast growth factor 20'

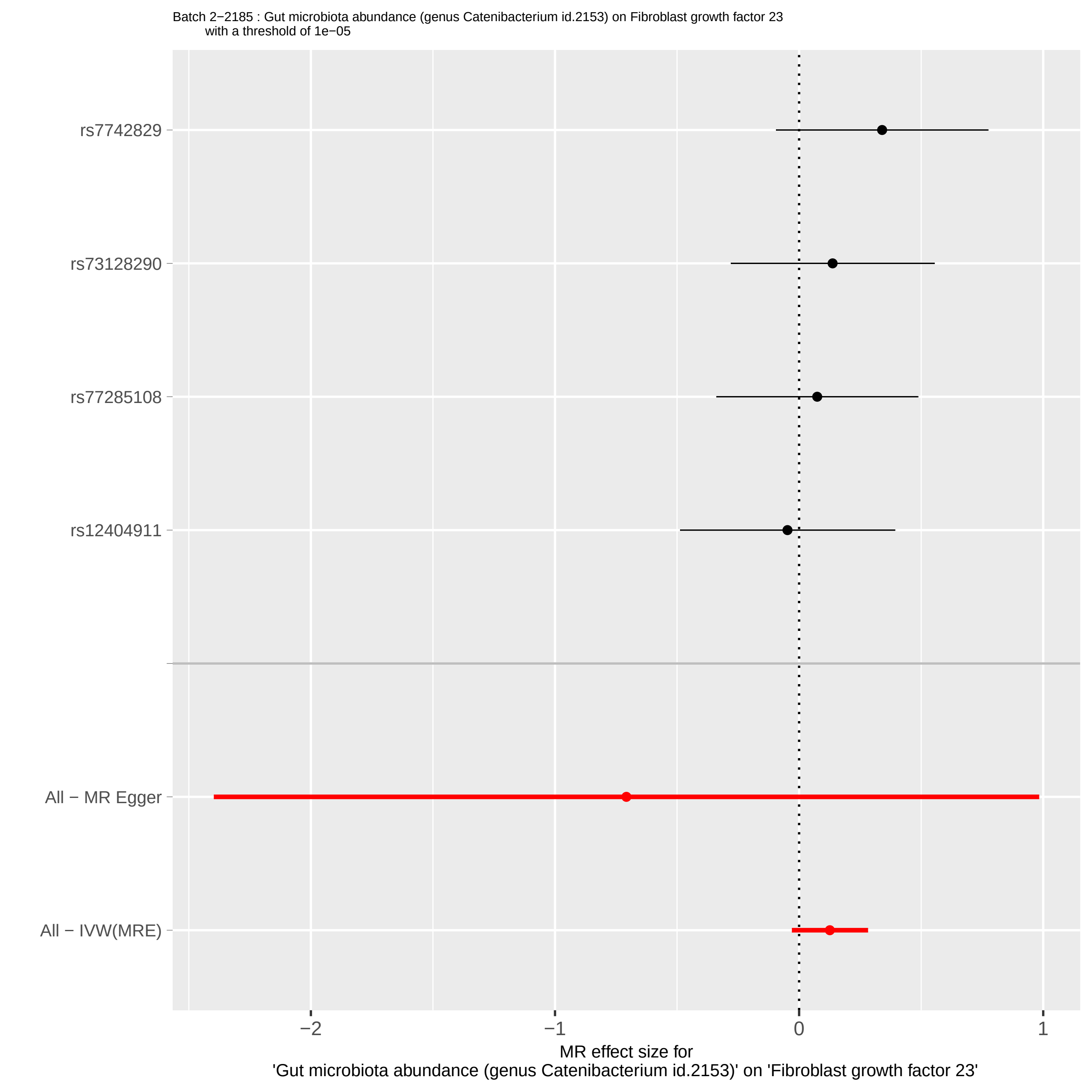
Batch 2−2185 : Gut microbiota abundance (genus Catenibacterium id.2153) on Fibroblast growth factor 23
with a threshold of 1e−05
rs7742829
rs73128290
rs77285108
rs12404911
All − MR Egger
All − IVW(MRE)
−2
−1
0
1
MR effect size for
'Gut microbiota abundance (genus Catenibacterium id.2153)' on 'Fibroblast growth factor 23'

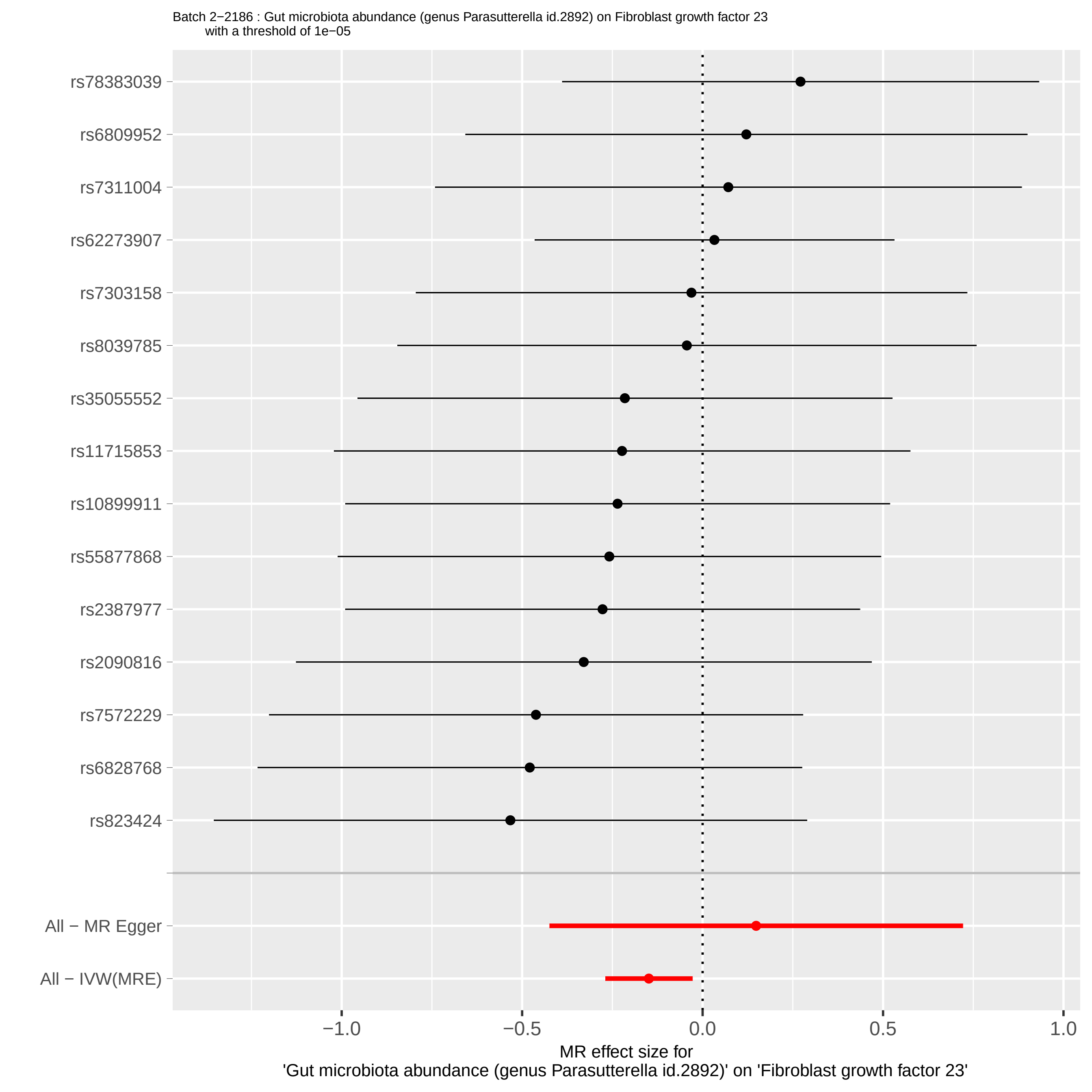

Batch 2−2186 : Gut microbiota abundance (genus Parasutterella id.2892) on Fibroblast growth factor 23
with a threshold of 1e−05
rs78383039
rs6809952
rs7311004
rs62273907
rs7303158
rs8039785
rs35055552
rs11715853
rs10899911
rs55877868
rs2387977
rs2090816
rs7572229
rs6828768
rs823424
All − MR Egger
All − IVW(MRE)
−1.0
−0.5
0.0
0.5
1.0
MR effect size for
'Gut microbiota abundance (genus Parasutterella id.2892)' on 'Fibroblast growth factor 23'

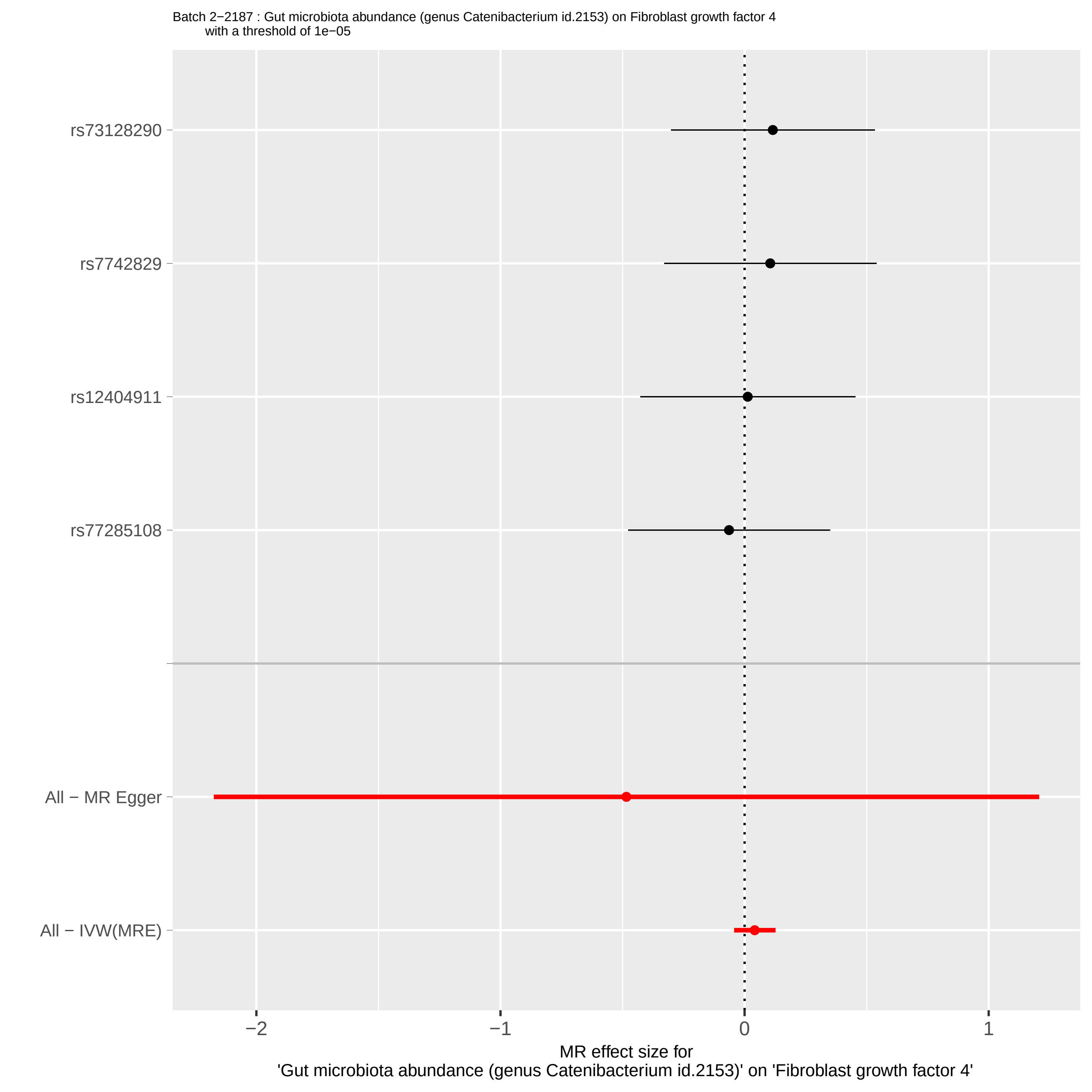
Batch 2−2187 : Gut microbiota abundance (genus Catenibacterium id.2153) on Fibroblast growth factor 4
with a threshold of 1e−05
rs73128290
rs7742829
rs12404911
rs77285108
All − MR Egger
All − IVW(MRE)
−2
−1
0
1
MR effect size for
'Gut microbiota abundance (genus Catenibacterium id.2153)' on 'Fibroblast growth factor 4'

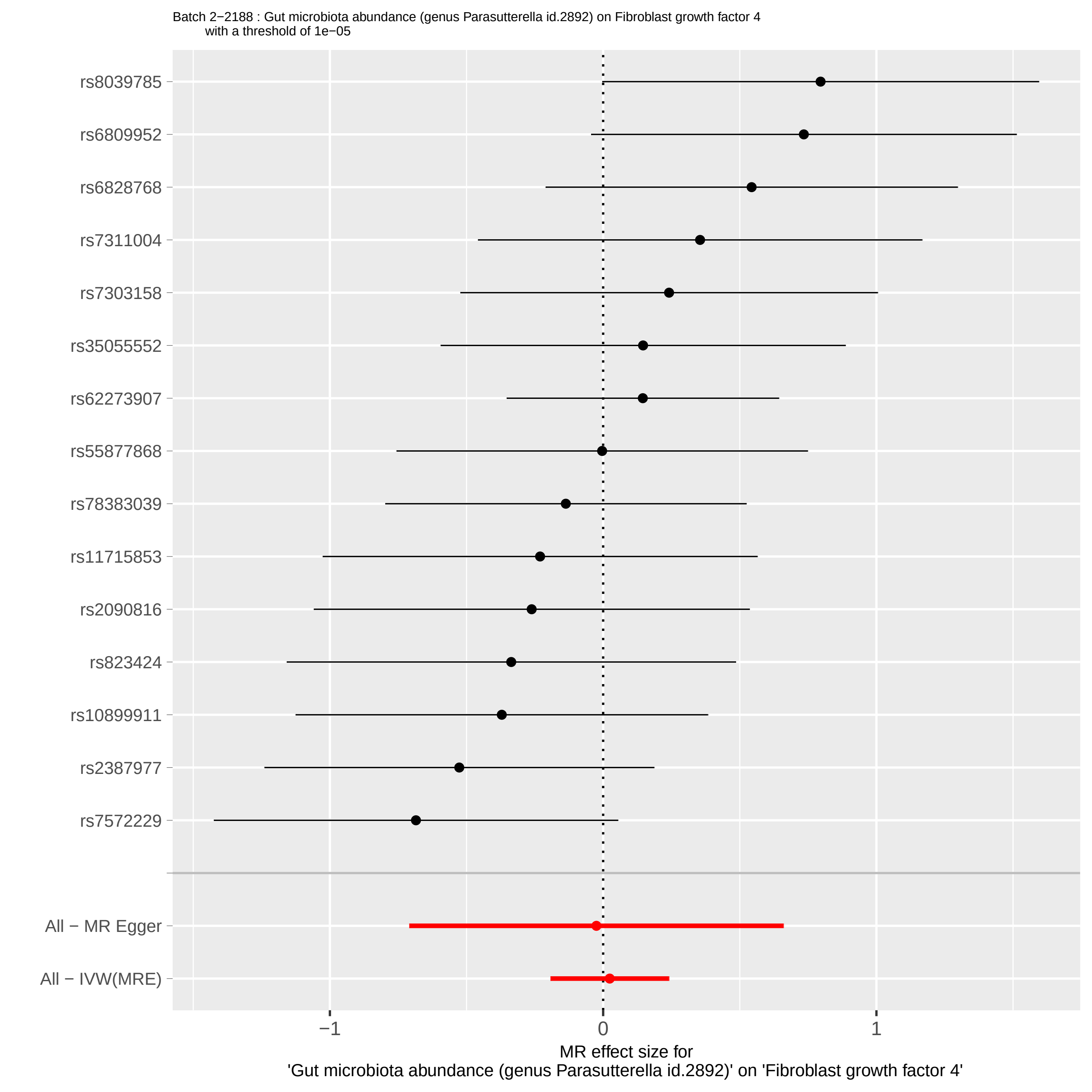
Batch 2−2188 : Gut microbiota abundance (genus Parasutterella id.2892) on Fibroblast growth factor 4
with a threshold of 1e−05
rs8039785
rs6809952
rs6828768
rs7311004
rs7303158
rs35055552
rs62273907
rs55877868
rs78383039
rs11715853
rs2090816
rs823424
rs10899911
rs2387977
rs7572229
All − MR Egger
All − IVW(MRE)
−1
0
1
MR effect size for
'Gut microbiota abundance (genus Parasutterella id.2892)' on 'Fibroblast growth factor 4'

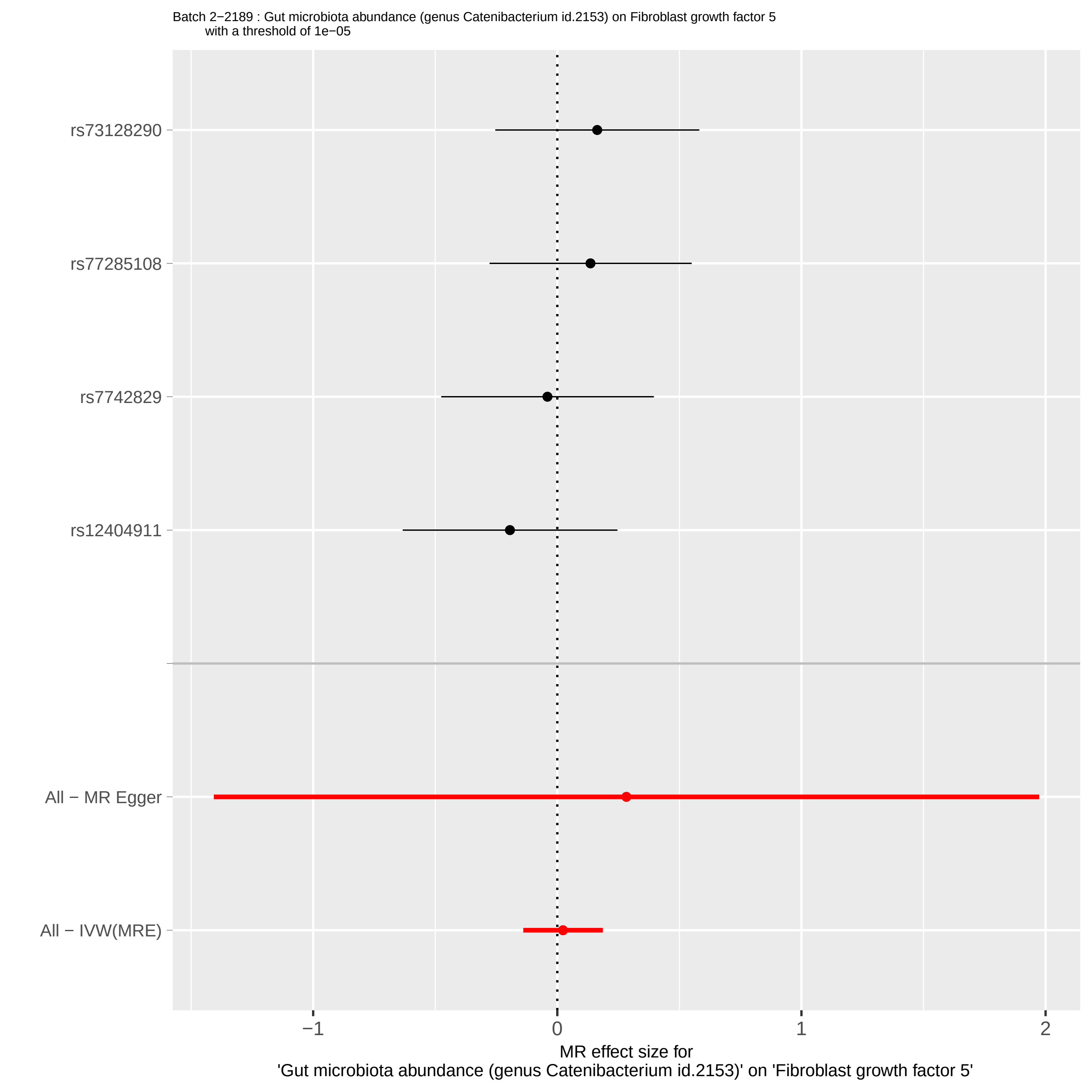

Batch 2−2189 : Gut microbiota abundance (genus Catenibacterium id.2153) on Fibroblast growth factor 5
with a threshold of 1e−05
rs73128290
rs77285108
rs7742829
rs12404911
All − MR Egger
All − IVW(MRE)
−1
0
1
2
MR effect size for
'Gut microbiota abundance (genus Catenibacterium id.2153)' on 'Fibroblast growth factor 5'

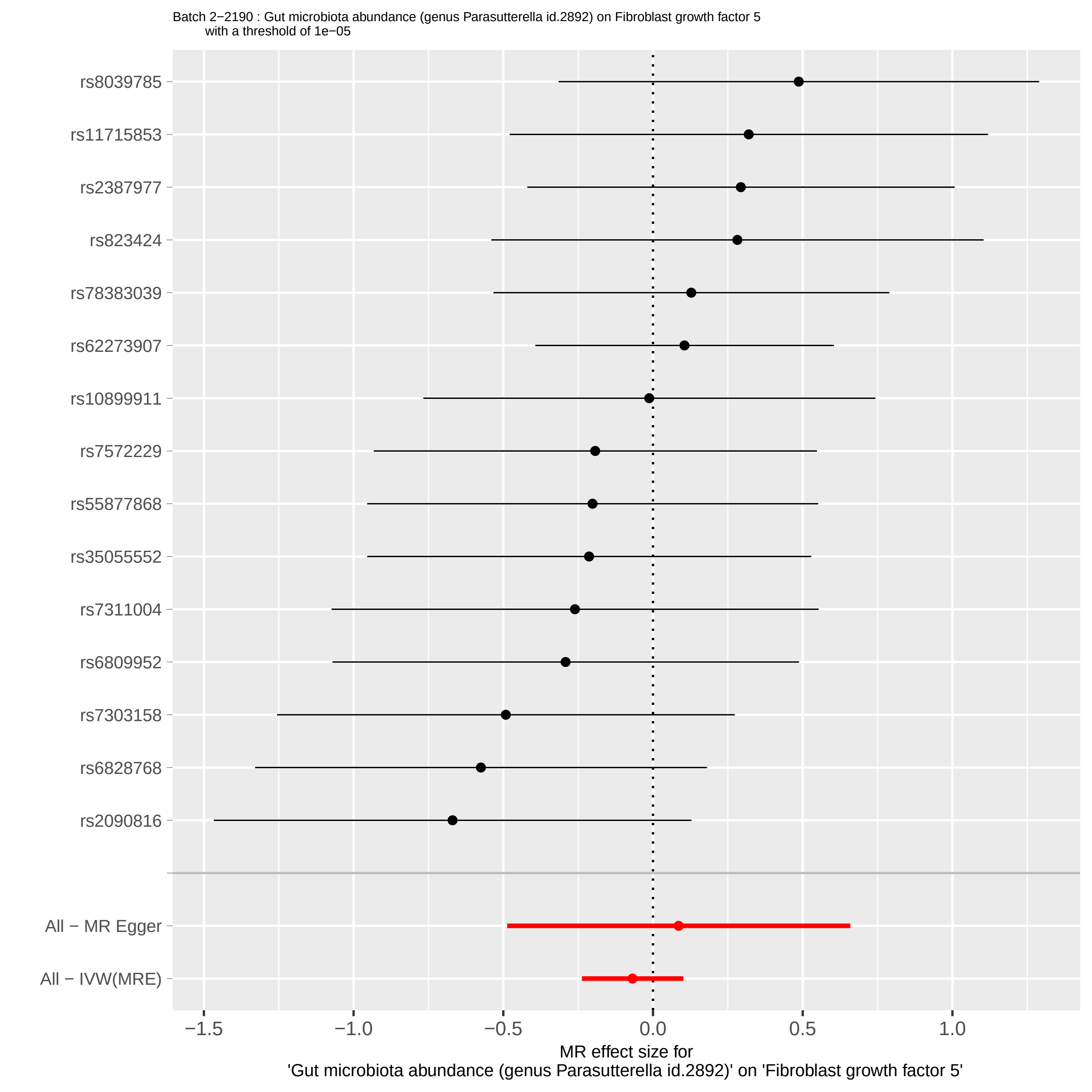

Batch 2−2190 : Gut microbiota abundance (genus Parasutterella id.2892) on Fibroblast growth factor 5
with a threshold of 1e−05
rs8039785
rs11715853
rs2387977
rs823424
rs78383039
rs62273907
rs10899911
rs7572229
rs55877868
rs35055552
rs7311004
rs6809952
rs7303158
rs6828768
rs2090816
All − MR Egger
All − IVW(MRE)
−1.5
−1.0
−0.5
0.0
0.5
1.0
MR effect size for
'Gut microbiota abundance (genus Parasutterella id.2892)' on 'Fibroblast growth factor 5'

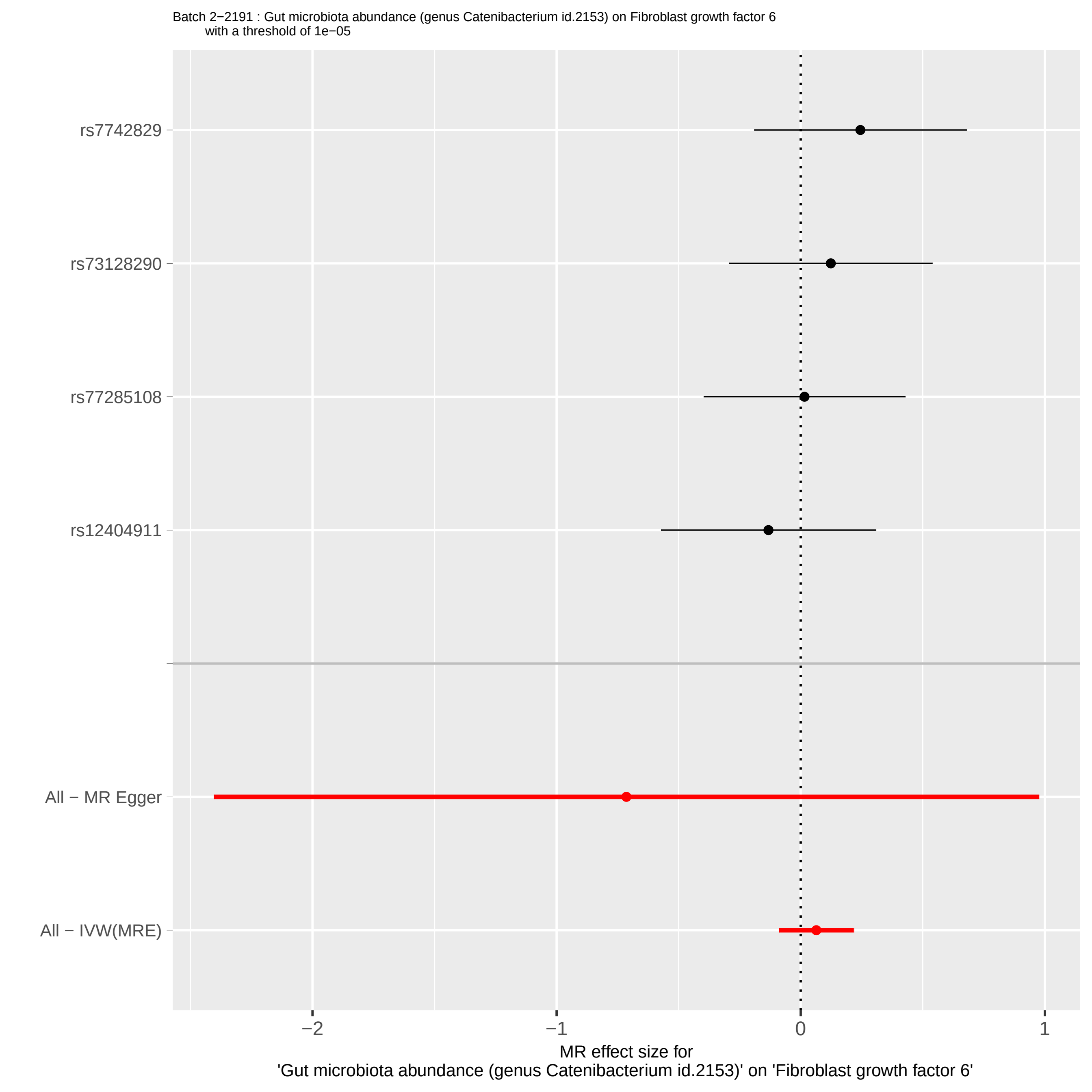

Batch 2−2191 : Gut microbiota abundance (genus Catenibacterium id.2153) on Fibroblast growth factor 6
with a threshold of 1e−05
rs7742829
rs73128290
rs77285108
rs12404911
All − MR Egger
All − IVW(MRE)
−2
−1
0
1
MR effect size for
'Gut microbiota abundance (genus Catenibacterium id.2153)' on 'Fibroblast growth factor 6'

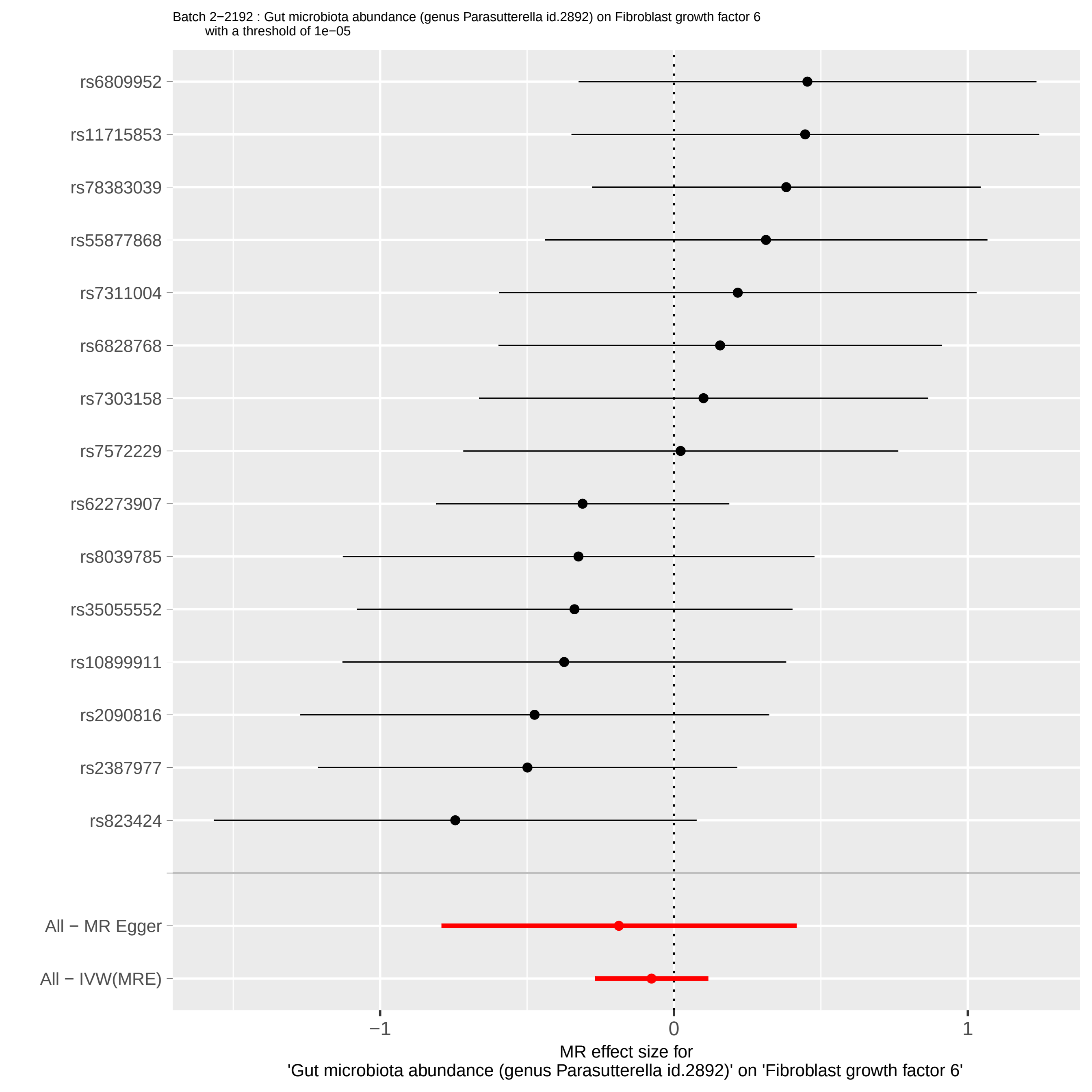

Batch 2−2192 : Gut microbiota abundance (genus Parasutterella id.2892) on Fibroblast growth factor 6
with a threshold of 1e−05
rs6809952
rs11715853
rs78383039
rs55877868
rs7311004
rs6828768
rs7303158
rs7572229
rs62273907
rs8039785
rs35055552
rs10899911
rs2090816
rs2387977
rs823424
All − MR Egger
All − IVW(MRE)
−1
0
1
MR effect size for
'Gut microbiota abundance (genus Parasutterella id.2892)' on 'Fibroblast growth factor 6'

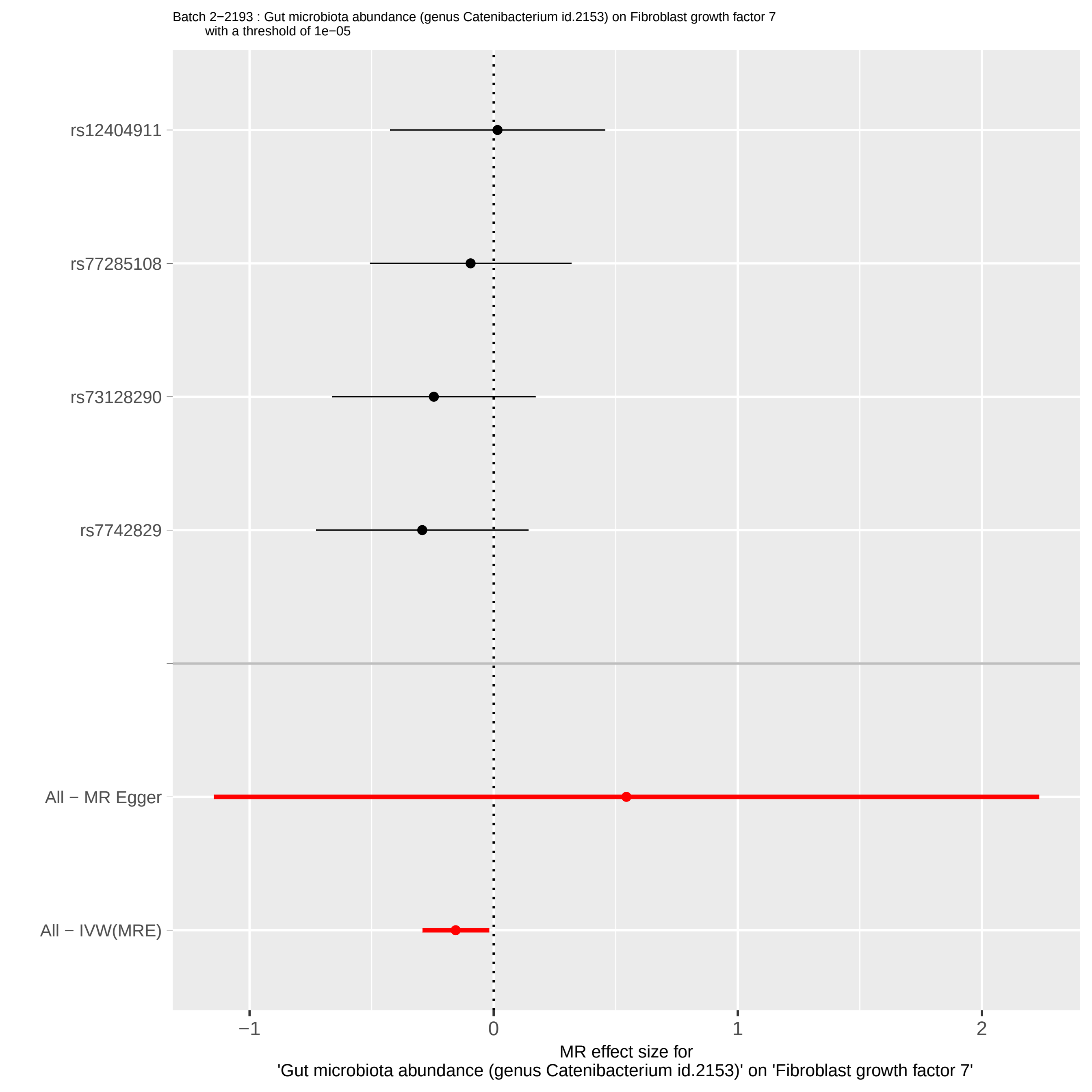

Batch 2−2193 : Gut microbiota abundance (genus Catenibacterium id.2153) on Fibroblast growth factor 7
with a threshold of 1e−05
rs12404911
rs77285108
rs73128290
rs7742829
All − MR Egger
All − IVW(MRE)
−1
0
1
2
MR effect size for
'Gut microbiota abundance (genus Catenibacterium id.2153)' on 'Fibroblast growth factor 7'

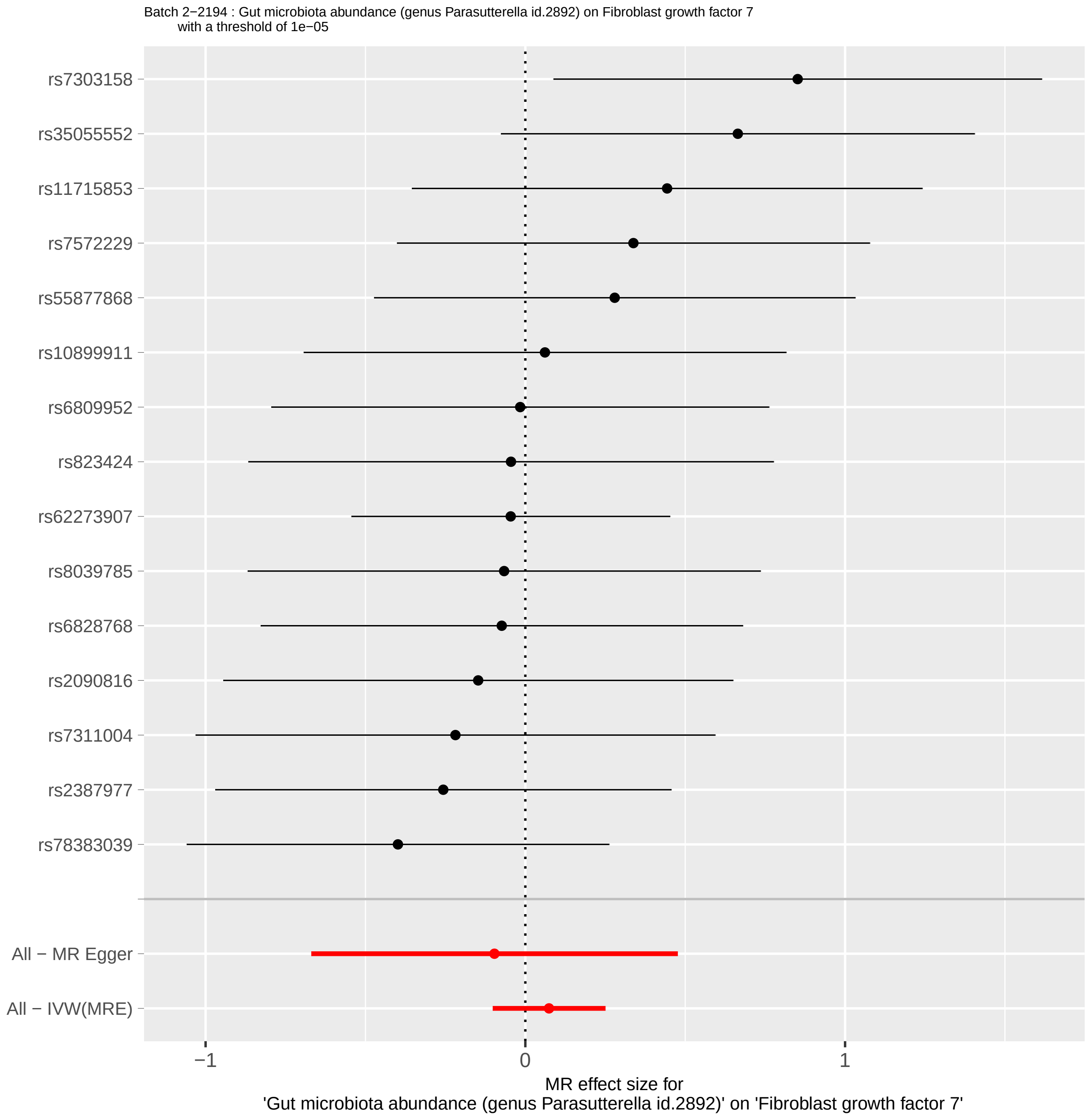
Batch 2−2194 : Gut microbiota abundance (genus Parasutterella id.2892) on Fibroblast growth factor 7
with a threshold of 1e−05
rs7303158
rs35055552
rs11715853
rs7572229
rs55877868
rs10899911
rs6809952
rs823424
rs62273907
rs8039785
rs6828768
rs2090816
rs7311004
rs2387977
rs78383039
All − MR Egger
All − IVW(MRE)
−1
0
1
MR effect size for
'Gut microbiota abundance (genus Parasutterella id.2892)' on 'Fibroblast growth factor 7'

Batch 2−2195 : Gut microbiota abundance (genus Catenibacterium id.2153) on Fibroblast growth factor 7
with a threshold of 1e−05

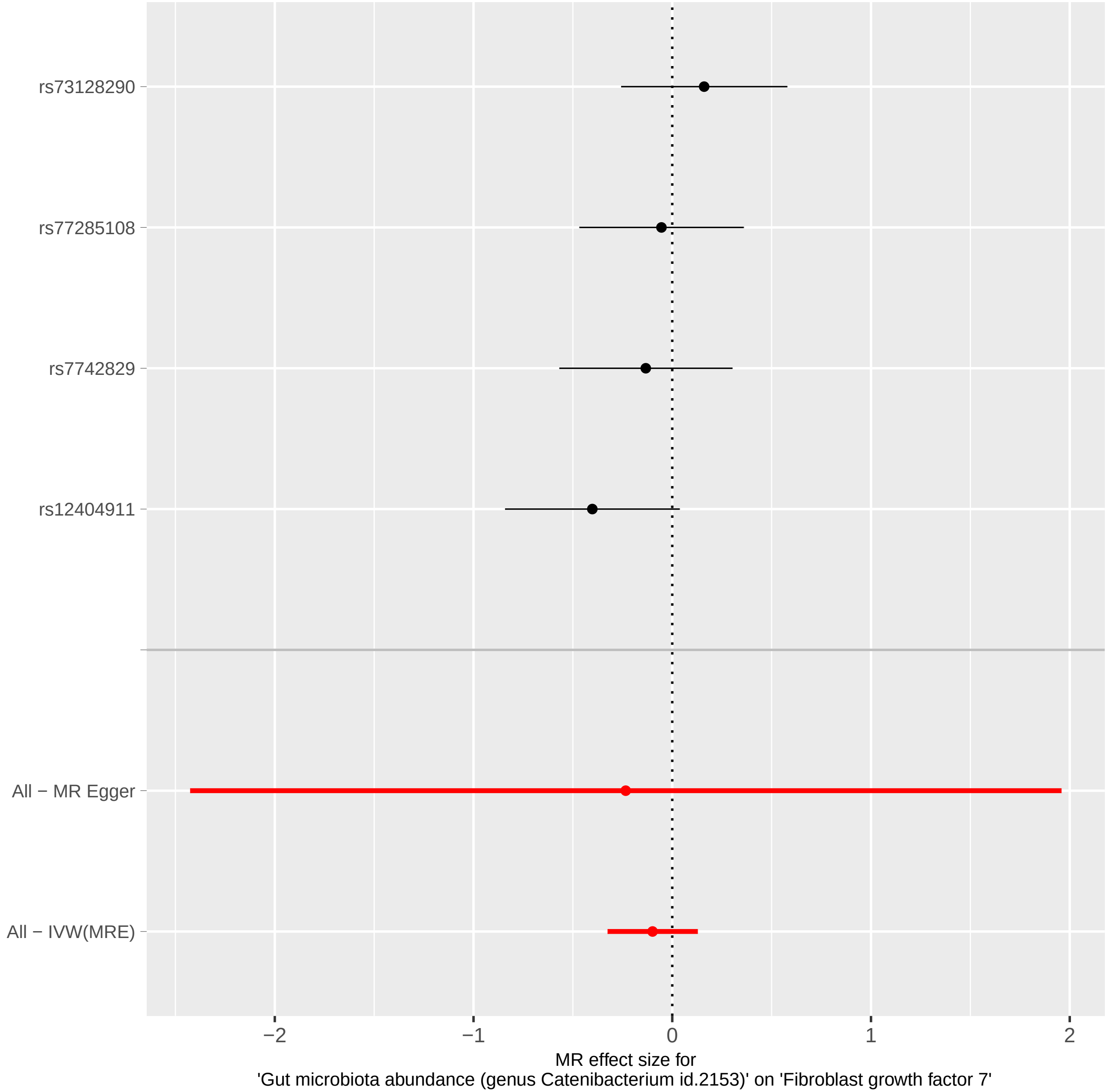

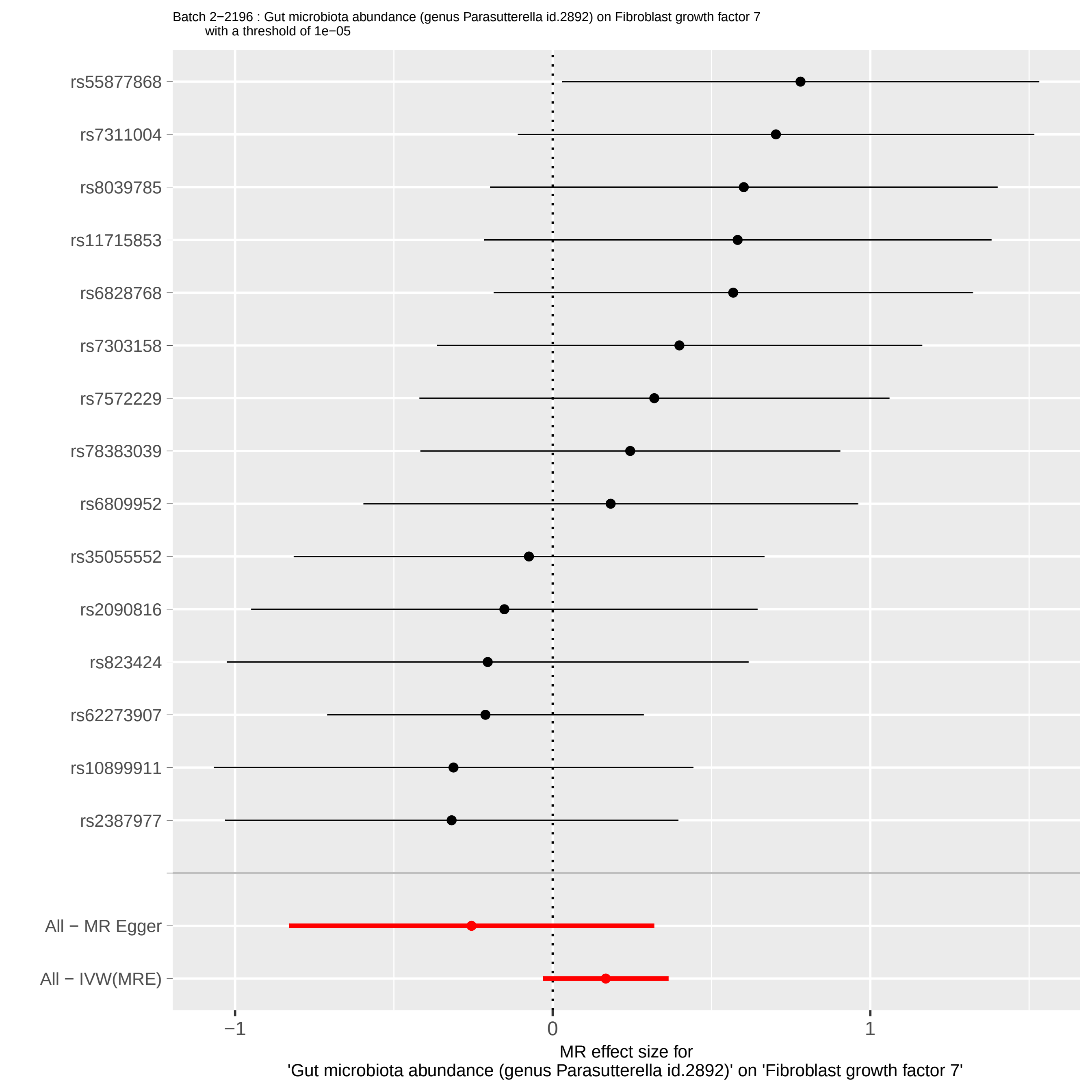

Batch 2−2196 : Gut microbiota abundance (genus Parasutterella id.2892) on Fibroblast growth factor 7
with a threshold of 1e−05
rs55877868
rs7311004
rs8039785
rs11715853
rs6828768
rs7303158
rs7572229
rs78383039
rs6809952
rs35055552
rs2090816
rs823424
rs62273907
rs10899911
rs2387977
All − MR Egger
All − IVW(MRE)
−1
0
1
MR effect size for
'Gut microbiota abundance (genus Parasutterella id.2892)' on 'Fibroblast growth factor 7'

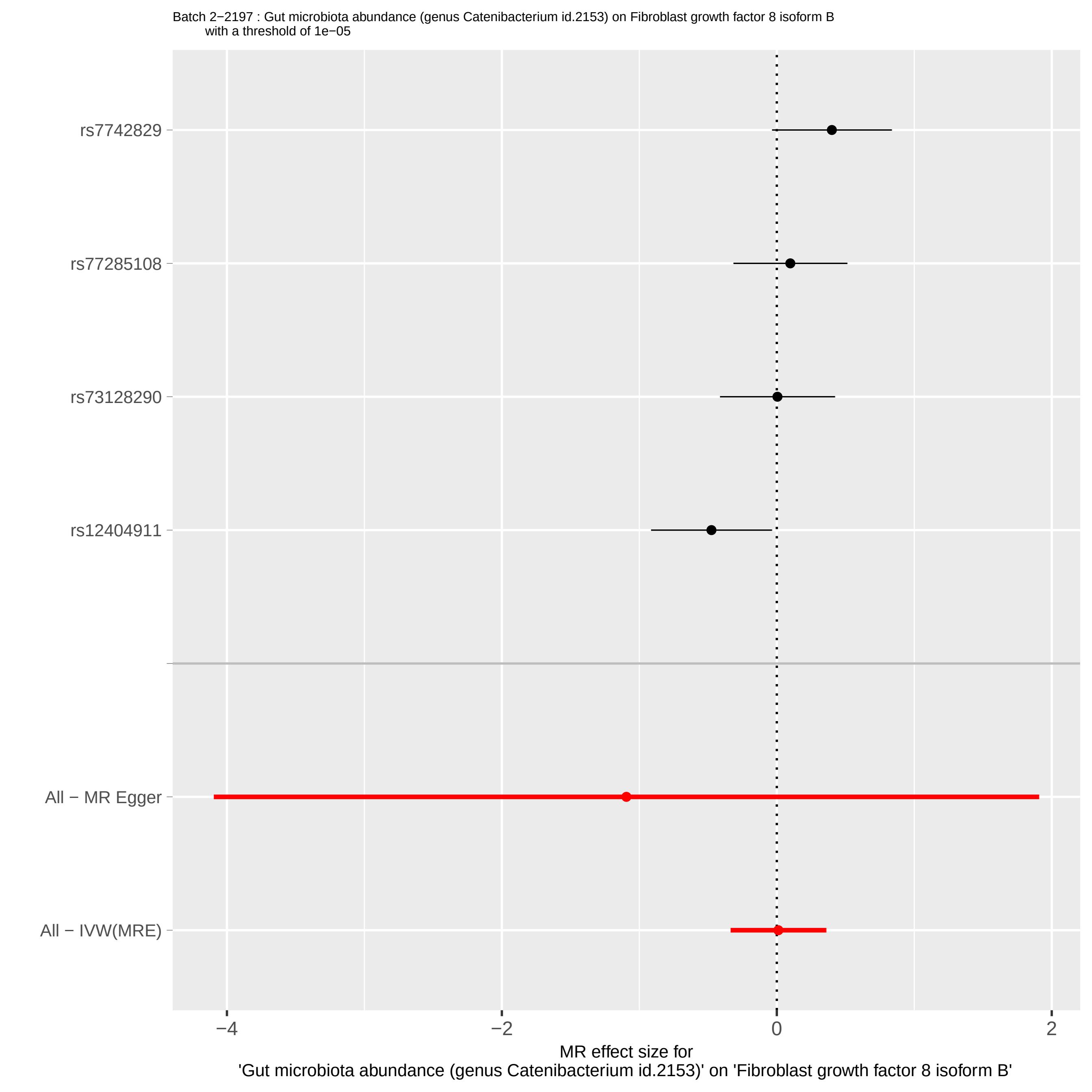

Batch 2−2197 : Gut microbiota abundance (genus Catenibacterium id.2153) on Fibroblast growth factor 8 isoform B
with a threshold of 1e−05
rs7742829
rs77285108
rs73128290
rs12404911
All − MR Egger
All − IVW(MRE)
−4
−2
0
2
MR effect size for
'Gut microbiota abundance (genus Catenibacterium id.2153)' on 'Fibroblast growth factor 8 isoform B'

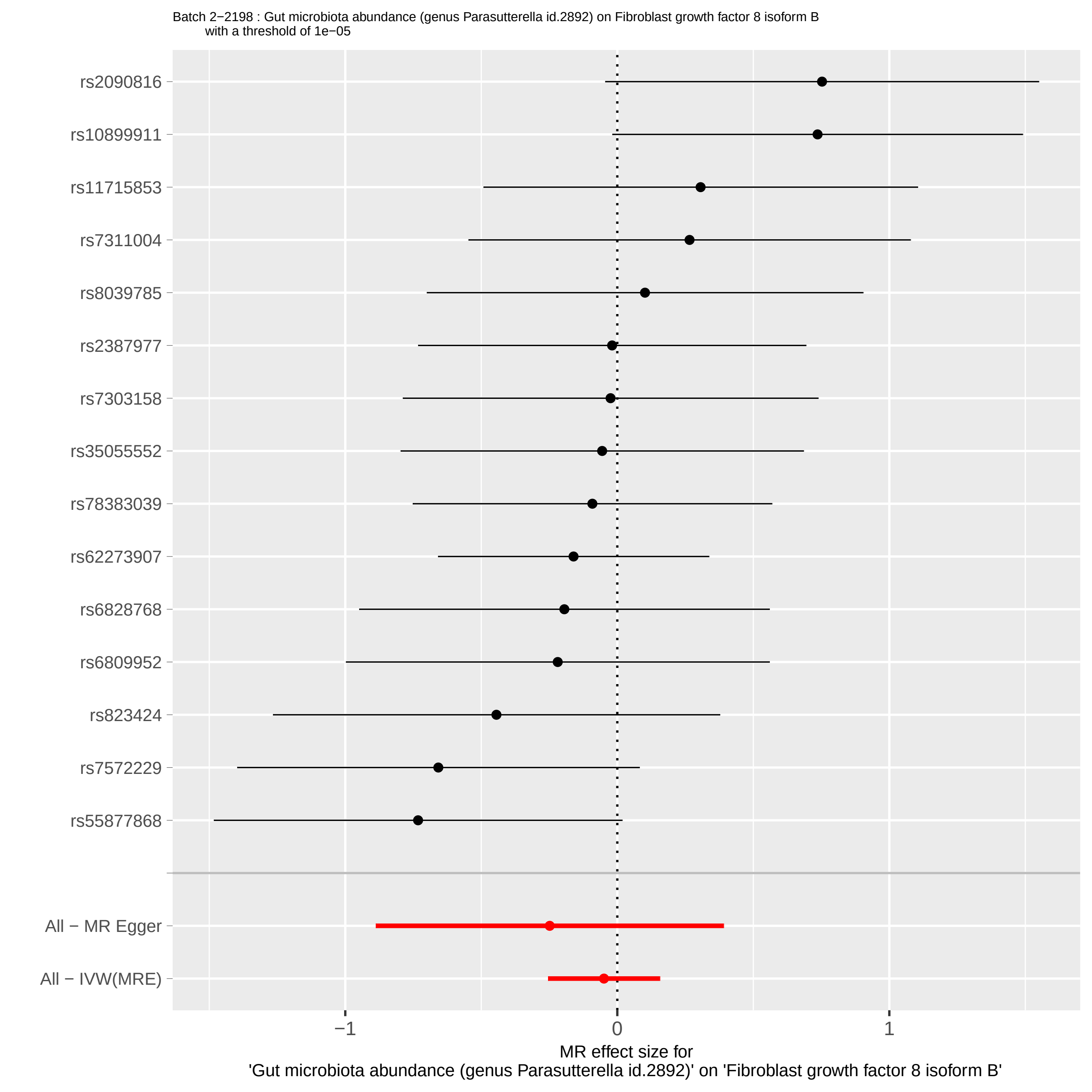

Batch 2−2198 : Gut microbiota abundance (genus Parasutterella id.2892) on Fibroblast growth factor 8 isoform B
with a threshold of 1e−05
rs2090816
rs10899911
rs11715853
rs7311004
rs8039785
rs2387977
rs7303158
rs35055552
rs78383039
rs62273907
rs6828768
rs6809952
rs823424
rs7572229
rs55877868
All − MR Egger
All − IVW(MRE)
−1
0
1
MR effect size for
'Gut microbiota abundance (genus Parasutterella id.2892)' on 'Fibroblast growth factor 8 isoform B'

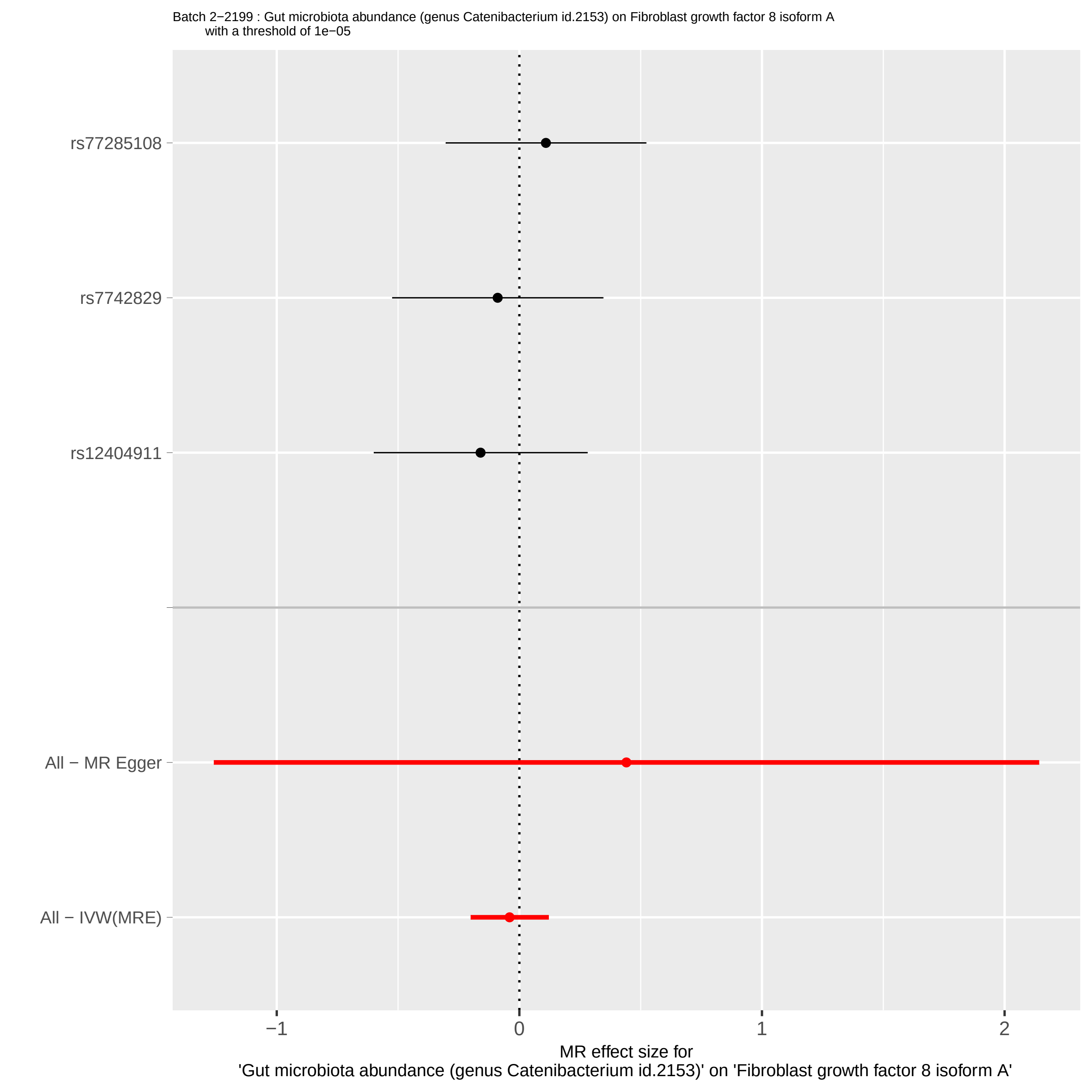

Batch 2−2199 : Gut microbiota abundance (genus Catenibacterium id.2153) on Fibroblast growth factor 8 isoform A
with a threshold of 1e−05
rs77285108
rs7742829
rs12404911
All − MR Egger
All − IVW(MRE)
−1
0
1
2
MR effect size for
'Gut microbiota abundance (genus Catenibacterium id.2153)' on 'Fibroblast growth factor 8 isoform A'

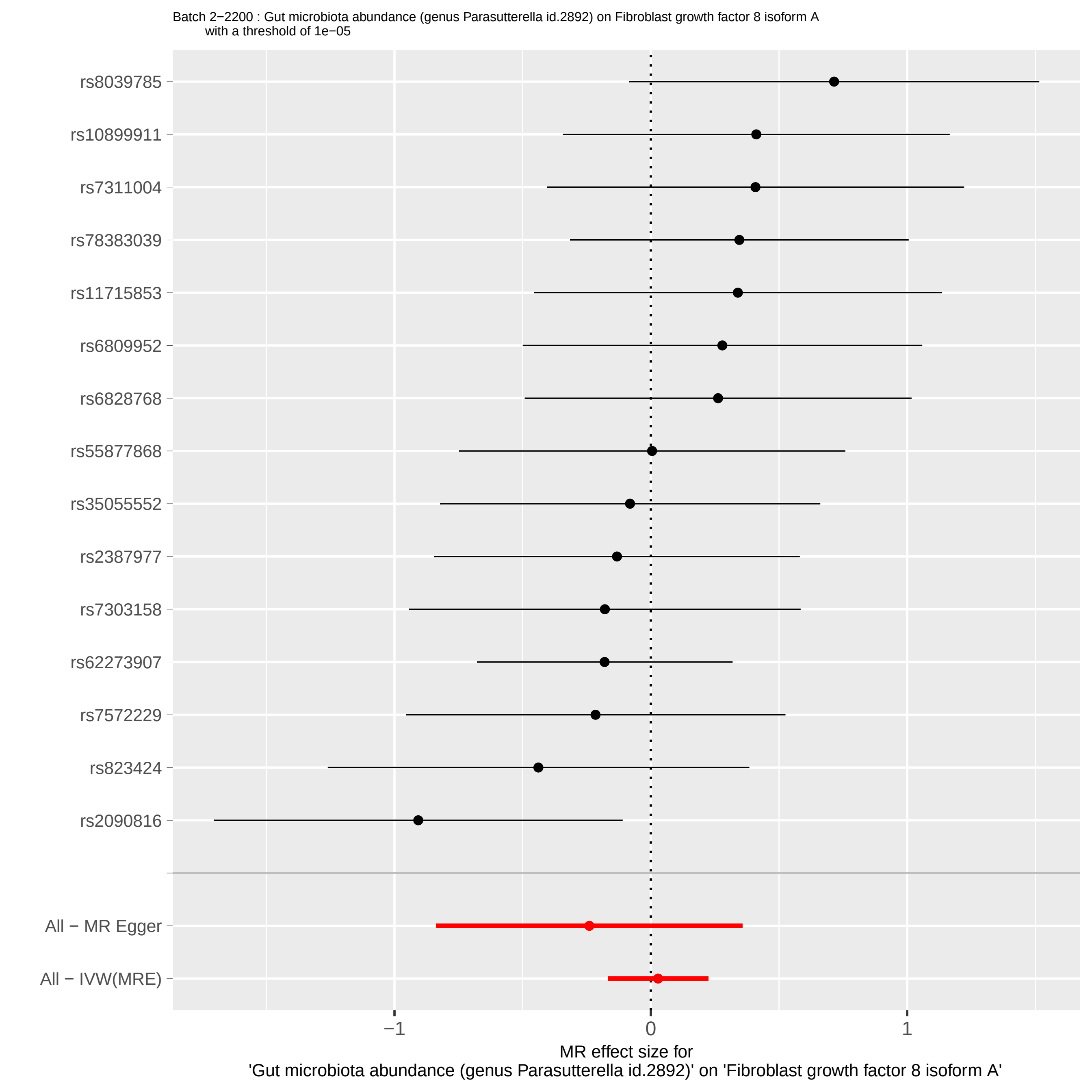
Batch 2−2200 : Gut microbiota abundance (genus Parasutterella id.2892) on Fibroblast growth factor 8 isoform A
with a threshold of 1e−05
rs8039785
rs10899911
rs7311004
rs78383039
rs11715853
rs6809952
rs6828768
rs55877868
rs35055552
rs2387977
rs7303158
rs62273907
rs7572229
rs823424
rs2090816
All − MR Egger
All − IVW(MRE)
−1
0
1
MR effect size for
'Gut microbiota abundance (genus Parasutterella id.2892)' on 'Fibroblast growth factor 8 isoform A'

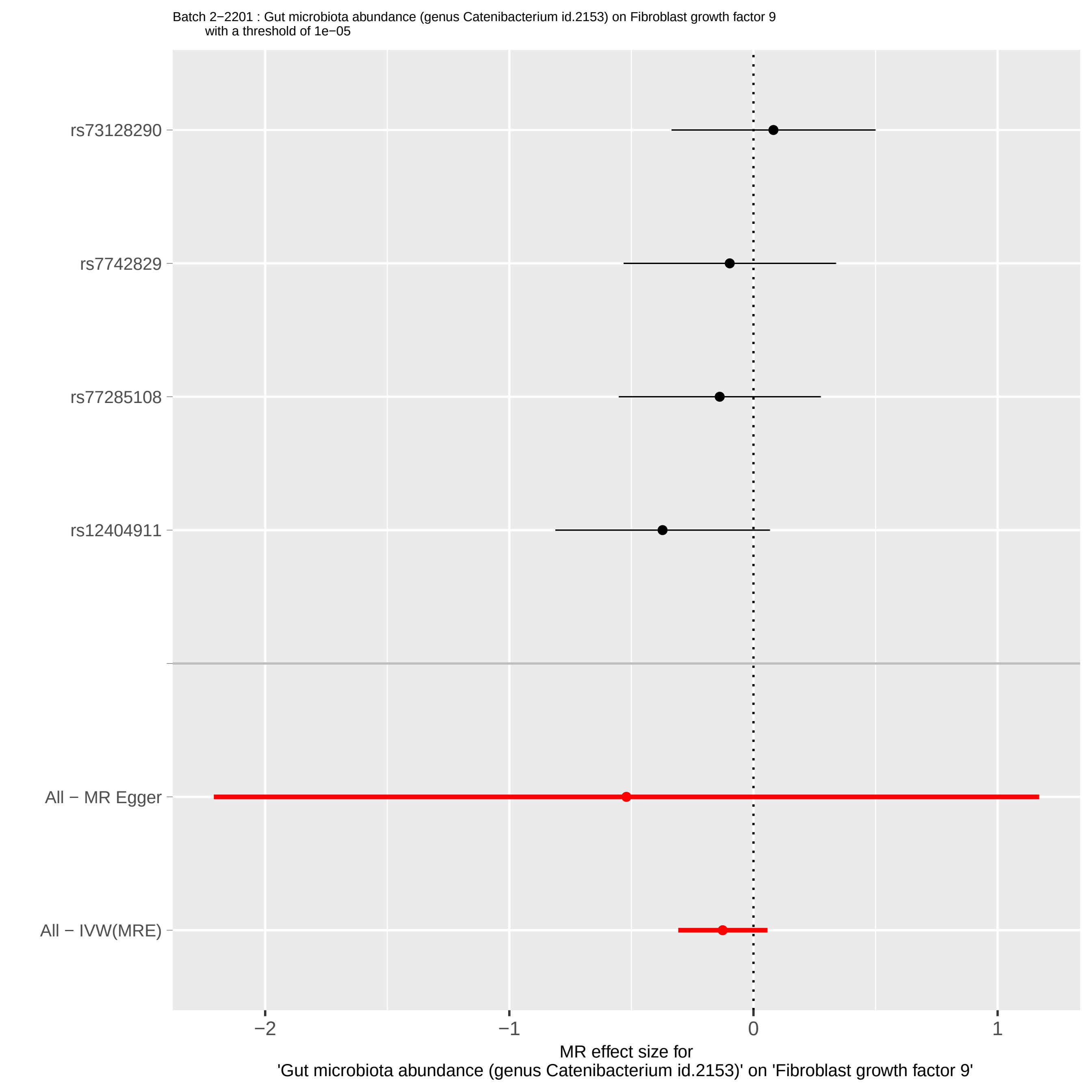

Batch 2−2201 : Gut microbiota abundance (genus Catenibacterium id.2153) on Fibroblast growth factor 9
with a threshold of 1e−05
rs73128290
rs7742829
rs77285108
rs12404911
All − MR Egger
All − IVW(MRE)
−2
−1
0
1
MR effect size for
'Gut microbiota abundance (genus Catenibacterium id.2153)' on 'Fibroblast growth factor 9'

Batch 2−2202 : Gut microbiota abundance (genus Parasutterella id.2892) on Fibroblast growth factor 9
with a threshold of 1e−05

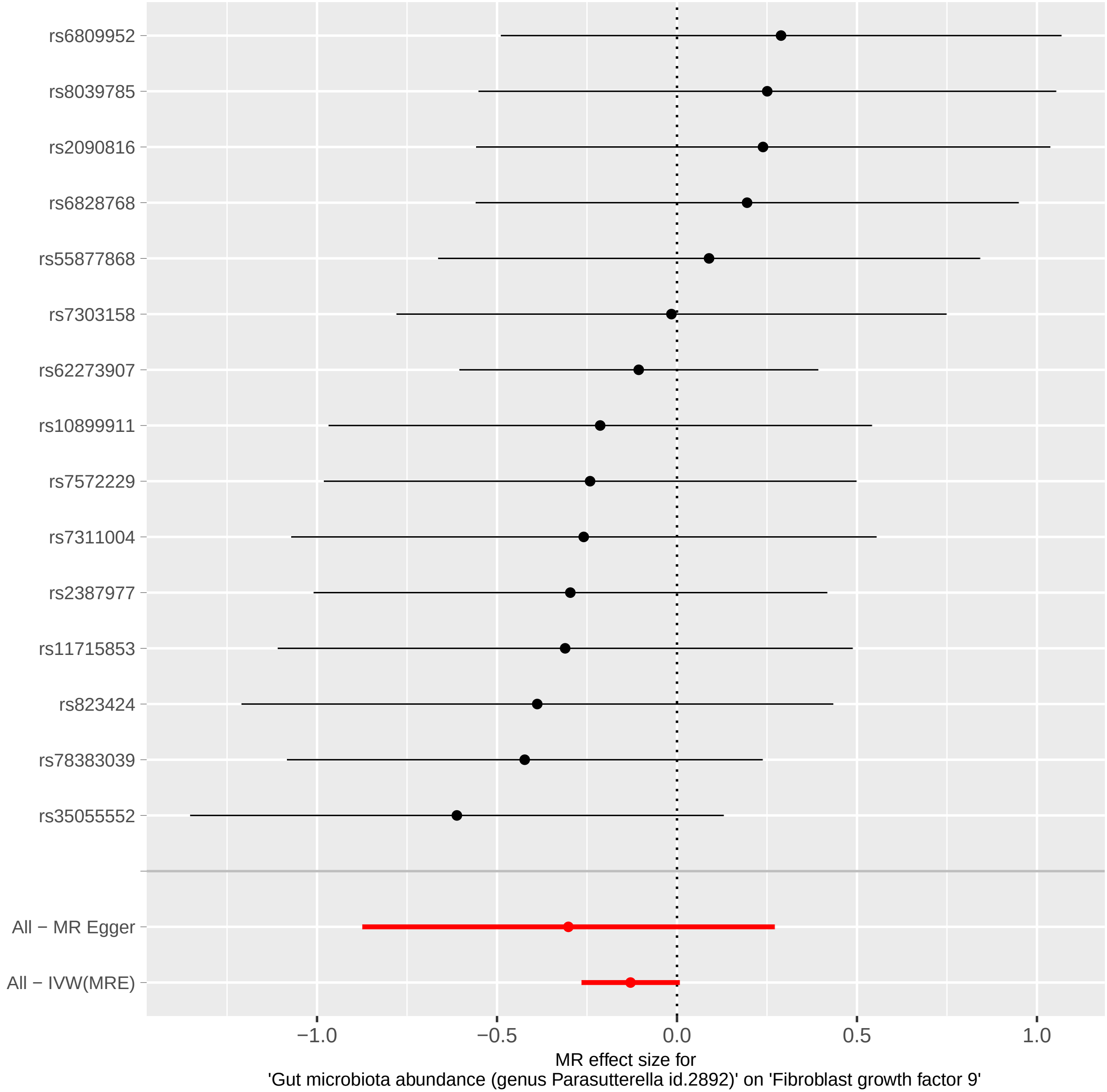

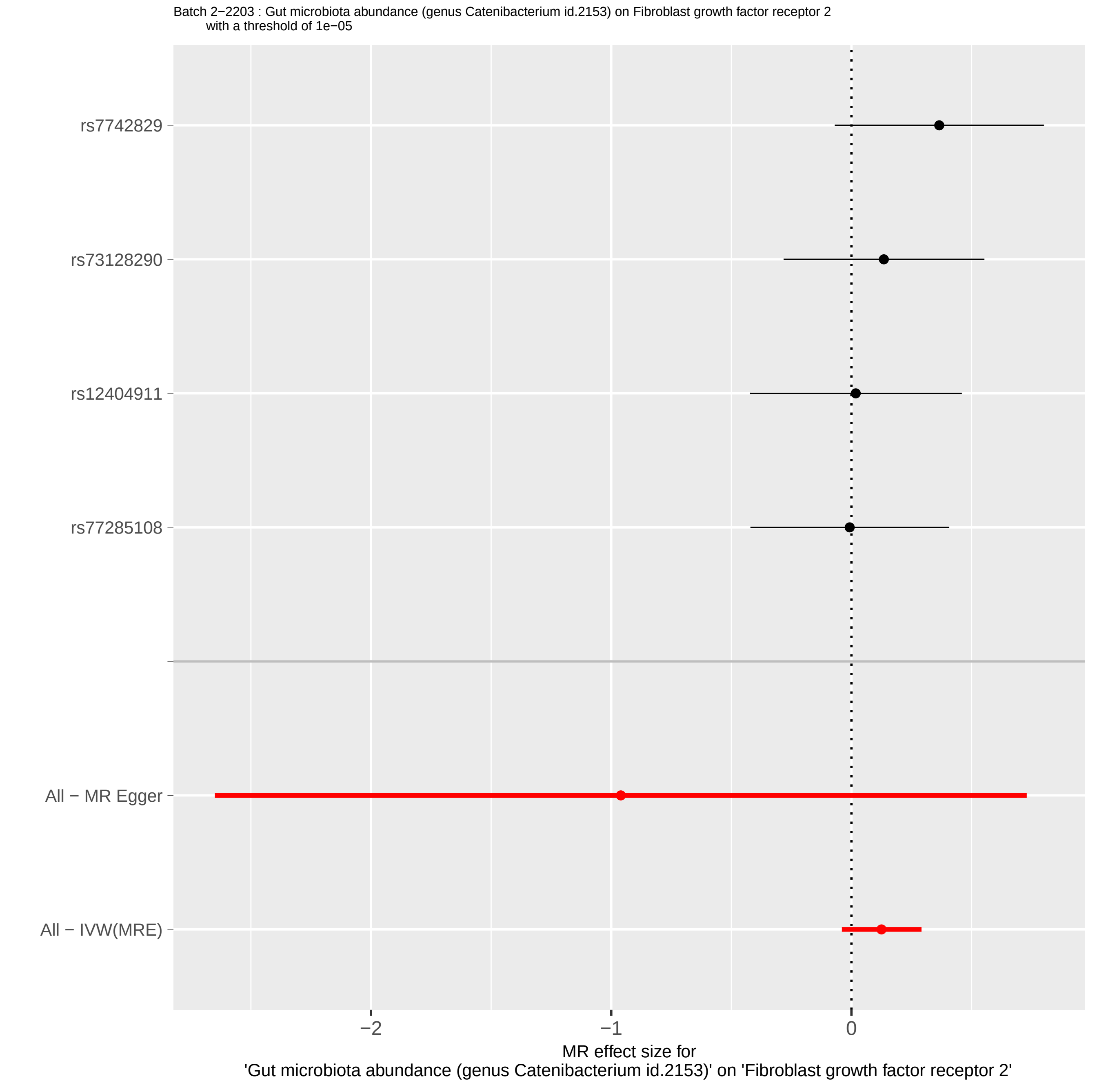

Batch 2−2203 : Gut microbiota abundance (genus Catenibacterium id.2153) on Fibroblast growth factor receptor 2
with a threshold of 1e−05
rs7742829
rs73128290
rs12404911
rs77285108
All − MR Egger
All − IVW(MRE)
−2
−1
0
MR effect size for
'Gut microbiota abundance (genus Catenibacterium id.2153)' on 'Fibroblast growth factor receptor 2'

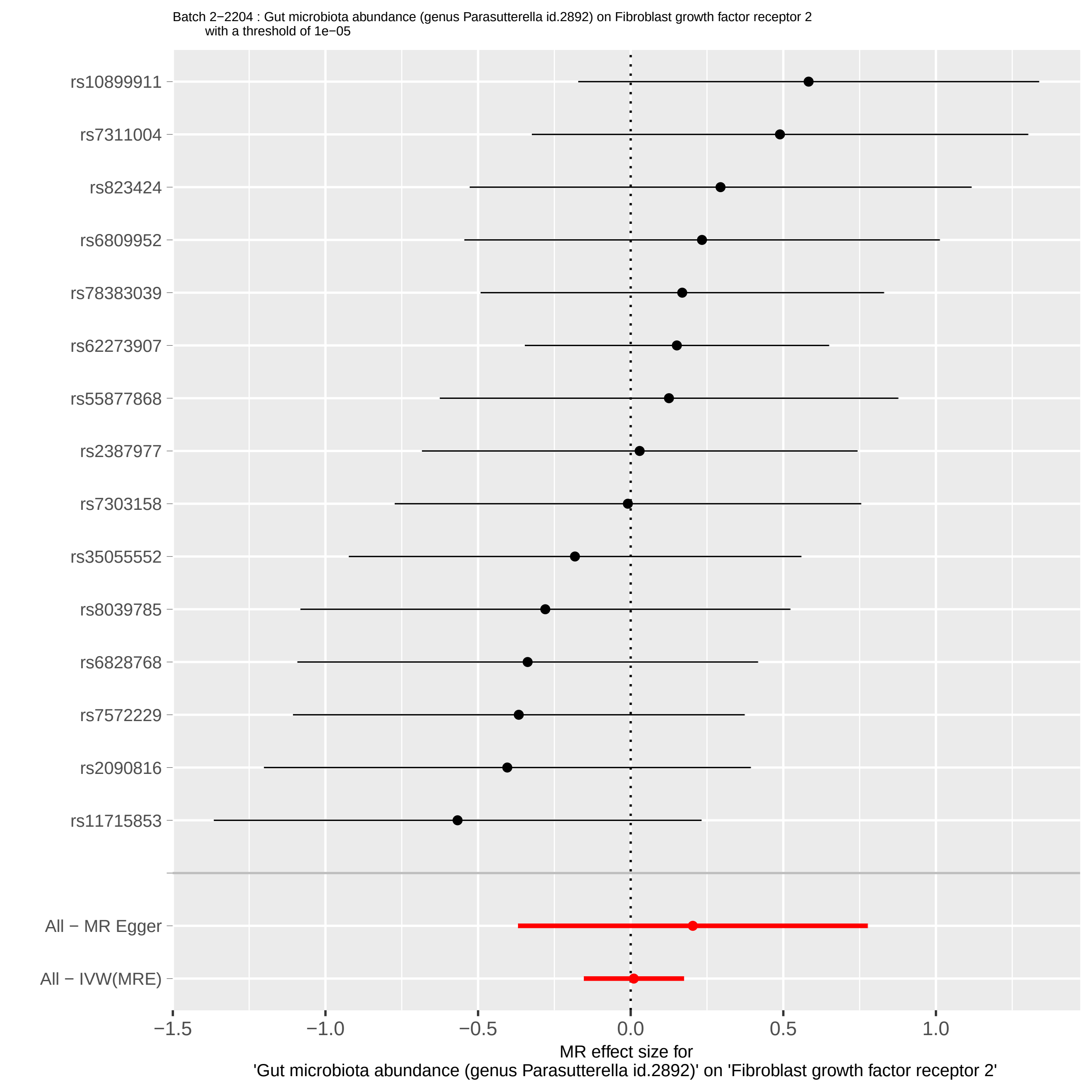

Batch 2−2204 : Gut microbiota abundance (genus Parasutterella id.2892) on Fibroblast growth factor receptor 2
with a threshold of 1e−05
rs10899911
rs7311004
rs823424
rs6809952
rs78383039
rs62273907
rs55877868
rs2387977
rs7303158
rs35055552
rs8039785
rs6828768
rs7572229
rs2090816
rs11715853
All − MR Egger
All − IVW(MRE)
−1.5
−1.0
−0.5
0.0
0.5
1.0
MR effect size for
'Gut microbiota abundance (genus Parasutterella id.2892)' on 'Fibroblast growth factor receptor 2'

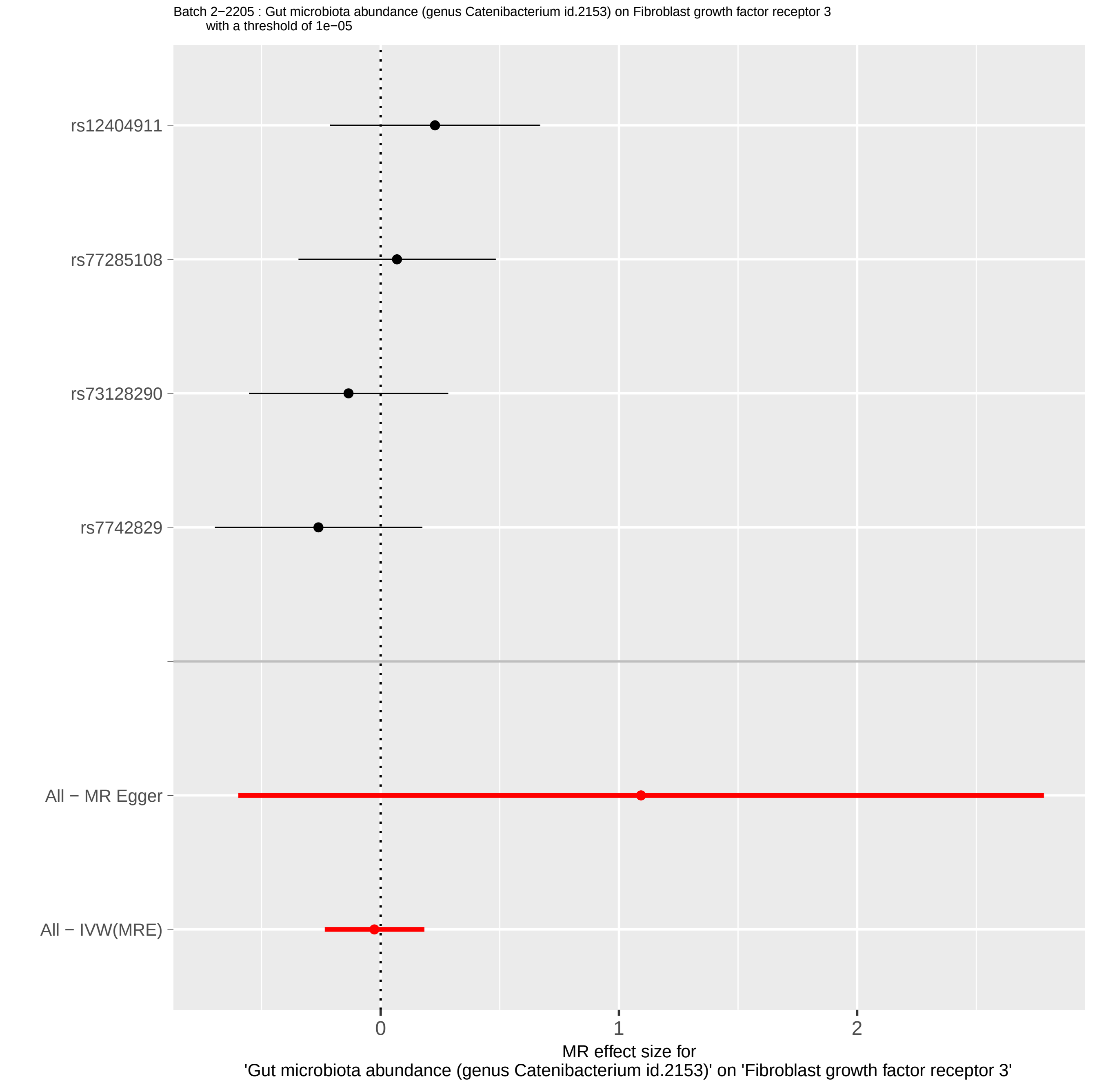
Batch 2−2205 : Gut microbiota abundance (genus Catenibacterium id.2153) on Fibroblast growth factor receptor 3
with a threshold of 1e−05
rs12404911
rs77285108
rs73128290
rs7742829
All − MR Egger
All − IVW(MRE)
0
1
2
MR effect size for
'Gut microbiota abundance (genus Catenibacterium id.2153)' on 'Fibroblast growth factor receptor 3'

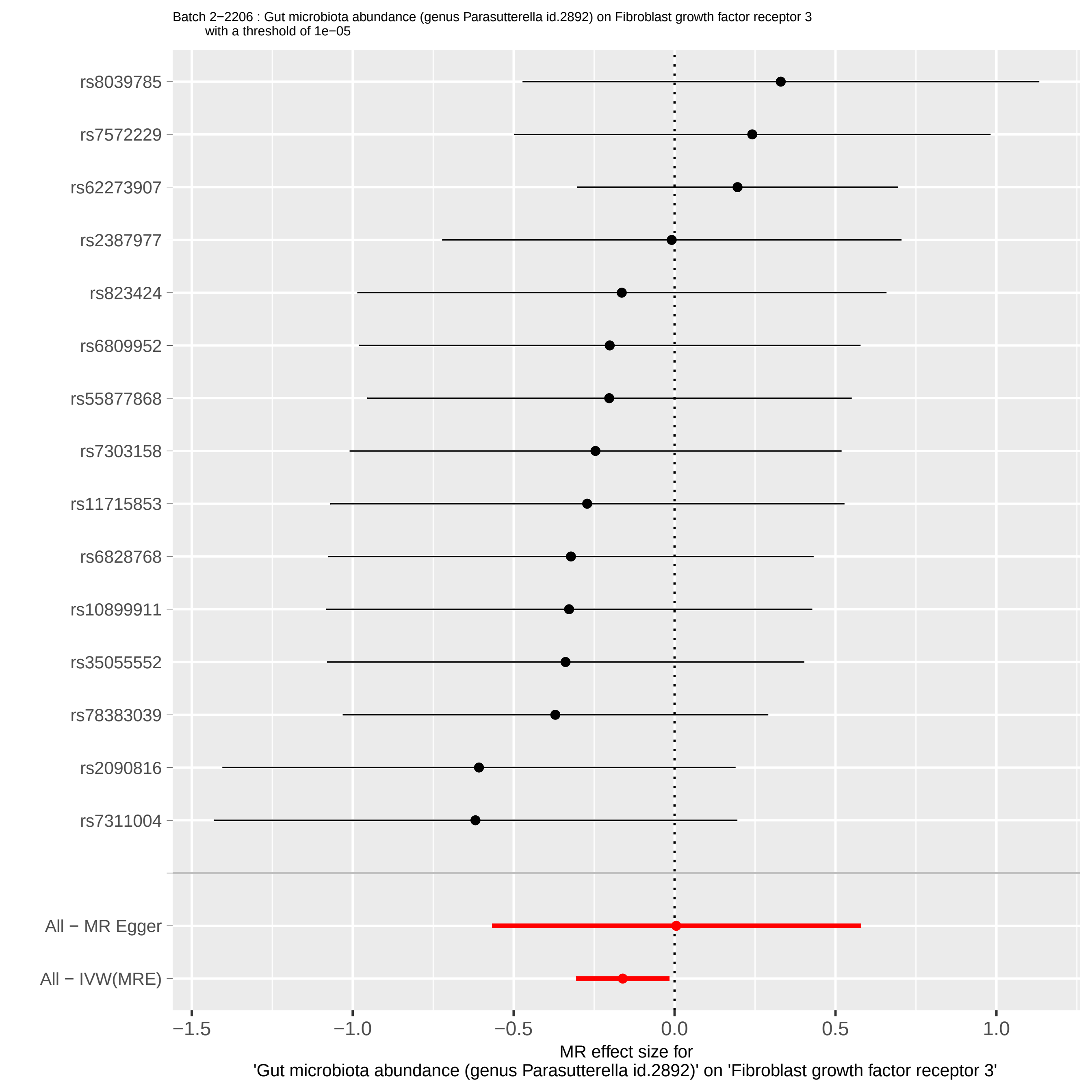
Batch 2−2206 : Gut microbiota abundance (genus Parasutterella id.2892) on Fibroblast growth factor receptor 3
with a threshold of 1e−05
rs8039785
rs7572229
rs62273907
rs2387977
rs823424
rs6809952
rs55877868
rs7303158
rs11715853
rs6828768
rs10899911
rs35055552
rs78383039
rs2090816
rs7311004
All − MR Egger
All − IVW(MRE)
−1.5
−1.0
−0.5
0.0
0.5
1.0
MR effect size for
'Gut microbiota abundance (genus Parasutterella id.2892)' on 'Fibroblast growth factor receptor 3'

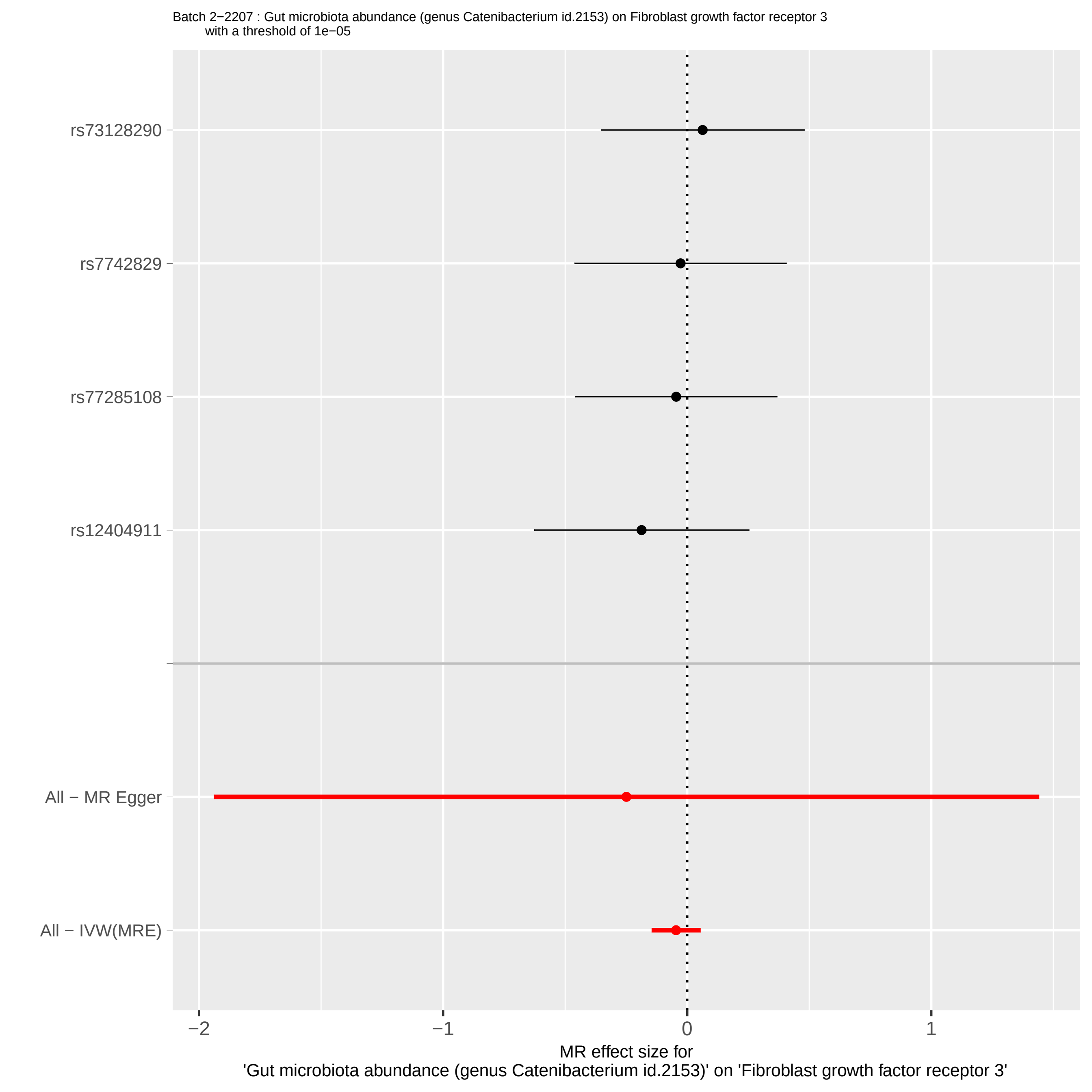

Batch 2−2207 : Gut microbiota abundance (genus Catenibacterium id.2153) on Fibroblast growth factor receptor 3
with a threshold of 1e−05
rs73128290
rs7742829
rs77285108
rs12404911
All − MR Egger
All − IVW(MRE)
−2
−1
0
1
MR effect size for
'Gut microbiota abundance (genus Catenibacterium id.2153)' on 'Fibroblast growth factor receptor 3'

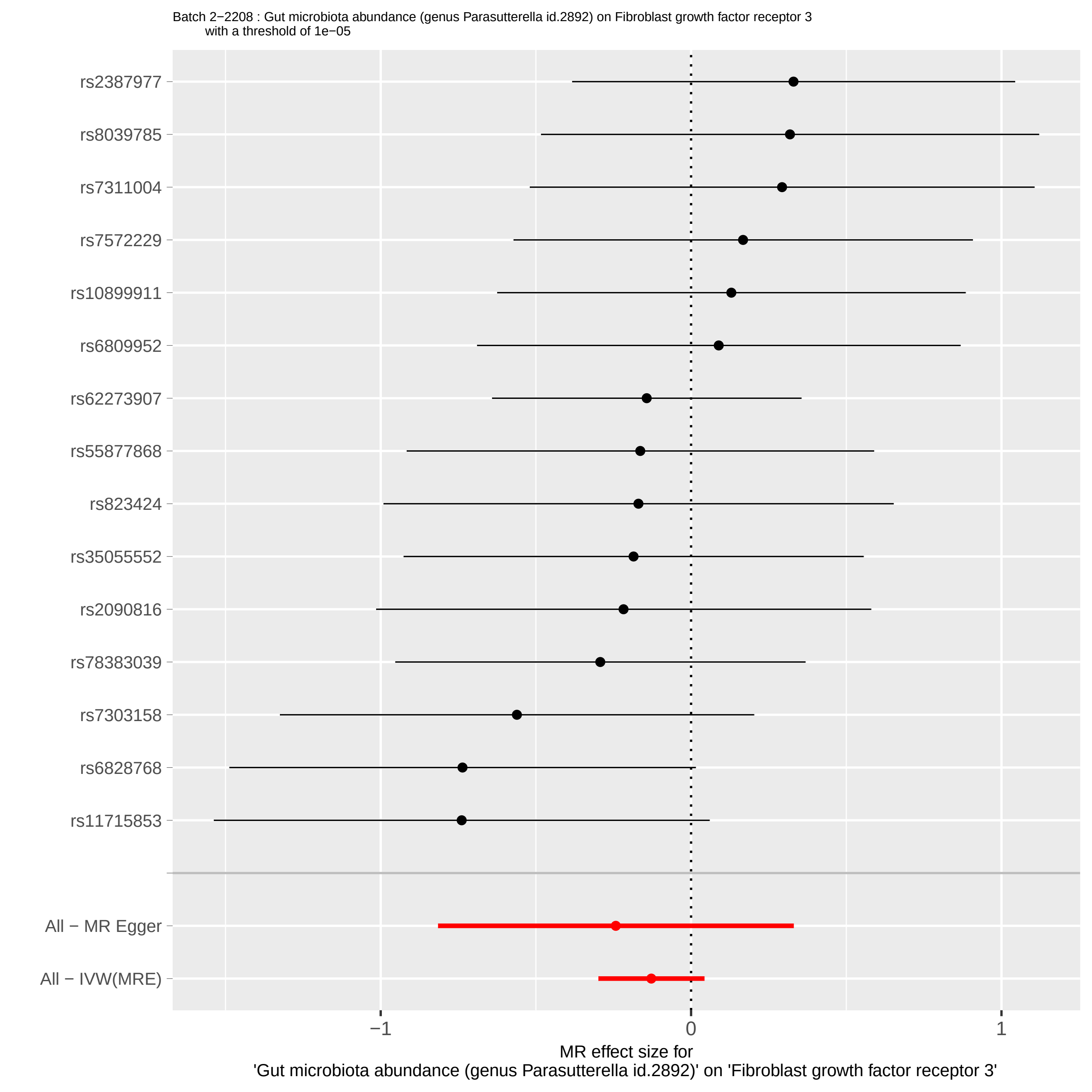

Batch 2−2208 : Gut microbiota abundance (genus Parasutterella id.2892) on Fibroblast growth factor receptor 3
with a threshold of 1e−05
rs2387977
rs8039785
rs7311004
rs7572229
rs10899911
rs6809952
rs62273907
rs55877868
rs823424
rs35055552
rs2090816
rs78383039
rs7303158
rs6828768
rs11715853
All − MR Egger
All − IVW(MRE)
−1
0
1
MR effect size for
'Gut microbiota abundance (genus Parasutterella id.2892)' on 'Fibroblast growth factor receptor 3'

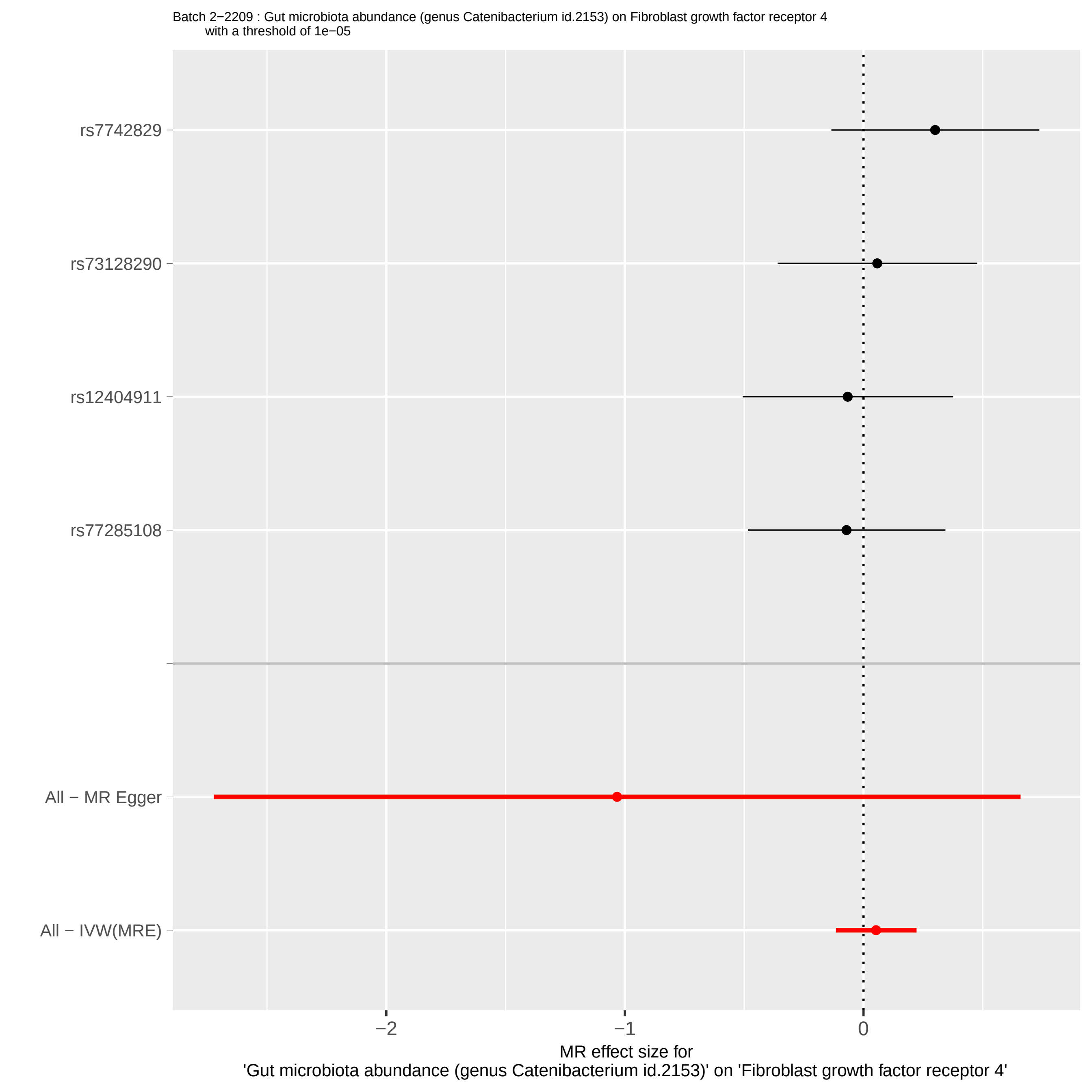

Batch 2−2209 : Gut microbiota abundance (genus Catenibacterium id.2153) on Fibroblast growth factor receptor 4
with a threshold of 1e−05
rs7742829
rs73128290
rs12404911
rs77285108
All − MR Egger
All − IVW(MRE)
−2
−1
0
MR effect size for
'Gut microbiota abundance (genus Catenibacterium id.2153)' on 'Fibroblast growth factor receptor 4'

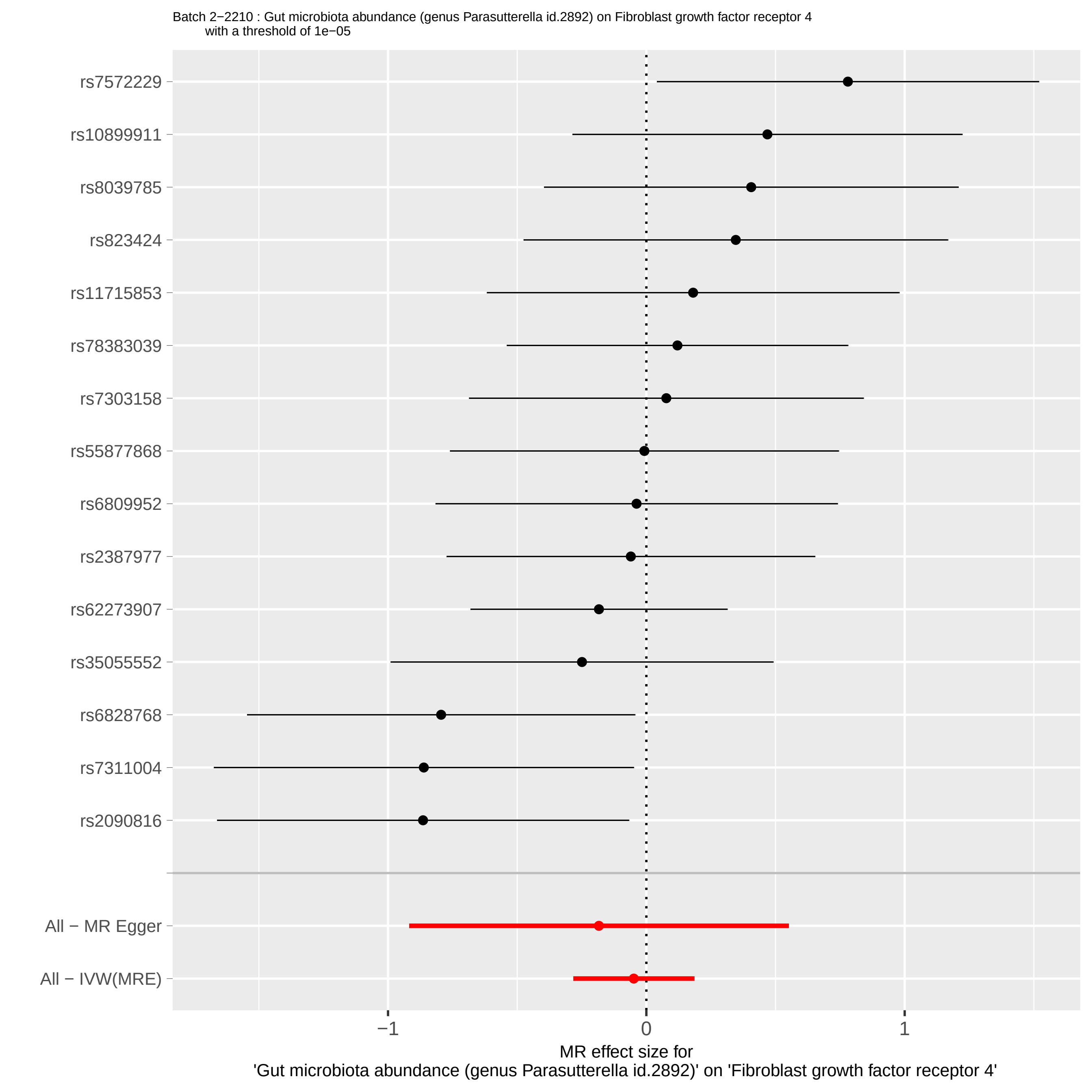

Batch 2−2210 : Gut microbiota abundance (genus Parasutterella id.2892) on Fibroblast growth factor receptor 4
with a threshold of 1e−05
rs7572229
rs10899911
rs8039785
rs823424
rs11715853
rs78383039
rs7303158
rs55877868
rs6809952
rs2387977
rs62273907
rs35055552
rs6828768
rs7311004
rs2090816
All − MR Egger
All − IVW(MRE)
−1
0
1
MR effect size for
'Gut microbiota abundance (genus Parasutterella id.2892)' on 'Fibroblast growth factor receptor 4'

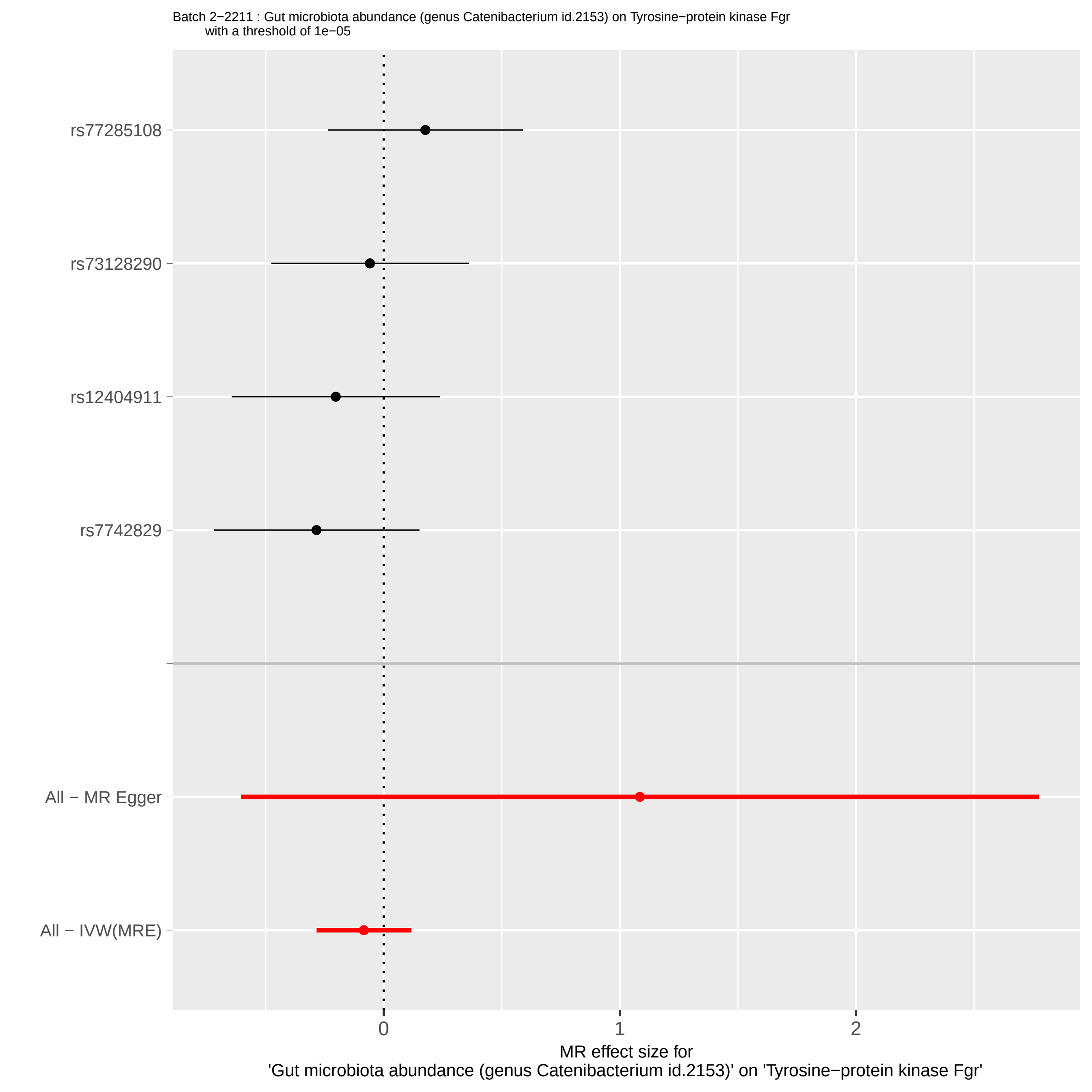

Batch 2−2211 : Gut microbiota abundance (genus Catenibacterium id.2153) on Tyrosine−protein kinase Fgr
with a threshold of 1e−05
rs77285108
rs73128290
rs12404911
rs7742829
All − MR Egger
All − IVW(MRE)
0
1
2
MR effect size for
'Gut microbiota abundance (genus Catenibacterium id.2153)' on 'Tyrosine−protein kinase Fgr'

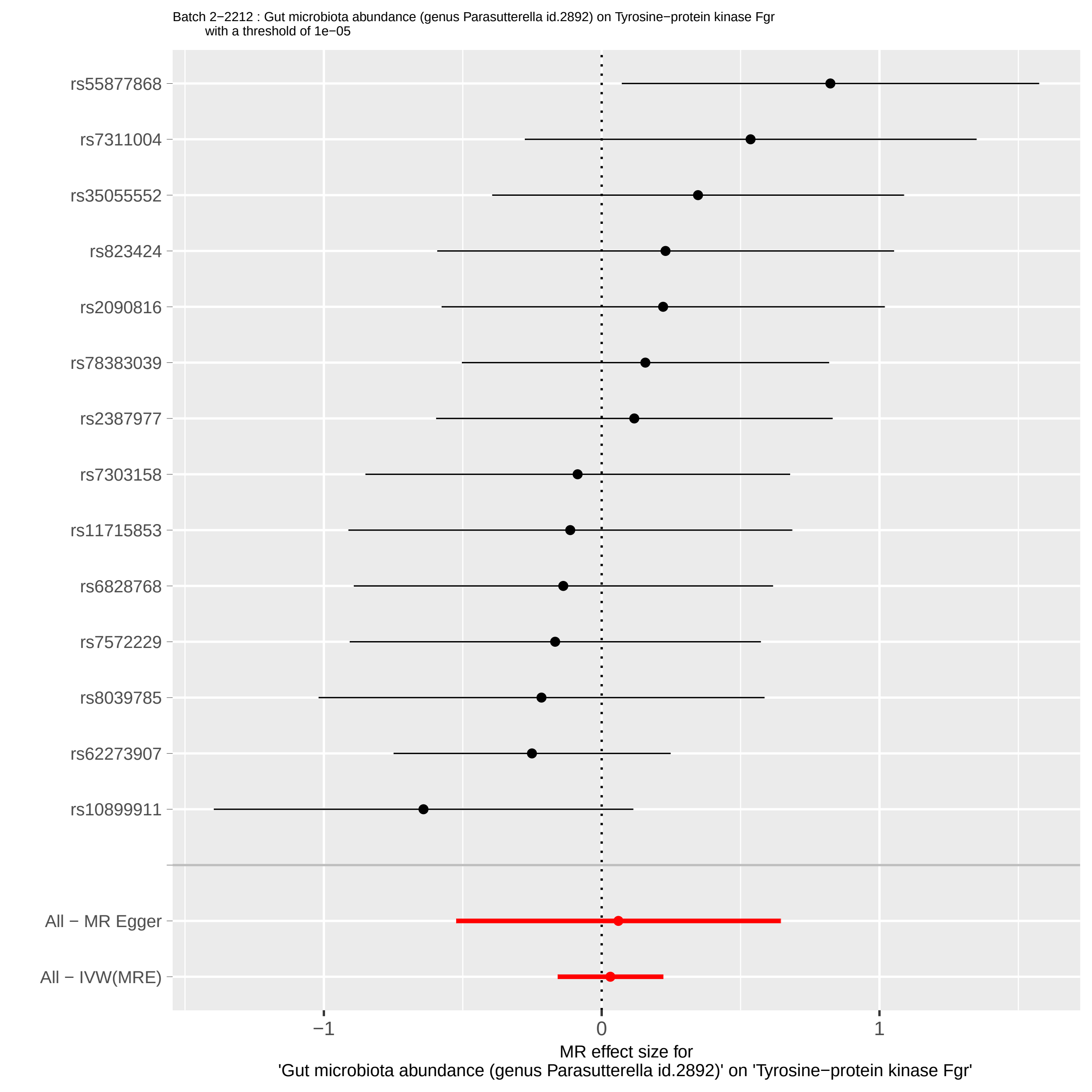

Batch 2−2212 : Gut microbiota abundance (genus Parasutterella id.2892) on Tyrosine−protein kinase Fgr
with a threshold of 1e−05
rs55877868
rs7311004
rs35055552
rs823424
rs2090816
rs78383039
rs2387977
rs7303158
rs11715853
rs6828768
rs7572229
rs8039785
rs62273907
rs10899911
All − MR Egger
All − IVW(MRE)
−1
0
1
MR effect size for
'Gut microbiota abundance (genus Parasutterella id.2892)' on 'Tyrosine−protein kinase Fgr'

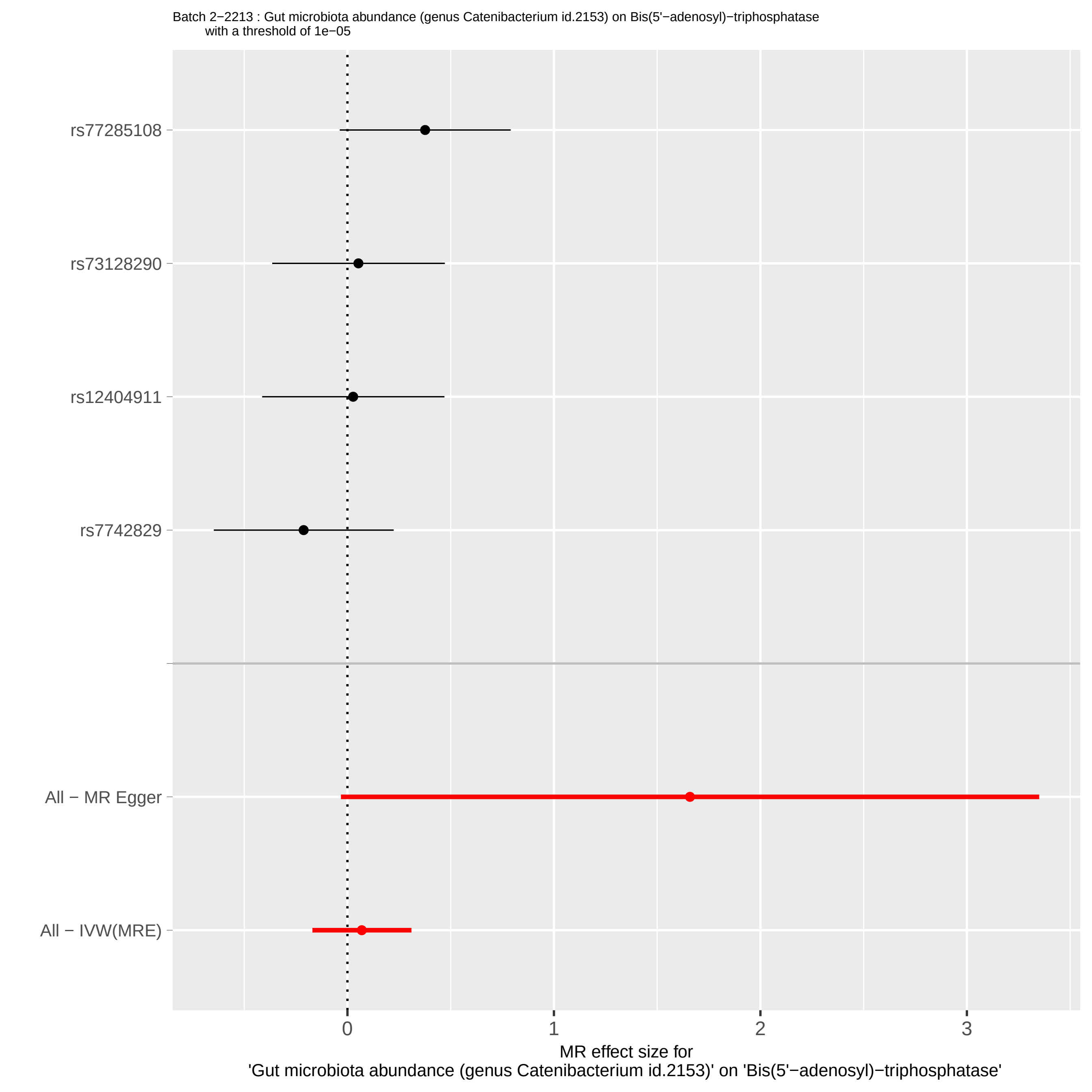
Batch 2−2213 : Gut microbiota abundance (genus Catenibacterium id.2153) on Bis(5'−adenosyl)−triphosphatase
with a threshold of 1e−05
rs77285108
rs73128290
rs12404911
rs7742829
All − MR Egger
All − IVW(MRE)
0
1
2
3
MR effect size for
'Gut microbiota abundance (genus Catenibacterium id.2153)' on 'Bis(5'−adenosyl)−triphosphatase'

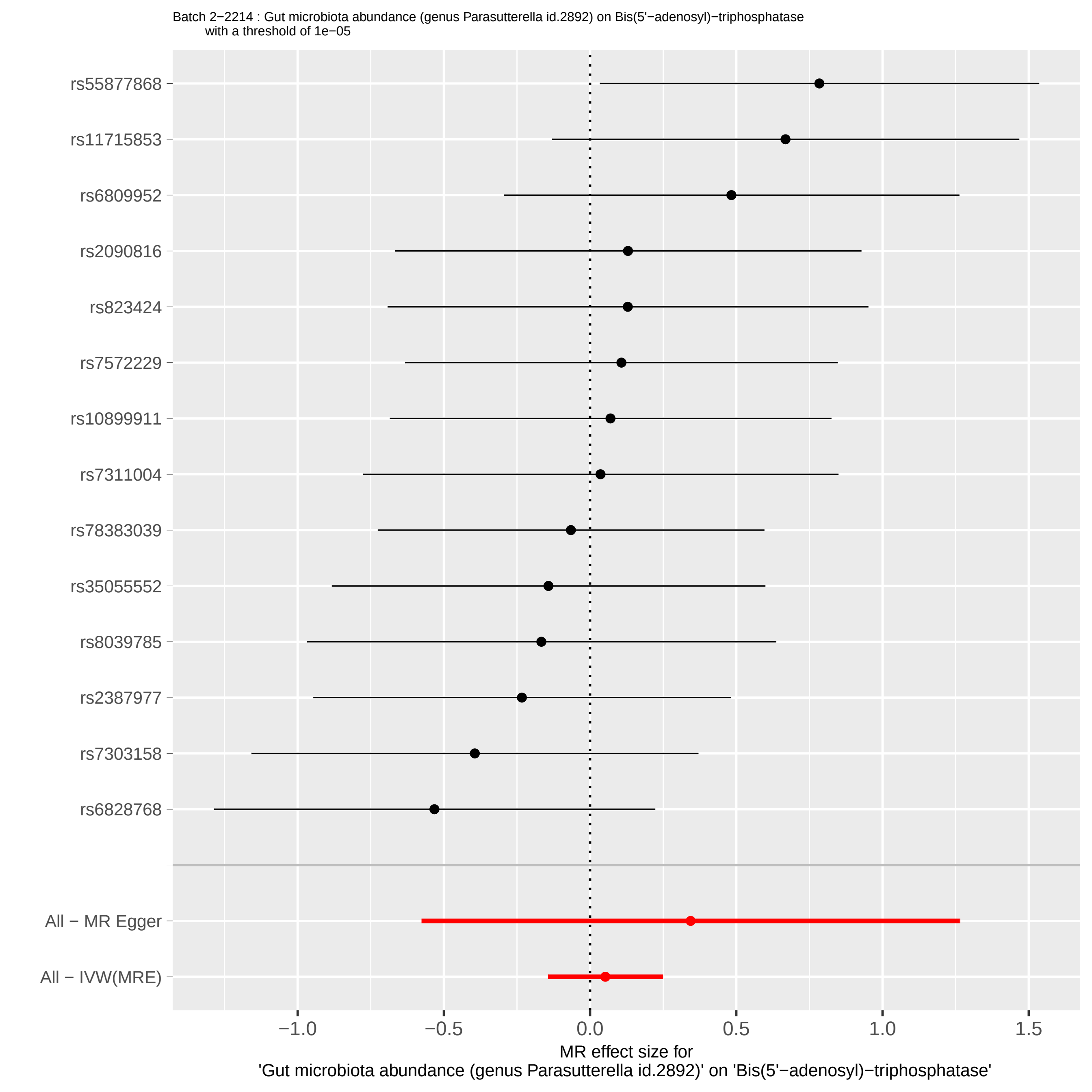
Batch 2−2214 : Gut microbiota abundance (genus Parasutterella id.2892) on Bis(5'−adenosyl)−triphosphatase
with a threshold of 1e−05
rs55877868
rs11715853
rs6809952
rs2090816
rs823424
rs7572229
rs10899911
rs7311004
rs78383039
rs35055552
rs8039785
rs2387977
rs7303158
rs6828768
All − MR Egger
All − IVW(MRE)
−1.0
−0.5
0.0
0.5
1.0
1.5
MR effect size for
'Gut microbiota abundance (genus Parasutterella id.2892)' on 'Bis(5'−adenosyl)−triphosphatase'

Batch 2−2215 : Gut microbiota abundance (genus Catenibacterium id.2153) on Fibrinogen C domain−containing protein 1
with a threshold of 1e−05

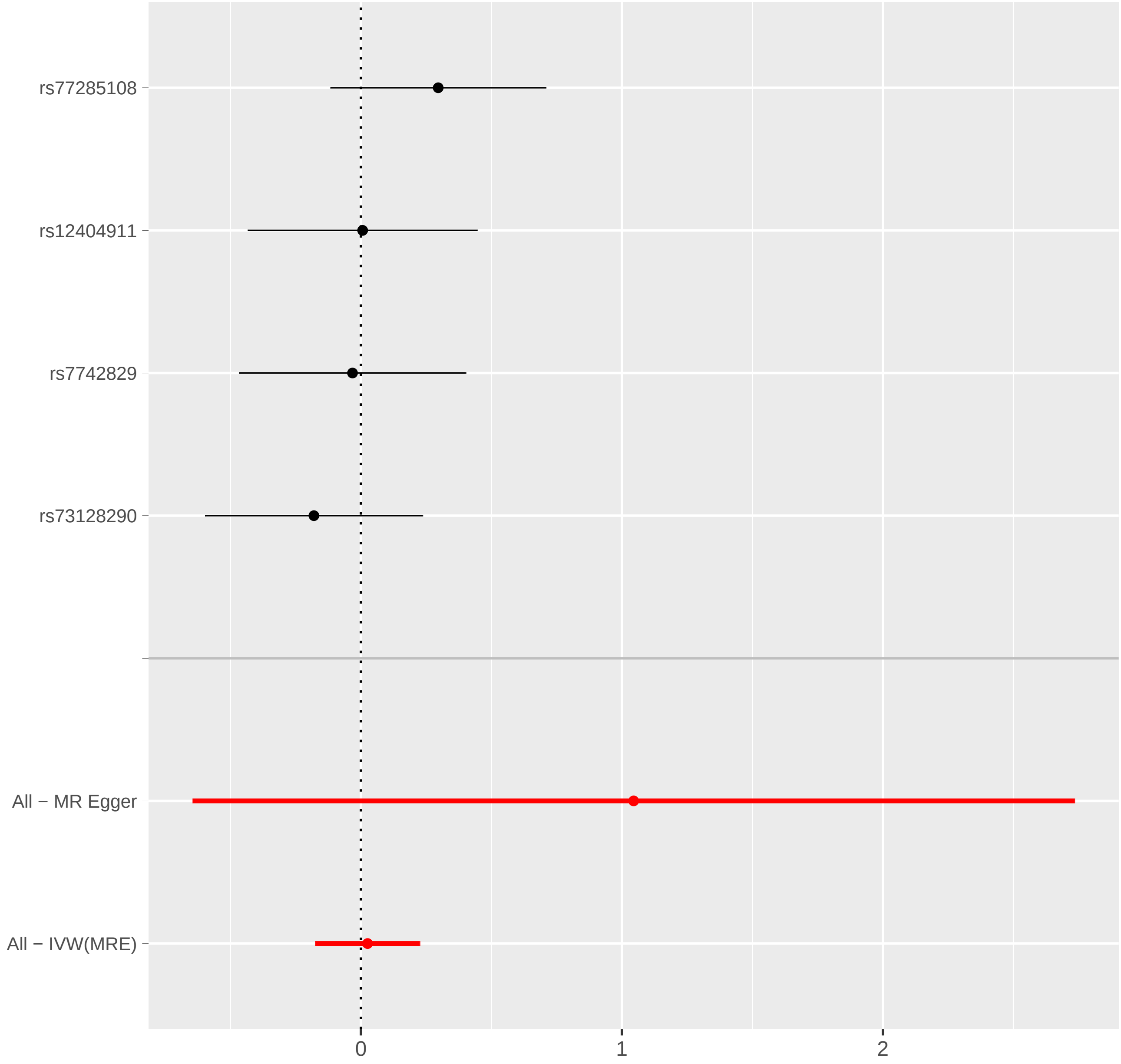

MR effect size for
'Gut microbiota abundance (genus Catenibacterium id.2153)' on 'Fibrinogen C domain−containing protein 1'

Batch 2−2216 : Gut microbiota abundance (genus Parasutterella id.2892) on Fibrinogen C domain−containing protein 1
with a threshold of 1e−05

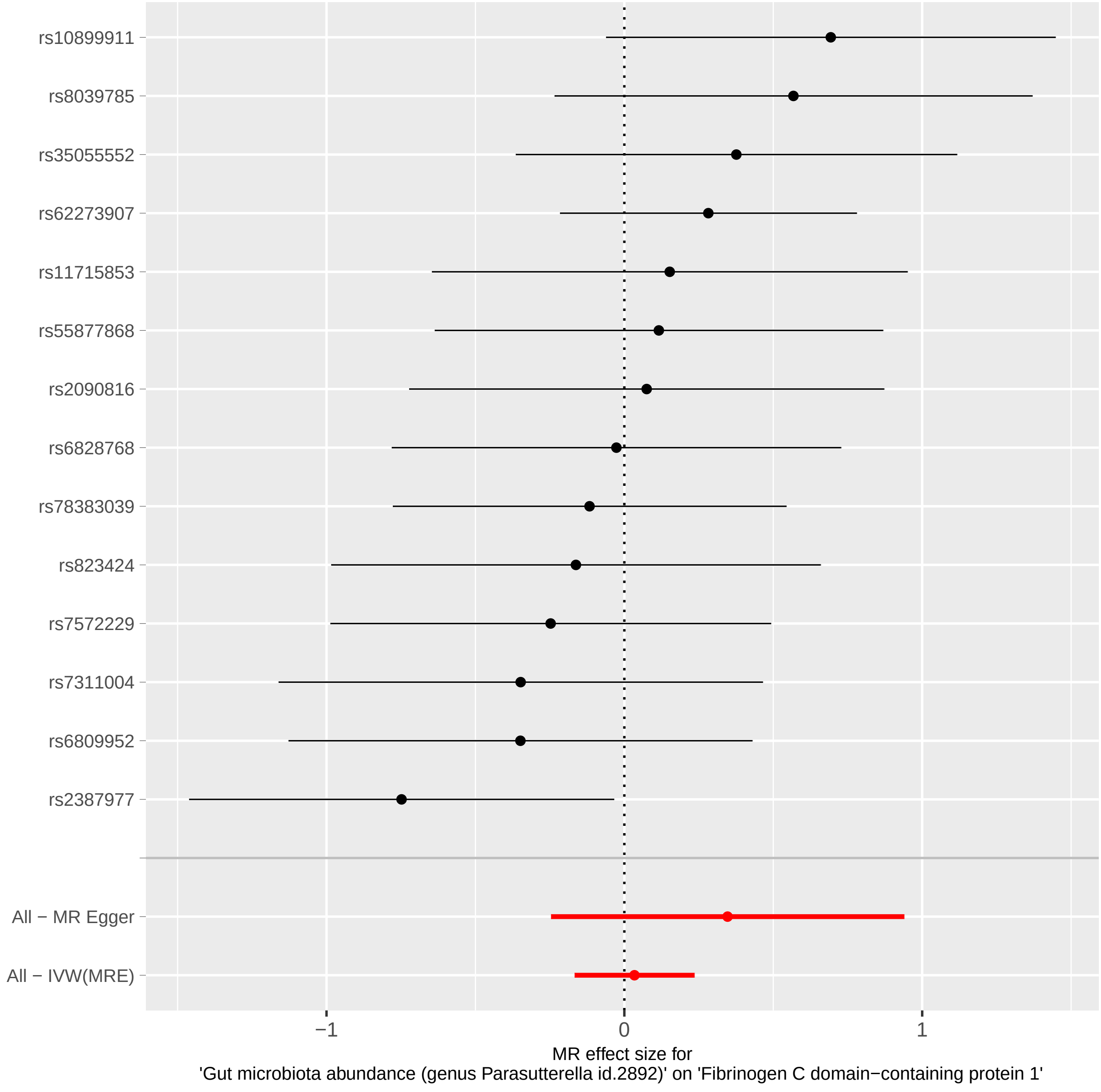

Batch 2−2217 : Gut microbiota abundance (genus Catenibacterium id.2153) on Polyphosphoinositide phosphatase
with a threshold of 1e−05

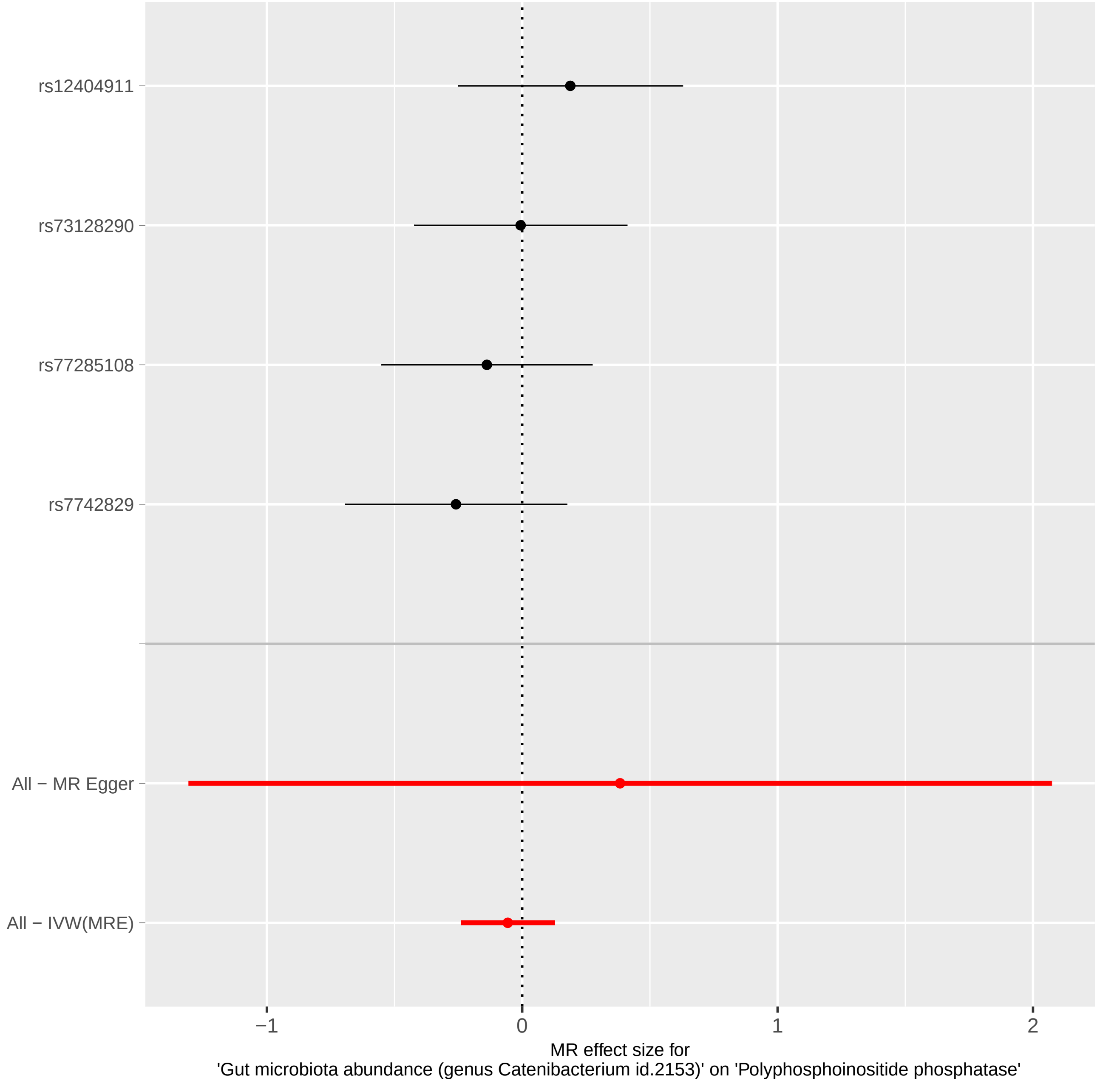

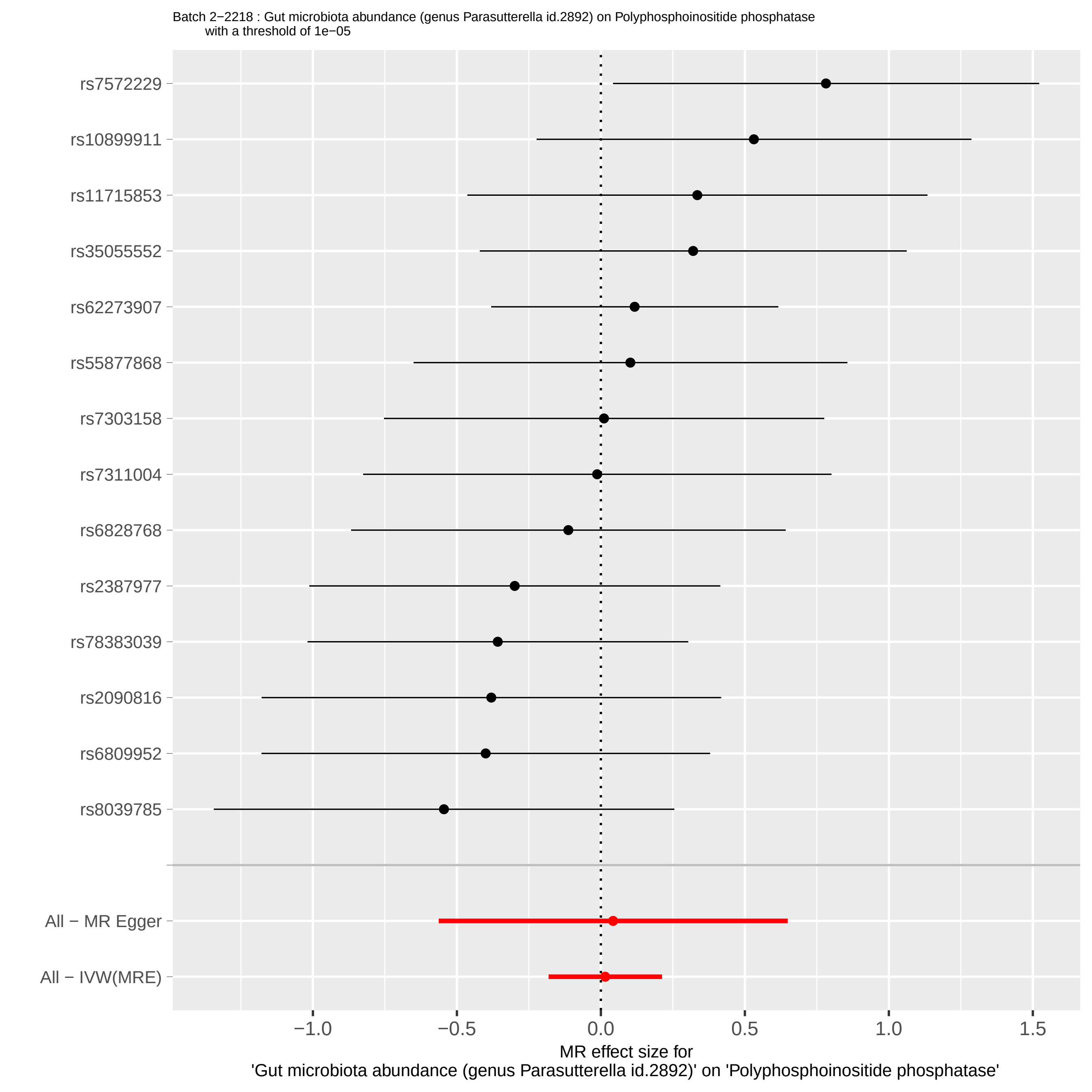

Batch 2−2218 : Gut microbiota abundance (genus Parasutterella id.2892) on Polyphosphoinositide phosphatase
with a threshold of 1e−05
rs7572229
rs10899911
rs11715853
rs35055552
rs62273907
rs55877868
rs7303158
rs7311004
rs6828768
rs2387977
rs78383039
rs2090816
rs6809952
rs8039785
All − MR Egger
All − IVW(MRE)
−1.0
−0.5
0.0
0.5
1.0
1.5
MR effect size for
'Gut microbiota abundance (genus Parasutterella id.2892)' on 'Polyphosphoinositide phosphatase'

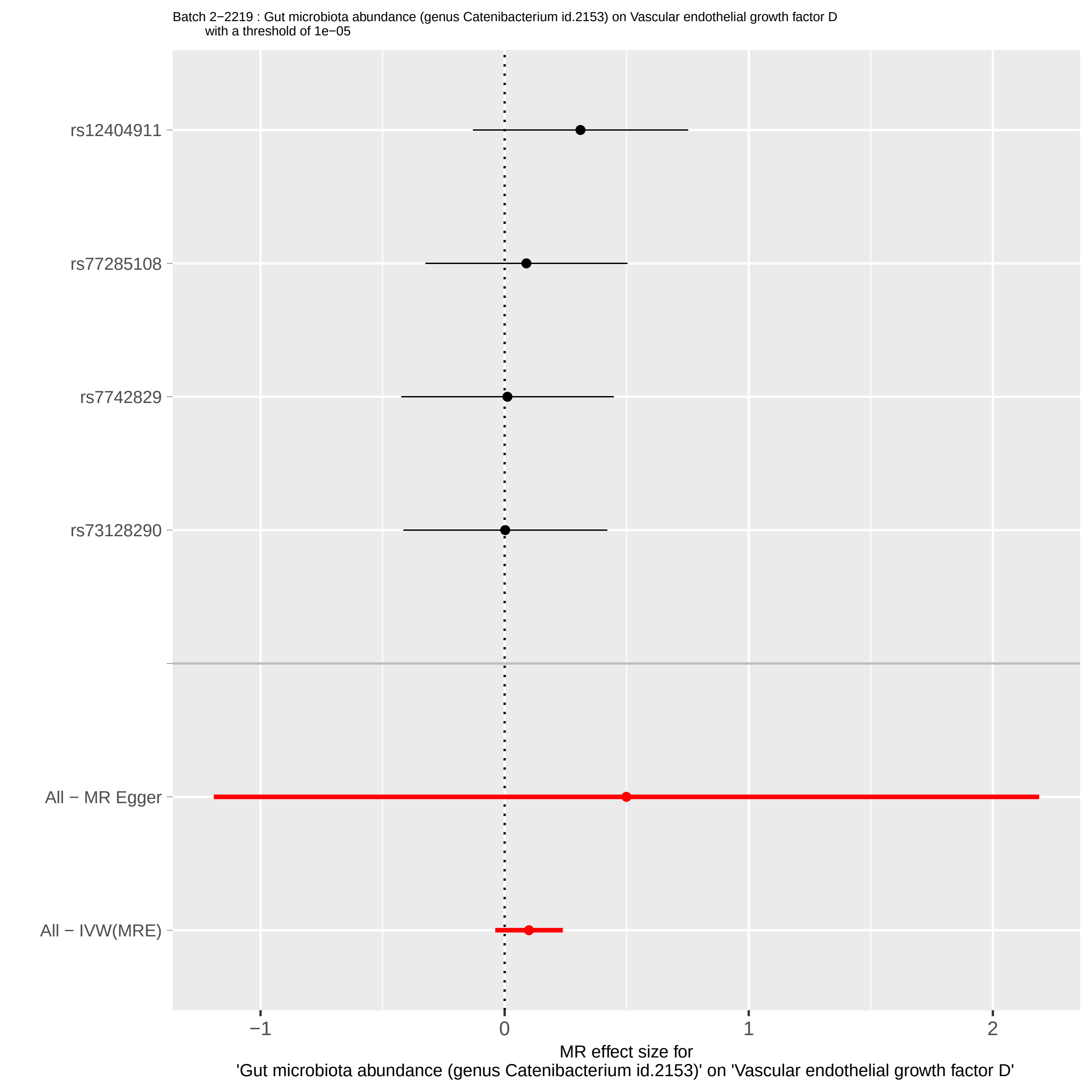

Batch 2−2219 : Gut microbiota abundance (genus Catenibacterium id.2153) on Vascular endothelial growth factor D
with a threshold of 1e−05
rs12404911
rs77285108
rs7742829
rs73128290
All − MR Egger
All − IVW(MRE)
−1
0
1
2
MR effect size for
'Gut microbiota abundance (genus Catenibacterium id.2153)' on 'Vascular endothelial growth factor D'

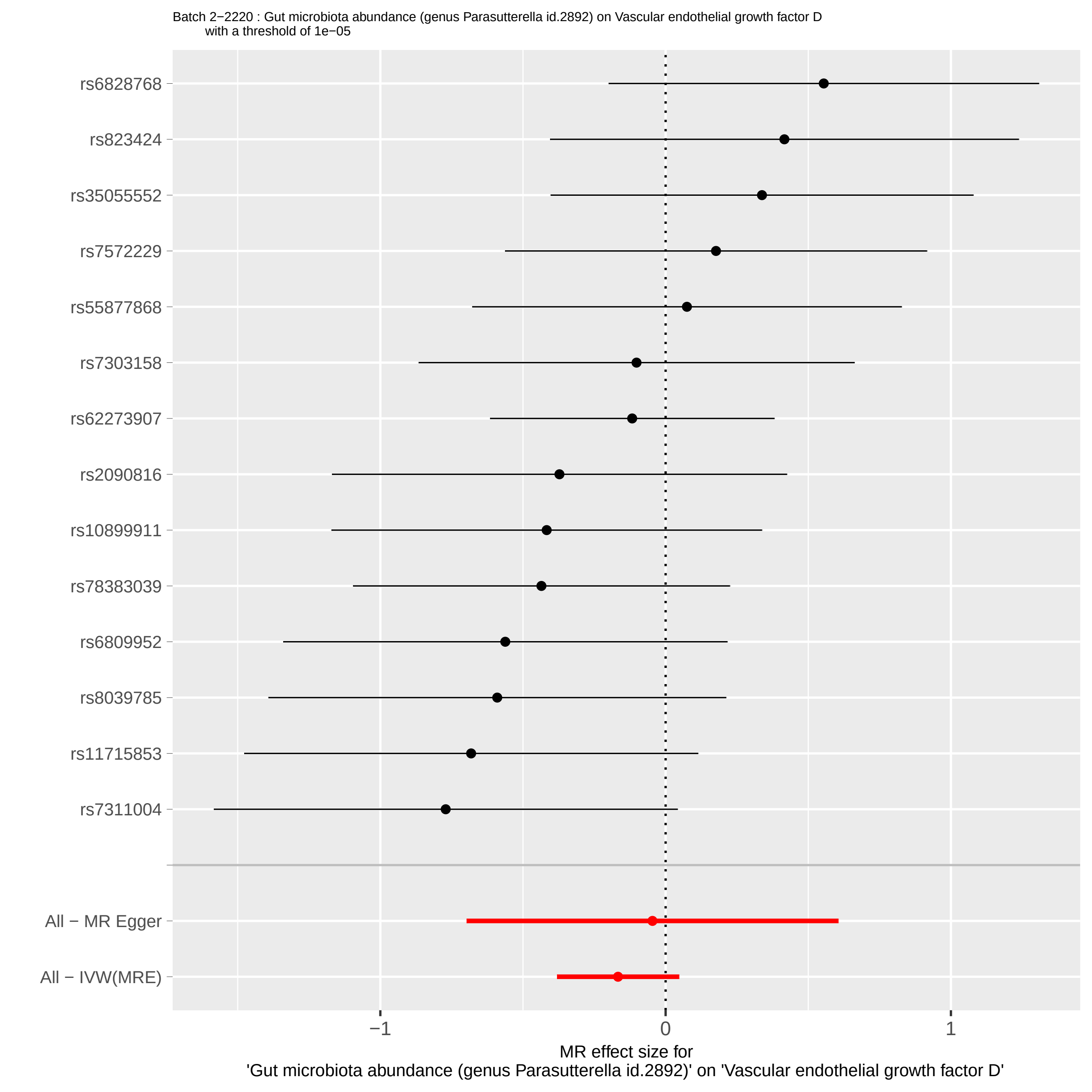

Batch 2−2220 : Gut microbiota abundance (genus Parasutterella id.2892) on Vascular endothelial growth factor D
with a threshold of 1e−05
rs6828768
rs823424
rs35055552
rs7572229
rs55877868
rs7303158
rs62273907
rs2090816
rs10899911
rs78383039
rs6809952
rs8039785
rs11715853
rs7311004
All − MR Egger
All − IVW(MRE)
−1
0
1
MR effect size for
'Gut microbiota abundance (genus Parasutterella id.2892)' on 'Vascular endothelial growth factor D'

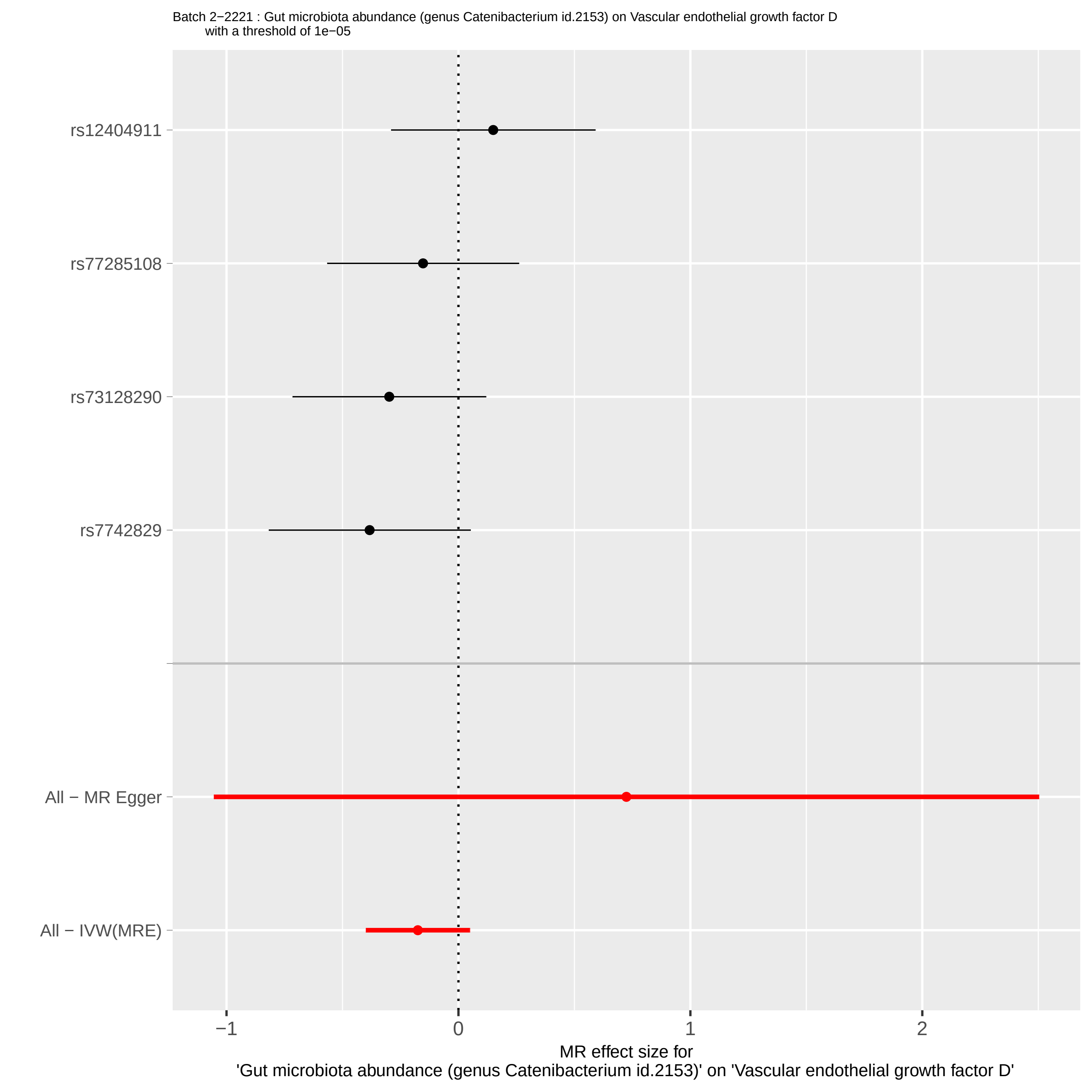

Batch 2−2221 : Gut microbiota abundance (genus Catenibacterium id.2153) on Vascular endothelial growth factor D
with a threshold of 1e−05
rs12404911
rs77285108
rs73128290
rs7742829
All − MR Egger
All − IVW(MRE)
−1
0
1
2
MR effect size for
'Gut microbiota abundance (genus Catenibacterium id.2153)' on 'Vascular endothelial growth factor D'

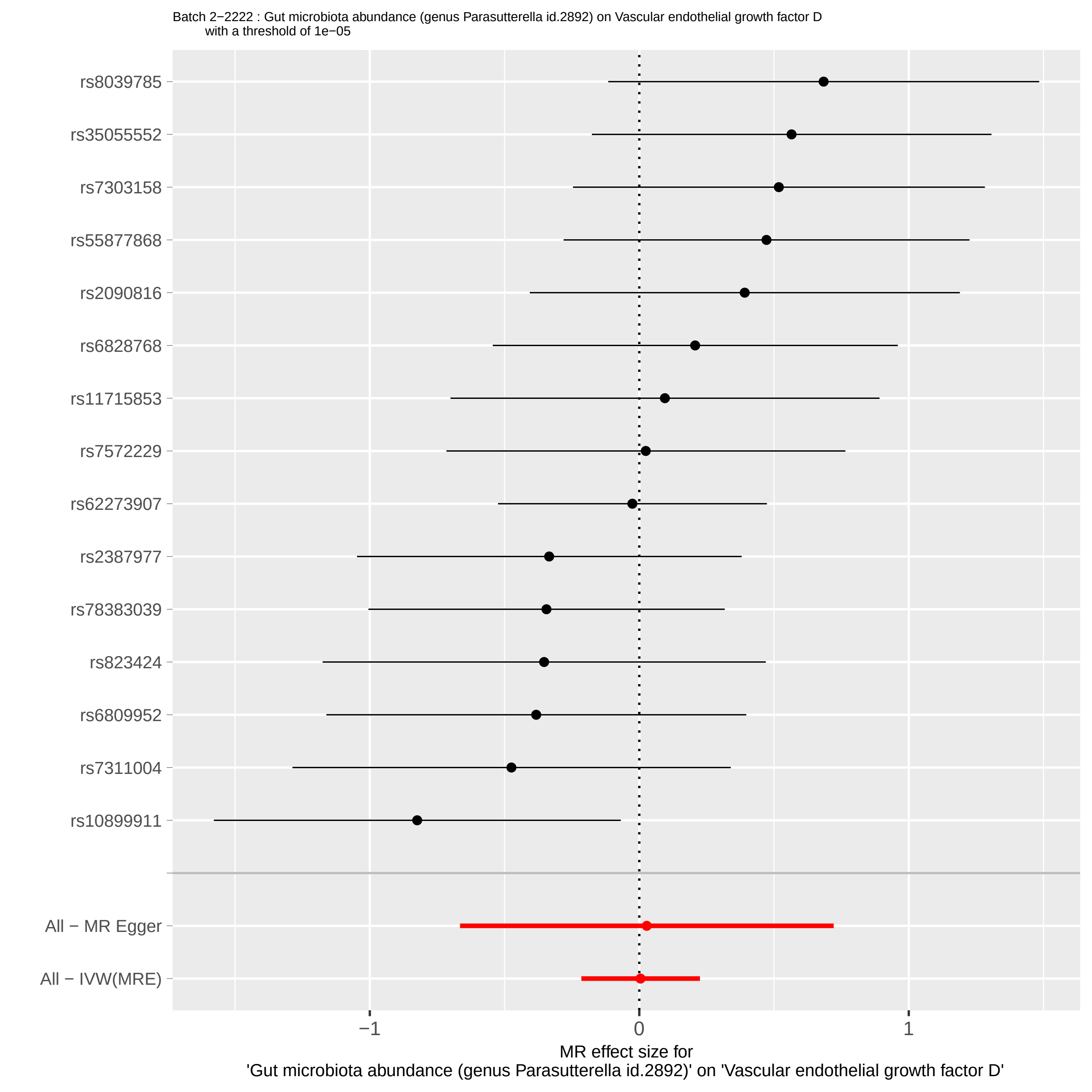

Batch 2−2222 : Gut microbiota abundance (genus Parasutterella id.2892) on Vascular endothelial growth factor D
with a threshold of 1e−05
rs8039785
rs35055552
rs7303158
rs55877868
rs2090816
rs6828768
rs11715853
rs7572229
rs62273907
rs2387977
rs78383039
rs823424
rs6809952
rs7311004
rs10899911
All − MR Egger
All − IVW(MRE)
−1
0
1
MR effect size for
'Gut microbiota abundance (genus Parasutterella id.2892)' on 'Vascular endothelial growth factor D'

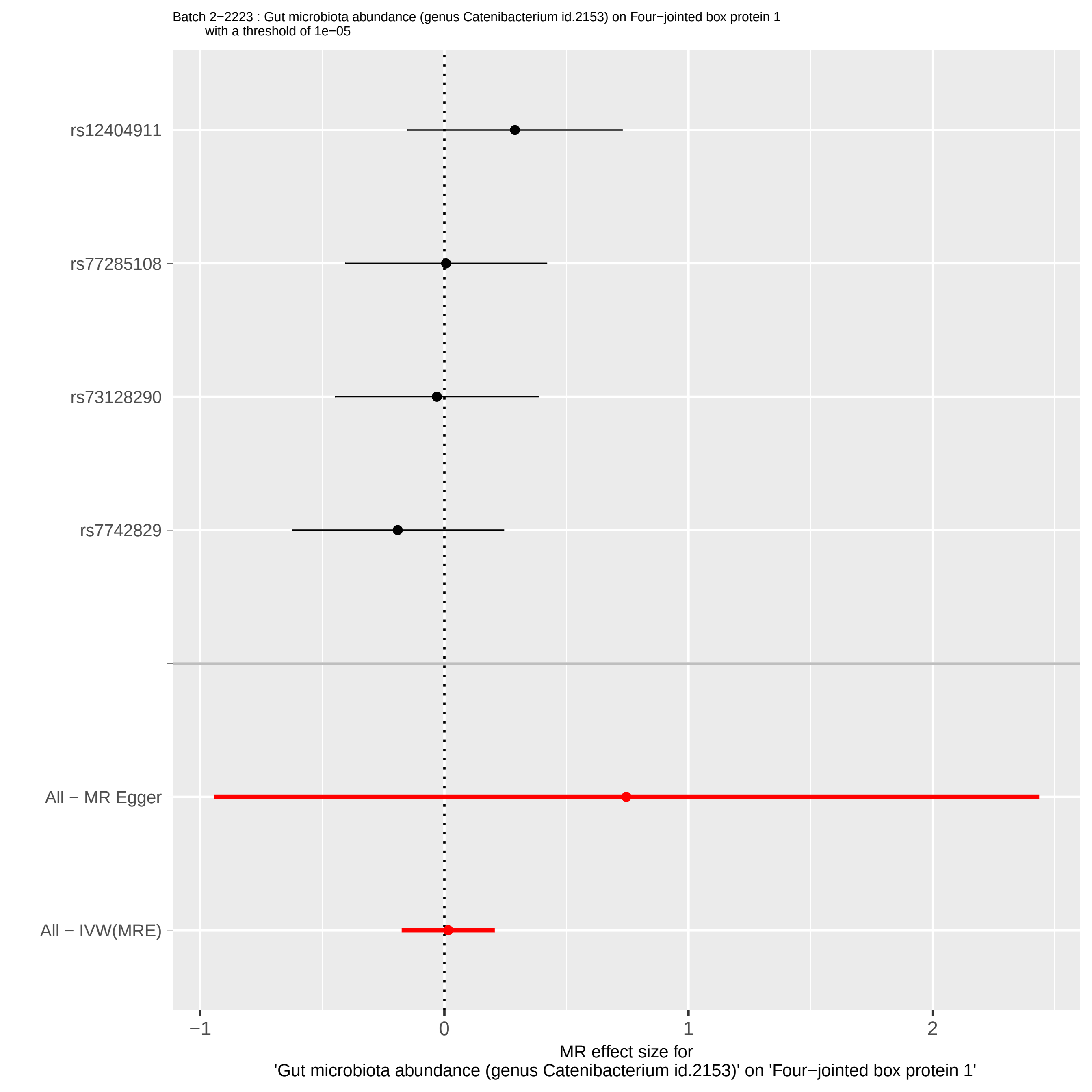

Batch 2−2223 : Gut microbiota abundance (genus Catenibacterium id.2153) on Four−jointed box protein 1
with a threshold of 1e−05
rs12404911
rs77285108
rs73128290
rs7742829
All − MR Egger
All − IVW(MRE)
−1
0
1
2
MR effect size for
'Gut microbiota abundance (genus Catenibacterium id.2153)' on 'Four−jointed box protein 1'

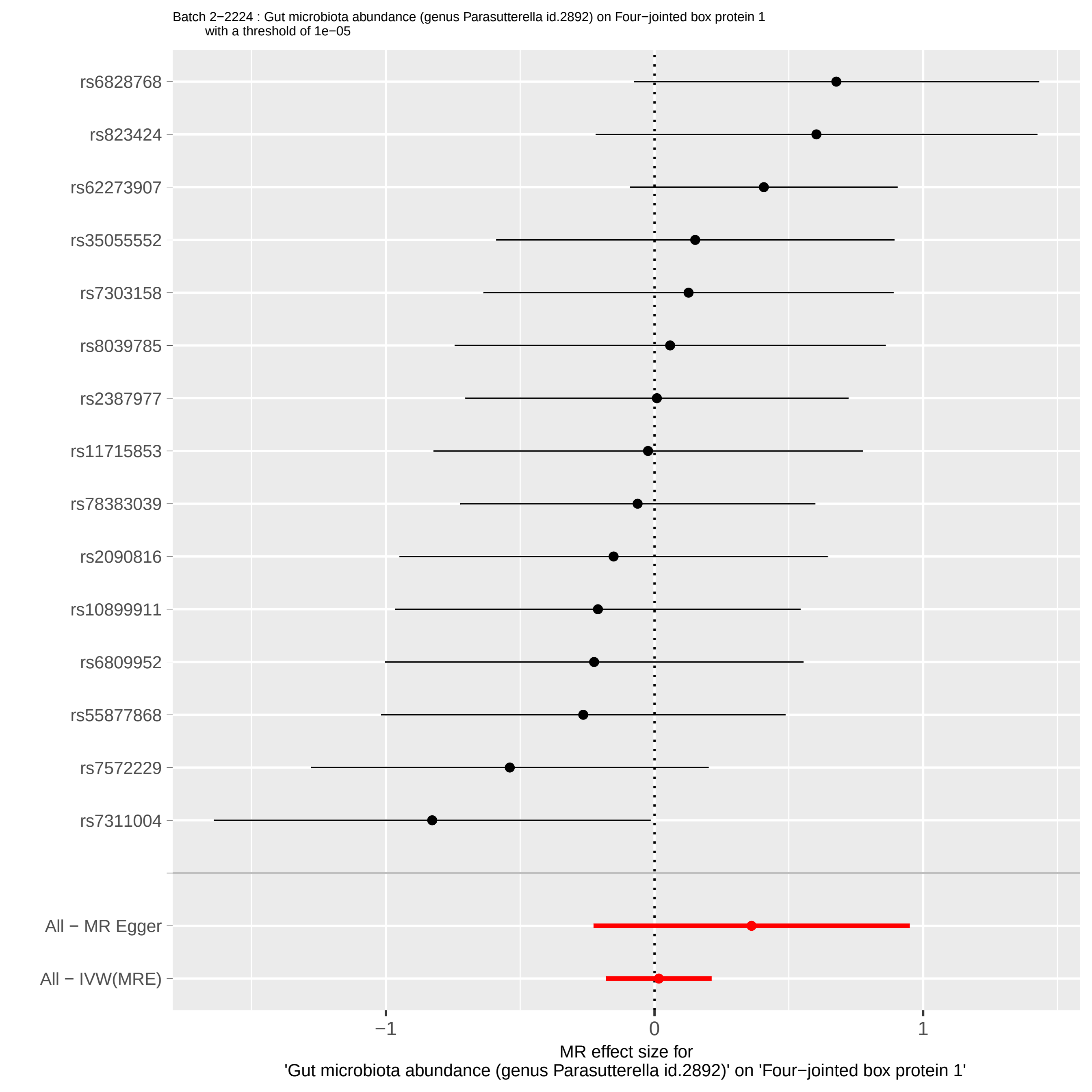

Batch 2−2224 : Gut microbiota abundance (genus Parasutterella id.2892) on Four−jointed box protein 1
with a threshold of 1e−05
rs6828768
rs823424
rs62273907
rs35055552
rs7303158
rs8039785
rs2387977
rs11715853
rs78383039
rs2090816
rs10899911
rs6809952
rs55877868
rs7572229
rs7311004
All − MR Egger
All − IVW(MRE)
−1
0
1
MR effect size for
'Gut microbiota abundance (genus Parasutterella id.2892)' on 'Four−jointed box protein 1'

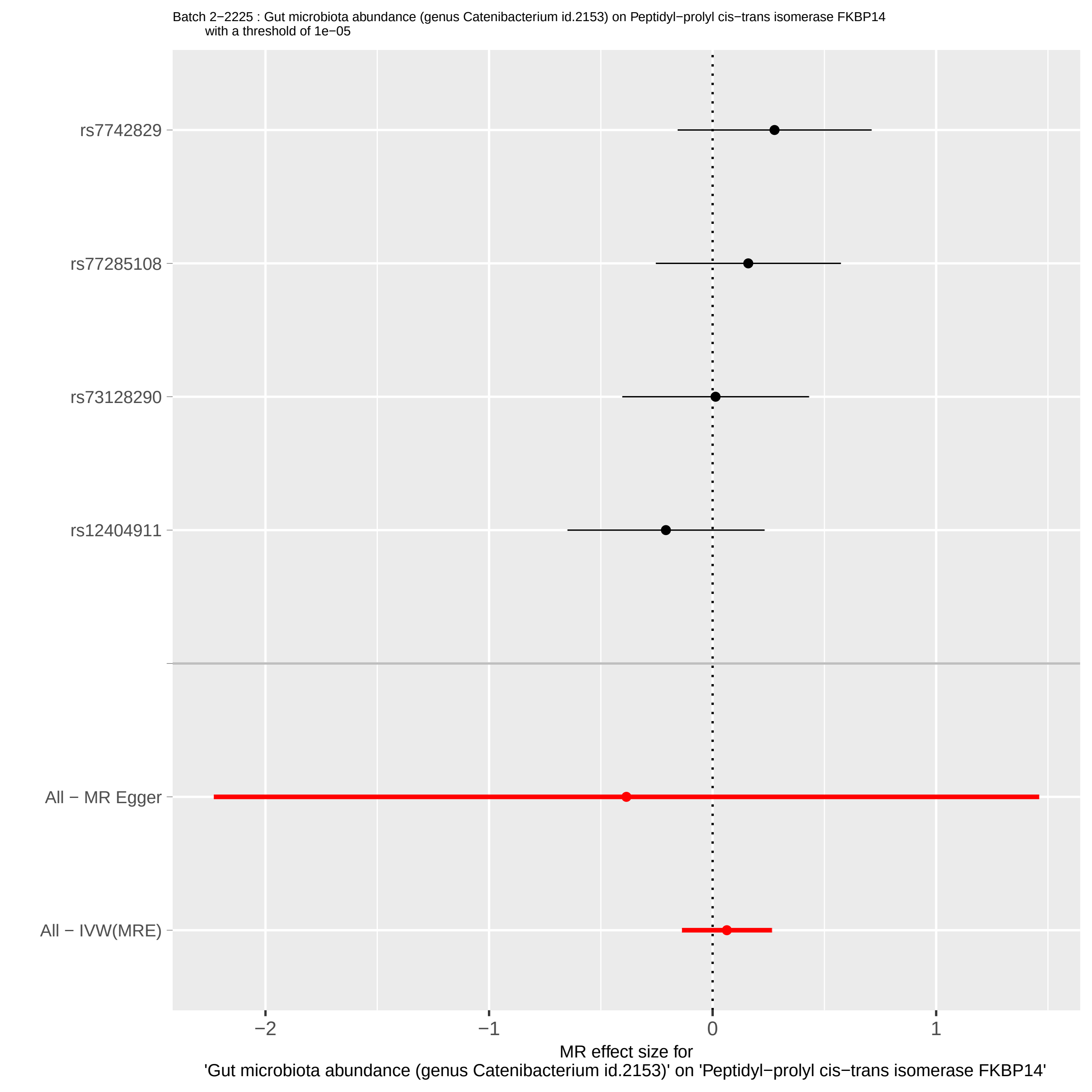

Batch 2−2225 : Gut microbiota abundance (genus Catenibacterium id.2153) on Peptidyl−prolyl cis−trans isomerase FKBP14
with a threshold of 1e−05
rs7742829
rs77285108
rs73128290
rs12404911
All − MR Egger
All − IVW(MRE)
−2
−1
0
1
MR effect size for
'Gut microbiota abundance (genus Catenibacterium id.2153)' on 'Peptidyl−prolyl cis−trans isomerase FKBP14'

Batch 2−2226 : Gut microbiota abundance (genus Parasutterella id.2892) on Peptidyl−prolyl cis−trans isomerase FKBP14 with a threshold of 1e−05

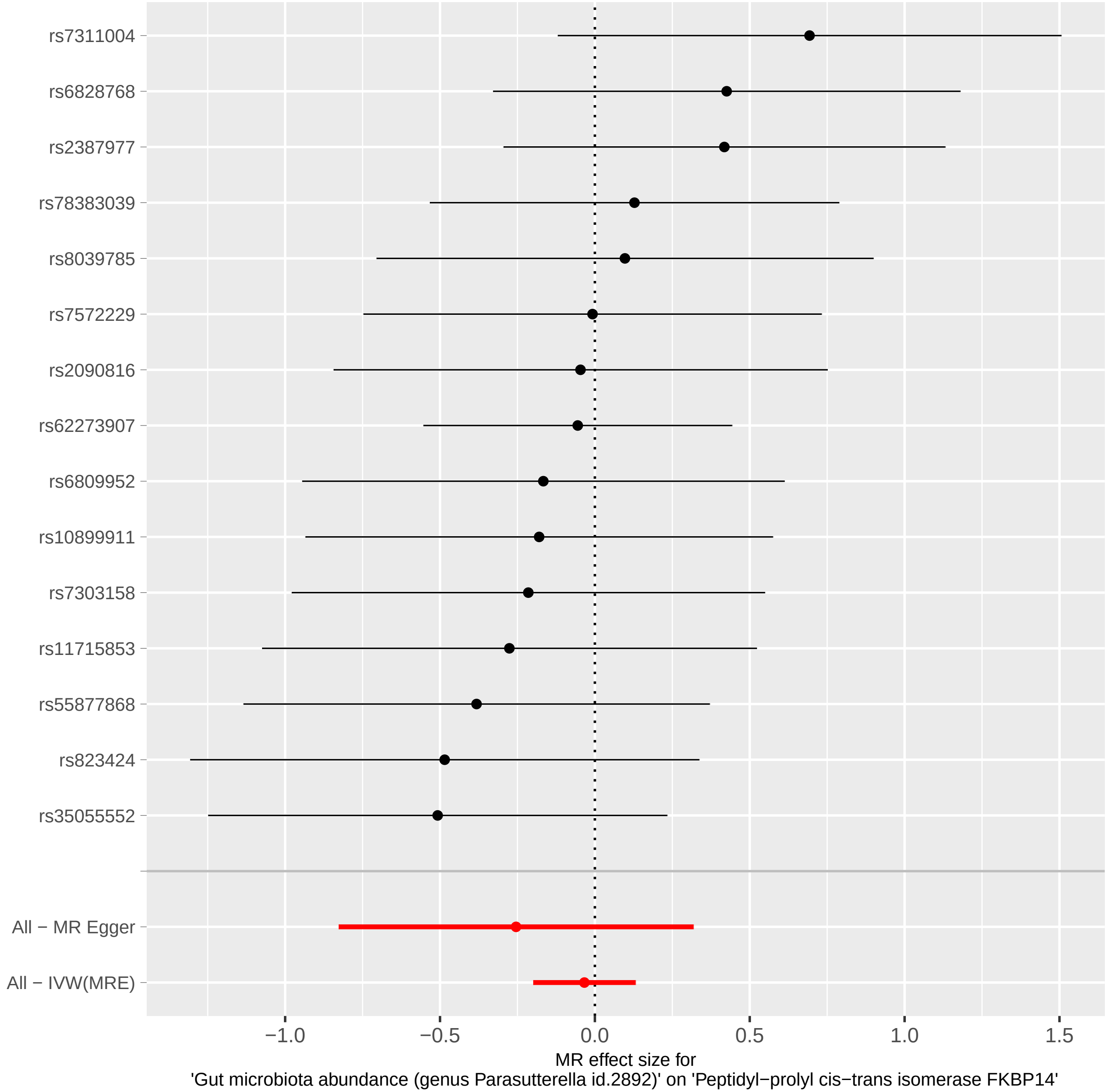

Batch 2−2227 : Gut microbiota abundance (genus Catenibacterium id.2153) on Peptidyl−prolyl cis−trans isomerase FKBP2
with a threshold of 1e−05

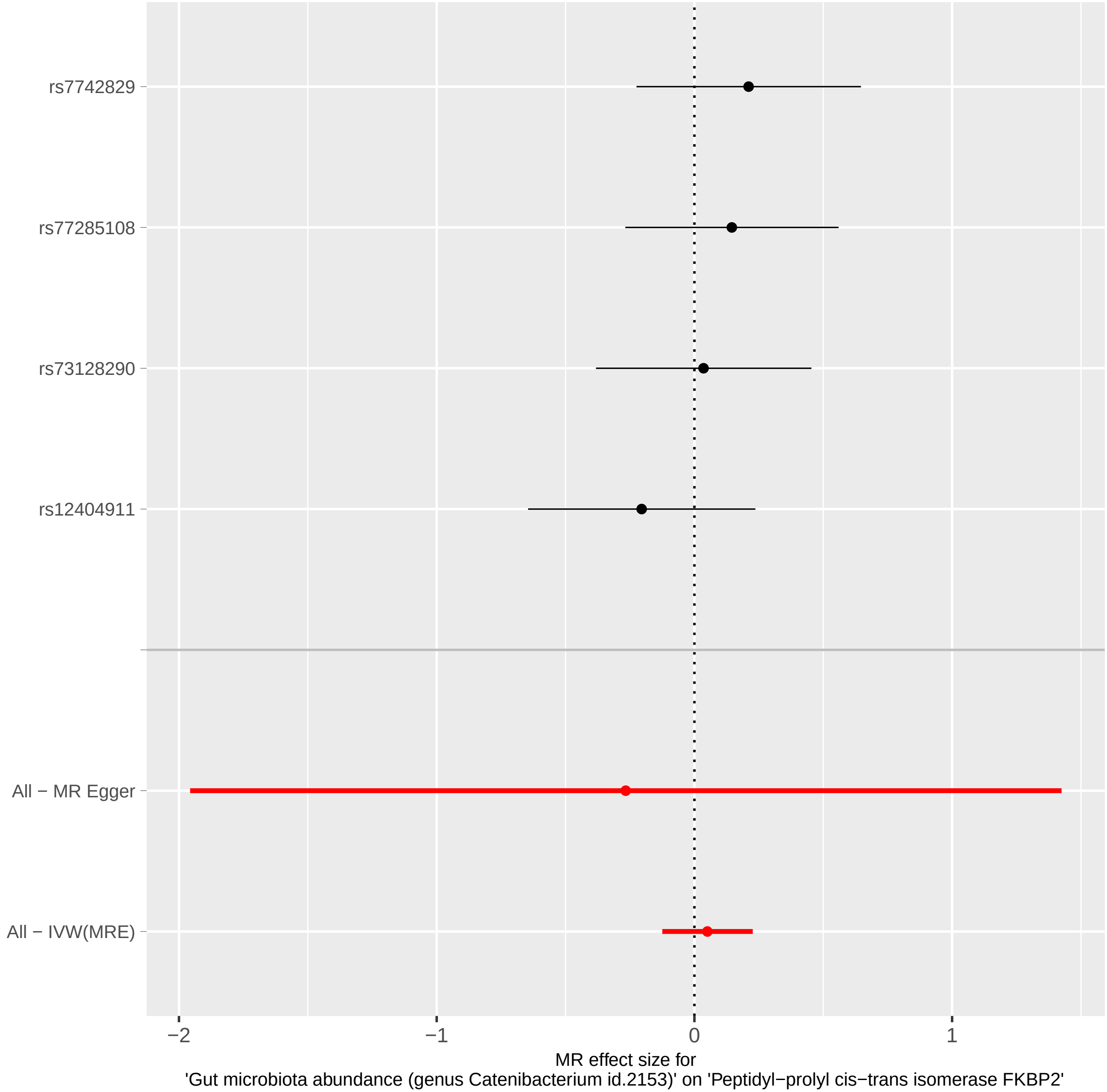

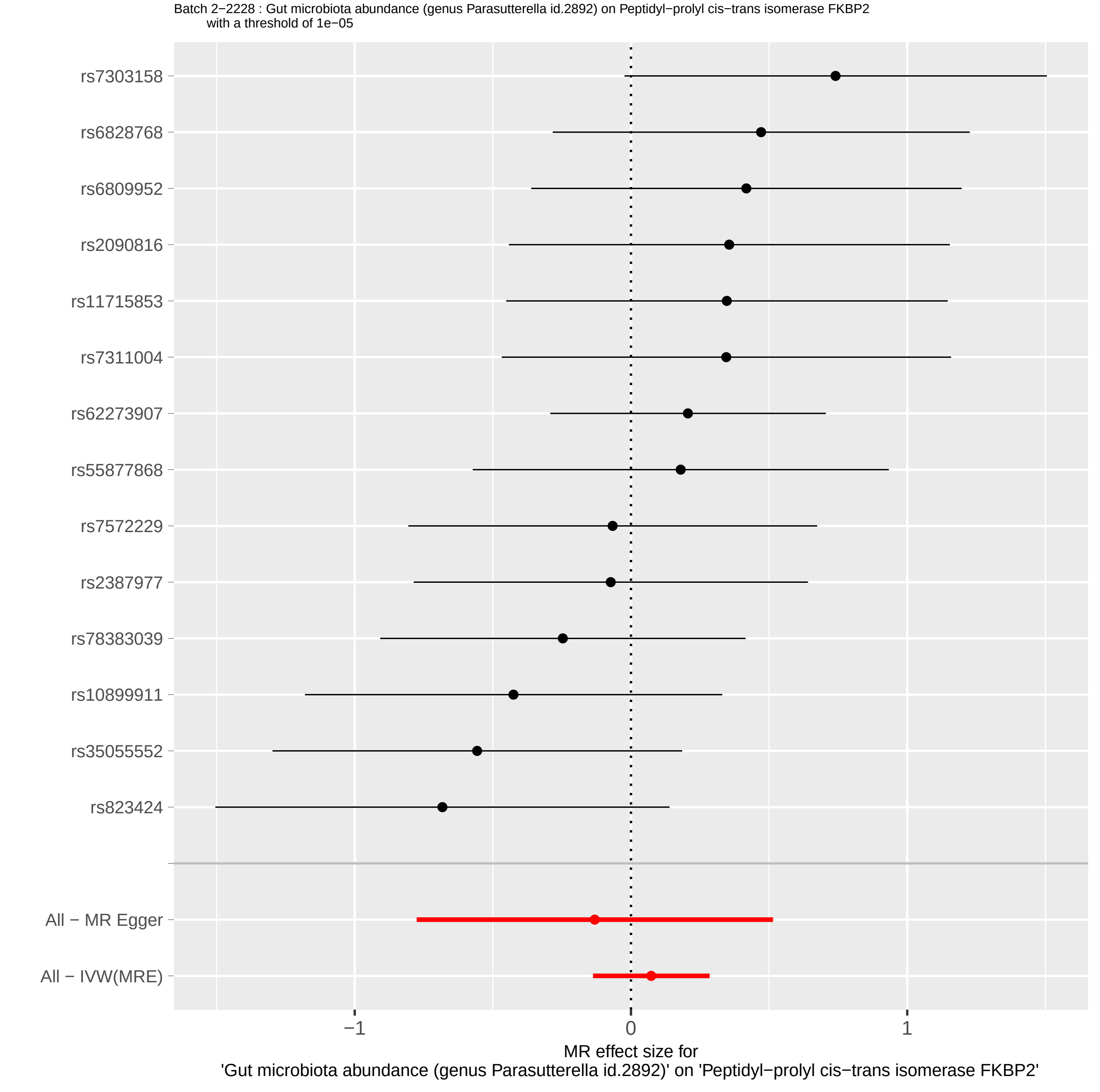
Batch 2−2228 : Gut microbiota abundance (genus Parasutterella id.2892) on Peptidyl−prolyl cis−trans isomerase FKBP2
with a threshold of 1e−05
rs7303158
rs6828768
rs6809952
rs2090816
rs11715853
rs7311004
rs62273907
rs55877868
rs7572229
rs2387977
rs78383039
rs10899911
rs35055552
rs823424
All − MR Egger
All − IVW(MRE)
−1
0
1
MR effect size for
'Gut microbiota abundance (genus Parasutterella id.2892)' on 'Peptidyl−prolyl cis−trans isomerase FKBP2'

Batch 2−2229 : Gut microbiota abundance (genus Catenibacterium id.2153) on Inactive peptidyl−prolyl cis−trans isomerase FKBP6 with a threshold of 1e−05

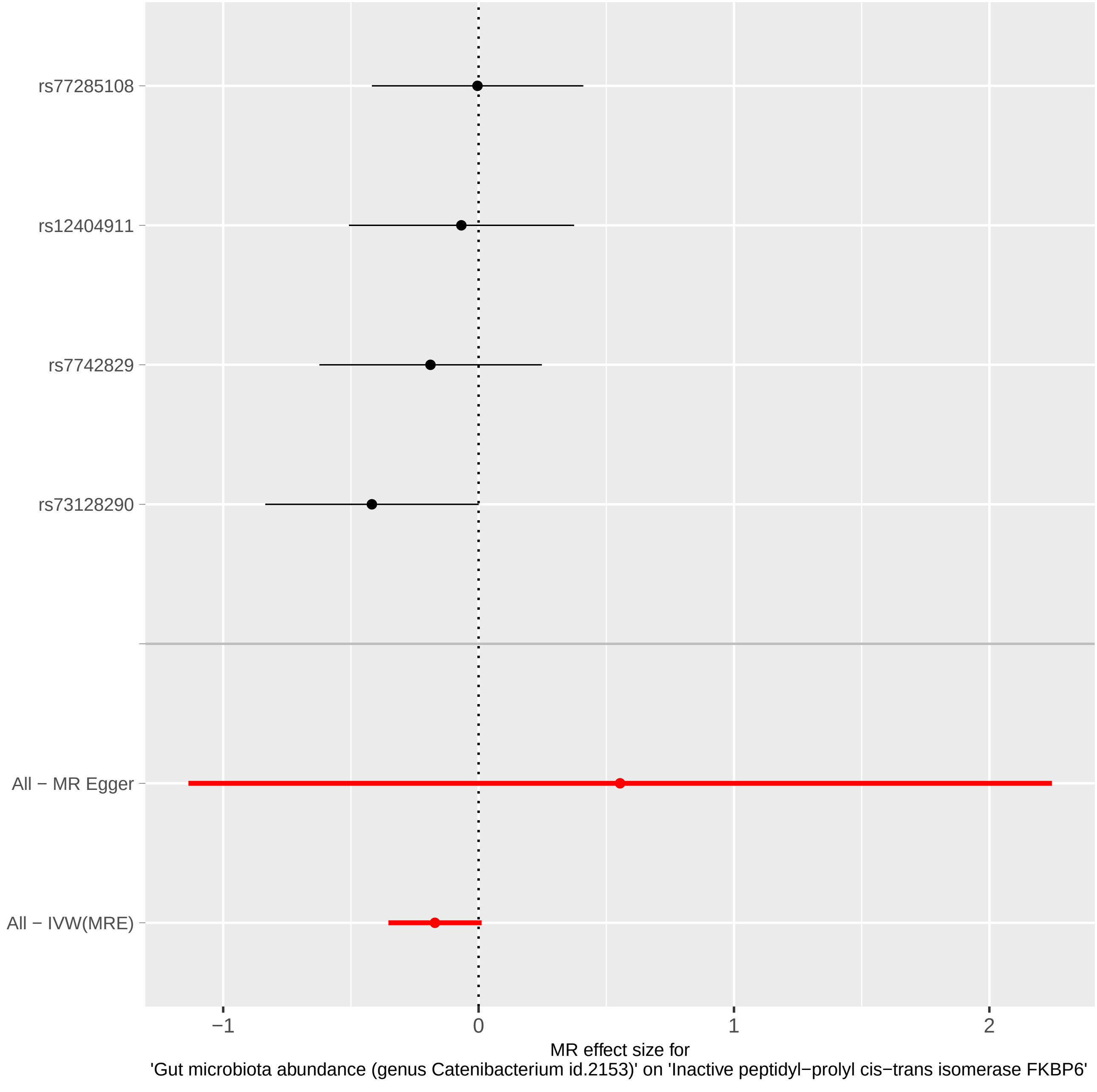

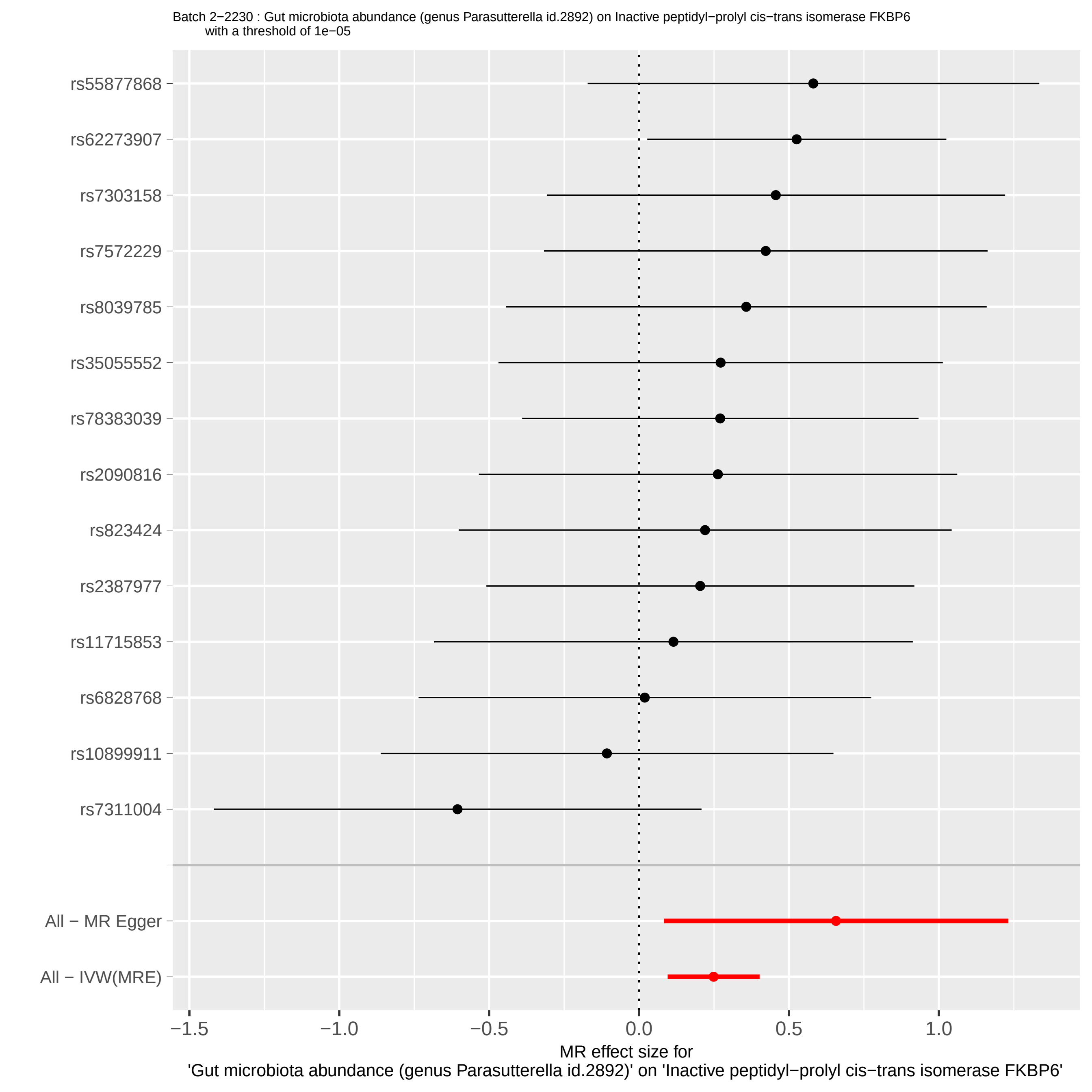

Batch 2−2230 : Gut microbiota abundance (genus Parasutterella id.2892) on Inactive peptidyl−prolyl cis−trans isomerase FKBP6
with a threshold of 1e−05
rs55877868
rs62273907
rs7303158
rs7572229
rs8039785
rs35055552
rs78383039
rs2090816
rs823424
rs2387977
rs11715853
rs6828768
rs10899911
rs7311004
All − MR Egger
All − IVW(MRE)
−1.5
−1.0
−0.5
0.0
0.5
1.0
MR effect size for
'Gut microbiota abundance (genus Parasutterella id.2892)' on 'Inactive peptidyl−prolyl cis−trans isomerase FKBP6'

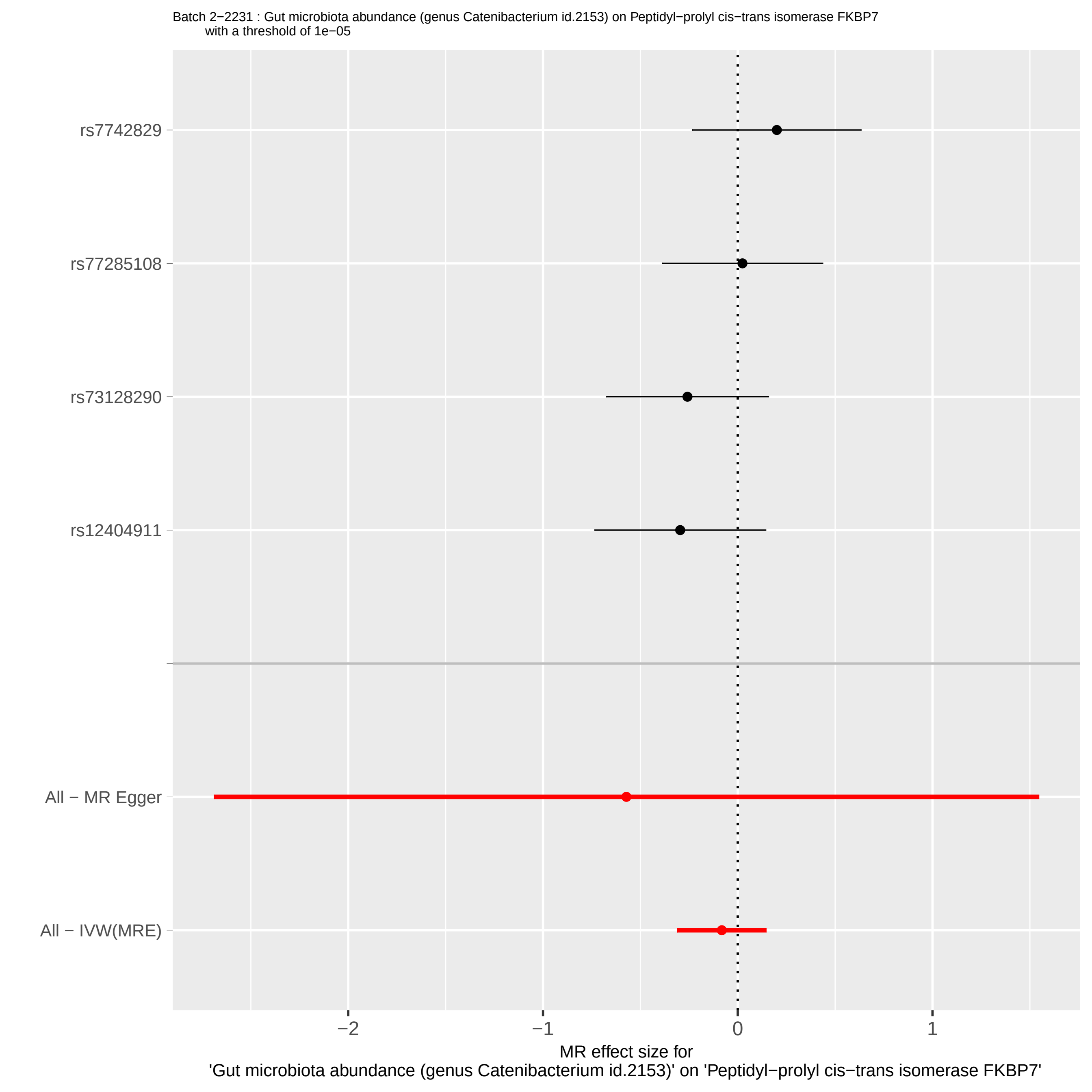

Batch 2−2231 : Gut microbiota abundance (genus Catenibacterium id.2153) on Peptidyl−prolyl cis−trans isomerase FKBP7
with a threshold of 1e−05
rs7742829
rs77285108
rs73128290
rs12404911
All − MR Egger
All − IVW(MRE)
−2
−1
0
1
MR effect size for
'Gut microbiota abundance (genus Catenibacterium id.2153)' on 'Peptidyl−prolyl cis−trans isomerase FKBP7'

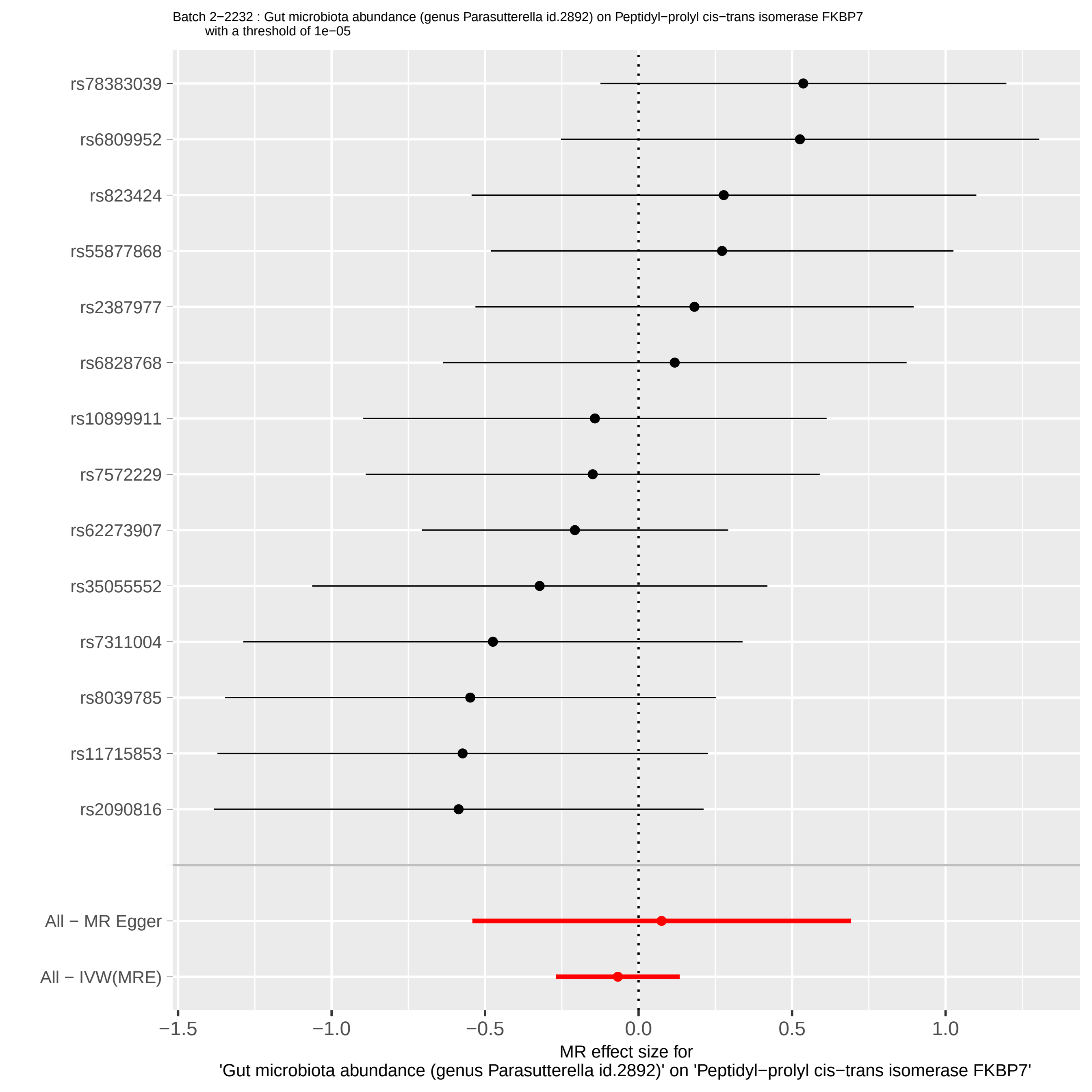

Batch 2−2232 : Gut microbiota abundance (genus Parasutterella id.2892) on Peptidyl−prolyl cis−trans isomerase FKBP7
with a threshold of 1e−05
rs78383039
rs6809952
rs823424
rs55877868
rs2387977
rs6828768
rs10899911
rs7572229
rs62273907
rs35055552
rs7311004
rs8039785
rs11715853
rs2090816
All − MR Egger
All − IVW(MRE)
−1.5
−1.0
−0.5
0.0
0.5
1.0
MR effect size for
'Gut microbiota abundance (genus Parasutterella id.2892)' on 'Peptidyl−prolyl cis−trans isomerase FKBP7'

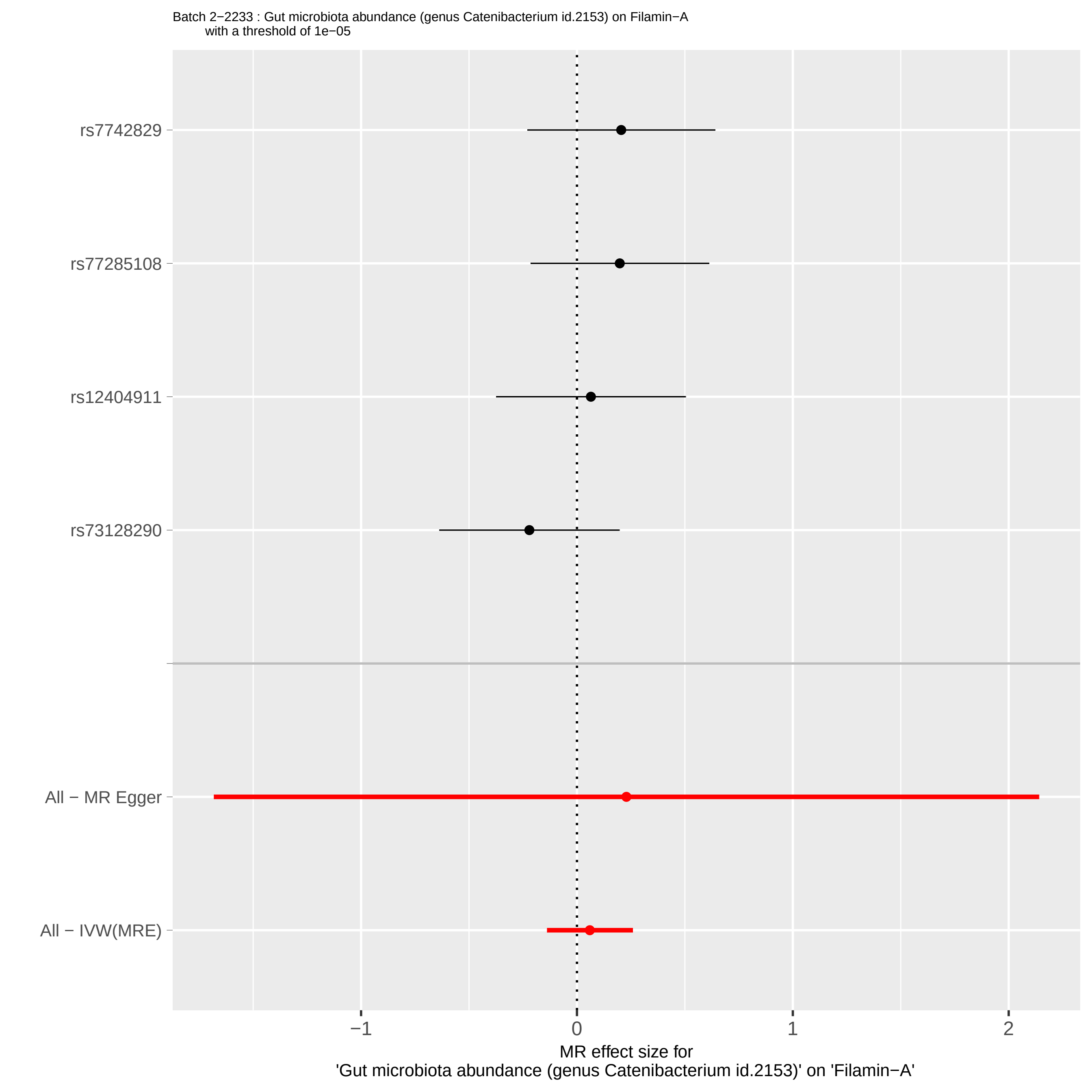

Batch 2−2233 : Gut microbiota abundance (genus Catenibacterium id.2153) on Filamin−A
with a threshold of 1e−05
rs7742829
rs77285108
rs12404911
rs73128290
All − MR Egger
All − IVW(MRE)
−1
0
1
2
MR effect size for
'Gut microbiota abundance (genus Catenibacterium id.2153)' on 'Filamin−A'

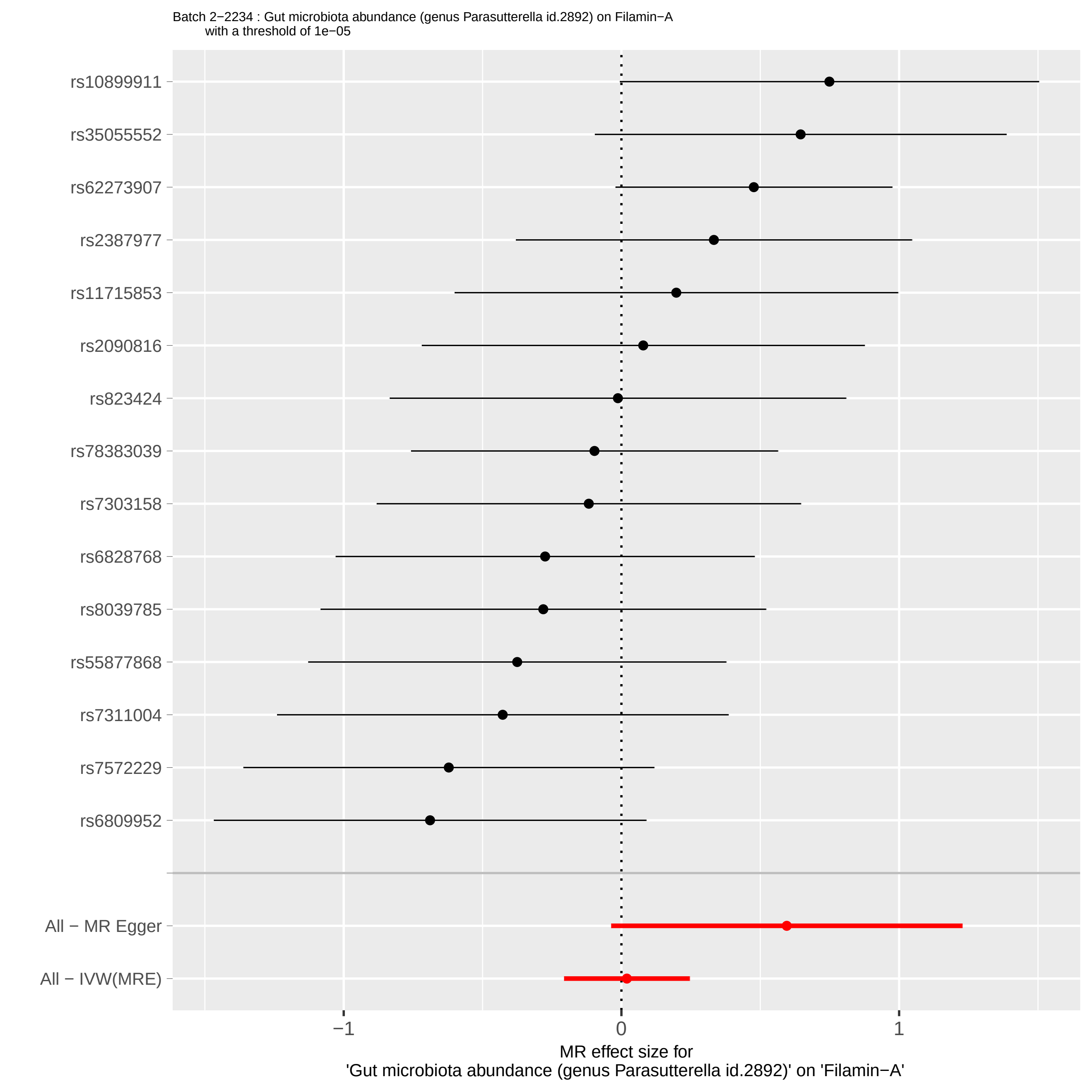

Batch 2−2234 : Gut microbiota abundance (genus Parasutterella id.2892) on Filamin−A
with a threshold of 1e−05
rs10899911
rs35055552
rs62273907
rs2387977
rs11715853
rs2090816
rs823424
rs78383039
rs7303158
rs6828768
rs8039785
rs55877868
rs7311004
rs7572229
rs6809952
All − MR Egger
All − IVW(MRE)
−1
0
1
MR effect size for
'Gut microbiota abundance (genus Parasutterella id.2892)' on 'Filamin−A'

Batch 2−2235 : Gut microbiota abundance (genus Catenibacterium id.2153) on Filamin−A
with a threshold of 1e−05

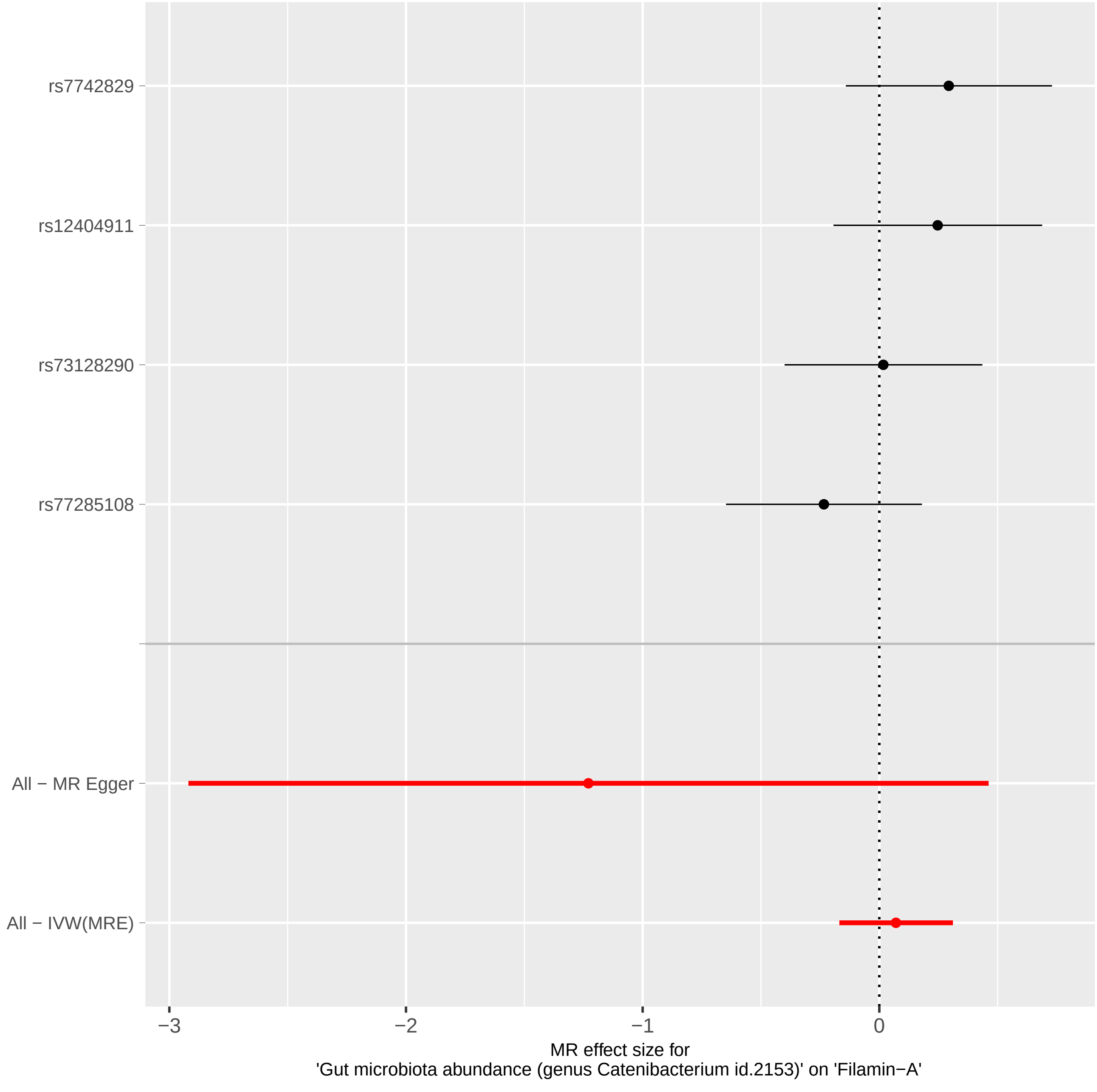

Batch 2−2236 : Gut microbiota abundance (genus Parasutterella id.2892) on Filamin−A
with a threshold of 1e−05

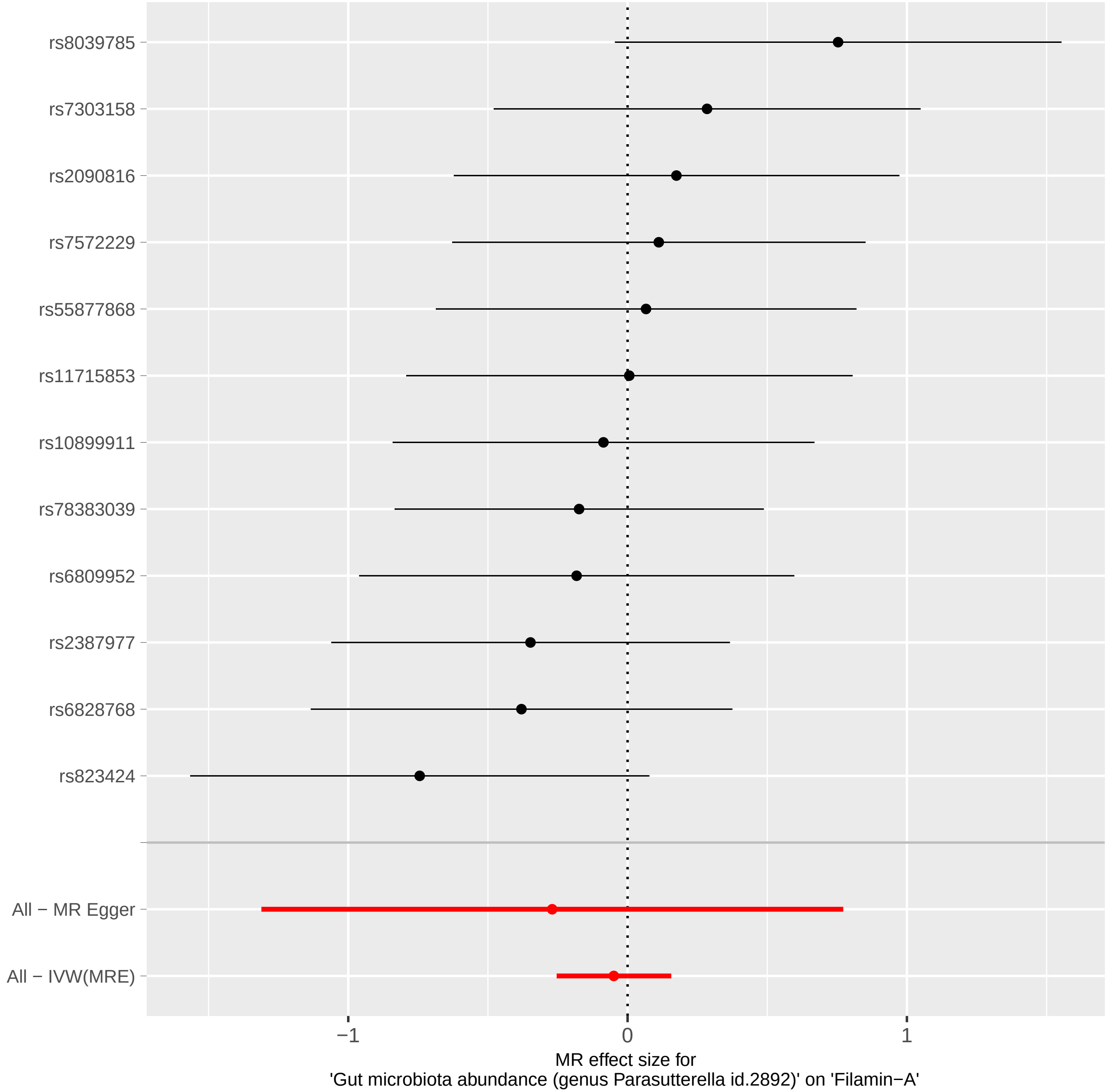

Batch 2−2237 : Gut microbiota abundance (genus Catenibacterium id.2153) on Filamin−A
with a threshold of 1e−05

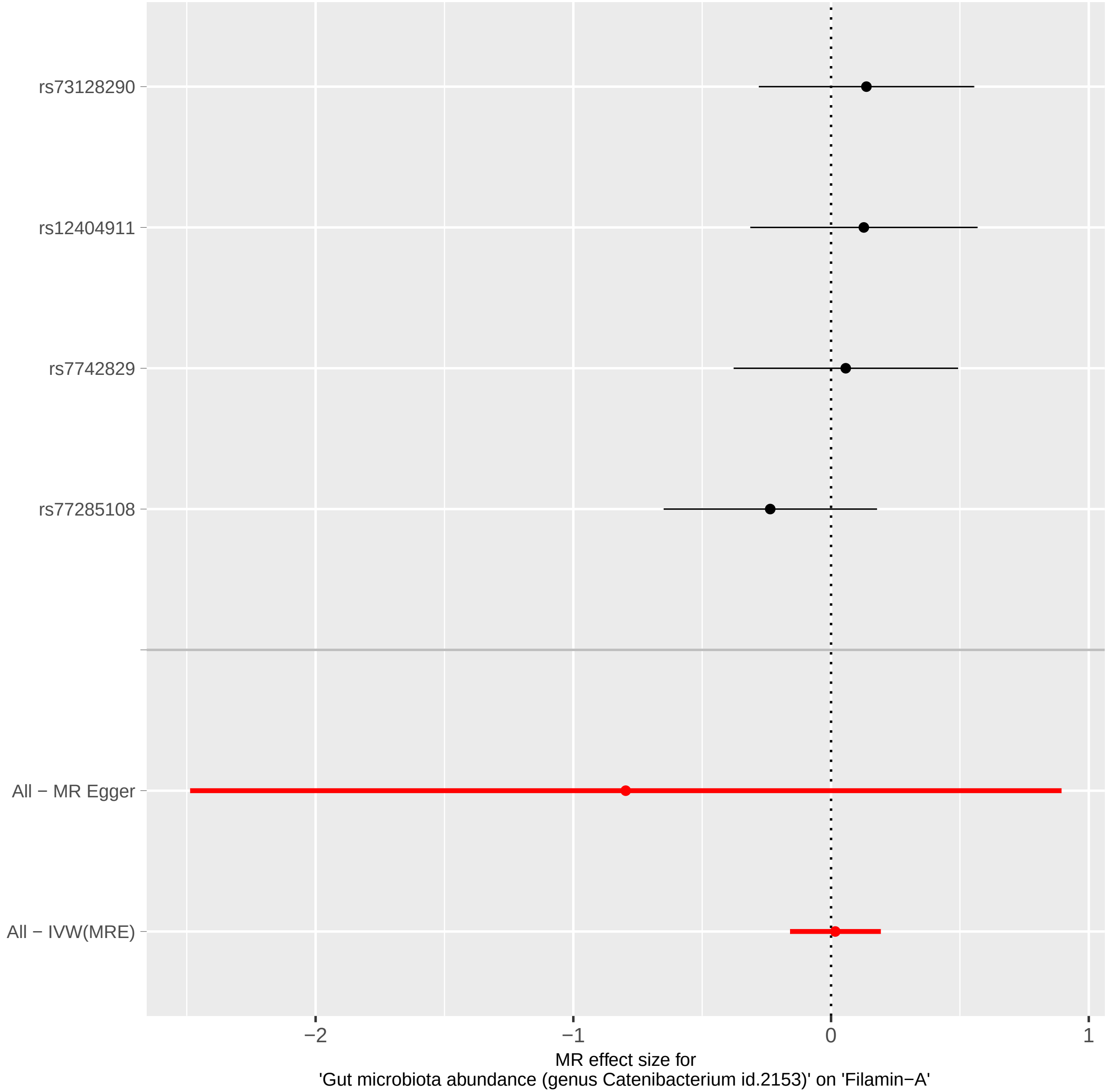

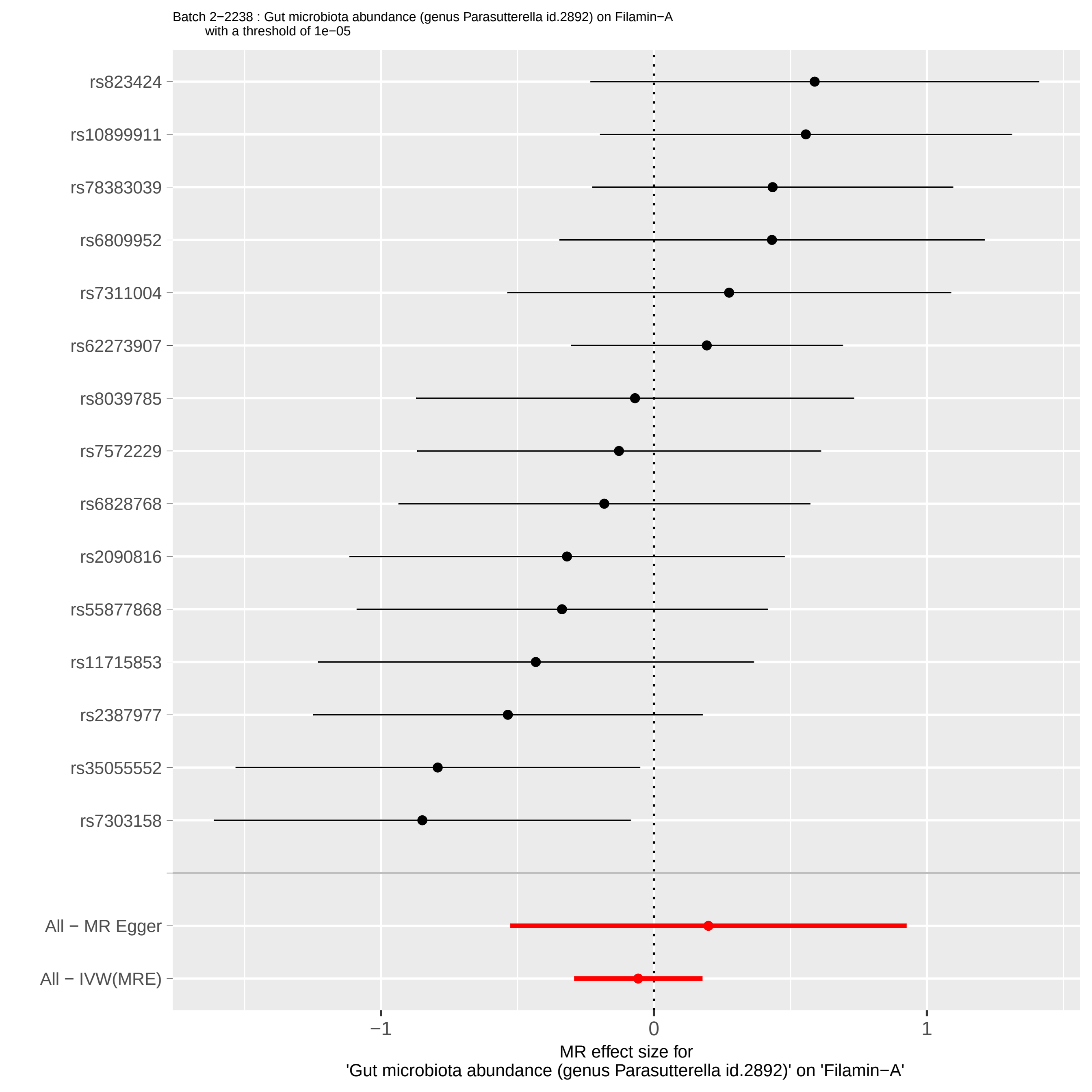

Batch 2−2238 : Gut microbiota abundance (genus Parasutterella id.2892) on Filamin−A
with a threshold of 1e−05
rs823424
rs10899911
rs78383039
rs6809952
rs7311004
rs62273907
rs8039785
rs7572229
rs6828768
rs2090816
rs55877868
rs11715853
rs2387977
rs35055552
rs7303158
All − MR Egger
All − IVW(MRE)
−1
0
1
MR effect size for
'Gut microbiota abundance (genus Parasutterella id.2892)' on 'Filamin−A'

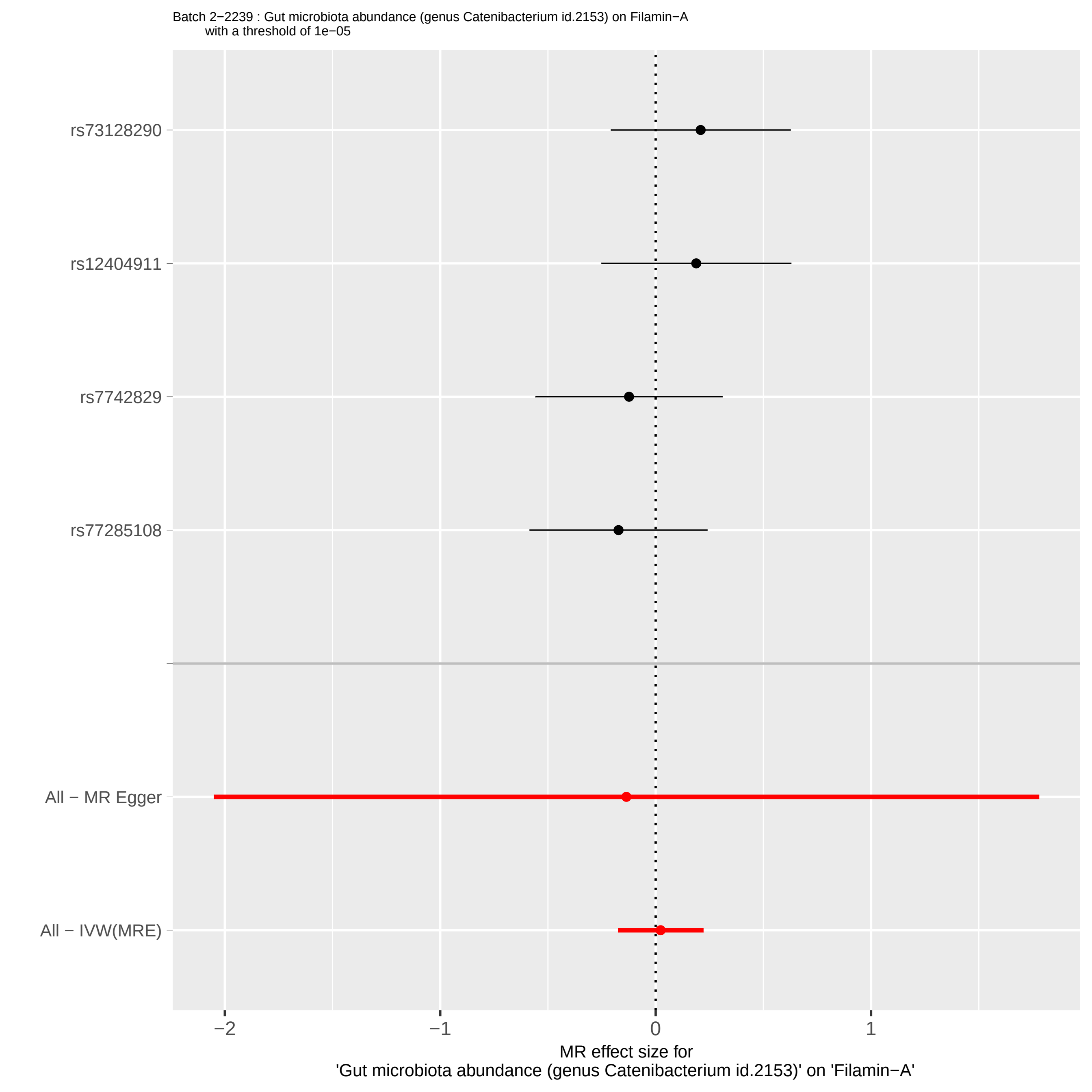

Batch 2−2239 : Gut microbiota abundance (genus Catenibacterium id.2153) on Filamin−A
with a threshold of 1e−05
rs73128290
rs12404911
rs7742829
rs77285108
All − MR Egger
All − IVW(MRE)
−2
−1
0
1
MR effect size for
'Gut microbiota abundance (genus Catenibacterium id.2153)' on 'Filamin−A'

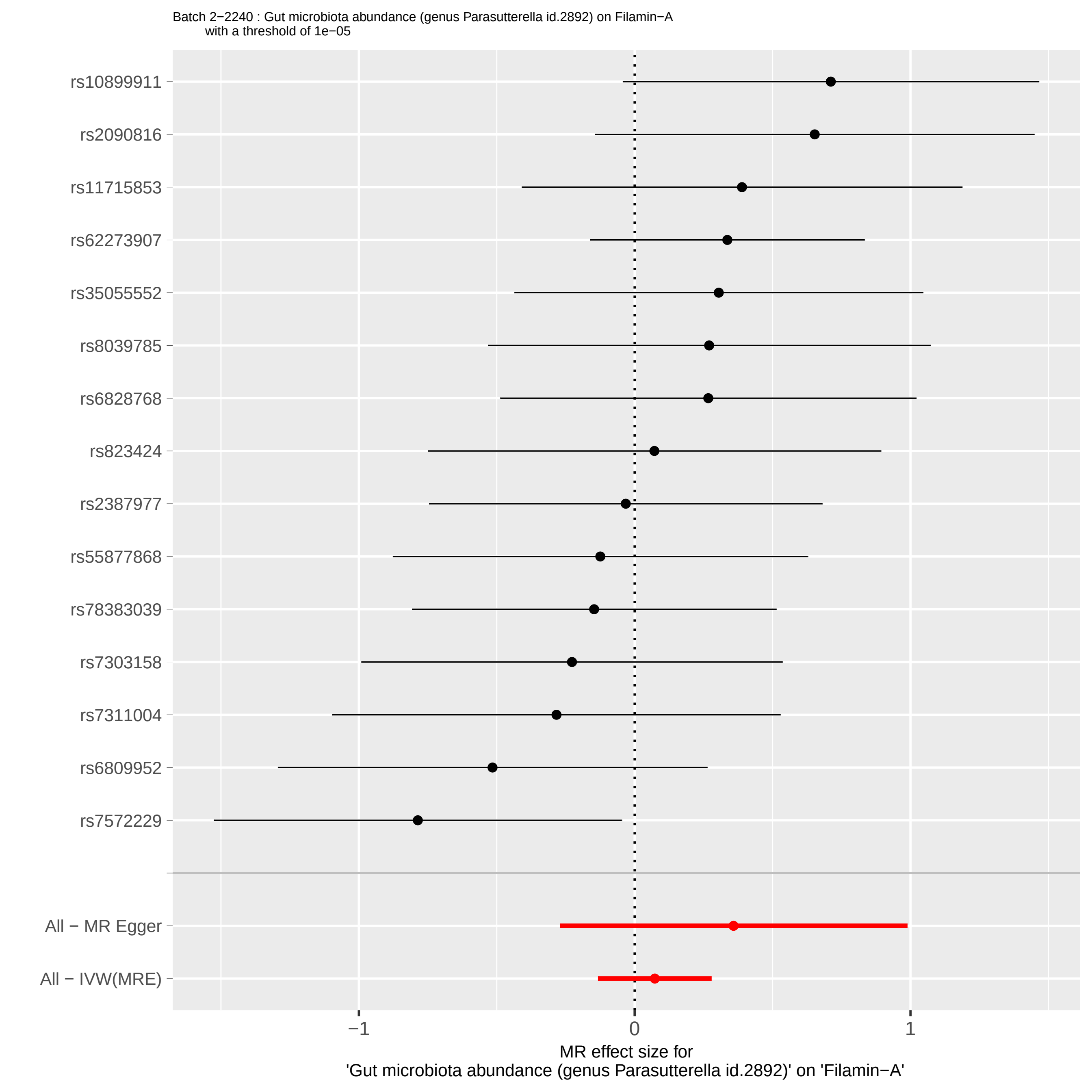
Batch 2−2240 : Gut microbiota abundance (genus Parasutterella id.2892) on Filamin−A
with a threshold of 1e−05
rs10899911
rs2090816
rs11715853
rs62273907
rs35055552
rs8039785
rs6828768
rs823424
rs2387977
rs55877868
rs78383039
rs7303158
rs7311004
rs6809952
rs7572229
All − MR Egger
All − IVW(MRE)
−1
0
1
MR effect size for
'Gut microbiota abundance (genus Parasutterella id.2892)' on 'Filamin−A'

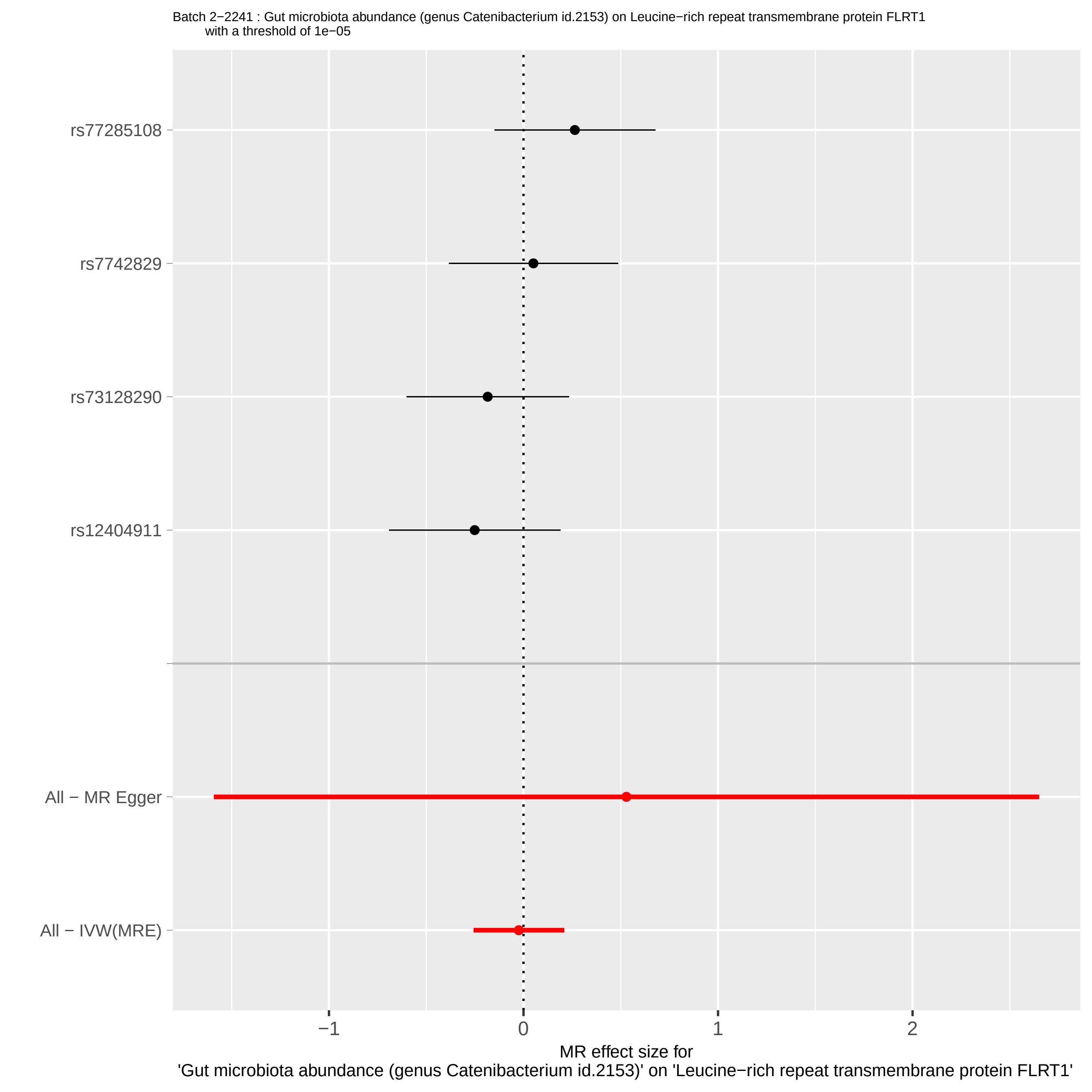

Batch 2−2241 : Gut microbiota abundance (genus Catenibacterium id.2153) on Leucine−rich repeat transmembrane protein FLRT1
with a threshold of 1e−05
rs77285108
rs7742829
rs73128290
rs12404911
All − MR Egger
All − IVW(MRE)
−1
0
1
2
MR effect size for
'Gut microbiota abundance (genus Catenibacterium id.2153)' on 'Leucine−rich repeat transmembrane protein FLRT1'

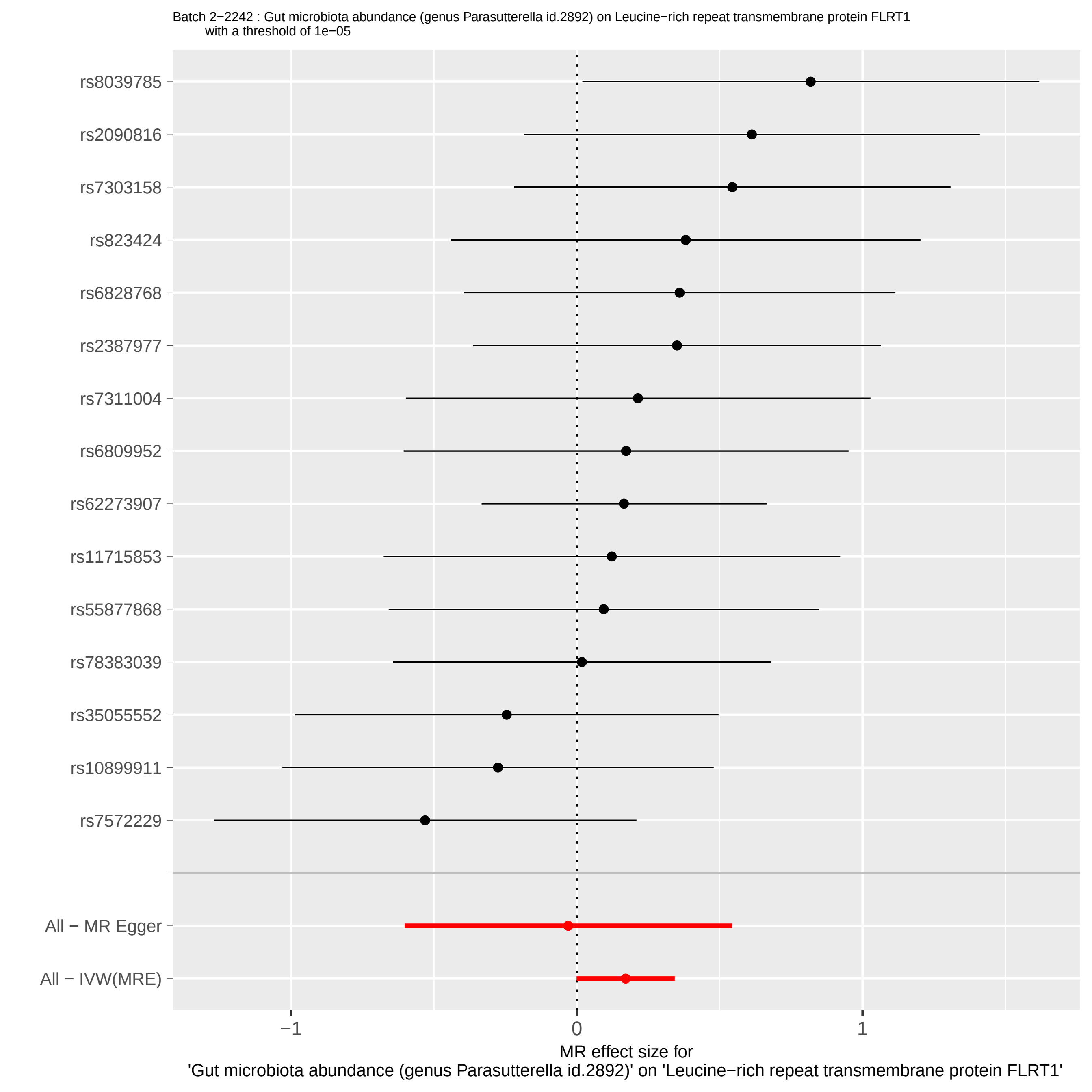
Batch 2−2242 : Gut microbiota abundance (genus Parasutterella id.2892) on Leucine−rich repeat transmembrane protein FLRT1
with a threshold of 1e−05
rs8039785
rs2090816
rs7303158
rs823424
rs6828768
rs2387977
rs7311004
rs6809952
rs62273907
rs11715853
rs55877868
rs78383039
rs35055552
rs10899911
rs7572229
All − MR Egger
All − IVW(MRE)
−1
0
1
MR effect size for
'Gut microbiota abundance (genus Parasutterella id.2892)' on 'Leucine−rich repeat transmembrane protein FLRT1'

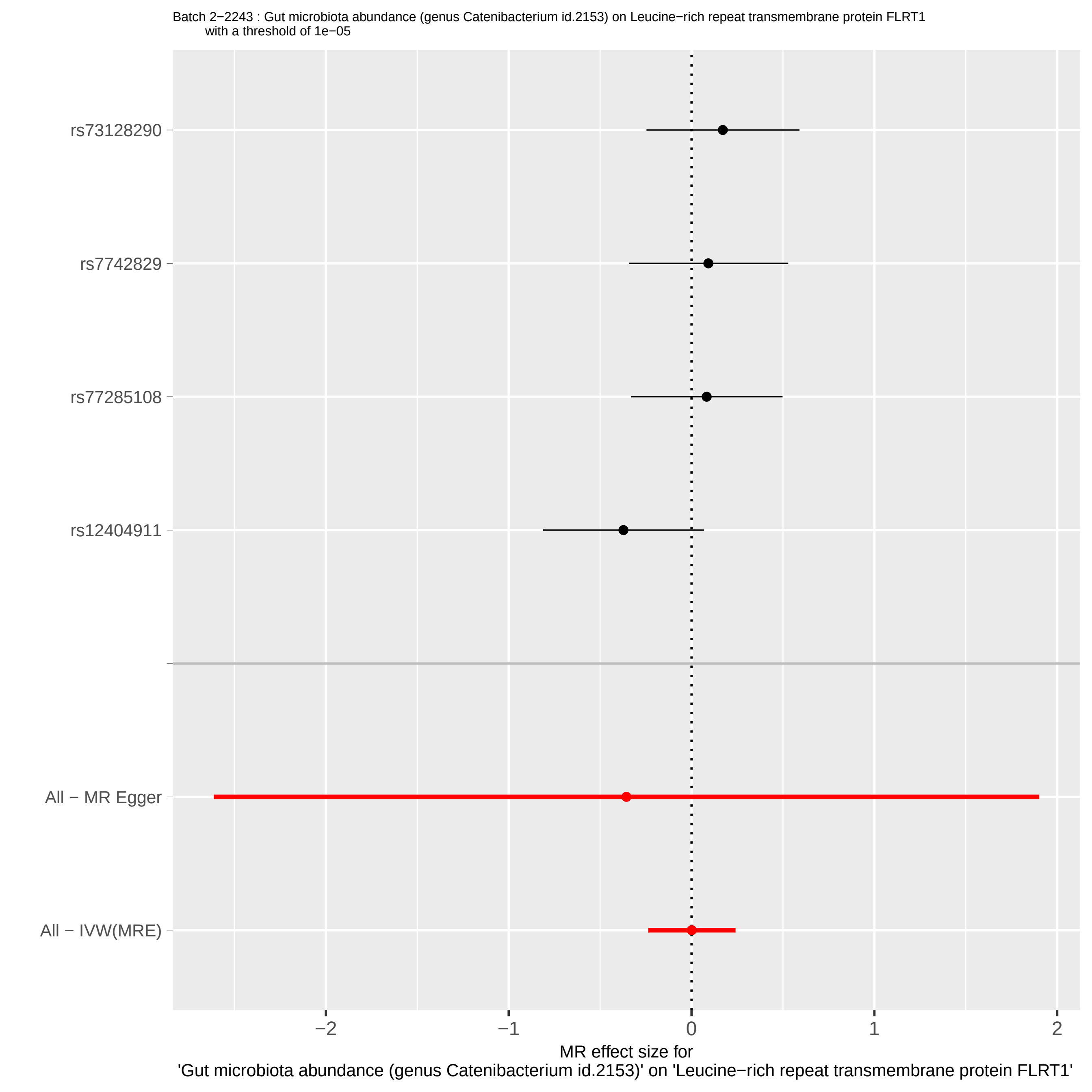

Batch 2−2243 : Gut microbiota abundance (genus Catenibacterium id.2153) on Leucine−rich repeat transmembrane protein FLRT1
with a threshold of 1e−05
rs73128290
rs7742829
rs77285108
rs12404911
All − MR Egger
All − IVW(MRE)
−2
−1
0
1
2
MR effect size for
'Gut microbiota abundance (genus Catenibacterium id.2153)' on 'Leucine−rich repeat transmembrane protein FLRT1'

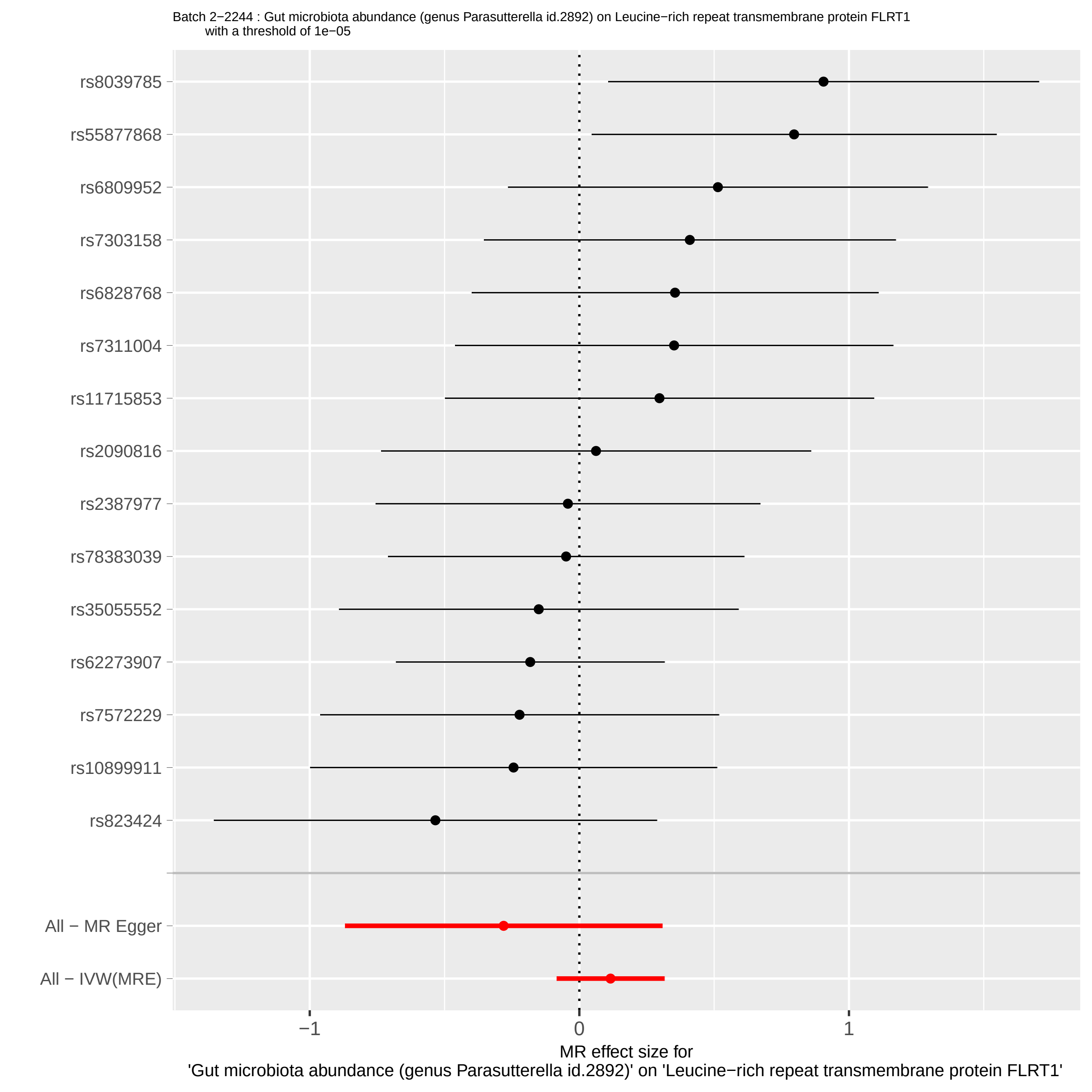
Batch 2−2244 : Gut microbiota abundance (genus Parasutterella id.2892) on Leucine−rich repeat transmembrane protein FLRT1
with a threshold of 1e−05
rs8039785
rs55877868
rs6809952
rs7303158
rs6828768
rs7311004
rs11715853
rs2090816
rs2387977
rs78383039
rs35055552
rs62273907
rs7572229
rs10899911
rs823424
All − MR Egger
All − IVW(MRE)
−1
0
1
MR effect size for
'Gut microbiota abundance (genus Parasutterella id.2892)' on 'Leucine−rich repeat transmembrane protein FLRT1'

Batch 2−2245 : Gut microbiota abundance (genus Catenibacterium id.2153) on Leucine−rich repeat transmembrane protein FLRT2
with a threshold of 1e−05

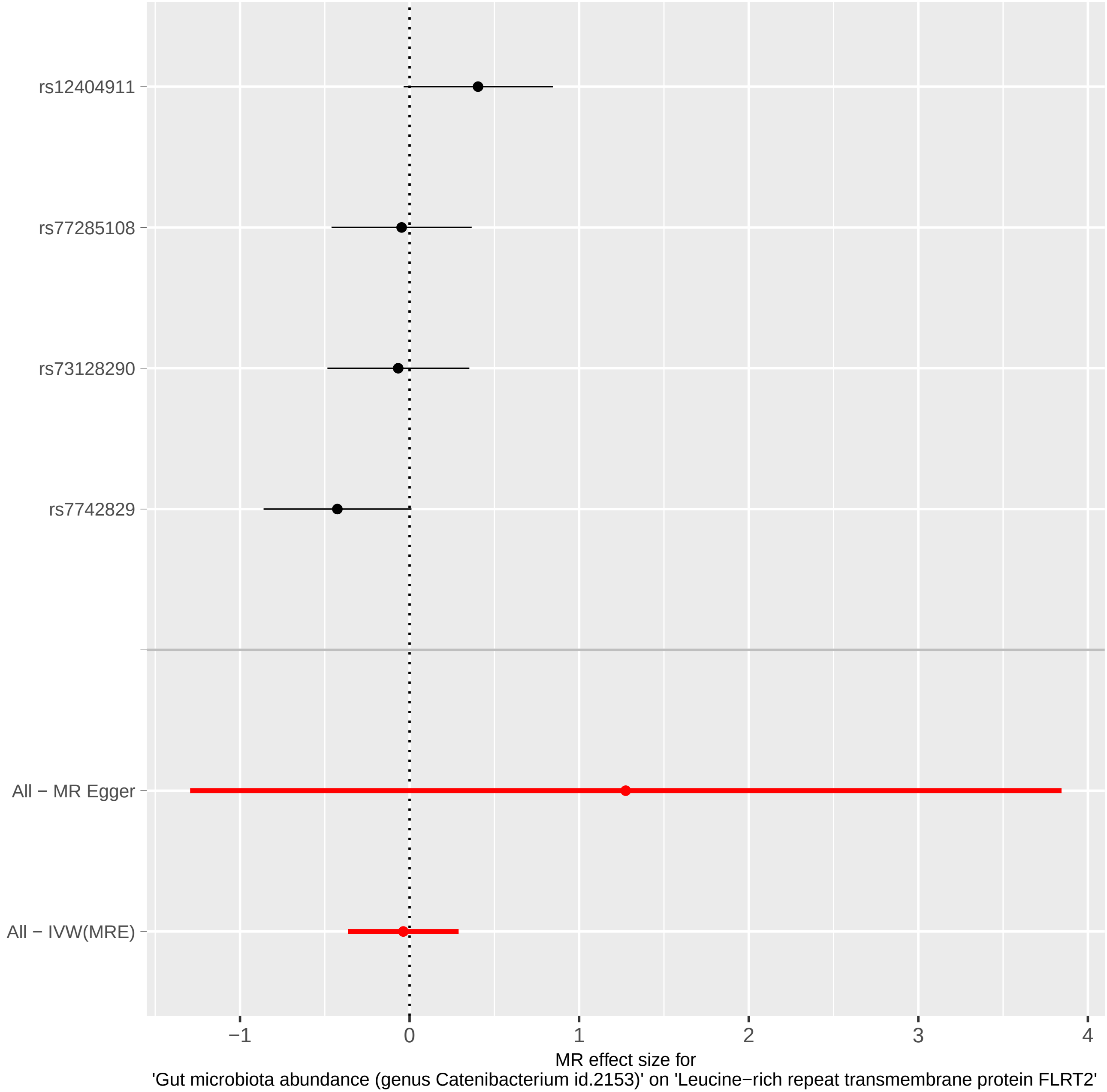

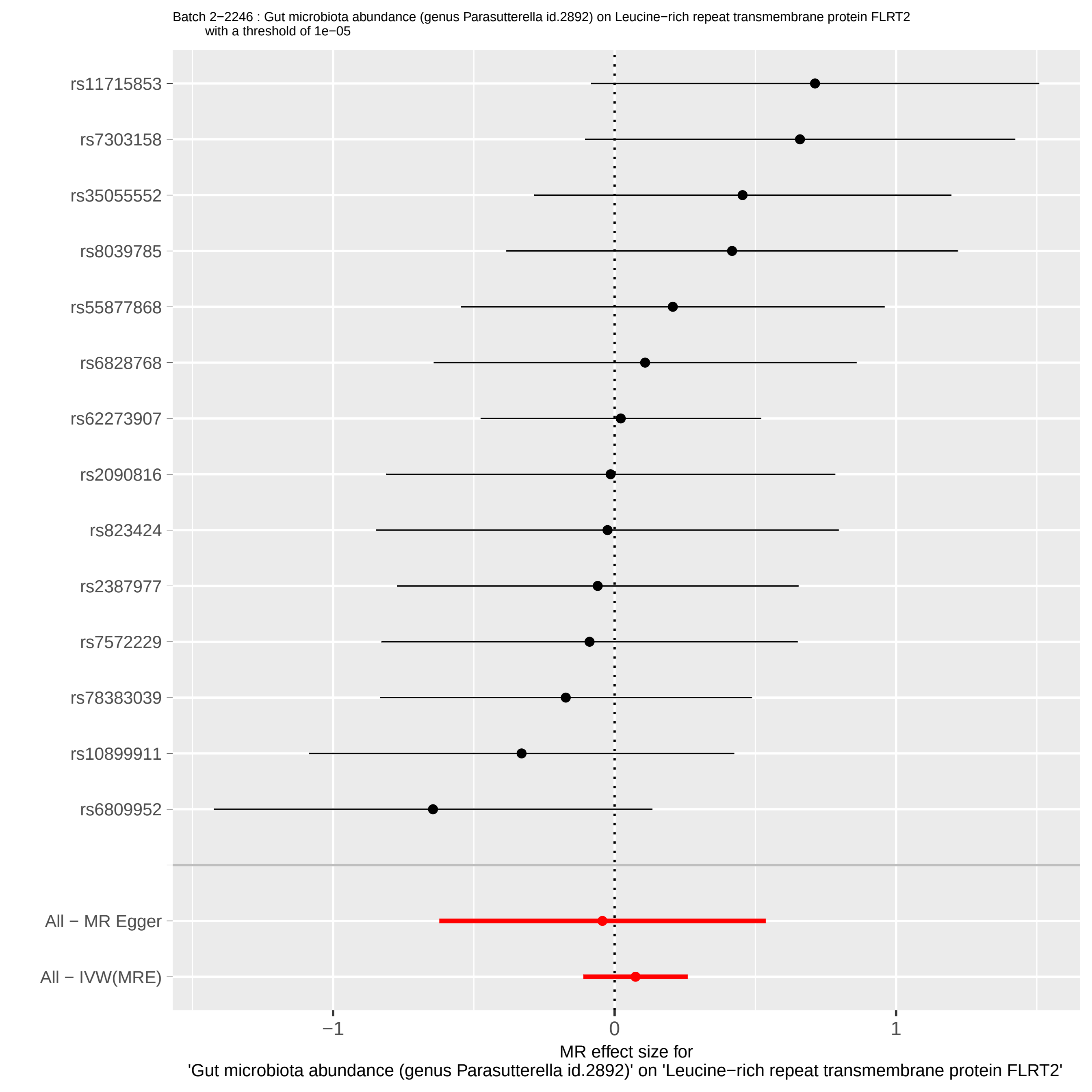
Batch 2−2246 : Gut microbiota abundance (genus Parasutterella id.2892) on Leucine−rich repeat transmembrane protein FLRT2
with a threshold of 1e−05
rs11715853
rs7303158
rs35055552
rs8039785
rs55877868
rs6828768
rs62273907
rs2090816
rs823424
rs2387977
rs7572229
rs78383039
rs10899911
rs6809952
All − MR Egger
All − IVW(MRE)
−1
0
1
MR effect size for
'Gut microbiota abundance (genus Parasutterella id.2892)' on 'Leucine−rich repeat transmembrane protein FLRT2'

Batch 2−2247 : Gut microbiota abundance (genus Catenibacterium id.2153) on Leucine−rich repeat transmembrane protein FLRT3
with a threshold of 1e−05

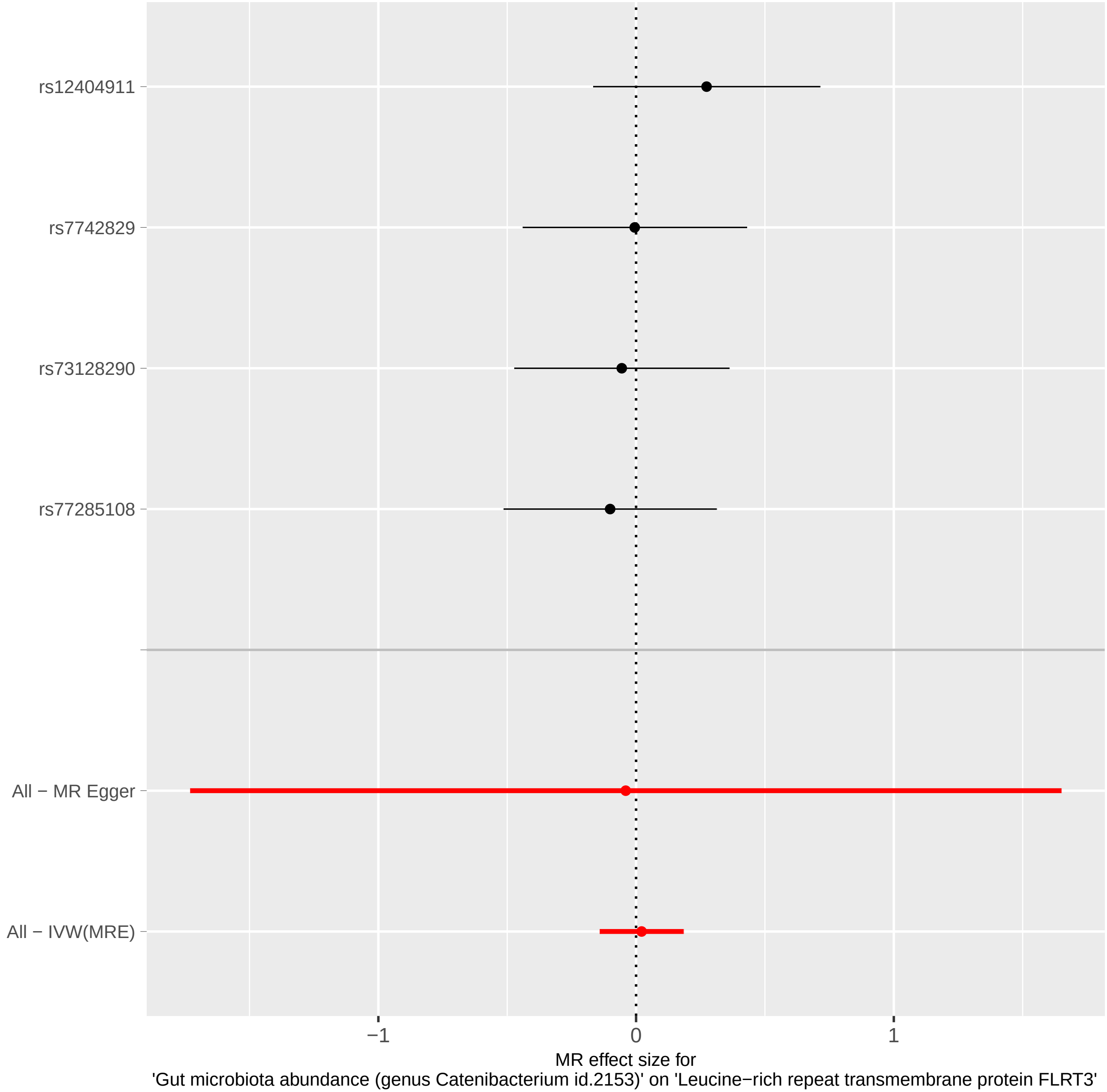

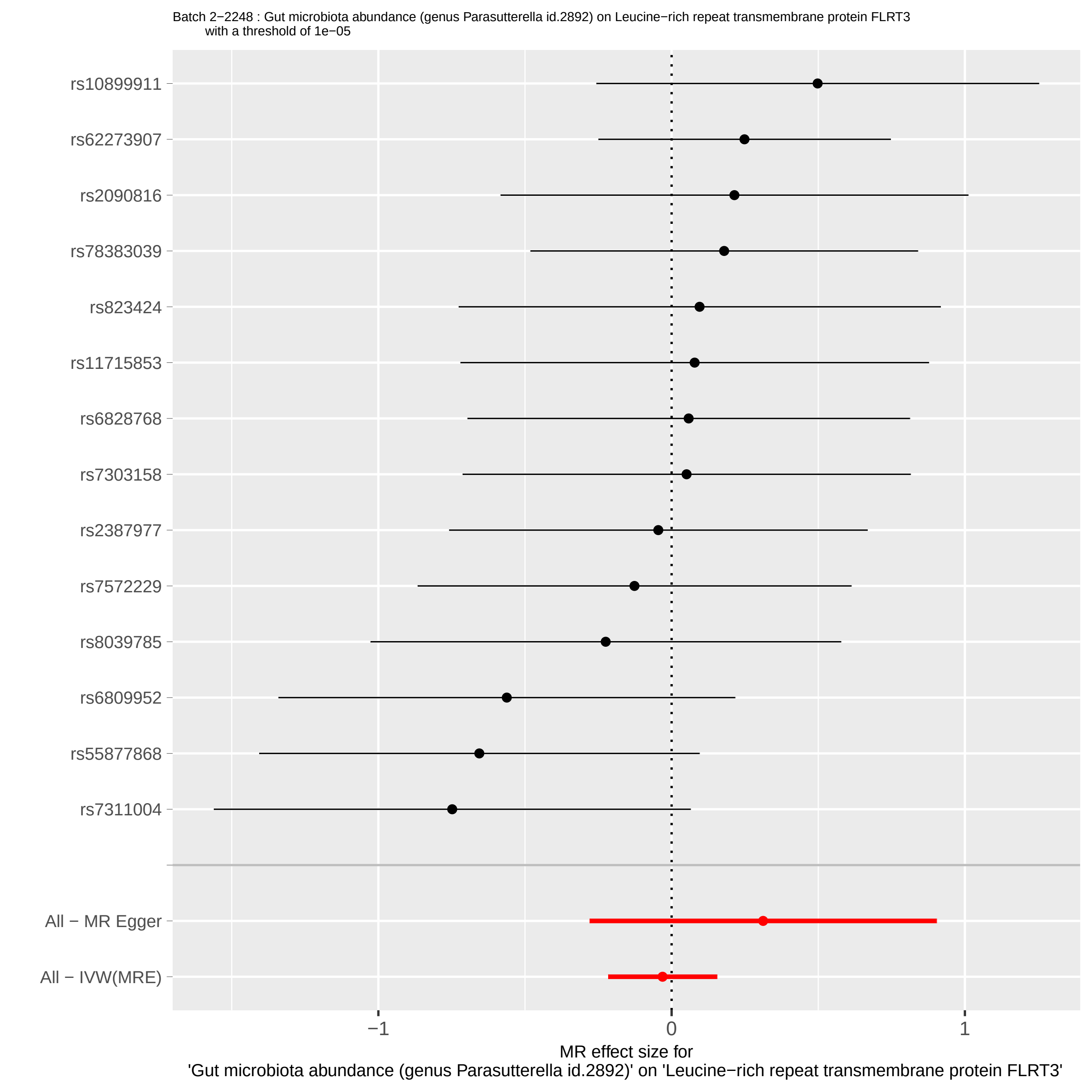
Batch 2−2248 : Gut microbiota abundance (genus Parasutterella id.2892) on Leucine−rich repeat transmembrane protein FLRT3
with a threshold of 1e−05
rs10899911
rs62273907
rs2090816
rs78383039
rs823424
rs11715853
rs6828768
rs7303158
rs2387977
rs7572229
rs8039785
rs6809952
rs55877868
rs7311004
All − MR Egger
All − IVW(MRE)
−1
0
1
MR effect size for
'Gut microbiota abundance (genus Parasutterella id.2892)' on 'Leucine−rich repeat transmembrane protein FLRT3'

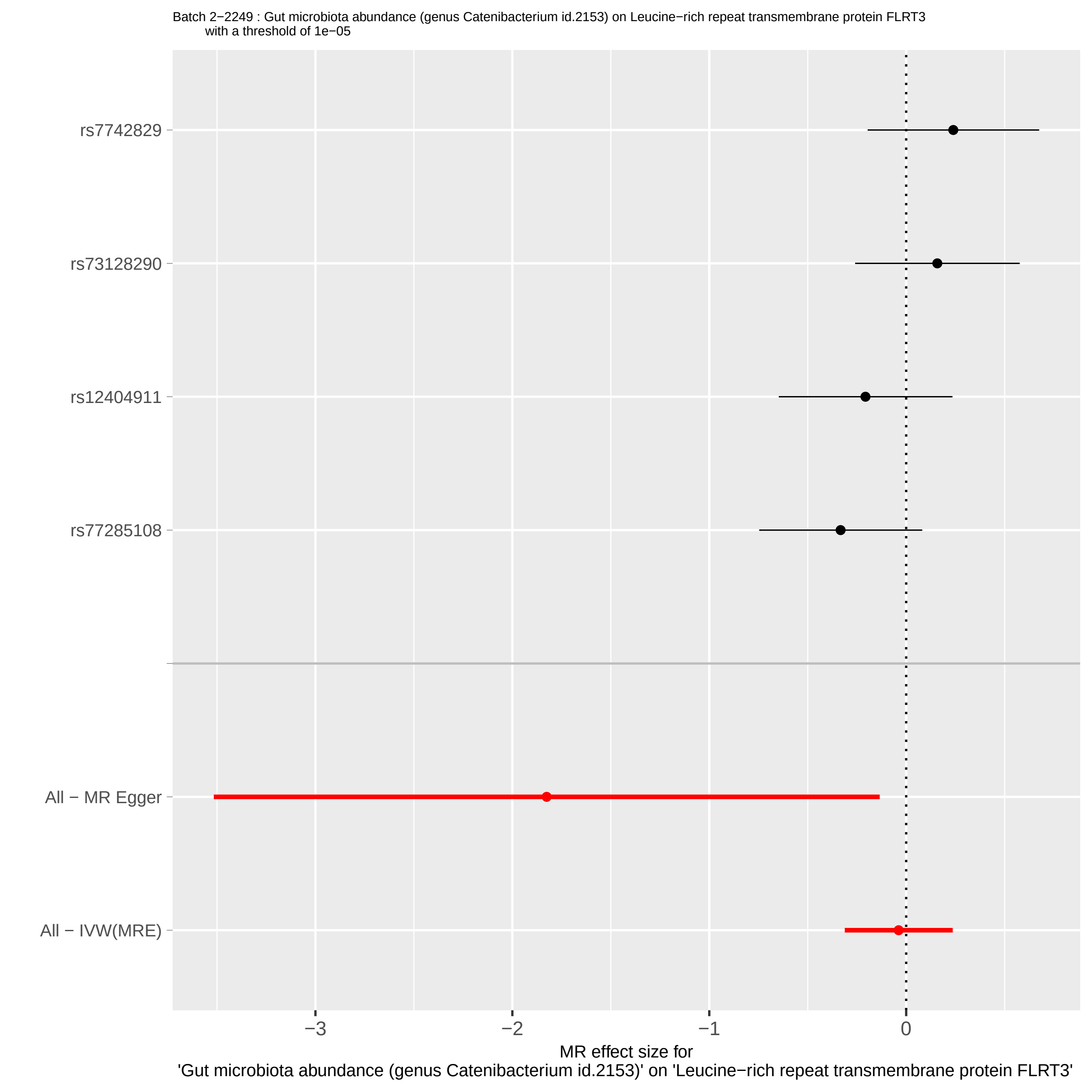
Batch 2−2249 : Gut microbiota abundance (genus Catenibacterium id.2153) on Leucine−rich repeat transmembrane protein FLRT3
with a threshold of 1e−05
rs7742829
rs73128290
rs12404911
rs77285108
All − MR Egger
All − IVW(MRE)
−3
−2
−1
0
MR effect size for
'Gut microbiota abundance (genus Catenibacterium id.2153)' on 'Leucine−rich repeat transmembrane protein FLRT3'

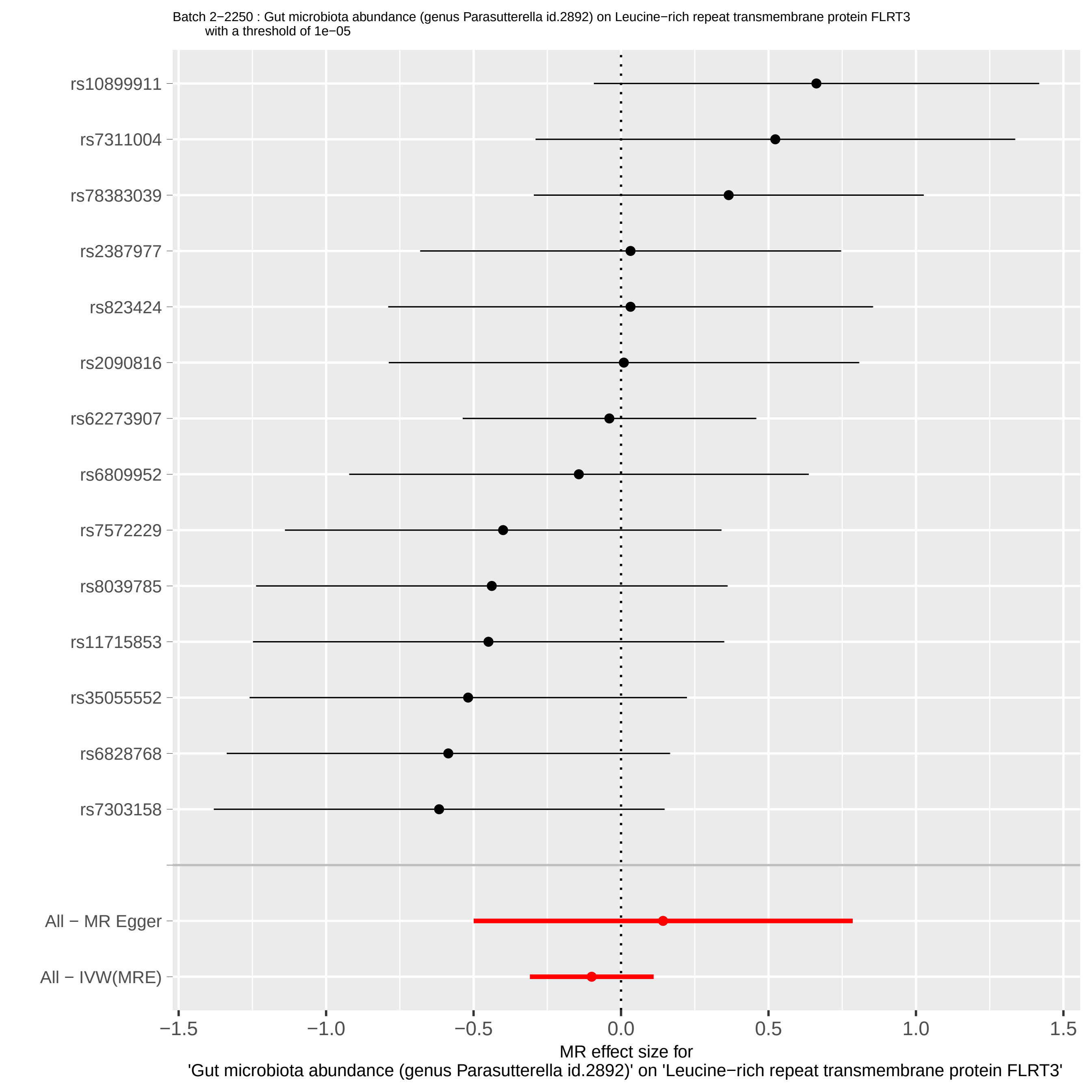

Batch 2−2250 : Gut microbiota abundance (genus Parasutterella id.2892) on Leucine−rich repeat transmembrane protein FLRT3
with a threshold of 1e−05
rs10899911
rs7311004
rs78383039
rs2387977
rs823424
rs2090816
rs62273907
rs6809952
rs7572229
rs8039785
rs11715853
rs35055552
rs6828768
rs7303158
All − MR Egger
All − IVW(MRE)
−1.5
−1.0
−0.5
0.0
0.5
1.0
1.5
MR effect size for
'Gut microbiota abundance (genus Parasutterella id.2892)' on 'Leucine−rich repeat transmembrane protein FLRT3'

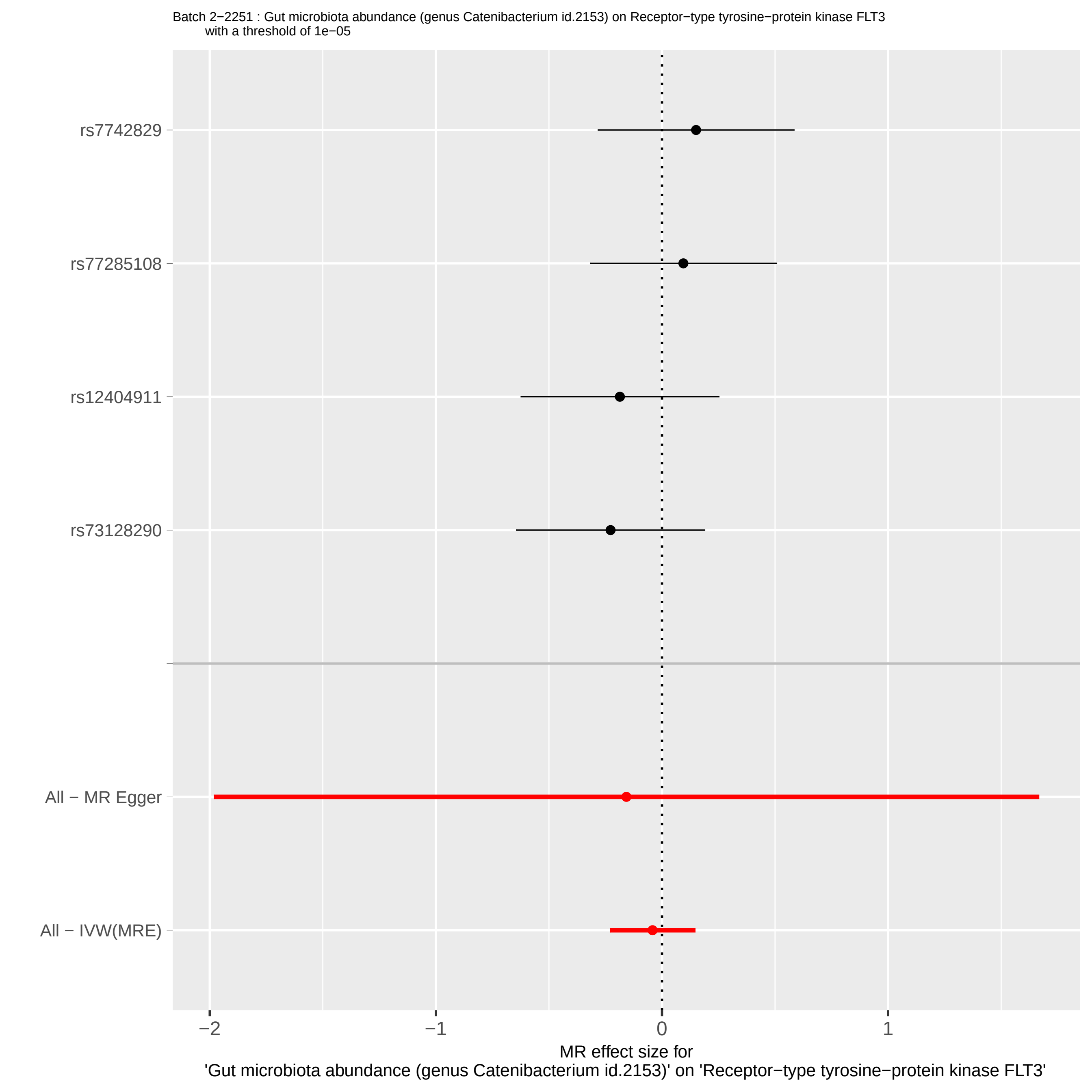

Batch 2−2251 : Gut microbiota abundance (genus Catenibacterium id.2153) on Receptor−type tyrosine−protein kinase FLT3
with a threshold of 1e−05
rs7742829
rs77285108
rs12404911
rs73128290
All − MR Egger
All − IVW(MRE)
−2
−1
0
1
MR effect size for
'Gut microbiota abundance (genus Catenibacterium id.2153)' on 'Receptor−type tyrosine−protein kinase FLT3'

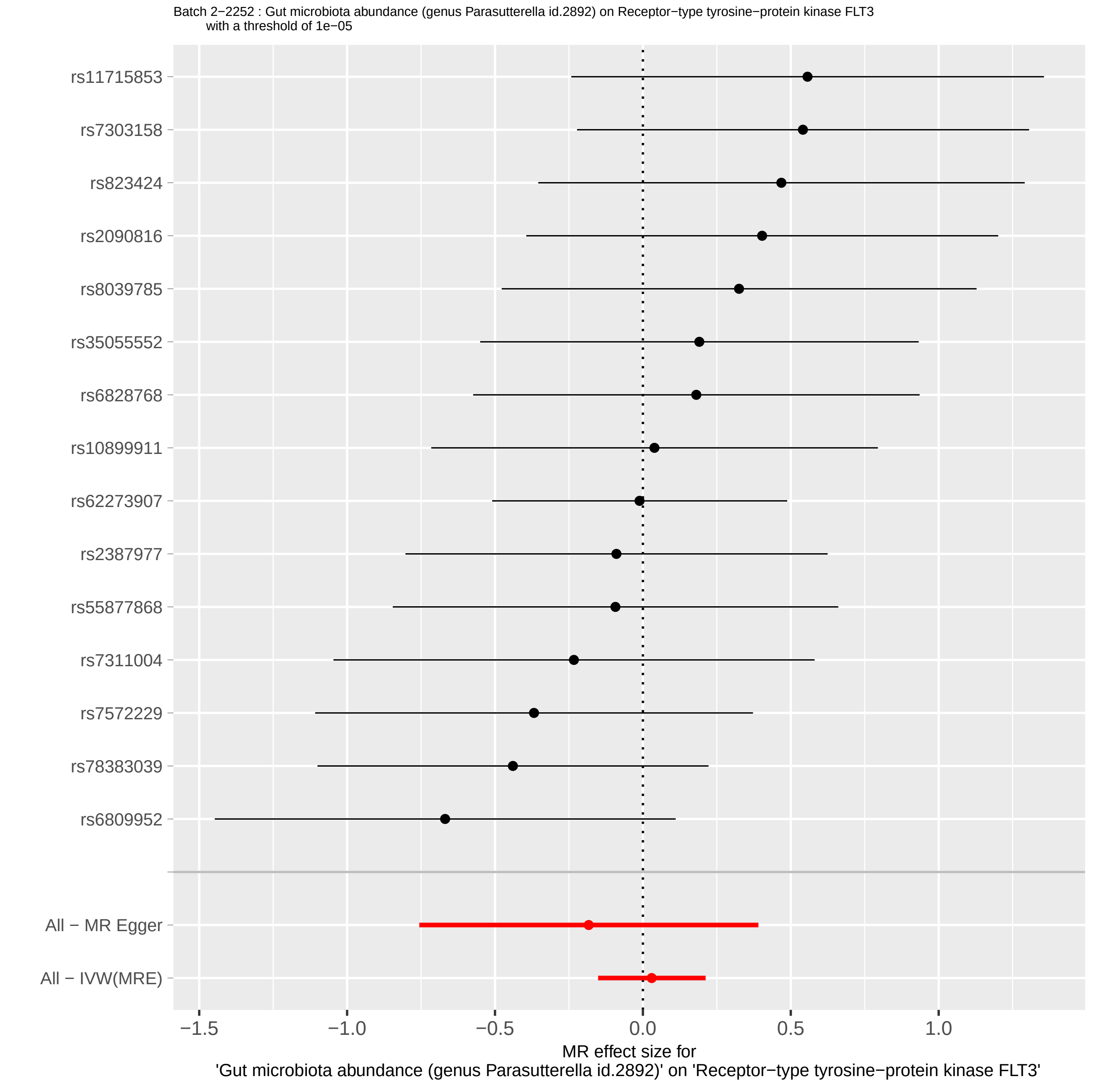

Batch 2−2252 : Gut microbiota abundance (genus Parasutterella id.2892) on Receptor−type tyrosine−protein kinase FLT3
with a threshold of 1e−05
rs11715853
rs7303158
rs823424
rs2090816
rs8039785
rs35055552
rs6828768
rs10899911
rs62273907
rs2387977
rs55877868
rs7311004
rs7572229
rs78383039
rs6809952
All − MR Egger
All − IVW(MRE)
−1.5
−1.0
−0.5
0.0
0.5
1.0
MR effect size for
'Gut microbiota abundance (genus Parasutterella id.2892)' on 'Receptor−type tyrosine−protein kinase FLT3'

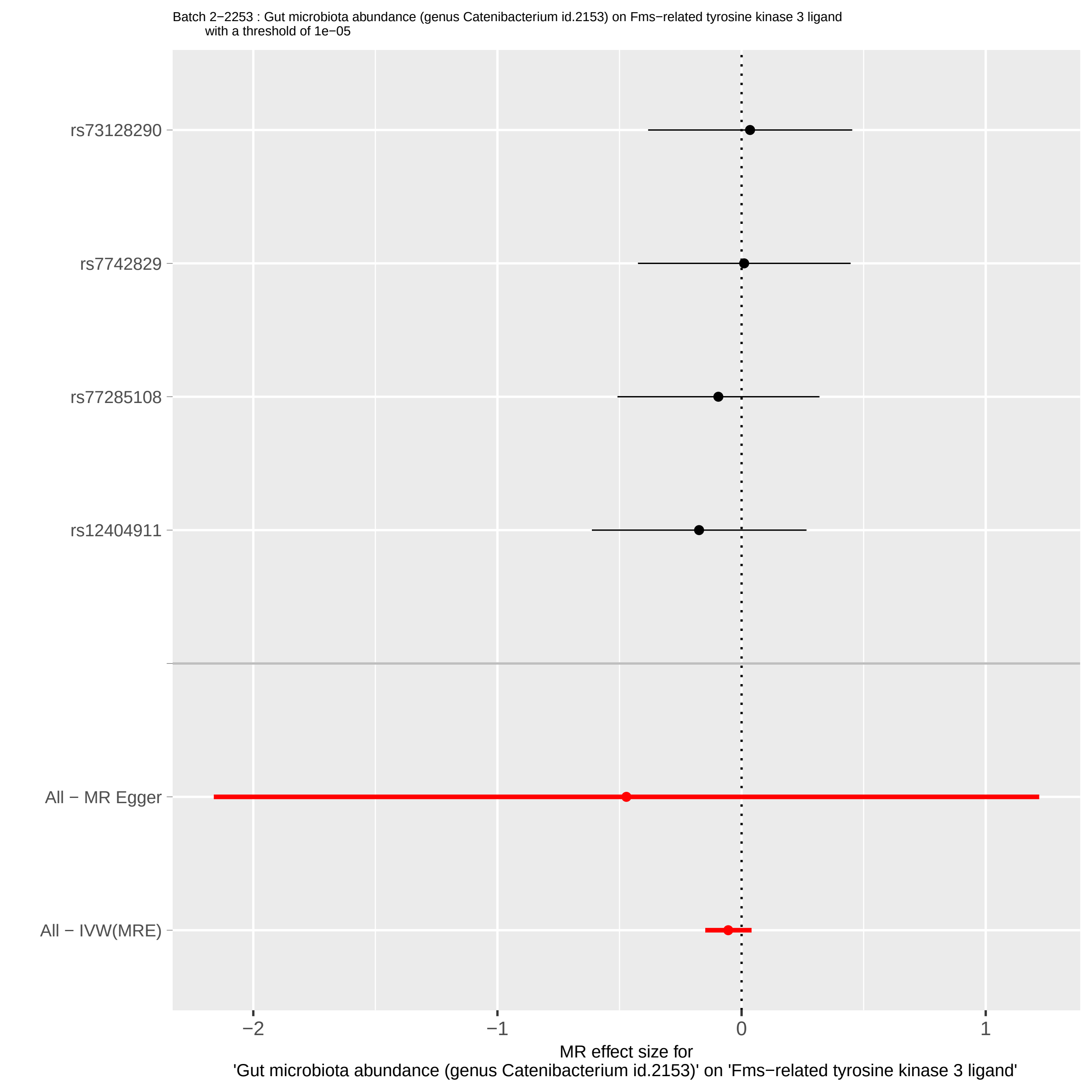

Batch 2−2253 : Gut microbiota abundance (genus Catenibacterium id.2153) on Fms−related tyrosine kinase 3 ligand
with a threshold of 1e−05
rs73128290
rs7742829
rs77285108
rs12404911
All − MR Egger
All − IVW(MRE)
−2
−1
0
1
MR effect size for
'Gut microbiota abundance (genus Catenibacterium id.2153)' on 'Fms−related tyrosine kinase 3 ligand'

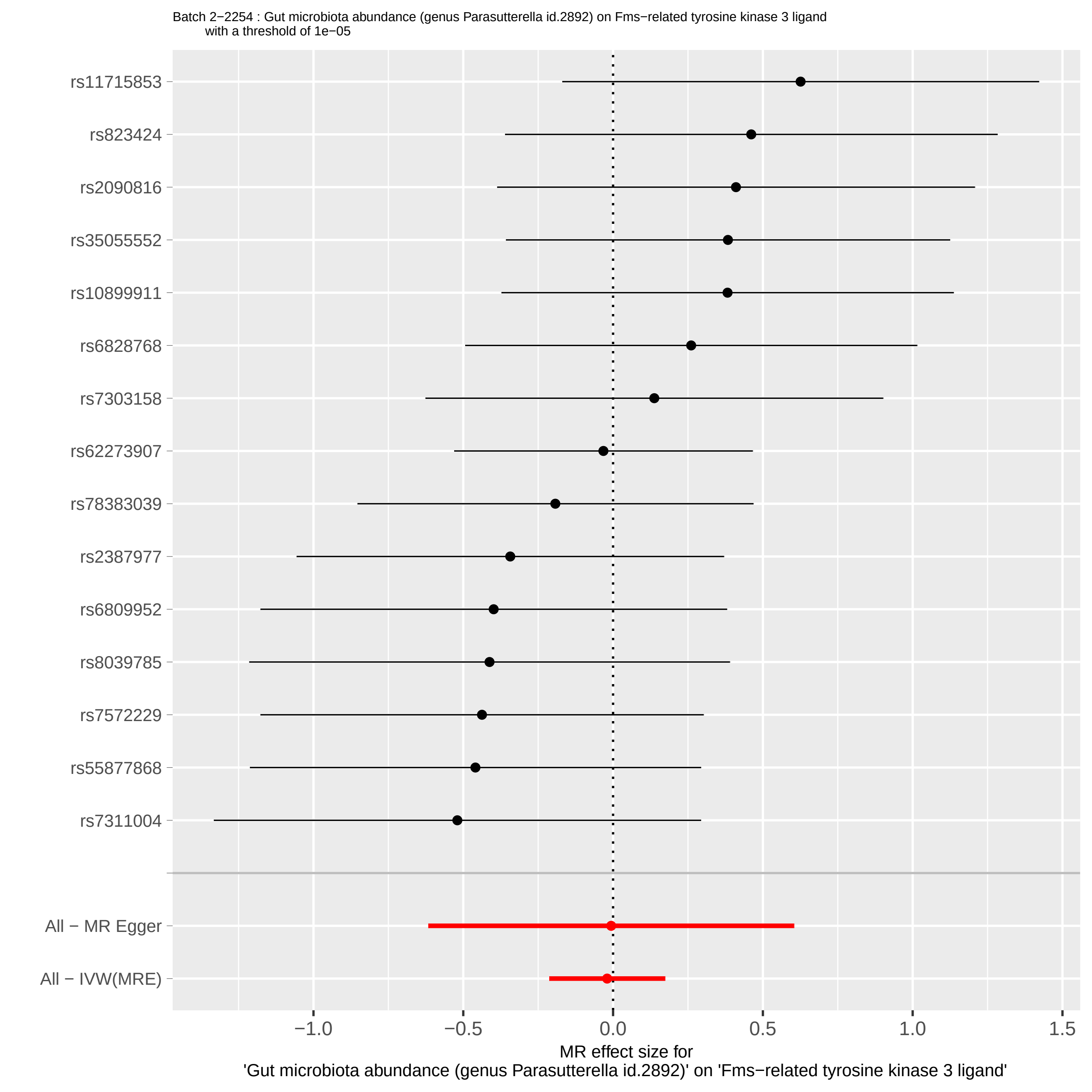

Batch 2−2254 : Gut microbiota abundance (genus Parasutterella id.2892) on Fms−related tyrosine kinase 3 ligand
with a threshold of 1e−05
rs11715853
rs823424
rs2090816
rs35055552
rs10899911
rs6828768
rs7303158
rs62273907
rs78383039
rs2387977
rs6809952
rs8039785
rs7572229
rs55877868
rs7311004
All − MR Egger
All − IVW(MRE)
−1.0
−0.5
0.0
0.5
1.0
1.5
MR effect size for
'Gut microbiota abundance (genus Parasutterella id.2892)' on 'Fms−related tyrosine kinase 3 ligand'

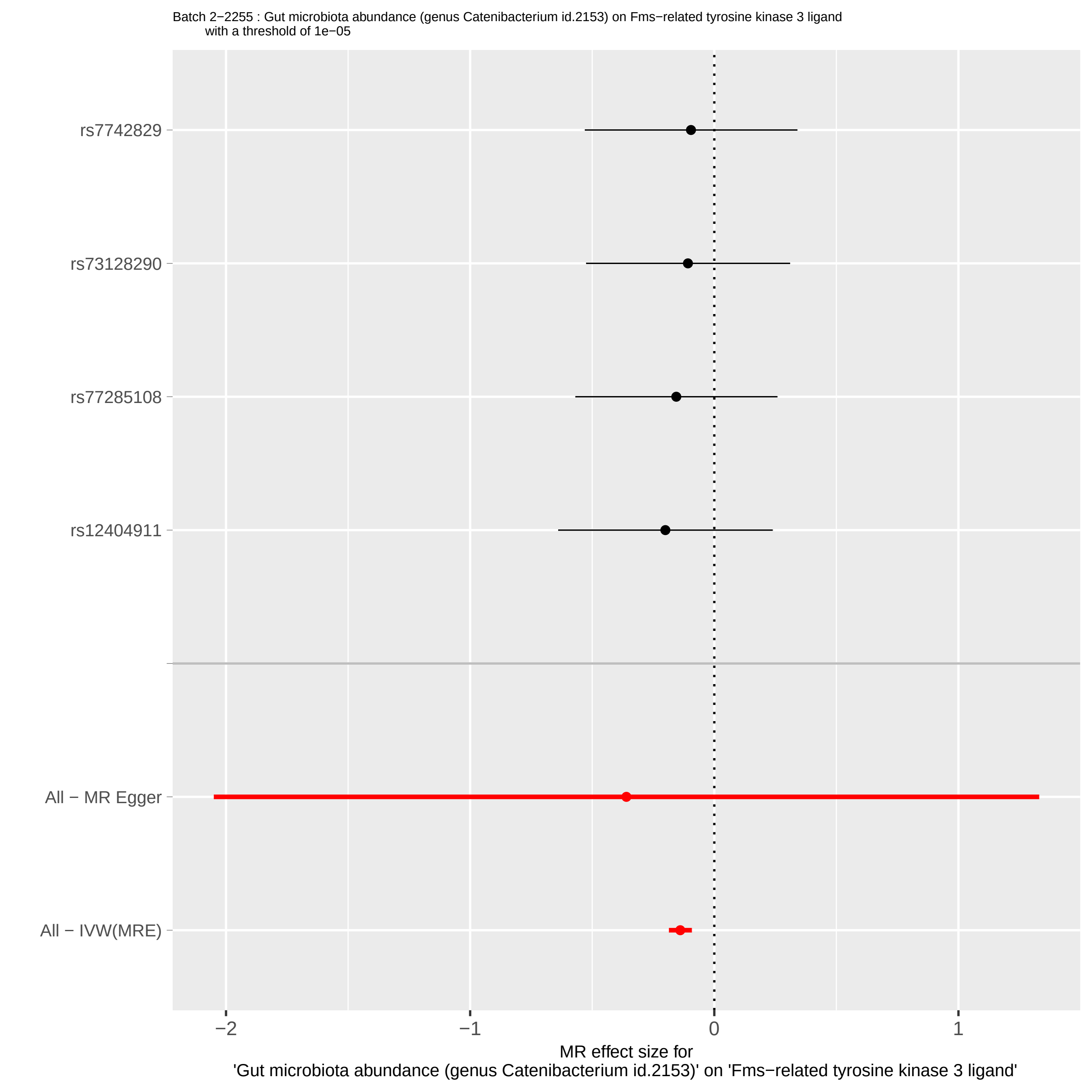
Batch 2−2255 : Gut microbiota abundance (genus Catenibacterium id.2153) on Fms−related tyrosine kinase 3 ligand
with a threshold of 1e−05
rs7742829
rs73128290
rs77285108
rs12404911
All − MR Egger
All − IVW(MRE)
−2
−1
0
1
MR effect size for
'Gut microbiota abundance (genus Catenibacterium id.2153)' on 'Fms−related tyrosine kinase 3 ligand'

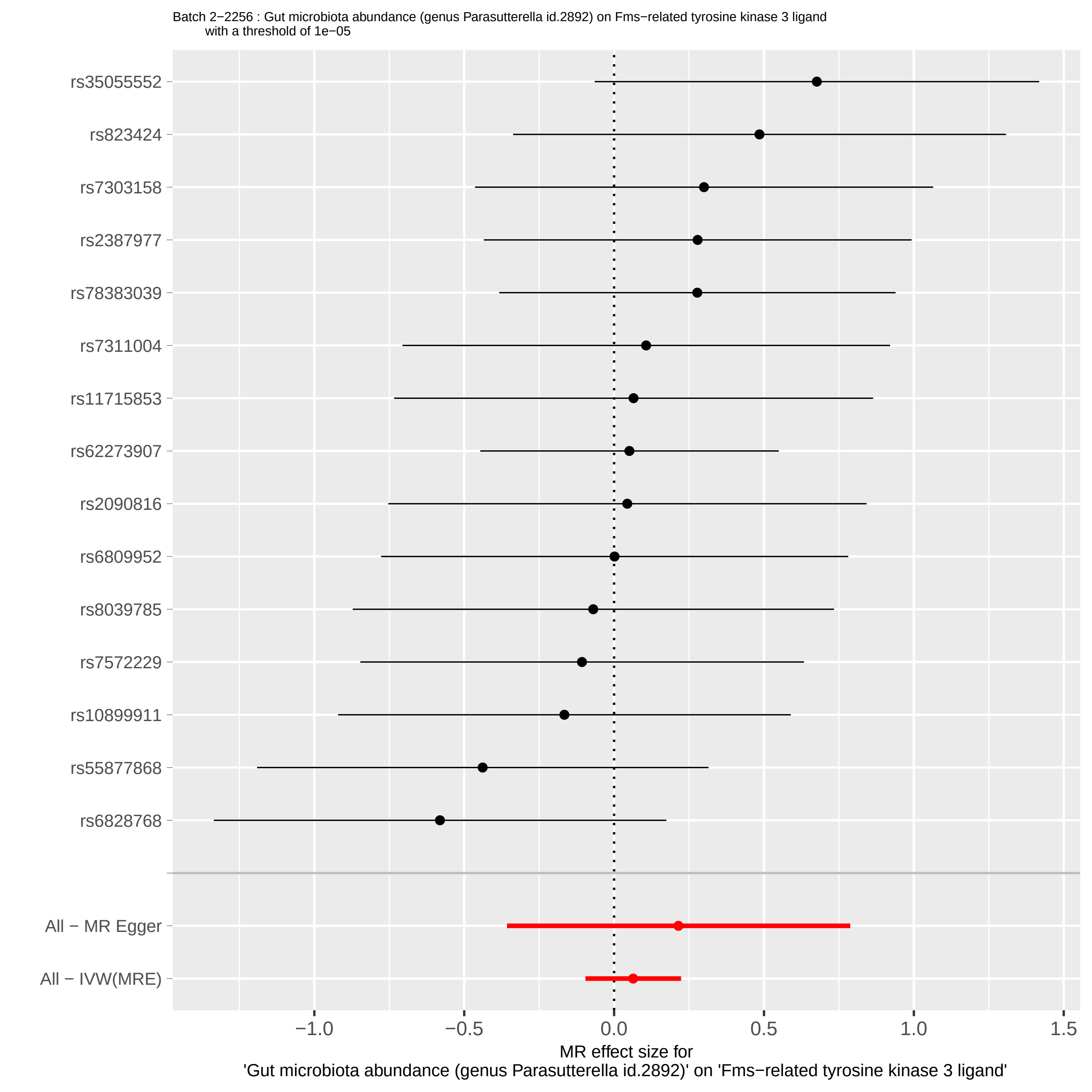

Batch 2−2256 : Gut microbiota abundance (genus Parasutterella id.2892) on Fms−related tyrosine kinase 3 ligand
with a threshold of 1e−05
rs35055552
rs823424
rs7303158
rs2387977
rs78383039
rs7311004
rs11715853
rs62273907
rs2090816
rs6809952
rs8039785
rs7572229
rs10899911
rs55877868
rs6828768
All − MR Egger
All − IVW(MRE)
−1.0
−0.5
0.0
0.5
1.0
1.5
MR effect size for
'Gut microbiota abundance (genus Parasutterella id.2892)' on 'Fms−related tyrosine kinase 3 ligand'

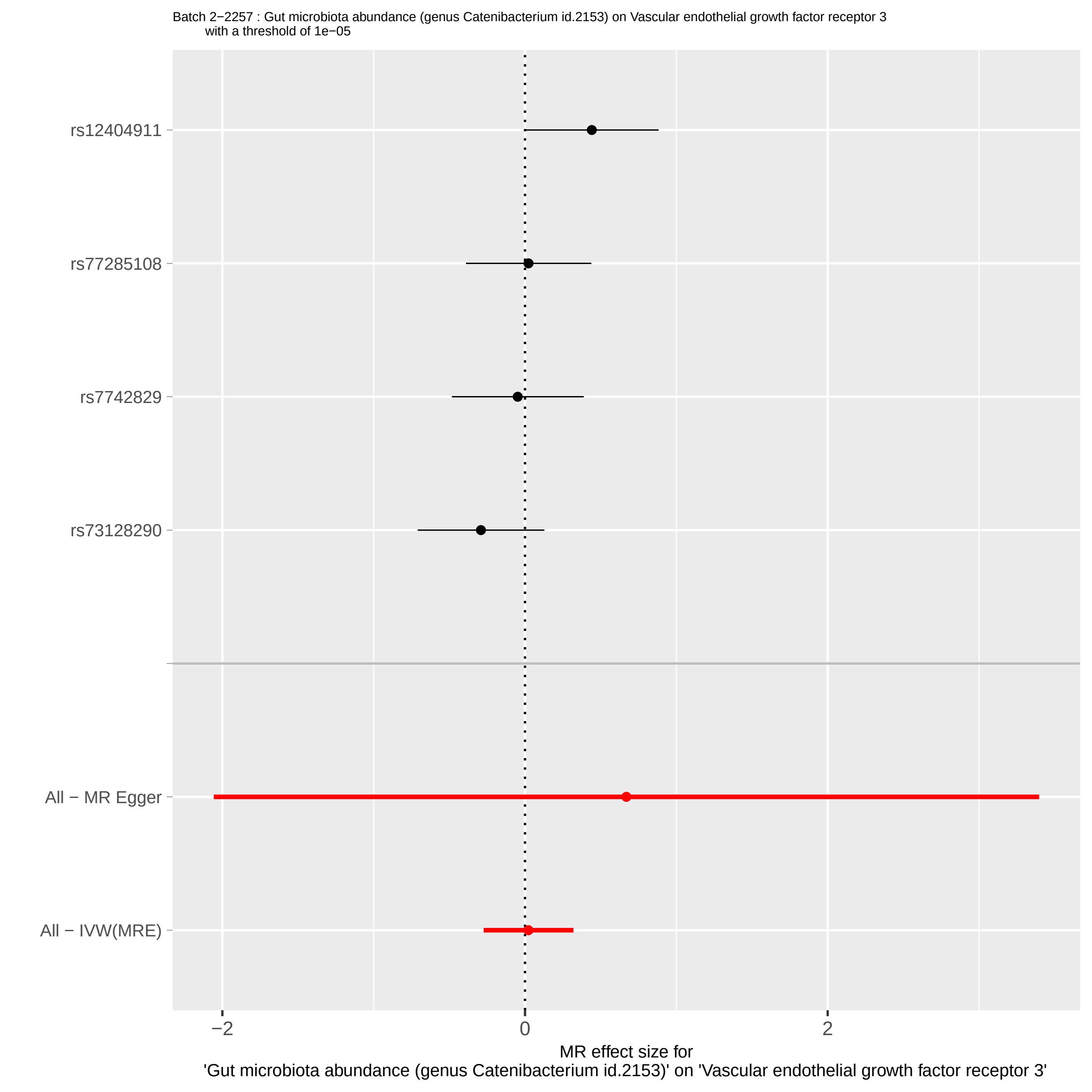

Batch 2−2257 : Gut microbiota abundance (genus Catenibacterium id.2153) on Vascular endothelial growth factor receptor 3
with a threshold of 1e−05
rs12404911
rs77285108
rs7742829
rs73128290
All − MR Egger
All − IVW(MRE)
−2
0
2
MR effect size for
'Gut microbiota abundance (genus Catenibacterium id.2153)' on 'Vascular endothelial growth factor receptor 3'

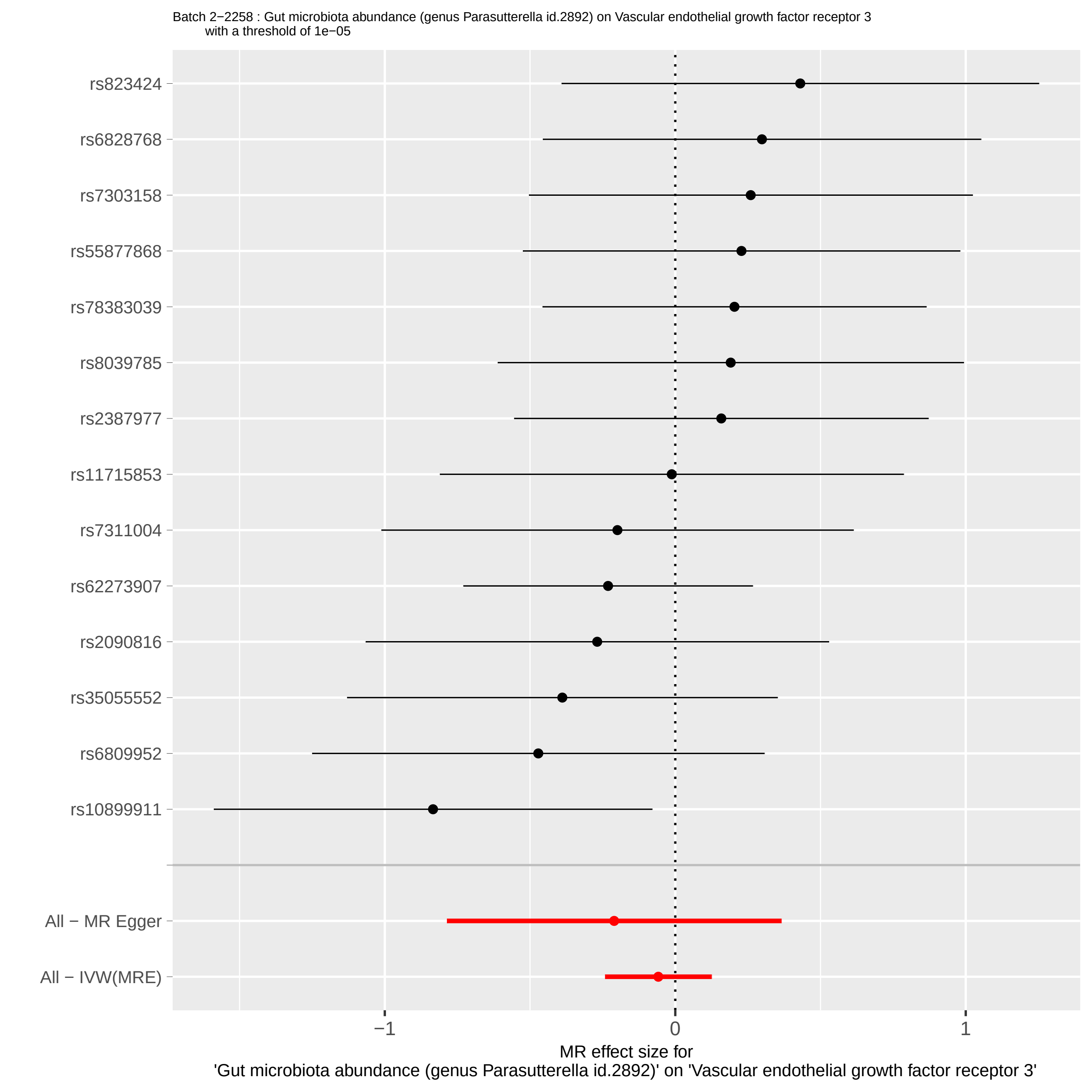
Batch 2−2258 : Gut microbiota abundance (genus Parasutterella id.2892) on Vascular endothelial growth factor receptor 3
with a threshold of 1e−05
rs823424
rs6828768
rs7303158
rs55877868
rs78383039
rs8039785
rs2387977
rs11715853
rs7311004
rs62273907
rs2090816
rs35055552
rs6809952
rs10899911
All − MR Egger
All − IVW(MRE)
−1
0
1
MR effect size for
'Gut microbiota abundance (genus Parasutterella id.2892)' on 'Vascular endothelial growth factor receptor 3'

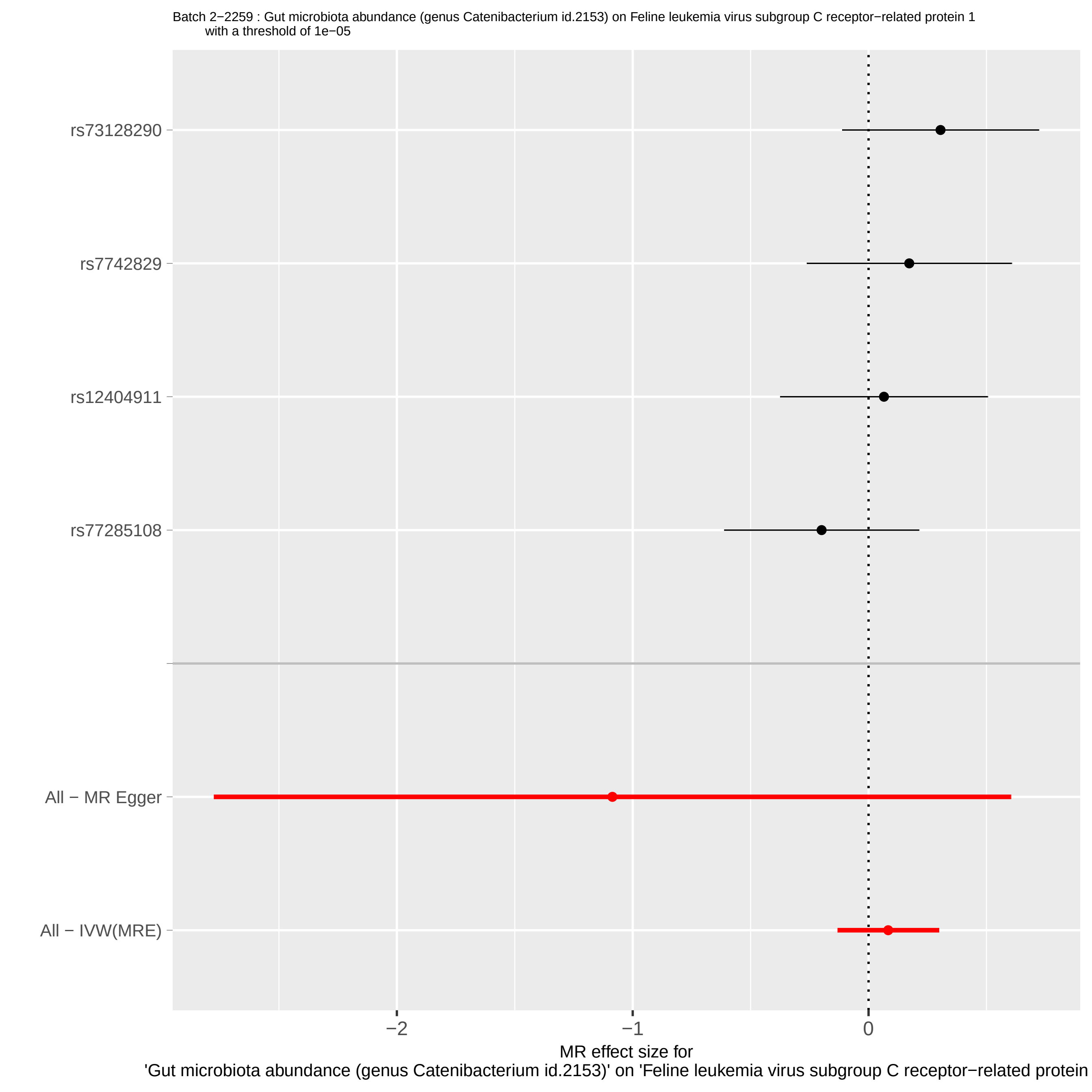

Batch 2−2259 : Gut microbiota abundance (genus Catenibacterium id.2153) on Feline leukemia virus subgroup C receptor−related protein 1 with a threshold of 1e−05
rs73128290
rs7742829
rs12404911
rs77285108
All − MR Egger
All − IVW(MRE)
−2
−1
0
MR effect size for
'Gut microbiota abundance (genus Catenibacterium id.2153)' on 'Feline leukemia virus subgroup C receptor−related protein

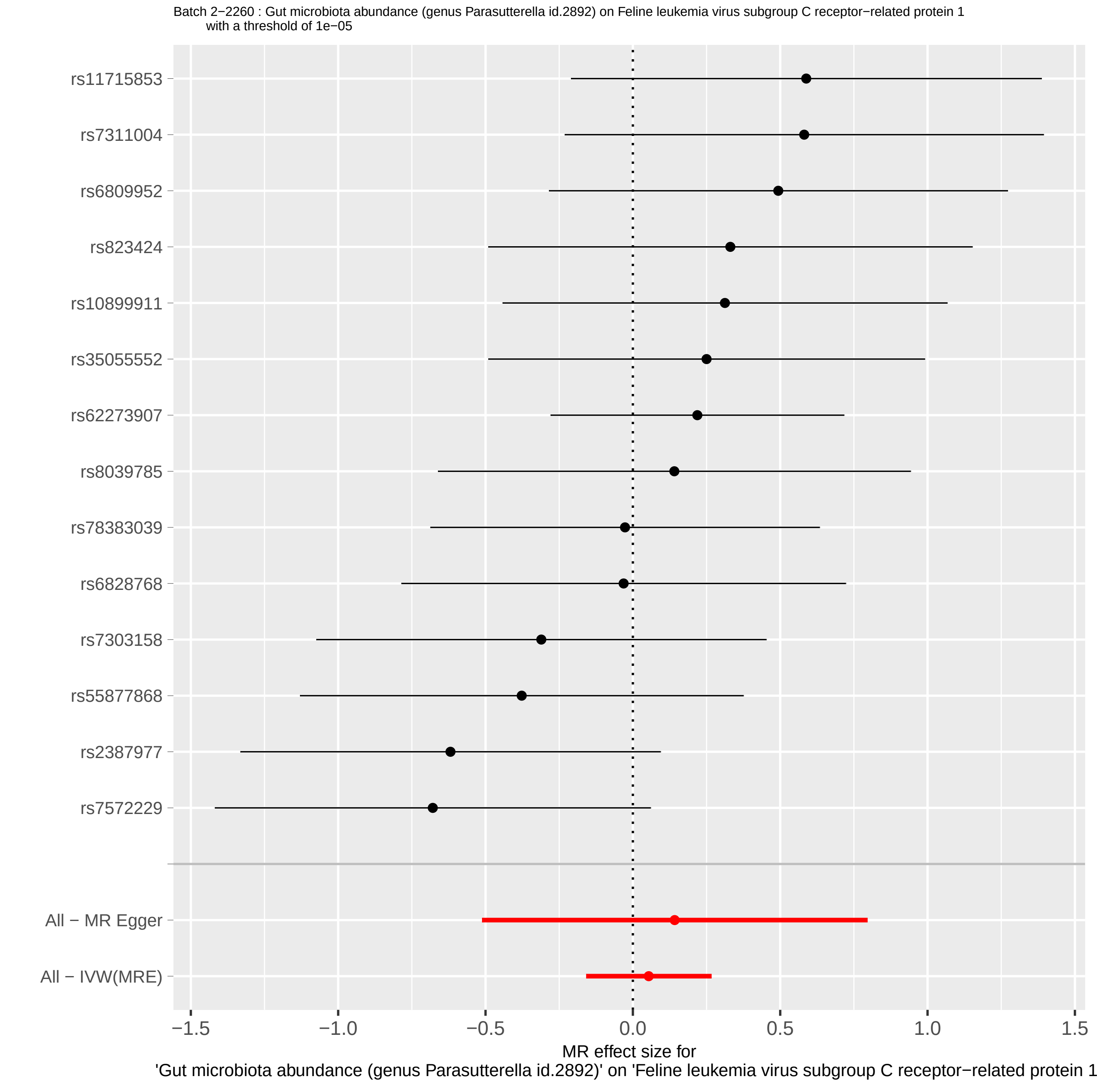

Batch 2−2260 : Gut microbiota abundance (genus Parasutterella id.2892) on Feline leukemia virus subgroup C receptor−related protein 1 with a threshold of 1e−05
rs11715853
rs7311004
rs6809952
rs823424
rs10899911
rs35055552
rs62273907
rs8039785
rs78383039
rs6828768
rs7303158
rs55877868
rs2387977
rs7572229
All − MR Egger
All − IVW(MRE)
−1.5
−1.0
−0.5
0.0
0.5
1.0
1.5
MR effect size for
'Gut microbiota abundance (genus Parasutterella id.2892)' on 'Feline leukemia virus subgroup C receptor−related protein 1

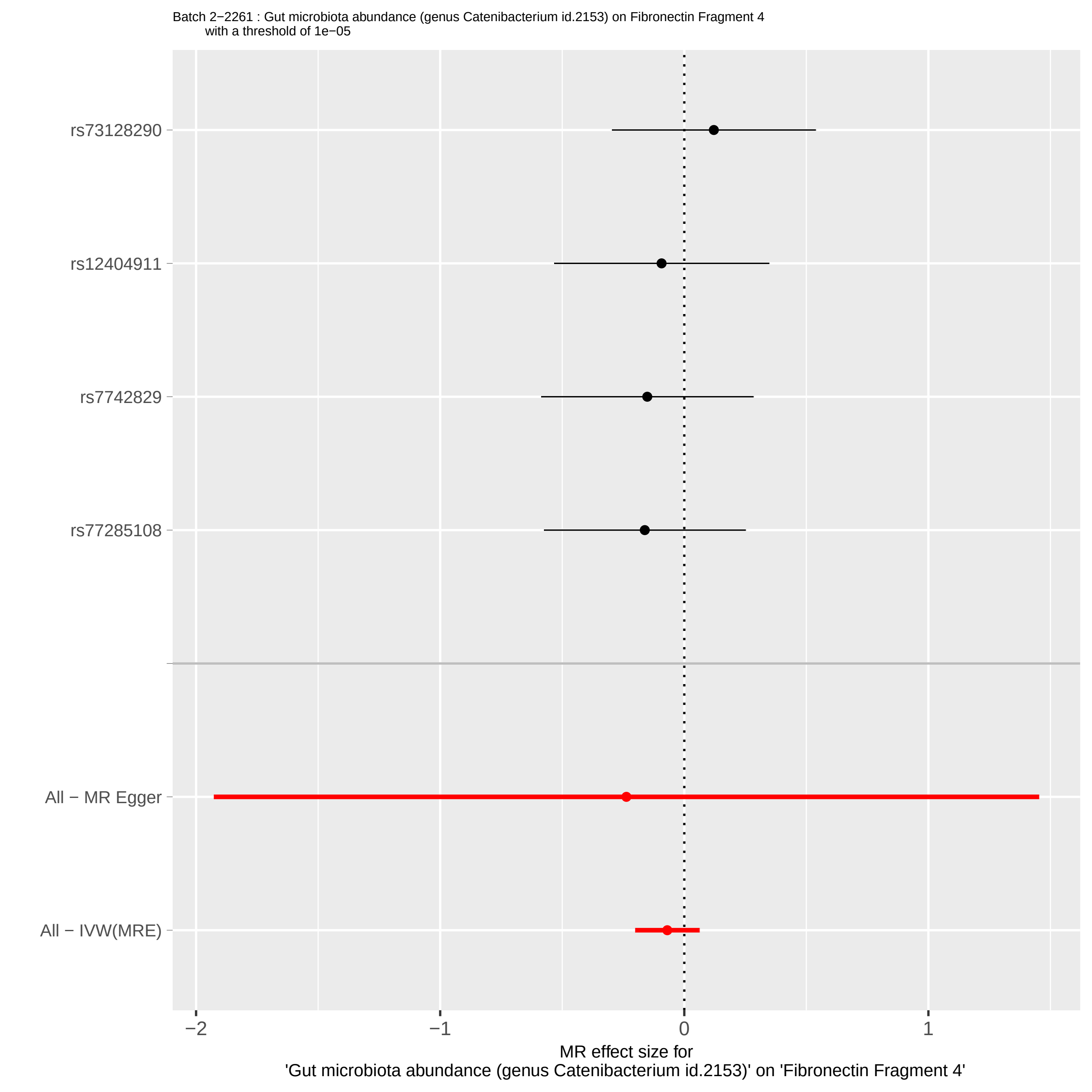

Batch 2−2261 : Gut microbiota abundance (genus Catenibacterium id.2153) on Fibronectin Fragment 4
with a threshold of 1e−05
rs73128290
rs12404911
rs7742829
rs77285108
All − MR Egger
All − IVW(MRE)
−2
−1
0
1
MR effect size for
'Gut microbiota abundance (genus Catenibacterium id.2153)' on 'Fibronectin Fragment 4'

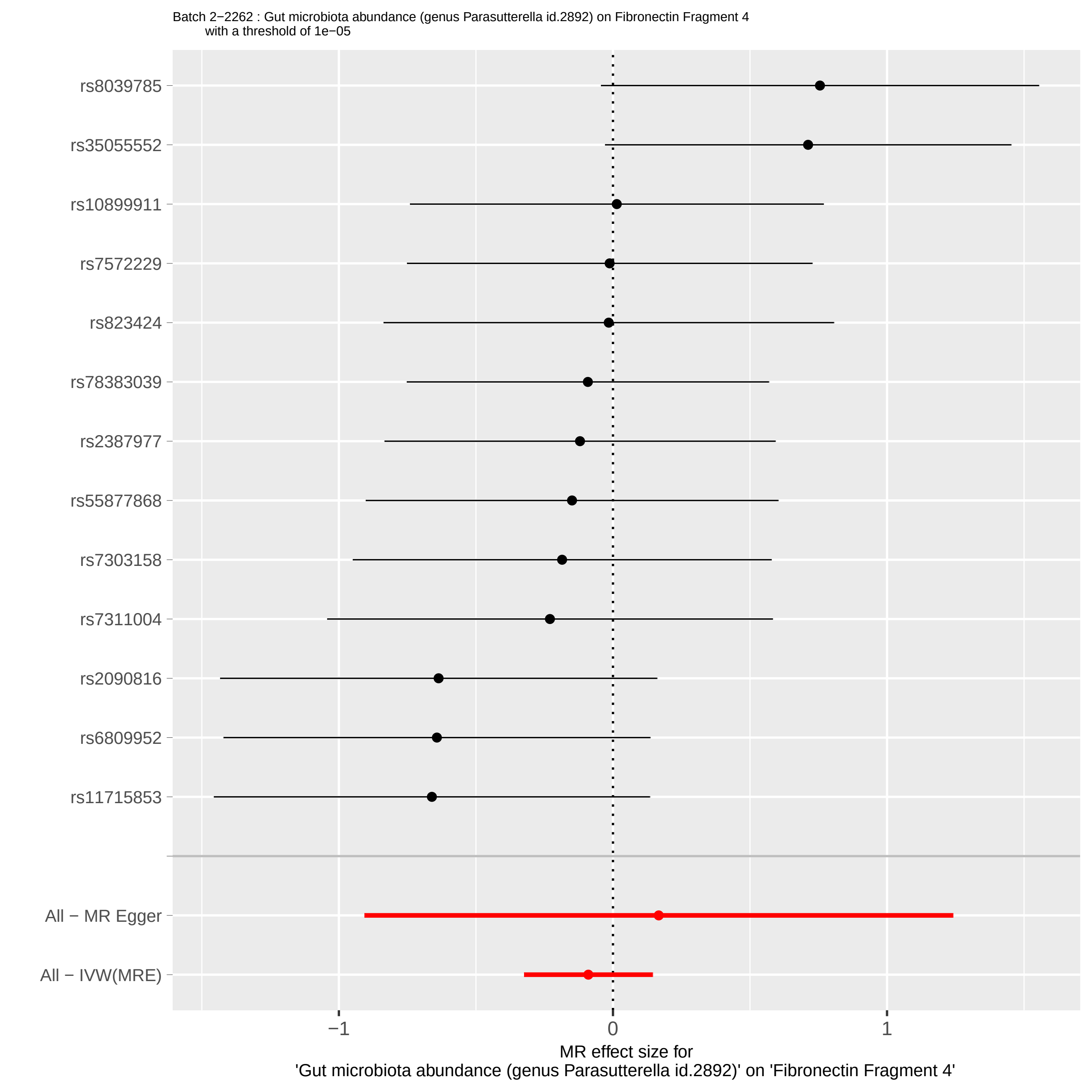
Batch 2−2262 : Gut microbiota abundance (genus Parasutterella id.2892) on Fibronectin Fragment 4
with a threshold of 1e−05
rs8039785
rs35055552
rs10899911
rs7572229
rs823424
rs78383039
rs2387977
rs55877868
rs7303158
rs7311004
rs2090816
rs6809952
rs11715853
All − MR Egger
All − IVW(MRE)
−1
0
1
MR effect size for
'Gut microbiota abundance (genus Parasutterella id.2892)' on 'Fibronectin Fragment 4'

Batch 2−2263 : Gut microbiota abundance (genus Catenibacterium id.2153) on Fibronectin type III domain−containing protein 4
with a threshold of 1e−05

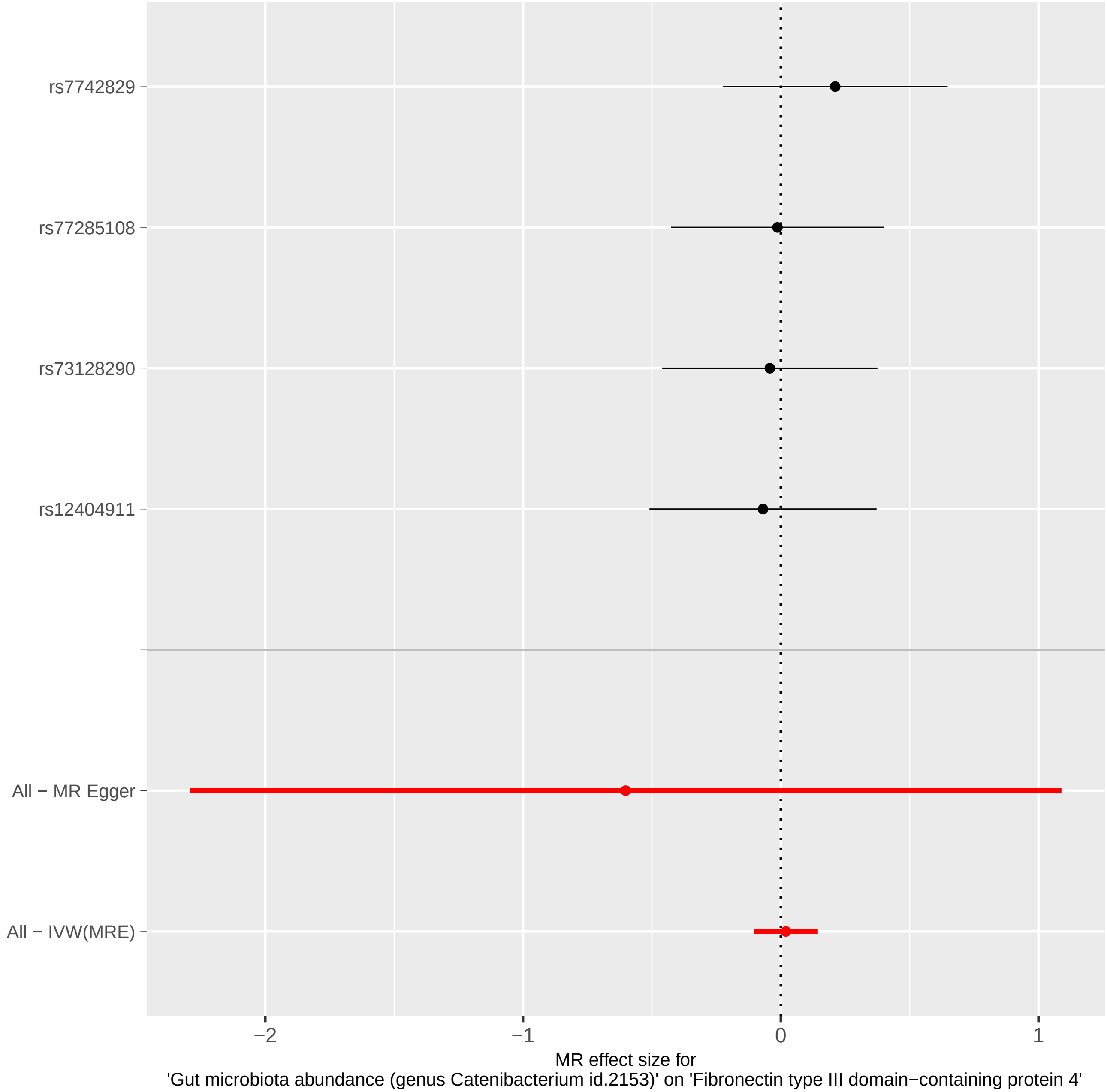

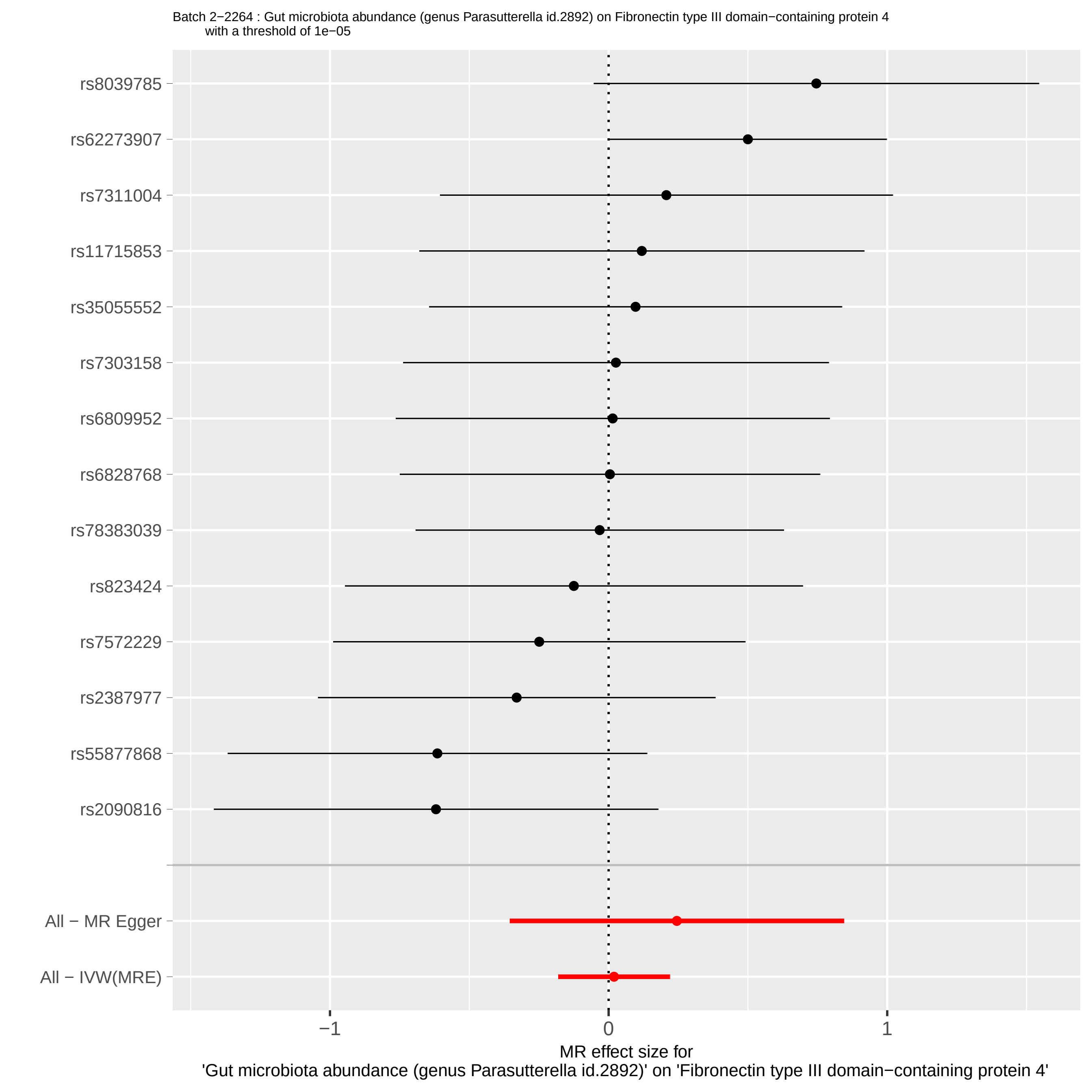
Batch 2−2264 : Gut microbiota abundance (genus Parasutterella id.2892) on Fibronectin type III domain−containing protein 4
with a threshold of 1e−05
rs8039785
rs62273907
rs7311004
rs11715853
rs35055552
rs7303158
rs6809952
rs6828768
rs78383039
rs823424
rs7572229
rs2387977
rs55877868
rs2090816
All − MR Egger
All − IVW(MRE)
−1
0
1
MR effect size for
'Gut microbiota abundance (genus Parasutterella id.2892)' on 'Fibronectin type III domain−containing protein 4'

Batch 2−2265 : Gut microbiota abundance (genus Catenibacterium id.2153) on Fibronectin type III domain−containing protein 5
with a threshold of 1e−05

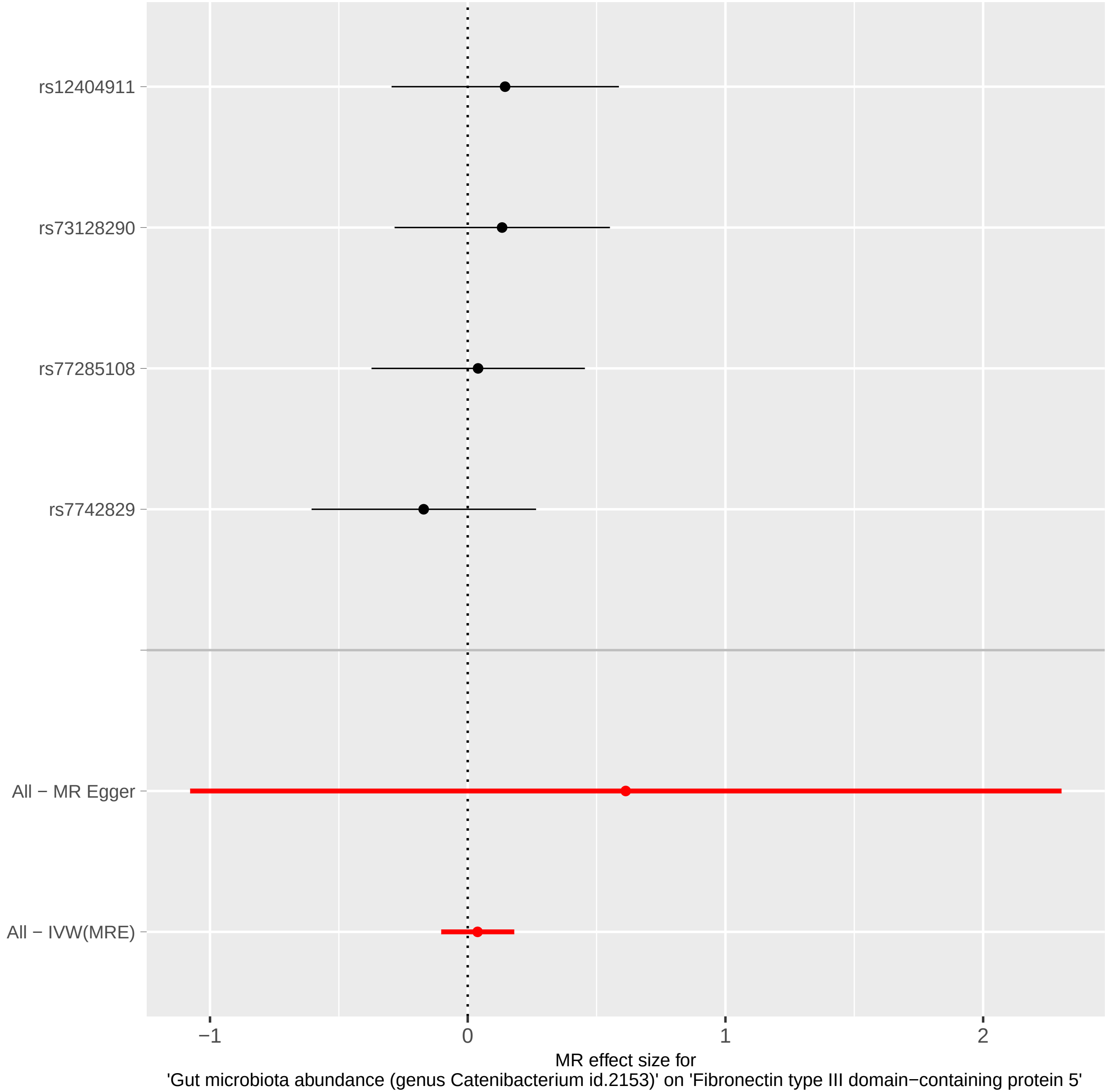

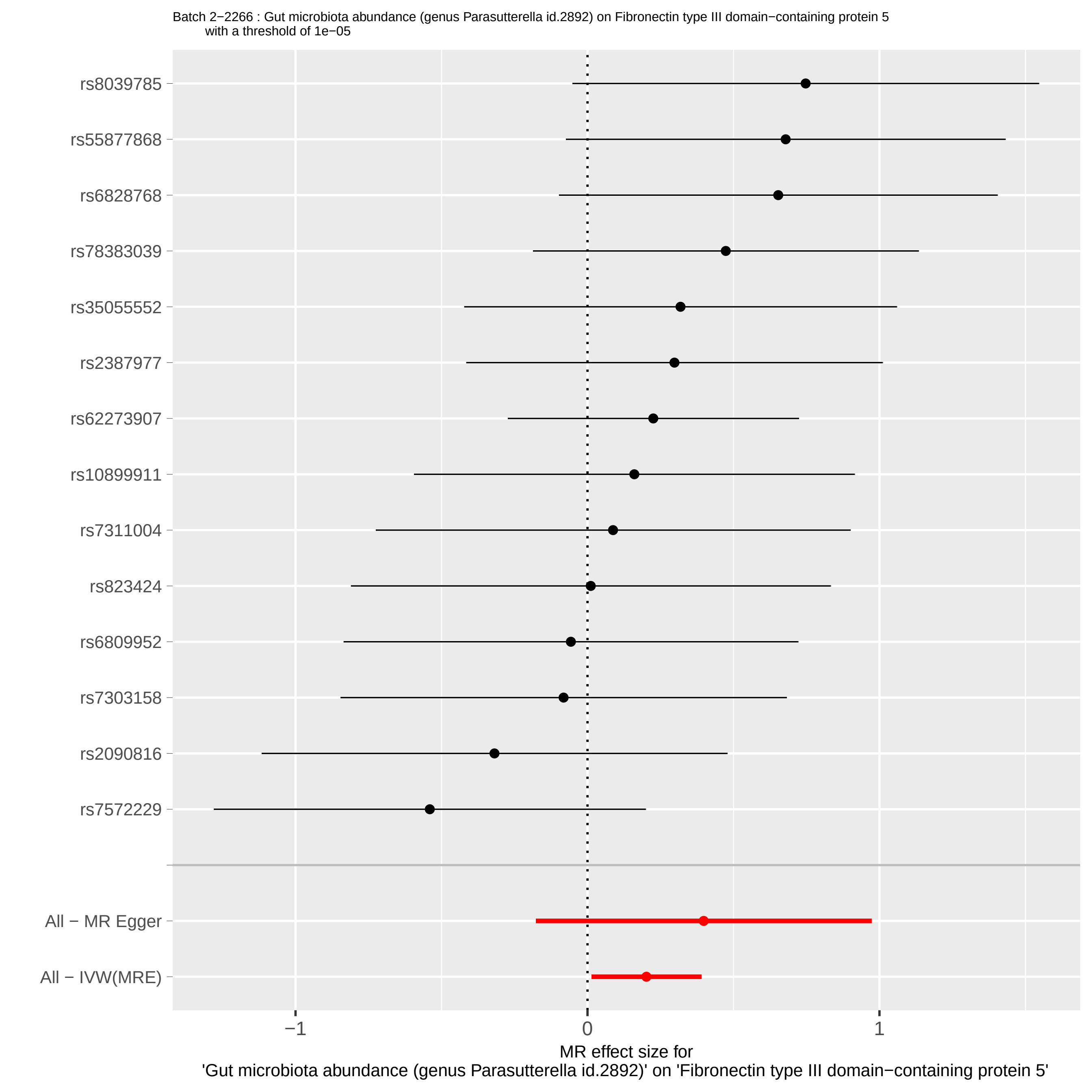

Batch 2−2266 : Gut microbiota abundance (genus Parasutterella id.2892) on Fibronectin type III domain−containing protein 5
with a threshold of 1e−05
rs8039785
rs55877868
rs6828768
rs78383039
rs35055552
rs2387977
rs62273907
rs10899911
rs7311004
rs823424
rs6809952
rs7303158
rs2090816
rs7572229
All − MR Egger
All − IVW(MRE)
−1
0
1
MR effect size for
'Gut microbiota abundance (genus Parasutterella id.2892)' on 'Fibronectin type III domain−containing protein 5'

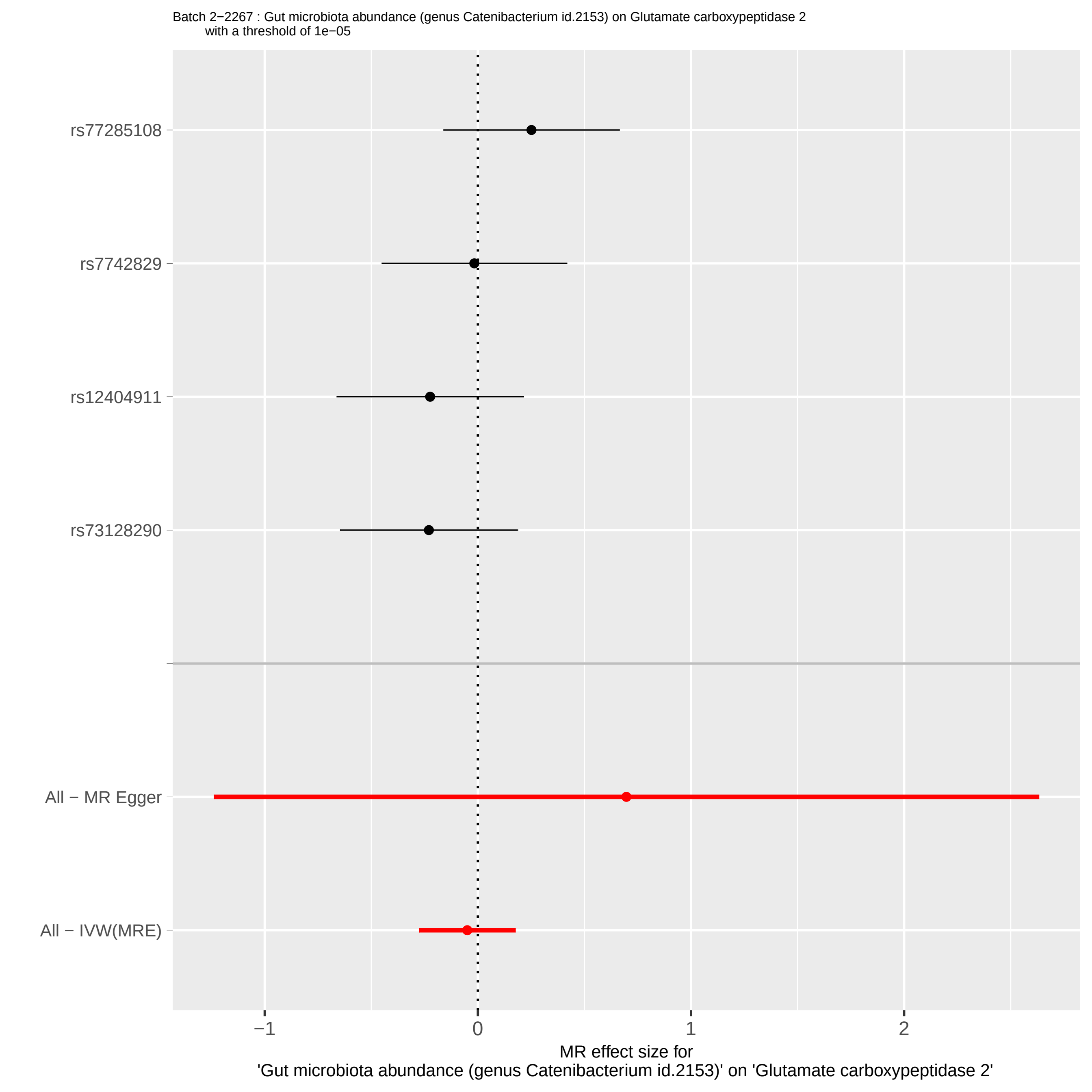
Batch 2−2267 : Gut microbiota abundance (genus Catenibacterium id.2153) on Glutamate carboxypeptidase 2
with a threshold of 1e−05
rs77285108
rs7742829
rs12404911
rs73128290
All − MR Egger
All − IVW(MRE)
−1
0
1
2
MR effect size for
'Gut microbiota abundance (genus Catenibacterium id.2153)' on 'Glutamate carboxypeptidase 2'

Batch 2−2268 : Gut microbiota abundance (genus Parasutterella id.2892) on Glutamate carboxypeptidase 2
with a threshold of 1e−05

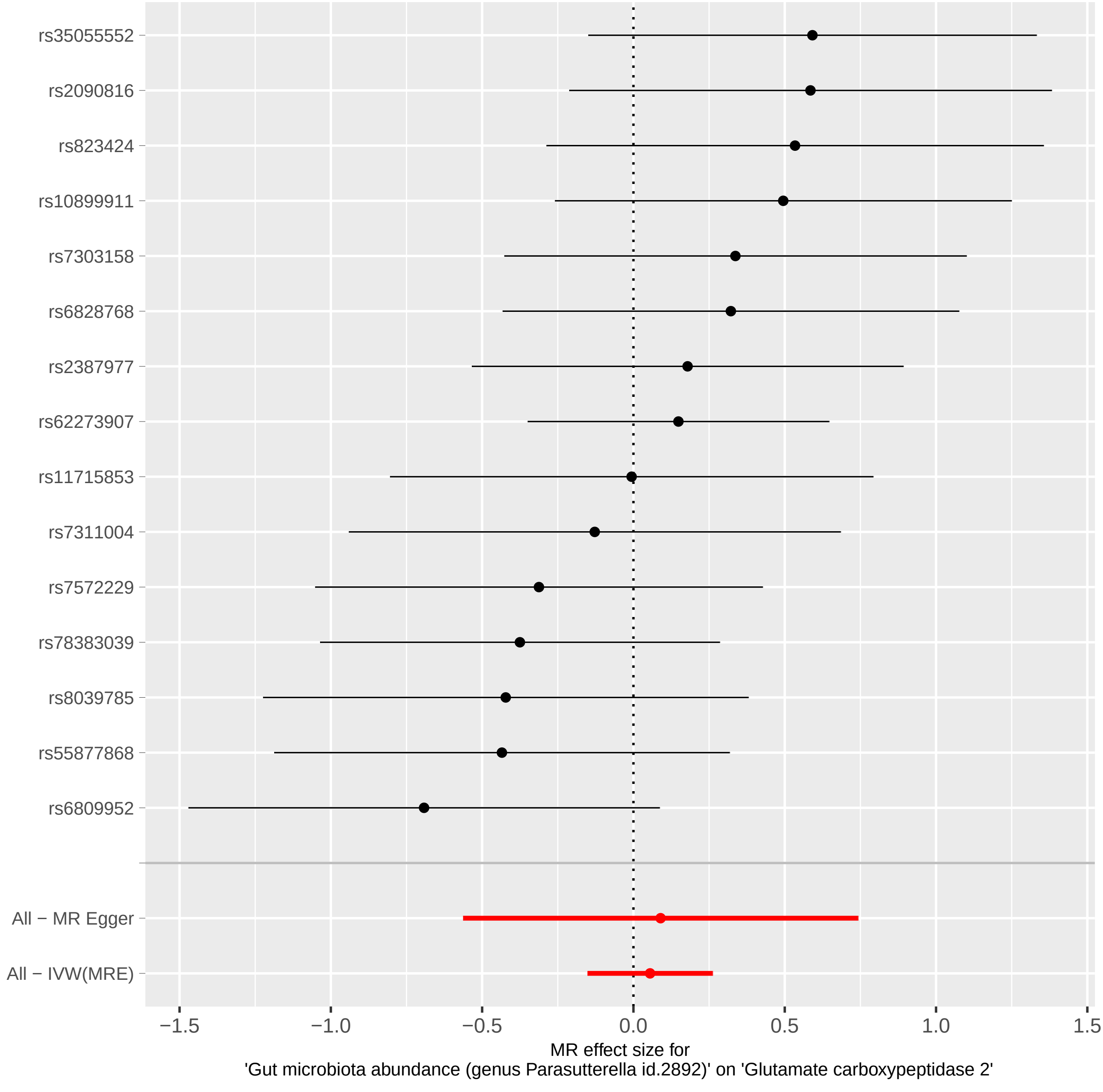

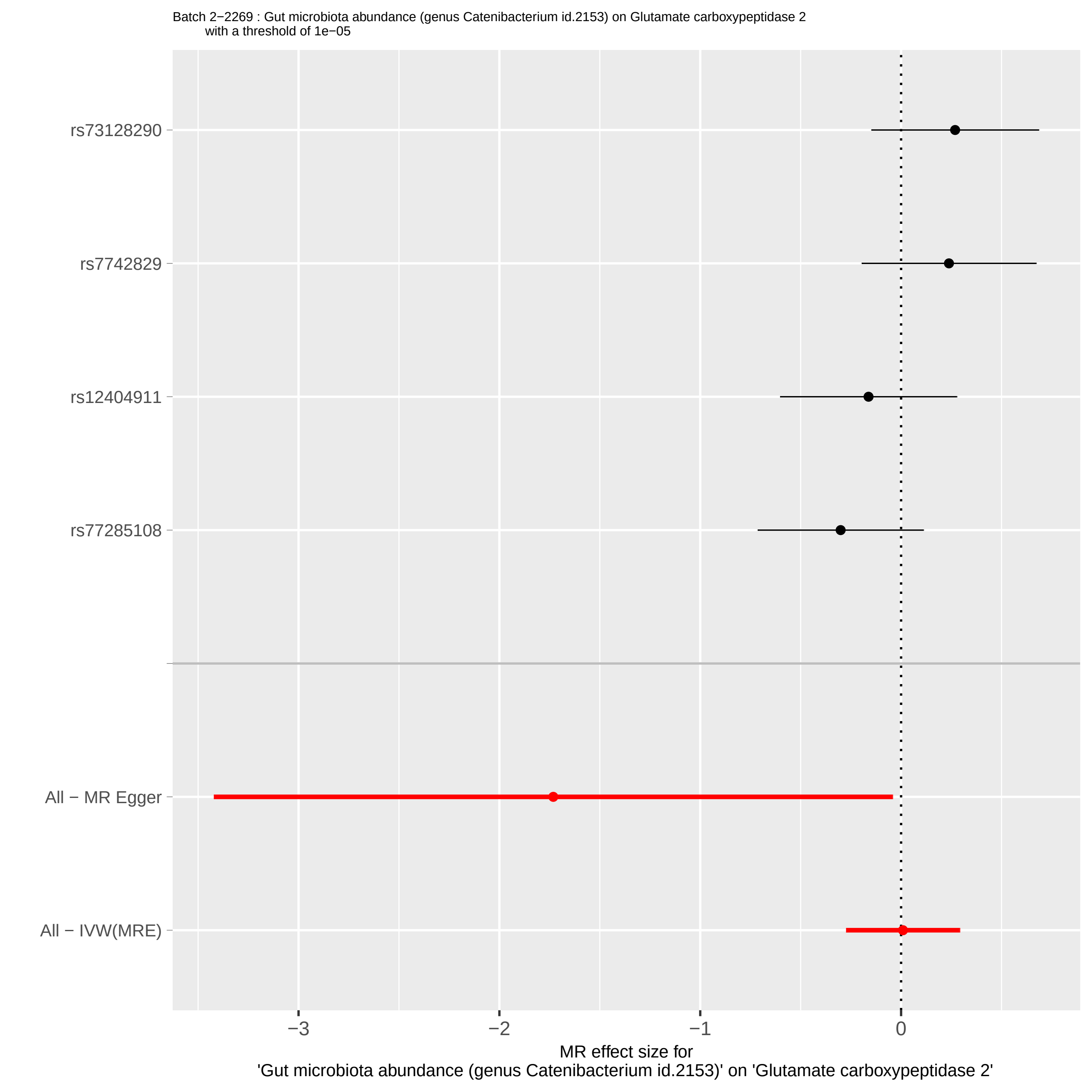
Batch 2−2269 : Gut microbiota abundance (genus Catenibacterium id.2153) on Glutamate carboxypeptidase 2
with a threshold of 1e−05
rs73128290
rs7742829
rs12404911
rs77285108
All − MR Egger
All − IVW(MRE)
−3
−2
−1
0
MR effect size for
'Gut microbiota abundance (genus Catenibacterium id.2153)' on 'Glutamate carboxypeptidase 2'

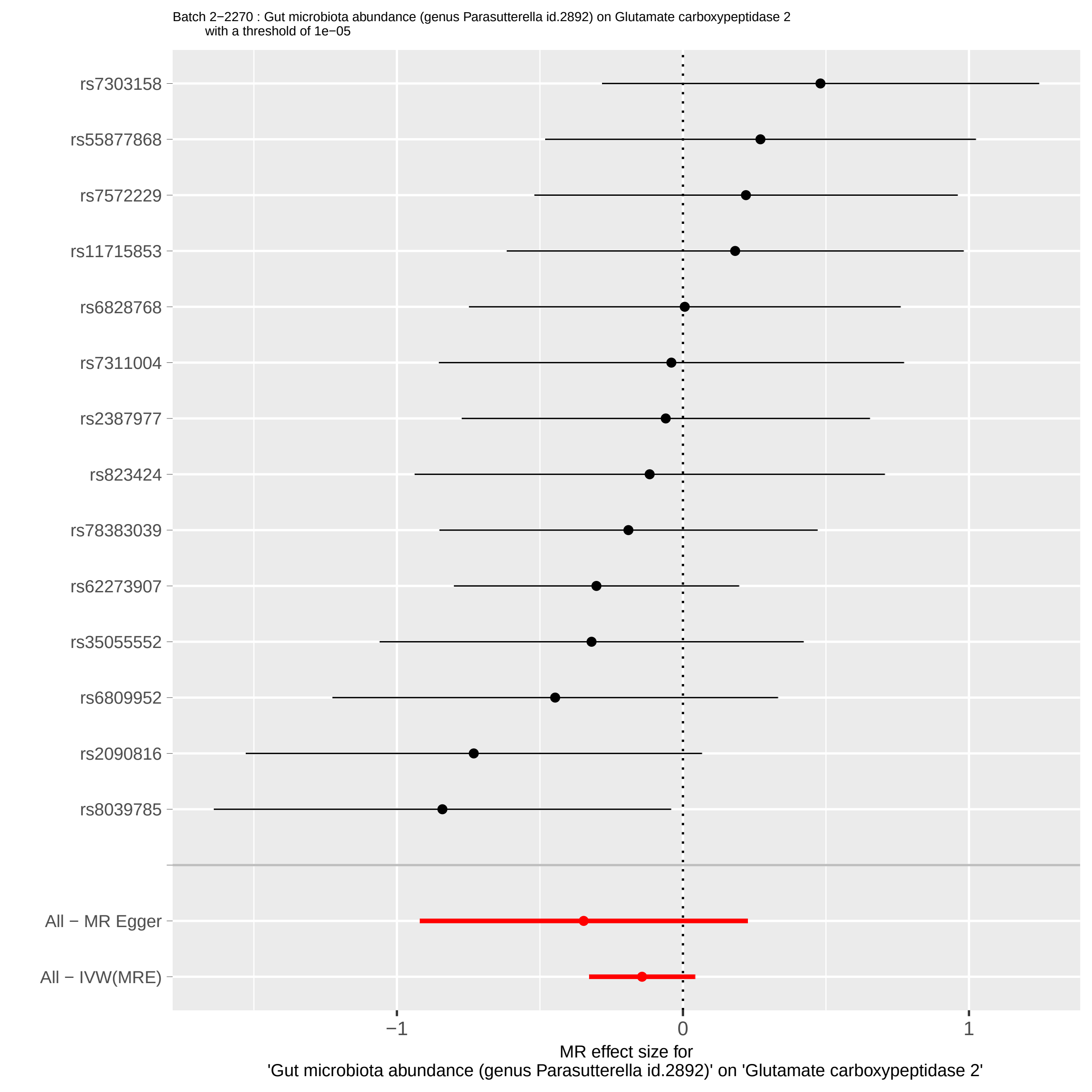

Batch 2−2270 : Gut microbiota abundance (genus Parasutterella id.2892) on Glutamate carboxypeptidase 2
with a threshold of 1e−05
rs7303158
rs55877868
rs7572229
rs11715853
rs6828768
rs7311004
rs2387977
rs823424
rs78383039
rs62273907
rs35055552
rs6809952
rs2090816
rs8039785
All − MR Egger
All − IVW(MRE)
−1
0
1
MR effect size for
'Gut microbiota abundance (genus Parasutterella id.2892)' on 'Glutamate carboxypeptidase 2'

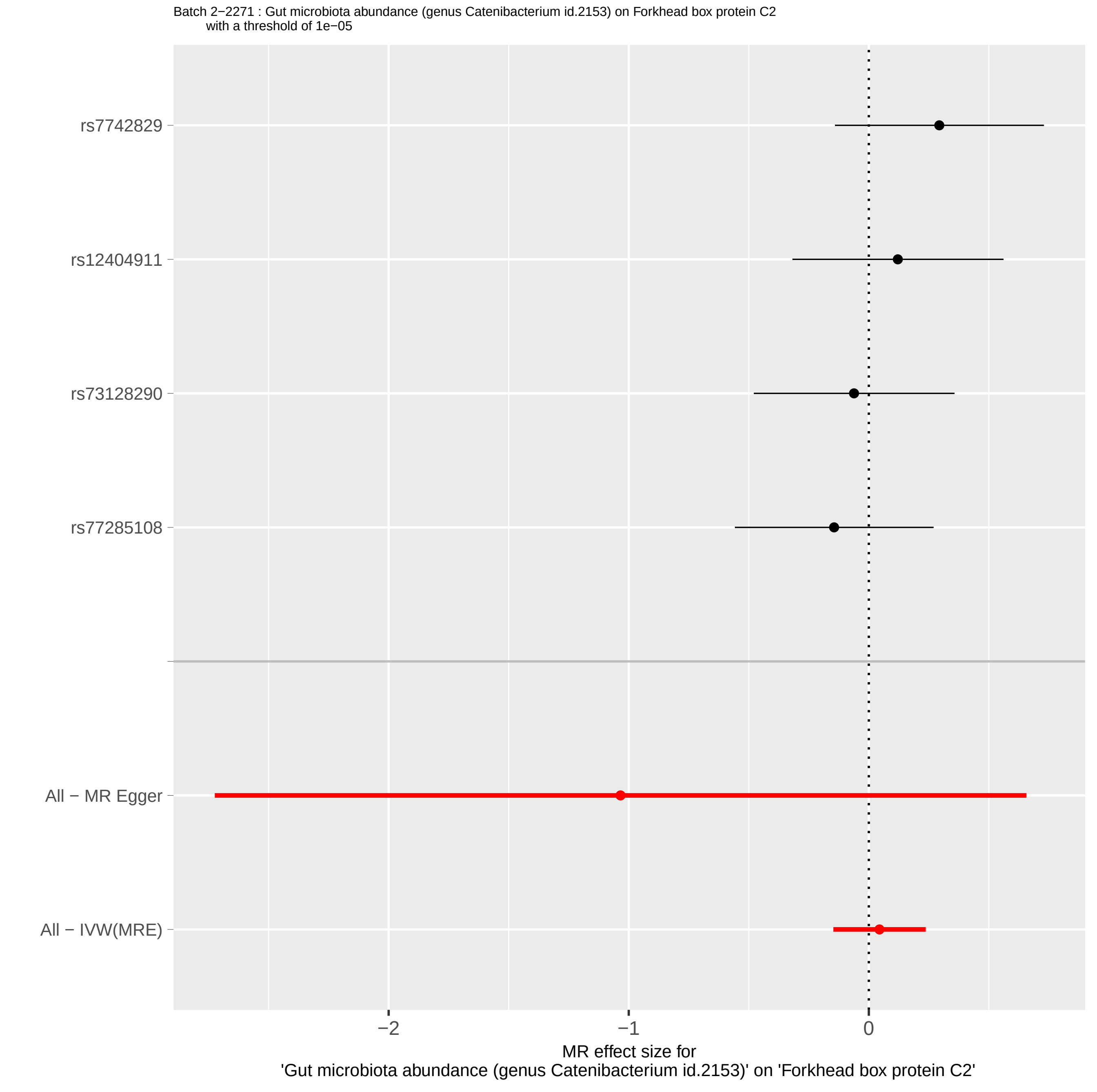

Batch 2−2271 : Gut microbiota abundance (genus Catenibacterium id.2153) on Forkhead box protein C2
with a threshold of 1e−05
rs7742829
rs12404911
rs73128290
rs77285108
All − MR Egger
All − IVW(MRE)
−2
−1
0
MR effect size for
'Gut microbiota abundance (genus Catenibacterium id.2153)' on 'Forkhead box protein C2'

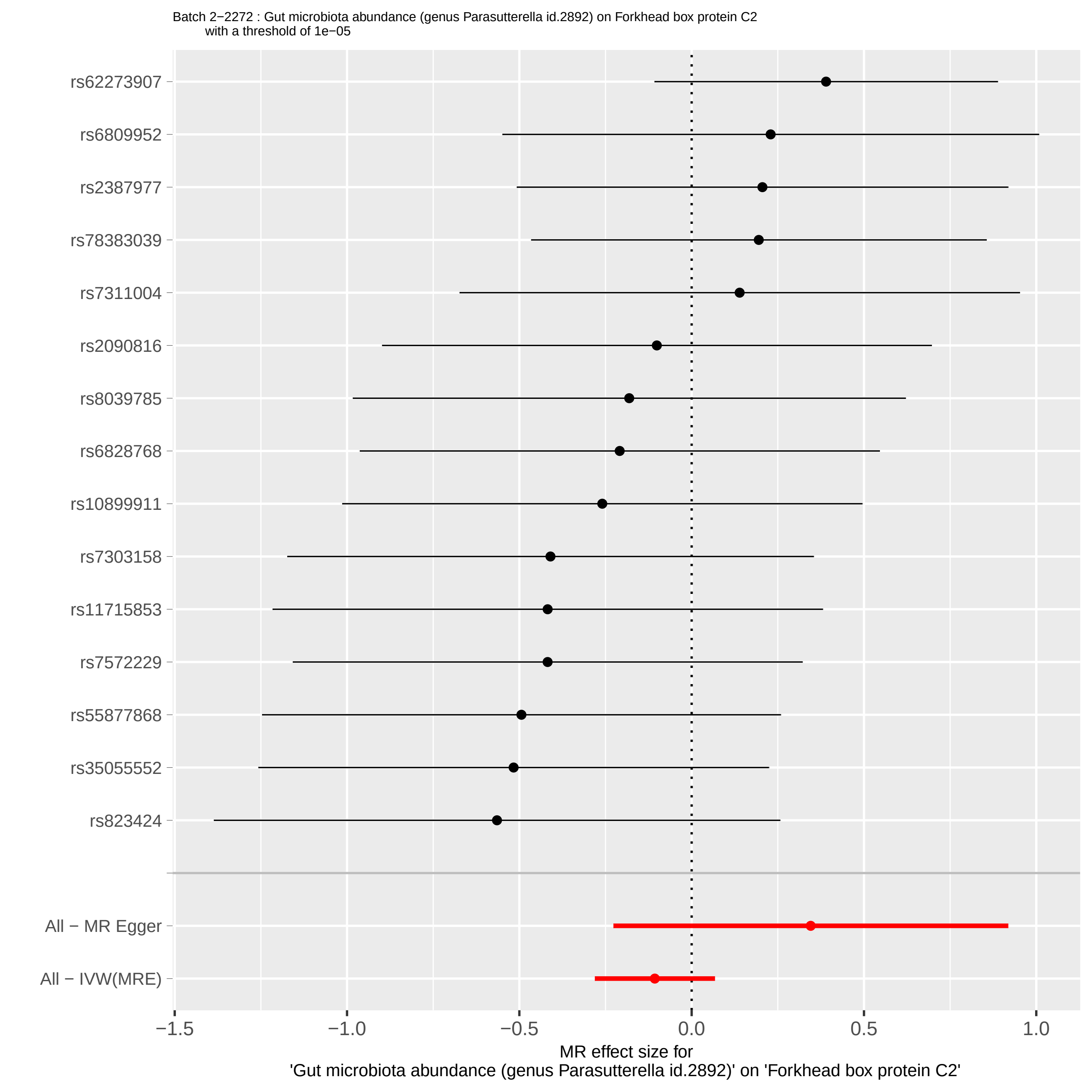

Batch 2−2272 : Gut microbiota abundance (genus Parasutterella id.2892) on Forkhead box protein C2
with a threshold of 1e−05
rs62273907
rs6809952
rs2387977
rs78383039
rs7311004
rs2090816
rs8039785
rs6828768
rs10899911
rs7303158
rs11715853
rs7572229
rs55877868
rs35055552
rs823424
All − MR Egger
All − IVW(MRE)
−1.5
−1.0
−0.5
0.0
0.5
1.0
MR effect size for
'Gut microbiota abundance (genus Parasutterella id.2892)' on 'Forkhead box protein C2'

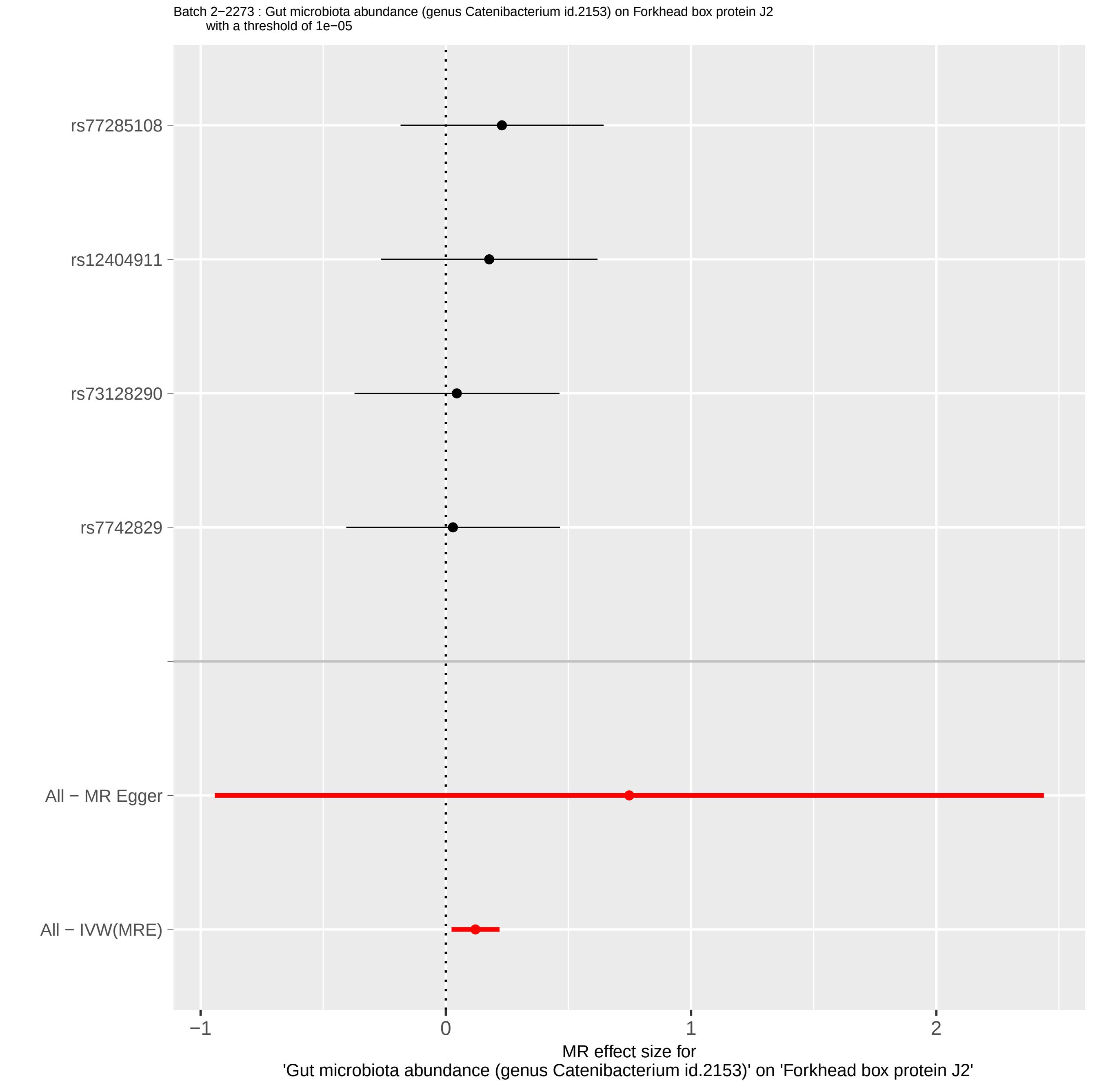

Batch 2−2273 : Gut microbiota abundance (genus Catenibacterium id.2153) on Forkhead box protein J2
with a threshold of 1e−05
rs77285108
rs12404911
rs73128290
rs7742829
All − MR Egger
All − IVW(MRE)
−1
0
1
2
MR effect size for
'Gut microbiota abundance (genus Catenibacterium id.2153)' on 'Forkhead box protein J2'

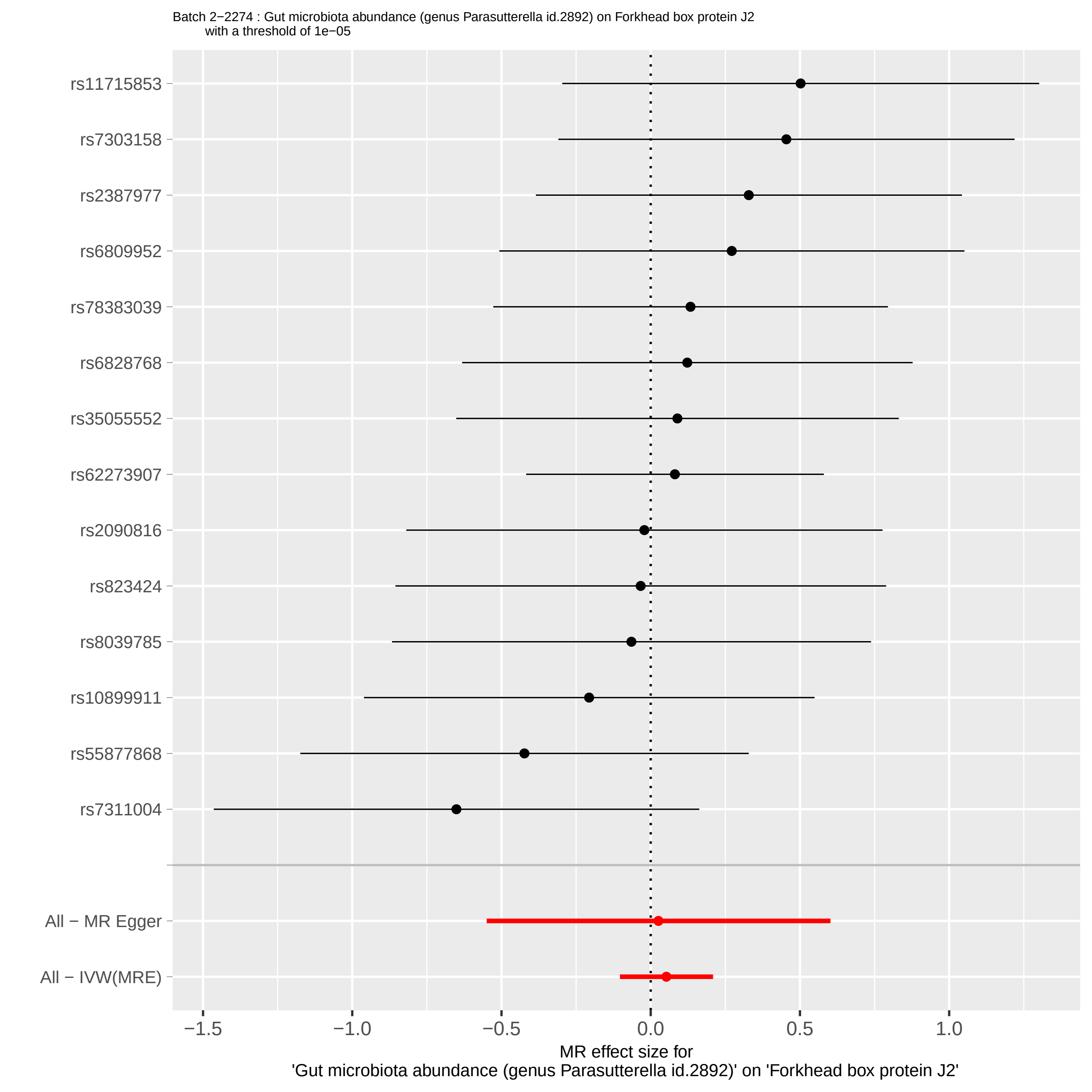
Batch 2−2274 : Gut microbiota abundance (genus Parasutterella id.2892) on Forkhead box protein J2
with a threshold of 1e−05
rs11715853
rs7303158
rs2387977
rs6809952
rs78383039
rs6828768
rs35055552
rs62273907
rs2090816
rs823424
rs8039785
rs10899911
rs55877868
rs7311004
All − MR Egger
All − IVW(MRE)
−1.5
−1.0
−0.5
0.0
0.5
1.0
MR effect size for
'Gut microbiota abundance (genus Parasutterella id.2892)' on 'Forkhead box protein J2'

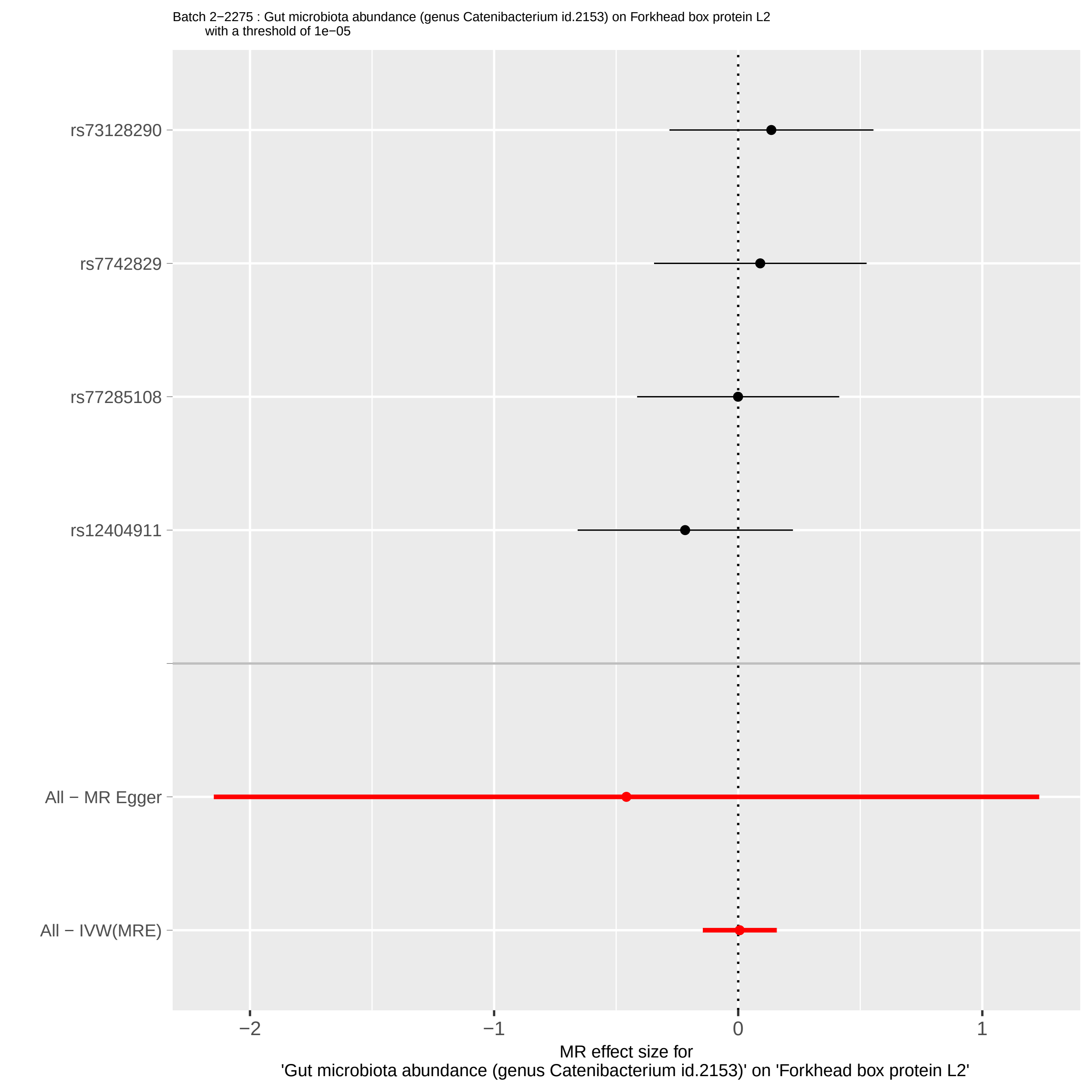
Batch 2−2275 : Gut microbiota abundance (genus Catenibacterium id.2153) on Forkhead box protein L2
with a threshold of 1e−05
rs73128290
rs7742829
rs77285108
rs12404911
All − MR Egger
All − IVW(MRE)
−2
−1
0
1
MR effect size for
'Gut microbiota abundance (genus Catenibacterium id.2153)' on 'Forkhead box protein L2'

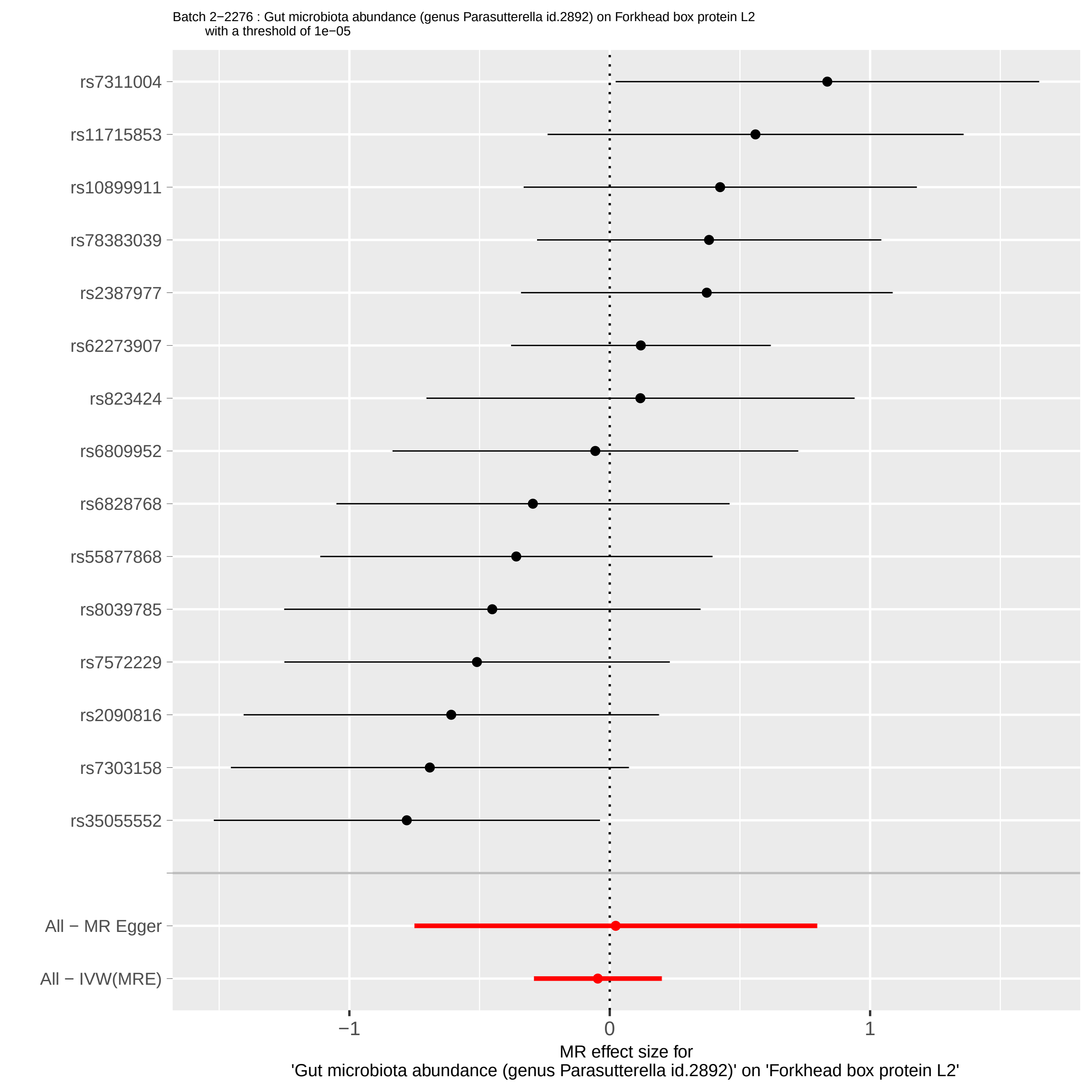

Batch 2−2276 : Gut microbiota abundance (genus Parasutterella id.2892) on Forkhead box protein L2
with a threshold of 1e−05
rs7311004
rs11715853
rs10899911
rs78383039
rs2387977
rs62273907
rs823424
rs6809952
rs6828768
rs55877868
rs8039785
rs7572229
rs2090816
rs7303158
rs35055552
All − MR Egger
All − IVW(MRE)
−1
0
1
MR effect size for
'Gut microbiota abundance (genus Parasutterella id.2892)' on 'Forkhead box protein L2'

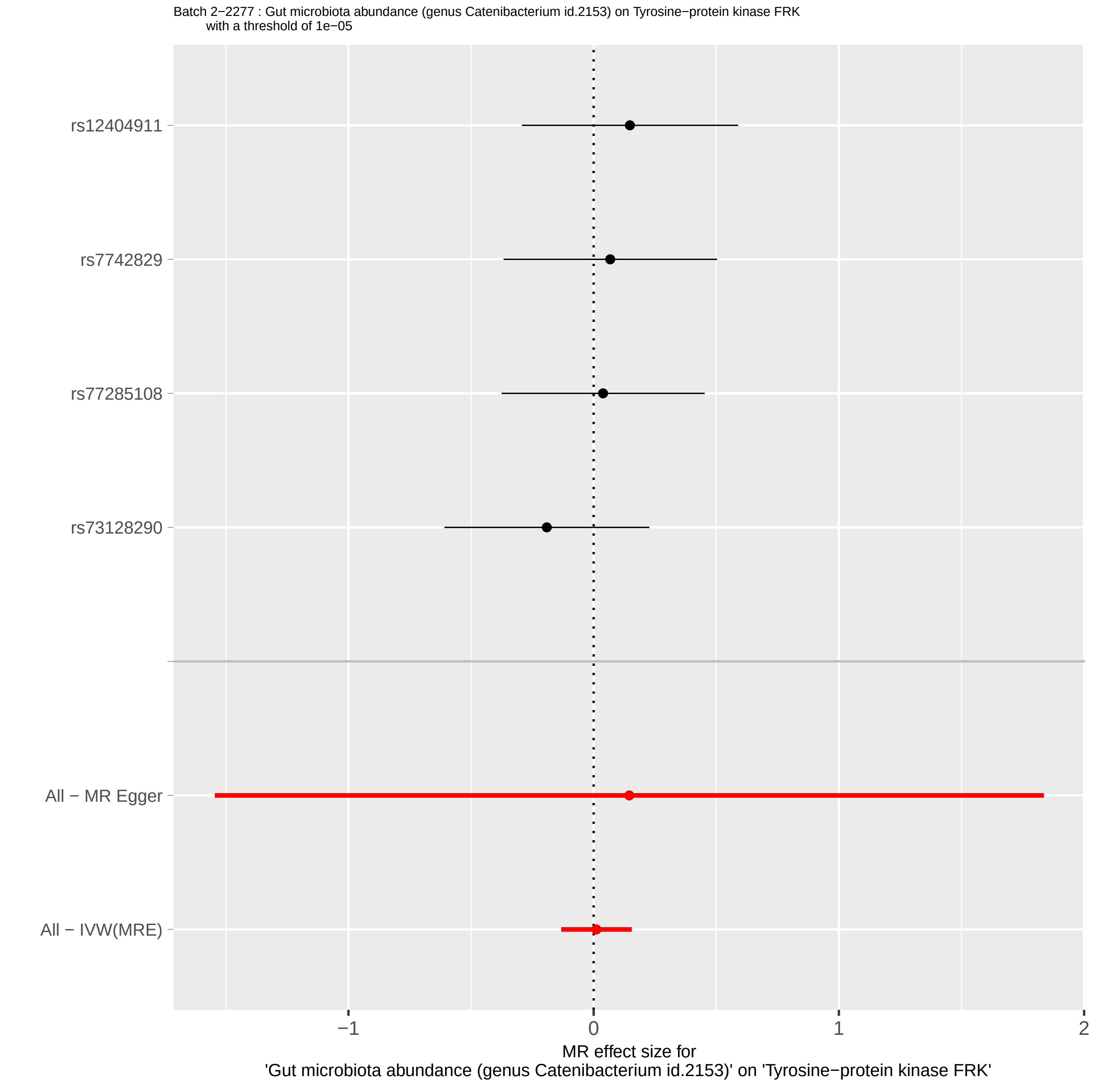

Batch 2−2277 : Gut microbiota abundance (genus Catenibacterium id.2153) on Tyrosine−protein kinase FRK
with a threshold of 1e−05
rs12404911
rs7742829
rs77285108
rs73128290
All − MR Egger
All − IVW(MRE)
−1
0
1
2
MR effect size for
'Gut microbiota abundance (genus Catenibacterium id.2153)' on 'Tyrosine−protein kinase FRK'

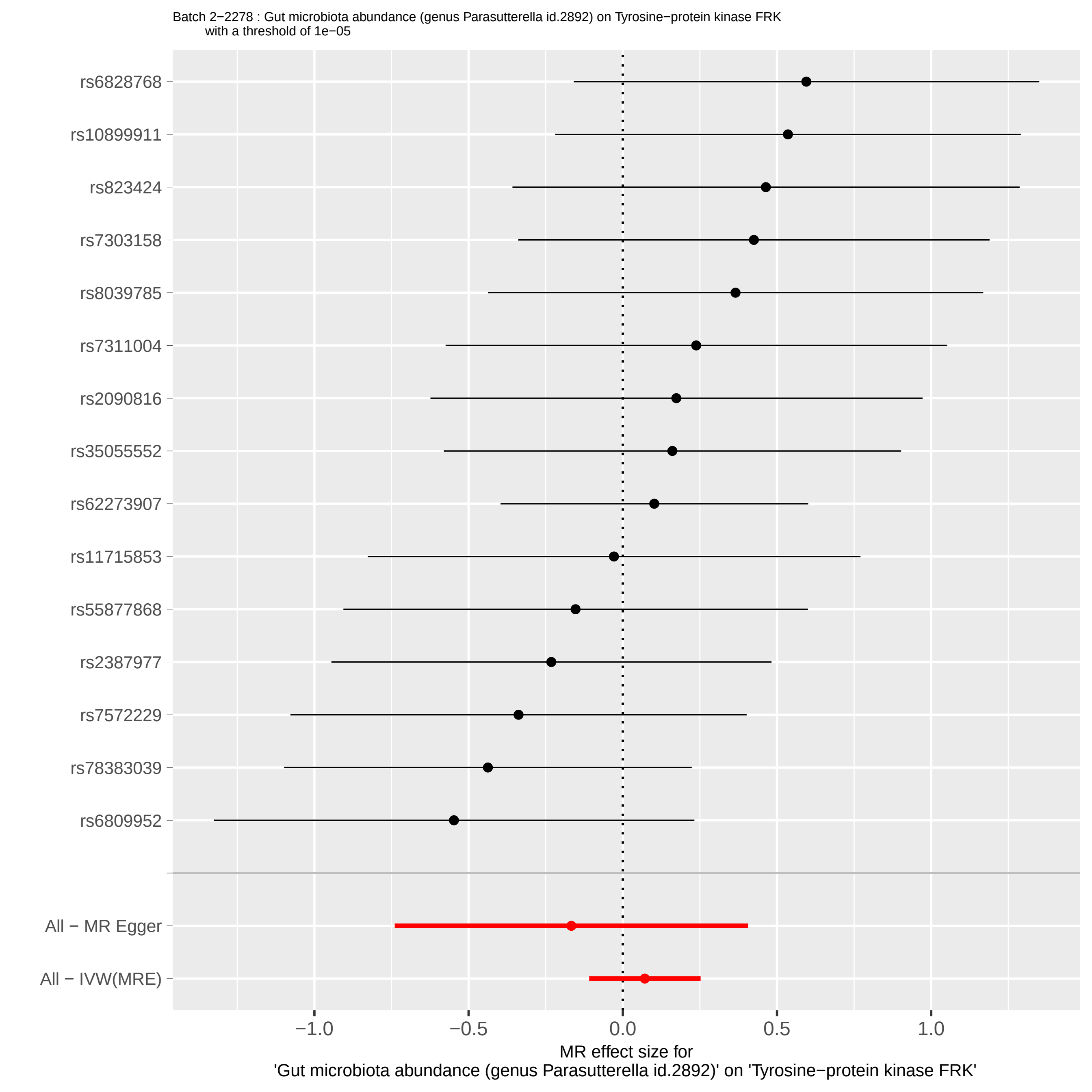

Batch 2−2278 : Gut microbiota abundance (genus Parasutterella id.2892) on Tyrosine−protein kinase FRK
with a threshold of 1e−05
rs6828768
rs10899911
rs823424
rs7303158
rs8039785
rs7311004
rs2090816
rs35055552
rs62273907
rs11715853
rs55877868
rs2387977
rs7572229
rs78383039
rs6809952
All − MR Egger
All − IVW(MRE)
−1.0
−0.5
0.0
0.5
1.0
MR effect size for
'Gut microbiota abundance (genus Parasutterella id.2892)' on 'Tyrosine−protein kinase FRK'

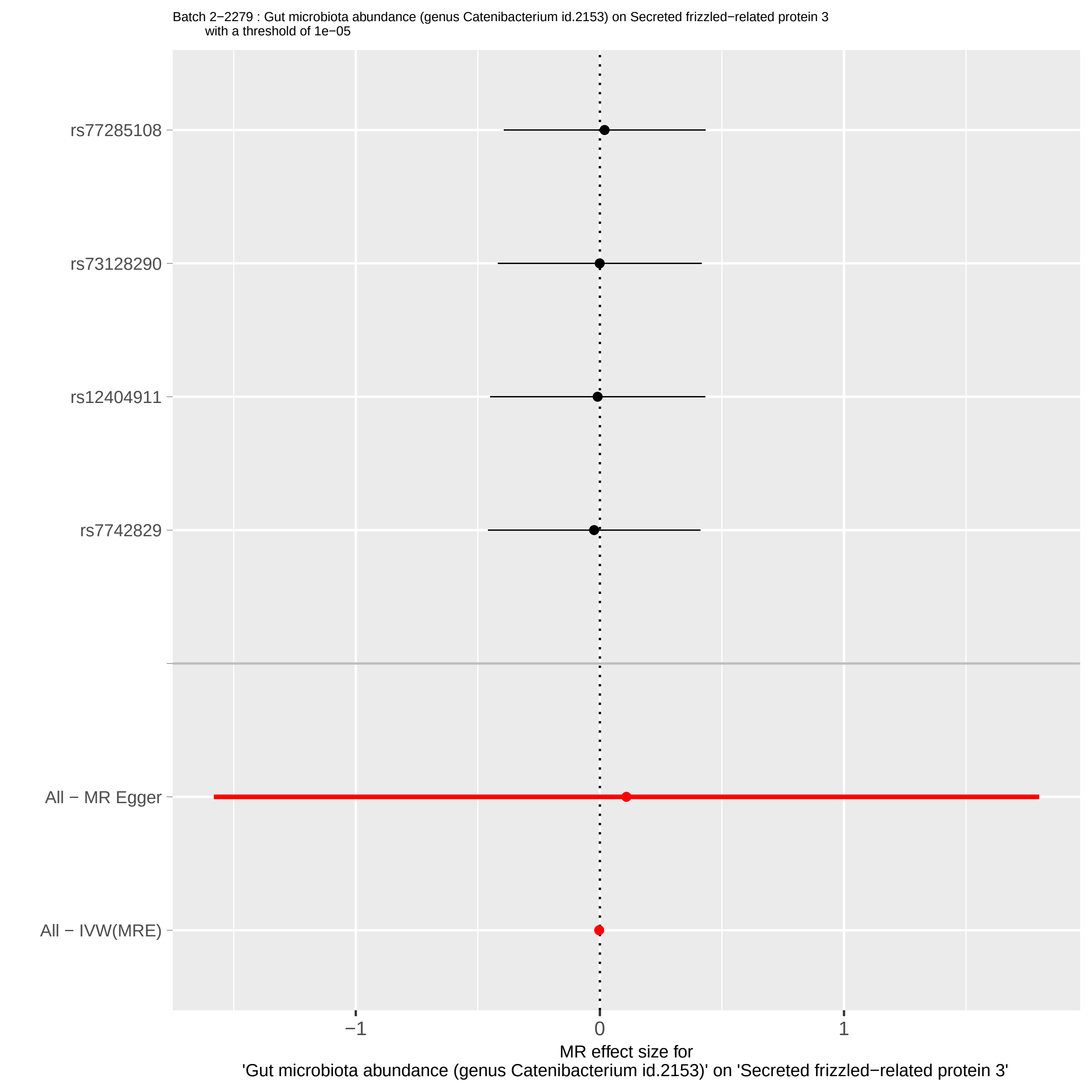
Batch 2−2279 : Gut microbiota abundance (genus Catenibacterium id.2153) on Secreted frizzled−related protein 3
with a threshold of 1e−05
rs77285108
rs73128290
rs12404911
rs7742829
All − MR Egger
All − IVW(MRE)
−1
0
1
MR effect size for
'Gut microbiota abundance (genus Catenibacterium id.2153)' on 'Secreted frizzled−related protein 3'

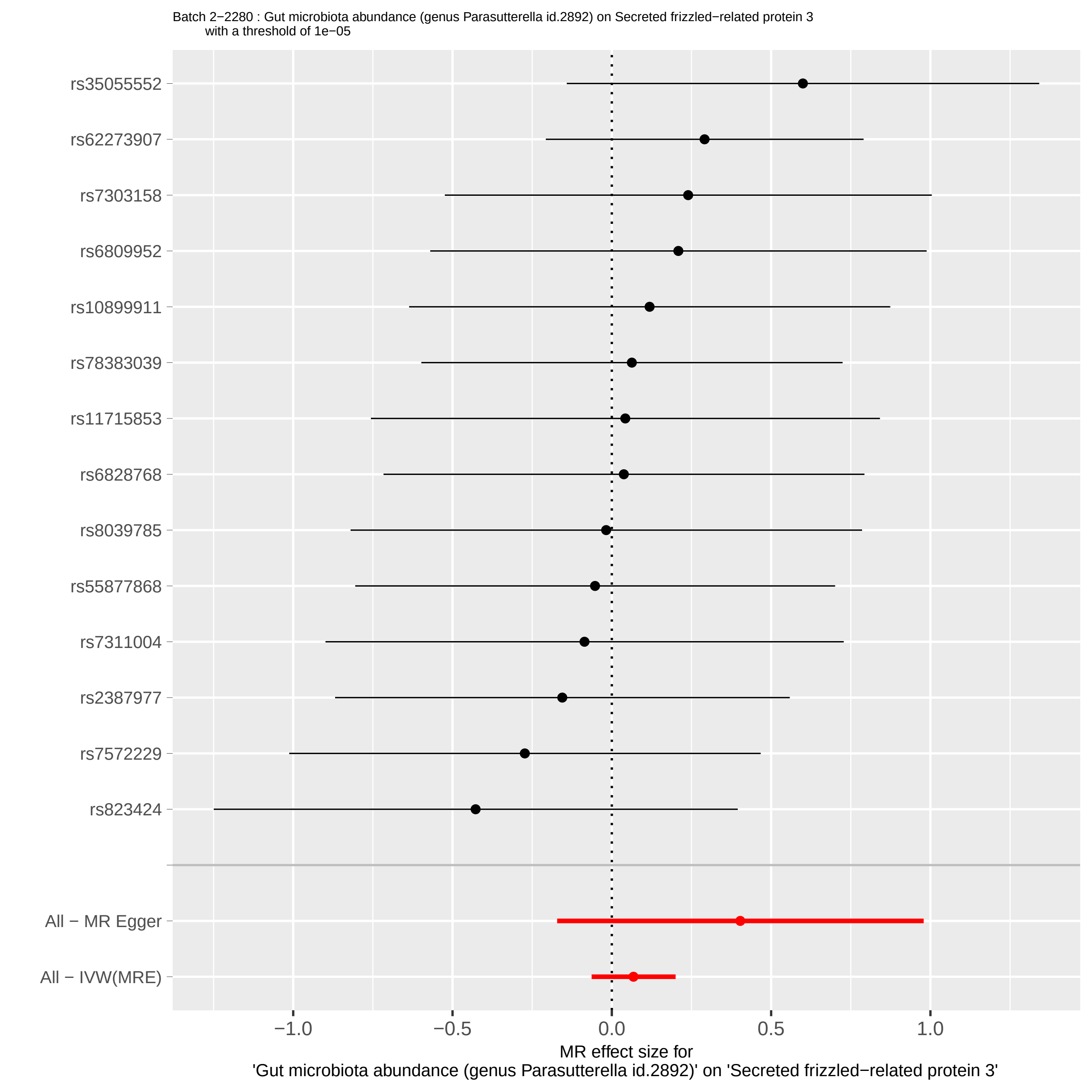

Batch 2−2280 : Gut microbiota abundance (genus Parasutterella id.2892) on Secreted frizzled−related protein 3
with a threshold of 1e−05
rs35055552
rs62273907
rs7303158
rs6809952
rs10899911
rs78383039
rs11715853
rs6828768
rs8039785
rs55877868
rs7311004
rs2387977
rs7572229
rs823424
All − MR Egger
All − IVW(MRE)
−1.0
−0.5
0.0
0.5
1.0
MR effect size for
'Gut microbiota abundance (genus Parasutterella id.2892)' on 'Secreted frizzled−related protein 3'

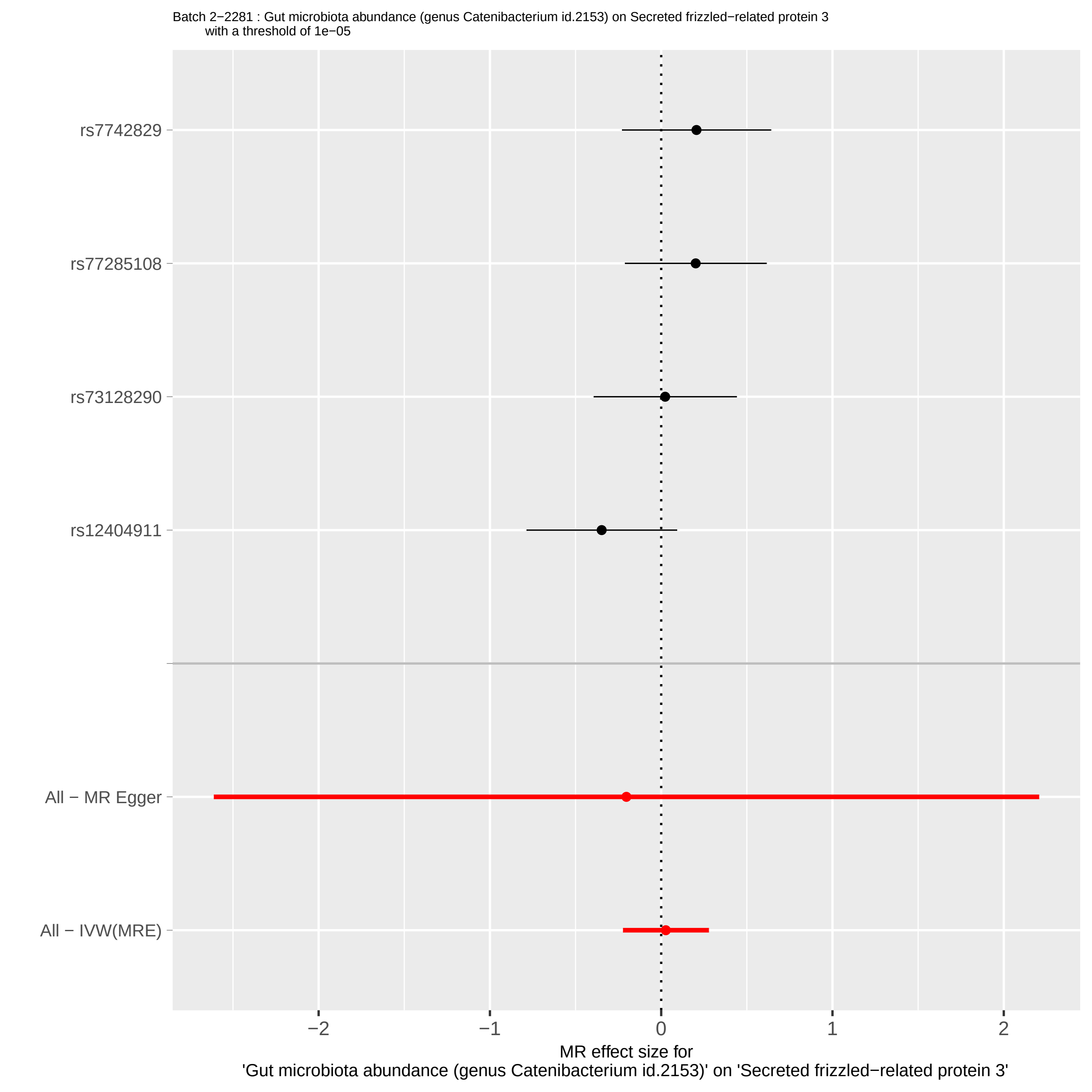
Batch 2−2281 : Gut microbiota abundance (genus Catenibacterium id.2153) on Secreted frizzled−related protein 3
with a threshold of 1e−05
rs7742829
rs77285108
rs73128290
rs12404911
All − MR Egger
All − IVW(MRE)
−2
−1
0
1
2
MR effect size for
'Gut microbiota abundance (genus Catenibacterium id.2153)' on 'Secreted frizzled−related protein 3'

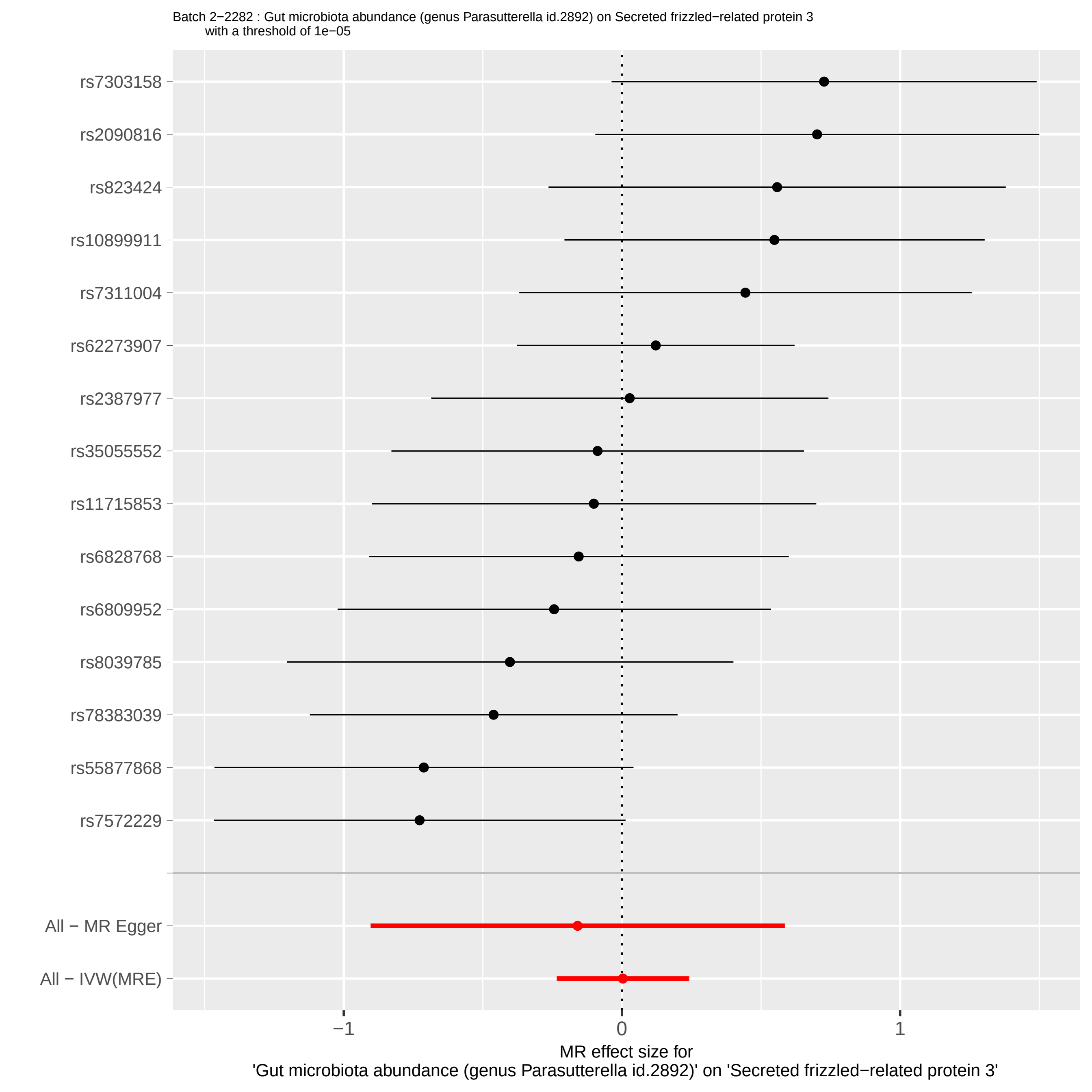

Batch 2−2282 : Gut microbiota abundance (genus Parasutterella id.2892) on Secreted frizzled−related protein 3
with a threshold of 1e−05
rs7303158
rs2090816
rs823424
rs10899911
rs7311004
rs62273907
rs2387977
rs35055552
rs11715853
rs6828768
rs6809952
rs8039785
rs78383039
rs55877868
rs7572229
All − MR Egger
All − IVW(MRE)
−1
0
1
MR effect size for
'Gut microbiota abundance (genus Parasutterella id.2892)' on 'Secreted frizzled−related protein 3'

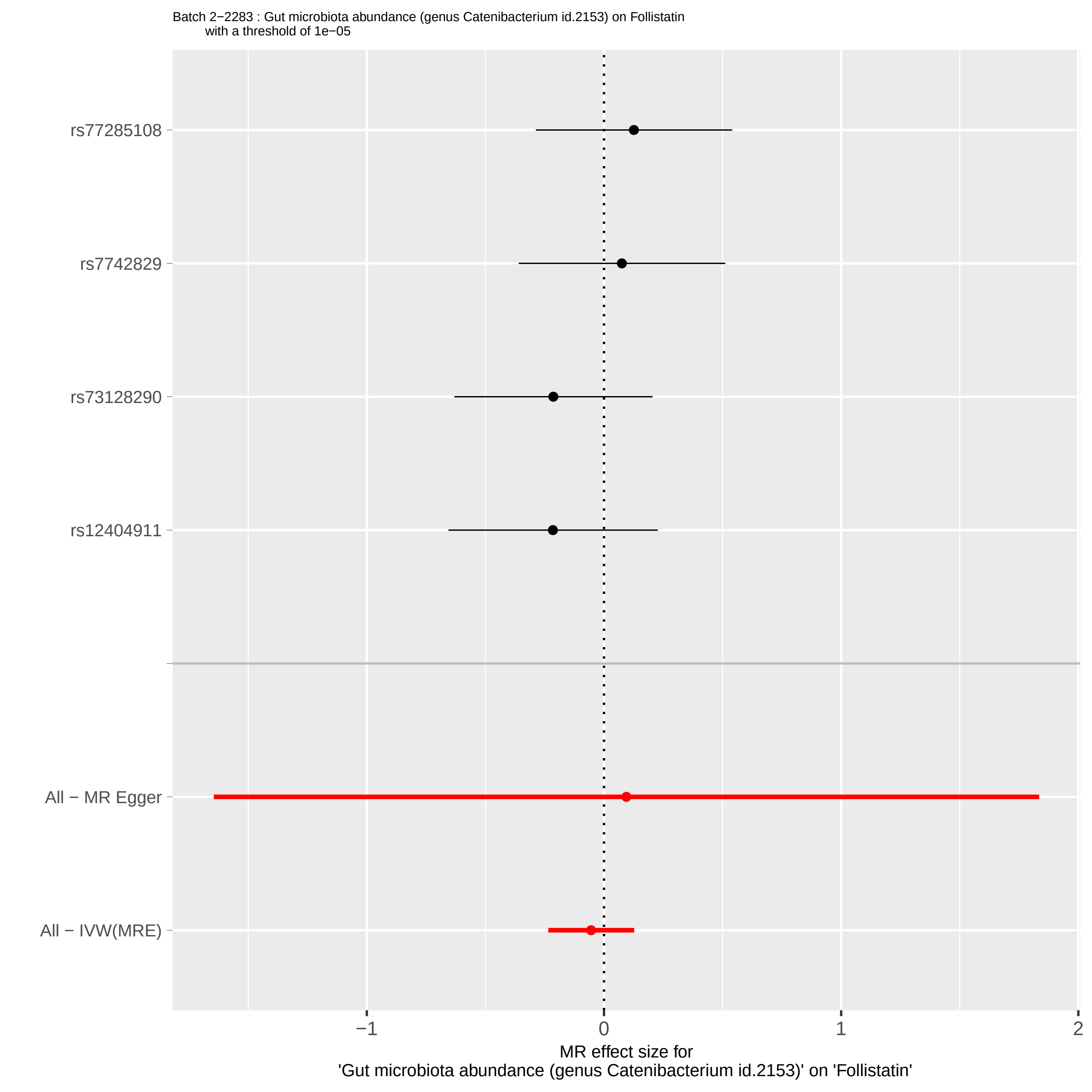
Batch 2−2283 : Gut microbiota abundance (genus Catenibacterium id.2153) on Follistatin
with a threshold of 1e−05
rs77285108
rs7742829
rs73128290
rs12404911
All − MR Egger
All − IVW(MRE)
−1
0
1
2
MR effect size for
'Gut microbiota abundance (genus Catenibacterium id.2153)' on 'Follistatin'

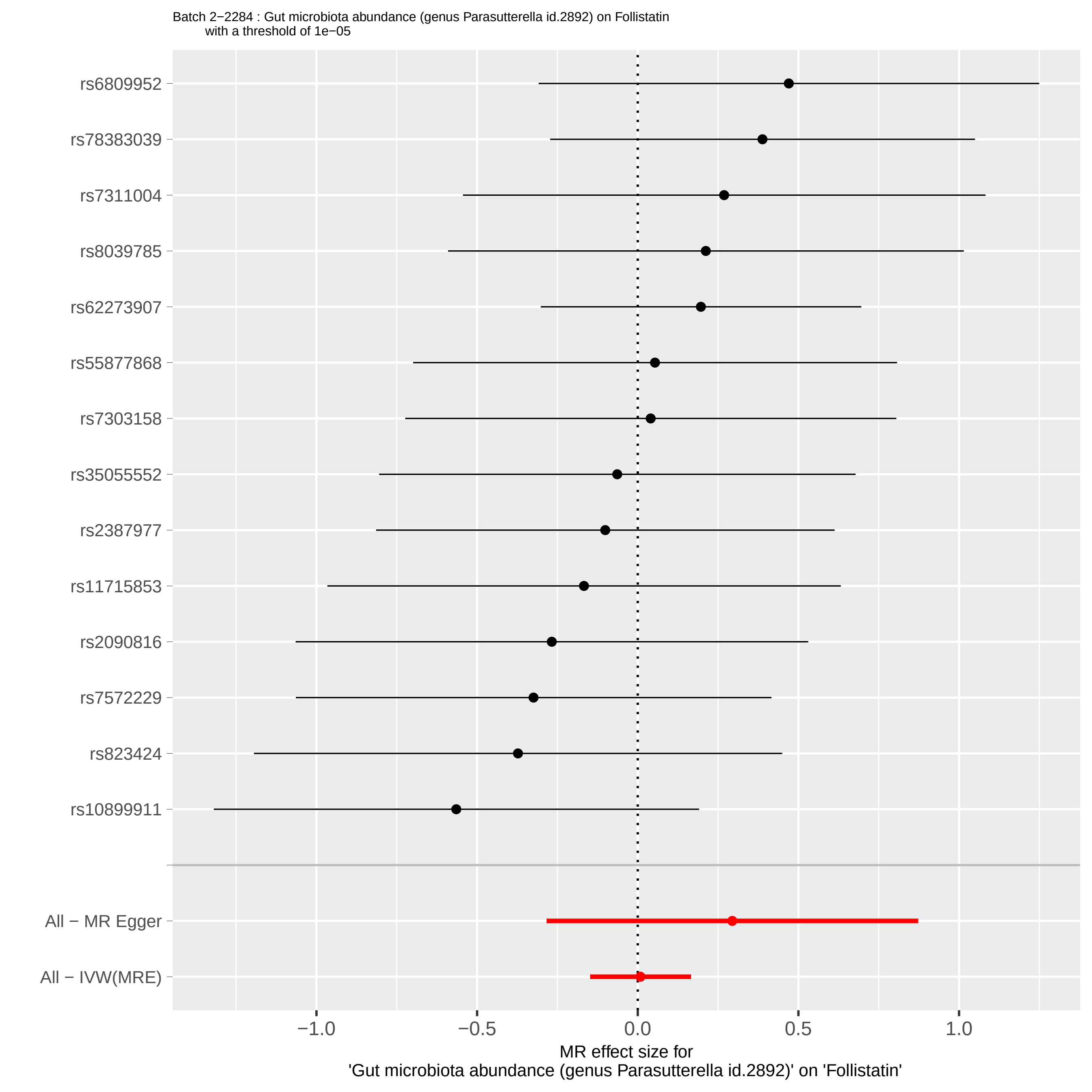

Batch 2−2284 : Gut microbiota abundance (genus Parasutterella id.2892) on Follistatin
with a threshold of 1e−05
rs6809952
rs78383039
rs7311004
rs8039785
rs62273907
rs55877868
rs7303158
rs35055552
rs2387977
rs11715853
rs2090816
rs7572229
rs823424
rs10899911
All − MR Egger
All − IVW(MRE)
−1.0
−0.5
0.0
0.5
1.0
MR effect size for
'Gut microbiota abundance (genus Parasutterella id.2892)' on 'Follistatin'

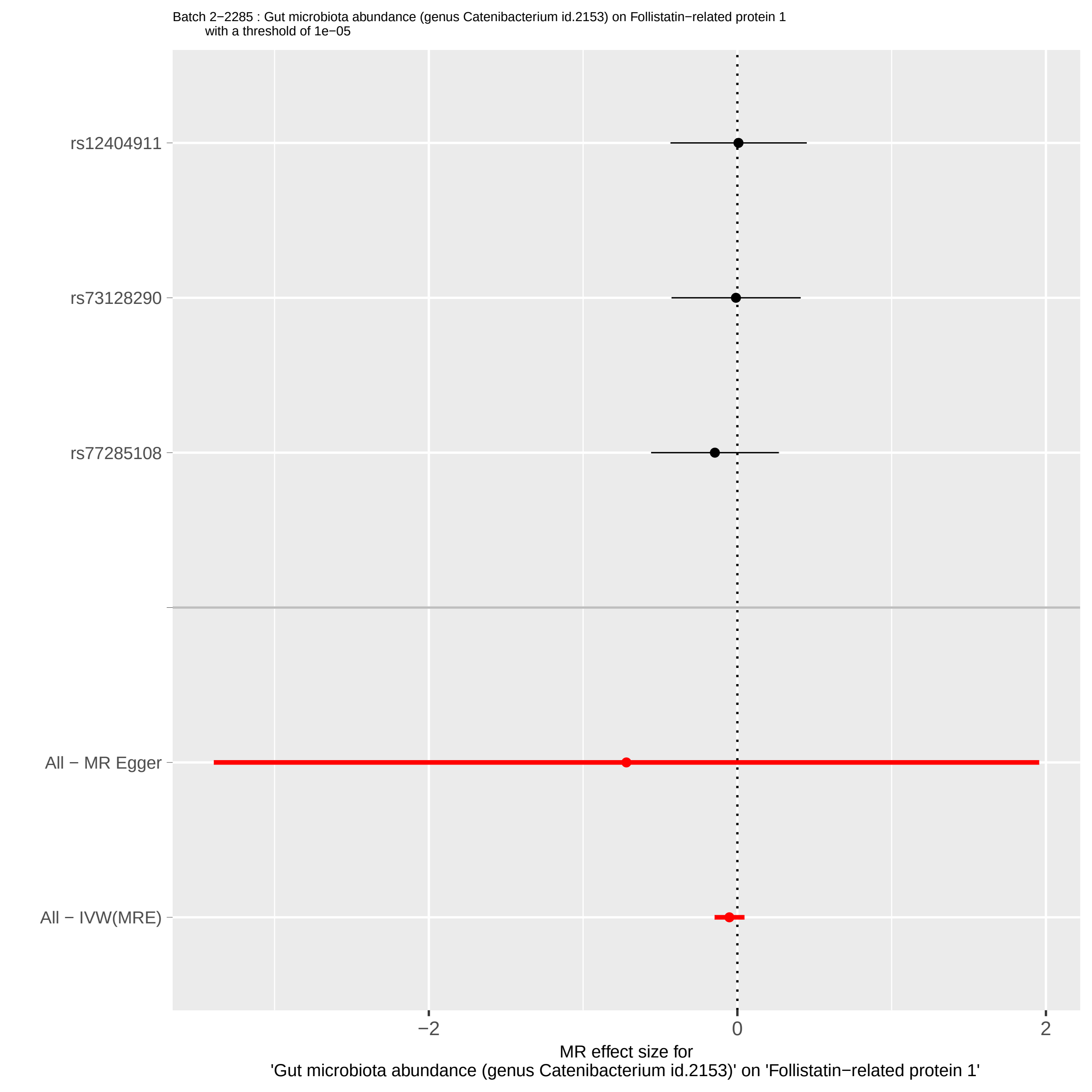

Batch 2−2285 : Gut microbiota abundance (genus Catenibacterium id.2153) on Follistatin−related protein 1
with a threshold of 1e−05
rs12404911
rs73128290
rs77285108
All − MR Egger
All − IVW(MRE)
−2
0
2
MR effect size for
'Gut microbiota abundance (genus Catenibacterium id.2153)' on 'Follistatin−related protein 1'

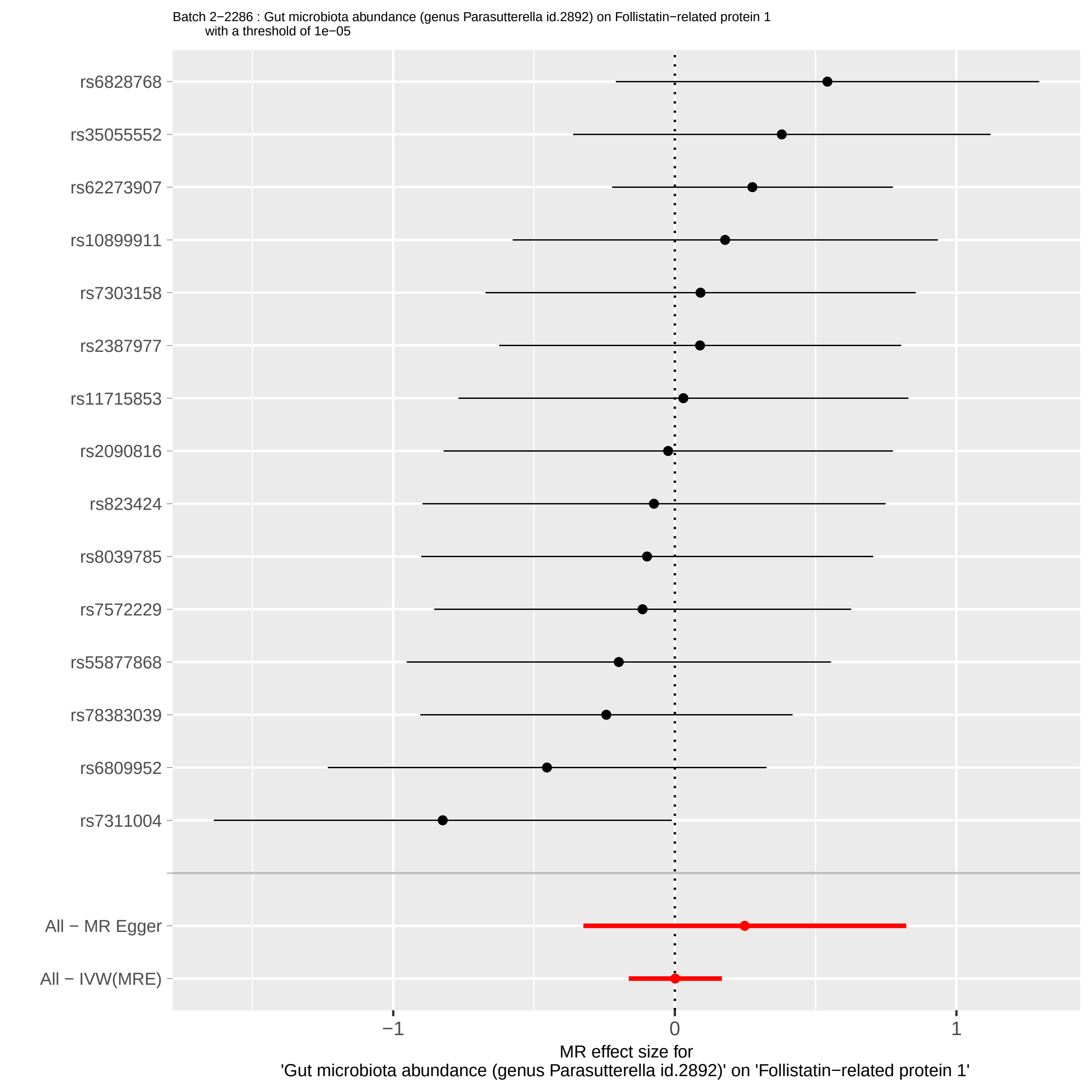
Batch 2−2286 : Gut microbiota abundance (genus Parasutterella id.2892) on Follistatin−related protein 1
with a threshold of 1e−05
rs6828768
rs35055552
rs62273907
rs10899911
rs7303158
rs2387977
rs11715853
rs2090816
rs823424
rs8039785
rs7572229
rs55877868
rs78383039
rs6809952
rs7311004
All − MR Egger
All − IVW(MRE)
−1
0
1
MR effect size for
'Gut microbiota abundance (genus Parasutterella id.2892)' on 'Follistatin−related protein 1'

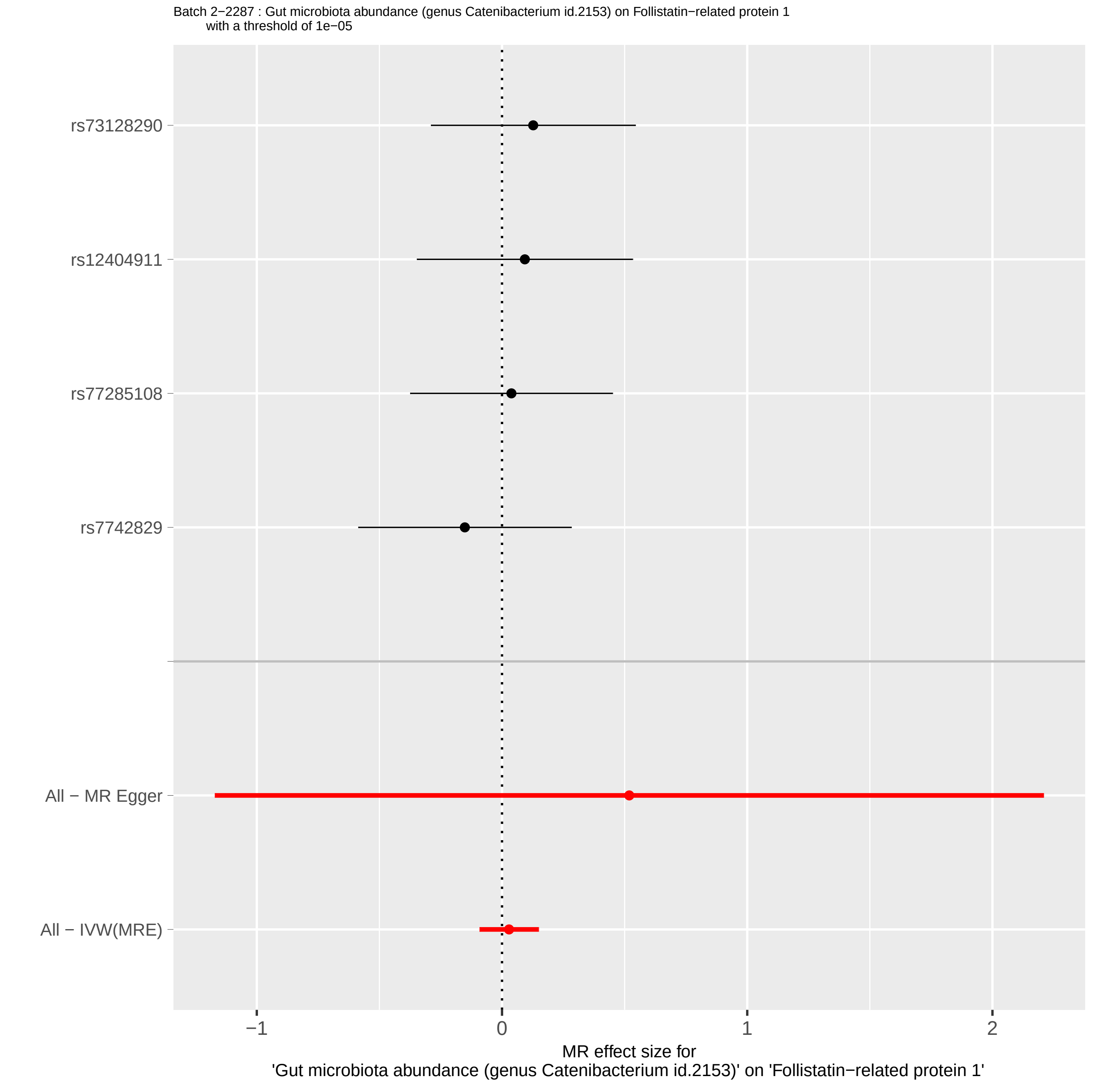

Batch 2−2287 : Gut microbiota abundance (genus Catenibacterium id.2153) on Follistatin−related protein 1
with a threshold of 1e−05
rs73128290
rs12404911
rs77285108
rs7742829
All − MR Egger
All − IVW(MRE)
−1
0
1
2
MR effect size for
'Gut microbiota abundance (genus Catenibacterium id.2153)' on 'Follistatin−related protein 1'

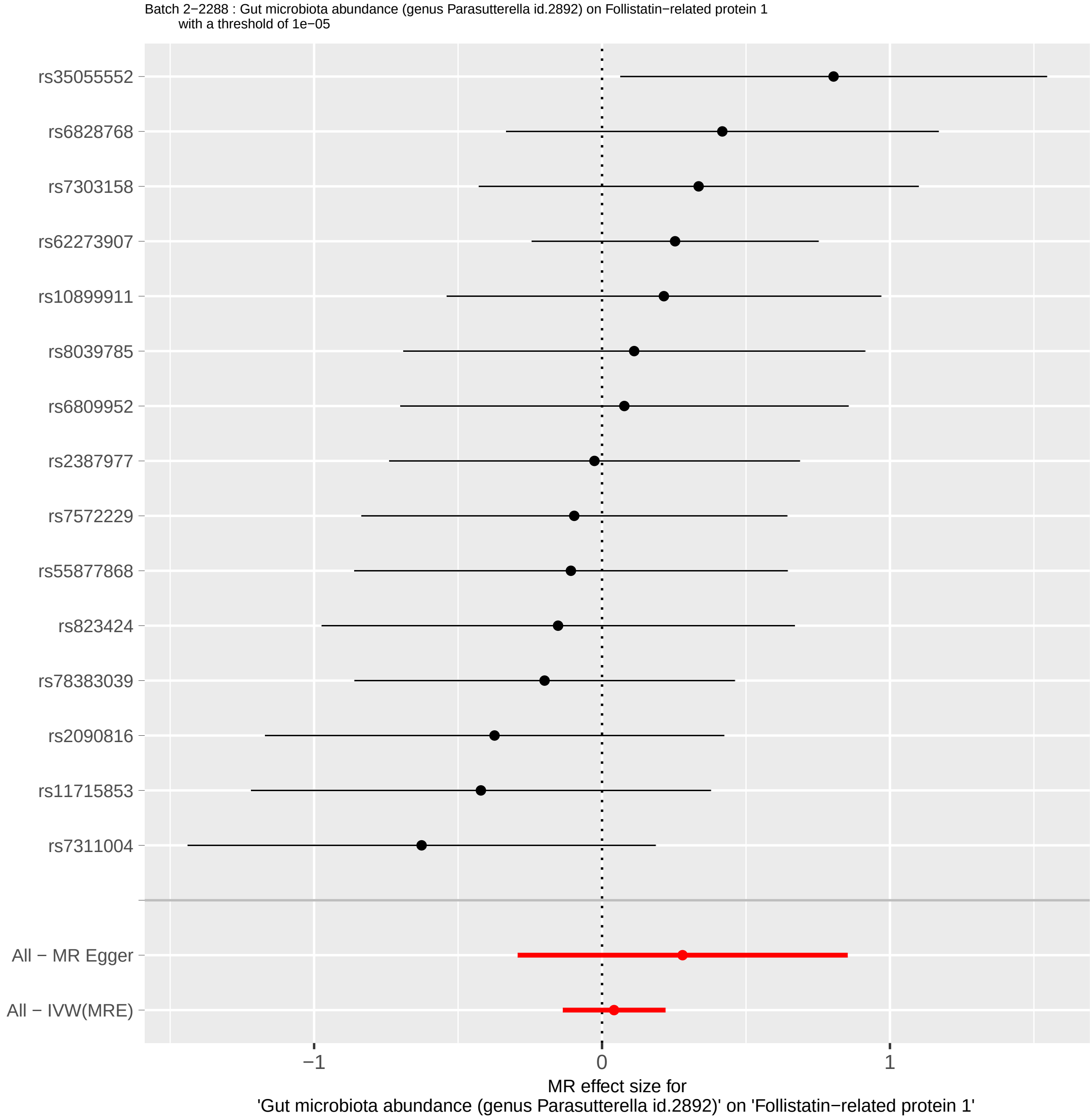

Batch 2−2288 : Gut microbiota abundance (genus Parasutterella id.2892) on Follistatin−related protein 1
with a threshold of 1e−05
rs35055552
rs6828768
rs7303158
rs62273907
rs10899911
rs8039785
rs6809952
rs2387977
rs7572229
rs55877868
rs823424
rs78383039
rs2090816
rs11715853
rs7311004
All − MR Egger
All − IVW(MRE)
−1
0
1
MR effect size for
'Gut microbiota abundance (genus Parasutterella id.2892)' on 'Follistatin−related protein 1'

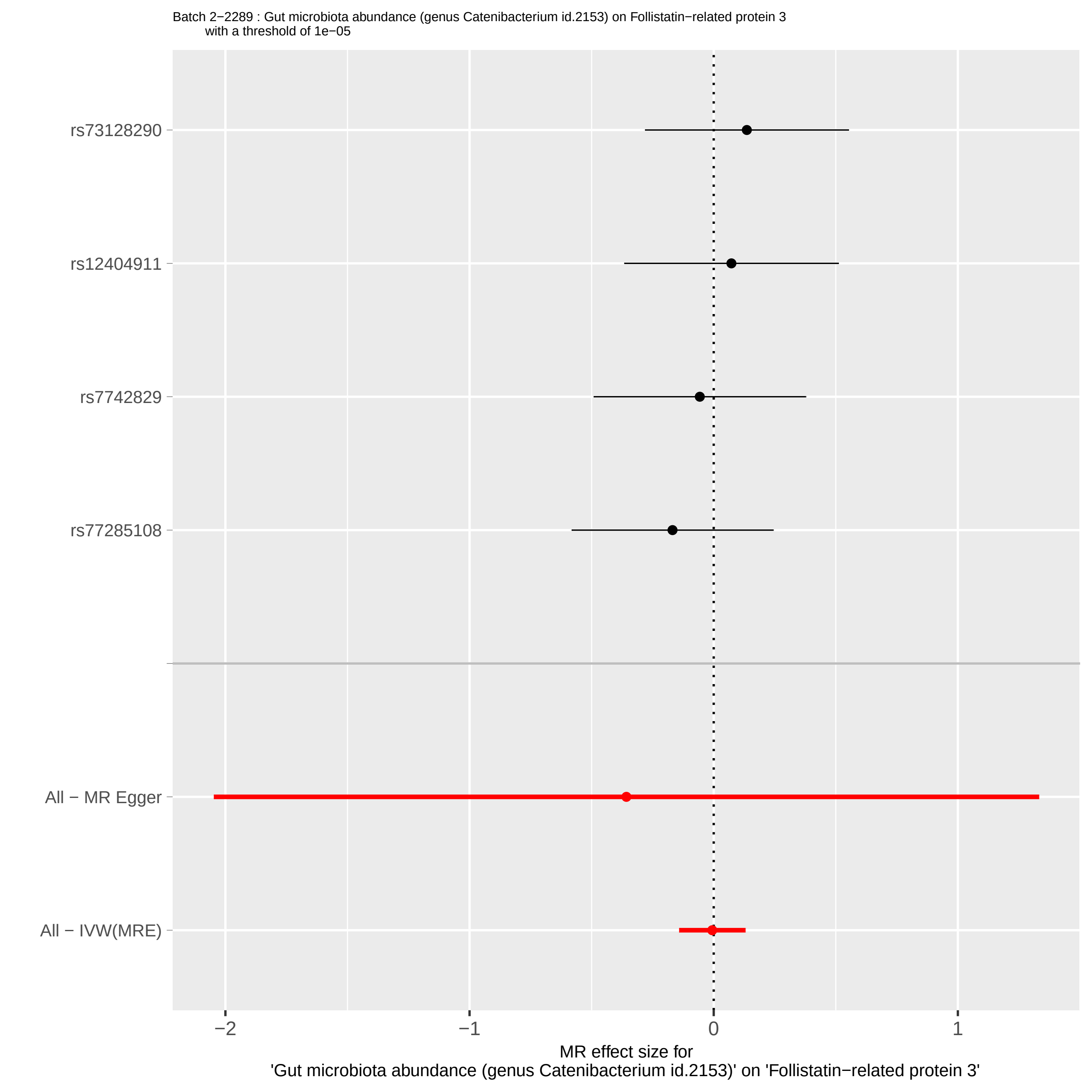

Batch 2−2289 : Gut microbiota abundance (genus Catenibacterium id.2153) on Follistatin−related protein 3
with a threshold of 1e−05
rs73128290
rs12404911
rs7742829
rs77285108
All − MR Egger
All − IVW(MRE)
−2
−1
0
1
MR effect size for
'Gut microbiota abundance (genus Catenibacterium id.2153)' on 'Follistatin−related protein 3'

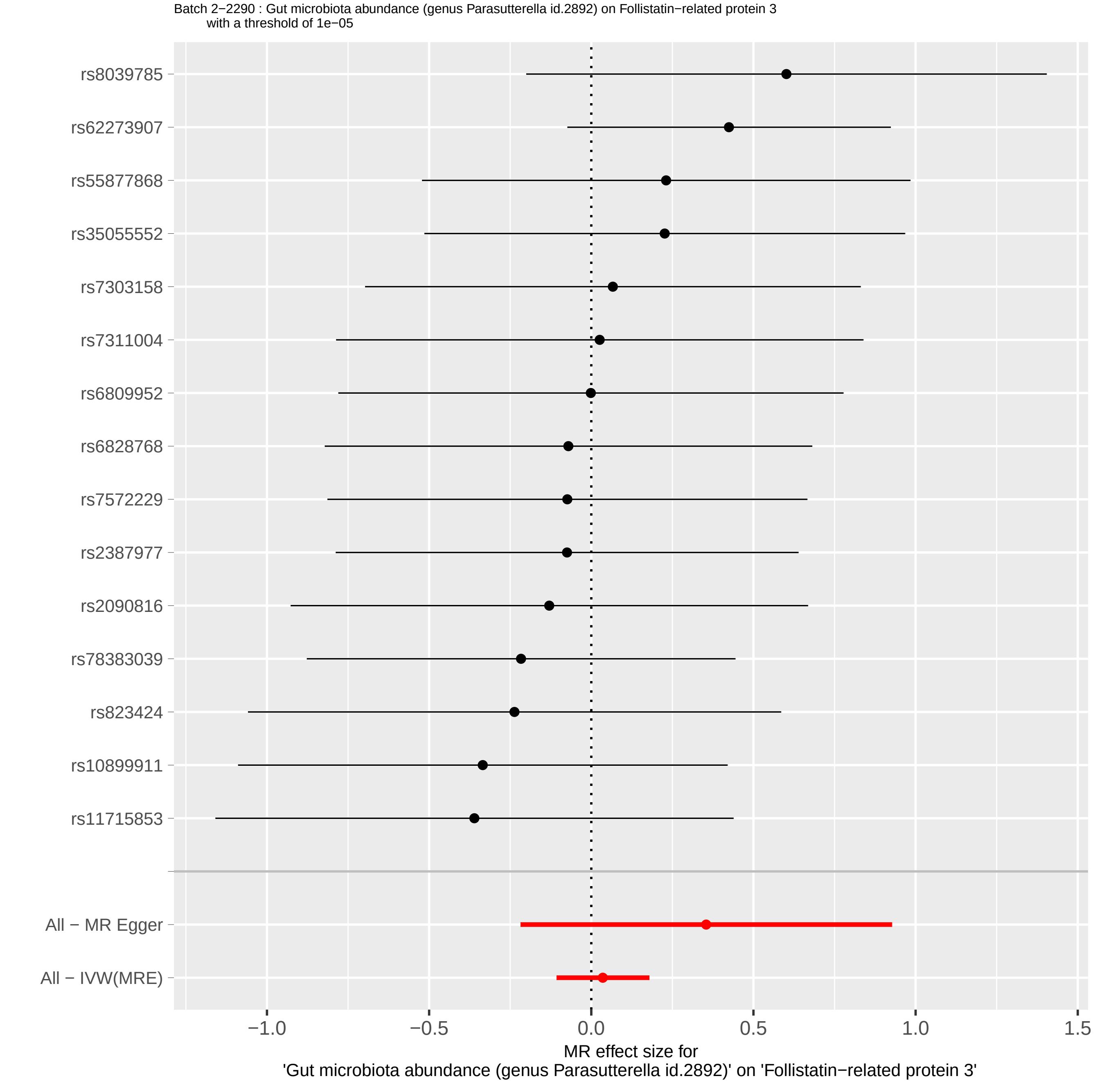

Batch 2−2290 : Gut microbiota abundance (genus Parasutterella id.2892) on Follistatin−related protein 3
with a threshold of 1e−05
rs8039785
rs62273907
rs55877868
rs35055552
rs7303158
rs7311004
rs6809952
rs6828768
rs7572229
rs2387977
rs2090816
rs78383039
rs823424
rs10899911
rs11715853
All − MR Egger
All − IVW(MRE)
−1.0
−0.5
0.0
0.5
1.0
1.5
MR effect size for
'Gut microbiota abundance (genus Parasutterella id.2892)' on 'Follistatin−related protein 3'

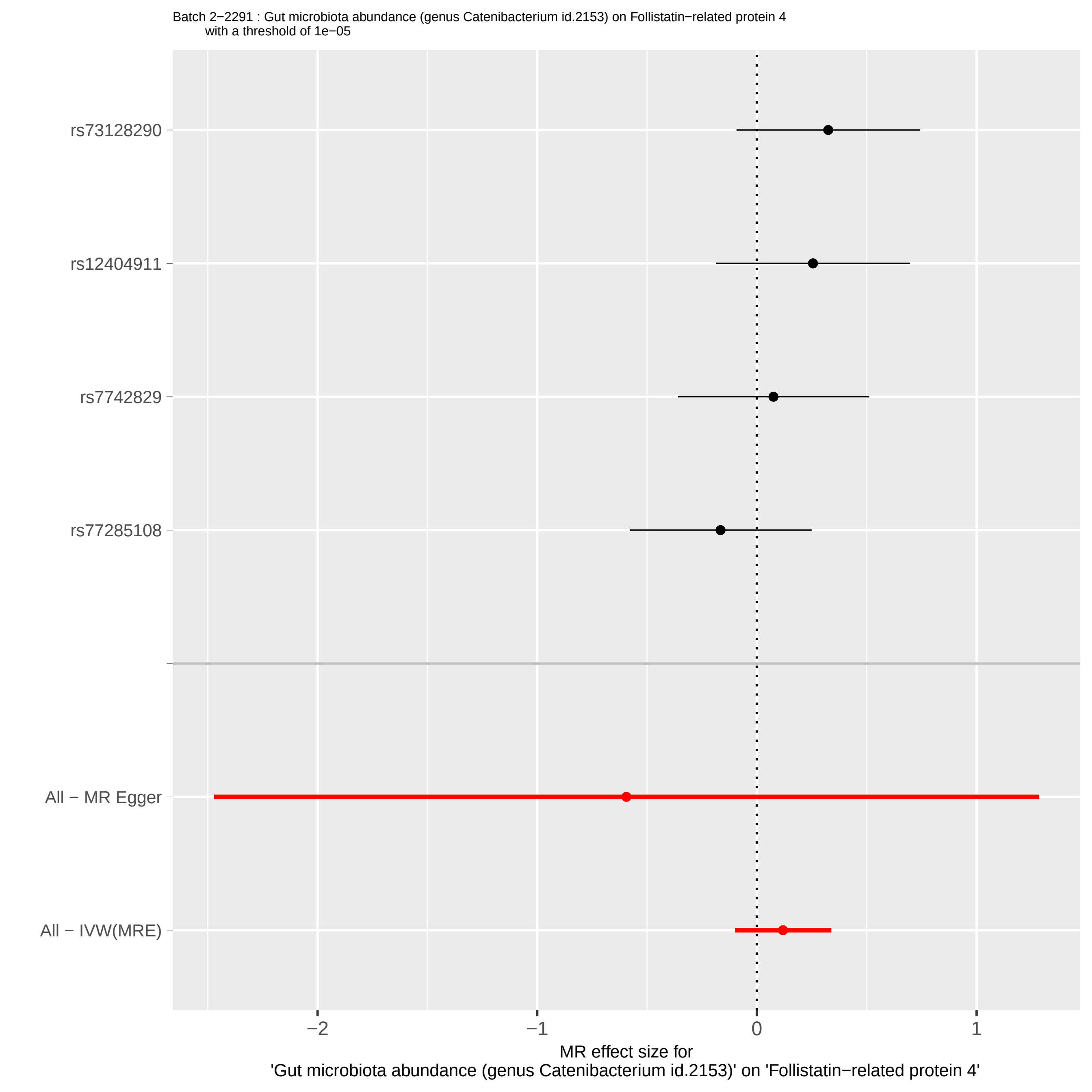

Batch 2−2291 : Gut microbiota abundance (genus Catenibacterium id.2153) on Follistatin−related protein 4
with a threshold of 1e−05
rs73128290
rs12404911
rs7742829
rs77285108
All − MR Egger
All − IVW(MRE)
−2
−1
0
1
MR effect size for
'Gut microbiota abundance (genus Catenibacterium id.2153)' on 'Follistatin−related protein 4'

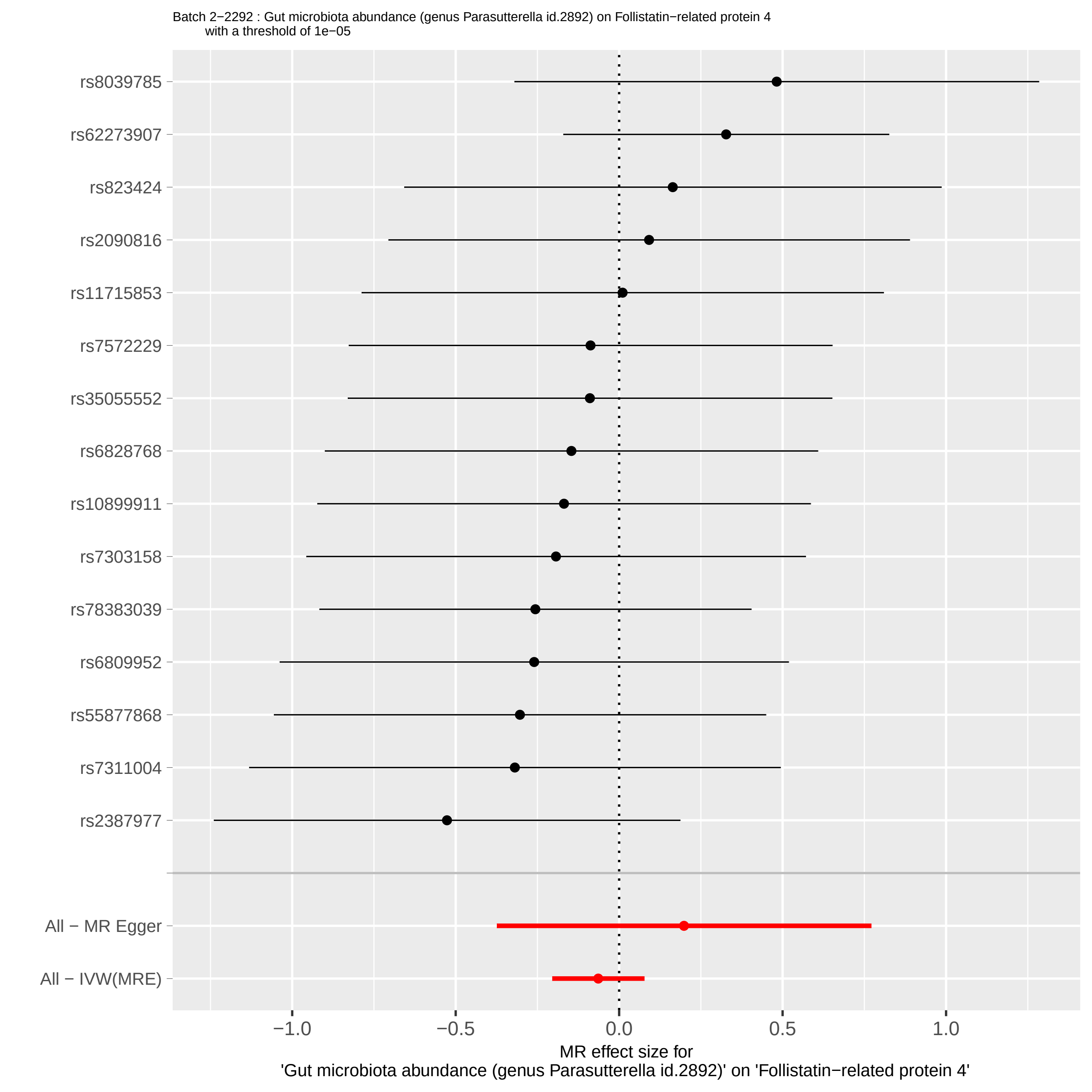

Batch 2−2292 : Gut microbiota abundance (genus Parasutterella id.2892) on Follistatin−related protein 4
with a threshold of 1e−05
rs8039785
rs62273907
rs823424
rs2090816
rs11715853
rs7572229
rs35055552
rs6828768
rs10899911
rs7303158
rs78383039
rs6809952
rs55877868
rs7311004
rs2387977
All − MR Egger
All − IVW(MRE)
−1.0
−0.5
0.0
0.5
1.0
MR effect size for
'Gut microbiota abundance (genus Parasutterella id.2892)' on 'Follistatin−related protein 4'

Batch 2−2293 : Gut microbiota abundance (genus Catenibacterium id.2153) on Follistatin−related protein 5
with a threshold of 1e−05

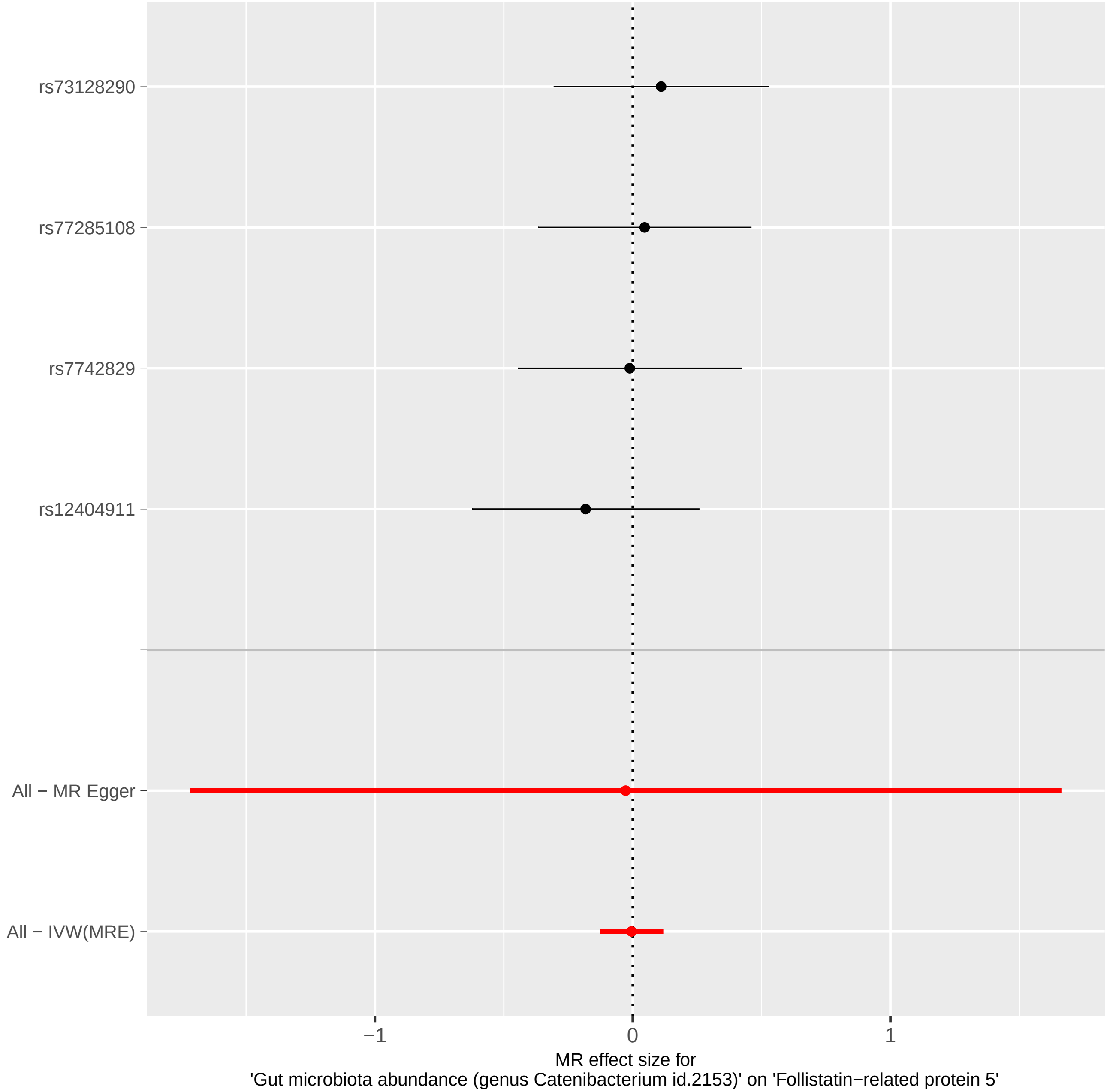

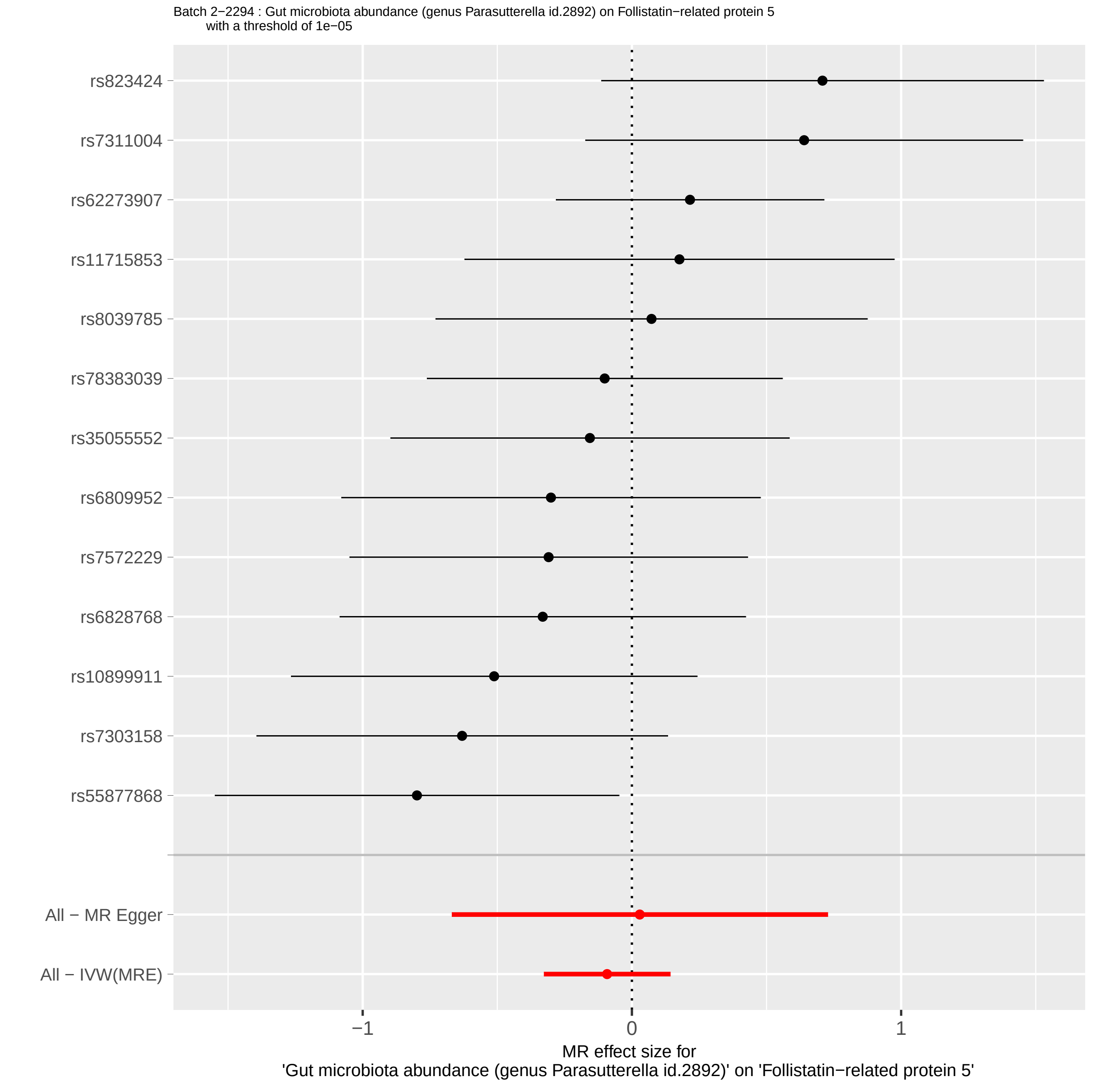
Batch 2−2294 : Gut microbiota abundance (genus Parasutterella id.2892) on Follistatin−related protein 5
with a threshold of 1e−05
rs823424
rs7311004
rs62273907
rs11715853
rs8039785
rs78383039
rs35055552
rs6809952
rs7572229
rs6828768
rs10899911
rs7303158
rs55877868
All − MR Egger
All − IVW(MRE)
−1
0
1
MR effect size for
'Gut microbiota abundance (genus Parasutterella id.2892)' on 'Follistatin−related protein 5'

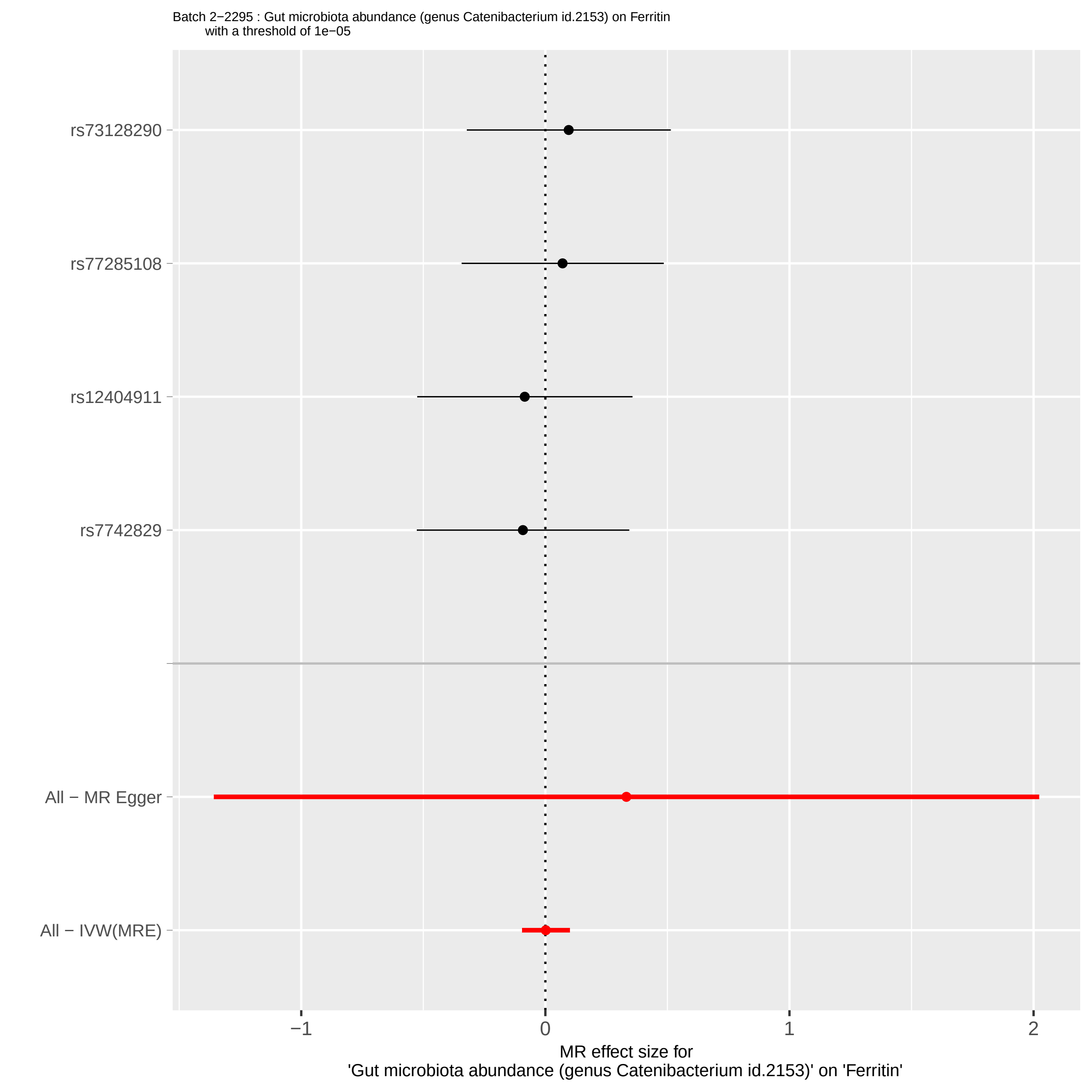

Batch 2−2295 : Gut microbiota abundance (genus Catenibacterium id.2153) on Ferritin
with a threshold of 1e−05
rs73128290
rs77285108
rs12404911
rs7742829
All − MR Egger
All − IVW(MRE)
−1
0
1
2
MR effect size for
'Gut microbiota abundance (genus Catenibacterium id.2153)' on 'Ferritin'

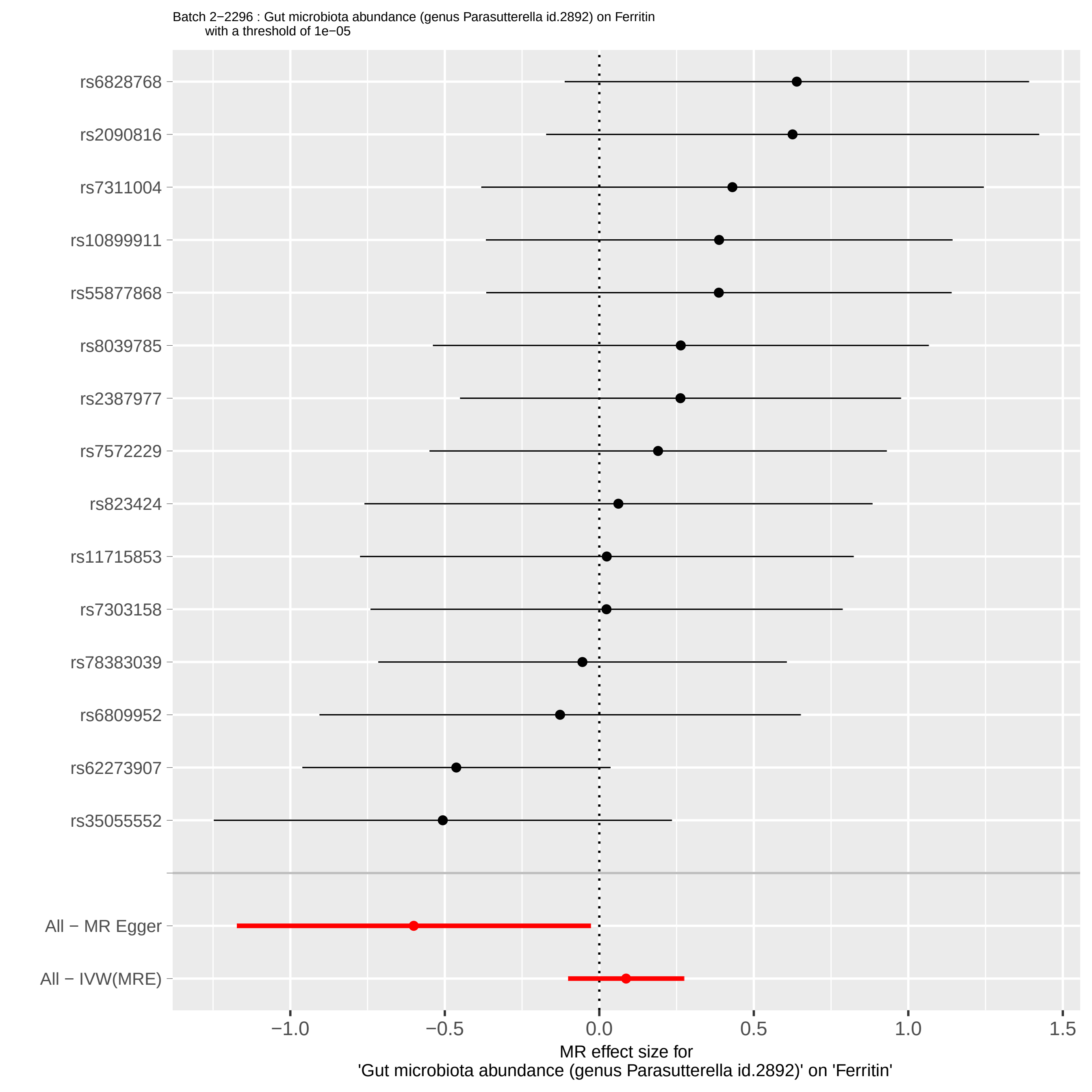

Batch 2−2296 : Gut microbiota abundance (genus Parasutterella id.2892) on Ferritin
with a threshold of 1e−05
rs6828768
rs2090816
rs7311004
rs10899911
rs55877868
rs8039785
rs2387977
rs7572229
rs823424
rs11715853
rs7303158
rs78383039
rs6809952
rs62273907
rs35055552
All − MR Egger
All − IVW(MRE)
−1.0
−0.5
0.0
0.5
1.0
1.5
MR effect size for
'Gut microbiota abundance (genus Parasutterella id.2892)' on 'Ferritin'

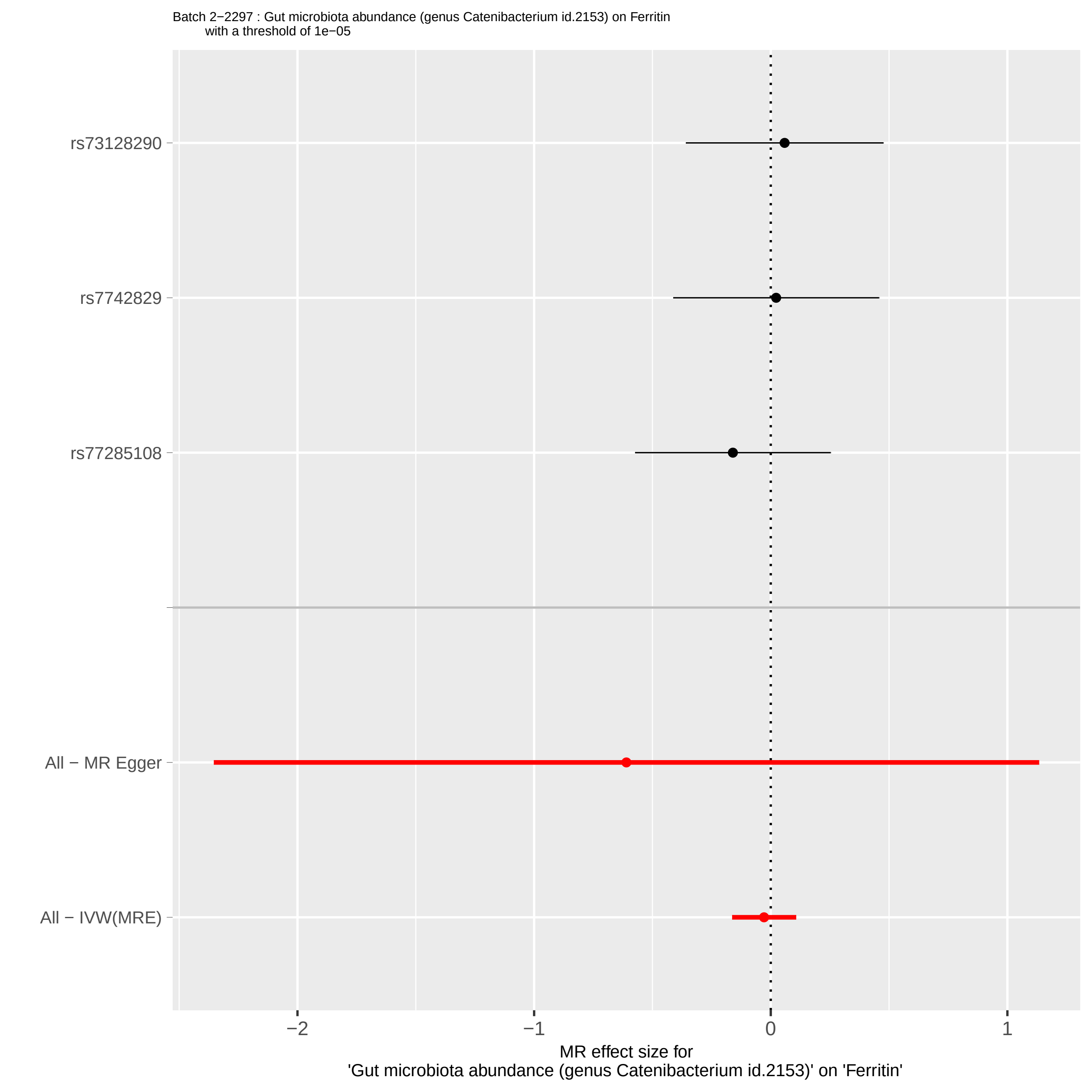

Batch 2−2297 : Gut microbiota abundance (genus Catenibacterium id.2153) on Ferritin
with a threshold of 1e−05
rs73128290
rs7742829
rs77285108
All − MR Egger
All − IVW(MRE)
−2
−1
0
1
MR effect size for
'Gut microbiota abundance (genus Catenibacterium id.2153)' on 'Ferritin'

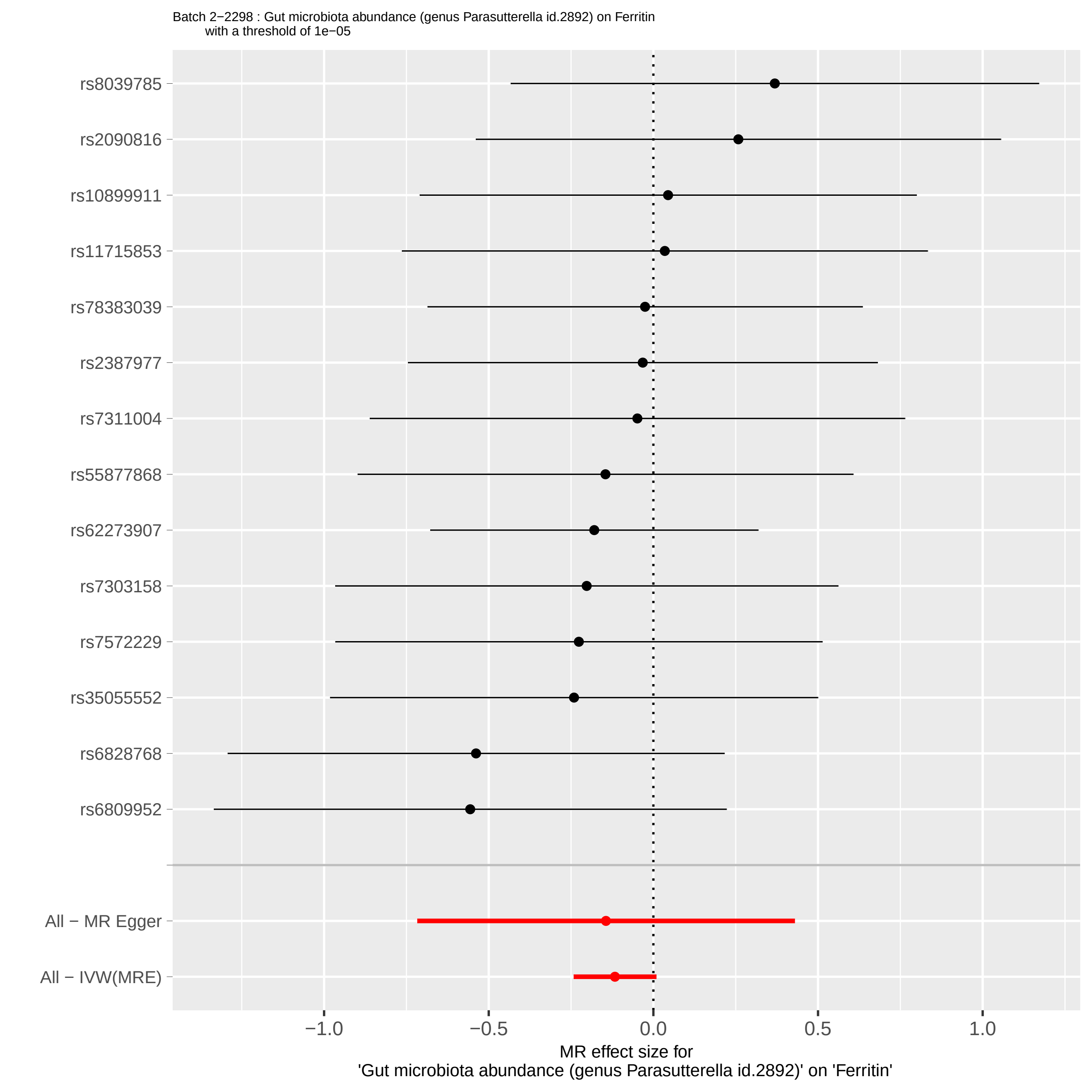

Batch 2−2298 : Gut microbiota abundance (genus Parasutterella id.2892) on Ferritin
with a threshold of 1e−05
rs8039785
rs2090816
rs10899911
rs11715853
rs78383039
rs2387977
rs7311004
rs55877868
rs62273907
rs7303158
rs7572229
rs35055552
rs6828768
rs6809952
All − MR Egger
All − IVW(MRE)
−1.0
−0.5
0.0
0.5
1.0
MR effect size for
'Gut microbiota abundance (genus Parasutterella id.2892)' on 'Ferritin'

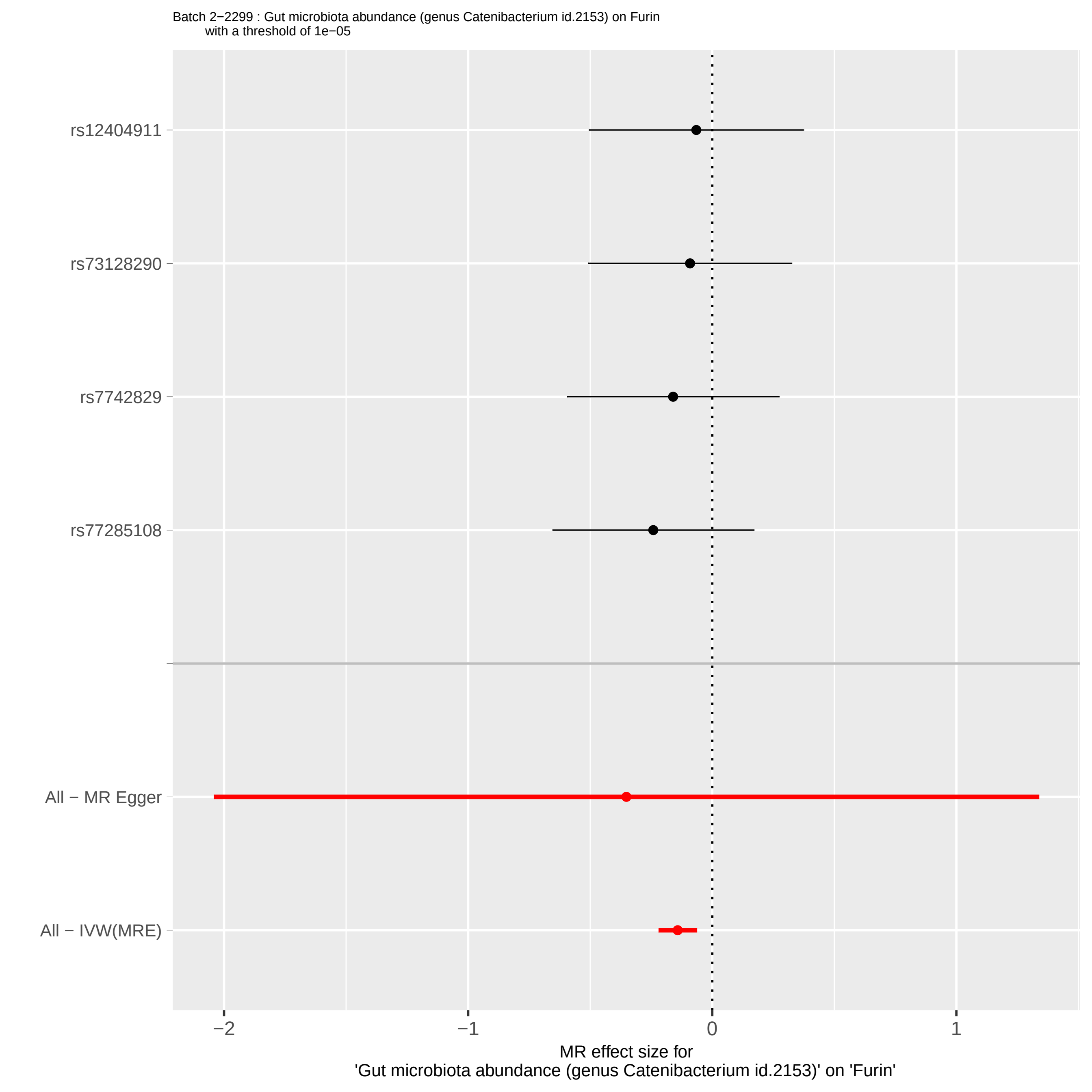
Batch 2−2299 : Gut microbiota abundance (genus Catenibacterium id.2153) on Furin
with a threshold of 1e−05
rs12404911
rs73128290
rs7742829
rs77285108
All − MR Egger
All − IVW(MRE)
−2
−1
0
1
MR effect size for
'Gut microbiota abundance (genus Catenibacterium id.2153)' on 'Furin'

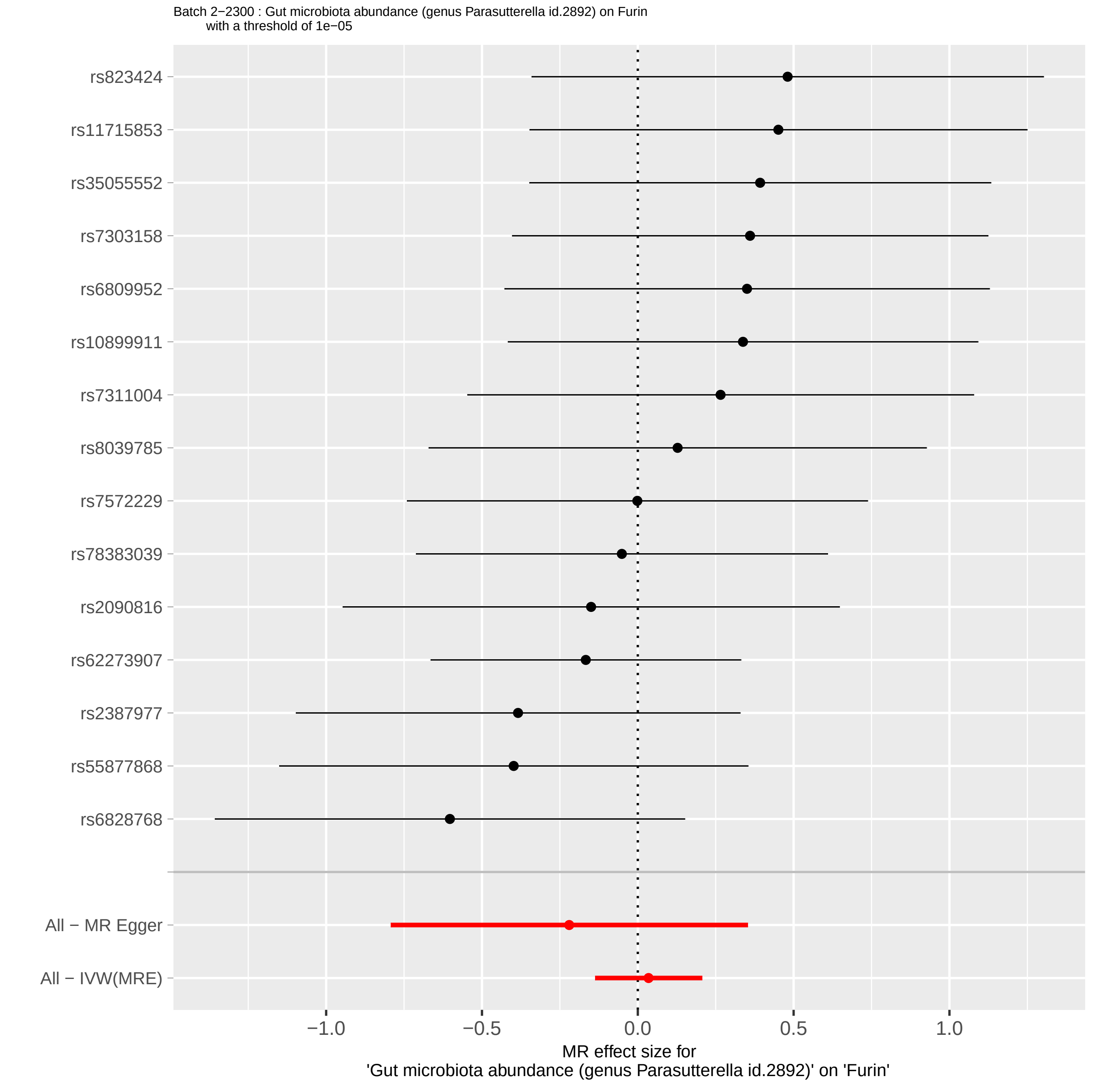

Batch 2−2300 : Gut microbiota abundance (genus Parasutterella id.2892) on Furin
with a threshold of 1e−05
rs823424
rs11715853
rs35055552
rs7303158
rs6809952
rs10899911
rs7311004
rs8039785
rs7572229
rs78383039
rs2090816
rs62273907
rs2387977
rs55877868
rs6828768
All − MR Egger
All − IVW(MRE)
−1.0
−0.5
0.0
0.5
1.0
MR effect size for
'Gut microbiota abundance (genus Parasutterella id.2892)' on 'Furin'

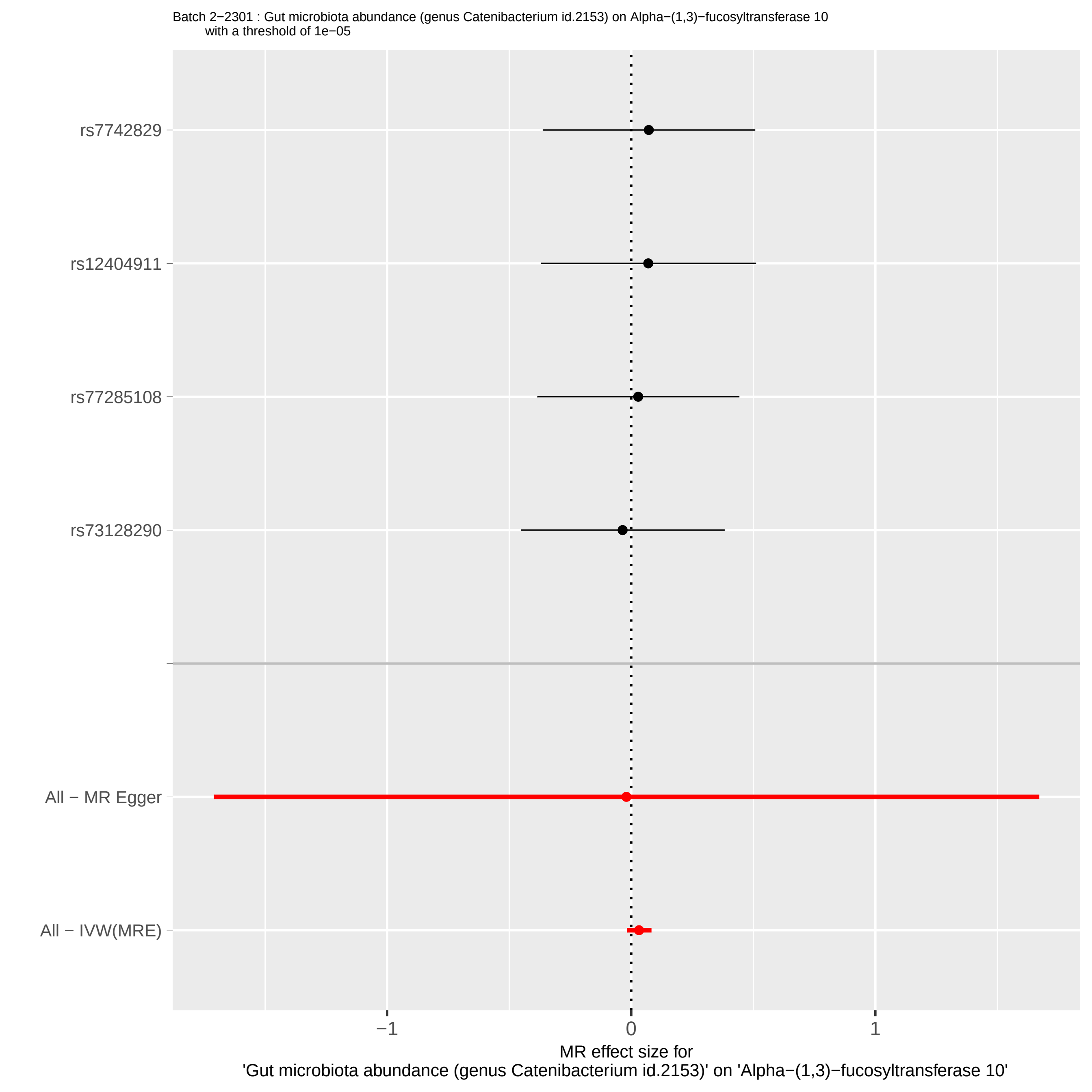
Batch 2−2301 : Gut microbiota abundance (genus Catenibacterium id.2153) on Alpha−(1,3)−fucosyltransferase 10
with a threshold of 1e−05
rs7742829
rs12404911
rs77285108
rs73128290
All − MR Egger
All − IVW(MRE)
−1
0
1
MR effect size for
'Gut microbiota abundance (genus Catenibacterium id.2153)' on 'Alpha−(1,3)−fucosyltransferase 10'

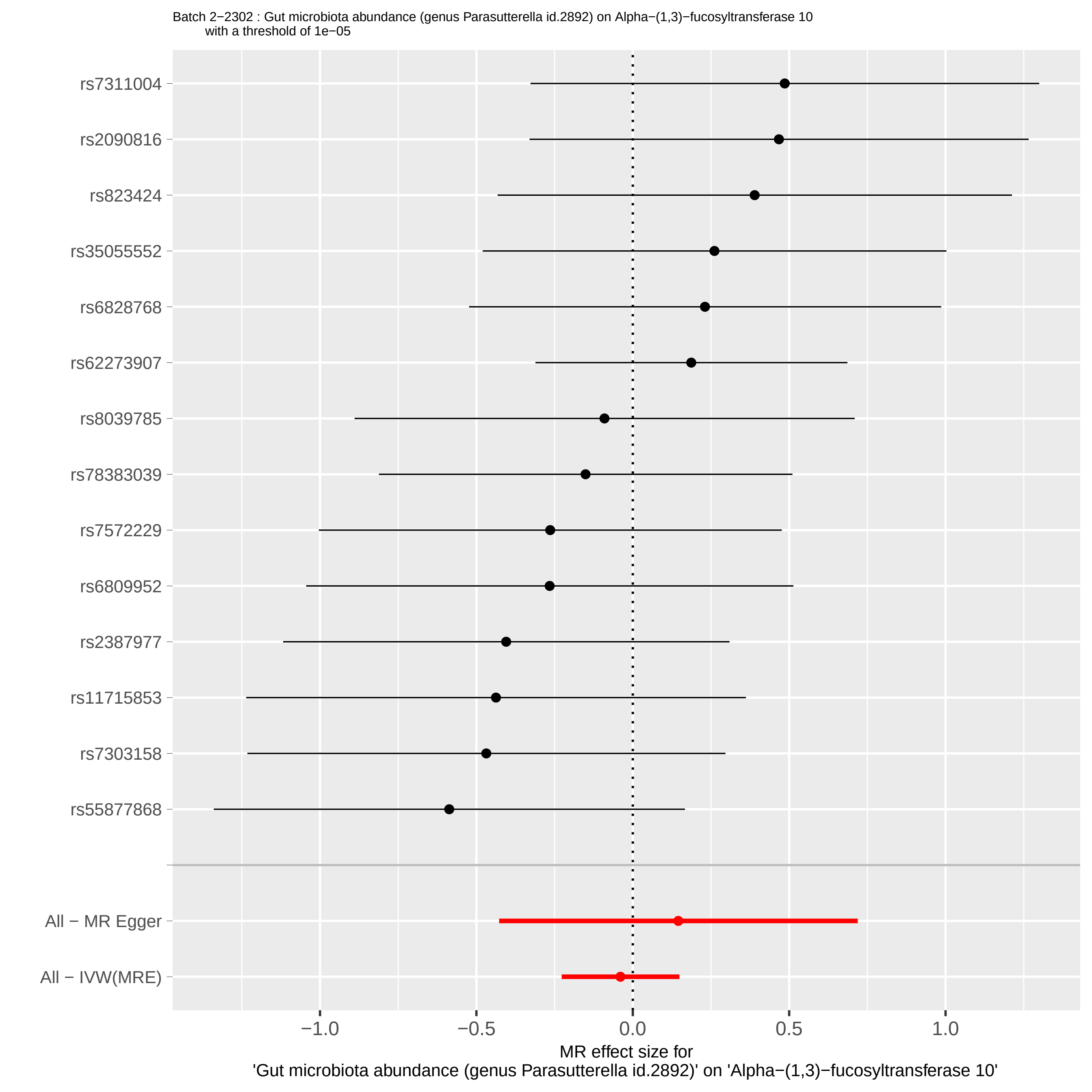

Batch 2−2302 : Gut microbiota abundance (genus Parasutterella id.2892) on Alpha−(1,3)−fucosyltransferase 10
with a threshold of 1e−05
rs7311004
rs2090816
rs823424
rs35055552
rs6828768
rs62273907
rs8039785
rs78383039
rs7572229
rs6809952
rs2387977
rs11715853
rs7303158
rs55877868
All − MR Egger
All − IVW(MRE)
−1.0
−0.5
0.0
0.5
1.0
MR effect size for
'Gut microbiota abundance (genus Parasutterella id.2892)' on 'Alpha−(1,3)−fucosyltransferase 10'

Batch 2−2303 : Gut microbiota abundance (genus Catenibacterium id.2153) on Galactoside 3(4)−L−fucosyltransferase
with a threshold of 1e−05

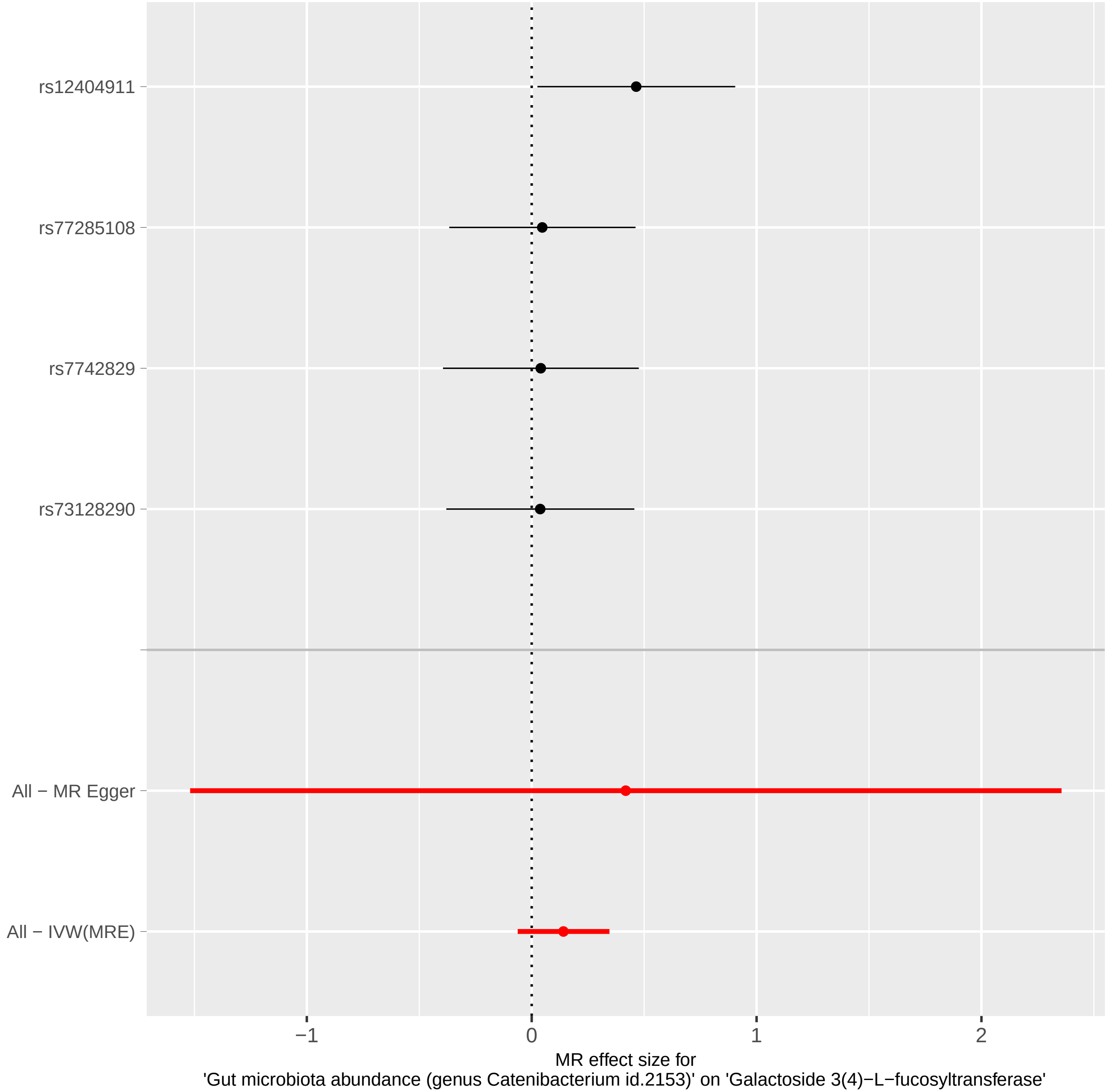

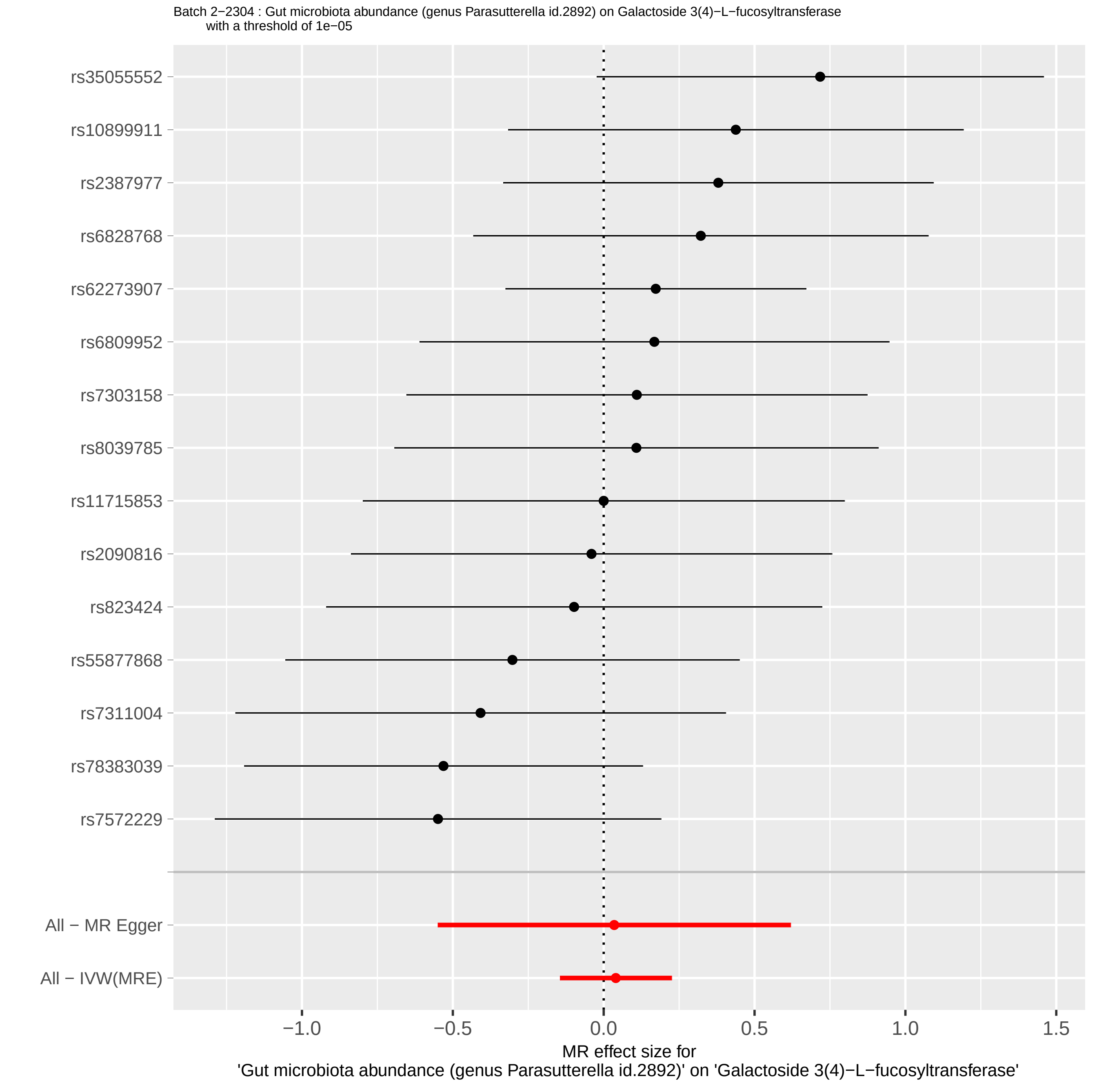

Batch 2−2304 : Gut microbiota abundance (genus Parasutterella id.2892) on Galactoside 3(4)−L−fucosyltransferase
with a threshold of 1e−05
rs35055552
rs10899911
rs2387977
rs6828768
rs62273907
rs6809952
rs7303158
rs8039785
rs11715853
rs2090816
rs823424
rs55877868
rs7311004
rs78383039
rs7572229
All − MR Egger
All − IVW(MRE)
−1.0
−0.5
0.0
0.5
1.0
1.5
MR effect size for
'Gut microbiota abundance (genus Parasutterella id.2892)' on 'Galactoside 3(4)−L−fucosyltransferase'

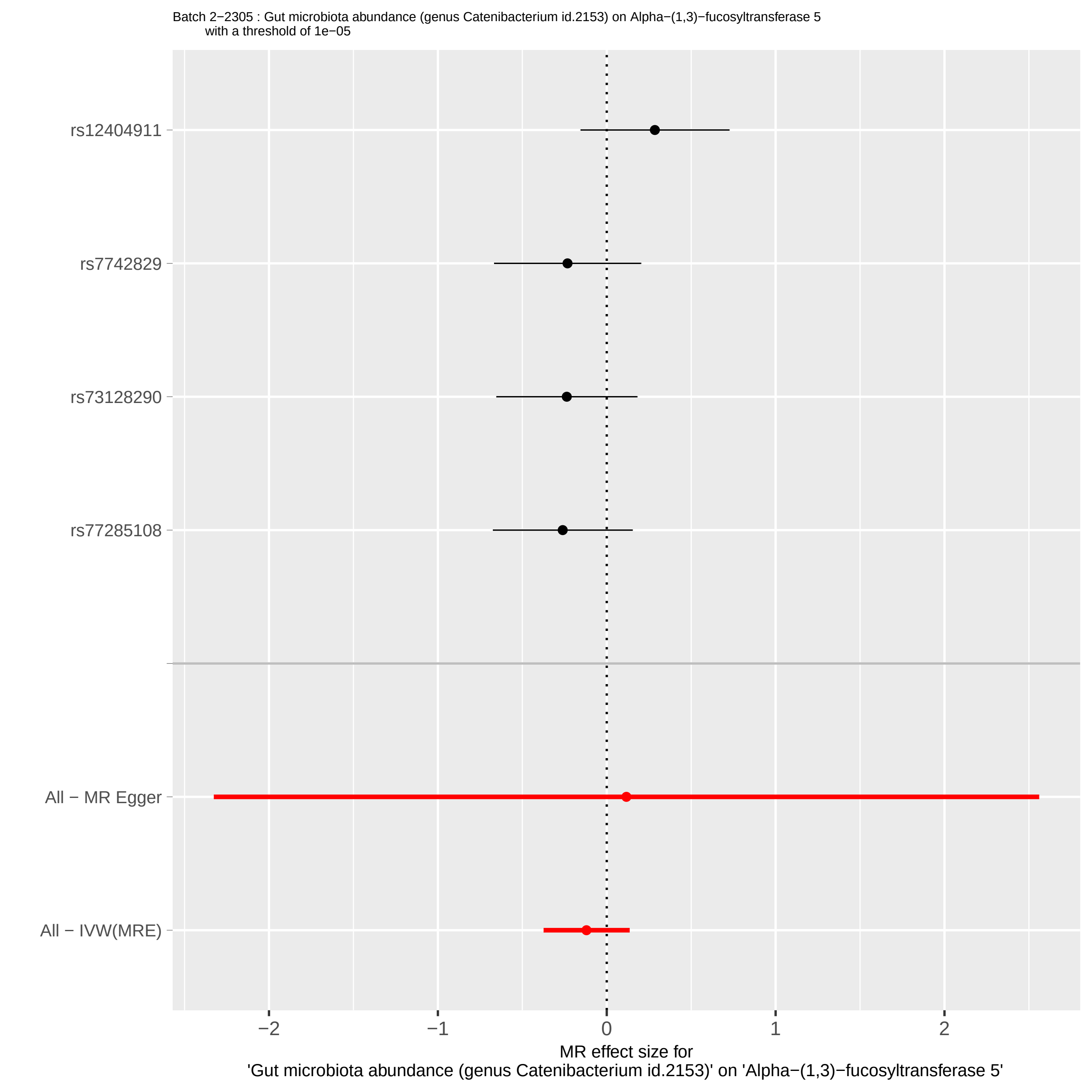
Batch 2−2305 : Gut microbiota abundance (genus Catenibacterium id.2153) on Alpha−(1,3)−fucosyltransferase 5
with a threshold of 1e−05
rs12404911
rs7742829
rs73128290
rs77285108
All − MR Egger
All − IVW(MRE)
−2
−1
0
1
2
MR effect size for
'Gut microbiota abundance (genus Catenibacterium id.2153)' on 'Alpha−(1,3)−fucosyltransferase 5'

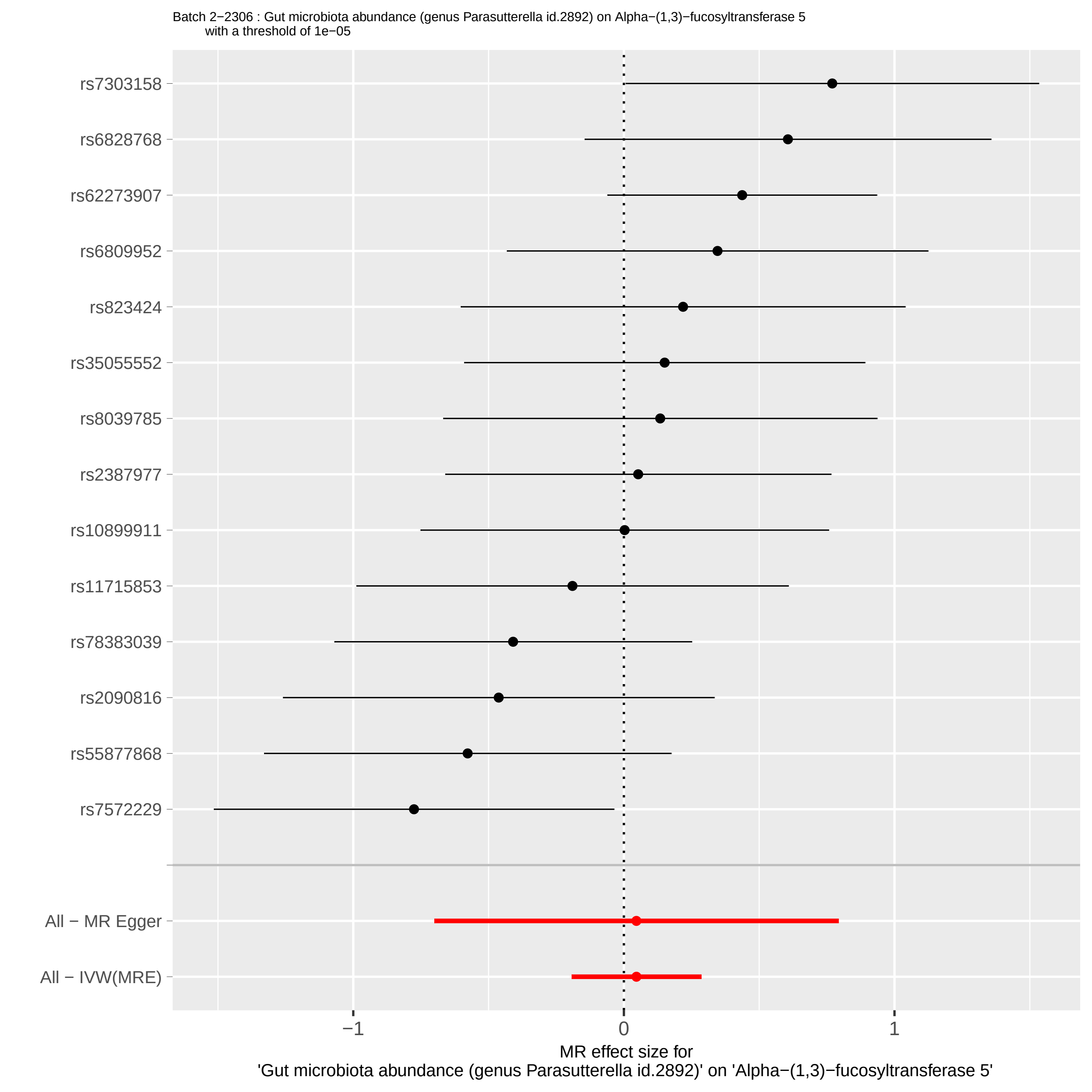

Batch 2−2306 : Gut microbiota abundance (genus Parasutterella id.2892) on Alpha−(1,3)−fucosyltransferase 5
with a threshold of 1e−05
rs7303158
rs6828768
rs62273907
rs6809952
rs823424
rs35055552
rs8039785
rs2387977
rs10899911
rs11715853
rs78383039
rs2090816
rs55877868
rs7572229
All − MR Egger
All − IVW(MRE)
−1
0
1
MR effect size for
'Gut microbiota abundance (genus Parasutterella id.2892)' on 'Alpha−(1,3)−fucosyltransferase 5'

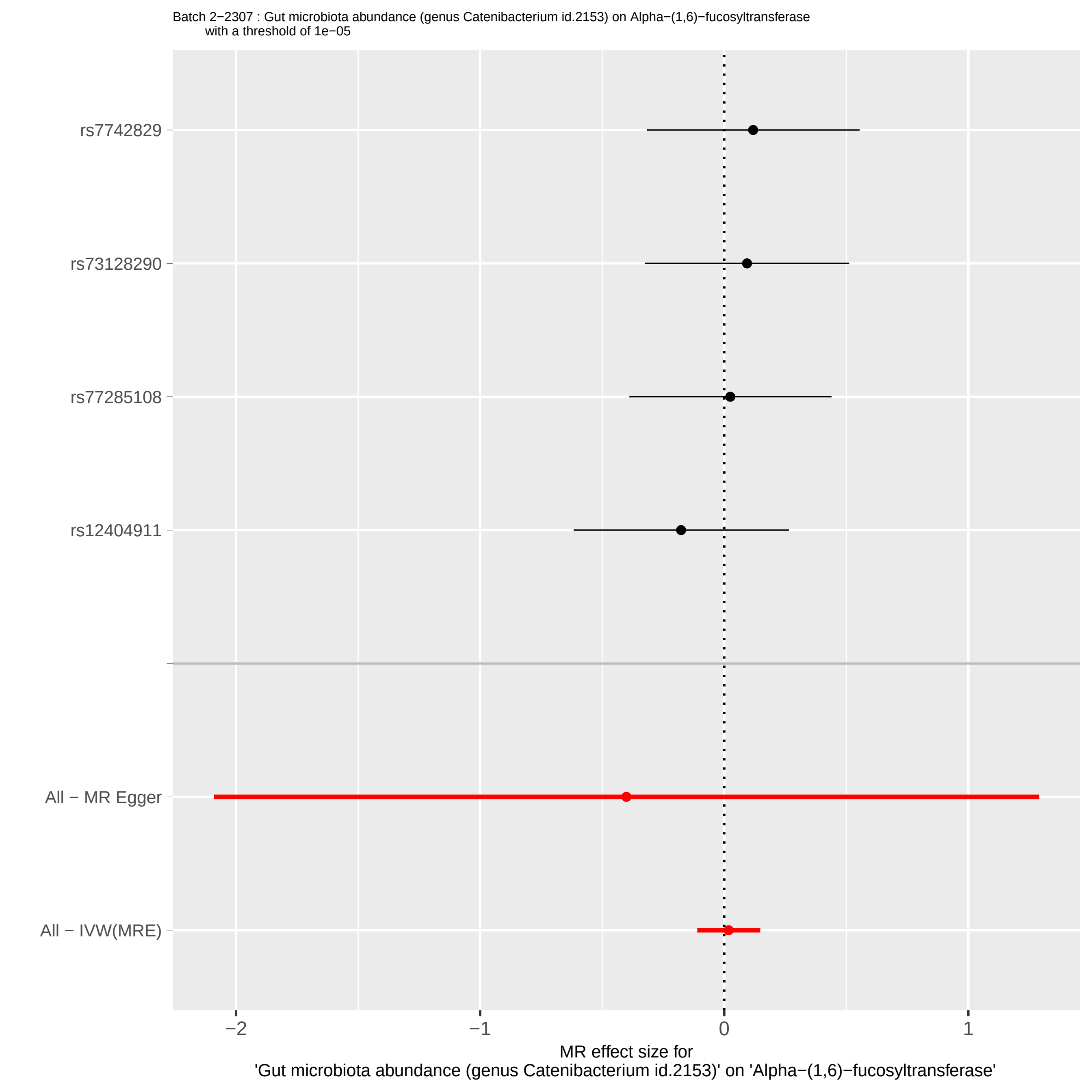
Batch 2−2307 : Gut microbiota abundance (genus Catenibacterium id.2153) on Alpha−(1,6)−fucosyltransferase
with a threshold of 1e−05
rs7742829
rs73128290
rs77285108
rs12404911
All − MR Egger
All − IVW(MRE)
−2
−1
0
1
MR effect size for
'Gut microbiota abundance (genus Catenibacterium id.2153)' on 'Alpha−(1,6)−fucosyltransferase'

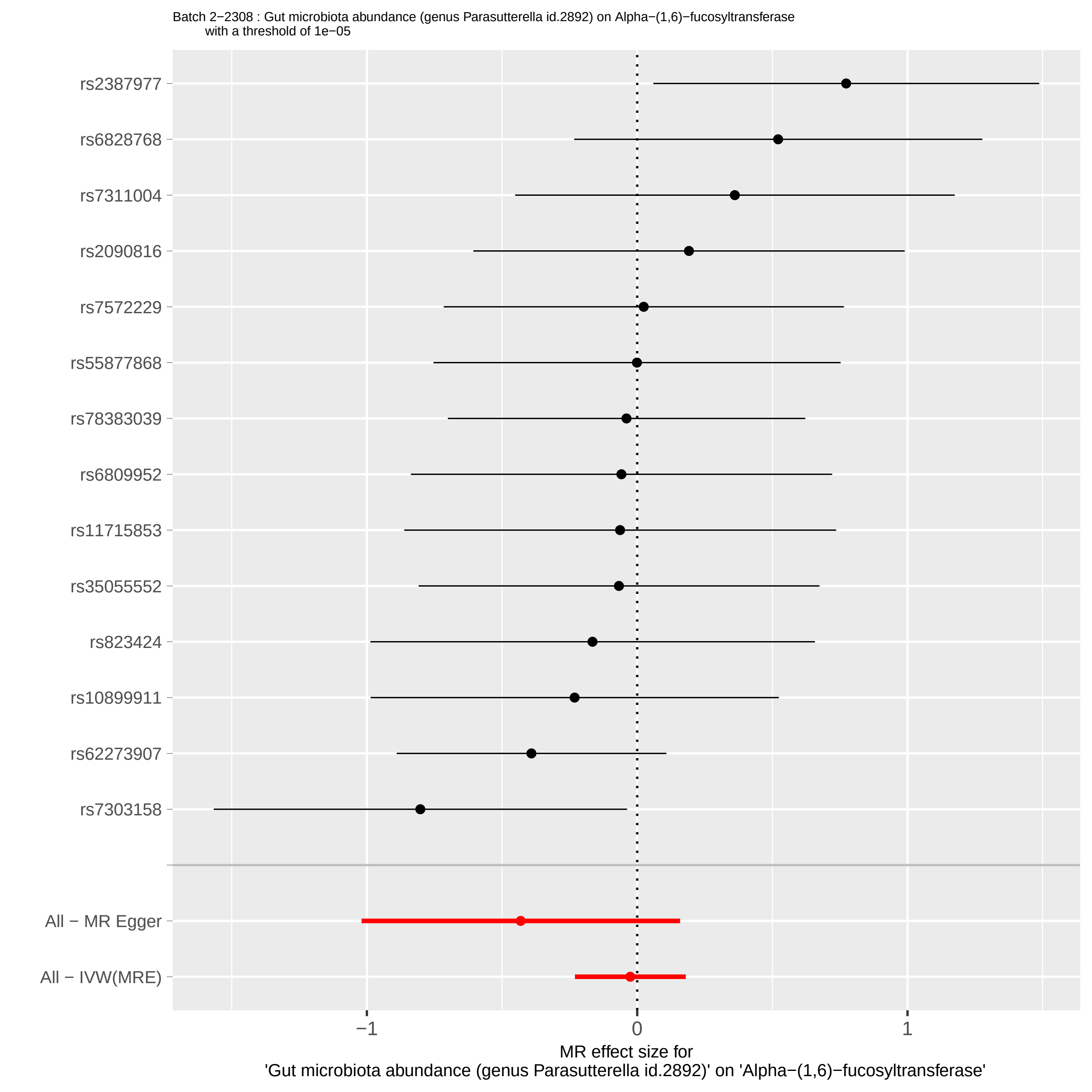

Batch 2−2308 : Gut microbiota abundance (genus Parasutterella id.2892) on Alpha−(1,6)−fucosyltransferase
with a threshold of 1e−05
rs2387977
rs6828768
rs7311004
rs2090816
rs7572229
rs55877868
rs78383039
rs6809952
rs11715853
rs35055552
rs823424
rs10899911
rs62273907
rs7303158
All − MR Egger
All − IVW(MRE)
−1
0
1
MR effect size for
'Gut microbiota abundance (genus Parasutterella id.2892)' on 'Alpha−(1,6)−fucosyltransferase'

Batch 2−2309 : Gut microbiota abundance (genus Catenibacterium id.2153) on Alpha−(1,3)−fucosyltransferase 9
with a threshold of 1e−05

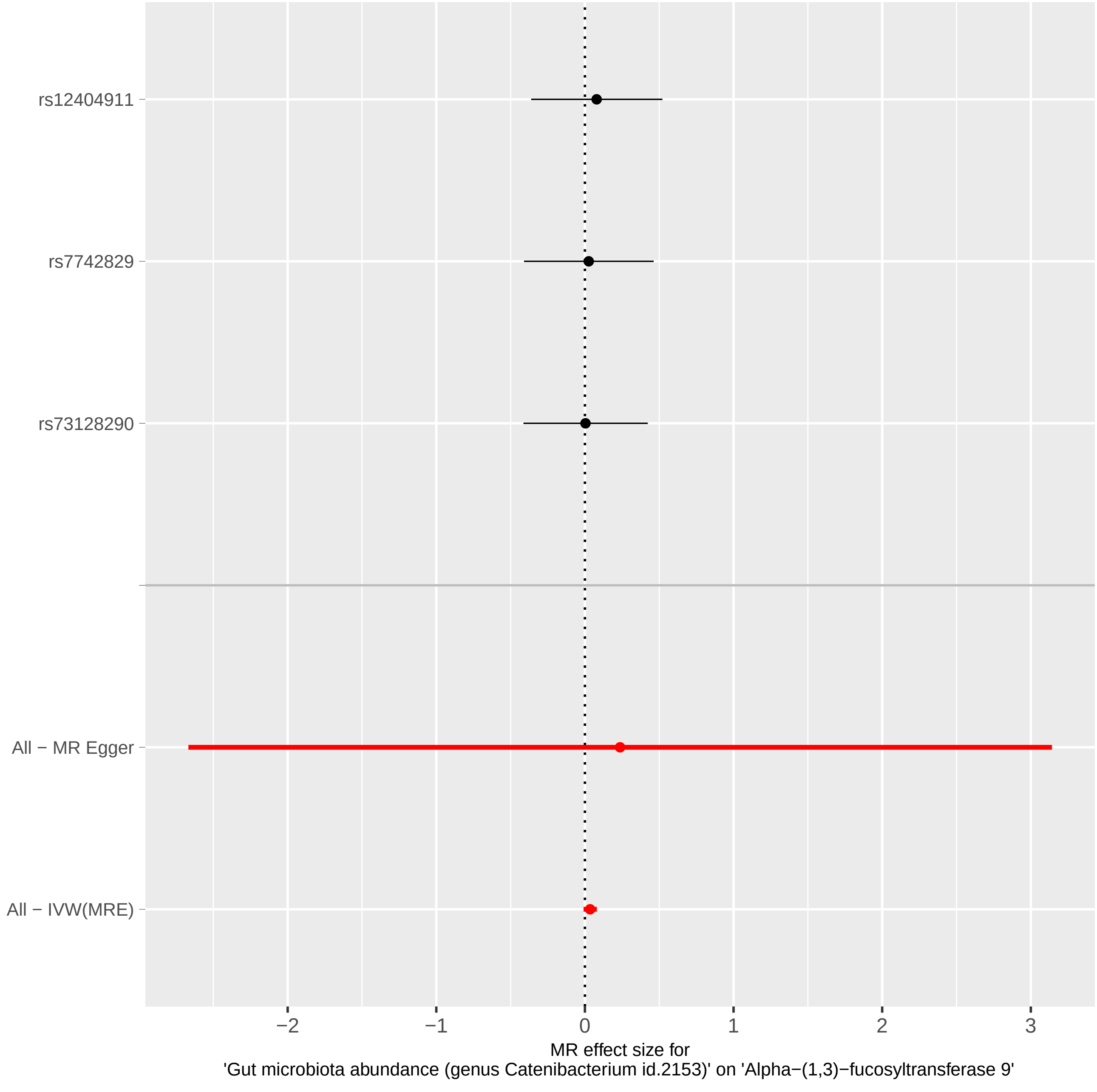

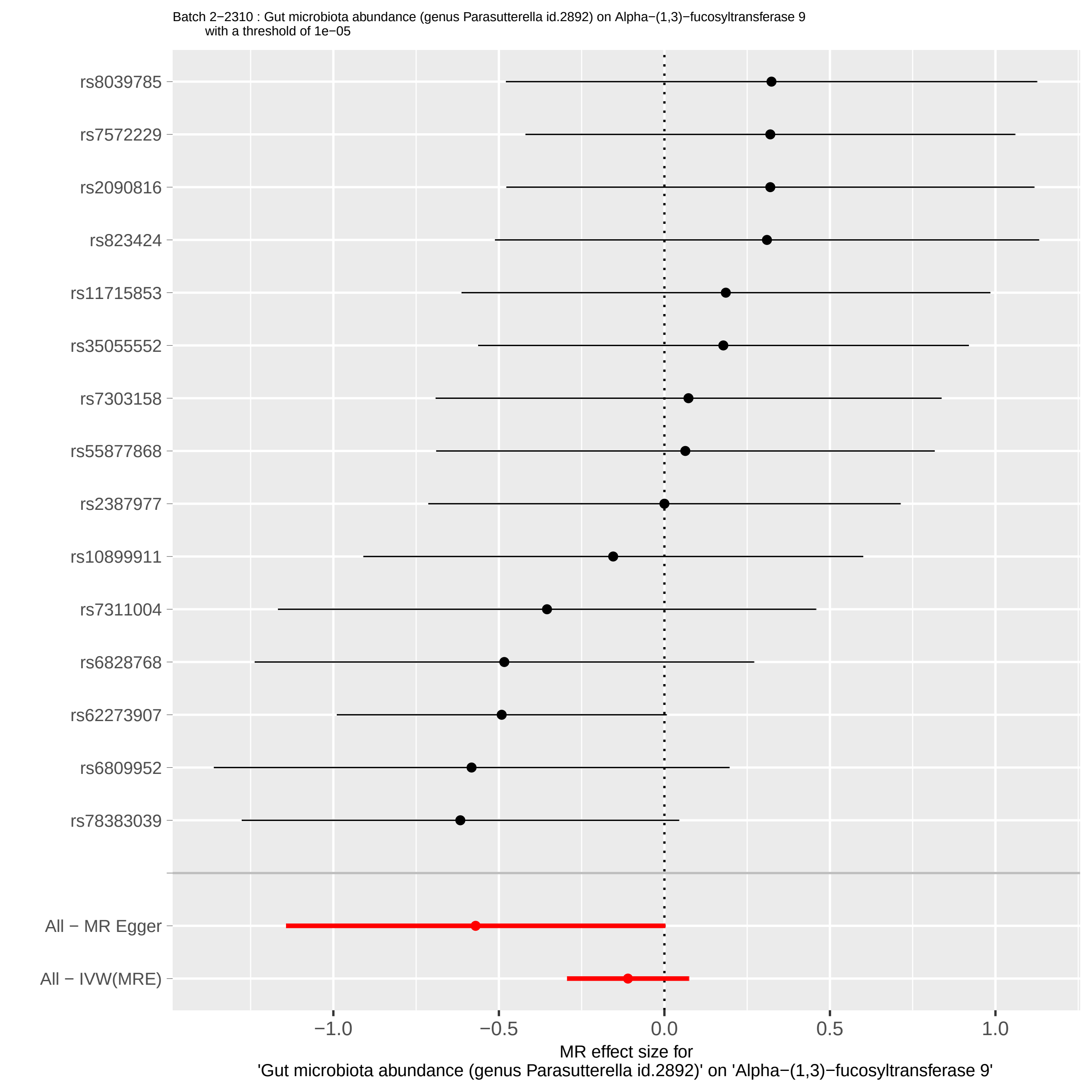
Batch 2−2310 : Gut microbiota abundance (genus Parasutterella id.2892) on Alpha−(1,3)−fucosyltransferase 9
with a threshold of 1e−05
rs8039785
rs7572229
rs2090816
rs823424
rs11715853
rs35055552
rs7303158
rs55877868
rs2387977
rs10899911
rs7311004
rs6828768
rs62273907
rs6809952
rs78383039
All − MR Egger
All − IVW(MRE)
−1.0
−0.5
0.0
0.5
1.0
MR effect size for
'Gut microbiota abundance (genus Parasutterella id.2892)' on 'Alpha−(1,3)−fucosyltransferase 9'

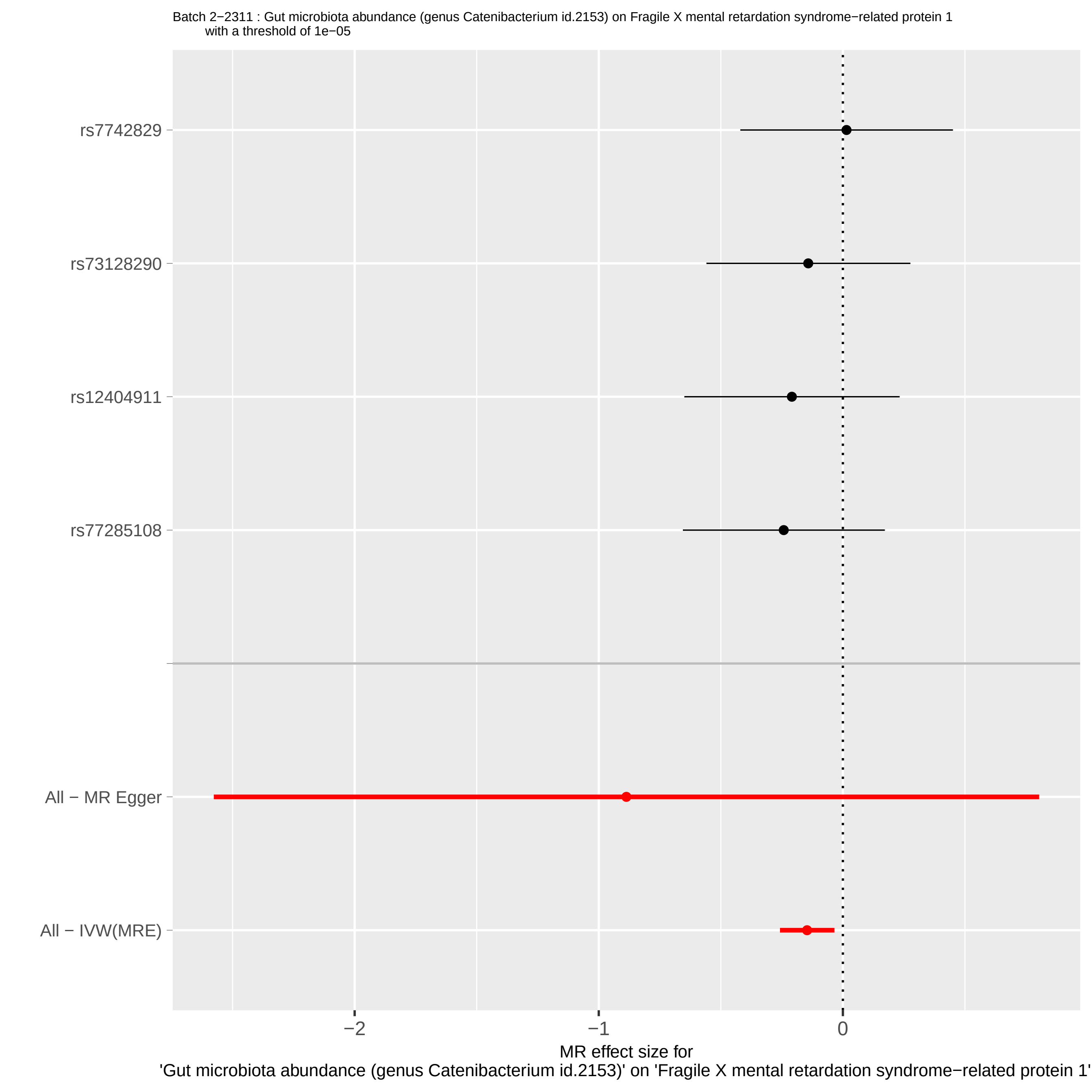
Batch 2−2311 : Gut microbiota abundance (genus Catenibacterium id.2153) on Fragile X mental retardation syndrome−related protein 1
with a threshold of 1e−05
rs7742829
rs73128290
rs12404911
rs77285108
All − MR Egger
All − IVW(MRE)
−2
−1
0
MR effect size for
'Gut microbiota abundance (genus Catenibacterium id.2153)' on 'Fragile X mental retardation syndrome−related protein 1'

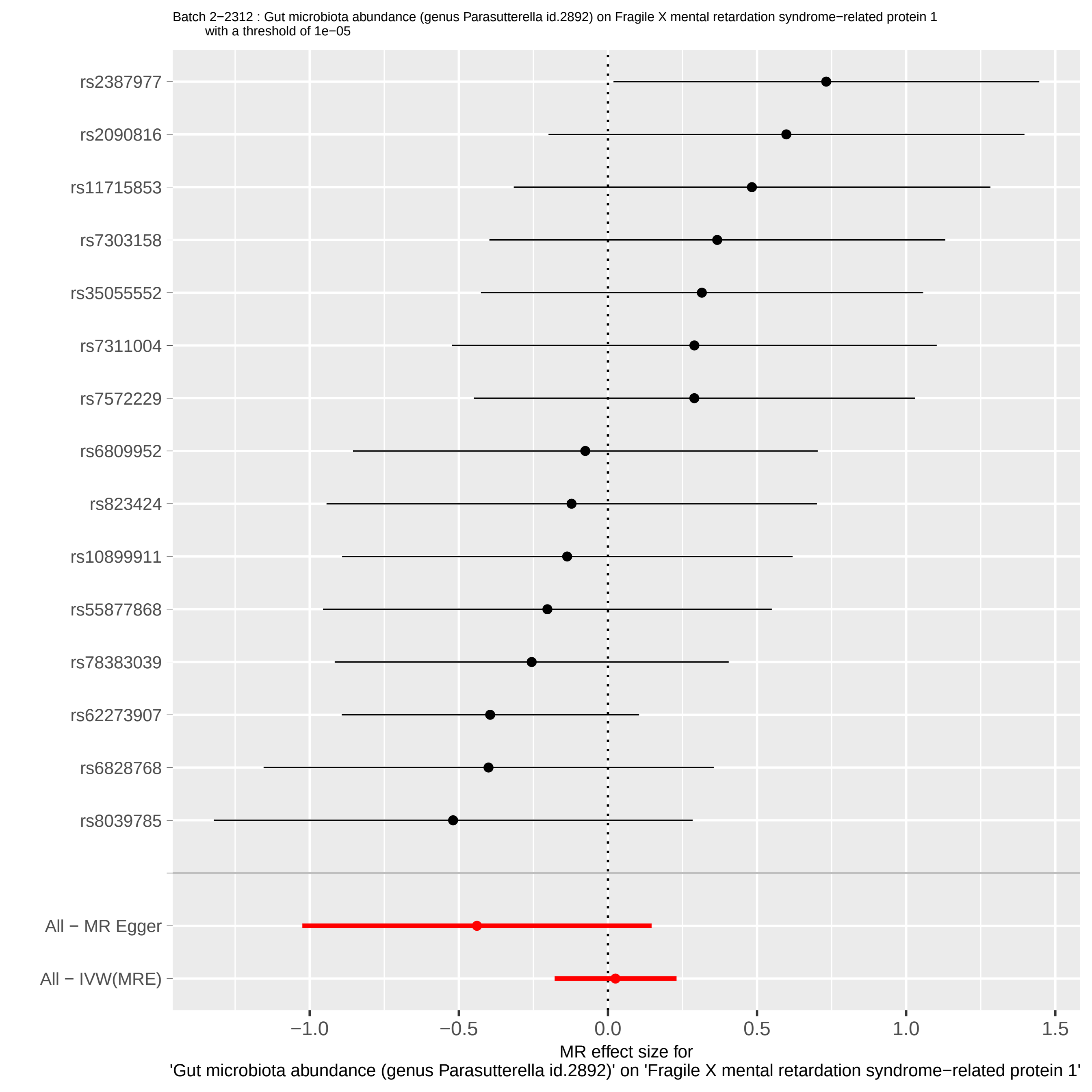

Batch 2−2312 : Gut microbiota abundance (genus Parasutterella id.2892) on Fragile X mental retardation syndrome−related protein 1
with a threshold of 1e−05
rs2387977
rs2090816
rs11715853
rs7303158
rs35055552
rs7311004
rs7572229
rs6809952
rs823424
rs10899911
rs55877868
rs78383039
rs62273907
rs6828768
rs8039785
All − MR Egger
All − IVW(MRE)
−1.0
−0.5
0.0
0.5
1.0
1.5
MR effect size for
'Gut microbiota abundance (genus Parasutterella id.2892)' on 'Fragile X mental retardation syndrome−related protein 1'

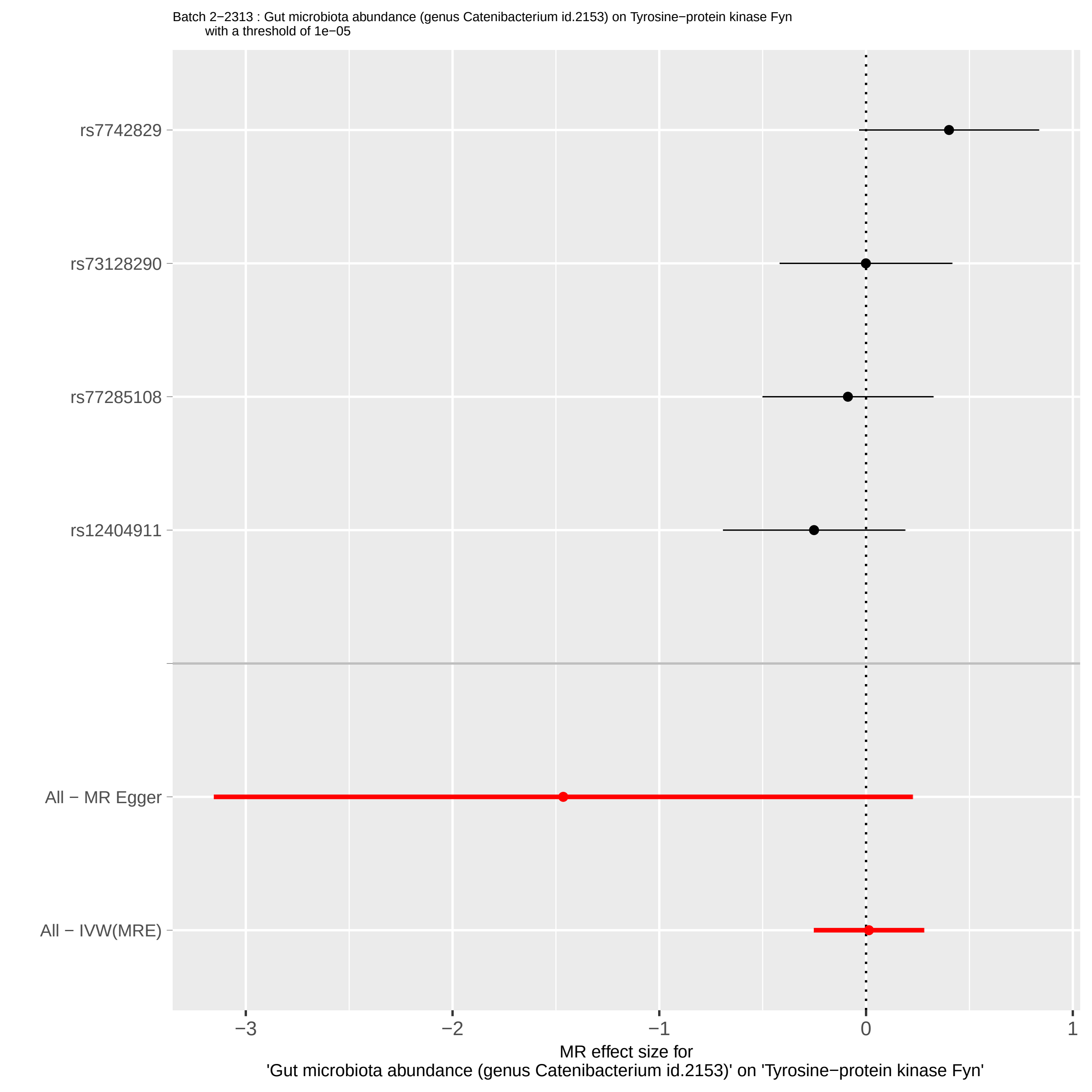

Batch 2−2313 : Gut microbiota abundance (genus Catenibacterium id.2153) on Tyrosine−protein kinase Fyn
with a threshold of 1e−05
rs7742829
rs73128290
rs77285108
rs12404911
All − MR Egger
All − IVW(MRE)
−3
−2
−1
0
1
MR effect size for
'Gut microbiota abundance (genus Catenibacterium id.2153)' on 'Tyrosine−protein kinase Fyn'

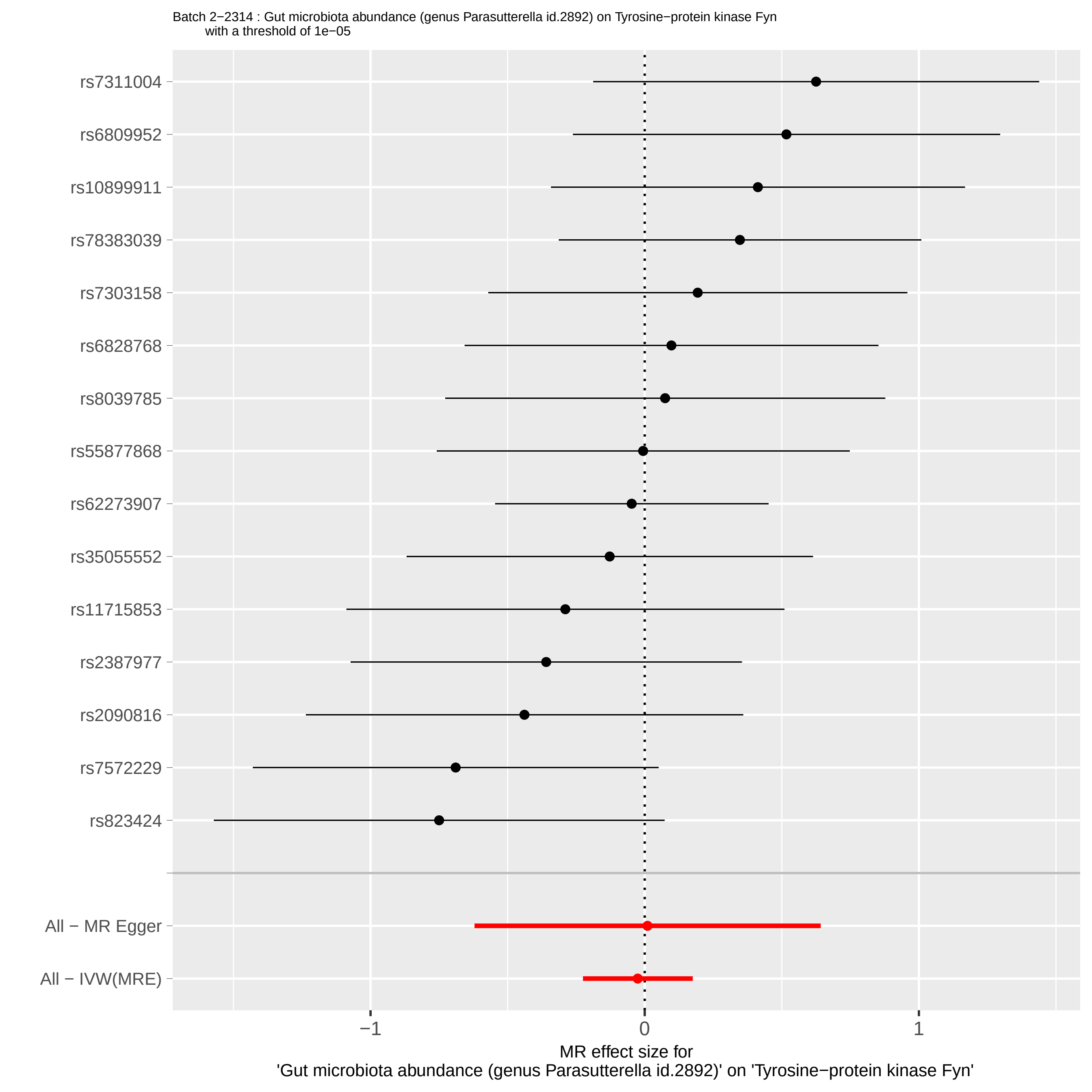

Batch 2−2314 : Gut microbiota abundance (genus Parasutterella id.2892) on Tyrosine−protein kinase Fyn
with a threshold of 1e−05
rs7311004
rs6809952
rs10899911
rs78383039
rs7303158
rs6828768
rs8039785
rs55877868
rs62273907
rs35055552
rs11715853
rs2387977
rs2090816
rs7572229
rs823424
All − MR Egger
All − IVW(MRE)
−1
0
1
MR effect size for
'Gut microbiota abundance (genus Parasutterella id.2892)' on 'Tyrosine−protein kinase Fyn'

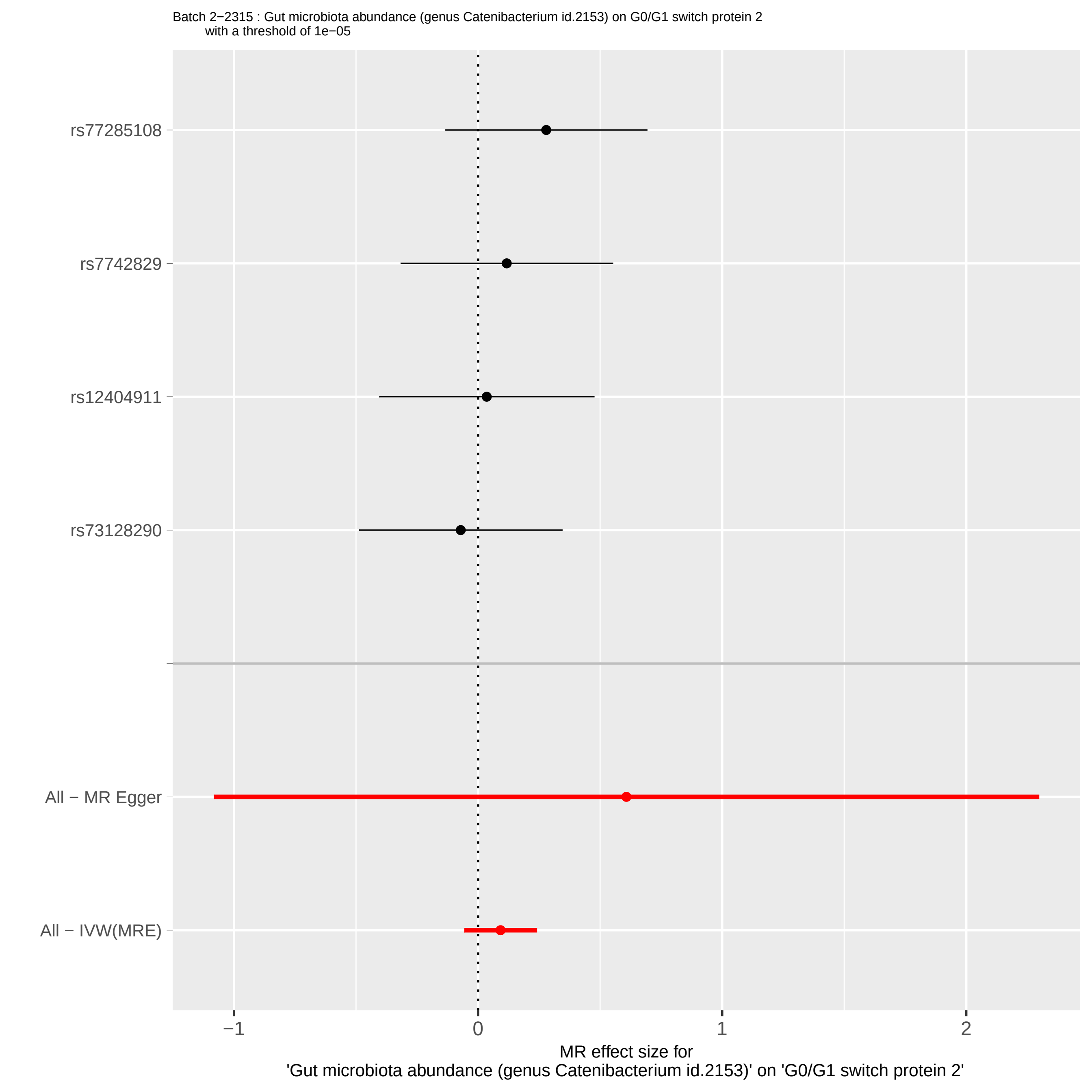

Batch 2−2315 : Gut microbiota abundance (genus Catenibacterium id.2153) on G0/G1 switch protein 2
with a threshold of 1e−05
rs77285108
rs7742829
rs12404911
rs73128290
All − MR Egger
All − IVW(MRE)
−1
0
1
2
MR effect size for
'Gut microbiota abundance (genus Catenibacterium id.2153)' on 'G0/G1 switch protein 2'

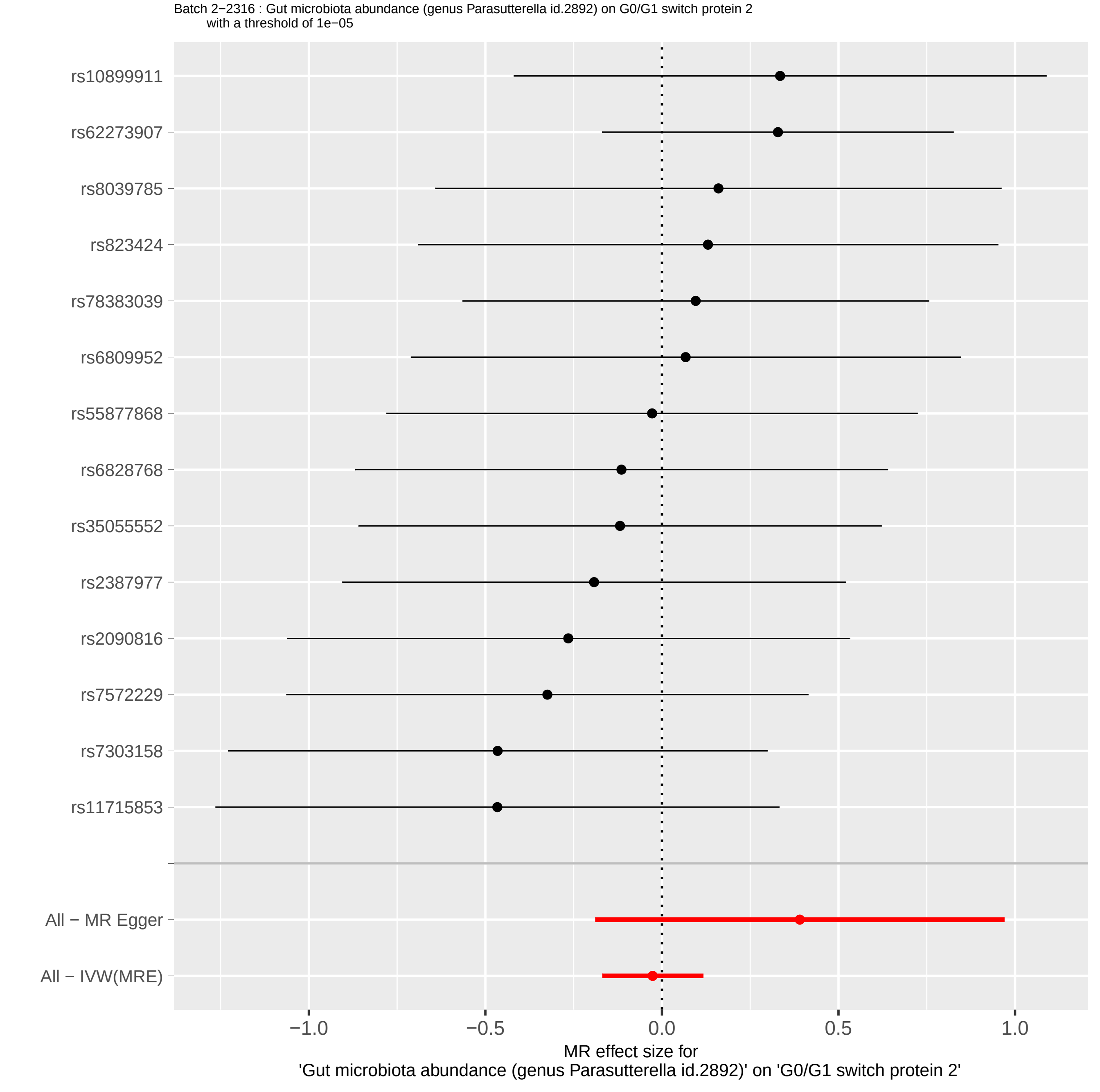

Batch 2−2316 : Gut microbiota abundance (genus Parasutterella id.2892) on G0/G1 switch protein 2
with a threshold of 1e−05
rs10899911
rs62273907
rs8039785
rs823424
rs78383039
rs6809952
rs55877868
rs6828768
rs35055552
rs2387977
rs2090816
rs7572229
rs7303158
rs11715853
All − MR Egger
All − IVW(MRE)
−1.0
−0.5
0.0
0.5
1.0
MR effect size for
'Gut microbiota abundance (genus Parasutterella id.2892)' on 'G0/G1 switch protein 2'

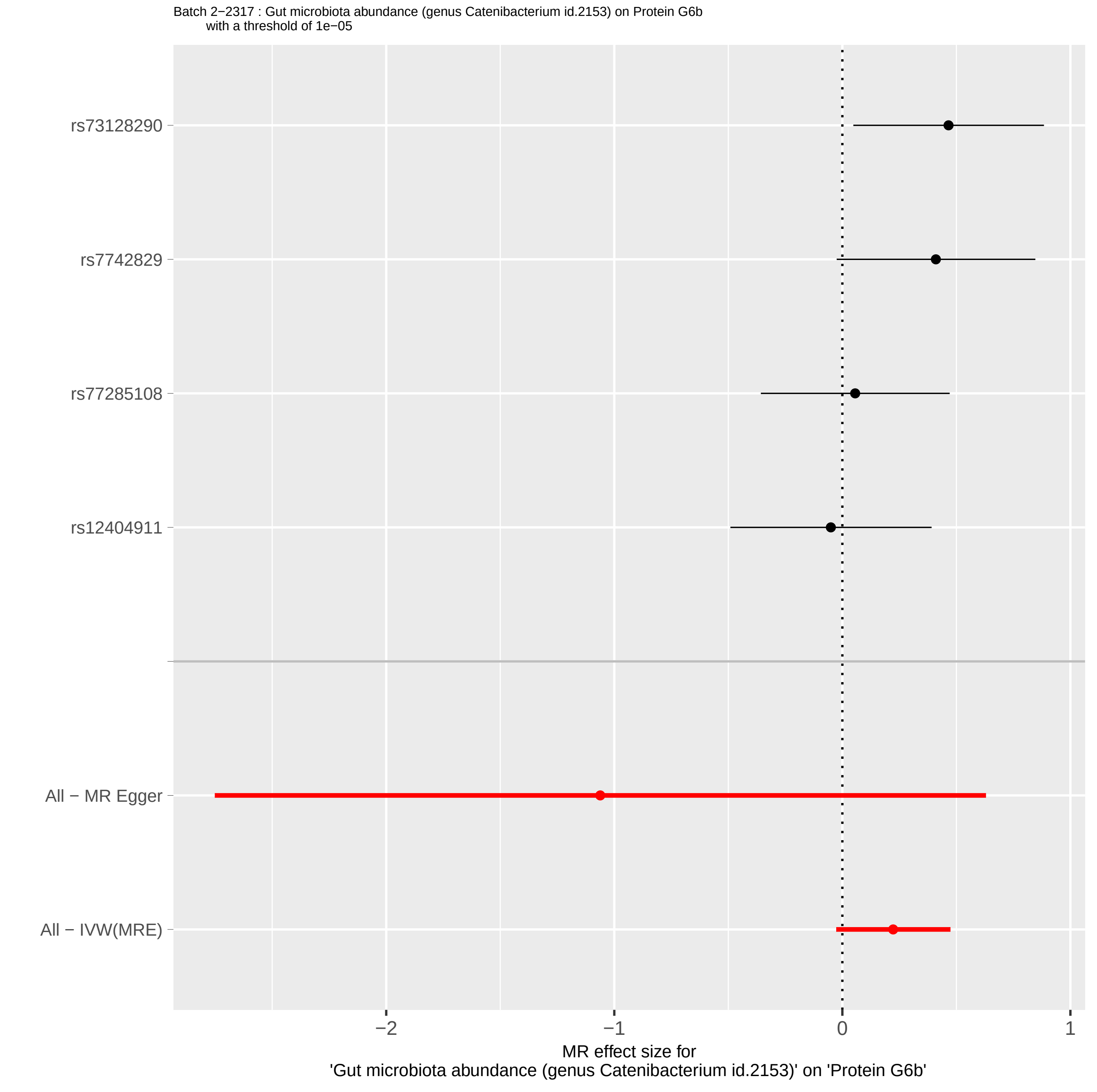

Batch 2−2317 : Gut microbiota abundance (genus Catenibacterium id.2153) on Protein G6b
with a threshold of 1e−05
rs73128290
rs7742829
rs77285108
rs12404911
All − MR Egger
All − IVW(MRE)
−2
−1
0
1
MR effect size for
'Gut microbiota abundance (genus Catenibacterium id.2153)' on 'Protein G6b'

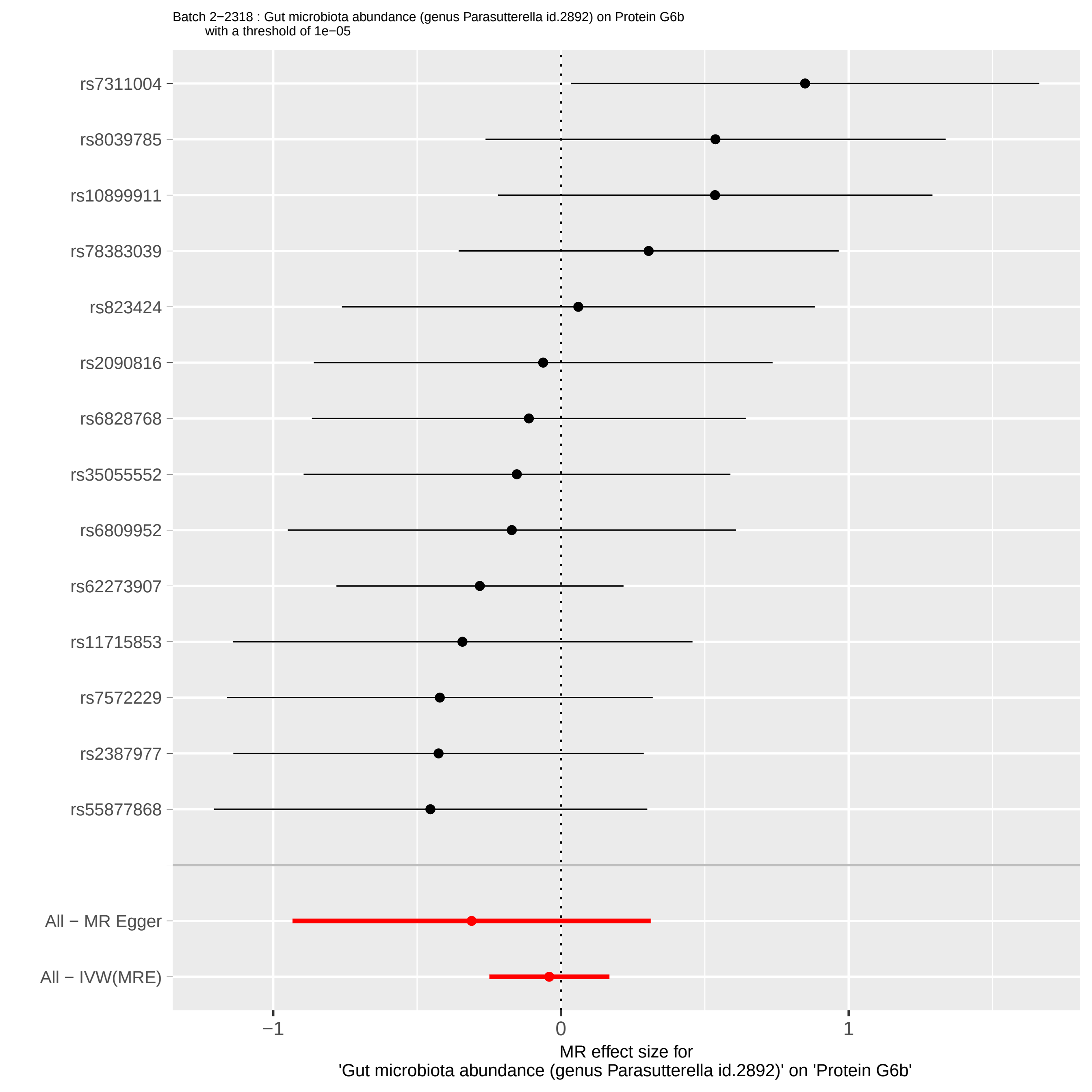

Batch 2−2318 : Gut microbiota abundance (genus Parasutterella id.2892) on Protein G6b
with a threshold of 1e−05
rs7311004
rs8039785
rs10899911
rs78383039
rs823424
rs2090816
rs6828768
rs35055552
rs6809952
rs62273907
rs11715853
rs7572229
rs2387977
rs55877868
All − MR Egger
All − IVW(MRE)
−1
0
1
MR effect size for
'Gut microbiota abundance (genus Parasutterella id.2892)' on 'Protein G6b'

Batch 2−2319 : Gut microbiota abundance (genus Catenibacterium id.2153) on Protein G6b
with a threshold of 1e−05

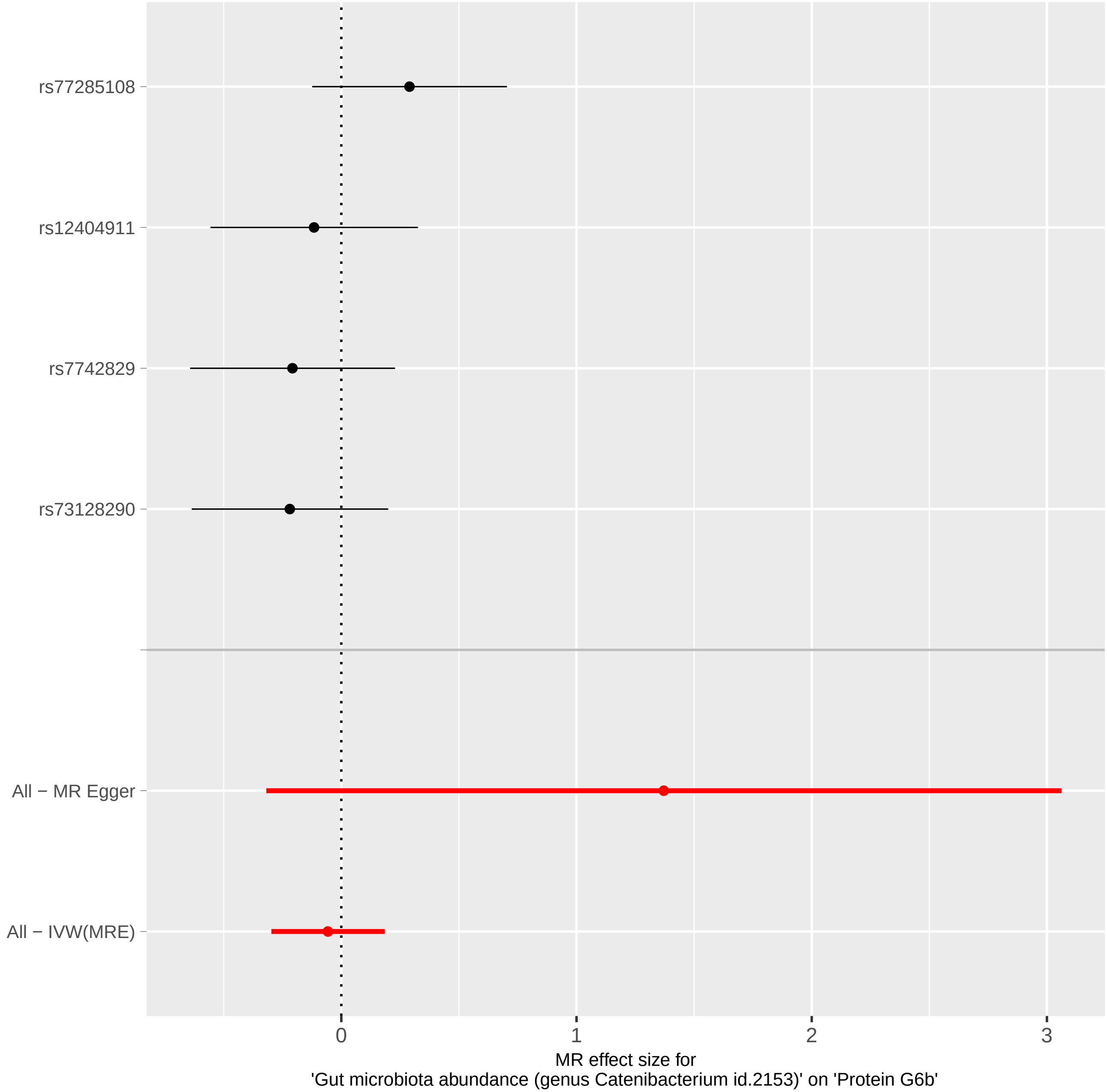

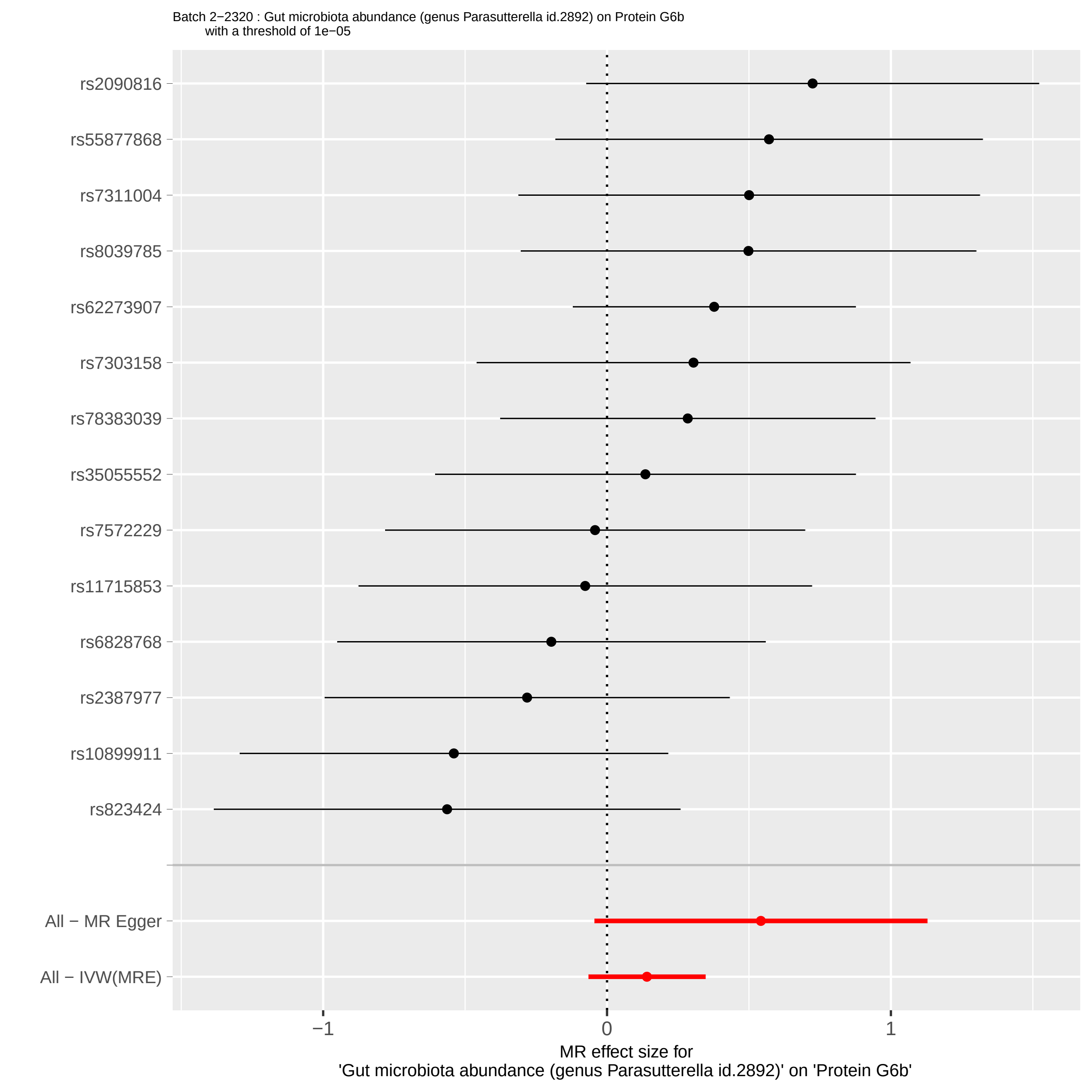
Batch 2−2320 : Gut microbiota abundance (genus Parasutterella id.2892) on Protein G6b
with a threshold of 1e−05
rs2090816
rs55877868
rs7311004
rs8039785
rs62273907
rs7303158
rs78383039
rs35055552
rs7572229
rs11715853
rs6828768
rs2387977
rs10899911
rs823424
All − MR Egger
All − IVW(MRE)
−1
0
1
MR effect size for
'Gut microbiota abundance (genus Parasutterella id.2892)' on 'Protein G6b'

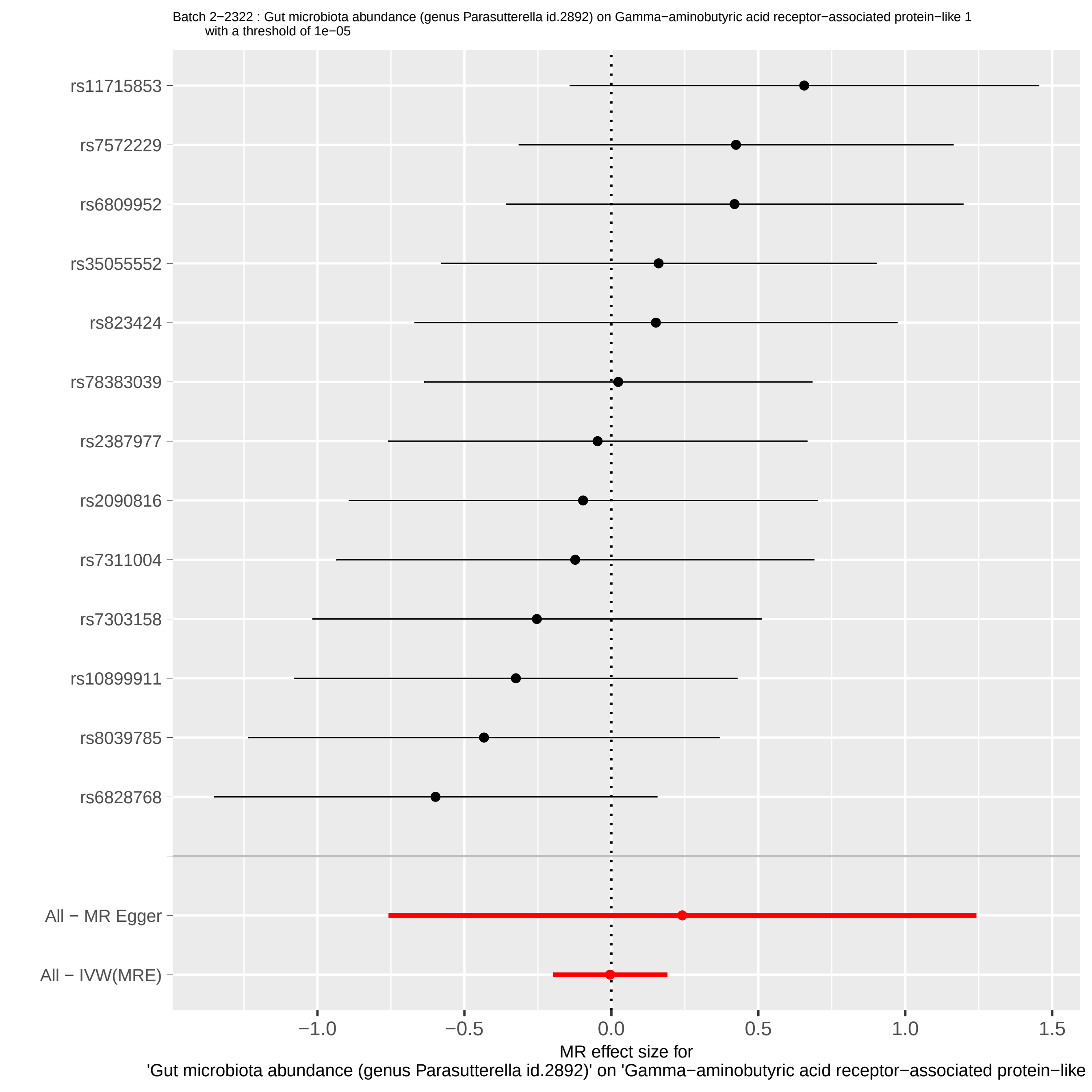

Batch 2−2322 : Gut microbiota abundance (genus Parasutterella id.2892) on Gamma−aminobutyric acid receptor−associated protein−like 1
with a threshold of 1e−05
rs11715853
rs7572229
rs6809952
rs35055552
rs823424
rs78383039
rs2387977
rs2090816
rs7311004
rs7303158
rs10899911
rs8039785
rs6828768
All − MR Egger
All − IVW(MRE)
−1.0
−0.5
0.0
0.5
1.0
1.5
MR effect size for
'Gut microbiota abundance (genus Parasutterella id.2892)' on 'Gamma−aminobutyric acid receptor−associated protein−like

Batch 2−2323 : Gut microbiota abundance (genus Catenibacterium id.2153) on Gamma−aminobutyric acid receptor−associated protein−like 2 with a threshold of 1e−05

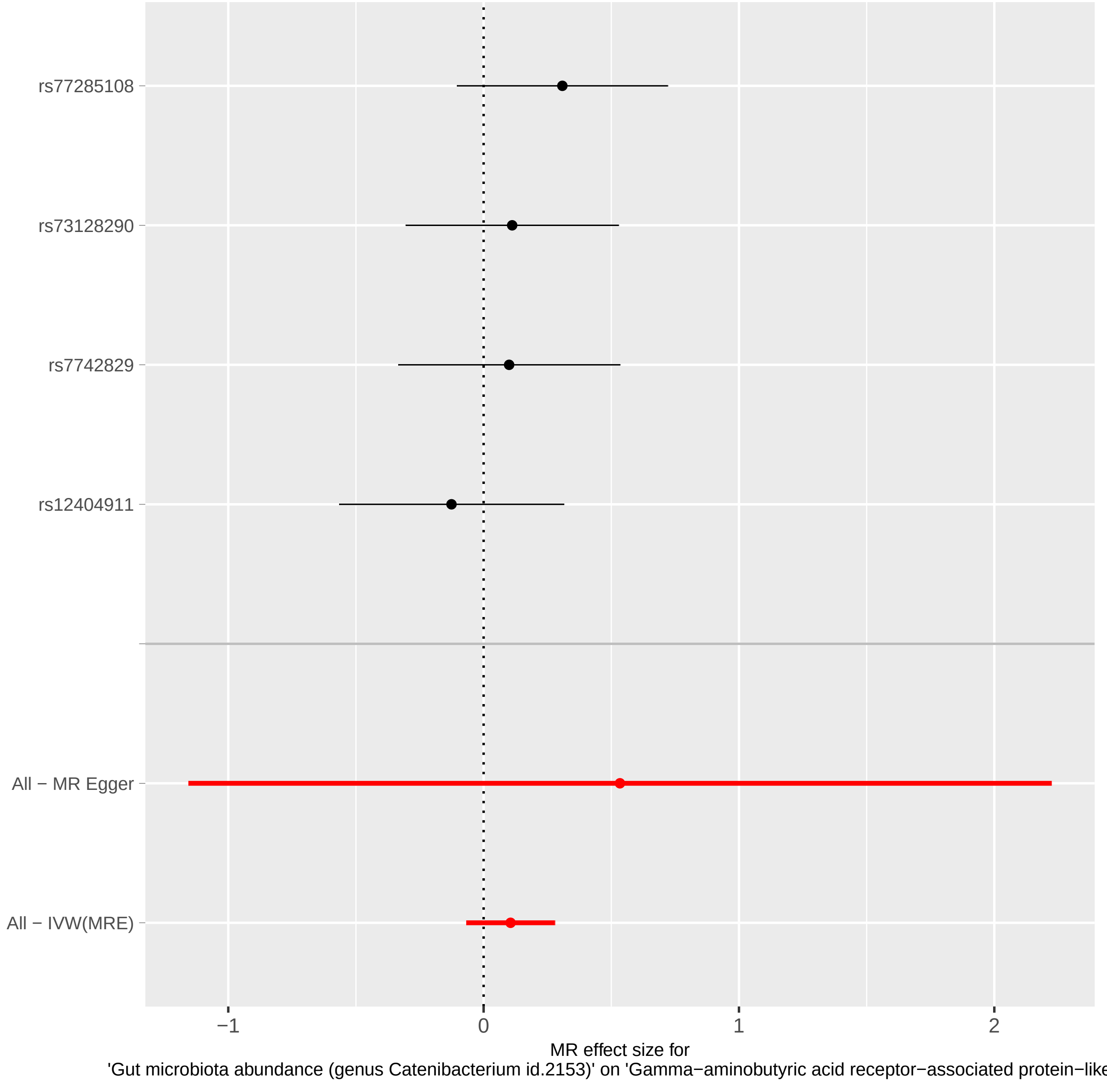

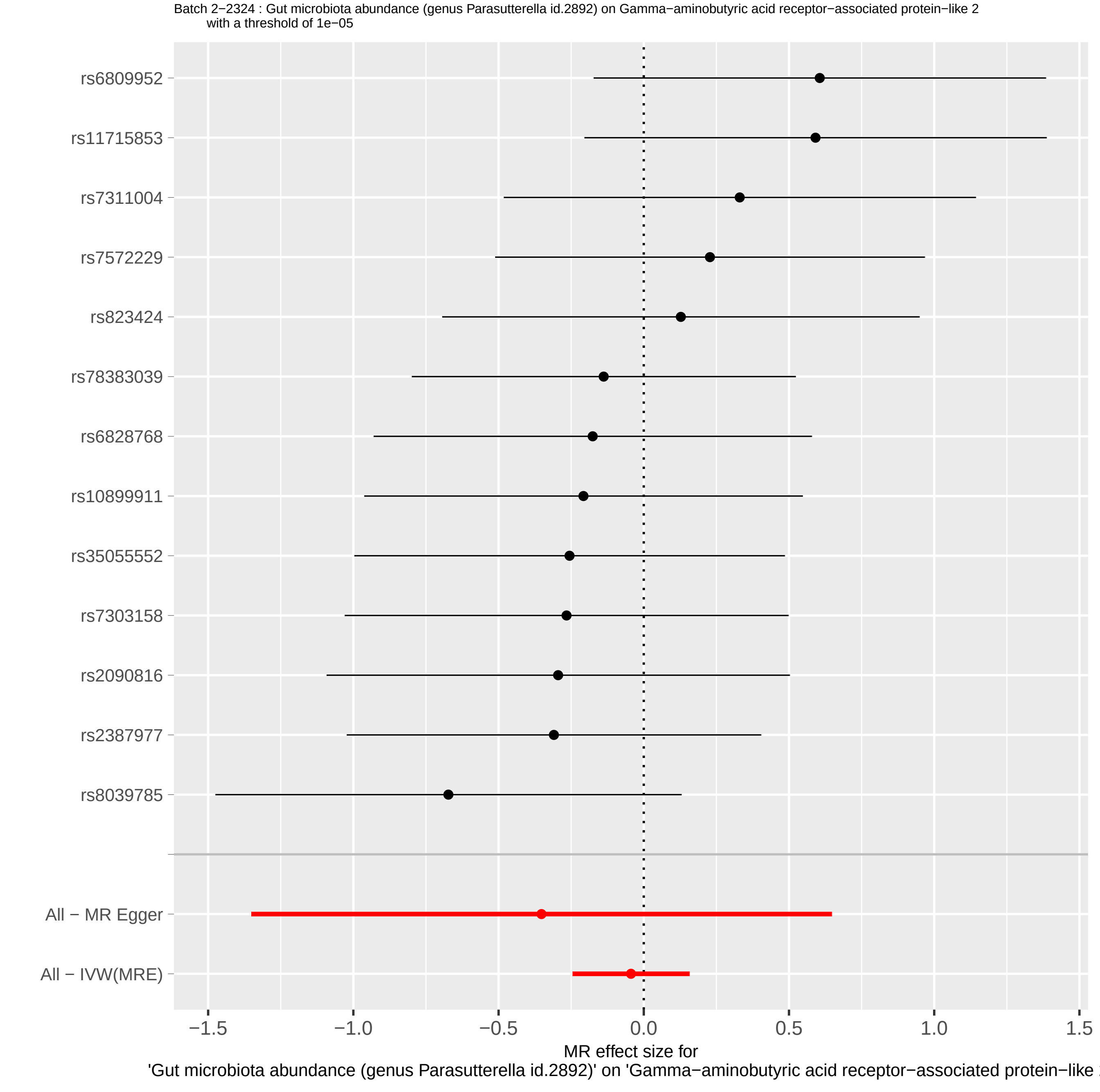

Batch 2−2324 : Gut microbiota abundance (genus Parasutterella id.2892) on Gamma−aminobutyric acid receptor−associated protein−like 2
with a threshold of 1e−05
rs6809952
rs11715853
rs7311004
rs7572229
rs823424
rs78383039
rs6828768
rs10899911
rs35055552
rs7303158
rs2090816
rs2387977
rs8039785
All − MR Egger
All − IVW(MRE)
−1.5
−1.0
−0.5
0.0
0.5
1.0
1.5
MR effect size for
'Gut microbiota abundance (genus Parasutterella id.2892)' on 'Gamma−aminobutyric acid receptor−associated protein−like

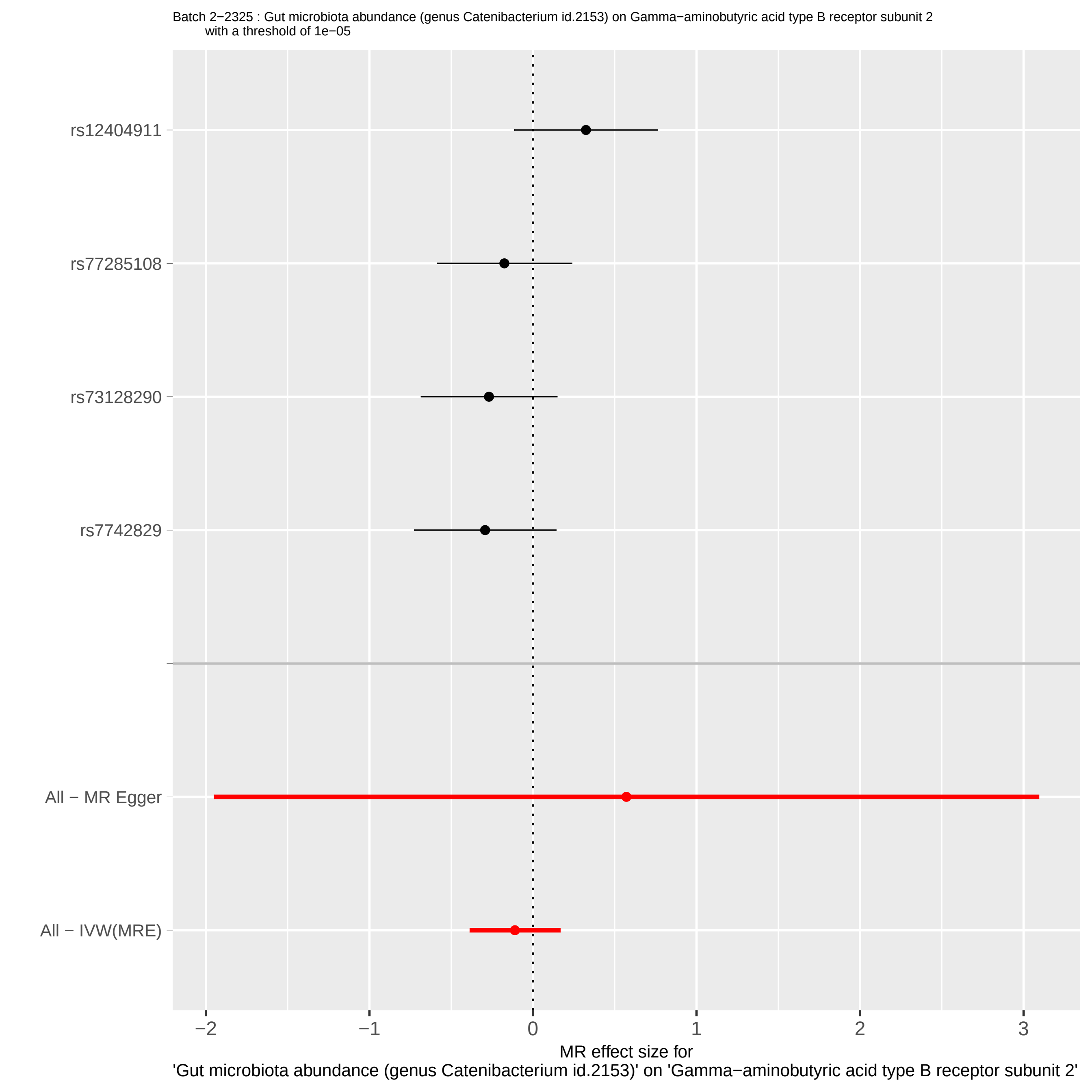
Batch 2−2325 : Gut microbiota abundance (genus Catenibacterium id.2153) on Gamma−aminobutyric acid type B receptor subunit 2
with a threshold of 1e−05
rs12404911
rs77285108
rs73128290
rs7742829
All − MR Egger
All − IVW(MRE)
−2
−1
0
1
2
3
MR effect size for
'Gut microbiota abundance (genus Catenibacterium id.2153)' on 'Gamma−aminobutyric acid type B receptor subunit 2'

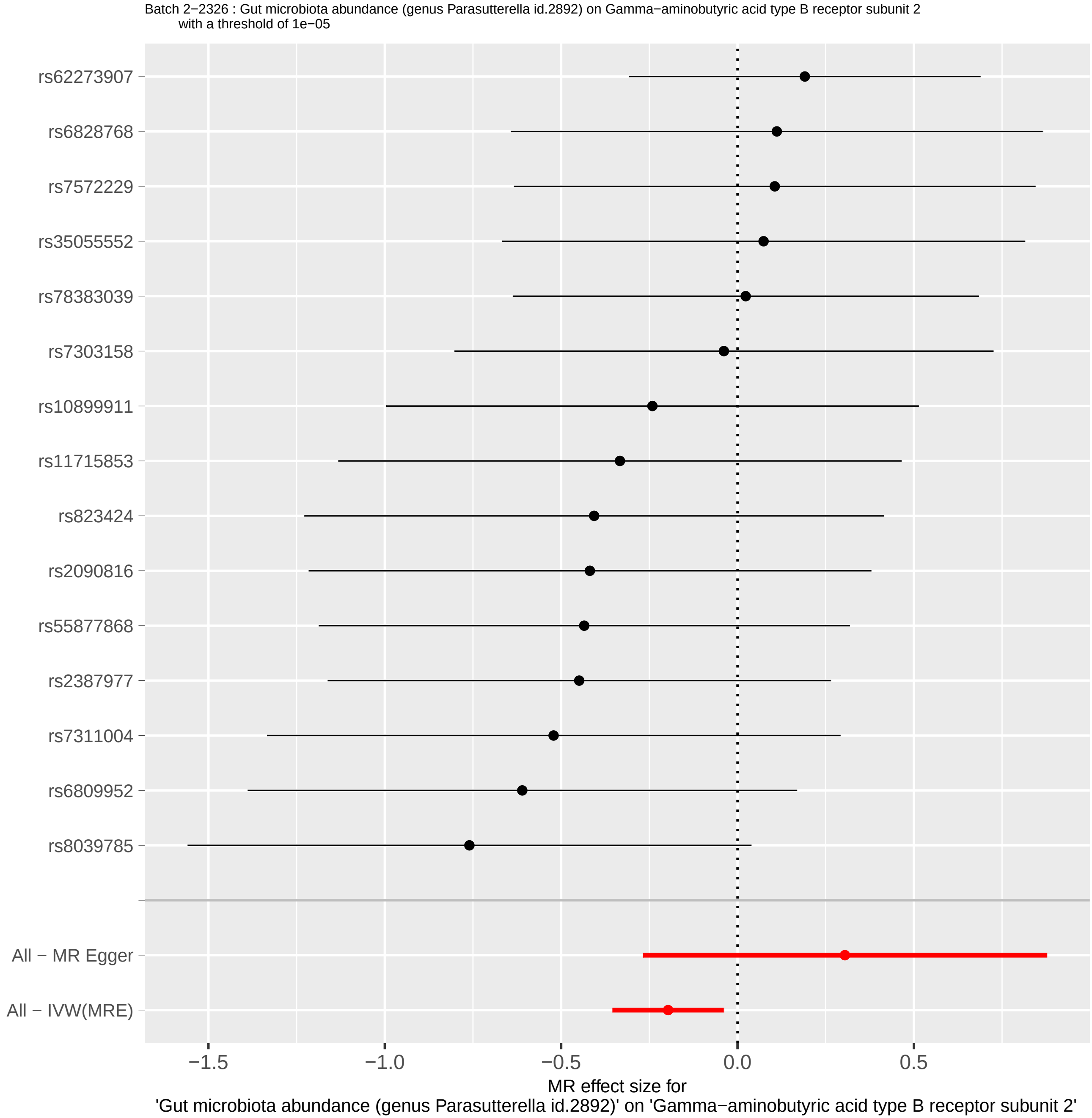
Batch 2−2326 : Gut microbiota abundance (genus Parasutterella id.2892) on Gamma−aminobutyric acid type B receptor subunit 2
with a threshold of 1e−05
rs62273907
rs6828768
rs7572229
rs35055552
rs78383039
rs7303158
rs10899911
rs11715853
rs823424
rs2090816
rs55877868
rs2387977
rs7311004
rs6809952
rs8039785
All − MR Egger
All − IVW(MRE)
−1.5
−1.0
−0.5
0.0
0.5
MR effect size for
'Gut microbiota abundance (genus Parasutterella id.2892)' on 'Gamma−aminobutyric acid type B receptor subunit 2'

Batch 2−2327 : Gut microbiota abundance (genus Catenibacterium id.2153) on Gamma−aminobutyric acid type B receptor subunit 2
with a threshold of 1e−05

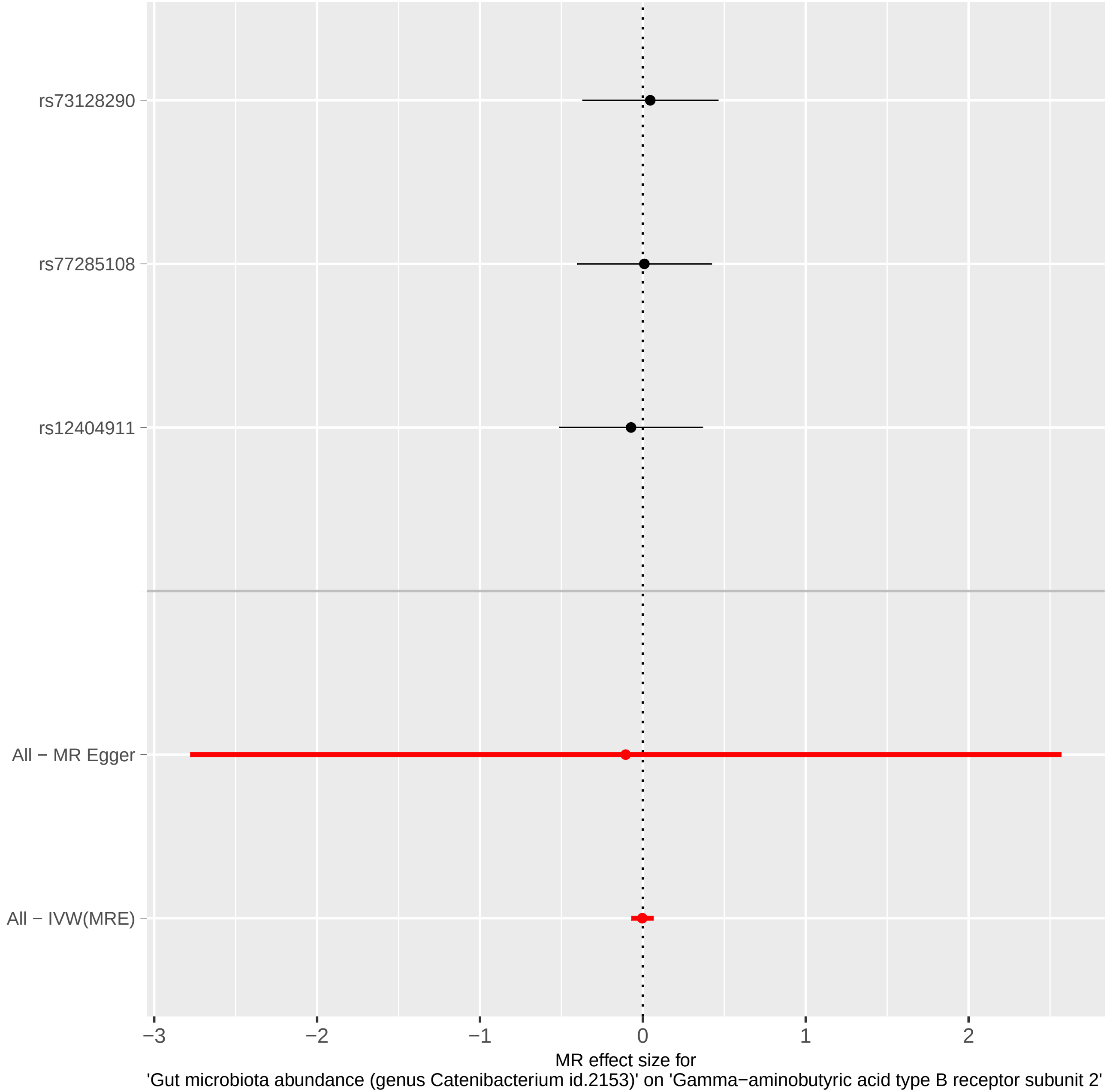

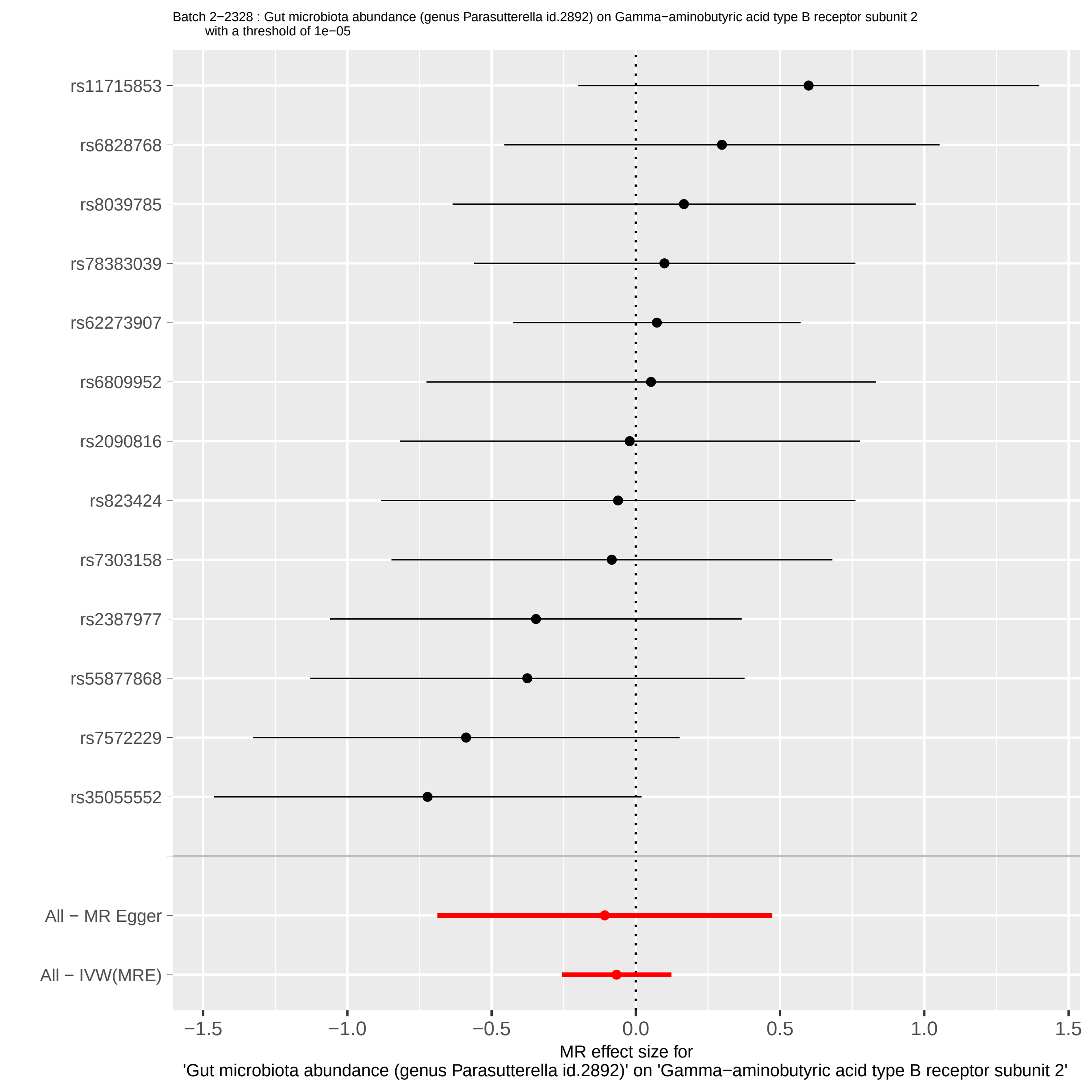

Batch 2−2328 : Gut microbiota abundance (genus Parasutterella id.2892) on Gamma−aminobutyric acid type B receptor subunit 2
with a threshold of 1e−05
rs11715853
rs6828768
rs8039785
rs78383039
rs62273907
rs6809952
rs2090816
rs823424
rs7303158
rs2387977
rs55877868
rs7572229
rs35055552
All − MR Egger
All − IVW(MRE)
−1.5
−1.0
−0.5
0.0
0.5
1.0
1.5
MR effect size for
'Gut microbiota abundance (genus Parasutterella id.2892)' on 'Gamma−aminobutyric acid type B receptor subunit 2'

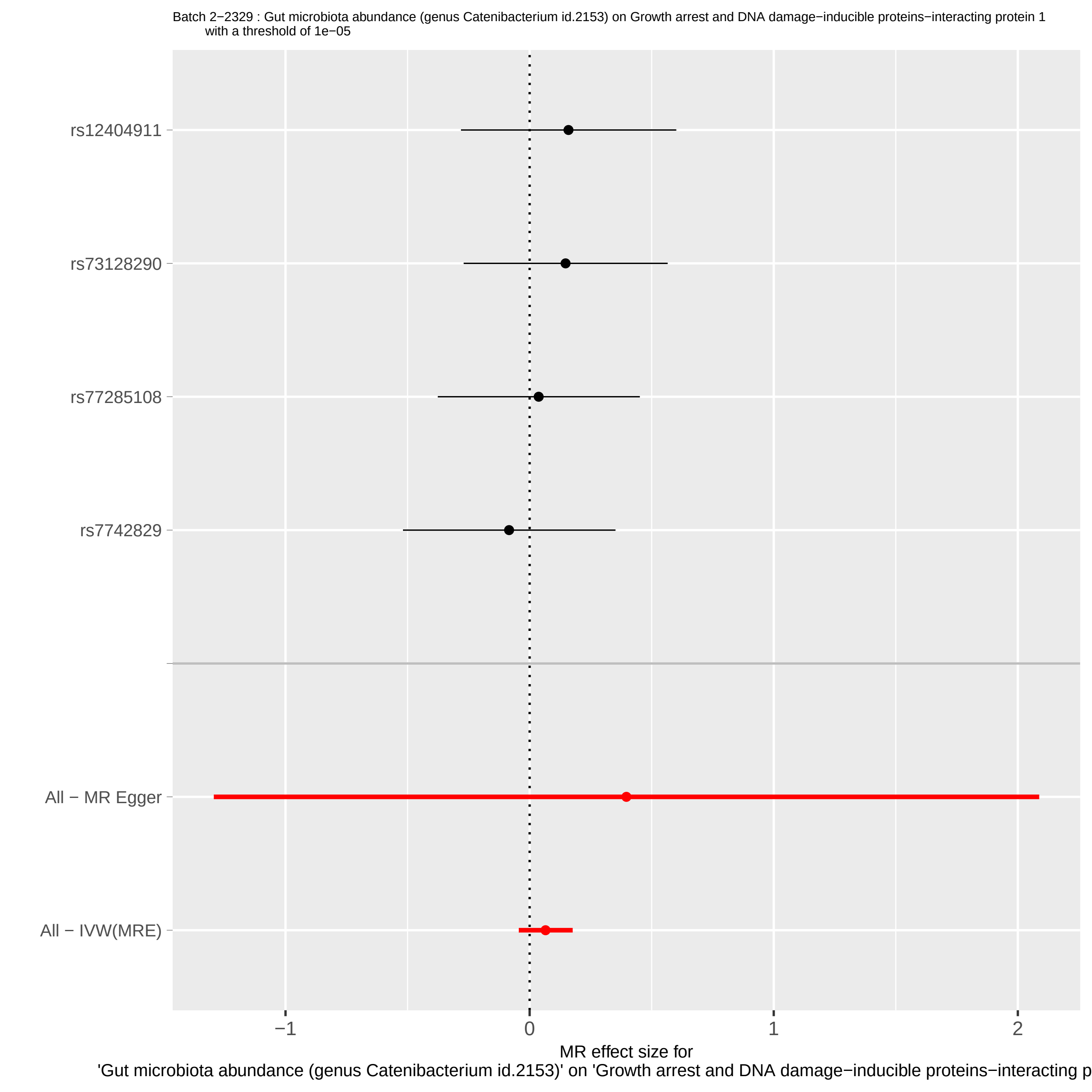

Batch 2−2329 : Gut microbiota abundance (genus Catenibacterium id.2153) on Growth arrest and DNA damage−inducible proteins−interacting protein 1 with a threshold of 1e−05
rs12404911
rs73128290
rs77285108
rs7742829
All − MR Egger
All − IVW(MRE)
−1
0
1
2
MR effect size for
'Gut microbiota abundance (genus Catenibacterium id.2153)' on 'Growth arrest and DNA damage−inducible proteins−interacting p

Batch 2−2330 : Gut microbiota abundance (genus Parasutterella id.2892) on Growth arrest and DNA damage−inducible proteins−interacting protein 1 with a threshold of 1e−05

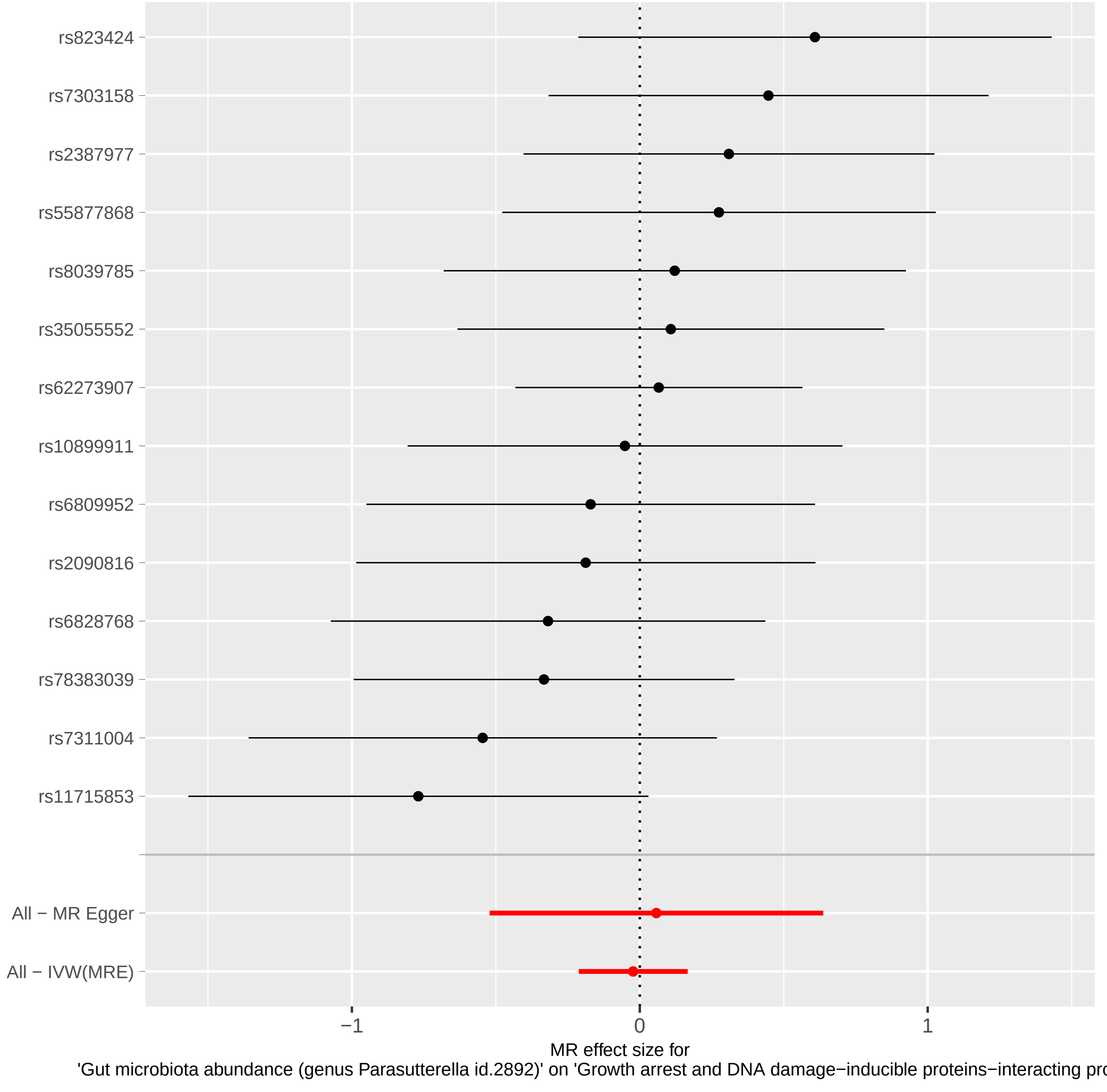

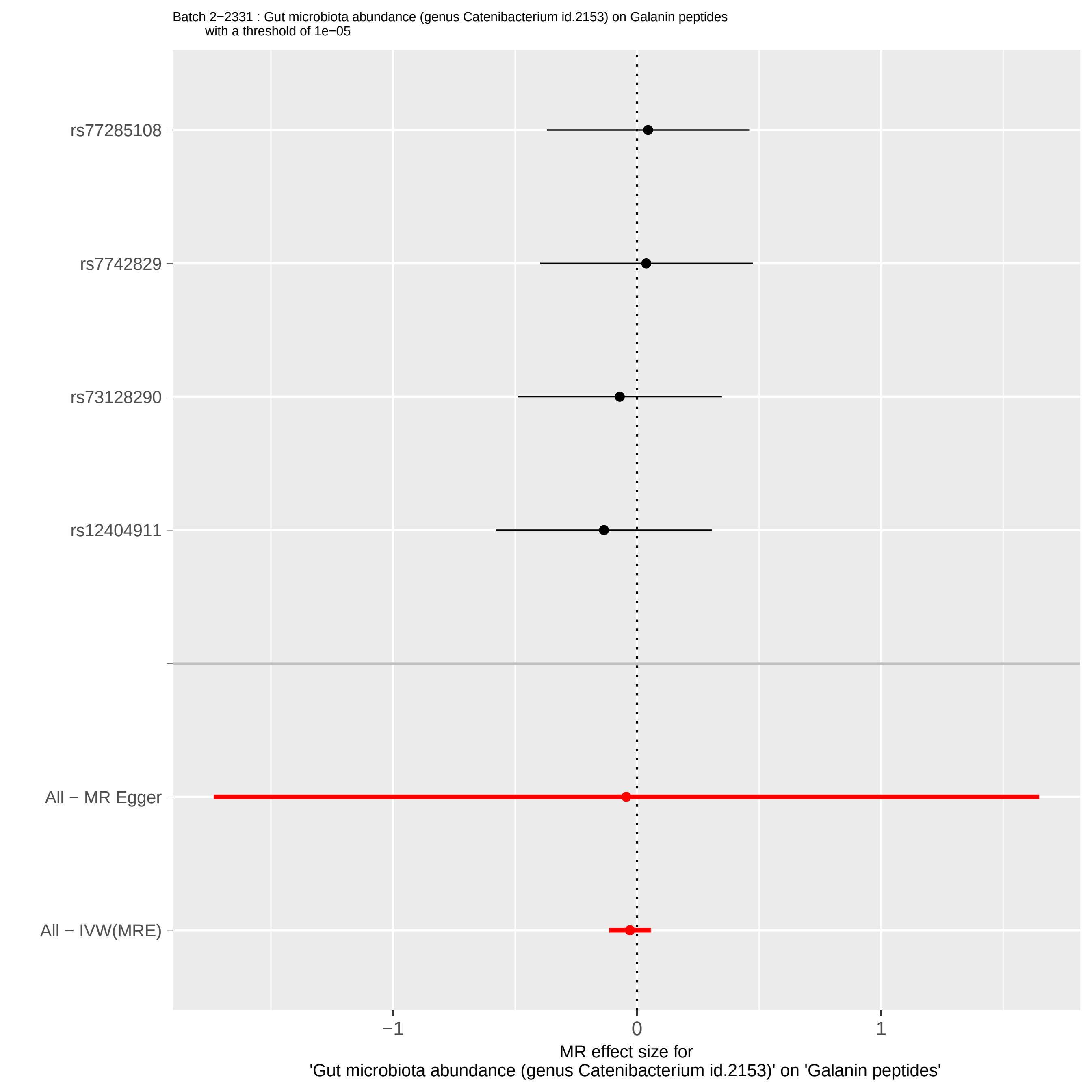

Batch 2−2331 : Gut microbiota abundance (genus Catenibacterium id.2153) on Galanin peptides
with a threshold of 1e−05
rs77285108
rs7742829
rs73128290
rs12404911
All − MR Egger
All − IVW(MRE)
−1
0
1
MR effect size for
'Gut microbiota abundance (genus Catenibacterium id.2153)' on 'Galanin peptides'

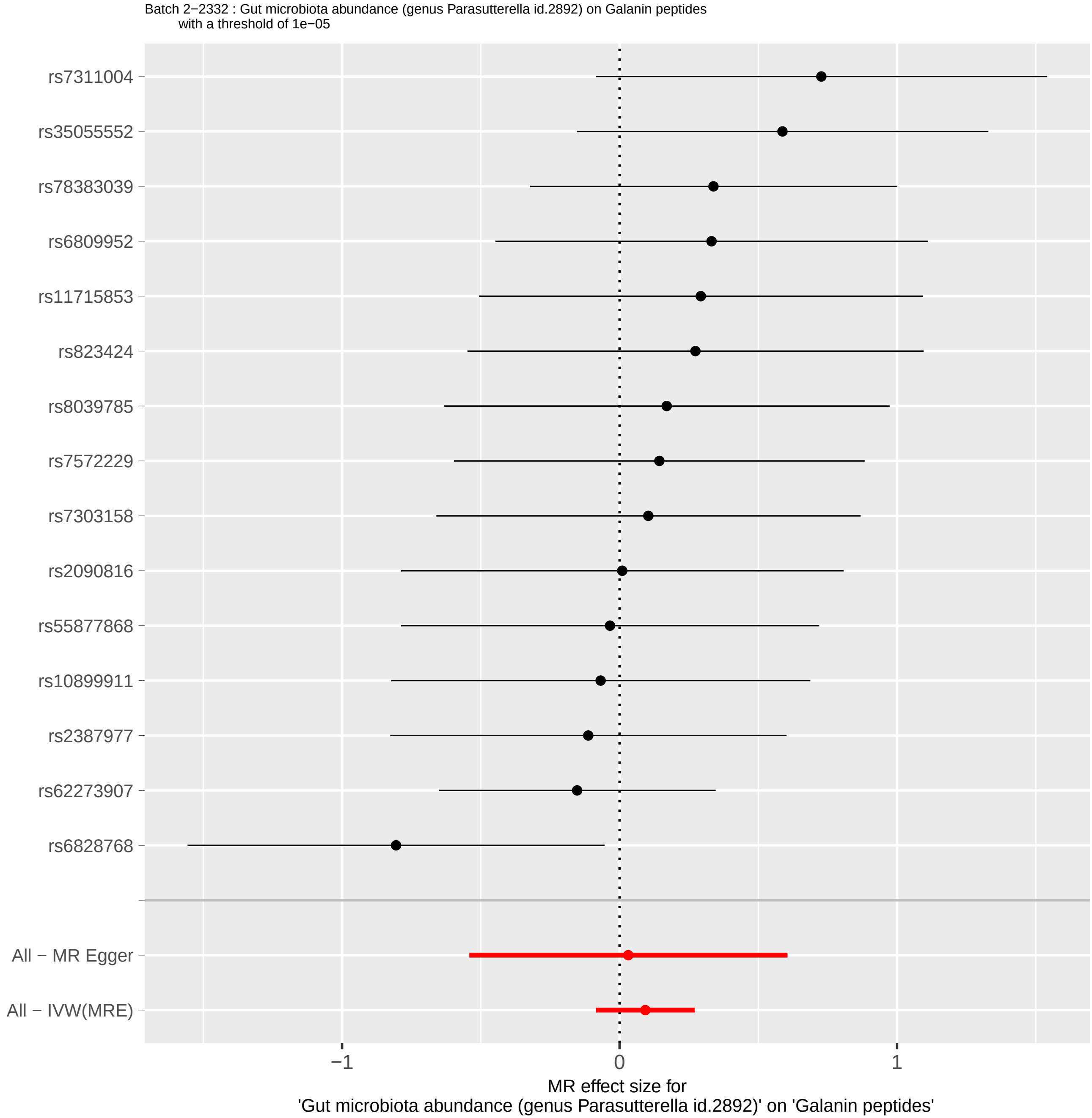

Batch 2−2332 : Gut microbiota abundance (genus Parasutterella id.2892) on Galanin peptides
with a threshold of 1e−05
rs7311004
rs35055552
rs78383039
rs6809952
rs11715853
rs823424
rs8039785
rs7572229
rs7303158
rs2090816
rs55877868
rs10899911
rs2387977
rs62273907
rs6828768
All − MR Egger
All − IVW(MRE)
−1
0
1
MR effect size for
'Gut microbiota abundance (genus Parasutterella id.2892)' on 'Galanin peptides'

Batch 2−2333 : Gut microbiota abundance (genus Catenibacterium id.2153) on Polypeptide N−acetylgalactosaminyltransferase 10
with a threshold of 1e−05

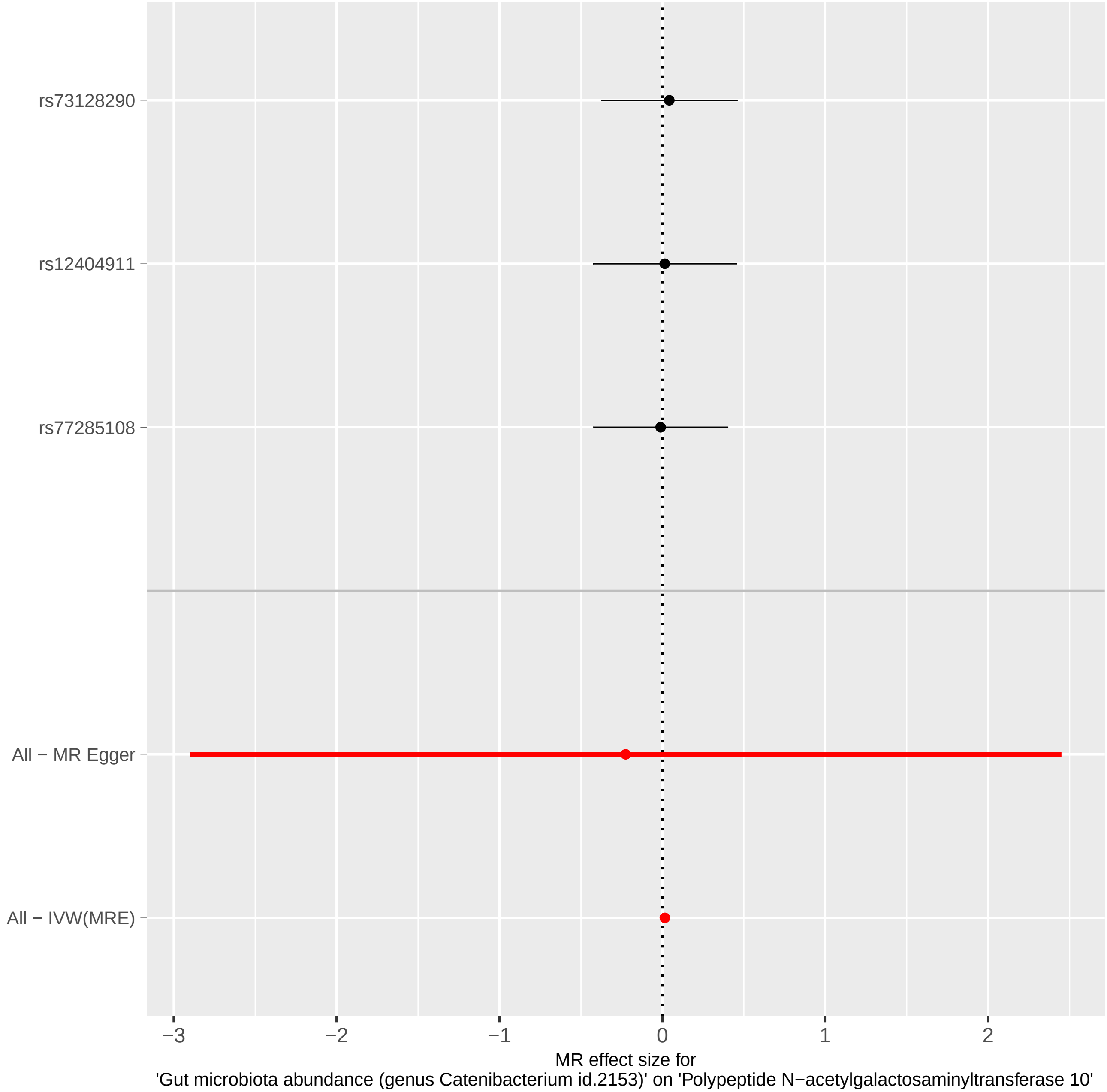

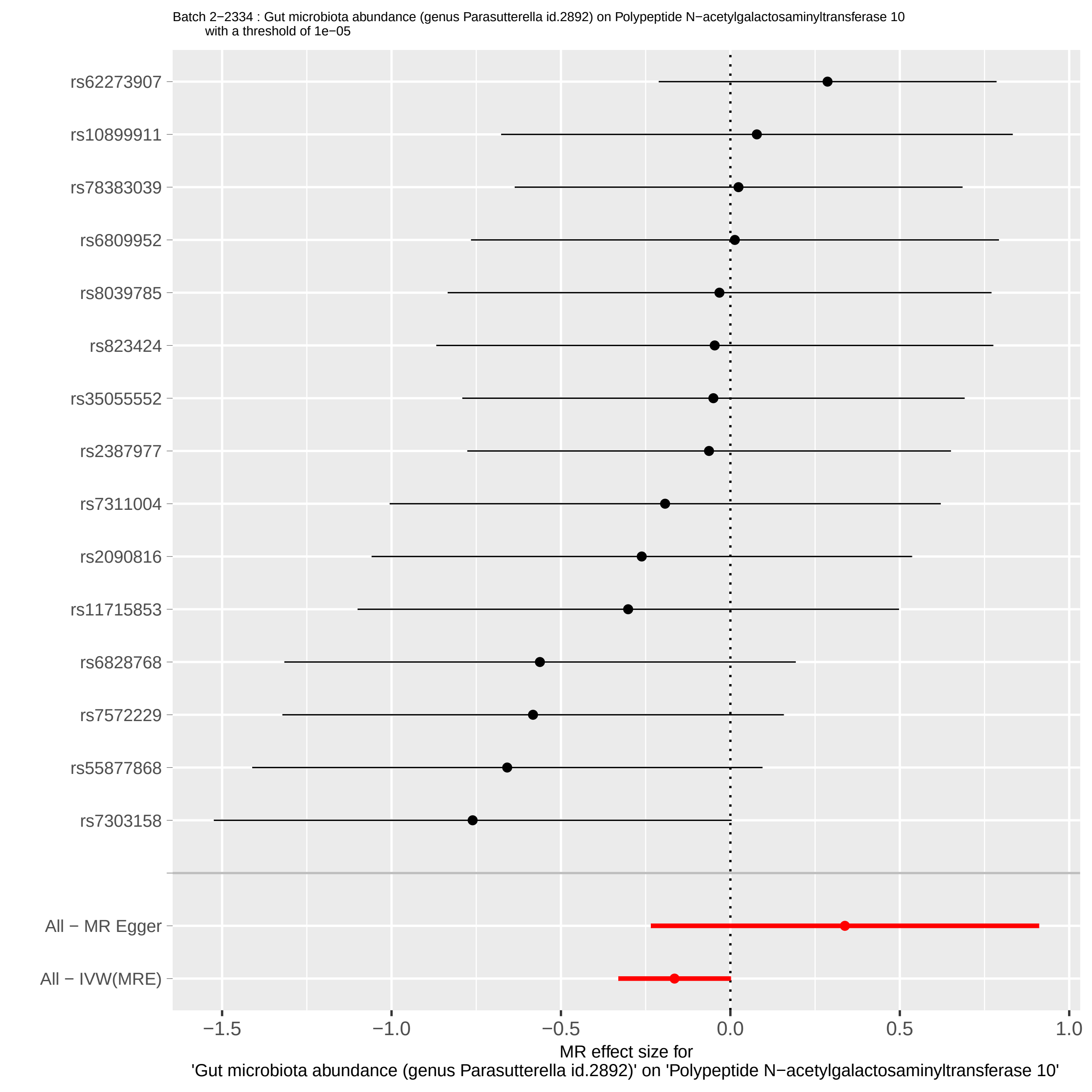

Batch 2−2334 : Gut microbiota abundance (genus Parasutterella id.2892) on Polypeptide N−acetylgalactosaminyltransferase 10
with a threshold of 1e−05
rs62273907
rs10899911
rs78383039
rs6809952
rs8039785
rs823424
rs35055552
rs2387977
rs7311004
rs2090816
rs11715853
rs6828768
rs7572229
rs55877868
rs7303158
All − MR Egger
All − IVW(MRE)
−1.5
−1.0
−0.5
0.0
0.5
1.0
MR effect size for
'Gut microbiota abundance (genus Parasutterella id.2892)' on 'Polypeptide N−acetylgalactosaminyltransferase 10'

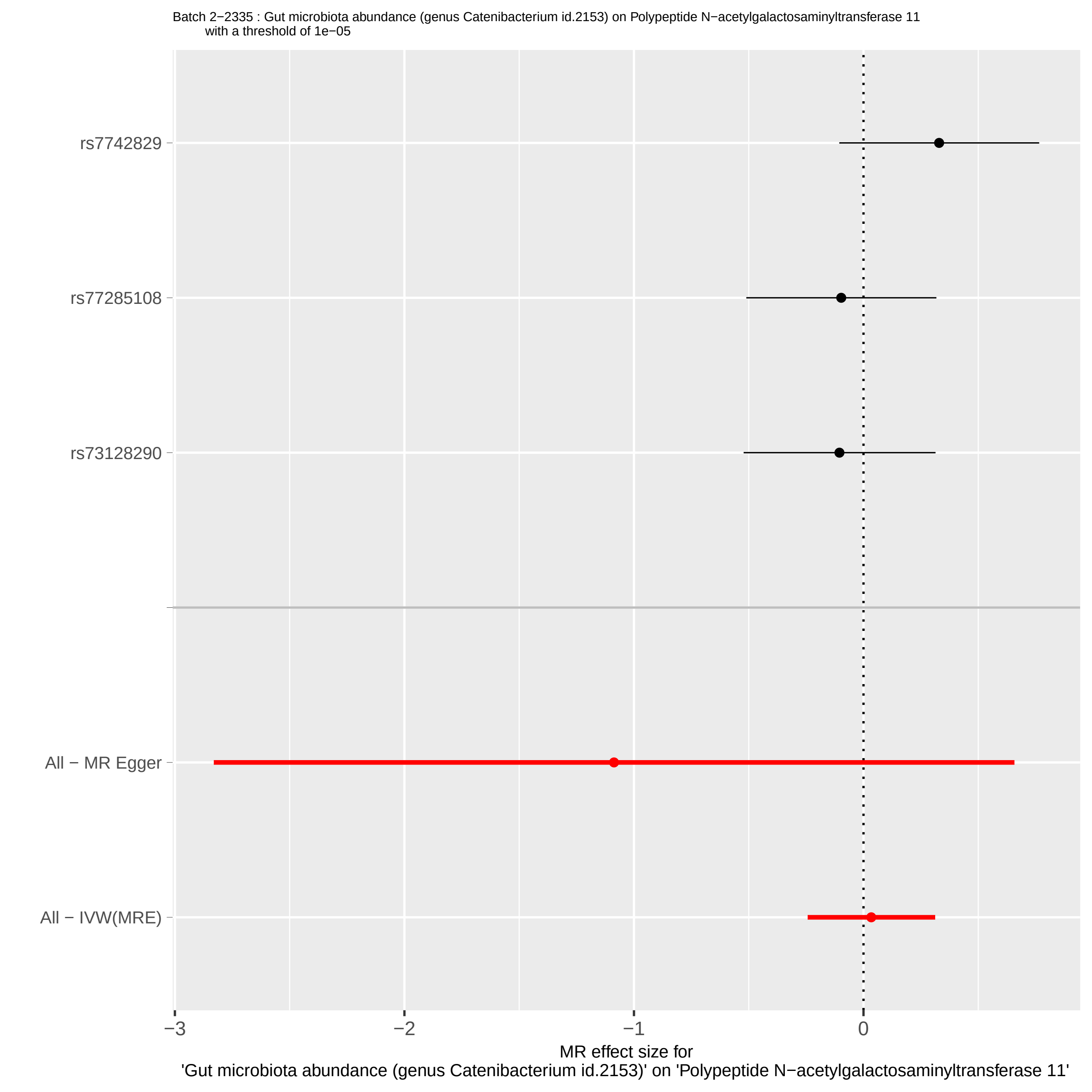

Batch 2−2335 : Gut microbiota abundance (genus Catenibacterium id.2153) on Polypeptide N−acetylgalactosaminyltransferase 11
with a threshold of 1e−05
rs7742829
rs77285108
rs73128290
All − MR Egger
All − IVW(MRE)
−3
−2
−1
0
MR effect size for
'Gut microbiota abundance (genus Catenibacterium id.2153)' on 'Polypeptide N−acetylgalactosaminyltransferase 11'

Batch 2−2336 : Gut microbiota abundance (genus Parasutterella id.2892) on Polypeptide N−acetylgalactosaminyltransferase 11
with a threshold of 1e−05

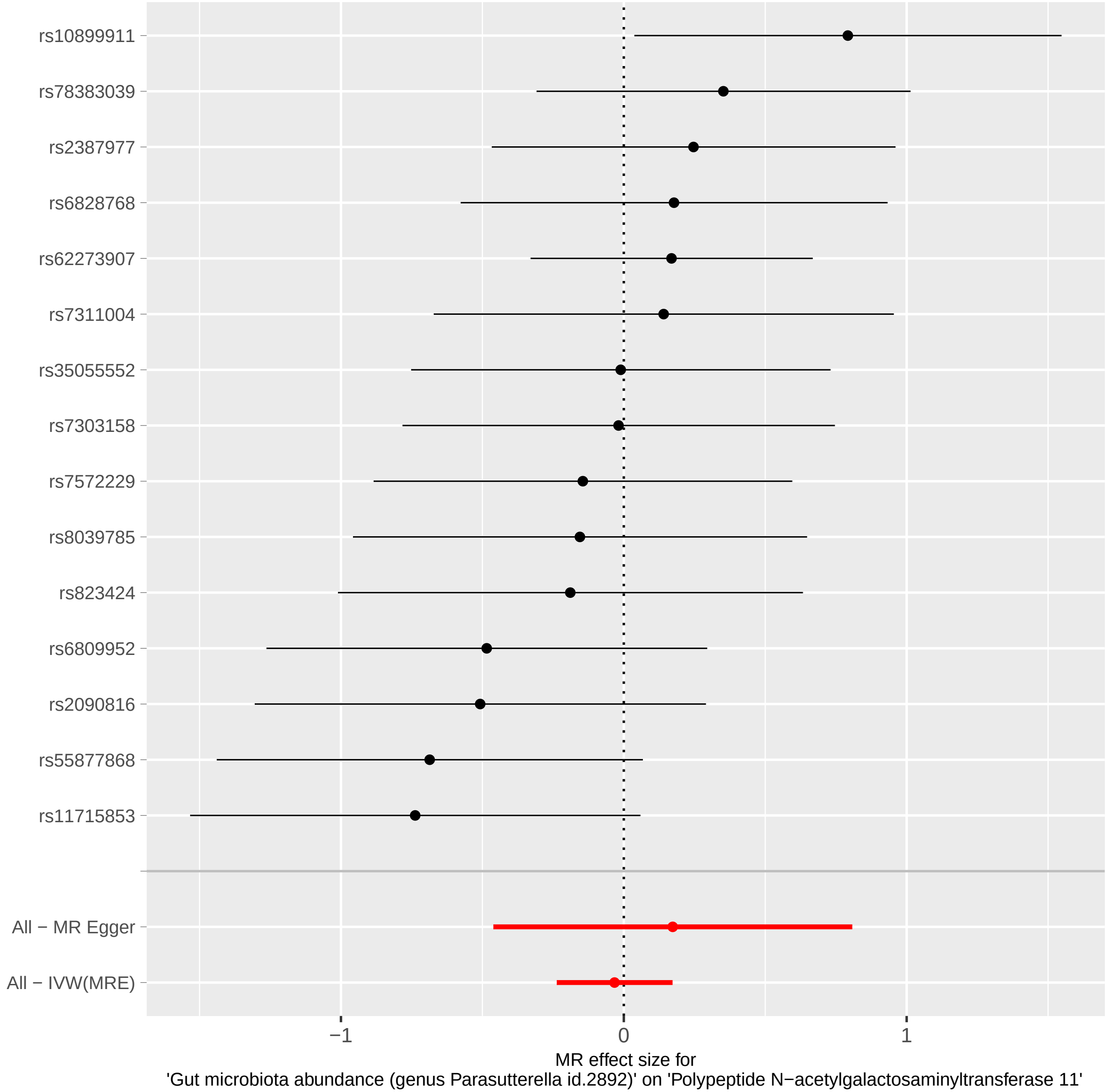

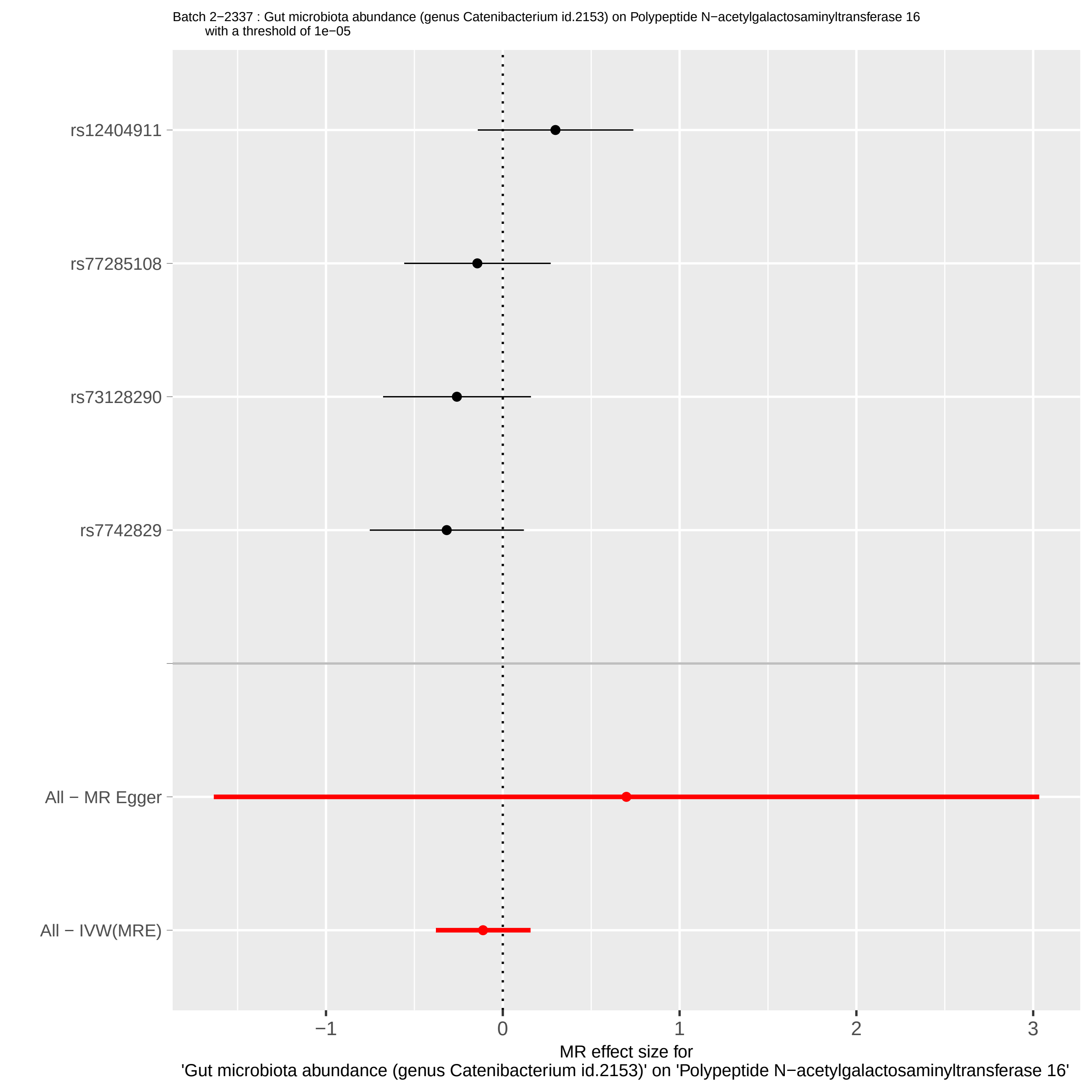
Batch 2−2337 : Gut microbiota abundance (genus Catenibacterium id.2153) on Polypeptide N−acetylgalactosaminyltransferase 16
with a threshold of 1e−05
rs12404911
rs77285108
rs73128290
rs7742829
All − MR Egger
All − IVW(MRE)
−1
0
1
2
3
MR effect size for
'Gut microbiota abundance (genus Catenibacterium id.2153)' on 'Polypeptide N−acetylgalactosaminyltransferase 16'

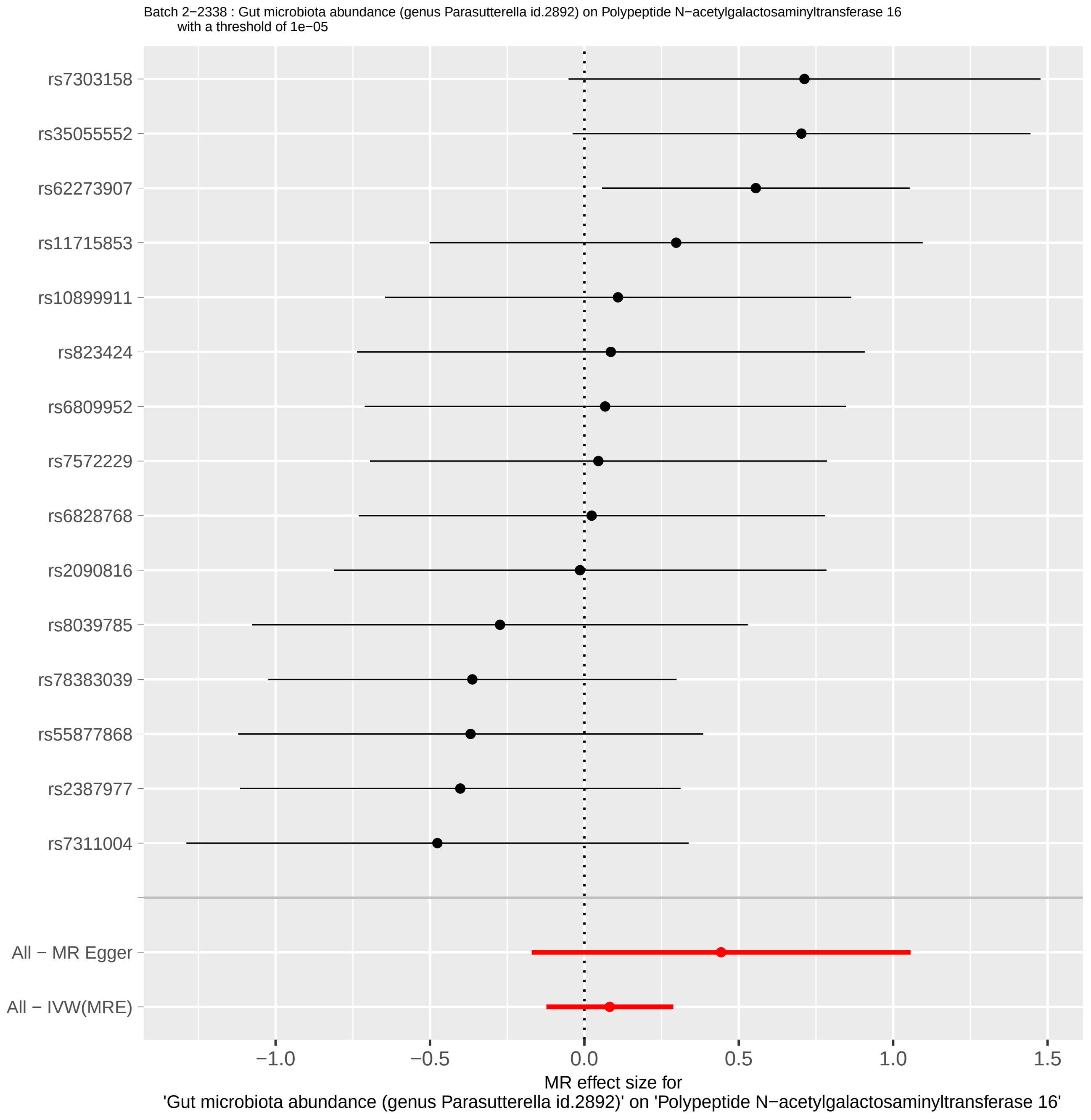
Batch 2−2338 : Gut microbiota abundance (genus Parasutterella id.2892) on Polypeptide N−acetylgalactosaminyltransferase 16
with a threshold of 1e−05
rs7303158
rs35055552
rs62273907
rs11715853
rs10899911
rs823424
rs6809952
rs7572229
rs6828768
rs2090816
rs8039785
rs78383039
rs55877868
rs2387977
rs7311004
All − MR Egger
All − IVW(MRE)
−1.0
−0.5
0.0
0.5
1.0
1.5
MR effect size for
'Gut microbiota abundance (genus Parasutterella id.2892)' on 'Polypeptide N−acetylgalactosaminyltransferase 16'

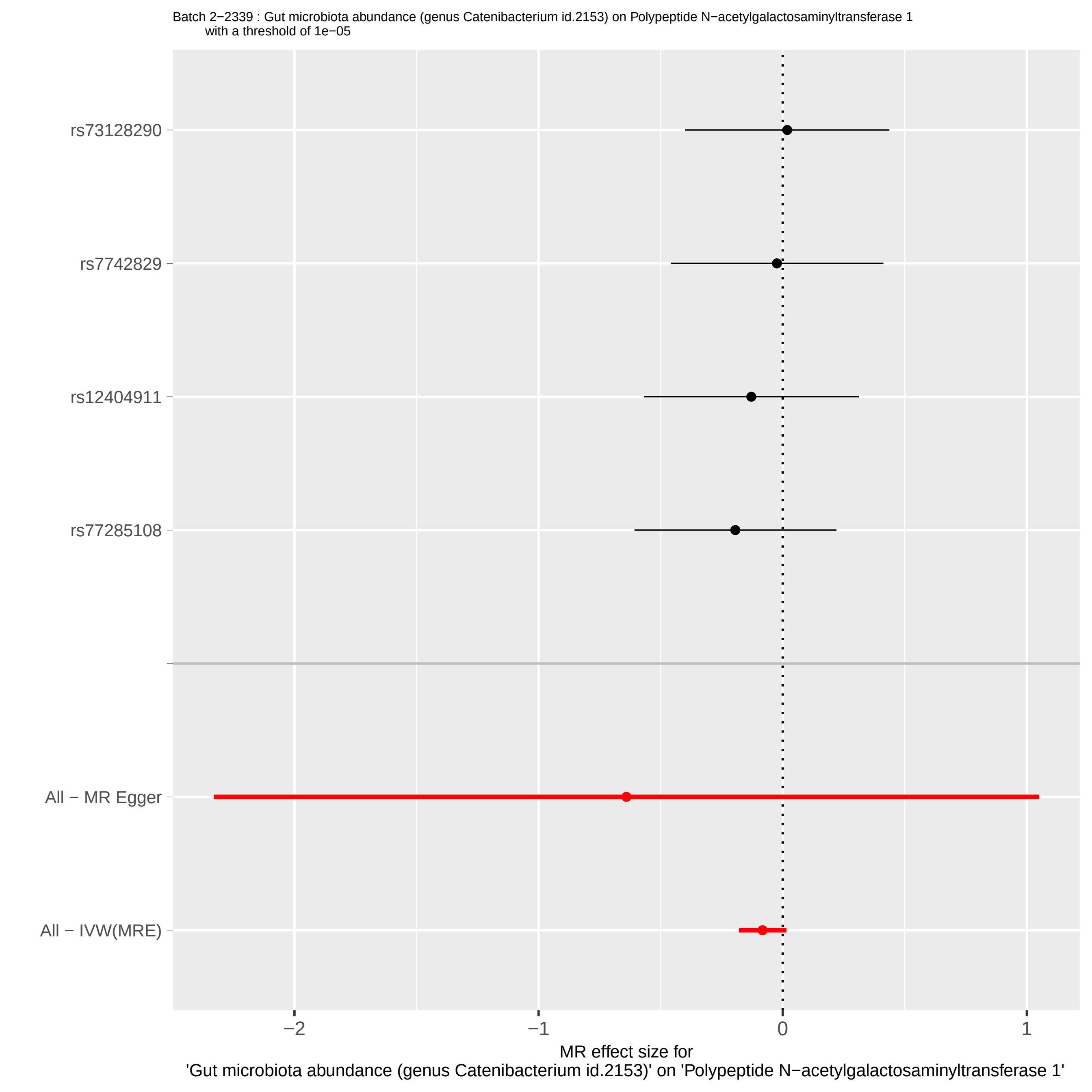

Batch 2−2339 : Gut microbiota abundance (genus Catenibacterium id.2153) on Polypeptide N−acetylgalactosaminyltransferase 1
with a threshold of 1e−05
rs73128290
rs7742829
rs12404911
rs77285108
All − MR Egger
All − IVW(MRE)
−2
−1
0
1
MR effect size for
'Gut microbiota abundance (genus Catenibacterium id.2153)' on 'Polypeptide N−acetylgalactosaminyltransferase 1'

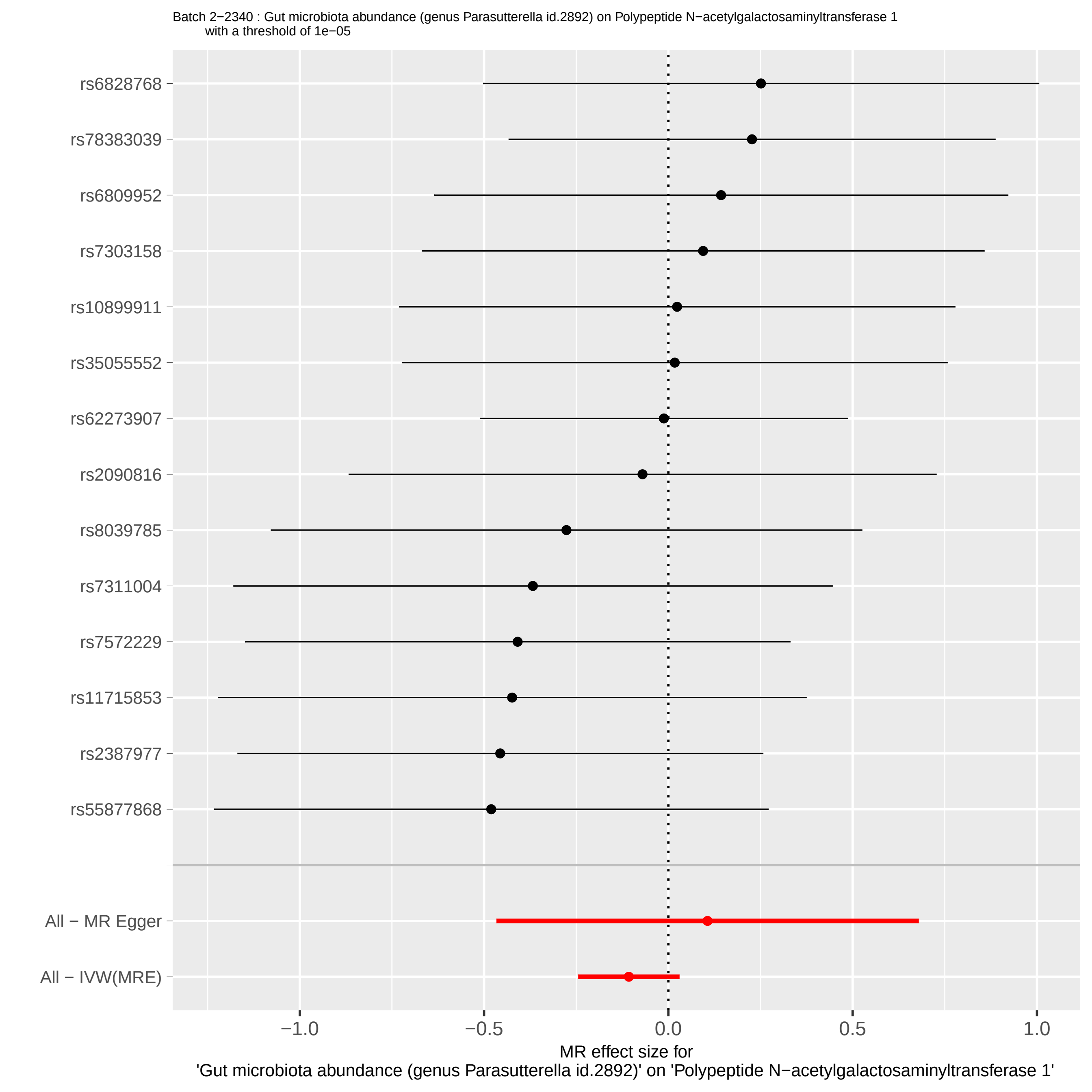

Batch 2−2340 : Gut microbiota abundance (genus Parasutterella id.2892) on Polypeptide N−acetylgalactosaminyltransferase 1
with a threshold of 1e−05
rs6828768
rs78383039
rs6809952
rs7303158
rs10899911
rs35055552
rs62273907
rs2090816
rs8039785
rs7311004
rs7572229
rs11715853
rs2387977
rs55877868
All − MR Egger
All − IVW(MRE)
−1.0
−0.5
0.0
0.5
1.0
MR effect size for
'Gut microbiota abundance (genus Parasutterella id.2892)' on 'Polypeptide N−acetylgalactosaminyltransferase 1'

Batch 2−2341 : Gut microbiota abundance (genus Catenibacterium id.2153) on Polypeptide N−acetylgalactosaminyltransferase 2
with a threshold of 1e−05

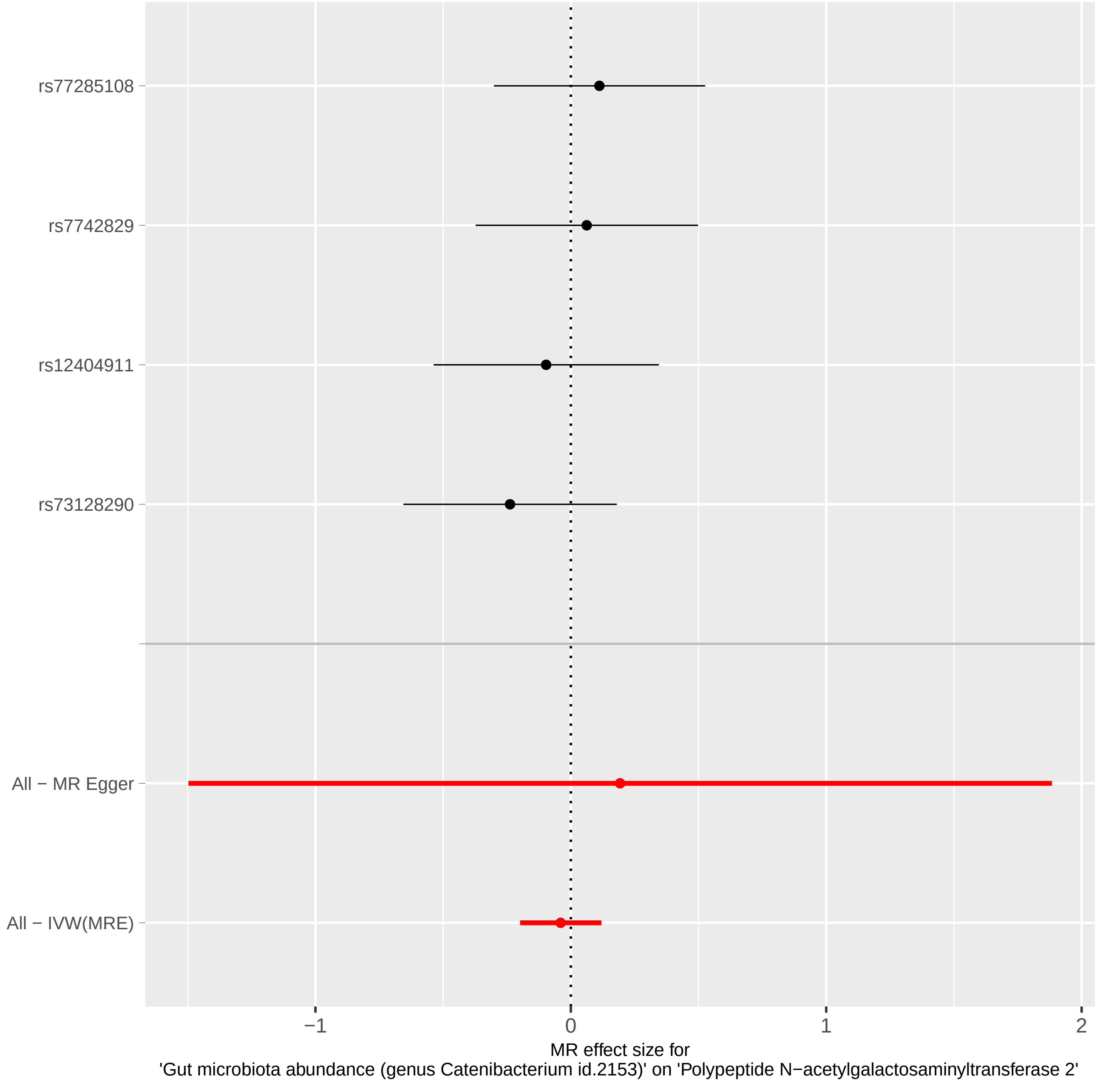

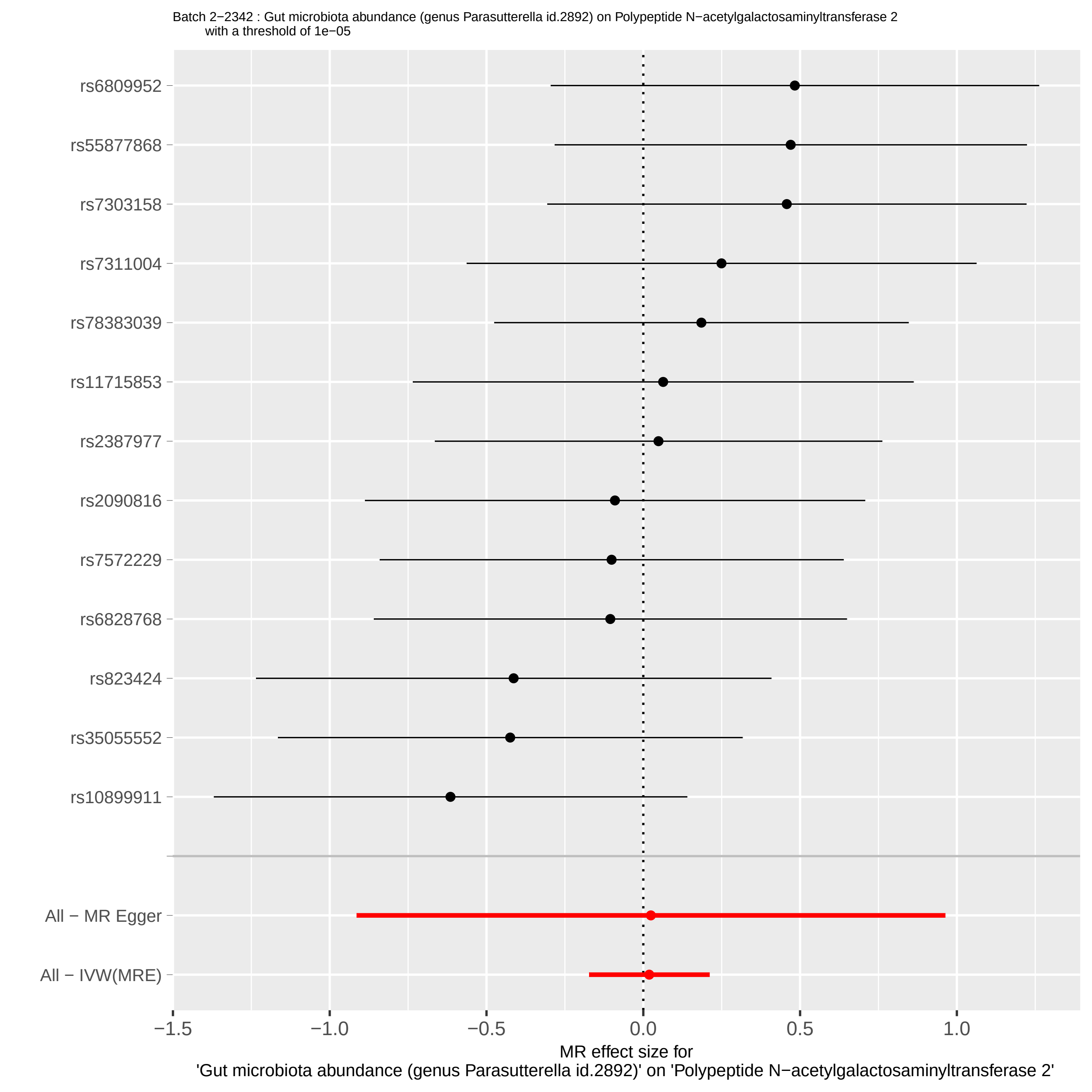

Batch 2−2342 : Gut microbiota abundance (genus Parasutterella id.2892) on Polypeptide N−acetylgalactosaminyltransferase 2
with a threshold of 1e−05
rs6809952
rs55877868
rs7303158
rs7311004
rs78383039
rs11715853
rs2387977
rs2090816
rs7572229
rs6828768
rs823424
rs35055552
rs10899911
All − MR Egger
All − IVW(MRE)
−1.5
−1.0
−0.5
0.0
0.5
1.0
MR effect size for
'Gut microbiota abundance (genus Parasutterella id.2892)' on 'Polypeptide N−acetylgalactosaminyltransferase 2'

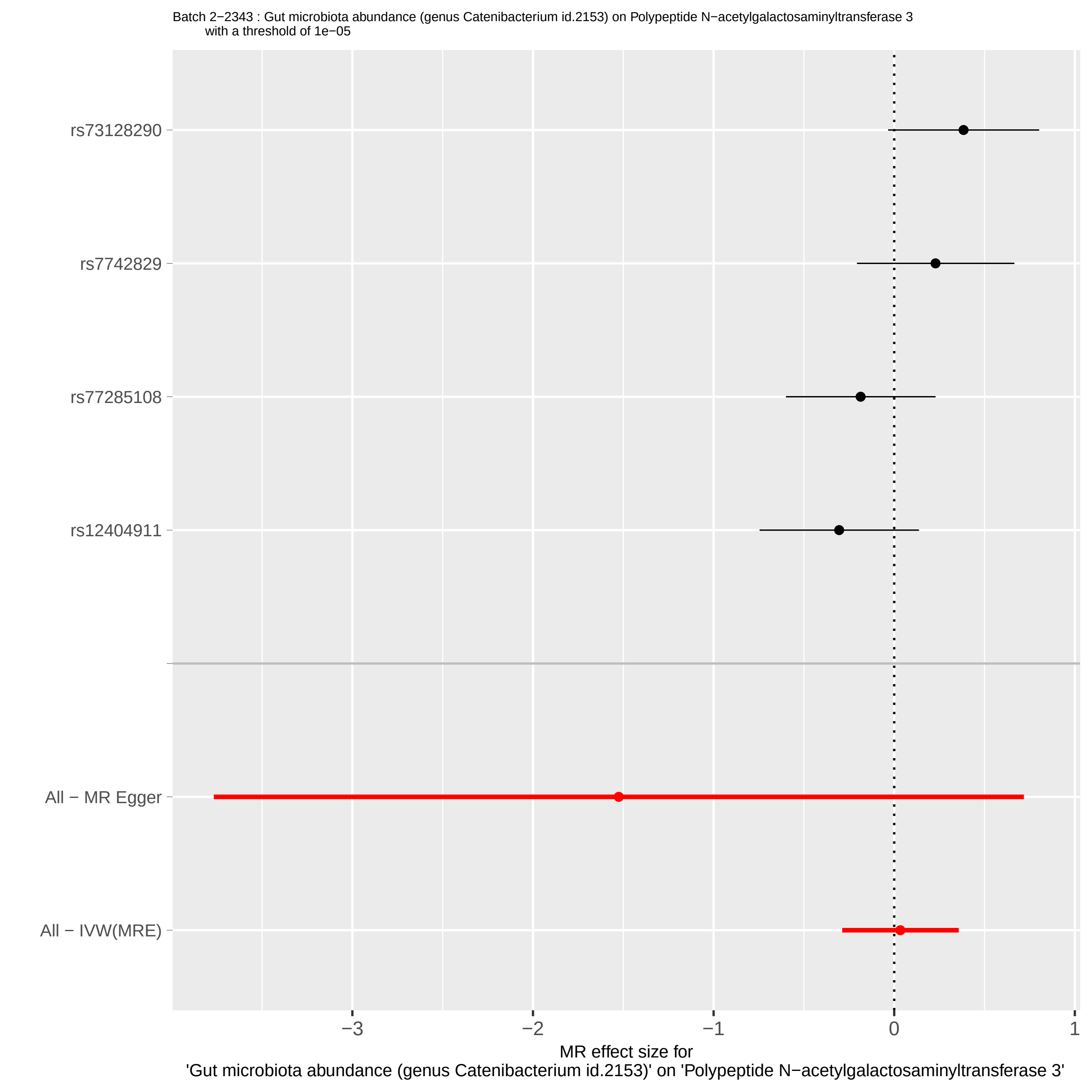
Batch 2−2343 : Gut microbiota abundance (genus Catenibacterium id.2153) on Polypeptide N−acetylgalactosaminyltransferase 3
with a threshold of 1e−05
rs73128290
rs7742829
rs77285108
rs12404911
All − MR Egger
All − IVW(MRE)
−3
−2
−1
0
1
MR effect size for
'Gut microbiota abundance (genus Catenibacterium id.2153)' on 'Polypeptide N−acetylgalactosaminyltransferase 3'

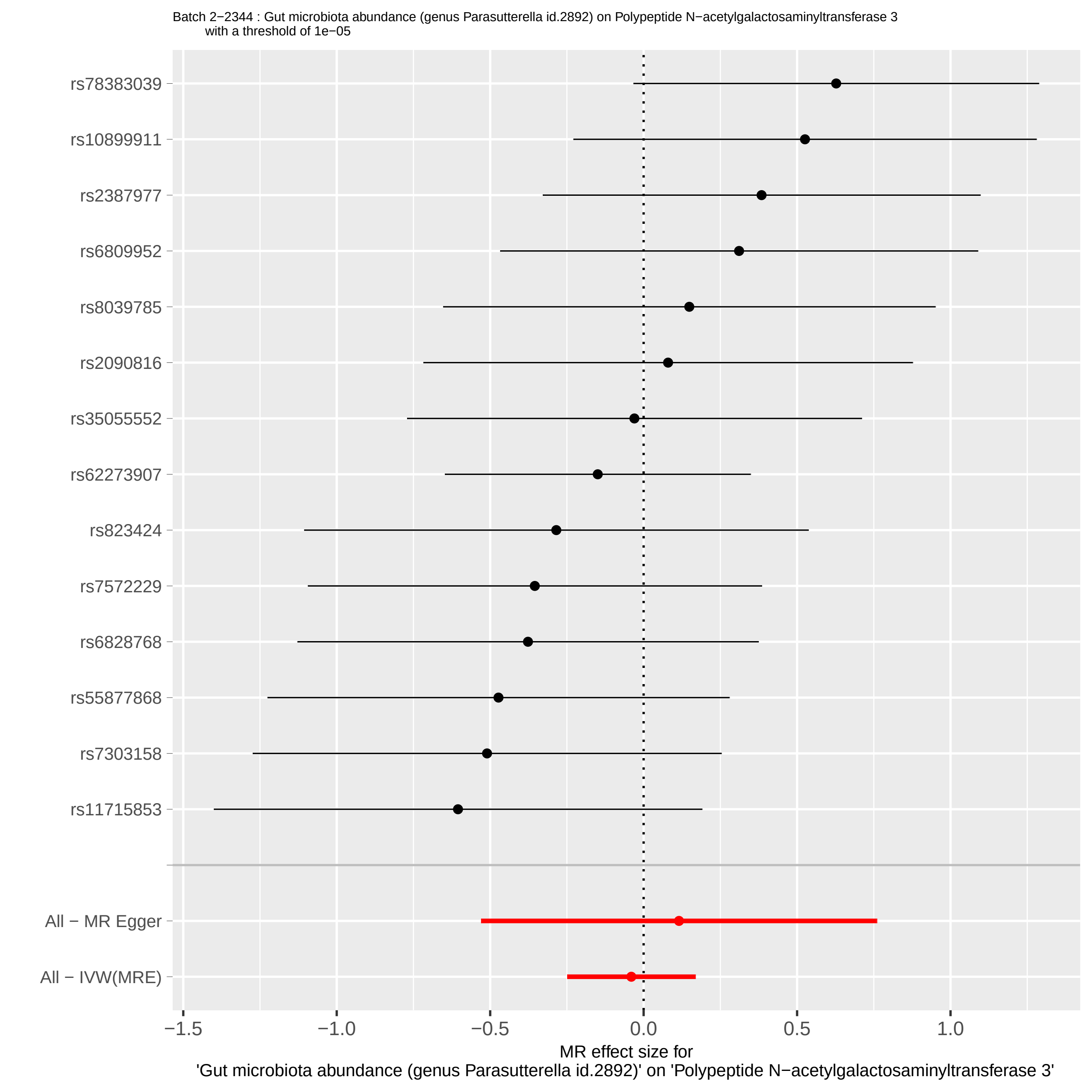

Batch 2−2344 : Gut microbiota abundance (genus Parasutterella id.2892) on Polypeptide N−acetylgalactosaminyltransferase 3
with a threshold of 1e−05
rs78383039
rs10899911
rs2387977
rs6809952
rs8039785
rs2090816
rs35055552
rs62273907
rs823424
rs7572229
rs6828768
rs55877868
rs7303158
rs11715853
All − MR Egger
All − IVW(MRE)
−1.5
−1.0
−0.5
0.0
0.5
1.0
MR effect size for
'Gut microbiota abundance (genus Parasutterella id.2892)' on 'Polypeptide N−acetylgalactosaminyltransferase 3'

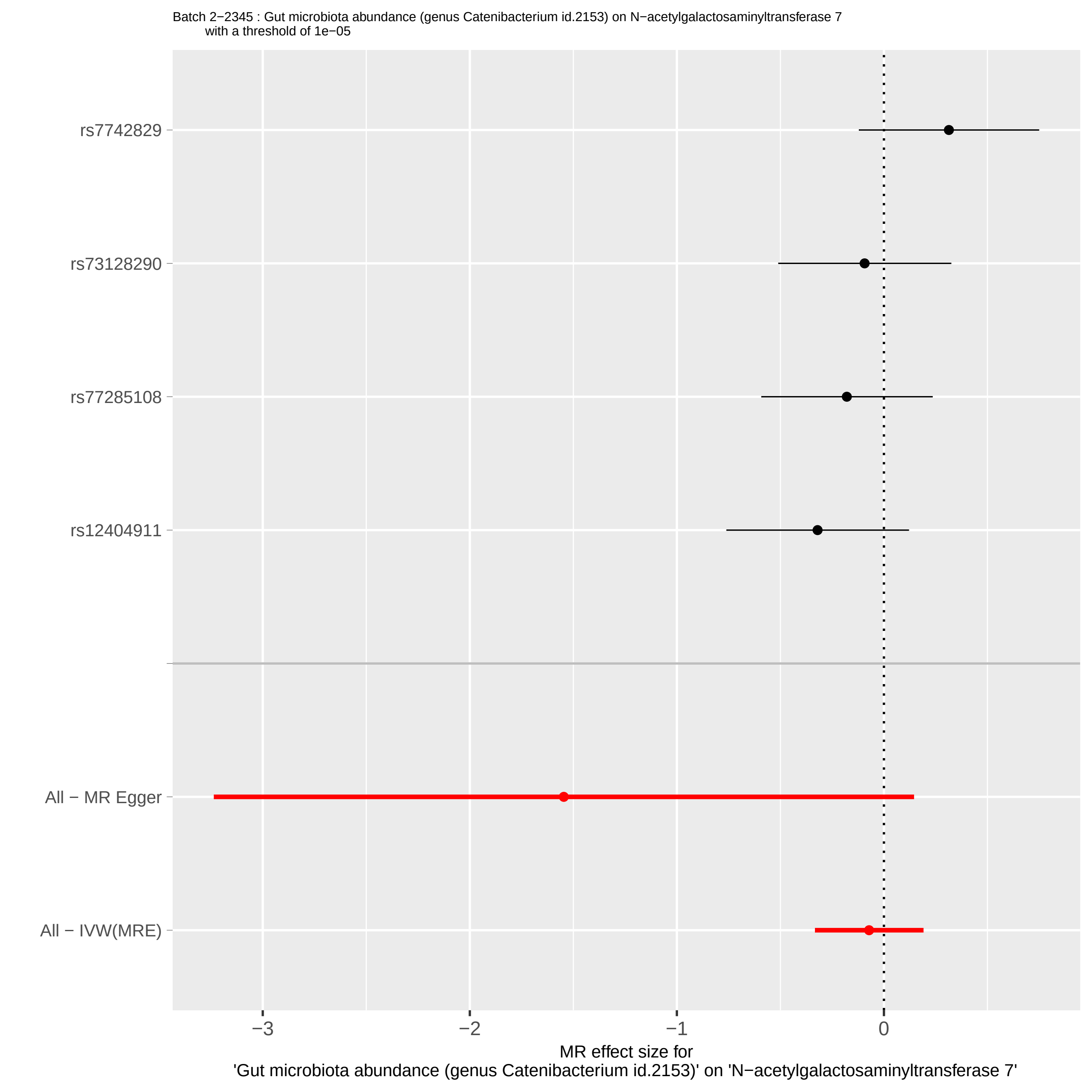
Batch 2−2345 : Gut microbiota abundance (genus Catenibacterium id.2153) on N−acetylgalactosaminyltransferase 7
with a threshold of 1e−05
rs7742829
rs73128290
rs77285108
rs12404911
All − MR Egger
All − IVW(MRE)
−3
−2
−1
0
MR effect size for
'Gut microbiota abundance (genus Catenibacterium id.2153)' on 'N−acetylgalactosaminyltransferase 7'

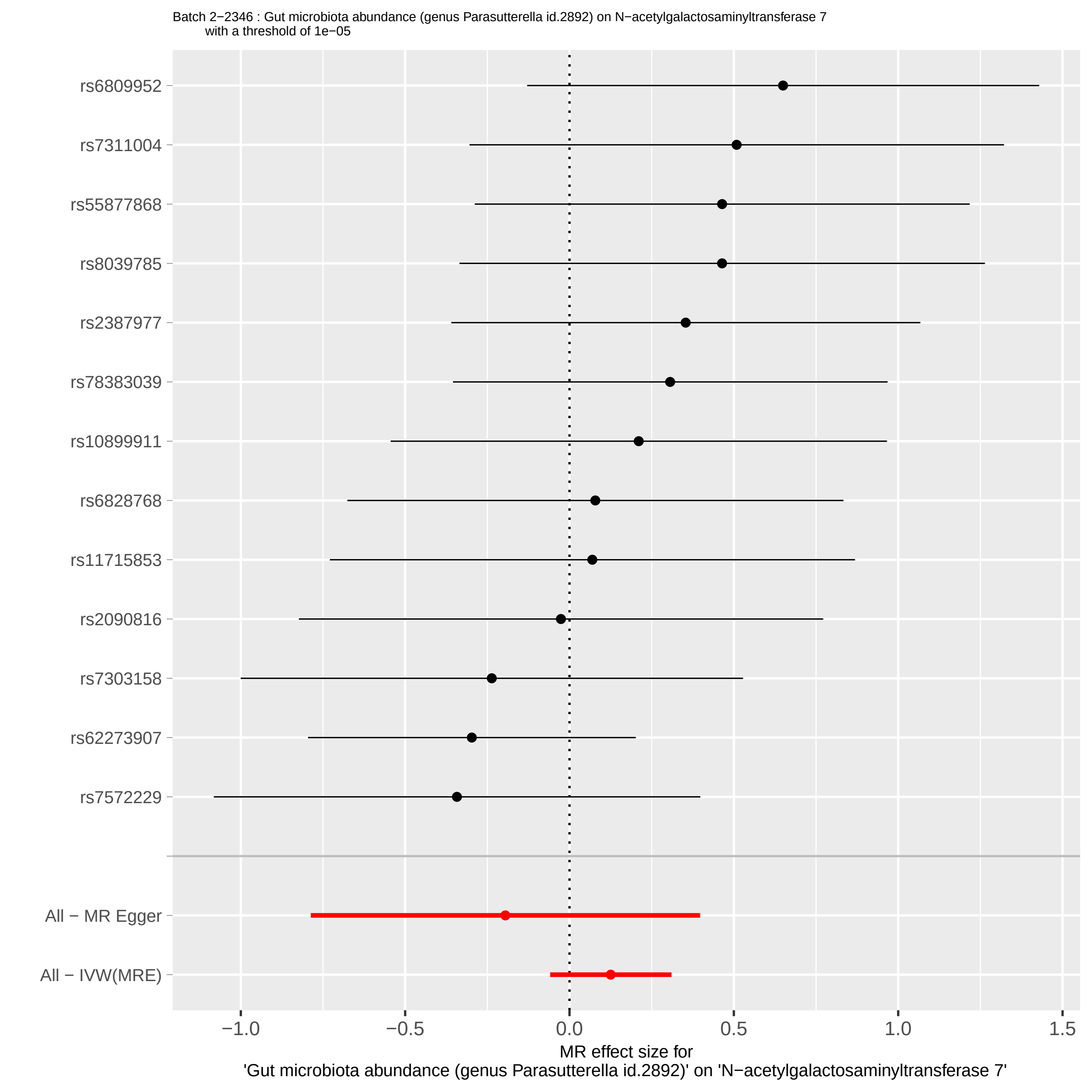

Batch 2−2346 : Gut microbiota abundance (genus Parasutterella id.2892) on N−acetylgalactosaminyltransferase 7
with a threshold of 1e−05
rs6809952
rs7311004
rs55877868
rs8039785
rs2387977
rs78383039
rs10899911
rs6828768
rs11715853
rs2090816
rs7303158
rs62273907
rs7572229
All − MR Egger
All − IVW(MRE)
−1.0
−0.5
0.0
0.5
1.0
1.5
MR effect size for
'Gut microbiota abundance (genus Parasutterella id.2892)' on 'N−acetylgalactosaminyltransferase 7'

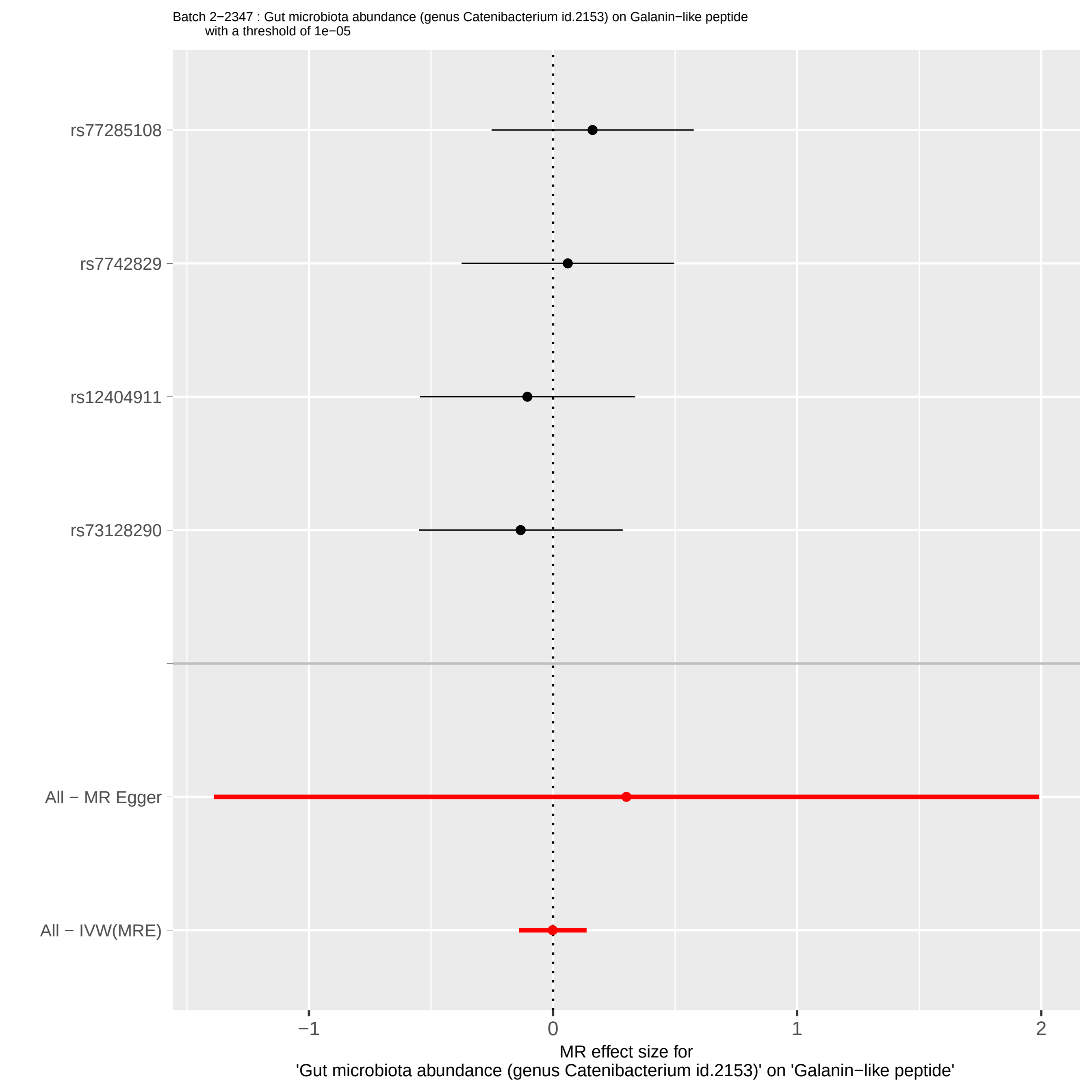

Batch 2−2347 : Gut microbiota abundance (genus Catenibacterium id.2153) on Galanin−like peptide
with a threshold of 1e−05
rs77285108
rs7742829
rs12404911
rs73128290
All − MR Egger
All − IVW(MRE)
−1
0
1
2
MR effect size for
'Gut microbiota abundance (genus Catenibacterium id.2153)' on 'Galanin−like peptide'

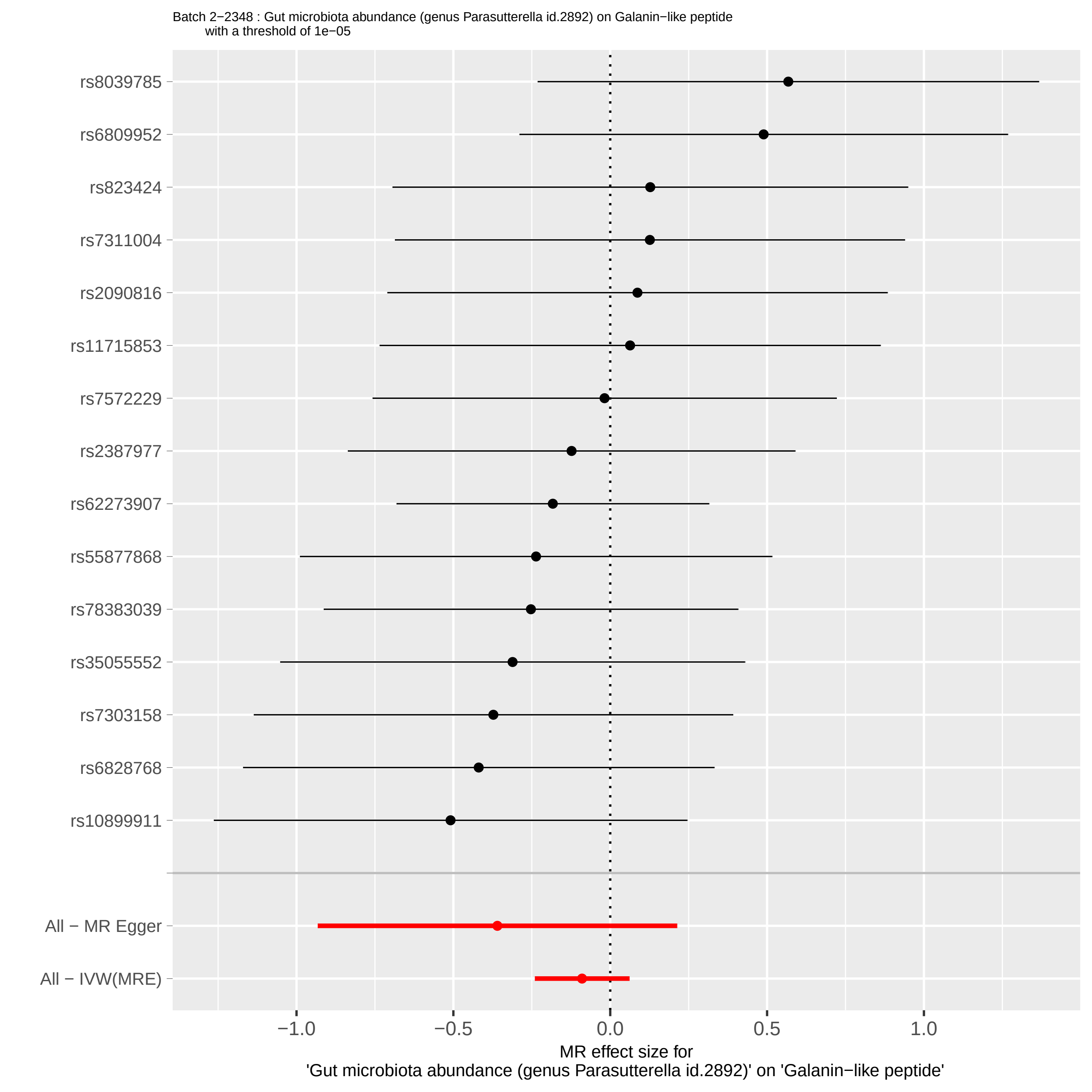

Batch 2−2348 : Gut microbiota abundance (genus Parasutterella id.2892) on Galanin−like peptide
with a threshold of 1e−05
rs8039785
rs6809952
rs823424
rs7311004
rs2090816
rs11715853
rs7572229
rs2387977
rs62273907
rs55877868
rs78383039
rs35055552
rs7303158
rs6828768
rs10899911
All − MR Egger
All − IVW(MRE)
−1.0
−0.5
0.0
0.5
1.0
MR effect size for
'Gut microbiota abundance (genus Parasutterella id.2892)' on 'Galanin−like peptide'

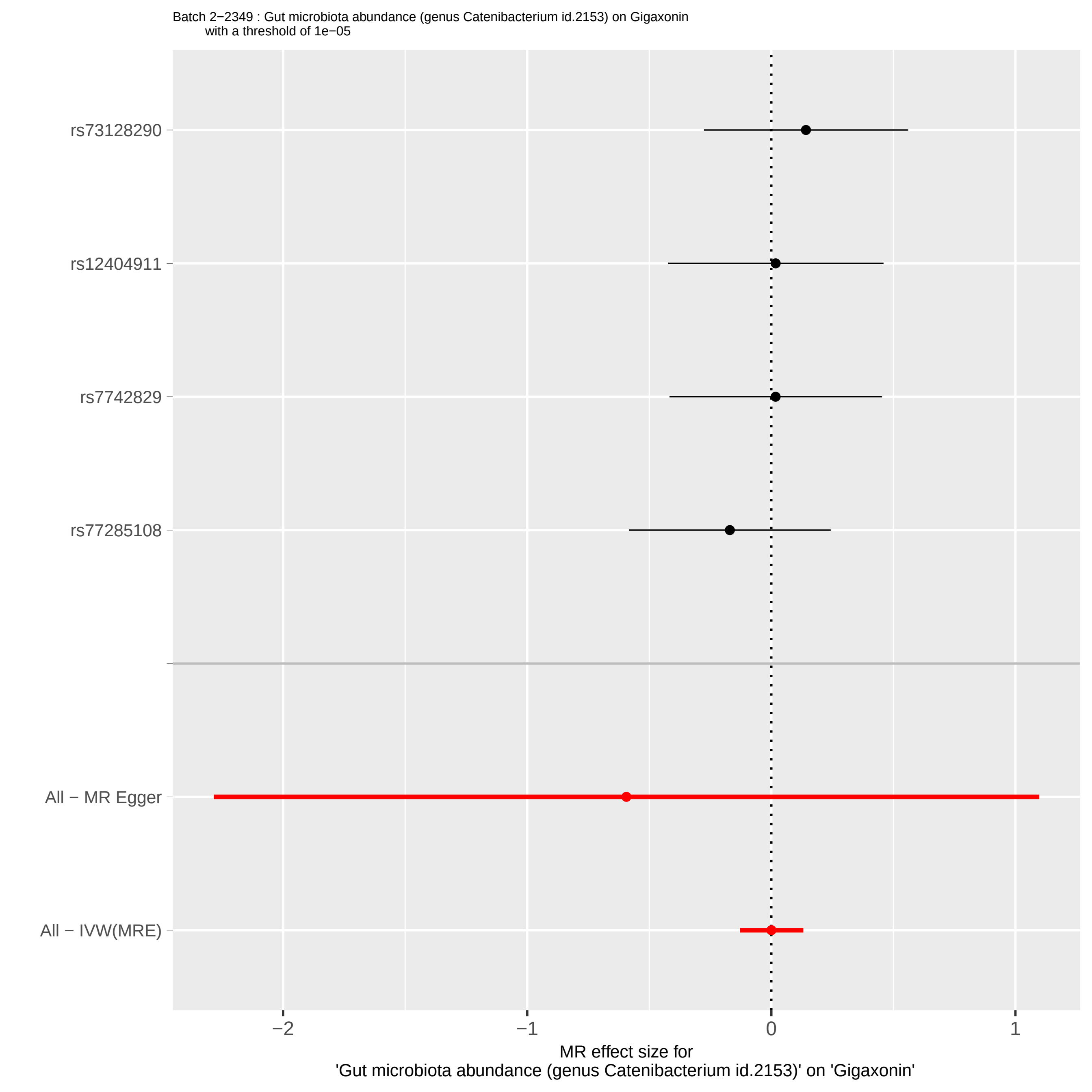
Batch 2−2349 : Gut microbiota abundance (genus Catenibacterium id.2153) on Gigaxonin
with a threshold of 1e−05
rs73128290
rs12404911
rs7742829
rs77285108
All − MR Egger
All − IVW(MRE)
−2
−1
0
1
MR effect size for
'Gut microbiota abundance (genus Catenibacterium id.2153)' on 'Gigaxonin'

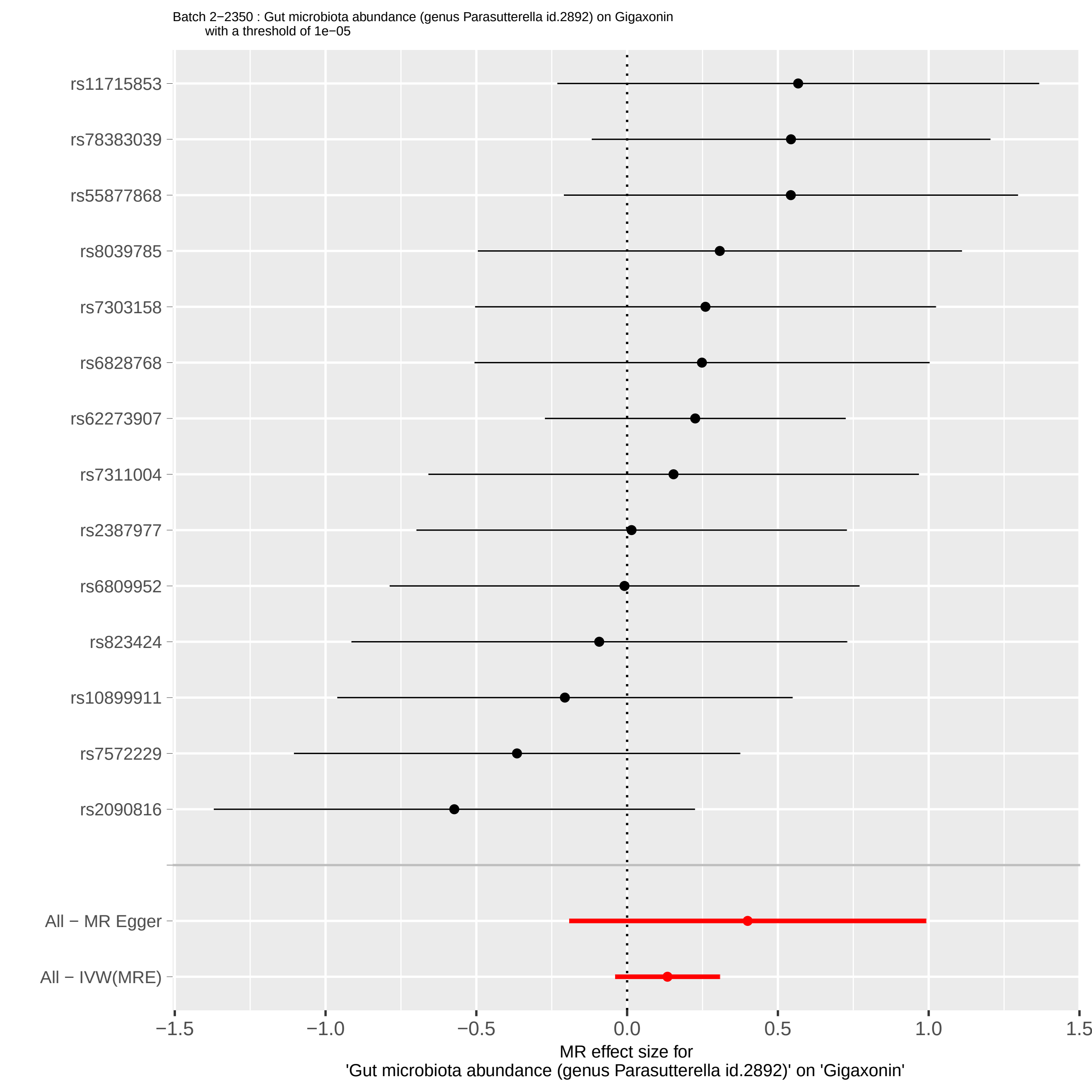

Batch 2−2350 : Gut microbiota abundance (genus Parasutterella id.2892) on Gigaxonin
with a threshold of 1e−05
rs11715853
rs78383039
rs55877868
rs8039785
rs7303158
rs6828768
rs62273907
rs7311004
rs2387977
rs6809952
rs823424
rs10899911
rs7572229
rs2090816
All − MR Egger
All − IVW(MRE)
−1.5
−1.0
−0.5
0.0
0.5
1.0
1.5
MR effect size for
'Gut microbiota abundance (genus Parasutterella id.2892)' on 'Gigaxonin'

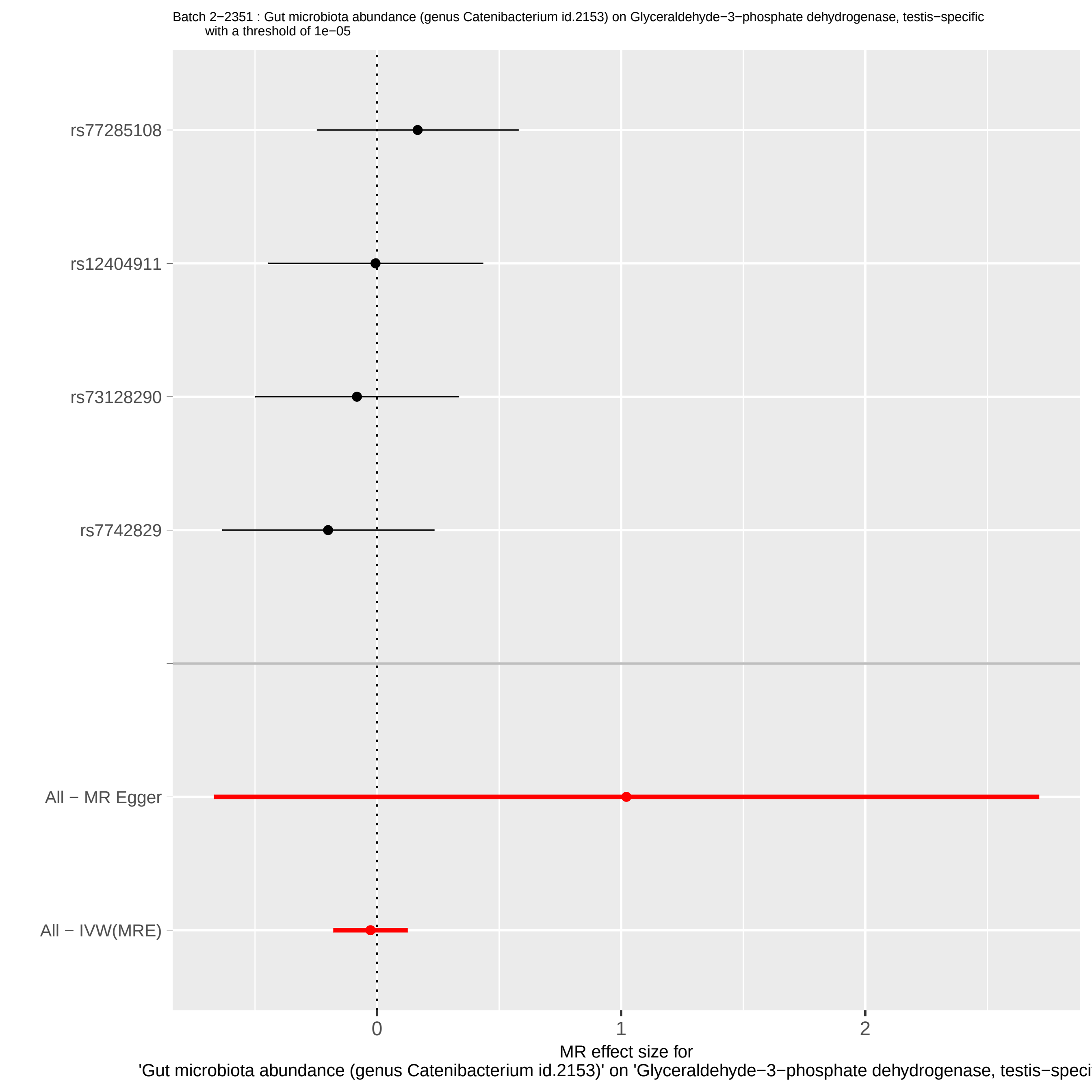

Batch 2−2351 : Gut microbiota abundance (genus Catenibacterium id.2153) on Glyceraldehyde−3−phosphate dehydrogenase, testis−specific
with a threshold of 1e−05
rs77285108
rs12404911
rs73128290
rs7742829
All − MR Egger
All − IVW(MRE)
0
1
2
MR effect size for
'Gut microbiota abundance (genus Catenibacterium id.2153)' on 'Glyceraldehyde−3−phosphate dehydrogenase, testis−speci

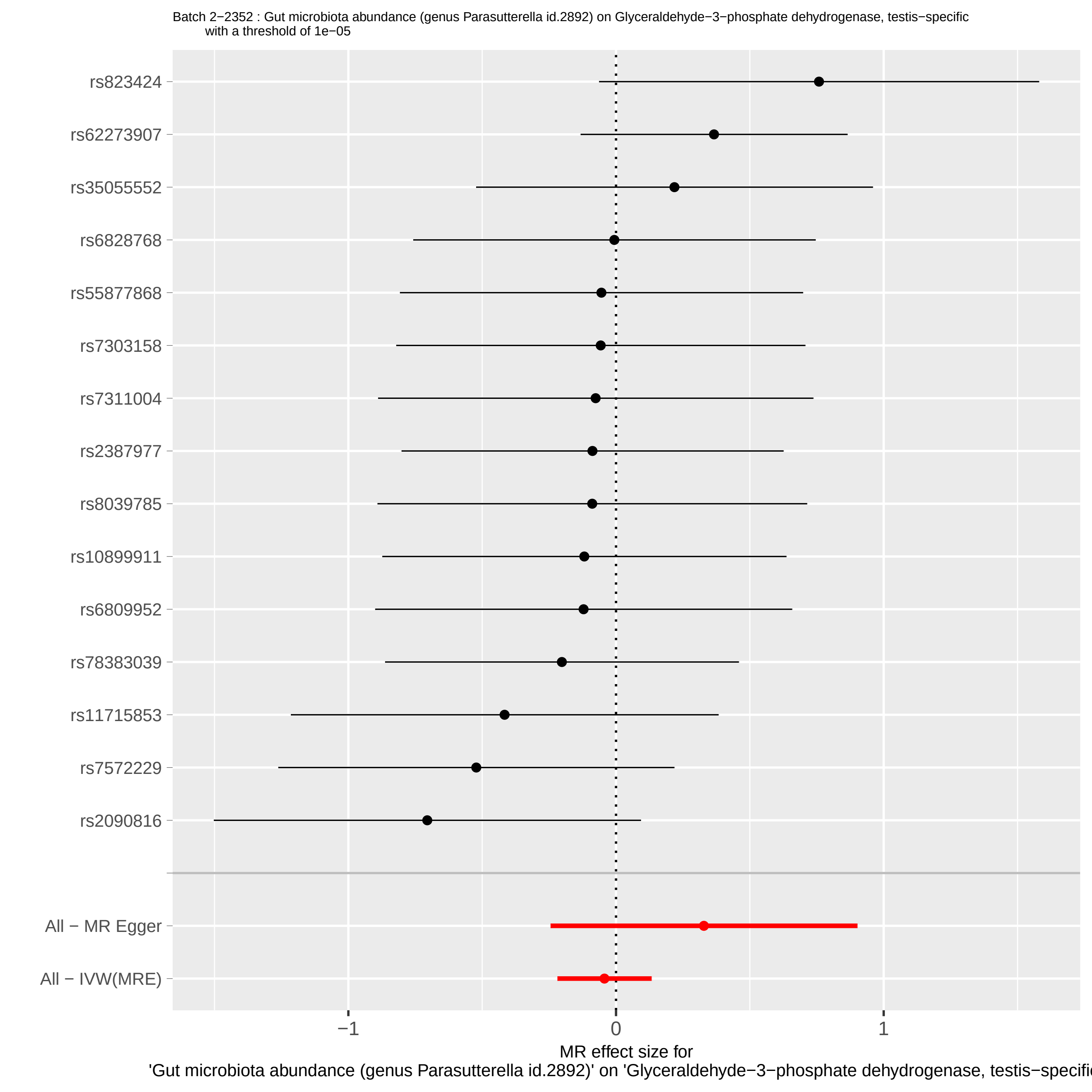
Batch 2−2352 : Gut microbiota abundance (genus Parasutterella id.2892) on Glyceraldehyde−3−phosphate dehydrogenase, testis−specific
with a threshold of 1e−05
rs823424
rs62273907
rs35055552
rs6828768
rs55877868
rs7303158
rs7311004
rs2387977
rs8039785
rs10899911
rs6809952
rs78383039
rs11715853
rs7572229
rs2090816
All − MR Egger
All − IVW(MRE)
−1
0
1
MR effect size for
'Gut microbiota abundance (genus Parasutterella id.2892)' on 'Glyceraldehyde−3−phosphate dehydrogenase, testis−specific

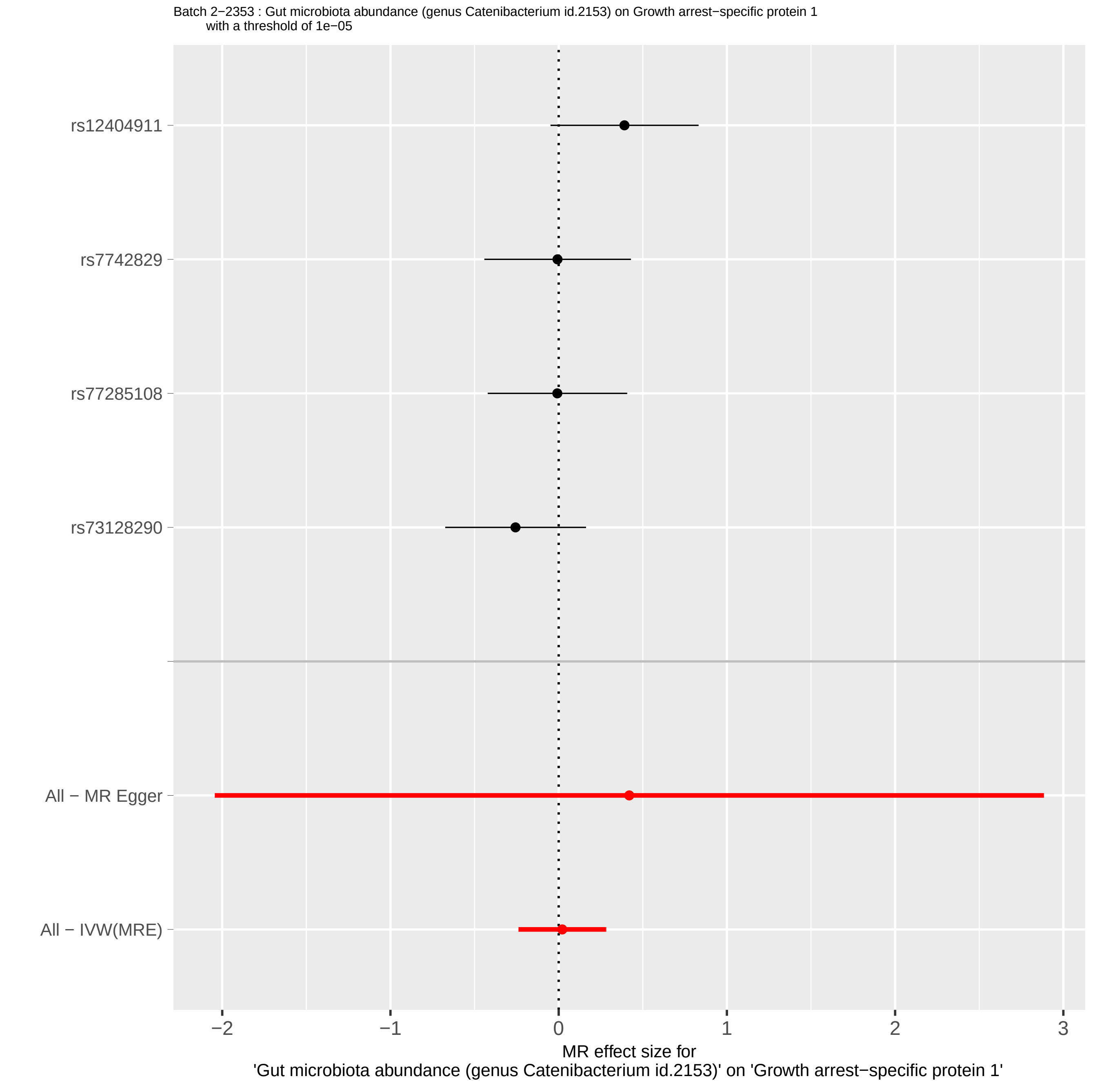
Batch 2−2353 : Gut microbiota abundance (genus Catenibacterium id.2153) on Growth arrest−specific protein 1
with a threshold of 1e−05
rs12404911
rs7742829
rs77285108
rs73128290
All − MR Egger
All − IVW(MRE)
−2
−1
0
1
2
3
MR effect size for
'Gut microbiota abundance (genus Catenibacterium id.2153)' on 'Growth arrest−specific protein 1'

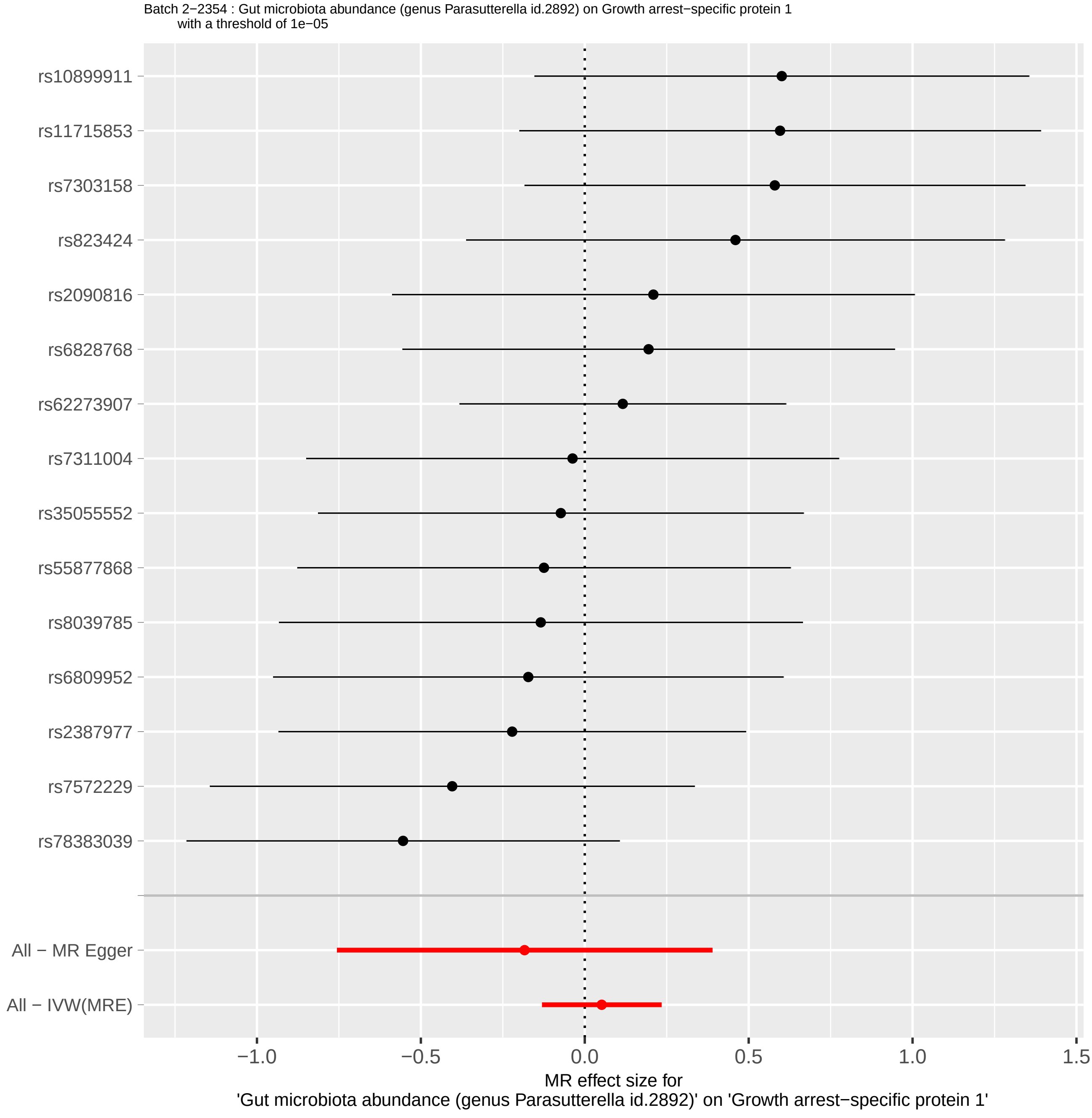
Batch 2−2354 : Gut microbiota abundance (genus Parasutterella id.2892) on Growth arrest−specific protein 1
with a threshold of 1e−05
rs10899911
rs11715853
rs7303158
rs823424
rs2090816
rs6828768
rs62273907
rs7311004
rs35055552
rs55877868
rs8039785
rs6809952
rs2387977
rs7572229
rs78383039
All − MR Egger
All − IVW(MRE)
−1.0
−0.5
0.0
0.5
1.0
1.5
MR effect size for
'Gut microbiota abundance (genus Parasutterella id.2892)' on 'Growth arrest−specific protein 1'

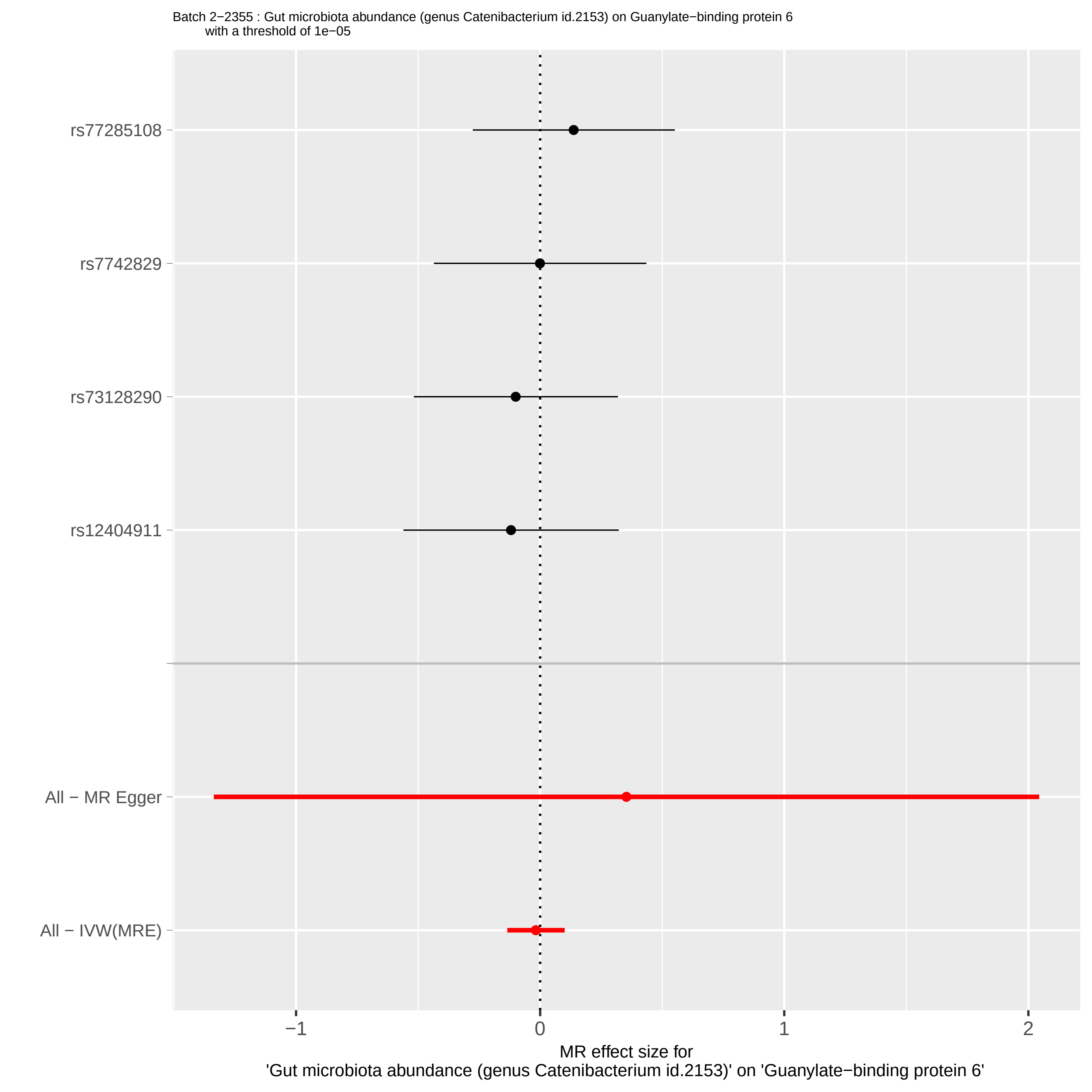
Batch 2−2355 : Gut microbiota abundance (genus Catenibacterium id.2153) on Guanylate−binding protein 6
with a threshold of 1e−05
rs77285108
rs7742829
rs73128290
rs12404911
All − MR Egger
All − IVW(MRE)
−1
0
1
2
MR effect size for
'Gut microbiota abundance (genus Catenibacterium id.2153)' on 'Guanylate−binding protein 6'

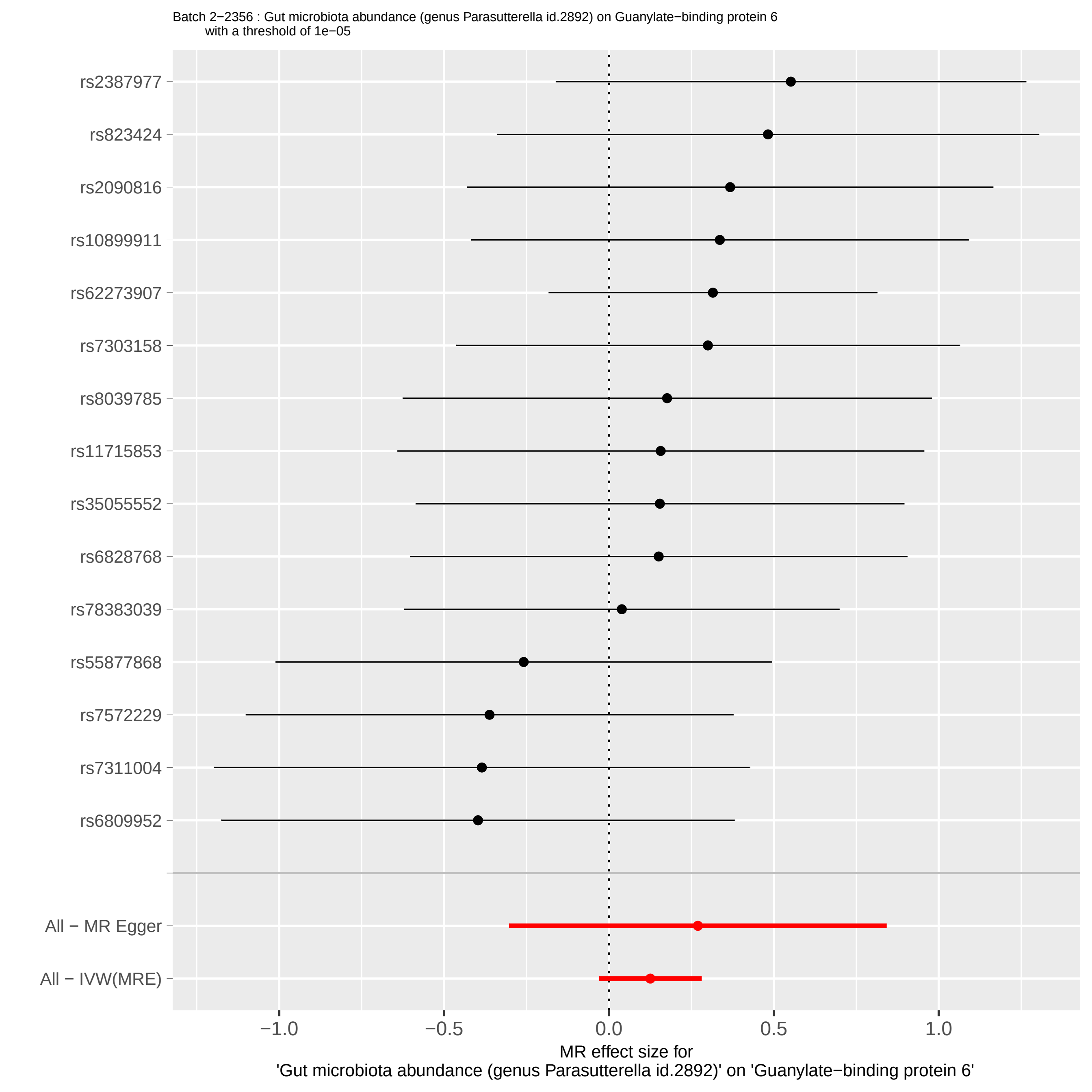

Batch 2−2356 : Gut microbiota abundance (genus Parasutterella id.2892) on Guanylate−binding protein 6
with a threshold of 1e−05
rs2387977
rs823424
rs2090816
rs10899911
rs62273907
rs7303158
rs8039785
rs11715853
rs35055552
rs6828768
rs78383039
rs55877868
rs7572229
rs7311004
rs6809952
All − MR Egger
All − IVW(MRE)
−1.0
−0.5
0.0
0.5
1.0
MR effect size for
'Gut microbiota abundance (genus Parasutterella id.2892)' on 'Guanylate−binding protein 6'

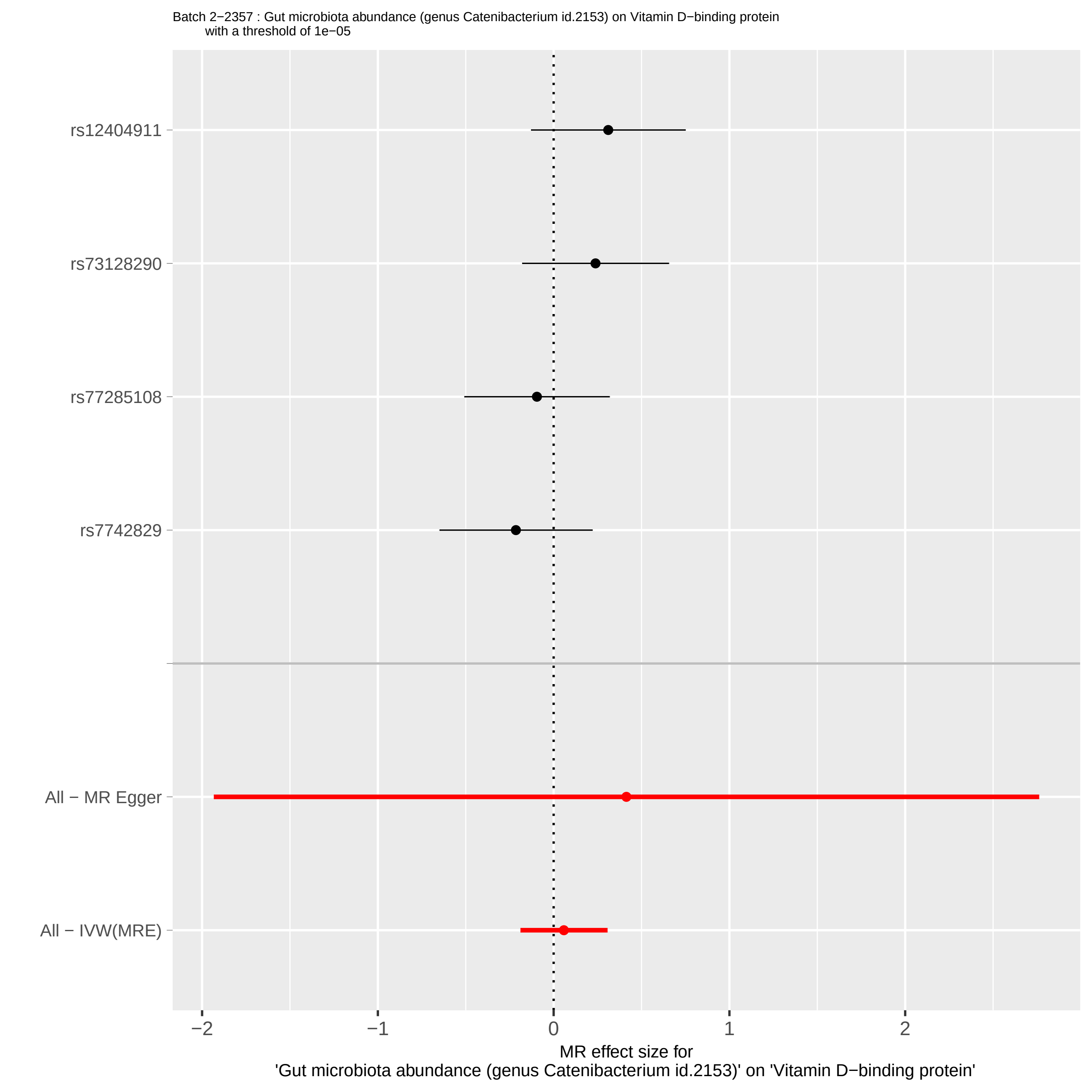

Batch 2−2357 : Gut microbiota abundance (genus Catenibacterium id.2153) on Vitamin D−binding protein
with a threshold of 1e−05
rs12404911
rs73128290
rs77285108
rs7742829
All − MR Egger
All − IVW(MRE)
−2
−1
0
1
2
MR effect size for
'Gut microbiota abundance (genus Catenibacterium id.2153)' on 'Vitamin D−binding protein'

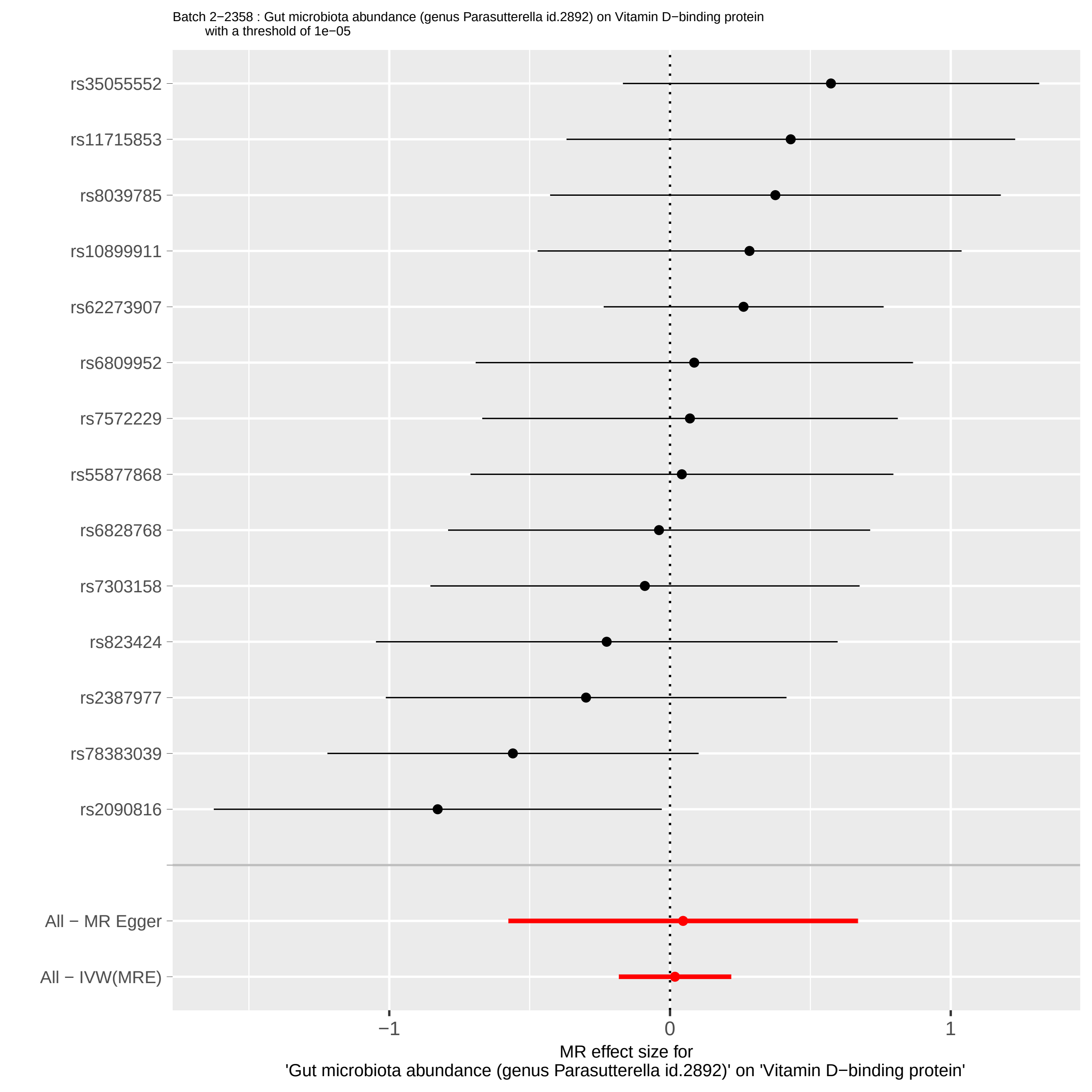

Batch 2−2358 : Gut microbiota abundance (genus Parasutterella id.2892) on Vitamin D−binding protein
with a threshold of 1e−05
rs35055552
rs11715853
rs8039785
rs10899911
rs62273907
rs6809952
rs7572229
rs55877868
rs6828768
rs7303158
rs823424
rs2387977
rs78383039
rs2090816
All − MR Egger
All − IVW(MRE)
−1
0
1
MR effect size for
'Gut microbiota abundance (genus Parasutterella id.2892)' on 'Vitamin D−binding protein'

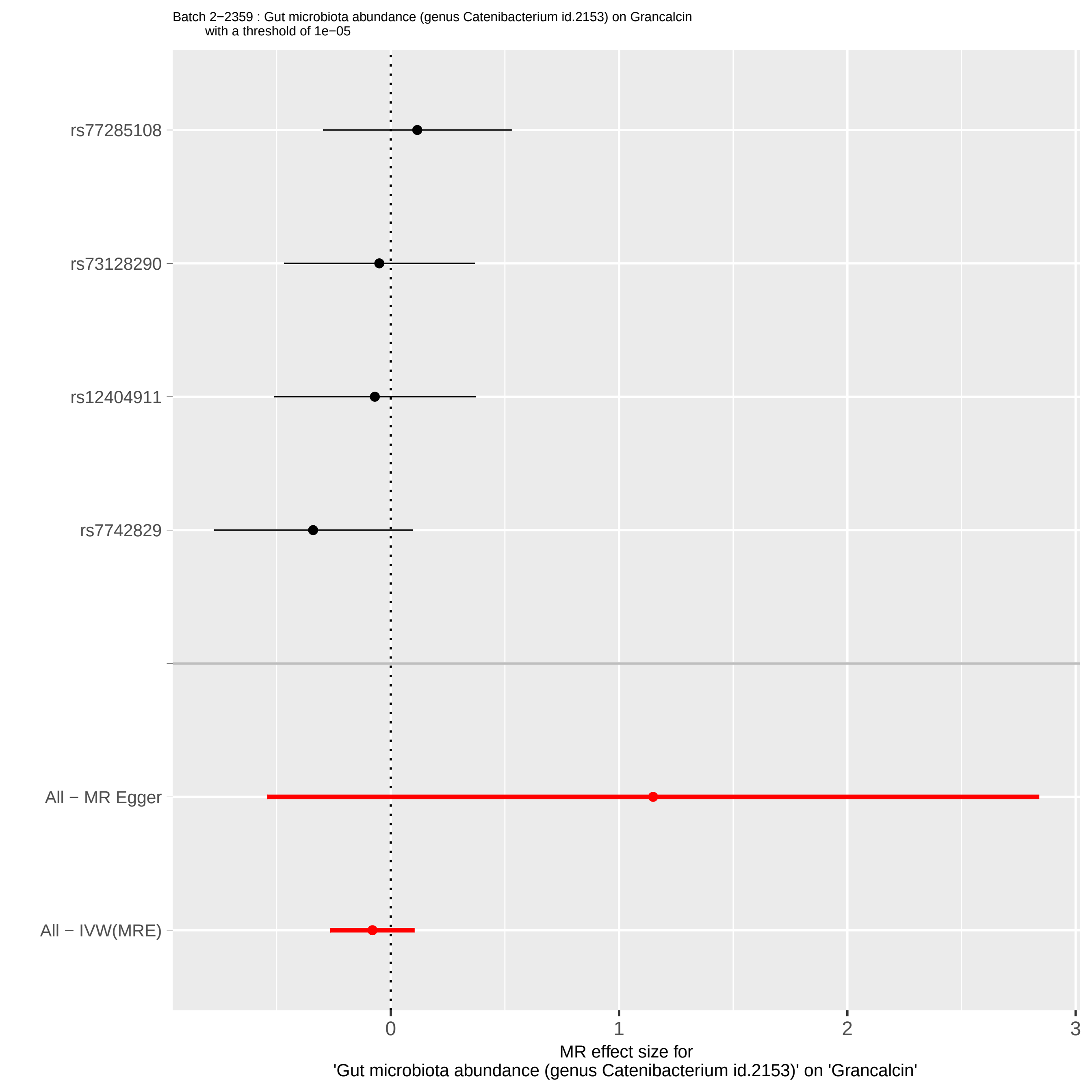
Batch 2−2359 : Gut microbiota abundance (genus Catenibacterium id.2153) on Grancalcin
with a threshold of 1e−05
rs77285108
rs73128290
rs12404911
rs7742829
All − MR Egger
All − IVW(MRE)
0
1
2
3
MR effect size for
'Gut microbiota abundance (genus Catenibacterium id.2153)' on 'Grancalcin'

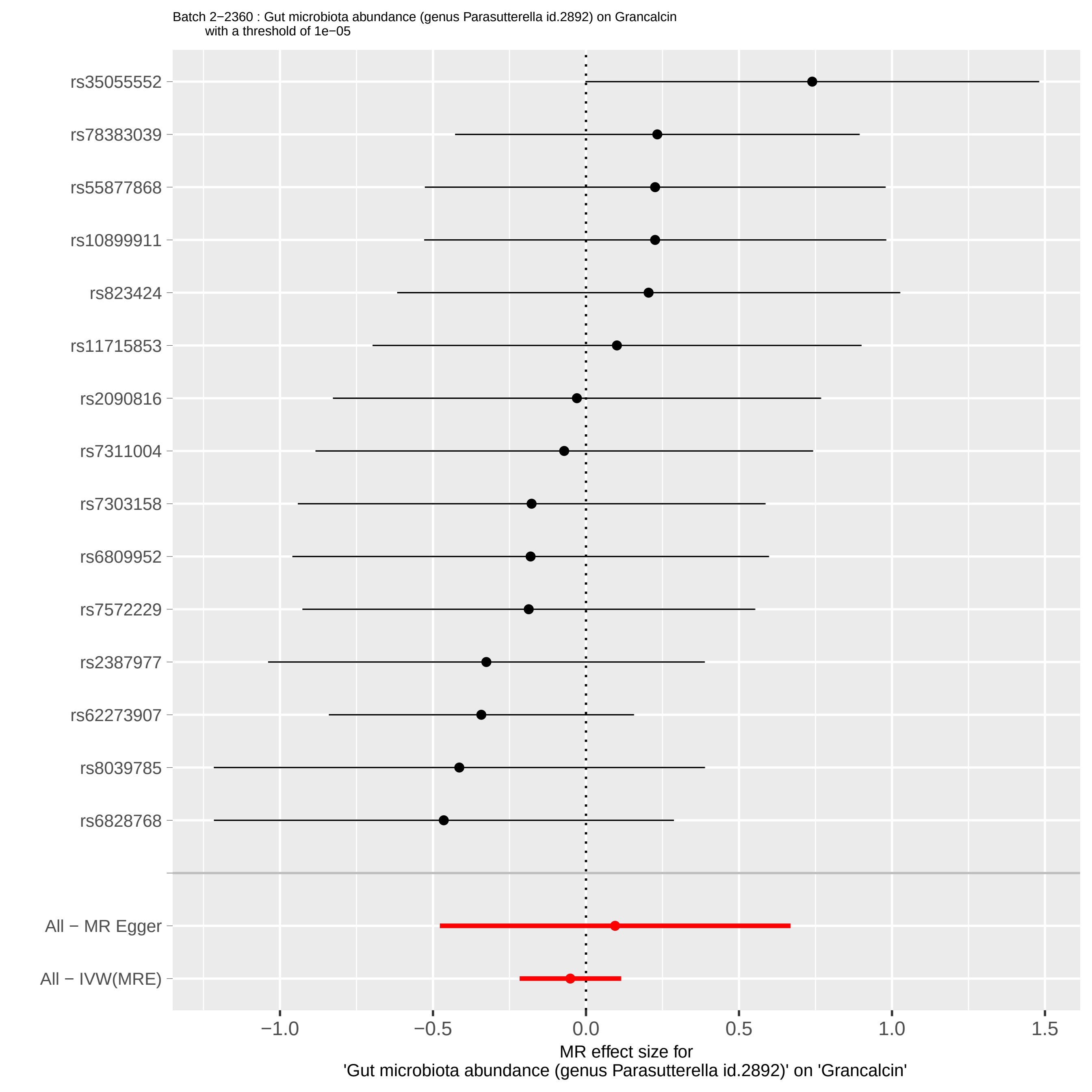

Batch 2−2360 : Gut microbiota abundance (genus Parasutterella id.2892) on Grancalcin
with a threshold of 1e−05
rs35055552
rs78383039
rs55877868
rs10899911
rs823424
rs11715853
rs2090816
rs7311004
rs7303158
rs6809952
rs7572229
rs2387977
rs62273907
rs8039785
rs6828768
All − MR Egger
All − IVW(MRE)
−1.0
−0.5
0.0
0.5
1.0
1.5
MR effect size for
'Gut microbiota abundance (genus Parasutterella id.2892)' on 'Grancalcin'

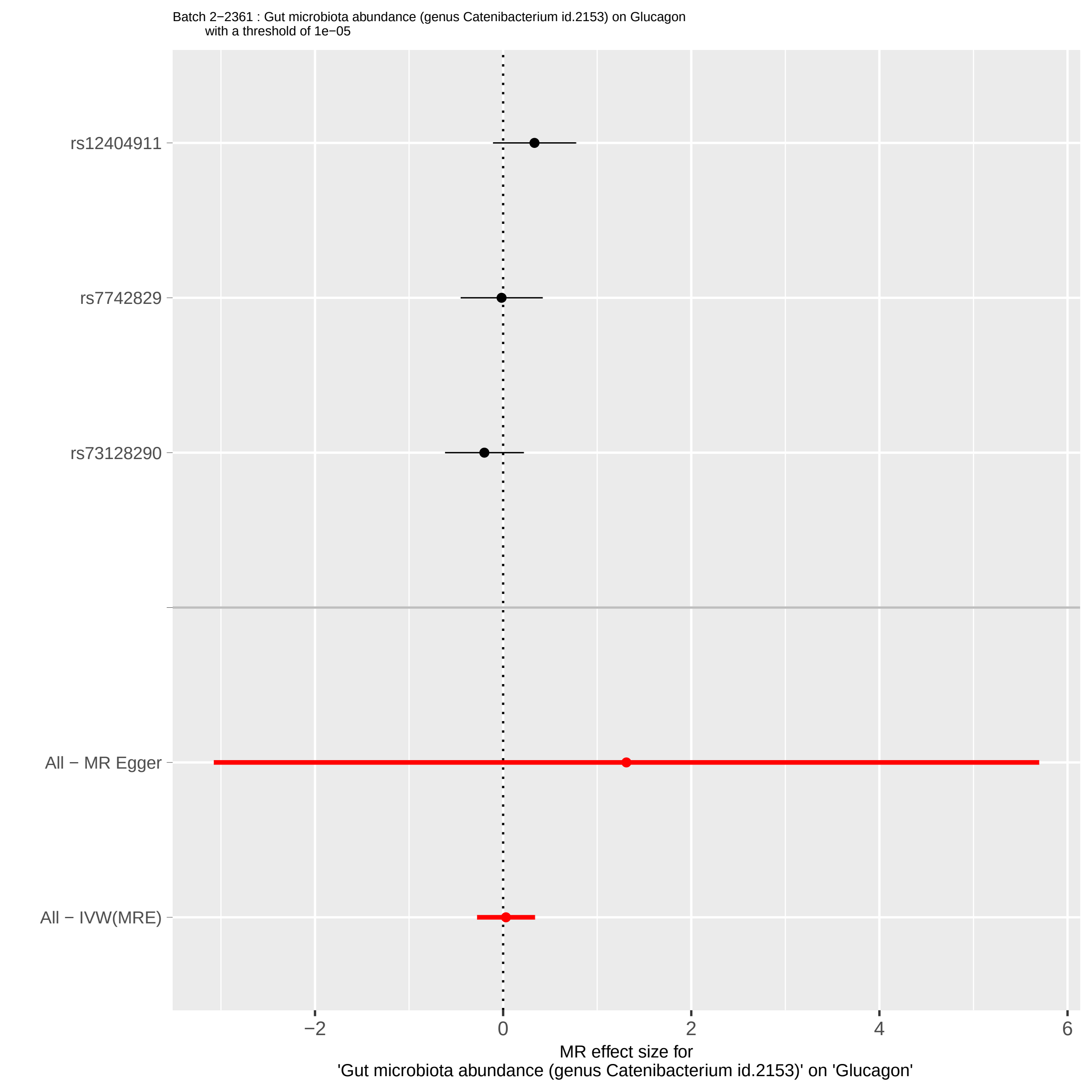

Batch 2−2361 : Gut microbiota abundance (genus Catenibacterium id.2153) on Glucagon
with a threshold of 1e−05
rs12404911
rs7742829
rs73128290
All − MR Egger
All − IVW(MRE)
−2
0
2
4
6
MR effect size for
'Gut microbiota abundance (genus Catenibacterium id.2153)' on 'Glucagon'

Batch 2−2362 : Gut microbiota abundance (genus Parasutterella id.2892) on Glucagon
with a threshold of 1e−05

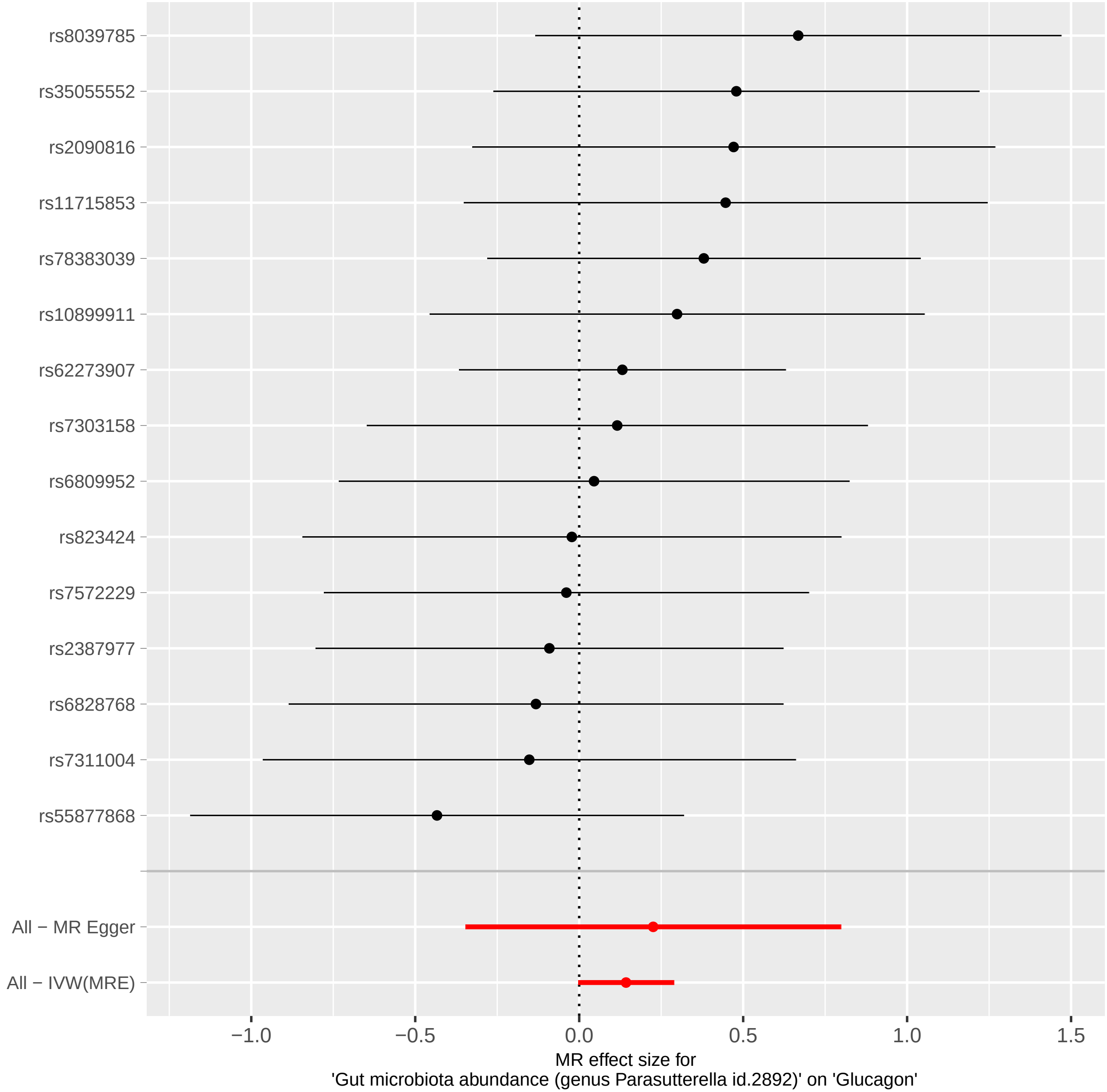

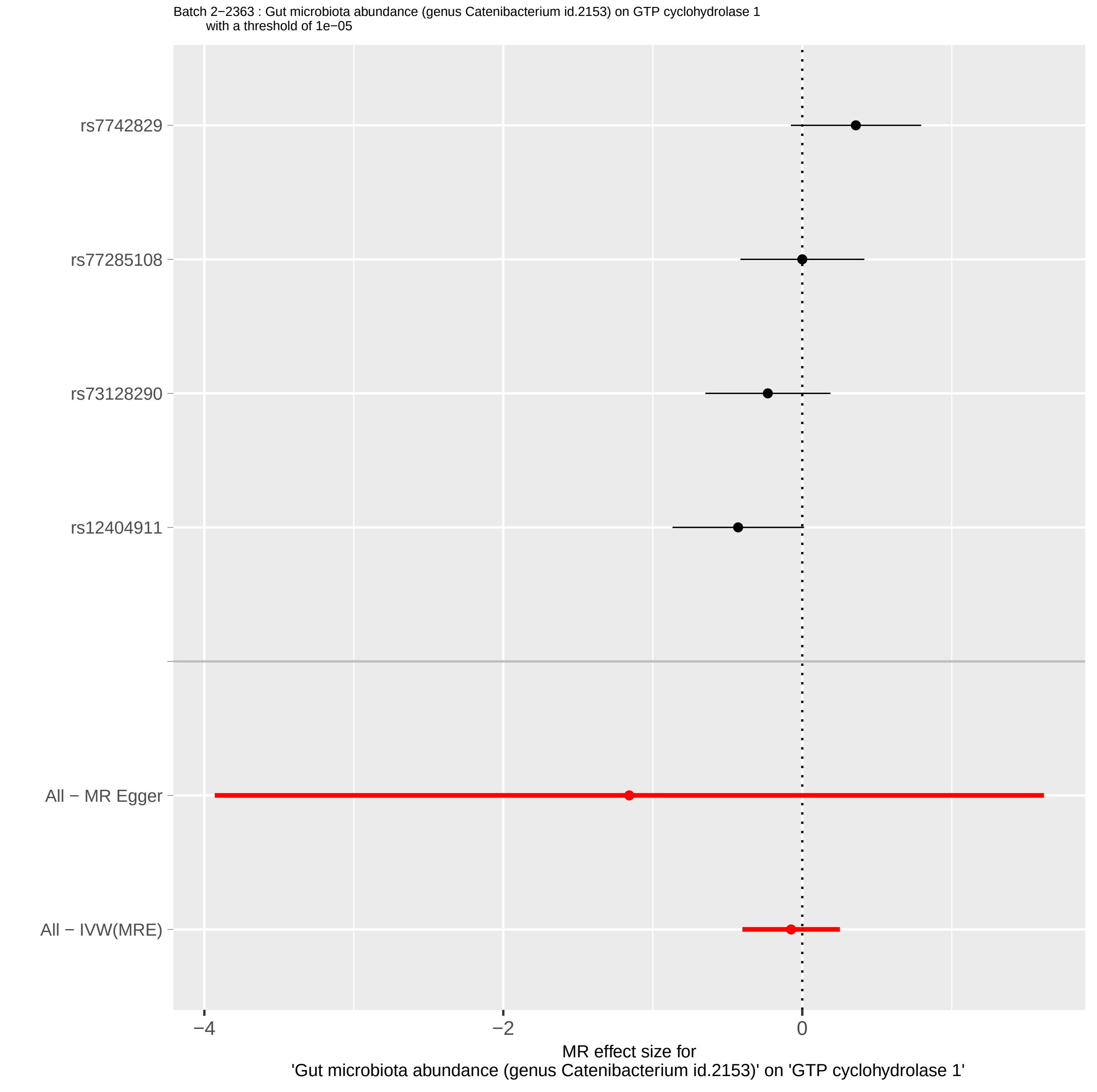

Batch 2−2363 : Gut microbiota abundance (genus Catenibacterium id.2153) on GTP cyclohydrolase 1
with a threshold of 1e−05
rs7742829
rs77285108
rs73128290
rs12404911
All − MR Egger
All − IVW(MRE)
−4
−2
0
MR effect size for
'Gut microbiota abundance (genus Catenibacterium id.2153)' on 'GTP cyclohydrolase 1'

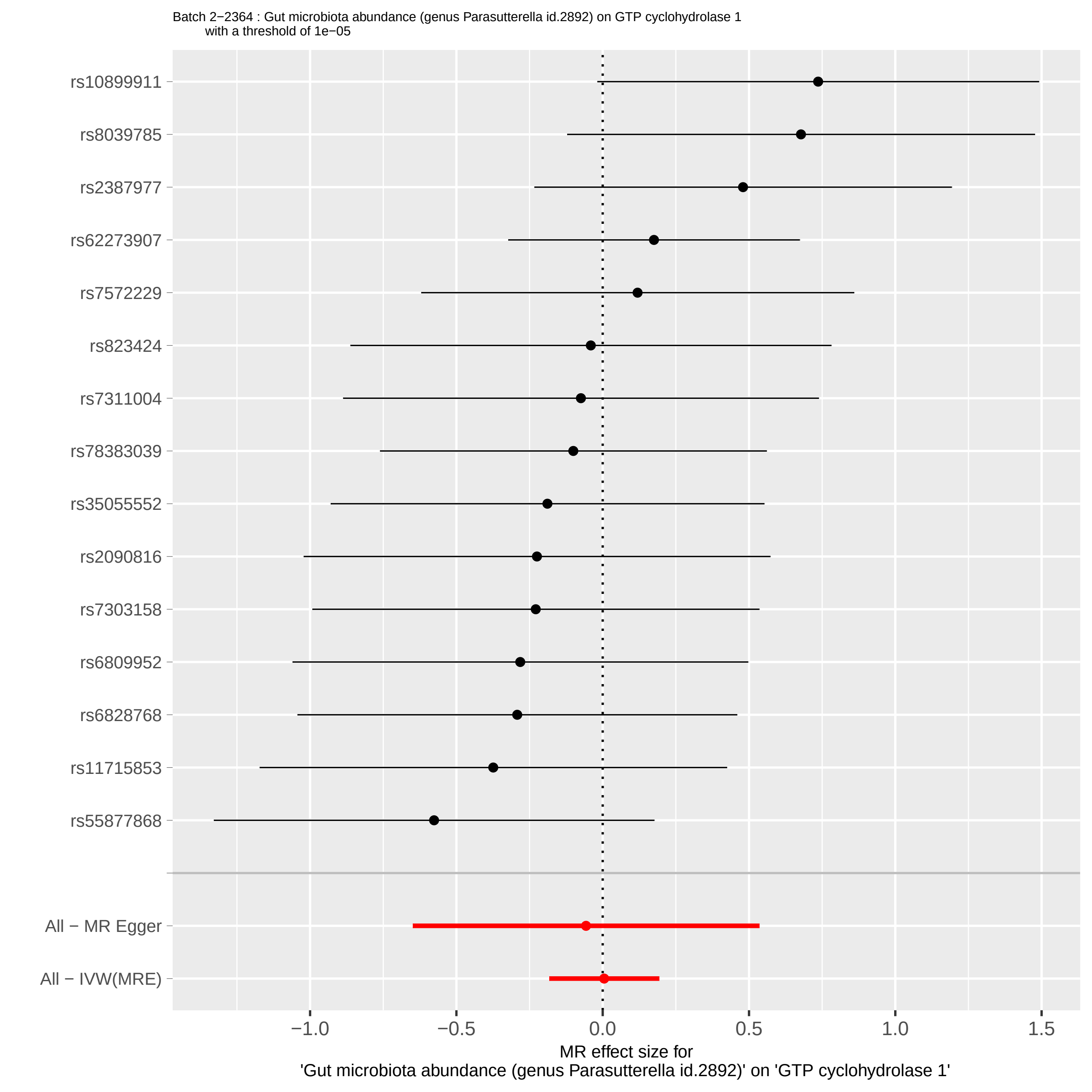

Batch 2−2364 : Gut microbiota abundance (genus Parasutterella id.2892) on GTP cyclohydrolase 1
with a threshold of 1e−05
rs10899911
rs8039785
rs2387977
rs62273907
rs7572229
rs823424
rs7311004
rs78383039
rs35055552
rs2090816
rs7303158
rs6809952
rs6828768
rs11715853
rs55877868
All − MR Egger
All − IVW(MRE)
−1.0
−0.5
0.0
0.5
1.0
1.5
MR effect size for
'Gut microbiota abundance (genus Parasutterella id.2892)' on 'GTP cyclohydrolase 1'

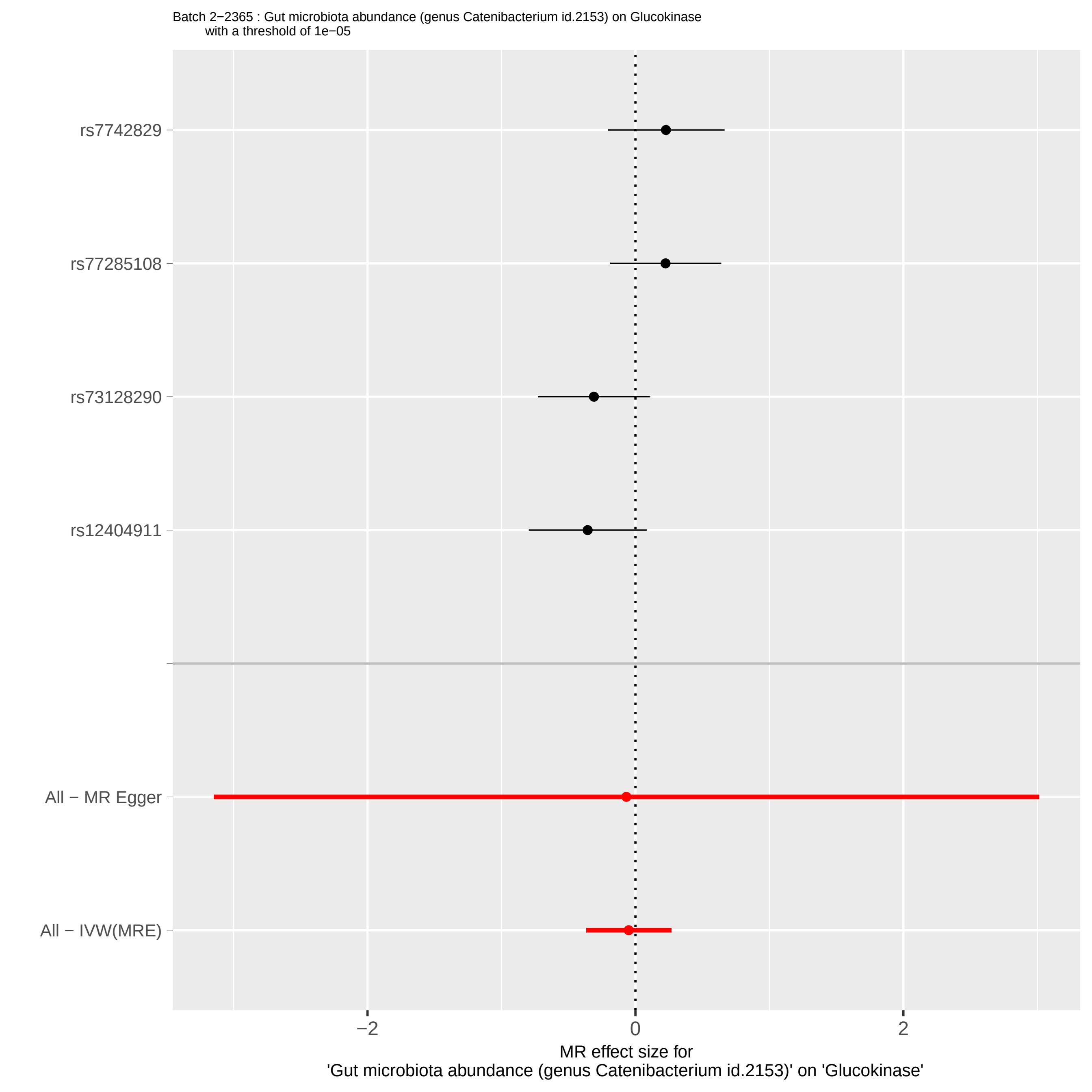

Batch 2−2365 : Gut microbiota abundance (genus Catenibacterium id.2153) on Glucokinase
with a threshold of 1e−05
rs7742829
rs77285108
rs73128290
rs12404911
All − MR Egger
All − IVW(MRE)
−2
0
2
MR effect size for
'Gut microbiota abundance (genus Catenibacterium id.2153)' on 'Glucokinase'

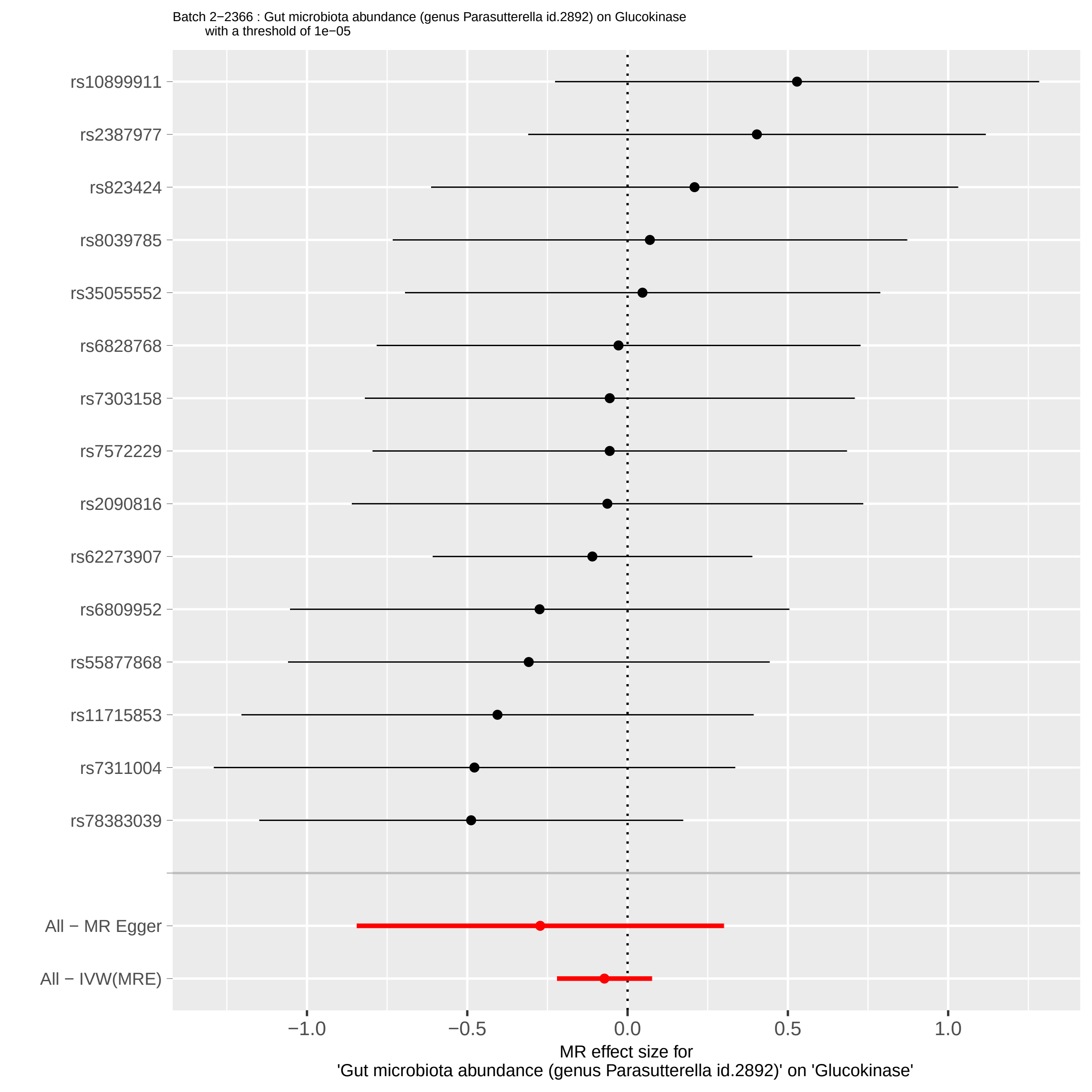

Batch 2−2366 : Gut microbiota abundance (genus Parasutterella id.2892) on Glucokinase
with a threshold of 1e−05
rs10899911
rs2387977
rs823424
rs8039785
rs35055552
rs6828768
rs7303158
rs7572229
rs2090816
rs62273907
rs6809952
rs55877868
rs11715853
rs7311004
rs78383039
All − MR Egger
All − IVW(MRE)
−1.0
−0.5
0.0
0.5
1.0
MR effect size for
'Gut microbiota abundance (genus Parasutterella id.2892)' on 'Glucokinase'

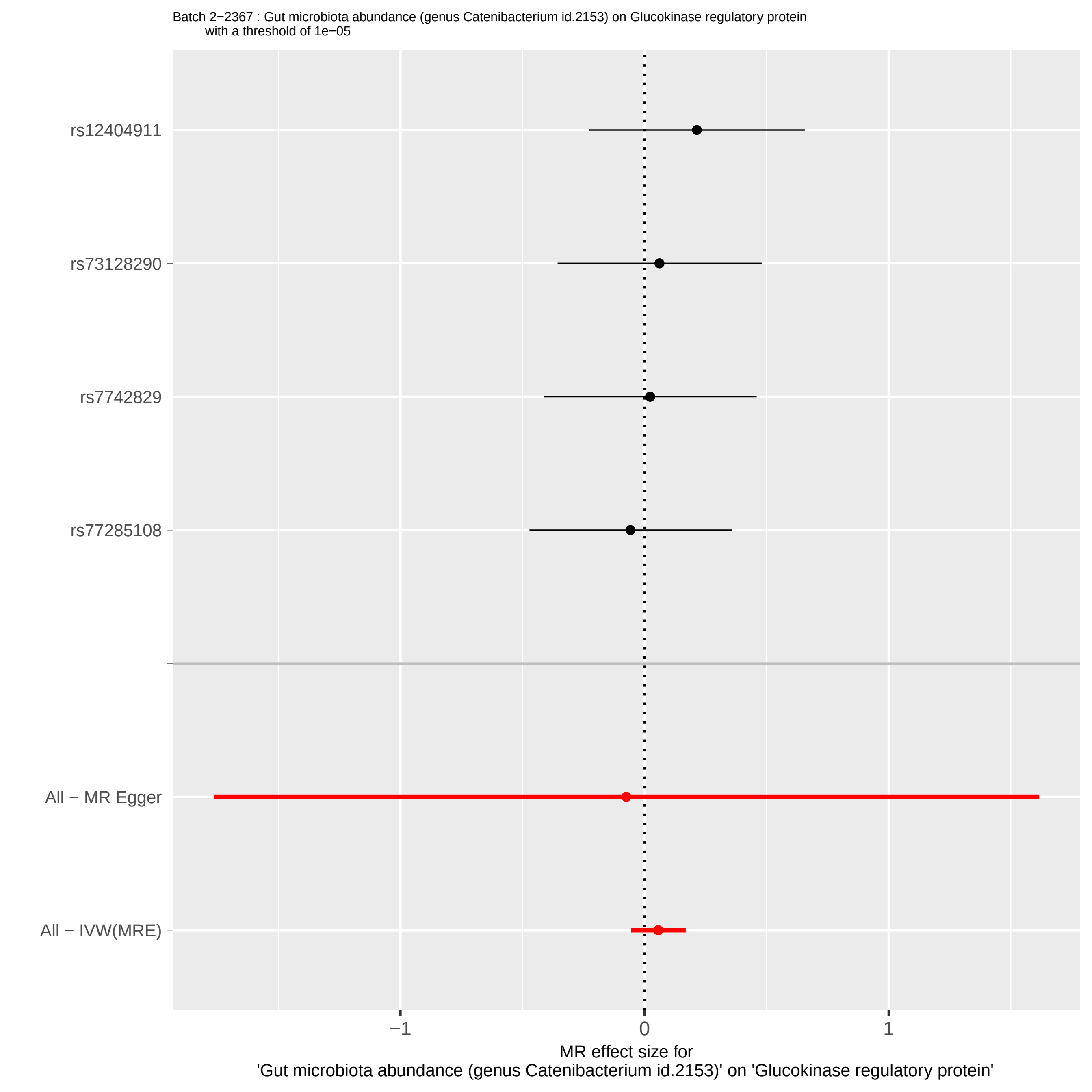
Batch 2−2367 : Gut microbiota abundance (genus Catenibacterium id.2153) on Glucokinase regulatory protein
with a threshold of 1e−05
rs12404911
rs73128290
rs7742829
rs77285108
All − MR Egger
All − IVW(MRE)
−1
0
1
MR effect size for
'Gut microbiota abundance (genus Catenibacterium id.2153)' on 'Glucokinase regulatory protein'

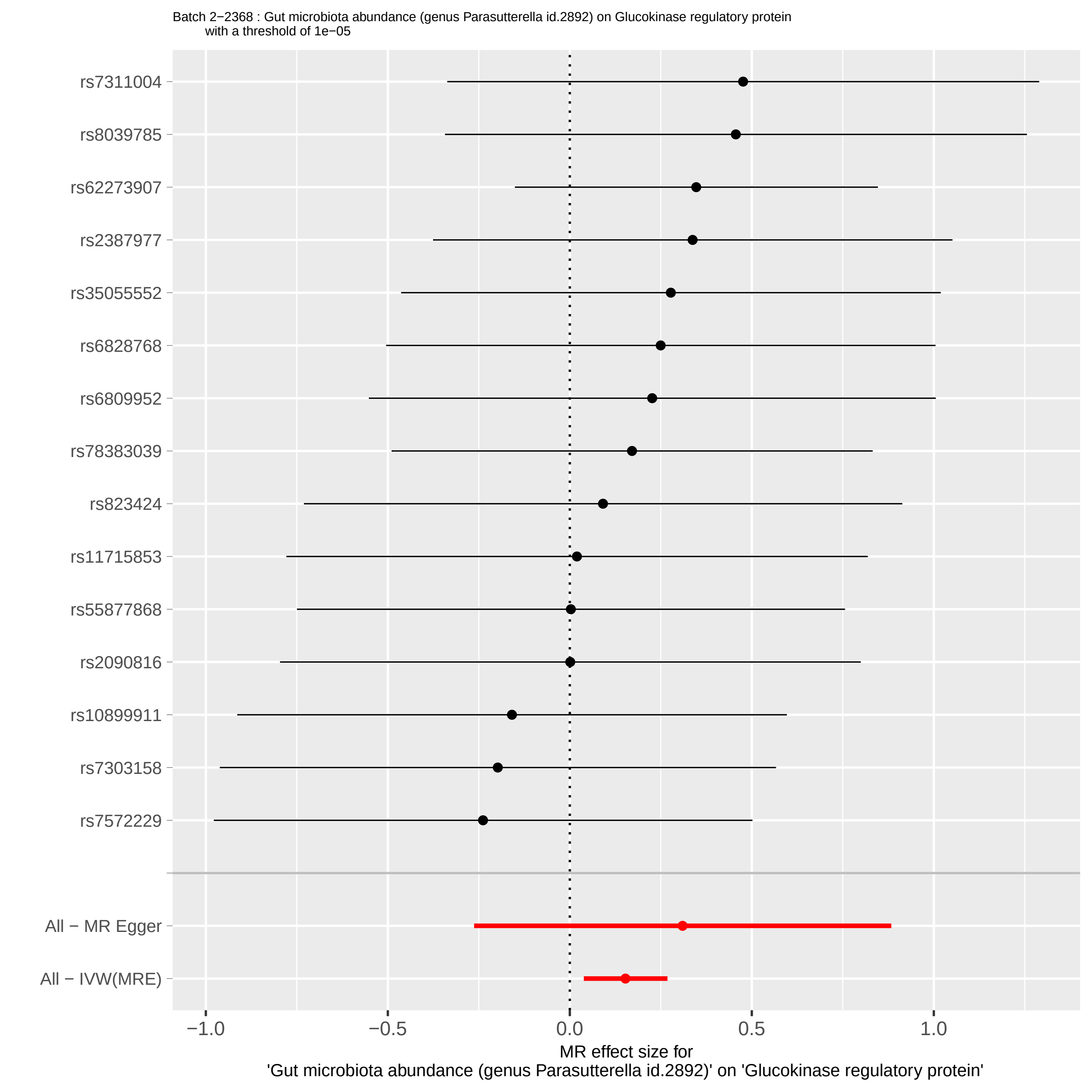

Batch 2−2368 : Gut microbiota abundance (genus Parasutterella id.2892) on Glucokinase regulatory protein
with a threshold of 1e−05
rs7311004
rs8039785
rs62273907
rs2387977
rs35055552
rs6828768
rs6809952
rs78383039
rs823424
rs11715853
rs55877868
rs2090816
rs10899911
rs7303158
rs7572229
All − MR Egger
All − IVW(MRE)
−1.0
−0.5
0.0
0.5
1.0
MR effect size for
'Gut microbiota abundance (genus Parasutterella id.2892)' on 'Glucokinase regulatory protein'

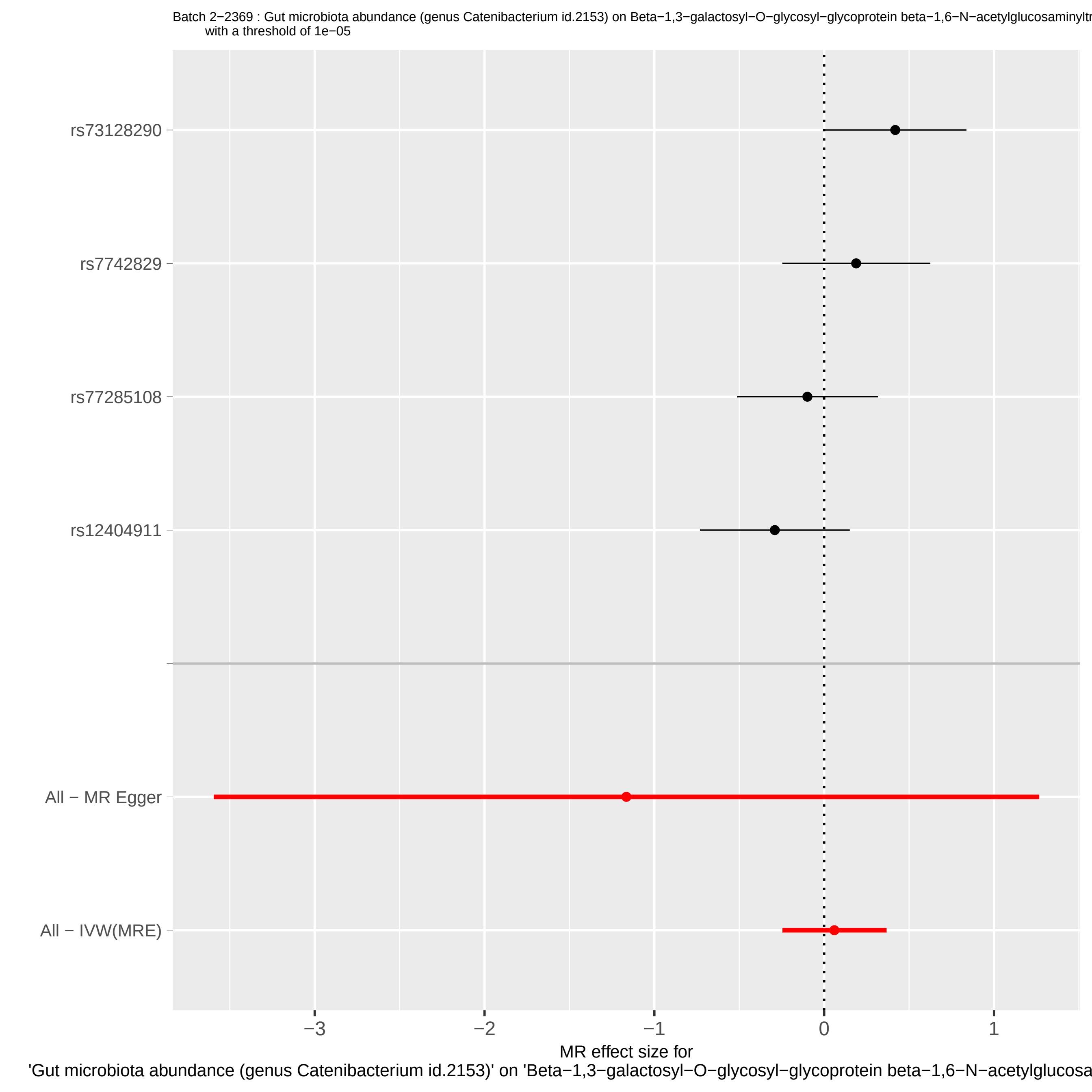

Batch 2−2369 : Gut microbiota abundance (genus Catenibacterium id.2153) on Beta−1,3−galactosyl−O−glycosyl−glycoprotein beta−1,6−N−acetylglucosaminyltr
with a threshold of 1e−05
rs73128290
rs7742829
rs77285108
rs12404911
All − MR Egger
All − IVW(MRE)
−3
−2
−1
0
1
MR effect size for
'Gut microbiota abundance (genus Catenibacterium id.2153)' on 'Beta−1,3−galactosyl−O−glycosyl−glycoprotein beta−1,6−N−acetylglucosa

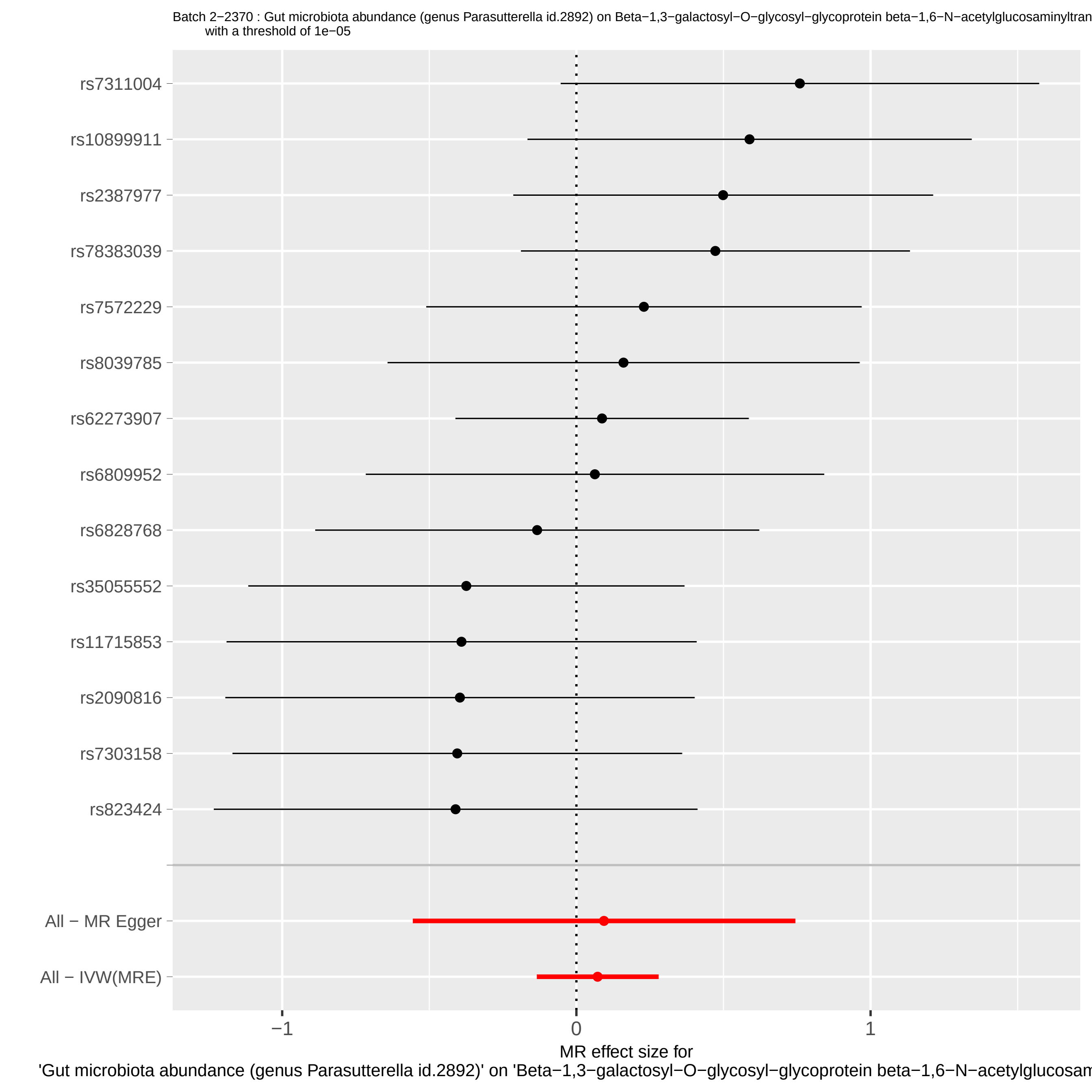

Batch 2−2370 : Gut microbiota abundance (genus Parasutterella id.2892) on Beta−1,3−galactosyl−O−glycosyl−glycoprotein beta−1,6−N−acetylglucosaminyltran
with a threshold of 1e−05
rs7311004
rs10899911
rs2387977
rs78383039
rs7572229
rs8039785
rs62273907
rs6809952
rs6828768
rs35055552
rs11715853
rs2090816
rs7303158
rs823424
All − MR Egger
All − IVW(MRE)
−1
0
1
MR effect size for
'Gut microbiota abundance (genus Parasutterella id.2892)' on 'Beta−1,3−galactosyl−O−glycosyl−glycoprotein beta−1,6−N−acetylglucosam

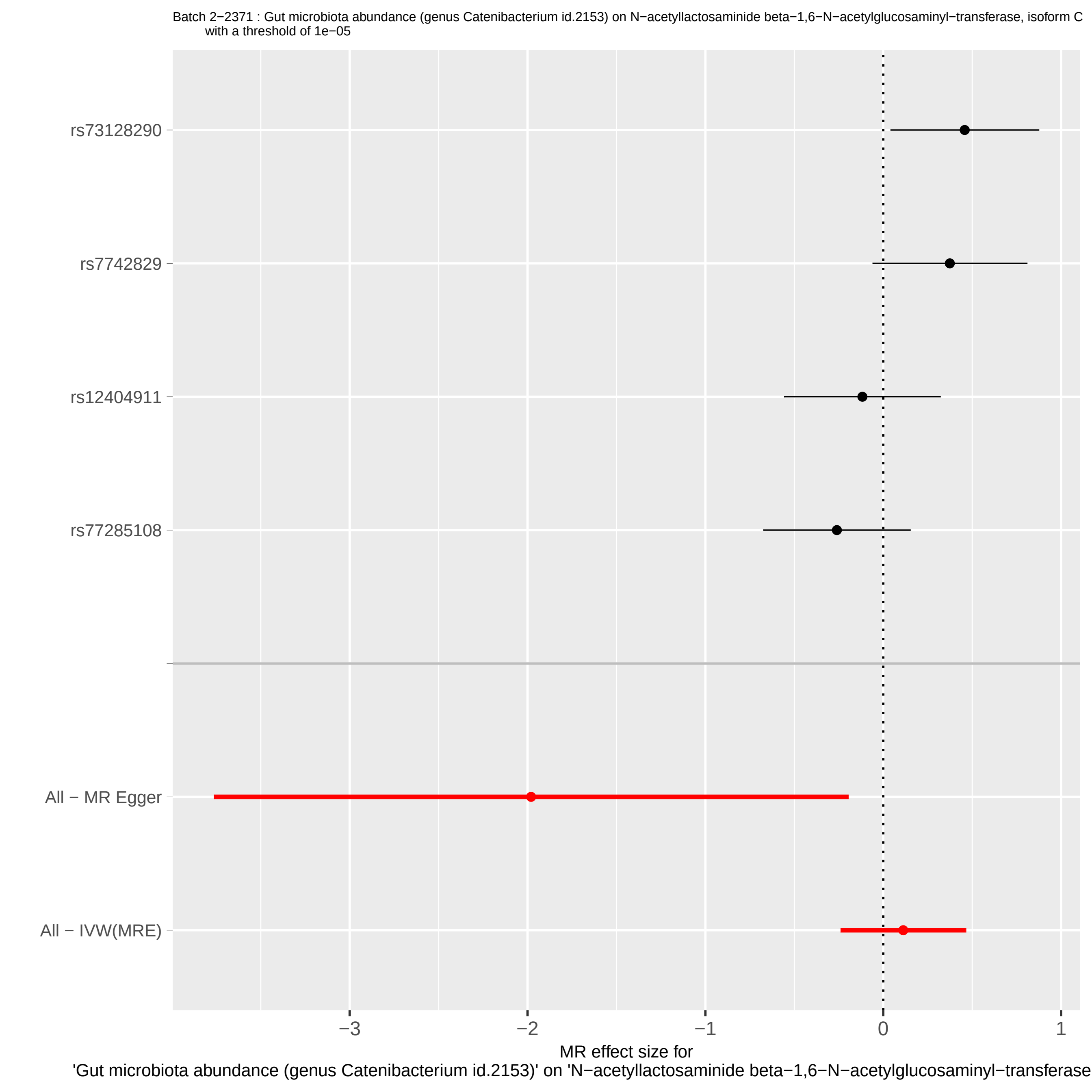
Batch 2−2371 : Gut microbiota abundance (genus Catenibacterium id.2153) on N−acetyllactosaminide beta−1,6−N−acetylglucosaminyl−transferase, isoform C
with a threshold of 1e−05
rs73128290
rs7742829
rs12404911
rs77285108
All − MR Egger
All − IVW(MRE)
−3
−2
−1
0
1
MR effect size for
'Gut microbiota abundance (genus Catenibacterium id.2153)' on 'N−acetyllactosaminide beta−1,6−N−acetylglucosaminyl−transferase

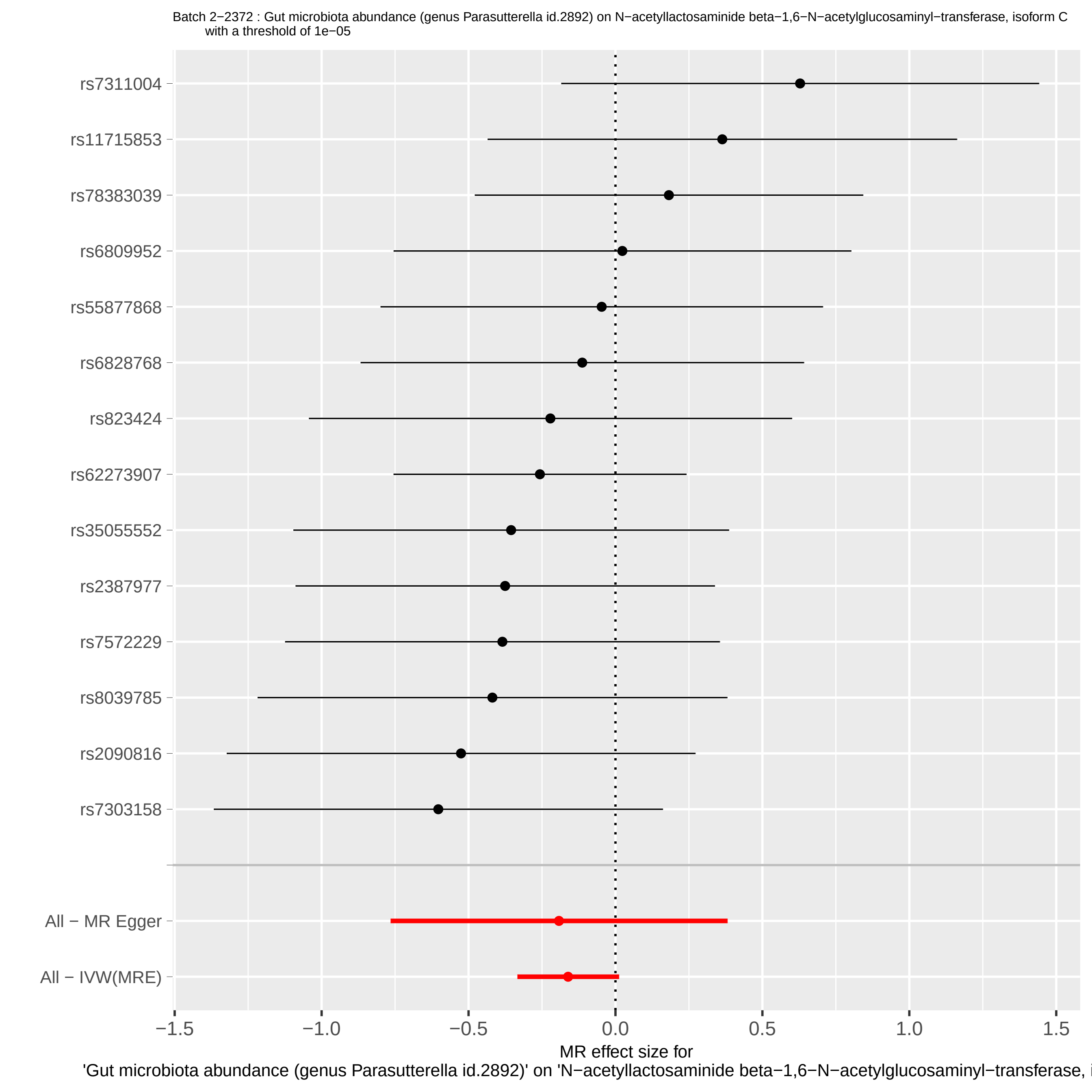

Batch 2−2372 : Gut microbiota abundance (genus Parasutterella id.2892) on N−acetyllactosaminide beta−1,6−N−acetylglucosaminyl−transferase, isoform C with a threshold of 1e−05
rs7311004
rs11715853
rs78383039
rs6809952
rs55877868
rs6828768
rs823424
rs62273907
rs35055552
rs2387977
rs7572229
rs8039785
rs2090816
rs7303158
All − MR Egger
All − IVW(MRE)
−1.5
−1.0
−0.5
0.0
0.5
1.0
1.5
MR effect size for
'Gut microbiota abundance (genus Parasutterella id.2892)' on 'N−acetyllactosaminide beta−1,6−N−acetylglucosaminyl−transferase, i

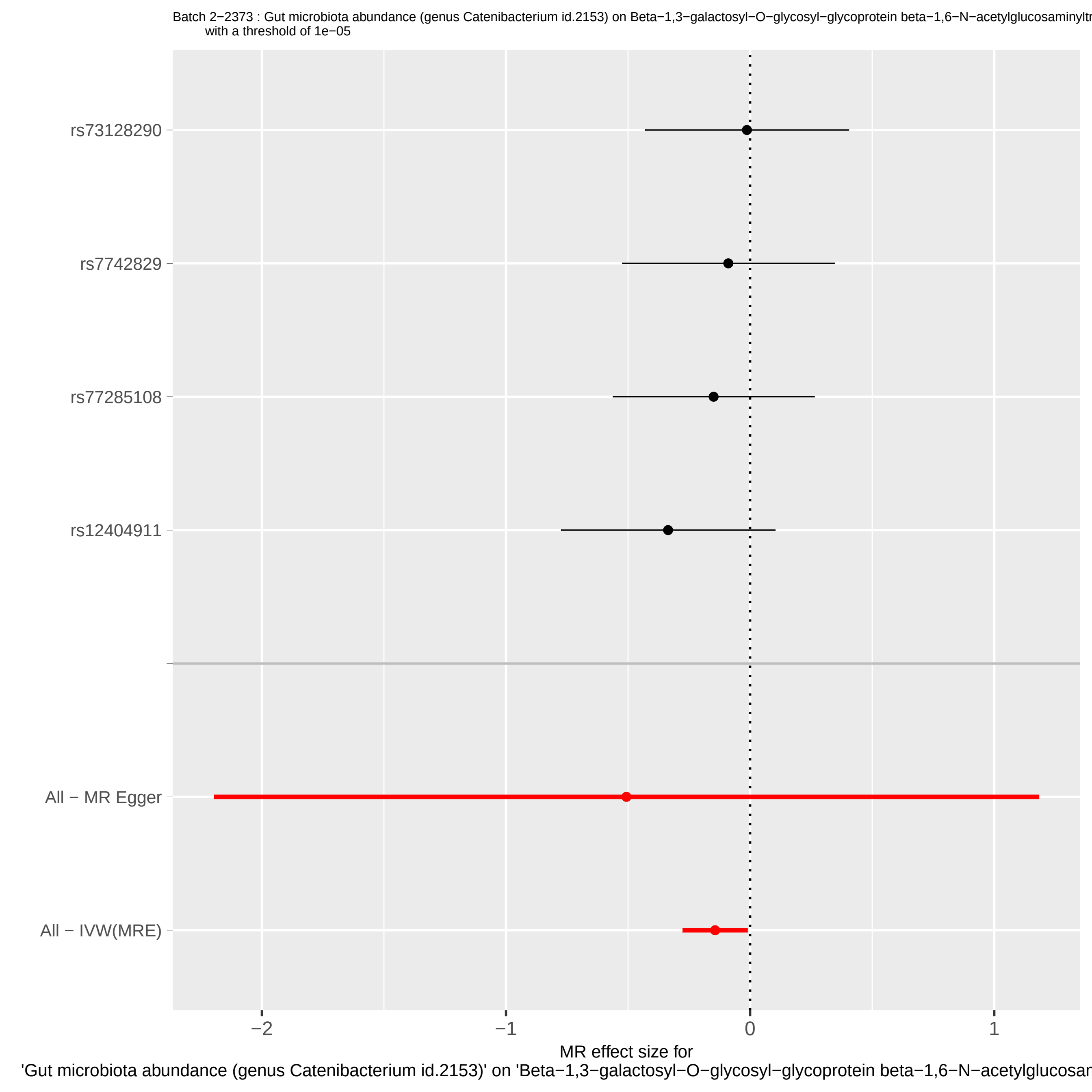

Batch 2−2373 : Gut microbiota abundance (genus Catenibacterium id.2153) on Beta−1,3−galactosyl−O−glycosyl−glycoprotein beta−1,6−N−acetylglucosaminyltr
with a threshold of 1e−05
rs73128290
rs7742829
rs77285108
rs12404911
All − MR Egger
All − IVW(MRE)
−2
−1
0
1
MR effect size for
'Gut microbiota abundance (genus Catenibacterium id.2153)' on 'Beta−1,3−galactosyl−O−glycosyl−glycoprotein beta−1,6−N−acetylglucosan

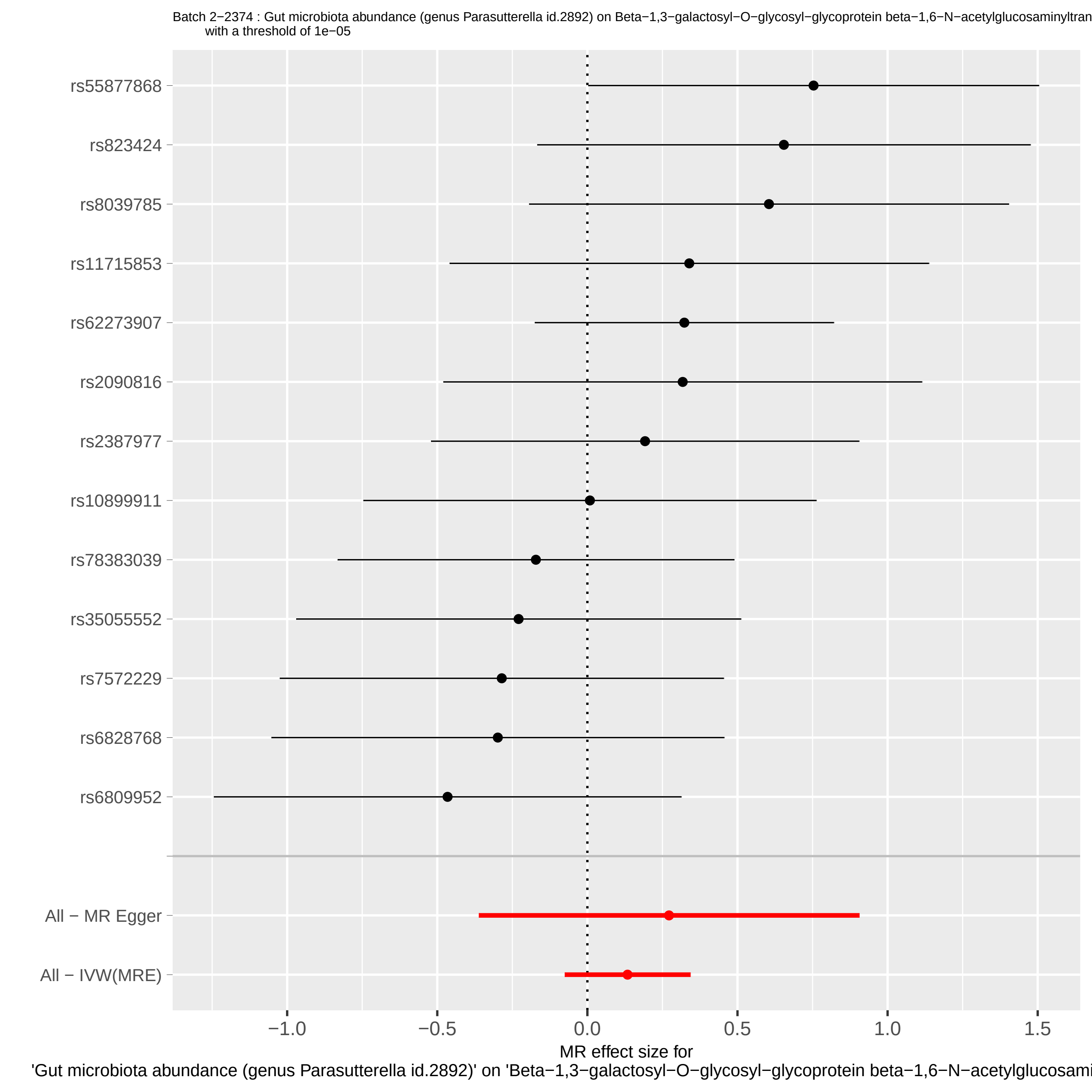

Batch 2−2374 : Gut microbiota abundance (genus Parasutterella id.2892) on Beta−1,3−galactosyl−O−glycosyl−glycoprotein beta−1,6−N−acetylglucosaminyltran
with a threshold of 1e−05
rs55877868
rs823424
rs8039785
rs11715853
rs62273907
rs2090816
rs2387977
rs10899911
rs78383039
rs35055552
rs7572229
rs6828768
rs6809952
All − MR Egger
All − IVW(MRE)
−1.0
−0.5
0.0
0.5
1.0
1.5
MR effect size for
'Gut microbiota abundance (genus Parasutterella id.2892)' on 'Beta−1,3−galactosyl−O−glycosyl−glycoprotein beta−1,6−N−acetylglucosami

Batch 2−2375 : Gut microbiota abundance (genus Catenibacterium id.2153) on Beta−1,3−galactosyl−O−glycosyl−glycoprotein beta−1,6−N−acetylglucosaminyltr
with a threshold of 1e−05

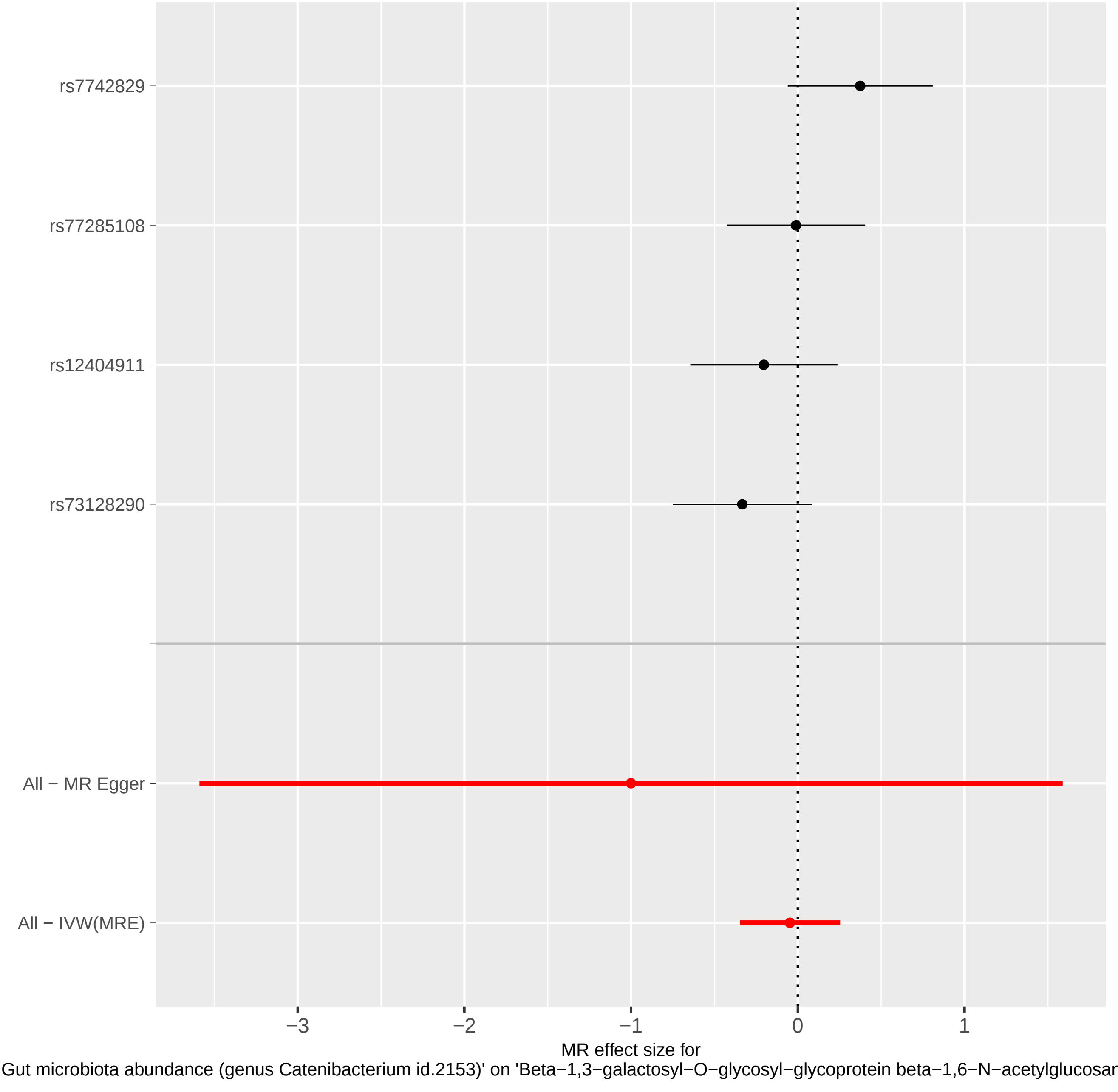

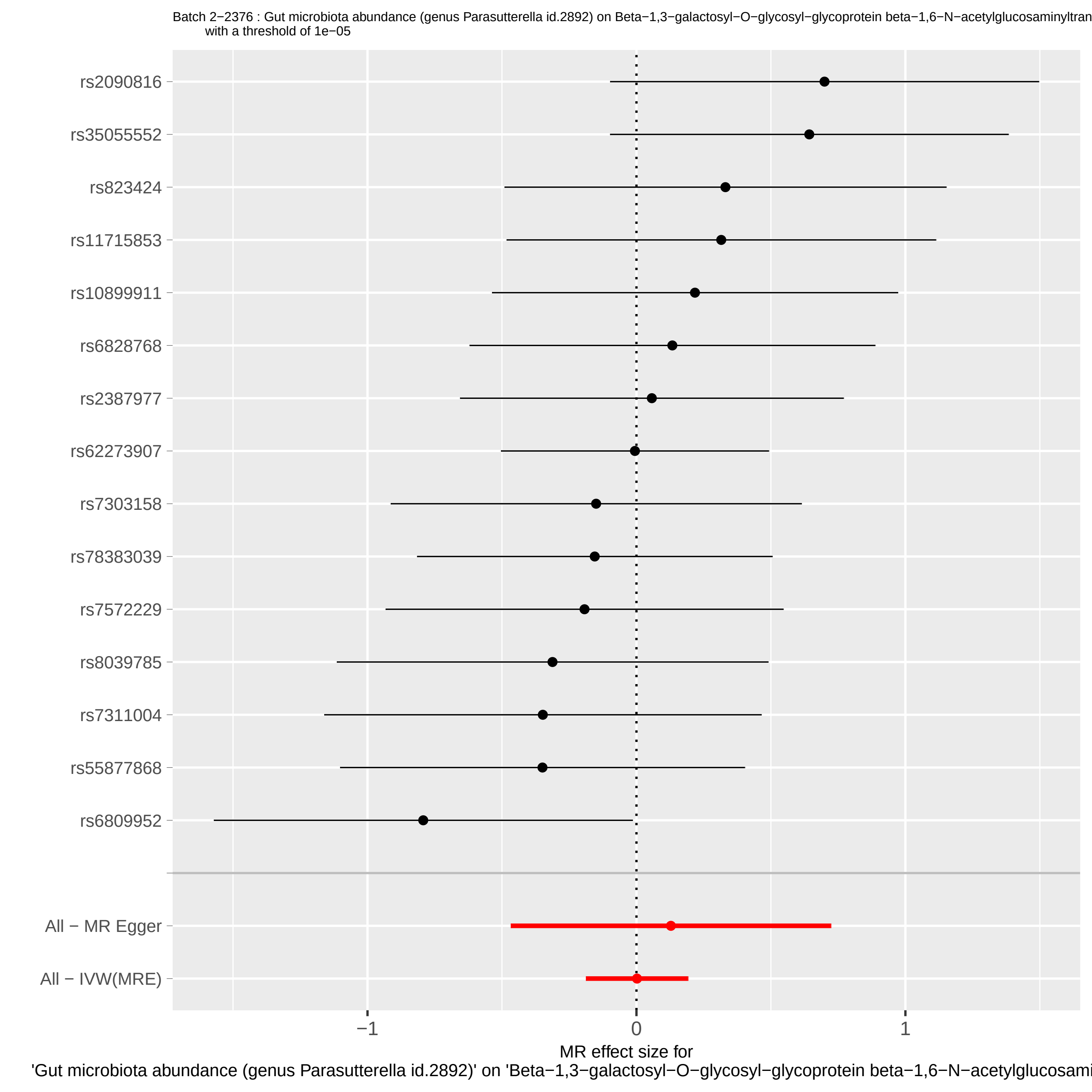

Batch 2−2376 : Gut microbiota abundance (genus Parasutterella id.2892) on Beta−1,3−galactosyl−O−glycosyl−glycoprotein beta−1,6−N−acetylglucosaminyltran
with a threshold of 1e−05
rs2090816
rs35055552
rs823424
rs11715853
rs10899911
rs6828768
rs2387977
rs62273907
rs7303158
rs78383039
rs7572229
rs8039785
rs7311004
rs55877868
rs6809952
All − MR Egger
All − IVW(MRE)
−1
0
1
MR effect size for
'Gut microbiota abundance (genus Parasutterella id.2892)' on 'Beta−1,3−galactosyl−O−glycosyl−glycoprotein beta−1,6−N−acetylglucosami

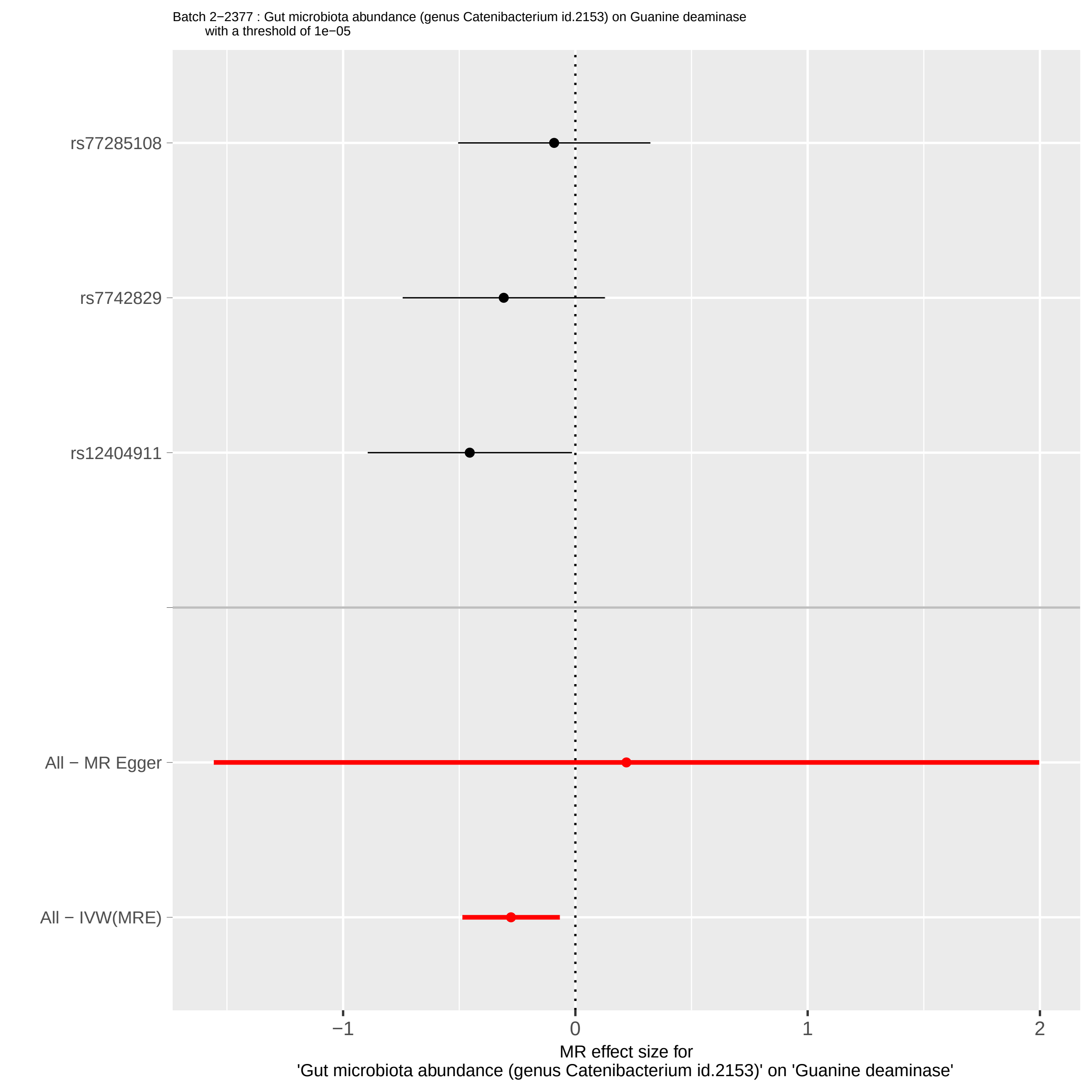

Batch 2−2377 : Gut microbiota abundance (genus Catenibacterium id.2153) on Guanine deaminase
with a threshold of 1e−05
rs77285108
rs7742829
rs12404911
All − MR Egger
All − IVW(MRE)
−1
0
1
2
MR effect size for
'Gut microbiota abundance (genus Catenibacterium id.2153)' on 'Guanine deaminase'

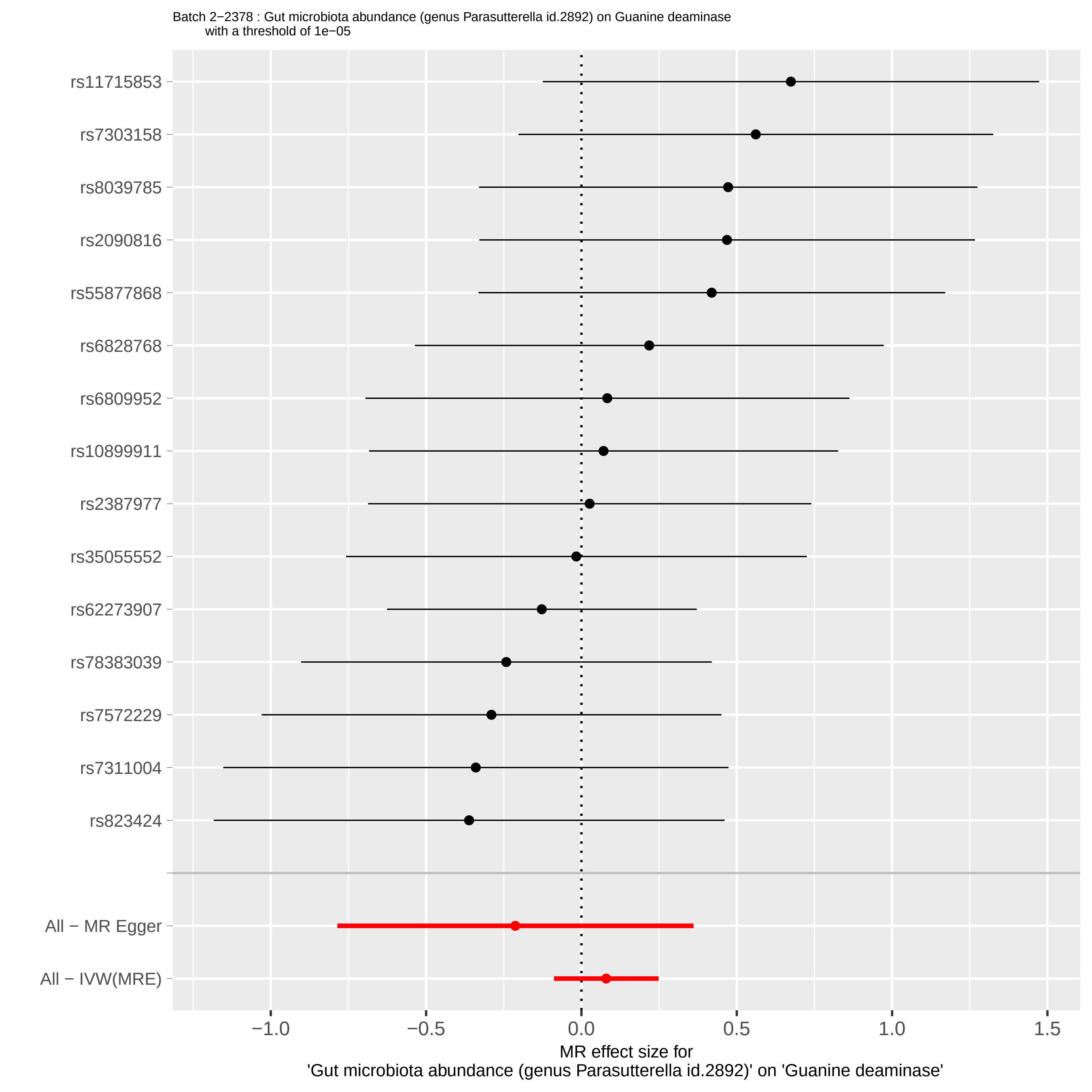
Batch 2−2378 : Gut microbiota abundance (genus Parasutterella id.2892) on Guanine deaminase
with a threshold of 1e−05
rs11715853
rs7303158
rs8039785
rs2090816
rs55877868
rs6828768
rs6809952
rs10899911
rs2387977
rs35055552
rs62273907
rs78383039
rs7572229
rs7311004
rs823424
All − MR Egger
All − IVW(MRE)
−1.0
−0.5
0.0
0.5
1.0
1.5
MR effect size for
'Gut microbiota abundance (genus Parasutterella id.2892)' on 'Guanine deaminase'

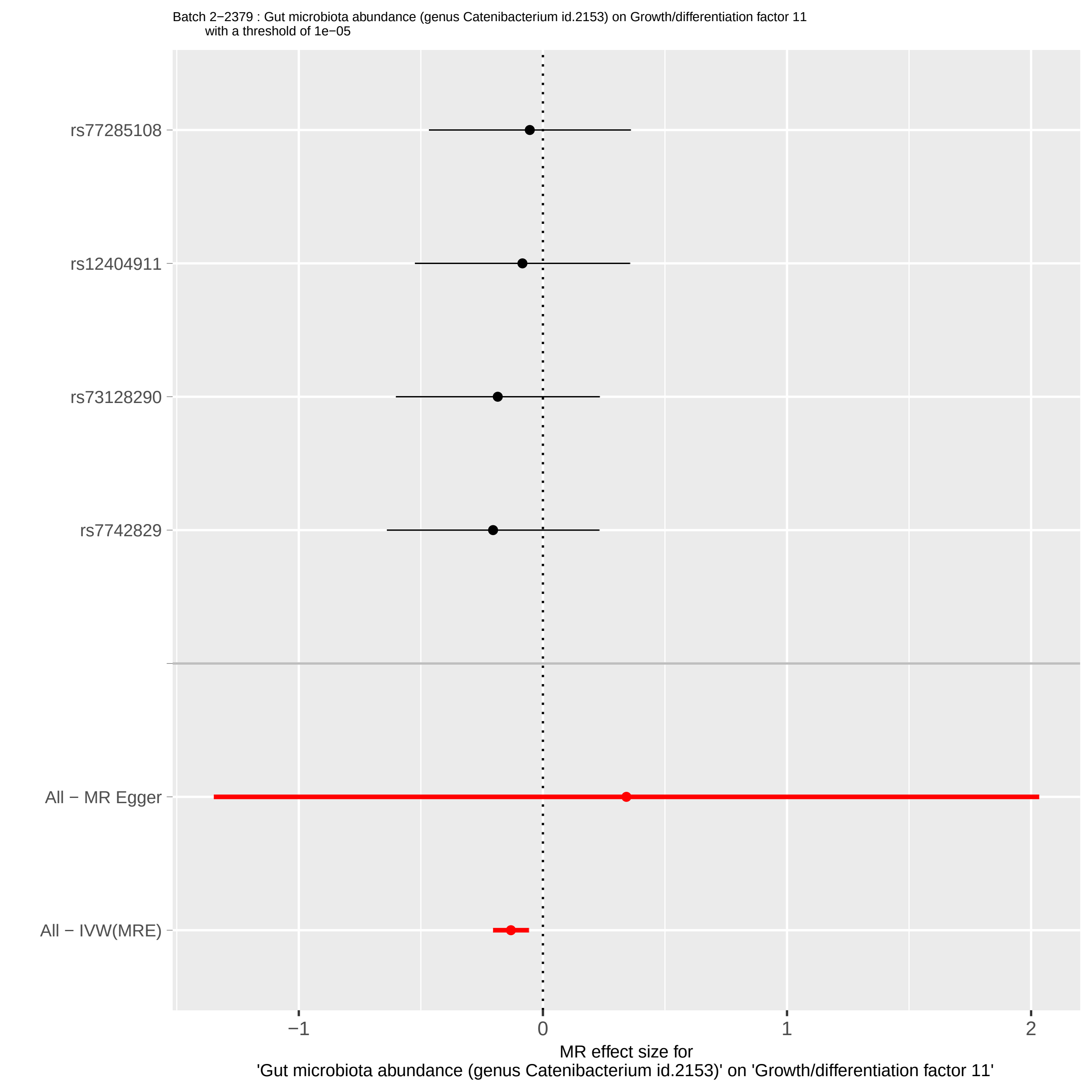

Batch 2−2379 : Gut microbiota abundance (genus Catenibacterium id.2153) on Growth/differentiation factor 11
with a threshold of 1e−05
rs77285108
rs12404911
rs73128290
rs7742829
All − MR Egger
All − IVW(MRE)
−1
0
1
2
MR effect size for
'Gut microbiota abundance (genus Catenibacterium id.2153)' on 'Growth/differentiation factor 11'

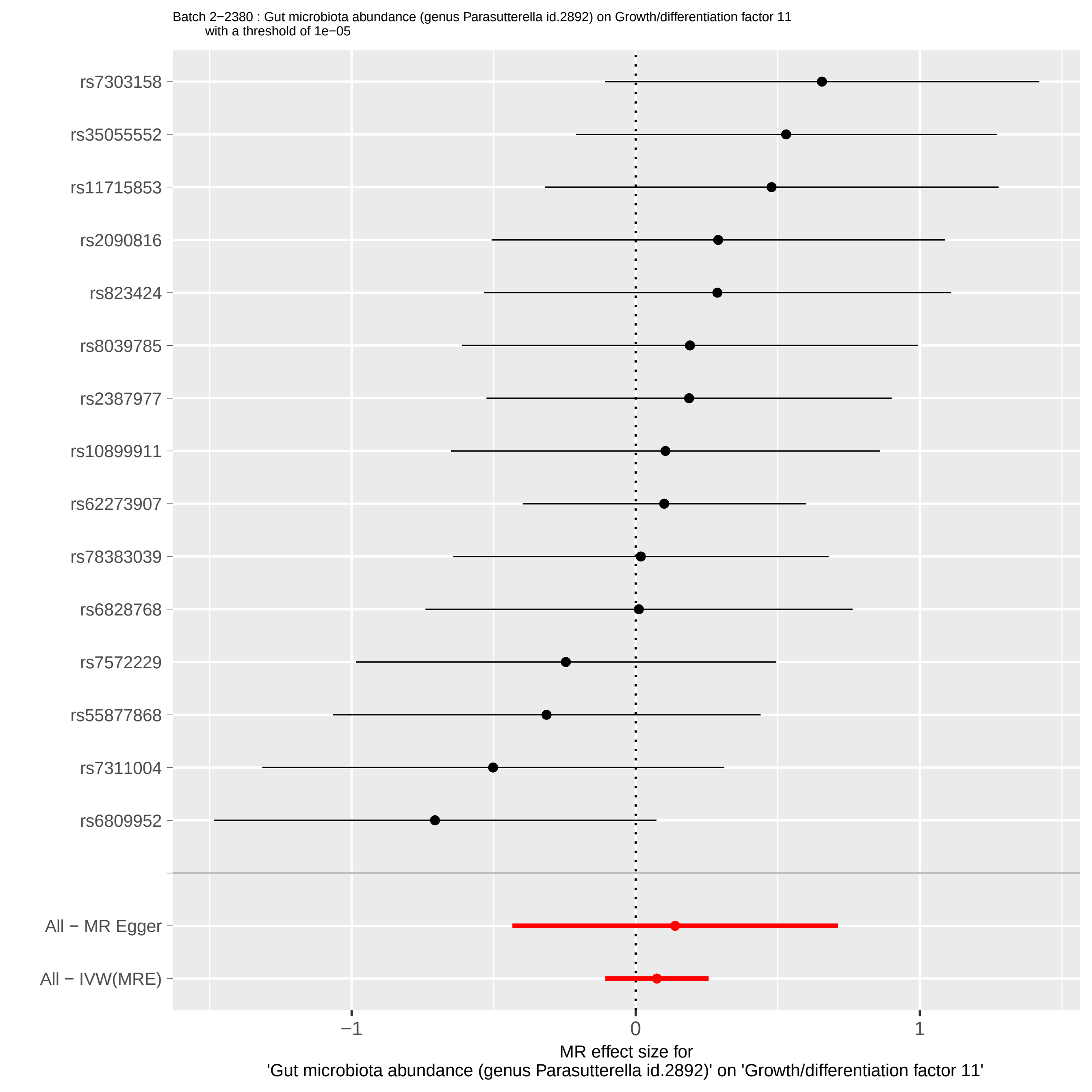

Batch 2−2380 : Gut microbiota abundance (genus Parasutterella id.2892) on Growth/differentiation factor 11
with a threshold of 1e−05
rs7303158
rs35055552
rs11715853
rs2090816
rs823424
rs8039785
rs2387977
rs10899911
rs62273907
rs78383039
rs6828768
rs7572229
rs55877868
rs7311004
rs6809952
All − MR Egger
All − IVW(MRE)
−1
0
1
MR effect size for
'Gut microbiota abundance (genus Parasutterella id.2892)' on 'Growth/differentiation factor 11'

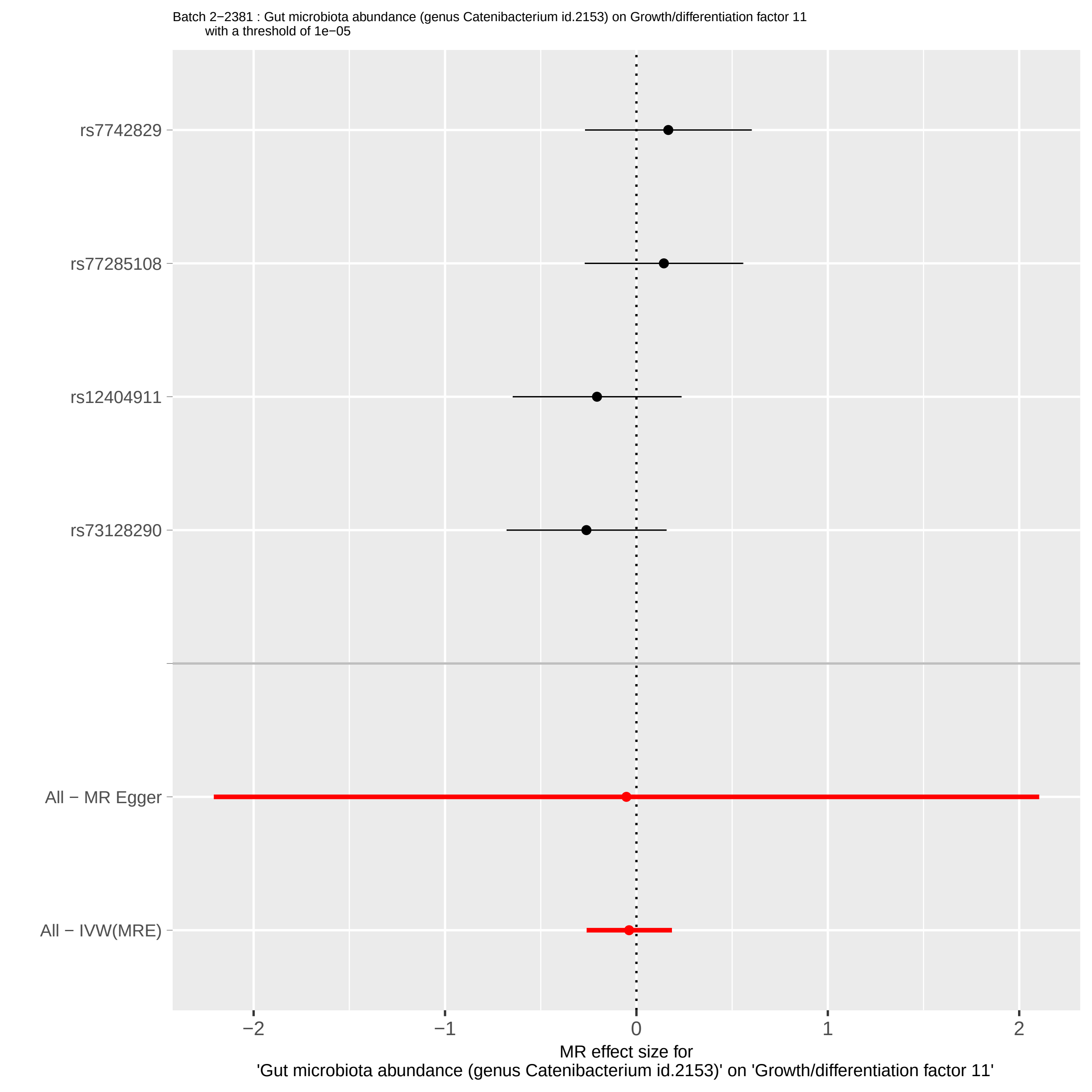
Batch 2−2381 : Gut microbiota abundance (genus Catenibacterium id.2153) on Growth/differentiation factor 11
with a threshold of 1e−05
rs7742829
rs77285108
rs12404911
rs73128290
All − MR Egger
All − IVW(MRE)
−2
−1
0
1
2
MR effect size for
'Gut microbiota abundance (genus Catenibacterium id.2153)' on 'Growth/differentiation factor 11'

Batch 2−2382 : Gut microbiota abundance (genus Parasutterella id.2892) on Growth/differentiation factor 11
with a threshold of 1e−05

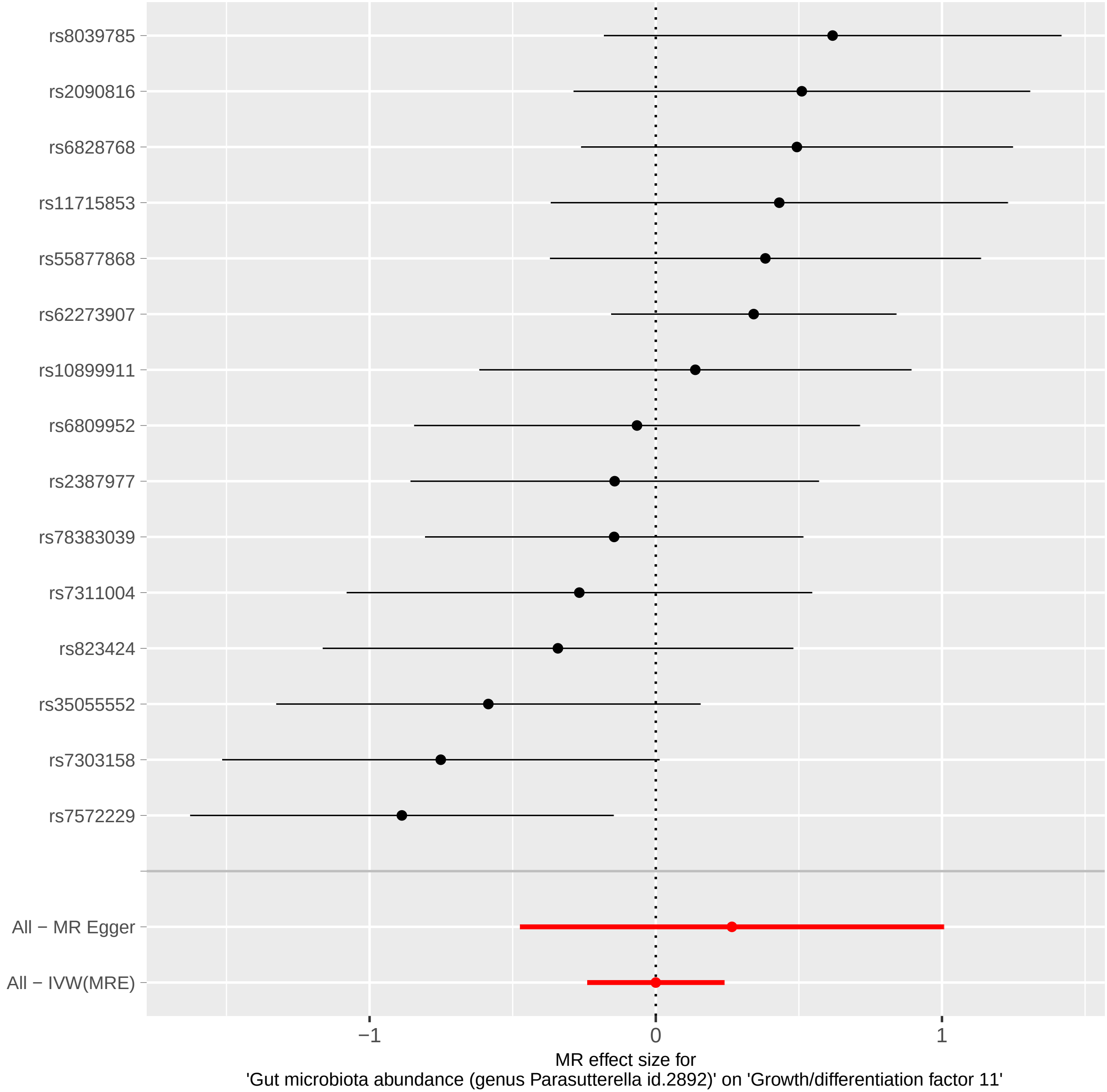

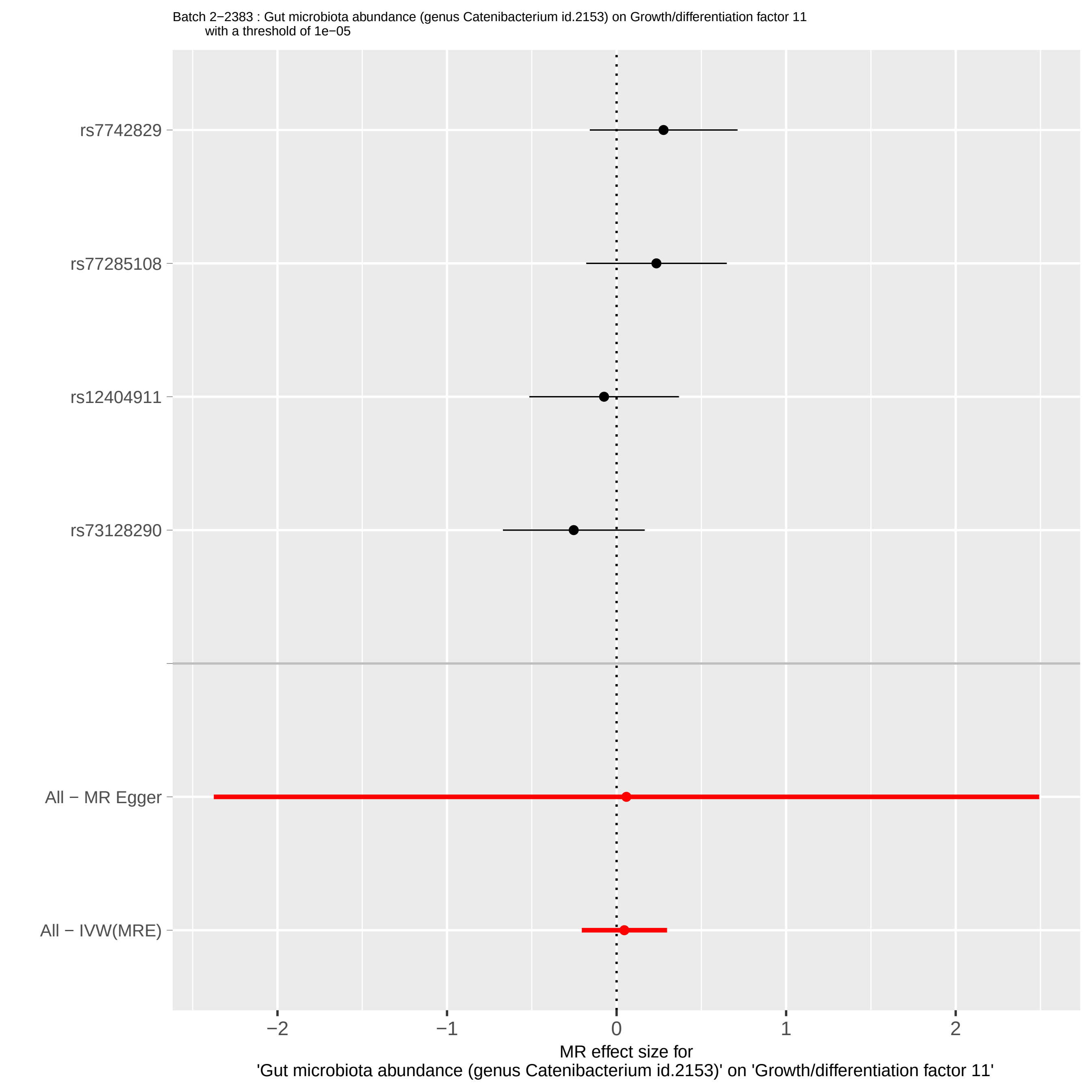
Batch 2−2383 : Gut microbiota abundance (genus Catenibacterium id.2153) on Growth/differentiation factor 11
with a threshold of 1e−05
rs7742829
rs77285108
rs12404911
rs73128290
All − MR Egger
All − IVW(MRE)
−2
−1
0
1
2
MR effect size for
'Gut microbiota abundance (genus Catenibacterium id.2153)' on 'Growth/differentiation factor 11'

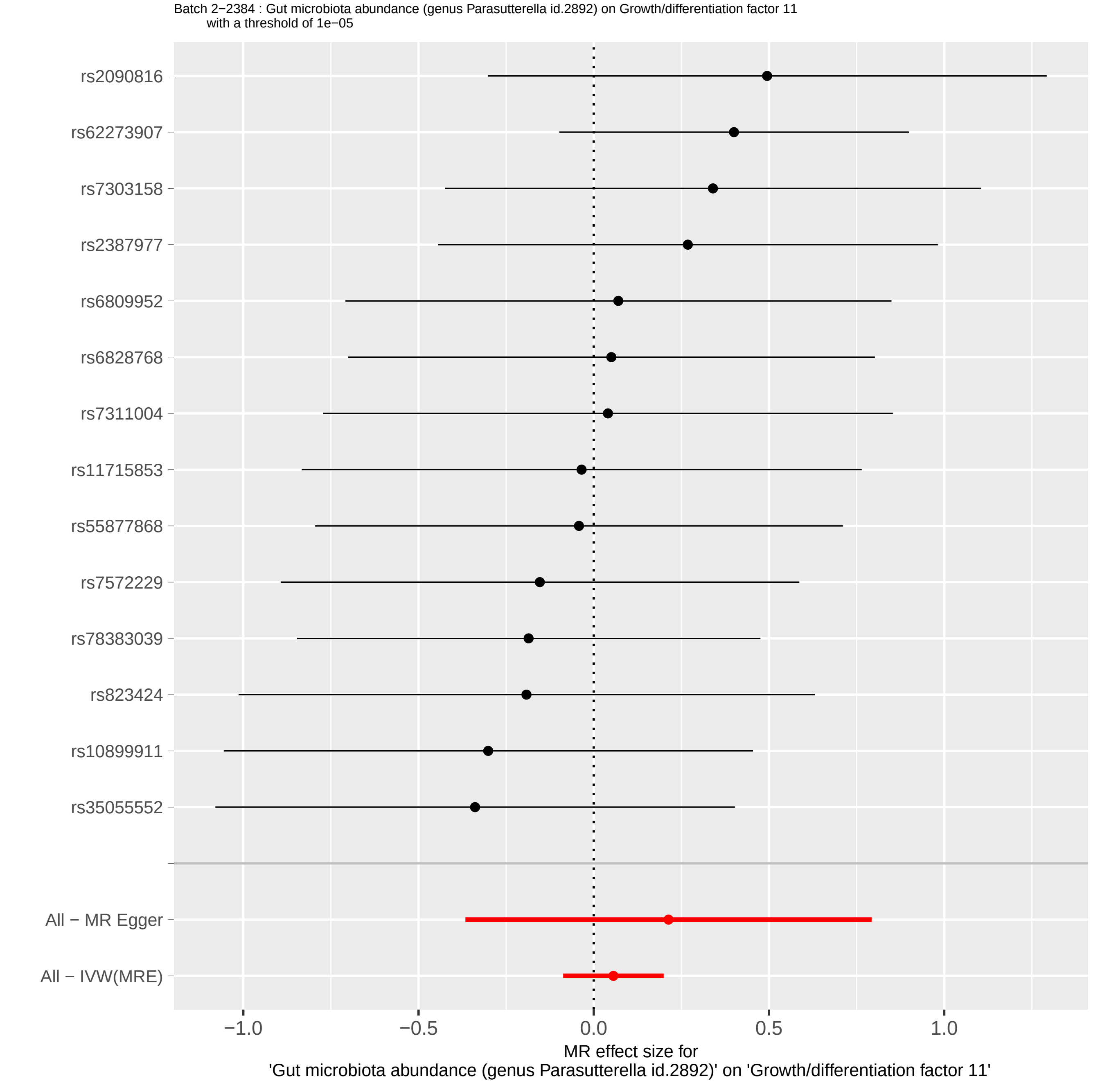

Batch 2−2384 : Gut microbiota abundance (genus Parasutterella id.2892) on Growth/differentiation factor 11
with a threshold of 1e−05
rs2090816
rs62273907
rs7303158
rs2387977
rs6809952
rs6828768
rs7311004
rs11715853
rs55877868
rs7572229
rs78383039
rs823424
rs10899911
rs35055552
All − MR Egger
All − IVW(MRE)
−1.0
−0.5
0.0
0.5
1.0
MR effect size for
'Gut microbiota abundance (genus Parasutterella id.2892)' on 'Growth/differentiation factor 11'

Batch 2−2385 : Gut microbiota abundance (genus Catenibacterium id.2153) on Growth/differentiation factor 11
with a threshold of 1e−05

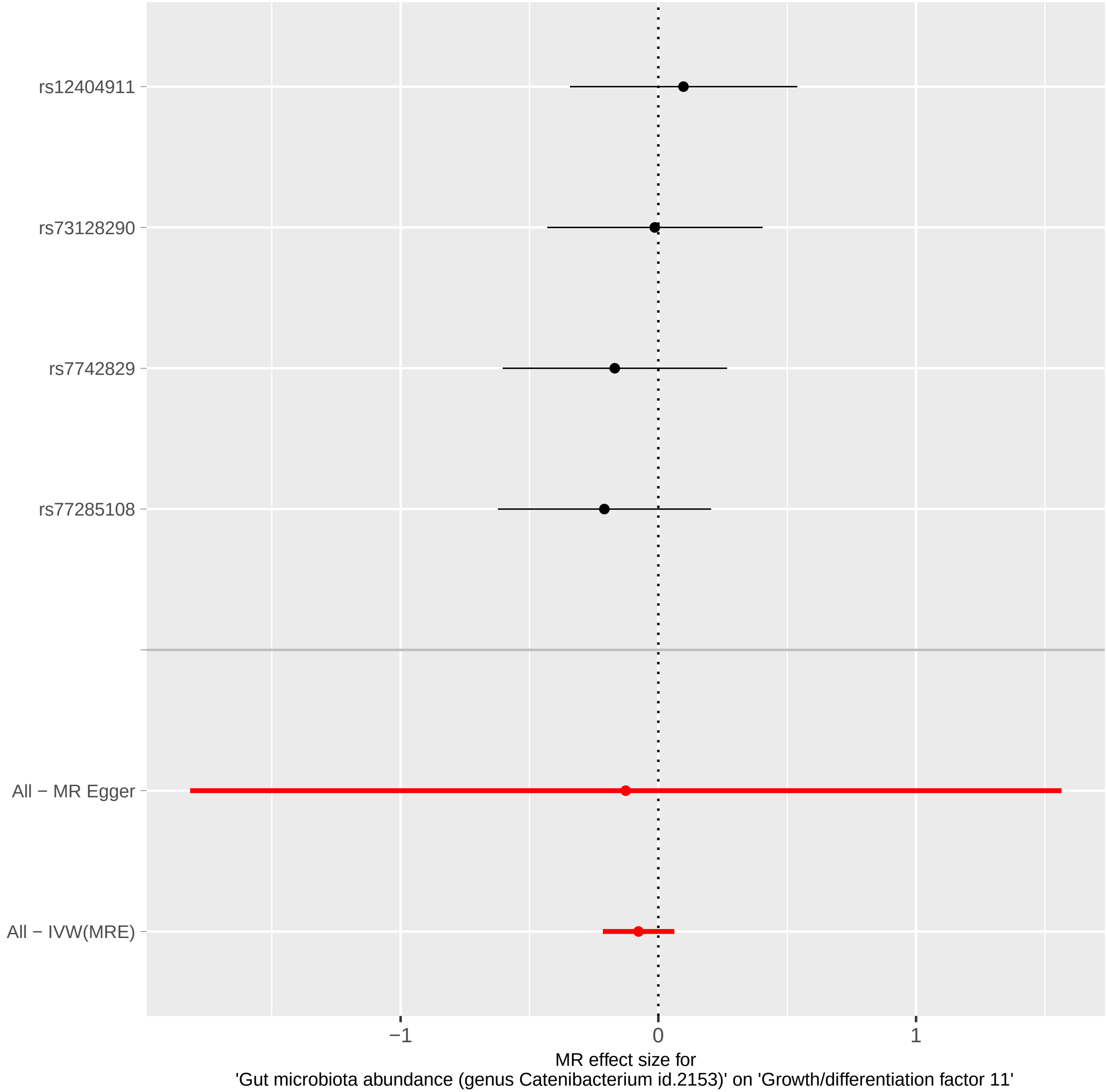

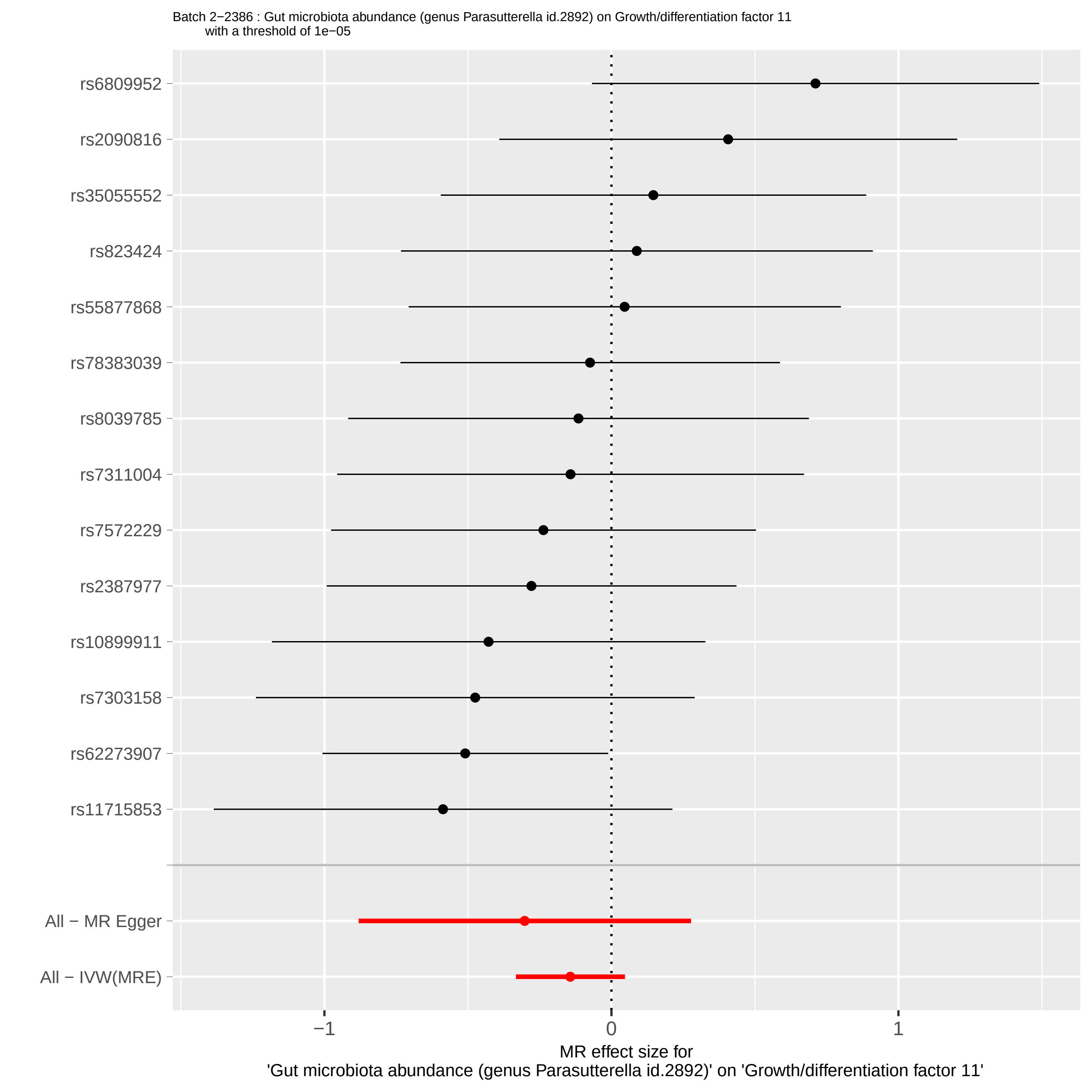

Batch 2−2386 : Gut microbiota abundance (genus Parasutterella id.2892) on Growth/differentiation factor 11
with a threshold of 1e−05
rs6809952
rs2090816
rs35055552
rs823424
rs55877868
rs78383039
rs8039785
rs7311004
rs7572229
rs2387977
rs10899911
rs7303158
rs62273907
rs11715853
All − MR Egger
All − IVW(MRE)
−1
0
1
MR effect size for
'Gut microbiota abundance (genus Parasutterella id.2892)' on 'Growth/differentiation factor 11'

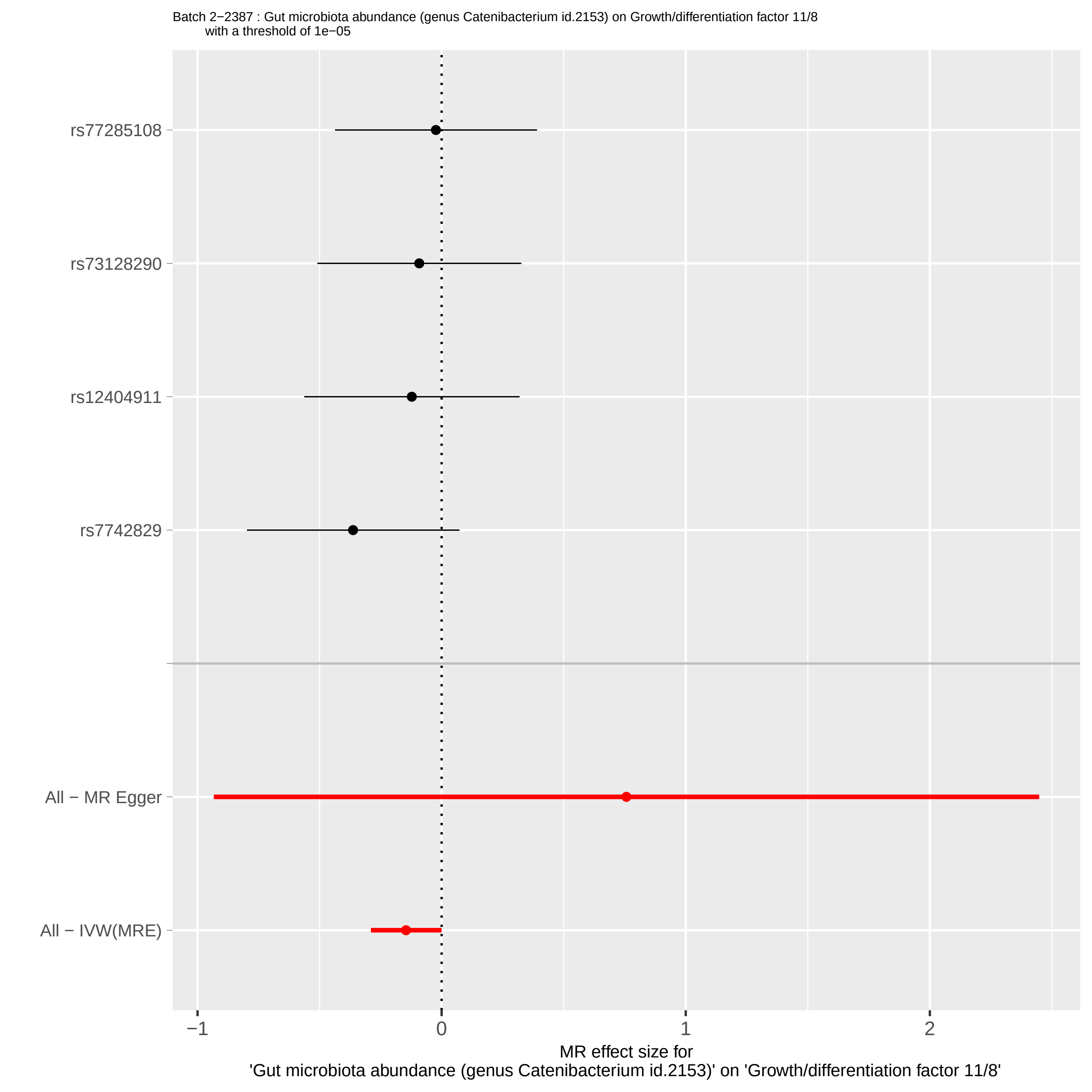

Batch 2−2387 : Gut microbiota abundance (genus Catenibacterium id.2153) on Growth/differentiation factor 11/8
with a threshold of 1e−05
rs77285108
rs73128290
rs12404911
rs7742829
All − MR Egger
All − IVW(MRE)
−1
0
1
2
MR effect size for
'Gut microbiota abundance (genus Catenibacterium id.2153)' on 'Growth/differentiation factor 11/8'

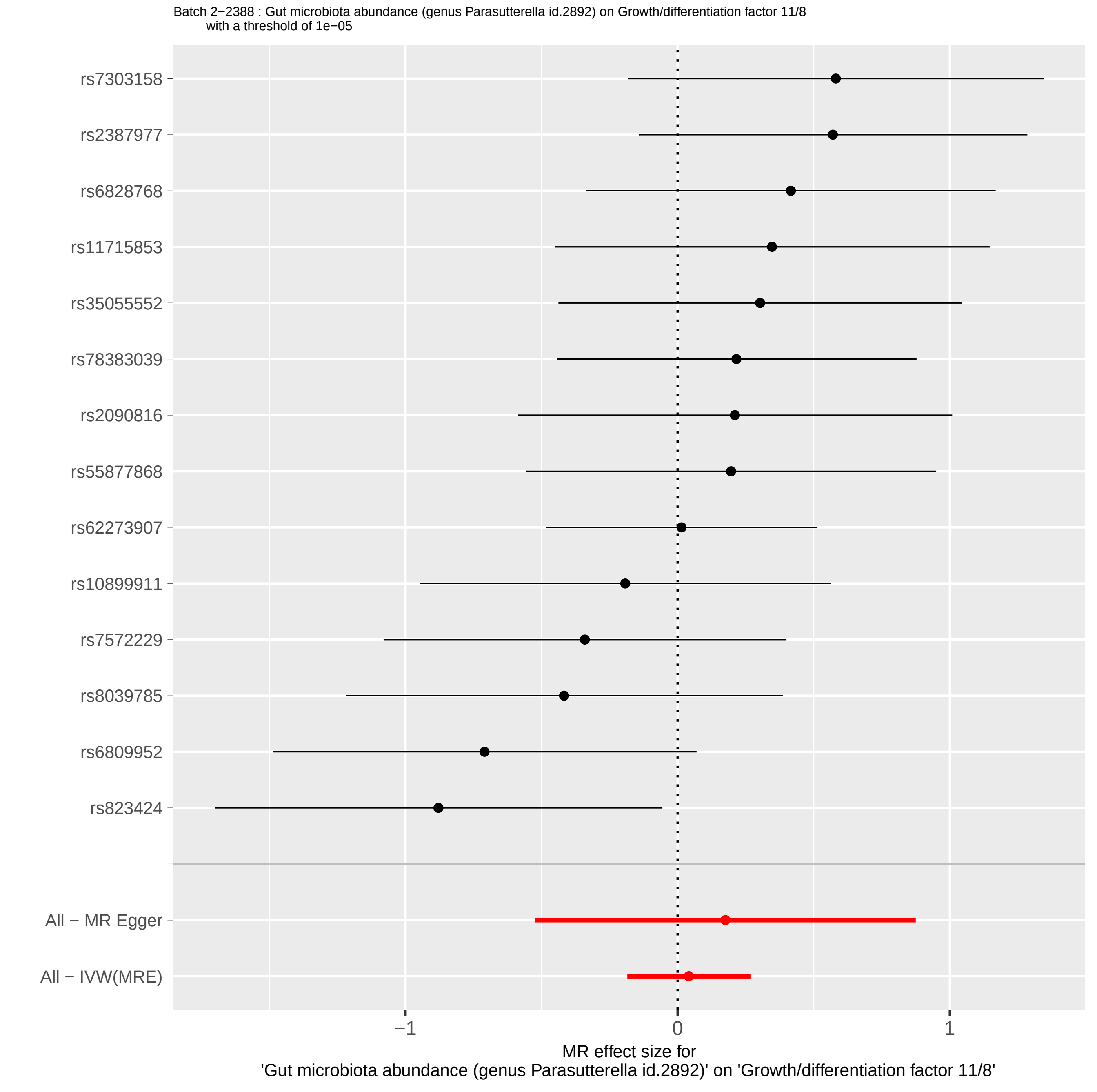

Batch 2−2388 : Gut microbiota abundance (genus Parasutterella id.2892) on Growth/differentiation factor 11/8
with a threshold of 1e−05
rs7303158
rs2387977
rs6828768
rs11715853
rs35055552
rs78383039
rs2090816
rs55877868
rs62273907
rs10899911
rs7572229
rs8039785
rs6809952
rs823424
All − MR Egger
All − IVW(MRE)
−1
0
1
MR effect size for
'Gut microbiota abundance (genus Parasutterella id.2892)' on 'Growth/differentiation factor 11/8'

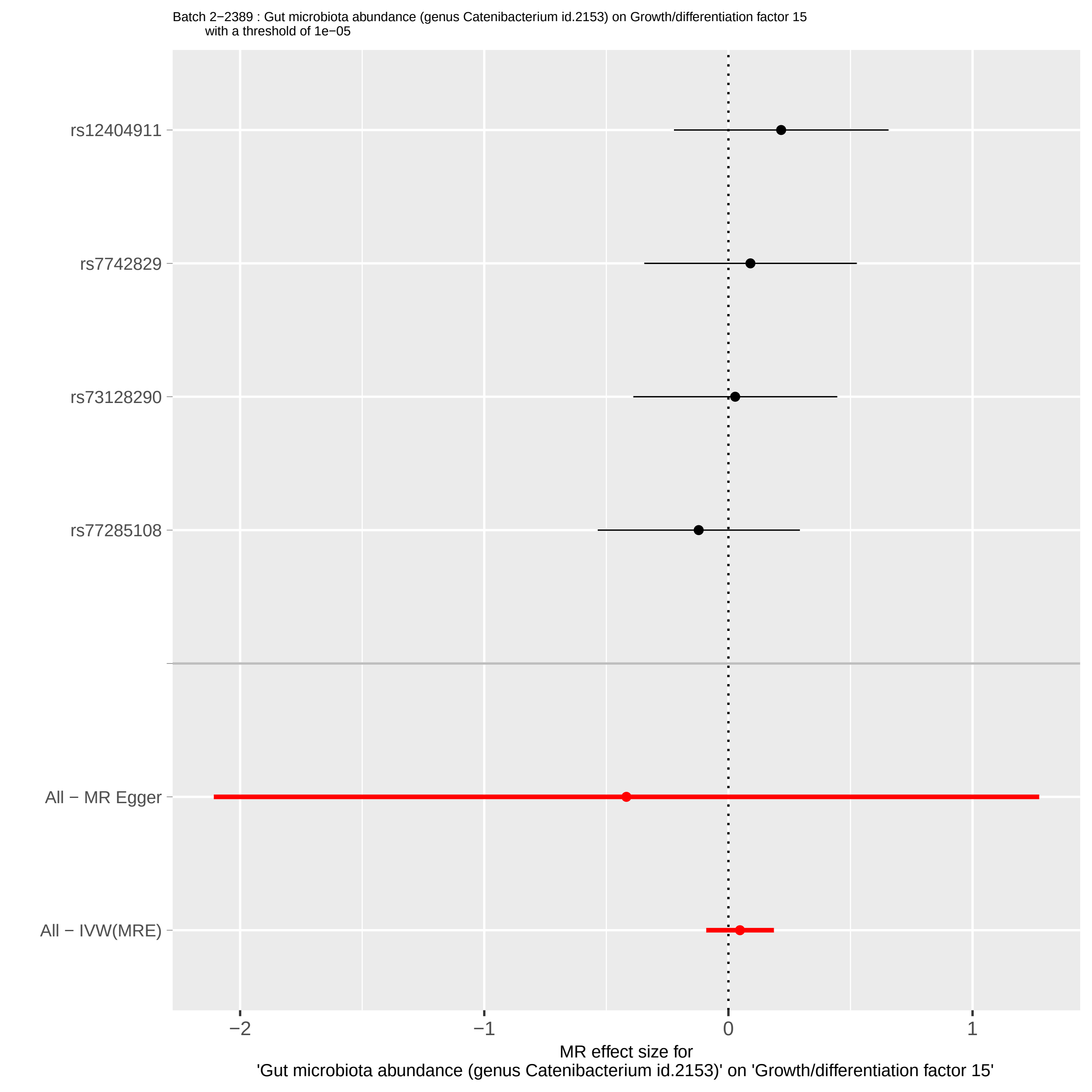
Batch 2−2389 : Gut microbiota abundance (genus Catenibacterium id.2153) on Growth/differentiation factor 15
with a threshold of 1e−05
rs12404911
rs7742829
rs73128290
rs77285108
All − MR Egger
All − IVW(MRE)
−2
−1
0
1
MR effect size for
'Gut microbiota abundance (genus Catenibacterium id.2153)' on 'Growth/differentiation factor 15'

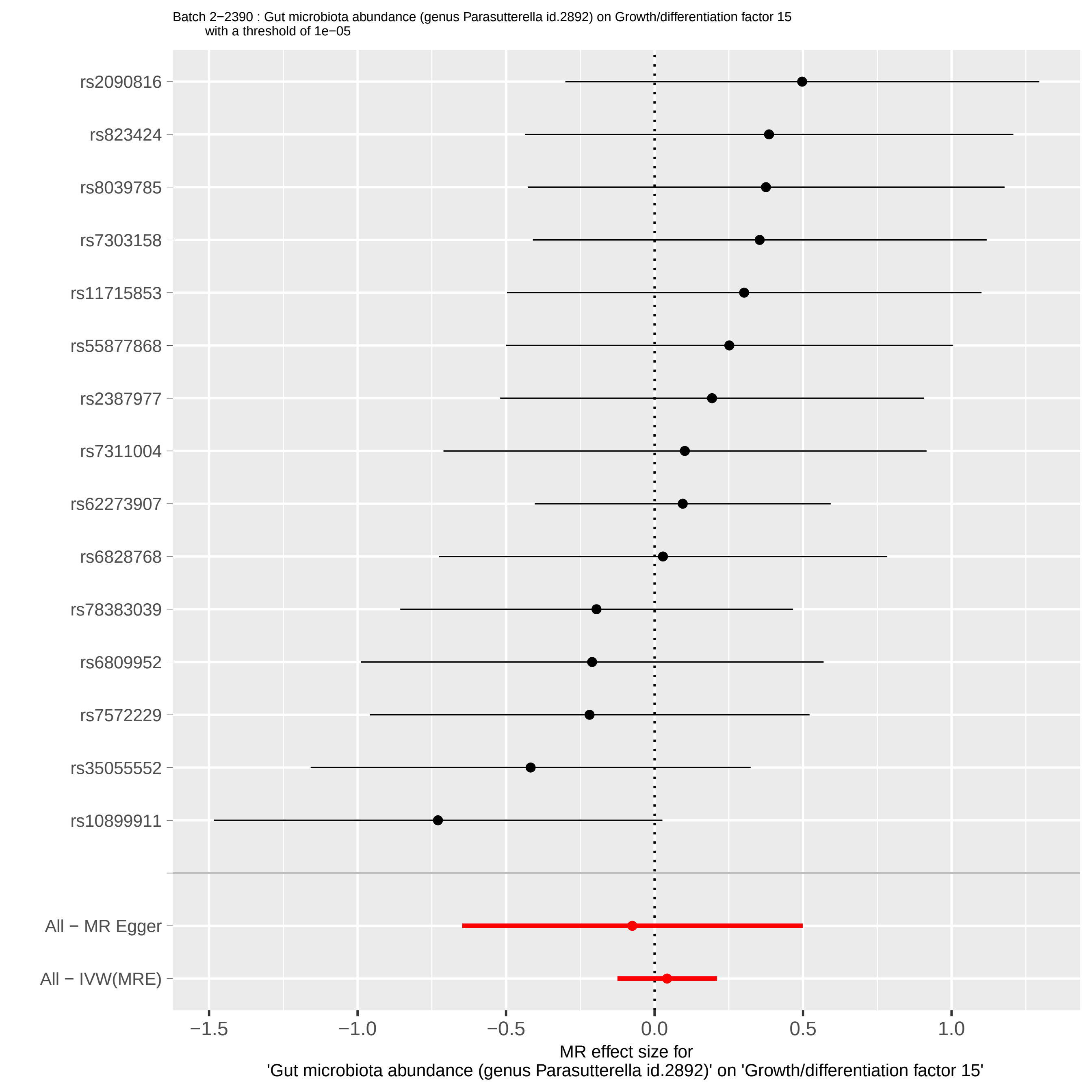
Batch 2−2390 : Gut microbiota abundance (genus Parasutterella id.2892) on Growth/differentiation factor 15
with a threshold of 1e−05
rs2090816
rs823424
rs8039785
rs7303158
rs11715853
rs55877868
rs2387977
rs7311004
rs62273907
rs6828768
rs78383039
rs6809952
rs7572229
rs35055552
rs10899911
All − MR Egger
All − IVW(MRE)
−1.5
−1.0
−0.5
0.0
0.5
1.0
MR effect size for
'Gut microbiota abundance (genus Parasutterella id.2892)' on 'Growth/differentiation factor 15'

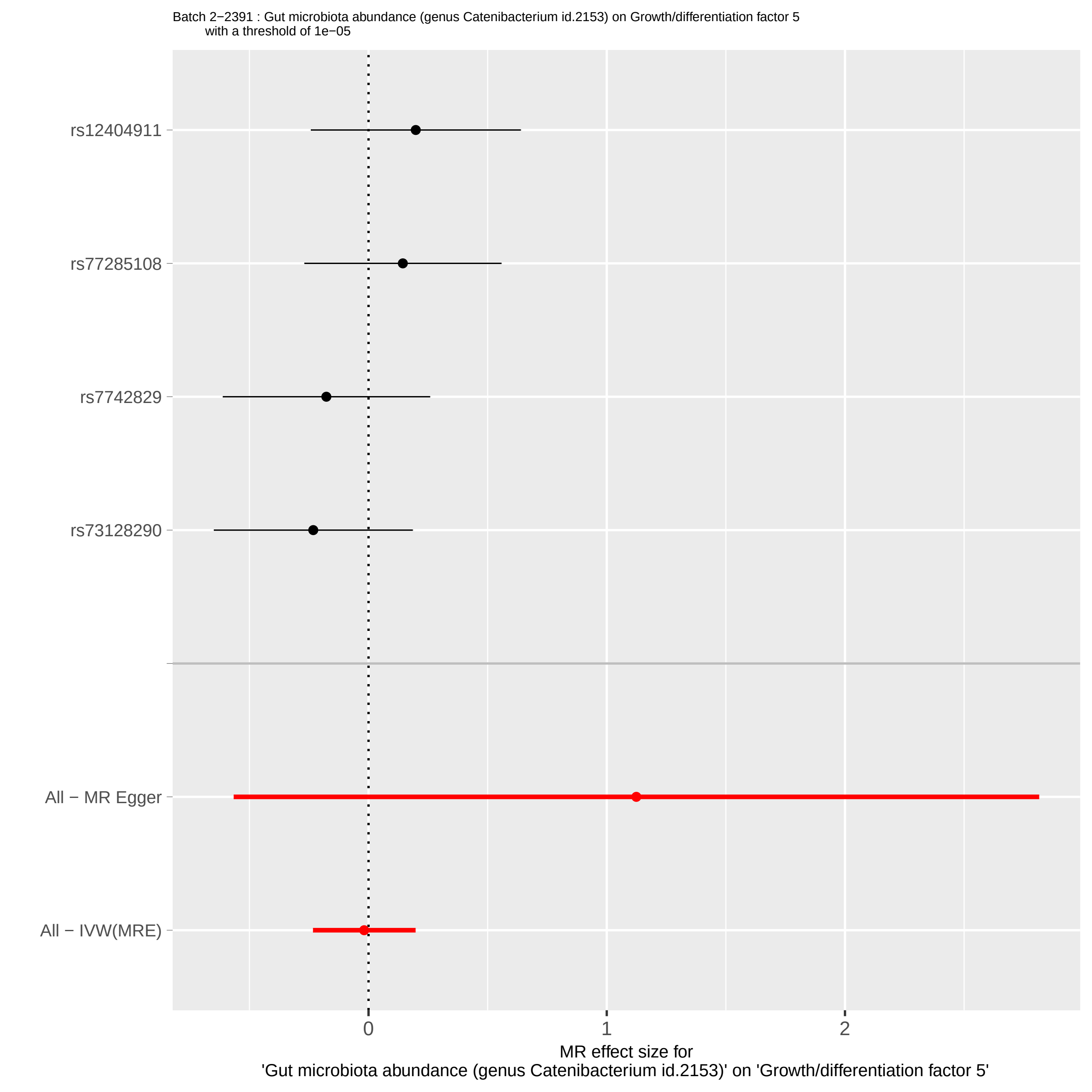
Batch 2−2391 : Gut microbiota abundance (genus Catenibacterium id.2153) on Growth/differentiation factor 5
with a threshold of 1e−05
rs12404911
rs77285108
rs7742829
rs73128290
All − MR Egger
All − IVW(MRE)
0
1
2
MR effect size for
'Gut microbiota abundance (genus Catenibacterium id.2153)' on 'Growth/differentiation factor 5'

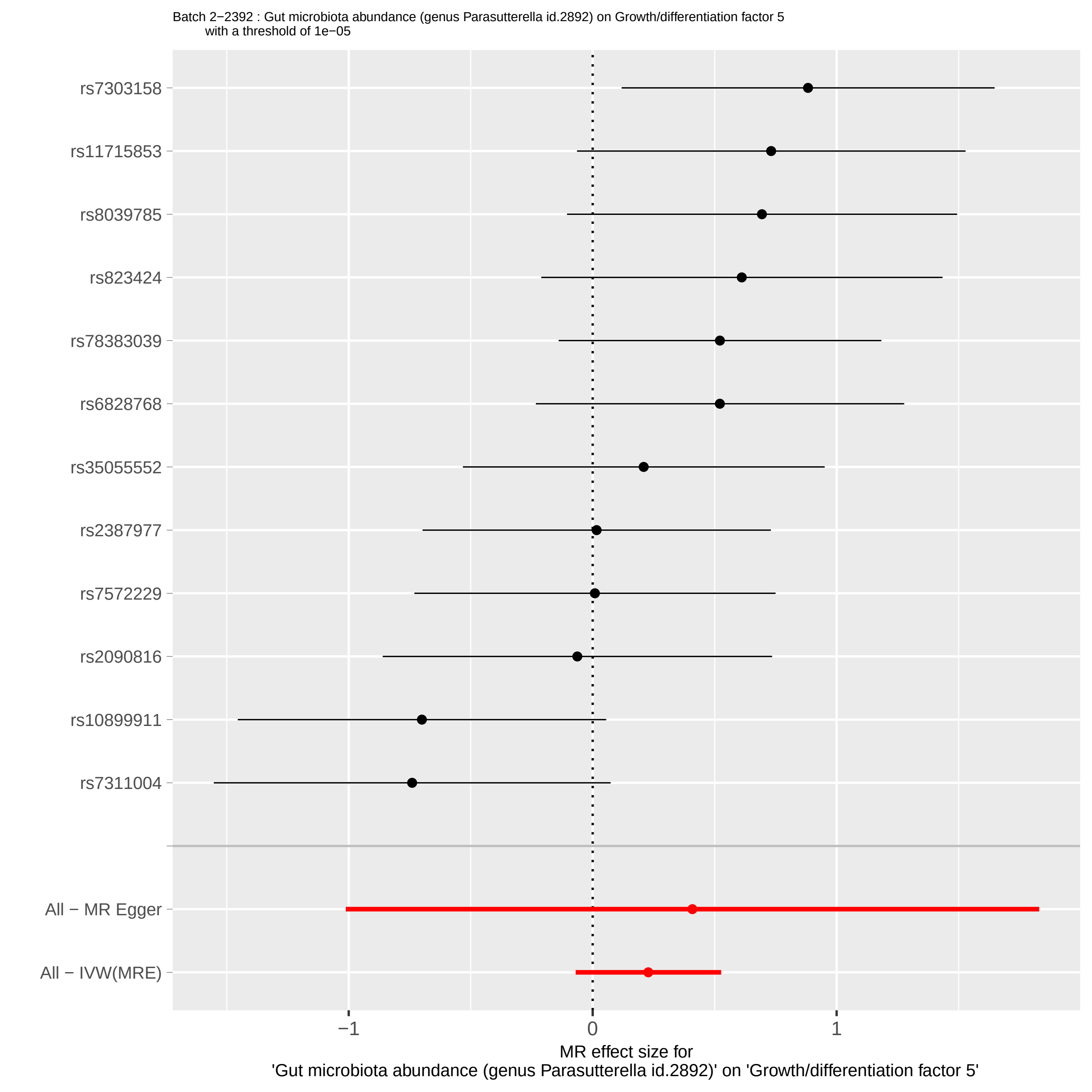

Batch 2−2392 : Gut microbiota abundance (genus Parasutterella id.2892) on Growth/differentiation factor 5
with a threshold of 1e−05
rs7303158
rs11715853
rs8039785
rs823424
rs78383039
rs6828768
rs35055552
rs2387977
rs7572229
rs2090816
rs10899911
rs7311004
All − MR Egger
All − IVW(MRE)
−1
0
1
MR effect size for
'Gut microbiota abundance (genus Parasutterella id.2892)' on 'Growth/differentiation factor 5'

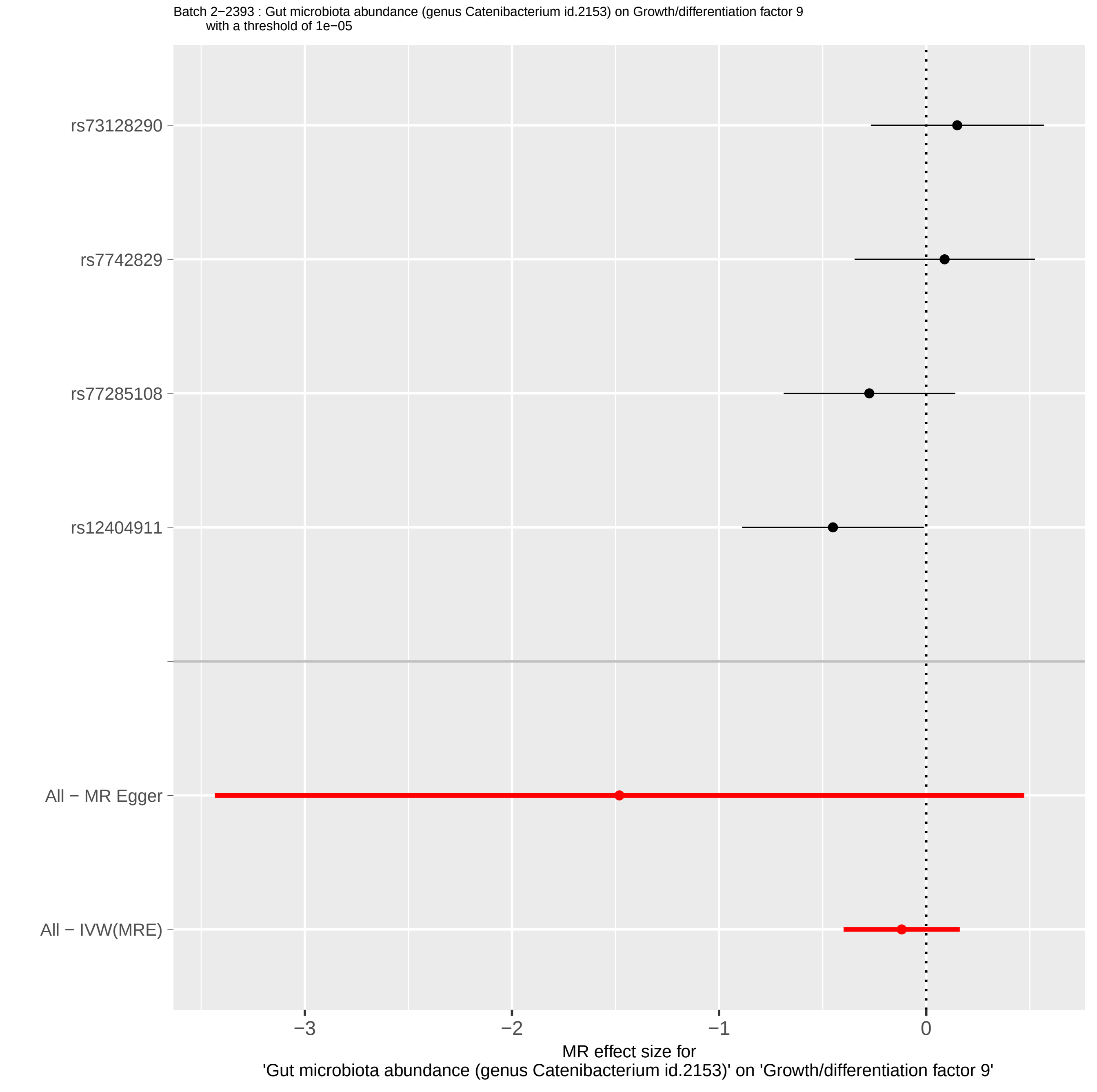

Batch 2−2393 : Gut microbiota abundance (genus Catenibacterium id.2153) on Growth/differentiation factor 9
with a threshold of 1e−05
rs73128290
rs7742829
rs77285108
rs12404911
All − MR Egger
All − IVW(MRE)
−3
−2
−1
0
MR effect size for
'Gut microbiota abundance (genus Catenibacterium id.2153)' on 'Growth/differentiation factor 9'

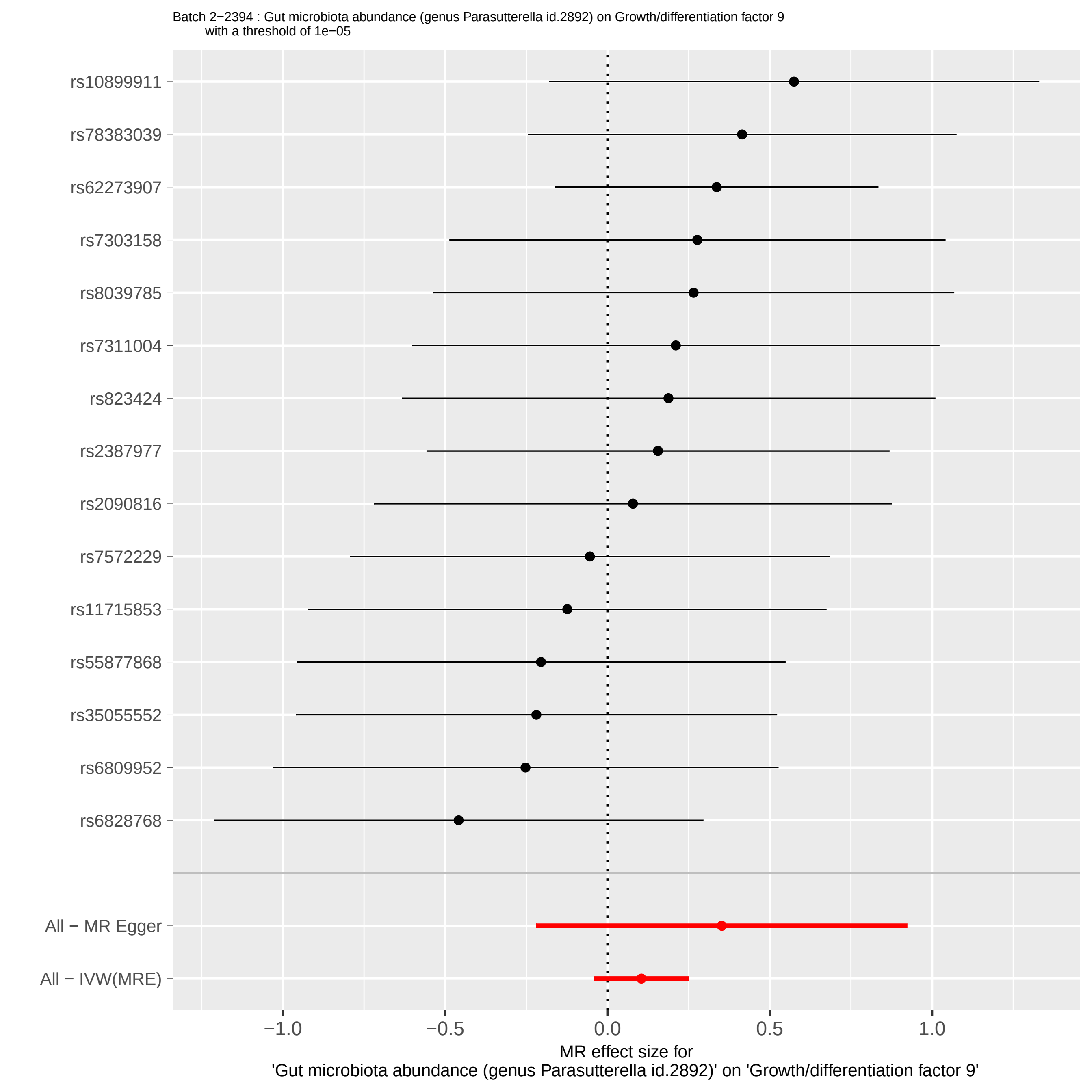

Batch 2−2394 : Gut microbiota abundance (genus Parasutterella id.2892) on Growth/differentiation factor 9
with a threshold of 1e−05
rs10899911
rs78383039
rs62273907
rs7303158
rs8039785
rs7311004
rs823424
rs2387977
rs2090816
rs7572229
rs11715853
rs55877868
rs35055552
rs6809952
rs6828768
All − MR Egger
All − IVW(MRE)
−1.0
−0.5
0.0
0.5
1.0
MR effect size for
'Gut microbiota abundance (genus Parasutterella id.2892)' on 'Growth/differentiation factor 9'

Batch 2−2395 : Gut microbiota abundance (genus Catenibacterium id.2153) on Glial cell line−derived neurotrophic factor
with a threshold of 1e−05

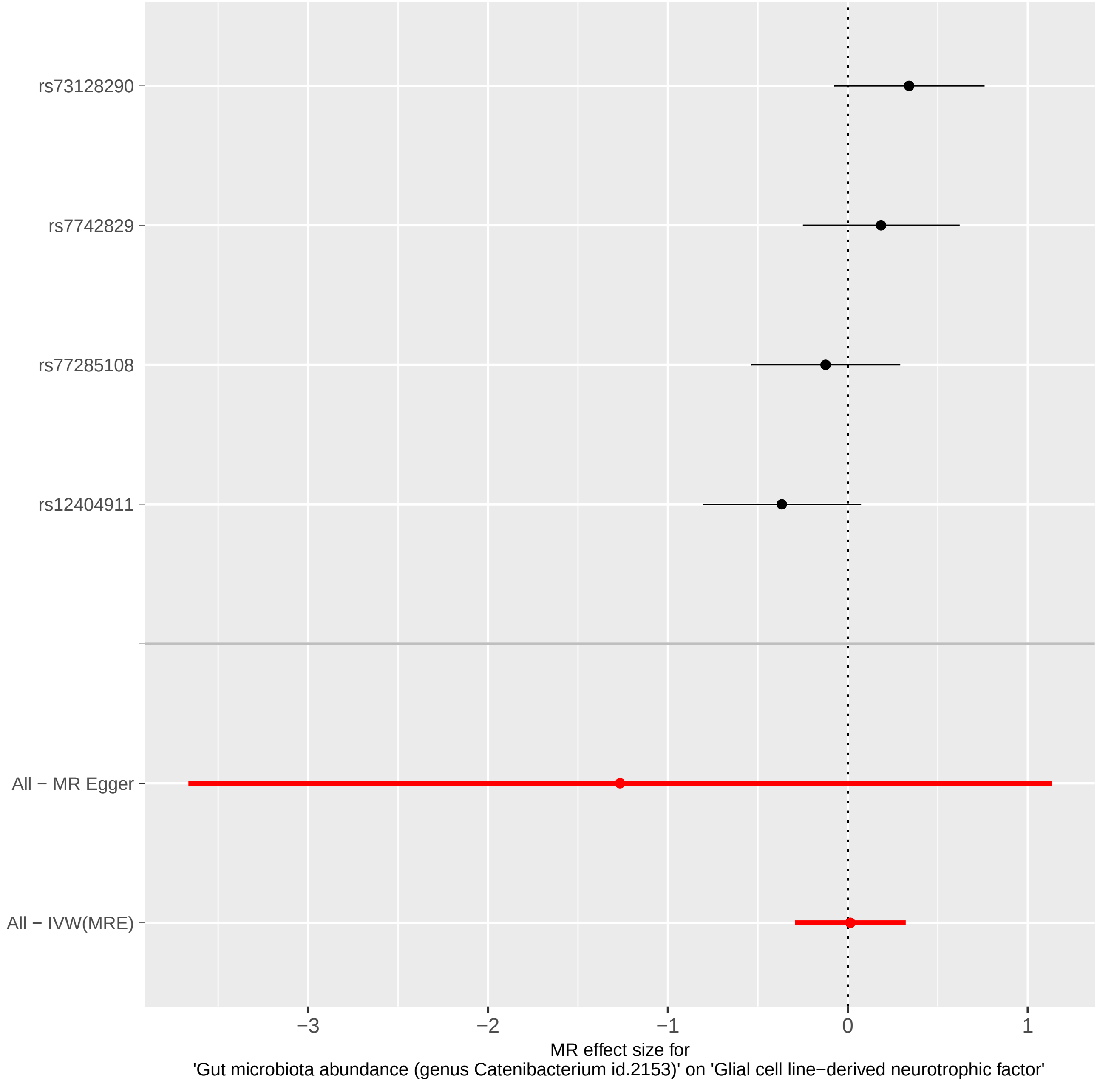

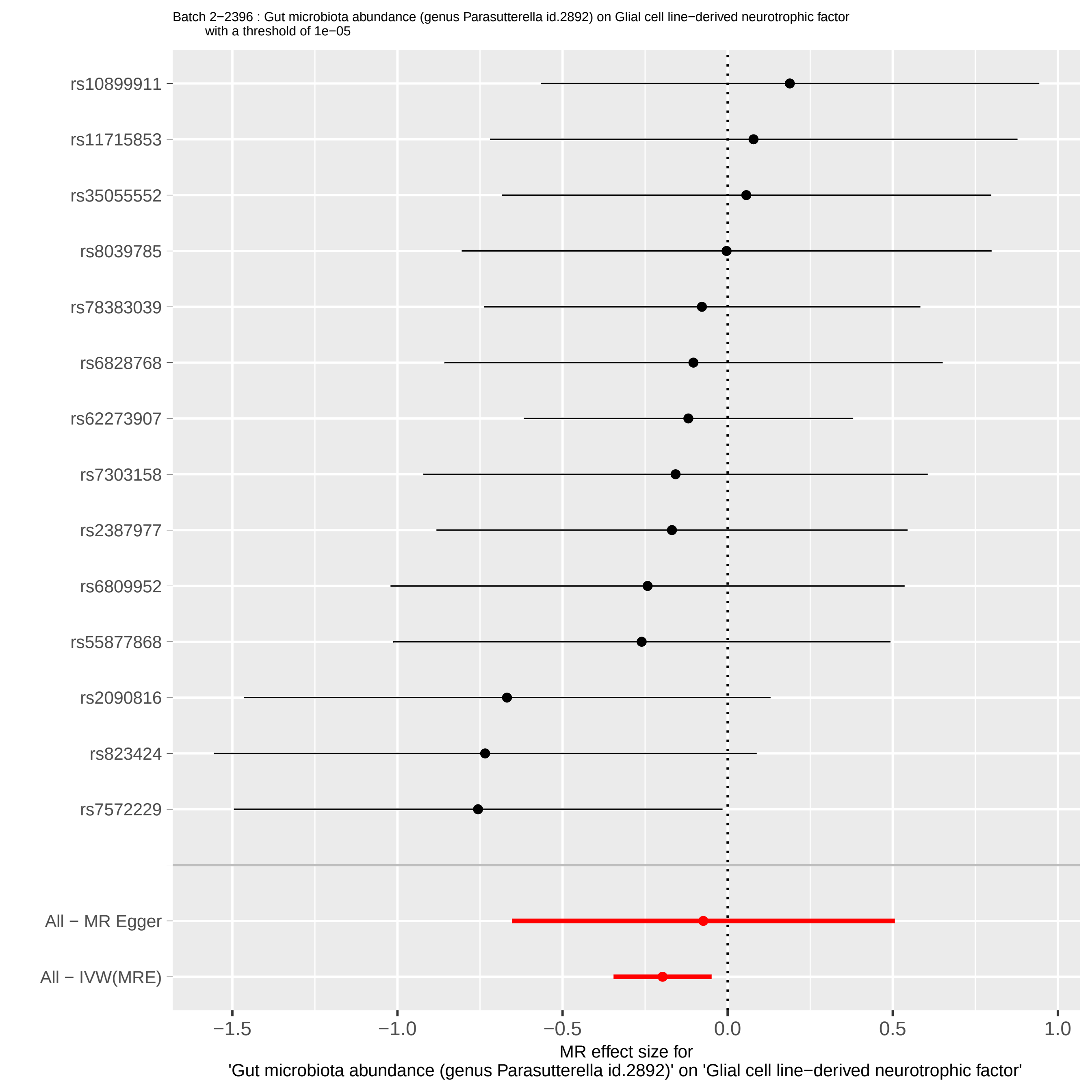

Batch 2−2396 : Gut microbiota abundance (genus Parasutterella id.2892) on Glial cell line−derived neurotrophic factor
with a threshold of 1e−05
rs10899911
rs11715853
rs35055552
rs8039785
rs78383039
rs6828768
rs62273907
rs7303158
rs2387977
rs6809952
rs55877868
rs2090816
rs823424
rs7572229
All − MR Egger
All − IVW(MRE)
−1.5
−1.0
−0.5
0.0
0.5
1.0
MR effect size for
'Gut microbiota abundance (genus Parasutterella id.2892)' on 'Glial cell line−derived neurotrophic factor'

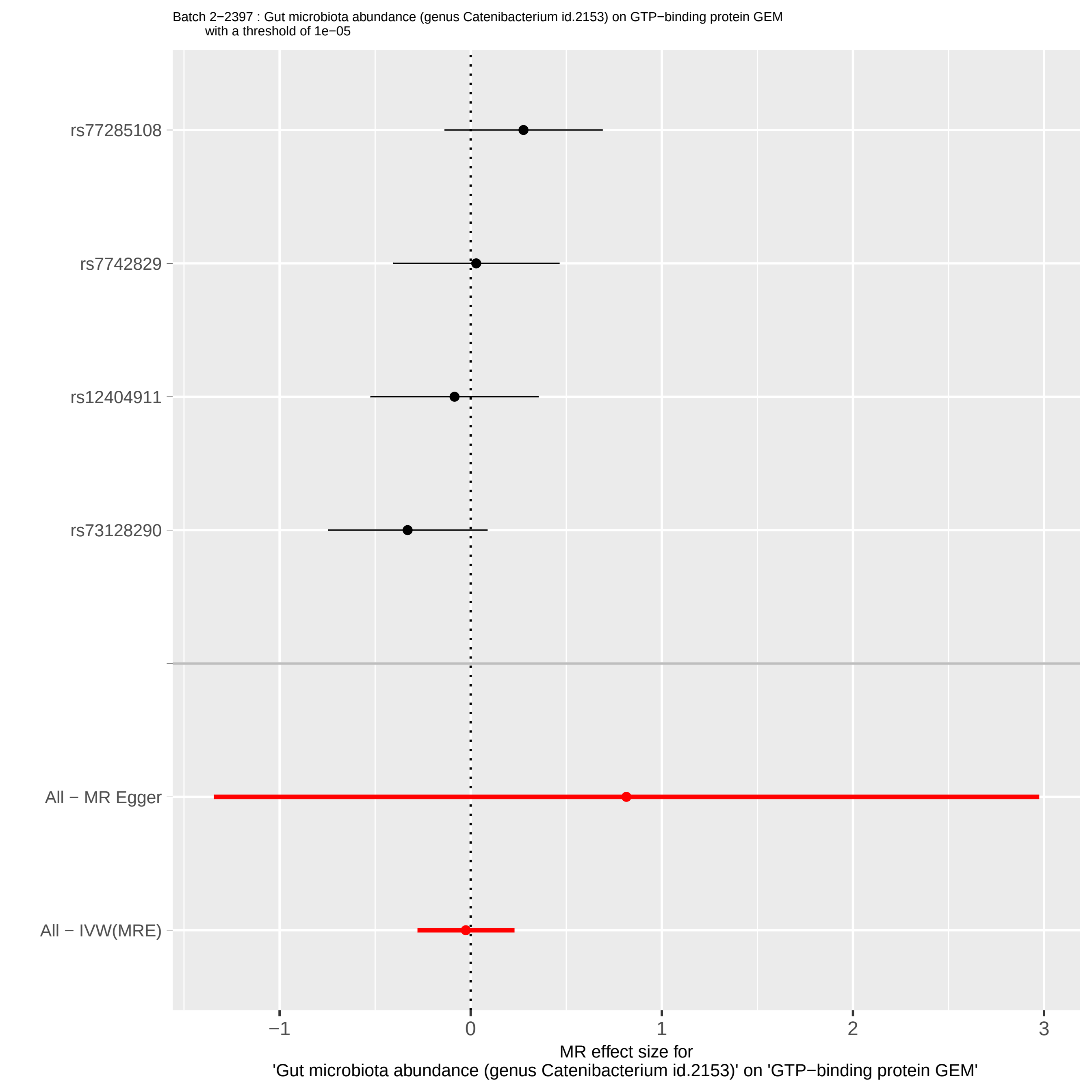
Batch 2−2397 : Gut microbiota abundance (genus Catenibacterium id.2153) on GTP−binding protein GEM
with a threshold of 1e−05
rs77285108
rs7742829
rs12404911
rs73128290
All − MR Egger
All − IVW(MRE)
−1
0
1
2
3
MR effect size for
'Gut microbiota abundance (genus Catenibacterium id.2153)' on 'GTP−binding protein GEM'

Batch 2−2398 : Gut microbiota abundance (genus Parasutterella id.2892) on GTP−binding protein GEM
with a threshold of 1e−05

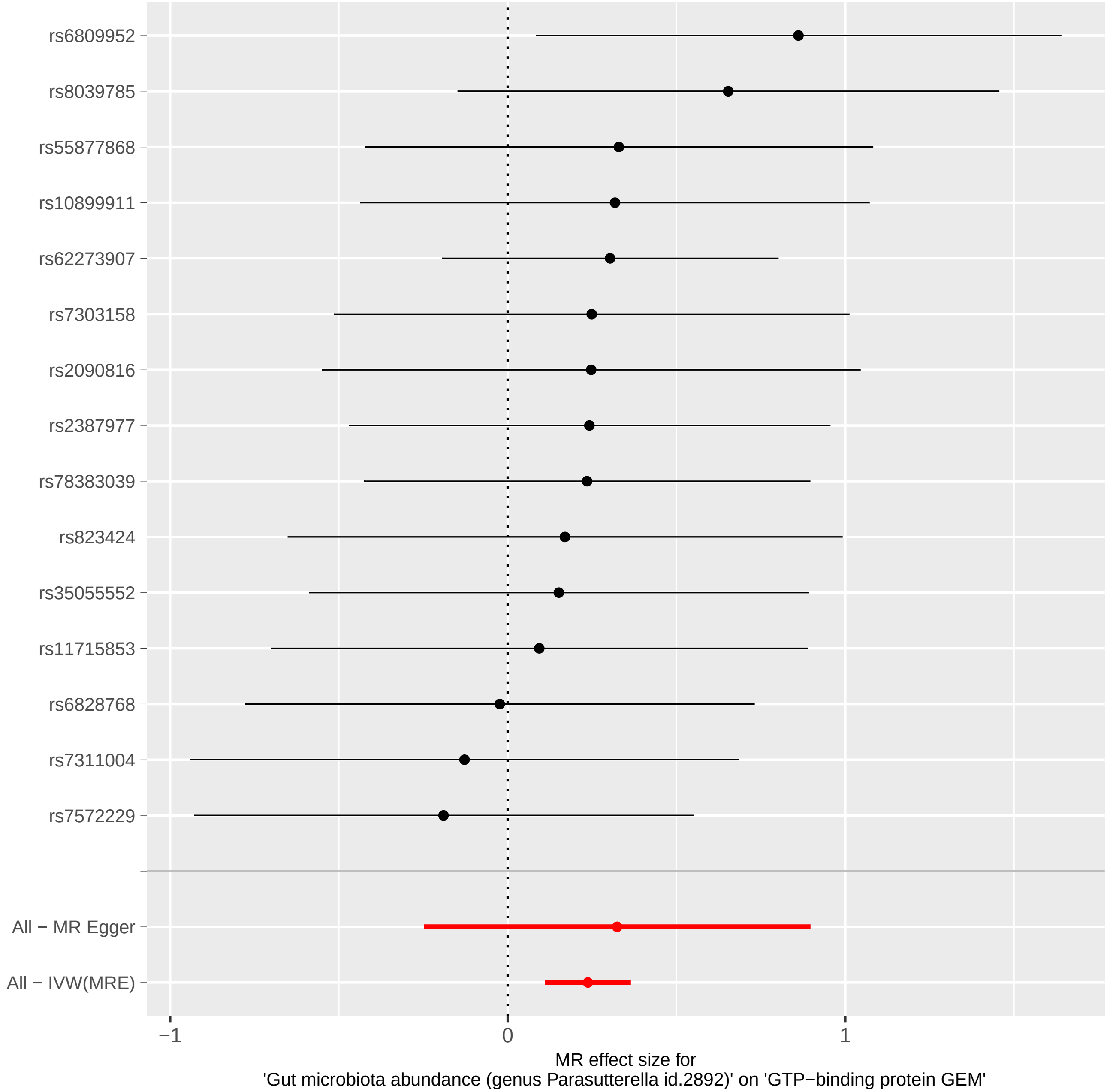

Batch 2−2399 : Gut microbiota abundance (genus Catenibacterium id.2153) on Glial fibrillary acidic protein
with a threshold of 1e−05

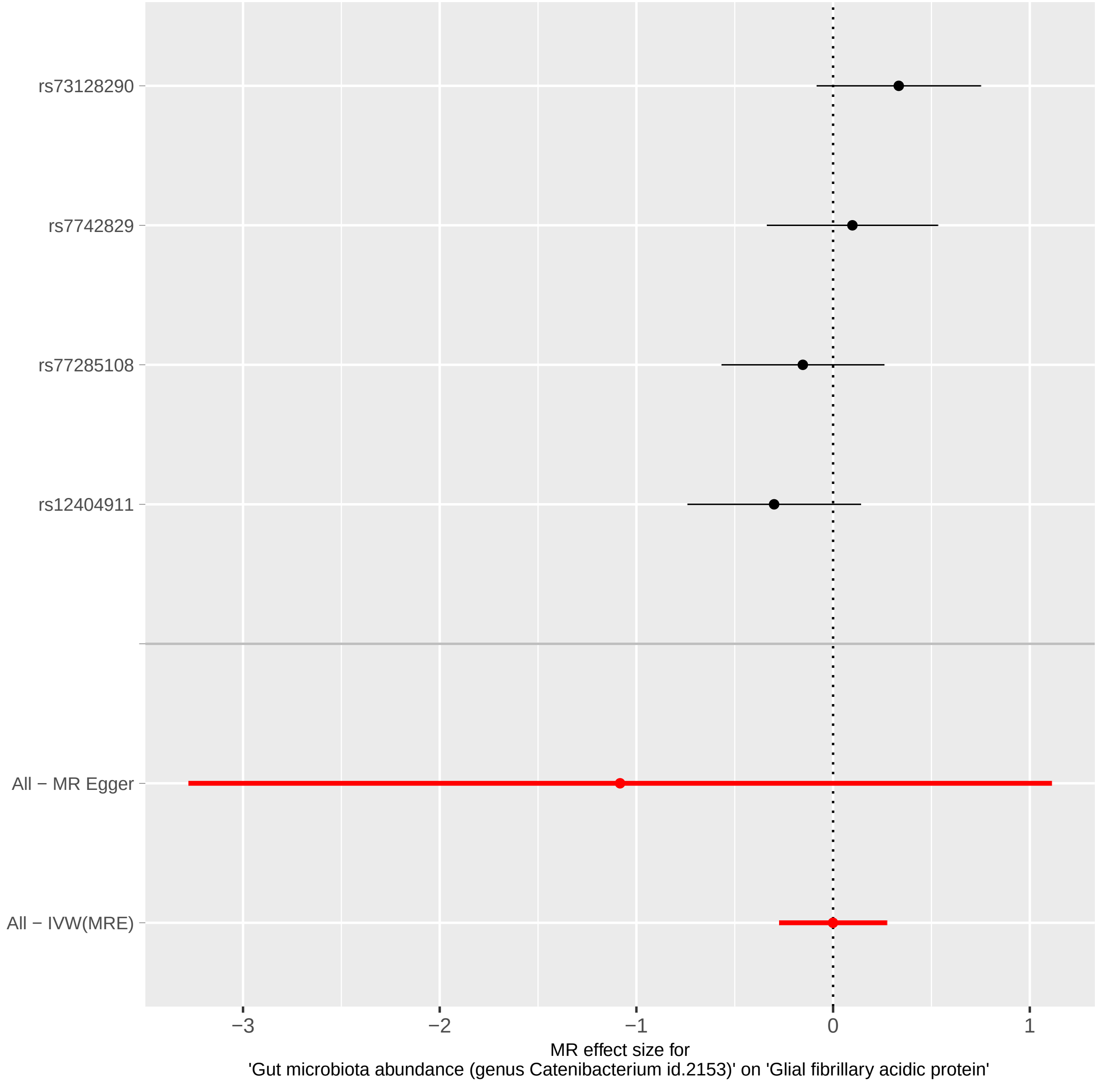

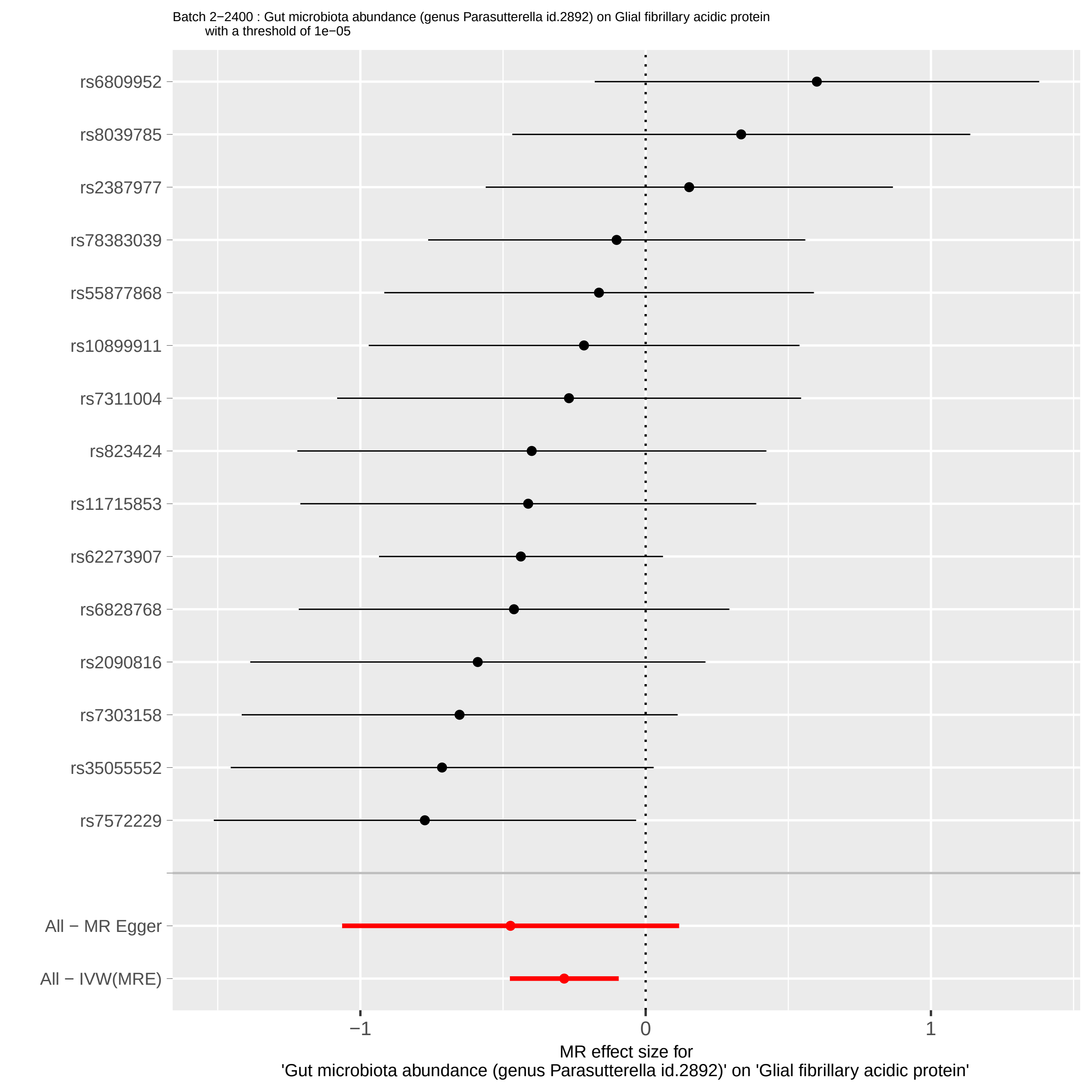

Batch 2−2400 : Gut microbiota abundance (genus Parasutterella id.2892) on Glial fibrillary acidic protein
with a threshold of 1e−05
rs6809952
rs8039785
rs2387977
rs78383039
rs55877868
rs10899911
rs7311004
rs823424
rs11715853
rs62273907
rs6828768
rs2090816
rs7303158
rs35055552
rs7572229
All − MR Egger
All − IVW(MRE)
−1
0
1
MR effect size for
'Gut microbiota abundance (genus Parasutterella id.2892)' on 'Glial fibrillary acidic protein'

Batch 2−2401 : Gut microbiota abundance (genus Catenibacterium id.2153) on GDNF family receptor alpha−1
with a threshold of 1e−05

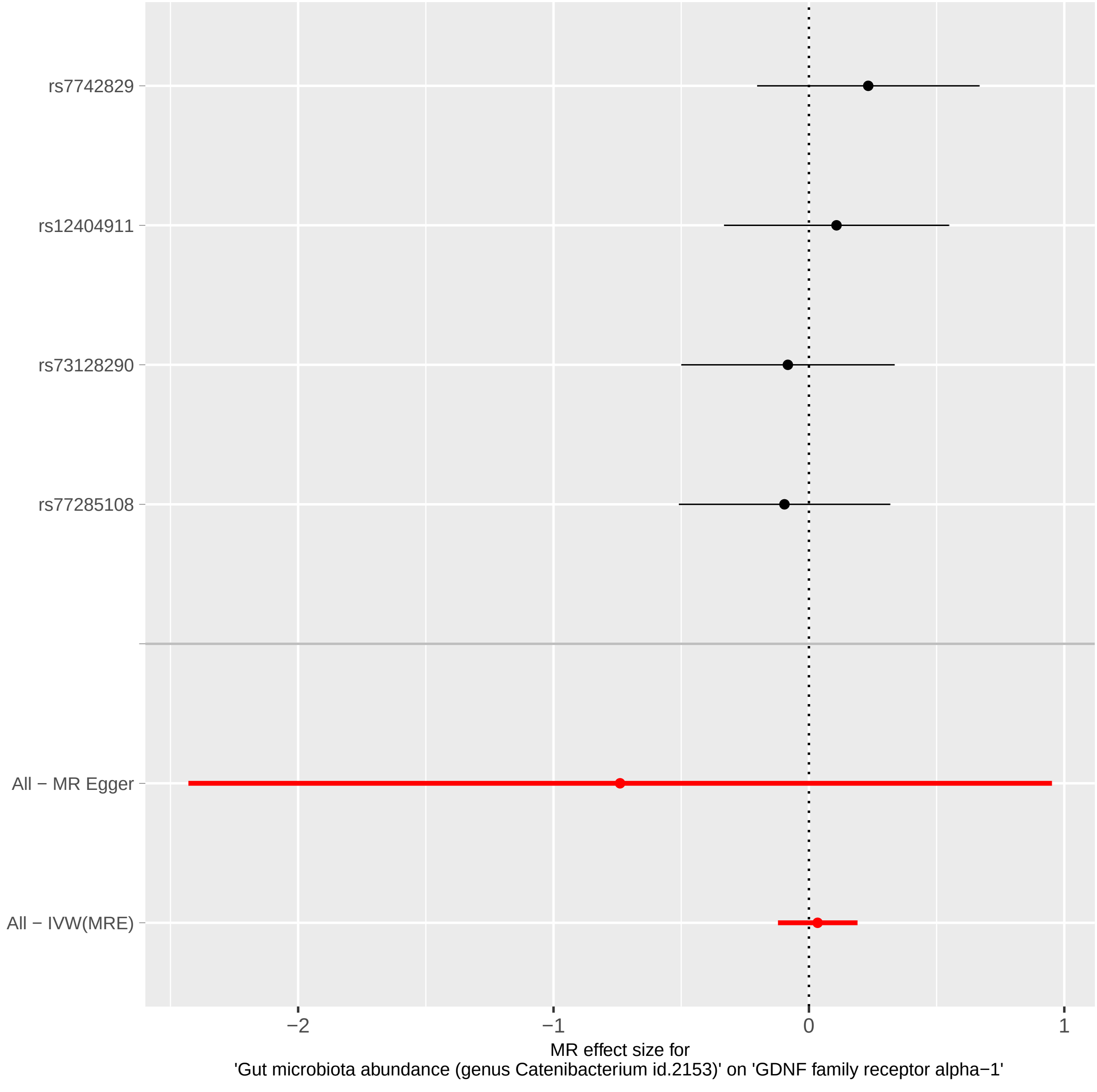

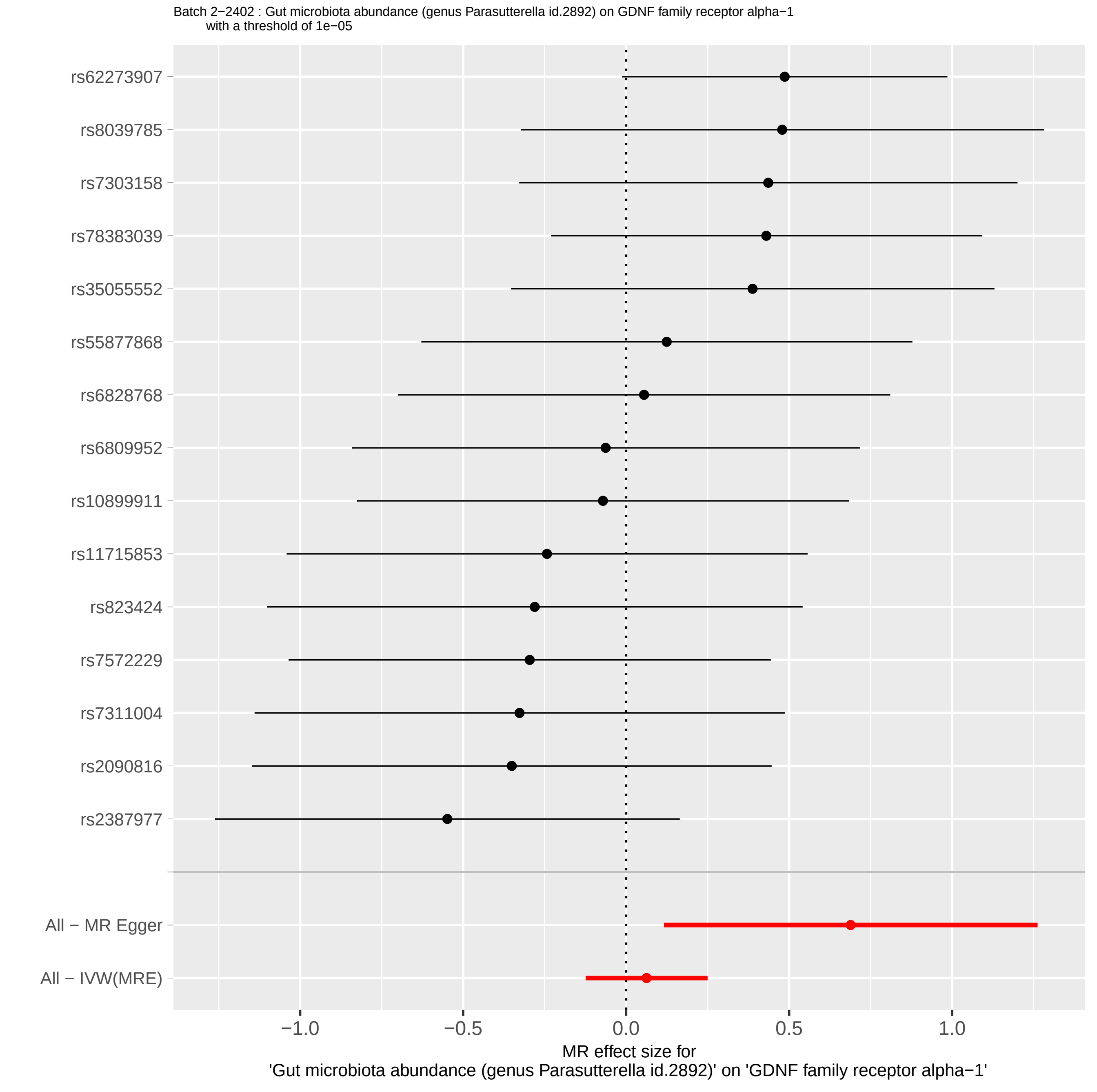

Batch 2−2402 : Gut microbiota abundance (genus Parasutterella id.2892) on GDNF family receptor alpha−1
with a threshold of 1e−05
rs62273907
rs8039785
rs7303158
rs78383039
rs35055552
rs55877868
rs6828768
rs6809952
rs10899911
rs11715853
rs823424
rs7572229
rs7311004
rs2090816
rs2387977
All − MR Egger
All − IVW(MRE)
−1.0
−0.5
0.0
0.5
1.0
MR effect size for
'Gut microbiota abundance (genus Parasutterella id.2892)' on 'GDNF family receptor alpha−1'

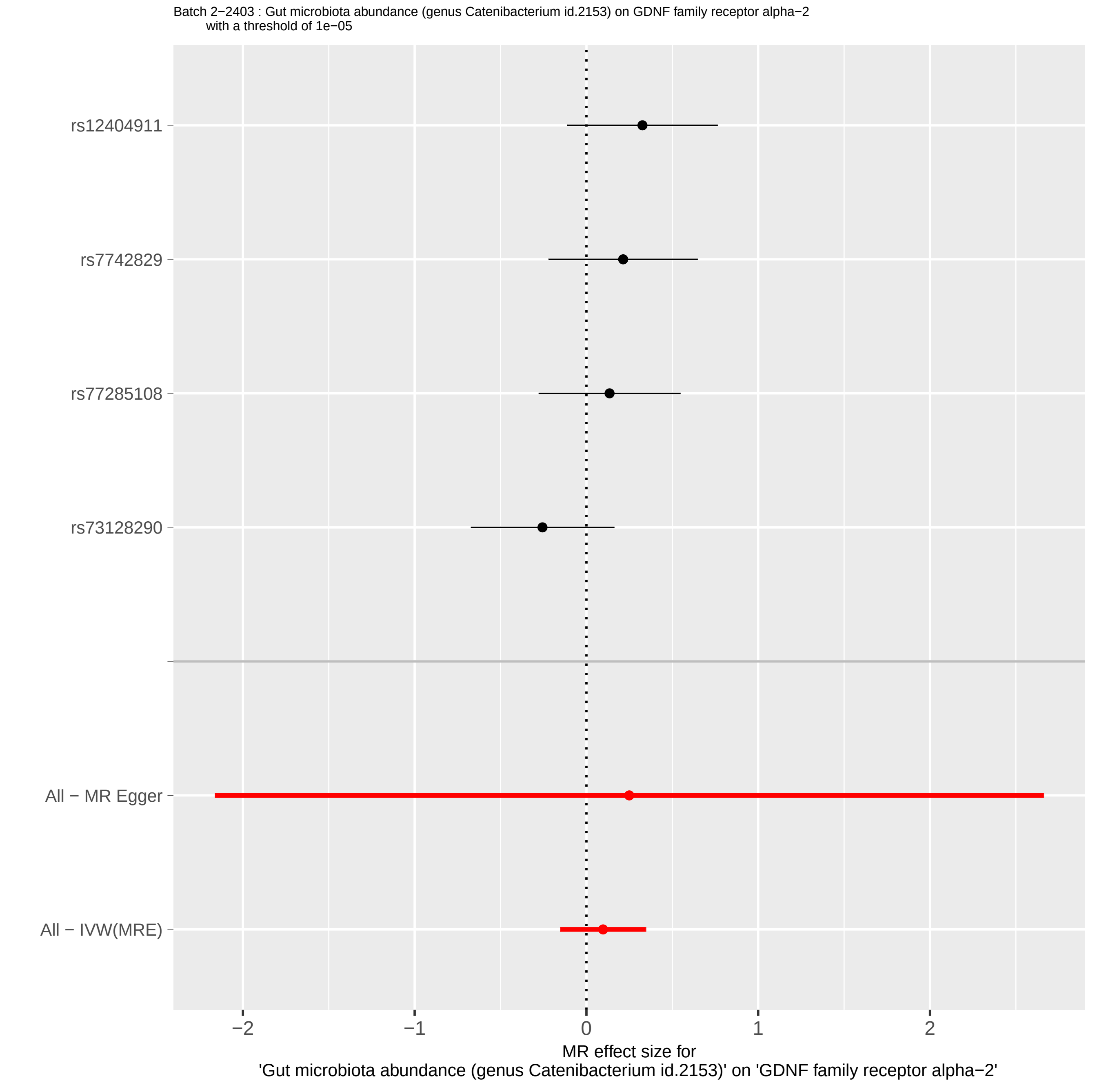

Batch 2−2403 : Gut microbiota abundance (genus Catenibacterium id.2153) on GDNF family receptor alpha−2
with a threshold of 1e−05
rs12404911
rs7742829
rs77285108
rs73128290
All − MR Egger
All − IVW(MRE)
−2
−1
0
1
2
MR effect size for
'Gut microbiota abundance (genus Catenibacterium id.2153)' on 'GDNF family receptor alpha−2'

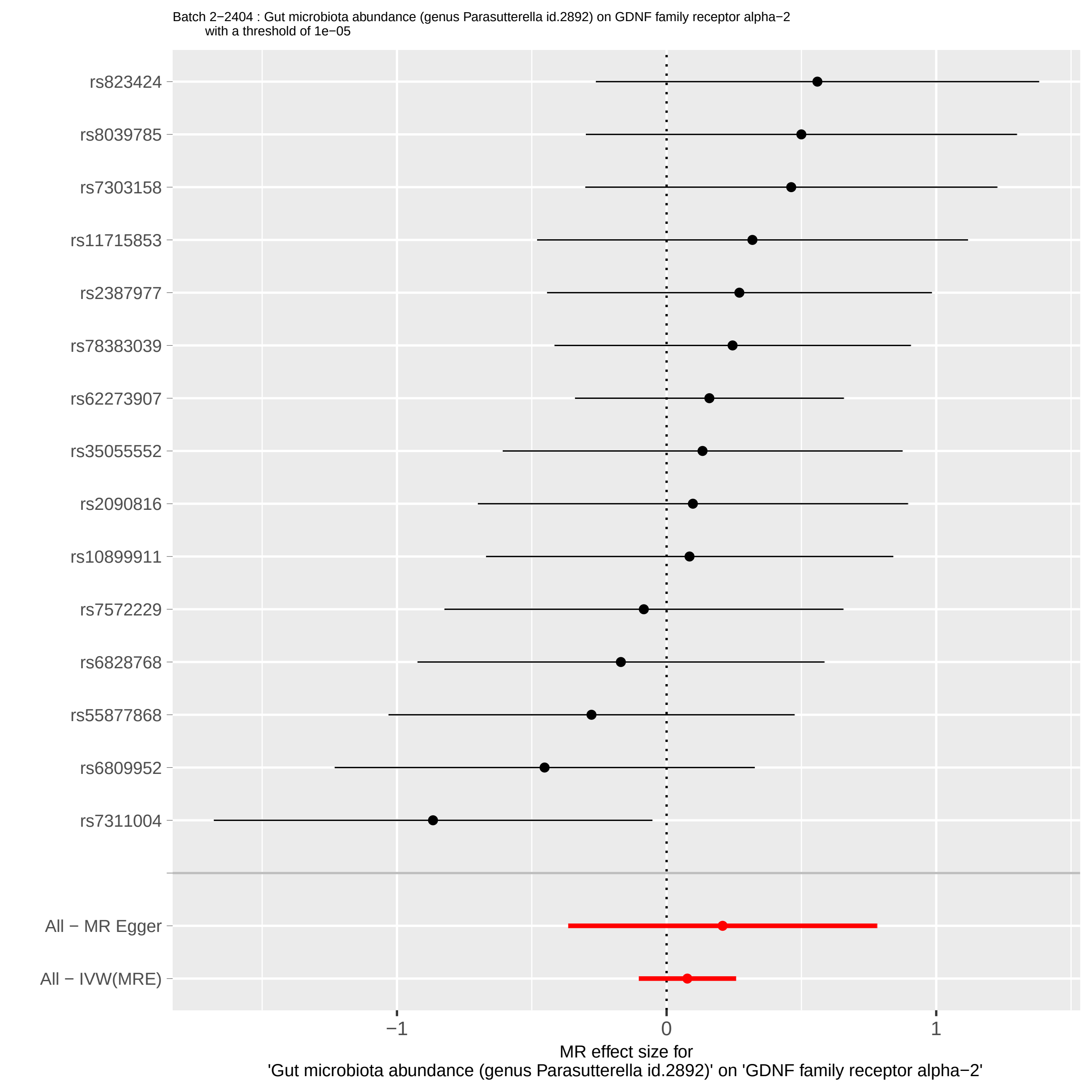
Batch 2−2404 : Gut microbiota abundance (genus Parasutterella id.2892) on GDNF family receptor alpha−2
with a threshold of 1e−05
rs823424
rs8039785
rs7303158
rs11715853
rs2387977
rs78383039
rs62273907
rs35055552
rs2090816
rs10899911
rs7572229
rs6828768
rs55877868
rs6809952
rs7311004
All − MR Egger
All − IVW(MRE)
−1
0
1
MR effect size for
'Gut microbiota abundance (genus Parasutterella id.2892)' on 'GDNF family receptor alpha−2'

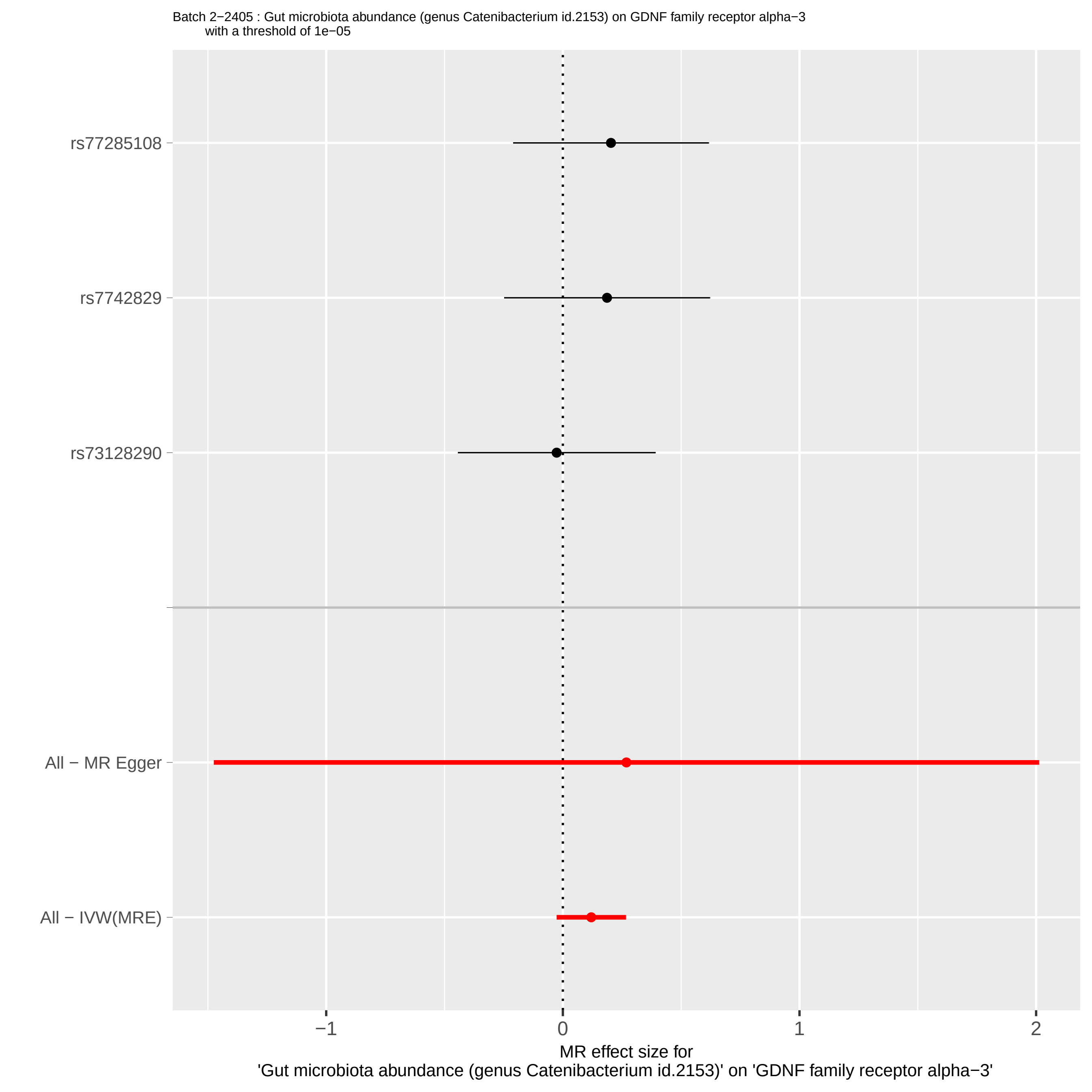
Batch 2−2405 : Gut microbiota abundance (genus Catenibacterium id.2153) on GDNF family receptor alpha−3
with a threshold of 1e−05
rs77285108
rs7742829
rs73128290
All − MR Egger
All − IVW(MRE)
−1
0
1
2
MR effect size for
'Gut microbiota abundance (genus Catenibacterium id.2153)' on 'GDNF family receptor alpha−3'

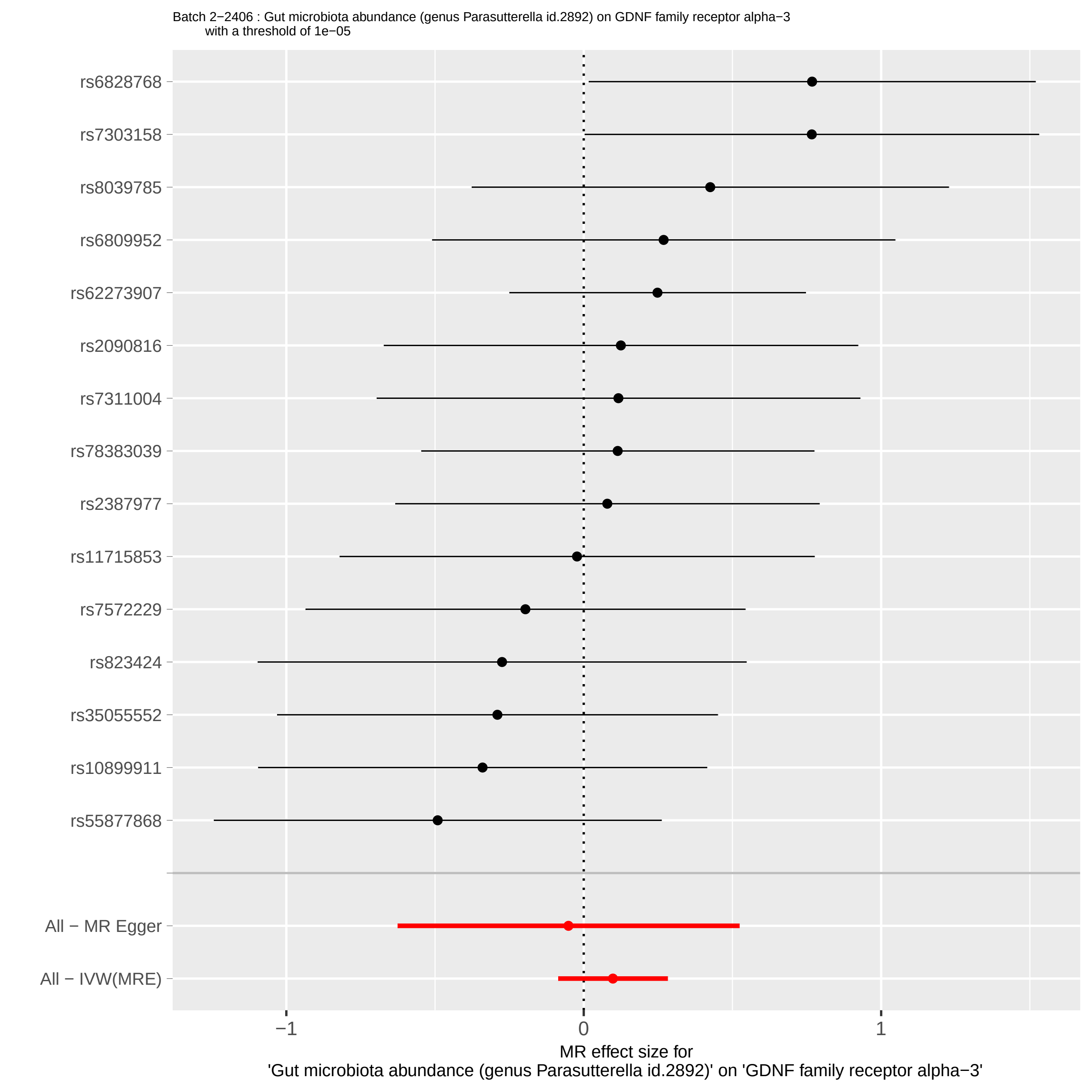
Batch 2−2406 : Gut microbiota abundance (genus Parasutterella id.2892) on GDNF family receptor alpha−3
with a threshold of 1e−05
rs6828768
rs7303158
rs8039785
rs6809952
rs62273907
rs2090816
rs7311004
rs78383039
rs2387977
rs11715853
rs7572229
rs823424
rs35055552
rs10899911
rs55877868
All − MR Egger
All − IVW(MRE)
−1
0
1
MR effect size for
'Gut microbiota abundance (genus Parasutterella id.2892)' on 'GDNF family receptor alpha−3'

Batch 2−2407 : Gut microbiota abundance (genus Catenibacterium id.2153) on GDNF family receptor alpha−like
with a threshold of 1e−05

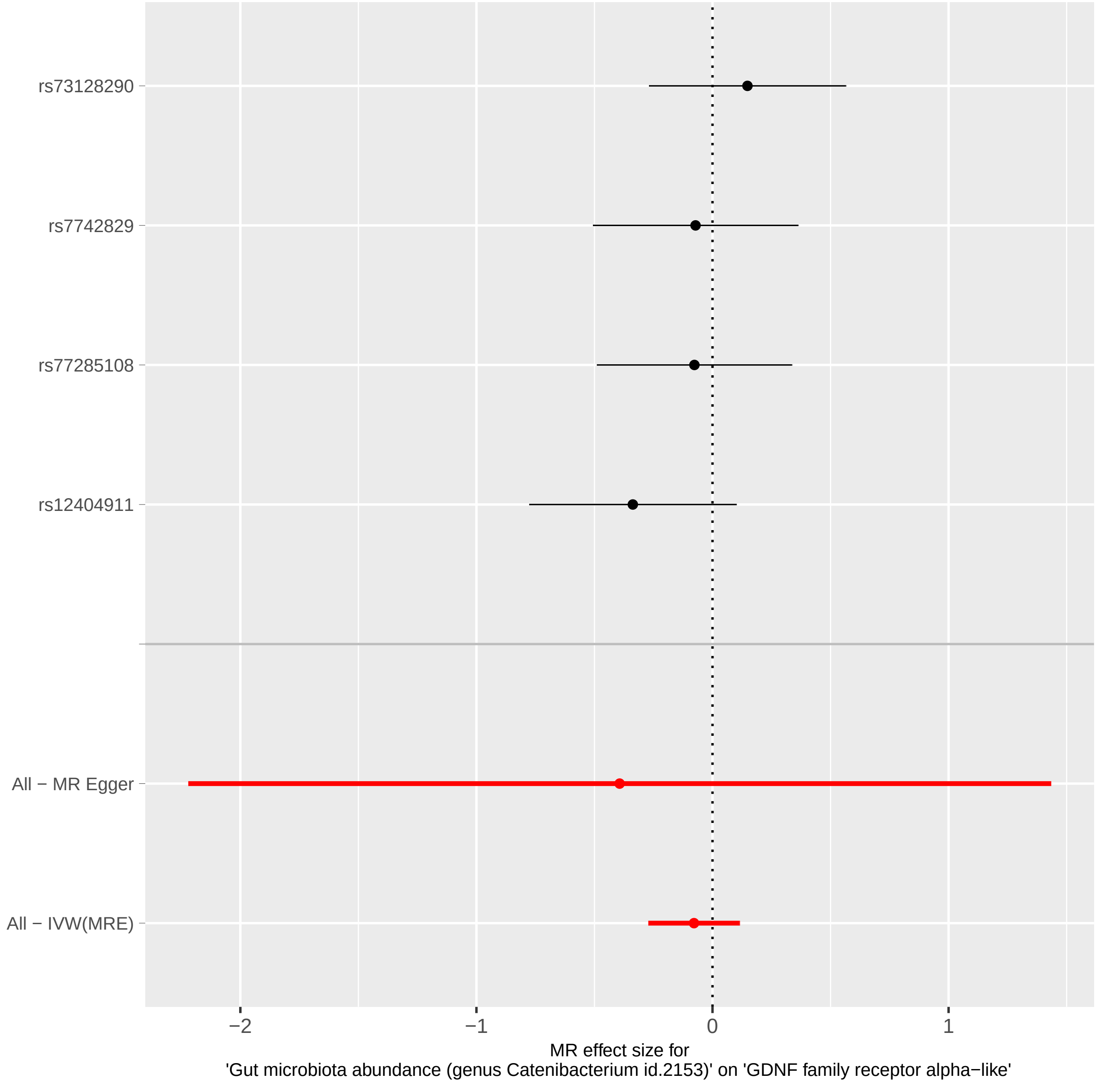

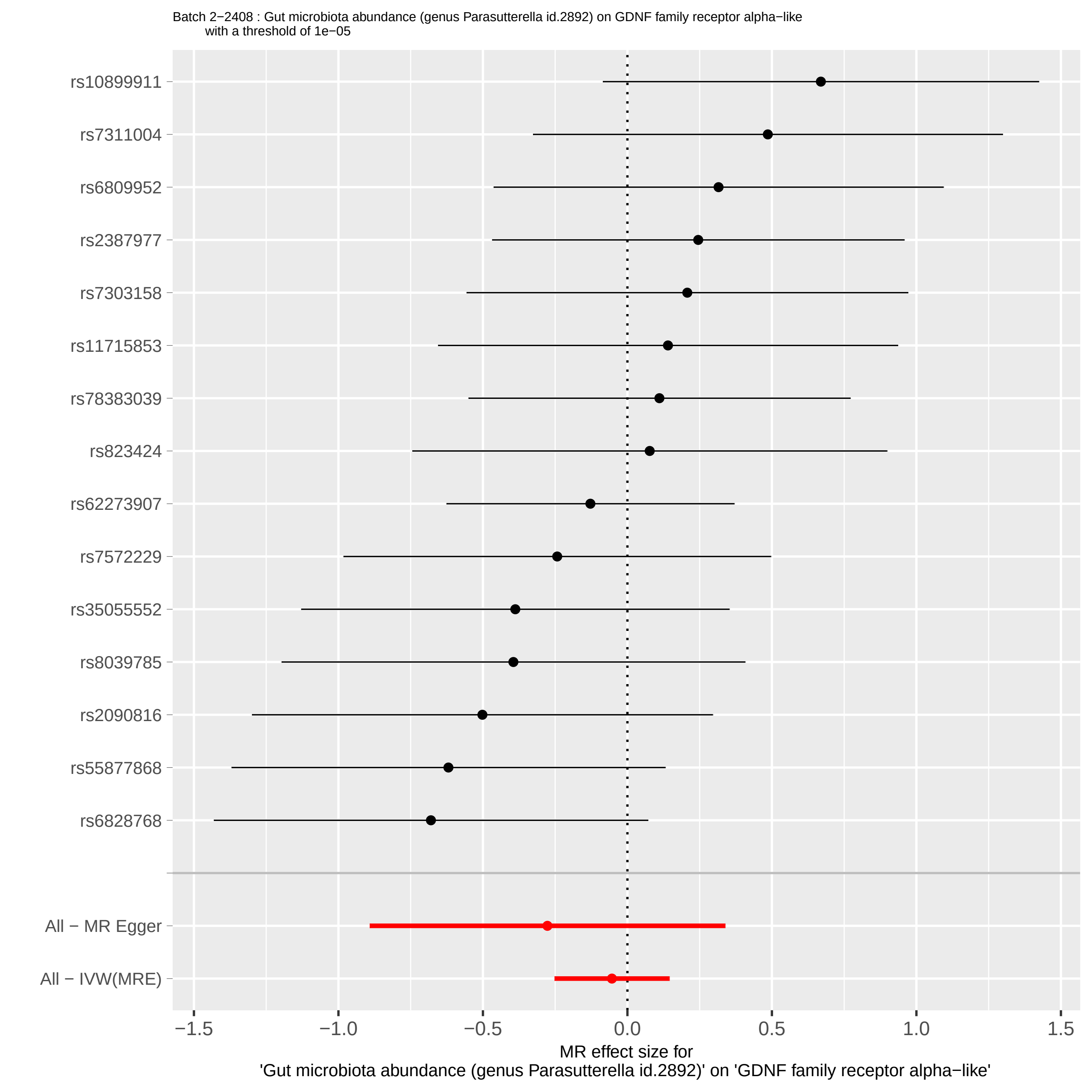
Batch 2−2408 : Gut microbiota abundance (genus Parasutterella id.2892) on GDNF family receptor alpha−like
with a threshold of 1e−05
rs10899911
rs7311004
rs6809952
rs2387977
rs7303158
rs11715853
rs78383039
rs823424
rs62273907
rs7572229
rs35055552
rs8039785
rs2090816
rs55877868
rs6828768
All − MR Egger
All − IVW(MRE)
−1.5
−1.0
−0.5
0.0
0.5
1.0
1.5
MR effect size for
'Gut microbiota abundance (genus Parasutterella id.2892)' on 'GDNF family receptor alpha−like'

Batch 2−2409 : Gut microbiota abundance (genus Catenibacterium id.2153) on ADP−ribosylation factor−binding protein GGA1
with a threshold of 1e−05

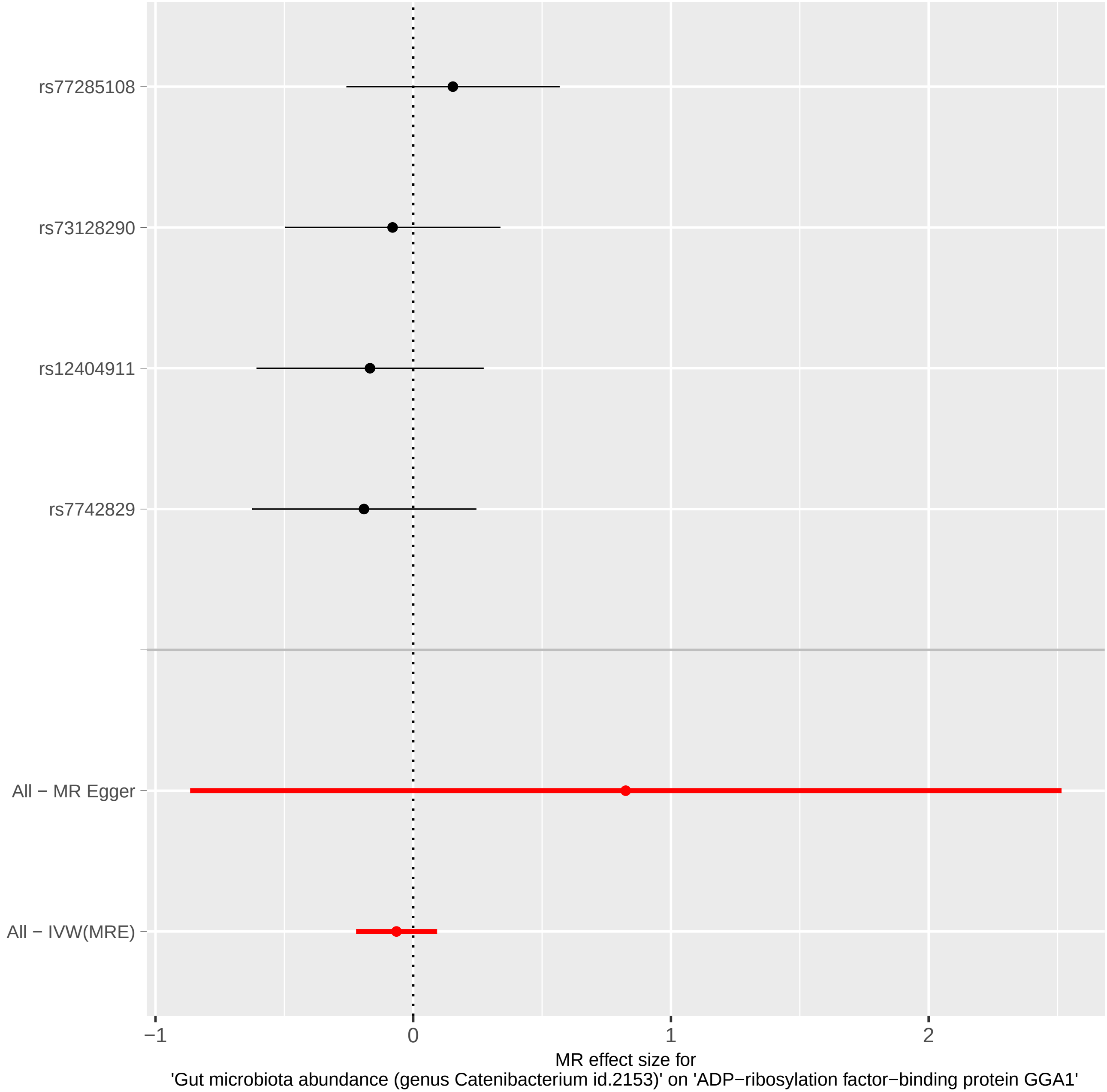

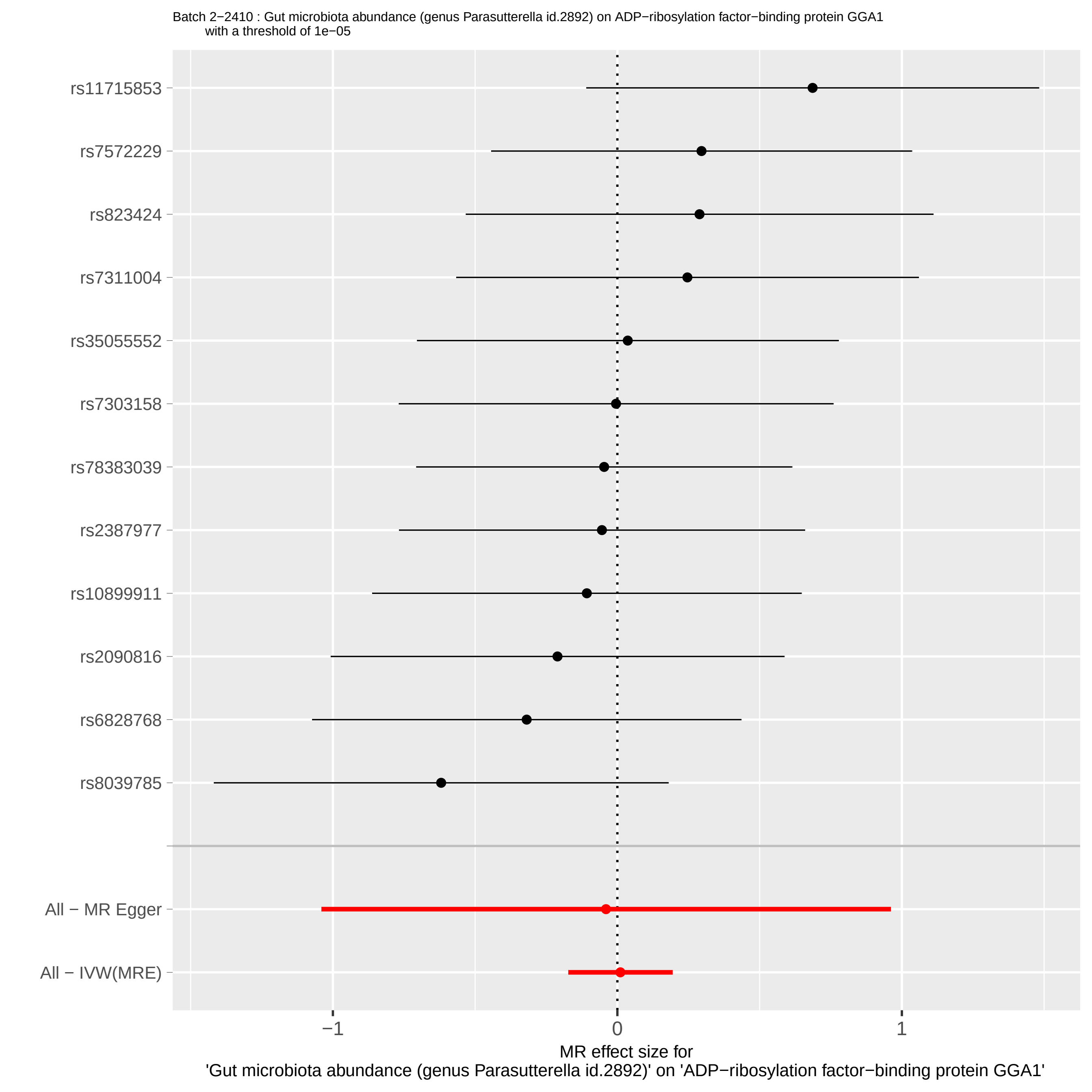

Batch 2−2410 : Gut microbiota abundance (genus Parasutterella id.2892) on ADP−ribosylation factor−binding protein GGA1
with a threshold of 1e−05
rs11715853
rs7572229
rs823424
rs7311004
rs35055552
rs7303158
rs78383039
rs2387977
rs10899911
rs2090816
rs6828768
rs8039785
All − MR Egger
All − IVW(MRE)
−1
0
1
MR effect size for
'Gut microbiota abundance (genus Parasutterella id.2892)' on 'ADP−ribosylation factor−binding protein GGA1'

Batch 2−2411 : Gut microbiota abundance (genus Catenibacterium id.2153) on ADP−ribosylation factor−binding protein GGA3 with a threshold of 1e−05

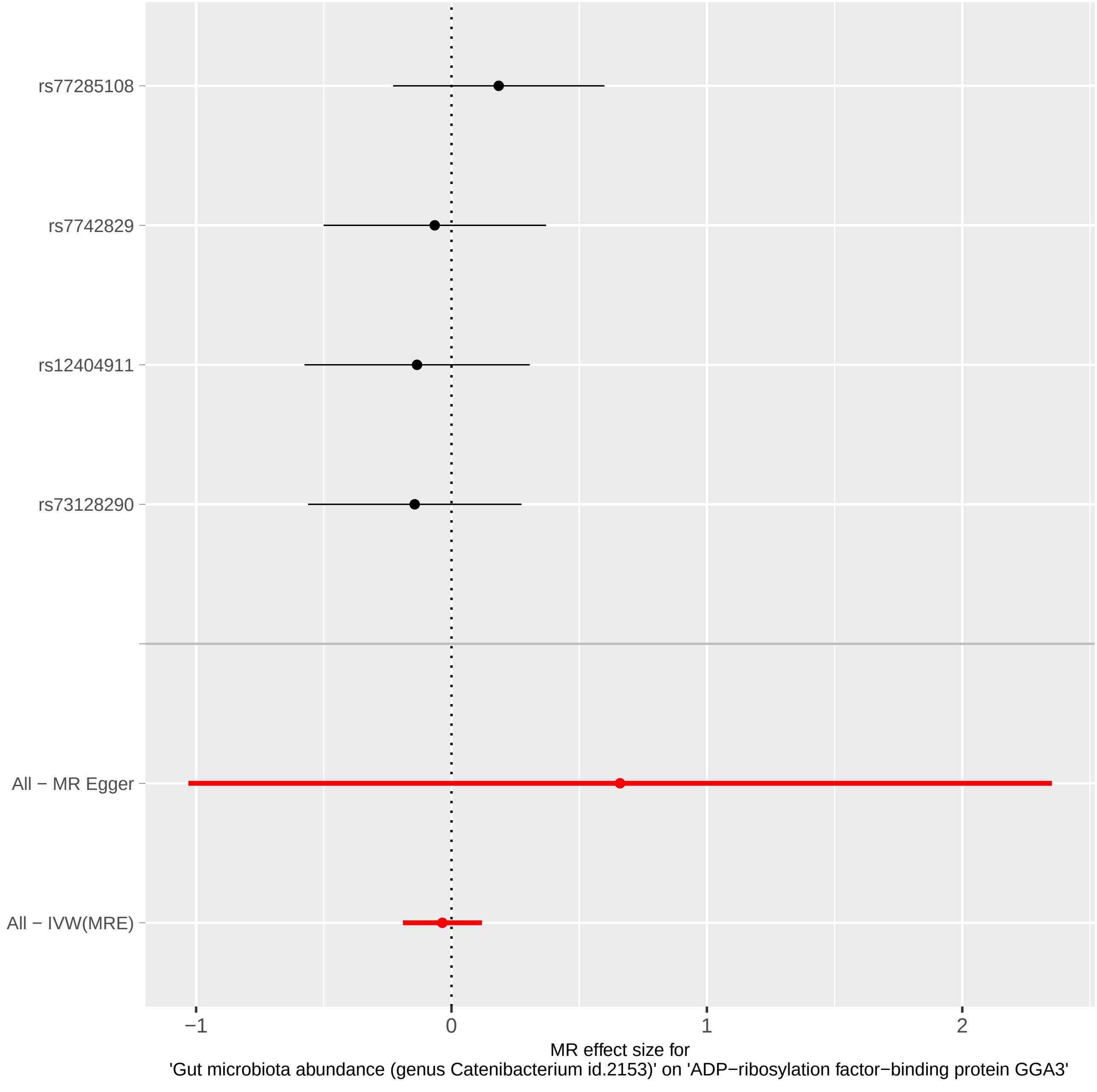

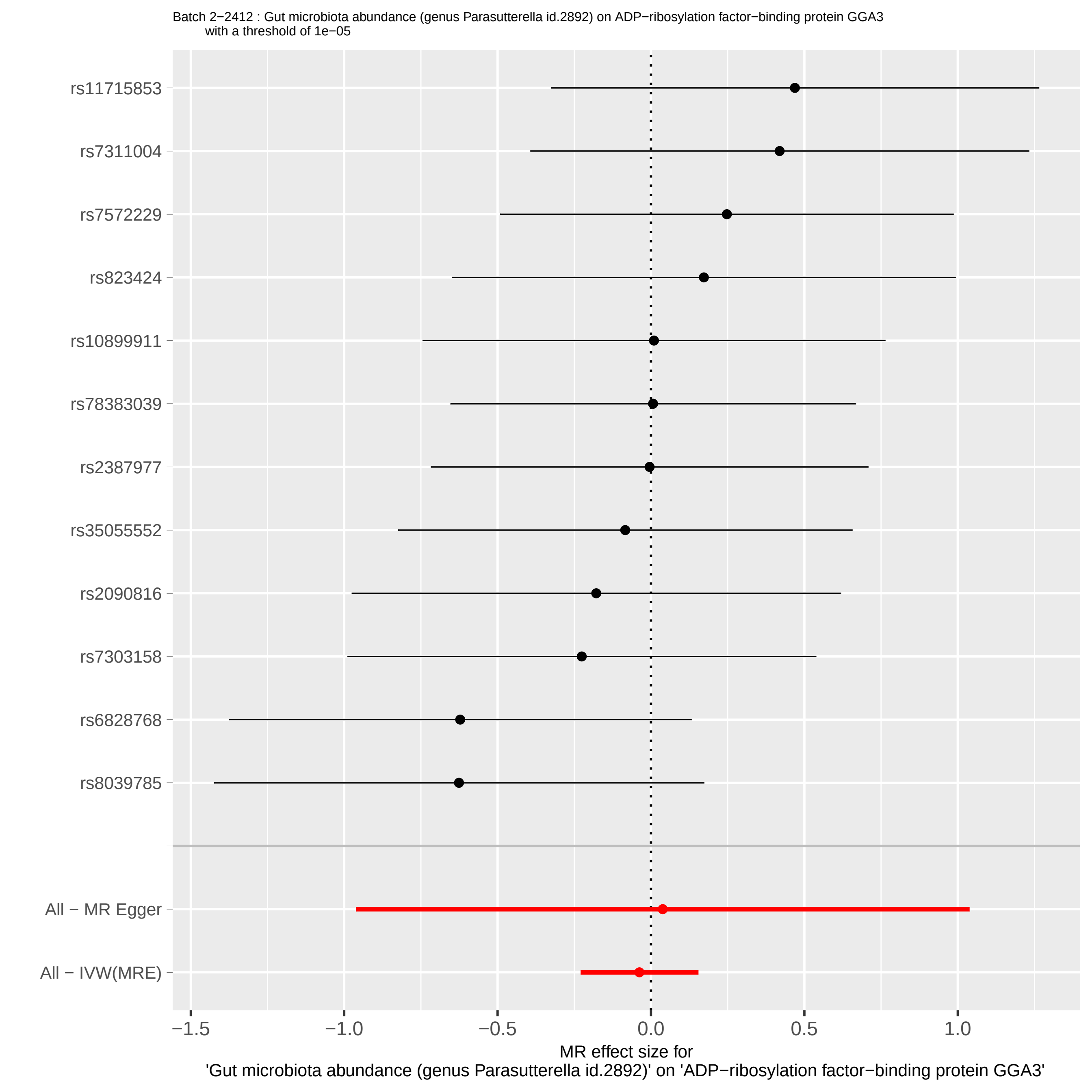

Batch 2−2412 : Gut microbiota abundance (genus Parasutterella id.2892) on ADP−ribosylation factor−binding protein GGA3
with a threshold of 1e−05
rs11715853
rs7311004
rs7572229
rs823424
rs10899911
rs78383039
rs2387977
rs35055552
rs2090816
rs7303158
rs6828768
rs8039785
All − MR Egger
All − IVW(MRE)
−1.5
−1.0
−0.5
0.0
0.5
1.0
MR effect size for
'Gut microbiota abundance (genus Parasutterella id.2892)' on 'ADP−ribosylation factor−binding protein GGA3'

Batch 2−2413 : Gut microbiota abundance (genus Catenibacterium id.2153) on Gamma−glutamyl hydrolase
with a threshold of 1e−05

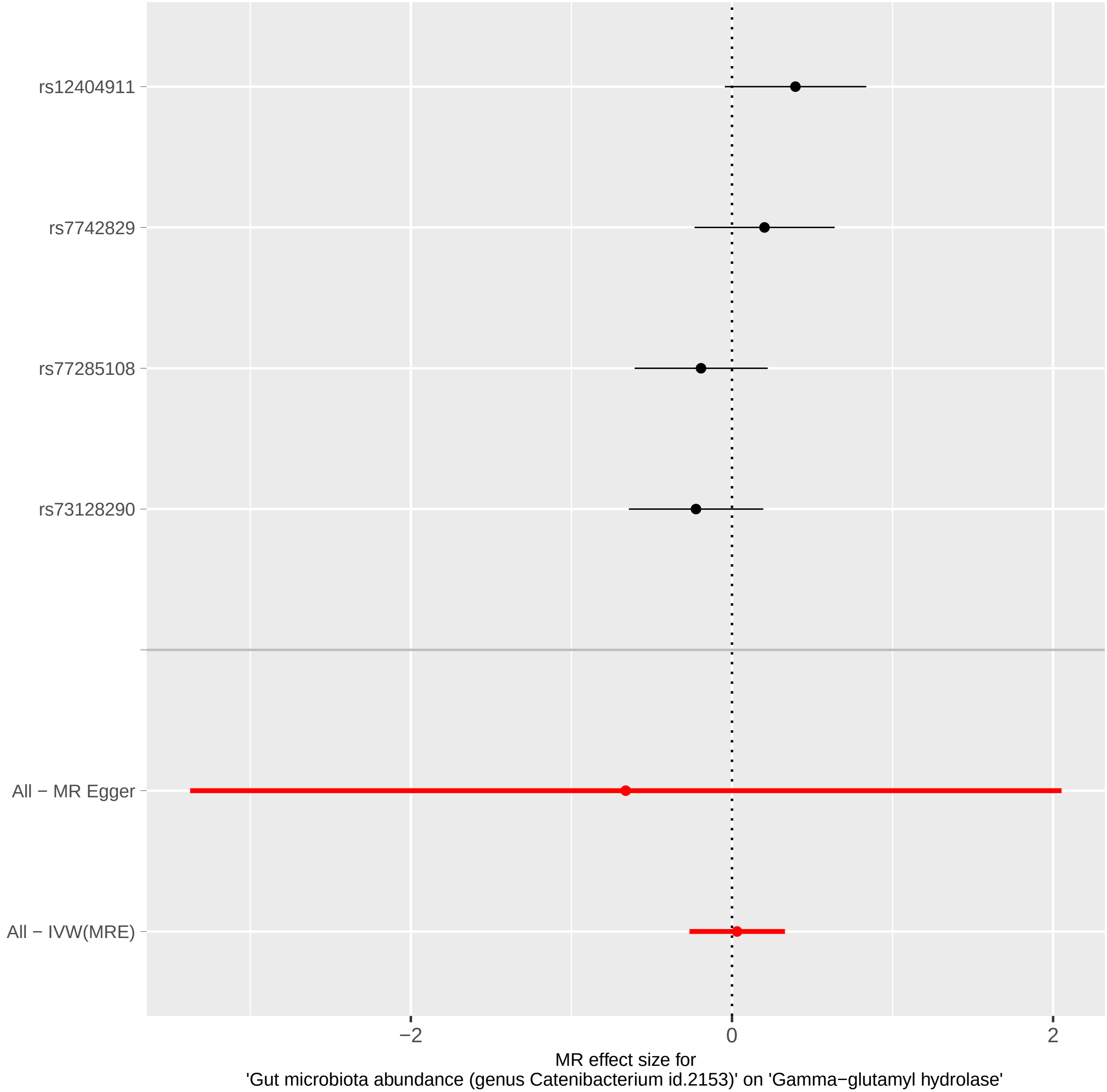

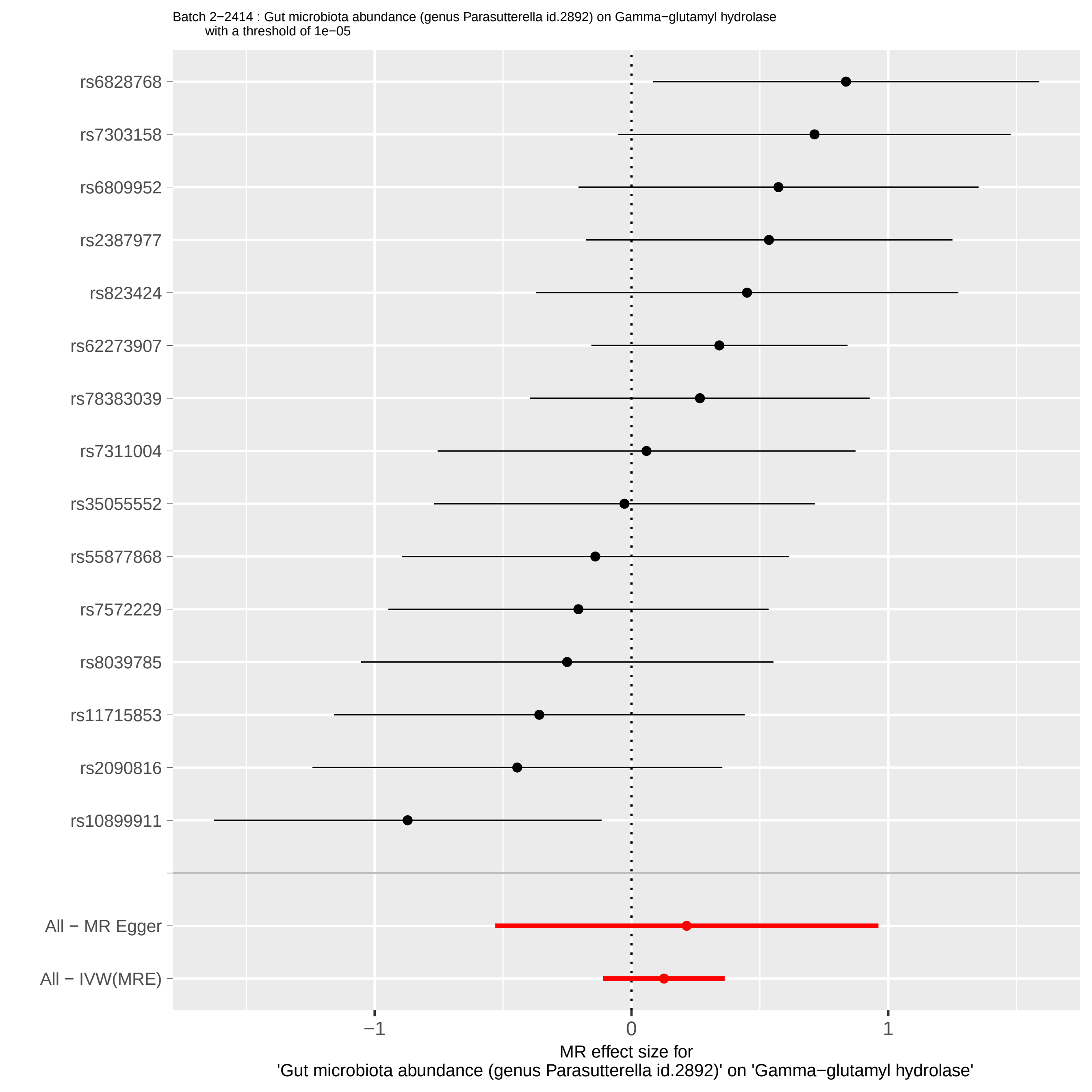
Batch 2−2414 : Gut microbiota abundance (genus Parasutterella id.2892) on Gamma−glutamyl hydrolase
with a threshold of 1e−05
rs6828768
rs7303158
rs6809952
rs2387977
rs823424
rs62273907
rs78383039
rs7311004
rs35055552
rs55877868
rs7572229
rs8039785
rs11715853
rs2090816
rs10899911
All − MR Egger
All − IVW(MRE)
−1
0
1
MR effect size for
'Gut microbiota abundance (genus Parasutterella id.2892)' on 'Gamma−glutamyl hydrolase'

Batch 2−2415 : Gut microbiota abundance (genus Catenibacterium id.2153) on Inactive gamma−glutamyltranspeptidase 2
with a threshold of 1e−05

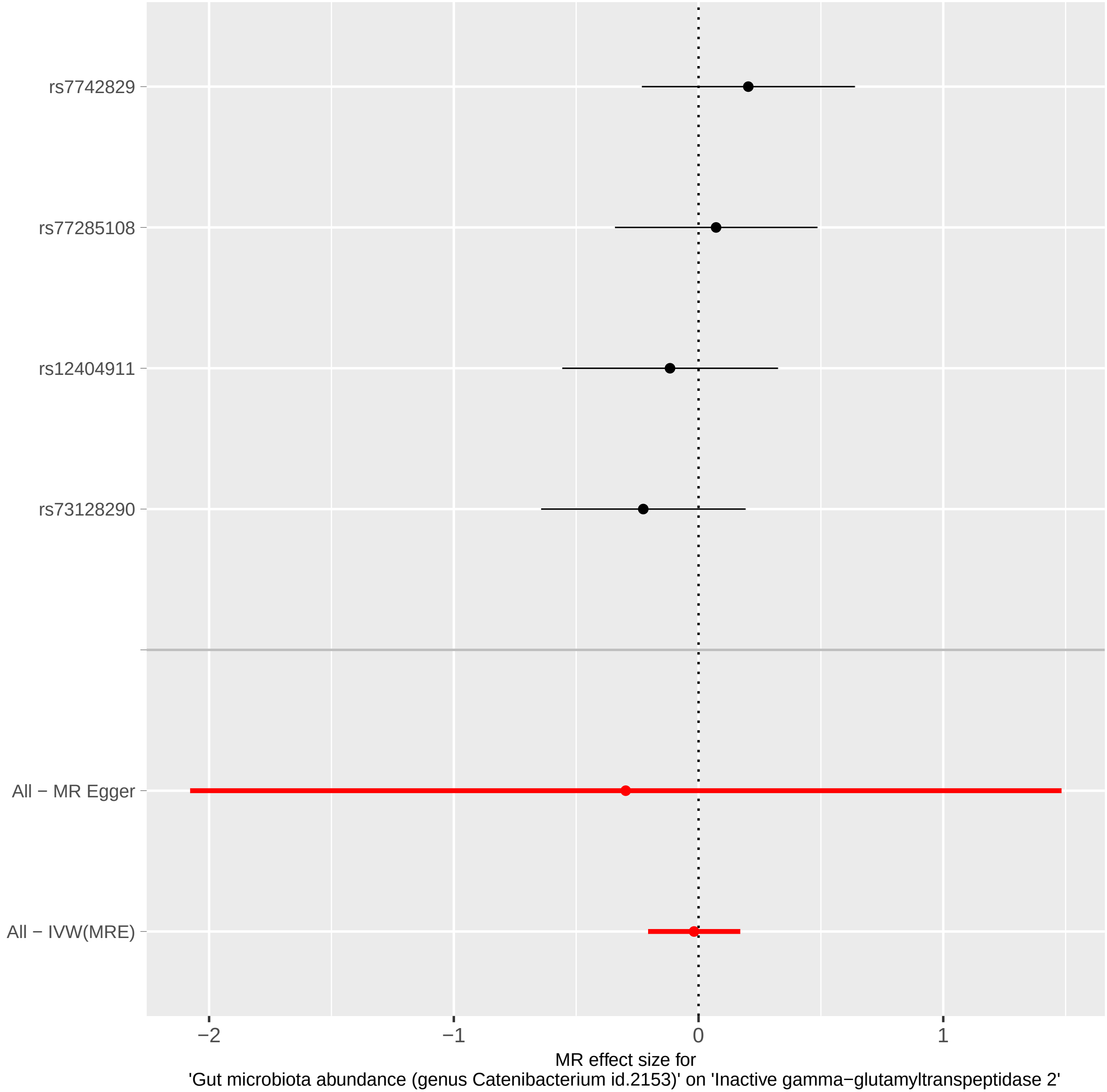

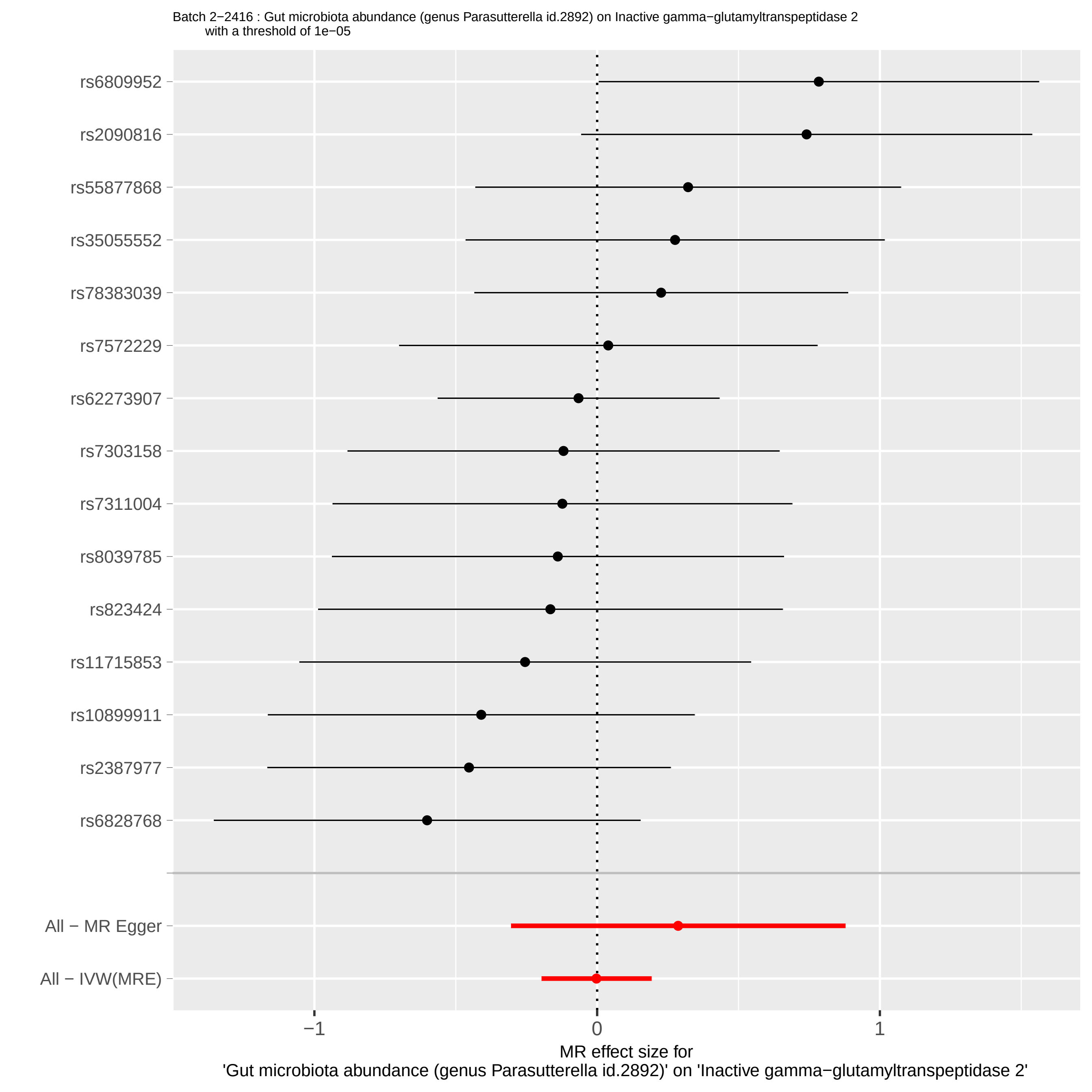
Batch 2−2416 : Gut microbiota abundance (genus Parasutterella id.2892) on Inactive gamma−glutamyltranspeptidase 2
with a threshold of 1e−05
rs6809952
rs2090816
rs55877868
rs35055552
rs78383039
rs7572229
rs62273907
rs7303158
rs7311004
rs8039785
rs823424
rs11715853
rs10899911
rs2387977
rs6828768
All − MR Egger
All − IVW(MRE)
−1
0
1
MR effect size for
'Gut microbiota abundance (genus Parasutterella id.2892)' on 'Inactive gamma−glutamyltranspeptidase 2'

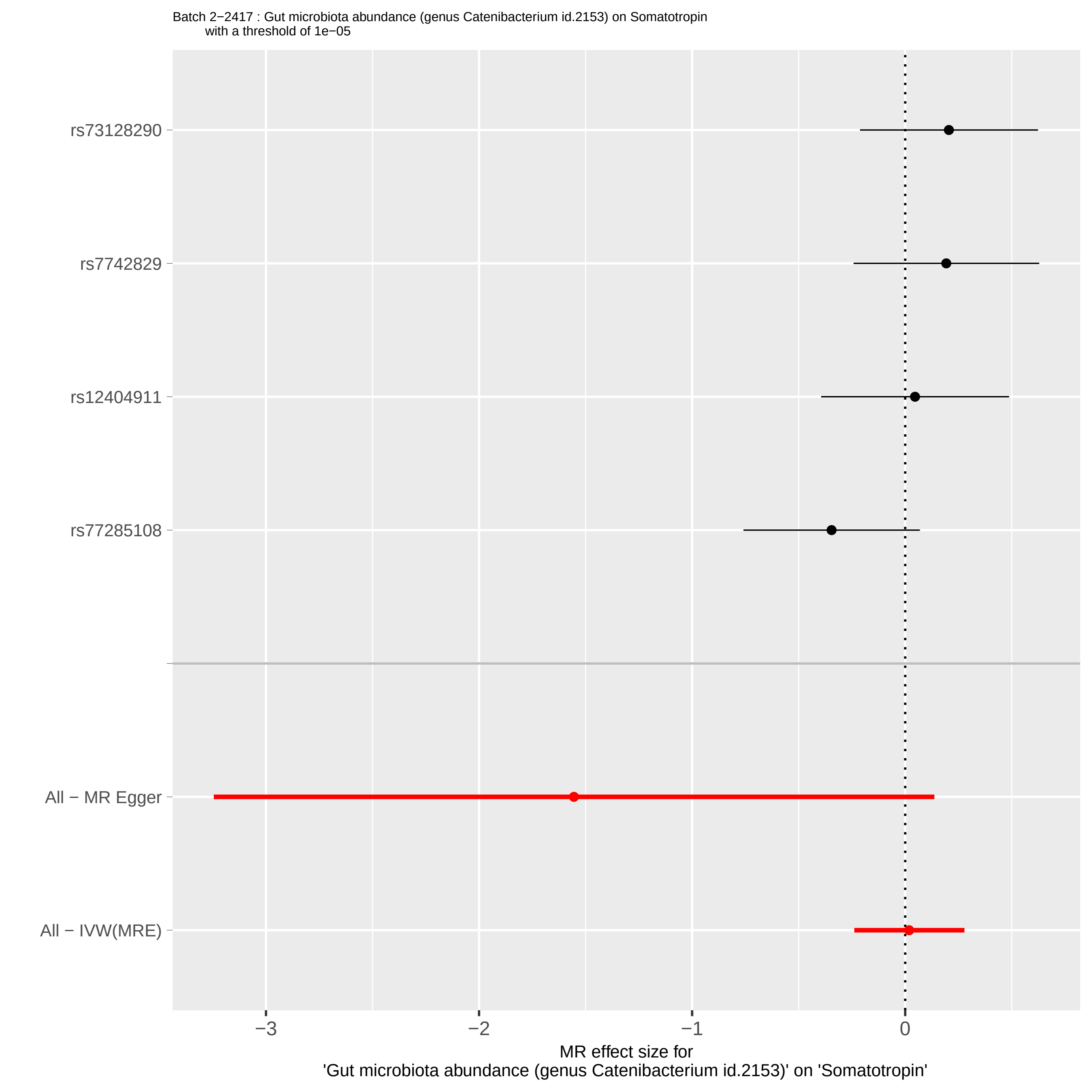
Batch 2−2417 : Gut microbiota abundance (genus Catenibacterium id.2153) on Somatotropin
with a threshold of 1e−05
rs73128290
rs7742829
rs12404911
rs77285108
All − MR Egger
All − IVW(MRE)
−3
−2
−1
0
MR effect size for
'Gut microbiota abundance (genus Catenibacterium id.2153)' on 'Somatotropin'

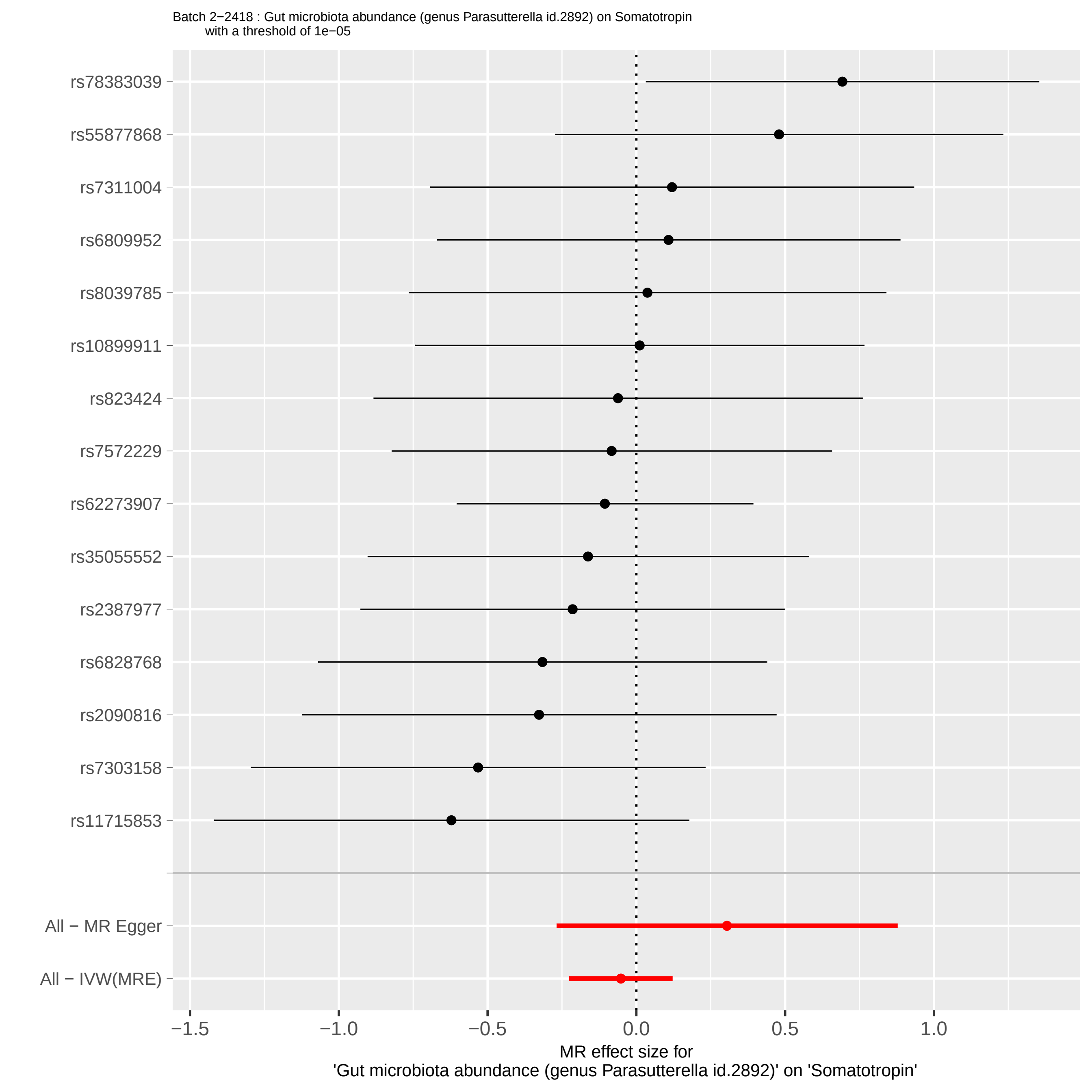
Batch 2−2418 : Gut microbiota abundance (genus Parasutterella id.2892) on Somatotropin
with a threshold of 1e−05
rs78383039
rs55877868
rs7311004
rs6809952
rs8039785
rs10899911
rs823424
rs7572229
rs62273907
rs35055552
rs2387977
rs6828768
rs2090816
rs7303158
rs11715853
All − MR Egger
All − IVW(MRE)
−1.5
−1.0
−0.5
0.0
0.5
1.0
MR effect size for
'Gut microbiota abundance (genus Parasutterella id.2892)' on 'Somatotropin'

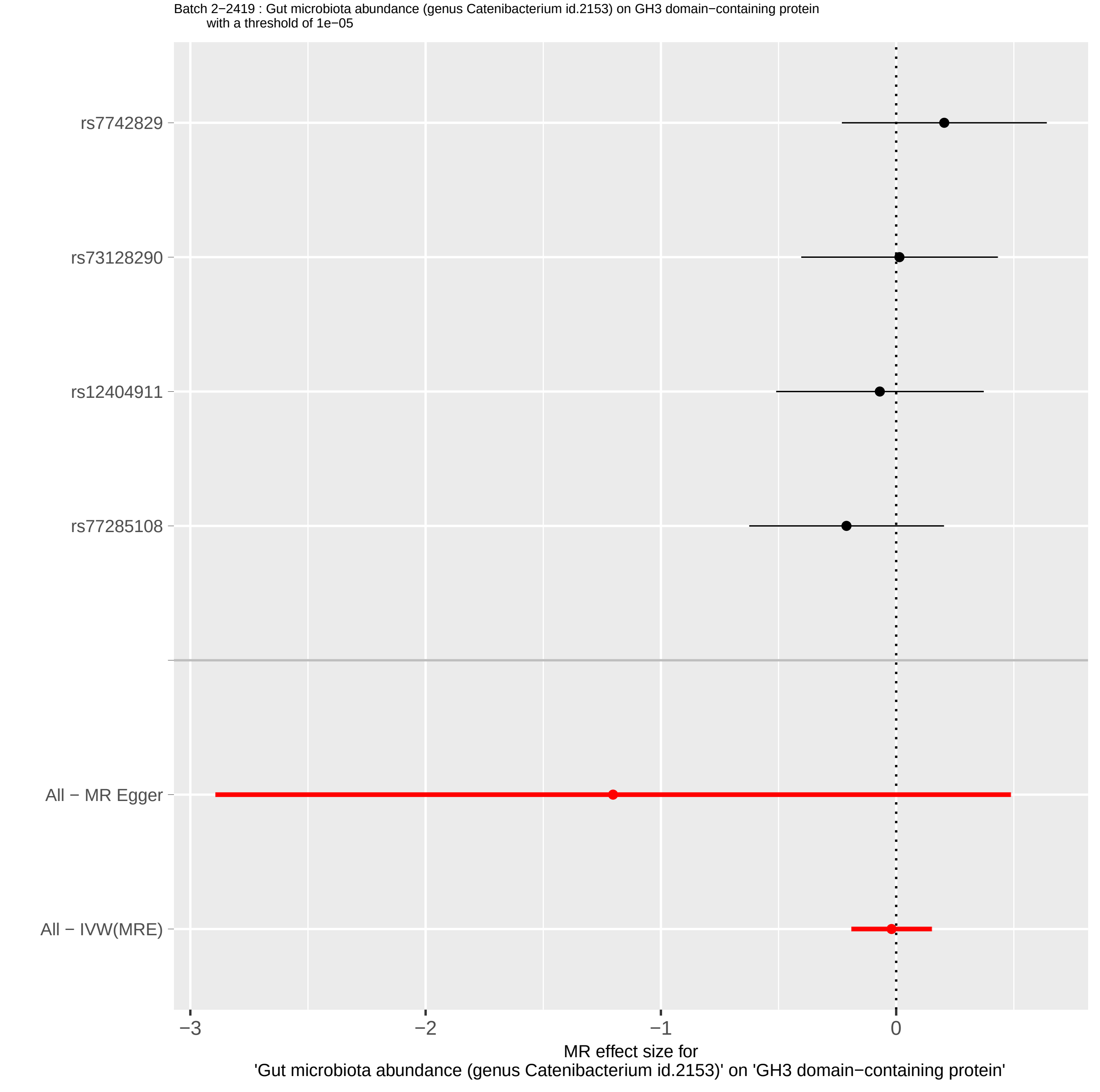

Batch 2−2419 : Gut microbiota abundance (genus Catenibacterium id.2153) on GH3 domain−containing protein
with a threshold of 1e−05
rs7742829
rs73128290
rs12404911
rs77285108
All − MR Egger
All − IVW(MRE)
−3
−2
−1
0
MR effect size for
'Gut microbiota abundance (genus Catenibacterium id.2153)' on 'GH3 domain−containing protein'

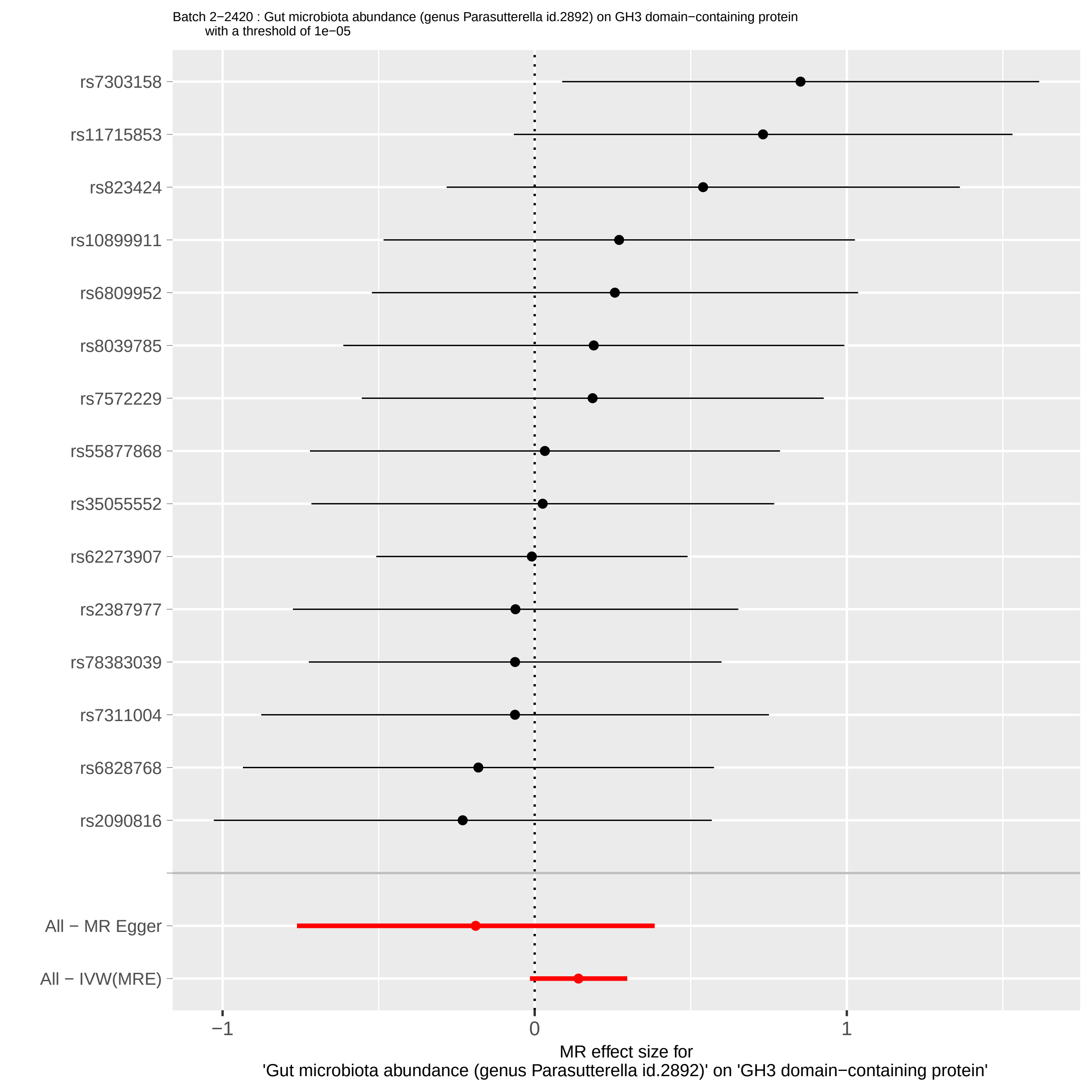

Batch 2−2420 : Gut microbiota abundance (genus Parasutterella id.2892) on GH3 domain−containing protein
with a threshold of 1e−05
rs7303158
rs11715853
rs823424
rs10899911
rs6809952
rs8039785
rs7572229
rs55877868
rs35055552
rs62273907
rs2387977
rs78383039
rs7311004
rs6828768
rs2090816
All − MR Egger
All − IVW(MRE)
−1
0
1
MR effect size for
'Gut microbiota abundance (genus Parasutterella id.2892)' on 'GH3 domain−containing protein'

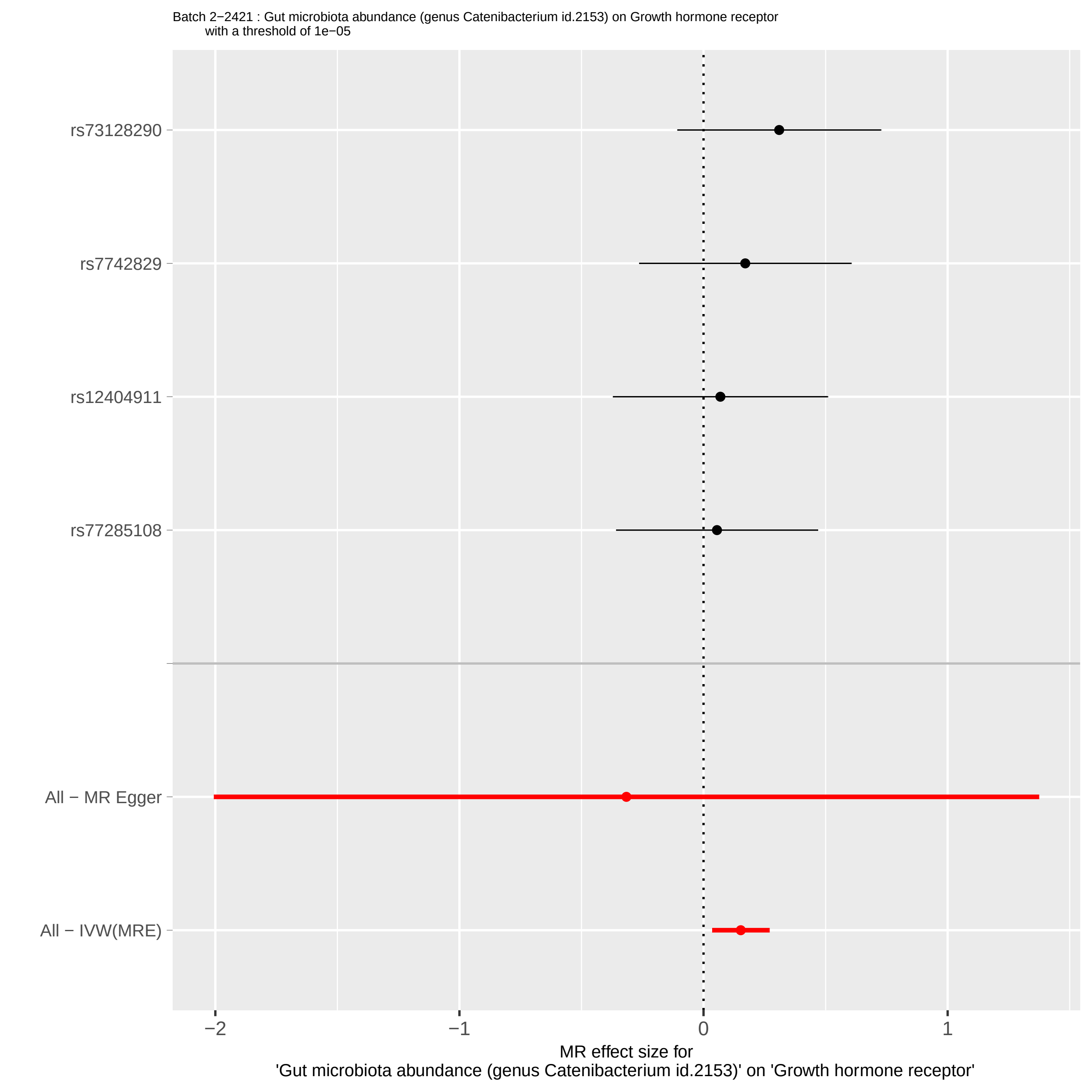

Batch 2−2421 : Gut microbiota abundance (genus Catenibacterium id.2153) on Growth hormone receptor
with a threshold of 1e−05
rs73128290
rs7742829
rs12404911
rs77285108
All − MR Egger
All − IVW(MRE)
−2
−1
0
1
MR effect size for
'Gut microbiota abundance (genus Catenibacterium id.2153)' on 'Growth hormone receptor'

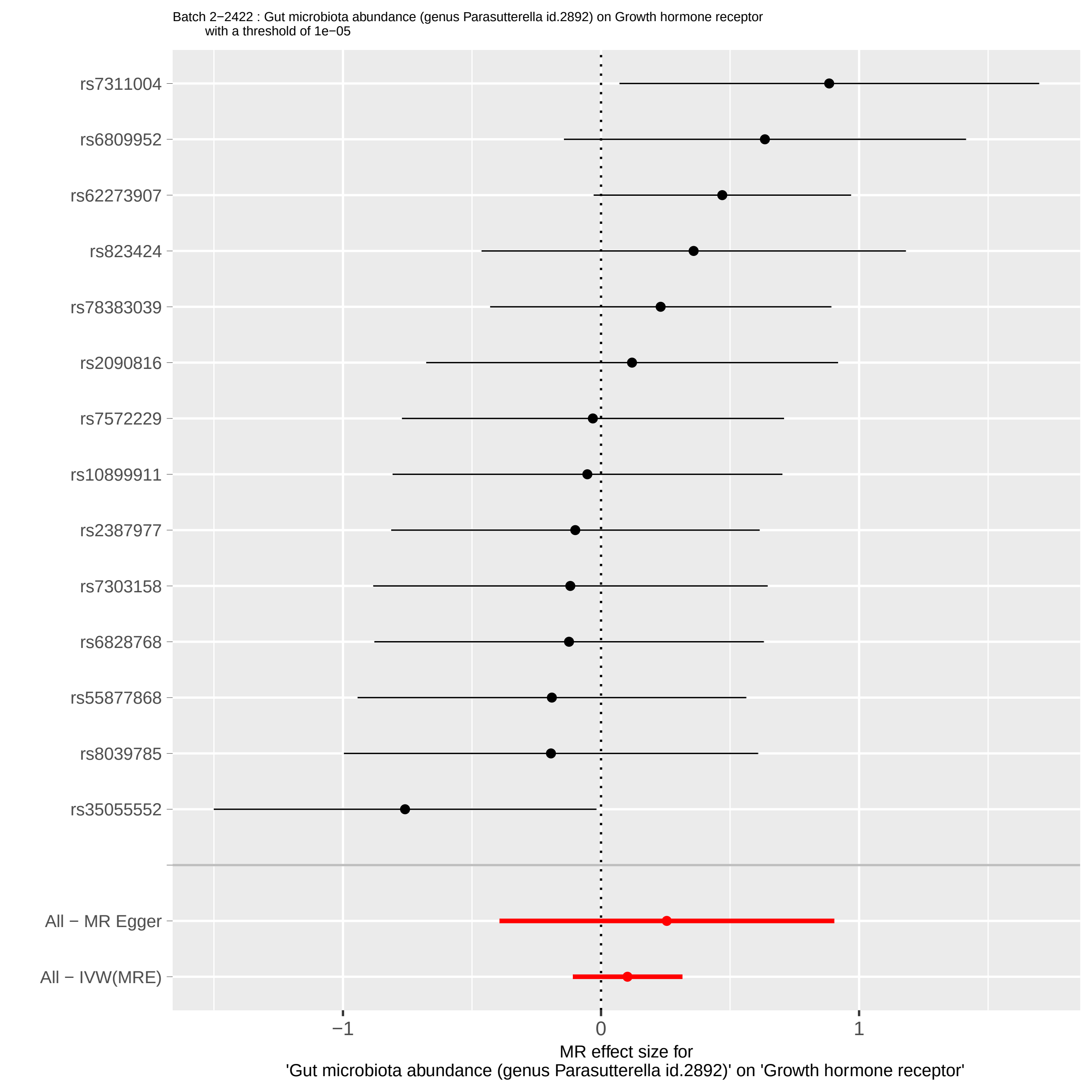

Batch 2−2422 : Gut microbiota abundance (genus Parasutterella id.2892) on Growth hormone receptor
with a threshold of 1e−05
rs7311004
rs6809952
rs62273907
rs823424
rs78383039
rs2090816
rs7572229
rs10899911
rs2387977
rs7303158
rs6828768
rs55877868
rs8039785
rs35055552
All − MR Egger
All − IVW(MRE)
−1
0
1
MR effect size for
'Gut microbiota abundance (genus Parasutterella id.2892)' on 'Growth hormone receptor'

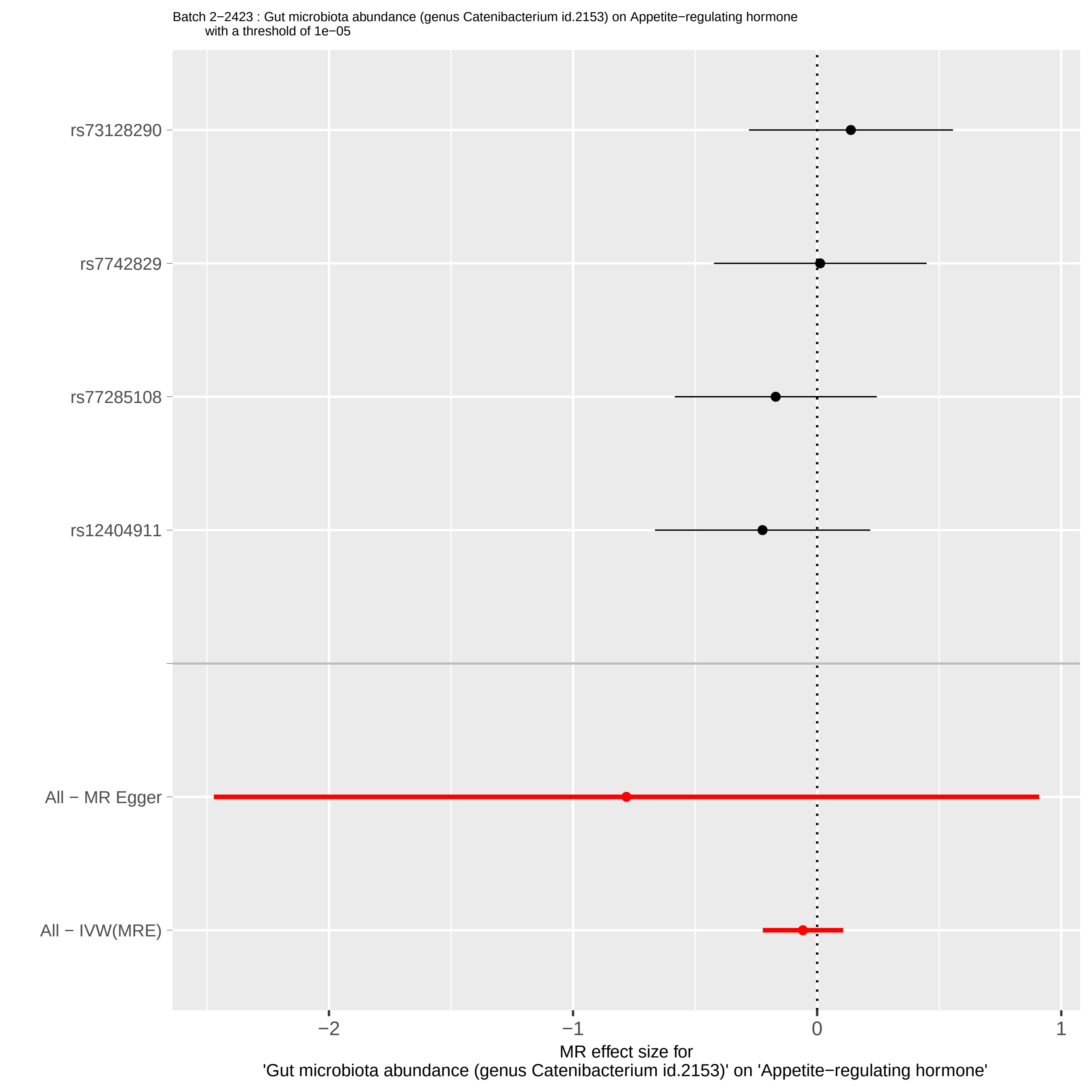
Batch 2−2423 : Gut microbiota abundance (genus Catenibacterium id.2153) on Appetite−regulating hormone
with a threshold of 1e−05
rs73128290
rs7742829
rs77285108
rs12404911
All − MR Egger
All − IVW(MRE)
−2
−1
0
1
MR effect size for
'Gut microbiota abundance (genus Catenibacterium id.2153)' on 'Appetite−regulating hormone'

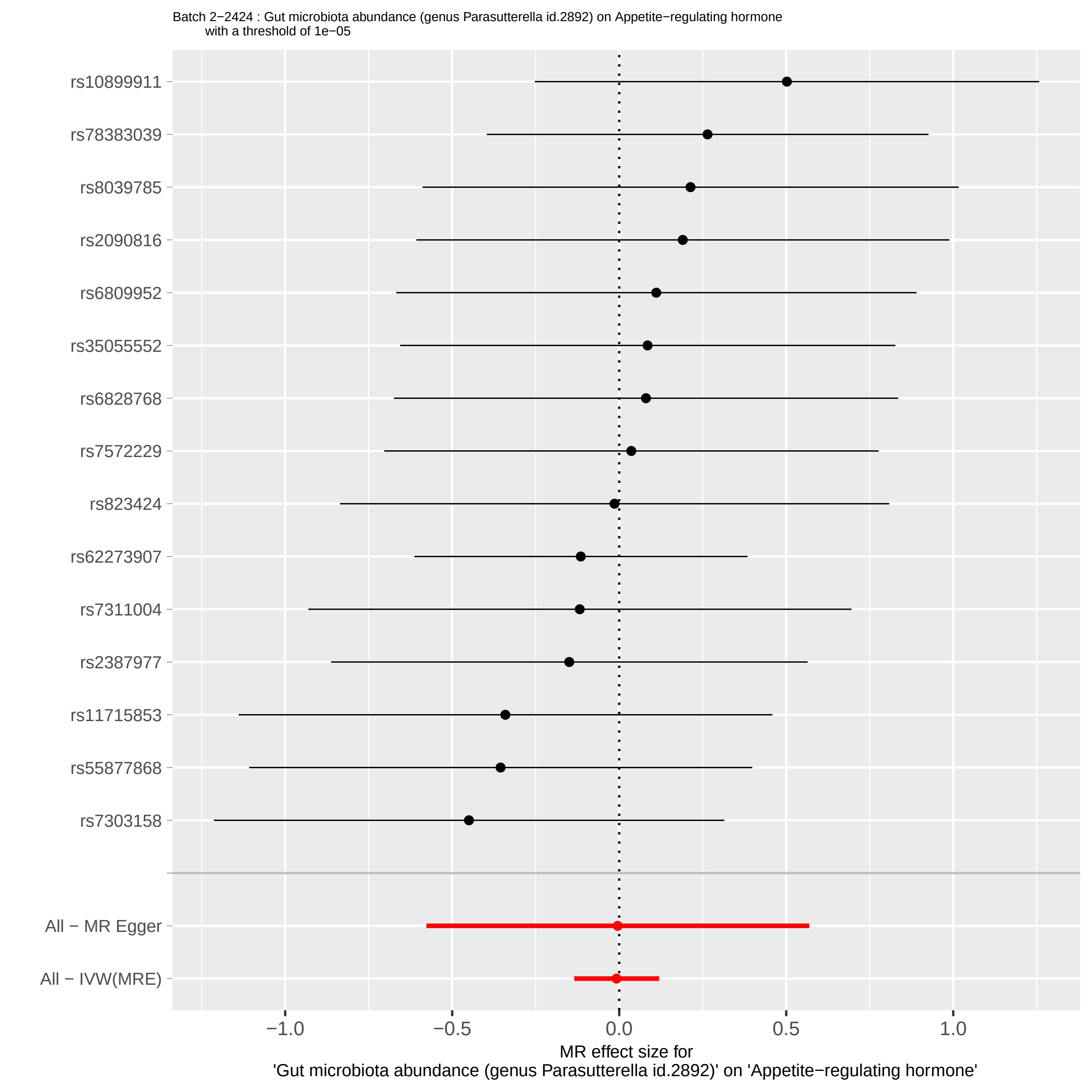

Batch 2−2424 : Gut microbiota abundance (genus Parasutterella id.2892) on Appetite−regulating hormone
with a threshold of 1e−05
rs10899911
rs78383039
rs8039785
rs2090816
rs6809952
rs35055552
rs6828768
rs7572229
rs823424
rs62273907
rs7311004
rs2387977
rs11715853
rs55877868
rs7303158
All − MR Egger
All − IVW(MRE)
−1.0
−0.5
0.0
0.5
1.0
MR effect size for
'Gut microbiota abundance (genus Parasutterella id.2892)' on 'Appetite−regulating hormone'

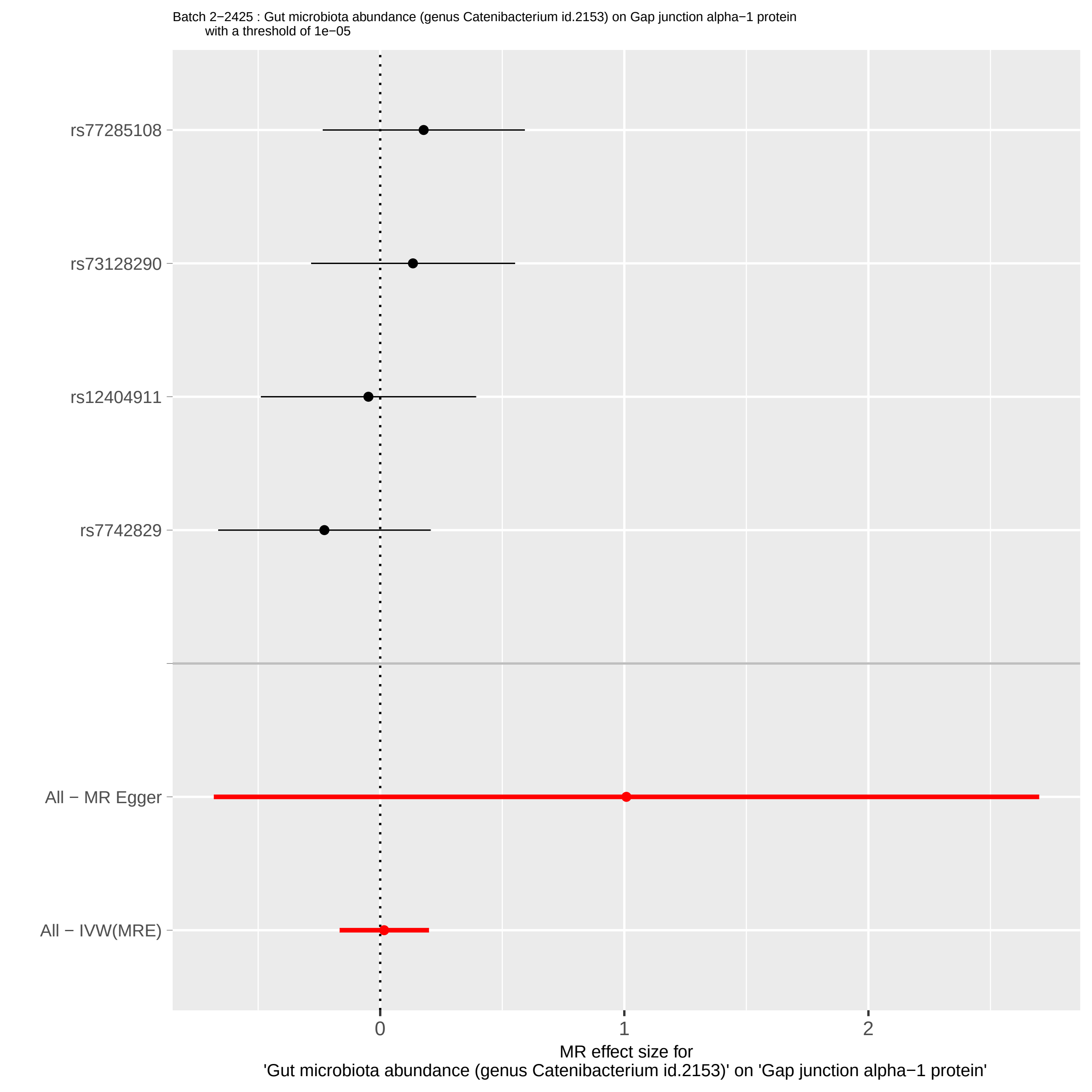

Batch 2−2425 : Gut microbiota abundance (genus Catenibacterium id.2153) on Gap junction alpha−1 protein
with a threshold of 1e−05
rs77285108
rs73128290
rs12404911
rs7742829
All − MR Egger
All − IVW(MRE)
0
1
2
MR effect size for
'Gut microbiota abundance (genus Catenibacterium id.2153)' on 'Gap junction alpha−1 protein'

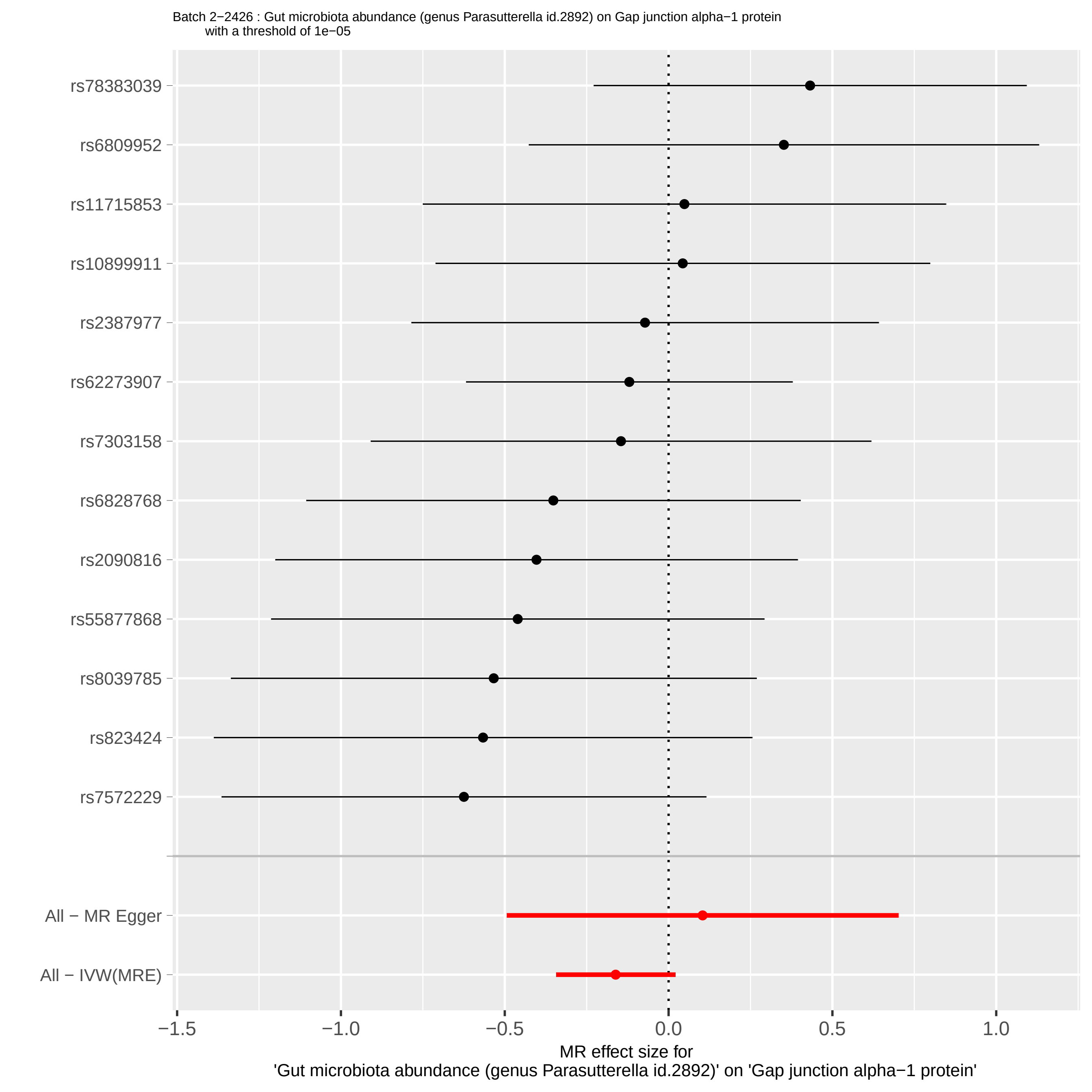
Batch 2−2426 : Gut microbiota abundance (genus Parasutterella id.2892) on Gap junction alpha−1 protein
with a threshold of 1e−05
rs78383039
rs6809952
rs11715853
rs10899911
rs2387977
rs62273907
rs7303158
rs6828768
rs2090816
rs55877868
rs8039785
rs823424
rs7572229
All − MR Egger
All − IVW(MRE)
−1.5
−1.0
−0.5
0.0
0.5
1.0
MR effect size for
'Gut microbiota abundance (genus Parasutterella id.2892)' on 'Gap junction alpha−1 protein'

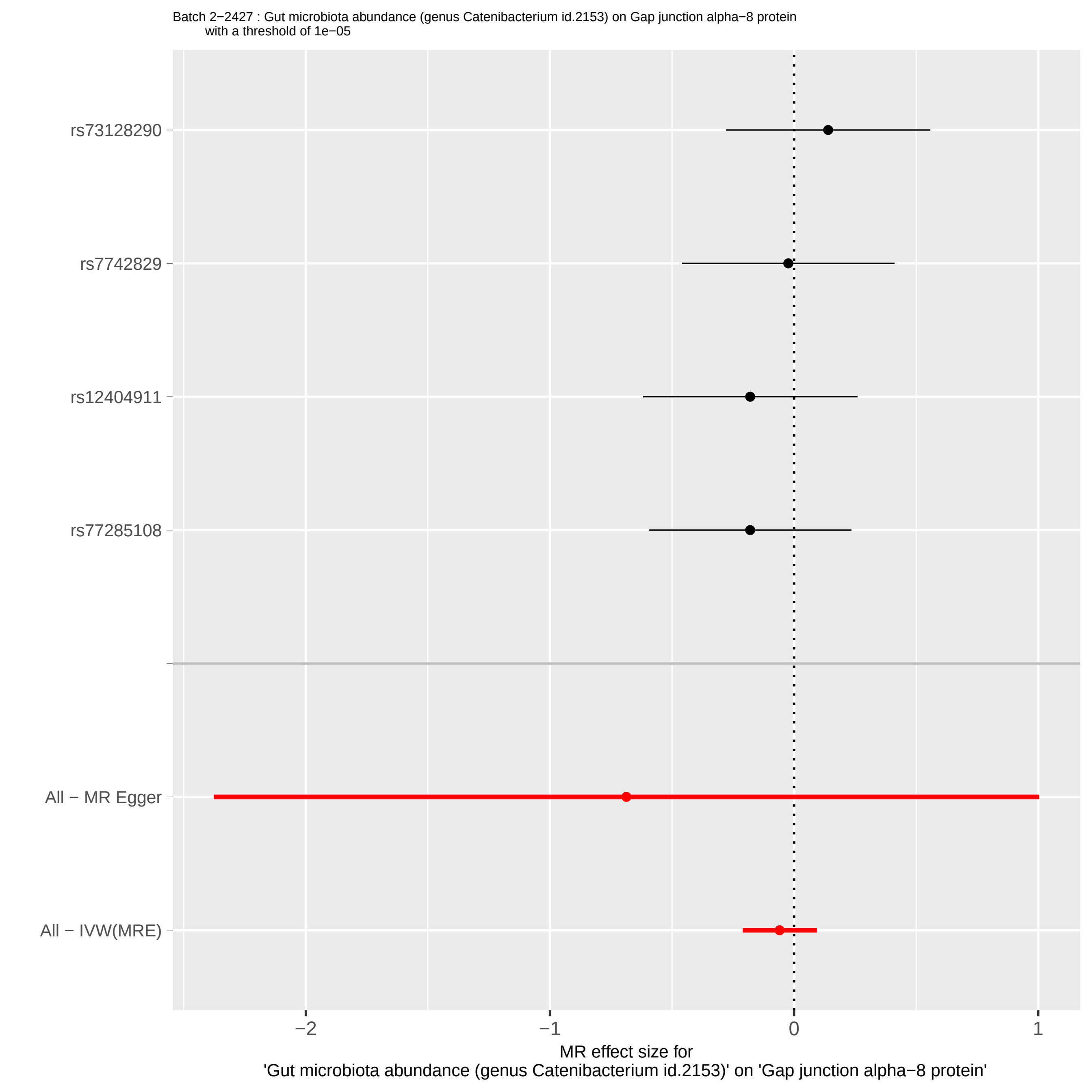

Batch 2−2427 : Gut microbiota abundance (genus Catenibacterium id.2153) on Gap junction alpha−8 protein
with a threshold of 1e−05
rs73128290
rs7742829
rs12404911
rs77285108
All − MR Egger
All − IVW(MRE)
−2
−1
0
1
MR effect size for
'Gut microbiota abundance (genus Catenibacterium id.2153)' on 'Gap junction alpha−8 protein'

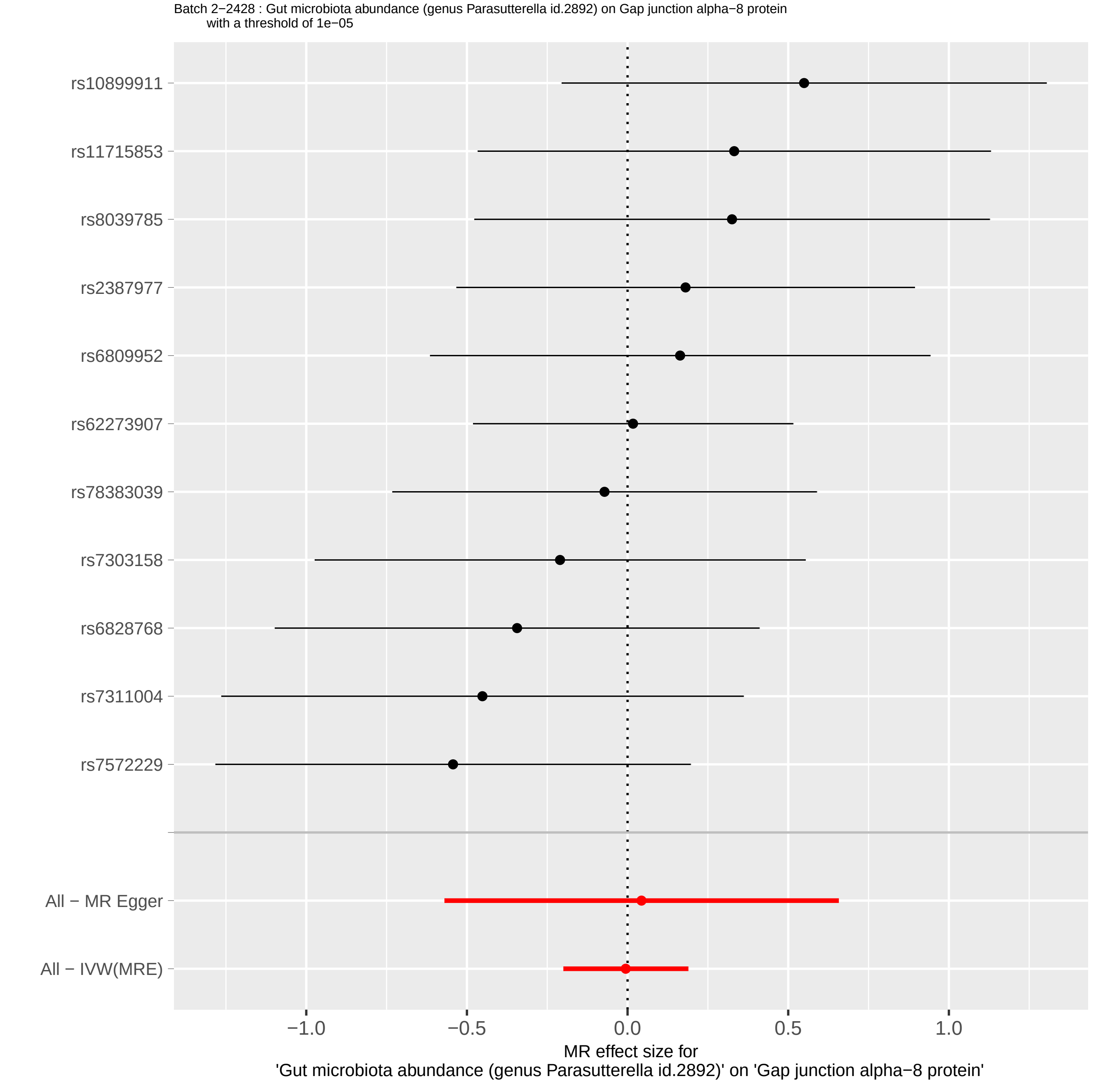

Batch 2−2428 : Gut microbiota abundance (genus Parasutterella id.2892) on Gap junction alpha−8 protein
with a threshold of 1e−05
rs10899911
rs11715853
rs8039785
rs2387977
rs6809952
rs62273907
rs78383039
rs7303158
rs6828768
rs7311004
rs7572229
All − MR Egger
All − IVW(MRE)
−1.0
−0.5
0.0
0.5
1.0
MR effect size for
'Gut microbiota abundance (genus Parasutterella id.2892)' on 'Gap junction alpha−8 protein'

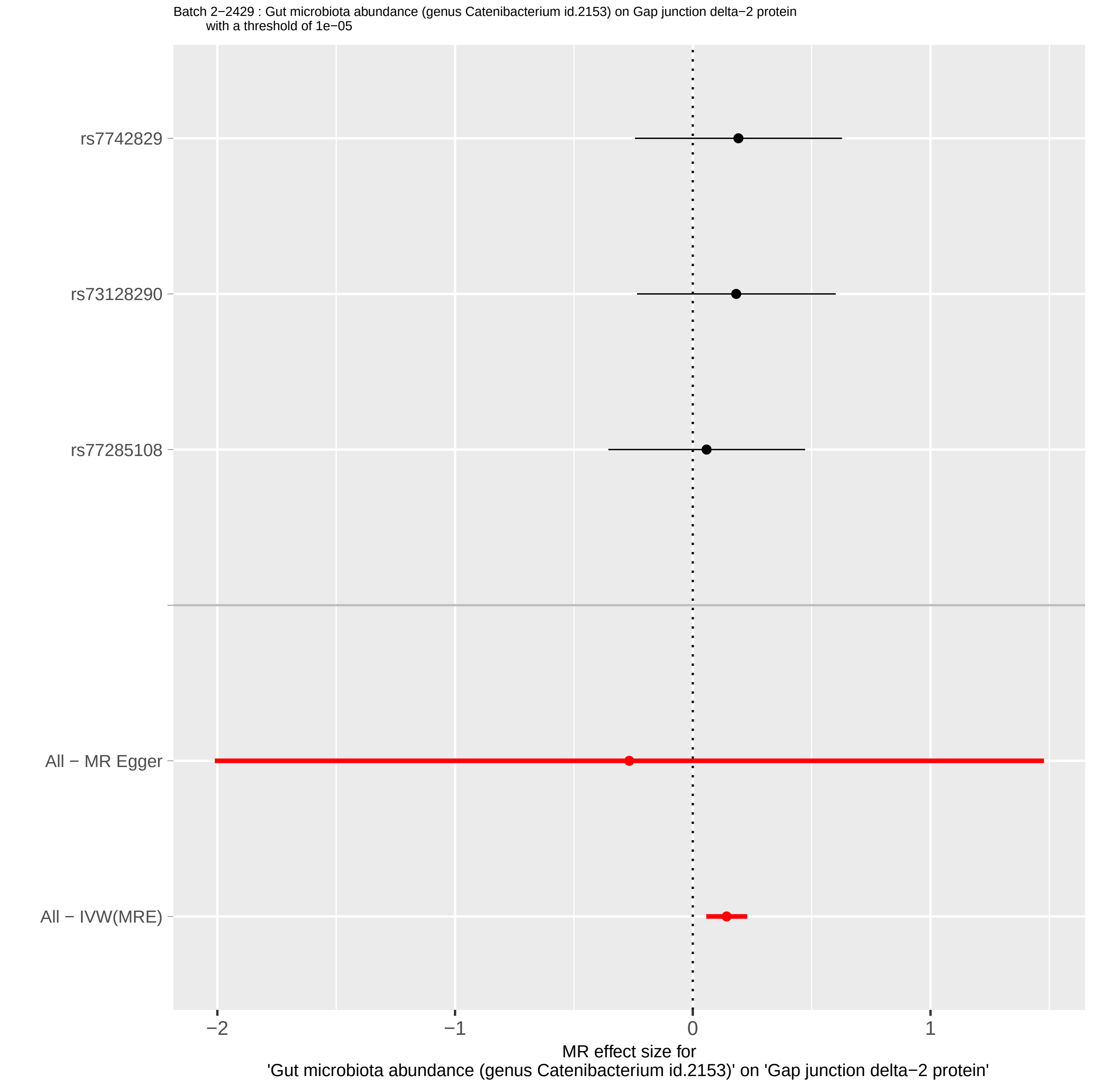

Batch 2−2429 : Gut microbiota abundance (genus Catenibacterium id.2153) on Gap junction delta−2 protein
with a threshold of 1e−05
rs7742829
rs73128290
rs77285108
All − MR Egger
All − IVW(MRE)
−2
−1
0
1
MR effect size for
'Gut microbiota abundance (genus Catenibacterium id.2153)' on 'Gap junction delta−2 protein'

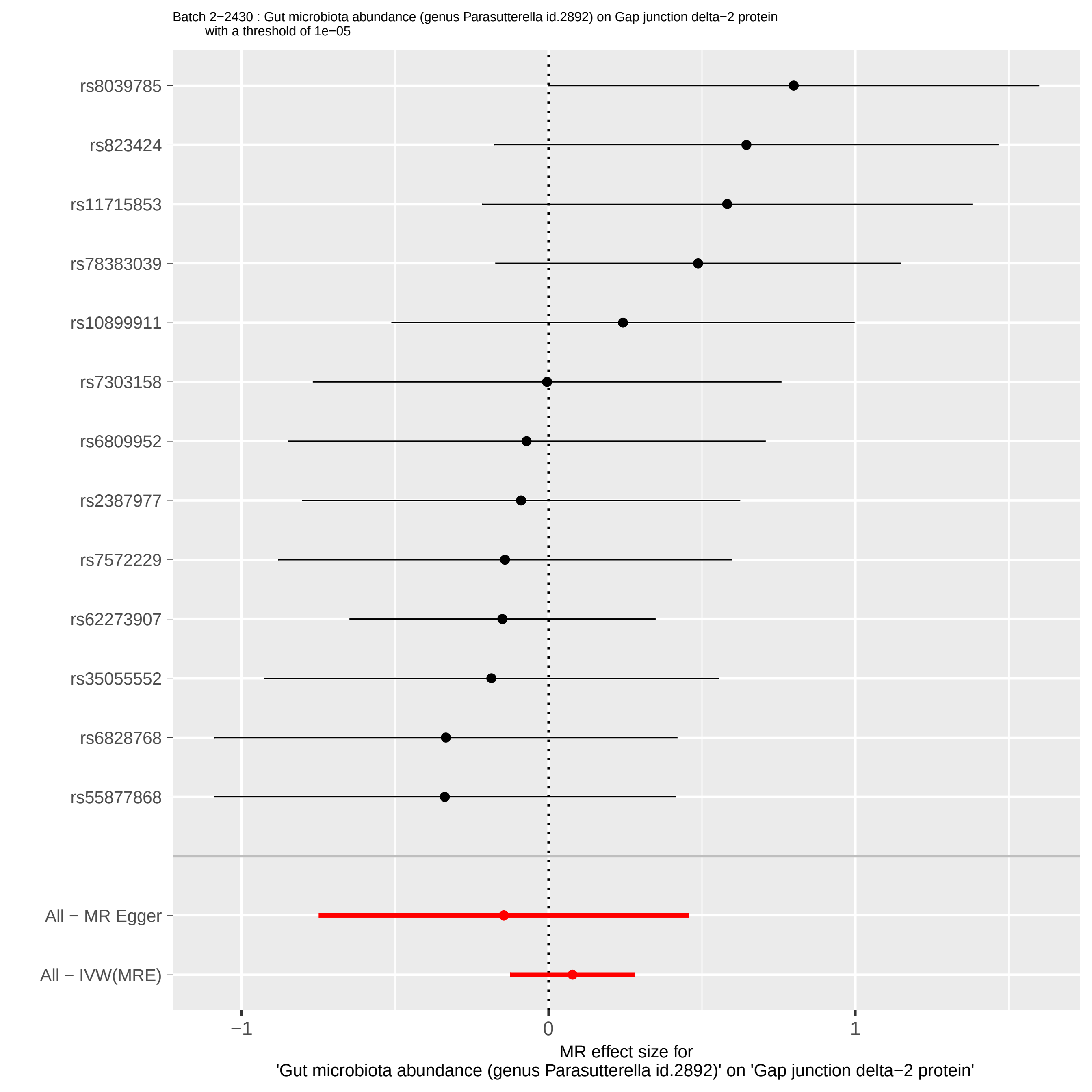

Batch 2−2430 : Gut microbiota abundance (genus Parasutterella id.2892) on Gap junction delta−2 protein
with a threshold of 1e−05
rs8039785
rs823424
rs11715853
rs78383039
rs10899911
rs7303158
rs6809952
rs2387977
rs7572229
rs62273907
rs35055552
rs6828768
rs55877868
All − MR Egger
All − IVW(MRE)
−1
0
1
MR effect size for
'Gut microbiota abundance (genus Parasutterella id.2892)' on 'Gap junction delta−2 protein'

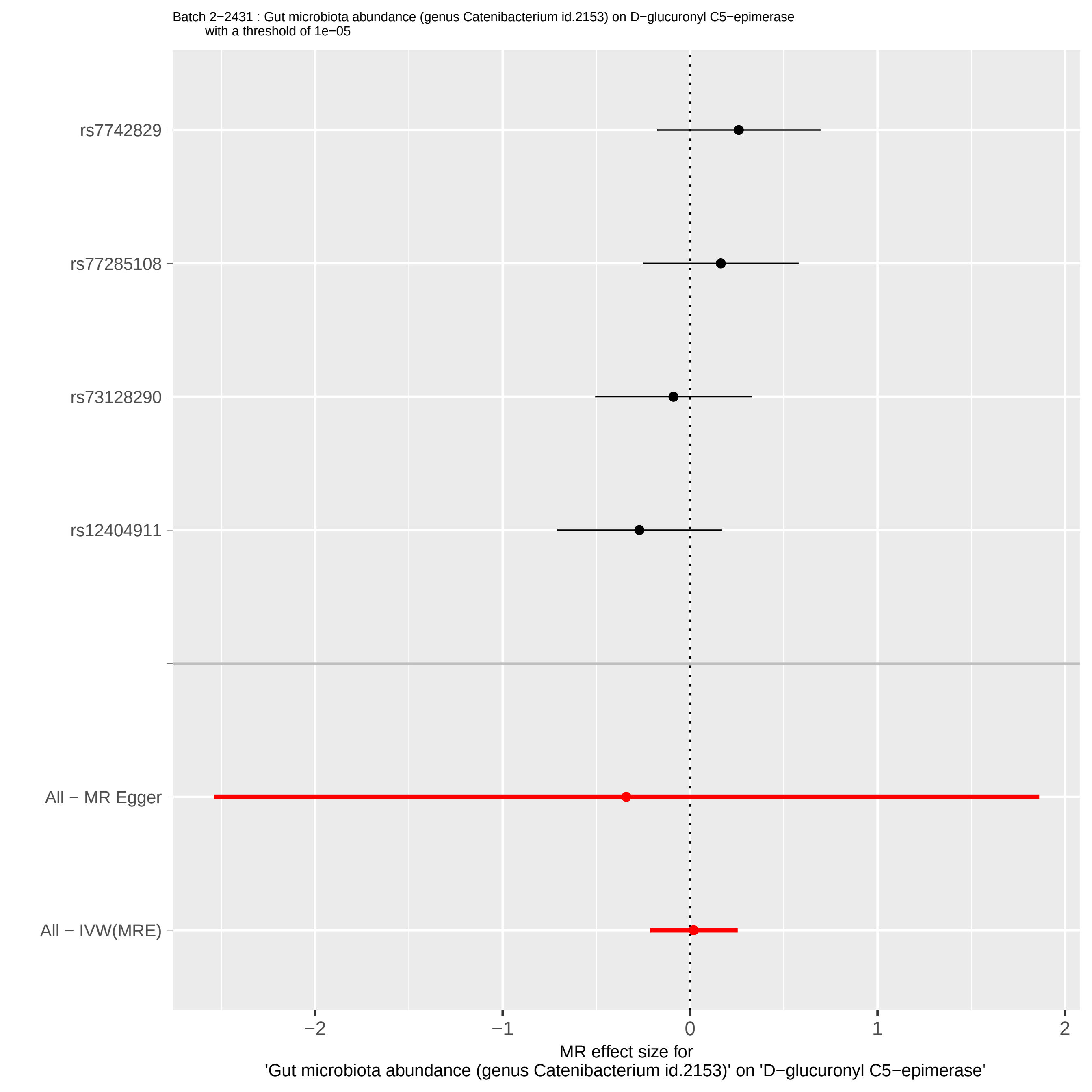

Batch 2−2431 : Gut microbiota abundance (genus Catenibacterium id.2153) on D−glucuronyl C5−epimerase
with a threshold of 1e−05
rs7742829
rs77285108
rs73128290
rs12404911
All − MR Egger
All − IVW(MRE)
−2
−1
0
1
2
MR effect size for
'Gut microbiota abundance (genus Catenibacterium id.2153)' on 'D−glucuronyl C5−epimerase'

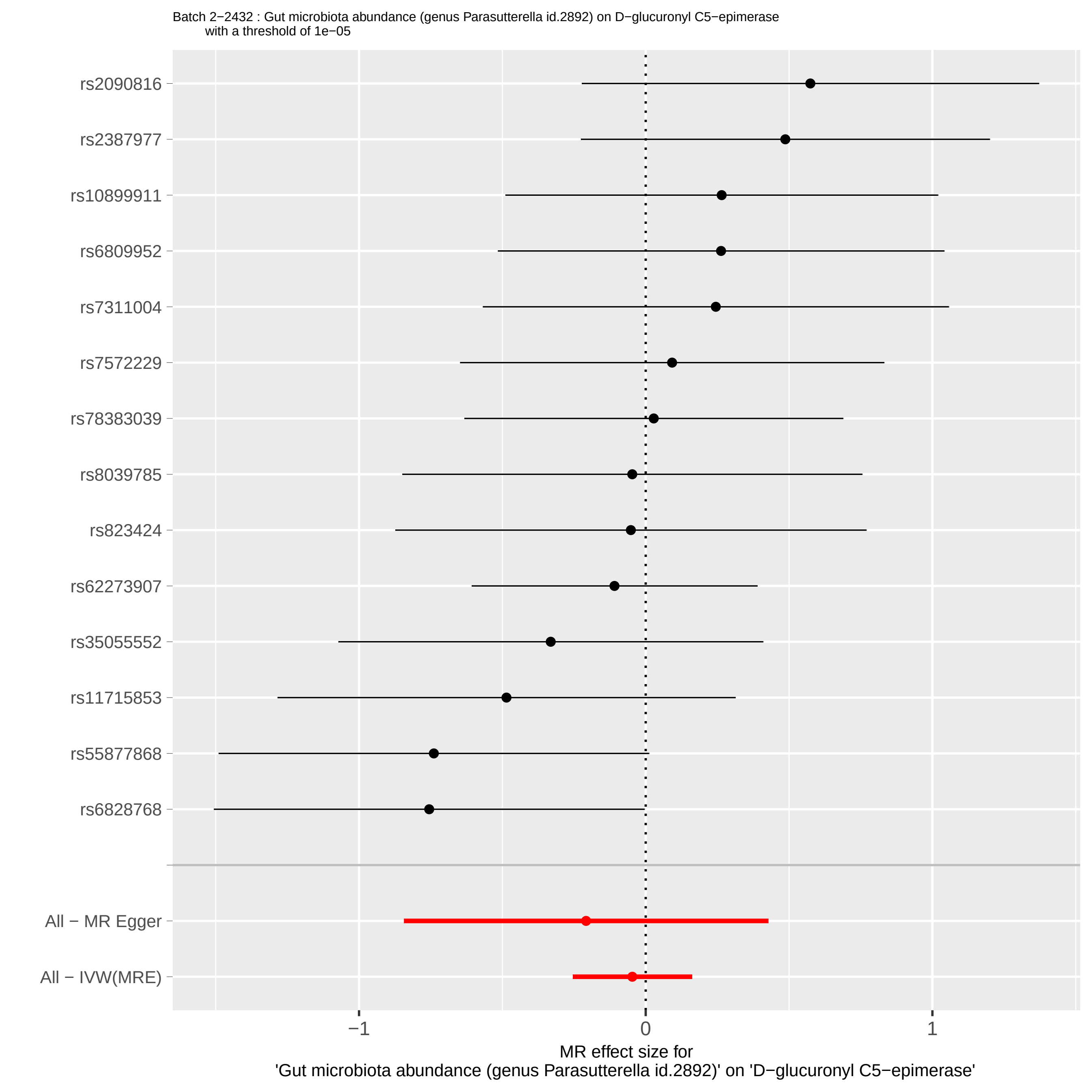

Batch 2−2432 : Gut microbiota abundance (genus Parasutterella id.2892) on D−glucuronyl C5−epimerase
with a threshold of 1e−05
rs2090816
rs2387977
rs10899911
rs6809952
rs7311004
rs7572229
rs78383039
rs8039785
rs823424
rs62273907
rs35055552
rs11715853
rs55877868
rs6828768
All − MR Egger
All − IVW(MRE)
−1
0
1
MR effect size for
'Gut microbiota abundance (genus Parasutterella id.2892)' on 'D−glucuronyl C5−epimerase'

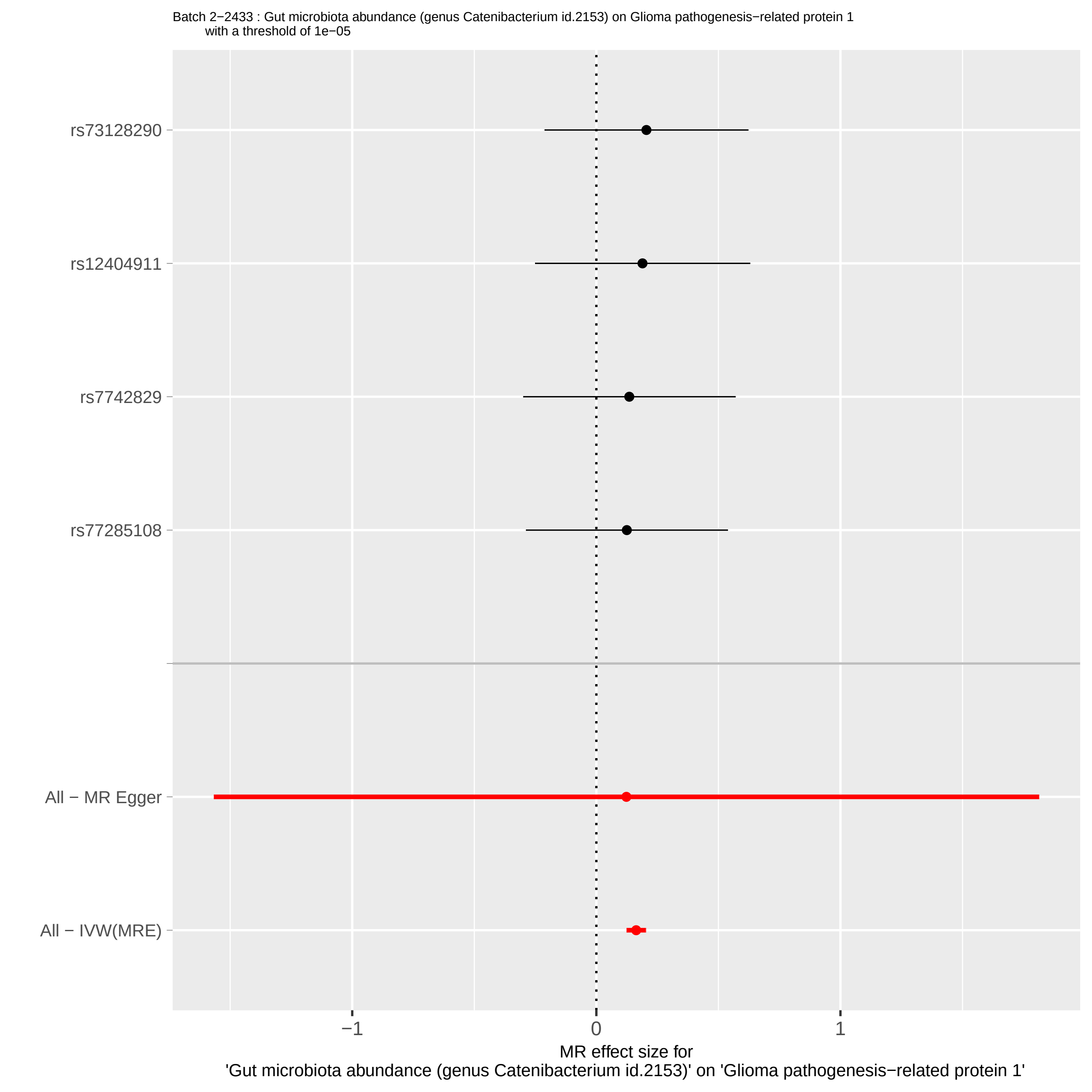
Batch 2−2433 : Gut microbiota abundance (genus Catenibacterium id.2153) on Glioma pathogenesis−related protein 1
with a threshold of 1e−05
rs73128290
rs12404911
rs7742829
rs77285108
All − MR Egger
All − IVW(MRE)
−1
0
1
MR effect size for
'Gut microbiota abundance (genus Catenibacterium id.2153)' on 'Glioma pathogenesis−related protein 1'

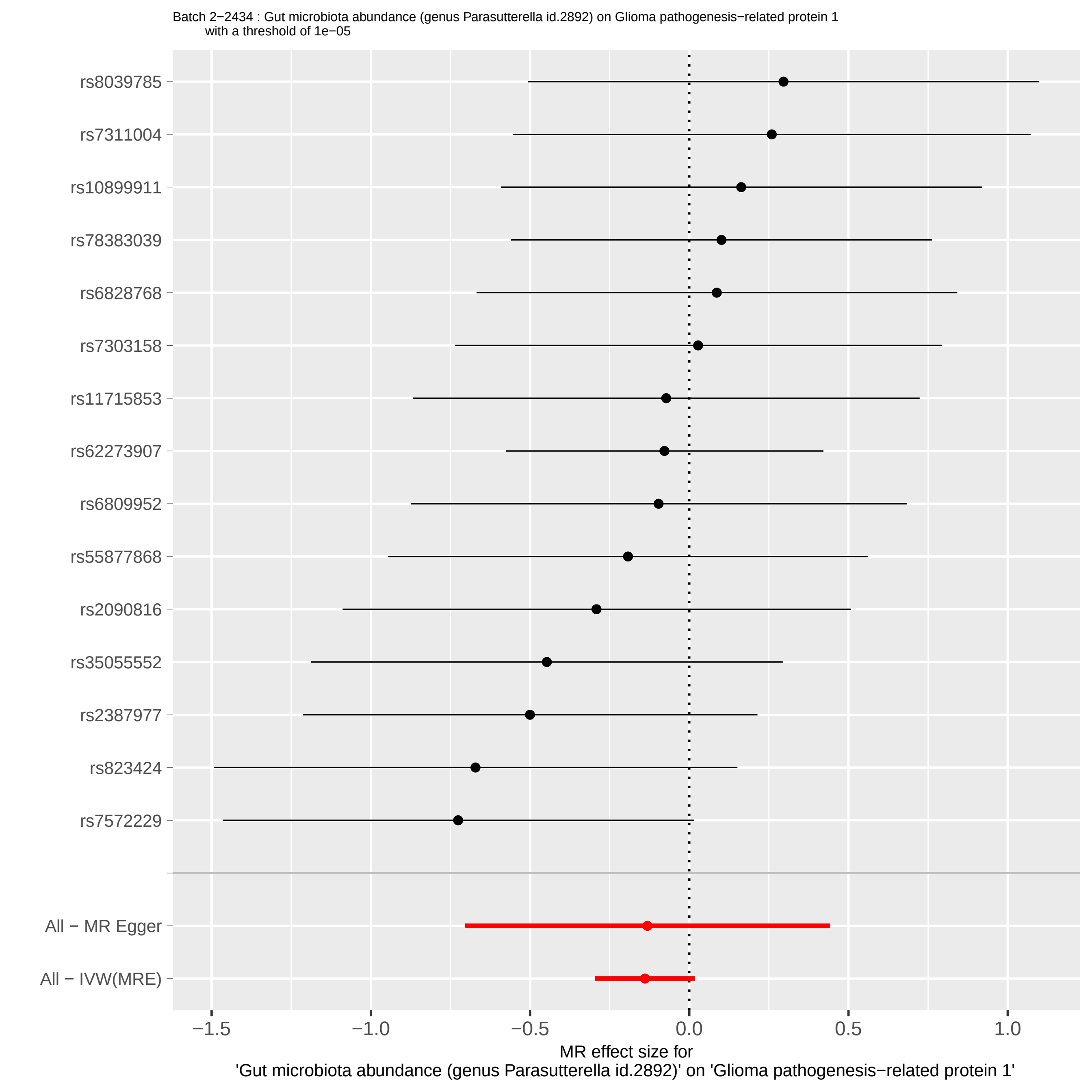

Batch 2−2434 : Gut microbiota abundance (genus Parasutterella id.2892) on Glioma pathogenesis−related protein 1
with a threshold of 1e−05
rs8039785
rs7311004
rs10899911
rs78383039
rs6828768
rs7303158
rs11715853
rs62273907
rs6809952
rs55877868
rs2090816
rs35055552
rs2387977
rs823424
rs7572229
All − MR Egger
All − IVW(MRE)
−1.5
−1.0
−0.5
0.0
0.5
1.0
MR effect size for
'Gut microbiota abundance (genus Parasutterella id.2892)' on 'Glioma pathogenesis−related protein 1'

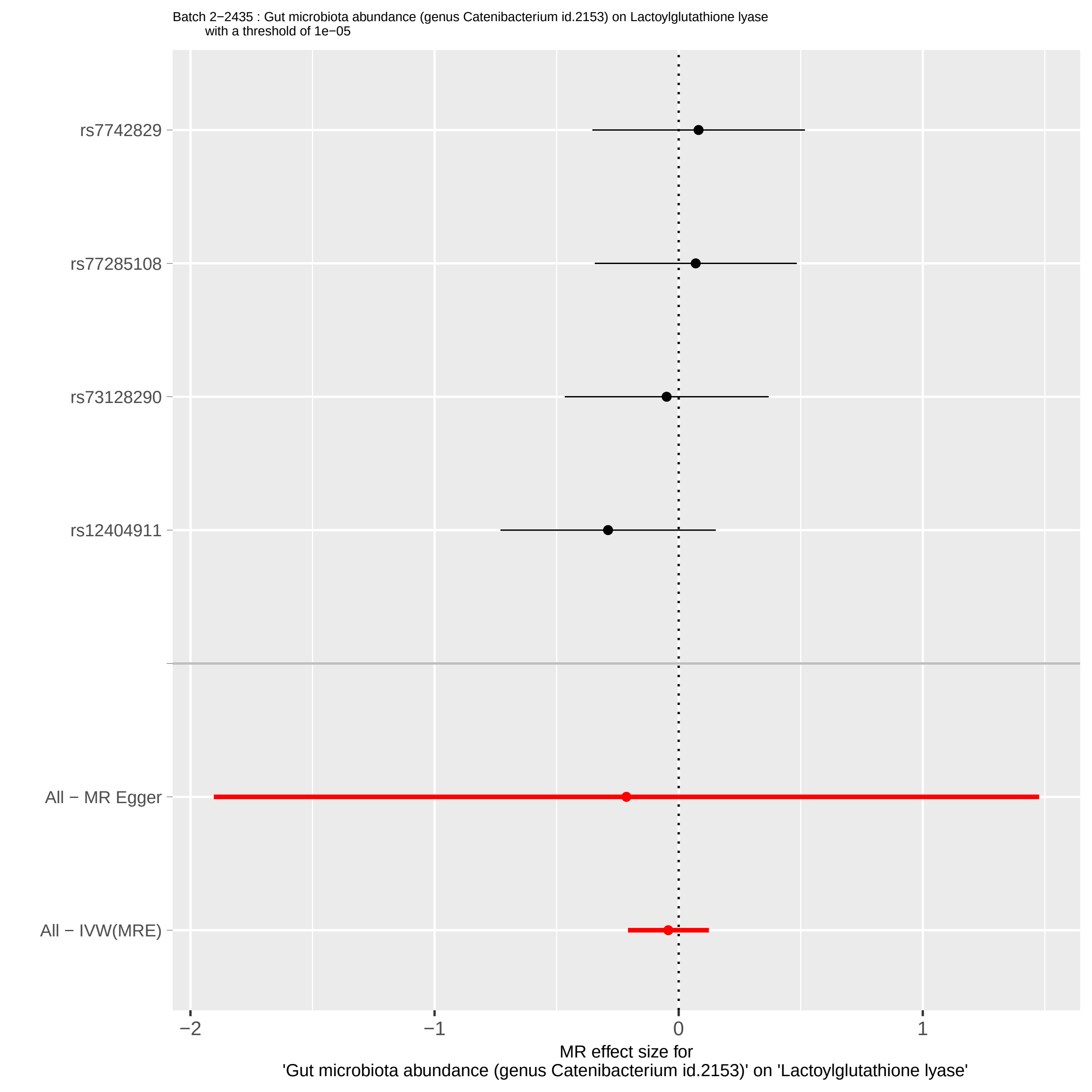

Batch 2−2435 : Gut microbiota abundance (genus Catenibacterium id.2153) on Lactoylglutathione lyase
with a threshold of 1e−05
rs7742829
rs77285108
rs73128290
rs12404911
All − MR Egger
All − IVW(MRE)
−2
−1
0
1
MR effect size for
'Gut microbiota abundance (genus Catenibacterium id.2153)' on 'Lactoylglutathione lyase'

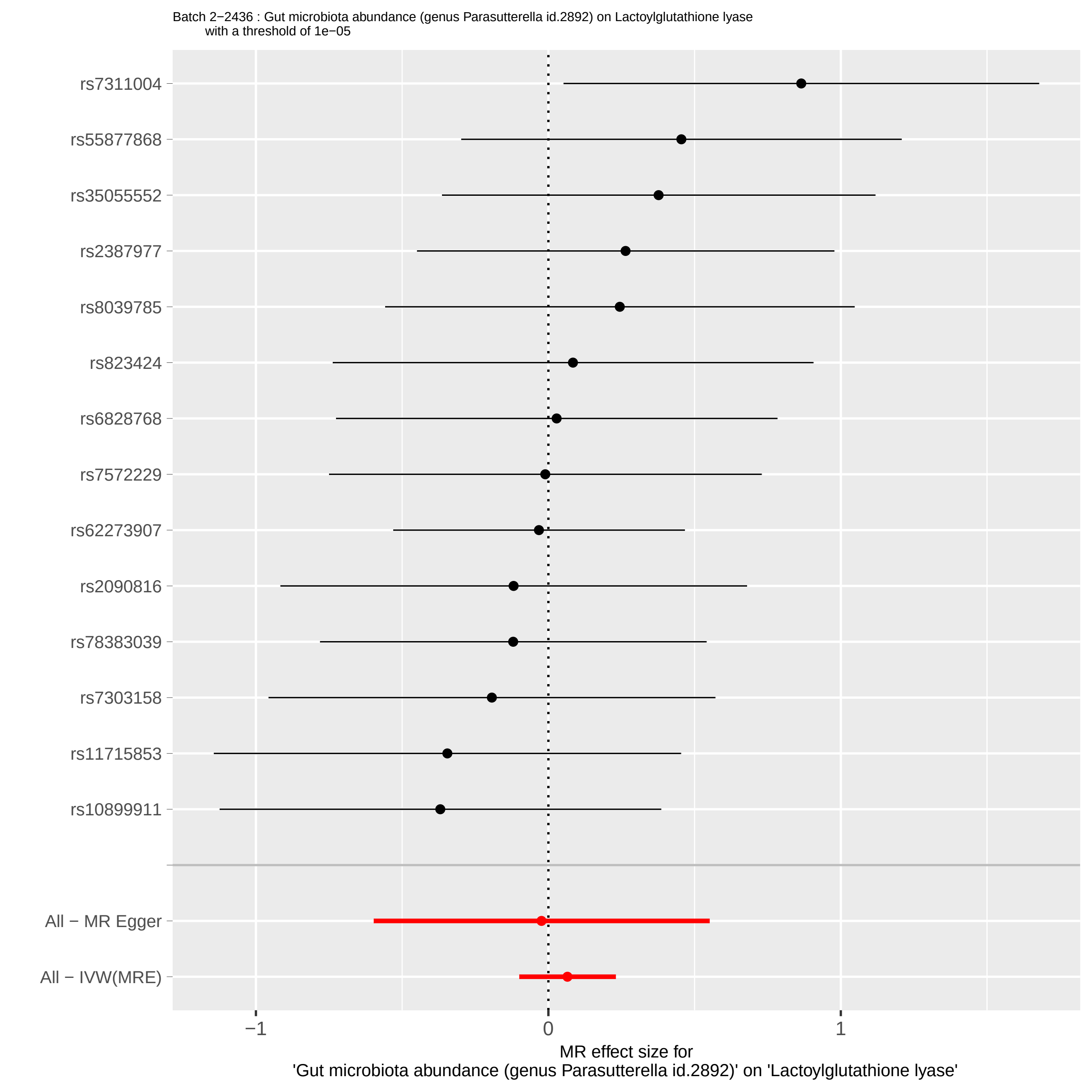
Batch 2−2436 : Gut microbiota abundance (genus Parasutterella id.2892) on Lactoylglutathione lyase
with a threshold of 1e−05
rs7311004
rs55877868
rs35055552
rs2387977
rs8039785
rs823424
rs6828768
rs7572229
rs62273907
rs2090816
rs78383039
rs7303158
rs11715853
rs10899911
All − MR Egger
All − IVW(MRE)
−1
0
1
MR effect size for
'Gut microbiota abundance (genus Parasutterella id.2892)' on 'Lactoylglutathione lyase'

Batch 2−2437 : Gut microbiota abundance (genus Catenibacterium id.2153) on Glucagon−like peptide 1 receptor
with a threshold of 1e−05

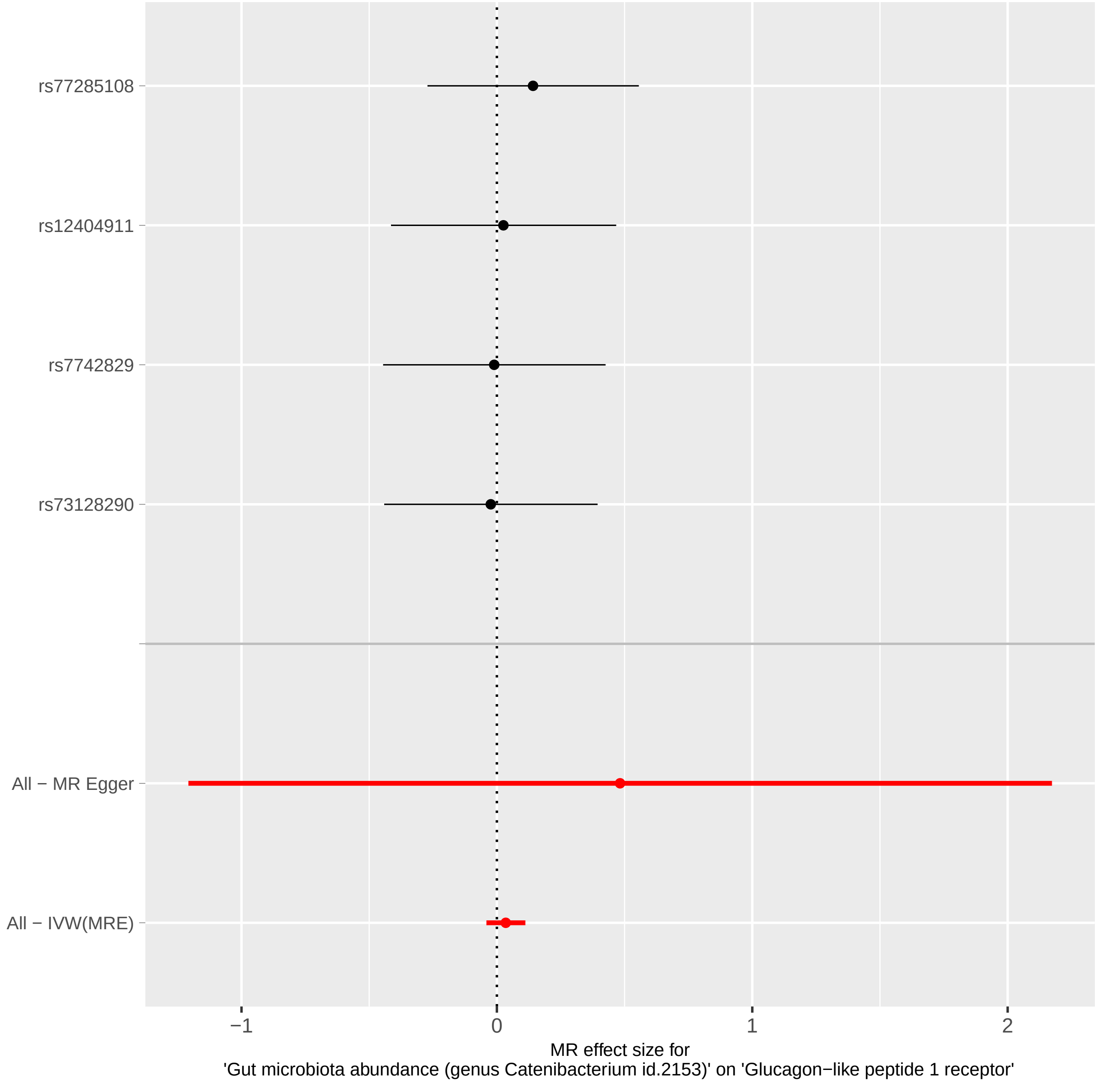

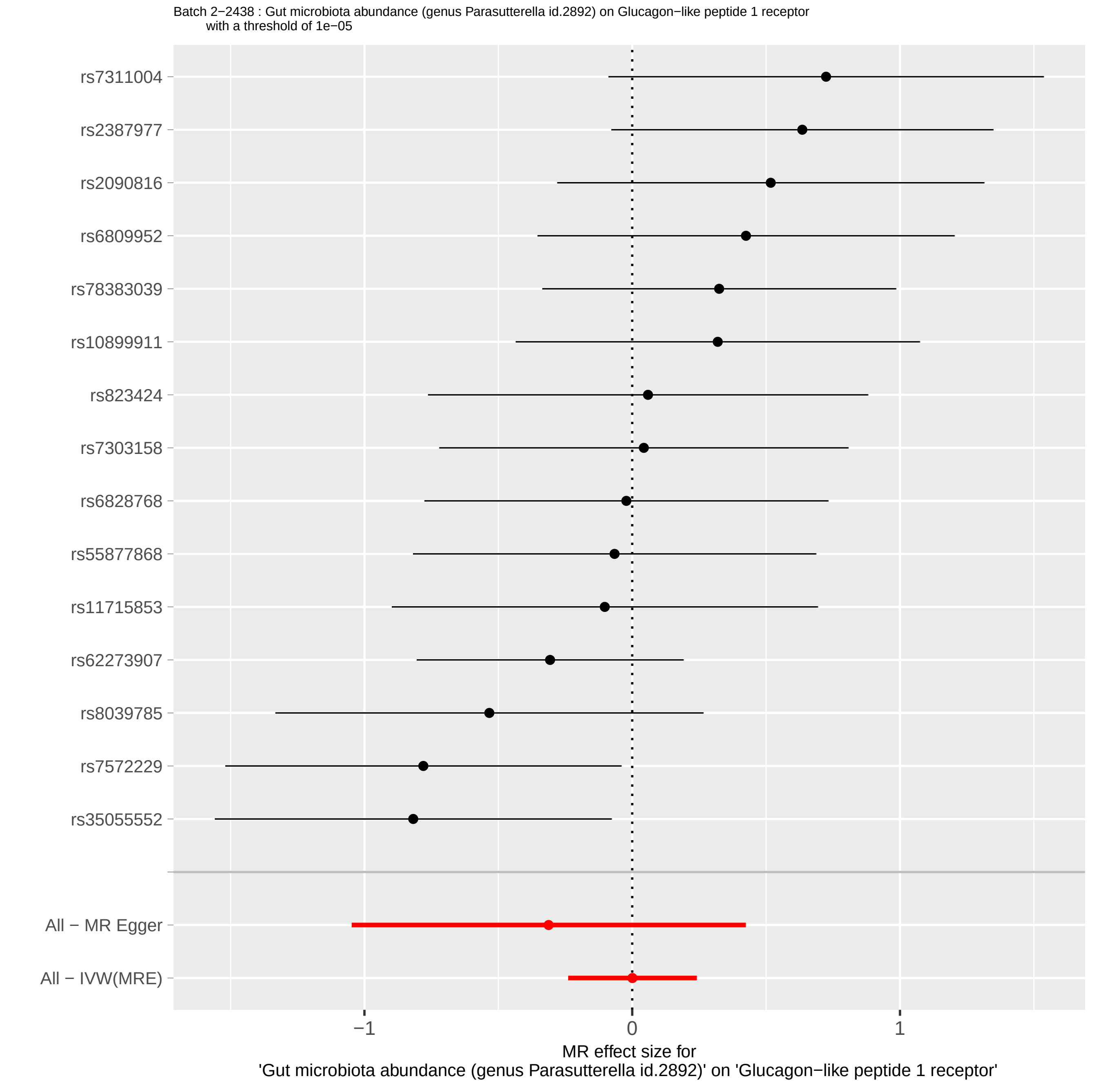
Batch 2−2438 : Gut microbiota abundance (genus Parasutterella id.2892) on Glucagon−like peptide 1 receptor
with a threshold of 1e−05
rs7311004
rs2387977
rs2090816
rs6809952
rs78383039
rs10899911
rs823424
rs7303158
rs6828768
rs55877868
rs11715853
rs62273907
rs8039785
rs7572229
rs35055552
All − MR Egger
All − IVW(MRE)
−1
0
1
MR effect size for
'Gut microbiota abundance (genus Parasutterella id.2892)' on 'Glucagon−like peptide 1 receptor'

Batch 2−2439 : Gut microbiota abundance (genus Catenibacterium id.2153) on Glutaredoxin−2, mitochondrial
with a threshold of 1e−05

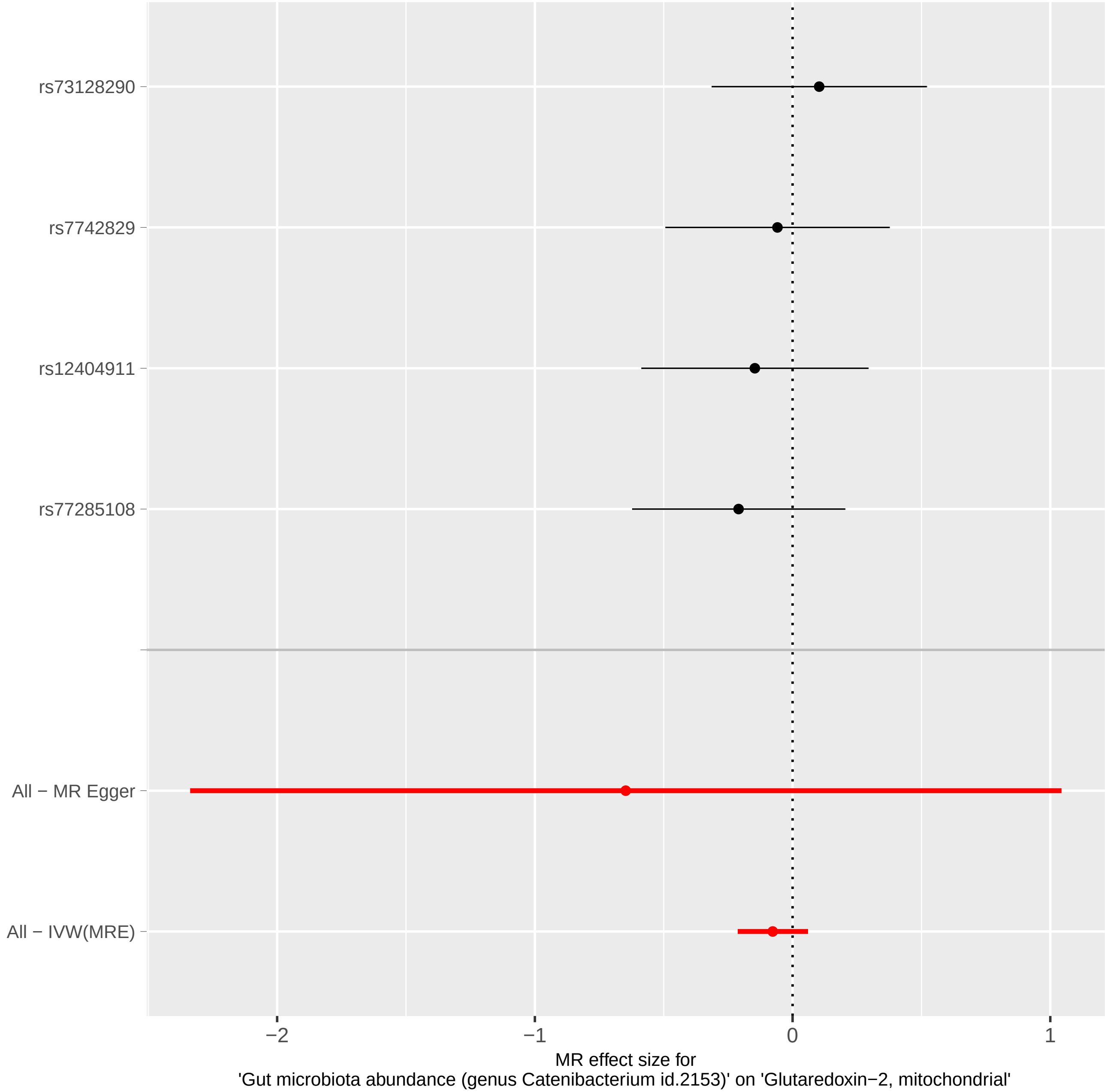

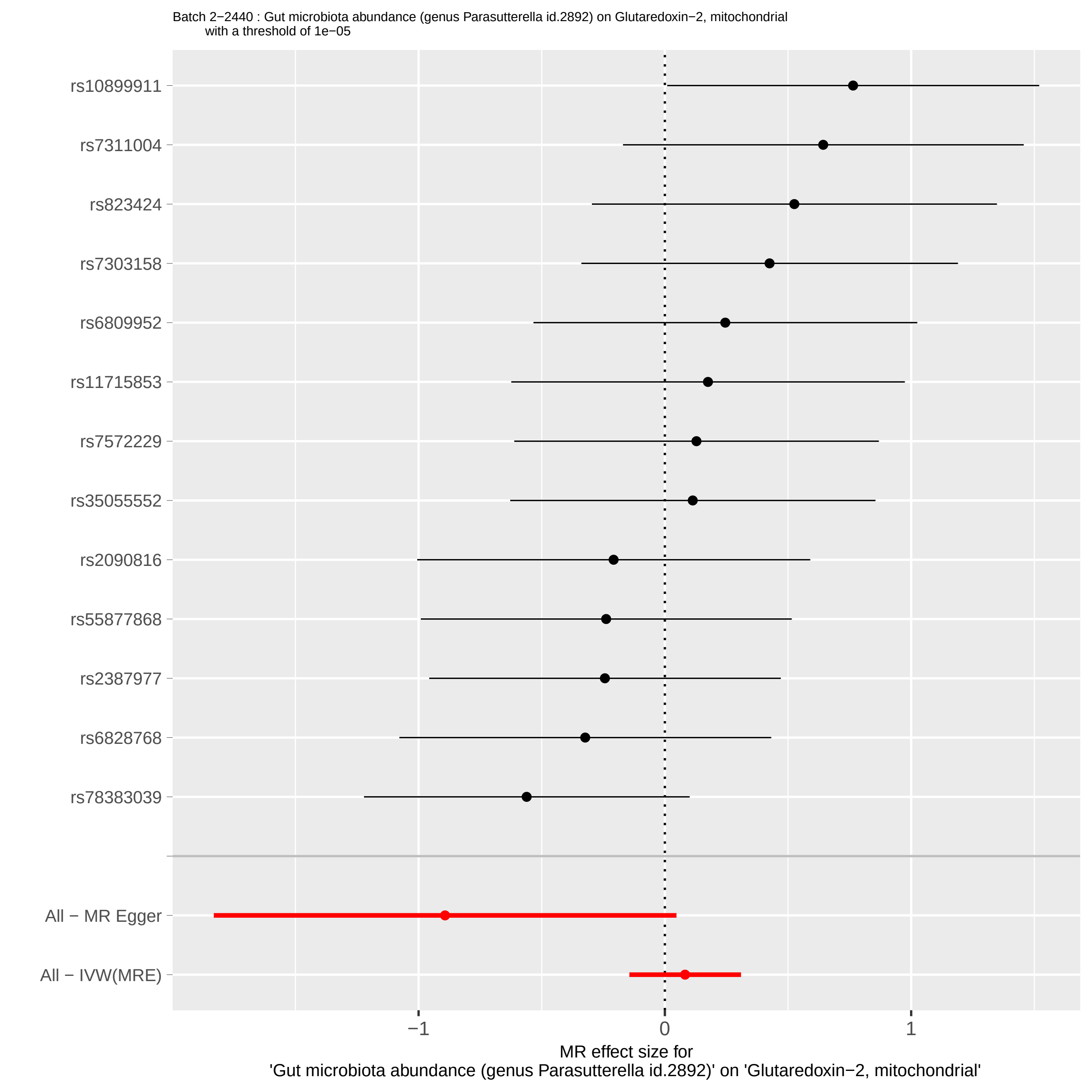

Batch 2−2440 : Gut microbiota abundance (genus Parasutterella id.2892) on Glutaredoxin−2, mitochondrial
with a threshold of 1e−05
rs10899911
rs7311004
rs823424
rs7303158
rs6809952
rs11715853
rs7572229
rs35055552
rs2090816
rs55877868
rs2387977
rs6828768
rs78383039
All − MR Egger
All − IVW(MRE)
−1
0
1
MR effect size for
'Gut microbiota abundance (genus Parasutterella id.2892)' on 'Glutaredoxin−2, mitochondrial'

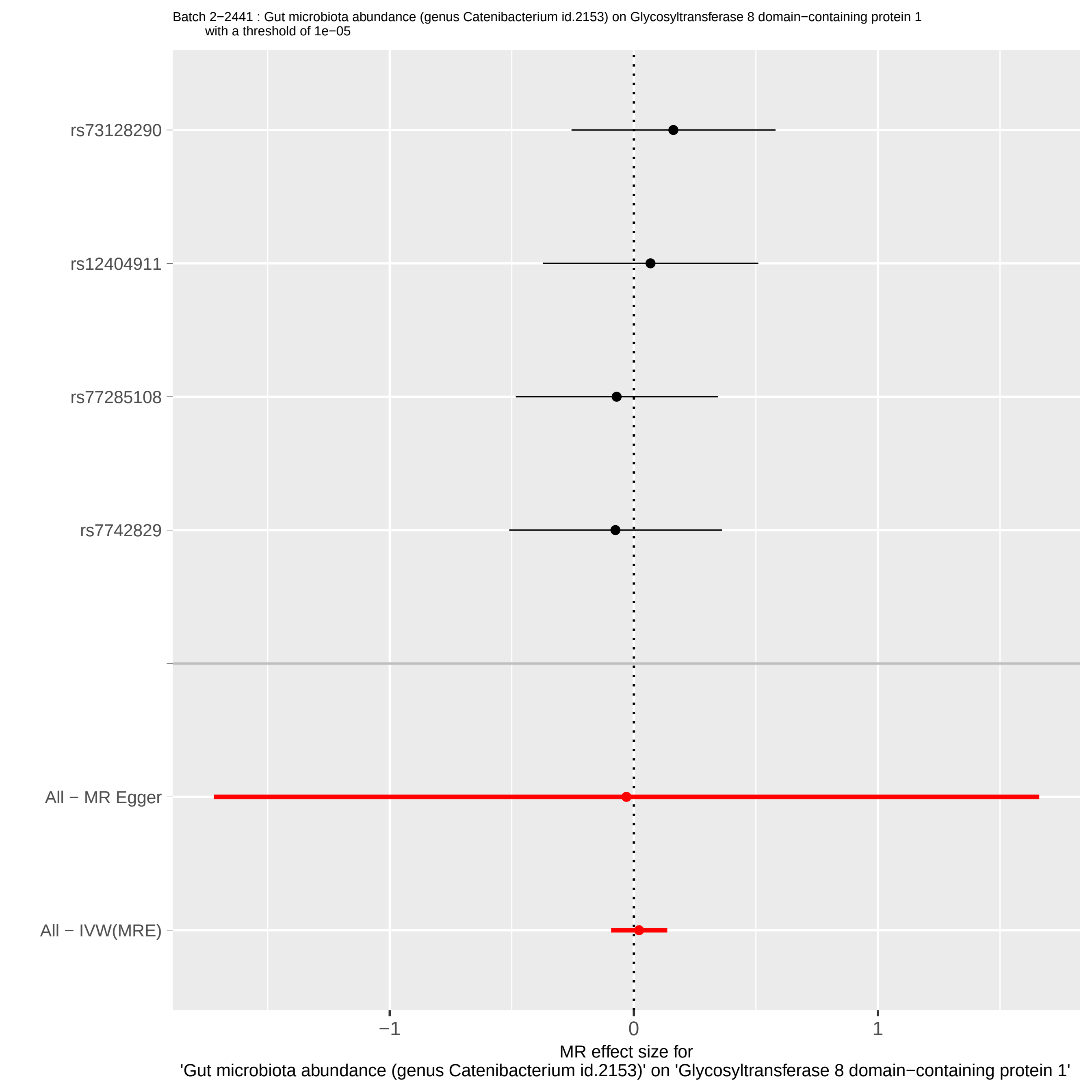

Batch 2−2441 : Gut microbiota abundance (genus Catenibacterium id.2153) on Glycosyltransferase 8 domain−containing protein 1
with a threshold of 1e−05
rs73128290
rs12404911
rs77285108
rs7742829
All − MR Egger
All − IVW(MRE)
−1
0
1
MR effect size for
'Gut microbiota abundance (genus Catenibacterium id.2153)' on 'Glycosyltransferase 8 domain−containing protein 1'

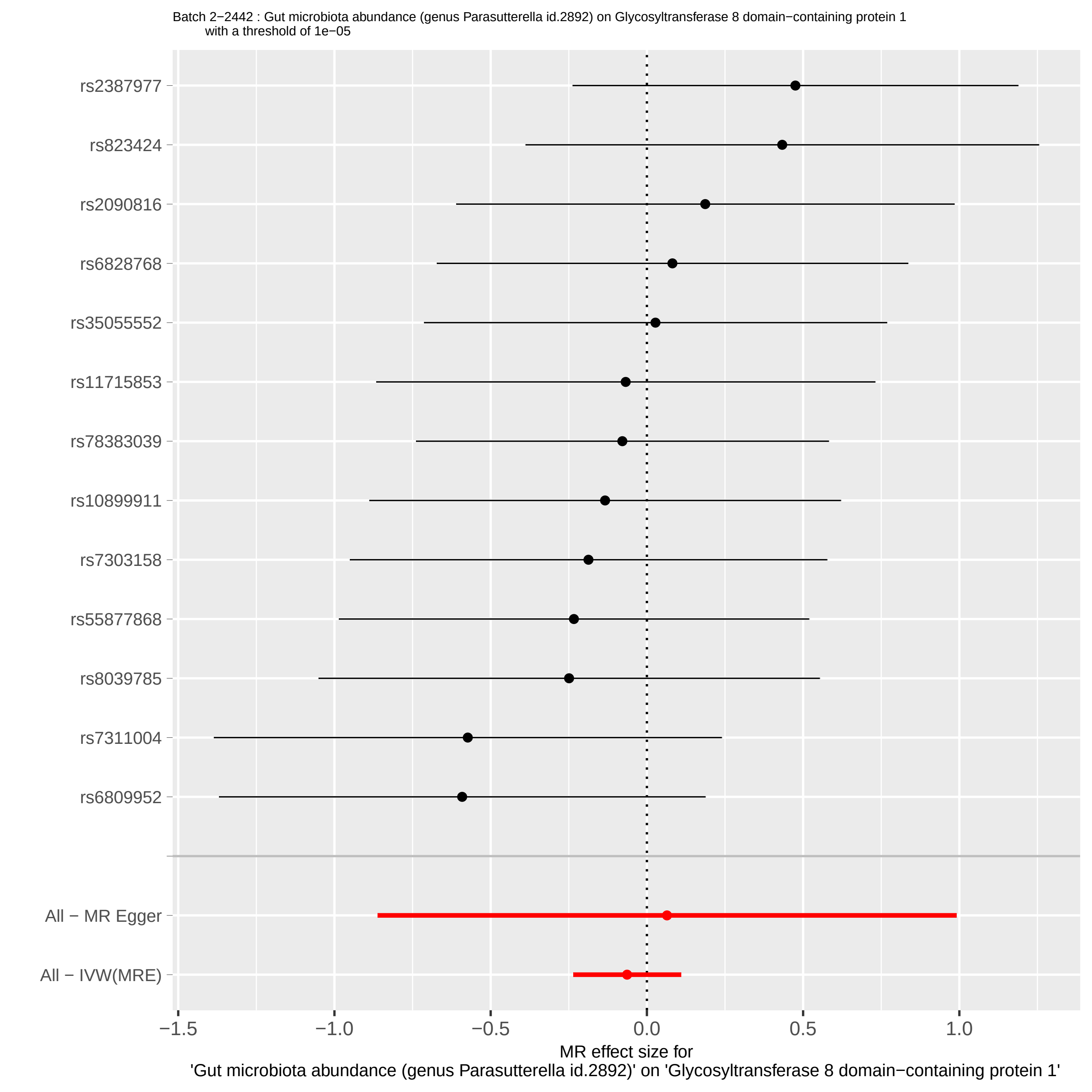

Batch 2−2442 : Gut microbiota abundance (genus Parasutterella id.2892) on Glycosyltransferase 8 domain−containing protein 1
with a threshold of 1e−05
rs2387977
rs823424
rs2090816
rs6828768
rs35055552
rs11715853
rs78383039
rs10899911
rs7303158
rs55877868
rs8039785
rs7311004
rs6809952
All − MR Egger
All − IVW(MRE)
−1.5
−1.0
−0.5
0.0
0.5
1.0
MR effect size for
'Gut microbiota abundance (genus Parasutterella id.2892)' on 'Glycosyltransferase 8 domain−containing protein 1'

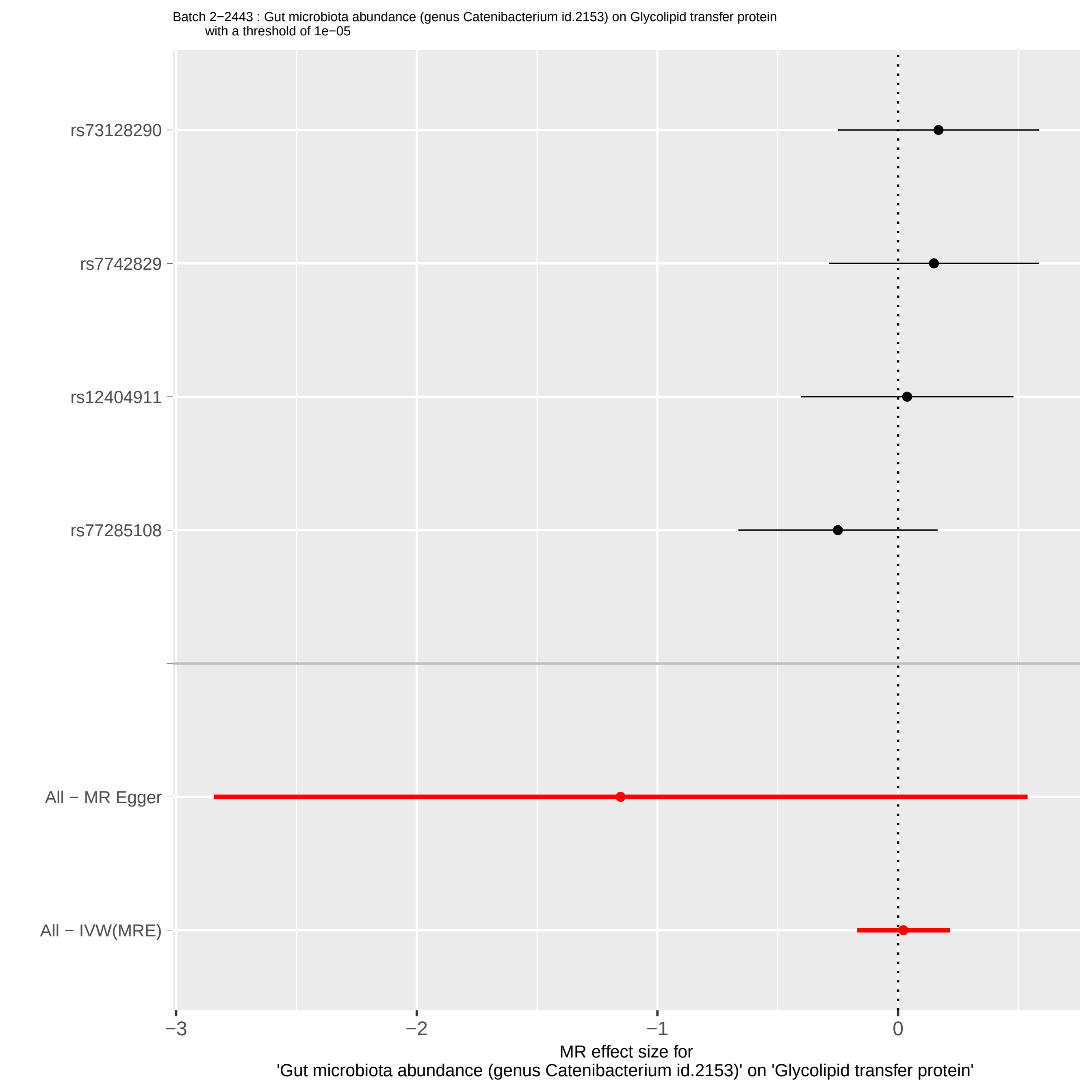

Batch 2−2443 : Gut microbiota abundance (genus Catenibacterium id.2153) on Glycolipid transfer protein
with a threshold of 1e−05
rs73128290
rs7742829
rs12404911
rs77285108
All − MR Egger
All − IVW(MRE)
−3
−2
−1
0
MR effect size for
'Gut microbiota abundance (genus Catenibacterium id.2153)' on 'Glycolipid transfer protein'

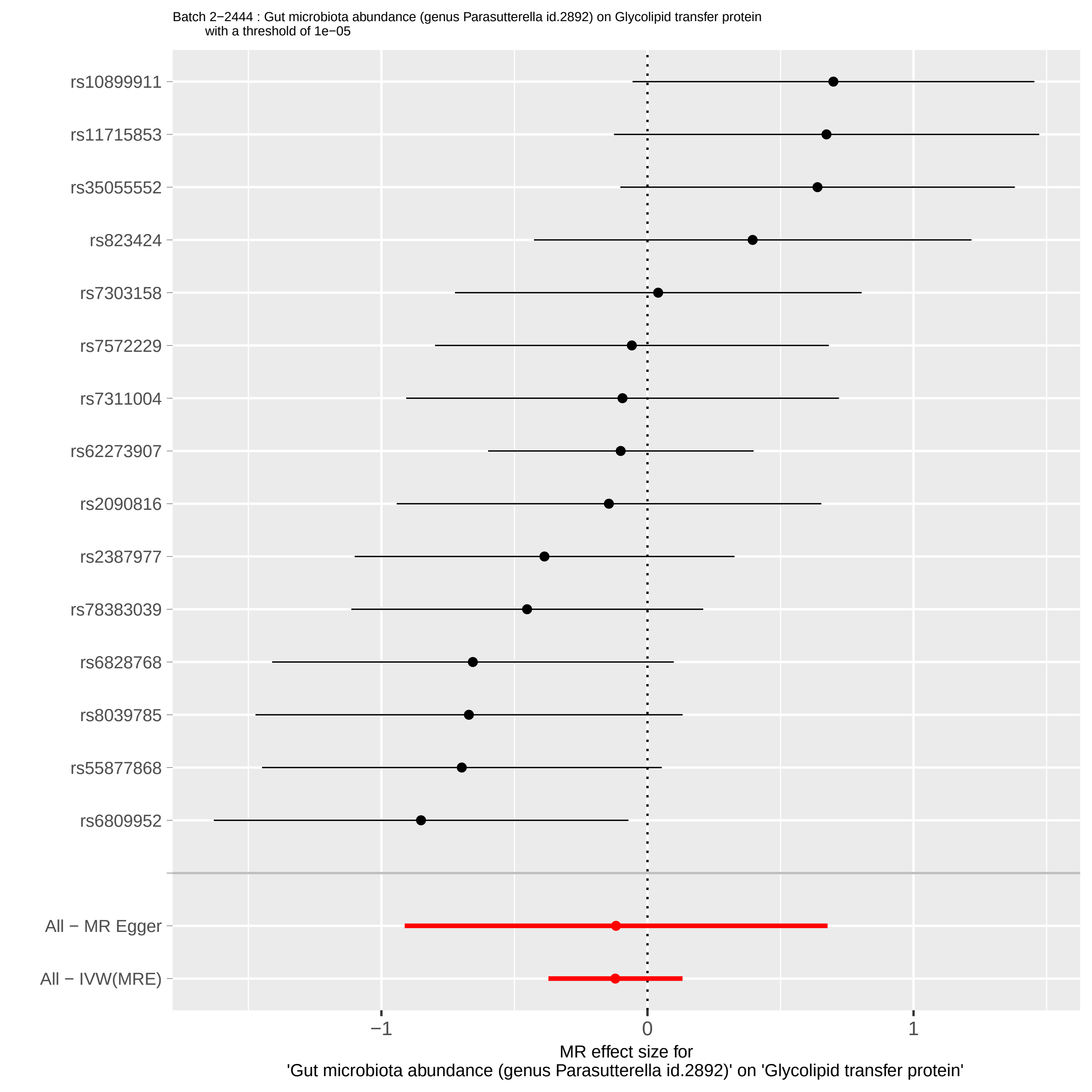

Batch 2−2444 : Gut microbiota abundance (genus Parasutterella id.2892) on Glycolipid transfer protein
with a threshold of 1e−05
rs10899911
rs11715853
rs35055552
rs823424
rs7303158
rs7572229
rs7311004
rs62273907
rs2090816
rs2387977
rs78383039
rs6828768
rs8039785
rs55877868
rs6809952
All − MR Egger
All − IVW(MRE)
−1
0
1
MR effect size for
'Gut microbiota abundance (genus Parasutterella id.2892)' on 'Glycolipid transfer protein'

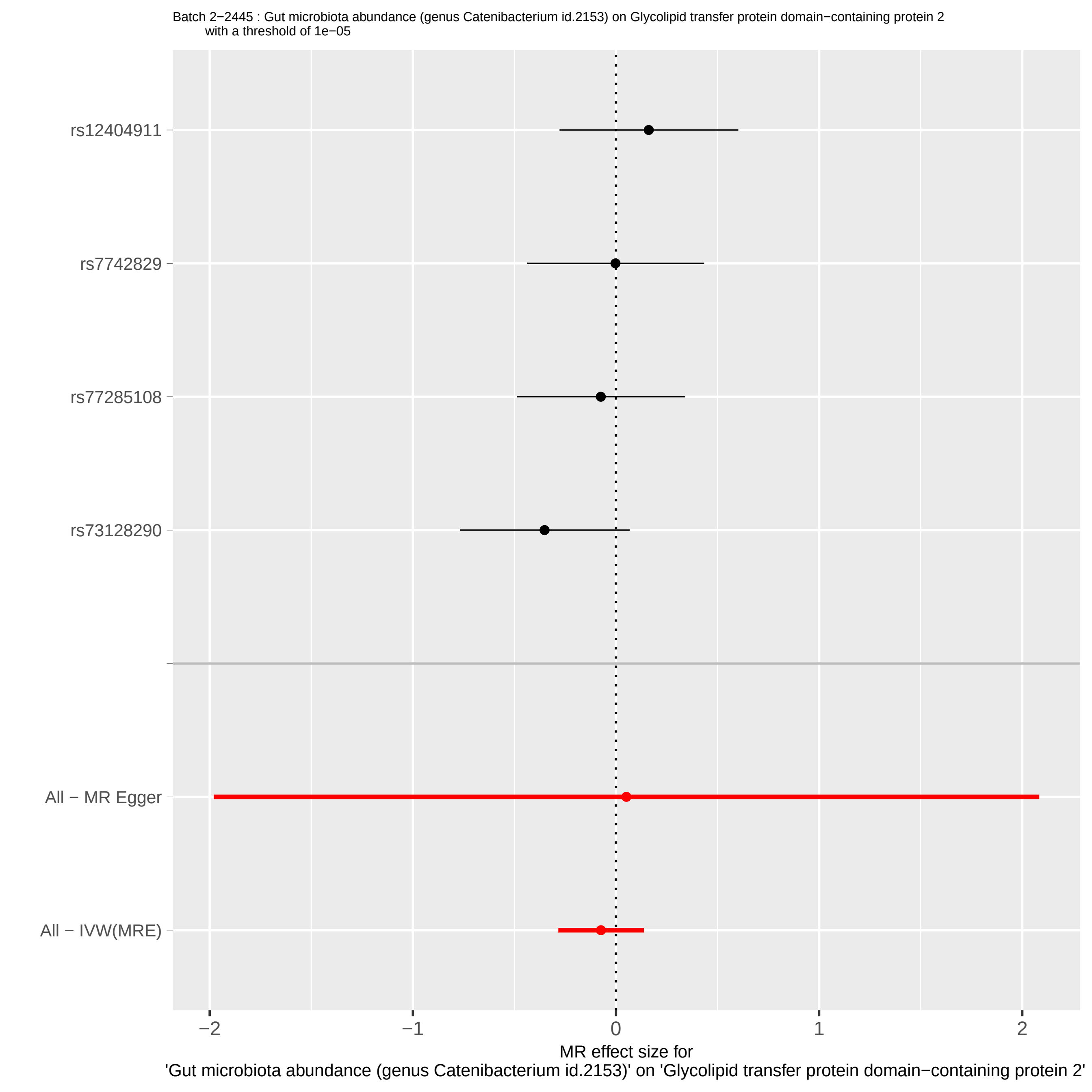

Batch 2−2445 : Gut microbiota abundance (genus Catenibacterium id.2153) on Glycolipid transfer protein domain−containing protein 2
with a threshold of 1e−05
rs12404911
rs7742829
rs77285108
rs73128290
All − MR Egger
All − IVW(MRE)
−2
−1
0
1
2
MR effect size for
'Gut microbiota abundance (genus Catenibacterium id.2153)' on 'Glycolipid transfer protein domain−containing protein 2'

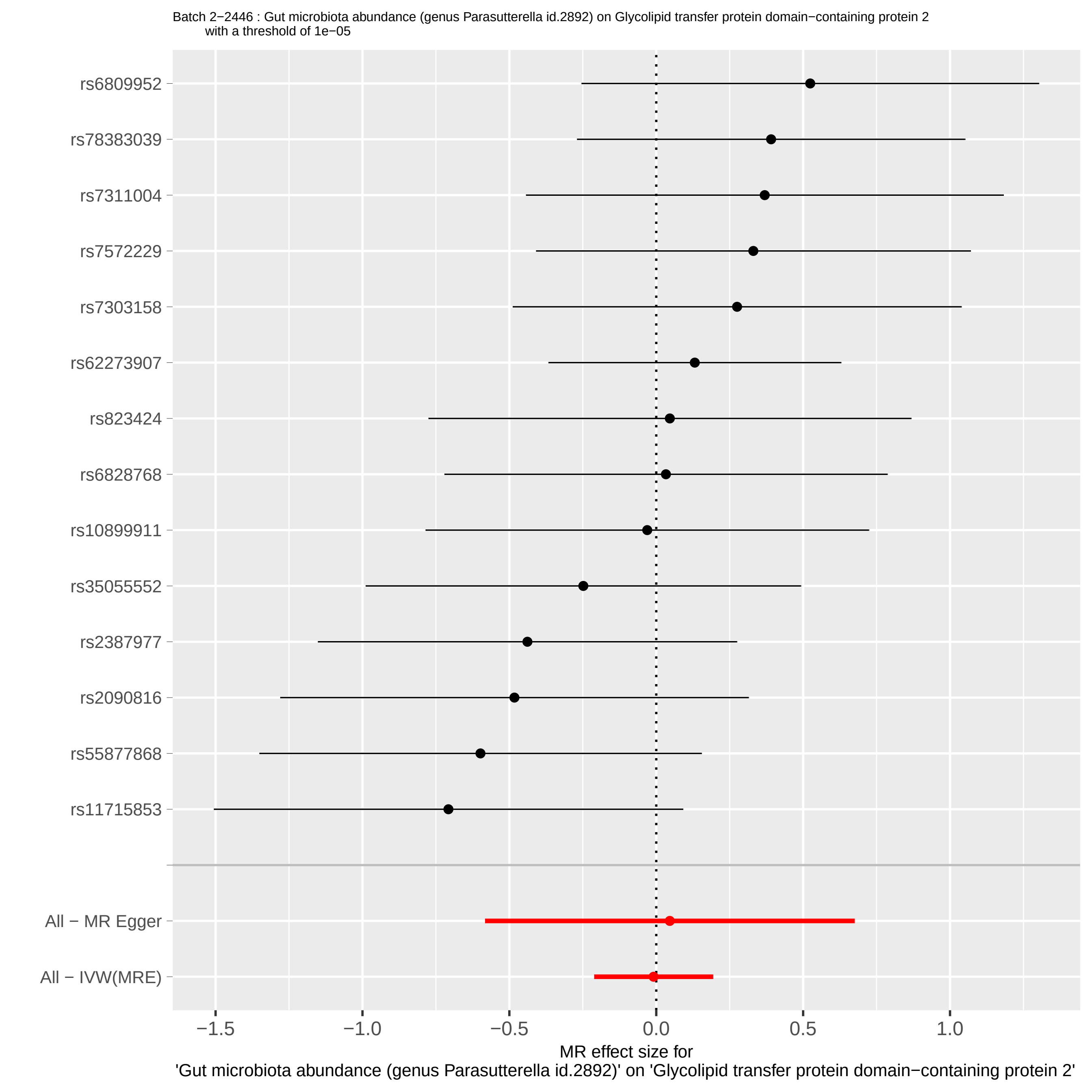
Batch 2−2446 : Gut microbiota abundance (genus Parasutterella id.2892) on Glycolipid transfer protein domain−containing protein 2
with a threshold of 1e−05
rs6809952
rs78383039
rs7311004
rs7572229
rs7303158
rs62273907
rs823424
rs6828768
rs10899911
rs35055552
rs2387977
rs2090816
rs55877868
rs11715853
All − MR Egger
All − IVW(MRE)
−1.5
−1.0
−0.5
0.0
0.5
1.0
MR effect size for
'Gut microbiota abundance (genus Parasutterella id.2892)' on 'Glycolipid transfer protein domain−containing protein 2'

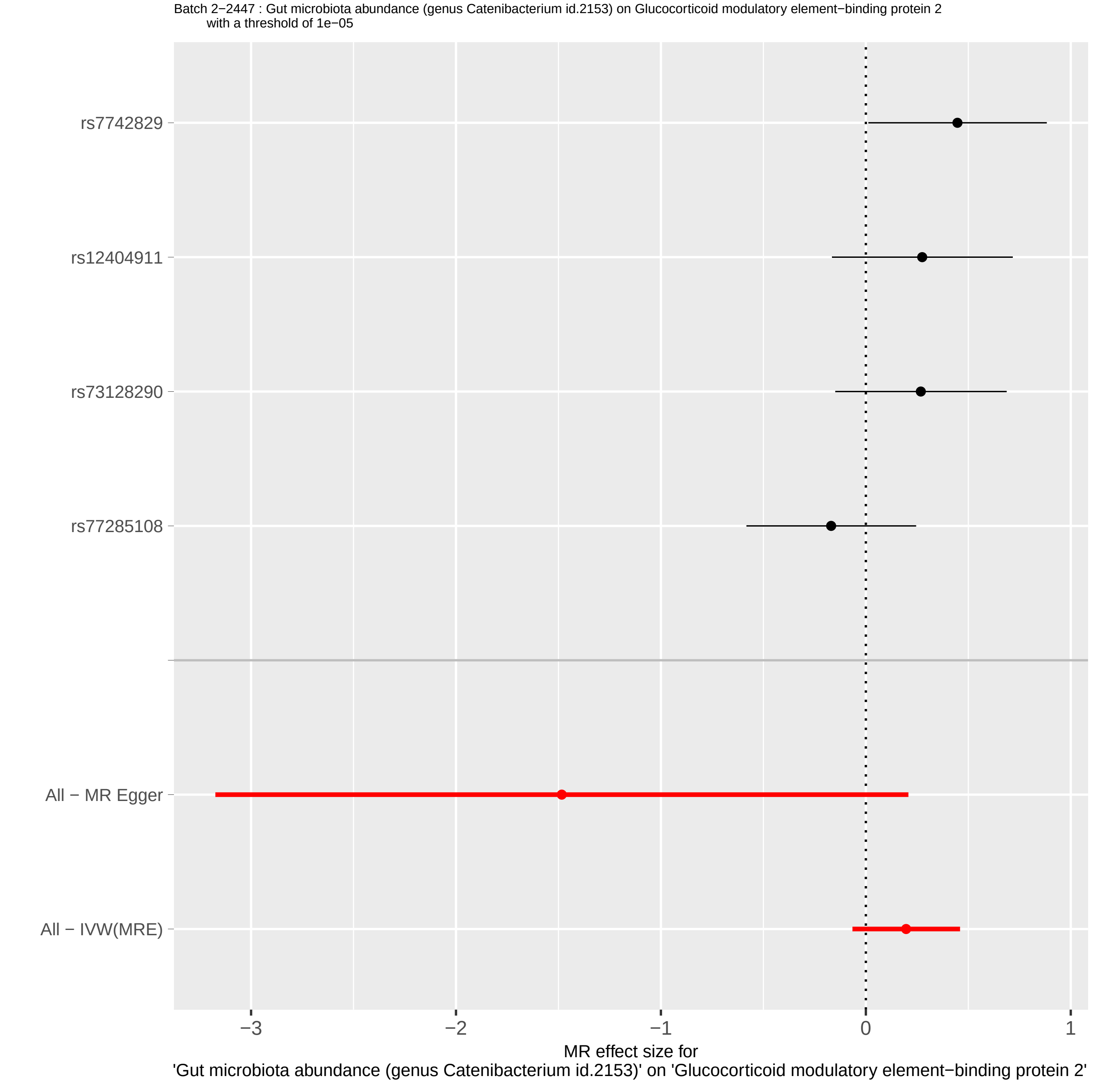

Batch 2−2447 : Gut microbiota abundance (genus Catenibacterium id.2153) on Glucocorticoid modulatory element−binding protein 2
with a threshold of 1e−05
rs7742829
rs12404911
rs73128290
rs77285108
All − MR Egger
All − IVW(MRE)
−3
−2
−1
0
1
MR effect size for
'Gut microbiota abundance (genus Catenibacterium id.2153)' on 'Glucocorticoid modulatory element−binding protein 2'

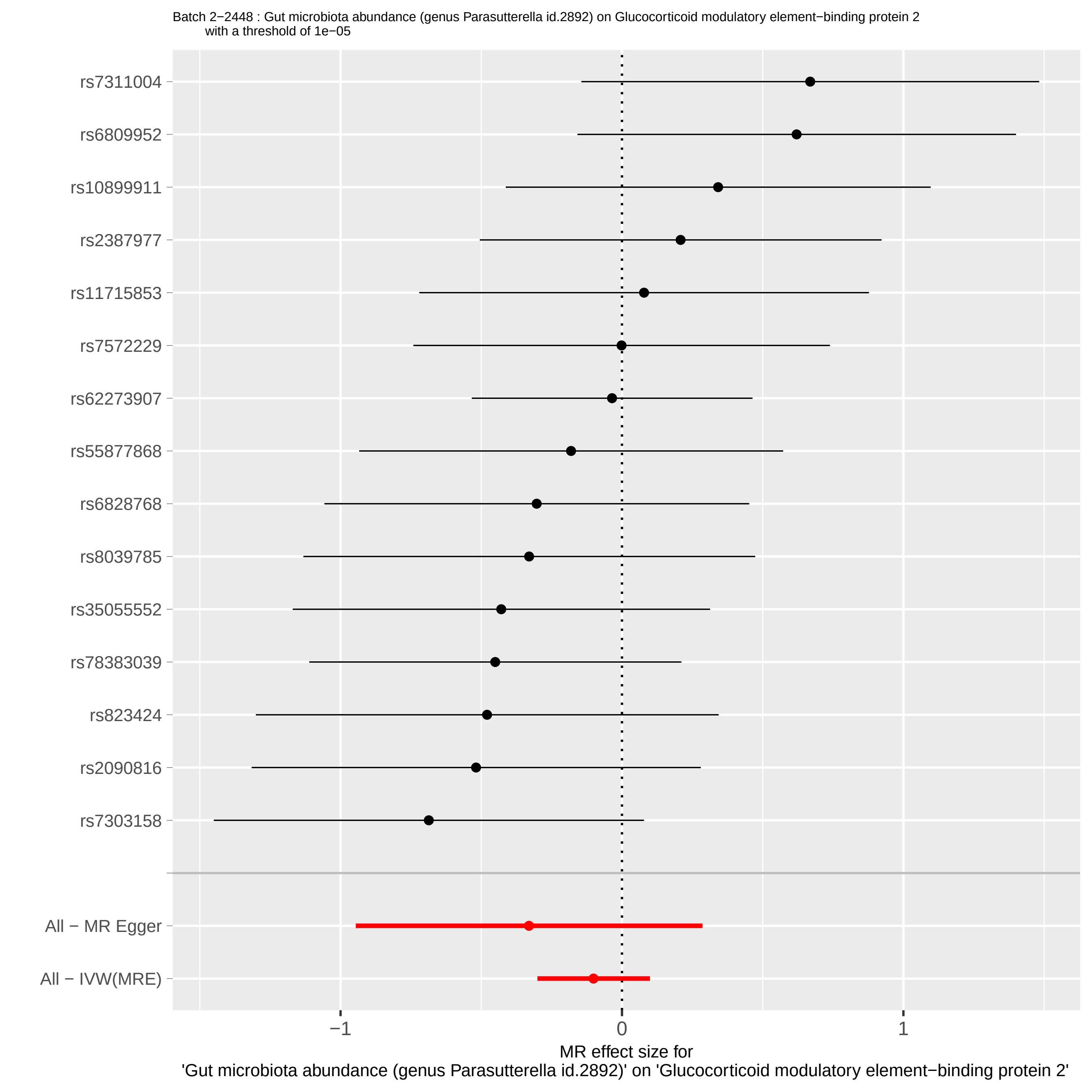
Batch 2−2448 : Gut microbiota abundance (genus Parasutterella id.2892) on Glucocorticoid modulatory element−binding protein 2
with a threshold of 1e−05
rs7311004
rs6809952
rs10899911
rs2387977
rs11715853
rs7572229
rs62273907
rs55877868
rs6828768
rs8039785
rs35055552
rs78383039
rs823424
rs2090816
rs7303158
All − MR Egger
All − IVW(MRE)
−1
0
1
MR effect size for
'Gut microbiota abundance (genus Parasutterella id.2892)' on 'Glucocorticoid modulatory element−binding protein 2'

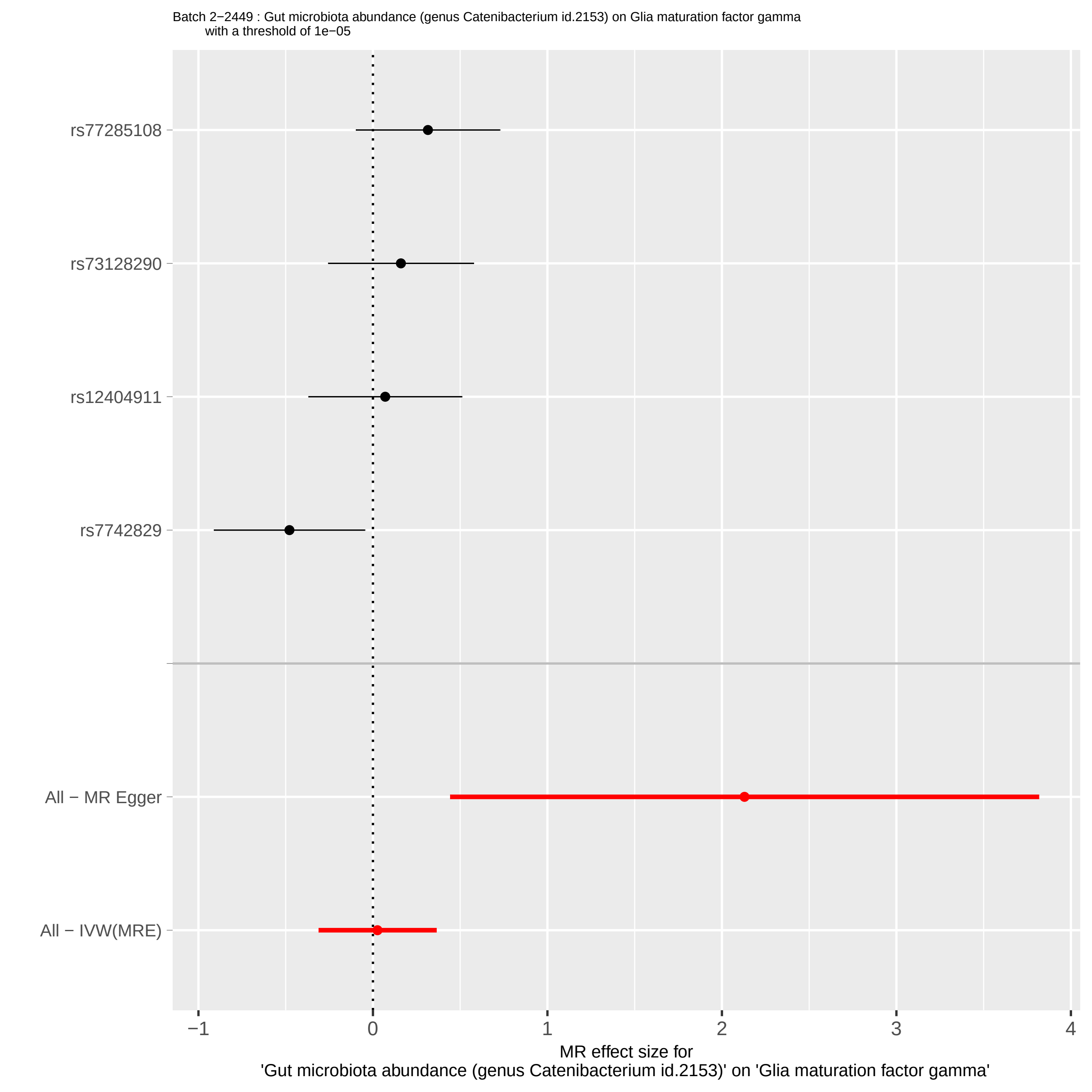
Batch 2−2449 : Gut microbiota abundance (genus Catenibacterium id.2153) on Glia maturation factor gamma
with a threshold of 1e−05
rs77285108
rs73128290
rs12404911
rs7742829
All − MR Egger
All − IVW(MRE)
−1
0
1
2
3
4
MR effect size for
'Gut microbiota abundance (genus Catenibacterium id.2153)' on 'Glia maturation factor gamma'

Batch 2−2450 : Gut microbiota abundance (genus Parasutterella id.2892) on Glia maturation factor gamma
with a threshold of 1e−05

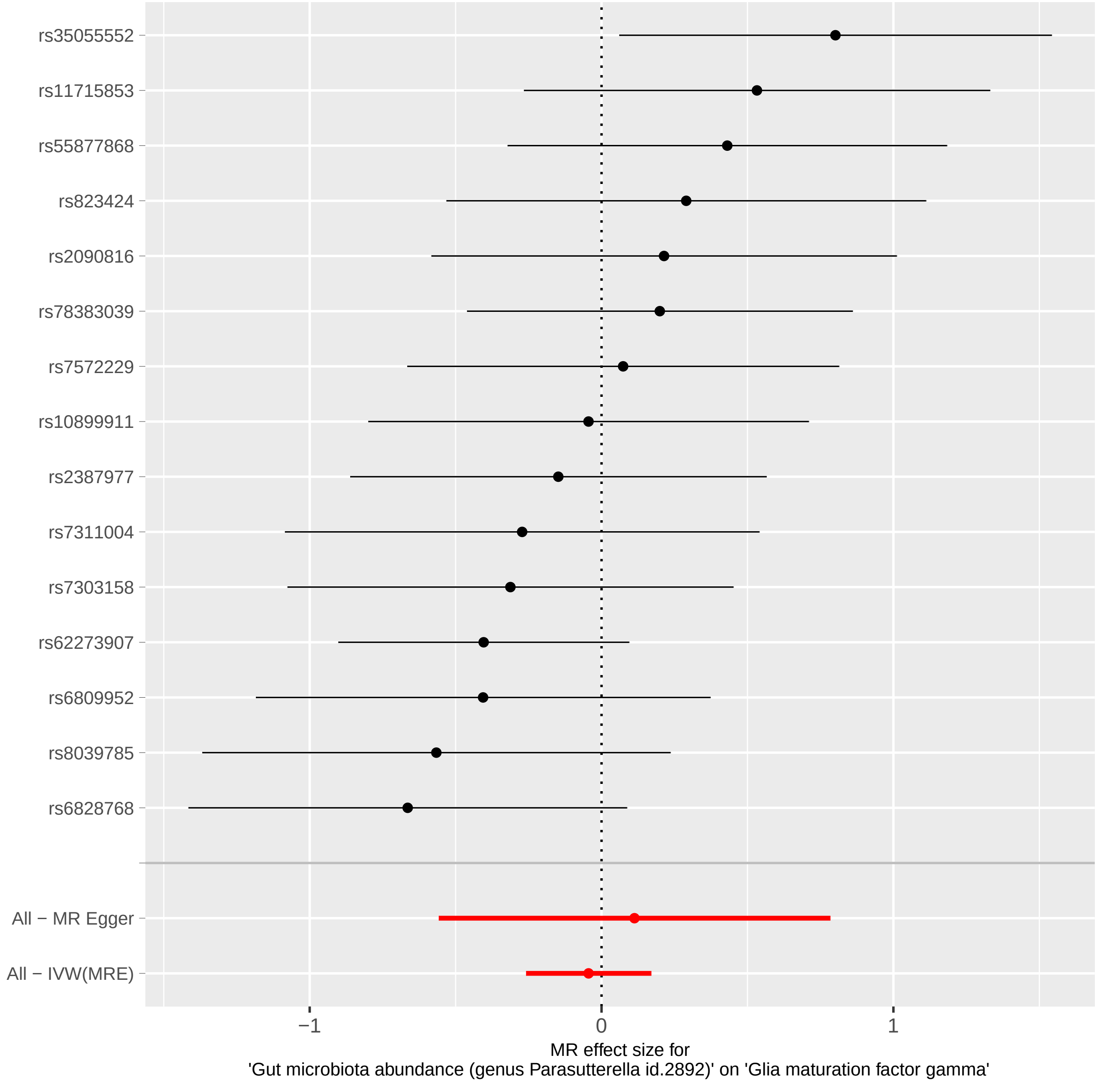

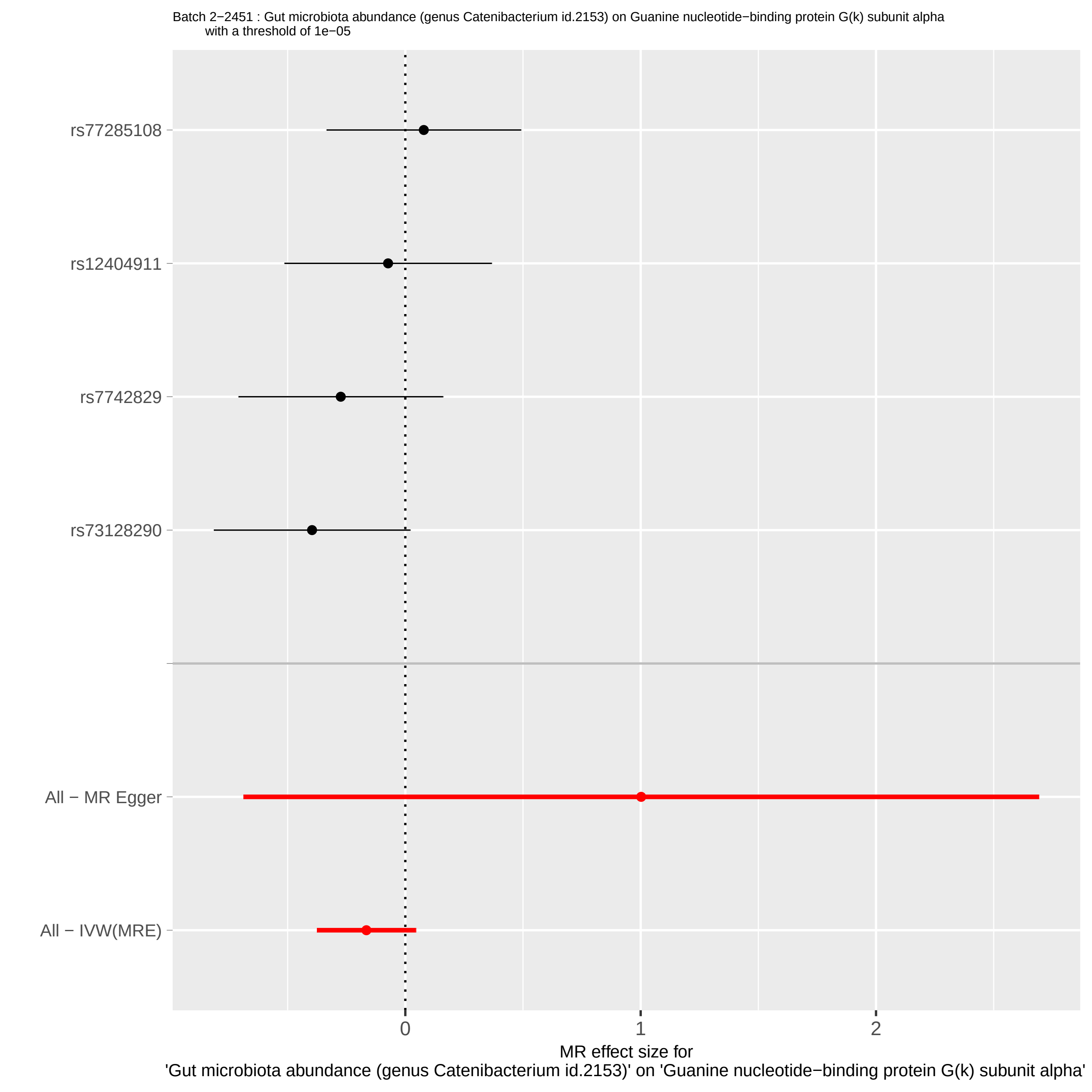

Batch 2−2451 : Gut microbiota abundance (genus Catenibacterium id.2153) on Guanine nucleotide−binding protein G(k) subunit alpha
with a threshold of 1e−05
rs77285108
rs12404911
rs7742829
rs73128290
All − MR Egger
All − IVW(MRE)
0
1
2
MR effect size for
'Gut microbiota abundance (genus Catenibacterium id.2153)' on 'Guanine nucleotide−binding protein G(k) subunit alpha'

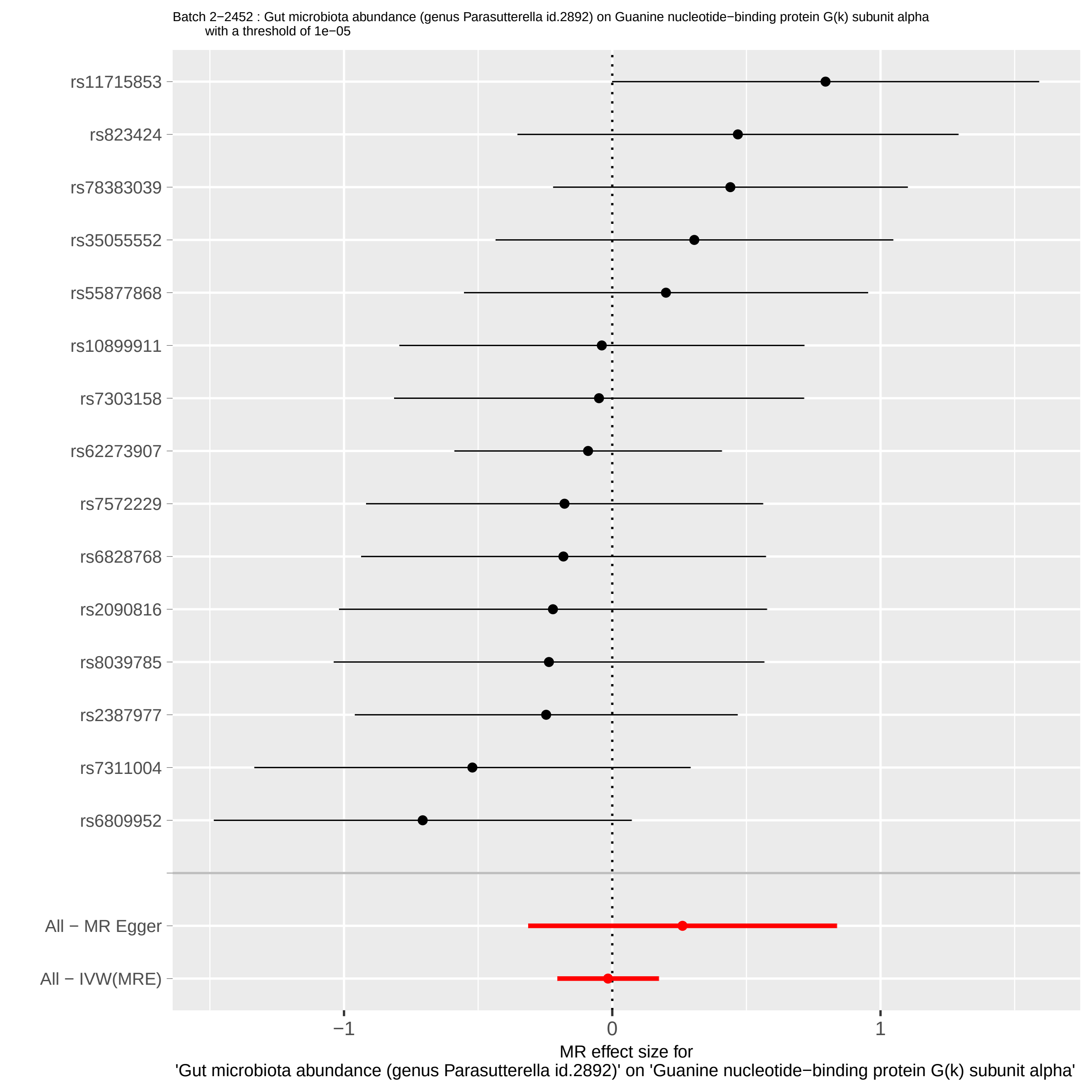

Batch 2−2452 : Gut microbiota abundance (genus Parasutterella id.2892) on Guanine nucleotide−binding protein G(k) subunit alpha
with a threshold of 1e−05
rs11715853
rs823424
rs78383039
rs35055552
rs55877868
rs10899911
rs7303158
rs62273907
rs7572229
rs6828768
rs2090816
rs8039785
rs2387977
rs7311004
rs6809952
All − MR Egger
All − IVW(MRE)
−1
0
1
MR effect size for
'Gut microbiota abundance (genus Parasutterella id.2892)' on 'Guanine nucleotide−binding protein G(k) subunit alpha'

Batch 2−2453 : Gut microbiota abundance (genus Catenibacterium id.2153) on Guanine nucleotide−binding protein G(I)/G(S)/G(O) subunit gamma−T2 with a threshold of 1e−05

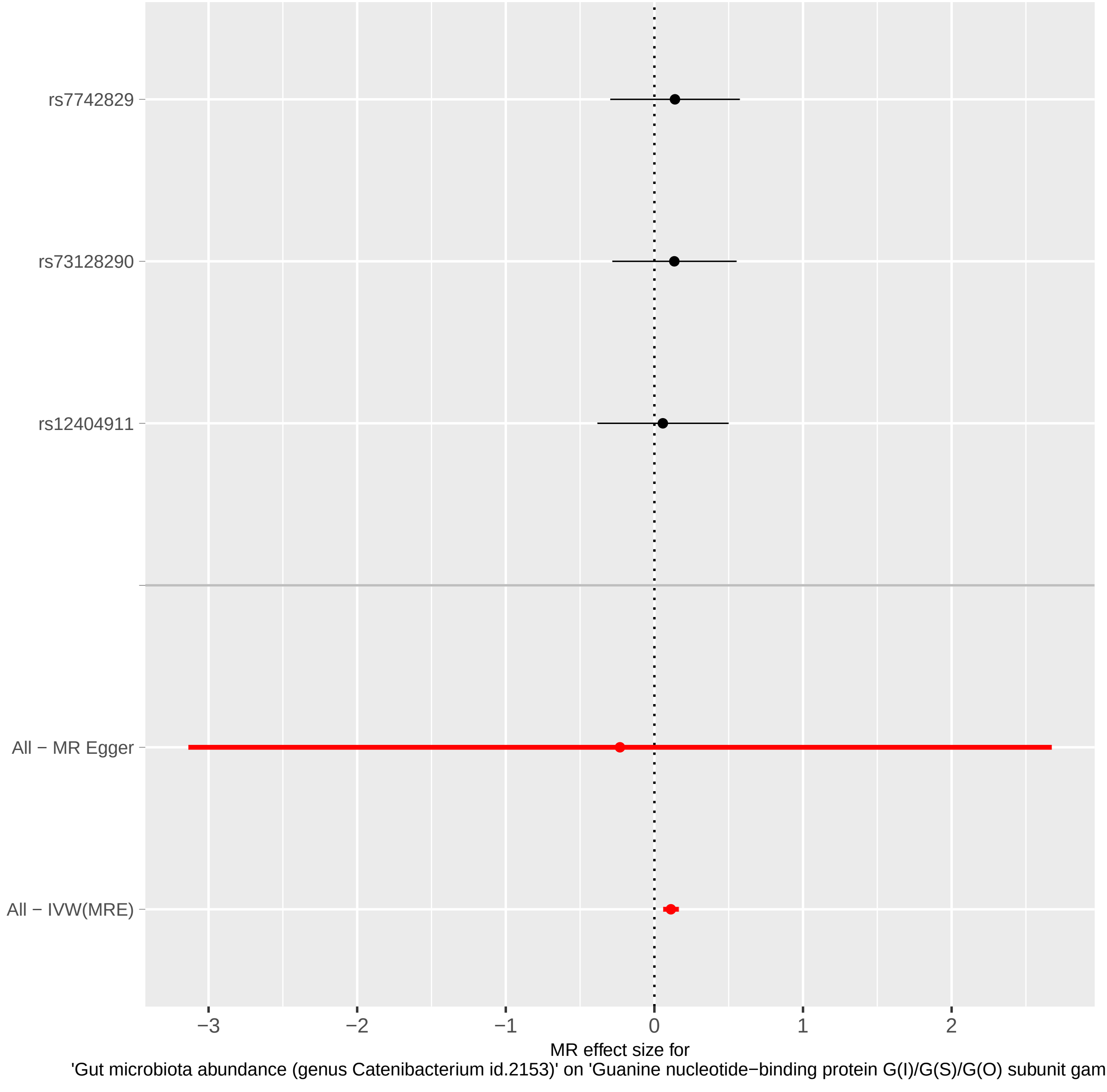

Batch 2−2454 : Gut microbiota abundance (genus Parasutterella id.2892) on Guanine nucleotide−binding protein G(I)/G(S)/G(O) subunit gamma−T2
with a threshold of 1e−05

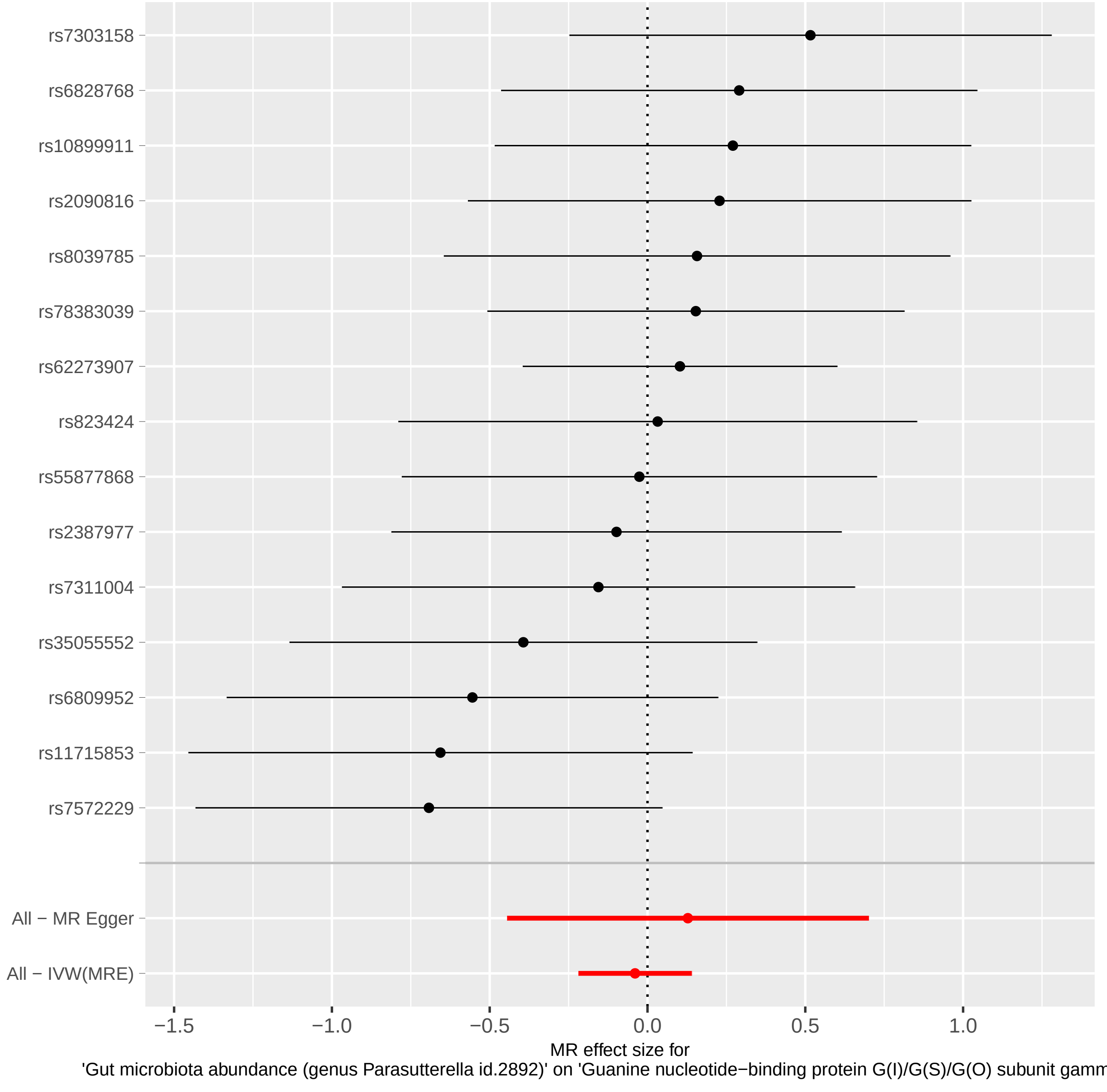

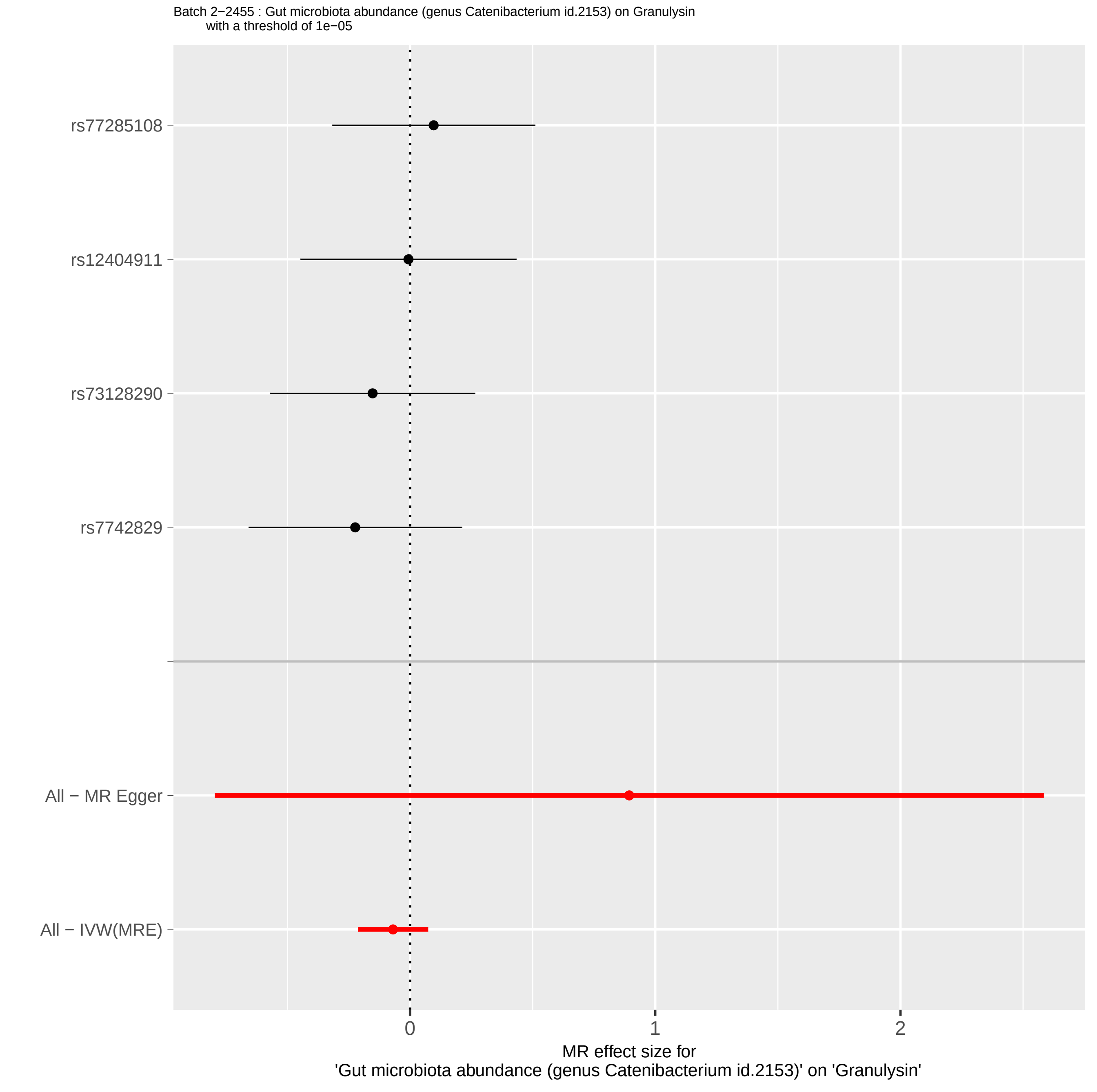
Batch 2−2455 : Gut microbiota abundance (genus Catenibacterium id.2153) on Granulysin
with a threshold of 1e−05
rs77285108
rs12404911
rs73128290
rs7742829
All − MR Egger
All − IVW(MRE)
0
1
2
MR effect size for
'Gut microbiota abundance (genus Catenibacterium id.2153)' on 'Granulysin'

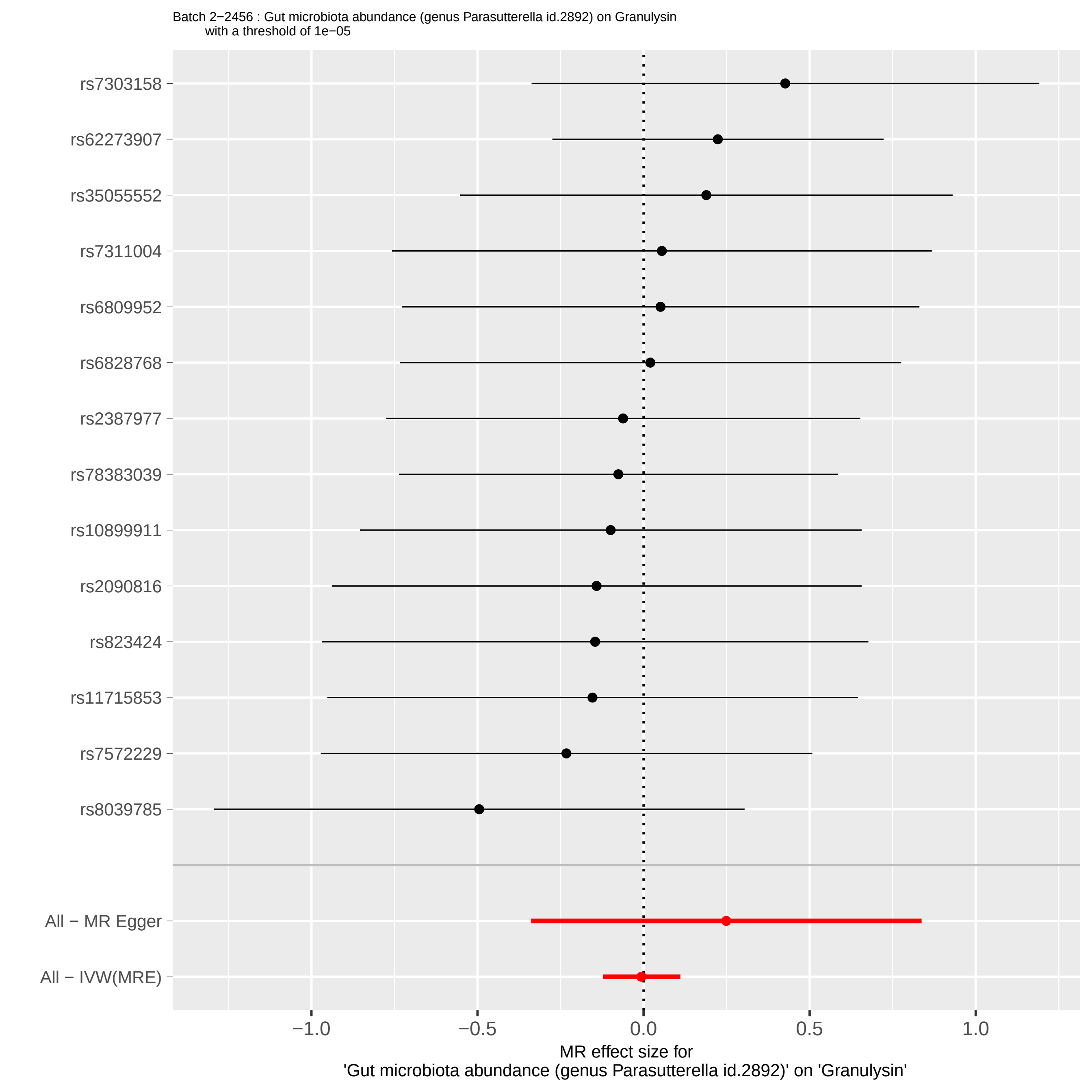
Batch 2−2456 : Gut microbiota abundance (genus Parasutterella id.2892) on Granulysin
with a threshold of 1e−05
rs7303158
rs62273907
rs35055552
rs7311004
rs6809952
rs6828768
rs2387977
rs78383039
rs10899911
rs2090816
rs823424
rs11715853
rs7572229
rs8039785
All − MR Egger
All − IVW(MRE)
−1.0
−0.5
0.0
0.5
1.0
MR effect size for
'Gut microbiota abundance (genus Parasutterella id.2892)' on 'Granulysin'

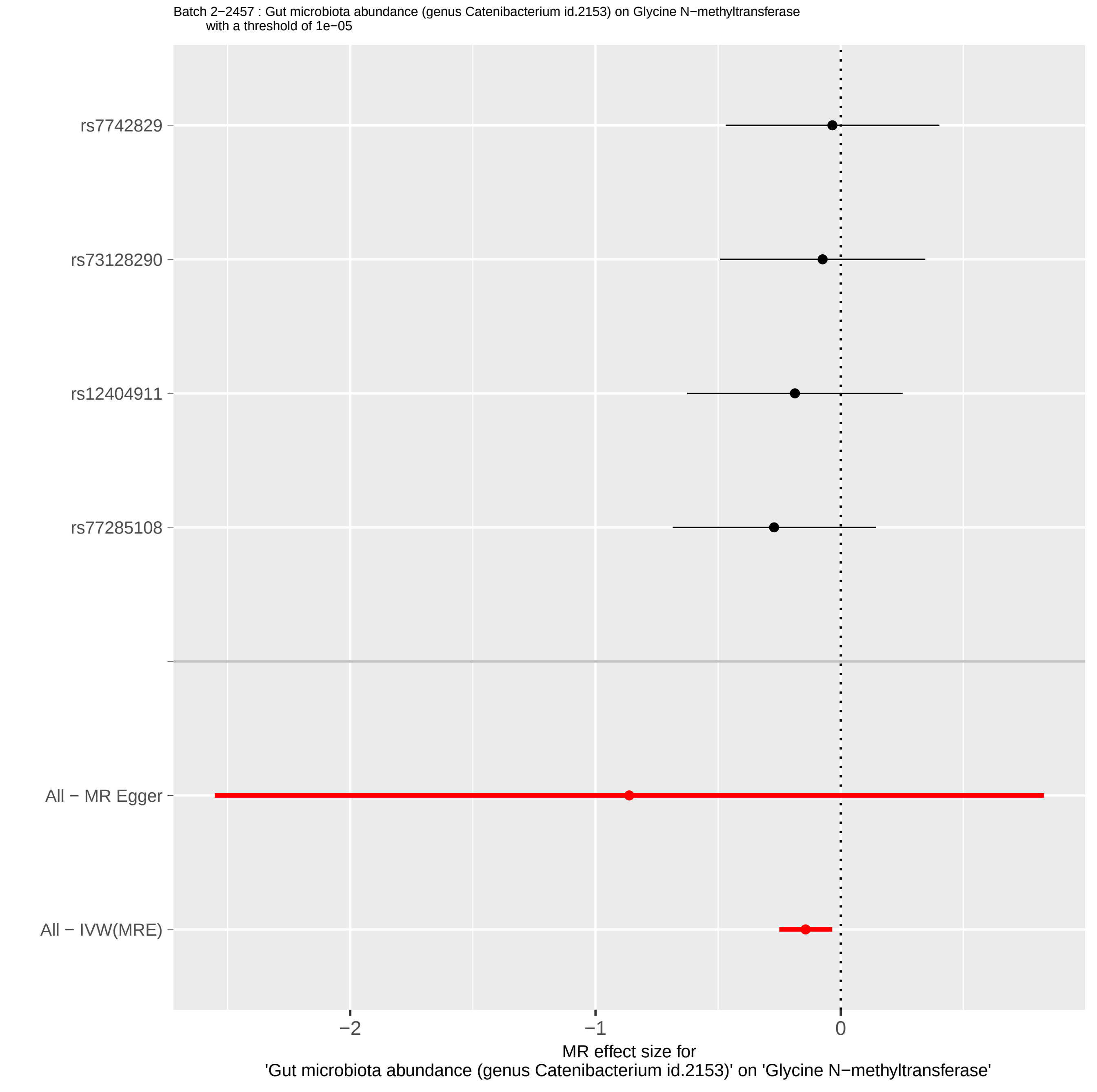

Batch 2−2457 : Gut microbiota abundance (genus Catenibacterium id.2153) on Glycine N−methyltransferase
with a threshold of 1e−05
rs7742829
rs73128290
rs12404911
rs77285108
All − MR Egger
All − IVW(MRE)
−2
−1
0
MR effect size for
'Gut microbiota abundance (genus Catenibacterium id.2153)' on 'Glycine N−methyltransferase'

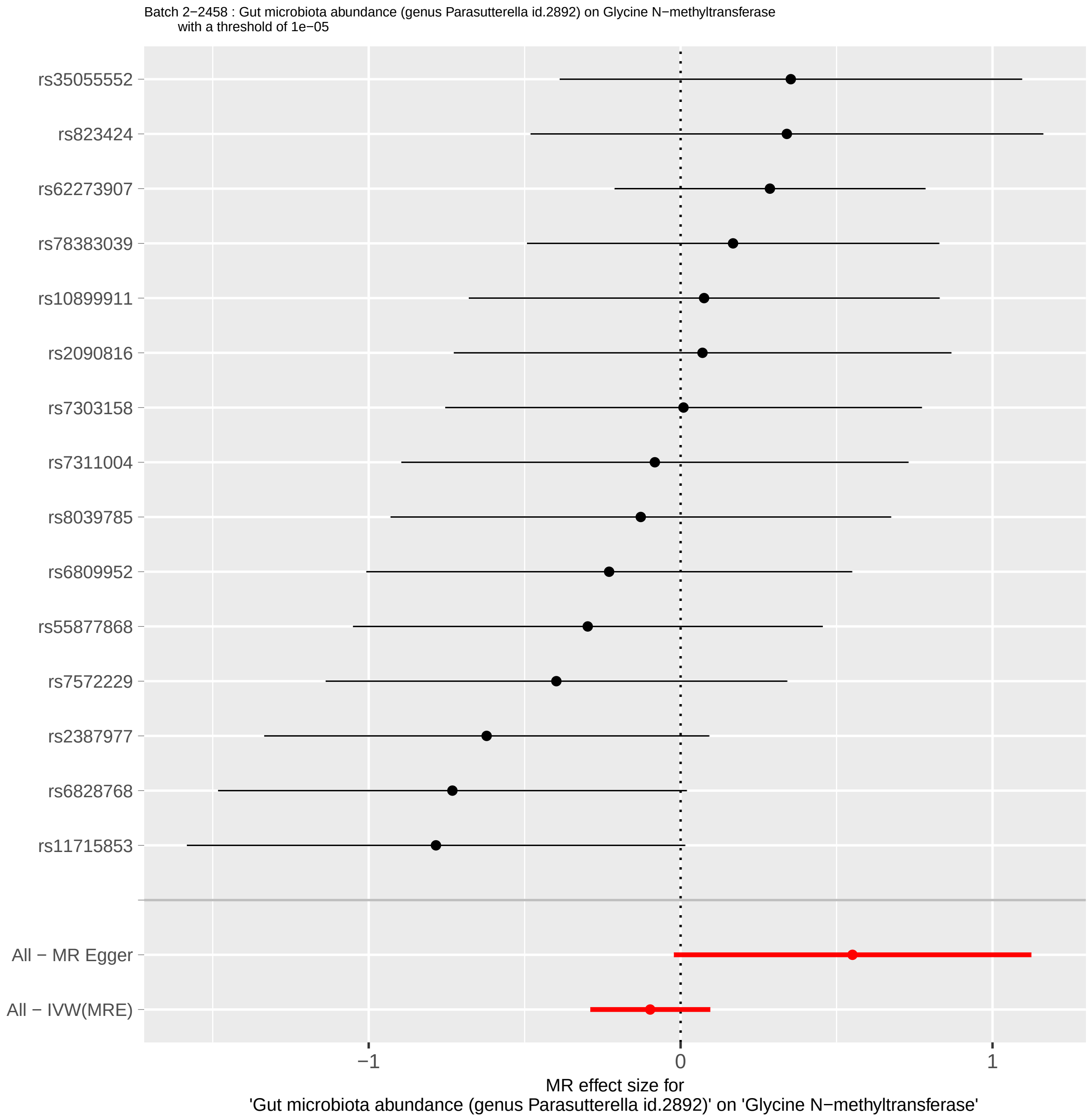

Batch 2−2458 : Gut microbiota abundance (genus Parasutterella id.2892) on Glycine N−methyltransferase
with a threshold of 1e−05
rs35055552
rs823424
rs62273907
rs78383039
rs10899911
rs2090816
rs7303158
rs7311004
rs8039785
rs6809952
rs55877868
rs7572229
rs2387977
rs6828768
rs11715853
All − MR Egger
All − IVW(MRE)
−1
0
1
MR effect size for
'Gut microbiota abundance (genus Parasutterella id.2892)' on 'Glycine N−methyltransferase'

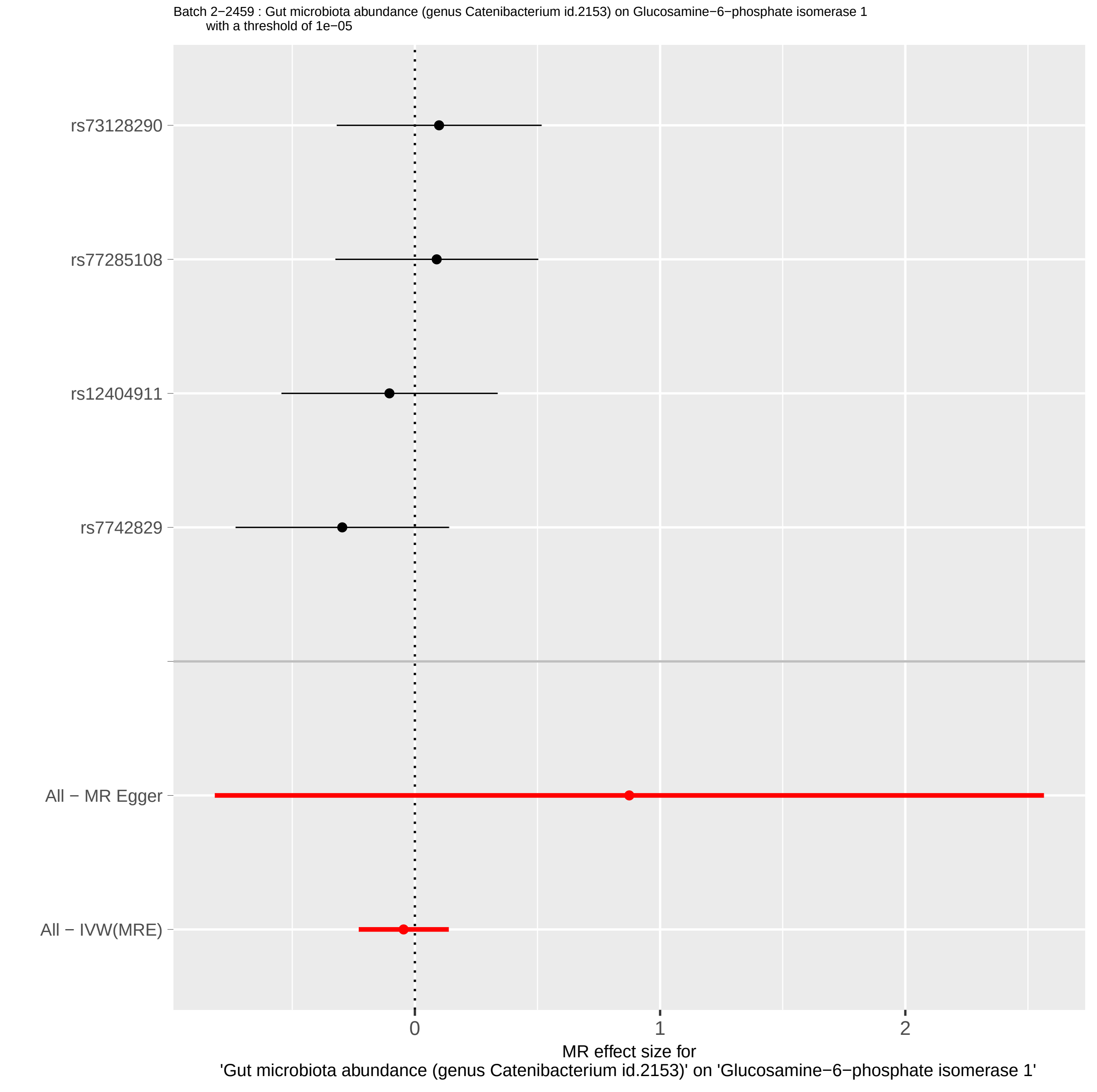
Batch 2−2459 : Gut microbiota abundance (genus Catenibacterium id.2153) on Glucosamine−6−phosphate isomerase 1
with a threshold of 1e−05
rs73128290
rs77285108
rs12404911
rs7742829
All − MR Egger
All − IVW(MRE)
0
1
2
MR effect size for
'Gut microbiota abundance (genus Catenibacterium id.2153)' on 'Glucosamine−6−phosphate isomerase 1'

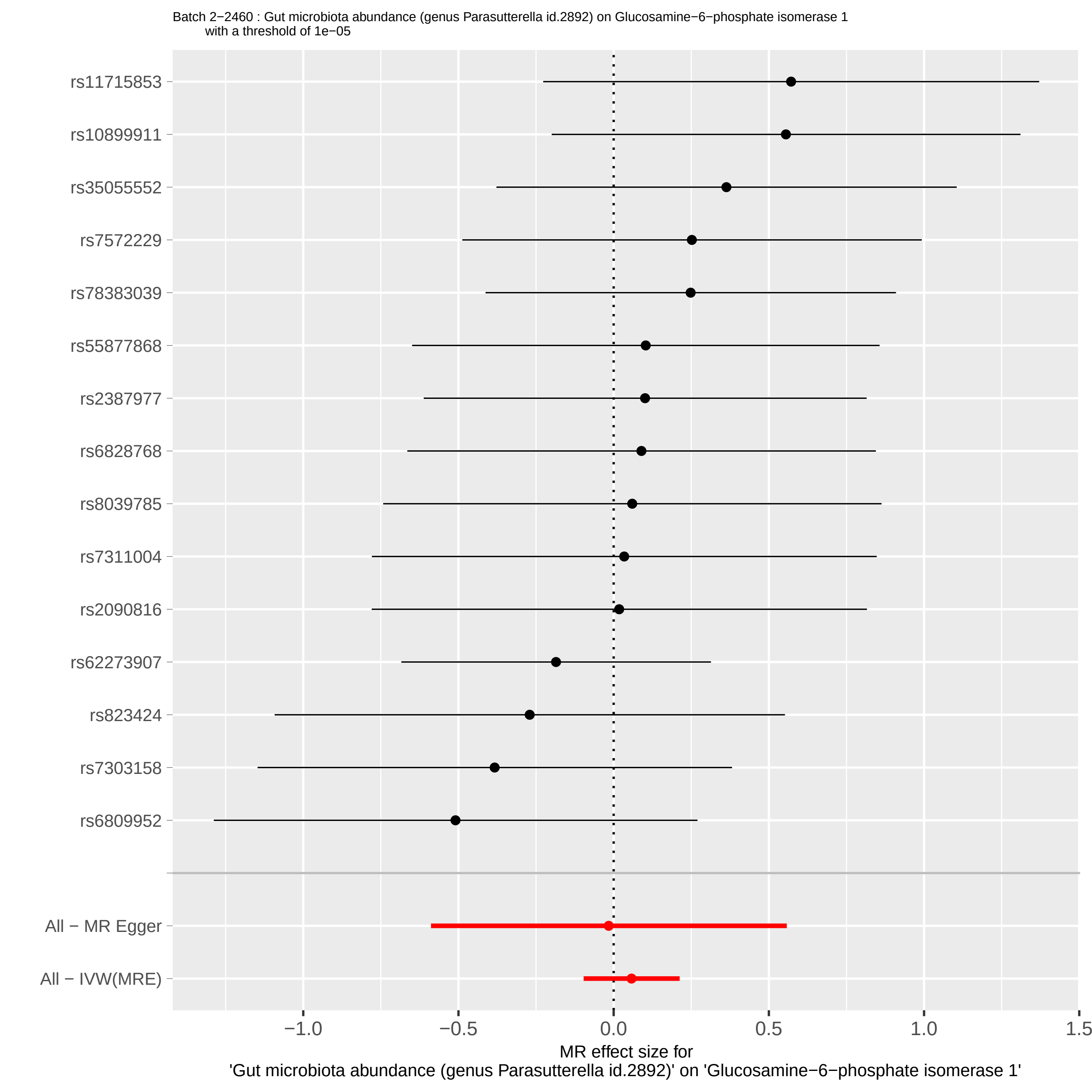

Batch 2−2460 : Gut microbiota abundance (genus Parasutterella id.2892) on Glucosamine−6−phosphate isomerase 1
with a threshold of 1e−05
rs11715853
rs10899911
rs35055552
rs7572229
rs78383039
rs55877868
rs2387977
rs6828768
rs8039785
rs7311004
rs2090816
rs62273907
rs823424
rs7303158
rs6809952
All − MR Egger
All − IVW(MRE)
−1.0
−0.5
0.0
0.5
1.0
1.5
MR effect size for
'Gut microbiota abundance (genus Parasutterella id.2892)' on 'Glucosamine−6−phosphate isomerase 1'

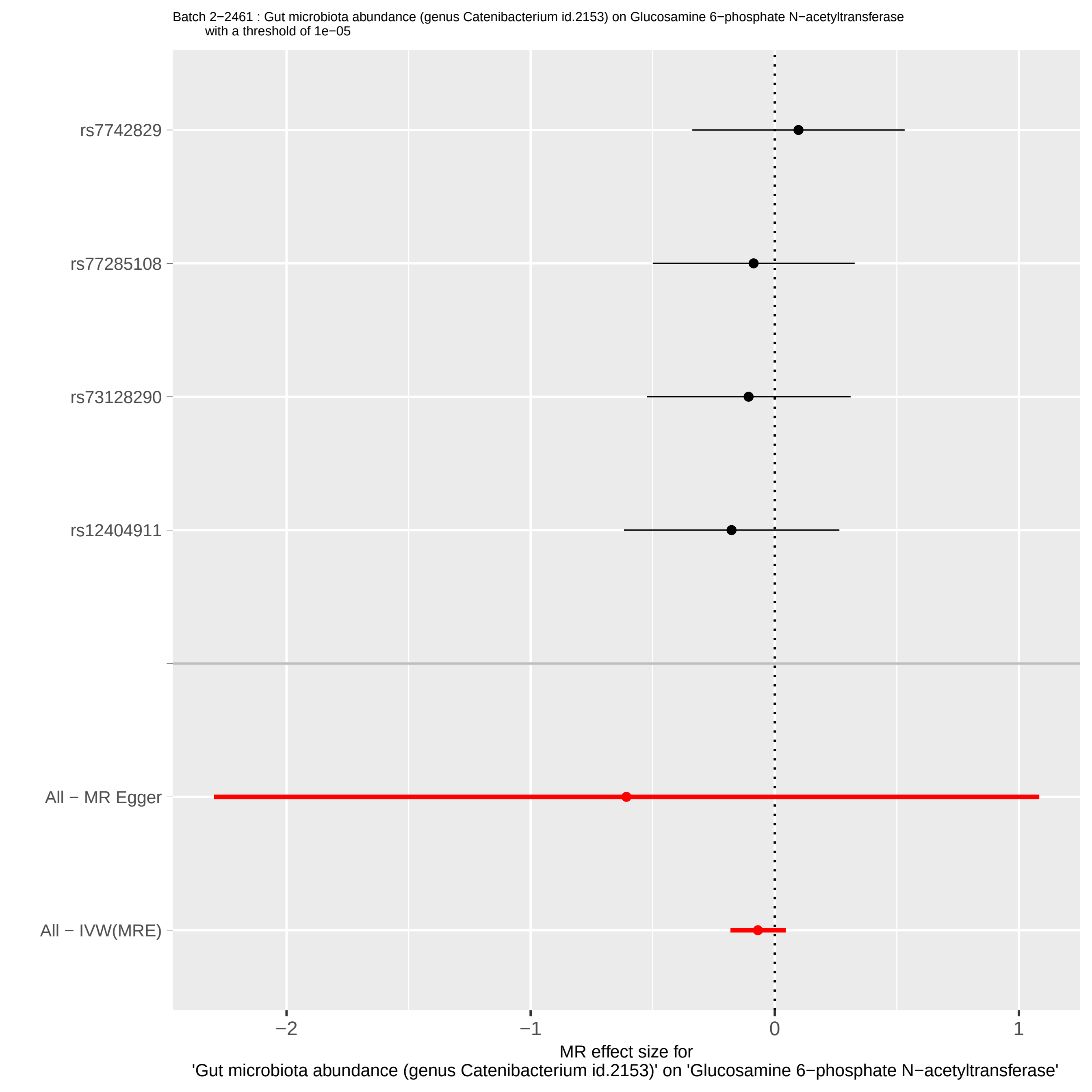
Batch 2−2461 : Gut microbiota abundance (genus Catenibacterium id.2153) on Glucosamine 6−phosphate N−acetyltransferase
with a threshold of 1e−05
rs7742829
rs77285108
rs73128290
rs12404911
All − MR Egger
All − IVW(MRE)
−2
−1
0
1
MR effect size for
'Gut microbiota abundance (genus Catenibacterium id.2153)' on 'Glucosamine 6−phosphate N−acetyltransferase'

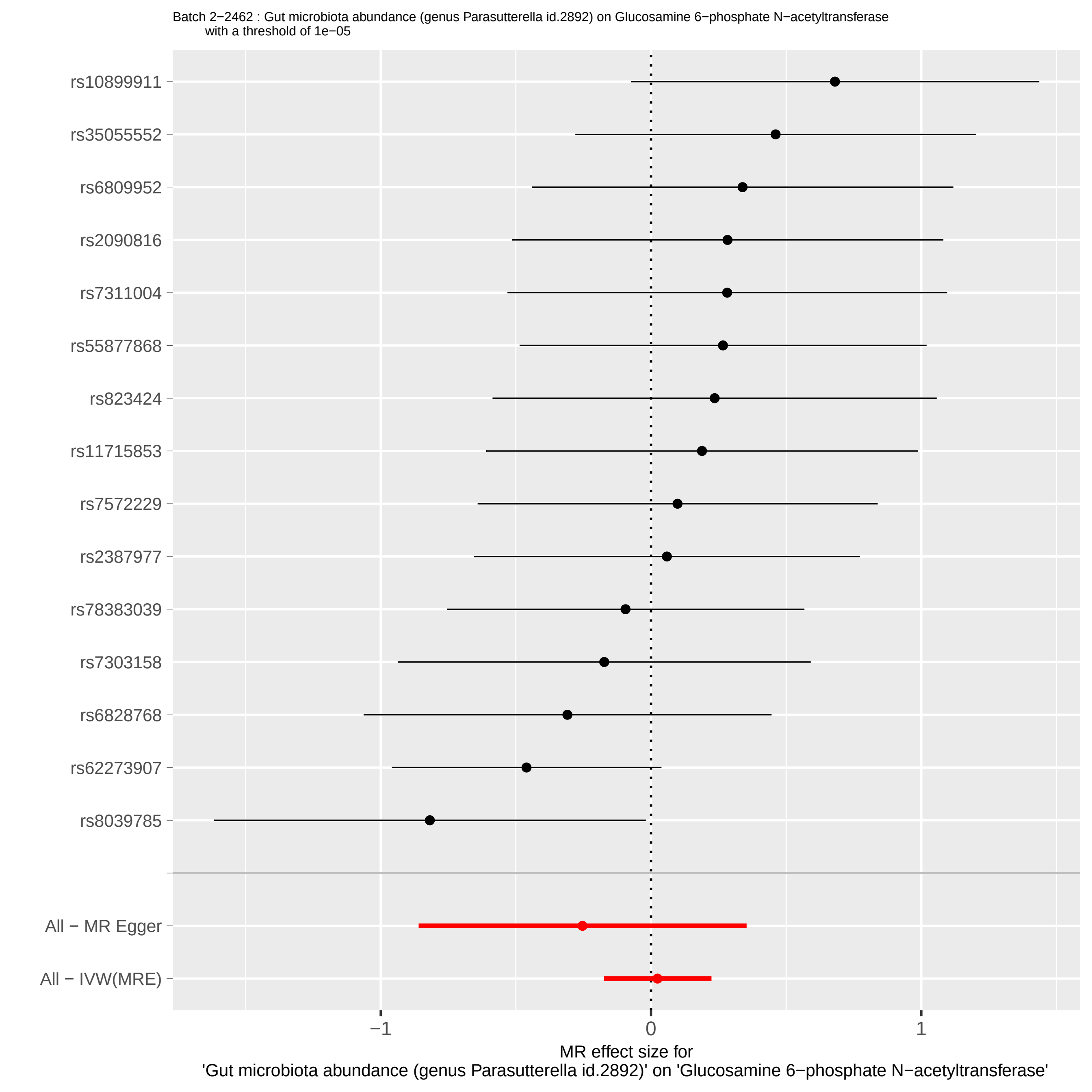
Batch 2−2462 : Gut microbiota abundance (genus Parasutterella id.2892) on Glucosamine 6−phosphate N−acetyltransferase
with a threshold of 1e−05
rs10899911
rs35055552
rs6809952
rs2090816
rs7311004
rs55877868
rs823424
rs11715853
rs7572229
rs2387977
rs78383039
rs7303158
rs6828768
rs62273907
rs8039785
All − MR Egger
All − IVW(MRE)
−1
0
1
MR effect size for
'Gut microbiota abundance (genus Parasutterella id.2892)' on 'Glucosamine 6−phosphate N−acetyltransferase'

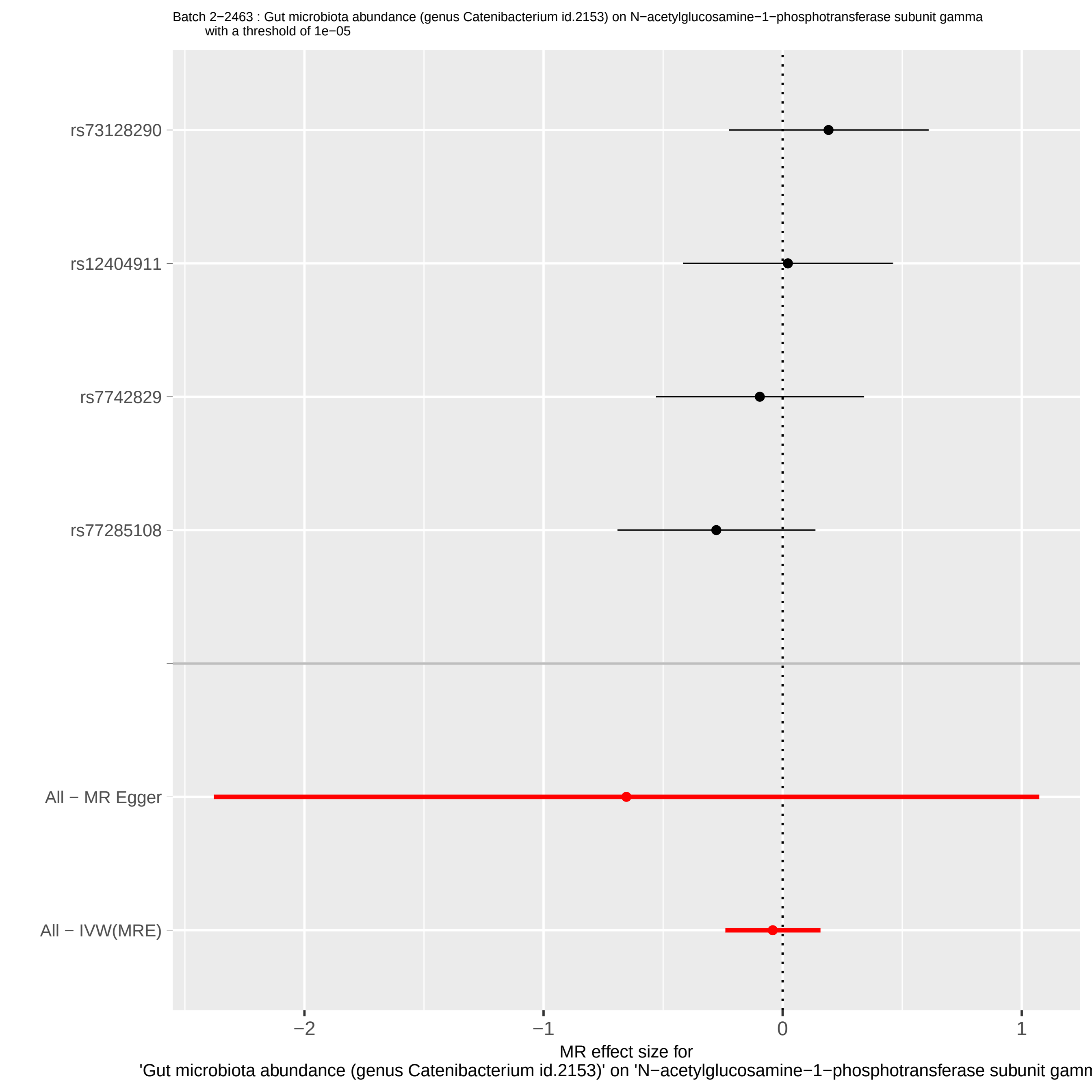

Batch 2−2463 : Gut microbiota abundance (genus Catenibacterium id.2153) on N−acetylglucosamine−1−phosphotransferase subunit gamma
with a threshold of 1e−05
rs73128290
rs12404911
rs7742829
rs77285108
All − MR Egger
All − IVW(MRE)
−2
−1
0
1
MR effect size for
'Gut microbiota abundance (genus Catenibacterium id.2153)' on 'N−acetylglucosamine−1−phosphotransferase subunit gamm

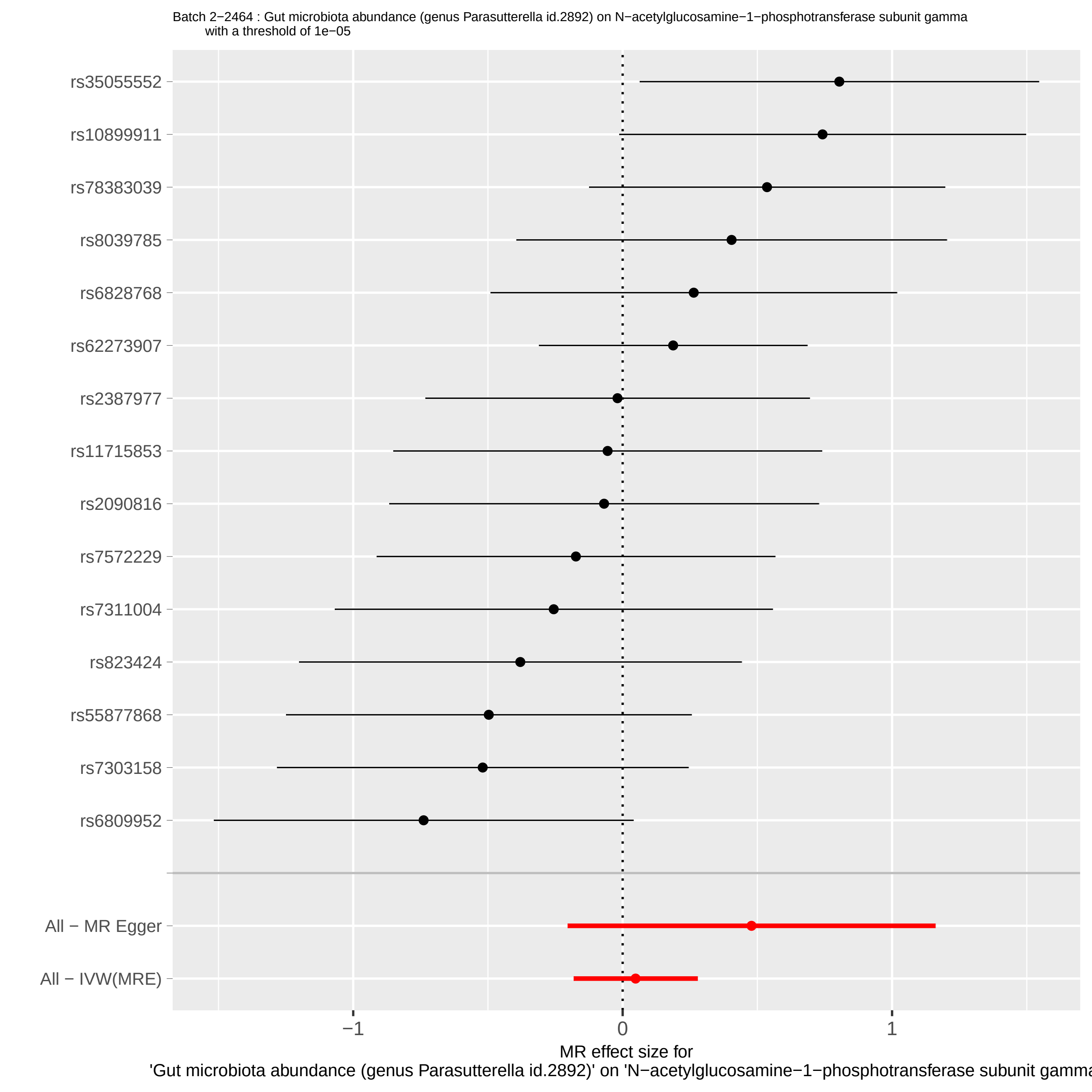

Batch 2−2464 : Gut microbiota abundance (genus Parasutterella id.2892) on N−acetylglucosamine−1−phosphotransferase subunit gamma
with a threshold of 1e−05
rs35055552
rs10899911
rs78383039
rs8039785
rs6828768
rs62273907
rs2387977
rs11715853
rs2090816
rs7572229
rs7311004
rs823424
rs55877868
rs7303158
rs6809952
All − MR Egger
All − IVW(MRE)
−1
0
1
MR effect size for
'Gut microbiota abundance (genus Parasutterella id.2892)' on 'N−acetylglucosamine−1−phosphotransferase subunit gamma

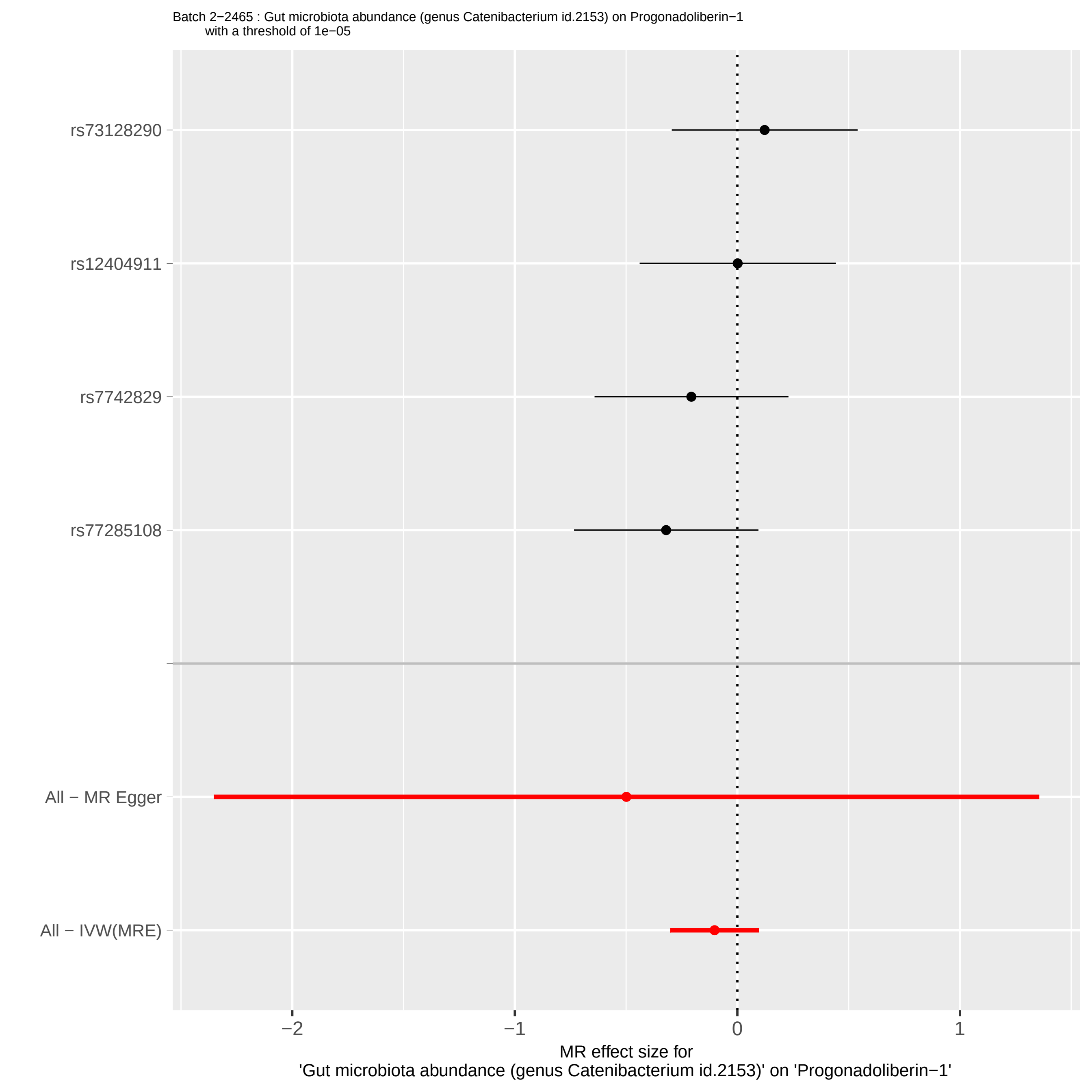
Batch 2−2465 : Gut microbiota abundance (genus Catenibacterium id.2153) on Progonadoliberin−1
with a threshold of 1e−05
rs73128290
rs12404911
rs7742829
rs77285108
All − MR Egger
All − IVW(MRE)
−2
−1
0
1
MR effect size for
'Gut microbiota abundance (genus Catenibacterium id.2153)' on 'Progonadoliberin−1'

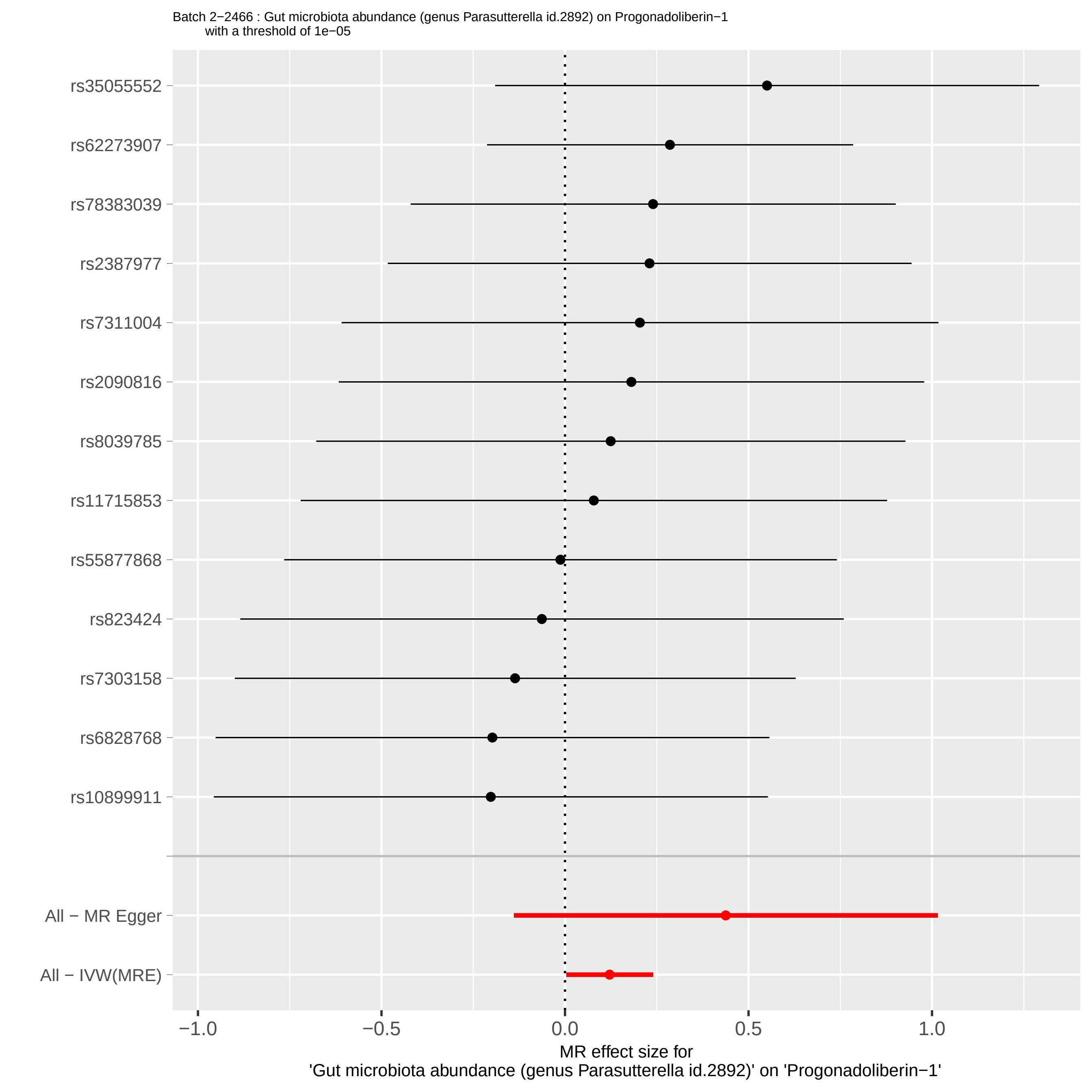

Batch 2−2466 : Gut microbiota abundance (genus Parasutterella id.2892) on Progonadoliberin−1
with a threshold of 1e−05
rs35055552
rs62273907
rs78383039
rs2387977
rs7311004
rs2090816
rs8039785
rs11715853
rs55877868
rs823424
rs7303158
rs6828768
rs10899911
All − MR Egger
All − IVW(MRE)
−1.0
−0.5
0.0
0.5
1.0
MR effect size for
'Gut microbiota abundance (genus Parasutterella id.2892)' on 'Progonadoliberin−1'

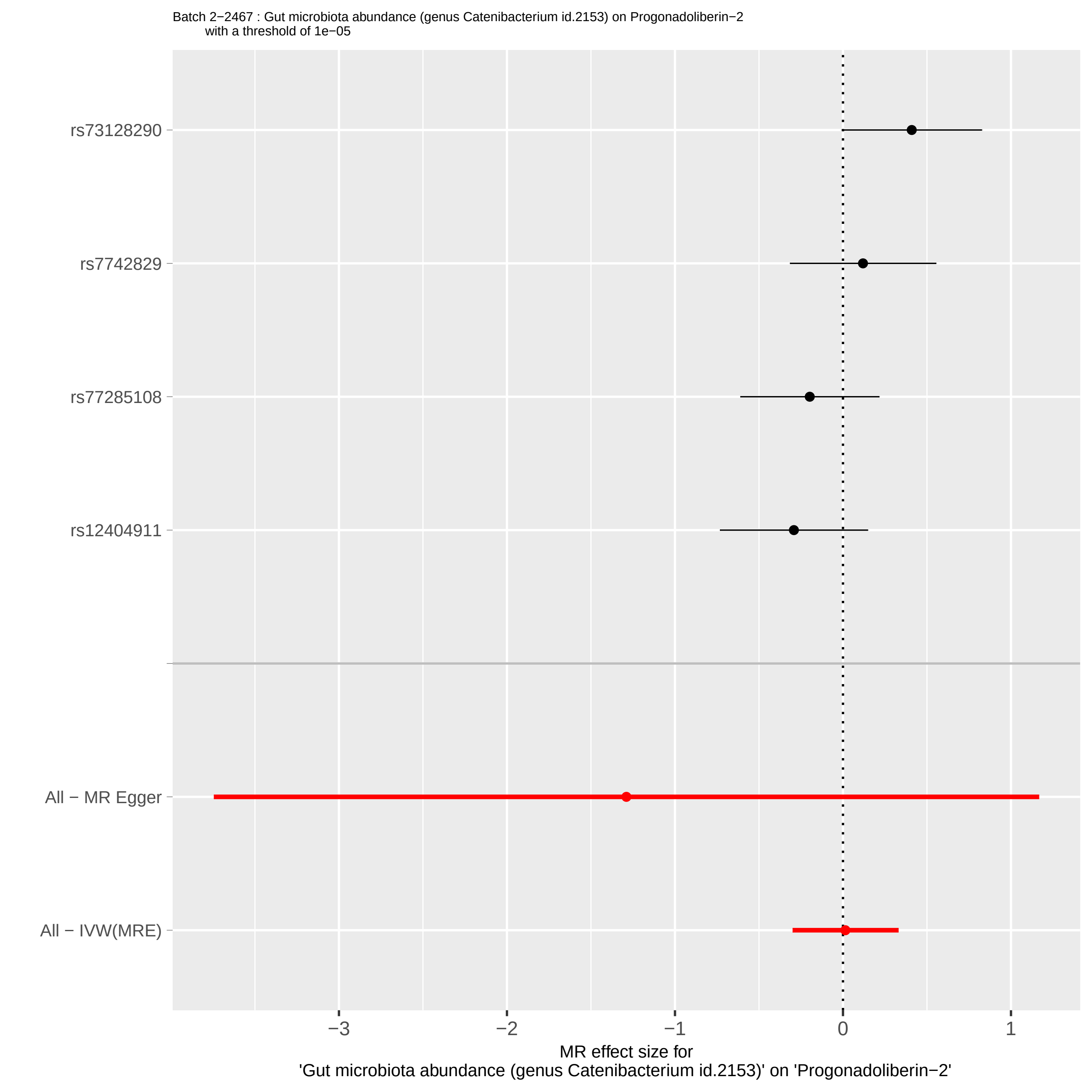
Batch 2−2467 : Gut microbiota abundance (genus Catenibacterium id.2153) on Progonadoliberin−2
with a threshold of 1e−05
rs73128290
rs7742829
rs77285108
rs12404911
All − MR Egger
All − IVW(MRE)
−3
−2
−1
0
1
MR effect size for
'Gut microbiota abundance (genus Catenibacterium id.2153)' on 'Progonadoliberin−2'

Batch 2−2468 : Gut microbiota abundance (genus Parasutterella id.2892) on Progonadoliberin−2
with a threshold of 1e−05

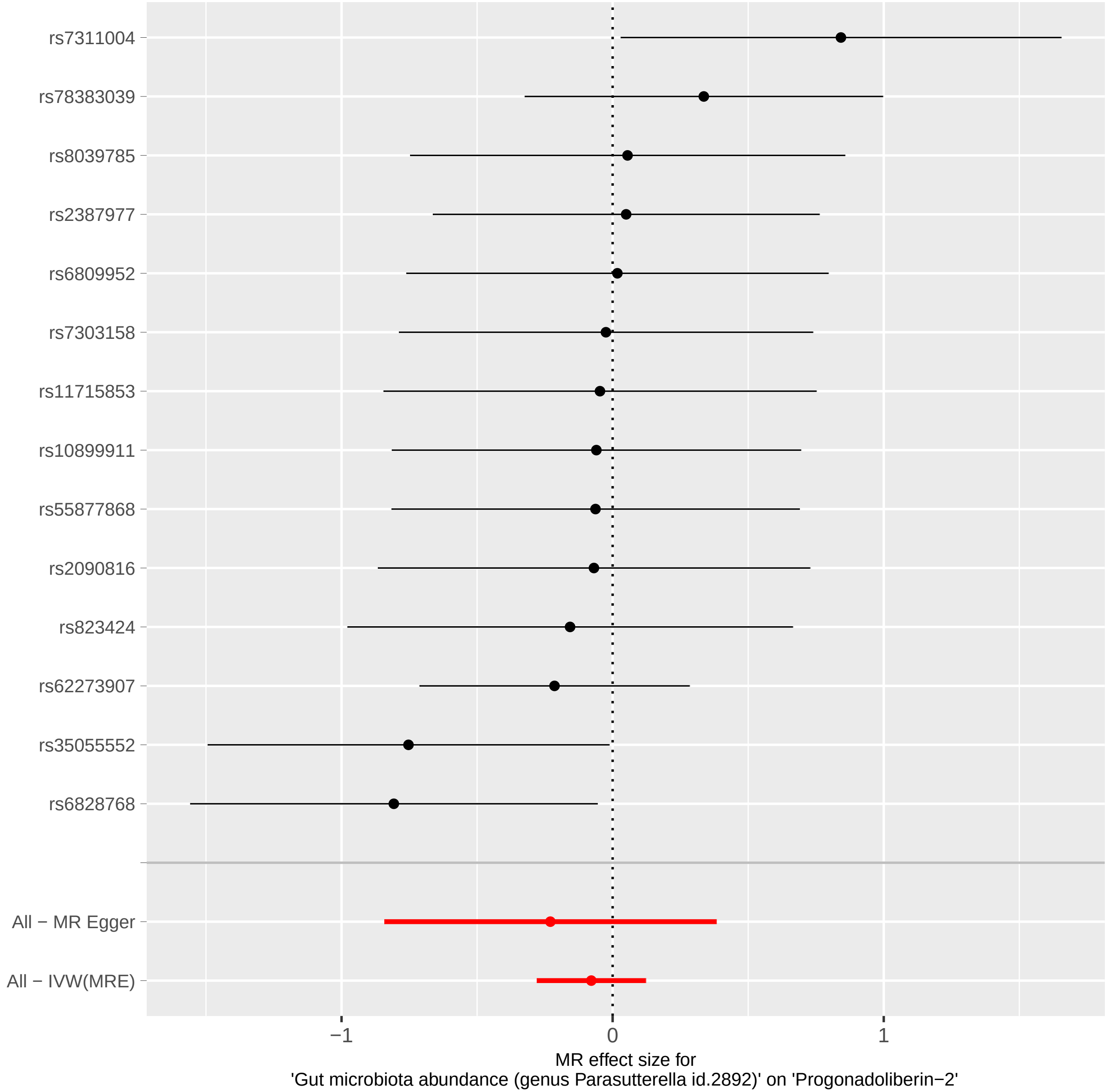

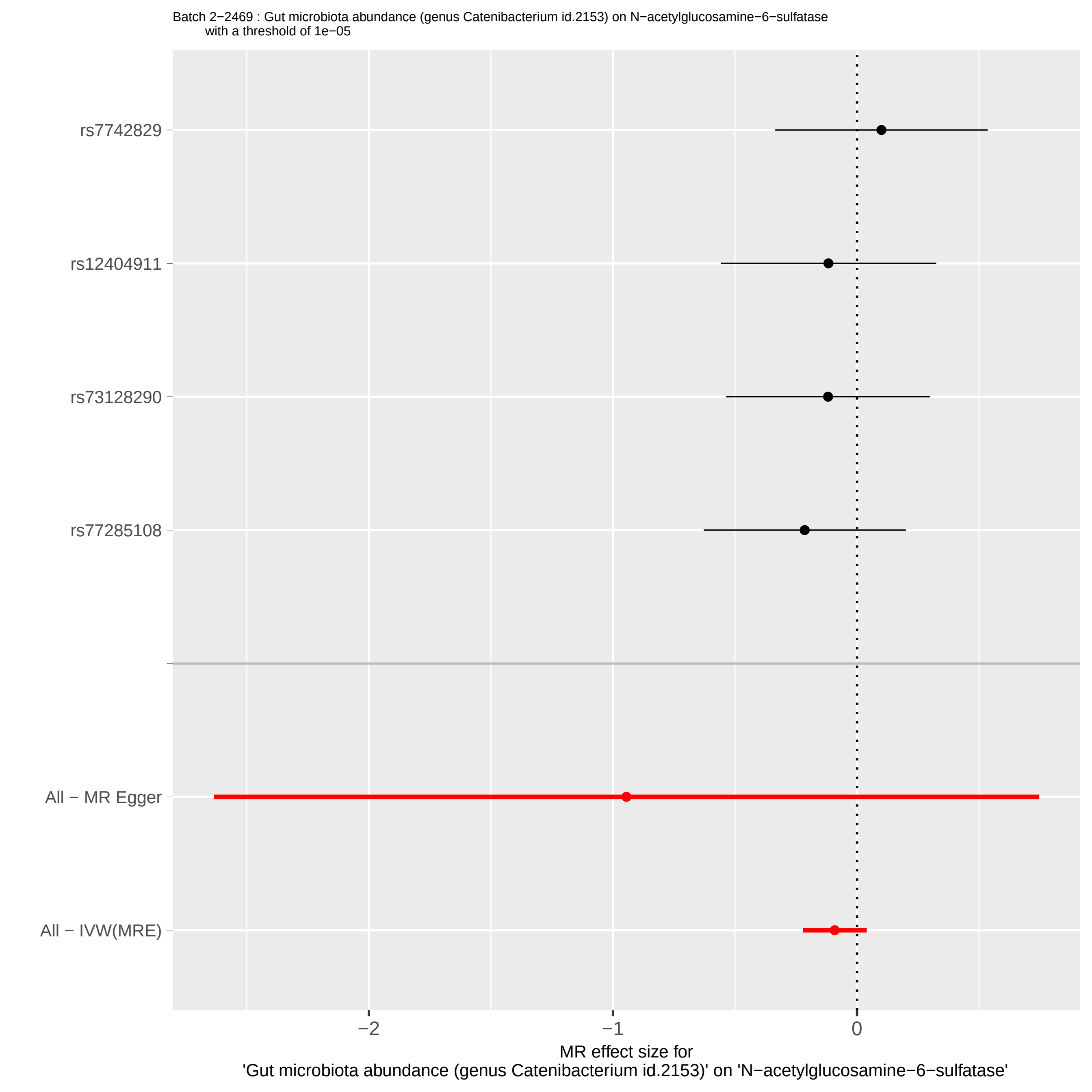

Batch 2−2469 : Gut microbiota abundance (genus Catenibacterium id.2153) on N−acetylglucosamine−6−sulfatase
with a threshold of 1e−05
rs7742829
rs12404911
rs73128290
rs77285108
All − MR Egger
All − IVW(MRE)
−2
−1
0
MR effect size for
'Gut microbiota abundance (genus Catenibacterium id.2153)' on 'N−acetylglucosamine−6−sulfatase'

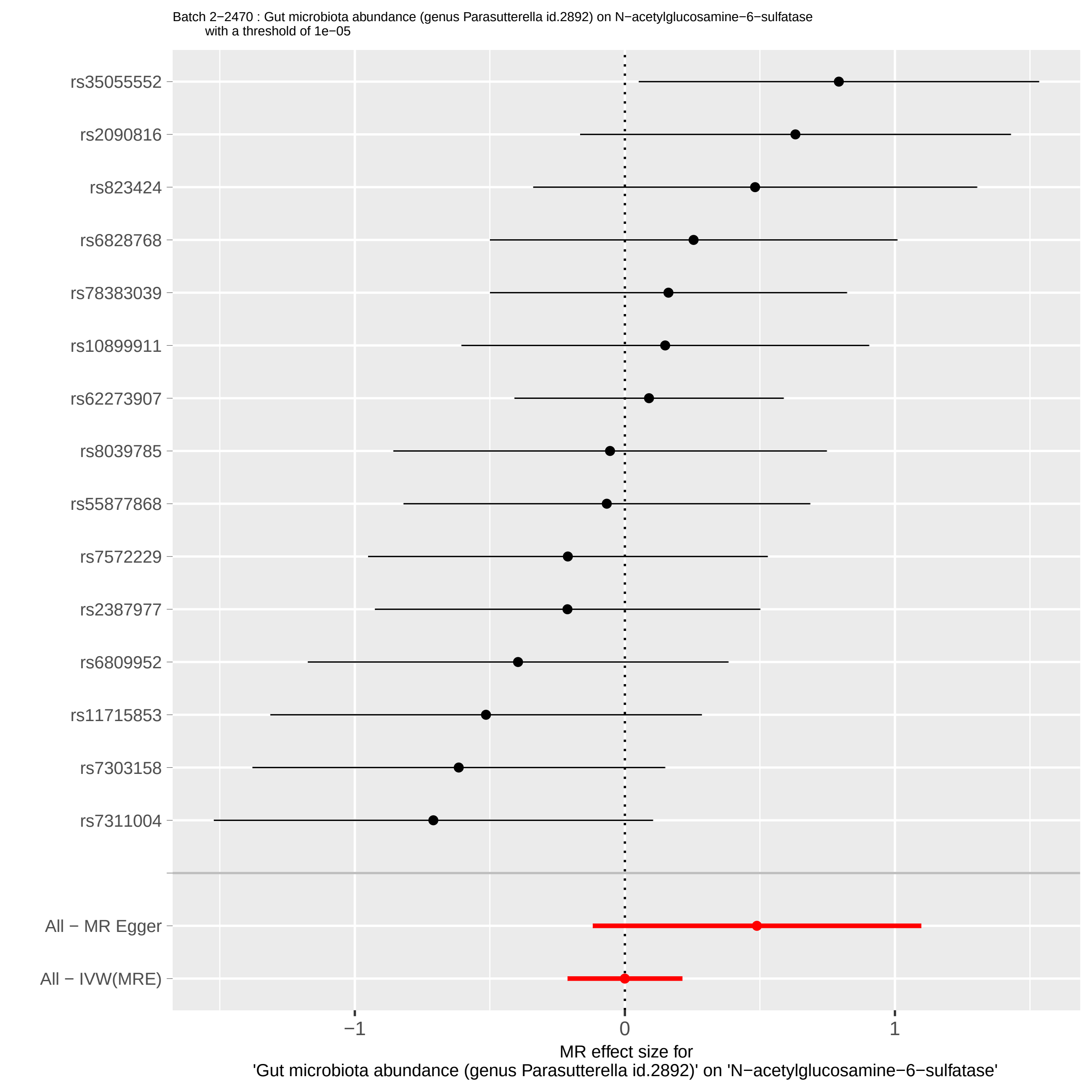
Batch 2−2470 : Gut microbiota abundance (genus Parasutterella id.2892) on N−acetylglucosamine−6−sulfatase
with a threshold of 1e−05
rs35055552
rs2090816
rs823424
rs6828768
rs78383039
rs10899911
rs62273907
rs8039785
rs55877868
rs7572229
rs2387977
rs6809952
rs11715853
rs7303158
rs7311004
All − MR Egger
All − IVW(MRE)
−1
0
1
MR effect size for
'Gut microbiota abundance (genus Parasutterella id.2892)' on 'N−acetylglucosamine−6−sulfatase'

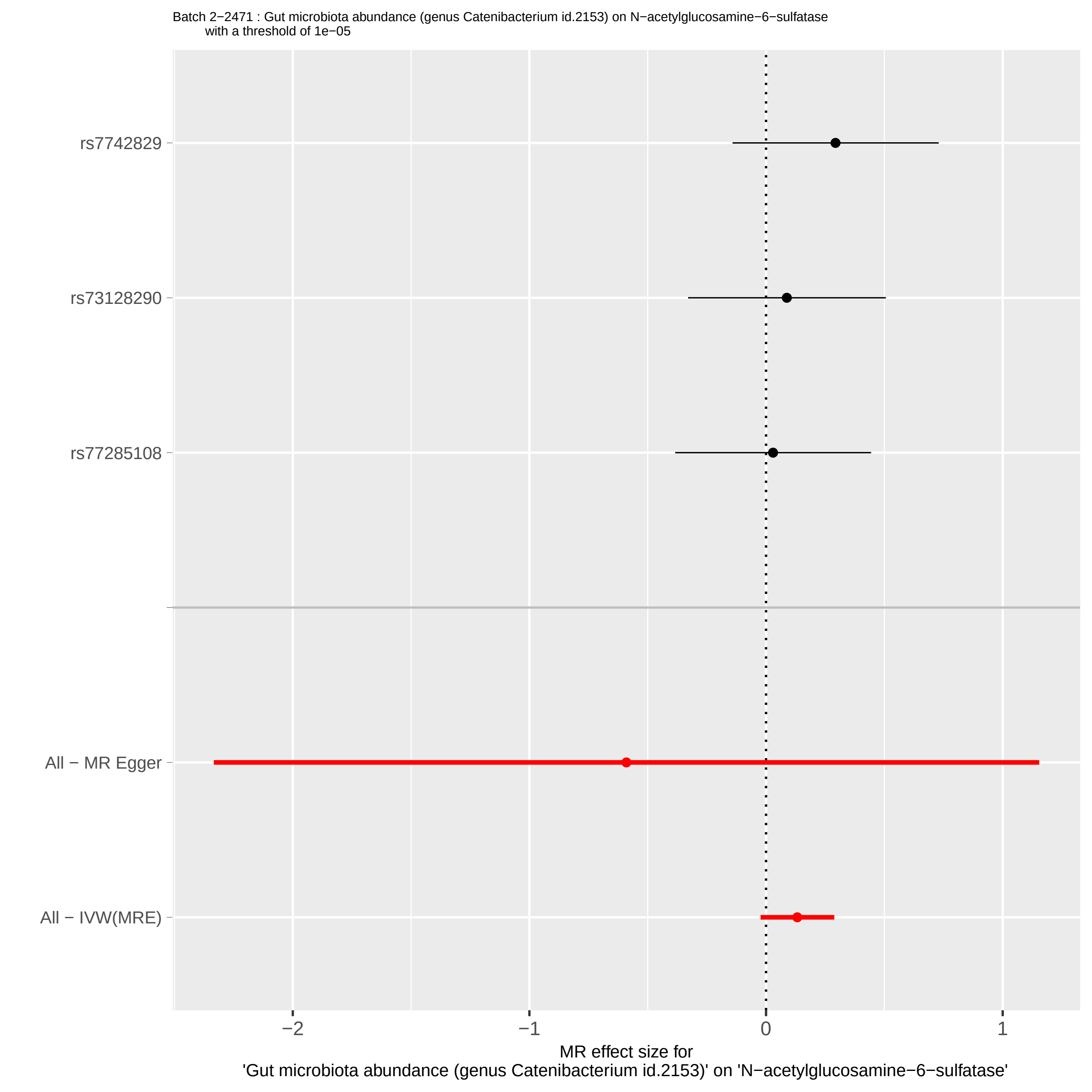

Batch 2−2471 : Gut microbiota abundance (genus Catenibacterium id.2153) on N−acetylglucosamine−6−sulfatase
with a threshold of 1e−05
rs7742829
rs73128290
rs77285108
All − MR Egger
All − IVW(MRE)
−2
−1
0
1
MR effect size for
'Gut microbiota abundance (genus Catenibacterium id.2153)' on 'N−acetylglucosamine−6−sulfatase'

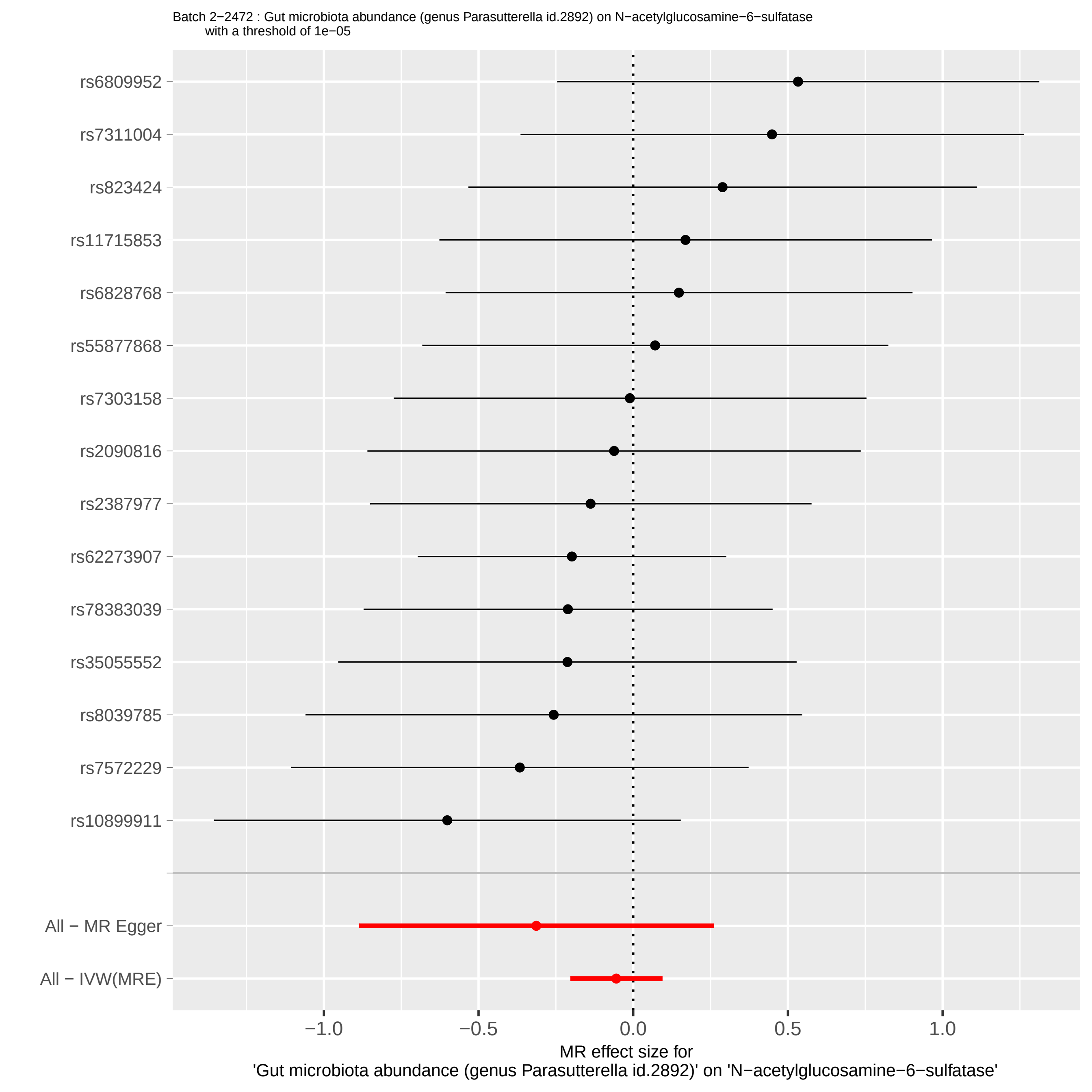

Batch 2−2472 : Gut microbiota abundance (genus Parasutterella id.2892) on N−acetylglucosamine−6−sulfatase
with a threshold of 1e−05
rs6809952
rs7311004
rs823424
rs11715853
rs6828768
rs55877868
rs7303158
rs2090816
rs2387977
rs62273907
rs78383039
rs35055552
rs8039785
rs7572229
rs10899911
All − MR Egger
All − IVW(MRE)
−1.0
−0.5
0.0
0.5
1.0
MR effect size for
'Gut microbiota abundance (genus Parasutterella id.2892)' on 'N−acetylglucosamine−6−sulfatase'

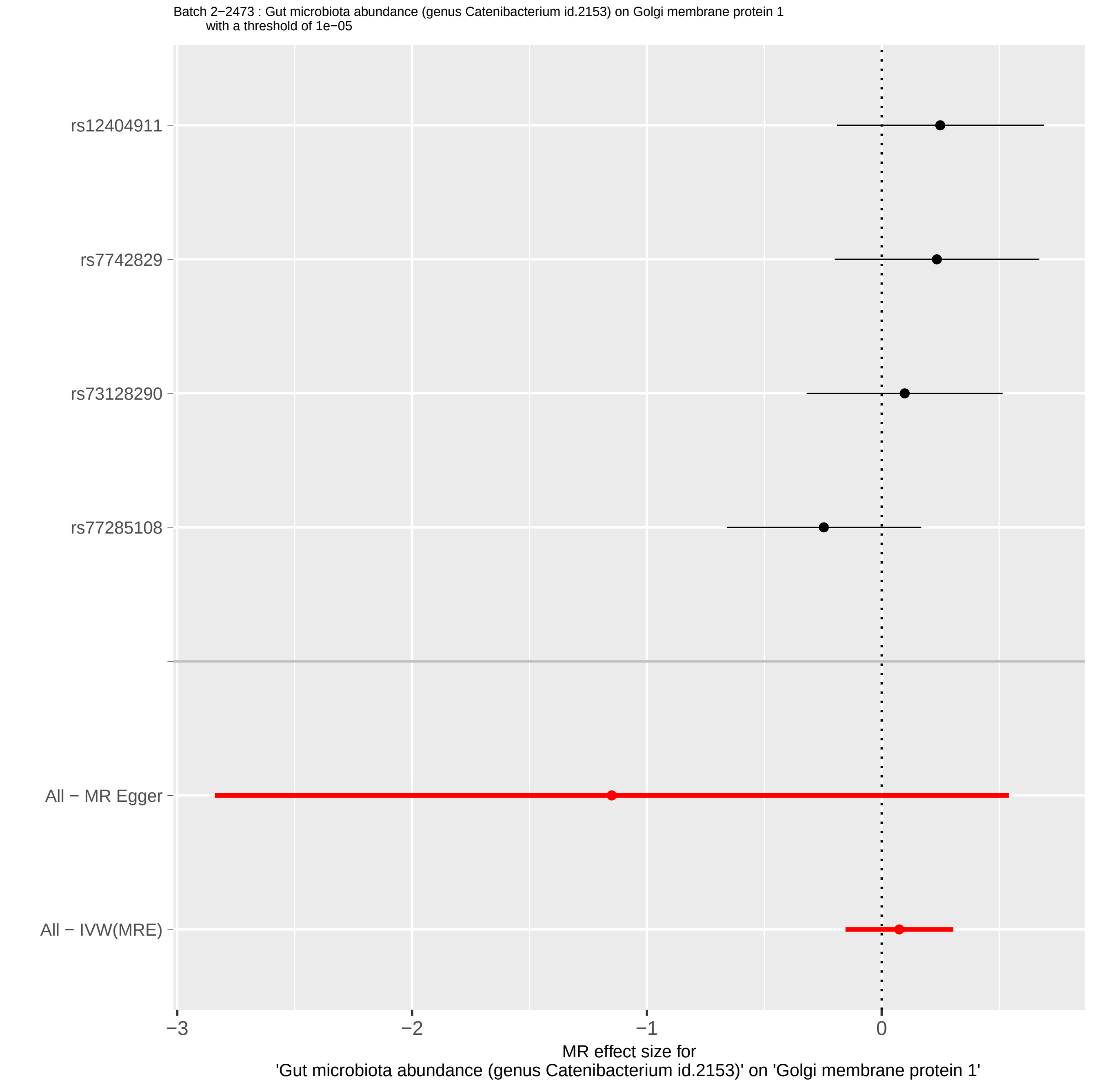

Batch 2−2473 : Gut microbiota abundance (genus Catenibacterium id.2153) on Golgi membrane protein 1
with a threshold of 1e−05
rs12404911
rs7742829
rs73128290
rs77285108
All − MR Egger
All − IVW(MRE)
−3
−2
−1
0
MR effect size for
'Gut microbiota abundance (genus Catenibacterium id.2153)' on 'Golgi membrane protein 1'

Batch 2−2474 : Gut microbiota abundance (genus Parasutterella id.2892) on Golgi membrane protein 1
with a threshold of 1e−05

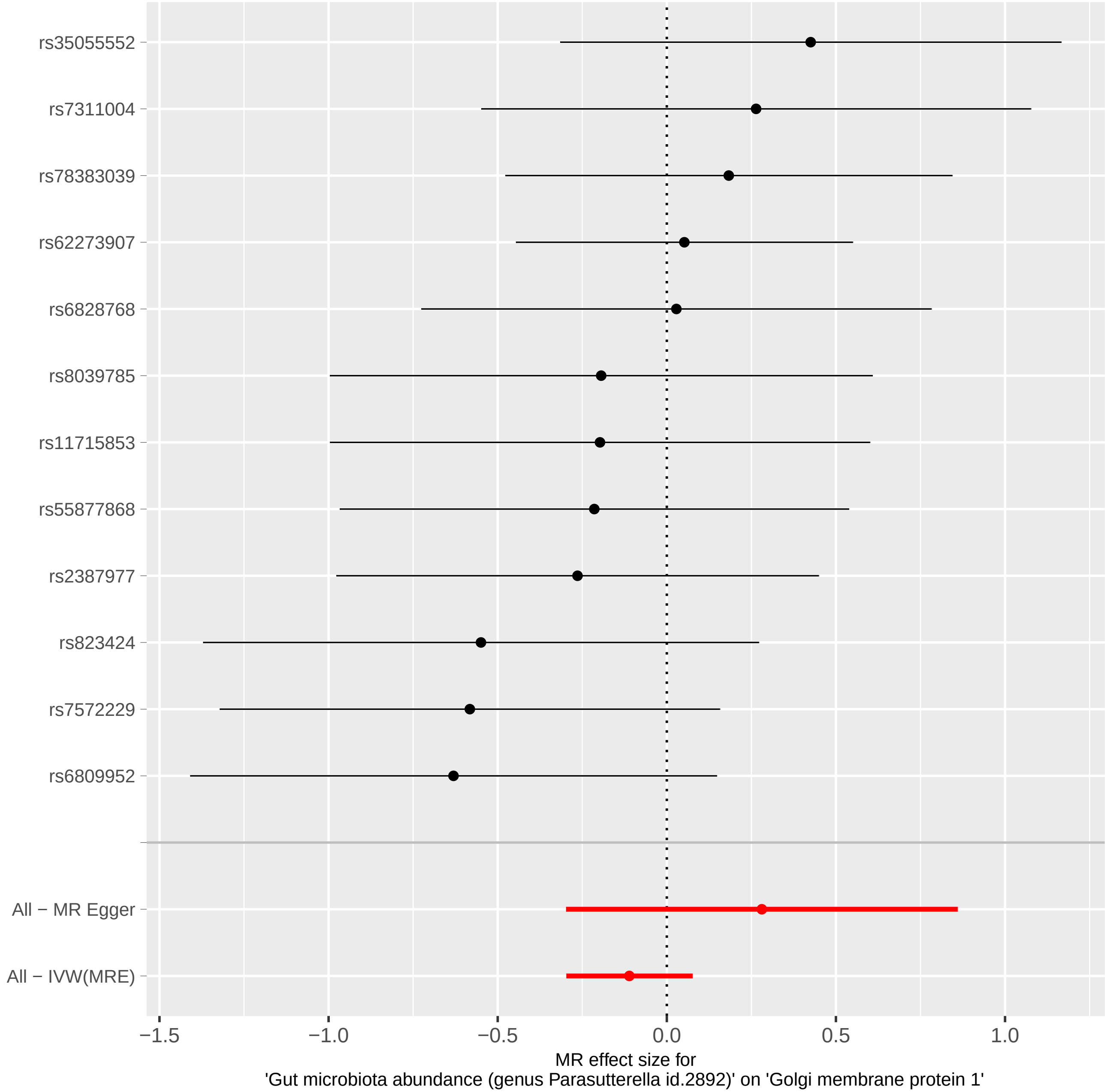

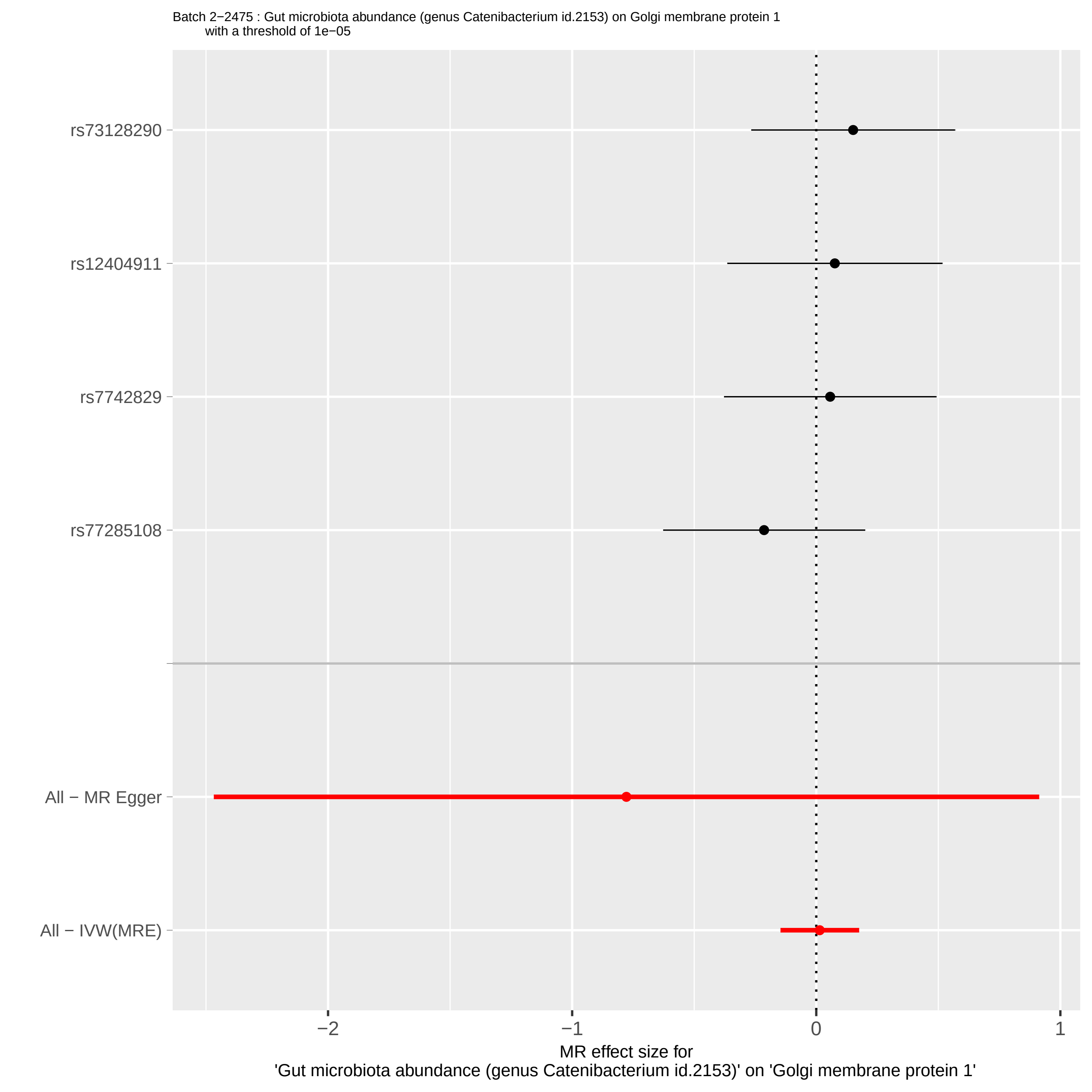

Batch 2−2475 : Gut microbiota abundance (genus Catenibacterium id.2153) on Golgi membrane protein 1
with a threshold of 1e−05
rs73128290
rs12404911
rs7742829
rs77285108
All − MR Egger
All − IVW(MRE)
−2
−1
0
1
MR effect size for
'Gut microbiota abundance (genus Catenibacterium id.2153)' on 'Golgi membrane protein 1'

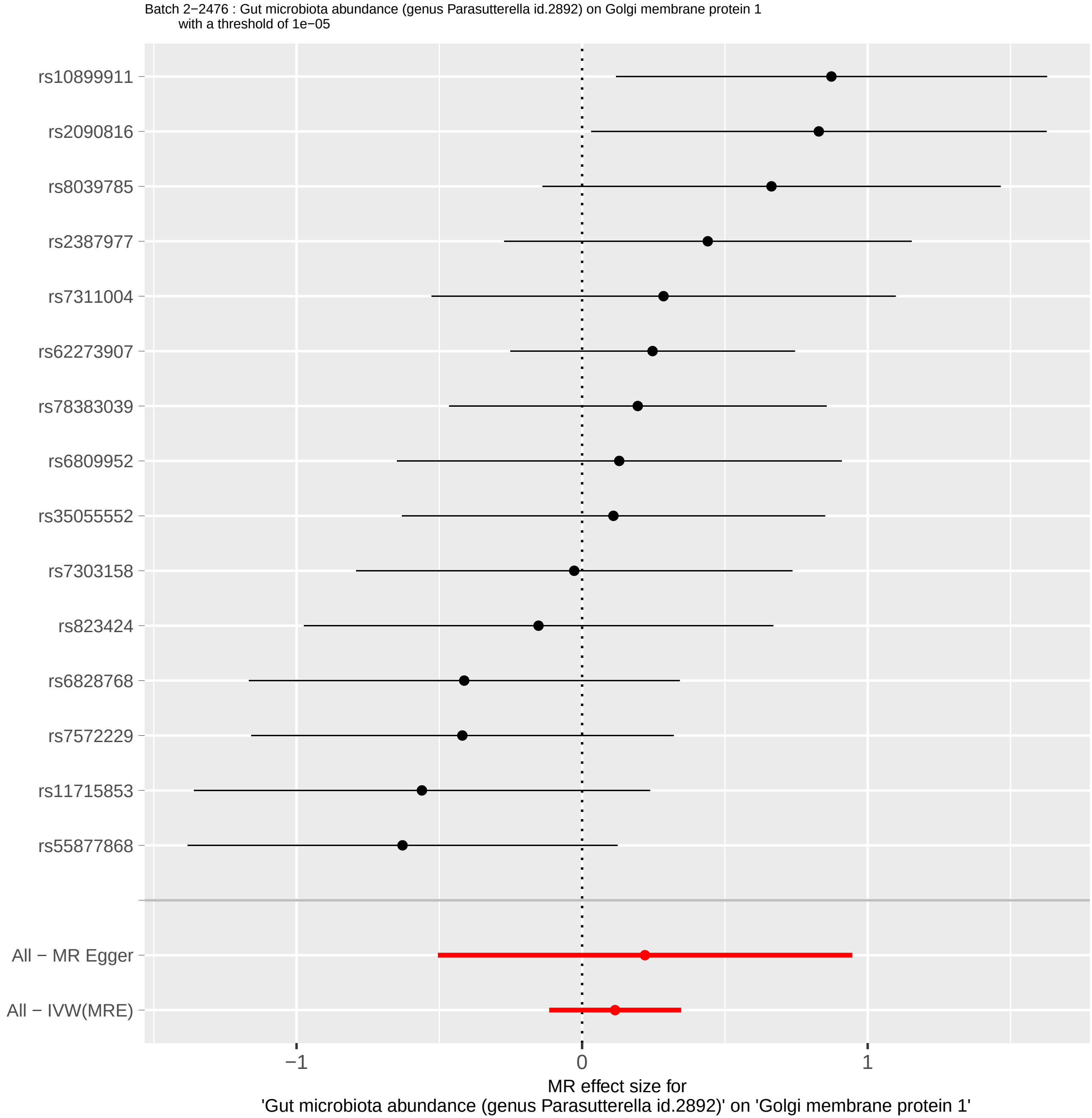
Batch 2−2476 : Gut microbiota abundance (genus Parasutterella id.2892) on Golgi membrane protein 1
with a threshold of 1e−05
rs10899911
rs2090816
rs8039785
rs2387977
rs7311004
rs62273907
rs78383039
rs6809952
rs35055552
rs7303158
rs823424
rs6828768
rs7572229
rs11715853
rs55877868
All − MR Egger
All − IVW(MRE)
−1
0
1
MR effect size for
'Gut microbiota abundance (genus Parasutterella id.2892)' on 'Golgi membrane protein 1'

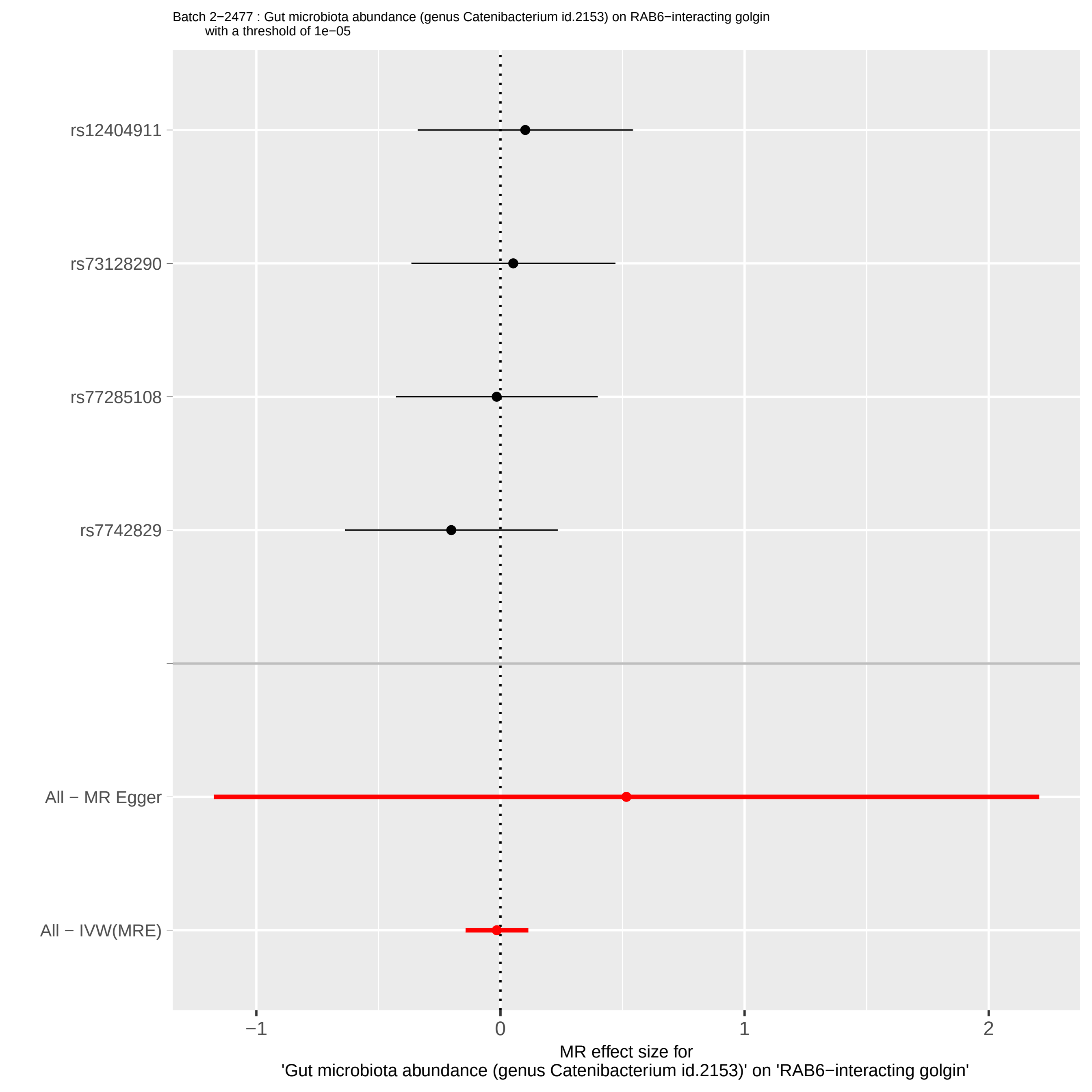

Batch 2−2477 : Gut microbiota abundance (genus Catenibacterium id.2153) on RAB6−interacting golgin
with a threshold of 1e−05
rs12404911
rs73128290
rs77285108
rs7742829
All − MR Egger
All − IVW(MRE)
−1
0
1
2
MR effect size for
'Gut microbiota abundance (genus Catenibacterium id.2153)' on 'RAB6−interacting golgin'

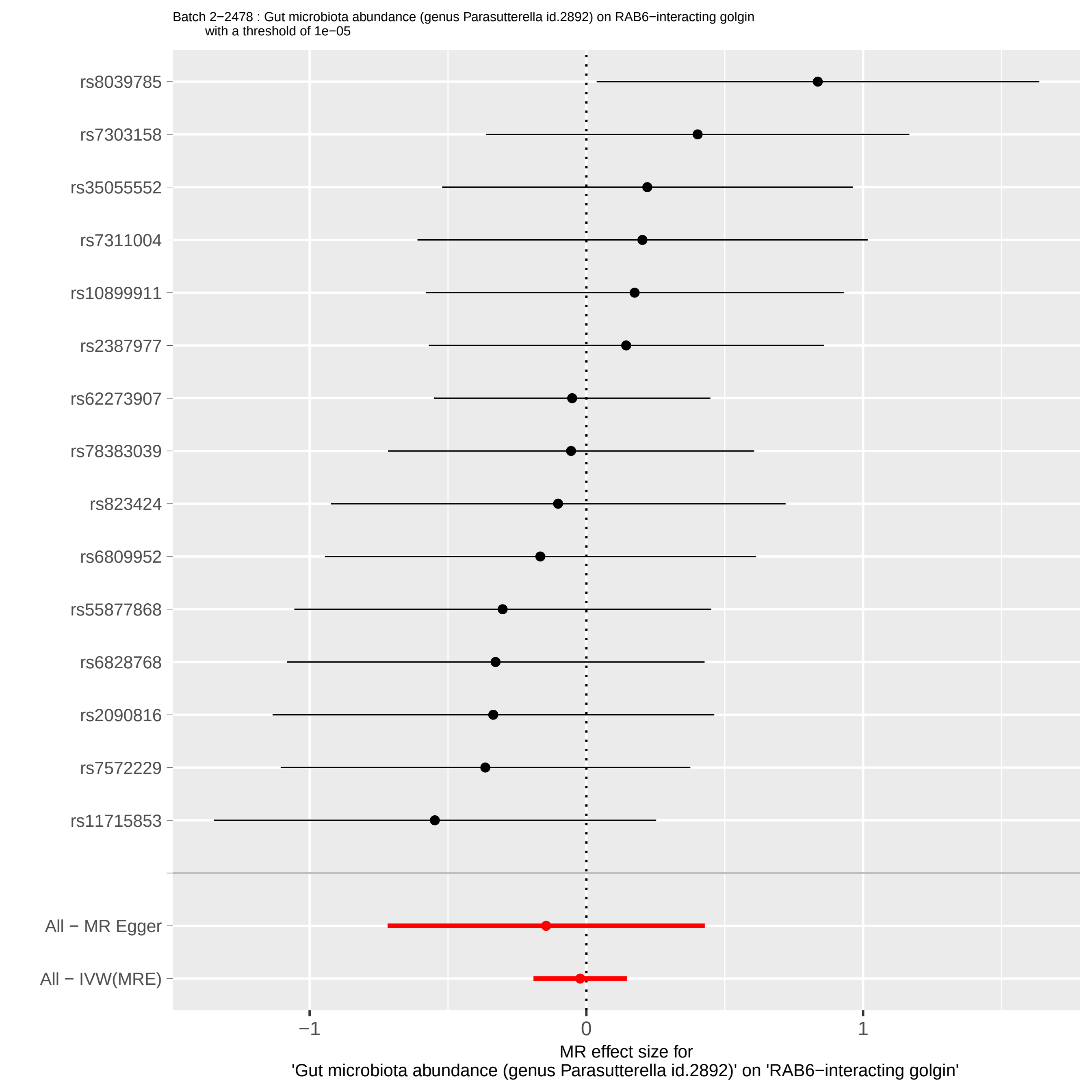
Batch 2−2478 : Gut microbiota abundance (genus Parasutterella id.2892) on RAB6−interacting golgin
with a threshold of 1e−05
rs8039785
rs7303158
rs35055552
rs7311004
rs10899911
rs2387977
rs62273907
rs78383039
rs823424
rs6809952
rs55877868
rs6828768
rs2090816
rs7572229
rs11715853
All − MR Egger
All − IVW(MRE)
−1
0
1
MR effect size for
'Gut microbiota abundance (genus Parasutterella id.2892)' on 'RAB6−interacting golgin'

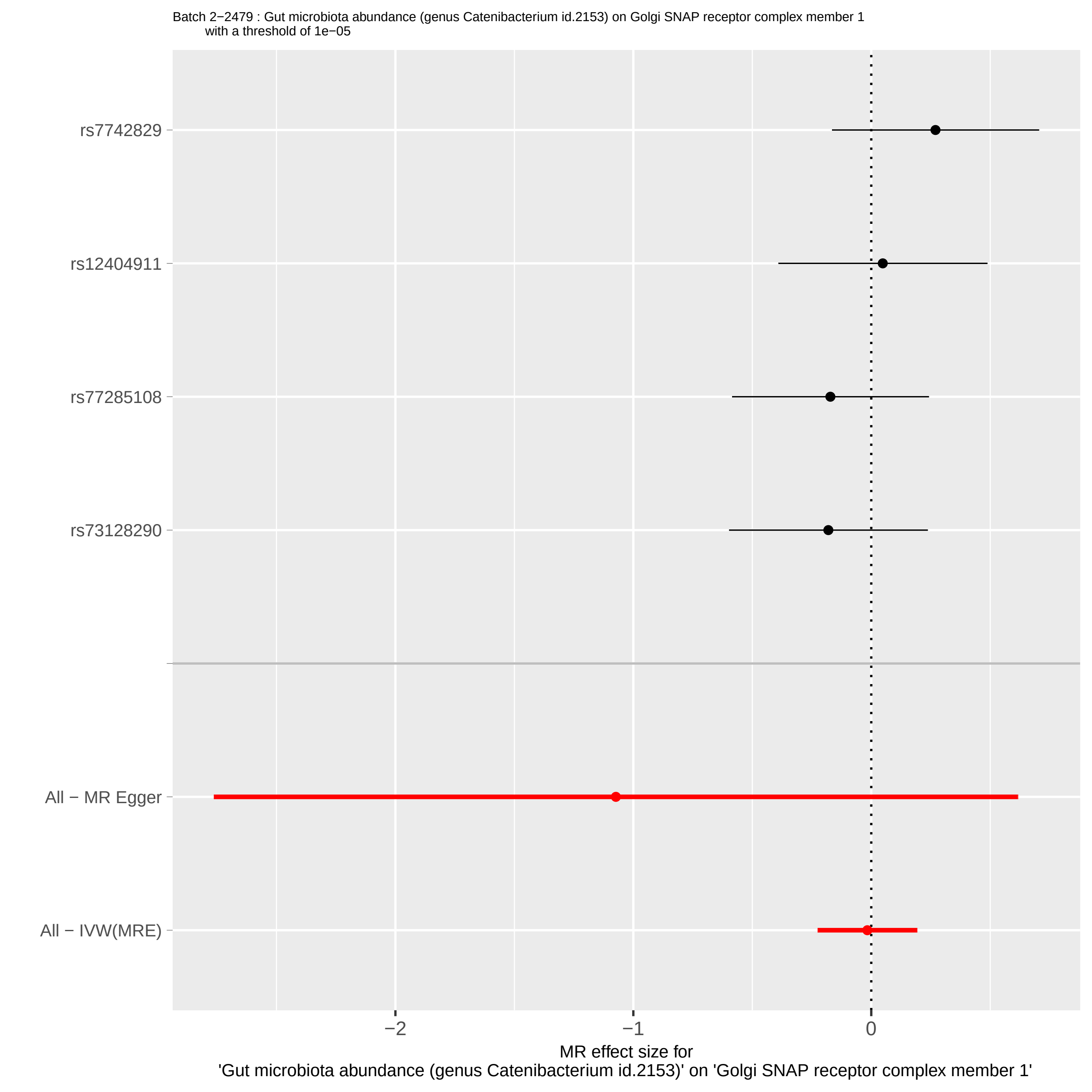
Batch 2−2479 : Gut microbiota abundance (genus Catenibacterium id.2153) on Golgi SNAP receptor complex member 1
with a threshold of 1e−05
rs7742829
rs12404911
rs77285108
rs73128290
All − MR Egger
All − IVW(MRE)
−2
−1
0
MR effect size for
'Gut microbiota abundance (genus Catenibacterium id.2153)' on 'Golgi SNAP receptor complex member 1'

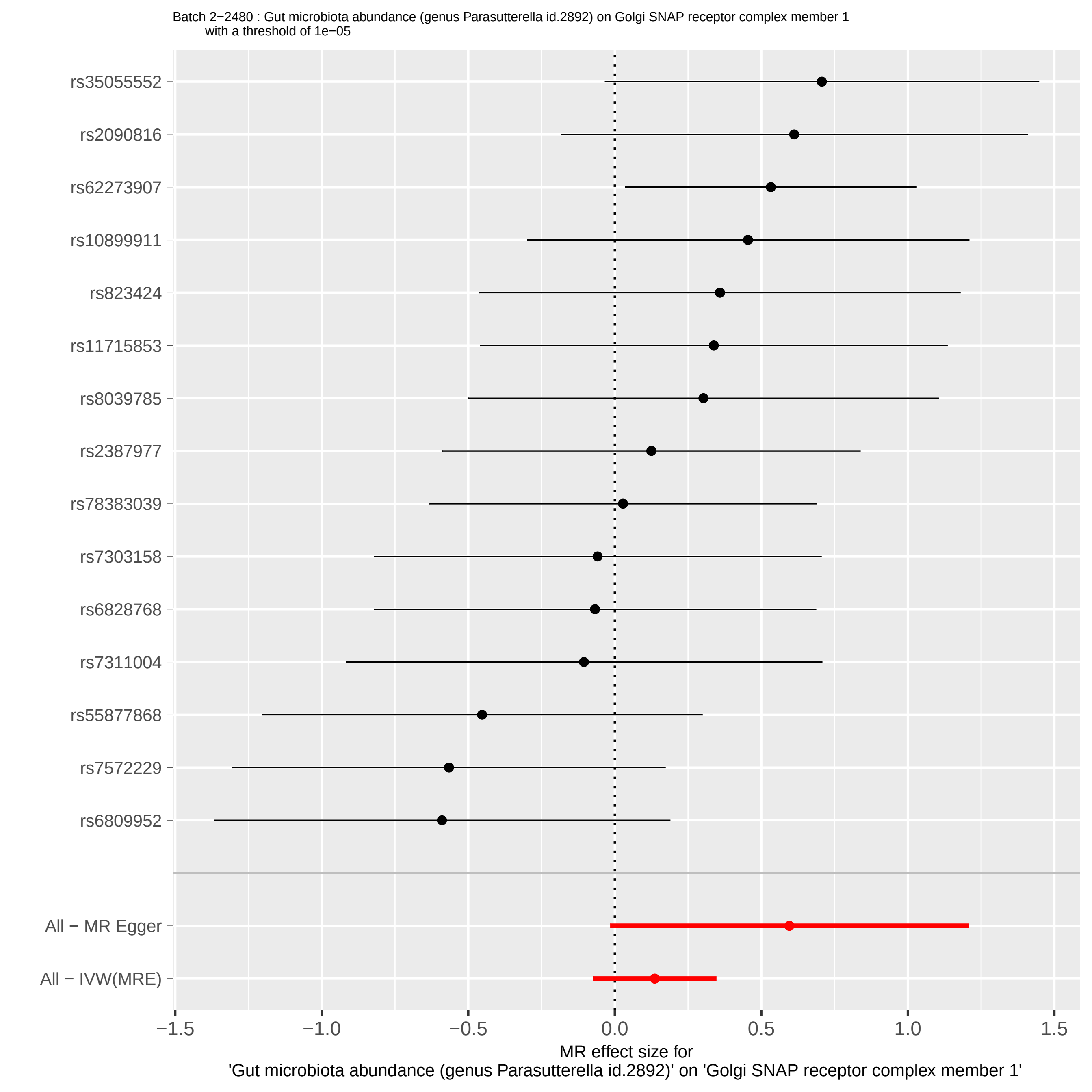
Batch 2−2480 : Gut microbiota abundance (genus Parasutterella id.2892) on Golgi SNAP receptor complex member 1
with a threshold of 1e−05
rs35055552
rs2090816
rs62273907
rs10899911
rs823424
rs11715853
rs8039785
rs2387977
rs78383039
rs7303158
rs6828768
rs7311004
rs55877868
rs7572229
rs6809952
All − MR Egger
All − IVW(MRE)
−1.5
−1.0
−0.5
0.0
0.5
1.0
1.5
MR effect size for
'Gut microbiota abundance (genus Parasutterella id.2892)' on 'Golgi SNAP receptor complex member 1'

Batch 2−2481 : Gut microbiota abundance (genus Catenibacterium id.2153) on Aspartate aminotransferase, cytoplasmic
with a threshold of 1e−05

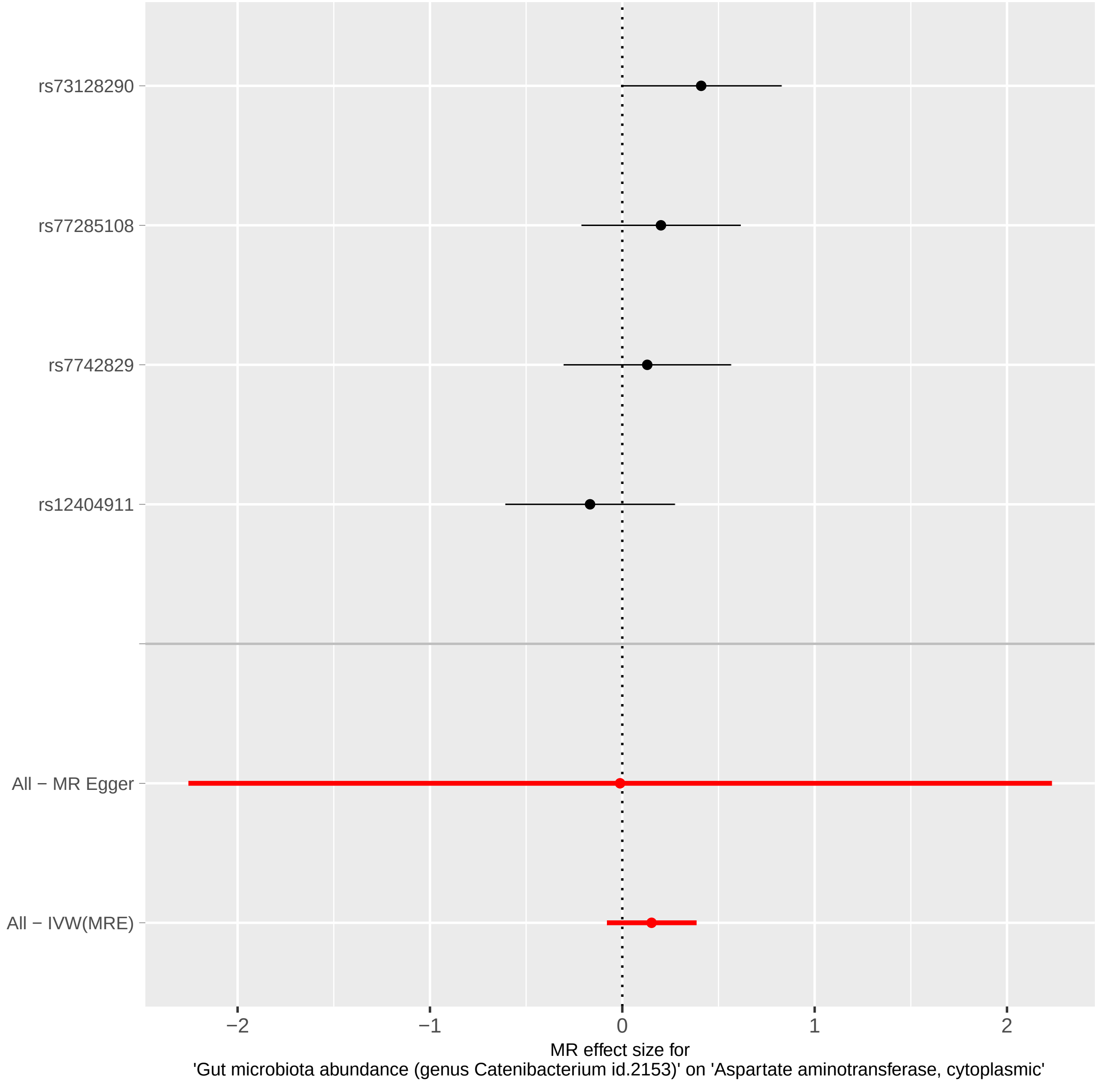

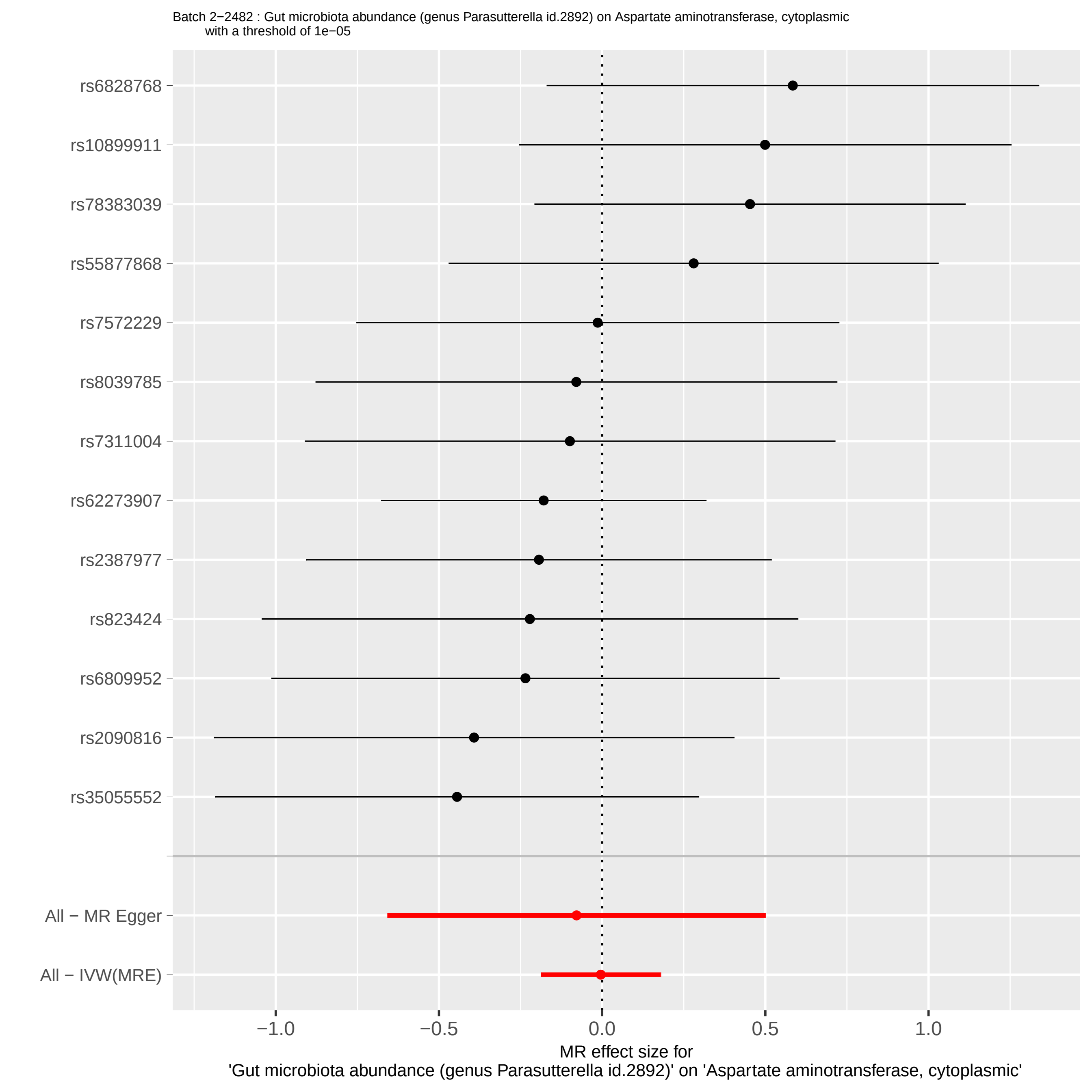
Batch 2−2482 : Gut microbiota abundance (genus Parasutterella id.2892) on Aspartate aminotransferase, cytoplasmic
with a threshold of 1e−05
rs6828768
rs10899911
rs78383039
rs55877868
rs7572229
rs8039785
rs7311004
rs62273907
rs2387977
rs823424
rs6809952
rs2090816
rs35055552
All − MR Egger
All − IVW(MRE)
−1.0
−0.5
0.0
0.5
1.0
MR effect size for
'Gut microbiota abundance (genus Parasutterella id.2892)' on 'Aspartate aminotransferase, cytoplasmic'

Batch 2−2483 : Gut microbiota abundance (genus Catenibacterium id.2153) on Platelet glycoprotein Ib alpha chain
with a threshold of 1e−05

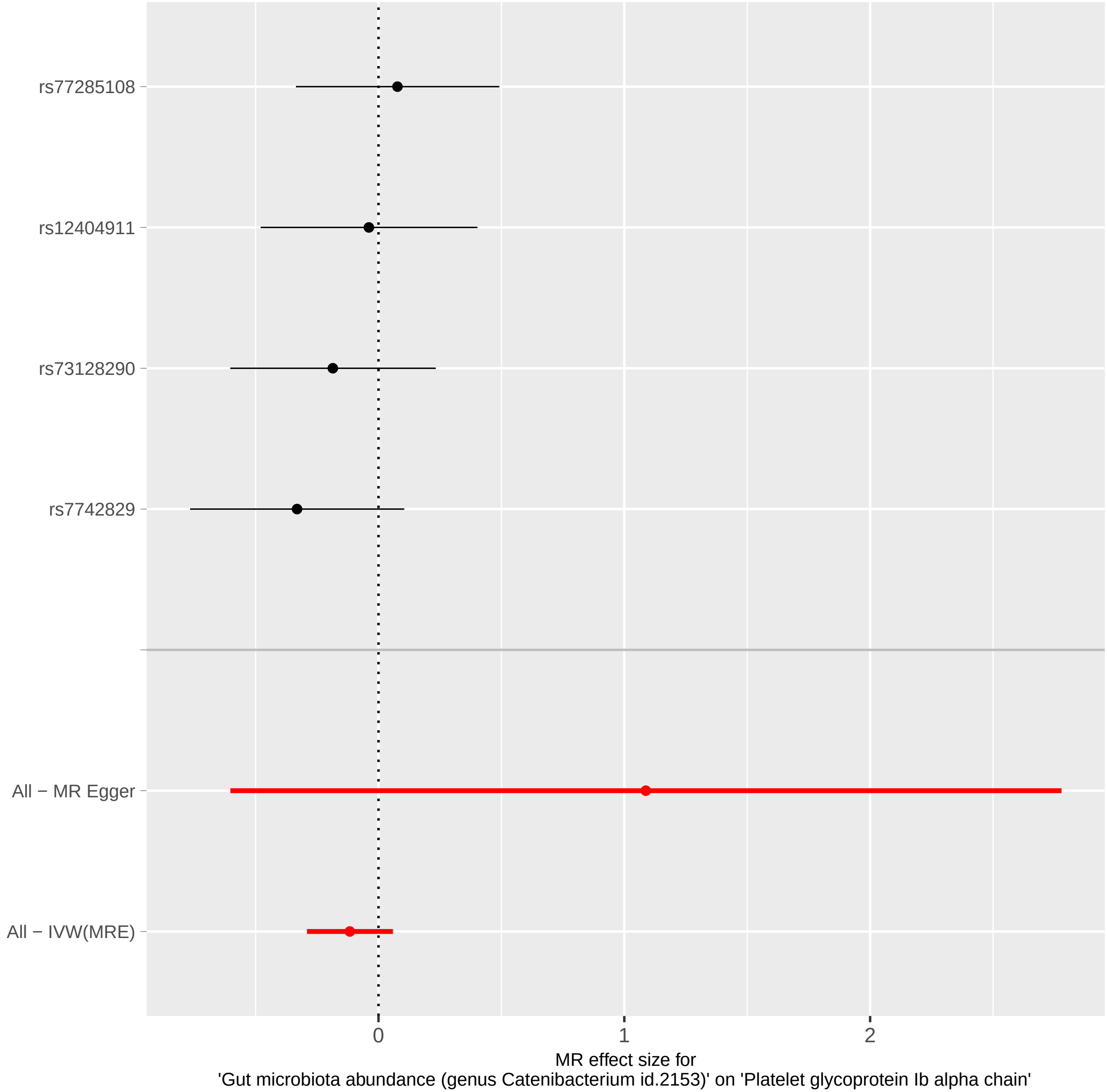

Batch 2−2484 : Gut microbiota abundance (genus Parasutterella id.2892) on Platelet glycoprotein Ib alpha chain
with a threshold of 1e−05

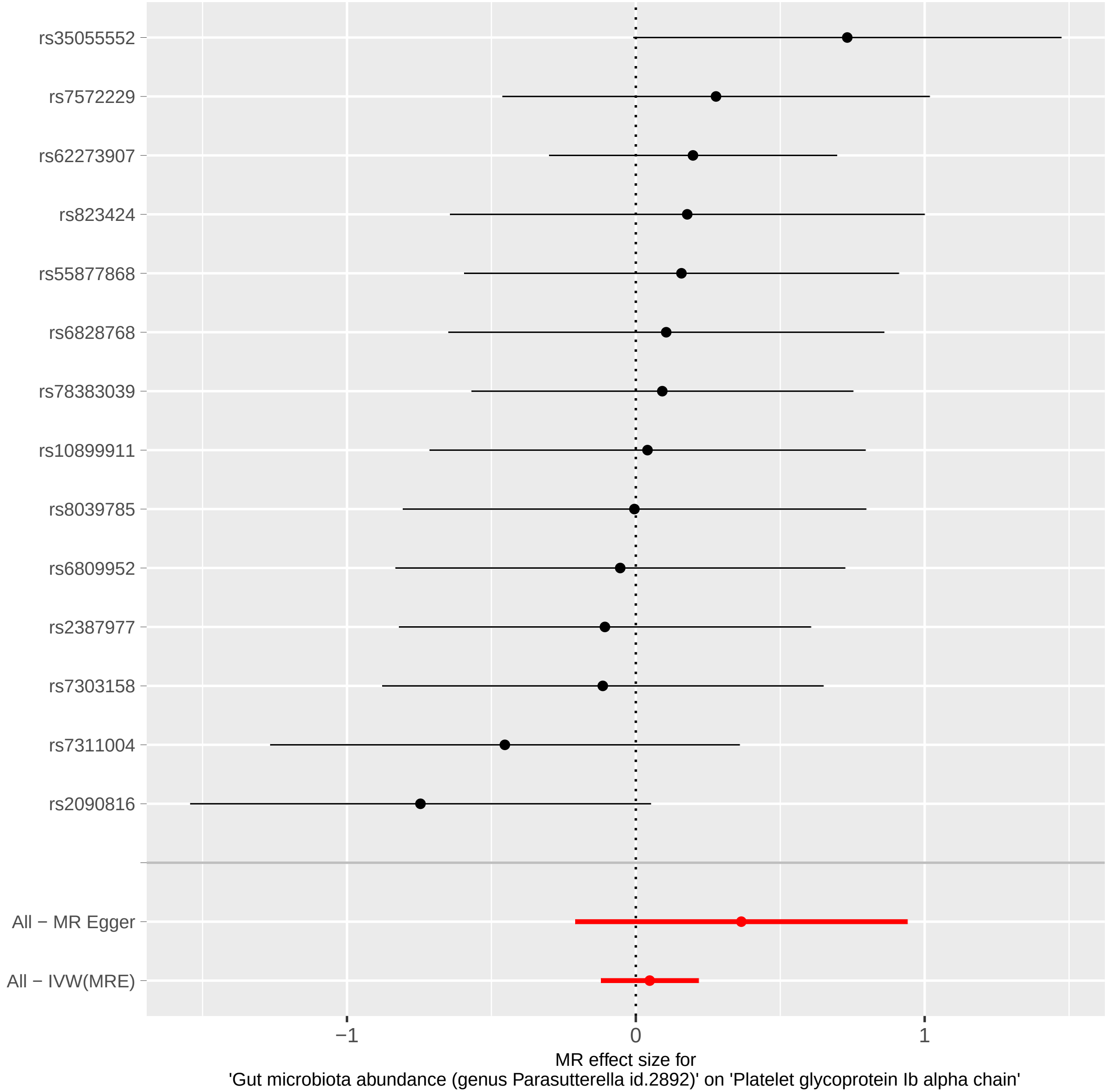

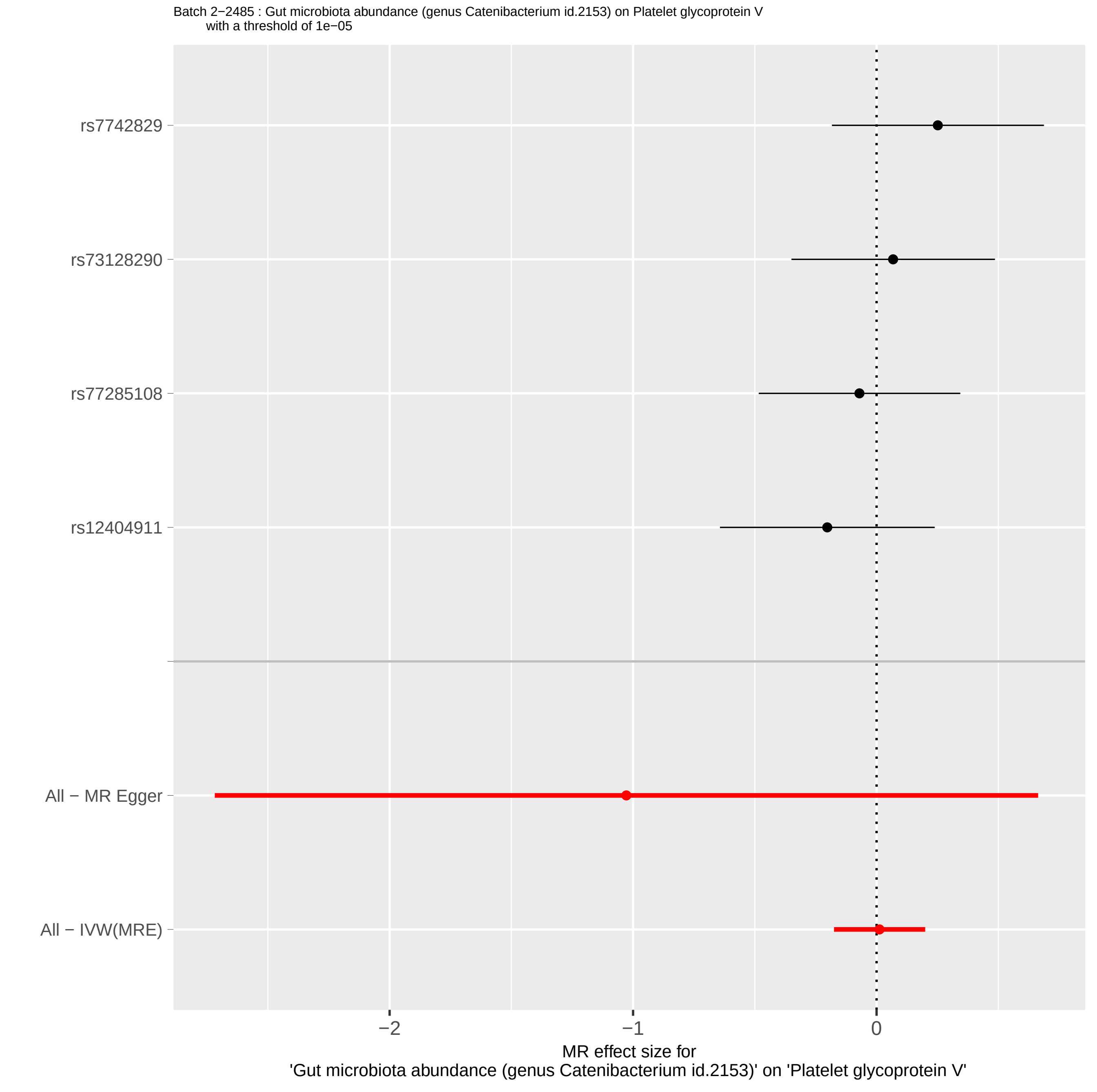

Batch 2−2485 : Gut microbiota abundance (genus Catenibacterium id.2153) on Platelet glycoprotein V
with a threshold of 1e−05
rs7742829
rs73128290
rs77285108
rs12404911
All − MR Egger
All − IVW(MRE)
−2
−1
0
MR effect size for
'Gut microbiota abundance (genus Catenibacterium id.2153)' on 'Platelet glycoprotein V'

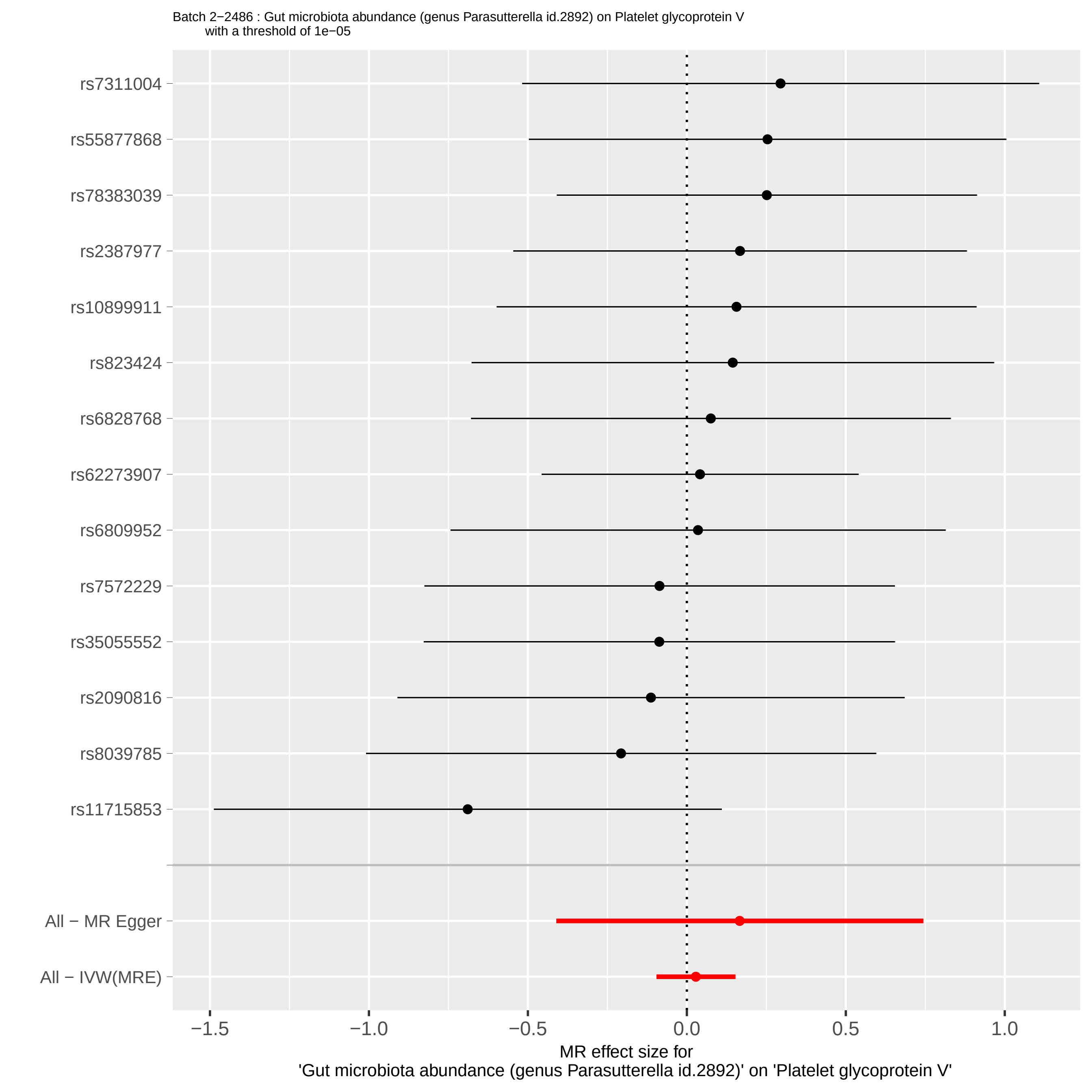
Batch 2−2486 : Gut microbiota abundance (genus Parasutterella id.2892) on Platelet glycoprotein V
with a threshold of 1e−05
rs7311004
rs55877868
rs78383039
rs2387977
rs10899911
rs823424
rs6828768
rs62273907
rs6809952
rs7572229
rs35055552
rs2090816
rs8039785
rs11715853
All − MR Egger
All − IVW(MRE)
−1.5
−1.0
−0.5
0.0
0.5
1.0
MR effect size for
'Gut microbiota abundance (genus Parasutterella id.2892)' on 'Platelet glycoprotein V'

Batch 2−2487 : Gut microbiota abundance (genus Catenibacterium id.2153) on Cell surface A33 antigen
with a threshold of 1e−05

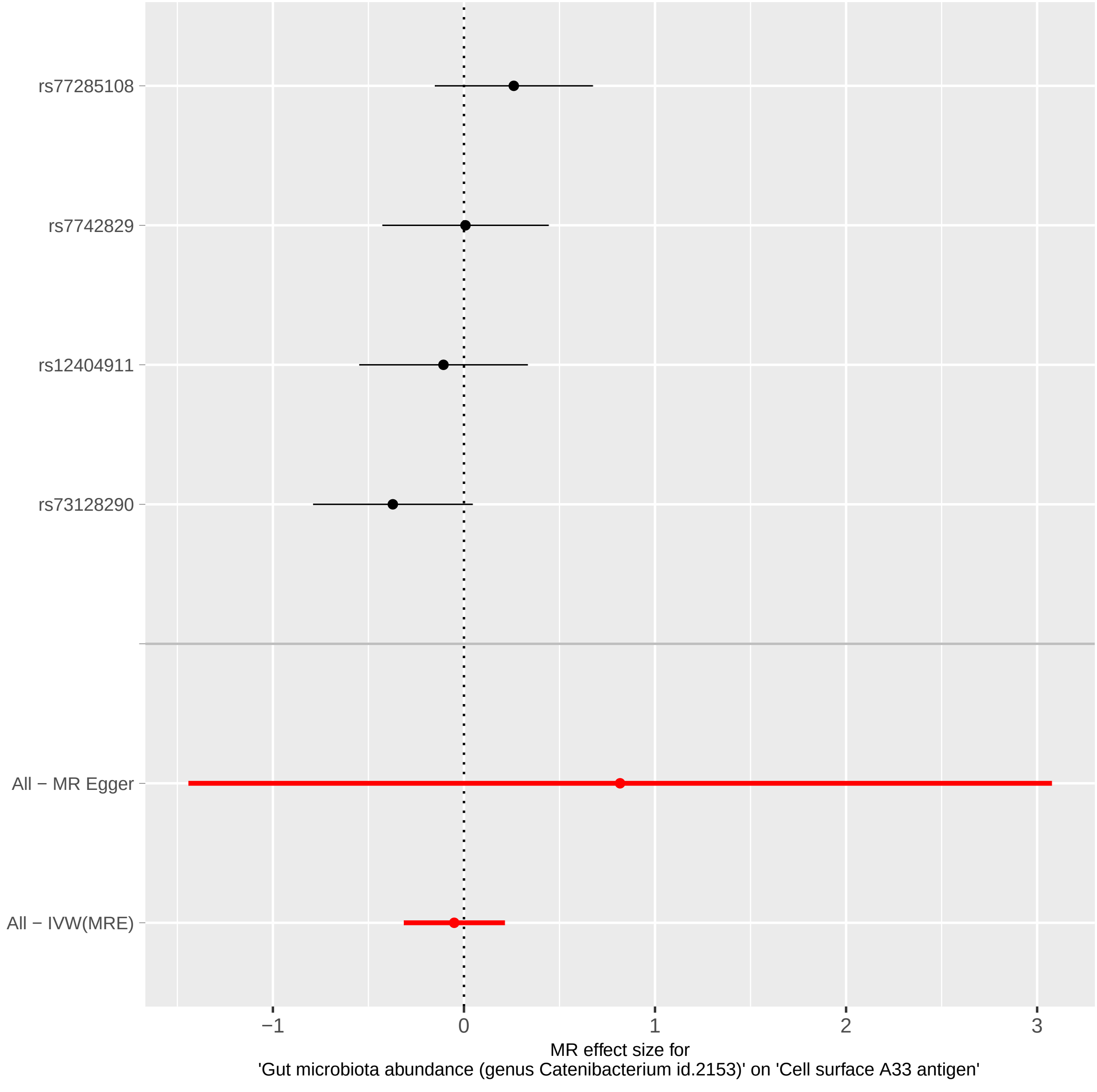

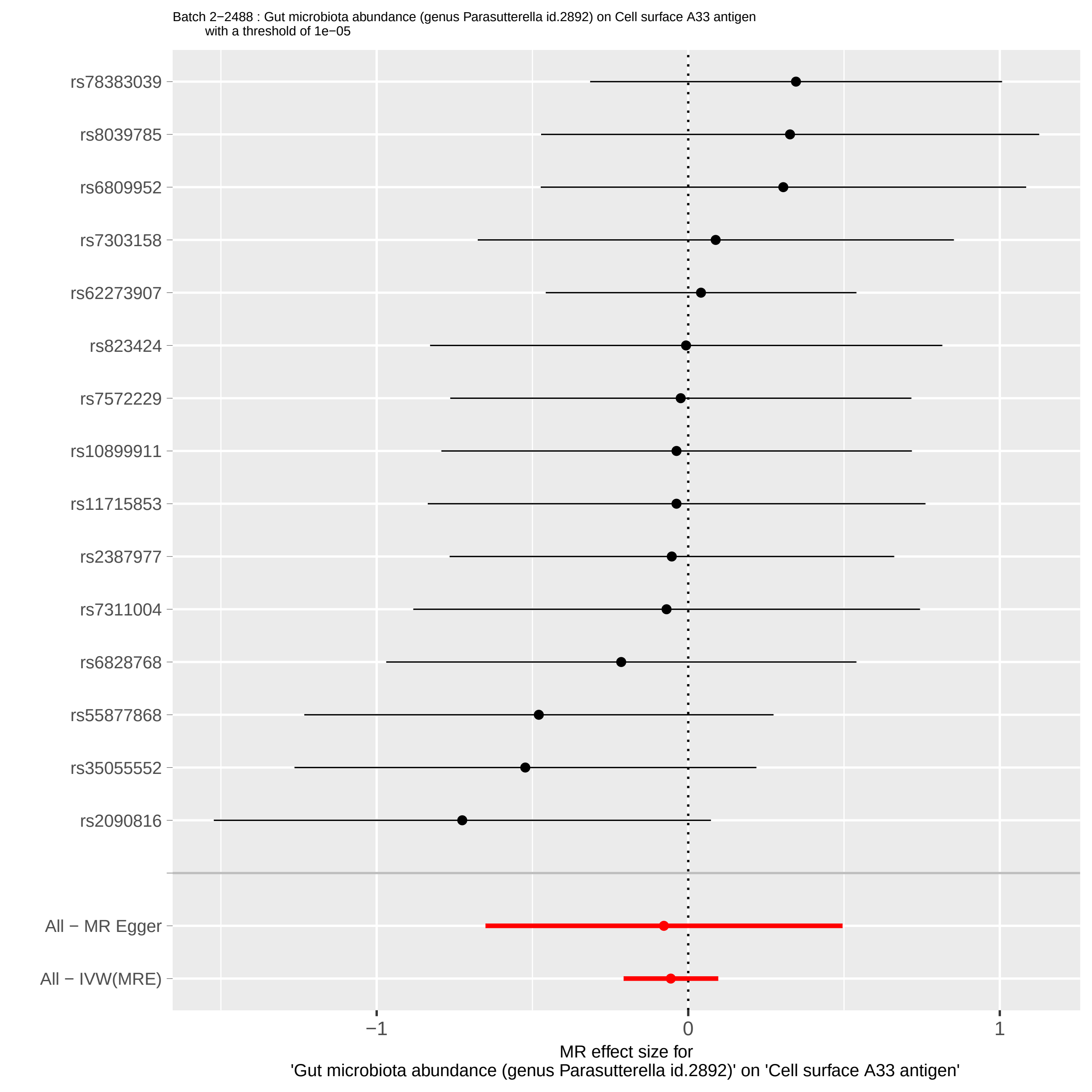

Batch 2−2488 : Gut microbiota abundance (genus Parasutterella id.2892) on Cell surface A33 antigen
with a threshold of 1e−05
rs78383039
rs8039785
rs6809952
rs7303158
rs62273907
rs823424
rs7572229
rs10899911
rs11715853
rs2387977
rs7311004
rs6828768
rs55877868
rs35055552
rs2090816
All − MR Egger
All − IVW(MRE)
−1
0
1
MR effect size for
'Gut microbiota abundance (genus Parasutterella id.2892)' on 'Cell surface A33 antigen'

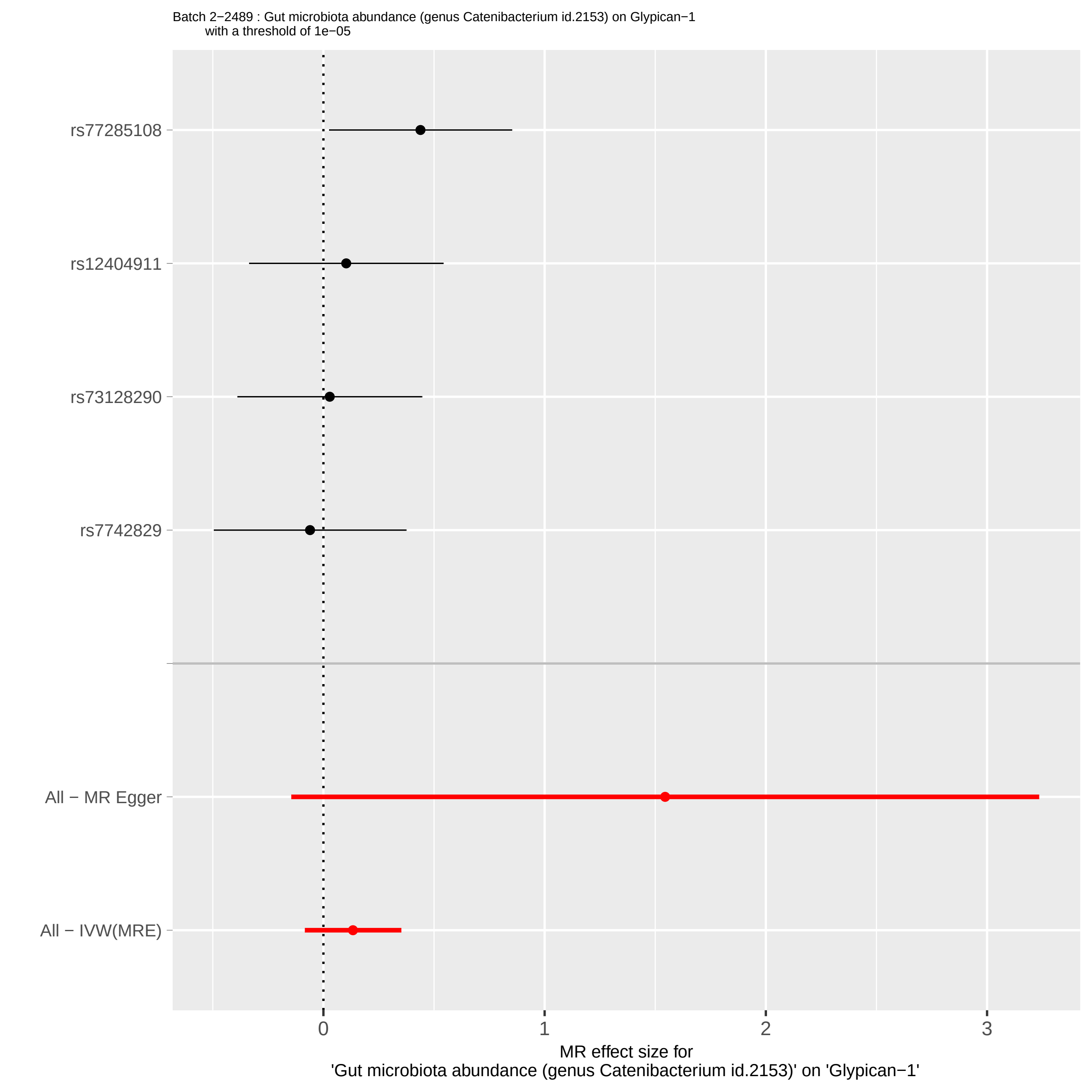

Batch 2−2489 : Gut microbiota abundance (genus Catenibacterium id.2153) on Glypican−1
with a threshold of 1e−05
rs77285108
rs12404911
rs73128290
rs7742829
All − MR Egger
All − IVW(MRE)
0
1
2
3
MR effect size for
'Gut microbiota abundance (genus Catenibacterium id.2153)' on 'Glypican−1'

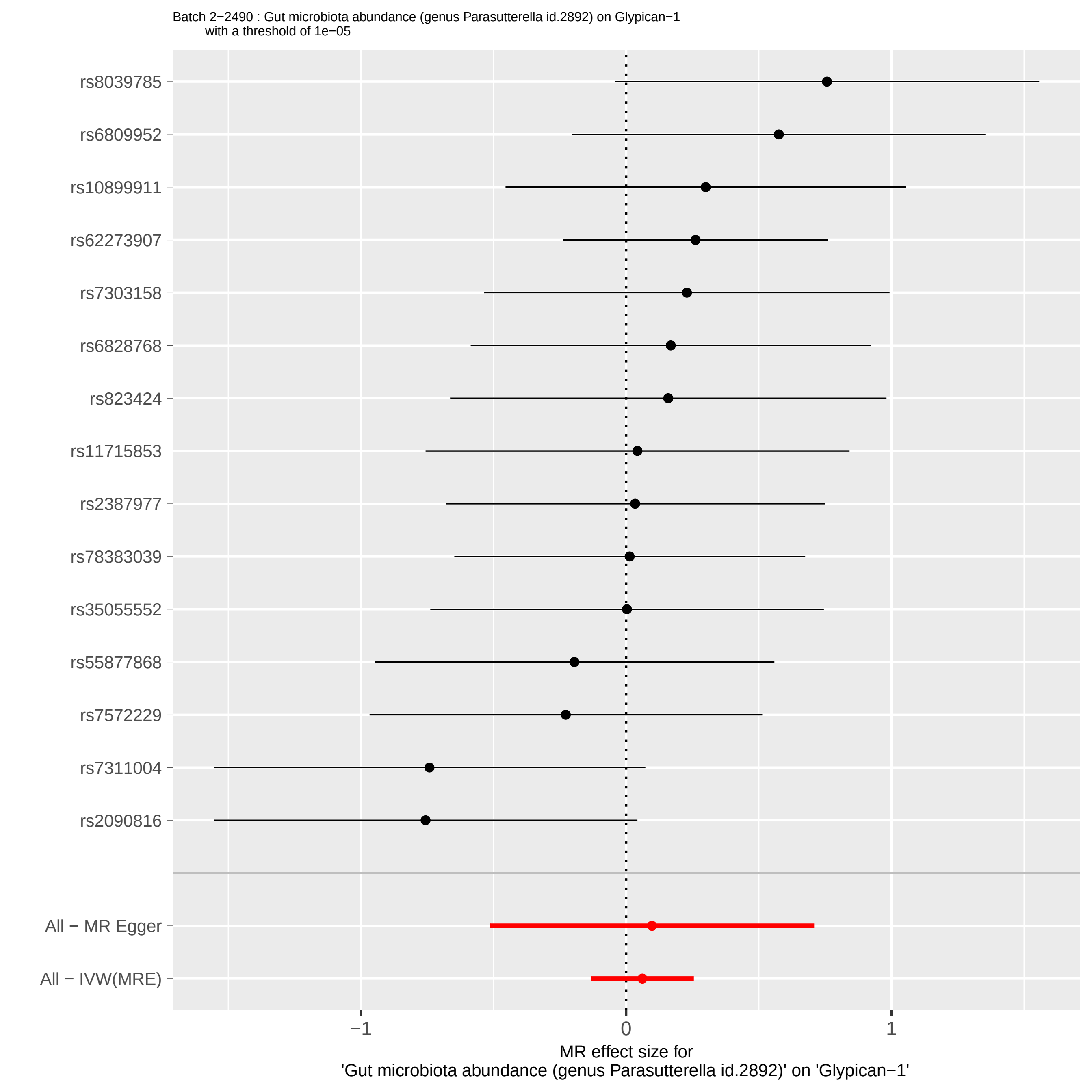

Batch 2−2490 : Gut microbiota abundance (genus Parasutterella id.2892) on Glypican−1
with a threshold of 1e−05
rs8039785
rs6809952
rs10899911
rs62273907
rs7303158
rs6828768
rs823424
rs11715853
rs2387977
rs78383039
rs35055552
rs55877868
rs7572229
rs7311004
rs2090816
All − MR Egger
All − IVW(MRE)
−1
0
1
MR effect size for
'Gut microbiota abundance (genus Parasutterella id.2892)' on 'Glypican−1'

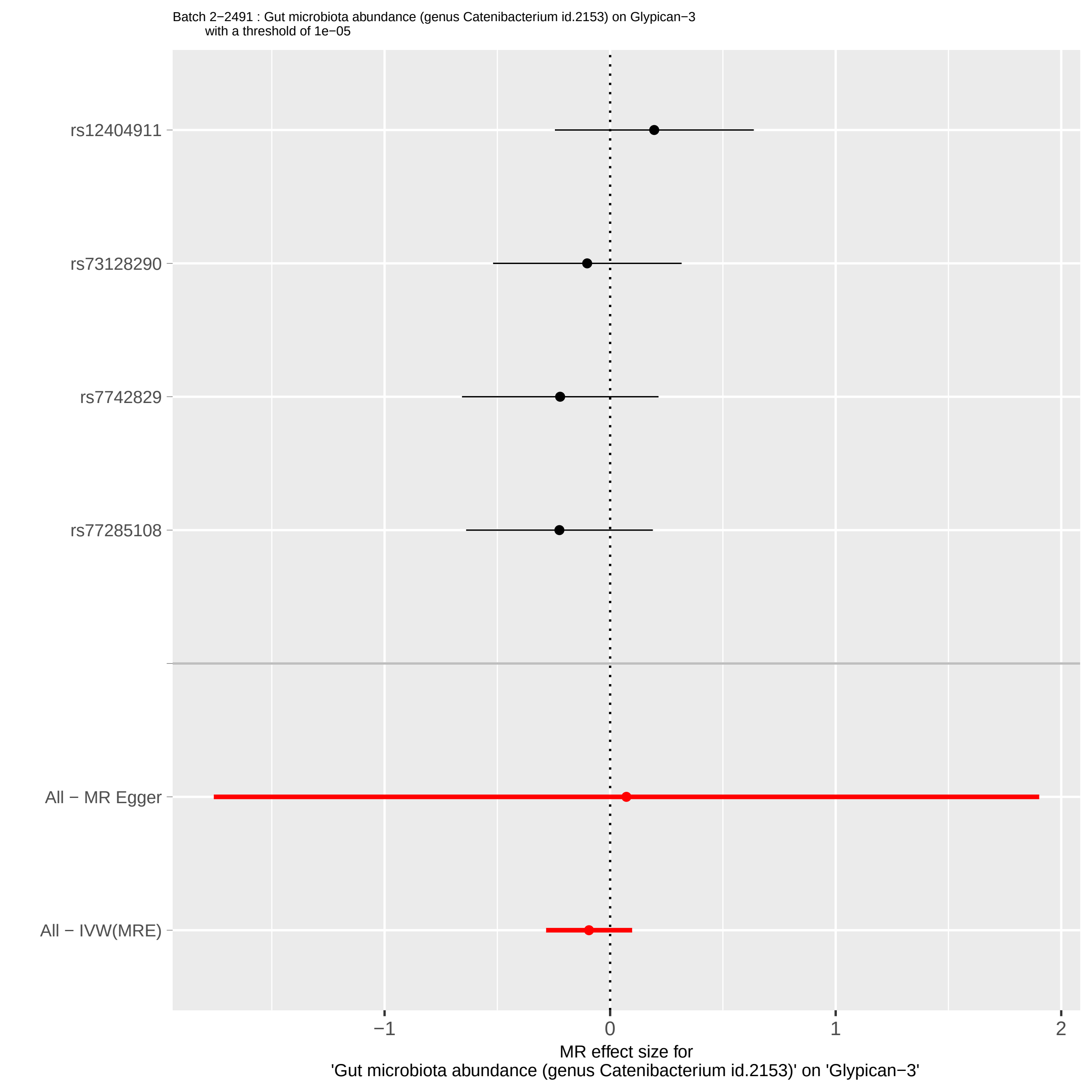
Batch 2−2491 : Gut microbiota abundance (genus Catenibacterium id.2153) on Glypican−3
with a threshold of 1e−05
rs12404911
rs73128290
rs7742829
rs77285108
All − MR Egger
All − IVW(MRE)
−1
0
1
2
MR effect size for
'Gut microbiota abundance (genus Catenibacterium id.2153)' on 'Glypican−3'

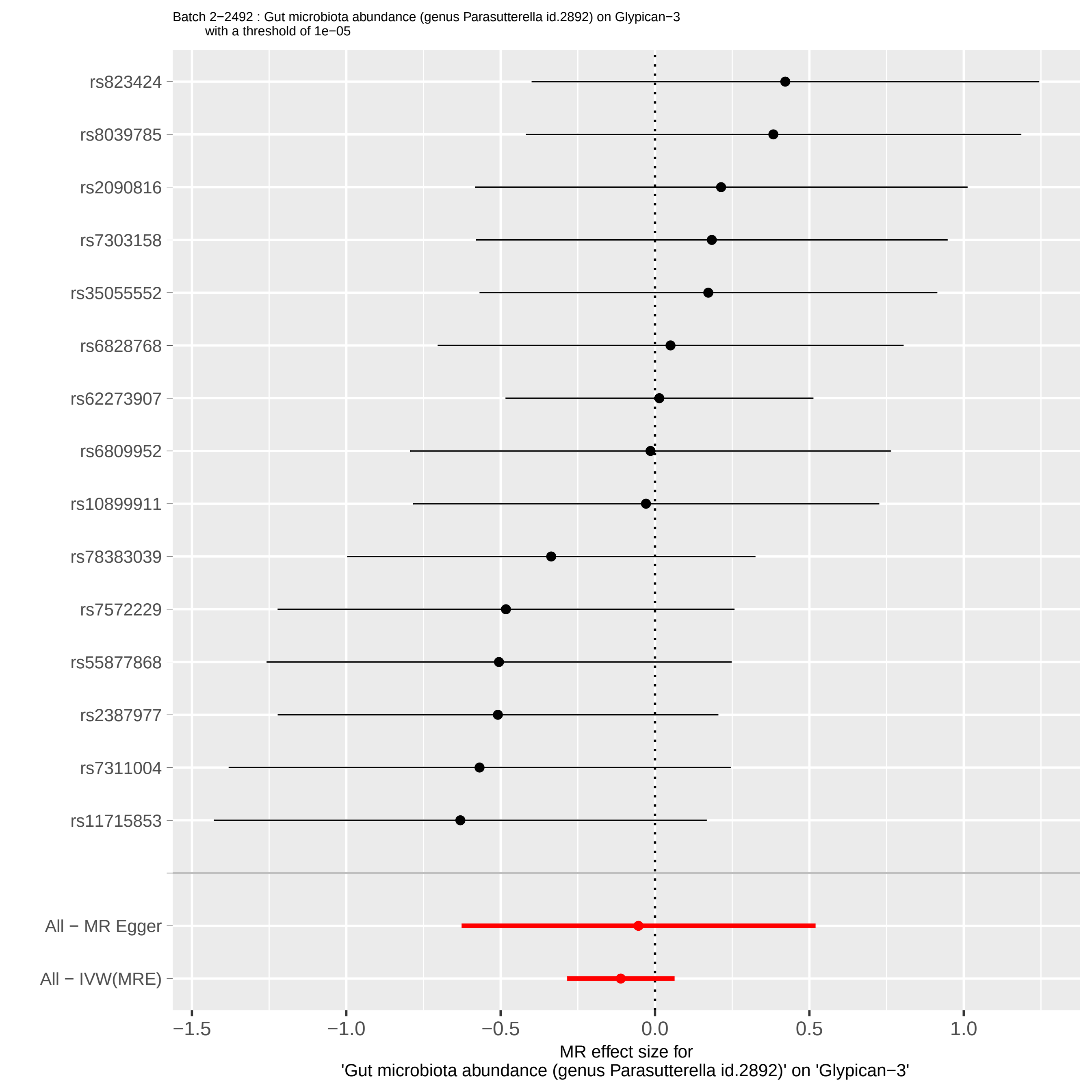
Batch 2−2492 : Gut microbiota abundance (genus Parasutterella id.2892) on Glypican−3
with a threshold of 1e−05
rs823424
rs8039785
rs2090816
rs7303158
rs35055552
rs6828768
rs62273907
rs6809952
rs10899911
rs78383039
rs7572229
rs55877868
rs2387977
rs7311004
rs11715853
All − MR Egger
All − IVW(MRE)
−1.5
−1.0
−0.5
0.0
0.5
1.0
MR effect size for
'Gut microbiota abundance (genus Parasutterella id.2892)' on 'Glypican−3'

Batch 2−2493 : Gut microbiota abundance (genus Catenibacterium id.2153) on Glypican−5
with a threshold of 1e−05

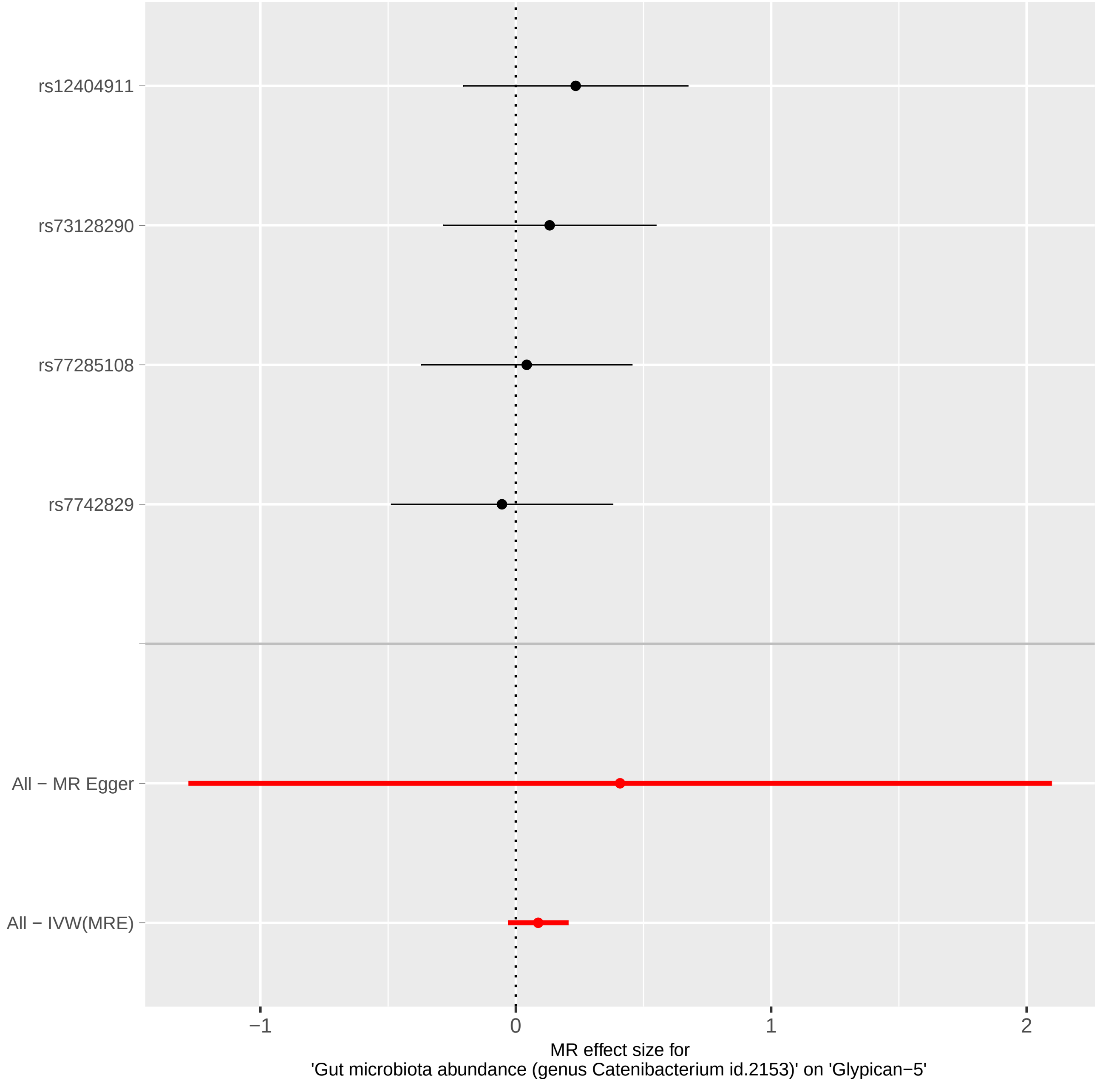

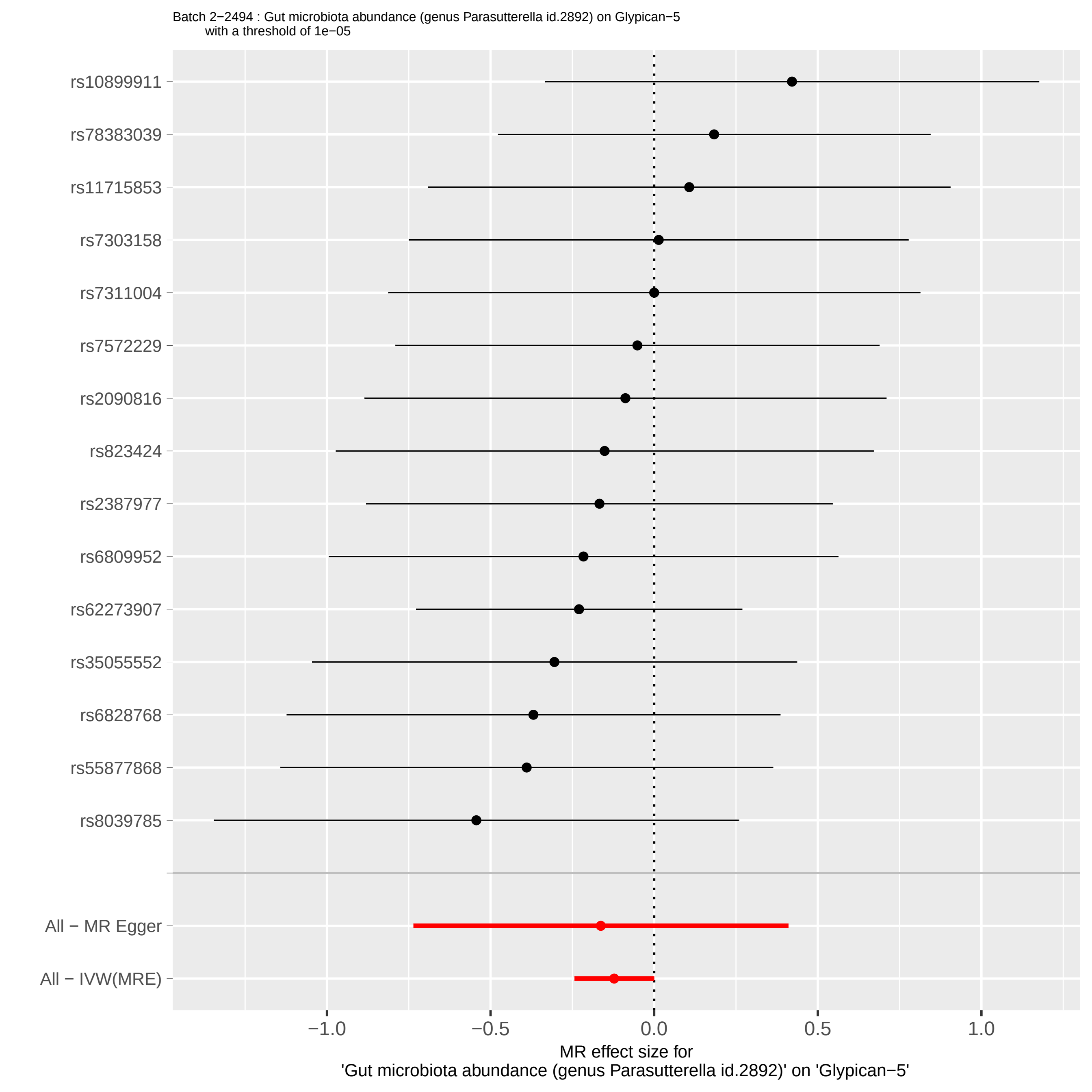
Batch 2−2494 : Gut microbiota abundance (genus Parasutterella id.2892) on Glypican−5
with a threshold of 1e−05
rs10899911
rs78383039
rs11715853
rs7303158
rs7311004
rs7572229
rs2090816
rs823424
rs2387977
rs6809952
rs62273907
rs35055552
rs6828768
rs55877868
rs8039785
All − MR Egger
All − IVW(MRE)
−1.0
−0.5
0.0
0.5
1.0
MR effect size for
'Gut microbiota abundance (genus Parasutterella id.2892)' on 'Glypican−5'

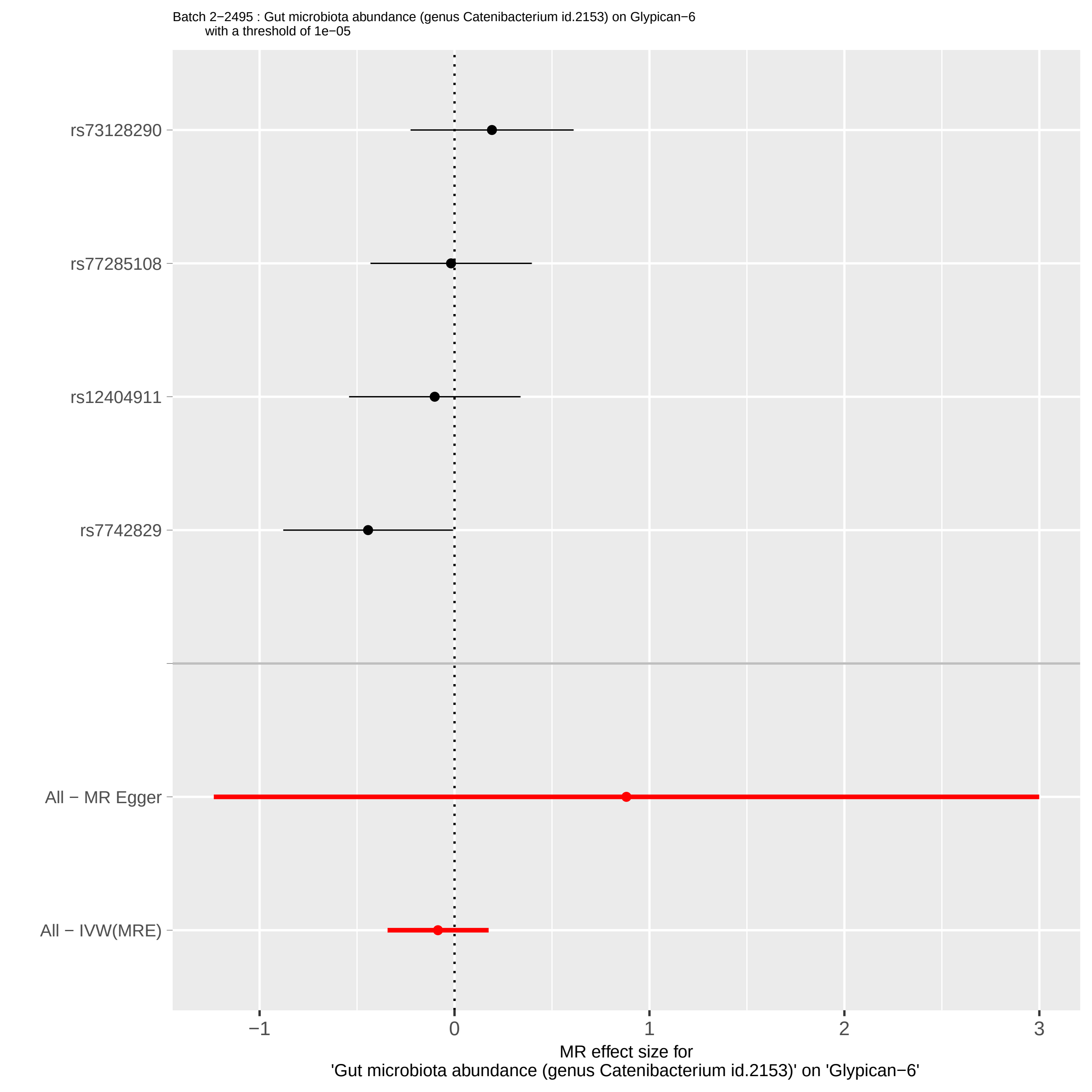
Batch 2−2495 : Gut microbiota abundance (genus Catenibacterium id.2153) on Glypican−6
with a threshold of 1e−05
rs73128290
rs77285108
rs12404911
rs7742829
All − MR Egger
All − IVW(MRE)
−1
0
1
2
3
MR effect size for
'Gut microbiota abundance (genus Catenibacterium id.2153)' on 'Glypican−6'

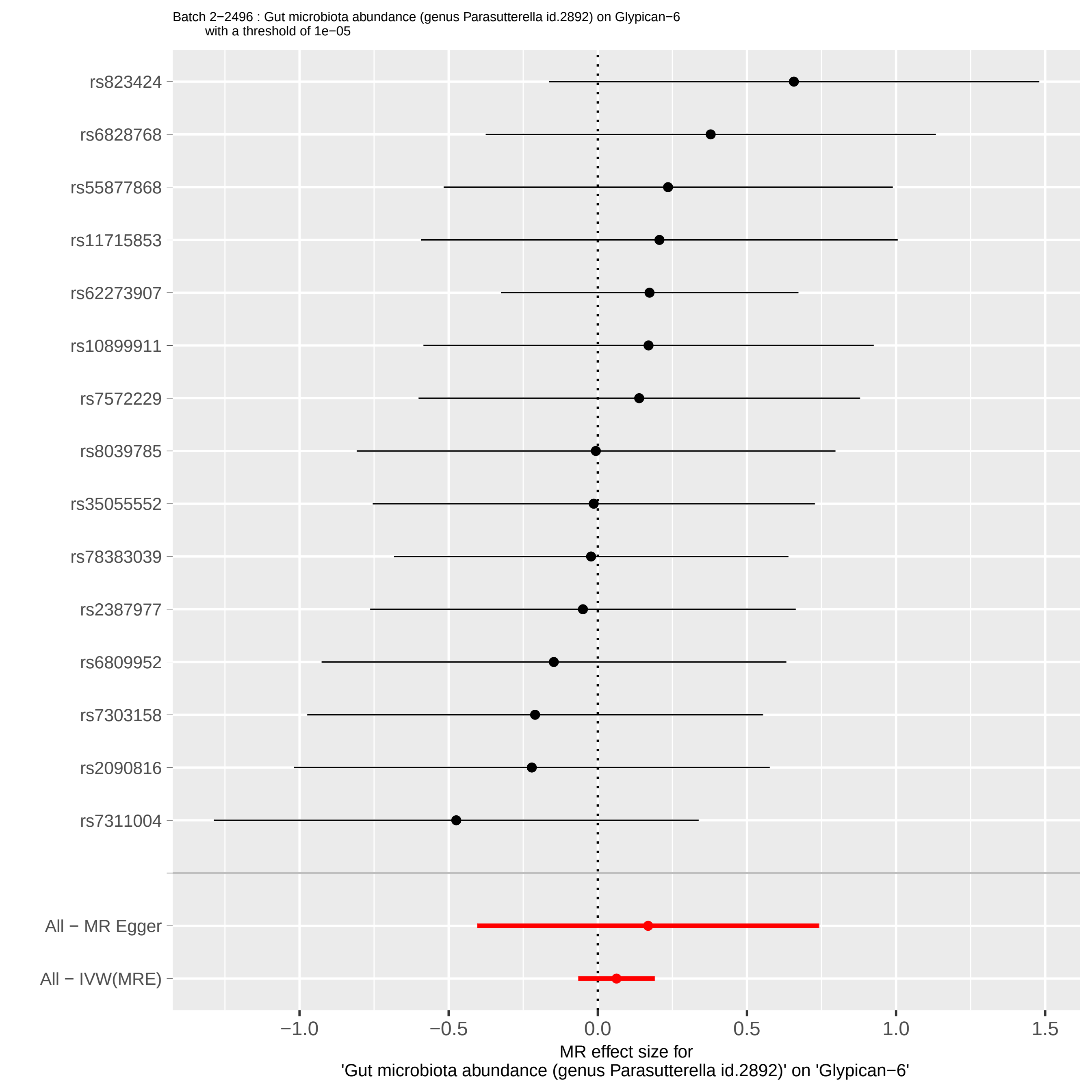
Batch 2−2496 : Gut microbiota abundance (genus Parasutterella id.2892) on Glypican−6
with a threshold of 1e−05
rs823424
rs6828768
rs55877868
rs11715853
rs62273907
rs10899911
rs7572229
rs8039785
rs35055552
rs78383039
rs2387977
rs6809952
rs7303158
rs2090816
rs7311004
All − MR Egger
All − IVW(MRE)
−1.0
−0.5
0.0
0.5
1.0
1.5
MR effect size for
'Gut microbiota abundance (genus Parasutterella id.2892)' on 'Glypican−6'

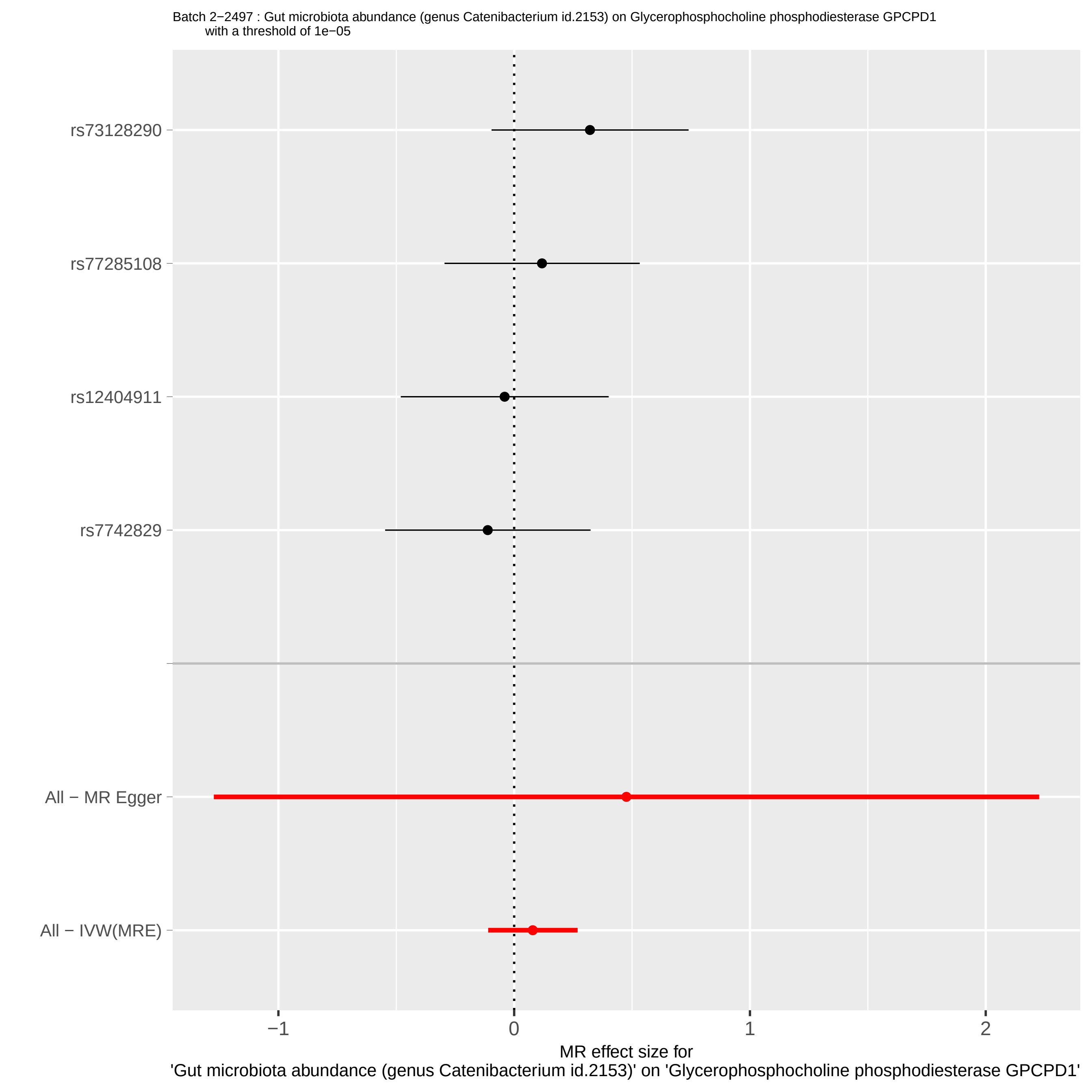
Batch 2−2497 : Gut microbiota abundance (genus Catenibacterium id.2153) on Glycerophosphocholine phosphodiesterase GPCPD1
with a threshold of 1e−05
rs73128290
rs77285108
rs12404911
rs7742829
All − MR Egger
All − IVW(MRE)
−1
0
1
2
MR effect size for
'Gut microbiota abundance (genus Catenibacterium id.2153)' on 'Glycerophosphocholine phosphodiesterase GPCPD1'

Batch 2−2498 : Gut microbiota abundance (genus Parasutterella id.2892) on Glycerophosphocholine phosphodiesterase GPCPD1
with a threshold of 1e−05

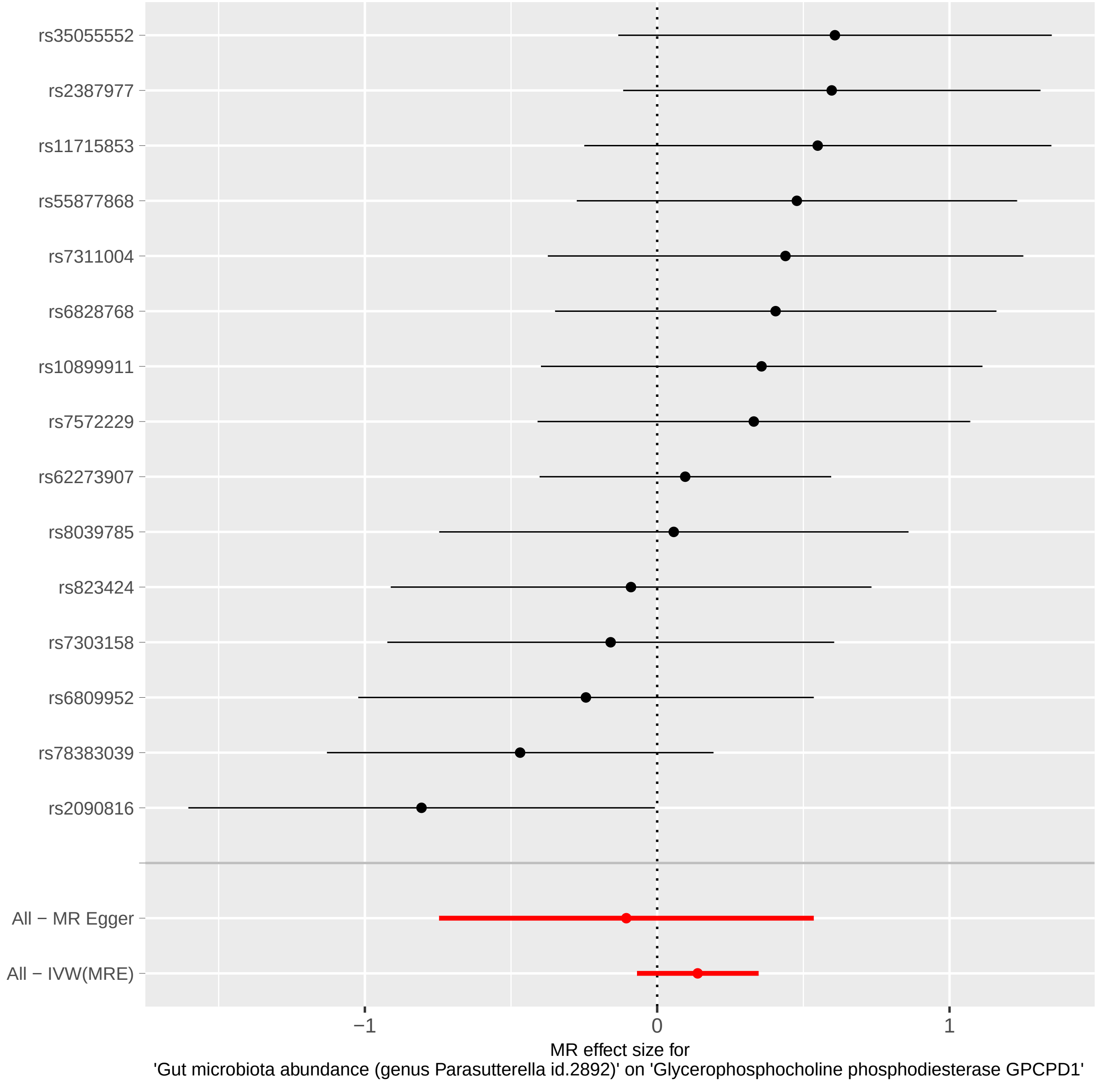

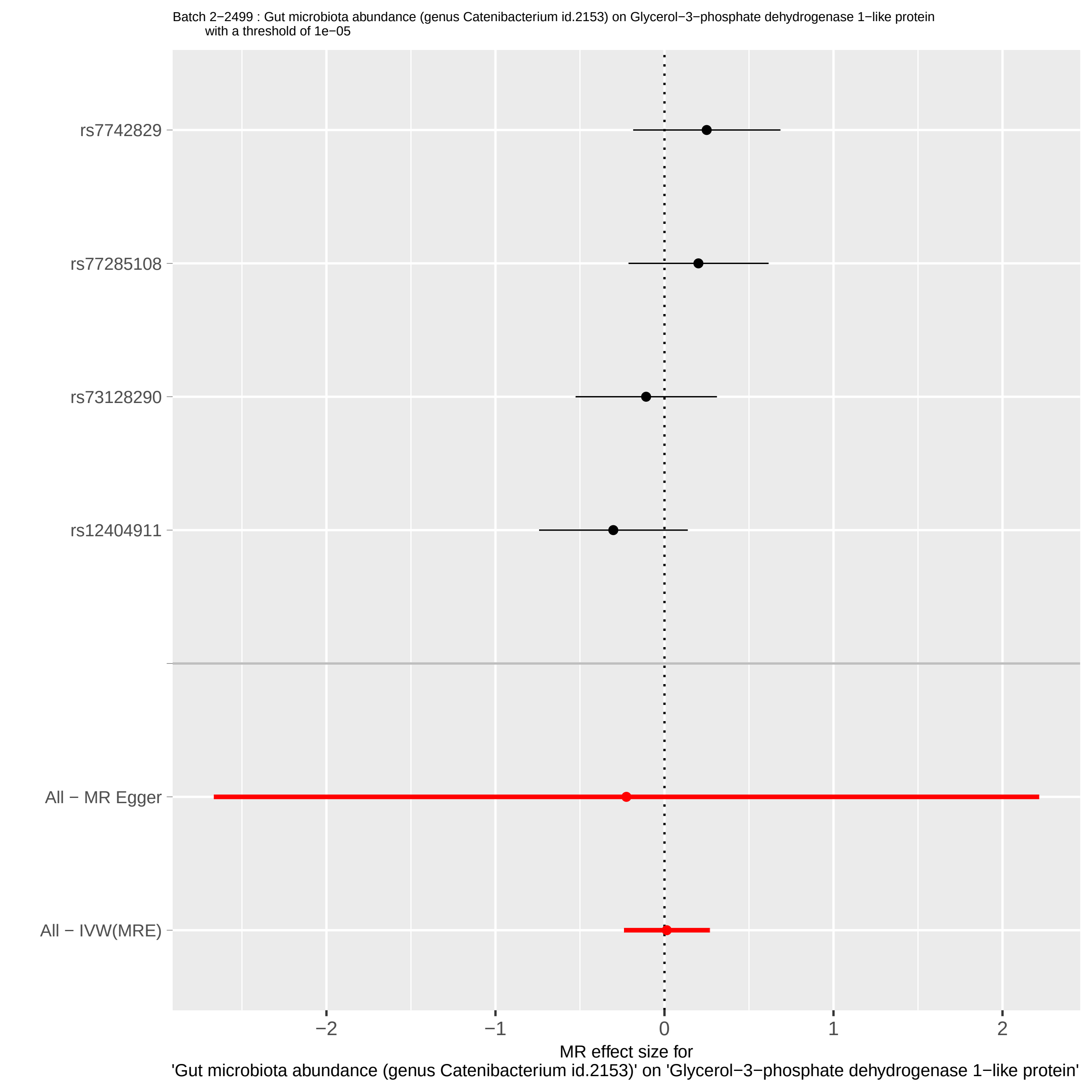

Batch 2−2499 : Gut microbiota abundance (genus Catenibacterium id.2153) on Glycerol−3−phosphate dehydrogenase 1−like protein
with a threshold of 1e−05
rs7742829
rs77285108
rs73128290
rs12404911
All − MR Egger
All − IVW(MRE)
−2
−1
0
1
2
MR effect size for
'Gut microbiota abundance (genus Catenibacterium id.2153)' on 'Glycerol−3−phosphate dehydrogenase 1−like protein'

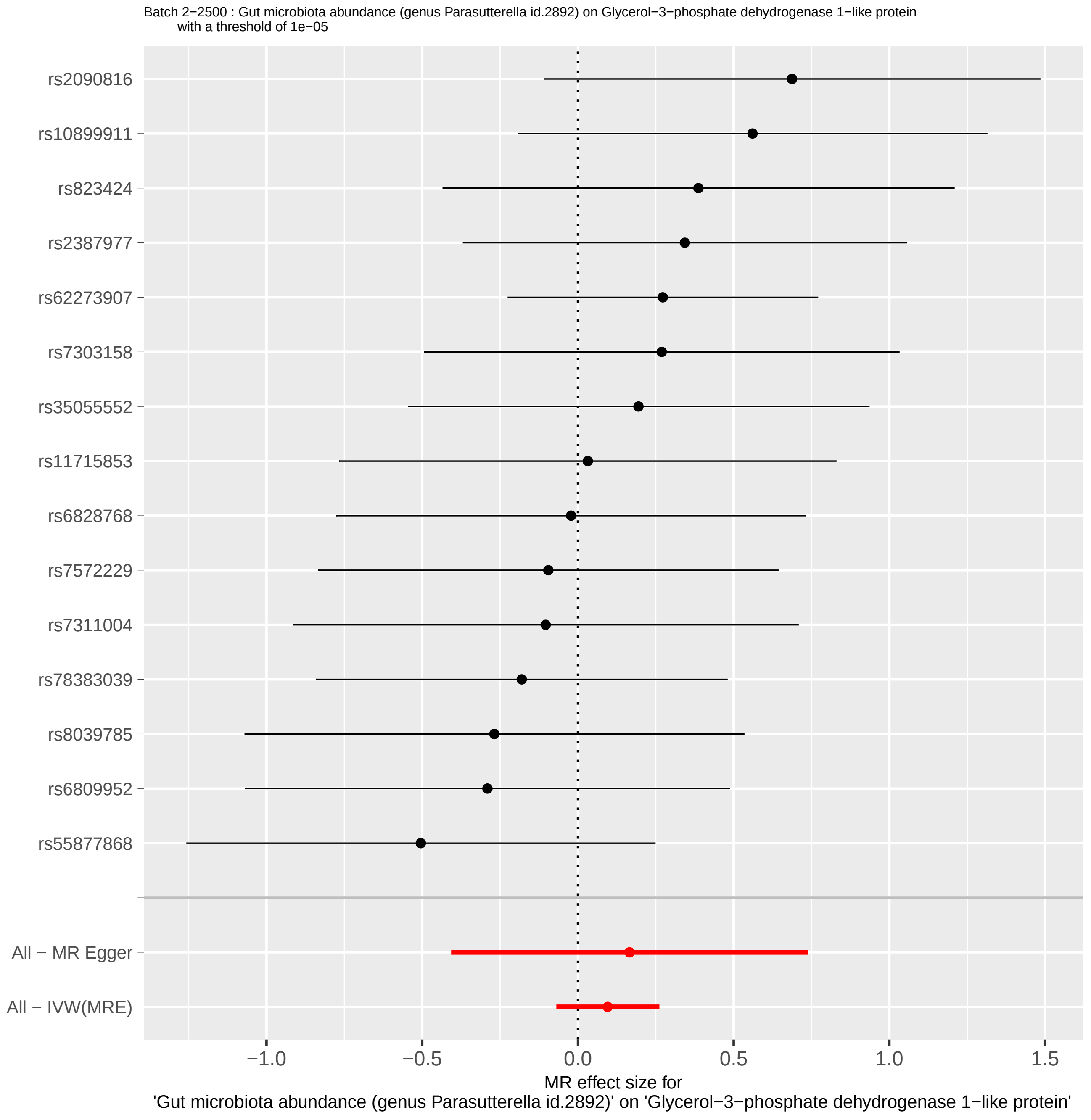

Batch 2−2500 : Gut microbiota abundance (genus Parasutterella id.2892) on Glycerol−3−phosphate dehydrogenase 1−like protein
with a threshold of 1e−05
rs2090816
rs10899911
rs823424
rs2387977
rs62273907
rs7303158
rs35055552
rs11715853
rs6828768
rs7572229
rs7311004
rs78383039
rs8039785
rs6809952
rs55877868
All − MR Egger
All − IVW(MRE)
−1.0
−0.5
0.0
0.5
1.0
1.5
MR effect size for
'Gut microbiota abundance (genus Parasutterella id.2892)' on 'Glycerol−3−phosphate dehydrogenase 1−like protein'

Batch 2−2501 : Gut microbiota abundance (genus Catenibacterium id.2153) on Glycoprotein hormone alpha−2
with a threshold of 1e−05

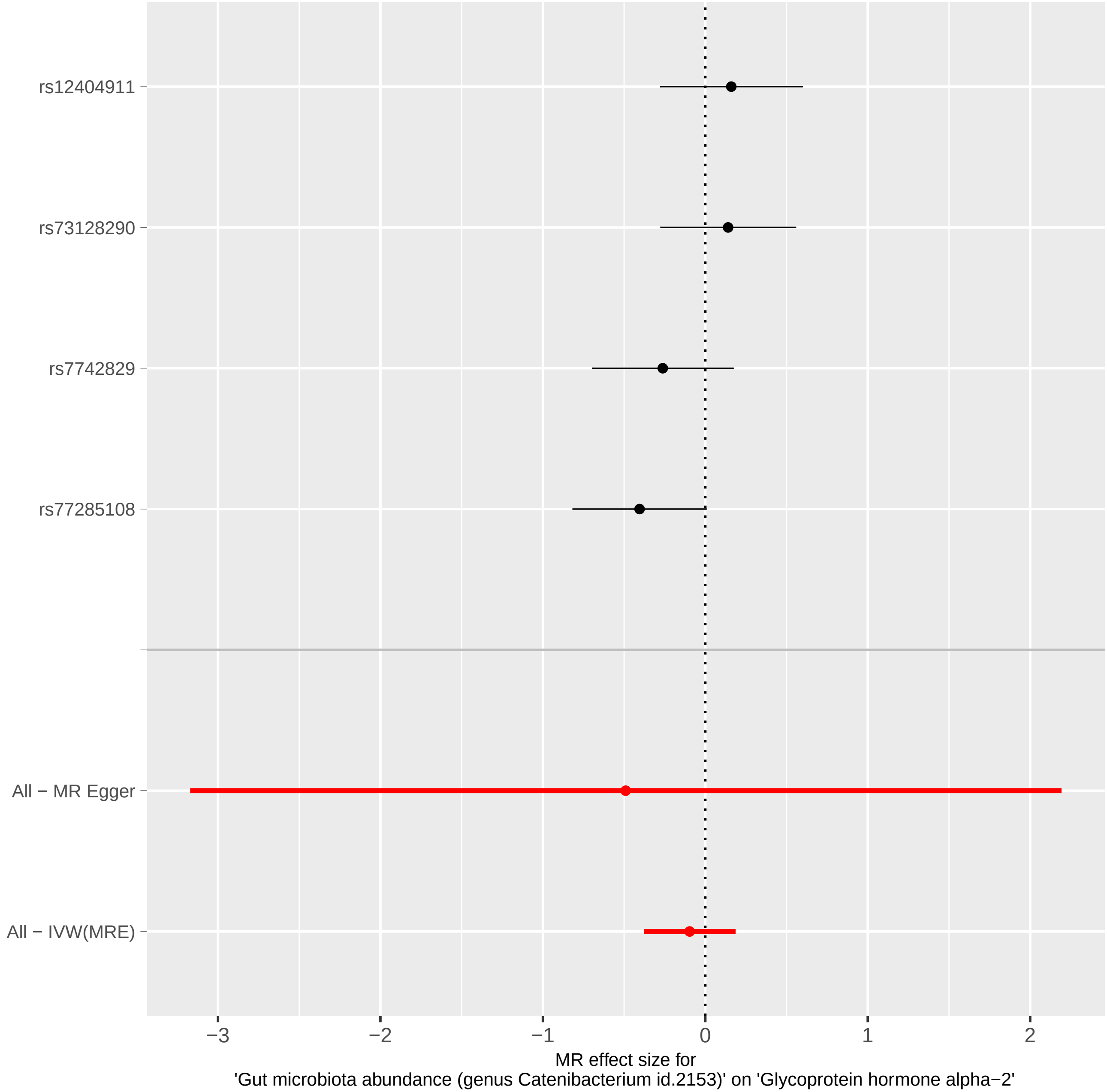

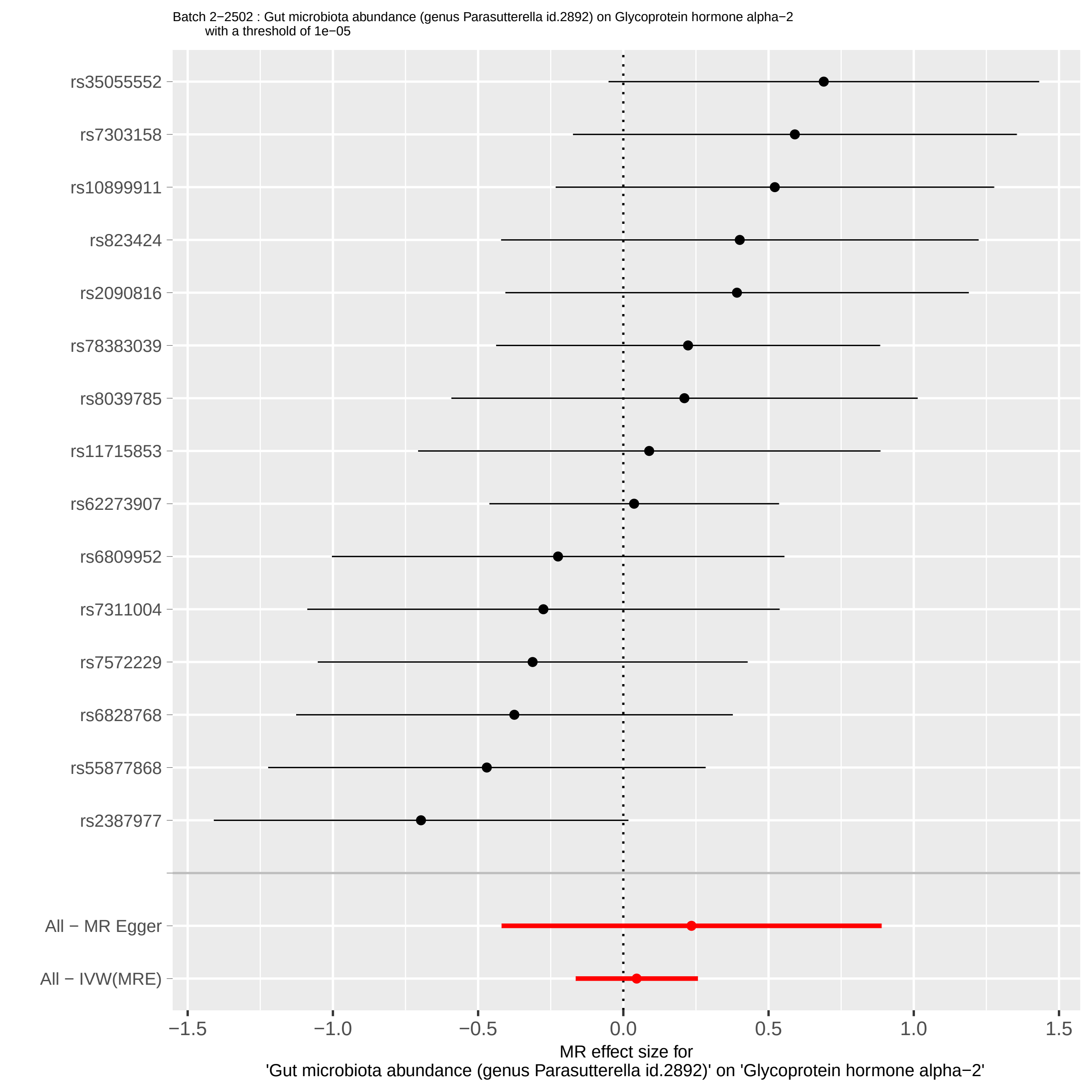

Batch 2−2502 : Gut microbiota abundance (genus Parasutterella id.2892) on Glycoprotein hormone alpha−2
with a threshold of 1e−05
rs35055552
rs7303158
rs10899911
rs823424
rs2090816
rs78383039
rs8039785
rs11715853
rs62273907
rs6809952
rs7311004
rs7572229
rs6828768
rs55877868
rs2387977
All − MR Egger
All − IVW(MRE)
−1.5
−1.0
−0.5
0.0
0.5
1.0
1.5
MR effect size for
'Gut microbiota abundance (genus Parasutterella id.2892)' on 'Glycoprotein hormone alpha−2'

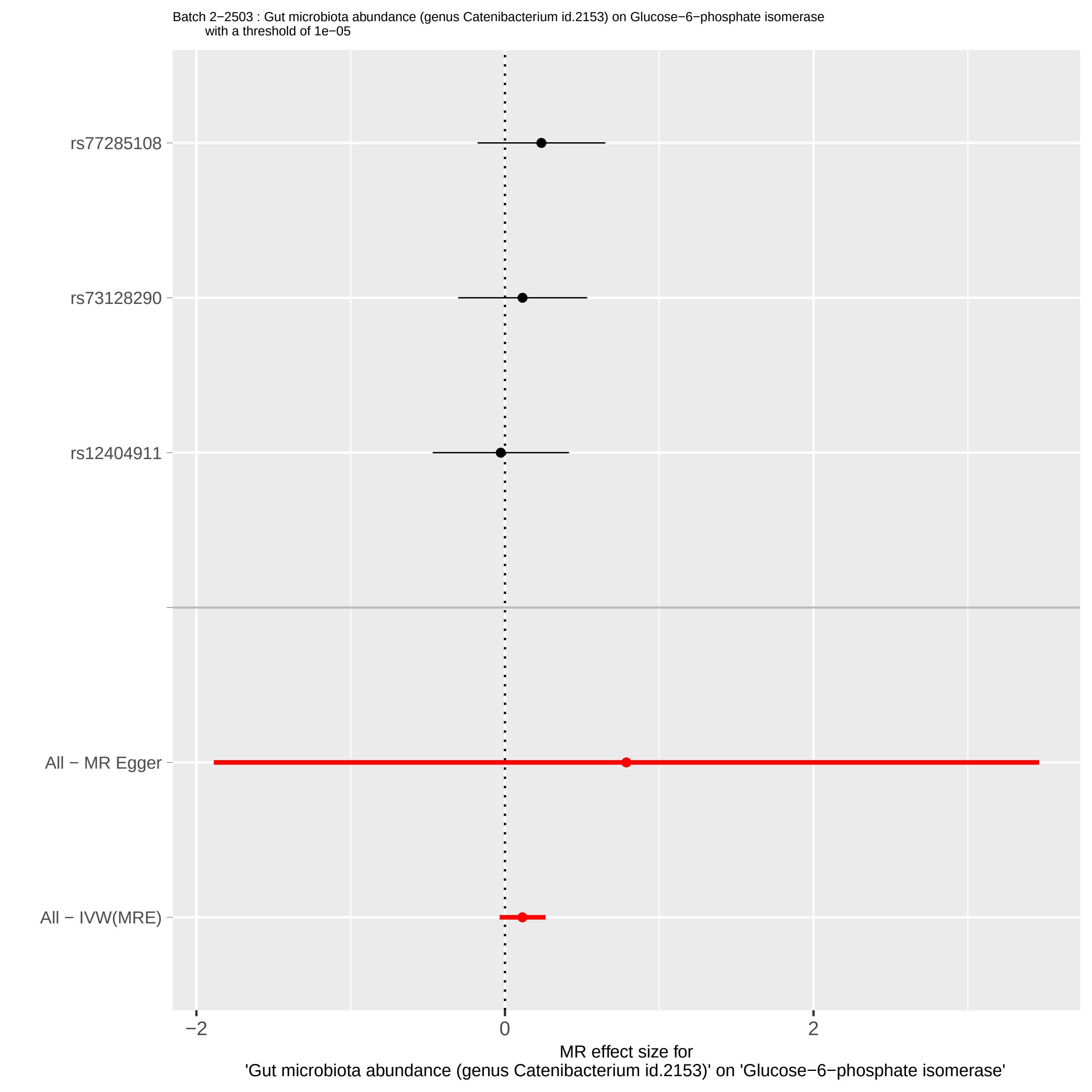
Batch 2−2503 : Gut microbiota abundance (genus Catenibacterium id.2153) on Glucose−6−phosphate isomerase
with a threshold of 1e−05
rs77285108
rs73128290
rs12404911
All − MR Egger
All − IVW(MRE)
−2
0
2
MR effect size for
'Gut microbiota abundance (genus Catenibacterium id.2153)' on 'Glucose−6−phosphate isomerase'

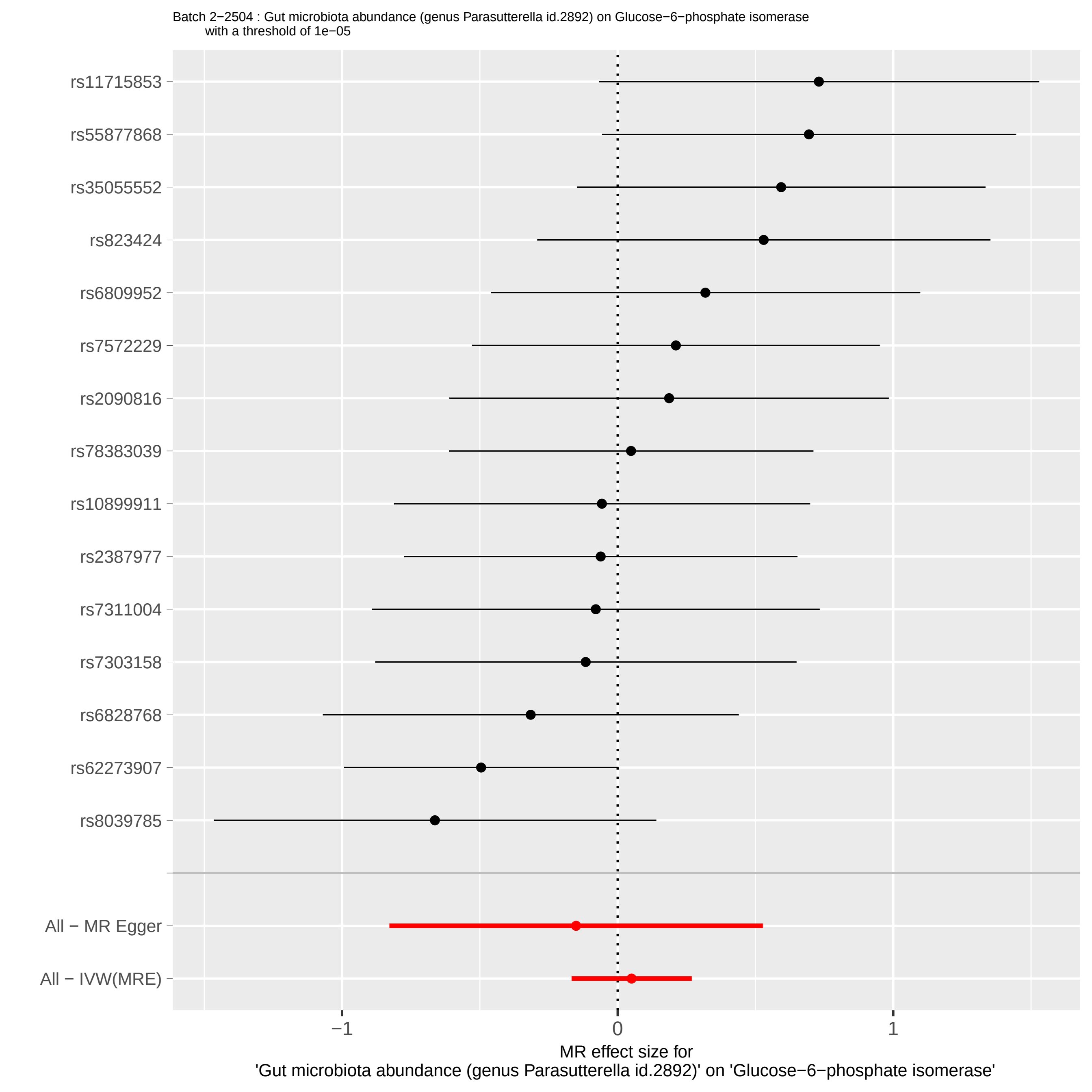

Batch 2−2504 : Gut microbiota abundance (genus Parasutterella id.2892) on Glucose−6−phosphate isomerase
with a threshold of 1e−05
rs11715853
rs55877868
rs35055552
rs823424
rs6809952
rs7572229
rs2090816
rs78383039
rs10899911
rs2387977
rs7311004
rs7303158
rs6828768
rs62273907
rs8039785
All − MR Egger
All − IVW(MRE)
−1
0
1
MR effect size for
'Gut microbiota abundance (genus Parasutterella id.2892)' on 'Glucose−6−phosphate isomerase'

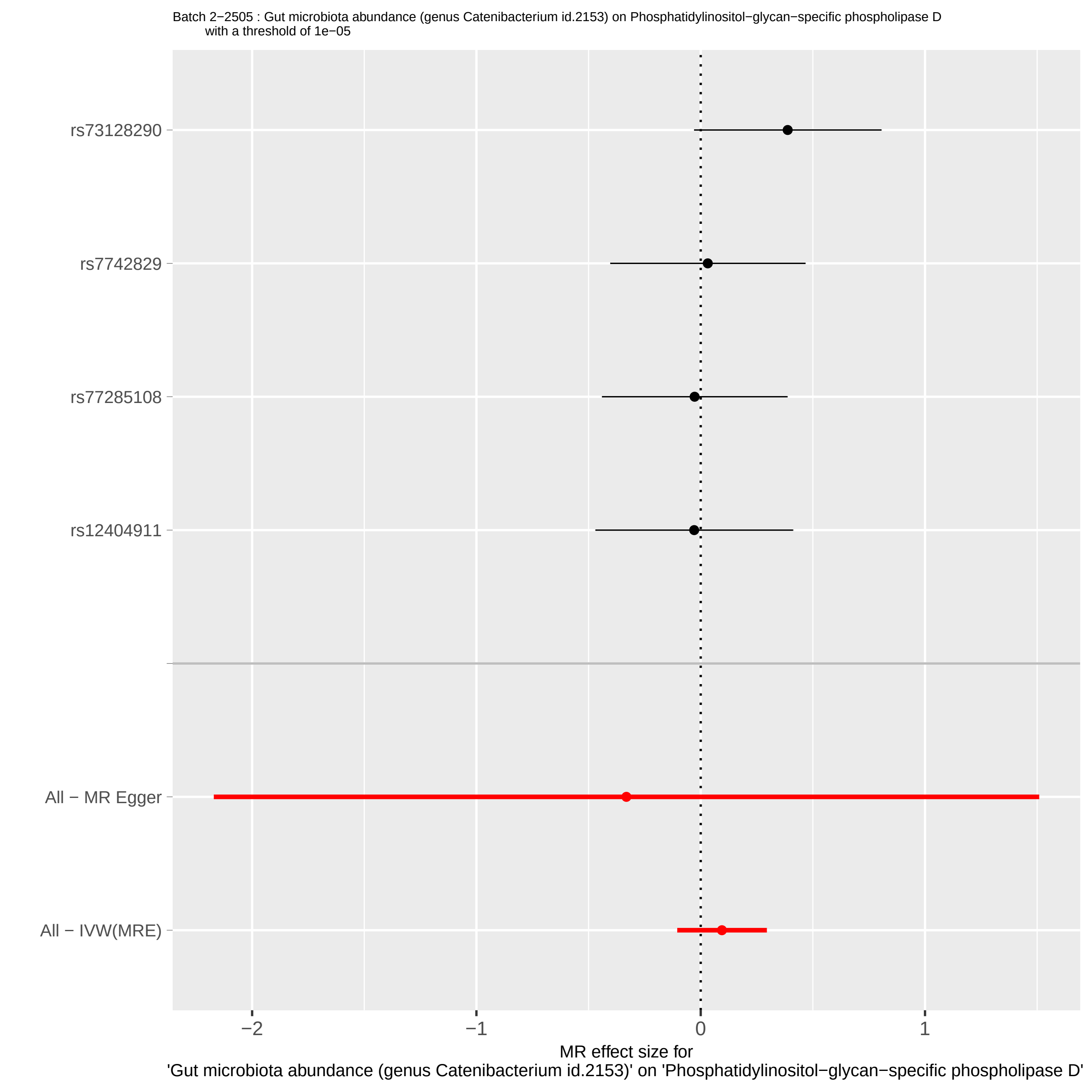

Batch 2−2505 : Gut microbiota abundance (genus Catenibacterium id.2153) on Phosphatidylinositol−glycan−specific phospholipase D
with a threshold of 1e−05
rs73128290
rs7742829
rs77285108
rs12404911
All − MR Egger
All − IVW(MRE)
−2
−1
0
1
MR effect size for
'Gut microbiota abundance (genus Catenibacterium id.2153)' on 'Phosphatidylinositol−glycan−specific phospholipase D'

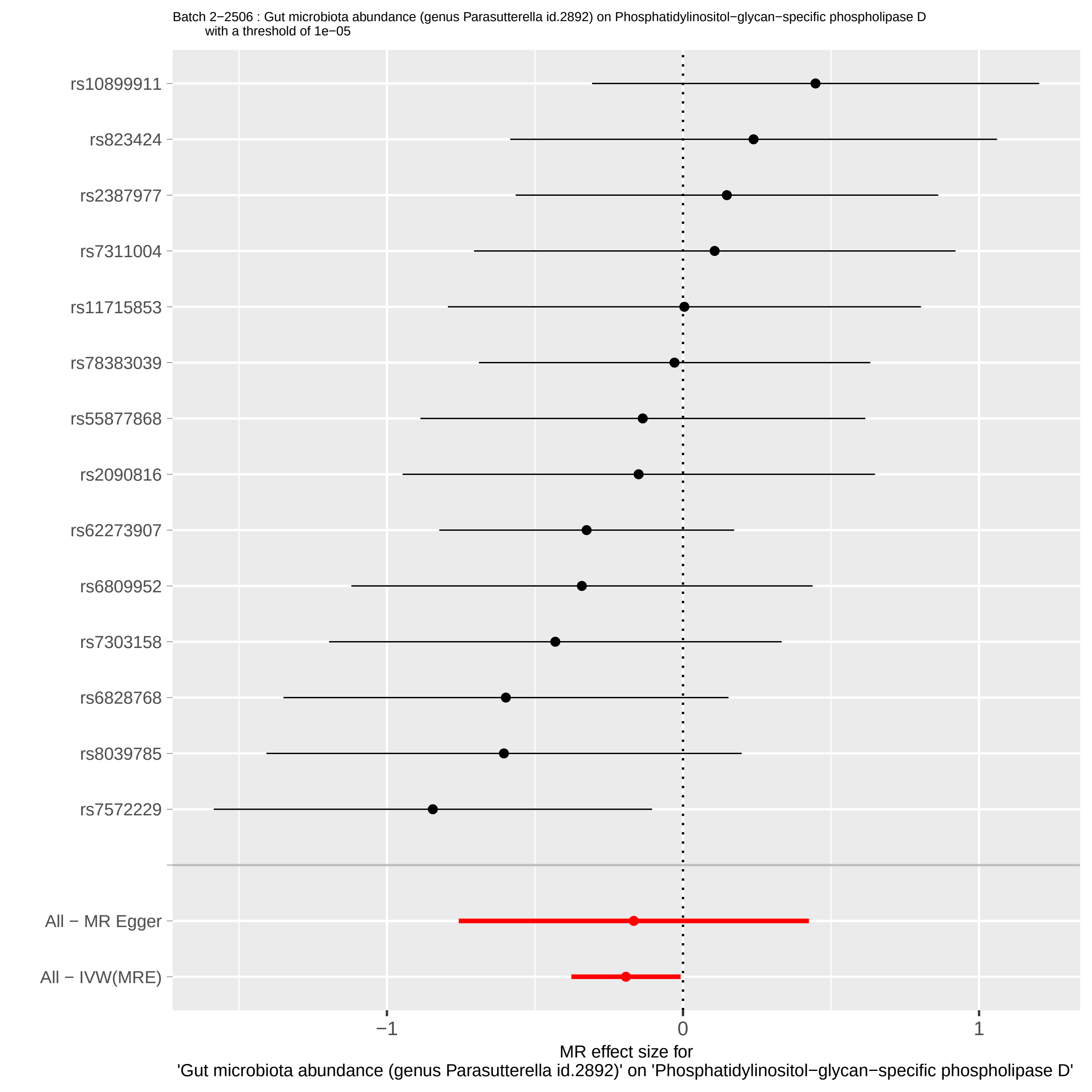

Batch 2−2506 : Gut microbiota abundance (genus Parasutterella id.2892) on Phosphatidylinositol−glycan−specific phospholipase D
with a threshold of 1e−05
rs10899911
rs823424
rs2387977
rs7311004
rs11715853
rs78383039
rs55877868
rs2090816
rs62273907
rs6809952
rs7303158
rs6828768
rs8039785
rs7572229
All − MR Egger
All − IVW(MRE)
−1
0
1
MR effect size for
'Gut microbiota abundance (genus Parasutterella id.2892)' on 'Phosphatidylinositol−glycan−specific phospholipase D'

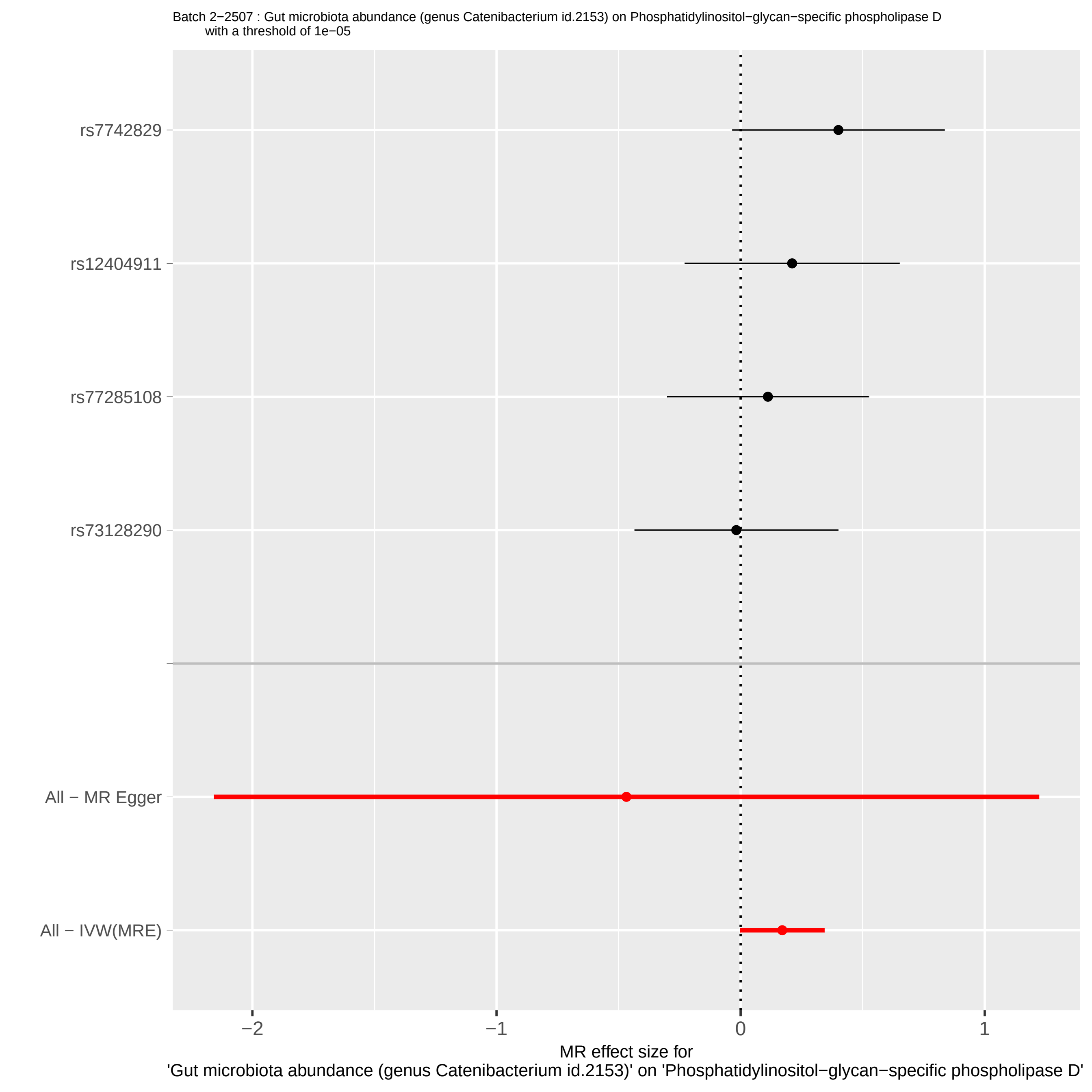

Batch 2−2507 : Gut microbiota abundance (genus Catenibacterium id.2153) on Phosphatidylinositol−glycan−specific phospholipase D
with a threshold of 1e−05
rs7742829
rs12404911
rs77285108
rs73128290
All − MR Egger
All − IVW(MRE)
−2
−1
0
1
MR effect size for
'Gut microbiota abundance (genus Catenibacterium id.2153)' on 'Phosphatidylinositol−glycan−specific phospholipase D'

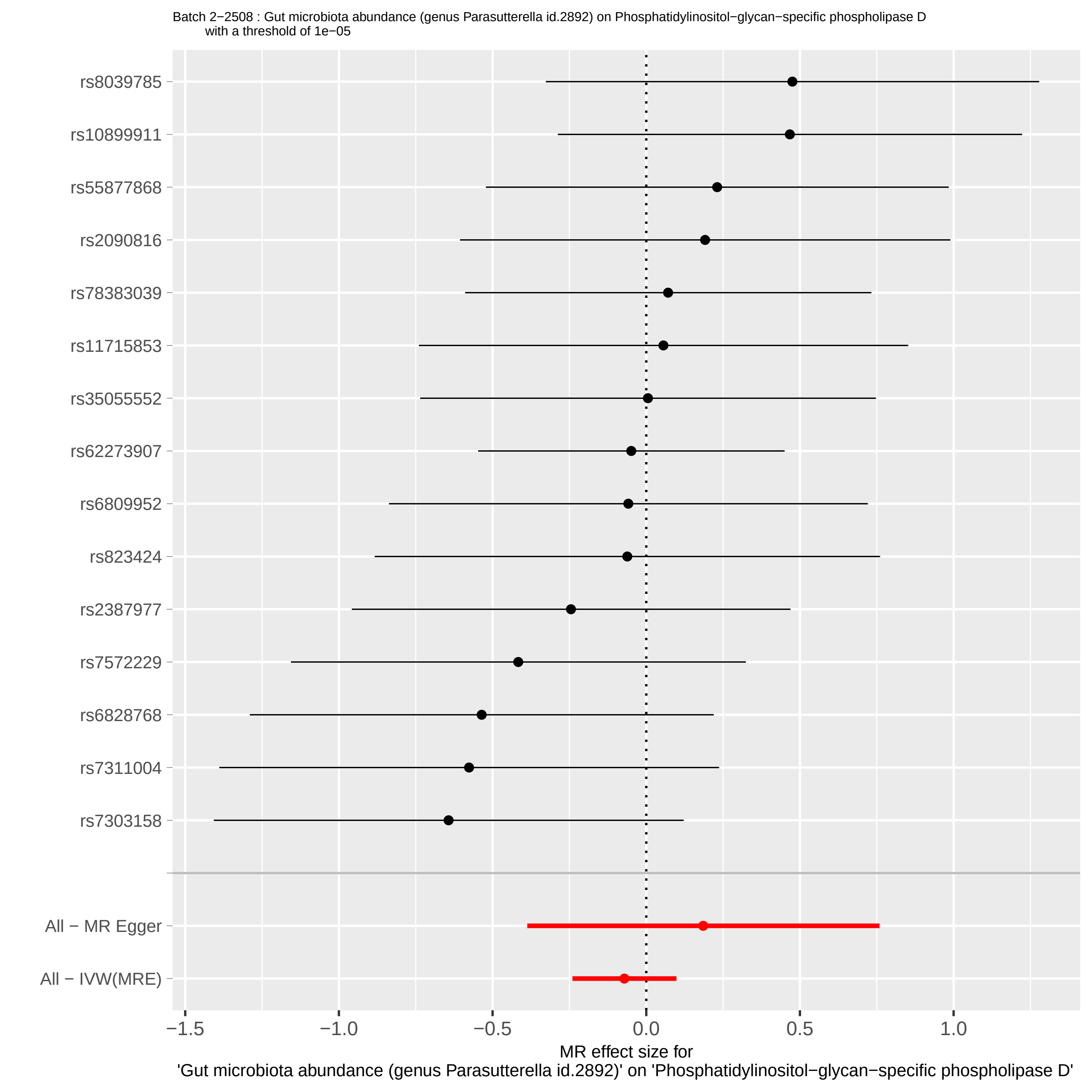
Batch 2−2508 : Gut microbiota abundance (genus Parasutterella id.2892) on Phosphatidylinositol−glycan−specific phospholipase D
with a threshold of 1e−05
rs8039785
rs10899911
rs55877868
rs2090816
rs78383039
rs11715853
rs35055552
rs62273907
rs6809952
rs823424
rs2387977
rs7572229
rs6828768
rs7311004
rs7303158
All − MR Egger
All − IVW(MRE)
−1.5
−1.0
−0.5
0.0
0.5
1.0
MR effect size for
'Gut microbiota abundance (genus Parasutterella id.2892)' on 'Phosphatidylinositol−glycan−specific phospholipase D'

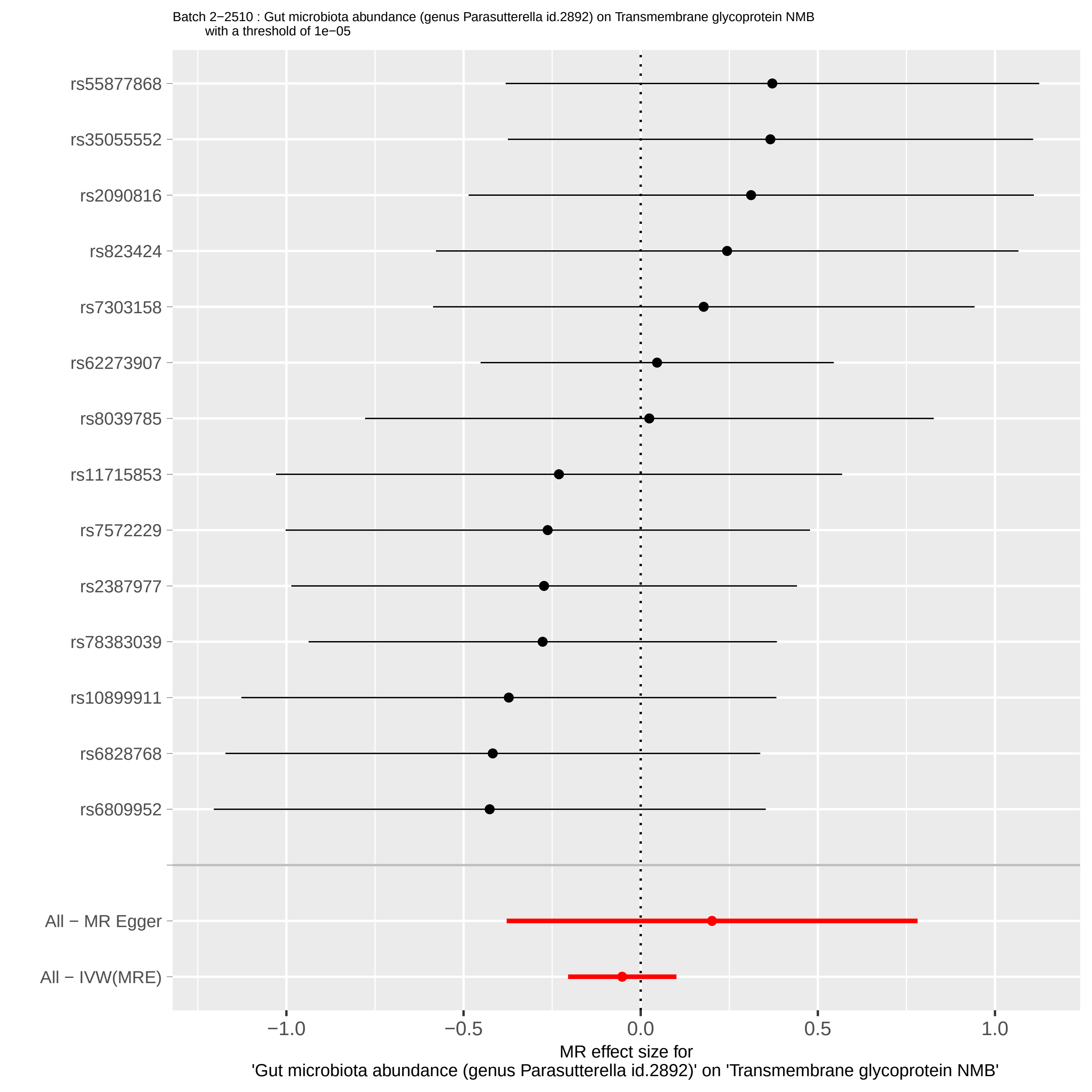

Batch 2−2510 : Gut microbiota abundance (genus Parasutterella id.2892) on Transmembrane glycoprotein NMB
with a threshold of 1e−05
rs55877868
rs35055552
rs2090816
rs823424
rs7303158
rs62273907
rs8039785
rs11715853
rs7572229
rs2387977
rs78383039
rs10899911
rs6828768
rs6809952
All − MR Egger
All − IVW(MRE)
−1.0
−0.5
0.0
0.5
1.0
MR effect size for
'Gut microbiota abundance (genus Parasutterella id.2892)' on 'Transmembrane glycoprotein NMB'

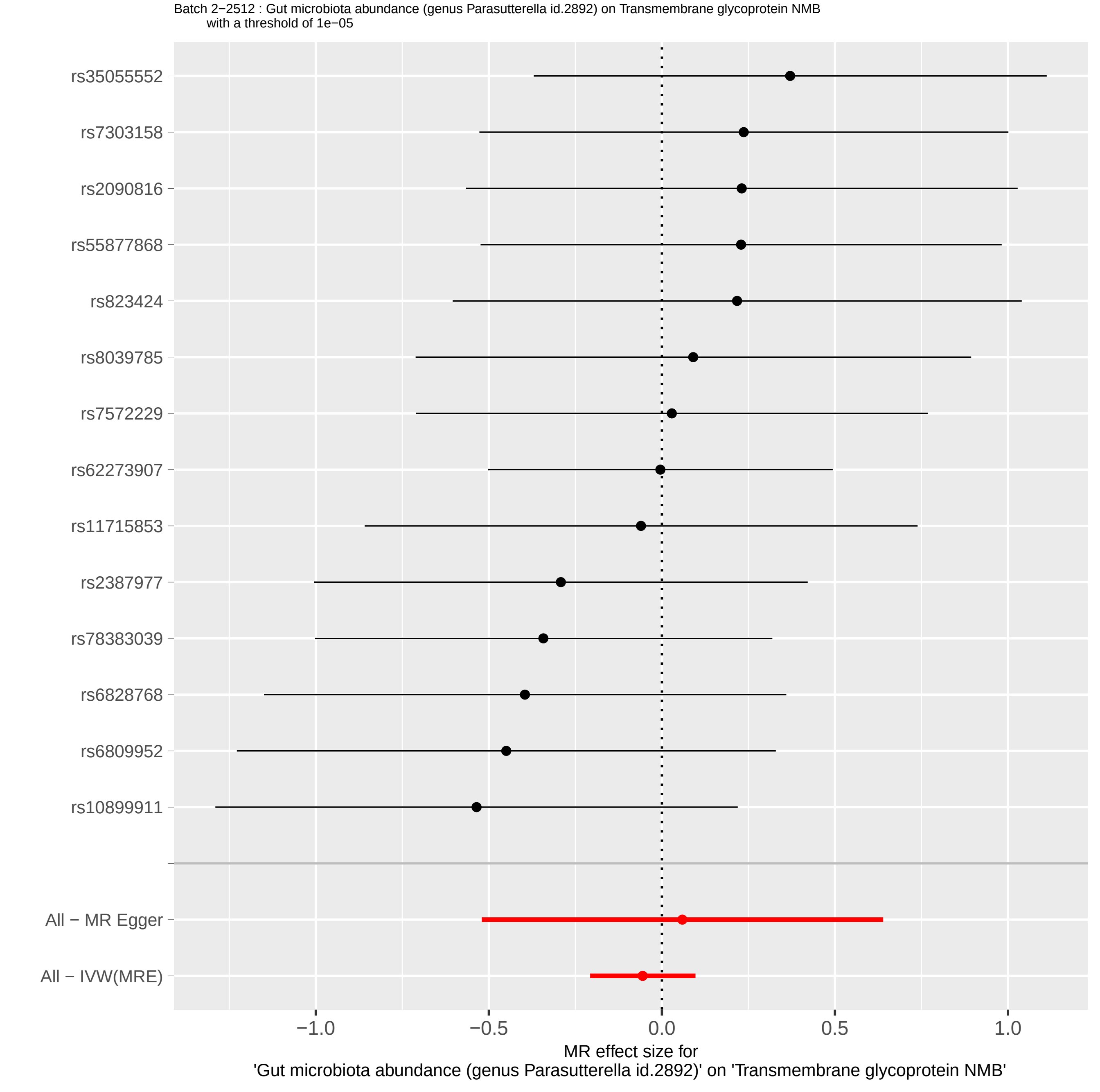

Batch 2−2512 : Gut microbiota abundance (genus Parasutterella id.2892) on Transmembrane glycoprotein NMB
with a threshold of 1e−05
rs35055552
rs7303158
rs2090816
rs55877868
rs823424
rs8039785
rs7572229
rs62273907
rs11715853
rs2387977
rs78383039
rs6828768
rs6809952
rs10899911
All − MR Egger
All − IVW(MRE)
−1.0
−0.5
0.0
0.5
1.0
MR effect size for
'Gut microbiota abundance (genus Parasutterella id.2892)' on 'Transmembrane glycoprotein NMB'

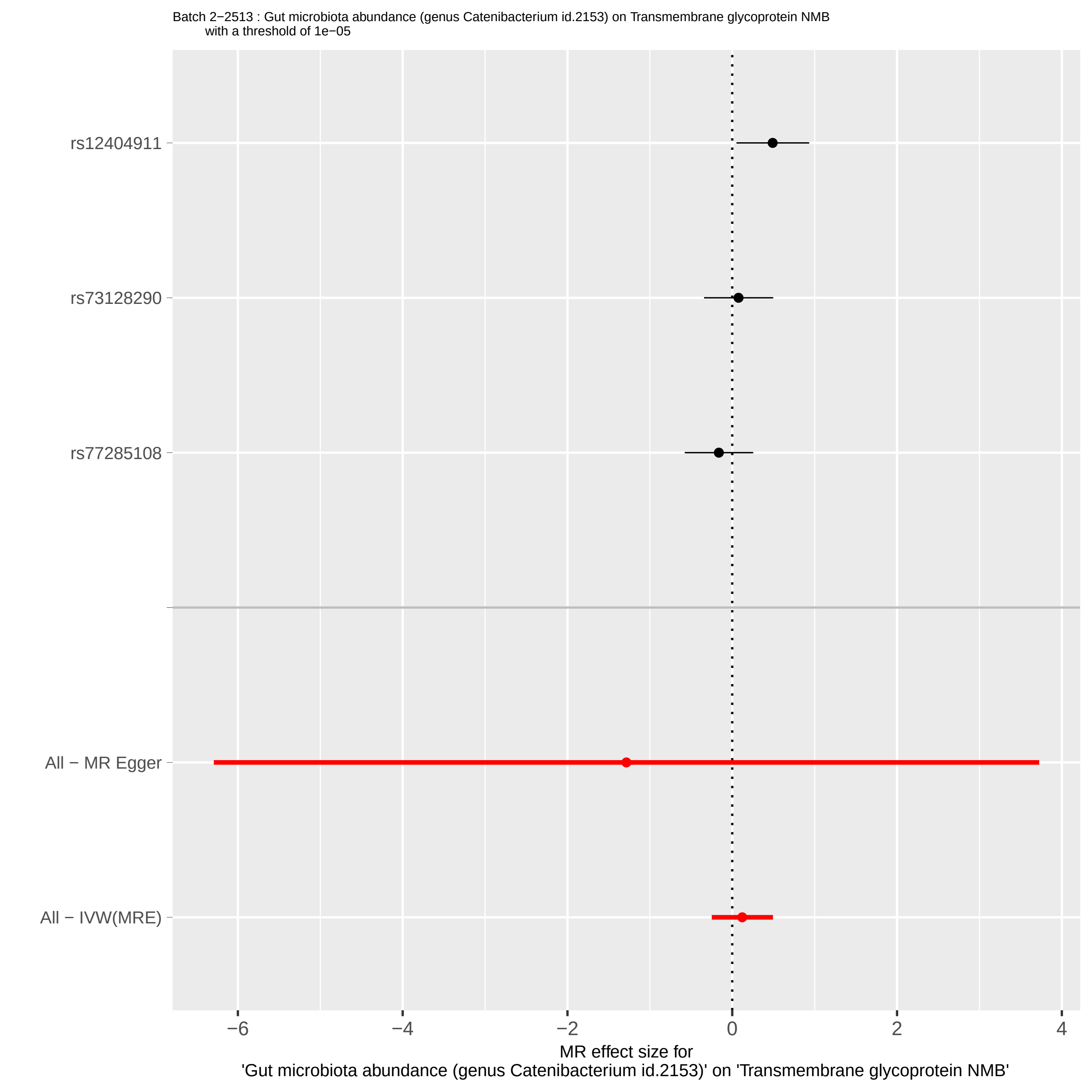
Batch 2−2513 : Gut microbiota abundance (genus Catenibacterium id.2153) on Transmembrane glycoprotein NMB
with a threshold of 1e−05
rs12404911
rs73128290
rs77285108
All − MR Egger
All − IVW(MRE)
−6
−4
−2
0
2
4
MR effect size for
'Gut microbiota abundance (genus Catenibacterium id.2153)' on 'Transmembrane glycoprotein NMB'

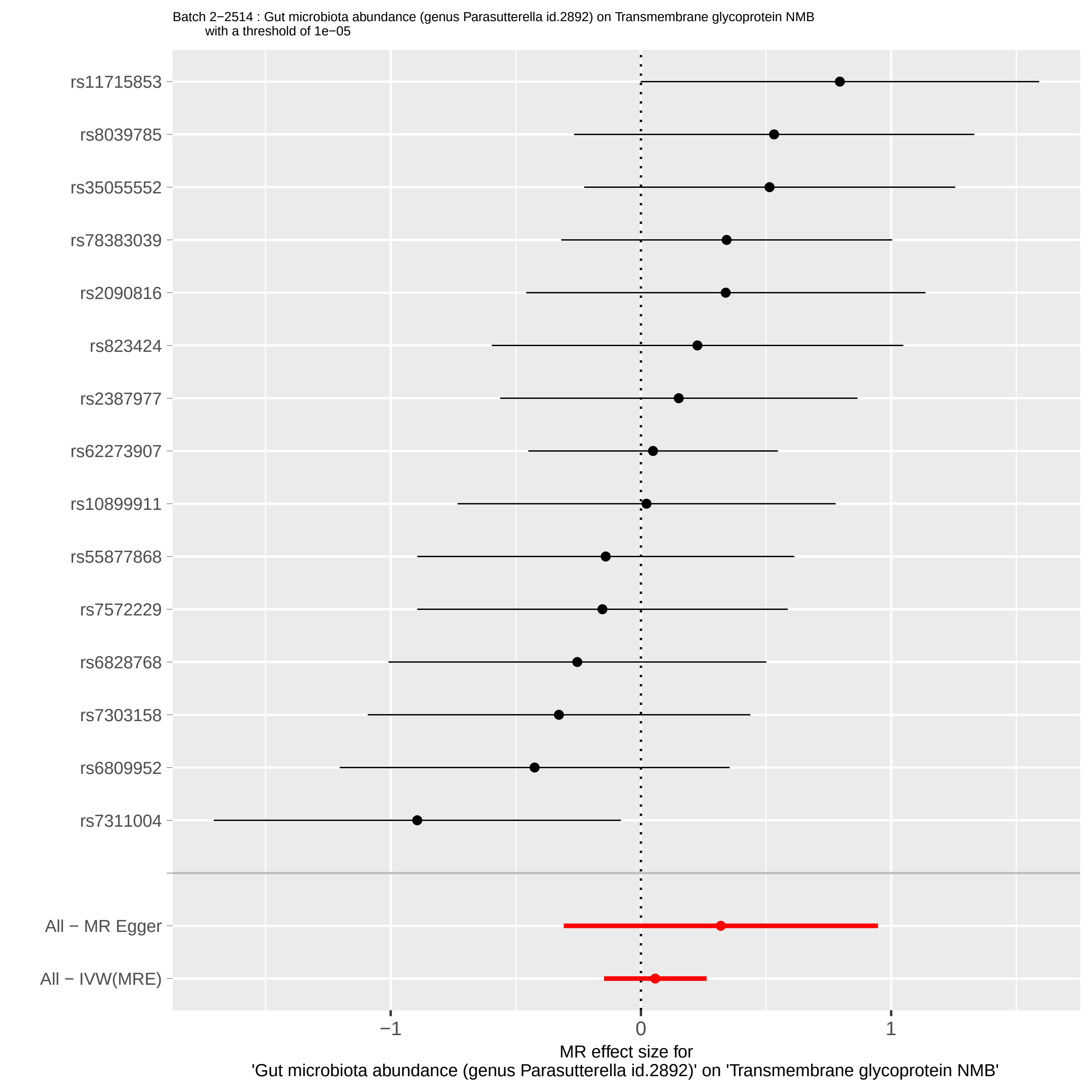

Batch 2−2514 : Gut microbiota abundance (genus Parasutterella id.2892) on Transmembrane glycoprotein NMB
with a threshold of 1e−05
rs11715853
rs8039785
rs35055552
rs78383039
rs2090816
rs823424
rs2387977
rs62273907
rs10899911
rs55877868
rs7572229
rs6828768
rs7303158
rs6809952
rs7311004
All − MR Egger
All − IVW(MRE)
−1
0
1
MR effect size for
'Gut microbiota abundance (genus Parasutterella id.2892)' on 'Transmembrane glycoprotein NMB'

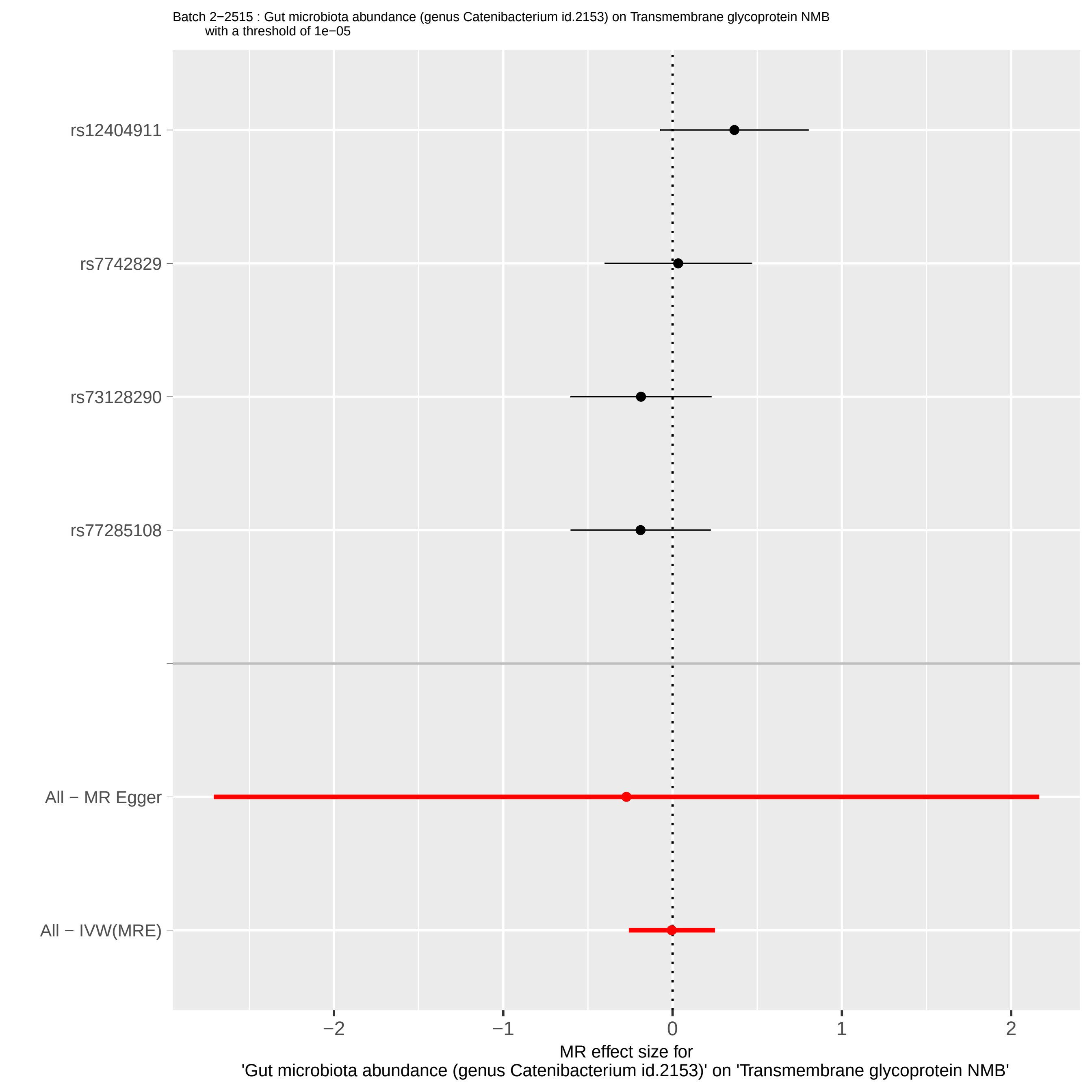

Batch 2−2515 : Gut microbiota abundance (genus Catenibacterium id.2153) on Transmembrane glycoprotein NMB
with a threshold of 1e−05
rs12404911
rs7742829
rs73128290
rs77285108
All − MR Egger
All − IVW(MRE)
−2
−1
0
1
2
MR effect size for
'Gut microbiota abundance (genus Catenibacterium id.2153)' on 'Transmembrane glycoprotein NMB'

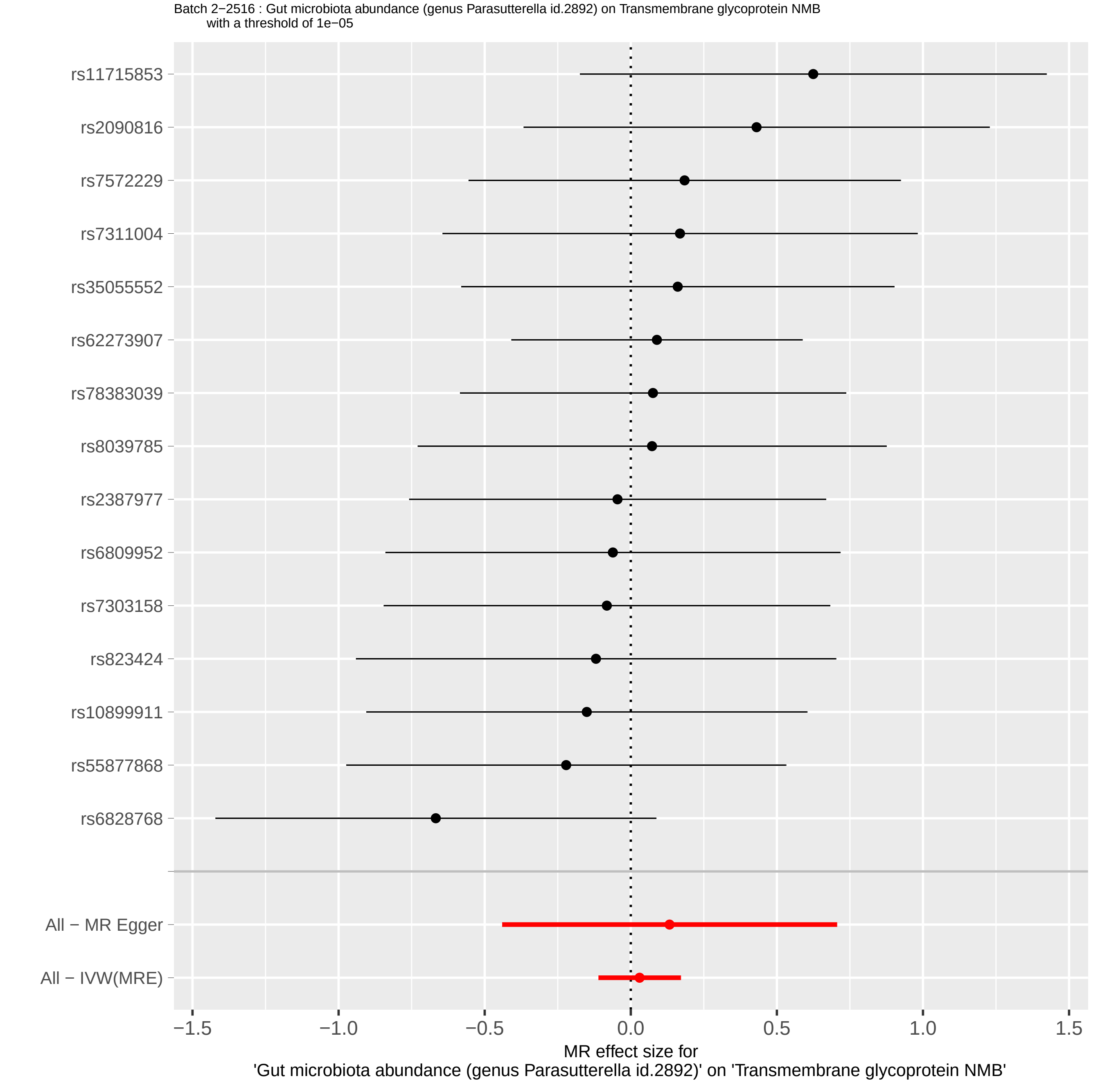

Batch 2−2516 : Gut microbiota abundance (genus Parasutterella id.2892) on Transmembrane glycoprotein NMB
with a threshold of 1e−05
rs11715853
rs2090816
rs7572229
rs7311004
rs35055552
rs62273907
rs78383039
rs8039785
rs2387977
rs6809952
rs7303158
rs823424
rs10899911
rs55877868
rs6828768
All − MR Egger
All − IVW(MRE)
−1.5
−1.0
−0.5
0.0
0.5
1.0
1.5
MR effect size for
'Gut microbiota abundance (genus Parasutterella id.2892)' on 'Transmembrane glycoprotein NMB'

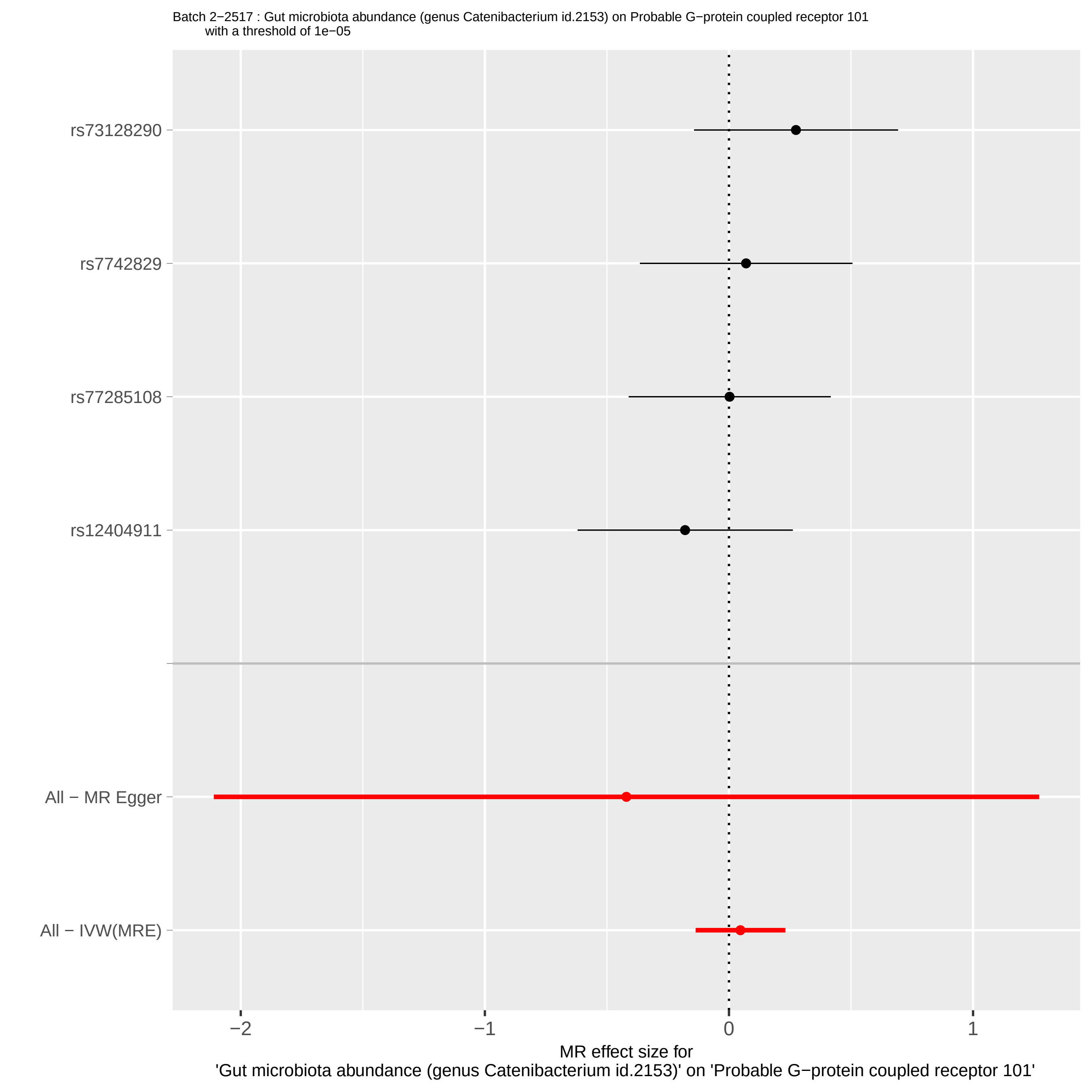

Batch 2−2517 : Gut microbiota abundance (genus Catenibacterium id.2153) on Probable G−protein coupled receptor 101
with a threshold of 1e−05
rs73128290
rs7742829
rs77285108
rs12404911
All − MR Egger
All − IVW(MRE)
−2
−1
0
1
MR effect size for
'Gut microbiota abundance (genus Catenibacterium id.2153)' on 'Probable G−protein coupled receptor 101'

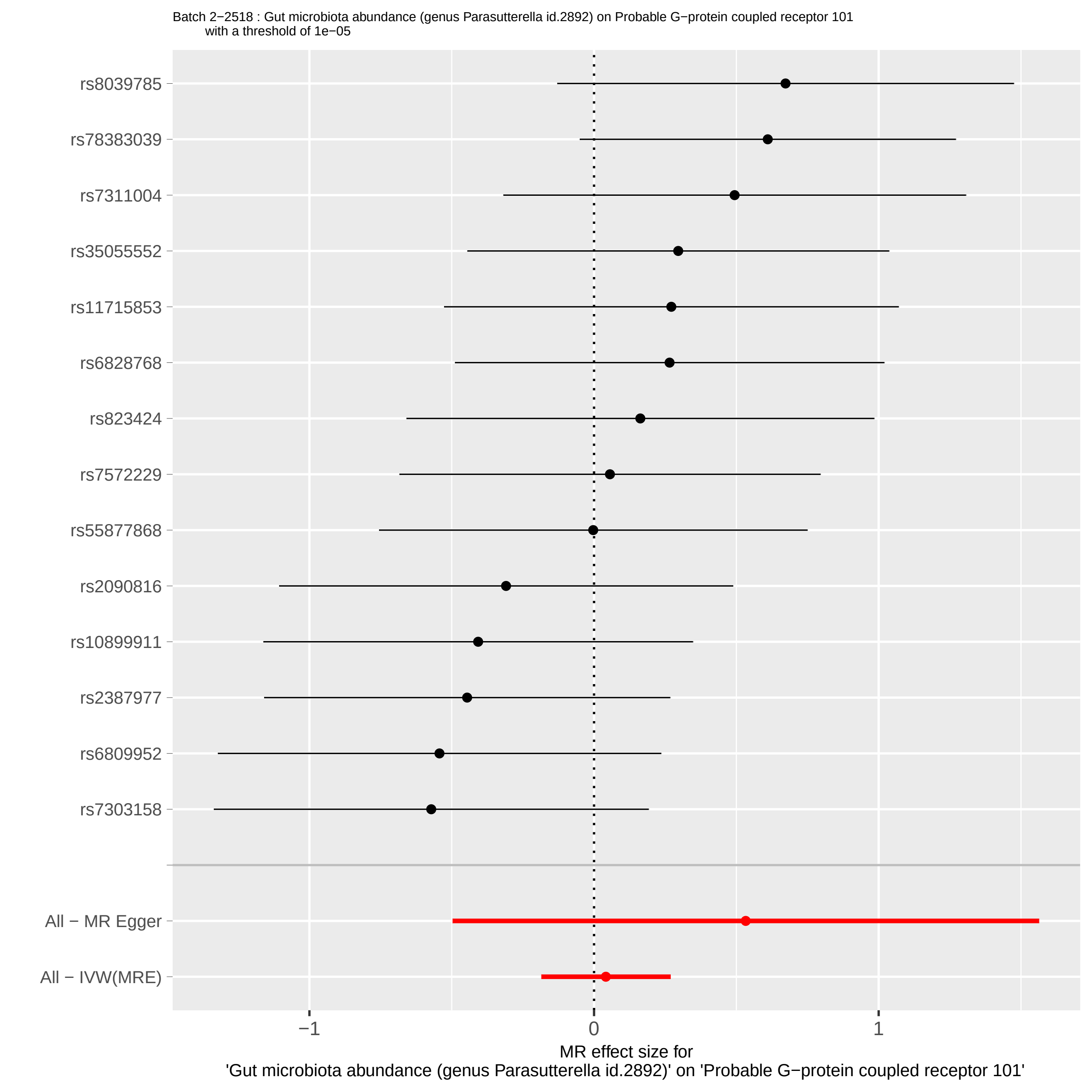

Batch 2−2518 : Gut microbiota abundance (genus Parasutterella id.2892) on Probable G−protein coupled receptor 101
with a threshold of 1e−05
rs8039785
rs78383039
rs7311004
rs35055552
rs11715853
rs6828768
rs823424
rs7572229
rs55877868
rs2090816
rs10899911
rs2387977
rs6809952
rs7303158
All − MR Egger
All − IVW(MRE)
−1
0
1
MR effect size for
'Gut microbiota abundance (genus Parasutterella id.2892)' on 'Probable G−protein coupled receptor 101'

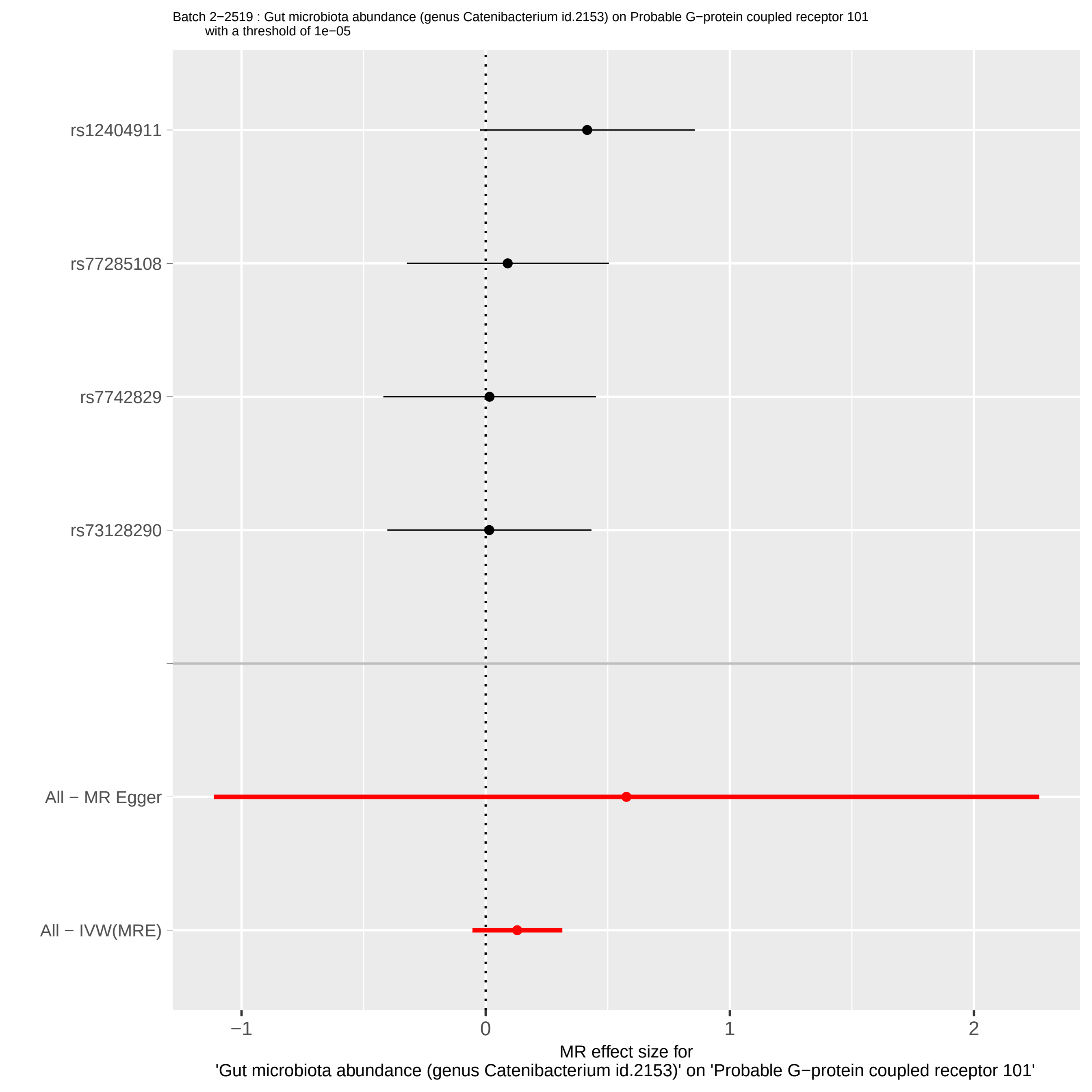
Batch 2−2519 : Gut microbiota abundance (genus Catenibacterium id.2153) on Probable G−protein coupled receptor 101
with a threshold of 1e−05
rs12404911
rs77285108
rs7742829
rs73128290
All − MR Egger
All − IVW(MRE)
−1
0
1
2
MR effect size for
'Gut microbiota abundance (genus Catenibacterium id.2153)' on 'Probable G−protein coupled receptor 101'

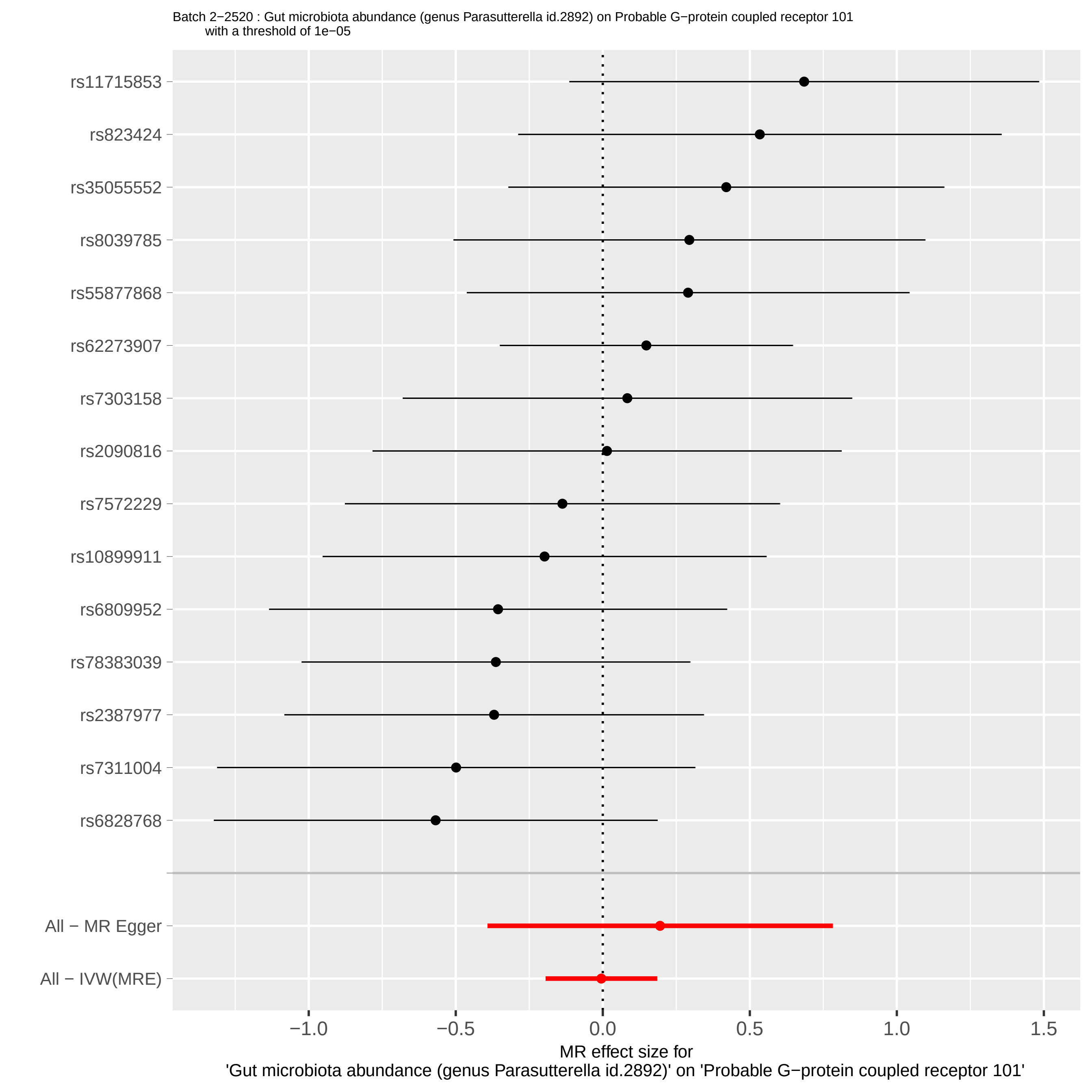
Batch 2−2520 : Gut microbiota abundance (genus Parasutterella id.2892) on Probable G−protein coupled receptor 101
with a threshold of 1e−05
rs11715853
rs823424
rs35055552
rs8039785
rs55877868
rs62273907
rs7303158
rs2090816
rs7572229
rs10899911
rs6809952
rs78383039
rs2387977
rs7311004
rs6828768
All − MR Egger
All − IVW(MRE)
−1.0
−0.5
0.0
0.5
1.0
1.5
MR effect size for
'Gut microbiota abundance (genus Parasutterella id.2892)' on 'Probable G−protein coupled receptor 101'

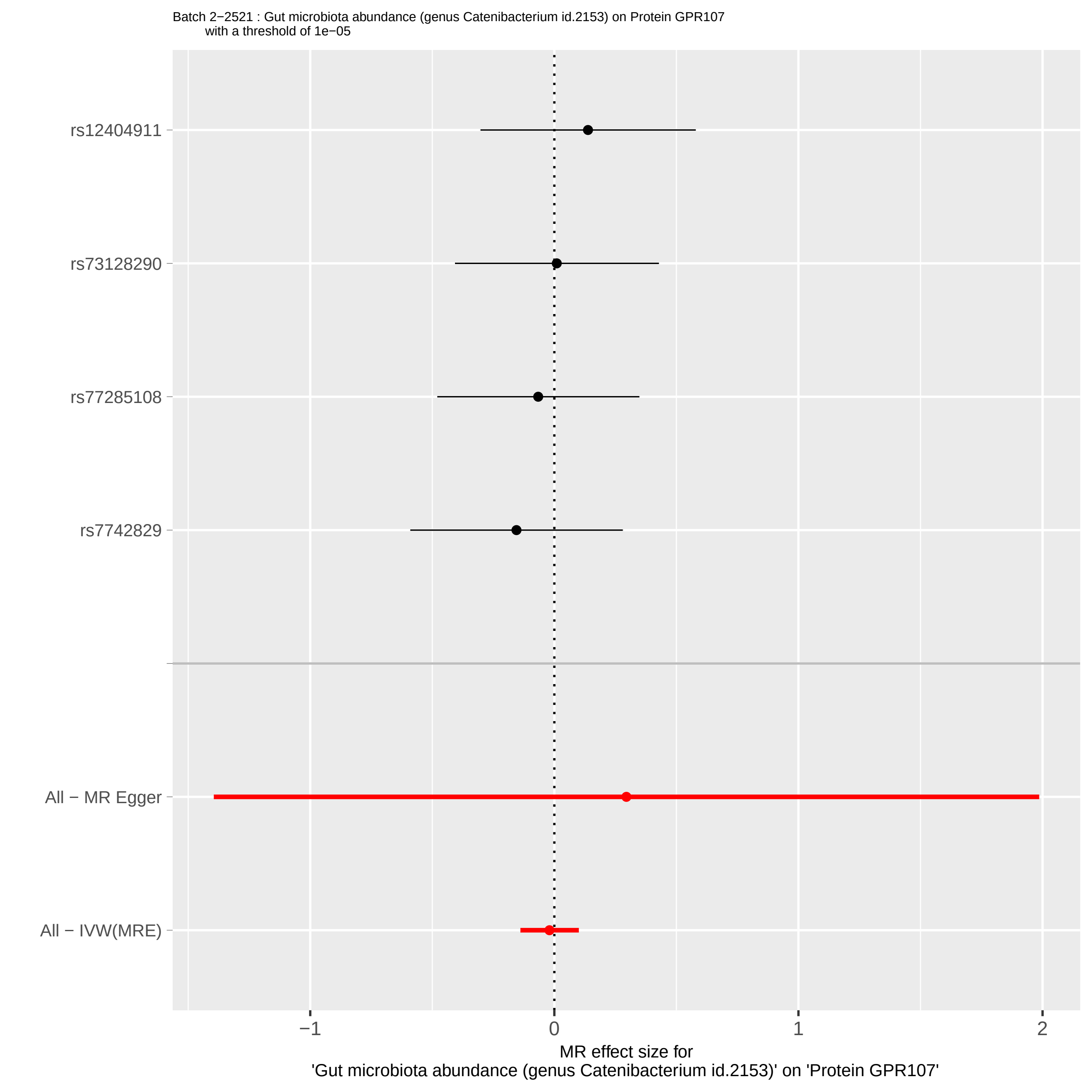

Batch 2−2521 : Gut microbiota abundance (genus Catenibacterium id.2153) on Protein GPR107
with a threshold of 1e−05
rs12404911
rs73128290
rs77285108
rs7742829
All − MR Egger
All − IVW(MRE)
−1
0
1
2
MR effect size for
'Gut microbiota abundance (genus Catenibacterium id.2153)' on 'Protein GPR107'

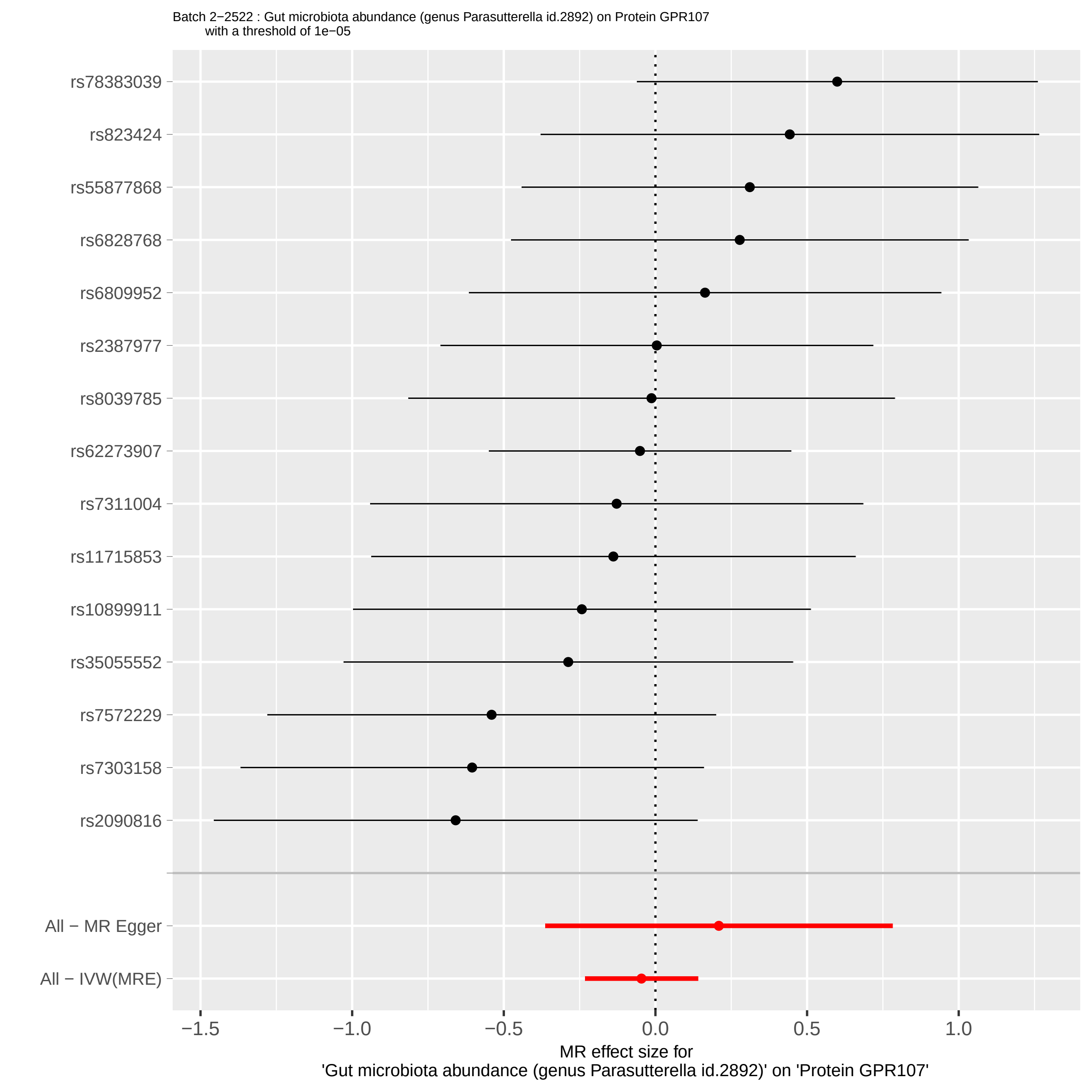

Batch 2−2522 : Gut microbiota abundance (genus Parasutterella id.2892) on Protein GPR107
with a threshold of 1e−05
rs78383039
rs823424
rs55877868
rs6828768
rs6809952
rs2387977
rs8039785
rs62273907
rs7311004
rs11715853
rs10899911
rs35055552
rs7572229
rs7303158
rs2090816
All − MR Egger
All − IVW(MRE)
−1.5
−1.0
−0.5
0.0
0.5
1.0
MR effect size for
'Gut microbiota abundance (genus Parasutterella id.2892)' on 'Protein GPR107'

Batch 2−2523 : Gut microbiota abundance (genus Catenibacterium id.2153) on Probable G−protein coupled receptor 135
with a threshold of 1e−05

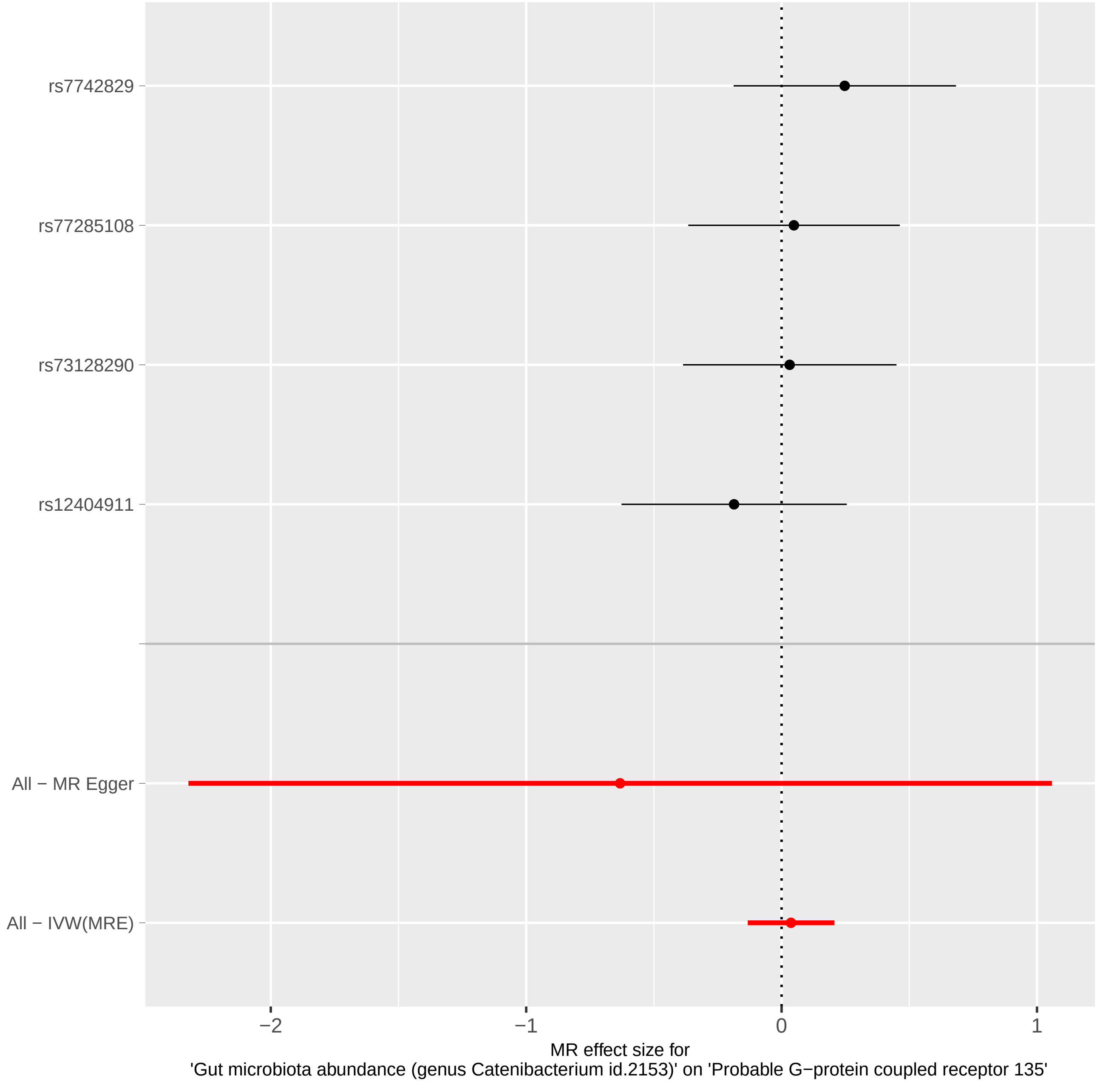

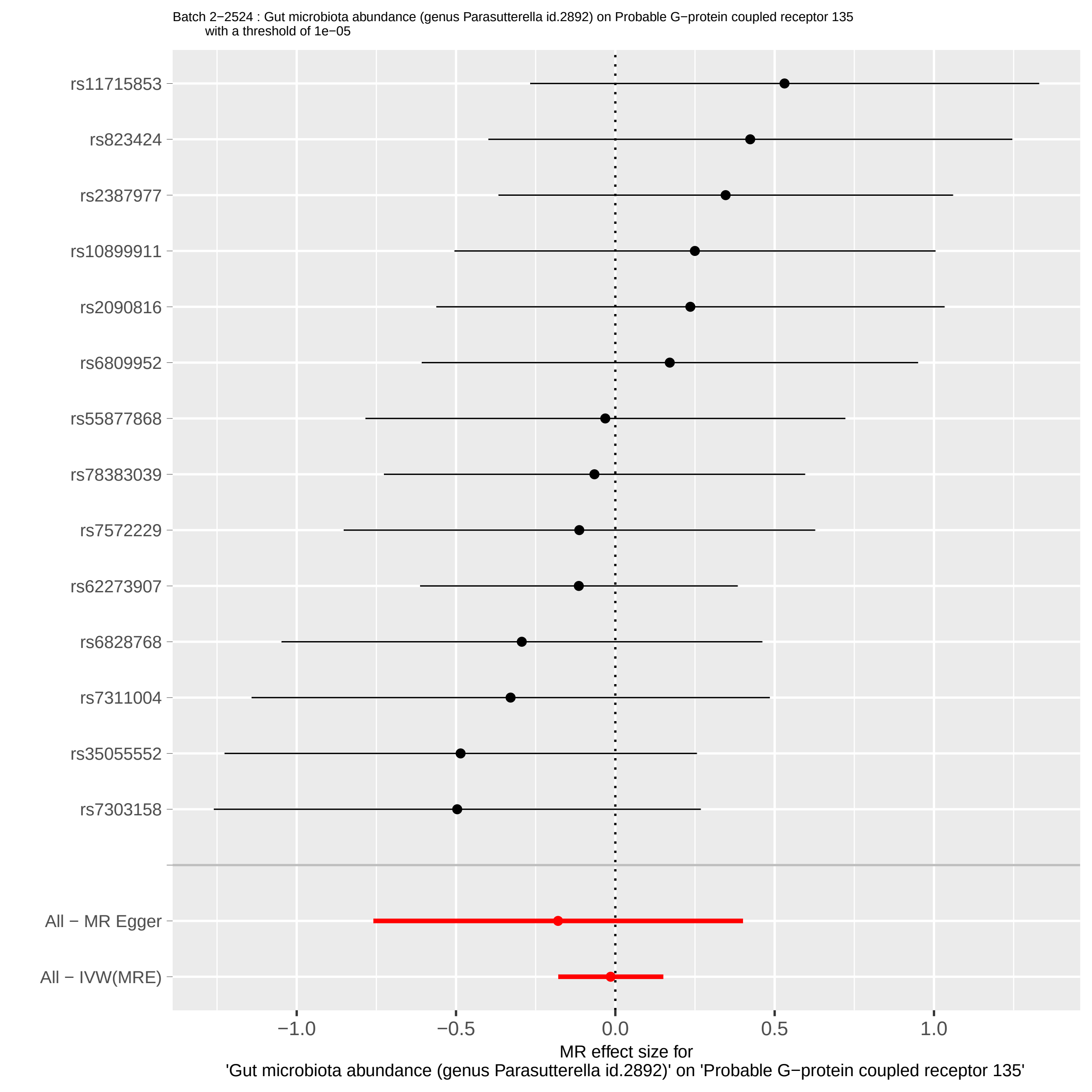

Batch 2−2524 : Gut microbiota abundance (genus Parasutterella id.2892) on Probable G−protein coupled receptor 135
with a threshold of 1e−05
rs11715853
rs823424
rs2387977
rs10899911
rs2090816
rs6809952
rs55877868
rs78383039
rs7572229
rs62273907
rs6828768
rs7311004
rs35055552
rs7303158
All − MR Egger
All − IVW(MRE)
−1.0
−0.5
0.0
0.5
1.0
MR effect size for
'Gut microbiota abundance (genus Parasutterella id.2892)' on 'Probable G−protein coupled receptor 135'

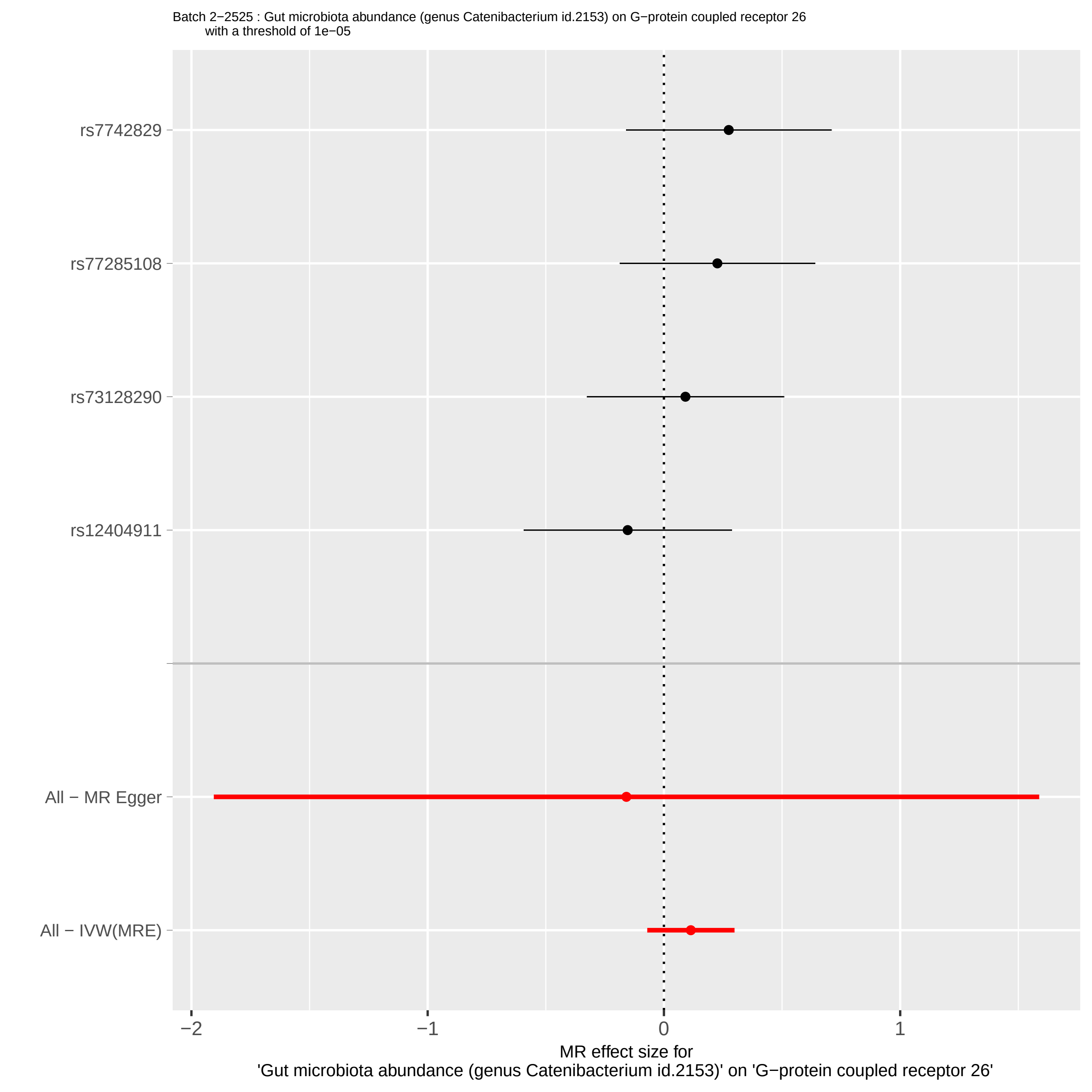
Batch 2−2525 : Gut microbiota abundance (genus Catenibacterium id.2153) on G−protein coupled receptor 26
with a threshold of 1e−05
rs7742829
rs77285108
rs73128290
rs12404911
All − MR Egger
All − IVW(MRE)
−2
−1
0
1
MR effect size for
'Gut microbiota abundance (genus Catenibacterium id.2153)' on 'G−protein coupled receptor 26'

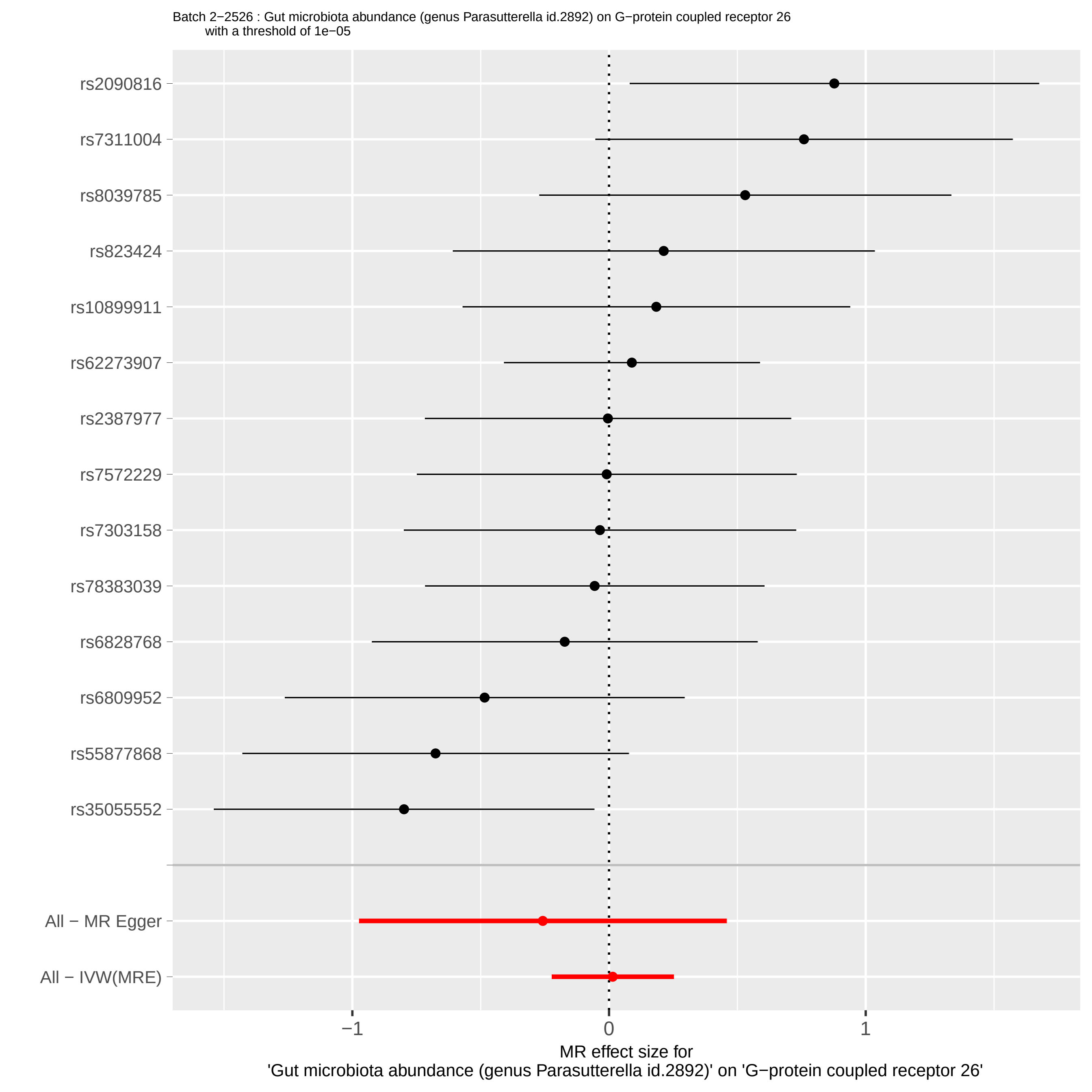

Batch 2−2526 : Gut microbiota abundance (genus Parasutterella id.2892) on G−protein coupled receptor 26
with a threshold of 1e−05
rs2090816
rs7311004
rs8039785
rs823424
rs10899911
rs62273907
rs2387977
rs7572229
rs7303158
rs78383039
rs6828768
rs6809952
rs55877868
rs35055552
All − MR Egger
All − IVW(MRE)
−1
0
1
MR effect size for
'Gut microbiota abundance (genus Parasutterella id.2892)' on 'G−protein coupled receptor 26'

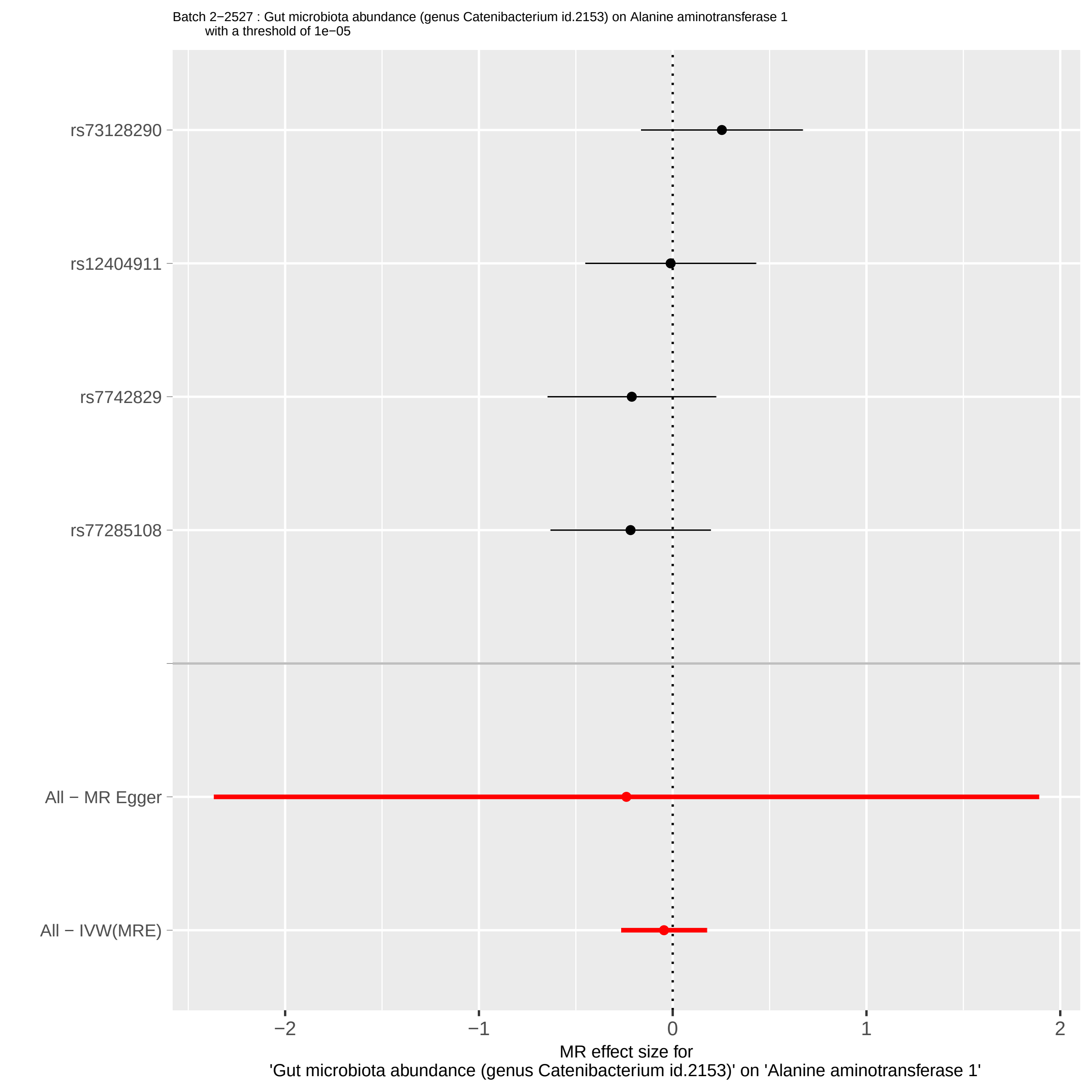

Batch 2−2527 : Gut microbiota abundance (genus Catenibacterium id.2153) on Alanine aminotransferase 1
with a threshold of 1e−05
rs73128290
rs12404911
rs7742829
rs77285108
All − MR Egger
All − IVW(MRE)
−2
−1
0
1
2
MR effect size for
'Gut microbiota abundance (genus Catenibacterium id.2153)' on 'Alanine aminotransferase 1'

Batch 2−2528 : Gut microbiota abundance (genus Parasutterella id.2892) on Alanine aminotransferase 1
with a threshold of 1e−05

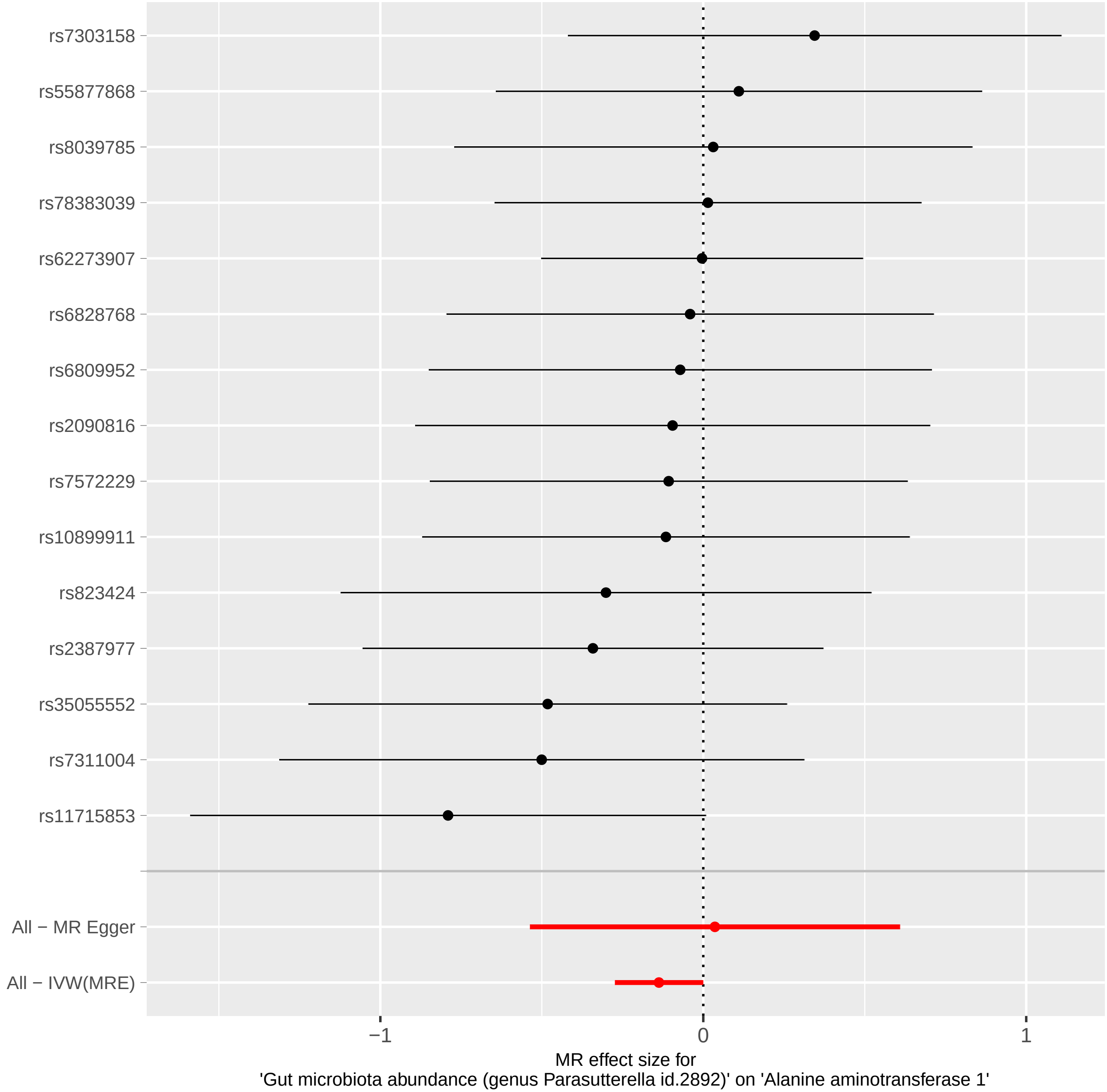

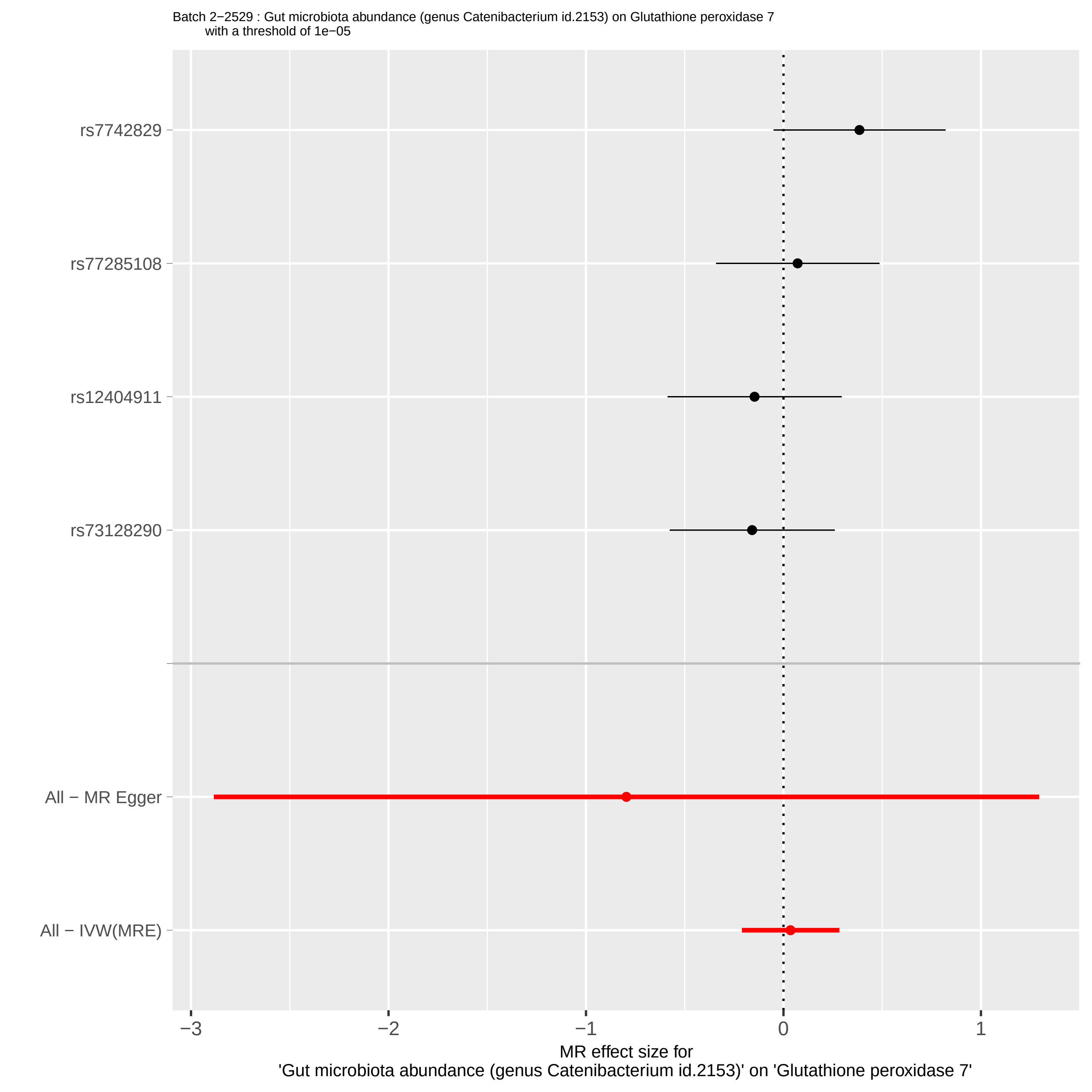
Batch 2−2529 : Gut microbiota abundance (genus Catenibacterium id.2153) on Glutathione peroxidase 7
with a threshold of 1e−05
rs7742829
rs77285108
rs12404911
rs73128290
All − MR Egger
All − IVW(MRE)
−3
−2
−1
0
1
MR effect size for
'Gut microbiota abundance (genus Catenibacterium id.2153)' on 'Glutathione peroxidase 7'

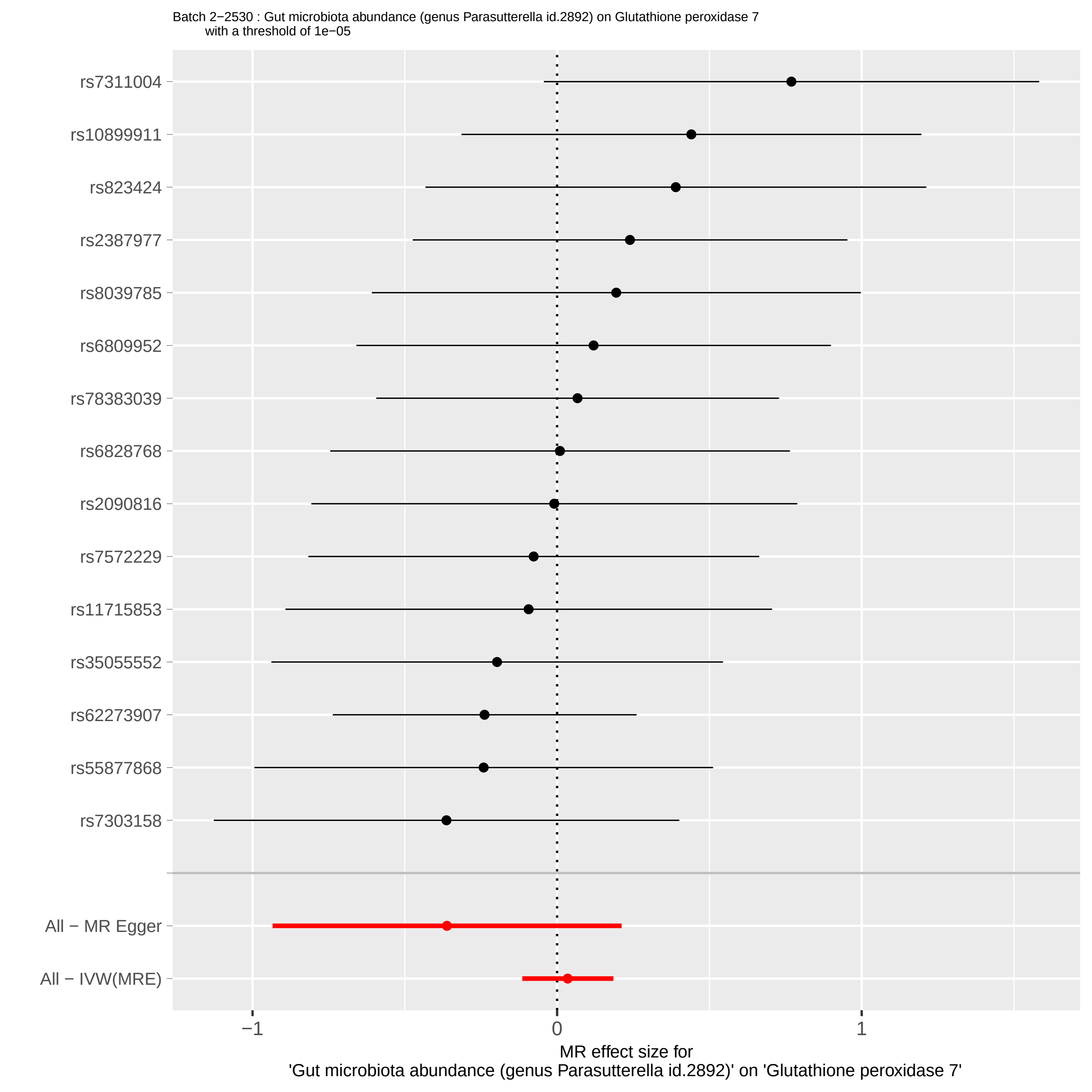

Batch 2−2530 : Gut microbiota abundance (genus Parasutterella id.2892) on Glutathione peroxidase 7
with a threshold of 1e−05
rs7311004
rs10899911
rs823424
rs2387977
rs8039785
rs6809952
rs78383039
rs6828768
rs2090816
rs7572229
rs11715853
rs35055552
rs62273907
rs55877868
rs7303158
All − MR Egger
All − IVW(MRE)
−1
0
1
MR effect size for
'Gut microbiota abundance (genus Parasutterella id.2892)' on 'Glutathione peroxidase 7'

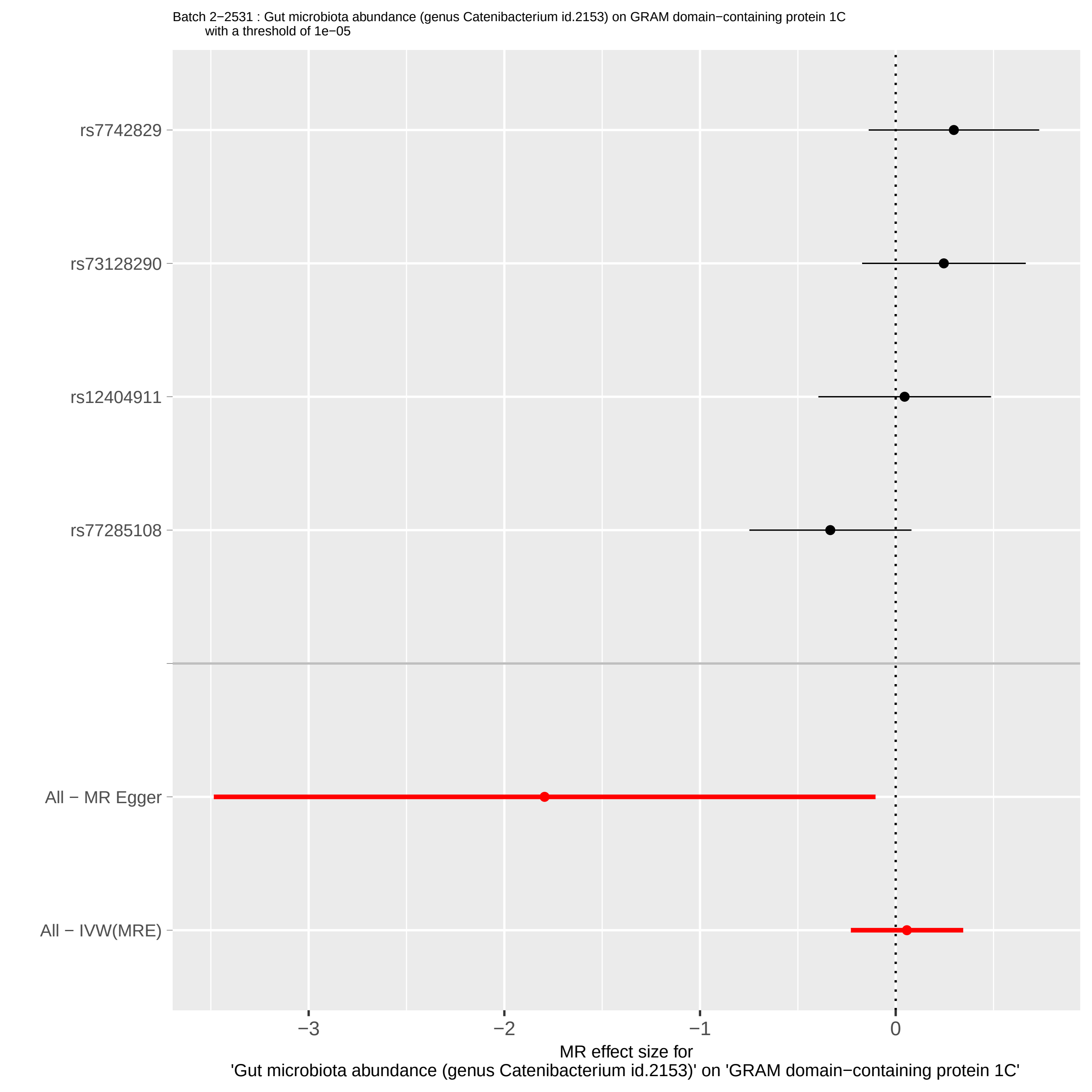

Batch 2−2531 : Gut microbiota abundance (genus Catenibacterium id.2153) on GRAM domain−containing protein 1C
with a threshold of 1e−05
rs7742829
rs73128290
rs12404911
rs77285108
All − MR Egger
All − IVW(MRE)
−3
−2
−1
0
MR effect size for
'Gut microbiota abundance (genus Catenibacterium id.2153)' on 'GRAM domain−containing protein 1C'

Batch 2−2532 : Gut microbiota abundance (genus Parasutterella id.2892) on GRAM domain−containing protein 1C
with a threshold of 1e−05

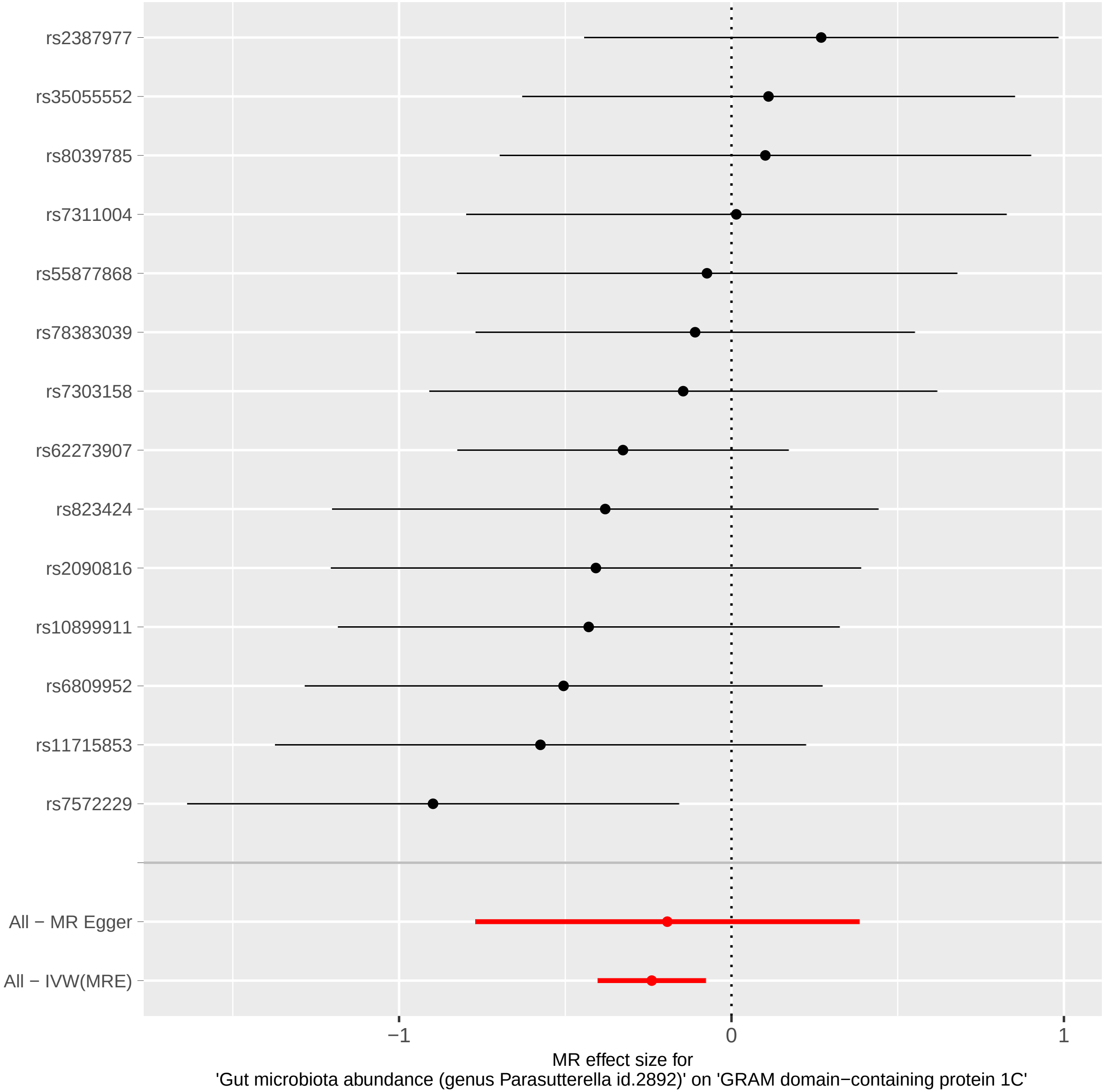

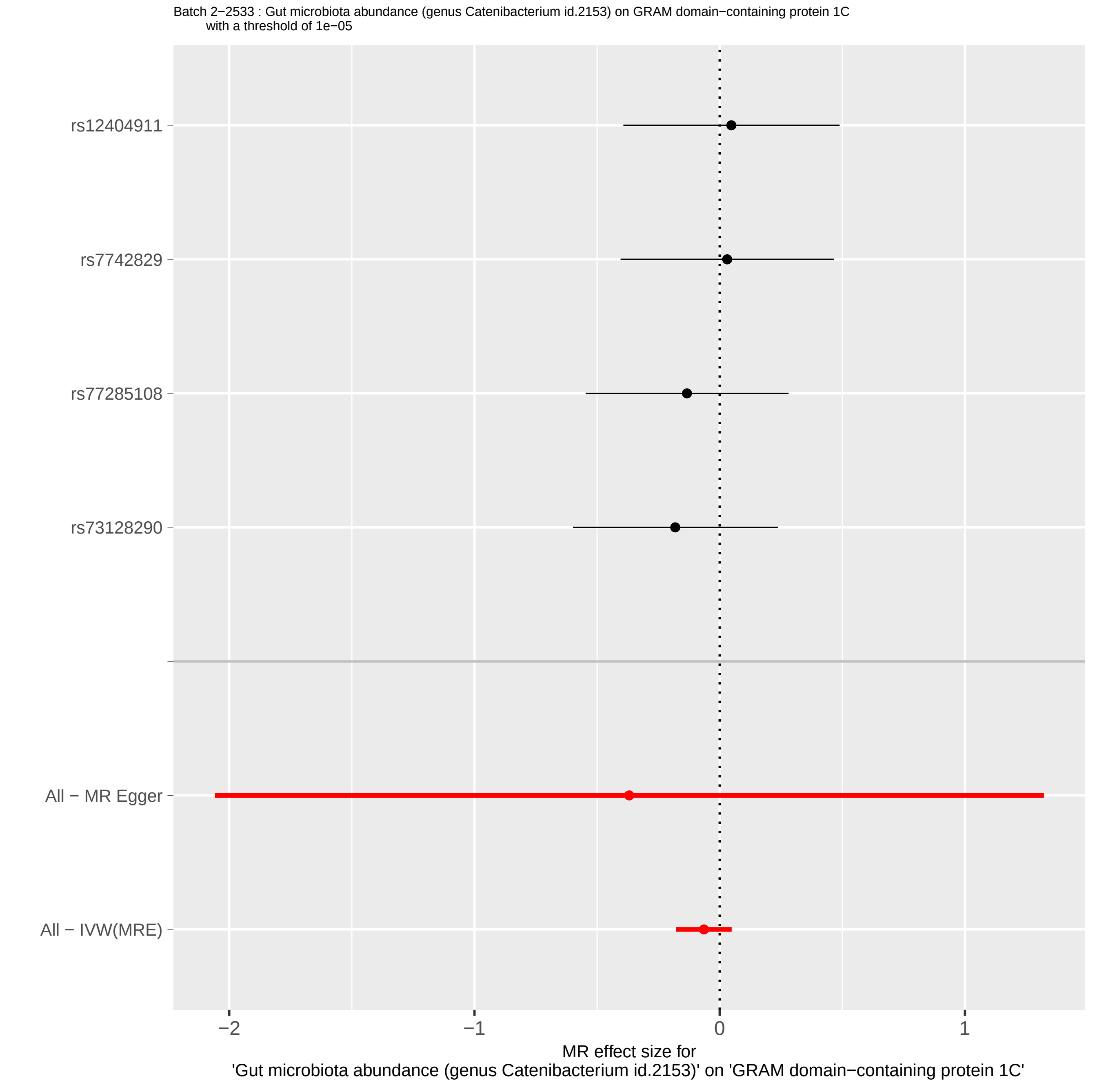
Batch 2−2533 : Gut microbiota abundance (genus Catenibacterium id.2153) on GRAM domain−containing protein 1C
with a threshold of 1e−05
rs12404911
rs7742829
rs77285108
rs73128290
All − MR Egger
All − IVW(MRE)
−2
−1
0
1
MR effect size for
'Gut microbiota abundance (genus Catenibacterium id.2153)' on 'GRAM domain−containing protein 1C'

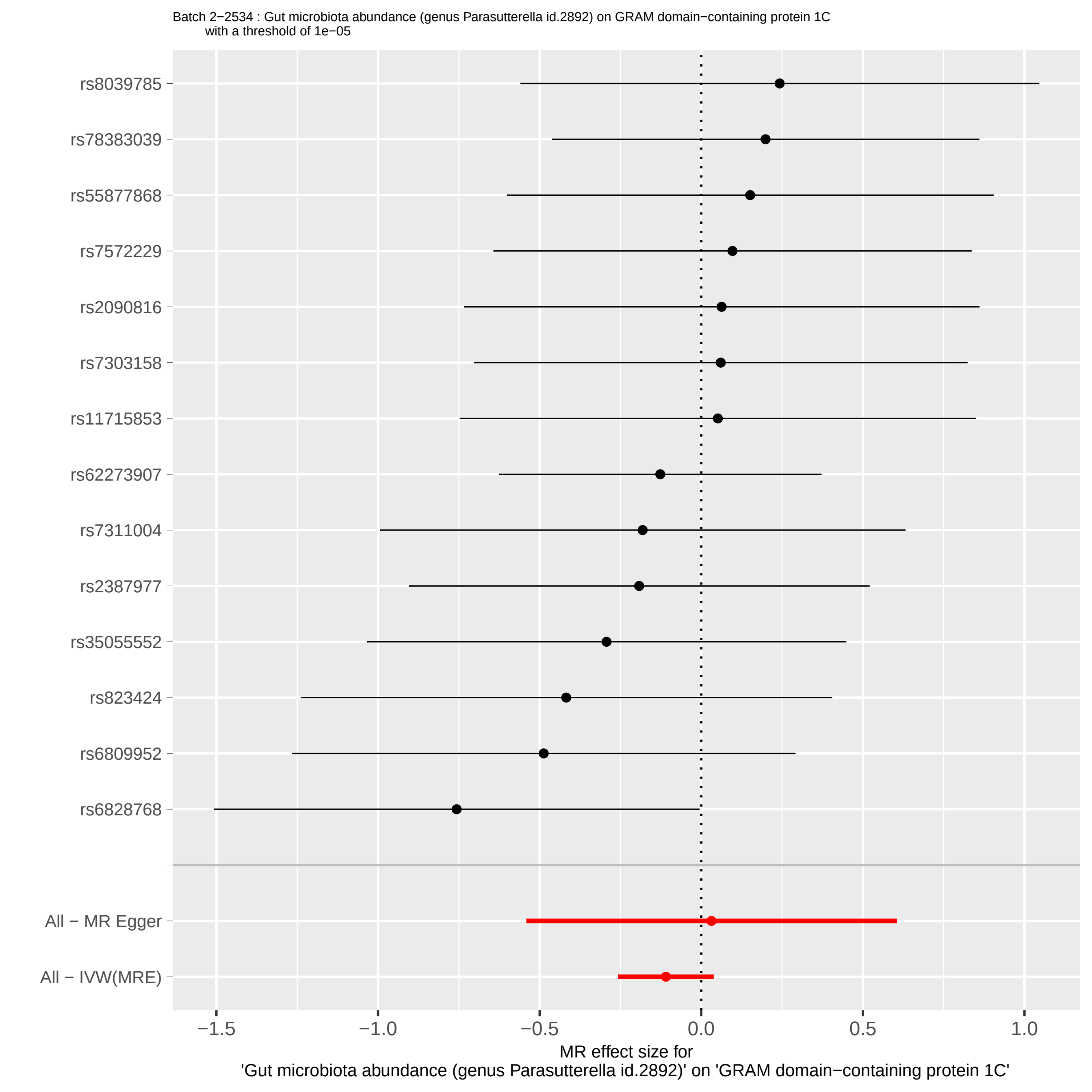
Batch 2−2534 : Gut microbiota abundance (genus Parasutterella id.2892) on GRAM domain−containing protein 1C
with a threshold of 1e−05
rs8039785
rs78383039
rs55877868
rs7572229
rs2090816
rs7303158
rs11715853
rs62273907
rs7311004
rs2387977
rs35055552
rs823424
rs6809952
rs6828768
All − MR Egger
All − IVW(MRE)
−1.5
−1.0
−0.5
0.0
0.5
1.0
MR effect size for
'Gut microbiota abundance (genus Parasutterella id.2892)' on 'GRAM domain−containing protein 1C'

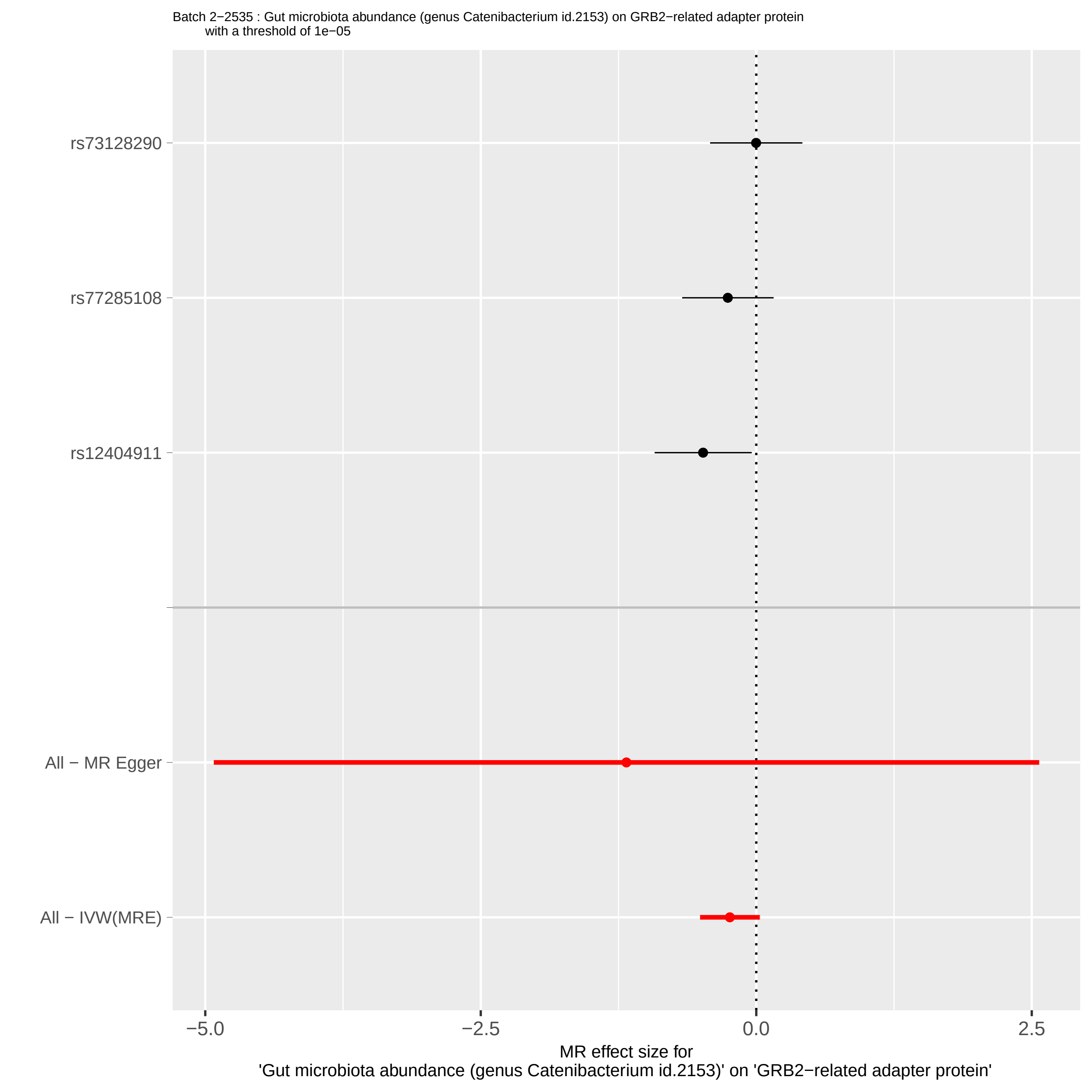

Batch 2−2535 : Gut microbiota abundance (genus Catenibacterium id.2153) on GRB2−related adapter protein
with a threshold of 1e−05
rs73128290
rs77285108
rs12404911
All − MR Egger
All − IVW(MRE)
−5.0
−2.5
0.0
2.5
MR effect size for
'Gut microbiota abundance (genus Catenibacterium id.2153)' on 'GRB2−related adapter protein'

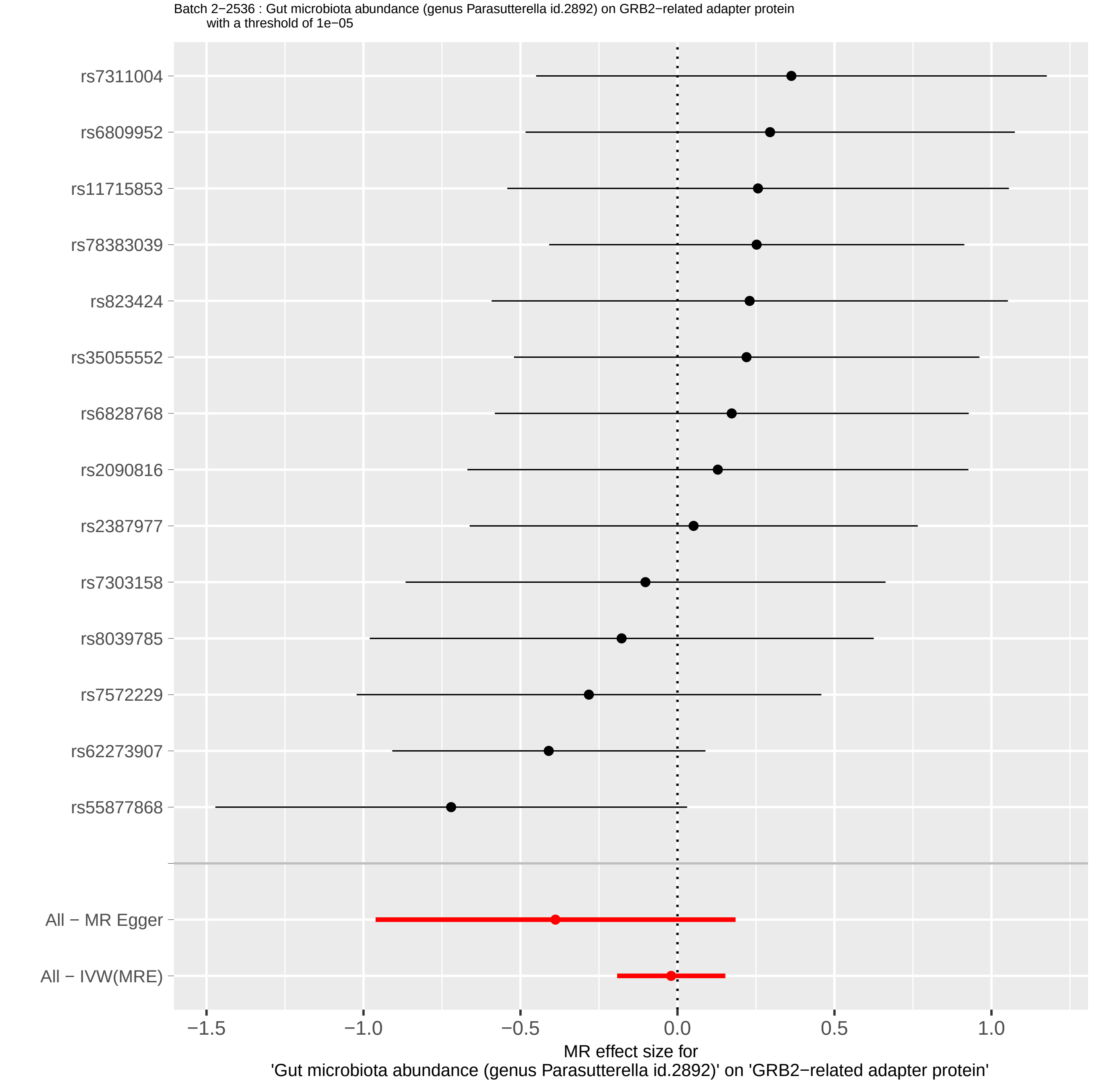

Batch 2−2536 : Gut microbiota abundance (genus Parasutterella id.2892) on GRB2−related adapter protein
with a threshold of 1e−05
rs7311004
rs6809952
rs11715853
rs78383039
rs823424
rs35055552
rs6828768
rs2090816
rs2387977
rs7303158
rs8039785
rs7572229
rs62273907
rs55877868
All − MR Egger
All − IVW(MRE)
−1.5
−1.0
−0.5
0.0
0.5
1.0
MR effect size for
'Gut microbiota abundance (genus Parasutterella id.2892)' on 'GRB2−related adapter protein'

Batch 2−2537 : Gut microbiota abundance (genus Catenibacterium id.2153) on GRB2−related adapter protein 2
with a threshold of 1e−05

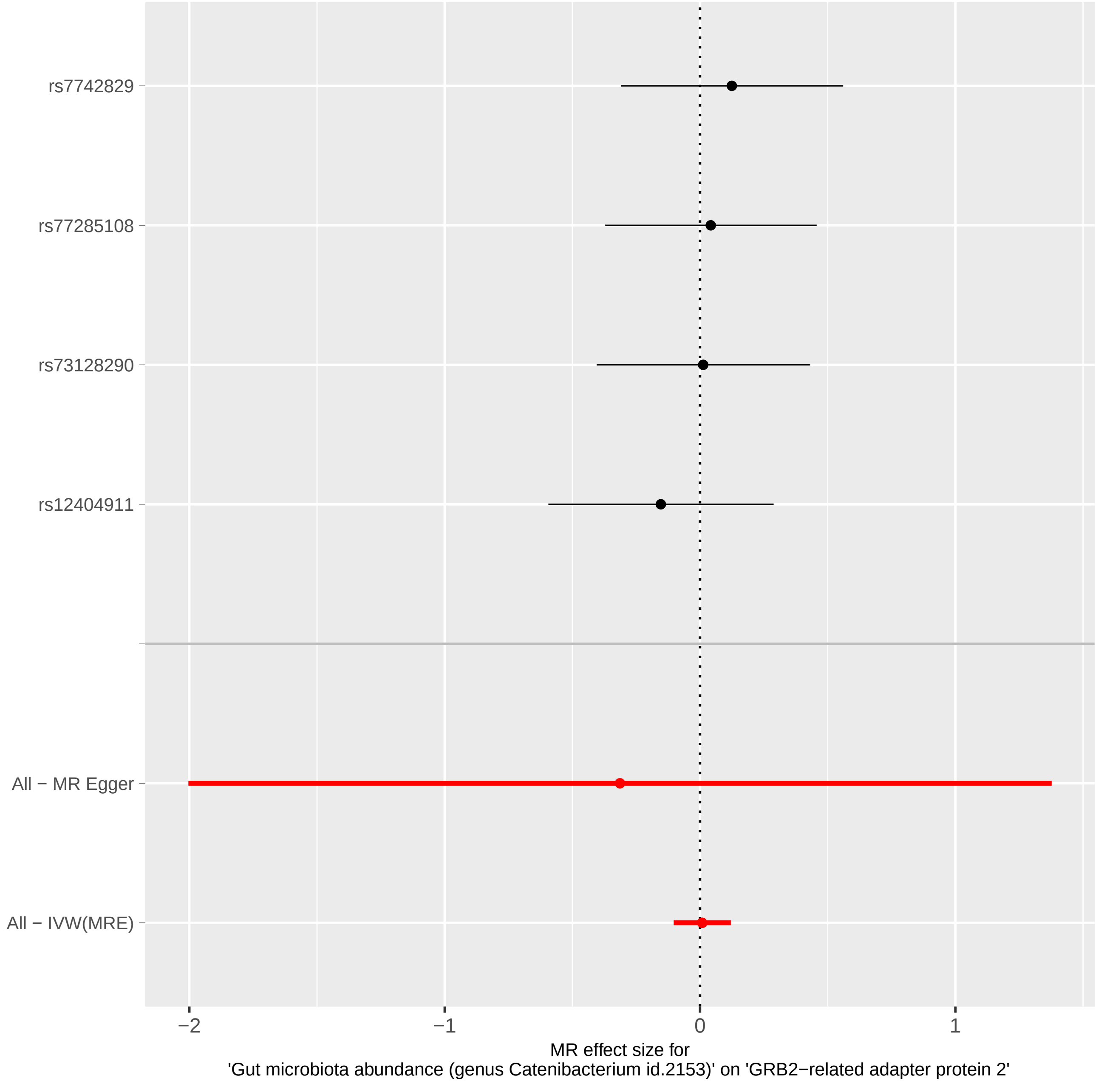

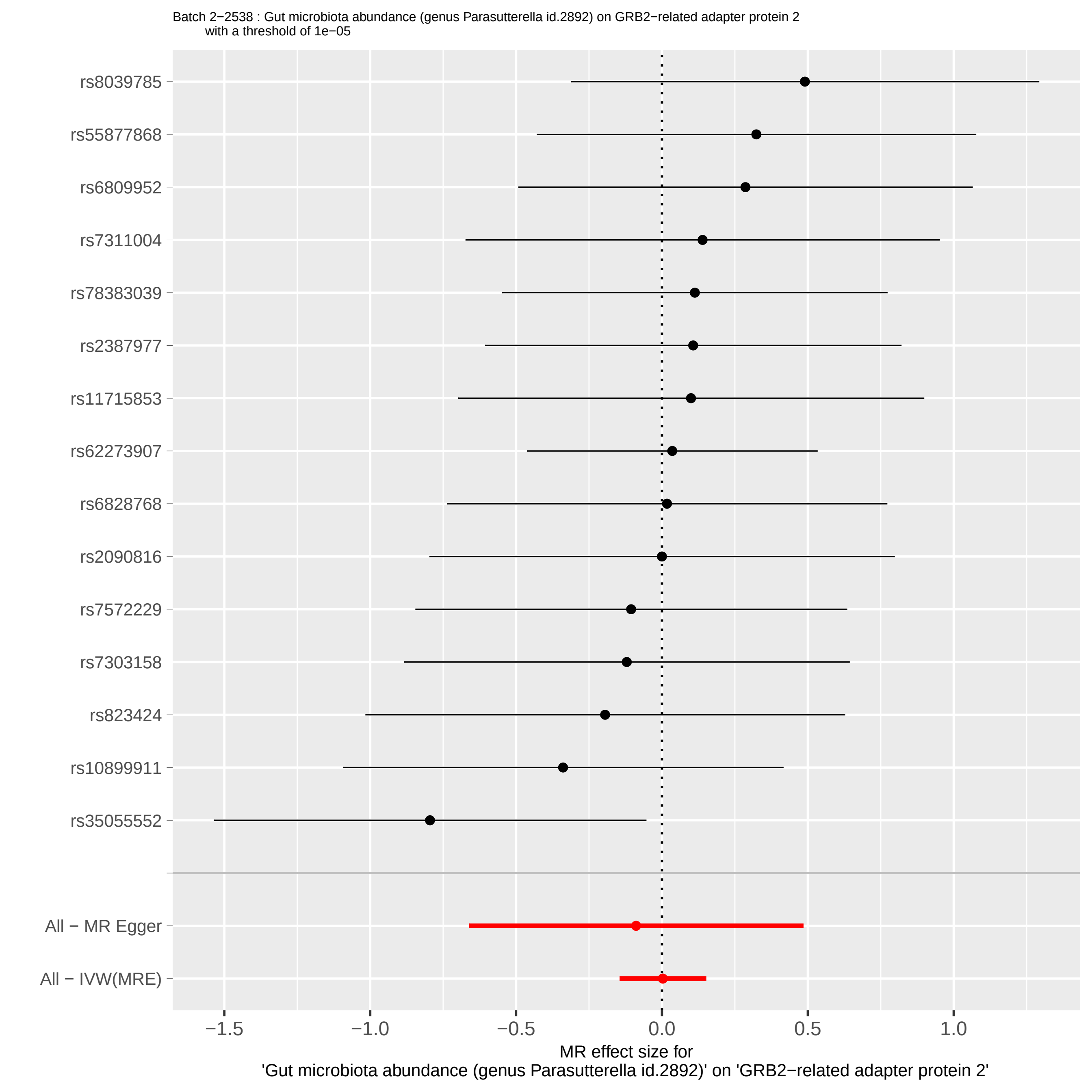

Batch 2−2538 : Gut microbiota abundance (genus Parasutterella id.2892) on GRB2−related adapter protein 2
with a threshold of 1e−05
rs8039785
rs55877868
rs6809952
rs7311004
rs78383039
rs2387977
rs11715853
rs62273907
rs6828768
rs2090816
rs7572229
rs7303158
rs823424
rs10899911
rs35055552
All − MR Egger
All − IVW(MRE)
−1.5
−1.0
−0.5
0.0
0.5
1.0
MR effect size for
'Gut microbiota abundance (genus Parasutterella id.2892)' on 'GRB2−related adapter protein 2'

Batch 2−2539 : Gut microbiota abundance (genus Catenibacterium id.2153) on Growth factor receptor−bound protein 14
with a threshold of 1e−05

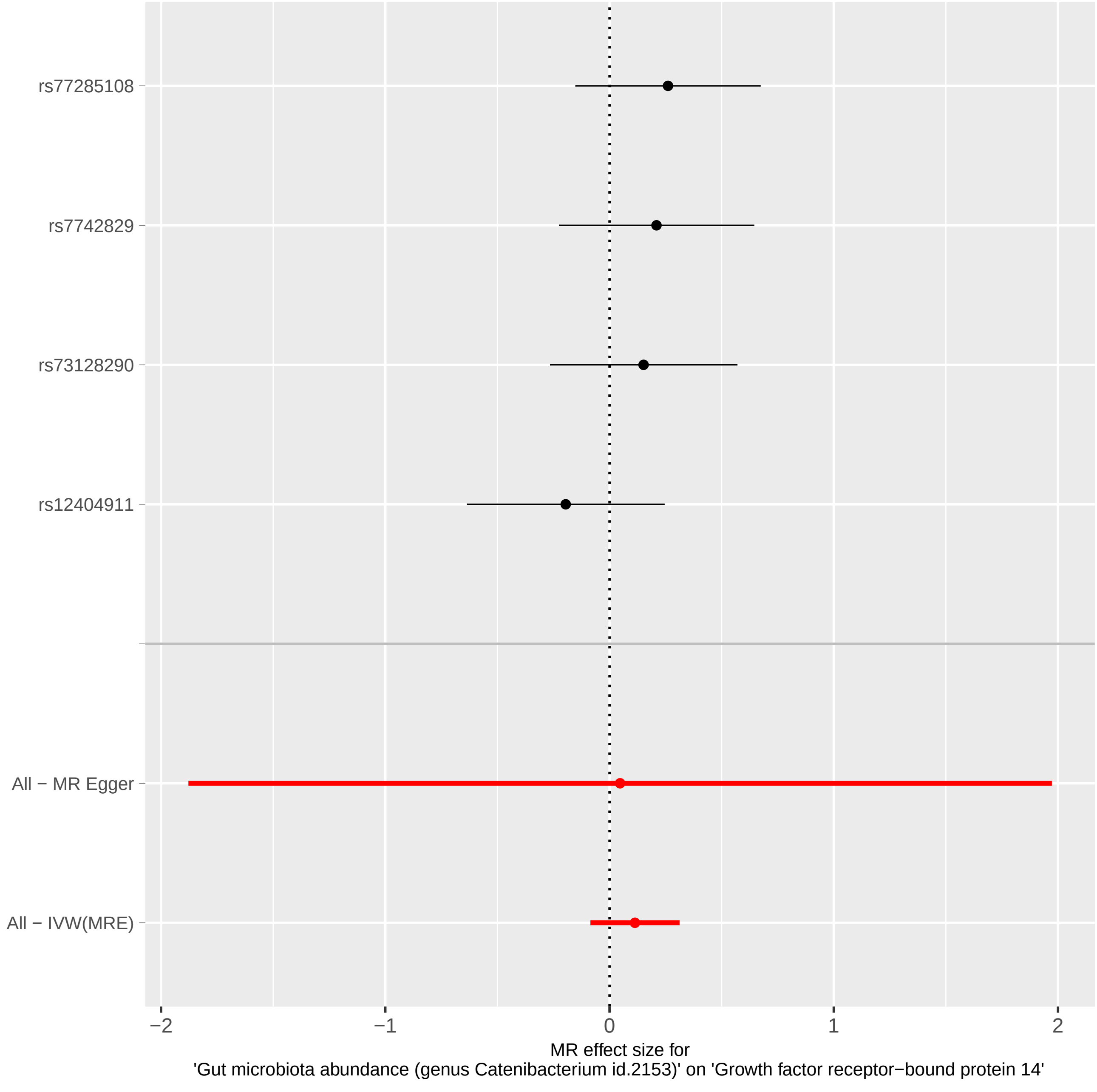

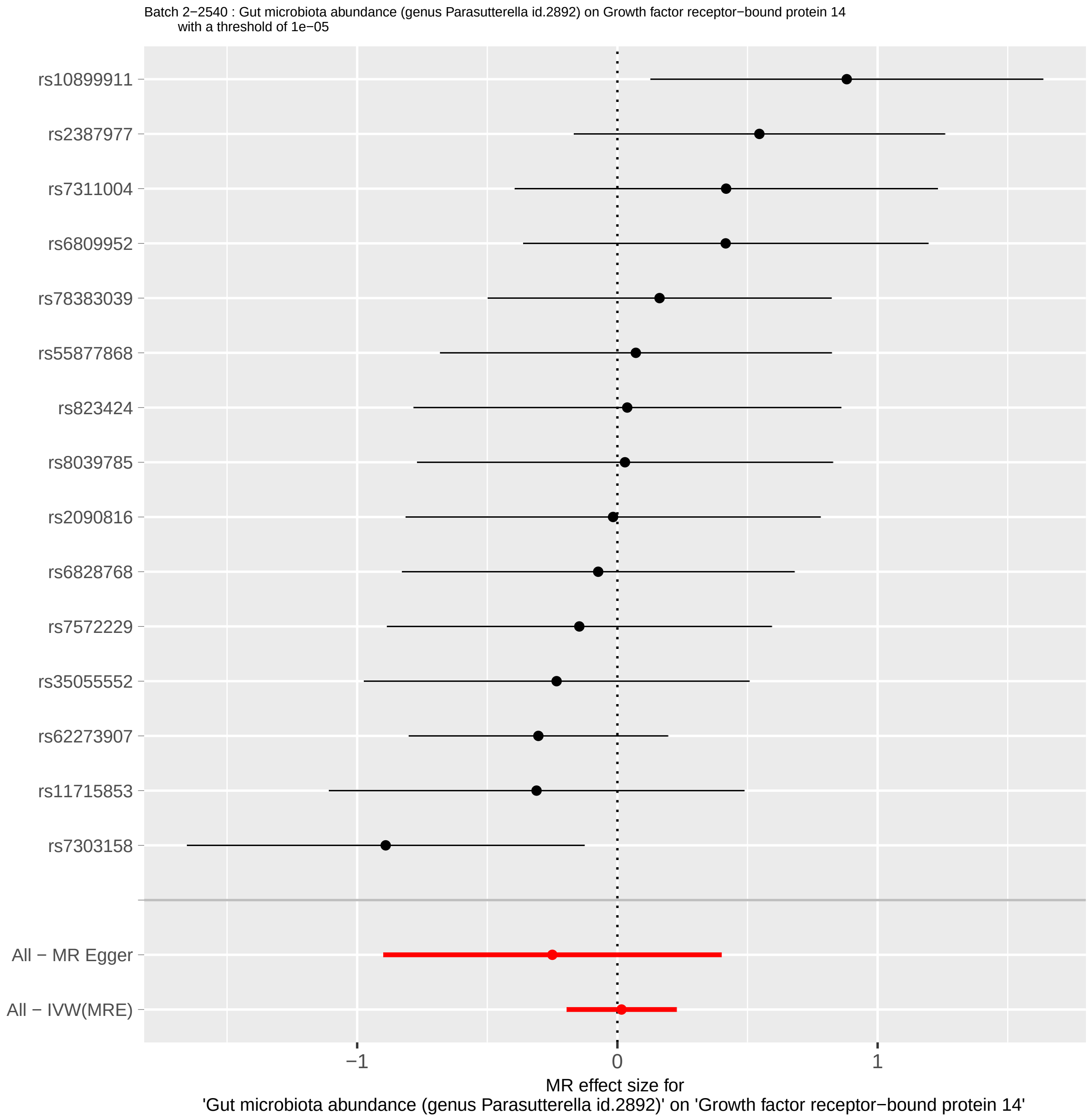

Batch 2−2540 : Gut microbiota abundance (genus Parasutterella id.2892) on Growth factor receptor−bound protein 14
with a threshold of 1e−05
rs10899911
rs2387977
rs7311004
rs6809952
rs78383039
rs55877868
rs823424
rs8039785
rs2090816
rs6828768
rs7572229
rs35055552
rs62273907
rs11715853
rs7303158
All − MR Egger
All − IVW(MRE)
−1
0
1
MR effect size for
'Gut microbiota abundance (genus Parasutterella id.2892)' on 'Growth factor receptor−bound protein 14'

Batch 2−2541 : Gut microbiota abundance (genus Catenibacterium id.2153) on Growth factor receptor−bound protein 2
with a threshold of 1e−05

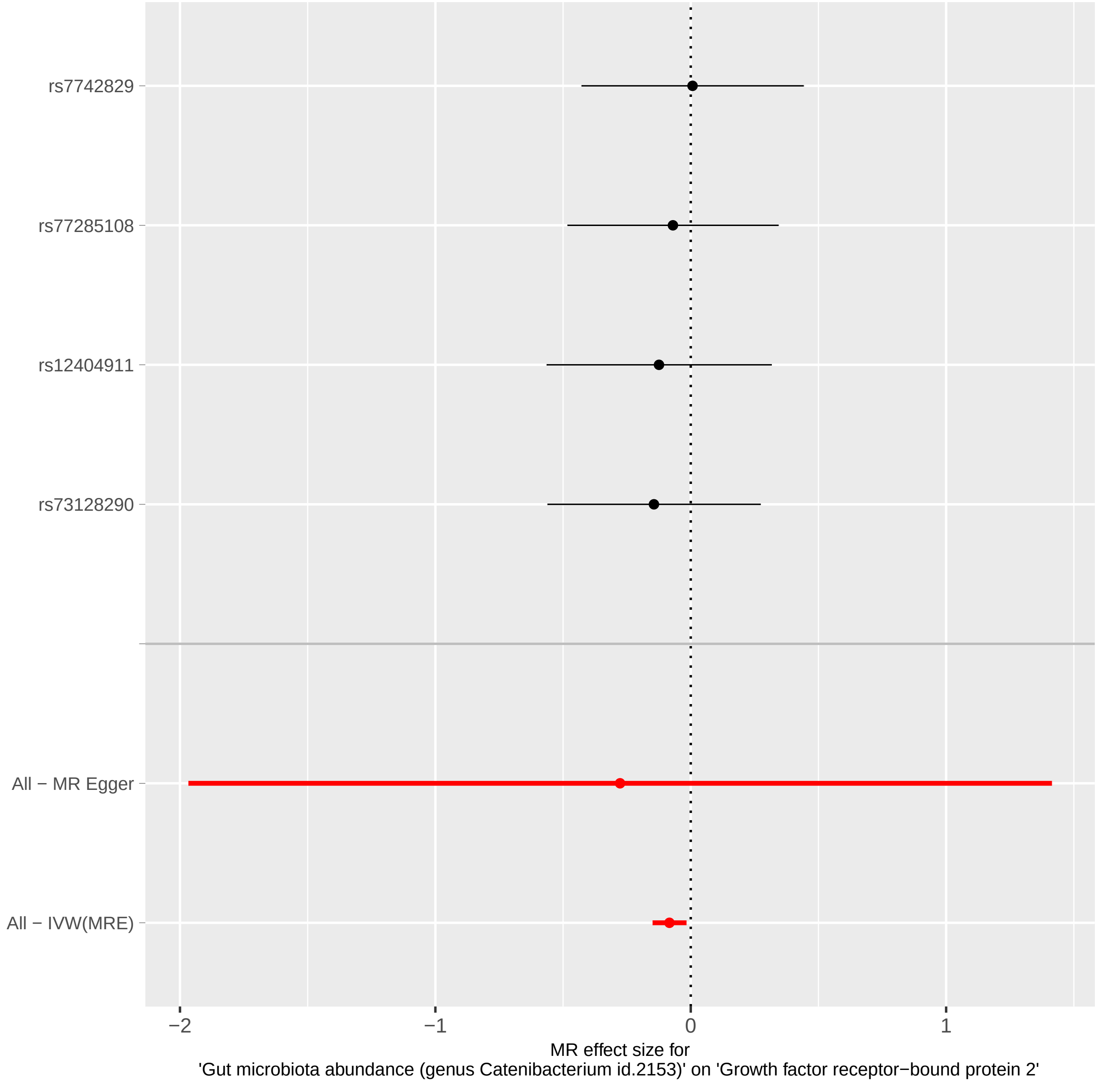

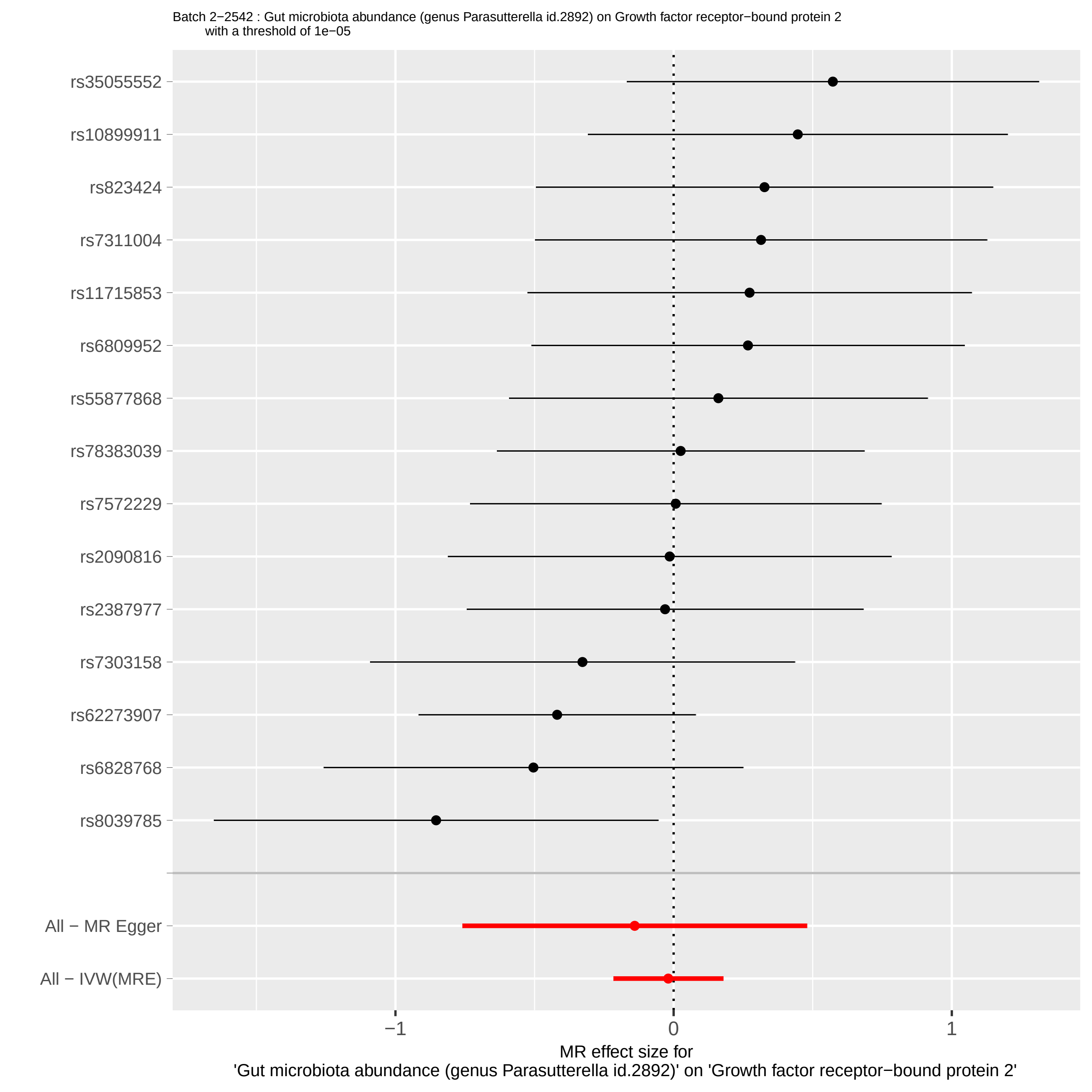
Batch 2−2542 : Gut microbiota abundance (genus Parasutterella id.2892) on Growth factor receptor−bound protein 2
with a threshold of 1e−05
rs35055552
rs10899911
rs823424
rs7311004
rs11715853
rs6809952
rs55877868
rs78383039
rs7572229
rs2090816
rs2387977
rs7303158
rs62273907
rs6828768
rs8039785
All − MR Egger
All − IVW(MRE)
−1
0
1
MR effect size for
'Gut microbiota abundance (genus Parasutterella id.2892)' on 'Growth factor receptor−bound protein 2'

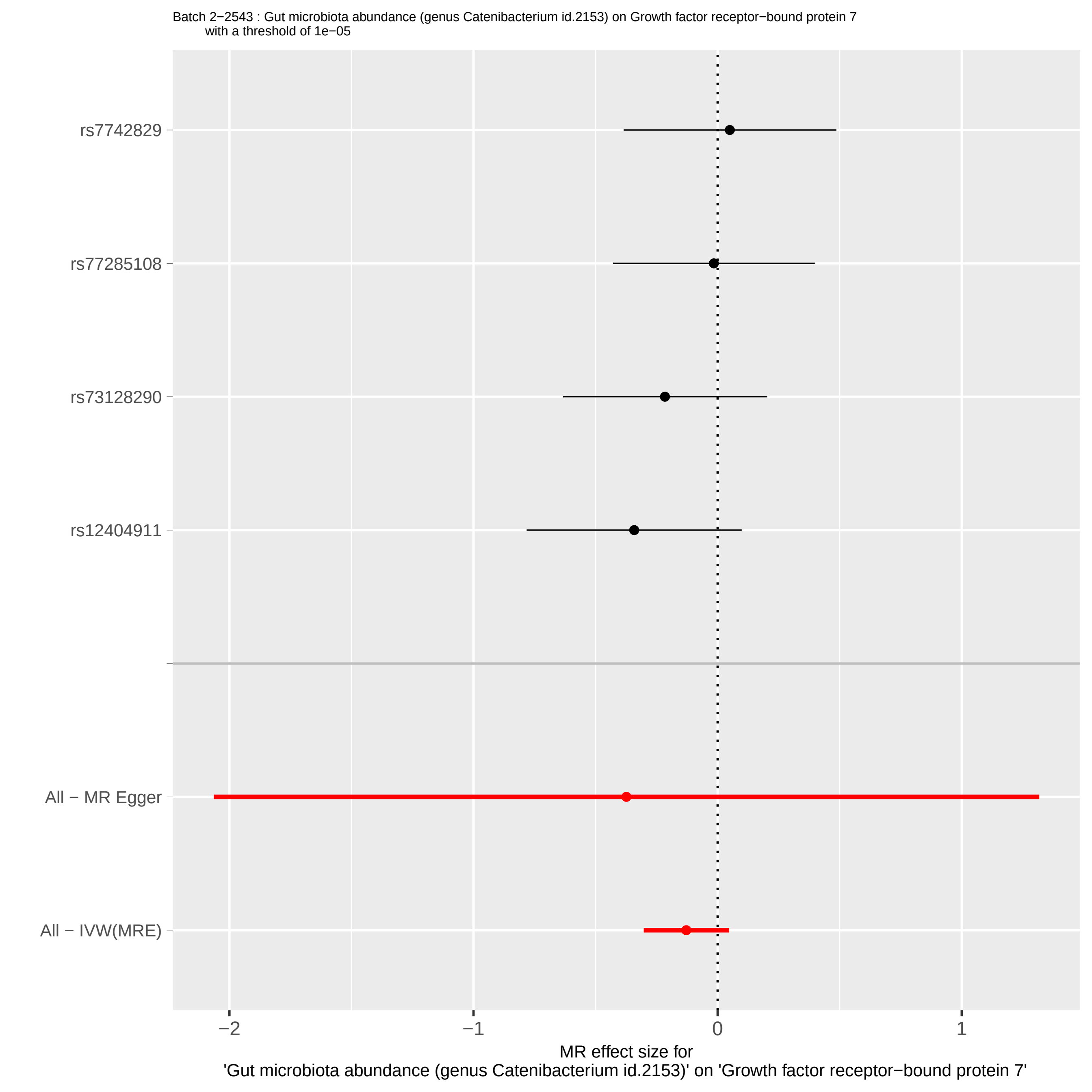

Batch 2−2543 : Gut microbiota abundance (genus Catenibacterium id.2153) on Growth factor receptor−bound protein 7
with a threshold of 1e−05
rs7742829
rs77285108
rs73128290
rs12404911
All − MR Egger
All − IVW(MRE)
−2
−1
0
1
MR effect size for
'Gut microbiota abundance (genus Catenibacterium id.2153)' on 'Growth factor receptor−bound protein 7'

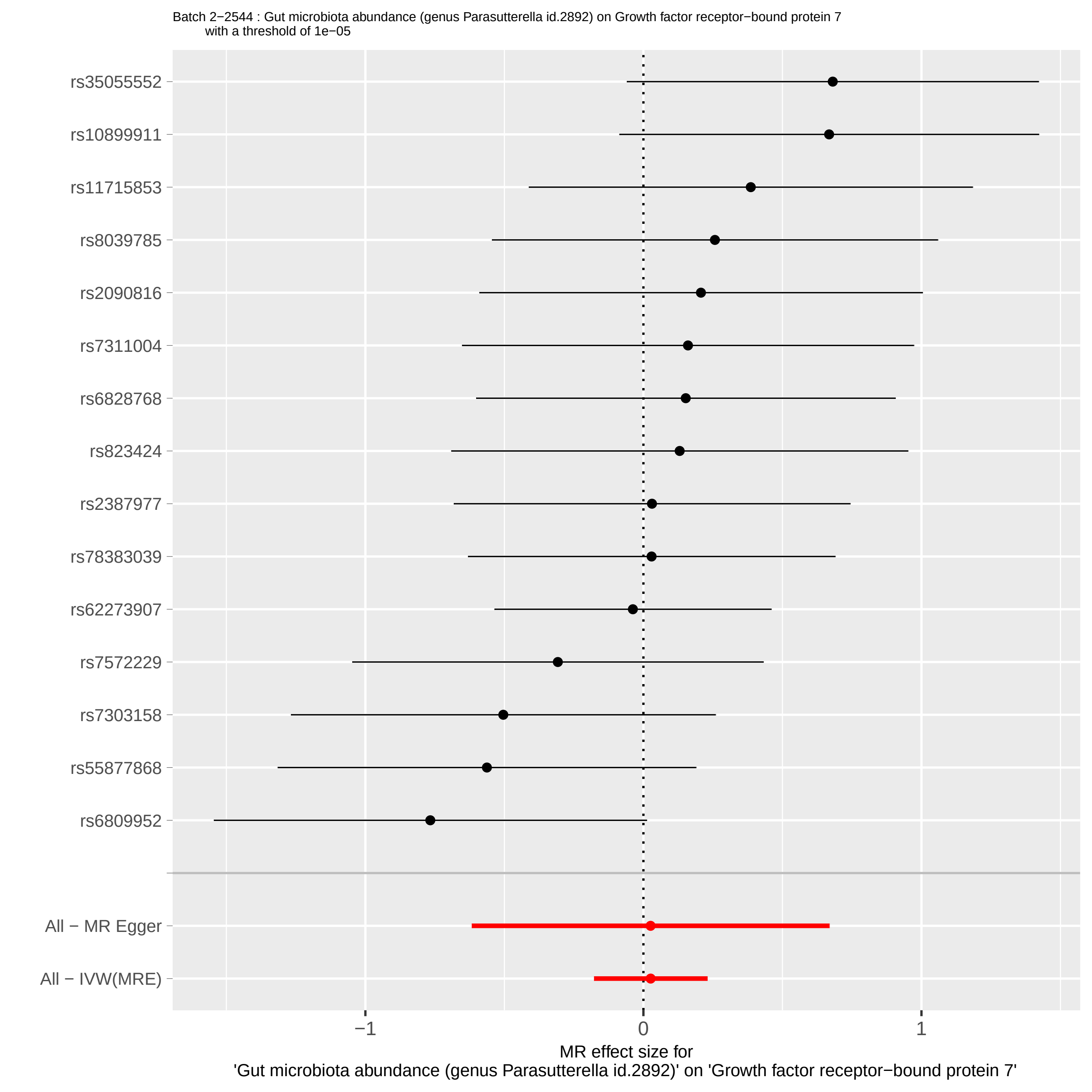

Batch 2−2544 : Gut microbiota abundance (genus Parasutterella id.2892) on Growth factor receptor−bound protein 7
with a threshold of 1e−05
rs35055552
rs10899911
rs11715853
rs8039785
rs2090816
rs7311004
rs6828768
rs823424
rs2387977
rs78383039
rs62273907
rs7572229
rs7303158
rs55877868
rs6809952
All − MR Egger
All − IVW(MRE)
−1
0
1
MR effect size for
'Gut microbiota abundance (genus Parasutterella id.2892)' on 'Growth factor receptor−bound protein 7'

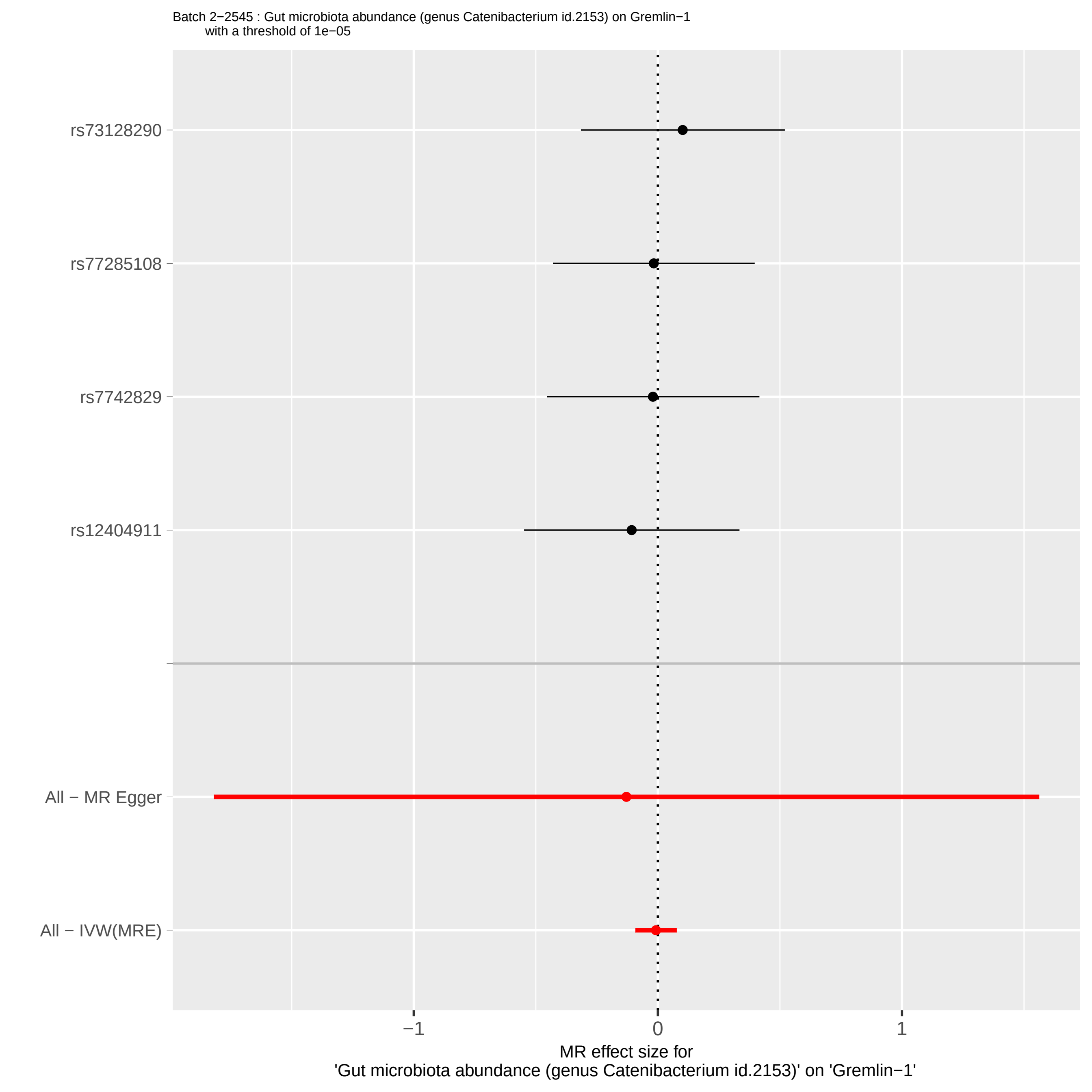

Batch 2−2545 : Gut microbiota abundance (genus Catenibacterium id.2153) on Gremlin−1
with a threshold of 1e−05
rs73128290
rs77285108
rs7742829
rs12404911
All − MR Egger
All − IVW(MRE)
−1
0
1
MR effect size for
'Gut microbiota abundance (genus Catenibacterium id.2153)' on 'Gremlin−1'

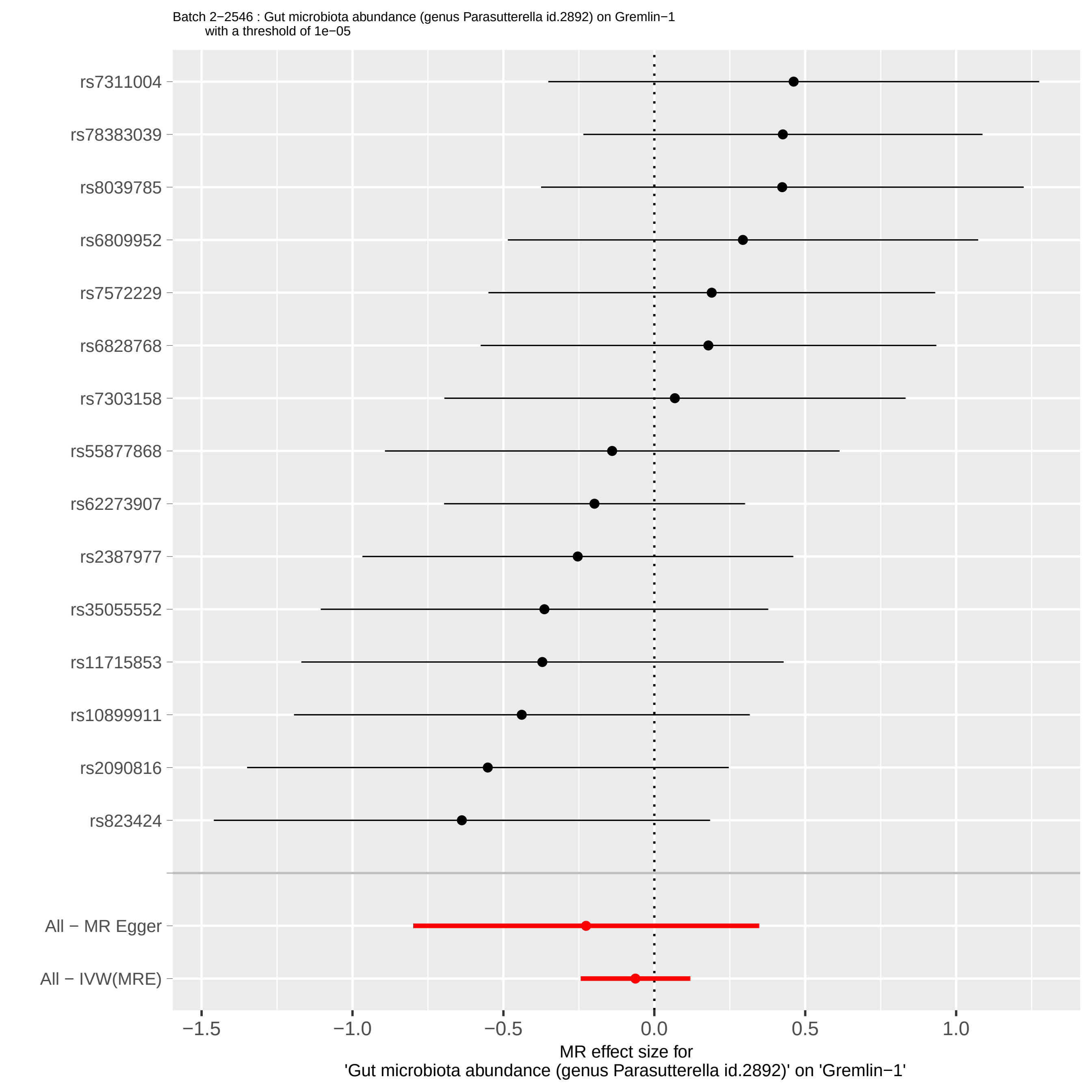

Batch 2−2546 : Gut microbiota abundance (genus Parasutterella id.2892) on Gremlin−1
with a threshold of 1e−05
rs7311004
rs78383039
rs8039785
rs6809952
rs7572229
rs6828768
rs7303158
rs55877868
rs62273907
rs2387977
rs35055552
rs11715853
rs10899911
rs2090816
rs823424
All − MR Egger
All − IVW(MRE)
−1.5
−1.0
−0.5
0.0
0.5
1.0
MR effect size for
'Gut microbiota abundance (genus Parasutterella id.2892)' on 'Gremlin−1'

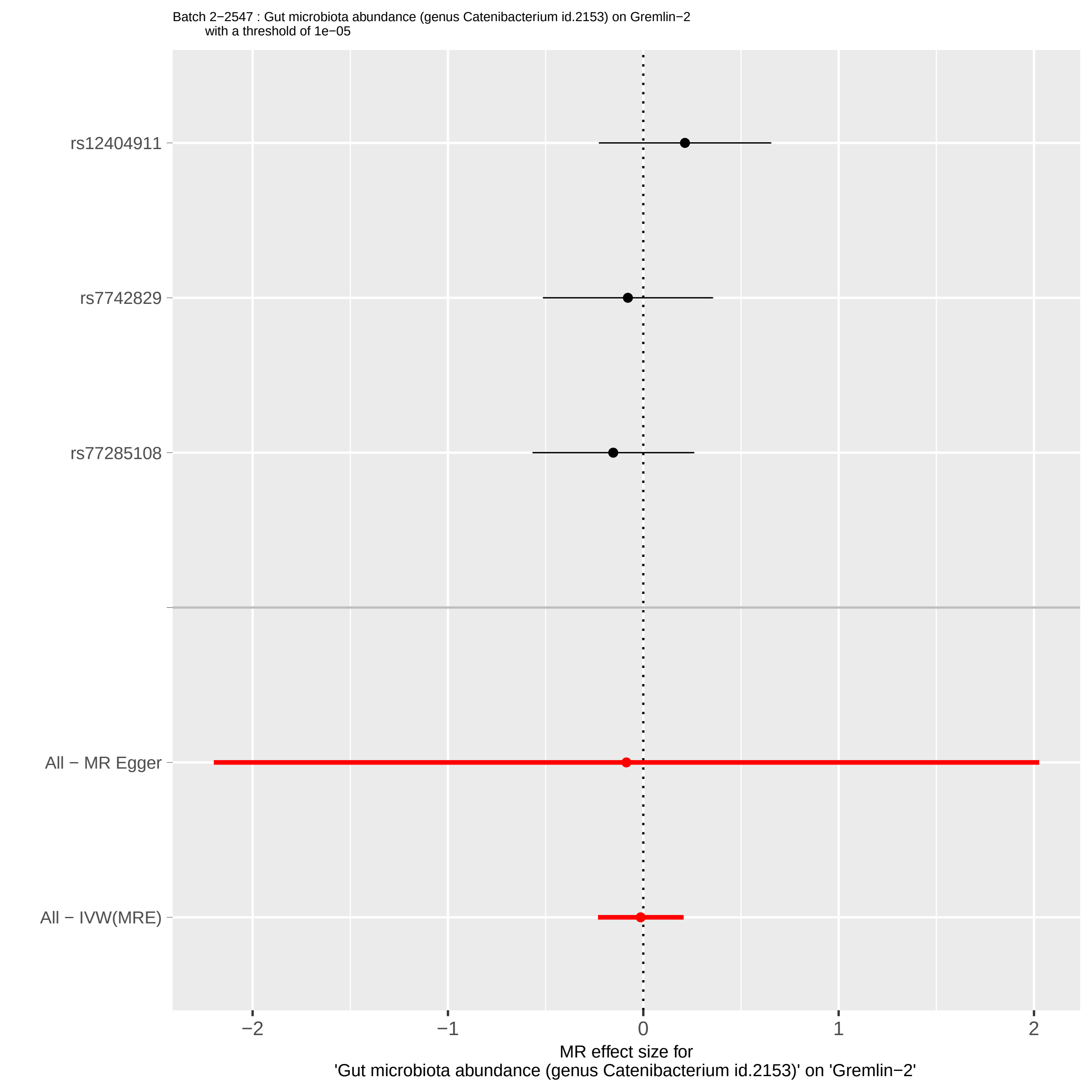

Batch 2−2547 : Gut microbiota abundance (genus Catenibacterium id.2153) on Gremlin−2
with a threshold of 1e−05
rs12404911
rs7742829
rs77285108
All − MR Egger
All − IVW(MRE)
−2
−1
0
1
2
MR effect size for
'Gut microbiota abundance (genus Catenibacterium id.2153)' on 'Gremlin−2'

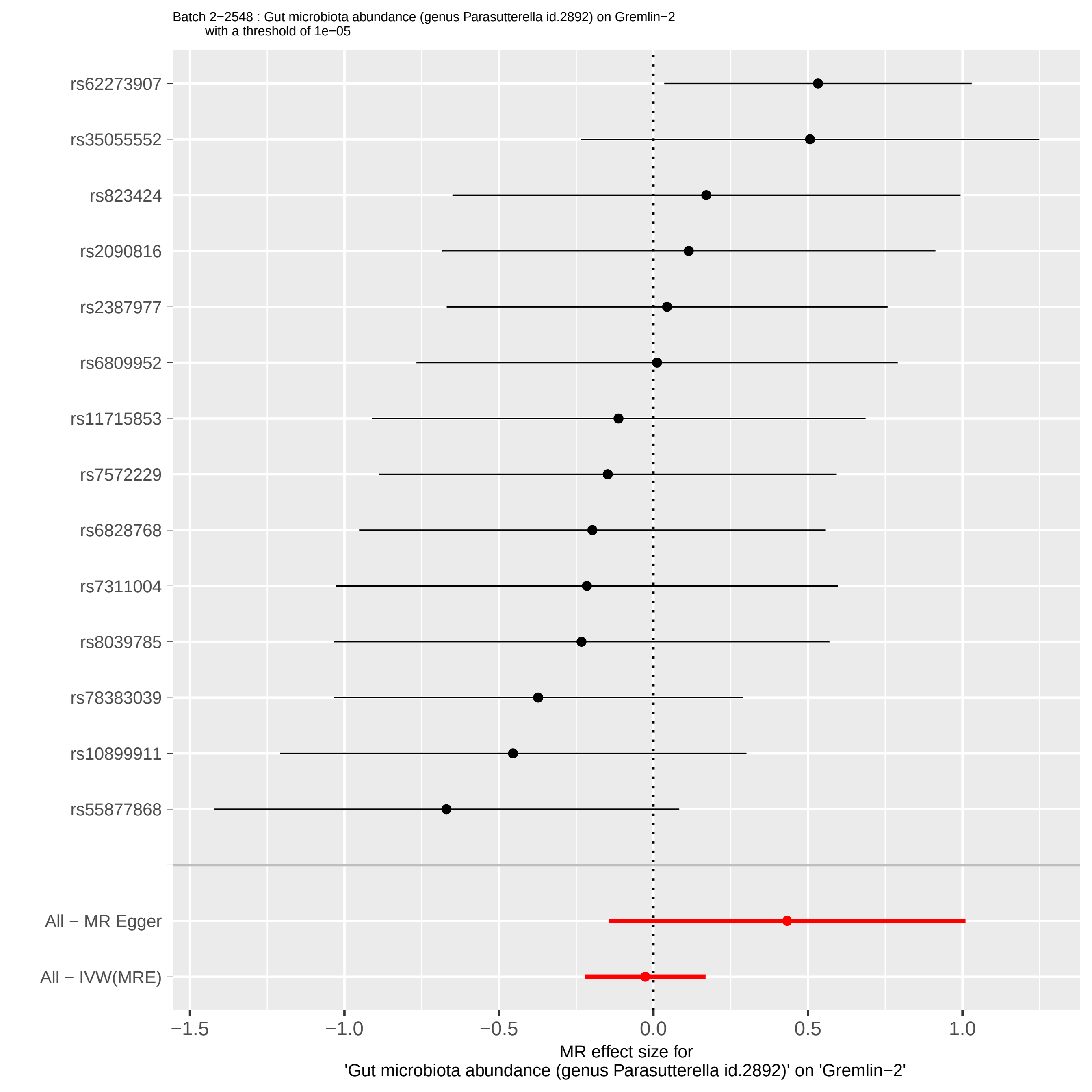

Batch 2−2548 : Gut microbiota abundance (genus Parasutterella id.2892) on Gremlin−2
with a threshold of 1e−05
rs62273907
rs35055552
rs823424
rs2090816
rs2387977
rs6809952
rs11715853
rs7572229
rs6828768
rs7311004
rs8039785
rs78383039
rs10899911
rs55877868
All − MR Egger
All − IVW(MRE)
−1.5
−1.0
−0.5
0.0
0.5
1.0
MR effect size for
'Gut microbiota abundance (genus Parasutterella id.2892)' on 'Gremlin−2'

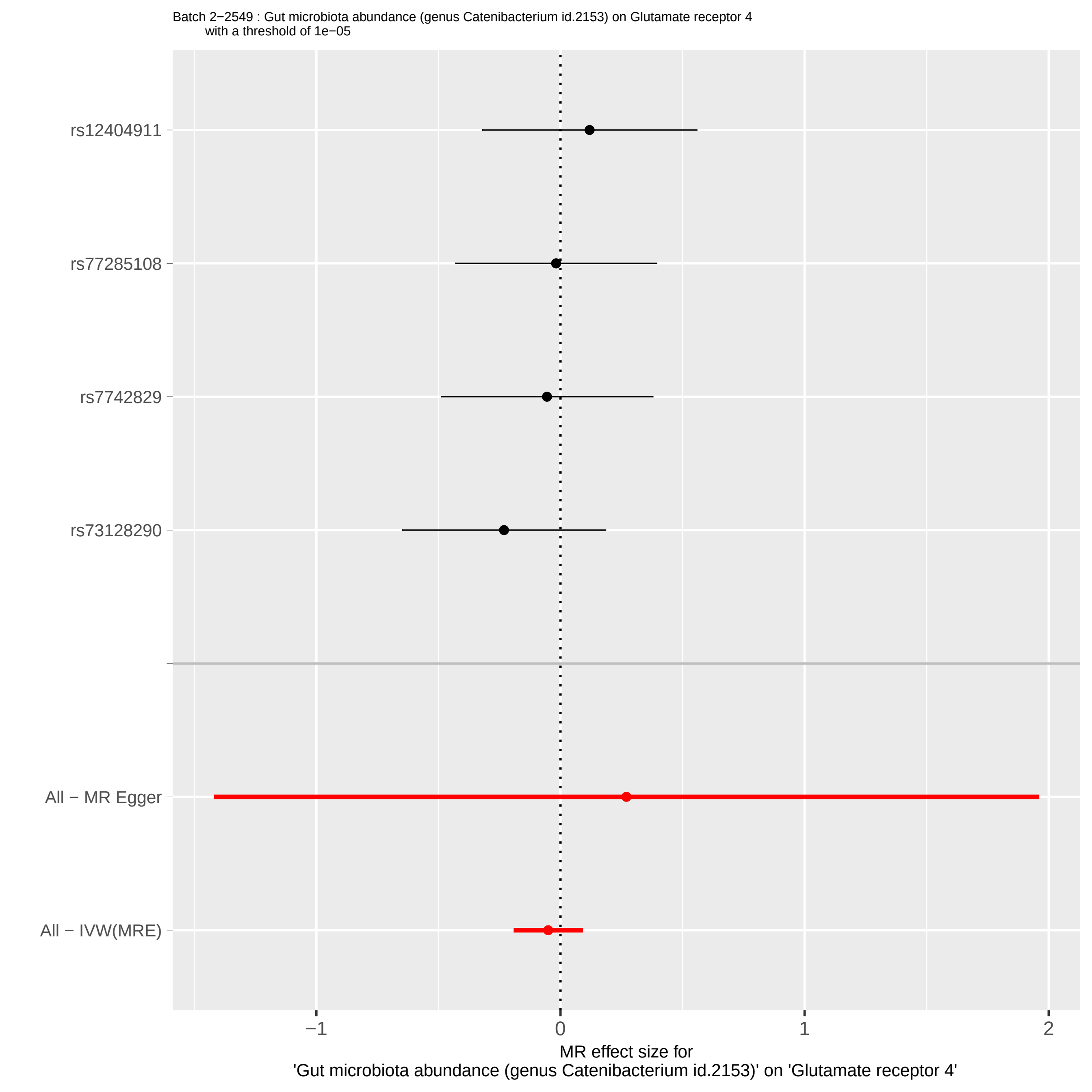
Batch 2−2549 : Gut microbiota abundance (genus Catenibacterium id.2153) on Glutamate receptor 4
with a threshold of 1e−05
rs12404911
rs77285108
rs7742829
rs73128290
All − MR Egger
All − IVW(MRE)
−1
0
1
2
MR effect size for
'Gut microbiota abundance (genus Catenibacterium id.2153)' on 'Glutamate receptor 4'

Batch 2−2550 : Gut microbiota abundance (genus Parasutterella id.2892) on Glutamate receptor 4
with a threshold of 1e−05

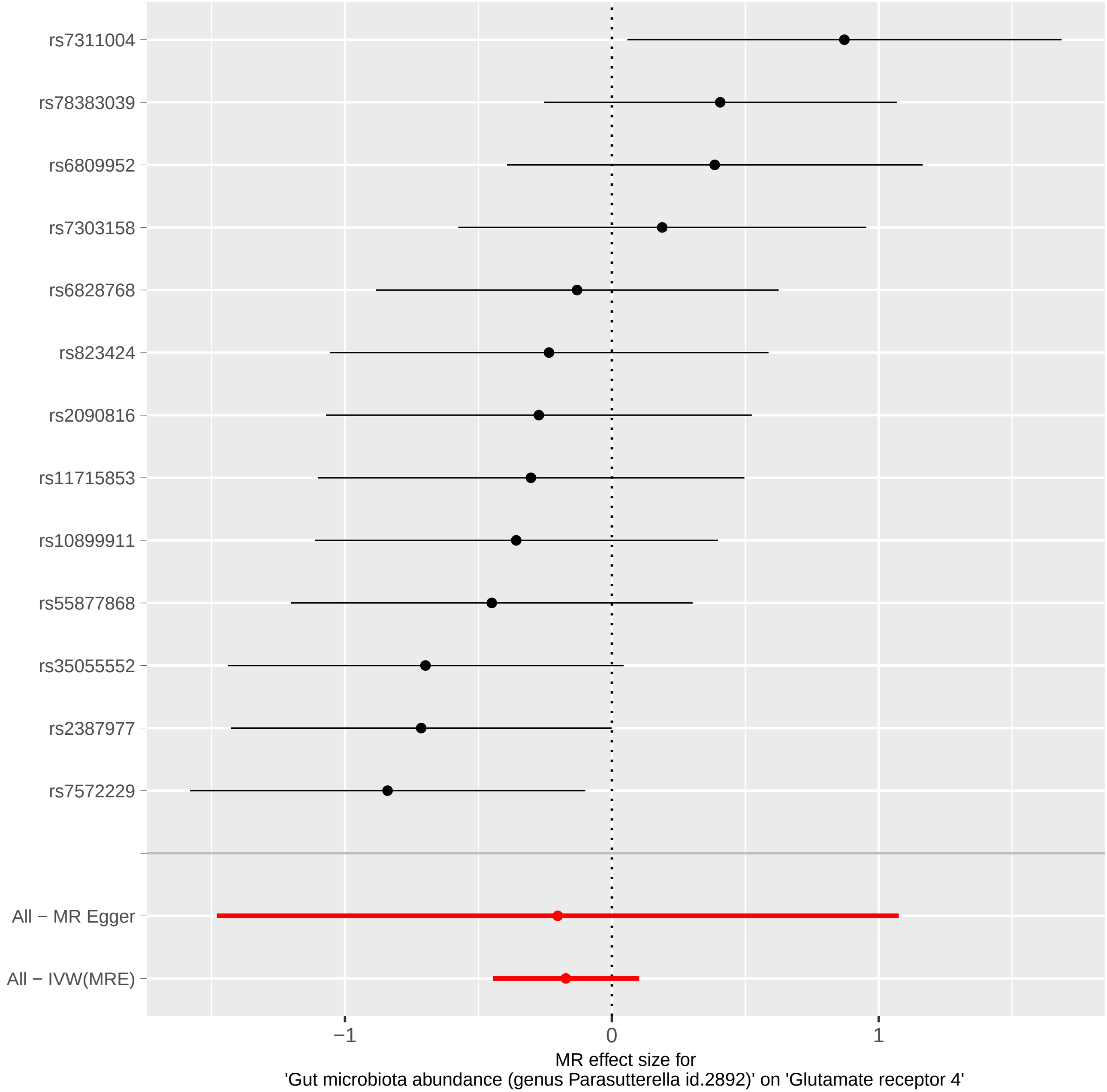

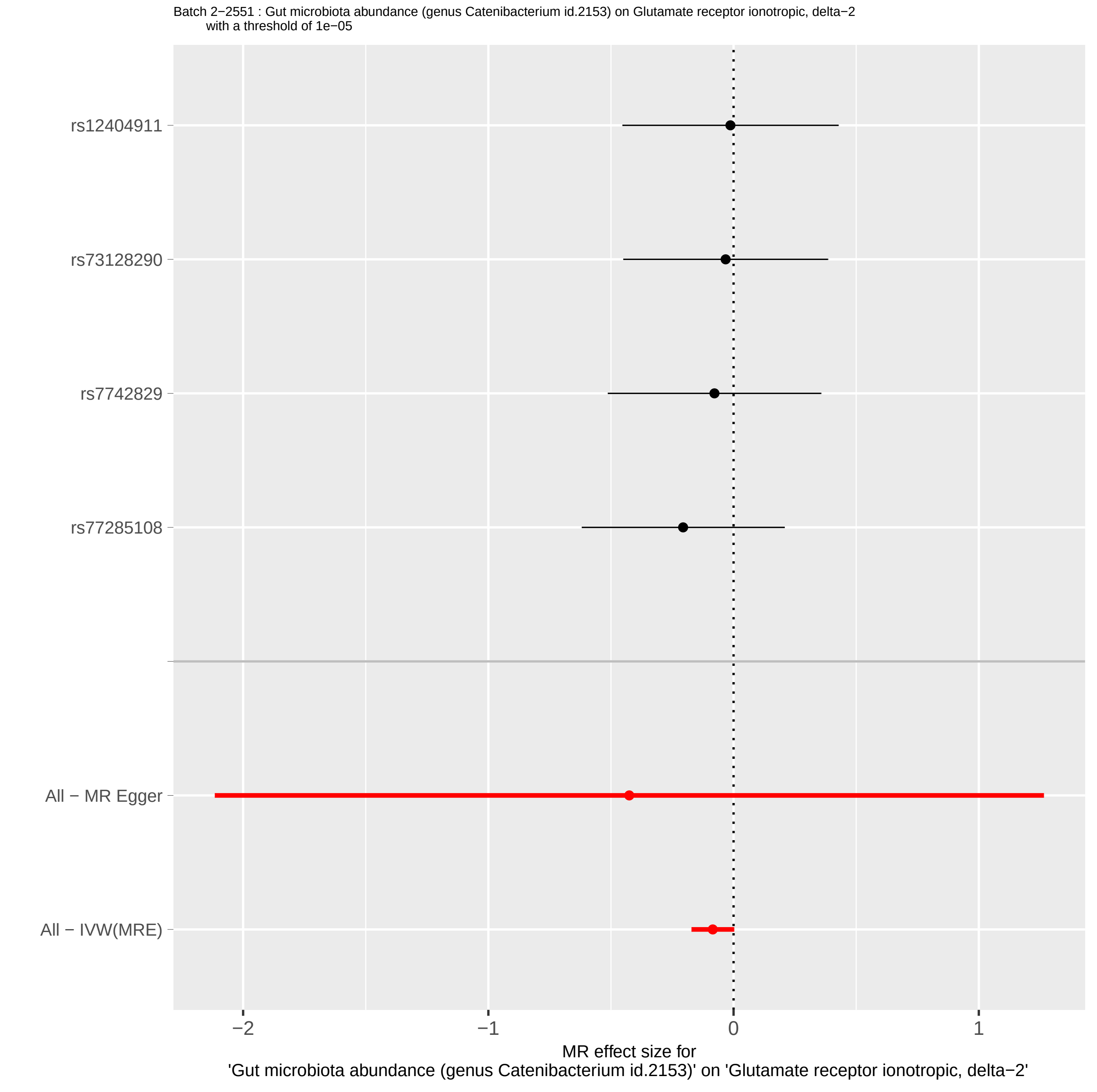

Batch 2−2551 : Gut microbiota abundance (genus Catenibacterium id.2153) on Glutamate receptor ionotropic, delta−2
with a threshold of 1e−05
rs12404911
rs73128290
rs7742829
rs77285108
All − MR Egger
All − IVW(MRE)
−2
−1
0
1
MR effect size for
'Gut microbiota abundance (genus Catenibacterium id.2153)' on 'Glutamate receptor ionotropic, delta−2'

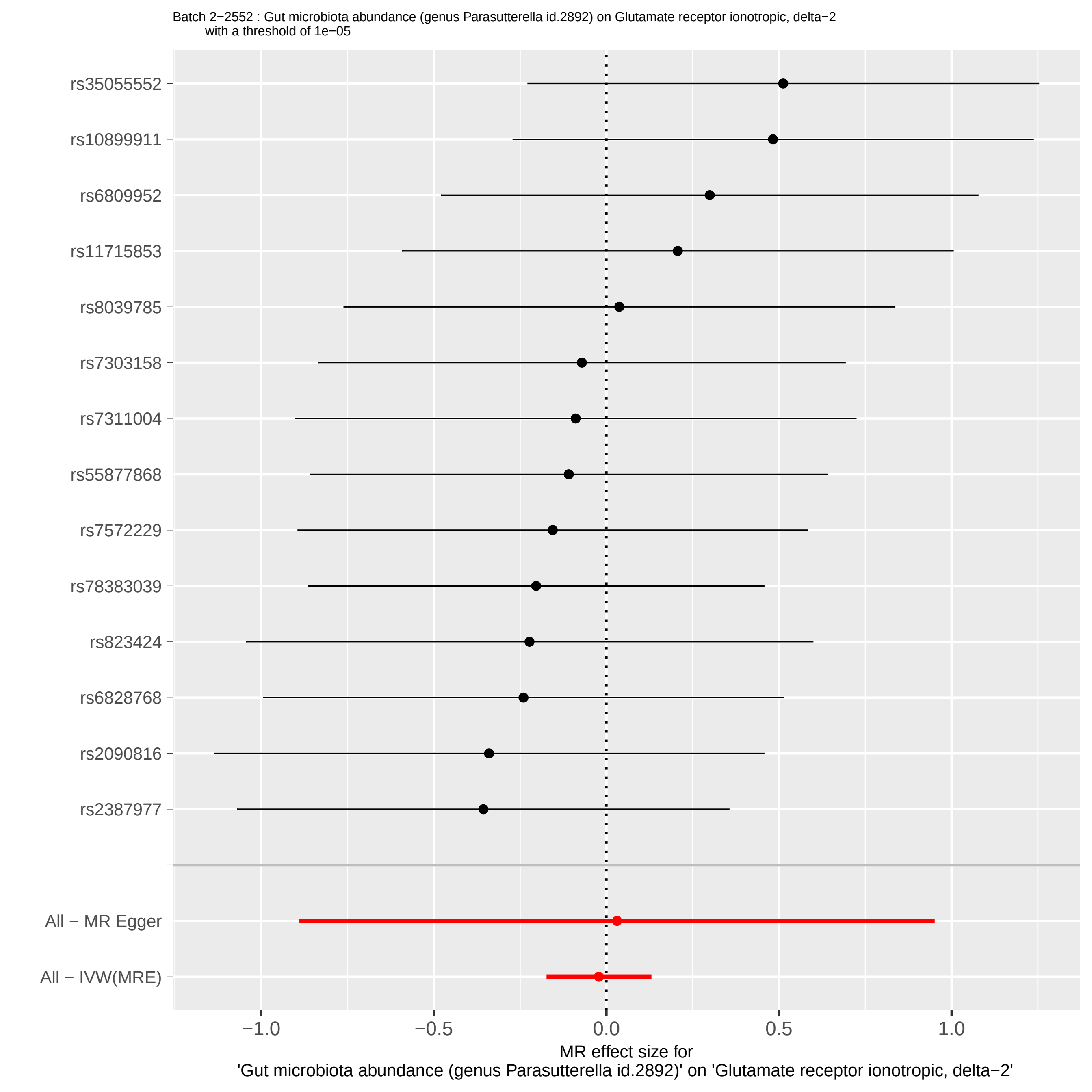
Batch 2−2552 : Gut microbiota abundance (genus Parasutterella id.2892) on Glutamate receptor ionotropic, delta−2
with a threshold of 1e−05
rs35055552
rs10899911
rs6809952
rs11715853
rs8039785
rs7303158
rs7311004
rs55877868
rs7572229
rs78383039
rs823424
rs6828768
rs2090816
rs2387977
All − MR Egger
All − IVW(MRE)
−1.0
−0.5
0.0
0.5
1.0
MR effect size for
'Gut microbiota abundance (genus Parasutterella id.2892)' on 'Glutamate receptor ionotropic, delta−2'

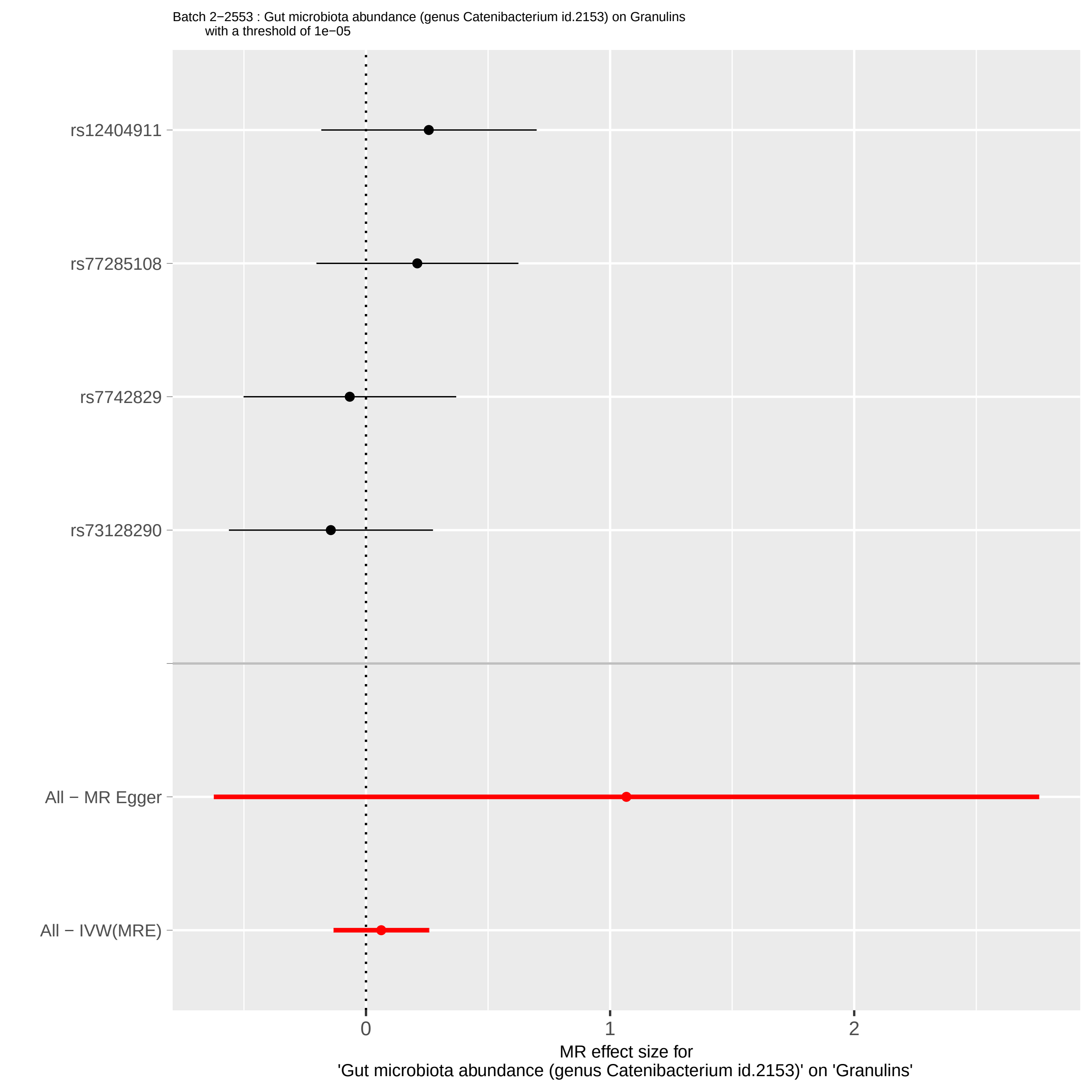
Batch 2−2553 : Gut microbiota abundance (genus Catenibacterium id.2153) on Granulins
with a threshold of 1e−05
rs12404911
rs77285108
rs7742829
rs73128290
All − MR Egger
All − IVW(MRE)
0
1
2
MR effect size for
'Gut microbiota abundance (genus Catenibacterium id.2153)' on 'Granulins'

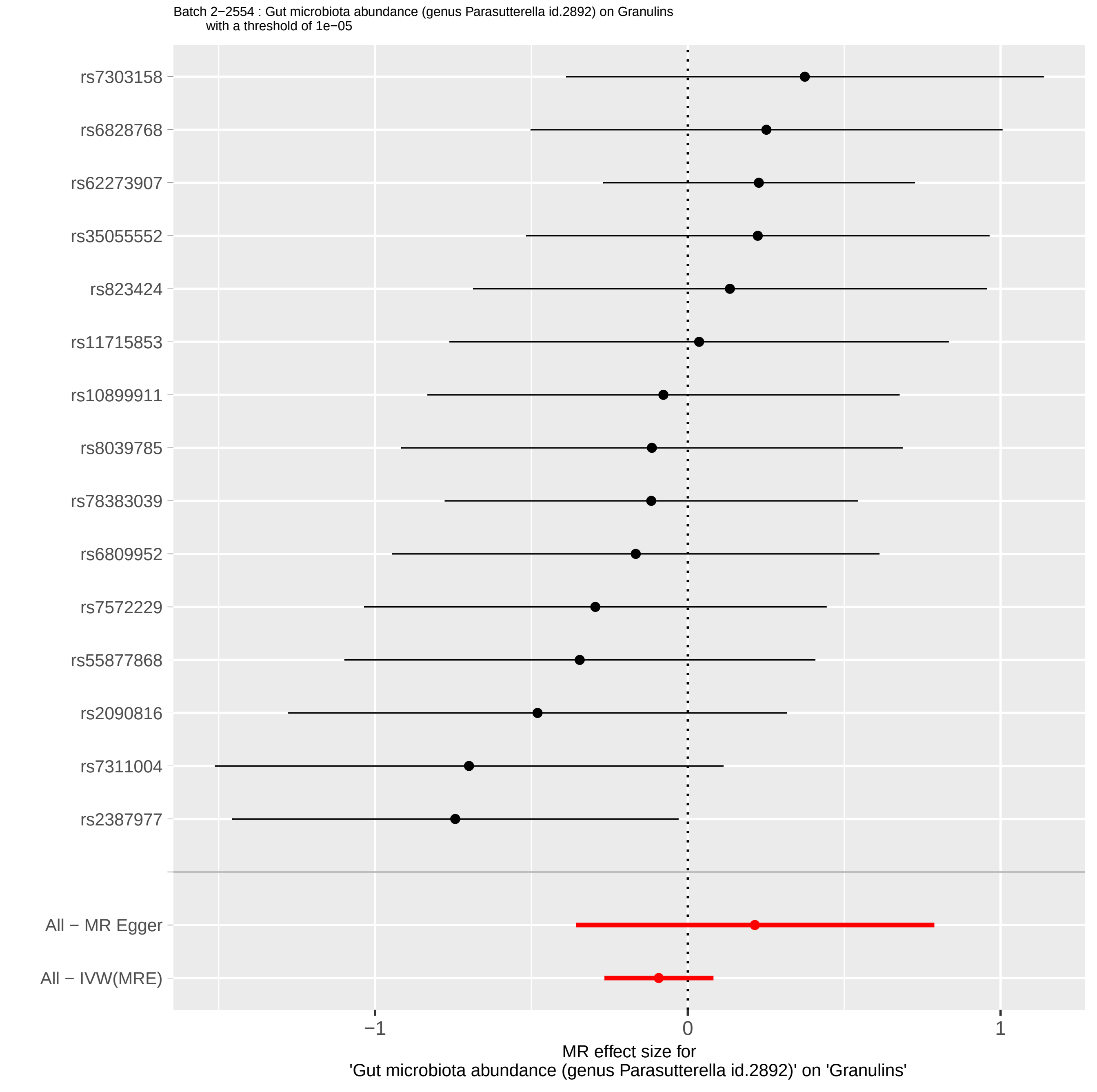

Batch 2−2554 : Gut microbiota abundance (genus Parasutterella id.2892) on Granulins
with a threshold of 1e−05
rs7303158
rs6828768
rs62273907
rs35055552
rs823424
rs11715853
rs10899911
rs8039785
rs78383039
rs6809952
rs7572229
rs55877868
rs2090816
rs7311004
rs2387977
All − MR Egger
All − IVW(MRE)
−1
0
1
MR effect size for
'Gut microbiota abundance (genus Parasutterella id.2892)' on 'Granulins'

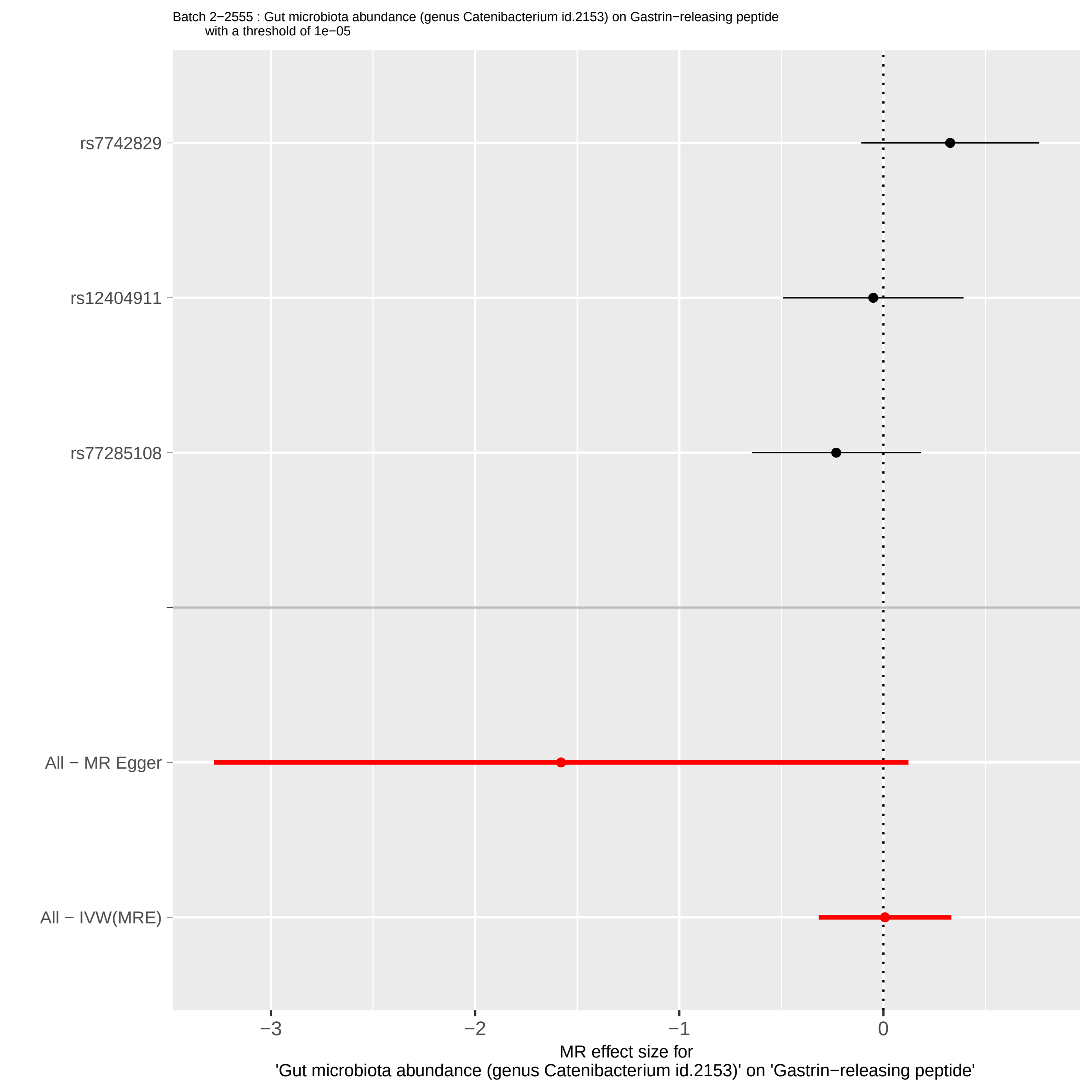
Batch 2−2555 : Gut microbiota abundance (genus Catenibacterium id.2153) on Gastrin−releasing peptide
with a threshold of 1e−05
rs7742829
rs12404911
rs77285108
All − MR Egger
All − IVW(MRE)
−3
−2
−1
0
MR effect size for
'Gut microbiota abundance (genus Catenibacterium id.2153)' on 'Gastrin−releasing peptide'

Batch 2−2556 : Gut microbiota abundance (genus Parasutterella id.2892) on Gastrin−releasing peptide
with a threshold of 1e−05

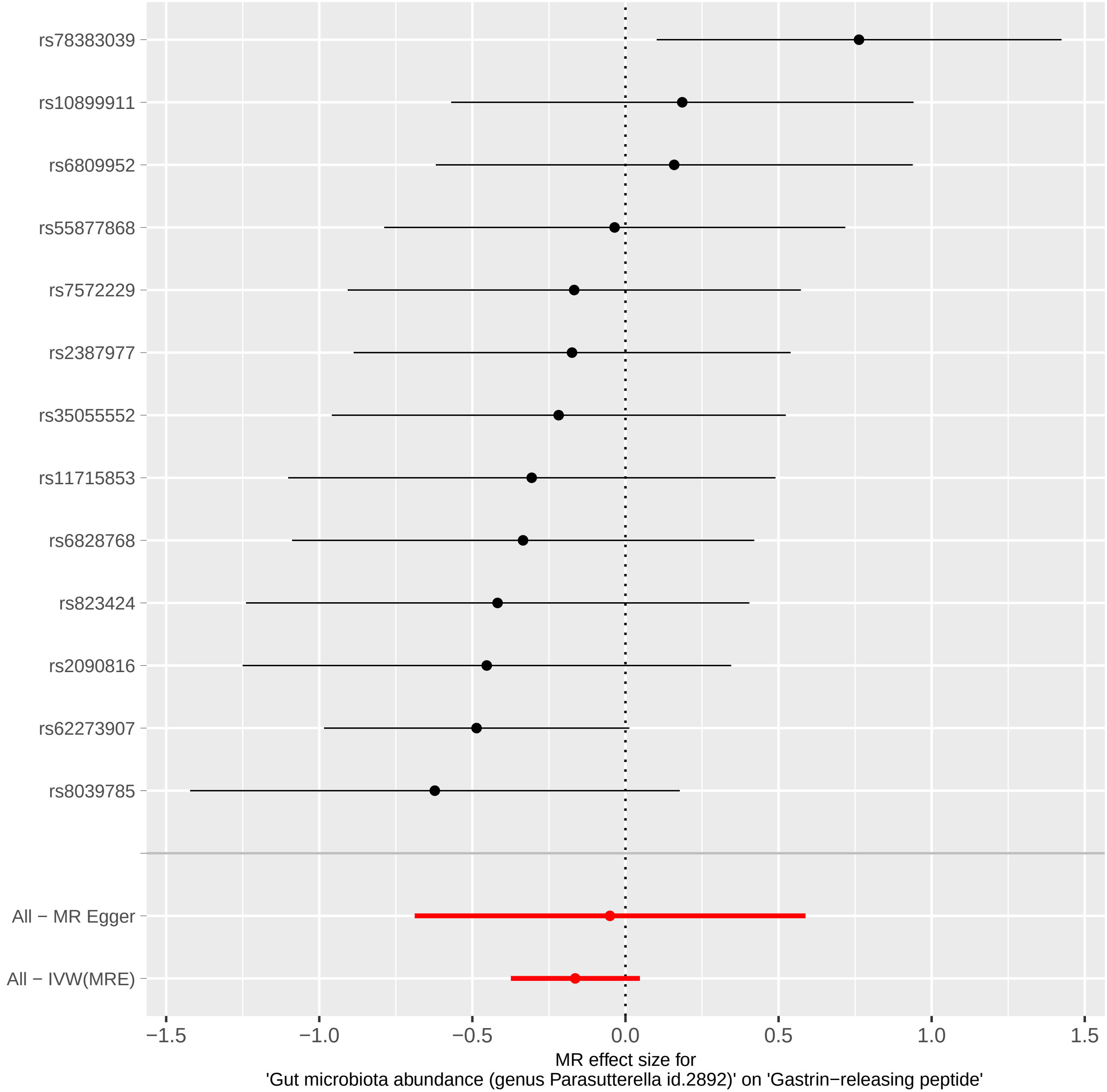

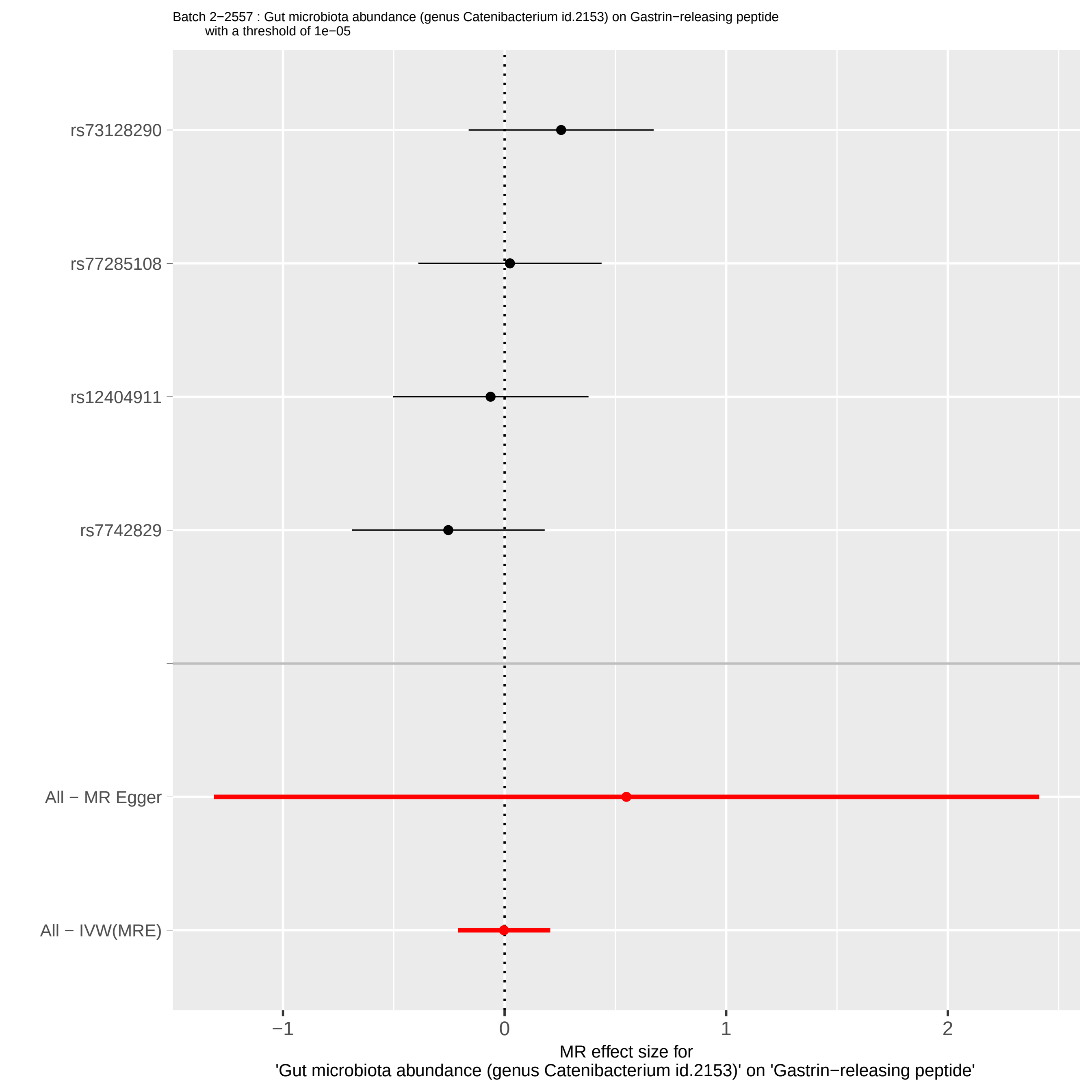
Batch 2−2557 : Gut microbiota abundance (genus Catenibacterium id.2153) on Gastrin−releasing peptide
with a threshold of 1e−05
rs73128290
rs77285108
rs12404911
rs7742829
All − MR Egger
All − IVW(MRE)
−1
0
1
2
MR effect size for
'Gut microbiota abundance (genus Catenibacterium id.2153)' on 'Gastrin−releasing peptide'

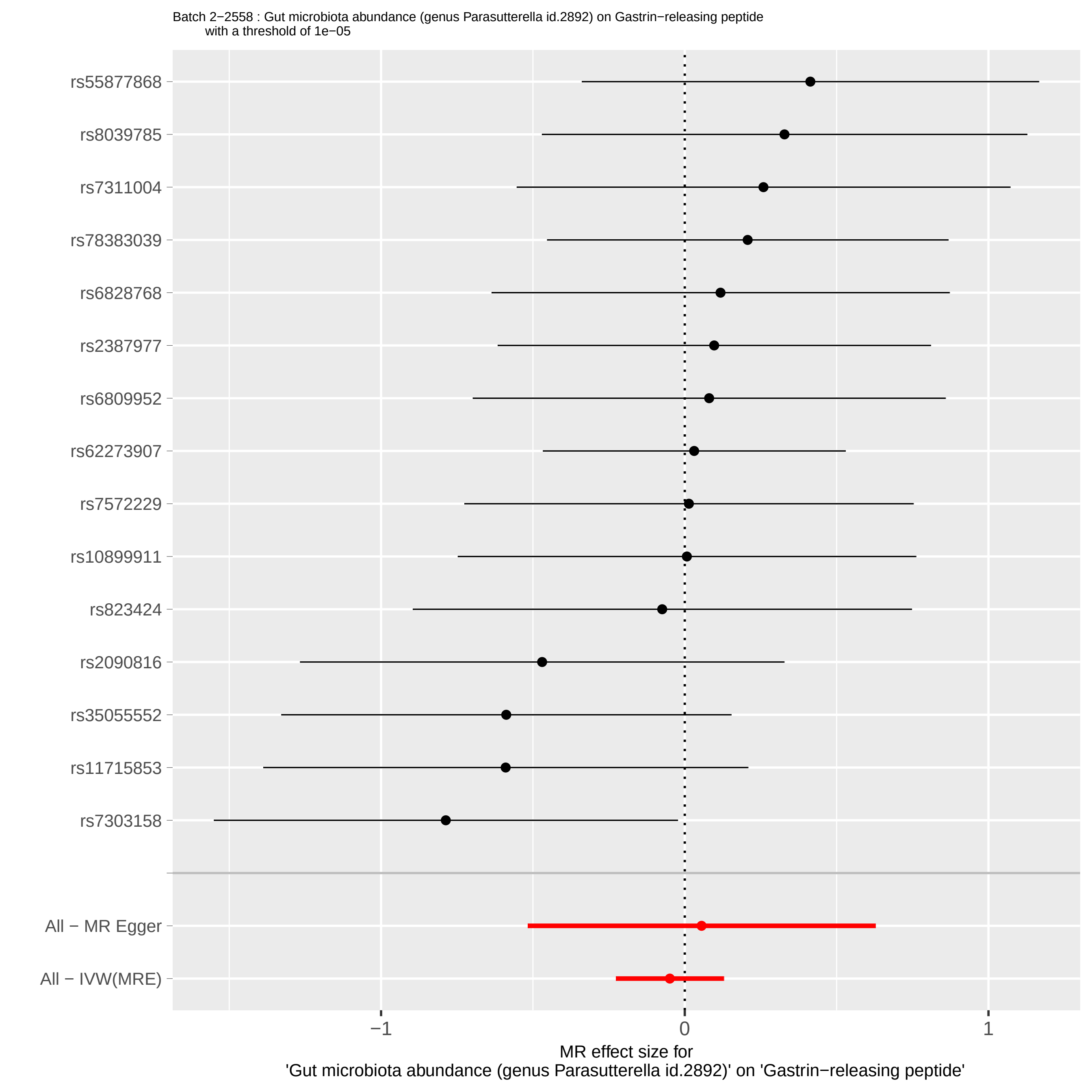

Batch 2−2558 : Gut microbiota abundance (genus Parasutterella id.2892) on Gastrin−releasing peptide
with a threshold of 1e−05
rs55877868
rs8039785
rs7311004
rs78383039
rs6828768
rs2387977
rs6809952
rs62273907
rs7572229
rs10899911
rs823424
rs2090816
rs35055552
rs11715853
rs7303158
All − MR Egger
All − IVW(MRE)
−1
0
1
MR effect size for
'Gut microbiota abundance (genus Parasutterella id.2892)' on 'Gastrin−releasing peptide'

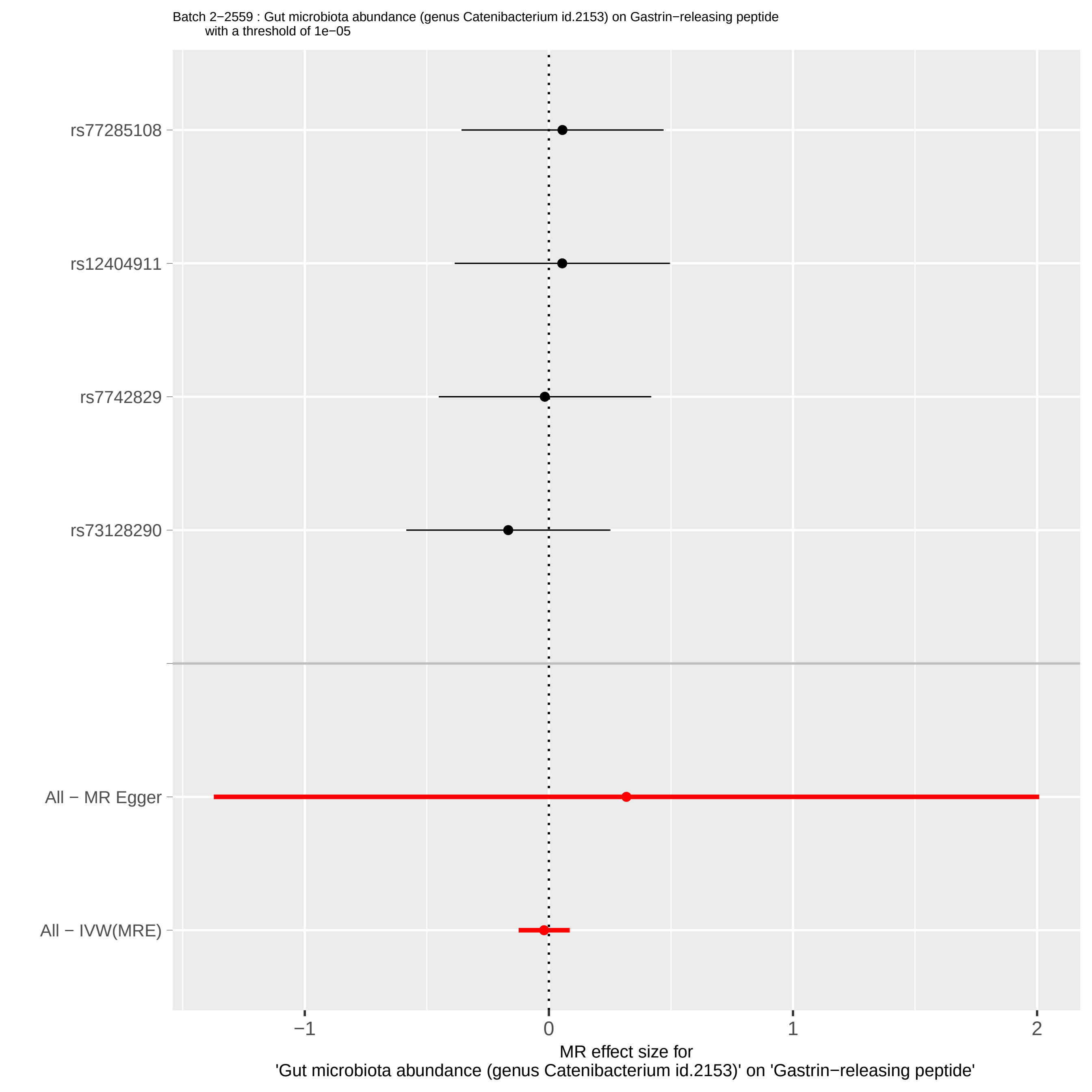

Batch 2−2559 : Gut microbiota abundance (genus Catenibacterium id.2153) on Gastrin−releasing peptide
with a threshold of 1e−05
rs77285108
rs12404911
rs7742829
rs73128290
All − MR Egger
All − IVW(MRE)
−1
0
1
2
MR effect size for
'Gut microbiota abundance (genus Catenibacterium id.2153)' on 'Gastrin−releasing peptide'

Batch 2−2560 : Gut microbiota abundance (genus Parasutterella id.2892) on Gastrin−releasing peptide
with a threshold of 1e−05

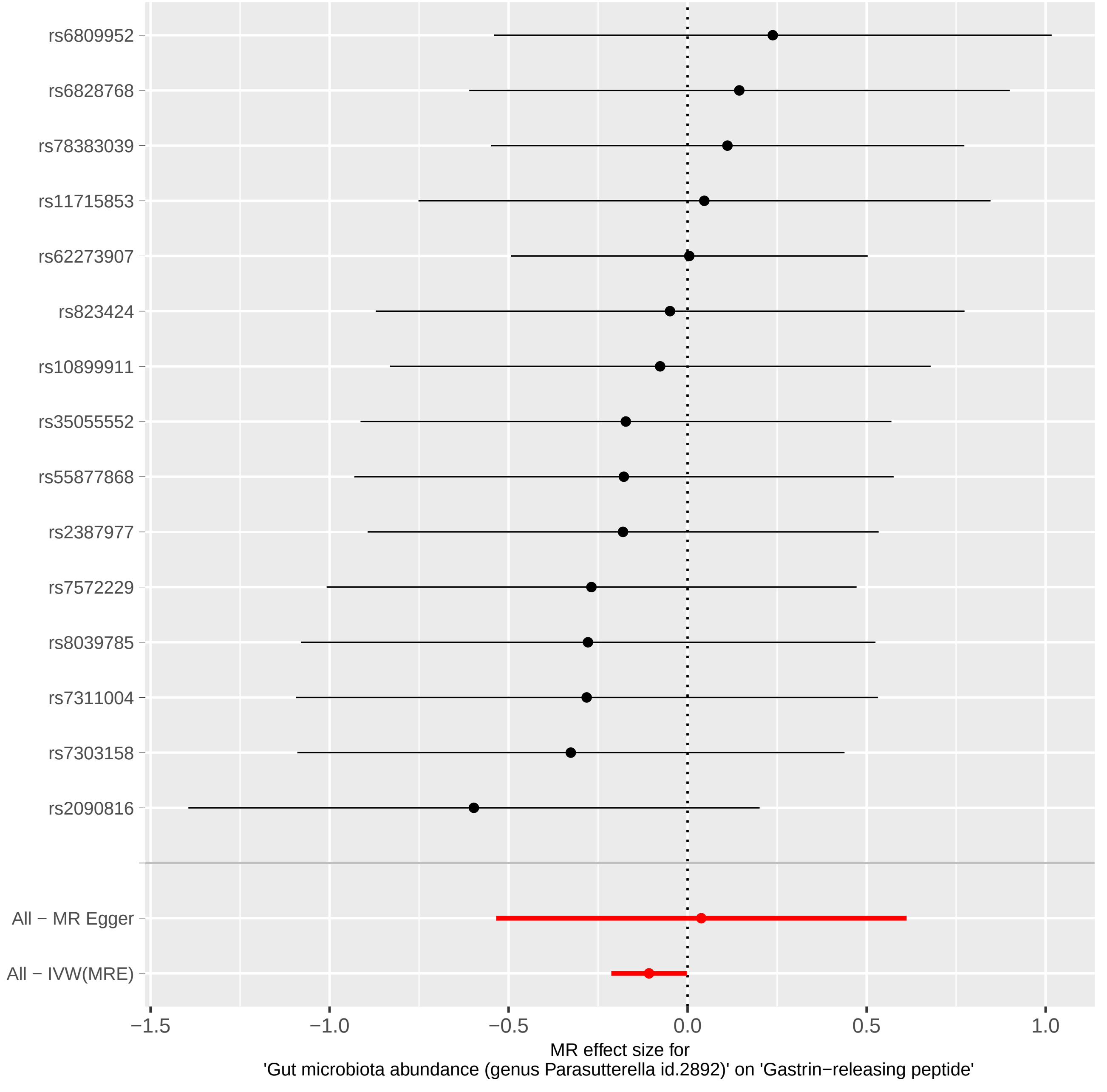

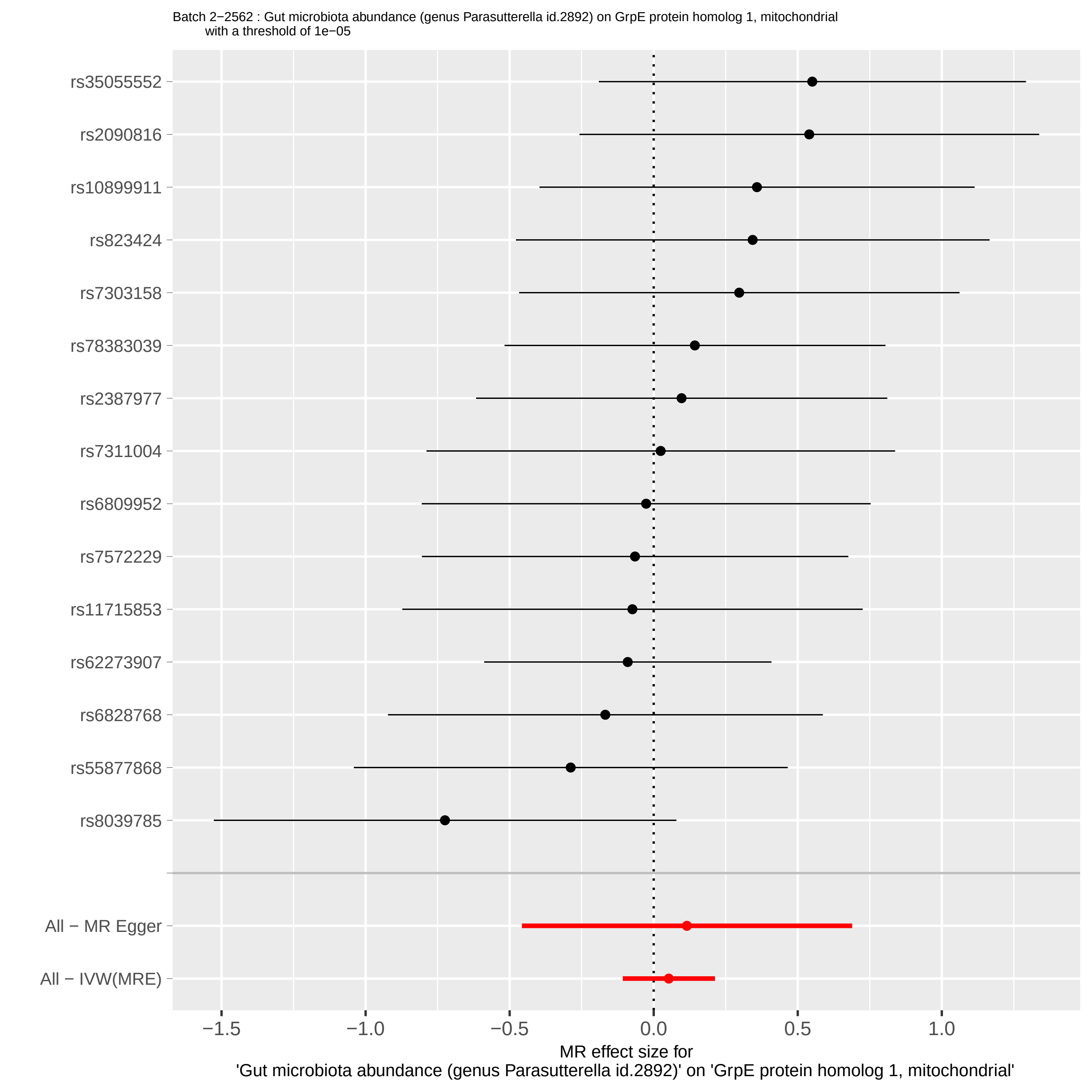
Batch 2−2562 : Gut microbiota abundance (genus Parasutterella id.2892) on GrpE protein homolog 1, mitochondrial
with a threshold of 1e−05
rs35055552
rs2090816
rs10899911
rs823424
rs7303158
rs78383039
rs2387977
rs7311004
rs6809952
rs7572229
rs11715853
rs62273907
rs6828768
rs55877868
rs8039785
All − MR Egger
All − IVW(MRE)
−1.5
−1.0
−0.5
0.0
0.5
1.0
MR effect size for
'Gut microbiota abundance (genus Parasutterella id.2892)' on 'GrpE protein homolog 1, mitochondrial'

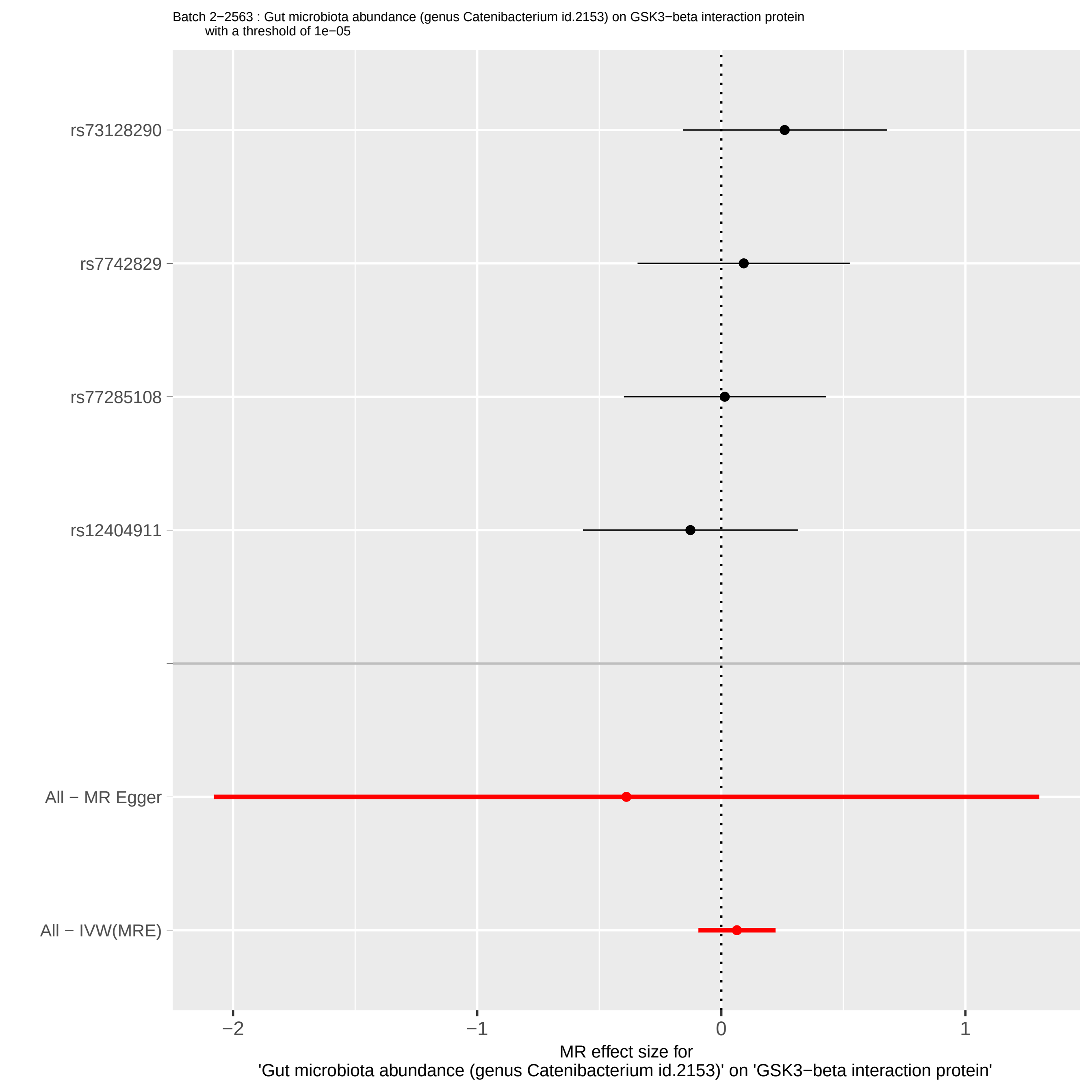

Batch 2−2563 : Gut microbiota abundance (genus Catenibacterium id.2153) on GSK3−beta interaction protein
with a threshold of 1e−05
rs73128290
rs7742829
rs77285108
rs12404911
All − MR Egger
All − IVW(MRE)
−2
−1
0
1
MR effect size for
'Gut microbiota abundance (genus Catenibacterium id.2153)' on 'GSK3−beta interaction protein'

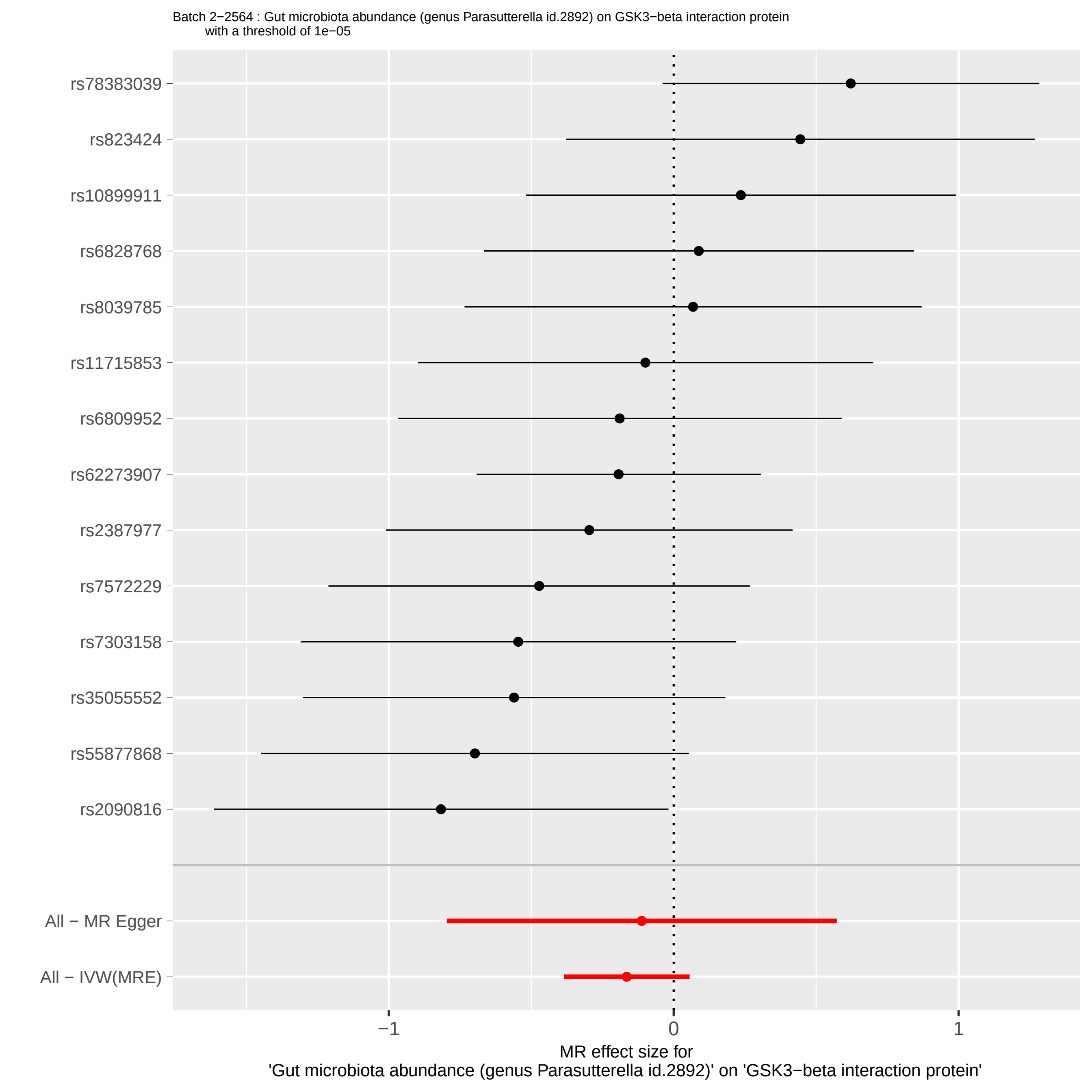

Batch 2−2564 : Gut microbiota abundance (genus Parasutterella id.2892) on GSK3−beta interaction protein
with a threshold of 1e−05
rs78383039
rs823424
rs10899911
rs6828768
rs8039785
rs11715853
rs6809952
rs62273907
rs2387977
rs7572229
rs7303158
rs35055552
rs55877868
rs2090816
All − MR Egger
All − IVW(MRE)
−1
0
1
MR effect size for
'Gut microbiota abundance (genus Parasutterella id.2892)' on 'GSK3−beta interaction protein'

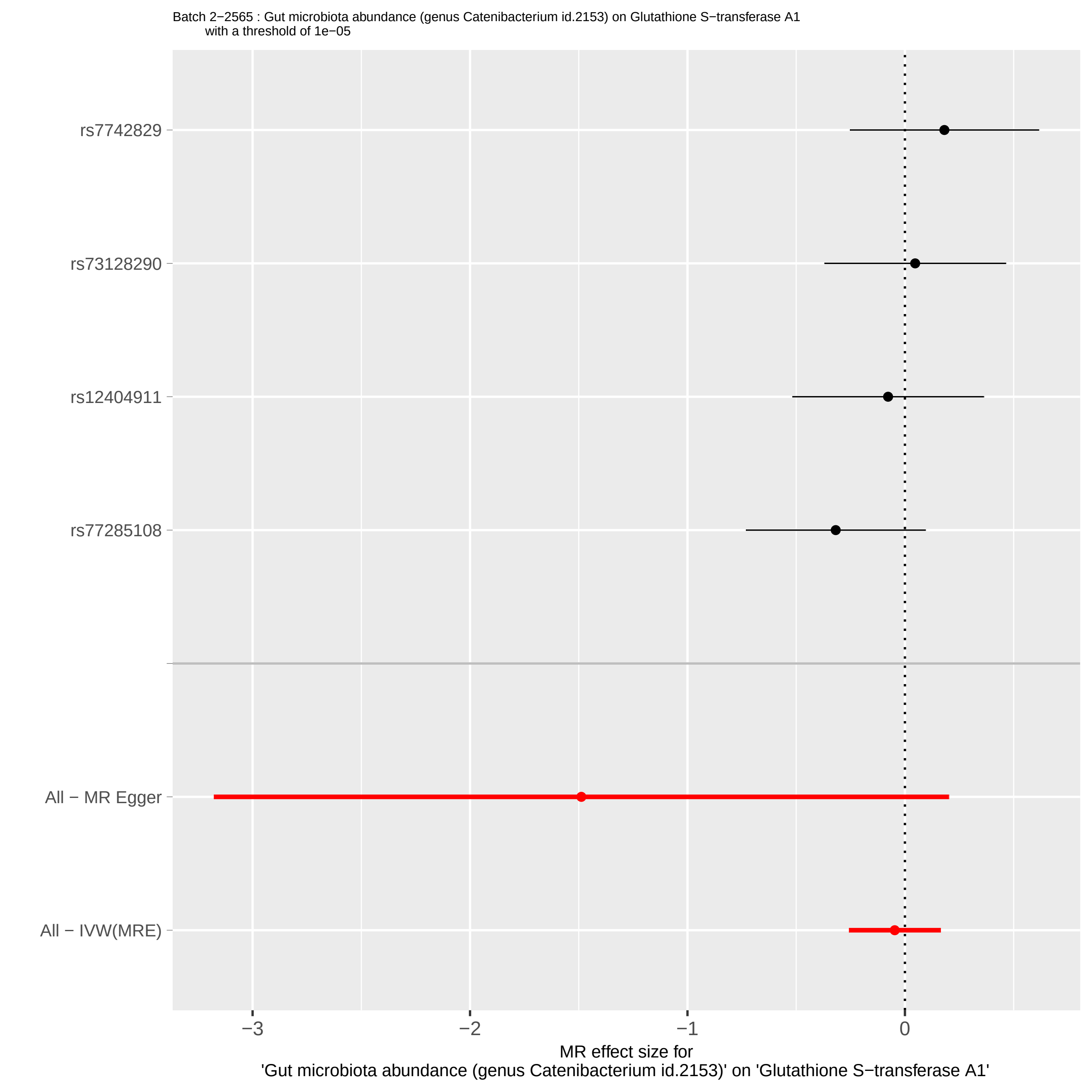

Batch 2−2565 : Gut microbiota abundance (genus Catenibacterium id.2153) on Glutathione S−transferase A1
with a threshold of 1e−05
rs7742829
rs73128290
rs12404911
rs77285108
All − MR Egger
All − IVW(MRE)
−3
−2
−1
0
MR effect size for
'Gut microbiota abundance (genus Catenibacterium id.2153)' on 'Glutathione S−transferase A1'

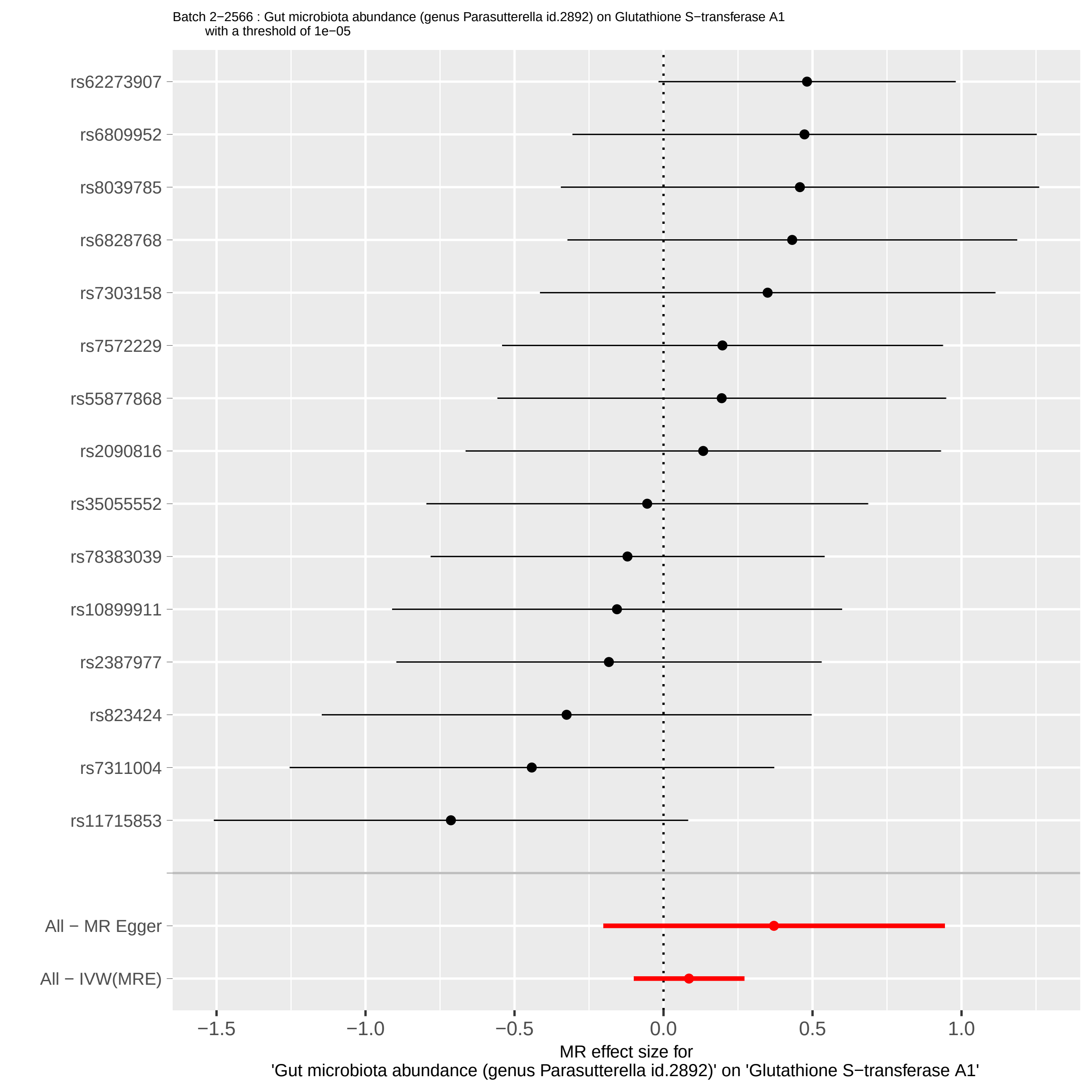

Batch 2−2566 : Gut microbiota abundance (genus Parasutterella id.2892) on Glutathione S−transferase A1
with a threshold of 1e−05
rs62273907
rs6809952
rs8039785
rs6828768
rs7303158
rs7572229
rs55877868
rs2090816
rs35055552
rs78383039
rs10899911
rs2387977
rs823424
rs7311004
rs11715853
All − MR Egger
All − IVW(MRE)
−1.5
−1.0
−0.5
0.0
0.5
1.0
MR effect size for
'Gut microbiota abundance (genus Parasutterella id.2892)' on 'Glutathione S−transferase A1'

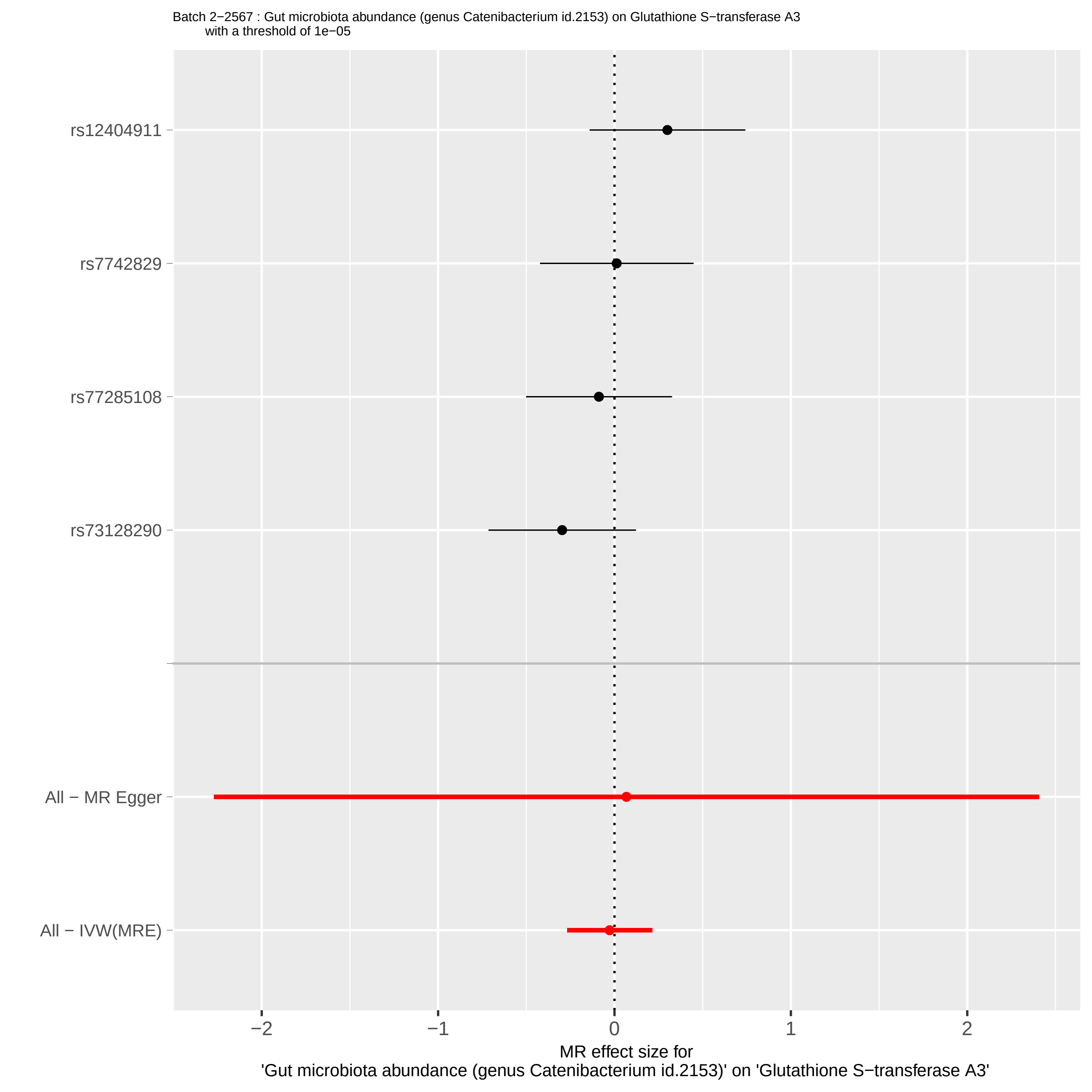
Batch 2−2567 : Gut microbiota abundance (genus Catenibacterium id.2153) on Glutathione S−transferase A3
with a threshold of 1e−05
rs12404911
rs7742829
rs77285108
rs73128290
All − MR Egger
All − IVW(MRE)
−2
−1
0
1
2
MR effect size for
'Gut microbiota abundance (genus Catenibacterium id.2153)' on 'Glutathione S−transferase A3'

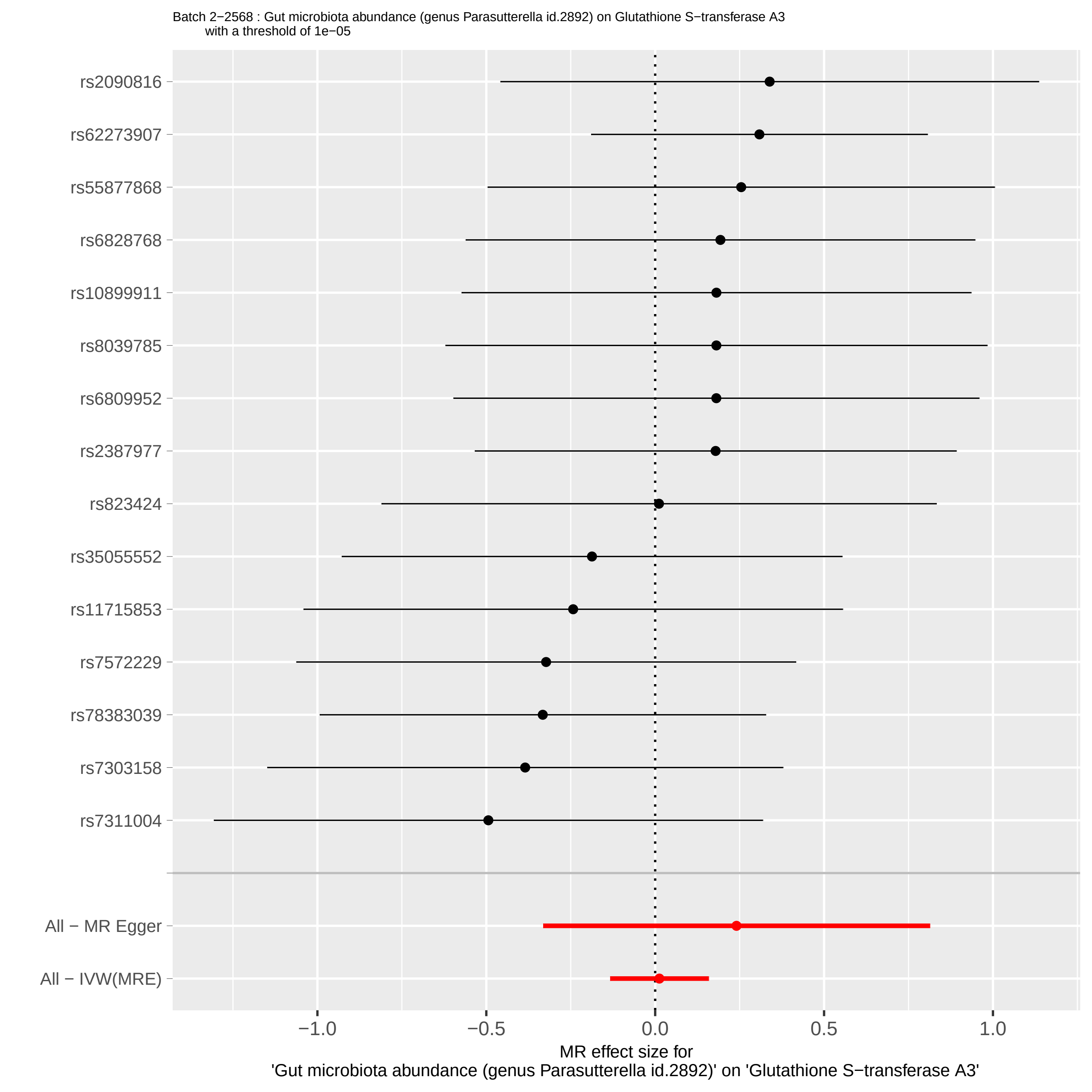
Batch 2−2568 : Gut microbiota abundance (genus Parasutterella id.2892) on Glutathione S−transferase A3
with a threshold of 1e−05
rs2090816
rs62273907
rs55877868
rs6828768
rs10899911
rs8039785
rs6809952
rs2387977
rs823424
rs35055552
rs11715853
rs7572229
rs78383039
rs7303158
rs7311004
All − MR Egger
All − IVW(MRE)
−1.0
−0.5
0.0
0.5
1.0
MR effect size for
'Gut microbiota abundance (genus Parasutterella id.2892)' on 'Glutathione S−transferase A3'

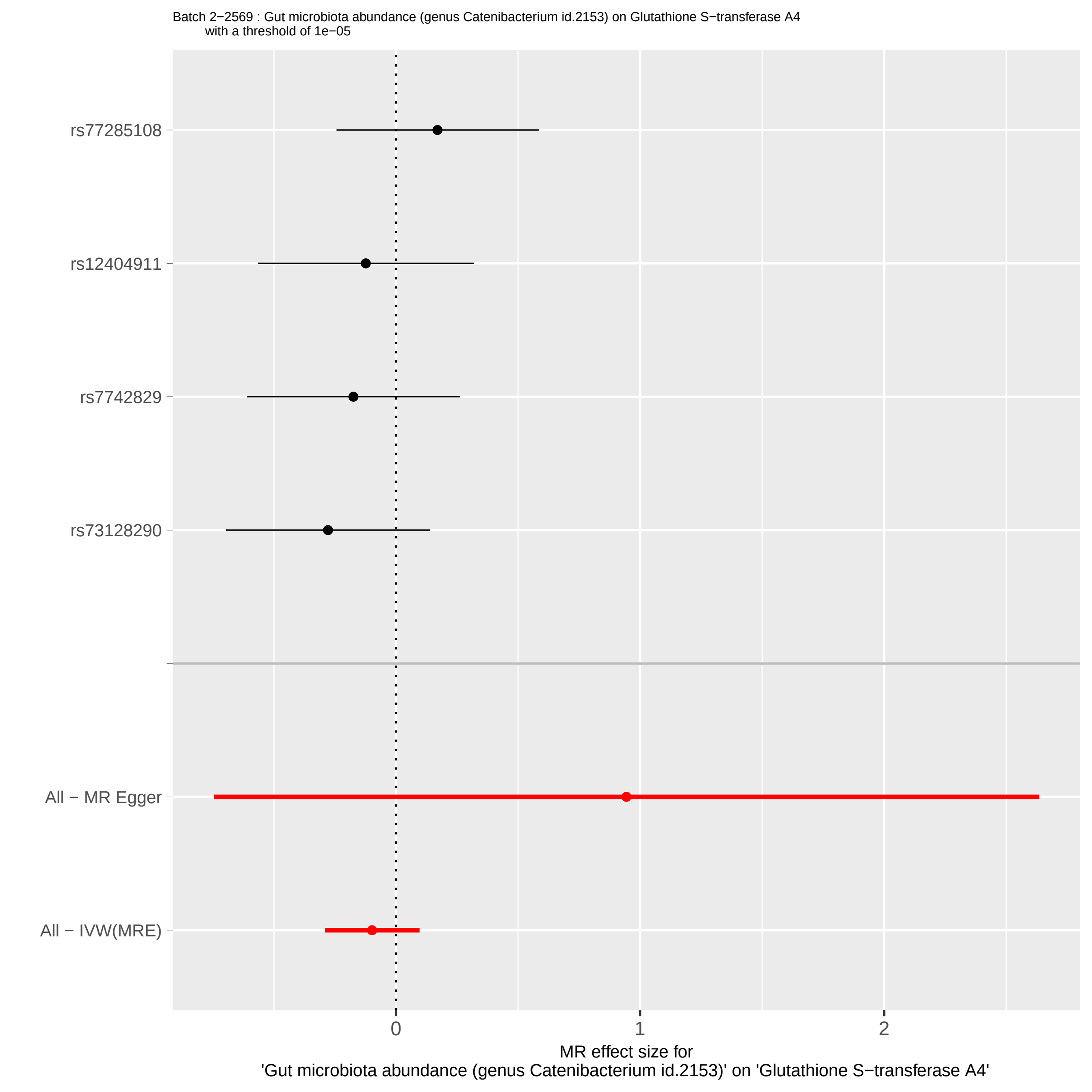
Batch 2−2569 : Gut microbiota abundance (genus Catenibacterium id.2153) on Glutathione S−transferase A4
with a threshold of 1e−05
rs77285108
rs12404911
rs7742829
rs73128290
All − MR Egger
All − IVW(MRE)
0
1
2
MR effect size for
'Gut microbiota abundance (genus Catenibacterium id.2153)' on 'Glutathione S−transferase A4'

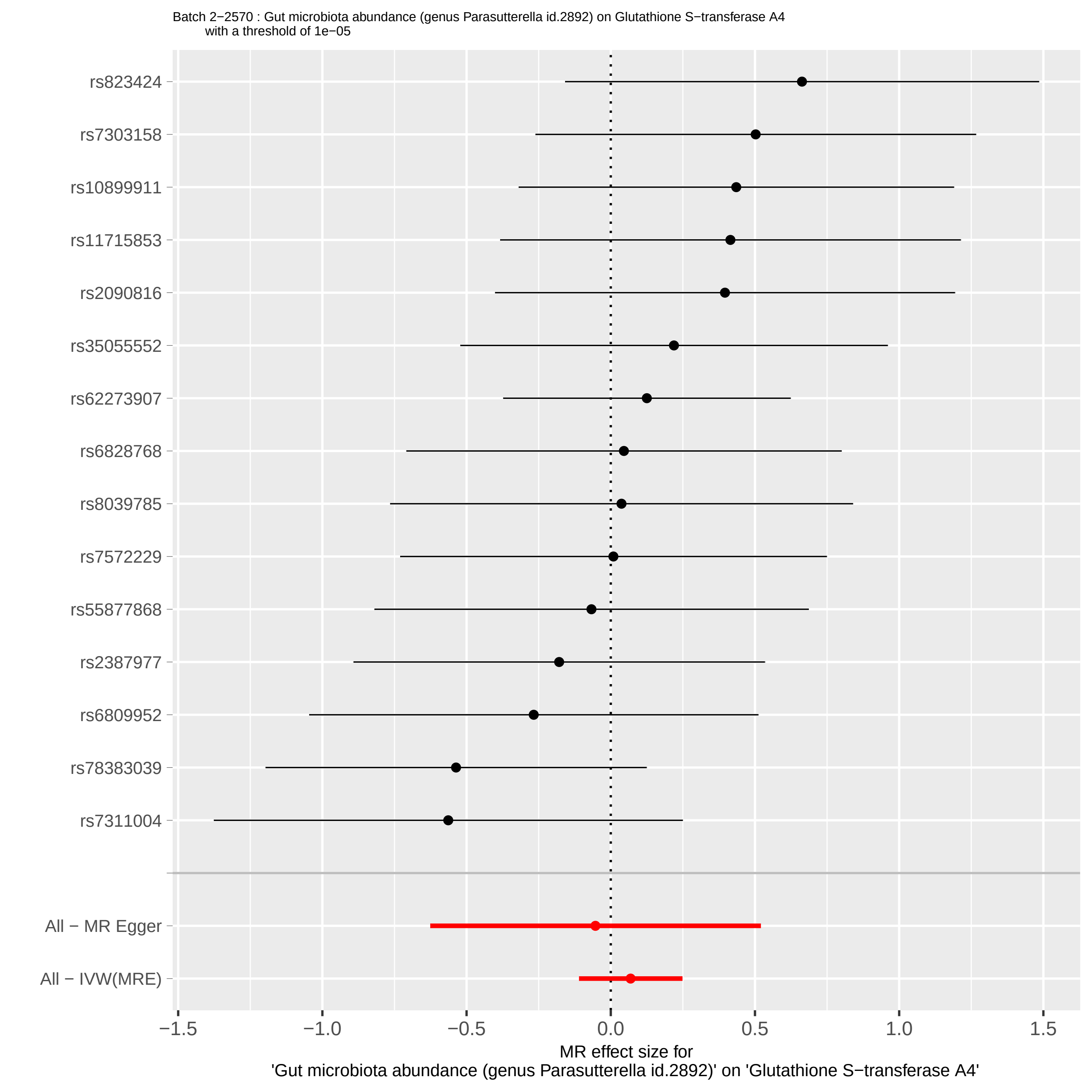

Batch 2−2570 : Gut microbiota abundance (genus Parasutterella id.2892) on Glutathione S−transferase A4
with a threshold of 1e−05
rs823424
rs7303158
rs10899911
rs11715853
rs2090816
rs35055552
rs62273907
rs6828768
rs8039785
rs7572229
rs55877868
rs2387977
rs6809952
rs78383039
rs7311004
All − MR Egger
All − IVW(MRE)
−1.5
−1.0
−0.5
0.0
0.5
1.0
1.5
MR effect size for
'Gut microbiota abundance (genus Parasutterella id.2892)' on 'Glutathione S−transferase A4'

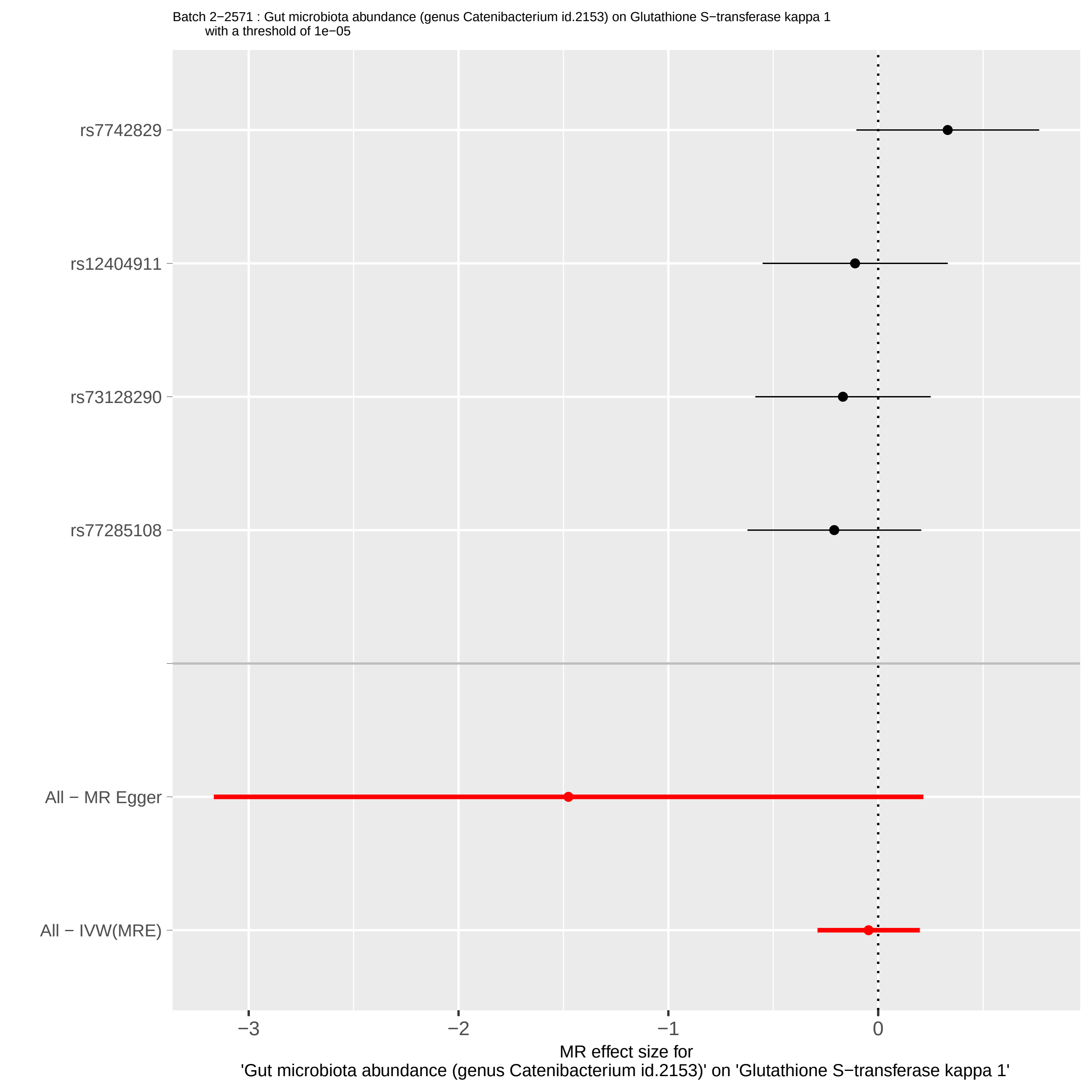

Batch 2−2571 : Gut microbiota abundance (genus Catenibacterium id.2153) on Glutathione S−transferase kappa 1
with a threshold of 1e−05
rs7742829
rs12404911
rs73128290
rs77285108
All − MR Egger
All − IVW(MRE)
−3
−2
−1
0
MR effect size for
'Gut microbiota abundance (genus Catenibacterium id.2153)' on 'Glutathione S−transferase kappa 1'

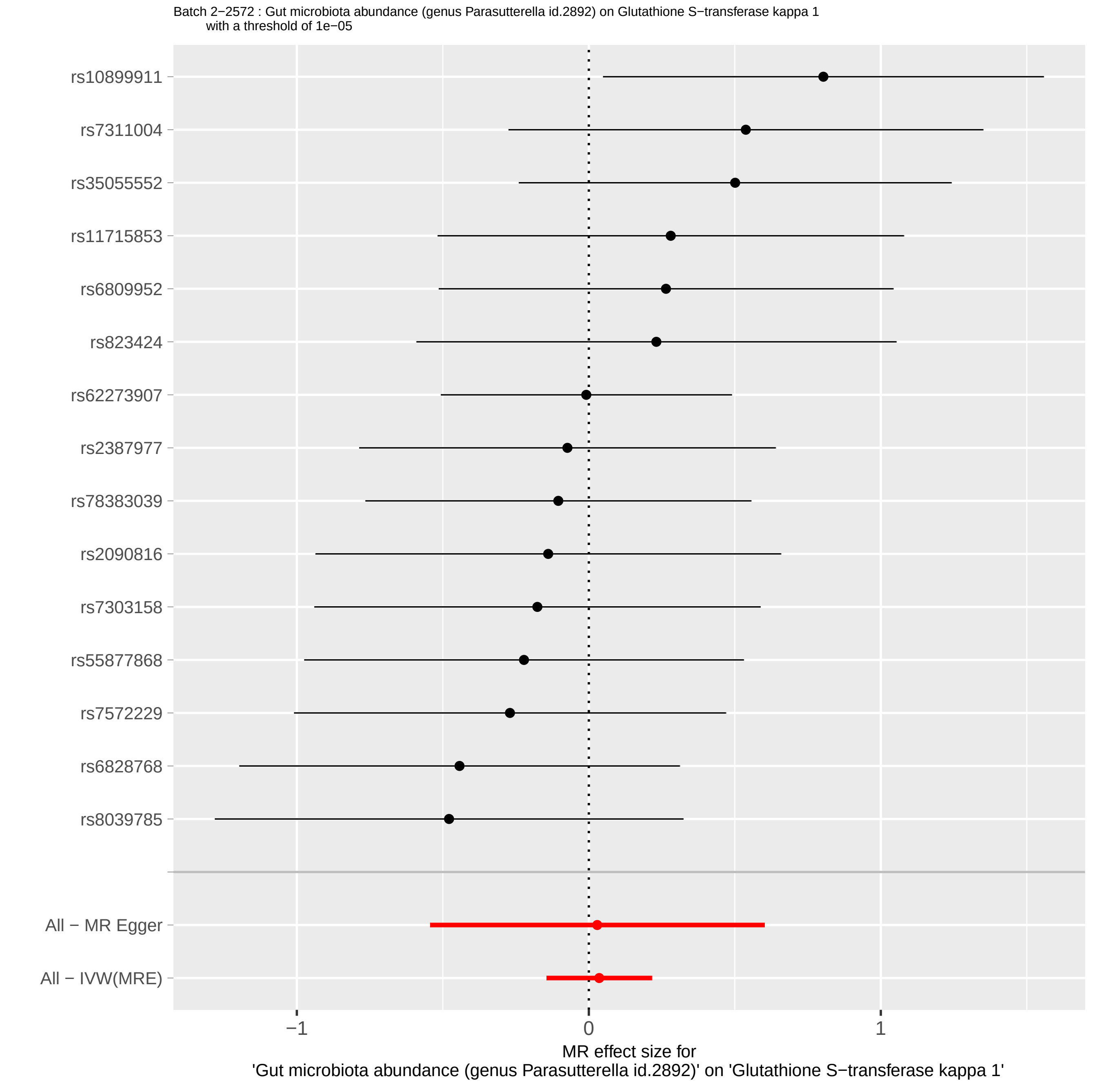

Batch 2−2572 : Gut microbiota abundance (genus Parasutterella id.2892) on Glutathione S−transferase kappa 1
with a threshold of 1e−05
rs10899911
rs7311004
rs35055552
rs11715853
rs6809952
rs823424
rs62273907
rs2387977
rs78383039
rs2090816
rs7303158
rs55877868
rs7572229
rs6828768
rs8039785
All − MR Egger
All − IVW(MRE)
−1
0
1
MR effect size for
'Gut microbiota abundance (genus Parasutterella id.2892)' on 'Glutathione S−transferase kappa 1'

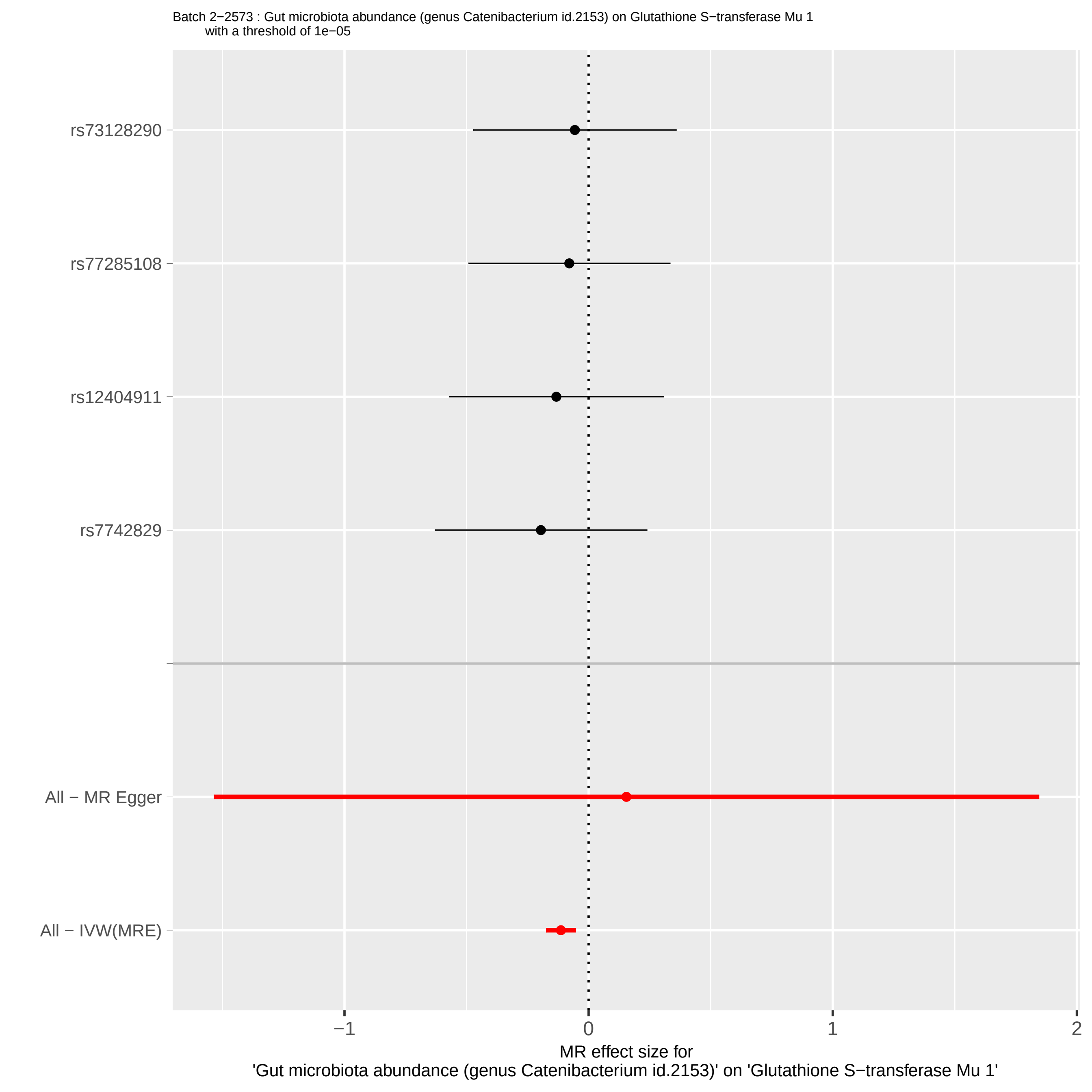

Batch 2−2573 : Gut microbiota abundance (genus Catenibacterium id.2153) on Glutathione S−transferase Mu 1
with a threshold of 1e−05
rs73128290
rs77285108
rs12404911
rs7742829
All − MR Egger
All − IVW(MRE)
−1
0
1
2
MR effect size for
'Gut microbiota abundance (genus Catenibacterium id.2153)' on 'Glutathione S−transferase Mu 1'

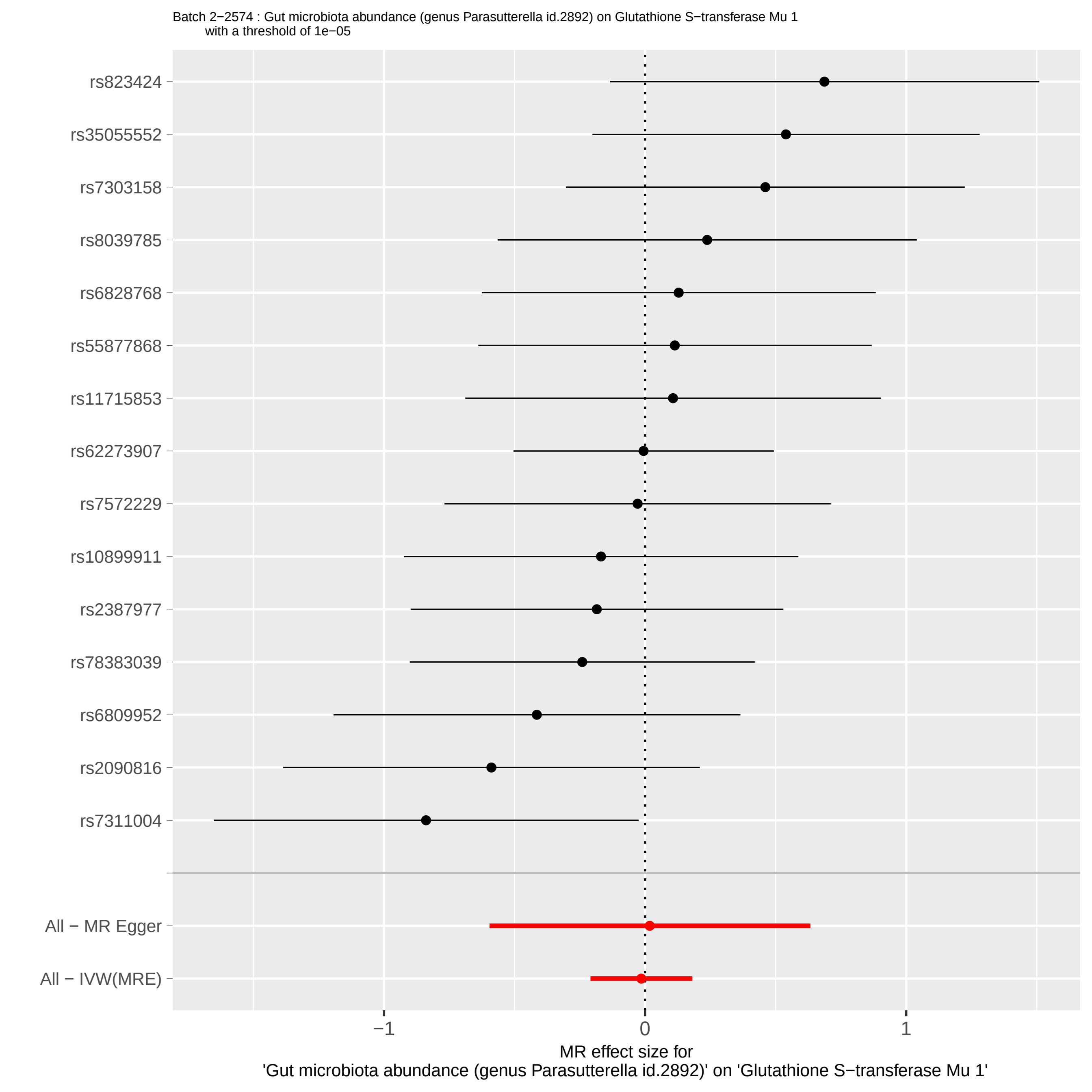
Batch 2−2574 : Gut microbiota abundance (genus Parasutterella id.2892) on Glutathione S−transferase Mu 1
with a threshold of 1e−05
rs823424
rs35055552
rs7303158
rs8039785
rs6828768
rs55877868
rs11715853
rs62273907
rs7572229
rs10899911
rs2387977
rs78383039
rs6809952
rs2090816
rs7311004
All − MR Egger
All − IVW(MRE)
−1
0
1
MR effect size for
'Gut microbiota abundance (genus Parasutterella id.2892)' on 'Glutathione S−transferase Mu 1'

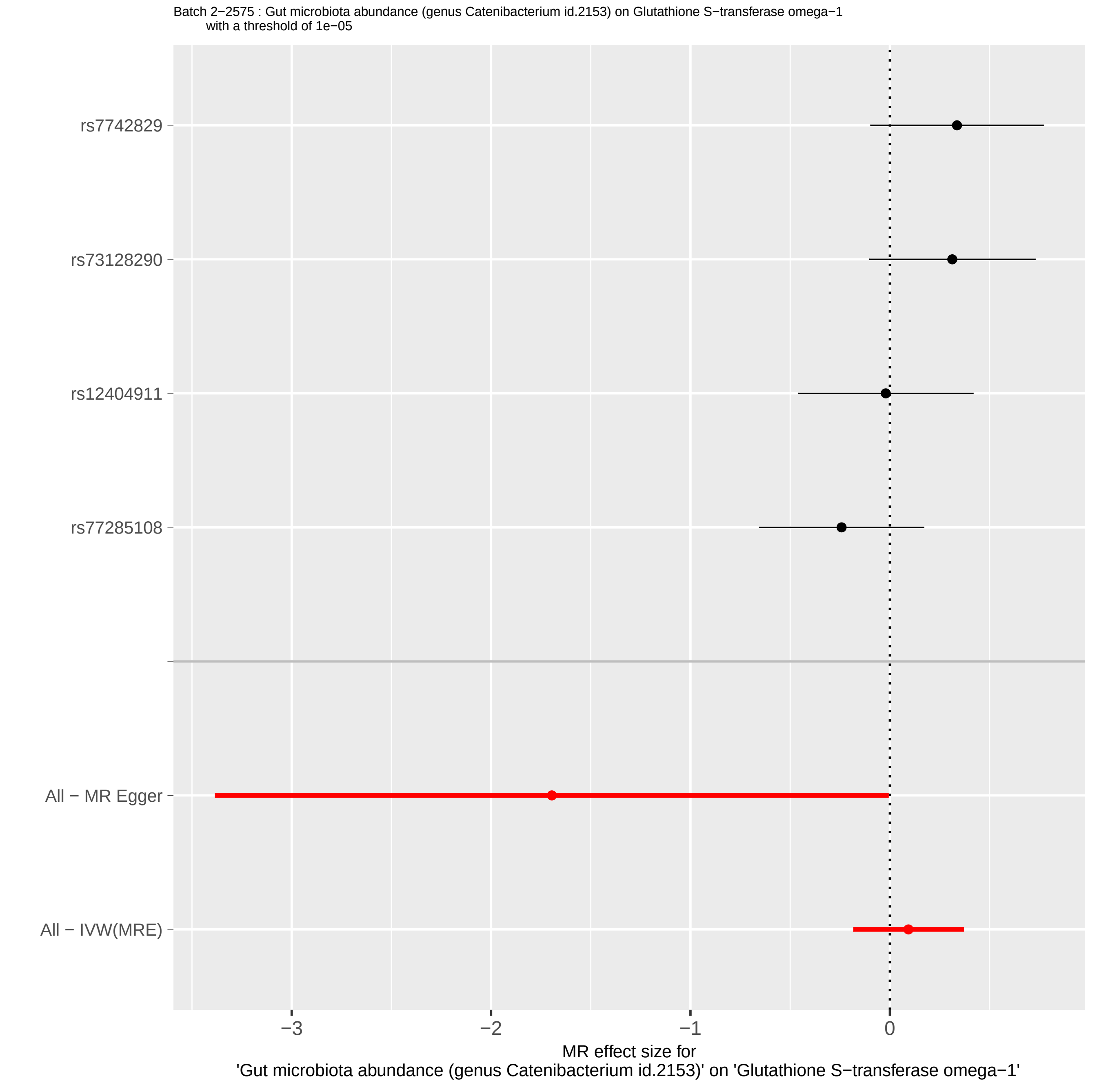

Batch 2−2575 : Gut microbiota abundance (genus Catenibacterium id.2153) on Glutathione S−transferase omega−1
with a threshold of 1e−05
rs7742829
rs73128290
rs12404911
rs77285108
All − MR Egger
All − IVW(MRE)
−3
−2
−1
0
MR effect size for
'Gut microbiota abundance (genus Catenibacterium id.2153)' on 'Glutathione S−transferase omega−1'

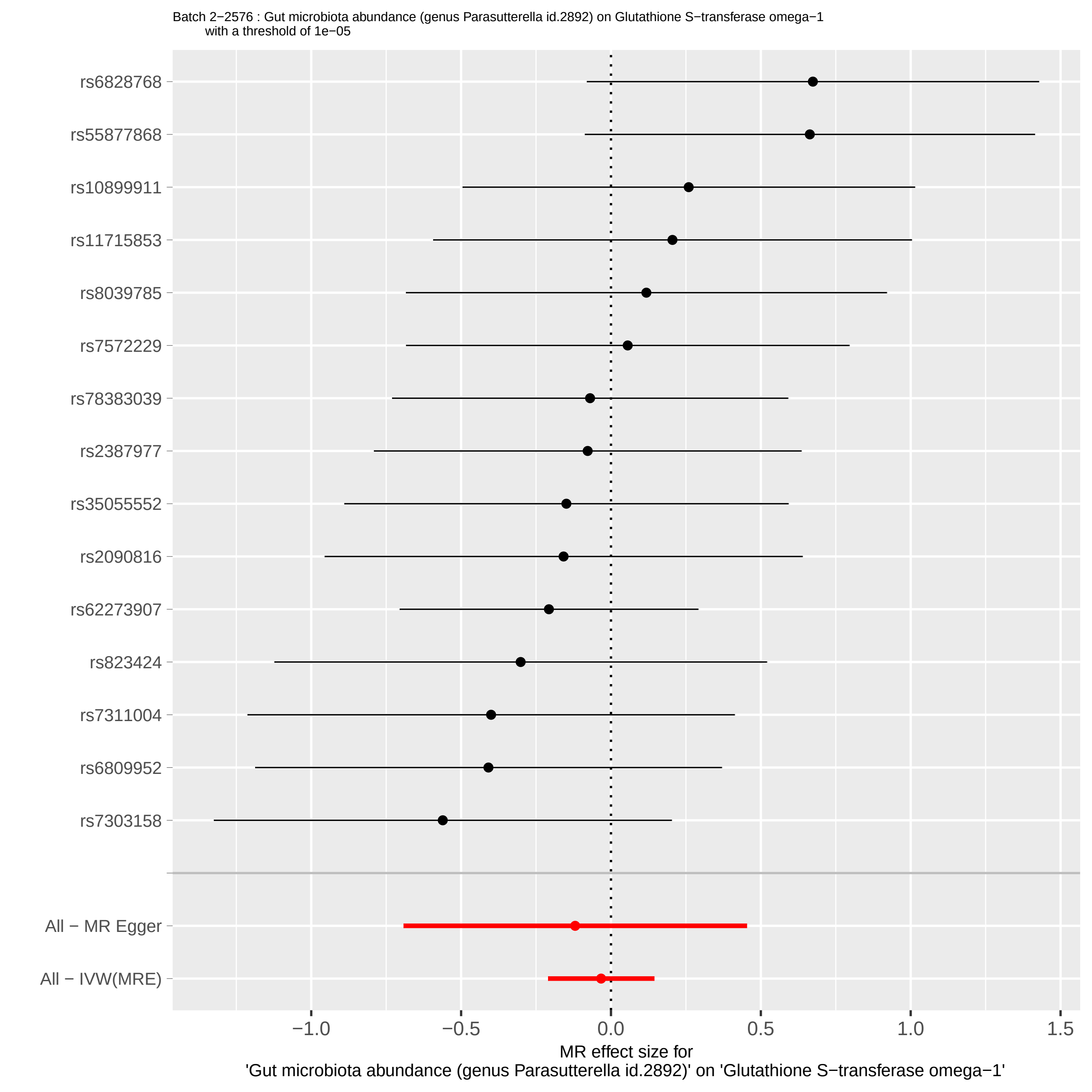

Batch 2−2576 : Gut microbiota abundance (genus Parasutterella id.2892) on Glutathione S−transferase omega−1
with a threshold of 1e−05
rs6828768
rs55877868
rs10899911
rs11715853
rs8039785
rs7572229
rs78383039
rs2387977
rs35055552
rs2090816
rs62273907
rs823424
rs7311004
rs6809952
rs7303158
All − MR Egger
All − IVW(MRE)
−1.0
−0.5
0.0
0.5
1.0
1.5
MR effect size for
'Gut microbiota abundance (genus Parasutterella id.2892)' on 'Glutathione S−transferase omega−1'

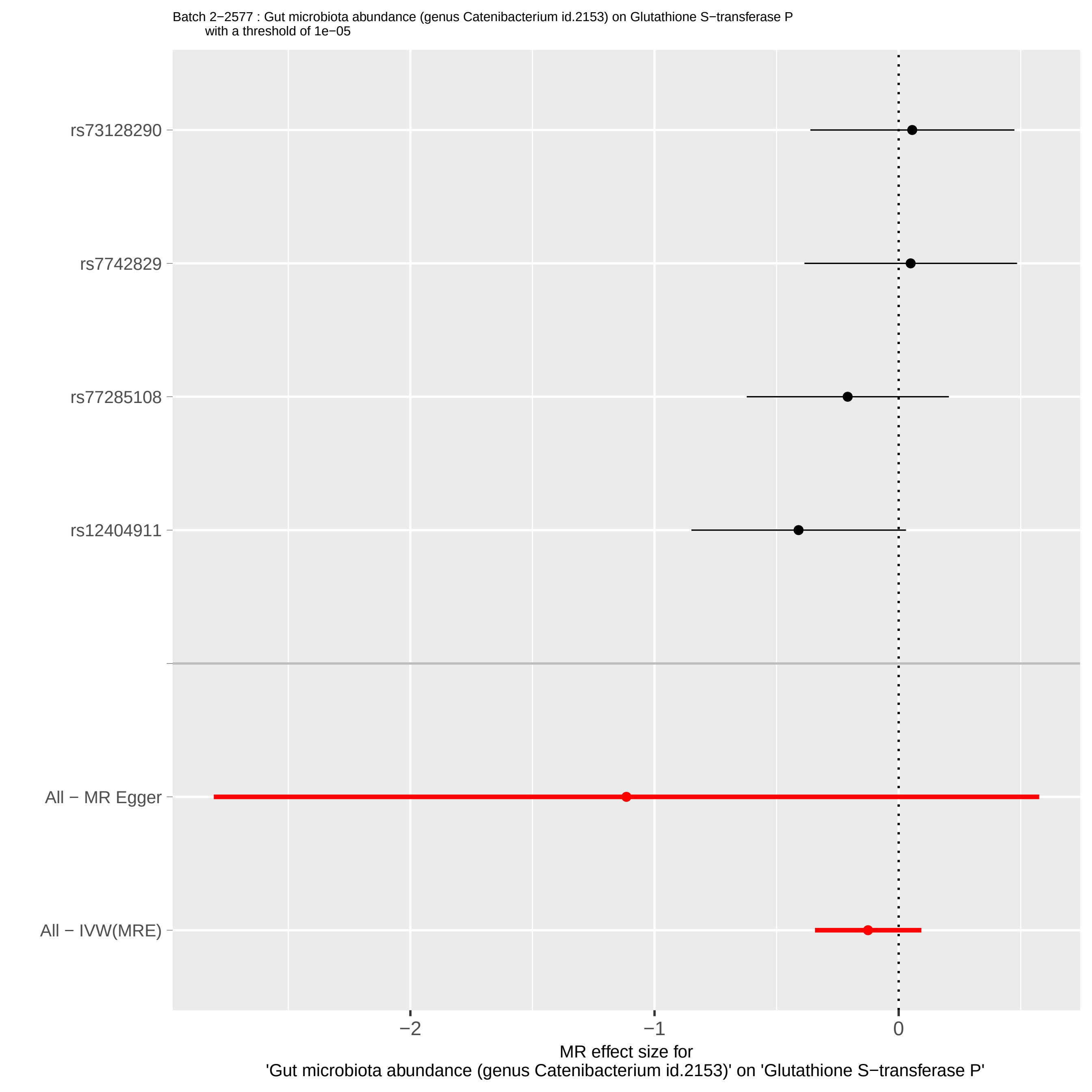
Batch 2−2577 : Gut microbiota abundance (genus Catenibacterium id.2153) on Glutathione S−transferase P
with a threshold of 1e−05
rs73128290
rs7742829
rs77285108
rs12404911
All − MR Egger
All − IVW(MRE)
−2
−1
0
MR effect size for
'Gut microbiota abundance (genus Catenibacterium id.2153)' on 'Glutathione S−transferase P'

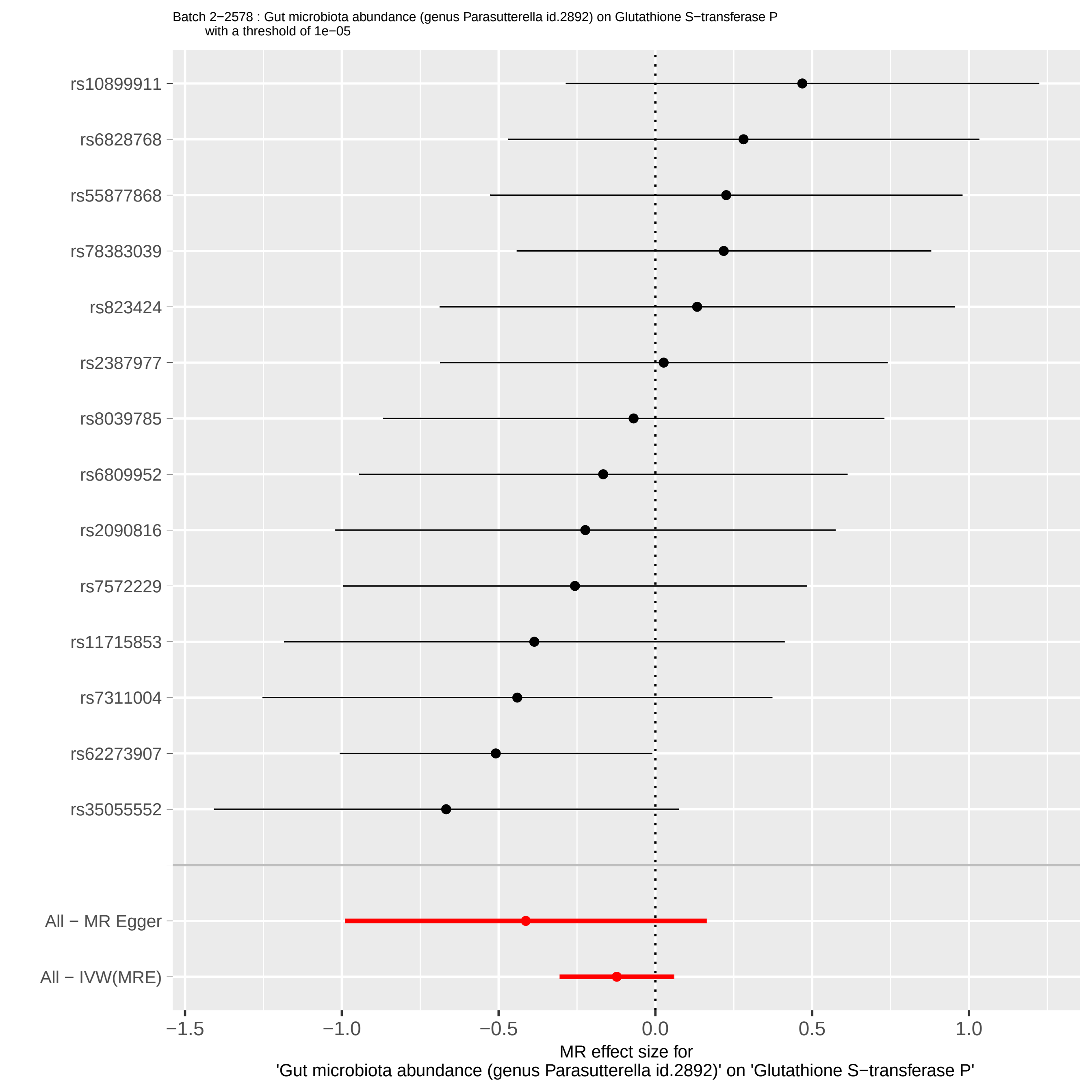

Batch 2−2578 : Gut microbiota abundance (genus Parasutterella id.2892) on Glutathione S−transferase P
with a threshold of 1e−05
rs10899911
rs6828768
rs55877868
rs78383039
rs823424
rs2387977
rs8039785
rs6809952
rs2090816
rs7572229
rs11715853
rs7311004
rs62273907
rs35055552
All − MR Egger
All − IVW(MRE)
−1.5
−1.0
−0.5
0.0
0.5
1.0
MR effect size for
'Gut microbiota abundance (genus Parasutterella id.2892)' on 'Glutathione S−transferase P'

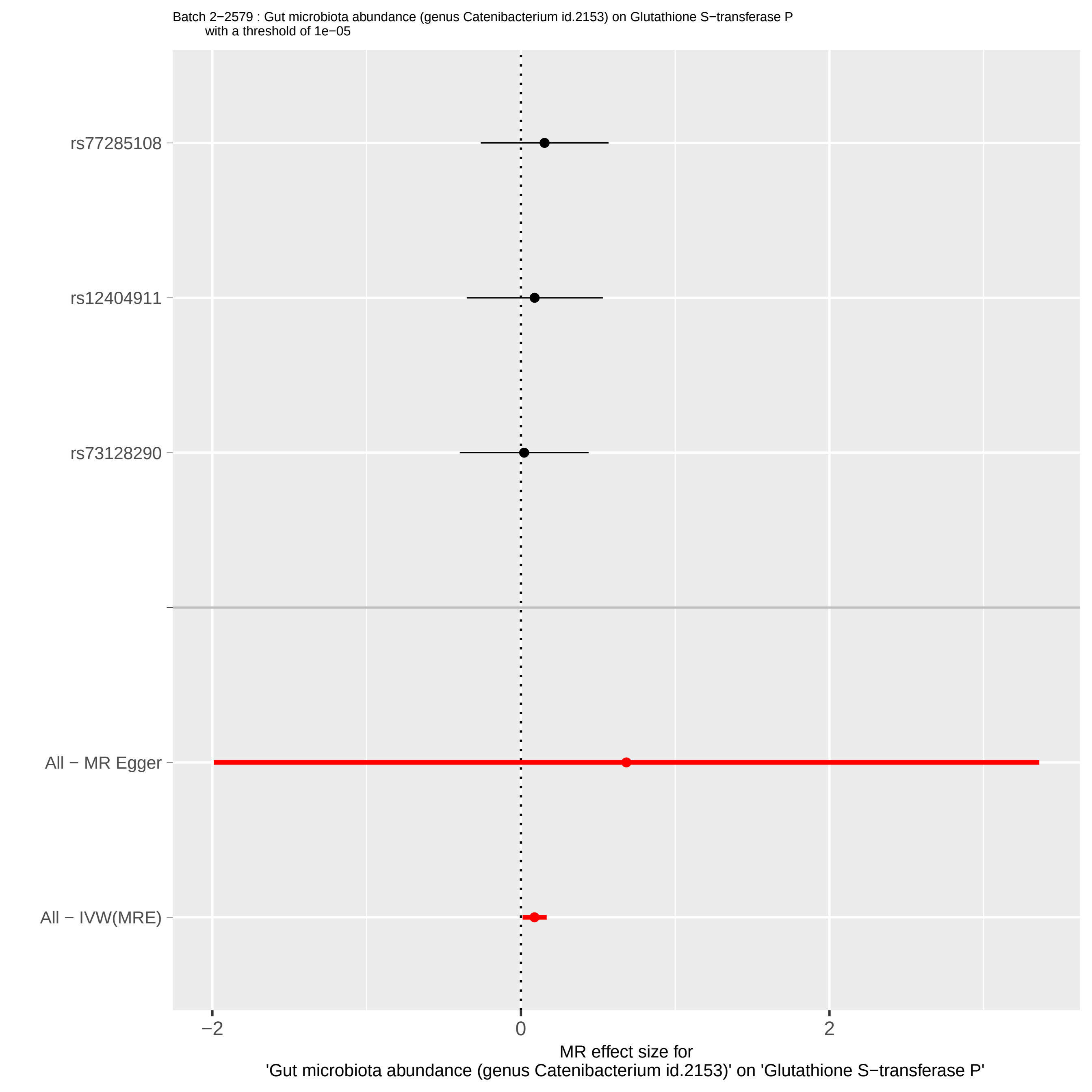

Batch 2−2579 : Gut microbiota abundance (genus Catenibacterium id.2153) on Glutathione S−transferase P
with a threshold of 1e−05
rs77285108
rs12404911
rs73128290
All − MR Egger
All − IVW(MRE)
−2
0
2
MR effect size for
'Gut microbiota abundance (genus Catenibacterium id.2153)' on 'Glutathione S−transferase P'

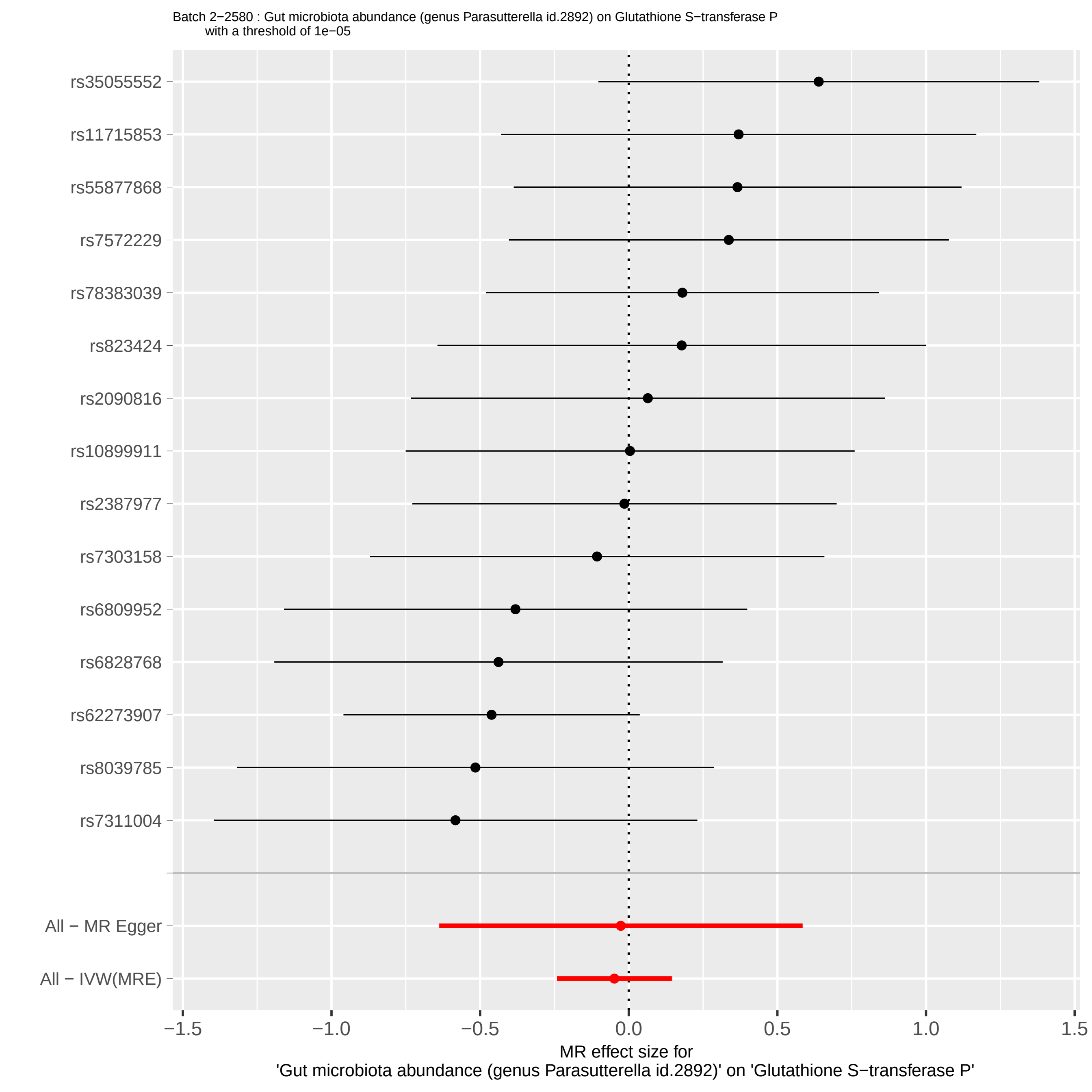
Batch 2−2580 : Gut microbiota abundance (genus Parasutterella id.2892) on Glutathione S−transferase P
with a threshold of 1e−05
rs35055552
rs11715853
rs55877868
rs7572229
rs78383039
rs823424
rs2090816
rs10899911
rs2387977
rs7303158
rs6809952
rs6828768
rs62273907
rs8039785
rs7311004
All − MR Egger
All − IVW(MRE)
−1.5
−1.0
−0.5
0.0
0.5
1.0
1.5
MR effect size for
'Gut microbiota abundance (genus Parasutterella id.2892)' on 'Glutathione S−transferase P'

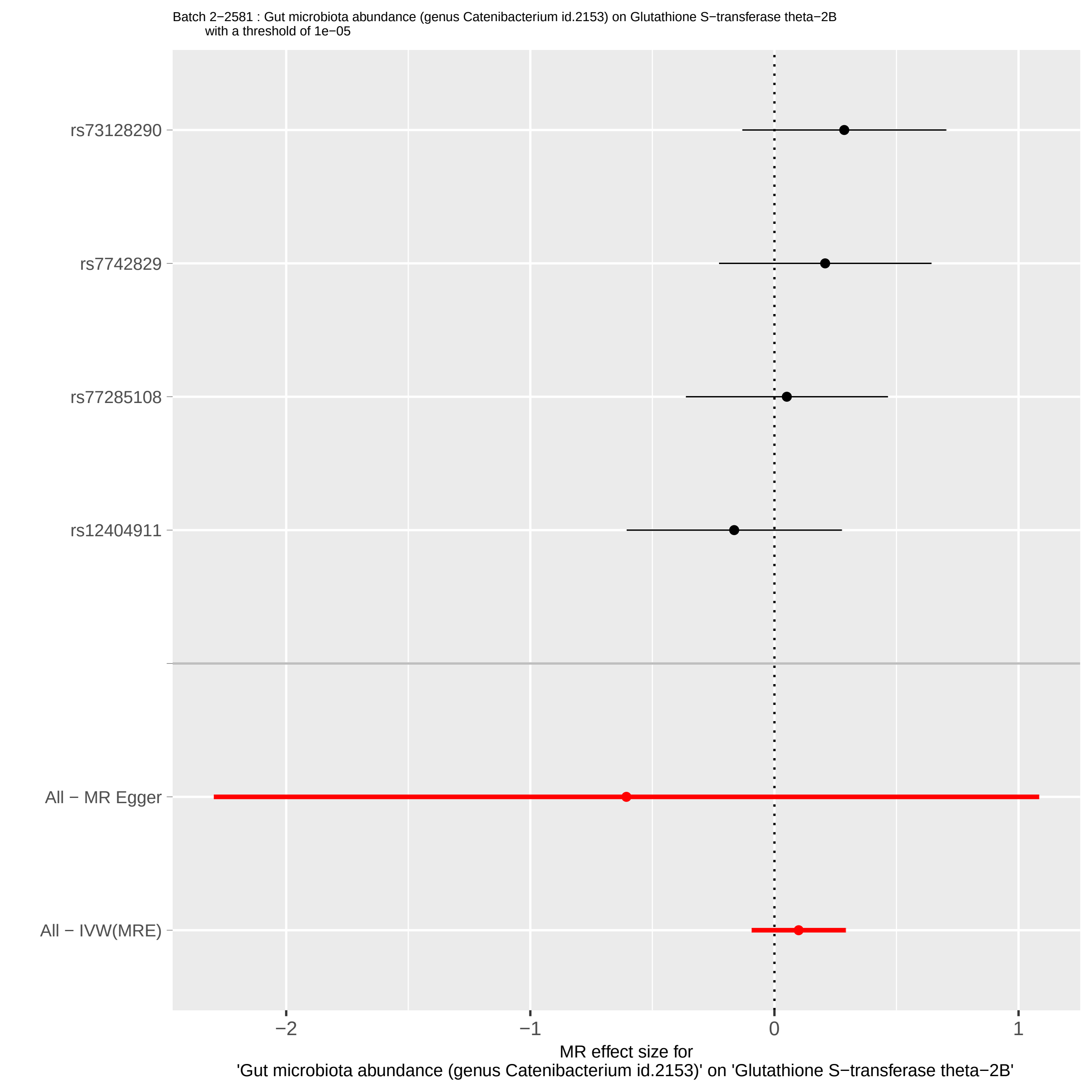
Batch 2−2581 : Gut microbiota abundance (genus Catenibacterium id.2153) on Glutathione S−transferase theta−2B
with a threshold of 1e−05
rs73128290
rs7742829
rs77285108
rs12404911
All − MR Egger
All − IVW(MRE)
−2
−1
0
1
MR effect size for
'Gut microbiota abundance (genus Catenibacterium id.2153)' on 'Glutathione S−transferase theta−2B'

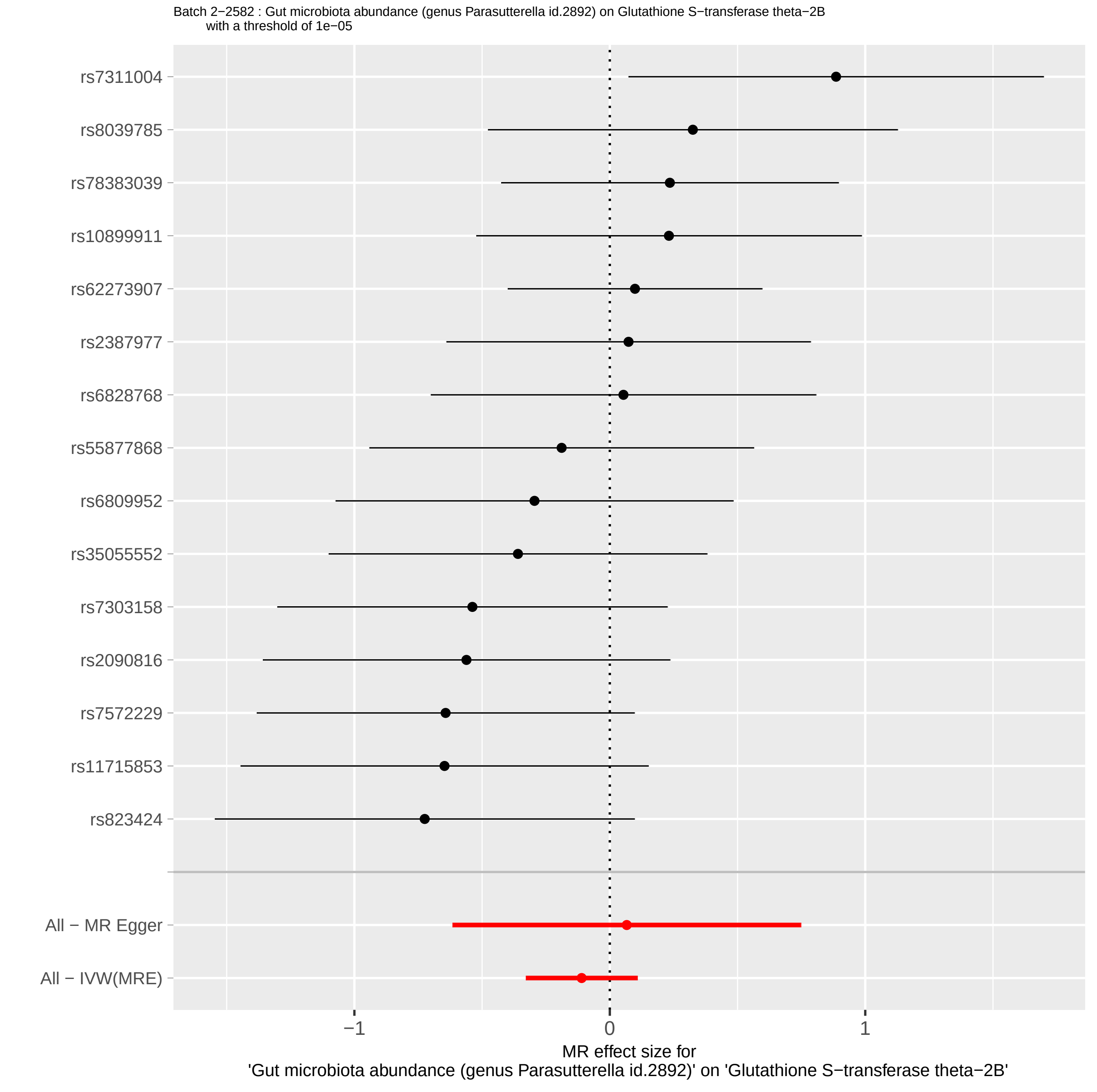

Batch 2−2582 : Gut microbiota abundance (genus Parasutterella id.2892) on Glutathione S−transferase theta−2B
with a threshold of 1e−05
rs7311004
rs8039785
rs78383039
rs10899911
rs62273907
rs2387977
rs6828768
rs55877868
rs6809952
rs35055552
rs7303158
rs2090816
rs7572229
rs11715853
rs823424
All − MR Egger
All − IVW(MRE)
−1
0
1
MR effect size for
'Gut microbiota abundance (genus Parasutterella id.2892)' on 'Glutathione S−transferase theta−2B'

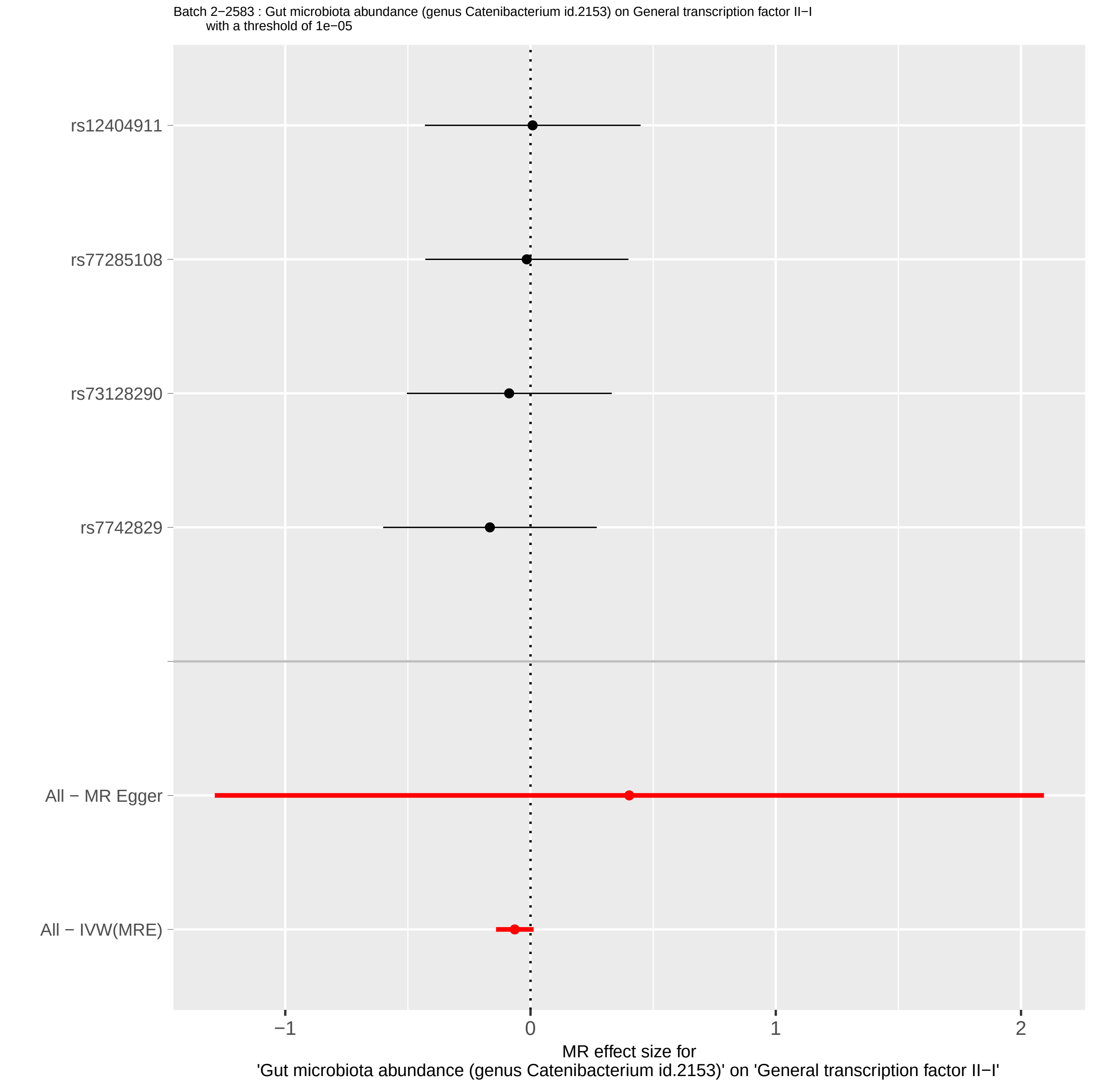

Batch 2−2583 : Gut microbiota abundance (genus Catenibacterium id.2153) on General transcription factor II−I
with a threshold of 1e−05
rs12404911
rs77285108
rs73128290
rs7742829
All − MR Egger
All − IVW(MRE)
−1
0
1
2
MR effect size for
'Gut microbiota abundance (genus Catenibacterium id.2153)' on 'General transcription factor II−I'

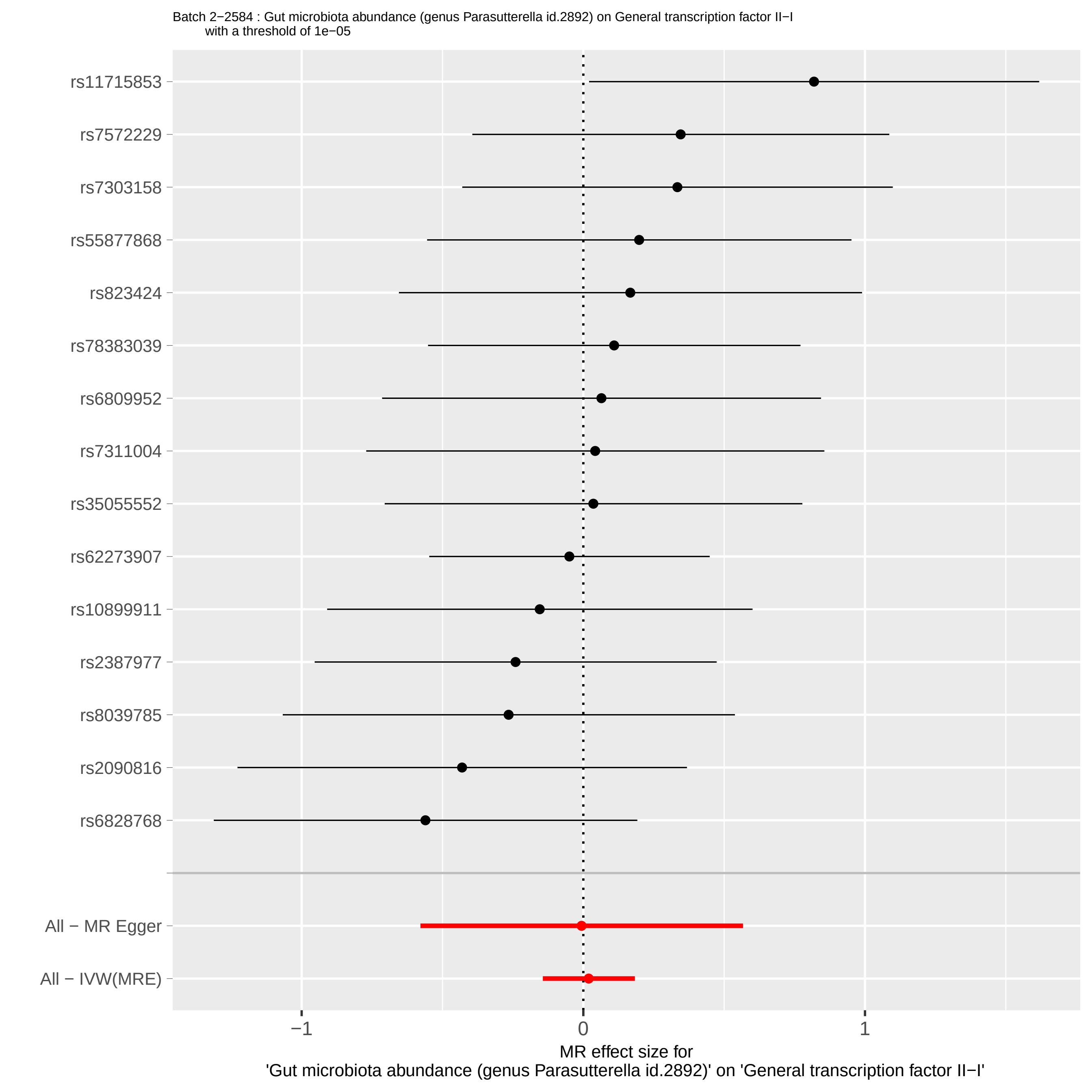

Batch 2−2584 : Gut microbiota abundance (genus Parasutterella id.2892) on General transcription factor II−I
with a threshold of 1e−05
rs11715853
rs7572229
rs7303158
rs55877868
rs823424
rs78383039
rs6809952
rs7311004
rs35055552
rs62273907
rs10899911
rs2387977
rs8039785
rs2090816
rs6828768
All − MR Egger
All − IVW(MRE)
−1
0
1
MR effect size for
'Gut microbiota abundance (genus Parasutterella id.2892)' on 'General transcription factor II−I'

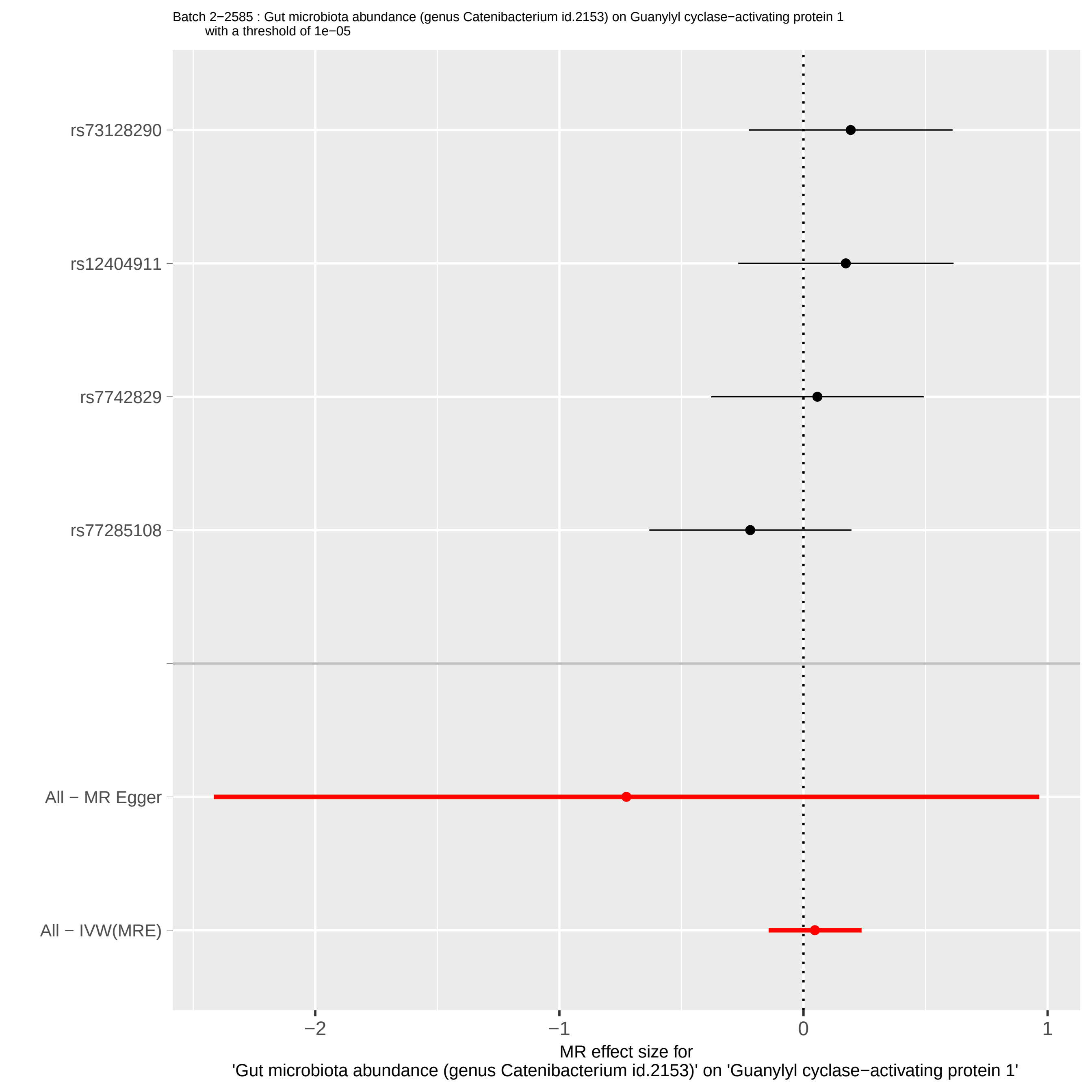
Batch 2−2585 : Gut microbiota abundance (genus Catenibacterium id.2153) on Guanylyl cyclase−activating protein 1
with a threshold of 1e−05
rs73128290
rs12404911
rs7742829
rs77285108
All − MR Egger
All − IVW(MRE)
−2
−1
0
1
MR effect size for
'Gut microbiota abundance (genus Catenibacterium id.2153)' on 'Guanylyl cyclase−activating protein 1'

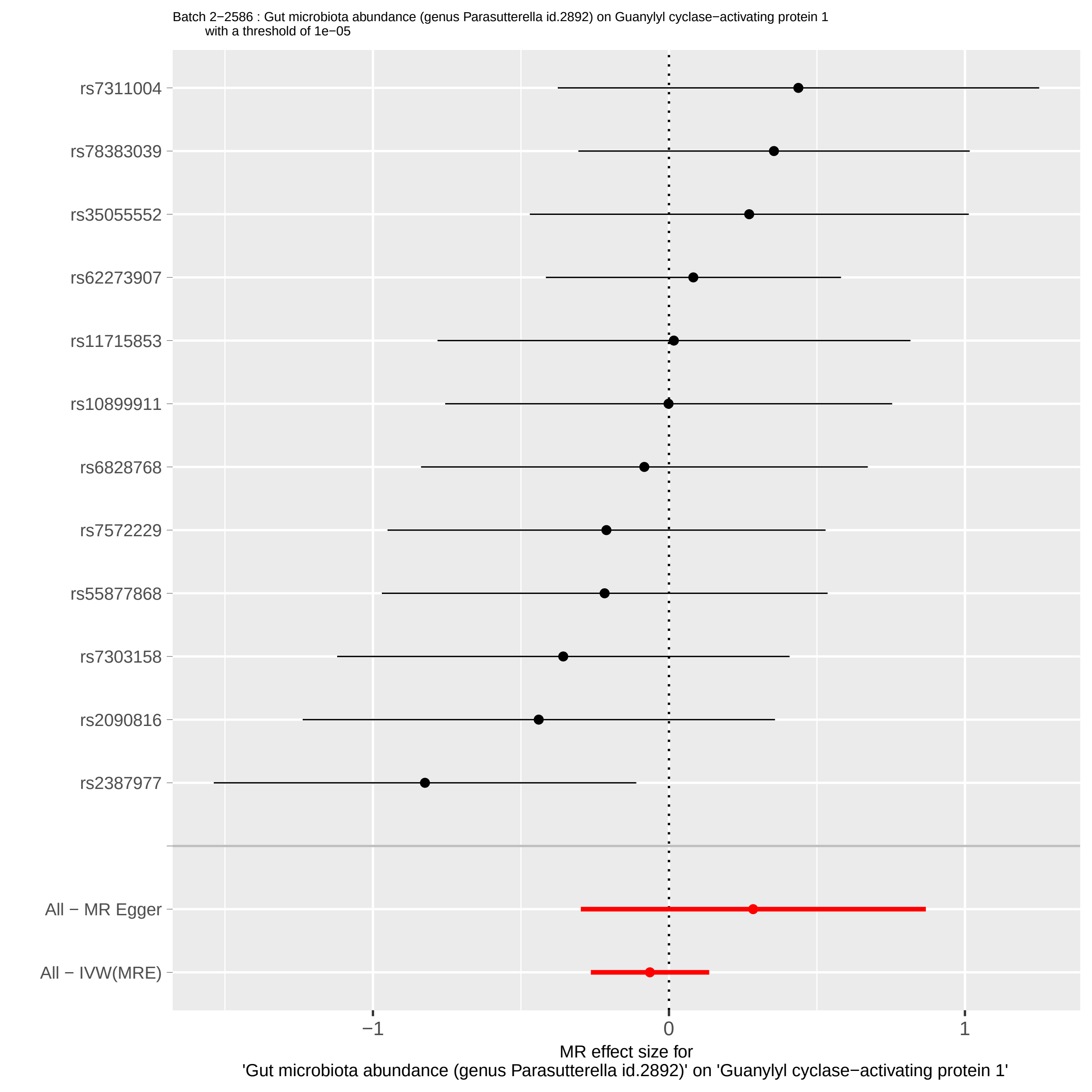

Batch 2−2586 : Gut microbiota abundance (genus Parasutterella id.2892) on Guanylyl cyclase−activating protein 1 with a threshold of 1e−05
rs7311004
rs78383039
rs35055552
rs62273907
rs11715853
rs10899911
rs6828768
rs7572229
rs55877868
rs7303158
rs2090816
rs2387977
All − MR Egger
All − IVW(MRE)
−1
0
1
MR effect size for
'Gut microbiota abundance (genus Parasutterella id.2892)' on 'Guanylyl cyclase−activating protein 1'

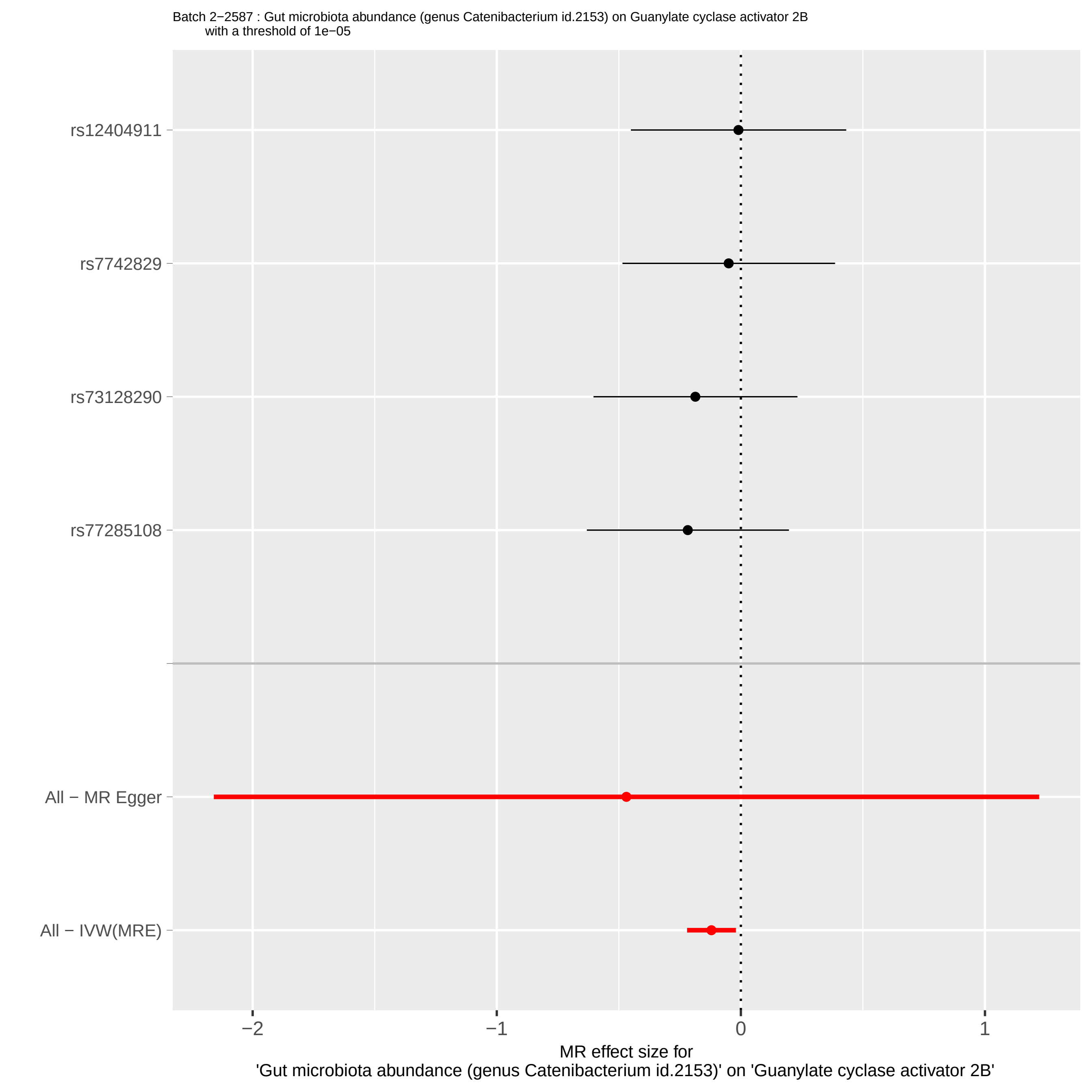
Batch 2−2587 : Gut microbiota abundance (genus Catenibacterium id.2153) on Guanylate cyclase activator 2B
with a threshold of 1e−05
rs12404911
rs7742829
rs73128290
rs77285108
All − MR Egger
All − IVW(MRE)
−2
−1
0
1
MR effect size for
'Gut microbiota abundance (genus Catenibacterium id.2153)' on 'Guanylate cyclase activator 2B'

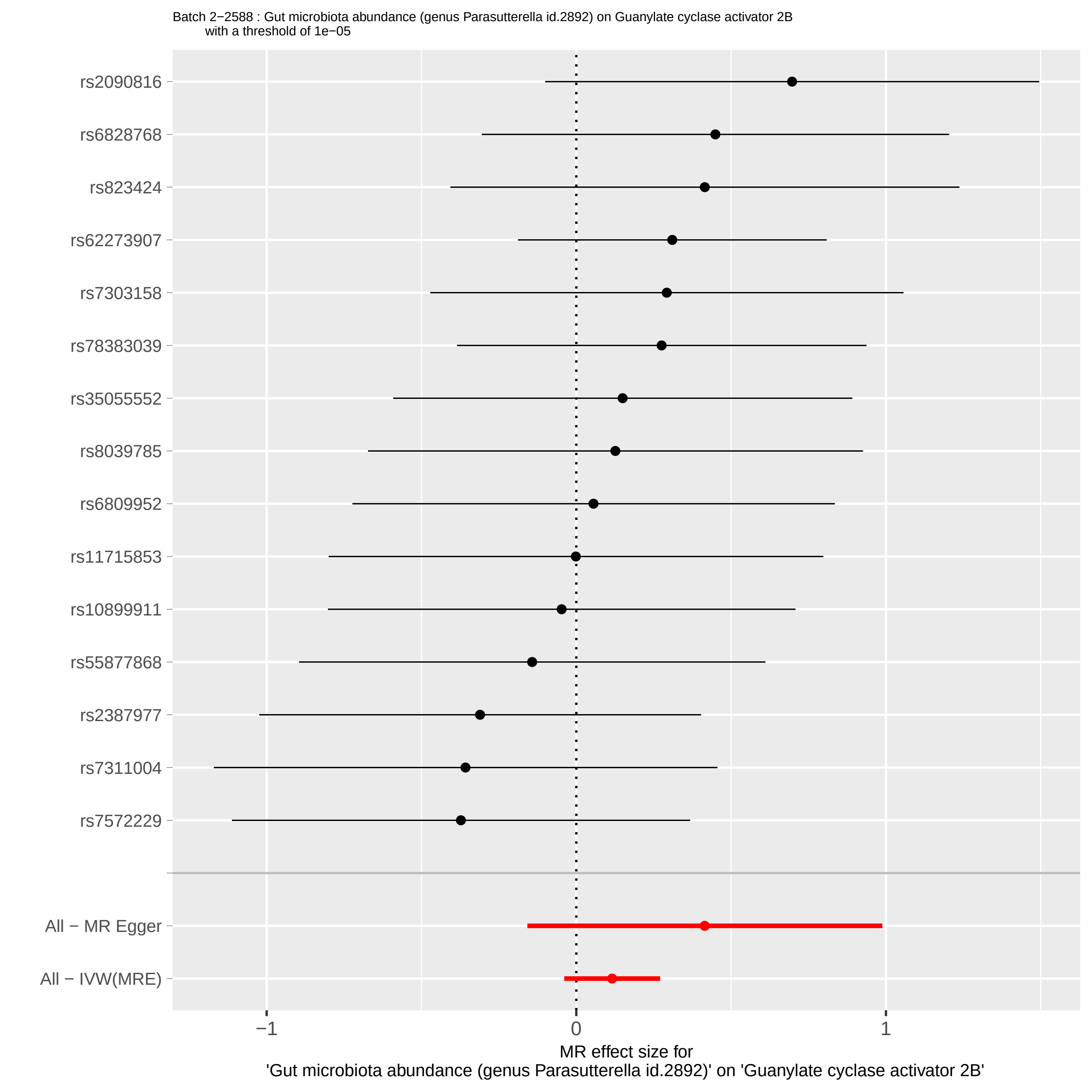
Batch 2−2588 : Gut microbiota abundance (genus Parasutterella id.2892) on Guanylate cyclase activator 2B
with a threshold of 1e−05
rs2090816
rs6828768
rs823424
rs62273907
rs7303158
rs78383039
rs35055552
rs8039785
rs6809952
rs11715853
rs10899911
rs55877868
rs2387977
rs7311004
rs7572229
All − MR Egger
All − IVW(MRE)
−1
0
1
MR effect size for
'Gut microbiota abundance (genus Parasutterella id.2892)' on 'Guanylate cyclase activator 2B'

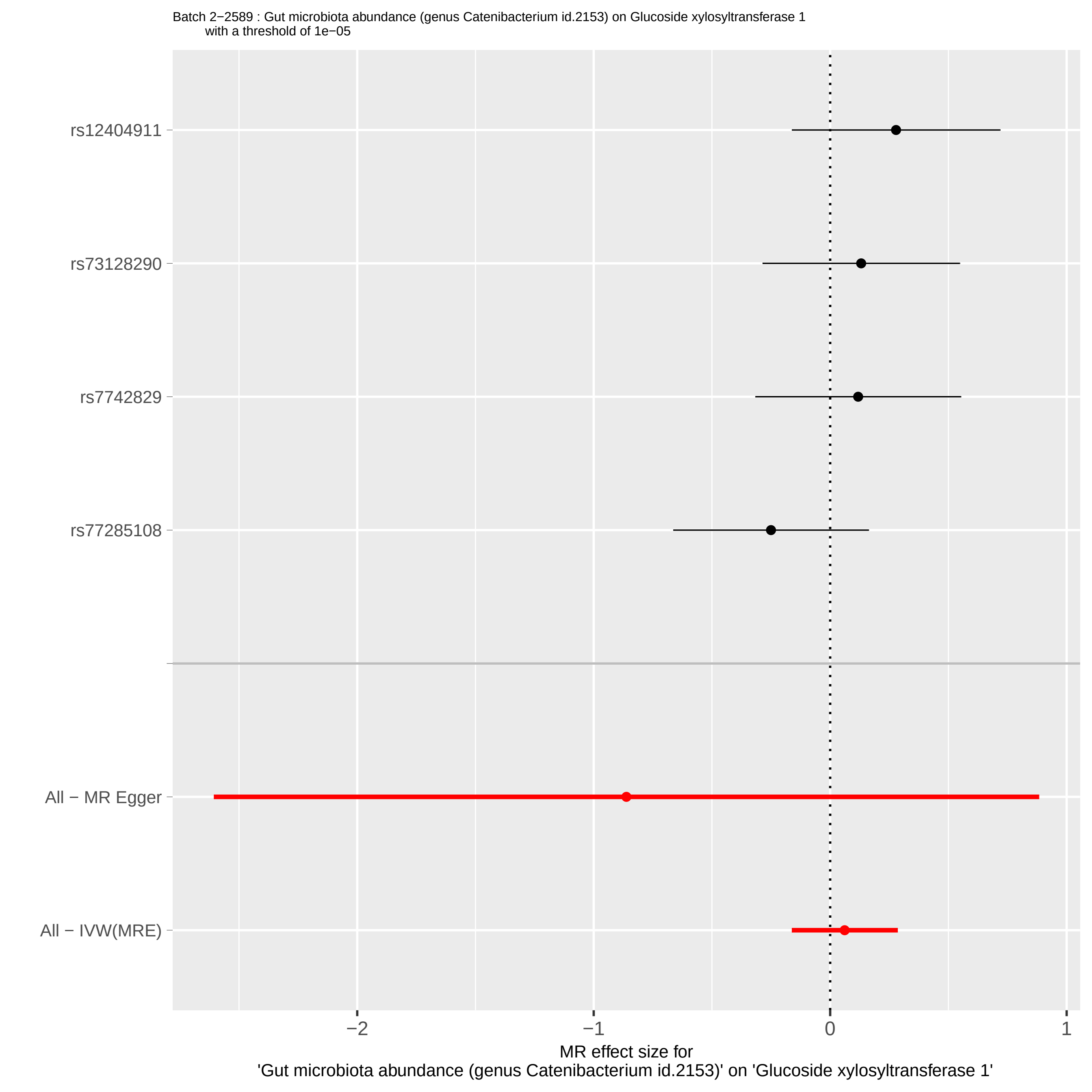

Batch 2−2589 : Gut microbiota abundance (genus Catenibacterium id.2153) on Glucoside xylosyltransferase 1
with a threshold of 1e−05
rs12404911
rs73128290
rs7742829
rs77285108
All − MR Egger
All − IVW(MRE)
−2
−1
0
1
MR effect size for
'Gut microbiota abundance (genus Catenibacterium id.2153)' on 'Glucoside xylosyltransferase 1'

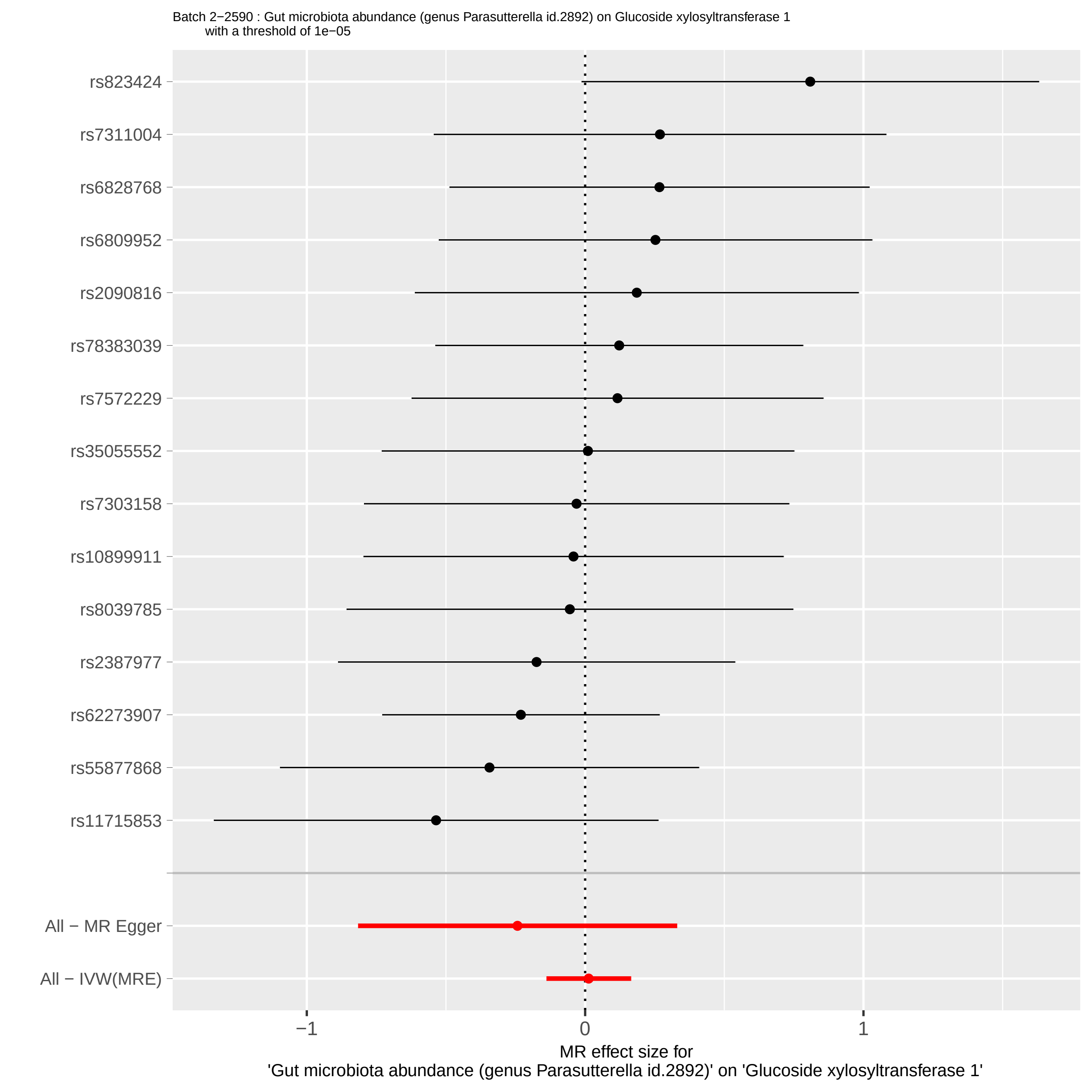

Batch 2−2590 : Gut microbiota abundance (genus Parasutterella id.2892) on Glucoside xylosyltransferase 1
with a threshold of 1e−05
rs823424
rs7311004
rs6828768
rs6809952
rs2090816
rs78383039
rs7572229
rs35055552
rs7303158
rs10899911
rs8039785
rs2387977
rs62273907
rs55877868
rs11715853
All − MR Egger
All − IVW(MRE)
−1
0
1
MR effect size for
'Gut microbiota abundance (genus Parasutterella id.2892)' on 'Glucoside xylosyltransferase 1'

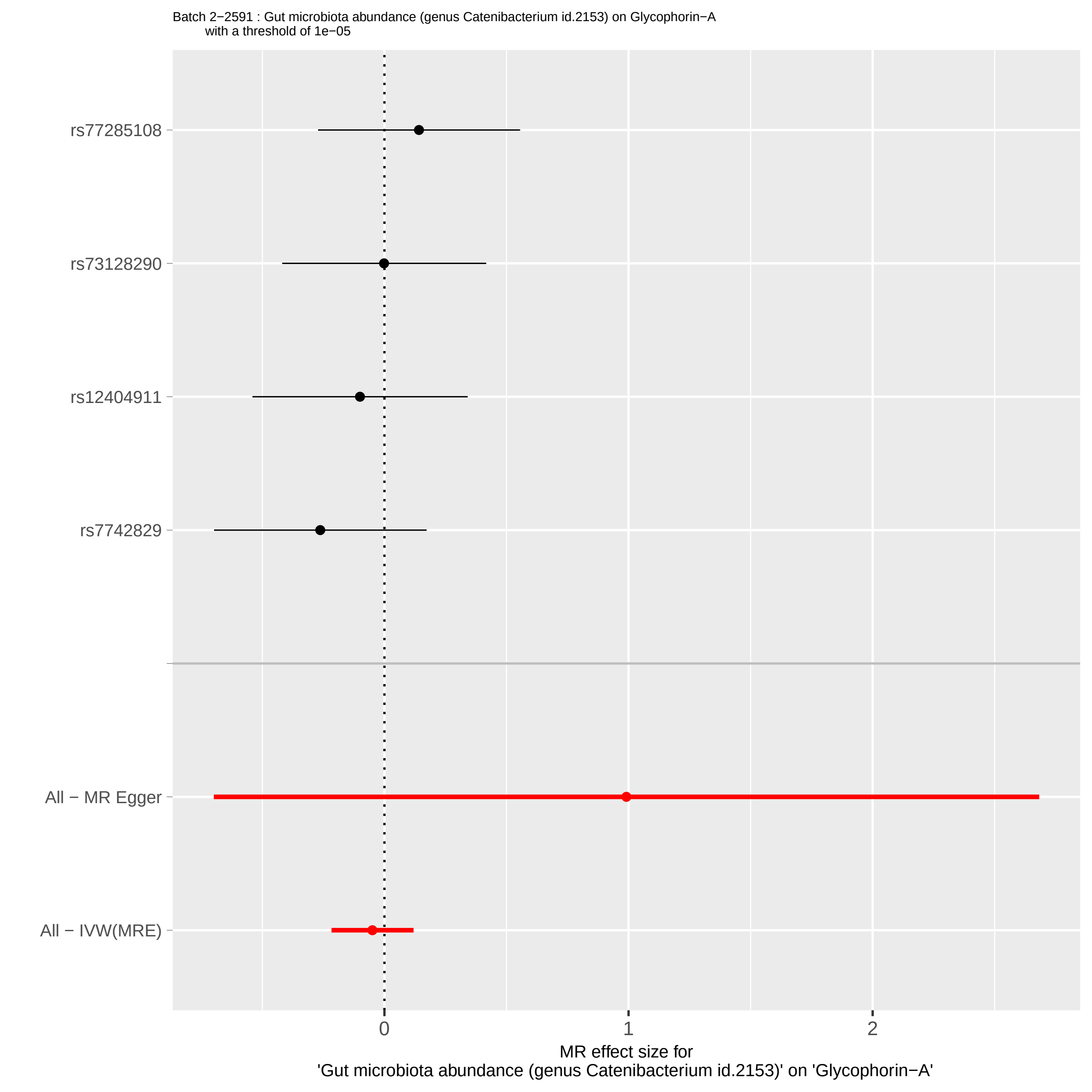

Batch 2−2591 : Gut microbiota abundance (genus Catenibacterium id.2153) on Glycophorin−A
with a threshold of 1e−05
rs77285108
rs73128290
rs12404911
rs7742829
All − MR Egger
All − IVW(MRE)
0
1
2
MR effect size for
'Gut microbiota abundance (genus Catenibacterium id.2153)' on 'Glycophorin−A'

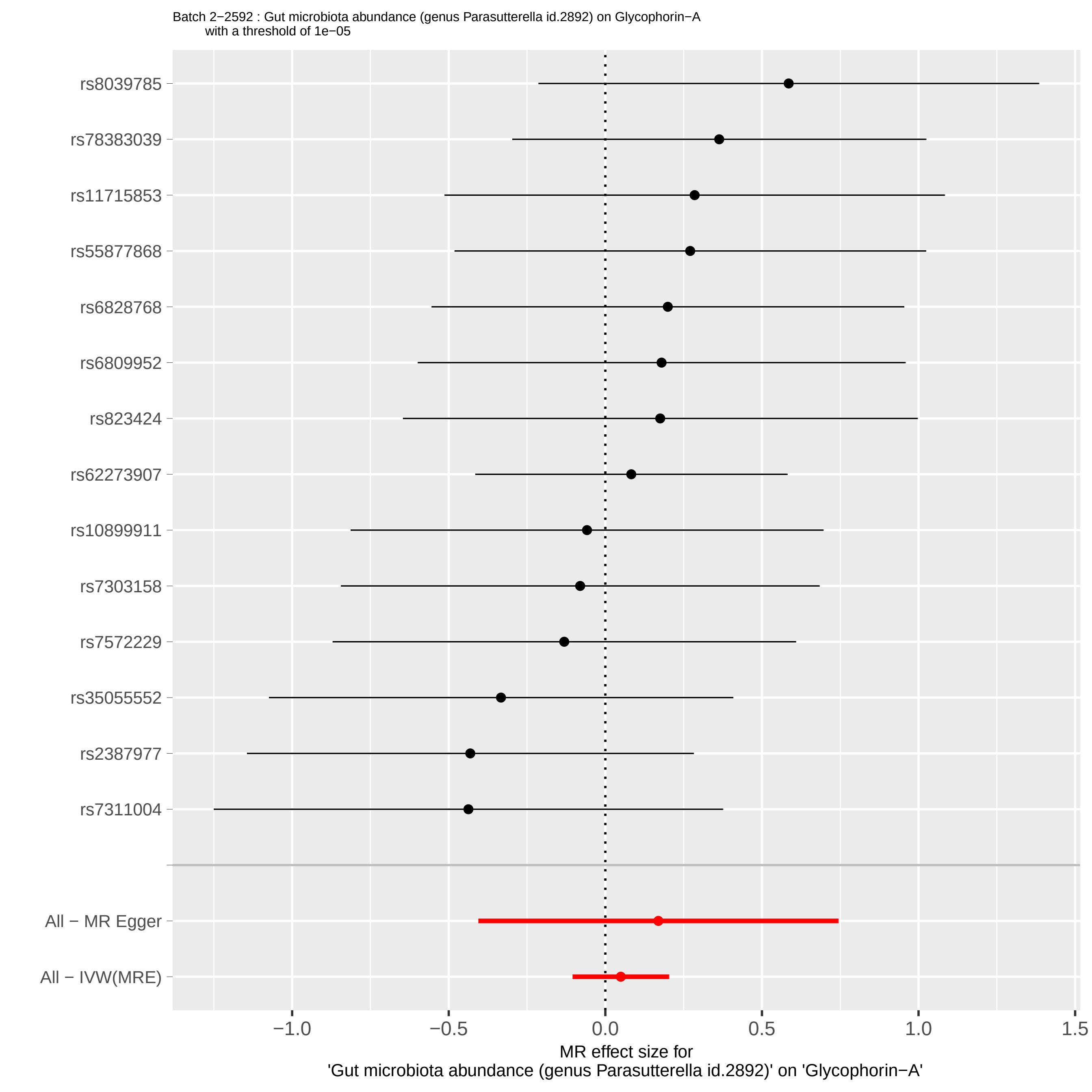

Batch 2−2592 : Gut microbiota abundance (genus Parasutterella id.2892) on Glycophorin−A
with a threshold of 1e−05
rs8039785
rs78383039
rs11715853
rs55877868
rs6828768
rs6809952
rs823424
rs62273907
rs10899911
rs7303158
rs7572229
rs35055552
rs2387977
rs7311004
All − MR Egger
All − IVW(MRE)
−1.0
−0.5
0.0
0.5
1.0
1.5
MR effect size for
'Gut microbiota abundance (genus Parasutterella id.2892)' on 'Glycophorin−A'

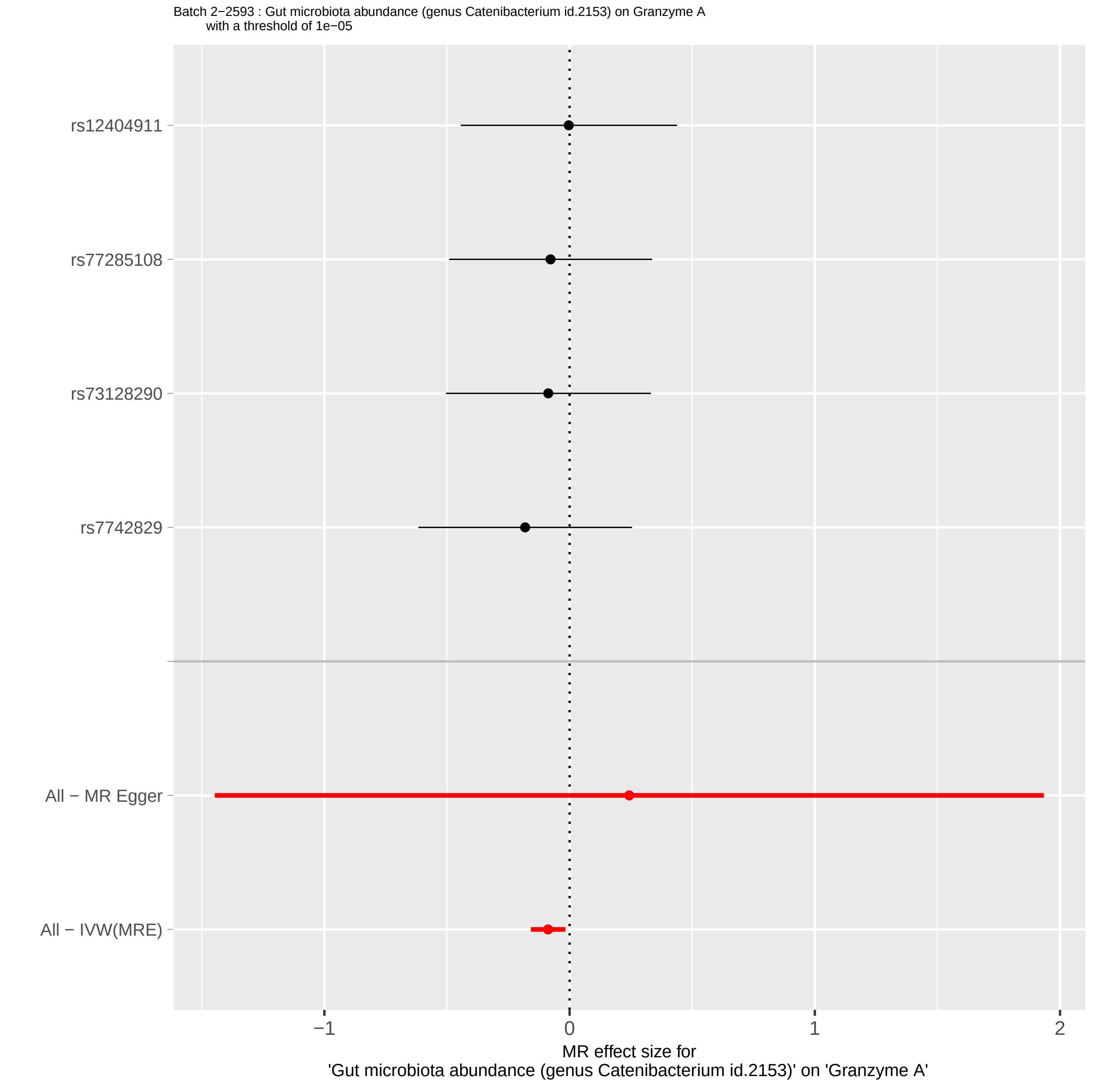
Batch 2−2593 : Gut microbiota abundance (genus Catenibacterium id.2153) on Granzyme A
with a threshold of 1e−05
rs12404911
rs77285108
rs73128290
rs7742829
All − MR Egger
All − IVW(MRE)
−1
0
1
2
MR effect size for
'Gut microbiota abundance (genus Catenibacterium id.2153)' on 'Granzyme A'

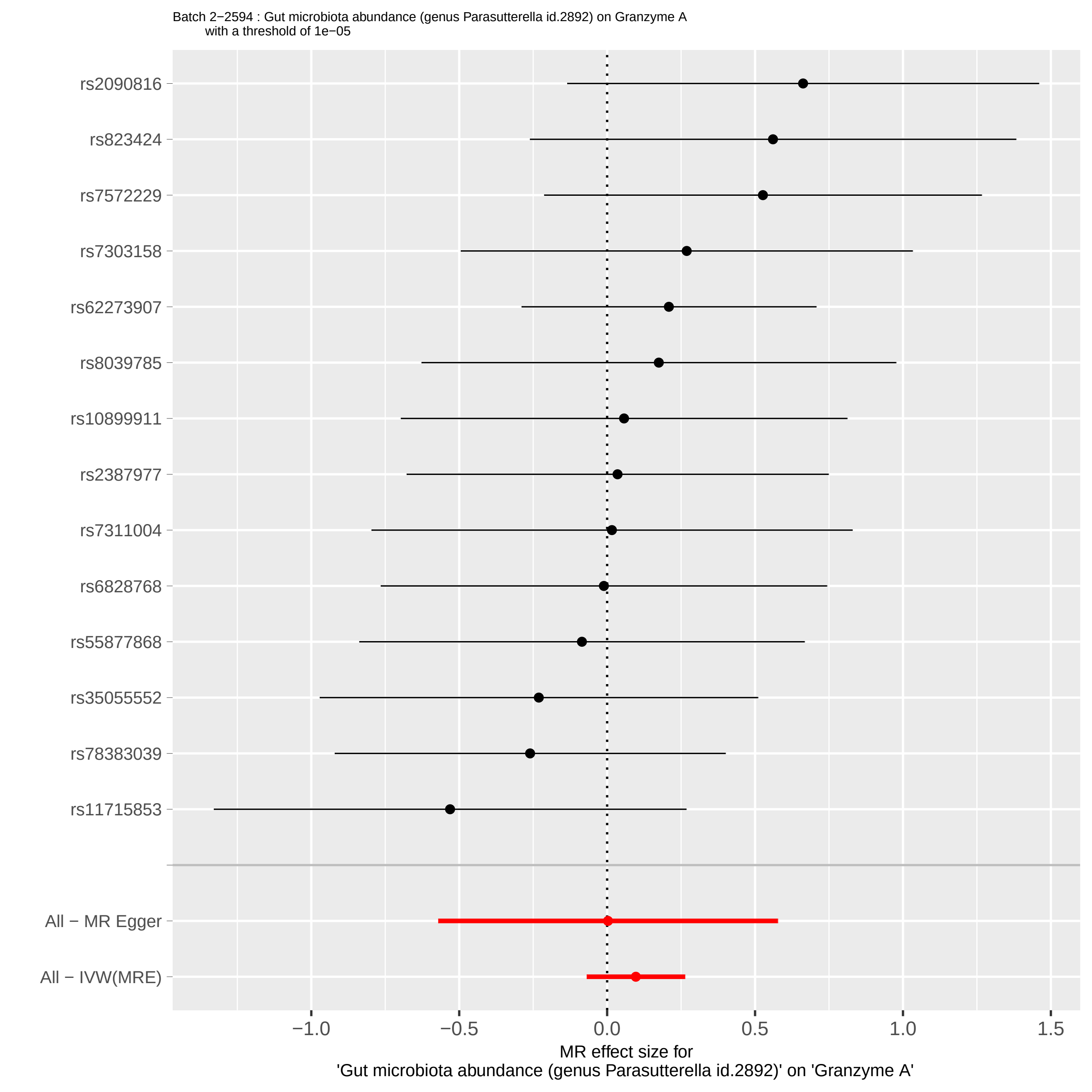

Batch 2−2594 : Gut microbiota abundance (genus Parasutterella id.2892) on Granzyme A
with a threshold of 1e−05
rs2090816
rs823424
rs7572229
rs7303158
rs62273907
rs8039785
rs10899911
rs2387977
rs7311004
rs6828768
rs55877868
rs35055552
rs78383039
rs11715853
All − MR Egger
All − IVW(MRE)
−1.0
−0.5
0.0
0.5
1.0
1.5
MR effect size for
'Gut microbiota abundance (genus Parasutterella id.2892)' on 'Granzyme A'

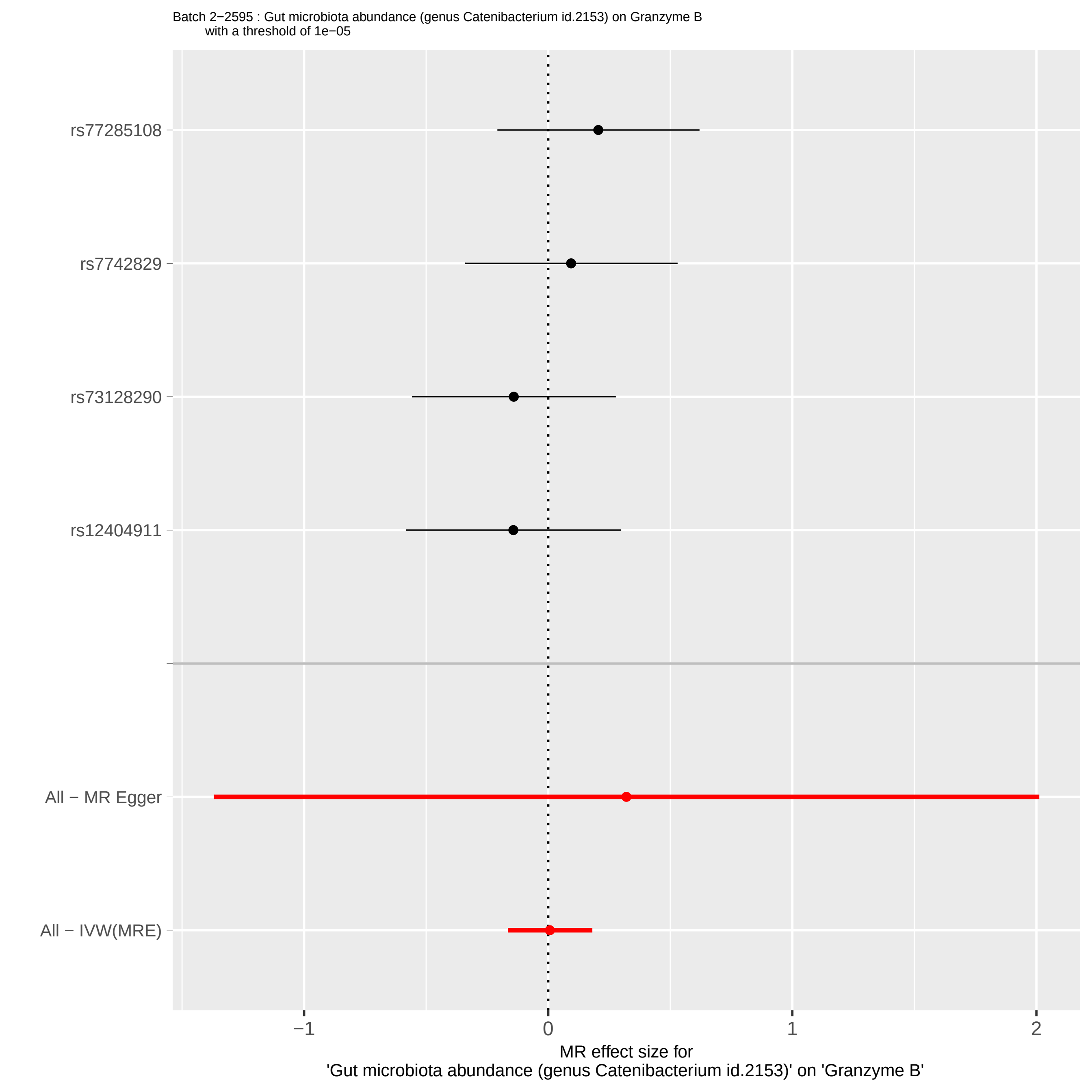
Batch 2−2595 : Gut microbiota abundance (genus Catenibacterium id.2153) on Granzyme B
with a threshold of 1e−05
rs77285108
rs7742829
rs73128290
rs12404911
All − MR Egger
All − IVW(MRE)
−1
0
1
2
MR effect size for
'Gut microbiota abundance (genus Catenibacterium id.2153)' on 'Granzyme B'

Batch 2−2596 : Gut microbiota abundance (genus Parasutterella id.2892) on Granzyme B
with a threshold of 1e−05

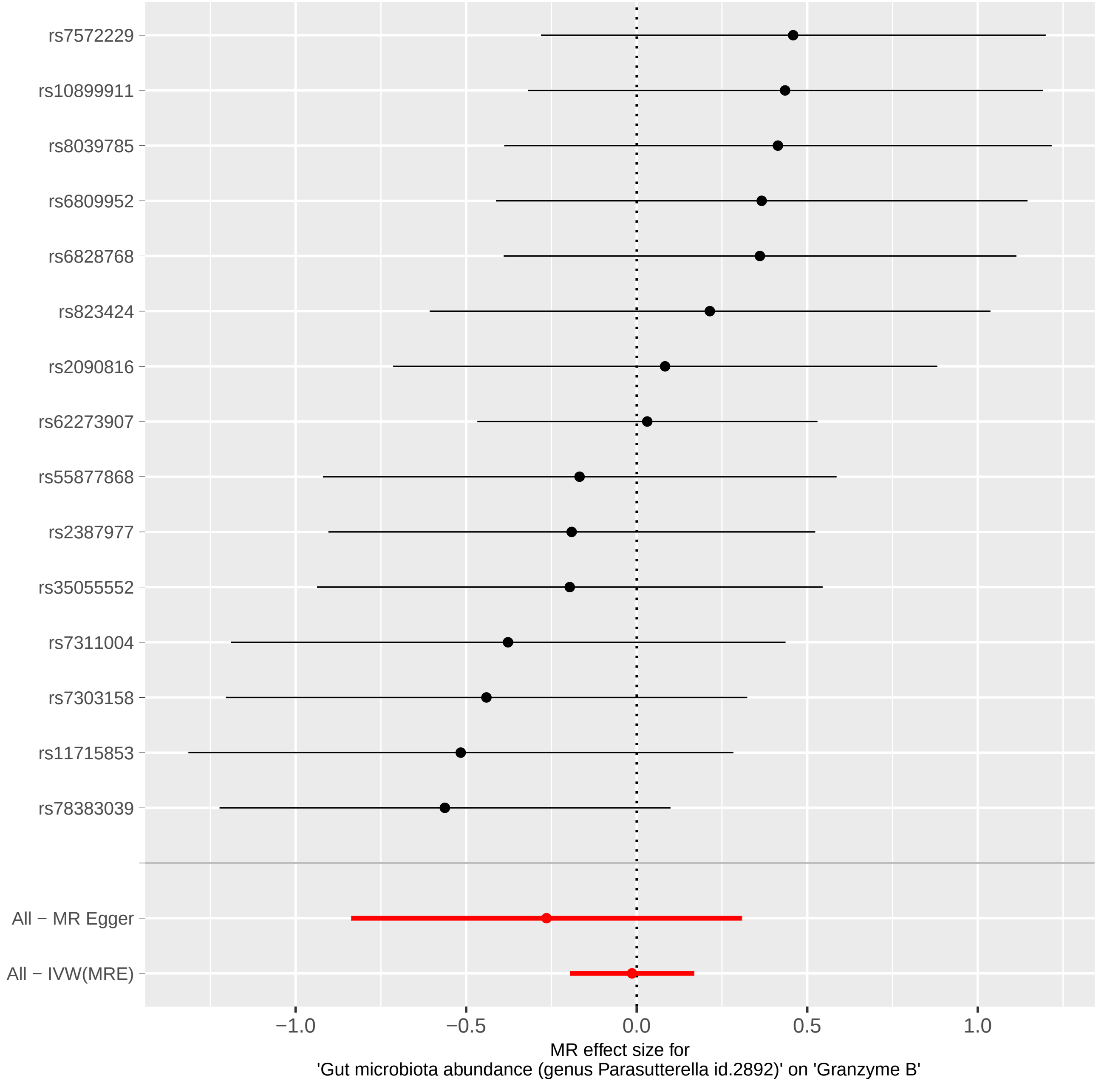

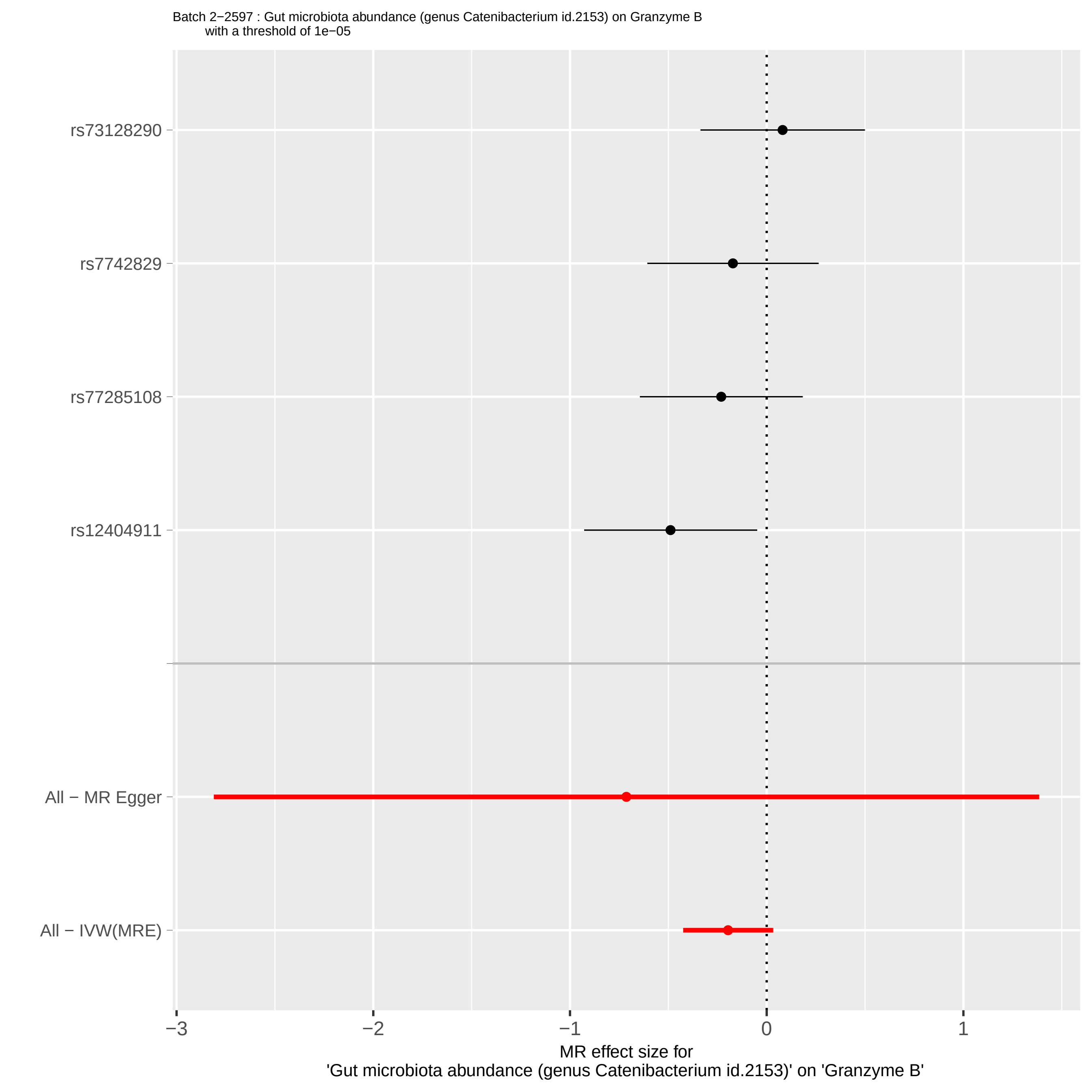
Batch 2−2597 : Gut microbiota abundance (genus Catenibacterium id.2153) on Granzyme B
with a threshold of 1e−05
rs73128290
rs7742829
rs77285108
rs12404911
All − MR Egger
All − IVW(MRE)
−3
−2
−1
0
1
MR effect size for
'Gut microbiota abundance (genus Catenibacterium id.2153)' on 'Granzyme B'

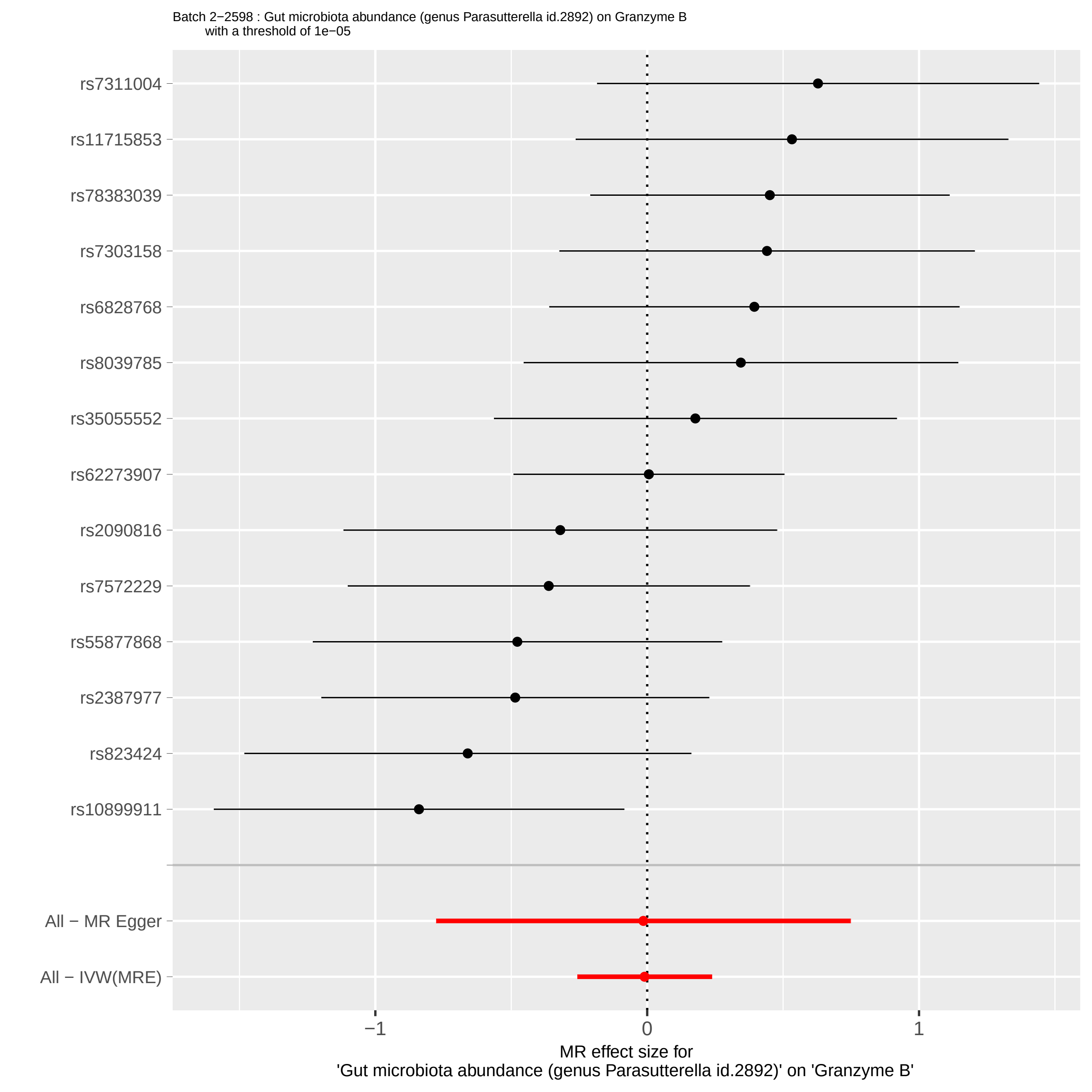

Batch 2−2598 : Gut microbiota abundance (genus Parasutterella id.2892) on Granzyme B
with a threshold of 1e−05
rs7311004
rs11715853
rs78383039
rs7303158
rs6828768
rs8039785
rs35055552
rs62273907
rs2090816
rs7572229
rs55877868
rs2387977
rs823424
rs10899911
All − MR Egger
All − IVW(MRE)
−1
0
1
MR effect size for
'Gut microbiota abundance (genus Parasutterella id.2892)' on 'Granzyme B'

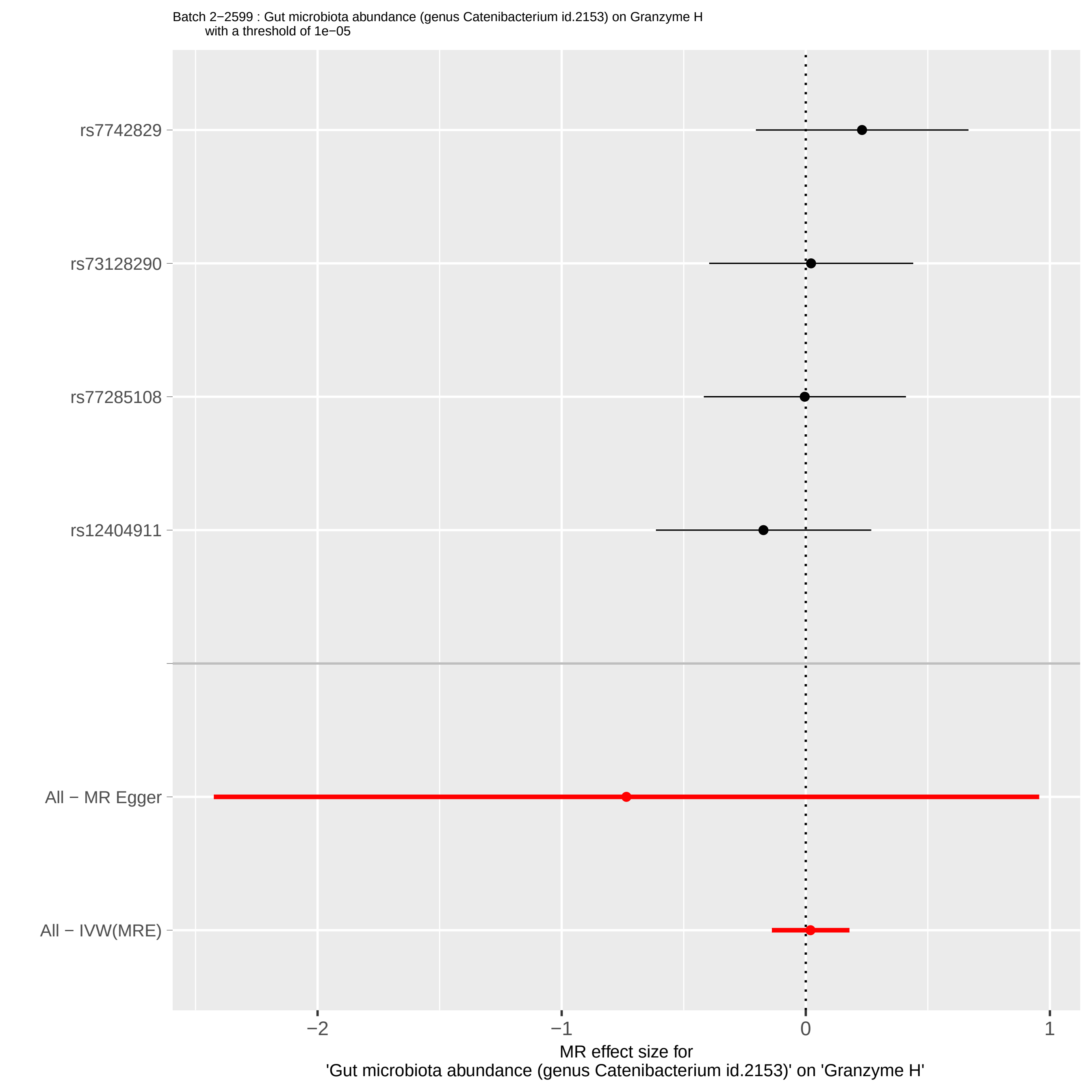

Batch 2−2599 : Gut microbiota abundance (genus Catenibacterium id.2153) on Granzyme H
with a threshold of 1e−05
rs7742829
rs73128290
rs77285108
rs12404911
All − MR Egger
All − IVW(MRE)
−2
−1
0
1
MR effect size for
'Gut microbiota abundance (genus Catenibacterium id.2153)' on 'Granzyme H'

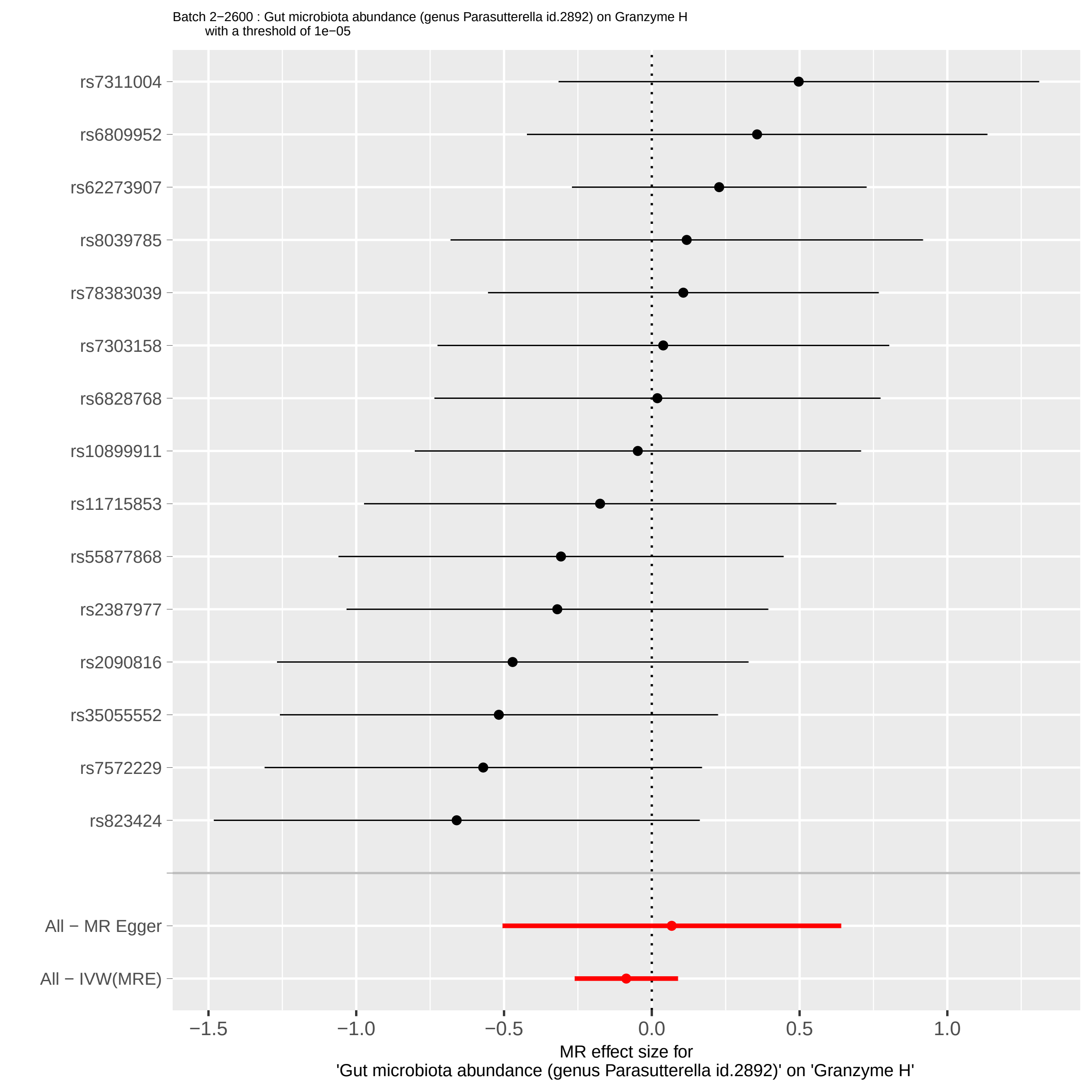
Batch 2−2600 : Gut microbiota abundance (genus Parasutterella id.2892) on Granzyme H
with a threshold of 1e−05
rs7311004
rs6809952
rs62273907
rs8039785
rs78383039
rs7303158
rs6828768
rs10899911
rs11715853
rs55877868
rs2387977
rs2090816
rs35055552
rs7572229
rs823424
All − MR Egger
All − IVW(MRE)
−1.5
−1.0
−0.5
0.0
0.5
1.0
MR effect size for
'Gut microbiota abundance (genus Parasutterella id.2892)' on 'Granzyme H'

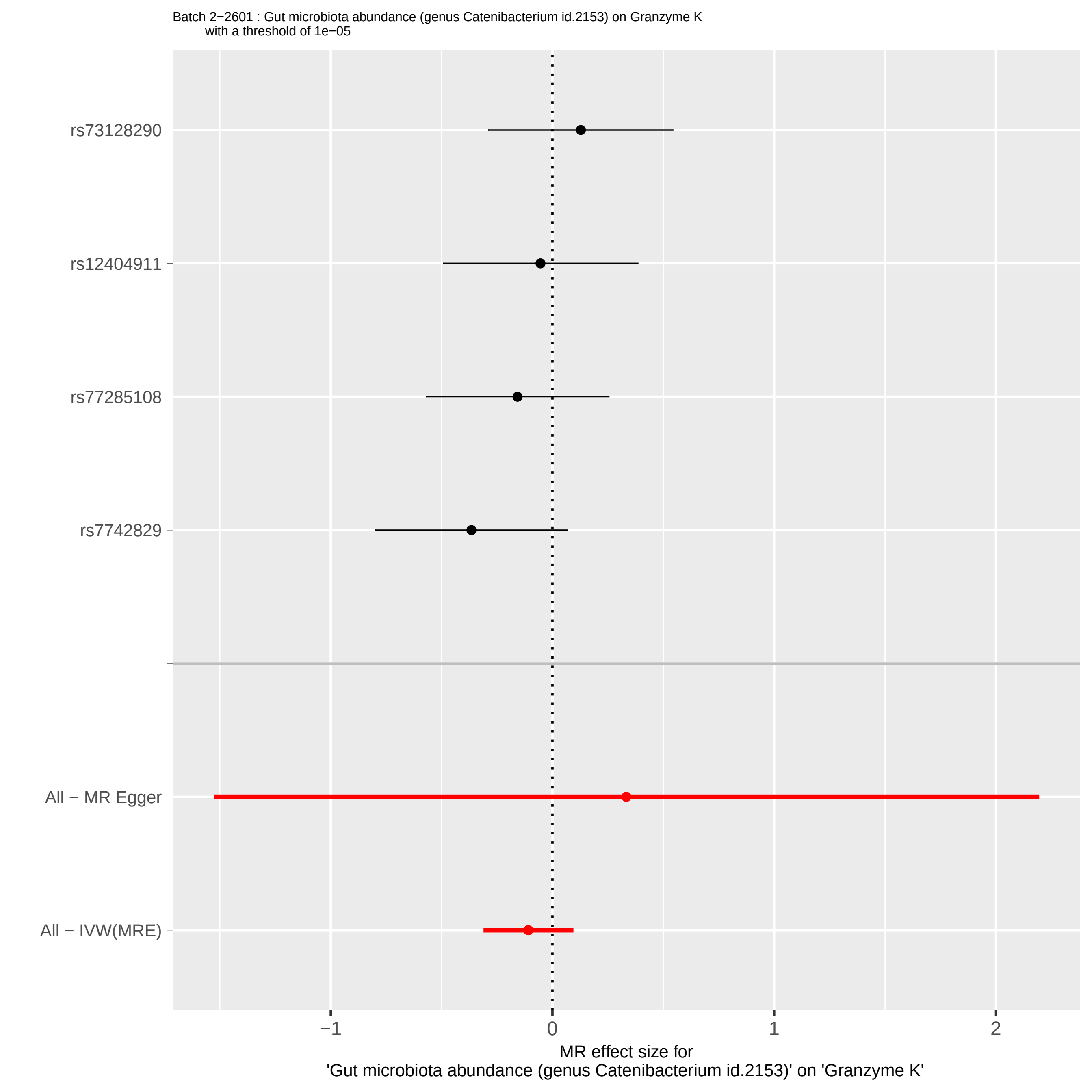

Batch 2−2601 : Gut microbiota abundance (genus Catenibacterium id.2153) on Granzyme K
with a threshold of 1e−05
rs73128290
rs12404911
rs77285108
rs7742829
All − MR Egger
All − IVW(MRE)
−1
0
1
2
MR effect size for
'Gut microbiota abundance (genus Catenibacterium id.2153)' on 'Granzyme K'

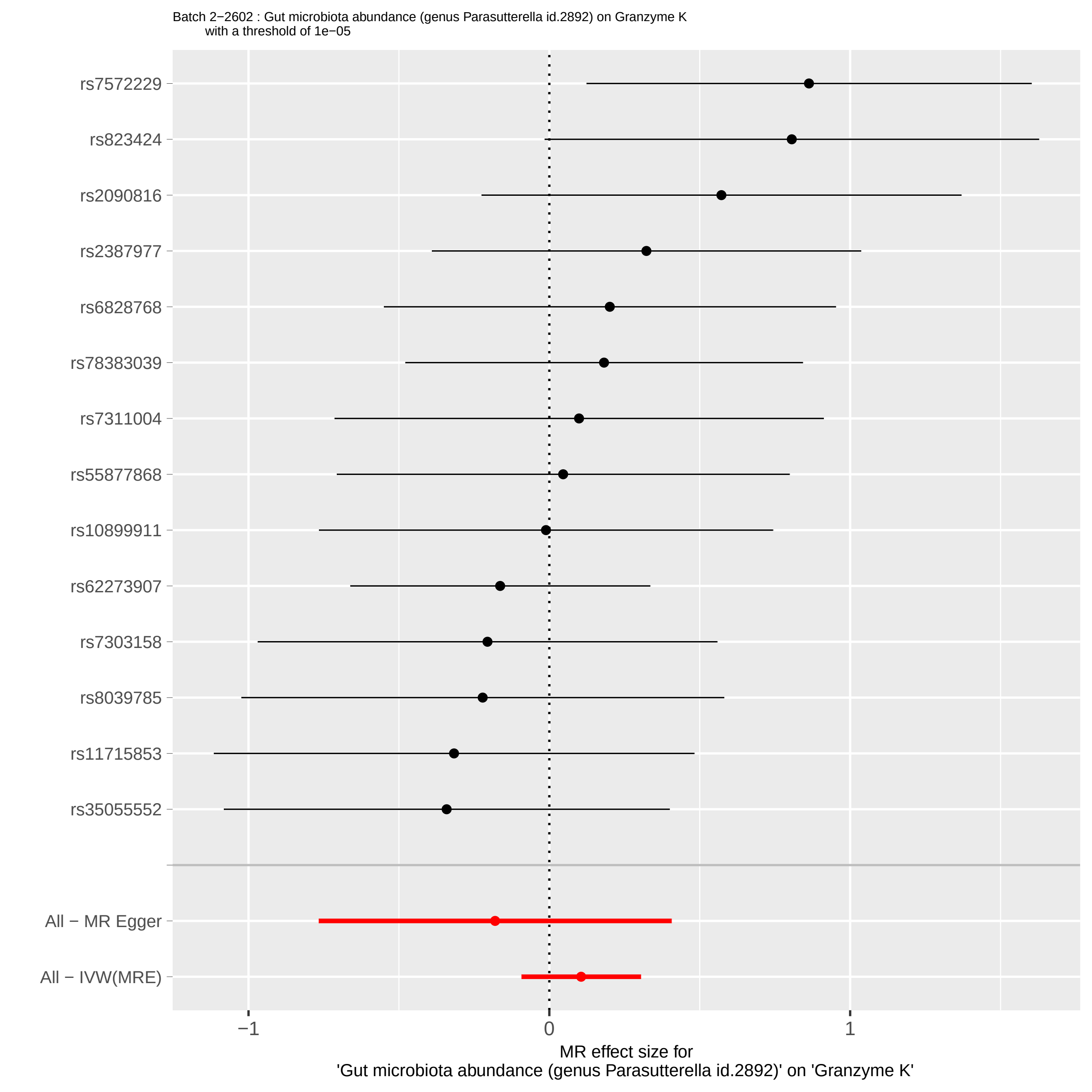

Batch 2−2602 : Gut microbiota abundance (genus Parasutterella id.2892) on Granzyme K
with a threshold of 1e−05
rs7572229
rs823424
rs2090816
rs2387977
rs6828768
rs78383039
rs7311004
rs55877868
rs10899911
rs62273907
rs7303158
rs8039785
rs11715853
rs35055552
All − MR Egger
All − IVW(MRE)
−1
0
1
MR effect size for
'Gut microbiota abundance (genus Parasutterella id.2892)' on 'Granzyme K'

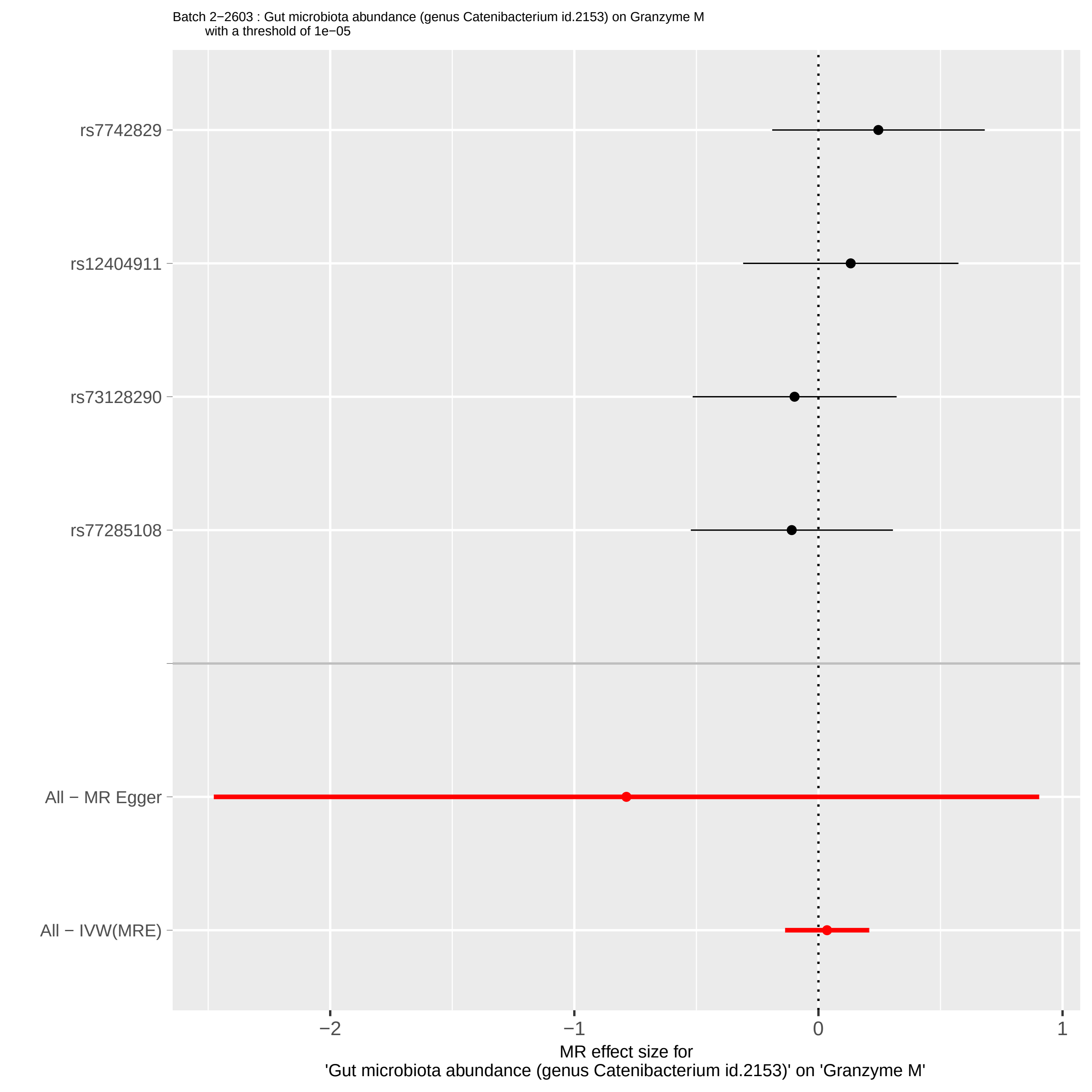
Batch 2−2603 : Gut microbiota abundance (genus Catenibacterium id.2153) on Granzyme M
with a threshold of 1e−05
rs7742829
rs12404911
rs73128290
rs77285108
All − MR Egger
All − IVW(MRE)
−2
−1
0
1
MR effect size for
'Gut microbiota abundance (genus Catenibacterium id.2153)' on 'Granzyme M'

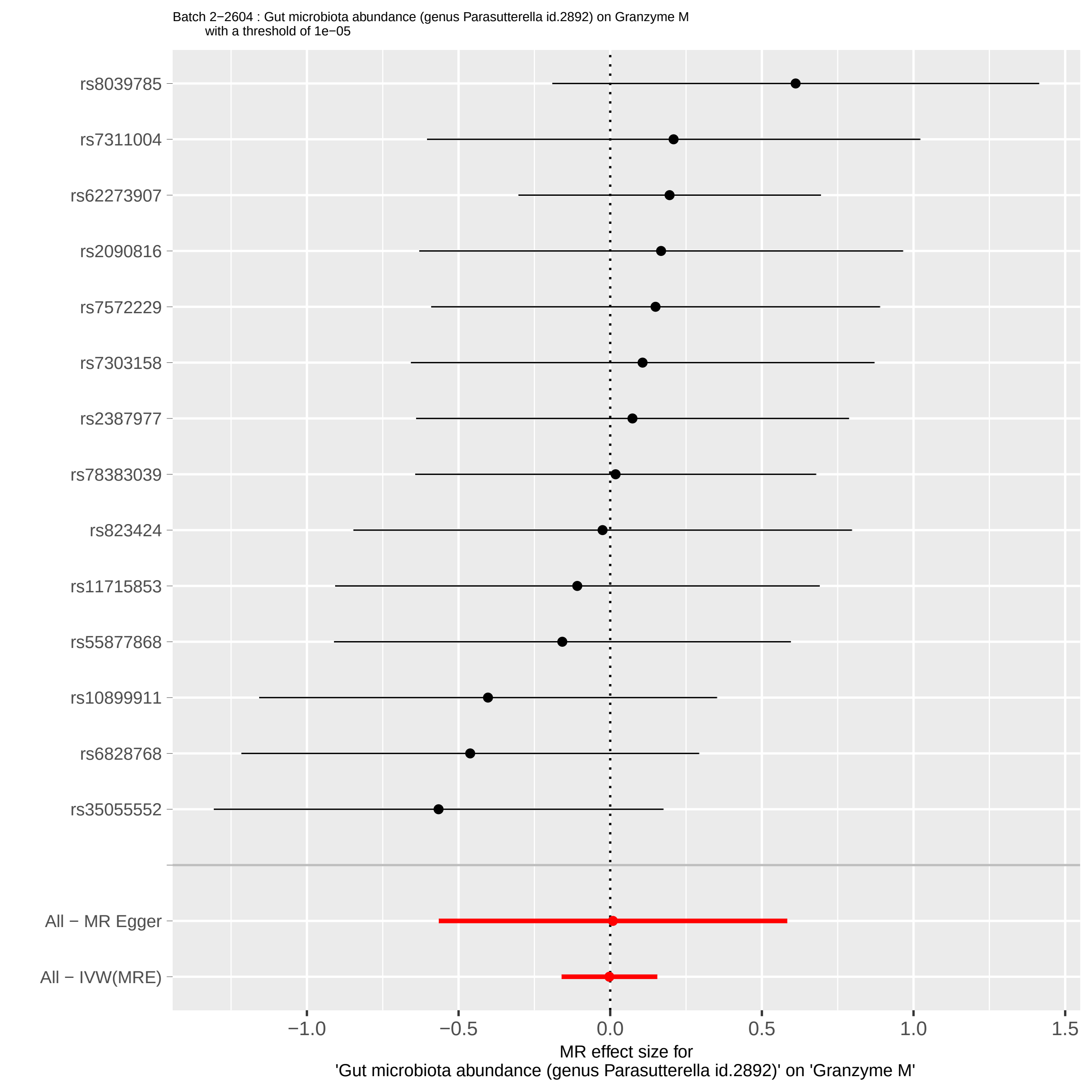
Batch 2−2604 : Gut microbiota abundance (genus Parasutterella id.2892) on Granzyme M
with a threshold of 1e−05
rs8039785
rs7311004
rs62273907
rs2090816
rs7572229
rs7303158
rs2387977
rs78383039
rs823424
rs11715853
rs55877868
rs10899911
rs6828768
rs35055552
All − MR Egger
All − IVW(MRE)
−1.0
−0.5
0.0
0.5
1.0
1.5
MR effect size for
'Gut microbiota abundance (genus Parasutterella id.2892)' on 'Granzyme M'

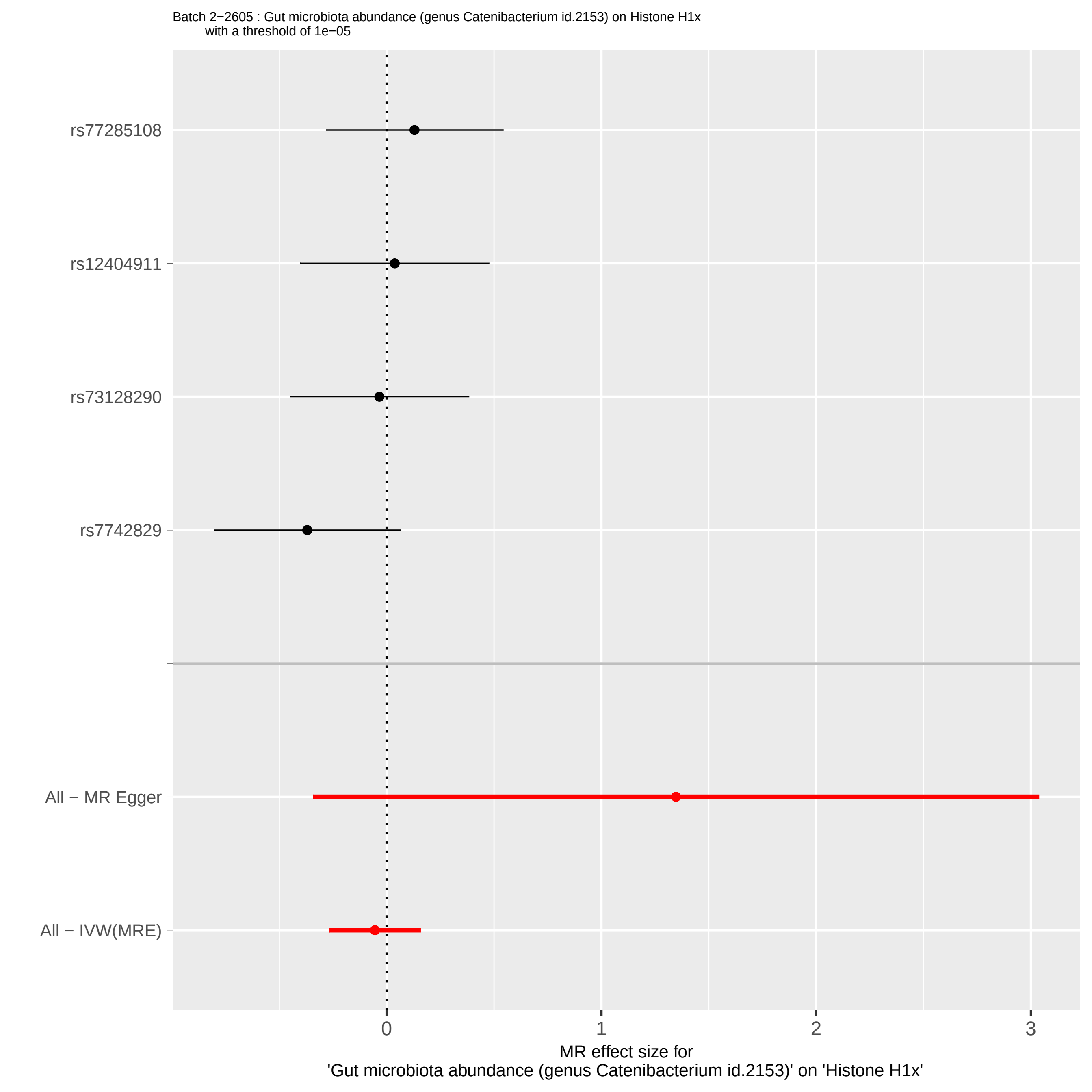

Batch 2−2605 : Gut microbiota abundance (genus Catenibacterium id.2153) on Histone H1x
with a threshold of 1e−05
rs77285108
rs12404911
rs73128290
rs7742829
All − MR Egger
All − IVW(MRE)
0
1
2
3
MR effect size for
'Gut microbiota abundance (genus Catenibacterium id.2153)' on 'Histone H1x'

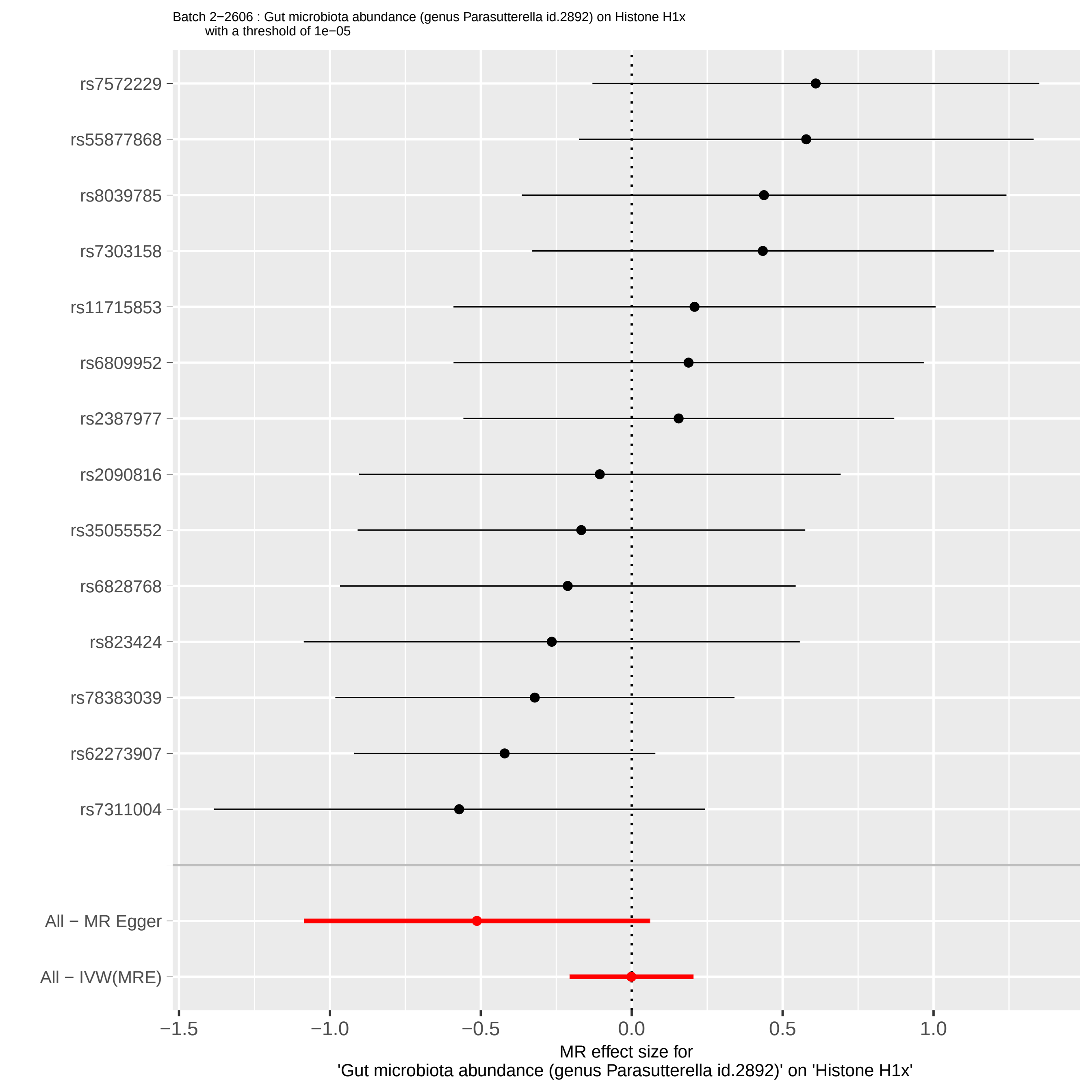

Batch 2−2606 : Gut microbiota abundance (genus Parasutterella id.2892) on Histone H1x
with a threshold of 1e−05
rs7572229
rs55877868
rs8039785
rs7303158
rs11715853
rs6809952
rs2387977
rs2090816
rs35055552
rs6828768
rs823424
rs78383039
rs62273907
rs7311004
All − MR Egger
All − IVW(MRE)
−1.5
−1.0
−0.5
0.0
0.5
1.0
MR effect size for
'Gut microbiota abundance (genus Parasutterella id.2892)' on 'Histone H1x'

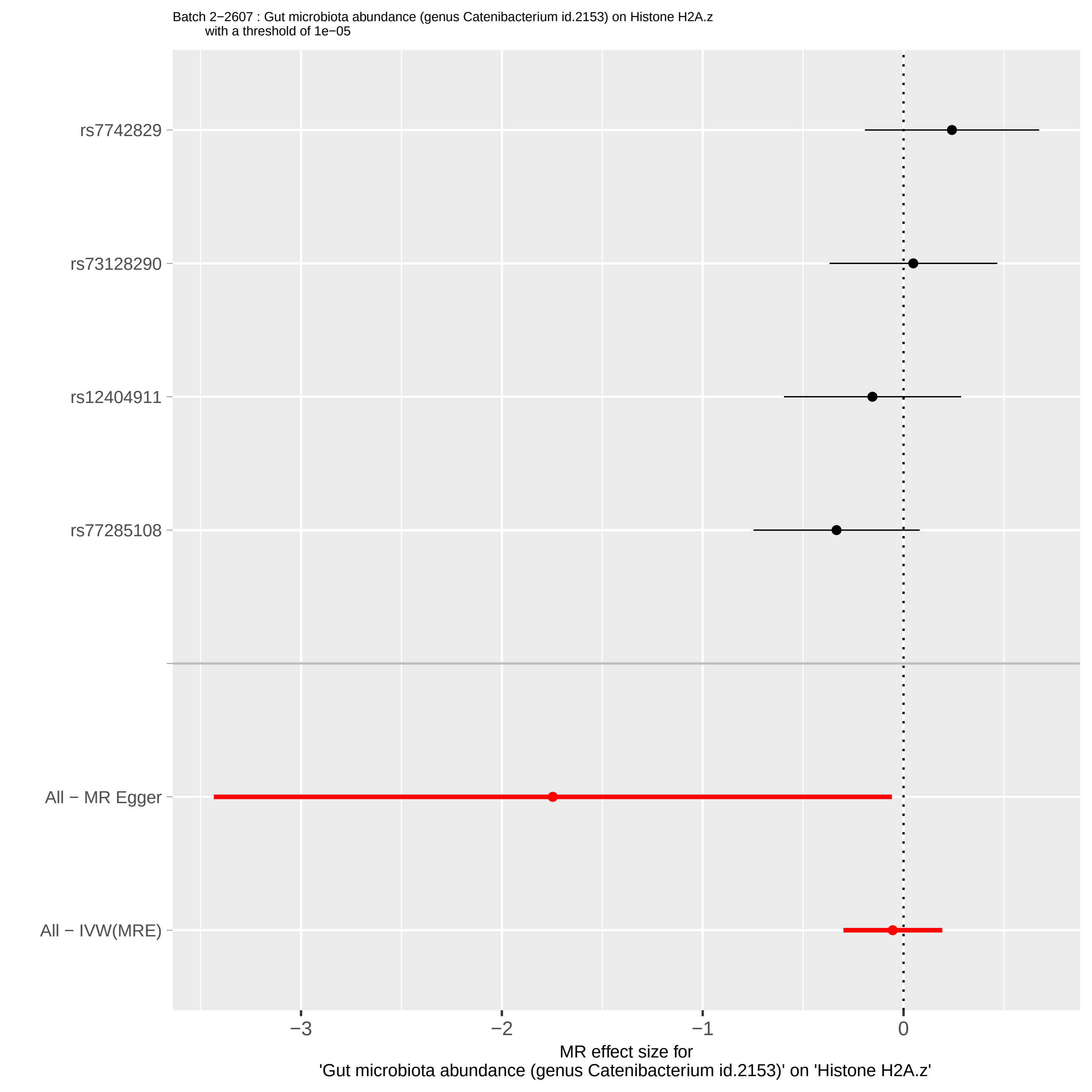
Batch 2−2607 : Gut microbiota abundance (genus Catenibacterium id.2153) on Histone H2A.z
with a threshold of 1e−05
rs7742829
rs73128290
rs12404911
rs77285108
All − MR Egger
All − IVW(MRE)
−3
−2
−1
0
MR effect size for
'Gut microbiota abundance (genus Catenibacterium id.2153)' on 'Histone H2A.z'

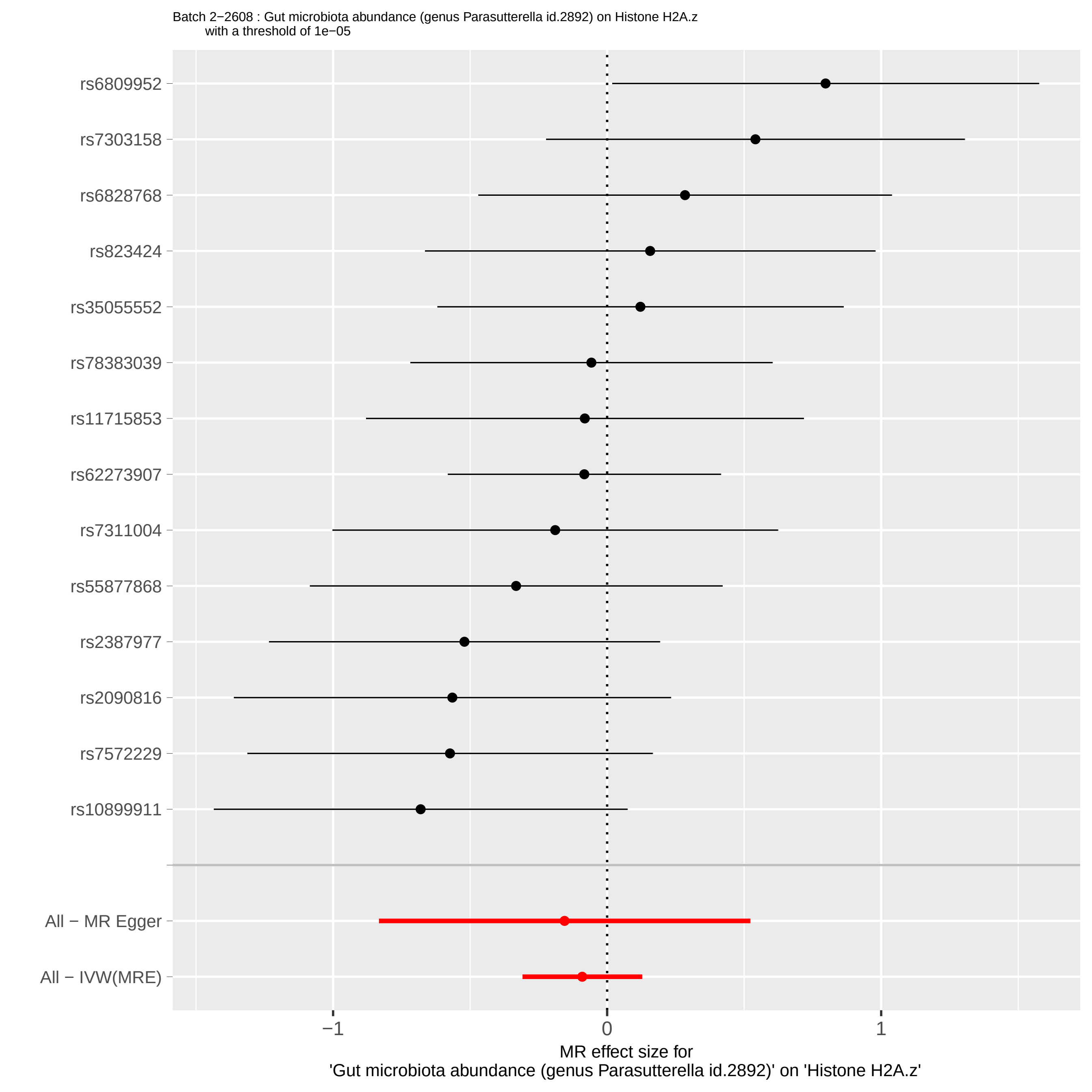
Batch 2−2608 : Gut microbiota abundance (genus Parasutterella id.2892) on Histone H2A.z
with a threshold of 1e−05
rs6809952
rs7303158
rs6828768
rs823424
rs35055552
rs78383039
rs11715853
rs62273907
rs7311004
rs55877868
rs2387977
rs2090816
rs7572229
rs10899911
All − MR Egger
All − IVW(MRE)
−1
0
1
MR effect size for
'Gut microbiota abundance (genus Parasutterella id.2892)' on 'Histone H2A.z'

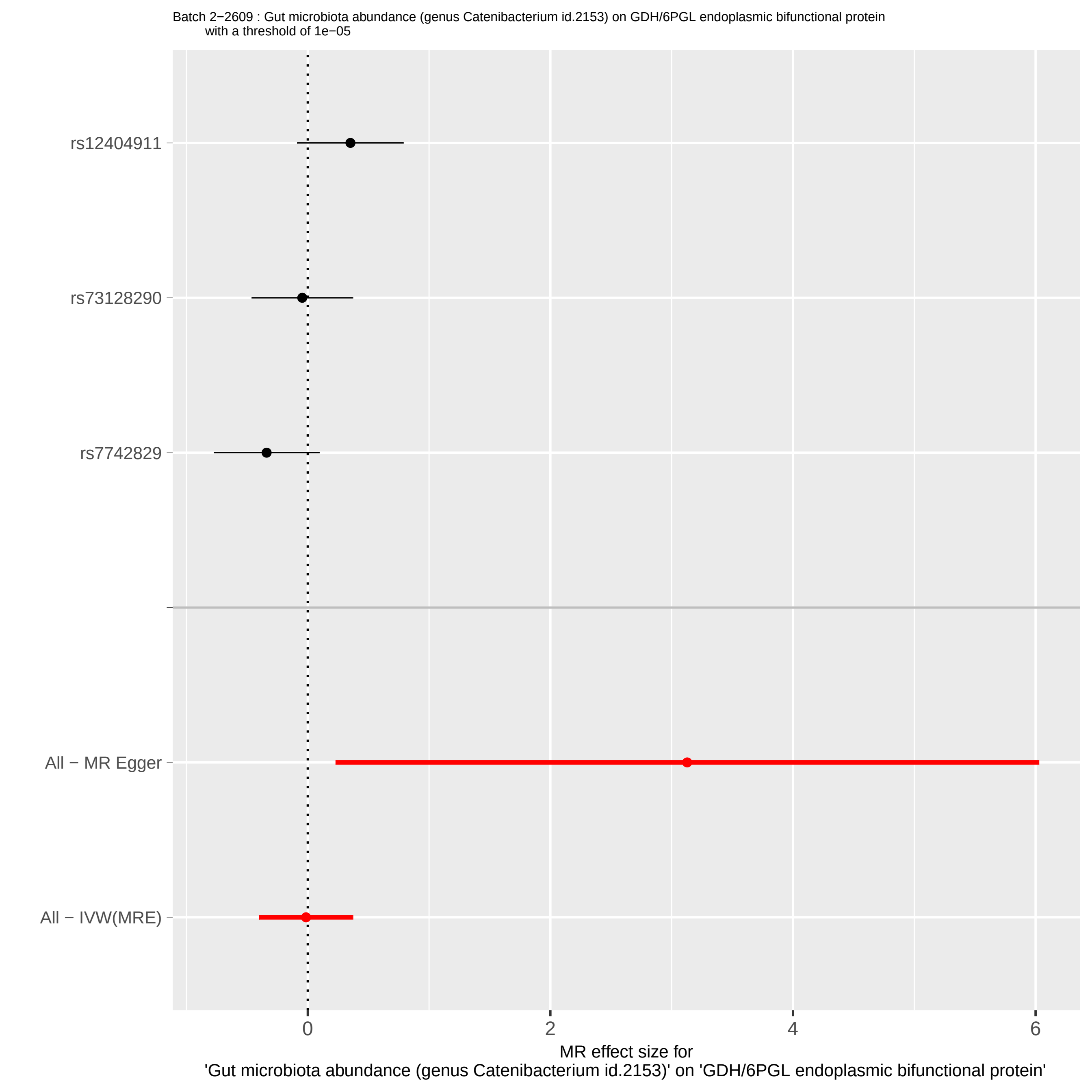

Batch 2−2609 : Gut microbiota abundance (genus Catenibacterium id.2153) on GDH/6PGL endoplasmic bifunctional protein
with a threshold of 1e−05
rs12404911
rs73128290
rs7742829
All − MR Egger
All − IVW(MRE)
0
2
4
6
MR effect size for
'Gut microbiota abundance (genus Catenibacterium id.2153)' on 'GDH/6PGL endoplasmic bifunctional protein'

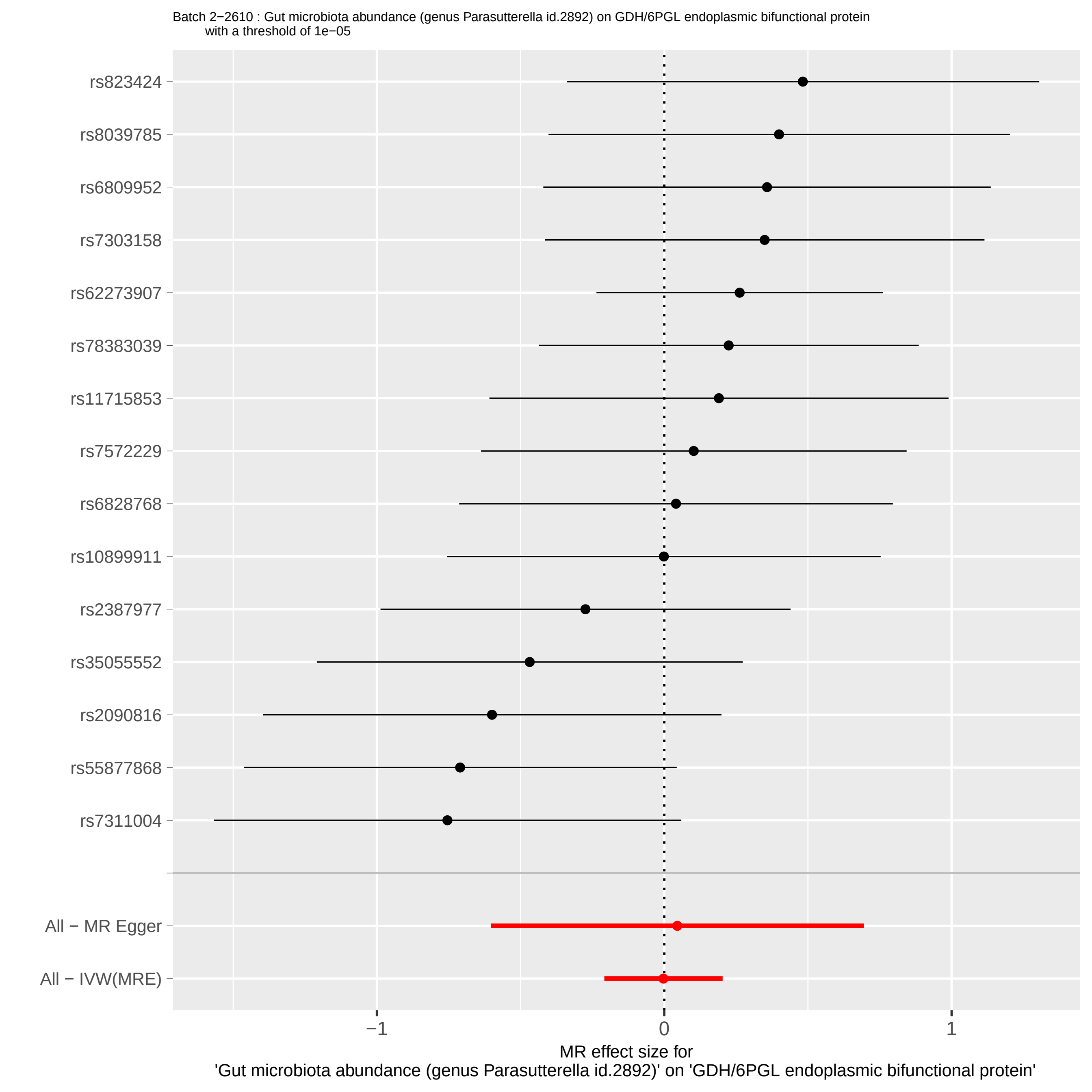
Batch 2−2610 : Gut microbiota abundance (genus Parasutterella id.2892) on GDH/6PGL endoplasmic bifunctional protein
with a threshold of 1e−05
rs823424
rs8039785
rs6809952
rs7303158
rs62273907
rs78383039
rs11715853
rs7572229
rs6828768
rs10899911
rs2387977
rs35055552
rs2090816
rs55877868
rs7311004
All − MR Egger
All − IVW(MRE)
−1
0
1
MR effect size for
'Gut microbiota abundance (genus Parasutterella id.2892)' on 'GDH/6PGL endoplasmic bifunctional protein'

Batch 2−2611 : Gut microbiota abundance (genus Catenibacterium id.2153) on 3−hydroxyanthranilate 3,4−dioxygenase
with a threshold of 1e−05

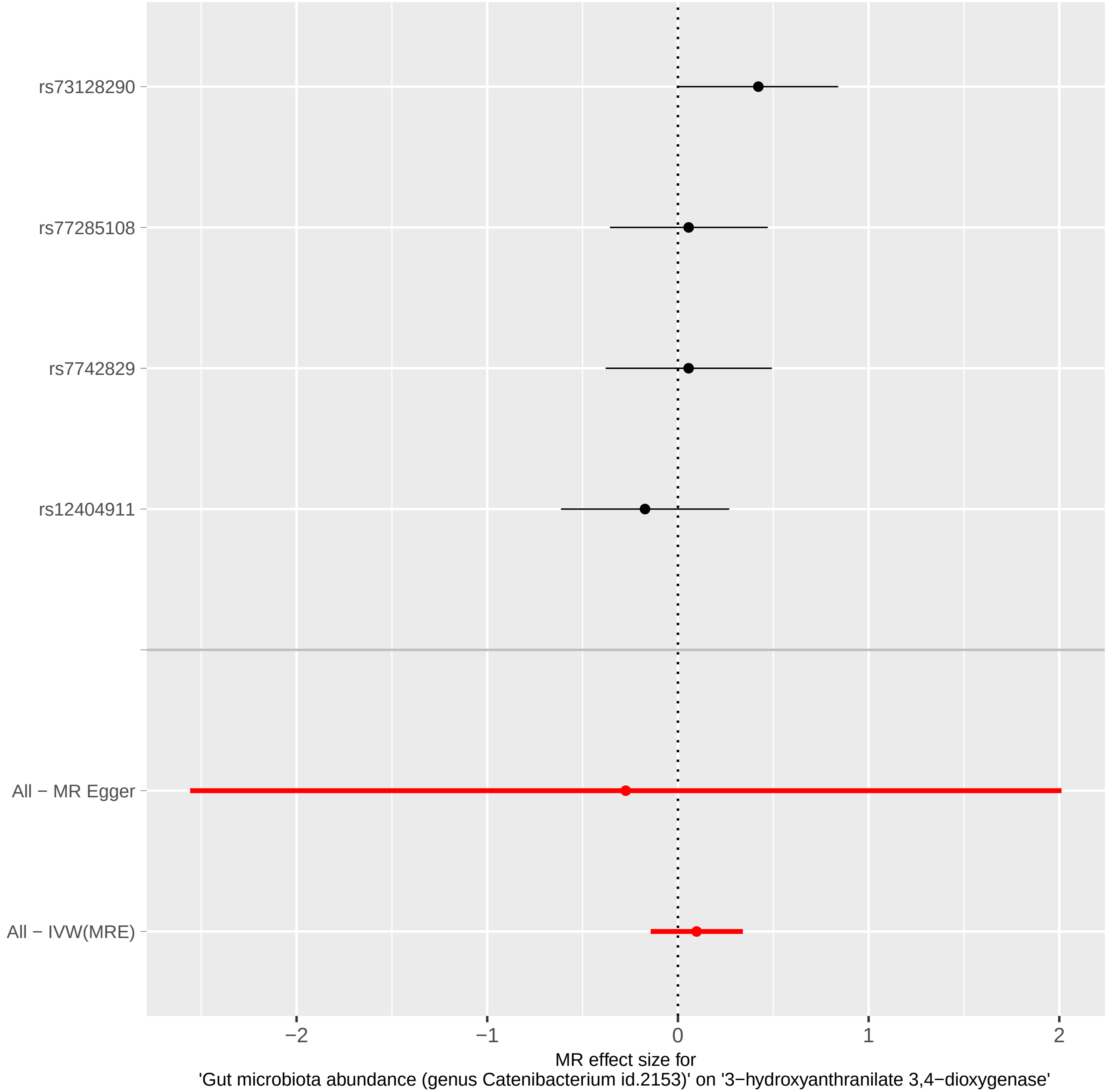

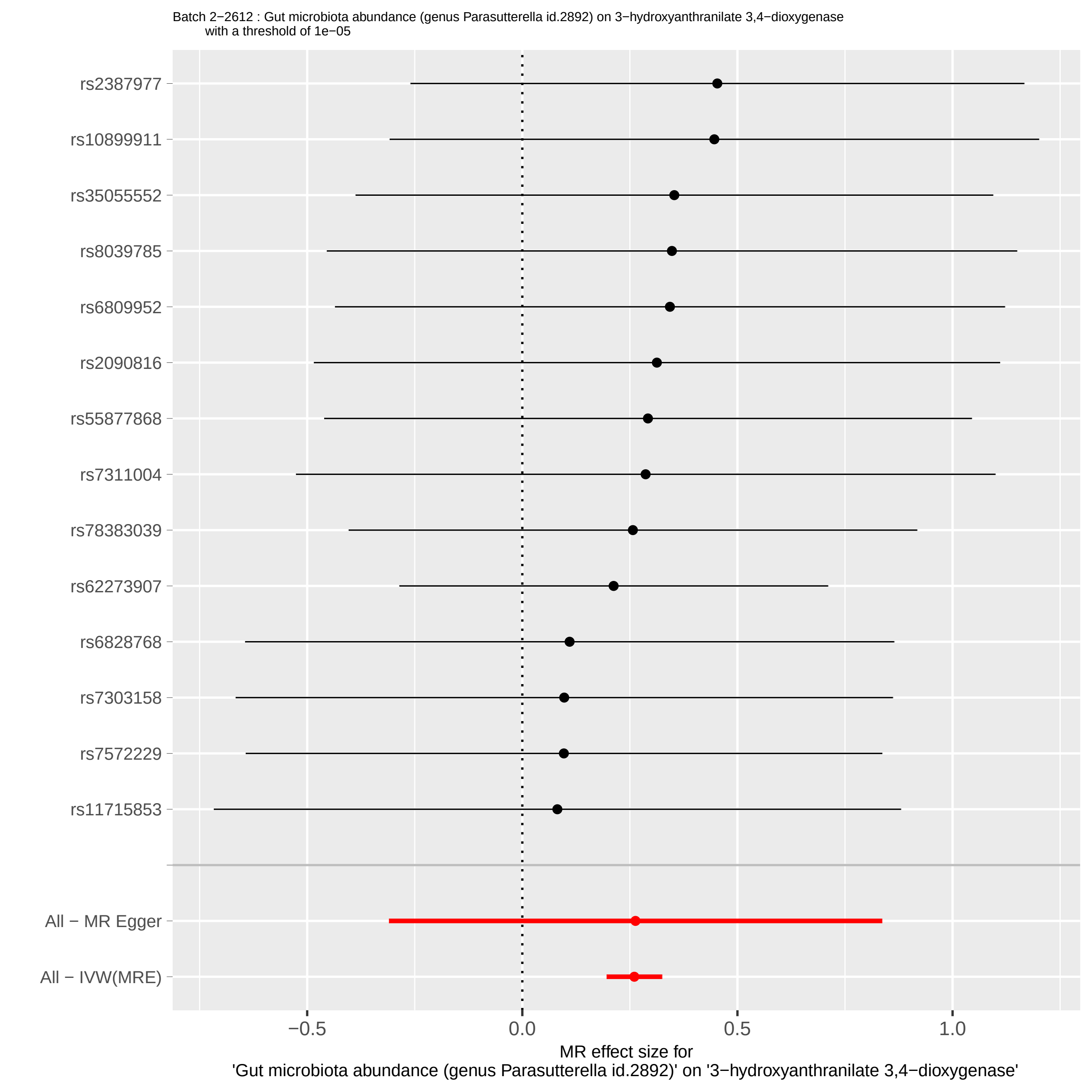

Batch 2−2612 : Gut microbiota abundance (genus Parasutterella id.2892) on 3−hydroxyanthranilate 3,4−dioxygenase
with a threshold of 1e−05
rs2387977
rs10899911
rs35055552
rs8039785
rs6809952
rs2090816
rs55877868
rs7311004
rs78383039
rs62273907
rs6828768
rs7303158
rs7572229
rs11715853
All − MR Egger
All − IVW(MRE)
−0.5
0.0
0.5
1.0
MR effect size for
'Gut microbiota abundance (genus Parasutterella id.2892)' on '3−hydroxyanthranilate 3,4−dioxygenase'

Batch 2−2613 : Gut microbiota abundance (genus Catenibacterium id.2153) on Hepcidin
with a threshold of 1e−05

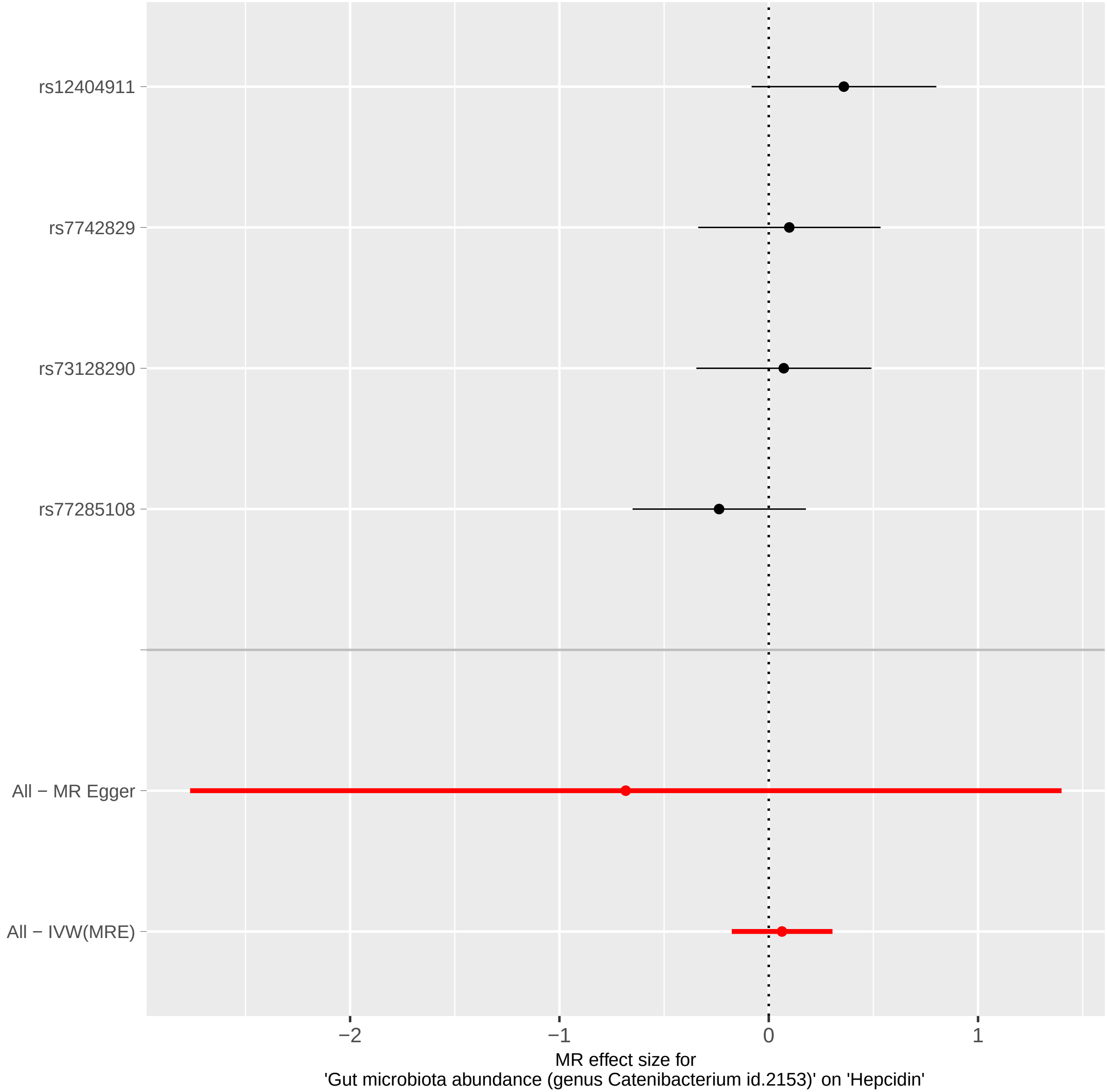

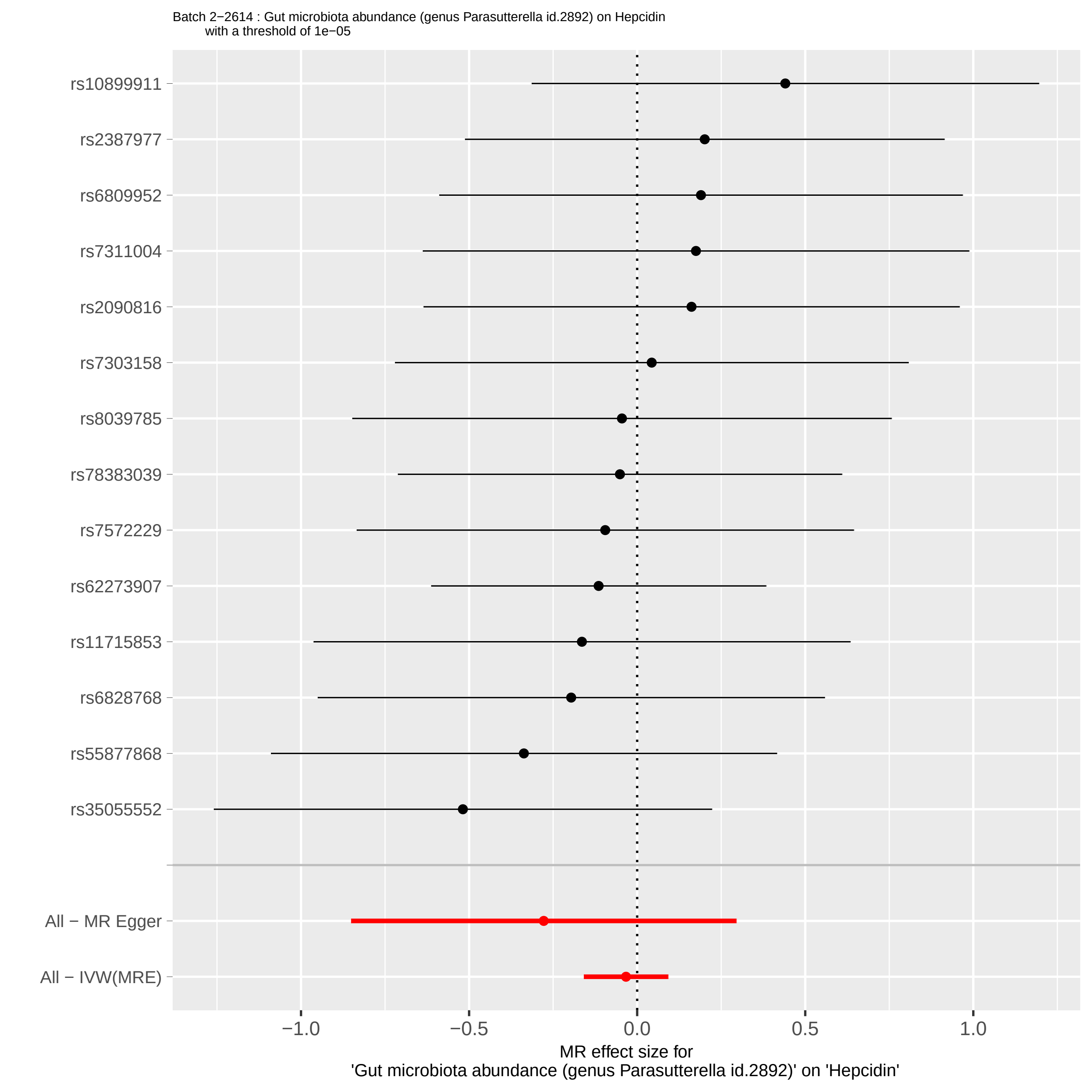
Batch 2−2614 : Gut microbiota abundance (genus Parasutterella id.2892) on Hepcidin
with a threshold of 1e−05
rs10899911
rs2387977
rs6809952
rs7311004
rs2090816
rs7303158
rs8039785
rs78383039
rs7572229
rs62273907
rs11715853
rs6828768
rs55877868
rs35055552
All − MR Egger
All − IVW(MRE)
−1.0
−0.5
0.0
0.5
1.0
MR effect size for
'Gut microbiota abundance (genus Parasutterella id.2892)' on 'Hepcidin'

Batch 2−2615 : Gut microbiota abundance (genus Catenibacterium id.2153) on Hyaluronan and proteoglycan link protein 1
with a threshold of 1e−05

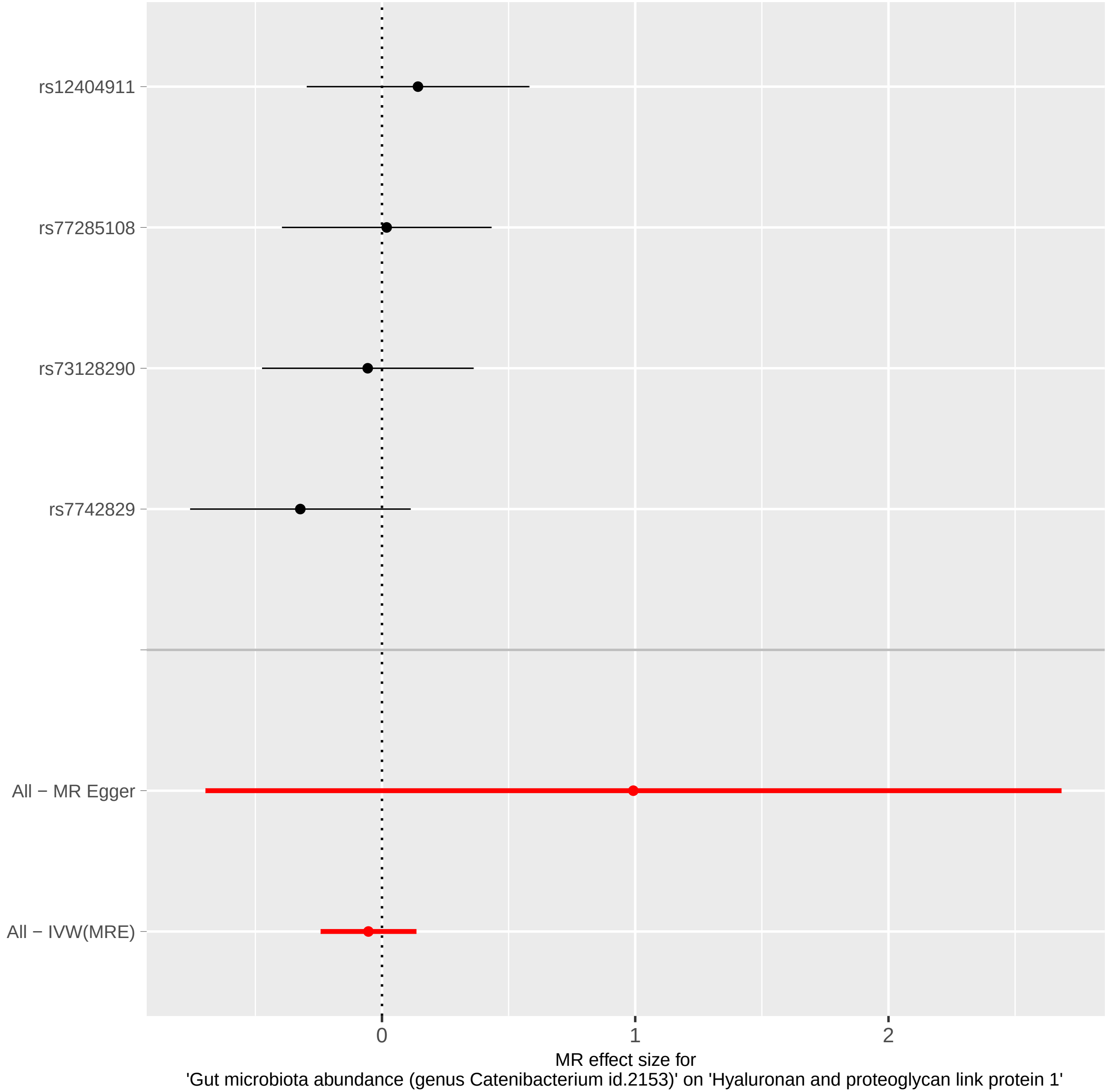

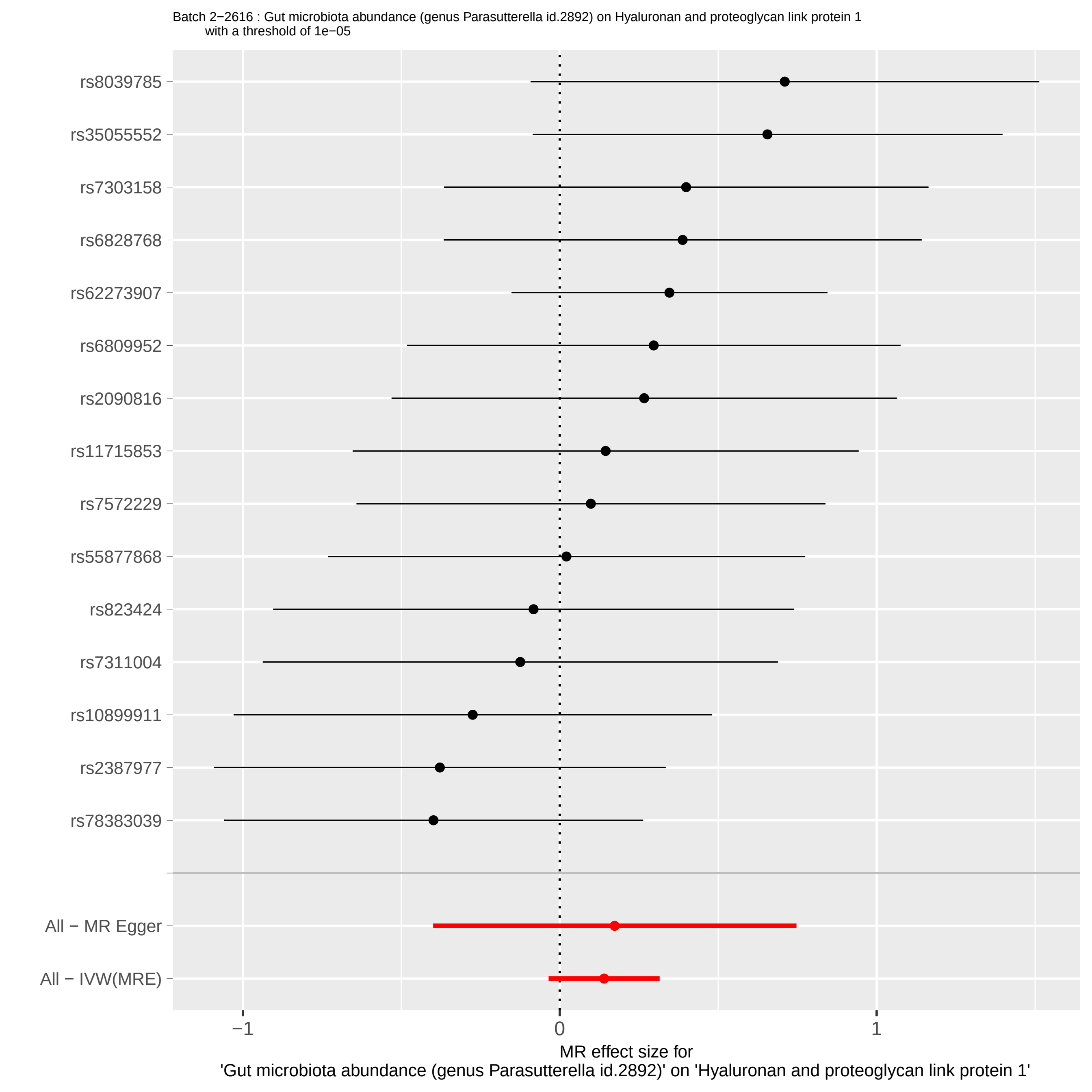
Batch 2−2616 : Gut microbiota abundance (genus Parasutterella id.2892) on Hyaluronan and proteoglycan link protein 1
with a threshold of 1e−05
rs8039785
rs35055552
rs7303158
rs6828768
rs62273907
rs6809952
rs2090816
rs11715853
rs7572229
rs55877868
rs823424
rs7311004
rs10899911
rs2387977
rs78383039
All − MR Egger
All − IVW(MRE)
−1
0
1
MR effect size for
'Gut microbiota abundance (genus Parasutterella id.2892)' on 'Hyaluronan and proteoglycan link protein 1'

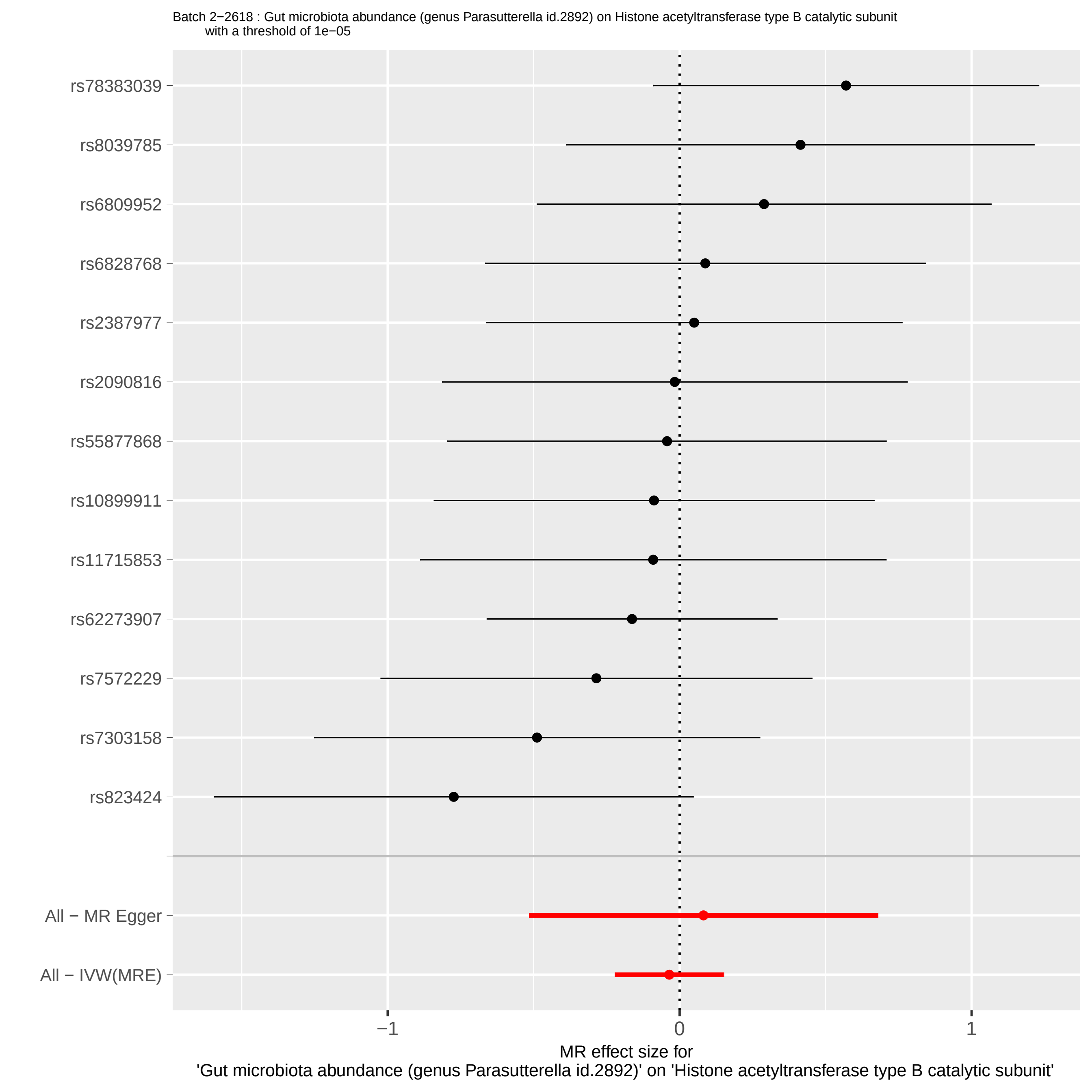
Batch 2−2618 : Gut microbiota abundance (genus Parasutterella id.2892) on Histone acetyltransferase type B catalytic subunit
with a threshold of 1e−05
rs78383039
rs8039785
rs6809952
rs6828768
rs2387977
rs2090816
rs55877868
rs10899911
rs11715853
rs62273907
rs7572229
rs7303158
rs823424
All − MR Egger
All − IVW(MRE)
−1
0
1
MR effect size for
'Gut microbiota abundance (genus Parasutterella id.2892)' on 'Histone acetyltransferase type B catalytic subunit'

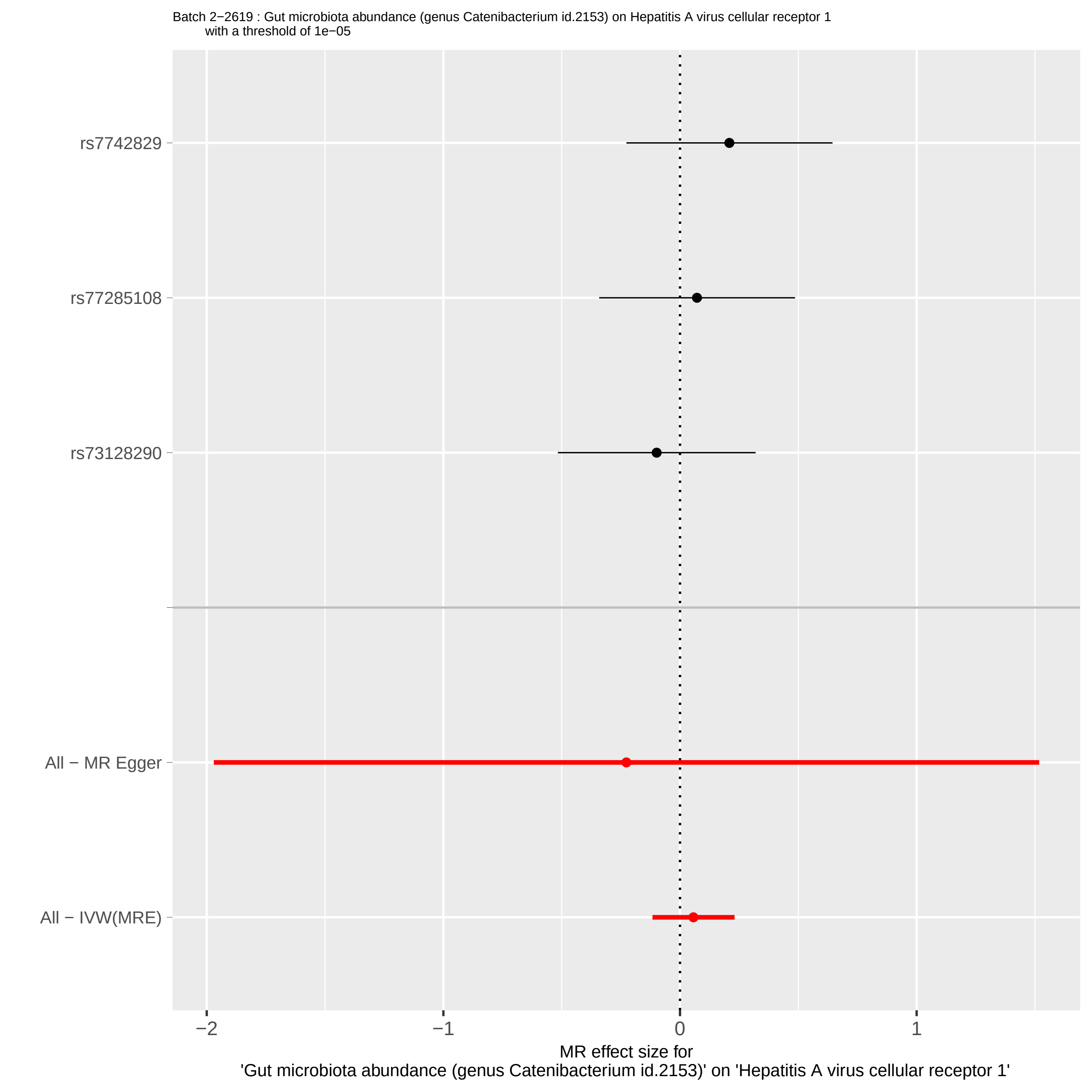
Batch 2−2619 : Gut microbiota abundance (genus Catenibacterium id.2153) on Hepatitis A virus cellular receptor 1
with a threshold of 1e−05
rs7742829
rs77285108
rs73128290
All − MR Egger
All − IVW(MRE)
−2
−1
0
1
MR effect size for
'Gut microbiota abundance (genus Catenibacterium id.2153)' on 'Hepatitis A virus cellular receptor 1'

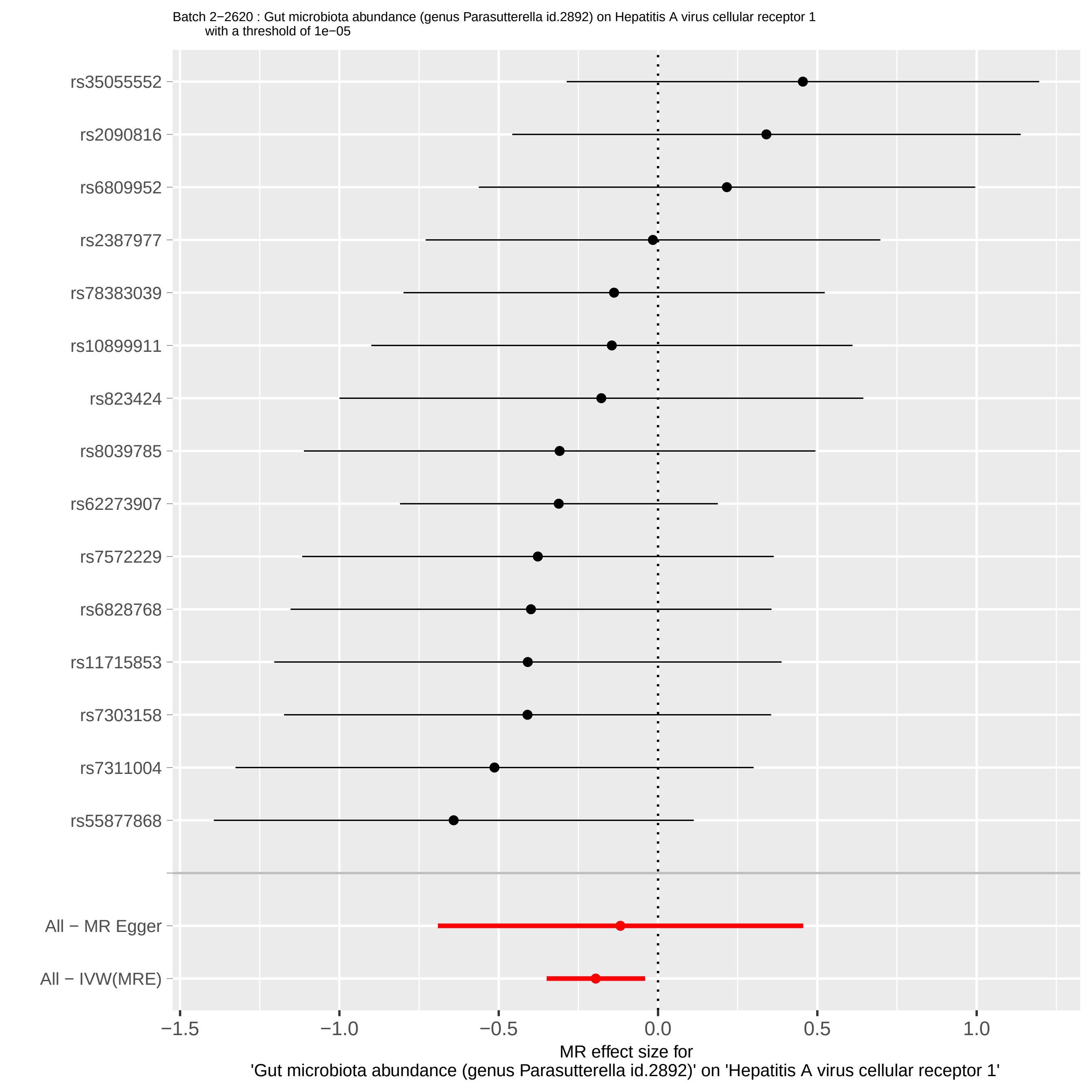
Batch 2−2620 : Gut microbiota abundance (genus Parasutterella id.2892) on Hepatitis A virus cellular receptor 1
with a threshold of 1e−05
rs35055552
rs2090816
rs6809952
rs2387977
rs78383039
rs10899911
rs823424
rs8039785
rs62273907
rs7572229
rs6828768
rs11715853
rs7303158
rs7311004
rs55877868
All − MR Egger
All − IVW(MRE)
−1.5
−1.0
−0.5
0.0
0.5
1.0
MR effect size for
'Gut microbiota abundance (genus Parasutterella id.2892)' on 'Hepatitis A virus cellular receptor 1'

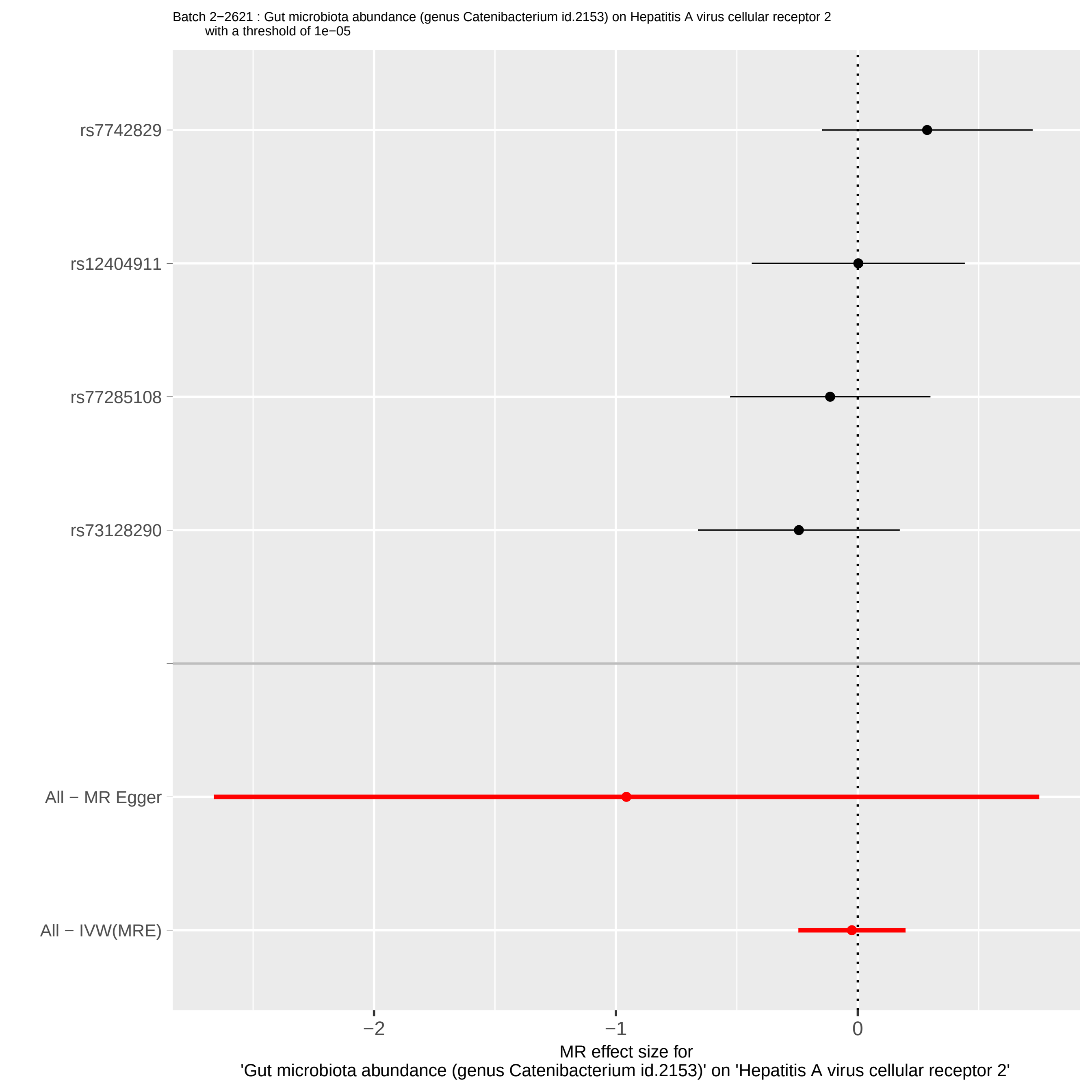

Batch 2−2621 : Gut microbiota abundance (genus Catenibacterium id.2153) on Hepatitis A virus cellular receptor 2
with a threshold of 1e−05
rs7742829
rs12404911
rs77285108
rs73128290
All − MR Egger
All − IVW(MRE)
−2
−1
0
MR effect size for
'Gut microbiota abundance (genus Catenibacterium id.2153)' on 'Hepatitis A virus cellular receptor 2'

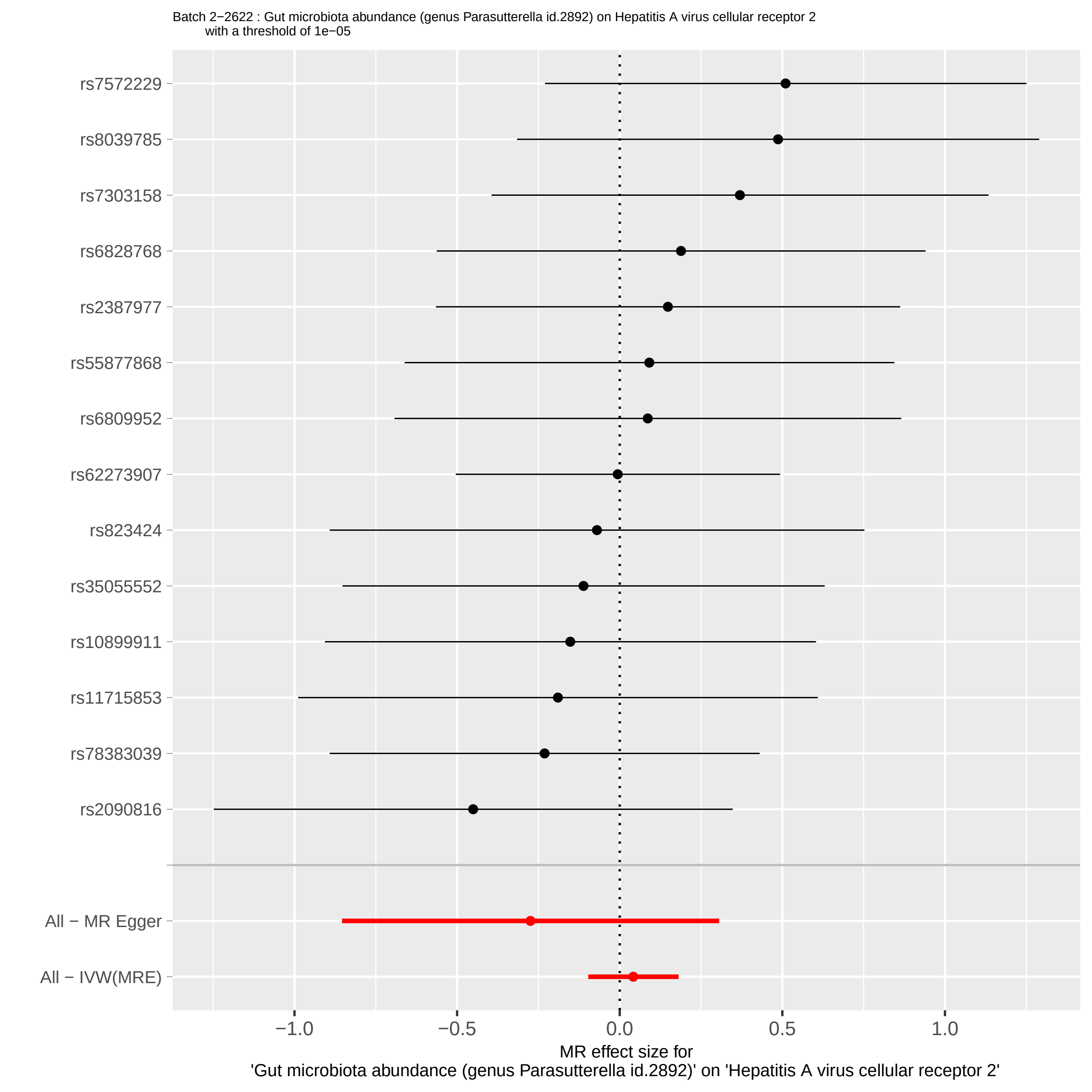

Batch 2−2622 : Gut microbiota abundance (genus Parasutterella id.2892) on Hepatitis A virus cellular receptor 2
with a threshold of 1e−05
rs7572229
rs8039785
rs7303158
rs6828768
rs2387977
rs55877868
rs6809952
rs62273907
rs823424
rs35055552
rs10899911
rs11715853
rs78383039
rs2090816
All − MR Egger
All − IVW(MRE)
−1.0
−0.5
0.0
0.5
1.0
MR effect size for
'Gut microbiota abundance (genus Parasutterella id.2892)' on 'Hepatitis A virus cellular receptor 2'

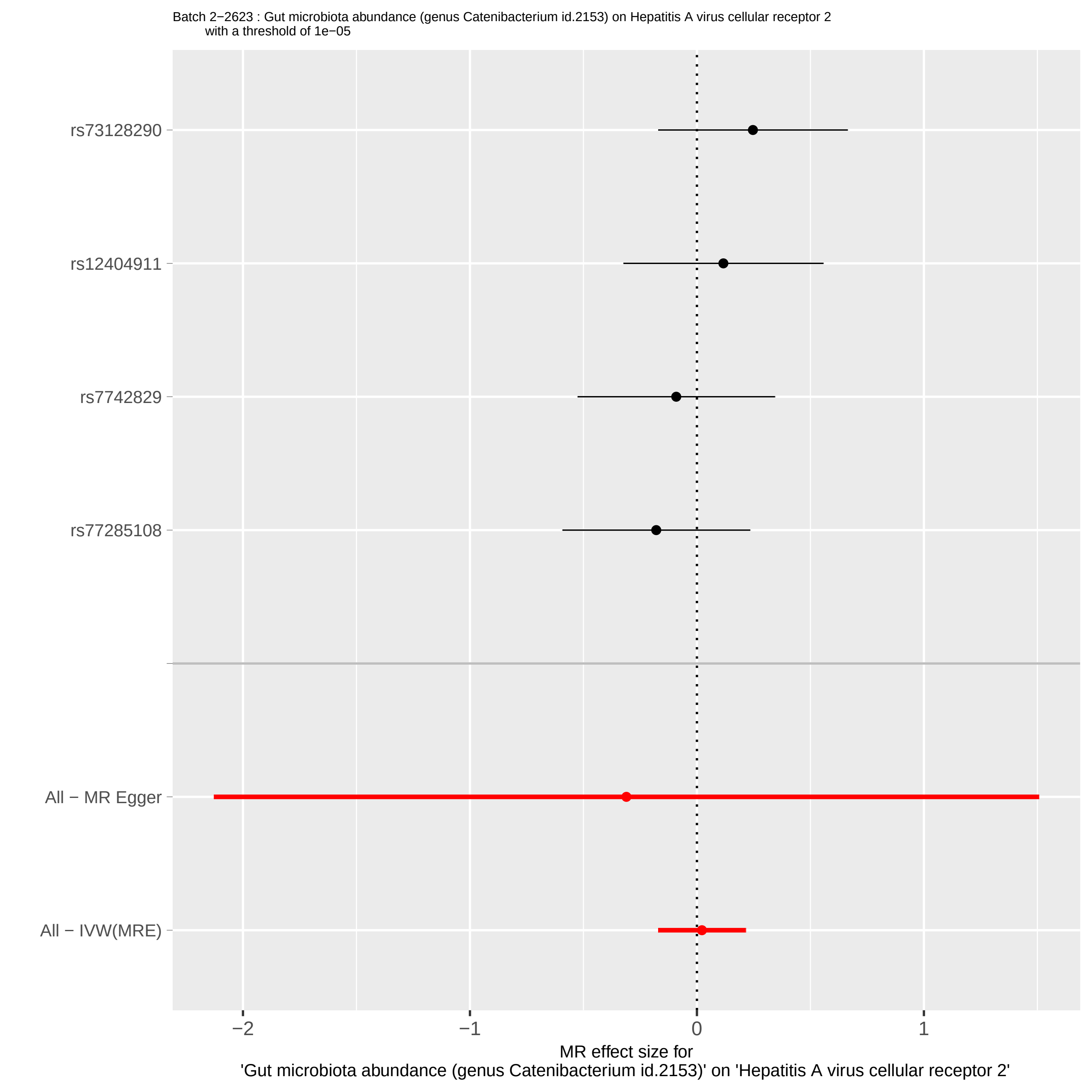

Batch 2−2623 : Gut microbiota abundance (genus Catenibacterium id.2153) on Hepatitis A virus cellular receptor 2
with a threshold of 1e−05
rs73128290
rs12404911
rs7742829
rs77285108
All − MR Egger
All − IVW(MRE)
−2
−1
0
1
MR effect size for
'Gut microbiota abundance (genus Catenibacterium id.2153)' on 'Hepatitis A virus cellular receptor 2'

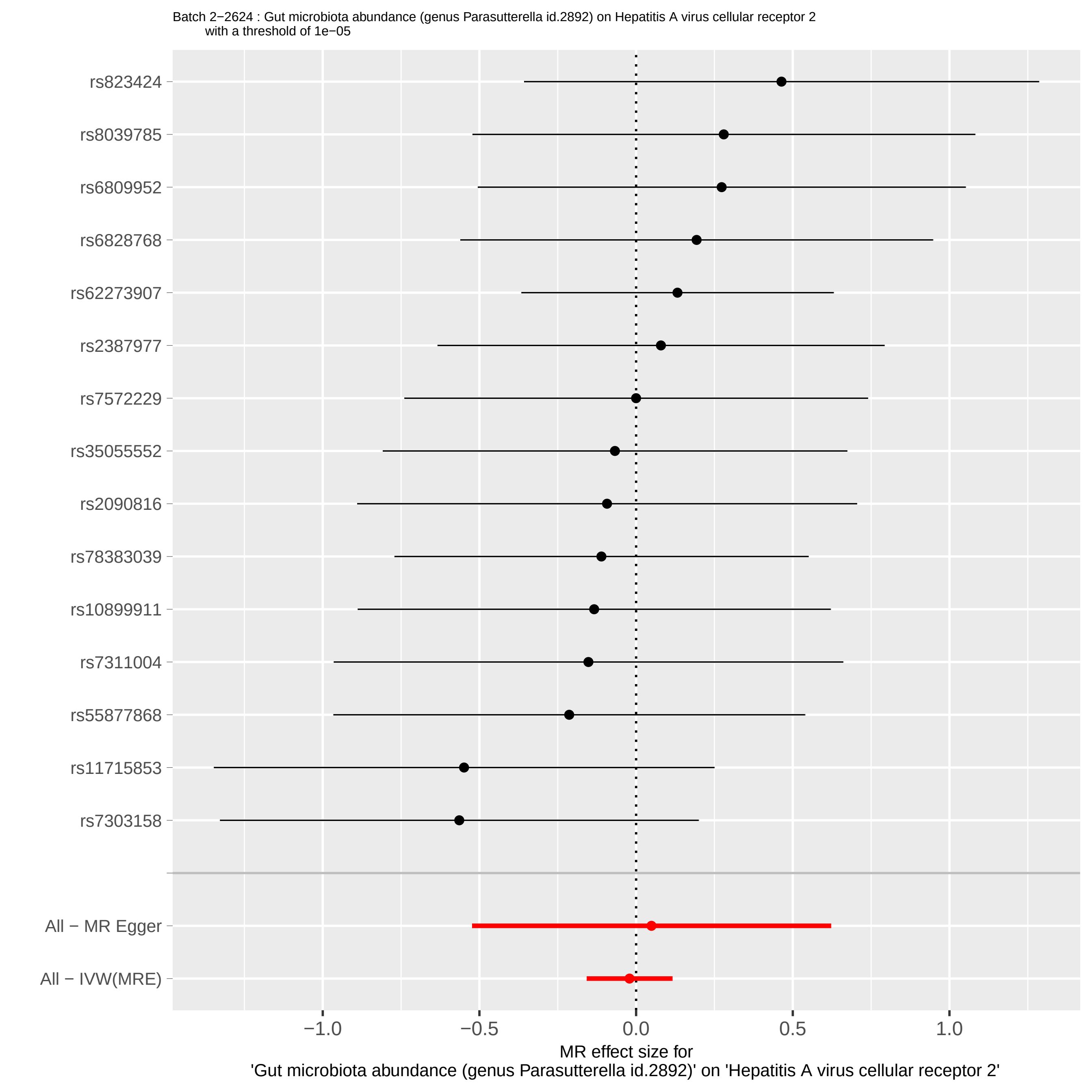
Batch 2−2624 : Gut microbiota abundance (genus Parasutterella id.2892) on Hepatitis A virus cellular receptor 2
with a threshold of 1e−05
rs823424
rs8039785
rs6809952
rs6828768
rs62273907
rs2387977
rs7572229
rs35055552
rs2090816
rs78383039
rs10899911
rs7311004
rs55877868
rs11715853
rs7303158
All − MR Egger
All − IVW(MRE)
−1.0
−0.5
0.0
0.5
1.0
MR effect size for
'Gut microbiota abundance (genus Parasutterella id.2892)' on 'Hepatitis A virus cellular receptor 2'

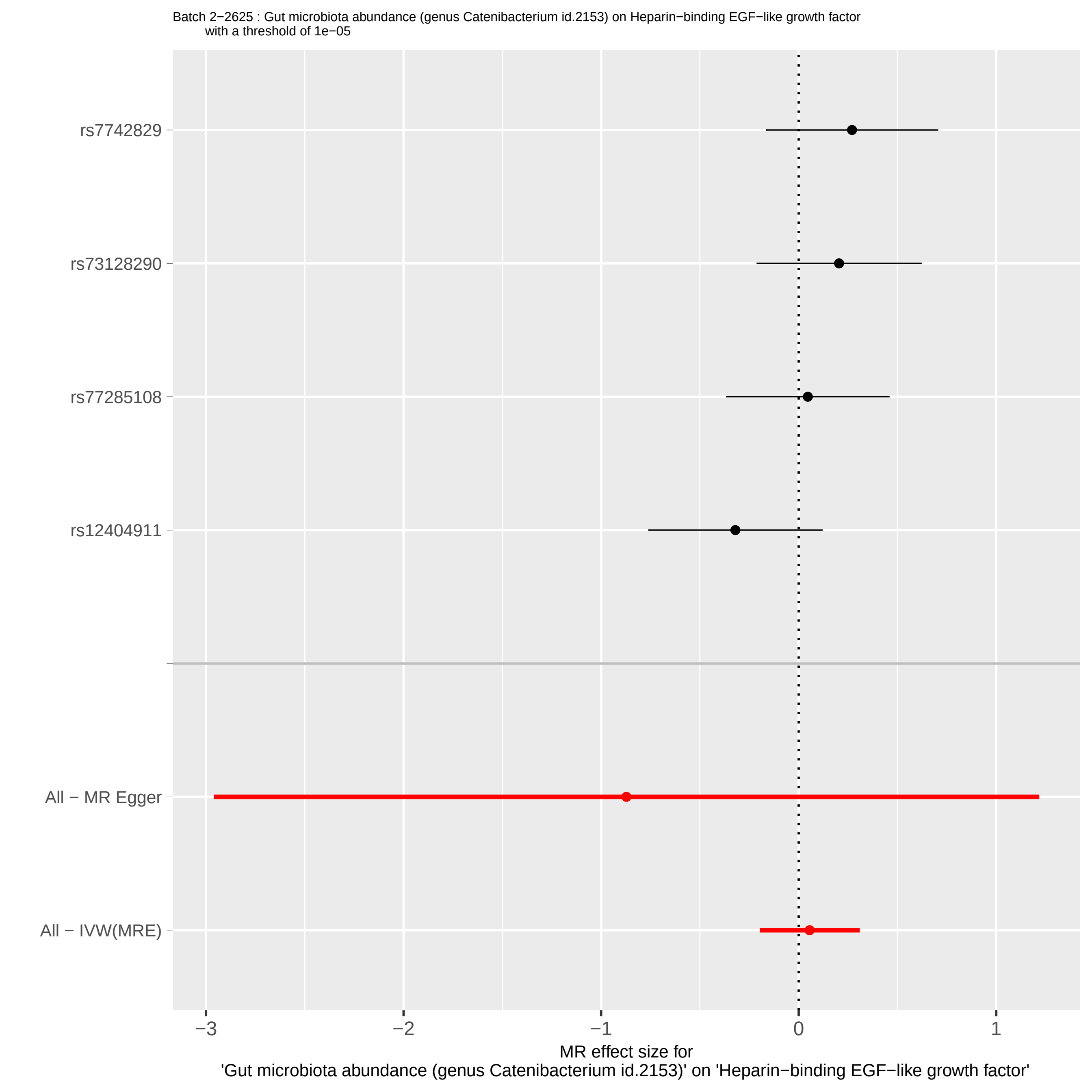

Batch 2−2625 : Gut microbiota abundance (genus Catenibacterium id.2153) on Heparin−binding EGF−like growth factor
with a threshold of 1e−05
rs7742829
rs73128290
rs77285108
rs12404911
All − MR Egger
All − IVW(MRE)
−3
−2
−1
0
1
MR effect size for
'Gut microbiota abundance (genus Catenibacterium id.2153)' on 'Heparin−binding EGF−like growth factor'

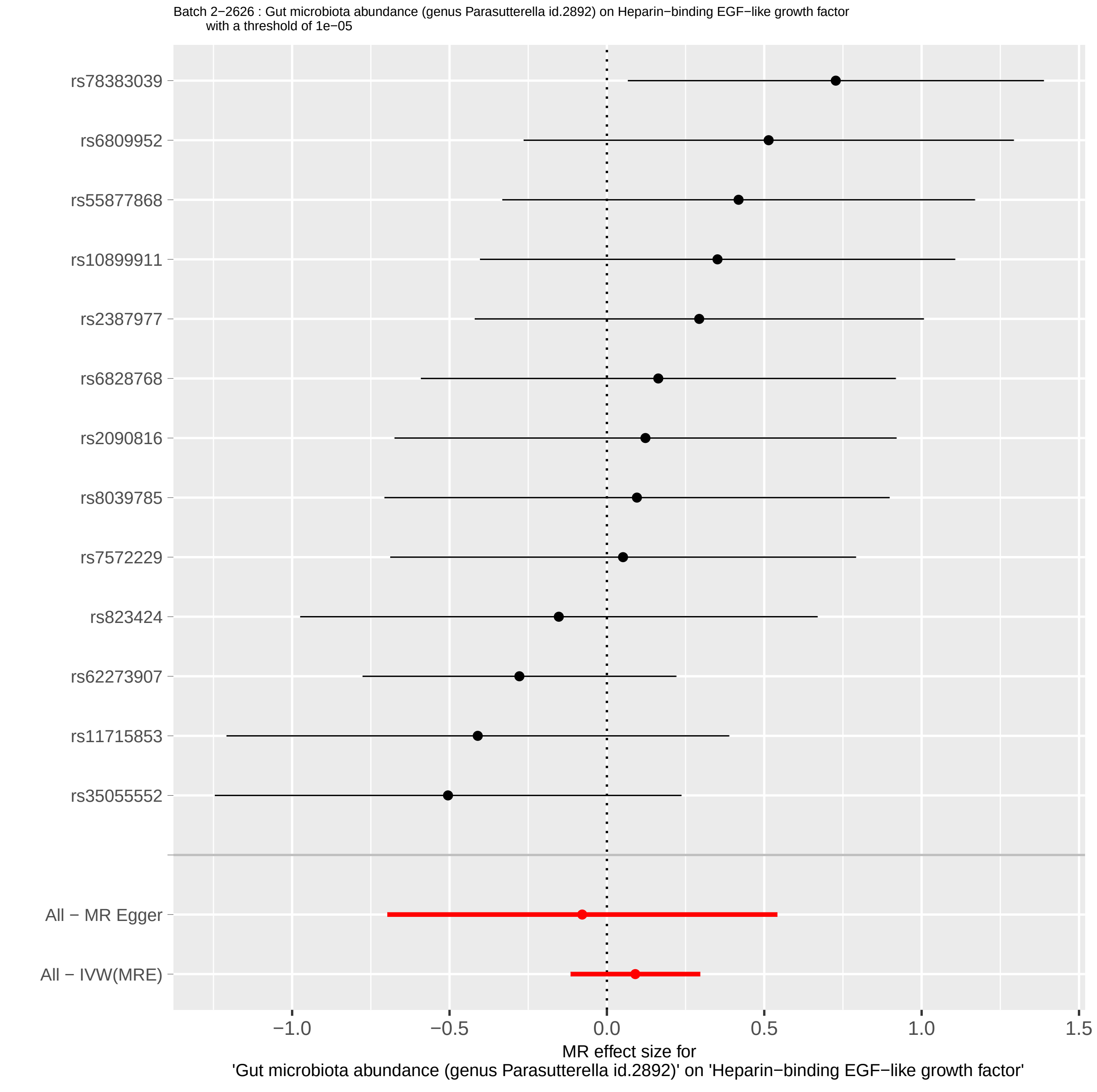

Batch 2−2626 : Gut microbiota abundance (genus Parasutterella id.2892) on Heparin−binding EGF−like growth factor
with a threshold of 1e−05
rs78383039
rs6809952
rs55877868
rs10899911
rs2387977
rs6828768
rs2090816
rs8039785
rs7572229
rs823424
rs62273907
rs11715853
rs35055552
All − MR Egger
All − IVW(MRE)
−1.0
−0.5
0.0
0.5
1.0
1.5
MR effect size for
'Gut microbiota abundance (genus Parasutterella id.2892)' on 'Heparin−binding EGF−like growth factor'

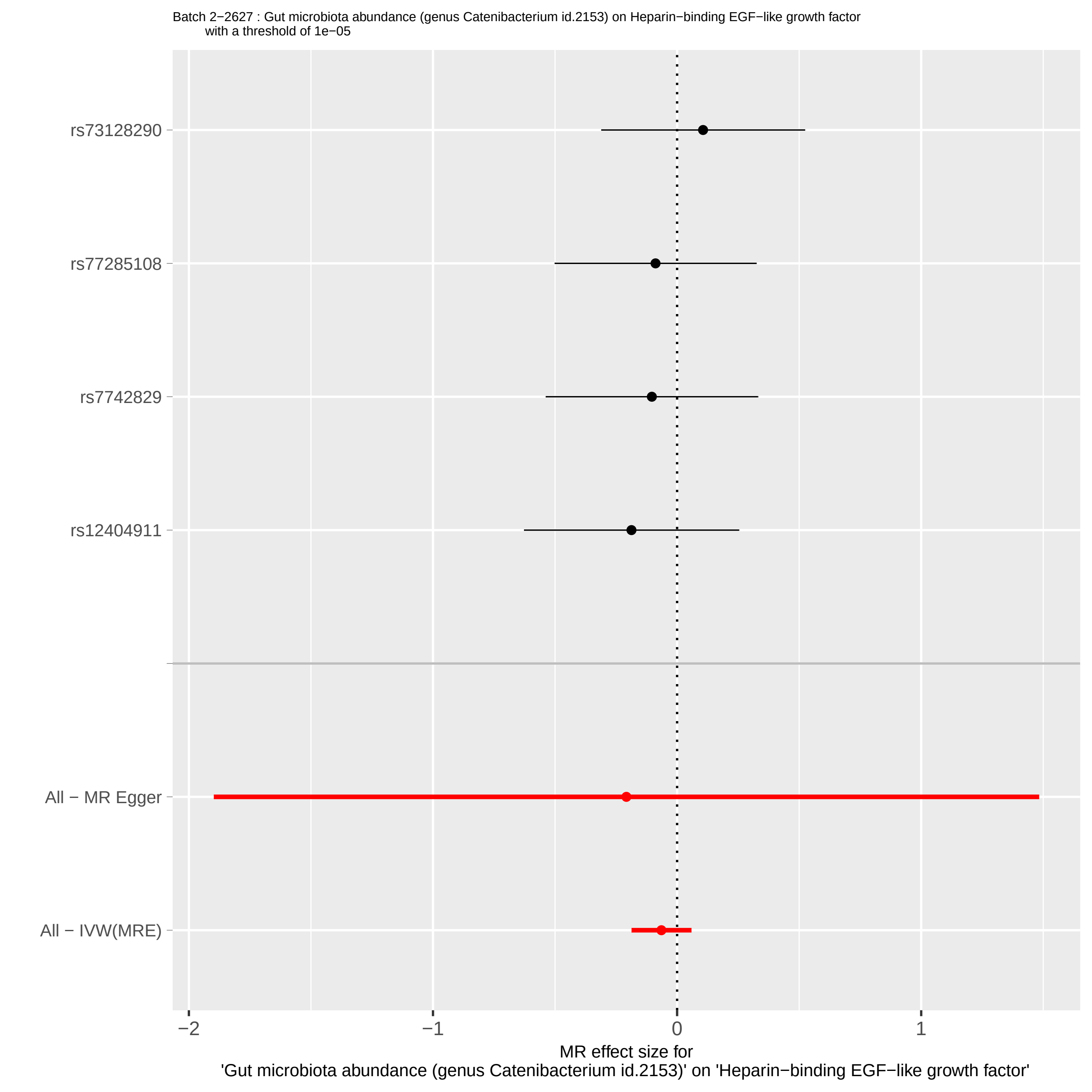

Batch 2−2627 : Gut microbiota abundance (genus Catenibacterium id.2153) on Heparin−binding EGF−like growth factor
with a threshold of 1e−05
rs73128290
rs77285108
rs7742829
rs12404911
All − MR Egger
All − IVW(MRE)
−2
−1
0
1
MR effect size for
'Gut microbiota abundance (genus Catenibacterium id.2153)' on 'Heparin−binding EGF−like growth factor'

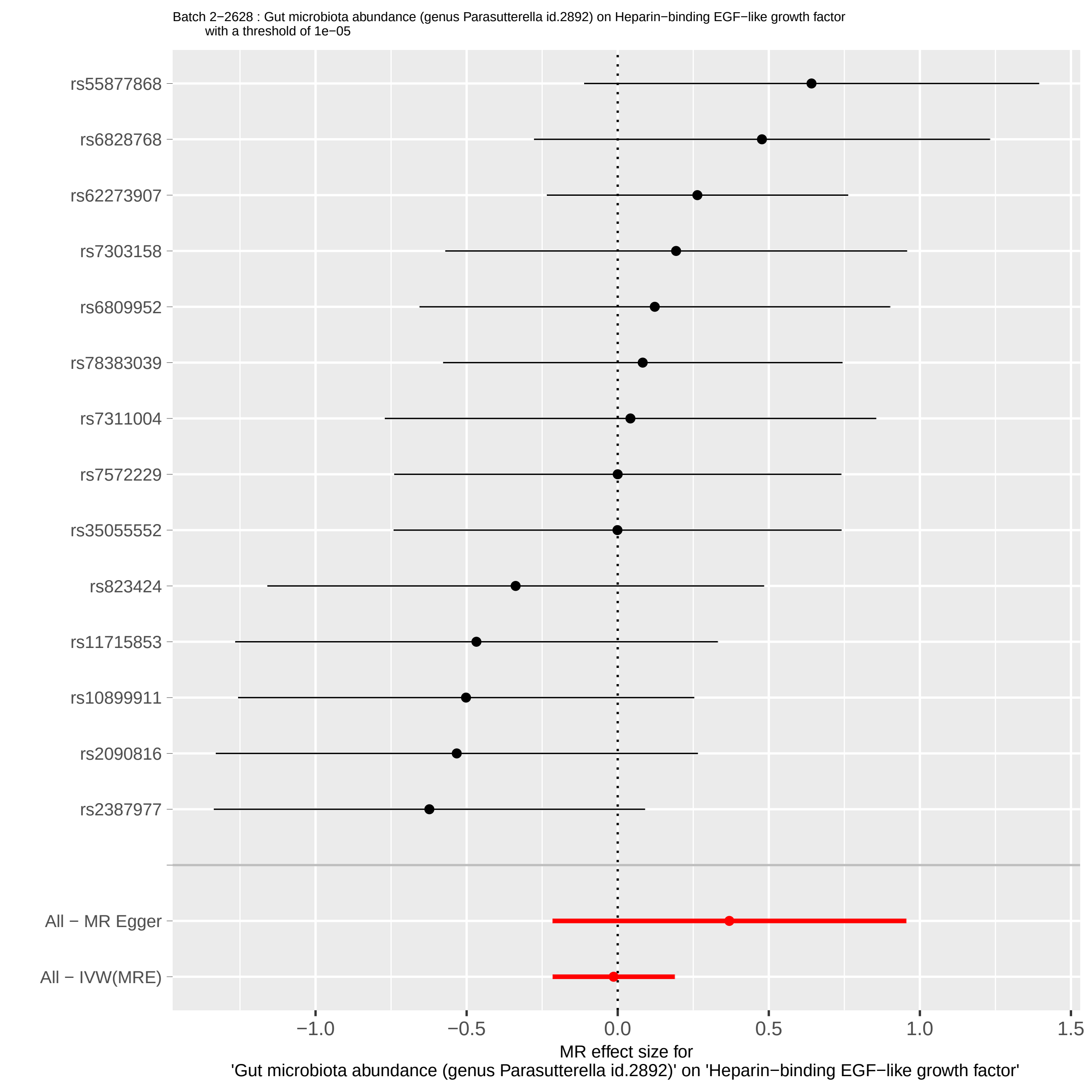

Batch 2−2628 : Gut microbiota abundance (genus Parasutterella id.2892) on Heparin−binding EGF−like growth factor
with a threshold of 1e−05
rs55877868
rs6828768
rs62273907
rs7303158
rs6809952
rs78383039
rs7311004
rs7572229
rs35055552
rs823424
rs11715853
rs10899911
rs2090816
rs2387977
All − MR Egger
All − IVW(MRE)
−1.0
−0.5
0.0
0.5
1.0
1.5
MR effect size for
'Gut microbiota abundance (genus Parasutterella id.2892)' on 'Heparin−binding EGF−like growth factor'

Batch 2−2629 : Gut microbiota abundance (genus Catenibacterium id.2153) on Hemoglobin subunit gamma−1
with a threshold of 1e−05

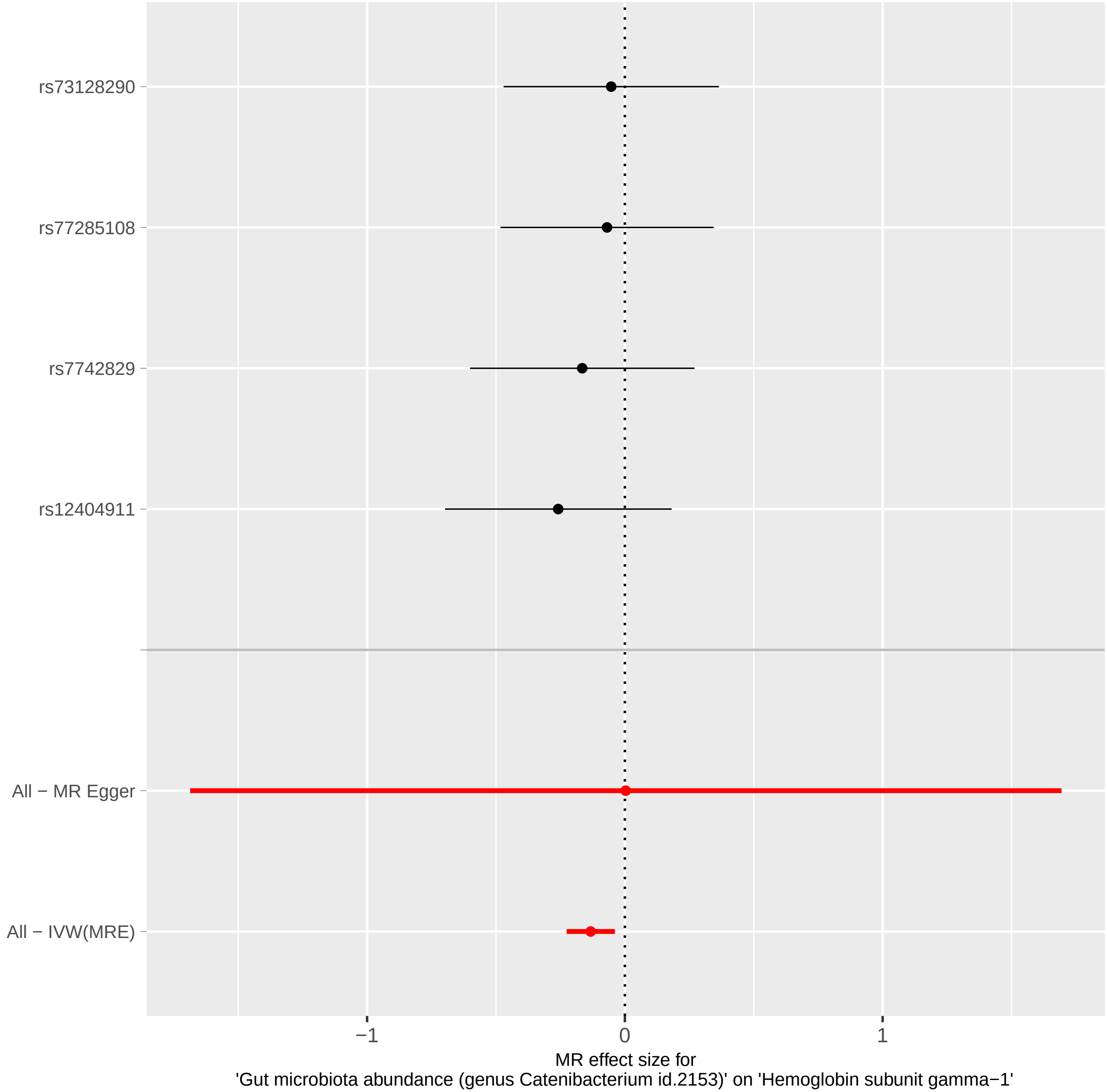

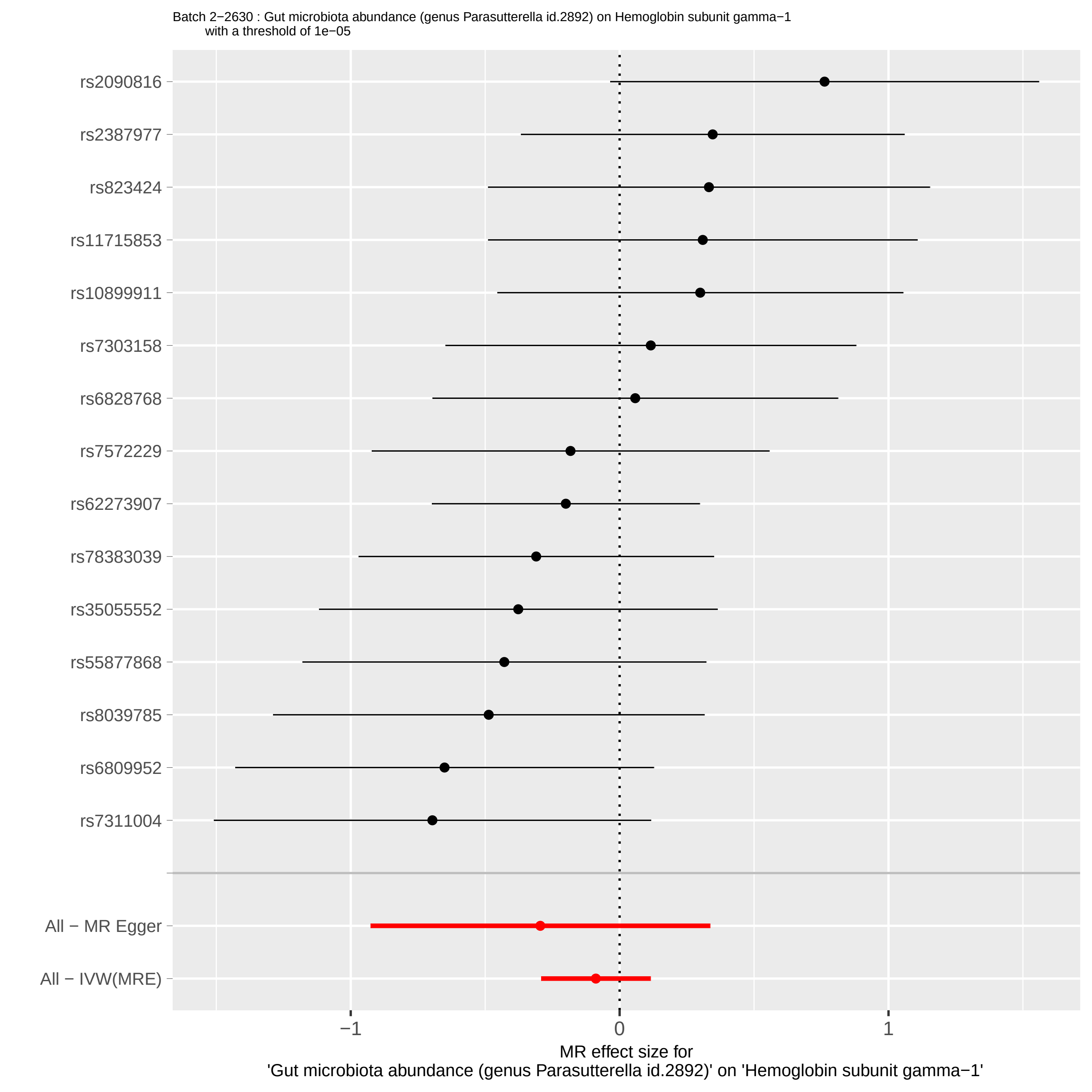
Batch 2−2630 : Gut microbiota abundance (genus Parasutterella id.2892) on Hemoglobin subunit gamma−1
with a threshold of 1e−05
rs2090816
rs2387977
rs823424
rs11715853
rs10899911
rs7303158
rs6828768
rs7572229
rs62273907
rs78383039
rs35055552
rs55877868
rs8039785
rs6809952
rs7311004
All − MR Egger
All − IVW(MRE)
−1
0
1
MR effect size for
'Gut microbiota abundance (genus Parasutterella id.2892)' on 'Hemoglobin subunit gamma−1'

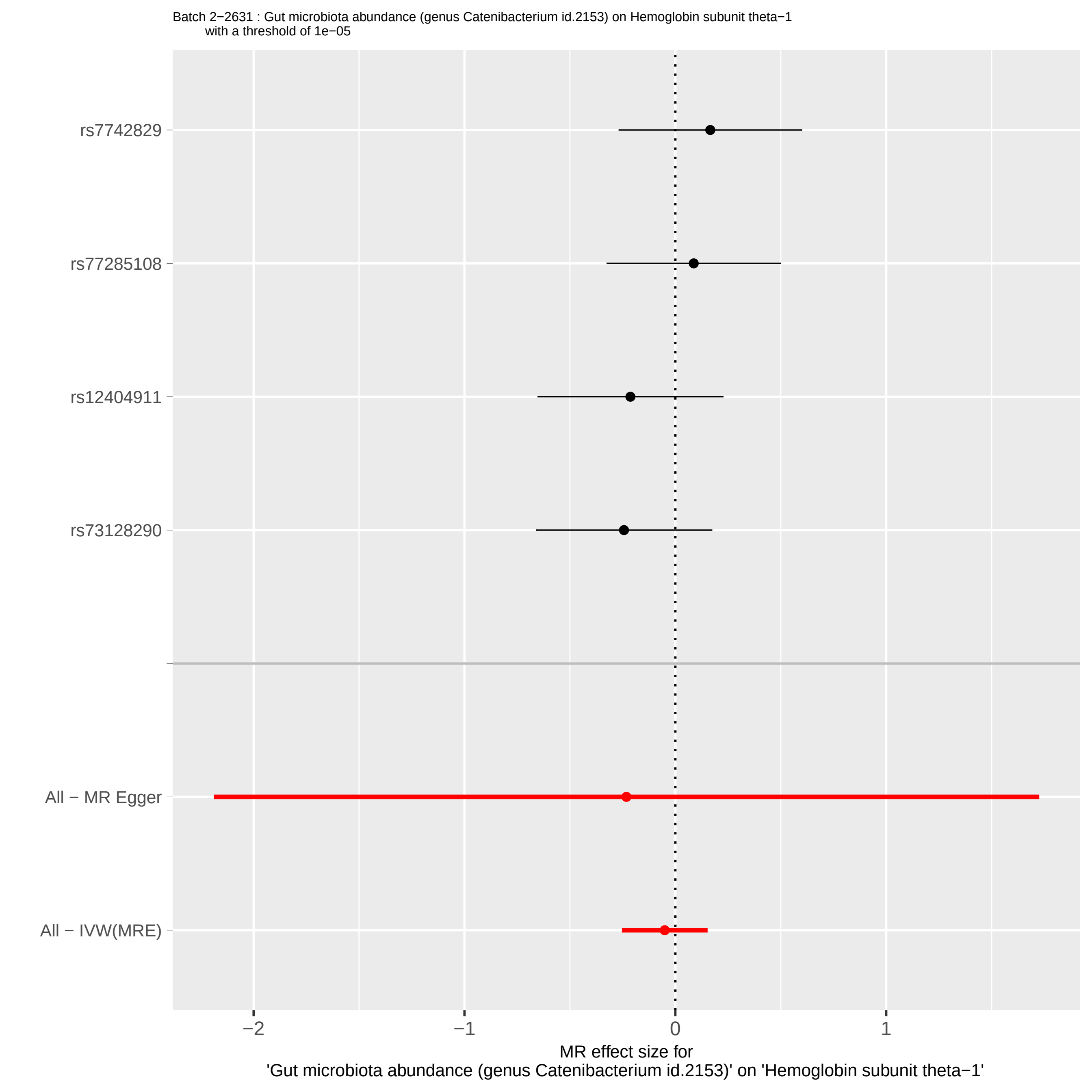

Batch 2−2631 : Gut microbiota abundance (genus Catenibacterium id.2153) on Hemoglobin subunit theta−1
with a threshold of 1e−05
rs7742829
rs77285108
rs12404911
rs73128290
All − MR Egger
All − IVW(MRE)
−2
−1
0
1
MR effect size for
'Gut microbiota abundance (genus Catenibacterium id.2153)' on 'Hemoglobin subunit theta−1'

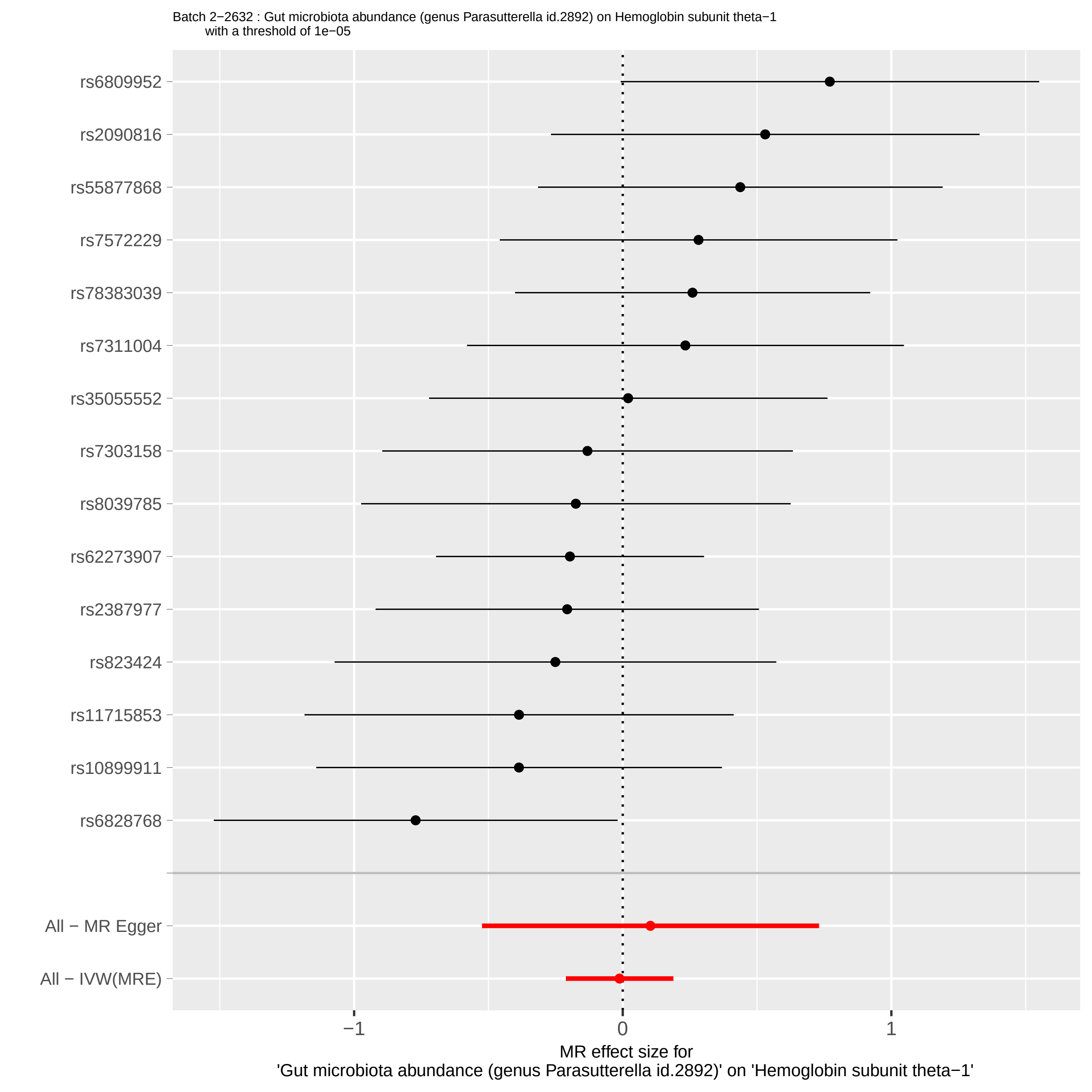

Batch 2−2632 : Gut microbiota abundance (genus Parasutterella id.2892) on Hemoglobin subunit theta−1
with a threshold of 1e−05
rs6809952
rs2090816
rs55877868
rs7572229
rs78383039
rs7311004
rs35055552
rs7303158
rs8039785
rs62273907
rs2387977
rs823424
rs11715853
rs10899911
rs6828768
All − MR Egger
All − IVW(MRE)
−1
0
1
MR effect size for
'Gut microbiota abundance (genus Parasutterella id.2892)' on 'Hemoglobin subunit theta−1'

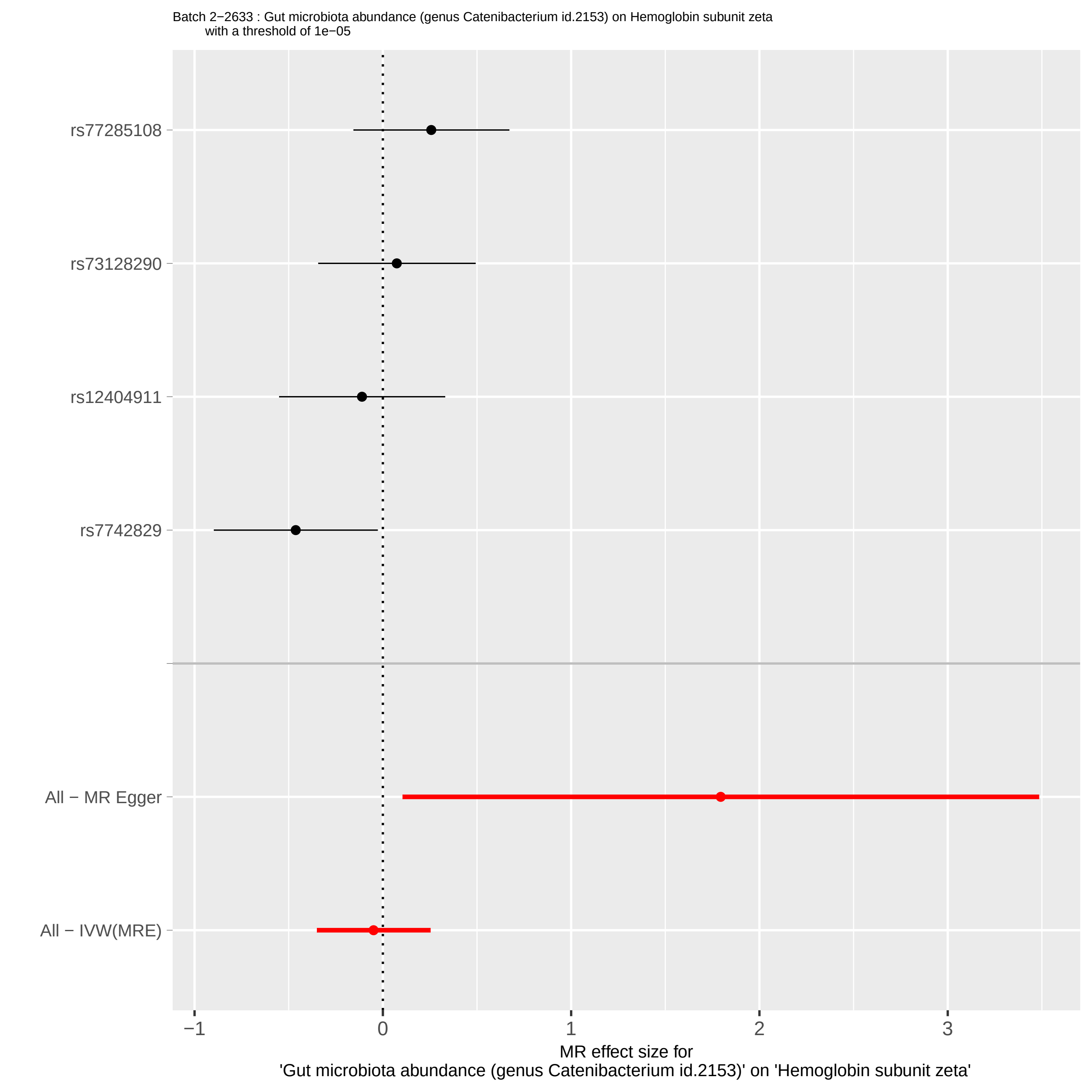

Batch 2−2633 : Gut microbiota abundance (genus Catenibacterium id.2153) on Hemoglobin subunit zeta
with a threshold of 1e−05
rs77285108
rs73128290
rs12404911
rs7742829
All − MR Egger
All − IVW(MRE)
−1
0
1
2
3
MR effect size for
'Gut microbiota abundance (genus Catenibacterium id.2153)' on 'Hemoglobin subunit zeta'

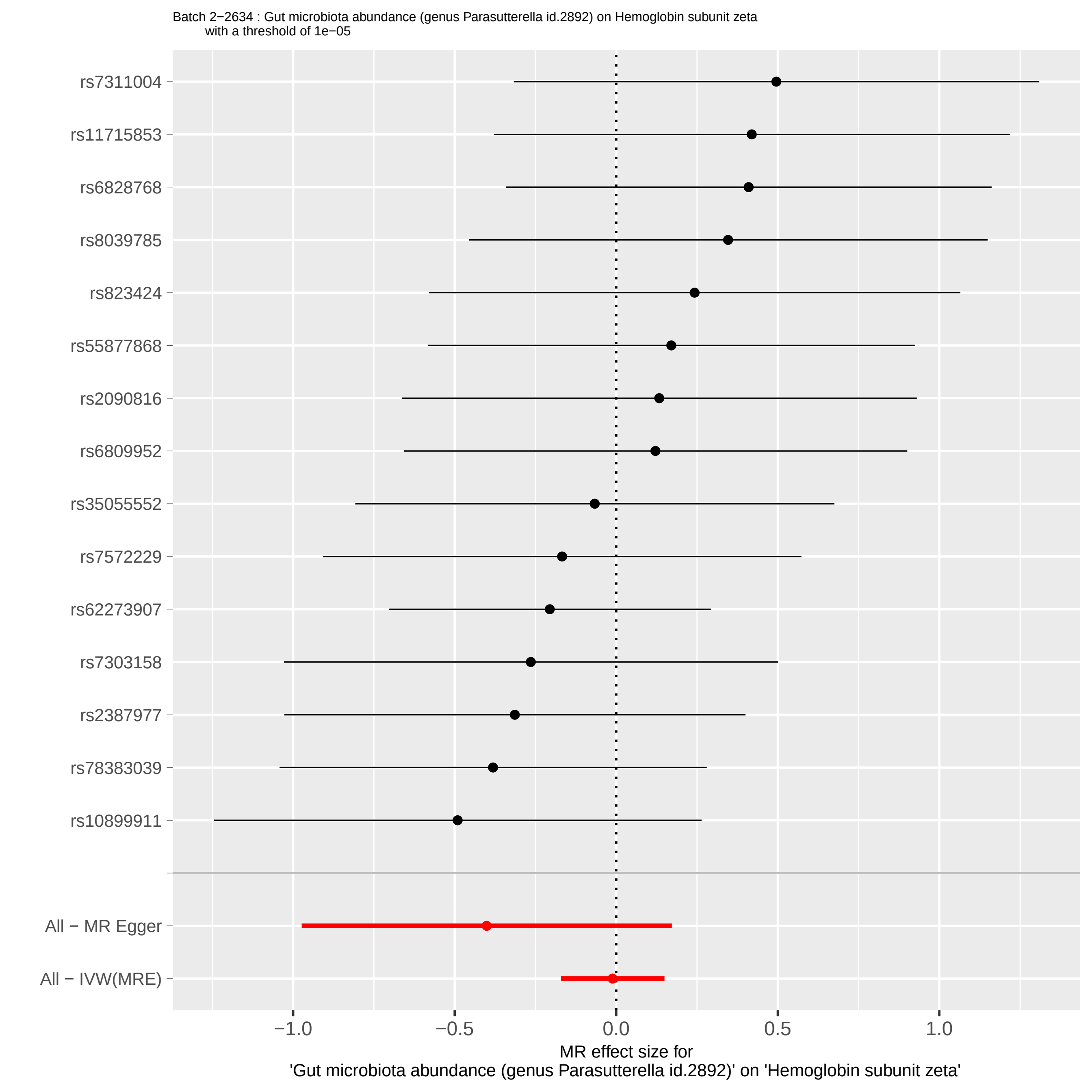

Batch 2−2634 : Gut microbiota abundance (genus Parasutterella id.2892) on Hemoglobin subunit zeta
with a threshold of 1e−05
rs7311004
rs11715853
rs6828768
rs8039785
rs823424
rs55877868
rs2090816
rs6809952
rs35055552
rs7572229
rs62273907
rs7303158
rs2387977
rs78383039
rs10899911
All − MR Egger
All − IVW(MRE)
−1.0
−0.5
0.0
0.5
1.0
MR effect size for
'Gut microbiota abundance (genus Parasutterella id.2892)' on 'Hemoglobin subunit zeta'

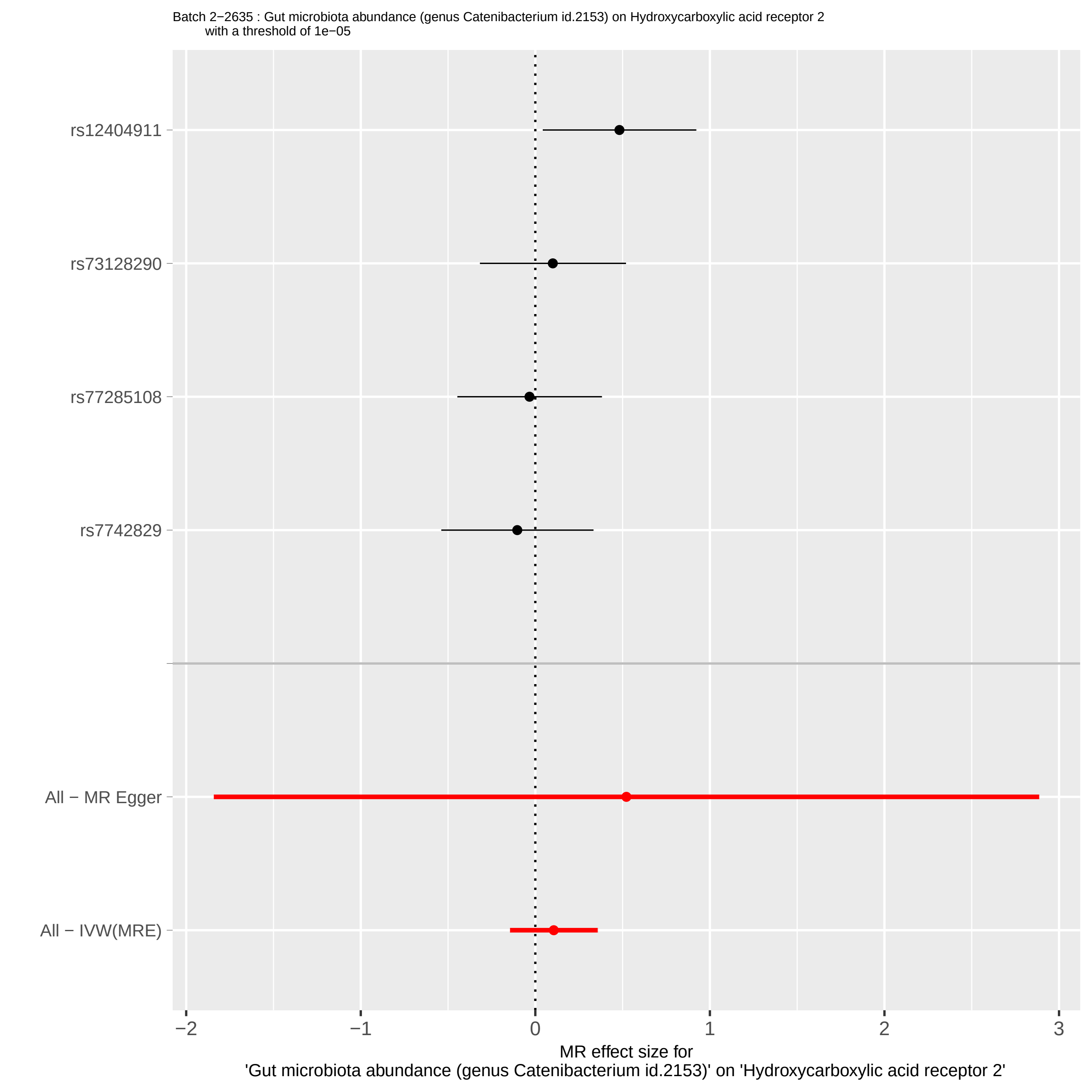

Batch 2−2635 : Gut microbiota abundance (genus Catenibacterium id.2153) on Hydroxycarboxylic acid receptor 2
with a threshold of 1e−05
rs12404911
rs73128290
rs77285108
rs7742829
All − MR Egger
All − IVW(MRE)
−2
−1
0
1
2
3
MR effect size for
'Gut microbiota abundance (genus Catenibacterium id.2153)' on 'Hydroxycarboxylic acid receptor 2'

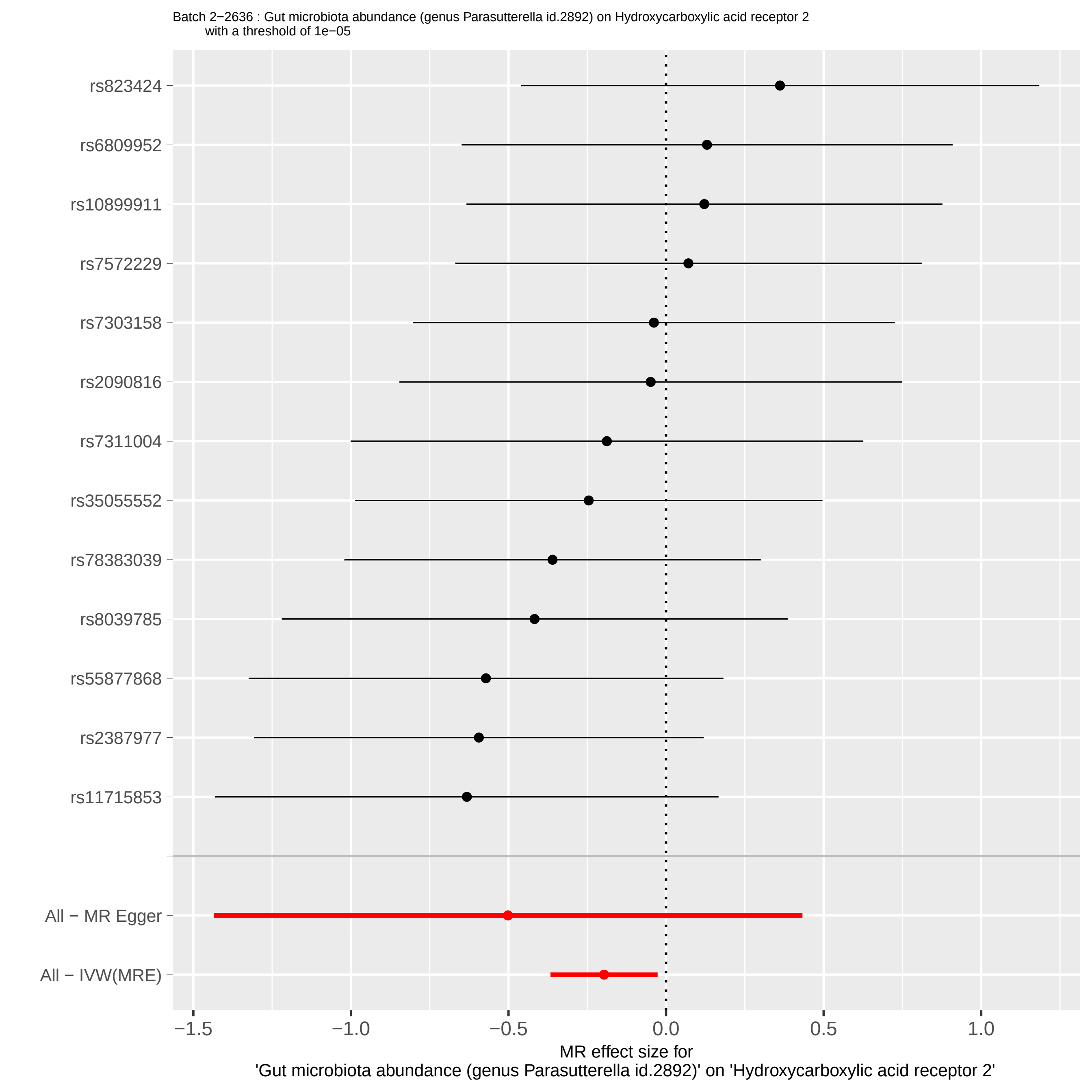
Batch 2−2636 : Gut microbiota abundance (genus Parasutterella id.2892) on Hydroxycarboxylic acid receptor 2
with a threshold of 1e−05
rs823424
rs6809952
rs10899911
rs7572229
rs7303158
rs2090816
rs7311004
rs35055552
rs78383039
rs8039785
rs55877868
rs2387977
rs11715853
All − MR Egger
All − IVW(MRE)
−1.5
−1.0
−0.5
0.0
0.5
1.0
MR effect size for
'Gut microbiota abundance (genus Parasutterella id.2892)' on 'Hydroxycarboxylic acid receptor 2'

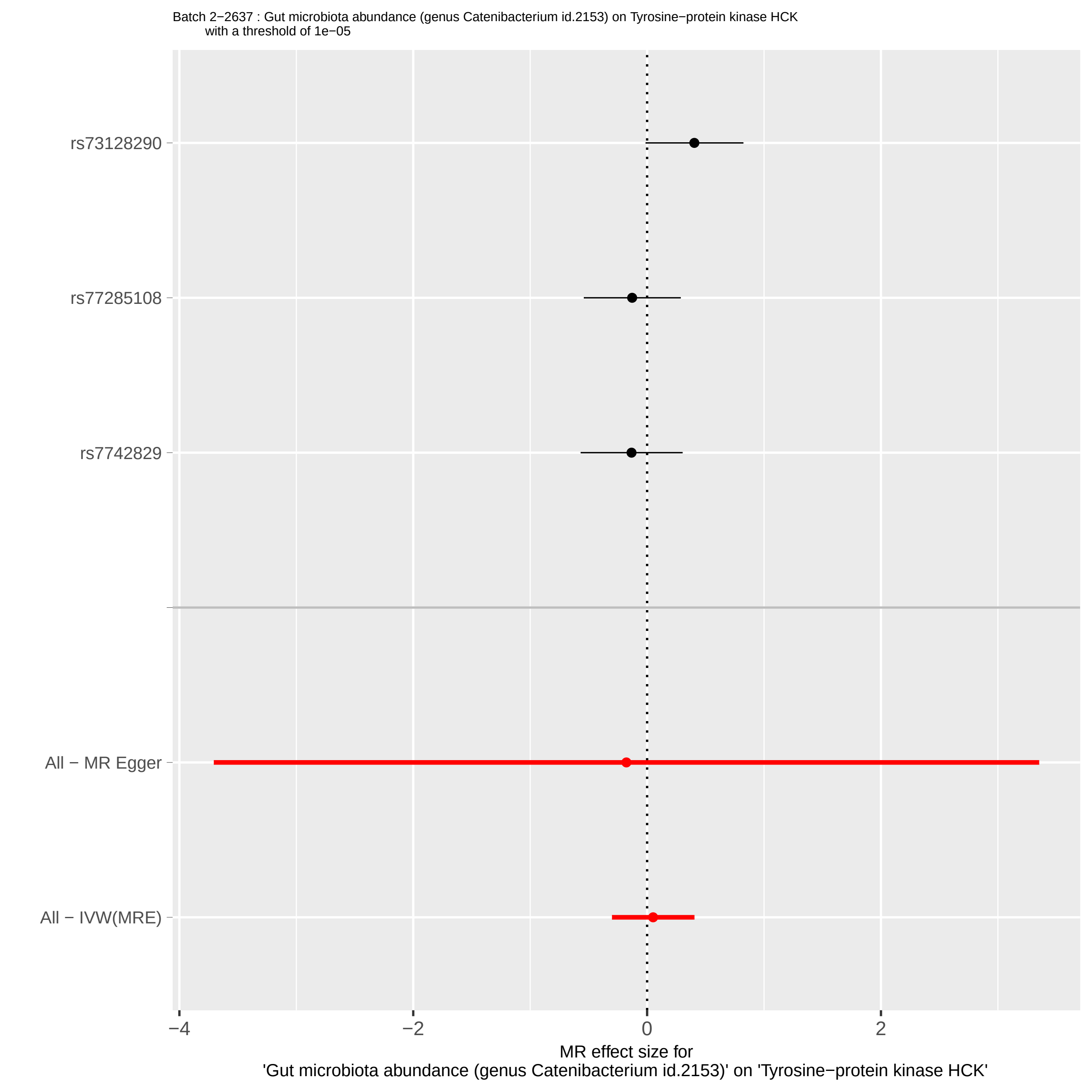

Batch 2−2637 : Gut microbiota abundance (genus Catenibacterium id.2153) on Tyrosine−protein kinase HCK
with a threshold of 1e−05
rs73128290
rs77285108
rs7742829
All − MR Egger
All − IVW(MRE)
−4
−2
0
2
MR effect size for
'Gut microbiota abundance (genus Catenibacterium id.2153)' on 'Tyrosine−protein kinase HCK'

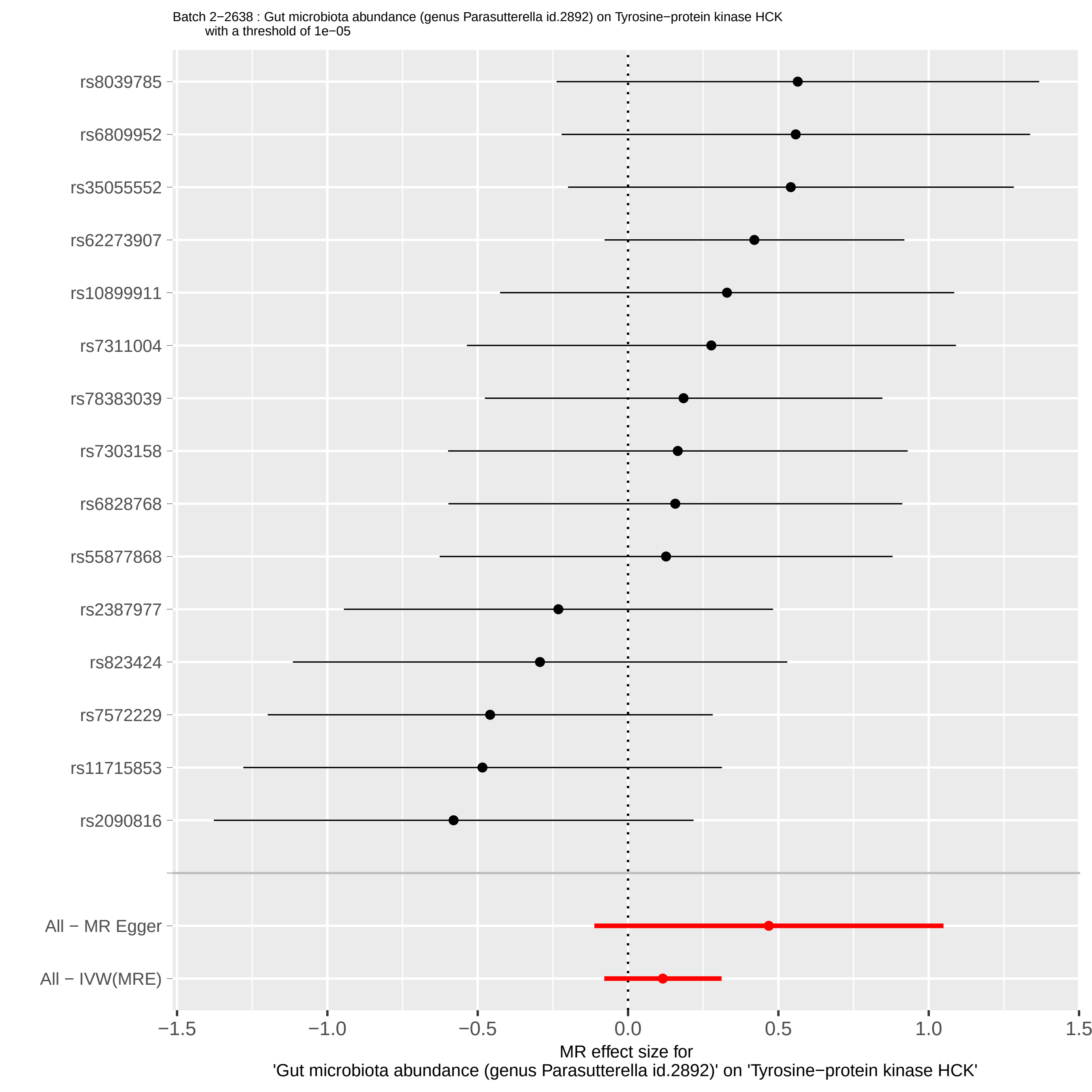
Batch 2−2638 : Gut microbiota abundance (genus Parasutterella id.2892) on Tyrosine−protein kinase HCK
with a threshold of 1e−05
rs8039785
rs6809952
rs35055552
rs62273907
rs10899911
rs7311004
rs78383039
rs7303158
rs6828768
rs55877868
rs2387977
rs823424
rs7572229
rs11715853
rs2090816
All − MR Egger
All − IVW(MRE)
−1.5
−1.0
−0.5
0.0
0.5
1.0
1.5
MR effect size for
'Gut microbiota abundance (genus Parasutterella id.2892)' on 'Tyrosine−protein kinase HCK'

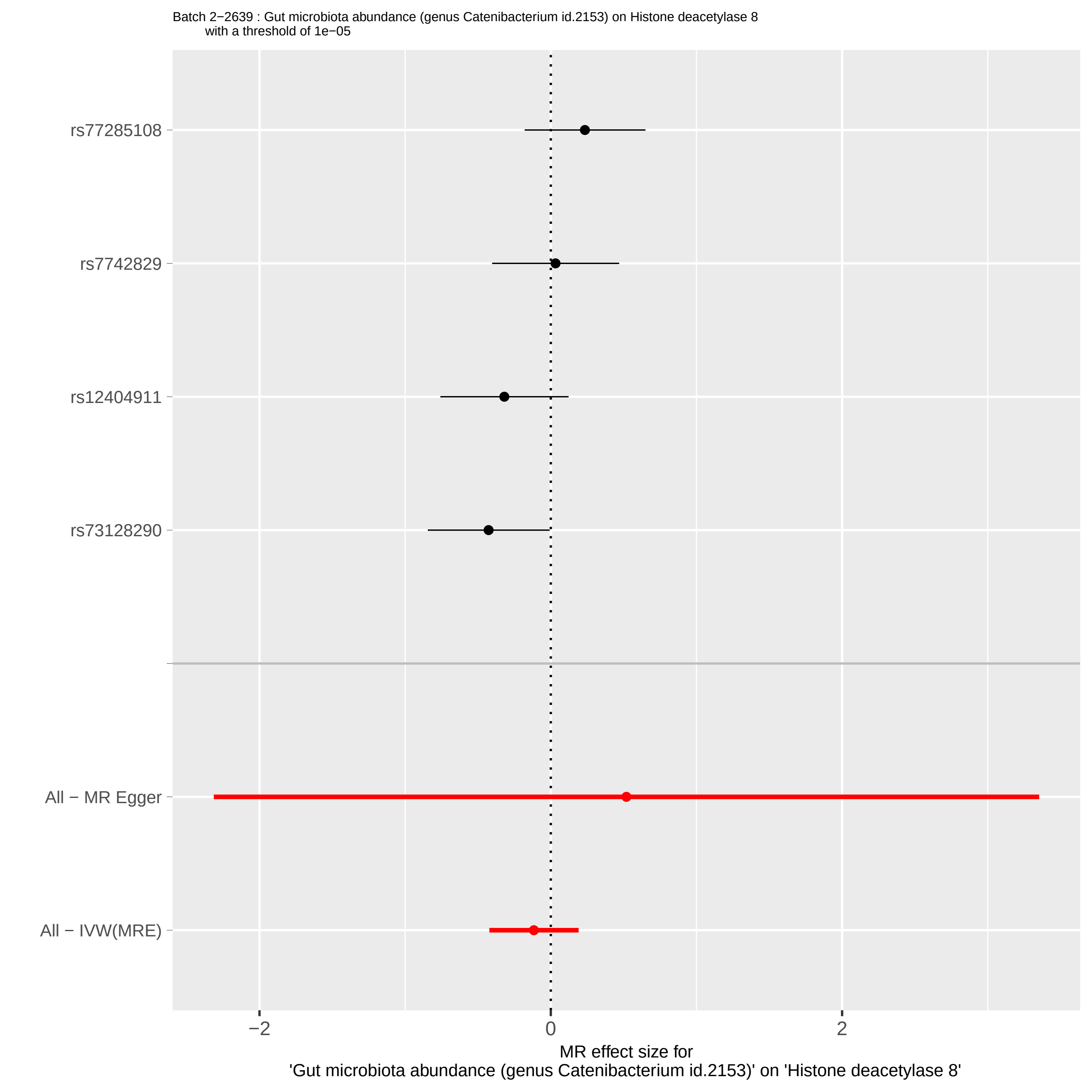

Batch 2−2639 : Gut microbiota abundance (genus Catenibacterium id.2153) on Histone deacetylase 8
with a threshold of 1e−05
rs77285108
rs7742829
rs12404911
rs73128290
All − MR Egger
All − IVW(MRE)
−2
0
2
MR effect size for
'Gut microbiota abundance (genus Catenibacterium id.2153)' on 'Histone deacetylase 8'

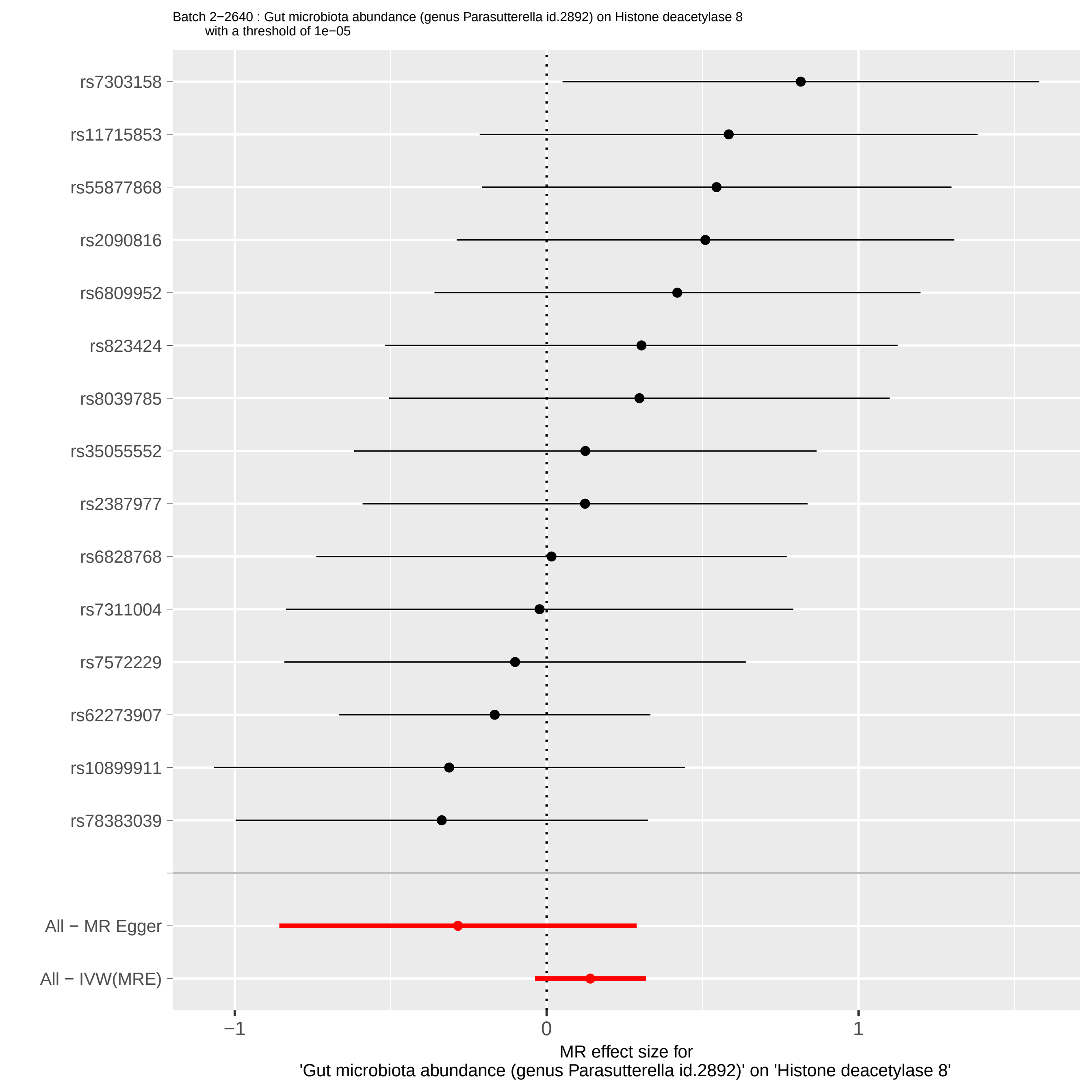

Batch 2−2640 : Gut microbiota abundance (genus Parasutterella id.2892) on Histone deacetylase 8
with a threshold of 1e−05
rs7303158
rs11715853
rs55877868
rs2090816
rs6809952
rs823424
rs8039785
rs35055552
rs2387977
rs6828768
rs7311004
rs7572229
rs62273907
rs10899911
rs78383039
All − MR Egger
All − IVW(MRE)
−1
0
1
MR effect size for
'Gut microbiota abundance (genus Parasutterella id.2892)' on 'Histone deacetylase 8'

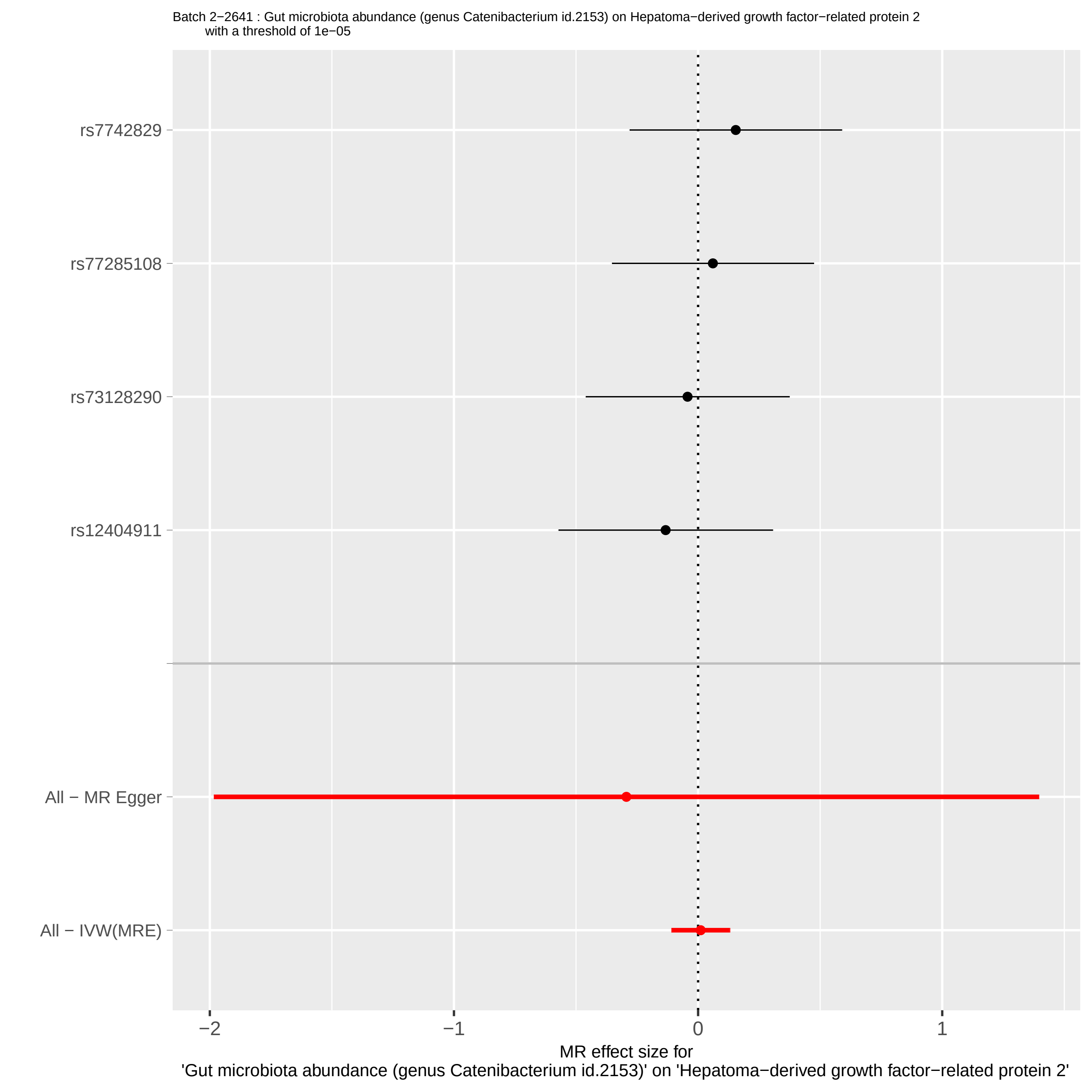

Batch 2−2641 : Gut microbiota abundance (genus Catenibacterium id.2153) on Hepatoma−derived growth factor−related protein 2
with a threshold of 1e−05
rs7742829
rs77285108
rs73128290
rs12404911
All − MR Egger
All − IVW(MRE)
−2
−1
0
1
MR effect size for
'Gut microbiota abundance (genus Catenibacterium id.2153)' on 'Hepatoma−derived growth factor−related protein 2'

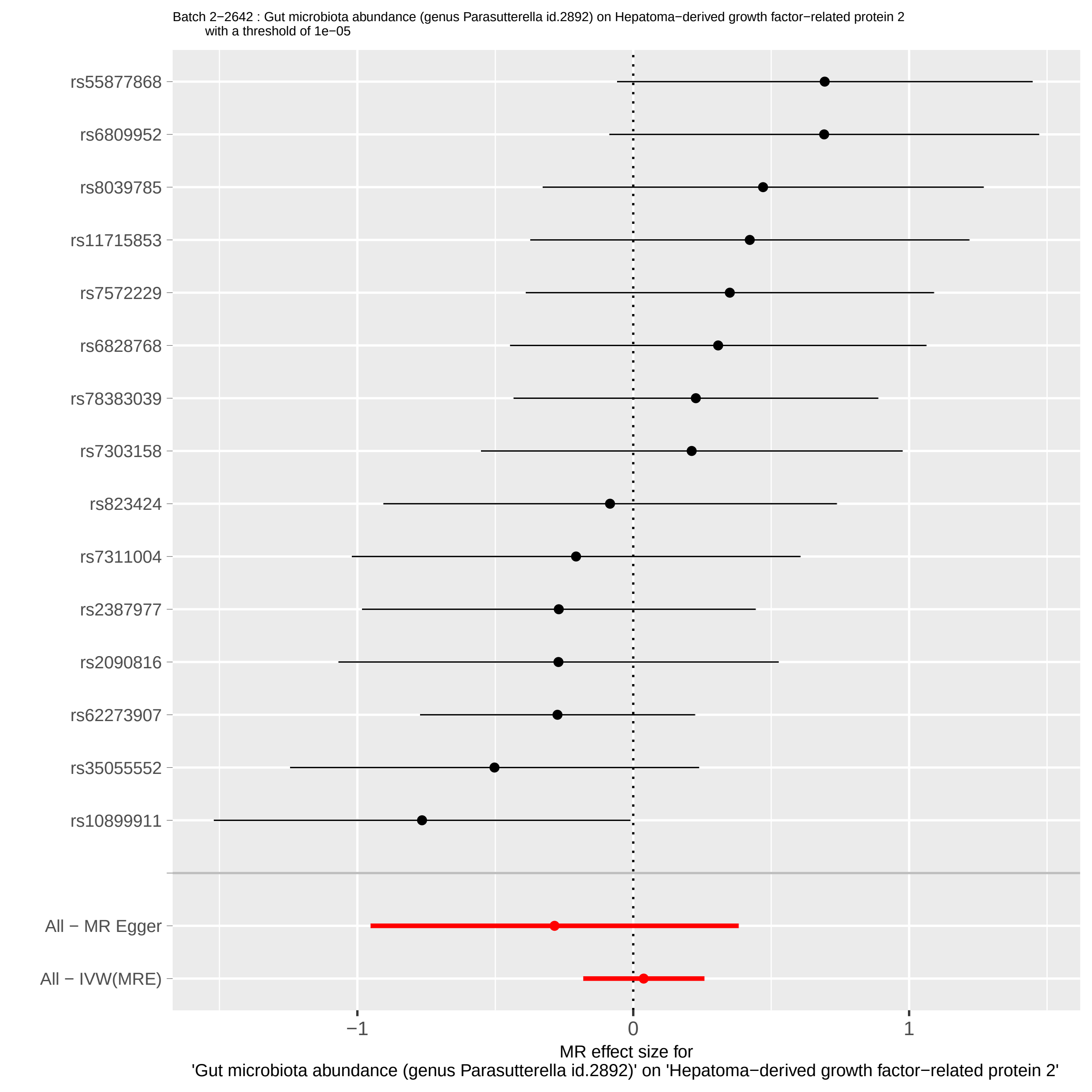

Batch 2−2642 : Gut microbiota abundance (genus Parasutterella id.2892) on Hepatoma−derived growth factor−related protein 2
with a threshold of 1e−05
rs55877868
rs6809952
rs8039785
rs11715853
rs7572229
rs6828768
rs78383039
rs7303158
rs823424
rs7311004
rs2387977
rs2090816
rs62273907
rs35055552
rs10899911
All − MR Egger
All − IVW(MRE)
−1
0
1
MR effect size for
'Gut microbiota abundance (genus Parasutterella id.2892)' on 'Hepatoma−derived growth factor−related protein 2'

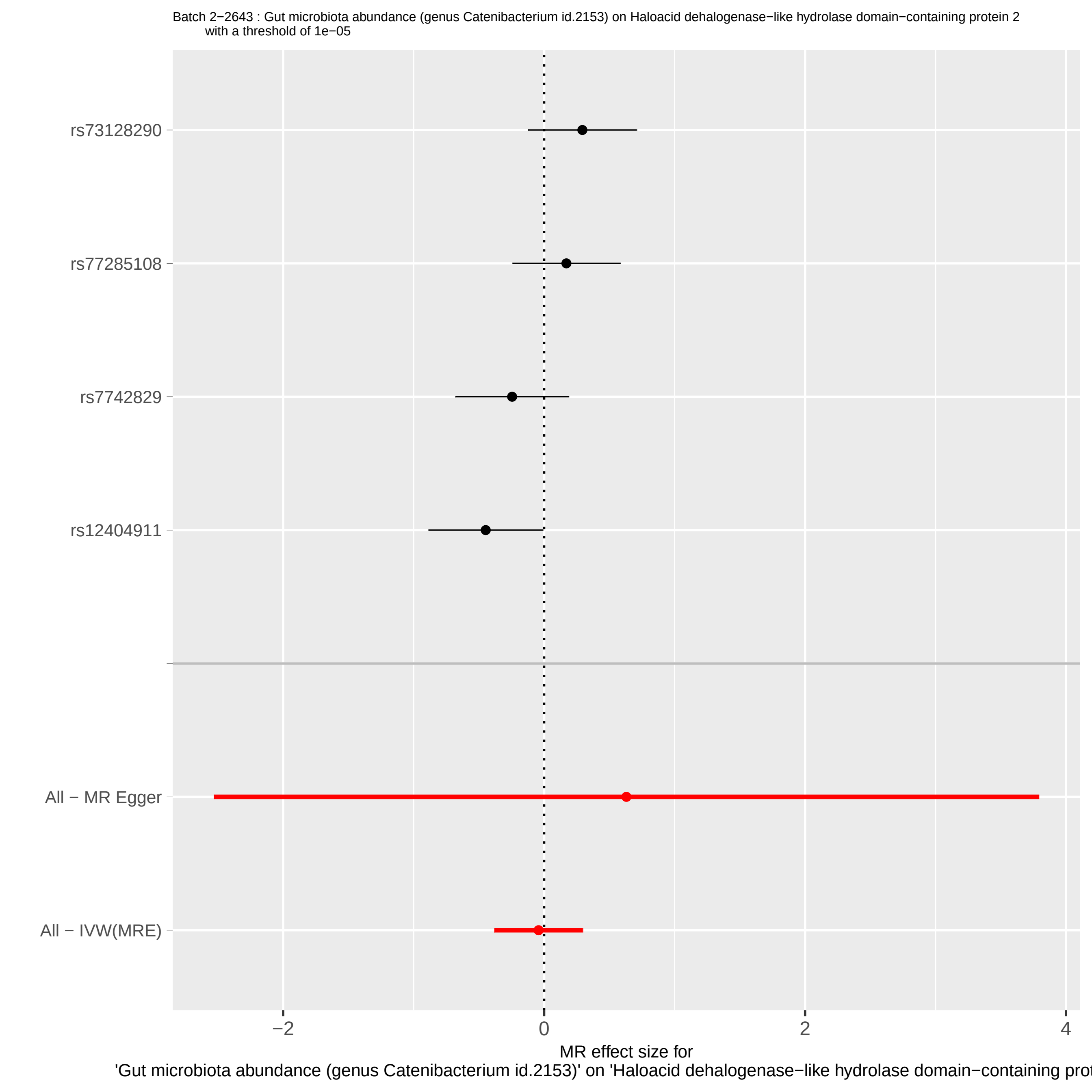

Batch 2−2643 : Gut microbiota abundance (genus Catenibacterium id.2153) on Haloacid dehalogenase−like hydrolase domain−containing protein 2
with a threshold of 1e−05
rs73128290
rs77285108
rs7742829
rs12404911
All − MR Egger
All − IVW(MRE)
−2
0
2
4
MR effect size for
'Gut microbiota abundance (genus Catenibacterium id.2153)' on 'Haloacid dehalogenase−like hydrolase domain−containing prot

Batch 2−2644 : Gut microbiota abundance (genus Parasutterella id.2892) on Haloacid dehalogenase−like hydrolase domain−containing protein 2 with a threshold of 1e−05

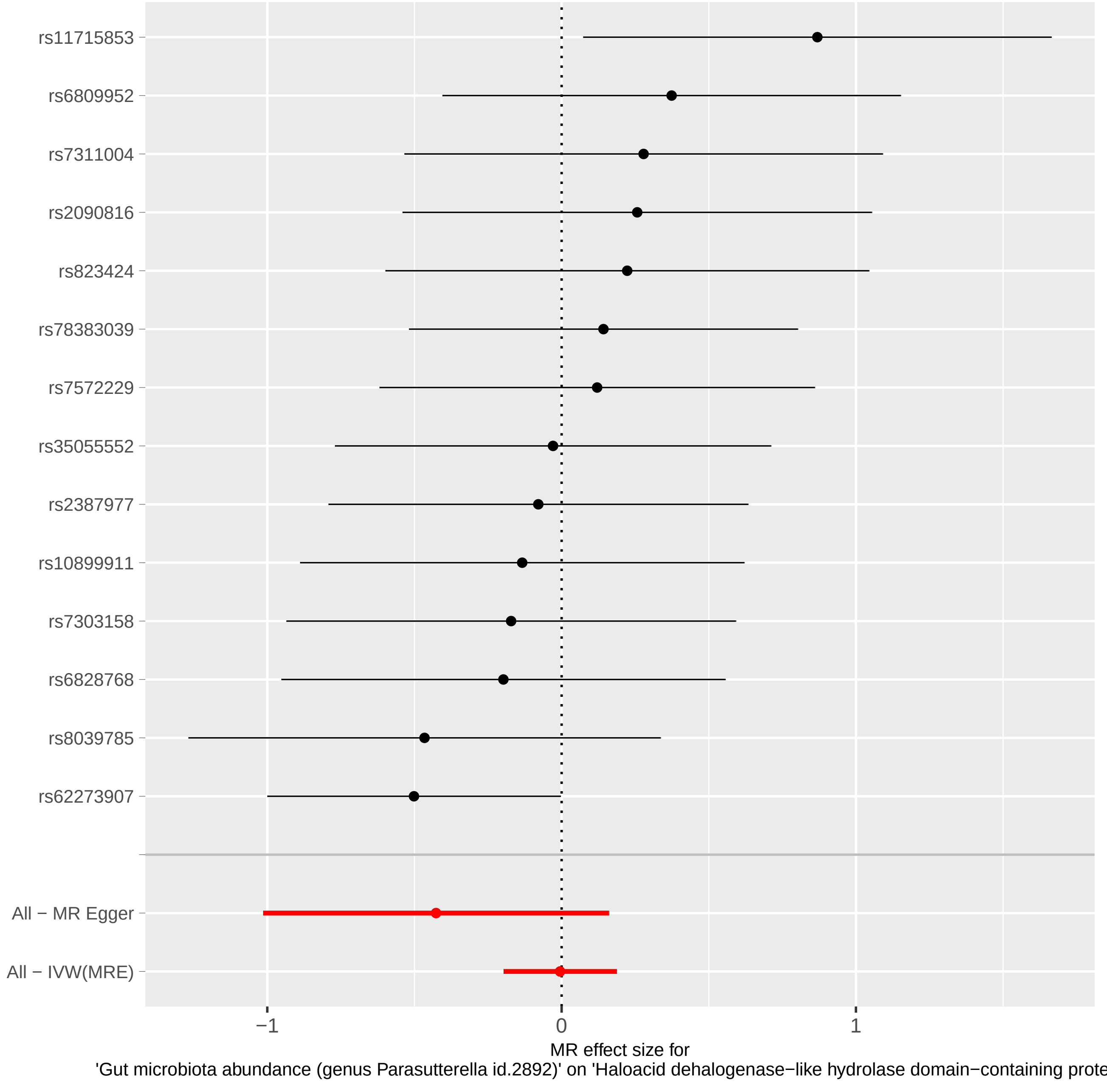

Batch 2−2645 : Gut microbiota abundance (genus Catenibacterium id.2153) on Vigilin
with a threshold of 1e−05

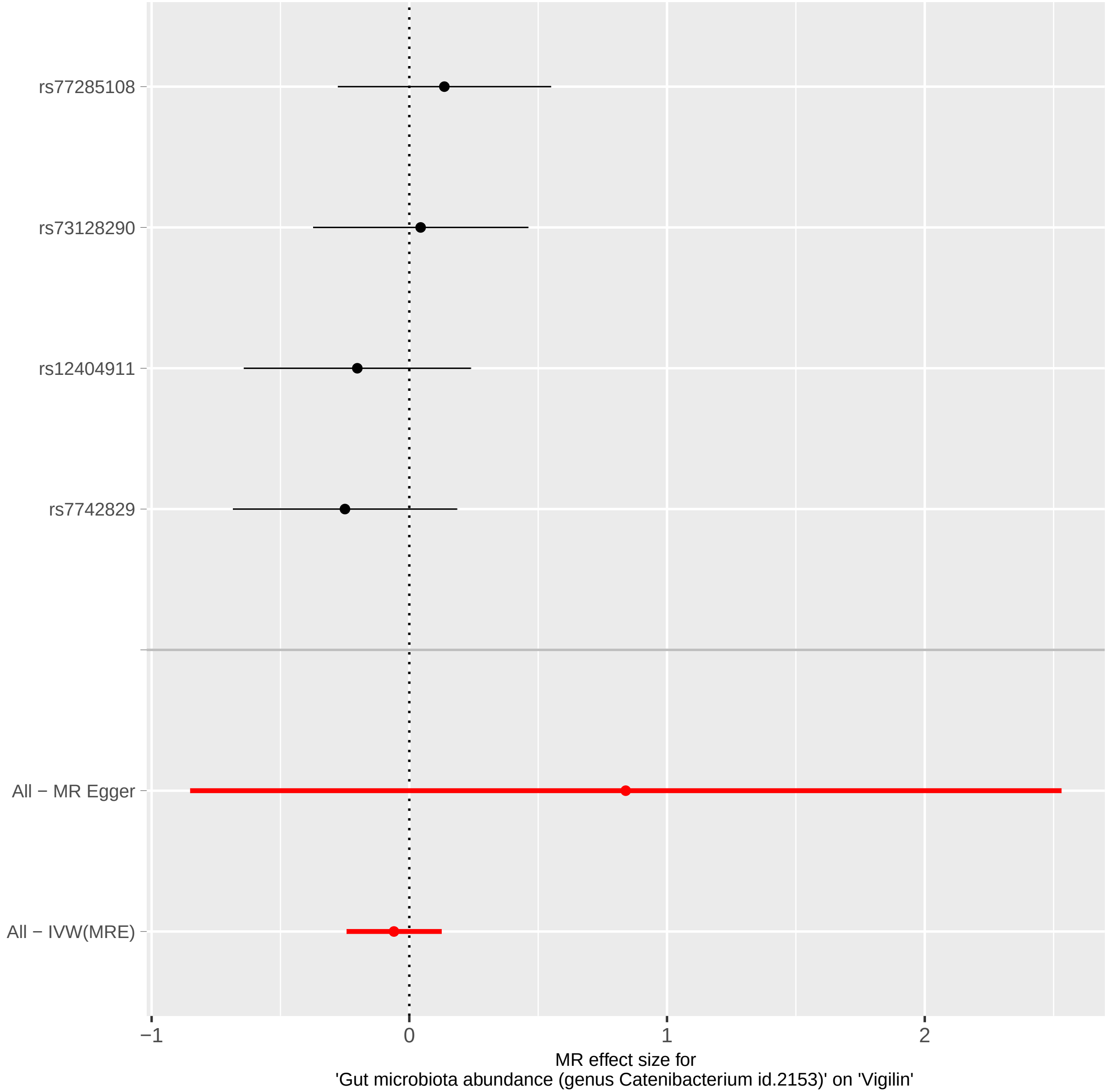

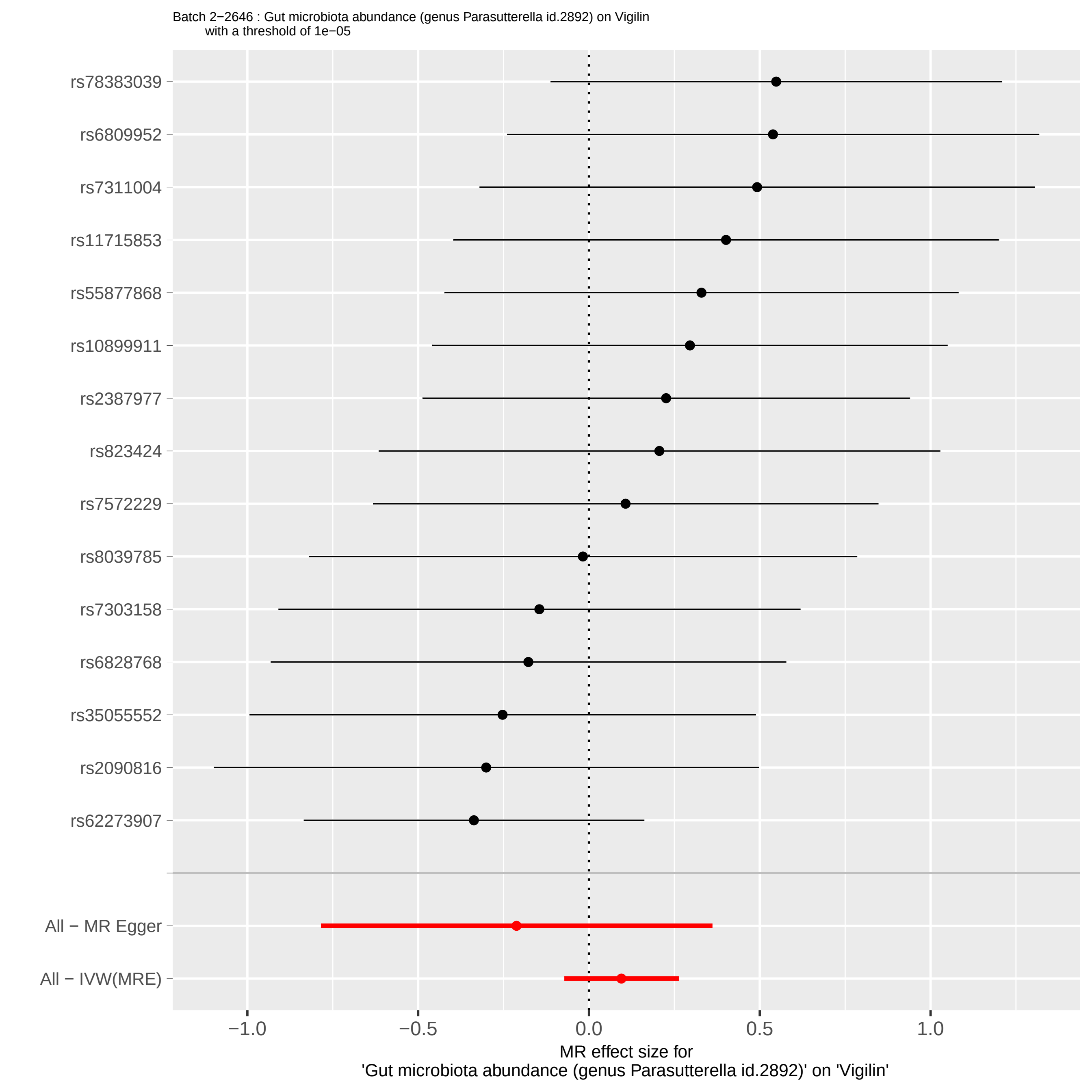
Batch 2−2646 : Gut microbiota abundance (genus Parasutterella id.2892) on Vigilin
with a threshold of 1e−05
rs78383039
rs6809952
rs7311004
rs11715853
rs55877868
rs10899911
rs2387977
rs823424
rs7572229
rs8039785
rs7303158
rs6828768
rs35055552
rs2090816
rs62273907
All − MR Egger
All − IVW(MRE)
−1.0
−0.5
0.0
0.5
1.0
MR effect size for
'Gut microbiota abundance (genus Parasutterella id.2892)' on 'Vigilin'

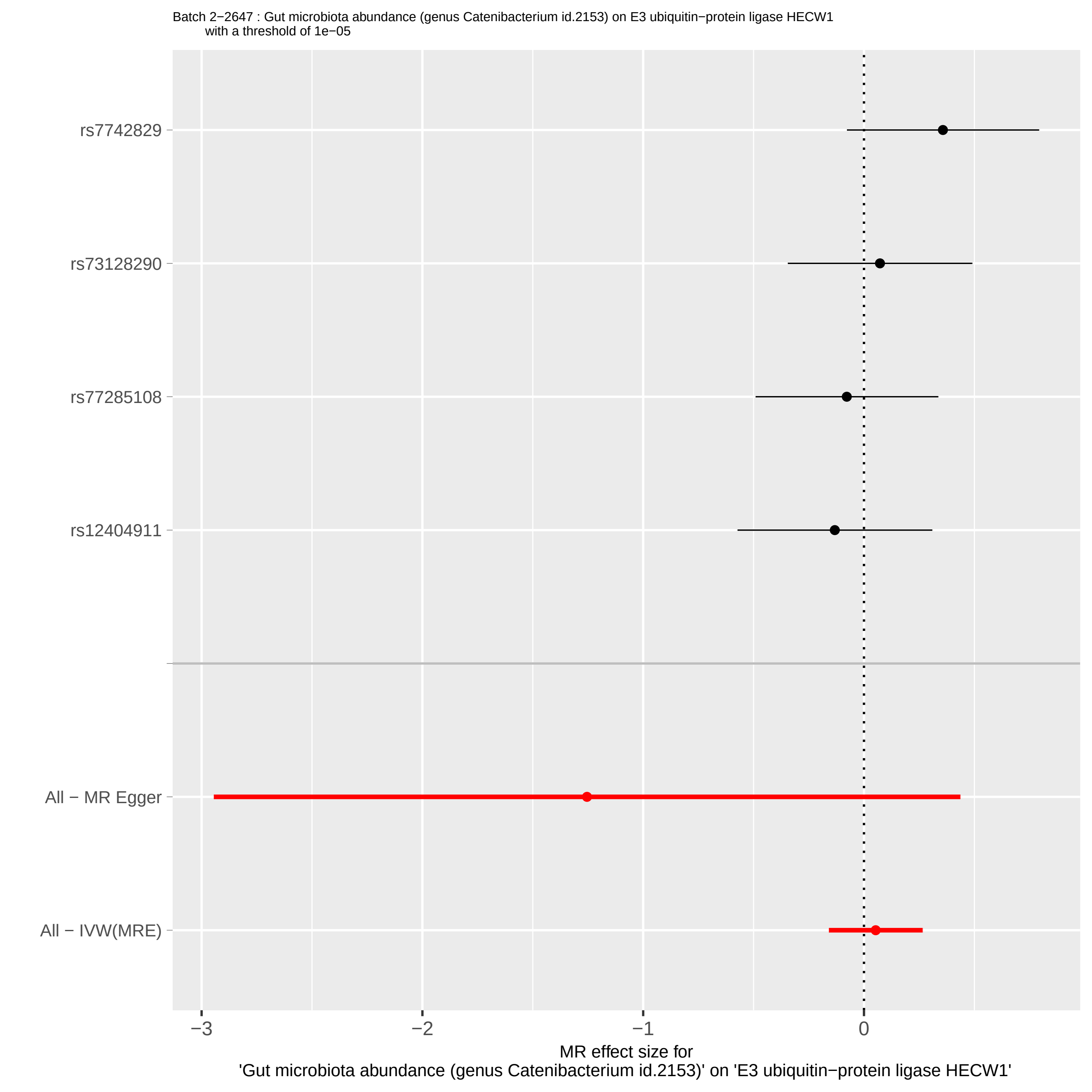

Batch 2−2647 : Gut microbiota abundance (genus Catenibacterium id.2153) on E3 ubiquitin−protein ligase HECW1
with a threshold of 1e−05
rs7742829
rs73128290
rs77285108
rs12404911
All − MR Egger
All − IVW(MRE)
−3
−2
−1
0
MR effect size for
'Gut microbiota abundance (genus Catenibacterium id.2153)' on 'E3 ubiquitin−protein ligase HECW1'

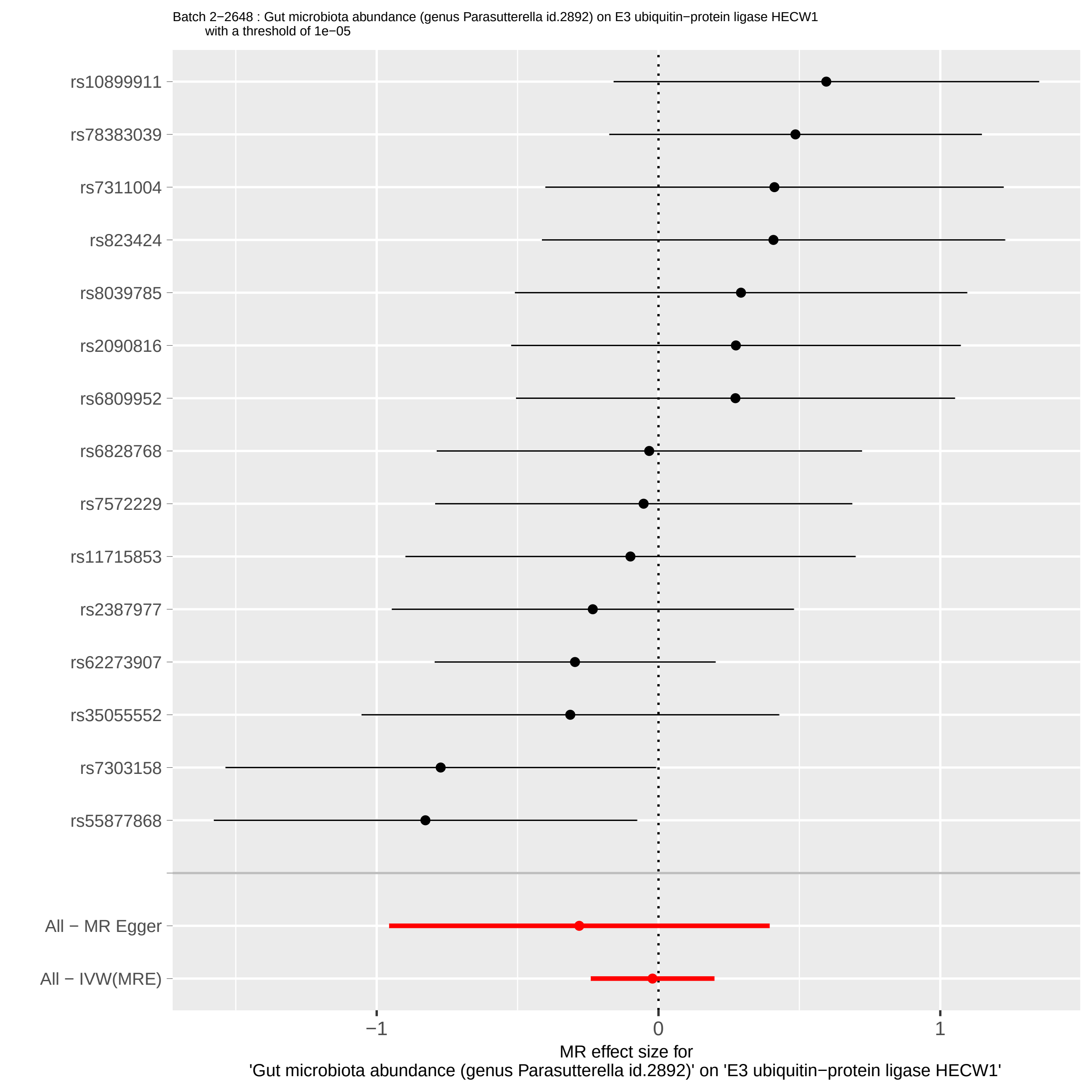

Batch 2−2648 : Gut microbiota abundance (genus Parasutterella id.2892) on E3 ubiquitin−protein ligase HECW1
with a threshold of 1e−05
rs10899911
rs78383039
rs7311004
rs823424
rs8039785
rs2090816
rs6809952
rs6828768
rs7572229
rs11715853
rs2387977
rs62273907
rs35055552
rs7303158
rs55877868
All − MR Egger
All − IVW(MRE)
−1
0
1
MR effect size for
'Gut microbiota abundance (genus Parasutterella id.2892)' on 'E3 ubiquitin−protein ligase HECW1'

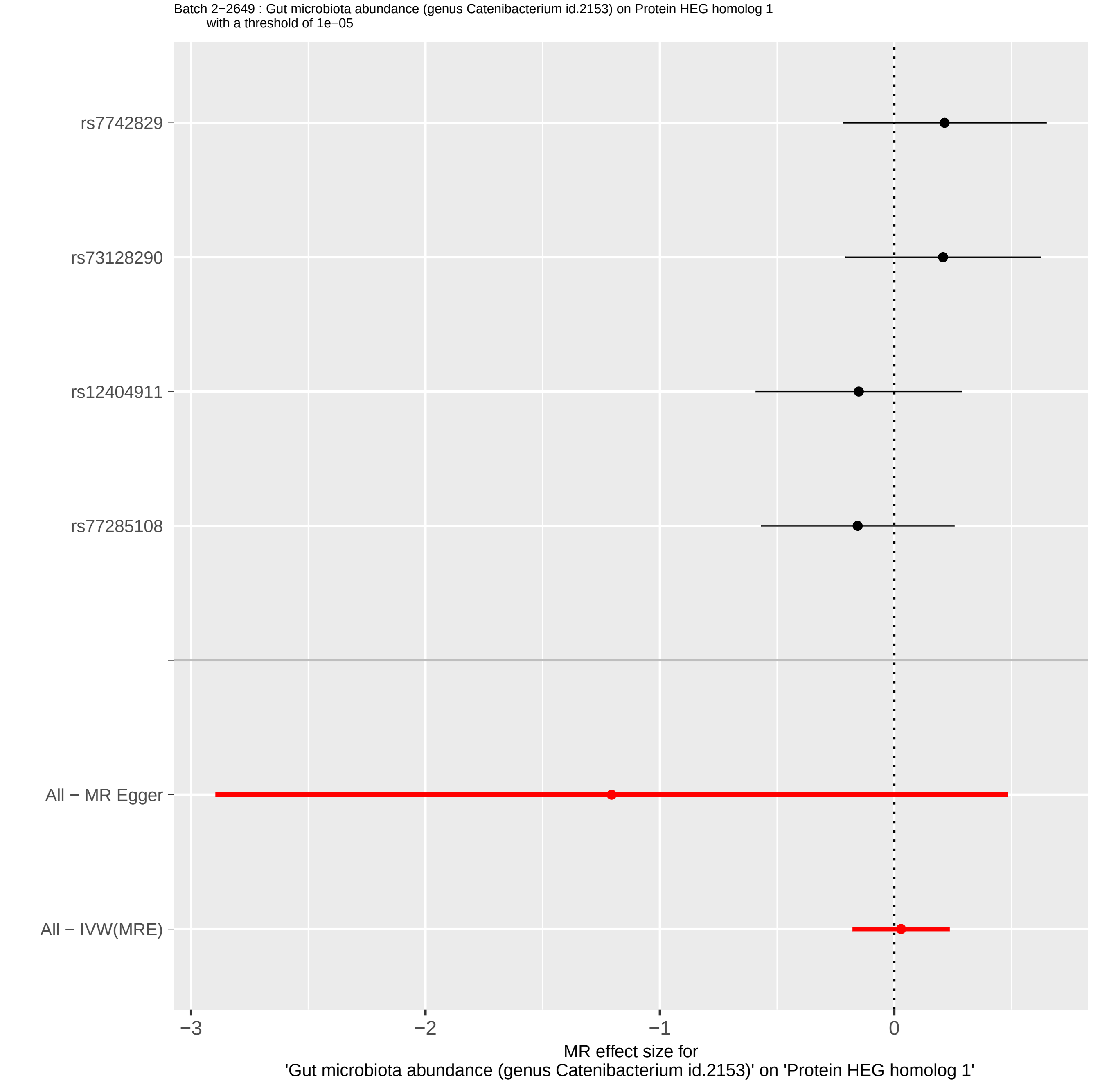
Batch 2−2649 : Gut microbiota abundance (genus Catenibacterium id.2153) on Protein HEG homolog 1
with a threshold of 1e−05
rs7742829
rs73128290
rs12404911
rs77285108
All − MR Egger
All − IVW(MRE)
−3
−2
−1
0
MR effect size for
'Gut microbiota abundance (genus Catenibacterium id.2153)' on 'Protein HEG homolog 1'

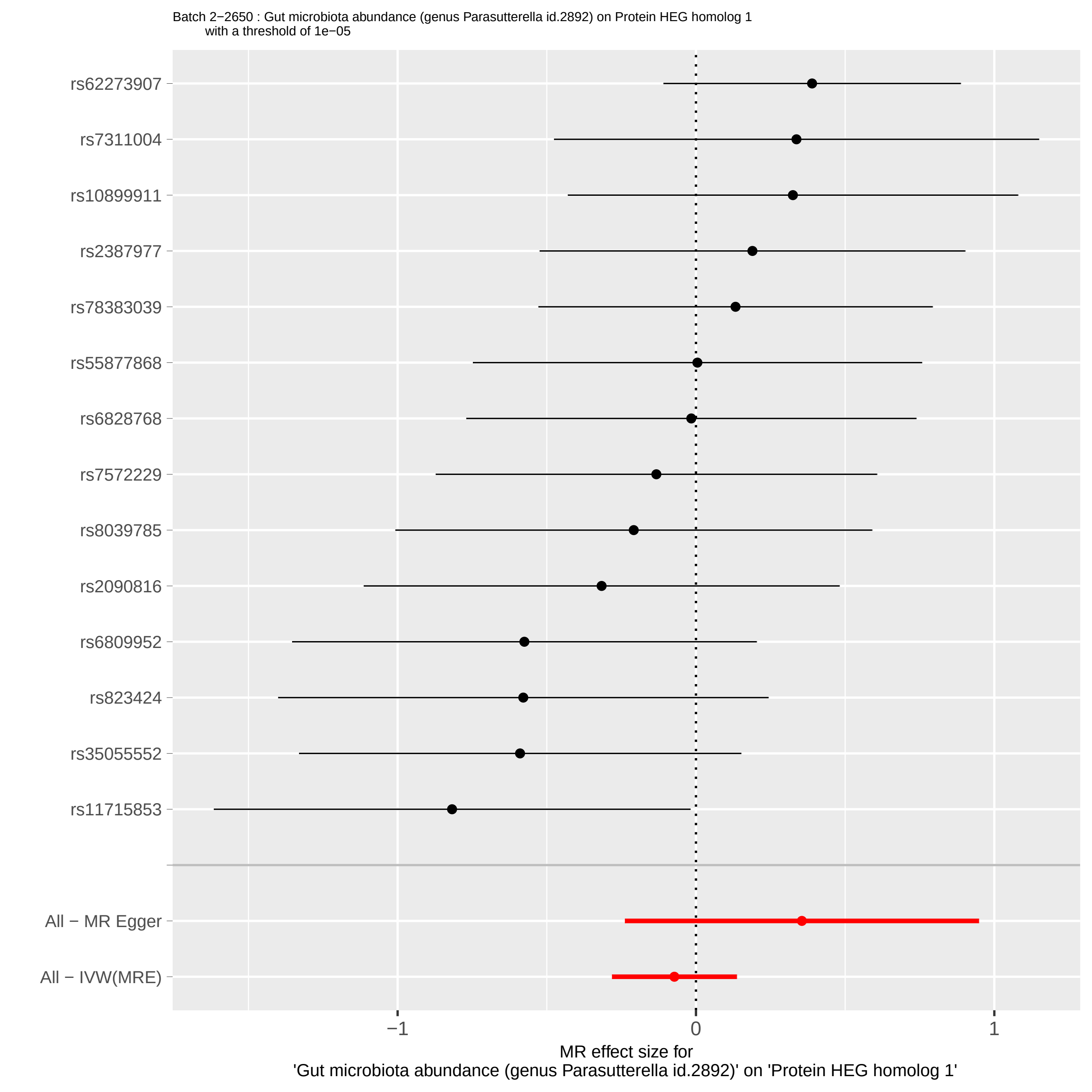

Batch 2−2650 : Gut microbiota abundance (genus Parasutterella id.2892) on Protein HEG homolog 1
with a threshold of 1e−05
rs62273907
rs7311004
rs10899911
rs2387977
rs78383039
rs55877868
rs6828768
rs7572229
rs8039785
rs2090816
rs6809952
rs823424
rs35055552
rs11715853
All − MR Egger
All − IVW(MRE)
−1
0
1
MR effect size for
'Gut microbiota abundance (genus Parasutterella id.2892)' on 'Protein HEG homolog 1'

Batch 2−2651 : Gut microbiota abundance (genus Catenibacterium id.2153) on HEPACAM family member 2
with a threshold of 1e−05

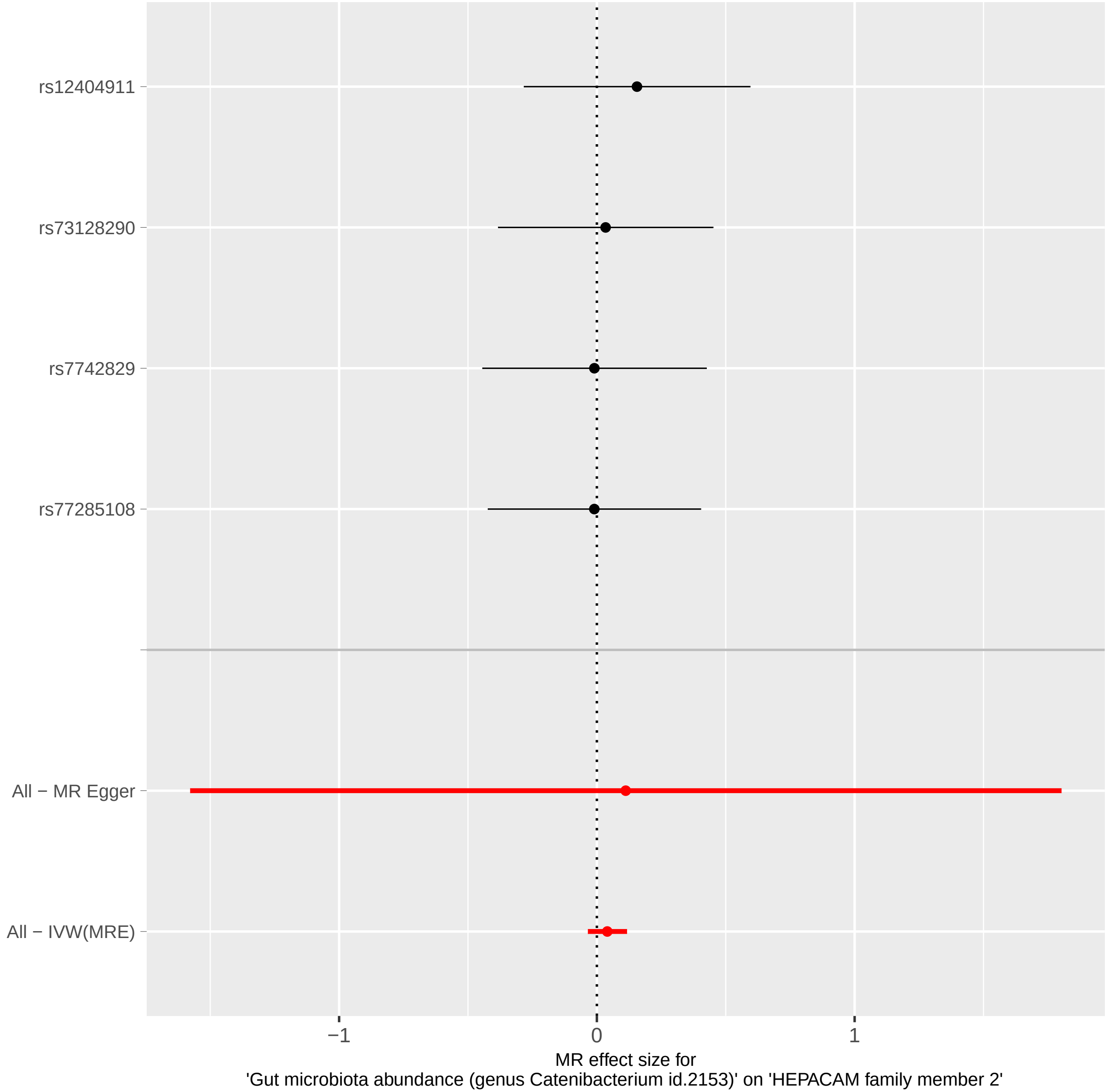

Batch 2−2652 : Gut microbiota abundance (genus Parasutterella id.2892) on HEPACAM family member 2
with a threshold of 1e−05

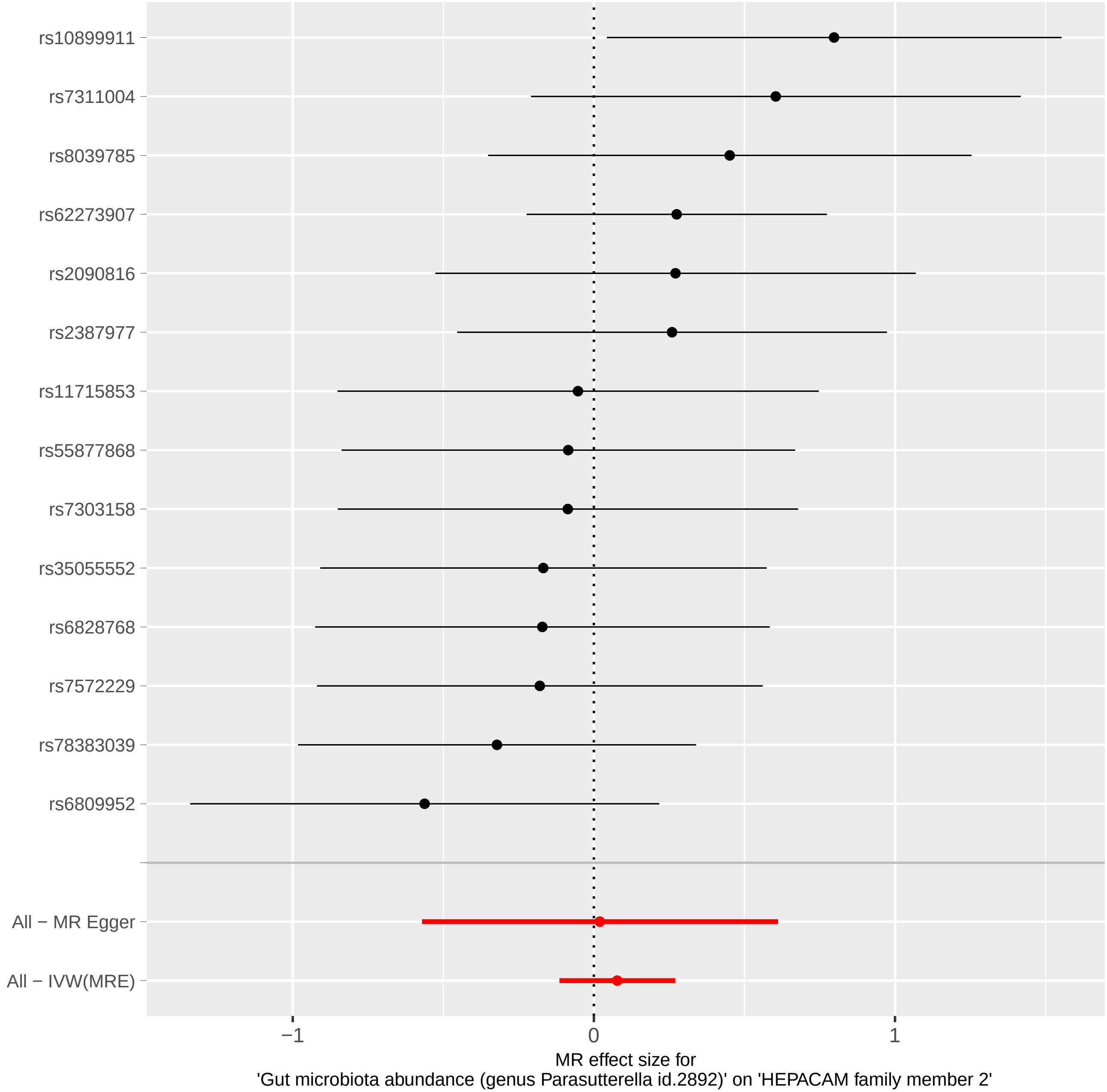

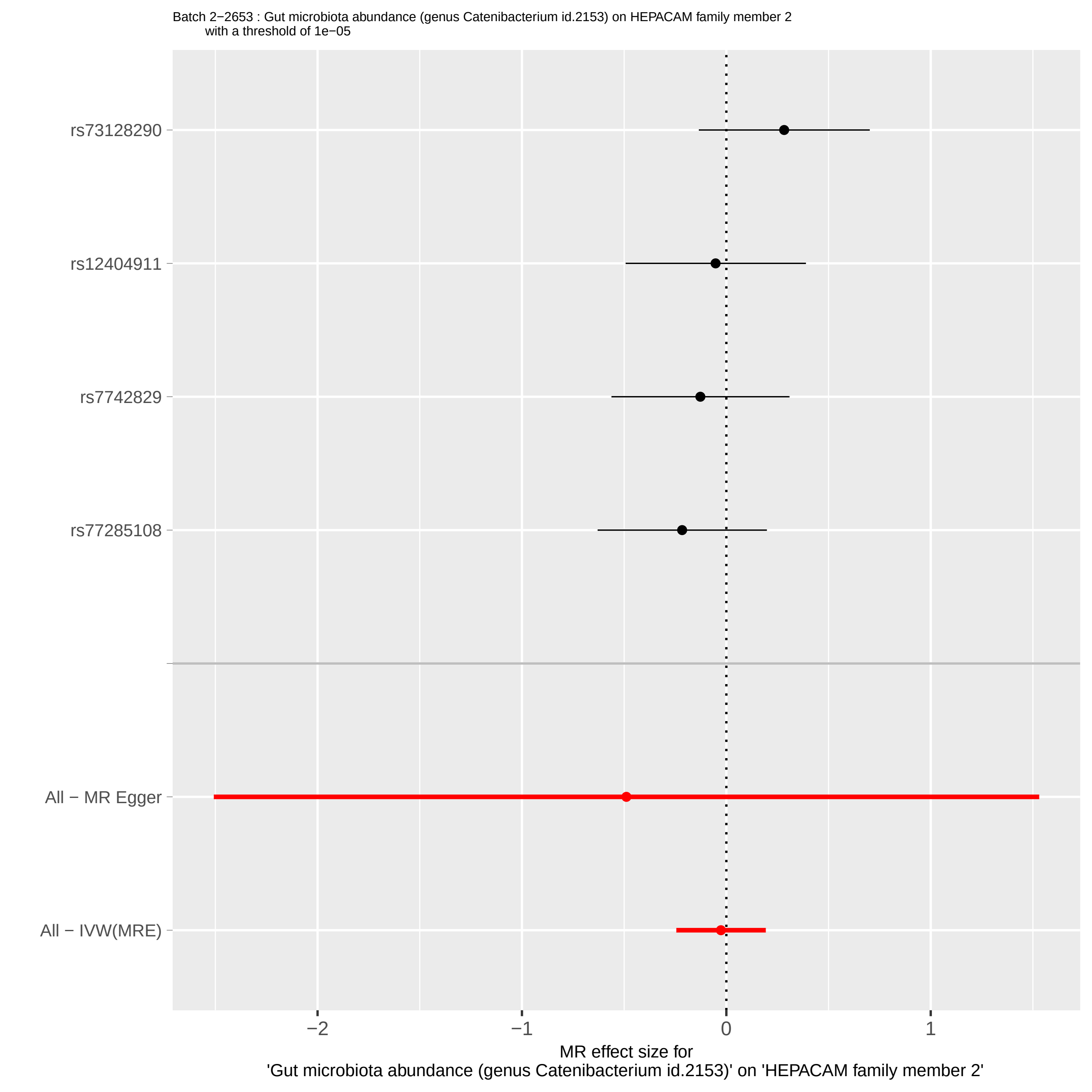

Batch 2−2653 : Gut microbiota abundance (genus Catenibacterium id.2153) on HEPACAM family member 2
with a threshold of 1e−05
rs73128290
rs12404911
rs7742829
rs77285108
All − MR Egger
All − IVW(MRE)
−2
−1
0
1
MR effect size for
'Gut microbiota abundance (genus Catenibacterium id.2153)' on 'HEPACAM family member 2'

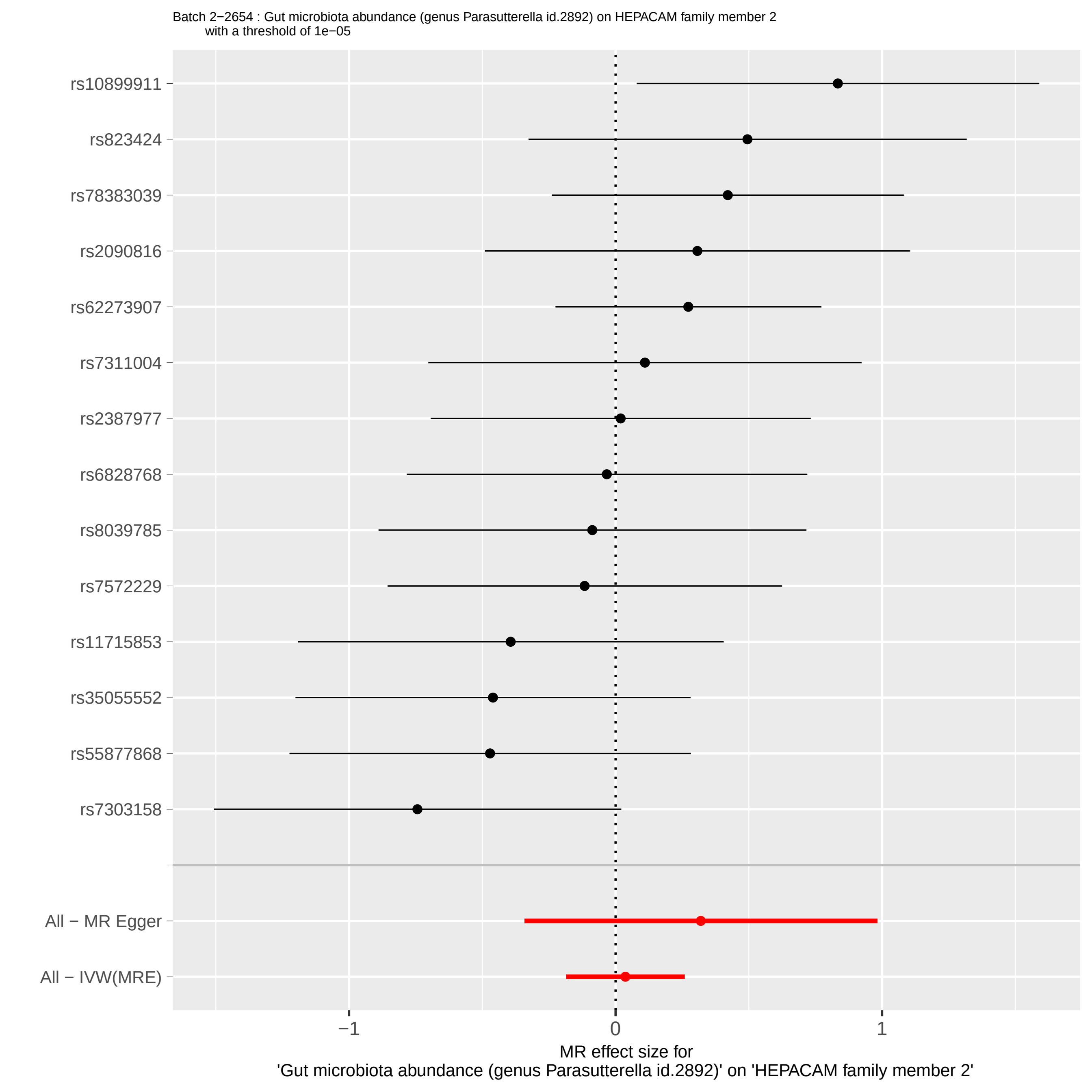

Batch 2−2654 : Gut microbiota abundance (genus Parasutterella id.2892) on HEPACAM family member 2
with a threshold of 1e−05
rs10899911
rs823424
rs78383039
rs2090816
rs62273907
rs7311004
rs2387977
rs6828768
rs8039785
rs7572229
rs11715853
rs35055552
rs55877868
rs7303158
All − MR Egger
All − IVW(MRE)
−1
0
1
MR effect size for
'Gut microbiota abundance (genus Parasutterella id.2892)' on 'HEPACAM family member 2'

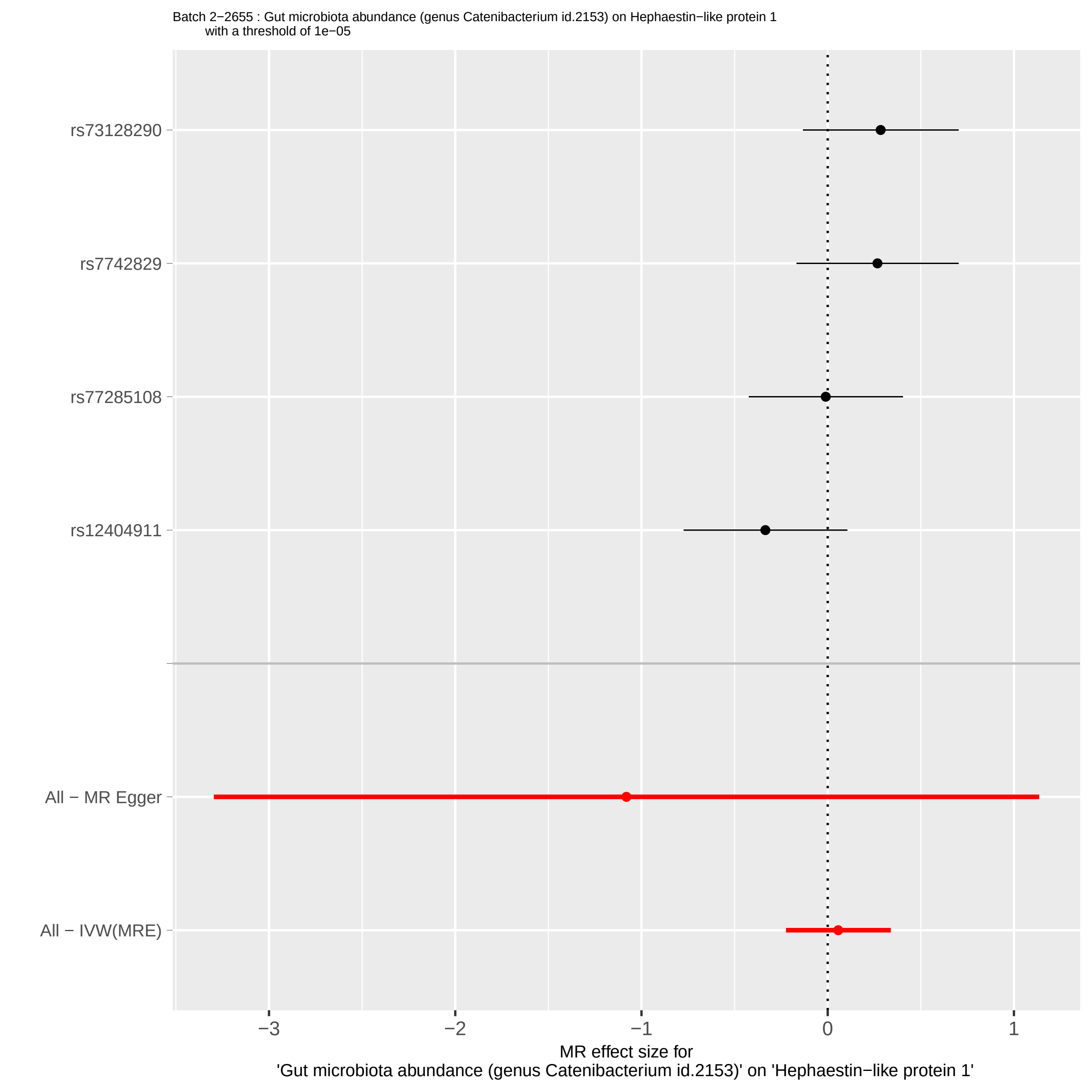
Batch 2−2655 : Gut microbiota abundance (genus Catenibacterium id.2153) on Hephaestin−like protein 1
with a threshold of 1e−05
rs73128290
rs7742829
rs77285108
rs12404911
All − MR Egger
All − IVW(MRE)
−3
−2
−1
0
1
MR effect size for
'Gut microbiota abundance (genus Catenibacterium id.2153)' on 'Hephaestin−like protein 1'

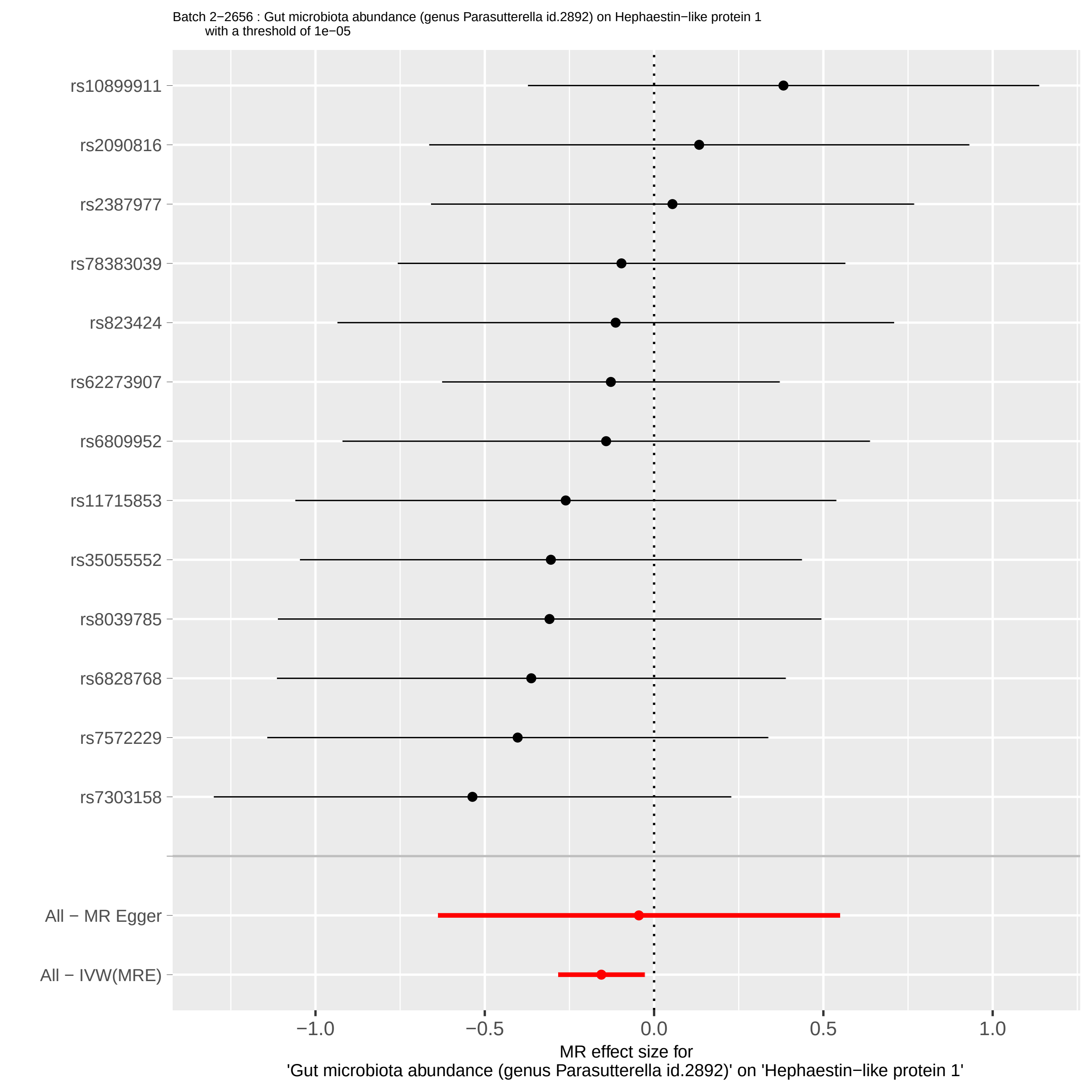

Batch 2−2656 : Gut microbiota abundance (genus Parasutterella id.2892) on Hephaestin−like protein 1
with a threshold of 1e−05
rs10899911
rs2090816
rs2387977
rs78383039
rs823424
rs62273907
rs6809952
rs11715853
rs35055552
rs8039785
rs6828768
rs7572229
rs7303158
All − MR Egger
All − IVW(MRE)
−1.0
−0.5
0.0
0.5
1.0
MR effect size for
'Gut microbiota abundance (genus Parasutterella id.2892)' on 'Hephaestin−like protein 1'

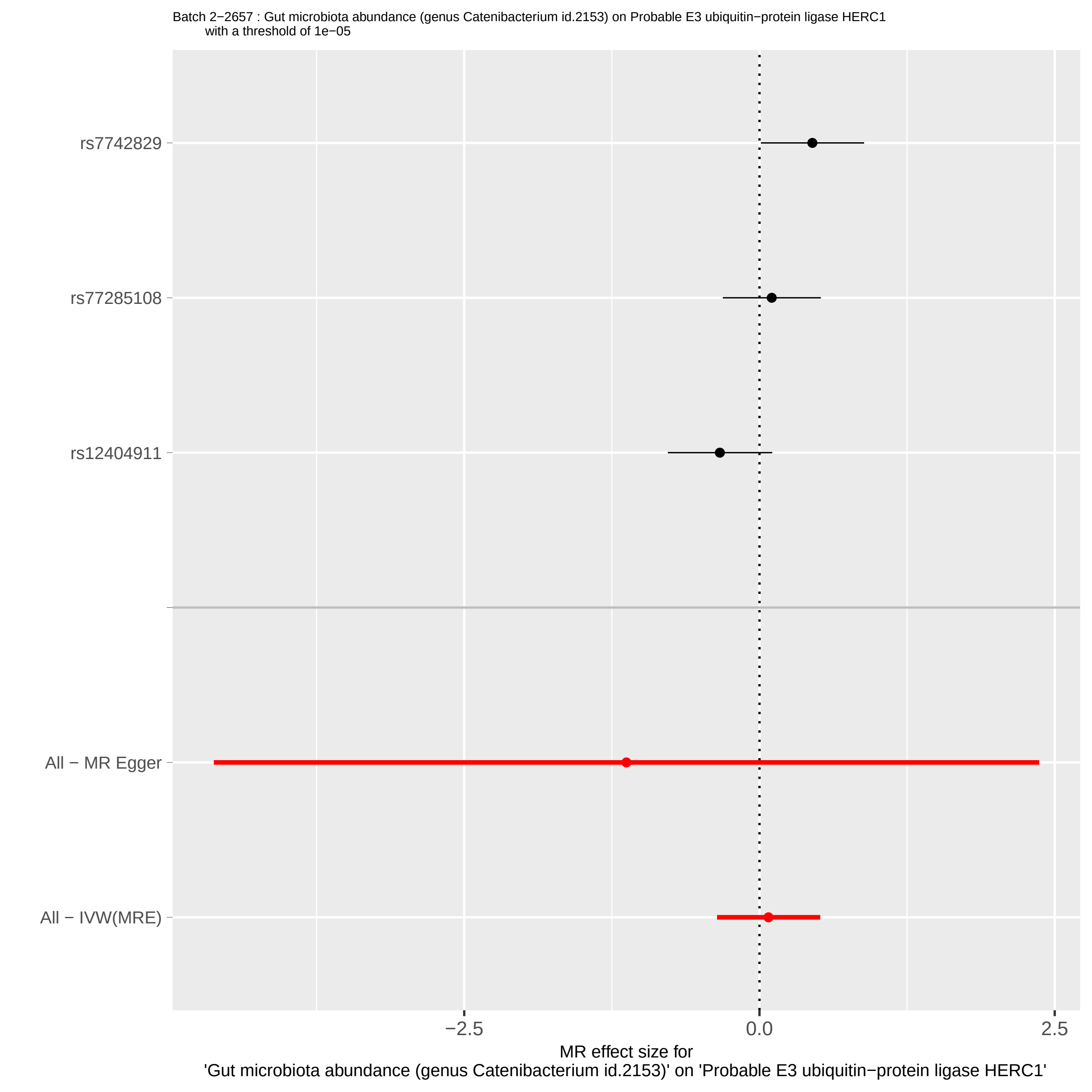
Batch 2−2657 : Gut microbiota abundance (genus Catenibacterium id.2153) on Probable E3 ubiquitin−protein ligase HERC1
with a threshold of 1e−05
rs7742829
rs77285108
rs12404911
All − MR Egger
All − IVW(MRE)
−2.5
0.0
2.5
MR effect size for
'Gut microbiota abundance (genus Catenibacterium id.2153)' on 'Probable E3 ubiquitin−protein ligase HERC1'

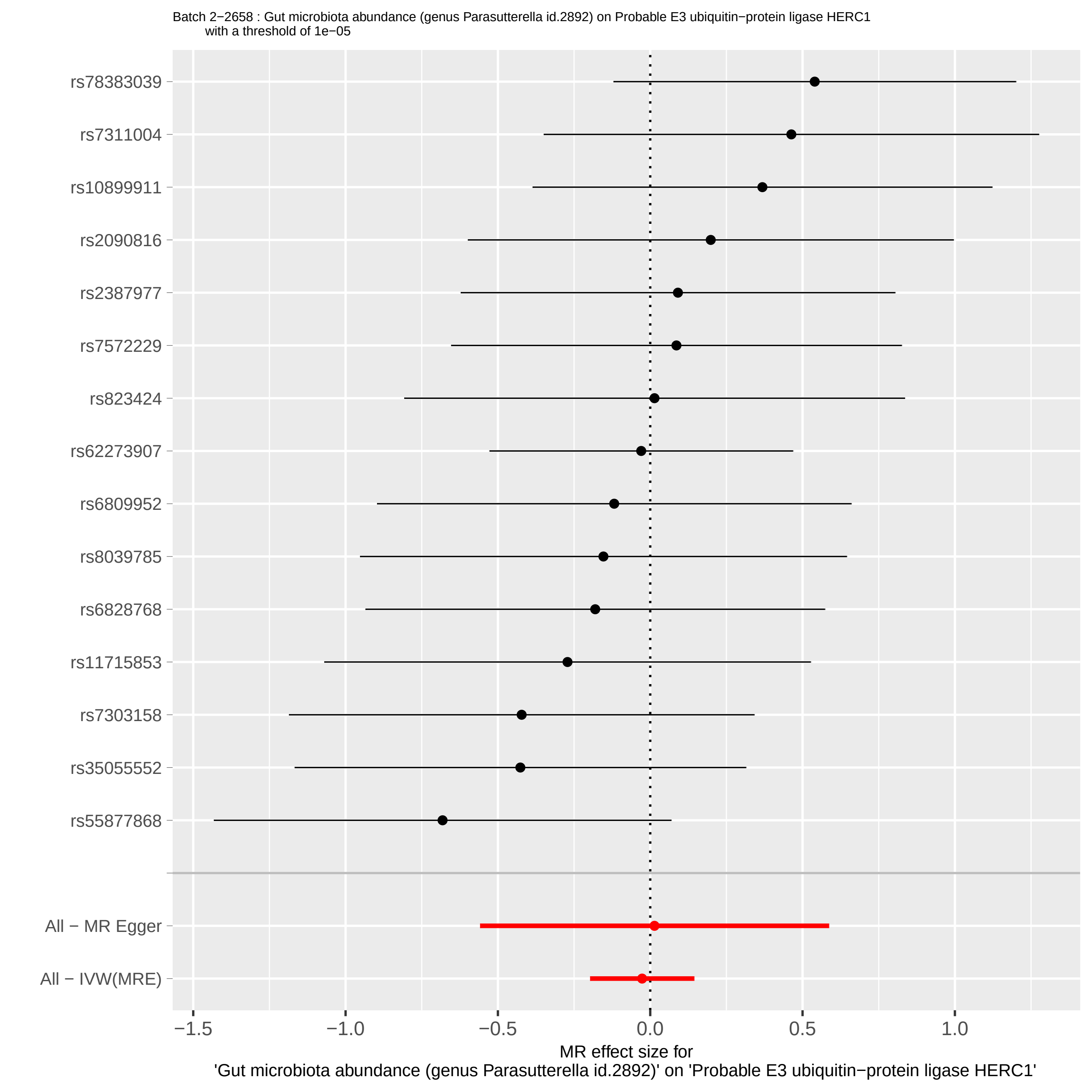

Batch 2−2658 : Gut microbiota abundance (genus Parasutterella id.2892) on Probable E3 ubiquitin−protein ligase HERC1
with a threshold of 1e−05
rs78383039
rs7311004
rs10899911
rs2090816
rs2387977
rs7572229
rs823424
rs62273907
rs6809952
rs8039785
rs6828768
rs11715853
rs7303158
rs35055552
rs55877868
All − MR Egger
All − IVW(MRE)
−1.5
−1.0
−0.5
0.0
0.5
1.0
MR effect size for
'Gut microbiota abundance (genus Parasutterella id.2892)' on 'Probable E3 ubiquitin−protein ligase HERC1'

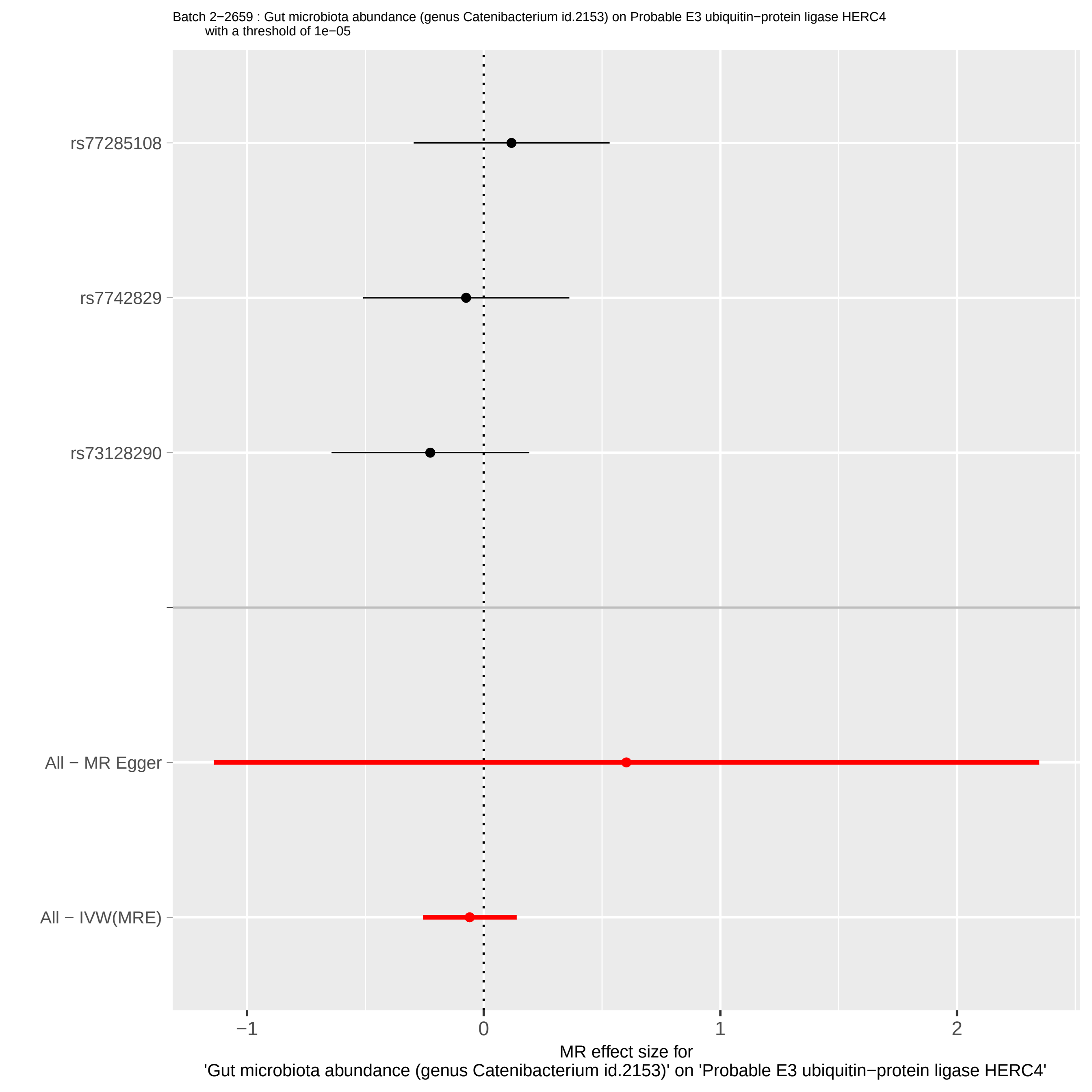

Batch 2−2659 : Gut microbiota abundance (genus Catenibacterium id.2153) on Probable E3 ubiquitin−protein ligase HERC4
with a threshold of 1e−05
rs77285108
rs7742829
rs73128290
All − MR Egger
All − IVW(MRE)
−1
0
1
2
MR effect size for
'Gut microbiota abundance (genus Catenibacterium id.2153)' on 'Probable E3 ubiquitin−protein ligase HERC4'

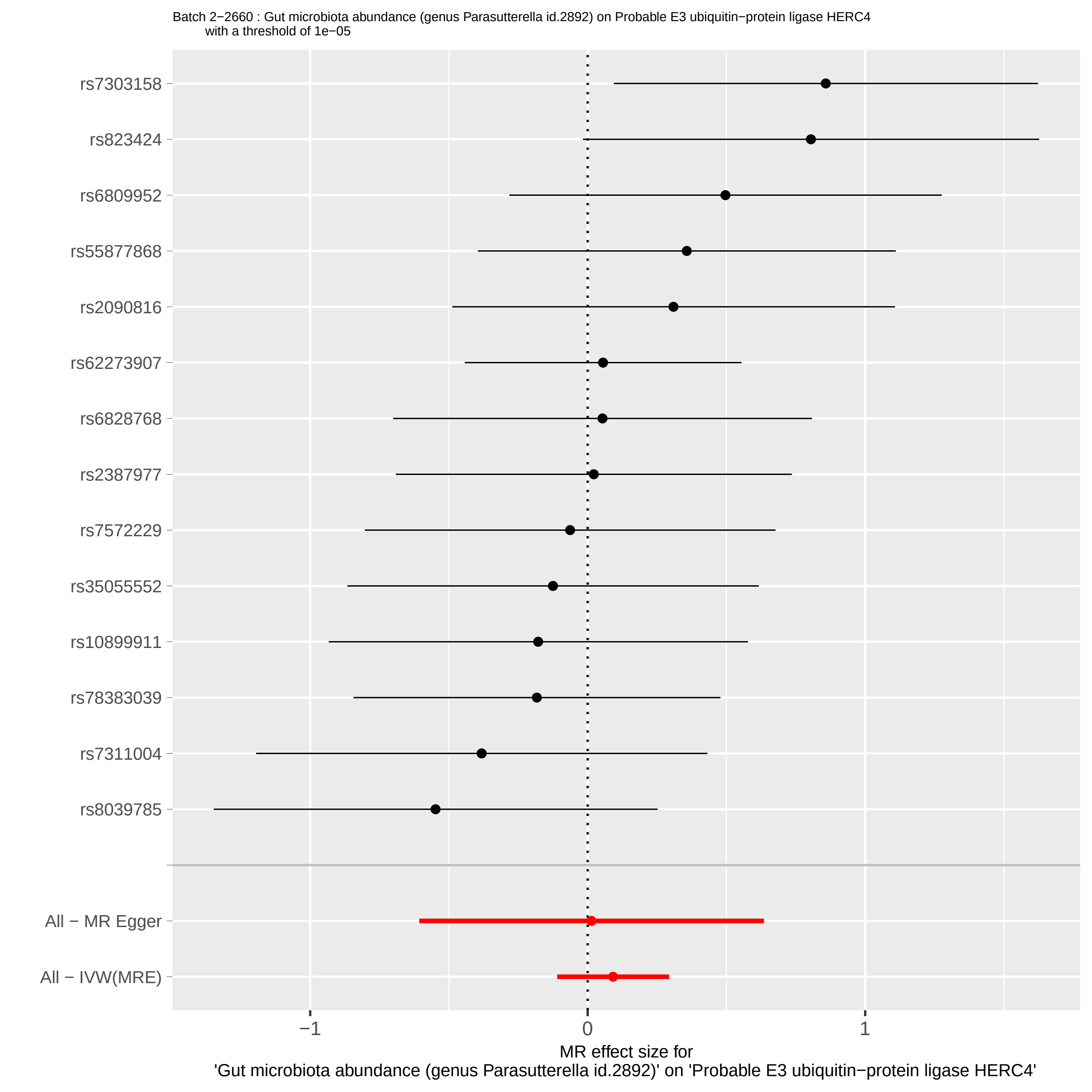

Batch 2−2660 : Gut microbiota abundance (genus Parasutterella id.2892) on Probable E3 ubiquitin−protein ligase HERC4
with a threshold of 1e−05
rs7303158
rs823424
rs6809952
rs55877868
rs2090816
rs62273907
rs6828768
rs2387977
rs7572229
rs35055552
rs10899911
rs78383039
rs7311004
rs8039785
All − MR Egger
All − IVW(MRE)
−1
0
1
MR effect size for
'Gut microbiota abundance (genus Parasutterella id.2892)' on 'Probable E3 ubiquitin−protein ligase HERC4'

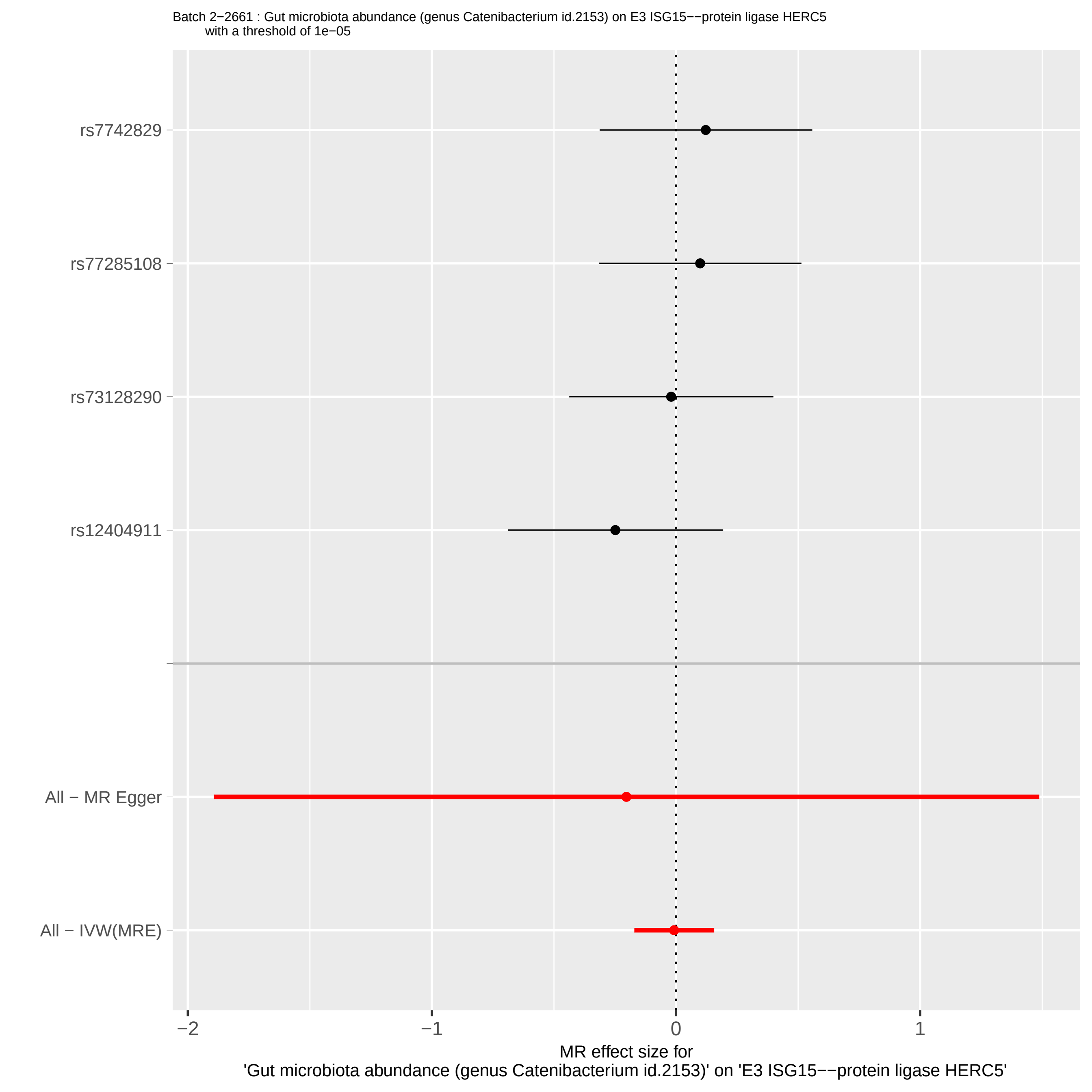

Batch 2−2661 : Gut microbiota abundance (genus Catenibacterium id.2153) on E3 ISG15−−protein ligase HERC5
with a threshold of 1e−05
rs7742829
rs77285108
rs73128290
rs12404911
All − MR Egger
All − IVW(MRE)
−2
−1
0
1
MR effect size for
'Gut microbiota abundance (genus Catenibacterium id.2153)' on 'E3 ISG15−−protein ligase HERC5'

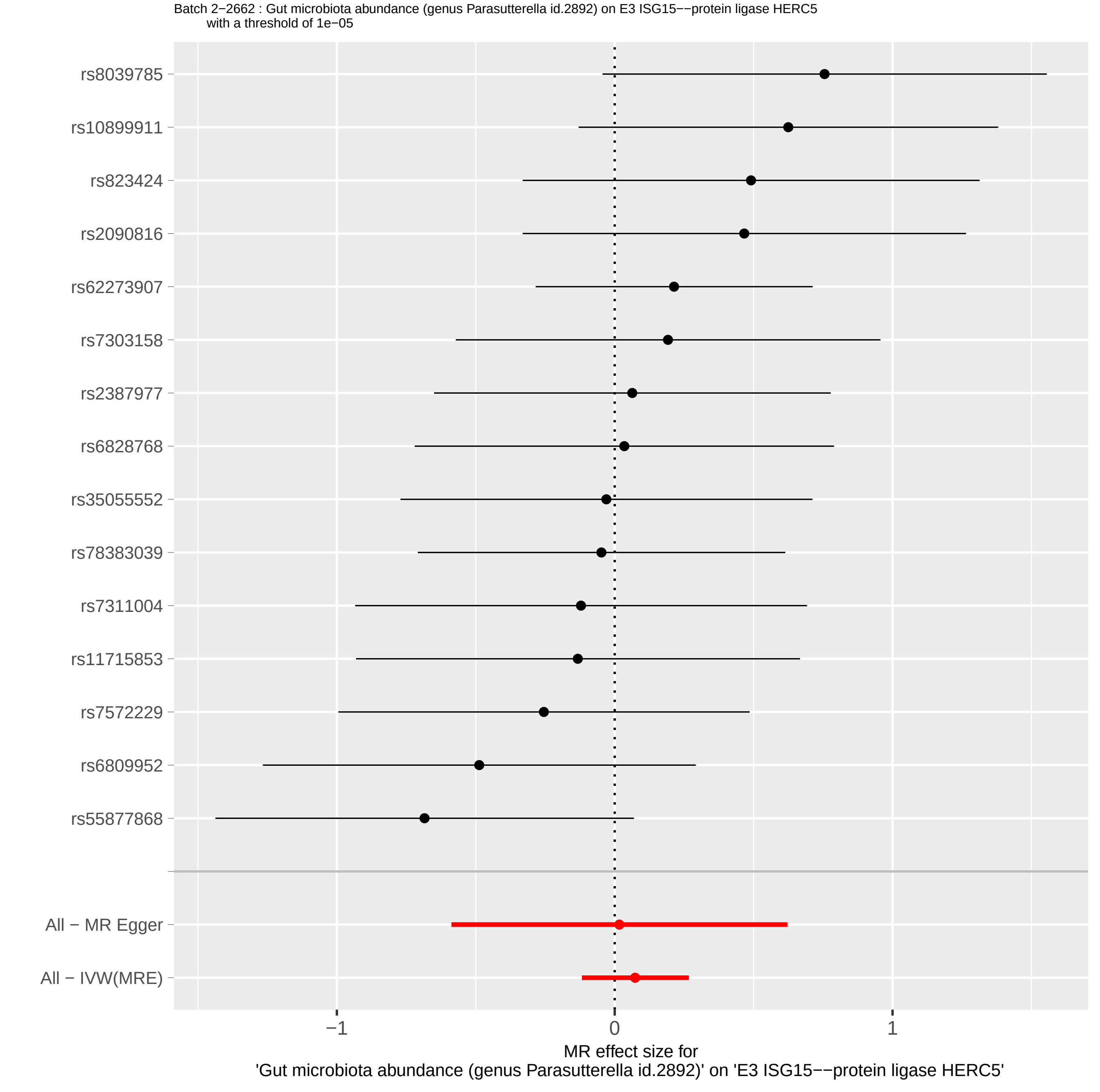

Batch 2−2662 : Gut microbiota abundance (genus Parasutterella id.2892) on E3 ISG15−−protein ligase HERC5
with a threshold of 1e−05
rs8039785
rs10899911
rs823424
rs2090816
rs62273907
rs7303158
rs2387977
rs6828768
rs35055552
rs78383039
rs7311004
rs11715853
rs7572229
rs6809952
rs55877868
All − MR Egger
All − IVW(MRE)
−1
0
1
MR effect size for
'Gut microbiota abundance (genus Parasutterella id.2892)' on 'E3 ISG15−−protein ligase HERC5'

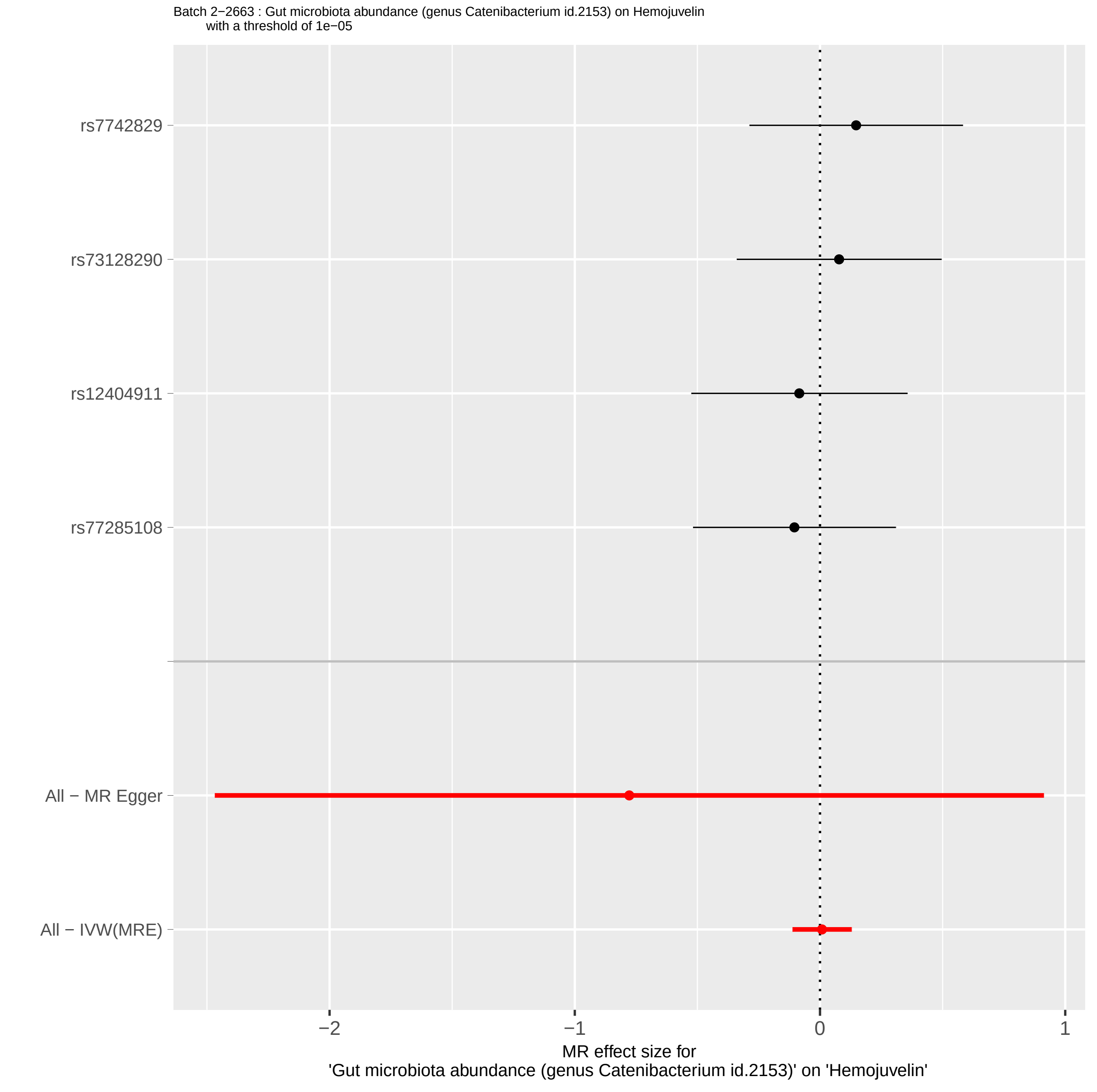

Batch 2−2663 : Gut microbiota abundance (genus Catenibacterium id.2153) on Hemojuvelin
with a threshold of 1e−05
rs7742829
rs73128290
rs12404911
rs77285108
All − MR Egger
All − IVW(MRE)
−2
−1
0
1
MR effect size for
'Gut microbiota abundance (genus Catenibacterium id.2153)' on 'Hemojuvelin'

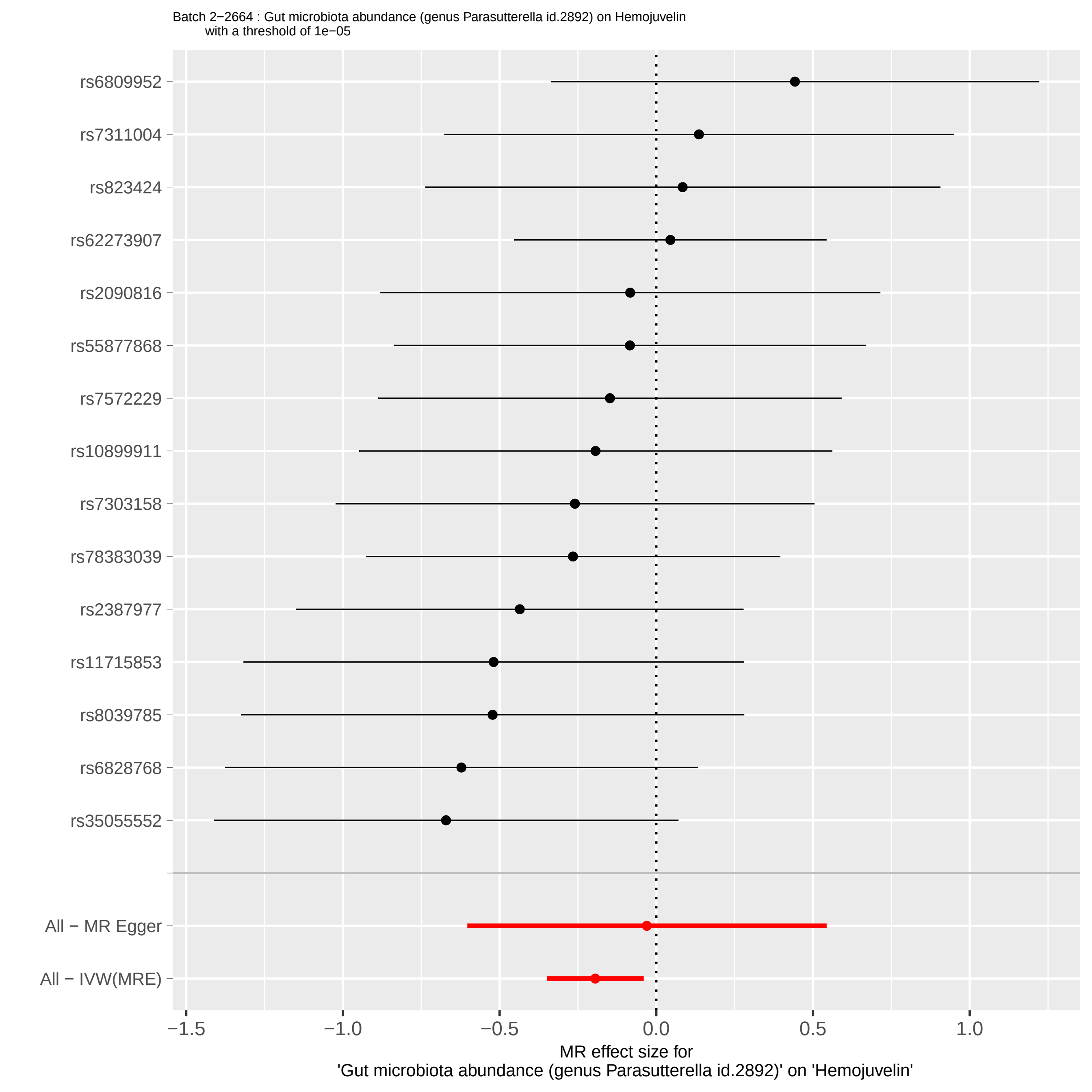

Batch 2−2664 : Gut microbiota abundance (genus Parasutterella id.2892) on Hemojuvelin
with a threshold of 1e−05
rs6809952
rs7311004
rs823424
rs62273907
rs2090816
rs55877868
rs7572229
rs10899911
rs7303158
rs78383039
rs2387977
rs11715853
rs8039785
rs6828768
rs35055552
All − MR Egger
All − IVW(MRE)
−1.5
−1.0
−0.5
0.0
0.5
1.0
MR effect size for
'Gut microbiota abundance (genus Parasutterella id.2892)' on 'Hemojuvelin'

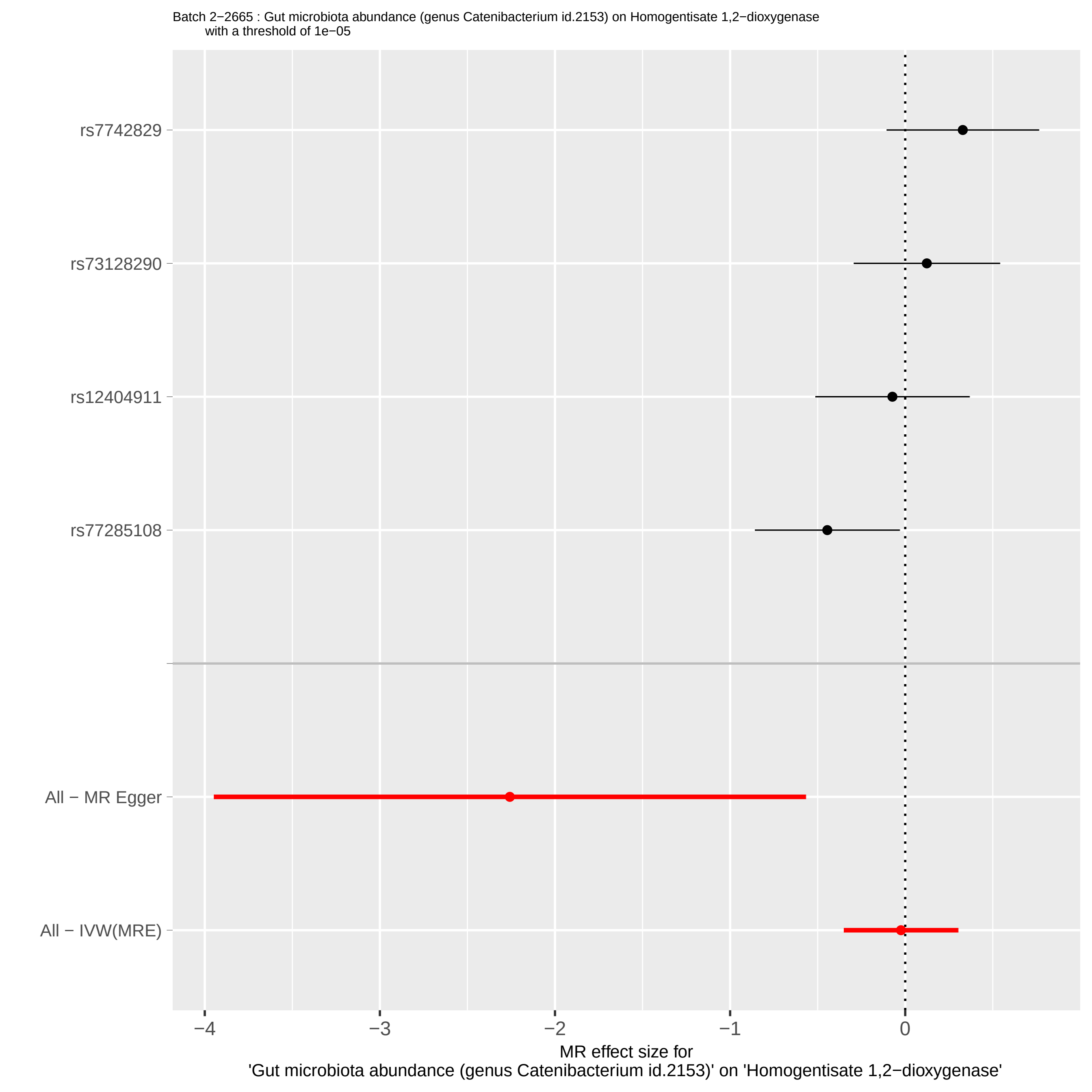

Batch 2−2665 : Gut microbiota abundance (genus Catenibacterium id.2153) on Homogentisate 1,2−dioxygenase
with a threshold of 1e−05
rs7742829
rs73128290
rs12404911
rs77285108
All − MR Egger
All − IVW(MRE)
−4
−3
−2
−1
0
MR effect size for
'Gut microbiota abundance (genus Catenibacterium id.2153)' on 'Homogentisate 1,2−dioxygenase'

Batch 2−2666 : Gut microbiota abundance (genus Parasutterella id.2892) on Homogentisate 1,2−dioxygenase
with a threshold of 1e−05

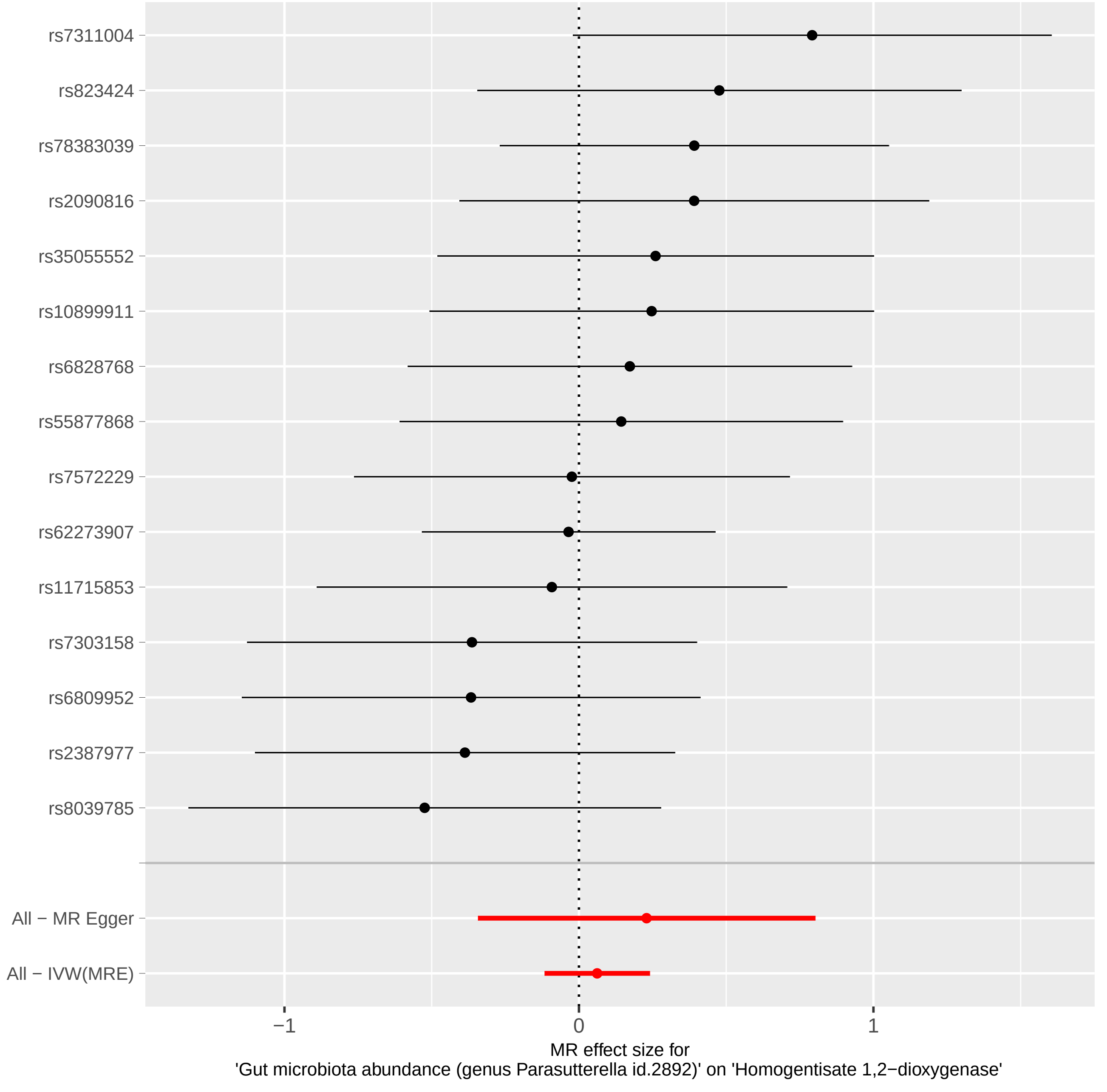

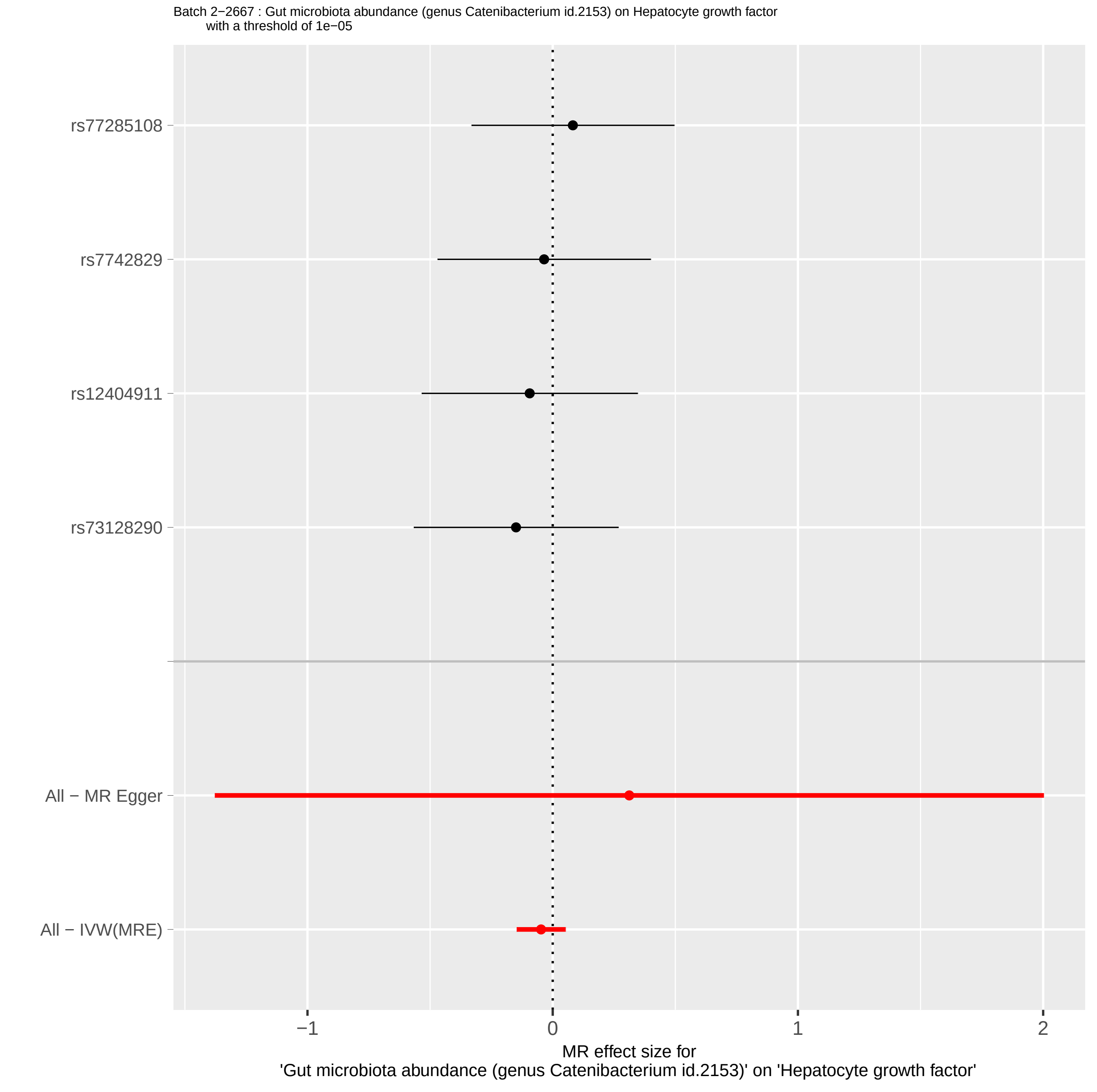

Batch 2−2667 : Gut microbiota abundance (genus Catenibacterium id.2153) on Hepatocyte growth factor
with a threshold of 1e−05
rs77285108
rs7742829
rs12404911
rs73128290
All − MR Egger
All − IVW(MRE)
−1
0
1
2
MR effect size for
'Gut microbiota abundance (genus Catenibacterium id.2153)' on 'Hepatocyte growth factor'

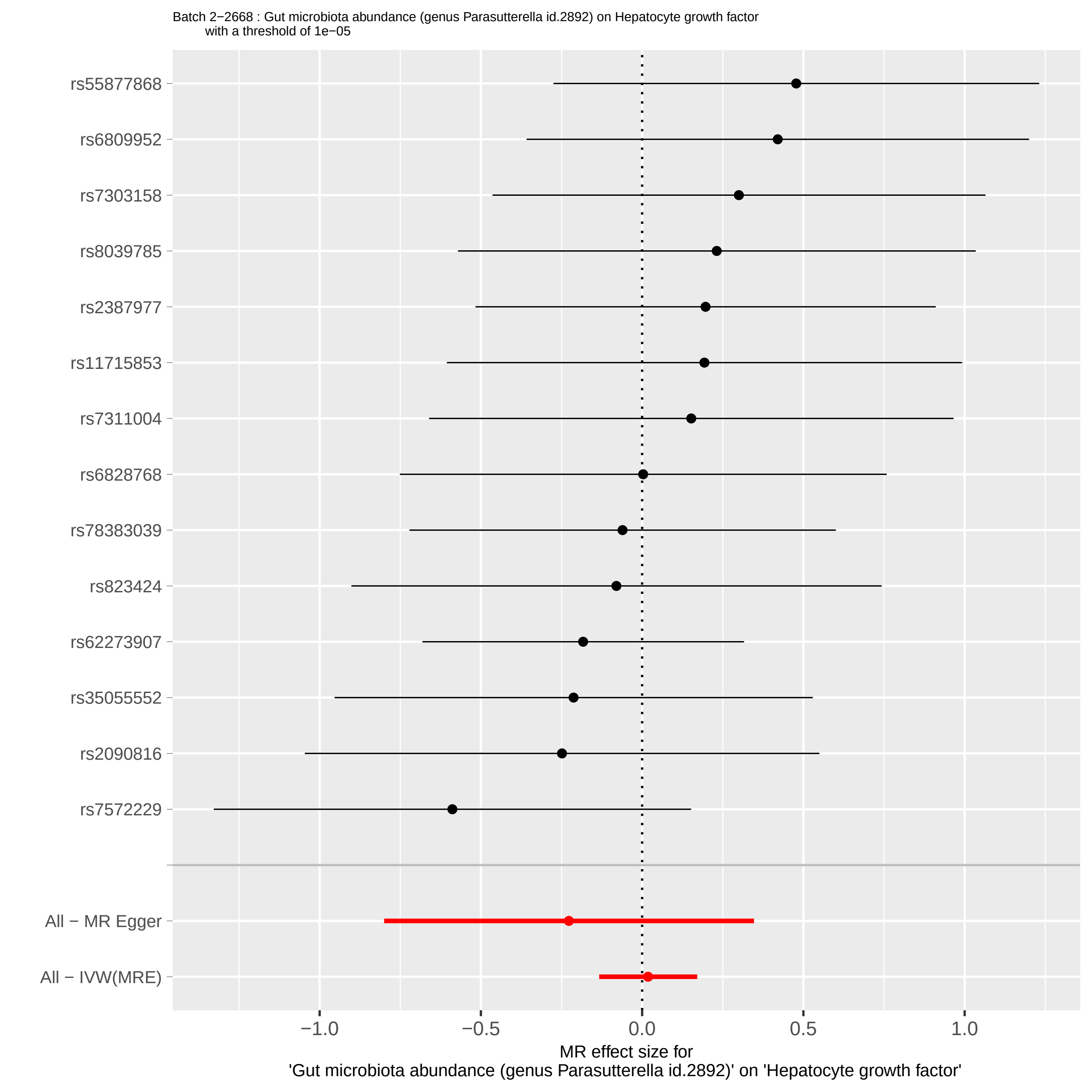
Batch 2−2668 : Gut microbiota abundance (genus Parasutterella id.2892) on Hepatocyte growth factor
with a threshold of 1e−05
rs55877868
rs6809952
rs7303158
rs8039785
rs2387977
rs11715853
rs7311004
rs6828768
rs78383039
rs823424
rs62273907
rs35055552
rs2090816
rs7572229
All − MR Egger
All − IVW(MRE)
−1.0
−0.5
0.0
0.5
1.0
MR effect size for
'Gut microbiota abundance (genus Parasutterella id.2892)' on 'Hepatocyte growth factor'

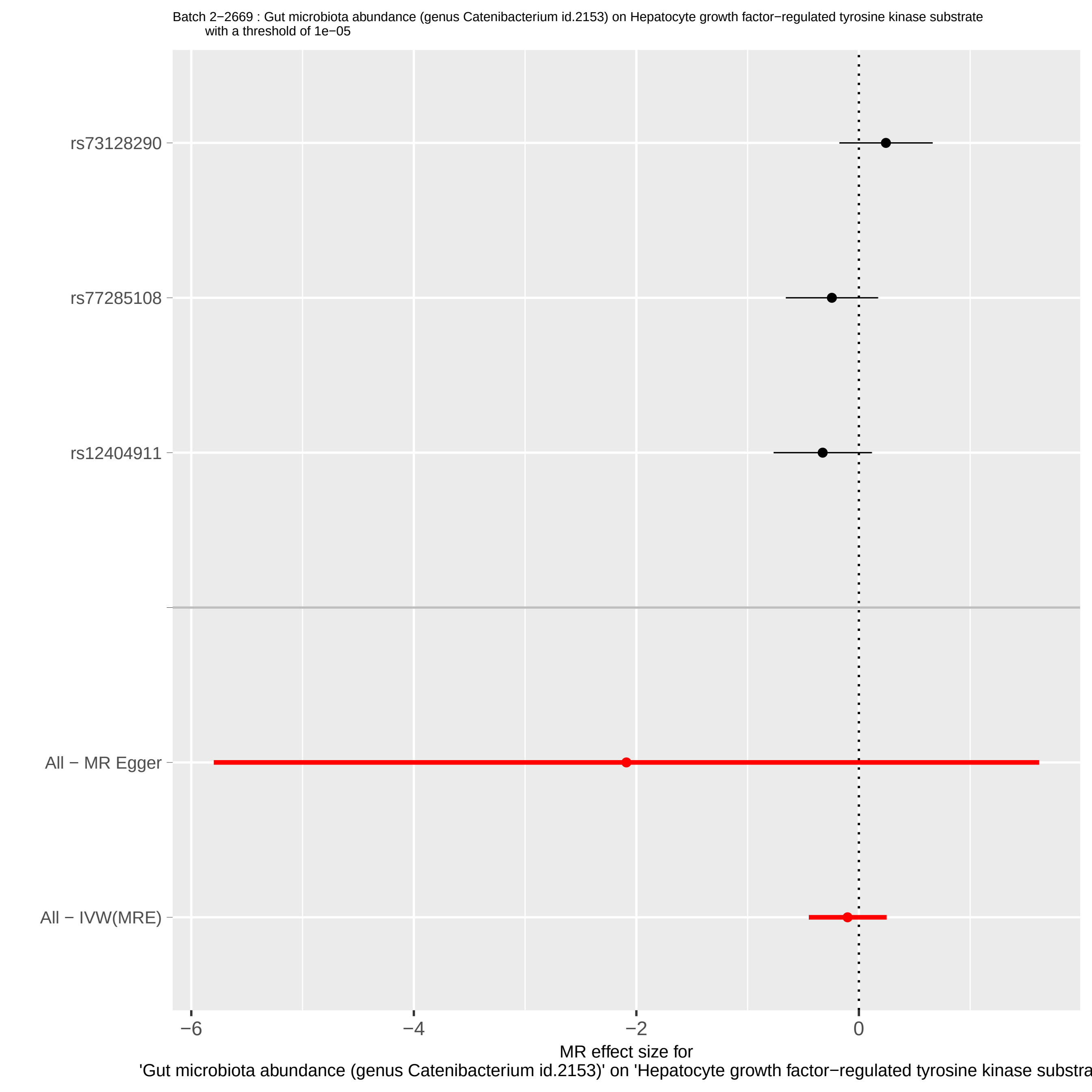

Batch 2−2669 : Gut microbiota abundance (genus Catenibacterium id.2153) on Hepatocyte growth factor−regulated tyrosine kinase substrate
with a threshold of 1e−05
rs73128290
rs77285108
rs12404911
All − MR Egger
All − IVW(MRE)
−6
−4
−2
0
MR effect size for
'Gut microbiota abundance (genus Catenibacterium id.2153)' on 'Hepatocyte growth factor−regulated tyrosine kinase substra

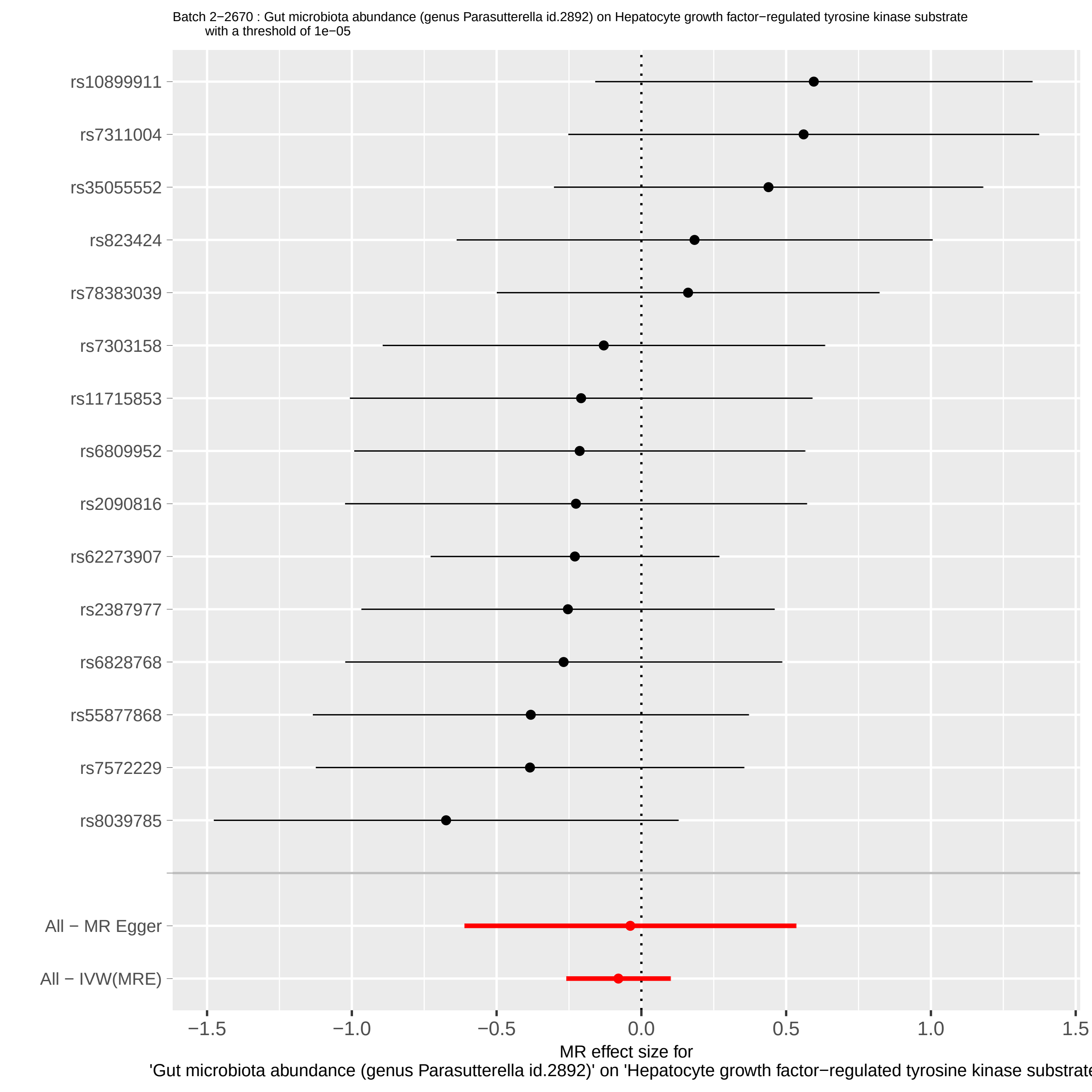

Batch 2−2670 : Gut microbiota abundance (genus Parasutterella id.2892) on Hepatocyte growth factor−regulated tyrosine kinase substrate
with a threshold of 1e−05
rs10899911
rs7311004
rs35055552
rs823424
rs78383039
rs7303158
rs11715853
rs6809952
rs2090816
rs62273907
rs2387977
rs6828768
rs55877868
rs7572229
rs8039785
All − MR Egger
All − IVW(MRE)
−1.5
−1.0
−0.5
0.0
0.5
1.0
1.5
MR effect size for
'Gut microbiota abundance (genus Parasutterella id.2892)' on 'Hepatocyte growth factor−regulated tyrosine kinase substrate

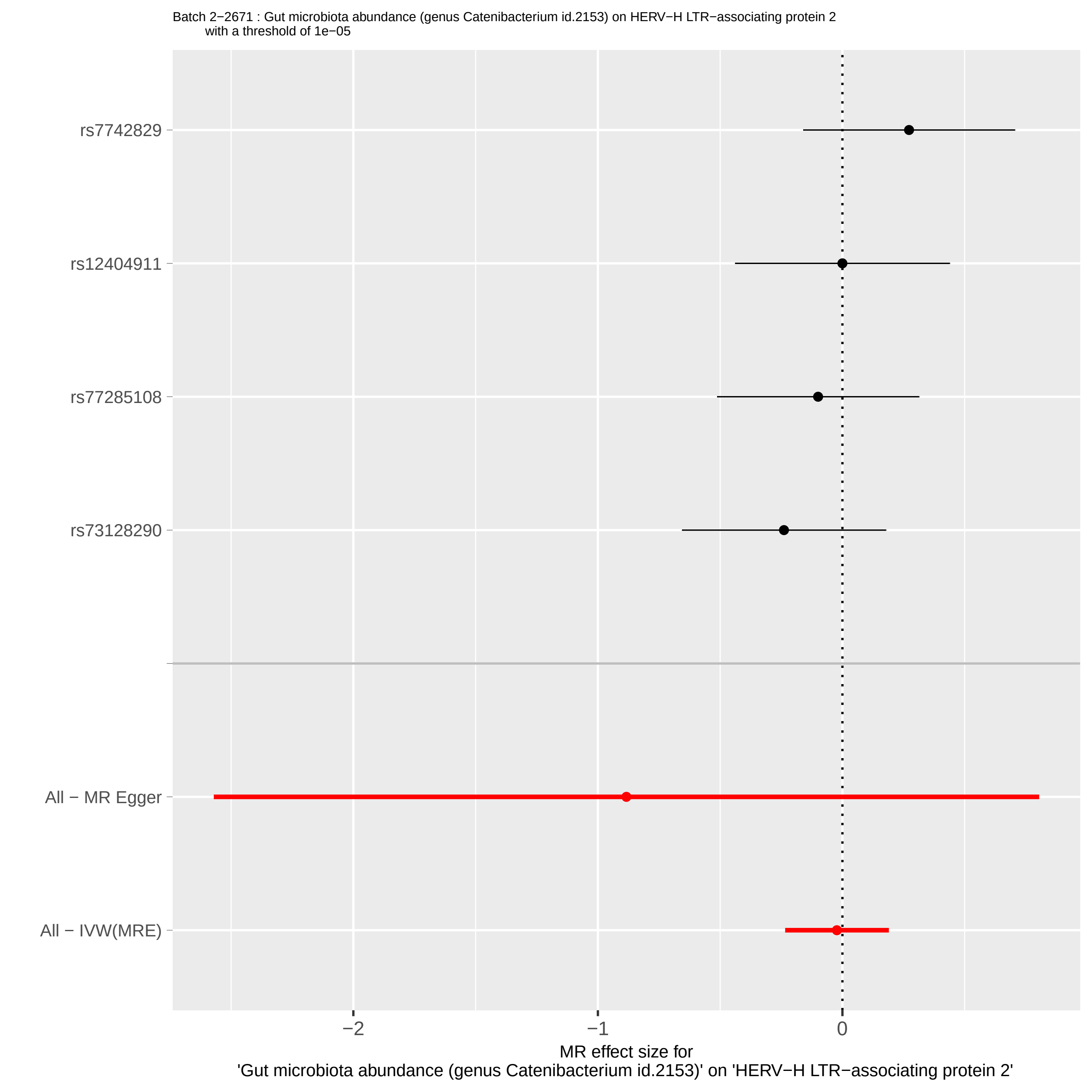
Batch 2−2671 : Gut microbiota abundance (genus Catenibacterium id.2153) on HERV−H LTR−associating protein 2
with a threshold of 1e−05
rs7742829
rs12404911
rs77285108
rs73128290
All − MR Egger
All − IVW(MRE)
−2
−1
0
MR effect size for
'Gut microbiota abundance (genus Catenibacterium id.2153)' on 'HERV−H LTR−associating protein 2'

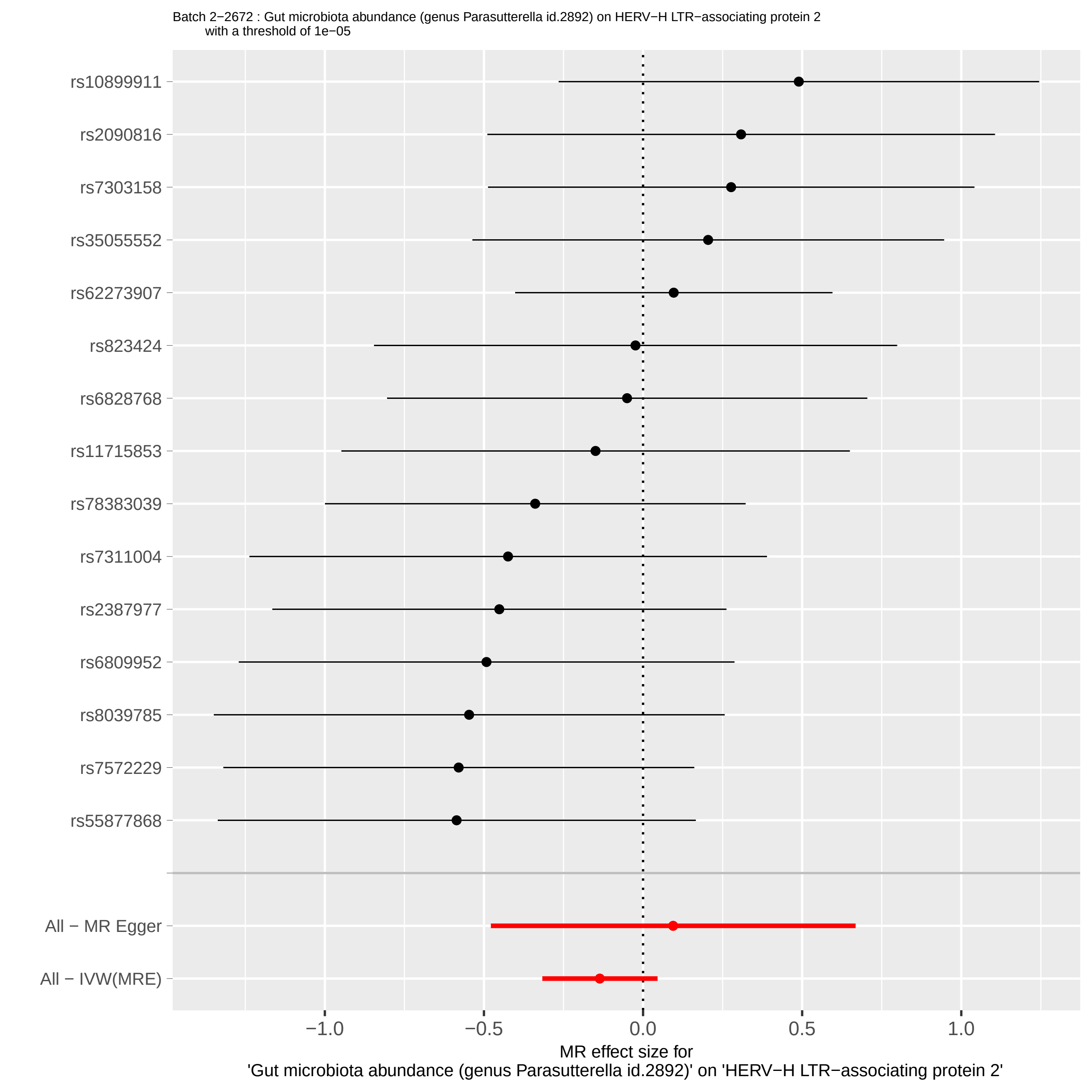

Batch 2−2672 : Gut microbiota abundance (genus Parasutterella id.2892) on HERV−H LTR−associating protein 2
with a threshold of 1e−05
rs10899911
rs2090816
rs7303158
rs35055552
rs62273907
rs823424
rs6828768
rs11715853
rs78383039
rs7311004
rs2387977
rs6809952
rs8039785
rs7572229
rs55877868
All − MR Egger
All − IVW(MRE)
−1.0
−0.5
0.0
0.5
1.0
MR effect size for
'Gut microbiota abundance (genus Parasutterella id.2892)' on 'HERV−H LTR−associating protein 2'

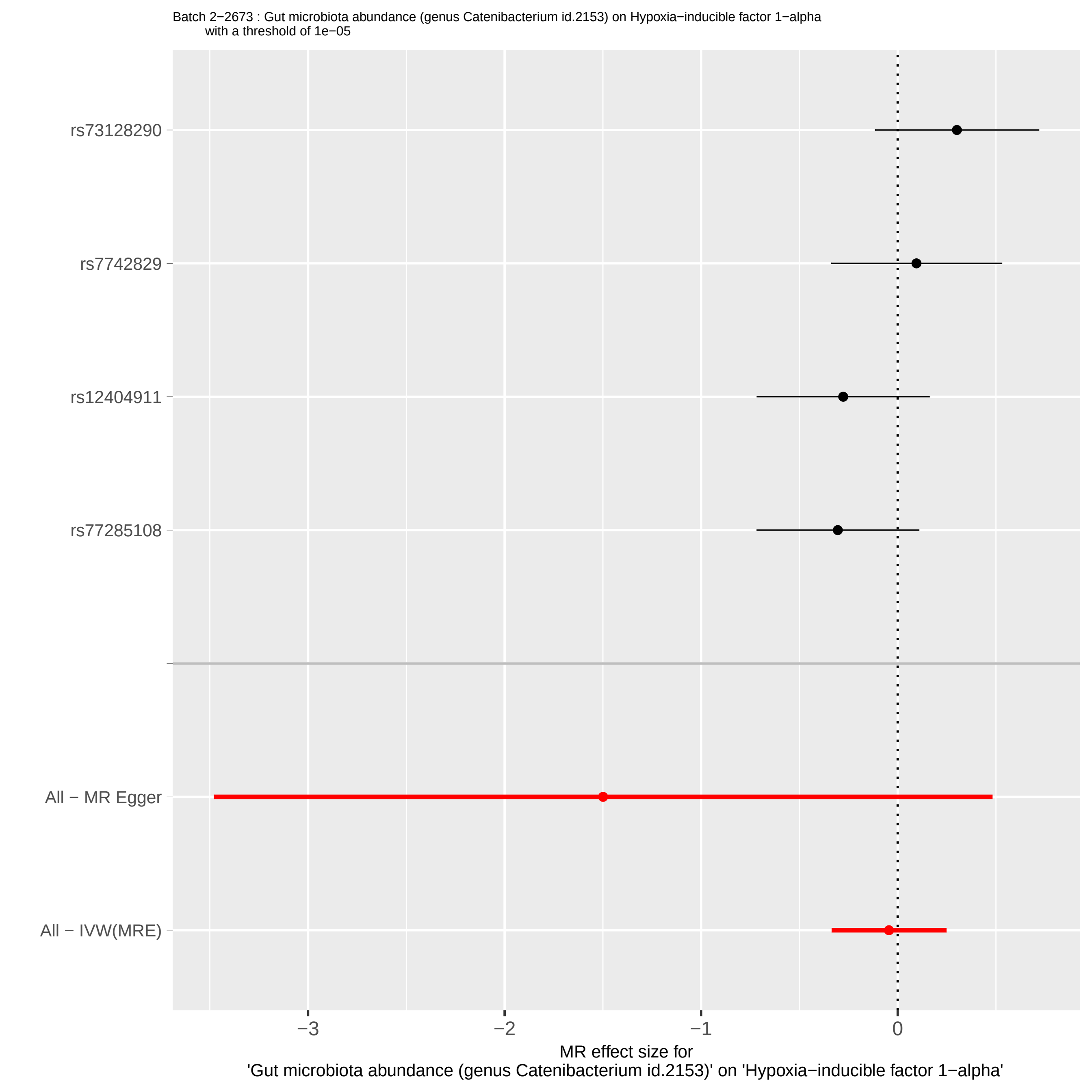

Batch 2−2673 : Gut microbiota abundance (genus Catenibacterium id.2153) on Hypoxia−inducible factor 1−alpha
with a threshold of 1e−05
rs73128290
rs7742829
rs12404911
rs77285108
All − MR Egger
All − IVW(MRE)
−3
−2
−1
0
MR effect size for
'Gut microbiota abundance (genus Catenibacterium id.2153)' on 'Hypoxia−inducible factor 1−alpha'

Batch 2−2674 : Gut microbiota abundance (genus Parasutterella id.2892) on Hypoxia−inducible factor 1−alpha
with a threshold of 1e−05

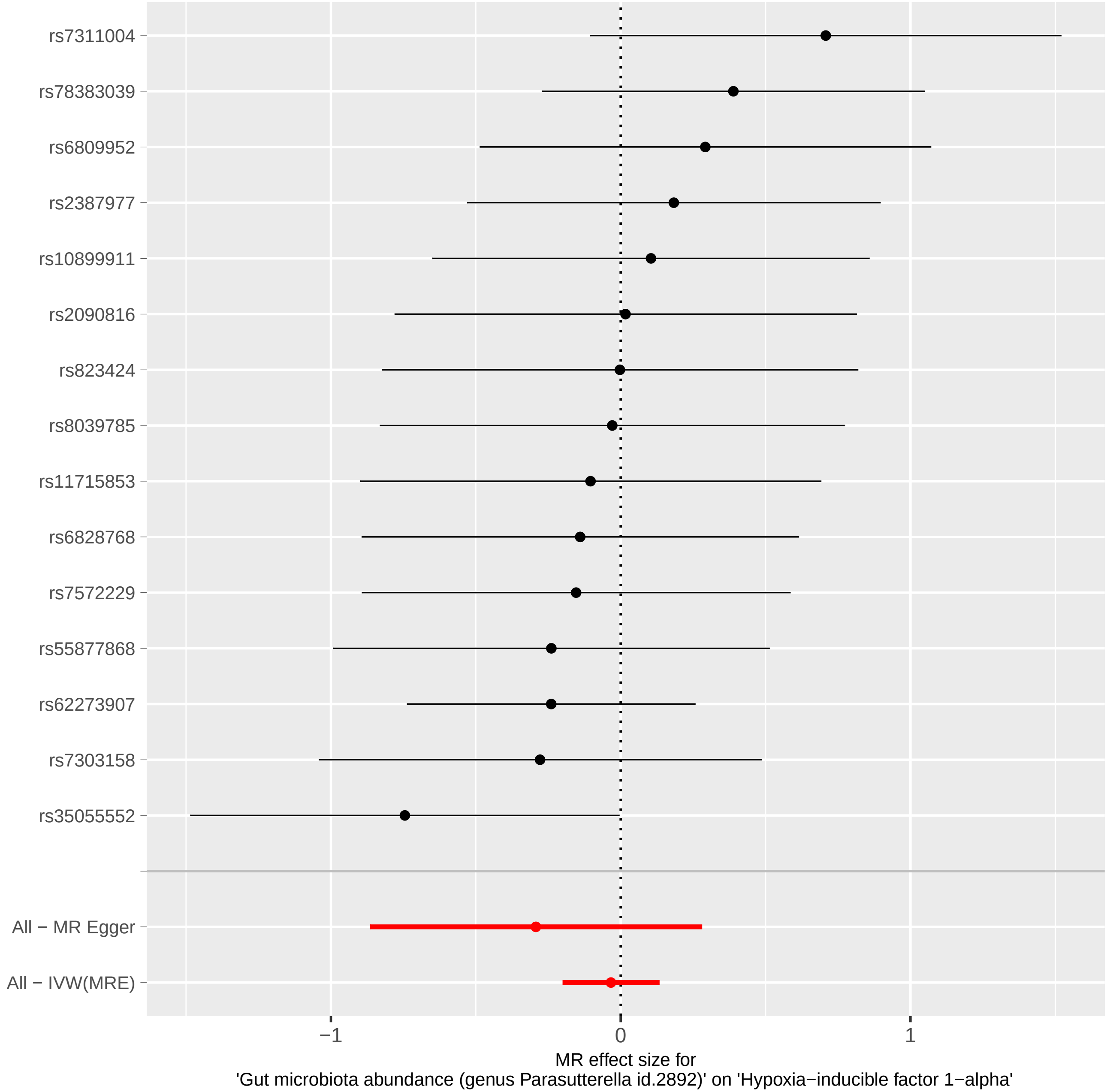

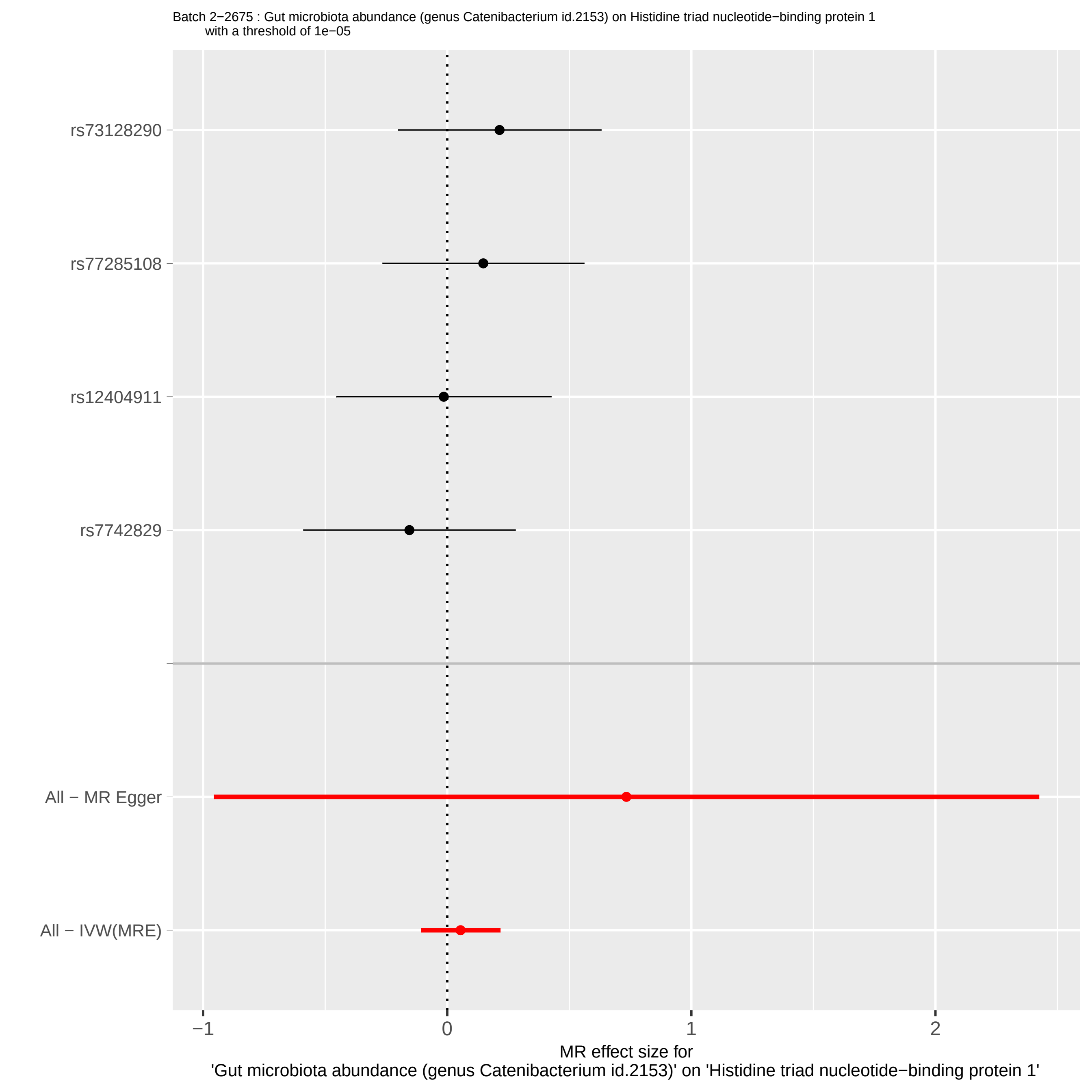

Batch 2−2675 : Gut microbiota abundance (genus Catenibacterium id.2153) on Histidine triad nucleotide−binding protein 1
with a threshold of 1e−05
rs73128290
rs77285108
rs12404911
rs7742829
All − MR Egger
All − IVW(MRE)
−1
0
1
2
MR effect size for
'Gut microbiota abundance (genus Catenibacterium id.2153)' on 'Histidine triad nucleotide−binding protein 1'

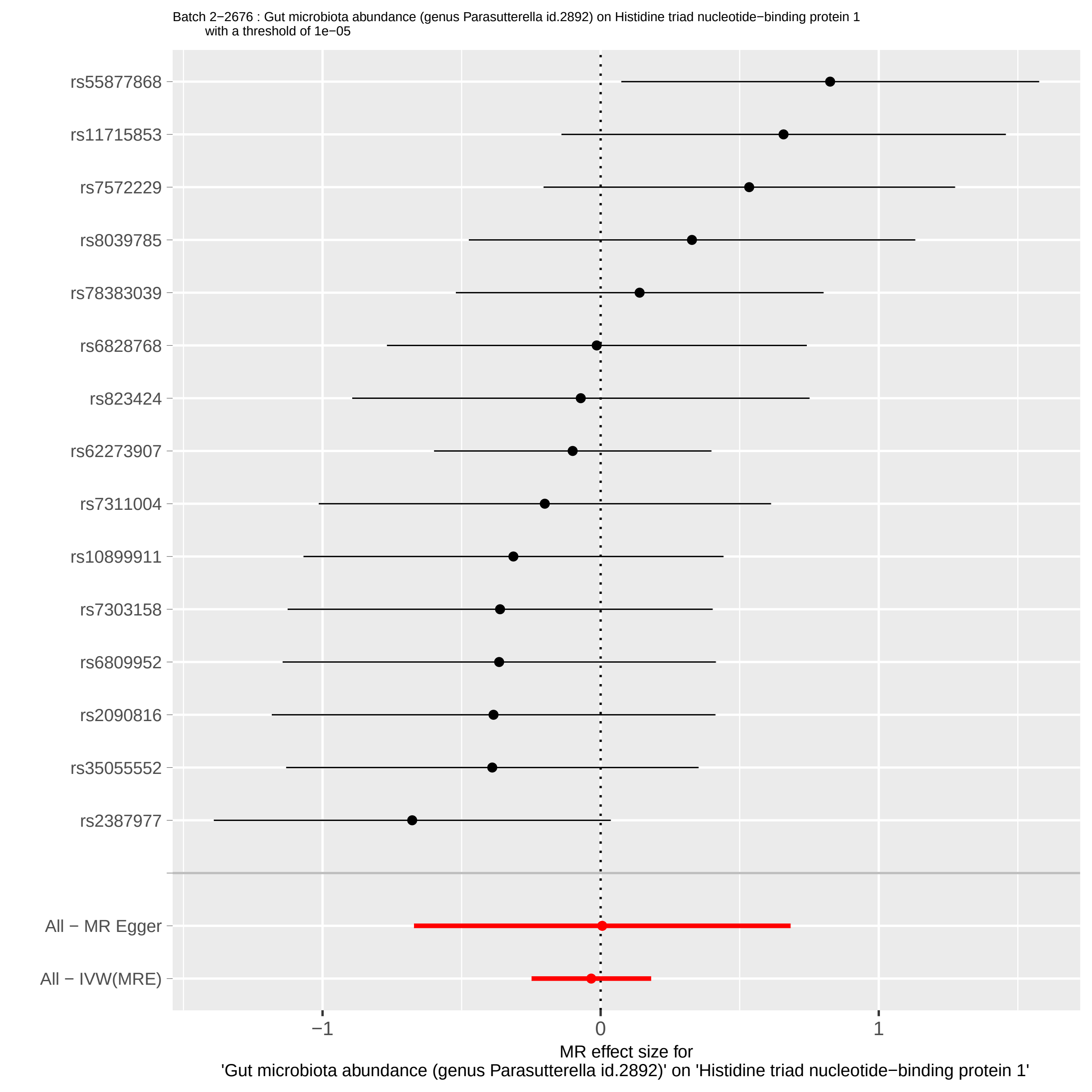

Batch 2−2676 : Gut microbiota abundance (genus Parasutterella id.2892) on Histidine triad nucleotide−binding protein 1
with a threshold of 1e−05
rs55877868
rs11715853
rs7572229
rs8039785
rs78383039
rs6828768
rs823424
rs62273907
rs7311004
rs10899911
rs7303158
rs6809952
rs2090816
rs35055552
rs2387977
All − MR Egger
All − IVW(MRE)
−1
0
1
MR effect size for
'Gut microbiota abundance (genus Parasutterella id.2892)' on 'Histidine triad nucleotide−binding protein 1'

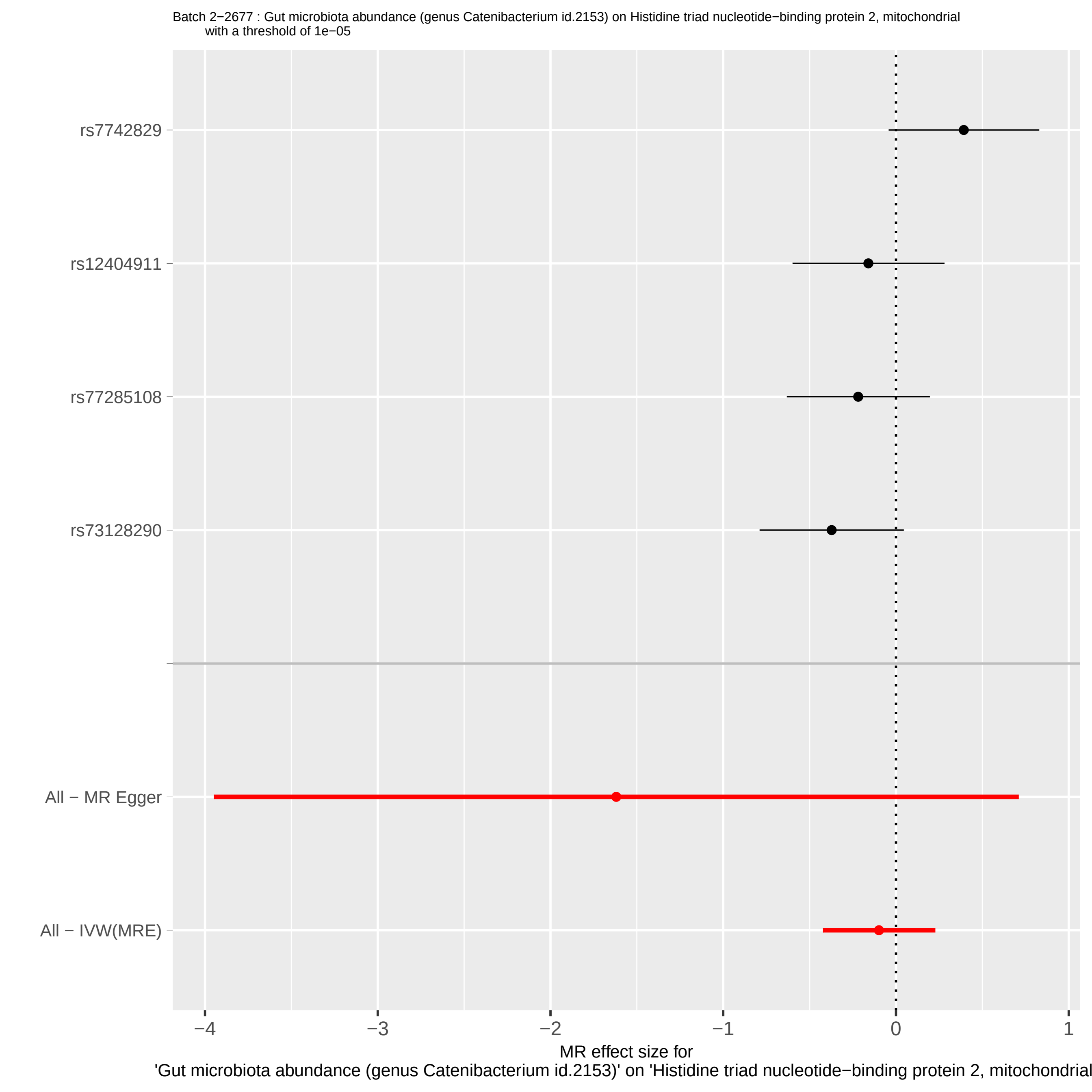

Batch 2−2677 : Gut microbiota abundance (genus Catenibacterium id.2153) on Histidine triad nucleotide−binding protein 2, mitochondrial
with a threshold of 1e−05
rs7742829
rs12404911
rs77285108
rs73128290
All − MR Egger
All − IVW(MRE)
−4
−3
−2
−1
0
1
MR effect size for
'Gut microbiota abundance (genus Catenibacterium id.2153)' on 'Histidine triad nucleotide−binding protein 2, mitochondrial'

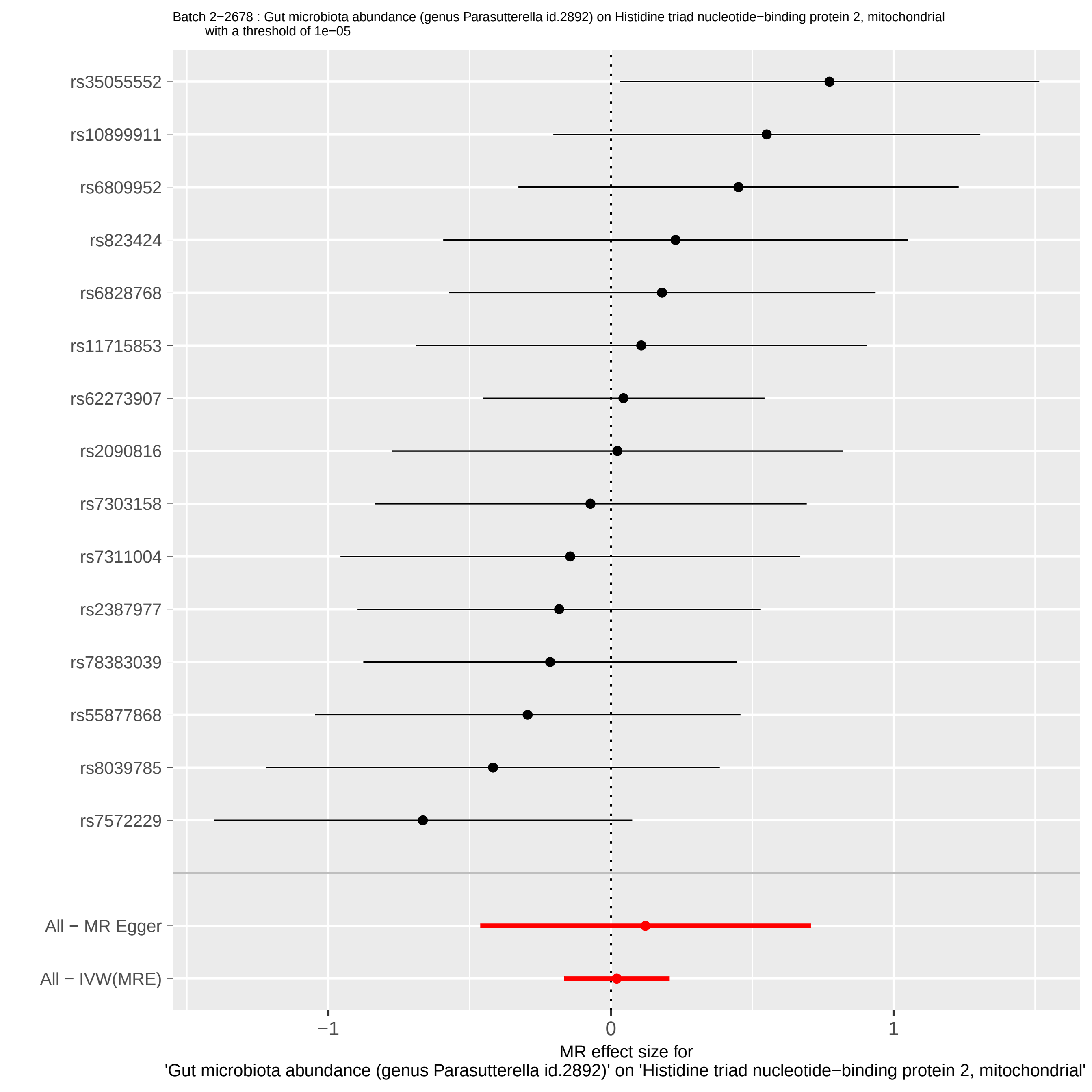

Batch 2−2678 : Gut microbiota abundance (genus Parasutterella id.2892) on Histidine triad nucleotide−binding protein 2, mitochondrial
with a threshold of 1e−05
rs35055552
rs10899911
rs6809952
rs823424
rs6828768
rs11715853
rs62273907
rs2090816
rs7303158
rs7311004
rs2387977
rs78383039
rs55877868
rs8039785
rs7572229
All − MR Egger
All − IVW(MRE)
−1
0
1
MR effect size for
'Gut microbiota abundance (genus Parasutterella id.2892)' on 'Histidine triad nucleotide−binding protein 2, mitochondrial'

Batch 2−2679 : Gut microbiota abundance (genus Catenibacterium id.2153) on Homeodomain−interacting protein kinase 3
with a threshold of 1e−05

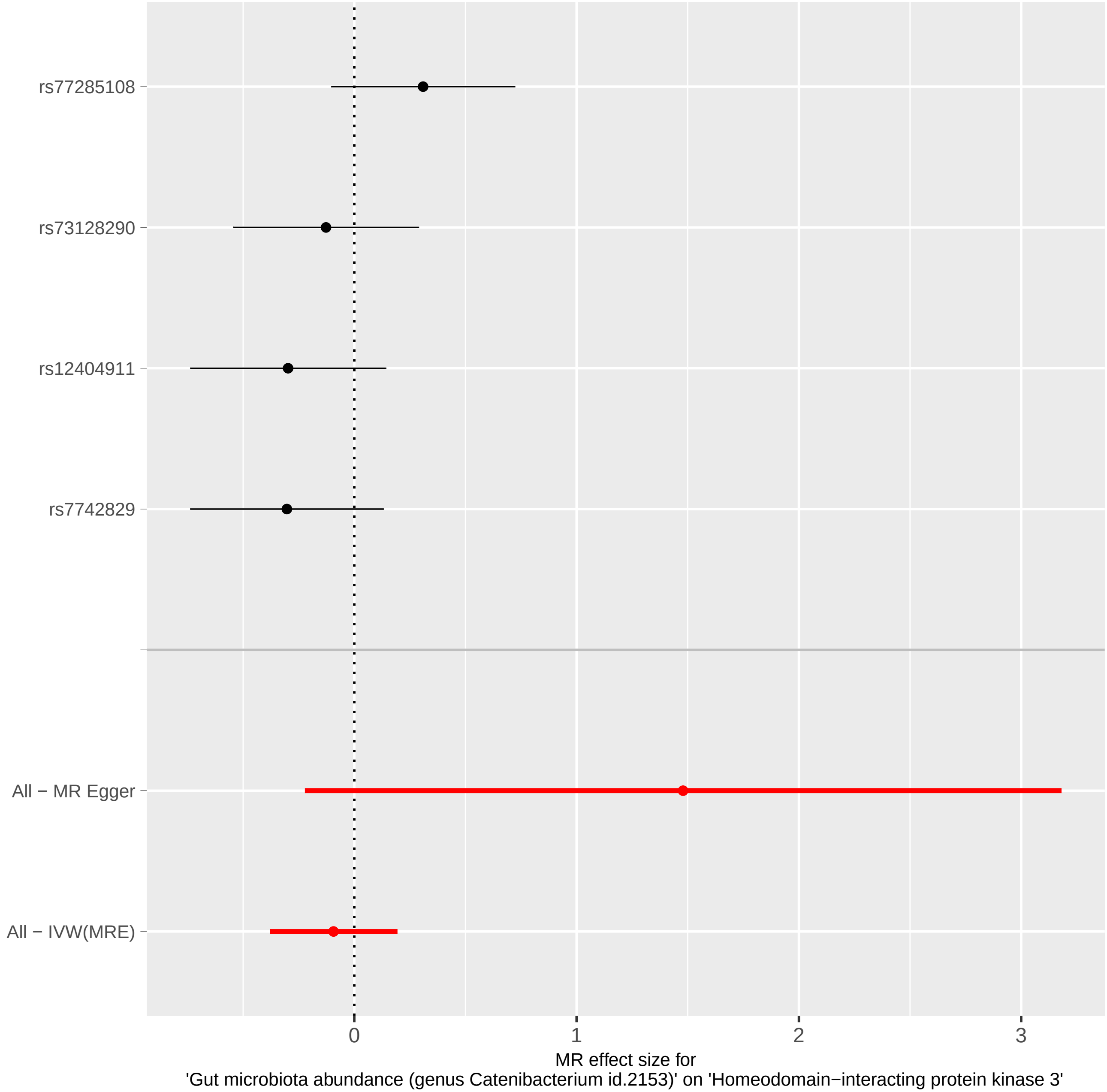

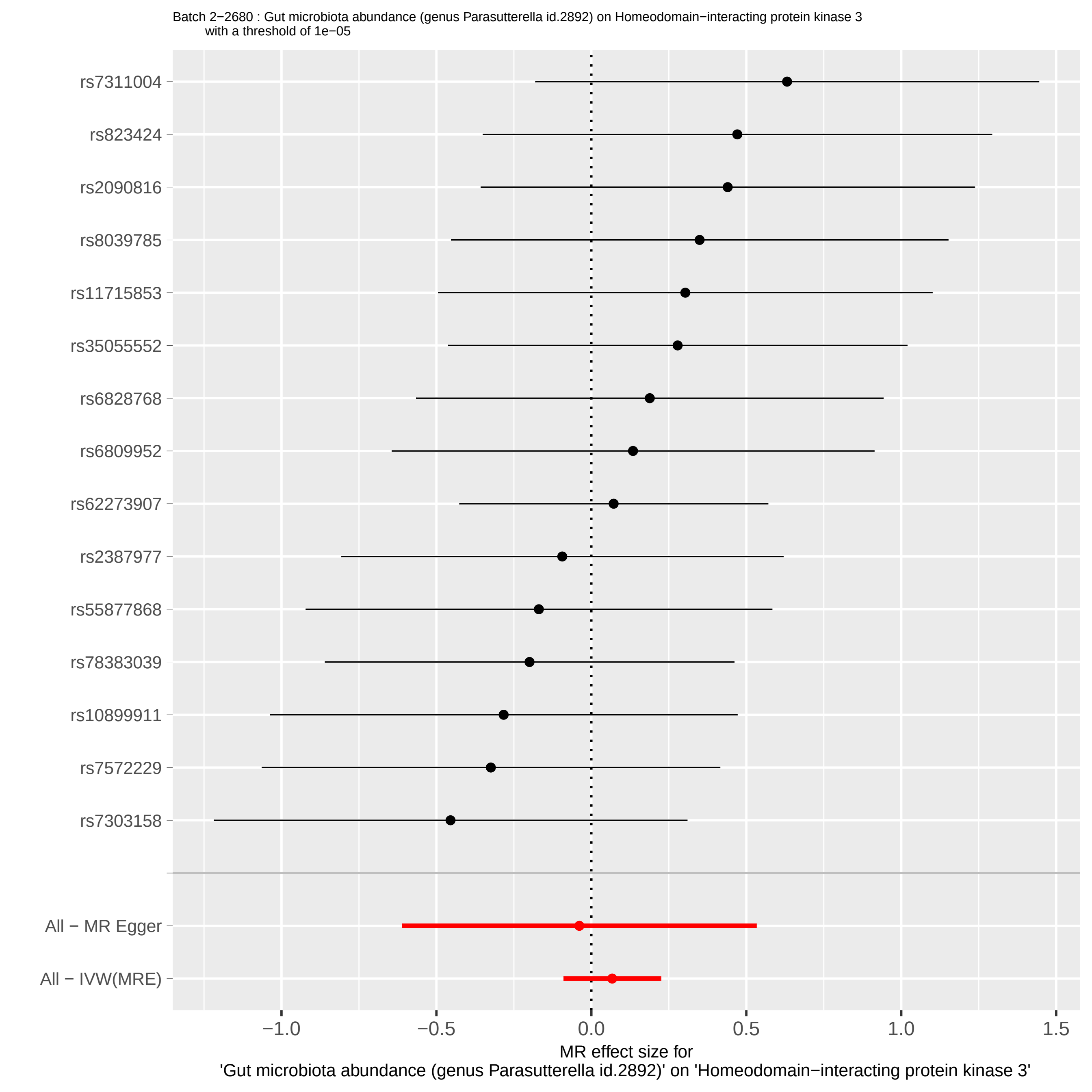
Batch 2−2680 : Gut microbiota abundance (genus Parasutterella id.2892) on Homeodomain−interacting protein kinase 3
with a threshold of 1e−05
rs7311004
rs823424
rs2090816
rs8039785
rs11715853
rs35055552
rs6828768
rs6809952
rs62273907
rs2387977
rs55877868
rs78383039
rs10899911
rs7572229
rs7303158
All − MR Egger
All − IVW(MRE)
−1.0
−0.5
0.0
0.5
1.0
1.5
MR effect size for
'Gut microbiota abundance (genus Parasutterella id.2892)' on 'Homeodomain−interacting protein kinase 3'

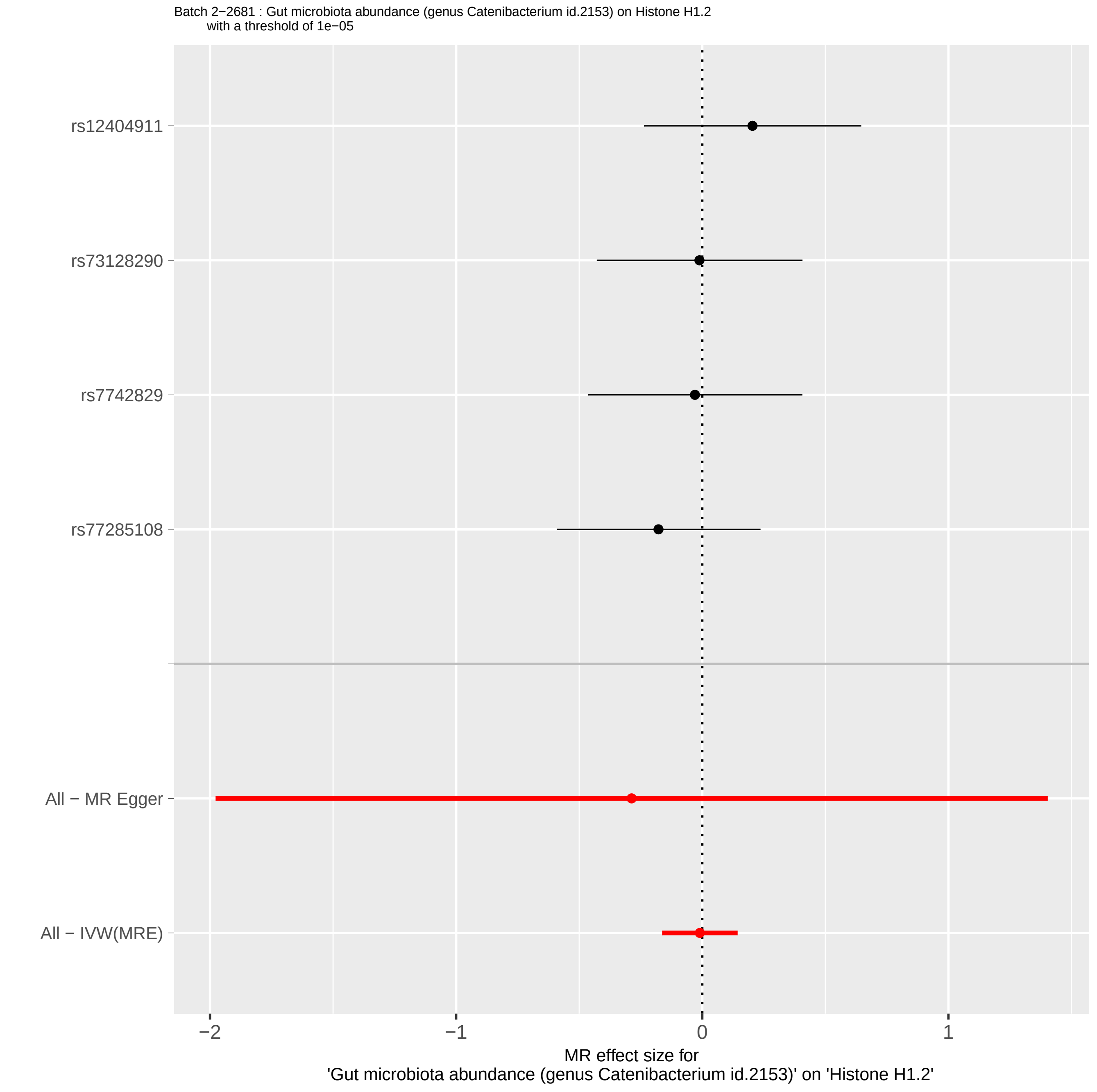

Batch 2−2681 : Gut microbiota abundance (genus Catenibacterium id.2153) on Histone H1.2
with a threshold of 1e−05
rs12404911
rs73128290
rs7742829
rs77285108
All − MR Egger
All − IVW(MRE)
−2
−1
0
1
MR effect size for
'Gut microbiota abundance (genus Catenibacterium id.2153)' on 'Histone H1.2'

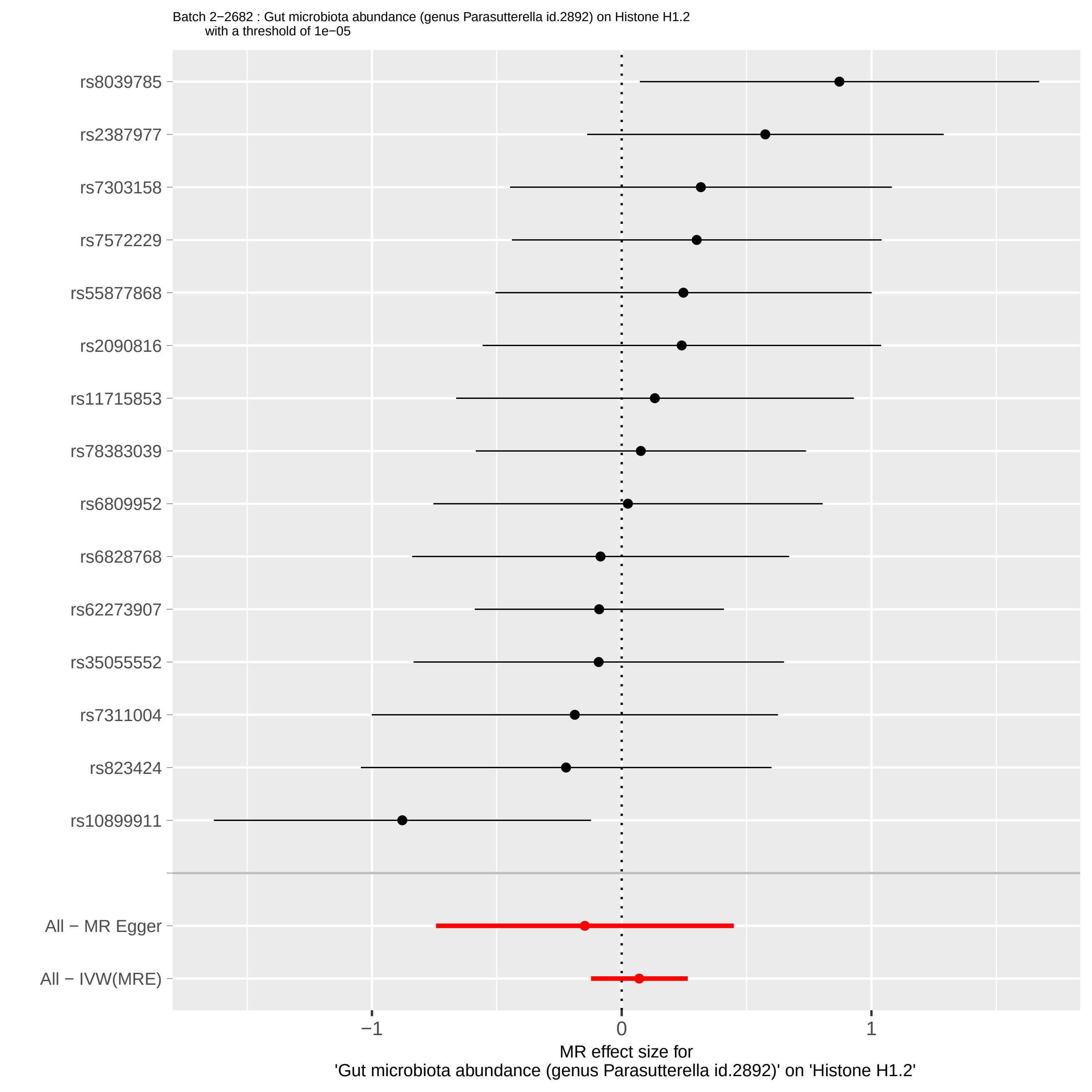

Batch 2−2682 : Gut microbiota abundance (genus Parasutterella id.2892) on Histone H1.2
with a threshold of 1e−05
rs8039785
rs2387977
rs7303158
rs7572229
rs55877868
rs2090816
rs11715853
rs78383039
rs6809952
rs6828768
rs62273907
rs35055552
rs7311004
rs823424
rs10899911
All − MR Egger
All − IVW(MRE)
−1
0
1
MR effect size for
'Gut microbiota abundance (genus Parasutterella id.2892)' on 'Histone H1.2'

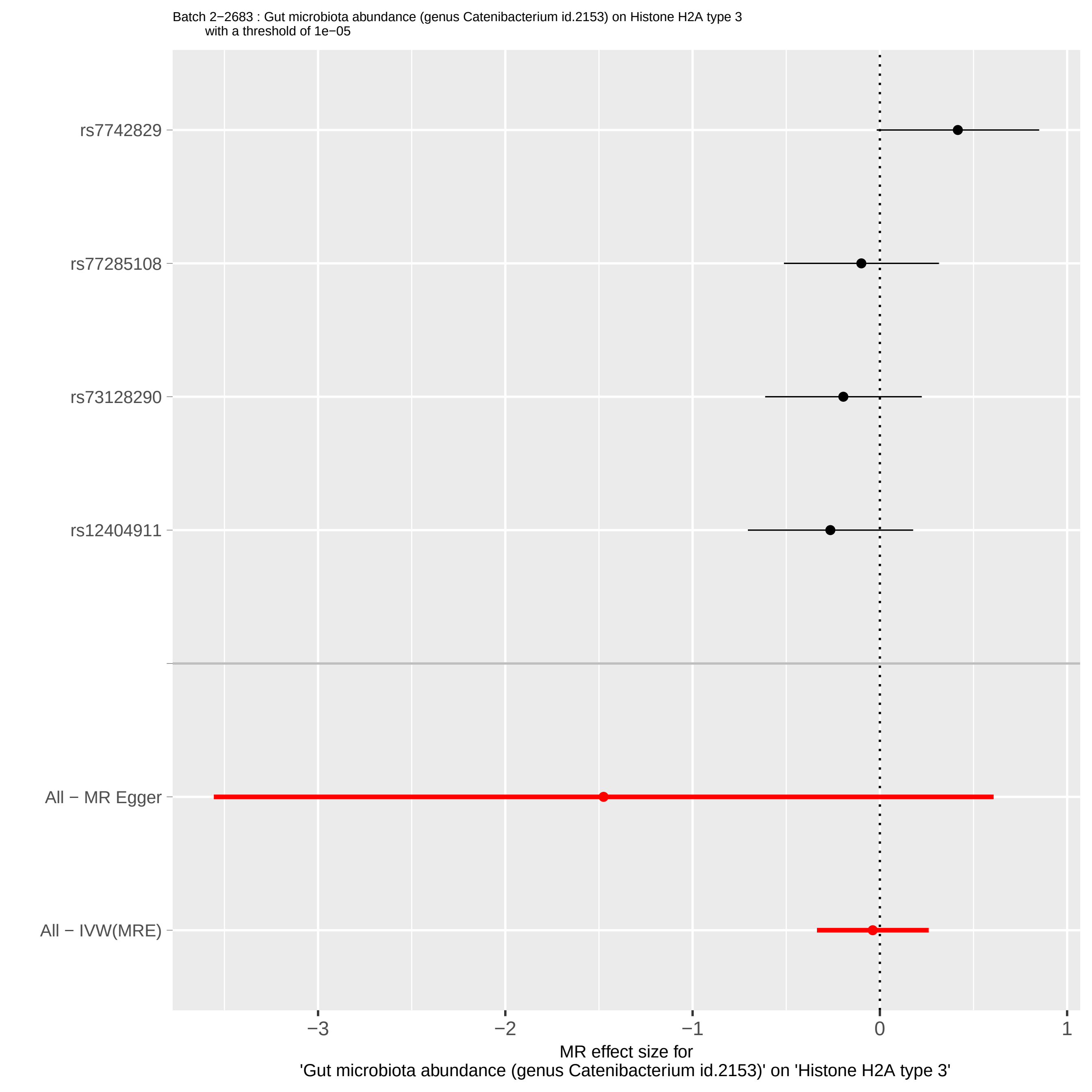

Batch 2−2683 : Gut microbiota abundance (genus Catenibacterium id.2153) on Histone H2A type 3
with a threshold of 1e−05
rs7742829
rs77285108
rs73128290
rs12404911
All − MR Egger
All − IVW(MRE)
−3
−2
−1
0
1
MR effect size for
'Gut microbiota abundance (genus Catenibacterium id.2153)' on 'Histone H2A type 3'

Batch 2−2684 : Gut microbiota abundance (genus Parasutterella id.2892) on Histone H2A type 3
with a threshold of 1e−05

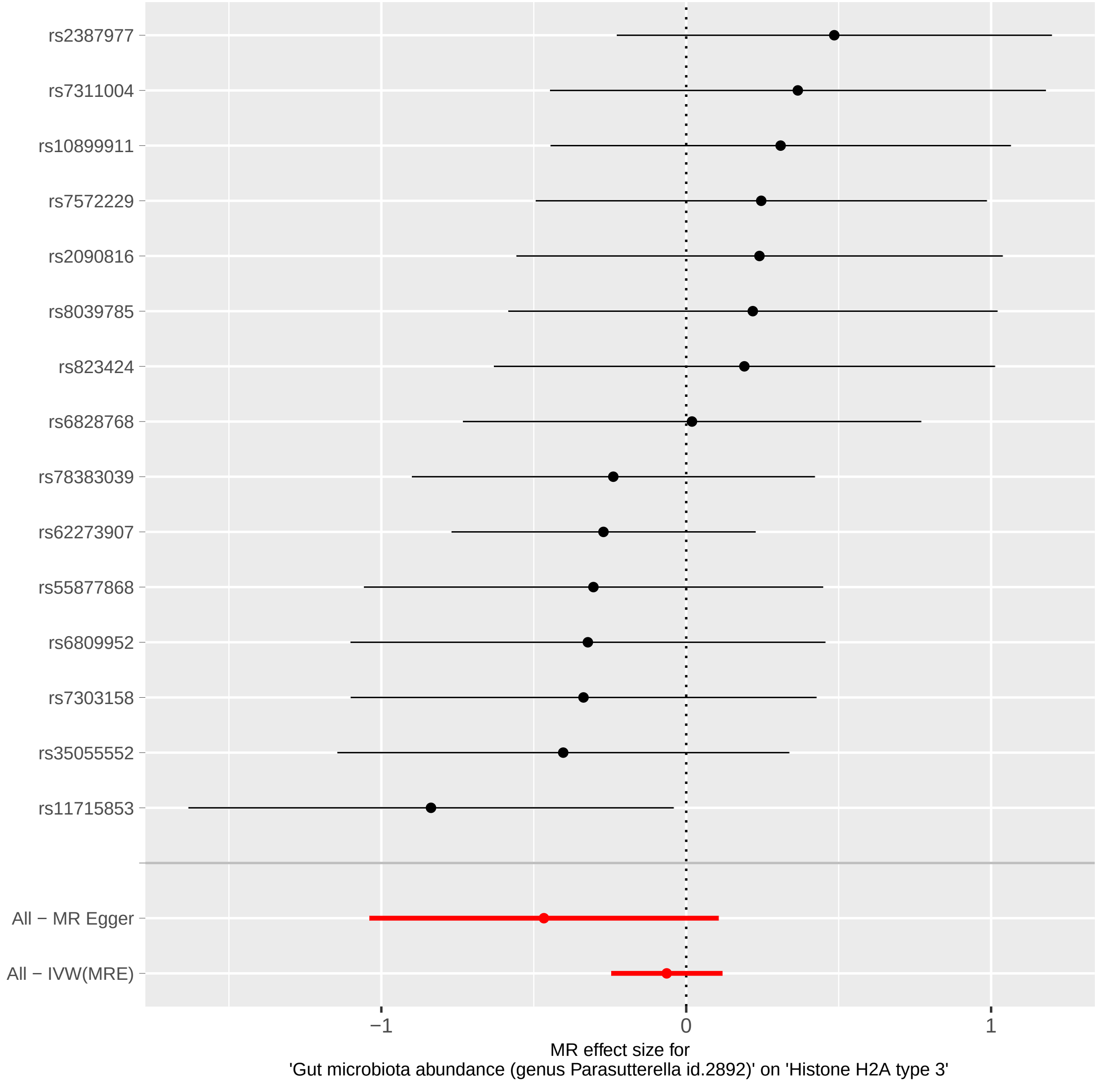

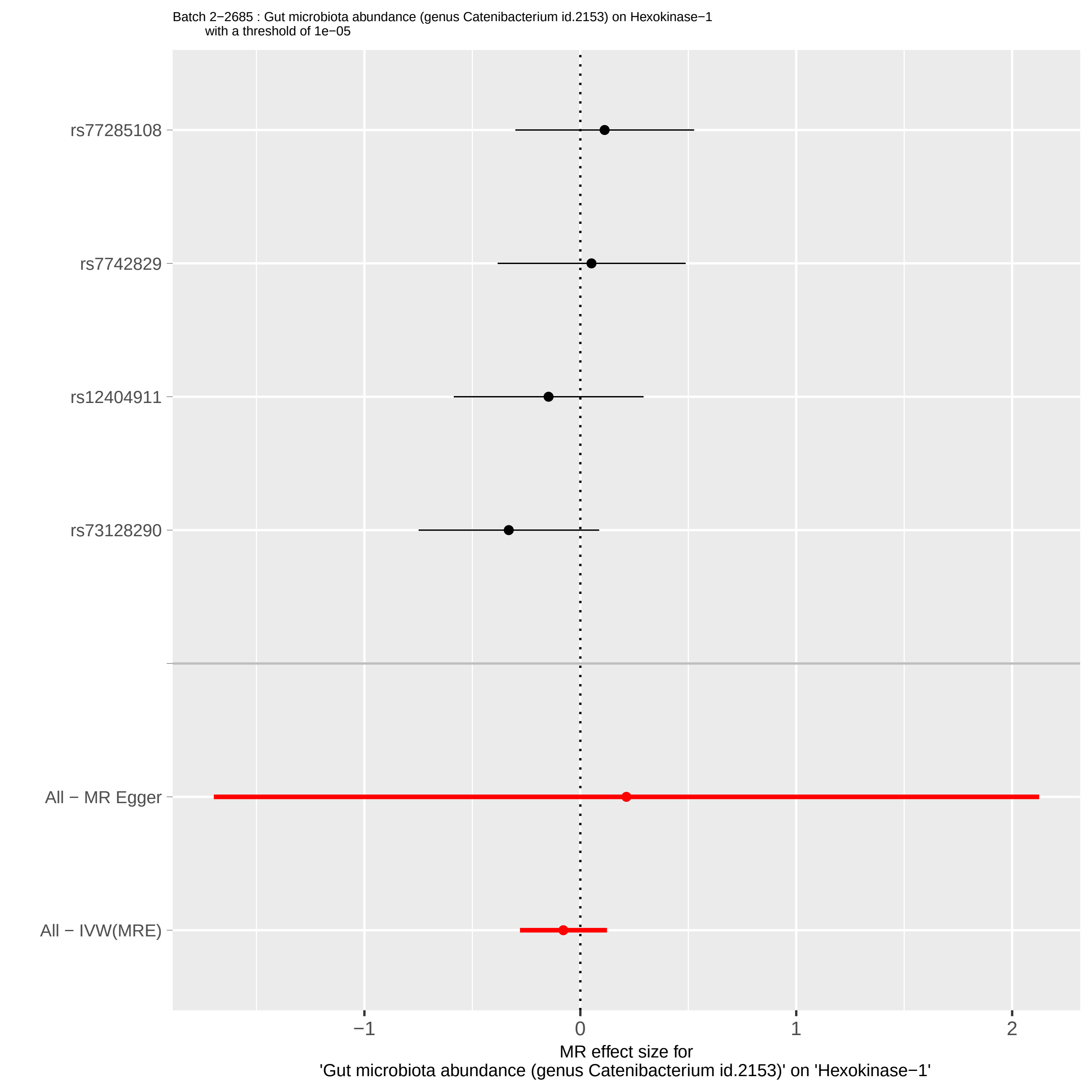

Batch 2−2685 : Gut microbiota abundance (genus Catenibacterium id.2153) on Hexokinase−1
with a threshold of 1e−05
rs77285108
rs7742829
rs12404911
rs73128290
All − MR Egger
All − IVW(MRE)
−1
0
1
2
MR effect size for
'Gut microbiota abundance (genus Catenibacterium id.2153)' on 'Hexokinase−1'

Batch 2−2686 : Gut microbiota abundance (genus Parasutterella id.2892) on Hexokinase−1
with a threshold of 1e−05

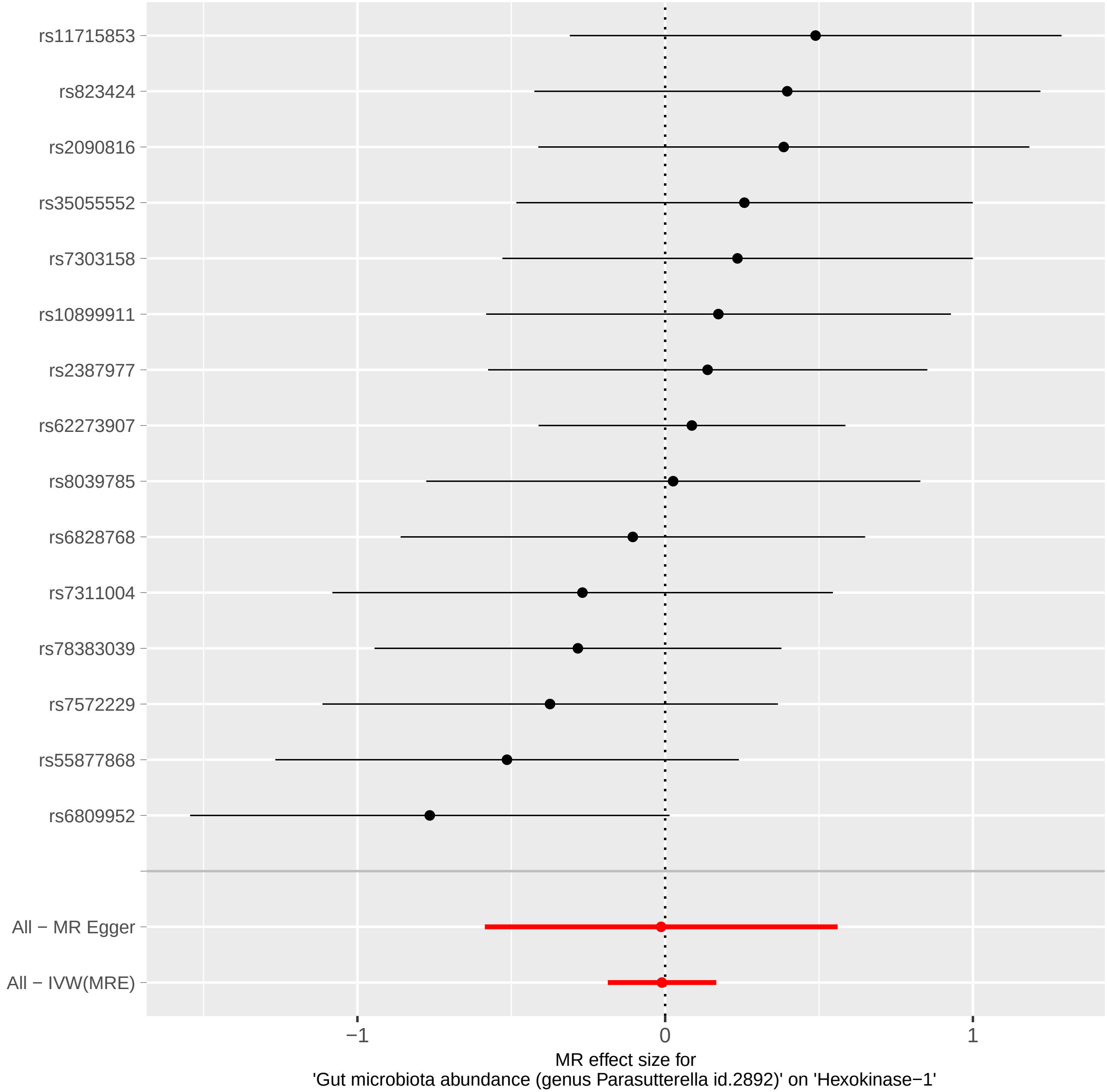

Batch 2−2687 : Gut microbiota abundance (genus Catenibacterium id.2153) on Hexokinase−2
with a threshold of 1e−05

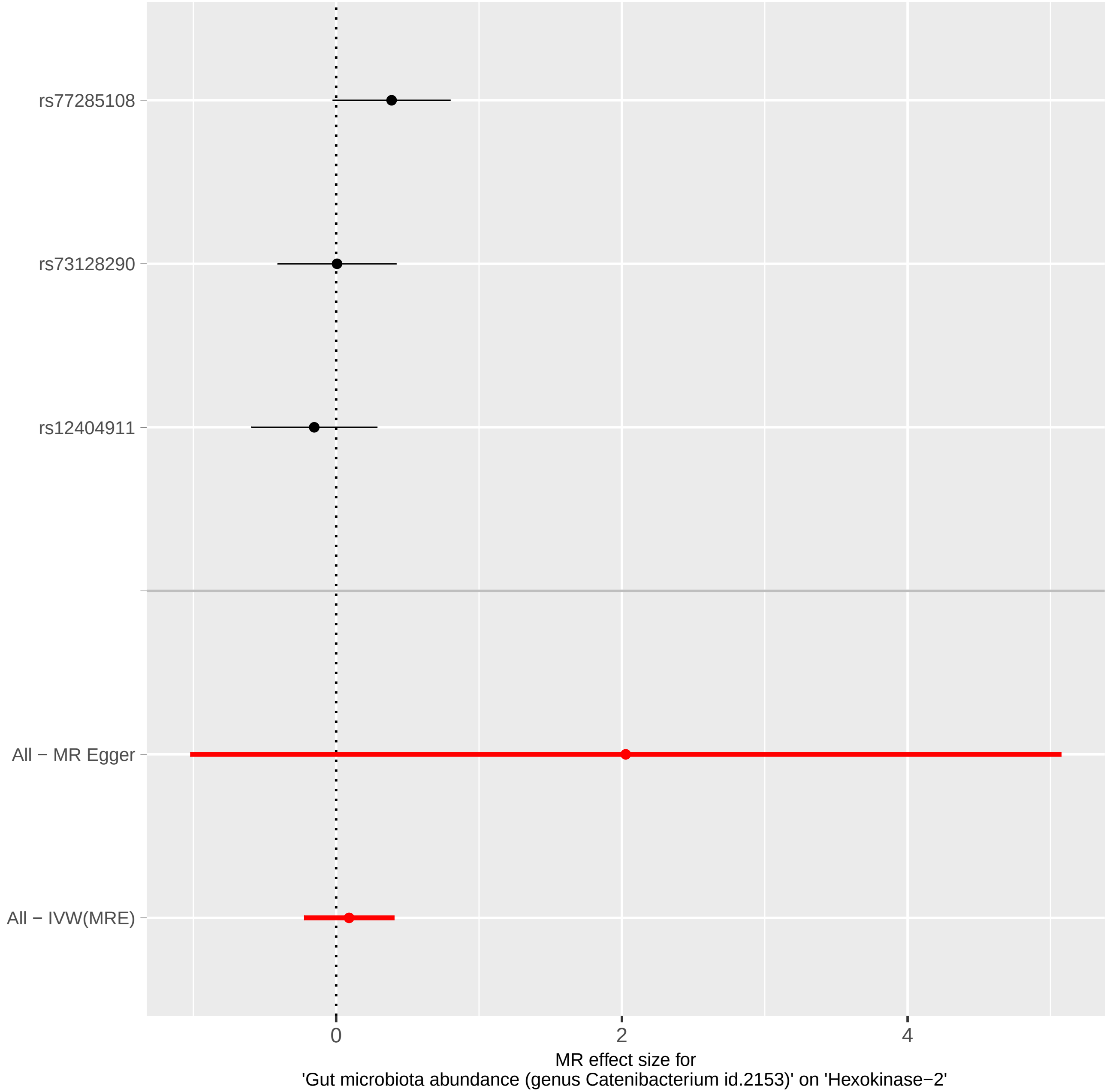

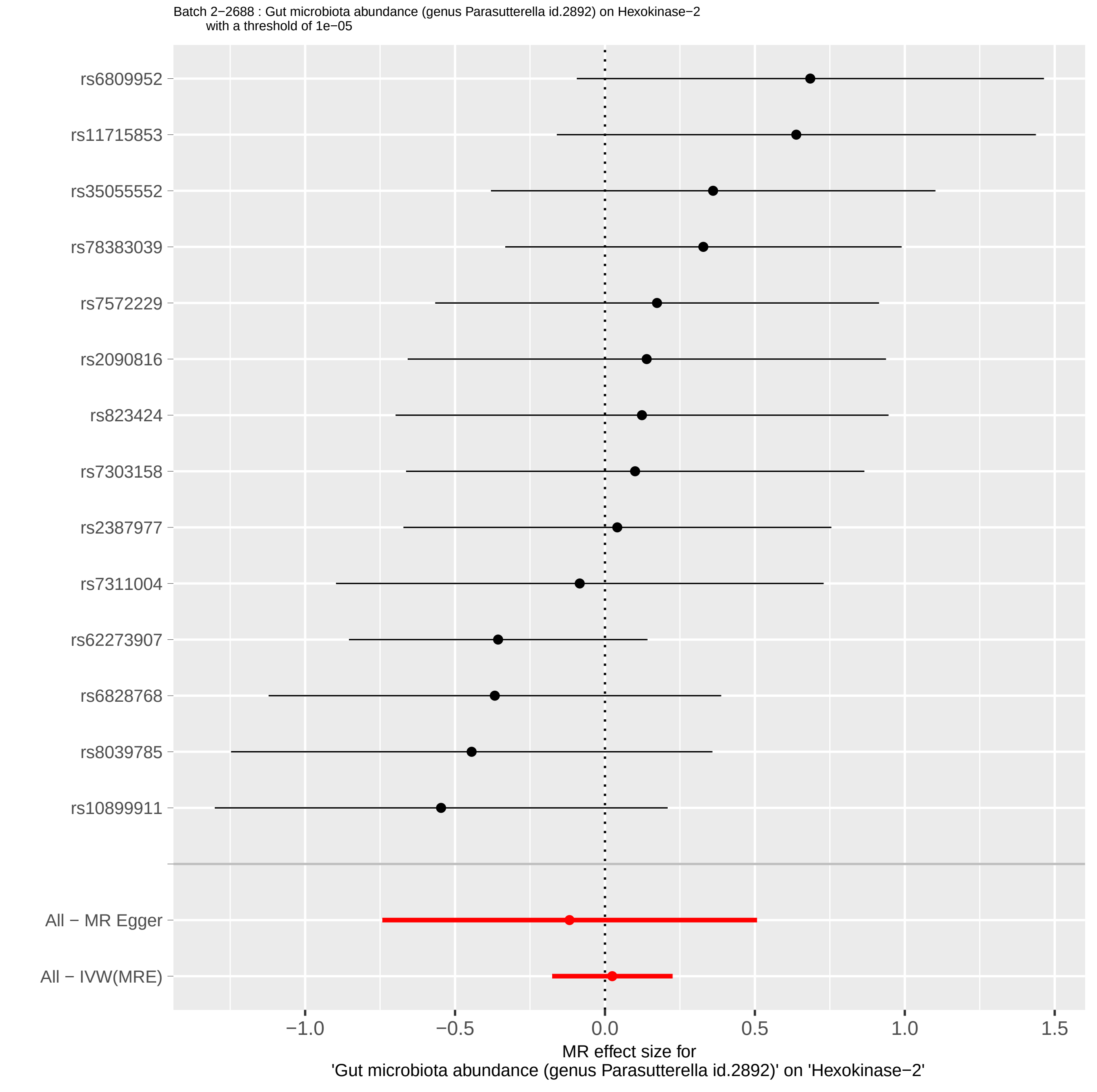
Batch 2−2688 : Gut microbiota abundance (genus Parasutterella id.2892) on Hexokinase−2
with a threshold of 1e−05
rs6809952
rs11715853
rs35055552
rs78383039
rs7572229
rs2090816
rs823424
rs7303158
rs2387977
rs7311004
rs62273907
rs6828768
rs8039785
rs10899911
All − MR Egger
All − IVW(MRE)
−1.0
−0.5
0.0
0.5
1.0
1.5
MR effect size for
'Gut microbiota abundance (genus Parasutterella id.2892)' on 'Hexokinase−2'

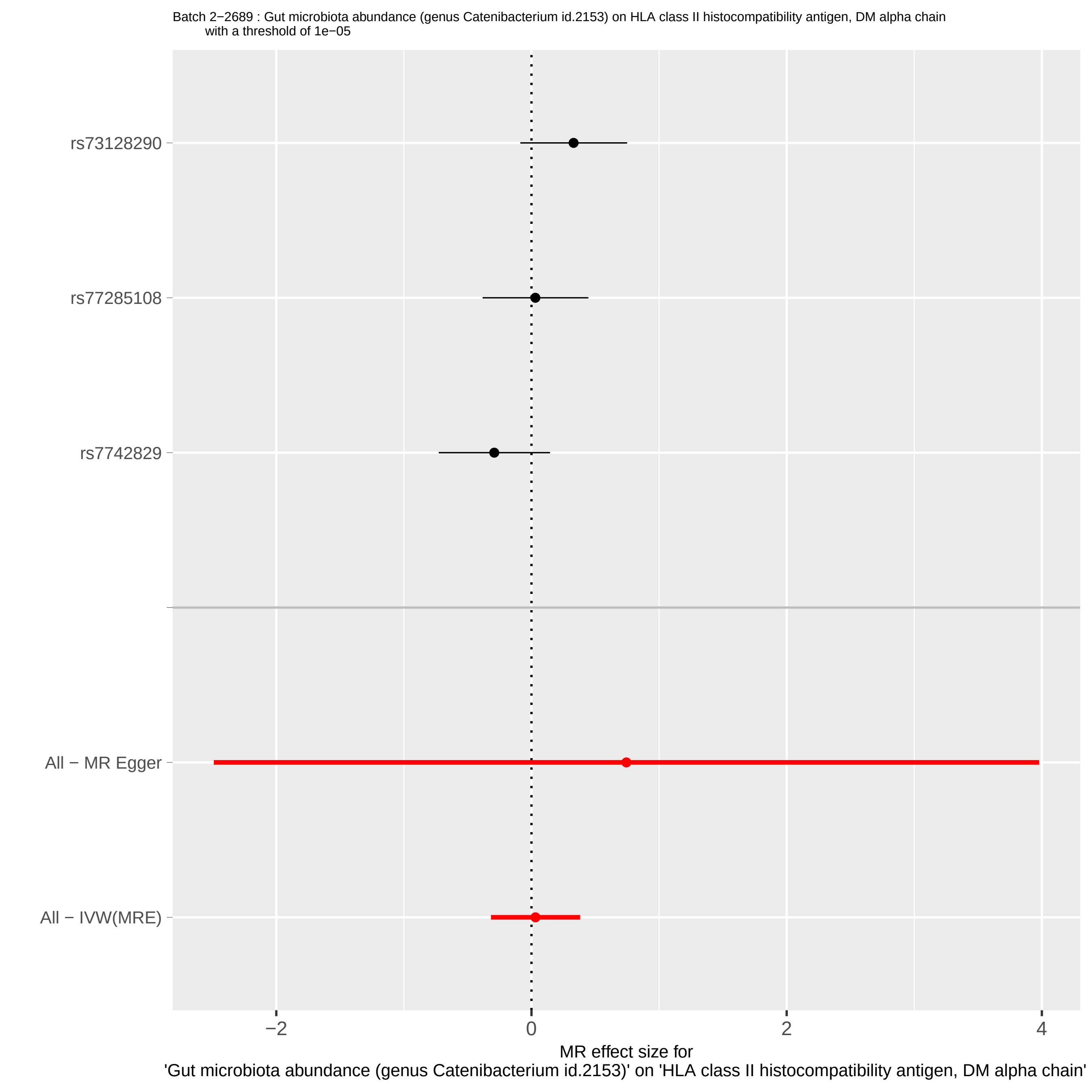

Batch 2−2689 : Gut microbiota abundance (genus Catenibacterium id.2153) on HLA class II histocompatibility antigen, DM alpha chain
with a threshold of 1e−05
rs73128290
rs77285108
rs7742829
All − MR Egger
All − IVW(MRE)
−2
0
2
4
MR effect size for
'Gut microbiota abundance (genus Catenibacterium id.2153)' on 'HLA class II histocompatibility antigen, DM alpha chain'

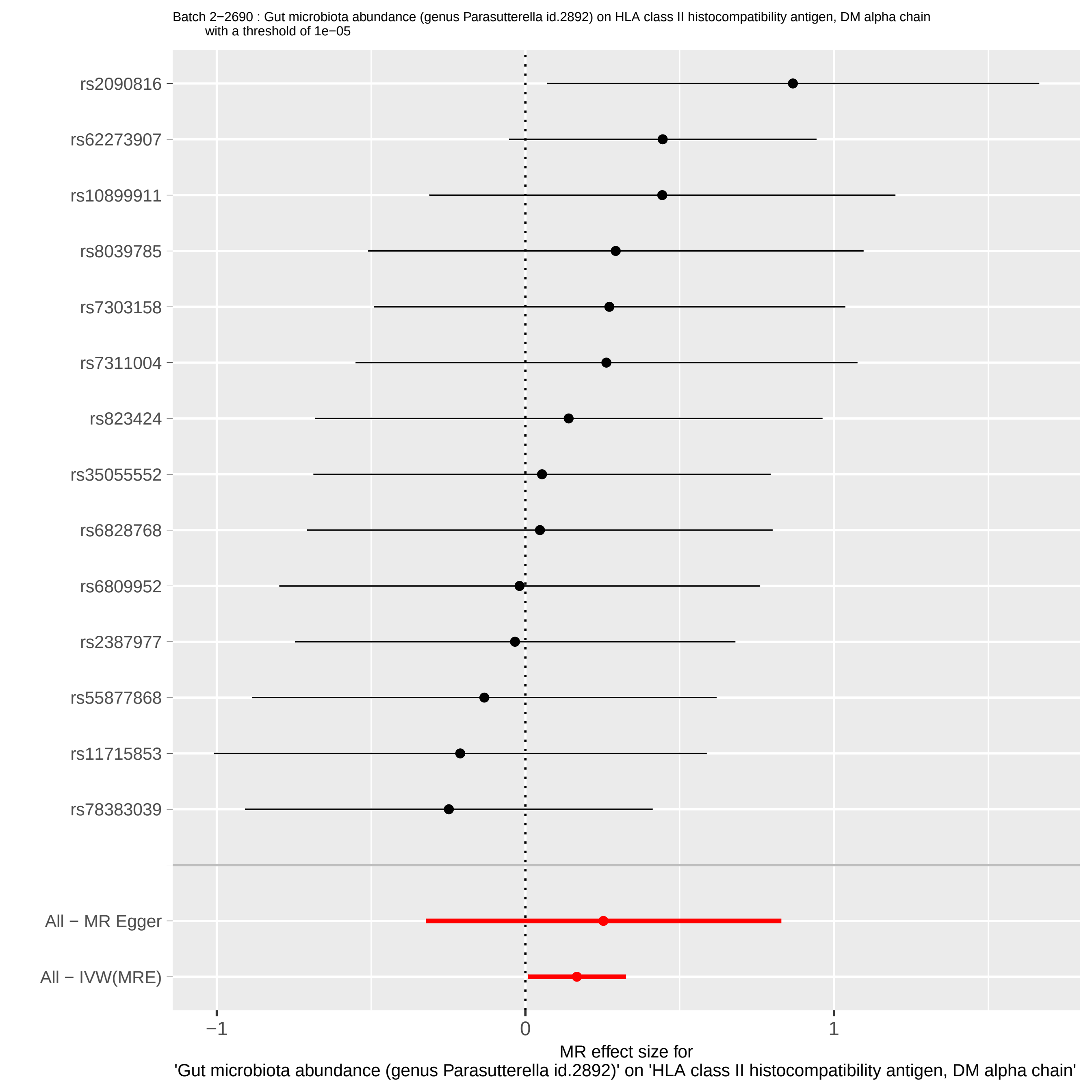

Batch 2−2690 : Gut microbiota abundance (genus Parasutterella id.2892) on HLA class II histocompatibility antigen, DM alpha chain
with a threshold of 1e−05
rs2090816
rs62273907
rs10899911
rs8039785
rs7303158
rs7311004
rs823424
rs35055552
rs6828768
rs6809952
rs2387977
rs55877868
rs11715853
rs78383039
All − MR Egger
All − IVW(MRE)
−1
0
1
MR effect size for
'Gut microbiota abundance (genus Parasutterella id.2892)' on 'HLA class II histocompatibility antigen, DM alpha chain'

Batch 2−2691 : Gut microbiota abundance (genus Catenibacterium id.2153) on HLA class II histocompatibility antigen, DP beta 1 chain with a threshold of 1e−05

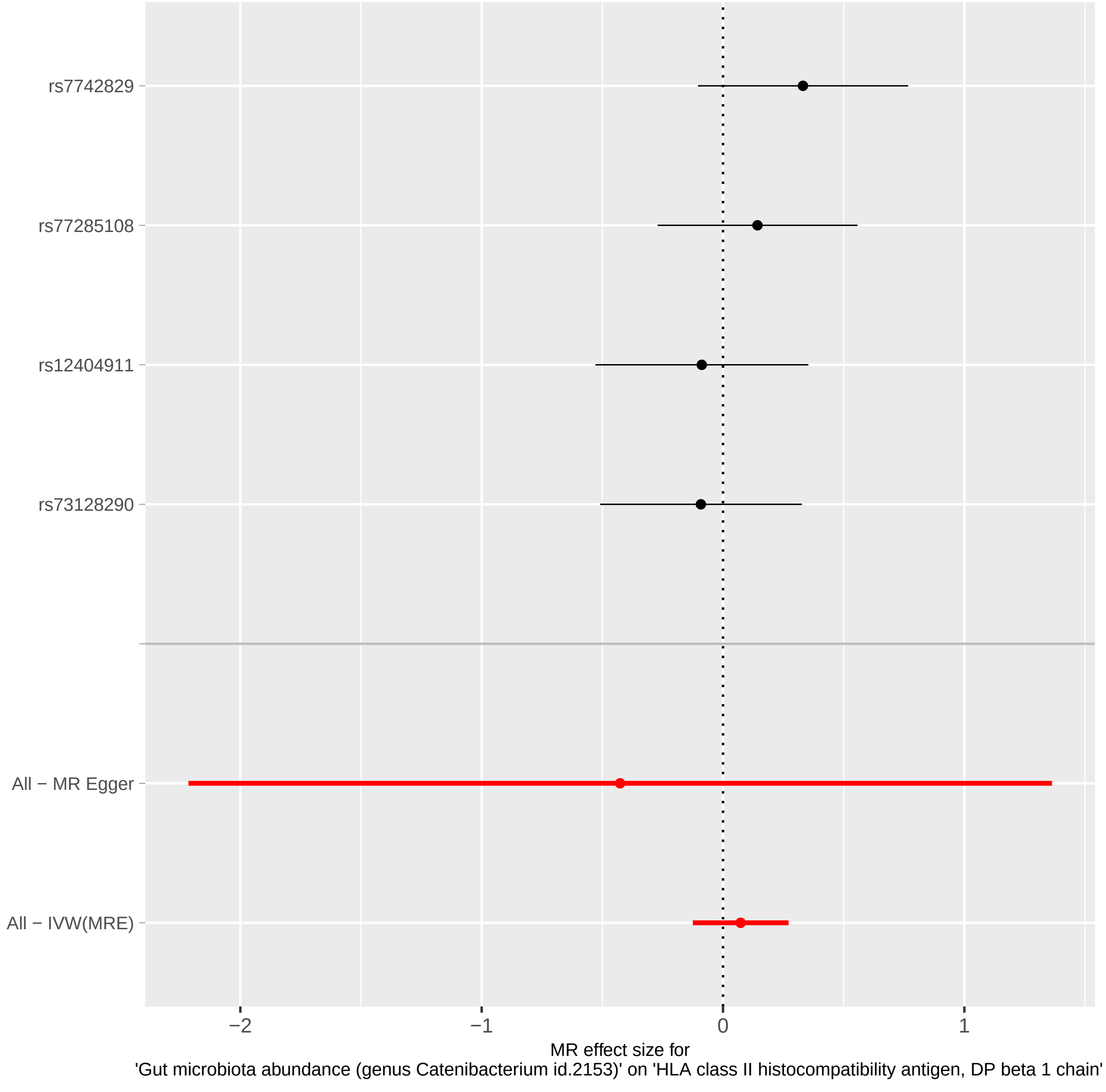

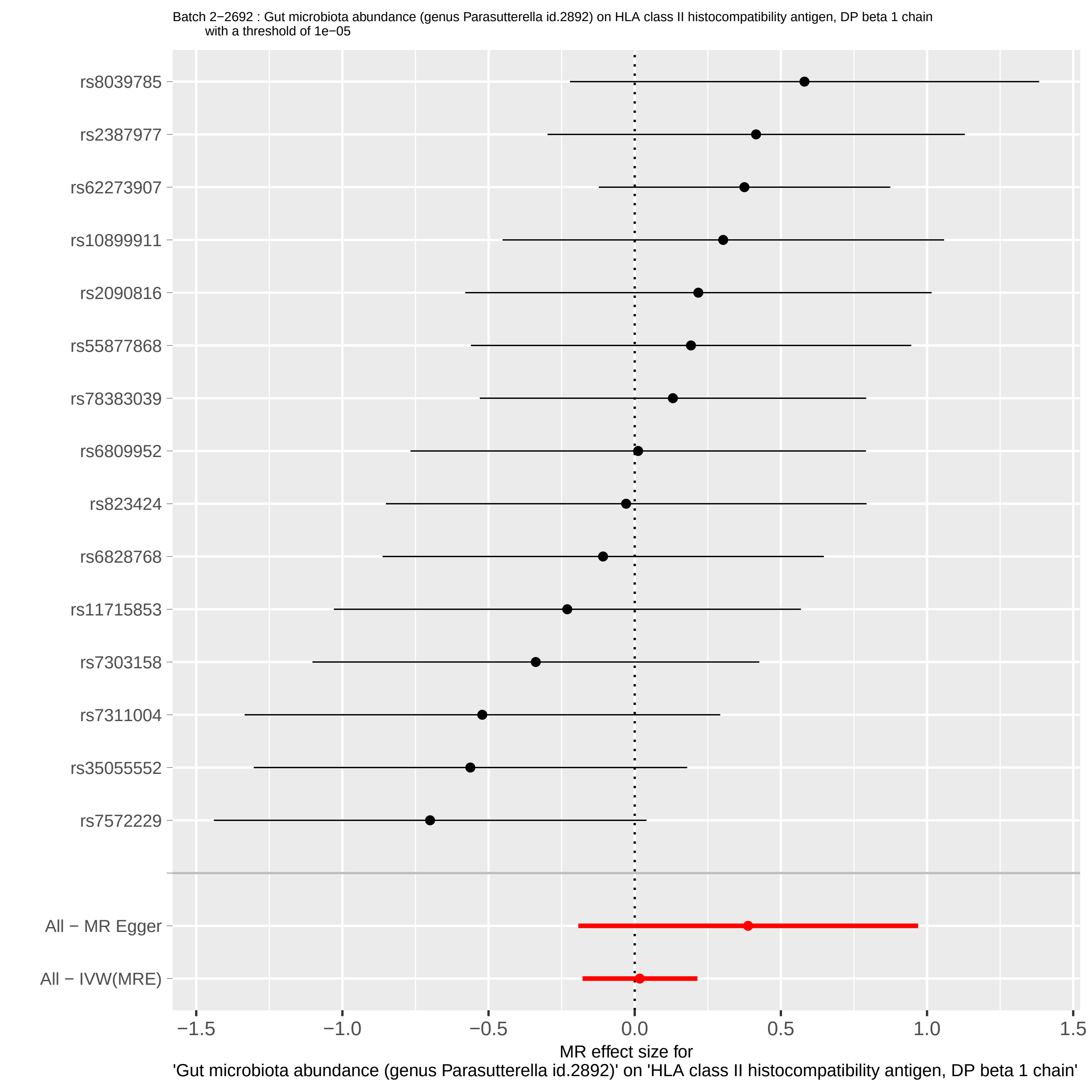
Batch 2−2692 : Gut microbiota abundance (genus Parasutterella id.2892) on HLA class II histocompatibility antigen, DP beta 1 chain
with a threshold of 1e−05
rs8039785
rs2387977
rs62273907
rs10899911
rs2090816
rs55877868
rs78383039
rs6809952
rs823424
rs6828768
rs11715853
rs7303158
rs7311004
rs35055552
rs7572229
All − MR Egger
All − IVW(MRE)
−1.5
−1.0
−0.5
0.0
0.5
1.0
1.5
MR effect size for
'Gut microbiota abundance (genus Parasutterella id.2892)' on 'HLA class II histocompatibility antigen, DP beta 1 chain'

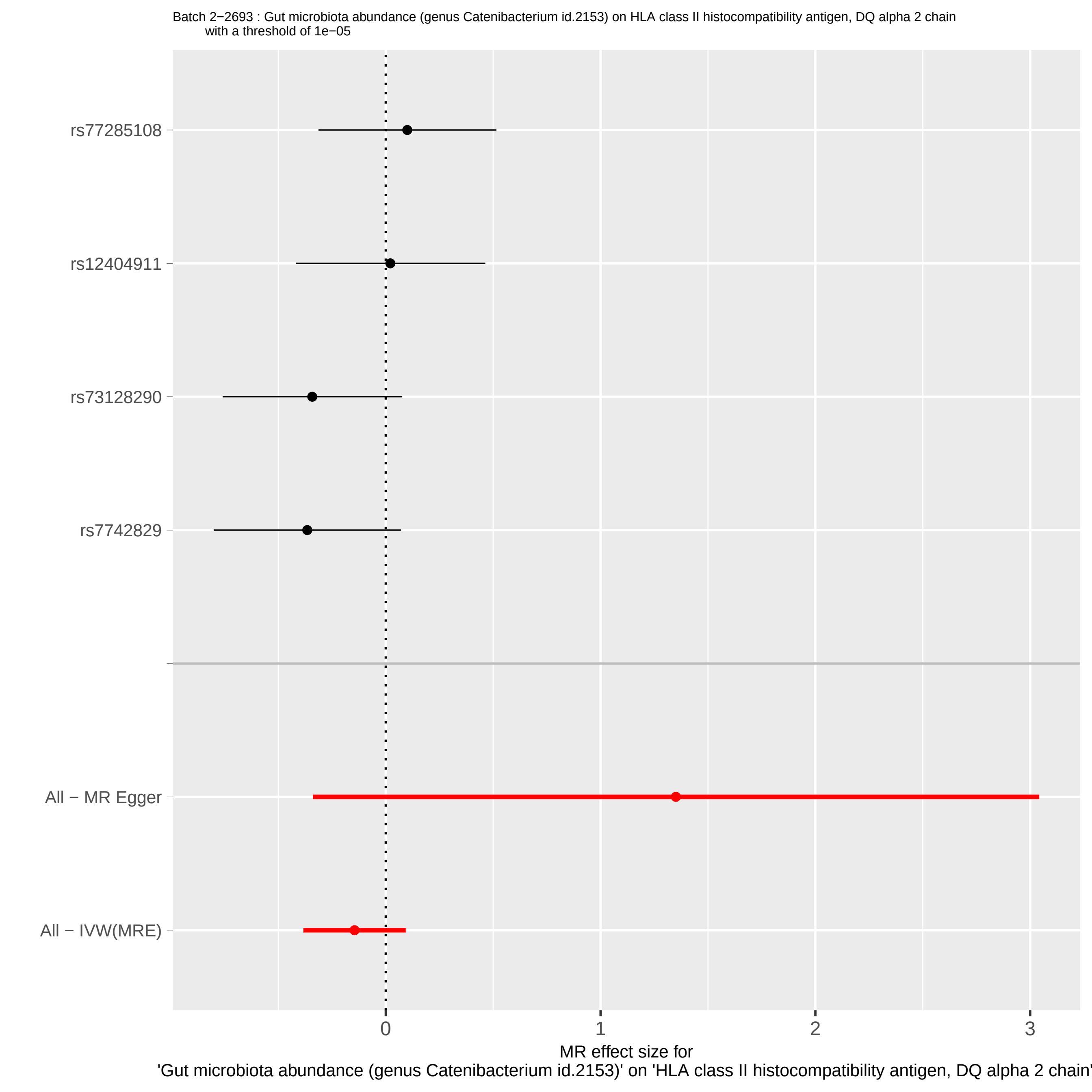
Batch 2−2693 : Gut microbiota abundance (genus Catenibacterium id.2153) on HLA class II histocompatibility antigen, DQ alpha 2 chain
with a threshold of 1e−05
rs77285108
rs12404911
rs73128290
rs7742829
All − MR Egger
All − IVW(MRE)
0
1
2
3
MR effect size for
'Gut microbiota abundance (genus Catenibacterium id.2153)' on 'HLA class II histocompatibility antigen, DQ alpha 2 chain'

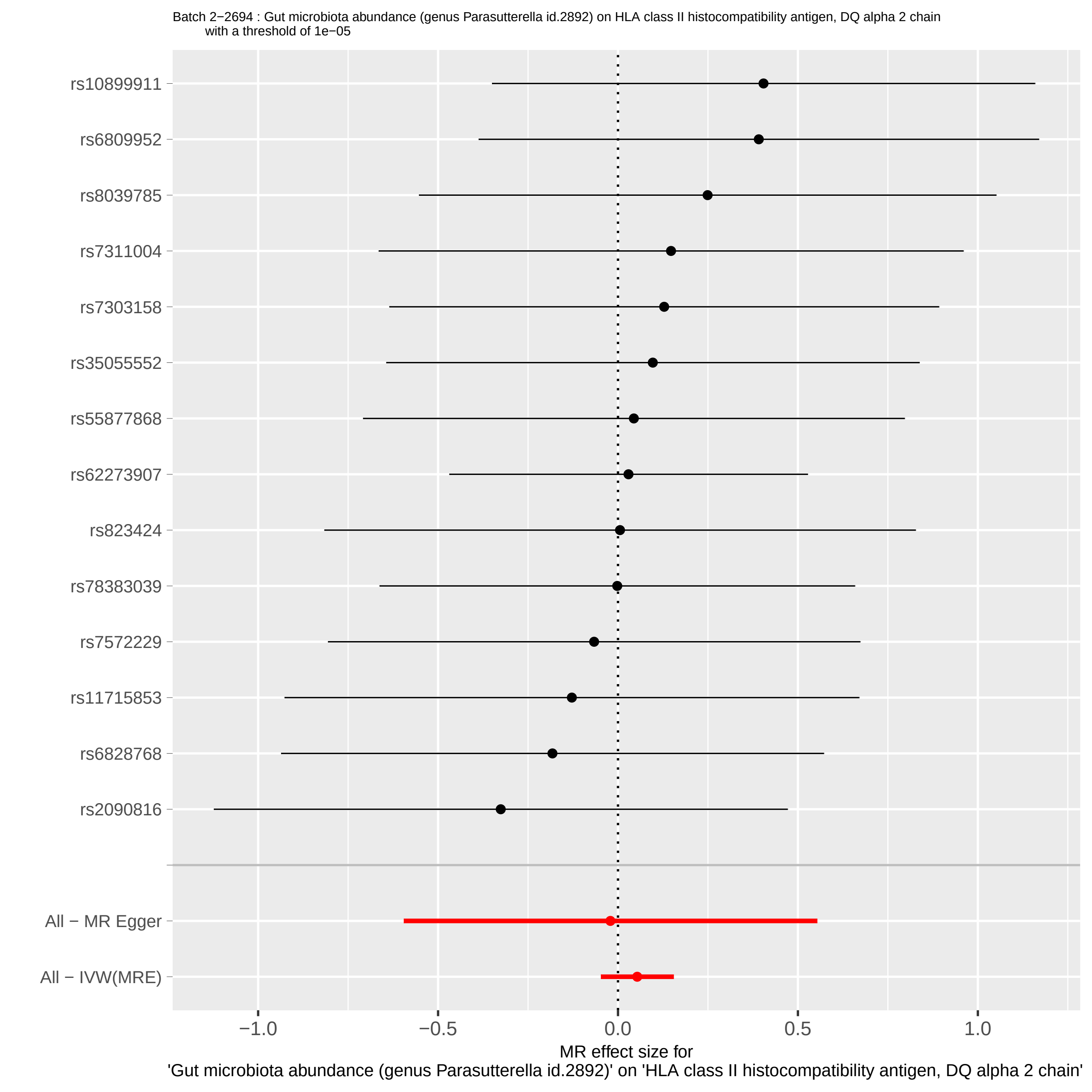

Batch 2−2694 : Gut microbiota abundance (genus Parasutterella id.2892) on HLA class II histocompatibility antigen, DQ alpha 2 chain
with a threshold of 1e−05
rs10899911
rs6809952
rs8039785
rs7311004
rs7303158
rs35055552
rs55877868
rs62273907
rs823424
rs78383039
rs7572229
rs11715853
rs6828768
rs2090816
All − MR Egger
All − IVW(MRE)
−1.0
−0.5
0.0
0.5
1.0
MR effect size for
'Gut microbiota abundance (genus Parasutterella id.2892)' on 'HLA class II histocompatibility antigen, DQ alpha 2 chain'

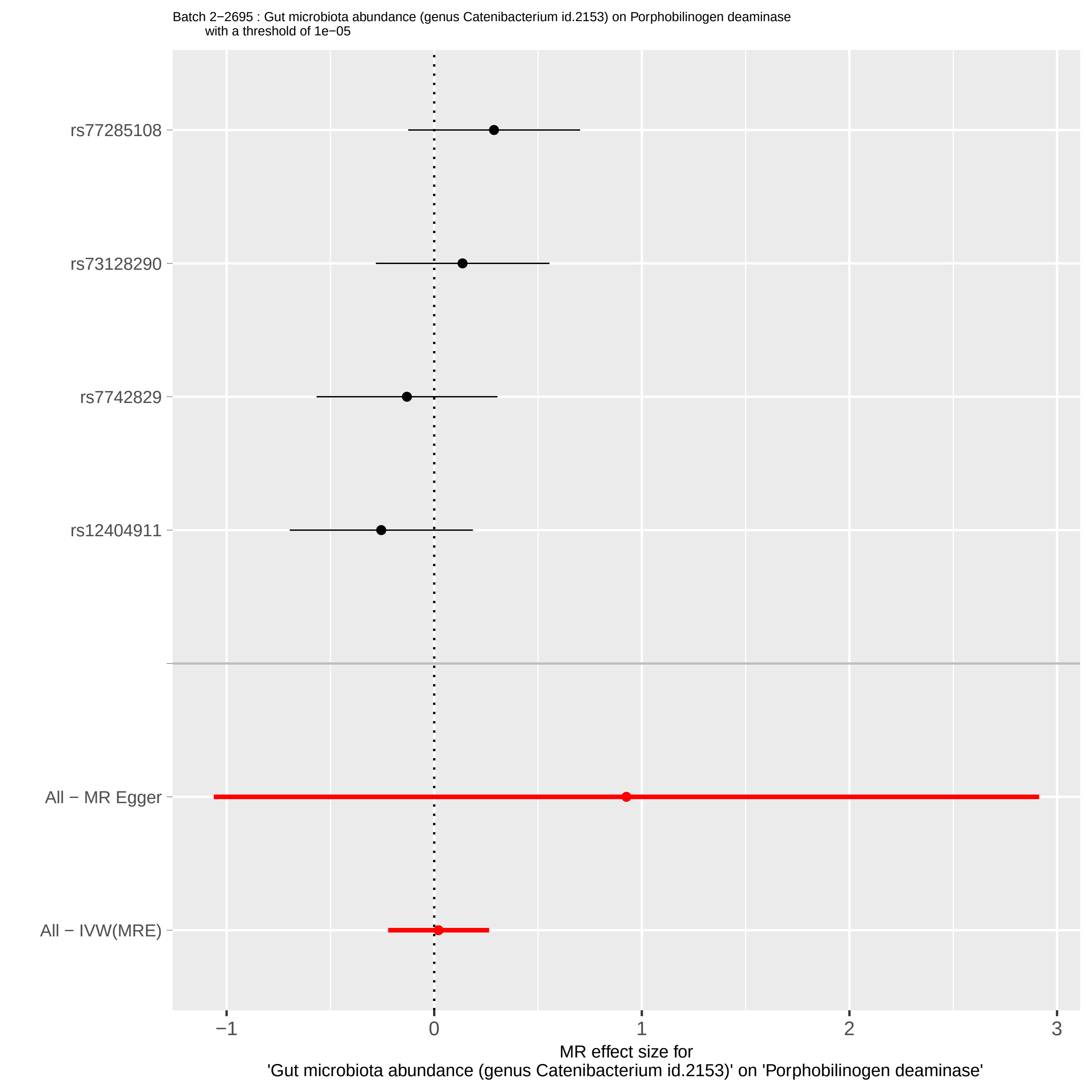

Batch 2−2695 : Gut microbiota abundance (genus Catenibacterium id.2153) on Porphobilinogen deaminase
with a threshold of 1e−05
rs77285108
rs73128290
rs7742829
rs12404911
All − MR Egger
All − IVW(MRE)
−1
0
1
2
3
MR effect size for
'Gut microbiota abundance (genus Catenibacterium id.2153)' on 'Porphobilinogen deaminase'

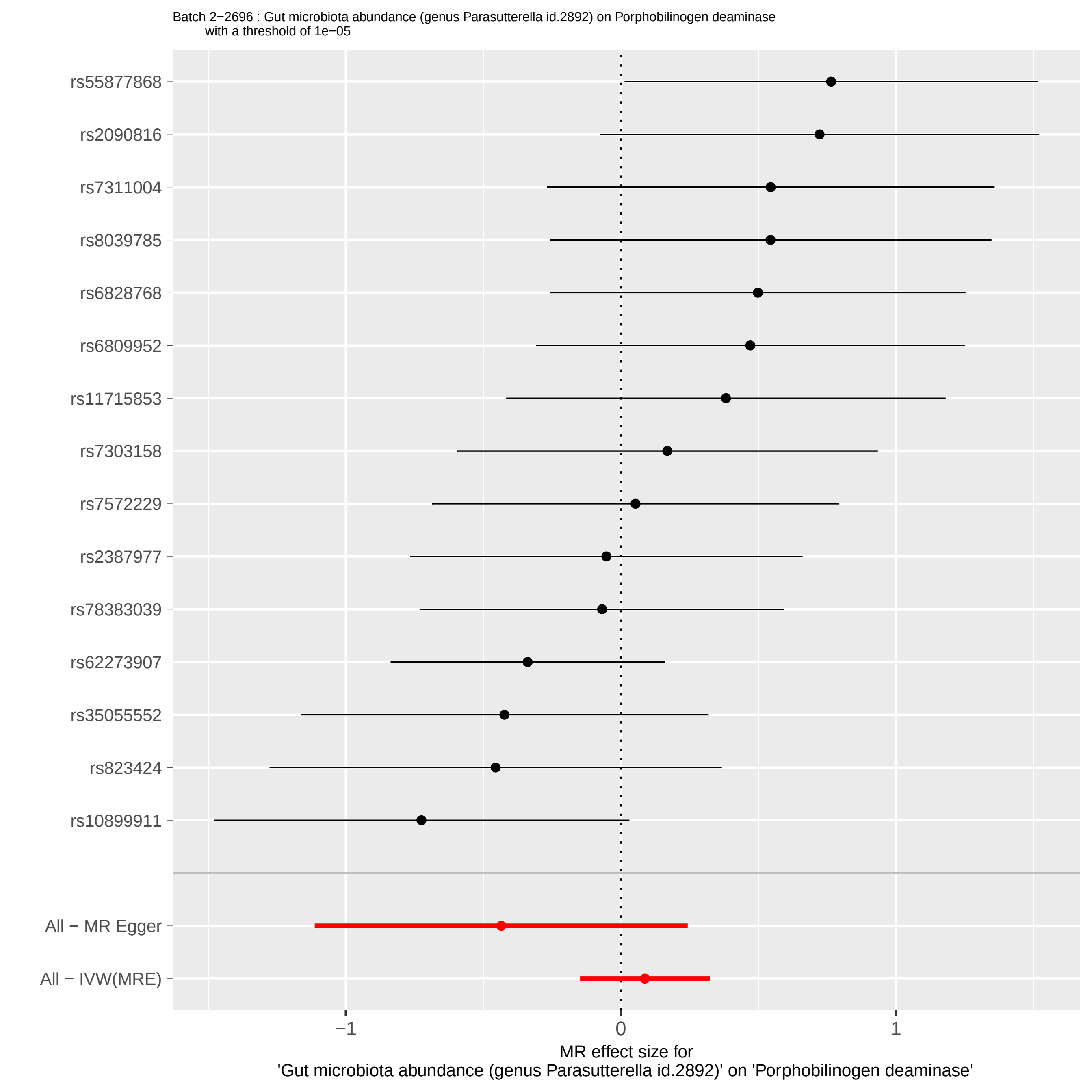
Batch 2−2696 : Gut microbiota abundance (genus Parasutterella id.2892) on Porphobilinogen deaminase
with a threshold of 1e−05
rs55877868
rs2090816
rs7311004
rs8039785
rs6828768
rs6809952
rs11715853
rs7303158
rs7572229
rs2387977
rs78383039
rs62273907
rs35055552
rs823424
rs10899911
All − MR Egger
All − IVW(MRE)
−1
0
1
MR effect size for
'Gut microbiota abundance (genus Parasutterella id.2892)' on 'Porphobilinogen deaminase'

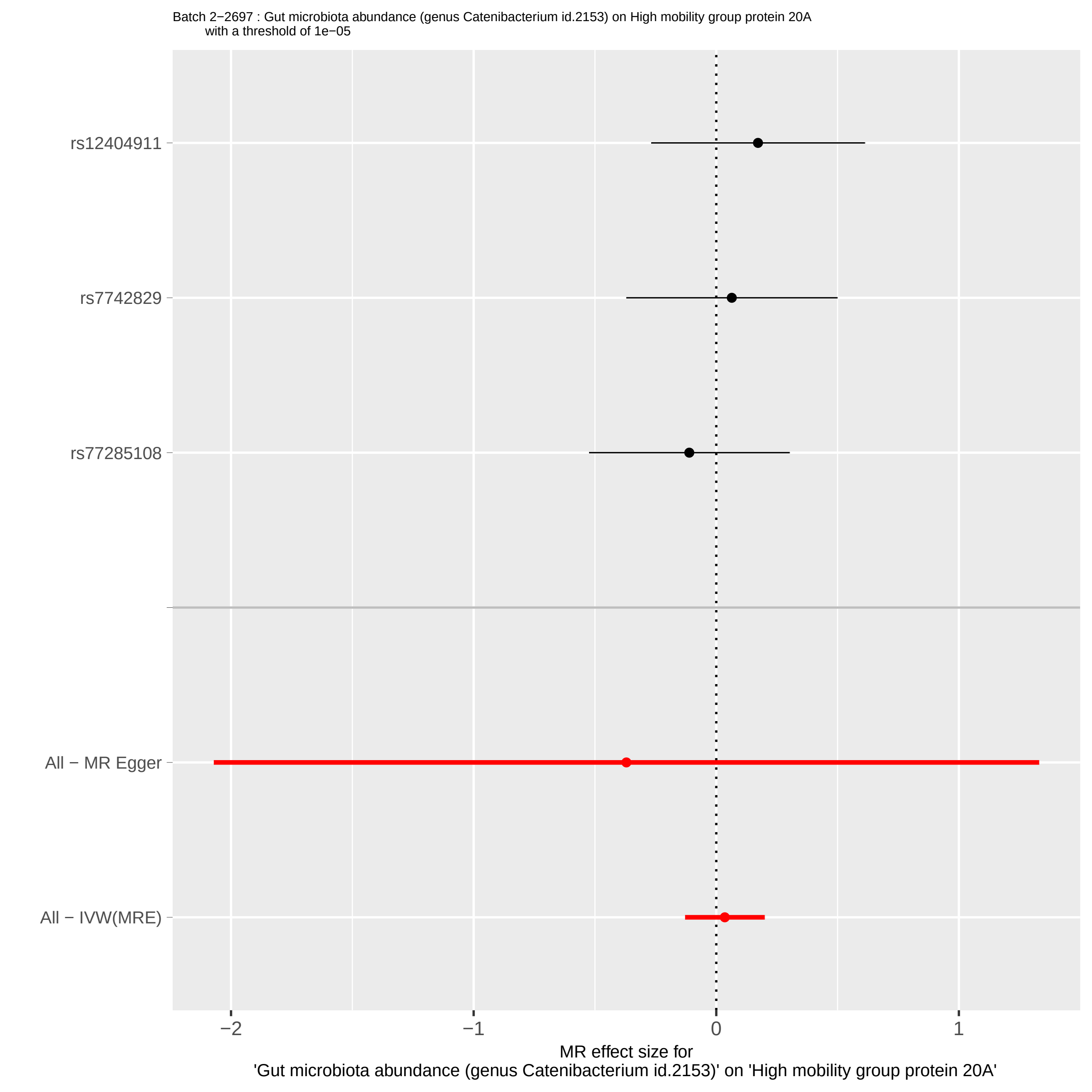

Batch 2−2697 : Gut microbiota abundance (genus Catenibacterium id.2153) on High mobility group protein 20A
with a threshold of 1e−05
rs12404911
rs7742829
rs77285108
All − MR Egger
All − IVW(MRE)
−2
−1
0
1
MR effect size for
'Gut microbiota abundance (genus Catenibacterium id.2153)' on 'High mobility group protein 20A'

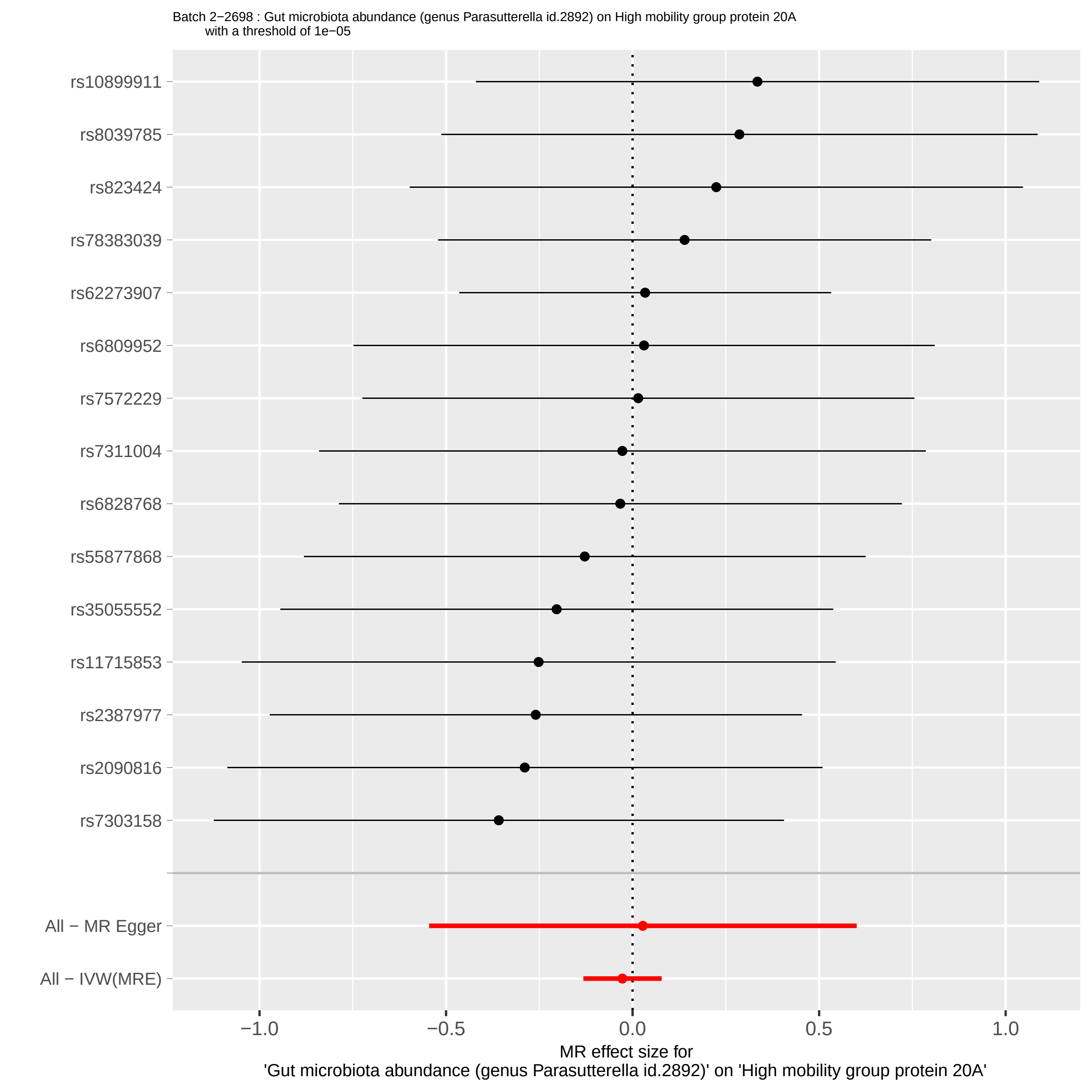
Batch 2−2698 : Gut microbiota abundance (genus Parasutterella id.2892) on High mobility group protein 20A
with a threshold of 1e−05
rs10899911
rs8039785
rs823424
rs78383039
rs62273907
rs6809952
rs7572229
rs7311004
rs6828768
rs55877868
rs35055552
rs11715853
rs2387977
rs2090816
rs7303158
All − MR Egger
All − IVW(MRE)
−1.0
−0.5
0.0
0.5
1.0
MR effect size for
'Gut microbiota abundance (genus Parasutterella id.2892)' on 'High mobility group protein 20A'

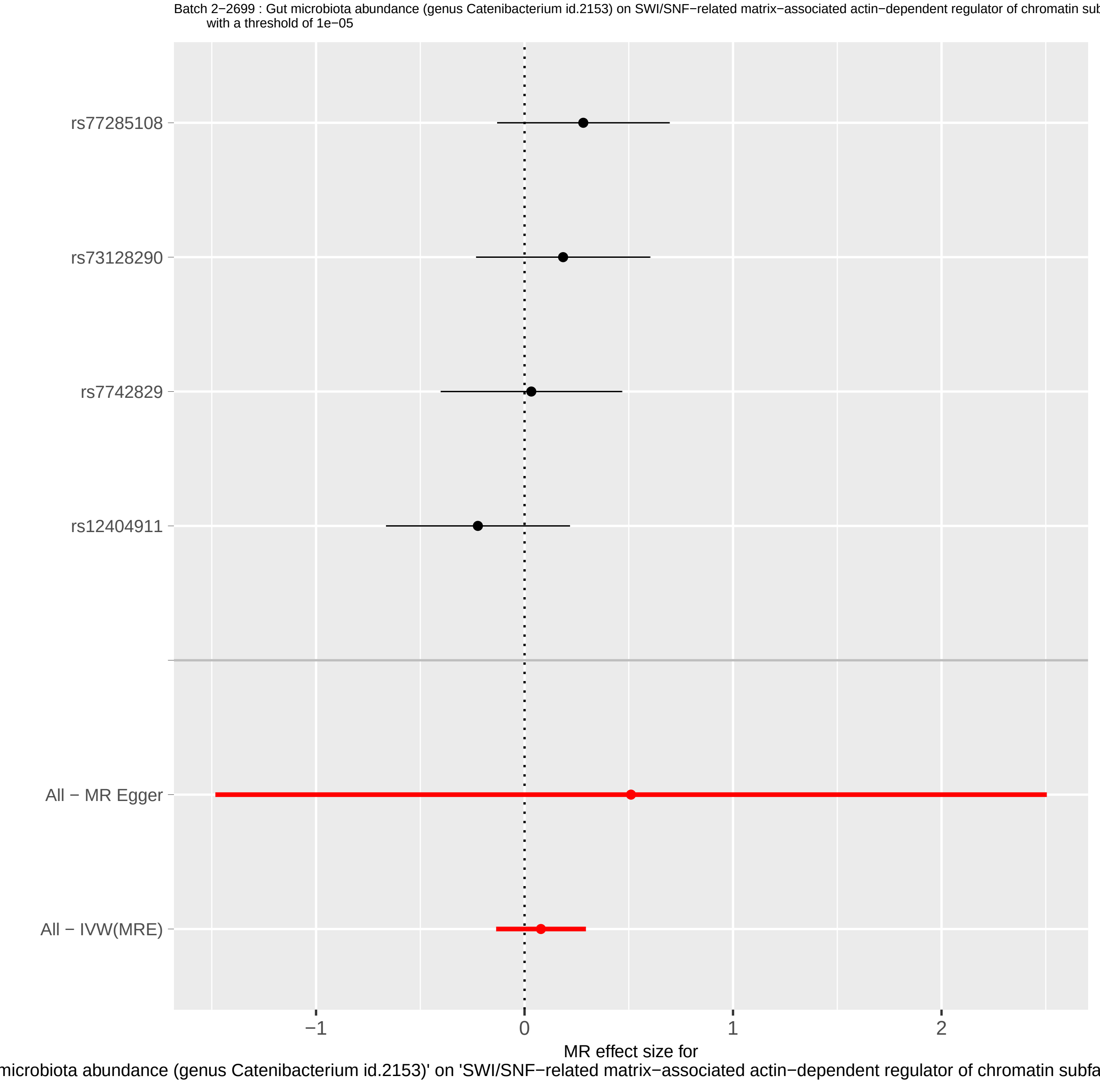

Batch 2−2699 : Gut microbiota abundance (genus Catenibacterium id.2153) on SWI/SNF−related matrix−associated actin−dependent regulator of chromatin sub
with a threshold of 1e−05
rs77285108
rs73128290
rs7742829
rs12404911
All − MR Egger
All − IVW(MRE)
−1
0
1
2
MR effect size for
microbiota abundance (genus Catenibacterium id.2153)' on 'SWI/SNF−related matrix−associated actin−dependent regulator of chromatin subfa

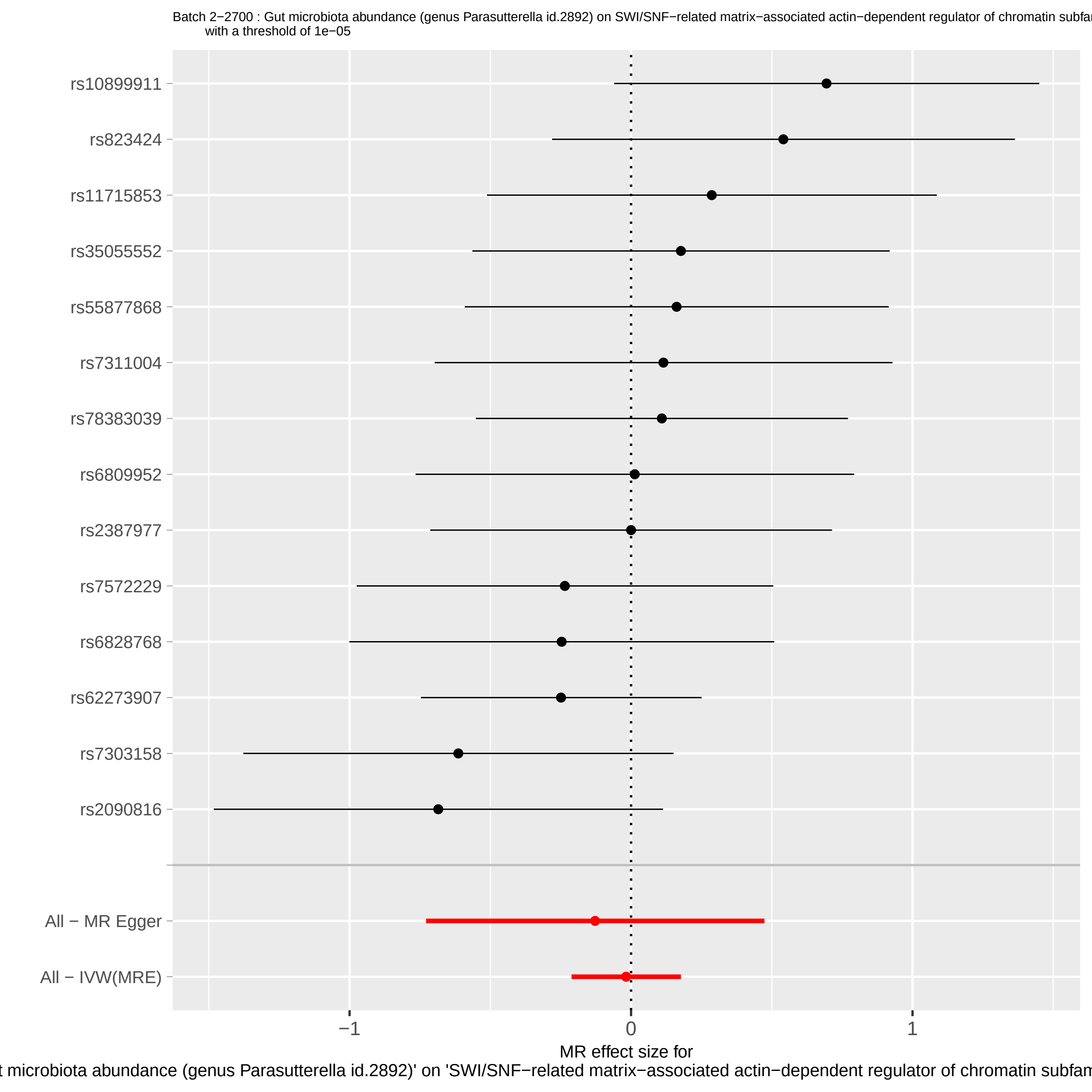
Batch 2−2700 : Gut microbiota abundance (genus Parasutterella id.2892) on SWI/SNF−related matrix−associated actin−dependent regulator of chromatin subfam
with a threshold of 1e−05
rs10899911
rs823424
rs11715853
rs35055552
rs55877868
rs7311004
rs78383039
rs6809952
rs2387977
rs7572229
rs6828768
rs62273907
rs7303158
rs2090816
All − MR Egger
All − IVW(MRE)
−1
0
1
MR effect size for
t microbiota abundance (genus Parasutterella id.2892)' on 'SWI/SNF−related matrix−associated actin−dependent regulator of chromatin subfam

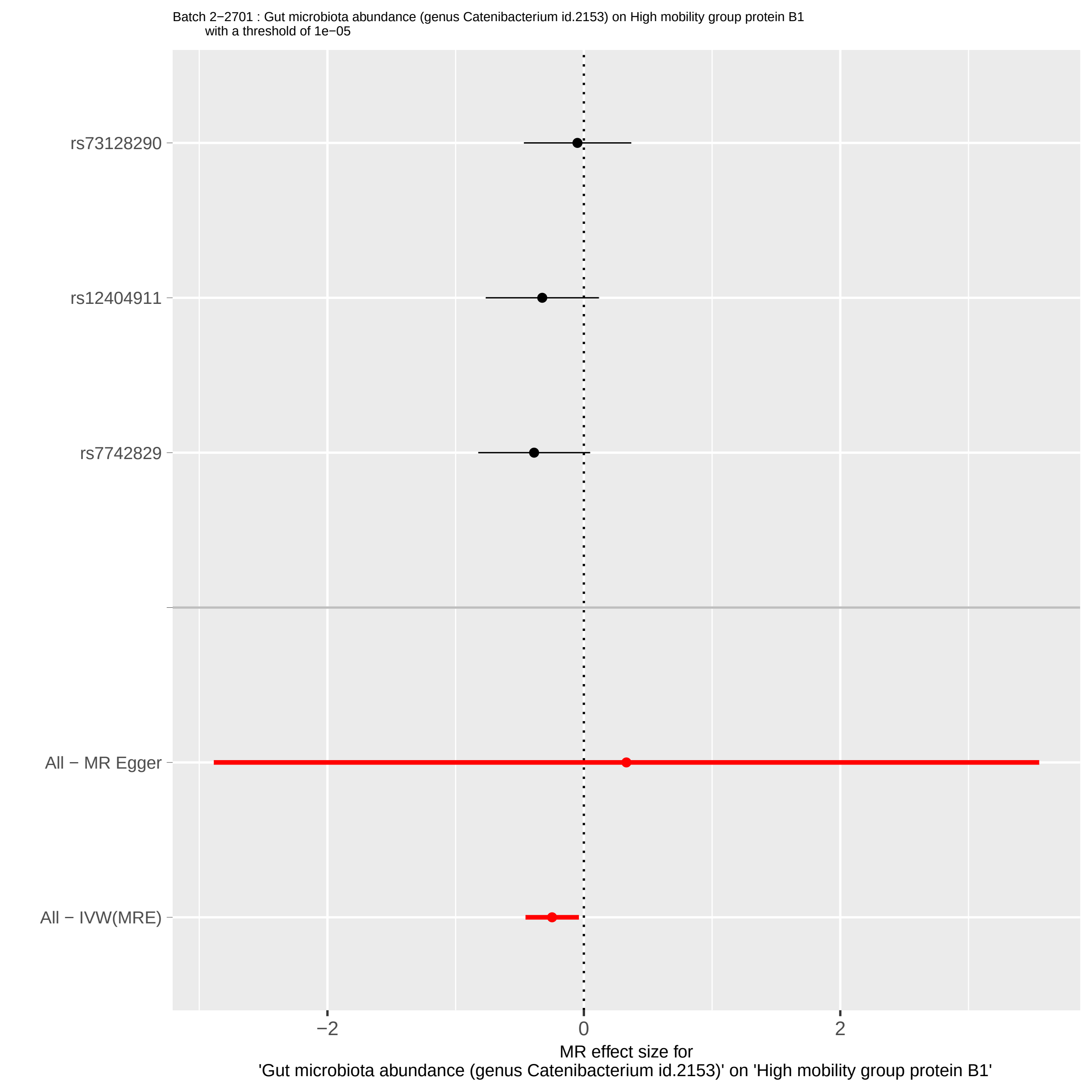

Batch 2−2701 : Gut microbiota abundance (genus Catenibacterium id.2153) on High mobility group protein B1
with a threshold of 1e−05
rs73128290
rs12404911
rs7742829
All − MR Egger
All − IVW(MRE)
−2
0
2
MR effect size for
'Gut microbiota abundance (genus Catenibacterium id.2153)' on 'High mobility group protein B1'

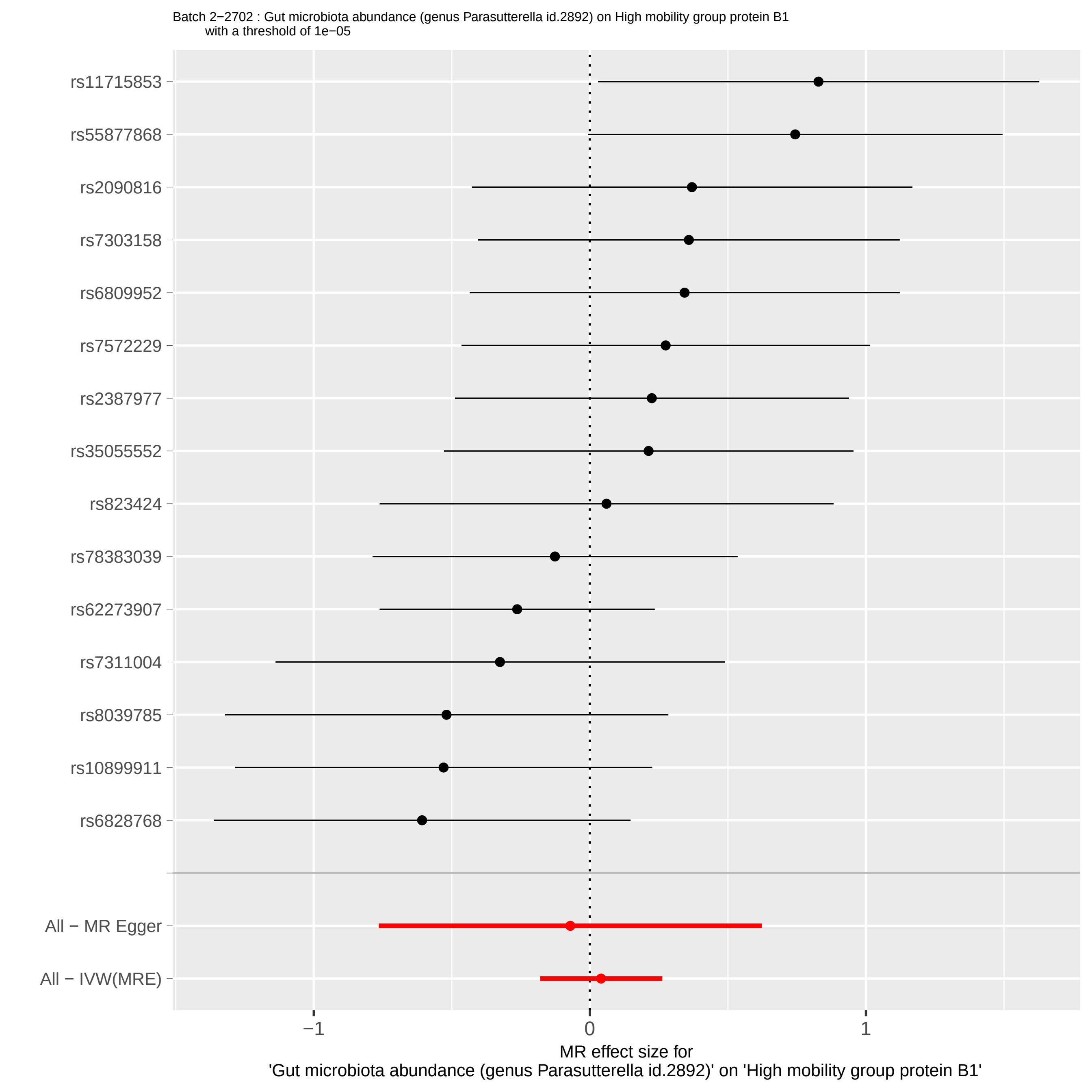
Batch 2−2702 : Gut microbiota abundance (genus Parasutterella id.2892) on High mobility group protein B1
with a threshold of 1e−05
rs11715853
rs55877868
rs2090816
rs7303158
rs6809952
rs7572229
rs2387977
rs35055552
rs823424
rs78383039
rs62273907
rs7311004
rs8039785
rs10899911
rs6828768
All − MR Egger
All − IVW(MRE)
−1
0
1
MR effect size for
'Gut microbiota abundance (genus Parasutterella id.2892)' on 'High mobility group protein B1'

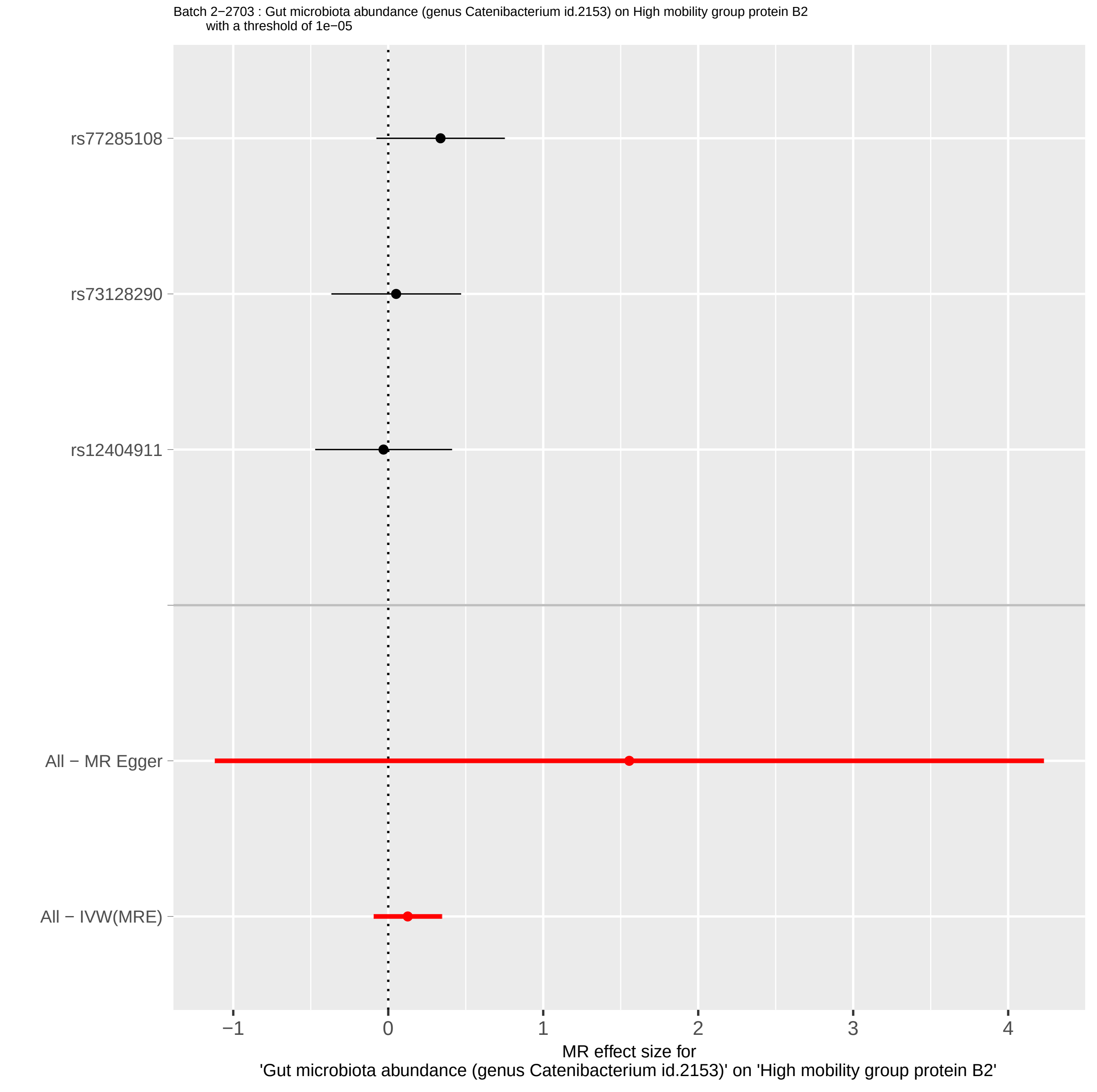

Batch 2−2703 : Gut microbiota abundance (genus Catenibacterium id.2153) on High mobility group protein B2
with a threshold of 1e−05
rs77285108
rs73128290
rs12404911
All − MR Egger
All − IVW(MRE)
−1
0
1
2
3
4
MR effect size for
'Gut microbiota abundance (genus Catenibacterium id.2153)' on 'High mobility group protein B2'

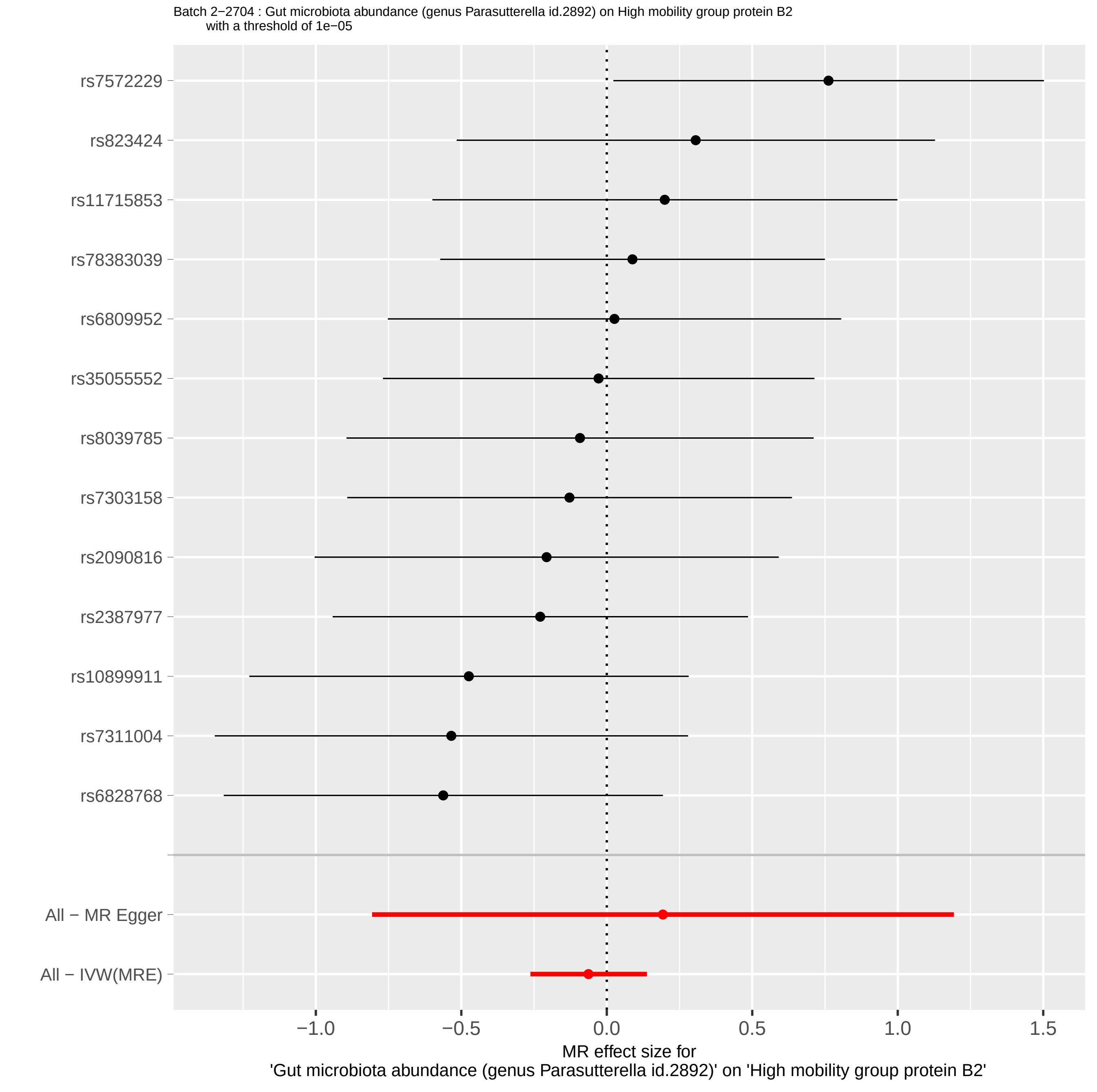

Batch 2−2704 : Gut microbiota abundance (genus Parasutterella id.2892) on High mobility group protein B2
with a threshold of 1e−05
rs7572229
rs823424
rs11715853
rs78383039
rs6809952
rs35055552
rs8039785
rs7303158
rs2090816
rs2387977
rs10899911
rs7311004
rs6828768
All − MR Egger
All − IVW(MRE)
−1.0
−0.5
0.0
0.5
1.0
1.5
MR effect size for
'Gut microbiota abundance (genus Parasutterella id.2892)' on 'High mobility group protein B2'

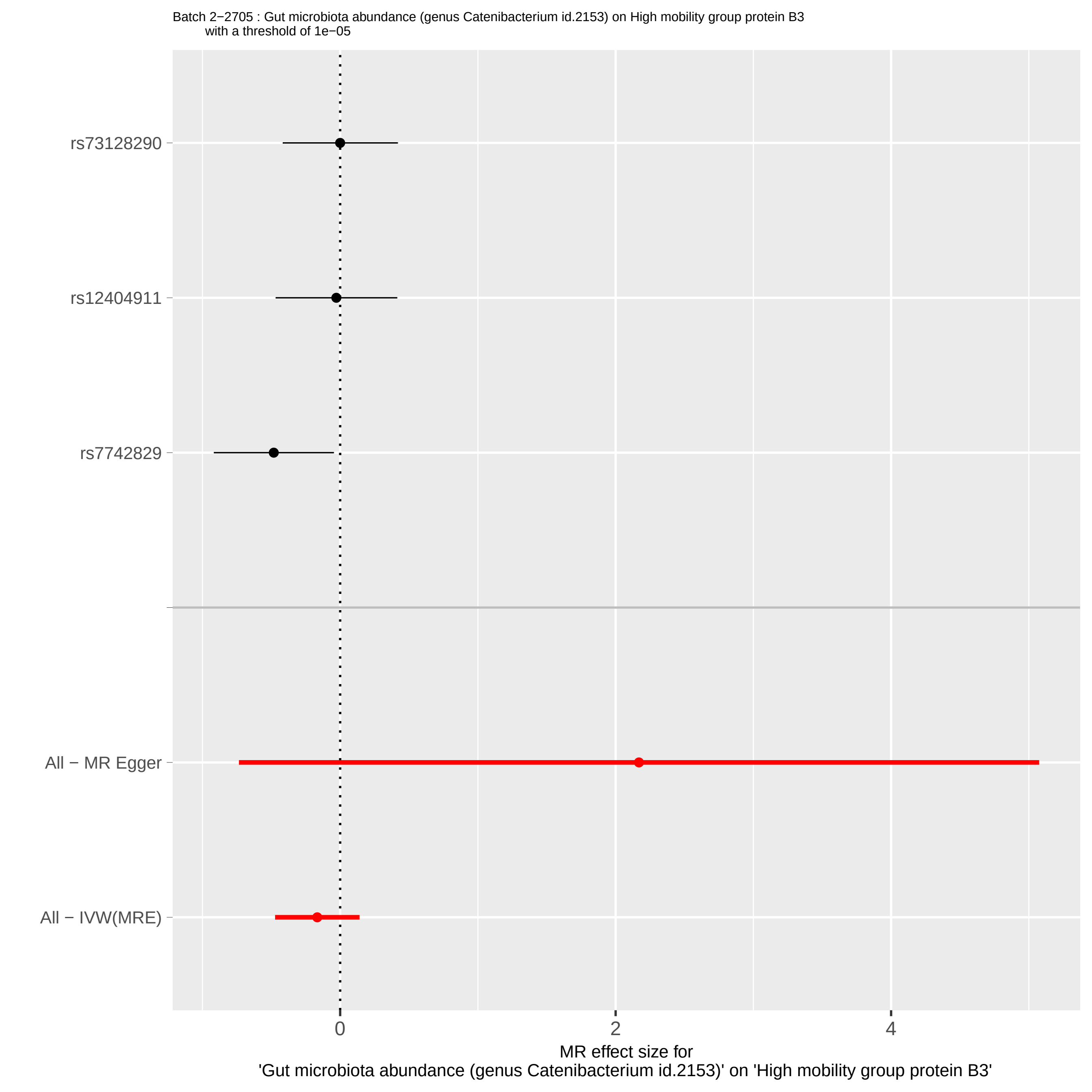
Batch 2−2705 : Gut microbiota abundance (genus Catenibacterium id.2153) on High mobility group protein B3
with a threshold of 1e−05
rs73128290
rs12404911
rs7742829
All − MR Egger
All − IVW(MRE)
0
2
4
MR effect size for
'Gut microbiota abundance (genus Catenibacterium id.2153)' on 'High mobility group protein B3'

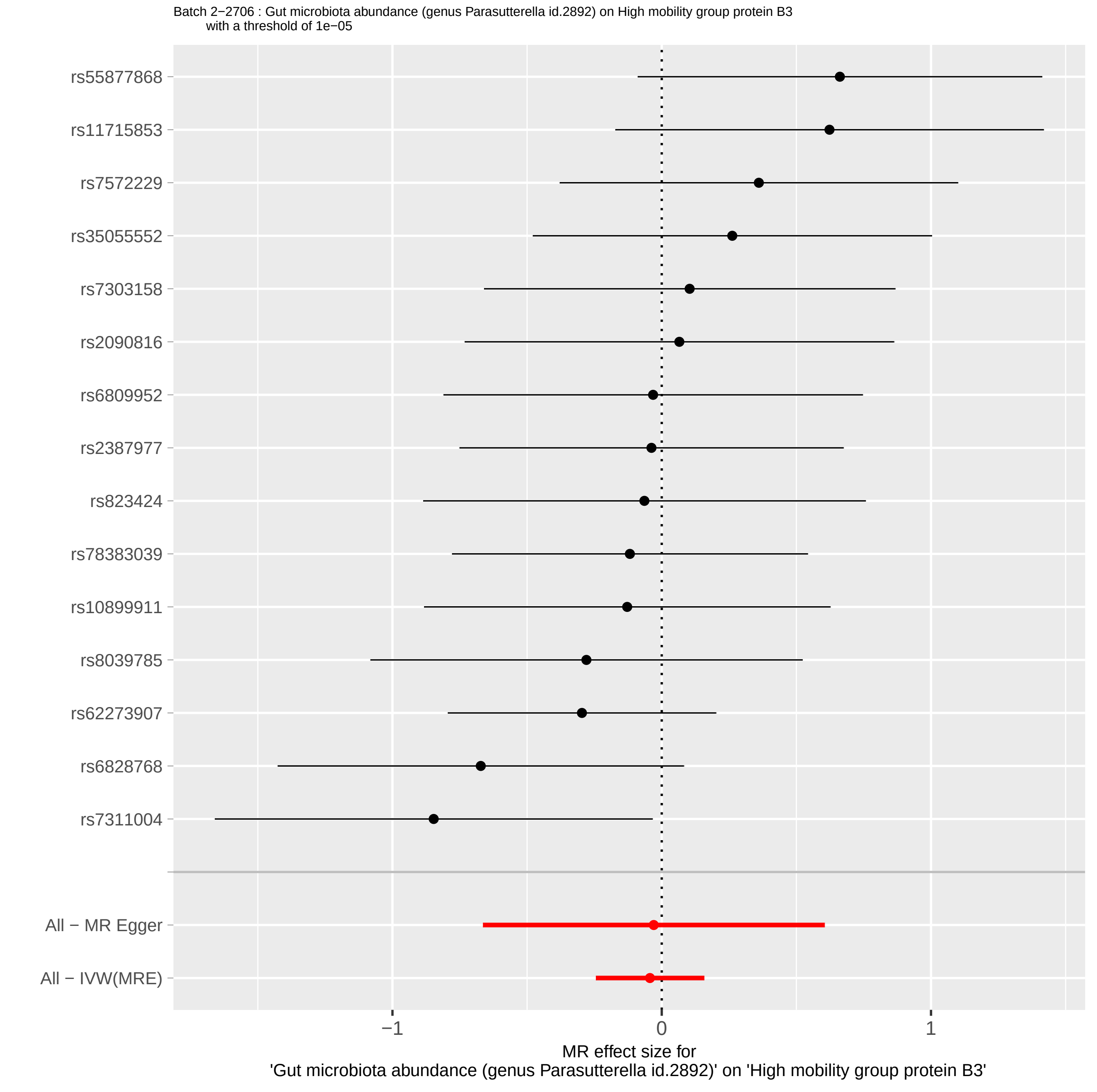

Batch 2−2706 : Gut microbiota abundance (genus Parasutterella id.2892) on High mobility group protein B3
with a threshold of 1e−05
rs55877868
rs11715853
rs7572229
rs35055552
rs7303158
rs2090816
rs6809952
rs2387977
rs823424
rs78383039
rs10899911
rs8039785
rs62273907
rs6828768
rs7311004
All − MR Egger
All − IVW(MRE)
−1
0
1
MR effect size for
'Gut microbiota abundance (genus Parasutterella id.2892)' on 'High mobility group protein B3'

Batch 2−2707 : Gut microbiota abundance (genus Catenibacterium id.2153) on 3−hydroxy−3−methylglutaryl−coenzyme A reductase
with a threshold of 1e−05

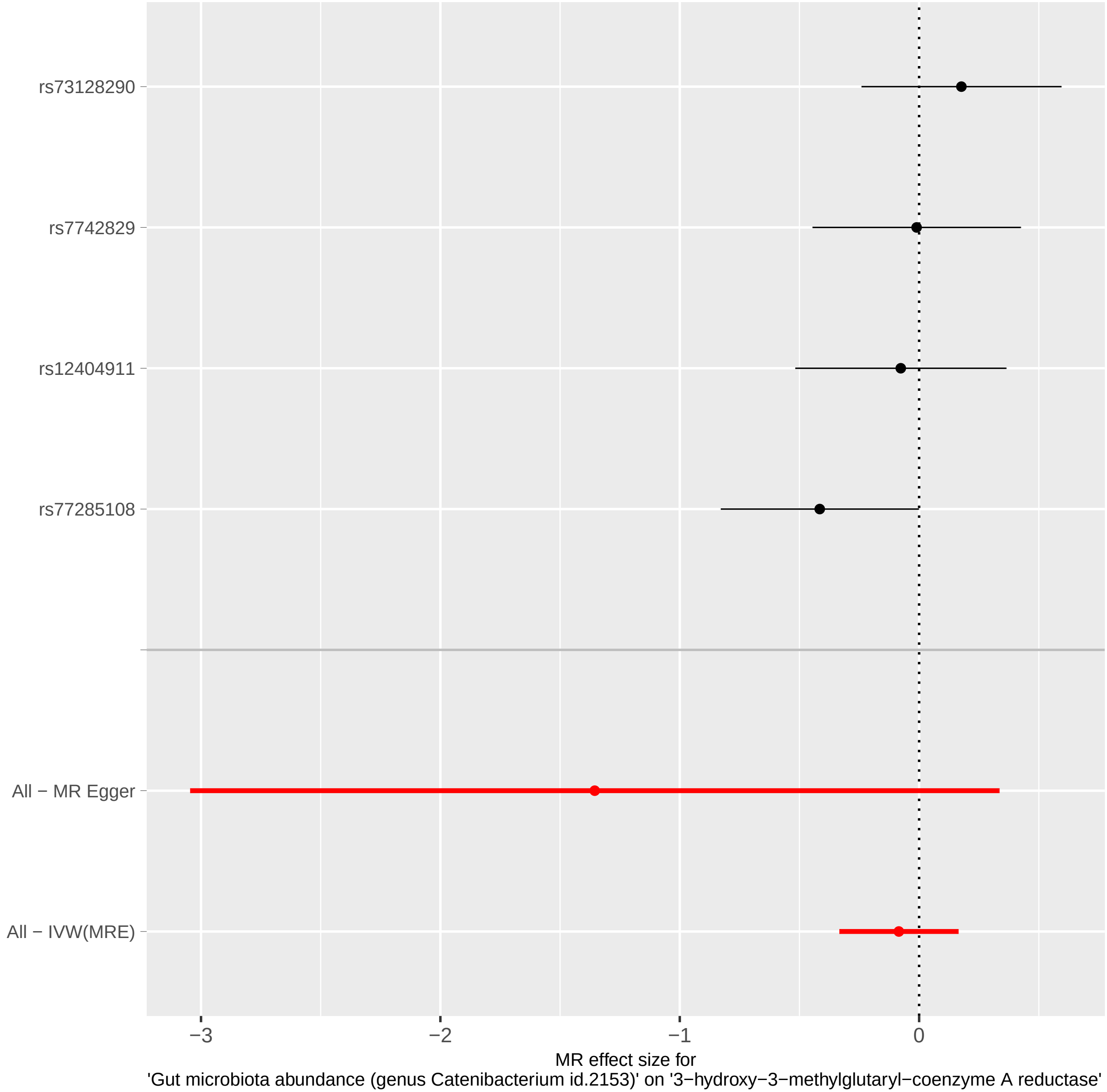

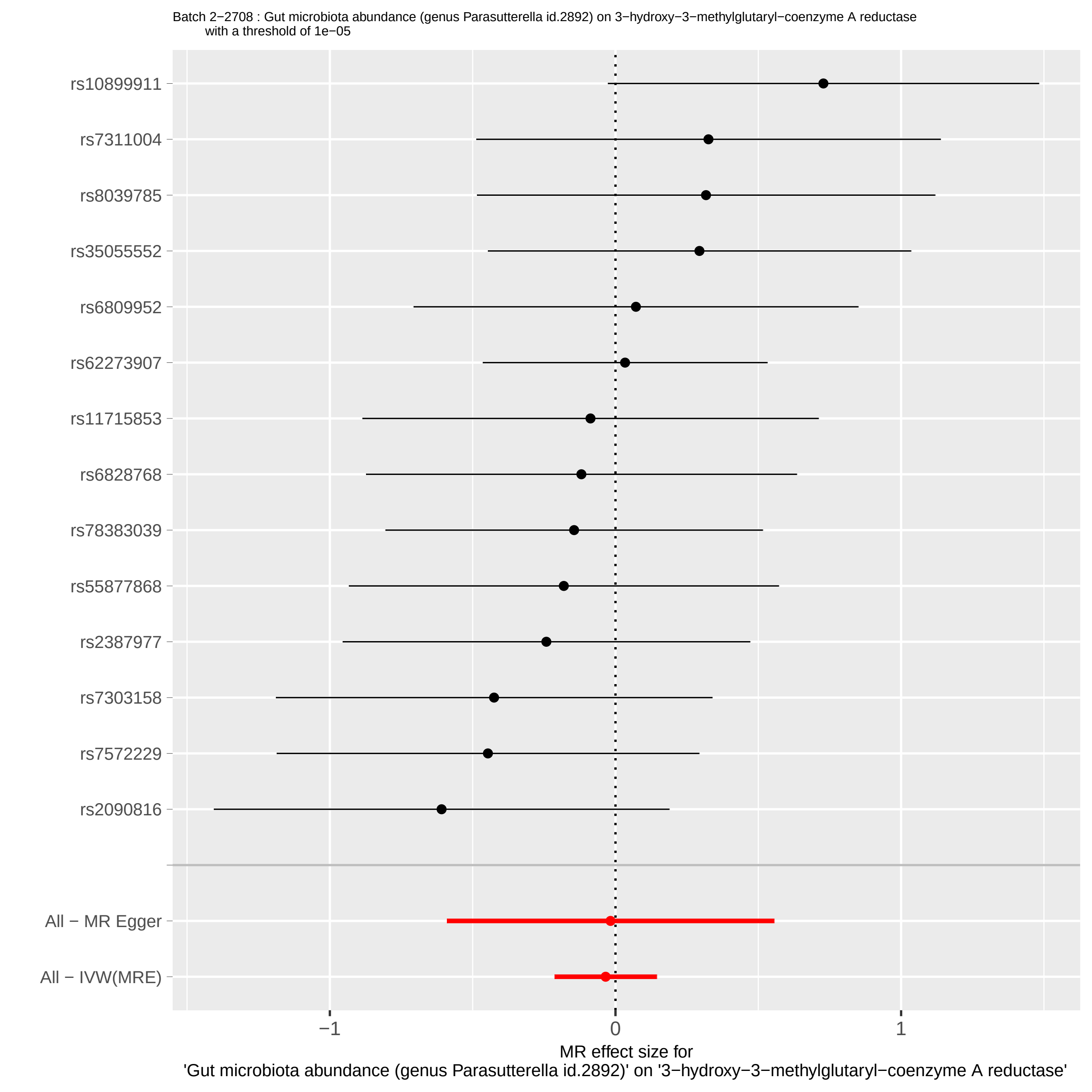

Batch 2−2708 : Gut microbiota abundance (genus Parasutterella id.2892) on 3−hydroxy−3−methylglutaryl−coenzyme A reductase
with a threshold of 1e−05
rs10899911
rs7311004
rs8039785
rs35055552
rs6809952
rs62273907
rs11715853
rs6828768
rs78383039
rs55877868
rs2387977
rs7303158
rs7572229
rs2090816
All − MR Egger
All − IVW(MRE)
−1
0
1
MR effect size for
'Gut microbiota abundance (genus Parasutterella id.2892)' on '3−hydroxy−3−methylglutaryl−coenzyme A reductase'

Batch 2−2709 : Gut microbiota abundance (genus Catenibacterium id.2153) on Hydroxymethylglutaryl−CoA synthase, cytoplasmic
with a threshold of 1e−05

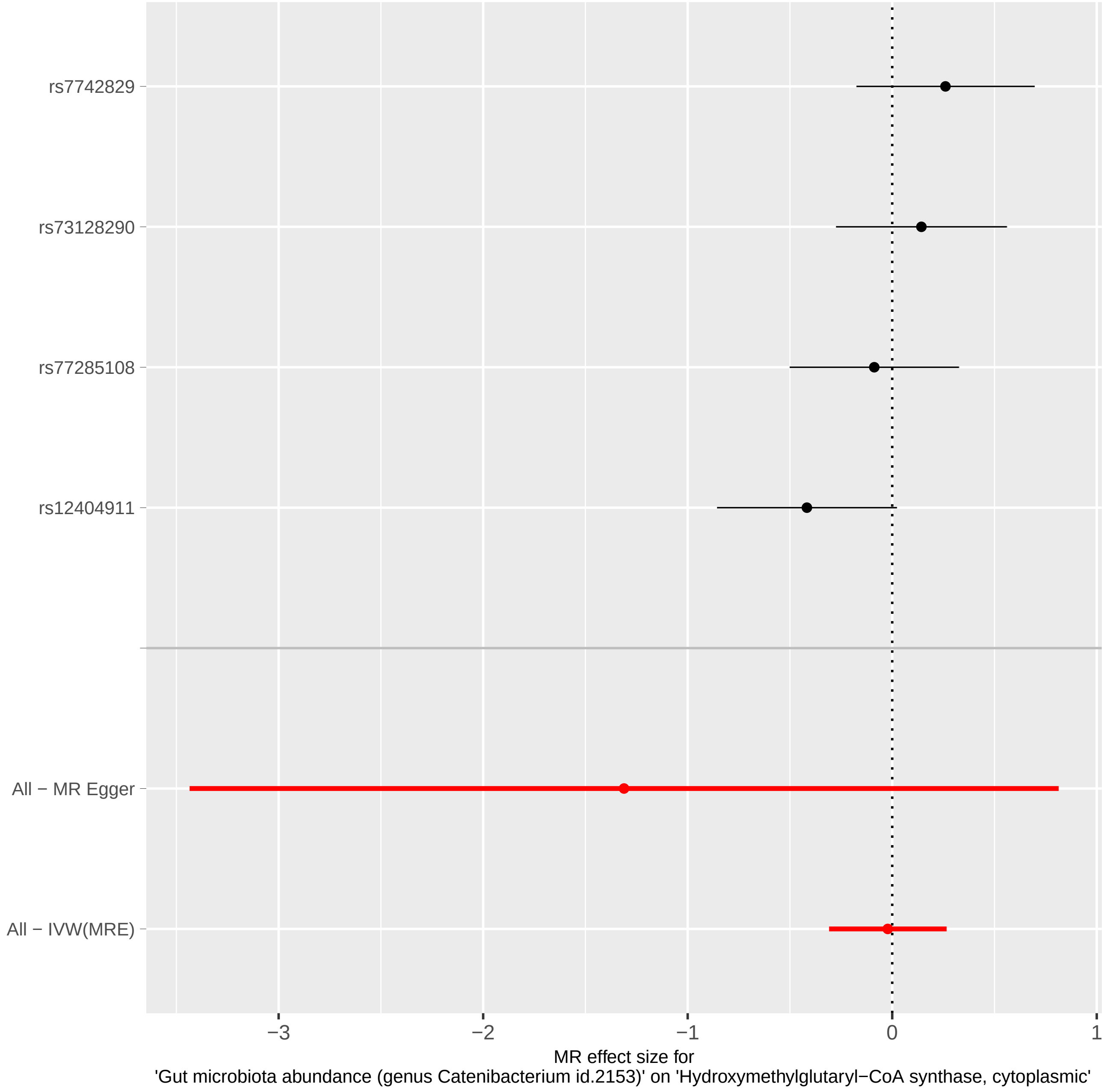

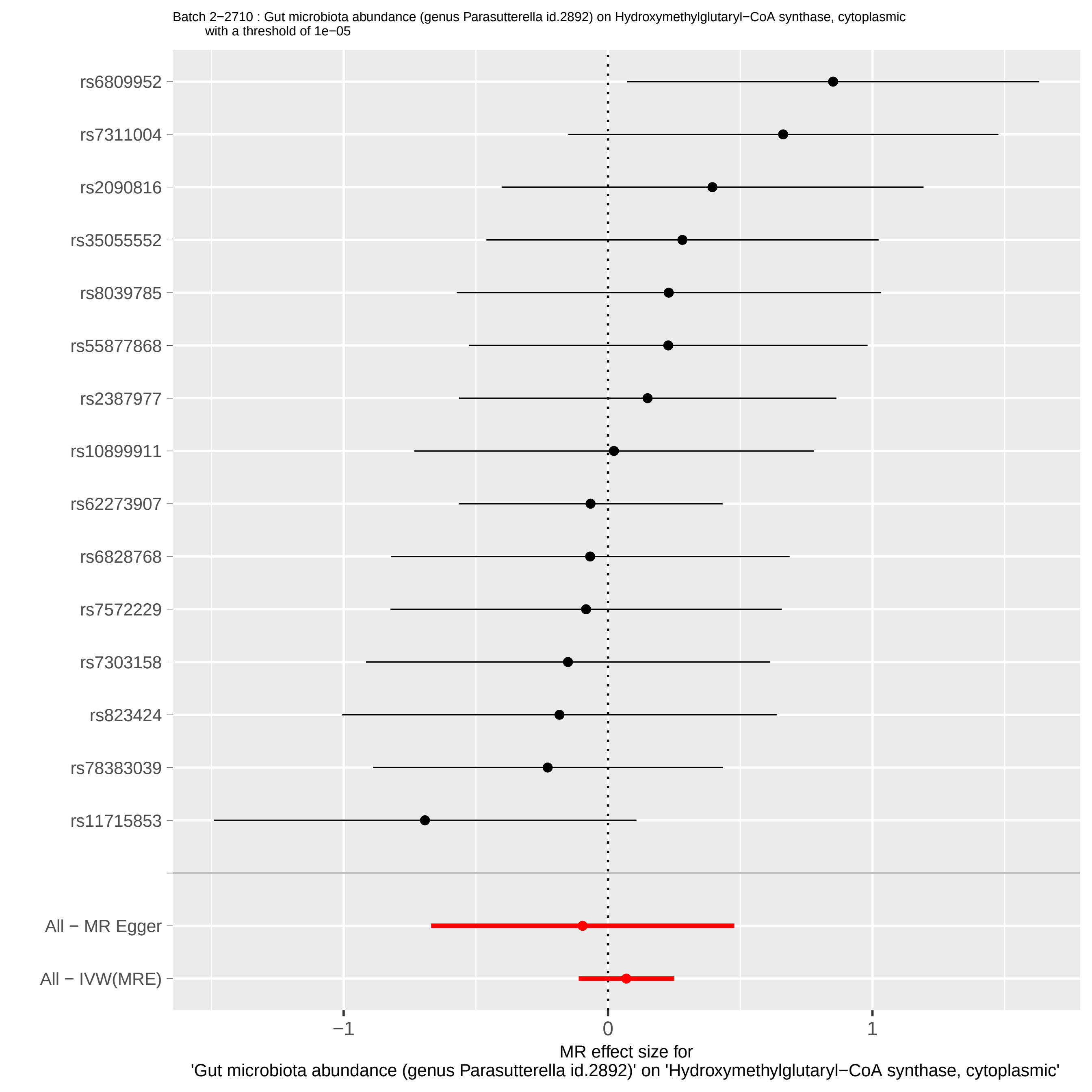

Batch 2−2710 : Gut microbiota abundance (genus Parasutterella id.2892) on Hydroxymethylglutaryl−CoA synthase, cytoplasmic
with a threshold of 1e−05
rs6809952
rs7311004
rs2090816
rs35055552
rs8039785
rs55877868
rs2387977
rs10899911
rs62273907
rs6828768
rs7572229
rs7303158
rs823424
rs78383039
rs11715853
All − MR Egger
All − IVW(MRE)
−1
0
1
MR effect size for
'Gut microbiota abundance (genus Parasutterella id.2892)' on 'Hydroxymethylglutaryl−CoA synthase, cytoplasmic'

Batch 2−2711 : Gut microbiota abundance (genus Catenibacterium id.2153) on Hydroxymethylglutaryl−CoA synthase, mitochondrial
with a threshold of 1e−05

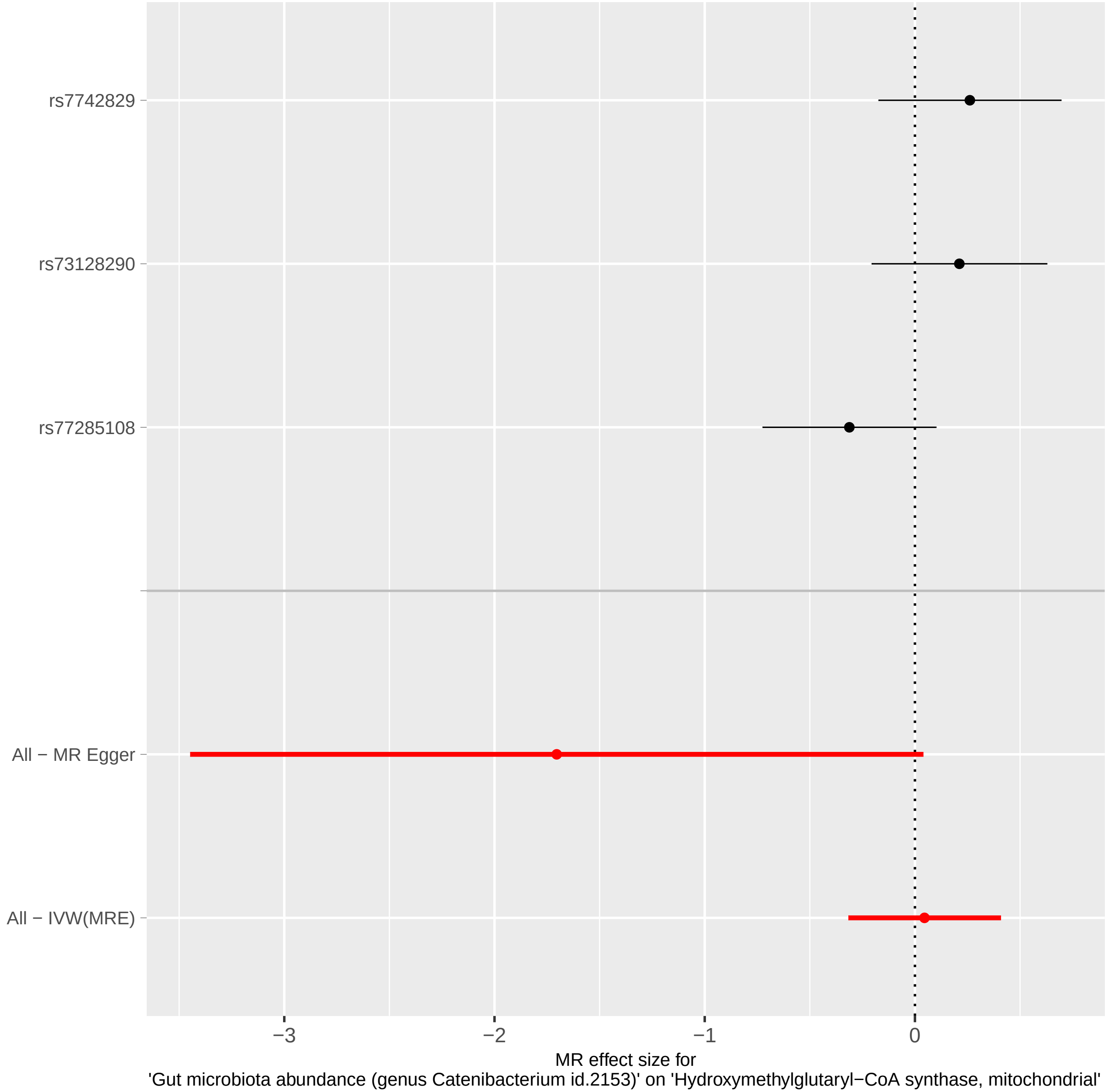

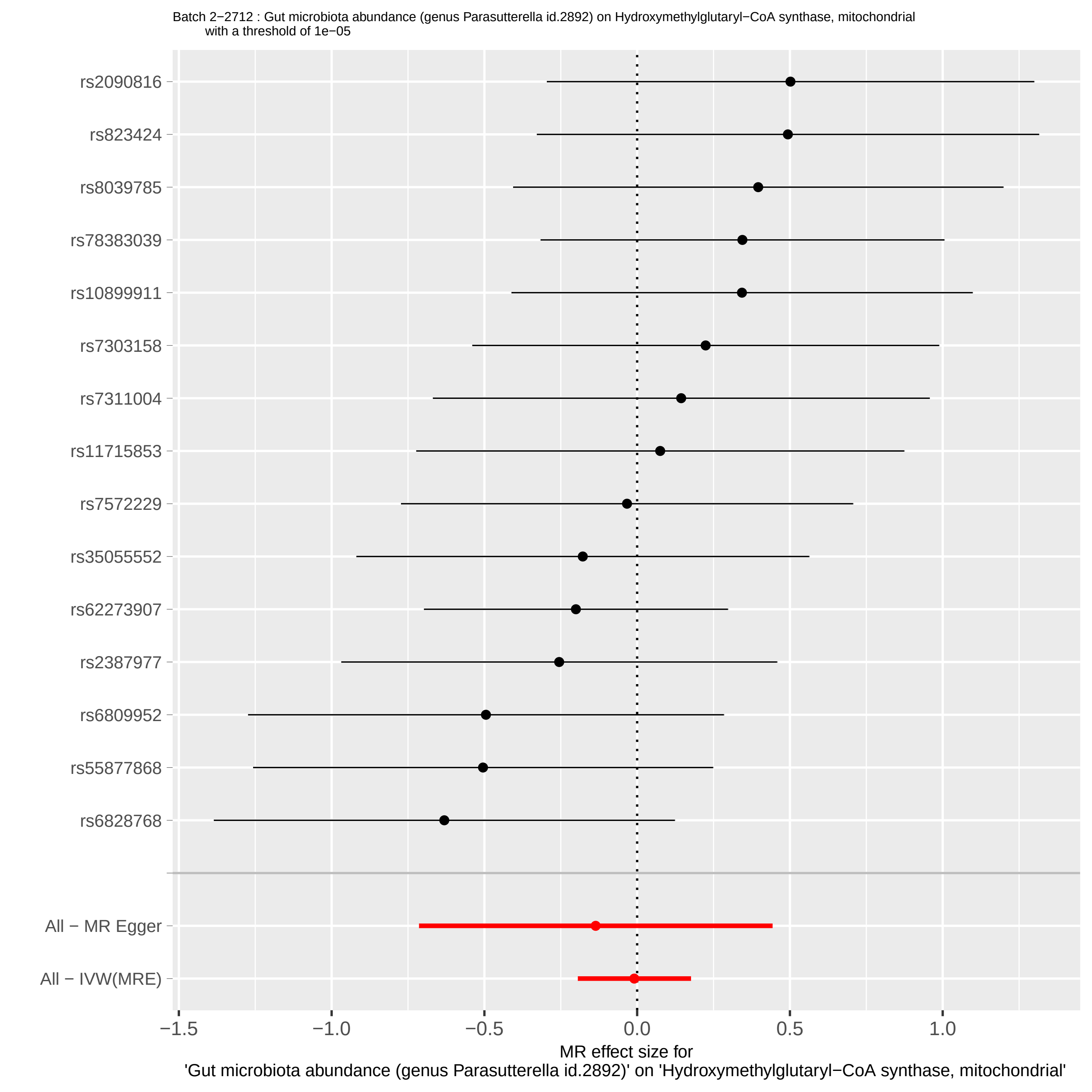
Batch 2−2712 : Gut microbiota abundance (genus Parasutterella id.2892) on Hydroxymethylglutaryl−CoA synthase, mitochondrial
with a threshold of 1e−05
rs2090816
rs823424
rs8039785
rs78383039
rs10899911
rs7303158
rs7311004
rs11715853
rs7572229
rs35055552
rs62273907
rs2387977
rs6809952
rs55877868
rs6828768
All − MR Egger
All − IVW(MRE)
−1.5
−1.0
−0.5
0.0
0.5
1.0
MR effect size for
'Gut microbiota abundance (genus Parasutterella id.2892)' on 'Hydroxymethylglutaryl−CoA synthase, mitochondrial'

Batch 2−2713 : Gut microbiota abundance (genus Catenibacterium id.2153) on Non−histone chromosomal protein HMG−14
with a threshold of 1e−05

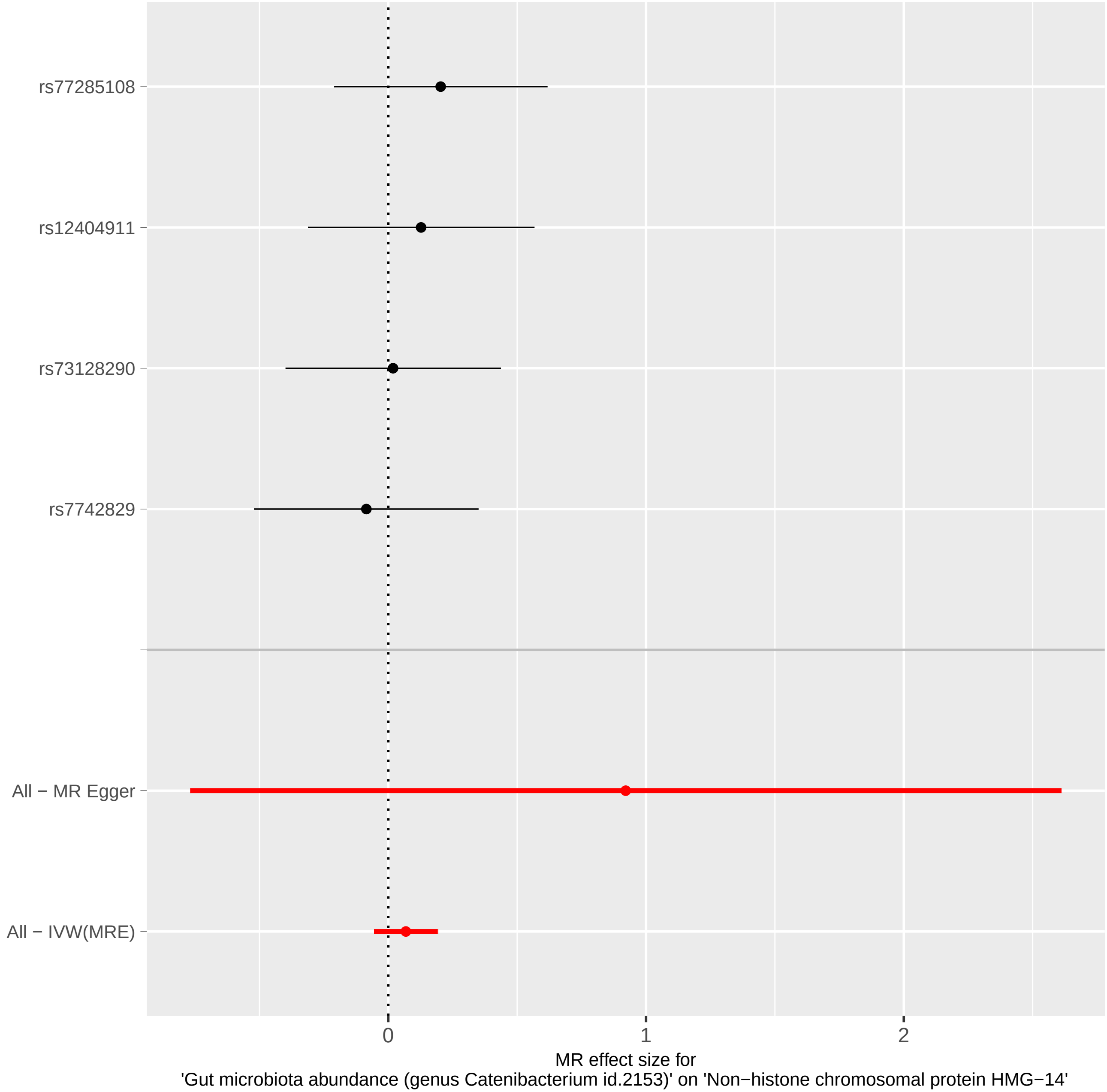

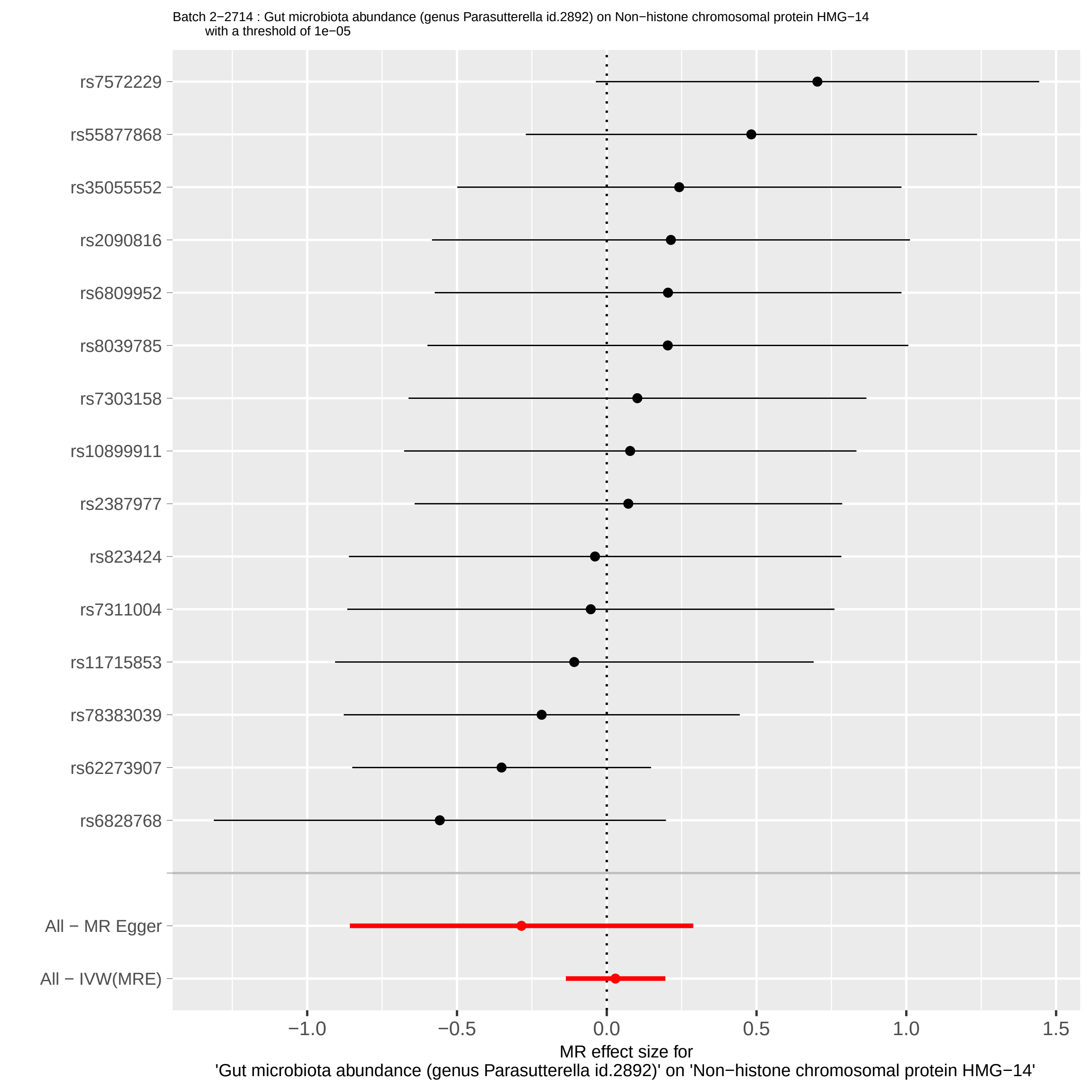
Batch 2−2714 : Gut microbiota abundance (genus Parasutterella id.2892) on Non−histone chromosomal protein HMG−14
with a threshold of 1e−05
rs7572229
rs55877868
rs35055552
rs2090816
rs6809952
rs8039785
rs7303158
rs10899911
rs2387977
rs823424
rs7311004
rs11715853
rs78383039
rs62273907
rs6828768
All − MR Egger
All − IVW(MRE)
−1.0
−0.5
0.0
0.5
1.0
1.5
MR effect size for
'Gut microbiota abundance (genus Parasutterella id.2892)' on 'Non−histone chromosomal protein HMG−14'

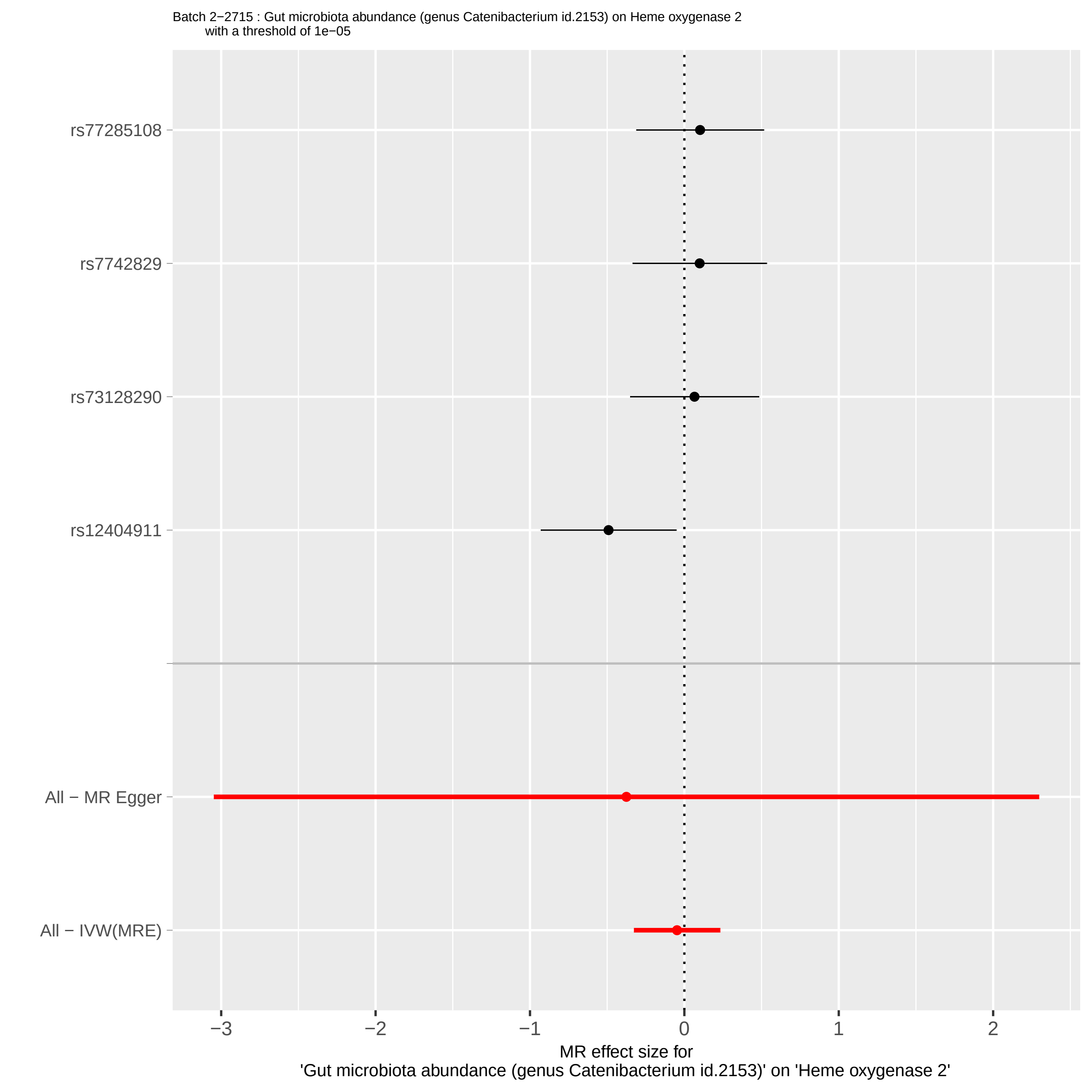

Batch 2−2715 : Gut microbiota abundance (genus Catenibacterium id.2153) on Heme oxygenase 2
with a threshold of 1e−05
rs77285108
rs7742829
rs73128290
rs12404911
All − MR Egger
All − IVW(MRE)
−3
−2
−1
0
1
2
MR effect size for
'Gut microbiota abundance (genus Catenibacterium id.2153)' on 'Heme oxygenase 2'

Batch 2−2716 : Gut microbiota abundance (genus Parasutterella id.2892) on Heme oxygenase 2
with a threshold of 1e−05

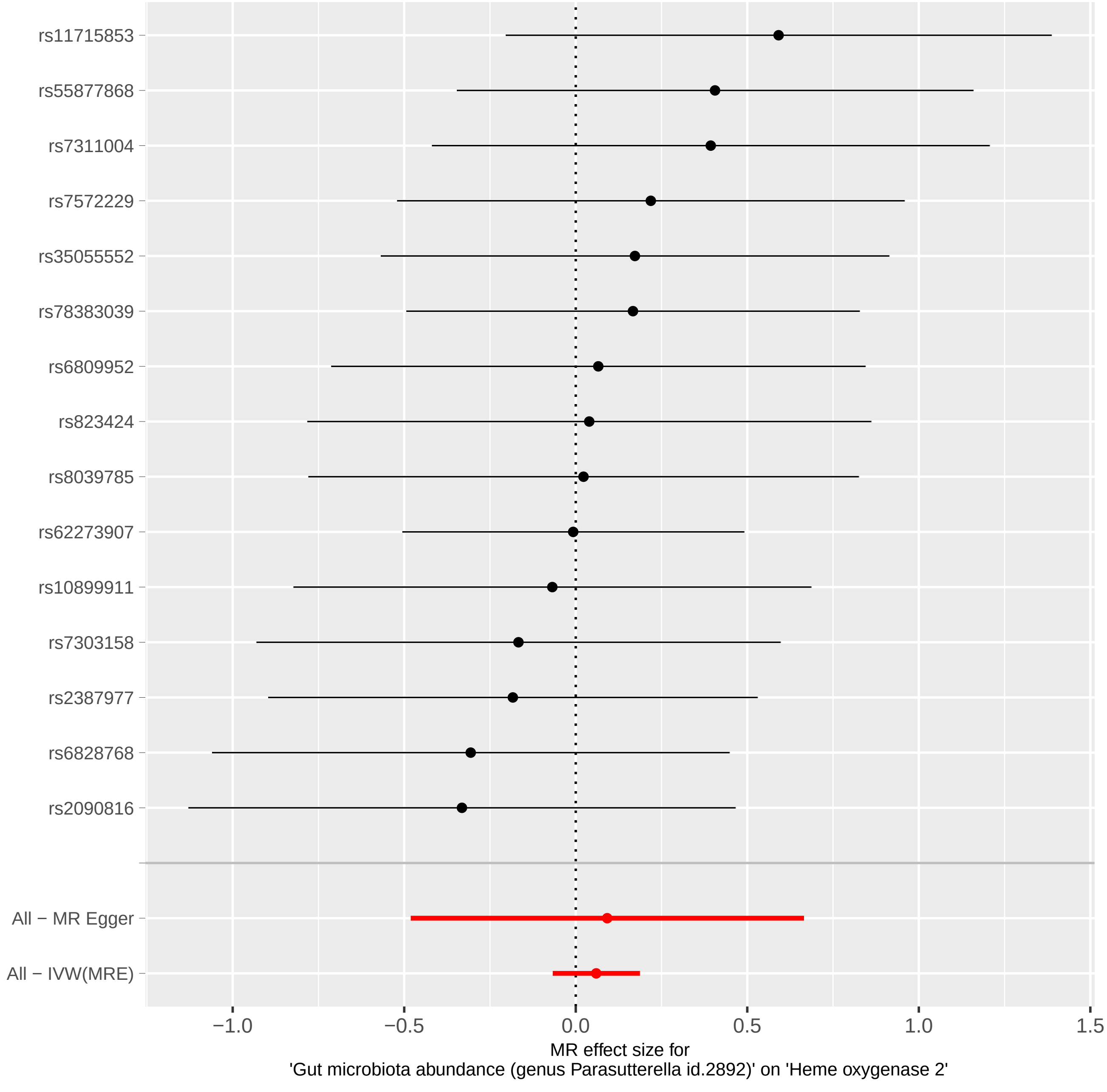

Batch 2−2717 : Gut microbiota abundance (genus Catenibacterium id.2153) on Hepatocyte nuclear factor 1−alpha
with a threshold of 1e−05

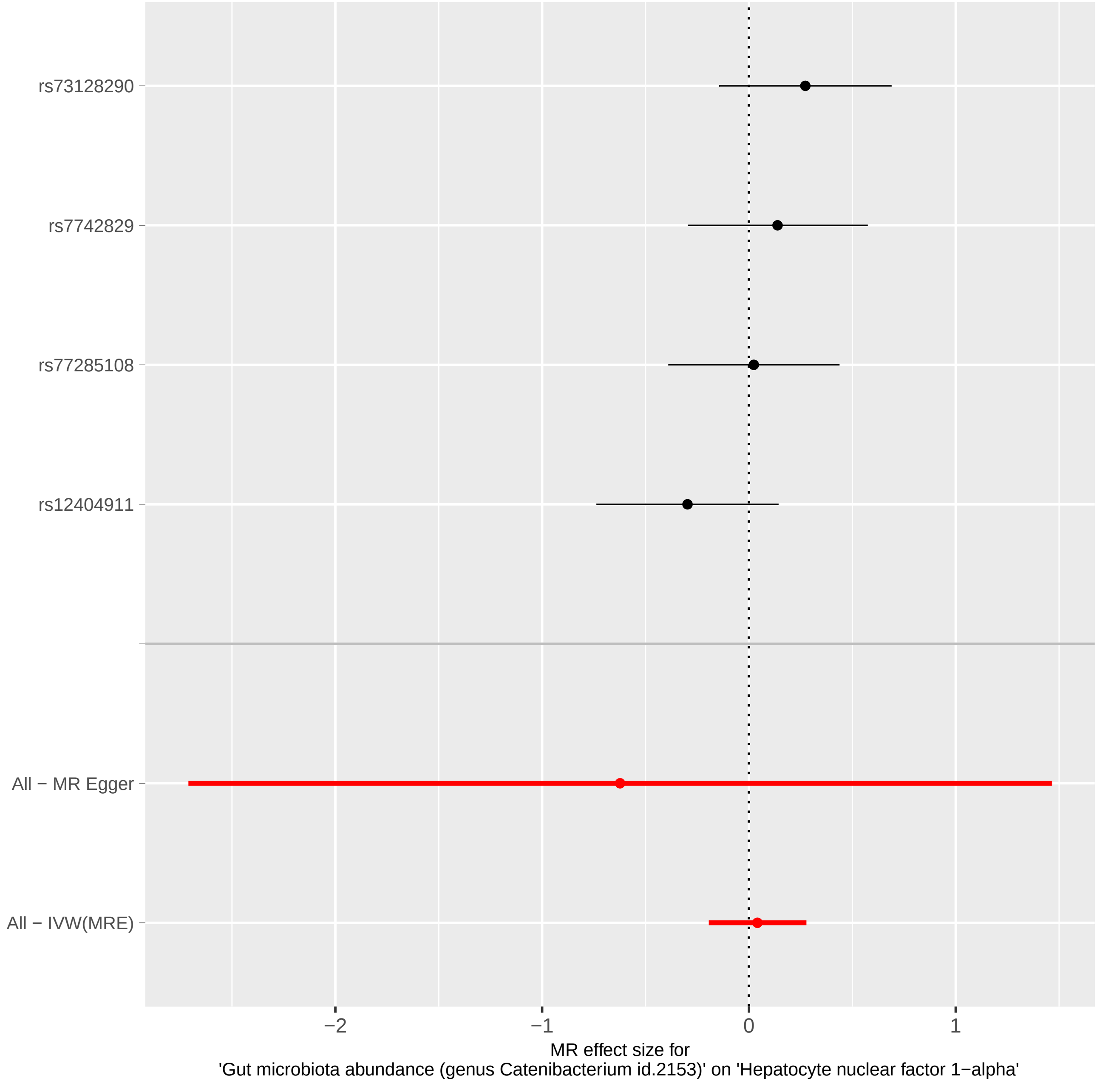

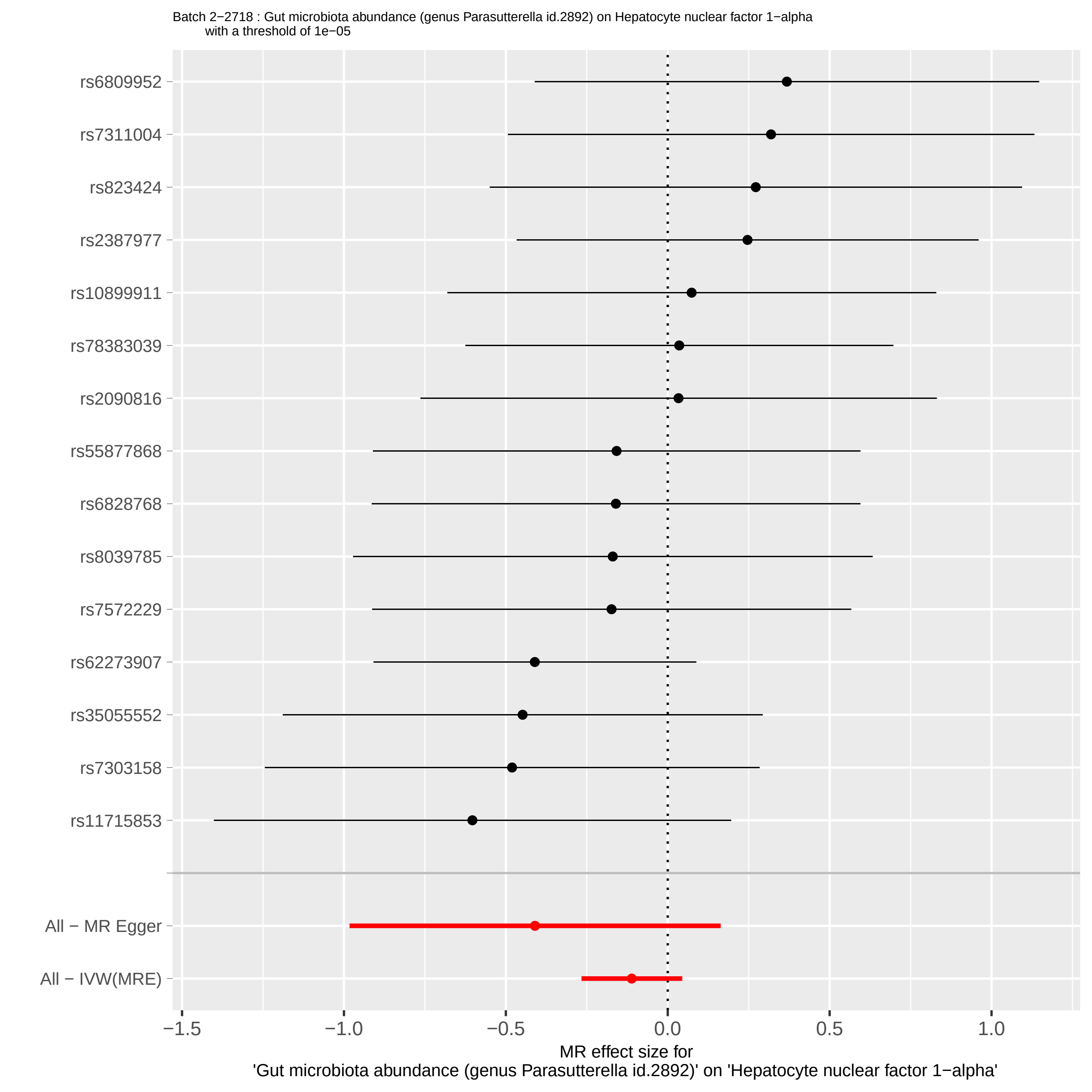

Batch 2−2718 : Gut microbiota abundance (genus Parasutterella id.2892) on Hepatocyte nuclear factor 1−alpha
with a threshold of 1e−05
rs6809952
rs7311004
rs823424
rs2387977
rs10899911
rs78383039
rs2090816
rs55877868
rs6828768
rs8039785
rs7572229
rs62273907
rs35055552
rs7303158
rs11715853
All − MR Egger
All − IVW(MRE)
−1.5
−1.0
−0.5
0.0
0.5
1.0
MR effect size for
'Gut microbiota abundance (genus Parasutterella id.2892)' on 'Hepatocyte nuclear factor 1−alpha'

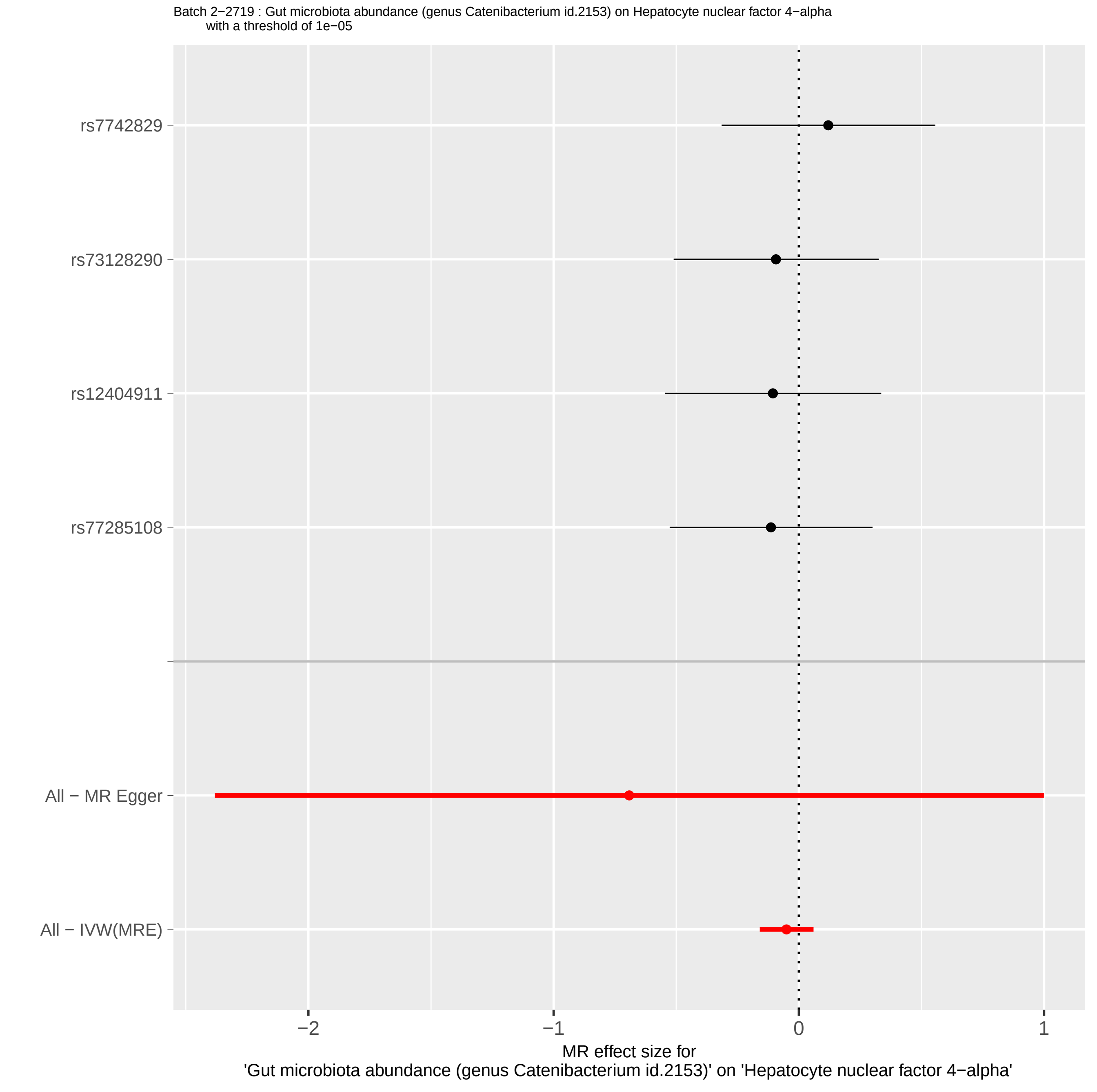

Batch 2−2719 : Gut microbiota abundance (genus Catenibacterium id.2153) on Hepatocyte nuclear factor 4−alpha
with a threshold of 1e−05
rs7742829
rs73128290
rs12404911
rs77285108
All − MR Egger
All − IVW(MRE)
−2
−1
0
1
MR effect size for
'Gut microbiota abundance (genus Catenibacterium id.2153)' on 'Hepatocyte nuclear factor 4−alpha'

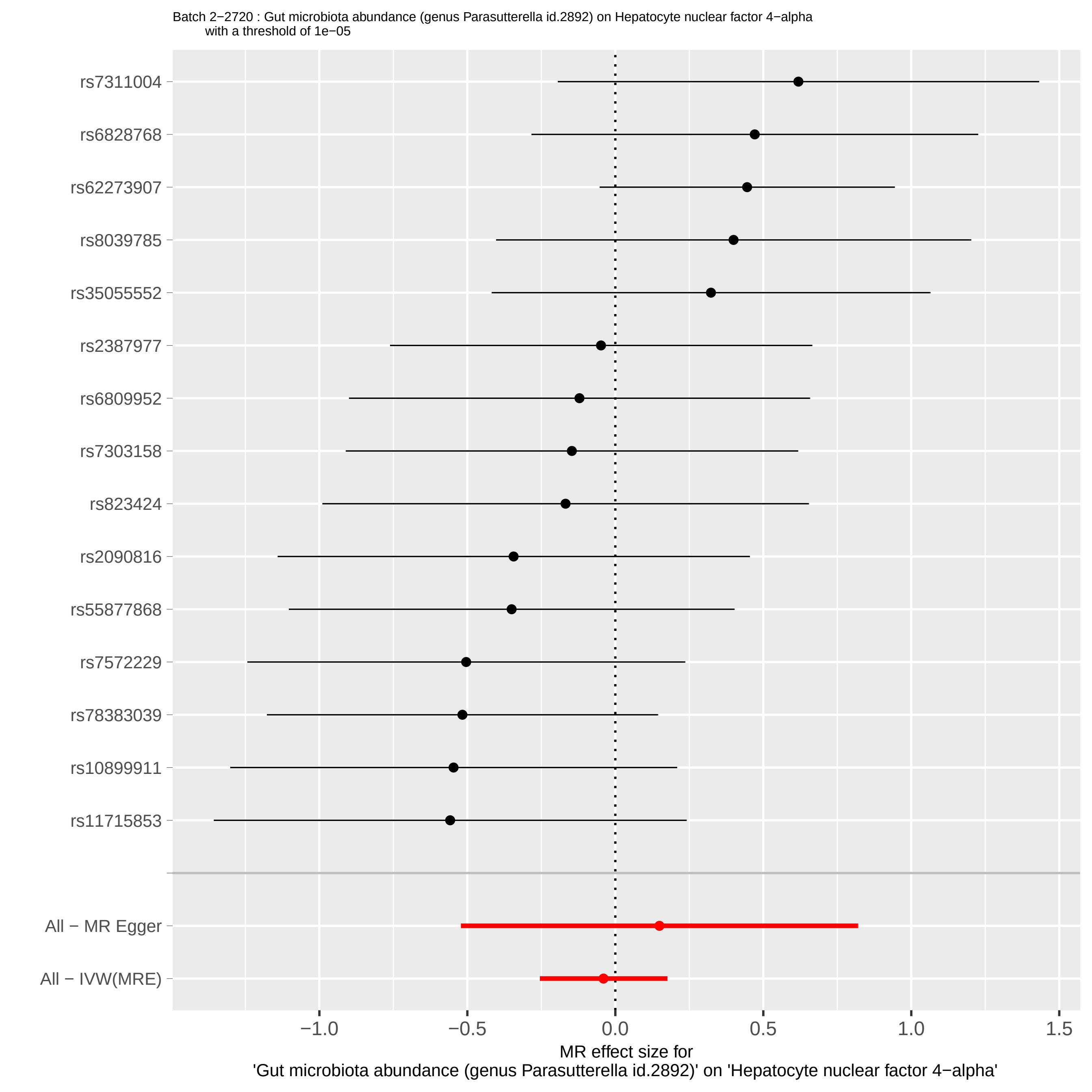
Batch 2−2720 : Gut microbiota abundance (genus Parasutterella id.2892) on Hepatocyte nuclear factor 4−alpha
with a threshold of 1e−05
rs7311004
rs6828768
rs62273907
rs8039785
rs35055552
rs2387977
rs6809952
rs7303158
rs823424
rs2090816
rs55877868
rs7572229
rs78383039
rs10899911
rs11715853
All − MR Egger
All − IVW(MRE)
−1.0
−0.5
0.0
0.5
1.0
1.5
MR effect size for
'Gut microbiota abundance (genus Parasutterella id.2892)' on 'Hepatocyte nuclear factor 4−alpha'

Batch 2−2721 : Gut microbiota abundance (genus Catenibacterium id.2153) on Heterogeneous nuclear ribonucleoproteins A2/B1
with a threshold of 1e−05

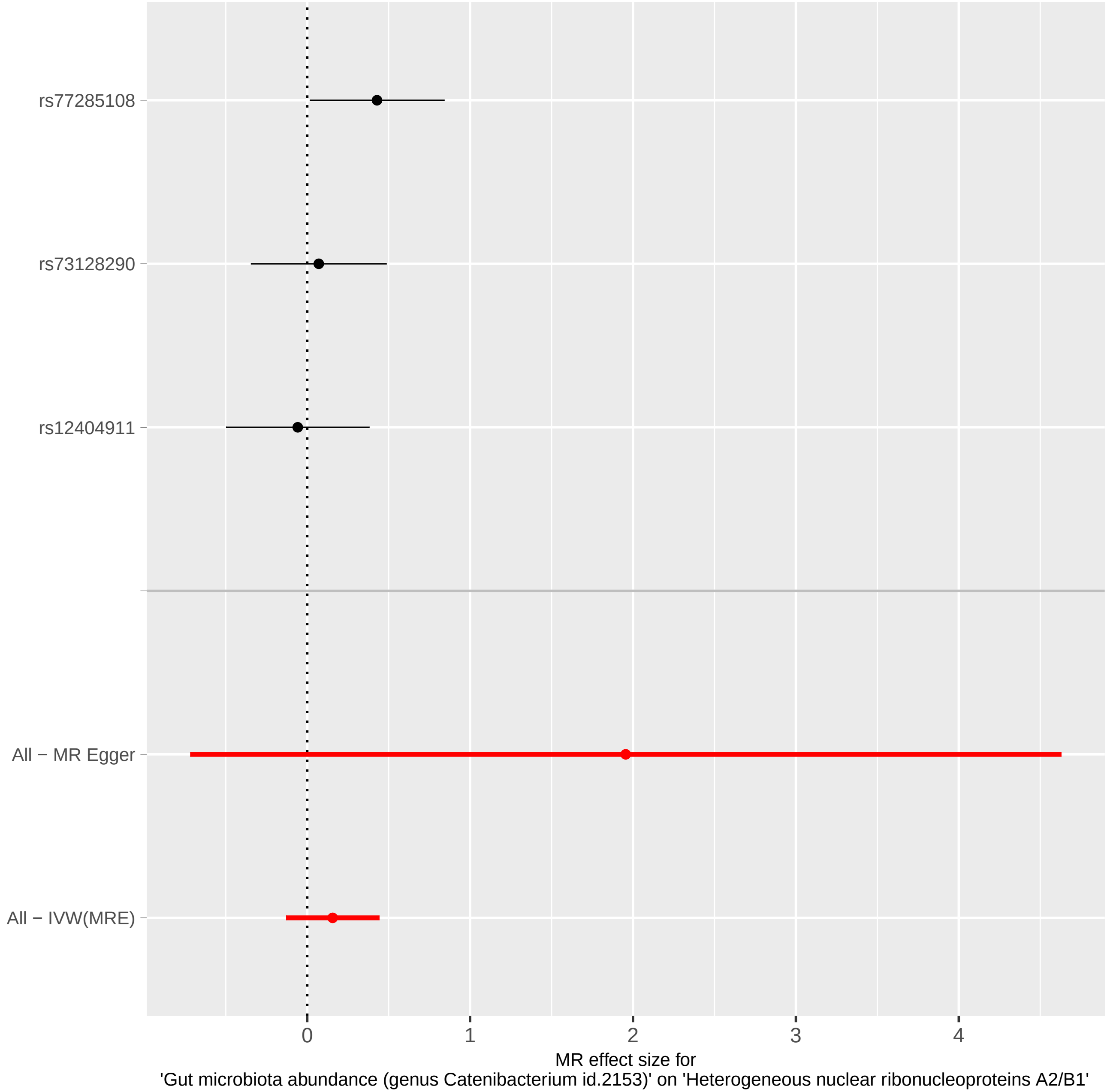

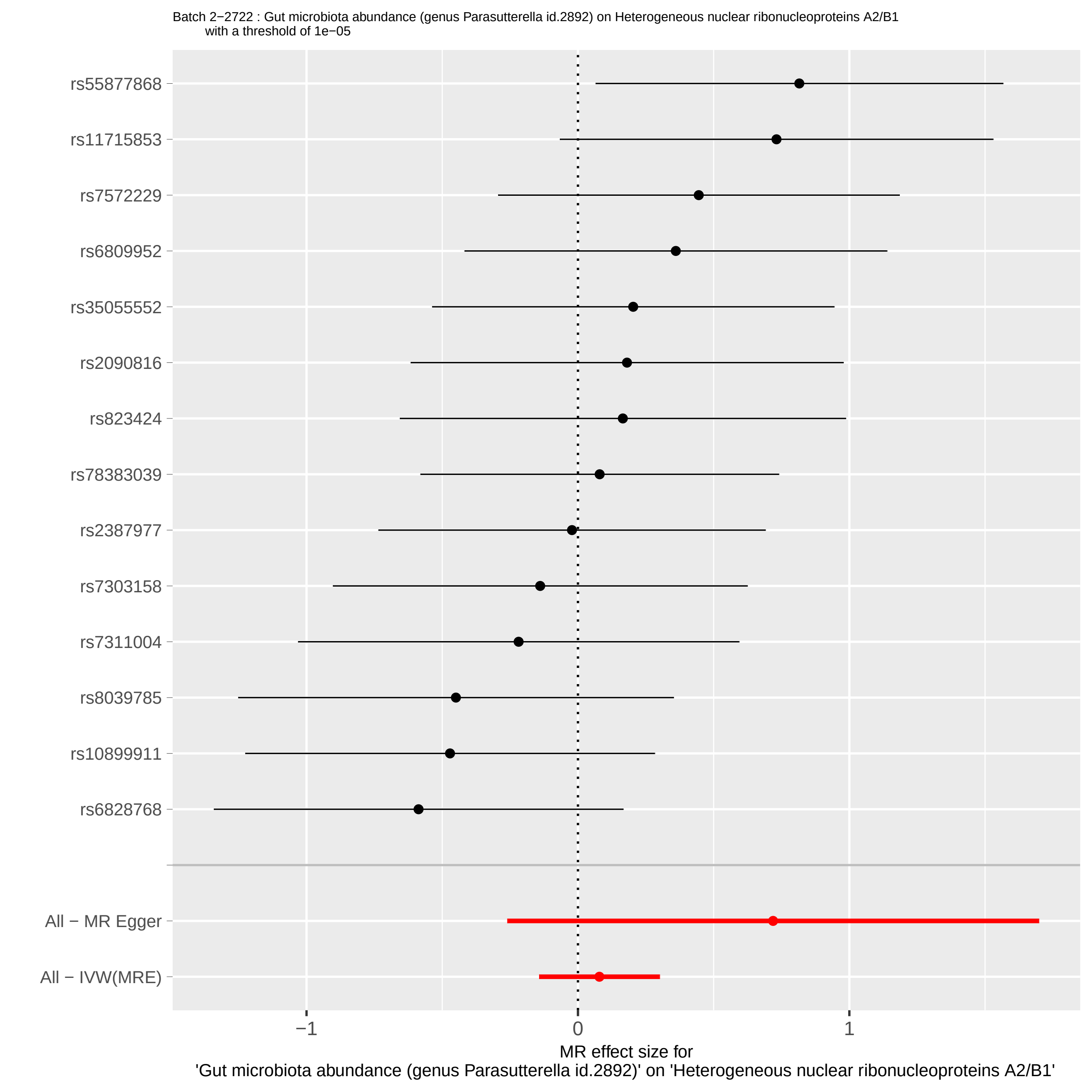

Batch 2−2722 : Gut microbiota abundance (genus Parasutterella id.2892) on Heterogeneous nuclear ribonucleoproteins A2/B1
with a threshold of 1e−05
rs55877868
rs11715853
rs7572229
rs6809952
rs35055552
rs2090816
rs823424
rs78383039
rs2387977
rs7303158
rs7311004
rs8039785
rs10899911
rs6828768
All − MR Egger
All − IVW(MRE)
−1
0
1
MR effect size for
'Gut microbiota abundance (genus Parasutterella id.2892)' on 'Heterogeneous nuclear ribonucleoproteins A2/B1'

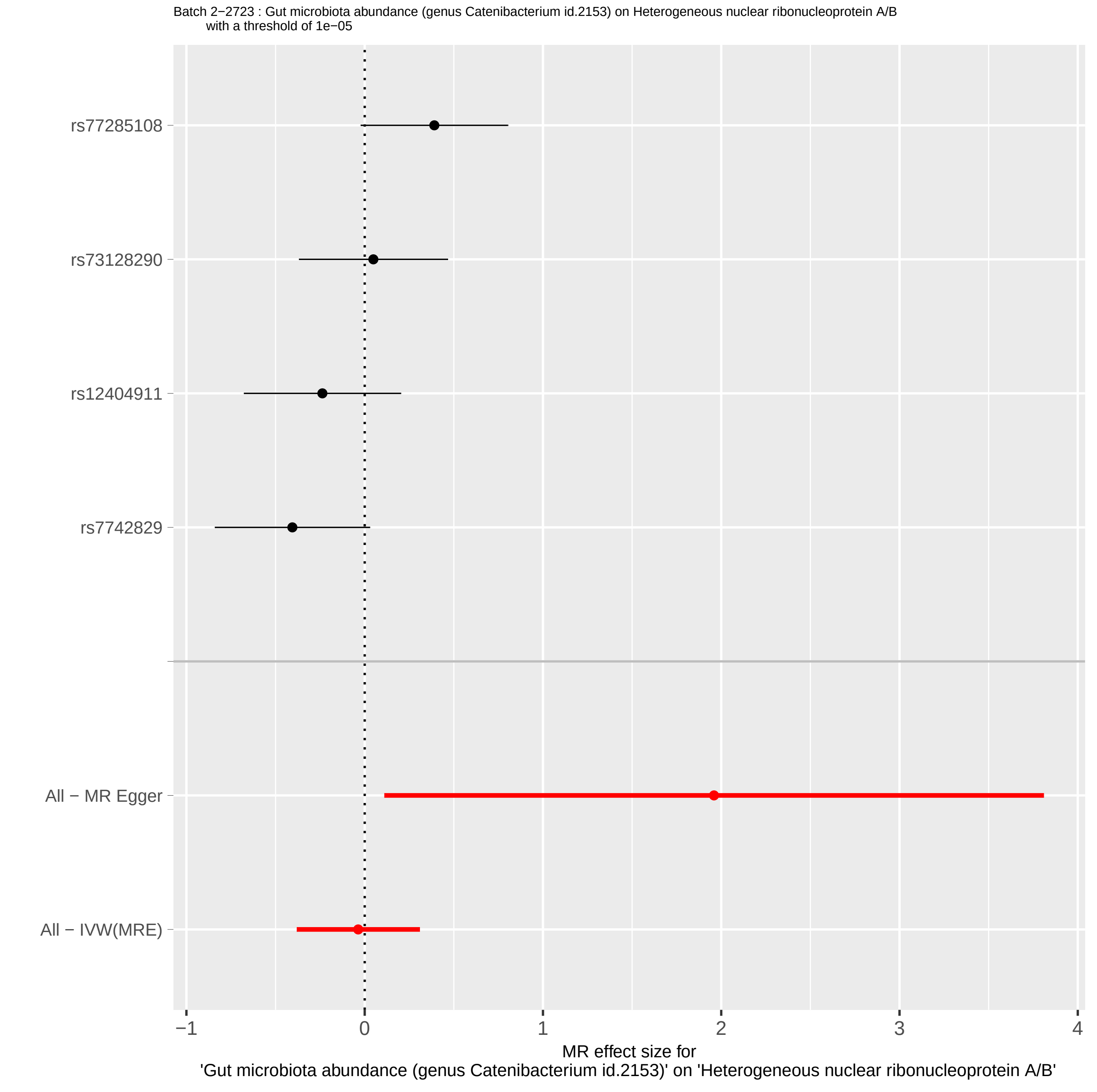
Batch 2−2723 : Gut microbiota abundance (genus Catenibacterium id.2153) on Heterogeneous nuclear ribonucleoprotein A/B
with a threshold of 1e−05
rs77285108
rs73128290
rs12404911
rs7742829
All − MR Egger
All − IVW(MRE)
−1
0
1
2
3
4
MR effect size for
'Gut microbiota abundance (genus Catenibacterium id.2153)' on 'Heterogeneous nuclear ribonucleoprotein A/B'

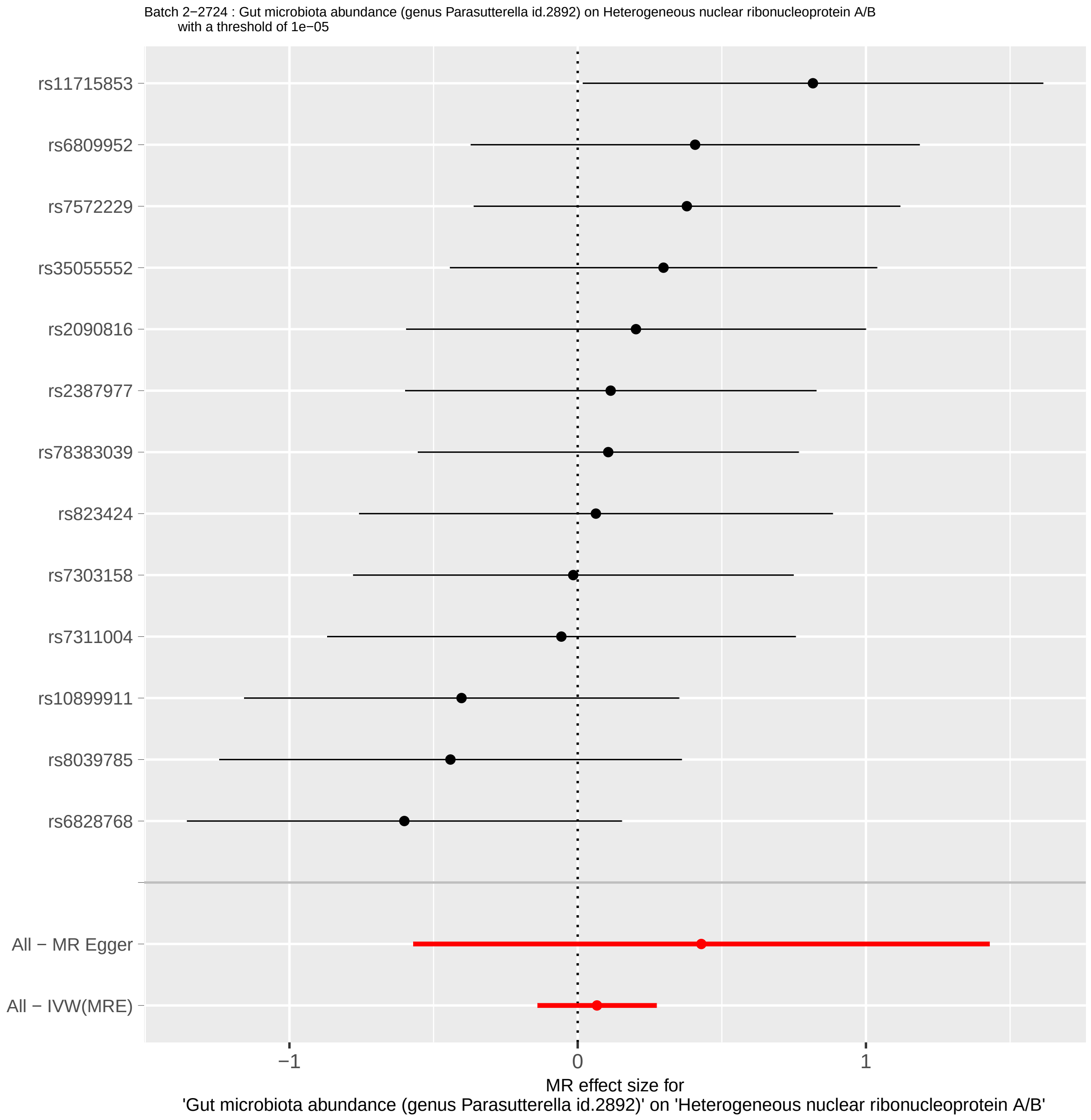

Batch 2−2724 : Gut microbiota abundance (genus Parasutterella id.2892) on Heterogeneous nuclear ribonucleoprotein A/B
with a threshold of 1e−05
rs11715853
rs6809952
rs7572229
rs35055552
rs2090816
rs2387977
rs78383039
rs823424
rs7303158
rs7311004
rs10899911
rs8039785
rs6828768
All − MR Egger
All − IVW(MRE)
−1
0
1
MR effect size for
'Gut microbiota abundance (genus Parasutterella id.2892)' on 'Heterogeneous nuclear ribonucleoprotein A/B'

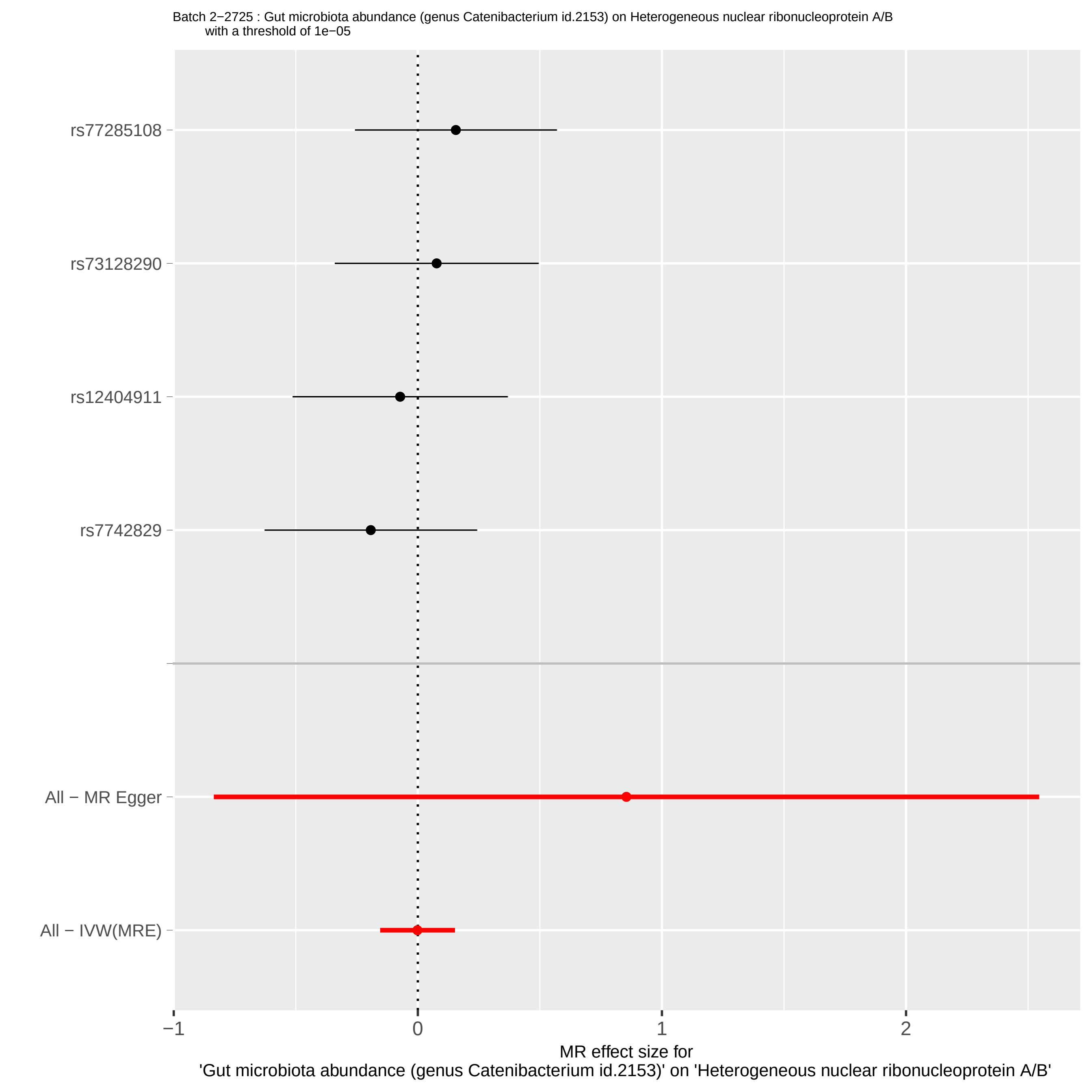
Batch 2−2725 : Gut microbiota abundance (genus Catenibacterium id.2153) on Heterogeneous nuclear ribonucleoprotein A/B
with a threshold of 1e−05
rs77285108
rs73128290
rs12404911
rs7742829
All − MR Egger
All − IVW(MRE)
−1
0
1
2
MR effect size for
'Gut microbiota abundance (genus Catenibacterium id.2153)' on 'Heterogeneous nuclear ribonucleoprotein A/B'

Batch 2−2726 : Gut microbiota abundance (genus Parasutterella id.2892) on Heterogeneous nuclear ribonucleoprotein A/B
with a threshold of 1e−05

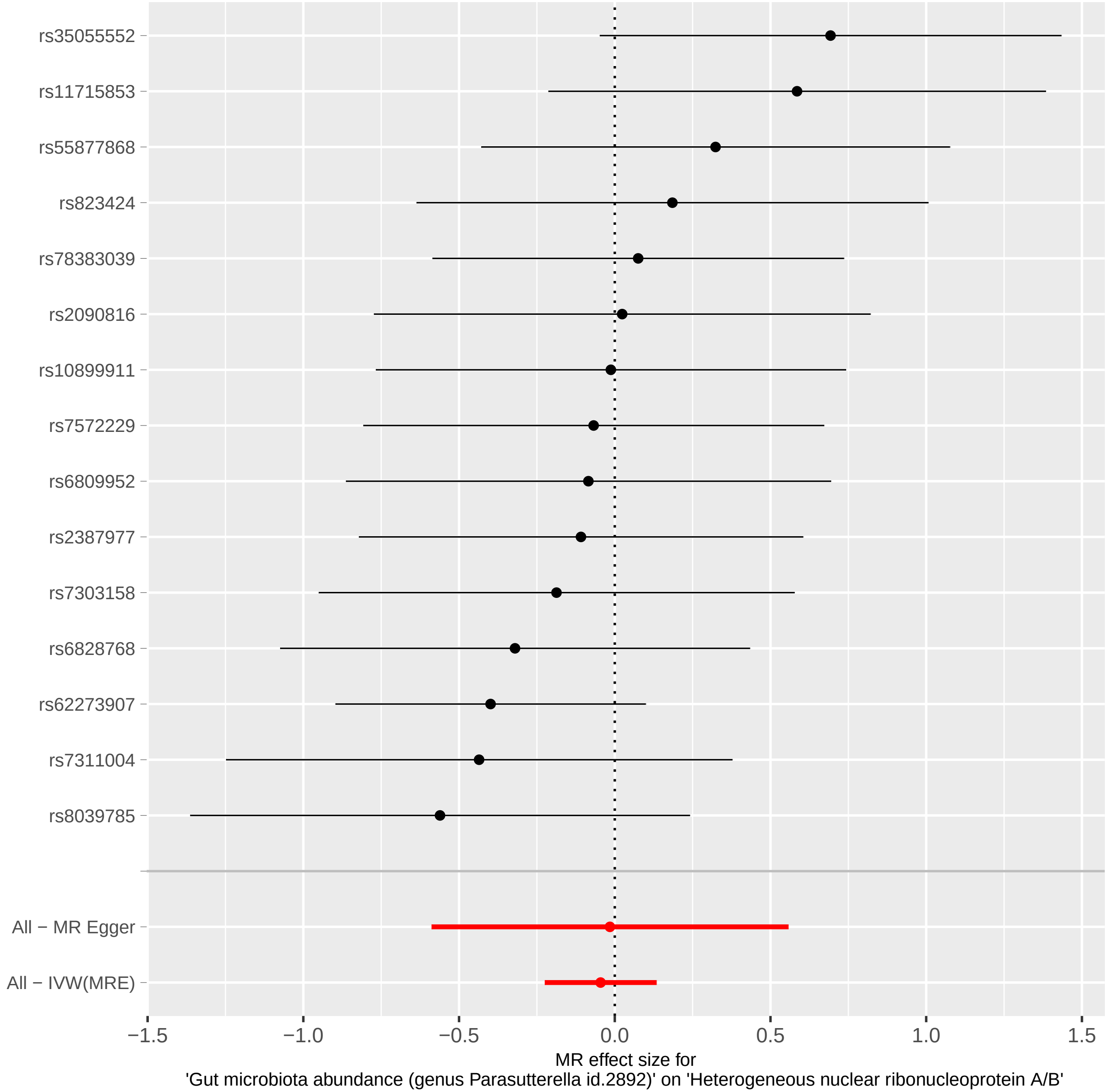

Batch 2−2727 : Gut microbiota abundance (genus Catenibacterium id.2153) on Heterogeneous nuclear ribonucleoproteins C1/C2
with a threshold of 1e−05

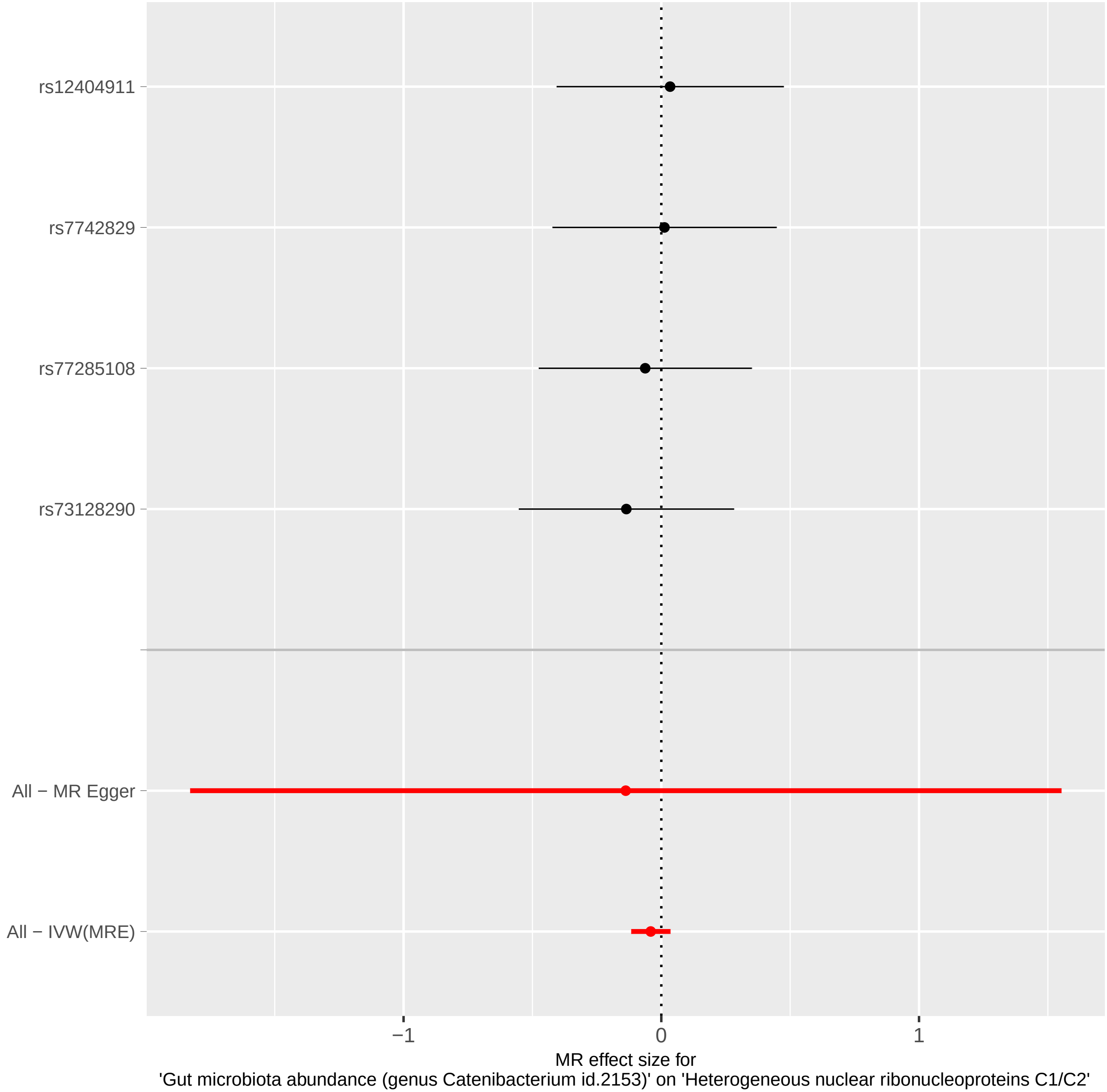

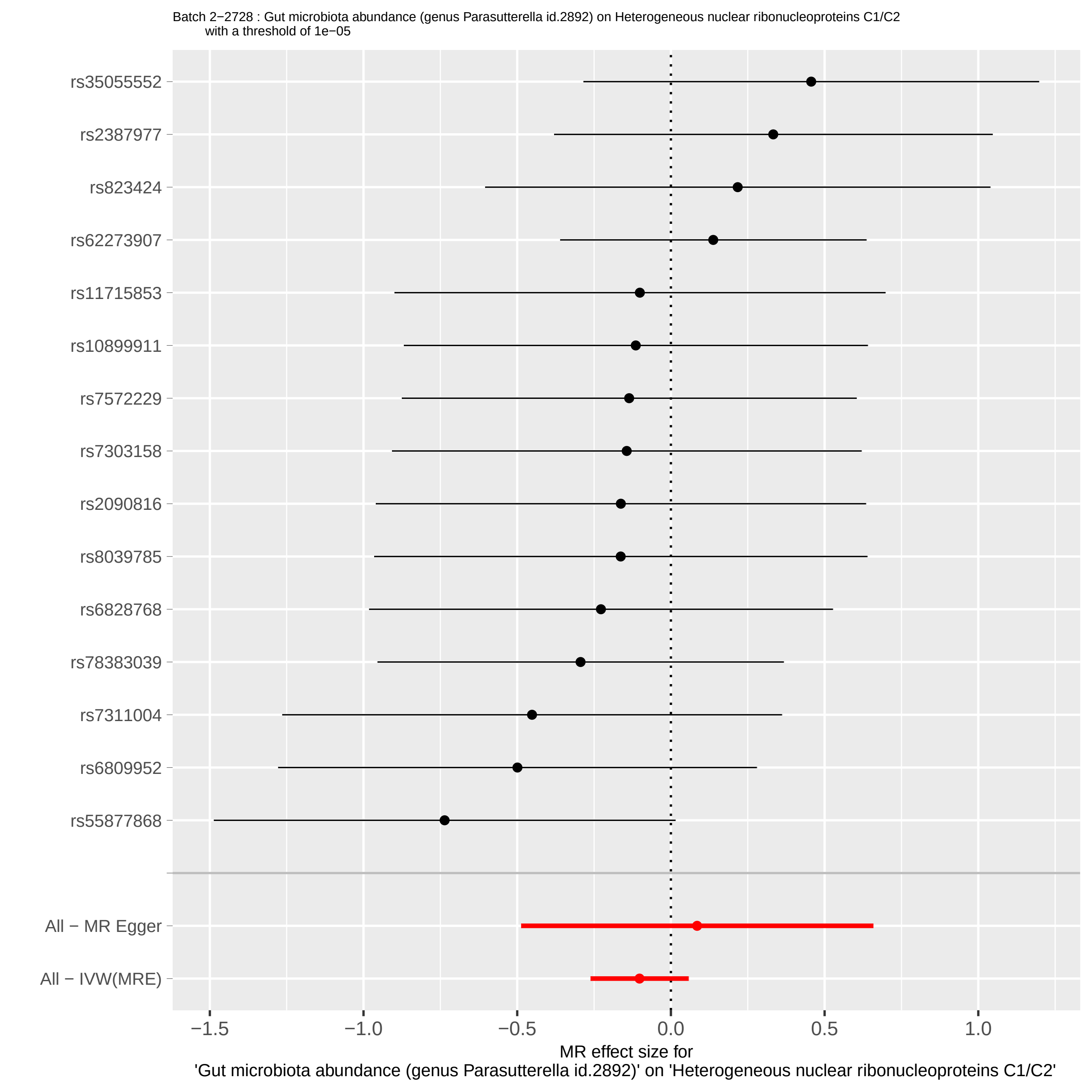

Batch 2−2728 : Gut microbiota abundance (genus Parasutterella id.2892) on Heterogeneous nuclear ribonucleoproteins C1/C2
with a threshold of 1e−05
rs35055552
rs2387977
rs823424
rs62273907
rs11715853
rs10899911
rs7572229
rs7303158
rs2090816
rs8039785
rs6828768
rs78383039
rs7311004
rs6809952
rs55877868
All − MR Egger
All − IVW(MRE)
−1.5
−1.0
−0.5
0.0
0.5
1.0
MR effect size for
'Gut microbiota abundance (genus Parasutterella id.2892)' on 'Heterogeneous nuclear ribonucleoproteins C1/C2'

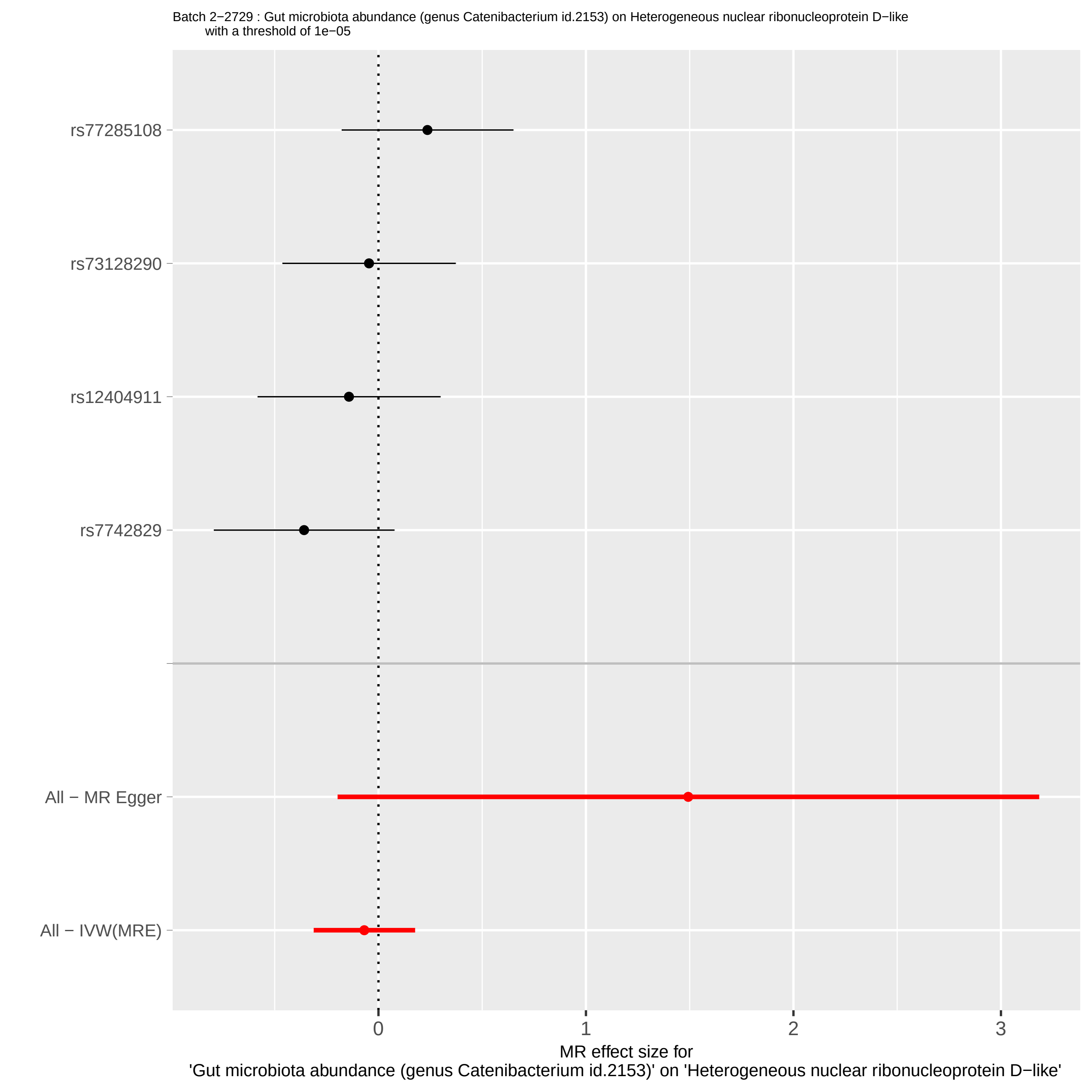

Batch 2−2729 : Gut microbiota abundance (genus Catenibacterium id.2153) on Heterogeneous nuclear ribonucleoprotein D−like
with a threshold of 1e−05
rs77285108
rs73128290
rs12404911
rs7742829
All − MR Egger
All − IVW(MRE)
0
1
2
3
MR effect size for
'Gut microbiota abundance (genus Catenibacterium id.2153)' on 'Heterogeneous nuclear ribonucleoprotein D−like'

Batch 2−2730 : Gut microbiota abundance (genus Parasutterella id.2892) on Heterogeneous nuclear ribonucleoprotein D−like with a threshold of 1e−05

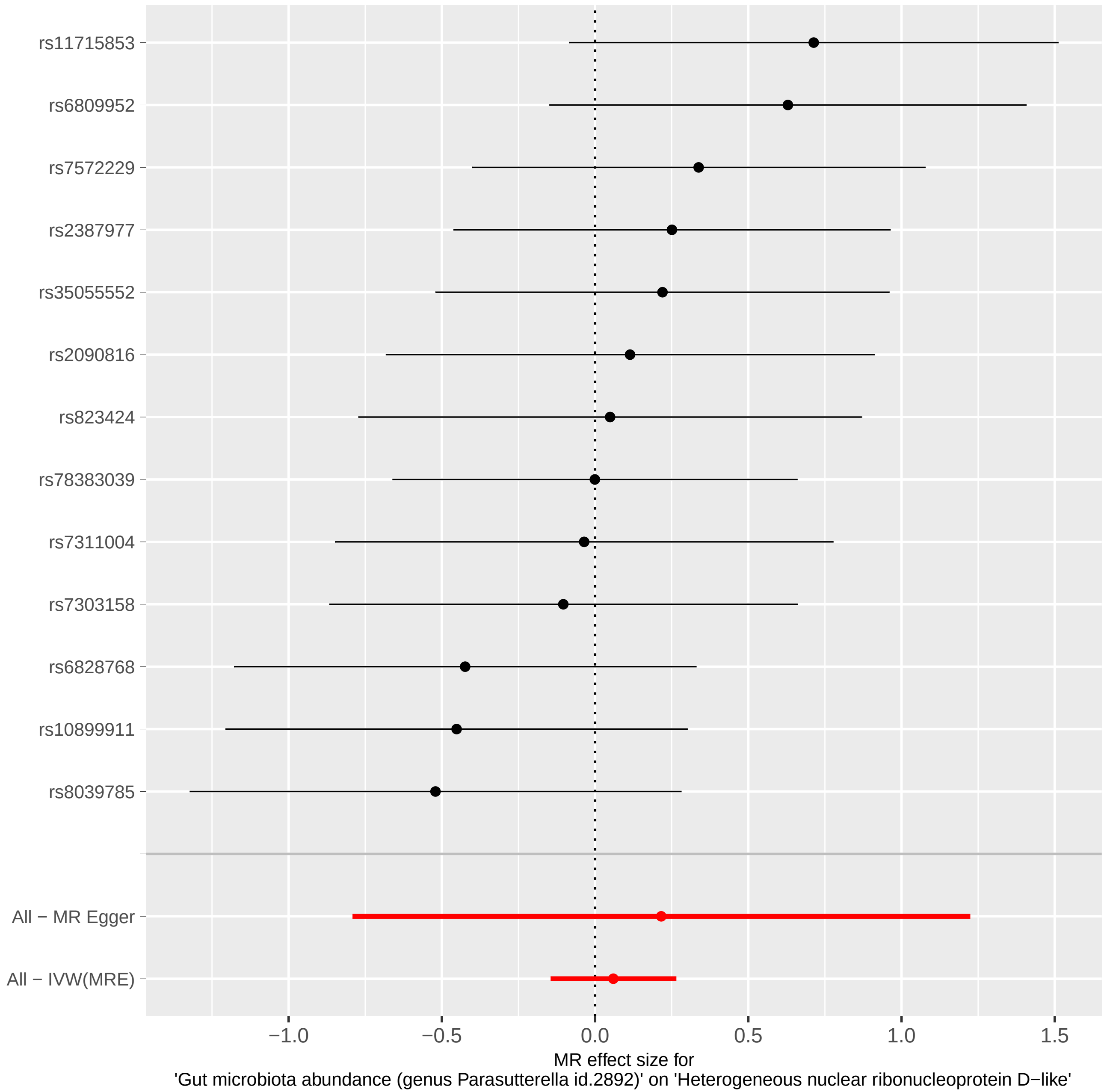

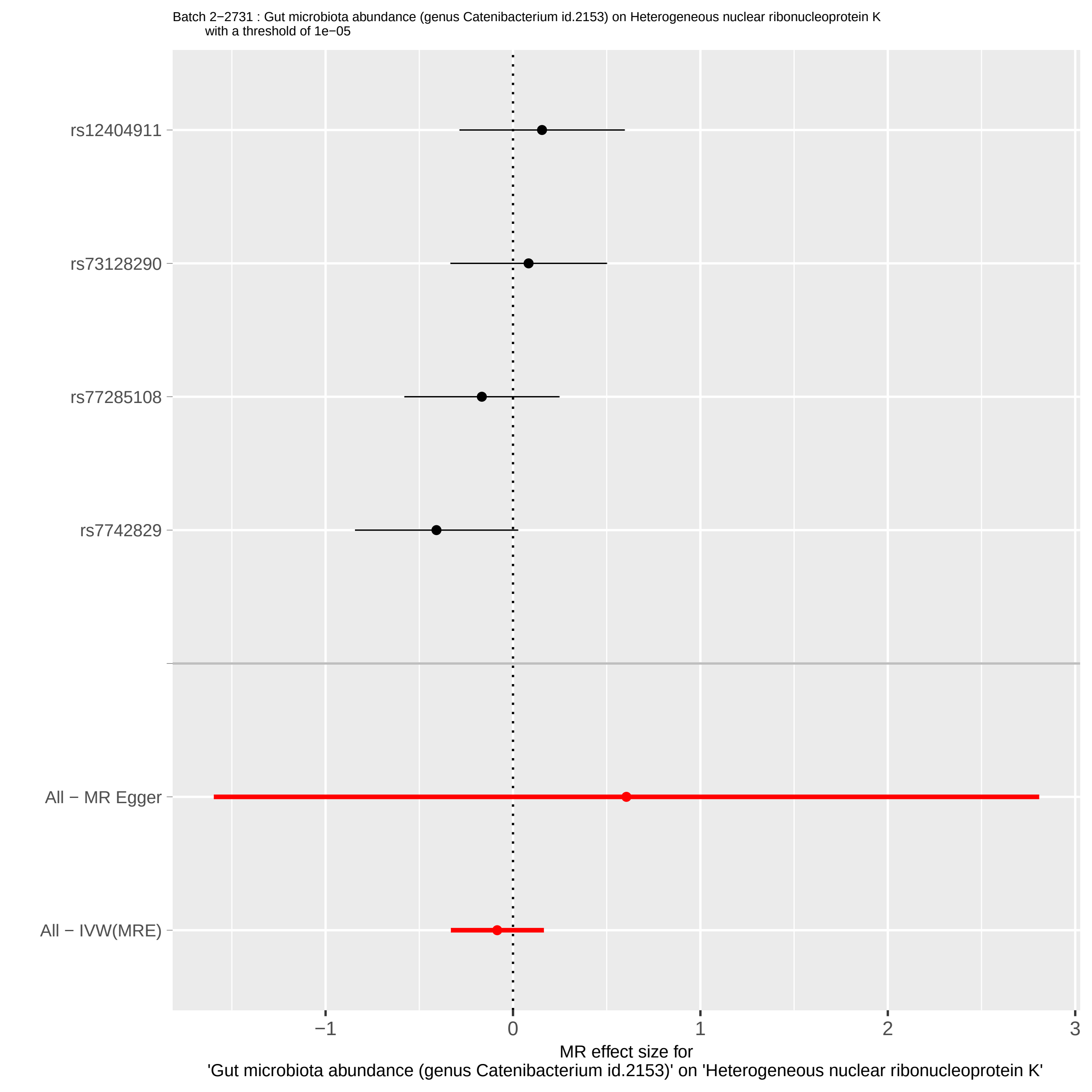

Batch 2−2731 : Gut microbiota abundance (genus Catenibacterium id.2153) on Heterogeneous nuclear ribonucleoprotein K
with a threshold of 1e−05
rs12404911
rs73128290
rs77285108
rs7742829
All − MR Egger
All − IVW(MRE)
−1
0
1
2
3
MR effect size for
'Gut microbiota abundance (genus Catenibacterium id.2153)' on 'Heterogeneous nuclear ribonucleoprotein K'

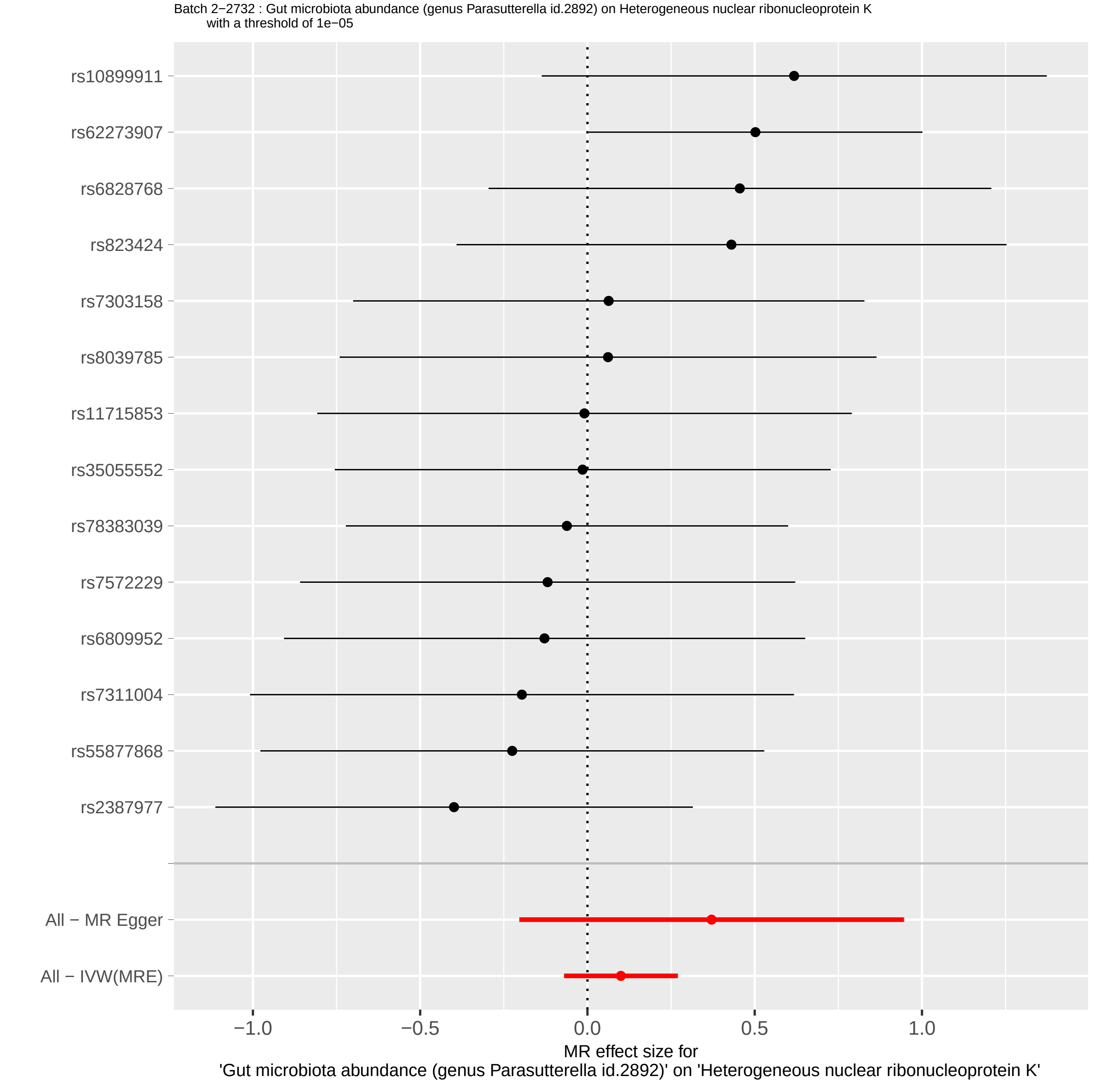
Batch 2−2732 : Gut microbiota abundance (genus Parasutterella id.2892) on Heterogeneous nuclear ribonucleoprotein K
with a threshold of 1e−05
rs10899911
rs62273907
rs6828768
rs823424
rs7303158
rs8039785
rs11715853
rs35055552
rs78383039
rs7572229
rs6809952
rs7311004
rs55877868
rs2387977
All − MR Egger
All − IVW(MRE)
−1.0
−0.5
0.0
0.5
1.0
MR effect size for
'Gut microbiota abundance (genus Parasutterella id.2892)' on 'Heterogeneous nuclear ribonucleoprotein K'

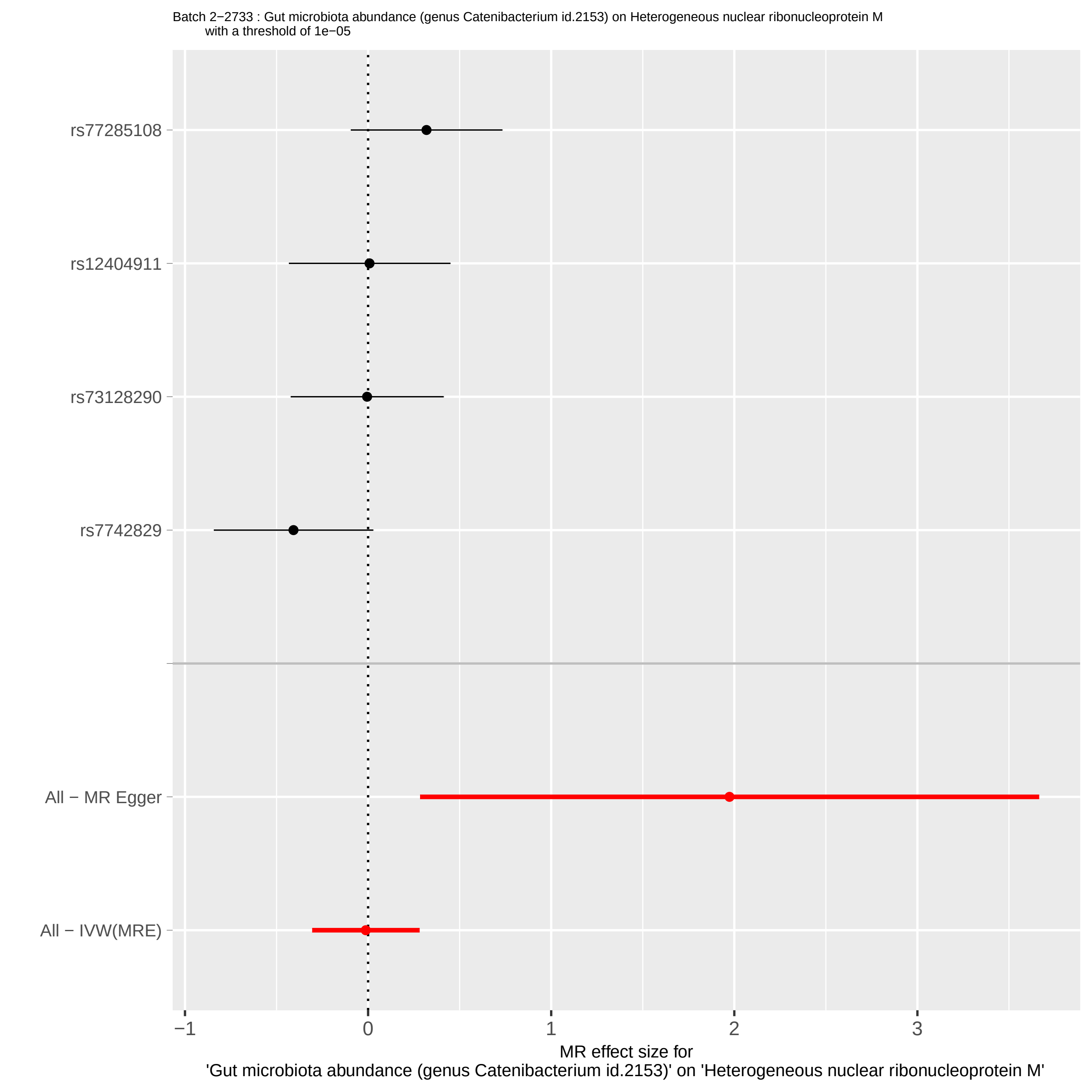

Batch 2−2733 : Gut microbiota abundance (genus Catenibacterium id.2153) on Heterogeneous nuclear ribonucleoprotein M
with a threshold of 1e−05
rs77285108
rs12404911
rs73128290
rs7742829
All − MR Egger
All − IVW(MRE)
−1
0
1
2
3
MR effect size for
'Gut microbiota abundance (genus Catenibacterium id.2153)' on 'Heterogeneous nuclear ribonucleoprotein M'

Batch 2−2734 : Gut microbiota abundance (genus Parasutterella id.2892) on Heterogeneous nuclear ribonucleoprotein M
with a threshold of 1e−05

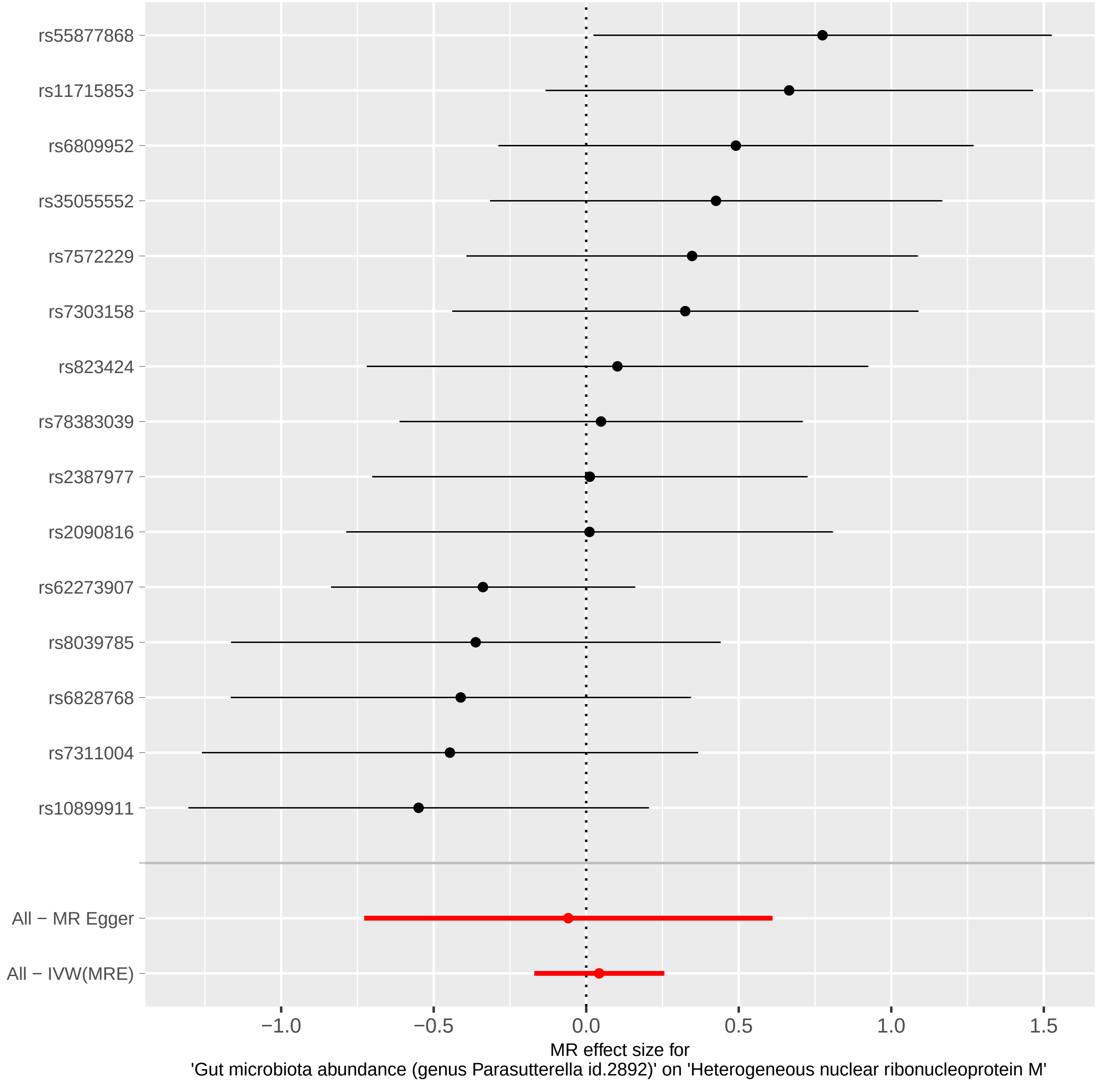

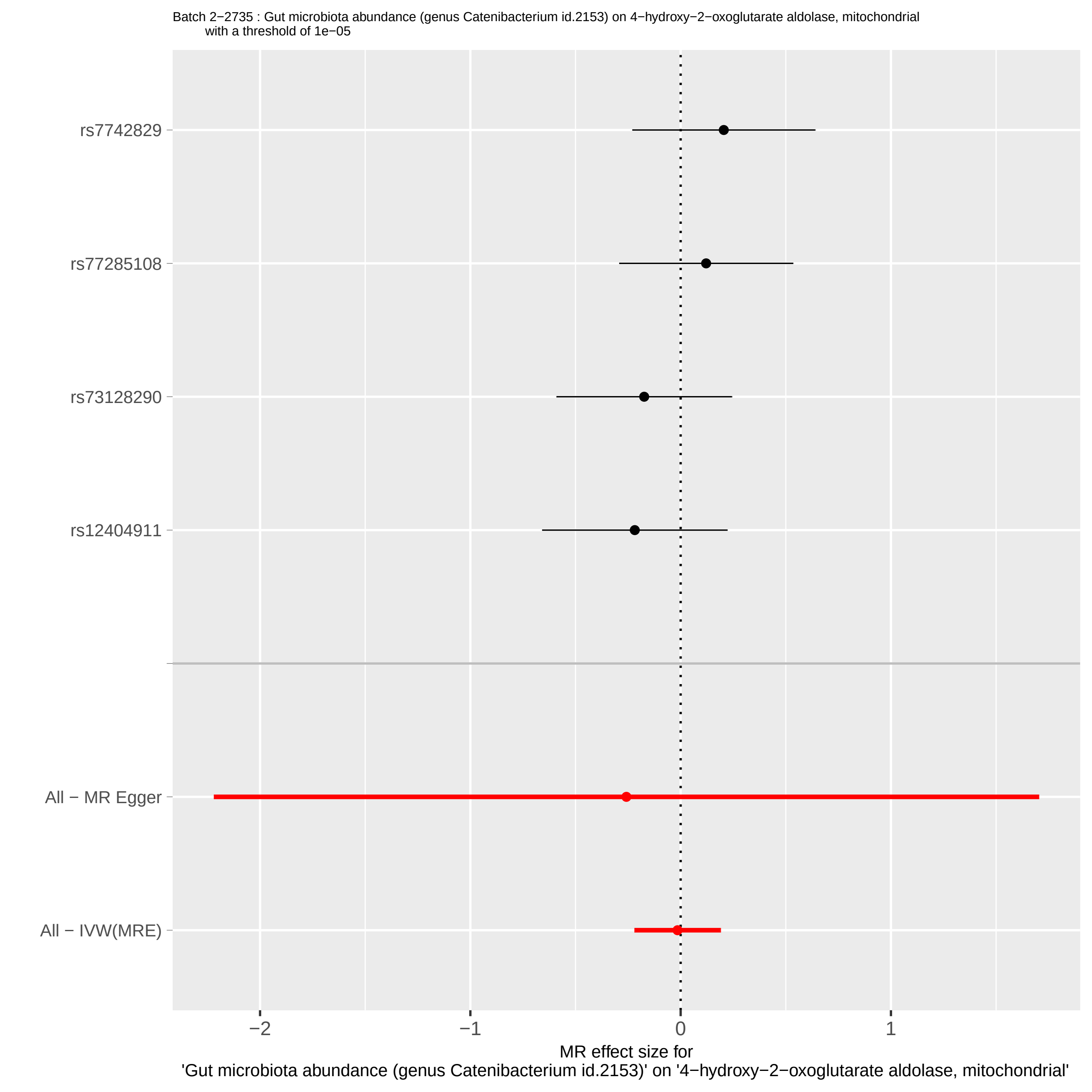

Batch 2−2735 : Gut microbiota abundance (genus Catenibacterium id.2153) on 4−hydroxy−2−oxoglutarate aldolase, mitochondrial with a threshold of 1e−05
rs7742829
rs77285108
rs73128290
rs12404911
All − MR Egger
All − IVW(MRE)
−2
−1
0
1
MR effect size for
'Gut microbiota abundance (genus Catenibacterium id.2153)' on '4−hydroxy−2−oxoglutarate aldolase, mitochondrial'

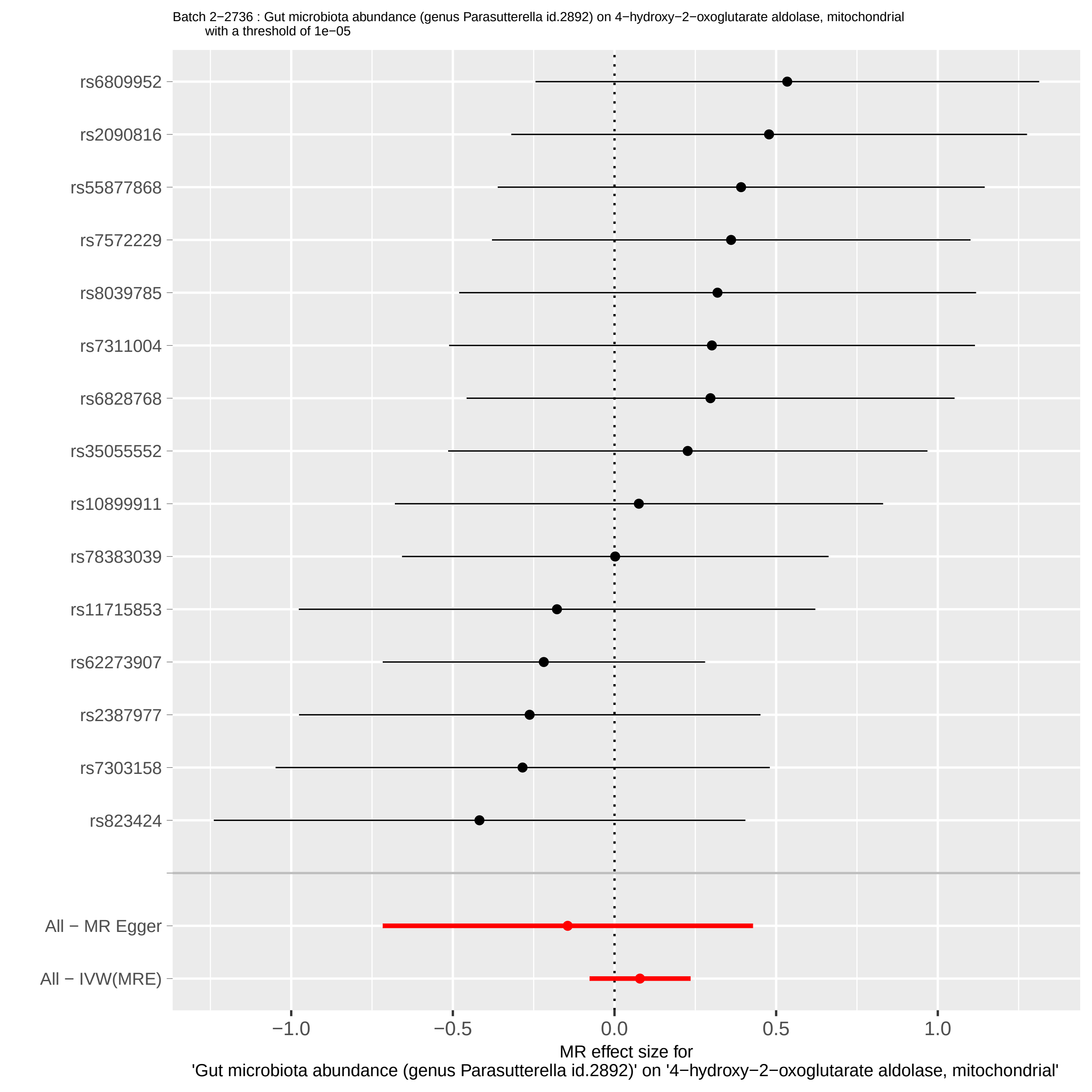

Batch 2−2736 : Gut microbiota abundance (genus Parasutterella id.2892) on 4−hydroxy−2−oxoglutarate aldolase, mitochondrial
with a threshold of 1e−05
rs6809952
rs2090816
rs55877868
rs7572229
rs8039785
rs7311004
rs6828768
rs35055552
rs10899911
rs78383039
rs11715853
rs62273907
rs2387977
rs7303158
rs823424
All − MR Egger
All − IVW(MRE)
−1.0
−0.5
0.0
0.5
1.0
MR effect size for
'Gut microbiota abundance (genus Parasutterella id.2892)' on '4−hydroxy−2−oxoglutarate aldolase, mitochondrial'

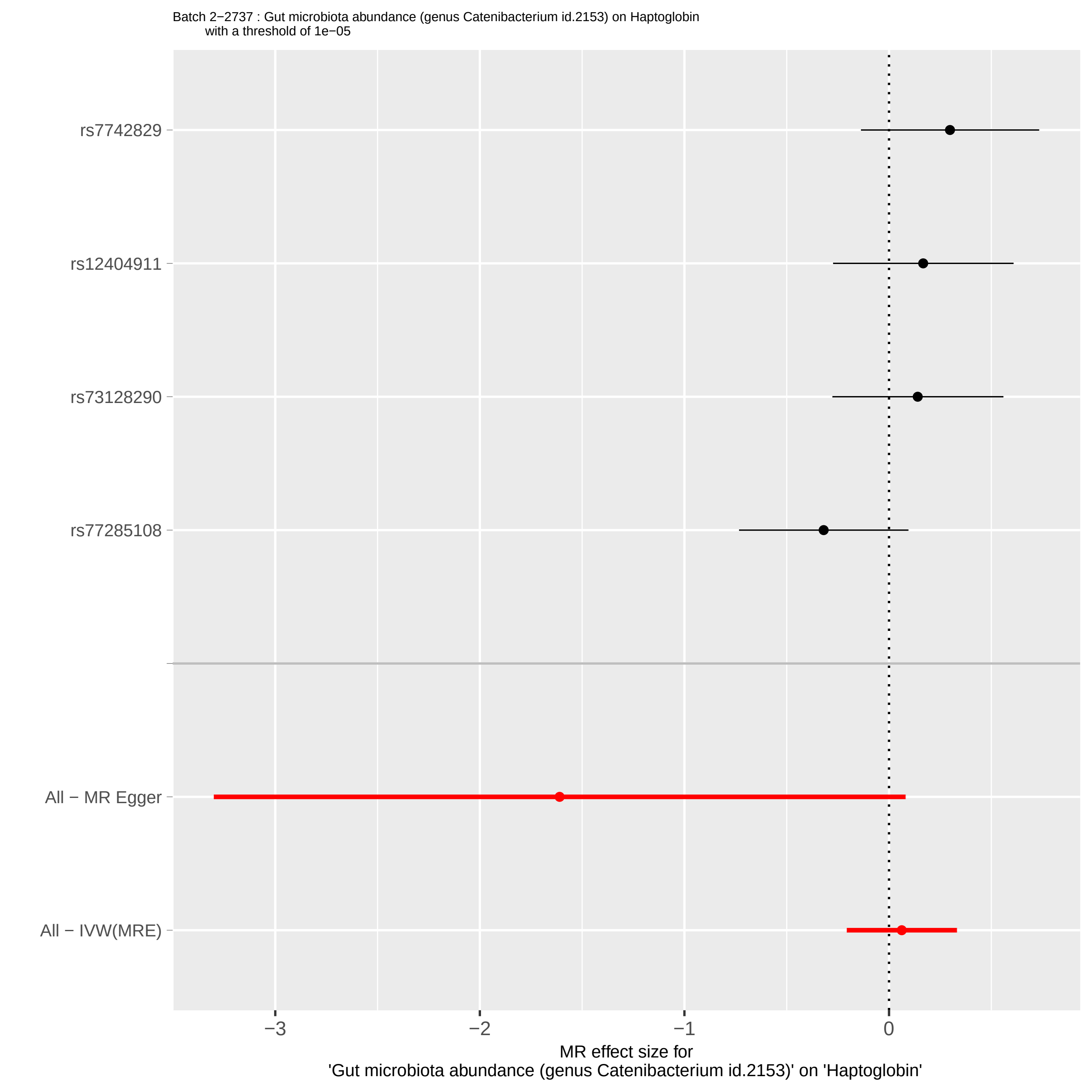

Batch 2−2737 : Gut microbiota abundance (genus Catenibacterium id.2153) on Haptoglobin
with a threshold of 1e−05
rs7742829
rs12404911
rs73128290
rs77285108
All − MR Egger
All − IVW(MRE)
−3
−2
−1
0
MR effect size for
'Gut microbiota abundance (genus Catenibacterium id.2153)' on 'Haptoglobin'

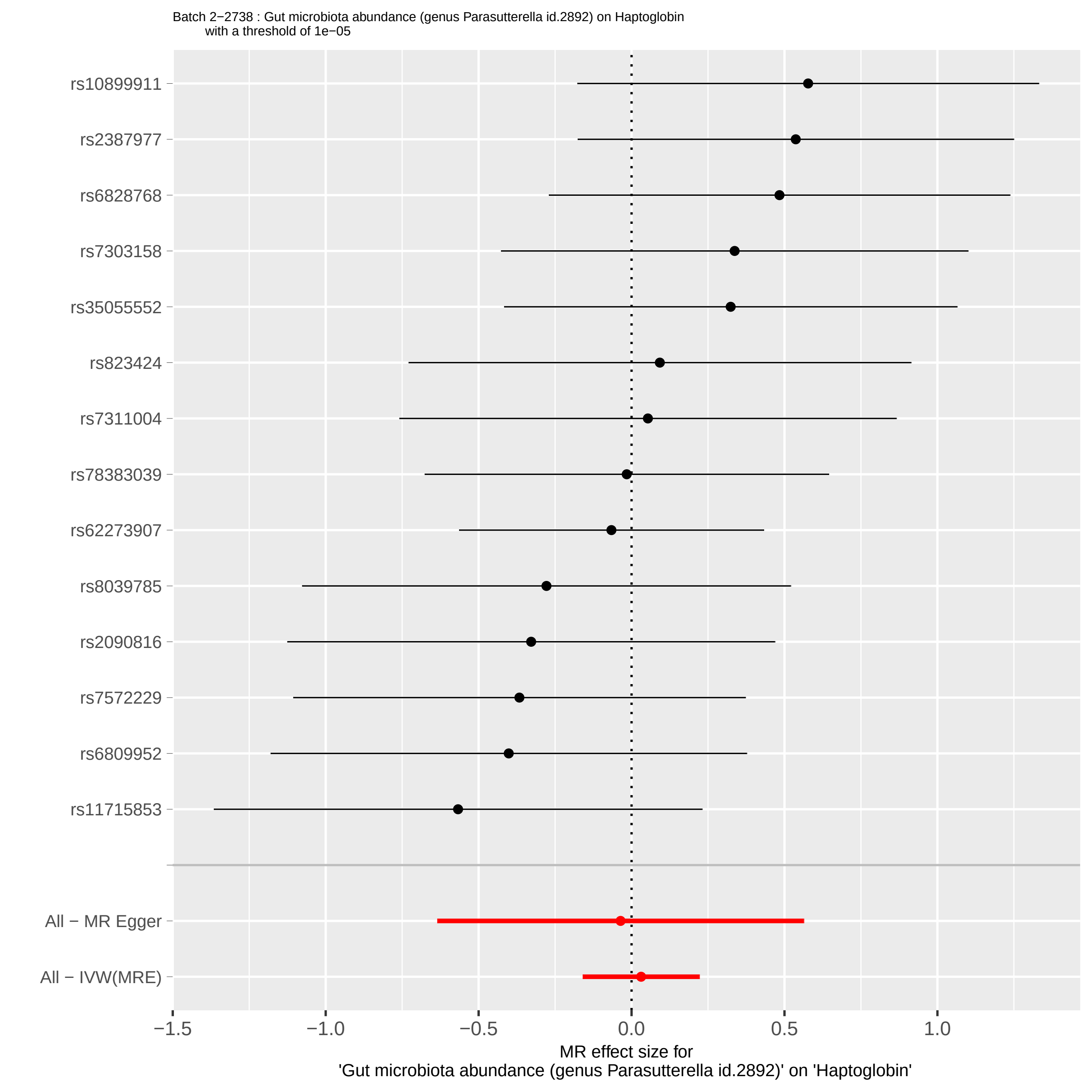

Batch 2−2738 : Gut microbiota abundance (genus Parasutterella id.2892) on Haptoglobin
with a threshold of 1e−05
rs10899911
rs2387977
rs6828768
rs7303158
rs35055552
rs823424
rs7311004
rs78383039
rs62273907
rs8039785
rs2090816
rs7572229
rs6809952
rs11715853
All − MR Egger
All − IVW(MRE)
−1.5
−1.0
−0.5
0.0
0.5
1.0
MR effect size for
'Gut microbiota abundance (genus Parasutterella id.2892)' on 'Haptoglobin'

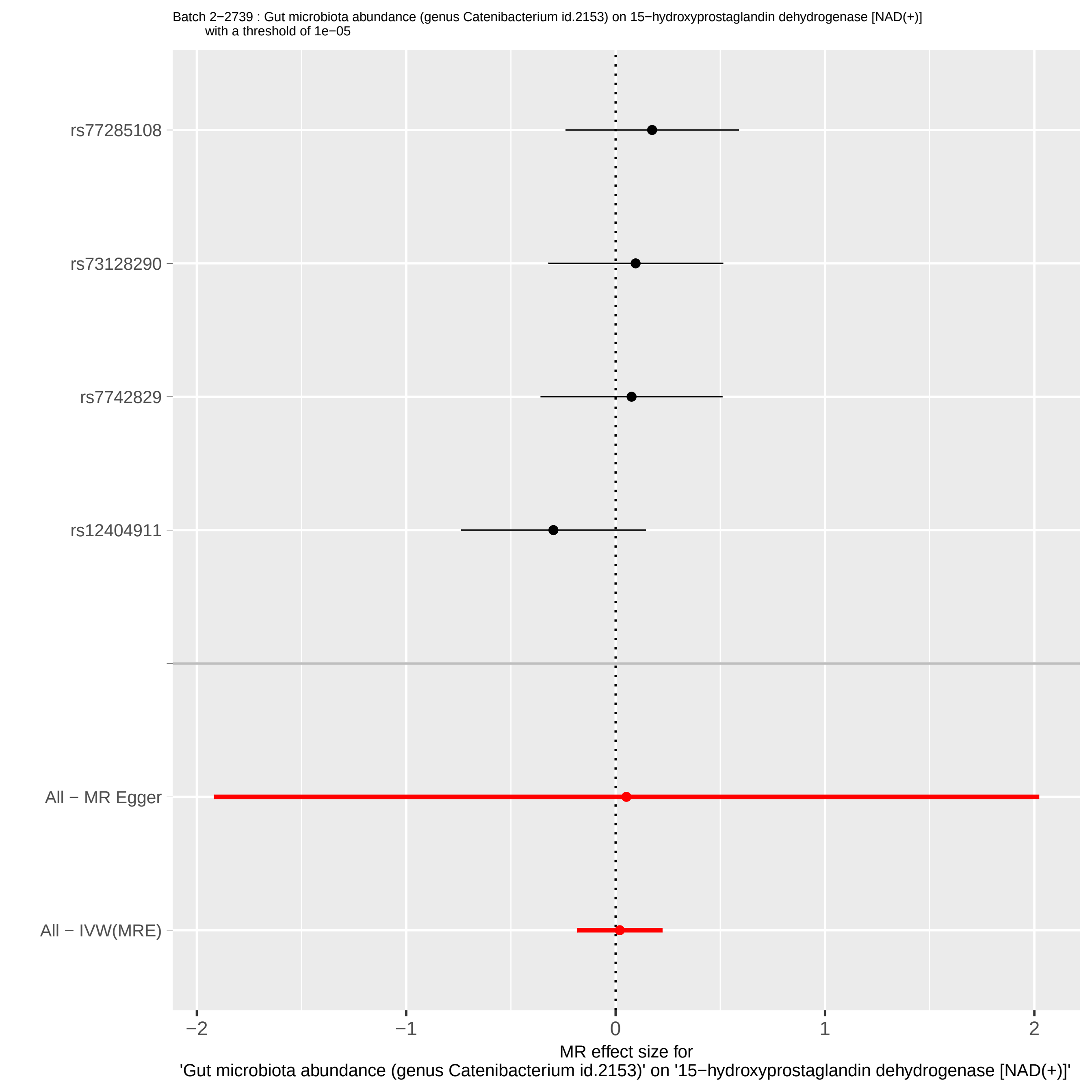

Batch 2−2739 : Gut microbiota abundance (genus Catenibacterium id.2153) on 15−hydroxyprostaglandin dehydrogenase [NAD(+)]
with a threshold of 1e−05
rs77285108
rs73128290
rs7742829
rs12404911
All − MR Egger
All − IVW(MRE)
−2
−1
0
1
2
MR effect size for
'Gut microbiota abundance (genus Catenibacterium id.2153)' on '15−hydroxyprostaglandin dehydrogenase [NAD(+)]'

Batch 2−2740 : Gut microbiota abundance (genus Parasutterella id.2892) on 15−hydroxyprostaglandin dehydrogenase [NAD(+)]
with a threshold of 1e−05

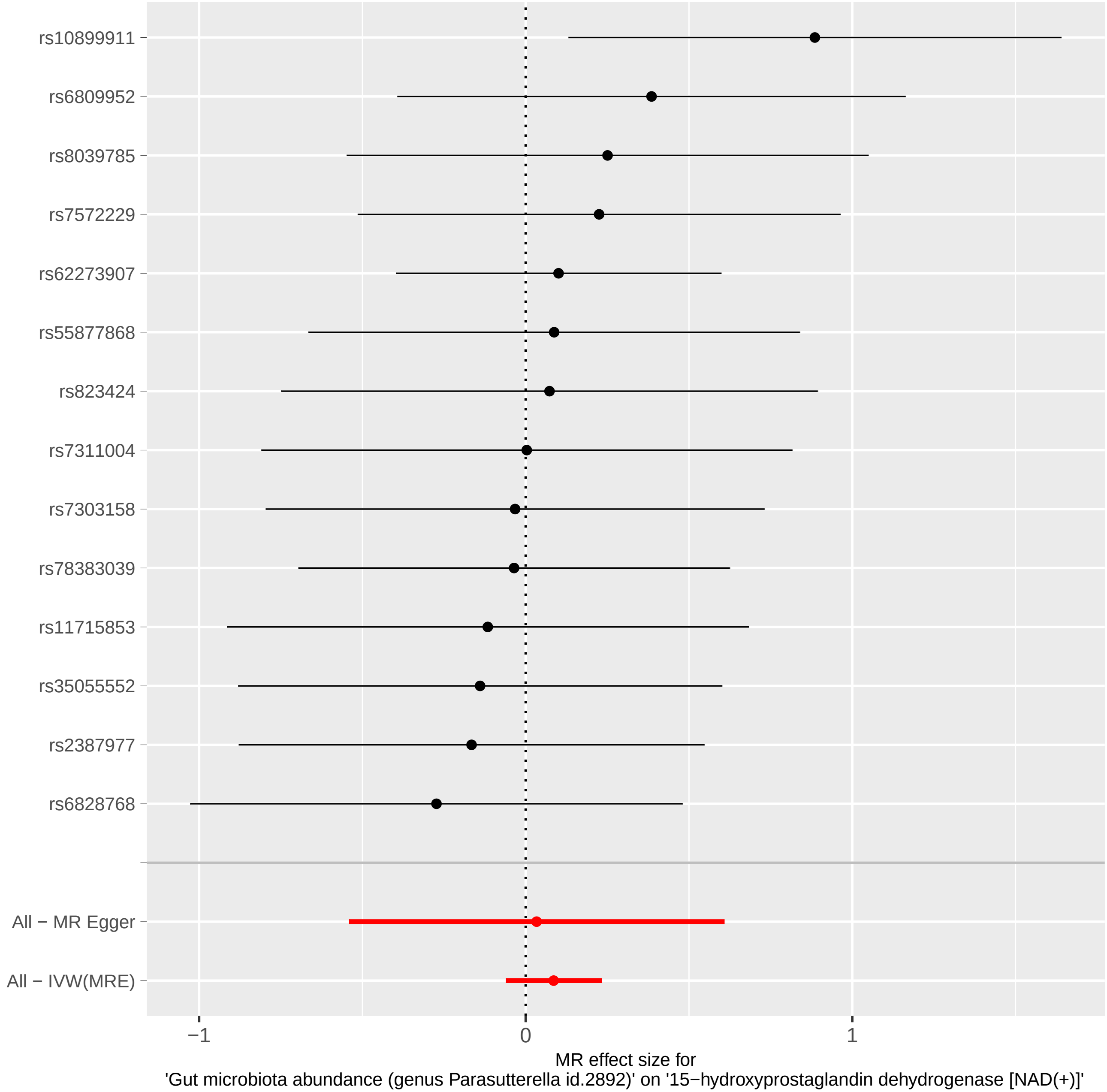

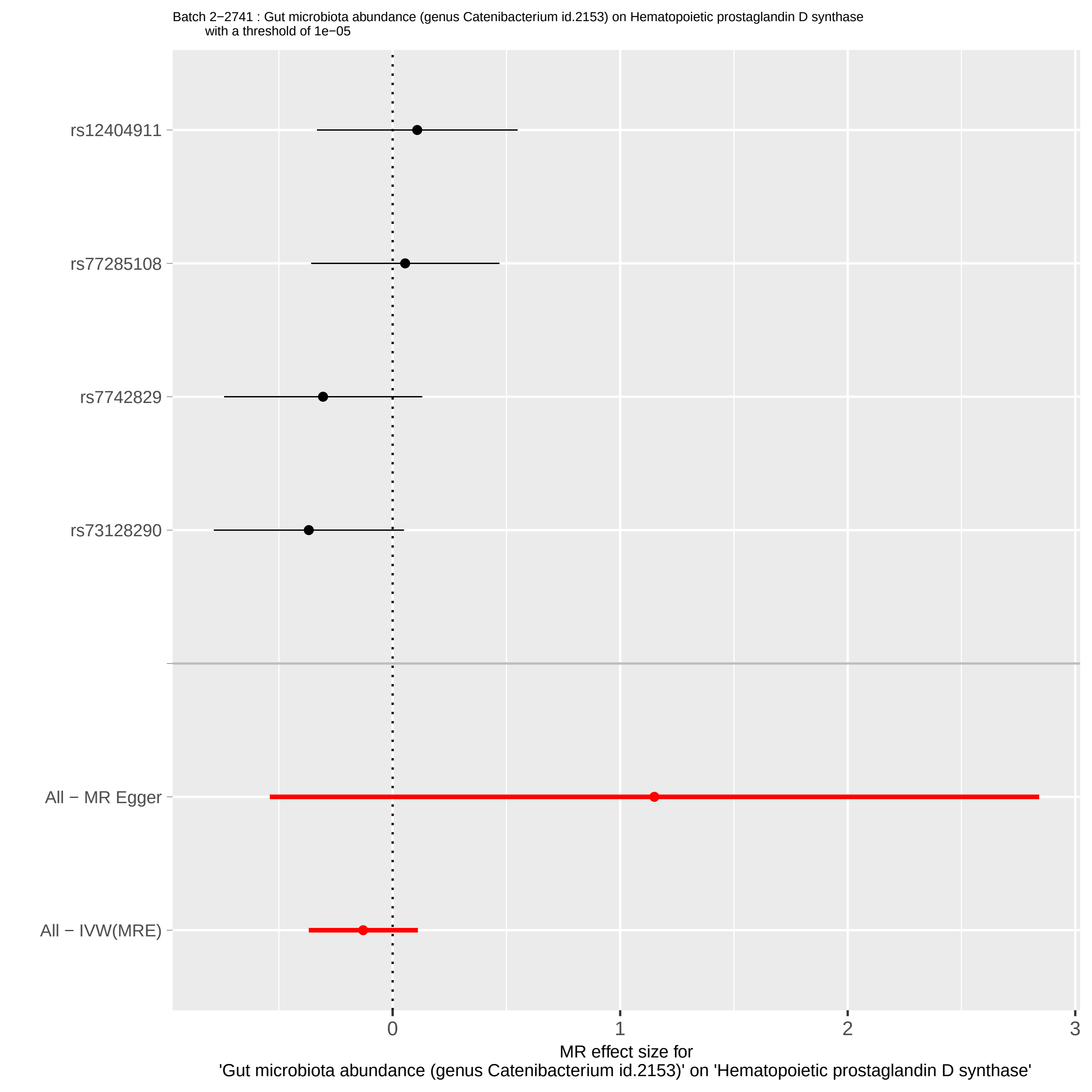

Batch 2−2741 : Gut microbiota abundance (genus Catenibacterium id.2153) on Hematopoietic prostaglandin D synthase
with a threshold of 1e−05
rs12404911
rs77285108
rs7742829
rs73128290
All − MR Egger
All − IVW(MRE)
0
1
2
3
MR effect size for
'Gut microbiota abundance (genus Catenibacterium id.2153)' on 'Hematopoietic prostaglandin D synthase'

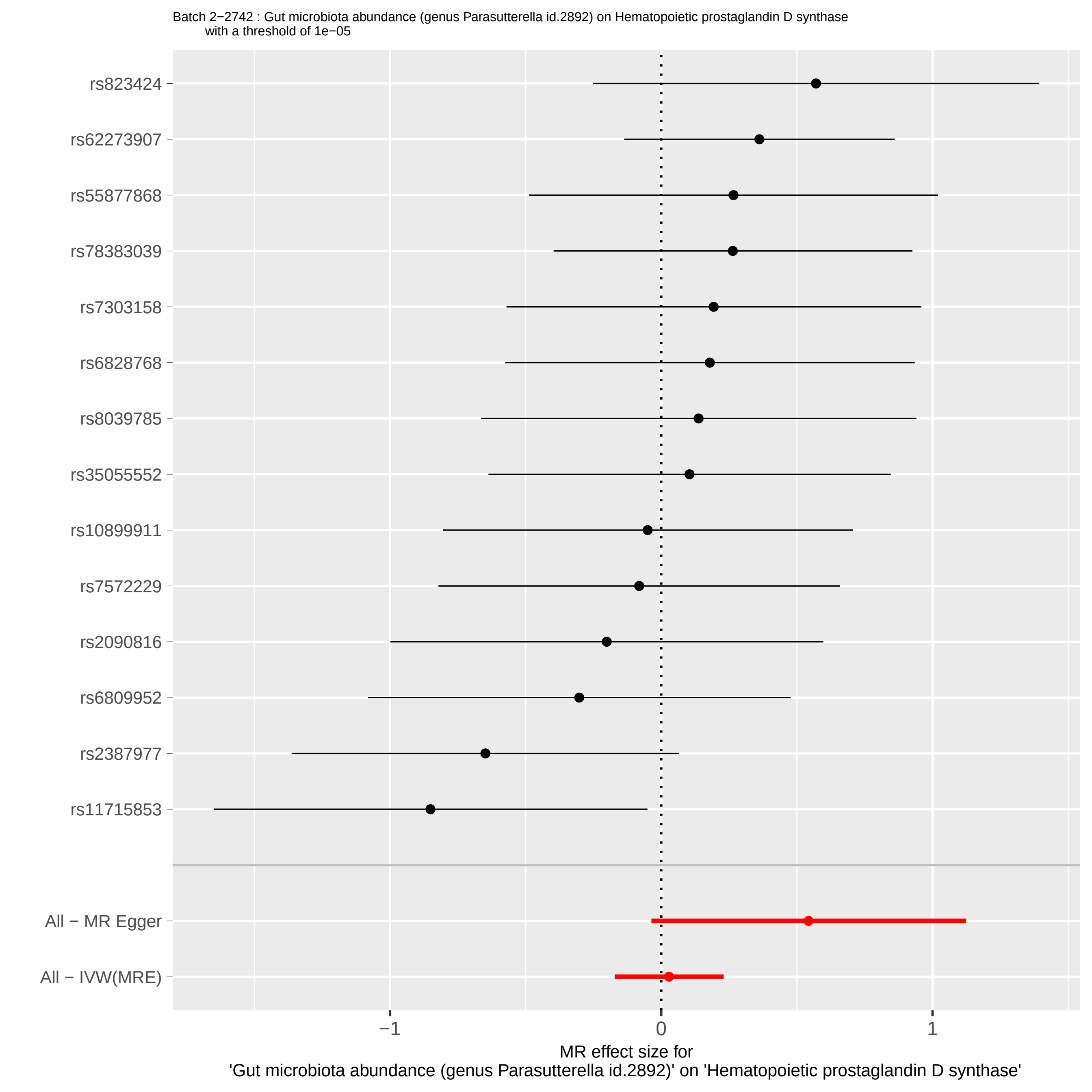
Batch 2−2742 : Gut microbiota abundance (genus Parasutterella id.2892) on Hematopoietic prostaglandin D synthase
with a threshold of 1e−05
rs823424
rs62273907
rs55877868
rs78383039
rs7303158
rs6828768
rs8039785
rs35055552
rs10899911
rs7572229
rs2090816
rs6809952
rs2387977
rs11715853
All − MR Egger
All − IVW(MRE)
−1
0
1
MR effect size for
'Gut microbiota abundance (genus Parasutterella id.2892)' on 'Hematopoietic prostaglandin D synthase'

Batch 2−2743 : Gut microbiota abundance (genus Catenibacterium id.2153) on HRAS−like suppressor 2
with a threshold of 1e−05

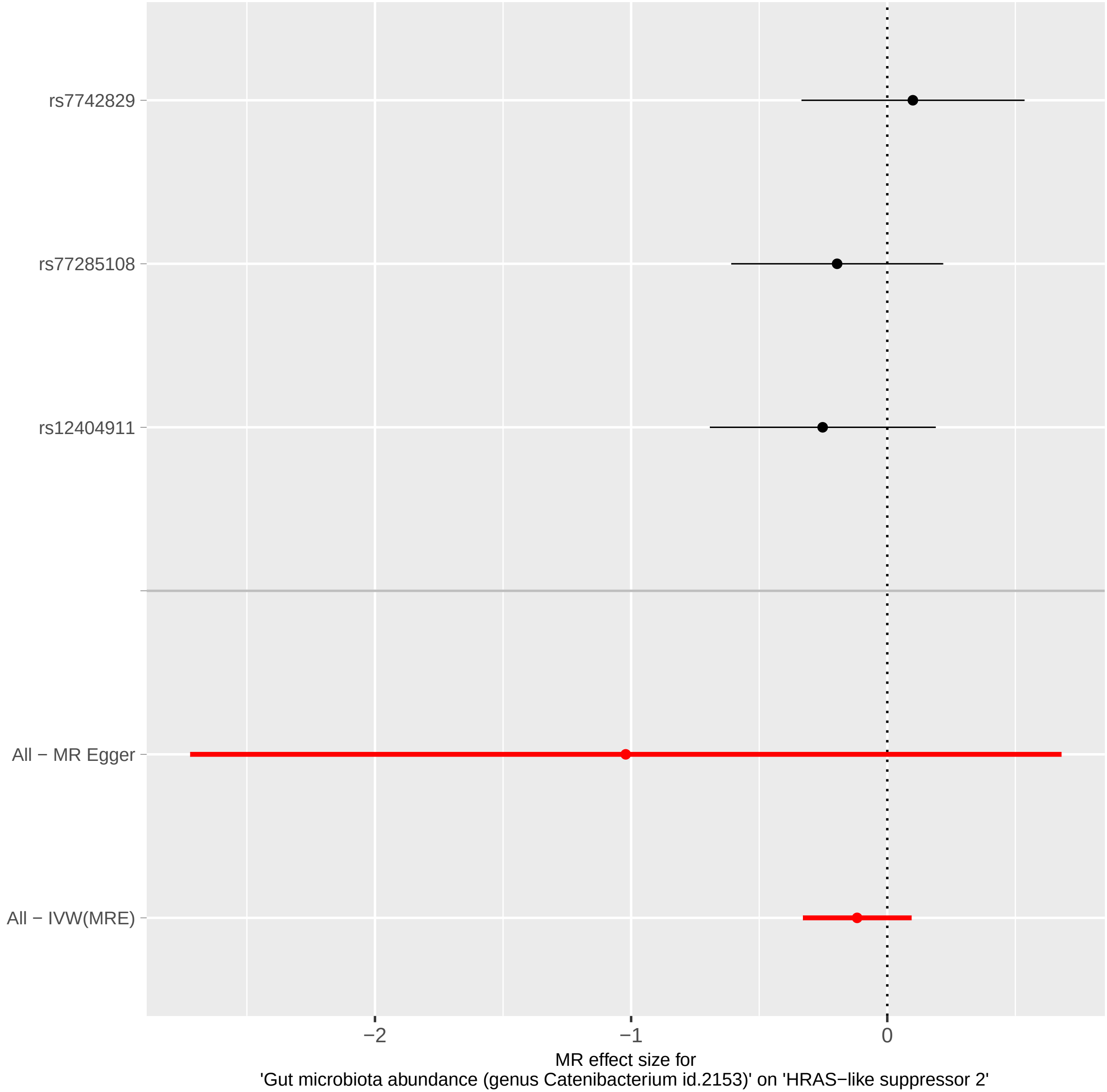

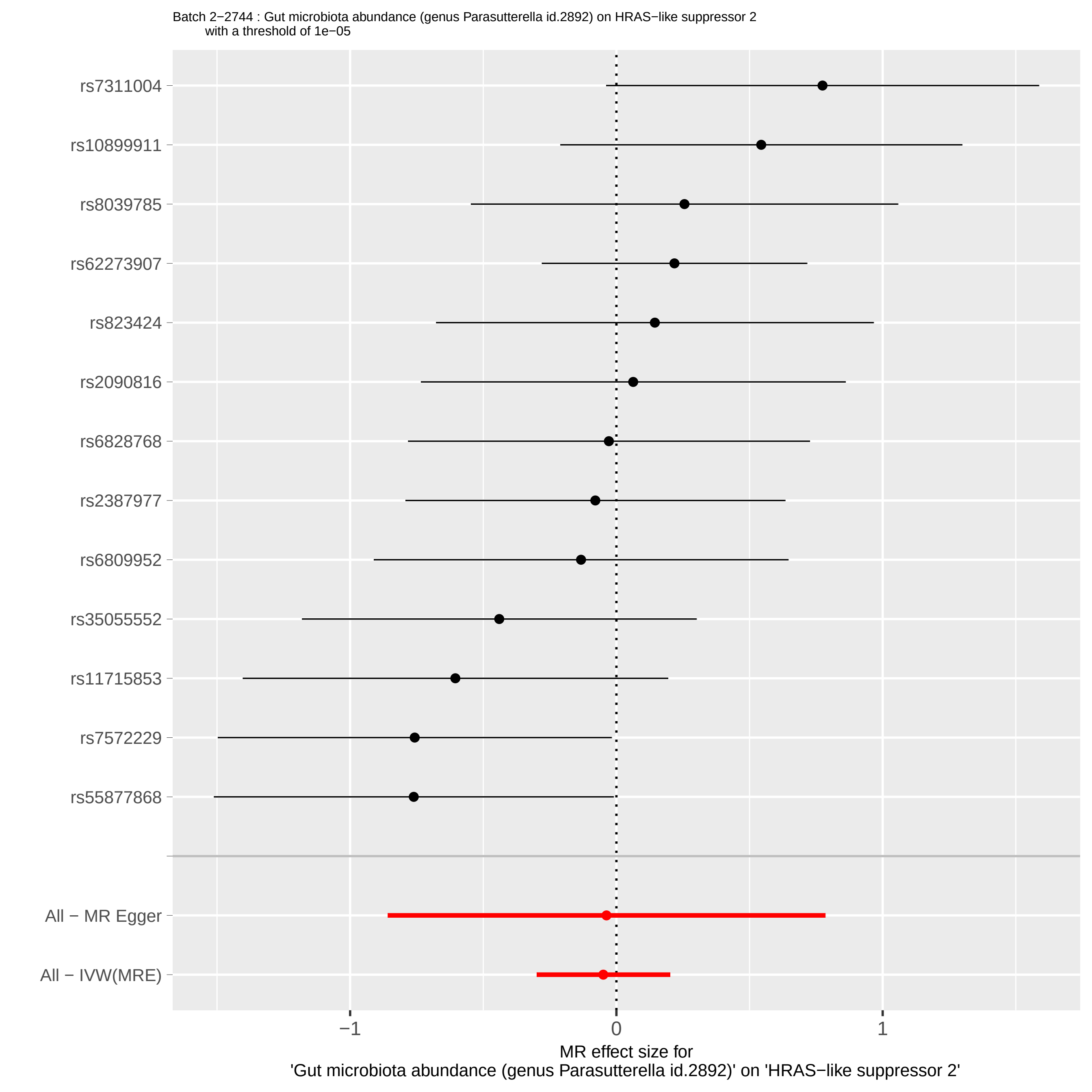
Batch 2−2744 : Gut microbiota abundance (genus Parasutterella id.2892) on HRAS−like suppressor 2
with a threshold of 1e−05
rs7311004
rs10899911
rs8039785
rs62273907
rs823424
rs2090816
rs6828768
rs2387977
rs6809952
rs35055552
rs11715853
rs7572229
rs55877868
All − MR Egger
All − IVW(MRE)
−1
0
1
MR effect size for
'Gut microbiota abundance (genus Parasutterella id.2892)' on 'HRAS−like suppressor 2'

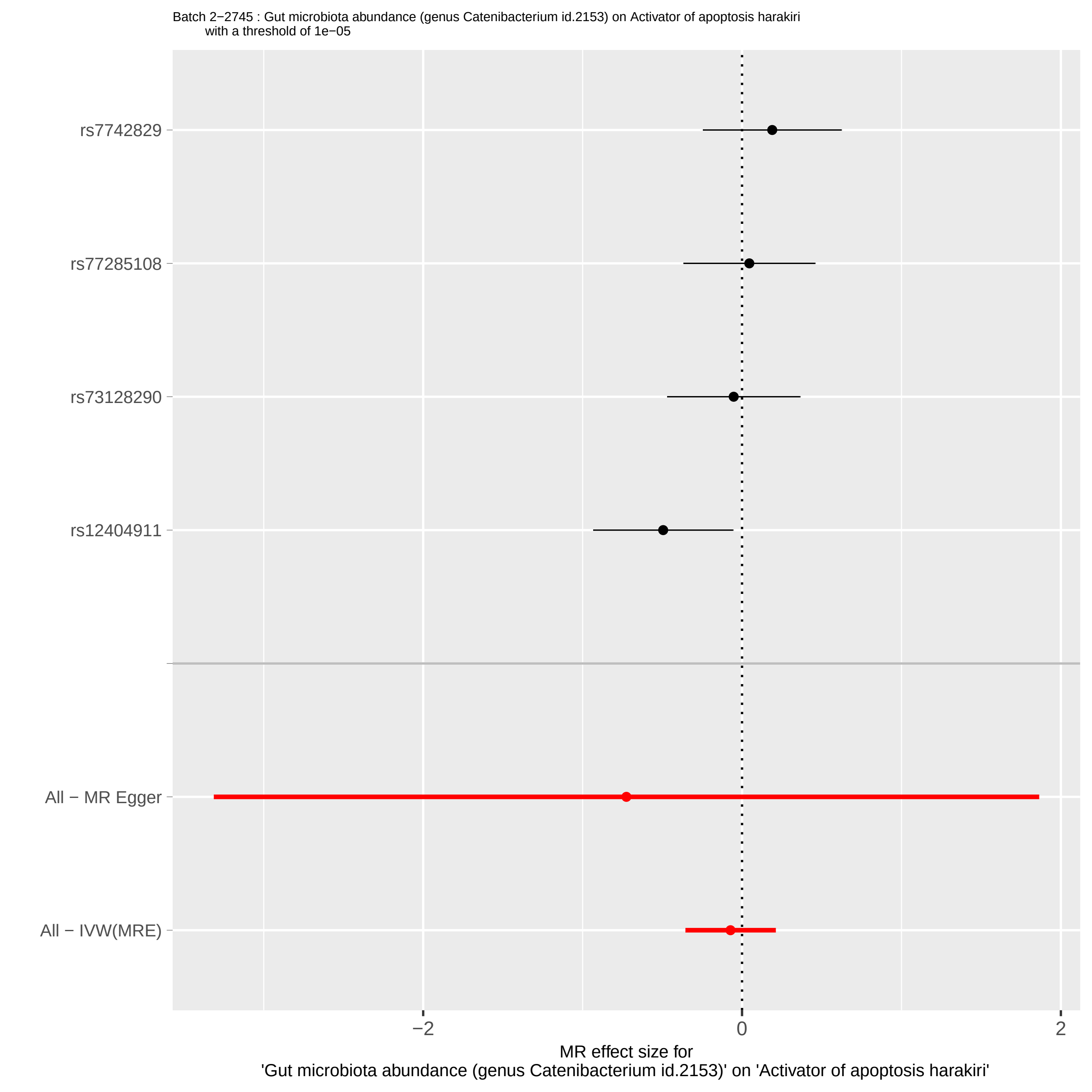

Batch 2−2745 : Gut microbiota abundance (genus Catenibacterium id.2153) on Activator of apoptosis harakiri
with a threshold of 1e−05
rs7742829
rs77285108
rs73128290
rs12404911
All − MR Egger
All − IVW(MRE)
−2
0
2
MR effect size for
'Gut microbiota abundance (genus Catenibacterium id.2153)' on 'Activator of apoptosis harakiri'

Batch 2−2746 : Gut microbiota abundance (genus Parasutterella id.2892) on Activator of apoptosis harakiri
with a threshold of 1e−05

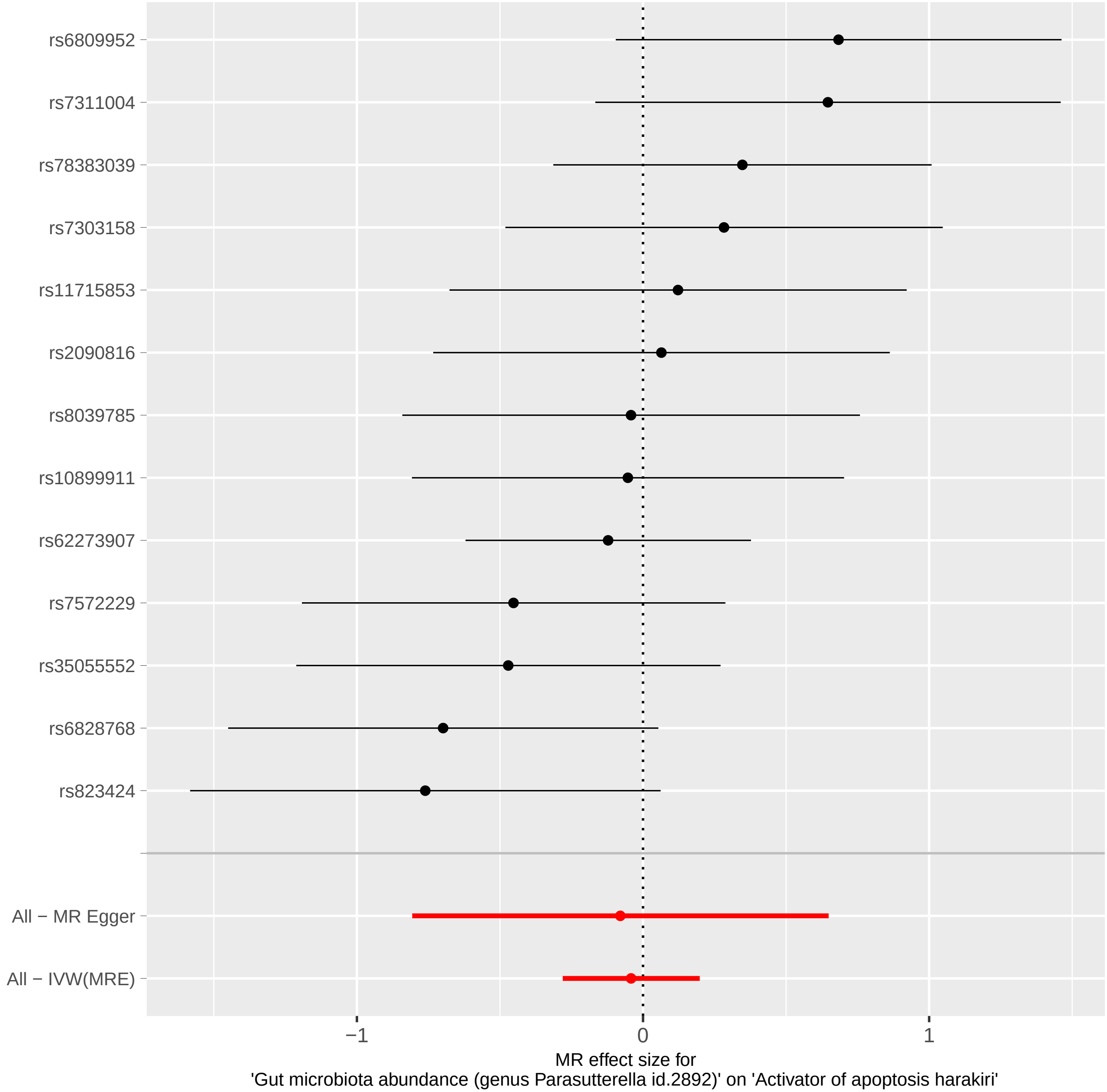

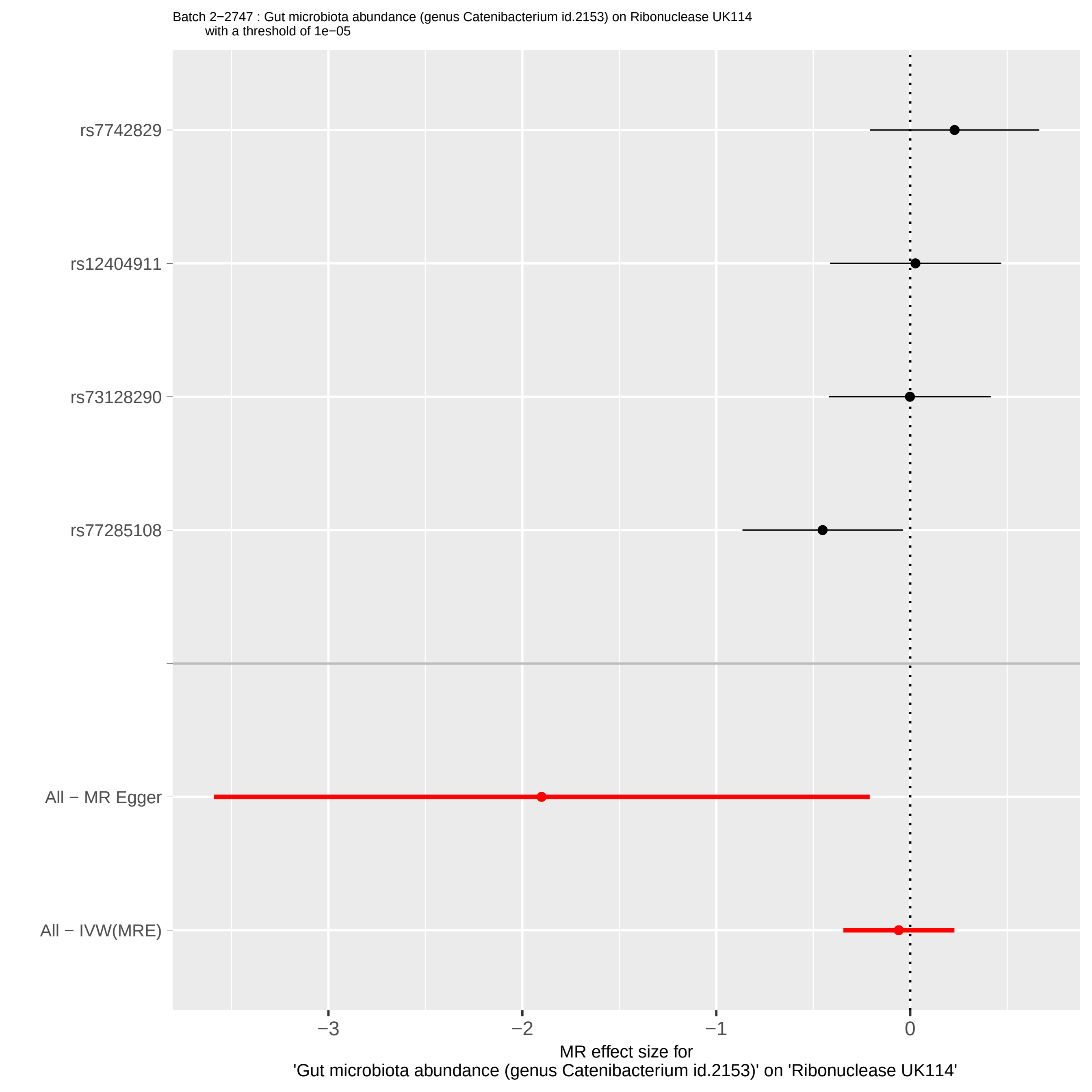
Batch 2−2747 : Gut microbiota abundance (genus Catenibacterium id.2153) on Ribonuclease UK114
with a threshold of 1e−05
rs7742829
rs12404911
rs73128290
rs77285108
All − MR Egger
All − IVW(MRE)
−3
−2
−1
0
MR effect size for
'Gut microbiota abundance (genus Catenibacterium id.2153)' on 'Ribonuclease UK114'

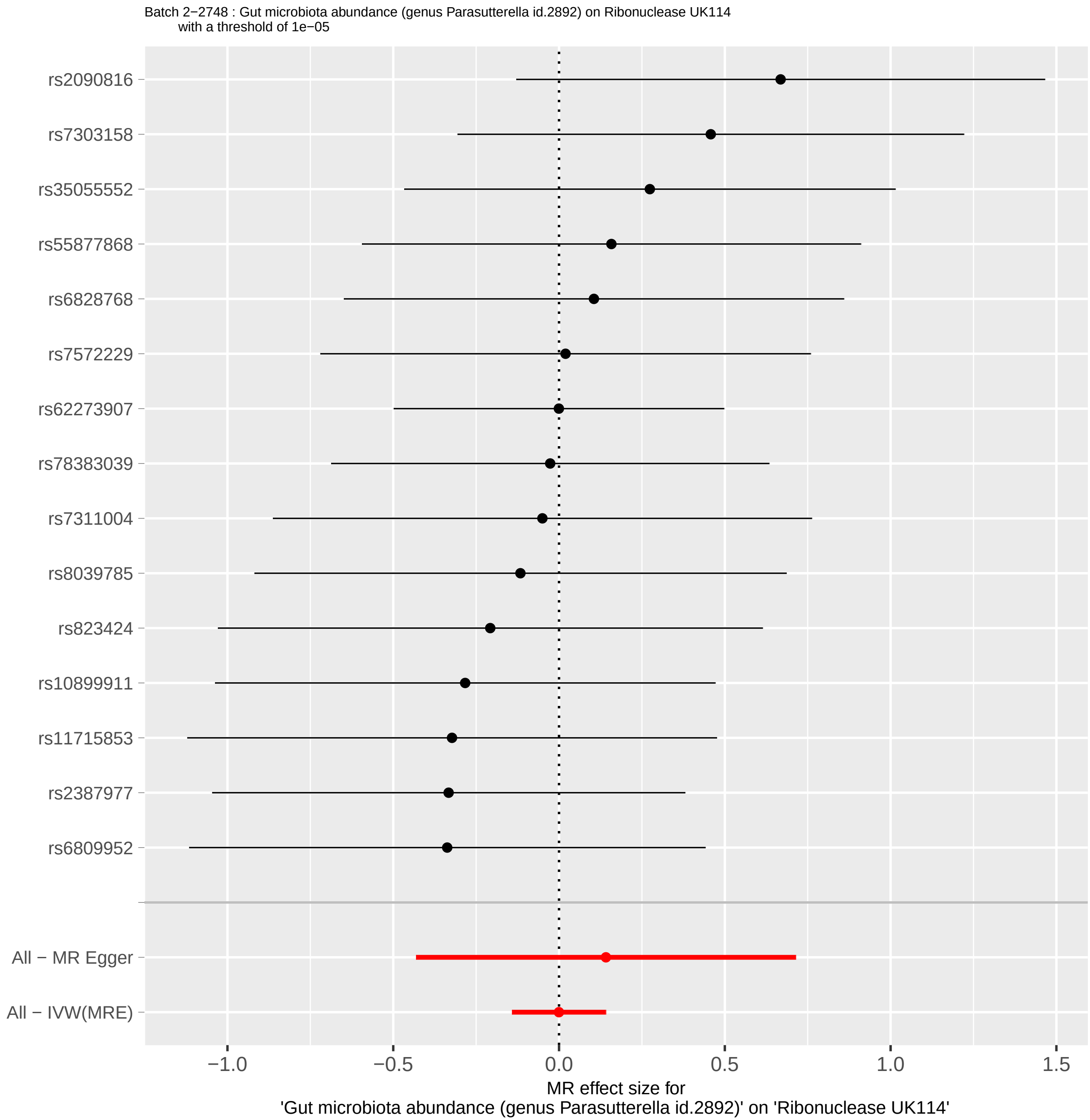

Batch 2−2748 : Gut microbiota abundance (genus Parasutterella id.2892) on Ribonuclease UK114
with a threshold of 1e−05
rs2090816
rs7303158
rs35055552
rs55877868
rs6828768
rs7572229
rs62273907
rs78383039
rs7311004
rs8039785
rs823424
rs10899911
rs11715853
rs2387977
rs6809952
All − MR Egger
All − IVW(MRE)
−1.0
−0.5
0.0
0.5
1.0
1.5
MR effect size for
'Gut microbiota abundance (genus Parasutterella id.2892)' on 'Ribonuclease UK114'

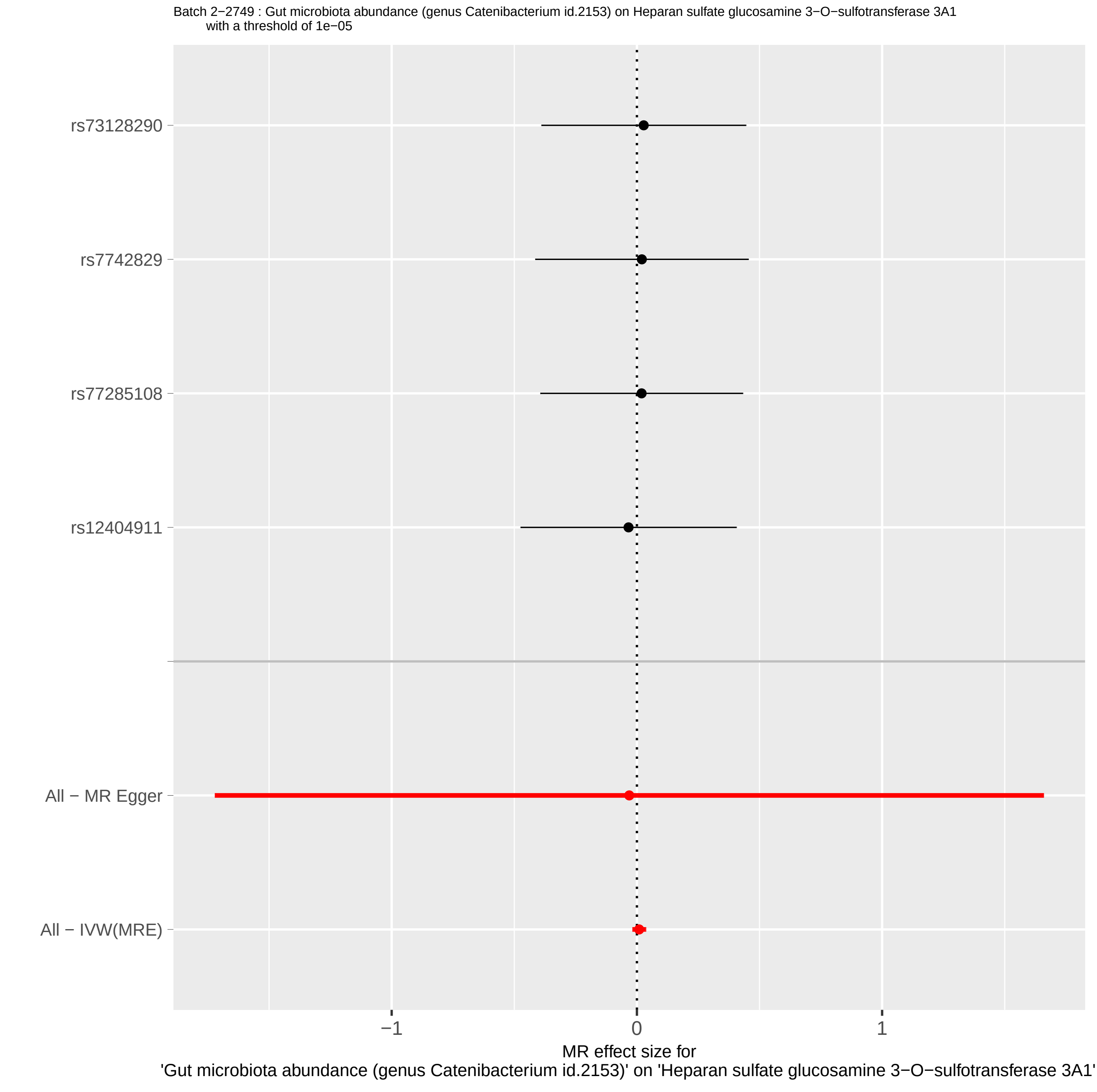
Batch 2−2749 : Gut microbiota abundance (genus Catenibacterium id.2153) on Heparan sulfate glucosamine 3−O−sulfotransferase 3A1
with a threshold of 1e−05
rs73128290
rs7742829
rs77285108
rs12404911
All − MR Egger
All − IVW(MRE)
−1
0
1
MR effect size for
'Gut microbiota abundance (genus Catenibacterium id.2153)' on 'Heparan sulfate glucosamine 3−O−sulfotransferase 3A1'

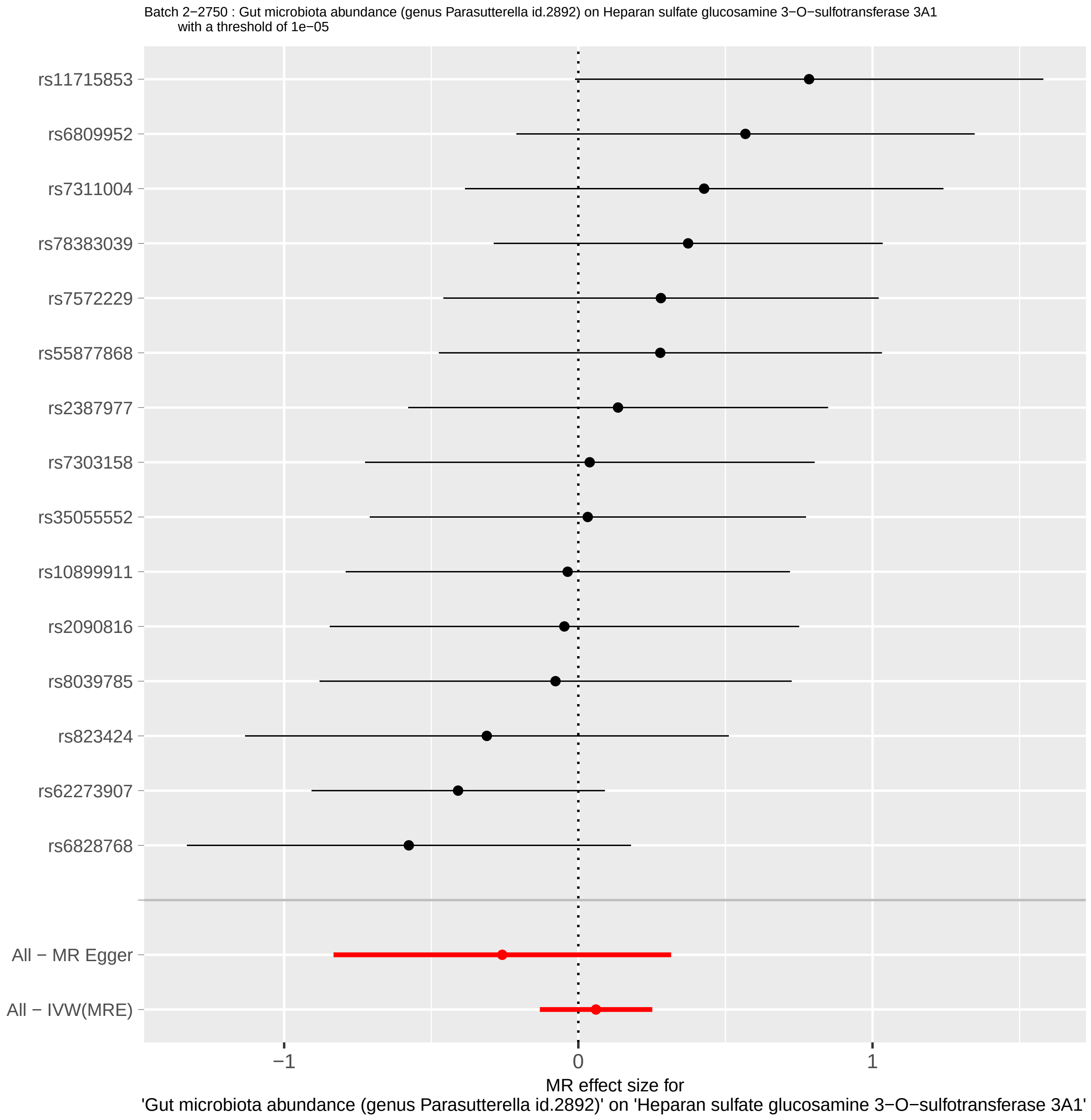

Batch 2−2750 : Gut microbiota abundance (genus Parasutterella id.2892) on Heparan sulfate glucosamine 3−O−sulfotransferase 3A1
with a threshold of 1e−05
rs11715853
rs6809952
rs7311004
rs78383039
rs7572229
rs55877868
rs2387977
rs7303158
rs35055552
rs10899911
rs2090816
rs8039785
rs823424
rs62273907
rs6828768
All − MR Egger
All − IVW(MRE)
−1
0
1
MR effect size for
'Gut microbiota abundance (genus Parasutterella id.2892)' on 'Heparan sulfate glucosamine 3−O−sulfotransferase 3A1'

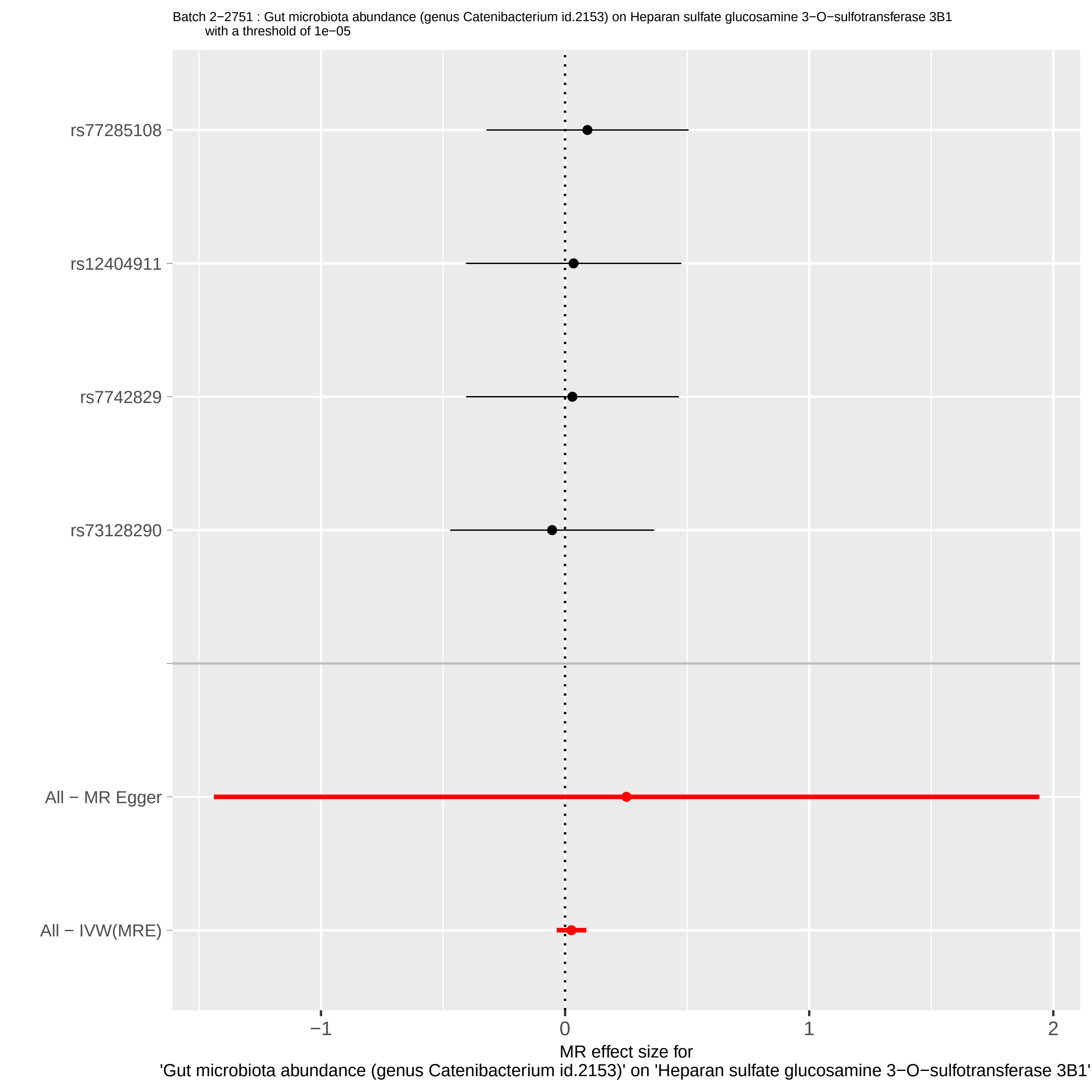

Batch 2−2751 : Gut microbiota abundance (genus Catenibacterium id.2153) on Heparan sulfate glucosamine 3−O−sulfotransferase 3B1
with a threshold of 1e−05
rs77285108
rs12404911
rs7742829
rs73128290
All − MR Egger
All − IVW(MRE)
−1
0
1
2
MR effect size for
'Gut microbiota abundance (genus Catenibacterium id.2153)' on 'Heparan sulfate glucosamine 3−O−sulfotransferase 3B1'

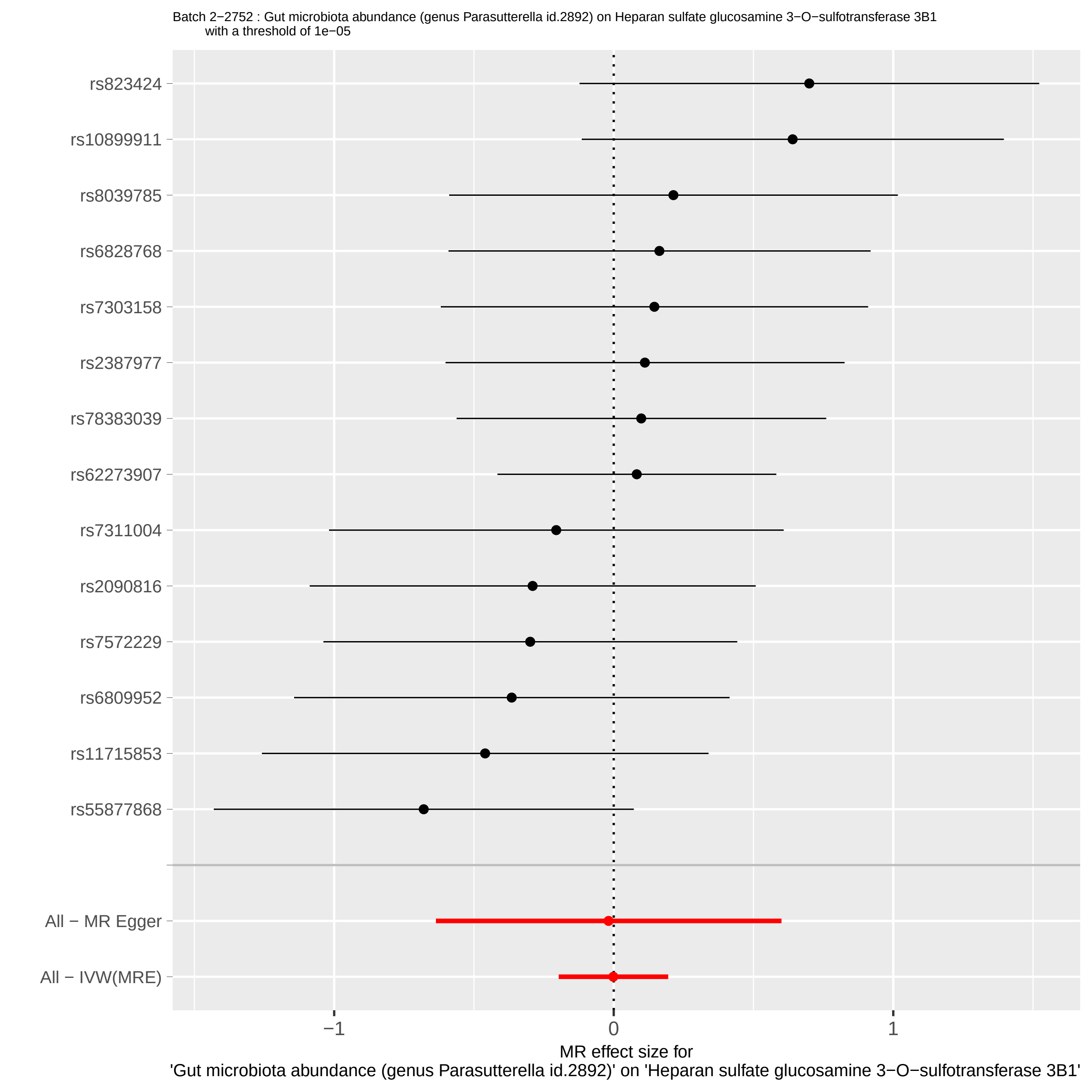

Batch 2−2752 : Gut microbiota abundance (genus Parasutterella id.2892) on Heparan sulfate glucosamine 3−O−sulfotransferase 3B1
with a threshold of 1e−05
rs823424
rs10899911
rs8039785
rs6828768
rs7303158
rs2387977
rs78383039
rs62273907
rs7311004
rs2090816
rs7572229
rs6809952
rs11715853
rs55877868
All − MR Egger
All − IVW(MRE)
−1
0
1
MR effect size for
'Gut microbiota abundance (genus Parasutterella id.2892)' on 'Heparan sulfate glucosamine 3−O−sulfotransferase 3B1'

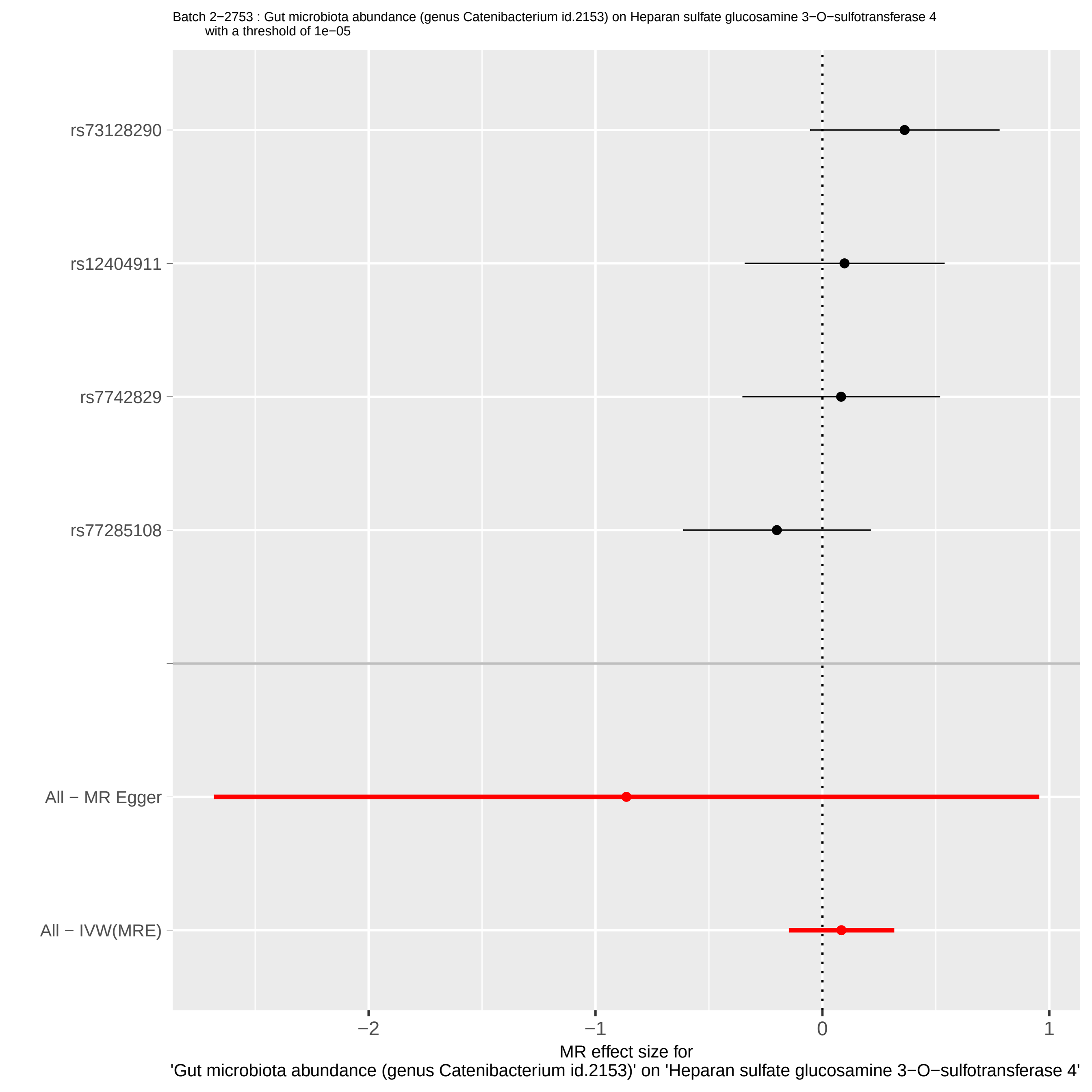

Batch 2−2753 : Gut microbiota abundance (genus Catenibacterium id.2153) on Heparan sulfate glucosamine 3−O−sulfotransferase 4
with a threshold of 1e−05
rs73128290
rs12404911
rs7742829
rs77285108
All − MR Egger
All − IVW(MRE)
−2
−1
0
1
MR effect size for
'Gut microbiota abundance (genus Catenibacterium id.2153)' on 'Heparan sulfate glucosamine 3−O−sulfotransferase 4'

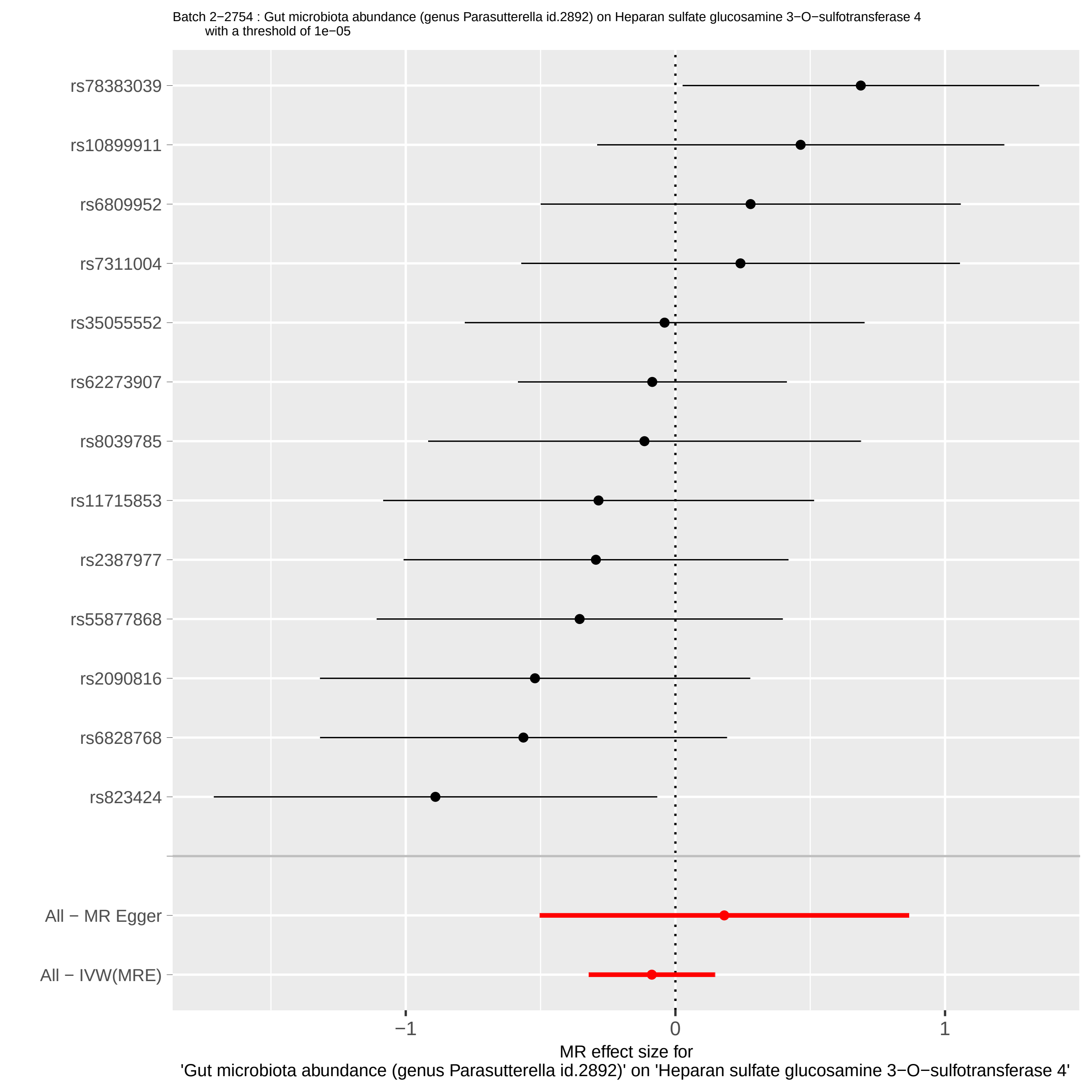
Batch 2−2754 : Gut microbiota abundance (genus Parasutterella id.2892) on Heparan sulfate glucosamine 3−O−sulfotransferase 4
with a threshold of 1e−05
rs78383039
rs10899911
rs6809952
rs7311004
rs35055552
rs62273907
rs8039785
rs11715853
rs2387977
rs55877868
rs2090816
rs6828768
rs823424
All − MR Egger
All − IVW(MRE)
−1
0
1
MR effect size for
'Gut microbiota abundance (genus Parasutterella id.2892)' on 'Heparan sulfate glucosamine 3−O−sulfotransferase 4'

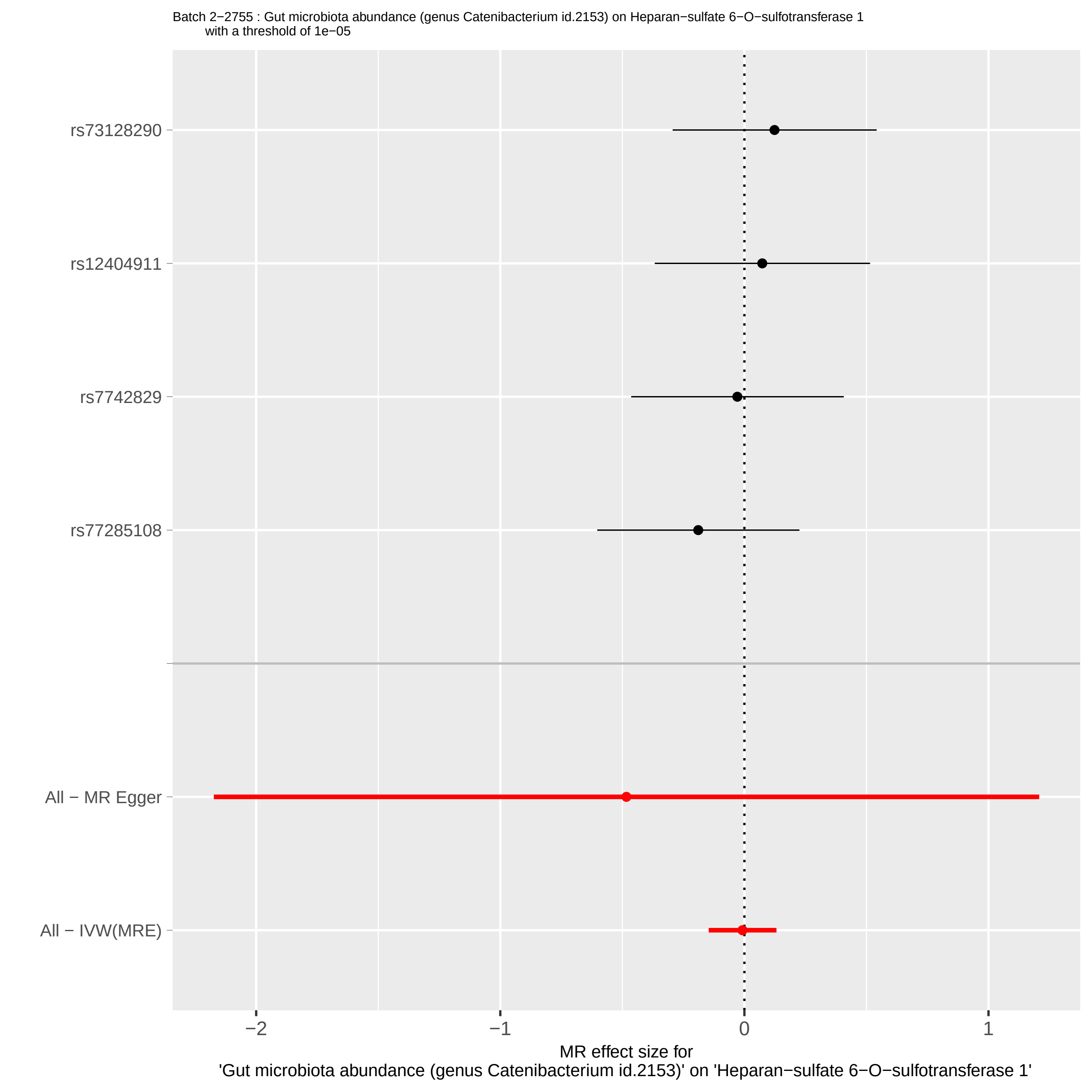
Batch 2−2755 : Gut microbiota abundance (genus Catenibacterium id.2153) on Heparan−sulfate 6−O−sulfotransferase 1
with a threshold of 1e−05
rs73128290
rs12404911
rs7742829
rs77285108
All − MR Egger
All − IVW(MRE)
−2
−1
0
1
MR effect size for
'Gut microbiota abundance (genus Catenibacterium id.2153)' on 'Heparan−sulfate 6−O−sulfotransferase 1'

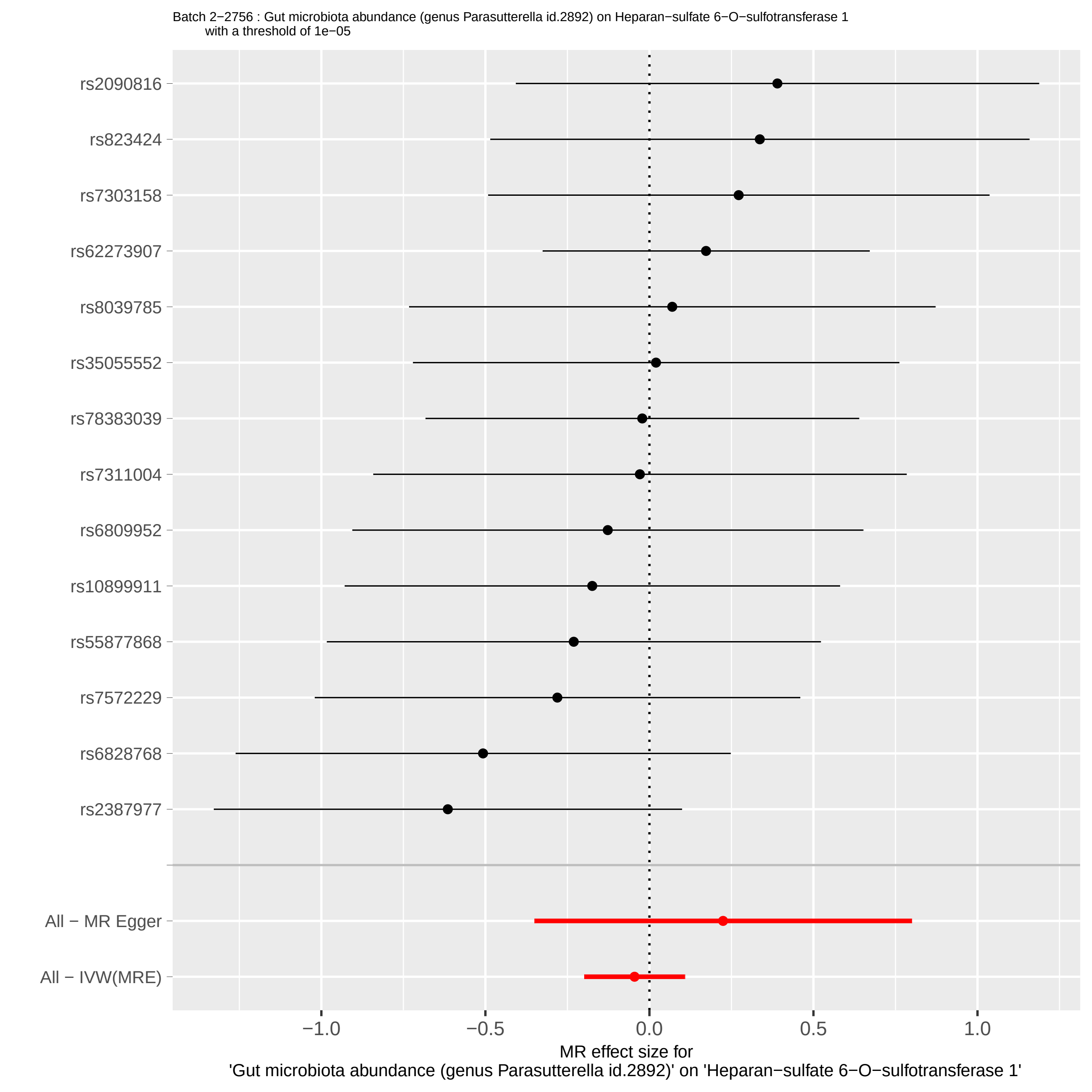

Batch 2−2756 : Gut microbiota abundance (genus Parasutterella id.2892) on Heparan−sulfate 6−O−sulfotransferase 1
with a threshold of 1e−05
rs2090816
rs823424
rs7303158
rs62273907
rs8039785
rs35055552
rs78383039
rs7311004
rs6809952
rs10899911
rs55877868
rs7572229
rs6828768
rs2387977
All − MR Egger
All − IVW(MRE)
−1.0
−0.5
0.0
0.5
1.0
MR effect size for
'Gut microbiota abundance (genus Parasutterella id.2892)' on 'Heparan−sulfate 6−O−sulfotransferase 1'

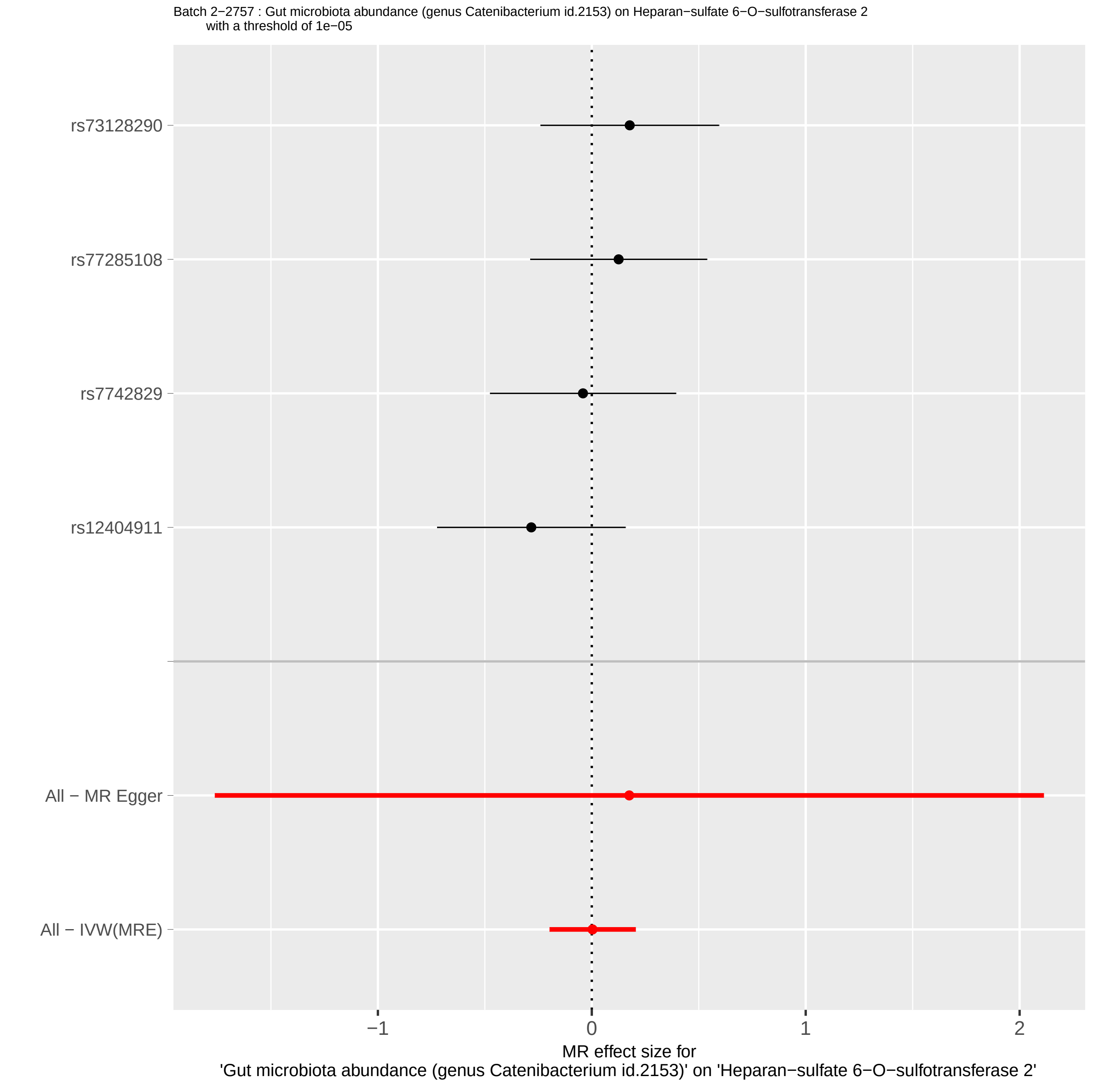

Batch 2−2757 : Gut microbiota abundance (genus Catenibacterium id.2153) on Heparan−sulfate 6−O−sulfotransferase 2
with a threshold of 1e−05
rs73128290
rs77285108
rs7742829
rs12404911
All − MR Egger
All − IVW(MRE)
−1
0
1
2
MR effect size for
'Gut microbiota abundance (genus Catenibacterium id.2153)' on 'Heparan−sulfate 6−O−sulfotransferase 2'

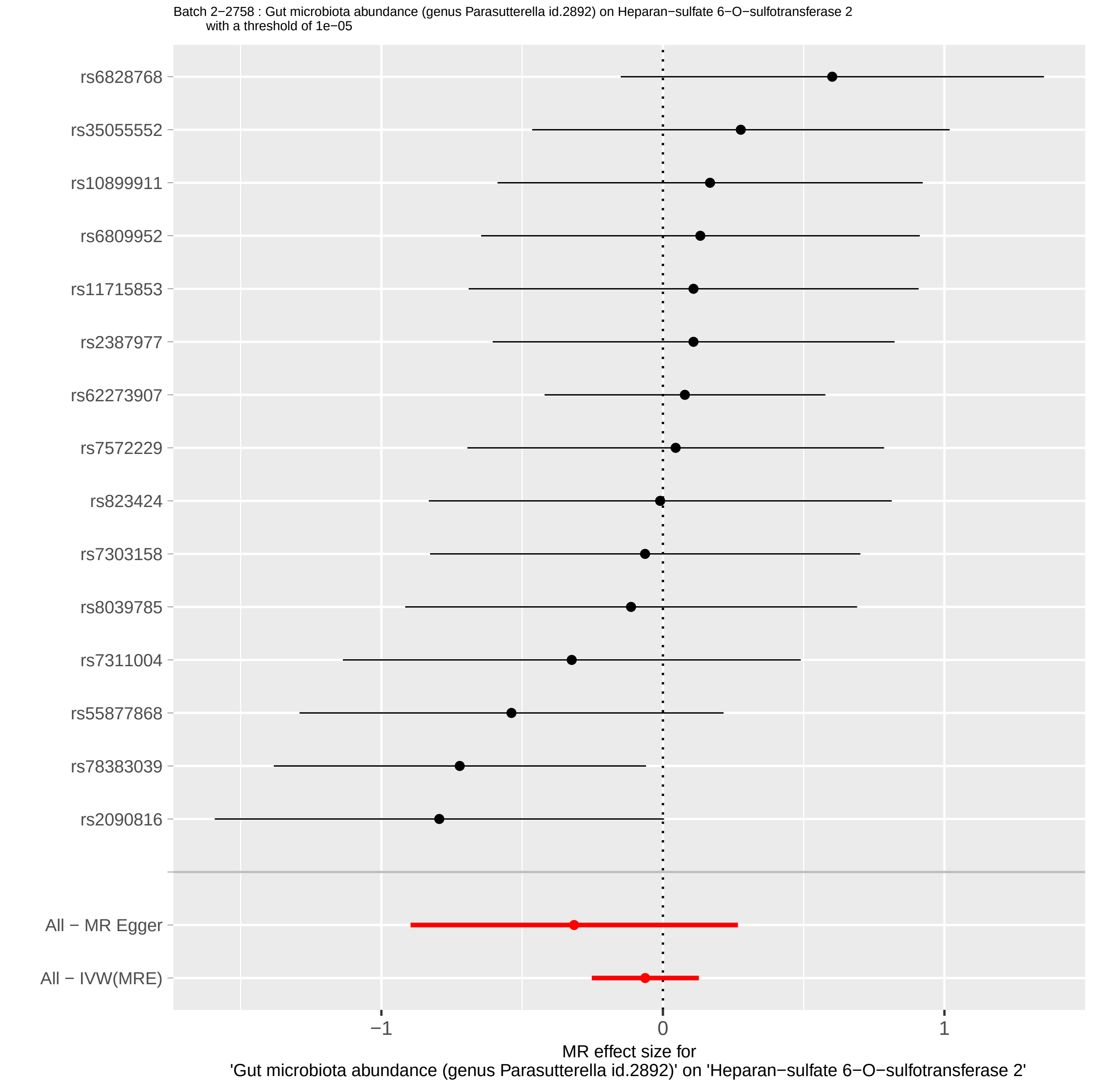

Batch 2−2758 : Gut microbiota abundance (genus Parasutterella id.2892) on Heparan−sulfate 6−O−sulfotransferase 2
with a threshold of 1e−05
rs6828768
rs35055552
rs10899911
rs6809952
rs11715853
rs2387977
rs62273907
rs7572229
rs823424
rs7303158
rs8039785
rs7311004
rs55877868
rs78383039
rs2090816
All − MR Egger
All − IVW(MRE)
−1
0
1
MR effect size for
'Gut microbiota abundance (genus Parasutterella id.2892)' on 'Heparan−sulfate 6−O−sulfotransferase 2'

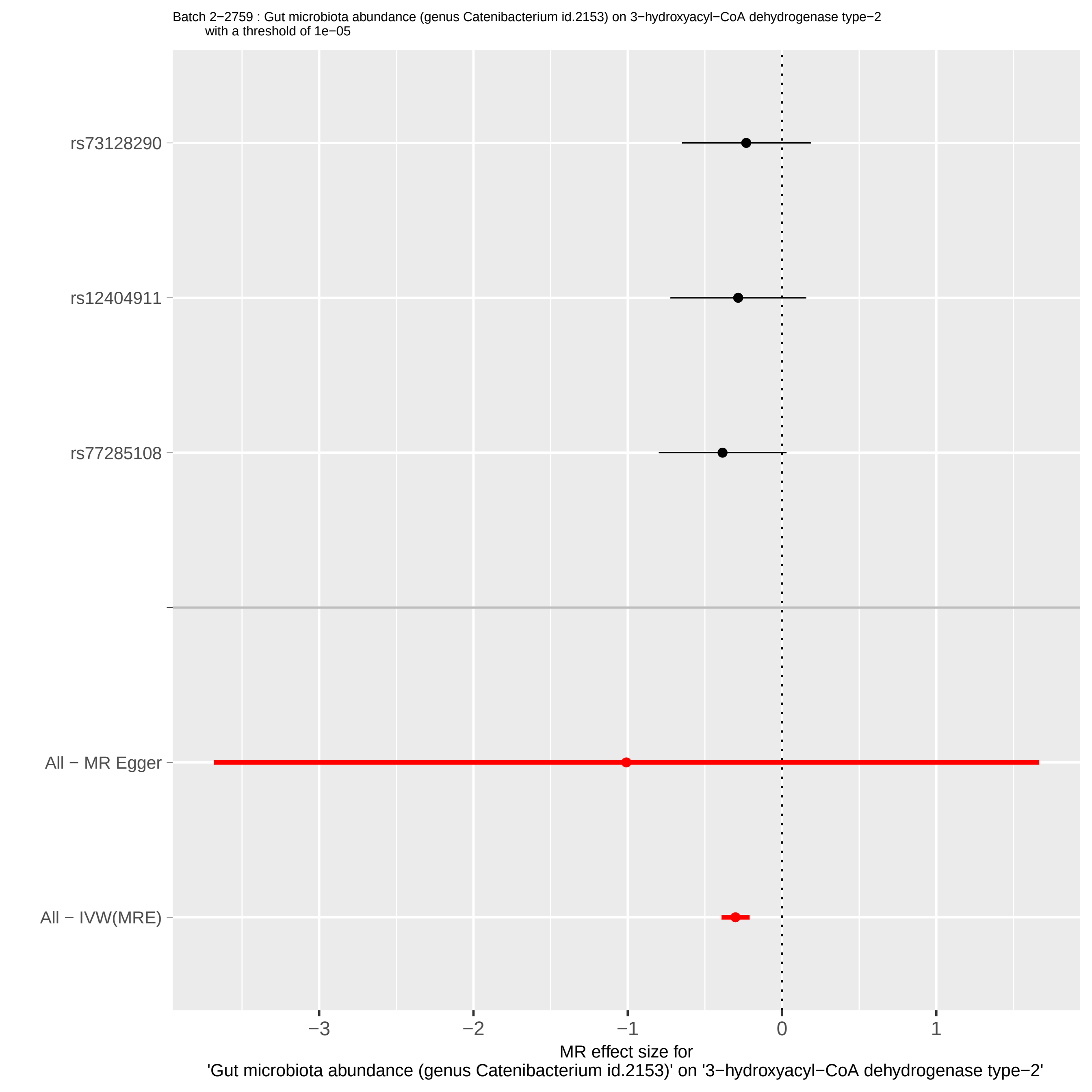

Batch 2−2759 : Gut microbiota abundance (genus Catenibacterium id.2153) on 3−hydroxyacyl−CoA dehydrogenase type−2
with a threshold of 1e−05
rs73128290
rs12404911
rs77285108
All − MR Egger
All − IVW(MRE)
−3
−2
−1
0
1
MR effect size for
'Gut microbiota abundance (genus Catenibacterium id.2153)' on '3−hydroxyacyl−CoA dehydrogenase type−2'

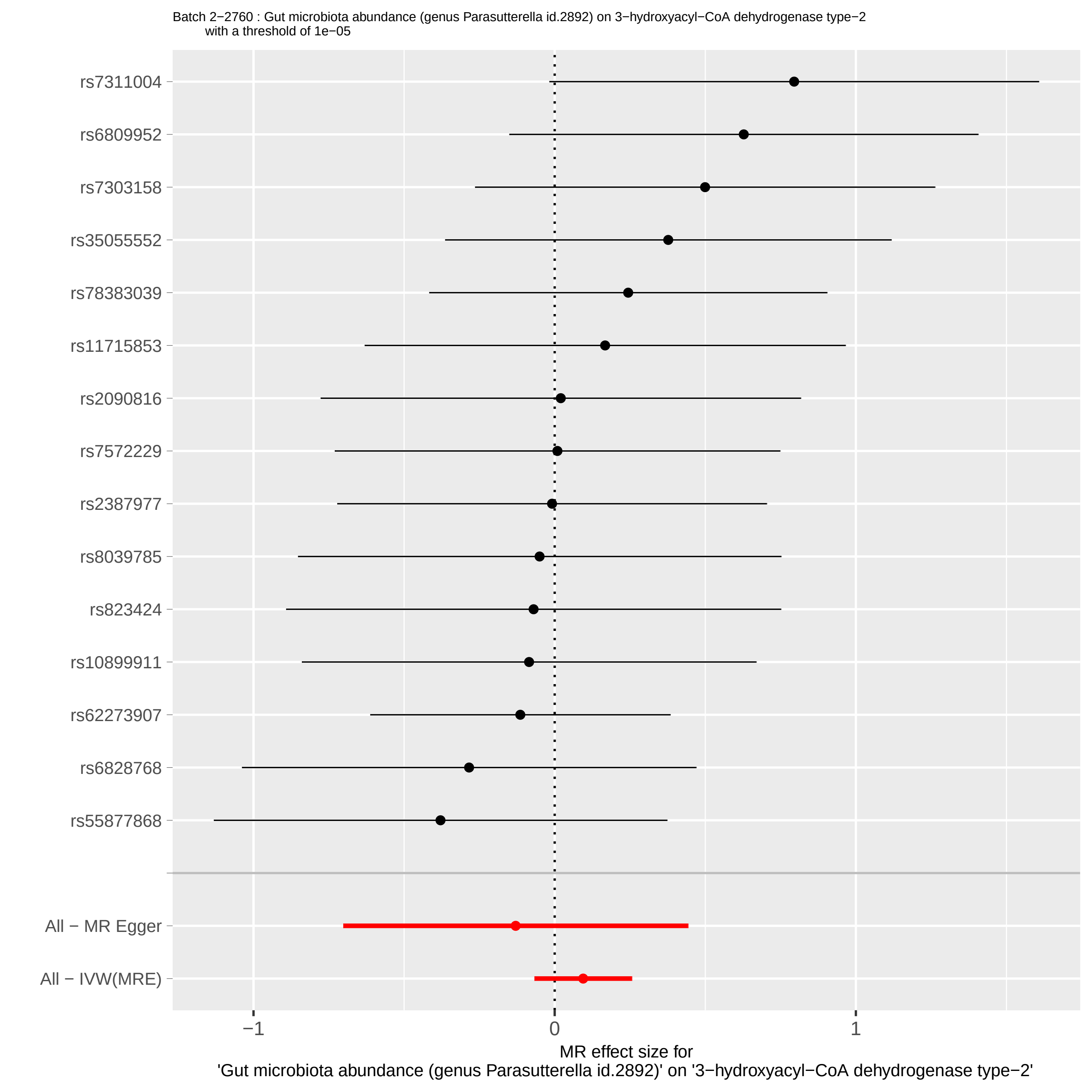
Batch 2−2760 : Gut microbiota abundance (genus Parasutterella id.2892) on 3−hydroxyacyl−CoA dehydrogenase type−2
with a threshold of 1e−05
rs7311004
rs6809952
rs7303158
rs35055552
rs78383039
rs11715853
rs2090816
rs7572229
rs2387977
rs8039785
rs823424
rs10899911
rs62273907
rs6828768
rs55877868
All − MR Egger
All − IVW(MRE)
−1
0
1
MR effect size for
'Gut microbiota abundance (genus Parasutterella id.2892)' on '3−hydroxyacyl−CoA dehydrogenase type−2'

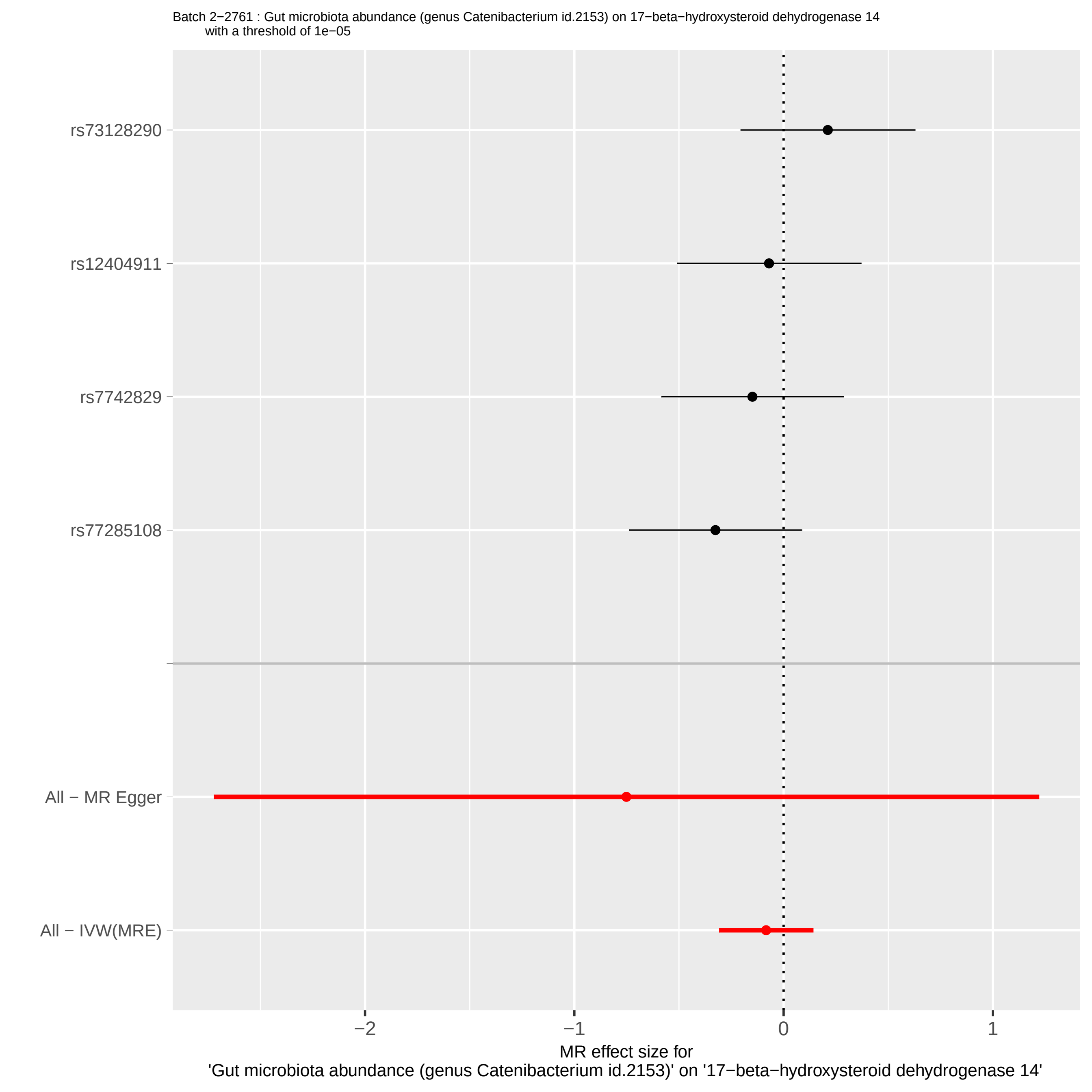

Batch 2−2761 : Gut microbiota abundance (genus Catenibacterium id.2153) on 17−beta−hydroxysteroid dehydrogenase 14
with a threshold of 1e−05
rs73128290
rs12404911
rs7742829
rs77285108
All − MR Egger
All − IVW(MRE)
−2
−1
0
1
MR effect size for
'Gut microbiota abundance (genus Catenibacterium id.2153)' on '17−beta−hydroxysteroid dehydrogenase 14'

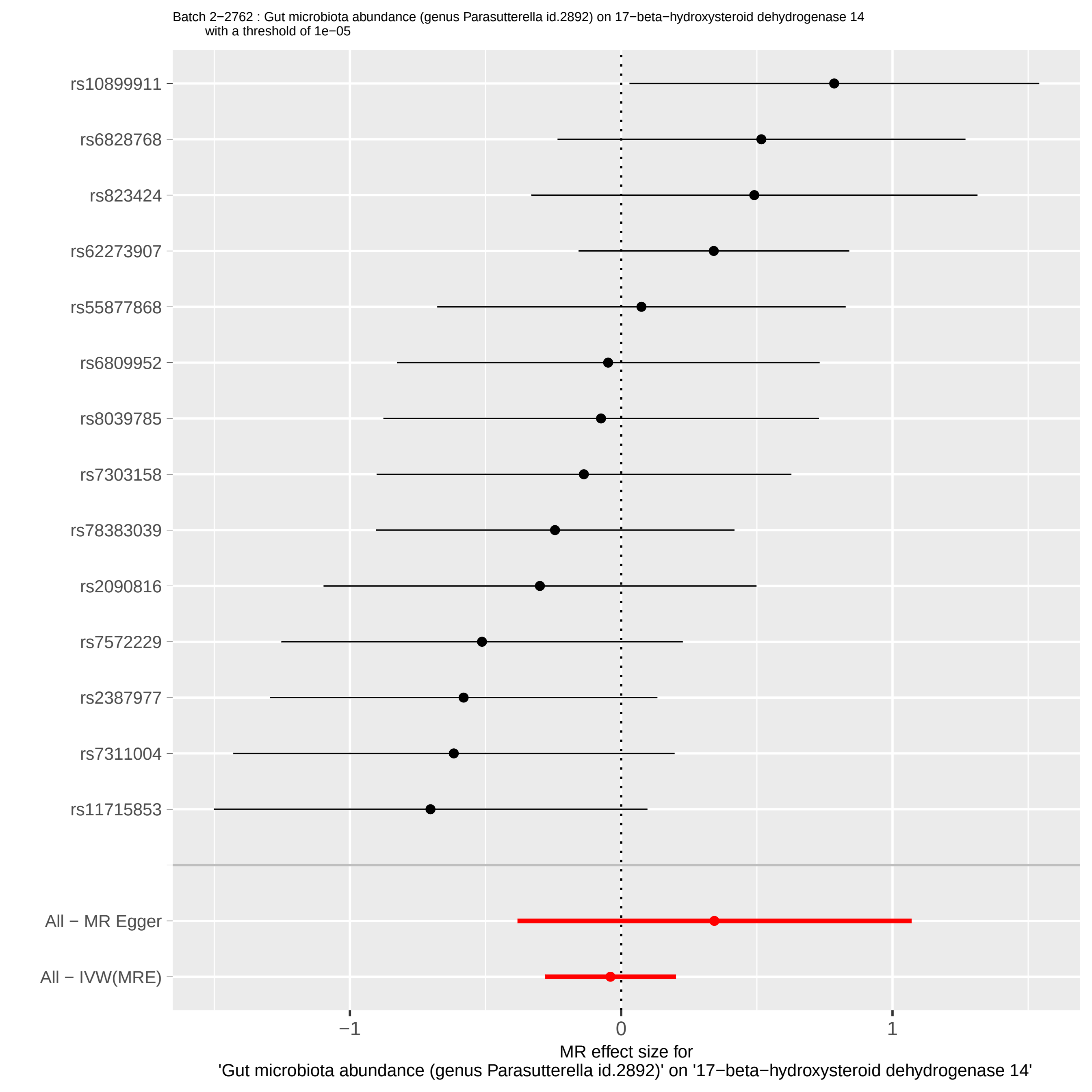

Batch 2−2762 : Gut microbiota abundance (genus Parasutterella id.2892) on 17−beta−hydroxysteroid dehydrogenase 14
with a threshold of 1e−05
rs10899911
rs6828768
rs823424
rs62273907
rs55877868
rs6809952
rs8039785
rs7303158
rs78383039
rs2090816
rs7572229
rs2387977
rs7311004
rs11715853
All − MR Egger
All − IVW(MRE)
−1
0
1
MR effect size for
'Gut microbiota abundance (genus Parasutterella id.2892)' on '17−beta−hydroxysteroid dehydrogenase 14'

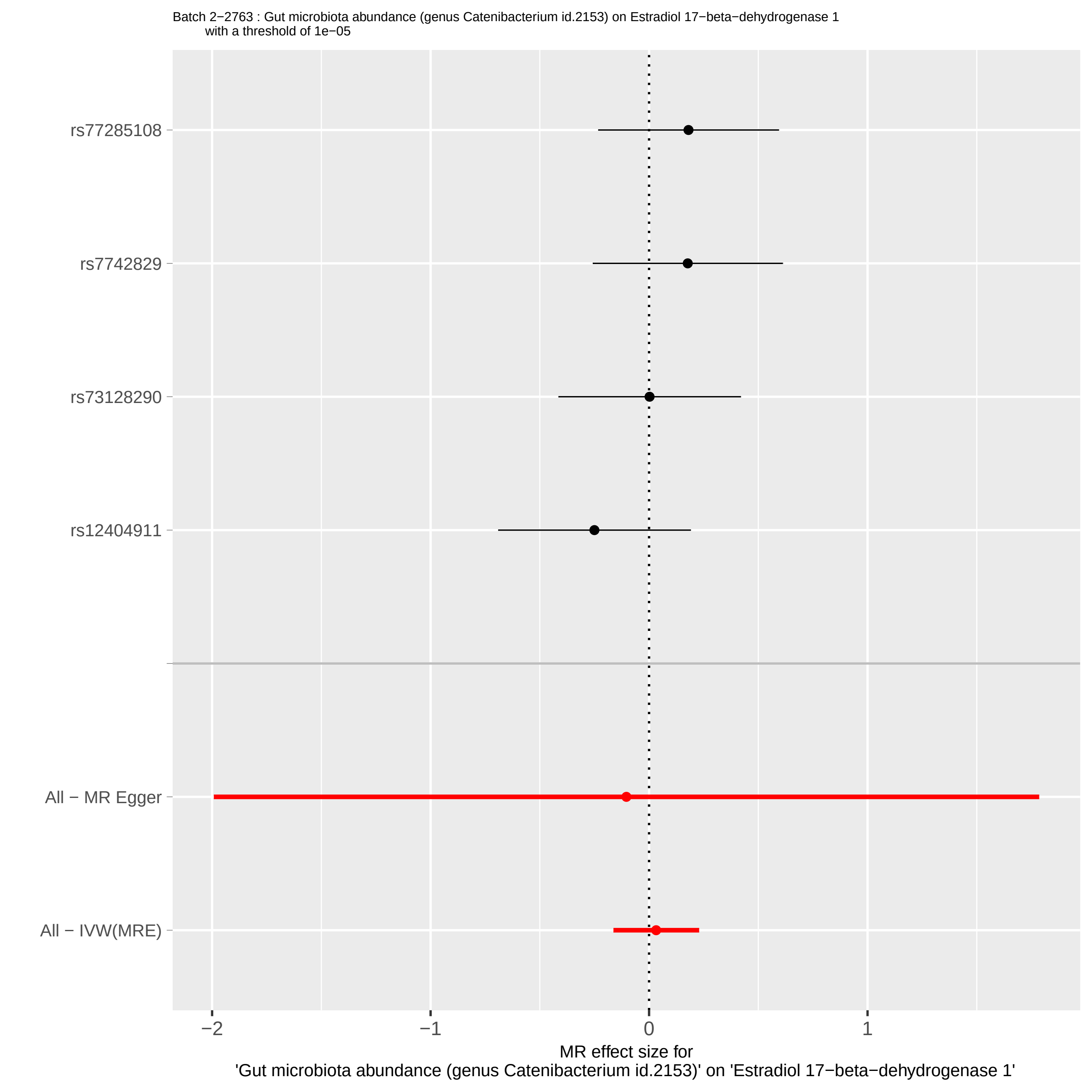

Batch 2−2763 : Gut microbiota abundance (genus Catenibacterium id.2153) on Estradiol 17−beta−dehydrogenase 1
with a threshold of 1e−05
rs77285108
rs7742829
rs73128290
rs12404911
All − MR Egger
All − IVW(MRE)
−2
−1
0
1
MR effect size for
'Gut microbiota abundance (genus Catenibacterium id.2153)' on 'Estradiol 17−beta−dehydrogenase 1'

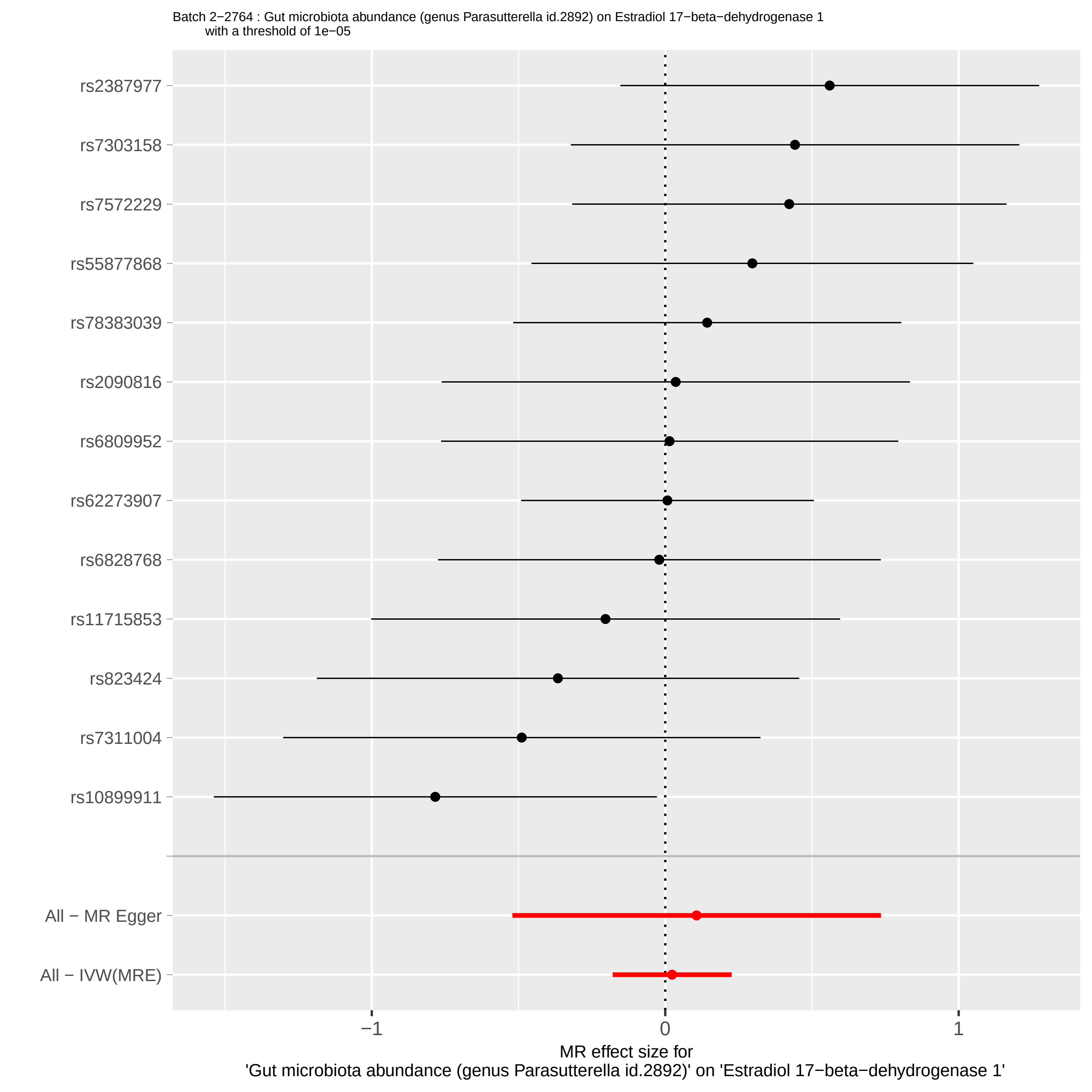

Batch 2−2764 : Gut microbiota abundance (genus Parasutterella id.2892) on Estradiol 17−beta−dehydrogenase 1
with a threshold of 1e−05
rs2387977
rs7303158
rs7572229
rs55877868
rs78383039
rs2090816
rs6809952
rs62273907
rs6828768
rs11715853
rs823424
rs7311004
rs10899911
All − MR Egger
All − IVW(MRE)
−1
0
1
MR effect size for
'Gut microbiota abundance (genus Parasutterella id.2892)' on 'Estradiol 17−beta−dehydrogenase 1'

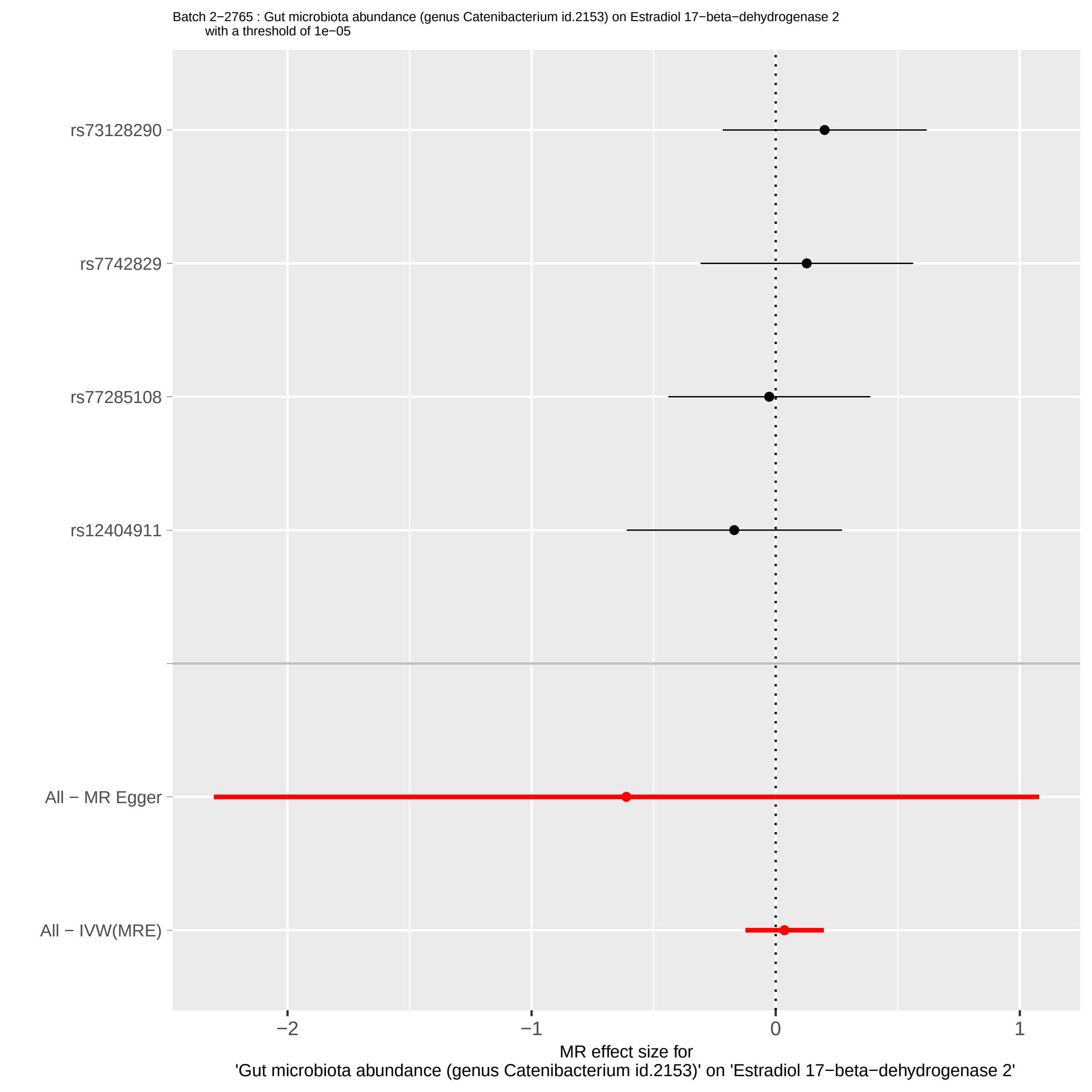
Batch 2−2765 : Gut microbiota abundance (genus Catenibacterium id.2153) on Estradiol 17−beta−dehydrogenase 2
with a threshold of 1e−05
rs73128290
rs7742829
rs77285108
rs12404911
All − MR Egger
All − IVW(MRE)
−2
−1
0
1
MR effect size for
'Gut microbiota abundance (genus Catenibacterium id.2153)' on 'Estradiol 17−beta−dehydrogenase 2'

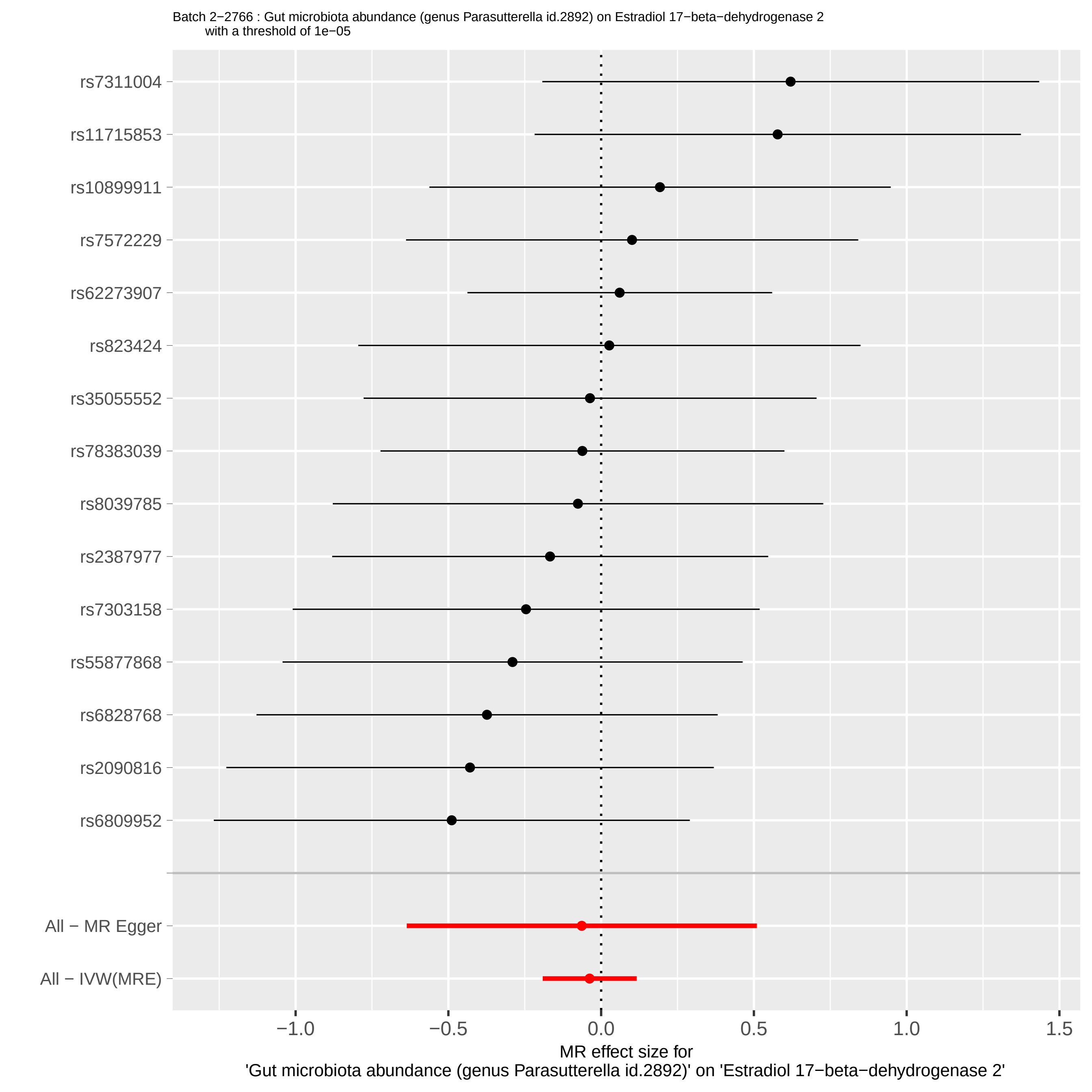
Batch 2−2766 : Gut microbiota abundance (genus Parasutterella id.2892) on Estradiol 17−beta−dehydrogenase 2
with a threshold of 1e−05
rs7311004
rs11715853
rs10899911
rs7572229
rs62273907
rs823424
rs35055552
rs78383039
rs8039785
rs2387977
rs7303158
rs55877868
rs6828768
rs2090816
rs6809952
All − MR Egger
All − IVW(MRE)
−1.0
−0.5
0.0
0.5
1.0
1.5
MR effect size for
'Gut microbiota abundance (genus Parasutterella id.2892)' on 'Estradiol 17−beta−dehydrogenase 2'

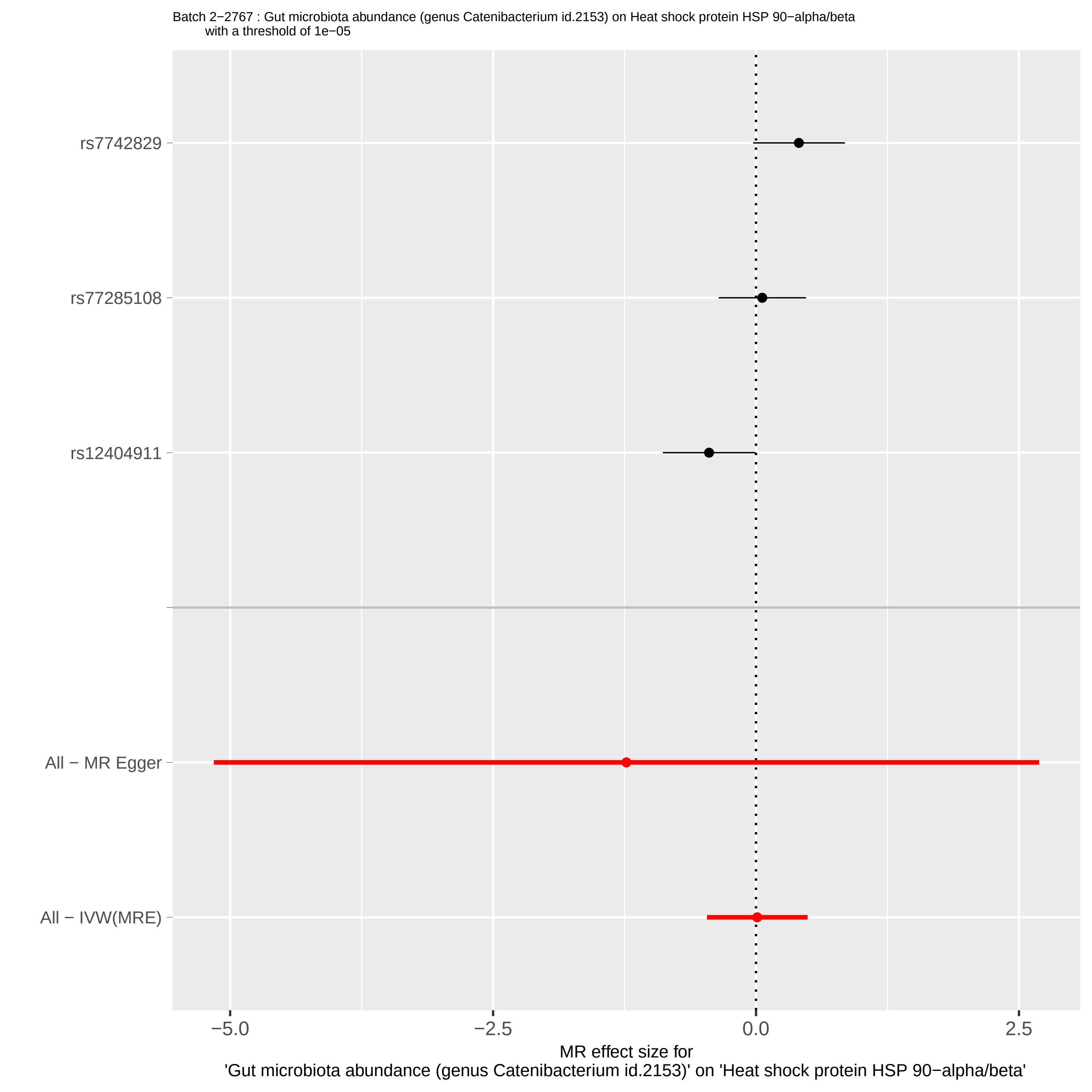

Batch 2−2767 : Gut microbiota abundance (genus Catenibacterium id.2153) on Heat shock protein HSP 90−alpha/beta
with a threshold of 1e−05
rs7742829
rs77285108
rs12404911
All − MR Egger
All − IVW(MRE)
−5.0
−2.5
0.0
2.5
MR effect size for
'Gut microbiota abundance (genus Catenibacterium id.2153)' on 'Heat shock protein HSP 90−alpha/beta'

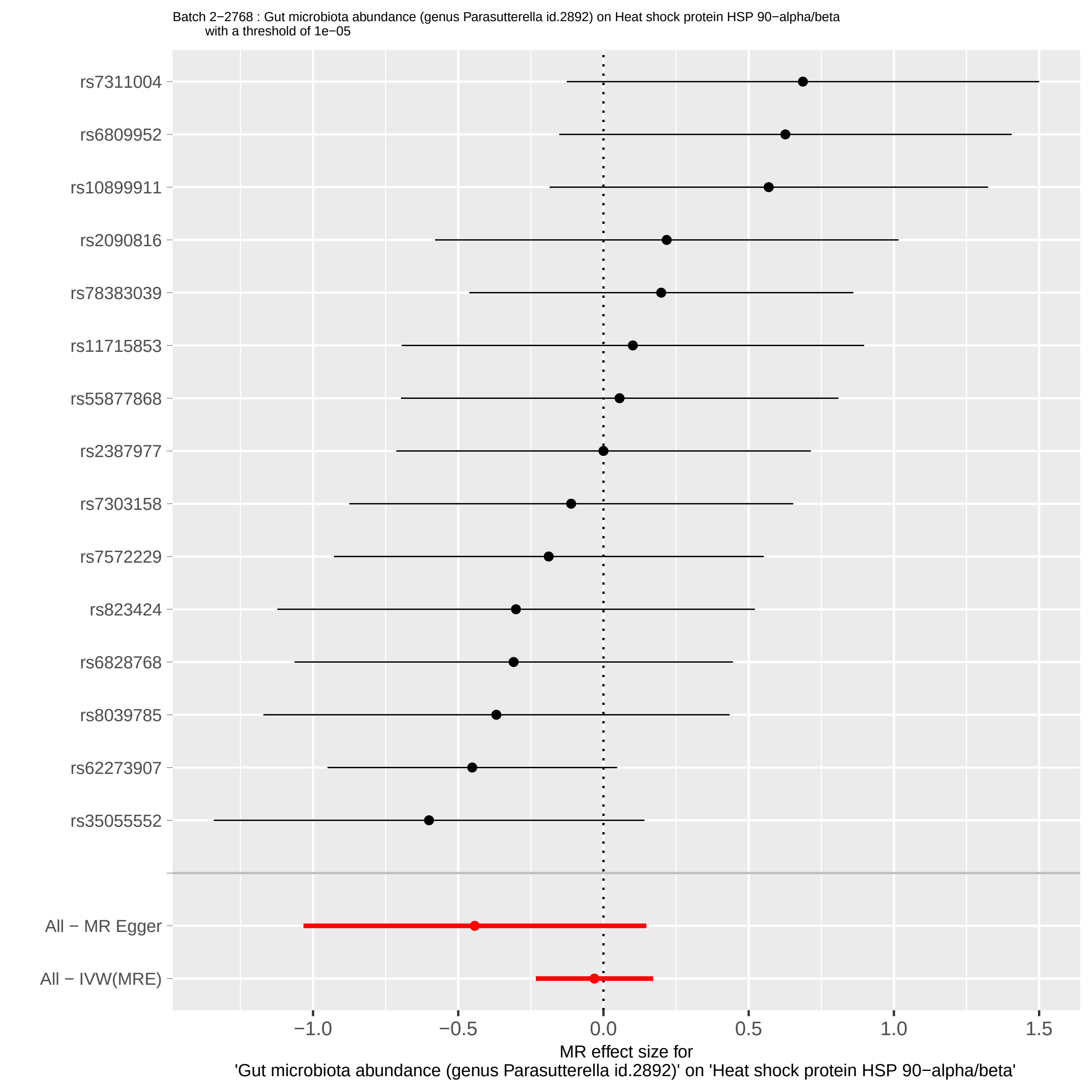
Batch 2−2768 : Gut microbiota abundance (genus Parasutterella id.2892) on Heat shock protein HSP 90−alpha/beta
with a threshold of 1e−05
rs7311004
rs6809952
rs10899911
rs2090816
rs78383039
rs11715853
rs55877868
rs2387977
rs7303158
rs7572229
rs823424
rs6828768
rs8039785
rs62273907
rs35055552
All − MR Egger
All − IVW(MRE)
−1.0
−0.5
0.0
0.5
1.0
1.5
MR effect size for
'Gut microbiota abundance (genus Parasutterella id.2892)' on 'Heat shock protein HSP 90−alpha/beta'

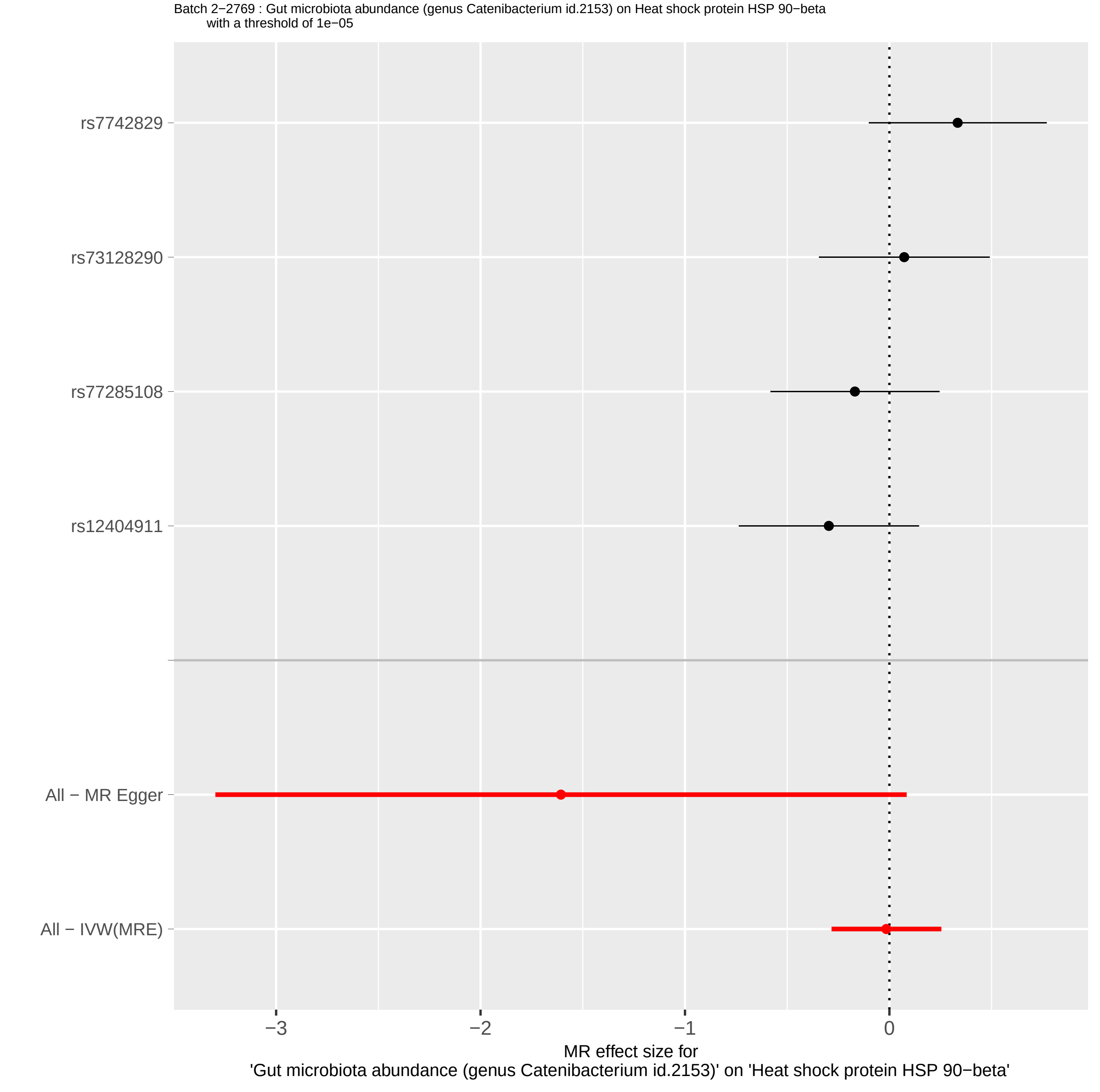
Batch 2−2769 : Gut microbiota abundance (genus Catenibacterium id.2153) on Heat shock protein HSP 90−beta
with a threshold of 1e−05
rs7742829
rs73128290
rs77285108
rs12404911
All − MR Egger
All − IVW(MRE)
−3
−2
−1
0
MR effect size for
'Gut microbiota abundance (genus Catenibacterium id.2153)' on 'Heat shock protein HSP 90−beta'

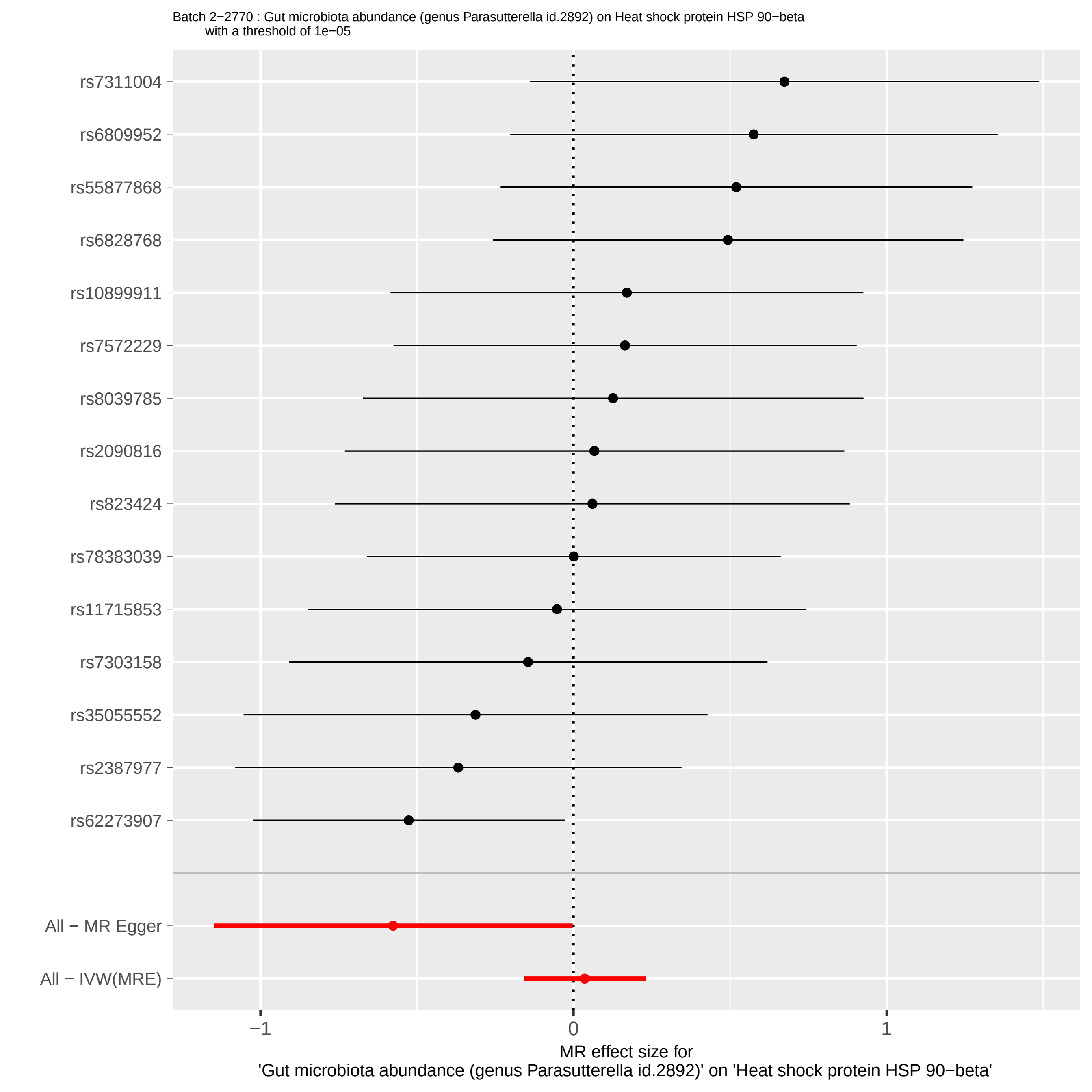

Batch 2−2770 : Gut microbiota abundance (genus Parasutterella id.2892) on Heat shock protein HSP 90−beta
with a threshold of 1e−05
rs7311004
rs6809952
rs55877868
rs6828768
rs10899911
rs7572229
rs8039785
rs2090816
rs823424
rs78383039
rs11715853
rs7303158
rs35055552
rs2387977
rs62273907
All − MR Egger
All − IVW(MRE)
−1
0
1
MR effect size for
'Gut microbiota abundance (genus Parasutterella id.2892)' on 'Heat shock protein HSP 90−beta'

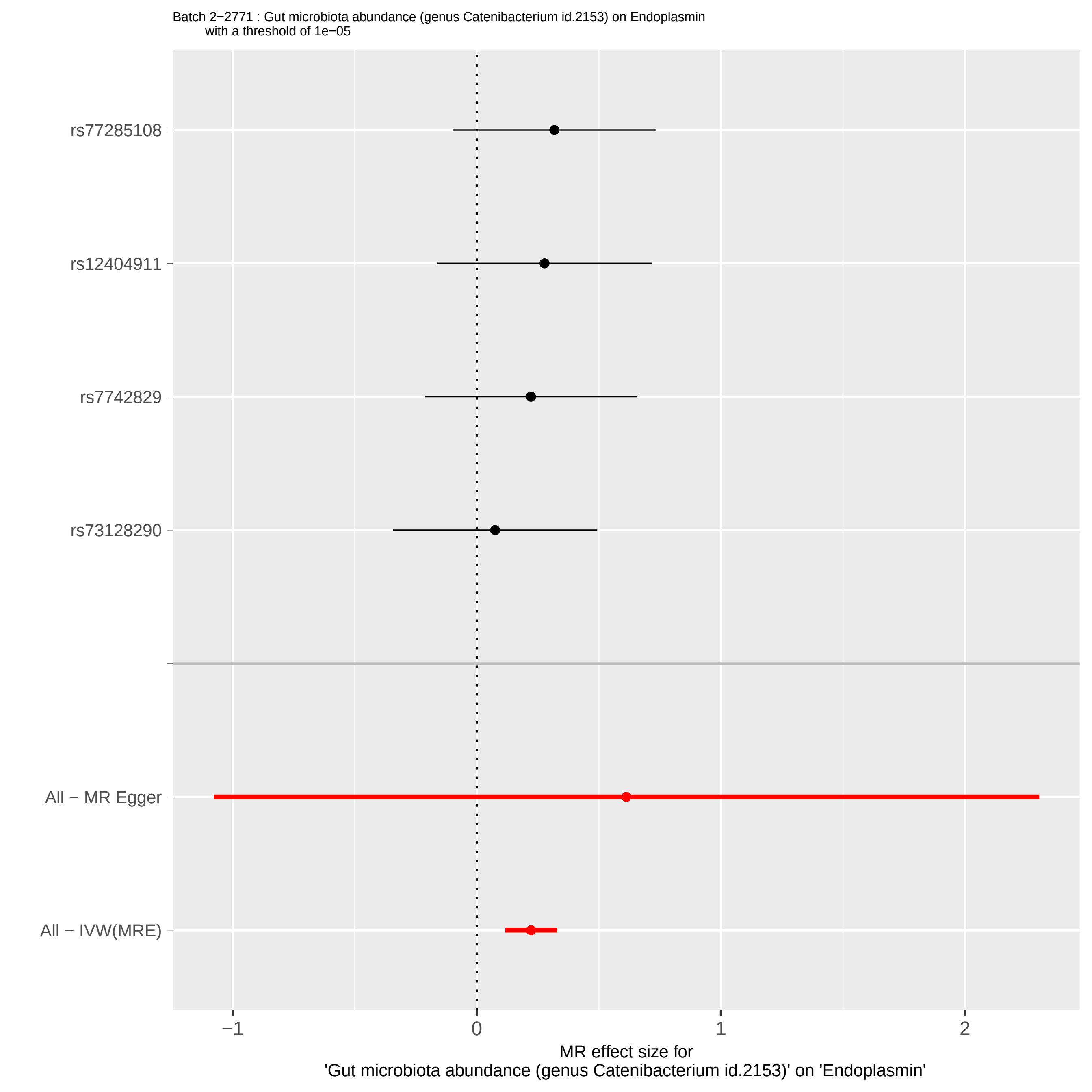

Batch 2−2771 : Gut microbiota abundance (genus Catenibacterium id.2153) on Endoplasmin
with a threshold of 1e−05
rs77285108
rs12404911
rs7742829
rs73128290
All − MR Egger
All − IVW(MRE)
−1
0
1
2
MR effect size for
'Gut microbiota abundance (genus Catenibacterium id.2153)' on 'Endoplasmin'

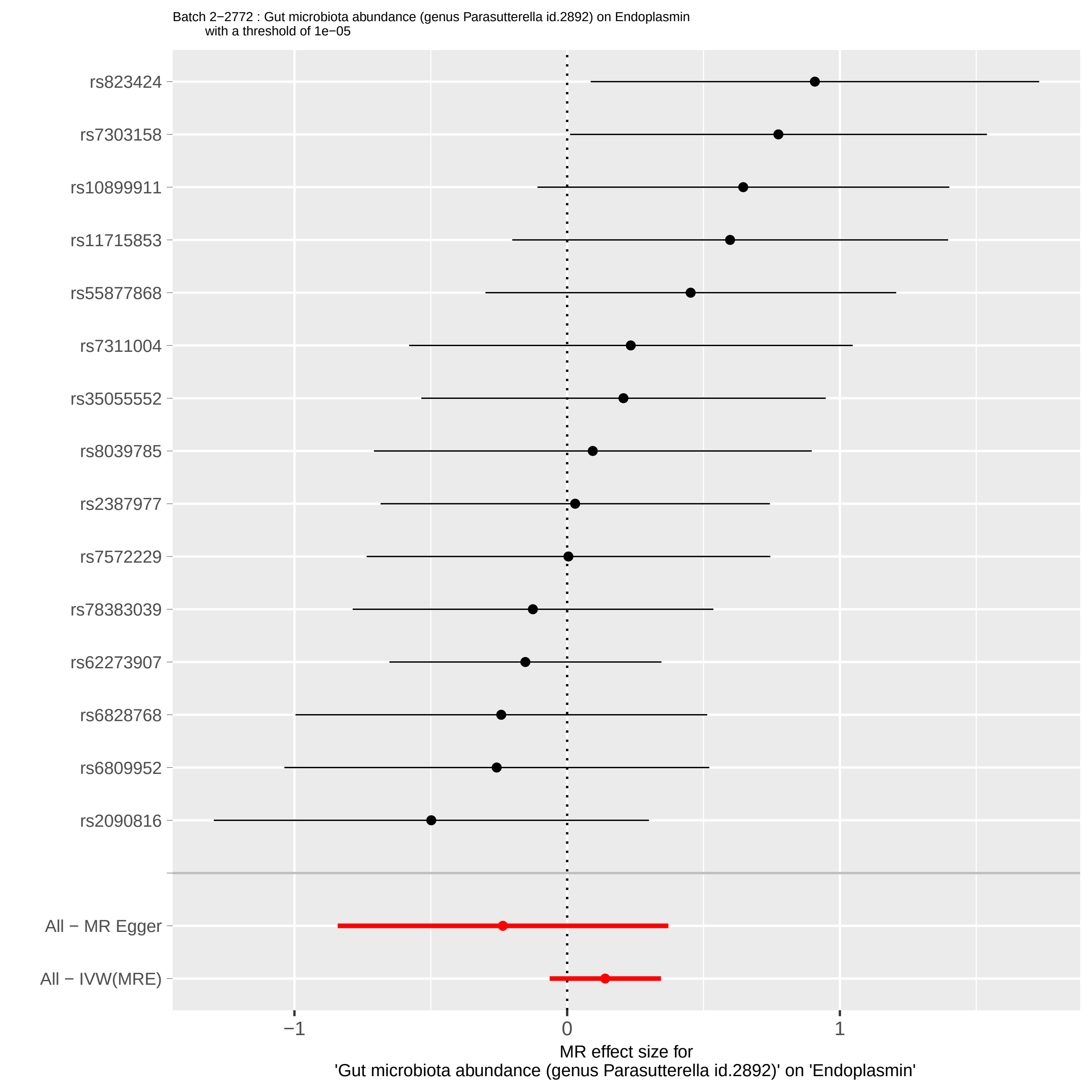
Batch 2−2772 : Gut microbiota abundance (genus Parasutterella id.2892) on Endoplasmin
with a threshold of 1e−05
rs823424
rs7303158
rs10899911
rs11715853
rs55877868
rs7311004
rs35055552
rs8039785
rs2387977
rs7572229
rs78383039
rs62273907
rs6828768
rs6809952
rs2090816
All − MR Egger
All − IVW(MRE)
−1
0
1
MR effect size for
'Gut microbiota abundance (genus Parasutterella id.2892)' on 'Endoplasmin'

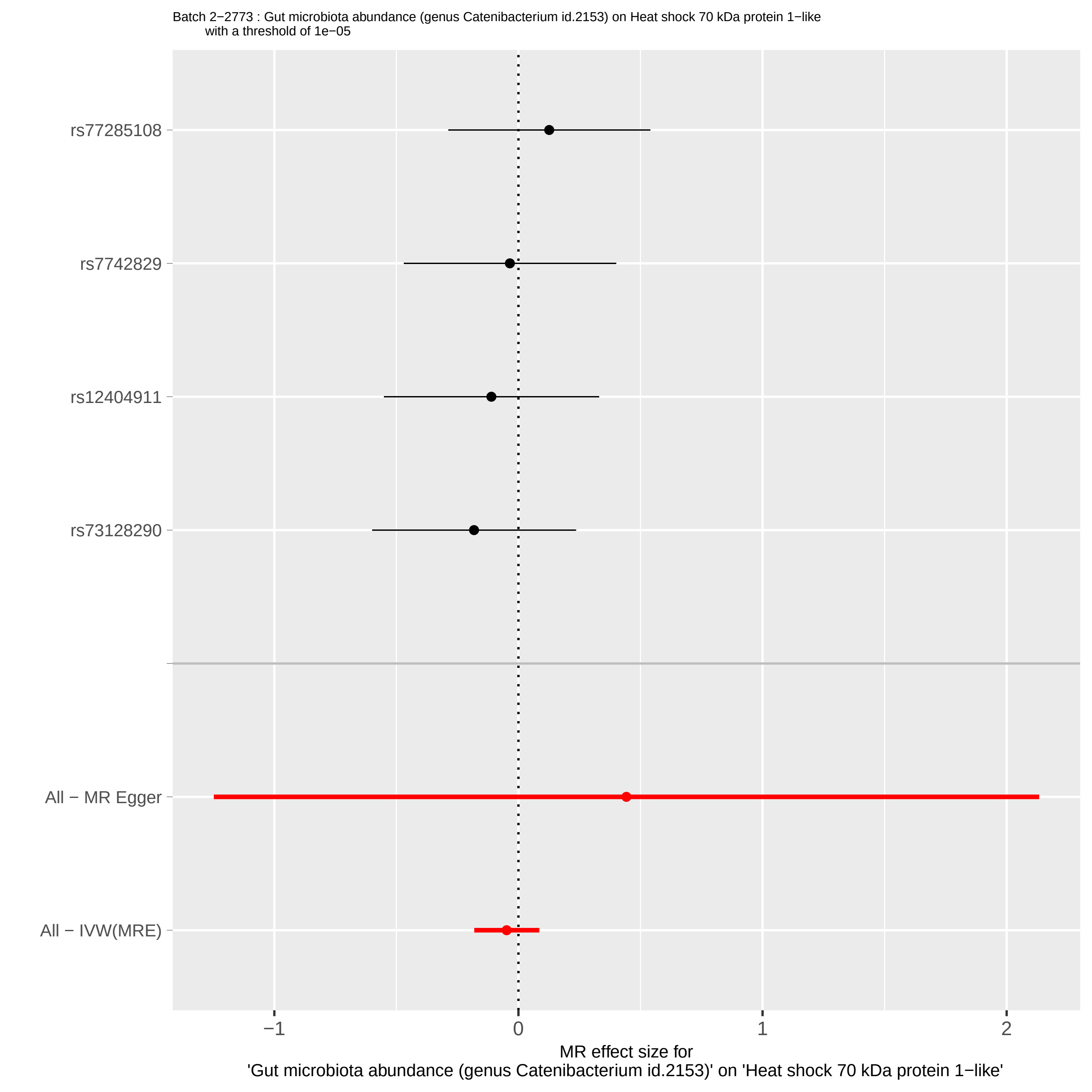

Batch 2−2773 : Gut microbiota abundance (genus Catenibacterium id.2153) on Heat shock 70 kDa protein 1−like
with a threshold of 1e−05
rs77285108
rs7742829
rs12404911
rs73128290
All − MR Egger
All − IVW(MRE)
−1
0
1
2
MR effect size for
'Gut microbiota abundance (genus Catenibacterium id.2153)' on 'Heat shock 70 kDa protein 1−like'

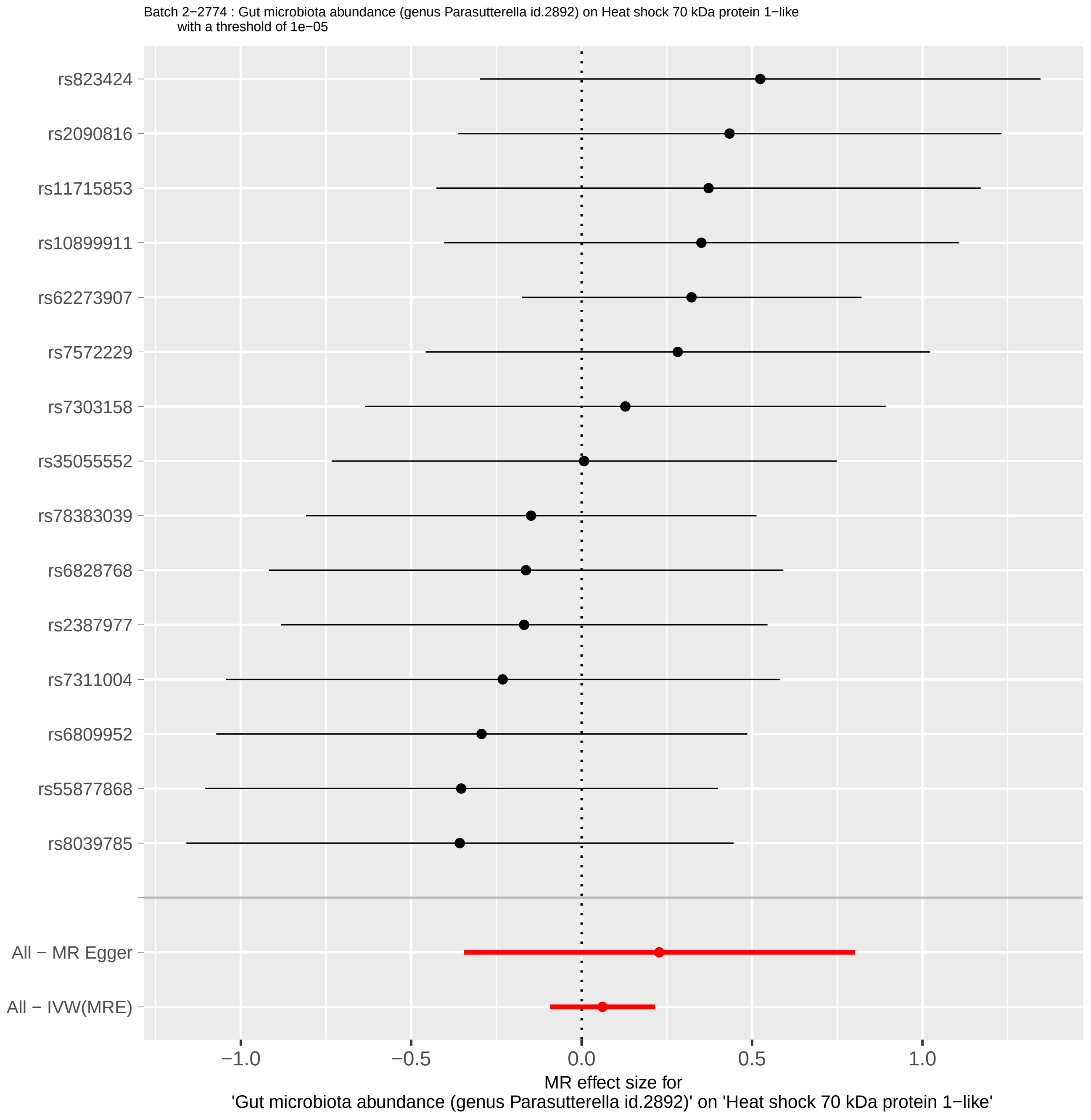

Batch 2−2774 : Gut microbiota abundance (genus Parasutterella id.2892) on Heat shock 70 kDa protein 1−like
with a threshold of 1e−05
rs823424
rs2090816
rs11715853
rs10899911
rs62273907
rs7572229
rs7303158
rs35055552
rs78383039
rs6828768
rs2387977
rs7311004
rs6809952
rs55877868
rs8039785
All − MR Egger
All − IVW(MRE)
−1.0
−0.5
0.0
0.5
1.0
MR effect size for
'Gut microbiota abundance (genus Parasutterella id.2892)' on 'Heat shock 70 kDa protein 1−like'

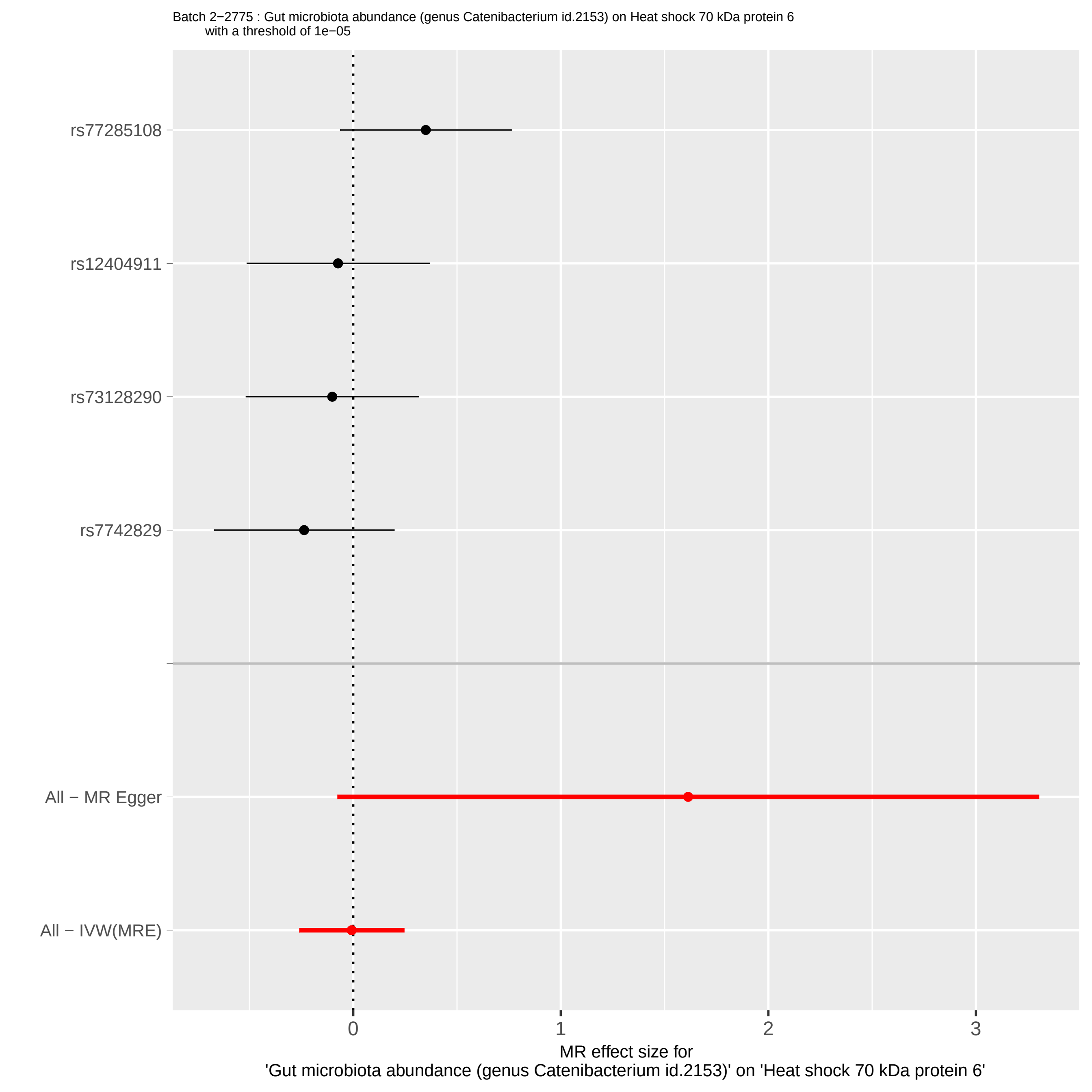

Batch 2−2775 : Gut microbiota abundance (genus Catenibacterium id.2153) on Heat shock 70 kDa protein 6
with a threshold of 1e−05
rs77285108
rs12404911
rs73128290
rs7742829
All − MR Egger
All − IVW(MRE)
0
1
2
3
MR effect size for
'Gut microbiota abundance (genus Catenibacterium id.2153)' on 'Heat shock 70 kDa protein 6'

Batch 2−2776 : Gut microbiota abundance (genus Parasutterella id.2892) on Heat shock 70 kDa protein 6
with a threshold of 1e−05

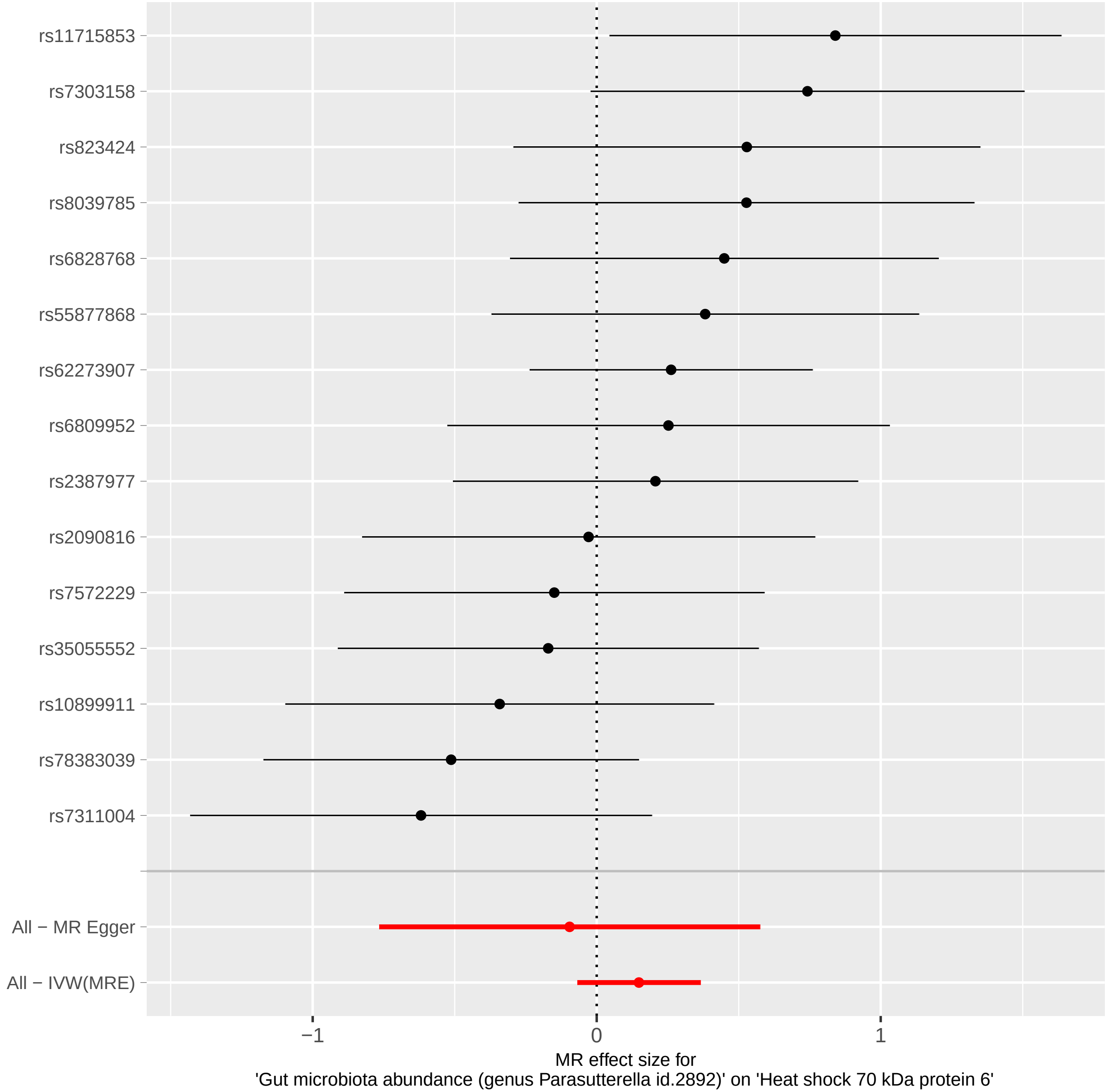

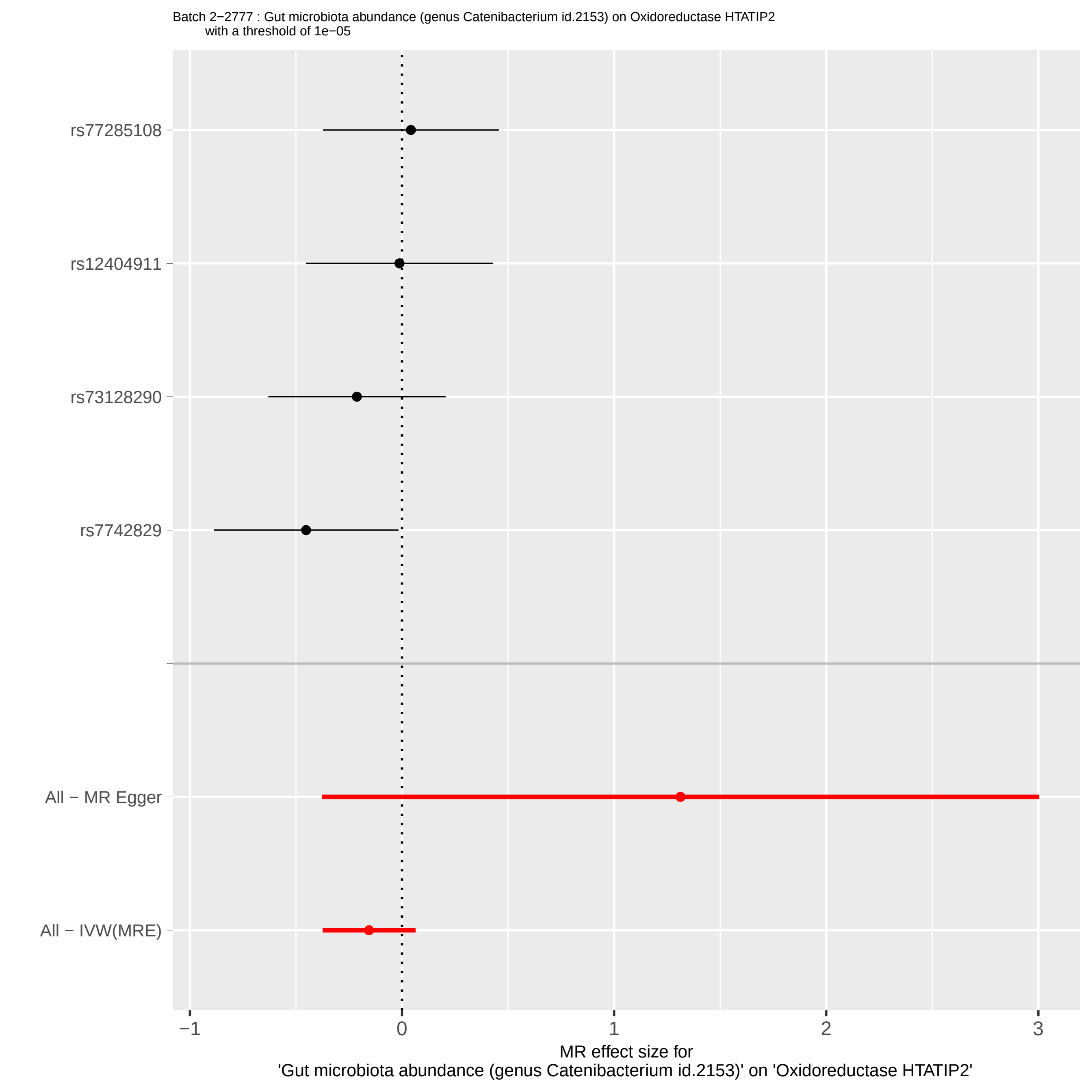

Batch 2−2777 : Gut microbiota abundance (genus Catenibacterium id.2153) on Oxidoreductase HTATIP2
with a threshold of 1e−05
rs77285108
rs12404911
rs73128290
rs7742829
All − MR Egger
All − IVW(MRE)
−1
0
1
2
3
MR effect size for
'Gut microbiota abundance (genus Catenibacterium id.2153)' on 'Oxidoreductase HTATIP2'

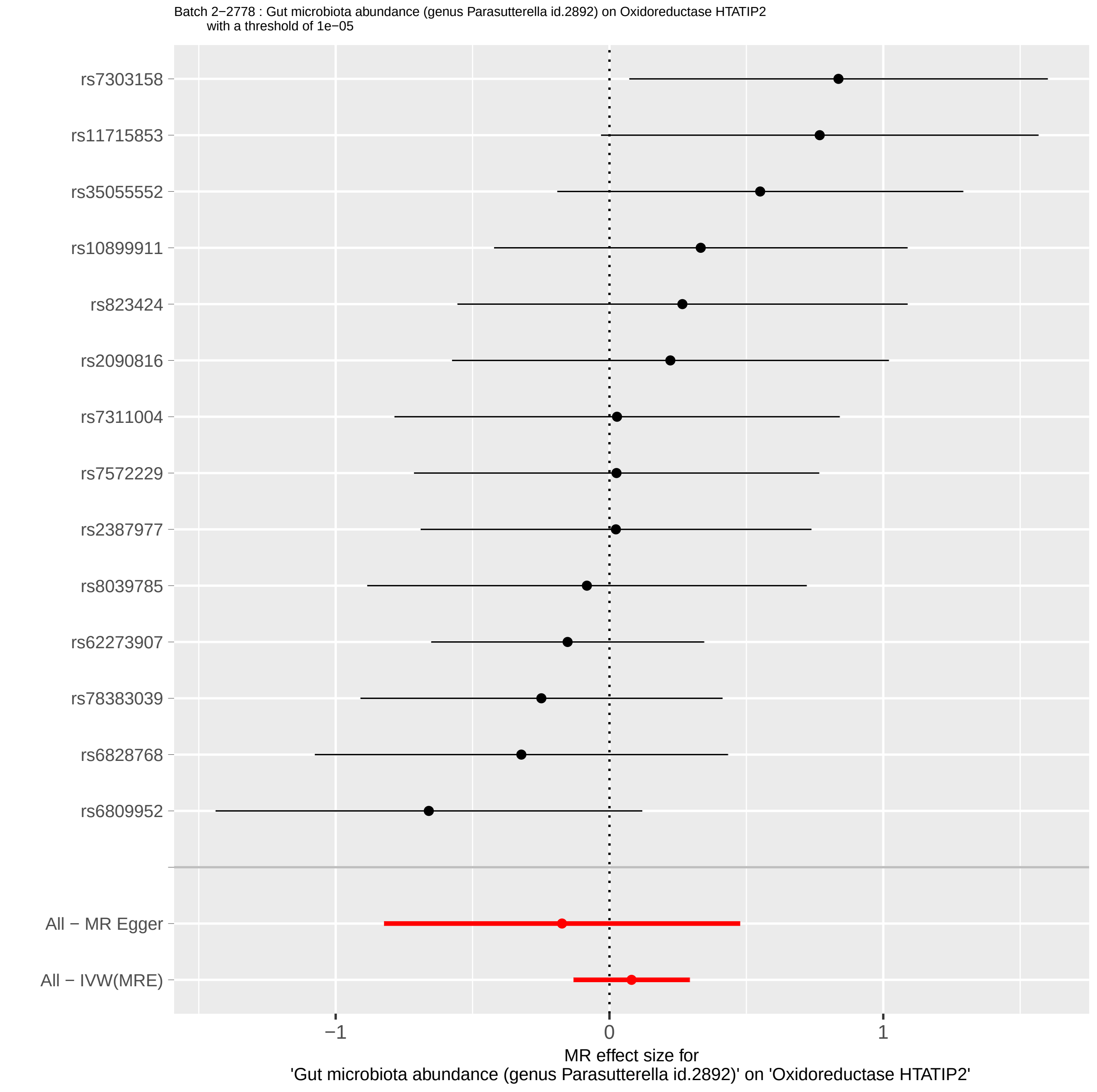

Batch 2−2778 : Gut microbiota abundance (genus Parasutterella id.2892) on Oxidoreductase HTATIP2
with a threshold of 1e−05
rs7303158
rs11715853
rs35055552
rs10899911
rs823424
rs2090816
rs7311004
rs7572229
rs2387977
rs8039785
rs62273907
rs78383039
rs6828768
rs6809952
All − MR Egger
All − IVW(MRE)
−1
0
1
MR effect size for
'Gut microbiota abundance (genus Parasutterella id.2892)' on 'Oxidoreductase HTATIP2'

Batch 2−2779 : Gut microbiota abundance (genus Catenibacterium id.2153) on Oxidoreductase HTATIP2
with a threshold of 1e−05

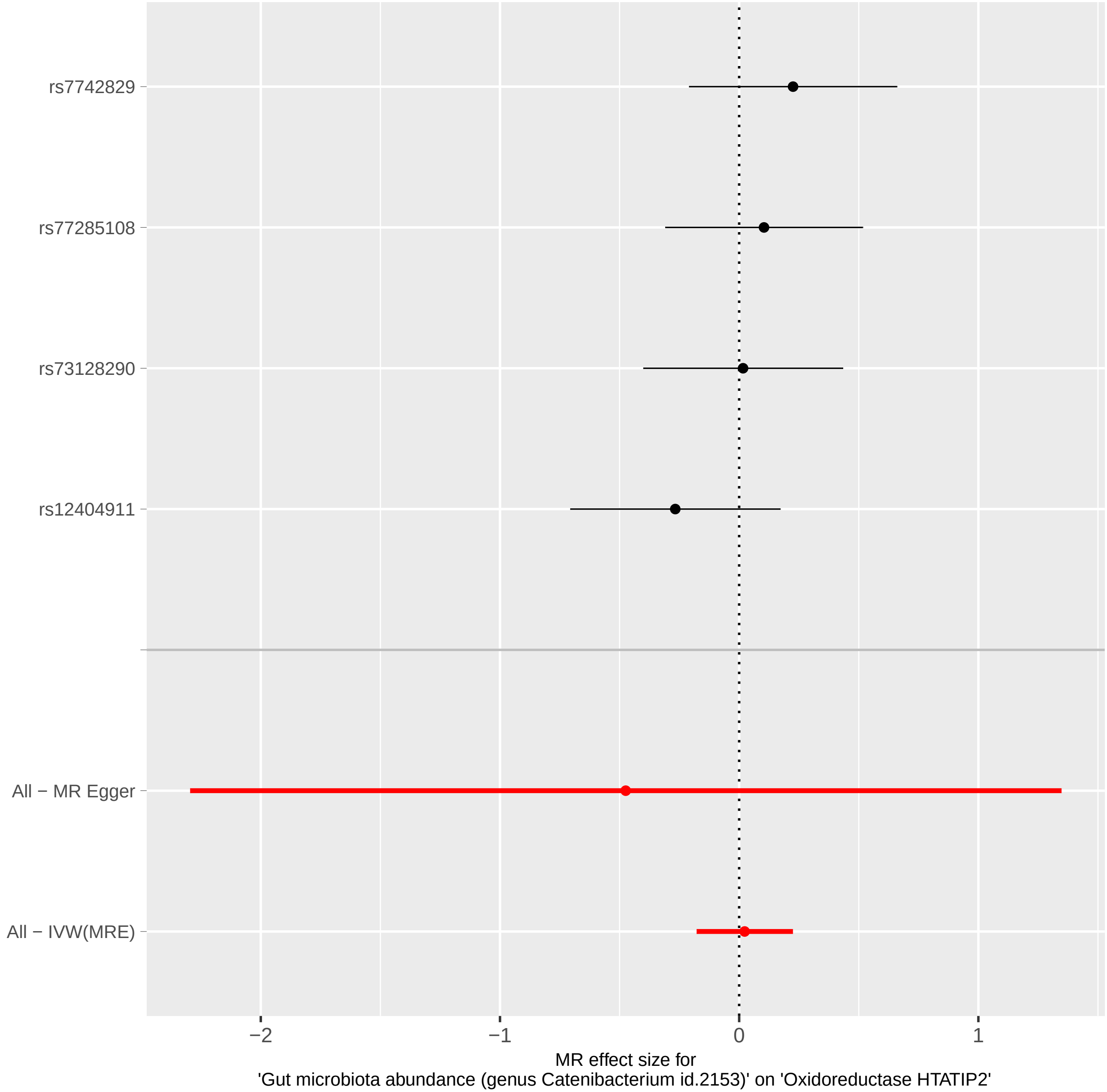

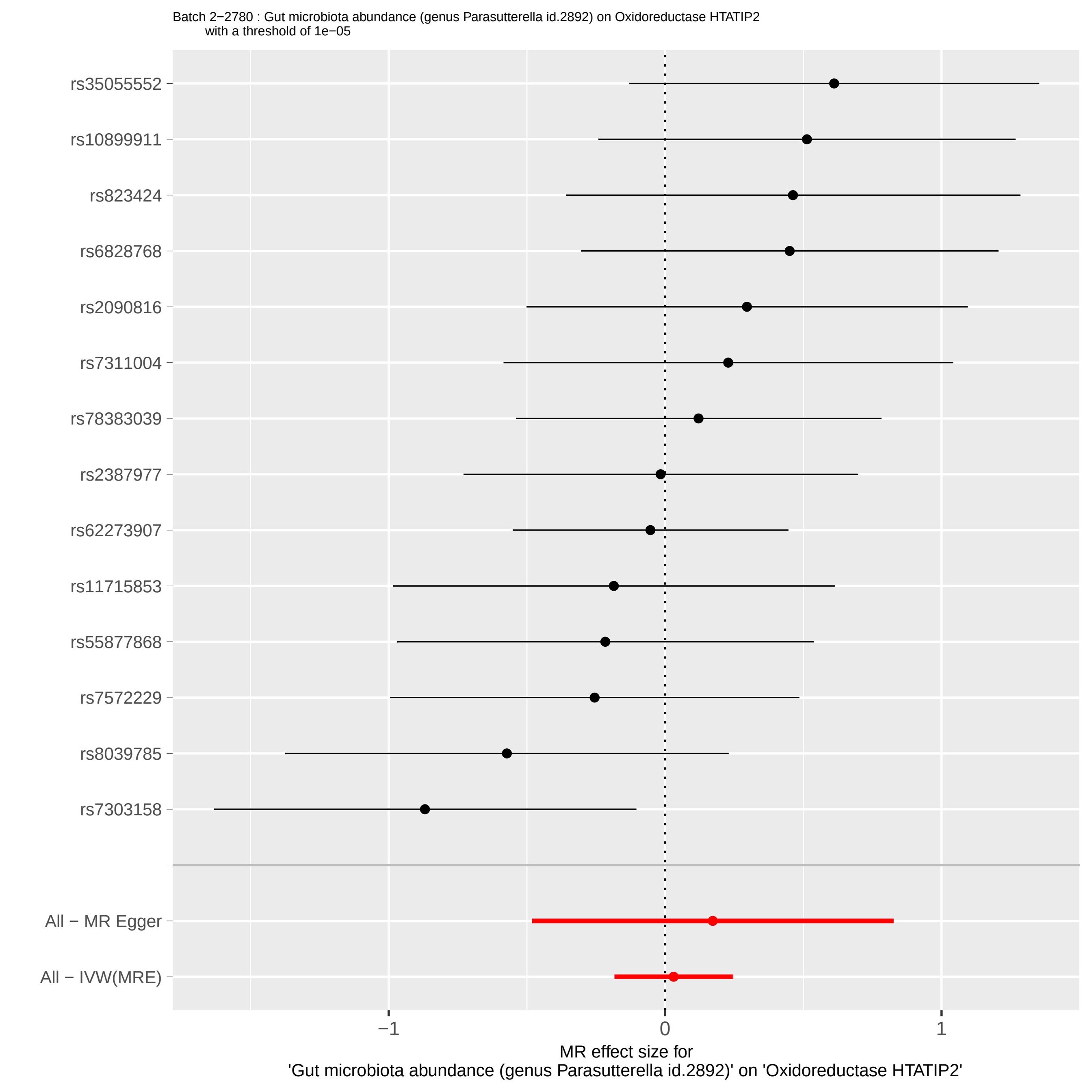

Batch 2−2780 : Gut microbiota abundance (genus Parasutterella id.2892) on Oxidoreductase HTATIP2
with a threshold of 1e−05
rs35055552
rs10899911
rs823424
rs6828768
rs2090816
rs7311004
rs78383039
rs2387977
rs62273907
rs11715853
rs55877868
rs7572229
rs8039785
rs7303158
All − MR Egger
All − IVW(MRE)
−1
0
1
MR effect size for
'Gut microbiota abundance (genus Parasutterella id.2892)' on 'Oxidoreductase HTATIP2'

Batch 2−2781 : Gut microbiota abundance (genus Catenibacterium id.2153) on Histatin−1
with a threshold of 1e−05

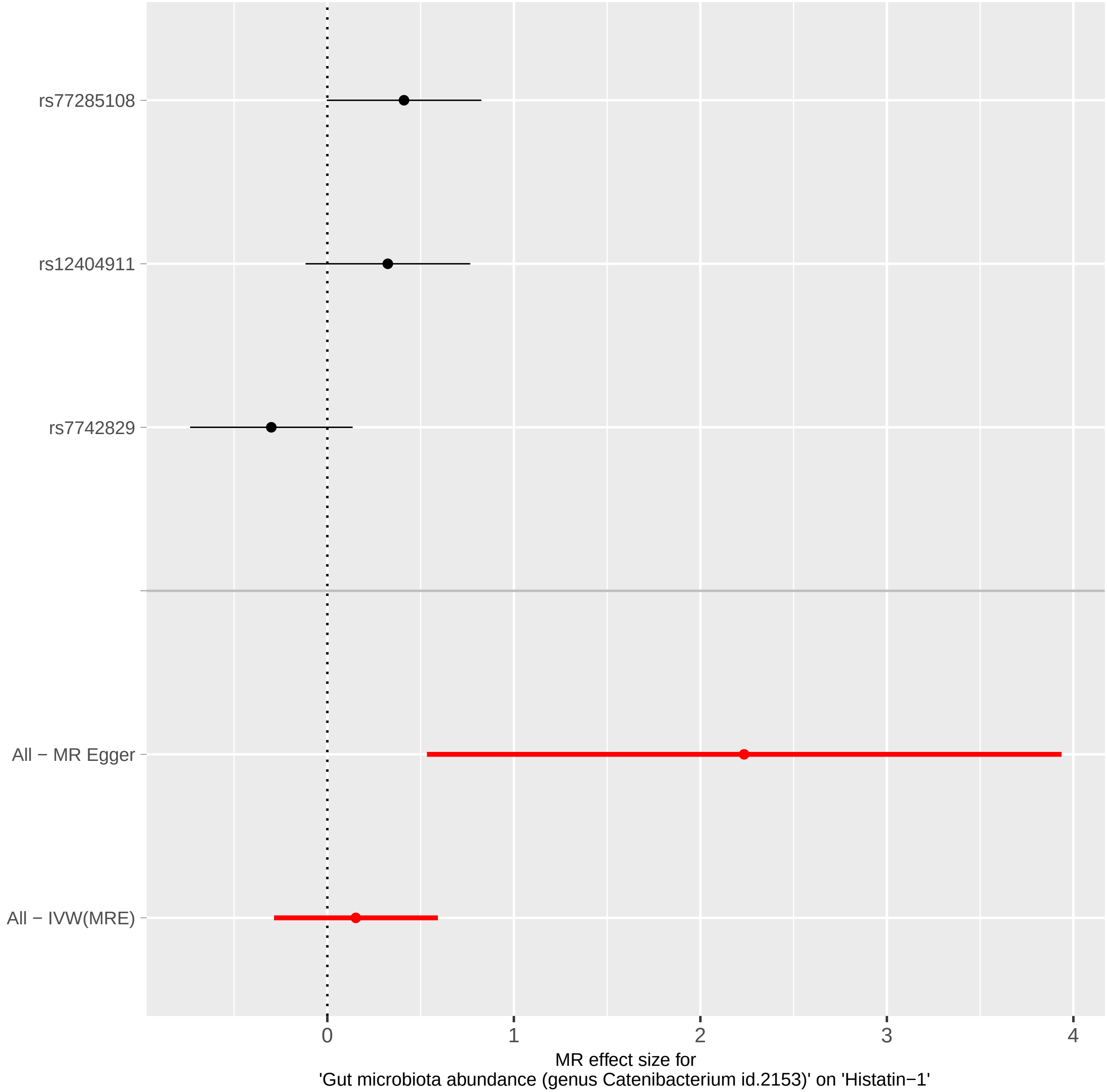

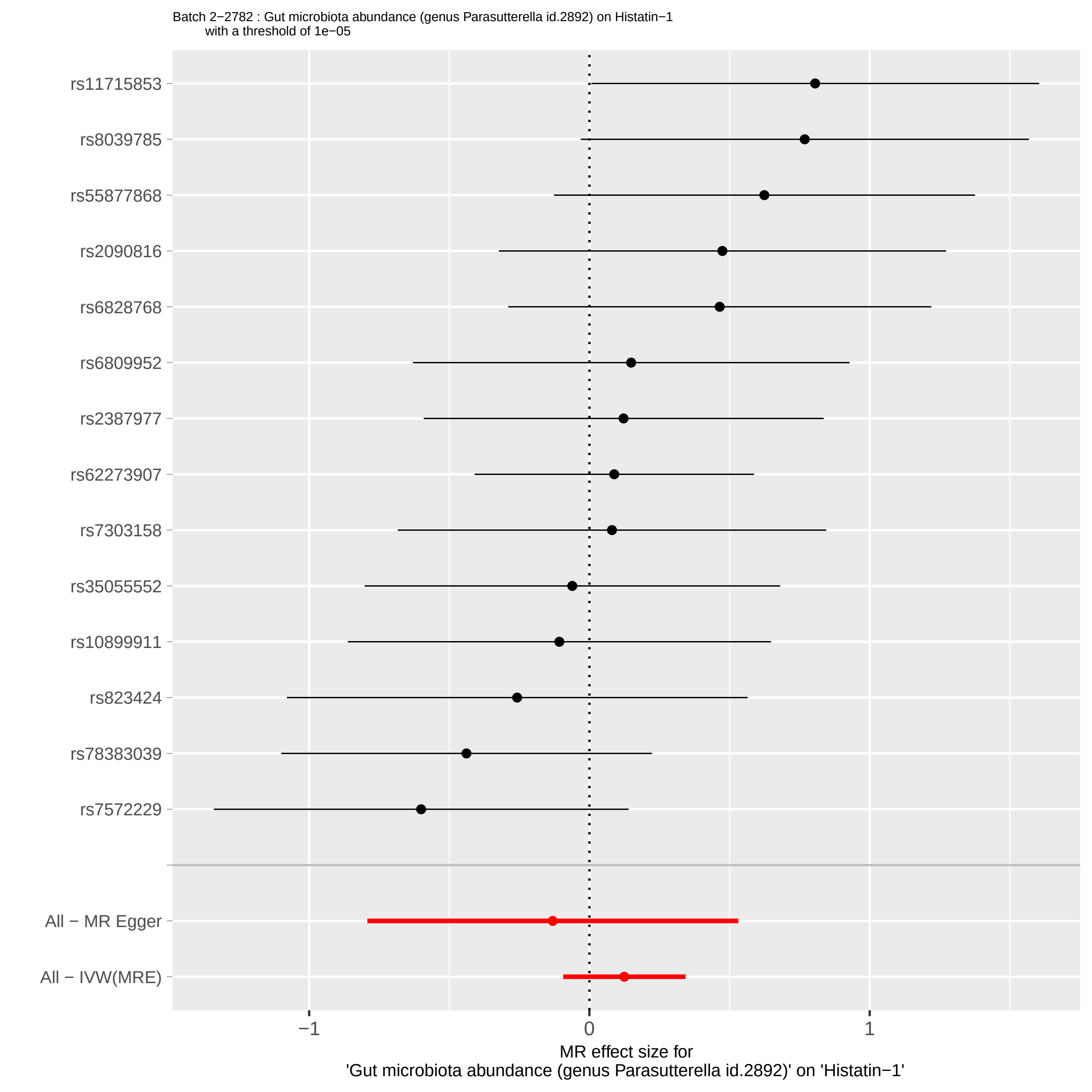

Batch 2−2782 : Gut microbiota abundance (genus Parasutterella id.2892) on Histatin−1
with a threshold of 1e−05
rs11715853
rs8039785
rs55877868
rs2090816
rs6828768
rs6809952
rs2387977
rs62273907
rs7303158
rs35055552
rs10899911
rs823424
rs78383039
rs7572229
All − MR Egger
All − IVW(MRE)
−1
0
1
MR effect size for
'Gut microbiota abundance (genus Parasutterella id.2892)' on 'Histatin−1'

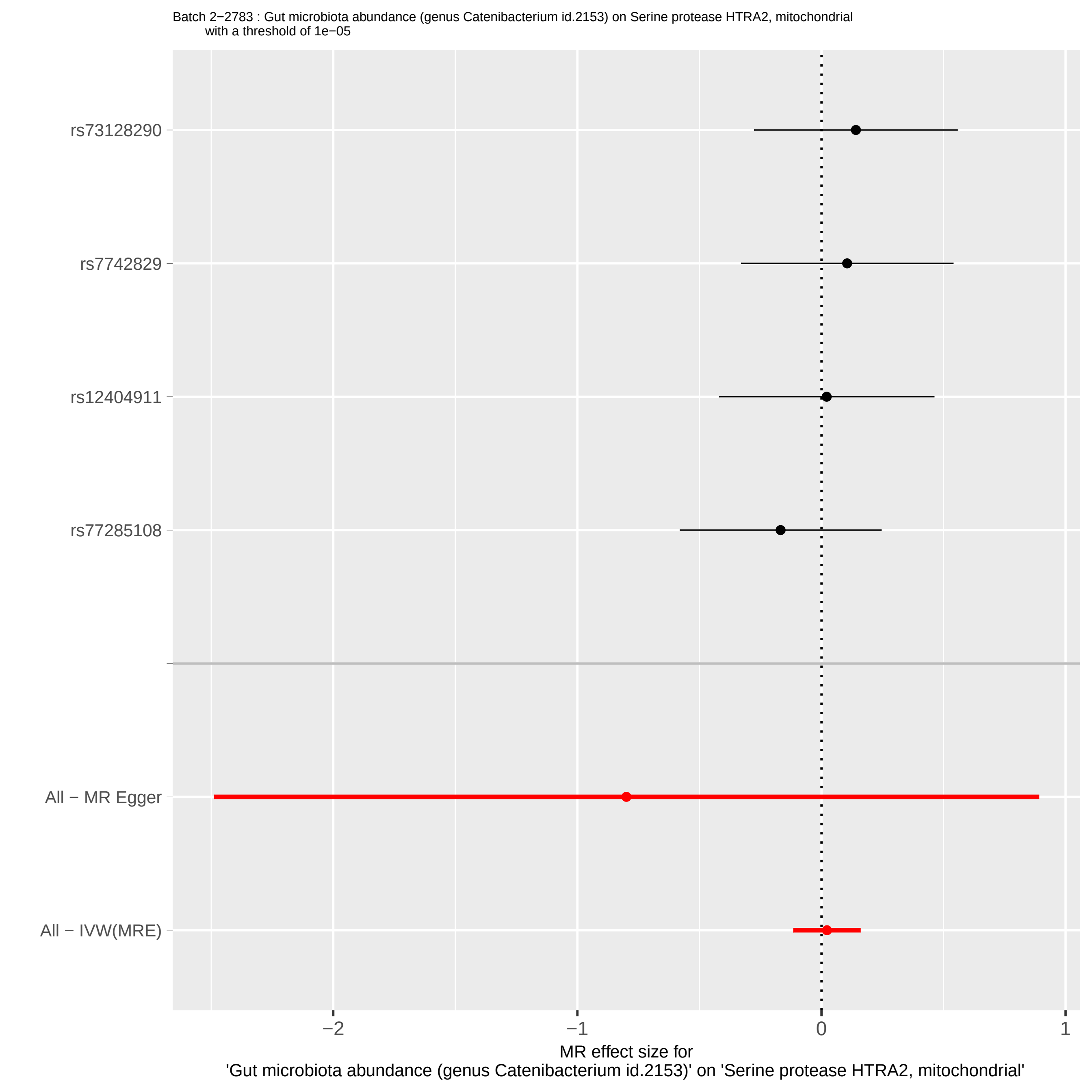

Batch 2−2783 : Gut microbiota abundance (genus Catenibacterium id.2153) on Serine protease HTRA2, mitochondrial
with a threshold of 1e−05
rs73128290
rs7742829
rs12404911
rs77285108
All − MR Egger
All − IVW(MRE)
−2
−1
0
1
MR effect size for
'Gut microbiota abundance (genus Catenibacterium id.2153)' on 'Serine protease HTRA2, mitochondrial'

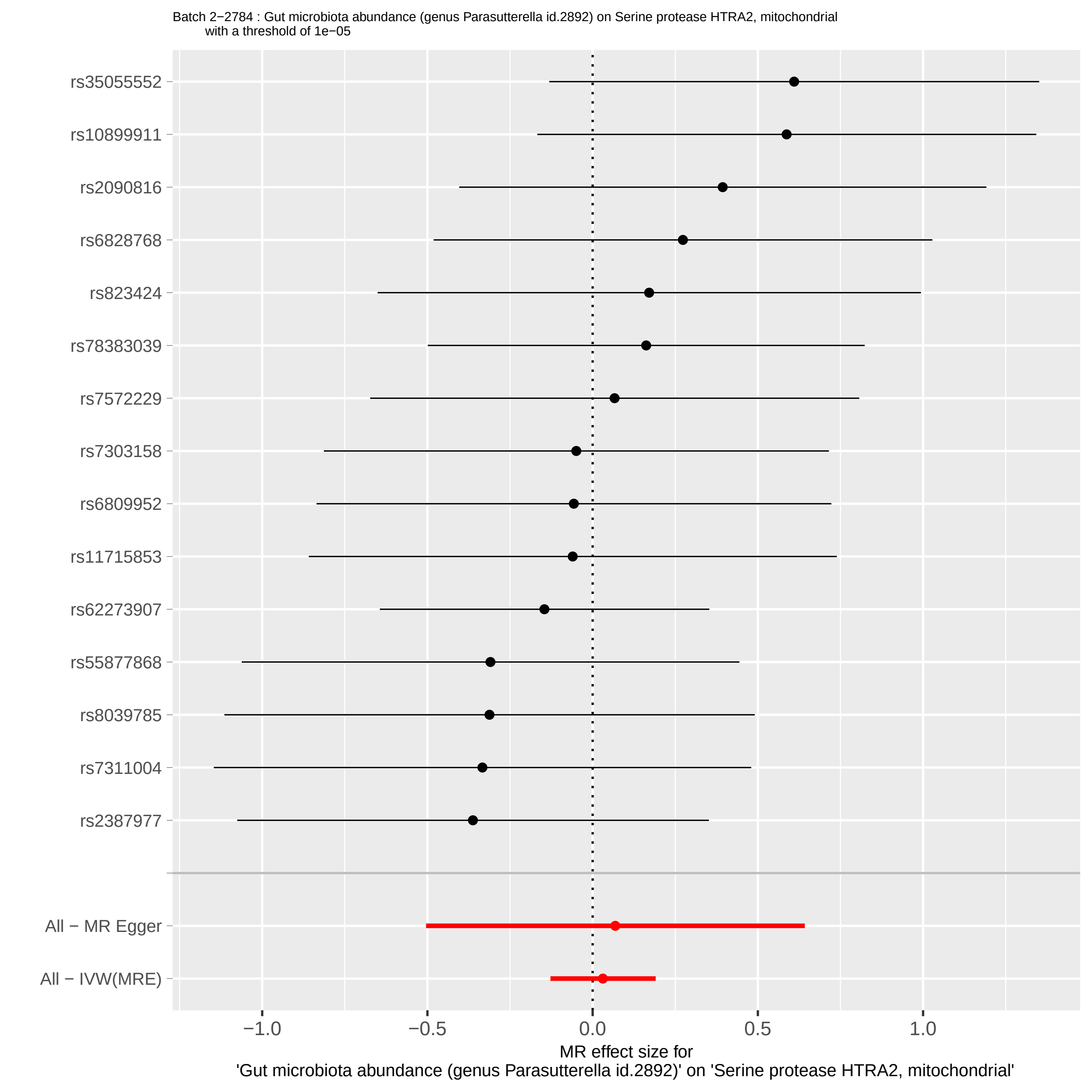

Batch 2−2784 : Gut microbiota abundance (genus Parasutterella id.2892) on Serine protease HTRA2, mitochondrial
with a threshold of 1e−05
rs35055552
rs10899911
rs2090816
rs6828768
rs823424
rs78383039
rs7572229
rs7303158
rs6809952
rs11715853
rs62273907
rs55877868
rs8039785
rs7311004
rs2387977
All − MR Egger
All − IVW(MRE)
−1.0
−0.5
0.0
0.5
1.0
MR effect size for
'Gut microbiota abundance (genus Parasutterella id.2892)' on 'Serine protease HTRA2, mitochondrial'

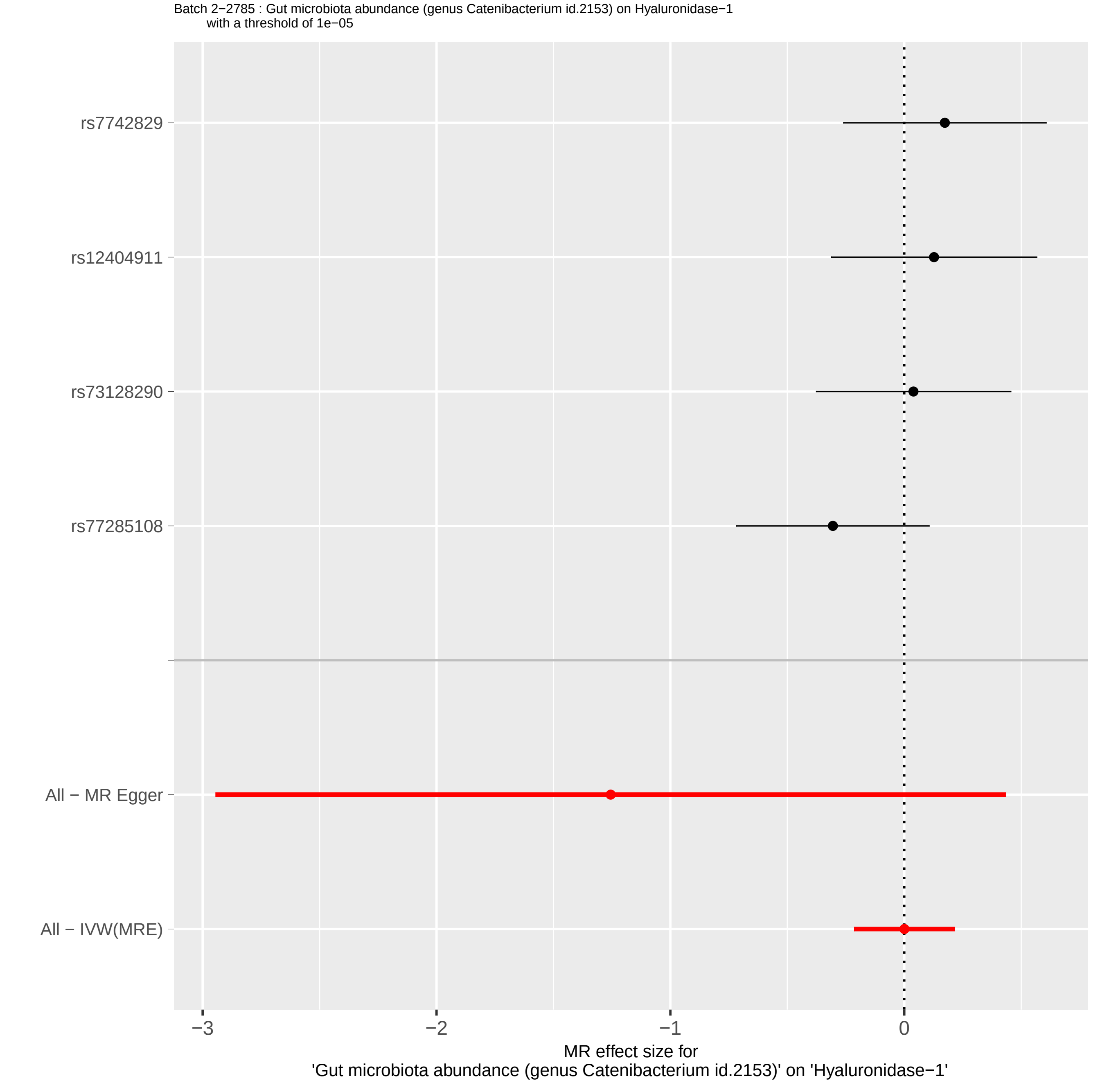

Batch 2−2785 : Gut microbiota abundance (genus Catenibacterium id.2153) on Hyaluronidase−1
with a threshold of 1e−05
rs7742829
rs12404911
rs73128290
rs77285108
All − MR Egger
All − IVW(MRE)
−3
−2
−1
0
MR effect size for
'Gut microbiota abundance (genus Catenibacterium id.2153)' on 'Hyaluronidase−1'

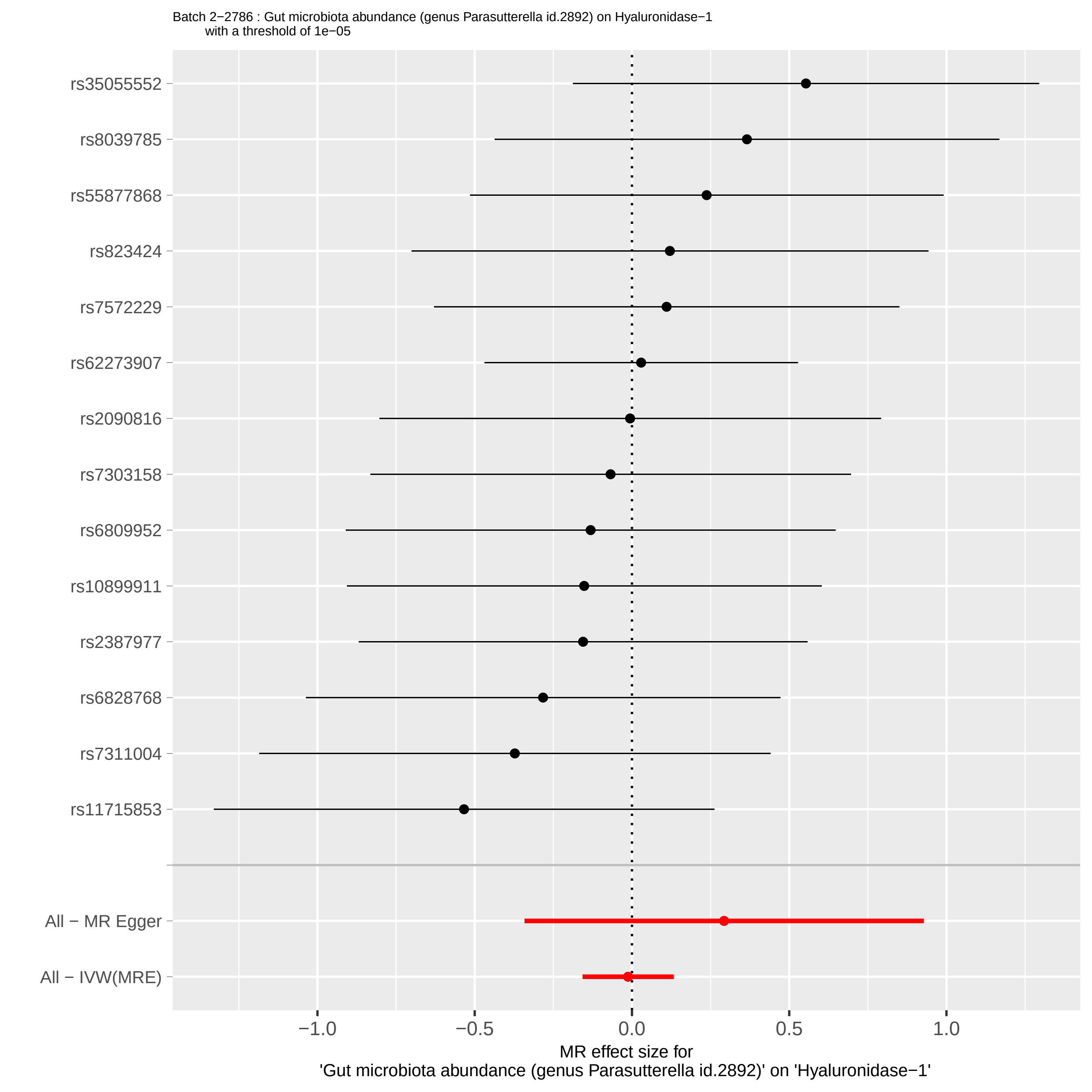

Batch 2−2786 : Gut microbiota abundance (genus Parasutterella id.2892) on Hyaluronidase−1
with a threshold of 1e−05
rs35055552
rs8039785
rs55877868
rs823424
rs7572229
rs62273907
rs2090816
rs7303158
rs6809952
rs10899911
rs2387977
rs6828768
rs7311004
rs11715853
All − MR Egger
All − IVW(MRE)
−1.0
−0.5
0.0
0.5
1.0
MR effect size for
'Gut microbiota abundance (genus Parasutterella id.2892)' on 'Hyaluronidase−1'

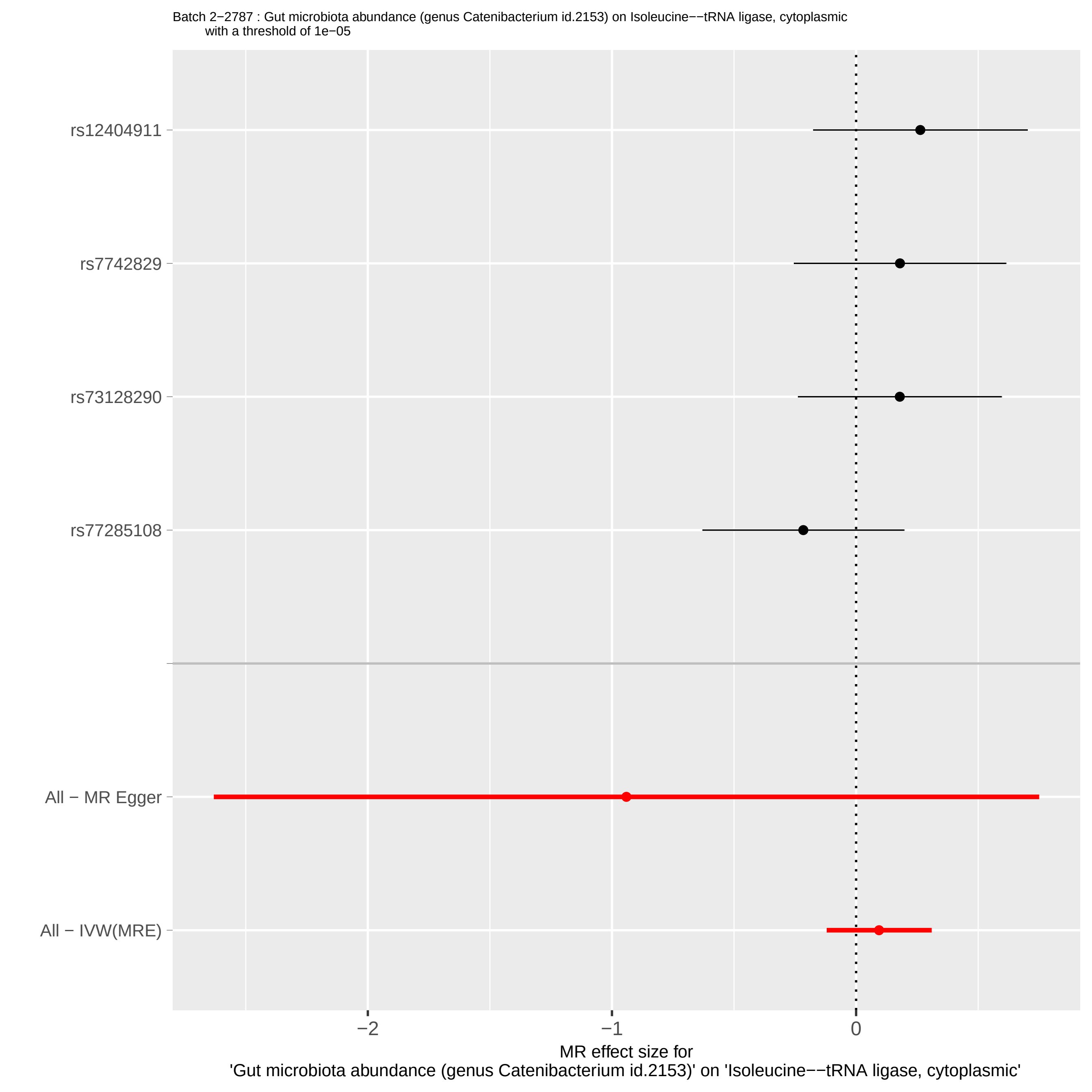
Batch 2−2787 : Gut microbiota abundance (genus Catenibacterium id.2153) on Isoleucine−−tRNA ligase, cytoplasmic
with a threshold of 1e−05
rs12404911
rs7742829
rs73128290
rs77285108
All − MR Egger
All − IVW(MRE)
−2
−1
0
MR effect size for
'Gut microbiota abundance (genus Catenibacterium id.2153)' on 'Isoleucine−−tRNA ligase, cytoplasmic'

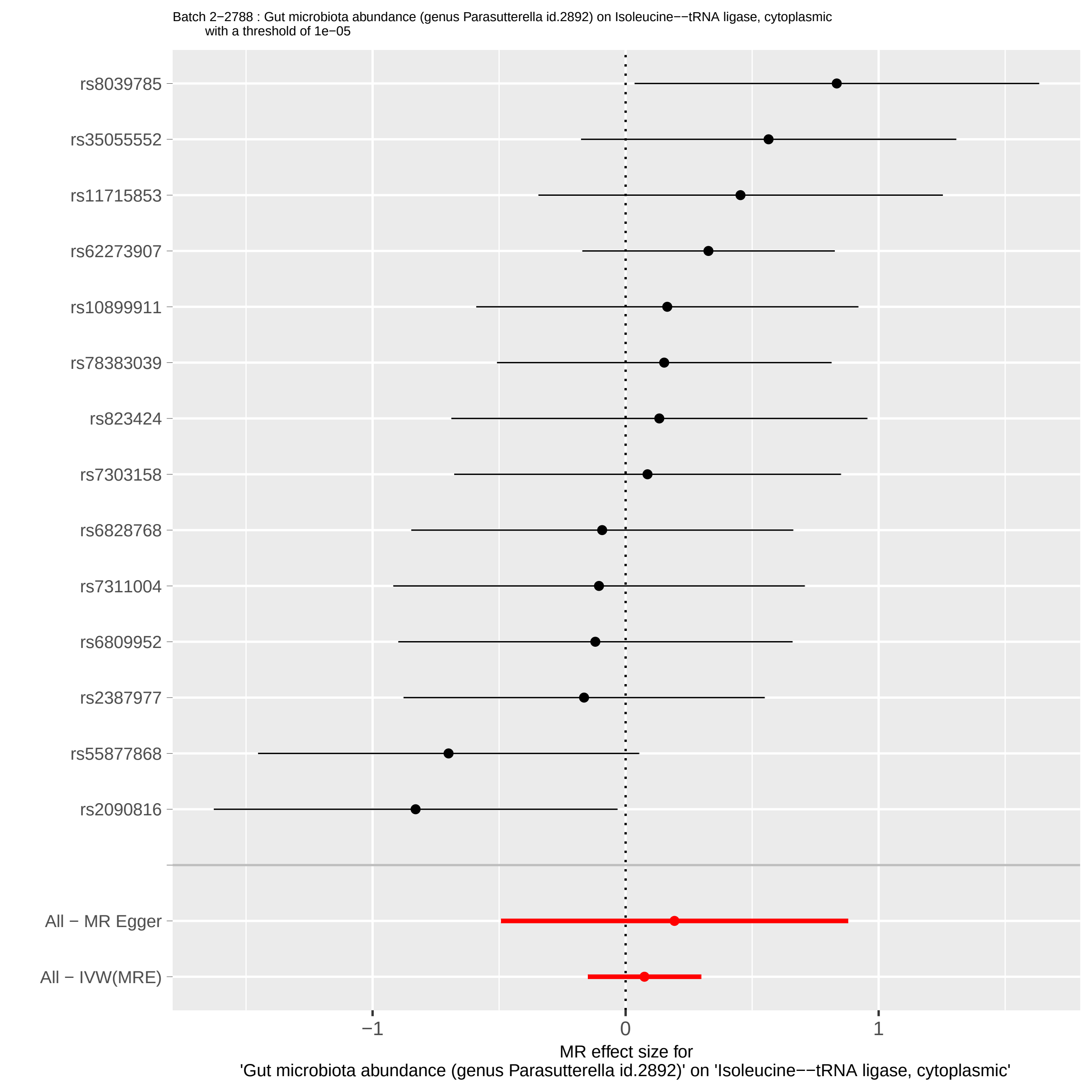

Batch 2−2788 : Gut microbiota abundance (genus Parasutterella id.2892) on Isoleucine−−tRNA ligase, cytoplasmic
with a threshold of 1e−05
rs8039785
rs35055552
rs11715853
rs62273907
rs10899911
rs78383039
rs823424
rs7303158
rs6828768
rs7311004
rs6809952
rs2387977
rs55877868
rs2090816
All − MR Egger
All − IVW(MRE)
−1
0
1
MR effect size for
'Gut microbiota abundance (genus Parasutterella id.2892)' on 'Isoleucine−−tRNA ligase, cytoplasmic'

Batch 2−2789 : Gut microbiota abundance (genus Catenibacterium id.2153) on Bone sialoprotein 2
with a threshold of 1e−05

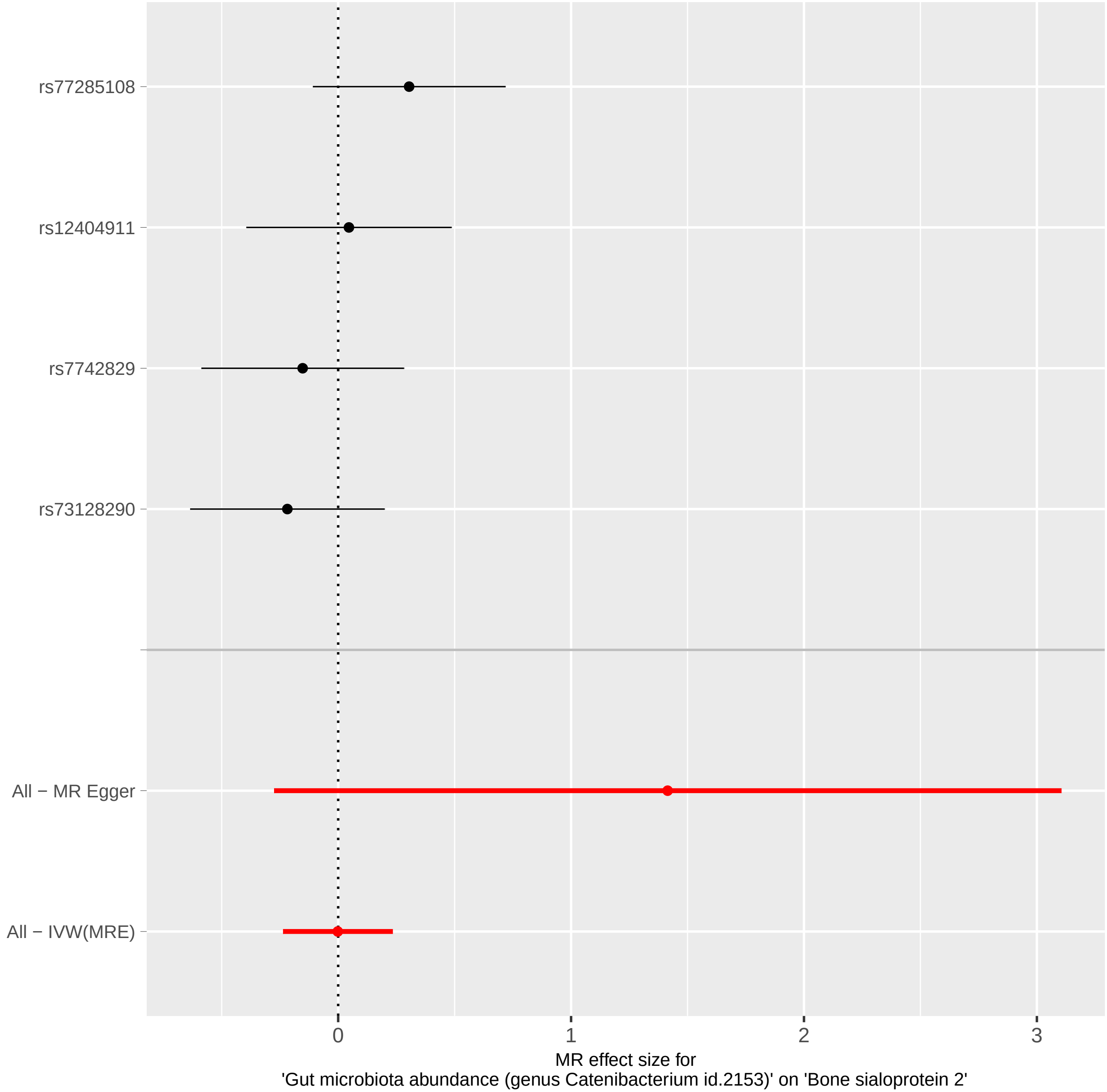

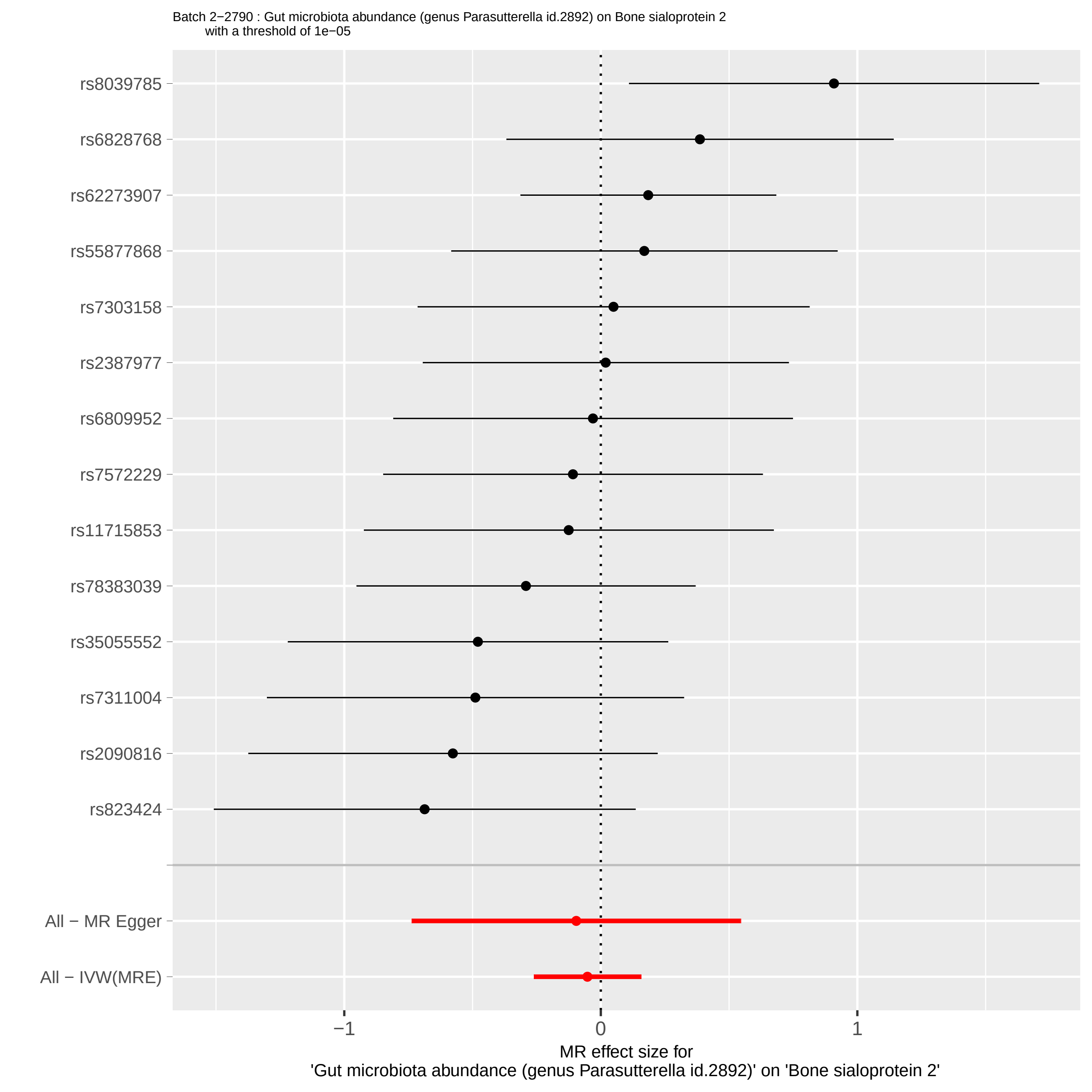
Batch 2−2790 : Gut microbiota abundance (genus Parasutterella id.2892) on Bone sialoprotein 2
with a threshold of 1e−05
rs8039785
rs6828768
rs62273907
rs55877868
rs7303158
rs2387977
rs6809952
rs7572229
rs11715853
rs78383039
rs35055552
rs7311004
rs2090816
rs823424
All − MR Egger
All − IVW(MRE)
−1
0
1
MR effect size for
'Gut microbiota abundance (genus Parasutterella id.2892)' on 'Bone sialoprotein 2'

Batch 2−2791 : Gut microbiota abundance (genus Catenibacterium id.2153) on Bone sialoprotein 2
with a threshold of 1e−05

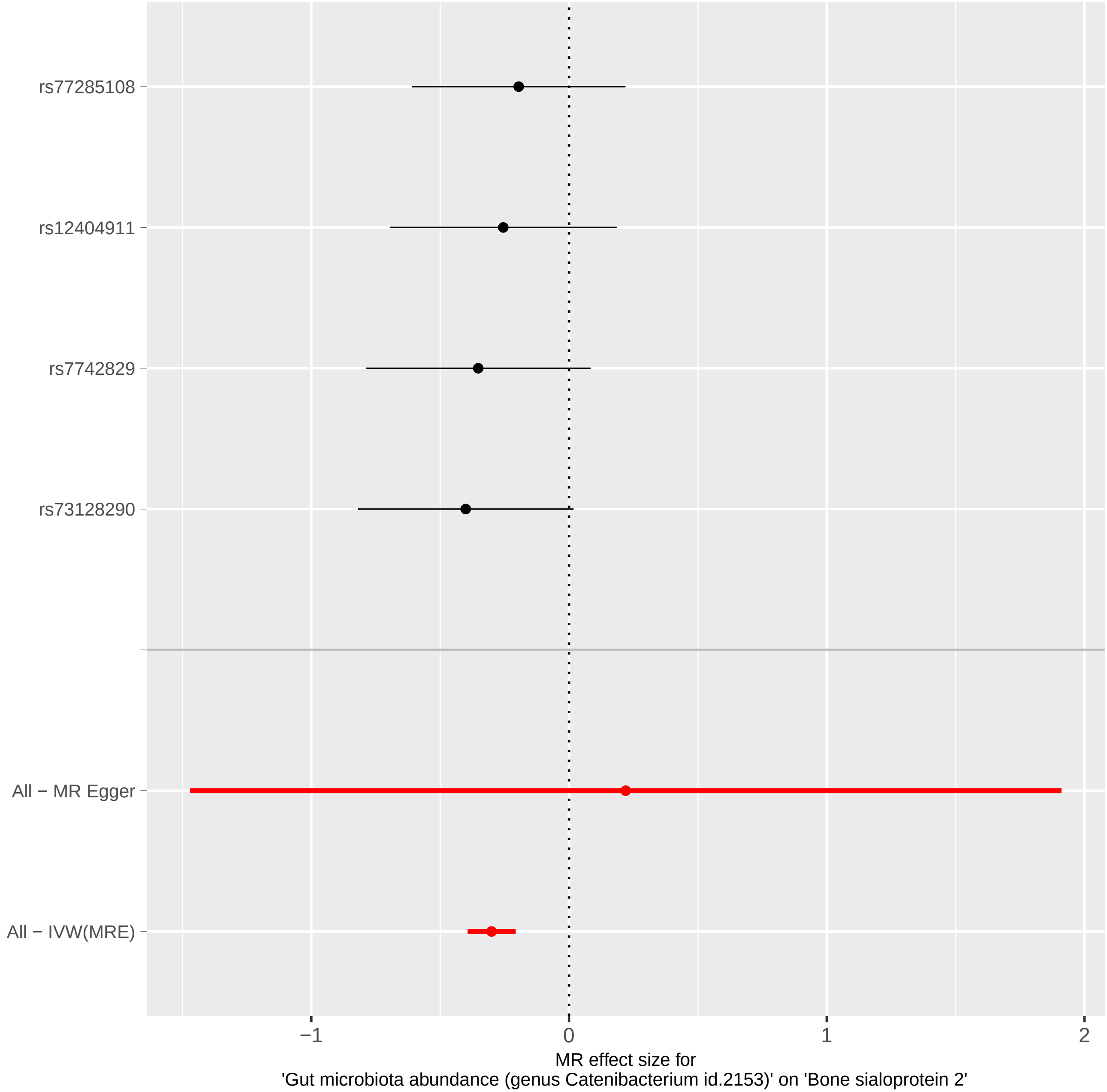

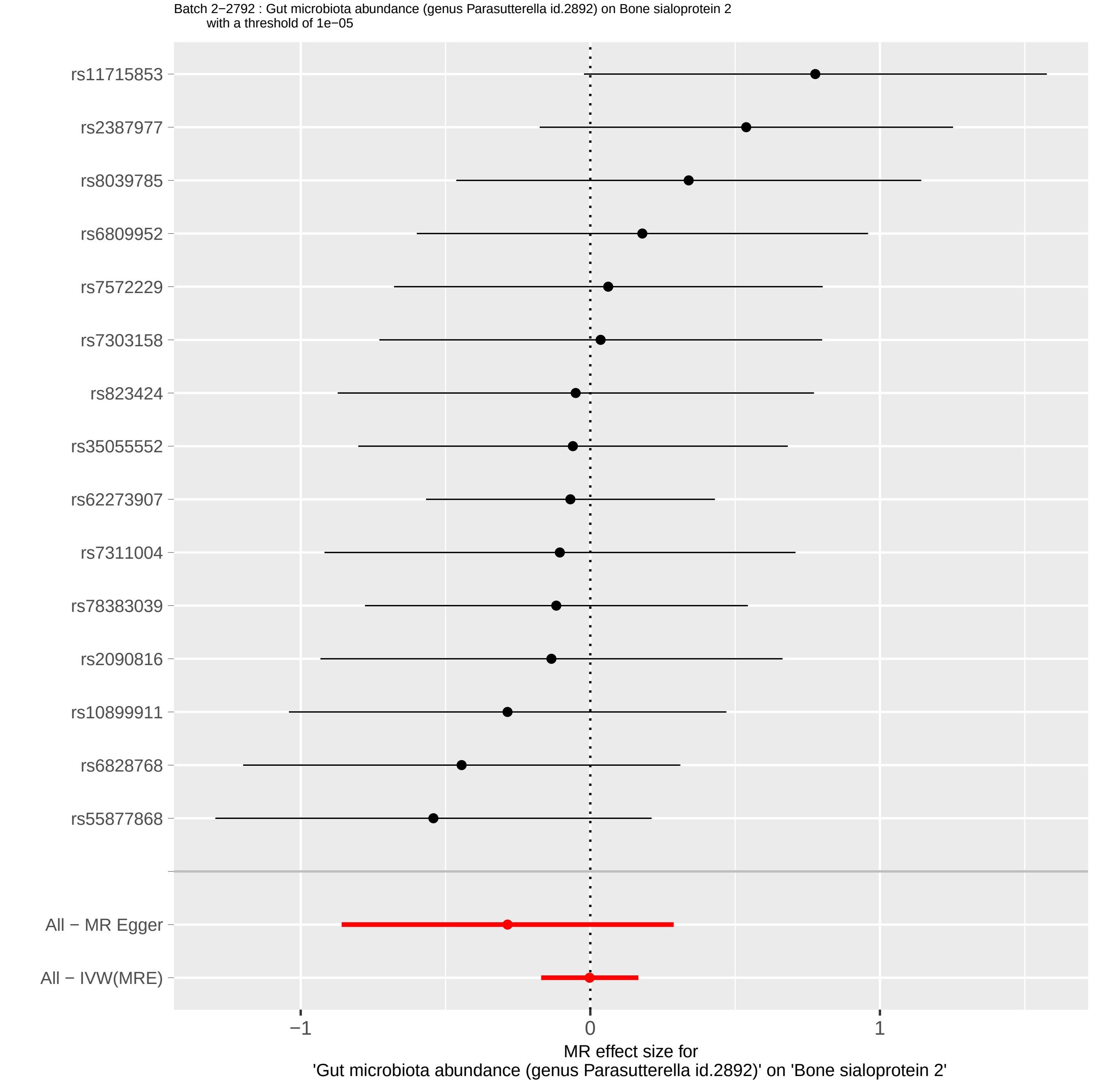
Batch 2−2792 : Gut microbiota abundance (genus Parasutterella id.2892) on Bone sialoprotein 2
with a threshold of 1e−05
rs11715853
rs2387977
rs8039785
rs6809952
rs7572229
rs7303158
rs823424
rs35055552
rs62273907
rs7311004
rs78383039
rs2090816
rs10899911
rs6828768
rs55877868
All − MR Egger
All − IVW(MRE)
−1
0
1
MR effect size for
'Gut microbiota abundance (genus Parasutterella id.2892)' on 'Bone sialoprotein 2'

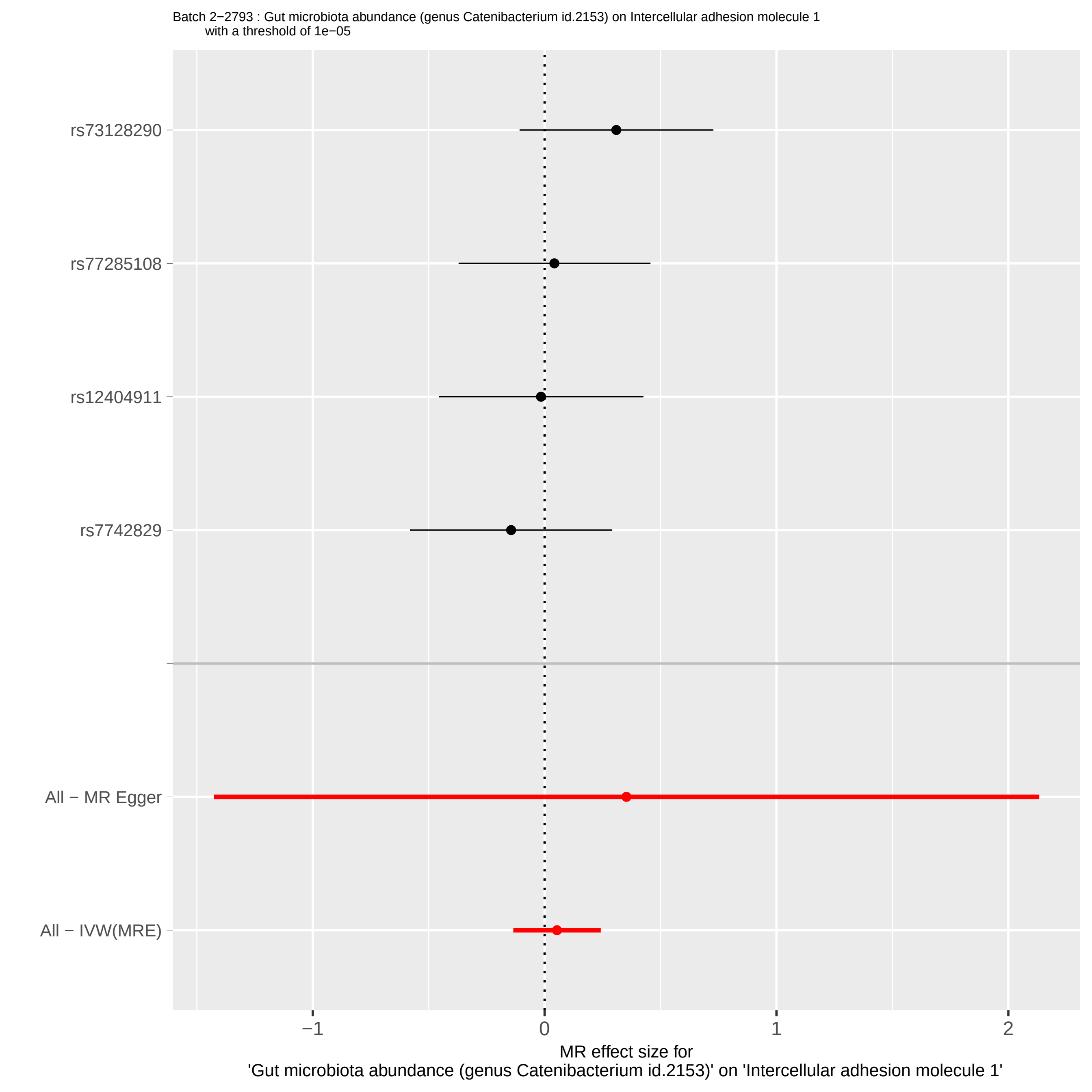
Batch 2−2793 : Gut microbiota abundance (genus Catenibacterium id.2153) on Intercellular adhesion molecule 1
with a threshold of 1e−05
rs73128290
rs77285108
rs12404911
rs7742829
All − MR Egger
All − IVW(MRE)
−1
0
1
2
MR effect size for
'Gut microbiota abundance (genus Catenibacterium id.2153)' on 'Intercellular adhesion molecule 1'

Batch 2−2794 : Gut microbiota abundance (genus Parasutterella id.2892) on Intercellular adhesion molecule 1
with a threshold of 1e−05

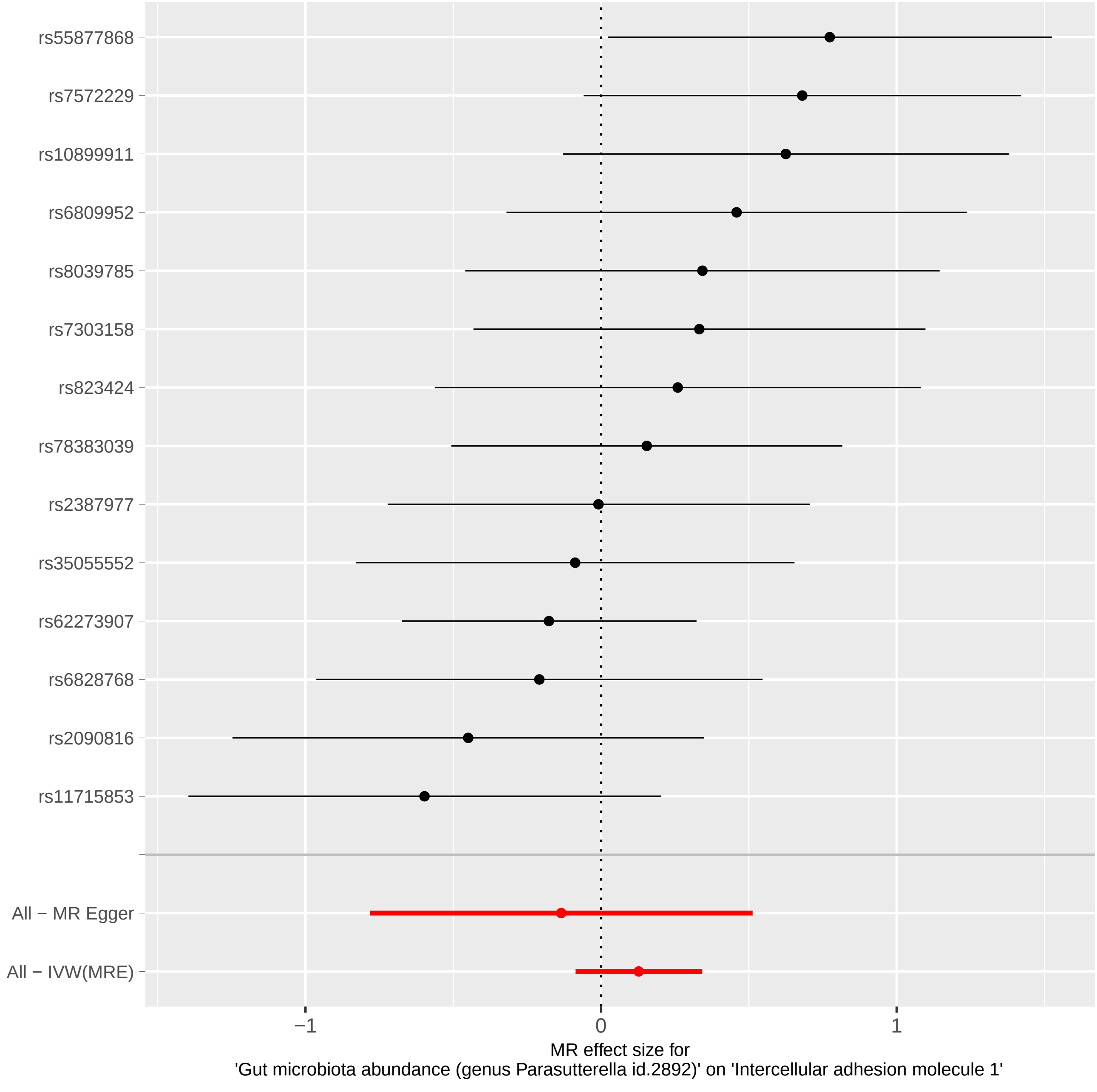

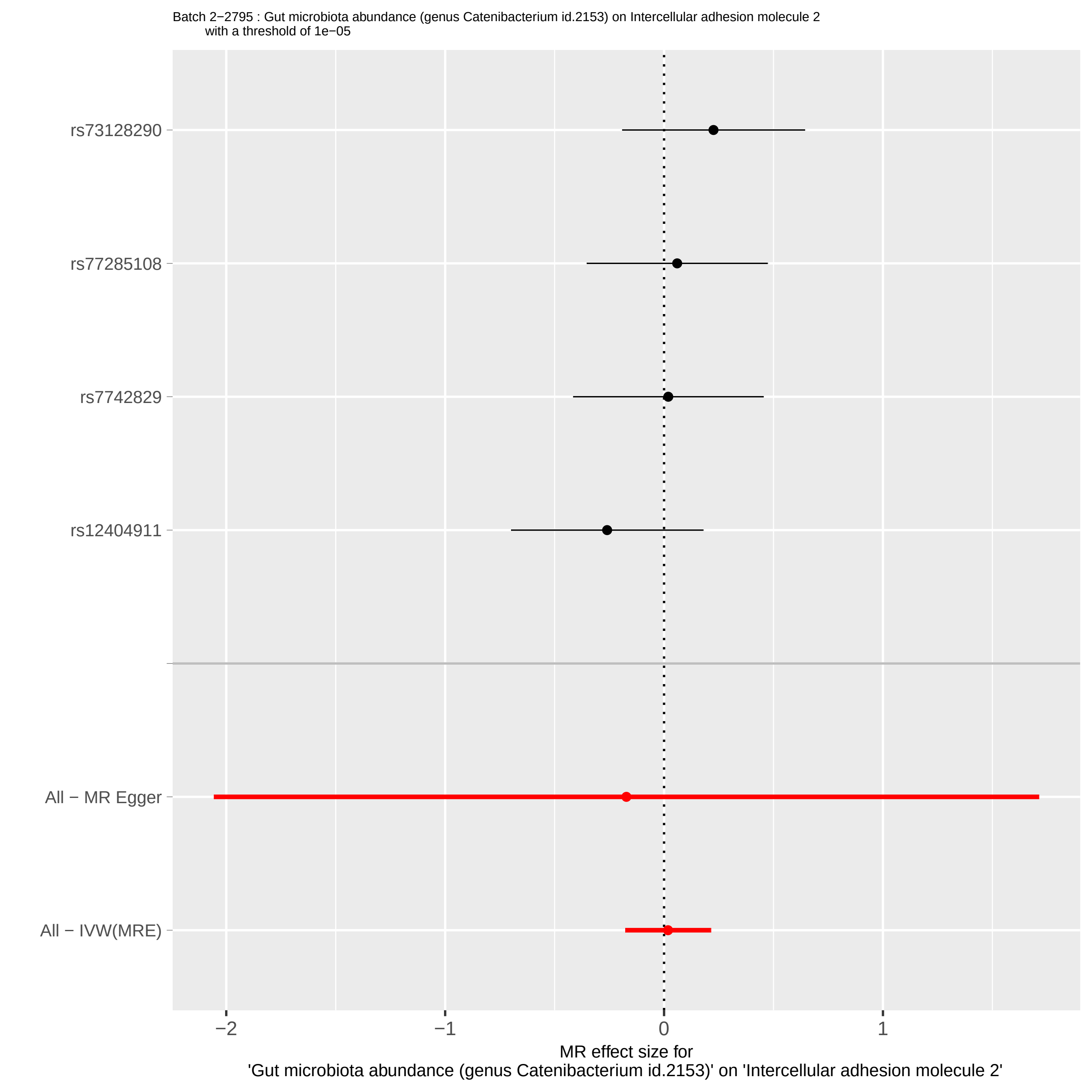
Batch 2−2795 : Gut microbiota abundance (genus Catenibacterium id.2153) on Intercellular adhesion molecule 2
with a threshold of 1e−05
rs73128290
rs77285108
rs7742829
rs12404911
All − MR Egger
All − IVW(MRE)
−2
−1
0
1
MR effect size for
'Gut microbiota abundance (genus Catenibacterium id.2153)' on 'Intercellular adhesion molecule 2'

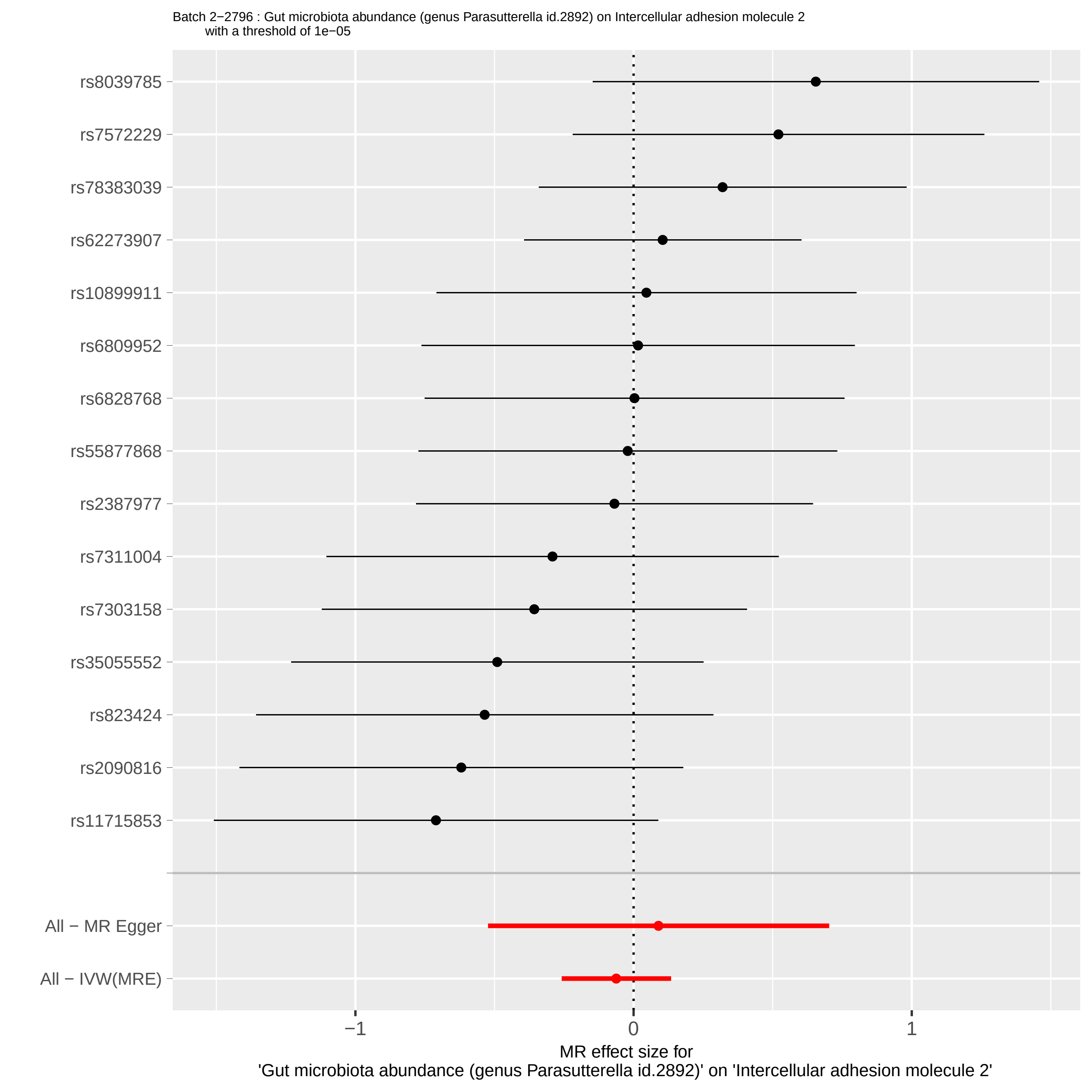
Batch 2−2796 : Gut microbiota abundance (genus Parasutterella id.2892) on Intercellular adhesion molecule 2
with a threshold of 1e−05
rs8039785
rs7572229
rs78383039
rs62273907
rs10899911
rs6809952
rs6828768
rs55877868
rs2387977
rs7311004
rs7303158
rs35055552
rs823424
rs2090816
rs11715853
All − MR Egger
All − IVW(MRE)
−1
0
1
MR effect size for
'Gut microbiota abundance (genus Parasutterella id.2892)' on 'Intercellular adhesion molecule 2'

Batch 2−2797 : Gut microbiota abundance (genus Catenibacterium id.2153) on Intercellular adhesion molecule 3
with a threshold of 1e−05

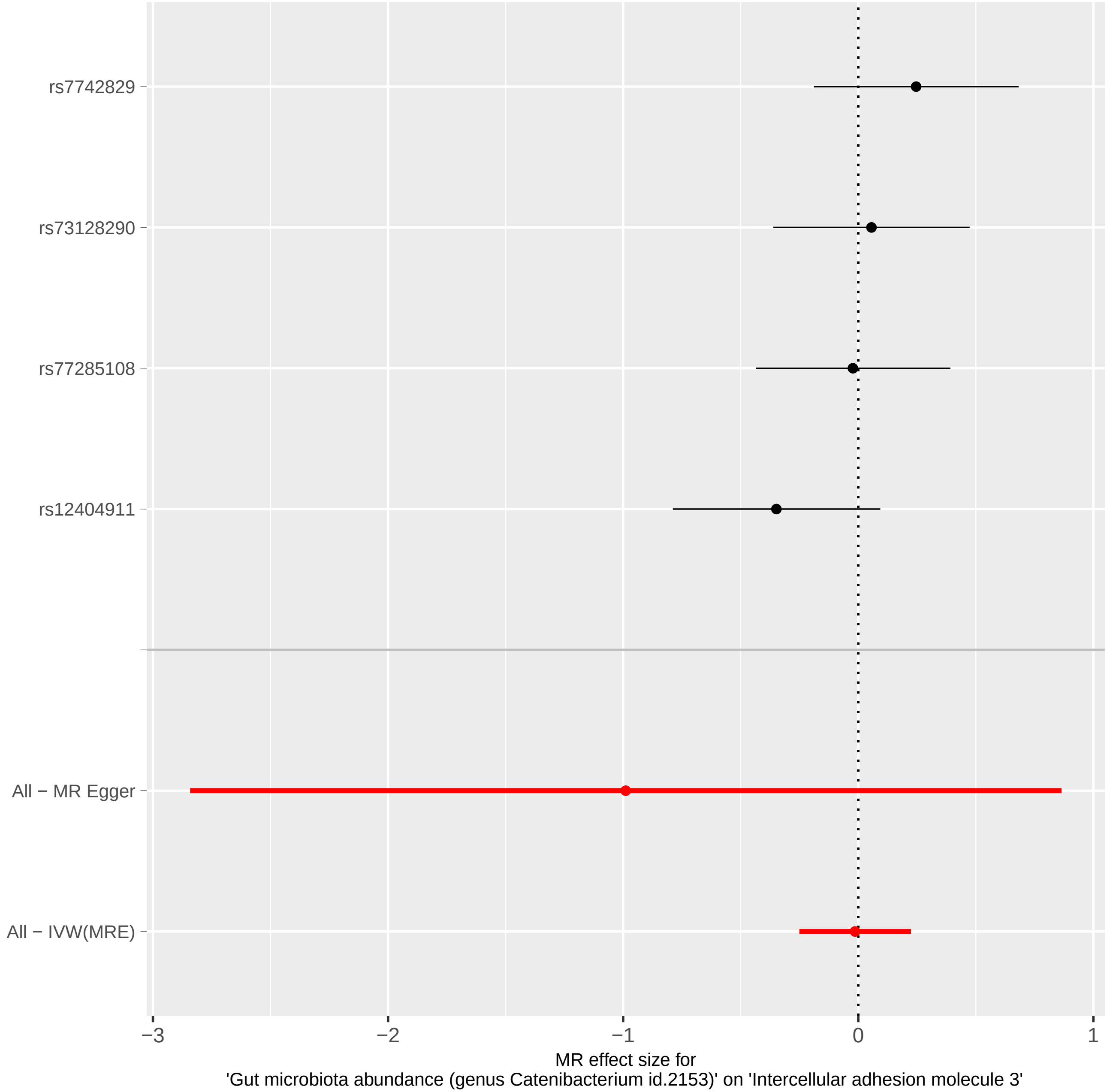

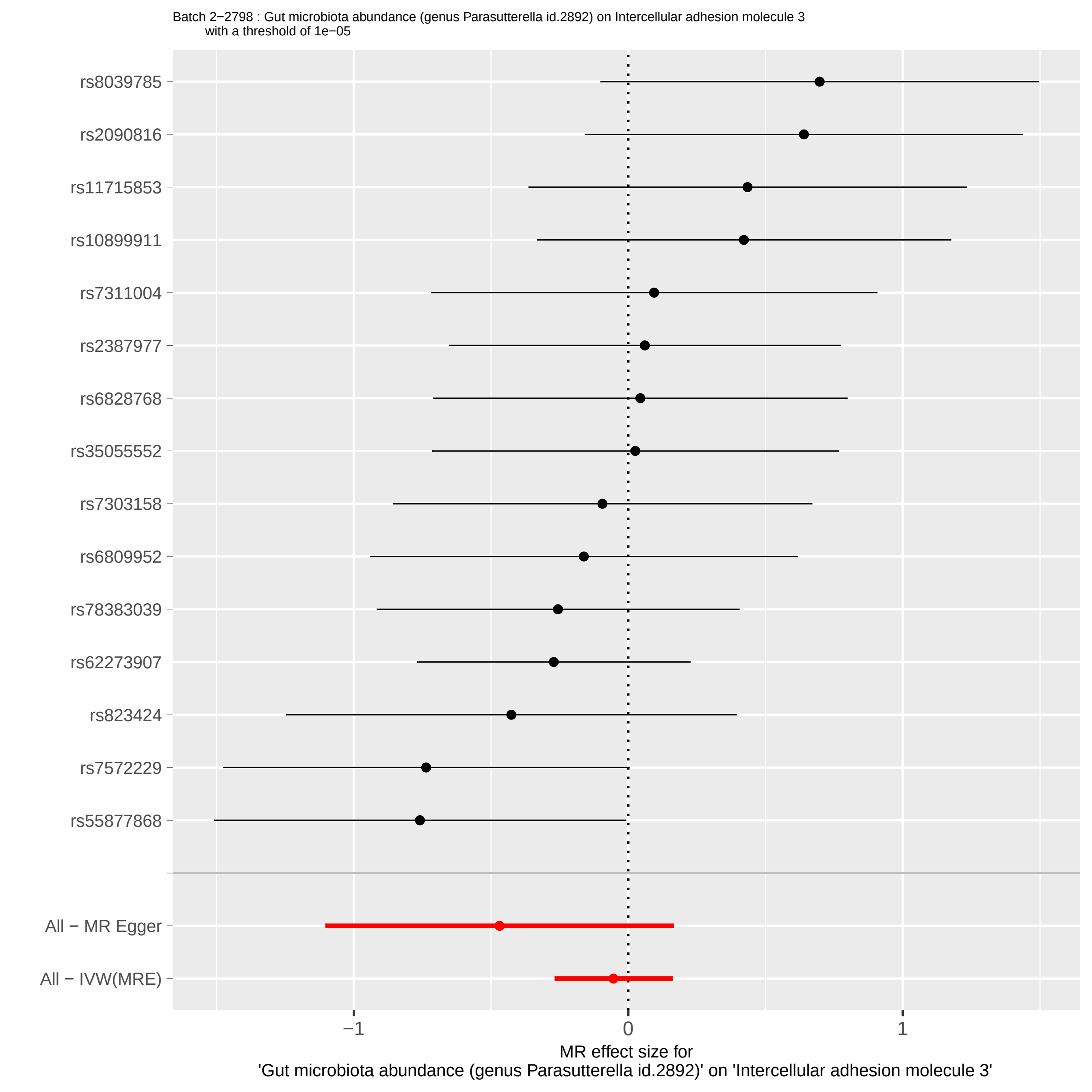

Batch 2−2798 : Gut microbiota abundance (genus Parasutterella id.2892) on Intercellular adhesion molecule 3
with a threshold of 1e−05
rs8039785
rs2090816
rs11715853
rs10899911
rs7311004
rs2387977
rs6828768
rs35055552
rs7303158
rs6809952
rs78383039
rs62273907
rs823424
rs7572229
rs55877868
All − MR Egger
All − IVW(MRE)
−1
0
1
MR effect size for
'Gut microbiota abundance (genus Parasutterella id.2892)' on 'Intercellular adhesion molecule 3'

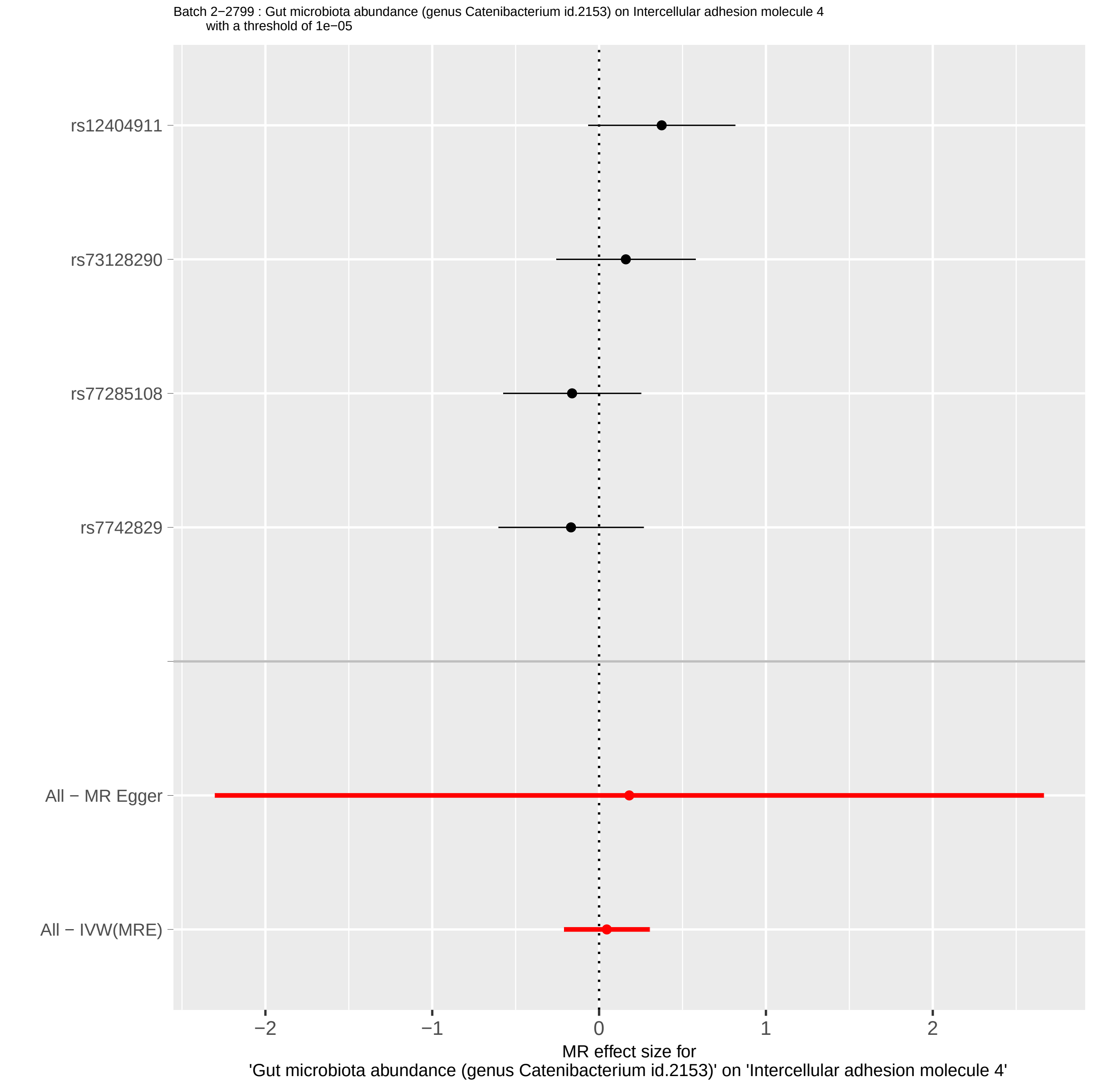

Batch 2−2799 : Gut microbiota abundance (genus Catenibacterium id.2153) on Intercellular adhesion molecule 4
with a threshold of 1e−05
rs12404911
rs73128290
rs77285108
rs7742829
All − MR Egger
All − IVW(MRE)
−2
−1
0
1
2
MR effect size for
'Gut microbiota abundance (genus Catenibacterium id.2153)' on 'Intercellular adhesion molecule 4'

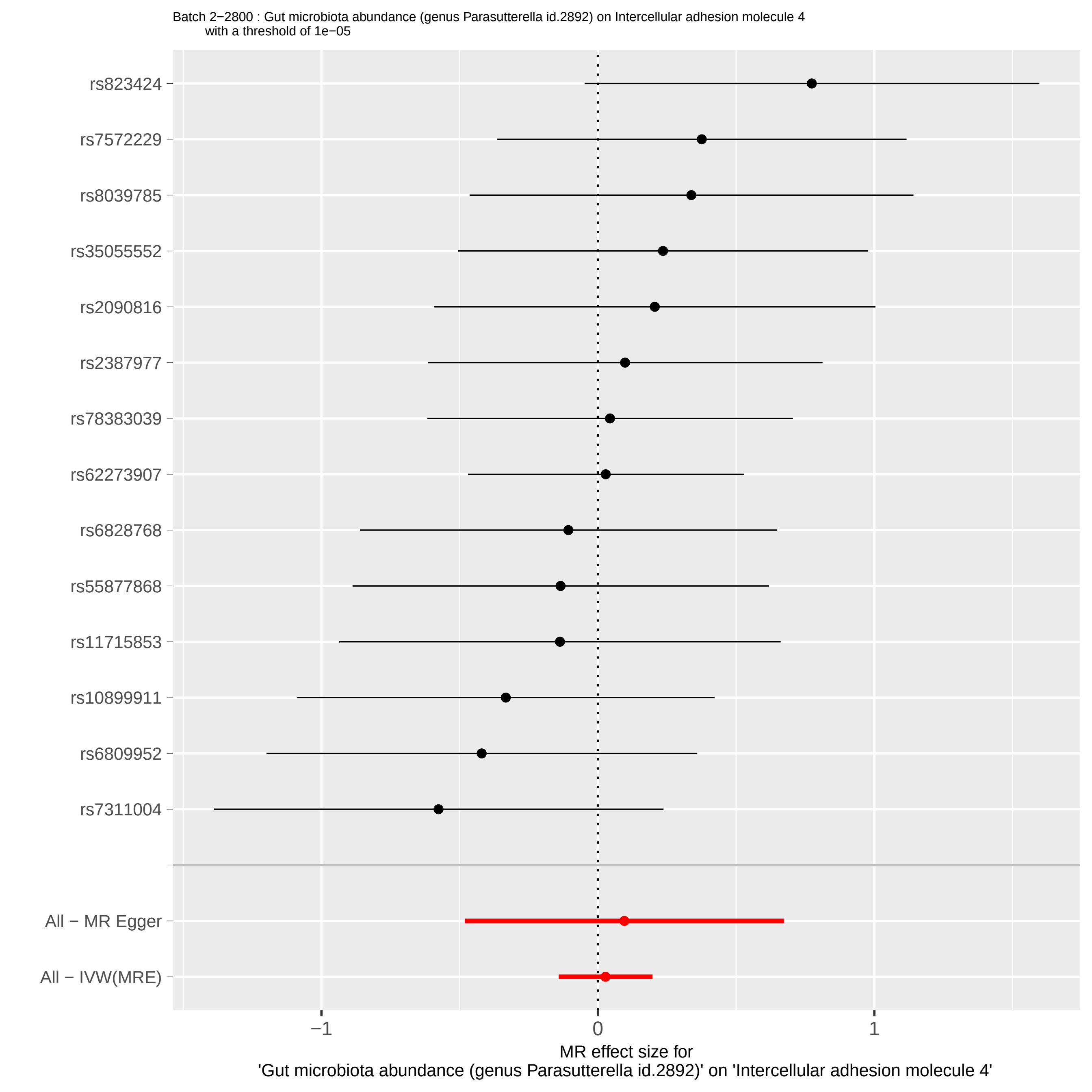

Batch 2−2800 : Gut microbiota abundance (genus Parasutterella id.2892) on Intercellular adhesion molecule 4
with a threshold of 1e−05
rs823424
rs7572229
rs8039785
rs35055552
rs2090816
rs2387977
rs78383039
rs62273907
rs6828768
rs55877868
rs11715853
rs10899911
rs6809952
rs7311004
All − MR Egger
All − IVW(MRE)
−1
0
1
MR effect size for
'Gut microbiota abundance (genus Parasutterella id.2892)' on 'Intercellular adhesion molecule 4'

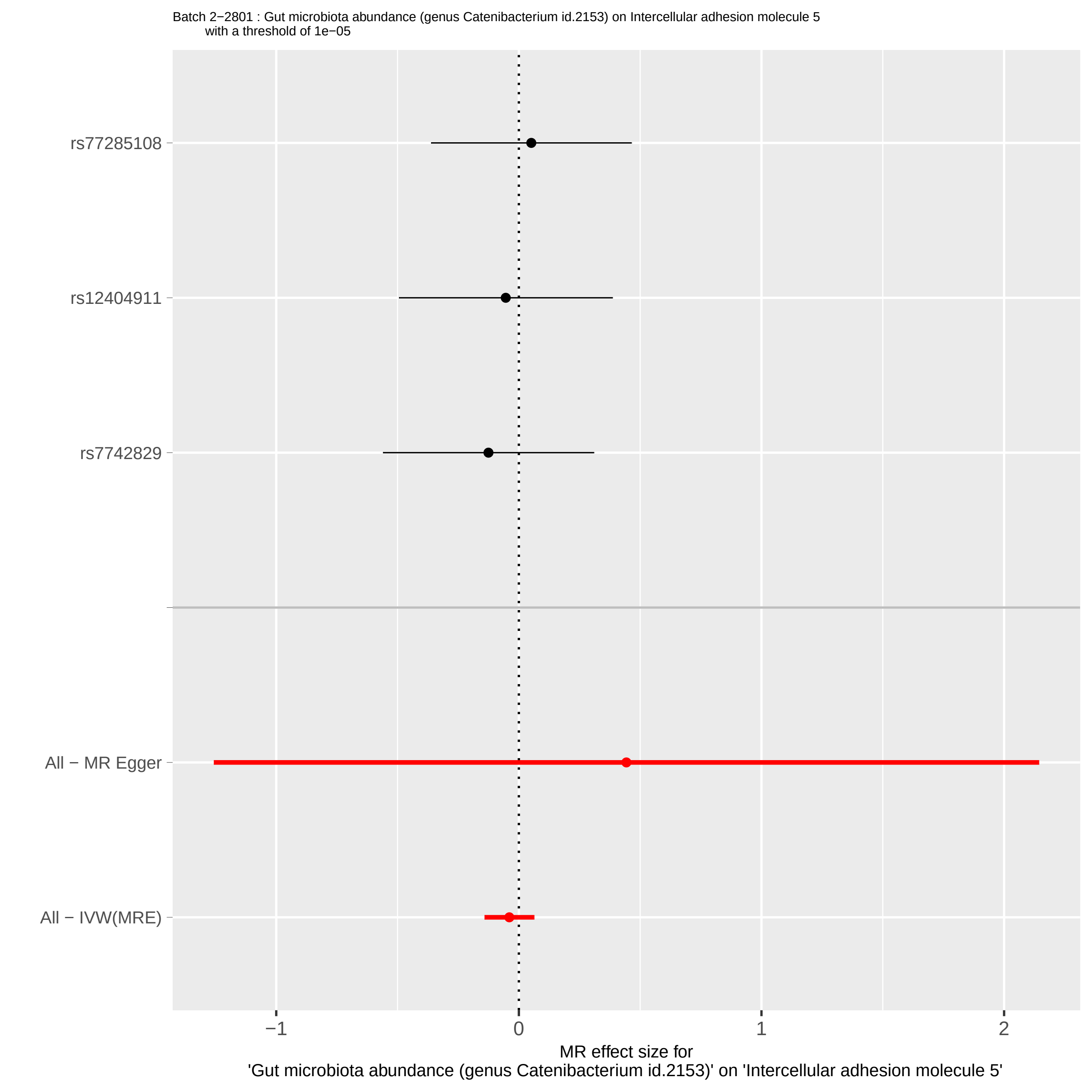

Batch 2−2801 : Gut microbiota abundance (genus Catenibacterium id.2153) on Intercellular adhesion molecule 5
with a threshold of 1e−05
rs77285108
rs12404911
rs7742829
All − MR Egger
All − IVW(MRE)
−1
0
1
2
MR effect size for
'Gut microbiota abundance (genus Catenibacterium id.2153)' on 'Intercellular adhesion molecule 5'

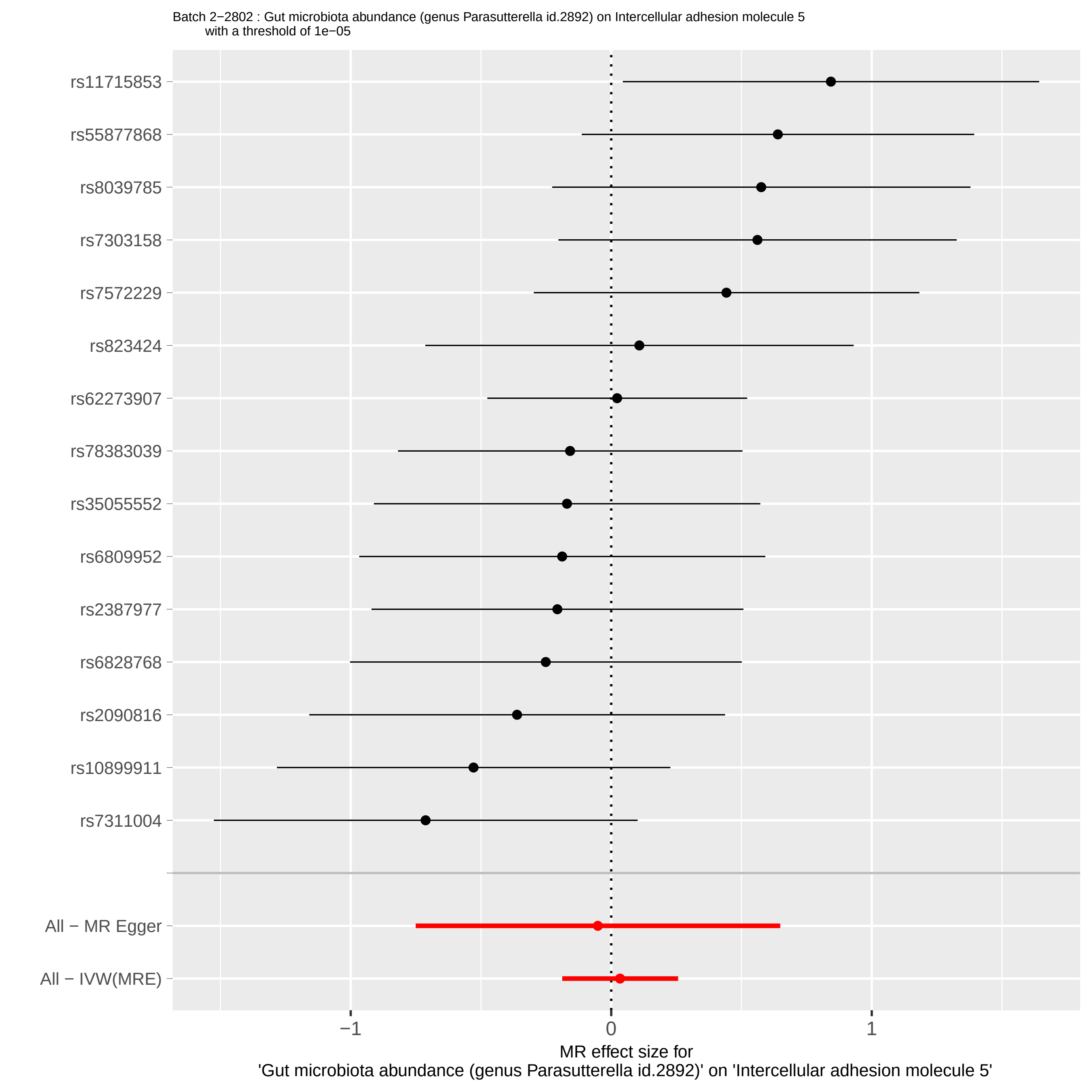

Batch 2−2802 : Gut microbiota abundance (genus Parasutterella id.2892) on Intercellular adhesion molecule 5
with a threshold of 1e−05
rs11715853
rs55877868
rs8039785
rs7303158
rs7572229
rs823424
rs62273907
rs78383039
rs35055552
rs6809952
rs2387977
rs6828768
rs2090816
rs10899911
rs7311004
All − MR Egger
All − IVW(MRE)
−1
0
1
MR effect size for
'Gut microbiota abundance (genus Parasutterella id.2892)' on 'Intercellular adhesion molecule 5'

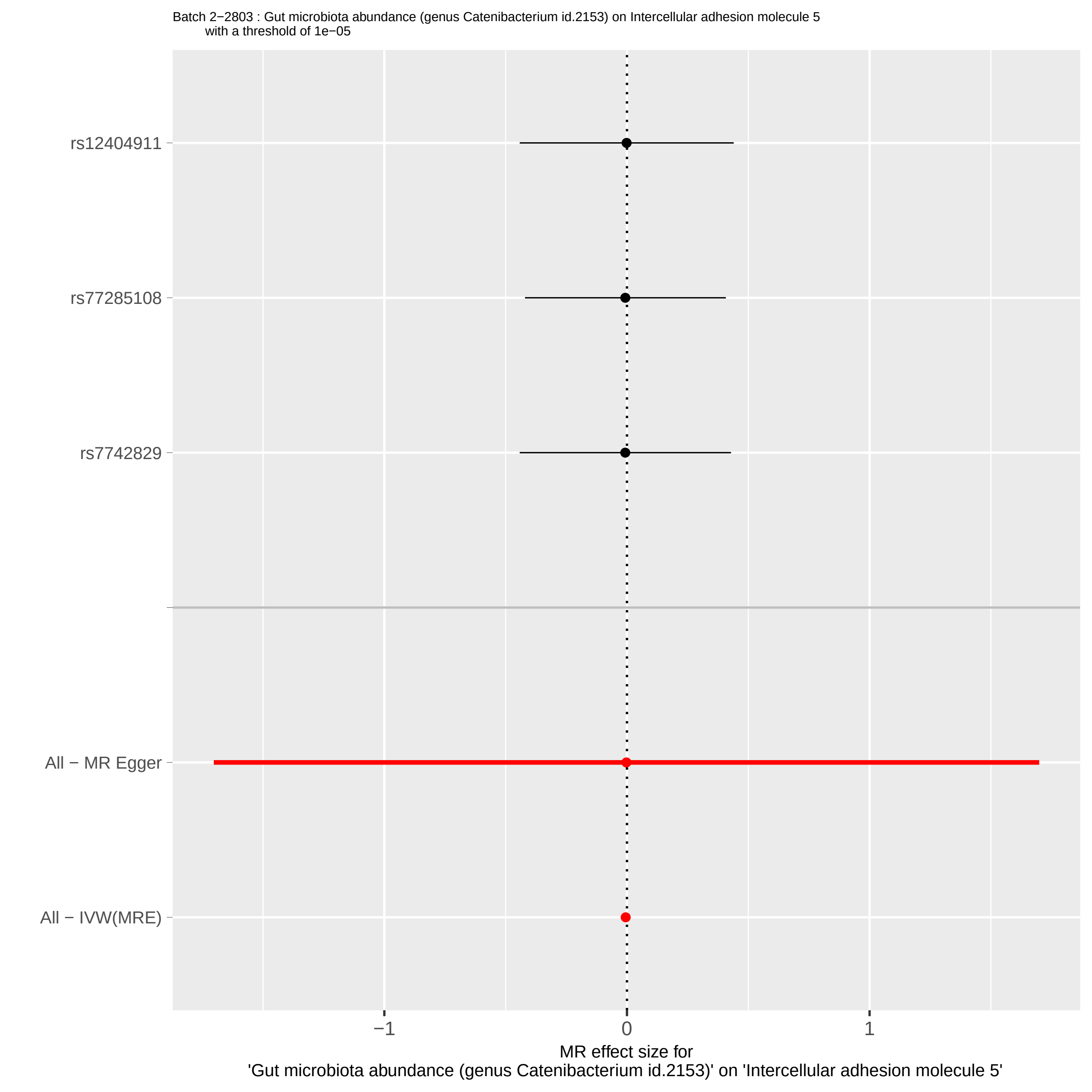
Batch 2−2803 : Gut microbiota abundance (genus Catenibacterium id.2153) on Intercellular adhesion molecule 5
with a threshold of 1e−05
rs12404911
rs77285108
rs7742829
All − MR Egger
All − IVW(MRE)
−1
0
1
MR effect size for
'Gut microbiota abundance (genus Catenibacterium id.2153)' on 'Intercellular adhesion molecule 5'

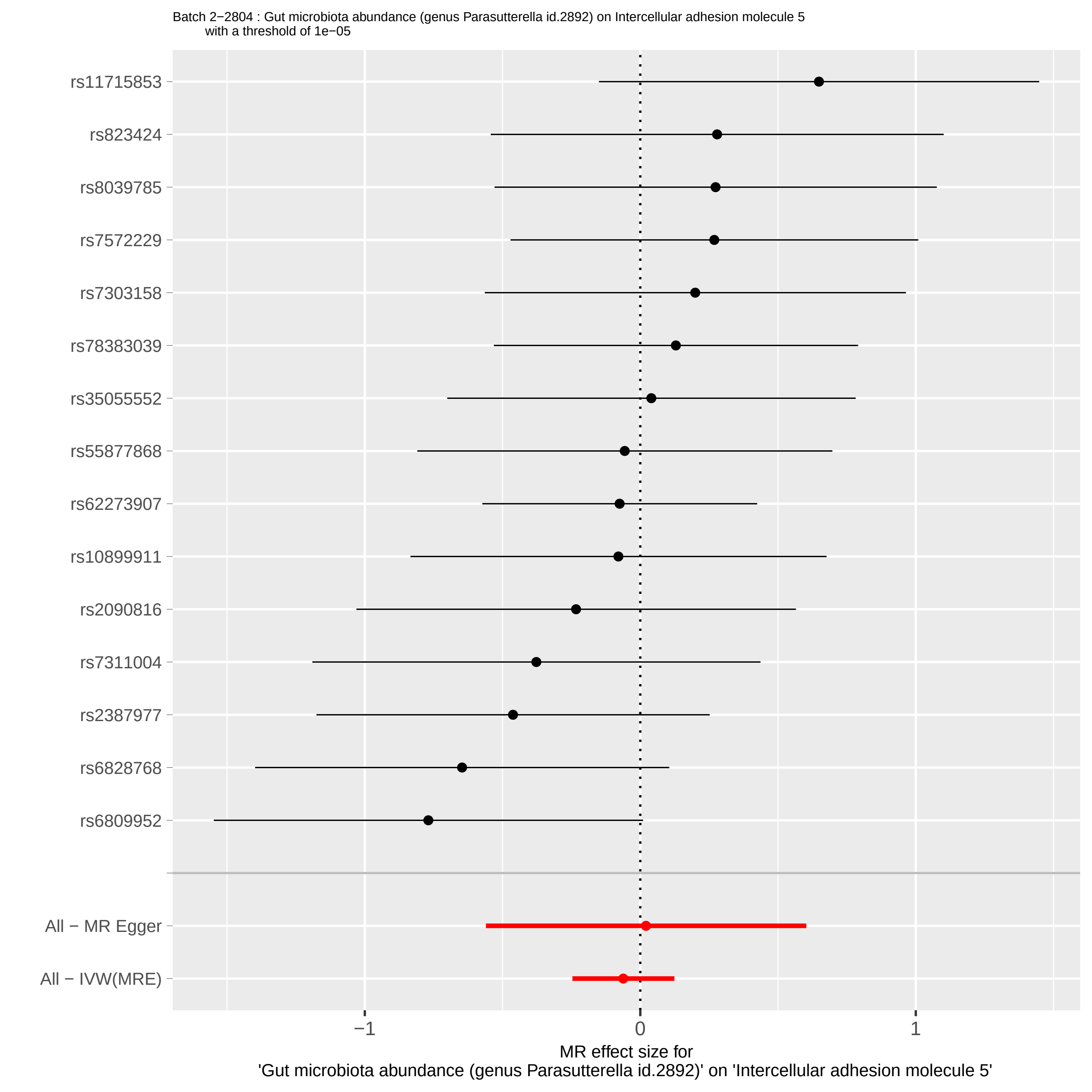

Batch 2−2804 : Gut microbiota abundance (genus Parasutterella id.2892) on Intercellular adhesion molecule 5
with a threshold of 1e−05
rs11715853
rs823424
rs8039785
rs7572229
rs7303158
rs78383039
rs35055552
rs55877868
rs62273907
rs10899911
rs2090816
rs7311004
rs2387977
rs6828768
rs6809952
All − MR Egger
All − IVW(MRE)
−1
0
1
MR effect size for
'Gut microbiota abundance (genus Parasutterella id.2892)' on 'Intercellular adhesion molecule 5'

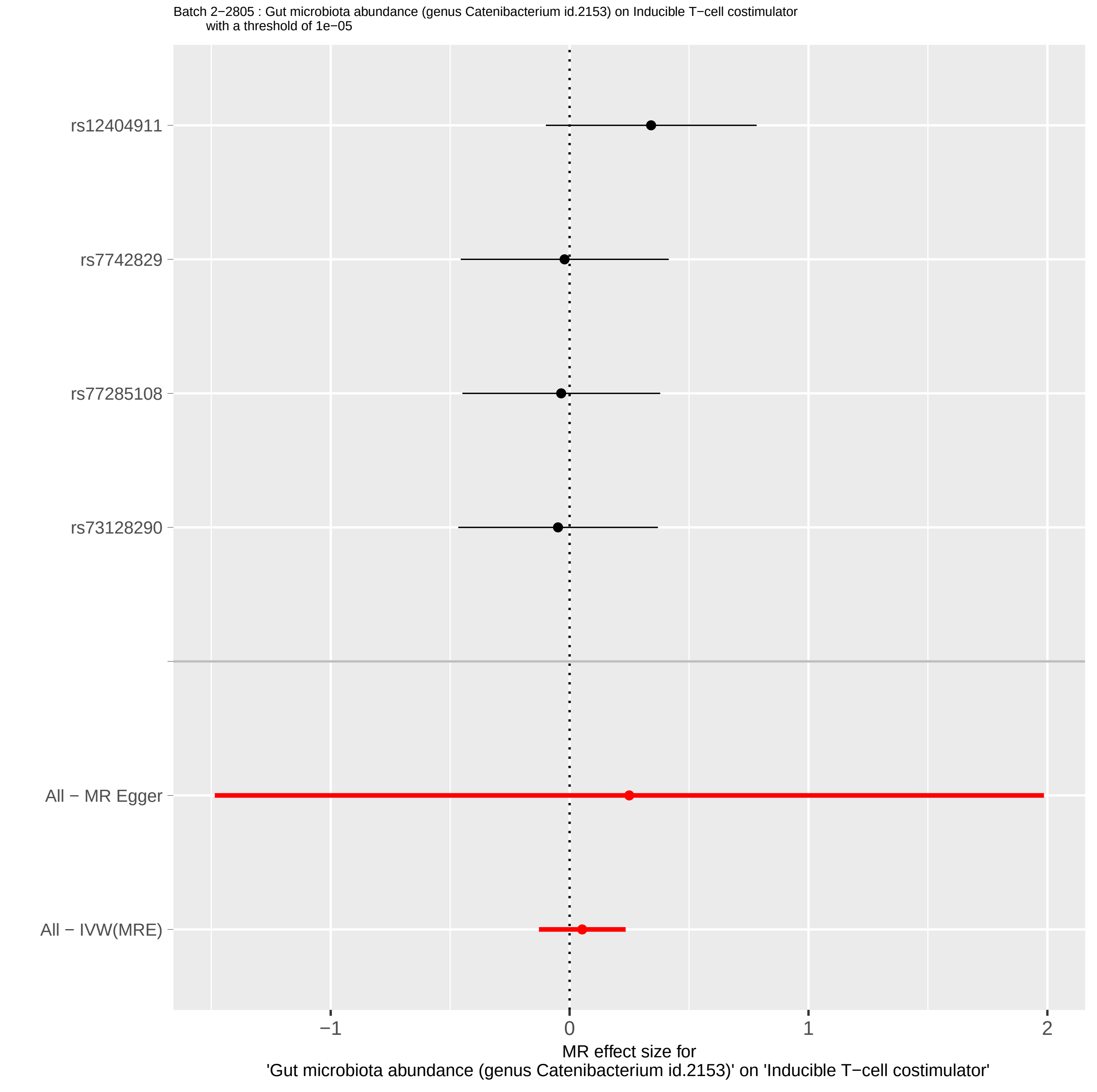

Batch 2−2805 : Gut microbiota abundance (genus Catenibacterium id.2153) on Inducible T−cell costimulator
with a threshold of 1e−05
rs12404911
rs7742829
rs77285108
rs73128290
All − MR Egger
All − IVW(MRE)
−1
0
1
2
MR effect size for
'Gut microbiota abundance (genus Catenibacterium id.2153)' on 'Inducible T−cell costimulator'

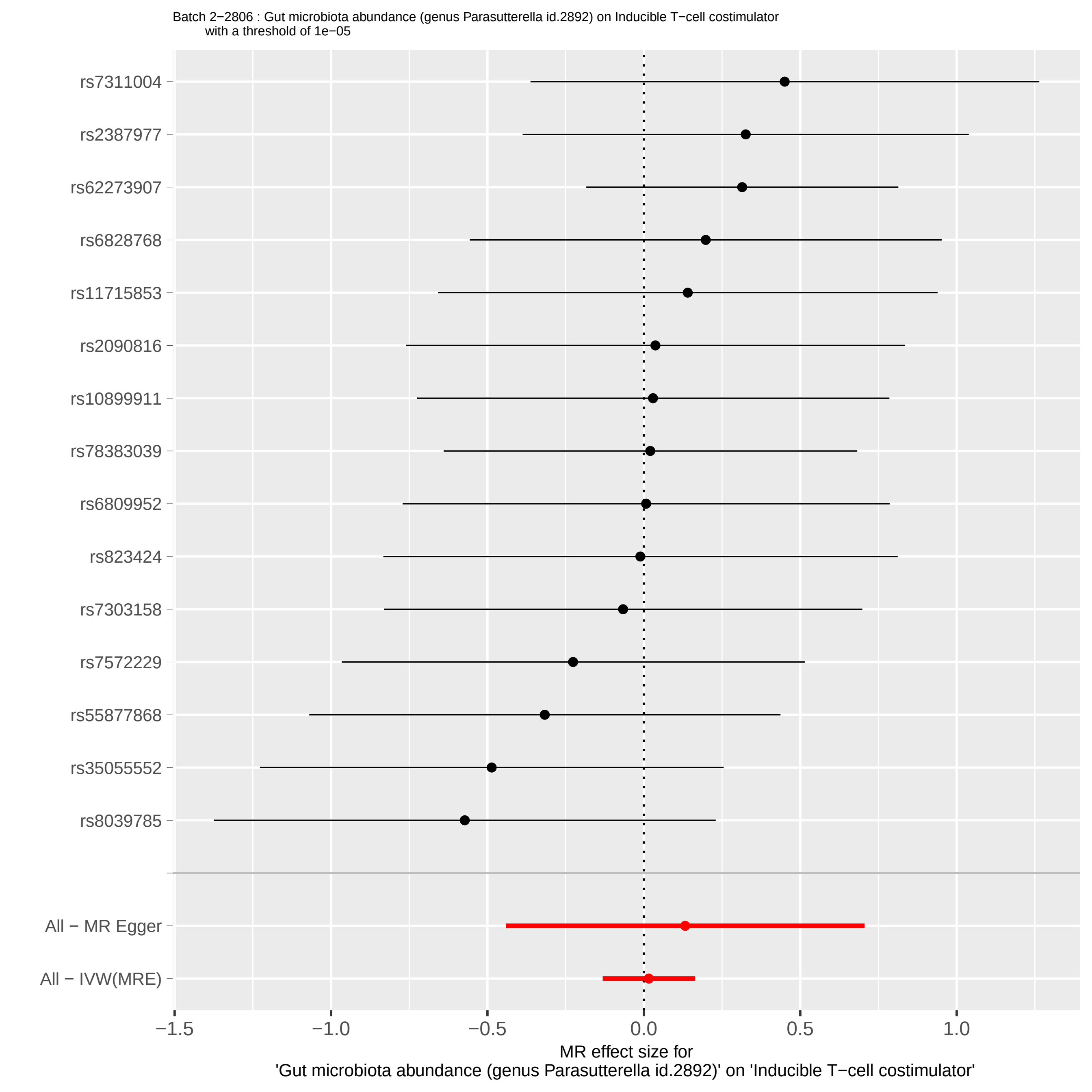

Batch 2−2806 : Gut microbiota abundance (genus Parasutterella id.2892) on Inducible T−cell costimulator
with a threshold of 1e−05
rs7311004
rs2387977
rs62273907
rs6828768
rs11715853
rs2090816
rs10899911
rs78383039
rs6809952
rs823424
rs7303158
rs7572229
rs55877868
rs35055552
rs8039785
All − MR Egger
All − IVW(MRE)
−1.5
−1.0
−0.5
0.0
0.5
1.0
MR effect size for
'Gut microbiota abundance (genus Parasutterella id.2892)' on 'Inducible T−cell costimulator'

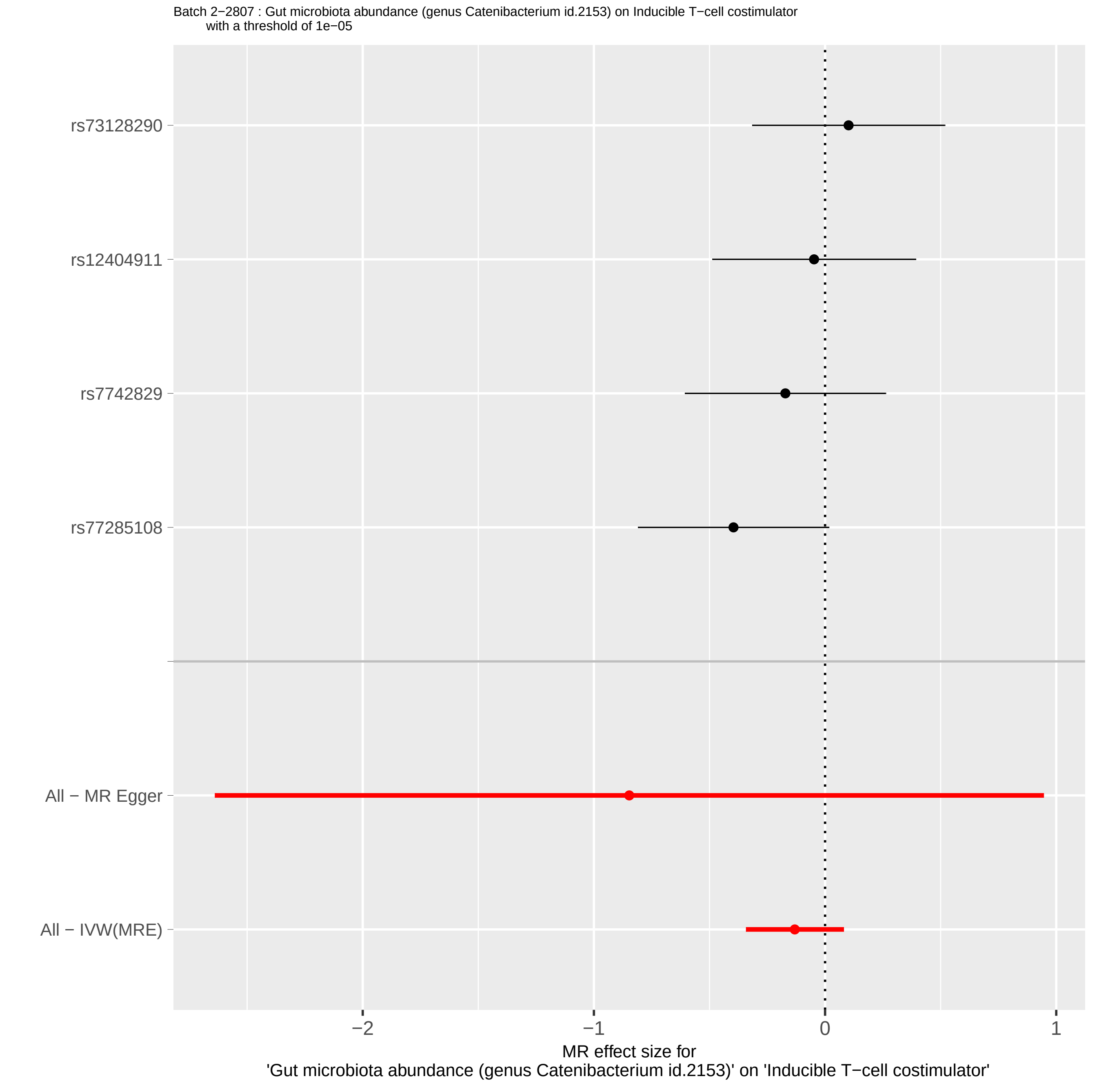
Batch 2−2807 : Gut microbiota abundance (genus Catenibacterium id.2153) on Inducible T−cell costimulator
with a threshold of 1e−05
rs73128290
rs12404911
rs7742829
rs77285108
All − MR Egger
All − IVW(MRE)
−2
−1
0
1
MR effect size for
'Gut microbiota abundance (genus Catenibacterium id.2153)' on 'Inducible T−cell costimulator'

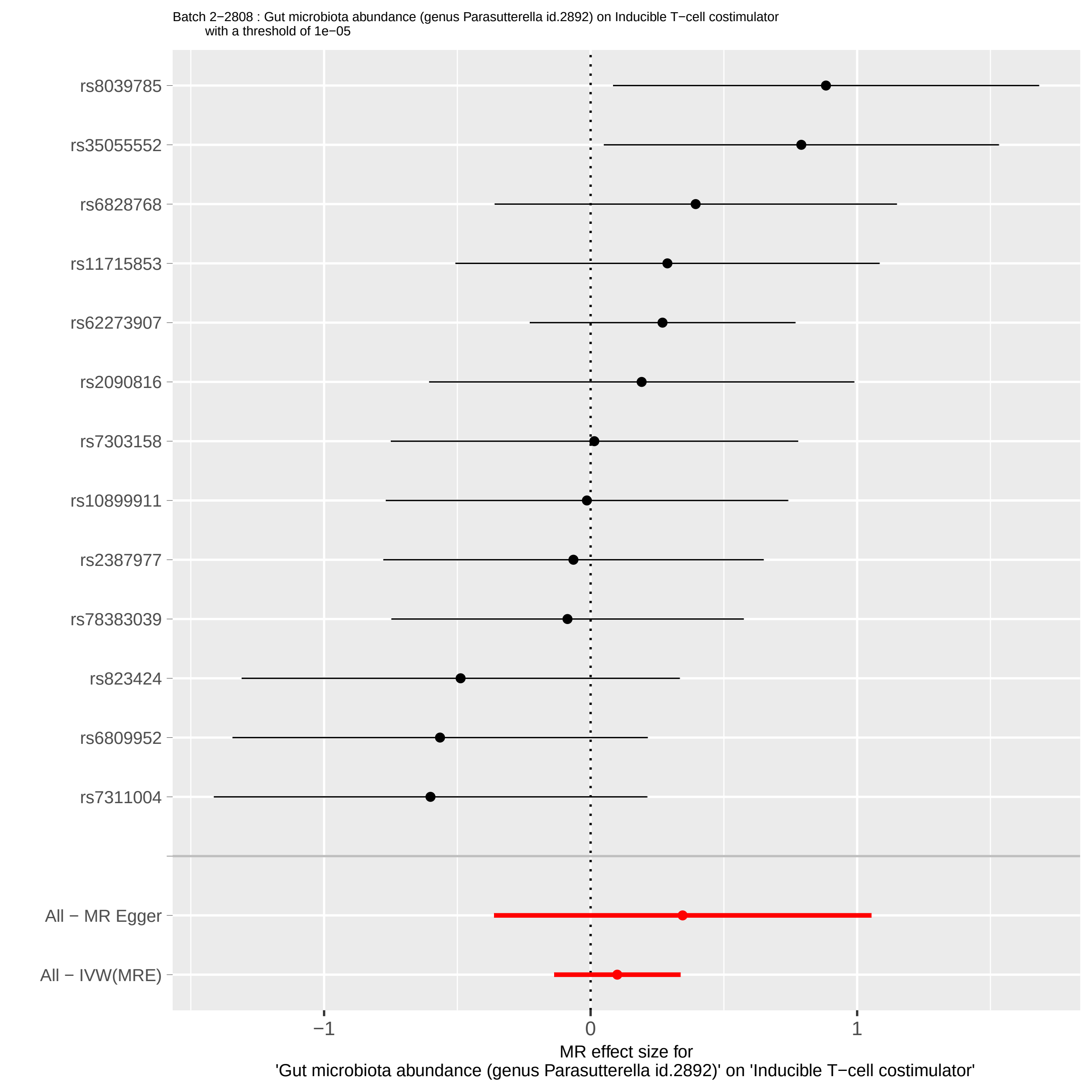

Batch 2−2808 : Gut microbiota abundance (genus Parasutterella id.2892) on Inducible T−cell costimulator
with a threshold of 1e−05
rs8039785
rs35055552
rs6828768
rs11715853
rs62273907
rs2090816
rs7303158
rs10899911
rs2387977
rs78383039
rs823424
rs6809952
rs7311004
All − MR Egger
All − IVW(MRE)
−1
0
1
MR effect size for
'Gut microbiota abundance (genus Parasutterella id.2892)' on 'Inducible T−cell costimulator'

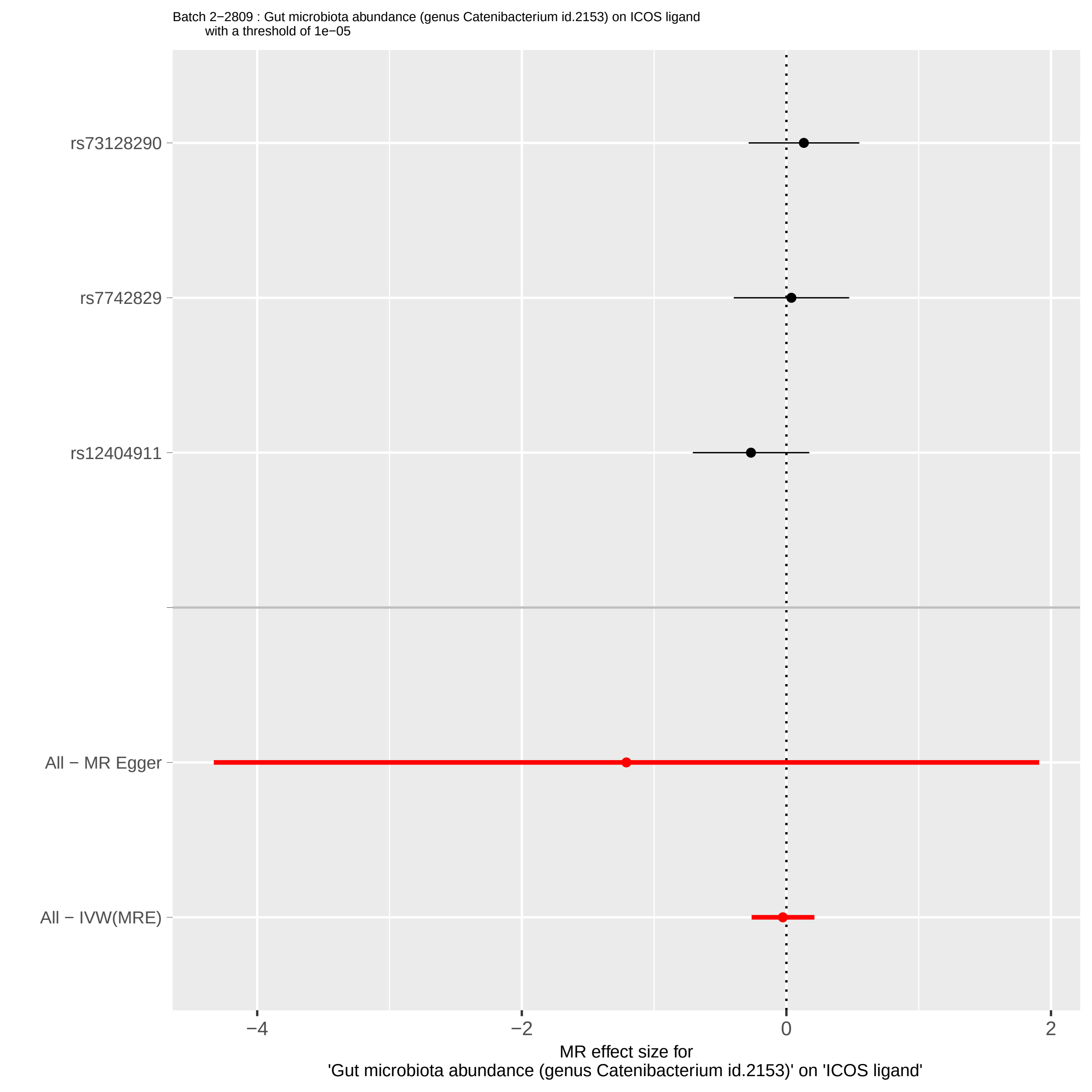

Batch 2−2809 : Gut microbiota abundance (genus Catenibacterium id.2153) on ICOS ligand
with a threshold of 1e−05
rs73128290
rs7742829
rs12404911
All − MR Egger
All − IVW(MRE)
−4
−2
0
2
MR effect size for
'Gut microbiota abundance (genus Catenibacterium id.2153)' on 'ICOS ligand'

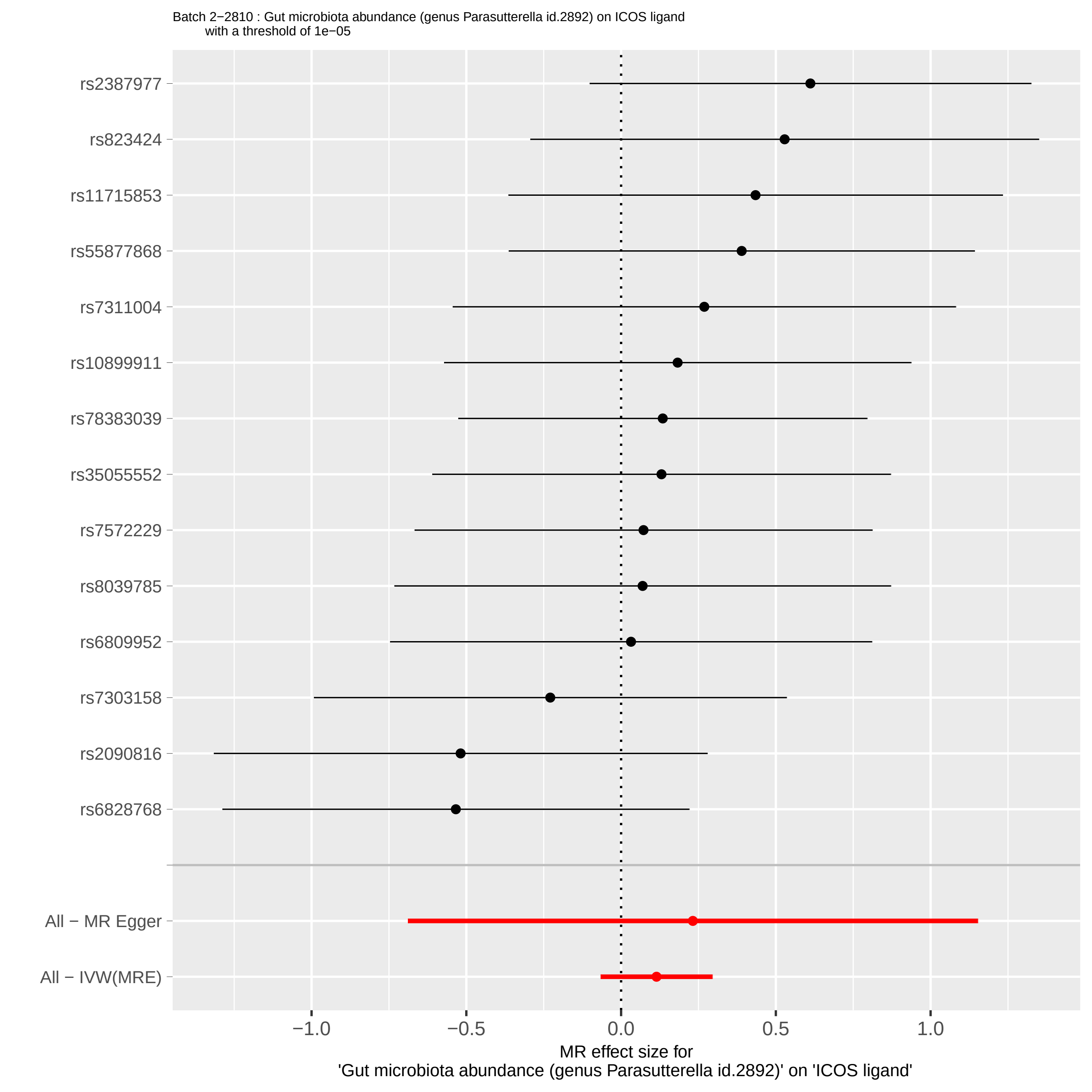
Batch 2−2810 : Gut microbiota abundance (genus Parasutterella id.2892) on ICOS ligand
with a threshold of 1e−05
rs2387977
rs823424
rs11715853
rs55877868
rs7311004
rs10899911
rs78383039
rs35055552
rs7572229
rs8039785
rs6809952
rs7303158
rs2090816
rs6828768
All − MR Egger
All − IVW(MRE)
−1.0
−0.5
0.0
0.5
1.0
MR effect size for
'Gut microbiota abundance (genus Parasutterella id.2892)' on 'ICOS ligand'

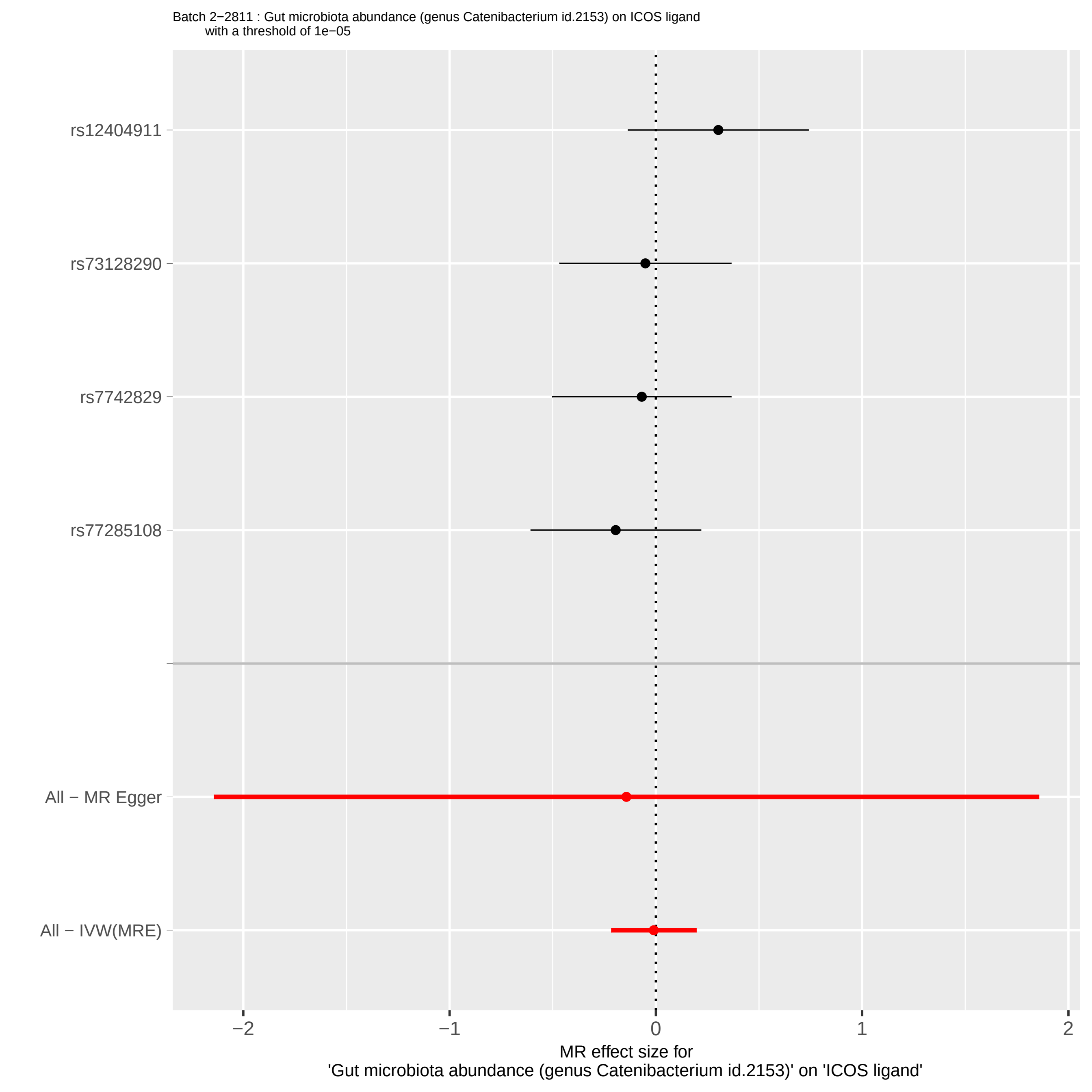
Batch 2−2811 : Gut microbiota abundance (genus Catenibacterium id.2153) on ICOS ligand
with a threshold of 1e−05
rs12404911
rs73128290
rs7742829
rs77285108
All − MR Egger
All − IVW(MRE)
−2
−1
0
1
2
MR effect size for
'Gut microbiota abundance (genus Catenibacterium id.2153)' on 'ICOS ligand'

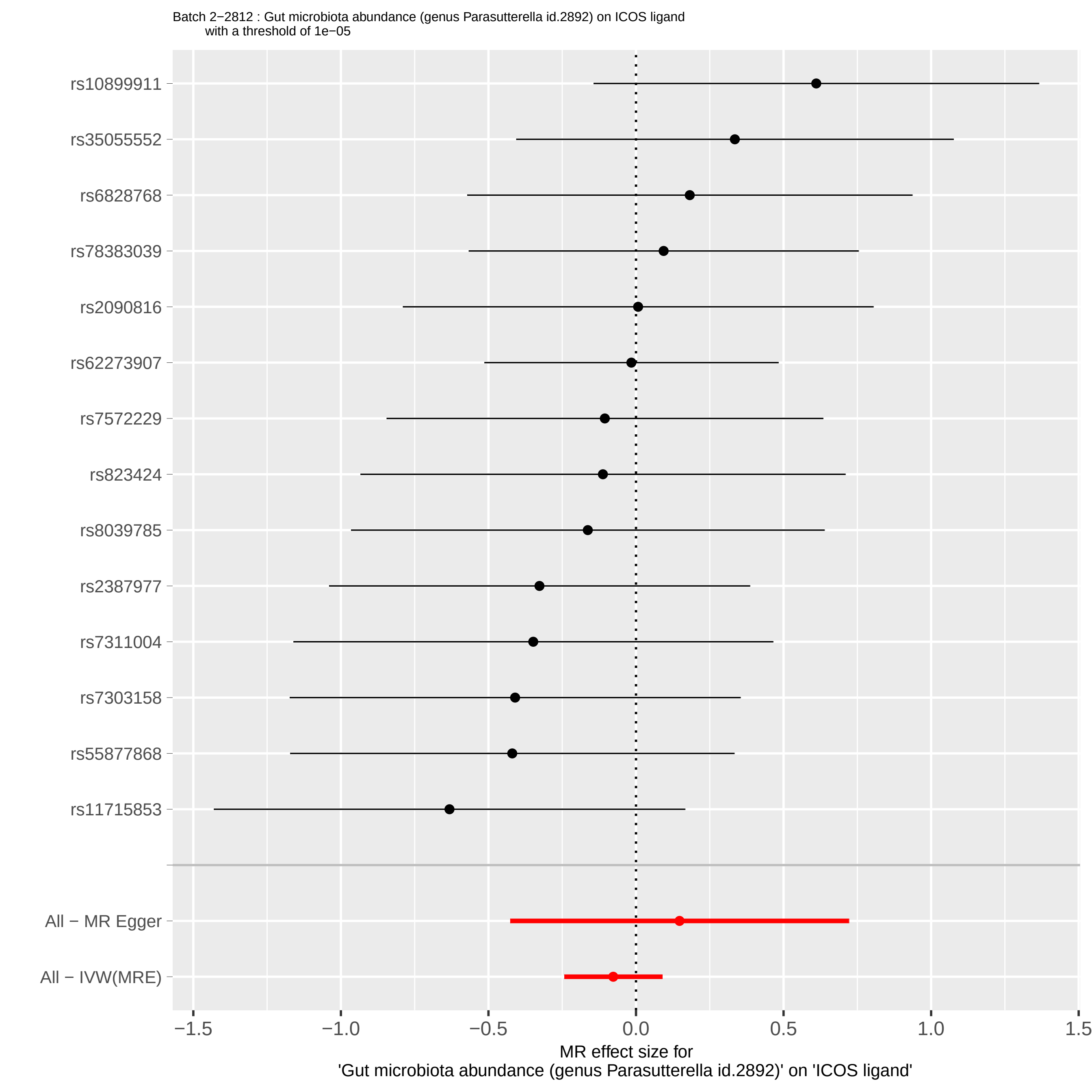

Batch 2−2812 : Gut microbiota abundance (genus Parasutterella id.2892) on ICOS ligand
with a threshold of 1e−05
rs10899911
rs35055552
rs6828768
rs78383039
rs2090816
rs62273907
rs7572229
rs823424
rs8039785
rs2387977
rs7311004
rs7303158
rs55877868
rs11715853
All − MR Egger
All − IVW(MRE)
−1.5
−1.0
−0.5
0.0
0.5
1.0
1.5
MR effect size for
'Gut microbiota abundance (genus Parasutterella id.2892)' on 'ICOS ligand'

Batch 2−2813 : Gut microbiota abundance (genus Catenibacterium id.2153) on DNA−binding protein inhibitor ID−1
with a threshold of 1e−05

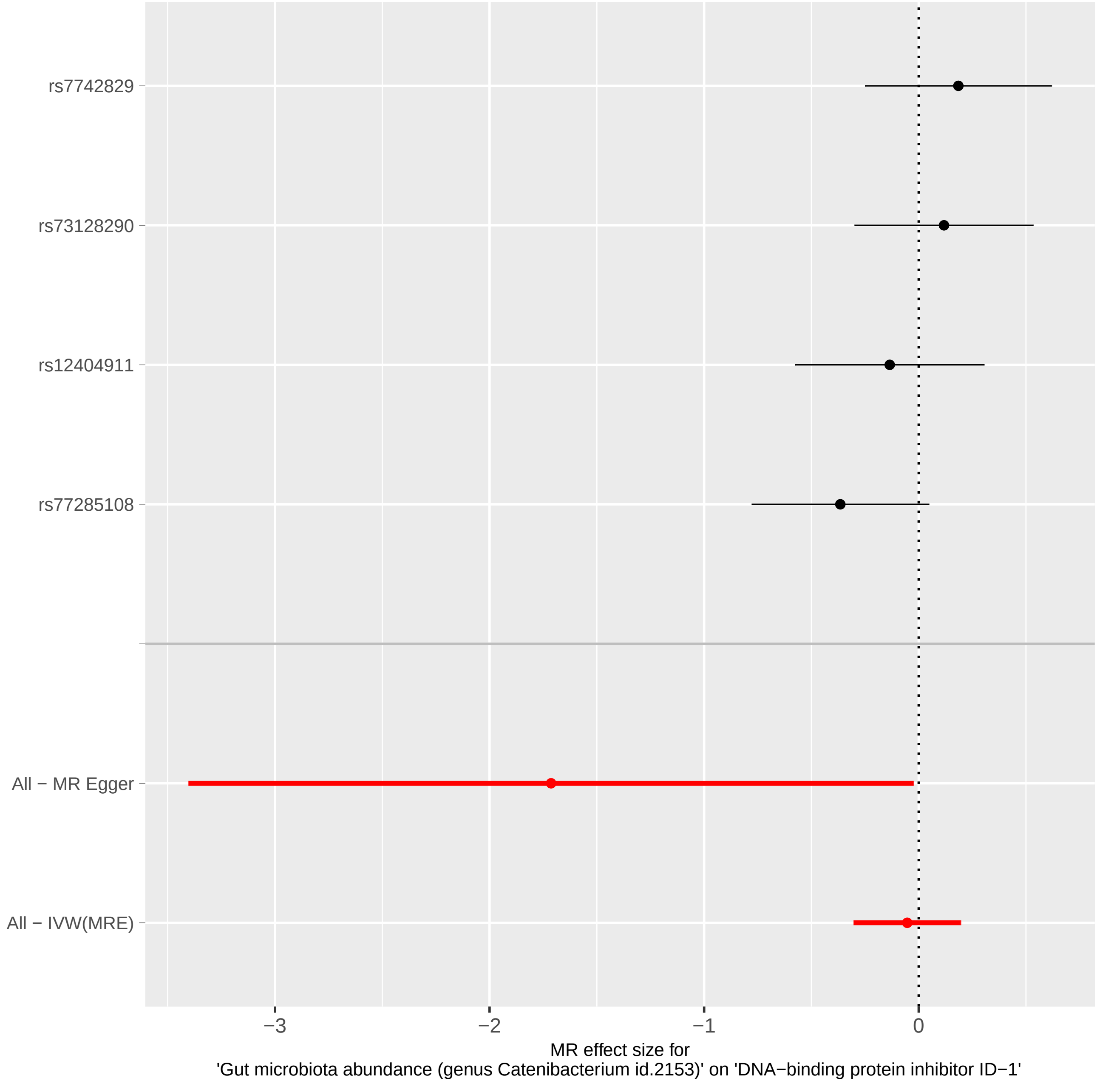

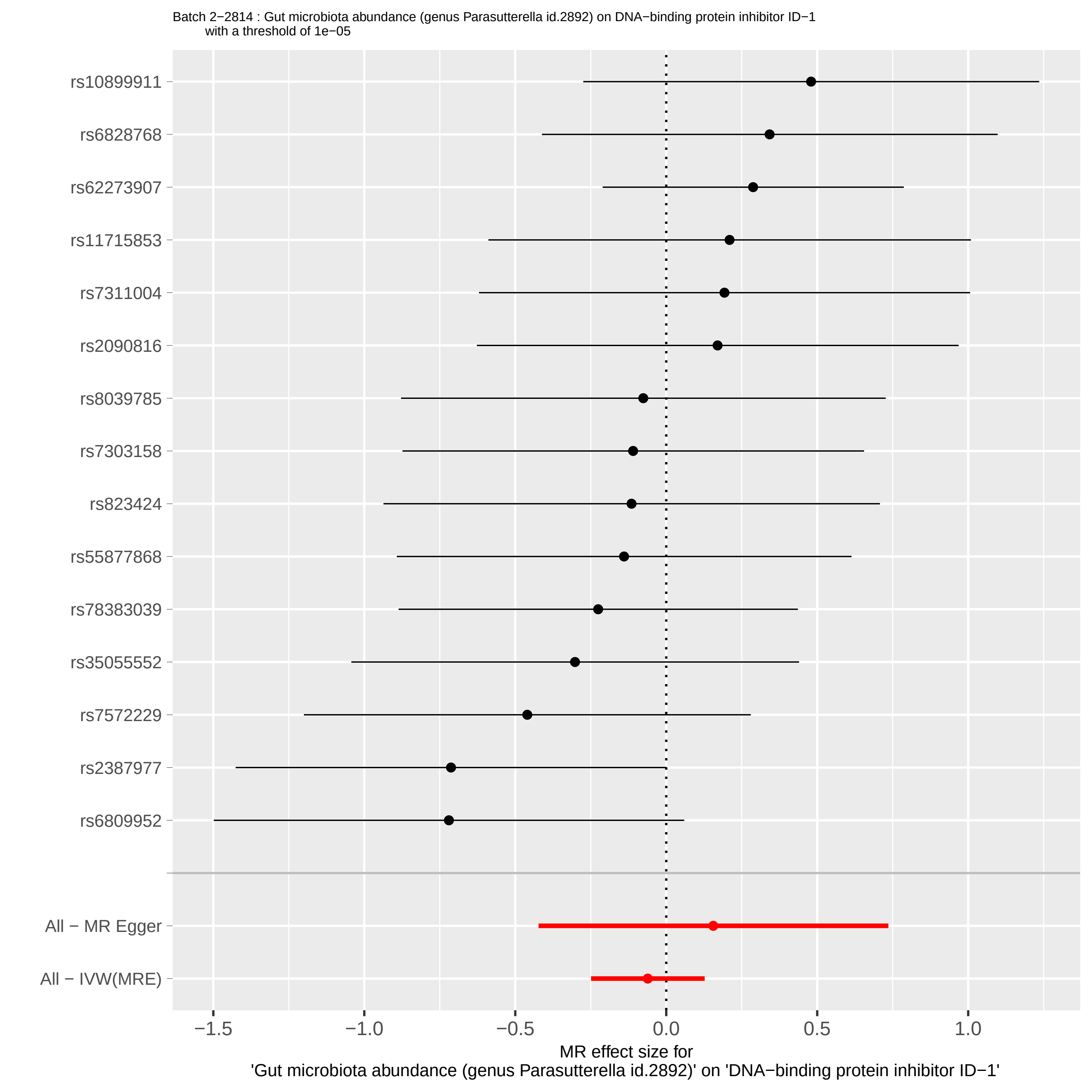
Batch 2−2814 : Gut microbiota abundance (genus Parasutterella id.2892) on DNA−binding protein inhibitor ID−1
with a threshold of 1e−05
rs10899911
rs6828768
rs62273907
rs11715853
rs7311004
rs2090816
rs8039785
rs7303158
rs823424
rs55877868
rs78383039
rs35055552
rs7572229
rs2387977
rs6809952
All − MR Egger
All − IVW(MRE)
−1.5
−1.0
−0.5
0.0
0.5
1.0
MR effect size for
'Gut microbiota abundance (genus Parasutterella id.2892)' on 'DNA−binding protein inhibitor ID−1'

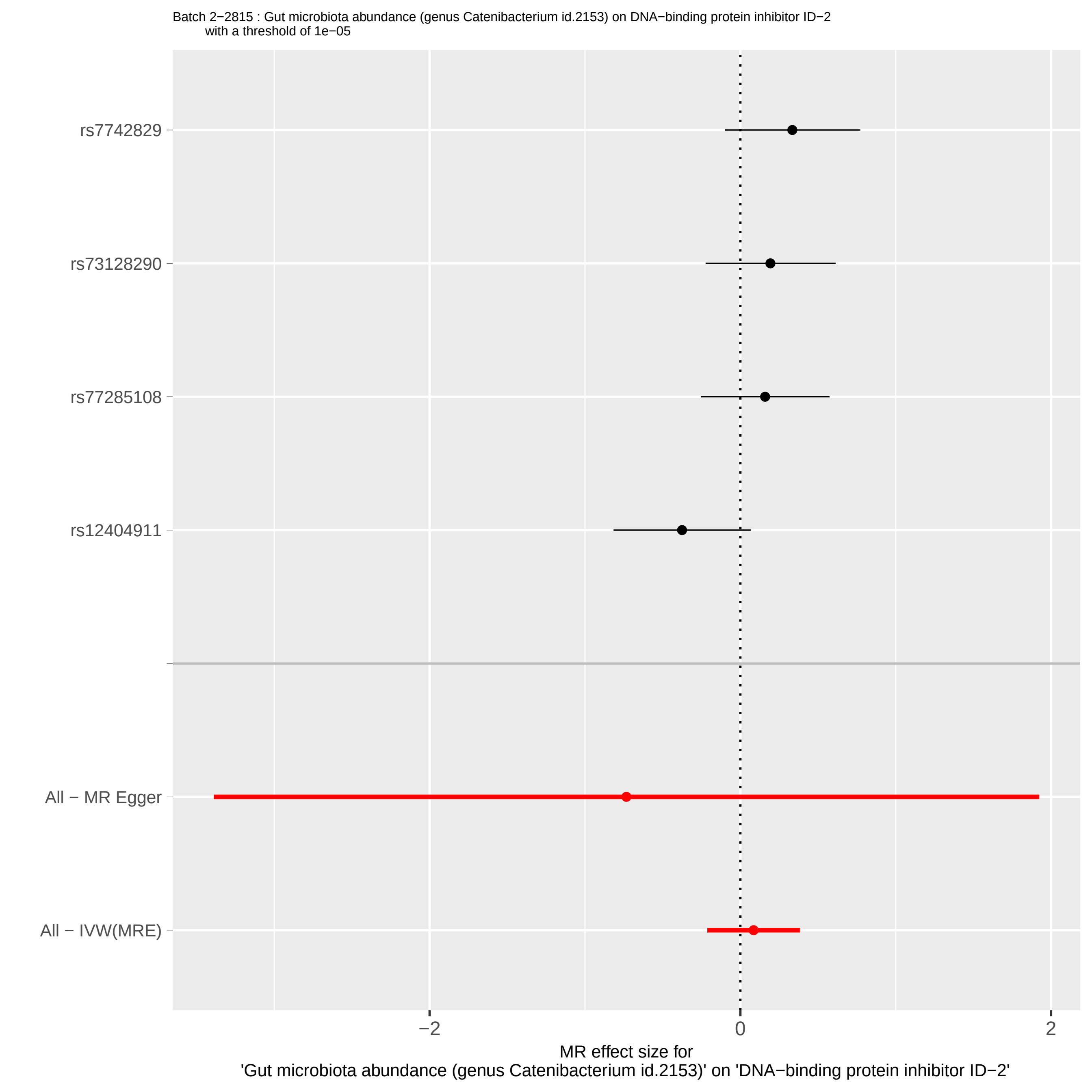

Batch 2−2815 : Gut microbiota abundance (genus Catenibacterium id.2153) on DNA−binding protein inhibitor ID−2
with a threshold of 1e−05
rs7742829
rs73128290
rs77285108
rs12404911
All − MR Egger
All − IVW(MRE)
−2
0
2
MR effect size for
'Gut microbiota abundance (genus Catenibacterium id.2153)' on 'DNA−binding protein inhibitor ID−2'

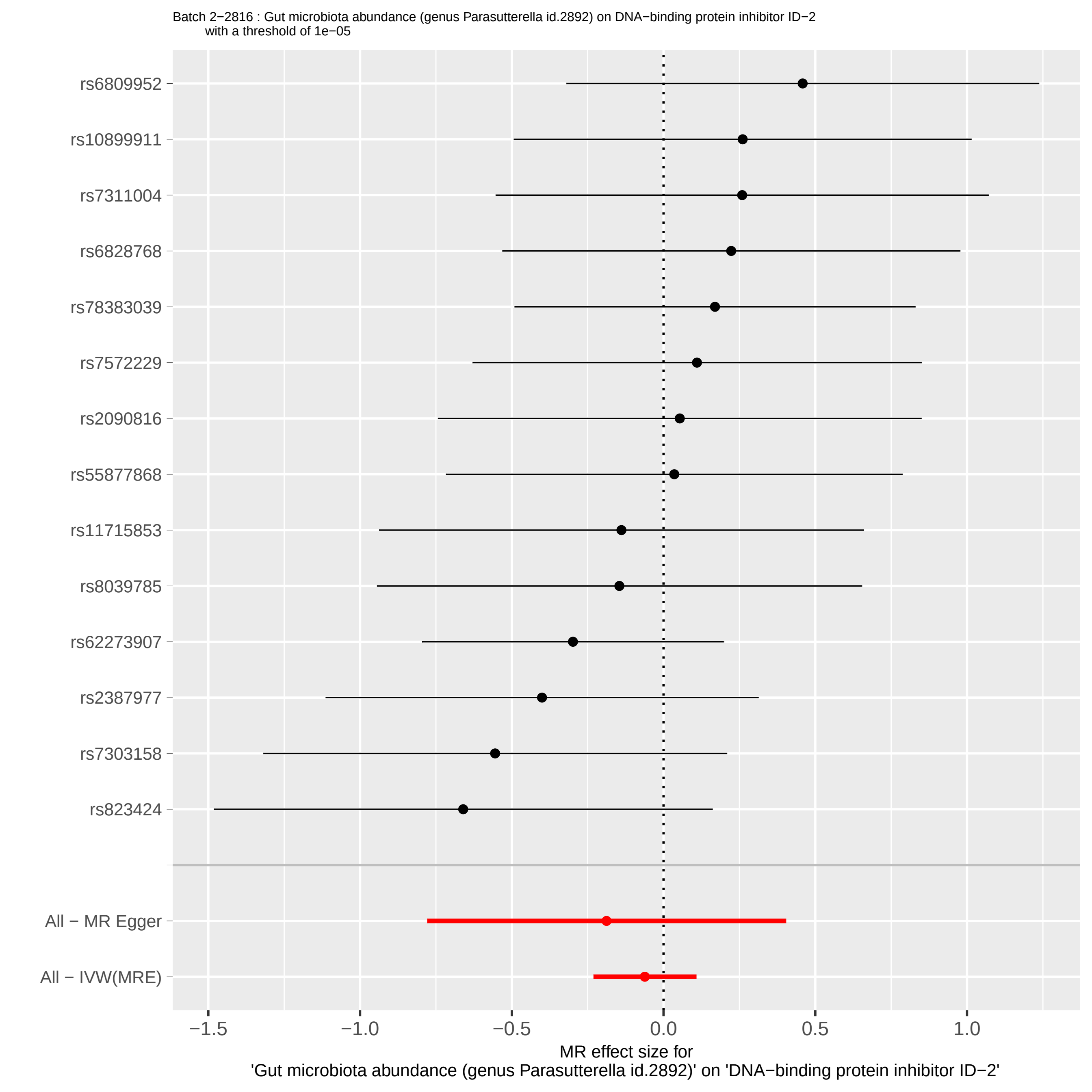

Batch 2−2816 : Gut microbiota abundance (genus Parasutterella id.2892) on DNA−binding protein inhibitor ID−2
with a threshold of 1e−05
rs6809952
rs10899911
rs7311004
rs6828768
rs78383039
rs7572229
rs2090816
rs55877868
rs11715853
rs8039785
rs62273907
rs2387977
rs7303158
rs823424
All − MR Egger
All − IVW(MRE)
−1.5
−1.0
−0.5
0.0
0.5
1.0
MR effect size for
'Gut microbiota abundance (genus Parasutterella id.2892)' on 'DNA−binding protein inhibitor ID−2'

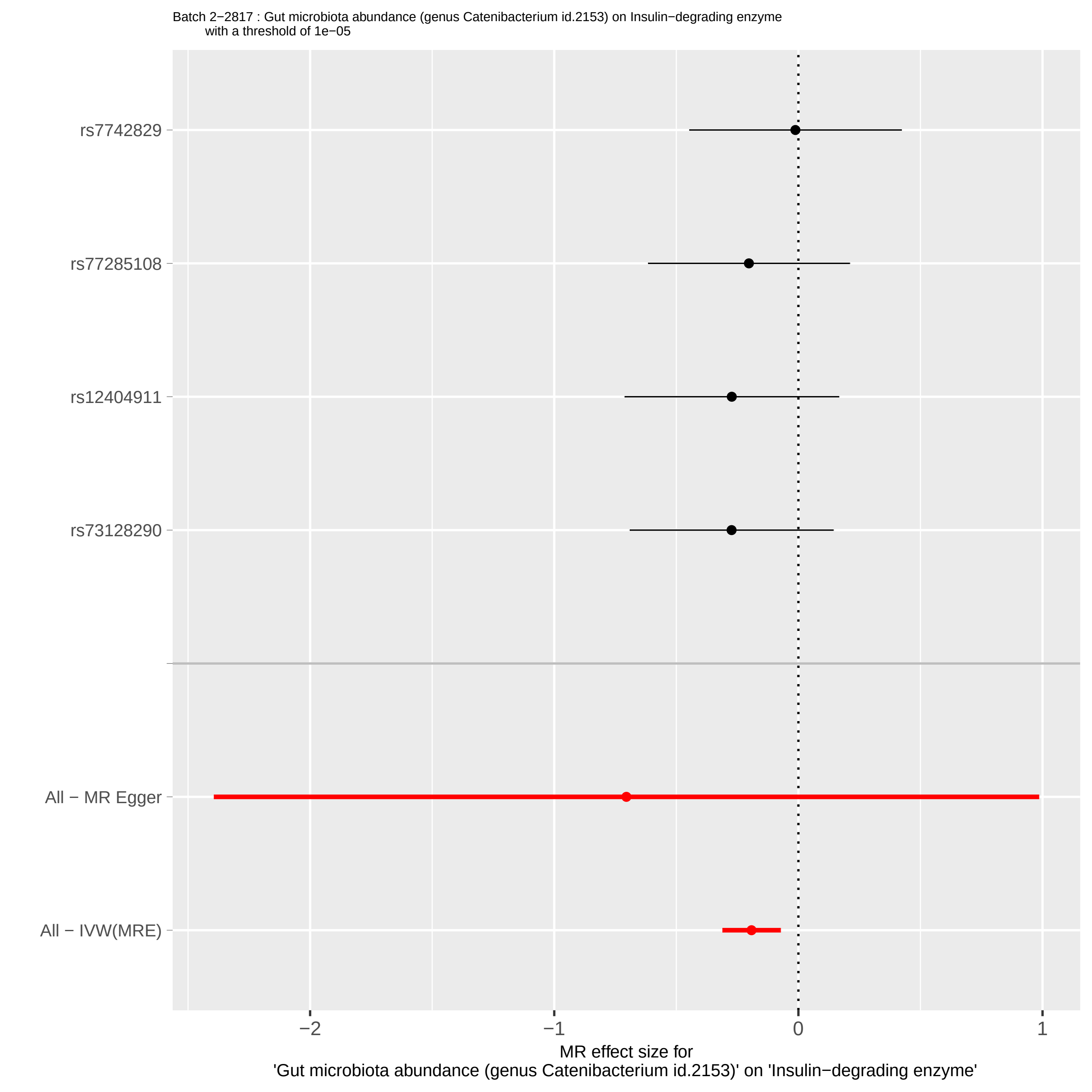
Batch 2−2817 : Gut microbiota abundance (genus Catenibacterium id.2153) on Insulin−degrading enzyme
with a threshold of 1e−05
rs7742829
rs77285108
rs12404911
rs73128290
All − MR Egger
All − IVW(MRE)
−2
−1
0
1
MR effect size for
'Gut microbiota abundance (genus Catenibacterium id.2153)' on 'Insulin−degrading enzyme'

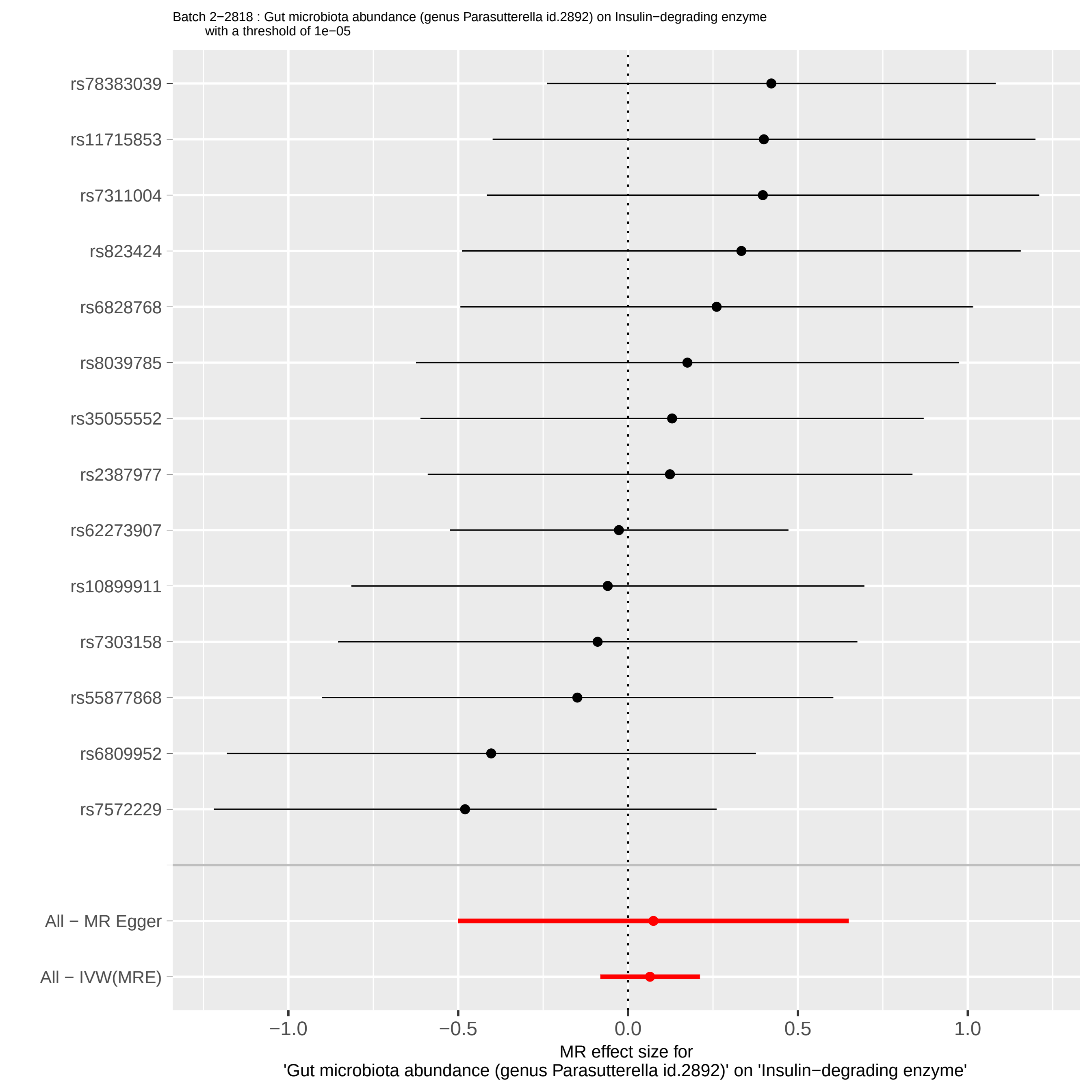

Batch 2−2818 : Gut microbiota abundance (genus Parasutterella id.2892) on Insulin−degrading enzyme
with a threshold of 1e−05
rs78383039
rs11715853
rs7311004
rs823424
rs6828768
rs8039785
rs35055552
rs2387977
rs62273907
rs10899911
rs7303158
rs55877868
rs6809952
rs7572229
All − MR Egger
All − IVW(MRE)
−1.0
−0.5
0.0
0.5
1.0
MR effect size for
'Gut microbiota abundance (genus Parasutterella id.2892)' on 'Insulin−degrading enzyme'

Batch 2−2819 : Gut microbiota abundance (genus Catenibacterium id.2153) on Indoleamine 2,3−dioxygenase 1
with a threshold of 1e−05

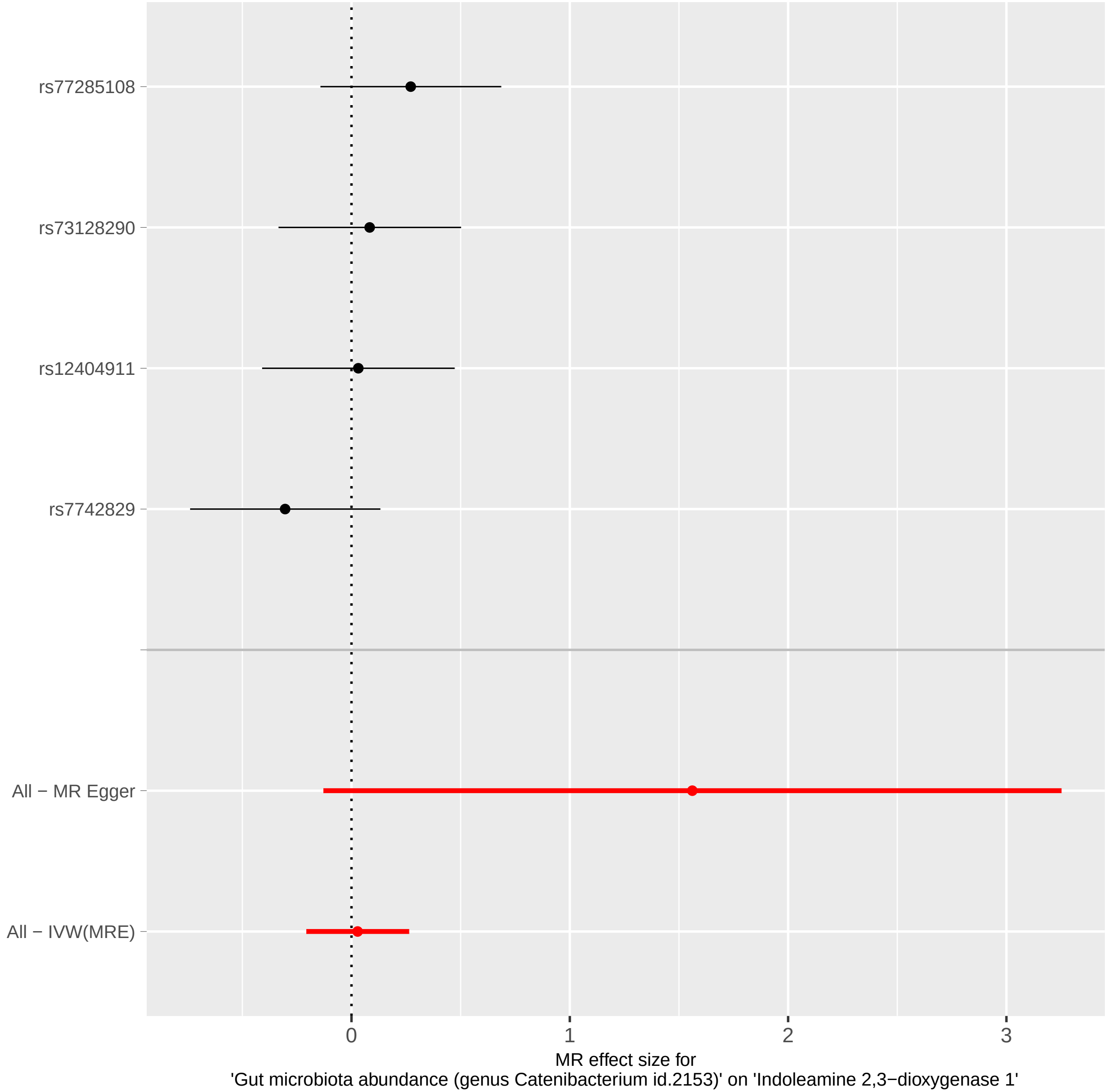

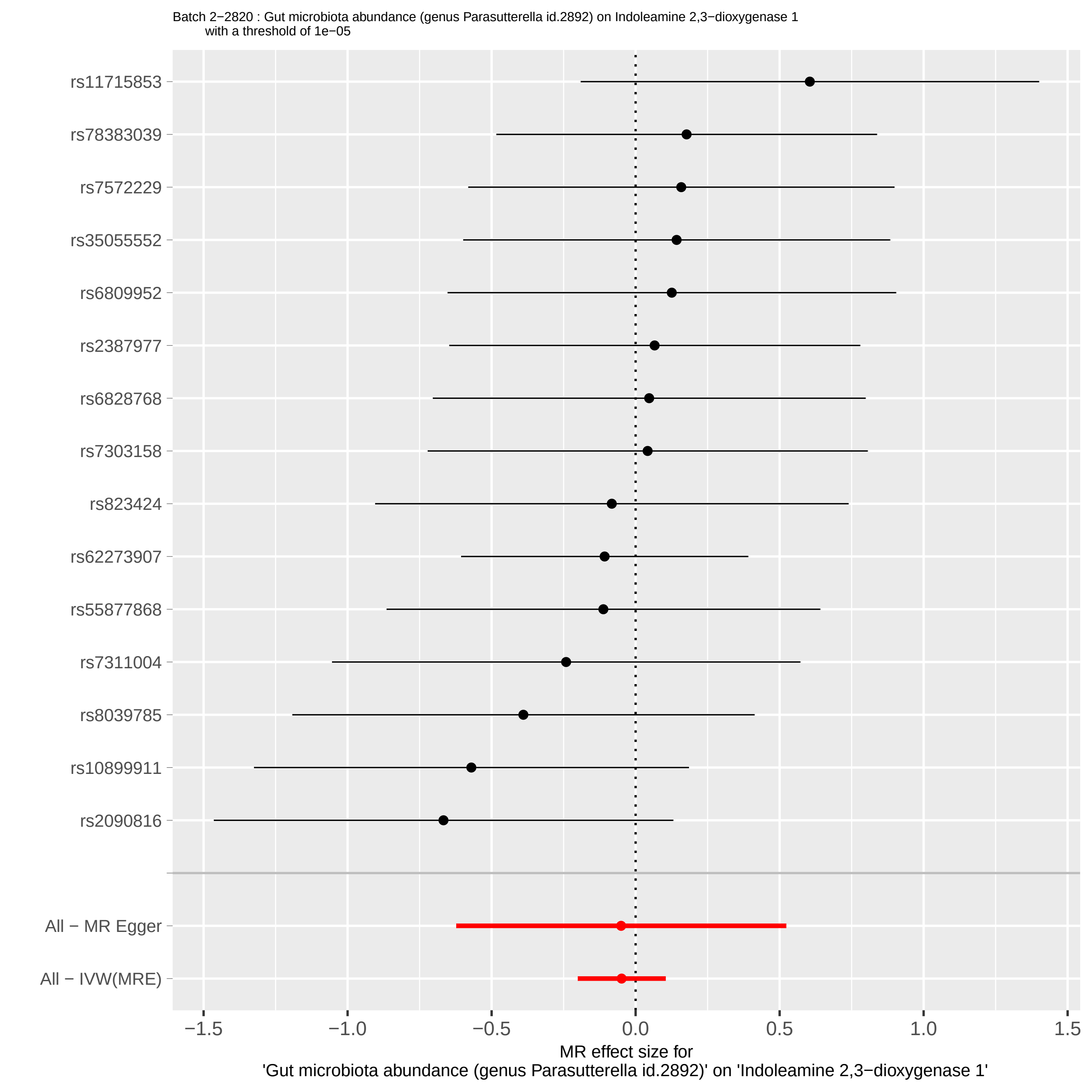

Batch 2−2820 : Gut microbiota abundance (genus Parasutterella id.2892) on Indoleamine 2,3−dioxygenase 1
with a threshold of 1e−05
rs11715853
rs78383039
rs7572229
rs35055552
rs6809952
rs2387977
rs6828768
rs7303158
rs823424
rs62273907
rs55877868
rs7311004
rs8039785
rs10899911
rs2090816
All − MR Egger
All − IVW(MRE)
−1.5
−1.0
−0.5
0.0
0.5
1.0
1.5
MR effect size for
'Gut microbiota abundance (genus Parasutterella id.2892)' on 'Indoleamine 2,3−dioxygenase 1'

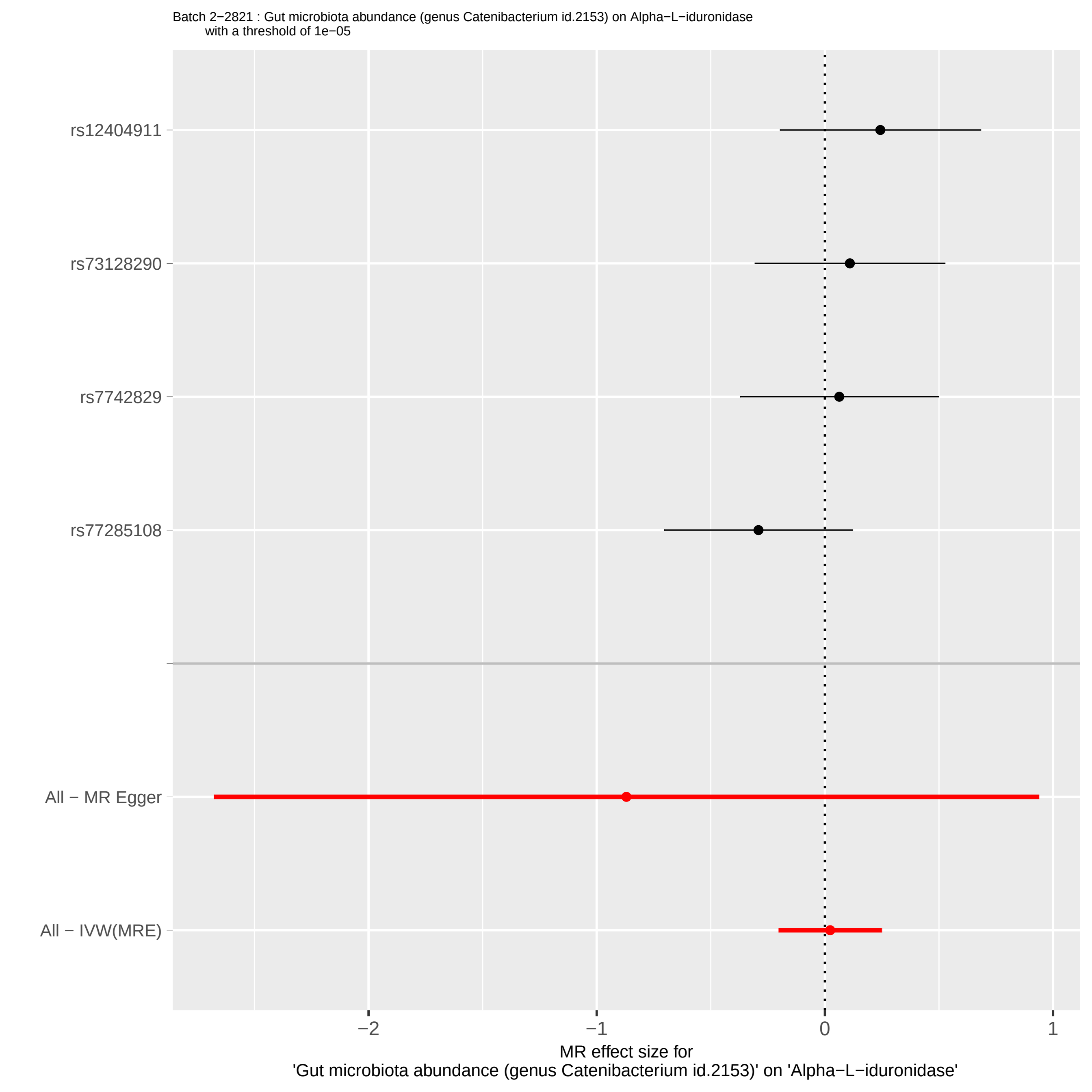

Batch 2−2821 : Gut microbiota abundance (genus Catenibacterium id.2153) on Alpha−L−iduronidase
with a threshold of 1e−05
rs12404911
rs73128290
rs7742829
rs77285108
All − MR Egger
All − IVW(MRE)
−2
−1
0
1
MR effect size for
'Gut microbiota abundance (genus Catenibacterium id.2153)' on 'Alpha−L−iduronidase'

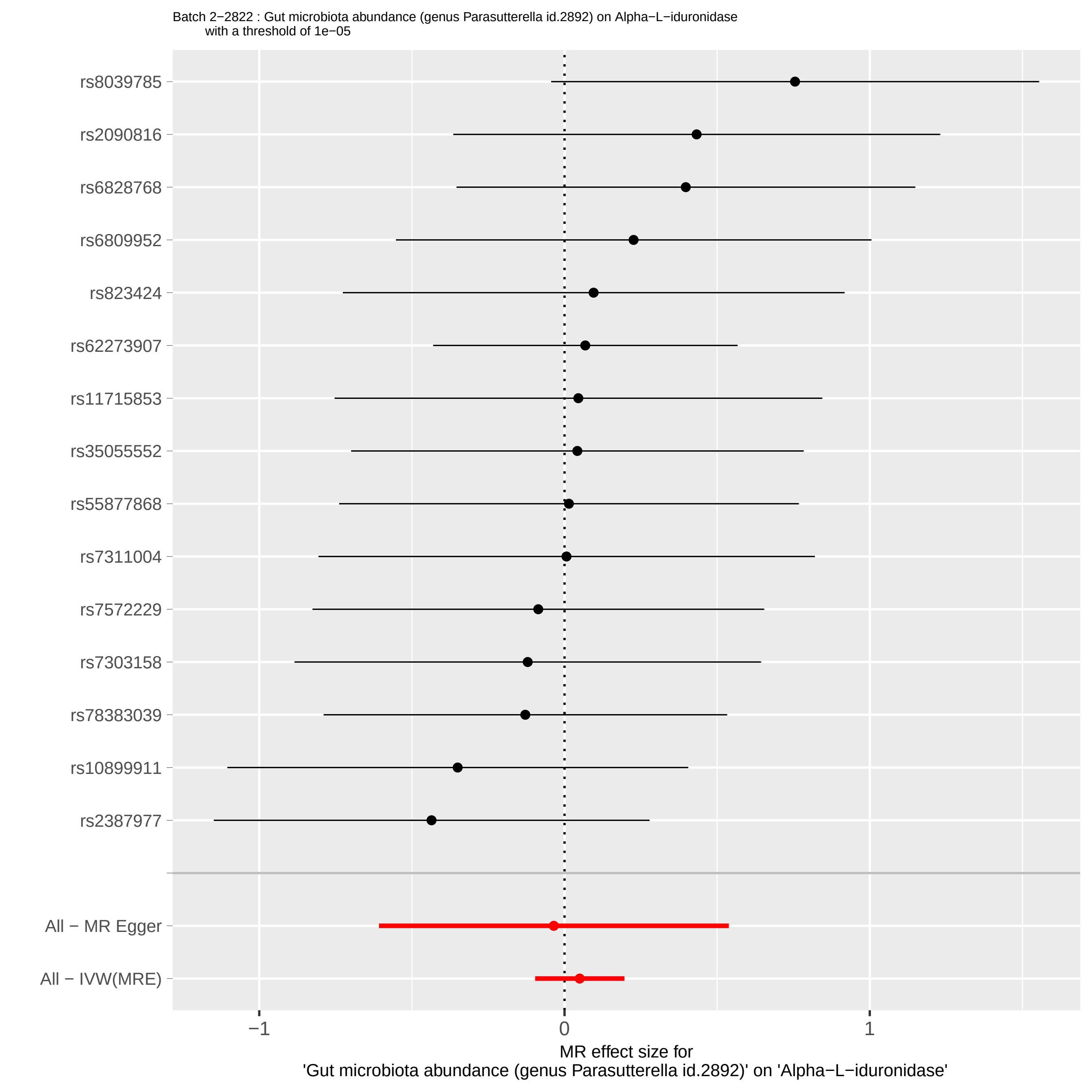
Batch 2−2822 : Gut microbiota abundance (genus Parasutterella id.2892) on Alpha−L−iduronidase
with a threshold of 1e−05
rs8039785
rs2090816
rs6828768
rs6809952
rs823424
rs62273907
rs11715853
rs35055552
rs55877868
rs7311004
rs7572229
rs7303158
rs78383039
rs10899911
rs2387977
All − MR Egger
All − IVW(MRE)
−1
0
1
MR effect size for
'Gut microbiota abundance (genus Parasutterella id.2892)' on 'Alpha−L−iduronidase'

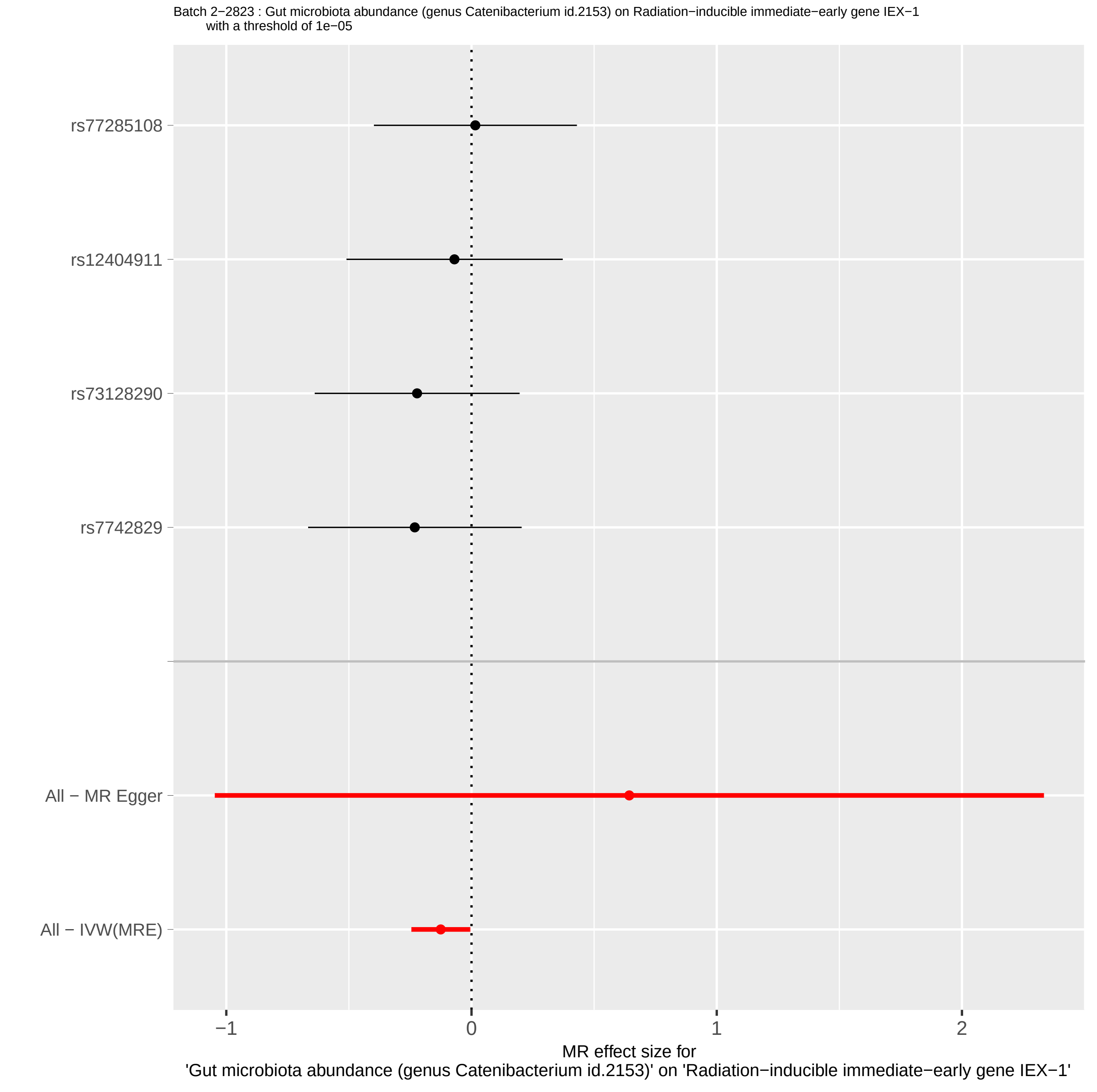

Batch 2−2823 : Gut microbiota abundance (genus Catenibacterium id.2153) on Radiation−inducible immediate−early gene IEX−1
with a threshold of 1e−05
rs77285108
rs12404911
rs73128290
rs7742829
All − MR Egger
All − IVW(MRE)
−1
0
1
2
MR effect size for
'Gut microbiota abundance (genus Catenibacterium id.2153)' on 'Radiation−inducible immediate−early gene IEX−1'

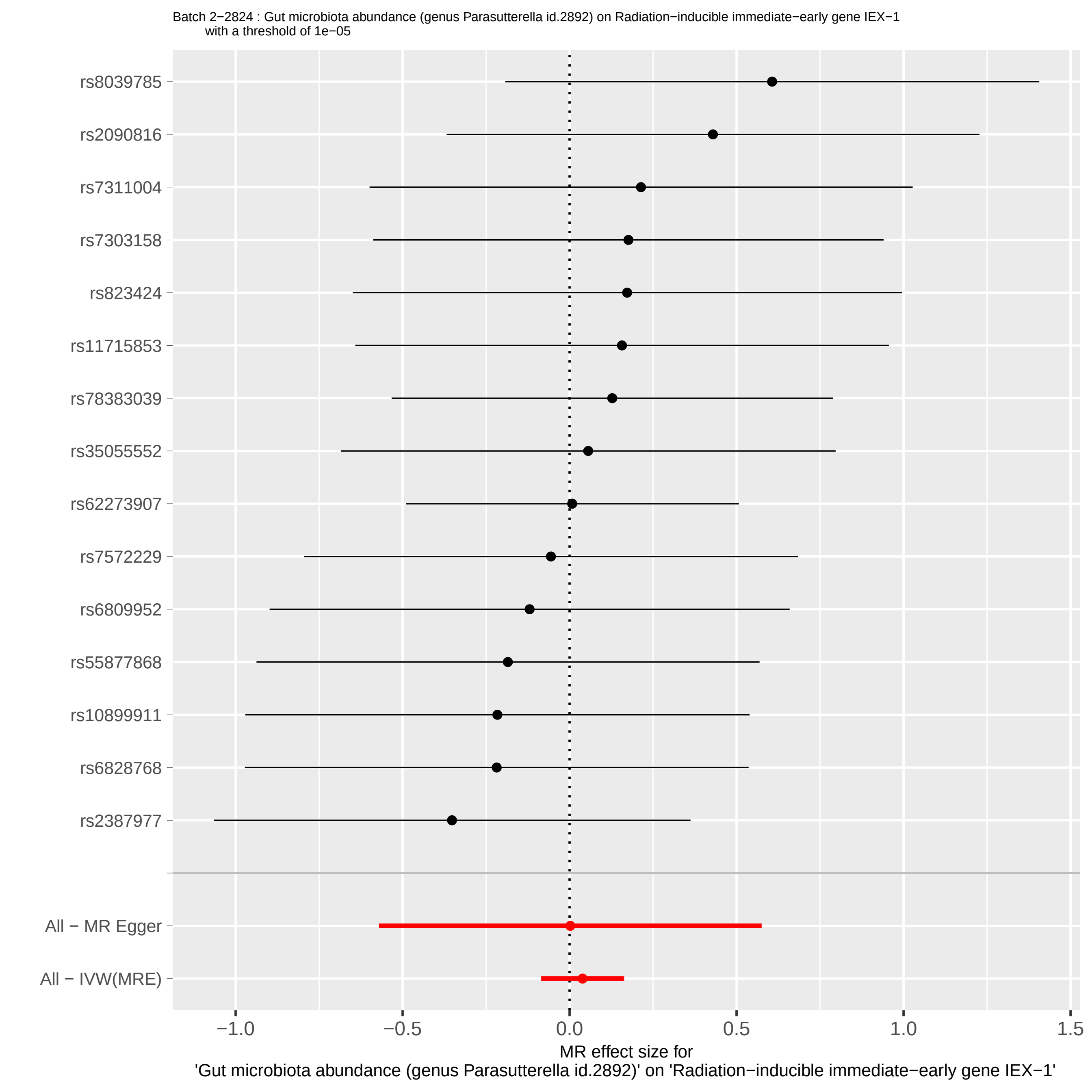
Batch 2−2824 : Gut microbiota abundance (genus Parasutterella id.2892) on Radiation−inducible immediate−early gene IEX−1
with a threshold of 1e−05
rs8039785
rs2090816
rs7311004
rs7303158
rs823424
rs11715853
rs78383039
rs35055552
rs62273907
rs7572229
rs6809952
rs55877868
rs10899911
rs6828768
rs2387977
All − MR Egger
All − IVW(MRE)
−1.0
−0.5
0.0
0.5
1.0
1.5
MR effect size for
'Gut microbiota abundance (genus Parasutterella id.2892)' on 'Radiation−inducible immediate−early gene IEX−1'

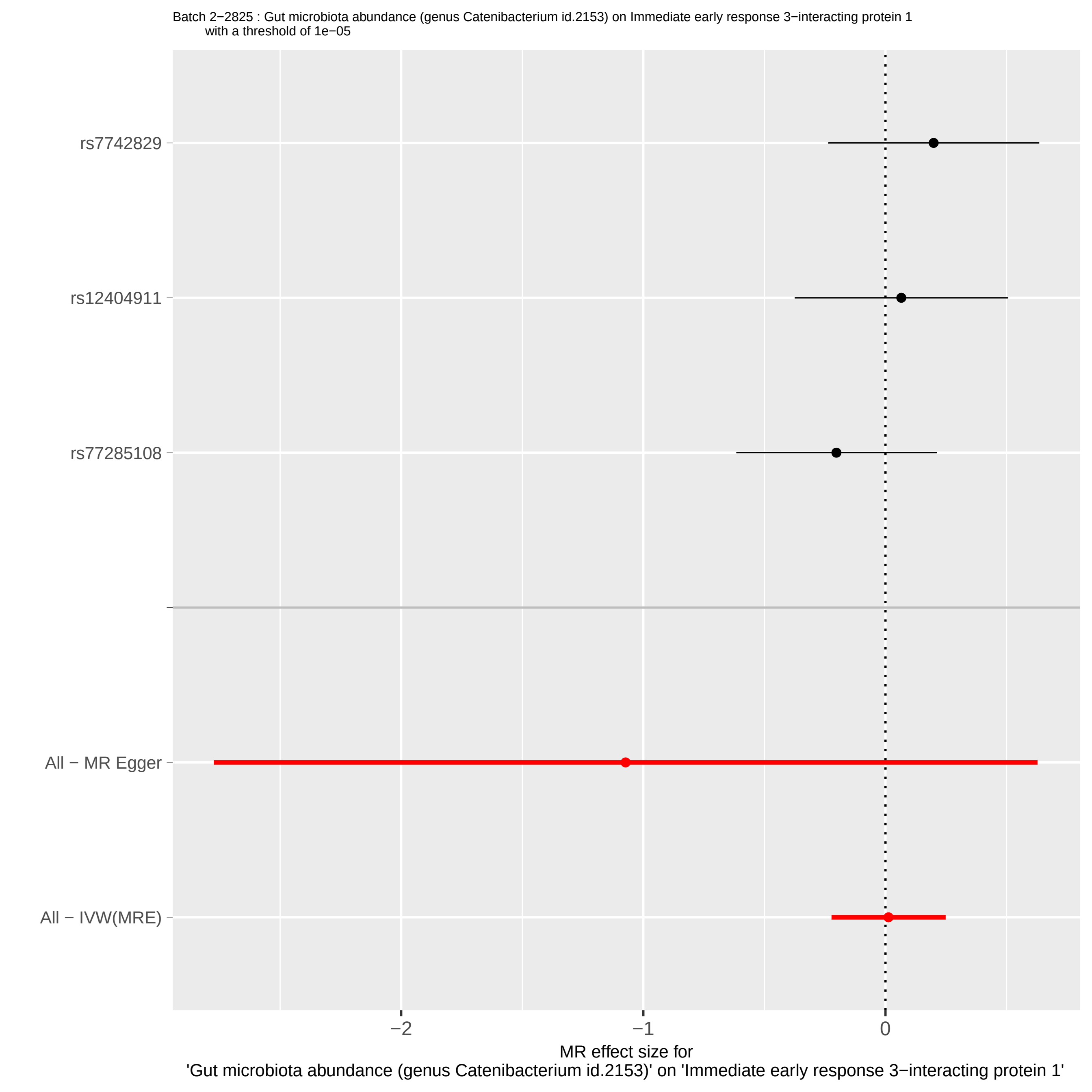
Batch 2−2825 : Gut microbiota abundance (genus Catenibacterium id.2153) on Immediate early response 3−interacting protein 1
with a threshold of 1e−05
rs7742829
rs12404911
rs77285108
All − MR Egger
All − IVW(MRE)
−2
−1
0
MR effect size for
'Gut microbiota abundance (genus Catenibacterium id.2153)' on 'Immediate early response 3−interacting protein 1'

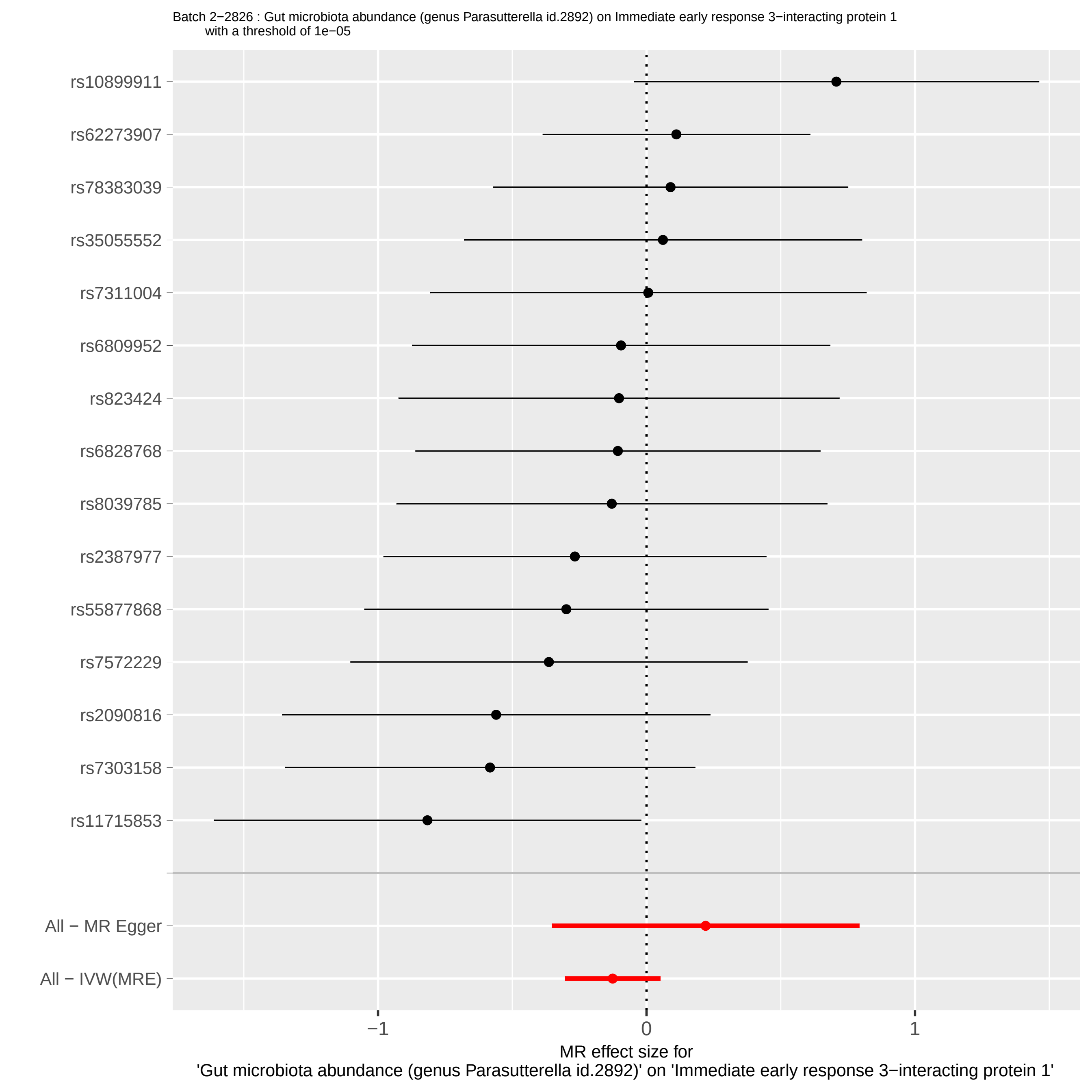

Batch 2−2826 : Gut microbiota abundance (genus Parasutterella id.2892) on Immediate early response 3−interacting protein 1
with a threshold of 1e−05
rs10899911
rs62273907
rs78383039
rs35055552
rs7311004
rs6809952
rs823424
rs6828768
rs8039785
rs2387977
rs55877868
rs7572229
rs2090816
rs7303158
rs11715853
All − MR Egger
All − IVW(MRE)
−1
0
1
MR effect size for
'Gut microbiota abundance (genus Parasutterella id.2892)' on 'Immediate early response 3−interacting protein 1'

Batch 2−2827 : Gut microbiota abundance (genus Catenibacterium id.2153) on Gamma−interferon−inducible protein 16
with a threshold of 1e−05

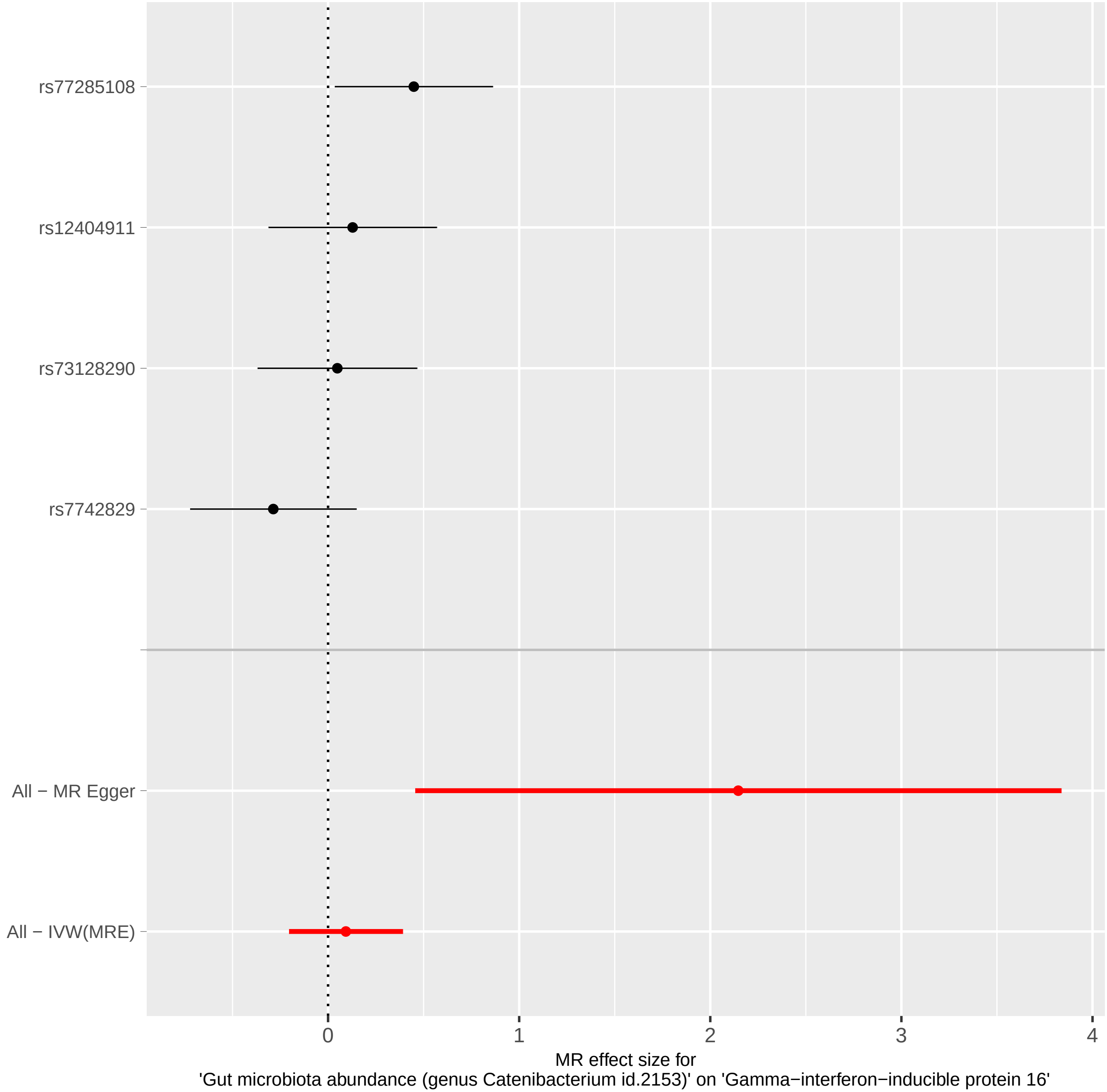

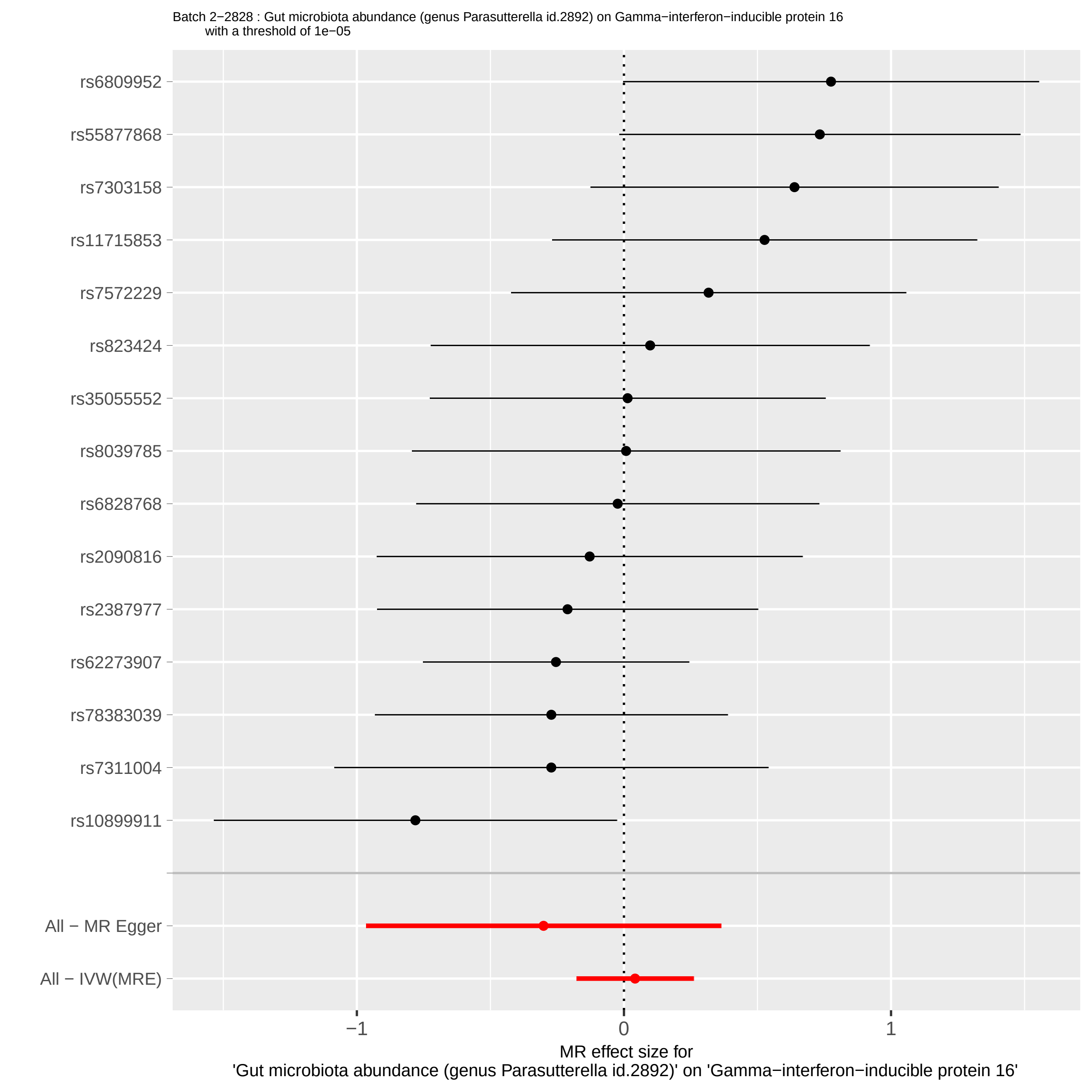
Batch 2−2828 : Gut microbiota abundance (genus Parasutterella id.2892) on Gamma−interferon−inducible protein 16
with a threshold of 1e−05
rs6809952
rs55877868
rs7303158
rs11715853
rs7572229
rs823424
rs35055552
rs8039785
rs6828768
rs2090816
rs2387977
rs62273907
rs78383039
rs7311004
rs10899911
All − MR Egger
All − IVW(MRE)
−1
0
1
MR effect size for
'Gut microbiota abundance (genus Parasutterella id.2892)' on 'Gamma−interferon−inducible protein 16'

Batch 2−2829 : Gut microbiota abundance (genus Catenibacterium id.2153) on Gamma−interferon−inducible protein 16
with a threshold of 1e−05

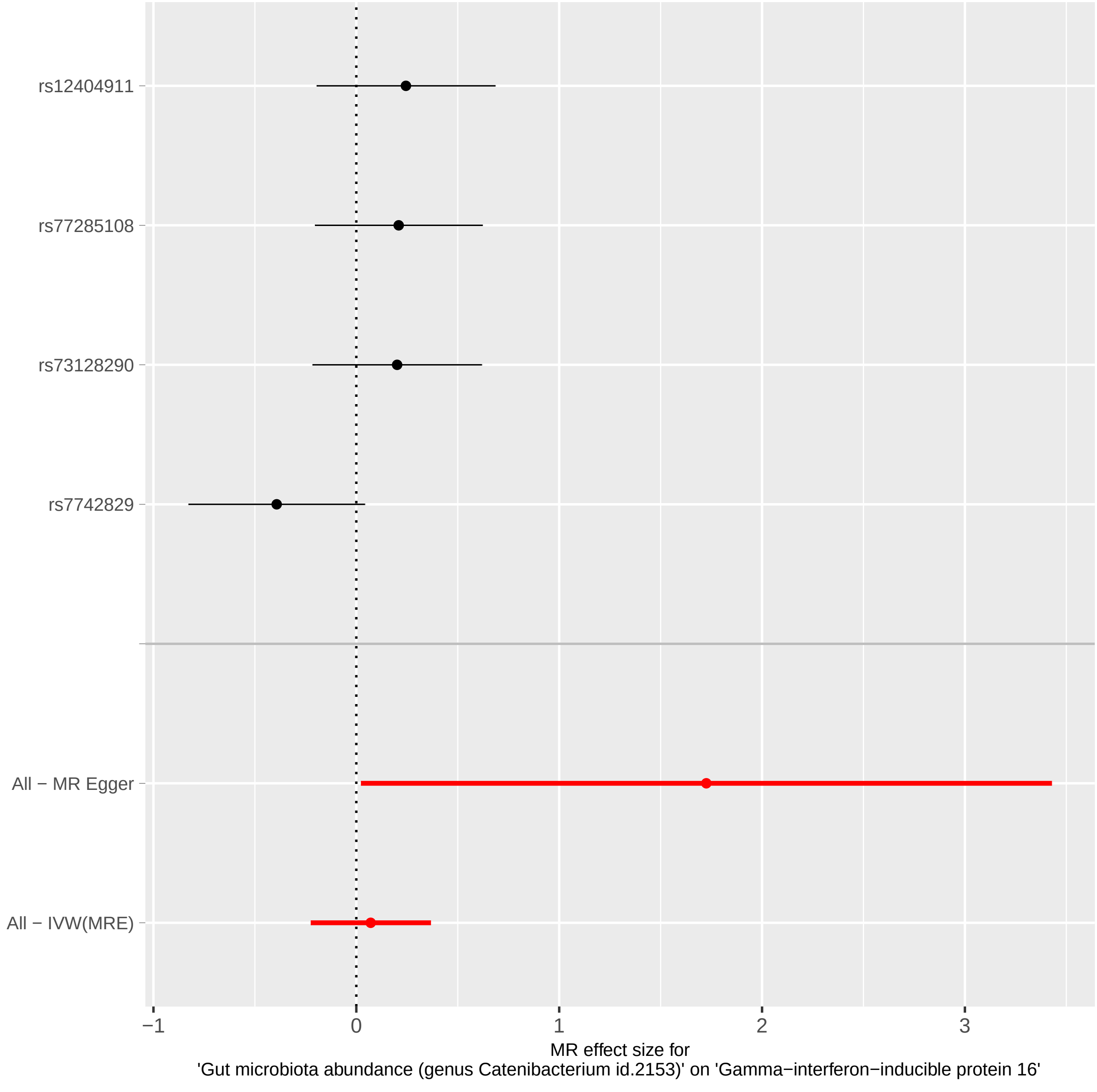

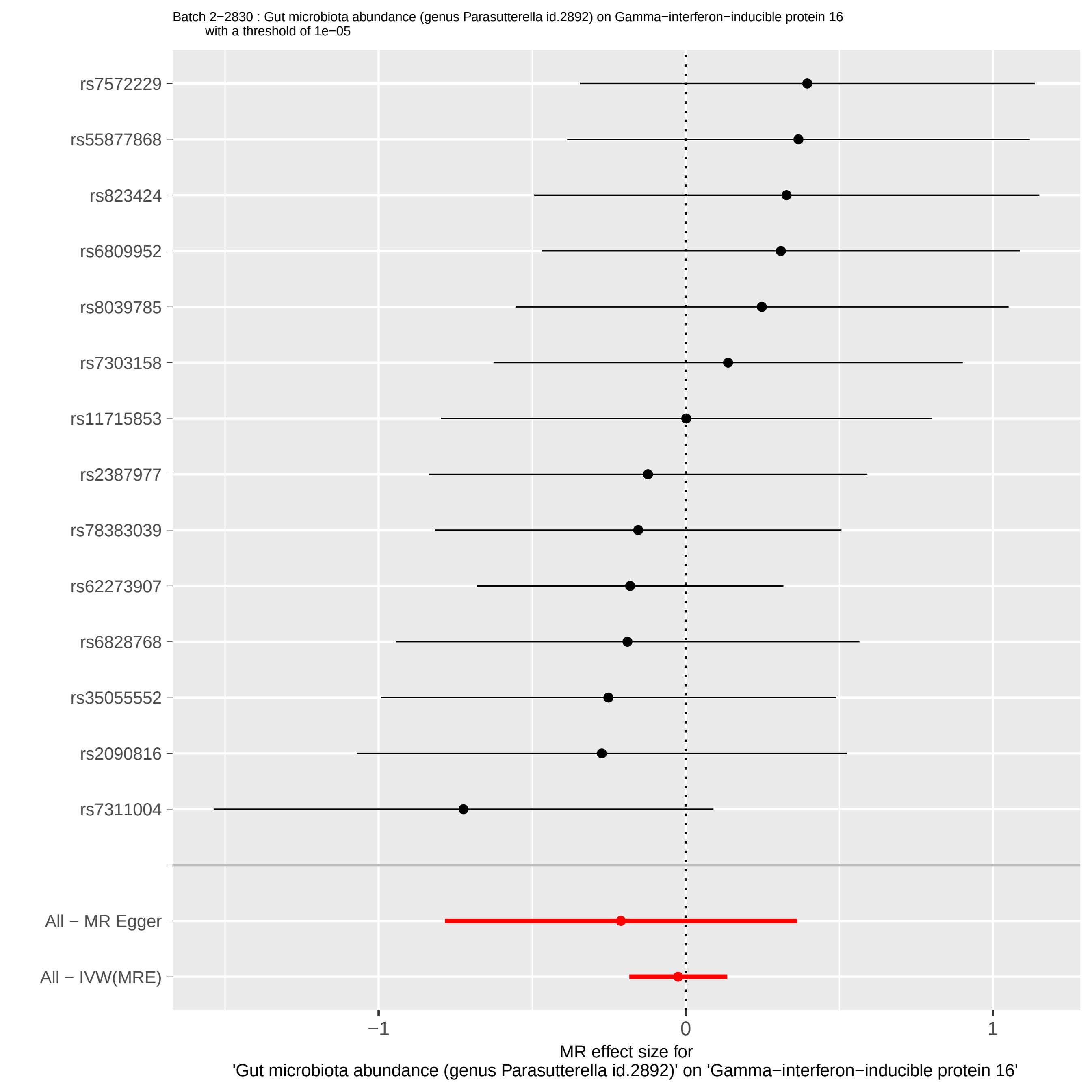

Batch 2−2830 : Gut microbiota abundance (genus Parasutterella id.2892) on Gamma−interferon−inducible protein 16
with a threshold of 1e−05
rs7572229
rs55877868
rs823424
rs6809952
rs8039785
rs7303158
rs11715853
rs2387977
rs78383039
rs62273907
rs6828768
rs35055552
rs2090816
rs7311004
All − MR Egger
All − IVW(MRE)
−1
0
1
MR effect size for
'Gut microbiota abundance (genus Parasutterella id.2892)' on 'Gamma−interferon−inducible protein 16'

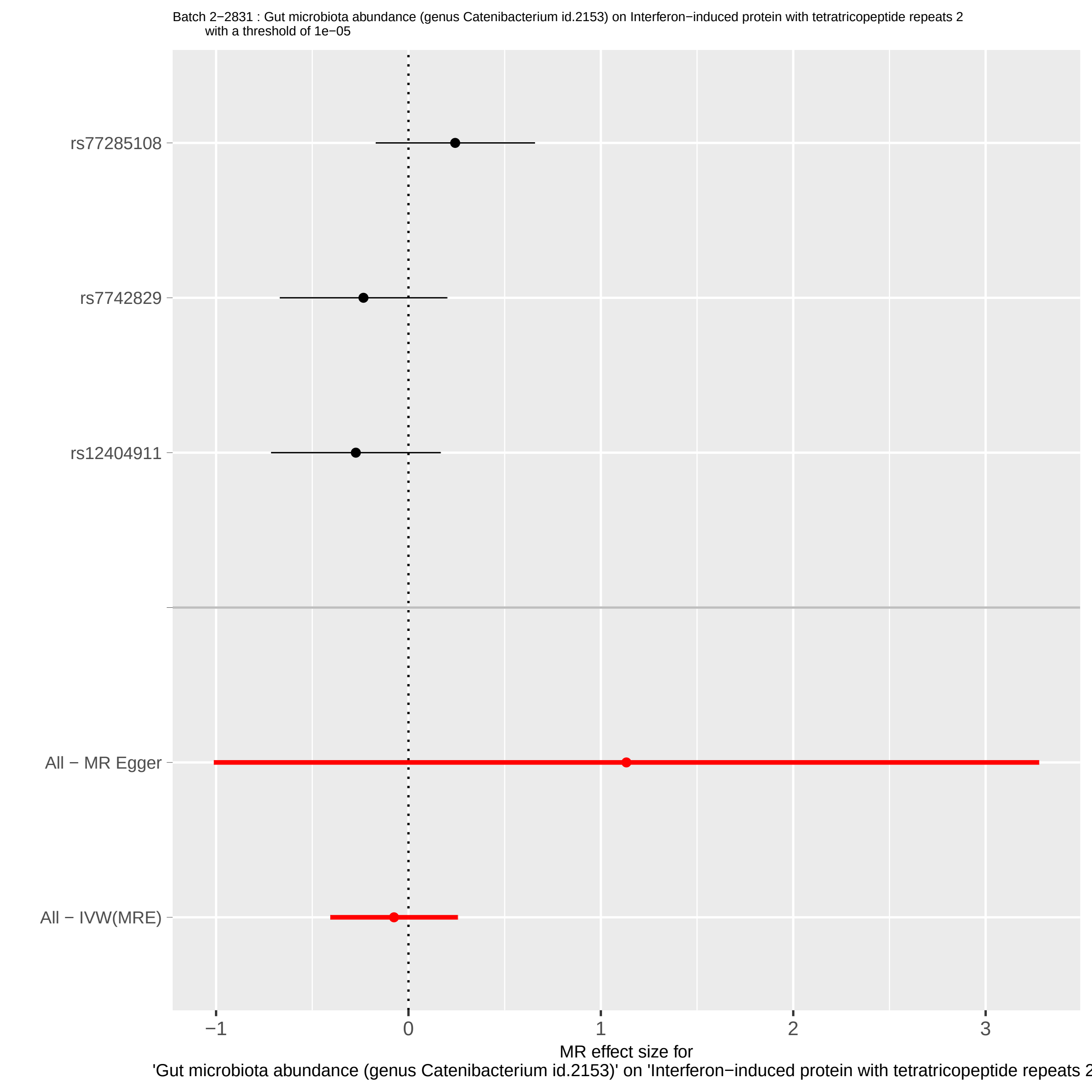

Batch 2−2831 : Gut microbiota abundance (genus Catenibacterium id.2153) on Interferon−induced protein with tetratricopeptide repeats 2
with a threshold of 1e−05
rs77285108
rs7742829
rs12404911
All − MR Egger
All − IVW(MRE)
−1
0
1
2
3
MR effect size for
'Gut microbiota abundance (genus Catenibacterium id.2153)' on 'Interferon−induced protein with tetratricopeptide repeats 2

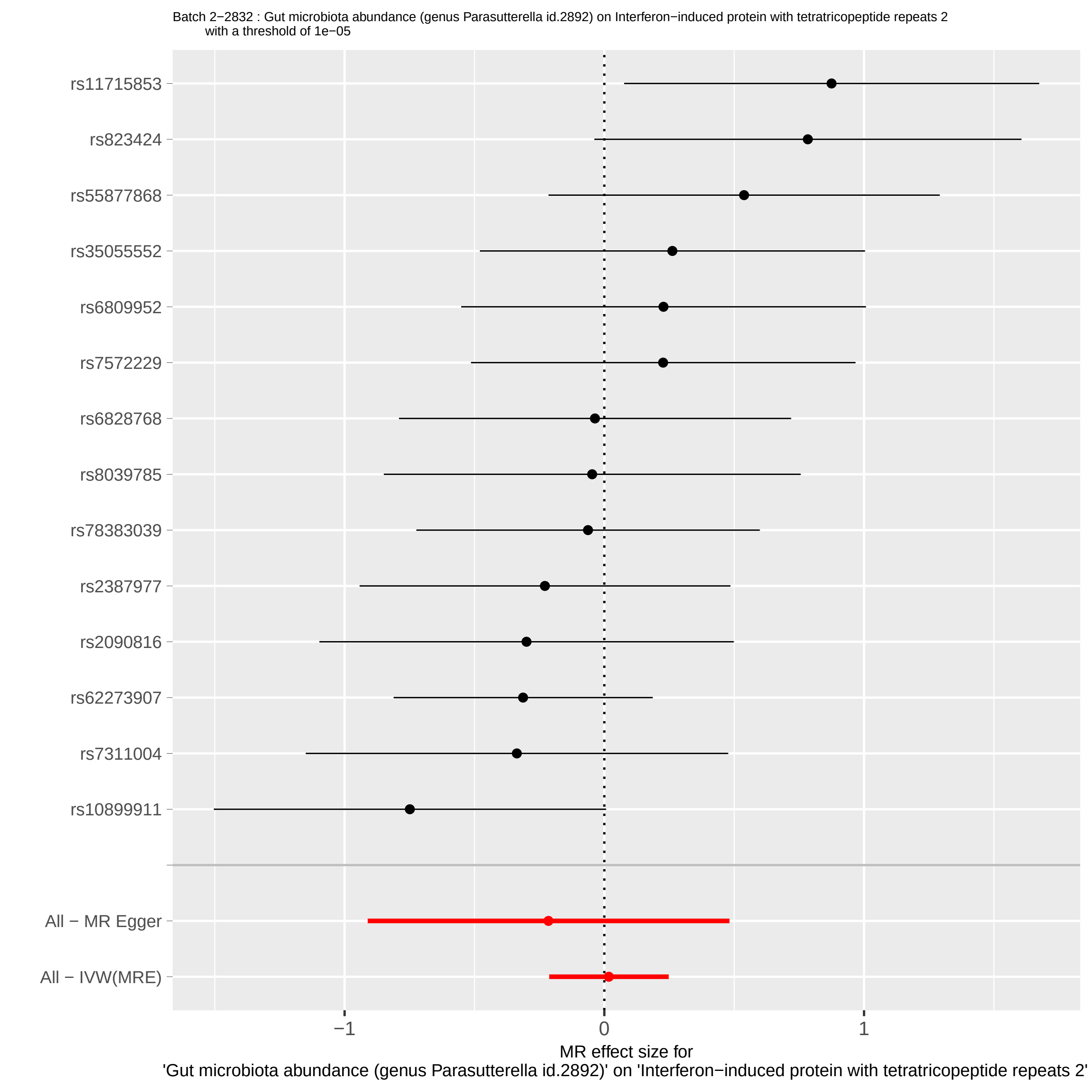

Batch 2−2832 : Gut microbiota abundance (genus Parasutterella id.2892) on Interferon−induced protein with tetratricopeptide repeats 2 with a threshold of 1e−05
rs11715853
rs823424
rs55877868
rs35055552
rs6809952
rs7572229
rs6828768
rs8039785
rs78383039
rs2387977
rs2090816
rs62273907
rs7311004
rs10899911
All − MR Egger
All − IVW(MRE)
−1
0
1
MR effect size for
'Gut microbiota abundance (genus Parasutterella id.2892)' on 'Interferon−induced protein with tetratricopeptide repeats 2'

Batch 2−2833 : Gut microbiota abundance (genus Catenibacterium id.2153) on Interferon−induced protein with tetratricopeptide repeats 3 with a threshold of 1e−05

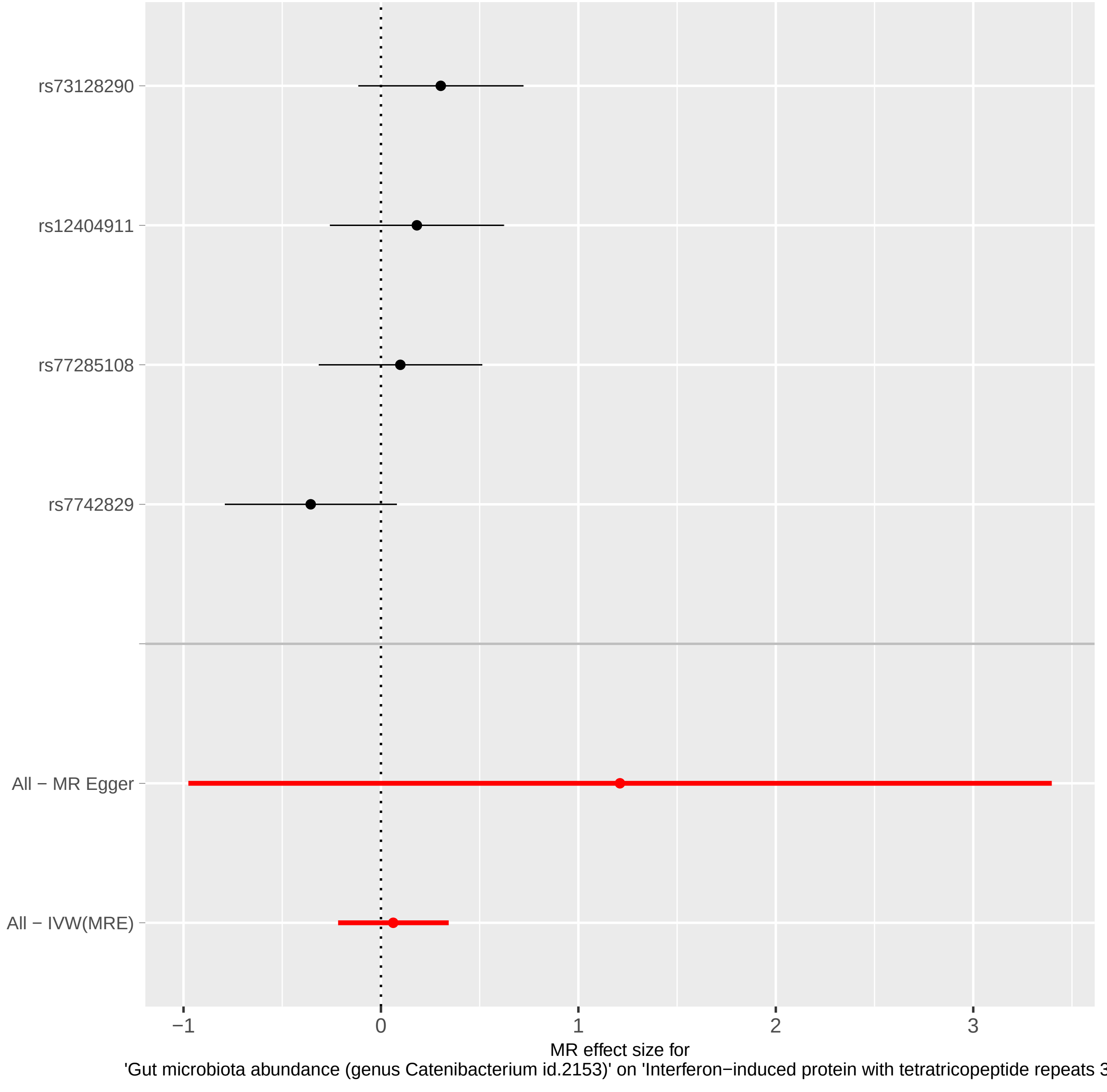

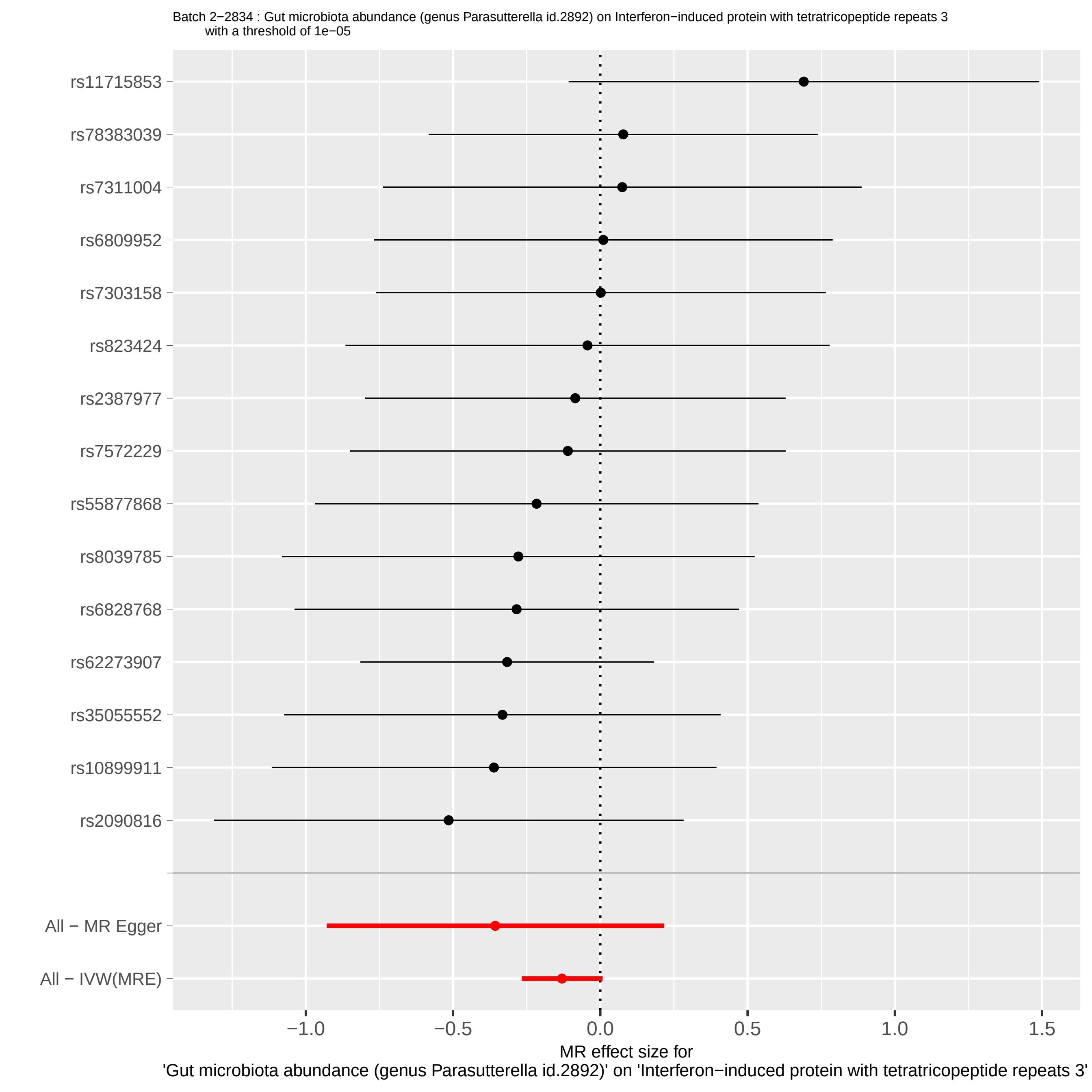
Batch 2−2834 : Gut microbiota abundance (genus Parasutterella id.2892) on Interferon−induced protein with tetratricopeptide repeats 3
with a threshold of 1e−05
rs11715853
rs78383039
rs7311004
rs6809952
rs7303158
rs823424
rs2387977
rs7572229
rs55877868
rs8039785
rs6828768
rs62273907
rs35055552
rs10899911
rs2090816
All − MR Egger
All − IVW(MRE)
−1.0
−0.5
0.0
0.5
1.0
1.5
MR effect size for
'Gut microbiota abundance (genus Parasutterella id.2892)' on 'Interferon−induced protein with tetratricopeptide repeats 3'

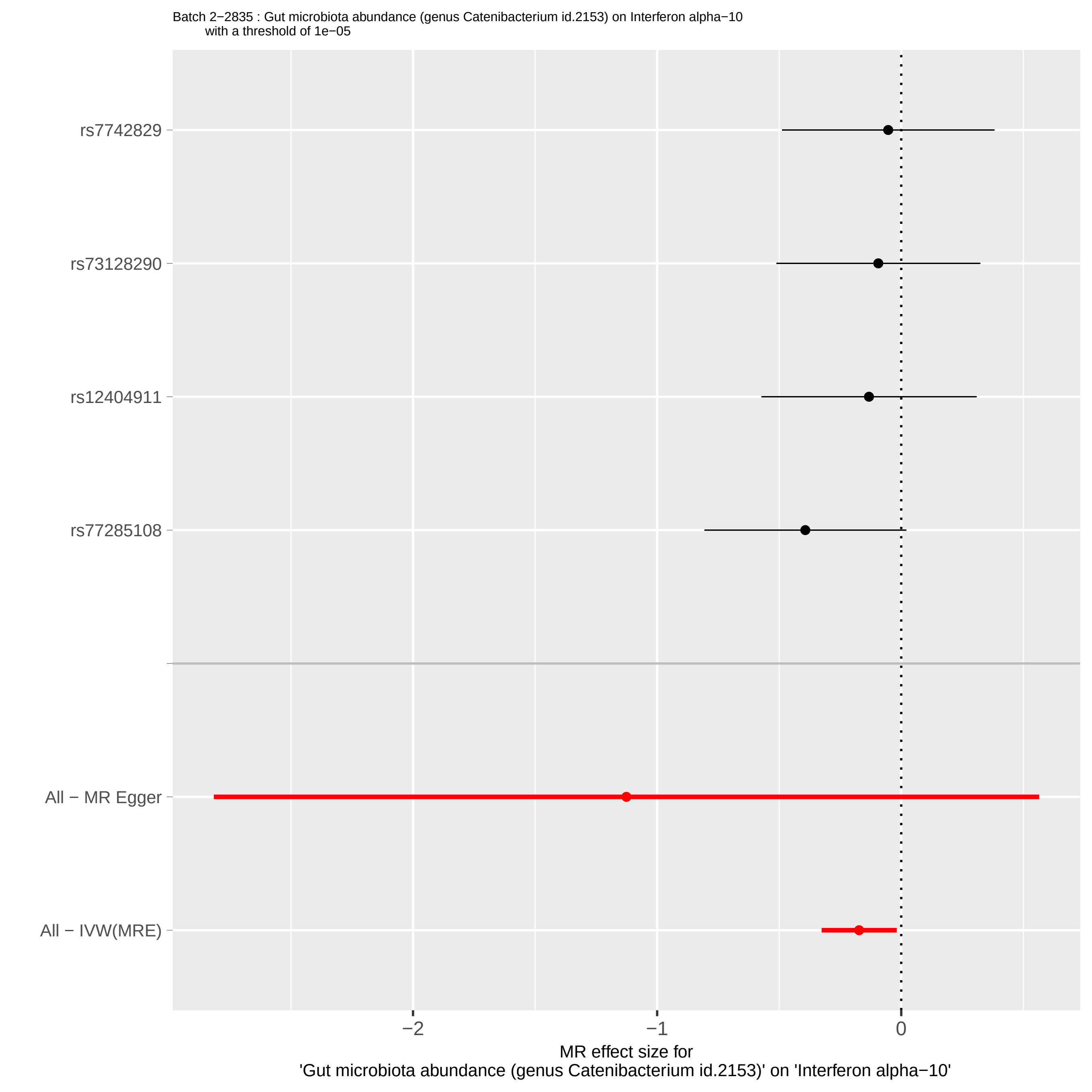
Batch 2−2835 : Gut microbiota abundance (genus Catenibacterium id.2153) on Interferon alpha−10
with a threshold of 1e−05
rs7742829
rs73128290
rs12404911
rs77285108
All − MR Egger
All − IVW(MRE)
−2
−1
0
MR effect size for
'Gut microbiota abundance (genus Catenibacterium id.2153)' on 'Interferon alpha−10'

Batch 2−2836 : Gut microbiota abundance (genus Parasutterella id.2892) on Interferon alpha−10
with a threshold of 1e−05

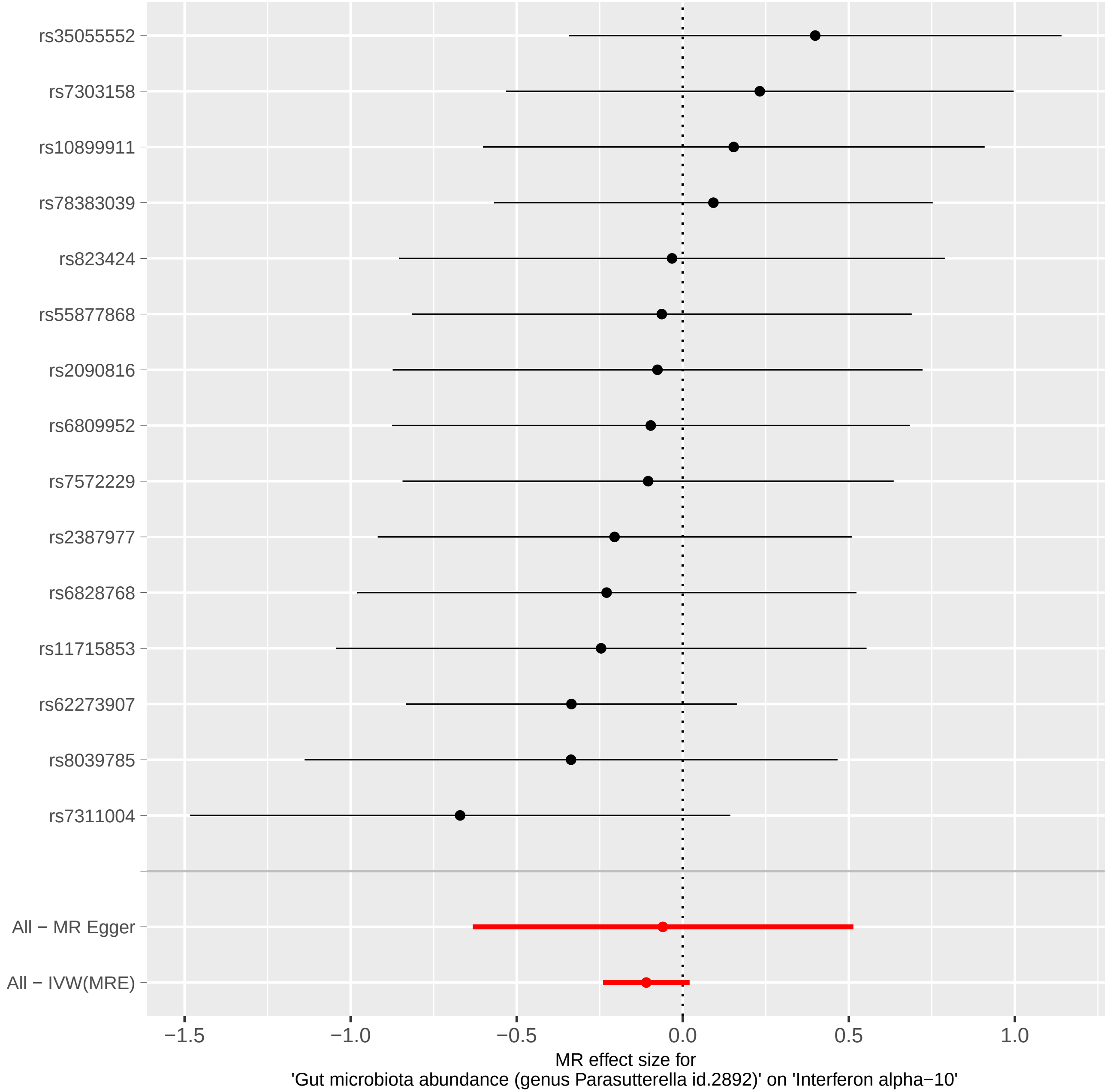

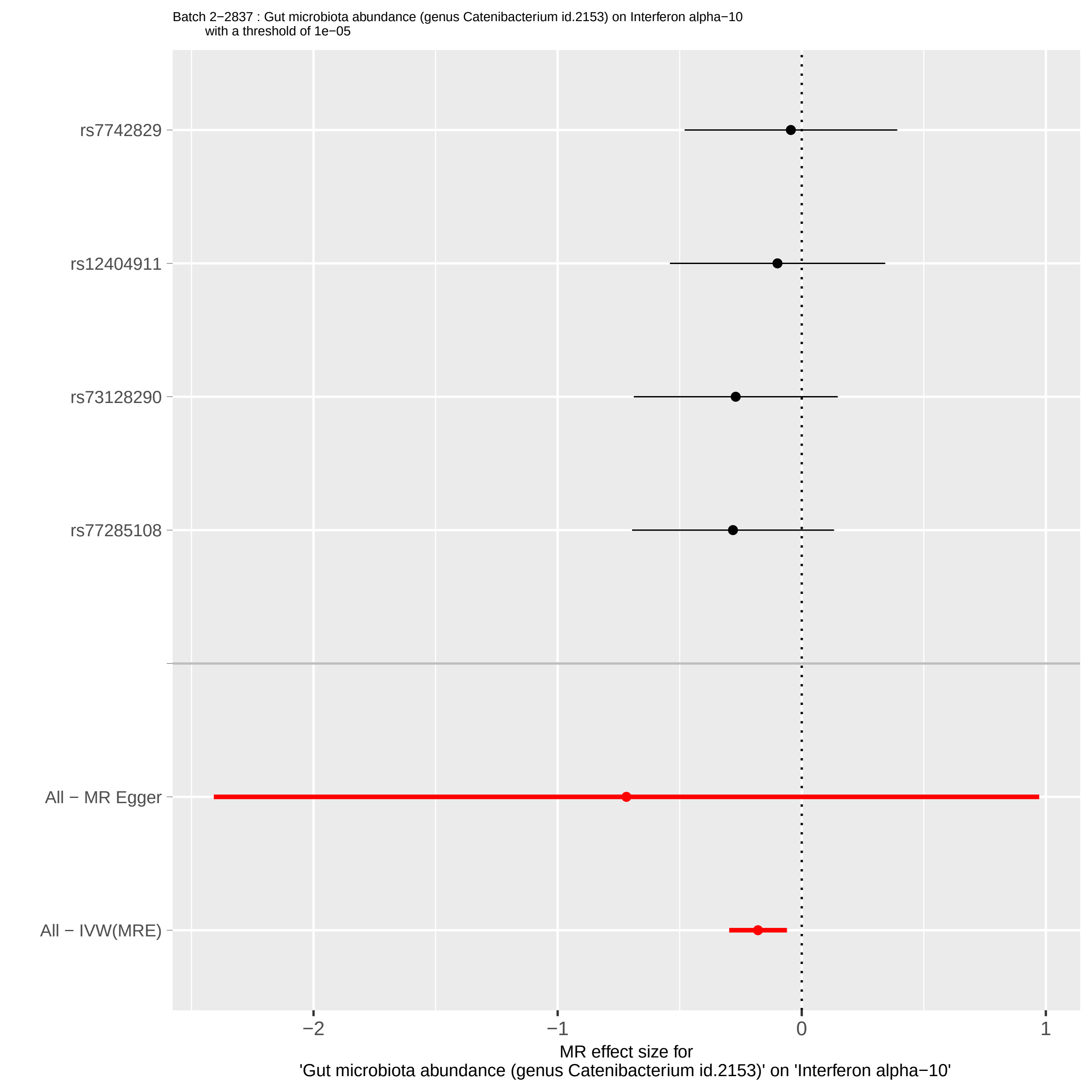
Batch 2−2837 : Gut microbiota abundance (genus Catenibacterium id.2153) on Interferon alpha−10
with a threshold of 1e−05
rs7742829
rs12404911
rs73128290
rs77285108
All − MR Egger
All − IVW(MRE)
−2
−1
0
1
MR effect size for
'Gut microbiota abundance (genus Catenibacterium id.2153)' on 'Interferon alpha−10'

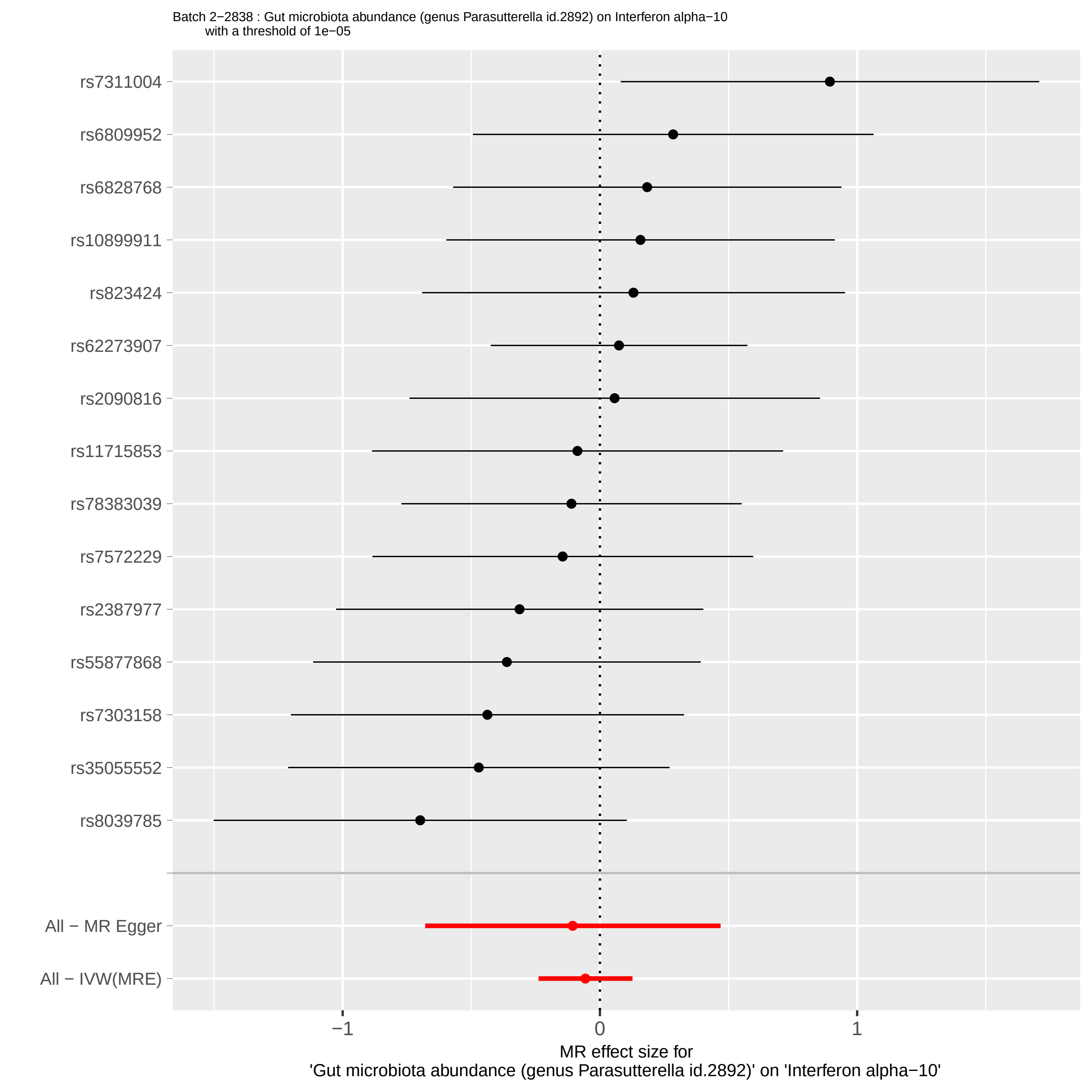

Batch 2−2838 : Gut microbiota abundance (genus Parasutterella id.2892) on Interferon alpha−10
with a threshold of 1e−05
rs7311004
rs6809952
rs6828768
rs10899911
rs823424
rs62273907
rs2090816
rs11715853
rs78383039
rs7572229
rs2387977
rs55877868
rs7303158
rs35055552
rs8039785
All − MR Egger
All − IVW(MRE)
−1
0
1
MR effect size for
'Gut microbiota abundance (genus Parasutterella id.2892)' on 'Interferon alpha−10'

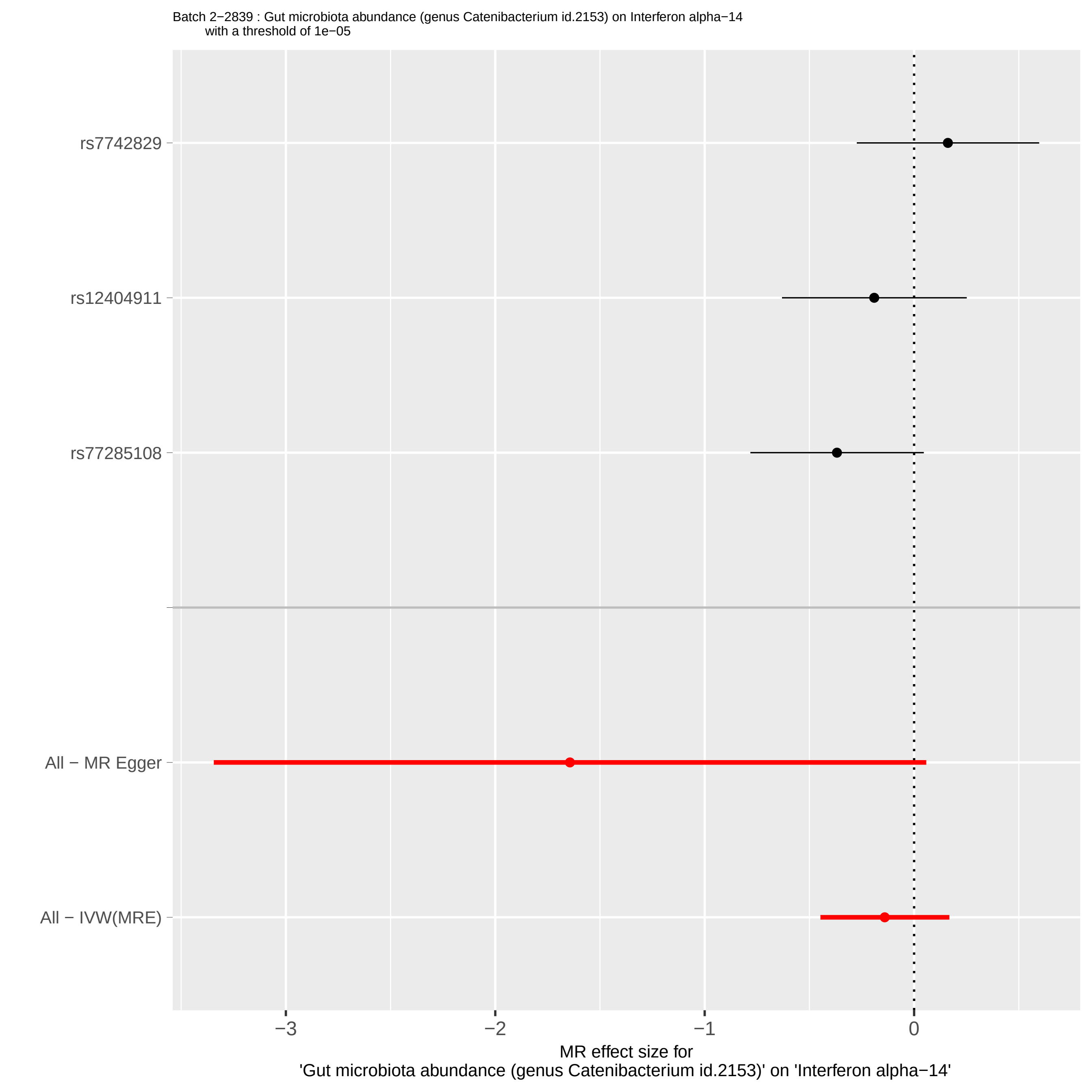
Batch 2−2839 : Gut microbiota abundance (genus Catenibacterium id.2153) on Interferon alpha−14
with a threshold of 1e−05
rs7742829
rs12404911
rs77285108
All − MR Egger
All − IVW(MRE)
−3
−2
−1
0
MR effect size for
'Gut microbiota abundance (genus Catenibacterium id.2153)' on 'Interferon alpha−14'

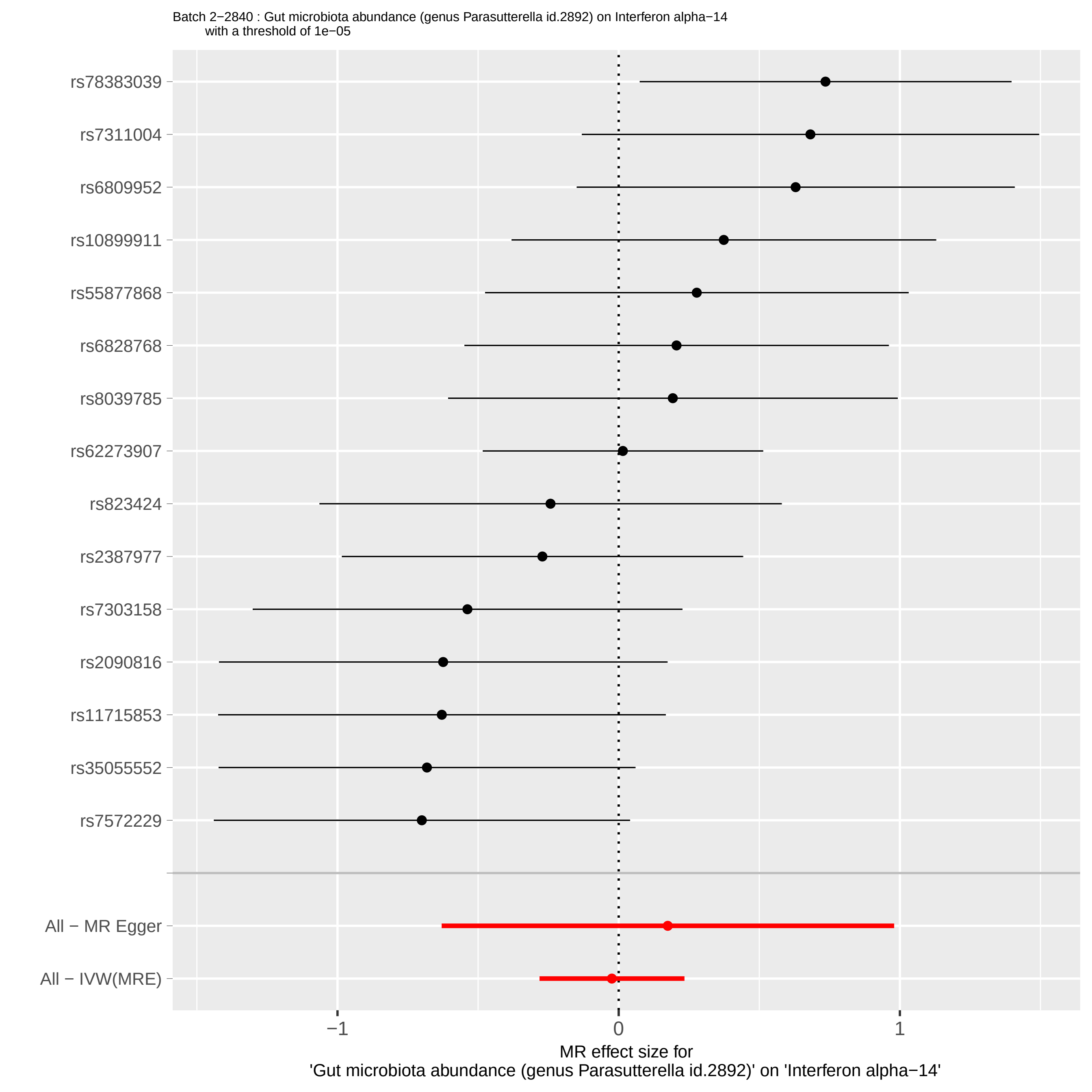

Batch 2−2840 : Gut microbiota abundance (genus Parasutterella id.2892) on Interferon alpha−14
with a threshold of 1e−05
rs78383039
rs7311004
rs6809952
rs10899911
rs55877868
rs6828768
rs8039785
rs62273907
rs823424
rs2387977
rs7303158
rs2090816
rs11715853
rs35055552
rs7572229
All − MR Egger
All − IVW(MRE)
−1
0
1
MR effect size for
'Gut microbiota abundance (genus Parasutterella id.2892)' on 'Interferon alpha−14'

Batch 2−2841 : Gut microbiota abundance (genus Catenibacterium id.2153) on Interferon alpha−4
with a threshold of 1e−05

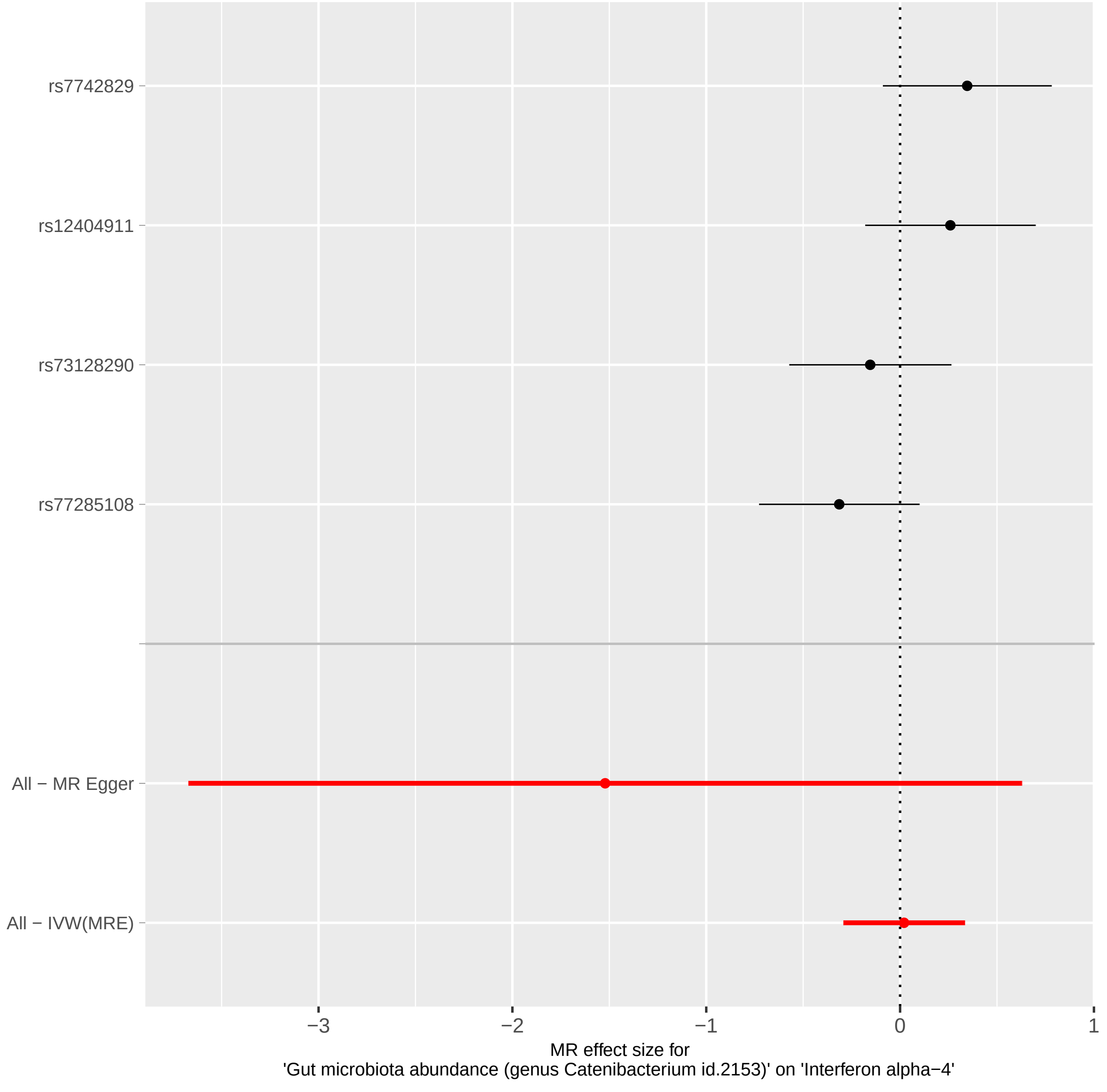

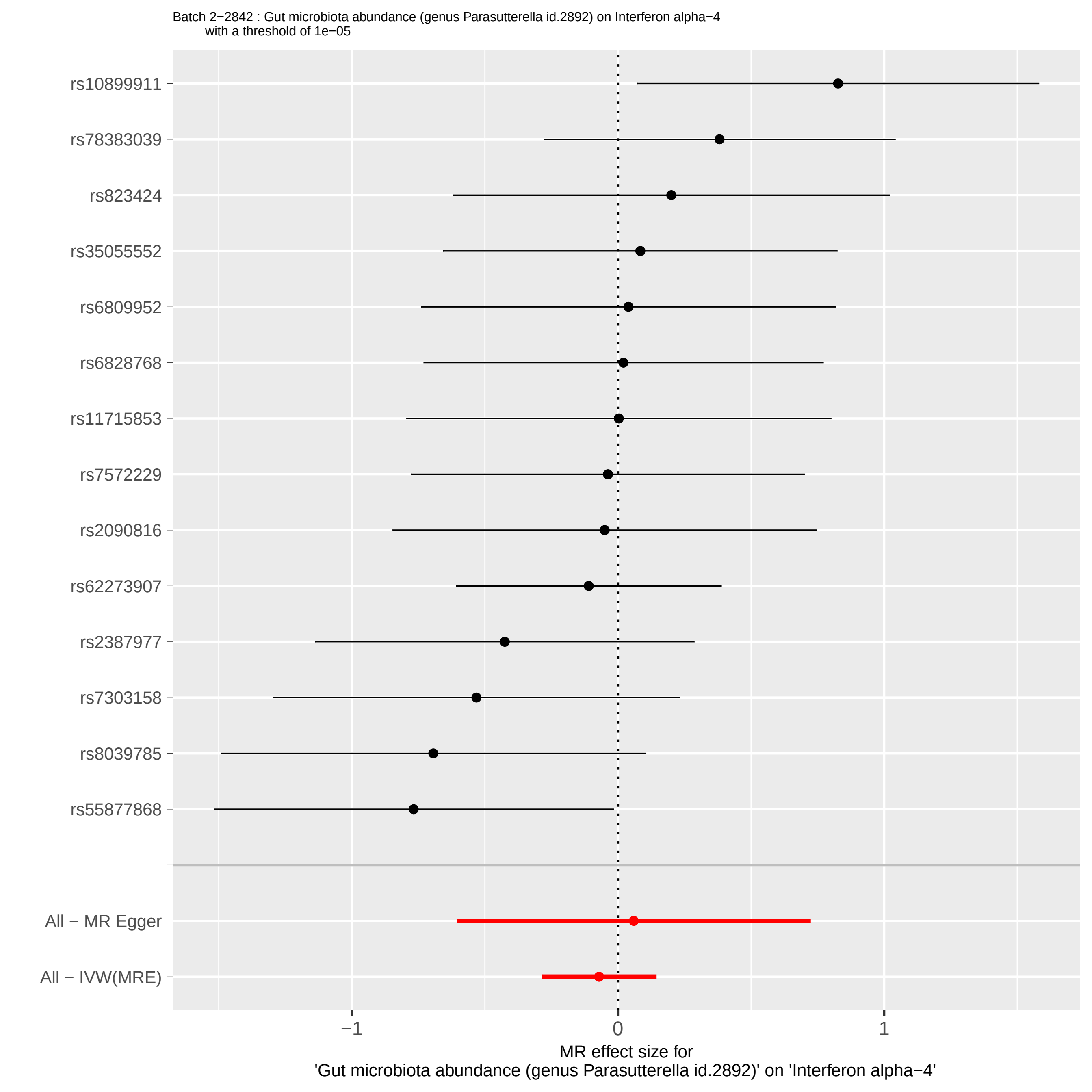

Batch 2−2842 : Gut microbiota abundance (genus Parasutterella id.2892) on Interferon alpha−4
with a threshold of 1e−05
rs10899911
rs78383039
rs823424
rs35055552
rs6809952
rs6828768
rs11715853
rs7572229
rs2090816
rs62273907
rs2387977
rs7303158
rs8039785
rs55877868
All − MR Egger
All − IVW(MRE)
−1
0
1
MR effect size for
'Gut microbiota abundance (genus Parasutterella id.2892)' on 'Interferon alpha−4'

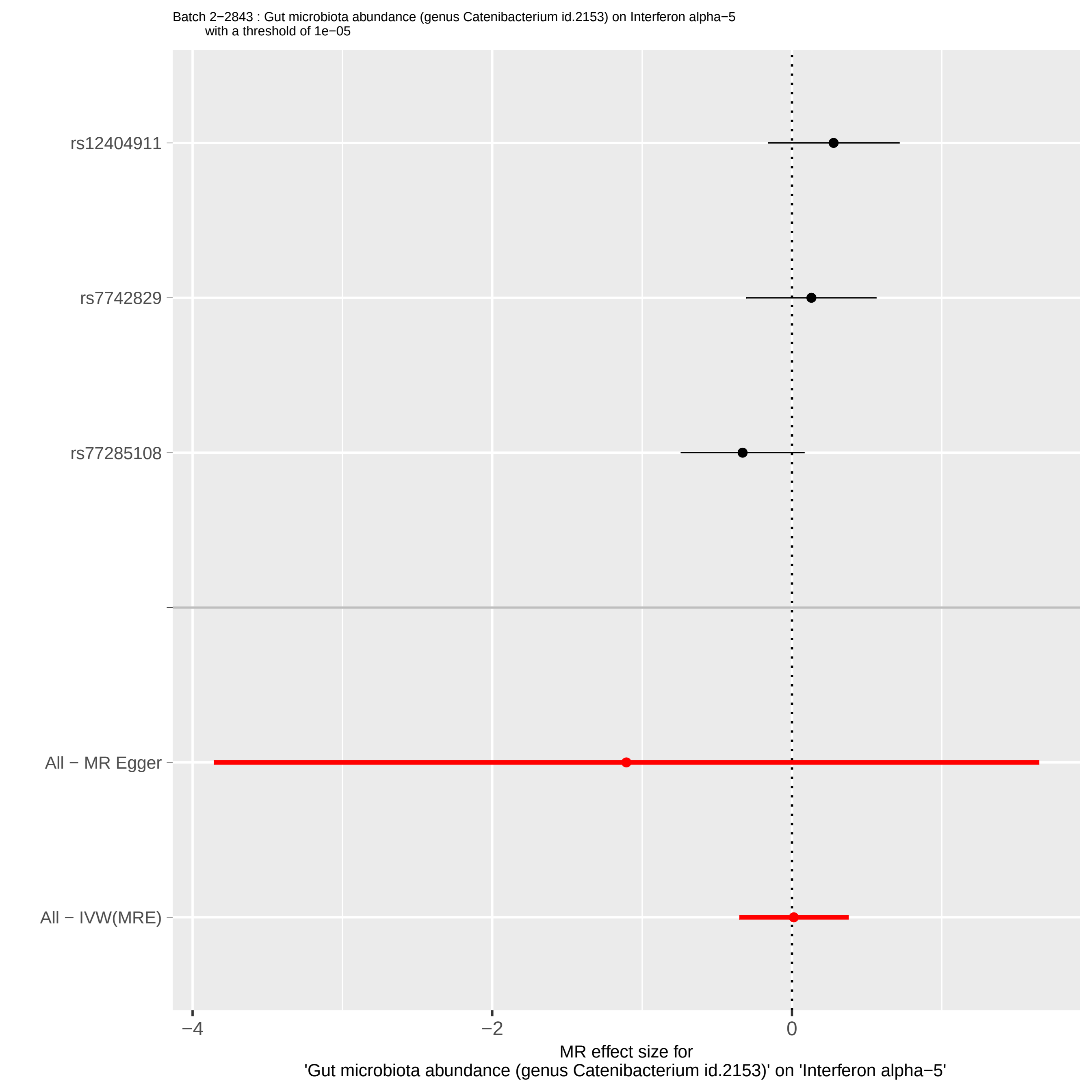

Batch 2−2843 : Gut microbiota abundance (genus Catenibacterium id.2153) on Interferon alpha−5
with a threshold of 1e−05
rs12404911
rs7742829
rs77285108
All − MR Egger
All − IVW(MRE)
−4
−2
0
MR effect size for
'Gut microbiota abundance (genus Catenibacterium id.2153)' on 'Interferon alpha−5'

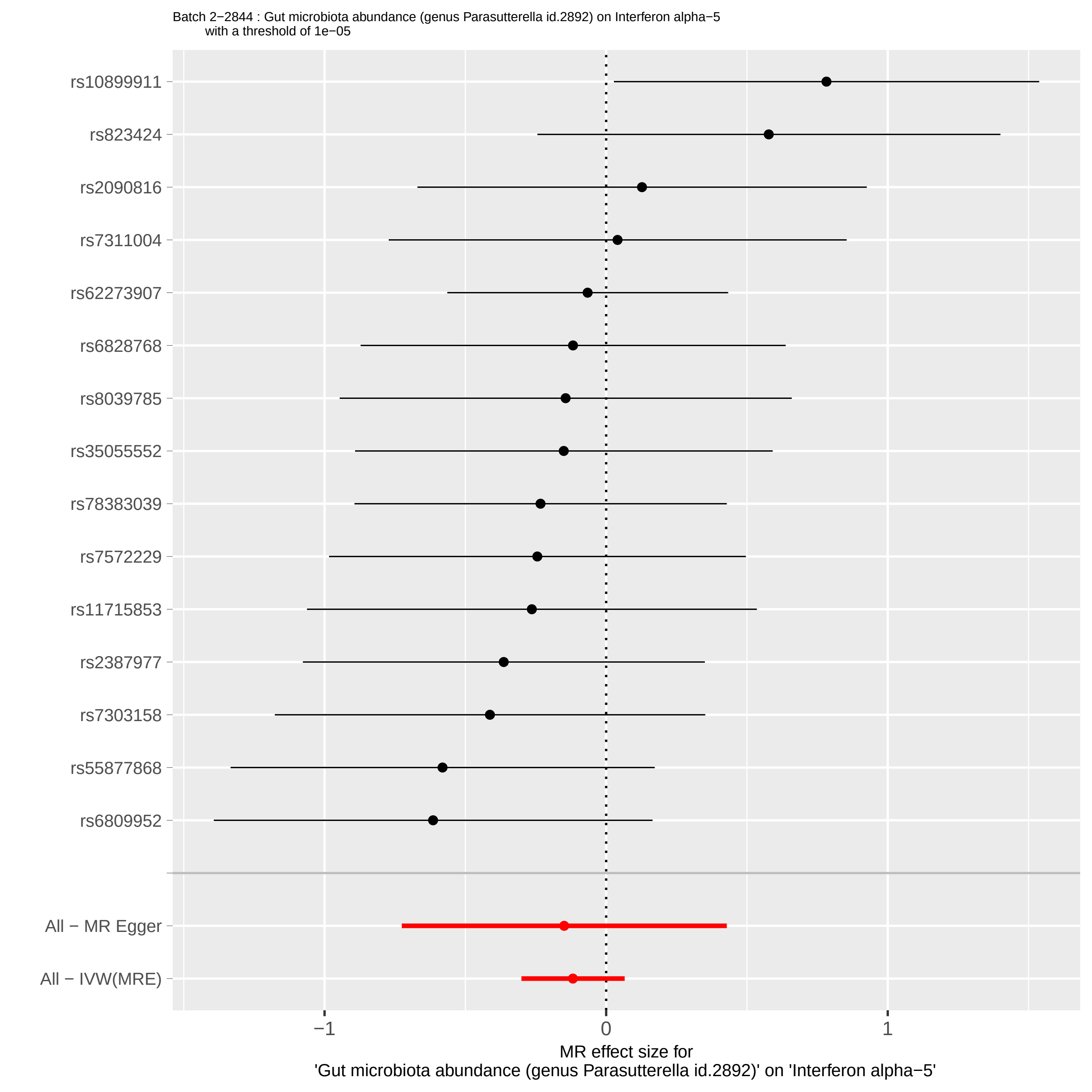

Batch 2−2844 : Gut microbiota abundance (genus Parasutterella id.2892) on Interferon alpha−5
with a threshold of 1e−05
rs10899911
rs823424
rs2090816
rs7311004
rs62273907
rs6828768
rs8039785
rs35055552
rs78383039
rs7572229
rs11715853
rs2387977
rs7303158
rs55877868
rs6809952
All − MR Egger
All − IVW(MRE)
−1
0
1
MR effect size for
'Gut microbiota abundance (genus Parasutterella id.2892)' on 'Interferon alpha−5'

Batch 2−2845 : Gut microbiota abundance (genus Catenibacterium id.2153) on Interferon alpha−6
with a threshold of 1e−05

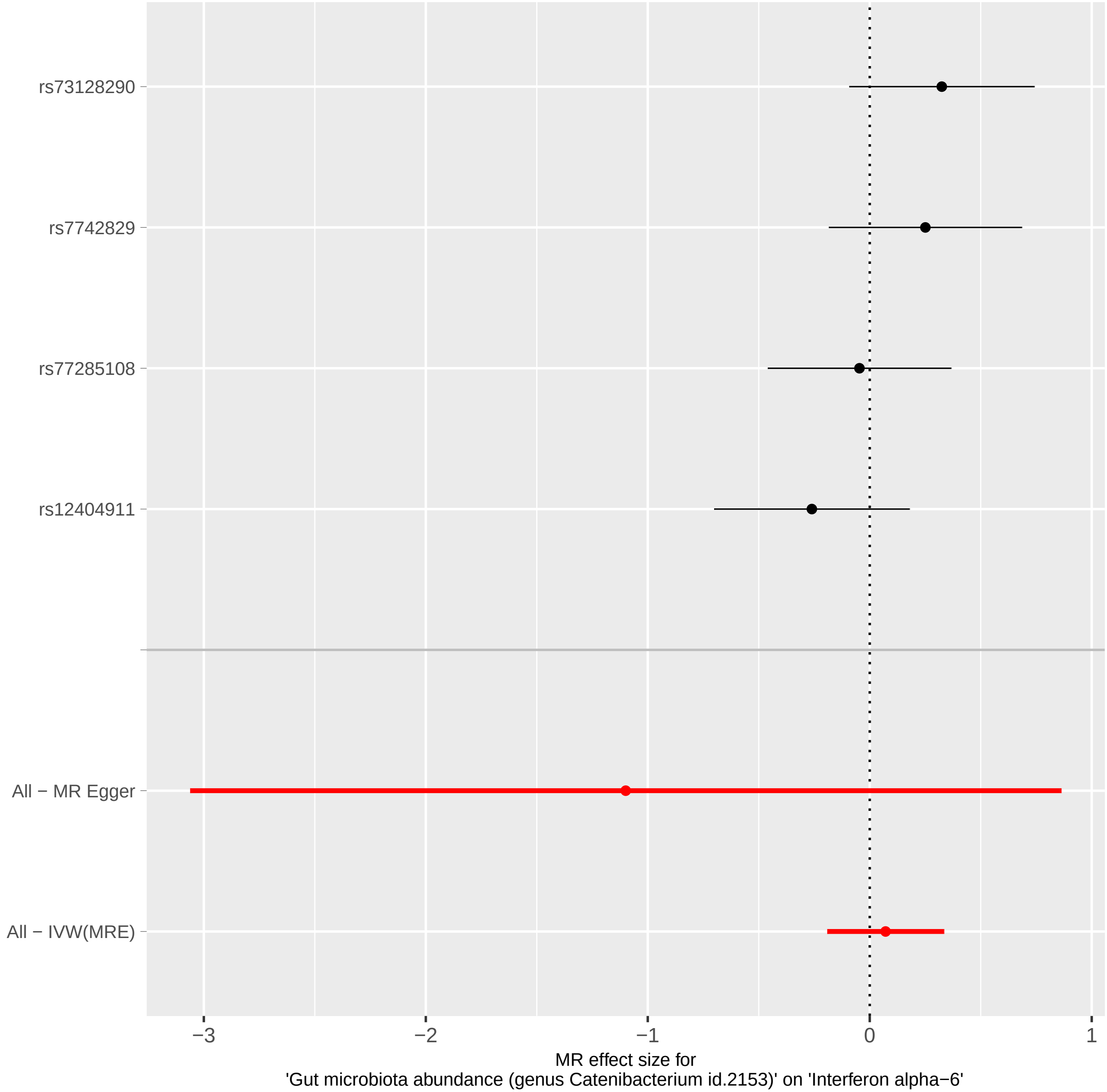

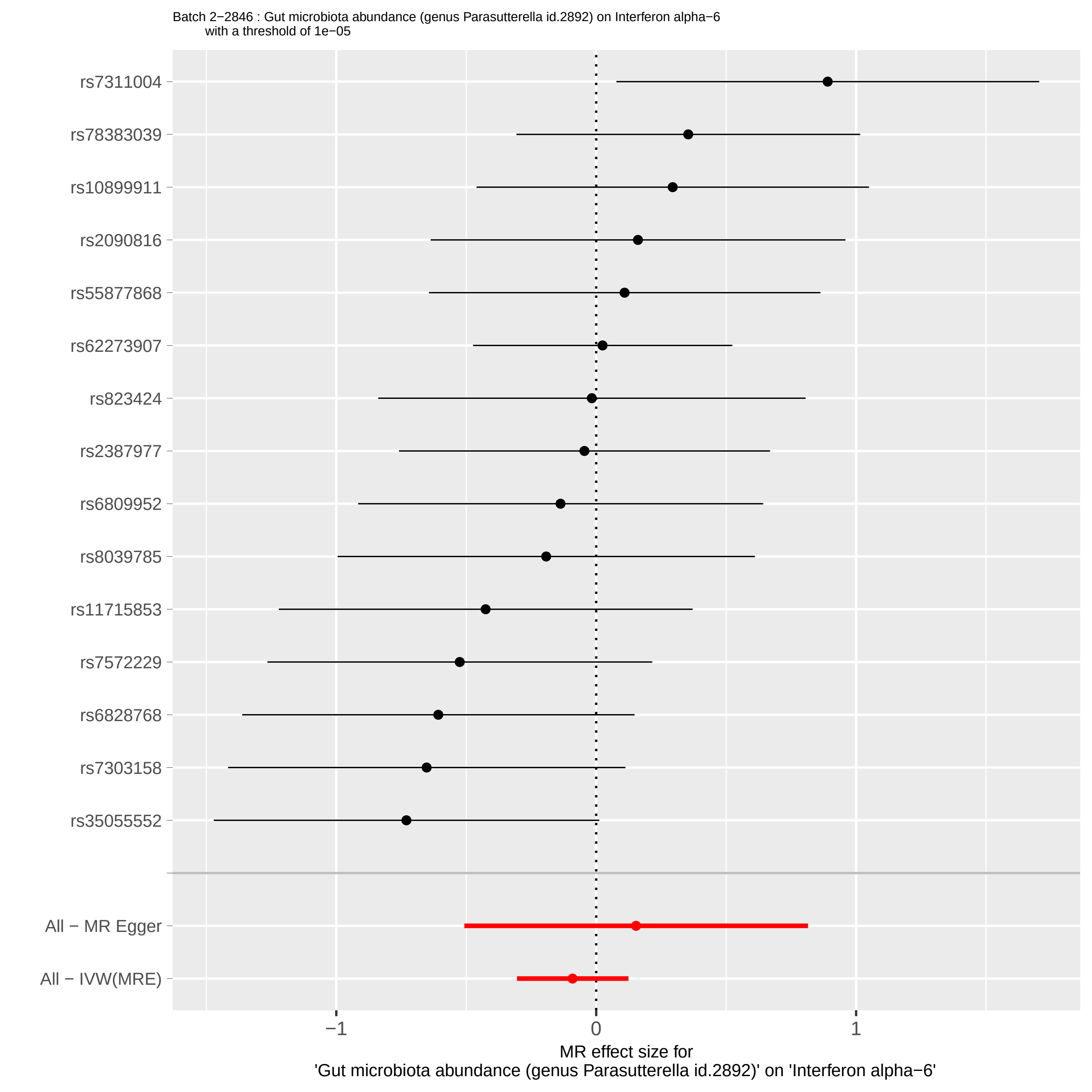
Batch 2−2846 : Gut microbiota abundance (genus Parasutterella id.2892) on Interferon alpha−6
with a threshold of 1e−05
rs7311004
rs78383039
rs10899911
rs2090816
rs55877868
rs62273907
rs823424
rs2387977
rs6809952
rs8039785
rs11715853
rs7572229
rs6828768
rs7303158
rs35055552
All − MR Egger
All − IVW(MRE)
−1
0
1
MR effect size for
'Gut microbiota abundance (genus Parasutterella id.2892)' on 'Interferon alpha−6'

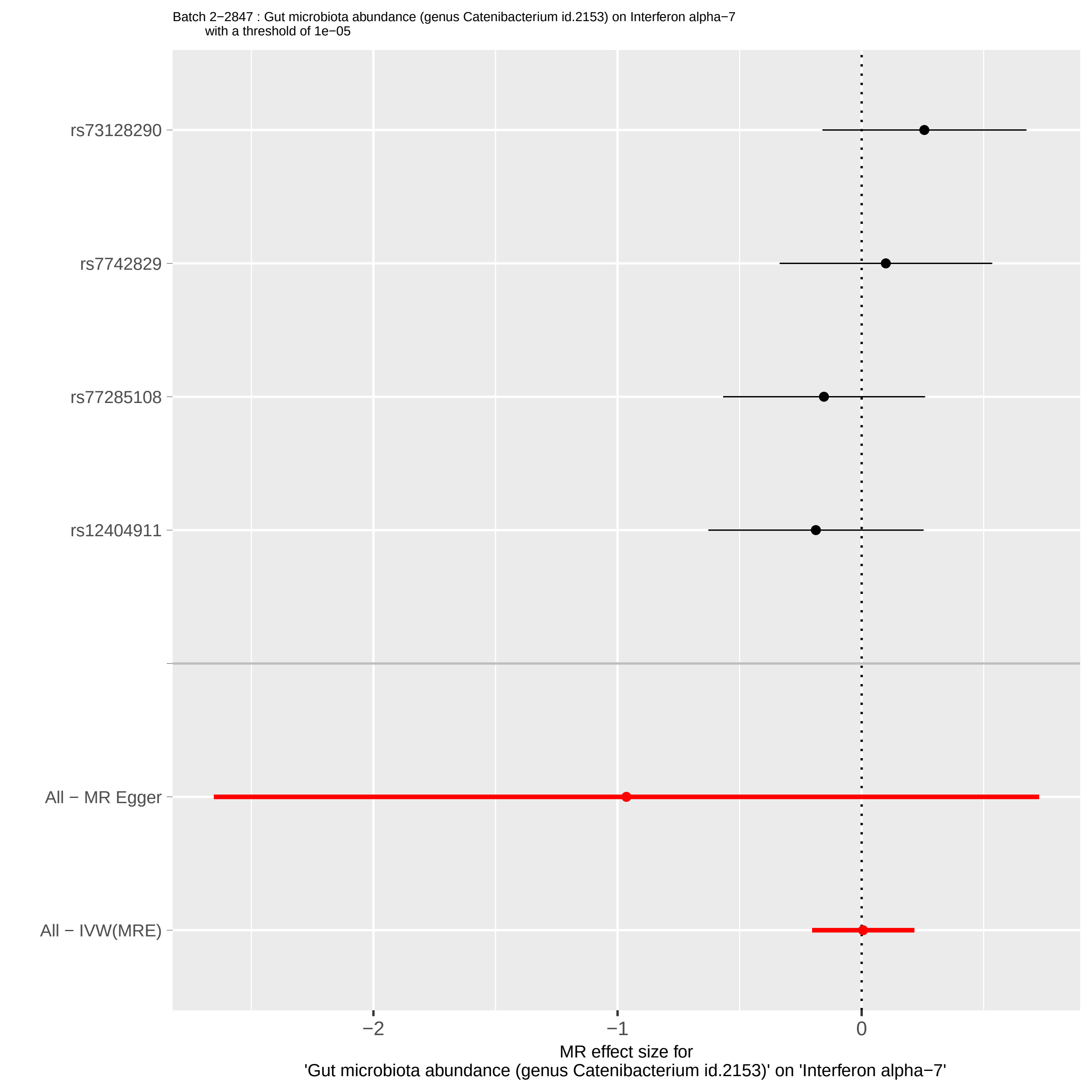
Batch 2−2847 : Gut microbiota abundance (genus Catenibacterium id.2153) on Interferon alpha−7
with a threshold of 1e−05
rs73128290
rs7742829
rs77285108
rs12404911
All − MR Egger
All − IVW(MRE)
−2
−1
0
MR effect size for
'Gut microbiota abundance (genus Catenibacterium id.2153)' on 'Interferon alpha−7'

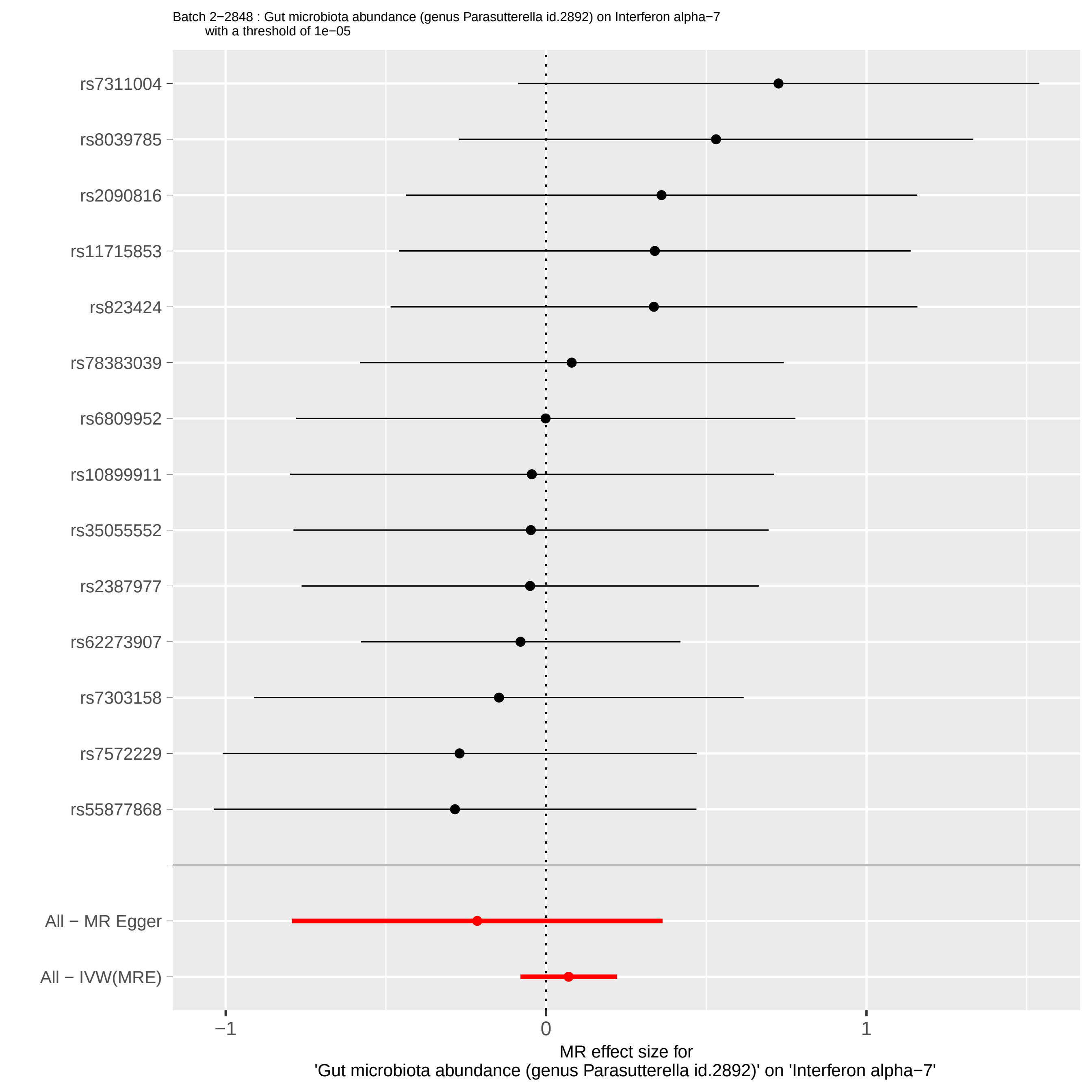
Batch 2−2848 : Gut microbiota abundance (genus Parasutterella id.2892) on Interferon alpha−7
with a threshold of 1e−05
rs7311004
rs8039785
rs2090816
rs11715853
rs823424
rs78383039
rs6809952
rs10899911
rs35055552
rs2387977
rs62273907
rs7303158
rs7572229
rs55877868
All − MR Egger
All − IVW(MRE)
−1
0
1
MR effect size for
'Gut microbiota abundance (genus Parasutterella id.2892)' on 'Interferon alpha−7'

Batch 2−2849 : Gut microbiota abundance (genus Catenibacterium id.2153) on Interferon alpha−8
with a threshold of 1e−05

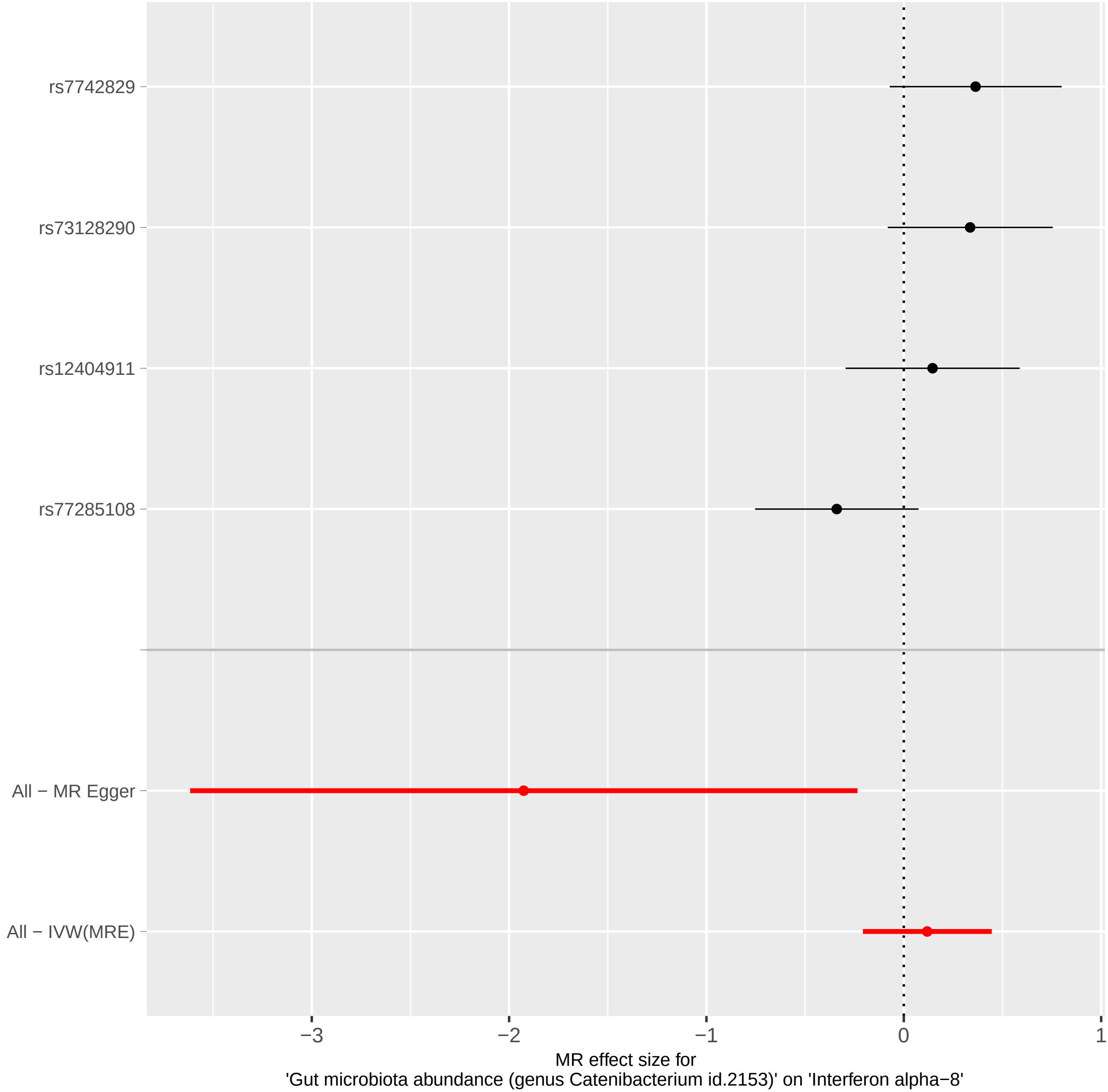

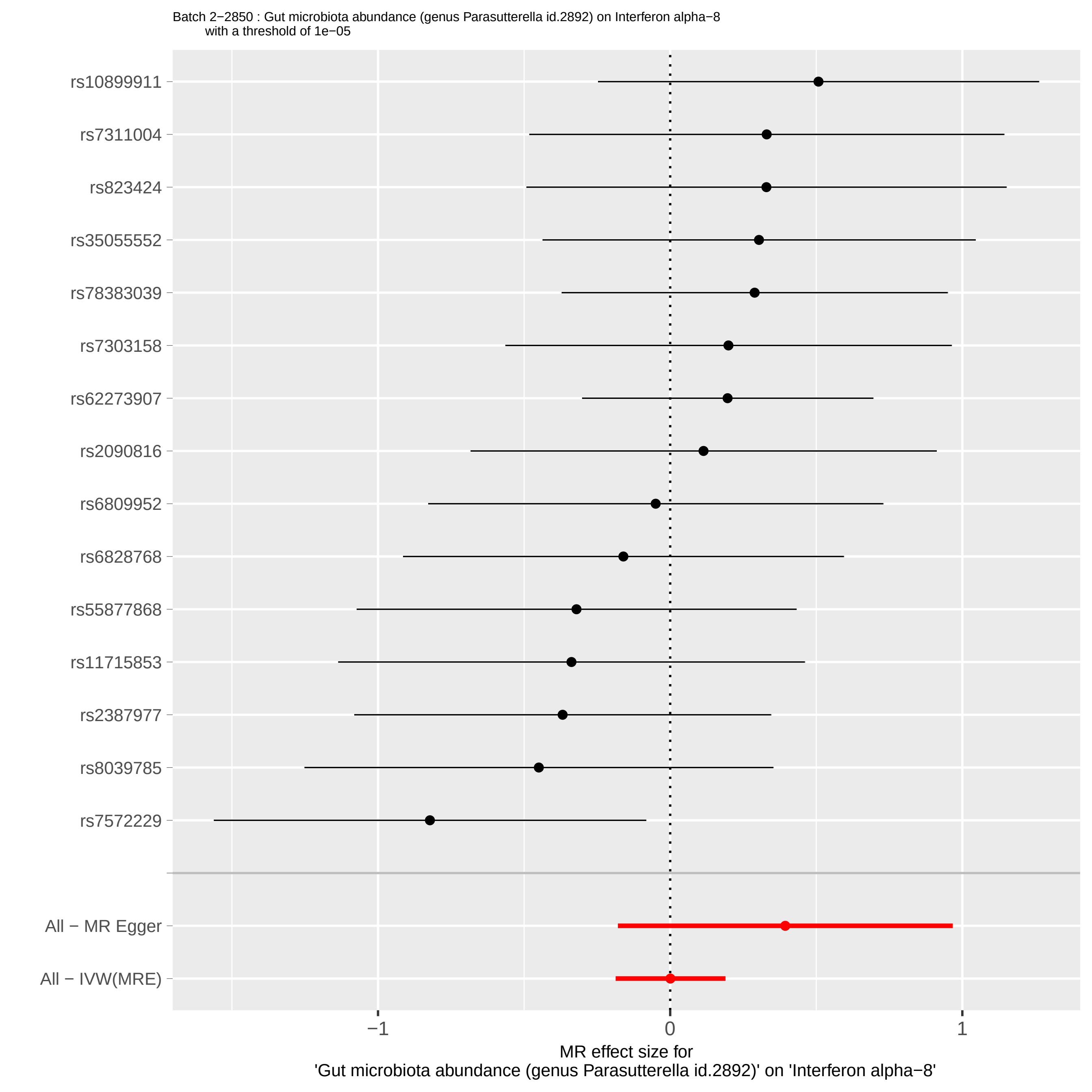
Batch 2−2850 : Gut microbiota abundance (genus Parasutterella id.2892) on Interferon alpha−8
with a threshold of 1e−05
rs10899911
rs7311004
rs823424
rs35055552
rs78383039
rs7303158
rs62273907
rs2090816
rs6809952
rs6828768
rs55877868
rs11715853
rs2387977
rs8039785
rs7572229
All − MR Egger
All − IVW(MRE)
−1
0
1
MR effect size for
'Gut microbiota abundance (genus Parasutterella id.2892)' on 'Interferon alpha−8'

Batch 2−2851 : Gut microbiota abundance (genus Catenibacterium id.2153) on Interferon alpha/beta receptor 1
with a threshold of 1e−05

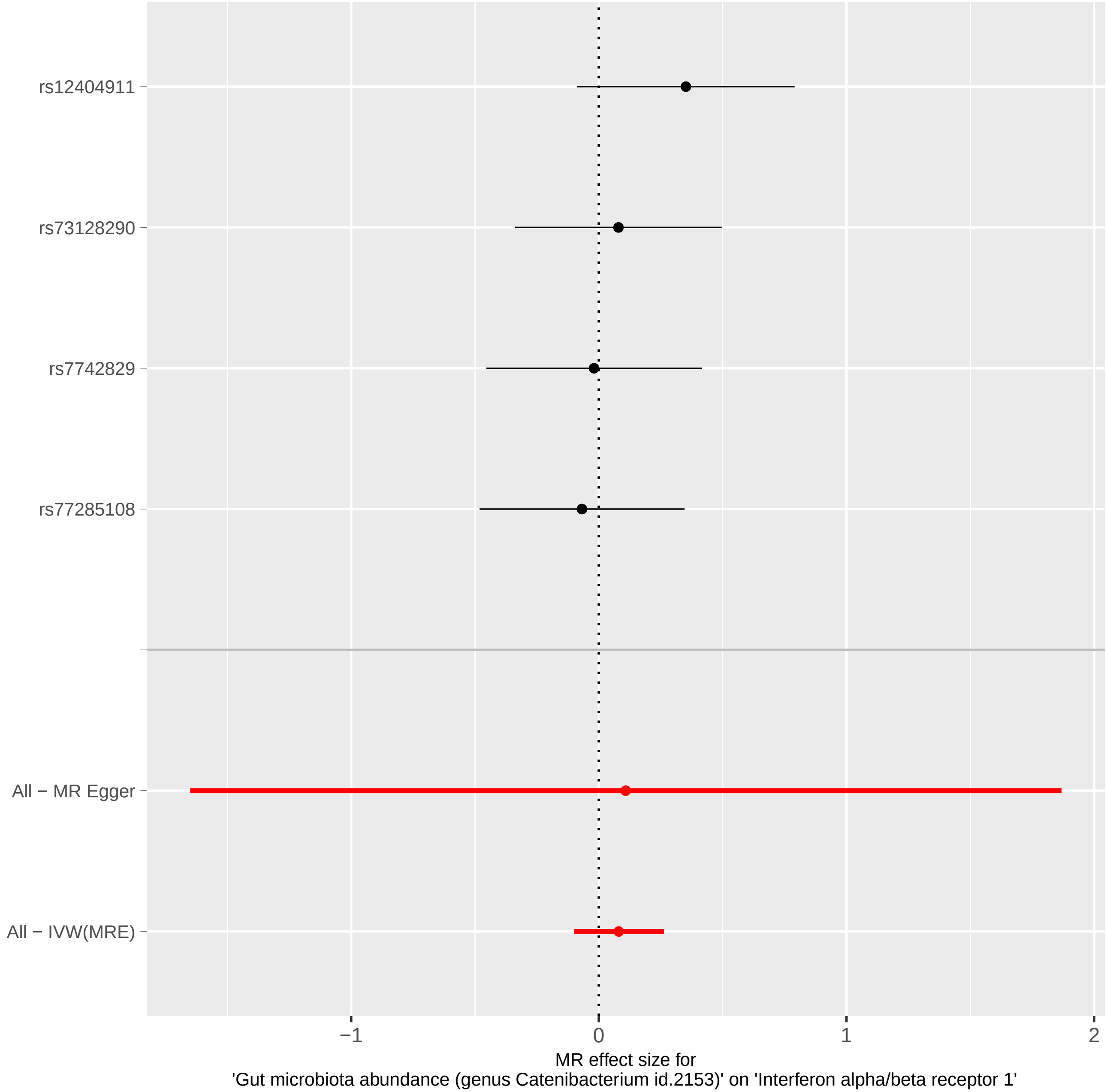

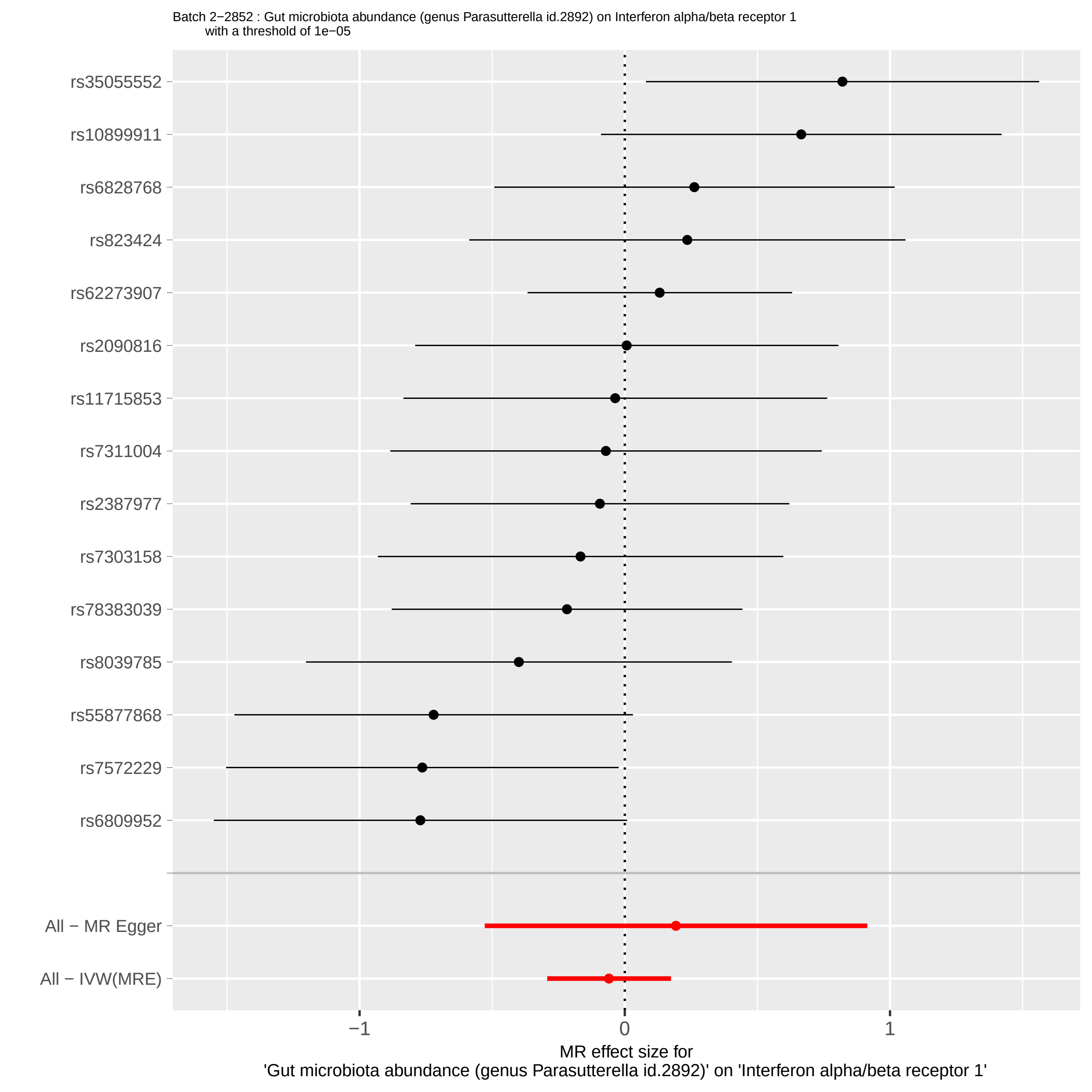

Batch 2−2852 : Gut microbiota abundance (genus Parasutterella id.2892) on Interferon alpha/beta receptor 1
with a threshold of 1e−05
rs35055552
rs10899911
rs6828768
rs823424
rs62273907
rs2090816
rs11715853
rs7311004
rs2387977
rs7303158
rs78383039
rs8039785
rs55877868
rs7572229
rs6809952
All − MR Egger
All − IVW(MRE)
−1
0
1
MR effect size for
'Gut microbiota abundance (genus Parasutterella id.2892)' on 'Interferon alpha/beta receptor 1'

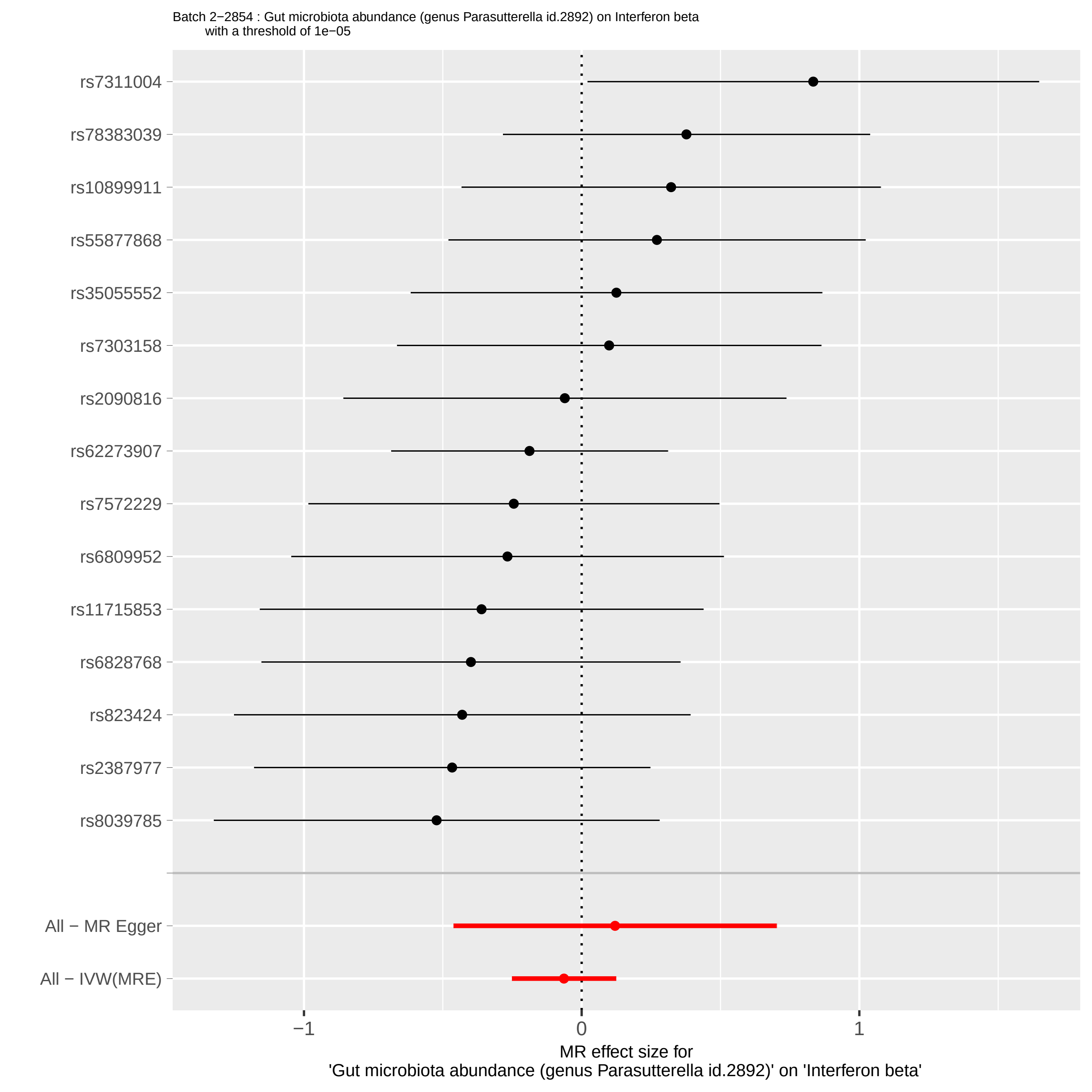

Batch 2−2854 : Gut microbiota abundance (genus Parasutterella id.2892) on Interferon beta
with a threshold of 1e−05
rs7311004
rs78383039
rs10899911
rs55877868
rs35055552
rs7303158
rs2090816
rs62273907
rs7572229
rs6809952
rs11715853
rs6828768
rs823424
rs2387977
rs8039785
All − MR Egger
All − IVW(MRE)
−1
0
1
MR effect size for
'Gut microbiota abundance (genus Parasutterella id.2892)' on 'Interferon beta'

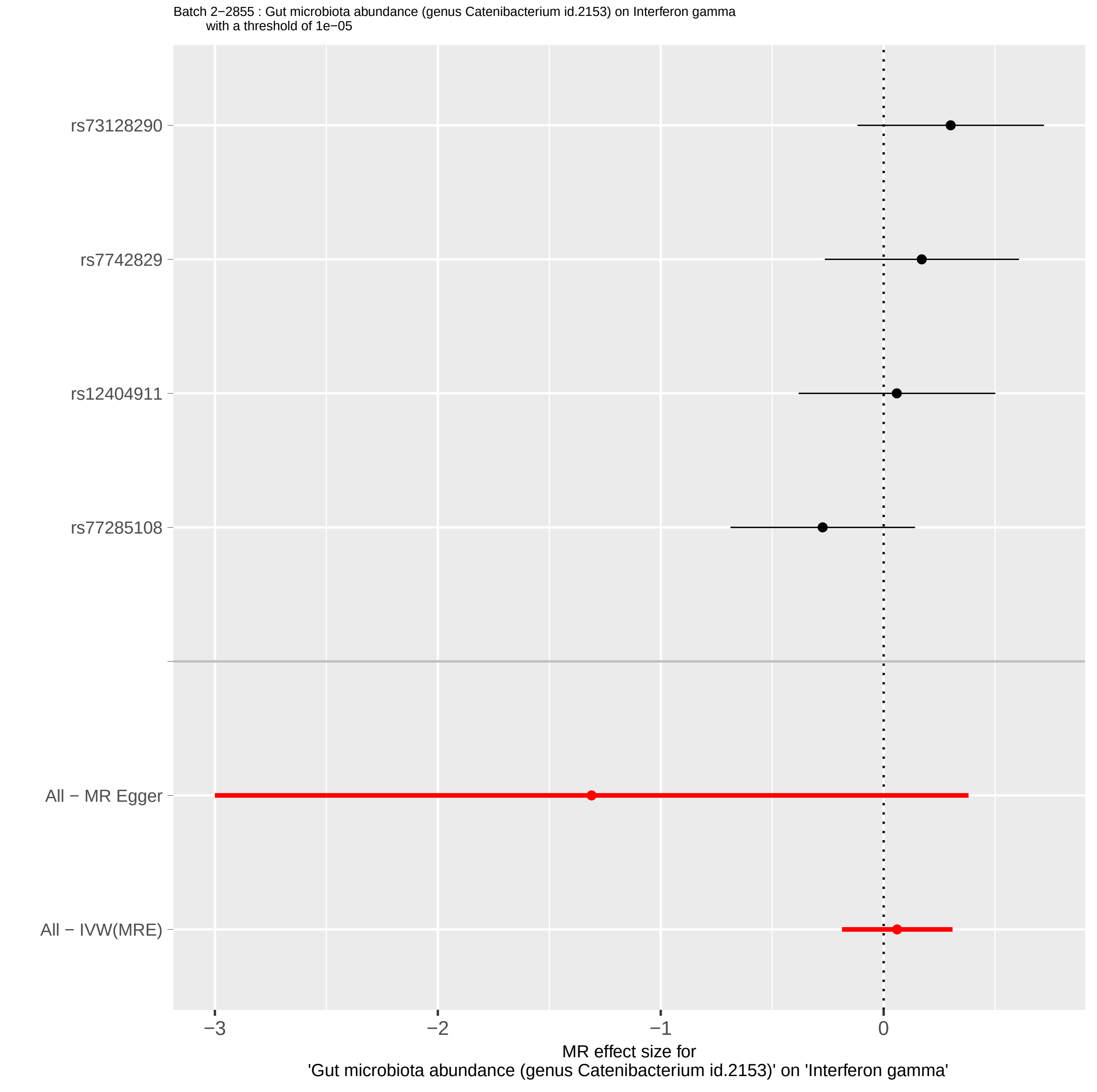

Batch 2−2855 : Gut microbiota abundance (genus Catenibacterium id.2153) on Interferon gamma
with a threshold of 1e−05
rs73128290
rs7742829
rs12404911
rs77285108
All − MR Egger
All − IVW(MRE)
−3
−2
−1
0
MR effect size for
'Gut microbiota abundance (genus Catenibacterium id.2153)' on 'Interferon gamma'

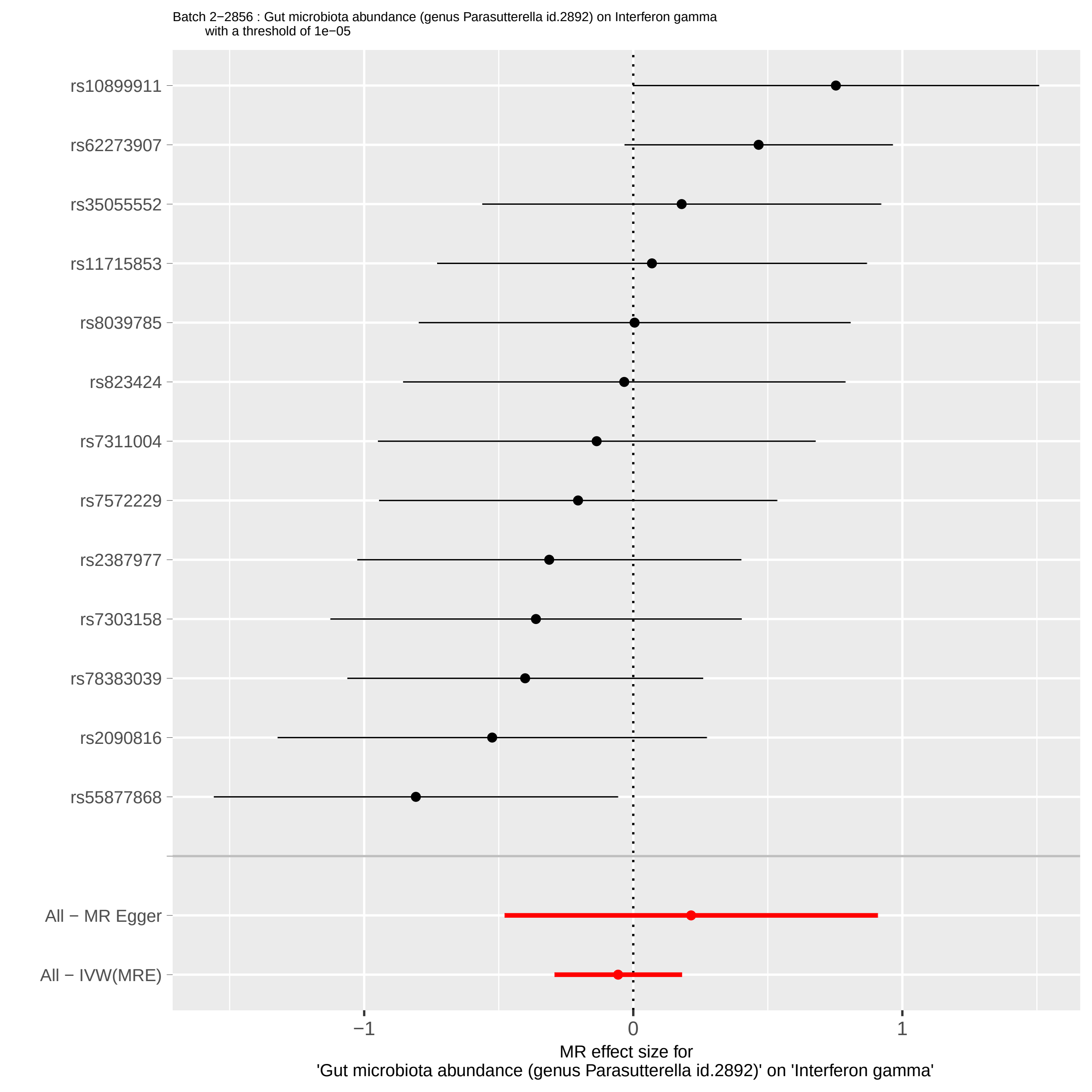

Batch 2−2856 : Gut microbiota abundance (genus Parasutterella id.2892) on Interferon gamma
with a threshold of 1e−05
rs10899911
rs62273907
rs35055552
rs11715853
rs8039785
rs823424
rs7311004
rs7572229
rs2387977
rs7303158
rs78383039
rs2090816
rs55877868
All − MR Egger
All − IVW(MRE)
−1
0
1
MR effect size for
'Gut microbiota abundance (genus Parasutterella id.2892)' on 'Interferon gamma'

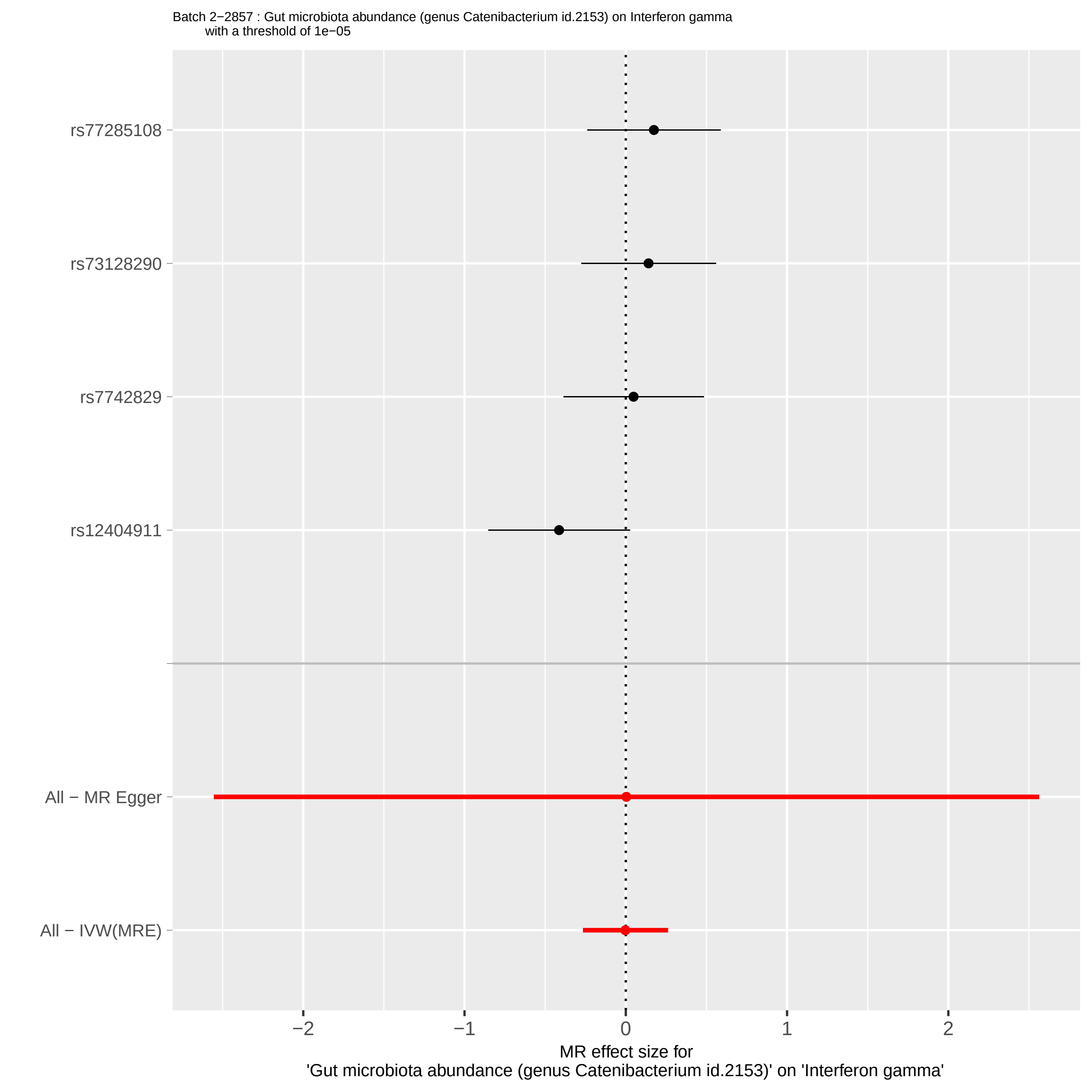

Batch 2−2857 : Gut microbiota abundance (genus Catenibacterium id.2153) on Interferon gamma
with a threshold of 1e−05
rs77285108
rs73128290
rs7742829
rs12404911
All − MR Egger
All − IVW(MRE)
−2
−1
0
1
2
MR effect size for
'Gut microbiota abundance (genus Catenibacterium id.2153)' on 'Interferon gamma'

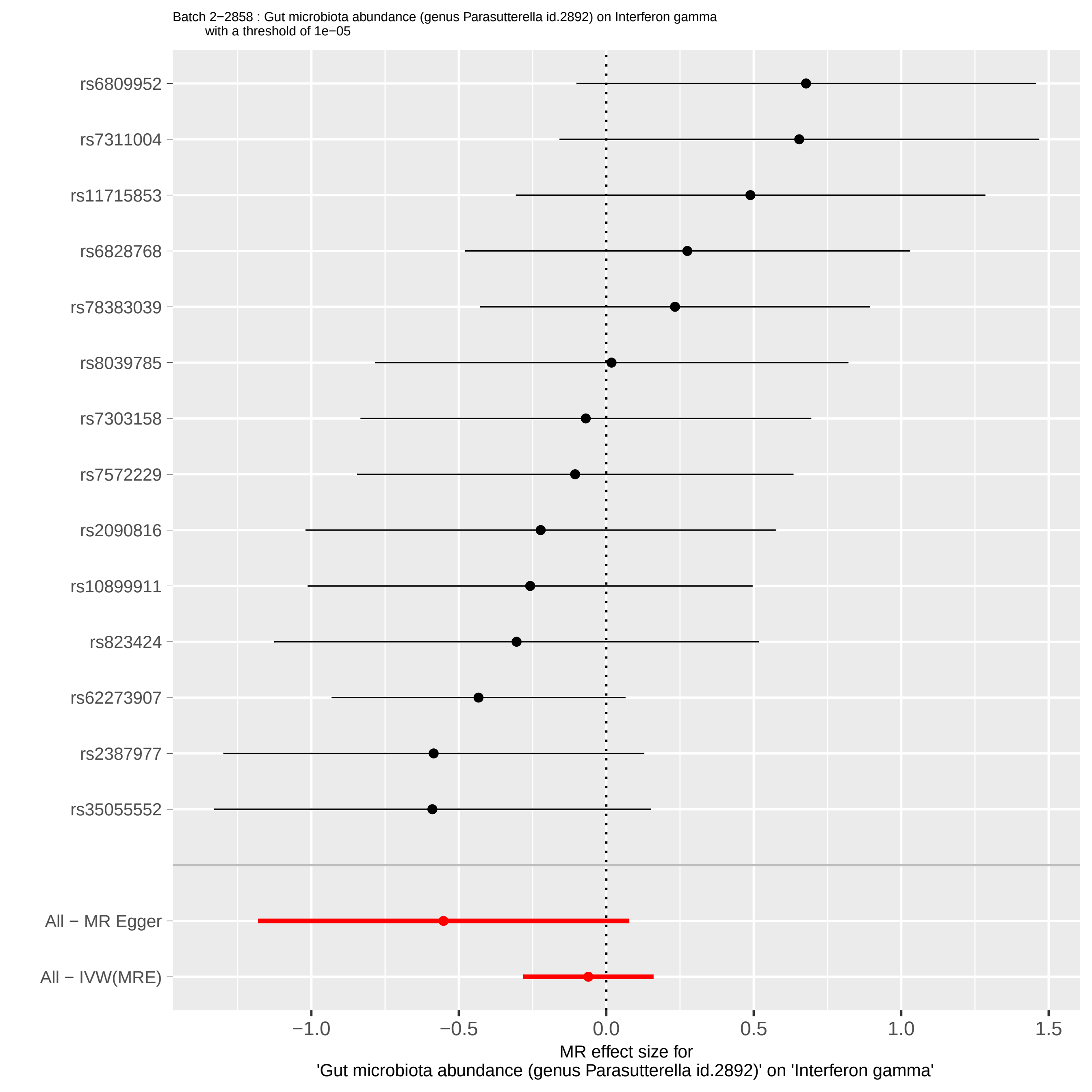

Batch 2−2858 : Gut microbiota abundance (genus Parasutterella id.2892) on Interferon gamma
with a threshold of 1e−05
rs6809952
rs7311004
rs11715853
rs6828768
rs78383039
rs8039785
rs7303158
rs7572229
rs2090816
rs10899911
rs823424
rs62273907
rs2387977
rs35055552
All − MR Egger
All − IVW(MRE)
−1.0
−0.5
0.0
0.5
1.0
1.5
MR effect size for
'Gut microbiota abundance (genus Parasutterella id.2892)' on 'Interferon gamma'

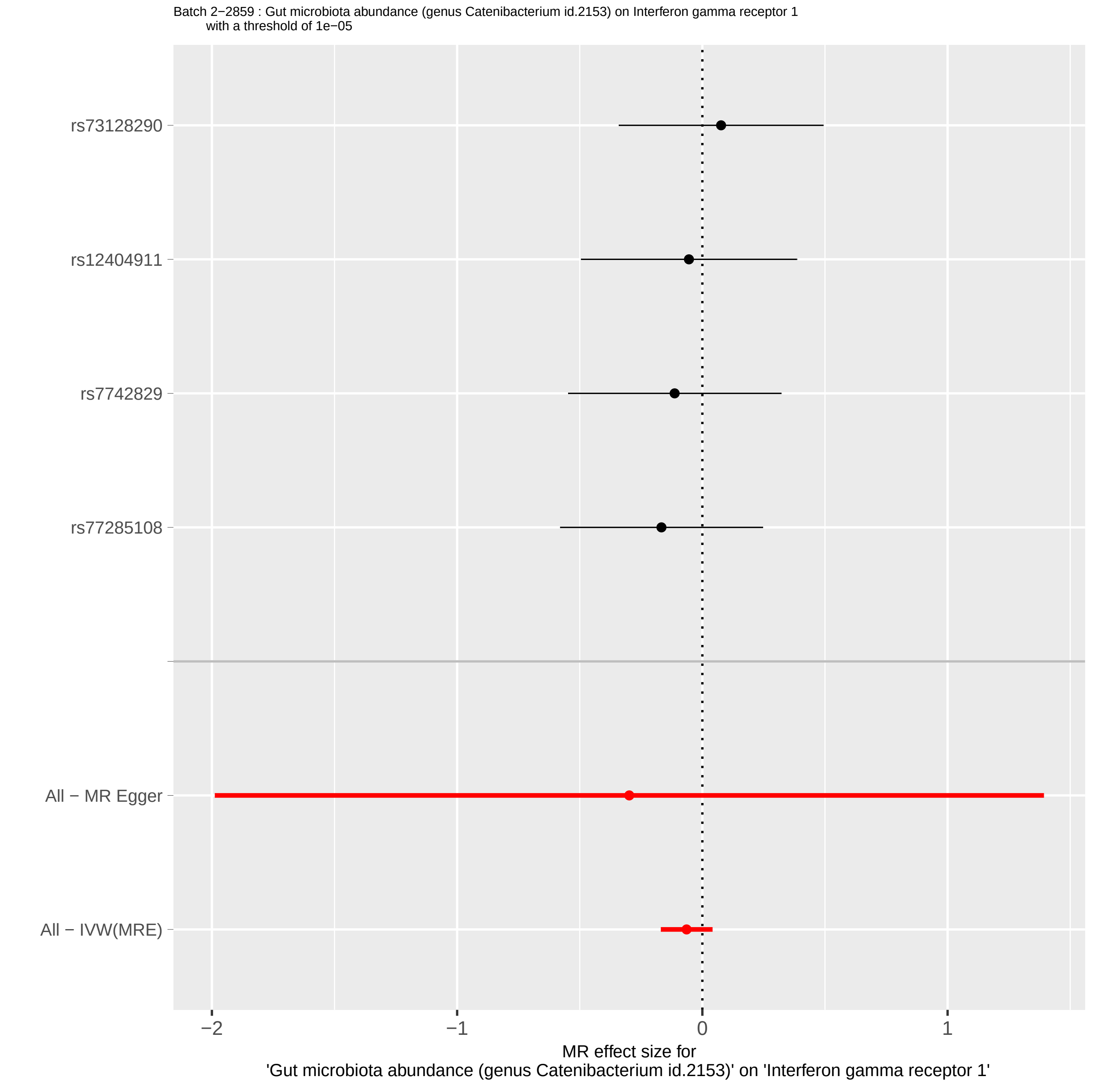

Batch 2−2859 : Gut microbiota abundance (genus Catenibacterium id.2153) on Interferon gamma receptor 1
with a threshold of 1e−05
rs73128290
rs12404911
rs7742829
rs77285108
All − MR Egger
All − IVW(MRE)
−2
−1
0
1
MR effect size for
'Gut microbiota abundance (genus Catenibacterium id.2153)' on 'Interferon gamma receptor 1'

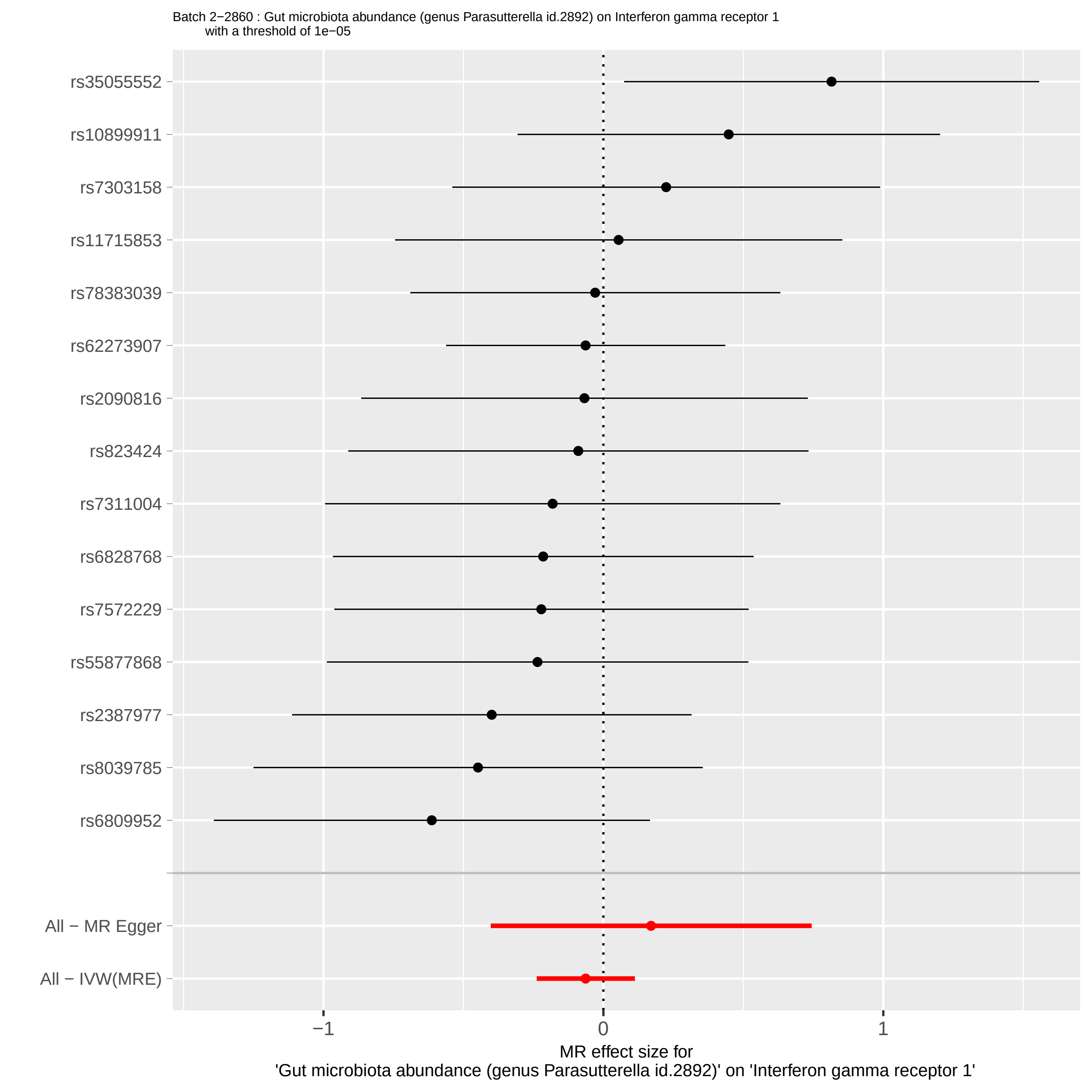

Batch 2−2860 : Gut microbiota abundance (genus Parasutterella id.2892) on Interferon gamma receptor 1
with a threshold of 1e−05
rs35055552
rs10899911
rs7303158
rs11715853
rs78383039
rs62273907
rs2090816
rs823424
rs7311004
rs6828768
rs7572229
rs55877868
rs2387977
rs8039785
rs6809952
All − MR Egger
All − IVW(MRE)
−1
0
1
MR effect size for
'Gut microbiota abundance (genus Parasutterella id.2892)' on 'Interferon gamma receptor 1'

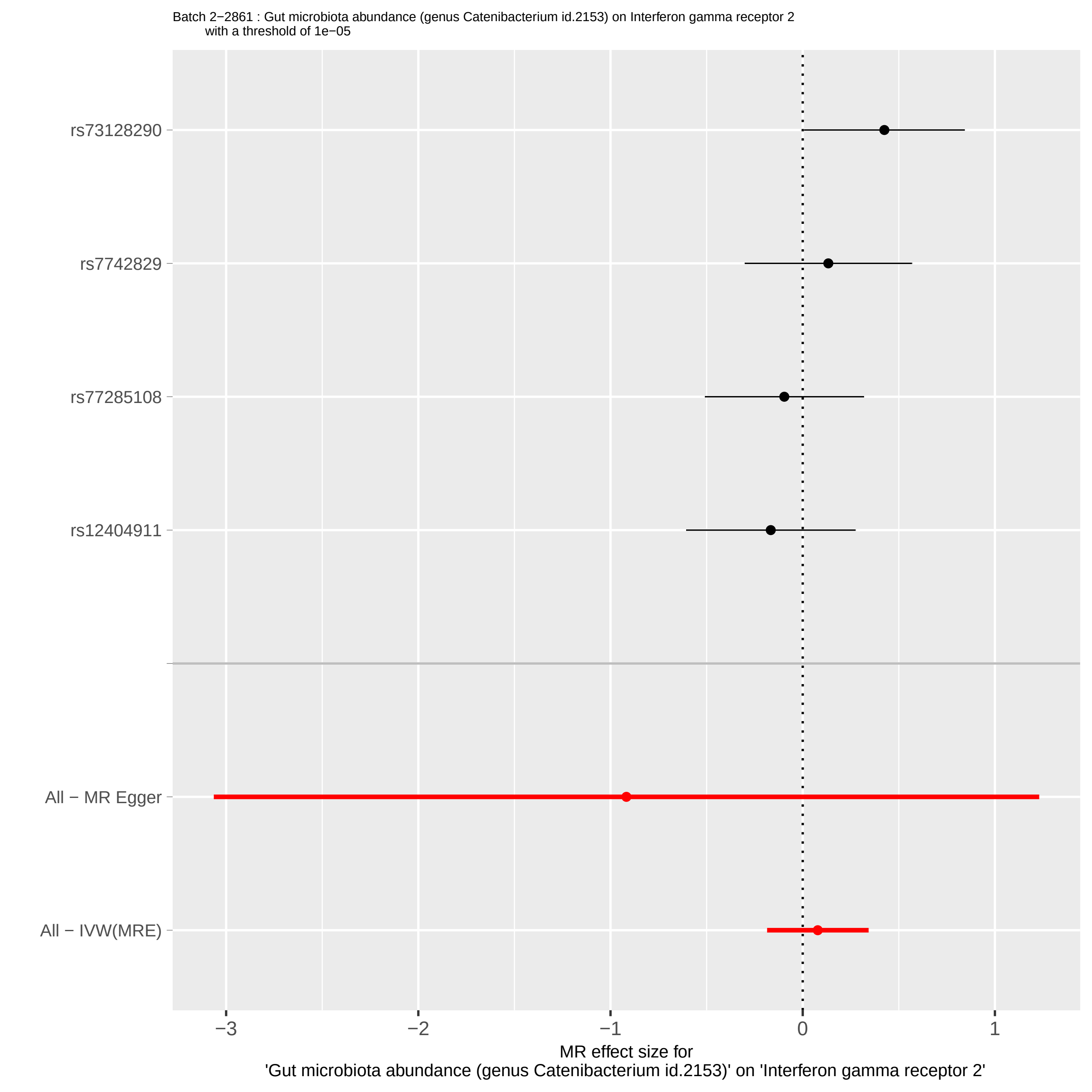

Batch 2−2861 : Gut microbiota abundance (genus Catenibacterium id.2153) on Interferon gamma receptor 2
with a threshold of 1e−05
rs73128290
rs7742829
rs77285108
rs12404911
All − MR Egger
All − IVW(MRE)
−3
−2
−1
0
1
MR effect size for
'Gut microbiota abundance (genus Catenibacterium id.2153)' on 'Interferon gamma receptor 2'

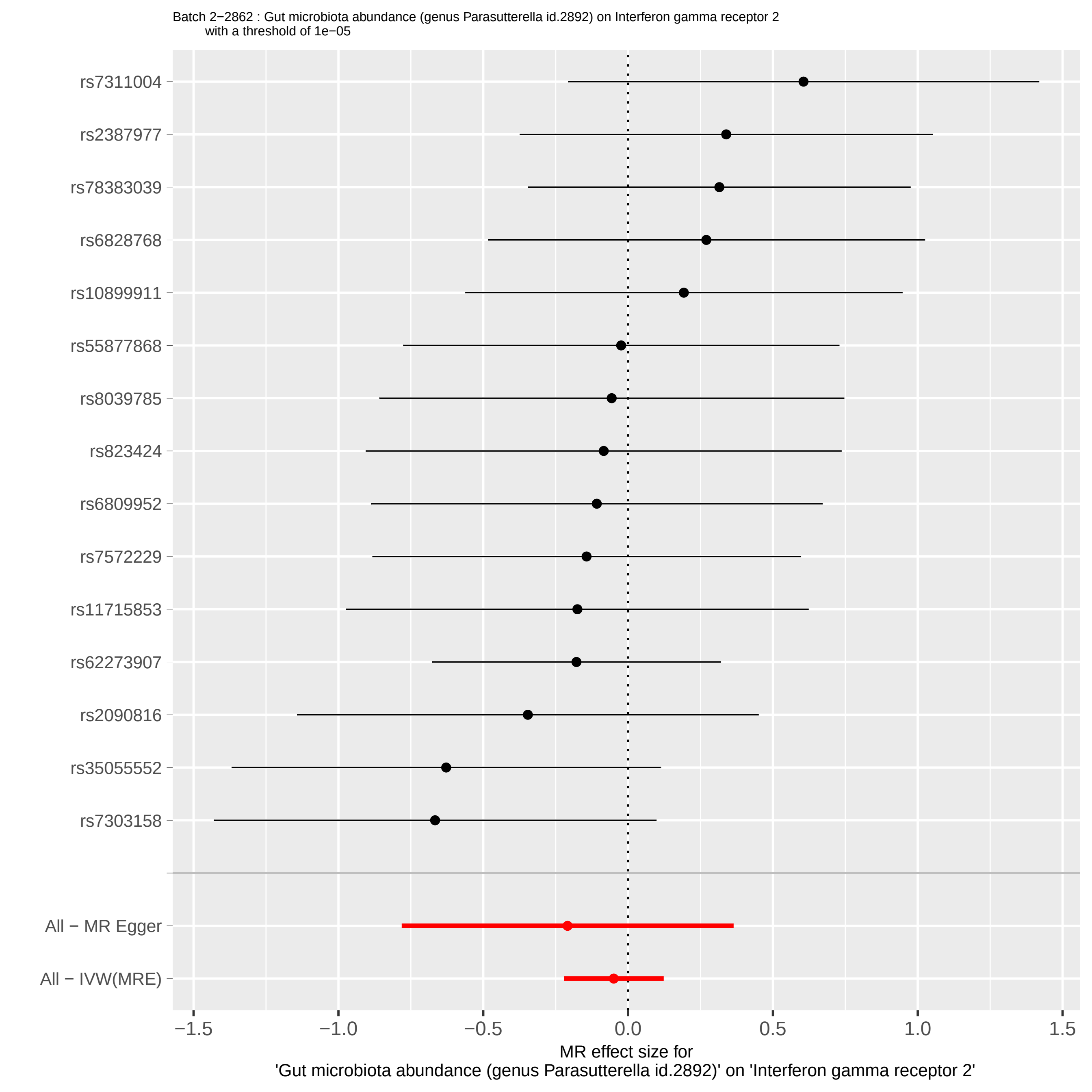

Batch 2−2862 : Gut microbiota abundance (genus Parasutterella id.2892) on Interferon gamma receptor 2
with a threshold of 1e−05
rs7311004
rs2387977
rs78383039
rs6828768
rs10899911
rs55877868
rs8039785
rs823424
rs6809952
rs7572229
rs11715853
rs62273907
rs2090816
rs35055552
rs7303158
All − MR Egger
All − IVW(MRE)
−1.5
−1.0
−0.5
0.0
0.5
1.0
1.5
MR effect size for
'Gut microbiota abundance (genus Parasutterella id.2892)' on 'Interferon gamma receptor 2'

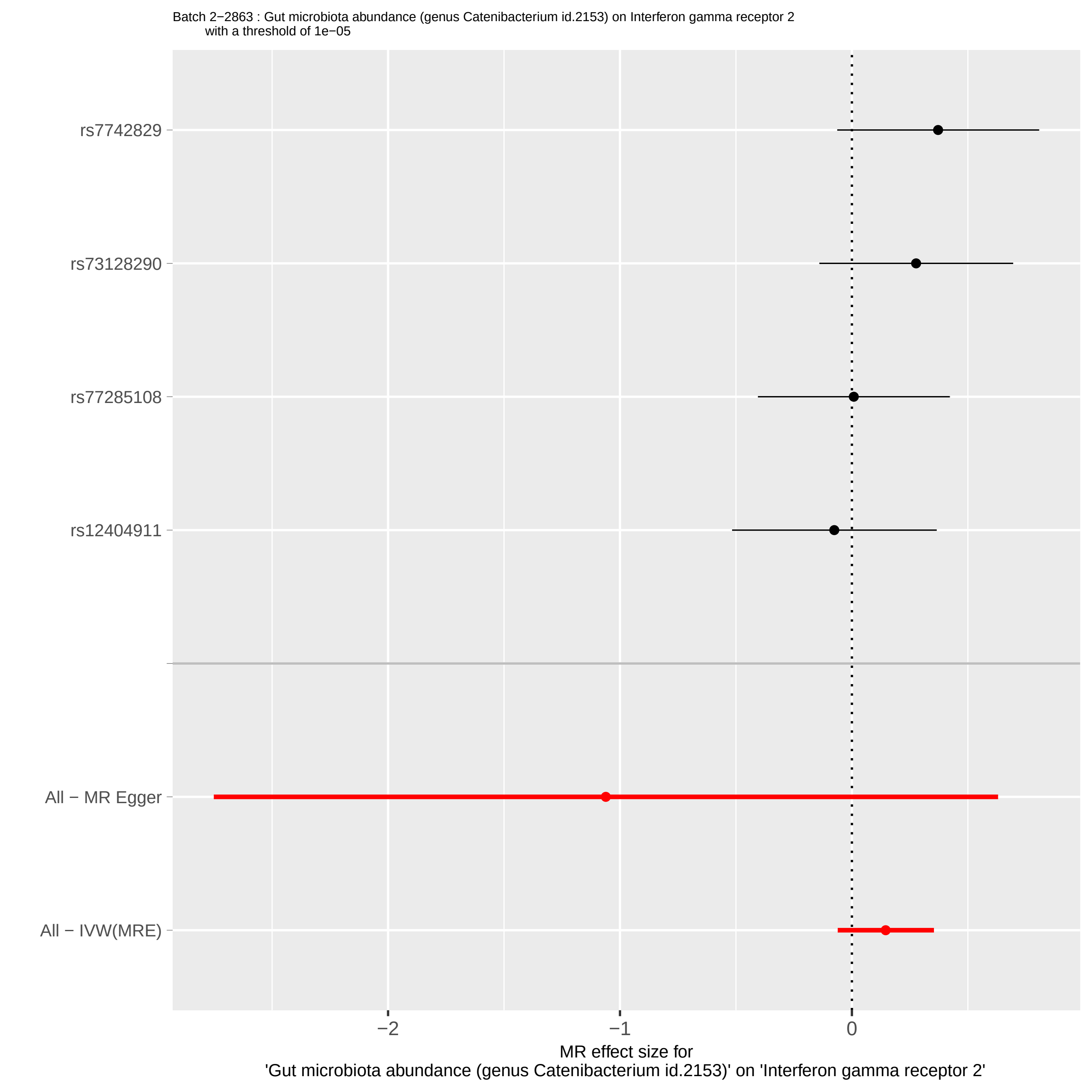
Batch 2−2863 : Gut microbiota abundance (genus Catenibacterium id.2153) on Interferon gamma receptor 2
with a threshold of 1e−05
rs7742829
rs73128290
rs77285108
rs12404911
All − MR Egger
All − IVW(MRE)
−2
−1
0
MR effect size for
'Gut microbiota abundance (genus Catenibacterium id.2153)' on 'Interferon gamma receptor 2'

Batch 2−2864 : Gut microbiota abundance (genus Parasutterella id.2892) on Interferon gamma receptor 2
with a threshold of 1e−05

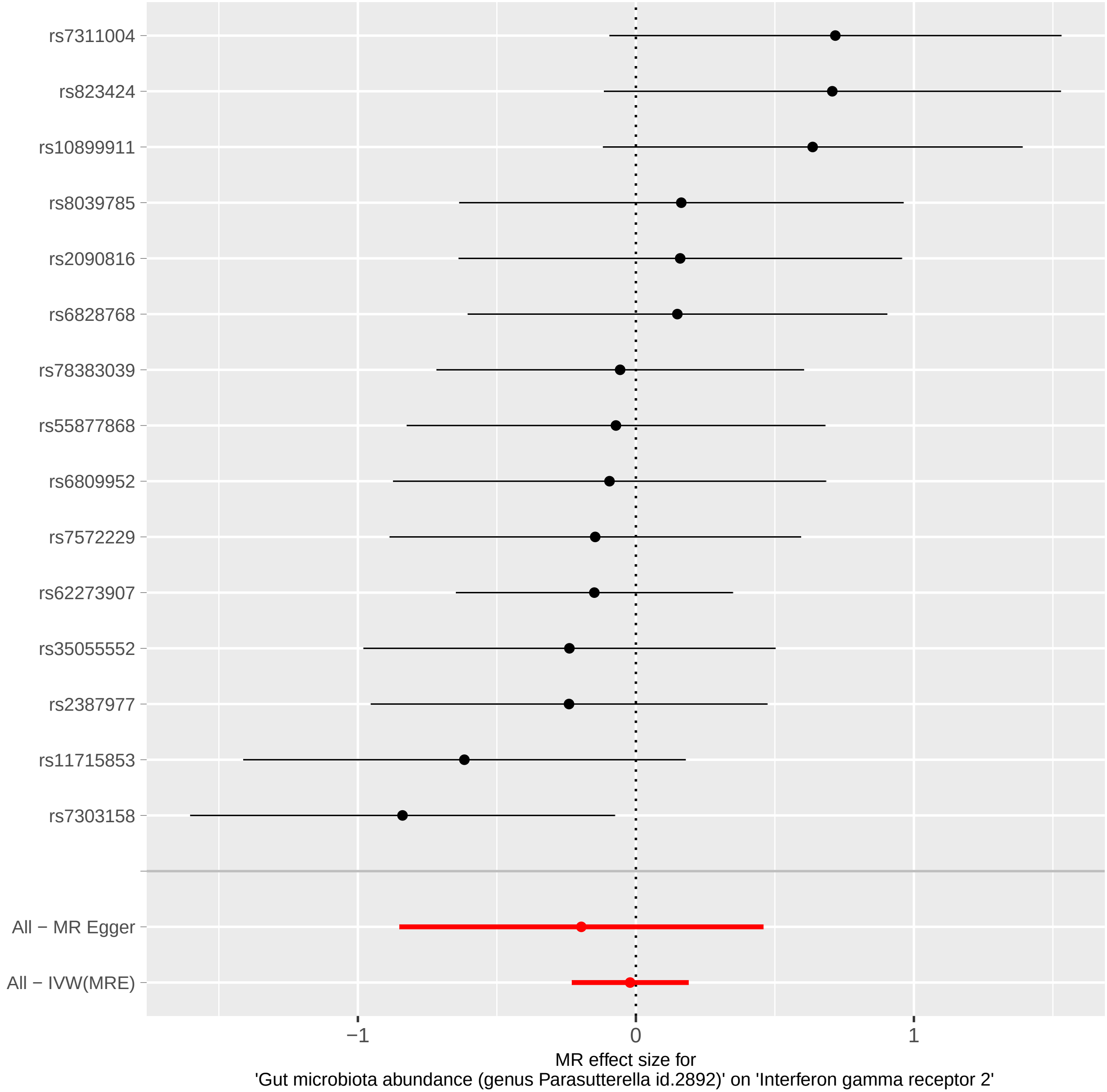

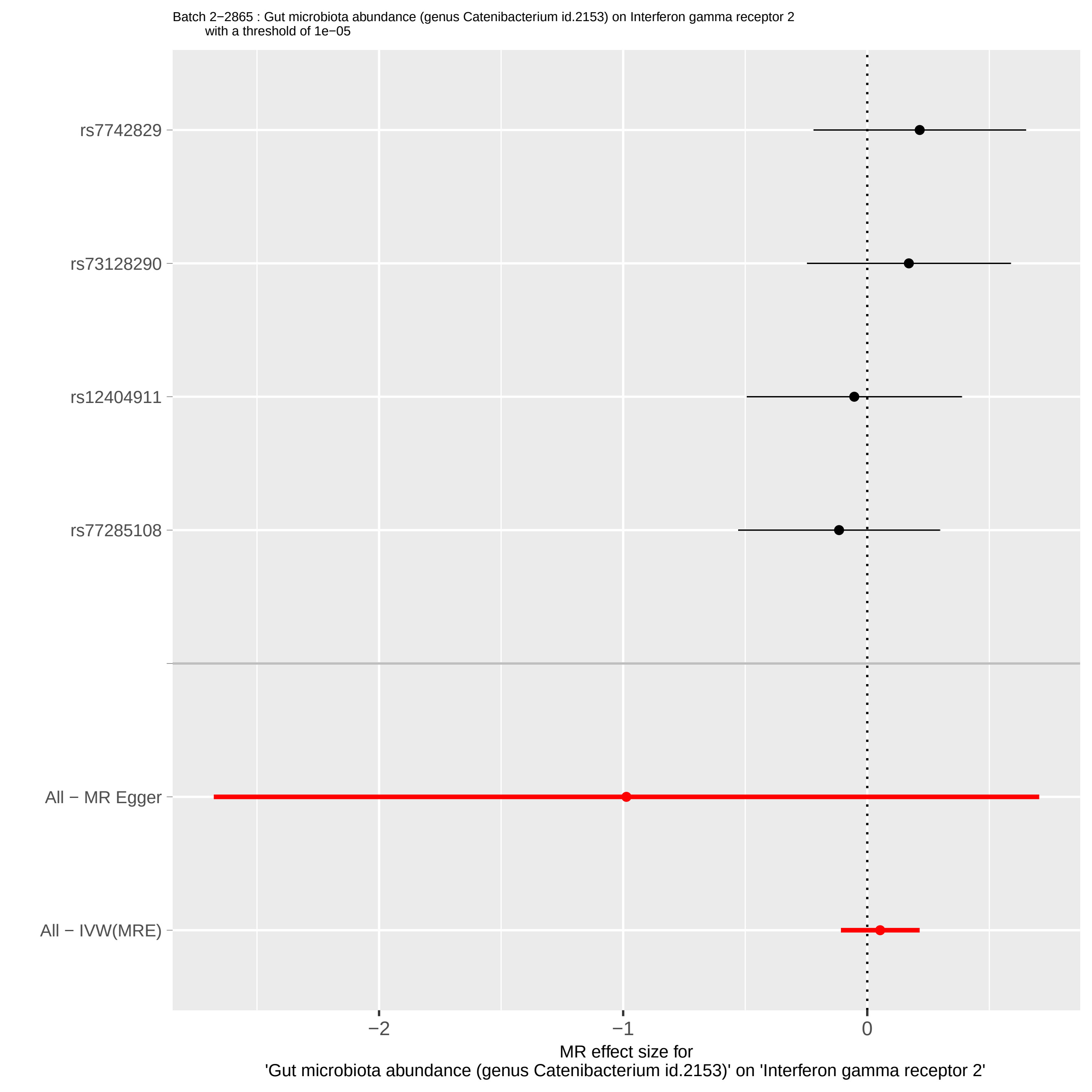

Batch 2−2865 : Gut microbiota abundance (genus Catenibacterium id.2153) on Interferon gamma receptor 2
with a threshold of 1e−05
rs7742829
rs73128290
rs12404911
rs77285108
All − MR Egger
All − IVW(MRE)
−2
−1
0
MR effect size for
'Gut microbiota abundance (genus Catenibacterium id.2153)' on 'Interferon gamma receptor 2'

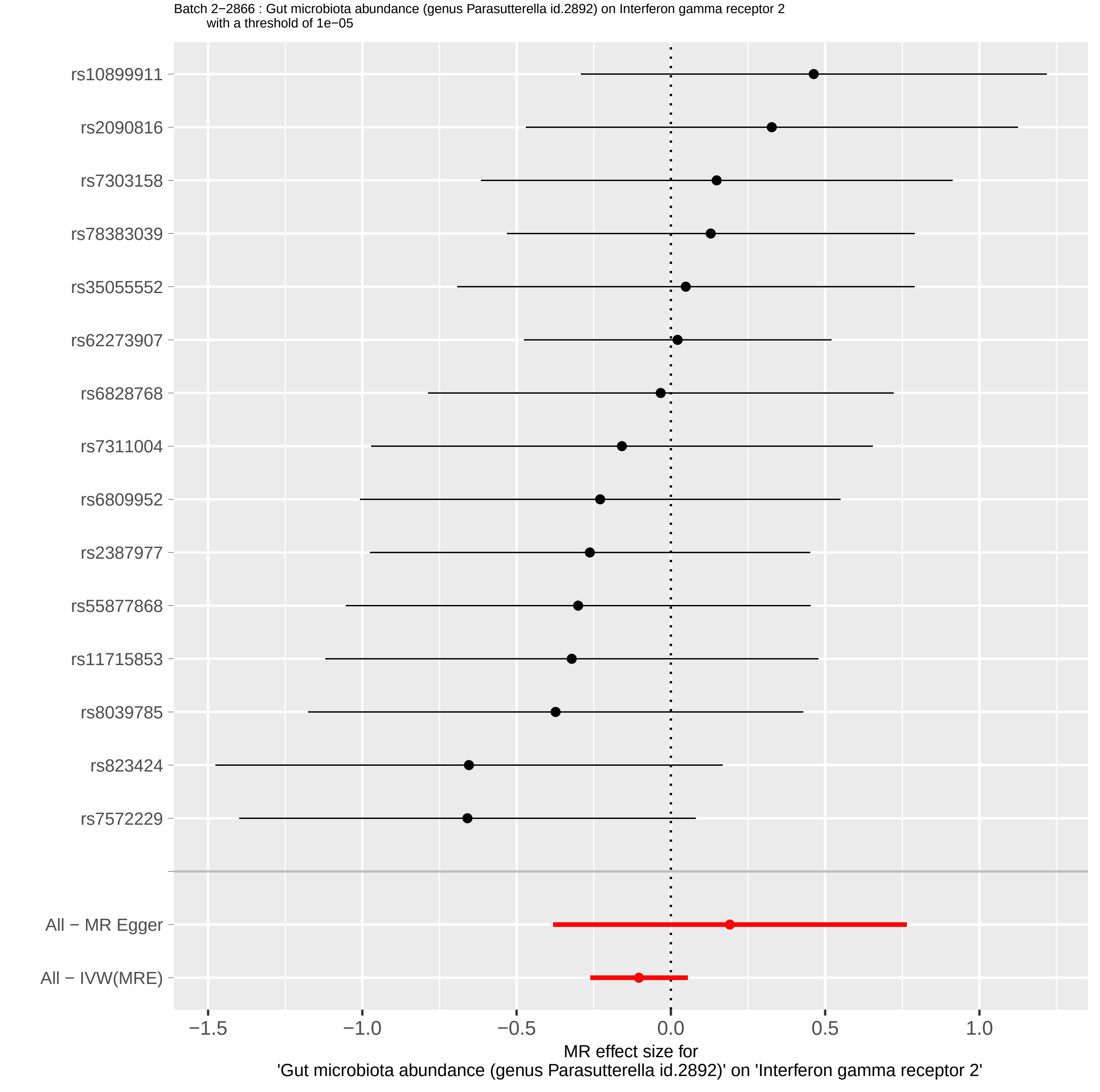

Batch 2−2866 : Gut microbiota abundance (genus Parasutterella id.2892) on Interferon gamma receptor 2
with a threshold of 1e−05
rs10899911
rs2090816
rs7303158
rs78383039
rs35055552
rs62273907
rs6828768
rs7311004
rs6809952
rs2387977
rs55877868
rs11715853
rs8039785
rs823424
rs7572229
All − MR Egger
All − IVW(MRE)
−1.5
−1.0
−0.5
0.0
0.5
1.0
MR effect size for
'Gut microbiota abundance (genus Parasutterella id.2892)' on 'Interferon gamma receptor 2'

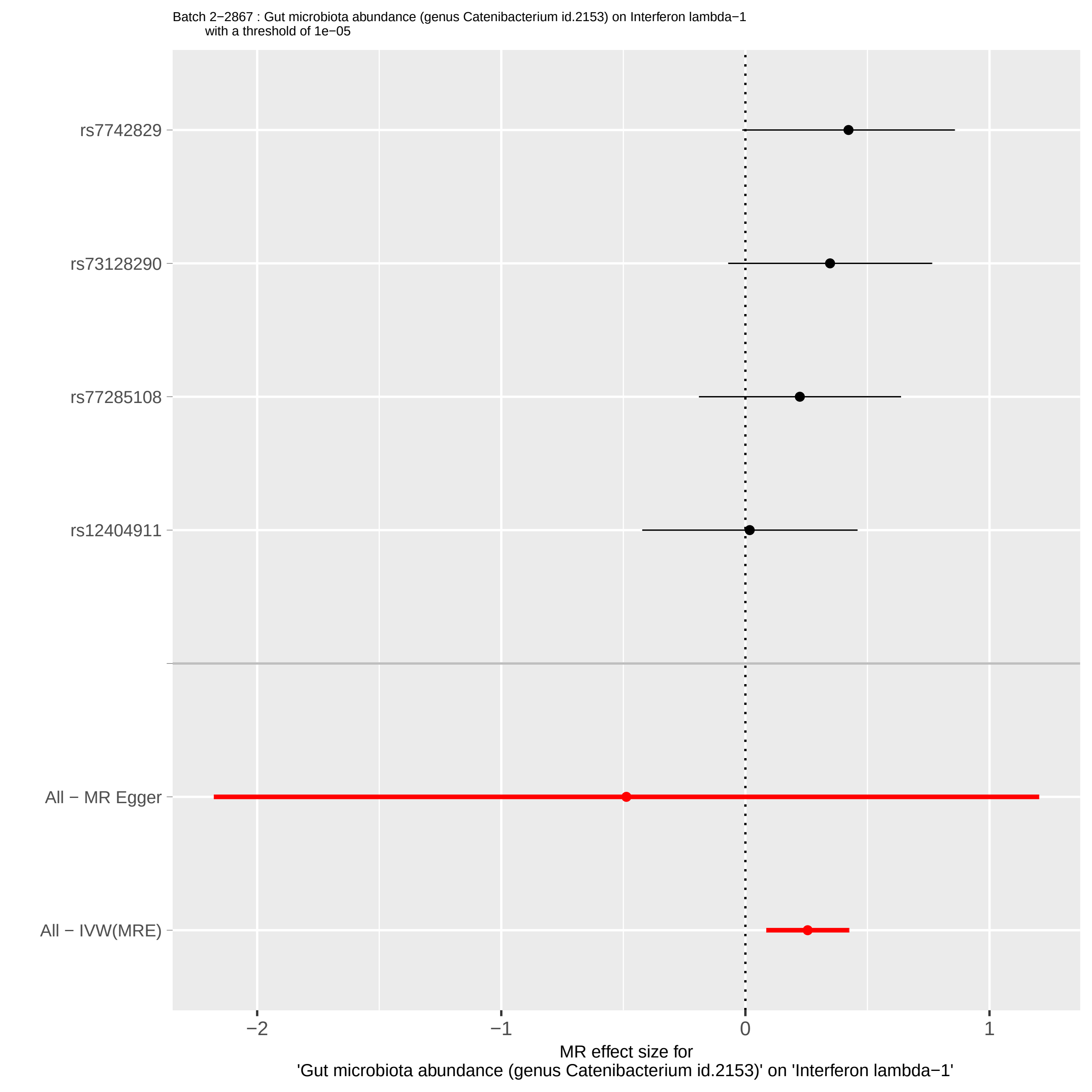
Batch 2−2867 : Gut microbiota abundance (genus Catenibacterium id.2153) on Interferon lambda−1
with a threshold of 1e−05
rs7742829
rs73128290
rs77285108
rs12404911
All − MR Egger
All − IVW(MRE)
−2
−1
0
1
MR effect size for
'Gut microbiota abundance (genus Catenibacterium id.2153)' on 'Interferon lambda−1'

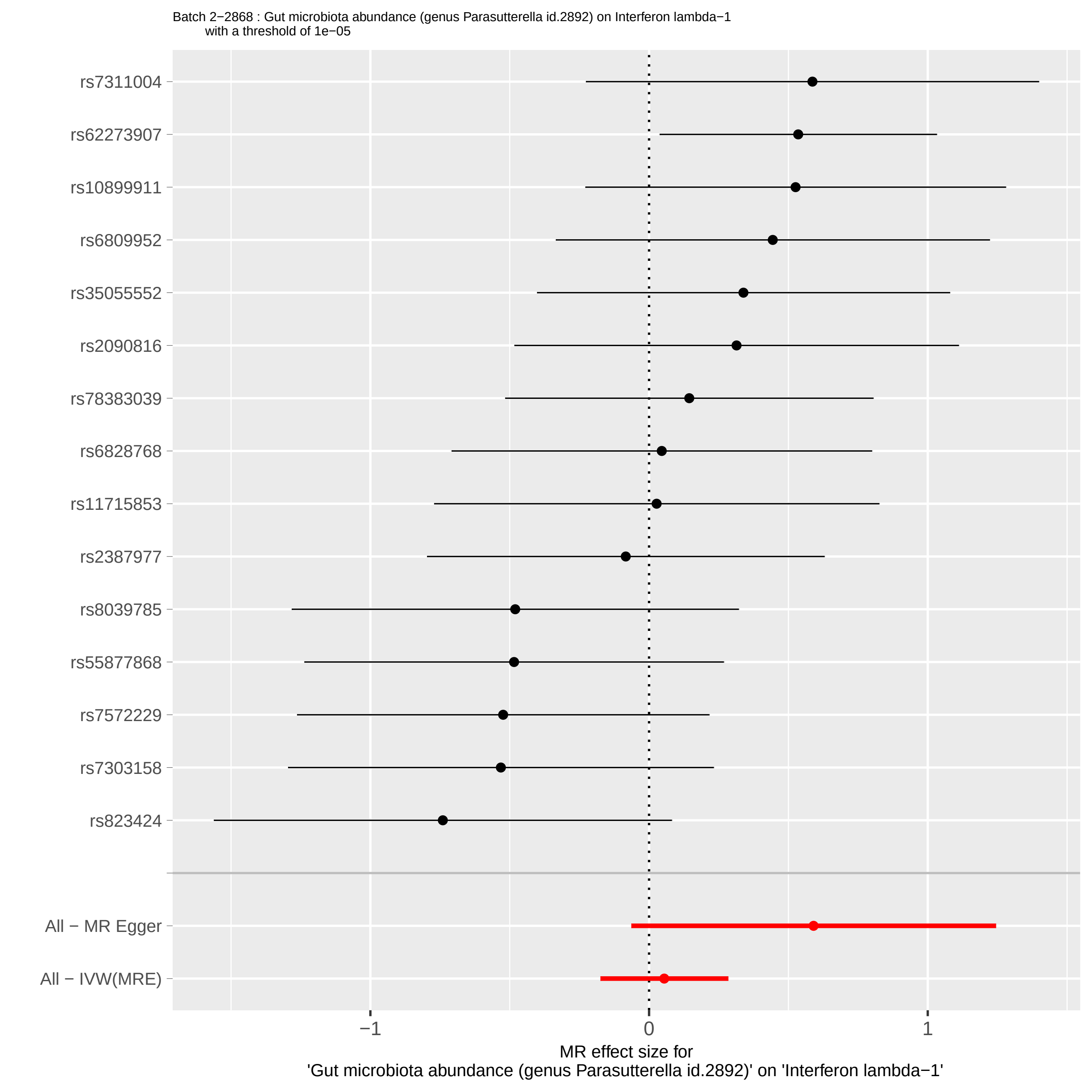
Batch 2−2868 : Gut microbiota abundance (genus Parasutterella id.2892) on Interferon lambda−1
with a threshold of 1e−05
rs7311004
rs62273907
rs10899911
rs6809952
rs35055552
rs2090816
rs78383039
rs6828768
rs11715853
rs2387977
rs8039785
rs55877868
rs7572229
rs7303158
rs823424
All − MR Egger
All − IVW(MRE)
−1
0
1
MR effect size for
'Gut microbiota abundance (genus Parasutterella id.2892)' on 'Interferon lambda−1'

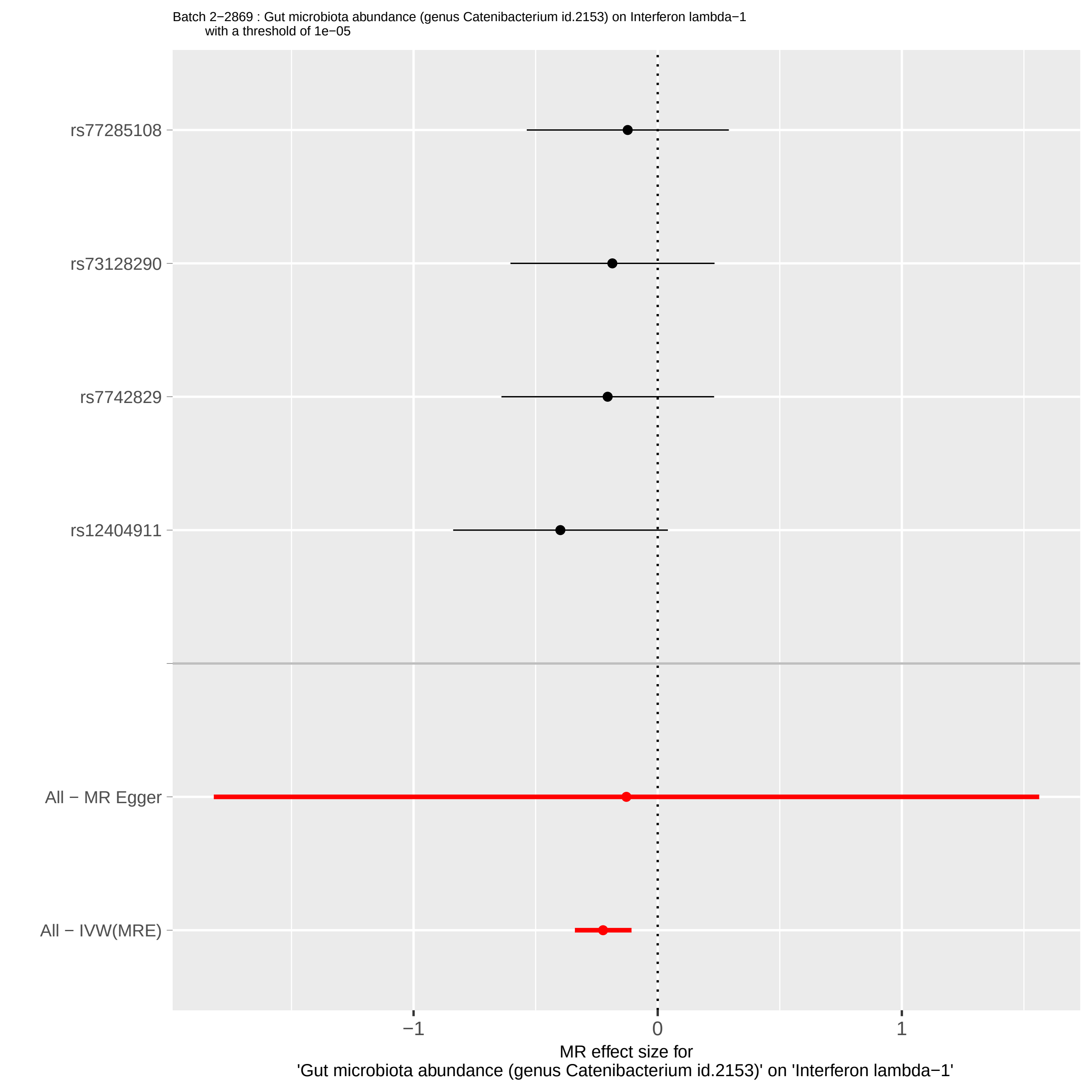

Batch 2−2869 : Gut microbiota abundance (genus Catenibacterium id.2153) on Interferon lambda−1
with a threshold of 1e−05
rs77285108
rs73128290
rs7742829
rs12404911
All − MR Egger
All − IVW(MRE)
−1
0
1
MR effect size for
'Gut microbiota abundance (genus Catenibacterium id.2153)' on 'Interferon lambda−1'

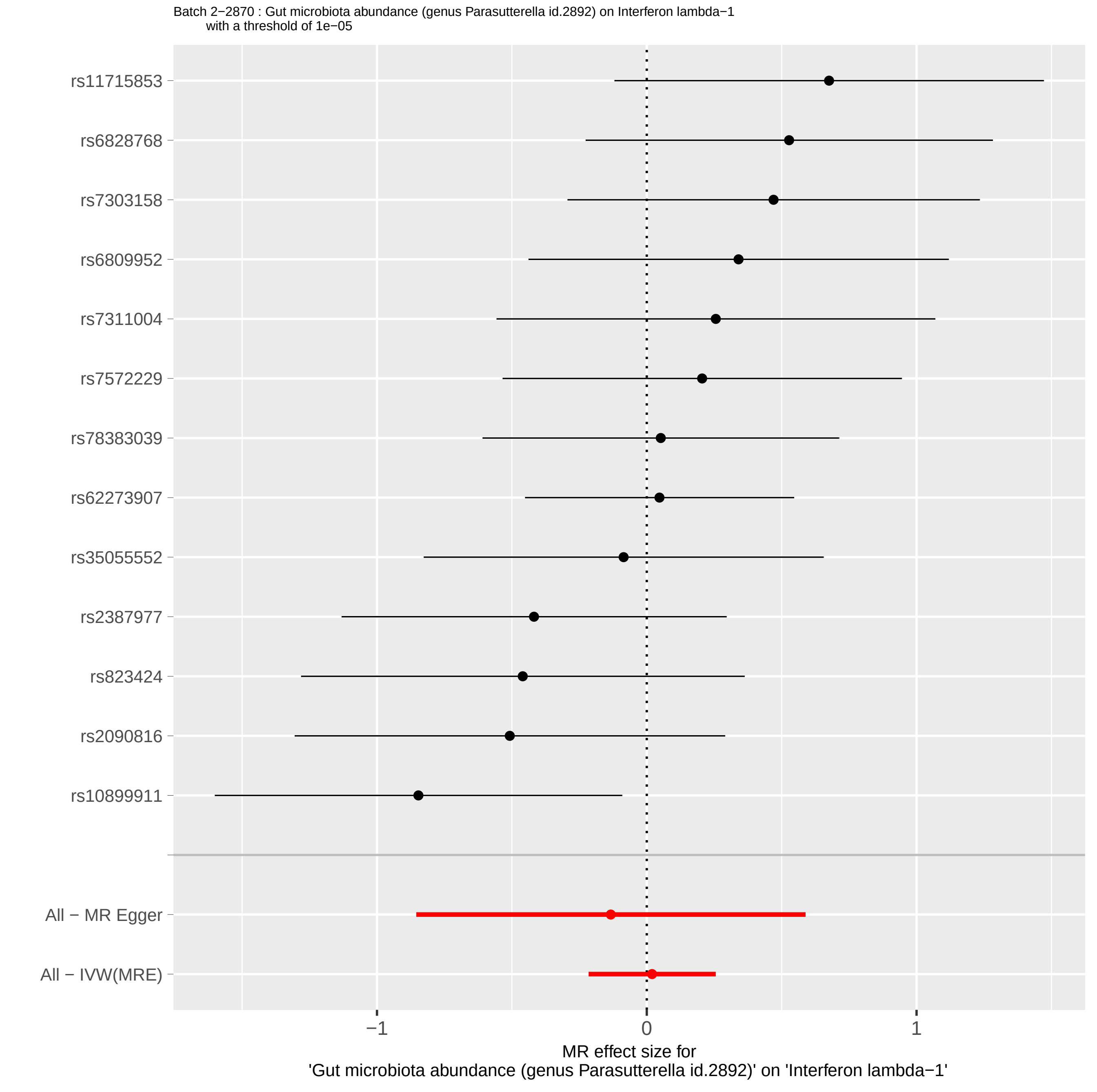

Batch 2−2870 : Gut microbiota abundance (genus Parasutterella id.2892) on Interferon lambda−1
with a threshold of 1e−05
rs11715853
rs6828768
rs7303158
rs6809952
rs7311004
rs7572229
rs78383039
rs62273907
rs35055552
rs2387977
rs823424
rs2090816
rs10899911
All − MR Egger
All − IVW(MRE)
−1
0
1
MR effect size for
'Gut microbiota abundance (genus Parasutterella id.2892)' on 'Interferon lambda−1'

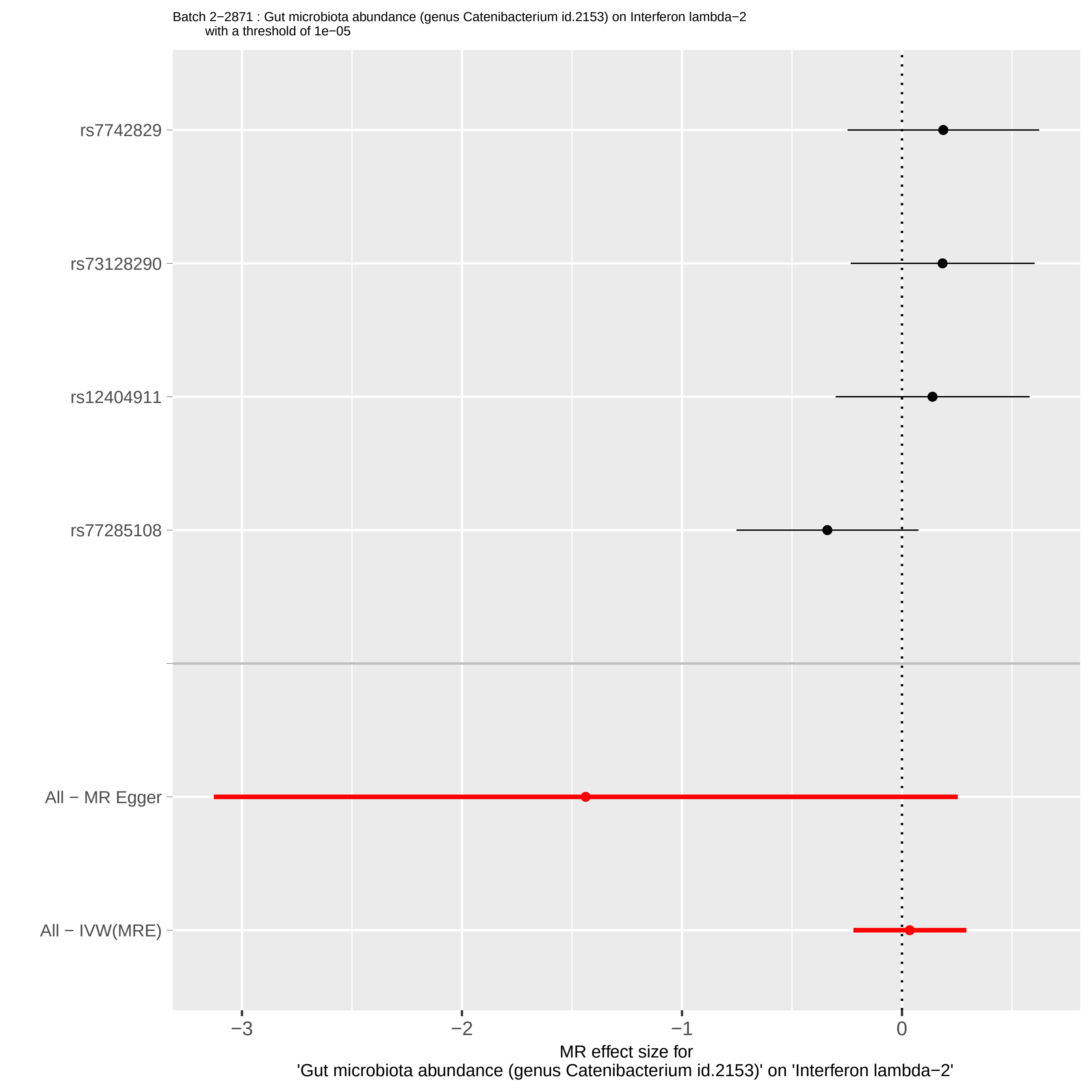
Batch 2−2871 : Gut microbiota abundance (genus Catenibacterium id.2153) on Interferon lambda−2
with a threshold of 1e−05
rs7742829
rs73128290
rs12404911
rs77285108
All − MR Egger
All − IVW(MRE)
−3
−2
−1
0
MR effect size for
'Gut microbiota abundance (genus Catenibacterium id.2153)' on 'Interferon lambda−2'

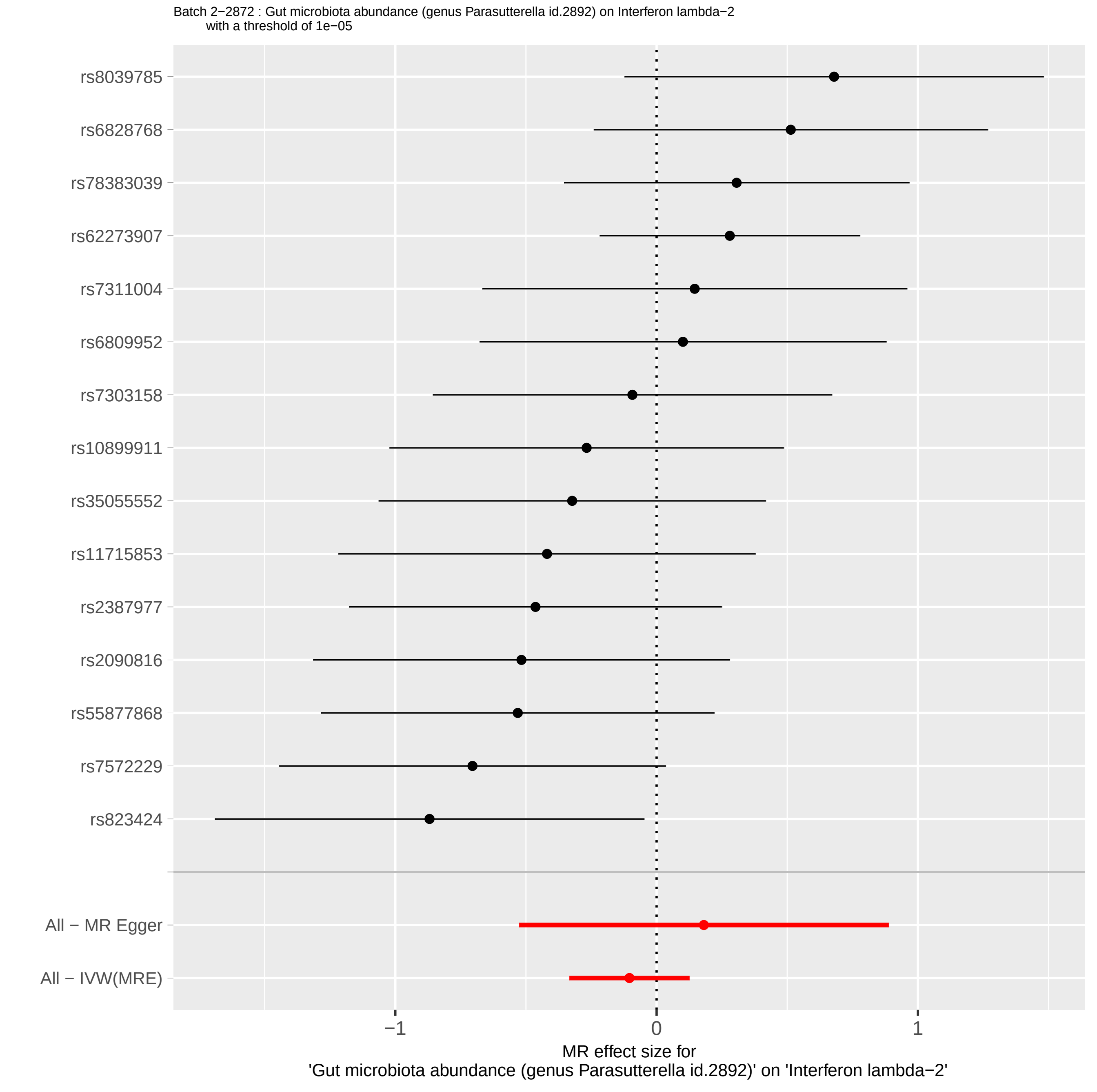

Batch 2−2872 : Gut microbiota abundance (genus Parasutterella id.2892) on Interferon lambda−2
with a threshold of 1e−05
rs8039785
rs6828768
rs78383039
rs62273907
rs7311004
rs6809952
rs7303158
rs10899911
rs35055552
rs11715853
rs2387977
rs2090816
rs55877868
rs7572229
rs823424
All − MR Egger
All − IVW(MRE)
−1
0
1
MR effect size for
'Gut microbiota abundance (genus Parasutterella id.2892)' on 'Interferon lambda−2'

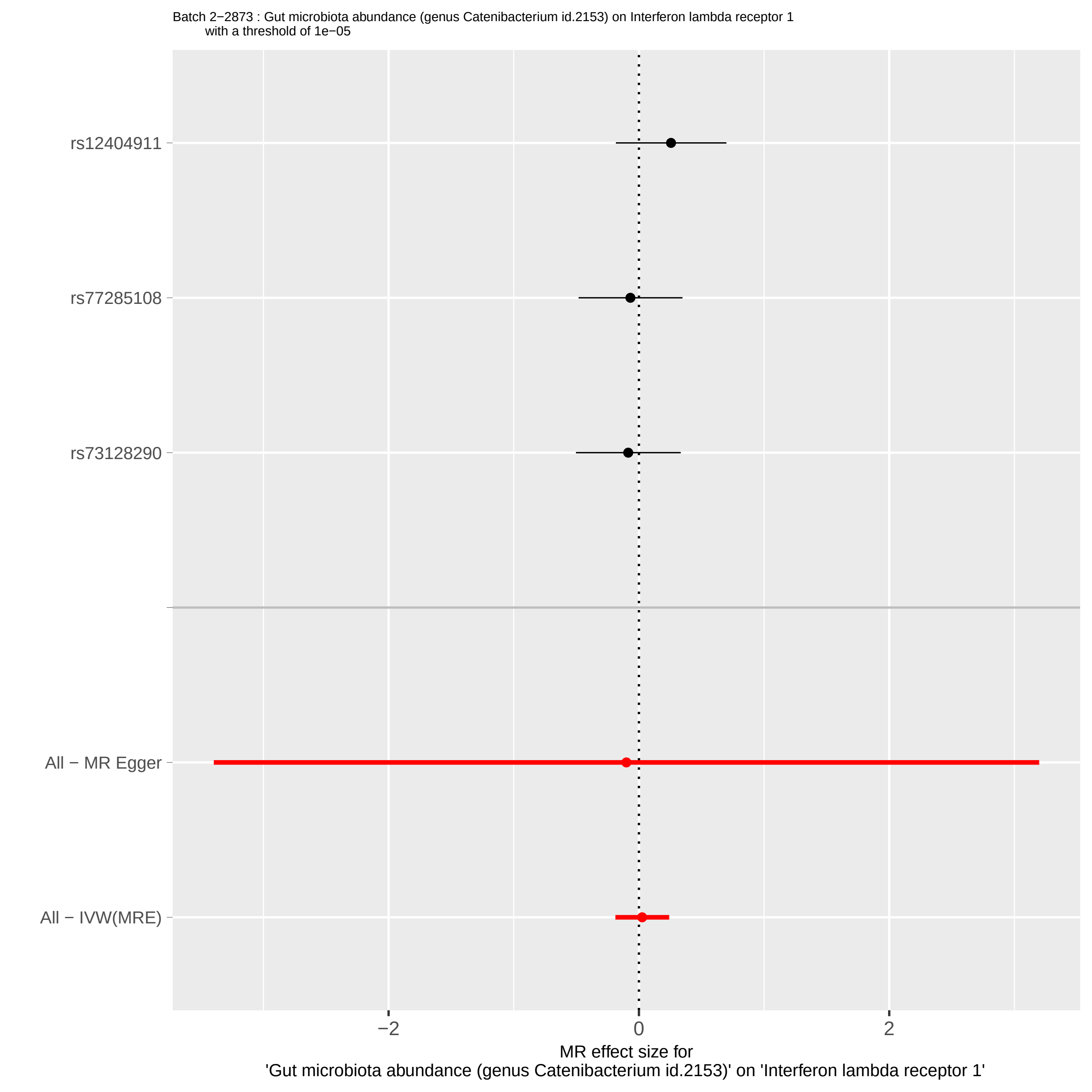
Batch 2−2873 : Gut microbiota abundance (genus Catenibacterium id.2153) on Interferon lambda receptor 1
with a threshold of 1e−05
rs12404911
rs77285108
rs73128290
All − MR Egger
All − IVW(MRE)
−2
0
2
MR effect size for
'Gut microbiota abundance (genus Catenibacterium id.2153)' on 'Interferon lambda receptor 1'

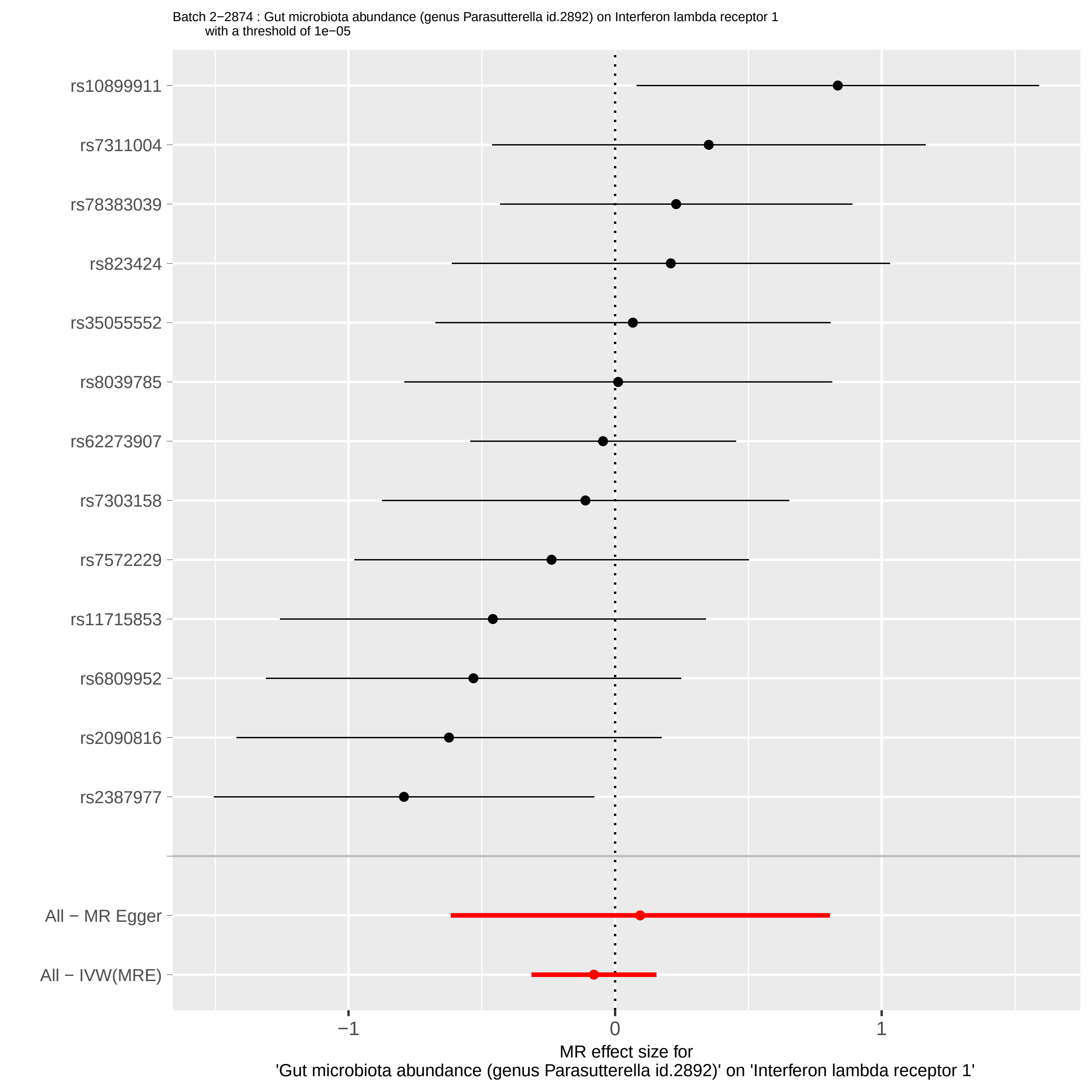

Batch 2−2874 : Gut microbiota abundance (genus Parasutterella id.2892) on Interferon lambda receptor 1
with a threshold of 1e−05
rs10899911
rs7311004
rs78383039
rs823424
rs35055552
rs8039785
rs62273907
rs7303158
rs7572229
rs11715853
rs6809952
rs2090816
rs2387977
All − MR Egger
All − IVW(MRE)
−1
0
1
MR effect size for
'Gut microbiota abundance (genus Parasutterella id.2892)' on 'Interferon lambda receptor 1'

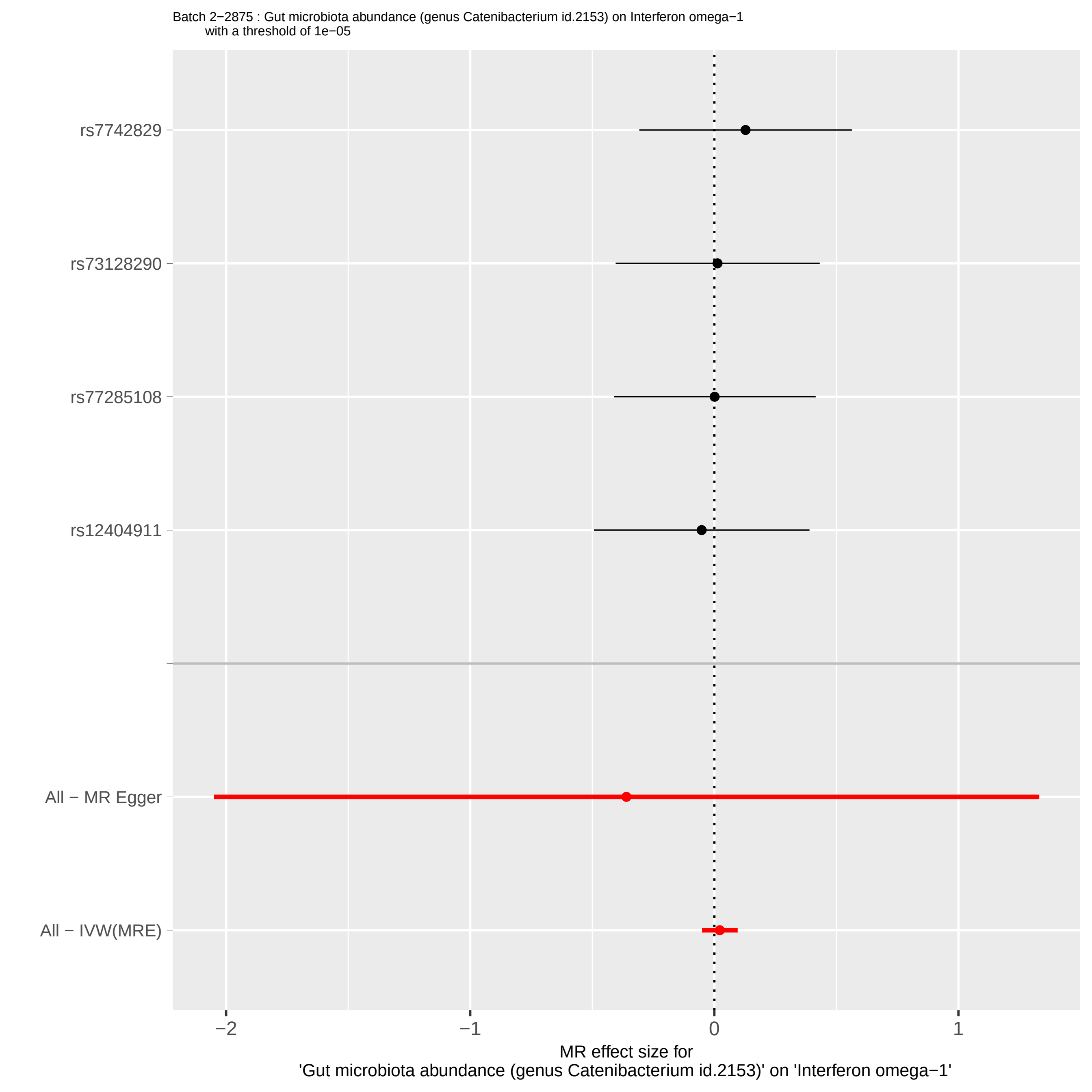
Batch 2−2875 : Gut microbiota abundance (genus Catenibacterium id.2153) on Interferon omega−1
with a threshold of 1e−05
rs7742829
rs73128290
rs77285108
rs12404911
All − MR Egger
All − IVW(MRE)
−2
−1
0
1
MR effect size for
'Gut microbiota abundance (genus Catenibacterium id.2153)' on 'Interferon omega−1'

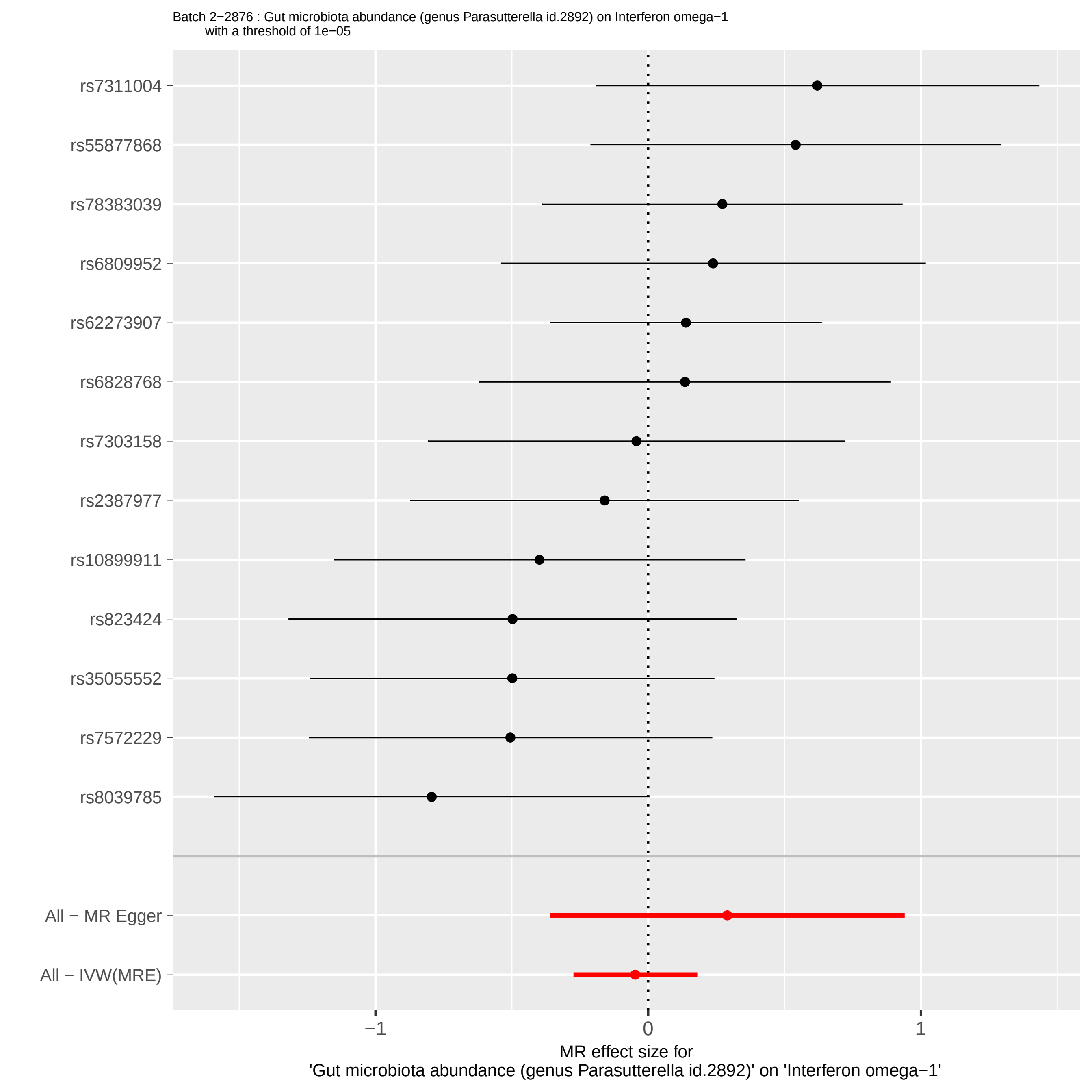

Batch 2−2876 : Gut microbiota abundance (genus Parasutterella id.2892) on Interferon omega−1
with a threshold of 1e−05
rs7311004
rs55877868
rs78383039
rs6809952
rs62273907
rs6828768
rs7303158
rs2387977
rs10899911
rs823424
rs35055552
rs7572229
rs8039785
All − MR Egger
All − IVW(MRE)
−1
0
1
MR effect size for
'Gut microbiota abundance (genus Parasutterella id.2892)' on 'Interferon omega−1'

Batch 2−2877 : Gut microbiota abundance (genus Catenibacterium id.2153) on Immunoglobulin−binding protein 1
with a threshold of 1e−05

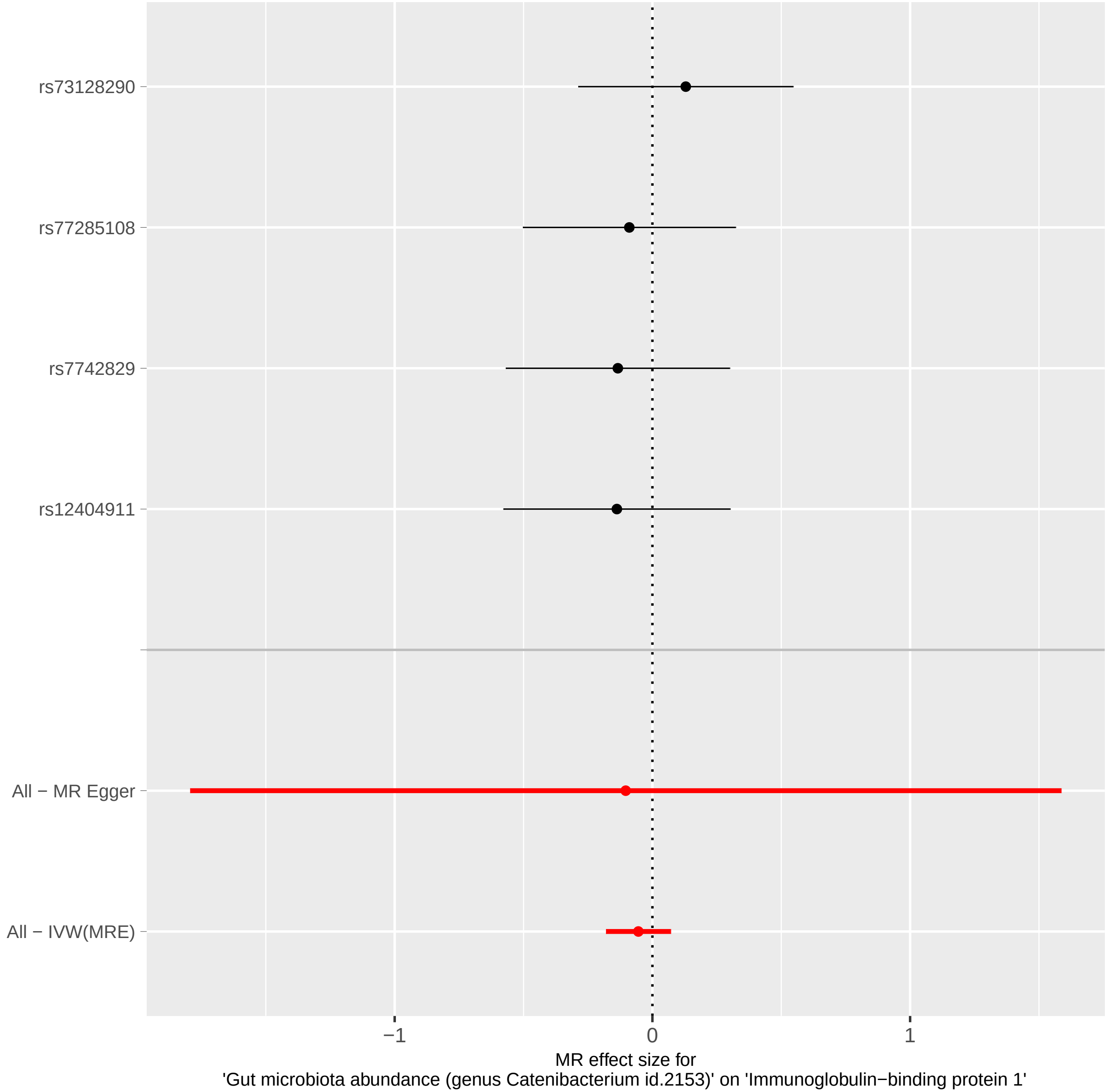

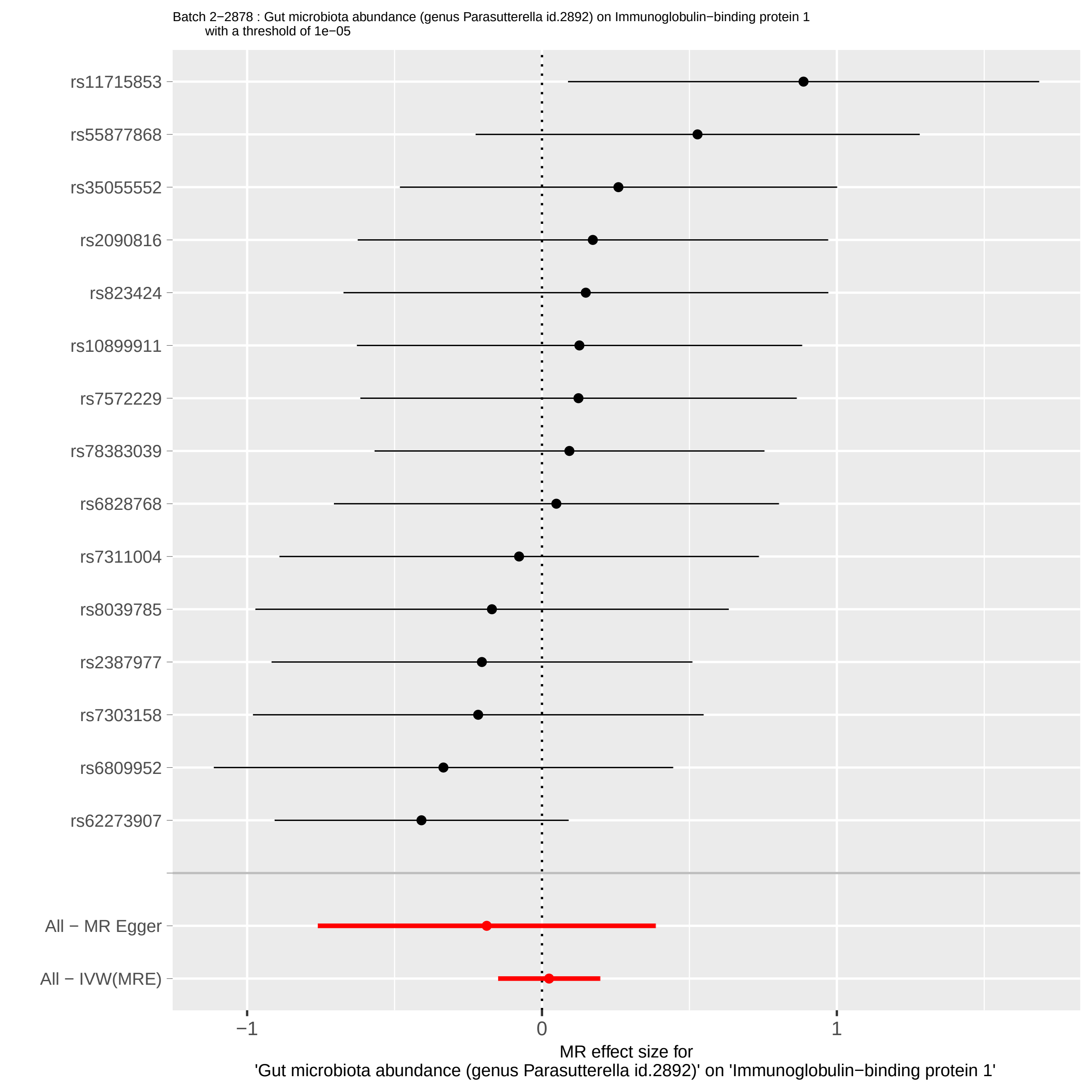

Batch 2−2878 : Gut microbiota abundance (genus Parasutterella id.2892) on Immunoglobulin−binding protein 1
with a threshold of 1e−05
rs11715853
rs55877868
rs35055552
rs2090816
rs823424
rs10899911
rs7572229
rs78383039
rs6828768
rs7311004
rs8039785
rs2387977
rs7303158
rs6809952
rs62273907
All − MR Egger
All − IVW(MRE)
−1
0
1
MR effect size for
'Gut microbiota abundance (genus Parasutterella id.2892)' on 'Immunoglobulin−binding protein 1'

Batch 2−2879 : Gut microbiota abundance (genus Catenibacterium id.2153) on Immunoglobulin superfamily DCC subclass member 3
with a threshold of 1e−05

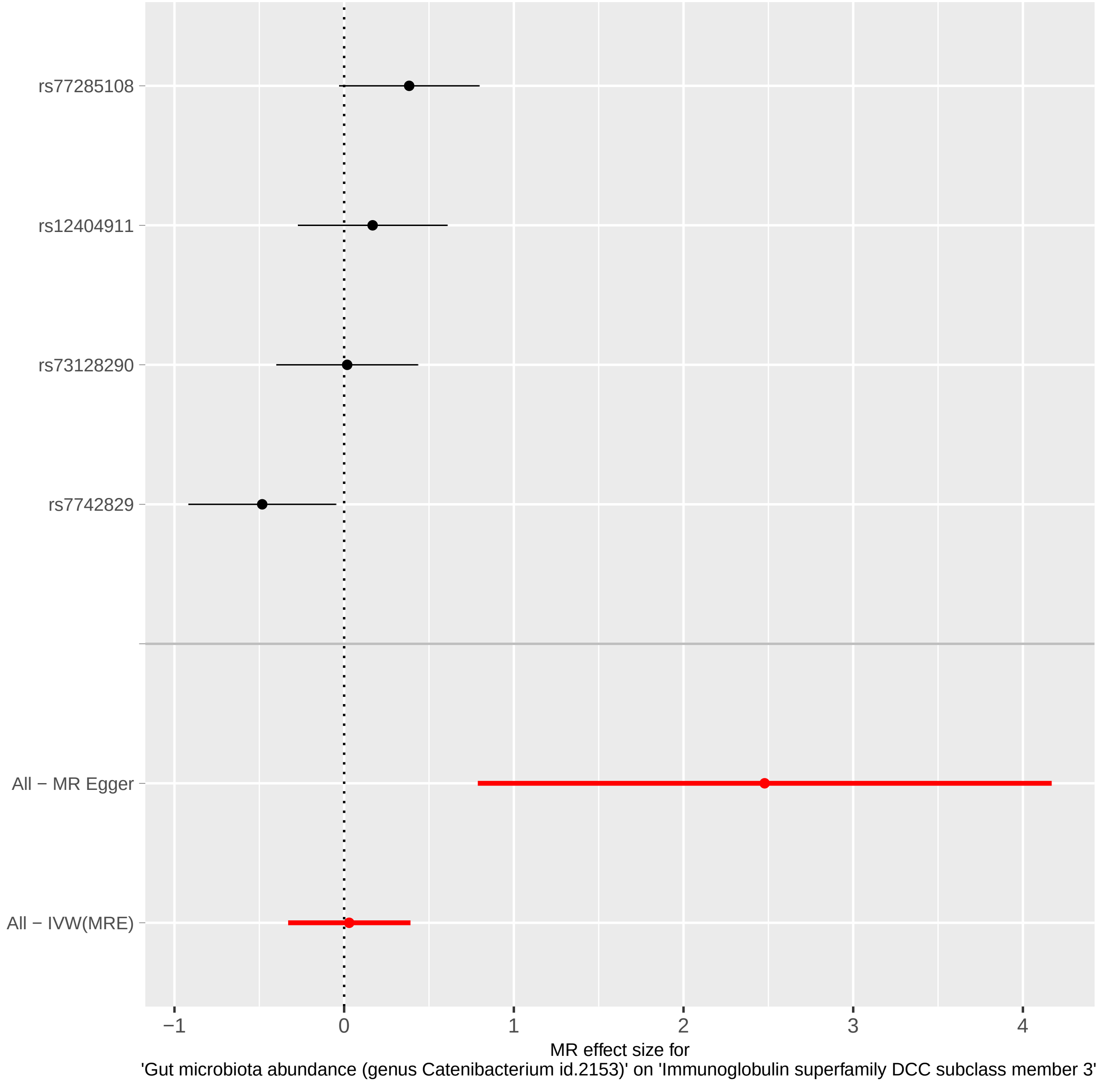

Batch 2−2880 : Gut microbiota abundance (genus Parasutterella id.2892) on Immunoglobulin superfamily DCC subclass member 3 with a threshold of 1e−05

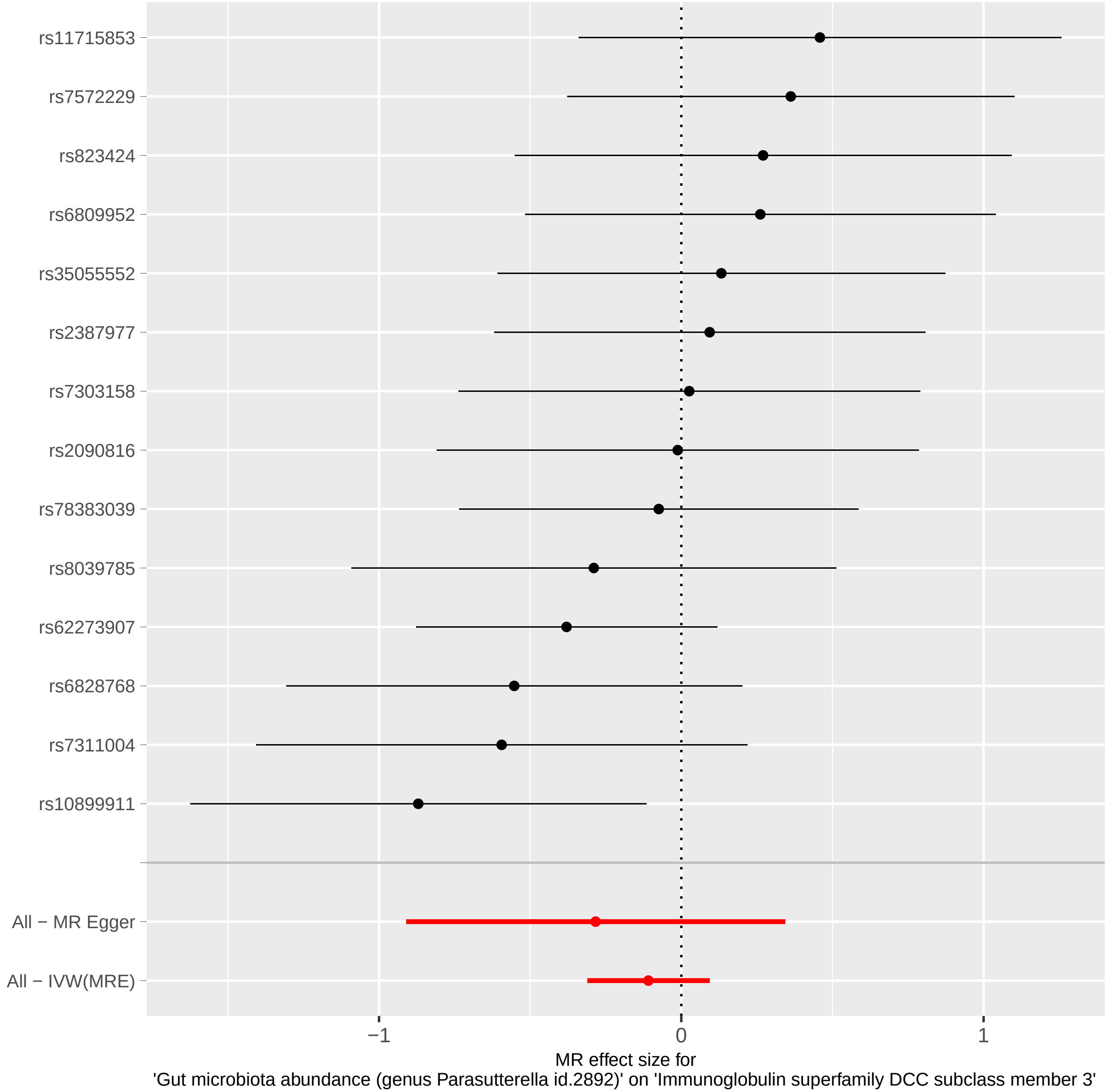

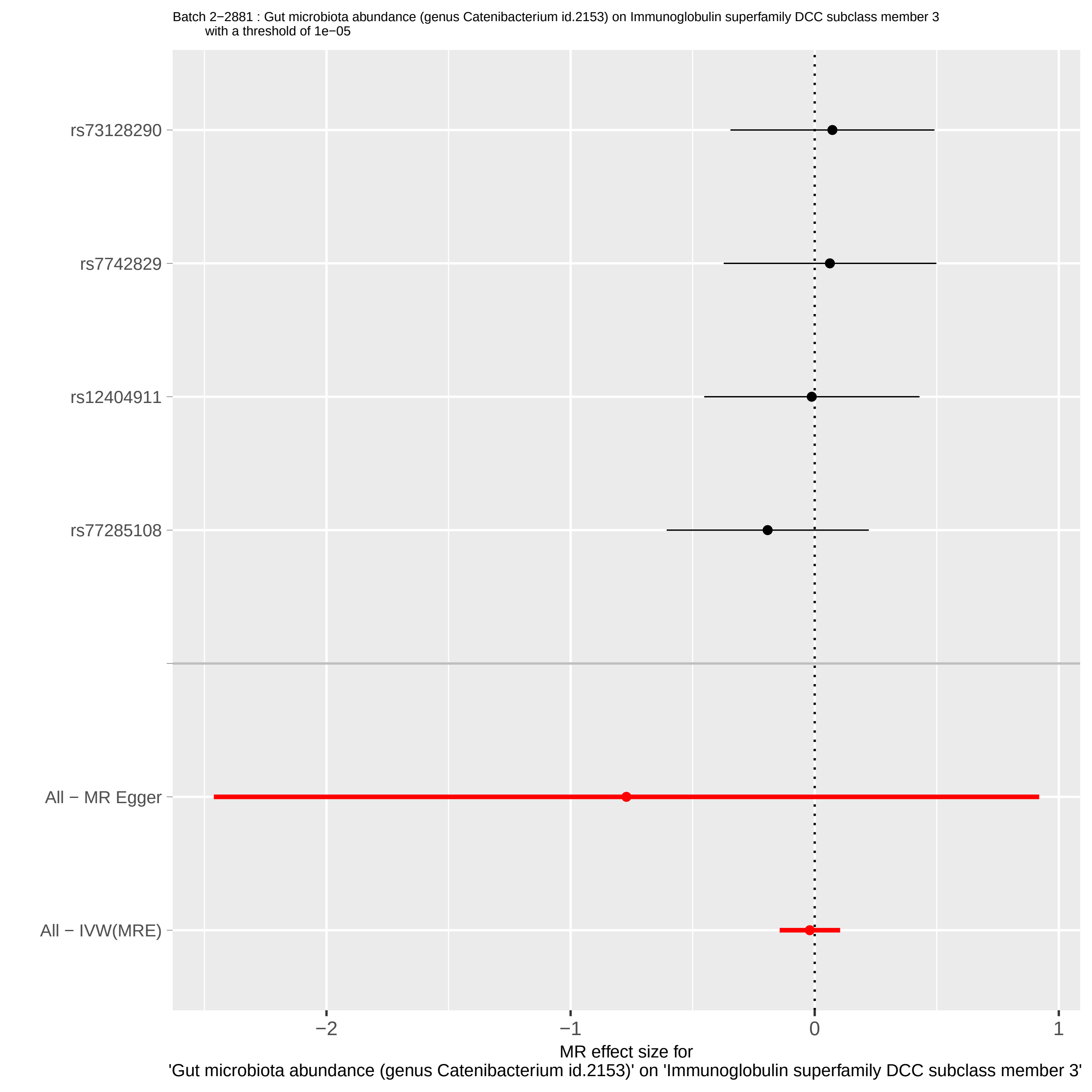

Batch 2−2881 : Gut microbiota abundance (genus Catenibacterium id.2153) on Immunoglobulin superfamily DCC subclass member 3
with a threshold of 1e−05
rs73128290
rs7742829
rs12404911
rs77285108
All − MR Egger
All − IVW(MRE)
−2
−1
0
1
MR effect size for
'Gut microbiota abundance (genus Catenibacterium id.2153)' on 'Immunoglobulin superfamily DCC subclass member 3'

Batch 2−2882 : Gut microbiota abundance (genus Parasutterella id.2892) on Immunoglobulin superfamily DCC subclass member 3 with a threshold of 1e−05

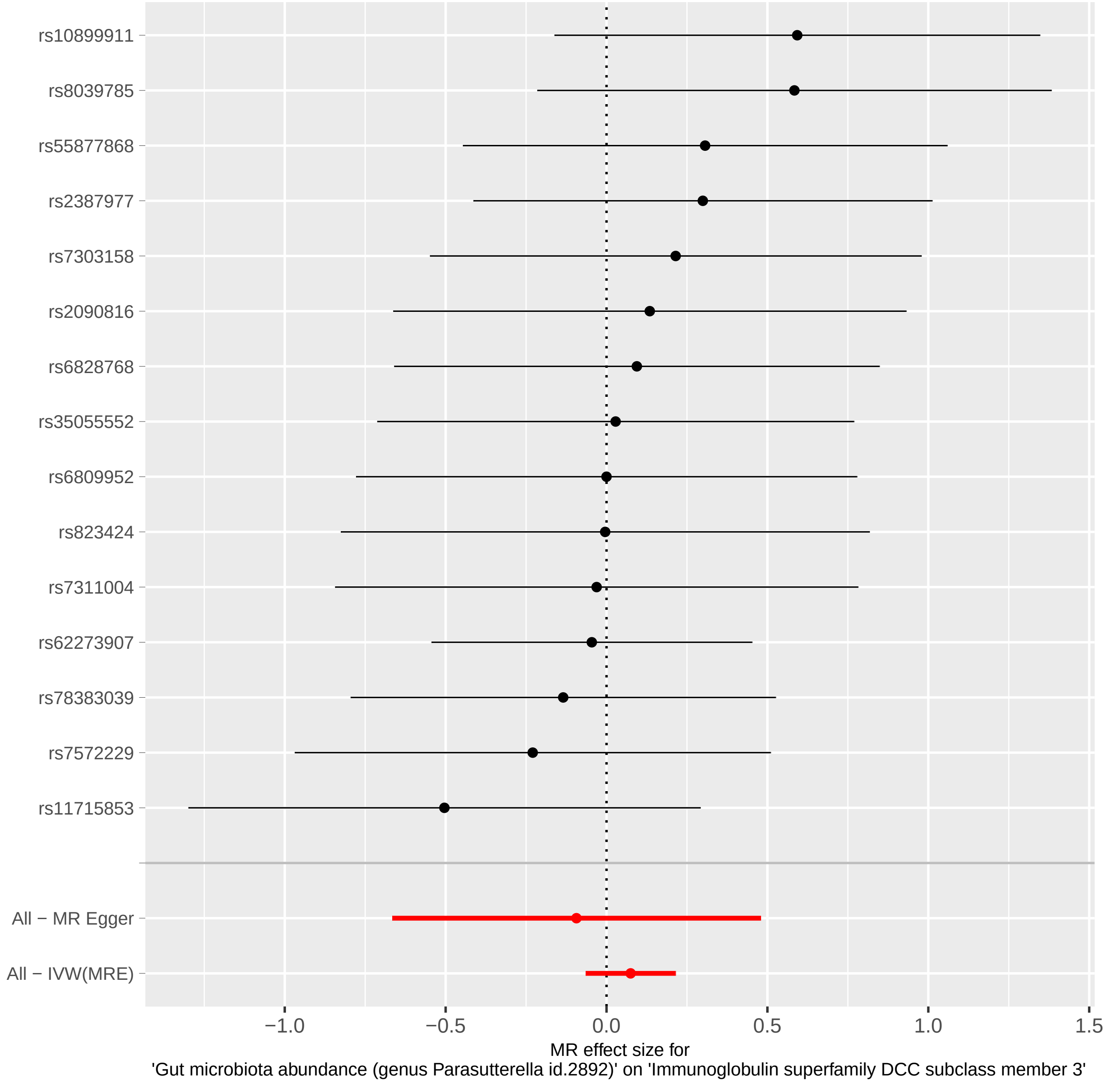

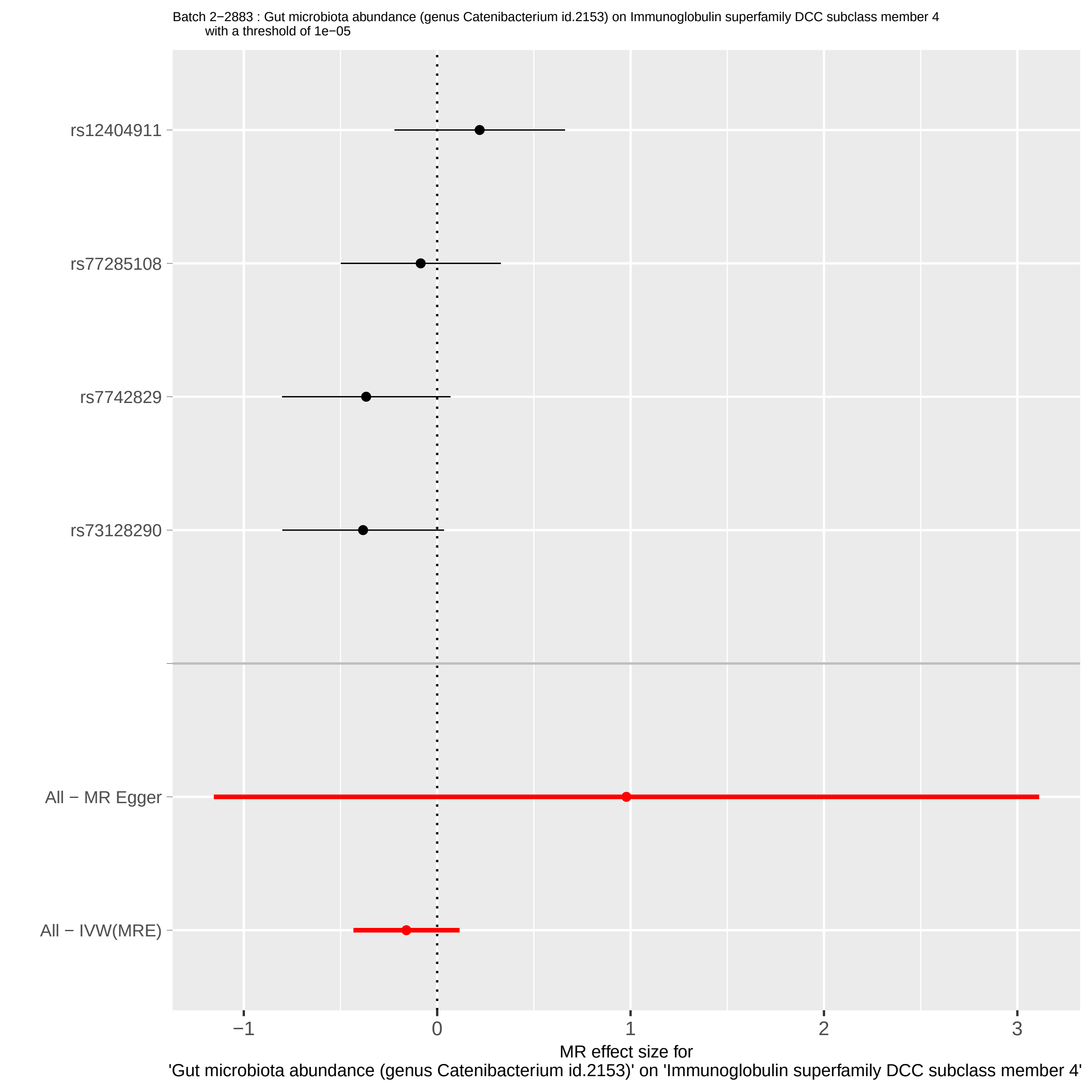

Batch 2−2883 : Gut microbiota abundance (genus Catenibacterium id.2153) on Immunoglobulin superfamily DCC subclass member 4
with a threshold of 1e−05
rs12404911
rs77285108
rs7742829
rs73128290
All − MR Egger
All − IVW(MRE)
−1
0
1
2
3
MR effect size for
'Gut microbiota abundance (genus Catenibacterium id.2153)' on 'Immunoglobulin superfamily DCC subclass member 4'

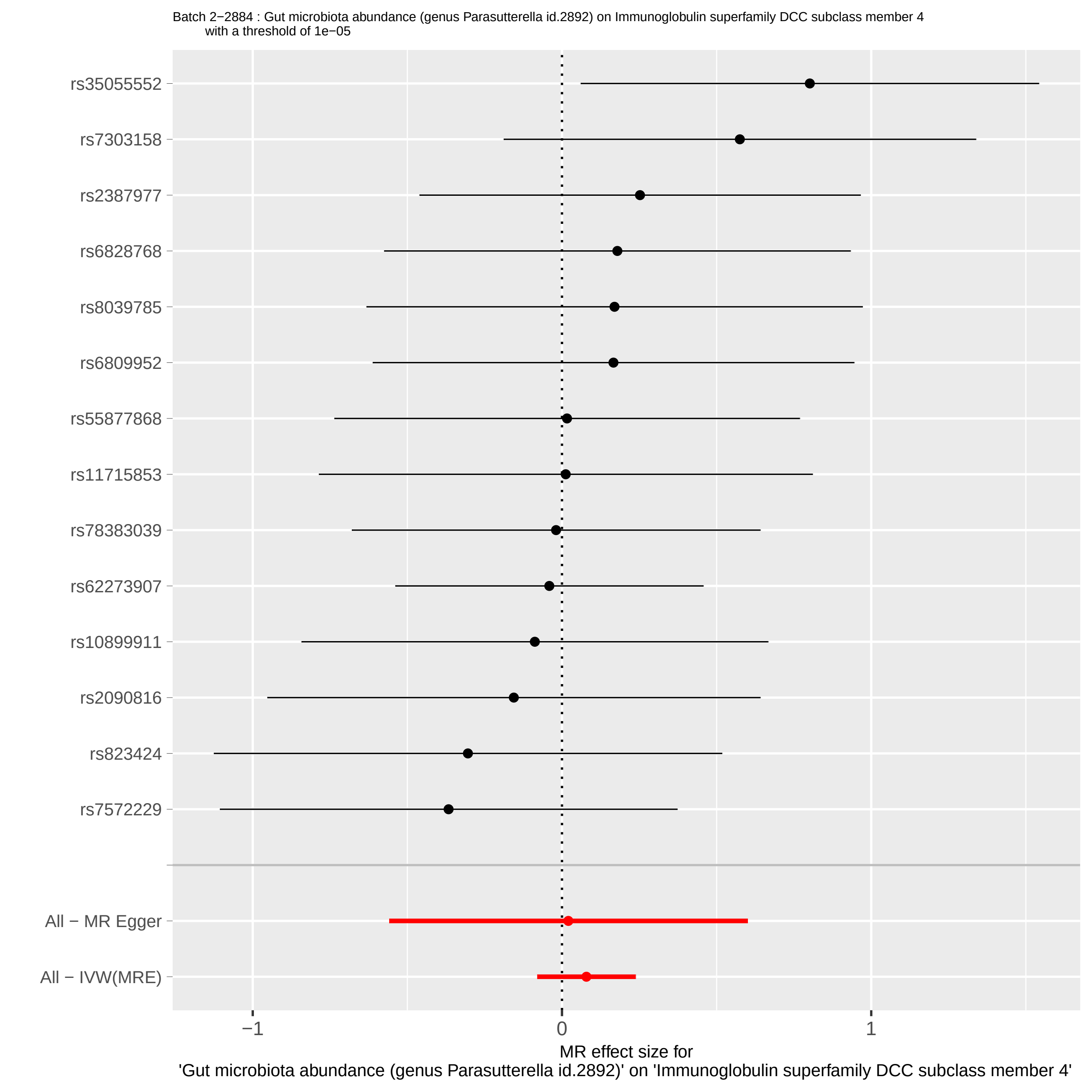
Batch 2−2884 : Gut microbiota abundance (genus Parasutterella id.2892) on Immunoglobulin superfamily DCC subclass member 4
with a threshold of 1e−05
rs35055552
rs7303158
rs2387977
rs6828768
rs8039785
rs6809952
rs55877868
rs11715853
rs78383039
rs62273907
rs10899911
rs2090816
rs823424
rs7572229
All − MR Egger
All − IVW(MRE)
−1
0
1
MR effect size for
'Gut microbiota abundance (genus Parasutterella id.2892)' on 'Immunoglobulin superfamily DCC subclass member 4'

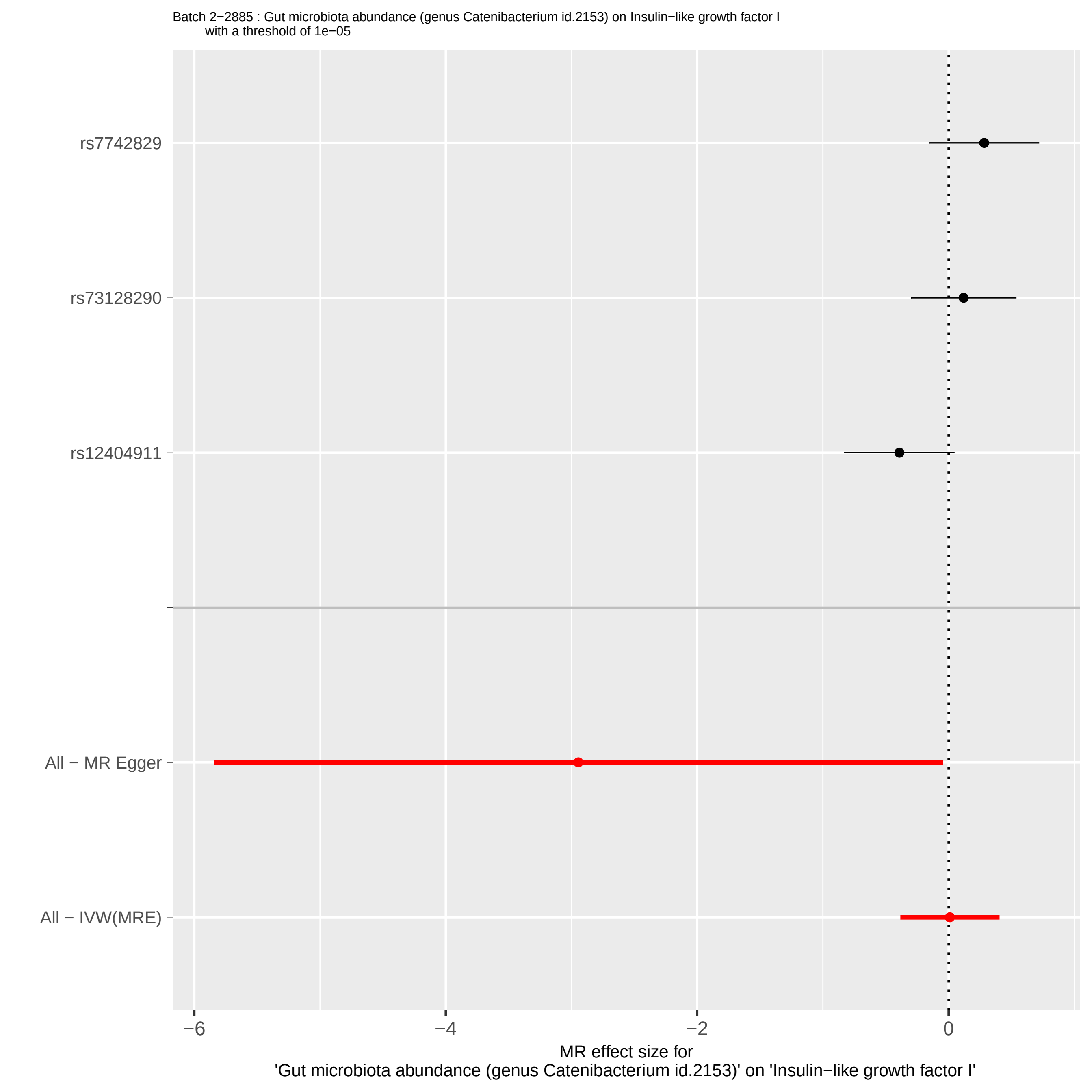

Batch 2−2885 : Gut microbiota abundance (genus Catenibacterium id.2153) on Insulin−like growth factor I
with a threshold of 1e−05
rs7742829
rs73128290
rs12404911
All − MR Egger
All − IVW(MRE)
−6
−4
−2
0
MR effect size for
'Gut microbiota abundance (genus Catenibacterium id.2153)' on 'Insulin−like growth factor I'

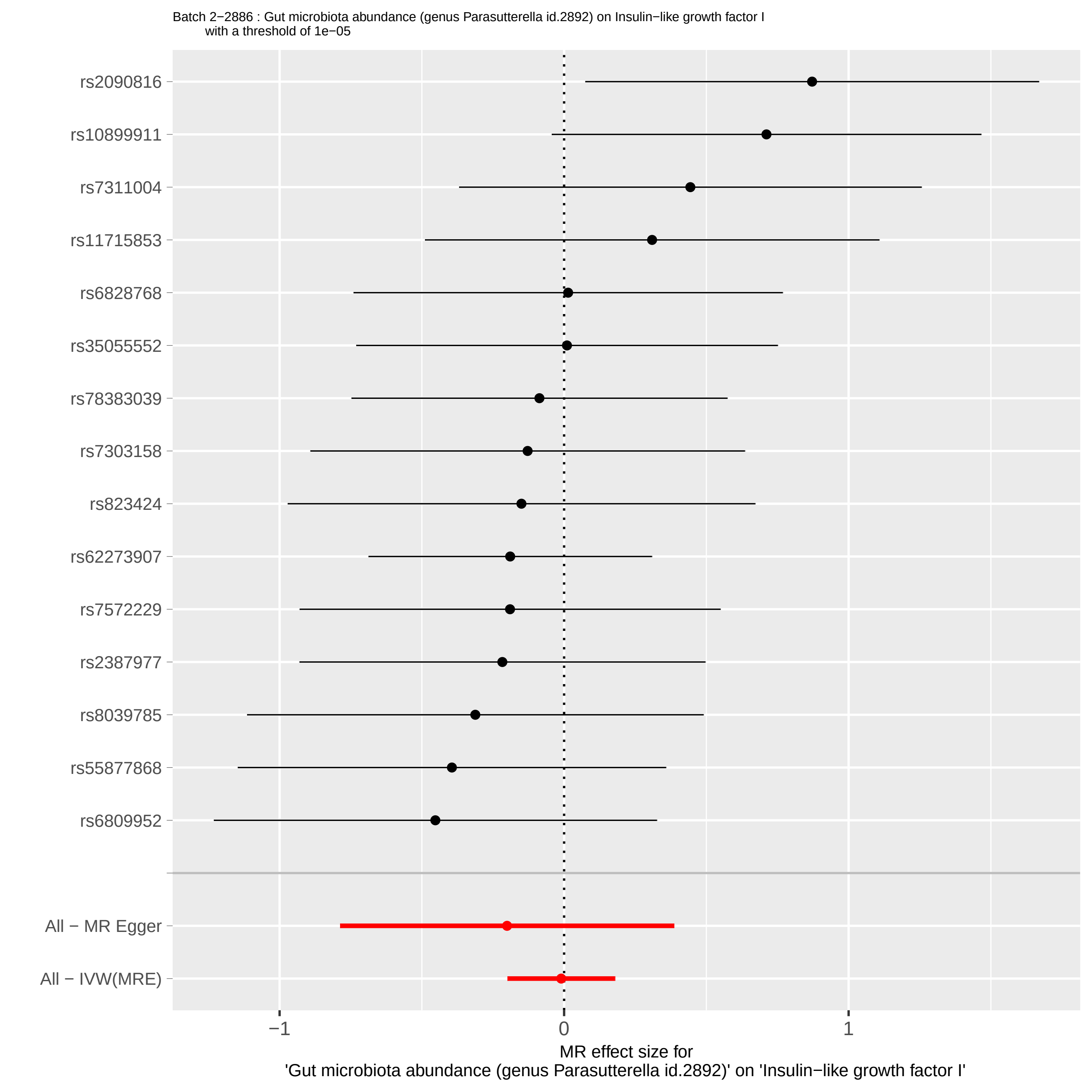

Batch 2−2886 : Gut microbiota abundance (genus Parasutterella id.2892) on Insulin−like growth factor I
with a threshold of 1e−05
rs2090816
rs10899911
rs7311004
rs11715853
rs6828768
rs35055552
rs78383039
rs7303158
rs823424
rs62273907
rs7572229
rs2387977
rs8039785
rs55877868
rs6809952
All − MR Egger
All − IVW(MRE)
−1
0
1
MR effect size for
'Gut microbiota abundance (genus Parasutterella id.2892)' on 'Insulin−like growth factor I'

Batch 2−2887 : Gut microbiota abundance (genus Catenibacterium id.2153) on Insulin−like growth factor 1 receptor
with a threshold of 1e−05

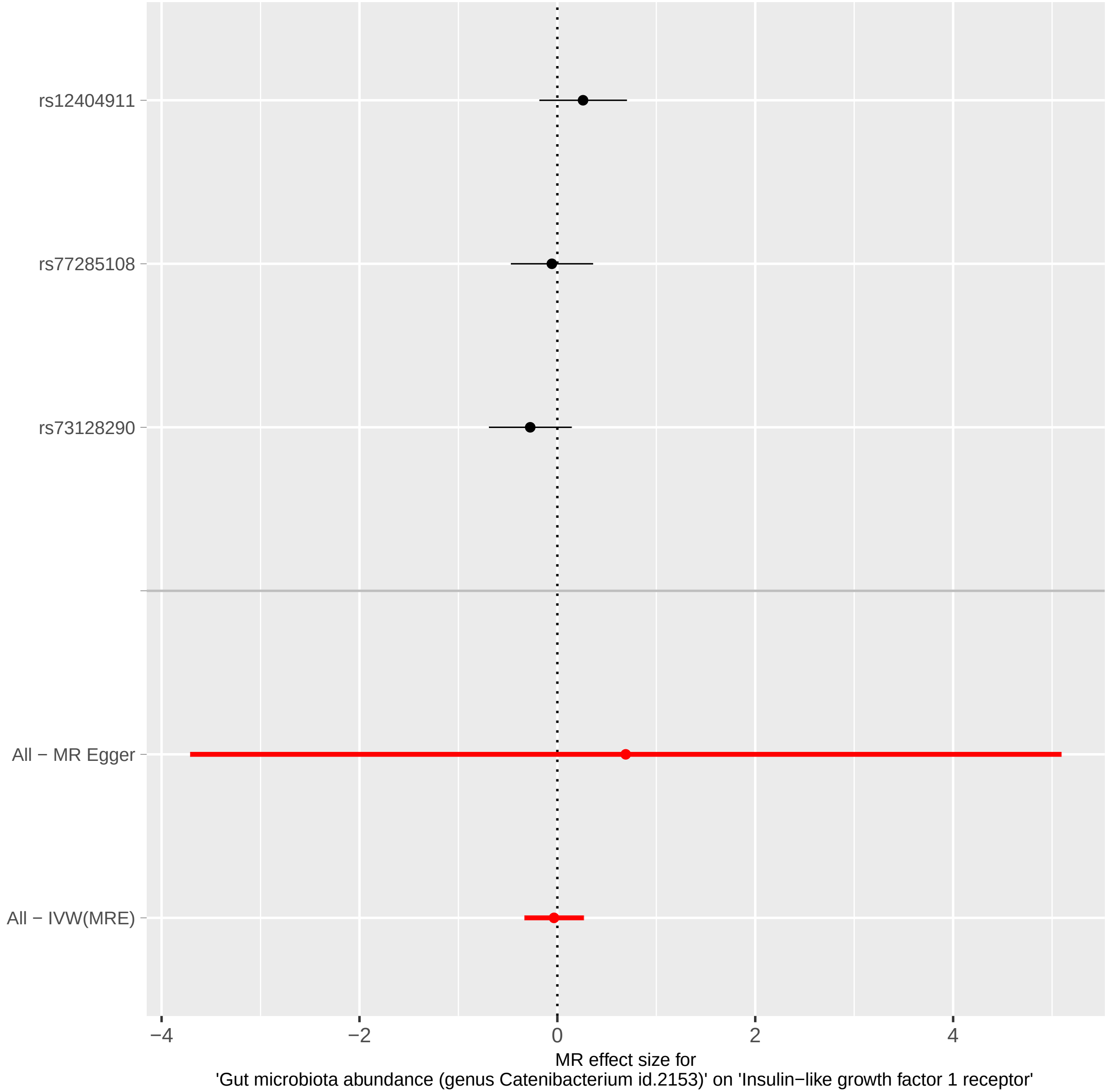

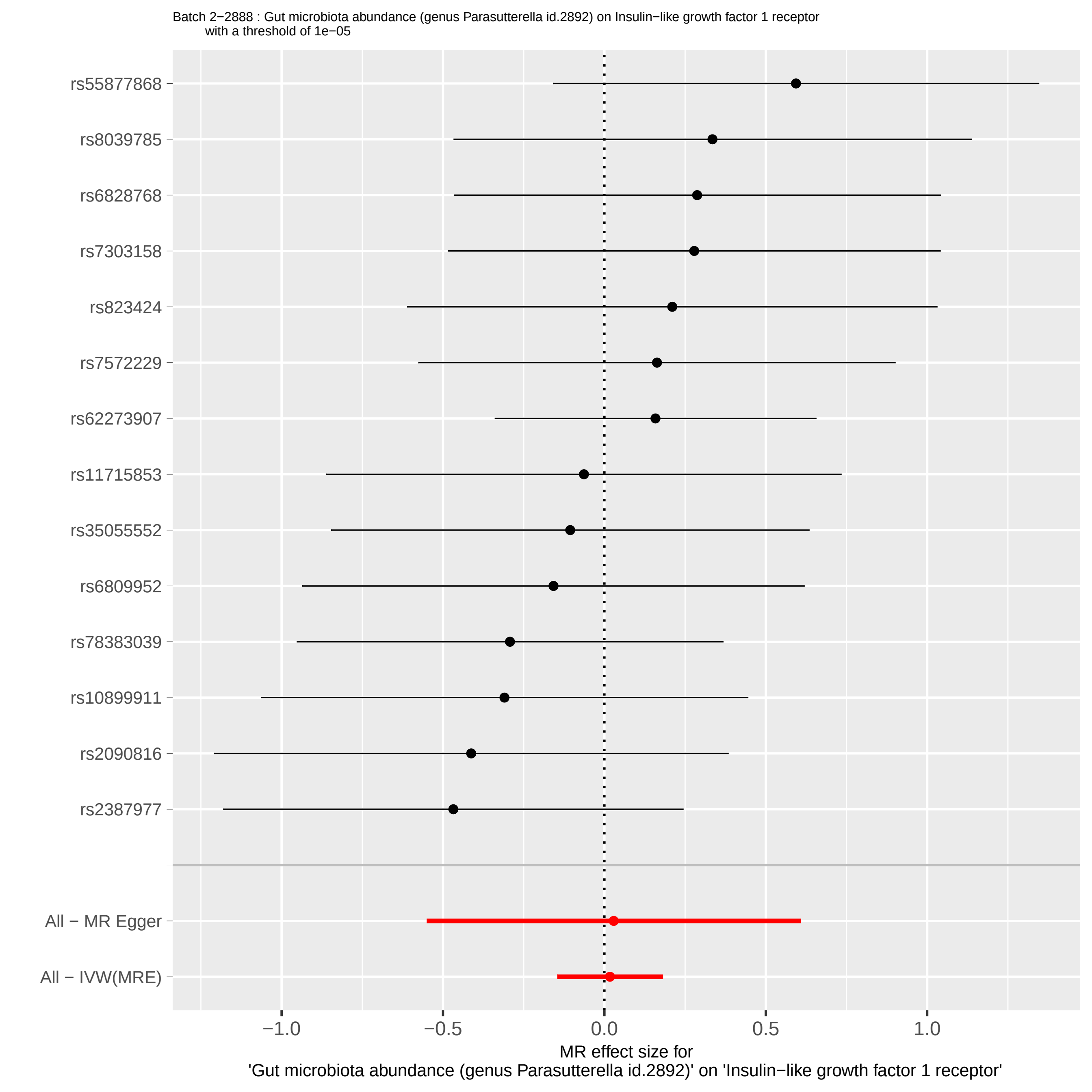

Batch 2−2888 : Gut microbiota abundance (genus Parasutterella id.2892) on Insulin−like growth factor 1 receptor
with a threshold of 1e−05
rs55877868
rs8039785
rs6828768
rs7303158
rs823424
rs7572229
rs62273907
rs11715853
rs35055552
rs6809952
rs78383039
rs10899911
rs2090816
rs2387977
All − MR Egger
All − IVW(MRE)
−1.0
−0.5
0.0
0.5
1.0
MR effect size for
'Gut microbiota abundance (genus Parasutterella id.2892)' on 'Insulin−like growth factor 1 receptor'

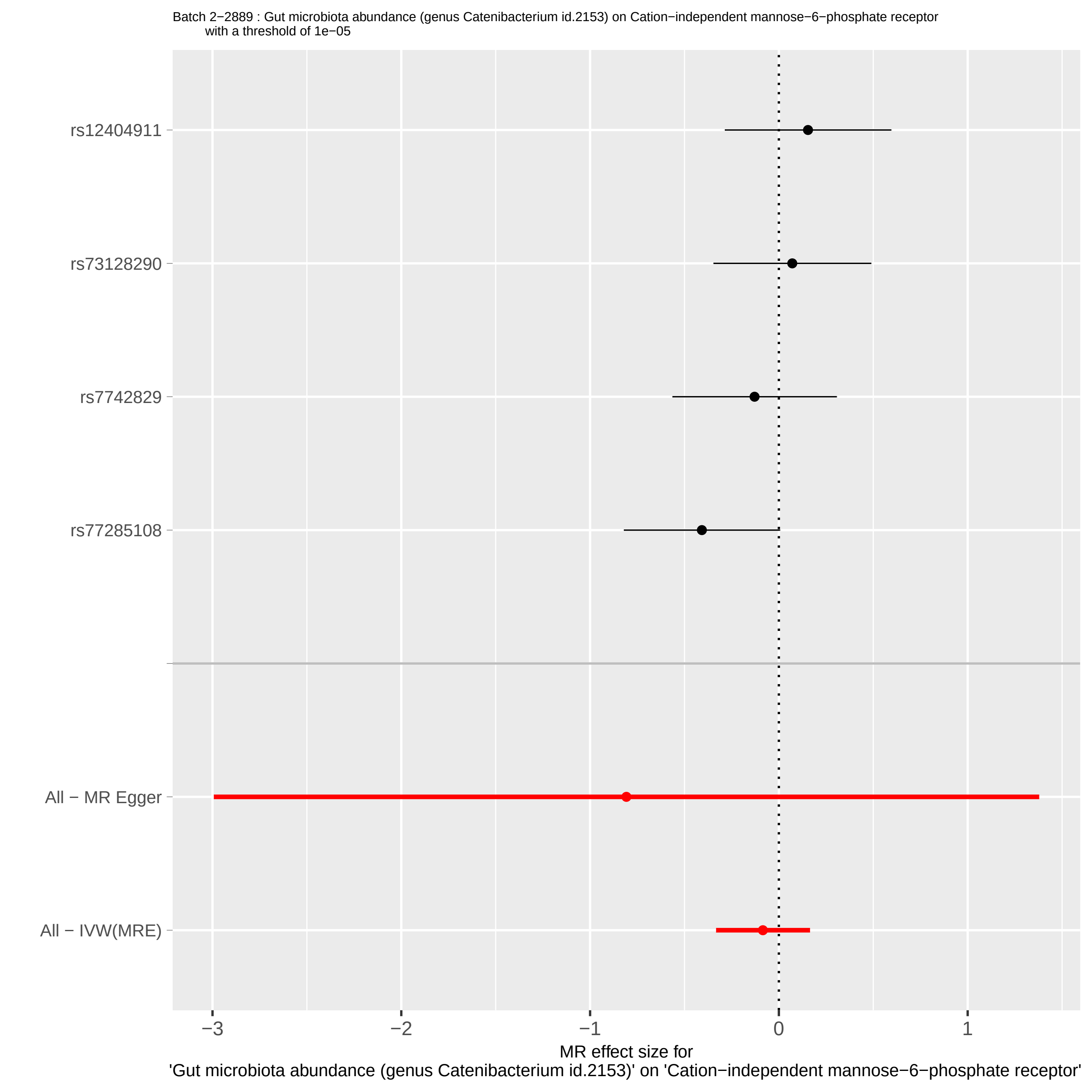
Batch 2−2889 : Gut microbiota abundance (genus Catenibacterium id.2153) on Cation−independent mannose−6−phosphate receptor
with a threshold of 1e−05
rs12404911
rs73128290
rs7742829
rs77285108
All − MR Egger
All − IVW(MRE)
−3
−2
−1
0
1
MR effect size for
'Gut microbiota abundance (genus Catenibacterium id.2153)' on 'Cation−independent mannose−6−phosphate receptor'

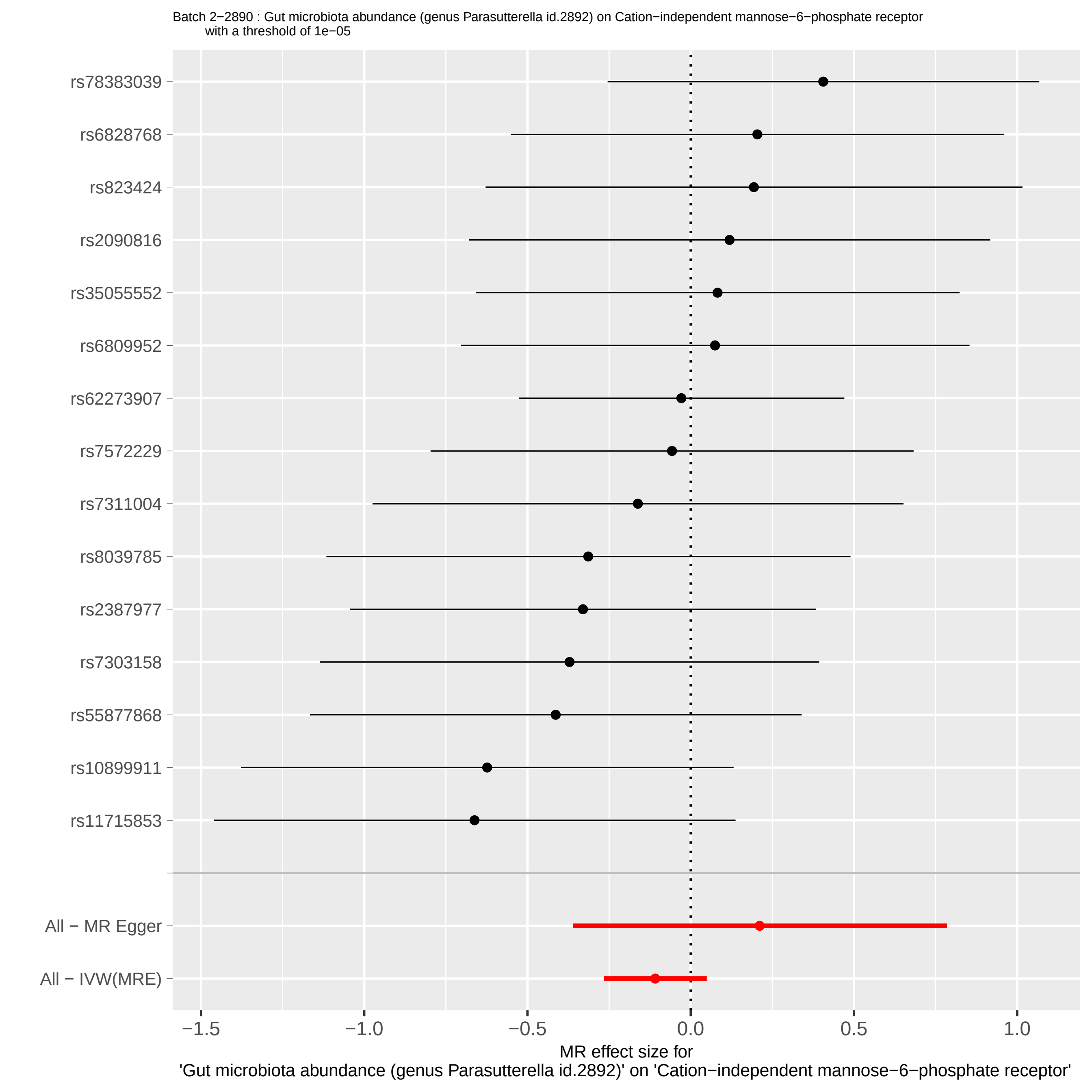

Batch 2−2890 : Gut microbiota abundance (genus Parasutterella id.2892) on Cation−independent mannose−6−phosphate receptor
with a threshold of 1e−05
rs78383039
rs6828768
rs823424
rs2090816
rs35055552
rs6809952
rs62273907
rs7572229
rs7311004
rs8039785
rs2387977
rs7303158
rs55877868
rs10899911
rs11715853
All − MR Egger
All − IVW(MRE)
−1.5
−1.0
−0.5
0.0
0.5
1.0
MR effect size for
'Gut microbiota abundance (genus Parasutterella id.2892)' on 'Cation−independent mannose−6−phosphate receptor'

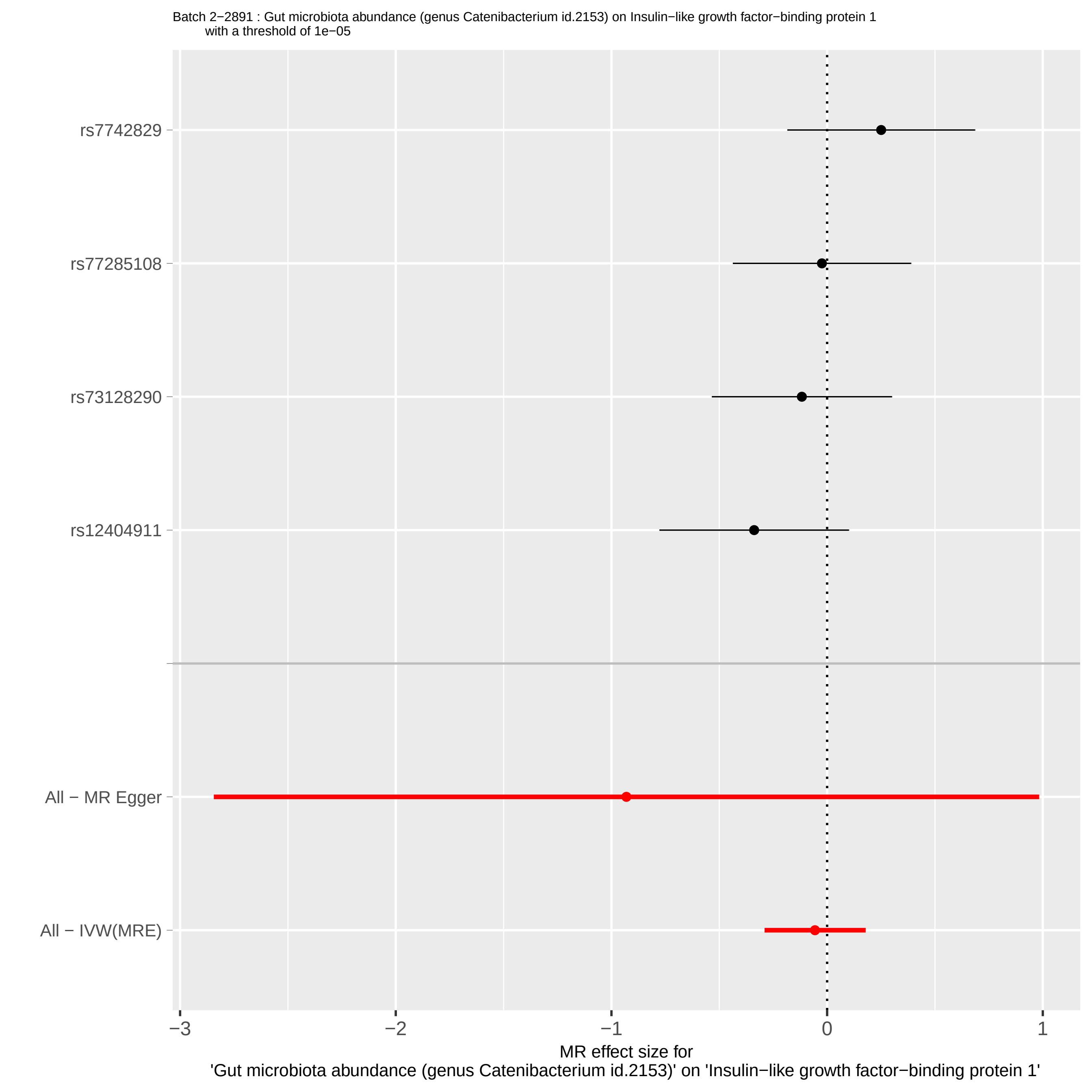
Batch 2−2891 : Gut microbiota abundance (genus Catenibacterium id.2153) on Insulin−like growth factor−binding protein 1
with a threshold of 1e−05
rs7742829
rs77285108
rs73128290
rs12404911
All − MR Egger
All − IVW(MRE)
−3
−2
−1
0
1
MR effect size for
'Gut microbiota abundance (genus Catenibacterium id.2153)' on 'Insulin−like growth factor−binding protein 1'

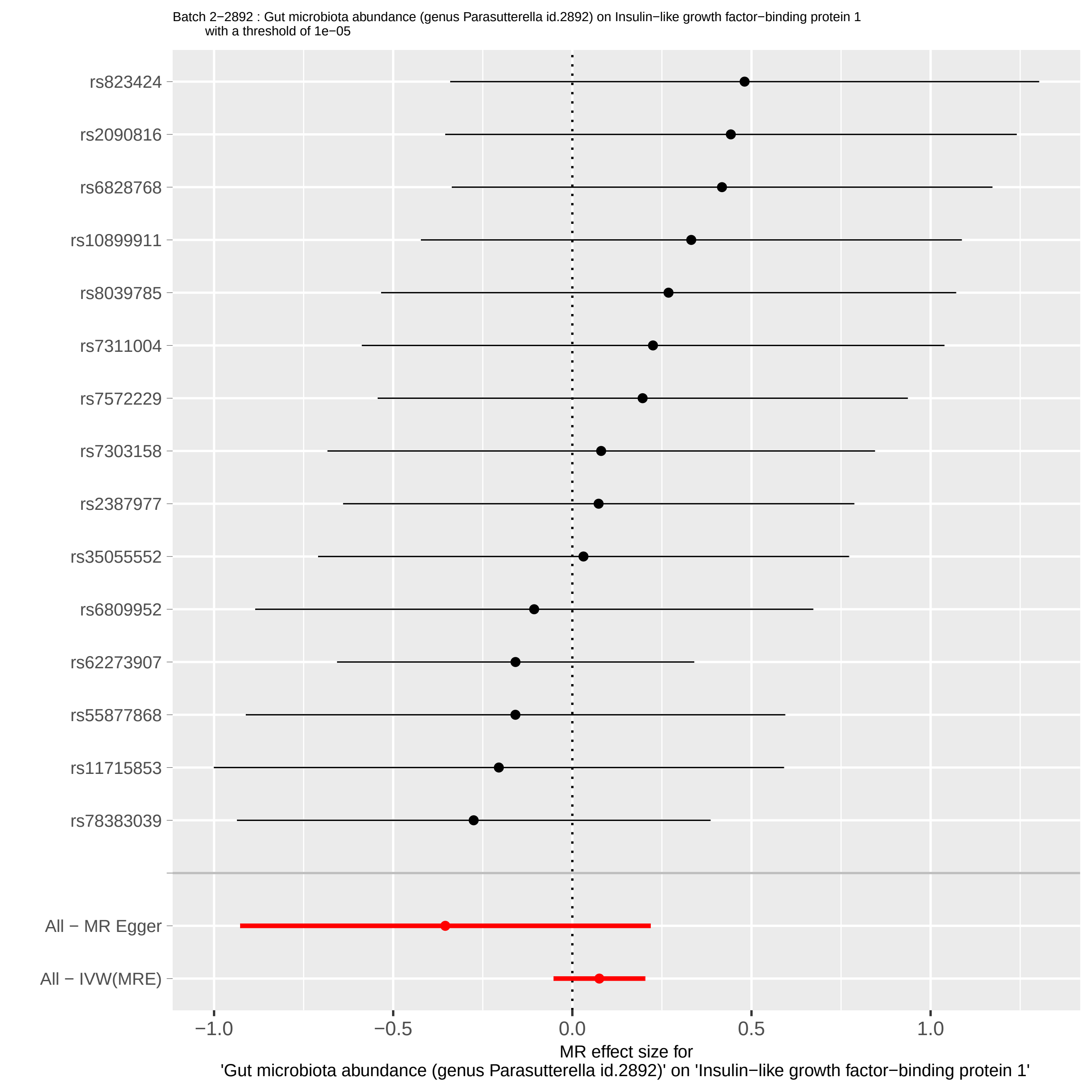

Batch 2−2892 : Gut microbiota abundance (genus Parasutterella id.2892) on Insulin−like growth factor−binding protein 1
with a threshold of 1e−05
rs823424
rs2090816
rs6828768
rs10899911
rs8039785
rs7311004
rs7572229
rs7303158
rs2387977
rs35055552
rs6809952
rs62273907
rs55877868
rs11715853
rs78383039
All − MR Egger
All − IVW(MRE)
−1.0
−0.5
0.0
0.5
1.0
MR effect size for
'Gut microbiota abundance (genus Parasutterella id.2892)' on 'Insulin−like growth factor−binding protein 1'

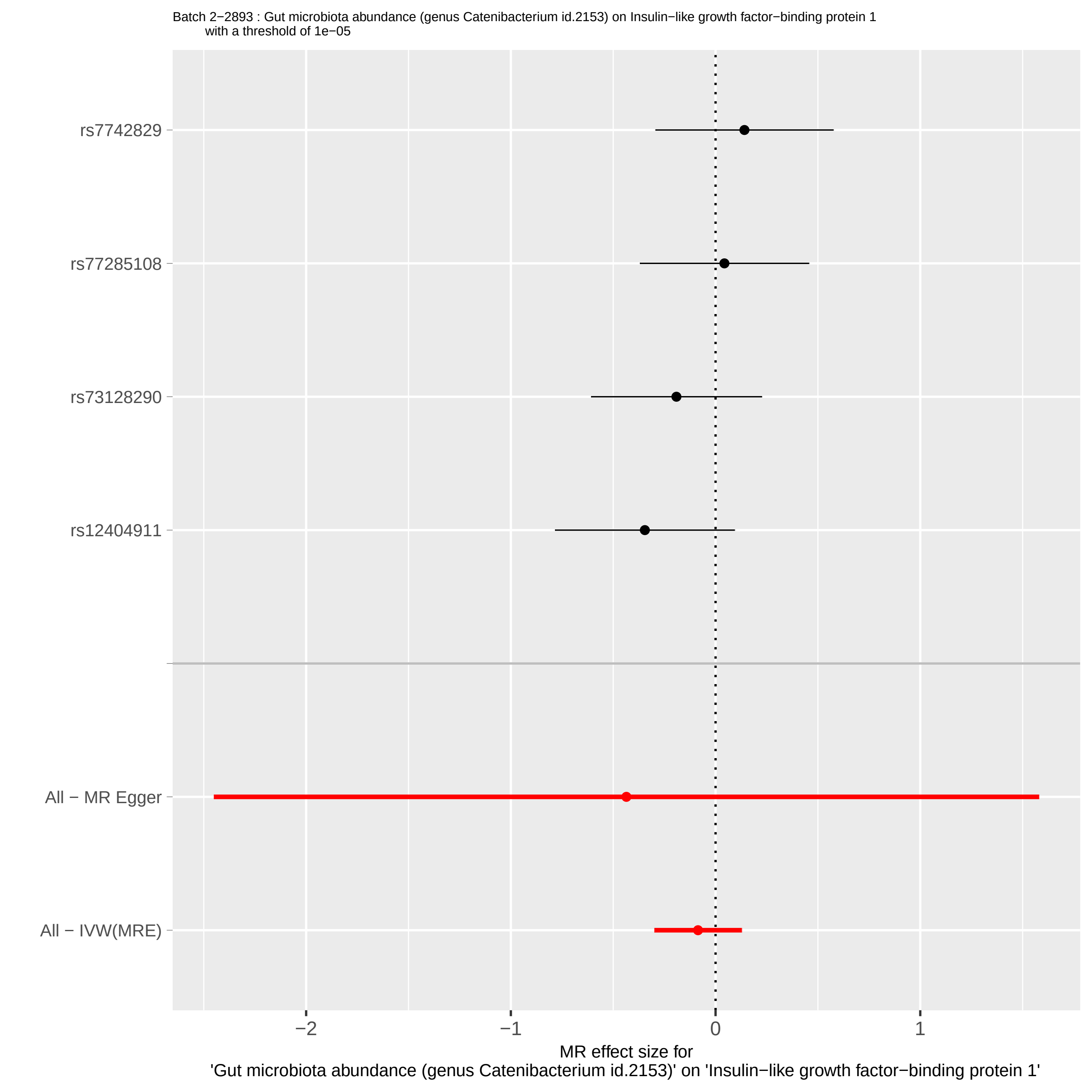
Batch 2−2893 : Gut microbiota abundance (genus Catenibacterium id.2153) on Insulin−like growth factor−binding protein 1
with a threshold of 1e−05
rs7742829
rs77285108
rs73128290
rs12404911
All − MR Egger
All − IVW(MRE)
−2
−1
0
1
MR effect size for
'Gut microbiota abundance (genus Catenibacterium id.2153)' on 'Insulin−like growth factor−binding protein 1'

Batch 2−2894 : Gut microbiota abundance (genus Parasutterella id.2892) on Insulin−like growth factor−binding protein 1
with a threshold of 1e−05

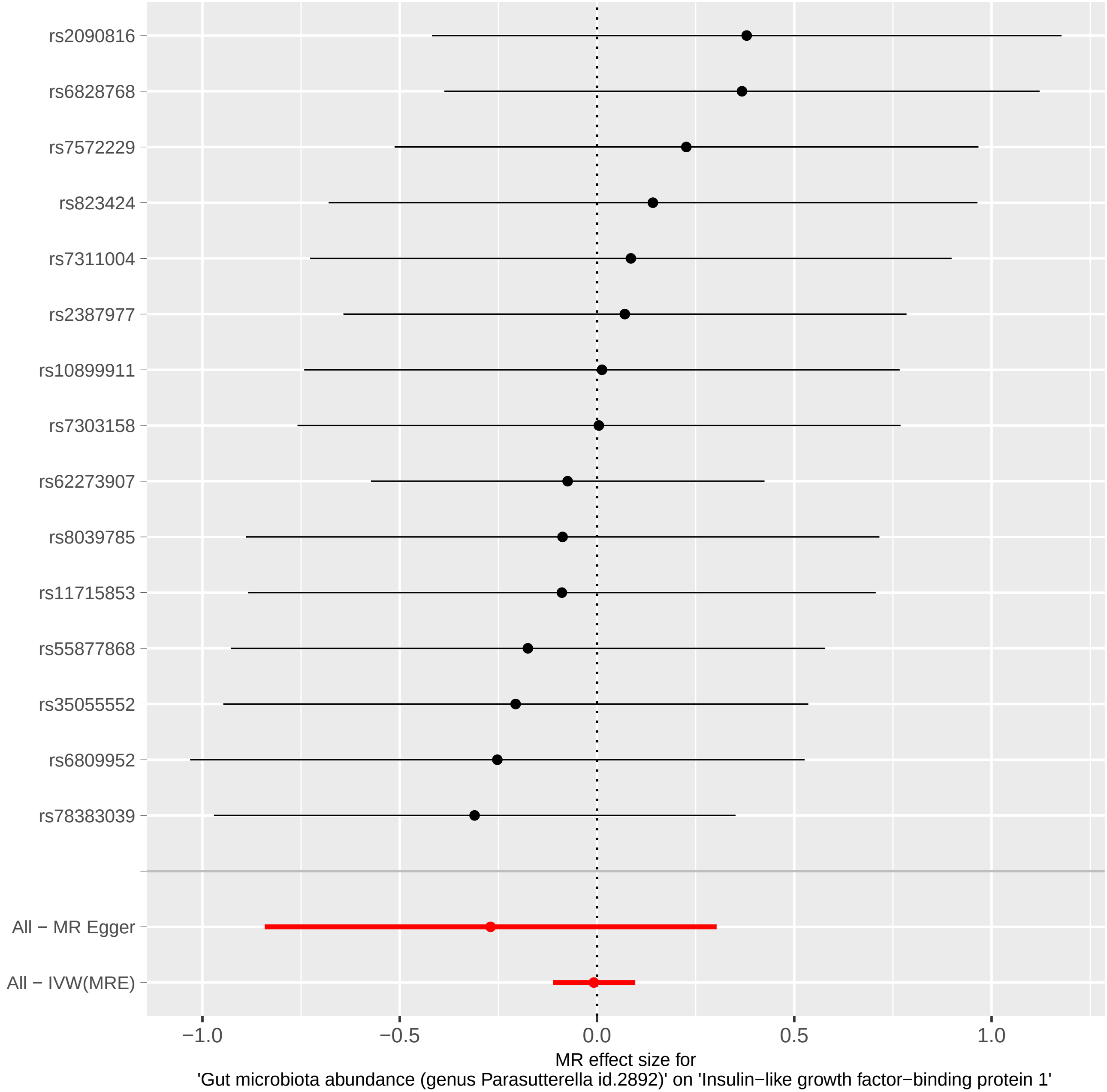

Batch 2−2895 : Gut microbiota abundance (genus Catenibacterium id.2153) on Insulin−like growth factor−binding protein 2
with a threshold of 1e−05

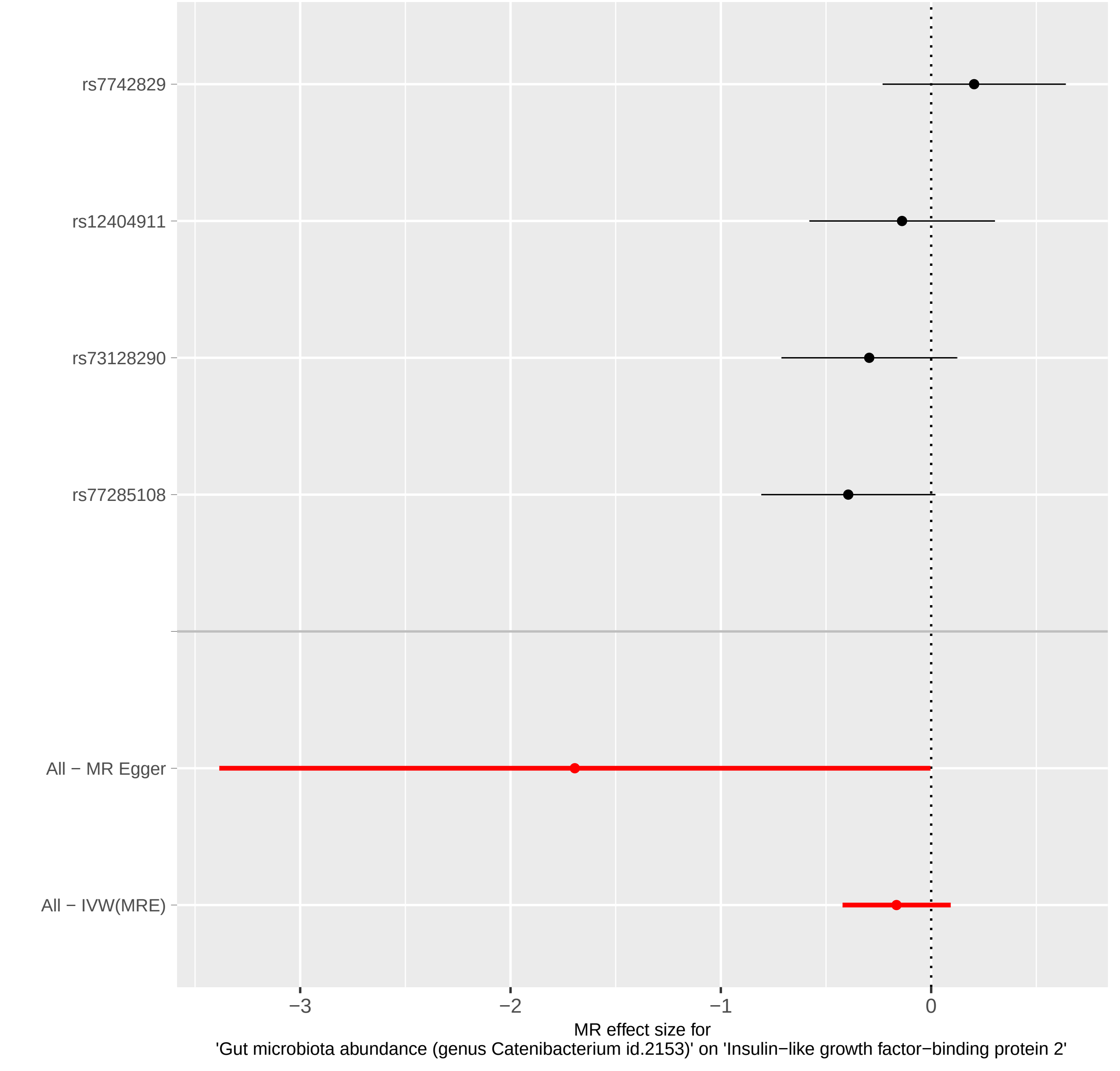

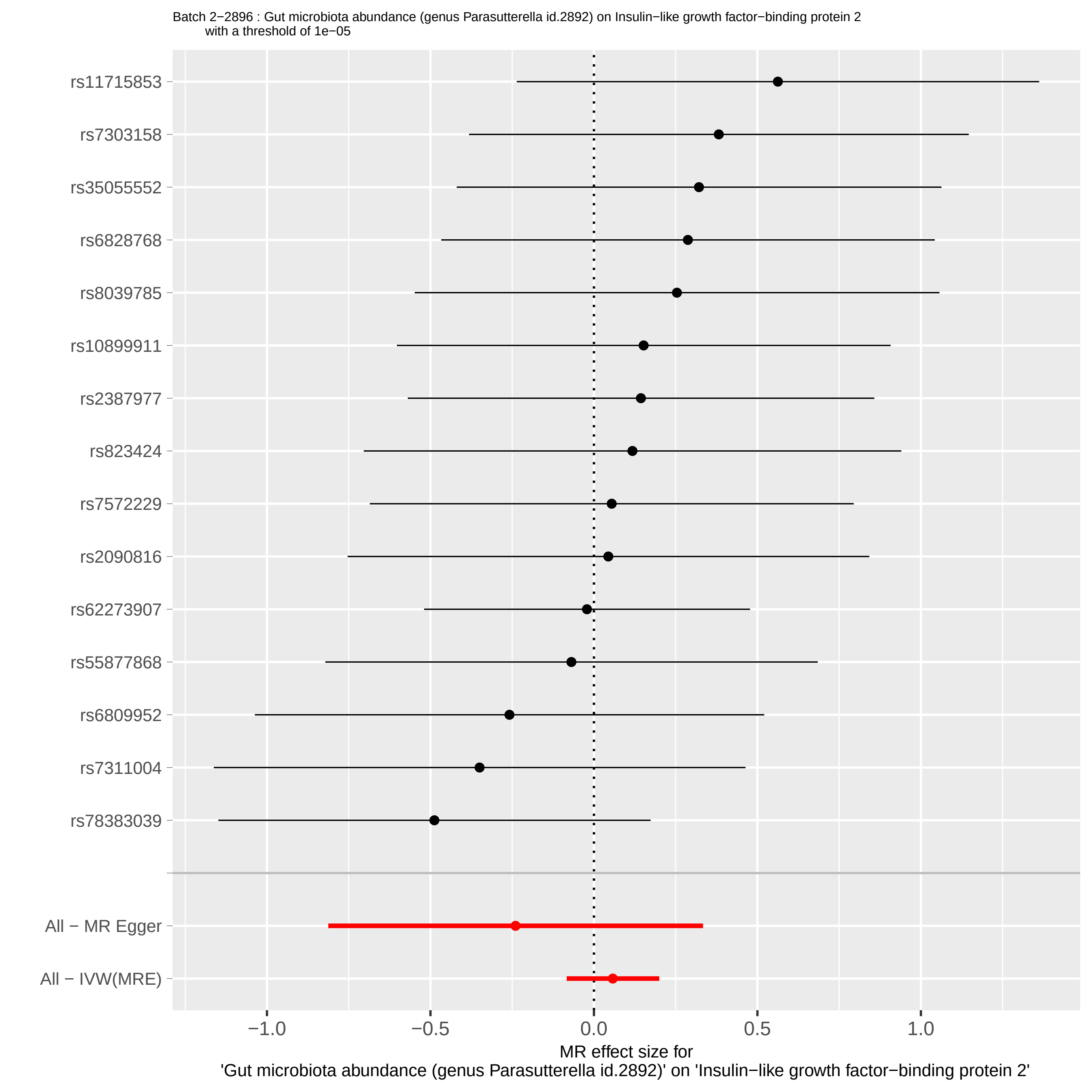

Batch 2−2896 : Gut microbiota abundance (genus Parasutterella id.2892) on Insulin−like growth factor−binding protein 2
with a threshold of 1e−05
rs11715853
rs7303158
rs35055552
rs6828768
rs8039785
rs10899911
rs2387977
rs823424
rs7572229
rs2090816
rs62273907
rs55877868
rs6809952
rs7311004
rs78383039
All − MR Egger
All − IVW(MRE)
−1.0
−0.5
0.0
0.5
1.0
MR effect size for
'Gut microbiota abundance (genus Parasutterella id.2892)' on 'Insulin−like growth factor−binding protein 2'

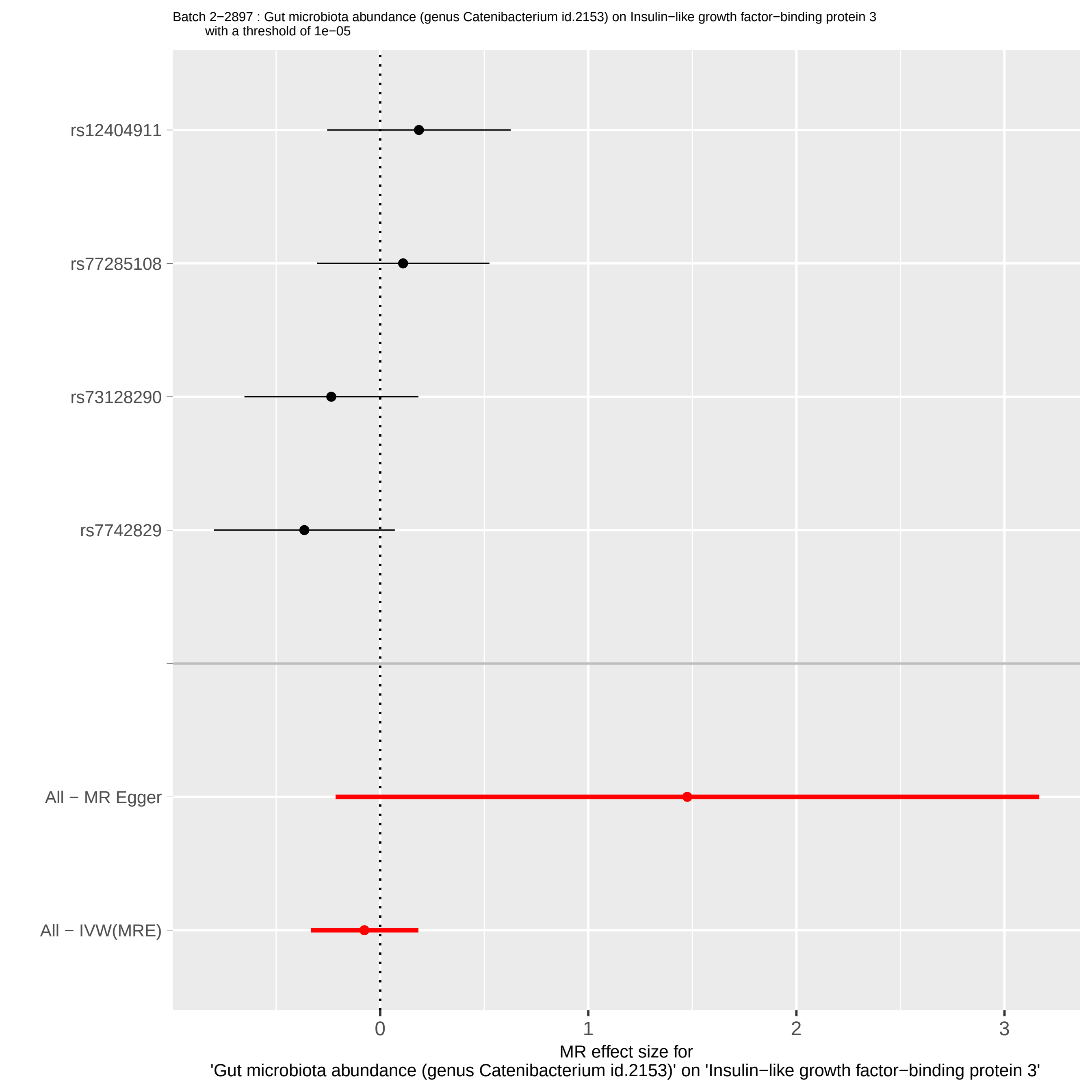
Batch 2−2897 : Gut microbiota abundance (genus Catenibacterium id.2153) on Insulin−like growth factor−binding protein 3
with a threshold of 1e−05
rs12404911
rs77285108
rs73128290
rs7742829
All − MR Egger
All − IVW(MRE)
0
1
2
3
MR effect size for
'Gut microbiota abundance (genus Catenibacterium id.2153)' on 'Insulin−like growth factor−binding protein 3'

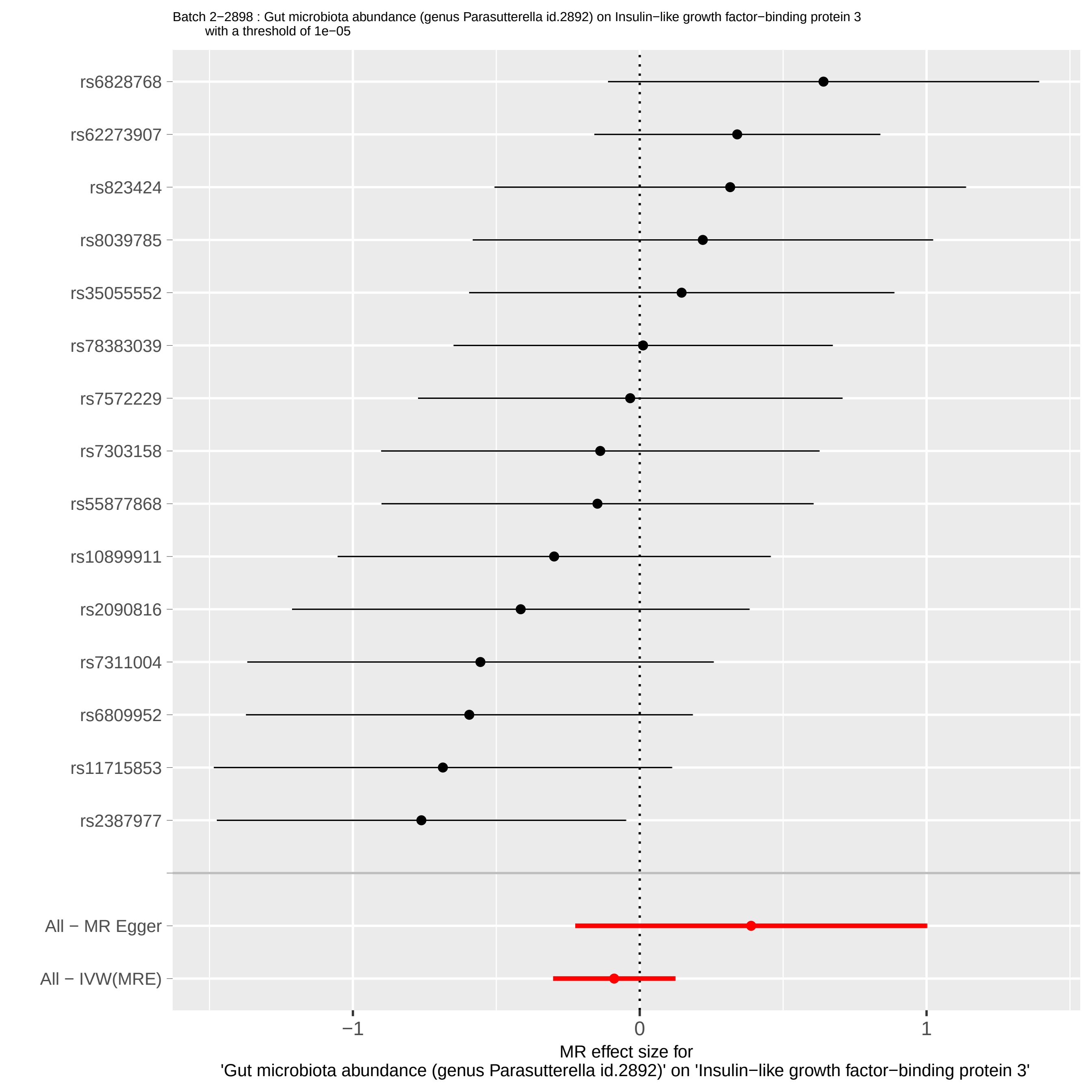
Batch 2−2898 : Gut microbiota abundance (genus Parasutterella id.2892) on Insulin−like growth factor−binding protein 3
with a threshold of 1e−05
rs6828768
rs62273907
rs823424
rs8039785
rs35055552
rs78383039
rs7572229
rs7303158
rs55877868
rs10899911
rs2090816
rs7311004
rs6809952
rs11715853
rs2387977
All − MR Egger
All − IVW(MRE)
−1
0
1
MR effect size for
'Gut microbiota abundance (genus Parasutterella id.2892)' on 'Insulin−like growth factor−binding protein 3'

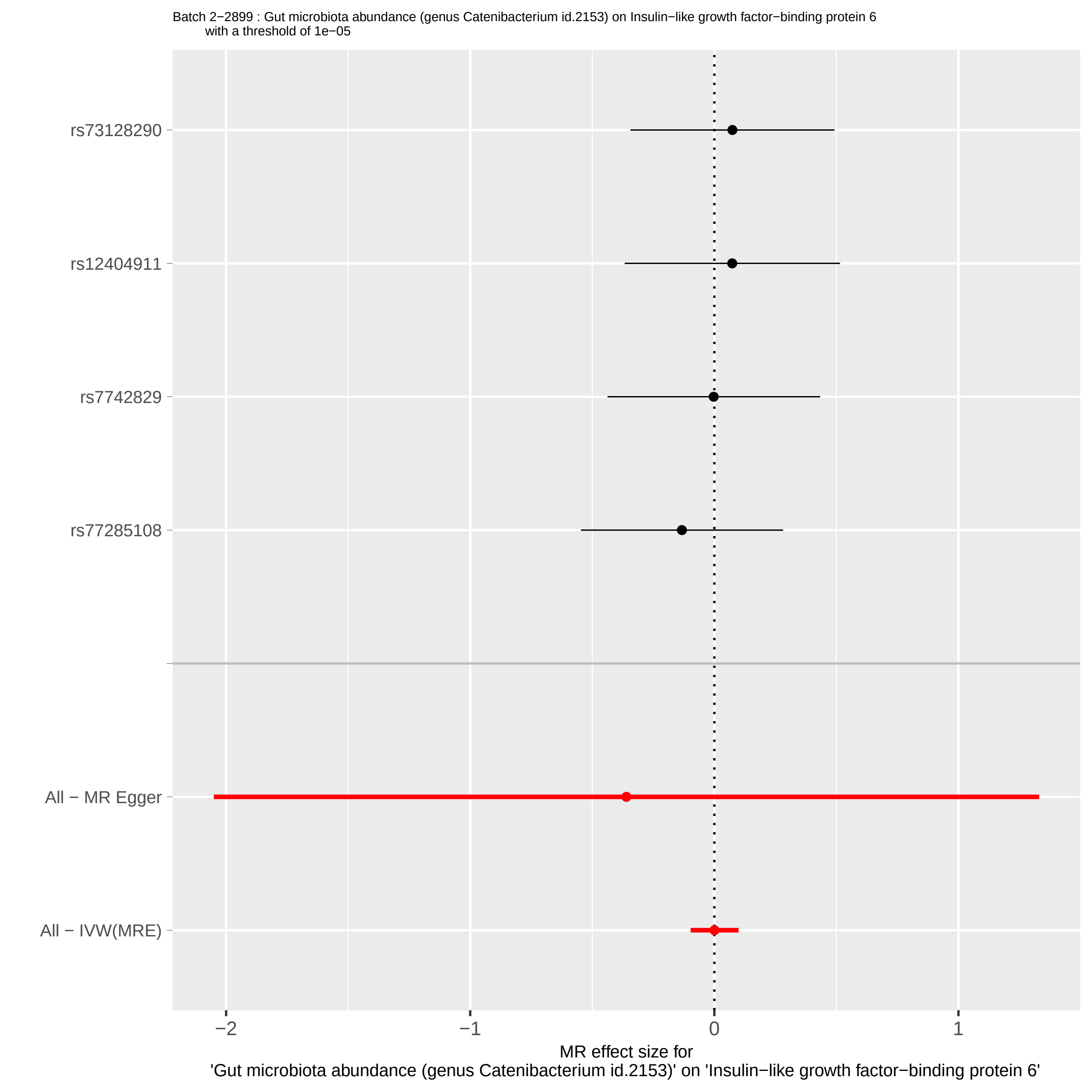
Batch 2−2899 : Gut microbiota abundance (genus Catenibacterium id.2153) on Insulin−like growth factor−binding protein 6
with a threshold of 1e−05
rs73128290
rs12404911
rs7742829
rs77285108
All − MR Egger
All − IVW(MRE)
−2
−1
0
1
MR effect size for
'Gut microbiota abundance (genus Catenibacterium id.2153)' on 'Insulin−like growth factor−binding protein 6'

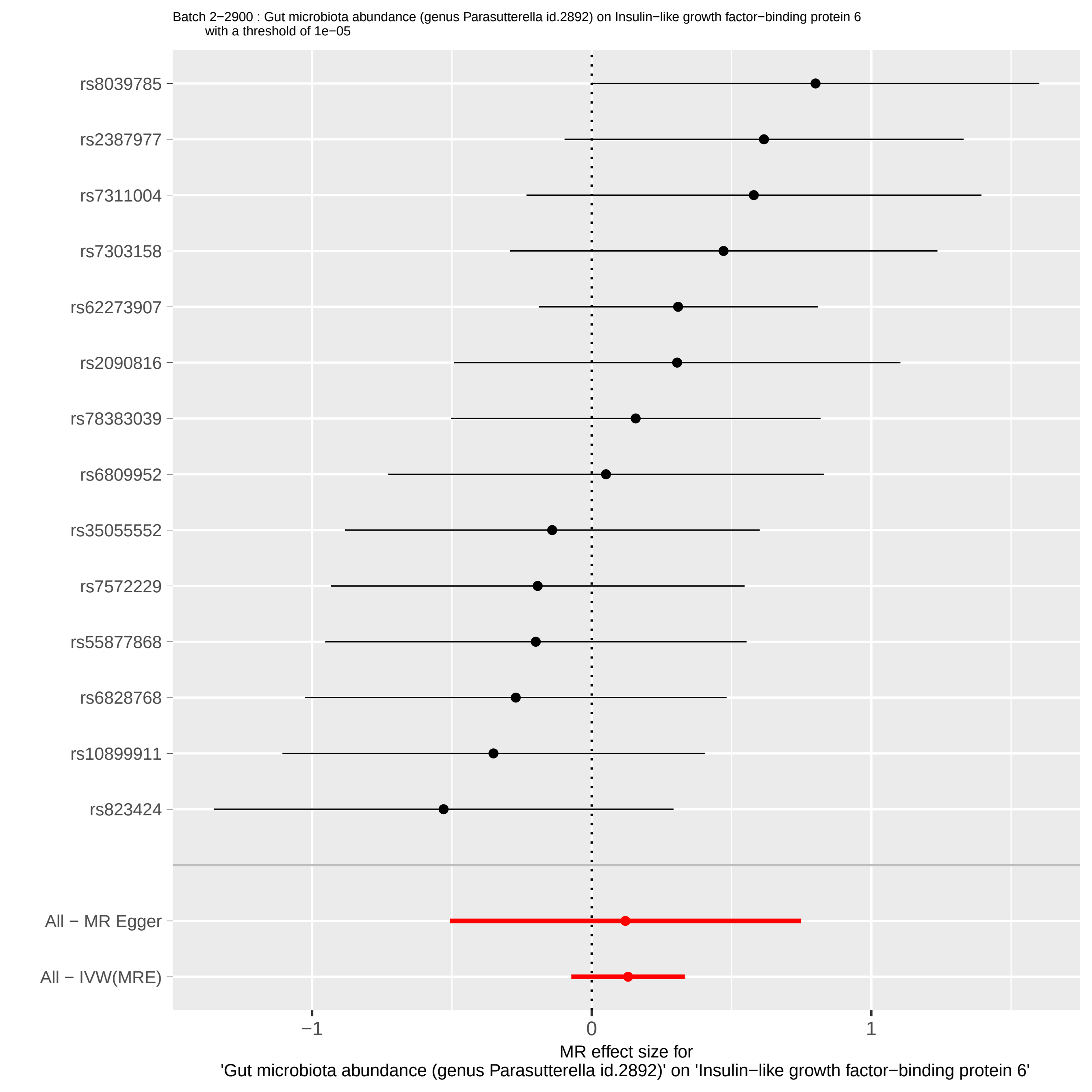

Batch 2−2900 : Gut microbiota abundance (genus Parasutterella id.2892) on Insulin−like growth factor−binding protein 6
with a threshold of 1e−05
rs8039785
rs2387977
rs7311004
rs7303158
rs62273907
rs2090816
rs78383039
rs6809952
rs35055552
rs7572229
rs55877868
rs6828768
rs10899911
rs823424
All − MR Egger
All − IVW(MRE)
−1
0
1
MR effect size for
'Gut microbiota abundance (genus Parasutterella id.2892)' on 'Insulin−like growth factor−binding protein 6'

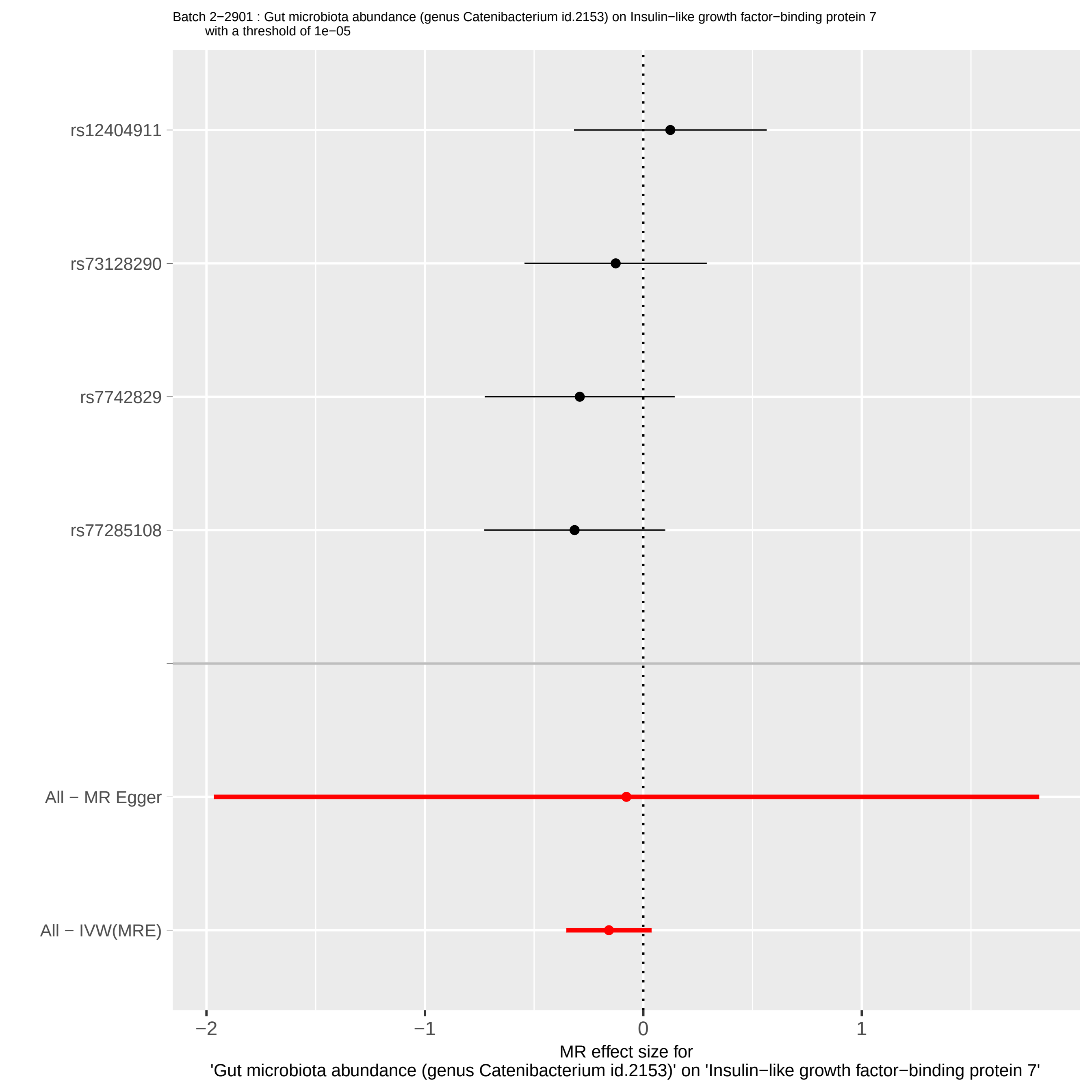

Batch 2−2901 : Gut microbiota abundance (genus Catenibacterium id.2153) on Insulin−like growth factor−binding protein 7
with a threshold of 1e−05
rs12404911
rs73128290
rs7742829
rs77285108
All − MR Egger
All − IVW(MRE)
−2
−1
0
1
MR effect size for
'Gut microbiota abundance (genus Catenibacterium id.2153)' on 'Insulin−like growth factor−binding protein 7'

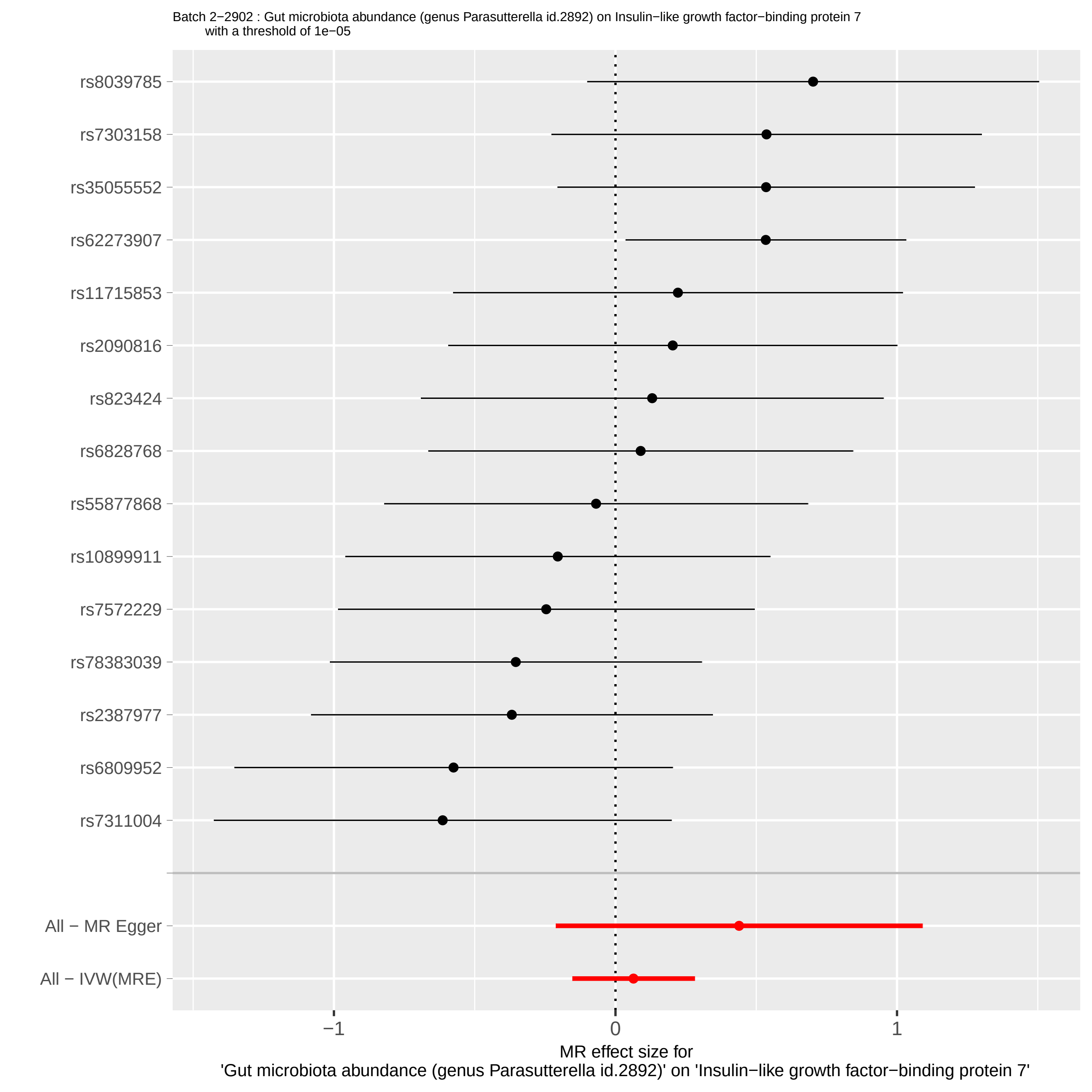

Batch 2−2902 : Gut microbiota abundance (genus Parasutterella id.2892) on Insulin−like growth factor−binding protein 7
with a threshold of 1e−05
rs8039785
rs7303158
rs35055552
rs62273907
rs11715853
rs2090816
rs823424
rs6828768
rs55877868
rs10899911
rs7572229
rs78383039
rs2387977
rs6809952
rs7311004
All − MR Egger
All − IVW(MRE)
−1
0
1
MR effect size for
'Gut microbiota abundance (genus Parasutterella id.2892)' on 'Insulin−like growth factor−binding protein 7'

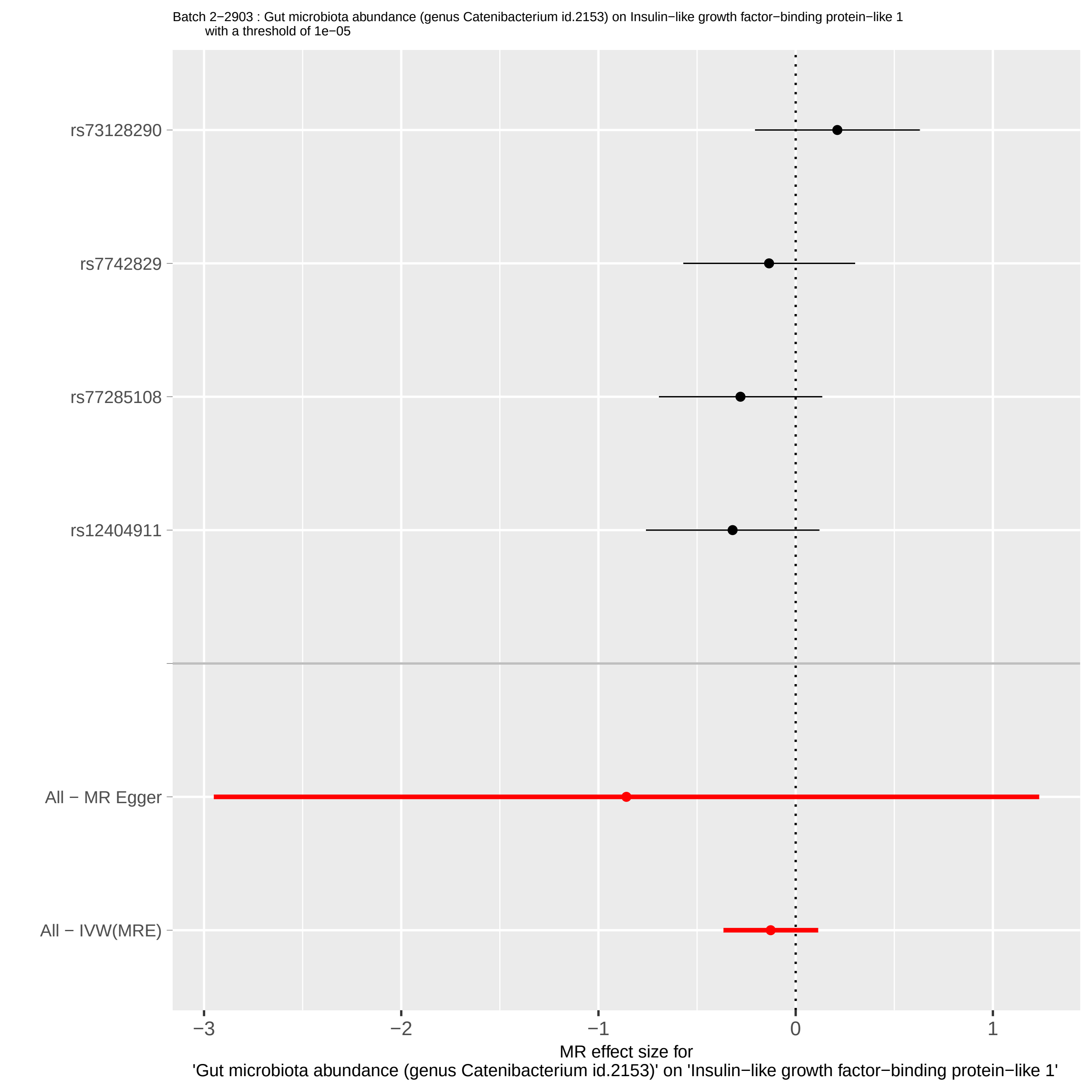

Batch 2−2903 : Gut microbiota abundance (genus Catenibacterium id.2153) on Insulin−like growth factor−binding protein−like 1
with a threshold of 1e−05
rs73128290
rs7742829
rs77285108
rs12404911
All − MR Egger
All − IVW(MRE)
−3
−2
−1
0
1
MR effect size for
'Gut microbiota abundance (genus Catenibacterium id.2153)' on 'Insulin−like growth factor−binding protein−like 1'

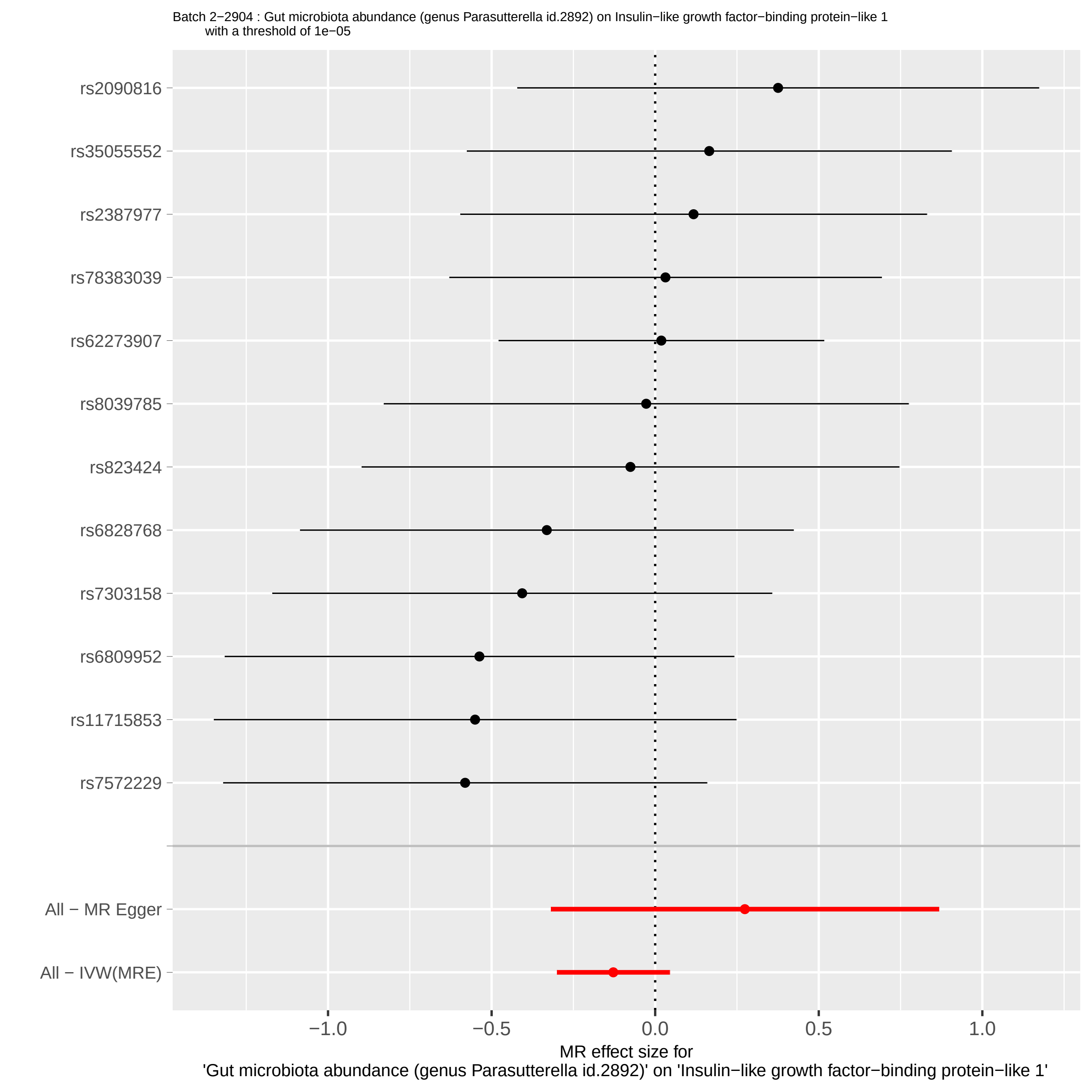

Batch 2−2904 : Gut microbiota abundance (genus Parasutterella id.2892) on Insulin−like growth factor−binding protein−like 1
with a threshold of 1e−05
rs2090816
rs35055552
rs2387977
rs78383039
rs62273907
rs8039785
rs823424
rs6828768
rs7303158
rs6809952
rs11715853
rs7572229
All − MR Egger
All − IVW(MRE)
−1.0
−0.5
0.0
0.5
1.0
MR effect size for
'Gut microbiota abundance (genus Parasutterella id.2892)' on 'Insulin−like growth factor−binding protein−like 1'

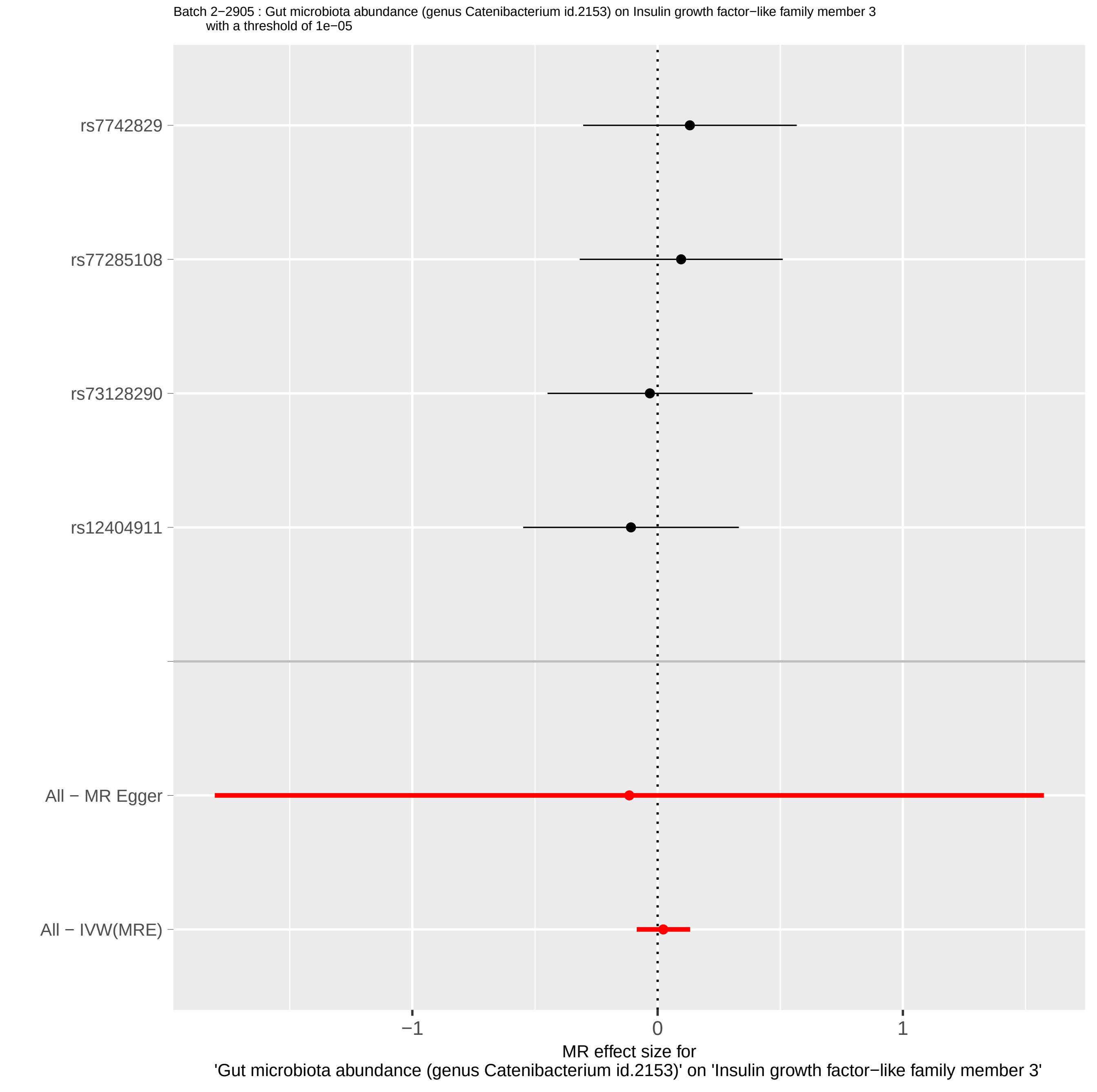

Batch 2−2905 : Gut microbiota abundance (genus Catenibacterium id.2153) on Insulin growth factor−like family member 3
with a threshold of 1e−05
rs7742829
rs77285108
rs73128290
rs12404911
All − MR Egger
All − IVW(MRE)
−1
0
1
MR effect size for
'Gut microbiota abundance (genus Catenibacterium id.2153)' on 'Insulin growth factor−like family member 3'

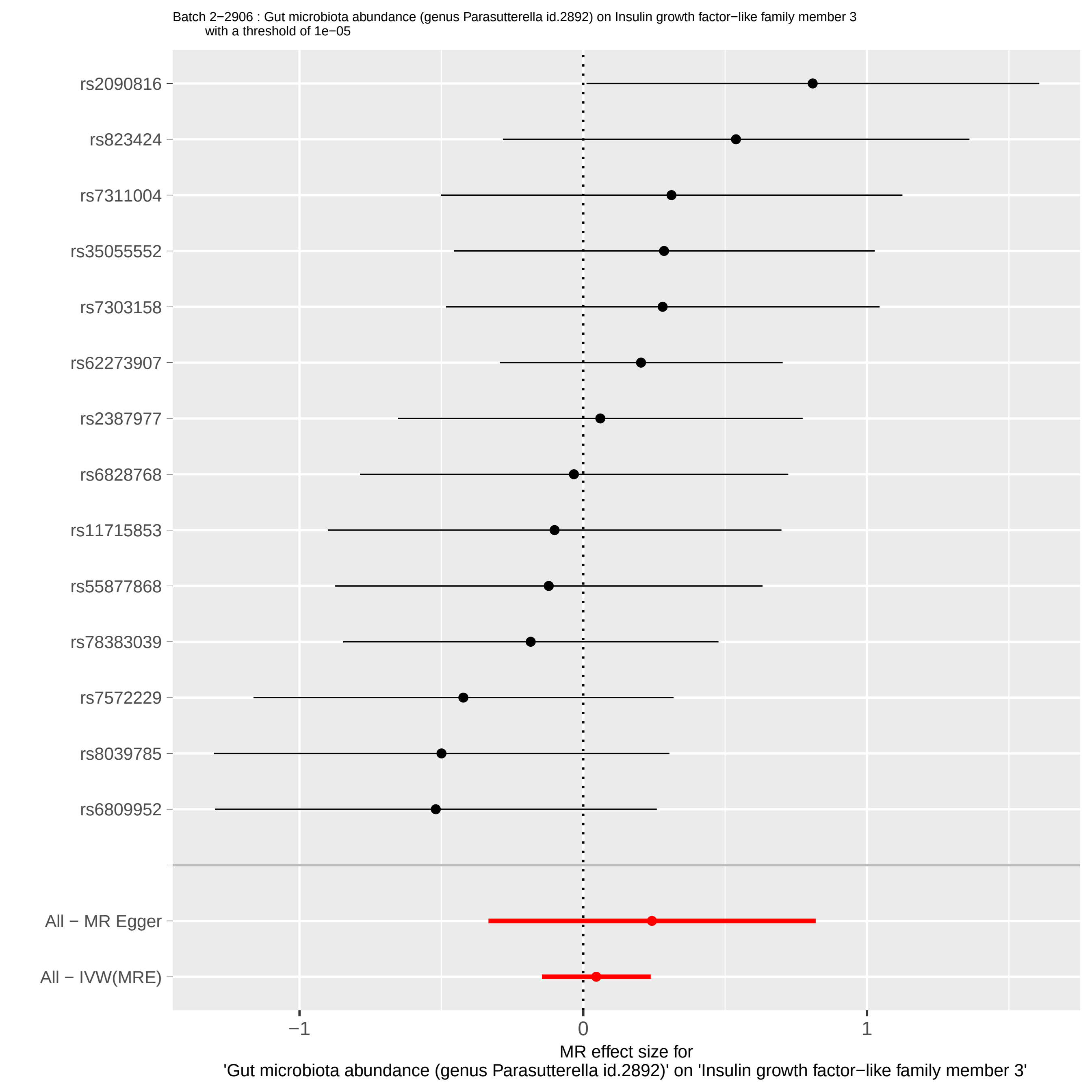

Batch 2−2906 : Gut microbiota abundance (genus Parasutterella id.2892) on Insulin growth factor−like family member 3
with a threshold of 1e−05
rs2090816
rs823424
rs7311004
rs35055552
rs7303158
rs62273907
rs2387977
rs6828768
rs11715853
rs55877868
rs78383039
rs7572229
rs8039785
rs6809952
All − MR Egger
All − IVW(MRE)
−1
0
1
MR effect size for
'Gut microbiota abundance (genus Parasutterella id.2892)' on 'Insulin growth factor−like family member 3'

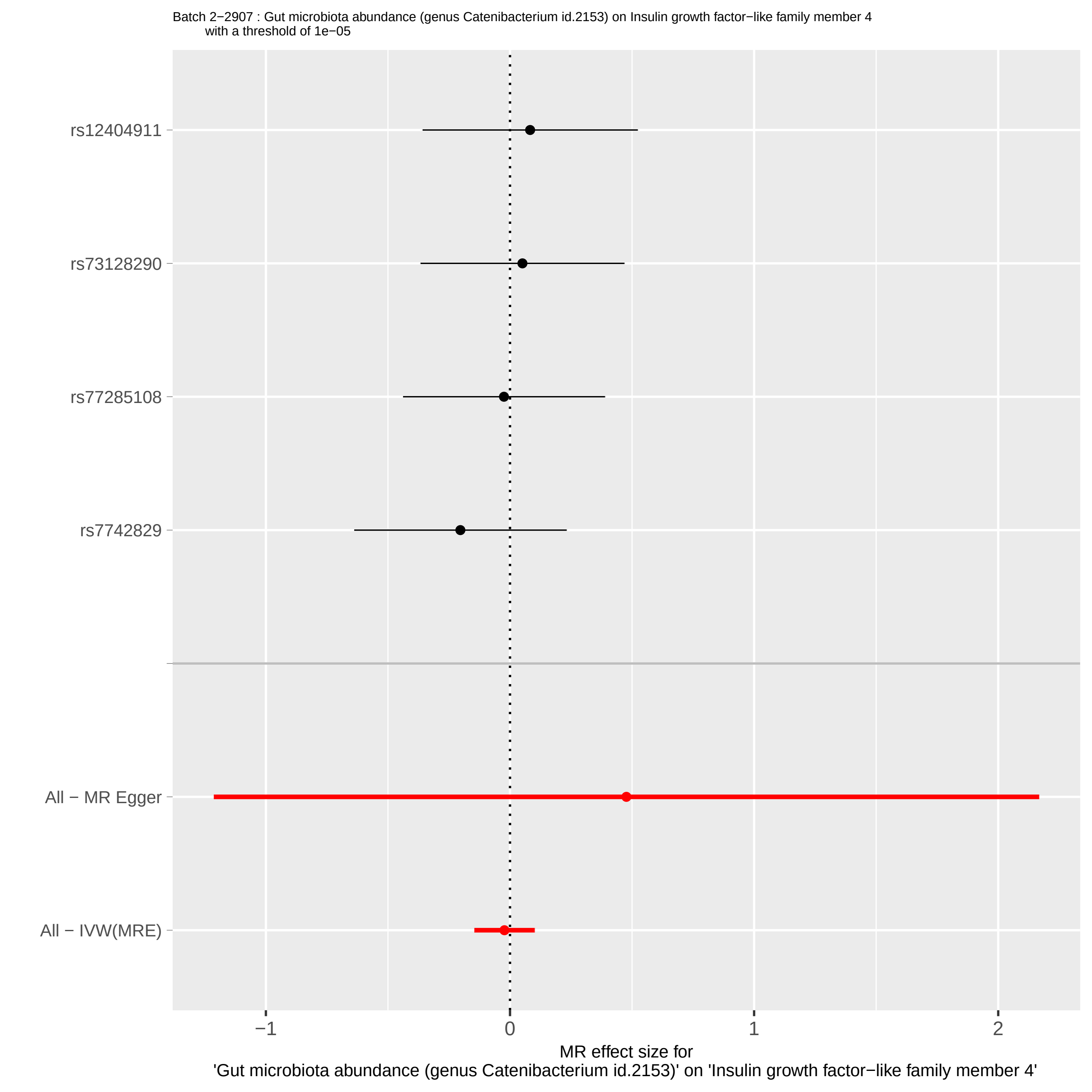
Batch 2−2907 : Gut microbiota abundance (genus Catenibacterium id.2153) on Insulin growth factor−like family member 4
with a threshold of 1e−05
rs12404911
rs73128290
rs77285108
rs7742829
All − MR Egger
All − IVW(MRE)
−1
0
1
2
MR effect size for
'Gut microbiota abundance (genus Catenibacterium id.2153)' on 'Insulin growth factor−like family member 4'

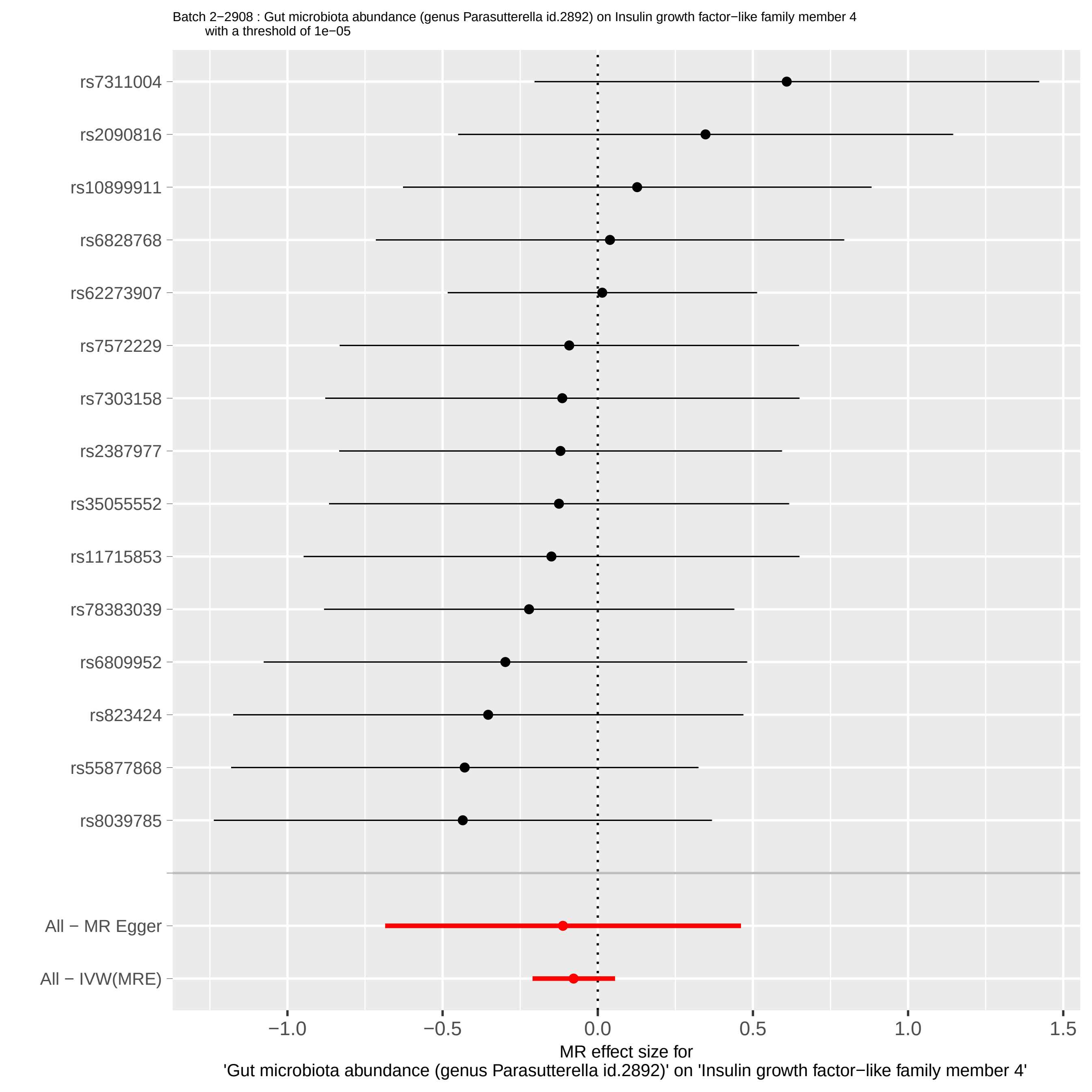

Batch 2−2908 : Gut microbiota abundance (genus Parasutterella id.2892) on Insulin growth factor−like family member 4
with a threshold of 1e−05
rs7311004
rs2090816
rs10899911
rs6828768
rs62273907
rs7572229
rs7303158
rs2387977
rs35055552
rs11715853
rs78383039
rs6809952
rs823424
rs55877868
rs8039785
All − MR Egger
All − IVW(MRE)
−1.0
−0.5
0.0
0.5
1.0
1.5
MR effect size for
'Gut microbiota abundance (genus Parasutterella id.2892)' on 'Insulin growth factor−like family member 4'

Batch 2−2909 : Gut microbiota abundance (genus Catenibacterium id.2153) on IGF−like family receptor 1
with a threshold of 1e−05

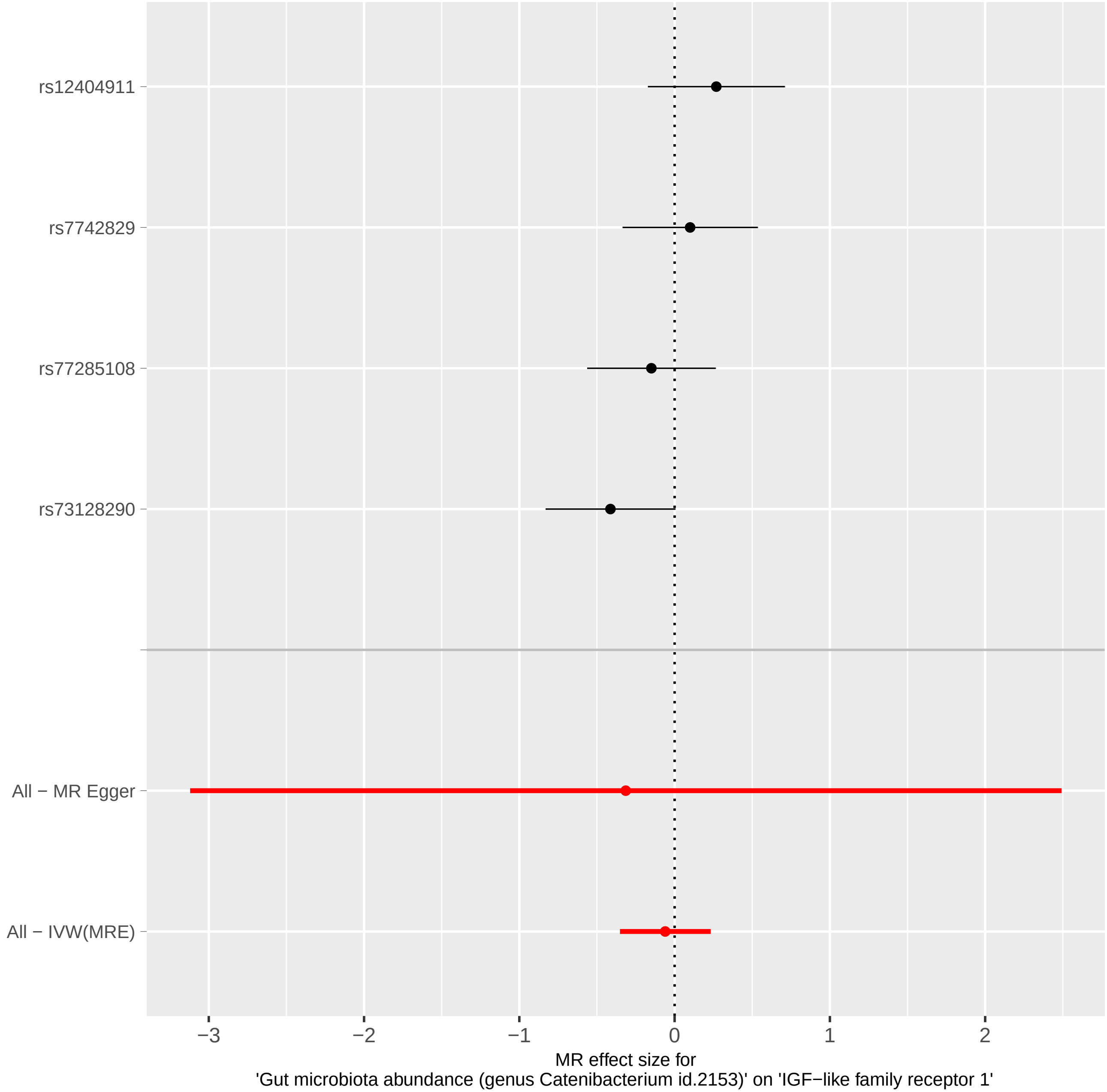

Batch 2−2910 : Gut microbiota abundance (genus Parasutterella id.2892) on IGF−like family receptor 1
with a threshold of 1e−05

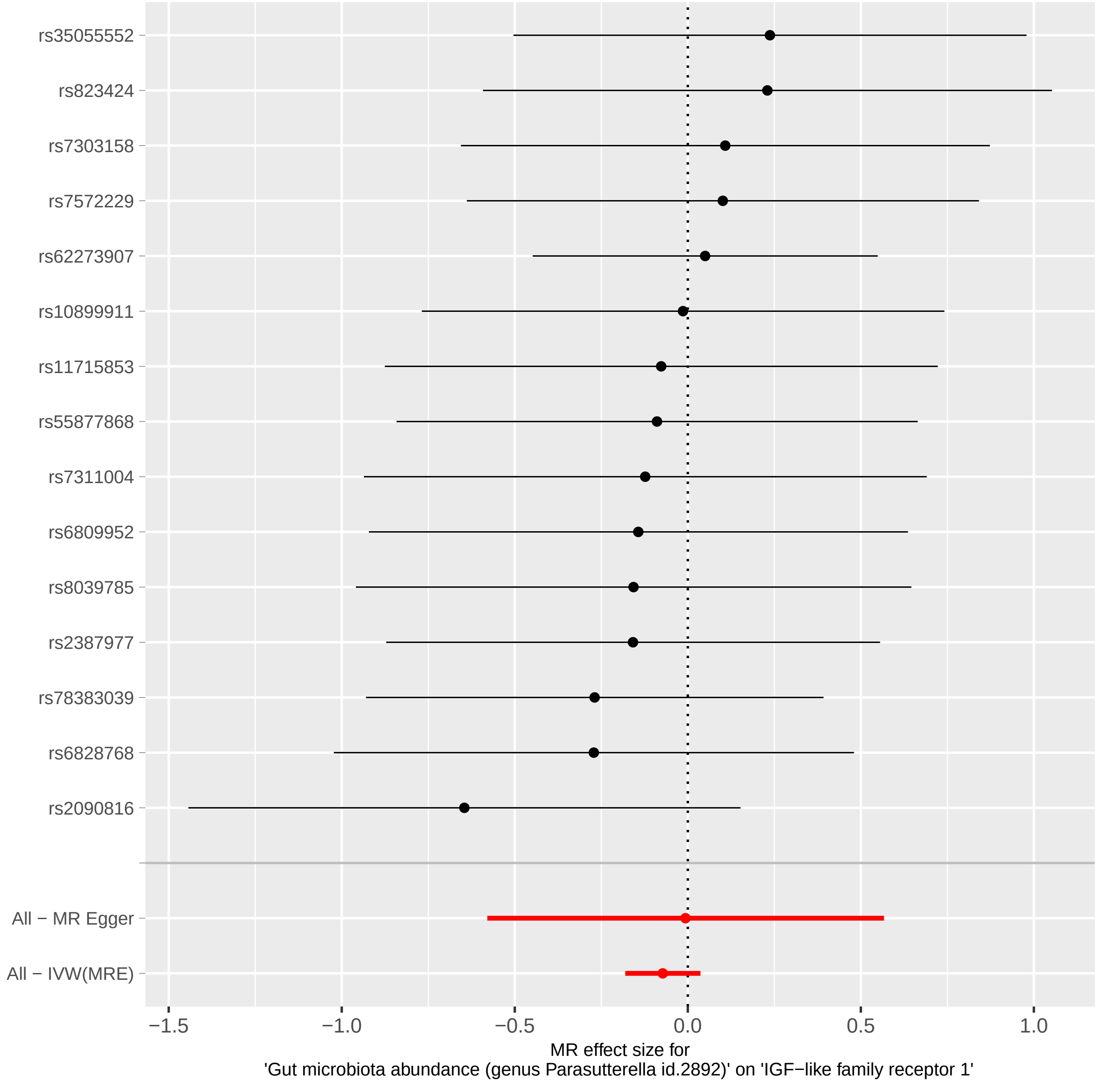

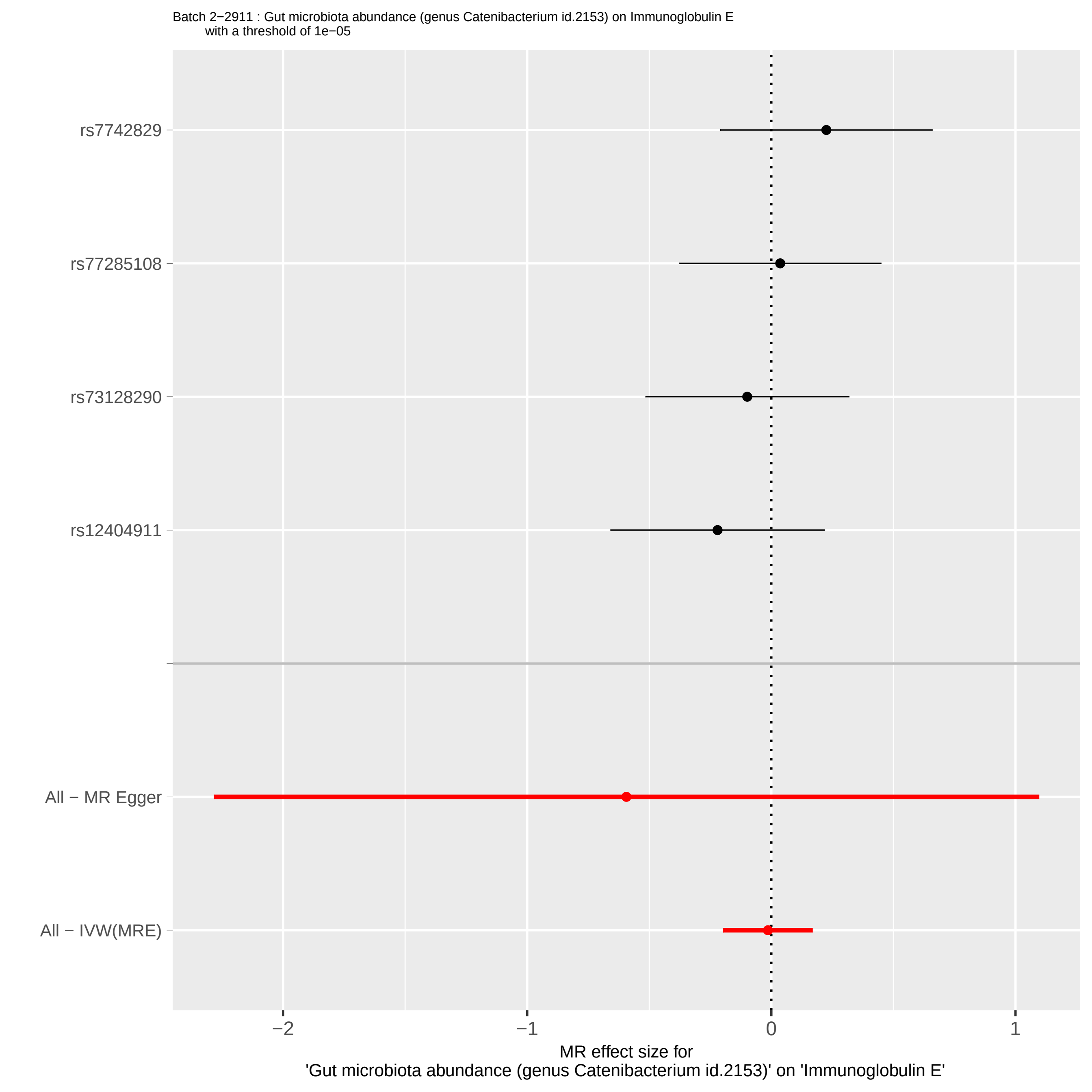
Batch 2−2911 : Gut microbiota abundance (genus Catenibacterium id.2153) on Immunoglobulin E
with a threshold of 1e−05
rs7742829
rs77285108
rs73128290
rs12404911
All − MR Egger
All − IVW(MRE)
−2
−1
0
1
MR effect size for
'Gut microbiota abundance (genus Catenibacterium id.2153)' on 'Immunoglobulin E'

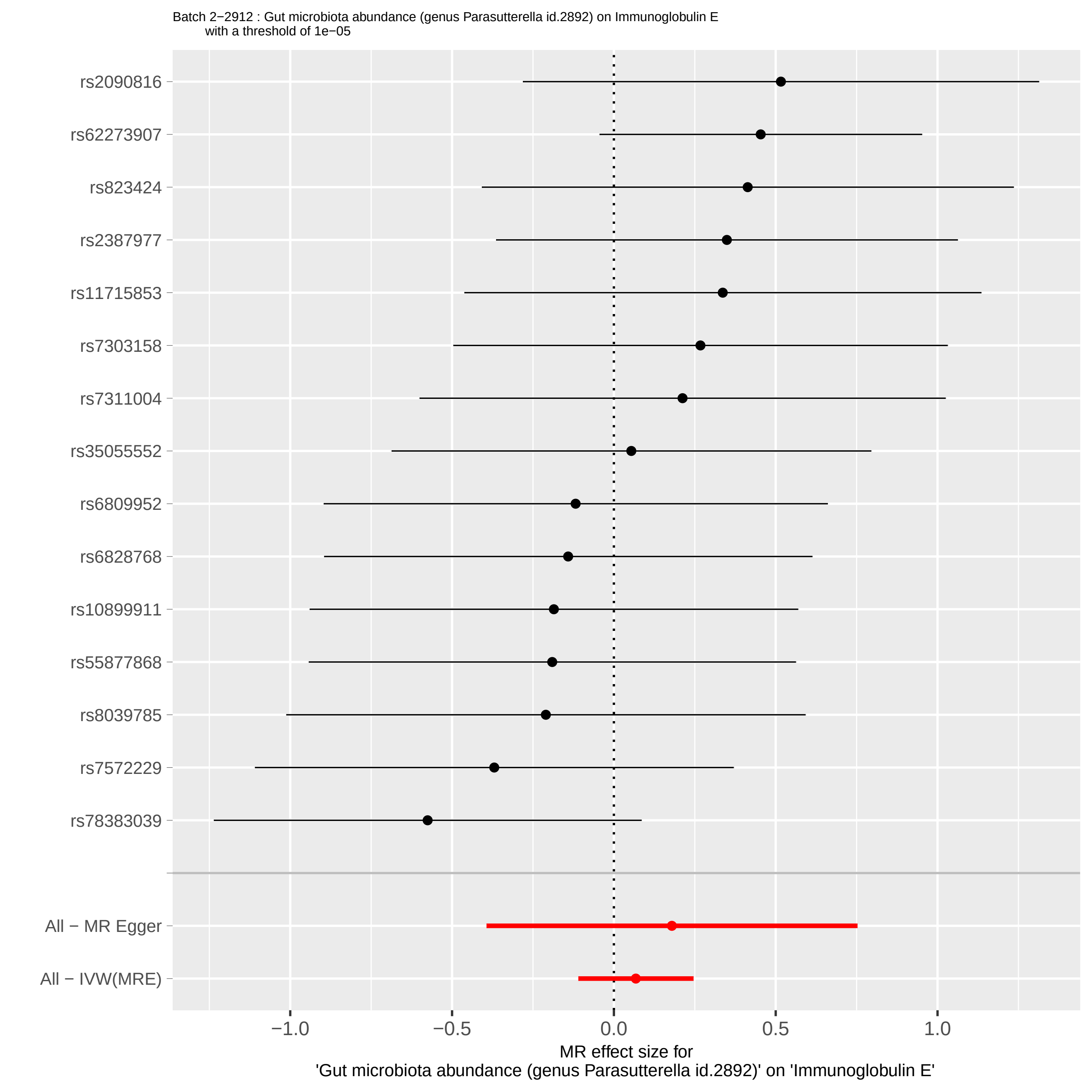
Batch 2−2912 : Gut microbiota abundance (genus Parasutterella id.2892) on Immunoglobulin E
with a threshold of 1e−05
rs2090816
rs62273907
rs823424
rs2387977
rs11715853
rs7303158
rs7311004
rs35055552
rs6809952
rs6828768
rs10899911
rs55877868
rs8039785
rs7572229
rs78383039
All − MR Egger
All − IVW(MRE)
−1.0
−0.5
0.0
0.5
1.0
MR effect size for
'Gut microbiota abundance (genus Parasutterella id.2892)' on 'Immunoglobulin E'

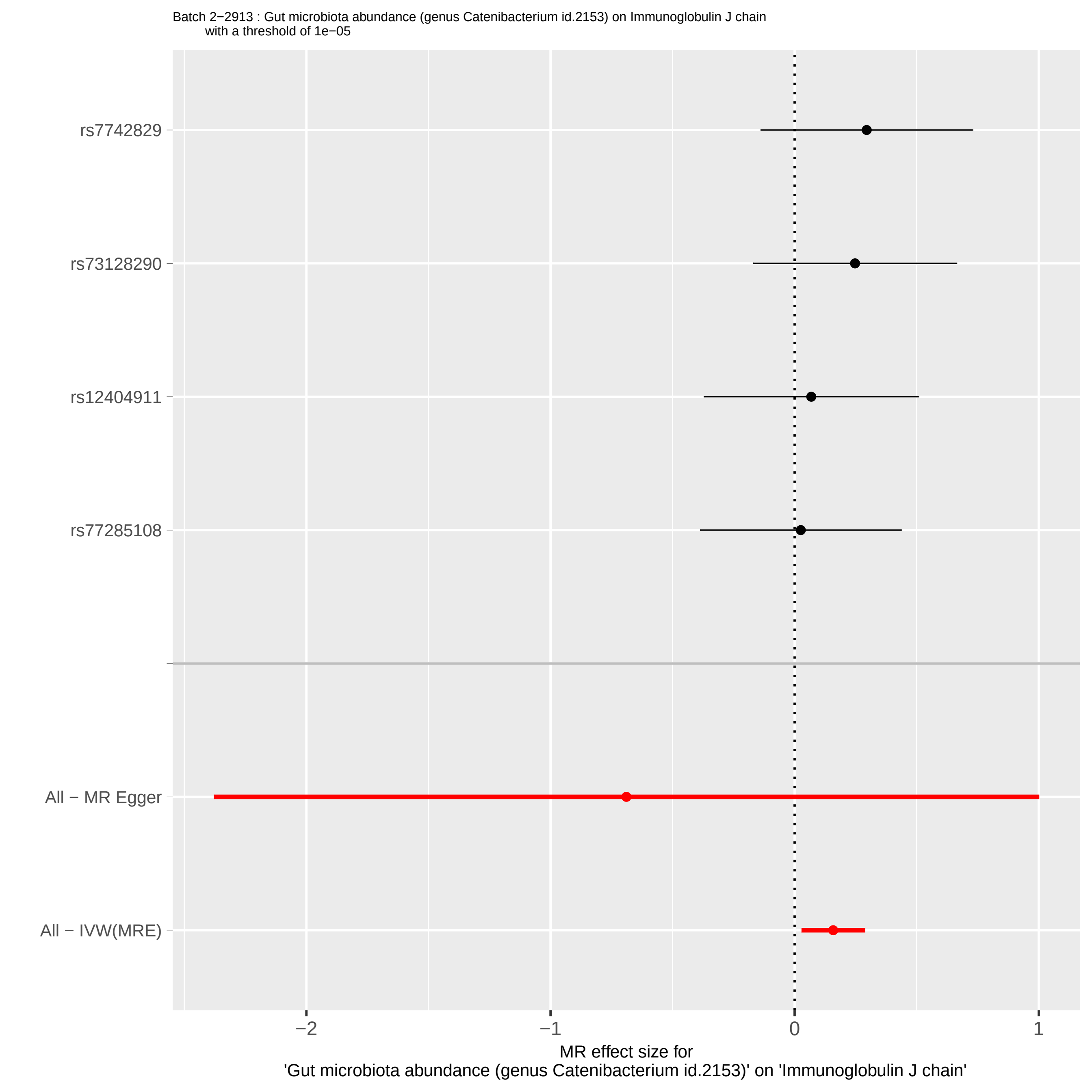
Batch 2−2913 : Gut microbiota abundance (genus Catenibacterium id.2153) on Immunoglobulin J chain
with a threshold of 1e−05
rs7742829
rs73128290
rs12404911
rs77285108
All − MR Egger
All − IVW(MRE)
−2
−1
0
1
MR effect size for
'Gut microbiota abundance (genus Catenibacterium id.2153)' on 'Immunoglobulin J chain'

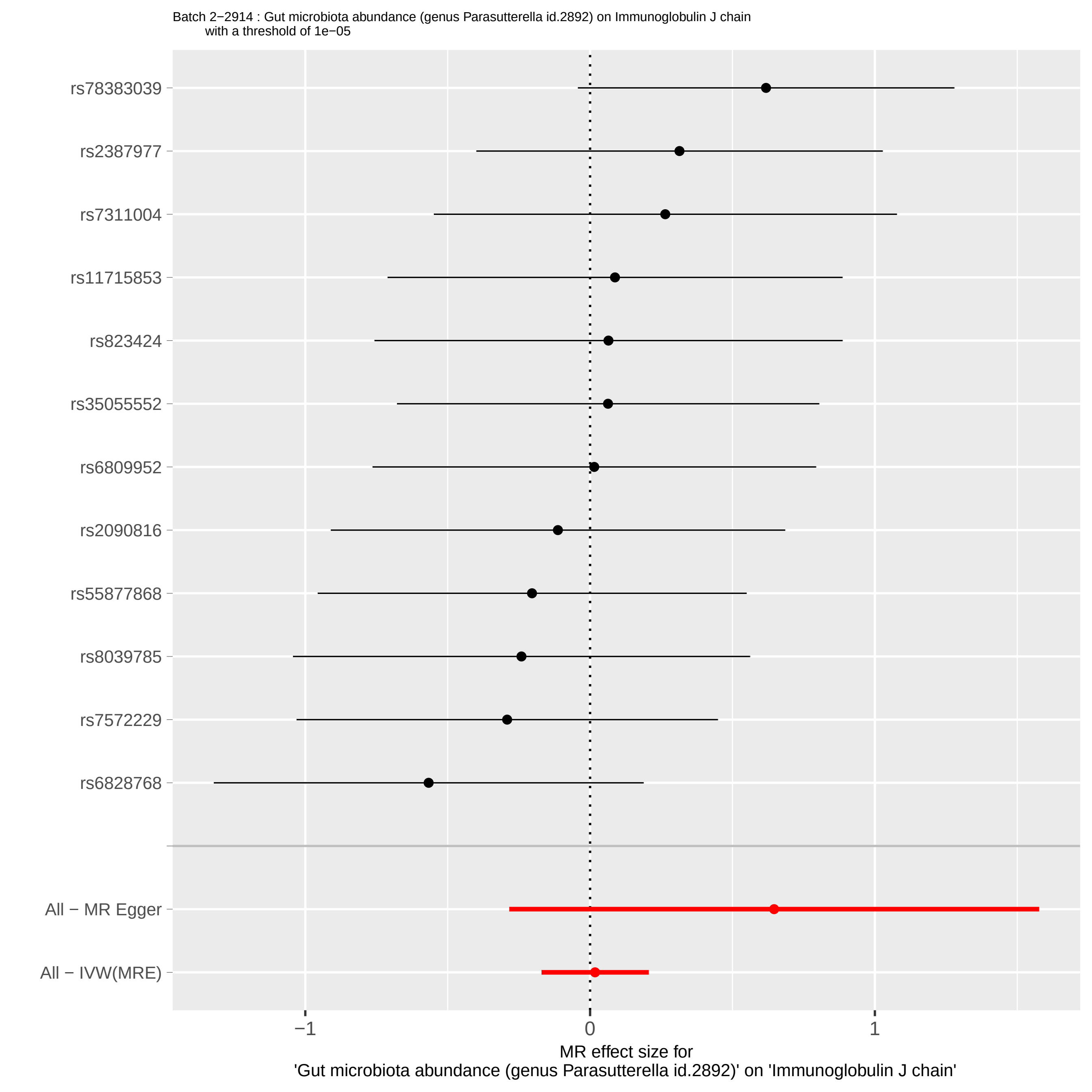

Batch 2−2914 : Gut microbiota abundance (genus Parasutterella id.2892) on Immunoglobulin J chain
with a threshold of 1e−05
rs78383039
rs2387977
rs7311004
rs11715853
rs823424
rs35055552
rs6809952
rs2090816
rs55877868
rs8039785
rs7572229
rs6828768
All − MR Egger
All − IVW(MRE)
−1
0
1
MR effect size for
'Gut microbiota abundance (genus Parasutterella id.2892)' on 'Immunoglobulin J chain'

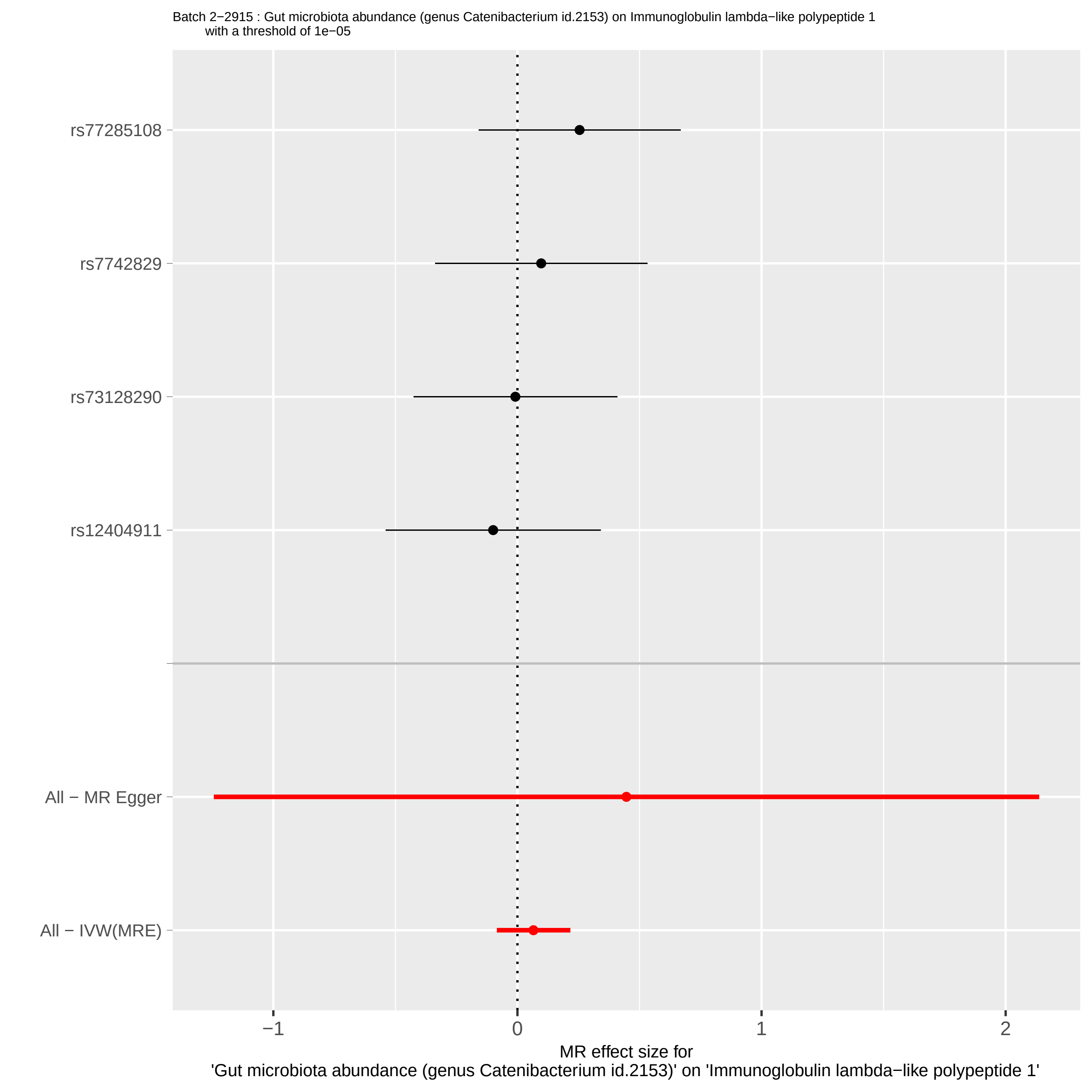
Batch 2−2915 : Gut microbiota abundance (genus Catenibacterium id.2153) on Immunoglobulin lambda−like polypeptide 1
with a threshold of 1e−05
rs77285108
rs7742829
rs73128290
rs12404911
All − MR Egger
All − IVW(MRE)
−1
0
1
2
MR effect size for
'Gut microbiota abundance (genus Catenibacterium id.2153)' on 'Immunoglobulin lambda−like polypeptide 1'

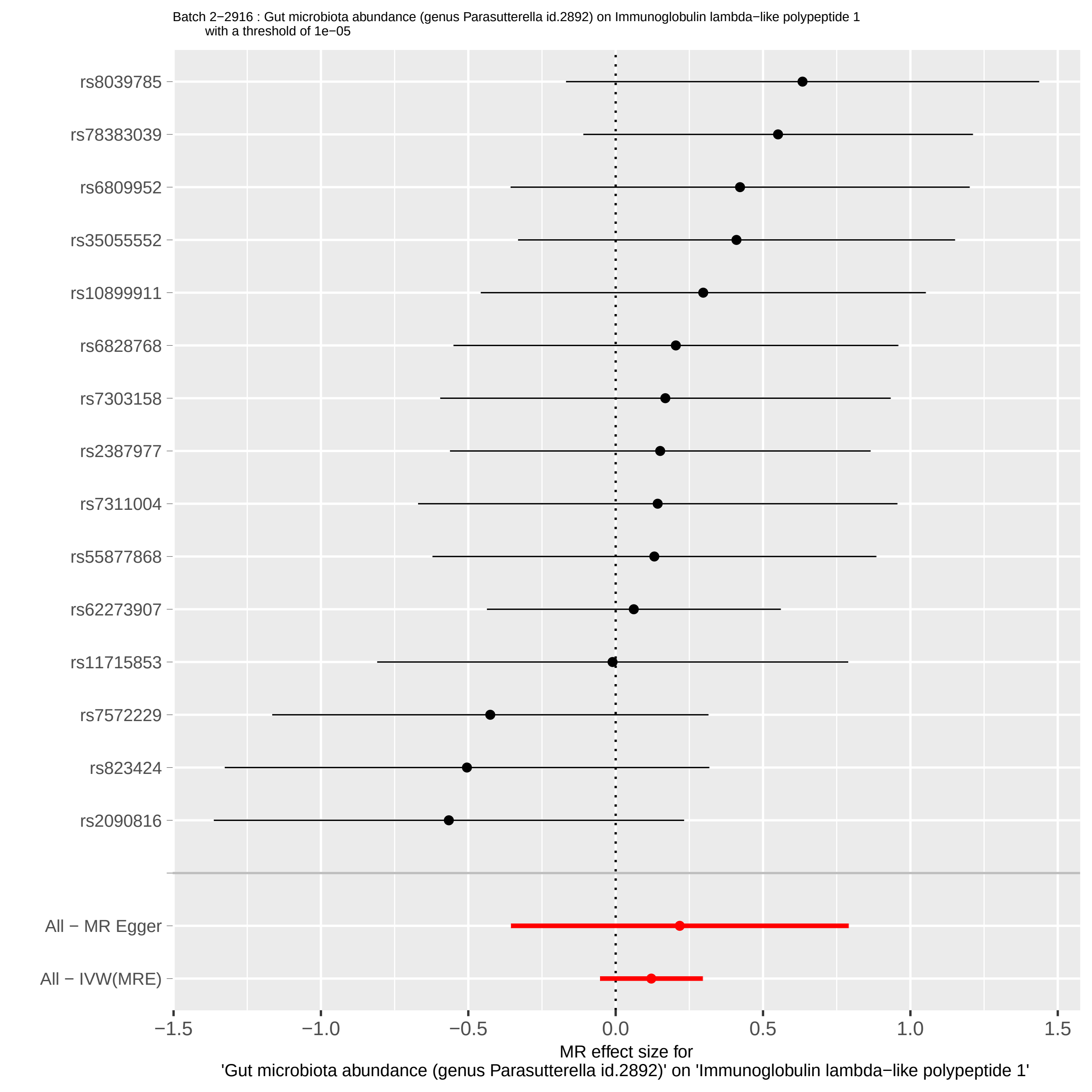
Batch 2−2916 : Gut microbiota abundance (genus Parasutterella id.2892) on Immunoglobulin lambda−like polypeptide 1
with a threshold of 1e−05
rs8039785
rs78383039
rs6809952
rs35055552
rs10899911
rs6828768
rs7303158
rs2387977
rs7311004
rs55877868
rs62273907
rs11715853
rs7572229
rs823424
rs2090816
All − MR Egger
All − IVW(MRE)
−1.5
−1.0
−0.5
0.0
0.5
1.0
1.5
MR effect size for
'Gut microbiota abundance (genus Parasutterella id.2892)' on 'Immunoglobulin lambda−like polypeptide 1'

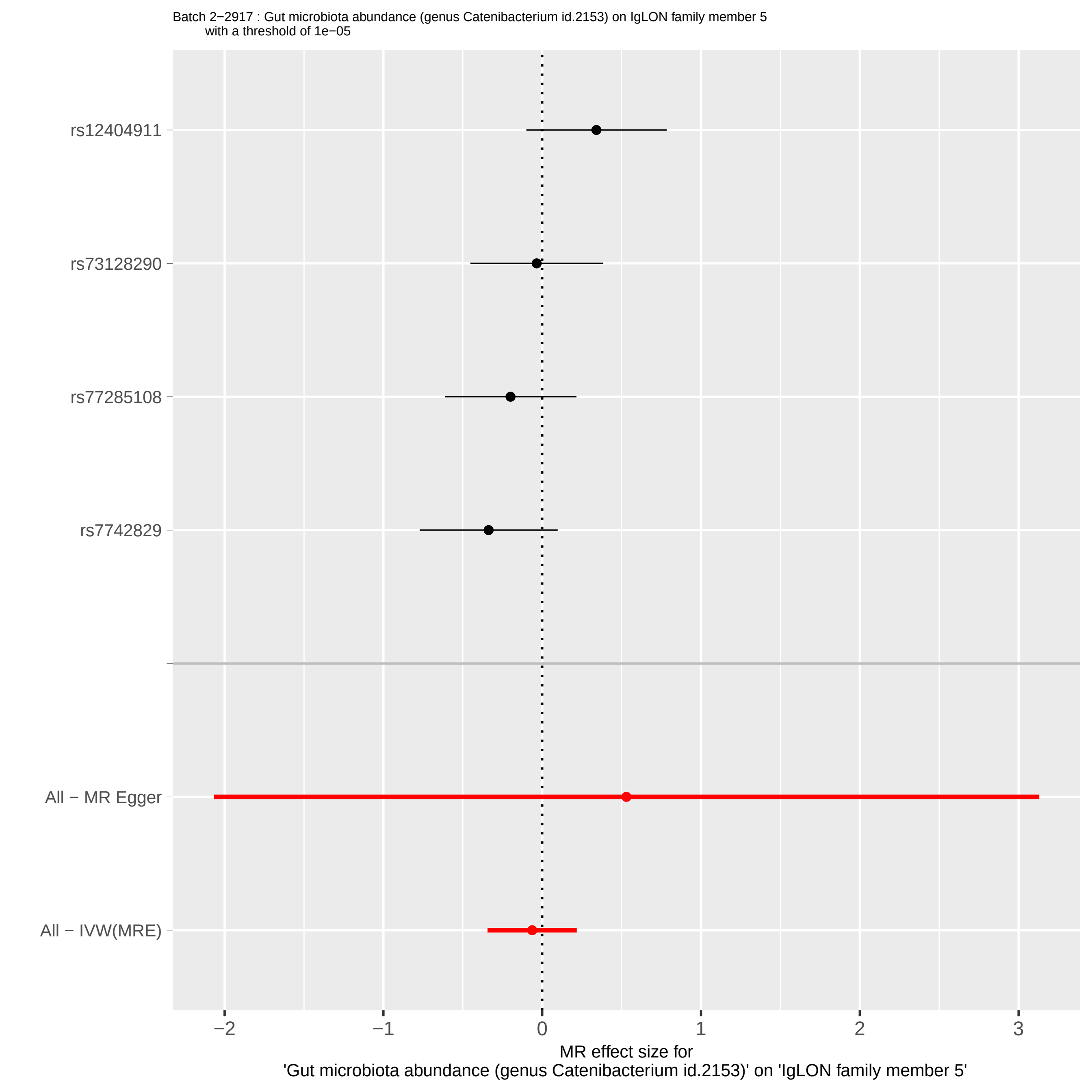
Batch 2−2917 : Gut microbiota abundance (genus Catenibacterium id.2153) on IgLON family member 5
with a threshold of 1e−05
rs12404911
rs73128290
rs77285108
rs7742829
All − MR Egger
All − IVW(MRE)
−2
−1
0
1
2
3
MR effect size for
'Gut microbiota abundance (genus Catenibacterium id.2153)' on 'IgLON family member 5'

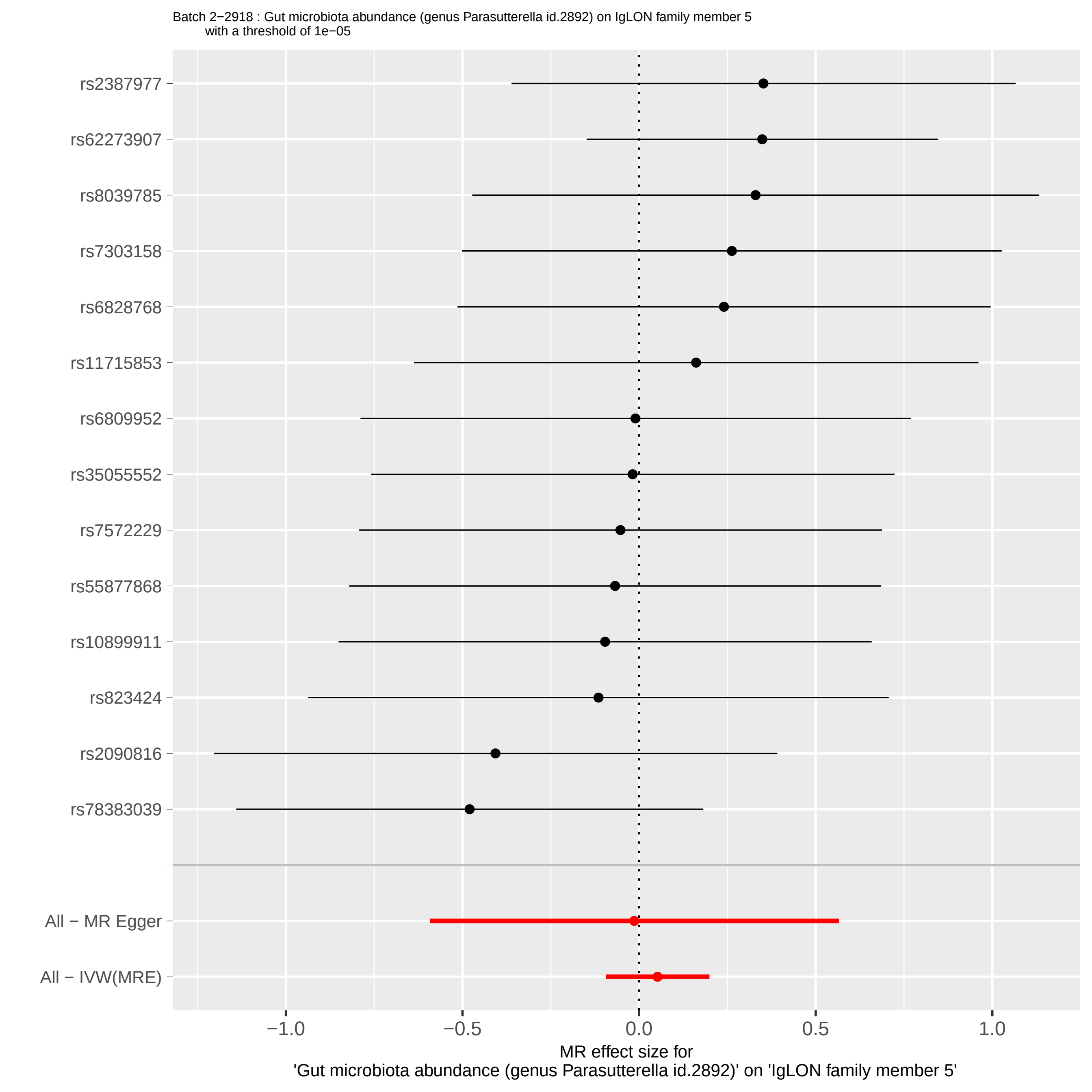
Batch 2−2918 : Gut microbiota abundance (genus Parasutterella id.2892) on IgLON family member 5
with a threshold of 1e−05
rs2387977
rs62273907
rs8039785
rs7303158
rs6828768
rs11715853
rs6809952
rs35055552
rs7572229
rs55877868
rs10899911
rs823424
rs2090816
rs78383039
All − MR Egger
All − IVW(MRE)
−1.0
−0.5
0.0
0.5
1.0
MR effect size for
'Gut microbiota abundance (genus Parasutterella id.2892)' on 'IgLON family member 5'

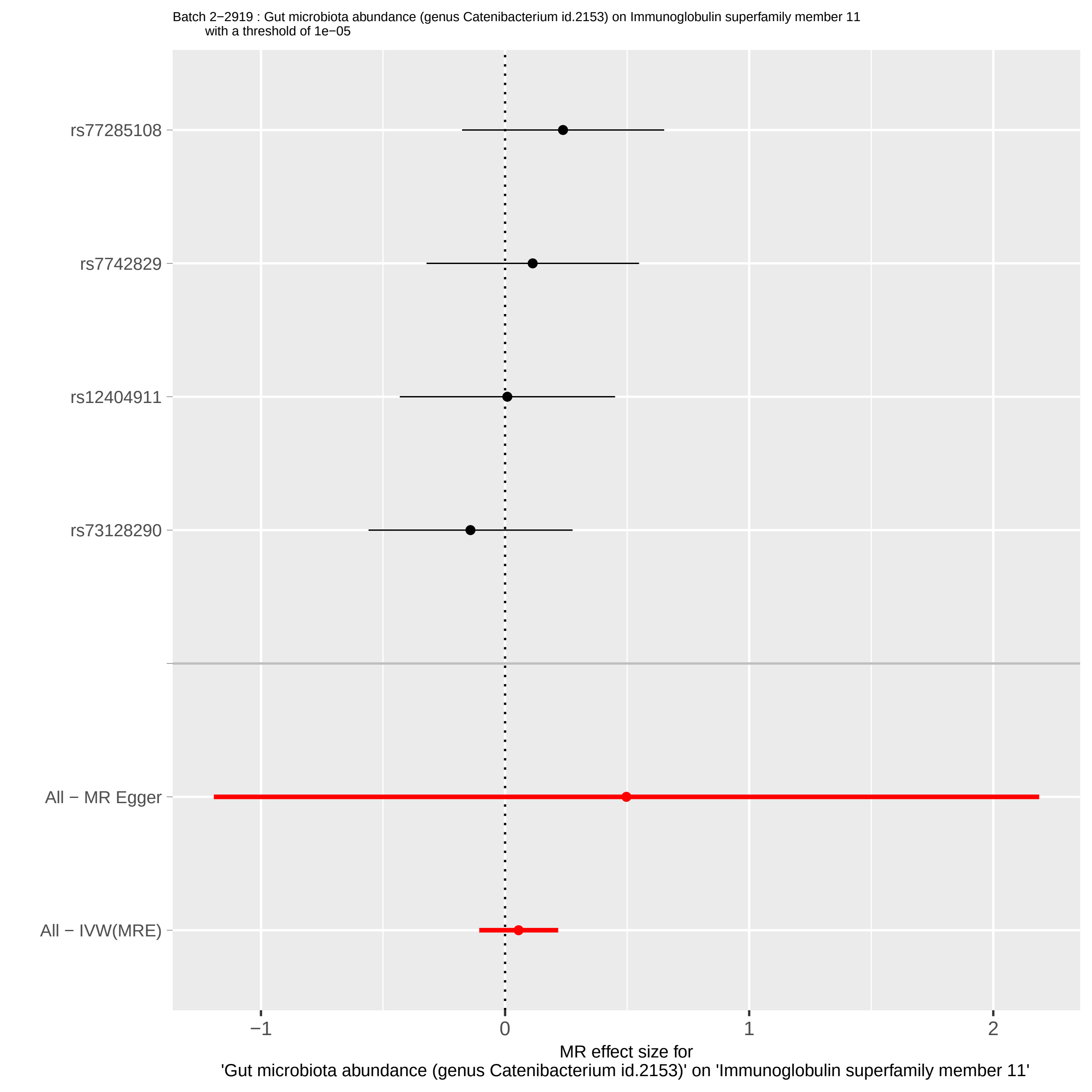

Batch 2−2919 : Gut microbiota abundance (genus Catenibacterium id.2153) on Immunoglobulin superfamily member 11
with a threshold of 1e−05
rs77285108
rs7742829
rs12404911
rs73128290
All − MR Egger
All − IVW(MRE)
−1
0
1
2
MR effect size for
'Gut microbiota abundance (genus Catenibacterium id.2153)' on 'Immunoglobulin superfamily member 11'

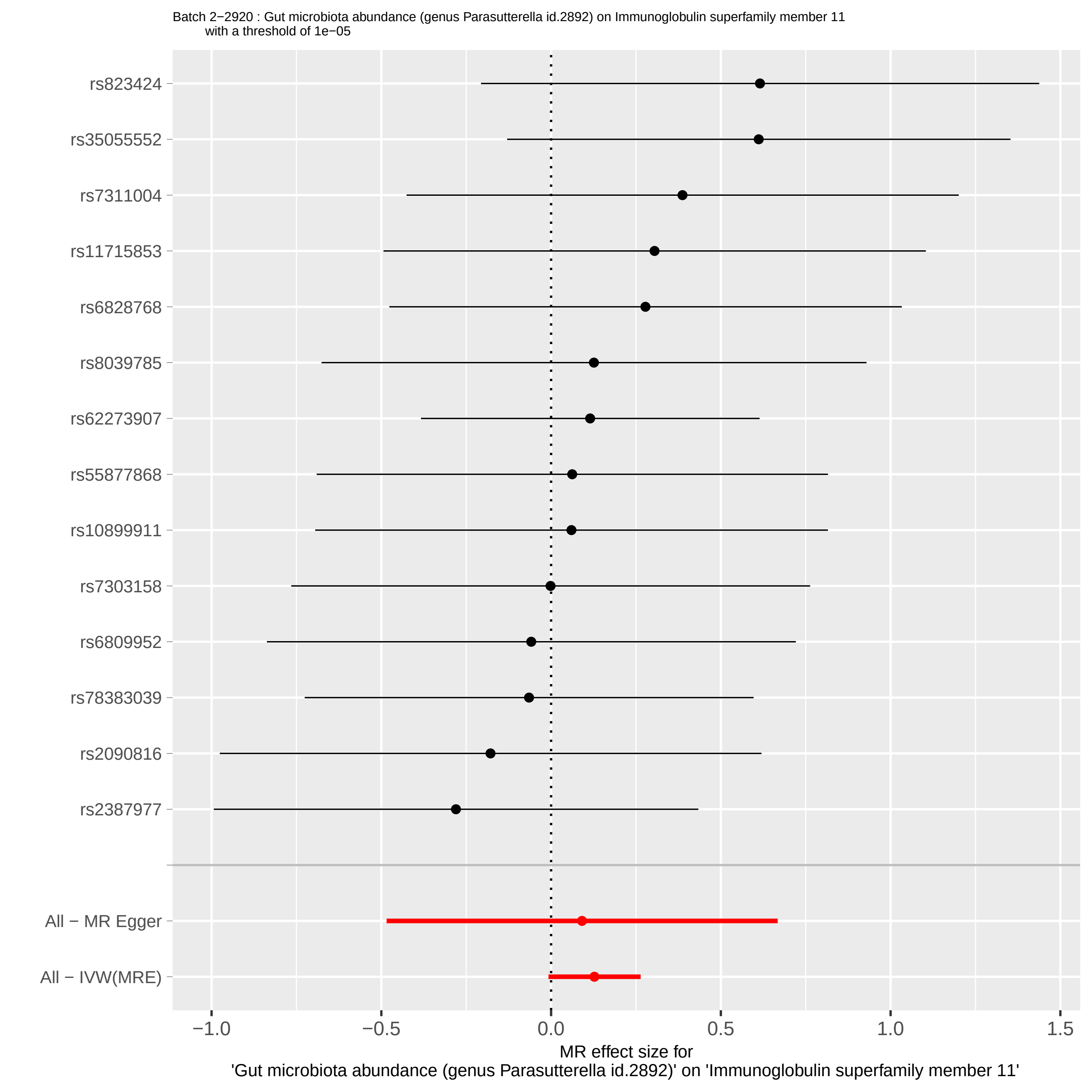

Batch 2−2920 : Gut microbiota abundance (genus Parasutterella id.2892) on Immunoglobulin superfamily member 11
with a threshold of 1e−05
rs823424
rs35055552
rs7311004
rs11715853
rs6828768
rs8039785
rs62273907
rs55877868
rs10899911
rs7303158
rs6809952
rs78383039
rs2090816
rs2387977
All − MR Egger
All − IVW(MRE)
−1.0
−0.5
0.0
0.5
1.0
1.5
MR effect size for
'Gut microbiota abundance (genus Parasutterella id.2892)' on 'Immunoglobulin superfamily member 11'

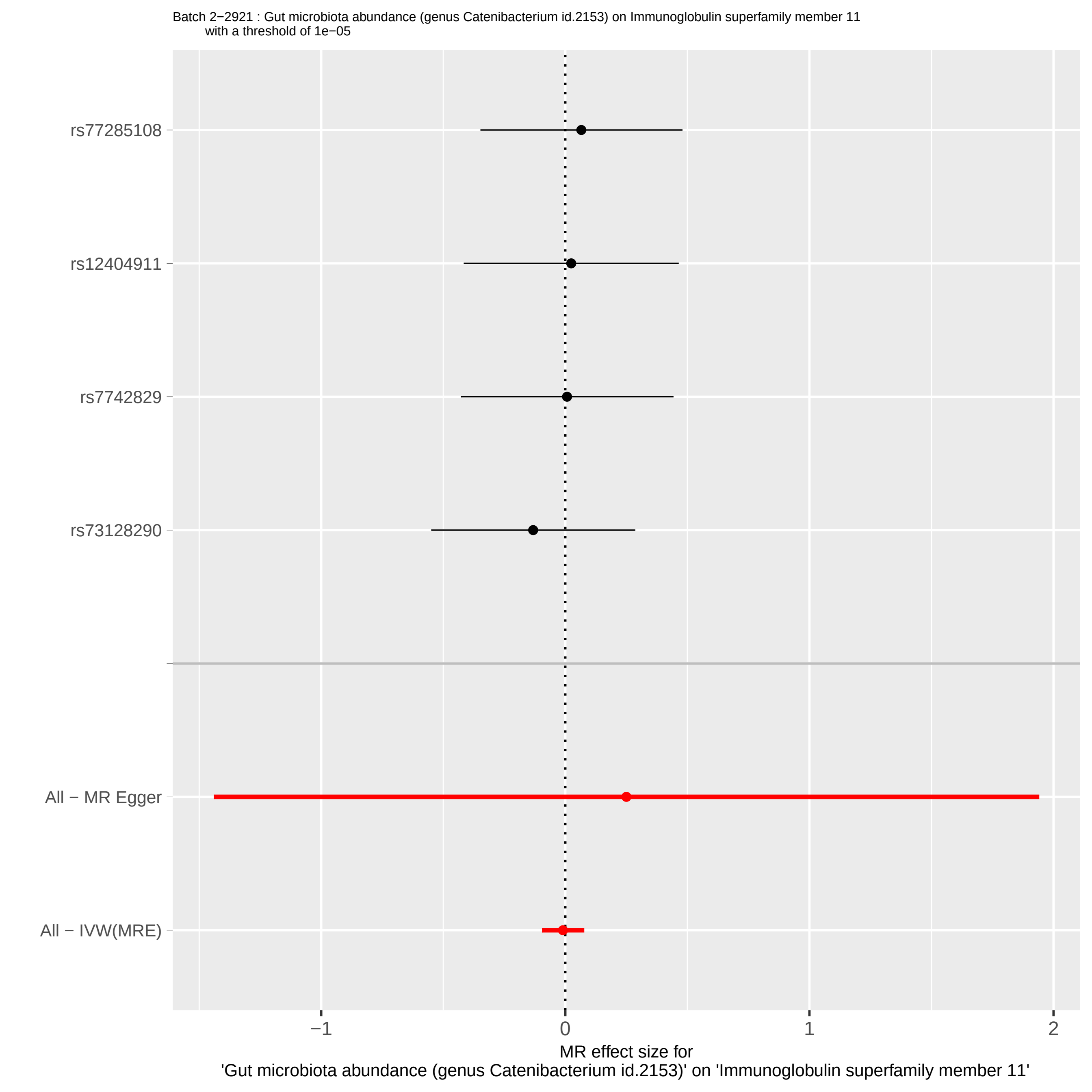
Batch 2−2921 : Gut microbiota abundance (genus Catenibacterium id.2153) on Immunoglobulin superfamily member 11
with a threshold of 1e−05
rs77285108
rs12404911
rs7742829
rs73128290
All − MR Egger
All − IVW(MRE)
−1
0
1
2
MR effect size for
'Gut microbiota abundance (genus Catenibacterium id.2153)' on 'Immunoglobulin superfamily member 11'

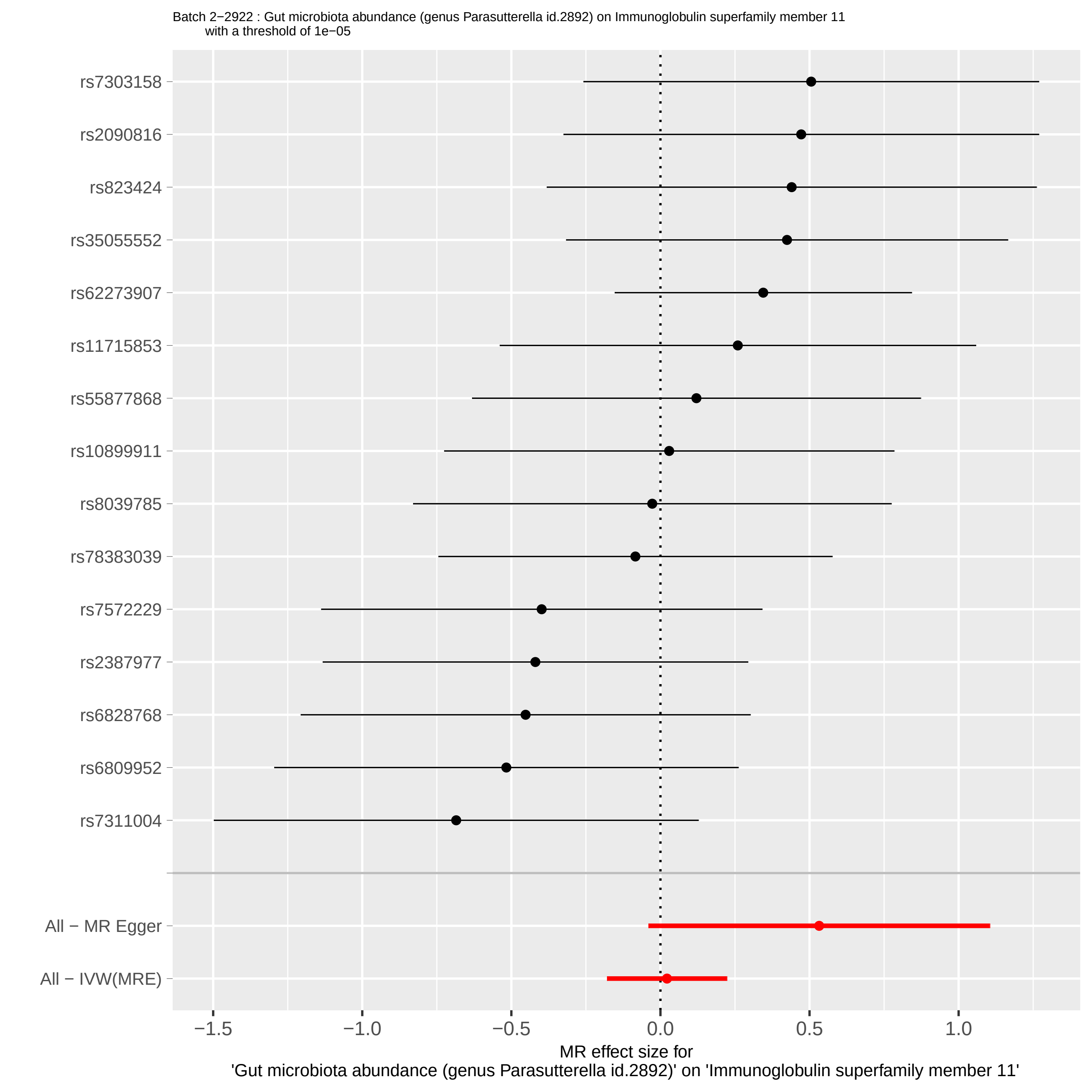
Batch 2−2922 : Gut microbiota abundance (genus Parasutterella id.2892) on Immunoglobulin superfamily member 11
with a threshold of 1e−05
rs7303158
rs2090816
rs823424
rs35055552
rs62273907
rs11715853
rs55877868
rs10899911
rs8039785
rs78383039
rs7572229
rs2387977
rs6828768
rs6809952
rs7311004
All − MR Egger
All − IVW(MRE)
−1.5
−1.0
−0.5
0.0
0.5
1.0
MR effect size for
'Gut microbiota abundance (genus Parasutterella id.2892)' on 'Immunoglobulin superfamily member 11'

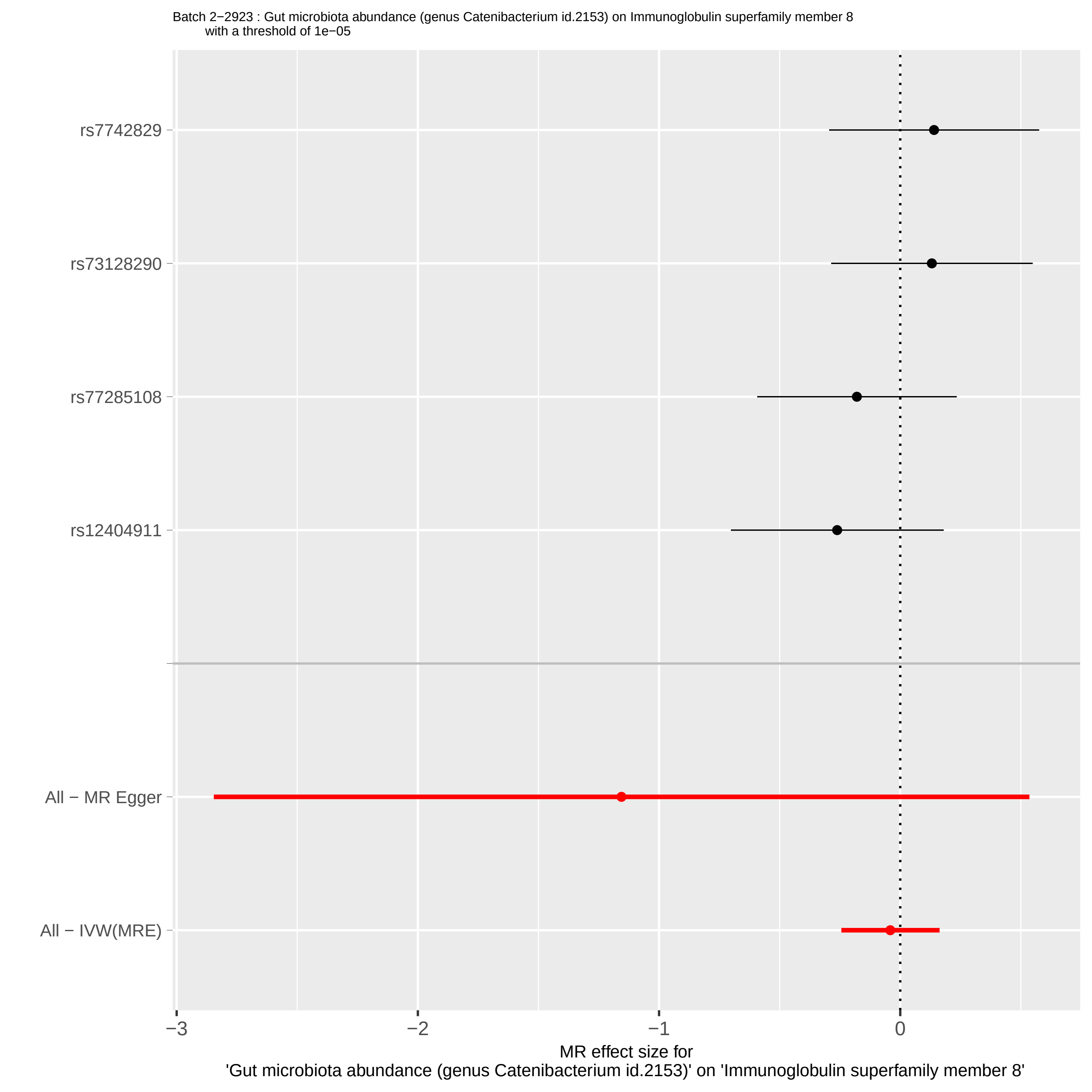
Batch 2−2923 : Gut microbiota abundance (genus Catenibacterium id.2153) on Immunoglobulin superfamily member 8
with a threshold of 1e−05
rs7742829
rs73128290
rs77285108
rs12404911
All − MR Egger
All − IVW(MRE)
−3
−2
−1
0
MR effect size for
'Gut microbiota abundance (genus Catenibacterium id.2153)' on 'Immunoglobulin superfamily member 8'

Batch 2−2924 : Gut microbiota abundance (genus Parasutterella id.2892) on Immunoglobulin superfamily member 8
with a threshold of 1e−05

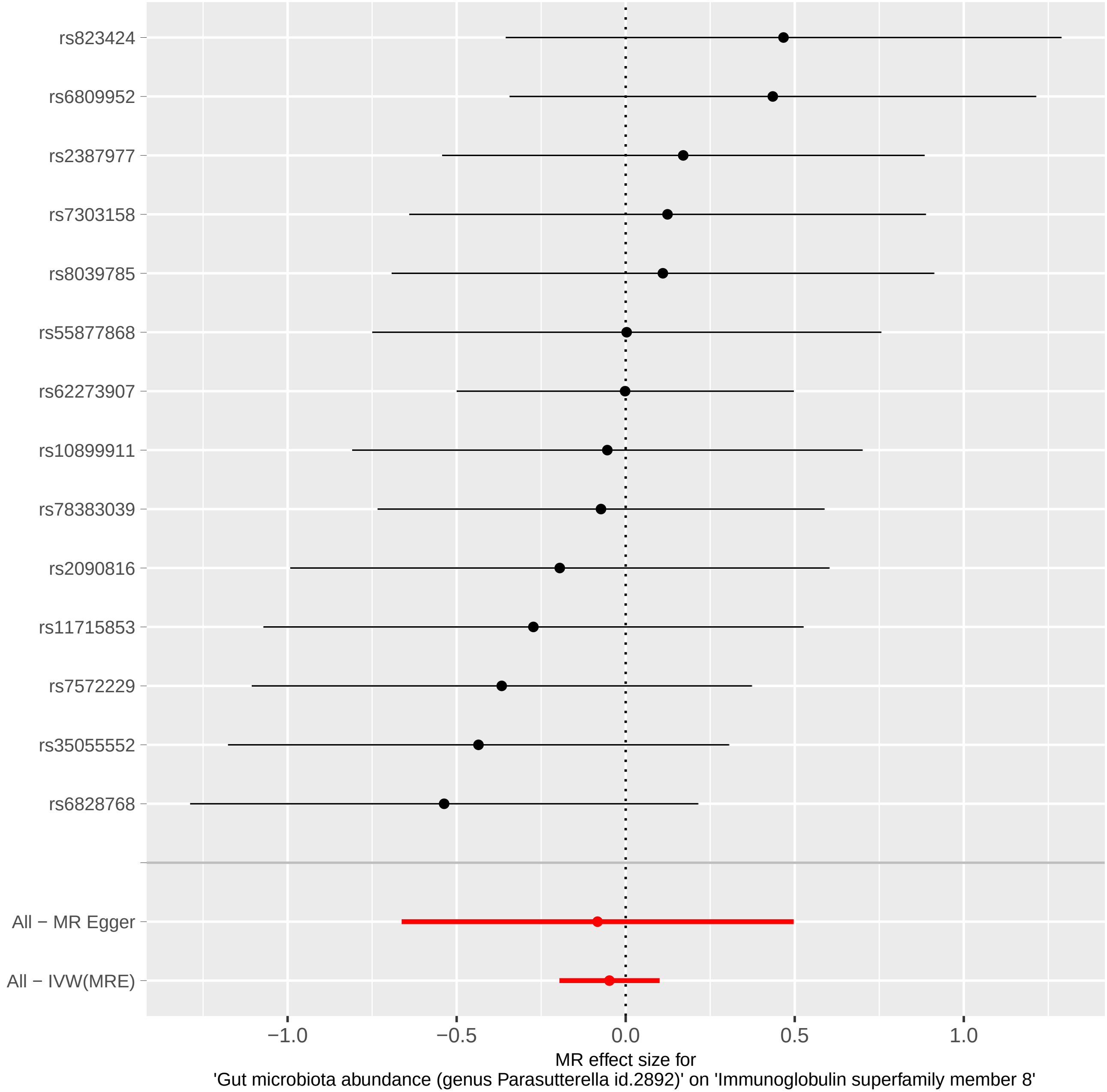

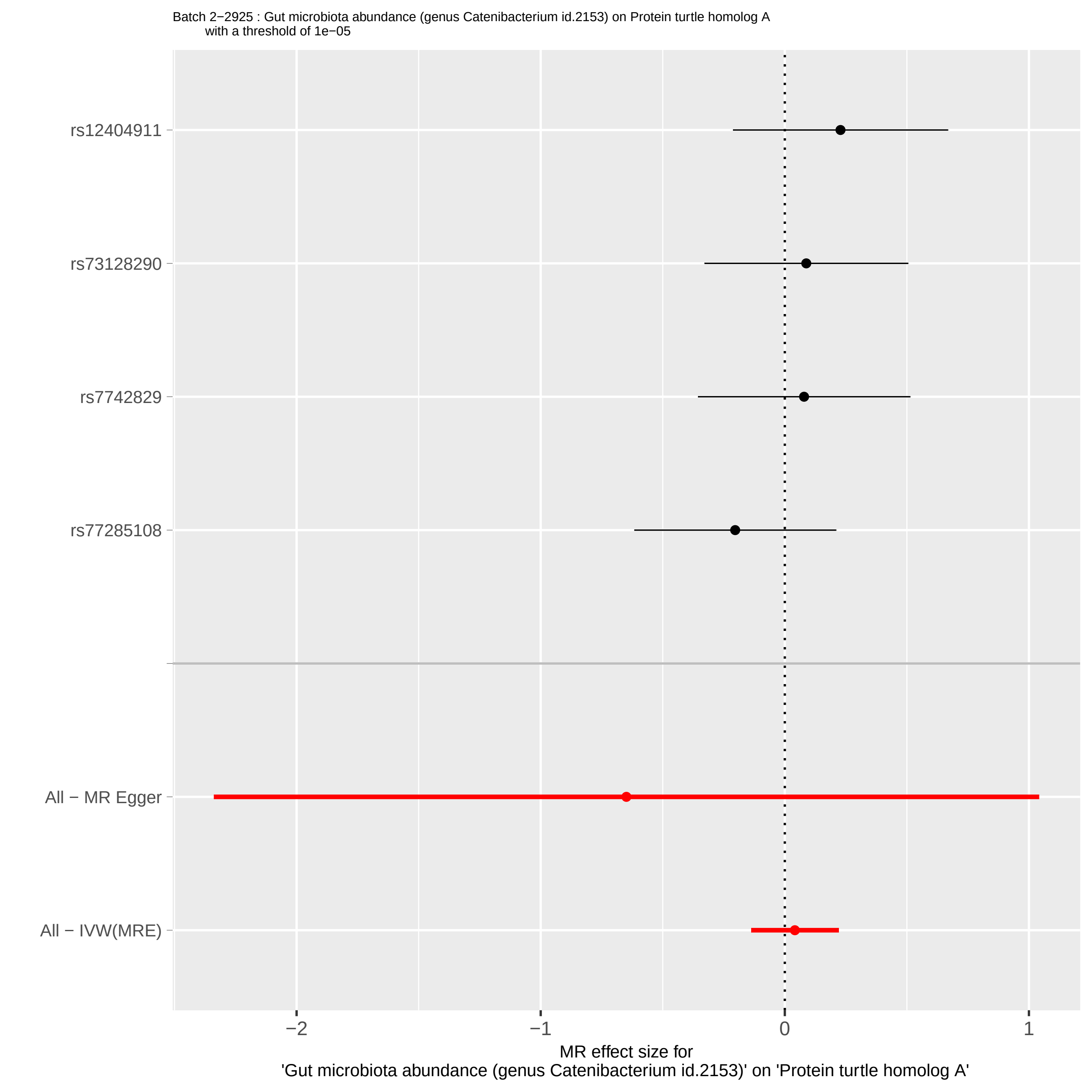

Batch 2−2925 : Gut microbiota abundance (genus Catenibacterium id.2153) on Protein turtle homolog A
with a threshold of 1e−05
rs12404911
rs73128290
rs7742829
rs77285108
All − MR Egger
All − IVW(MRE)
−2
−1
0
1
MR effect size for
'Gut microbiota abundance (genus Catenibacterium id.2153)' on 'Protein turtle homolog A'

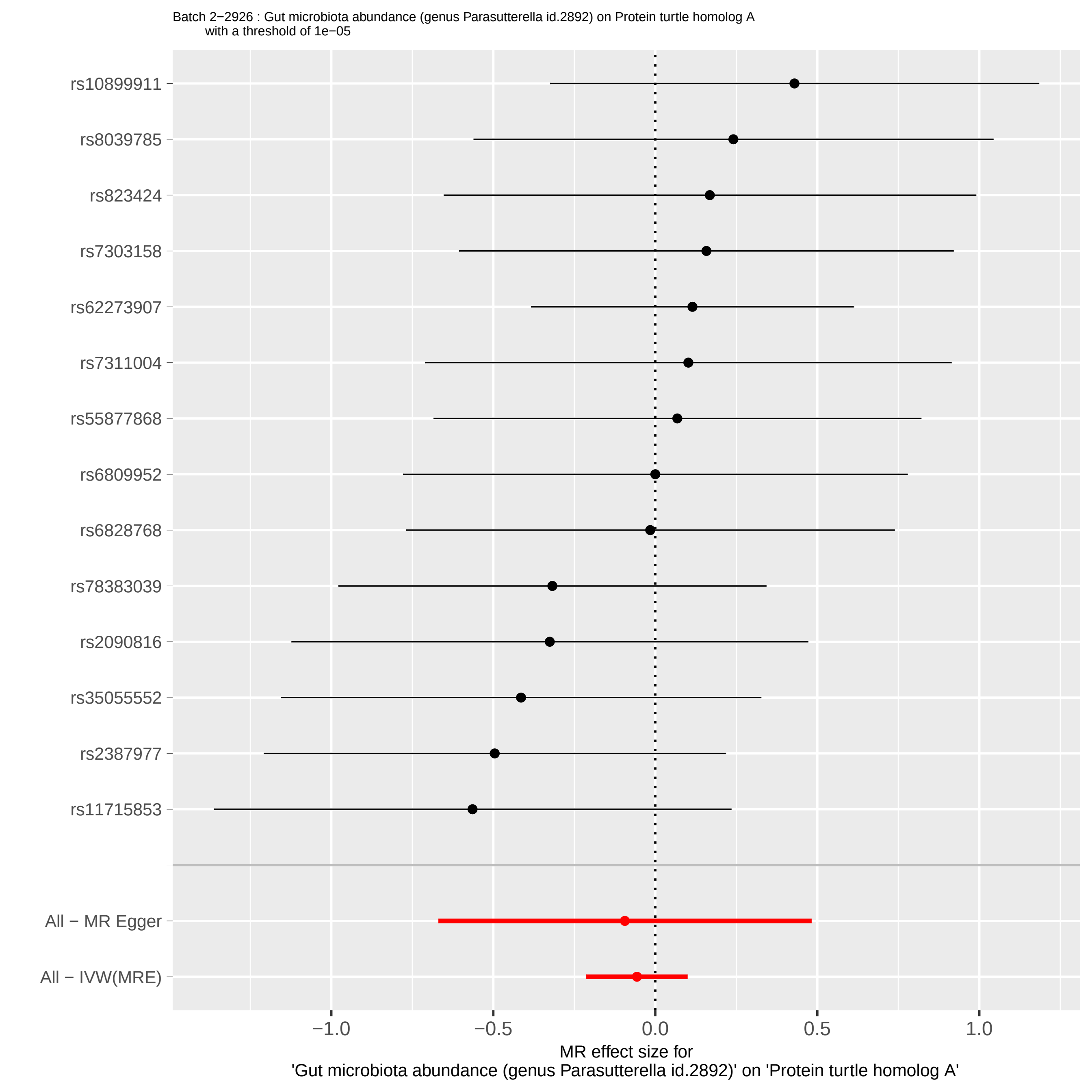

Batch 2−2926 : Gut microbiota abundance (genus Parasutterella id.2892) on Protein turtle homolog A with a threshold of 1e−05
rs10899911
rs8039785
rs823424
rs7303158
rs62273907
rs7311004
rs55877868
rs6809952
rs6828768
rs78383039
rs2090816
rs35055552
rs2387977
rs11715853
All − MR Egger
All − IVW(MRE)
−1.0
−0.5
0.0
0.5
1.0
MR effect size for
'Gut microbiota abundance (genus Parasutterella id.2892)' on 'Protein turtle homolog A'

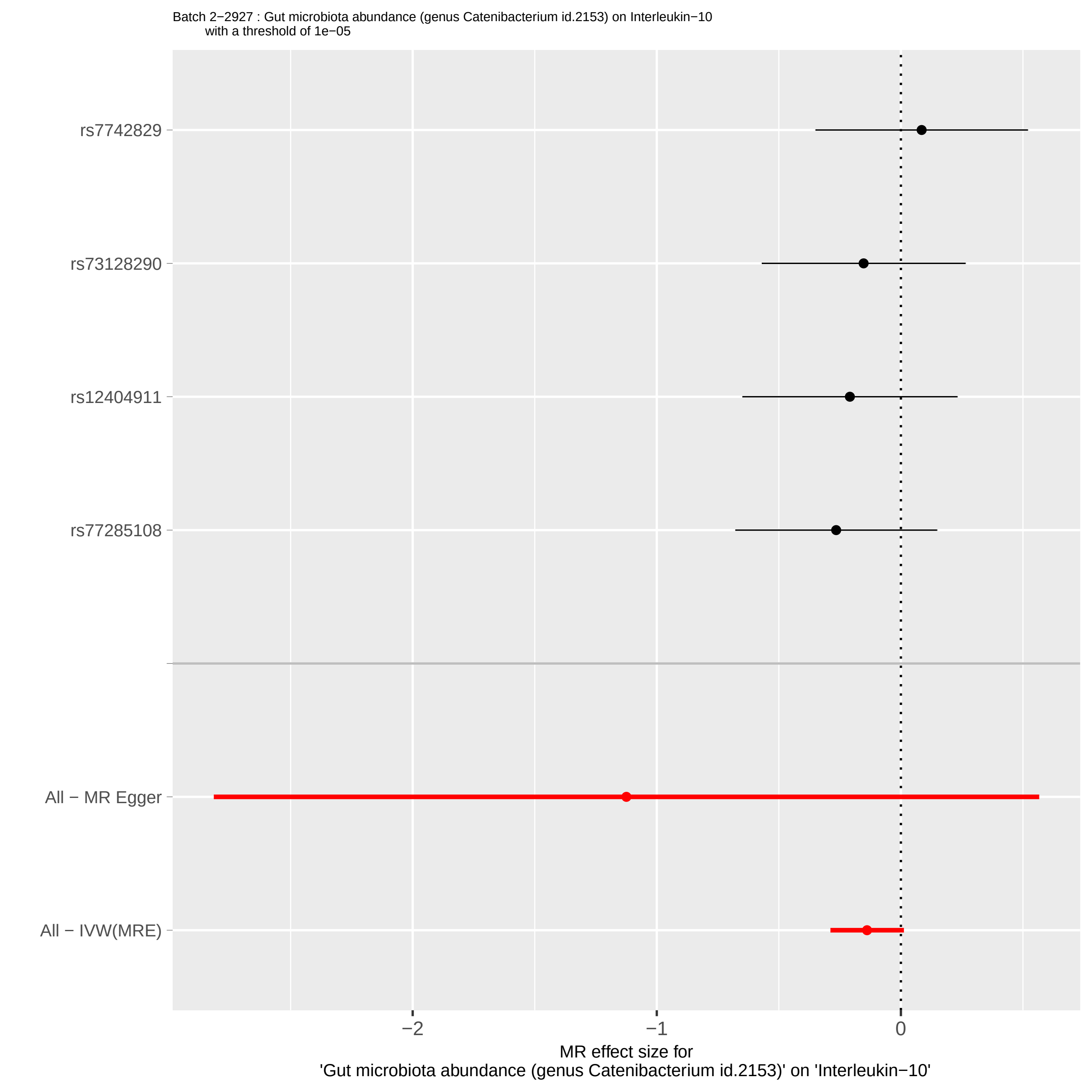
Batch 2−2927 : Gut microbiota abundance (genus Catenibacterium id.2153) on Interleukin−10
with a threshold of 1e−05
rs7742829
rs73128290
rs12404911
rs77285108
All − MR Egger
All − IVW(MRE)
−2
−1
0
MR effect size for
'Gut microbiota abundance (genus Catenibacterium id.2153)' on 'Interleukin−10'

Batch 2−2928 : Gut microbiota abundance (genus Parasutterella id.2892) on Interleukin−10
with a threshold of 1e−05

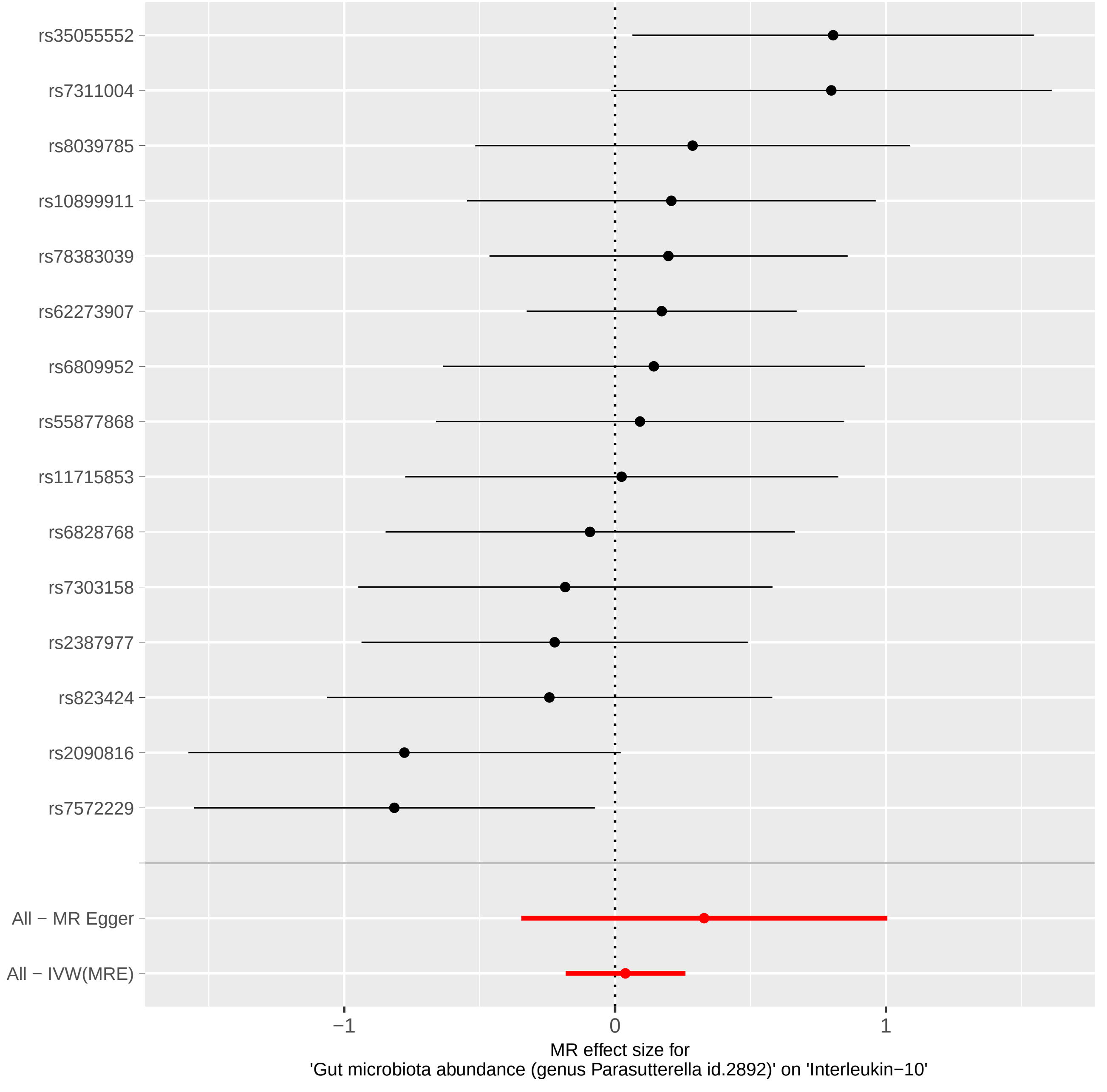

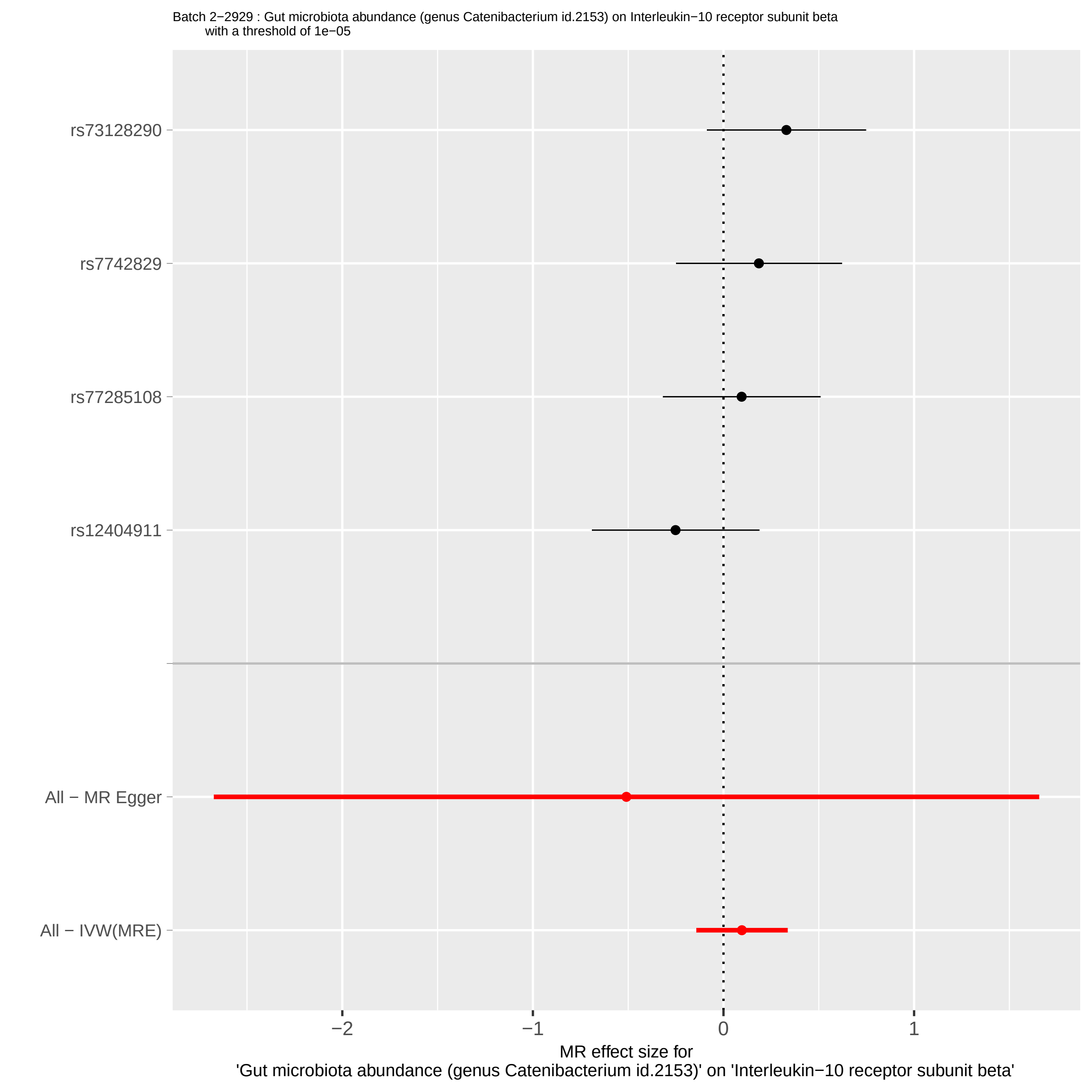
Batch 2−2929 : Gut microbiota abundance (genus Catenibacterium id.2153) on Interleukin−10 receptor subunit beta
with a threshold of 1e−05
rs73128290
rs7742829
rs77285108
rs12404911
All − MR Egger
All − IVW(MRE)
−2
−1
0
1
MR effect size for
'Gut microbiota abundance (genus Catenibacterium id.2153)' on 'Interleukin−10 receptor subunit beta'

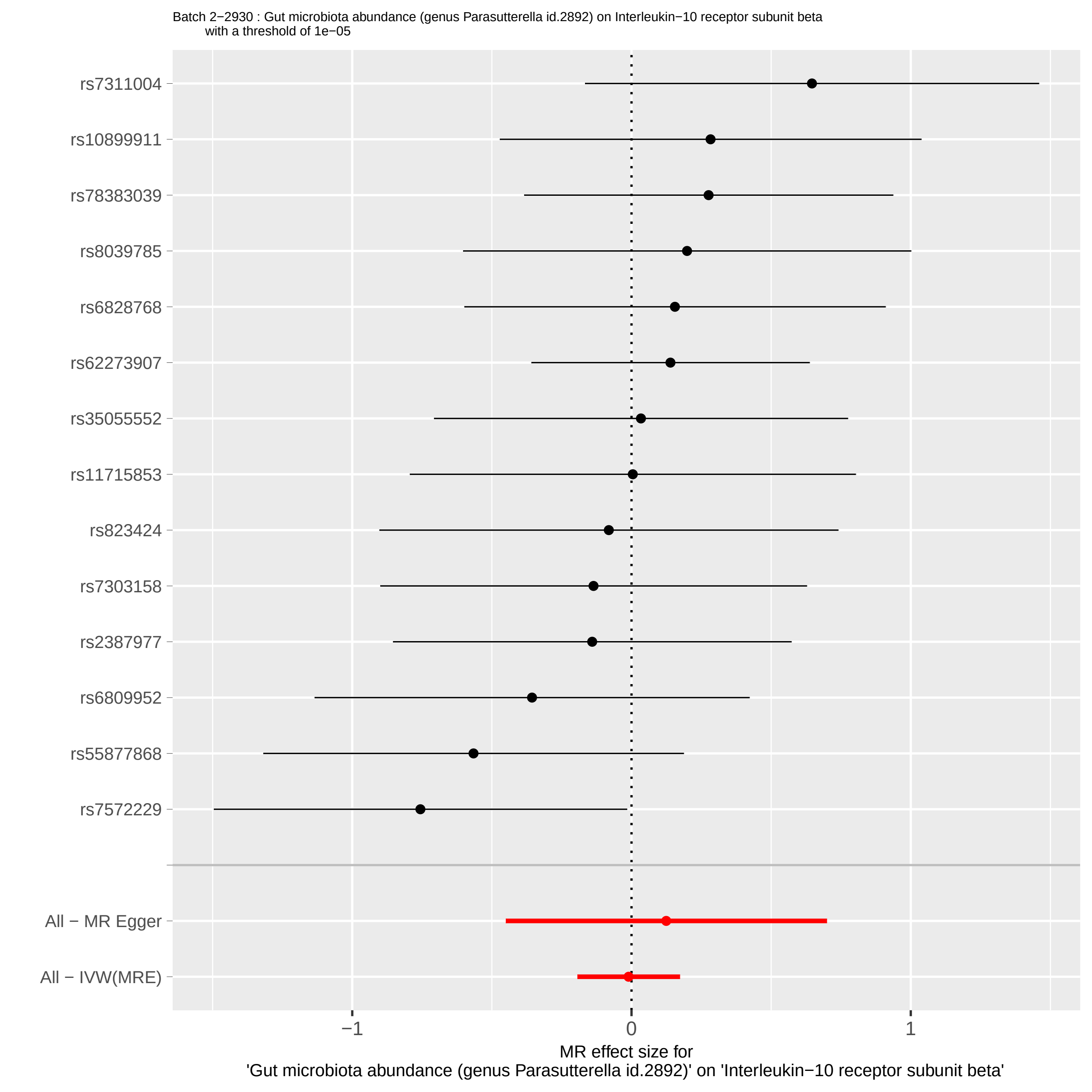
Batch 2−2930 : Gut microbiota abundance (genus Parasutterella id.2892) on Interleukin−10 receptor subunit beta
with a threshold of 1e−05
rs7311004
rs10899911
rs78383039
rs8039785
rs6828768
rs62273907
rs35055552
rs11715853
rs823424
rs7303158
rs2387977
rs6809952
rs55877868
rs7572229
All − MR Egger
All − IVW(MRE)
−1
0
1
MR effect size for
'Gut microbiota abundance (genus Parasutterella id.2892)' on 'Interleukin−10 receptor subunit beta'

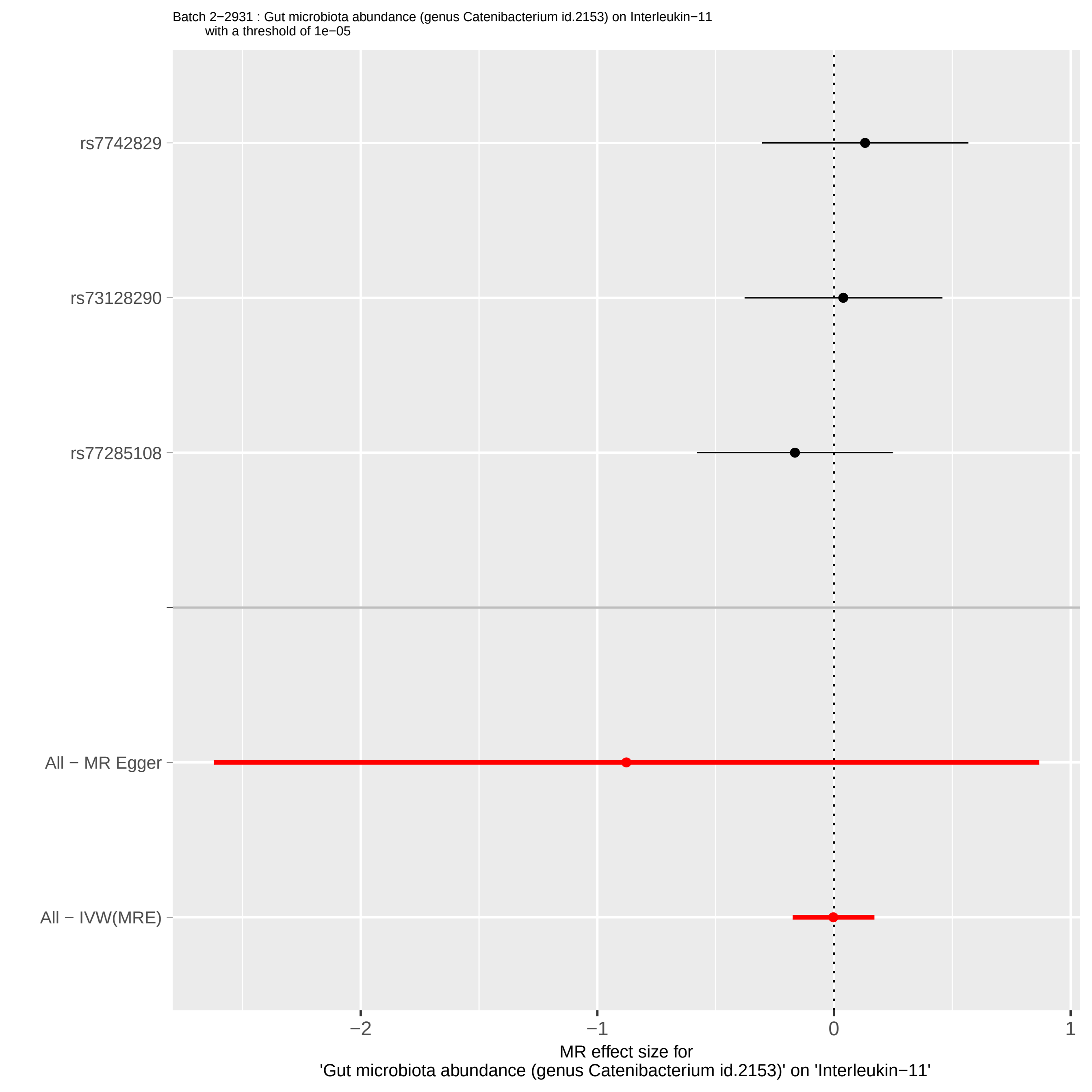

Batch 2−2931 : Gut microbiota abundance (genus Catenibacterium id.2153) on Interleukin−11
with a threshold of 1e−05
rs7742829
rs73128290
rs77285108
All − MR Egger
All − IVW(MRE)
−2
−1
0
1
MR effect size for
'Gut microbiota abundance (genus Catenibacterium id.2153)' on 'Interleukin−11'

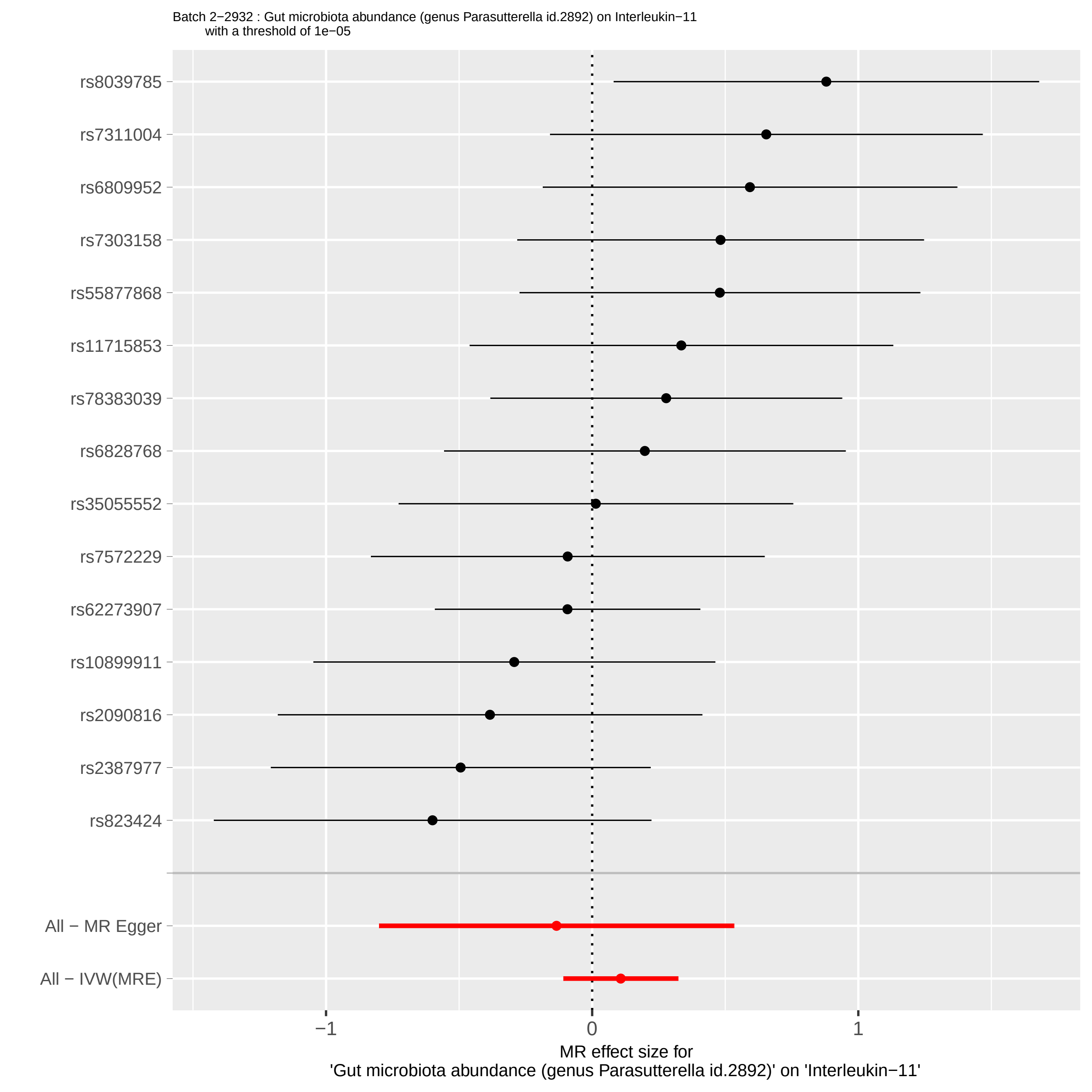
Batch 2−2932 : Gut microbiota abundance (genus Parasutterella id.2892) on Interleukin−11
with a threshold of 1e−05
rs8039785
rs7311004
rs6809952
rs7303158
rs55877868
rs11715853
rs78383039
rs6828768
rs35055552
rs7572229
rs62273907
rs10899911
rs2090816
rs2387977
rs823424
All − MR Egger
All − IVW(MRE)
−1
0
1
MR effect size for
'Gut microbiota abundance (genus Parasutterella id.2892)' on 'Interleukin−11'

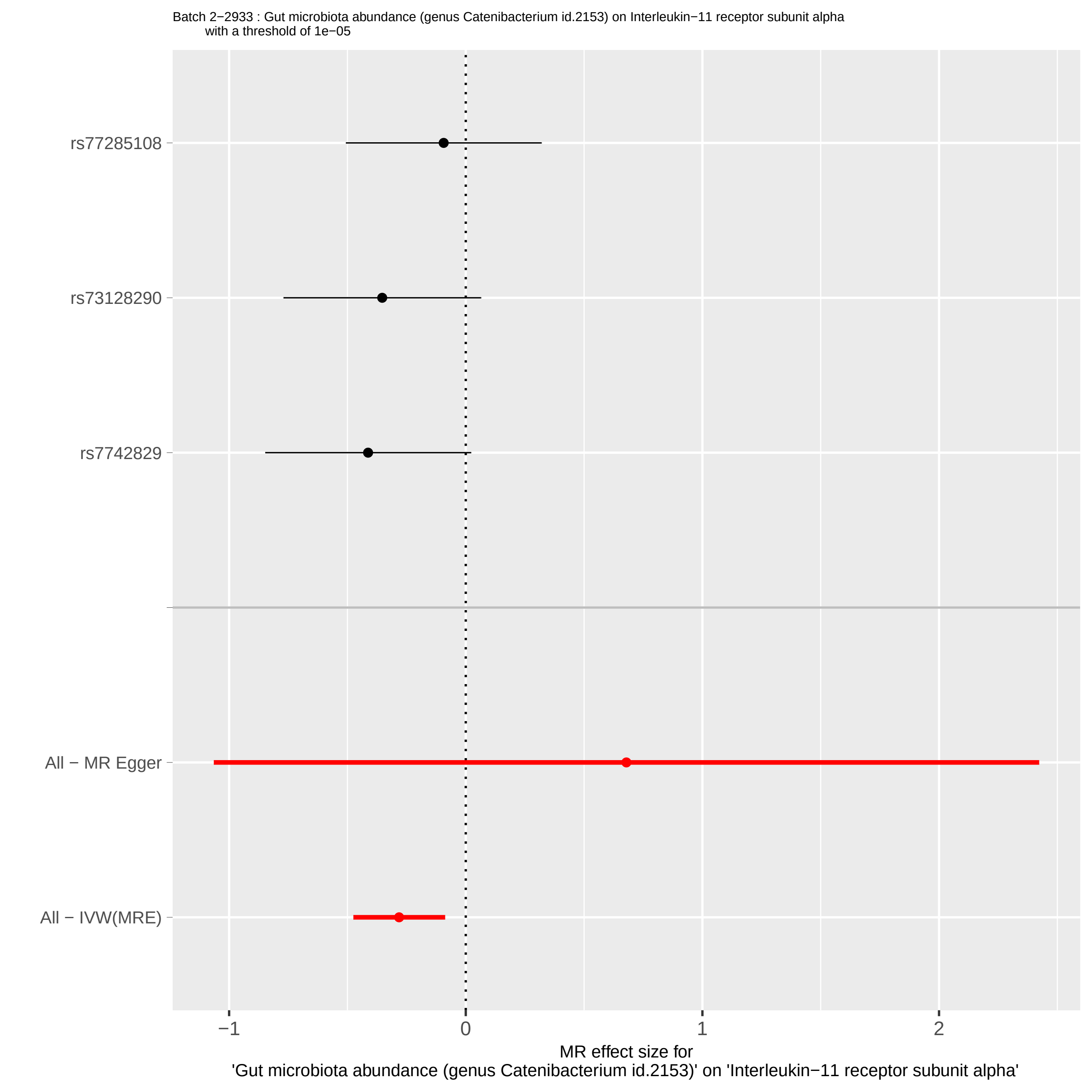
Batch 2−2933 : Gut microbiota abundance (genus Catenibacterium id.2153) on Interleukin−11 receptor subunit alpha
with a threshold of 1e−05
rs77285108
rs73128290
rs7742829
All − MR Egger
All − IVW(MRE)
−1
0
1
2
MR effect size for
'Gut microbiota abundance (genus Catenibacterium id.2153)' on 'Interleukin−11 receptor subunit alpha'

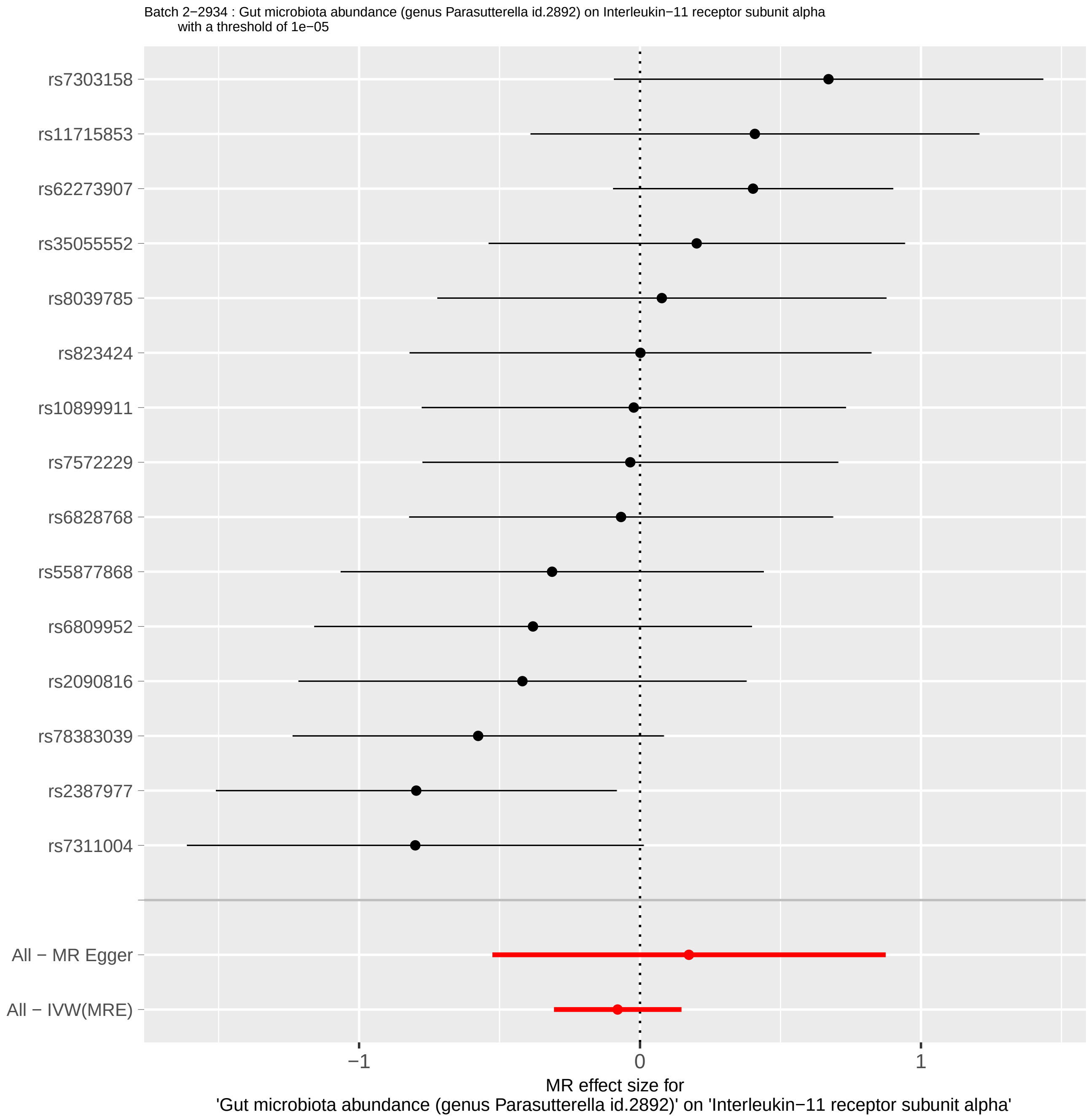

Batch 2−2934 : Gut microbiota abundance (genus Parasutterella id.2892) on Interleukin−11 receptor subunit alpha
with a threshold of 1e−05
rs7303158
rs11715853
rs62273907
rs35055552
rs8039785
rs823424
rs10899911
rs7572229
rs6828768
rs55877868
rs6809952
rs2090816
rs78383039
rs2387977
rs7311004
All − MR Egger
All − IVW(MRE)
−1
0
1
MR effect size for
'Gut microbiota abundance (genus Parasutterella id.2892)' on 'Interleukin−11 receptor subunit alpha'

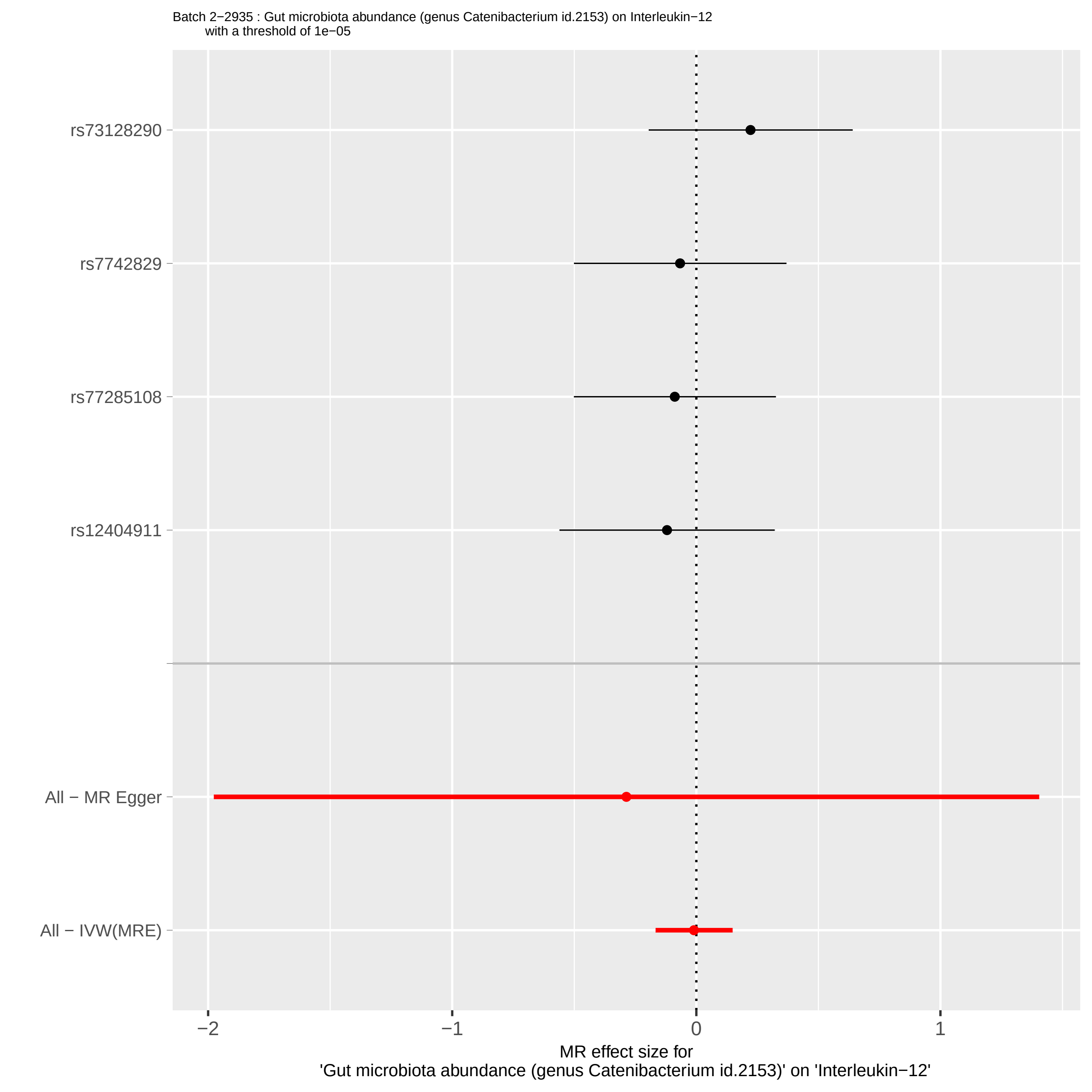
Batch 2−2935 : Gut microbiota abundance (genus Catenibacterium id.2153) on Interleukin−12
with a threshold of 1e−05
rs73128290
rs7742829
rs77285108
rs12404911
All − MR Egger
All − IVW(MRE)
−2
−1
0
1
MR effect size for
'Gut microbiota abundance (genus Catenibacterium id.2153)' on 'Interleukin−12'

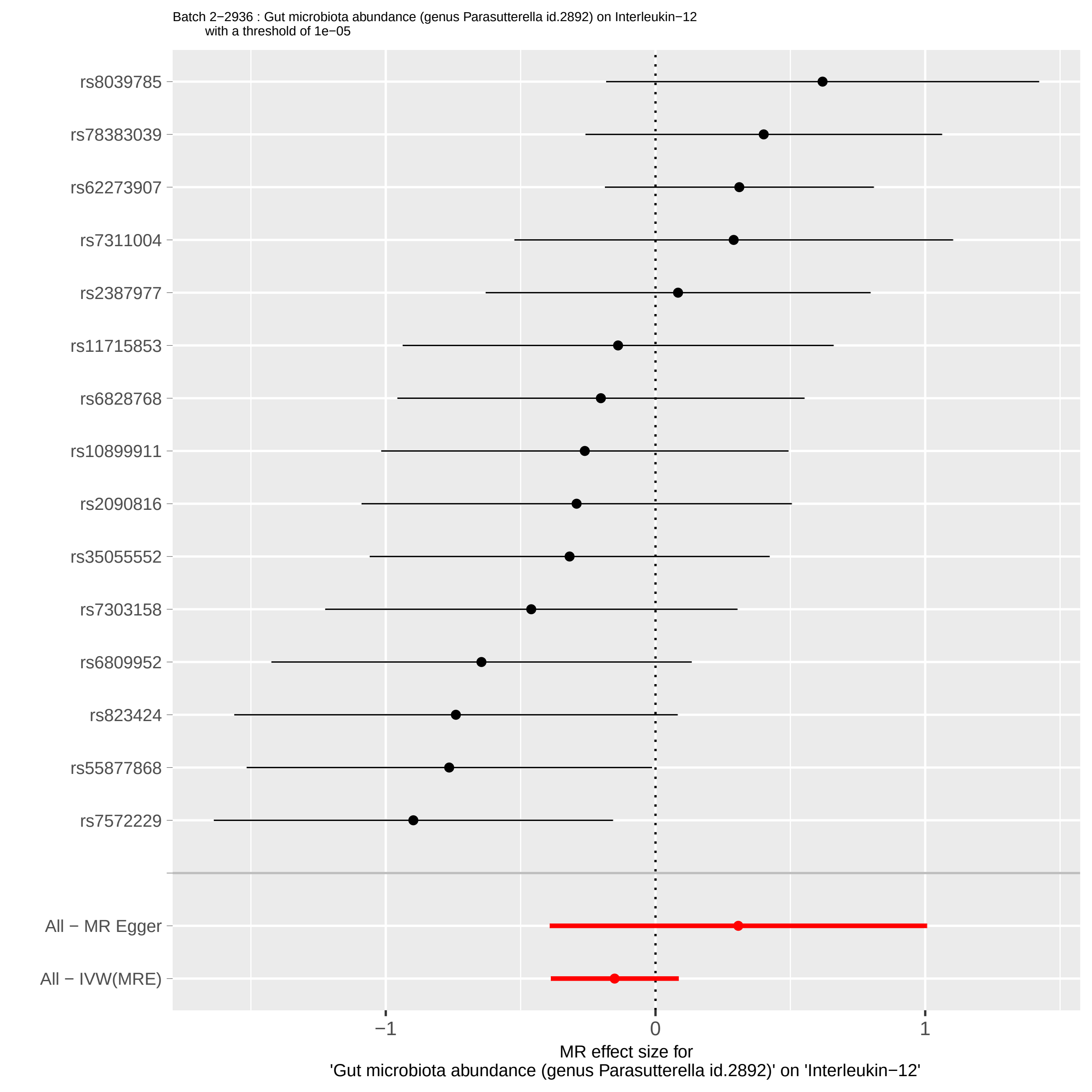
Batch 2−2936 : Gut microbiota abundance (genus Parasutterella id.2892) on Interleukin−12
with a threshold of 1e−05
rs8039785
rs78383039
rs62273907
rs7311004
rs2387977
rs11715853
rs6828768
rs10899911
rs2090816
rs35055552
rs7303158
rs6809952
rs823424
rs55877868
rs7572229
All − MR Egger
All − IVW(MRE)
−1
0
1
MR effect size for
'Gut microbiota abundance (genus Parasutterella id.2892)' on 'Interleukin−12'

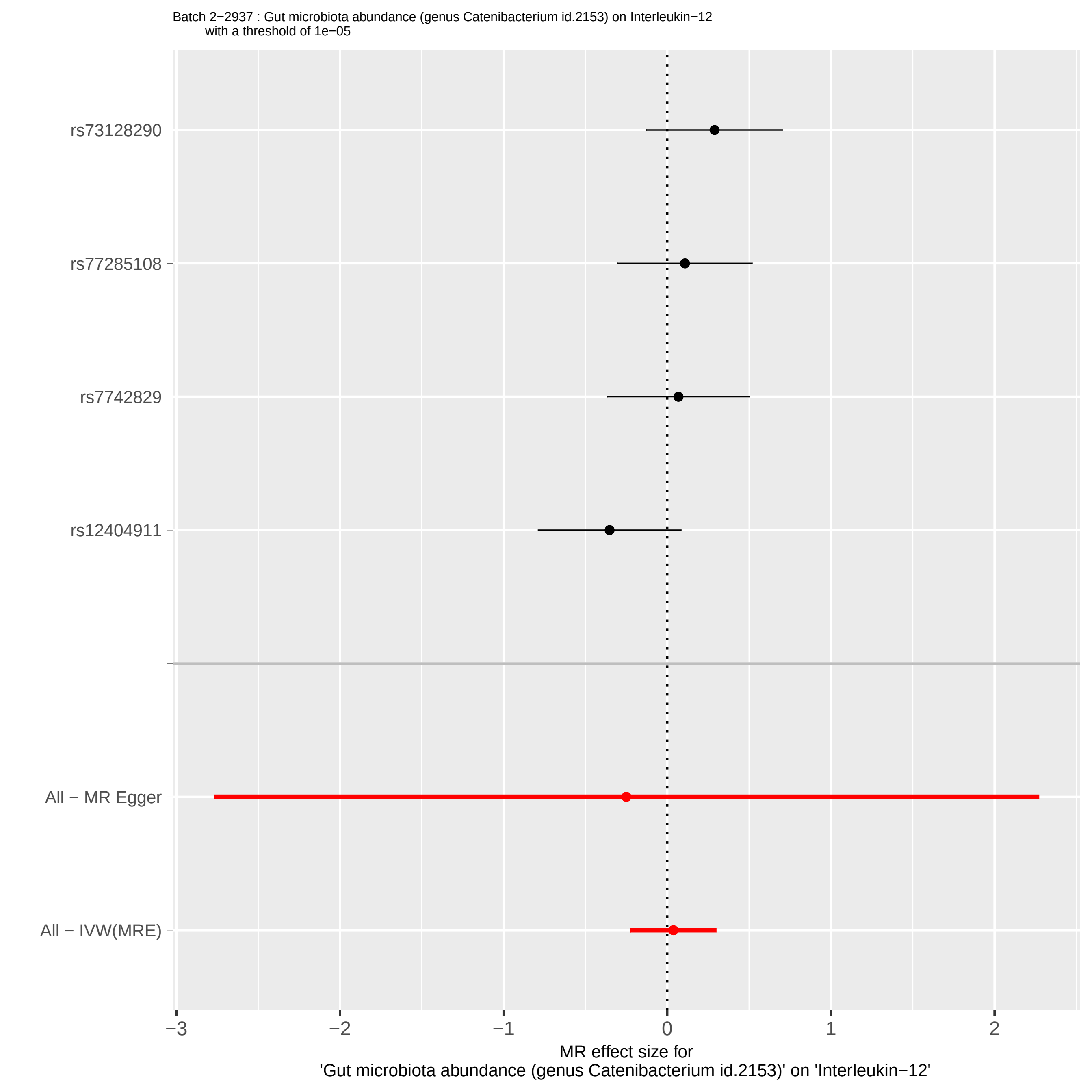
Batch 2−2937 : Gut microbiota abundance (genus Catenibacterium id.2153) on Interleukin−12
with a threshold of 1e−05
rs73128290
rs77285108
rs7742829
rs12404911
All − MR Egger
All − IVW(MRE)
−3
−2
−1
0
1
2
MR effect size for
'Gut microbiota abundance (genus Catenibacterium id.2153)' on 'Interleukin−12'

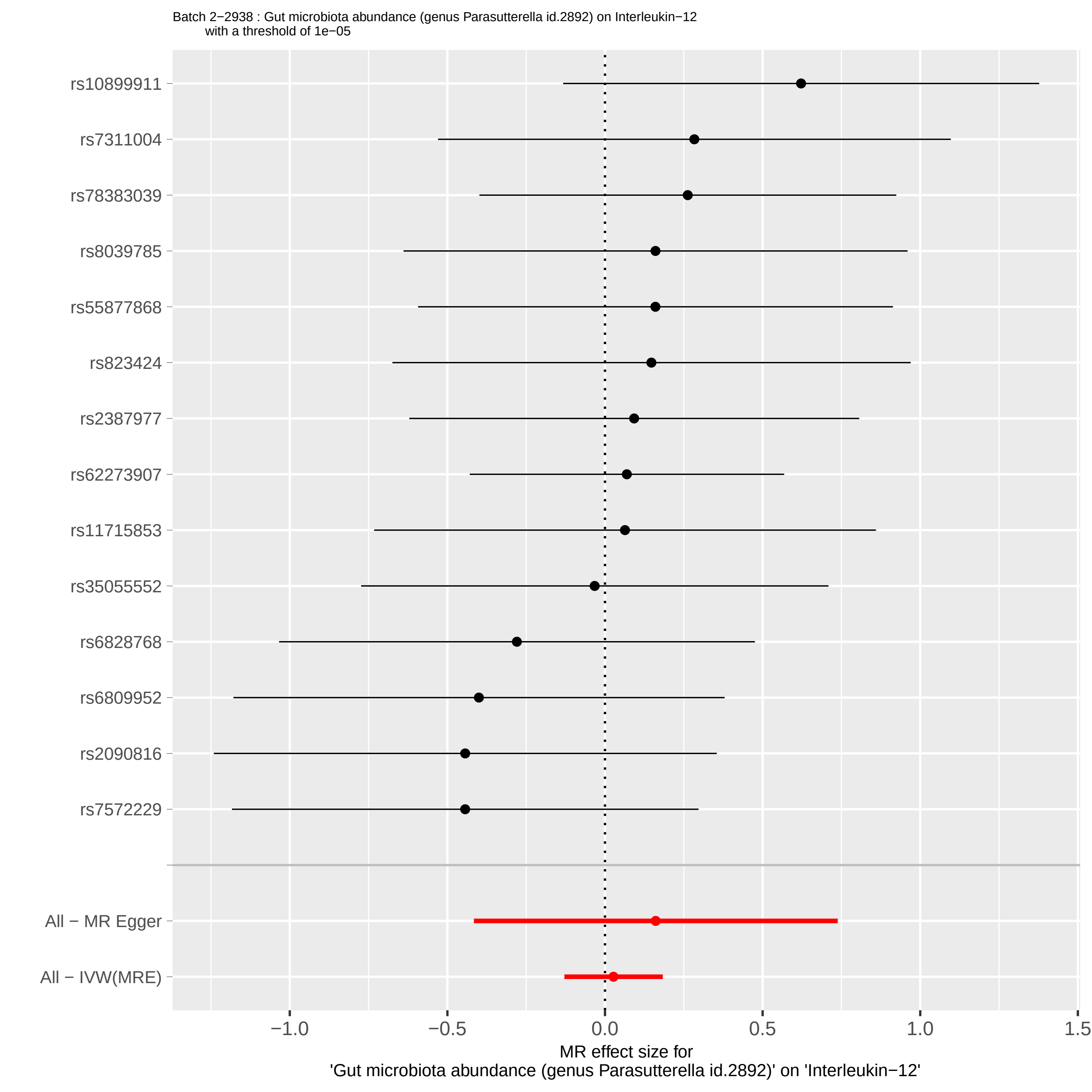

Batch 2−2938 : Gut microbiota abundance (genus Parasutterella id.2892) on Interleukin−12
with a threshold of 1e−05
rs10899911
rs7311004
rs78383039
rs8039785
rs55877868
rs823424
rs2387977
rs62273907
rs11715853
rs35055552
rs6828768
rs6809952
rs2090816
rs7572229
All − MR Egger
All − IVW(MRE)
−1.0
−0.5
0.0
0.5
1.0
1.5
MR effect size for
'Gut microbiota abundance (genus Parasutterella id.2892)' on 'Interleukin−12'

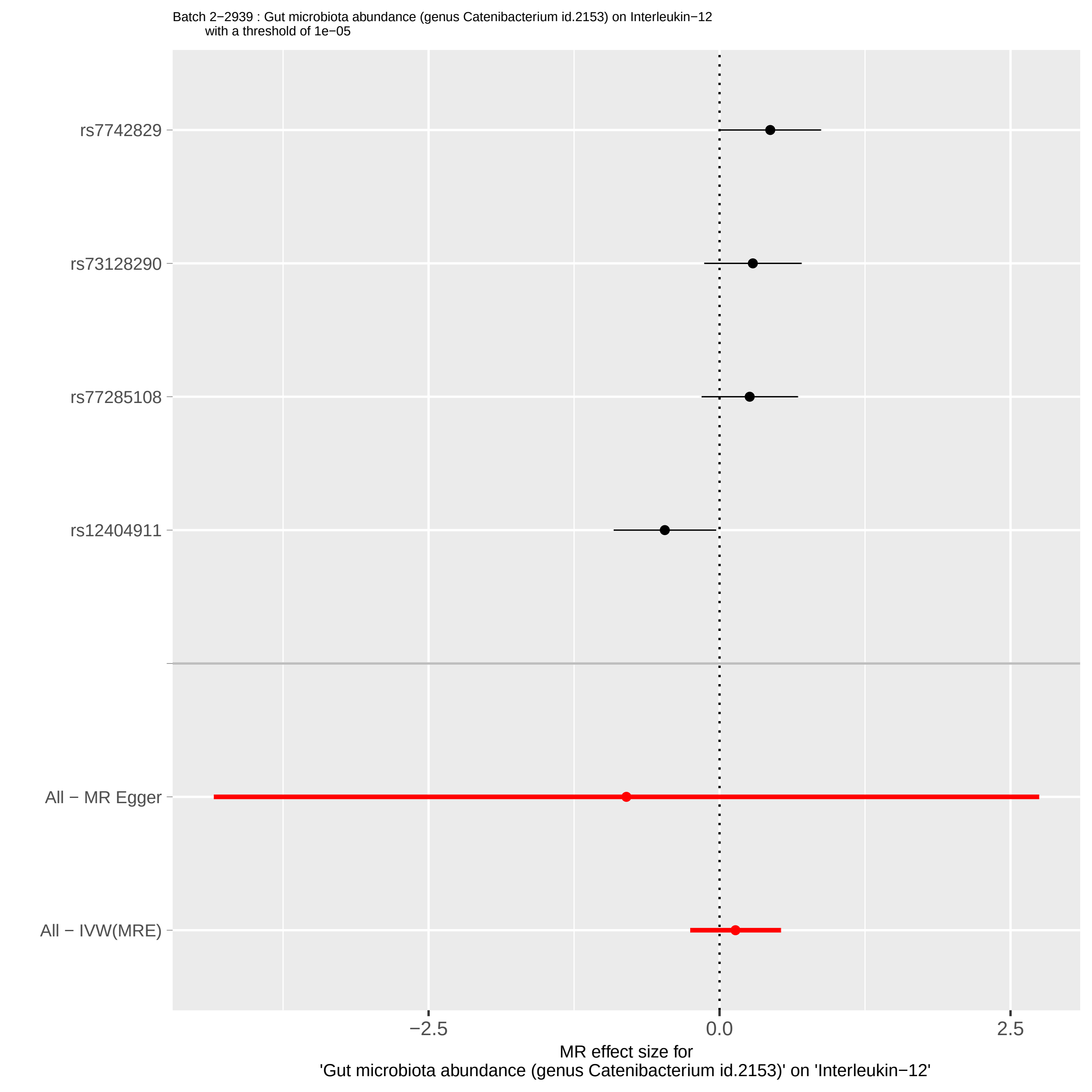
Batch 2−2939 : Gut microbiota abundance (genus Catenibacterium id.2153) on Interleukin−12
with a threshold of 1e−05
rs7742829
rs73128290
rs77285108
rs12404911
All − MR Egger
All − IVW(MRE)
−2.5
0.0
2.5
MR effect size for
'Gut microbiota abundance (genus Catenibacterium id.2153)' on 'Interleukin−12'

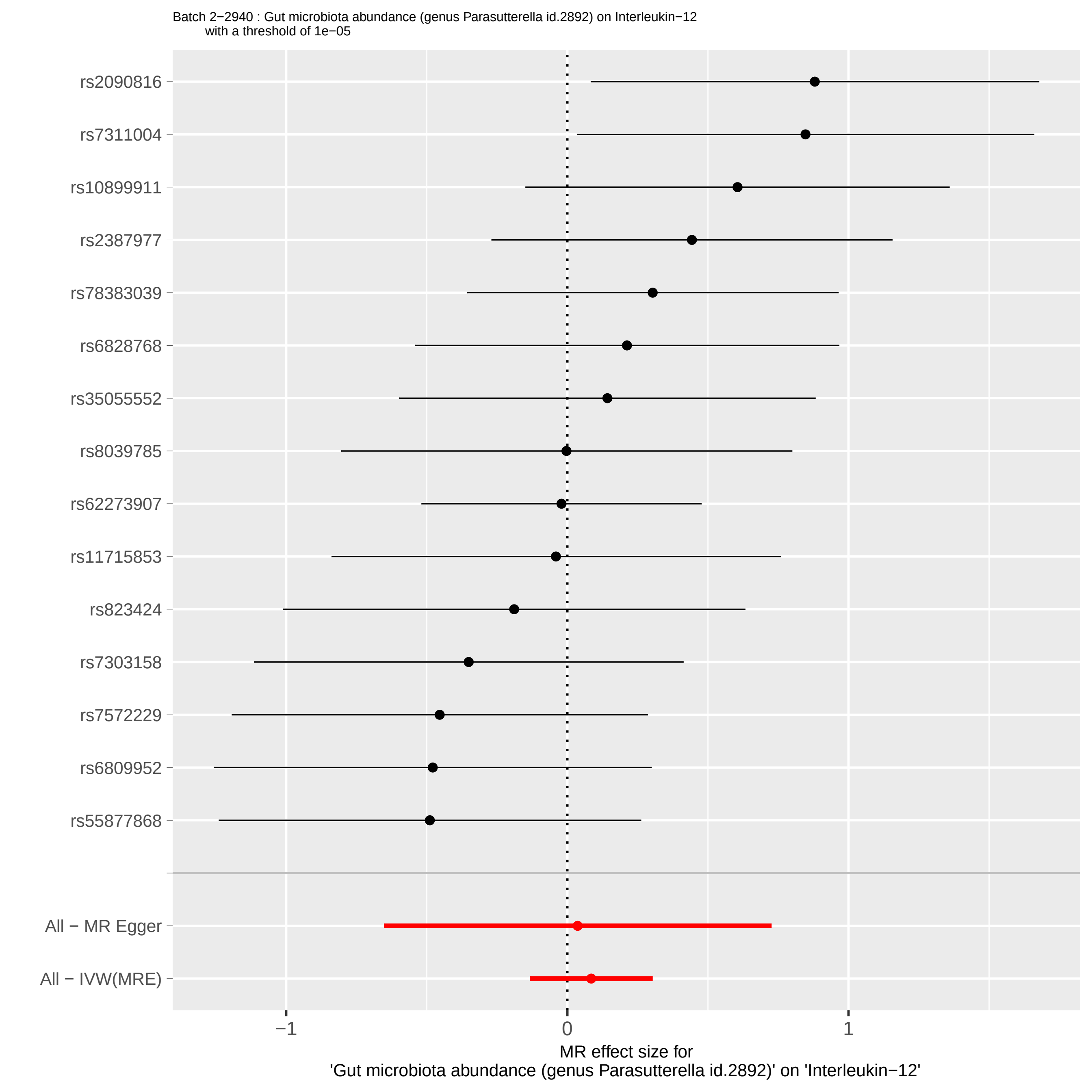
Batch 2−2940 : Gut microbiota abundance (genus Parasutterella id.2892) on Interleukin−12
with a threshold of 1e−05
rs2090816
rs7311004
rs10899911
rs2387977
rs78383039
rs6828768
rs35055552
rs8039785
rs62273907
rs11715853
rs823424
rs7303158
rs7572229
rs6809952
rs55877868
All − MR Egger
All − IVW(MRE)
−1
0
1
MR effect size for
'Gut microbiota abundance (genus Parasutterella id.2892)' on 'Interleukin−12'

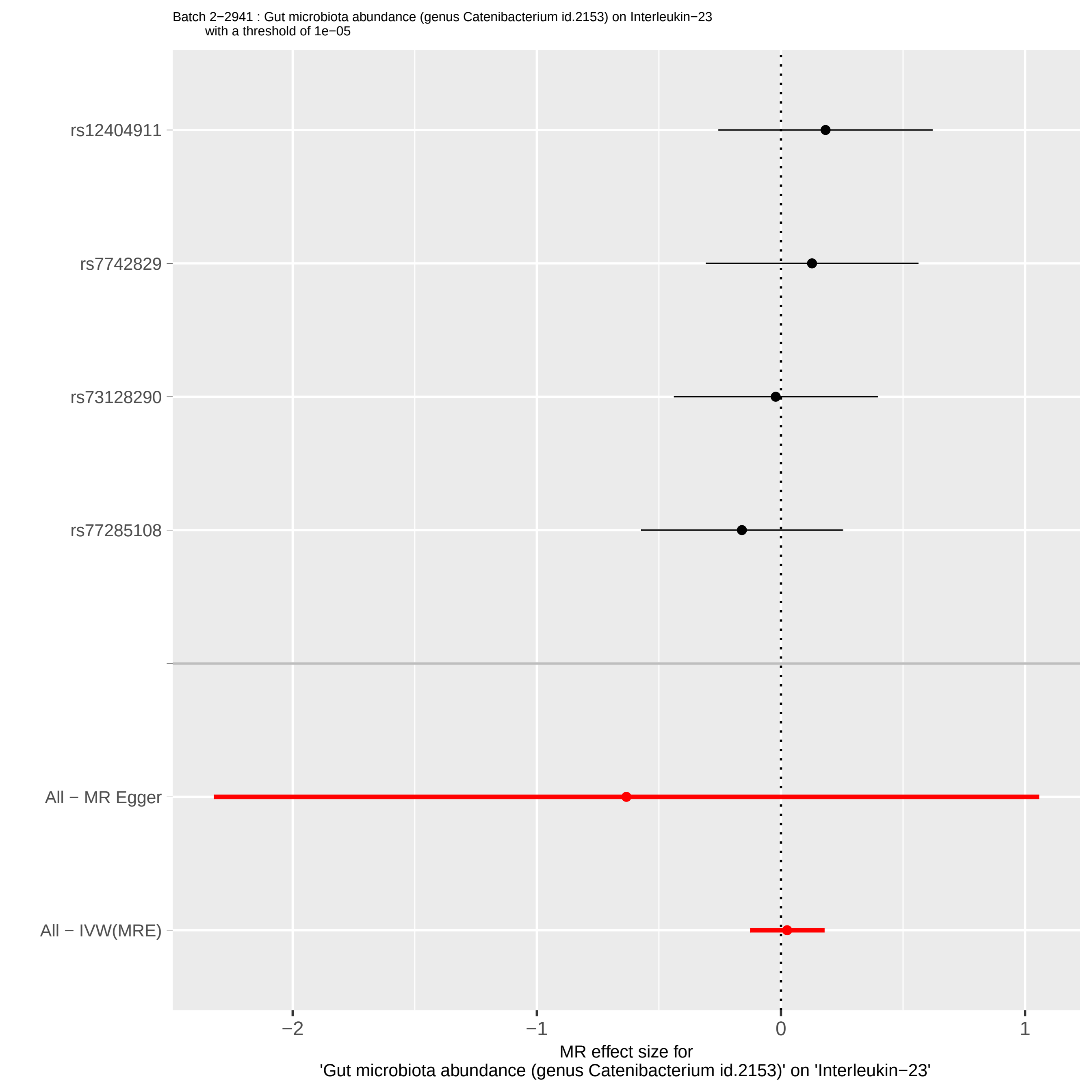

Batch 2−2941 : Gut microbiota abundance (genus Catenibacterium id.2153) on Interleukin−23
with a threshold of 1e−05
rs12404911
rs7742829
rs73128290
rs77285108
All − MR Egger
All − IVW(MRE)
−2
−1
0
1
MR effect size for
'Gut microbiota abundance (genus Catenibacterium id.2153)' on 'Interleukin−23'

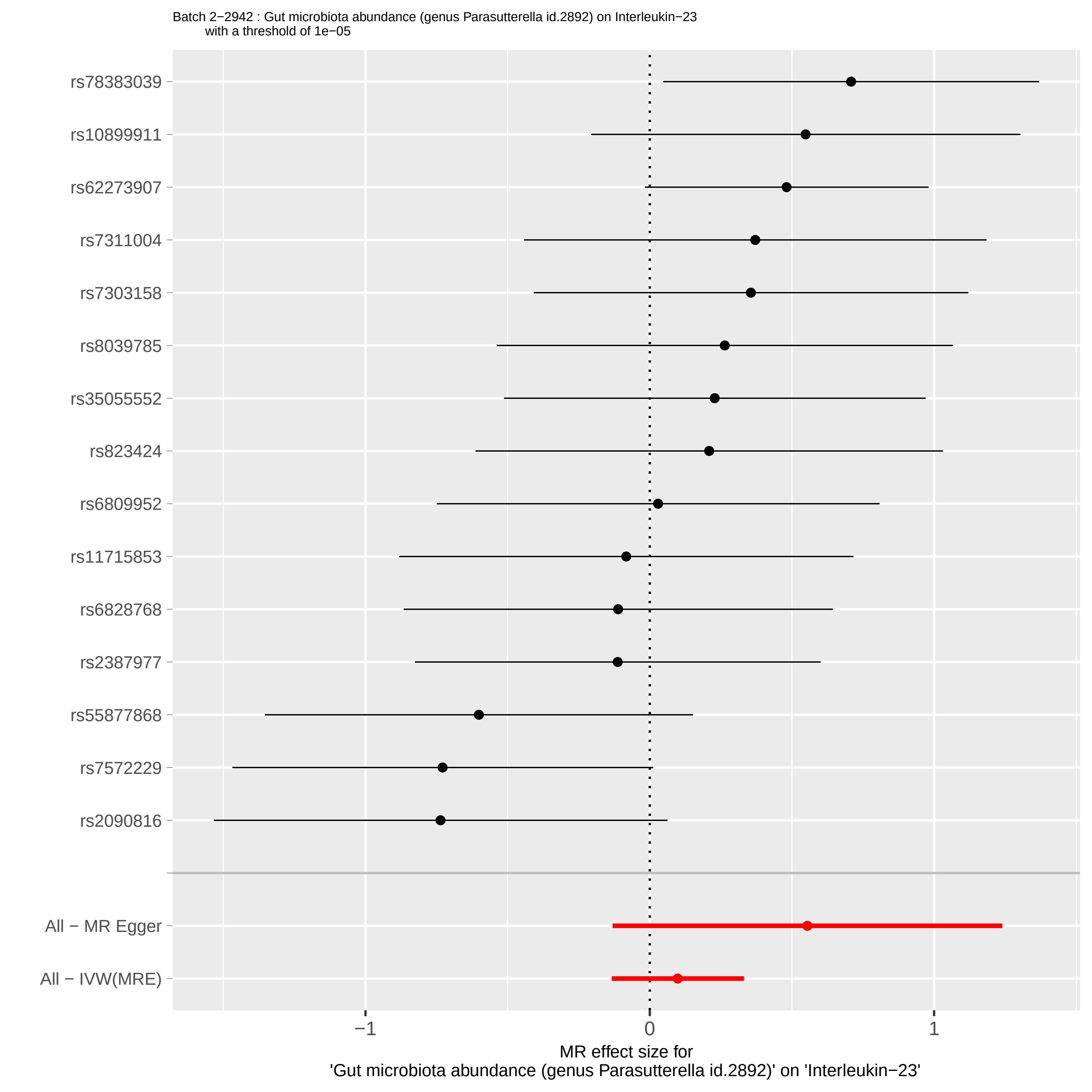

Batch 2−2942 : Gut microbiota abundance (genus Parasutterella id.2892) on Interleukin−23
with a threshold of 1e−05
rs78383039
rs10899911
rs62273907
rs7311004
rs7303158
rs8039785
rs35055552
rs823424
rs6809952
rs11715853
rs6828768
rs2387977
rs55877868
rs7572229
rs2090816
All − MR Egger
All − IVW(MRE)
−1
0
1
MR effect size for
'Gut microbiota abundance (genus Parasutterella id.2892)' on 'Interleukin−23'

Batch 2−2943 : Gut microbiota abundance (genus Catenibacterium id.2153) on Interleukin−23
with a threshold of 1e−05

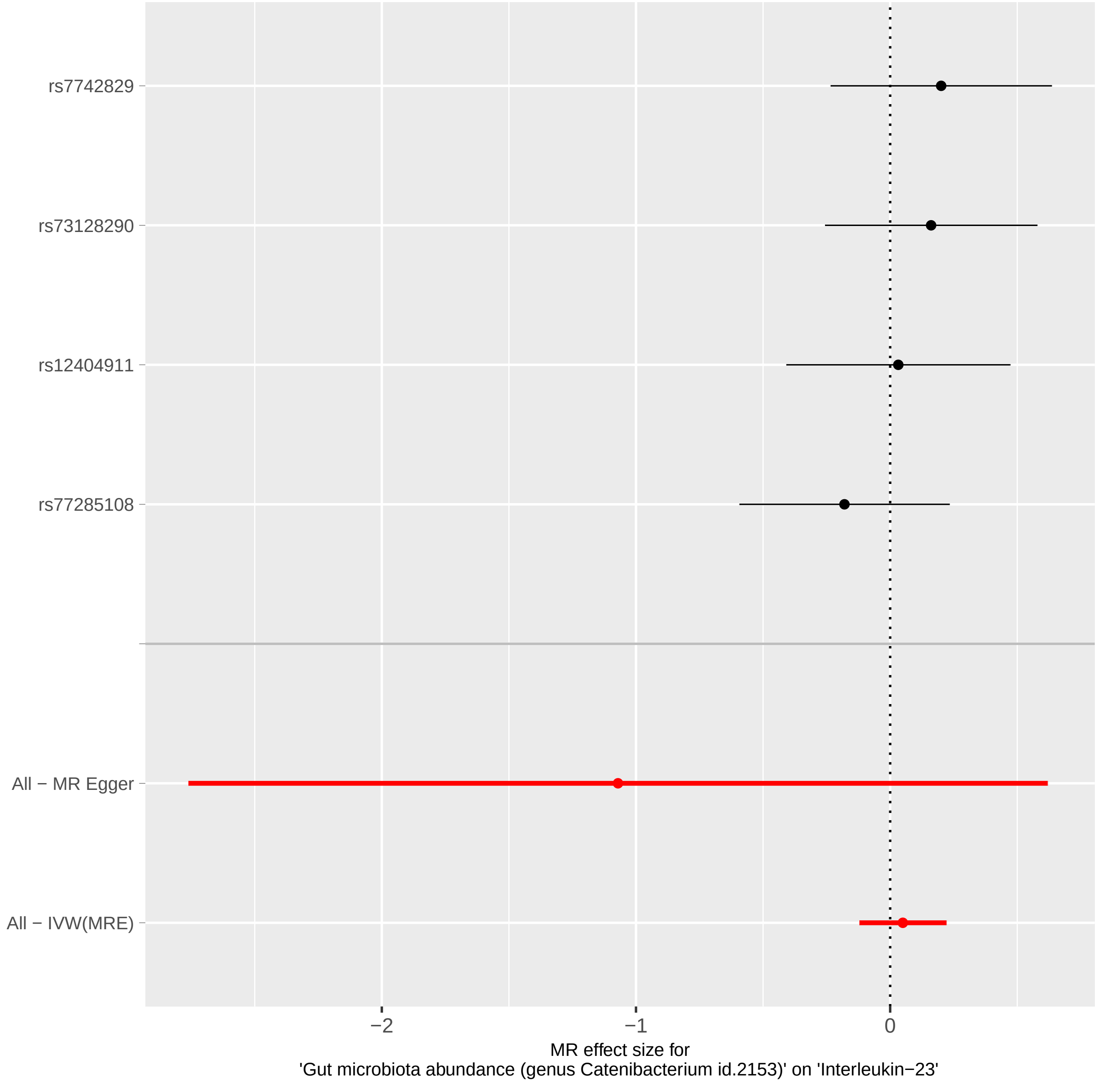

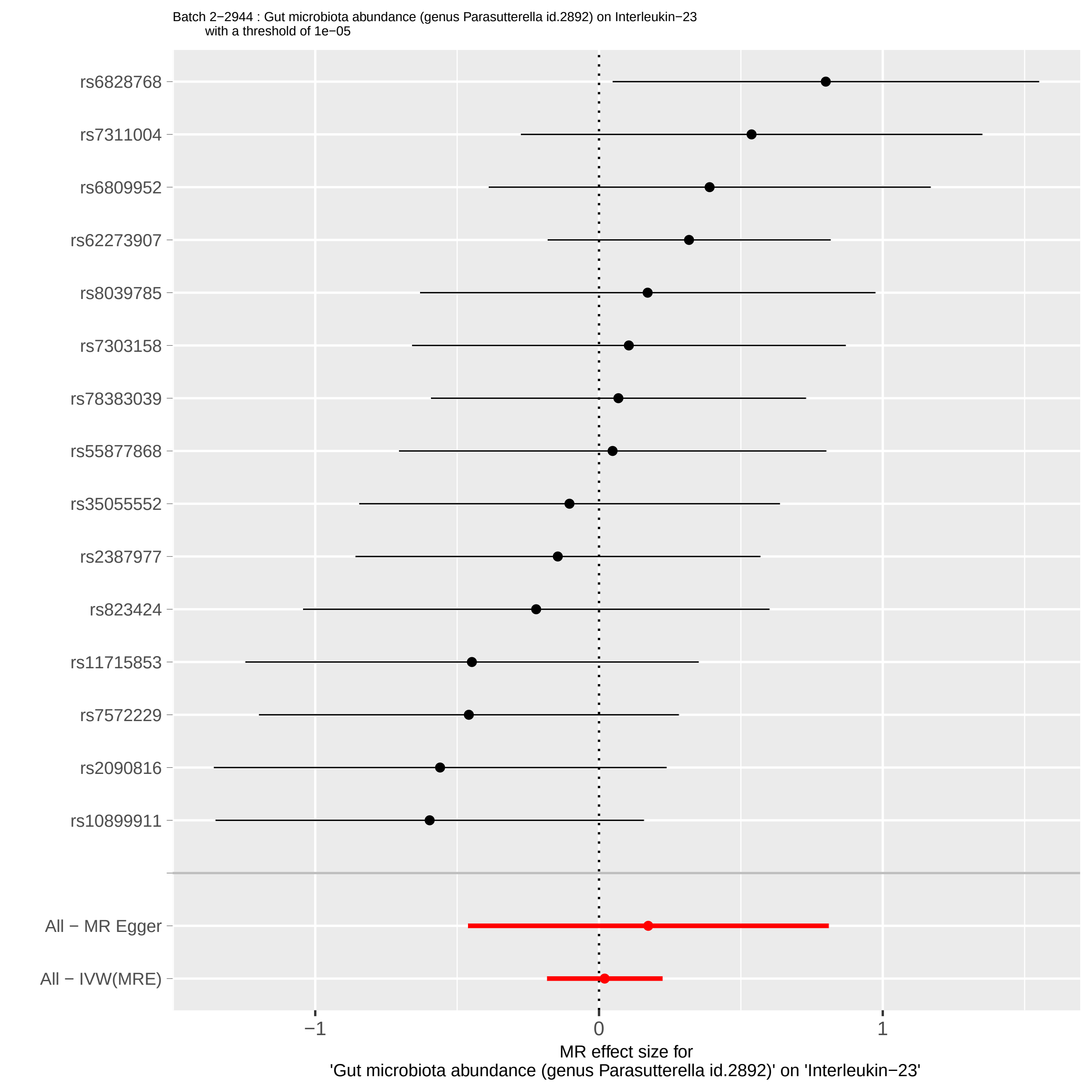

Batch 2−2944 : Gut microbiota abundance (genus Parasutterella id.2892) on Interleukin−23
with a threshold of 1e−05
rs6828768
rs7311004
rs6809952
rs62273907
rs8039785
rs7303158
rs78383039
rs55877868
rs35055552
rs2387977
rs823424
rs11715853
rs7572229
rs2090816
rs10899911
All − MR Egger
All − IVW(MRE)
−1
0
1
MR effect size for
'Gut microbiota abundance (genus Parasutterella id.2892)' on 'Interleukin−23'

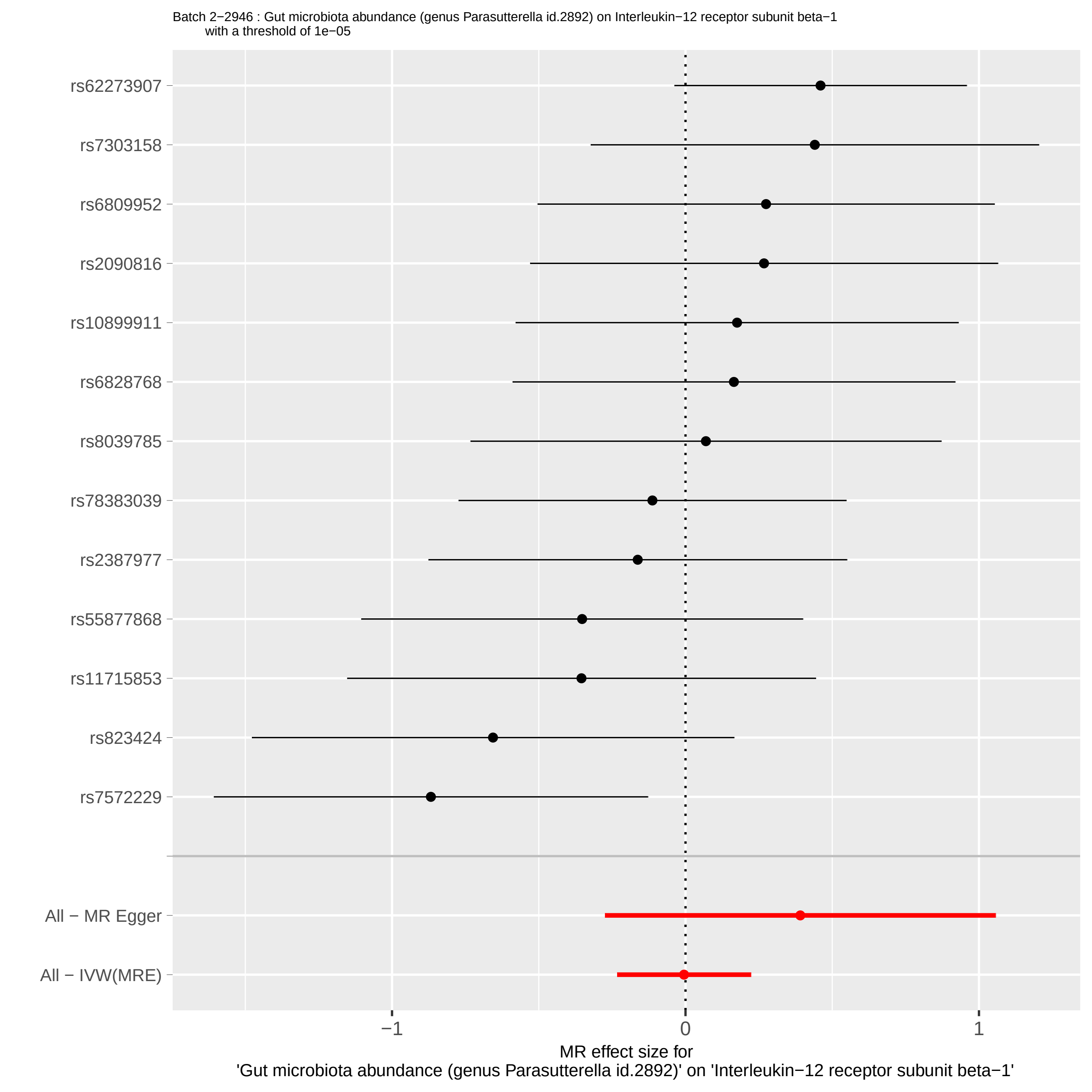

Batch 2−2946 : Gut microbiota abundance (genus Parasutterella id.2892) on Interleukin−12 receptor subunit beta−1
with a threshold of 1e−05
rs62273907
rs7303158
rs6809952
rs2090816
rs10899911
rs6828768
rs8039785
rs78383039
rs2387977
rs55877868
rs11715853
rs823424
rs7572229
All − MR Egger
All − IVW(MRE)
−1
0
1
MR effect size for
'Gut microbiota abundance (genus Parasutterella id.2892)' on 'Interleukin−12 receptor subunit beta−1'

Batch 2−2947 : Gut microbiota abundance (genus Catenibacterium id.2153) on Interleukin−12 receptor subunit beta−2
with a threshold of 1e−05

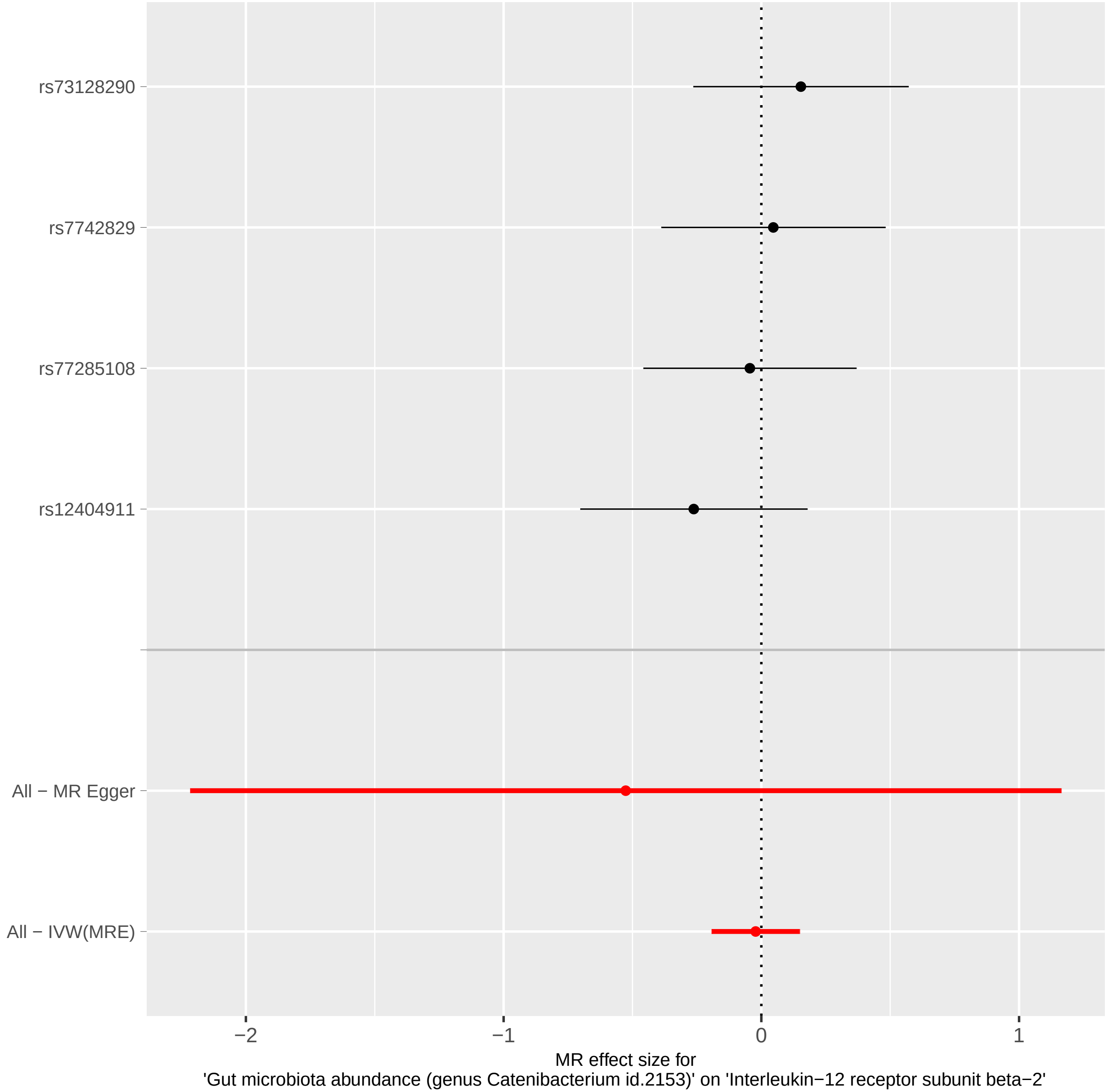

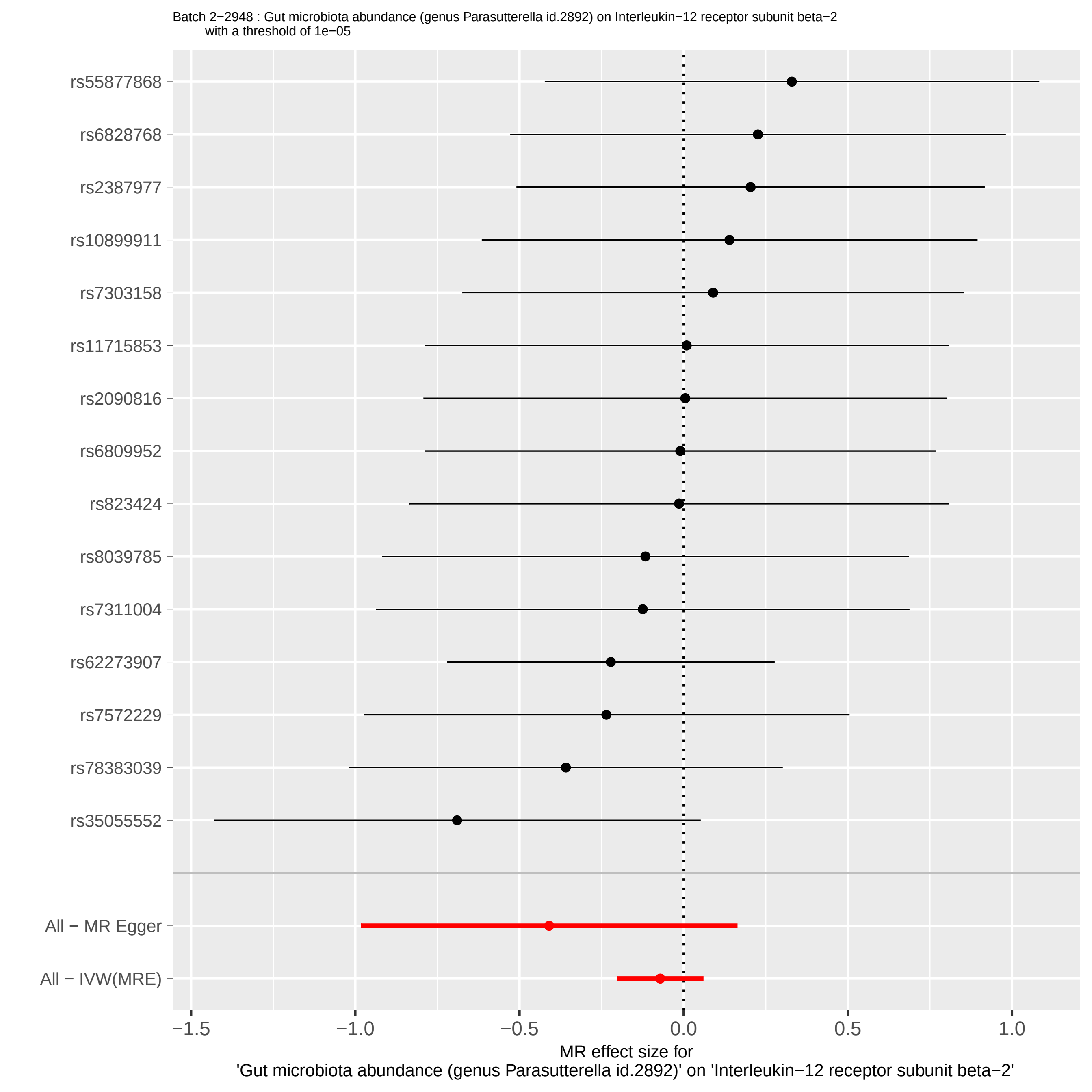
Batch 2−2948 : Gut microbiota abundance (genus Parasutterella id.2892) on Interleukin−12 receptor subunit beta−2
with a threshold of 1e−05
rs55877868
rs6828768
rs2387977
rs10899911
rs7303158
rs11715853
rs2090816
rs6809952
rs823424
rs8039785
rs7311004
rs62273907
rs7572229
rs78383039
rs35055552
All − MR Egger
All − IVW(MRE)
−1.5
−1.0
−0.5
0.0
0.5
1.0
MR effect size for
'Gut microbiota abundance (genus Parasutterella id.2892)' on 'Interleukin−12 receptor subunit beta−2'

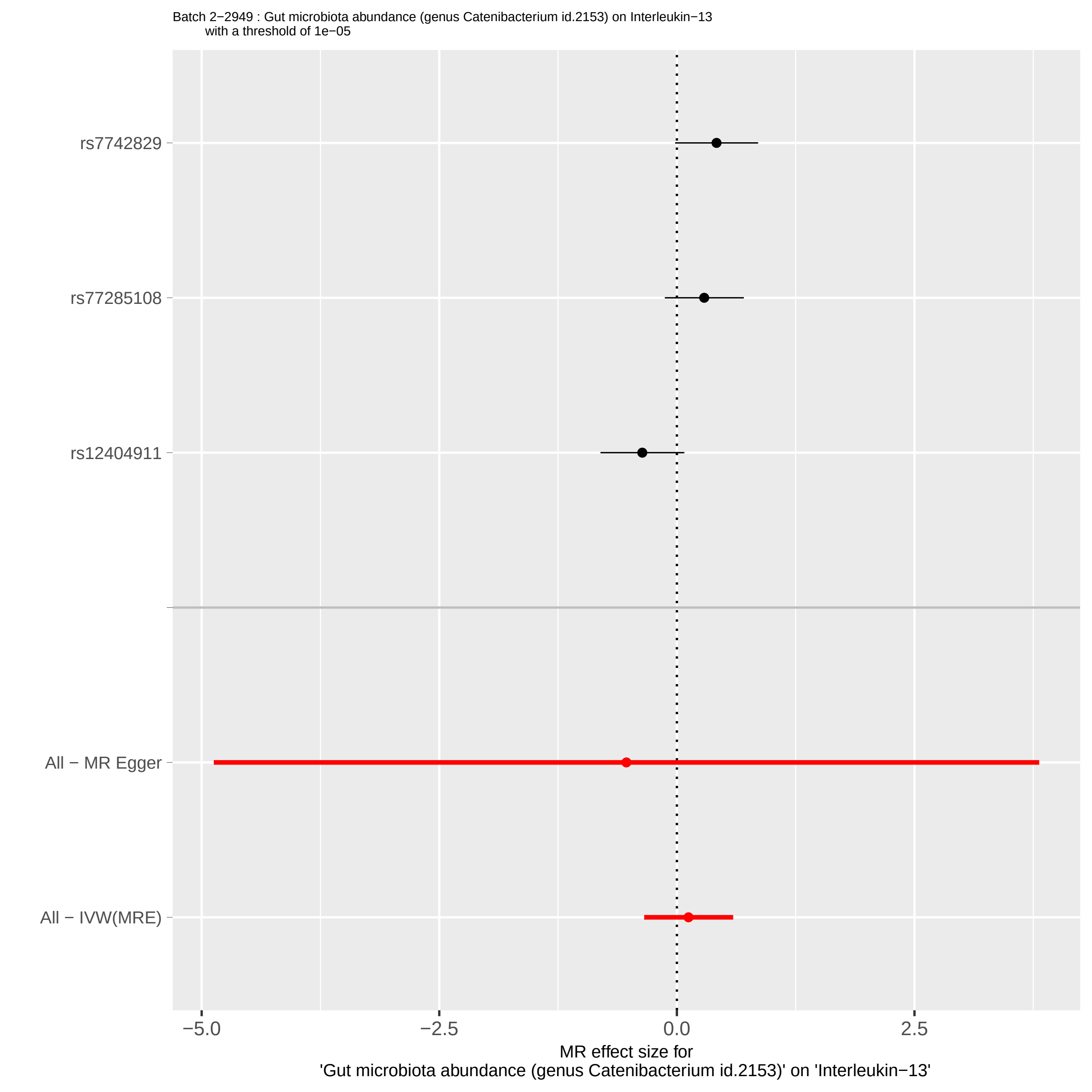

Batch 2−2949 : Gut microbiota abundance (genus Catenibacterium id.2153) on Interleukin−13
with a threshold of 1e−05
rs7742829
rs77285108
rs12404911
All − MR Egger
All − IVW(MRE)
−5.0
−2.5
0.0
2.5
MR effect size for
'Gut microbiota abundance (genus Catenibacterium id.2153)' on 'Interleukin−13'

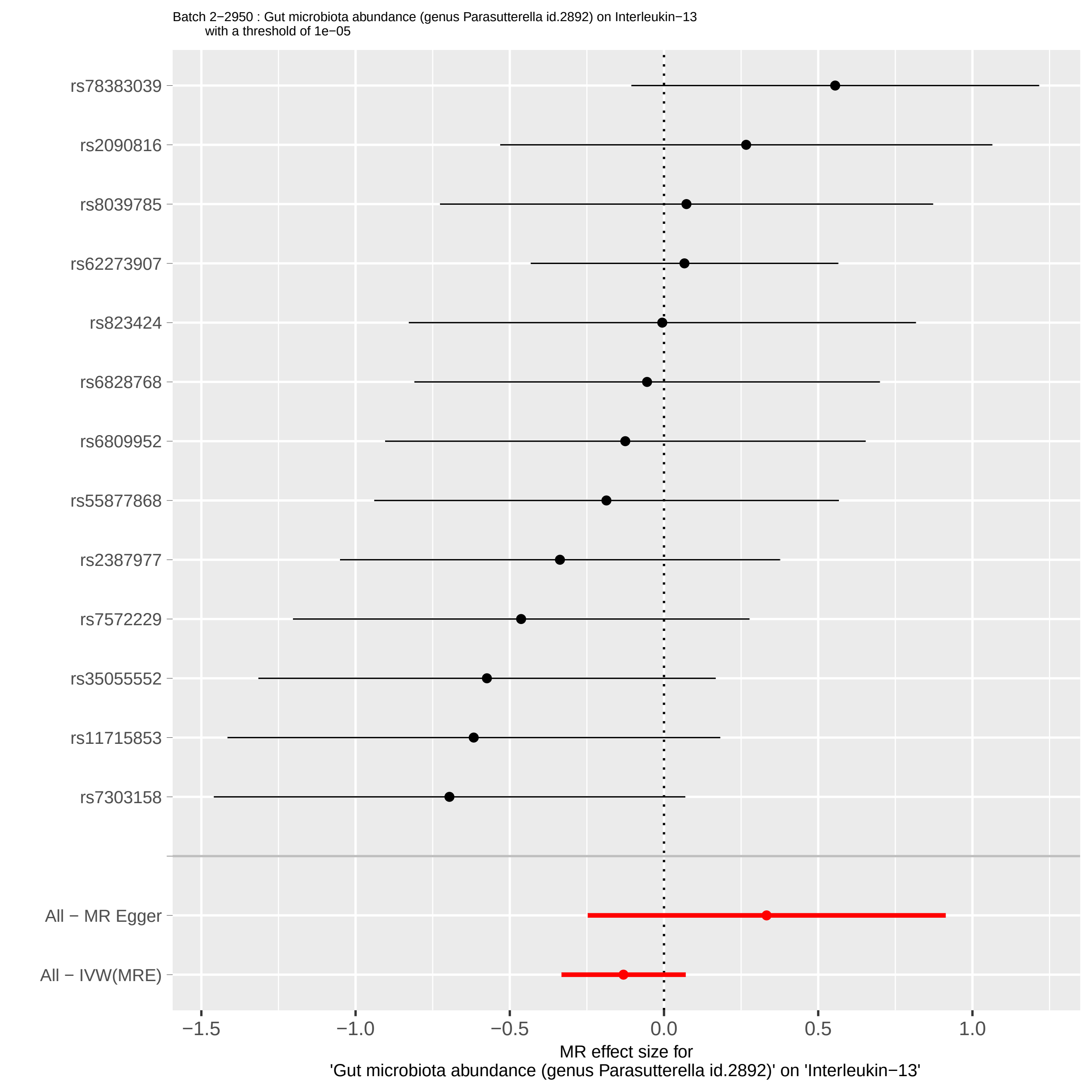
Batch 2−2950 : Gut microbiota abundance (genus Parasutterella id.2892) on Interleukin−13
with a threshold of 1e−05
rs78383039
rs2090816
rs8039785
rs62273907
rs823424
rs6828768
rs6809952
rs55877868
rs2387977
rs7572229
rs35055552
rs11715853
rs7303158
All − MR Egger
All − IVW(MRE)
−1.5
−1.0
−0.5
0.0
0.5
1.0
MR effect size for
'Gut microbiota abundance (genus Parasutterella id.2892)' on 'Interleukin−13'

Batch 2−2951 : Gut microbiota abundance (genus Catenibacterium id.2153) on Interleukin−13 receptor subunit alpha−1
with a threshold of 1e−05

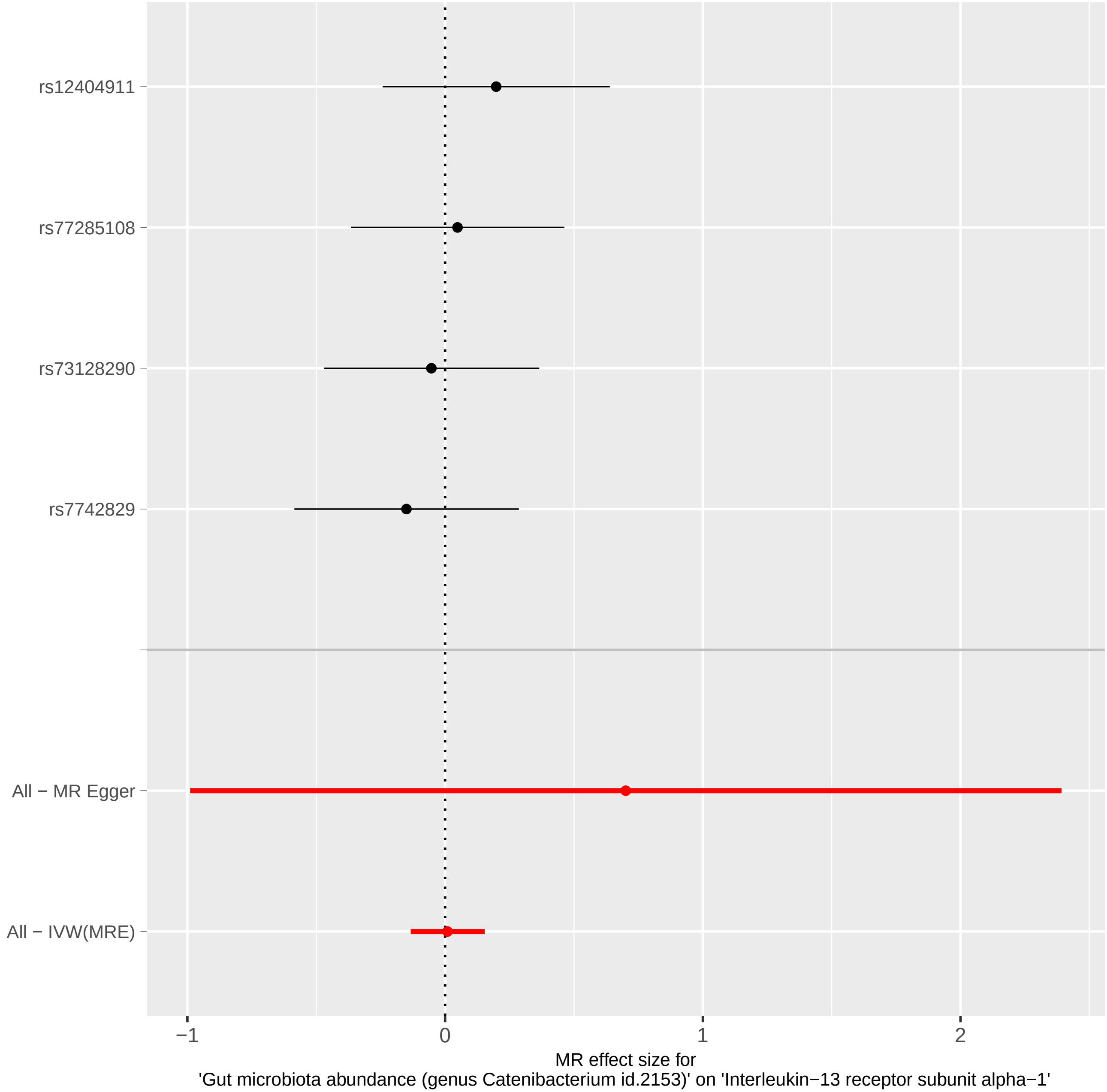

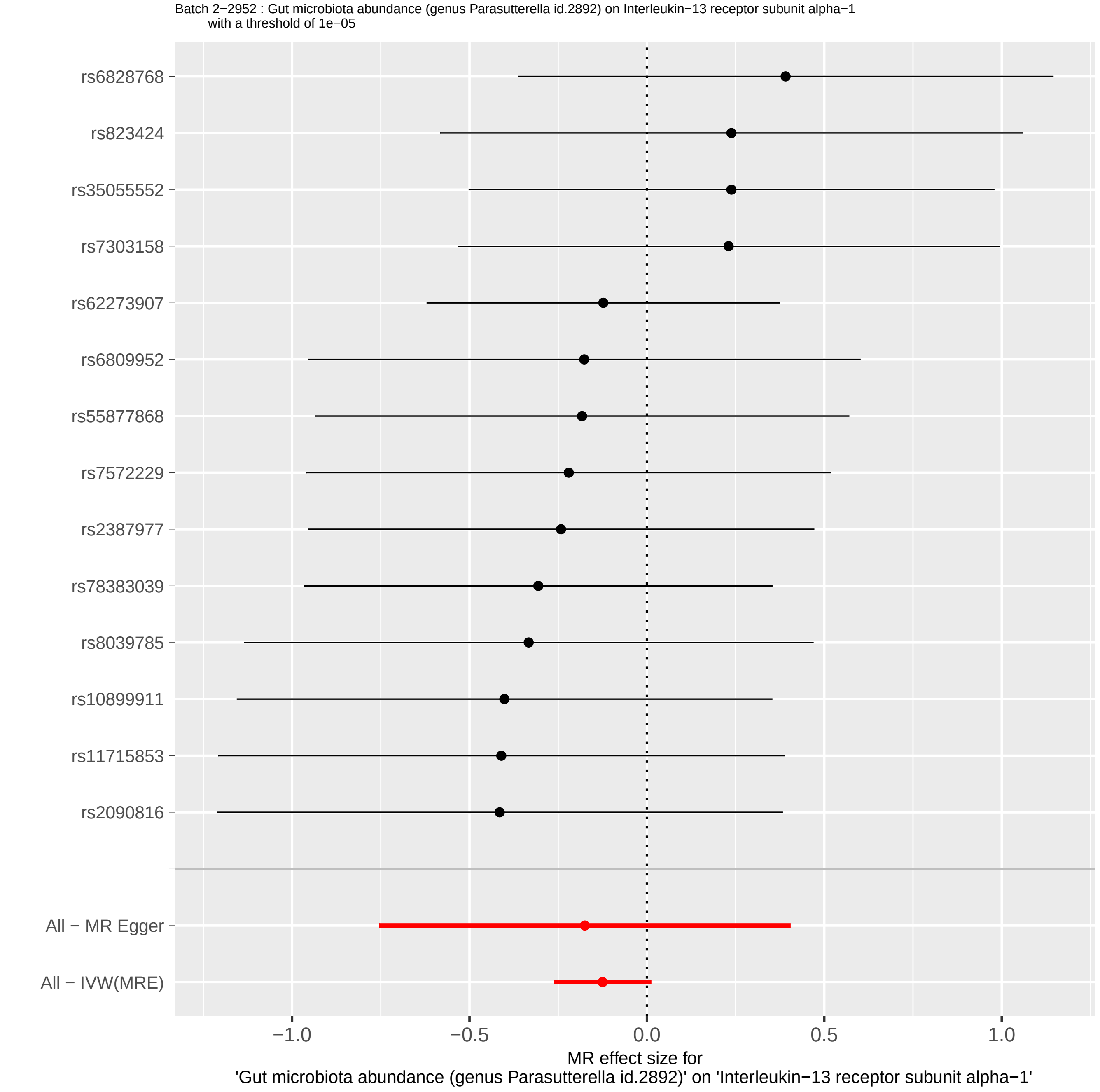
Batch 2−2952 : Gut microbiota abundance (genus Parasutterella id.2892) on Interleukin−13 receptor subunit alpha−1
with a threshold of 1e−05
rs6828768
rs823424
rs35055552
rs7303158
rs62273907
rs6809952
rs55877868
rs7572229
rs2387977
rs78383039
rs8039785
rs10899911
rs11715853
rs2090816
All − MR Egger
All − IVW(MRE)
−1.0
−0.5
0.0
0.5
1.0
MR effect size for
'Gut microbiota abundance (genus Parasutterella id.2892)' on 'Interleukin−13 receptor subunit alpha−1'

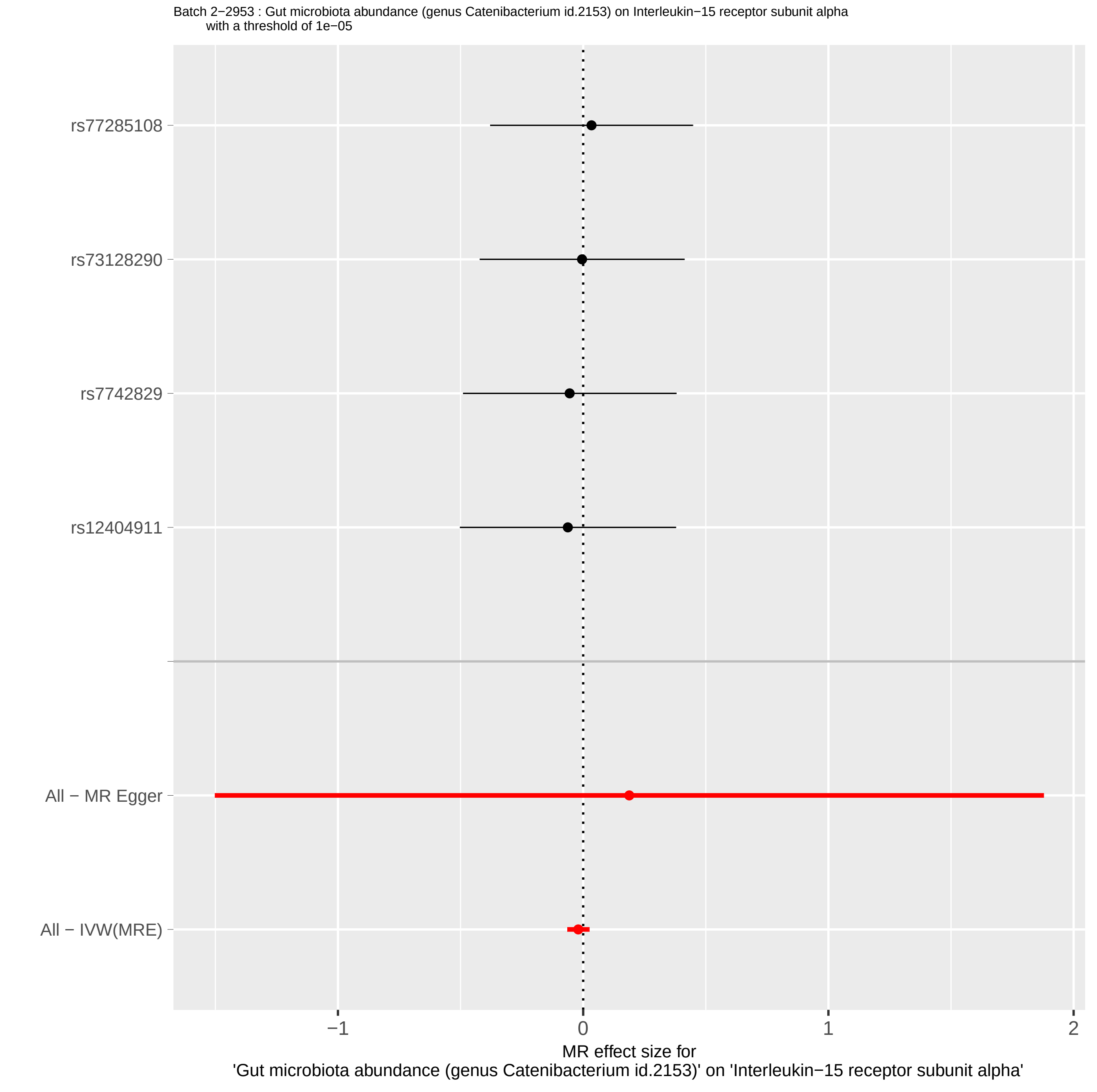

Batch 2−2953 : Gut microbiota abundance (genus Catenibacterium id.2153) on Interleukin−15 receptor subunit alpha
with a threshold of 1e−05
rs77285108
rs73128290
rs7742829
rs12404911
All − MR Egger
All − IVW(MRE)
−1
0
1
2
MR effect size for
'Gut microbiota abundance (genus Catenibacterium id.2153)' on 'Interleukin−15 receptor subunit alpha'

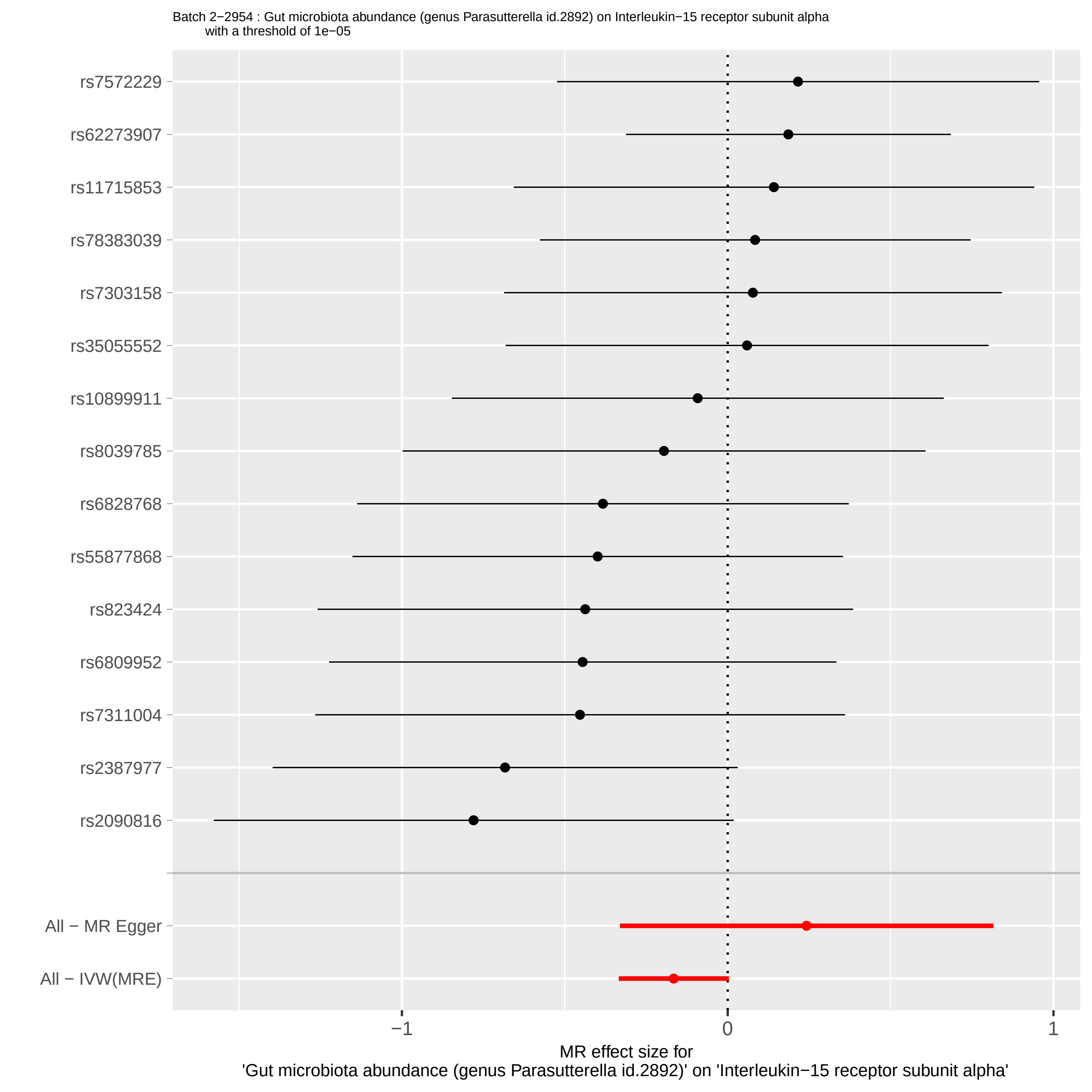
Batch 2−2954 : Gut microbiota abundance (genus Parasutterella id.2892) on Interleukin−15 receptor subunit alpha
with a threshold of 1e−05
rs7572229
rs62273907
rs11715853
rs78383039
rs7303158
rs35055552
rs10899911
rs8039785
rs6828768
rs55877868
rs823424
rs6809952
rs7311004
rs2387977
rs2090816
All − MR Egger
All − IVW(MRE)
−1
0
1
MR effect size for
'Gut microbiota abundance (genus Parasutterella id.2892)' on 'Interleukin−15 receptor subunit alpha'

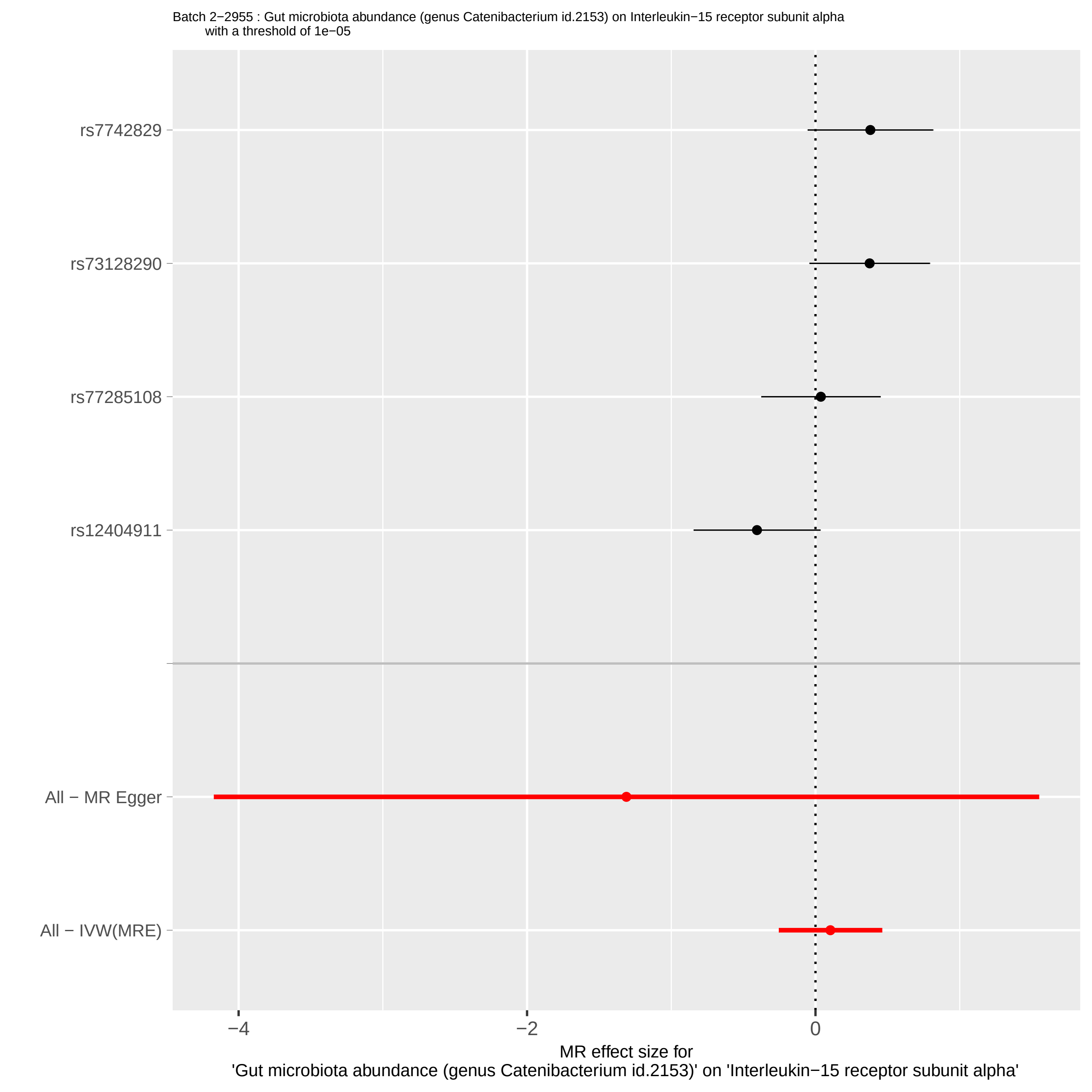
Batch 2−2955 : Gut microbiota abundance (genus Catenibacterium id.2153) on Interleukin−15 receptor subunit alpha
with a threshold of 1e−05
rs7742829
rs73128290
rs77285108
rs12404911
All − MR Egger
All − IVW(MRE)
−4
−2
0
MR effect size for
'Gut microbiota abundance (genus Catenibacterium id.2153)' on 'Interleukin−15 receptor subunit alpha'

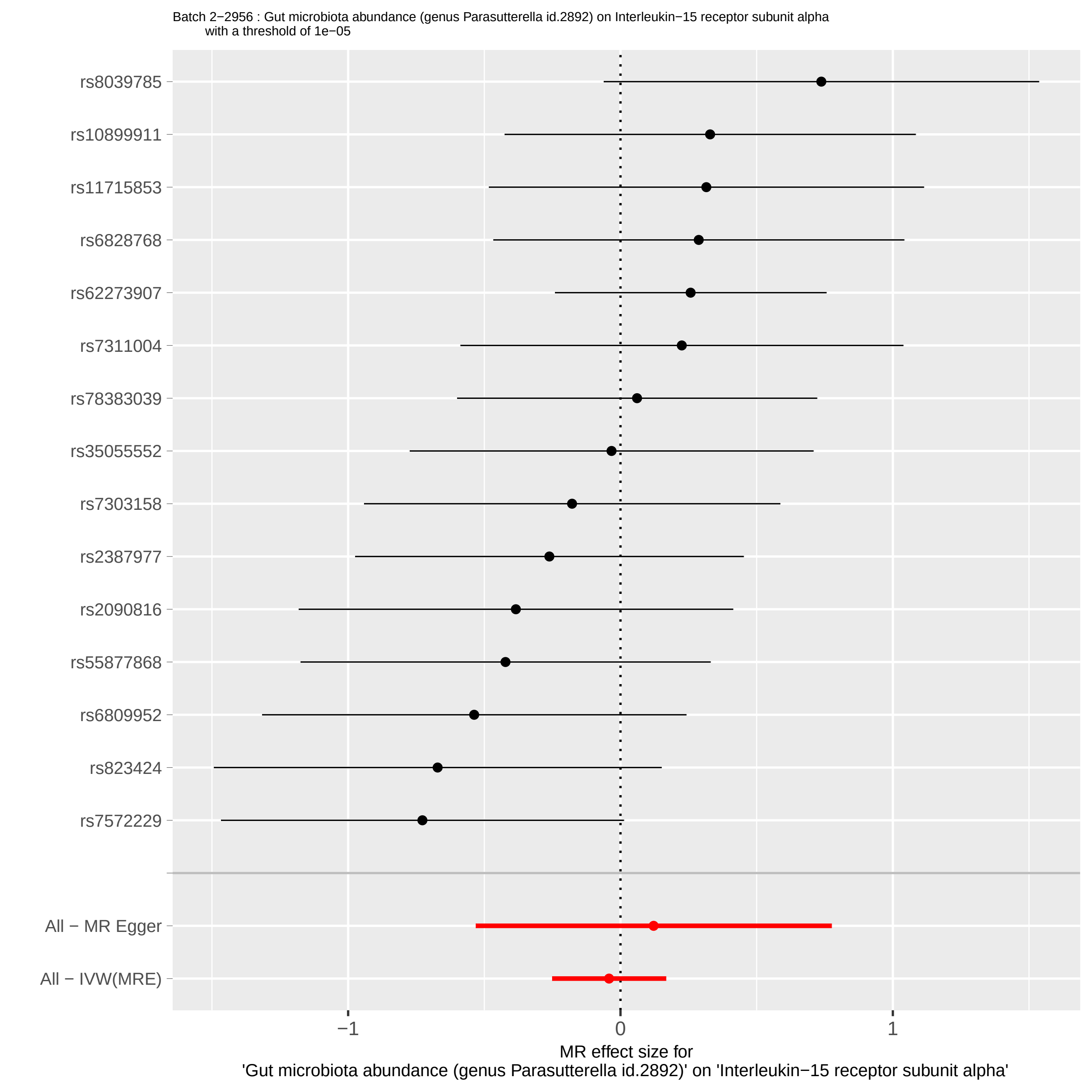
Batch 2−2956 : Gut microbiota abundance (genus Parasutterella id.2892) on Interleukin−15 receptor subunit alpha
with a threshold of 1e−05
rs8039785
rs10899911
rs11715853
rs6828768
rs62273907
rs7311004
rs78383039
rs35055552
rs7303158
rs2387977
rs2090816
rs55877868
rs6809952
rs823424
rs7572229
All − MR Egger
All − IVW(MRE)
−1
0
1
MR effect size for
'Gut microbiota abundance (genus Parasutterella id.2892)' on 'Interleukin−15 receptor subunit alpha'

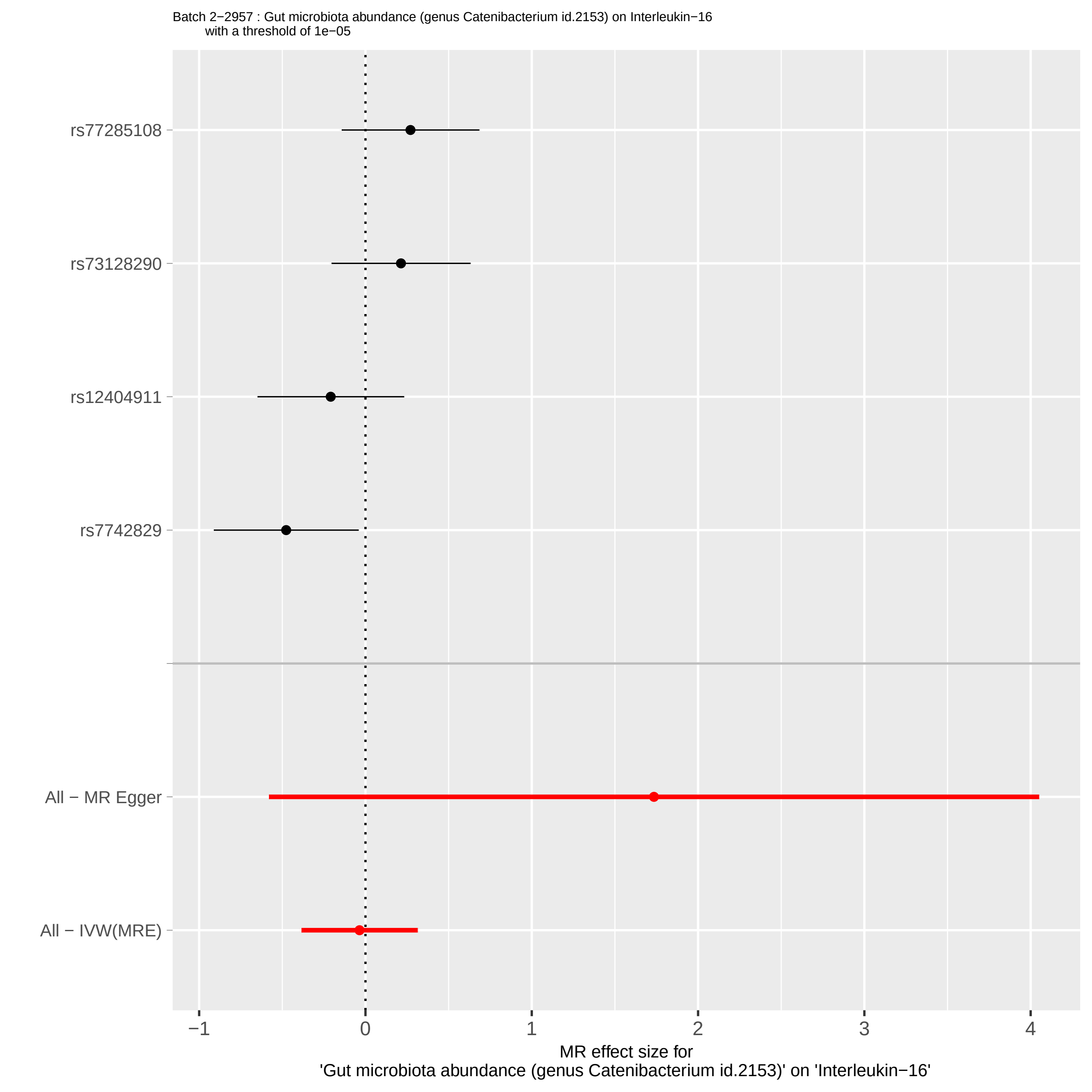

Batch 2−2957 : Gut microbiota abundance (genus Catenibacterium id.2153) on Interleukin−16
with a threshold of 1e−05
rs77285108
rs73128290
rs12404911
rs7742829
All − MR Egger
All − IVW(MRE)
−1
0
1
2
3
4
MR effect size for
'Gut microbiota abundance (genus Catenibacterium id.2153)' on 'Interleukin−16'

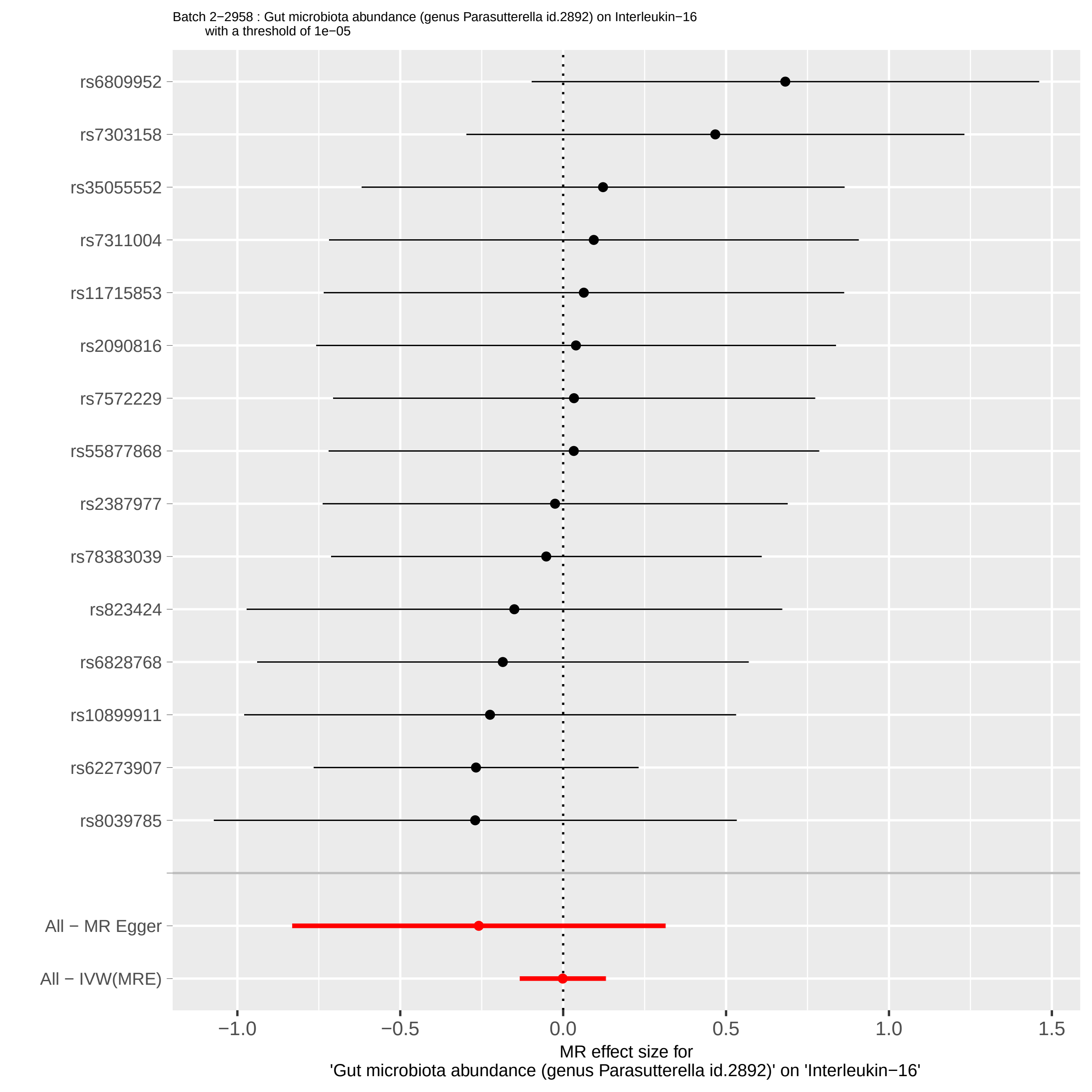

Batch 2−2958 : Gut microbiota abundance (genus Parasutterella id.2892) on Interleukin−16
with a threshold of 1e−05
rs6809952
rs7303158
rs35055552
rs7311004
rs11715853
rs2090816
rs7572229
rs55877868
rs2387977
rs78383039
rs823424
rs6828768
rs10899911
rs62273907
rs8039785
All − MR Egger
All − IVW(MRE)
−1.0
−0.5
0.0
0.5
1.0
1.5
MR effect size for
'Gut microbiota abundance (genus Parasutterella id.2892)' on 'Interleukin−16'

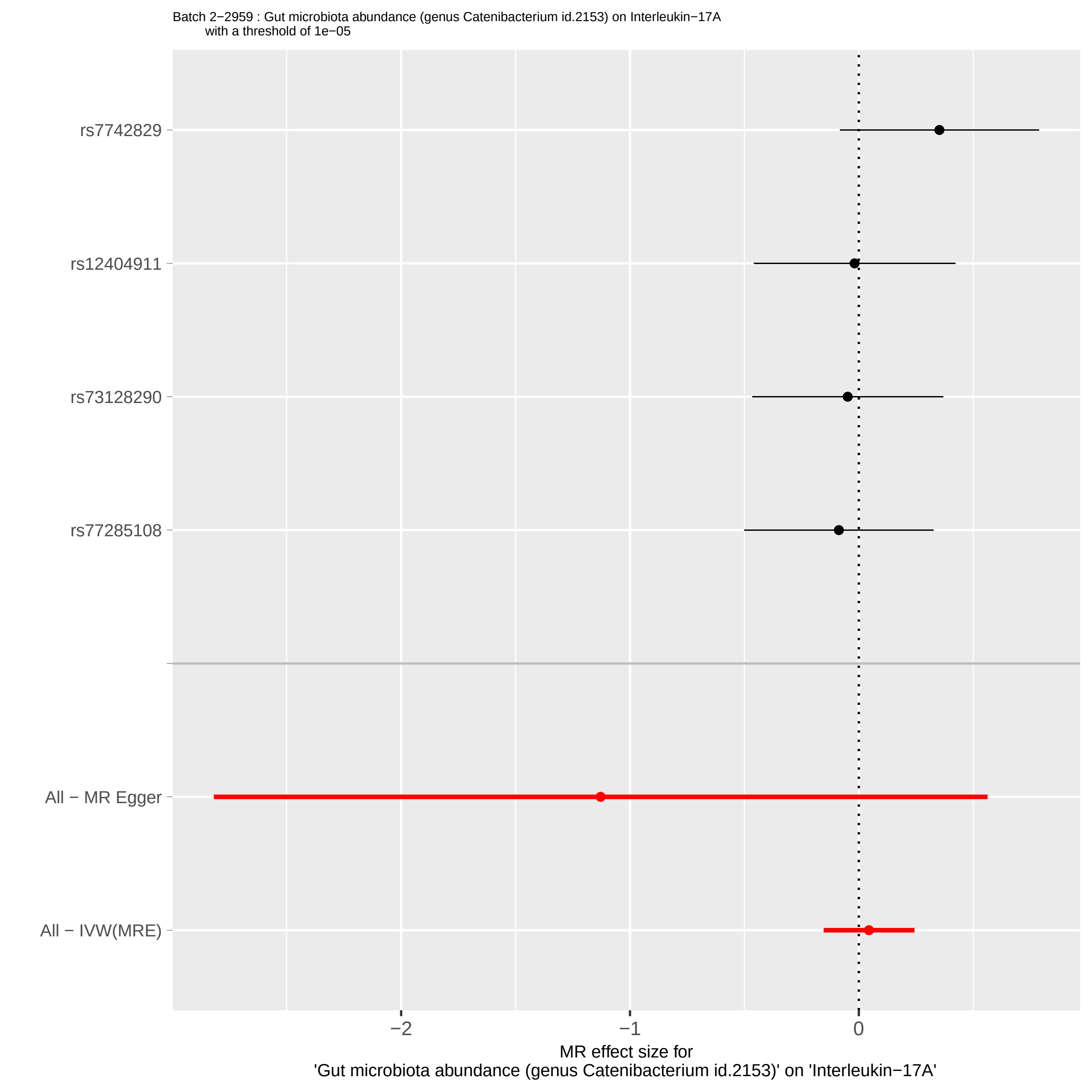
Batch 2−2959 : Gut microbiota abundance (genus Catenibacterium id.2153) on Interleukin−17A
with a threshold of 1e−05
rs7742829
rs12404911
rs73128290
rs77285108
All − MR Egger
All − IVW(MRE)
−2
−1
0
MR effect size for
'Gut microbiota abundance (genus Catenibacterium id.2153)' on 'Interleukin−17A'

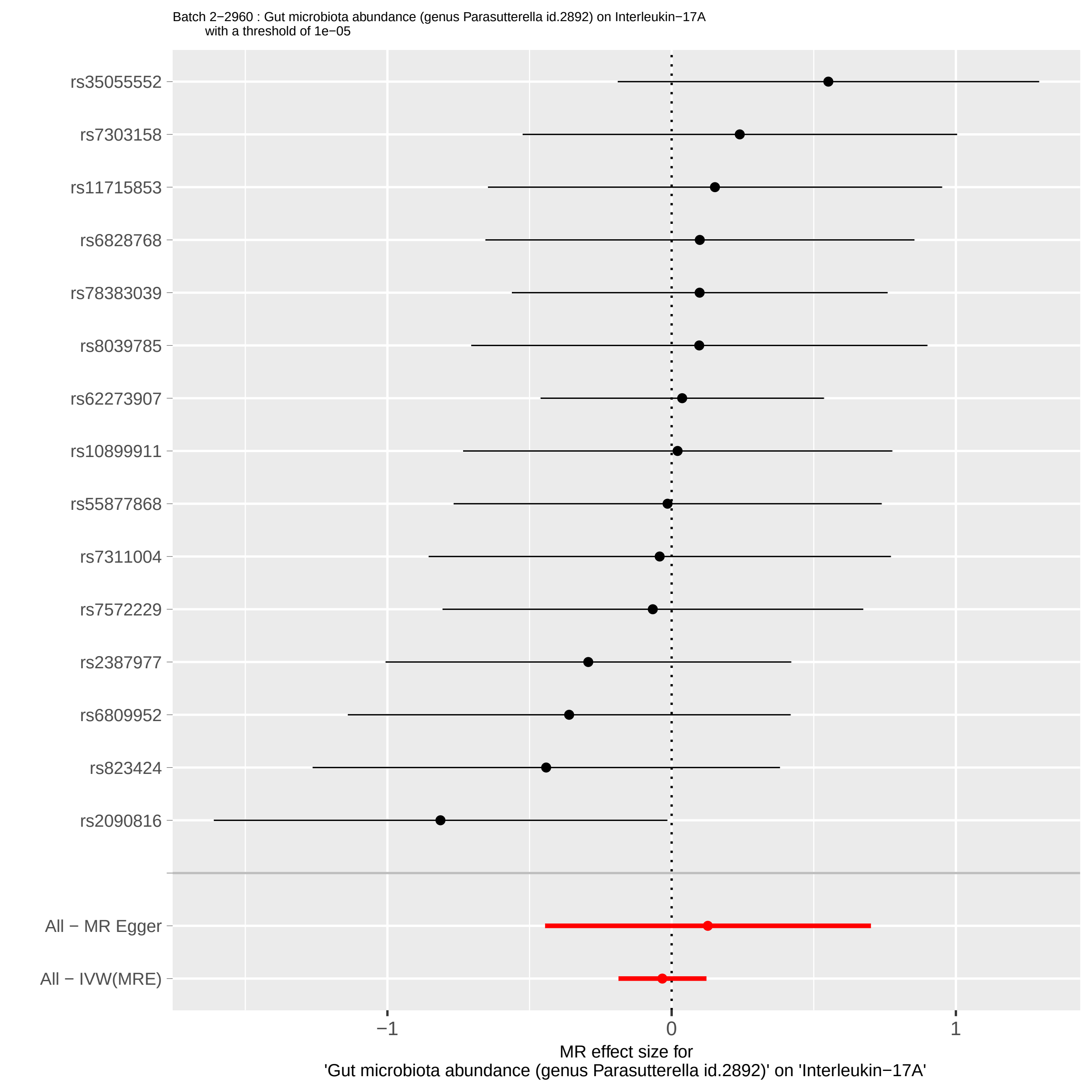
Batch 2−2960 : Gut microbiota abundance (genus Parasutterella id.2892) on Interleukin−17A
with a threshold of 1e−05
rs35055552
rs7303158
rs11715853
rs6828768
rs78383039
rs8039785
rs62273907
rs10899911
rs55877868
rs7311004
rs7572229
rs2387977
rs6809952
rs823424
rs2090816
All − MR Egger
All − IVW(MRE)
−1
0
1
MR effect size for
'Gut microbiota abundance (genus Parasutterella id.2892)' on 'Interleukin−17A'

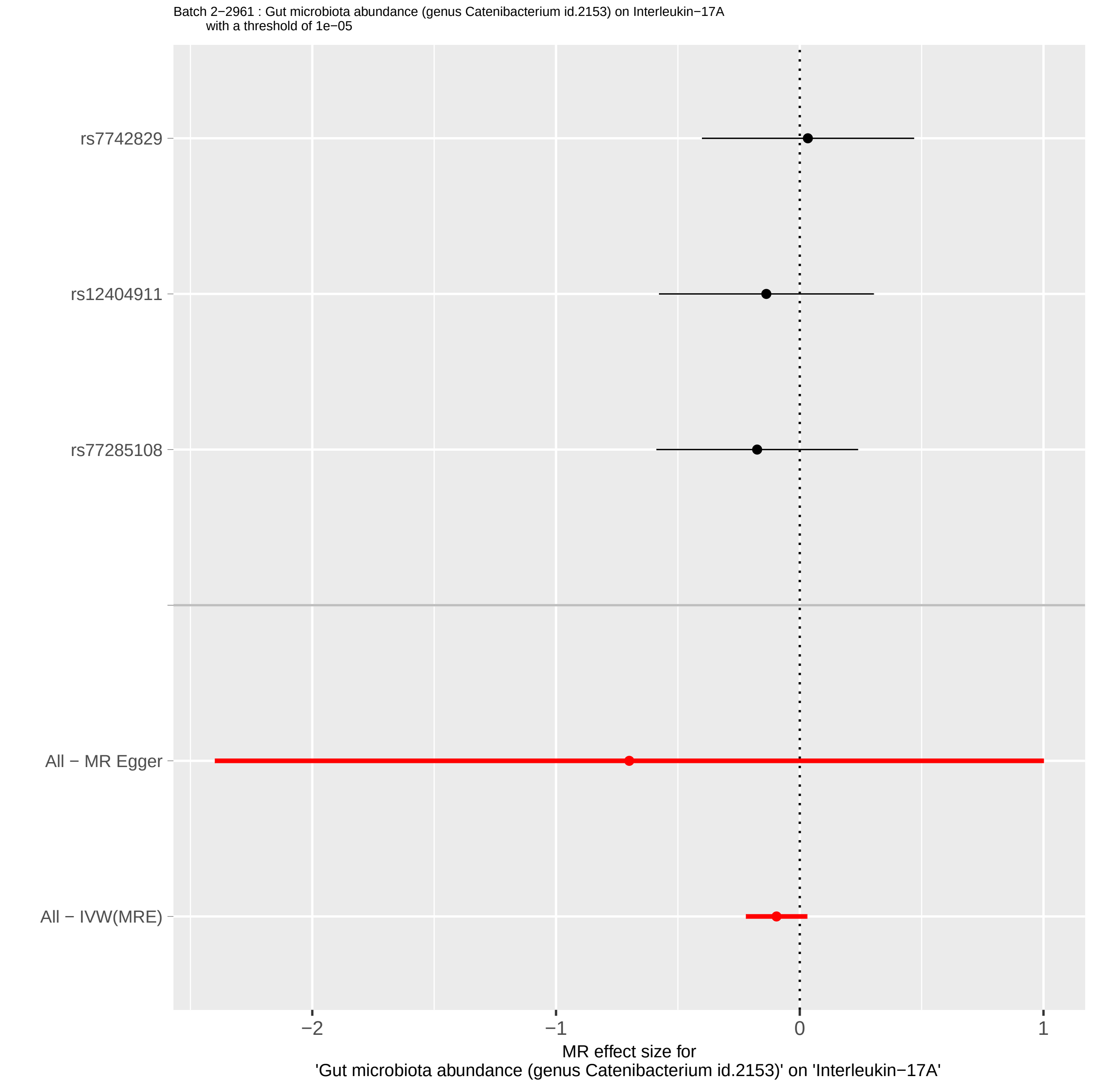

Batch 2−2961 : Gut microbiota abundance (genus Catenibacterium id.2153) on Interleukin−17A
with a threshold of 1e−05
rs7742829
rs12404911
rs77285108
All − MR Egger
All − IVW(MRE)
−2
−1
0
1
MR effect size for
'Gut microbiota abundance (genus Catenibacterium id.2153)' on 'Interleukin−17A'

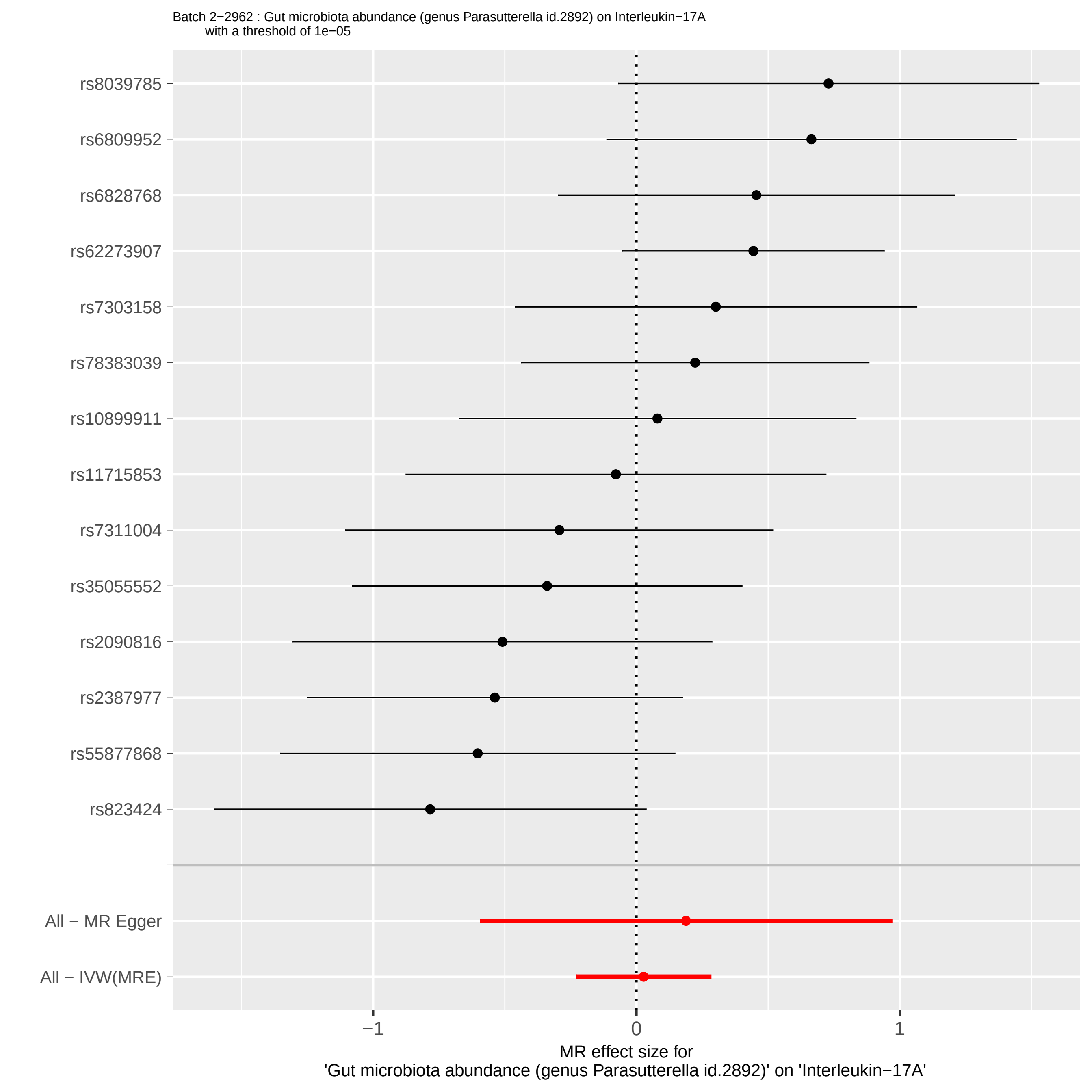
Batch 2−2962 : Gut microbiota abundance (genus Parasutterella id.2892) on Interleukin−17A
with a threshold of 1e−05
rs8039785
rs6809952
rs6828768
rs62273907
rs7303158
rs78383039
rs10899911
rs11715853
rs7311004
rs35055552
rs2090816
rs2387977
rs55877868
rs823424
All − MR Egger
All − IVW(MRE)
−1
0
1
MR effect size for
'Gut microbiota abundance (genus Parasutterella id.2892)' on 'Interleukin−17A'

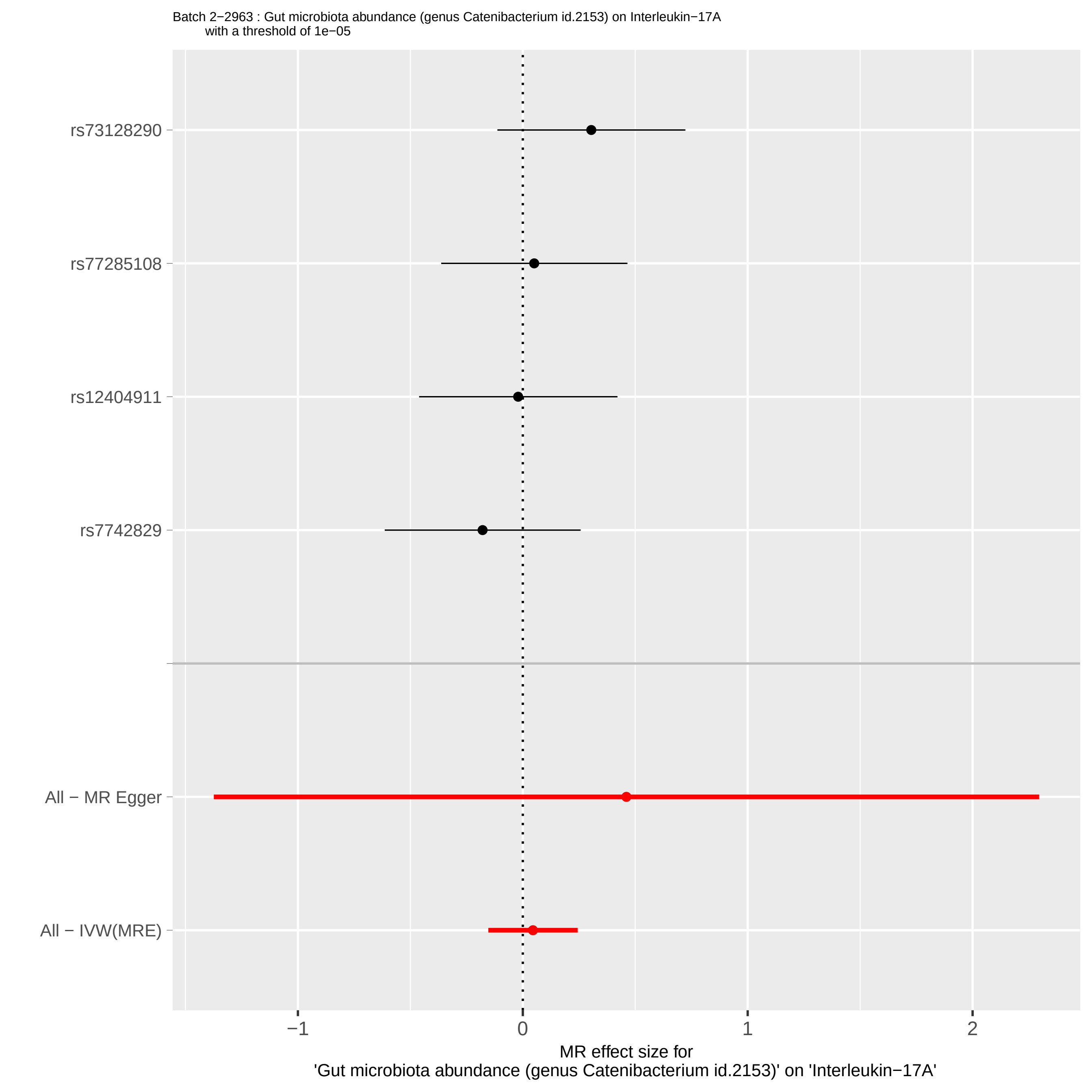
Batch 2−2963 : Gut microbiota abundance (genus Catenibacterium id.2153) on Interleukin−17A
with a threshold of 1e−05
rs73128290
rs77285108
rs12404911
rs7742829
All − MR Egger
All − IVW(MRE)
−1
0
1
2
MR effect size for
'Gut microbiota abundance (genus Catenibacterium id.2153)' on 'Interleukin−17A'

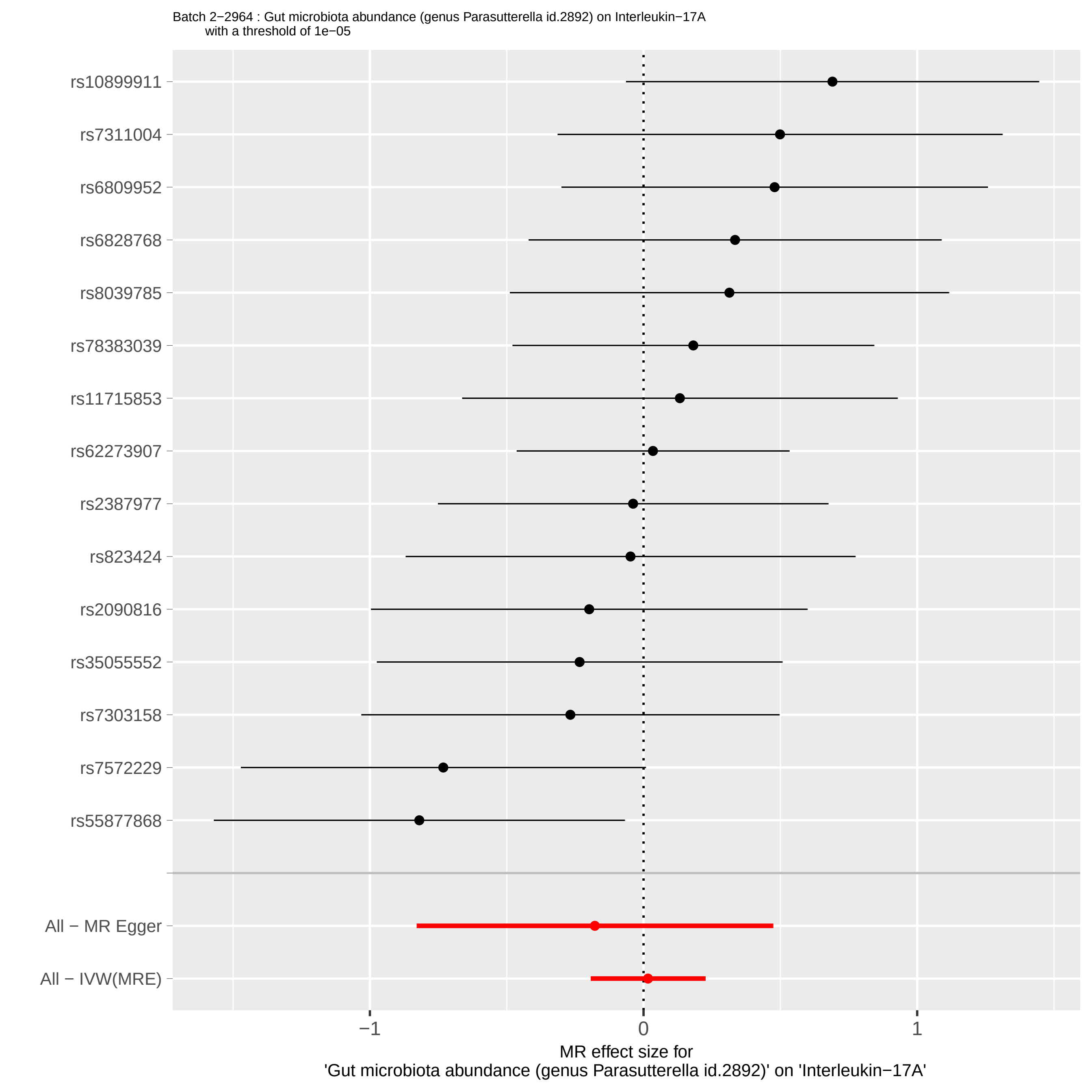
Batch 2−2964 : Gut microbiota abundance (genus Parasutterella id.2892) on Interleukin−17A
with a threshold of 1e−05
rs10899911
rs7311004
rs6809952
rs6828768
rs8039785
rs78383039
rs11715853
rs62273907
rs2387977
rs823424
rs2090816
rs35055552
rs7303158
rs7572229
rs55877868
All − MR Egger
All − IVW(MRE)
−1
0
1
MR effect size for
'Gut microbiota abundance (genus Parasutterella id.2892)' on 'Interleukin−17A'

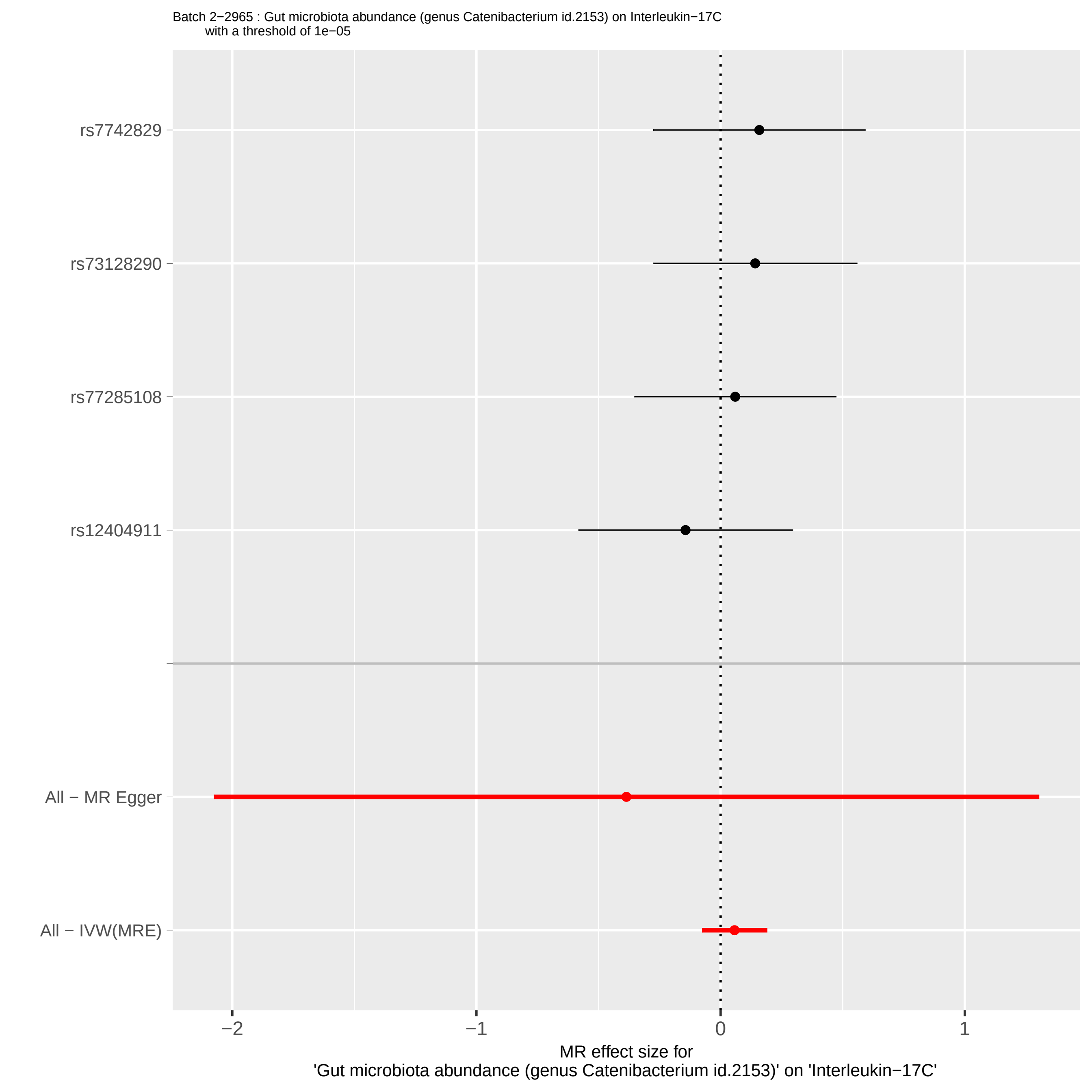

Batch 2−2965 : Gut microbiota abundance (genus Catenibacterium id.2153) on Interleukin−17C
with a threshold of 1e−05
rs7742829
rs73128290
rs77285108
rs12404911
All − MR Egger
All − IVW(MRE)
−2
−1
0
1
MR effect size for
'Gut microbiota abundance (genus Catenibacterium id.2153)' on 'Interleukin−17C'

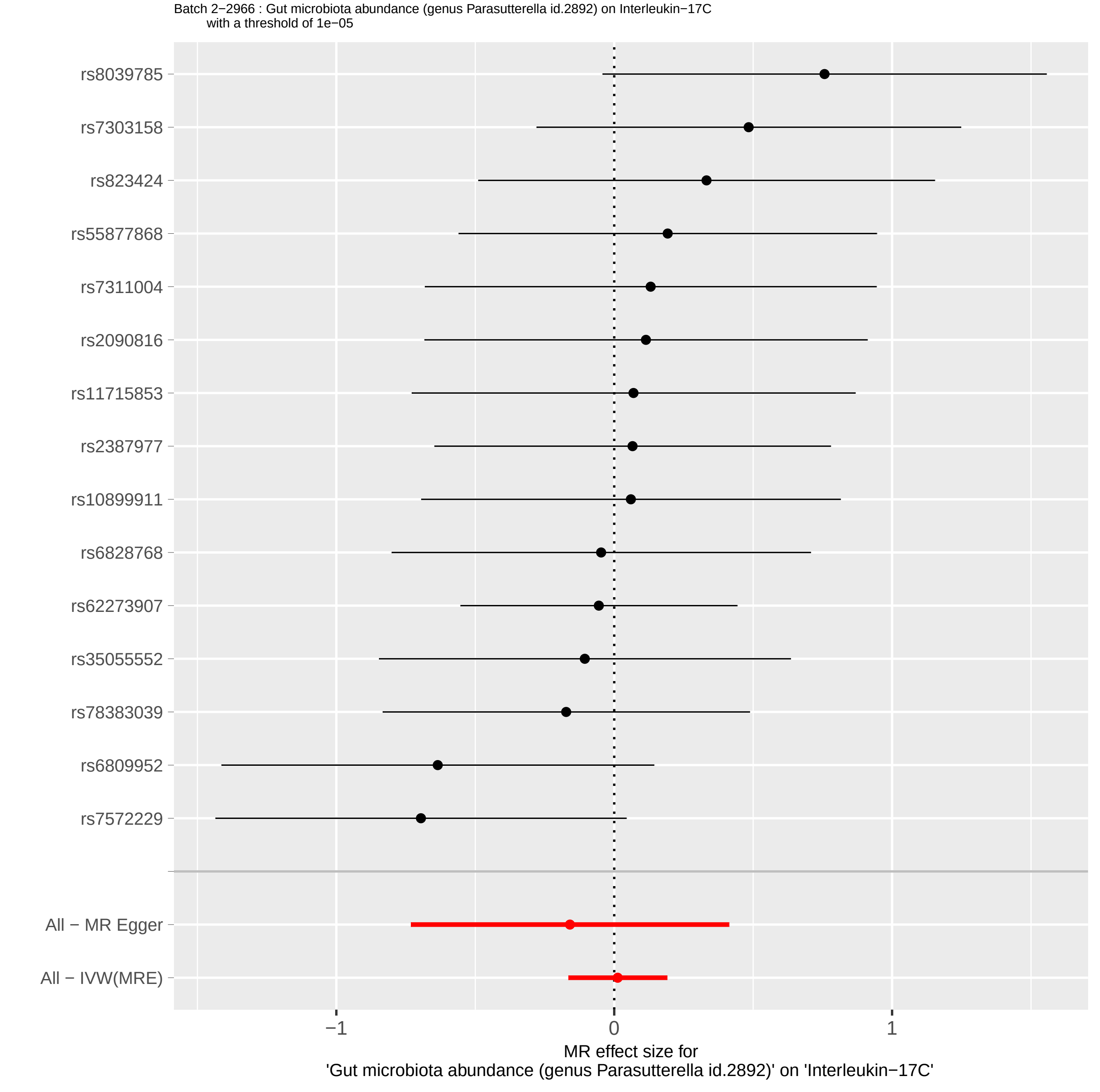

Batch 2−2966 : Gut microbiota abundance (genus Parasutterella id.2892) on Interleukin−17C
with a threshold of 1e−05
rs8039785
rs7303158
rs823424
rs55877868
rs7311004
rs2090816
rs11715853
rs2387977
rs10899911
rs6828768
rs62273907
rs35055552
rs78383039
rs6809952
rs7572229
All − MR Egger
All − IVW(MRE)
−1
0
1
MR effect size for
'Gut microbiota abundance (genus Parasutterella id.2892)' on 'Interleukin−17C'

Batch 2−2967 : Gut microbiota abundance (genus Catenibacterium id.2153) on Interleukin−17D
with a threshold of 1e−05

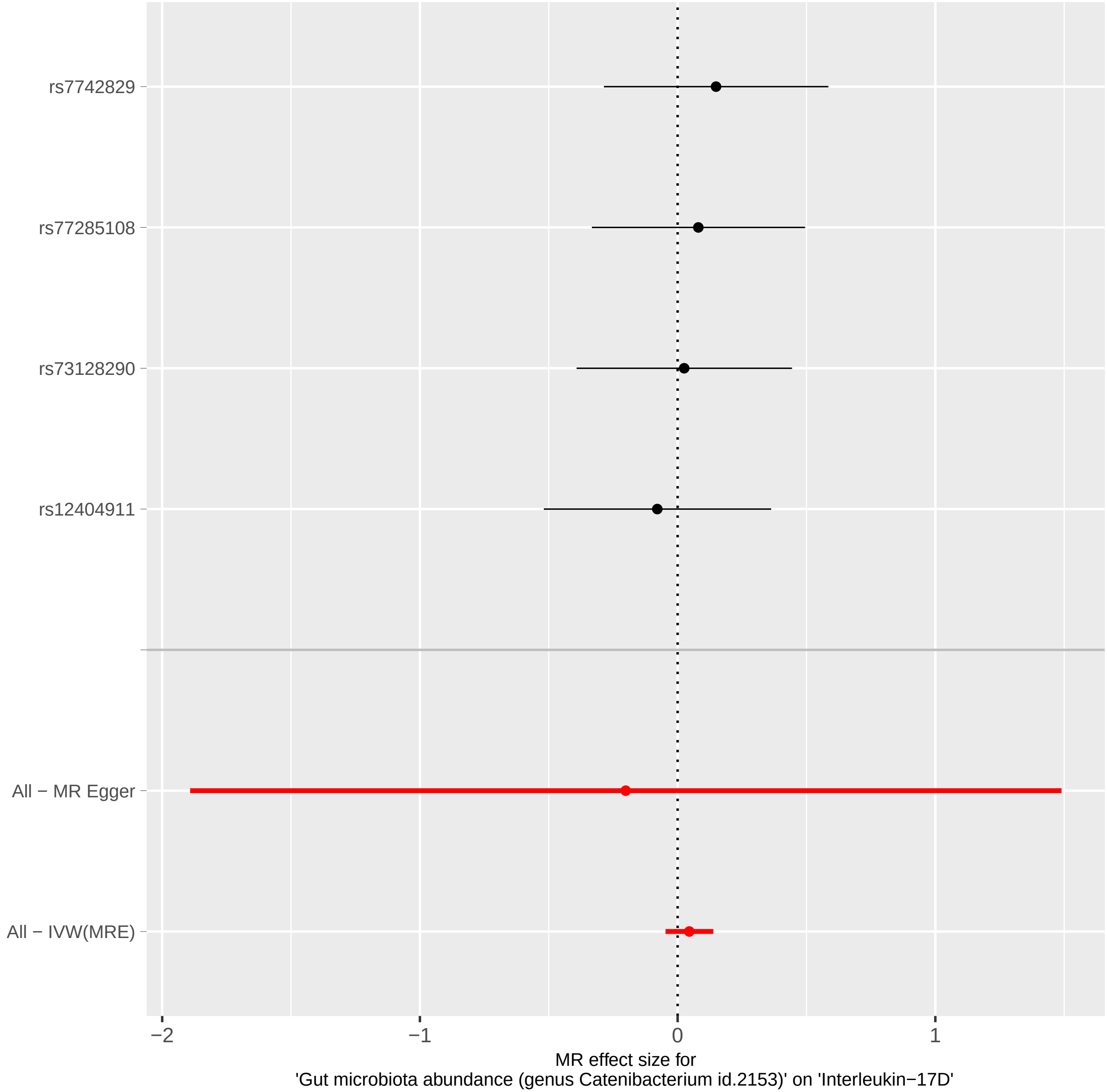

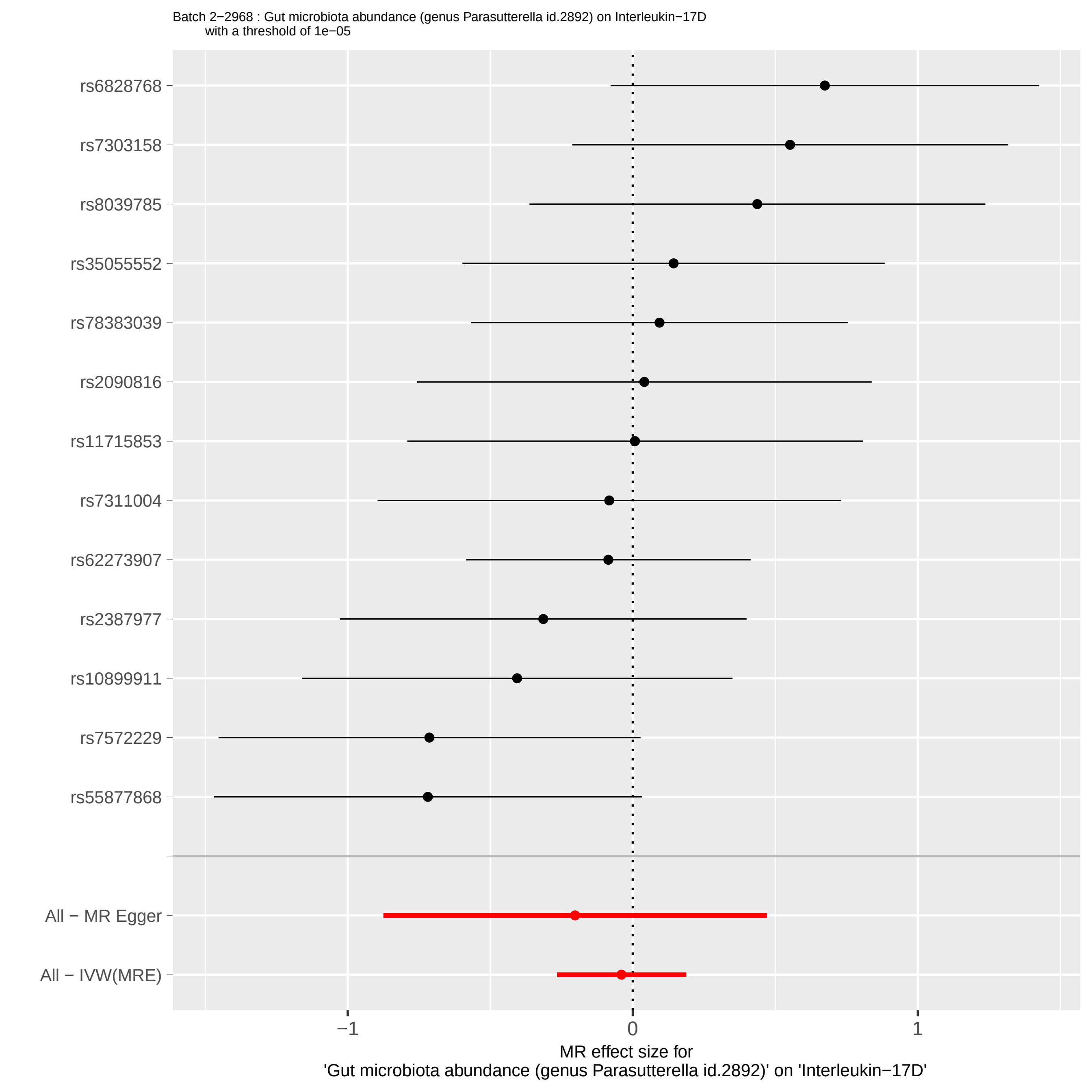
Batch 2−2968 : Gut microbiota abundance (genus Parasutterella id.2892) on Interleukin−17D
with a threshold of 1e−05
rs6828768
rs7303158
rs8039785
rs35055552
rs78383039
rs2090816
rs11715853
rs7311004
rs62273907
rs2387977
rs10899911
rs7572229
rs55877868
All − MR Egger
All − IVW(MRE)
−1
0
1
MR effect size for
'Gut microbiota abundance (genus Parasutterella id.2892)' on 'Interleukin−17D'

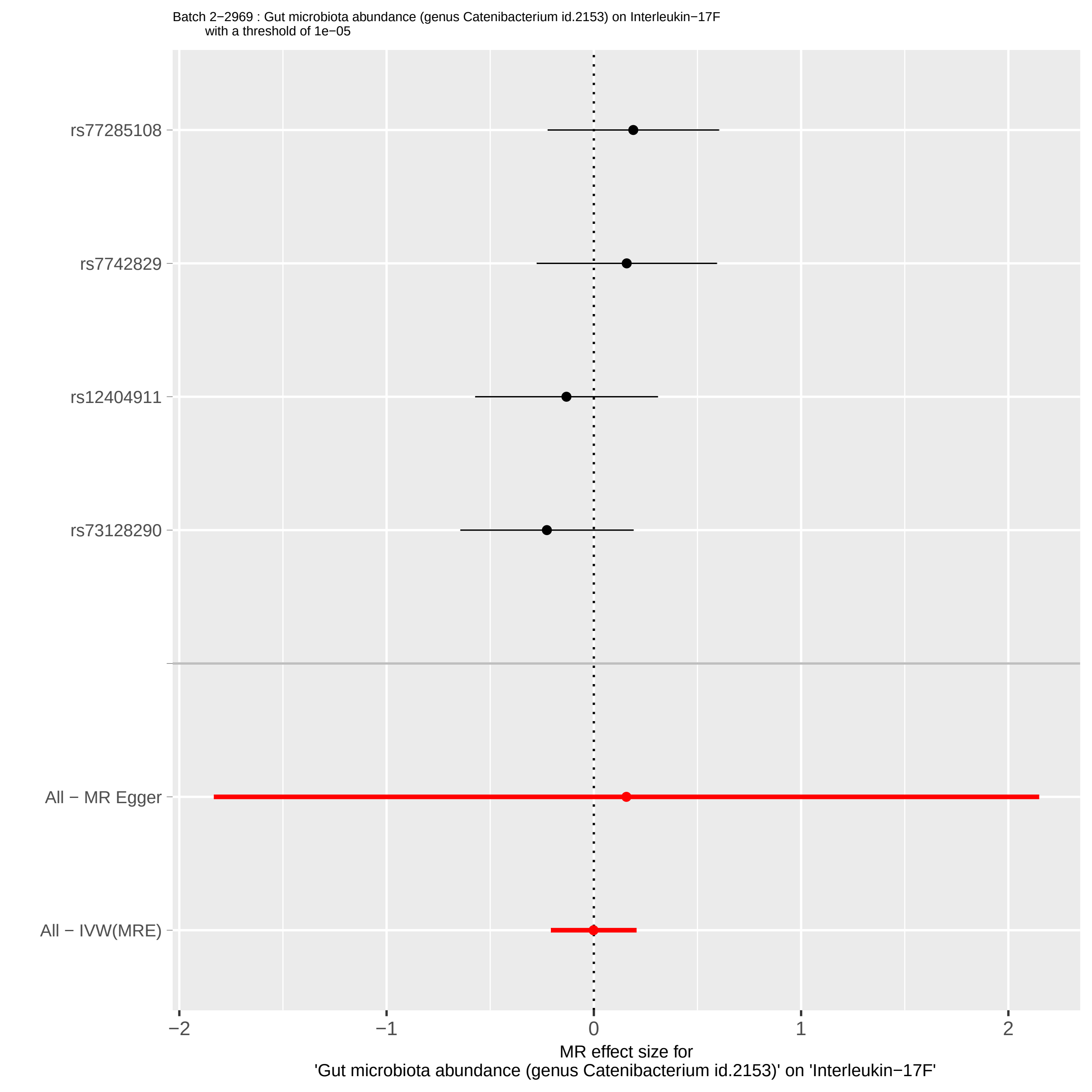

Batch 2−2969 : Gut microbiota abundance (genus Catenibacterium id.2153) on Interleukin−17F
with a threshold of 1e−05
rs77285108
rs7742829
rs12404911
rs73128290
All − MR Egger
All − IVW(MRE)
−2
−1
0
1
2
MR effect size for
'Gut microbiota abundance (genus Catenibacterium id.2153)' on 'Interleukin−17F'

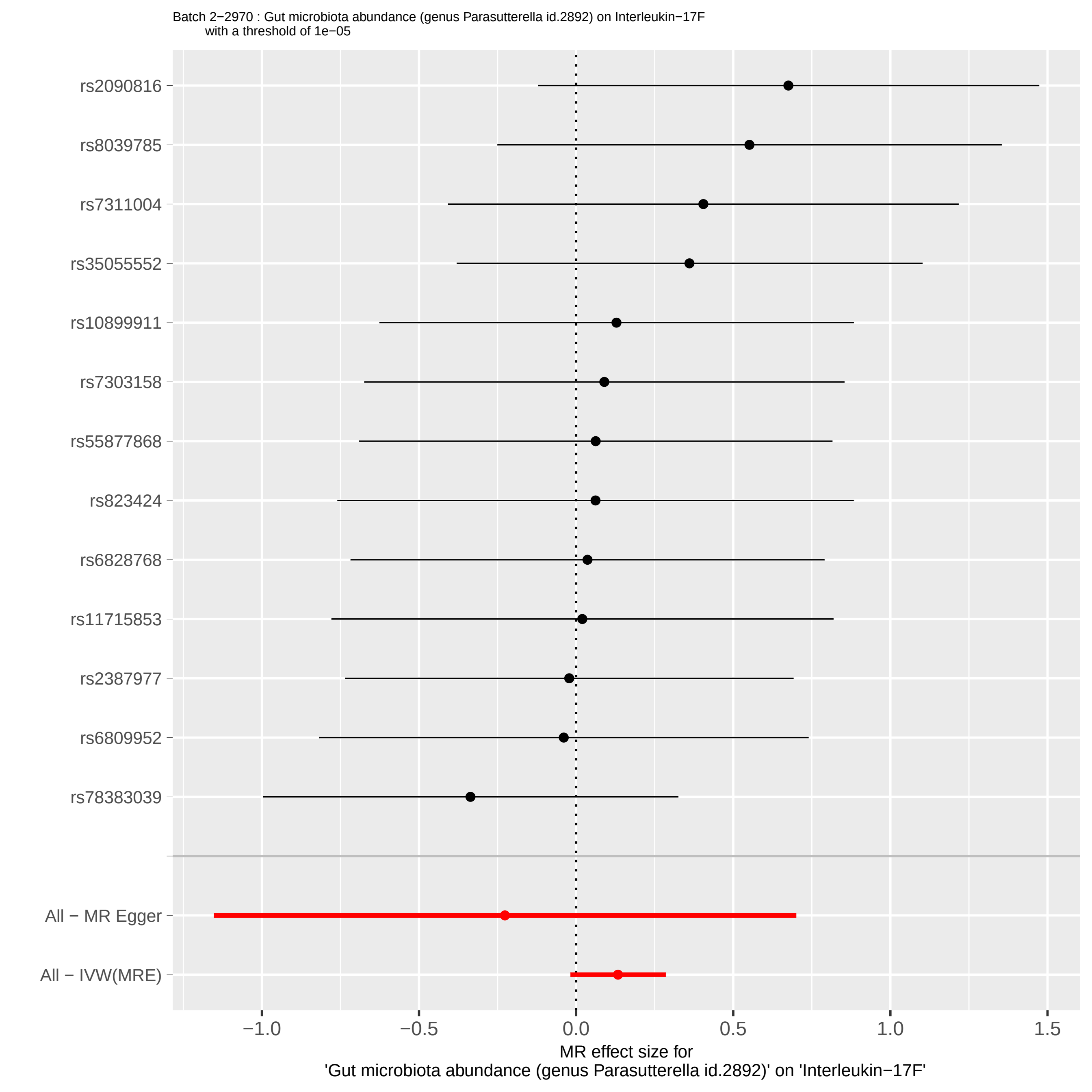

Batch 2−2970 : Gut microbiota abundance (genus Parasutterella id.2892) on Interleukin−17F
with a threshold of 1e−05
rs2090816
rs8039785
rs7311004
rs35055552
rs10899911
rs7303158
rs55877868
rs823424
rs6828768
rs11715853
rs2387977
rs6809952
rs78383039
All − MR Egger
All − IVW(MRE)
−1.0
−0.5
0.0
0.5
1.0
1.5
MR effect size for
'Gut microbiota abundance (genus Parasutterella id.2892)' on 'Interleukin−17F'

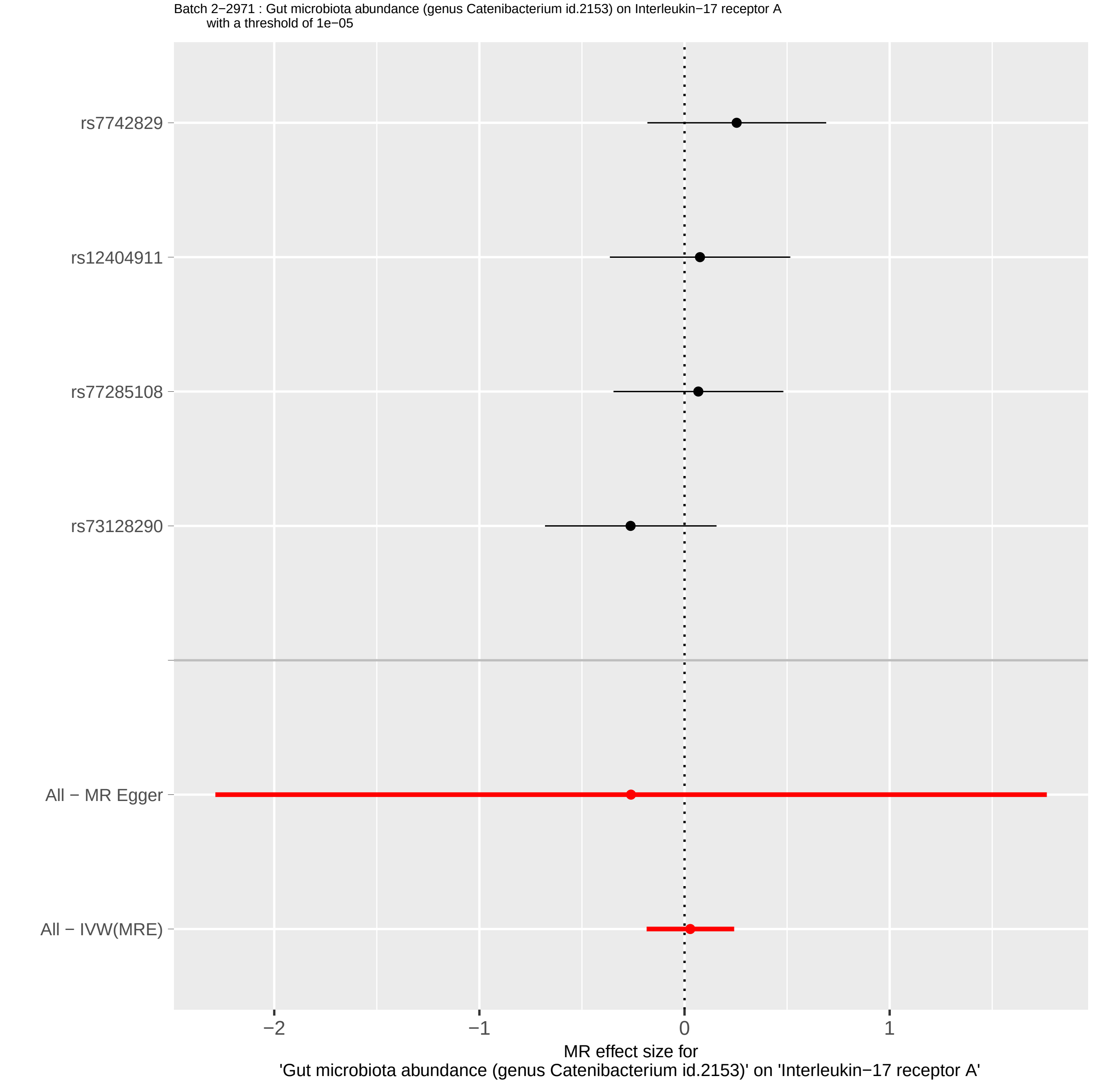
Batch 2−2971 : Gut microbiota abundance (genus Catenibacterium id.2153) on Interleukin−17 receptor A
with a threshold of 1e−05
rs7742829
rs12404911
rs77285108
rs73128290
All − MR Egger
All − IVW(MRE)
−2
−1
0
1
MR effect size for
'Gut microbiota abundance (genus Catenibacterium id.2153)' on 'Interleukin−17 receptor A'

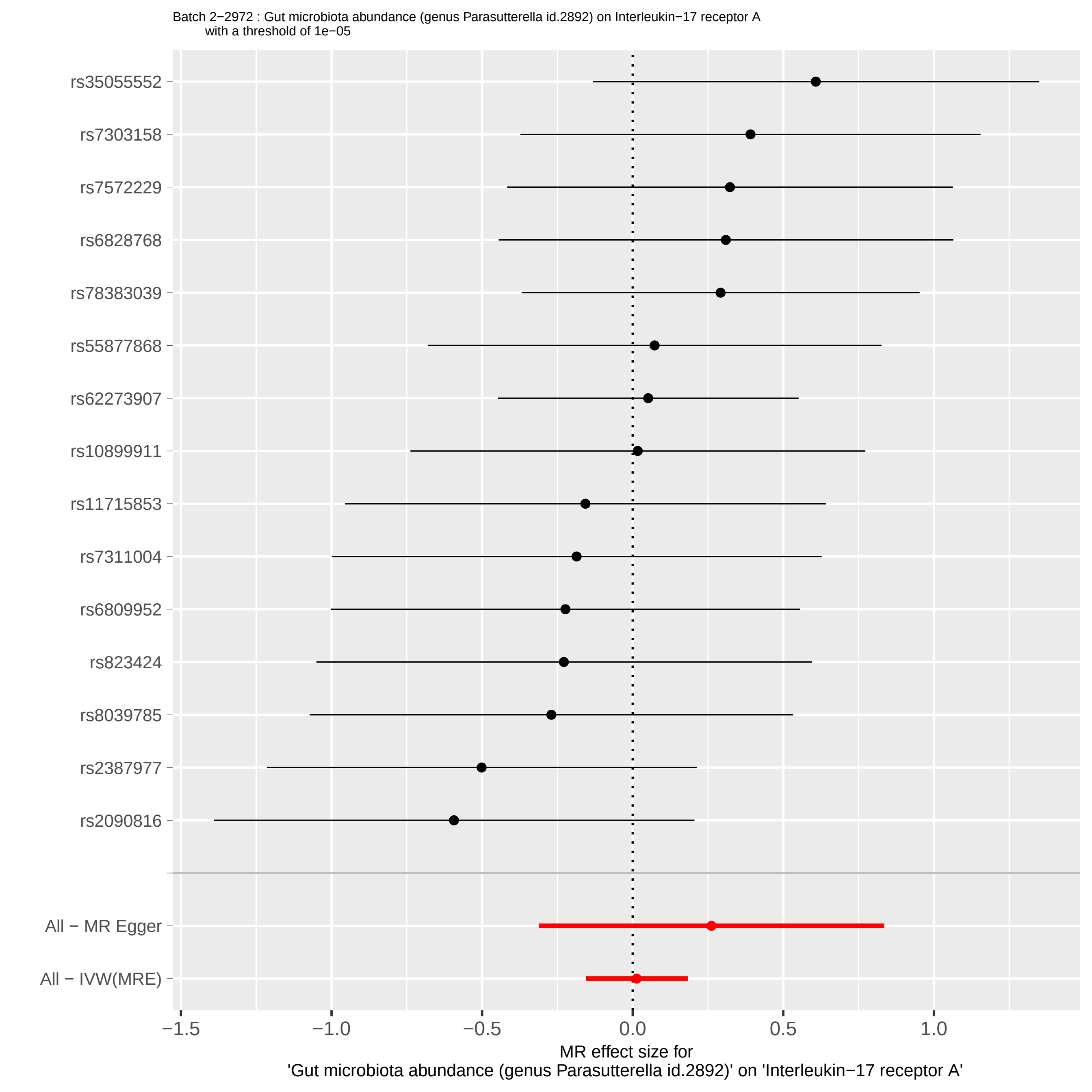

Batch 2−2972 : Gut microbiota abundance (genus Parasutterella id.2892) on Interleukin−17 receptor A
with a threshold of 1e−05
rs35055552
rs7303158
rs7572229
rs6828768
rs78383039
rs55877868
rs62273907
rs10899911
rs11715853
rs7311004
rs6809952
rs823424
rs8039785
rs2387977
rs2090816
All − MR Egger
All − IVW(MRE)
−1.5
−1.0
−0.5
0.0
0.5
1.0
MR effect size for
'Gut microbiota abundance (genus Parasutterella id.2892)' on 'Interleukin−17 receptor A'

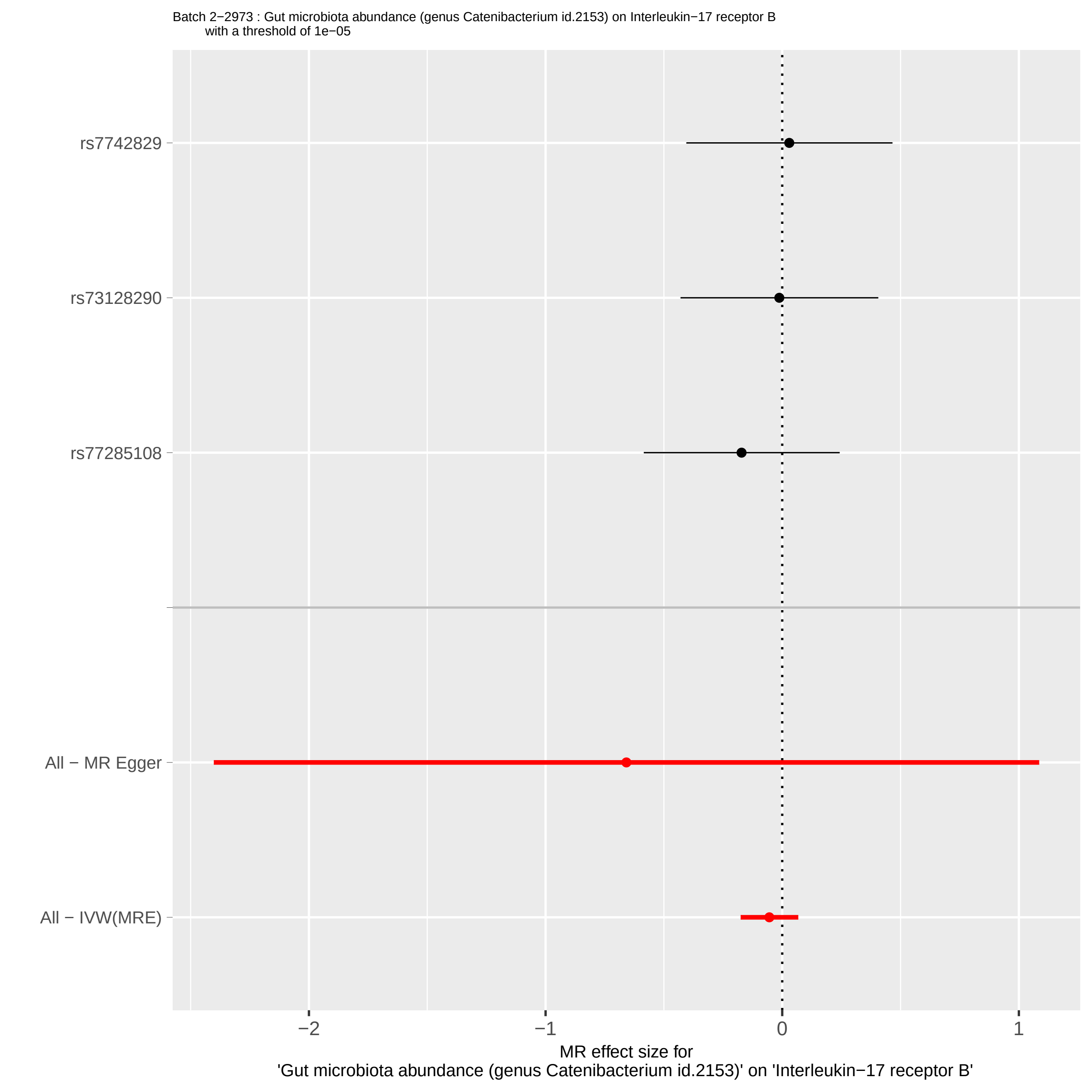
Batch 2−2973 : Gut microbiota abundance (genus Catenibacterium id.2153) on Interleukin−17 receptor B
with a threshold of 1e−05
rs7742829
rs73128290
rs77285108
All − MR Egger
All − IVW(MRE)
−2
−1
0
1
MR effect size for
'Gut microbiota abundance (genus Catenibacterium id.2153)' on 'Interleukin−17 receptor B'

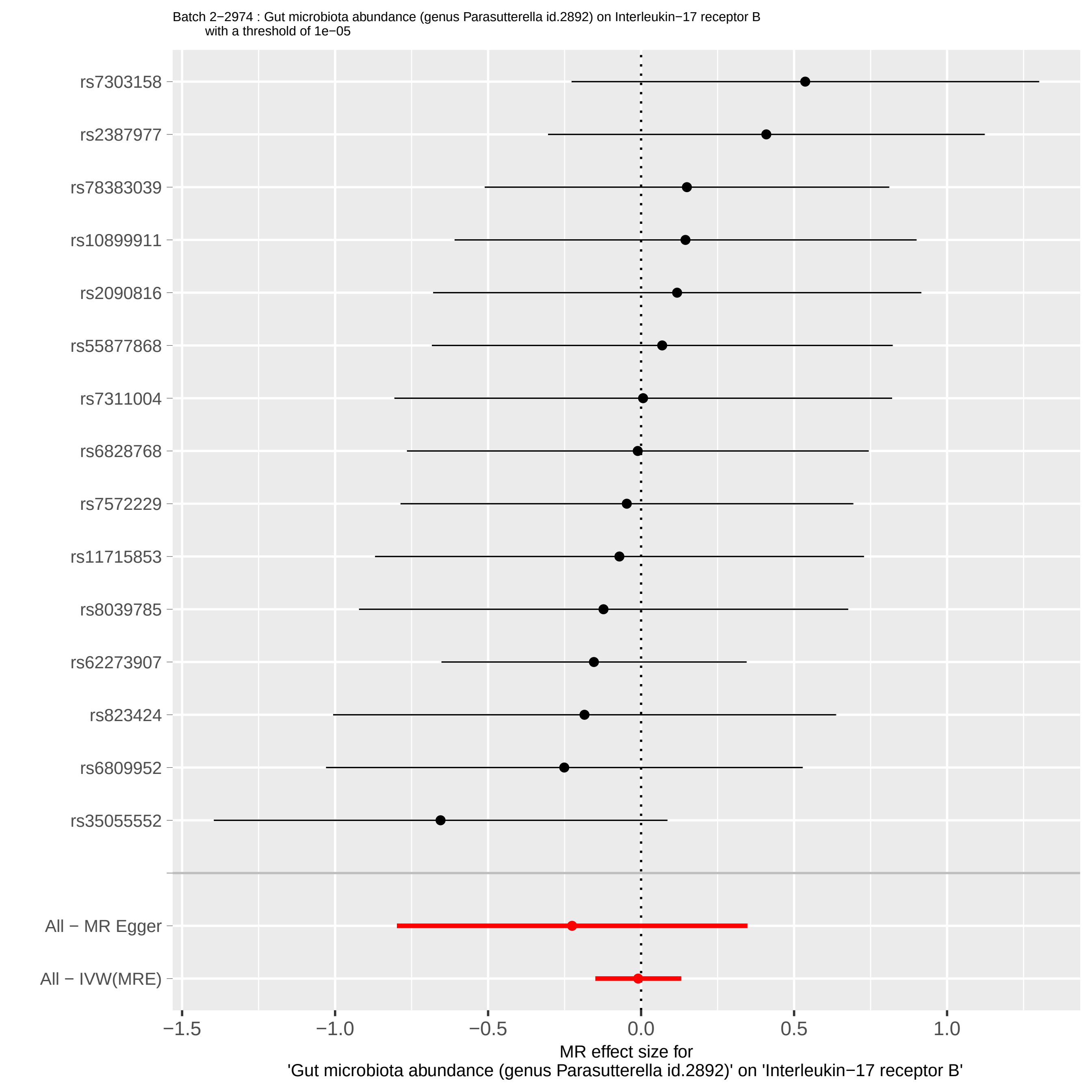
Batch 2−2974 : Gut microbiota abundance (genus Parasutterella id.2892) on Interleukin−17 receptor B
with a threshold of 1e−05
rs7303158
rs2387977
rs78383039
rs10899911
rs2090816
rs55877868
rs7311004
rs6828768
rs7572229
rs11715853
rs8039785
rs62273907
rs823424
rs6809952
rs35055552
All − MR Egger
All − IVW(MRE)
−1.5
−1.0
−0.5
0.0
0.5
1.0
MR effect size for
'Gut microbiota abundance (genus Parasutterella id.2892)' on 'Interleukin−17 receptor B'

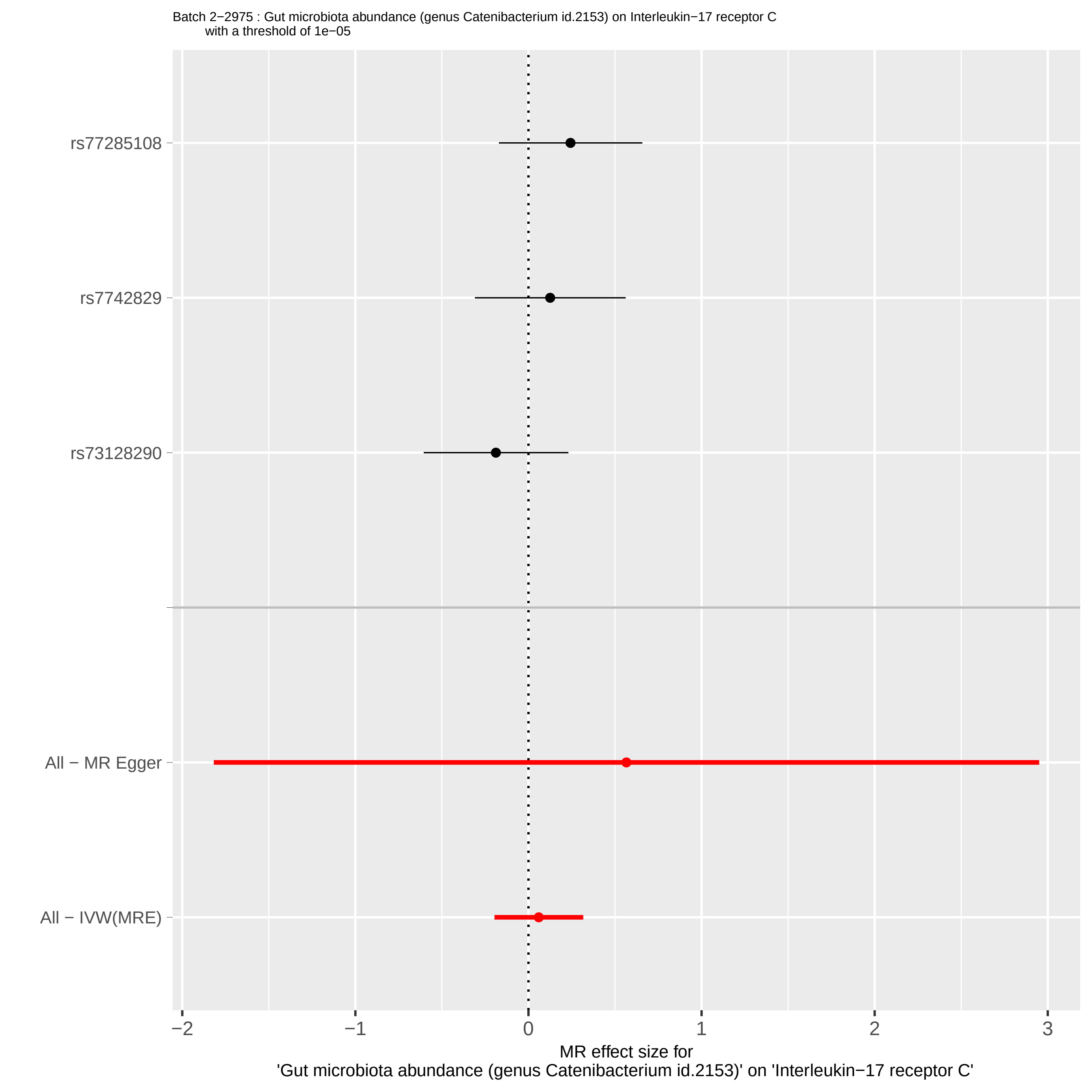
Batch 2−2975 : Gut microbiota abundance (genus Catenibacterium id.2153) on Interleukin−17 receptor C
with a threshold of 1e−05
rs77285108
rs7742829
rs73128290
All − MR Egger
All − IVW(MRE)
−2
−1
0
1
2
3
MR effect size for
'Gut microbiota abundance (genus Catenibacterium id.2153)' on 'Interleukin−17 receptor C'

Batch 2−2976 : Gut microbiota abundance (genus Parasutterella id.2892) on Interleukin−17 receptor C
with a threshold of 1e−05

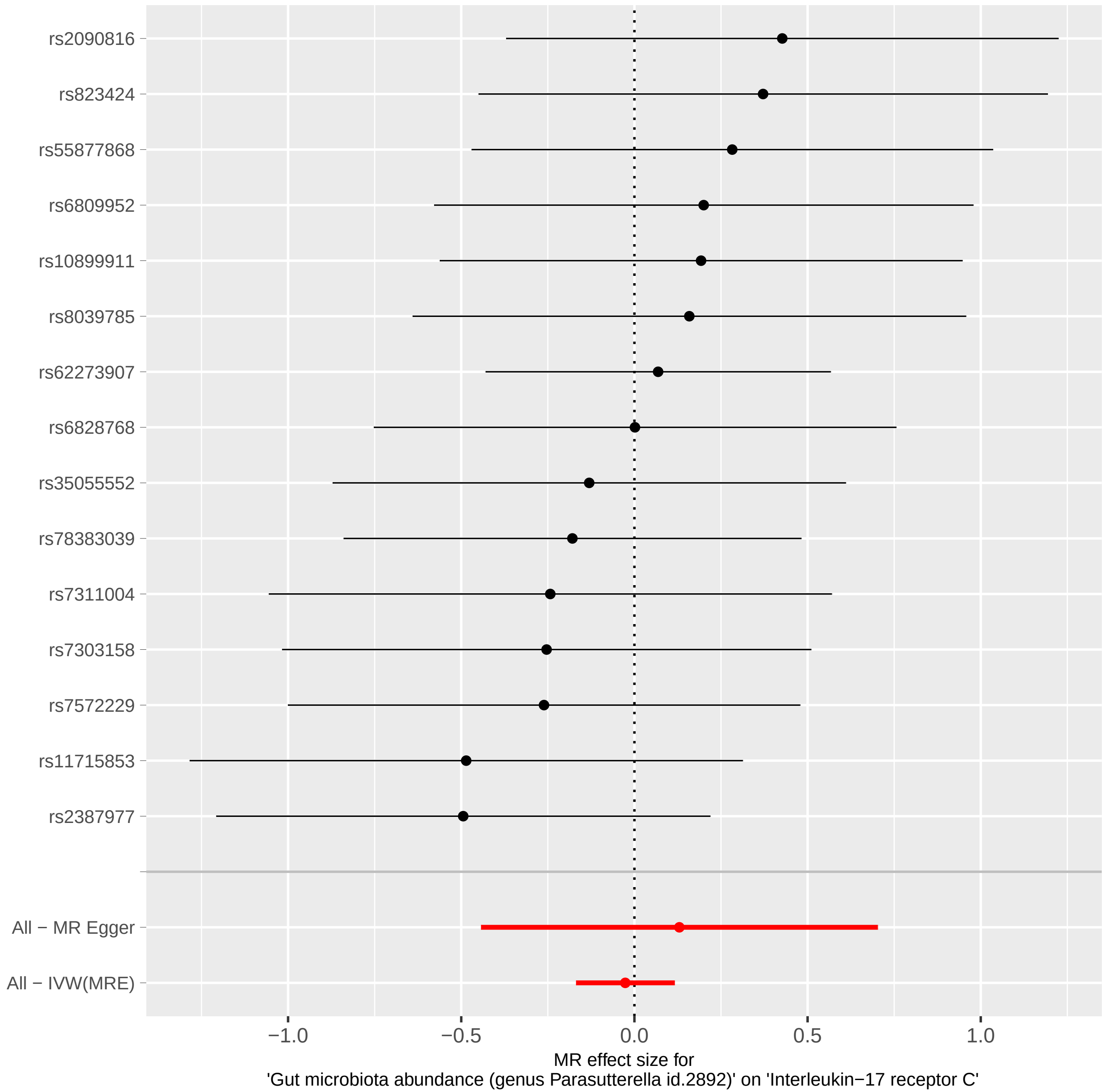

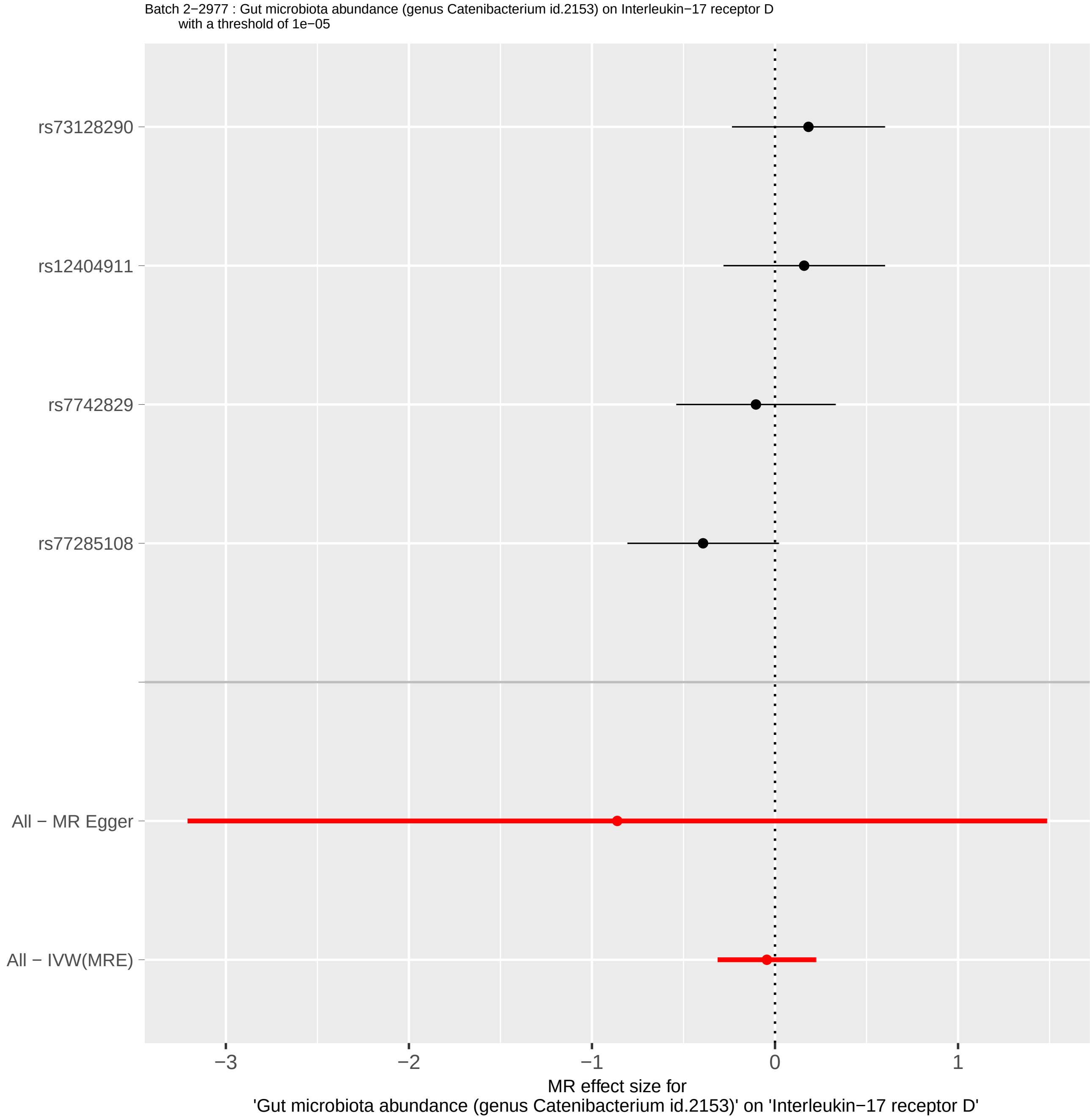

Batch 2−2977 : Gut microbiota abundance (genus Catenibacterium id.2153) on Interleukin−17 receptor D
with a threshold of 1e−05
rs73128290
rs12404911
rs7742829
rs77285108
All − MR Egger
All − IVW(MRE)
−3
−2
−1
0
1
MR effect size for
'Gut microbiota abundance (genus Catenibacterium id.2153)' on 'Interleukin−17 receptor D'

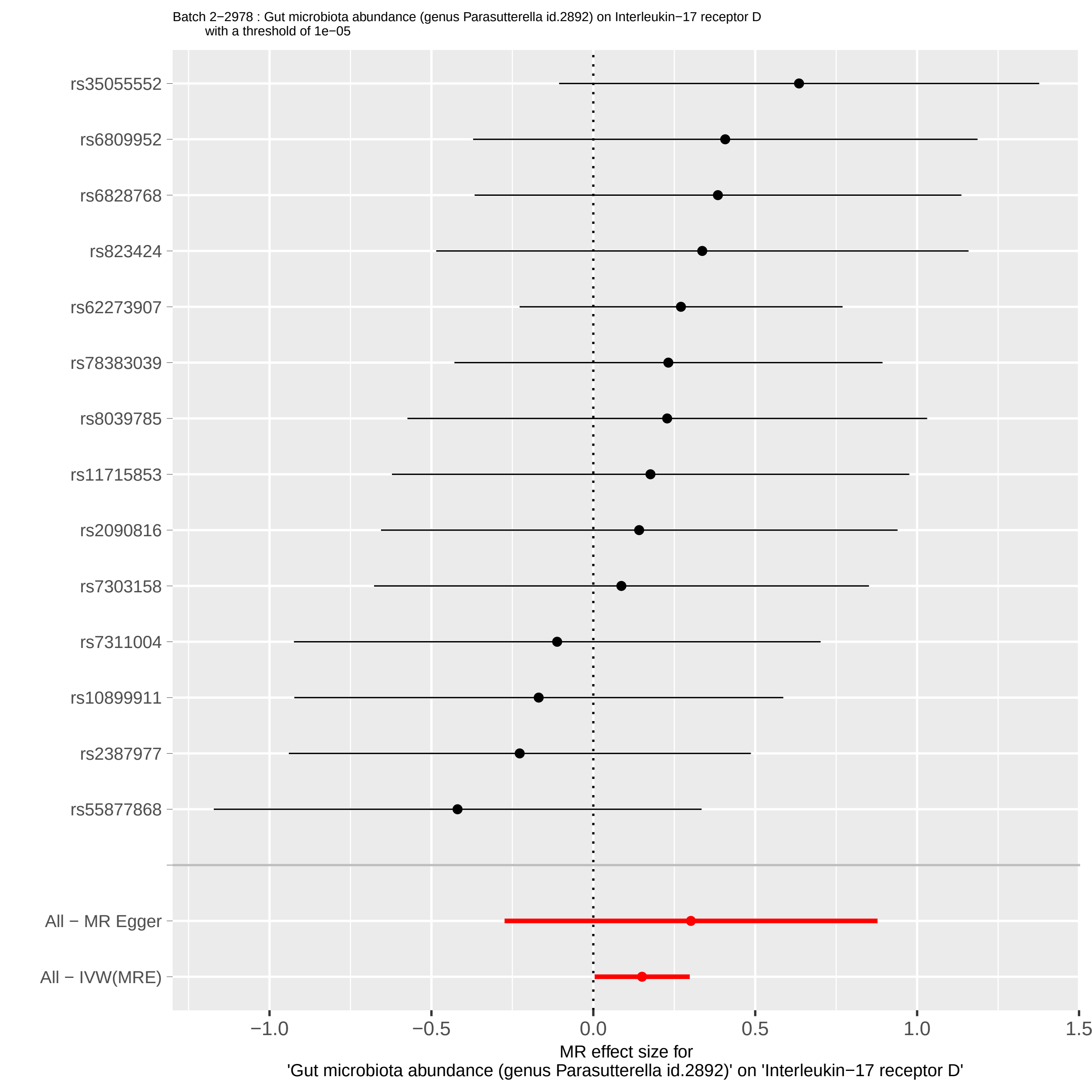

Batch 2−2978 : Gut microbiota abundance (genus Parasutterella id.2892) on Interleukin−17 receptor D
with a threshold of 1e−05
rs35055552
rs6809952
rs6828768
rs823424
rs62273907
rs78383039
rs8039785
rs11715853
rs2090816
rs7303158
rs7311004
rs10899911
rs2387977
rs55877868
All − MR Egger
All − IVW(MRE)
−1.0
−0.5
0.0
0.5
1.0
1.5
MR effect size for
'Gut microbiota abundance (genus Parasutterella id.2892)' on 'Interleukin−17 receptor D'

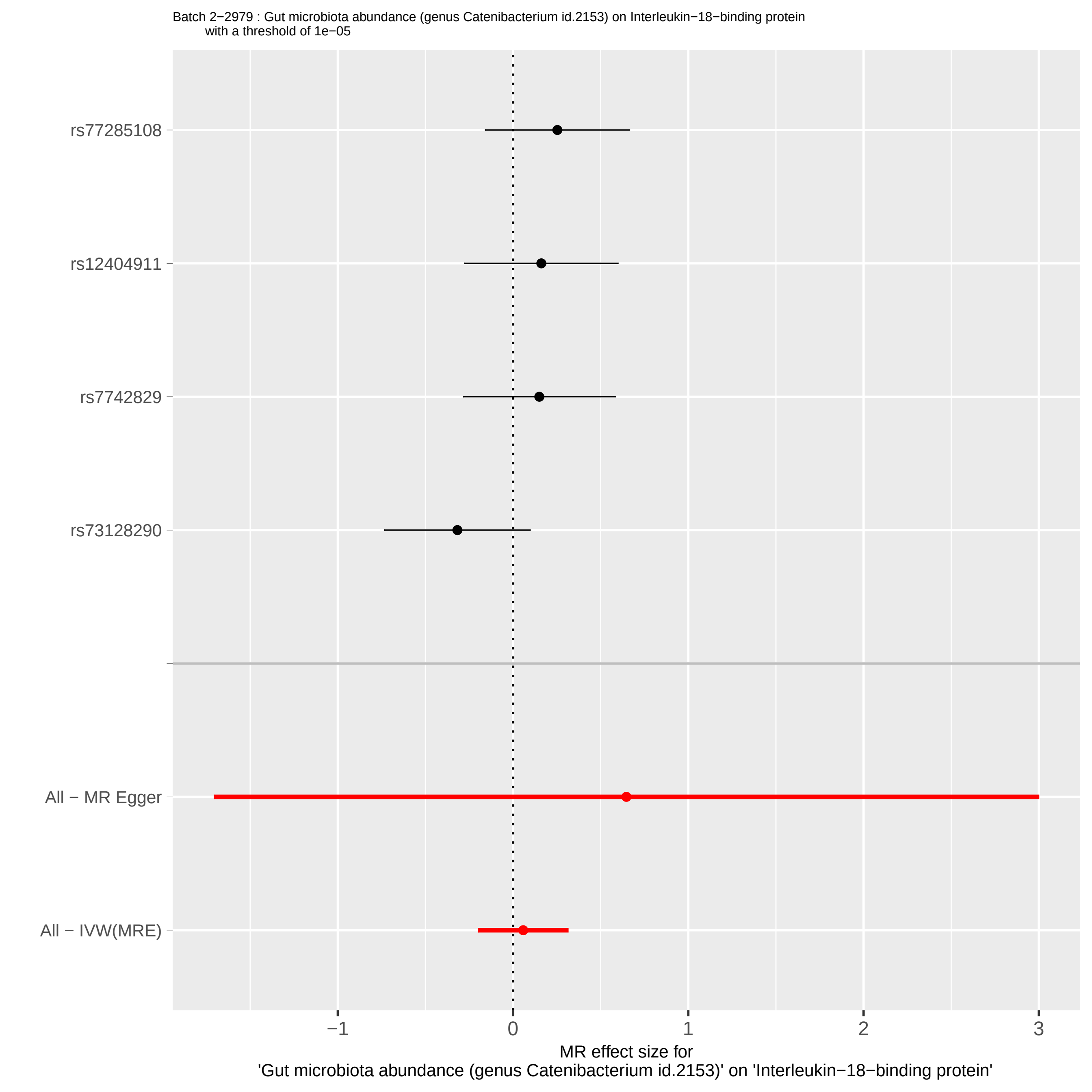
Batch 2−2979 : Gut microbiota abundance (genus Catenibacterium id.2153) on Interleukin−18−binding protein
with a threshold of 1e−05
rs77285108
rs12404911
rs7742829
rs73128290
All − MR Egger
All − IVW(MRE)
−1
0
1
2
3
MR effect size for
'Gut microbiota abundance (genus Catenibacterium id.2153)' on 'Interleukin−18−binding protein'

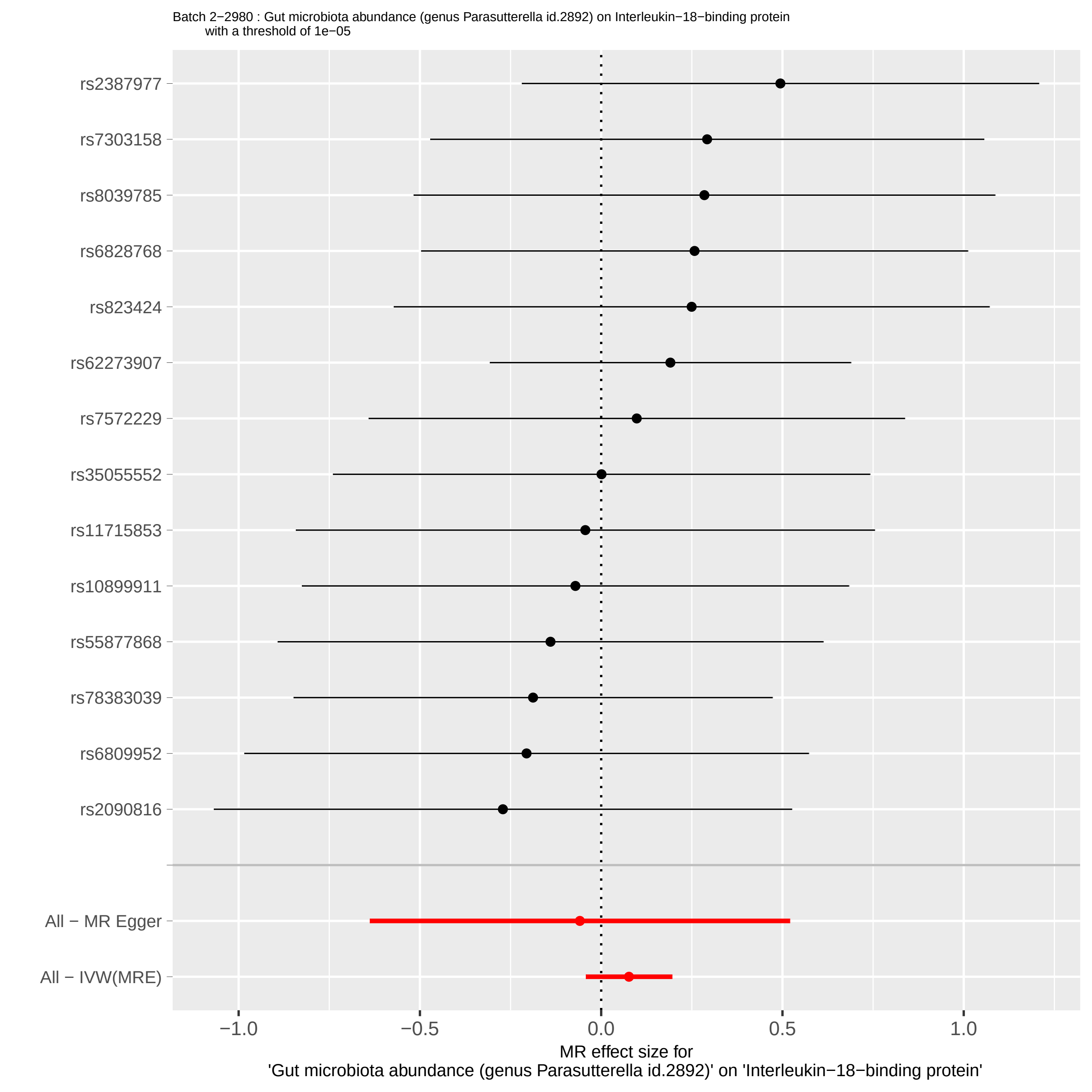

Batch 2−2980 : Gut microbiota abundance (genus Parasutterella id.2892) on Interleukin−18−binding protein
with a threshold of 1e−05
rs2387977
rs7303158
rs8039785
rs6828768
rs823424
rs62273907
rs7572229
rs35055552
rs11715853
rs10899911
rs55877868
rs78383039
rs6809952
rs2090816
All − MR Egger
All − IVW(MRE)
−1.0
−0.5
0.0
0.5
1.0
MR effect size for
'Gut microbiota abundance (genus Parasutterella id.2892)' on 'Interleukin−18−binding protein'

Batch 2−2981 : Gut microbiota abundance (genus Catenibacterium id.2153) on Interleukin−18 receptor 1
with a threshold of 1e−05

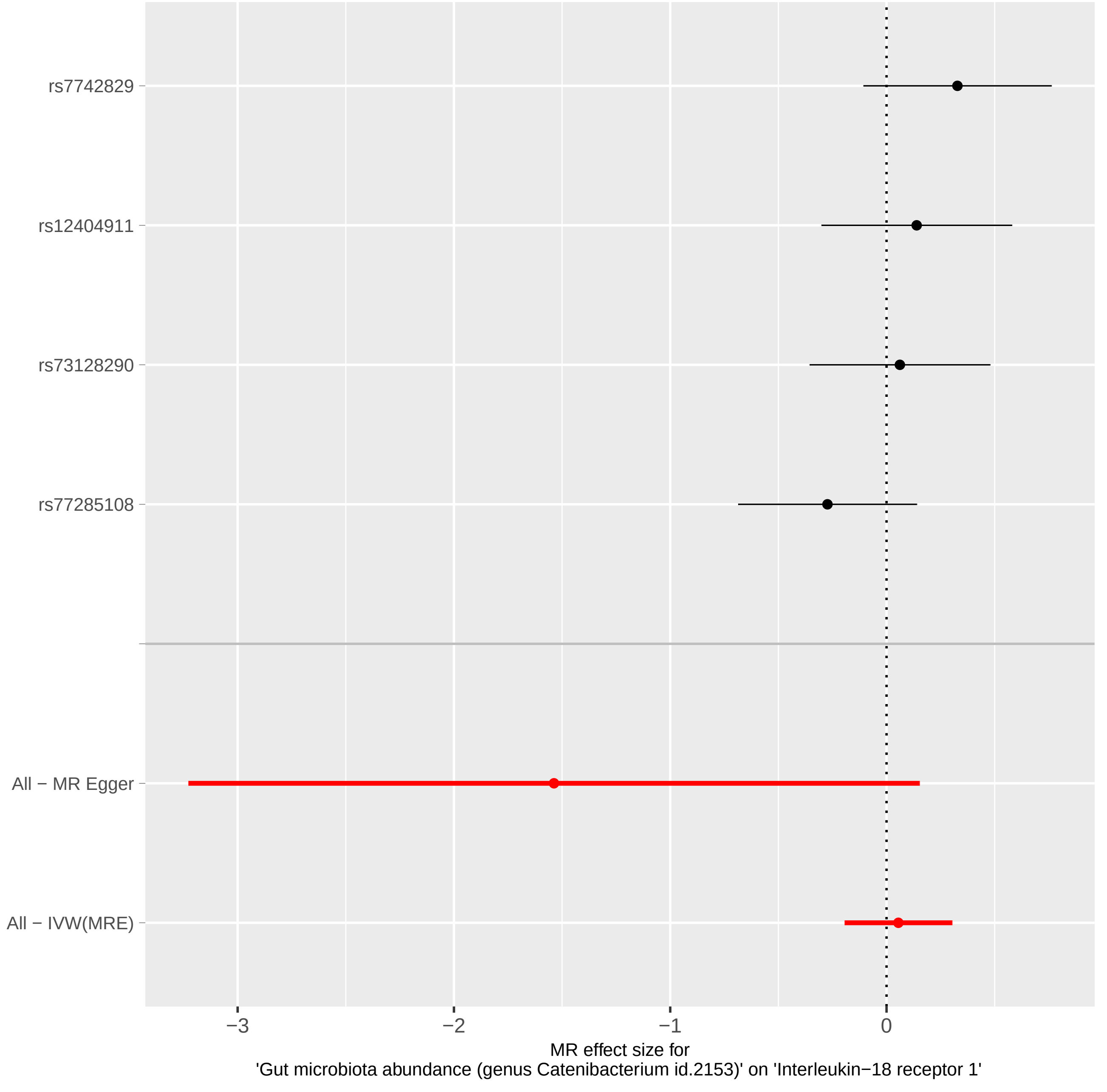

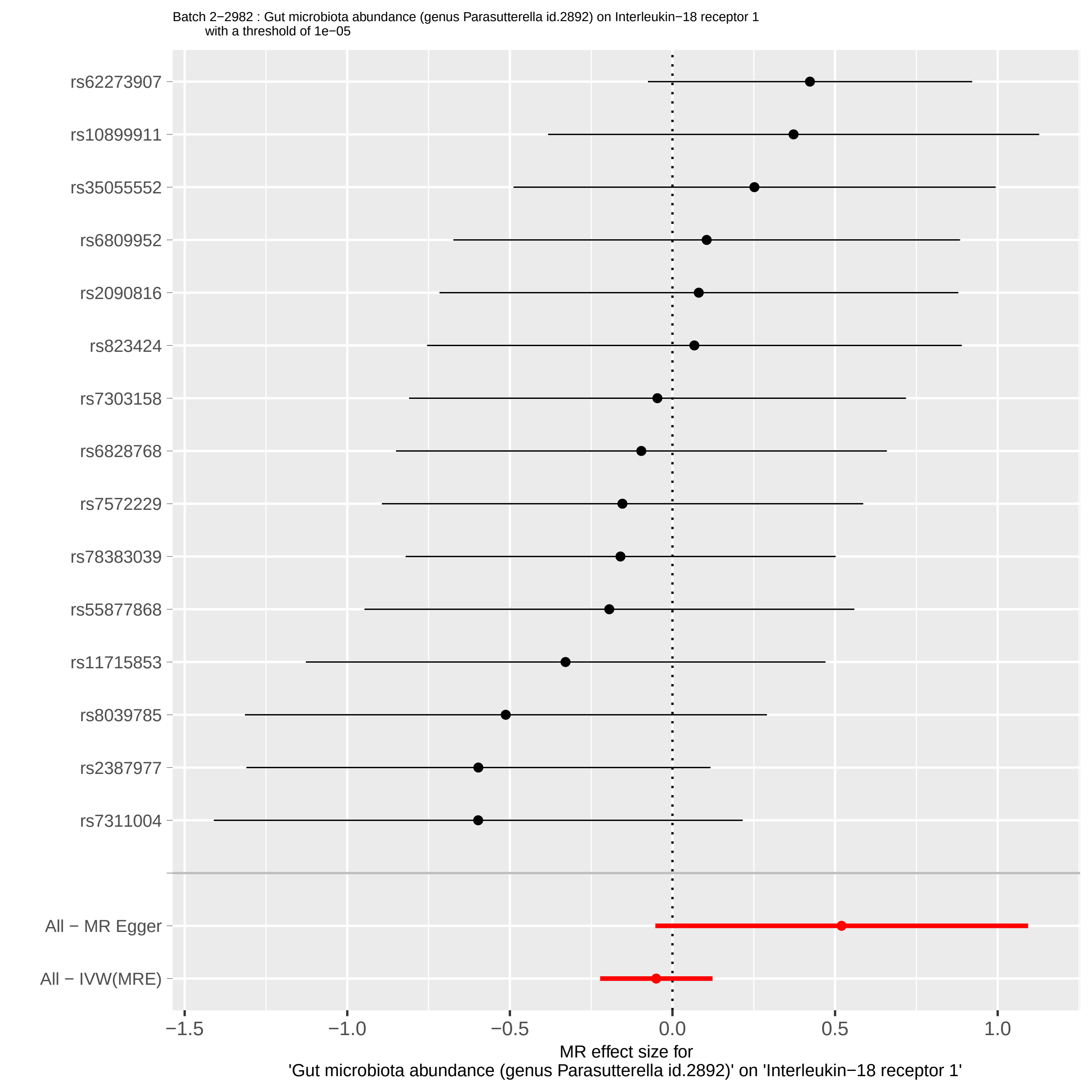

Batch 2−2982 : Gut microbiota abundance (genus Parasutterella id.2892) on Interleukin−18 receptor 1
with a threshold of 1e−05
rs62273907
rs10899911
rs35055552
rs6809952
rs2090816
rs823424
rs7303158
rs6828768
rs7572229
rs78383039
rs55877868
rs11715853
rs8039785
rs2387977
rs7311004
All − MR Egger
All − IVW(MRE)
−1.5
−1.0
−0.5
0.0
0.5
1.0
MR effect size for
'Gut microbiota abundance (genus Parasutterella id.2892)' on 'Interleukin−18 receptor 1'

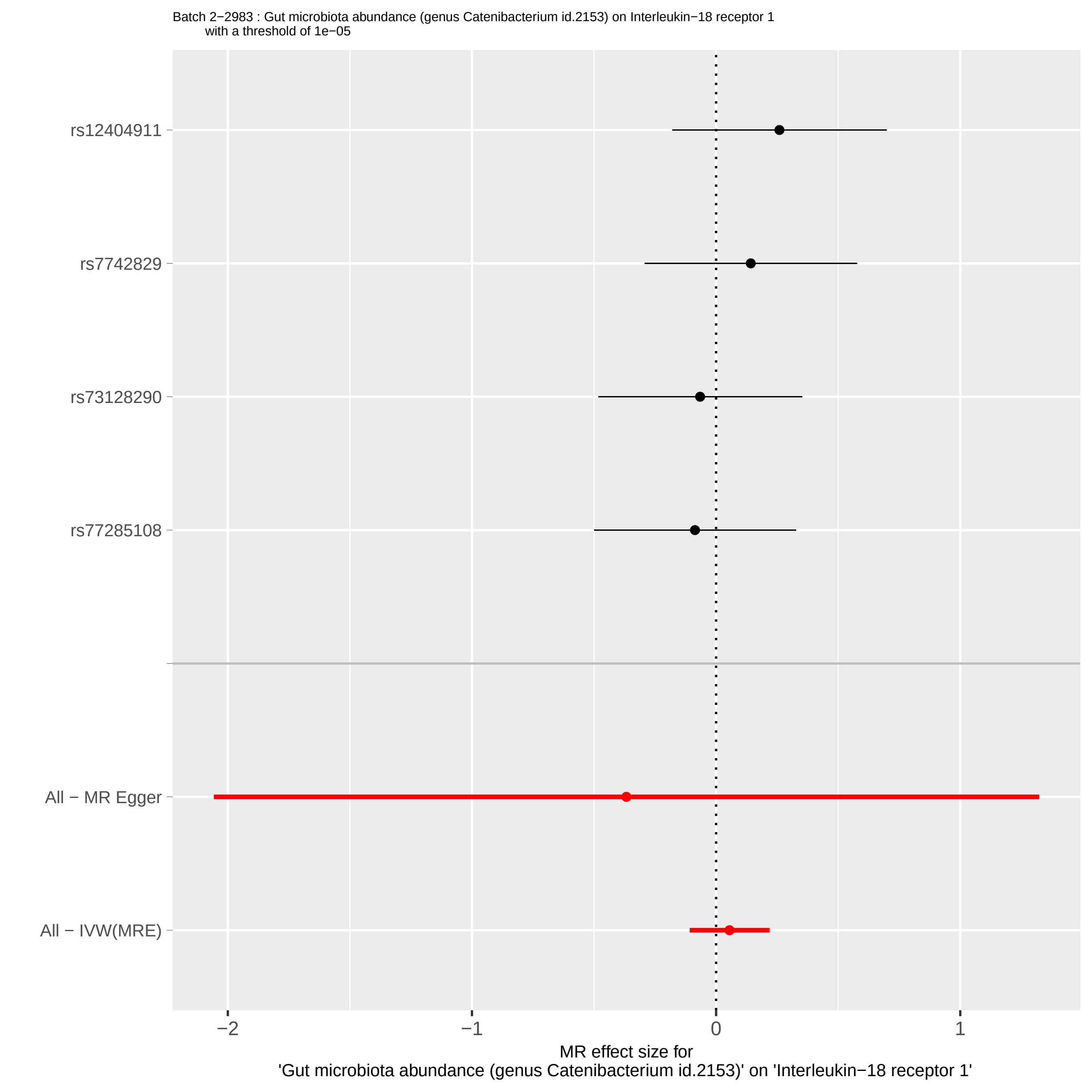

Batch 2−2983 : Gut microbiota abundance (genus Catenibacterium id.2153) on Interleukin−18 receptor 1
with a threshold of 1e−05
rs12404911
rs7742829
rs73128290
rs77285108
All − MR Egger
All − IVW(MRE)
−2
−1
0
1
MR effect size for
'Gut microbiota abundance (genus Catenibacterium id.2153)' on 'Interleukin−18 receptor 1'

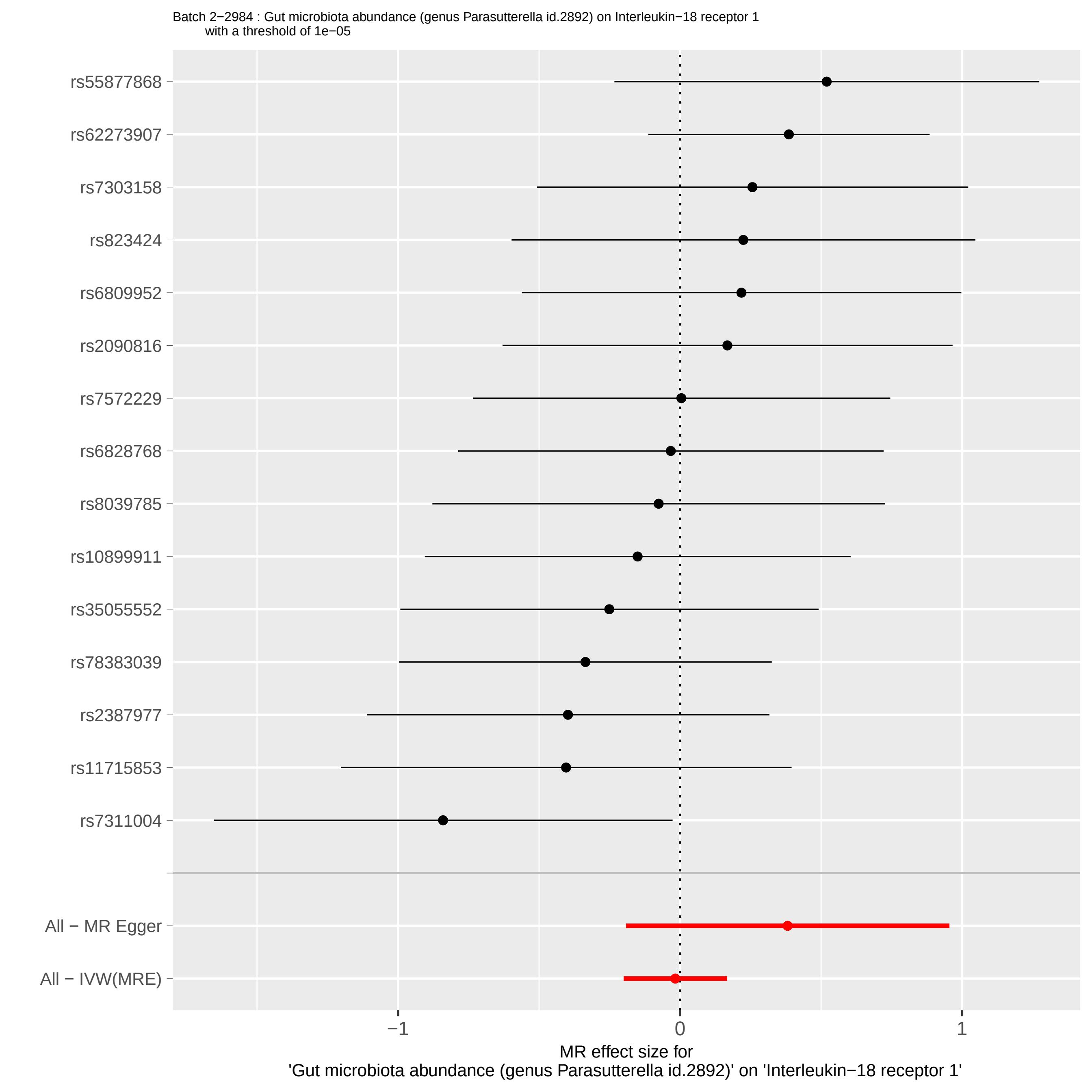
Batch 2−2984 : Gut microbiota abundance (genus Parasutterella id.2892) on Interleukin−18 receptor 1
with a threshold of 1e−05
rs55877868
rs62273907
rs7303158
rs823424
rs6809952
rs2090816
rs7572229
rs6828768
rs8039785
rs10899911
rs35055552
rs78383039
rs2387977
rs11715853
rs7311004
All − MR Egger
All − IVW(MRE)
−1
0
1
MR effect size for
'Gut microbiota abundance (genus Parasutterella id.2892)' on 'Interleukin−18 receptor 1'

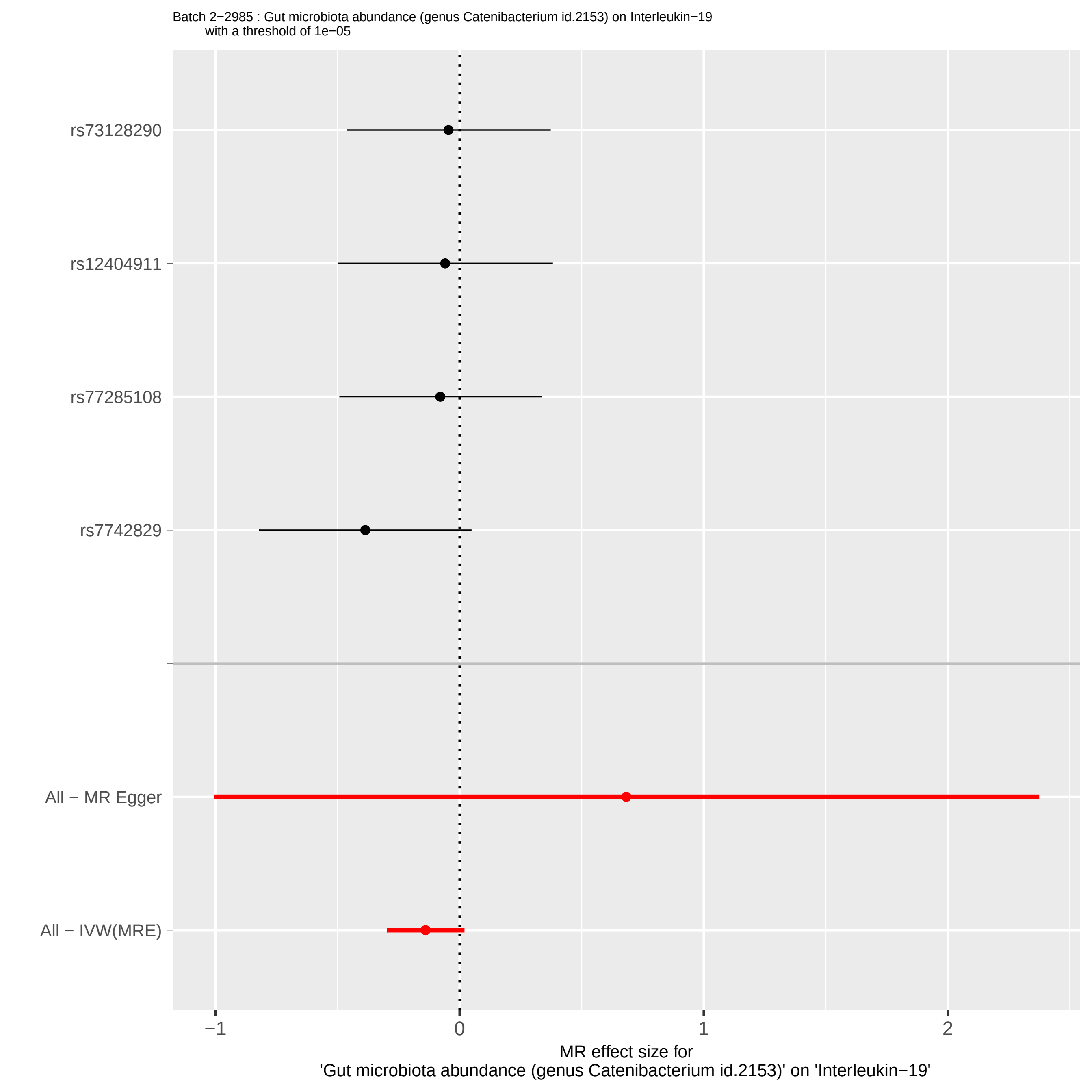
Batch 2−2985 : Gut microbiota abundance (genus Catenibacterium id.2153) on Interleukin−19
with a threshold of 1e−05
rs73128290
rs12404911
rs77285108
rs7742829
All − MR Egger
All − IVW(MRE)
−1
0
1
2
MR effect size for
'Gut microbiota abundance (genus Catenibacterium id.2153)' on 'Interleukin−19'

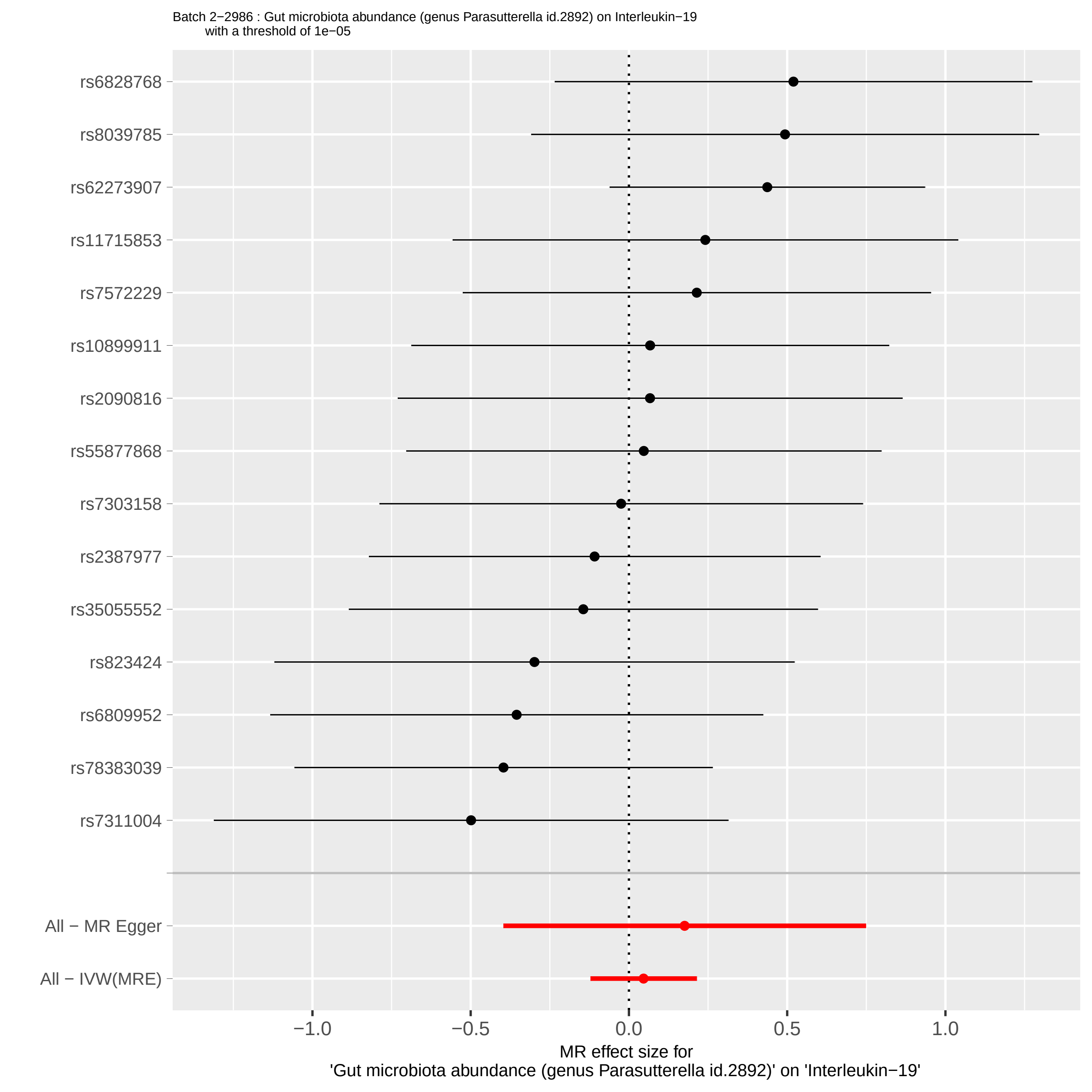

Batch 2−2986 : Gut microbiota abundance (genus Parasutterella id.2892) on Interleukin−19
with a threshold of 1e−05
rs6828768
rs8039785
rs62273907
rs11715853
rs7572229
rs10899911
rs2090816
rs55877868
rs7303158
rs2387977
rs35055552
rs823424
rs6809952
rs78383039
rs7311004
All − MR Egger
All − IVW(MRE)
−1.0
−0.5
0.0
0.5
1.0
MR effect size for
'Gut microbiota abundance (genus Parasutterella id.2892)' on 'Interleukin−19'

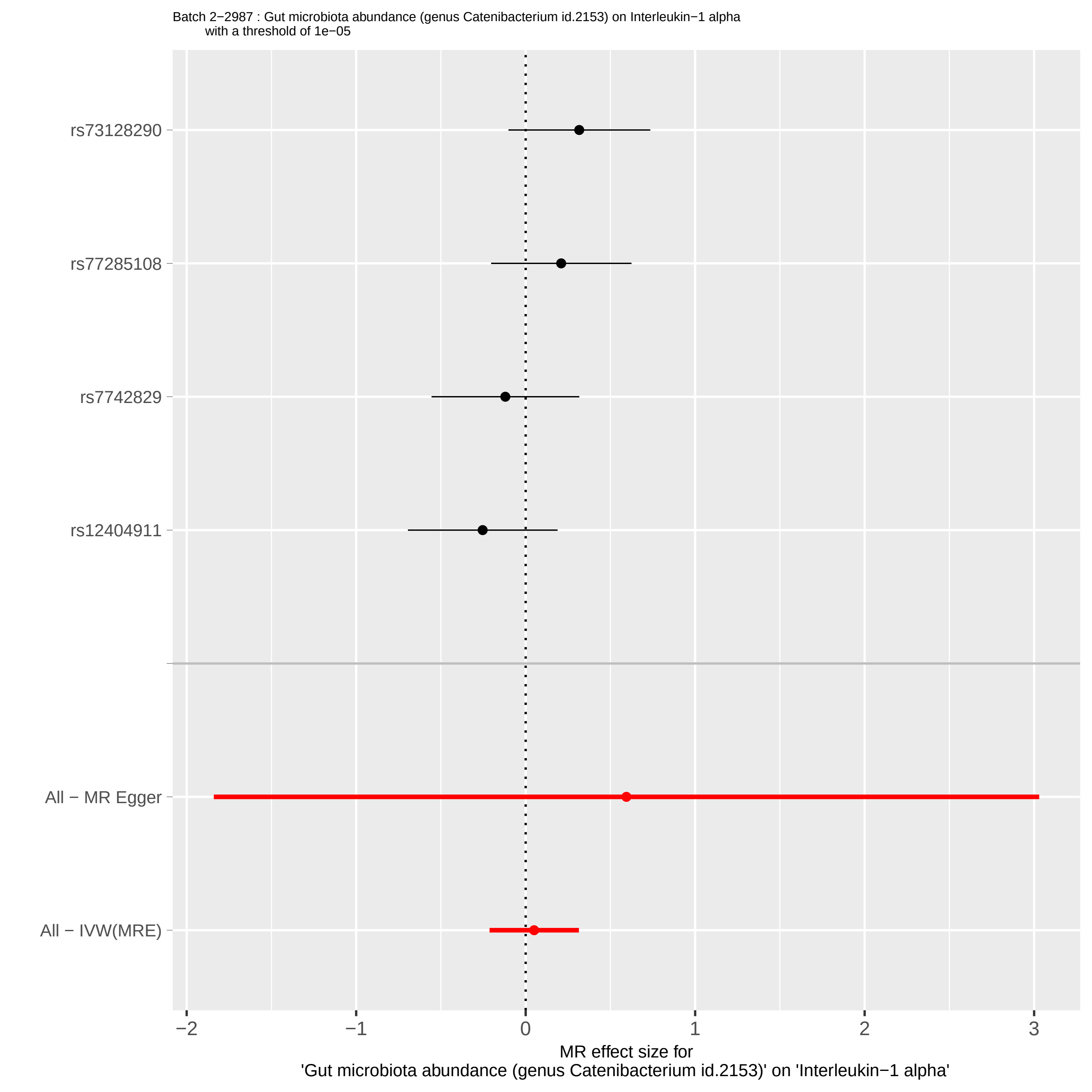
Batch 2−2987 : Gut microbiota abundance (genus Catenibacterium id.2153) on Interleukin−1 alpha
with a threshold of 1e−05
rs73128290
rs77285108
rs7742829
rs12404911
All − MR Egger
All − IVW(MRE)
−2
−1
0
1
2
3
MR effect size for
'Gut microbiota abundance (genus Catenibacterium id.2153)' on 'Interleukin−1 alpha'

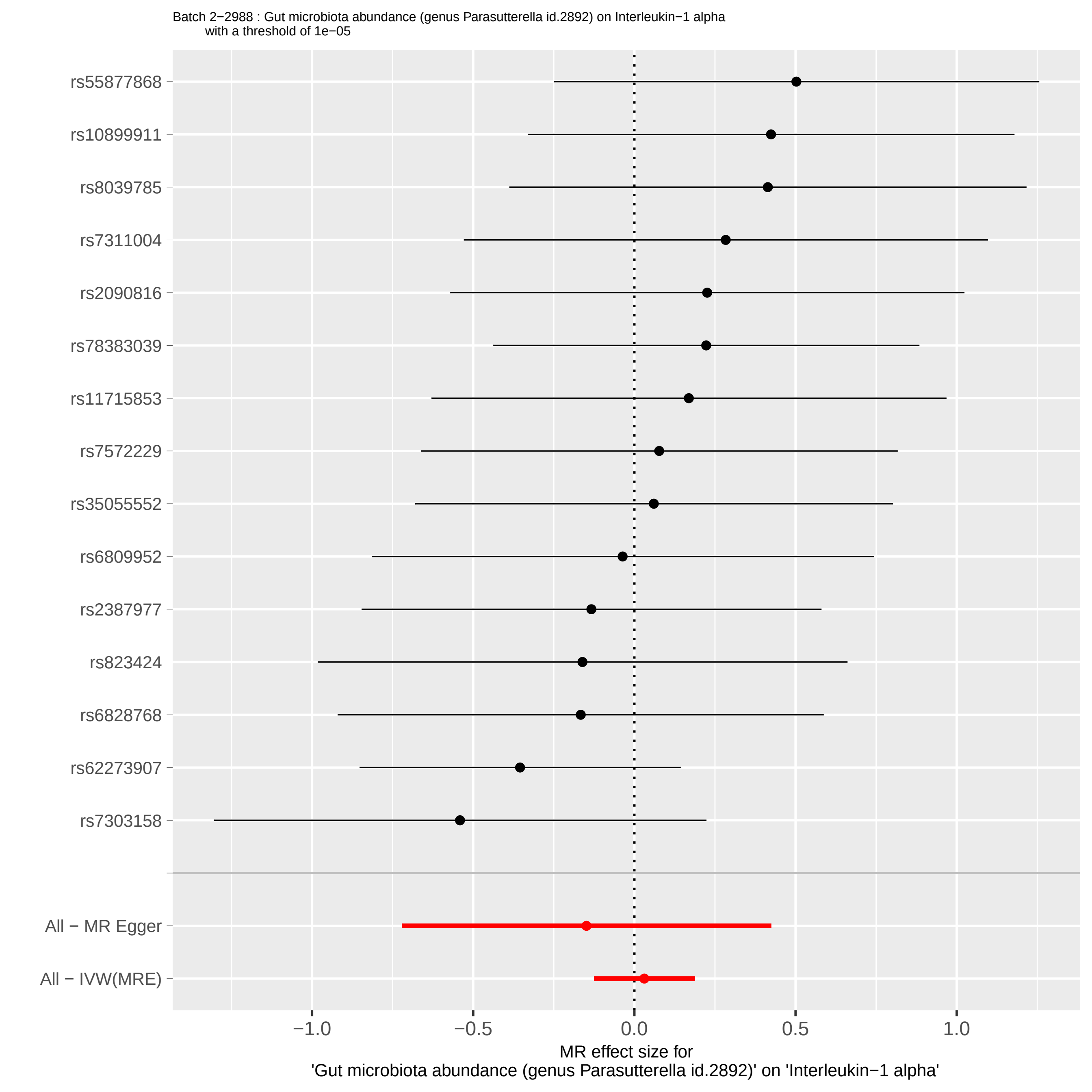
Batch 2−2988 : Gut microbiota abundance (genus Parasutterella id.2892) on Interleukin−1 alpha
with a threshold of 1e−05
rs55877868
rs10899911
rs8039785
rs7311004
rs2090816
rs78383039
rs11715853
rs7572229
rs35055552
rs6809952
rs2387977
rs823424
rs6828768
rs62273907
rs7303158
All − MR Egger
All − IVW(MRE)
−1.0
−0.5
0.0
0.5
1.0
MR effect size for
'Gut microbiota abundance (genus Parasutterella id.2892)' on 'Interleukin−1 alpha'

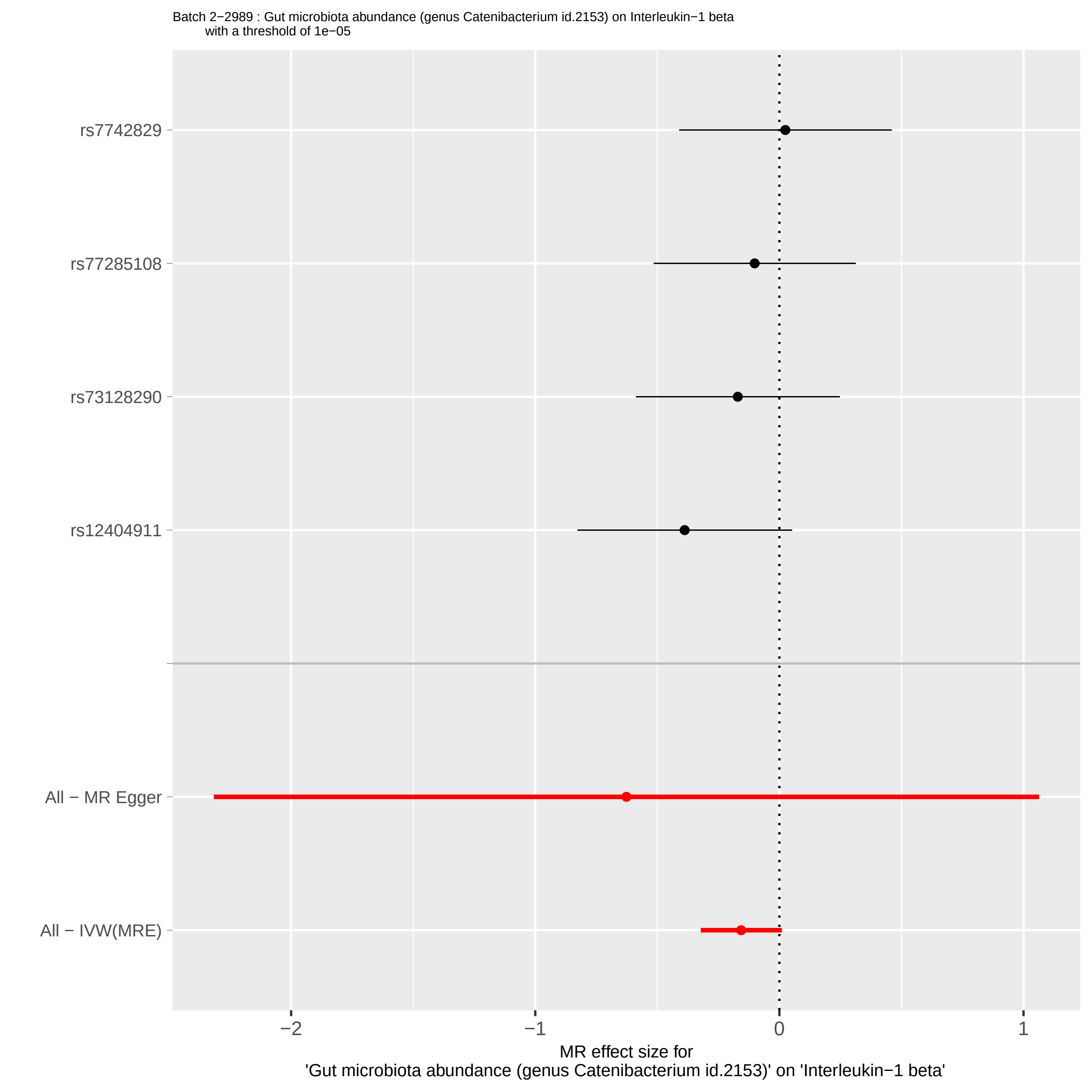
Batch 2−2989 : Gut microbiota abundance (genus Catenibacterium id.2153) on Interleukin−1 beta
with a threshold of 1e−05
rs7742829
rs77285108
rs73128290
rs12404911
All − MR Egger
All − IVW(MRE)
−2
−1
0
1
MR effect size for
'Gut microbiota abundance (genus Catenibacterium id.2153)' on 'Interleukin−1 beta'

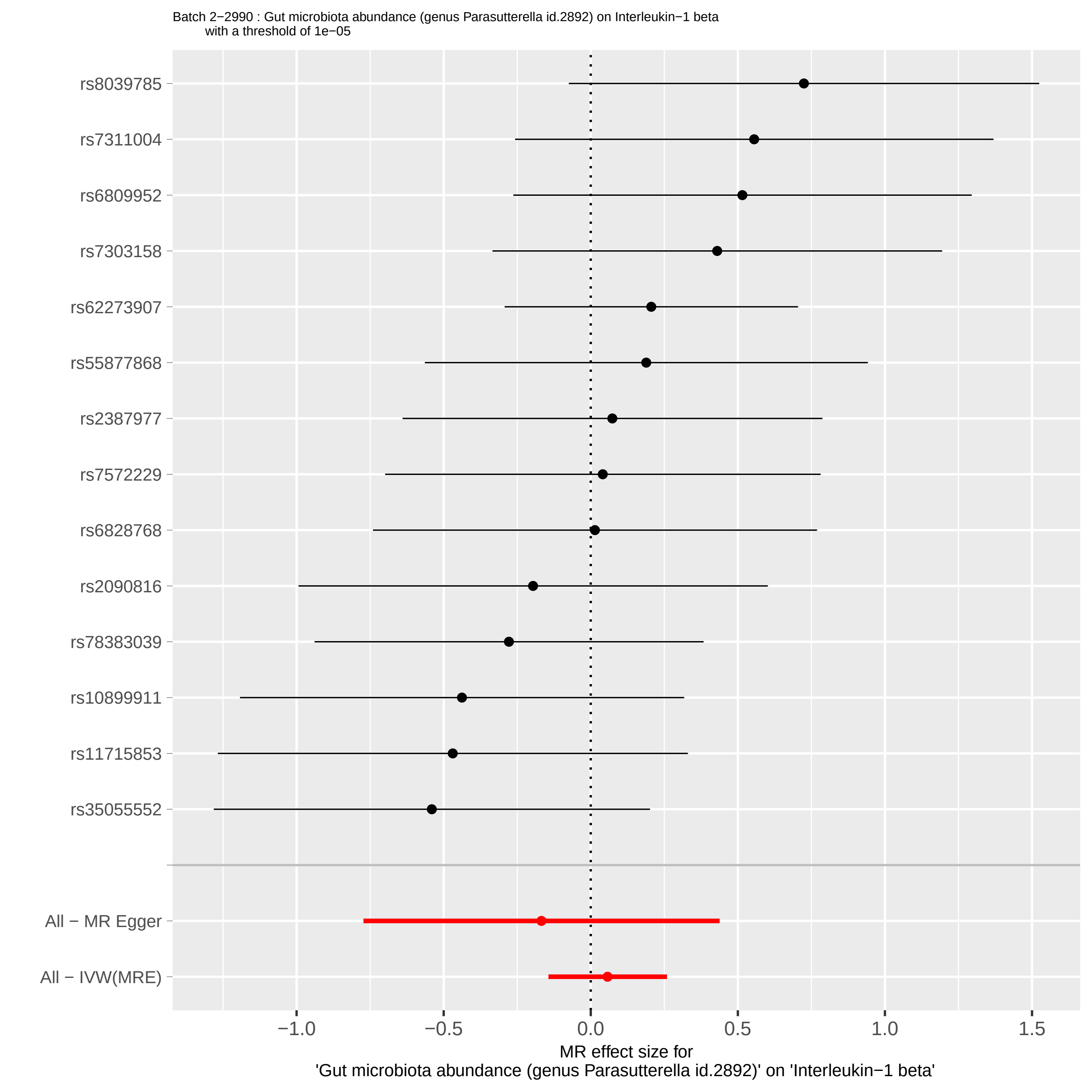
Batch 2−2990 : Gut microbiota abundance (genus Parasutterella id.2892) on Interleukin−1 beta
with a threshold of 1e−05
rs8039785
rs7311004
rs6809952
rs7303158
rs62273907
rs55877868
rs2387977
rs7572229
rs6828768
rs2090816
rs78383039
rs10899911
rs11715853
rs35055552
All − MR Egger
All − IVW(MRE)
−1.0
−0.5
0.0
0.5
1.0
1.5
MR effect size for
'Gut microbiota abundance (genus Parasutterella id.2892)' on 'Interleukin−1 beta'

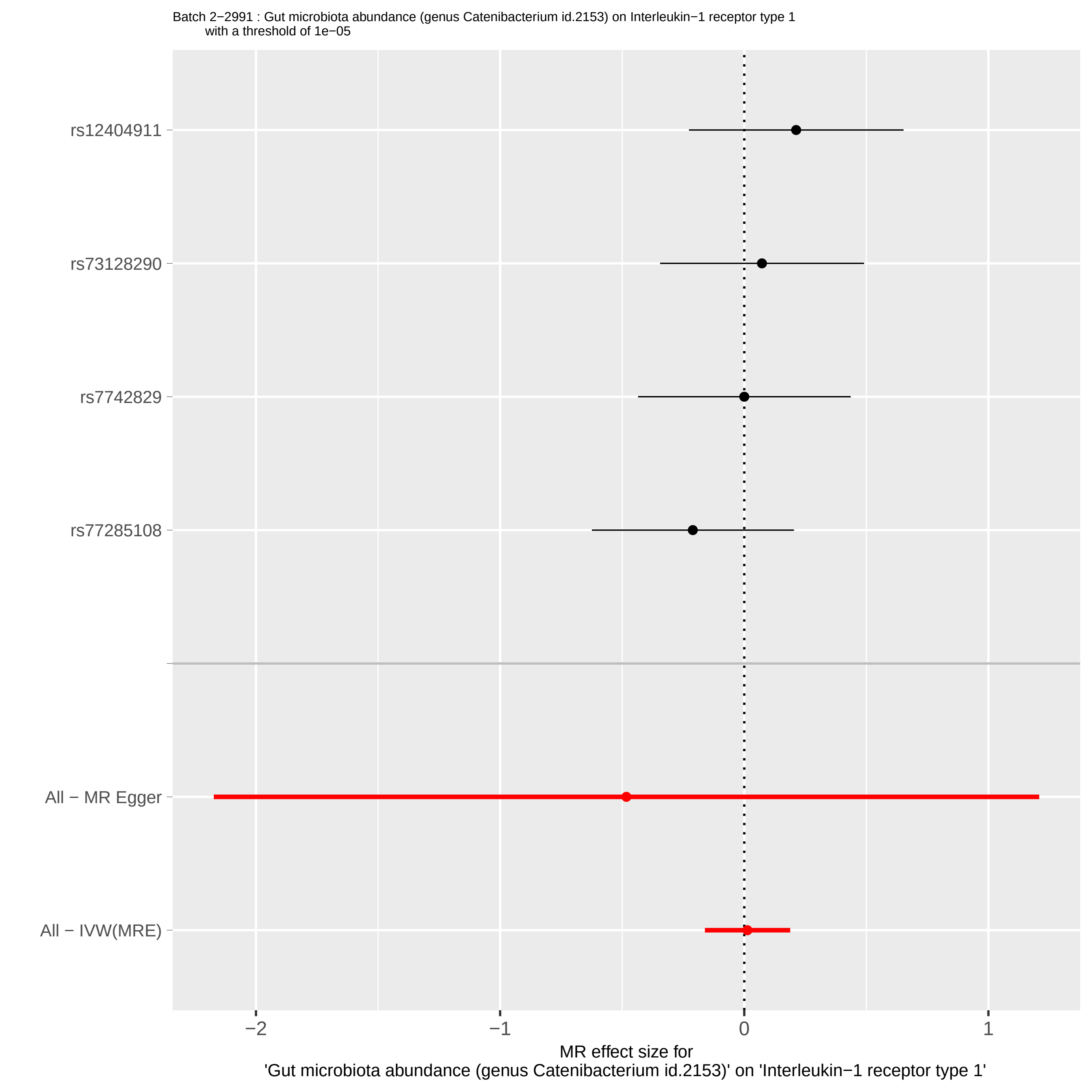
Batch 2−2991 : Gut microbiota abundance (genus Catenibacterium id.2153) on Interleukin−1 receptor type 1
with a threshold of 1e−05
rs12404911
rs73128290
rs7742829
rs77285108
All − MR Egger
All − IVW(MRE)
−2
−1
0
1
MR effect size for
'Gut microbiota abundance (genus Catenibacterium id.2153)' on 'Interleukin−1 receptor type 1'

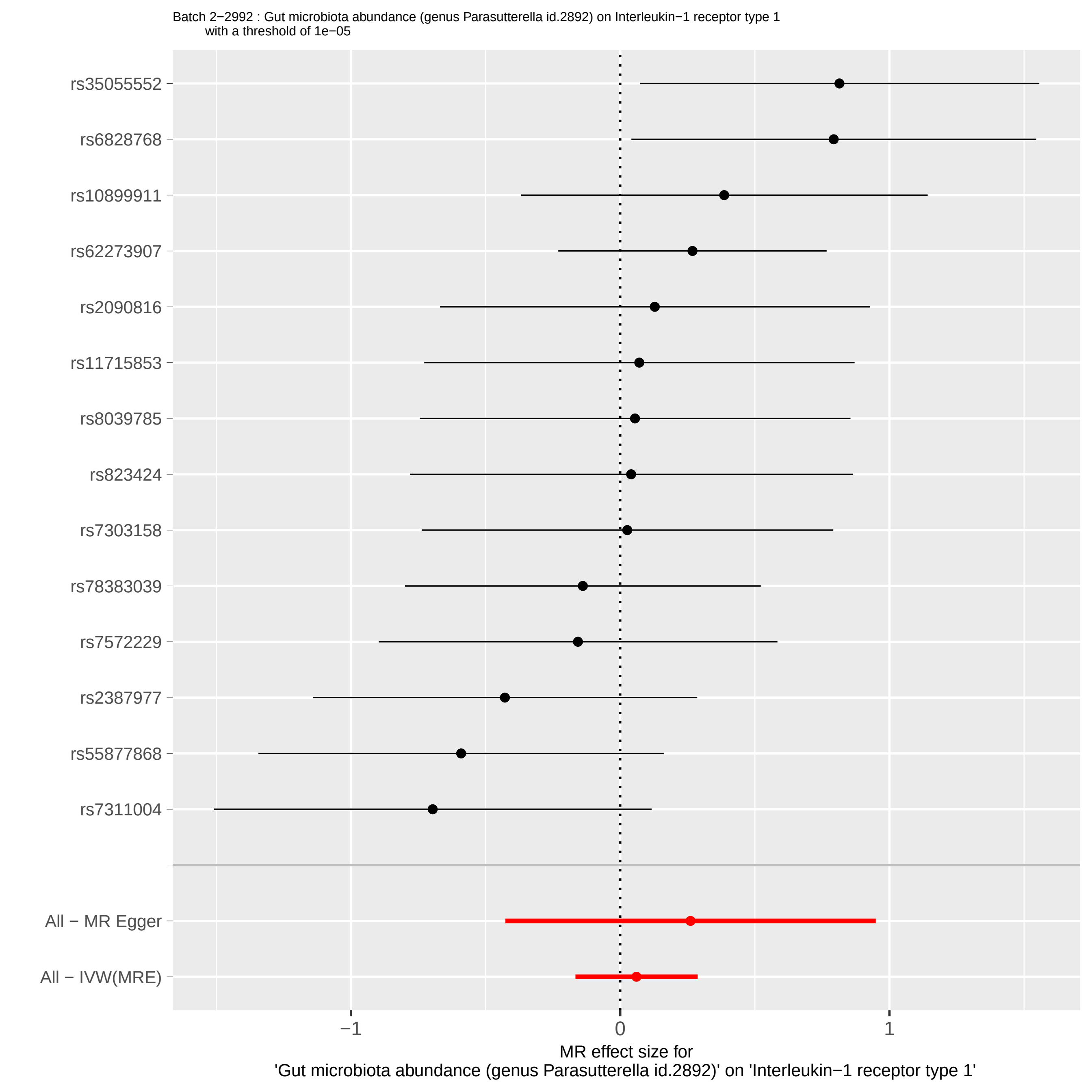

Batch 2−2992 : Gut microbiota abundance (genus Parasutterella id.2892) on Interleukin−1 receptor type 1
with a threshold of 1e−05
rs35055552
rs6828768
rs10899911
rs62273907
rs2090816
rs11715853
rs8039785
rs823424
rs7303158
rs78383039
rs7572229
rs2387977
rs55877868
rs7311004
All − MR Egger
All − IVW(MRE)
−1
0
1
MR effect size for
'Gut microbiota abundance (genus Parasutterella id.2892)' on 'Interleukin−1 receptor type 1'

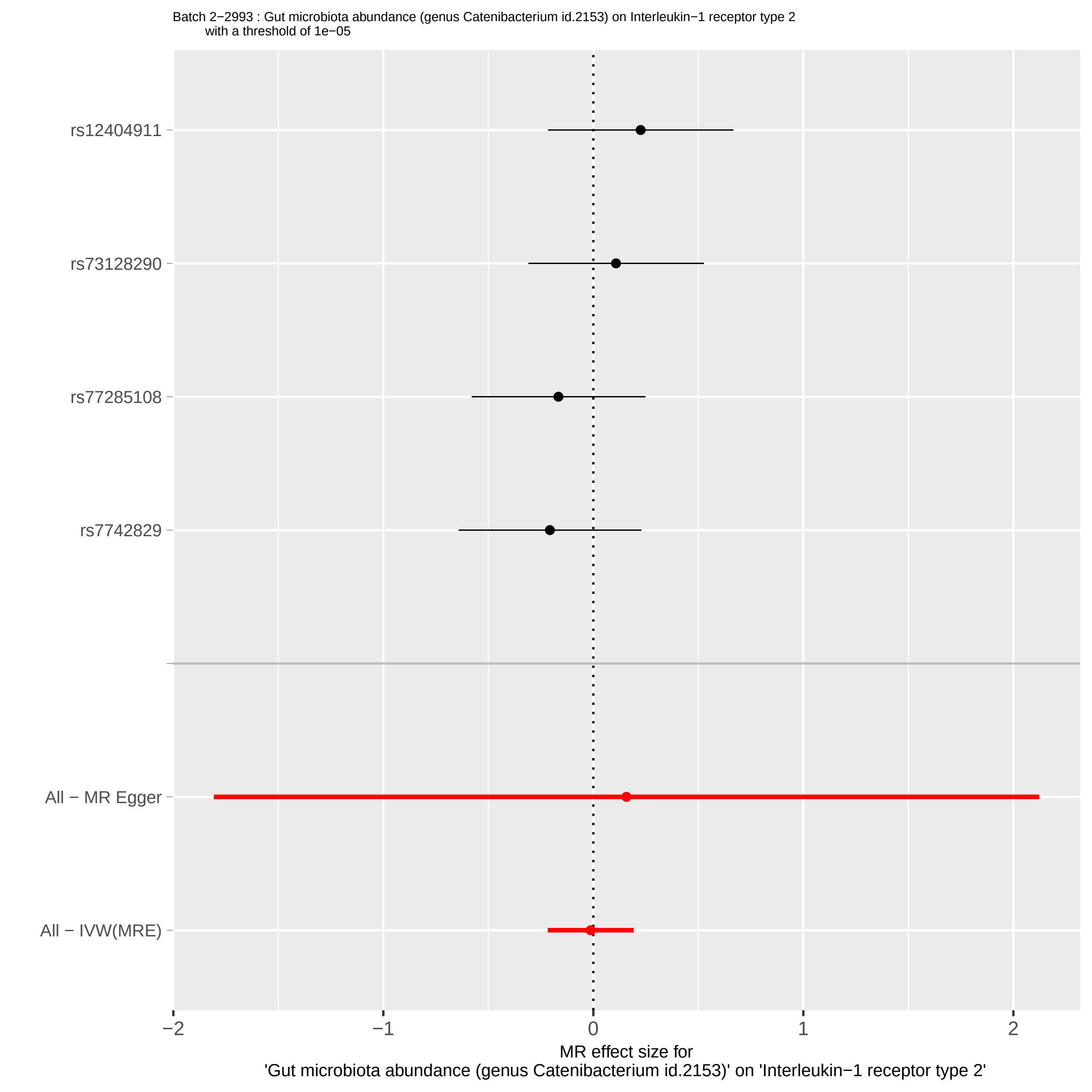
Batch 2−2993 : Gut microbiota abundance (genus Catenibacterium id.2153) on Interleukin−1 receptor type 2
with a threshold of 1e−05
rs12404911
rs73128290
rs77285108
rs7742829
All − MR Egger
All − IVW(MRE)
−2
−1
0
1
2
MR effect size for
'Gut microbiota abundance (genus Catenibacterium id.2153)' on 'Interleukin−1 receptor type 2'

Batch 2−2994 : Gut microbiota abundance (genus Parasutterella id.2892) on Interleukin−1 receptor type 2
with a threshold of 1e−05

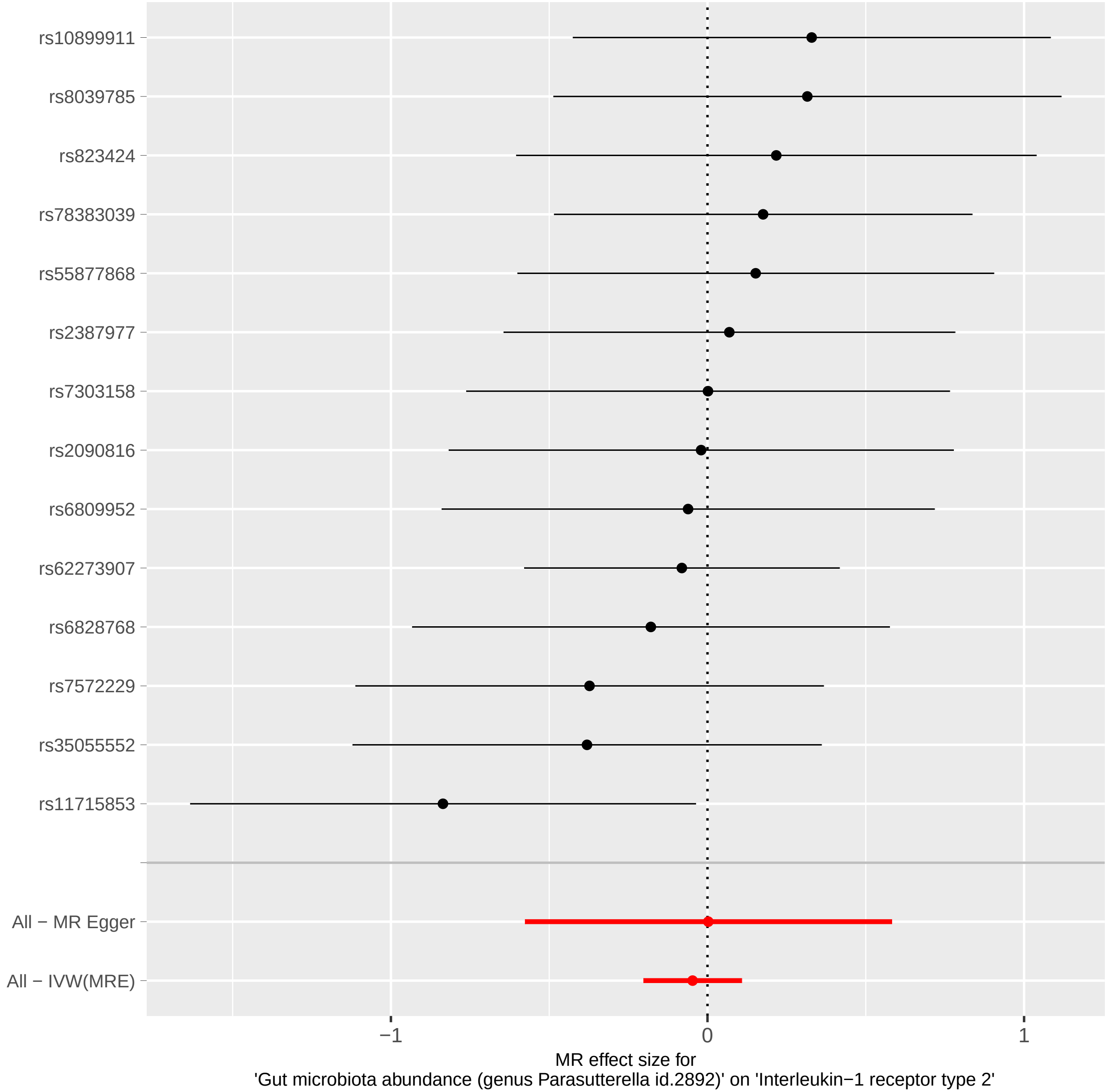

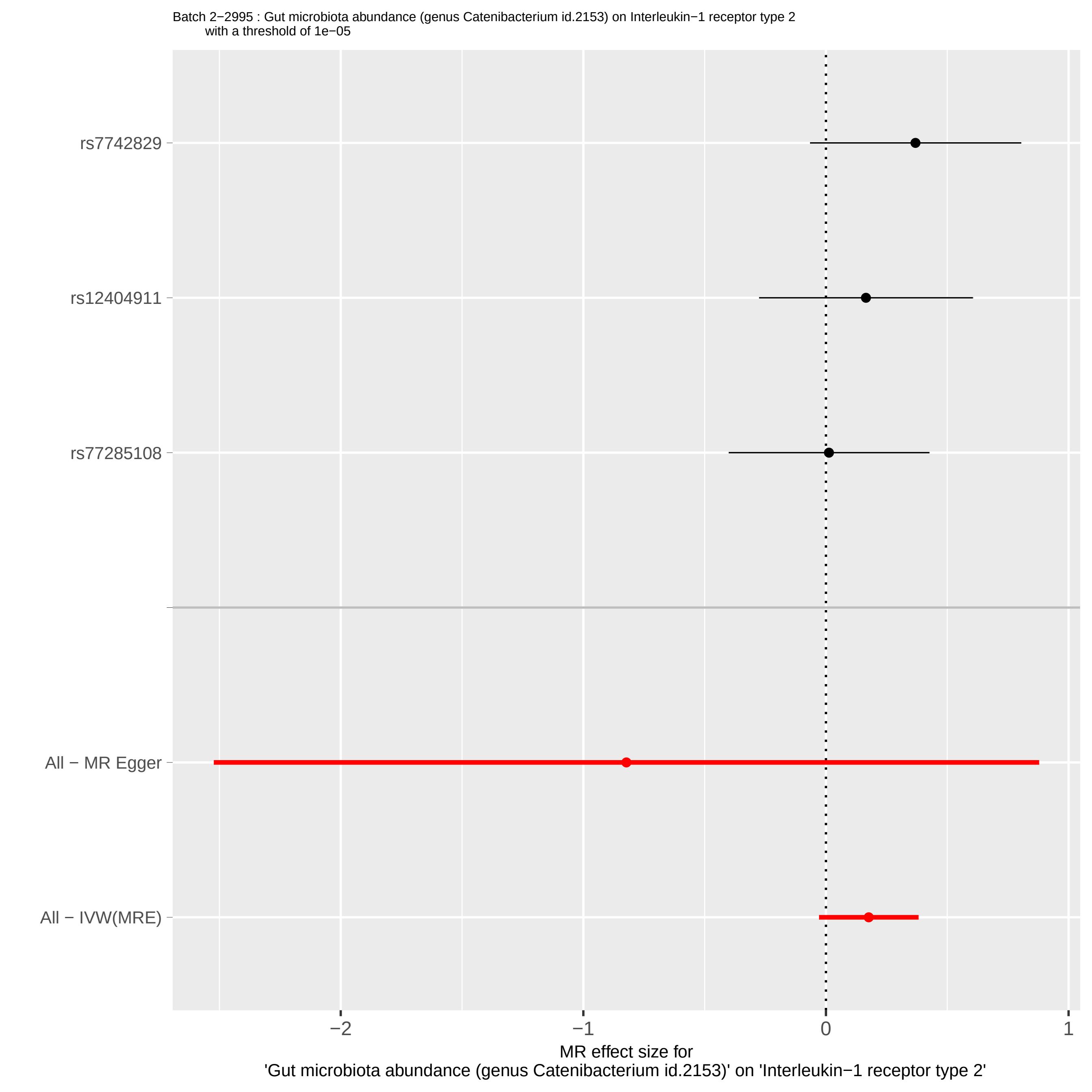

Batch 2−2995 : Gut microbiota abundance (genus Catenibacterium id.2153) on Interleukin−1 receptor type 2
with a threshold of 1e−05
rs7742829
rs12404911
rs77285108
All − MR Egger
All − IVW(MRE)
−2
−1
0
1
MR effect size for
'Gut microbiota abundance (genus Catenibacterium id.2153)' on 'Interleukin−1 receptor type 2'

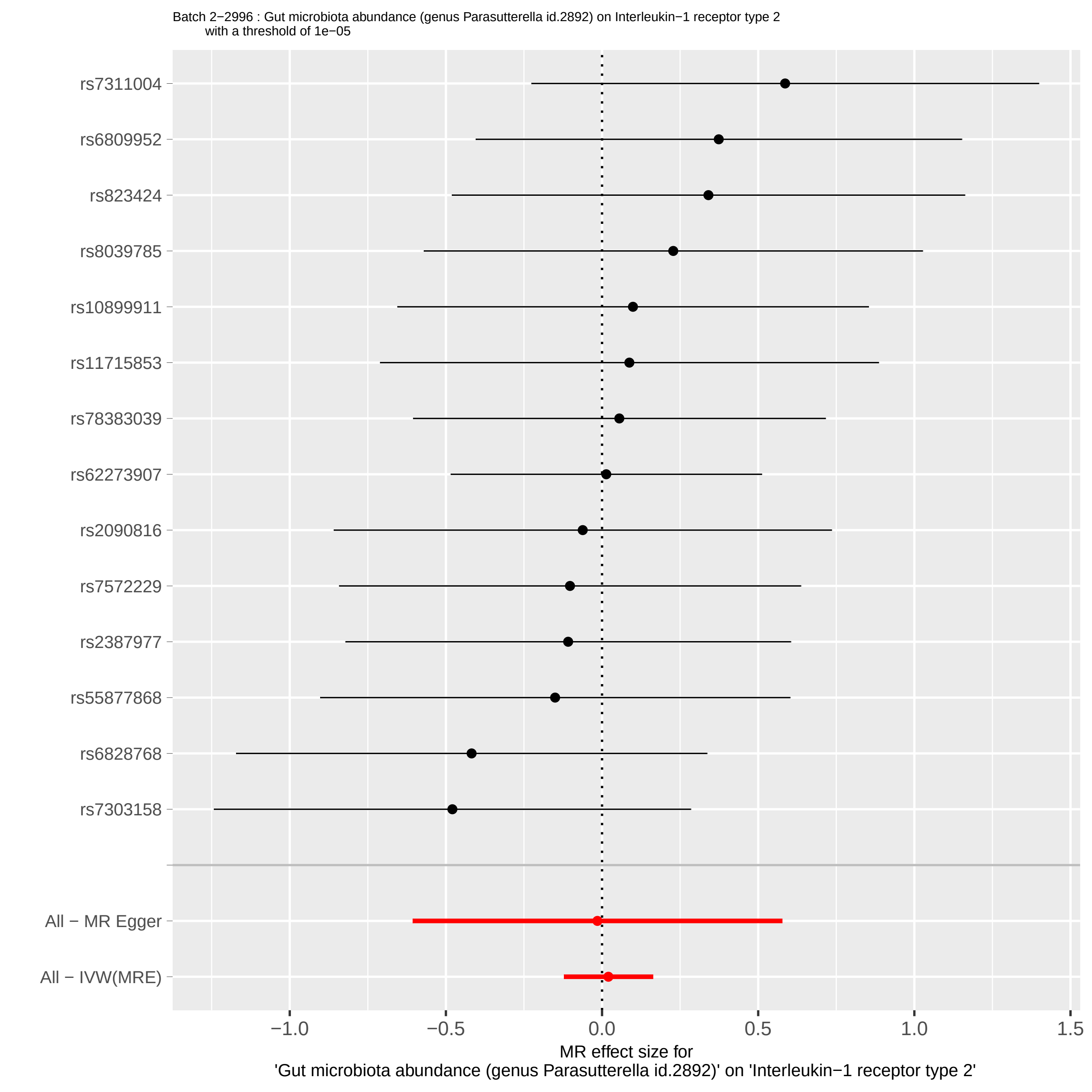

Batch 2−2996 : Gut microbiota abundance (genus Parasutterella id.2892) on Interleukin−1 receptor type 2
with a threshold of 1e−05
rs7311004
rs6809952
rs823424
rs8039785
rs10899911
rs11715853
rs78383039
rs62273907
rs2090816
rs7572229
rs2387977
rs55877868
rs6828768
rs7303158
All − MR Egger
All − IVW(MRE)
−1.0
−0.5
0.0
0.5
1.0
1.5
MR effect size for
'Gut microbiota abundance (genus Parasutterella id.2892)' on 'Interleukin−1 receptor type 2'

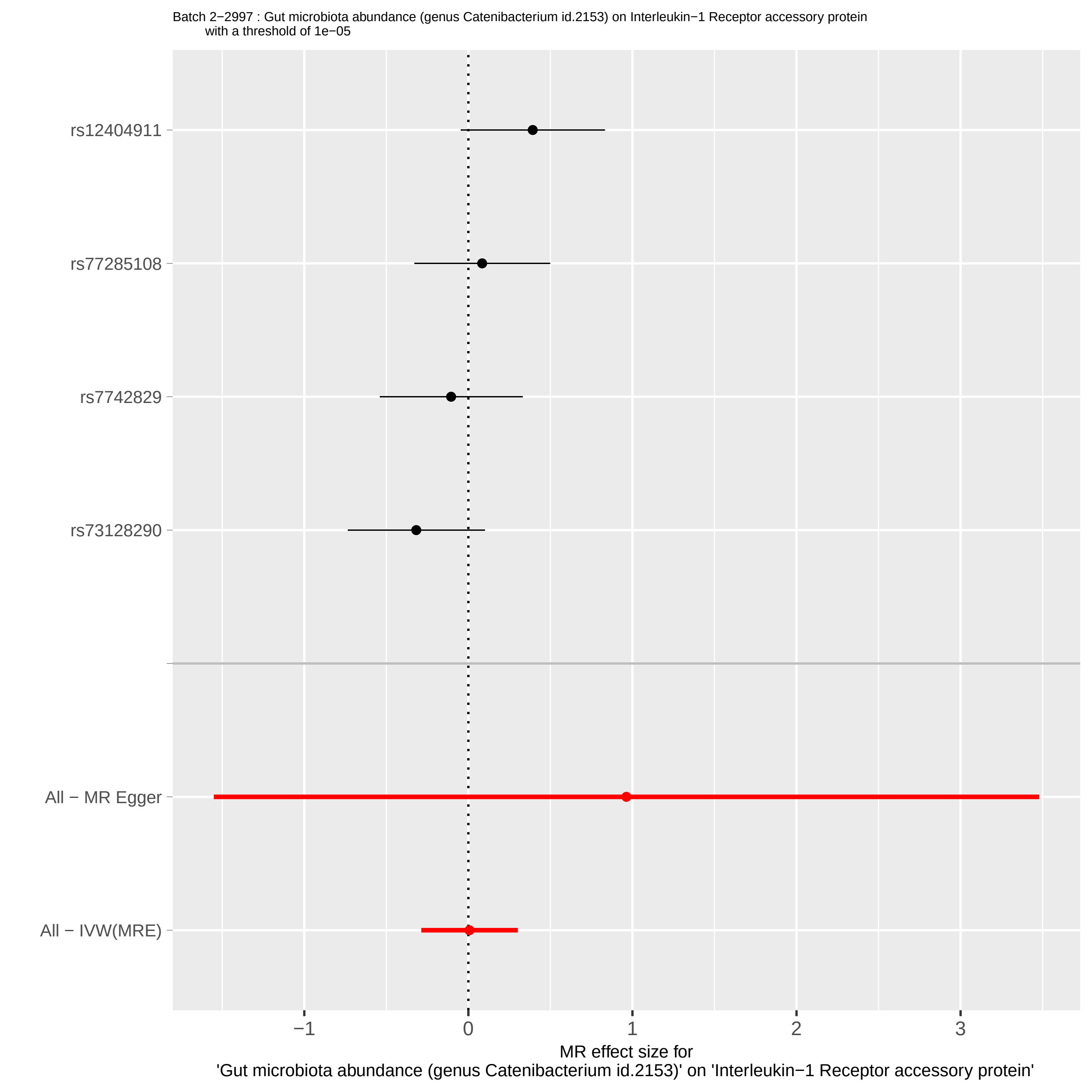

Batch 2−2997 : Gut microbiota abundance (genus Catenibacterium id.2153) on Interleukin−1 Receptor accessory protein
with a threshold of 1e−05
rs12404911
rs77285108
rs7742829
rs73128290
All − MR Egger
All − IVW(MRE)
−1
0
1
2
3
MR effect size for
'Gut microbiota abundance (genus Catenibacterium id.2153)' on 'Interleukin−1 Receptor accessory protein'

Batch 2−2998 : Gut microbiota abundance (genus Parasutterella id.2892) on Interleukin−1 Receptor accessory protein
with a threshold of 1e−05

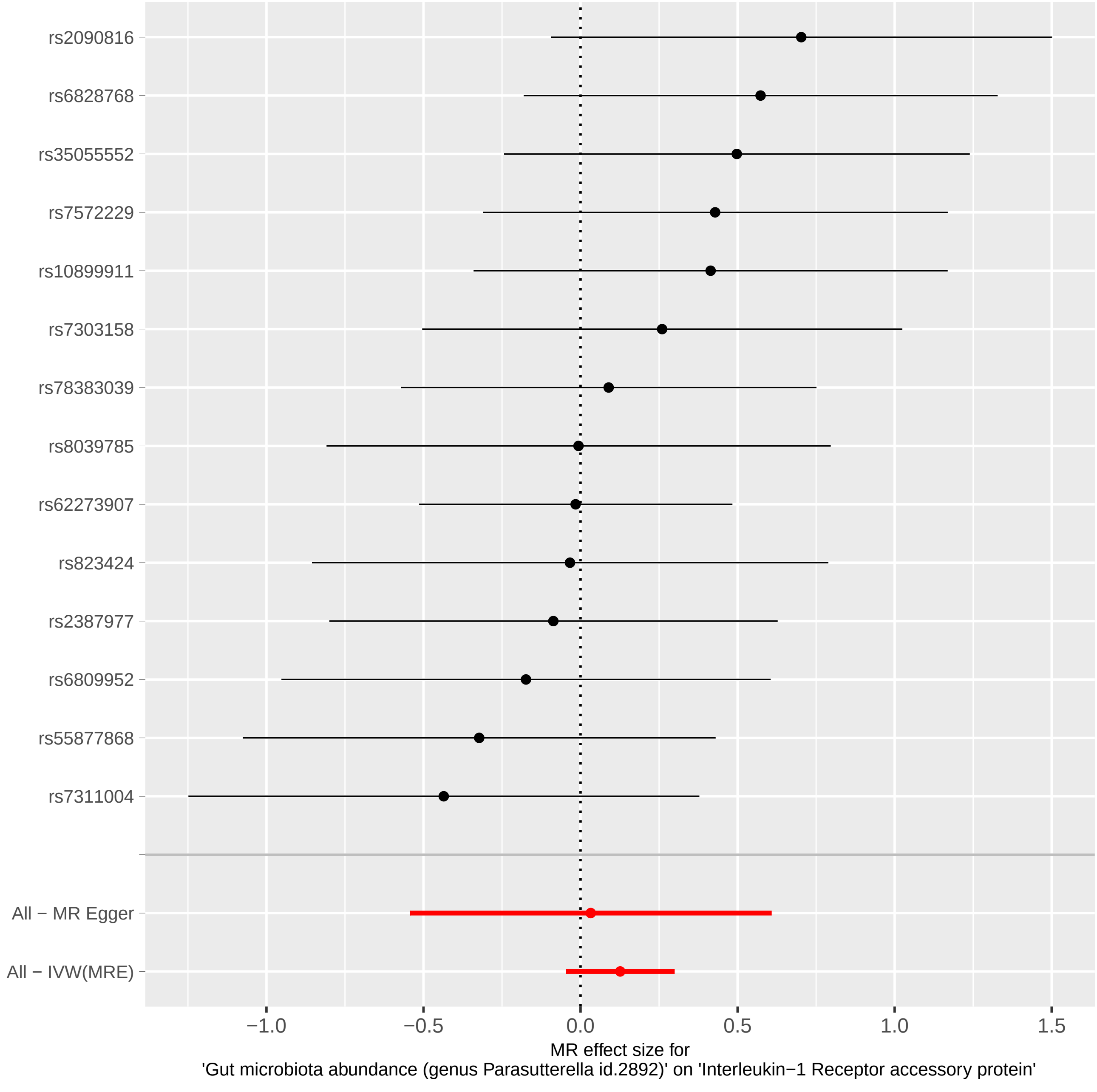

Batch 2−2999 : Gut microbiota abundance (genus Catenibacterium id.2153) on Interleukin−1 Receptor accessory protein
with a threshold of 1e−05

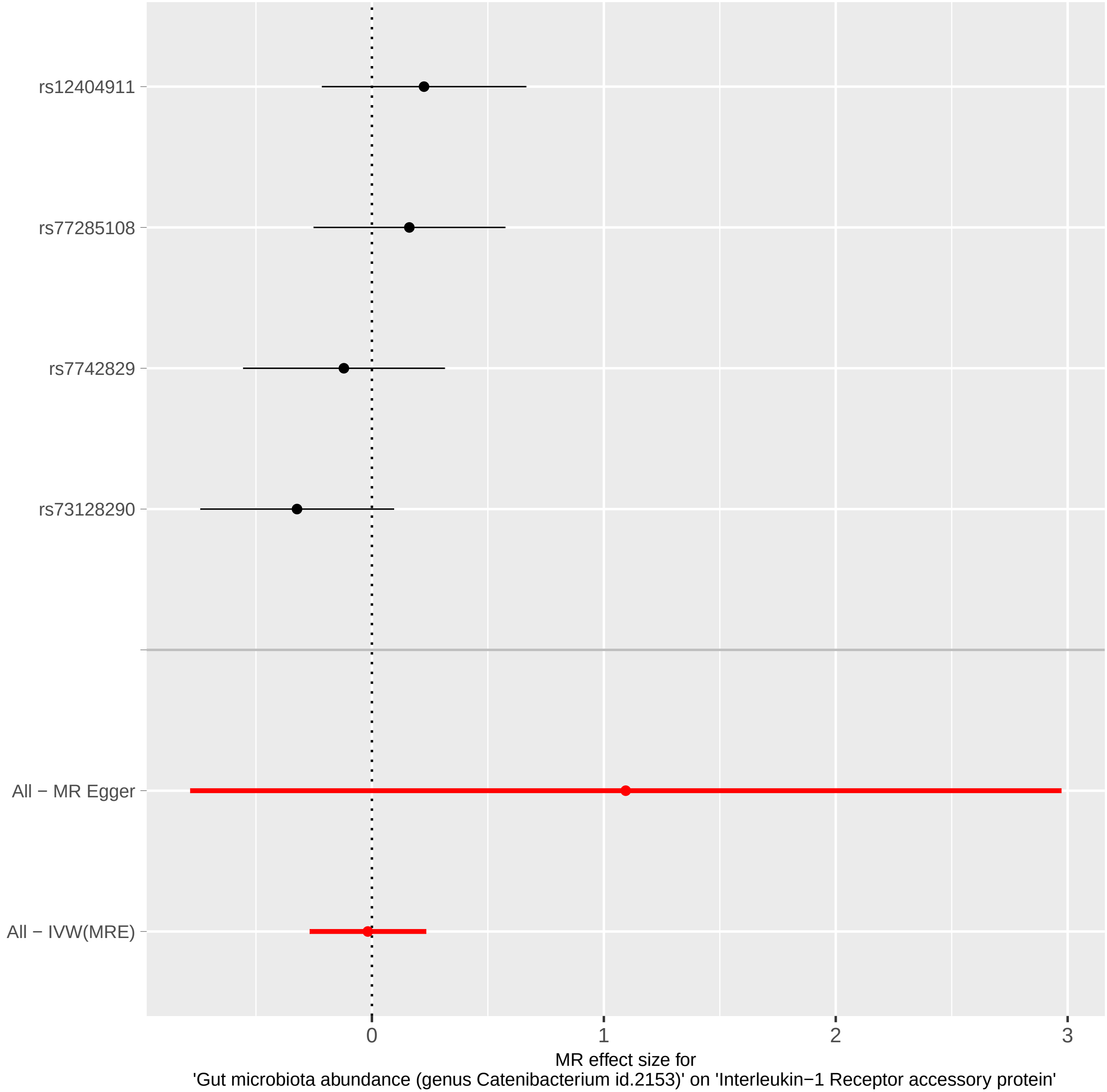

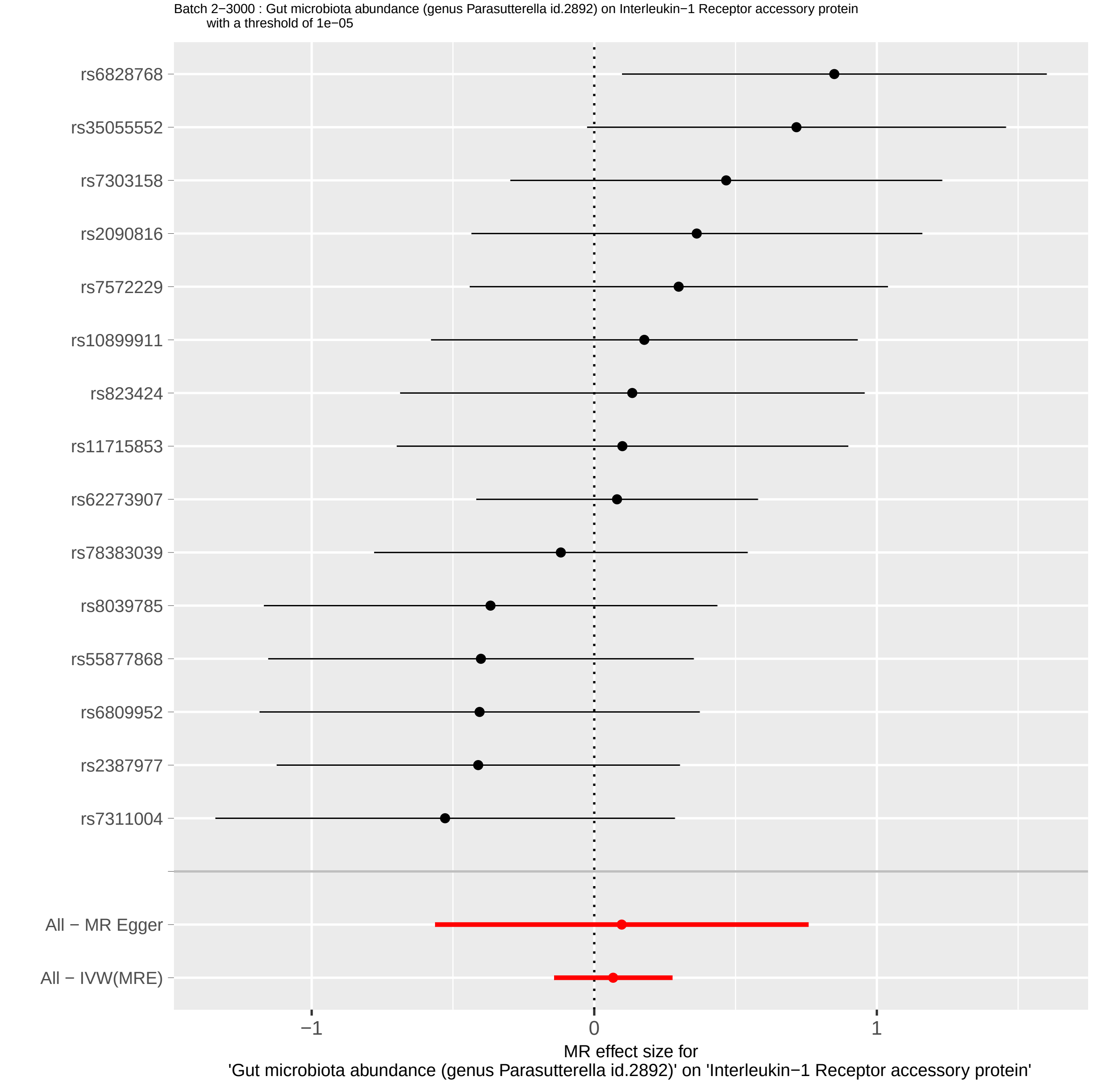
Batch 2−3000 : Gut microbiota abundance (genus Parasutterella id.2892) on Interleukin−1 Receptor accessory protein
with a threshold of 1e−05
rs6828768
rs35055552
rs7303158
rs2090816
rs7572229
rs10899911
rs823424
rs11715853
rs62273907
rs78383039
rs8039785
rs55877868
rs6809952
rs2387977
rs7311004
All − MR Egger
All − IVW(MRE)
−1
0
1
MR effect size for
'Gut microbiota abundance (genus Parasutterella id.2892)' on 'Interleukin−1 Receptor accessory protein'

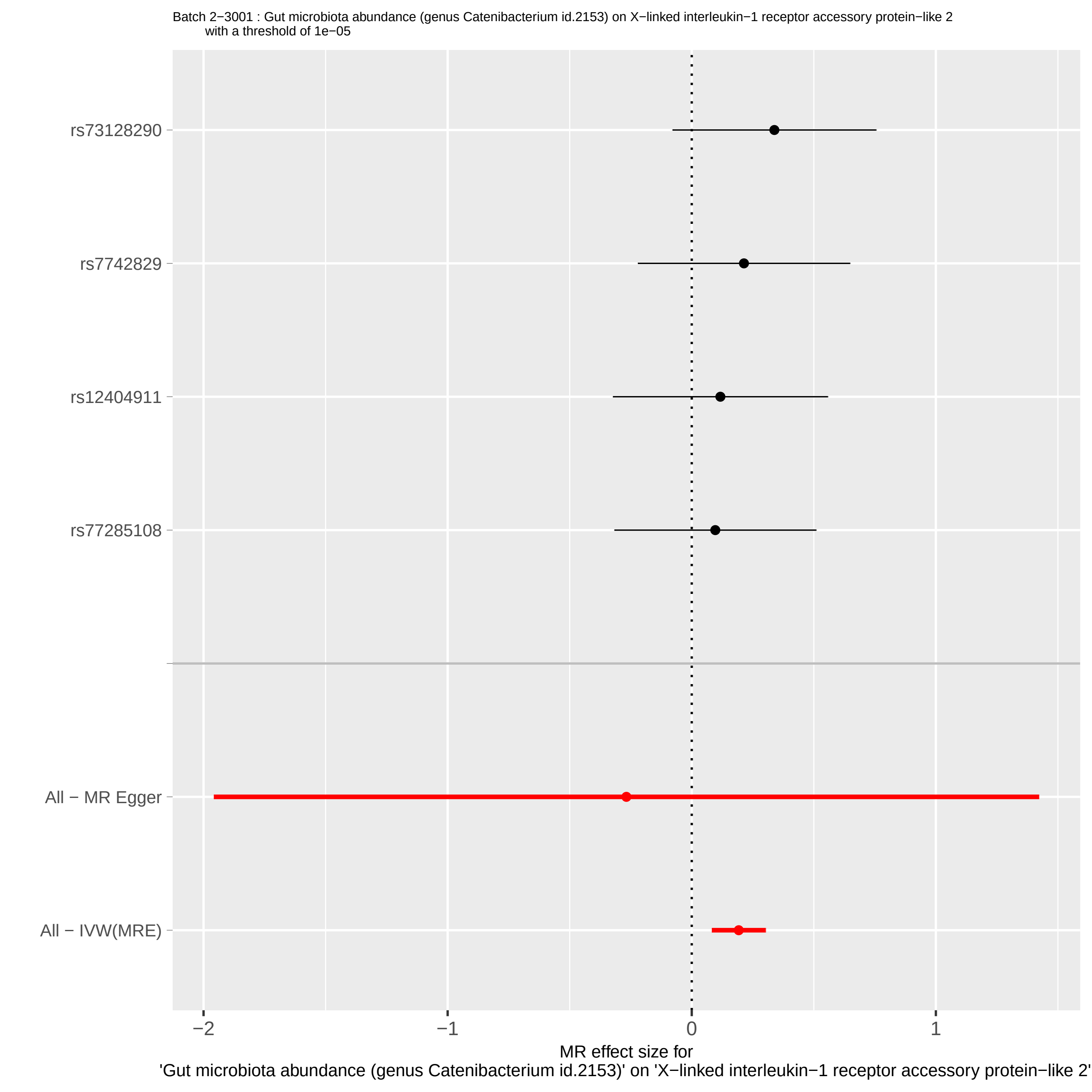
Batch 2−3001 : Gut microbiota abundance (genus Catenibacterium id.2153) on X−linked interleukin−1 receptor accessory protein−like 2
with a threshold of 1e−05
rs73128290
rs7742829
rs12404911
rs77285108
All − MR Egger
All − IVW(MRE)
−2
−1
0
1
MR effect size for
'Gut microbiota abundance (genus Catenibacterium id.2153)' on 'X−linked interleukin−1 receptor accessory protein−like 2'

Batch 2−3002 : Gut microbiota abundance (genus Parasutterella id.2892) on X−linked interleukin−1 receptor accessory protein−like 2 with a threshold of 1e−05

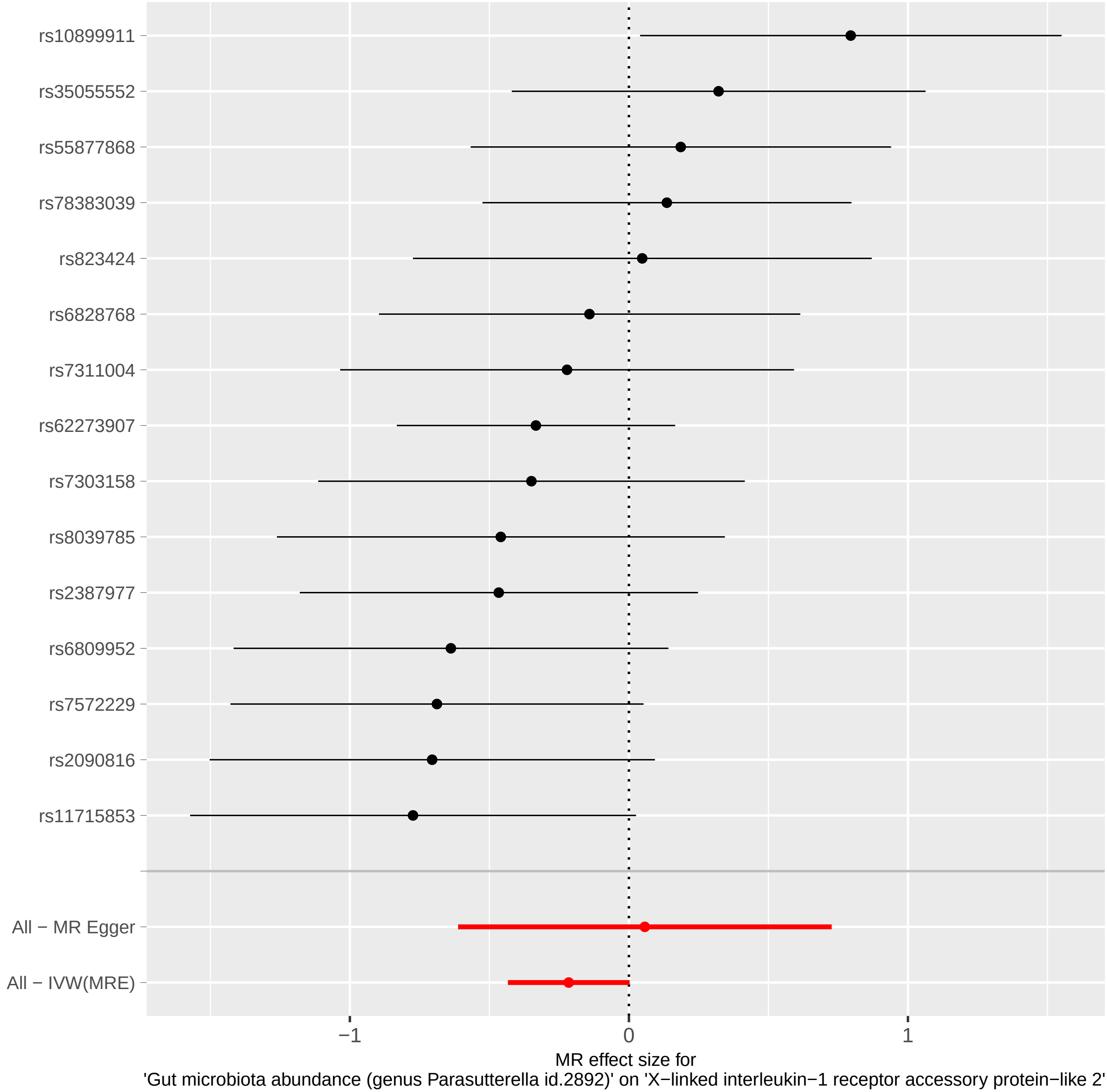

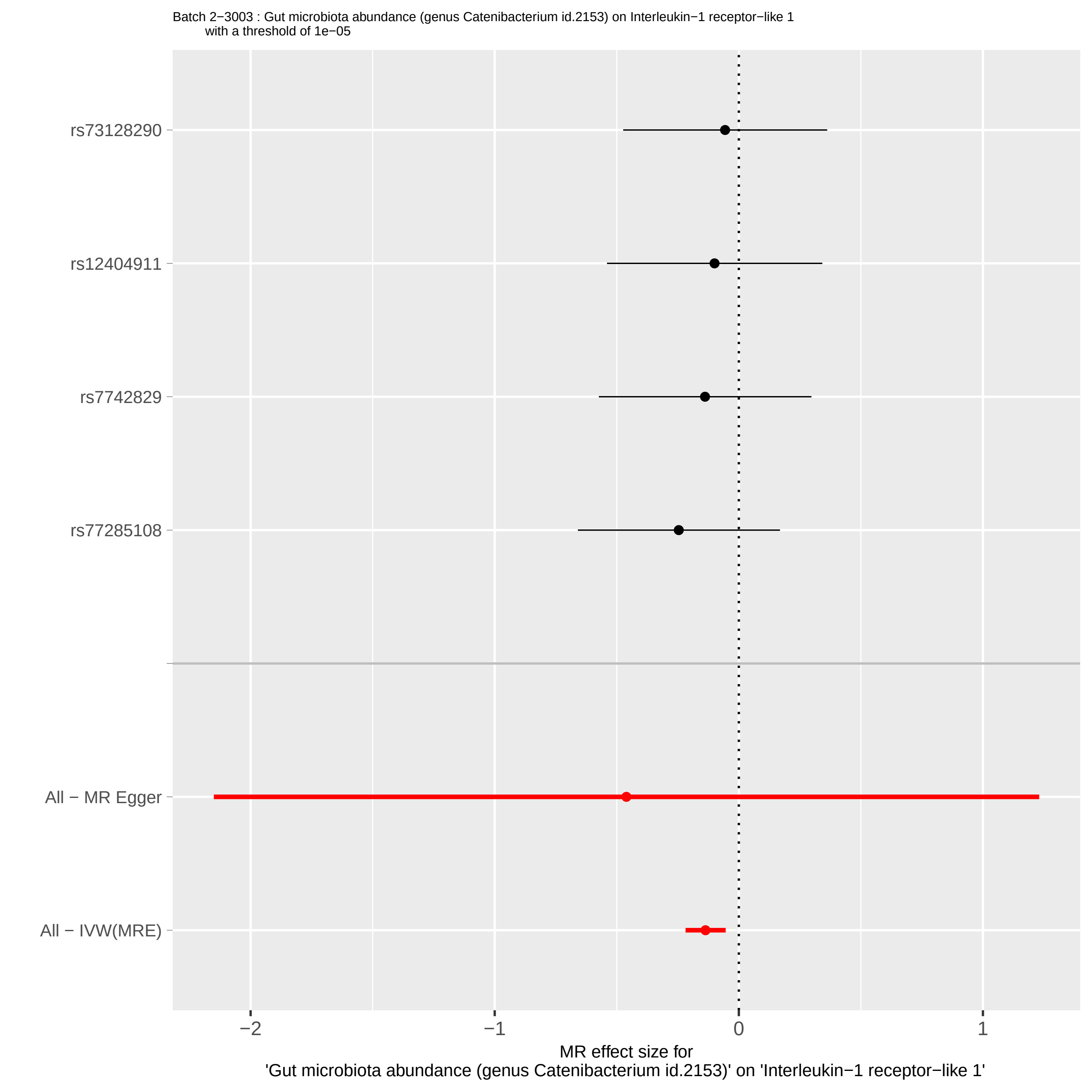
Batch 2−3003 : Gut microbiota abundance (genus Catenibacterium id.2153) on Interleukin−1 receptor−like 1
with a threshold of 1e−05
rs73128290
rs12404911
rs7742829
rs77285108
All − MR Egger
All − IVW(MRE)
−2
−1
0
1
MR effect size for
'Gut microbiota abundance (genus Catenibacterium id.2153)' on 'Interleukin−1 receptor−like 1'

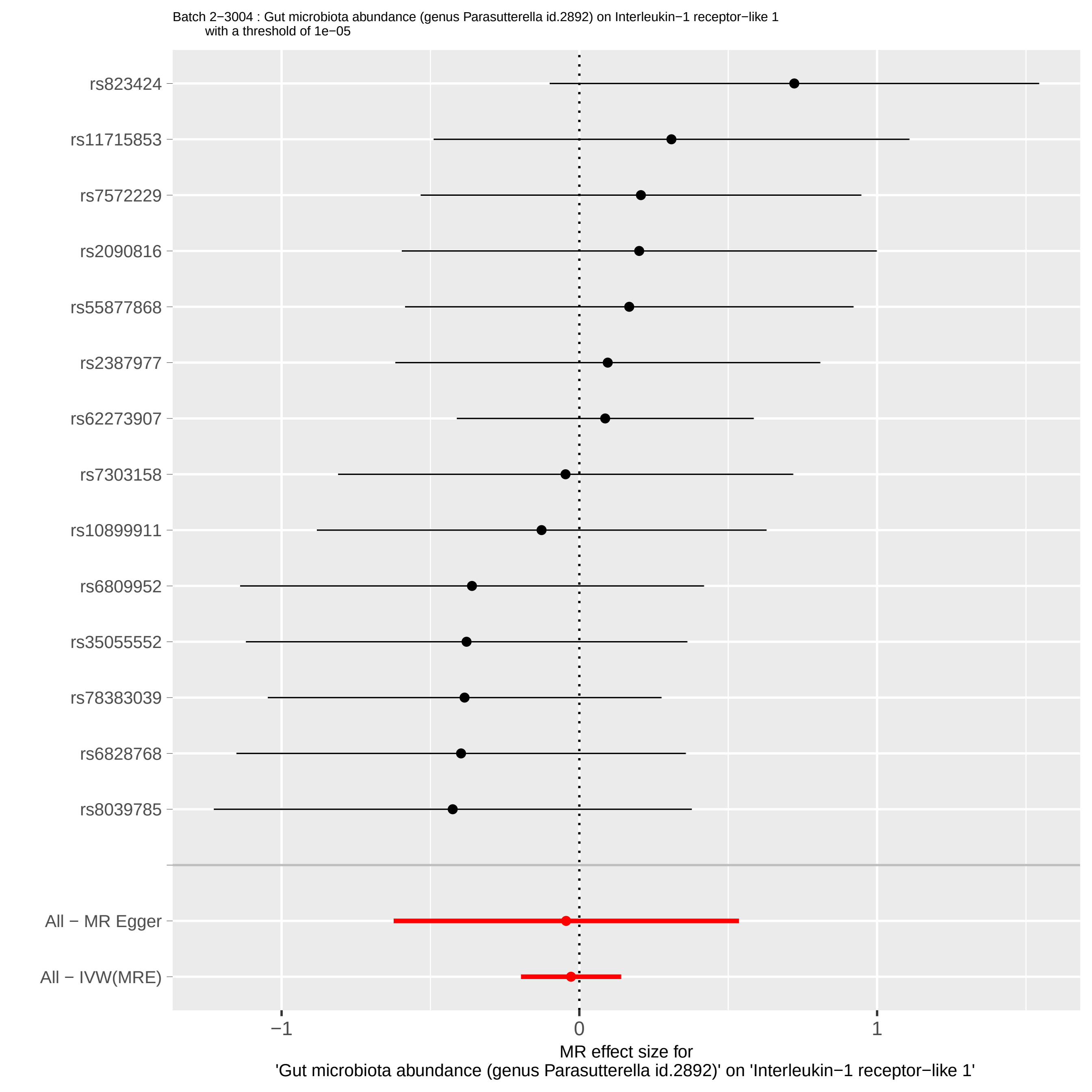

Batch 2−3004 : Gut microbiota abundance (genus Parasutterella id.2892) on Interleukin−1 receptor−like 1
with a threshold of 1e−05
rs823424
rs11715853
rs7572229
rs2090816
rs55877868
rs2387977
rs62273907
rs7303158
rs10899911
rs6809952
rs35055552
rs78383039
rs6828768
rs8039785
All − MR Egger
All − IVW(MRE)
−1
0
1
MR effect size for
'Gut microbiota abundance (genus Parasutterella id.2892)' on 'Interleukin−1 receptor−like 1'

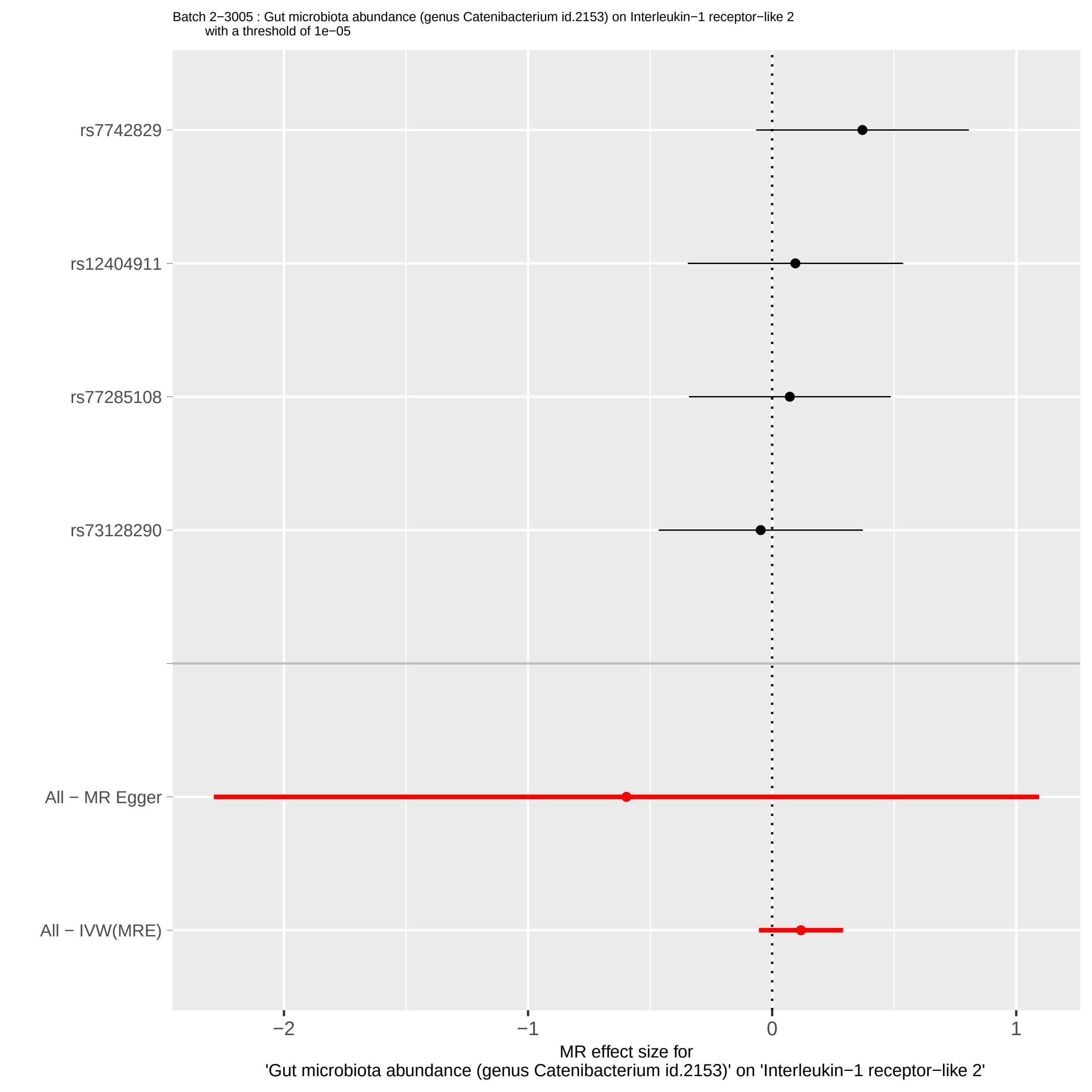
Batch 2−3005 : Gut microbiota abundance (genus Catenibacterium id.2153) on Interleukin−1 receptor−like 2
with a threshold of 1e−05
rs7742829
rs12404911
rs77285108
rs73128290
All − MR Egger
All − IVW(MRE)
−2
−1
0
1
MR effect size for
'Gut microbiota abundance (genus Catenibacterium id.2153)' on 'Interleukin−1 receptor−like 2'

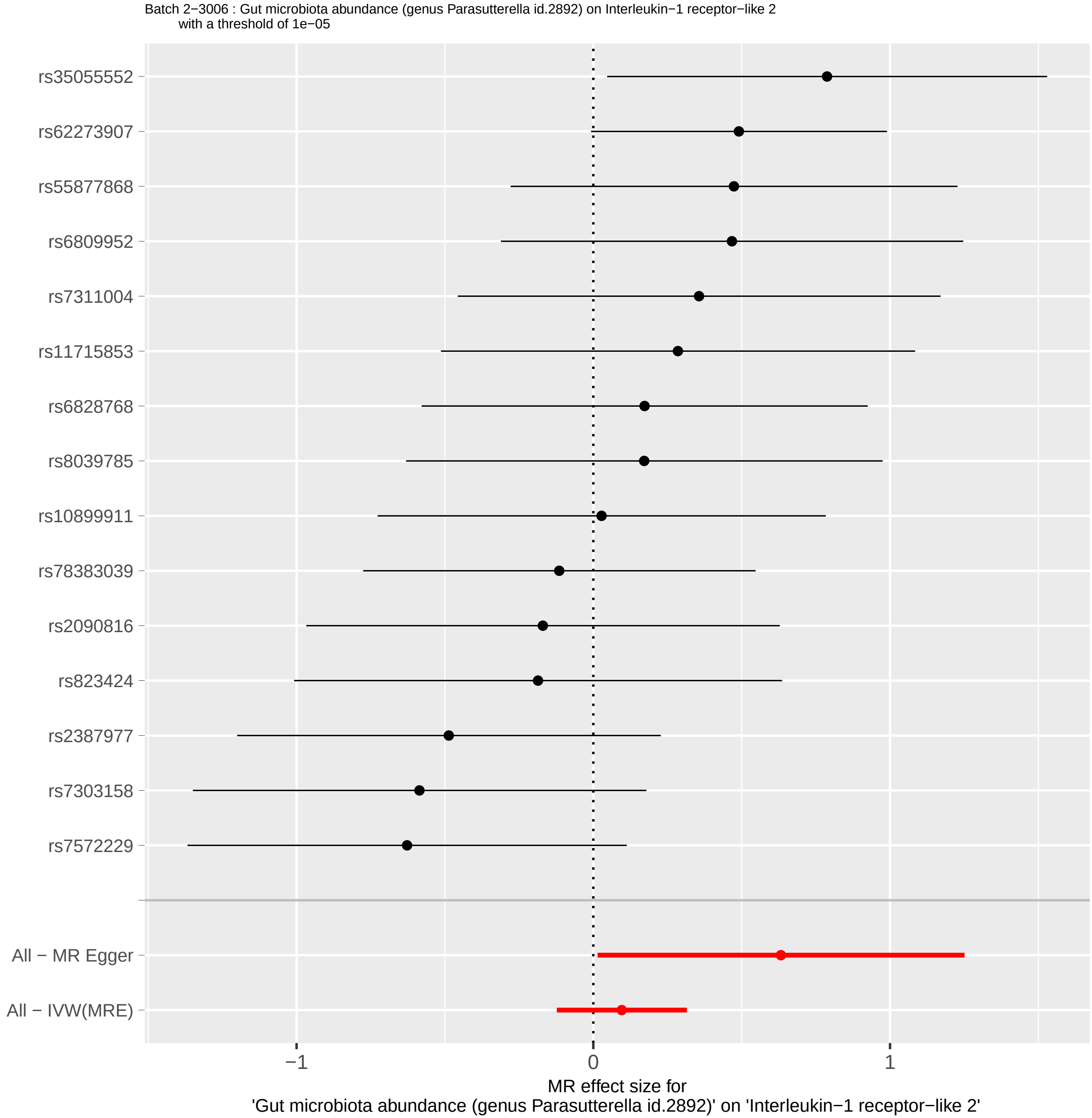
Batch 2−3006 : Gut microbiota abundance (genus Parasutterella id.2892) on Interleukin−1 receptor−like 2
with a threshold of 1e−05
rs35055552
rs62273907
rs55877868
rs6809952
rs7311004
rs11715853
rs6828768
rs8039785
rs10899911
rs78383039
rs2090816
rs823424
rs2387977
rs7303158
rs7572229
All − MR Egger
All − IVW(MRE)
−1
0
1
MR effect size for
'Gut microbiota abundance (genus Parasutterella id.2892)' on 'Interleukin−1 receptor−like 2'

Batch 2−3007 : Gut microbiota abundance (genus Catenibacterium id.2153) on Interleukin−1 receptor antagonist protein
with a threshold of 1e−05

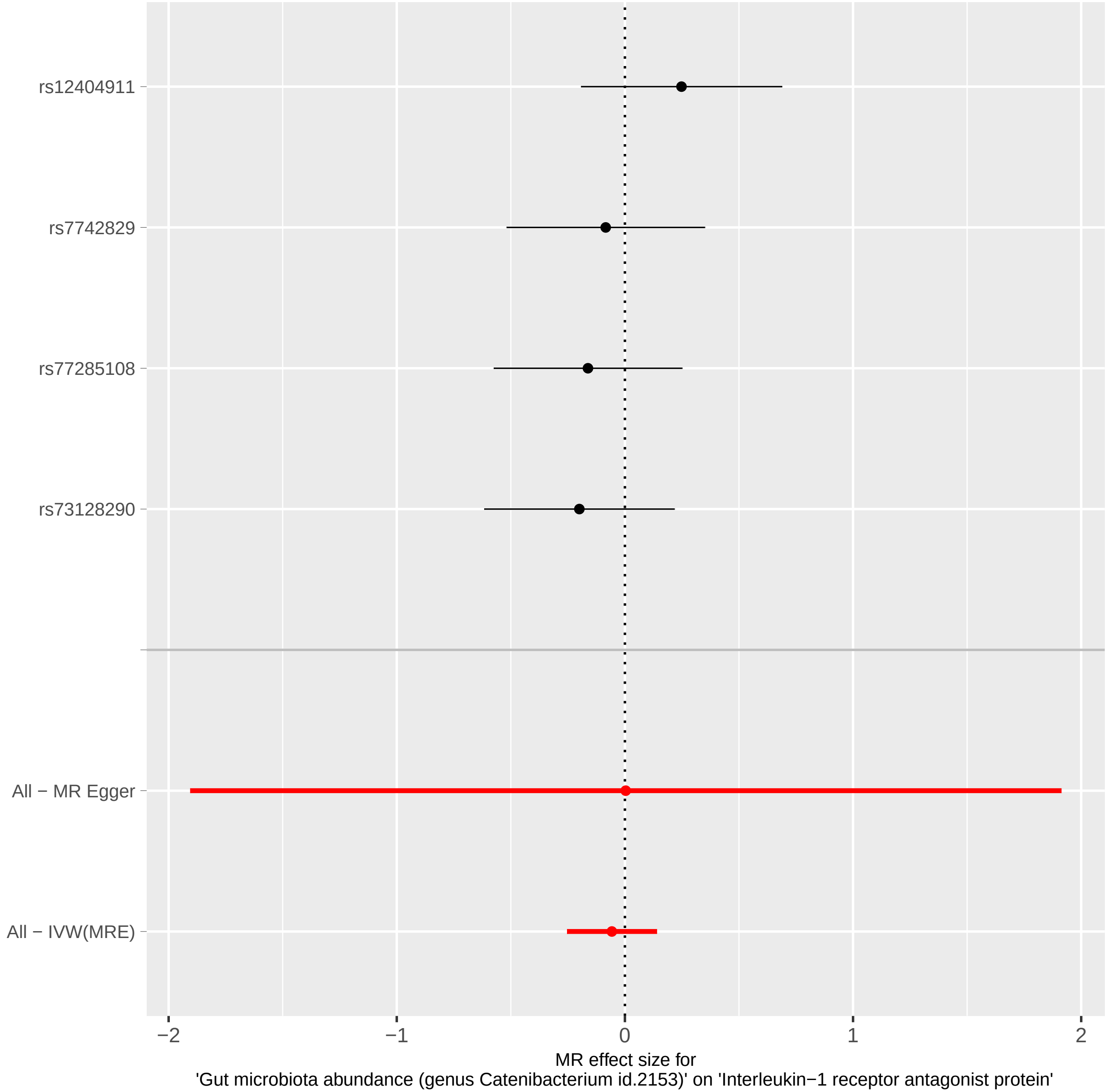

Batch 2−3008 : Gut microbiota abundance (genus Parasutterella id.2892) on Interleukin−1 receptor antagonist protein
with a threshold of 1e−05

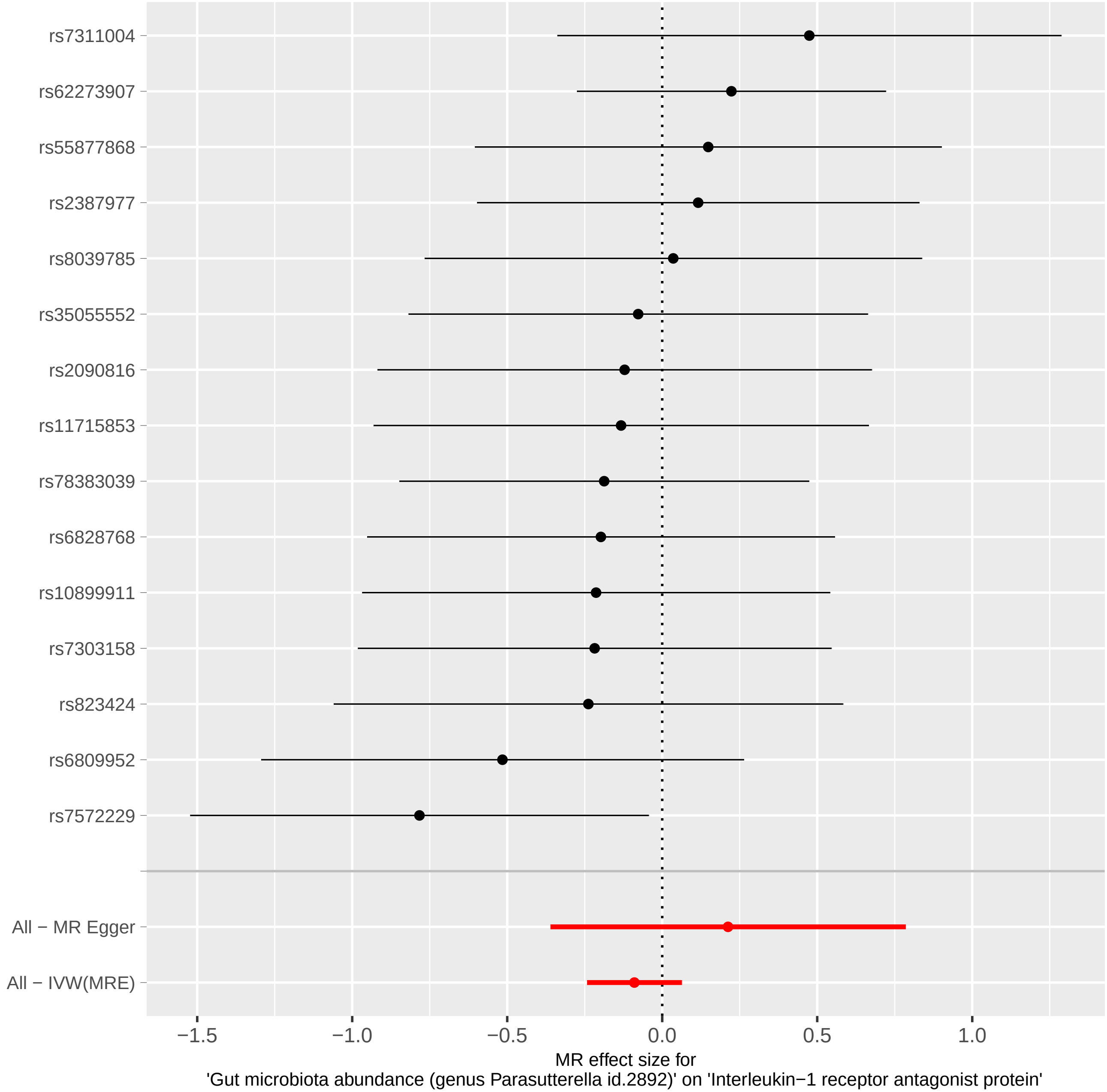

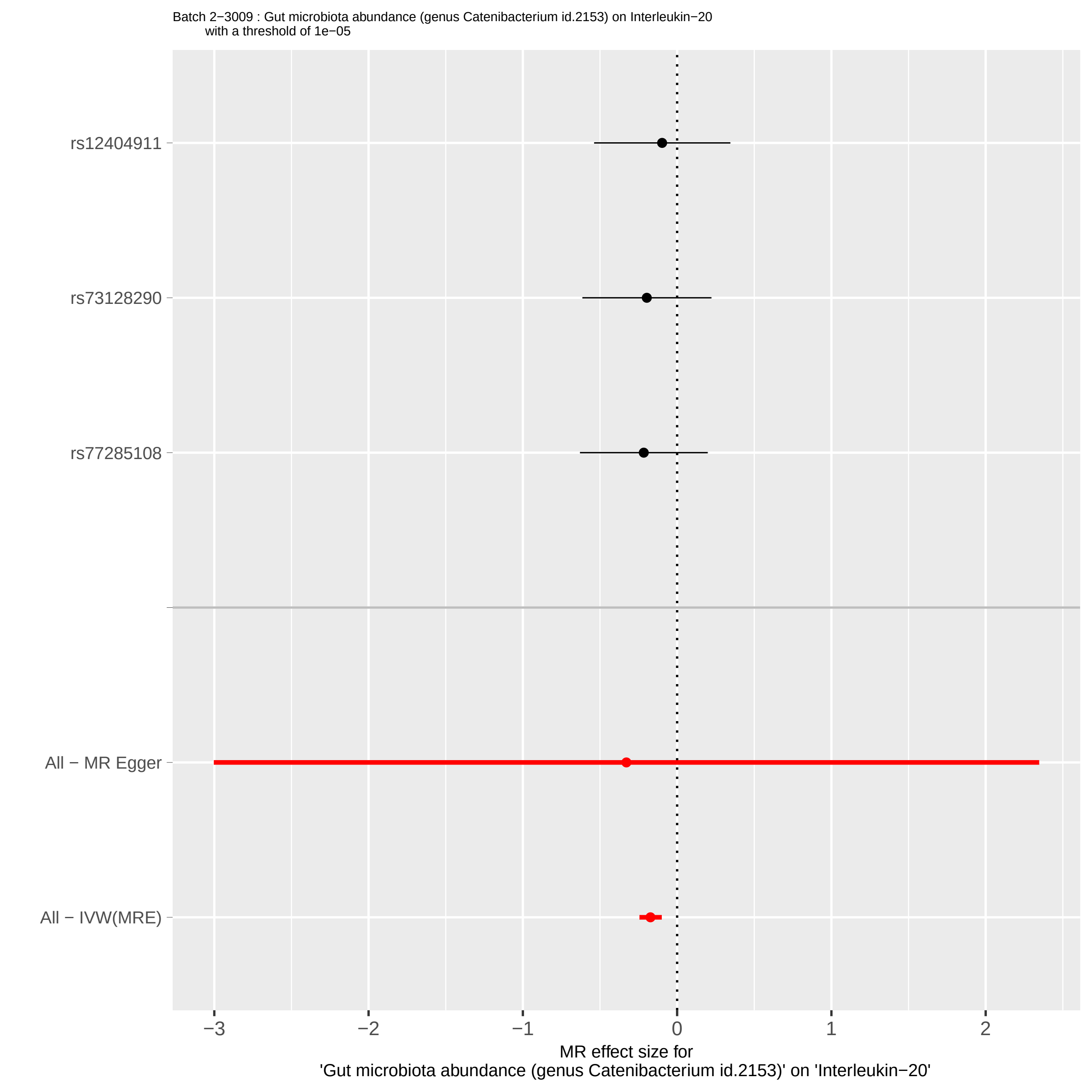

Batch 2−3009 : Gut microbiota abundance (genus Catenibacterium id.2153) on Interleukin−20
with a threshold of 1e−05
rs12404911
rs73128290
rs77285108
All − MR Egger
All − IVW(MRE)
−3
−2
−1
0
1
2
MR effect size for
'Gut microbiota abundance (genus Catenibacterium id.2153)' on 'Interleukin−20'

Batch 2−3010 : Gut microbiota abundance (genus Parasutterella id.2892) on Interleukin−20
with a threshold of 1e−05

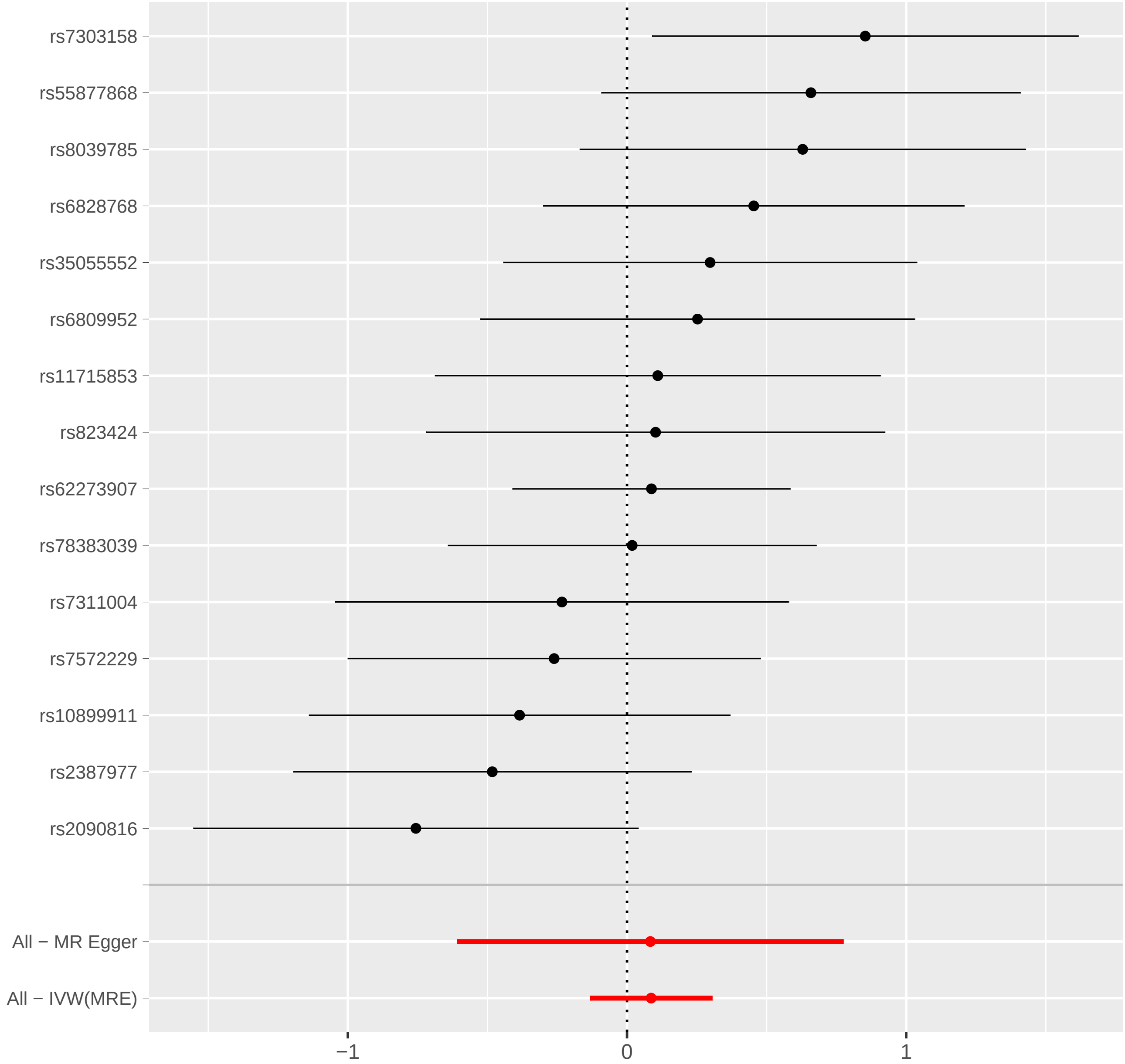

MR effect size for
'Gut microbiota abundance (genus Parasutterella id.2892)' on 'Interleukin−20'

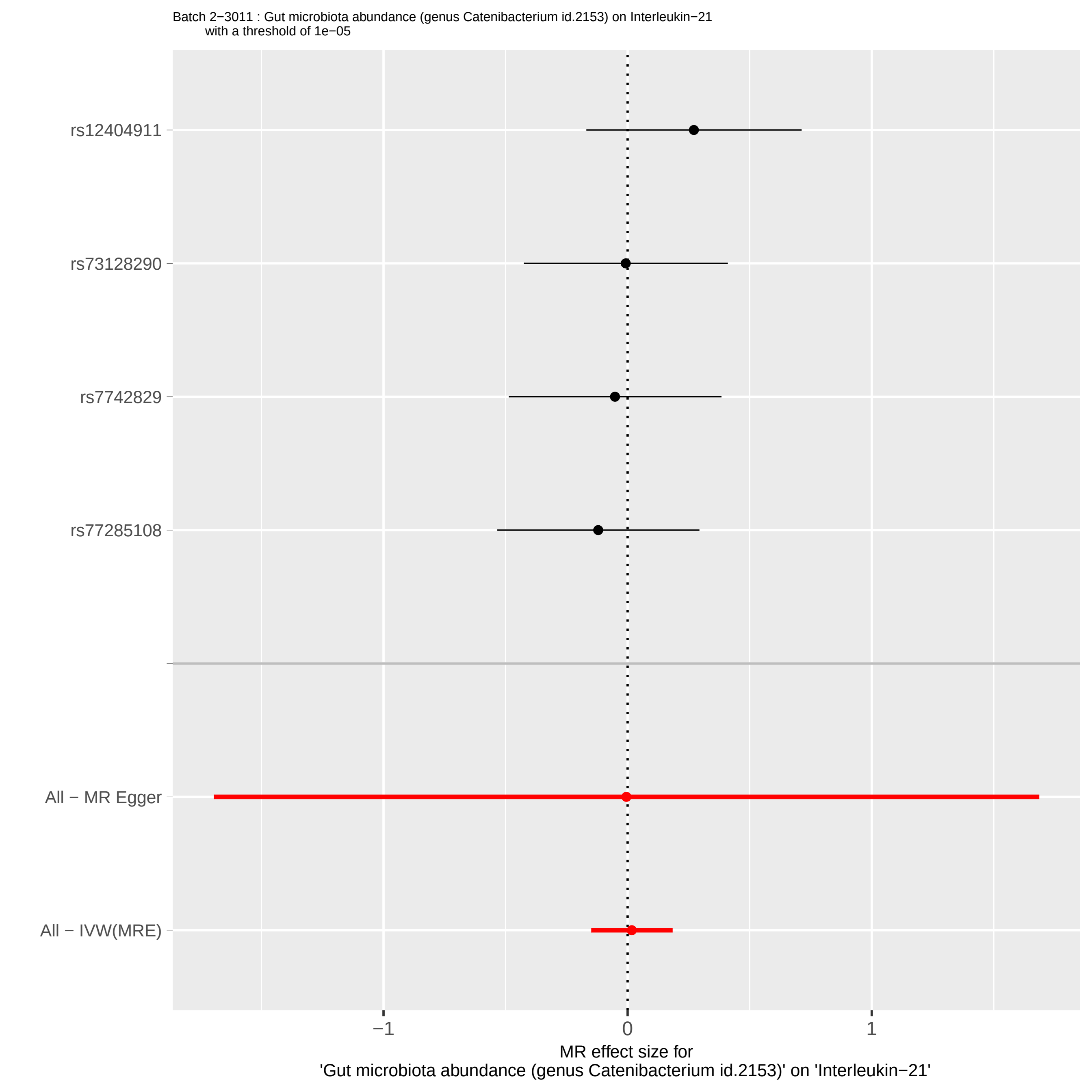

Batch 2−3011 : Gut microbiota abundance (genus Catenibacterium id.2153) on Interleukin−21
with a threshold of 1e−05
rs12404911
rs73128290
rs7742829
rs77285108
All − MR Egger
All − IVW(MRE)
−1
0
1
MR effect size for
'Gut microbiota abundance (genus Catenibacterium id.2153)' on 'Interleukin−21'

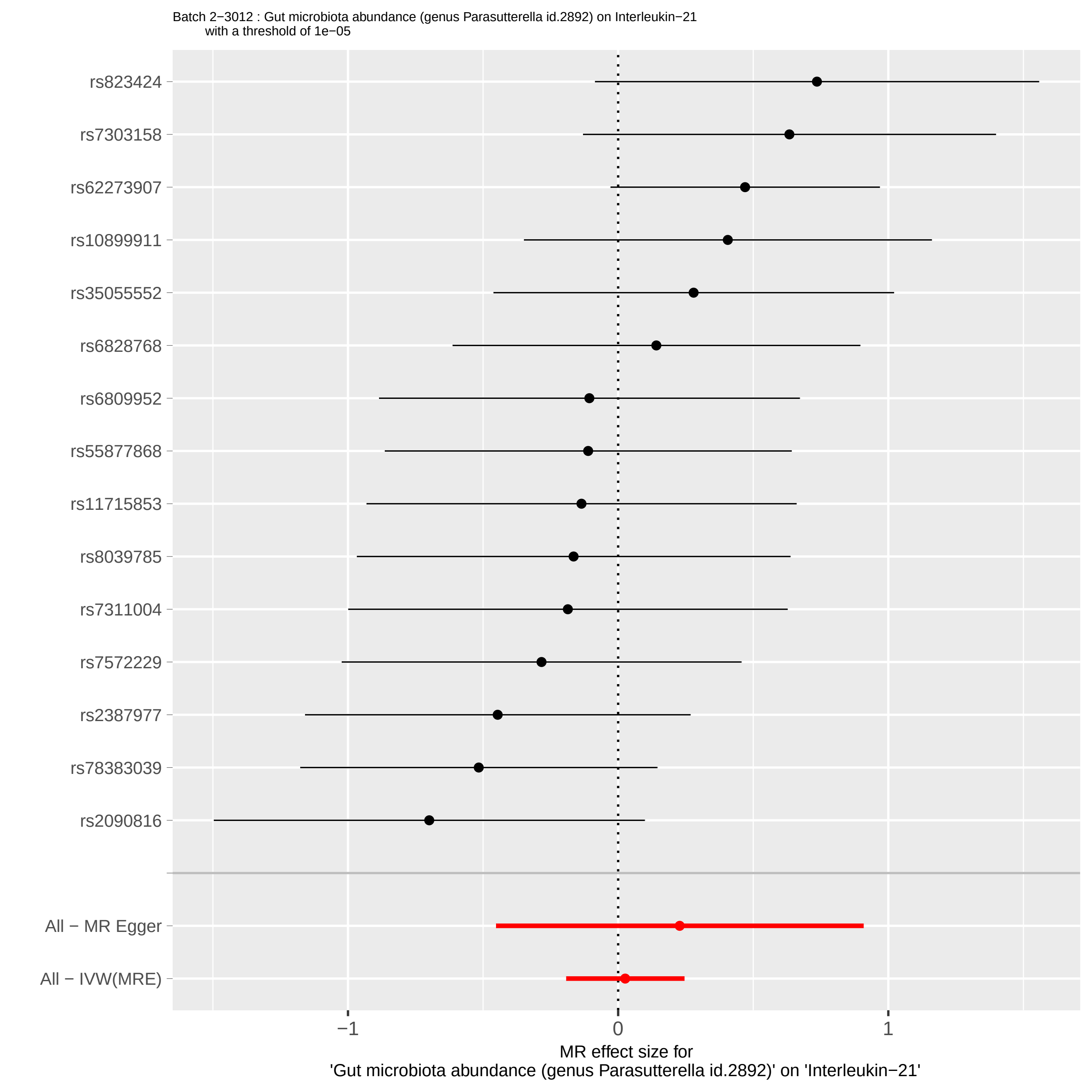
Batch 2−3012 : Gut microbiota abundance (genus Parasutterella id.2892) on Interleukin−21
with a threshold of 1e−05
rs823424
rs7303158
rs62273907
rs10899911
rs35055552
rs6828768
rs6809952
rs55877868
rs11715853
rs8039785
rs7311004
rs7572229
rs2387977
rs78383039
rs2090816
All − MR Egger
All − IVW(MRE)
−1
0
1
MR effect size for
'Gut microbiota abundance (genus Parasutterella id.2892)' on 'Interleukin−21'

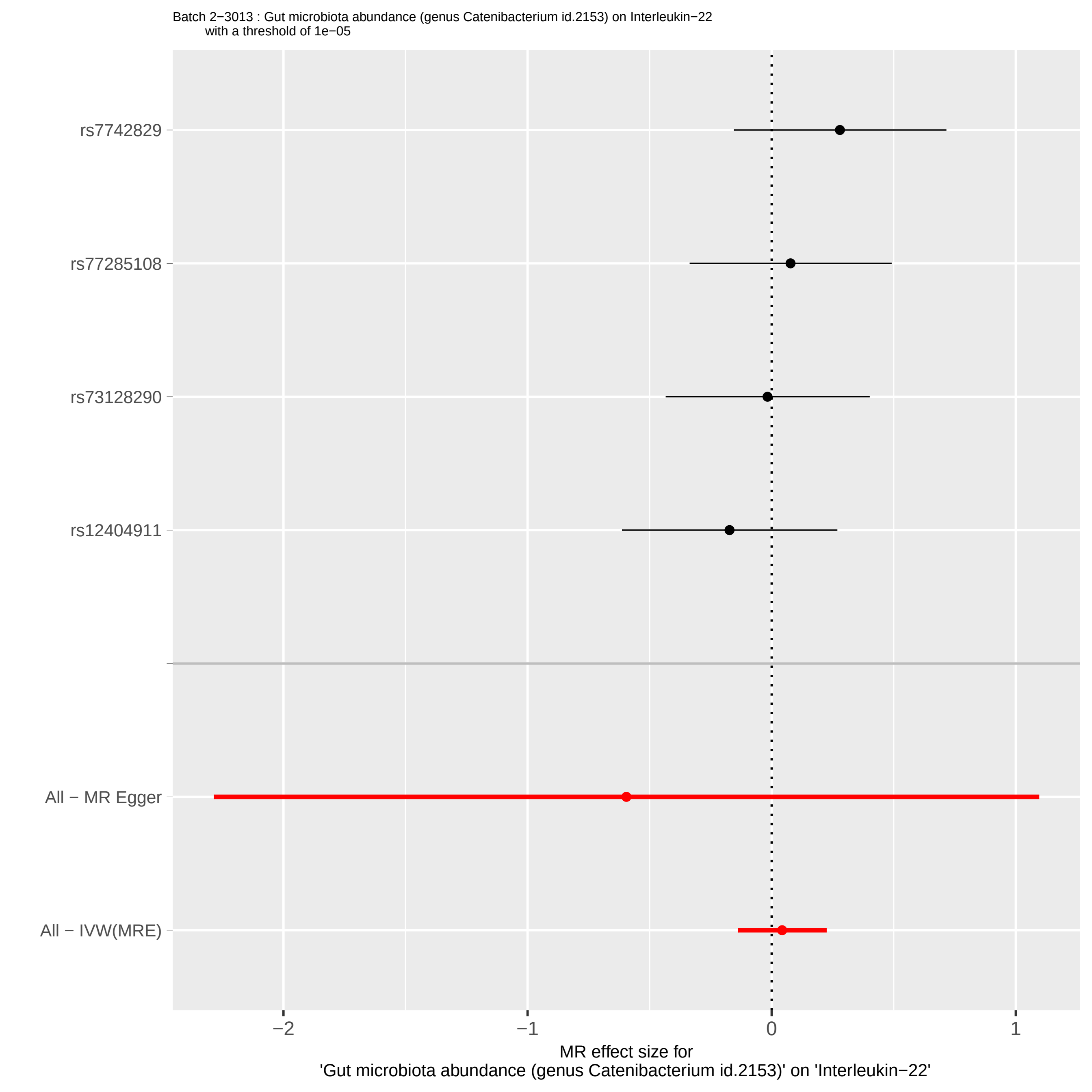

Batch 2−3013 : Gut microbiota abundance (genus Catenibacterium id.2153) on Interleukin−22
with a threshold of 1e−05
rs7742829
rs77285108
rs73128290
rs12404911
All − MR Egger
All − IVW(MRE)
−2
−1
0
1
MR effect size for
'Gut microbiota abundance (genus Catenibacterium id.2153)' on 'Interleukin−22'

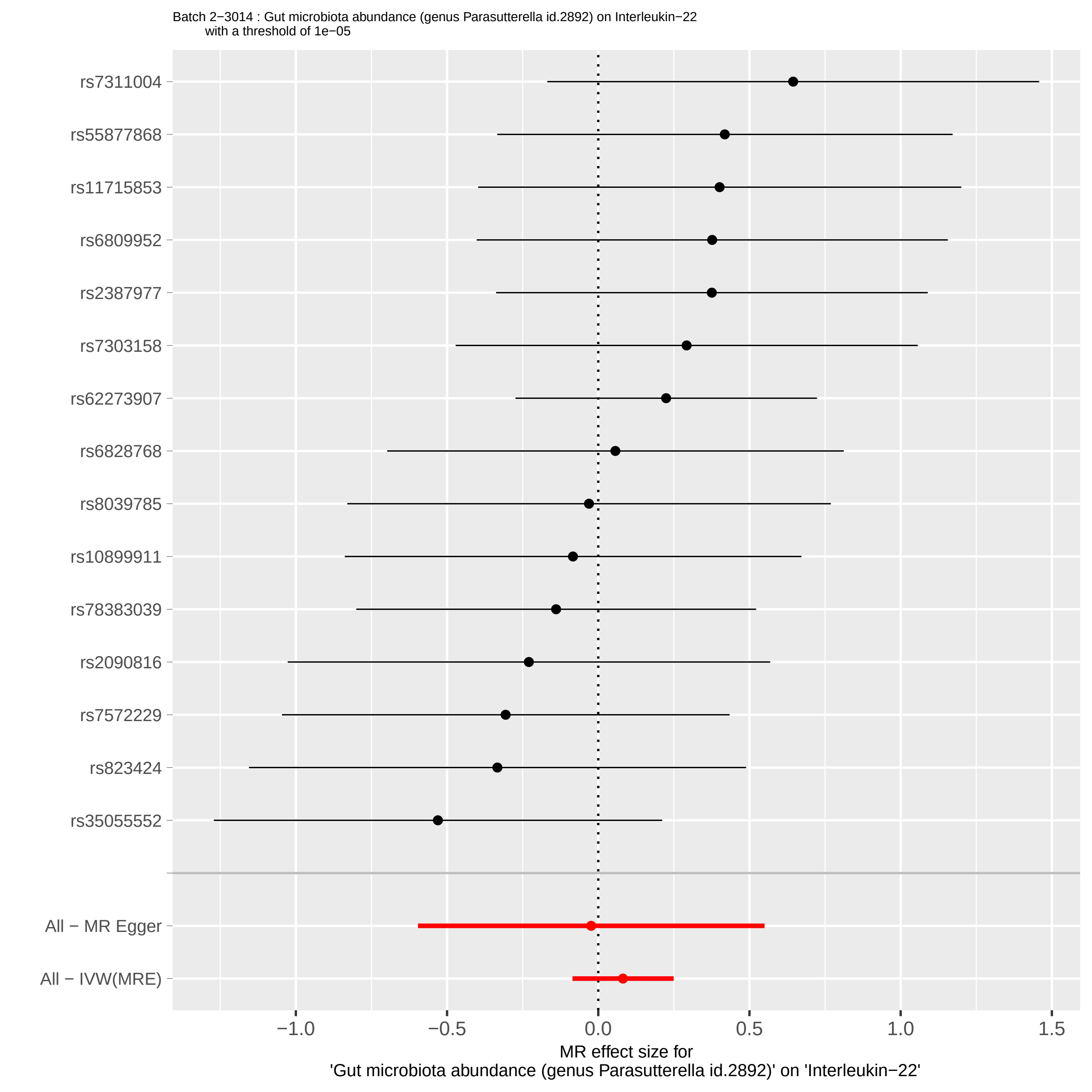

Batch 2−3014 : Gut microbiota abundance (genus Parasutterella id.2892) on Interleukin−22
with a threshold of 1e−05
rs7311004
rs55877868
rs11715853
rs6809952
rs2387977
rs7303158
rs62273907
rs6828768
rs8039785
rs10899911
rs78383039
rs2090816
rs7572229
rs823424
rs35055552
All − MR Egger
All − IVW(MRE)
−1.0
−0.5
0.0
0.5
1.0
1.5
MR effect size for
'Gut microbiota abundance (genus Parasutterella id.2892)' on 'Interleukin−22'

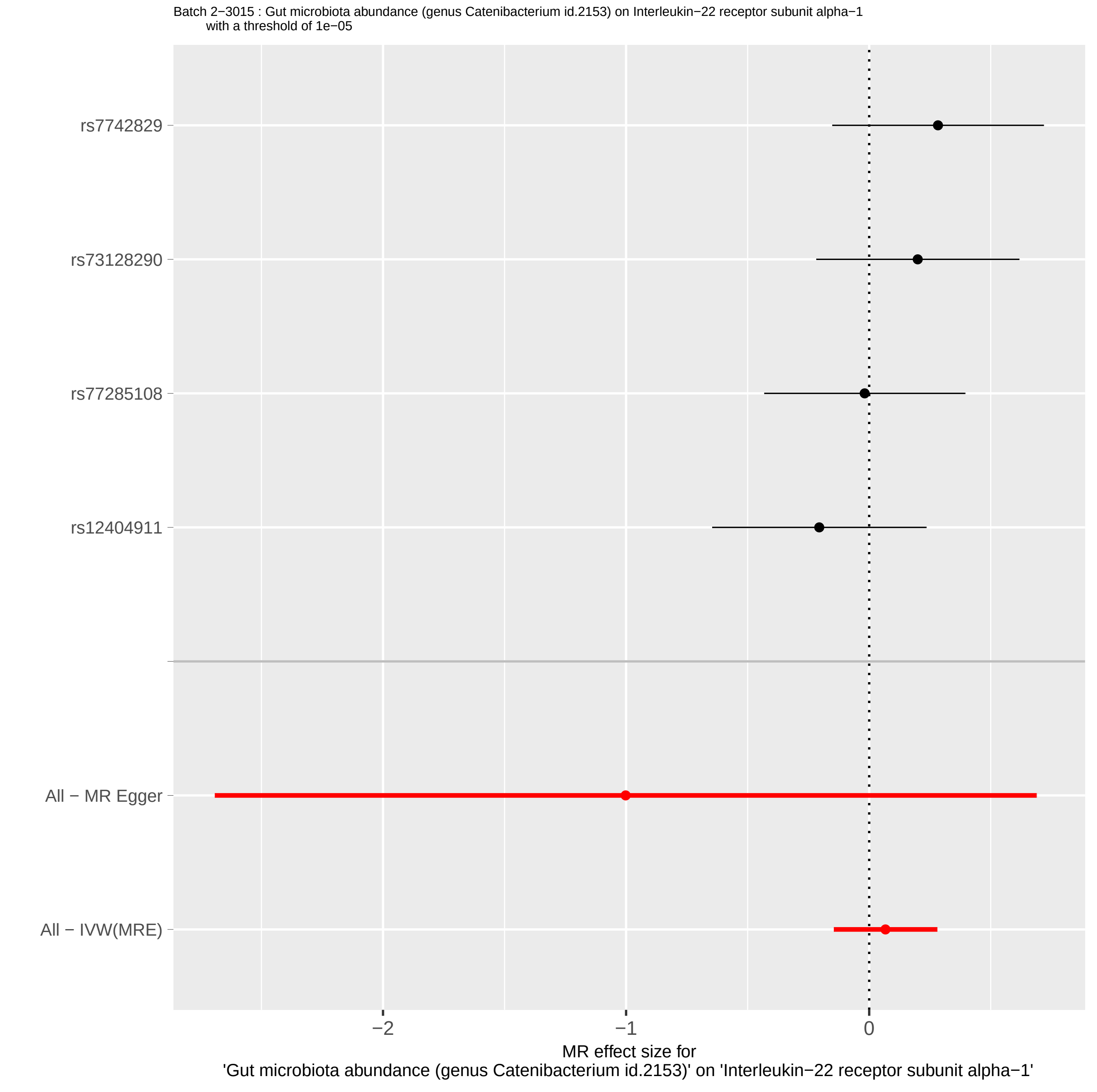
Batch 2−3015 : Gut microbiota abundance (genus Catenibacterium id.2153) on Interleukin−22 receptor subunit alpha−1
with a threshold of 1e−05
rs7742829
rs73128290
rs77285108
rs12404911
All − MR Egger
All − IVW(MRE)
−2
−1
0
MR effect size for
'Gut microbiota abundance (genus Catenibacterium id.2153)' on 'Interleukin−22 receptor subunit alpha−1'

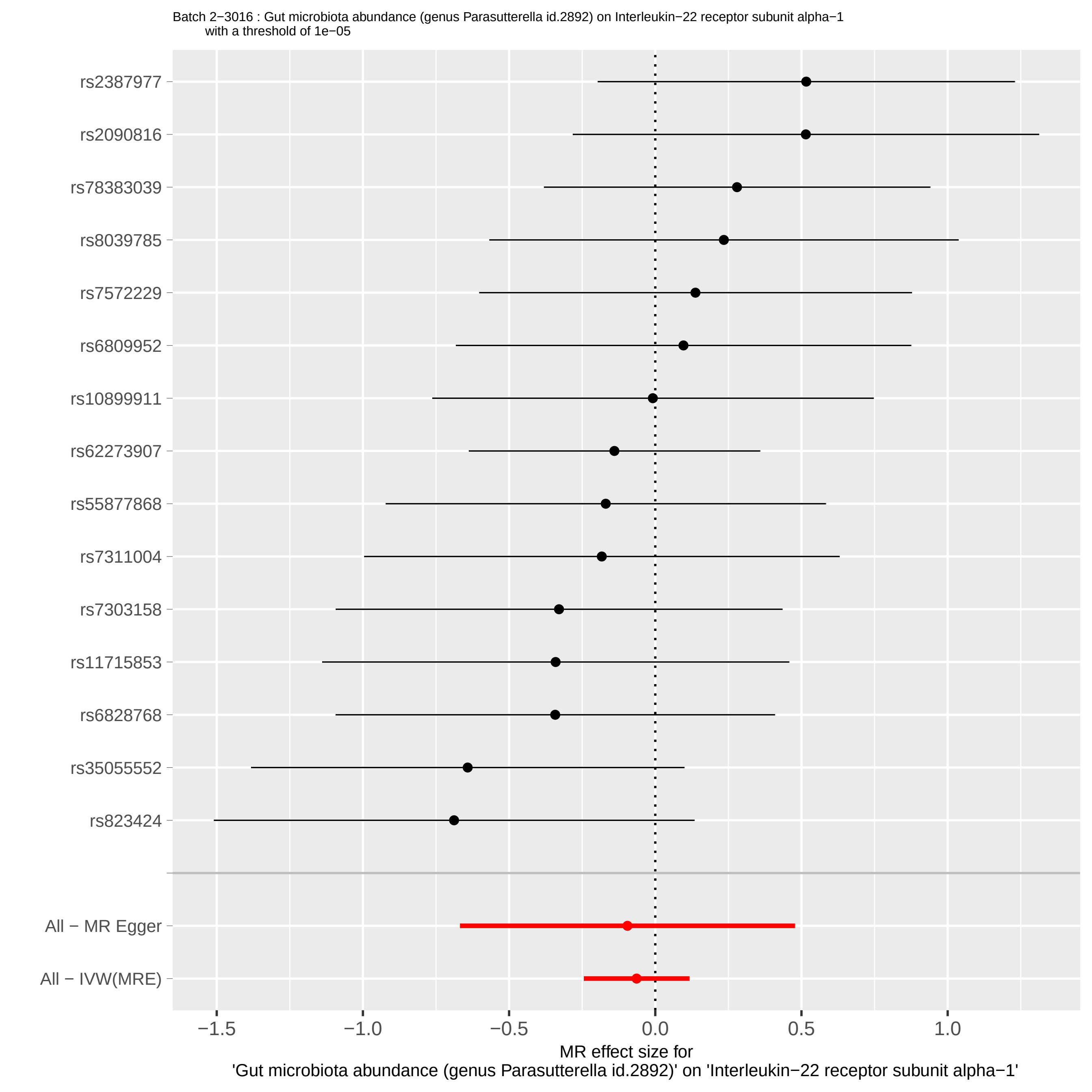
Batch 2−3016 : Gut microbiota abundance (genus Parasutterella id.2892) on Interleukin−22 receptor subunit alpha−1
with a threshold of 1e−05
rs2387977
rs2090816
rs78383039
rs8039785
rs7572229
rs6809952
rs10899911
rs62273907
rs55877868
rs7311004
rs7303158
rs11715853
rs6828768
rs35055552
rs823424
All − MR Egger
All − IVW(MRE)
−1.5
−1.0
−0.5
0.0
0.5
1.0
MR effect size for
'Gut microbiota abundance (genus Parasutterella id.2892)' on 'Interleukin−22 receptor subunit alpha−1'

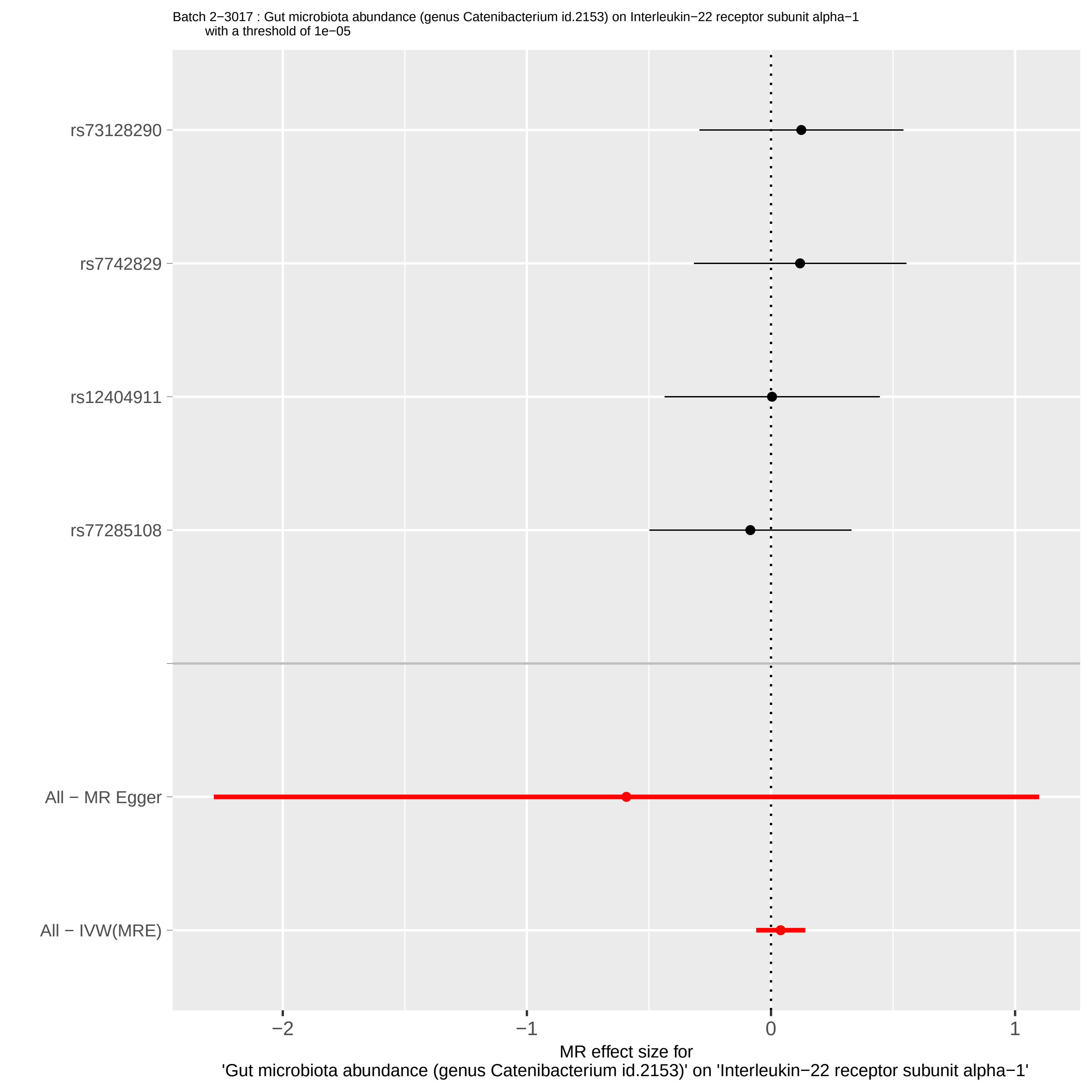

Batch 2−3017 : Gut microbiota abundance (genus Catenibacterium id.2153) on Interleukin−22 receptor subunit alpha−1
with a threshold of 1e−05
rs73128290
rs7742829
rs12404911
rs77285108
All − MR Egger
All − IVW(MRE)
−2
−1
0
1
MR effect size for
'Gut microbiota abundance (genus Catenibacterium id.2153)' on 'Interleukin−22 receptor subunit alpha−1'

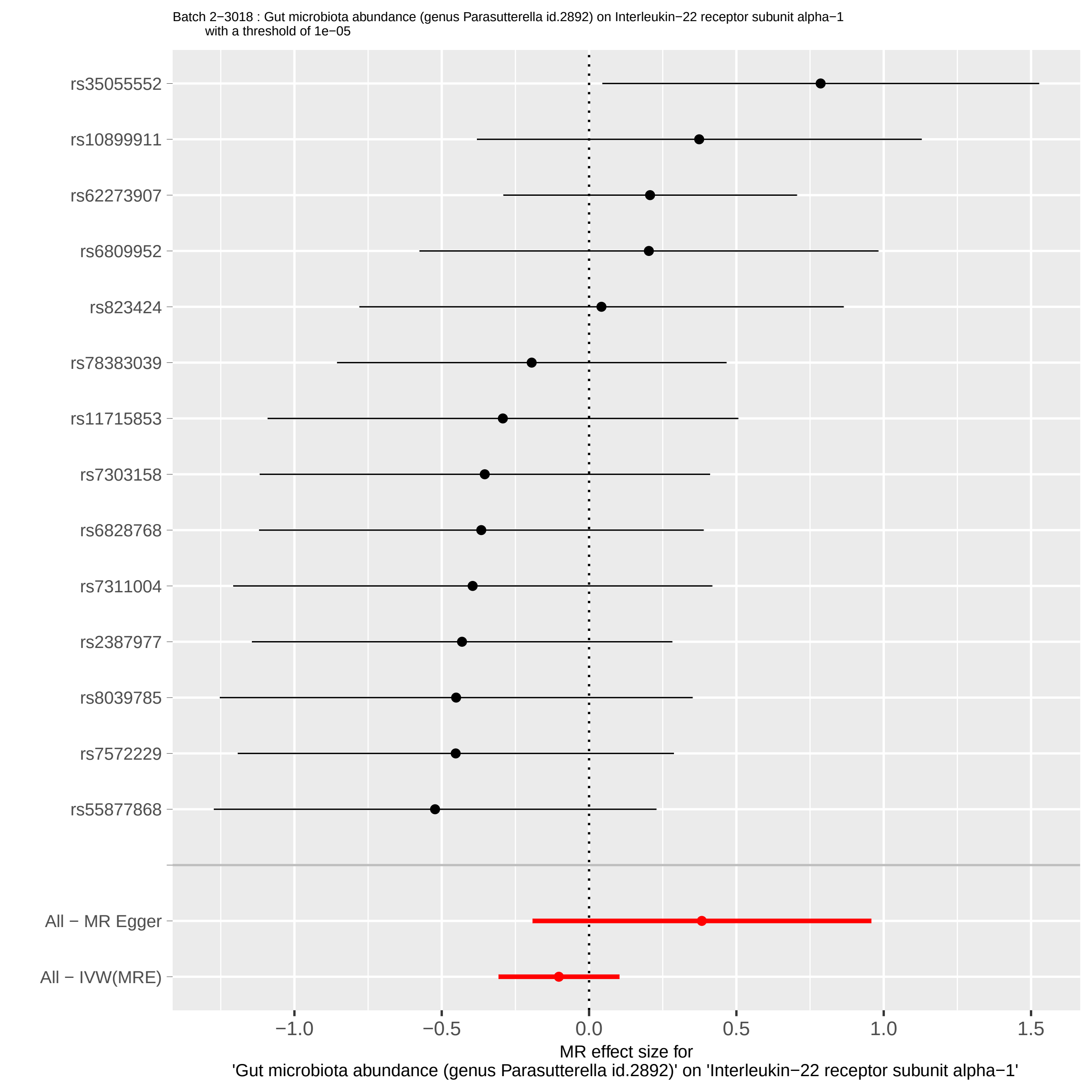
Batch 2−3018 : Gut microbiota abundance (genus Parasutterella id.2892) on Interleukin−22 receptor subunit alpha−1
with a threshold of 1e−05
rs35055552
rs10899911
rs62273907
rs6809952
rs823424
rs78383039
rs11715853
rs7303158
rs6828768
rs7311004
rs2387977
rs8039785
rs7572229
rs55877868
All − MR Egger
All − IVW(MRE)
−1.0
−0.5
0.0
0.5
1.0
1.5
MR effect size for
'Gut microbiota abundance (genus Parasutterella id.2892)' on 'Interleukin−22 receptor subunit alpha−1'

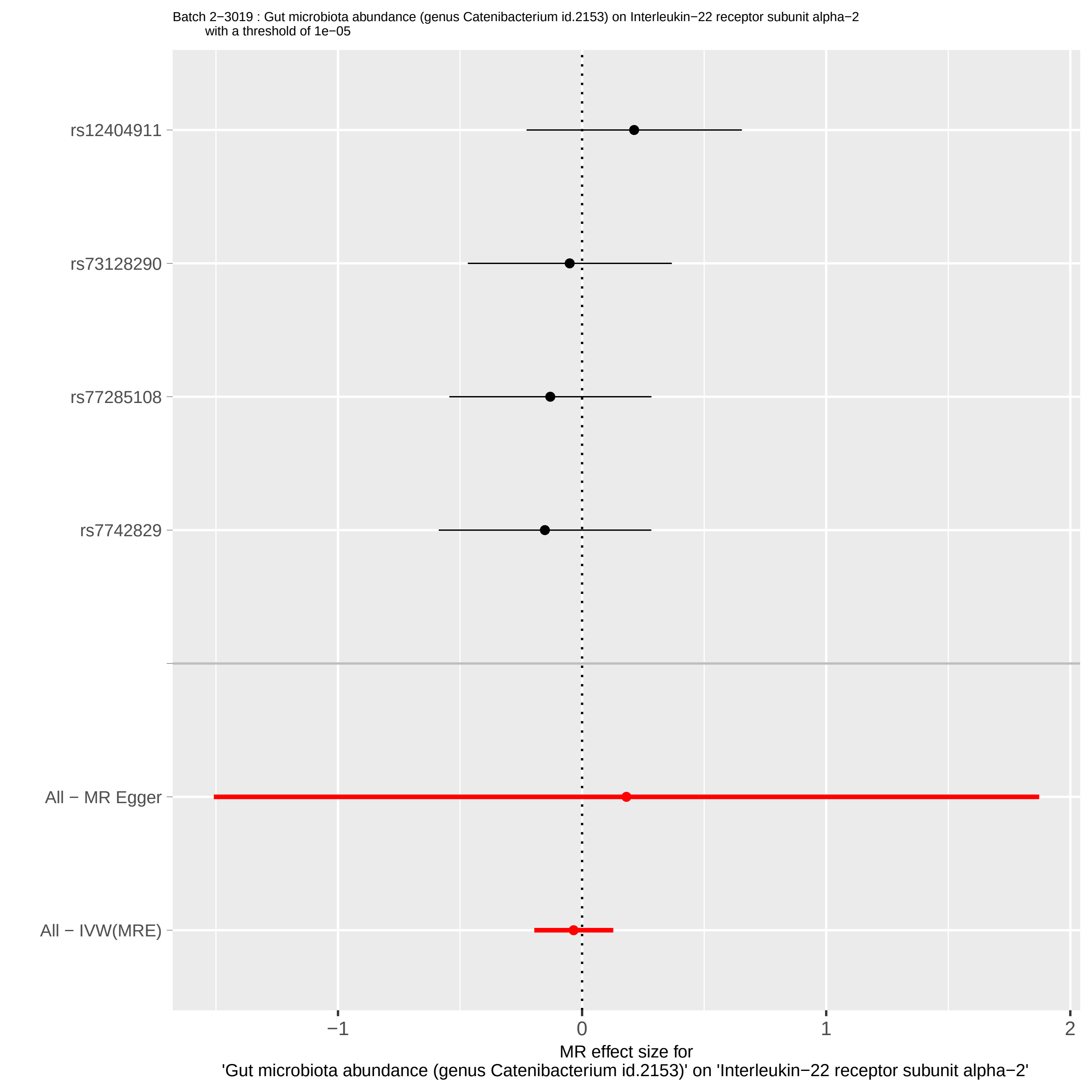

Batch 2−3019 : Gut microbiota abundance (genus Catenibacterium id.2153) on Interleukin−22 receptor subunit alpha−2
with a threshold of 1e−05
rs12404911
rs73128290
rs77285108
rs7742829
All − MR Egger
All − IVW(MRE)
−1
0
1
2
MR effect size for
'Gut microbiota abundance (genus Catenibacterium id.2153)' on 'Interleukin−22 receptor subunit alpha−2'

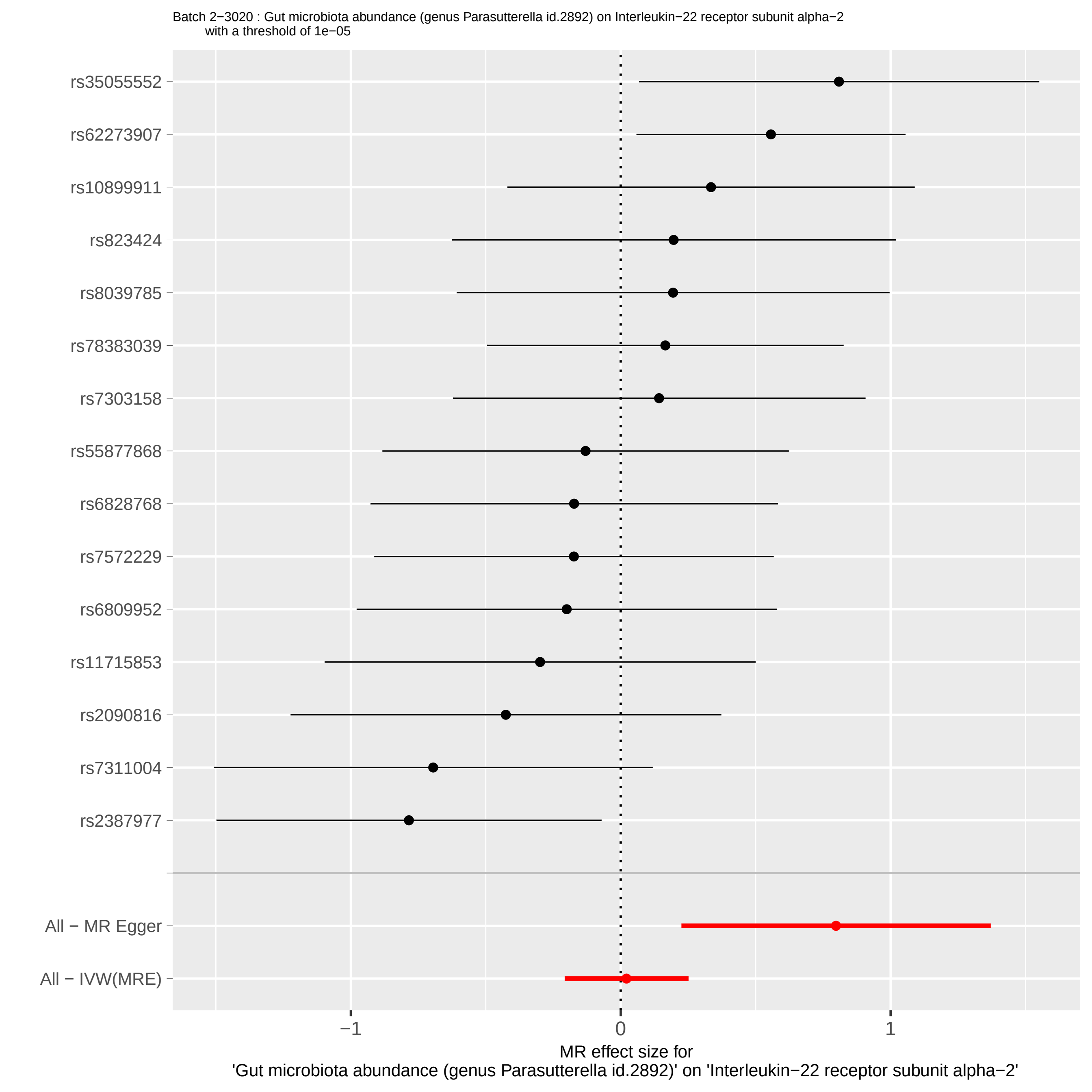

Batch 2−3020 : Gut microbiota abundance (genus Parasutterella id.2892) on Interleukin−22 receptor subunit alpha−2
with a threshold of 1e−05
rs35055552
rs62273907
rs10899911
rs823424
rs8039785
rs78383039
rs7303158
rs55877868
rs6828768
rs7572229
rs6809952
rs11715853
rs2090816
rs7311004
rs2387977
All − MR Egger
All − IVW(MRE)
−1
0
1
MR effect size for
'Gut microbiota abundance (genus Parasutterella id.2892)' on 'Interleukin−22 receptor subunit alpha−2'

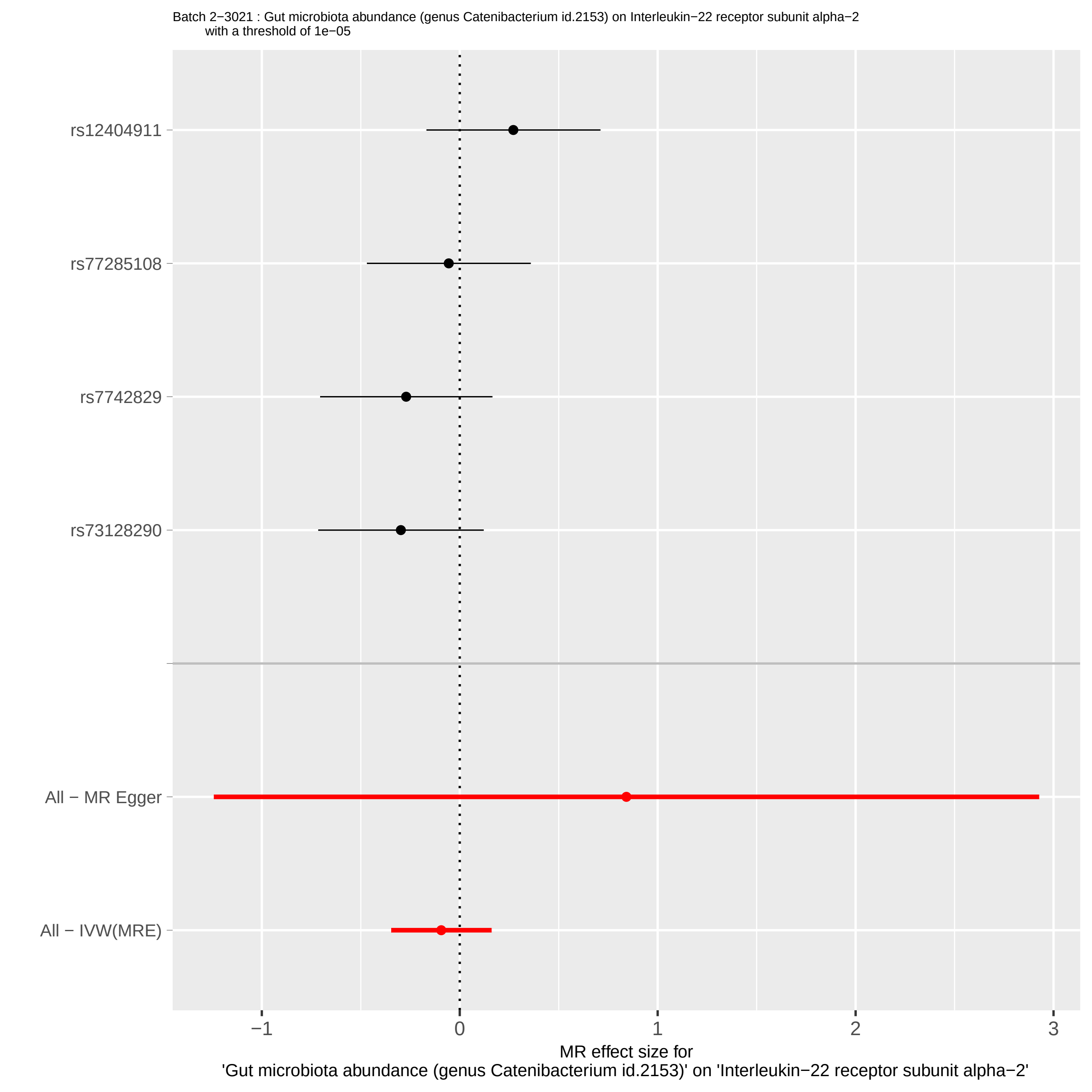

Batch 2−3021 : Gut microbiota abundance (genus Catenibacterium id.2153) on Interleukin−22 receptor subunit alpha−2
with a threshold of 1e−05
rs12404911
rs77285108
rs7742829
rs73128290
All − MR Egger
All − IVW(MRE)
−1
0
1
2
3
MR effect size for
'Gut microbiota abundance (genus Catenibacterium id.2153)' on 'Interleukin−22 receptor subunit alpha−2'

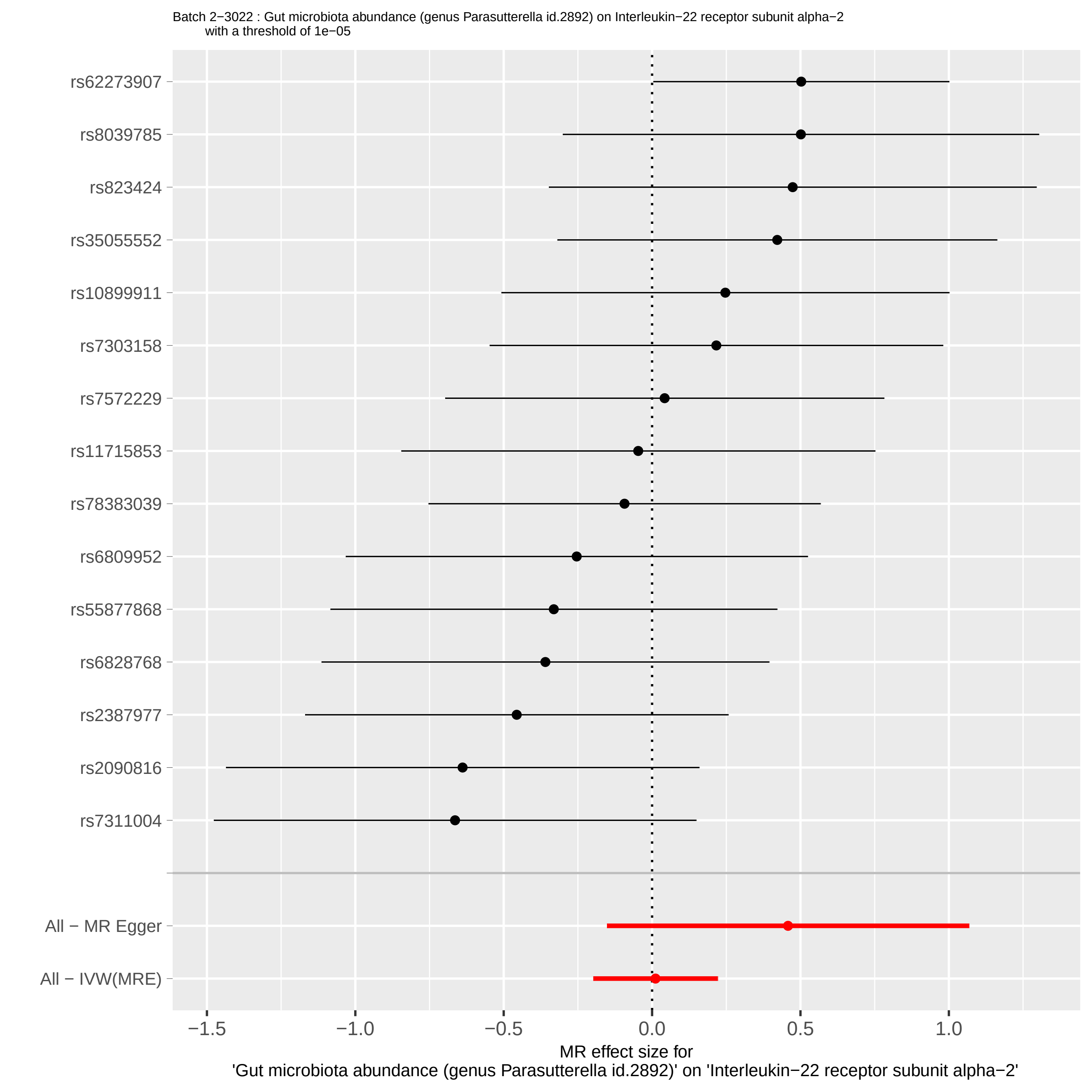

Batch 2−3022 : Gut microbiota abundance (genus Parasutterella id.2892) on Interleukin−22 receptor subunit alpha−2
with a threshold of 1e−05
rs62273907
rs8039785
rs823424
rs35055552
rs10899911
rs7303158
rs7572229
rs11715853
rs78383039
rs6809952
rs55877868
rs6828768
rs2387977
rs2090816
rs7311004
All − MR Egger
All − IVW(MRE)
−1.5
−1.0
−0.5
0.0
0.5
1.0
MR effect size for
'Gut microbiota abundance (genus Parasutterella id.2892)' on 'Interleukin−22 receptor subunit alpha−2'

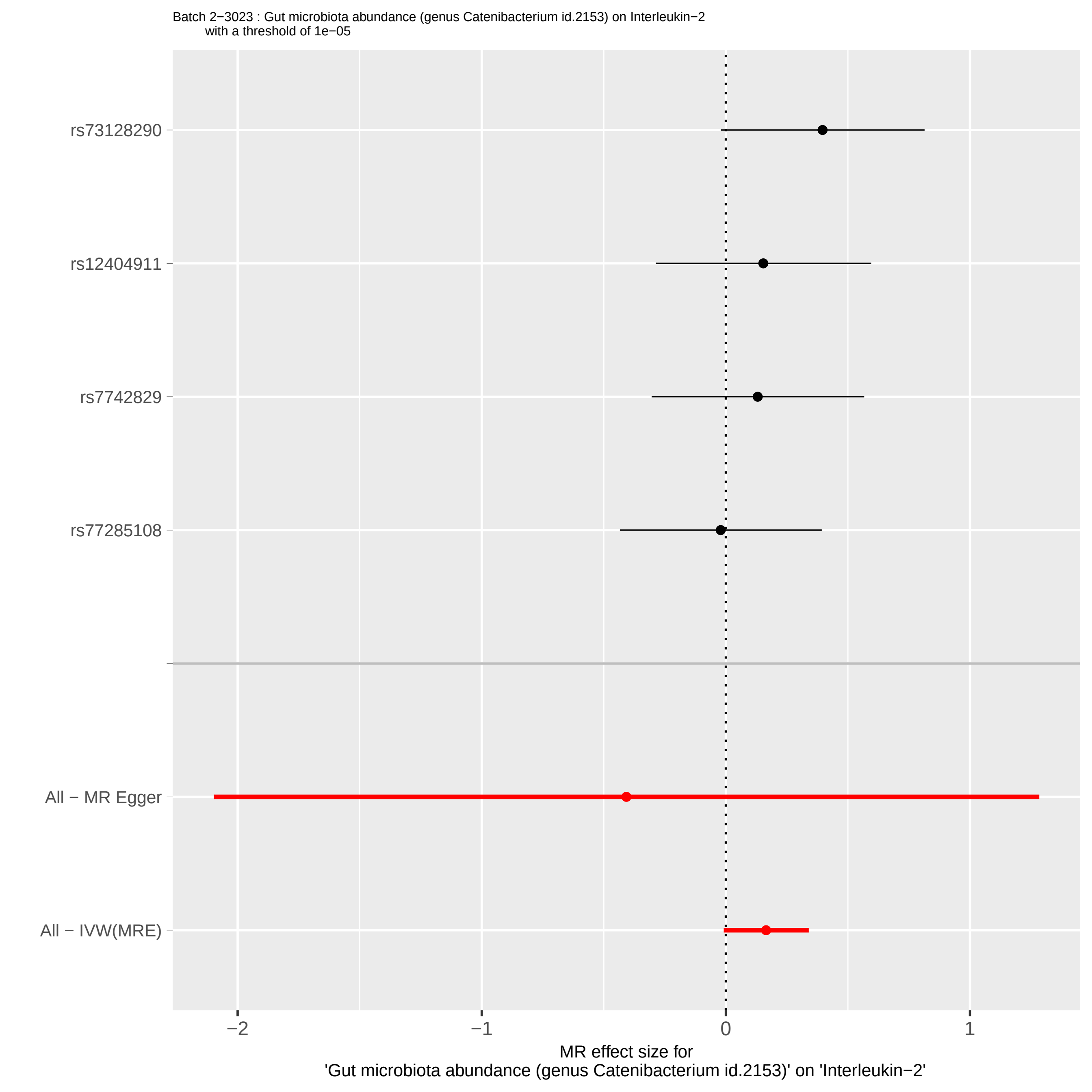

Batch 2−3023 : Gut microbiota abundance (genus Catenibacterium id.2153) on Interleukin−2
with a threshold of 1e−05
rs73128290
rs12404911
rs7742829
rs77285108
All − MR Egger
All − IVW(MRE)
−2
−1
0
1
MR effect size for
'Gut microbiota abundance (genus Catenibacterium id.2153)' on 'Interleukin−2'

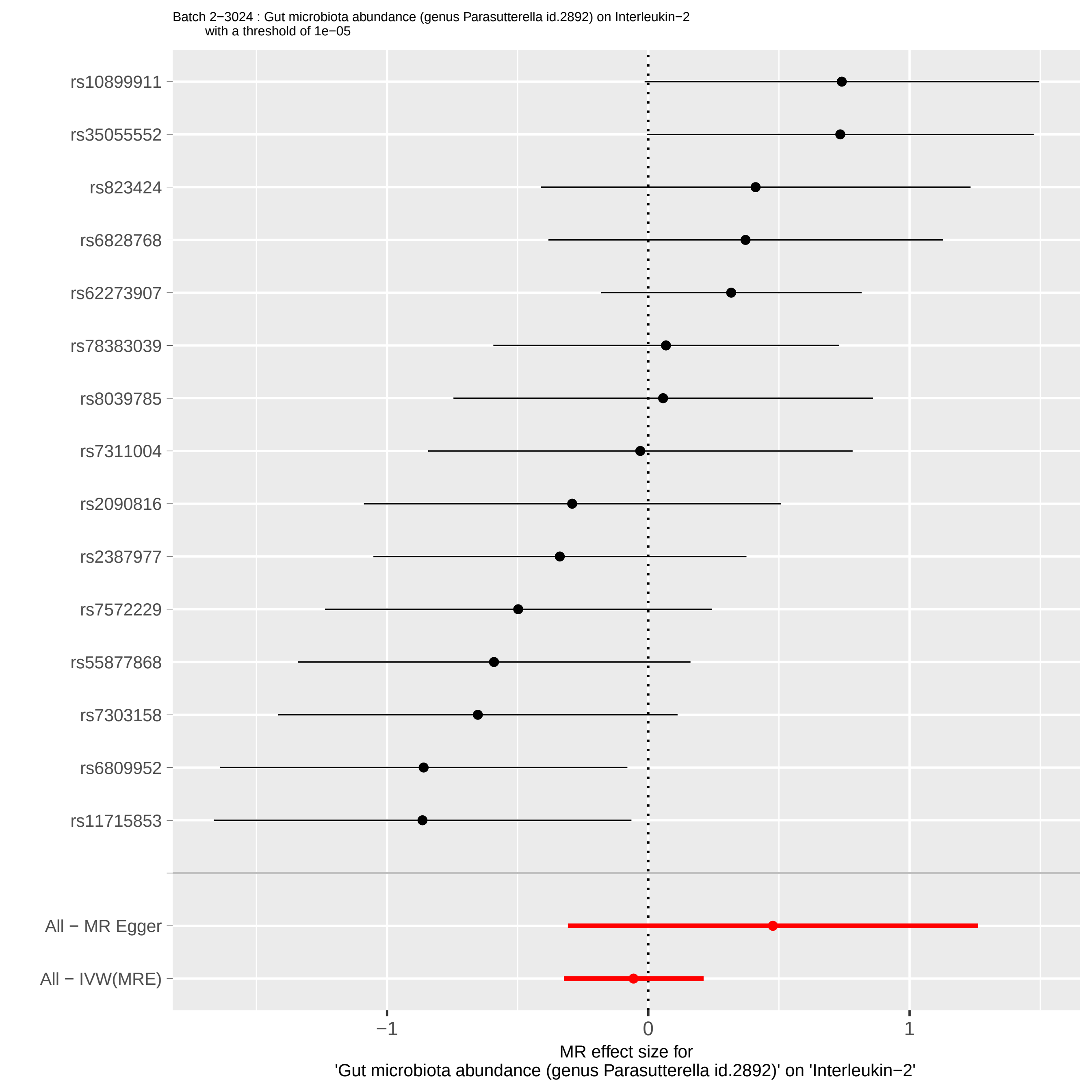

Batch 2−3024 : Gut microbiota abundance (genus Parasutterella id.2892) on Interleukin−2
with a threshold of 1e−05
rs10899911
rs35055552
rs823424
rs6828768
rs62273907
rs78383039
rs8039785
rs7311004
rs2090816
rs2387977
rs7572229
rs55877868
rs7303158
rs6809952
rs11715853
All − MR Egger
All − IVW(MRE)
−1
0
1
MR effect size for
'Gut microbiota abundance (genus Parasutterella id.2892)' on 'Interleukin−2'

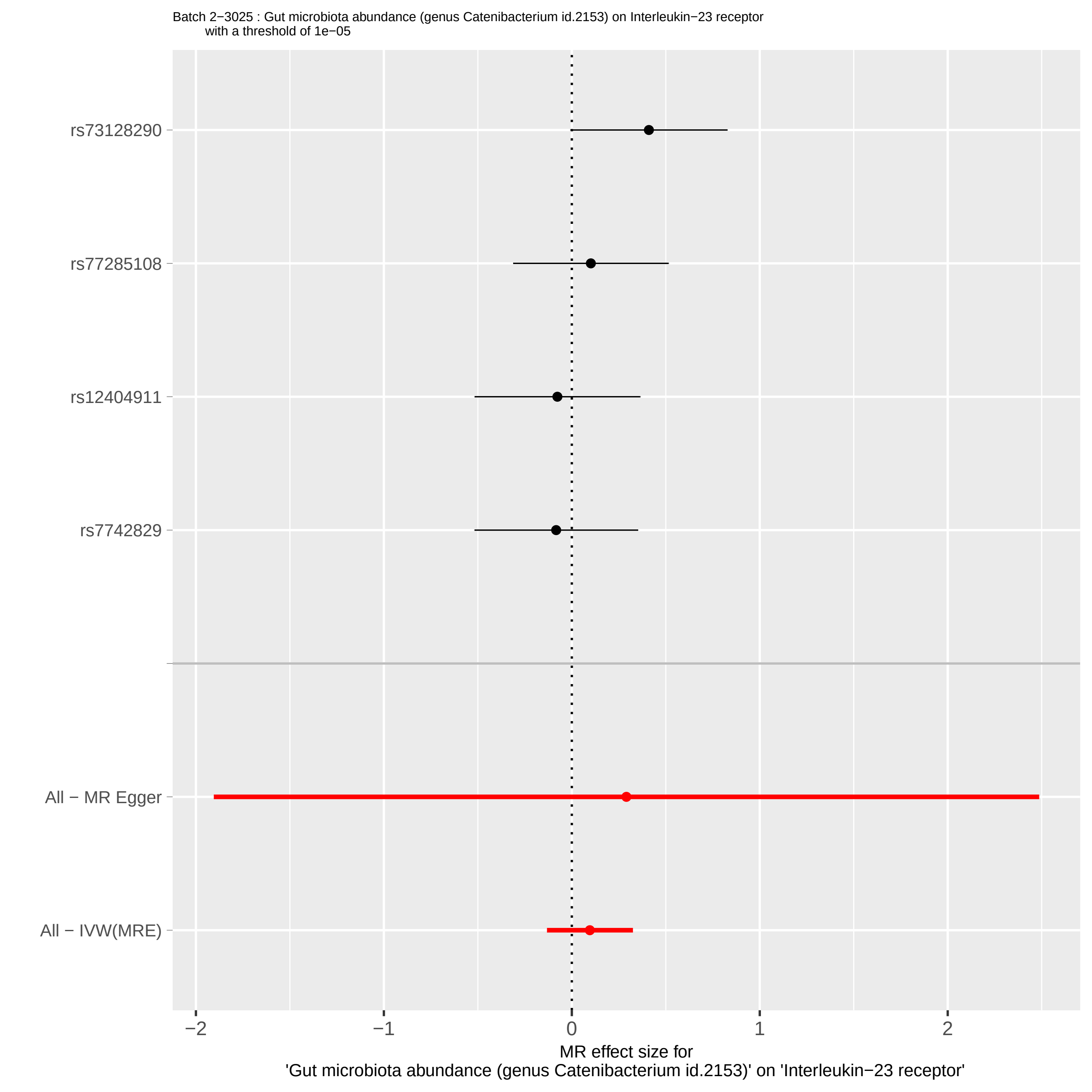

Batch 2−3025 : Gut microbiota abundance (genus Catenibacterium id.2153) on Interleukin−23 receptor
with a threshold of 1e−05
rs73128290
rs77285108
rs12404911
rs7742829
All − MR Egger
All − IVW(MRE)
−2
−1
0
1
2
MR effect size for
'Gut microbiota abundance (genus Catenibacterium id.2153)' on 'Interleukin−23 receptor'

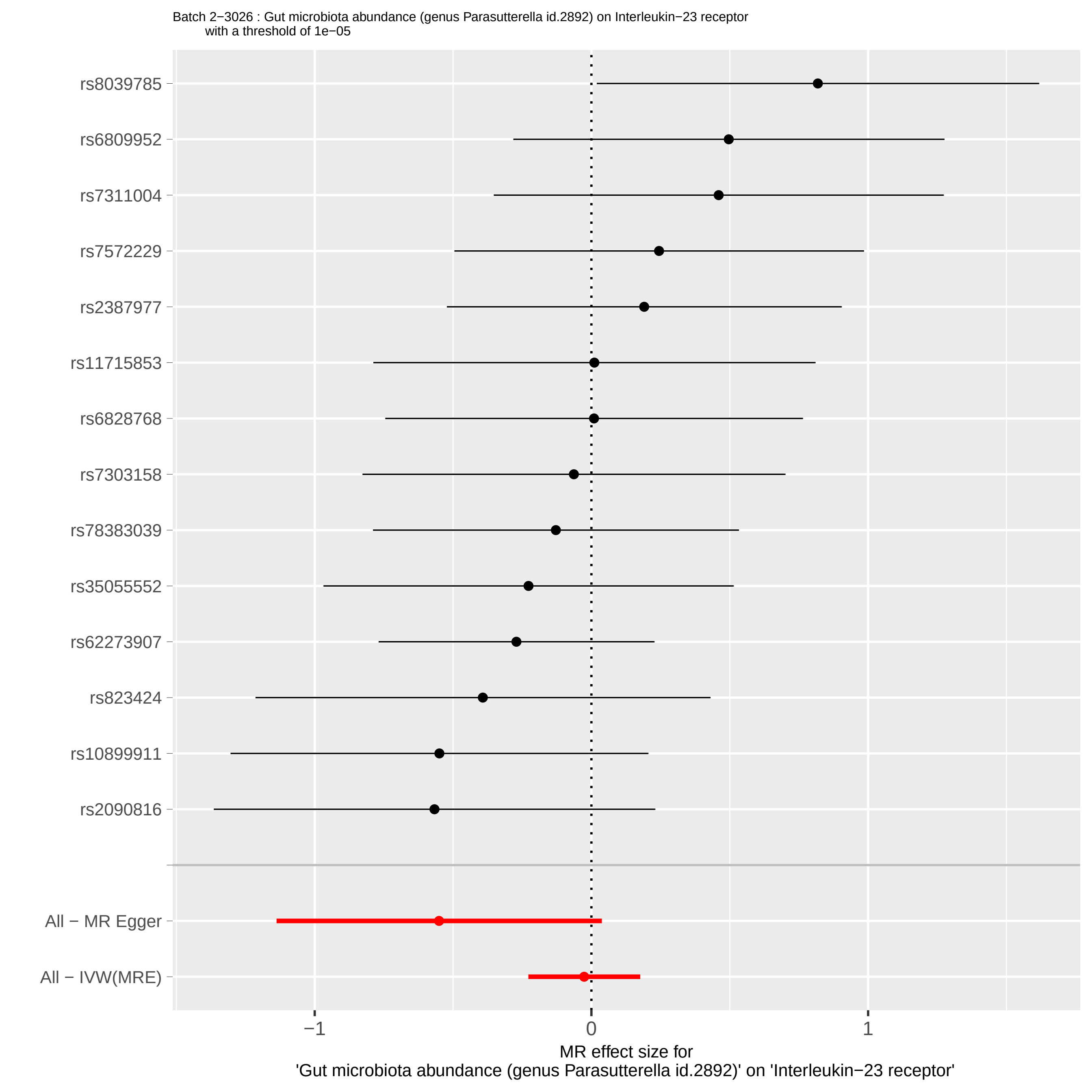

Batch 2−3026 : Gut microbiota abundance (genus Parasutterella id.2892) on Interleukin−23 receptor
with a threshold of 1e−05
rs8039785
rs6809952
rs7311004
rs7572229
rs2387977
rs11715853
rs6828768
rs7303158
rs78383039
rs35055552
rs62273907
rs823424
rs10899911
rs2090816
All − MR Egger
All − IVW(MRE)
−1
0
1
MR effect size for
'Gut microbiota abundance (genus Parasutterella id.2892)' on 'Interleukin−23 receptor'

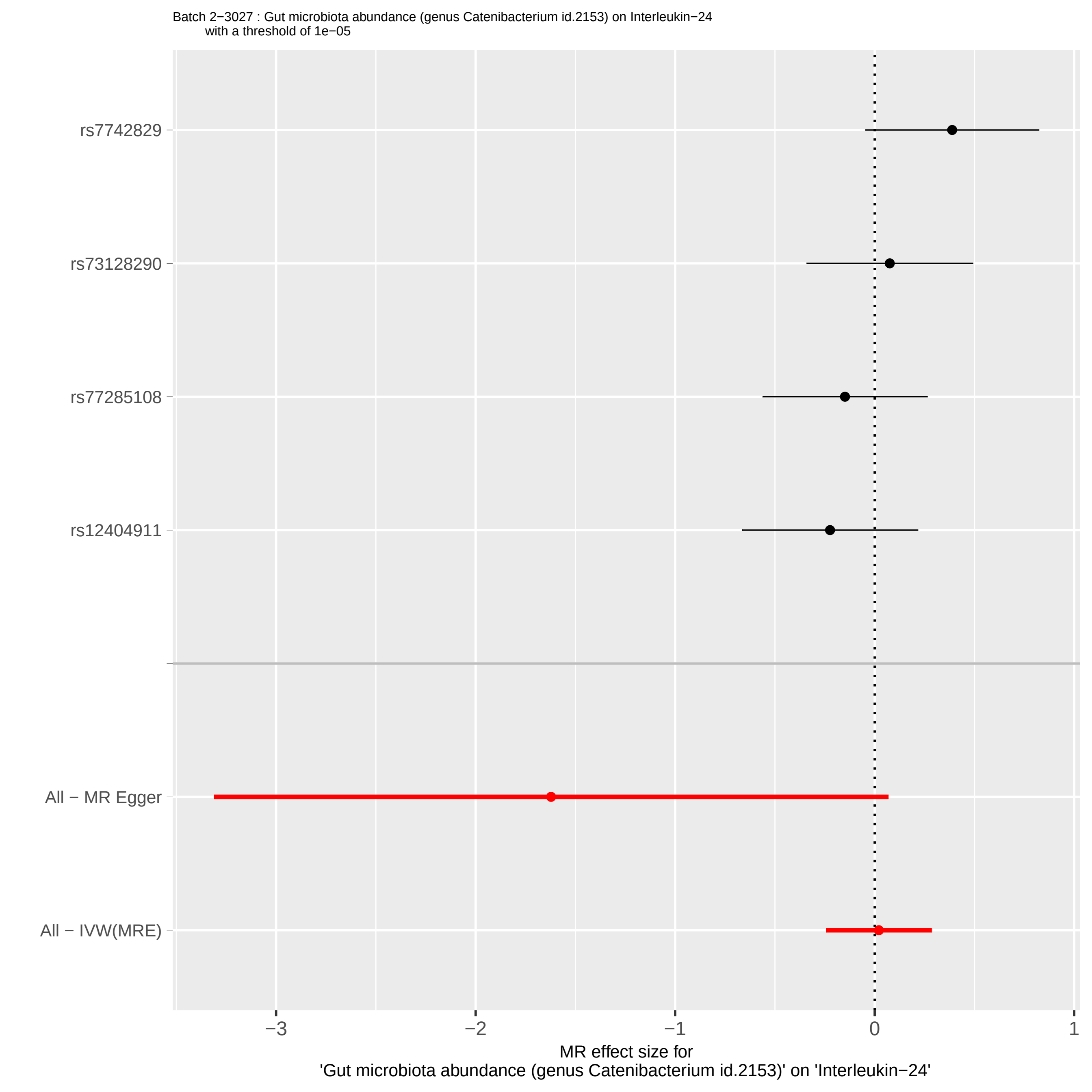

Batch 2−3027 : Gut microbiota abundance (genus Catenibacterium id.2153) on Interleukin−24
with a threshold of 1e−05
rs7742829
rs73128290
rs77285108
rs12404911
All − MR Egger
All − IVW(MRE)
−3
−2
−1
0
1
MR effect size for
'Gut microbiota abundance (genus Catenibacterium id.2153)' on 'Interleukin−24'

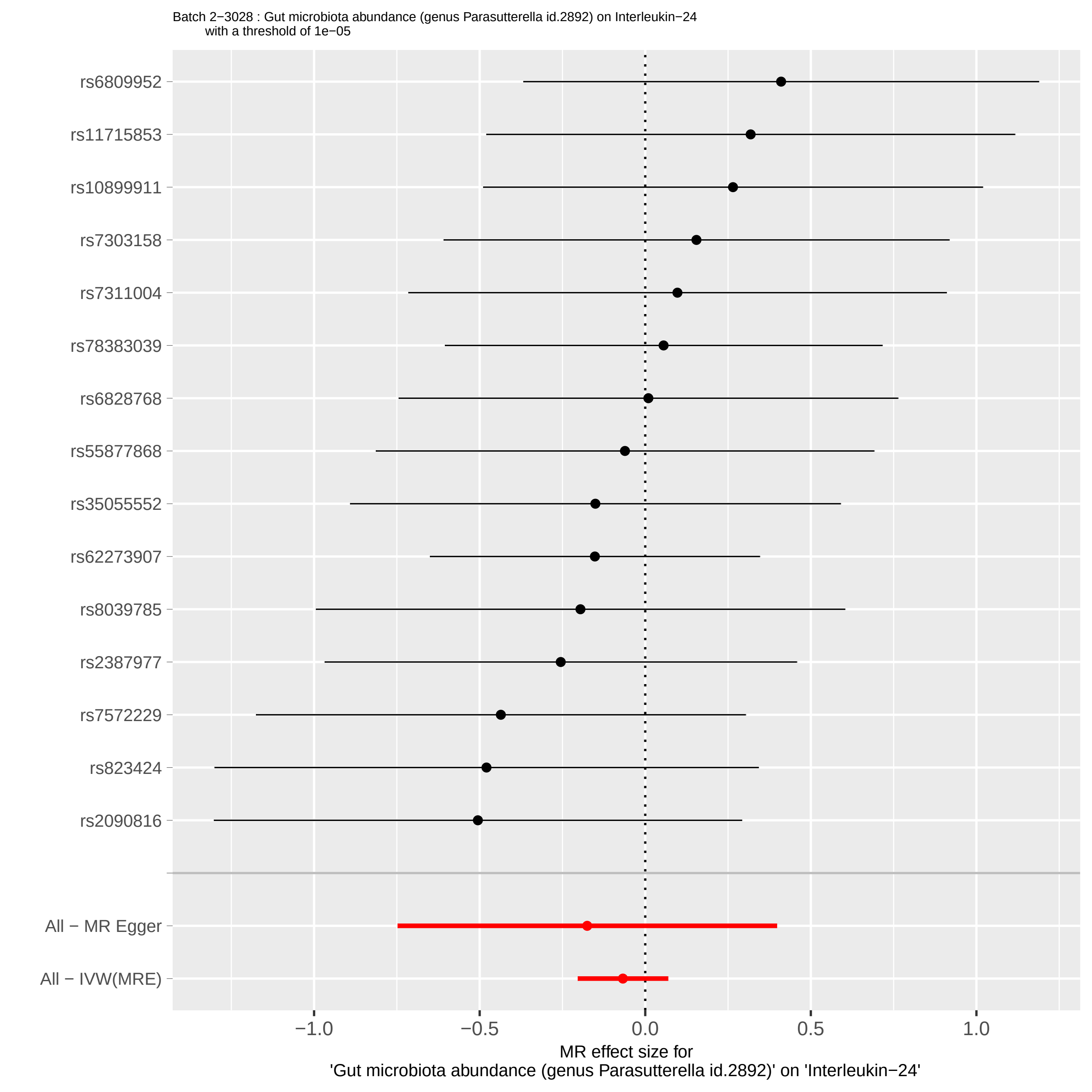
Batch 2−3028 : Gut microbiota abundance (genus Parasutterella id.2892) on Interleukin−24
with a threshold of 1e−05
rs6809952
rs11715853
rs10899911
rs7303158
rs7311004
rs78383039
rs6828768
rs55877868
rs35055552
rs62273907
rs8039785
rs2387977
rs7572229
rs823424
rs2090816
All − MR Egger
All − IVW(MRE)
−1.0
−0.5
0.0
0.5
1.0
MR effect size for
'Gut microbiota abundance (genus Parasutterella id.2892)' on 'Interleukin−24'

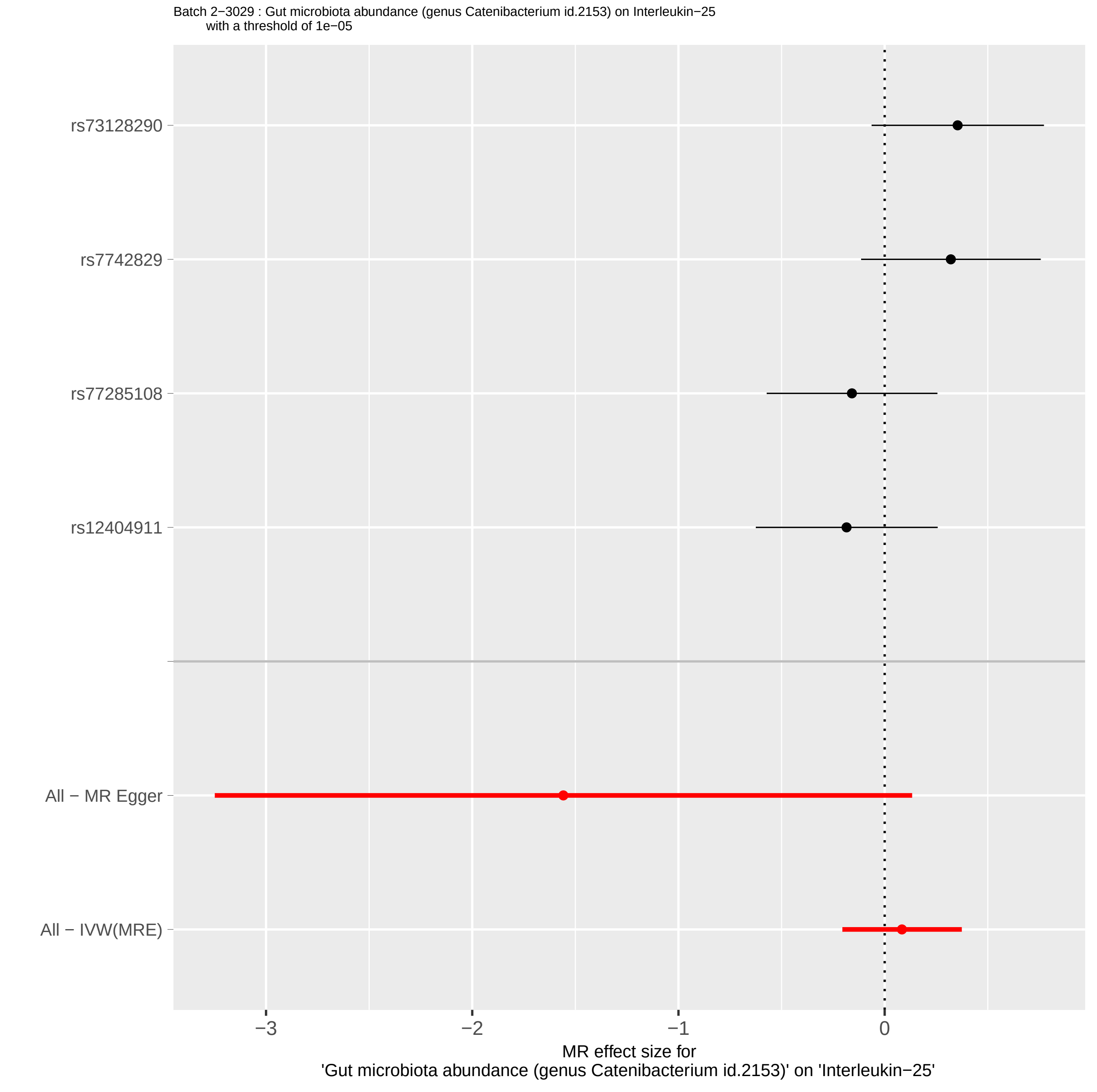

Batch 2−3029 : Gut microbiota abundance (genus Catenibacterium id.2153) on Interleukin−25
with a threshold of 1e−05
rs73128290
rs7742829
rs77285108
rs12404911
All − MR Egger
All − IVW(MRE)
−3
−2
−1
0
MR effect size for
'Gut microbiota abundance (genus Catenibacterium id.2153)' on 'Interleukin−25'

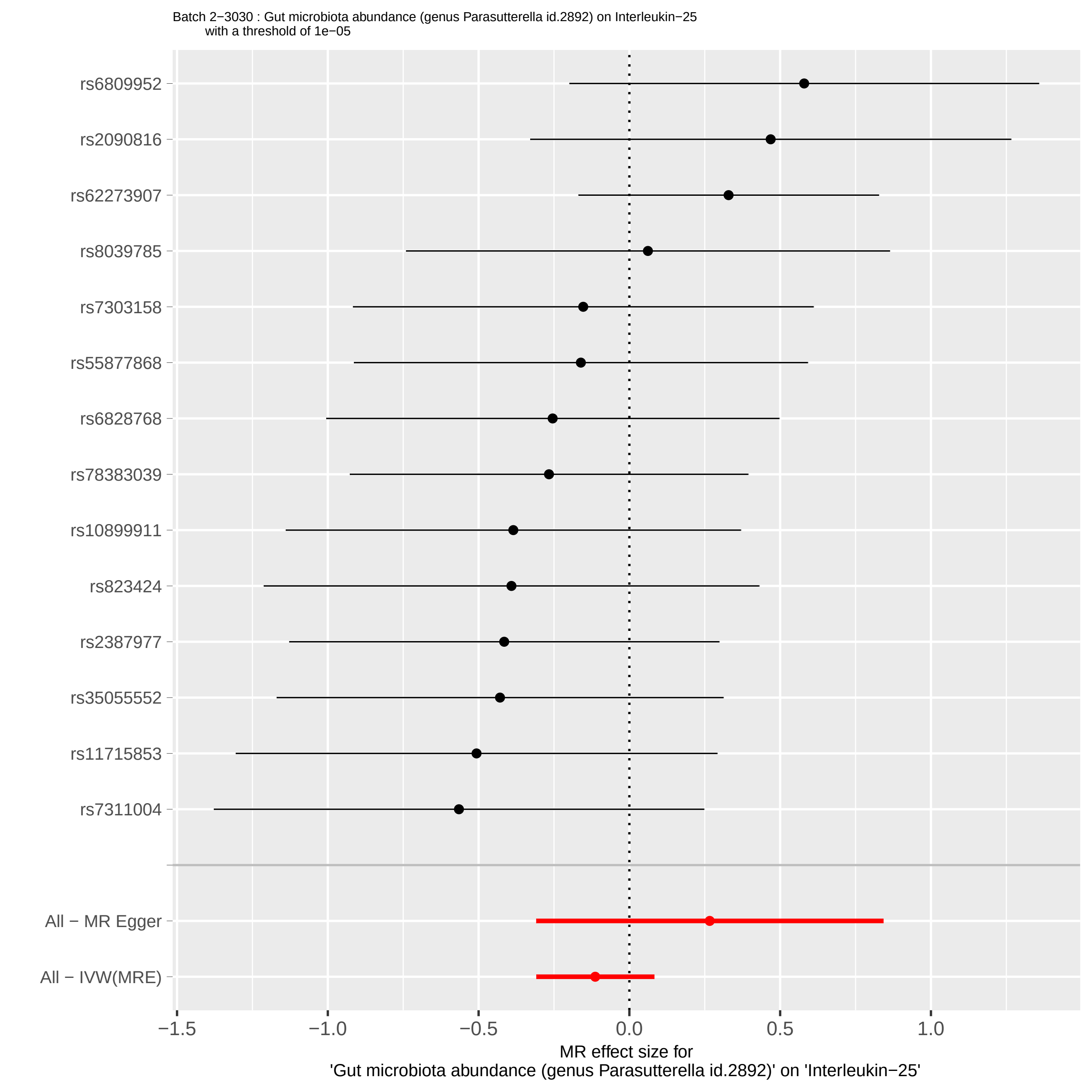

Batch 2−3030 : Gut microbiota abundance (genus Parasutterella id.2892) on Interleukin−25
with a threshold of 1e−05
rs6809952
rs2090816
rs62273907
rs8039785
rs7303158
rs55877868
rs6828768
rs78383039
rs10899911
rs823424
rs2387977
rs35055552
rs11715853
rs7311004
All − MR Egger
All − IVW(MRE)
−1.5
−1.0
−0.5
0.0
0.5
1.0
MR effect size for
'Gut microbiota abundance (genus Parasutterella id.2892)' on 'Interleukin−25'

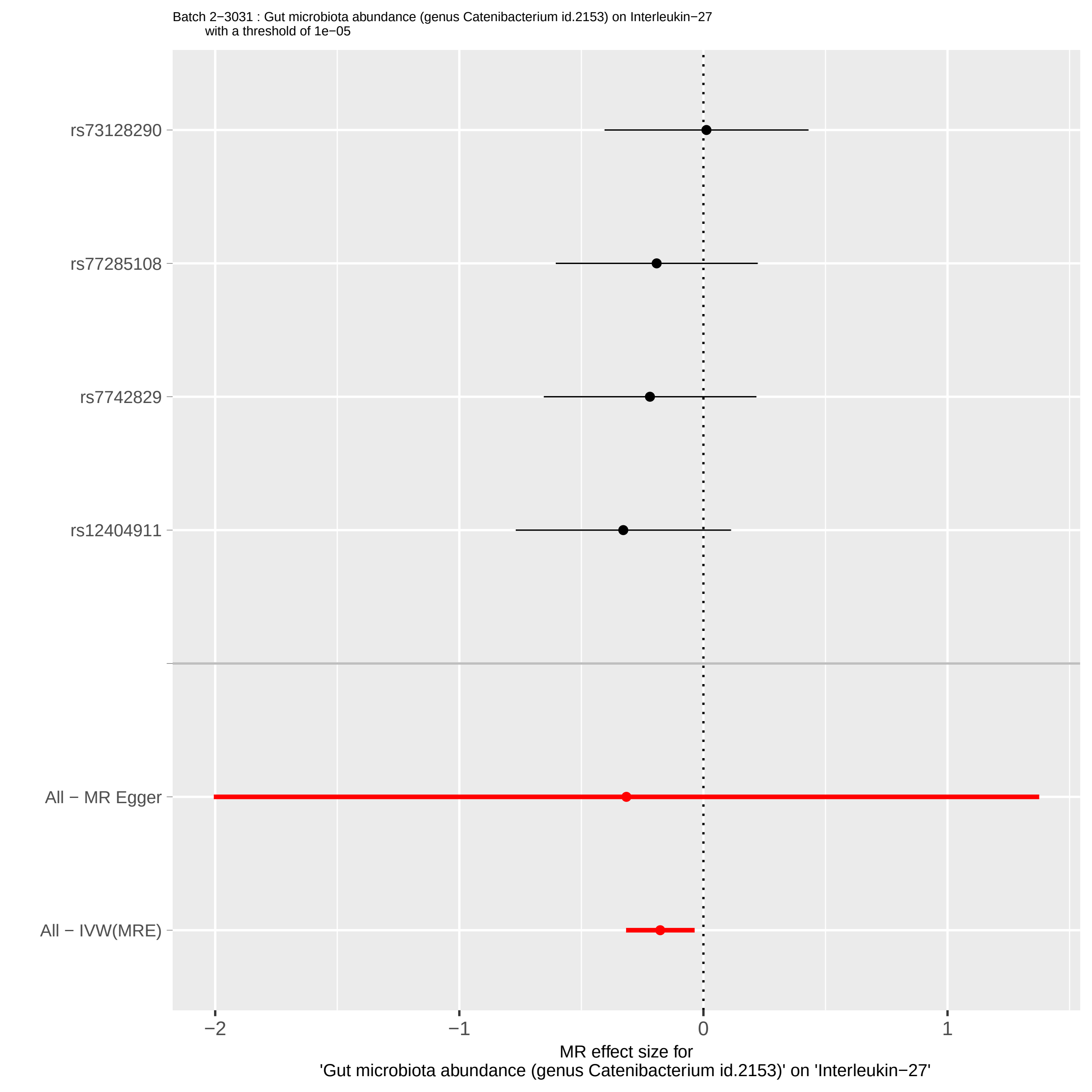
Batch 2−3031 : Gut microbiota abundance (genus Catenibacterium id.2153) on Interleukin−27
with a threshold of 1e−05
rs73128290
rs77285108
rs7742829
rs12404911
All − MR Egger
All − IVW(MRE)
−2
−1
0
1
MR effect size for
'Gut microbiota abundance (genus Catenibacterium id.2153)' on 'Interleukin−27'

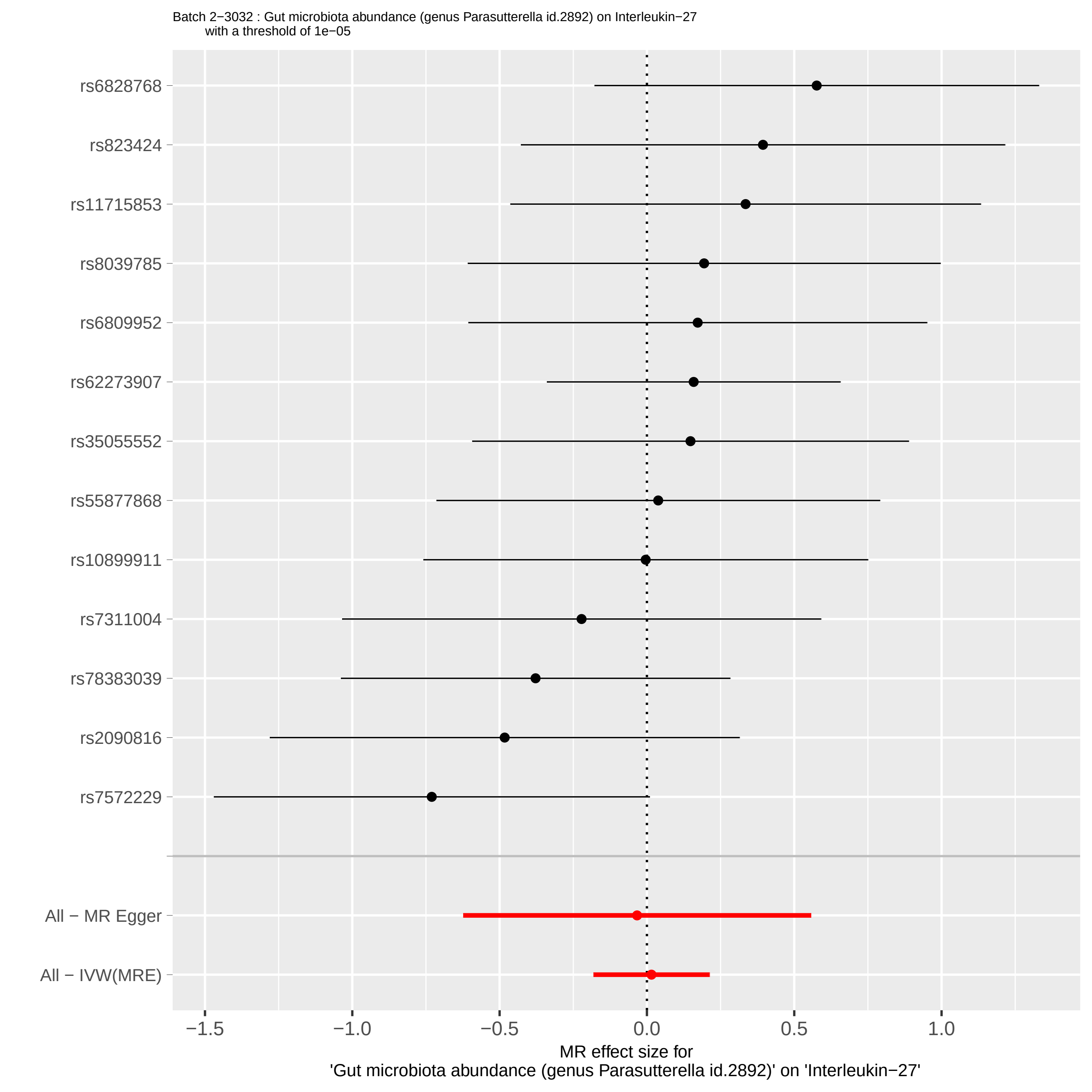
Batch 2−3032 : Gut microbiota abundance (genus Parasutterella id.2892) on Interleukin−27
with a threshold of 1e−05
rs6828768
rs823424
rs11715853
rs8039785
rs6809952
rs62273907
rs35055552
rs55877868
rs10899911
rs7311004
rs78383039
rs2090816
rs7572229
All − MR Egger
All − IVW(MRE)
−1.5
−1.0
−0.5
0.0
0.5
1.0
MR effect size for
'Gut microbiota abundance (genus Parasutterella id.2892)' on 'Interleukin−27'

Batch 2−3033 : Gut microbiota abundance (genus Catenibacterium id.2153) on Interleukin−27 receptor subunit alpha
with a threshold of 1e−05

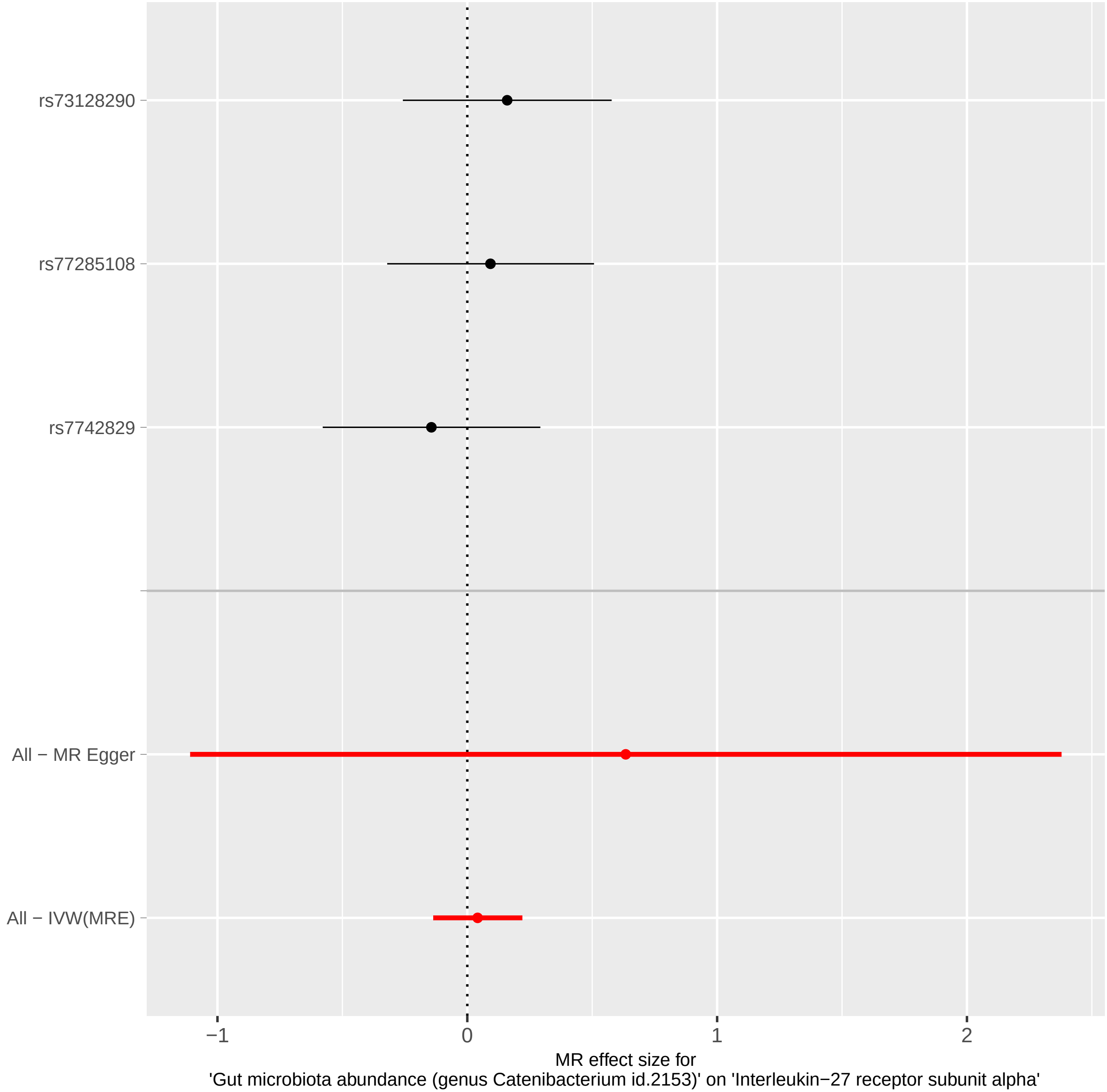

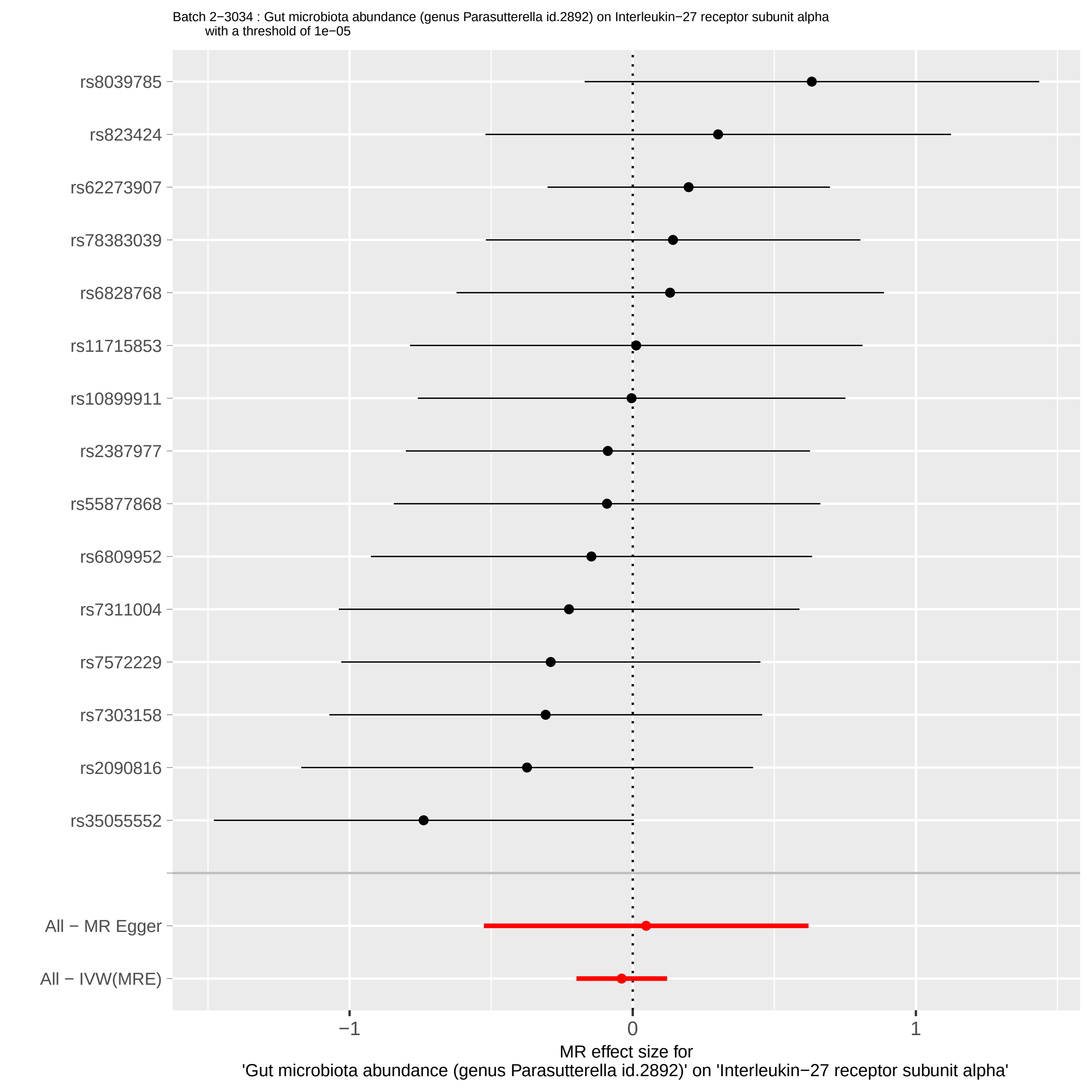
Batch 2−3034 : Gut microbiota abundance (genus Parasutterella id.2892) on Interleukin−27 receptor subunit alpha
with a threshold of 1e−05
rs8039785
rs823424
rs62273907
rs78383039
rs6828768
rs11715853
rs10899911
rs2387977
rs55877868
rs6809952
rs7311004
rs7572229
rs7303158
rs2090816
rs35055552
All − MR Egger
All − IVW(MRE)
−1
0
1
MR effect size for
'Gut microbiota abundance (genus Parasutterella id.2892)' on 'Interleukin−27 receptor subunit alpha'

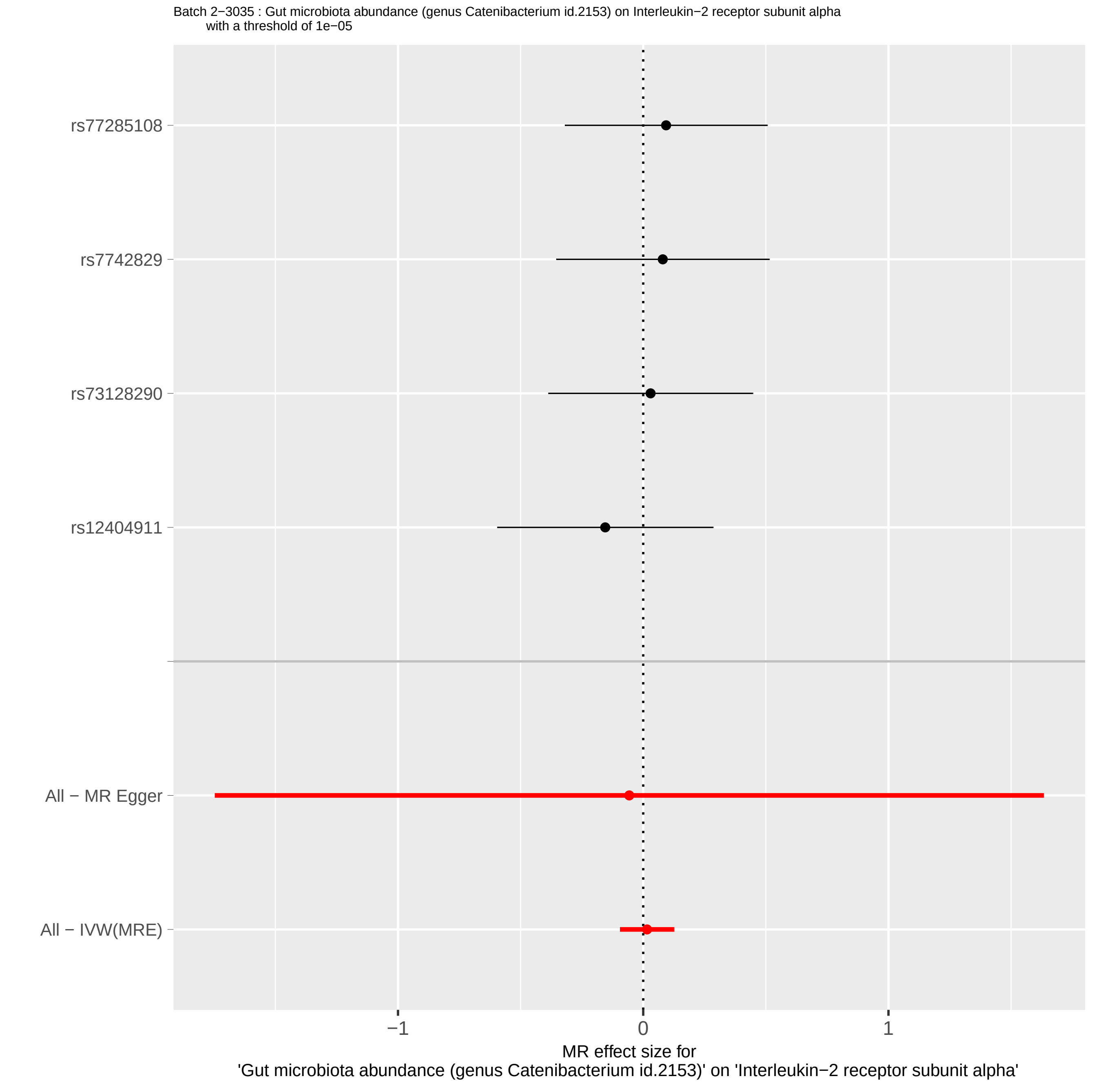

Batch 2−3035 : Gut microbiota abundance (genus Catenibacterium id.2153) on Interleukin−2 receptor subunit alpha
with a threshold of 1e−05
rs77285108
rs7742829
rs73128290
rs12404911
All − MR Egger
All − IVW(MRE)
−1
0
1
MR effect size for
'Gut microbiota abundance (genus Catenibacterium id.2153)' on 'Interleukin−2 receptor subunit alpha'

Batch 2−3036 : Gut microbiota abundance (genus Parasutterella id.2892) on Interleukin−2 receptor subunit alpha
with a threshold of 1e−05

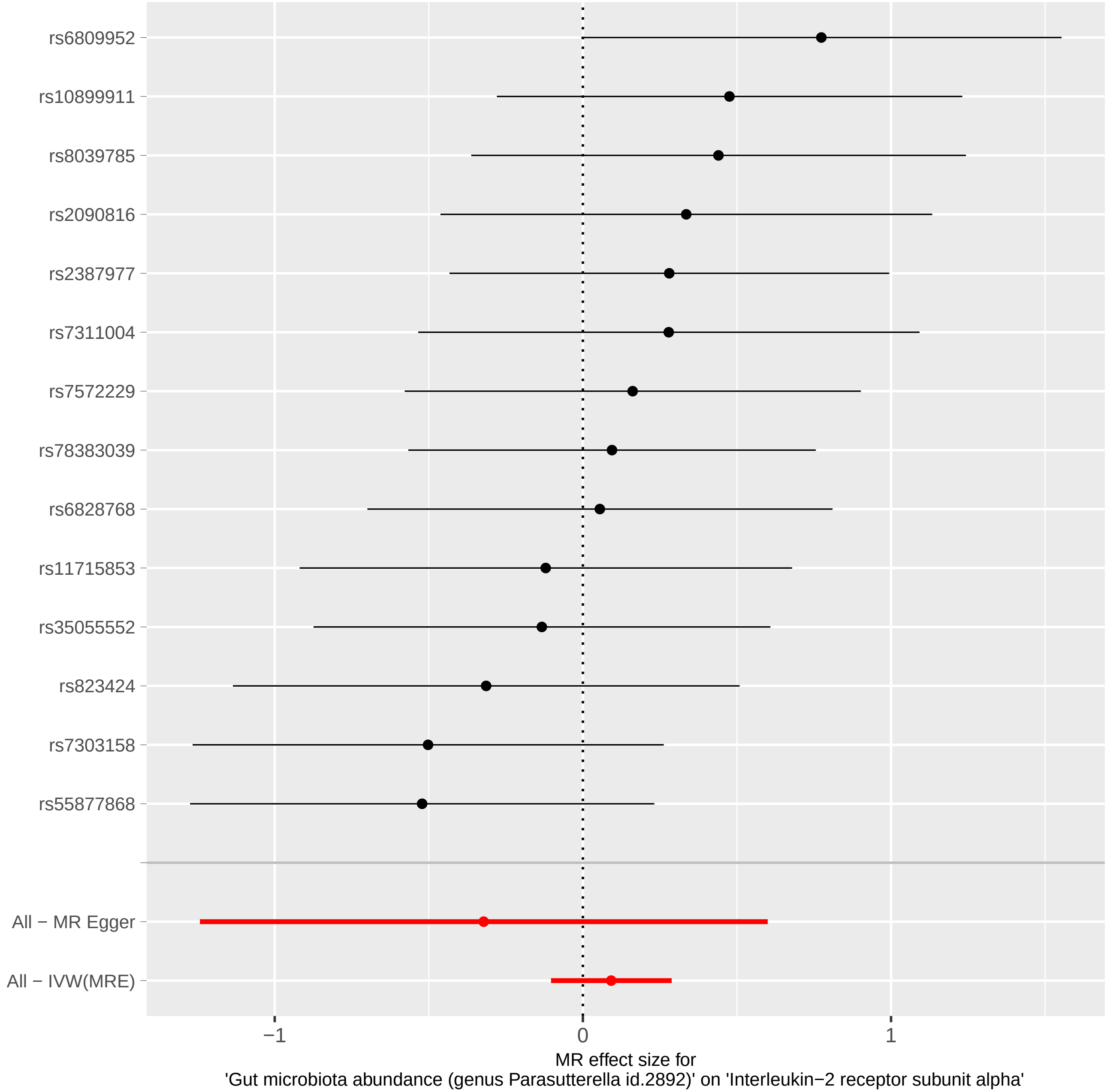

Batch 2−3037 : Gut microbiota abundance (genus Catenibacterium id.2153) on Interleukin−2 receptor subunit beta
with a threshold of 1e−05

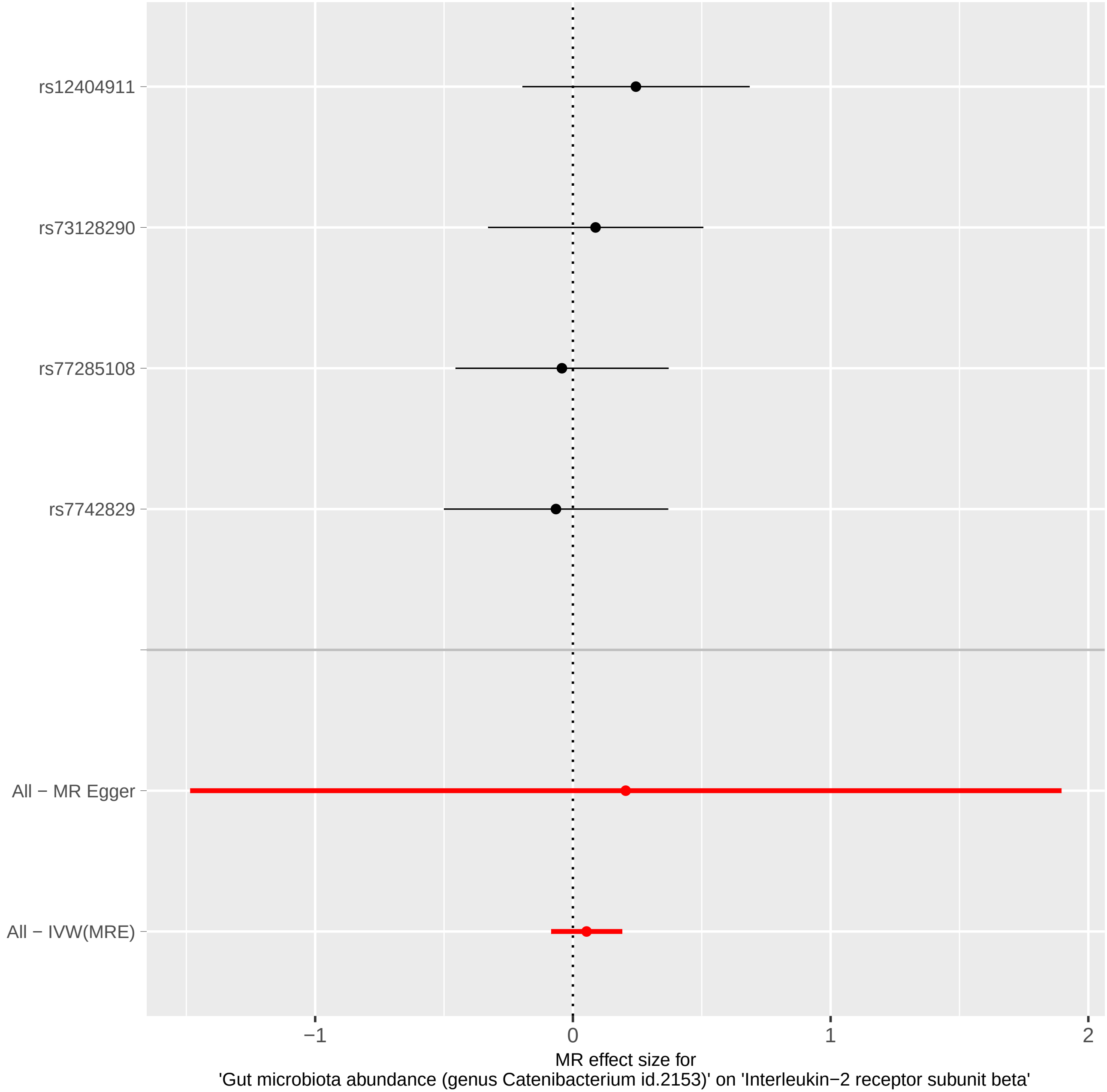

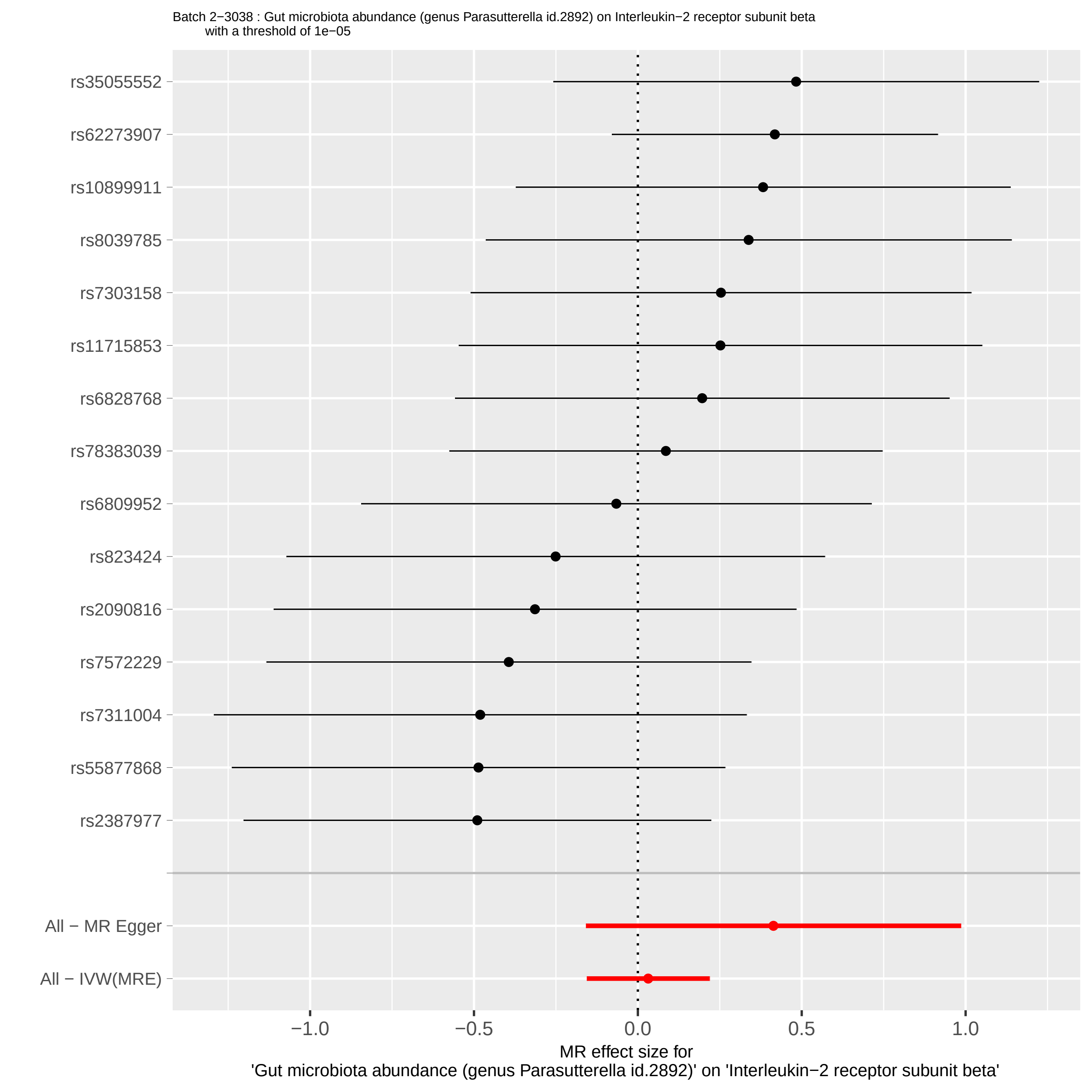
Batch 2−3038 : Gut microbiota abundance (genus Parasutterella id.2892) on Interleukin−2 receptor subunit beta
with a threshold of 1e−05
rs35055552
rs62273907
rs10899911
rs8039785
rs7303158
rs11715853
rs6828768
rs78383039
rs6809952
rs823424
rs2090816
rs7572229
rs7311004
rs55877868
rs2387977
All − MR Egger
All − IVW(MRE)
−1.0
−0.5
0.0
0.5
1.0
MR effect size for
'Gut microbiota abundance (genus Parasutterella id.2892)' on 'Interleukin−2 receptor subunit beta'

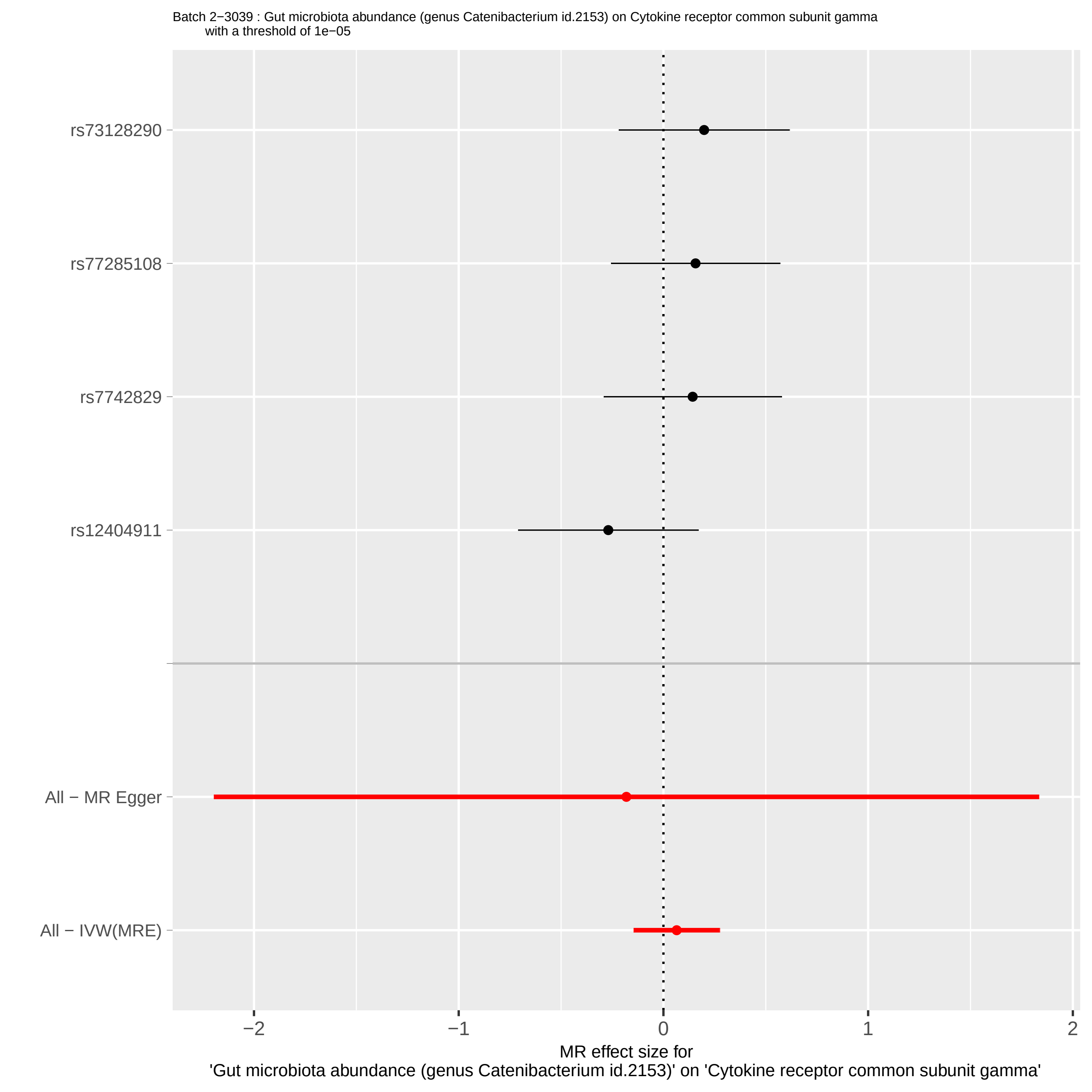
Batch 2−3039 : Gut microbiota abundance (genus Catenibacterium id.2153) on Cytokine receptor common subunit gamma
with a threshold of 1e−05
rs73128290
rs77285108
rs7742829
rs12404911
All − MR Egger
All − IVW(MRE)
−2
−1
0
1
2
MR effect size for
'Gut microbiota abundance (genus Catenibacterium id.2153)' on 'Cytokine receptor common subunit gamma'

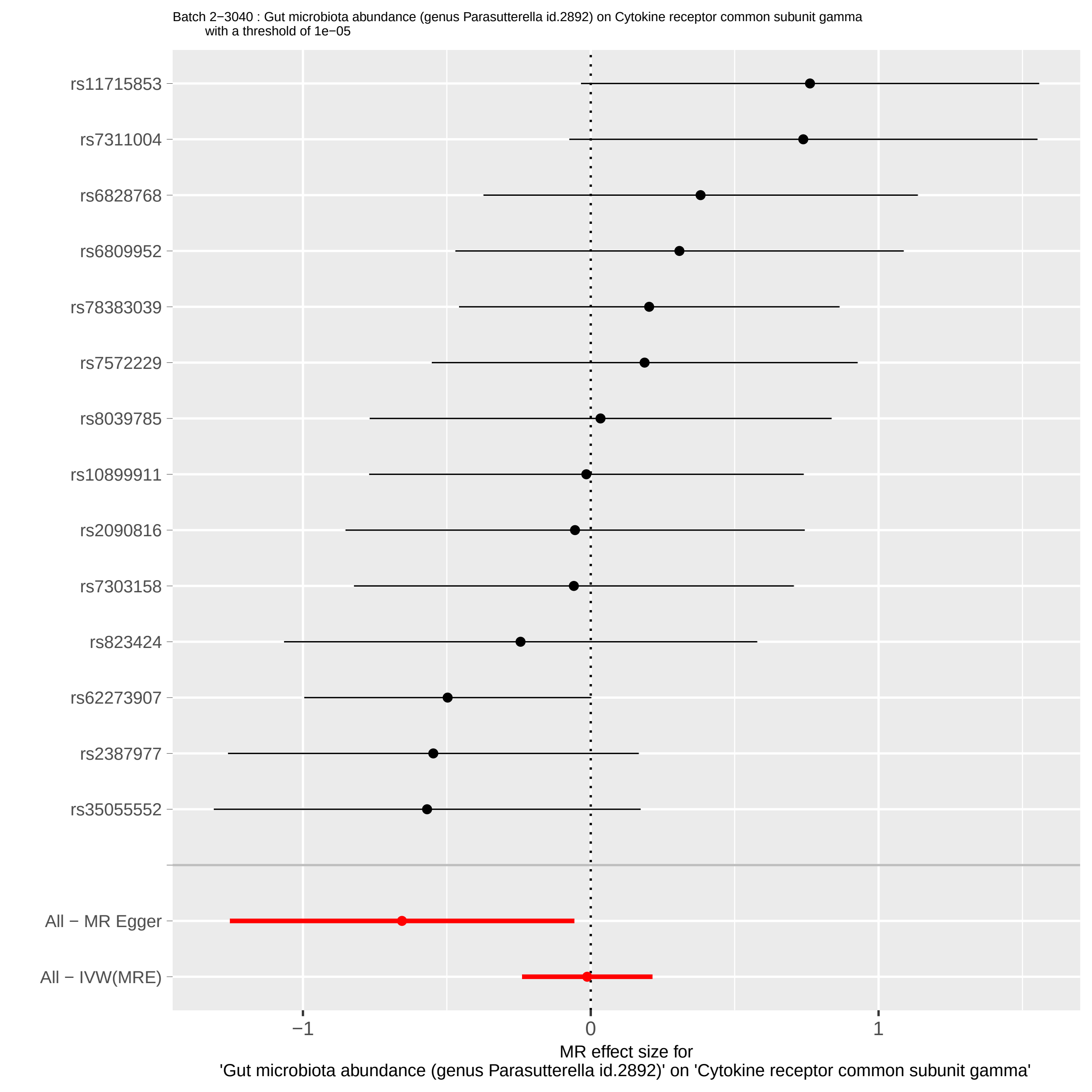

Batch 2−3040 : Gut microbiota abundance (genus Parasutterella id.2892) on Cytokine receptor common subunit gamma
with a threshold of 1e−05
rs11715853
rs7311004
rs6828768
rs6809952
rs78383039
rs7572229
rs8039785
rs10899911
rs2090816
rs7303158
rs823424
rs62273907
rs2387977
rs35055552
All − MR Egger
All − IVW(MRE)
−1
0
1
MR effect size for
'Gut microbiota abundance (genus Parasutterella id.2892)' on 'Cytokine receptor common subunit gamma'

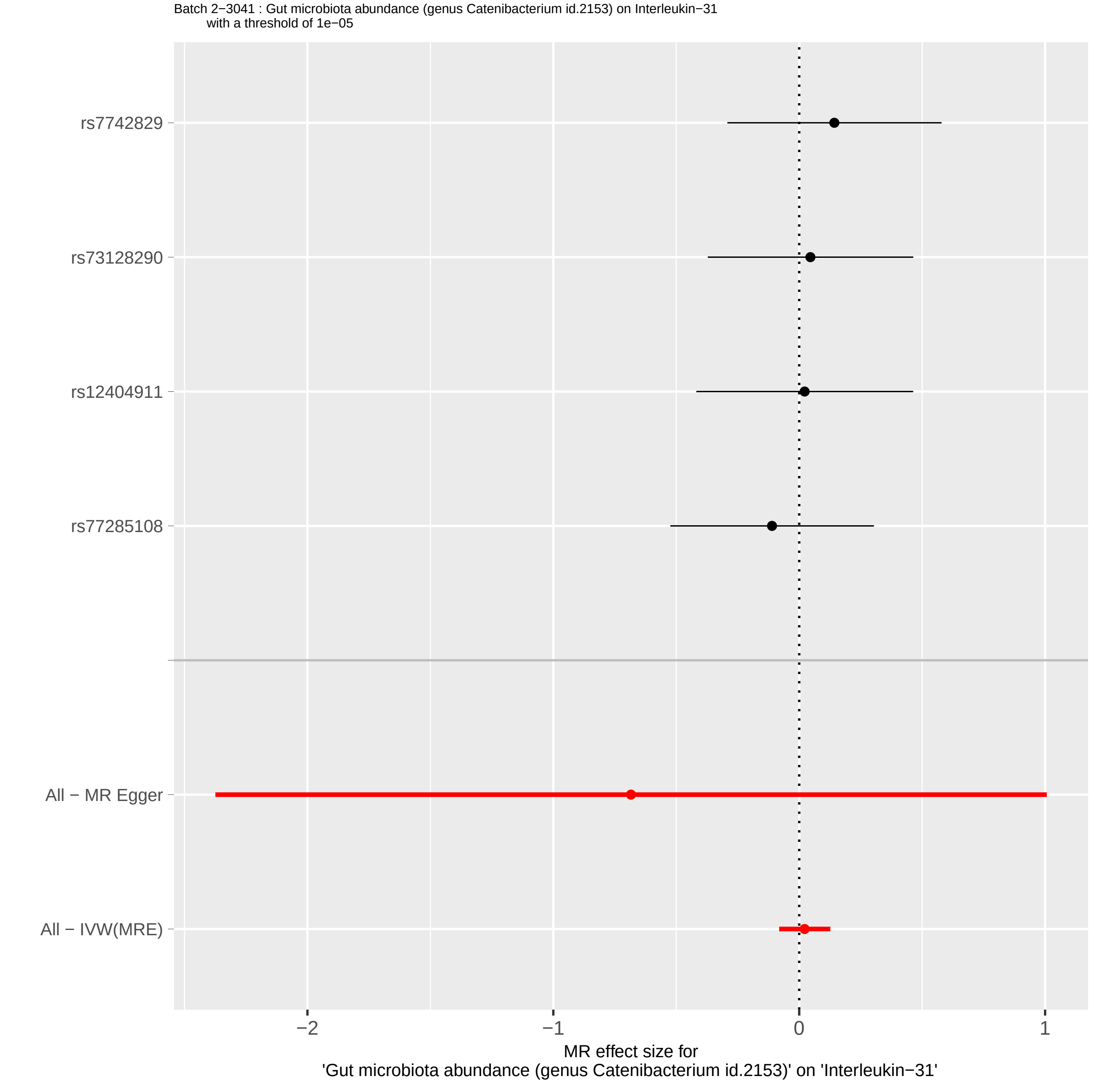
Batch 2−3041 : Gut microbiota abundance (genus Catenibacterium id.2153) on Interleukin−31
with a threshold of 1e−05
rs7742829
rs73128290
rs12404911
rs77285108
All − MR Egger
All − IVW(MRE)
−2
−1
0
1
MR effect size for
'Gut microbiota abundance (genus Catenibacterium id.2153)' on 'Interleukin−31'

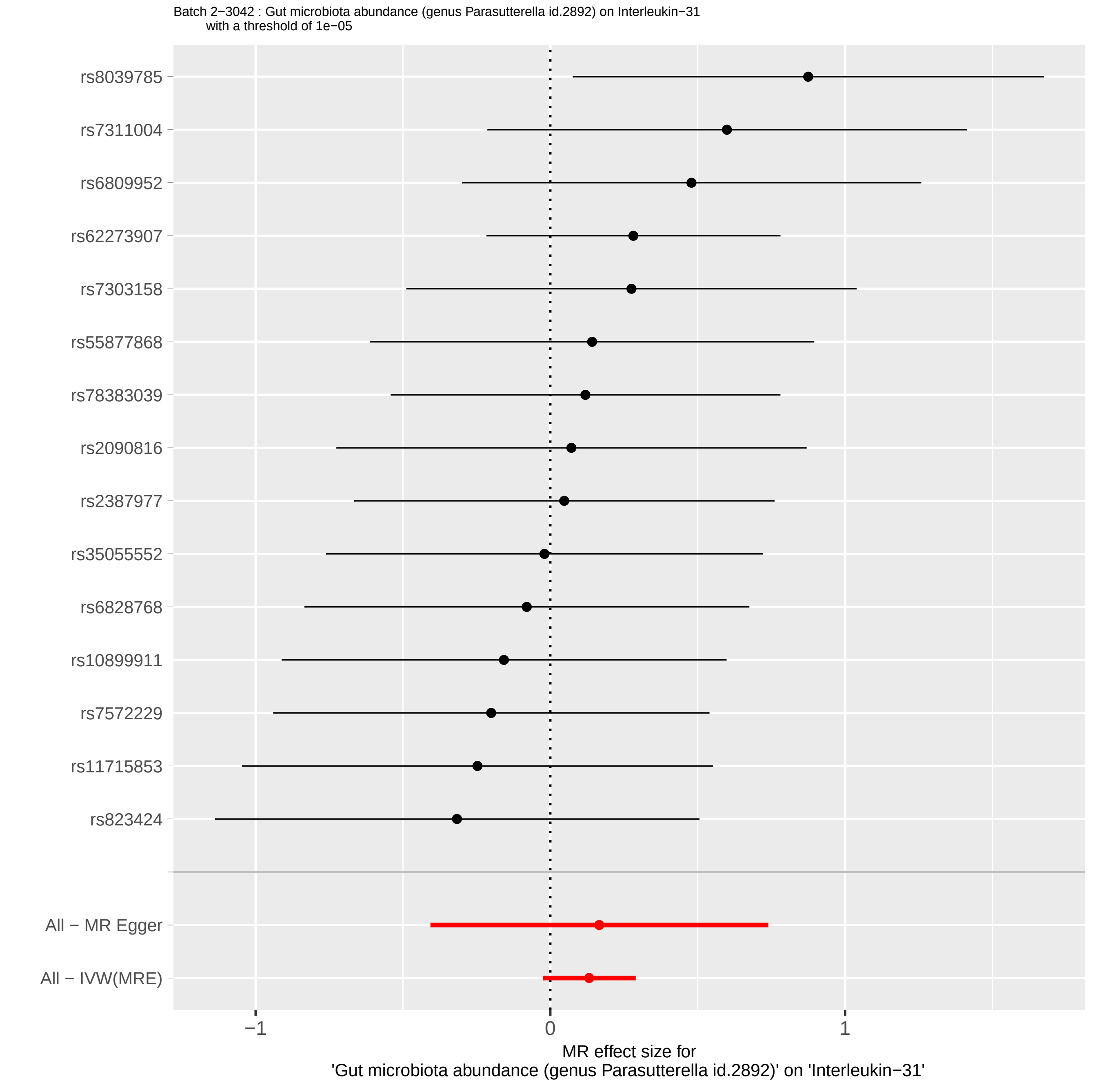

Batch 2−3042 : Gut microbiota abundance (genus Parasutterella id.2892) on Interleukin−31
with a threshold of 1e−05
rs8039785
rs7311004
rs6809952
rs62273907
rs7303158
rs55877868
rs78383039
rs2090816
rs2387977
rs35055552
rs6828768
rs10899911
rs7572229
rs11715853
rs823424
All − MR Egger
All − IVW(MRE)
−1
0
1
MR effect size for
'Gut microbiota abundance (genus Parasutterella id.2892)' on 'Interleukin−31'

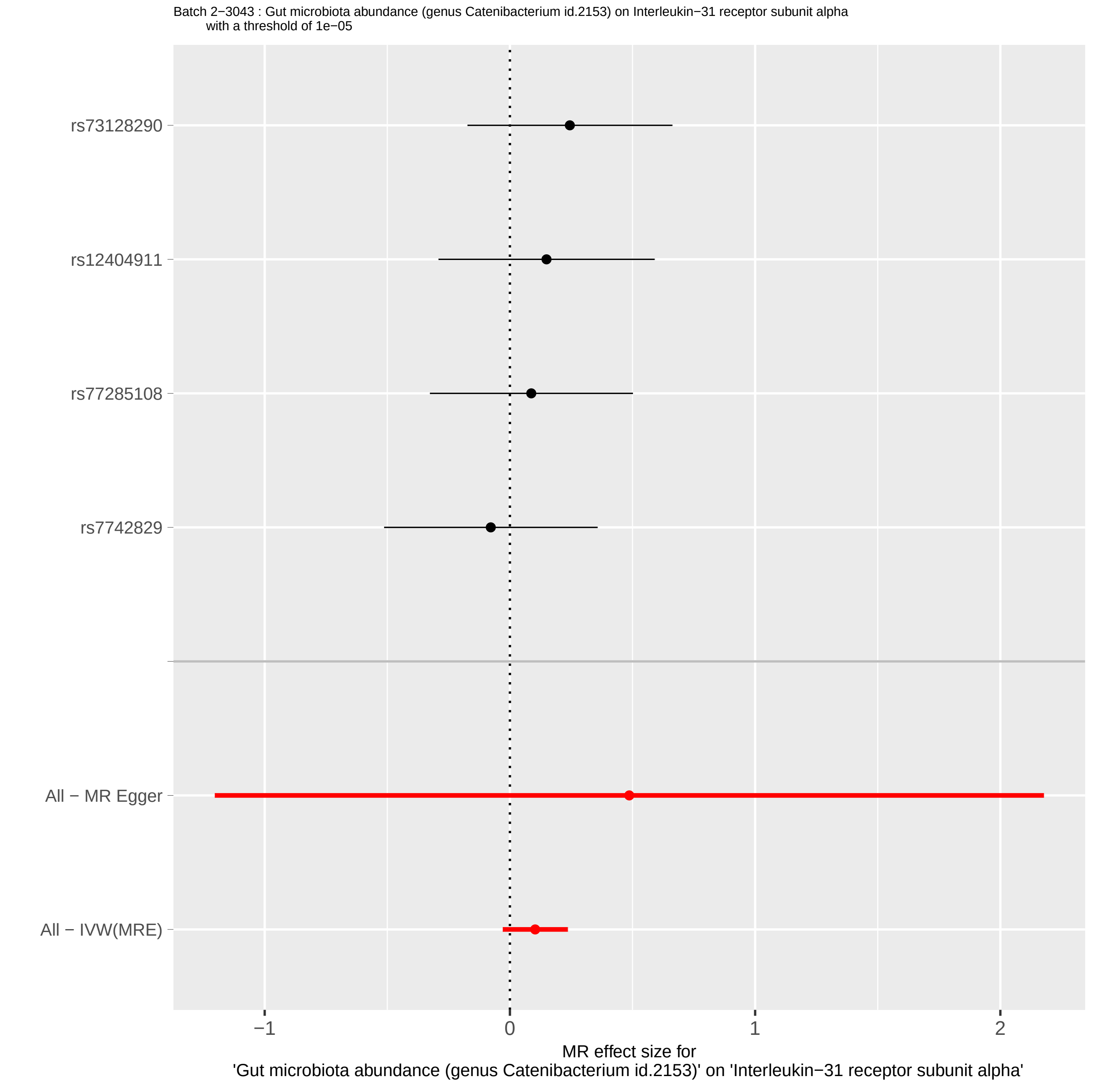

Batch 2−3043 : Gut microbiota abundance (genus Catenibacterium id.2153) on Interleukin−31 receptor subunit alpha
with a threshold of 1e−05
rs73128290
rs12404911
rs77285108
rs7742829
All − MR Egger
All − IVW(MRE)
−1
0
1
2
MR effect size for
'Gut microbiota abundance (genus Catenibacterium id.2153)' on 'Interleukin−31 receptor subunit alpha'

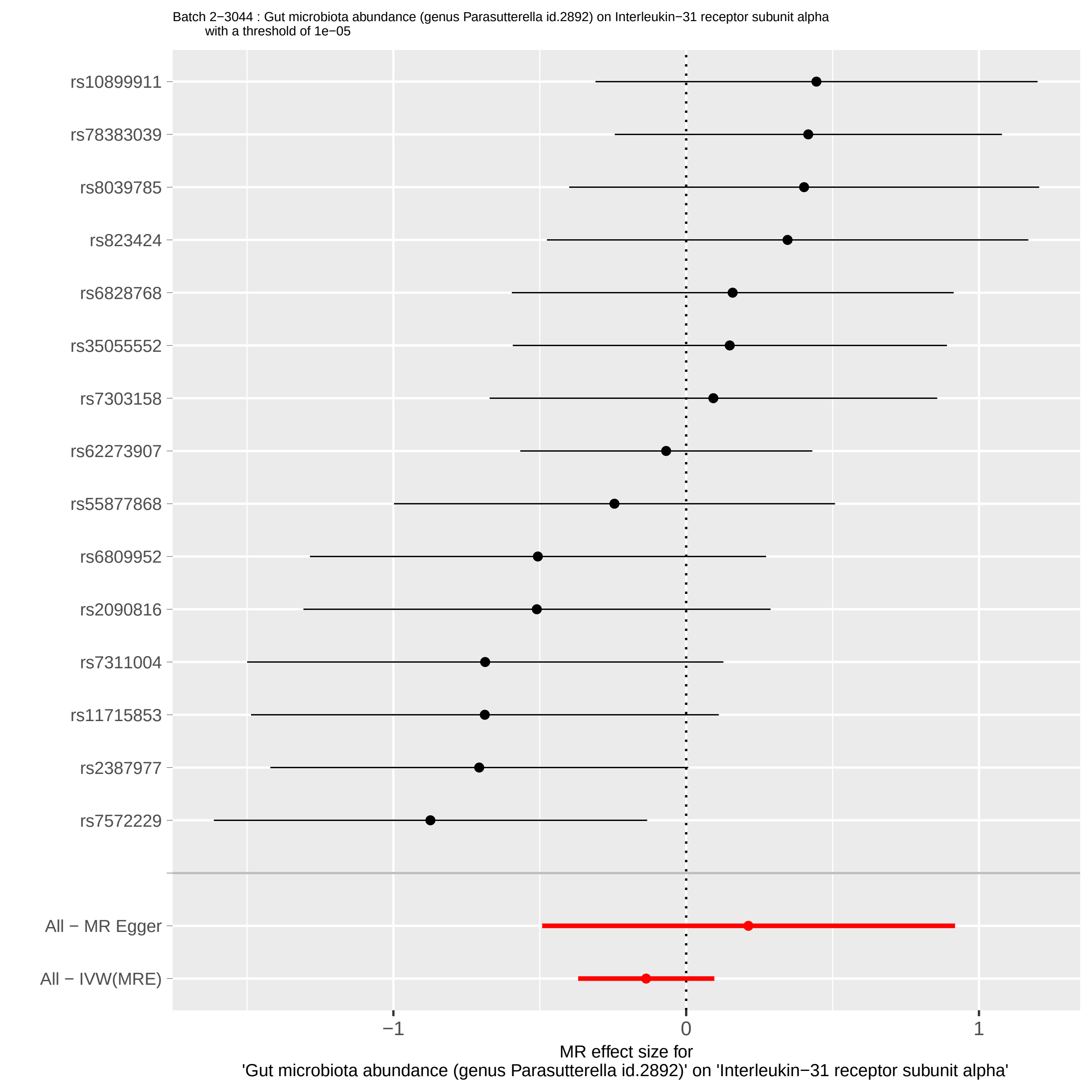

Batch 2−3044 : Gut microbiota abundance (genus Parasutterella id.2892) on Interleukin−31 receptor subunit alpha
with a threshold of 1e−05
rs10899911
rs78383039
rs8039785
rs823424
rs6828768
rs35055552
rs7303158
rs62273907
rs55877868
rs6809952
rs2090816
rs7311004
rs11715853
rs2387977
rs7572229
All − MR Egger
All − IVW(MRE)
−1
0
1
MR effect size for
'Gut microbiota abundance (genus Parasutterella id.2892)' on 'Interleukin−31 receptor subunit alpha'

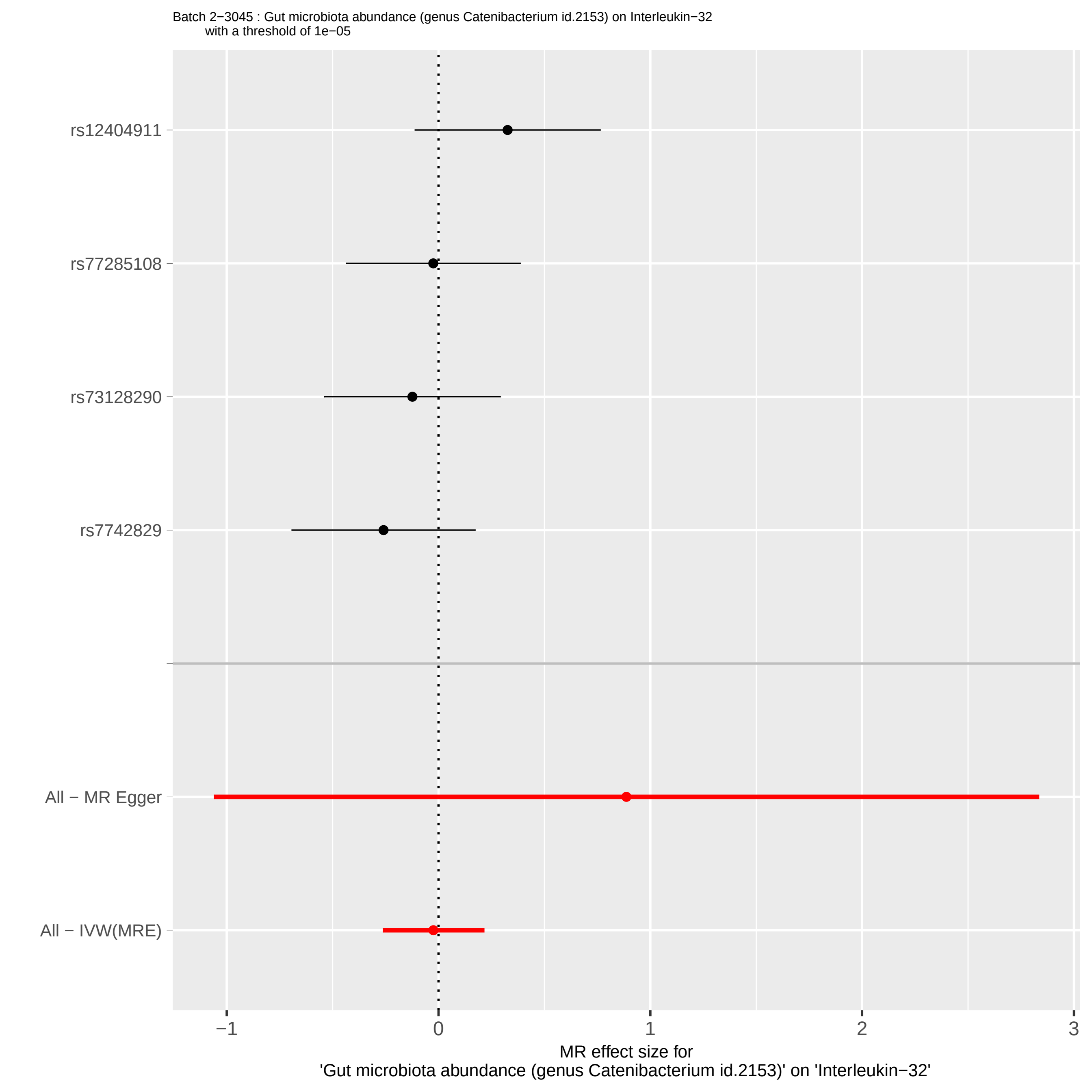

Batch 2−3045 : Gut microbiota abundance (genus Catenibacterium id.2153) on Interleukin−32
with a threshold of 1e−05
rs12404911
rs77285108
rs73128290
rs7742829
All − MR Egger
All − IVW(MRE)
−1
0
1
2
3
MR effect size for
'Gut microbiota abundance (genus Catenibacterium id.2153)' on 'Interleukin−32'

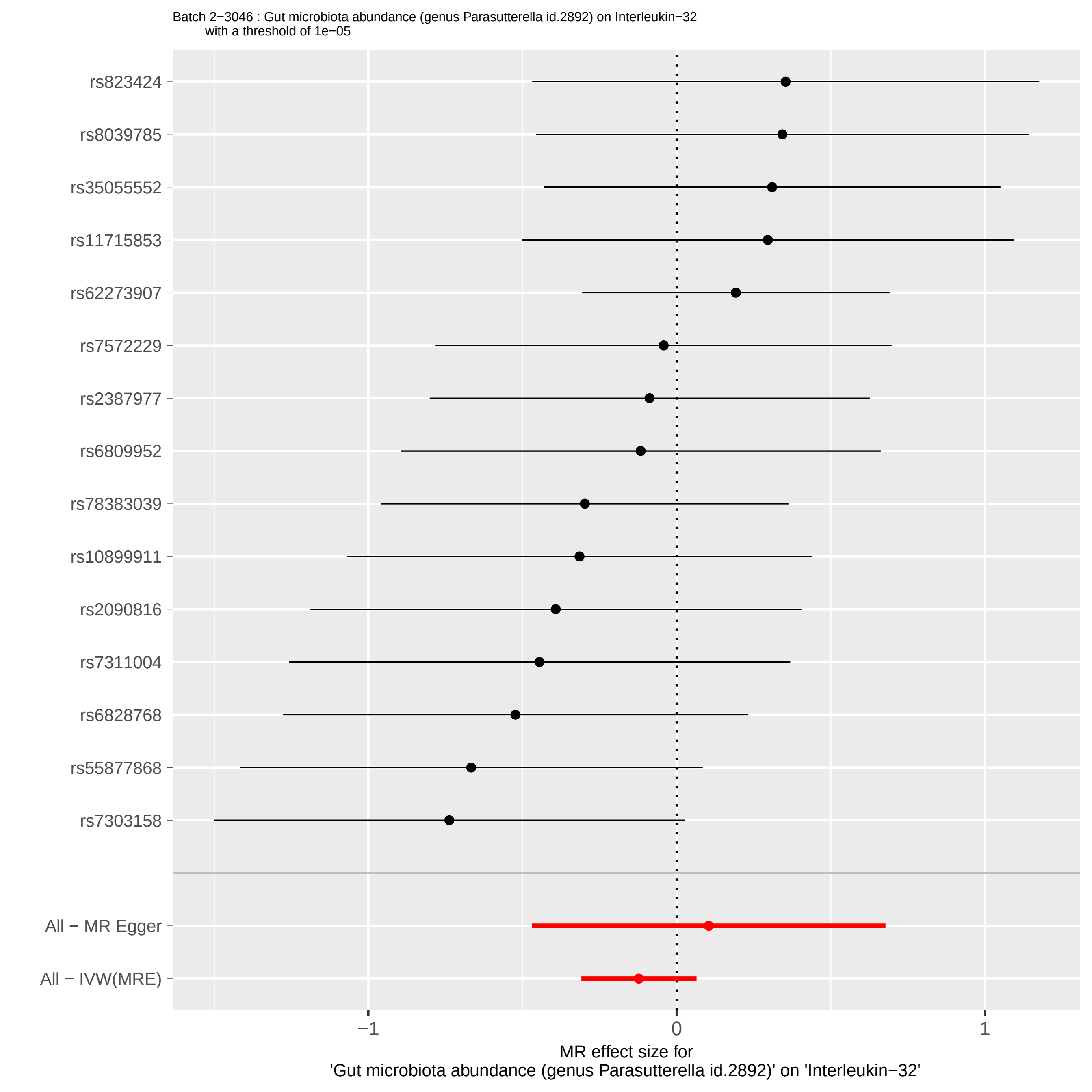
Batch 2−3046 : Gut microbiota abundance (genus Parasutterella id.2892) on Interleukin−32
with a threshold of 1e−05
rs823424
rs8039785
rs35055552
rs11715853
rs62273907
rs7572229
rs2387977
rs6809952
rs78383039
rs10899911
rs2090816
rs7311004
rs6828768
rs55877868
rs7303158
All − MR Egger
All − IVW(MRE)
−1
0
1
MR effect size for
'Gut microbiota abundance (genus Parasutterella id.2892)' on 'Interleukin−32'

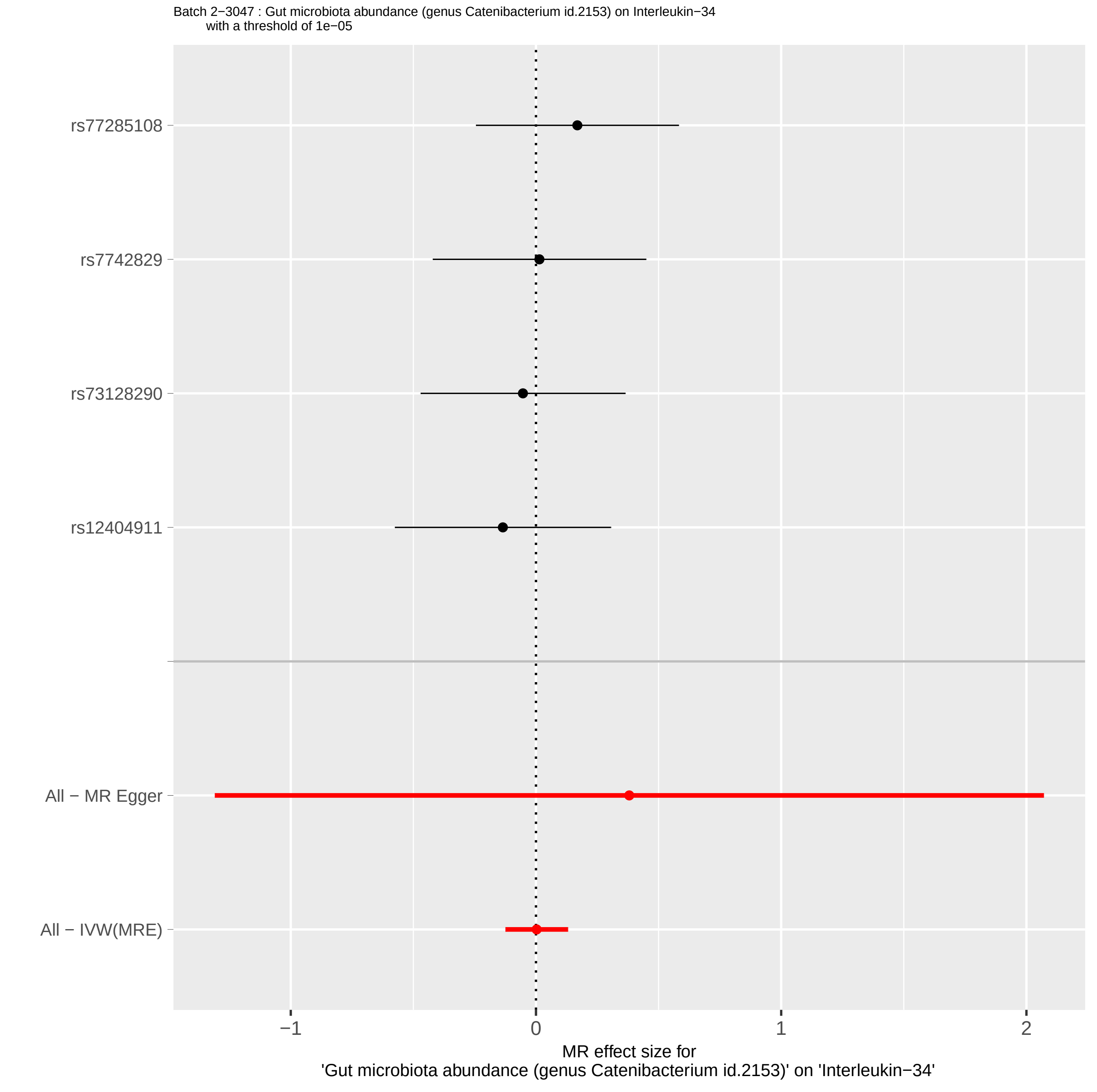
Batch 2−3047 : Gut microbiota abundance (genus Catenibacterium id.2153) on Interleukin−34
with a threshold of 1e−05
rs77285108
rs7742829
rs73128290
rs12404911
All − MR Egger
All − IVW(MRE)
−1
0
1
2
MR effect size for
'Gut microbiota abundance (genus Catenibacterium id.2153)' on 'Interleukin−34'

Batch 2−3048 : Gut microbiota abundance (genus Parasutterella id.2892) on Interleukin−34
with a threshold of 1e−05

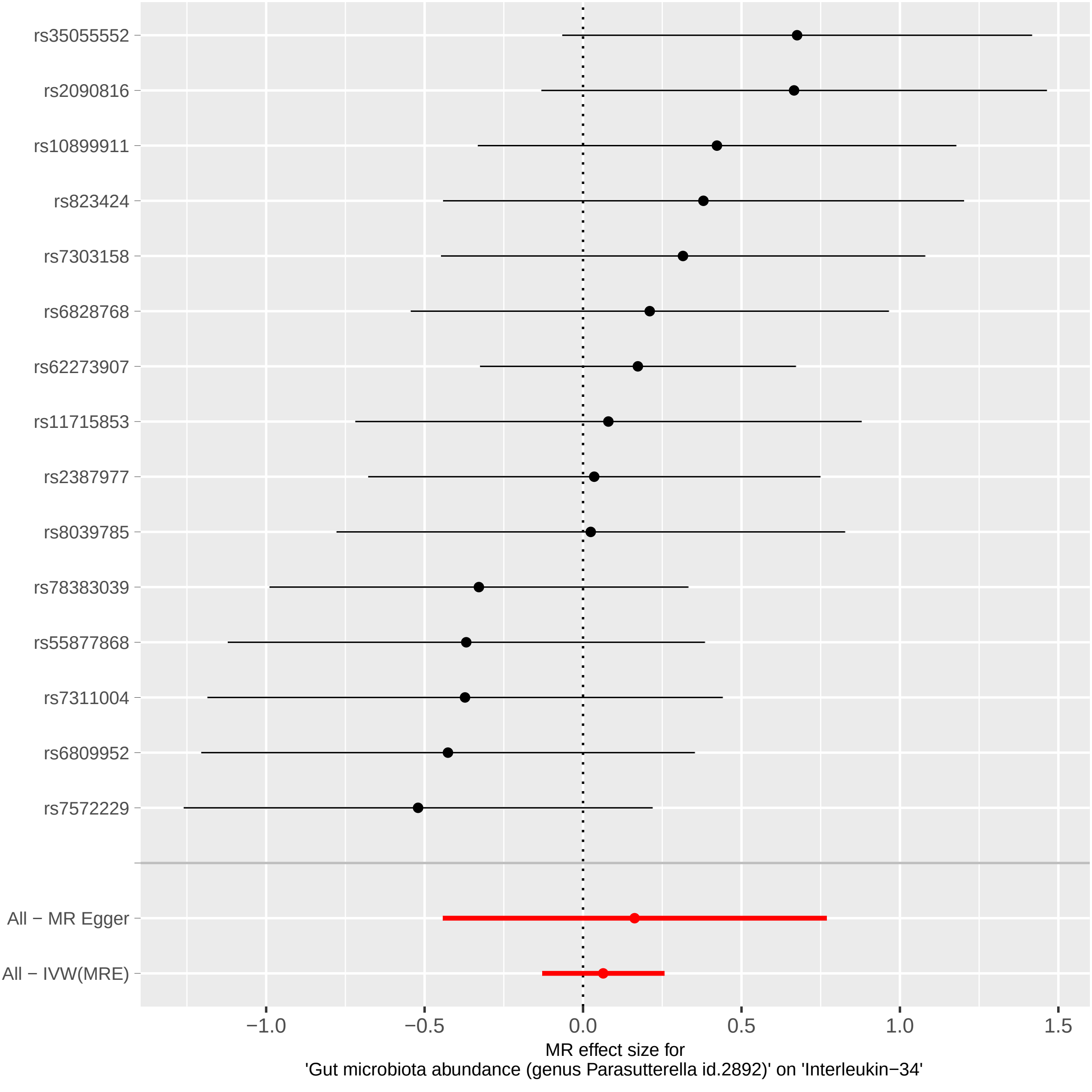

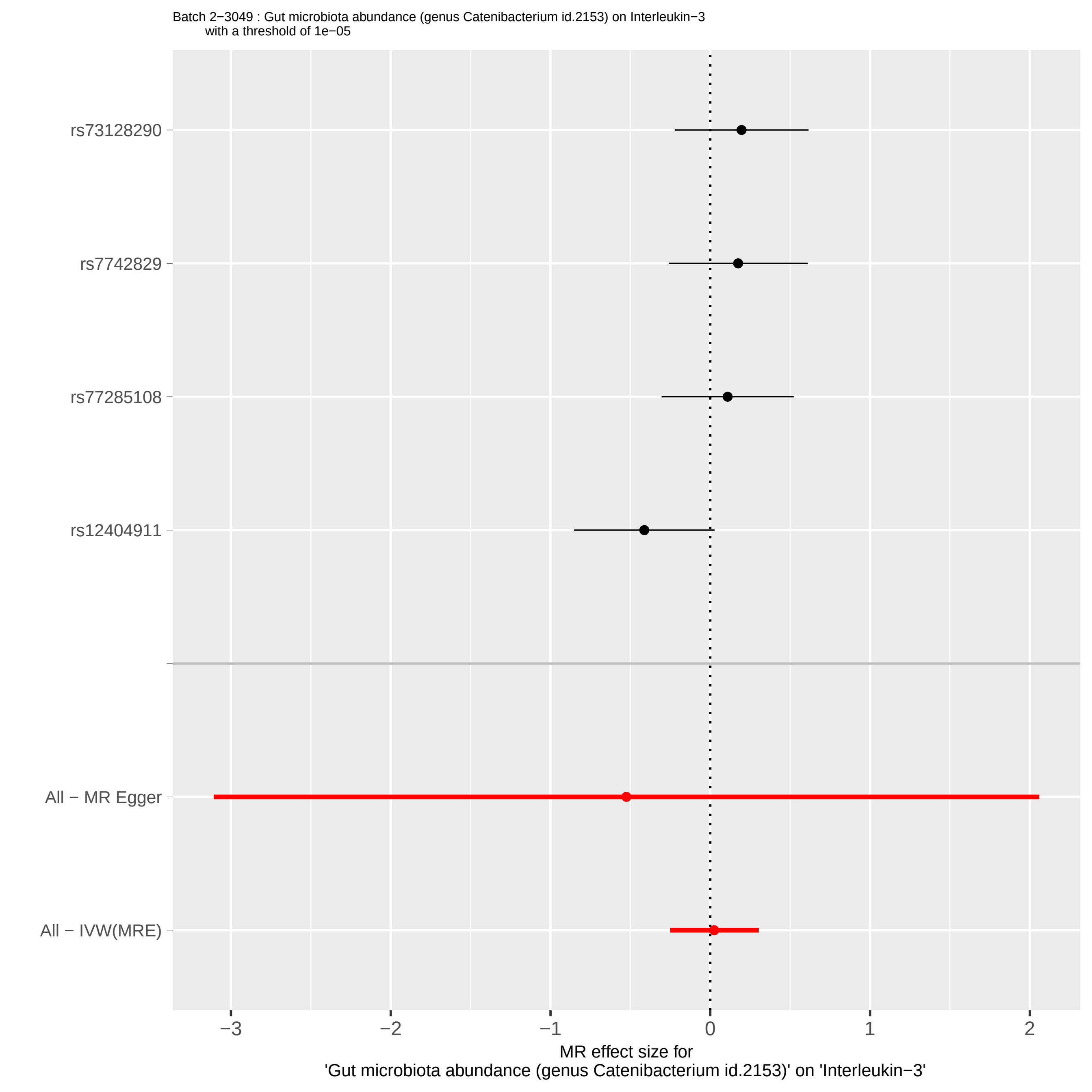
Batch 2−3049 : Gut microbiota abundance (genus Catenibacterium id.2153) on Interleukin−3
with a threshold of 1e−05
rs73128290
rs7742829
rs77285108
rs12404911
All − MR Egger
All − IVW(MRE)
−3
−2
−1
0
1
2
MR effect size for
'Gut microbiota abundance (genus Catenibacterium id.2153)' on 'Interleukin−3'

Batch 2−3050 : Gut microbiota abundance (genus Parasutterella id.2892) on Interleukin−3
with a threshold of 1e−05

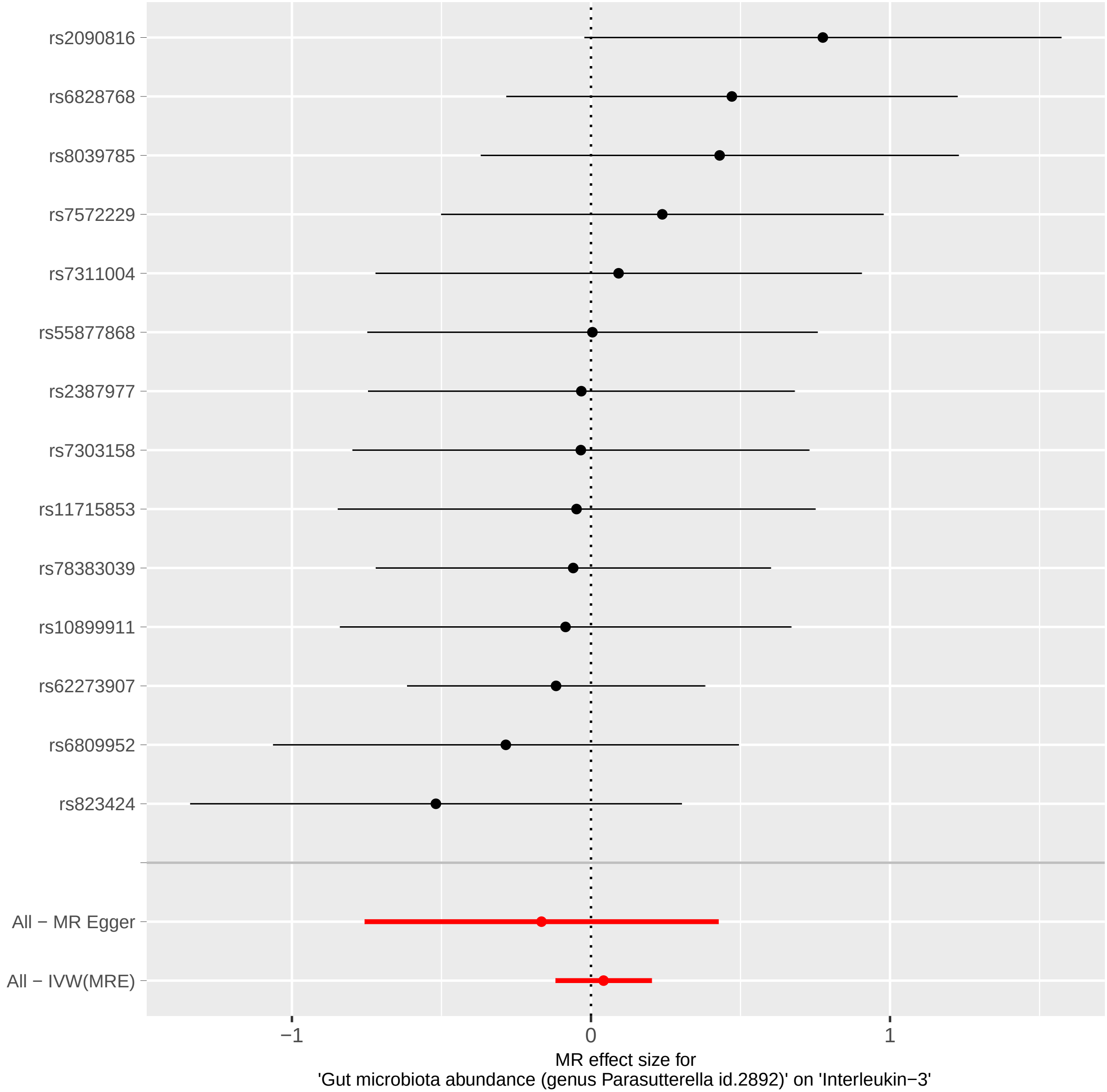

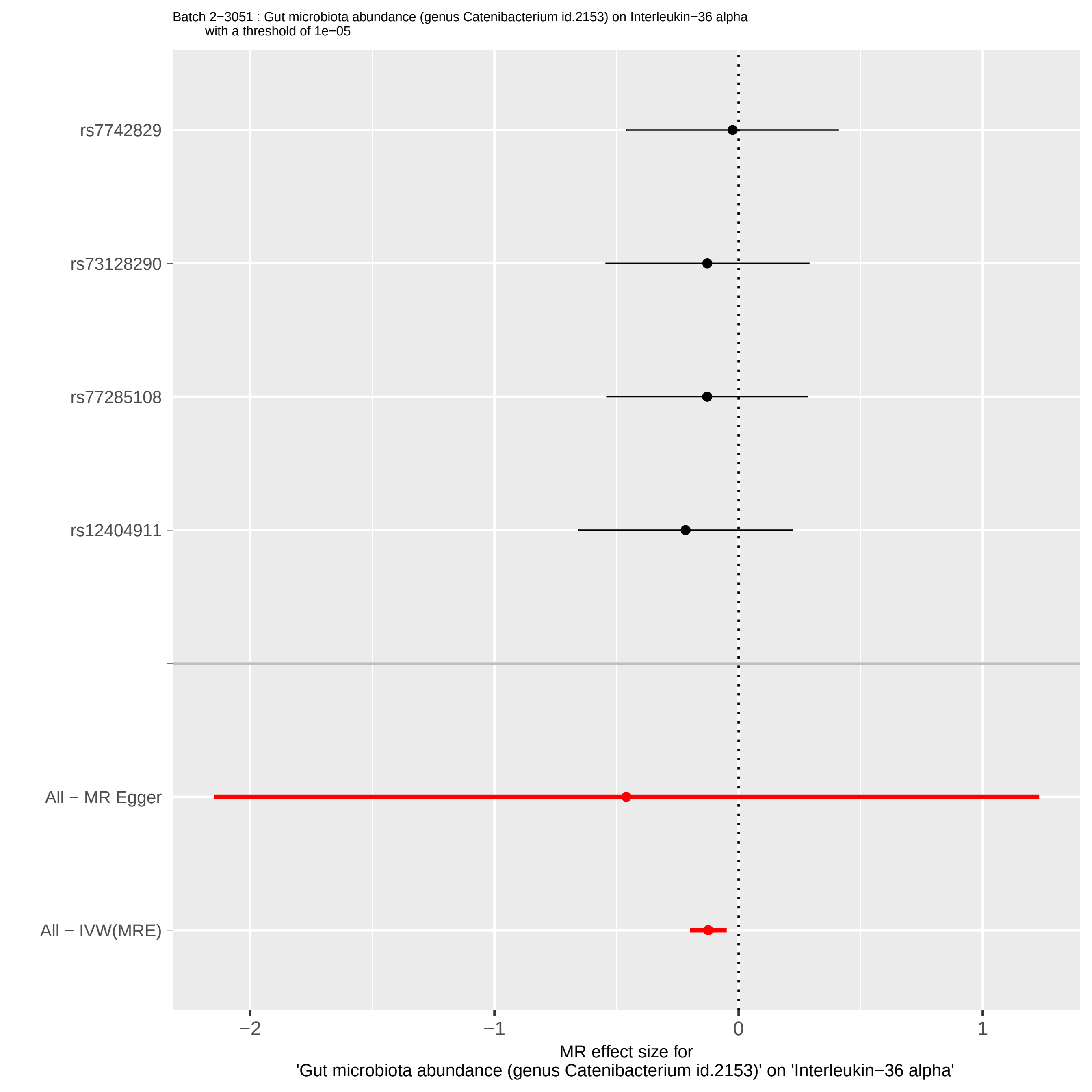

Batch 2−3051 : Gut microbiota abundance (genus Catenibacterium id.2153) on Interleukin−36 alpha
with a threshold of 1e−05
rs7742829
rs73128290
rs77285108
rs12404911
All − MR Egger
All − IVW(MRE)
−2
−1
0
1
MR effect size for
'Gut microbiota abundance (genus Catenibacterium id.2153)' on 'Interleukin−36 alpha'

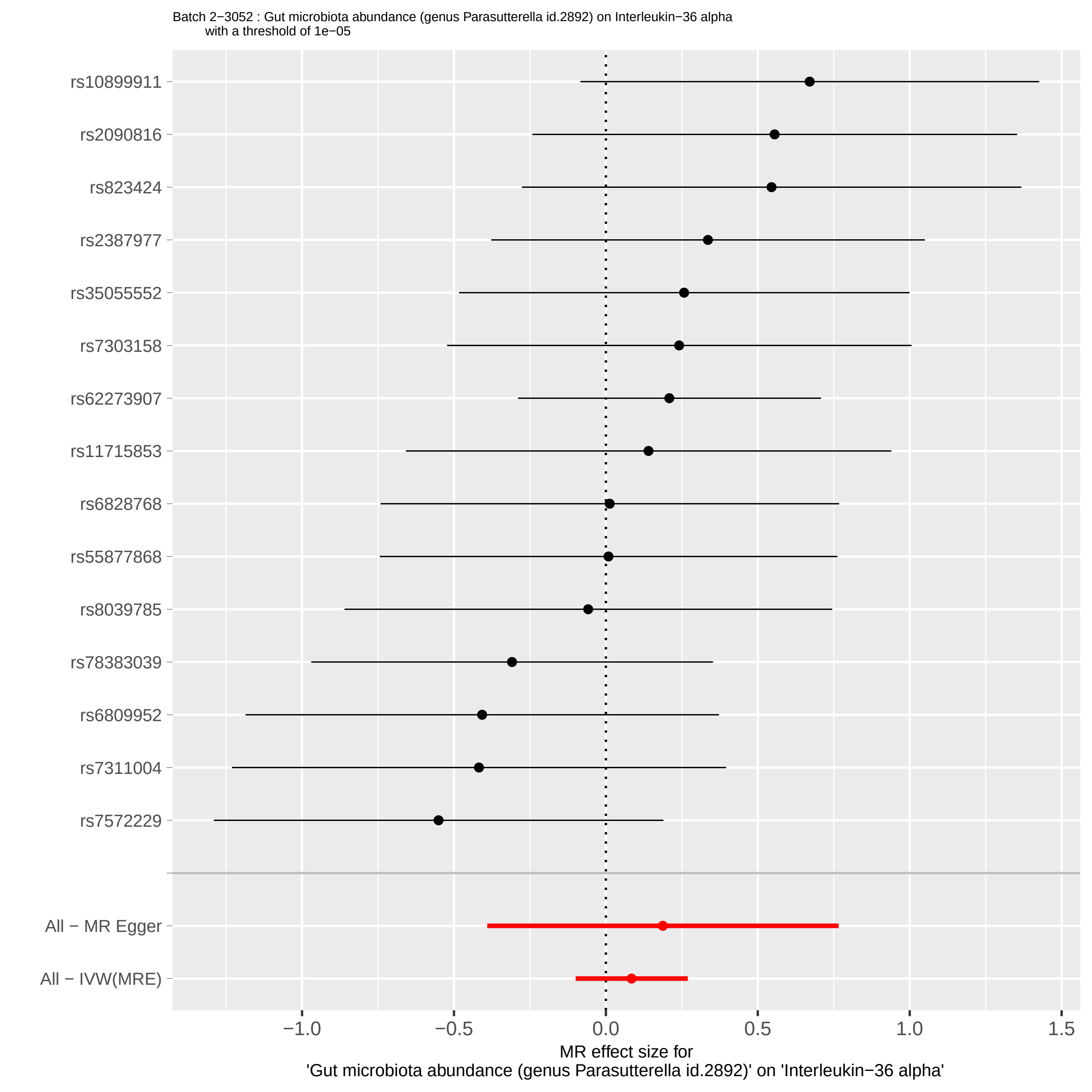
Batch 2−3052 : Gut microbiota abundance (genus Parasutterella id.2892) on Interleukin−36 alpha
with a threshold of 1e−05
rs10899911
rs2090816
rs823424
rs2387977
rs35055552
rs7303158
rs62273907
rs11715853
rs6828768
rs55877868
rs8039785
rs78383039
rs6809952
rs7311004
rs7572229
All − MR Egger
All − IVW(MRE)
−1.0
−0.5
0.0
0.5
1.0
1.5
MR effect size for
'Gut microbiota abundance (genus Parasutterella id.2892)' on 'Interleukin−36 alpha'

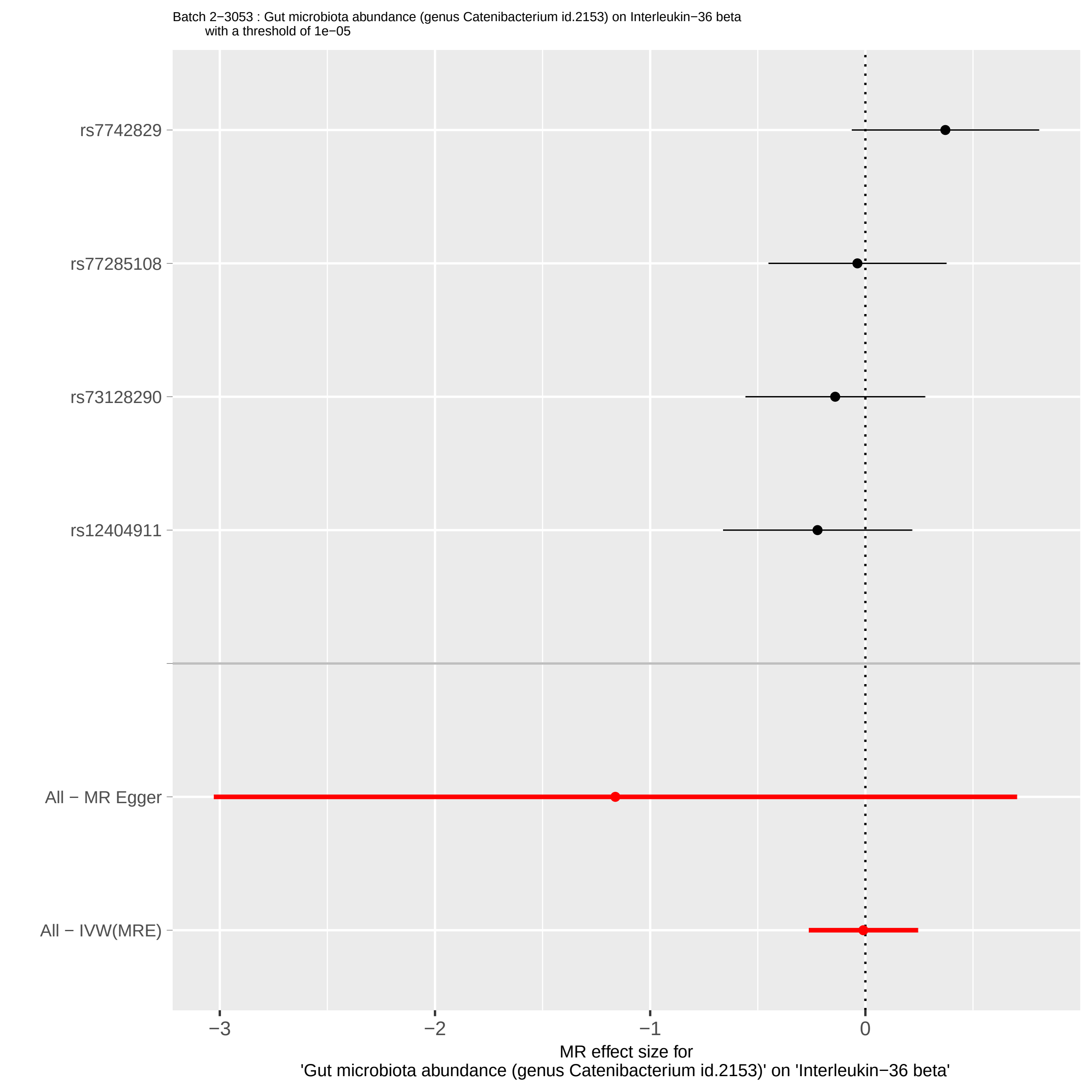
Batch 2−3053 : Gut microbiota abundance (genus Catenibacterium id.2153) on Interleukin−36 beta
with a threshold of 1e−05
rs7742829
rs77285108
rs73128290
rs12404911
All − MR Egger
All − IVW(MRE)
−3
−2
−1
0
MR effect size for
'Gut microbiota abundance (genus Catenibacterium id.2153)' on 'Interleukin−36 beta'

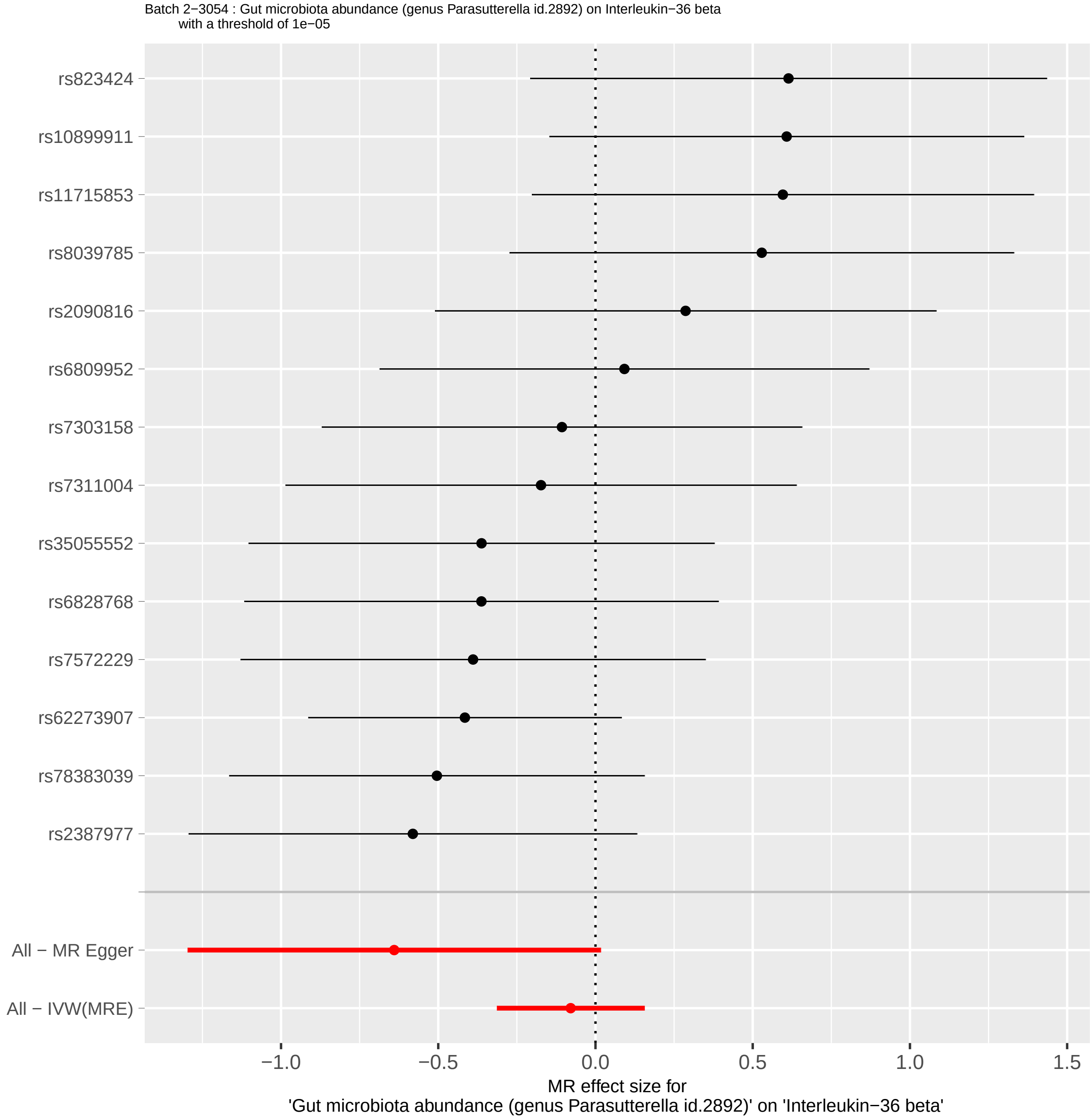

Batch 2−3054 : Gut microbiota abundance (genus Parasutterella id.2892) on Interleukin−36 beta
with a threshold of 1e−05
rs823424
rs10899911
rs11715853
rs8039785
rs2090816
rs6809952
rs7303158
rs7311004
rs35055552
rs6828768
rs7572229
rs62273907
rs78383039
rs2387977
All − MR Egger
All − IVW(MRE)
−1.0
−0.5
0.0
0.5
1.0
1.5
MR effect size for
'Gut microbiota abundance (genus Parasutterella id.2892)' on 'Interleukin−36 beta'

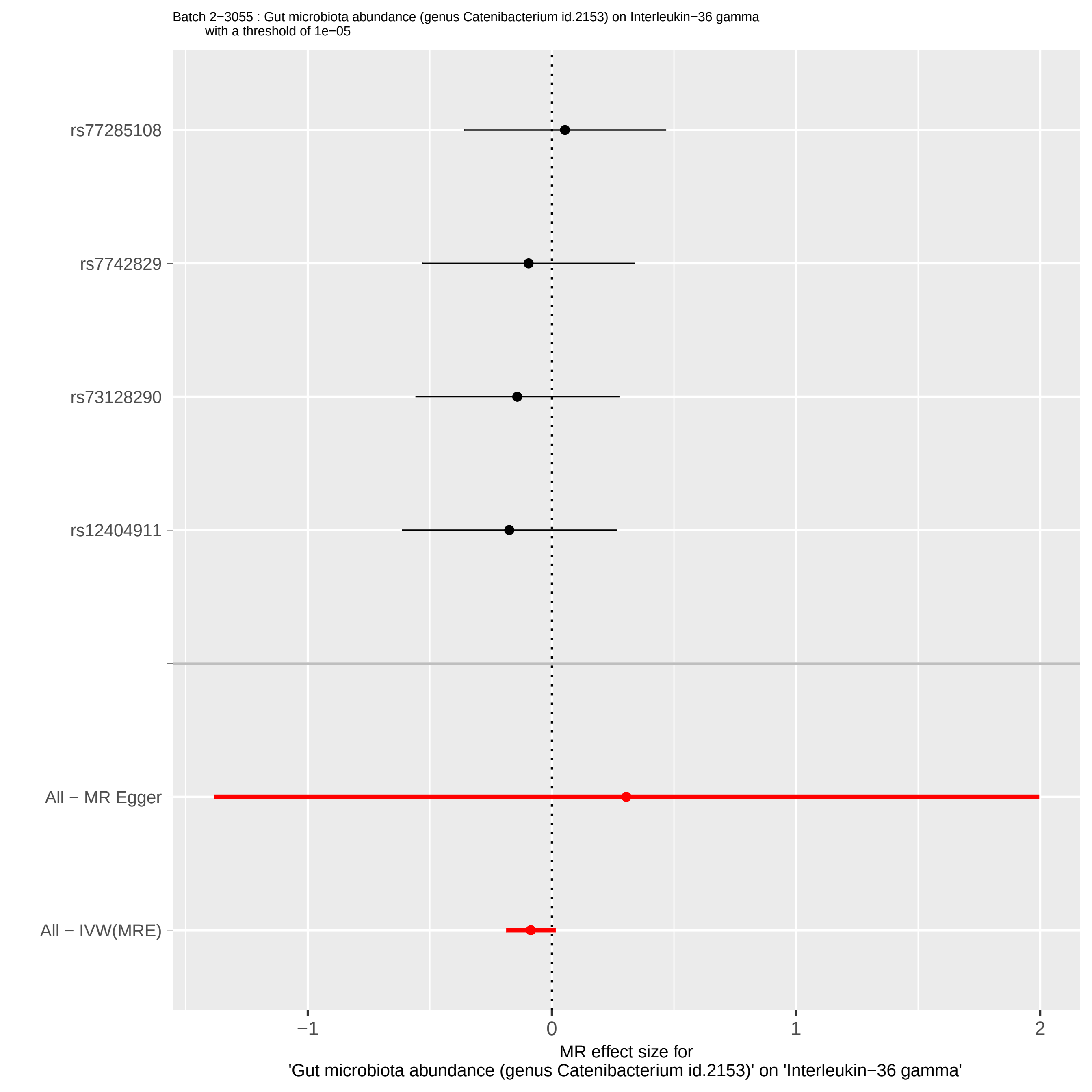

Batch 2−3055 : Gut microbiota abundance (genus Catenibacterium id.2153) on Interleukin−36 gamma
with a threshold of 1e−05
rs77285108
rs7742829
rs73128290
rs12404911
All − MR Egger
All − IVW(MRE)
−1
0
1
2
MR effect size for
'Gut microbiota abundance (genus Catenibacterium id.2153)' on 'Interleukin−36 gamma'

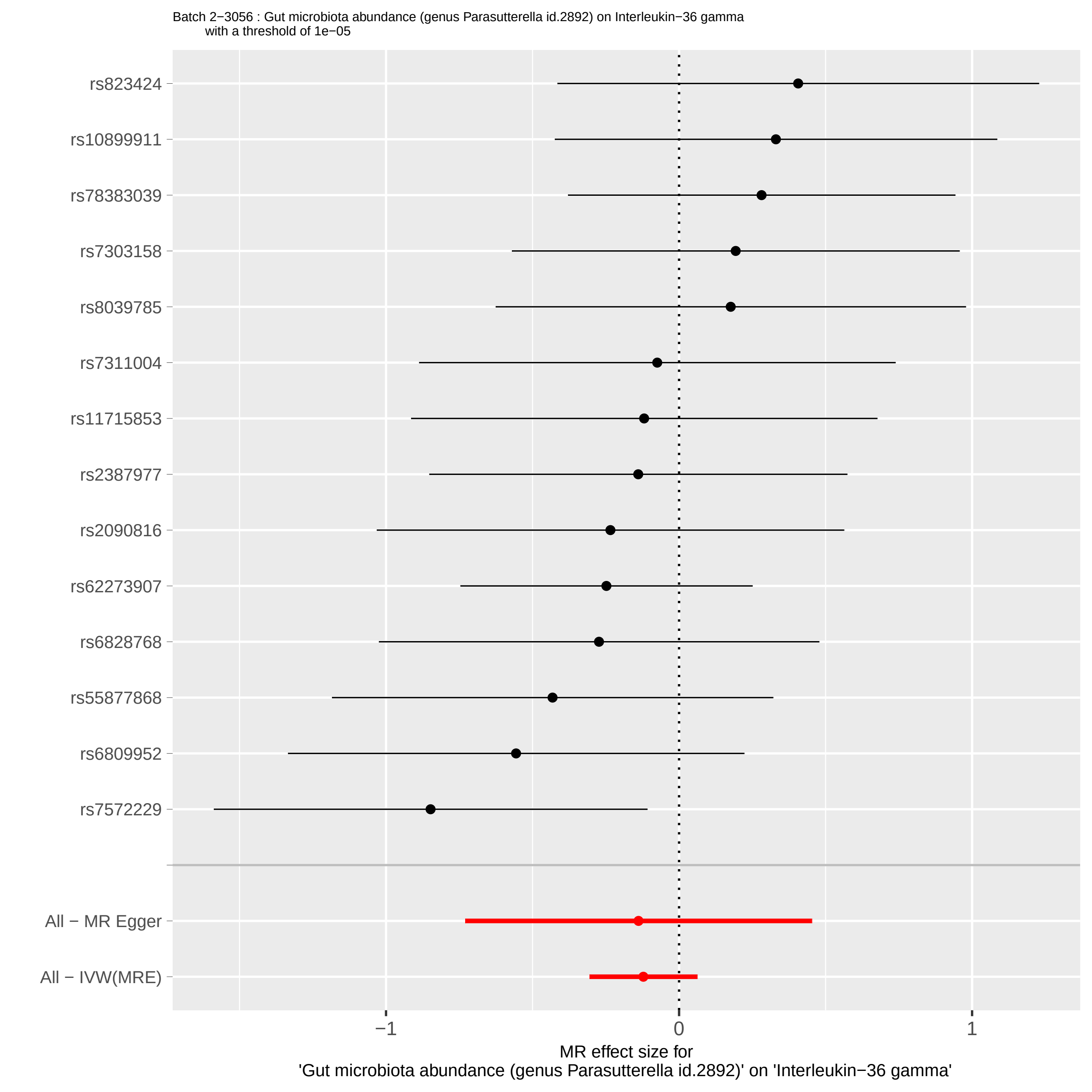

Batch 2−3056 : Gut microbiota abundance (genus Parasutterella id.2892) on Interleukin−36 gamma
with a threshold of 1e−05
rs823424
rs10899911
rs78383039
rs7303158
rs8039785
rs7311004
rs11715853
rs2387977
rs2090816
rs62273907
rs6828768
rs55877868
rs6809952
rs7572229
All − MR Egger
All − IVW(MRE)
−1
0
1
MR effect size for
'Gut microbiota abundance (genus Parasutterella id.2892)' on 'Interleukin−36 gamma'

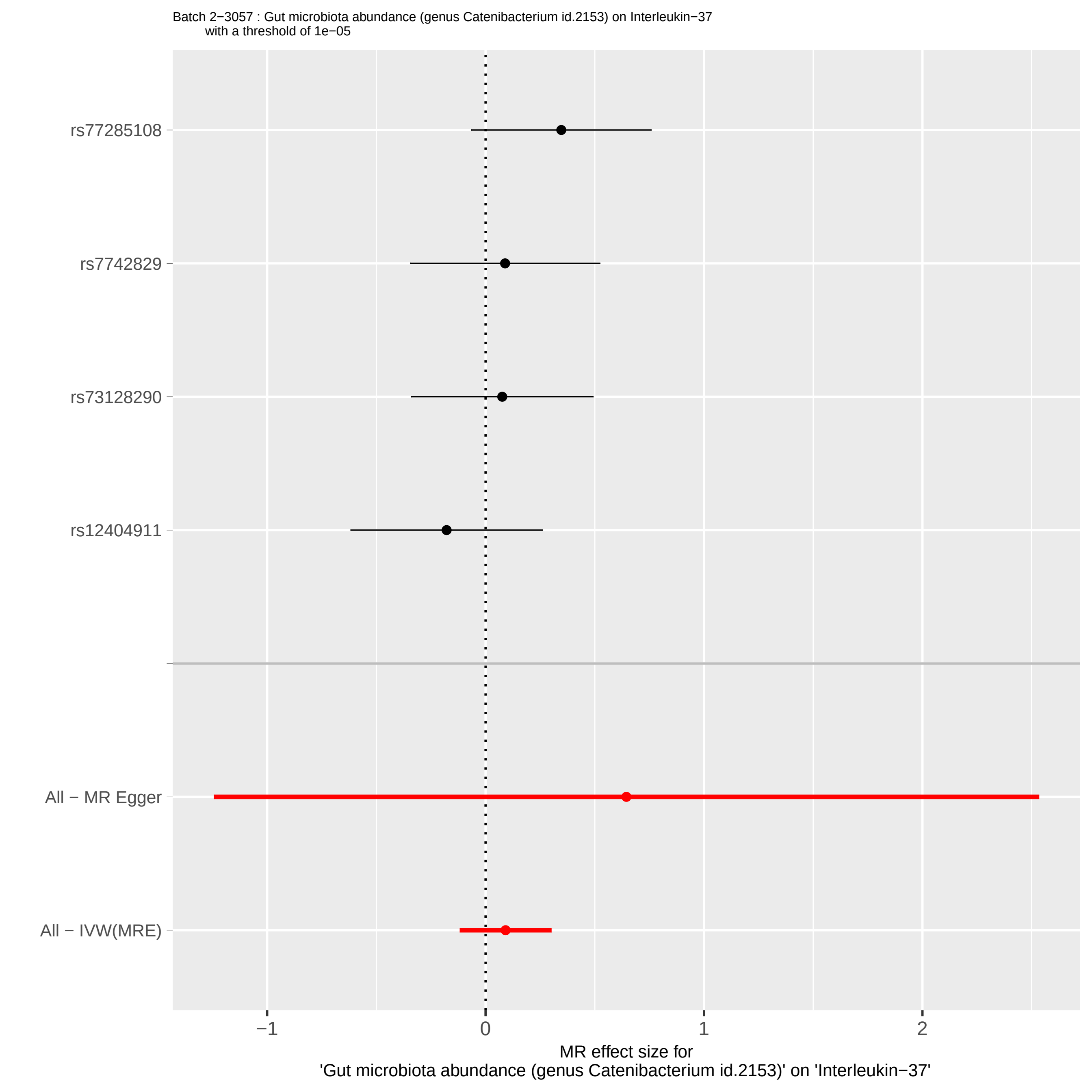
Batch 2−3057 : Gut microbiota abundance (genus Catenibacterium id.2153) on Interleukin−37
with a threshold of 1e−05
rs77285108
rs7742829
rs73128290
rs12404911
All − MR Egger
All − IVW(MRE)
−1
0
1
2
MR effect size for
'Gut microbiota abundance (genus Catenibacterium id.2153)' on 'Interleukin−37'

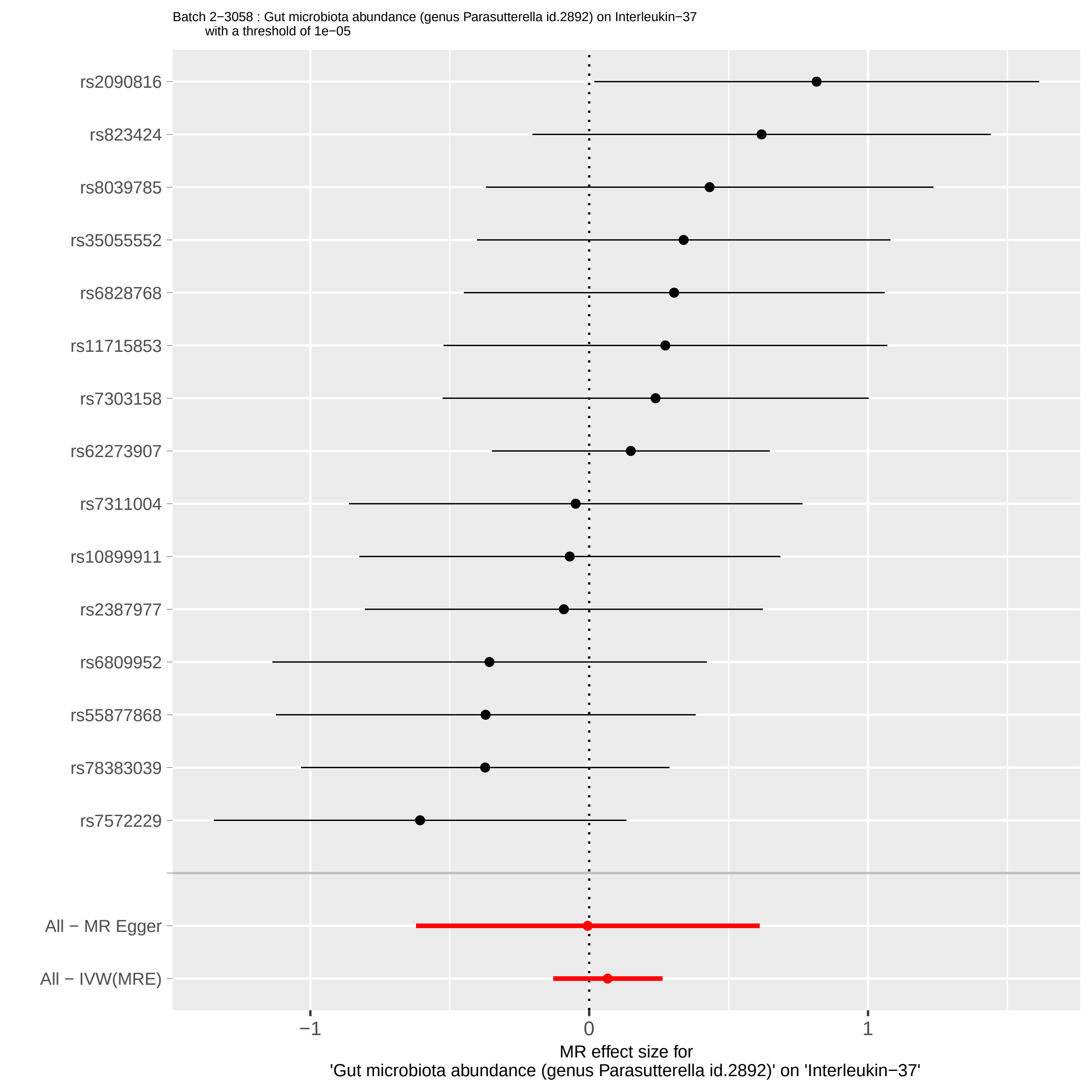
Batch 2−3058 : Gut microbiota abundance (genus Parasutterella id.2892) on Interleukin−37
with a threshold of 1e−05
rs2090816
rs823424
rs8039785
rs35055552
rs6828768
rs11715853
rs7303158
rs62273907
rs7311004
rs10899911
rs2387977
rs6809952
rs55877868
rs78383039
rs7572229
All − MR Egger
All − IVW(MRE)
−1
0
1
MR effect size for
'Gut microbiota abundance (genus Parasutterella id.2892)' on 'Interleukin−37'

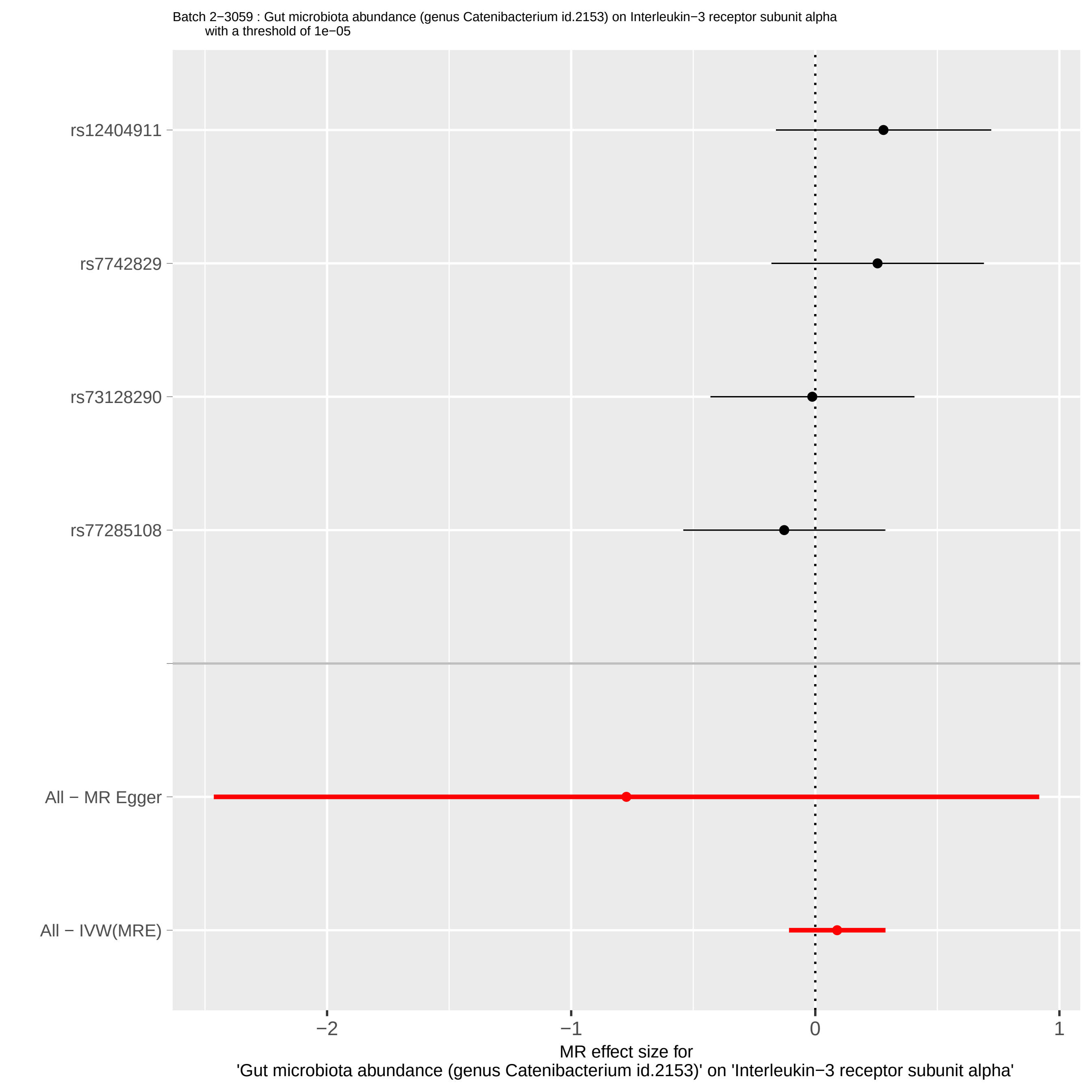

Batch 2−3059 : Gut microbiota abundance (genus Catenibacterium id.2153) on Interleukin−3 receptor subunit alpha
with a threshold of 1e−05
rs12404911
rs7742829
rs73128290
rs77285108
All − MR Egger
All − IVW(MRE)
−2
−1
0
1
MR effect size for
'Gut microbiota abundance (genus Catenibacterium id.2153)' on 'Interleukin−3 receptor subunit alpha'

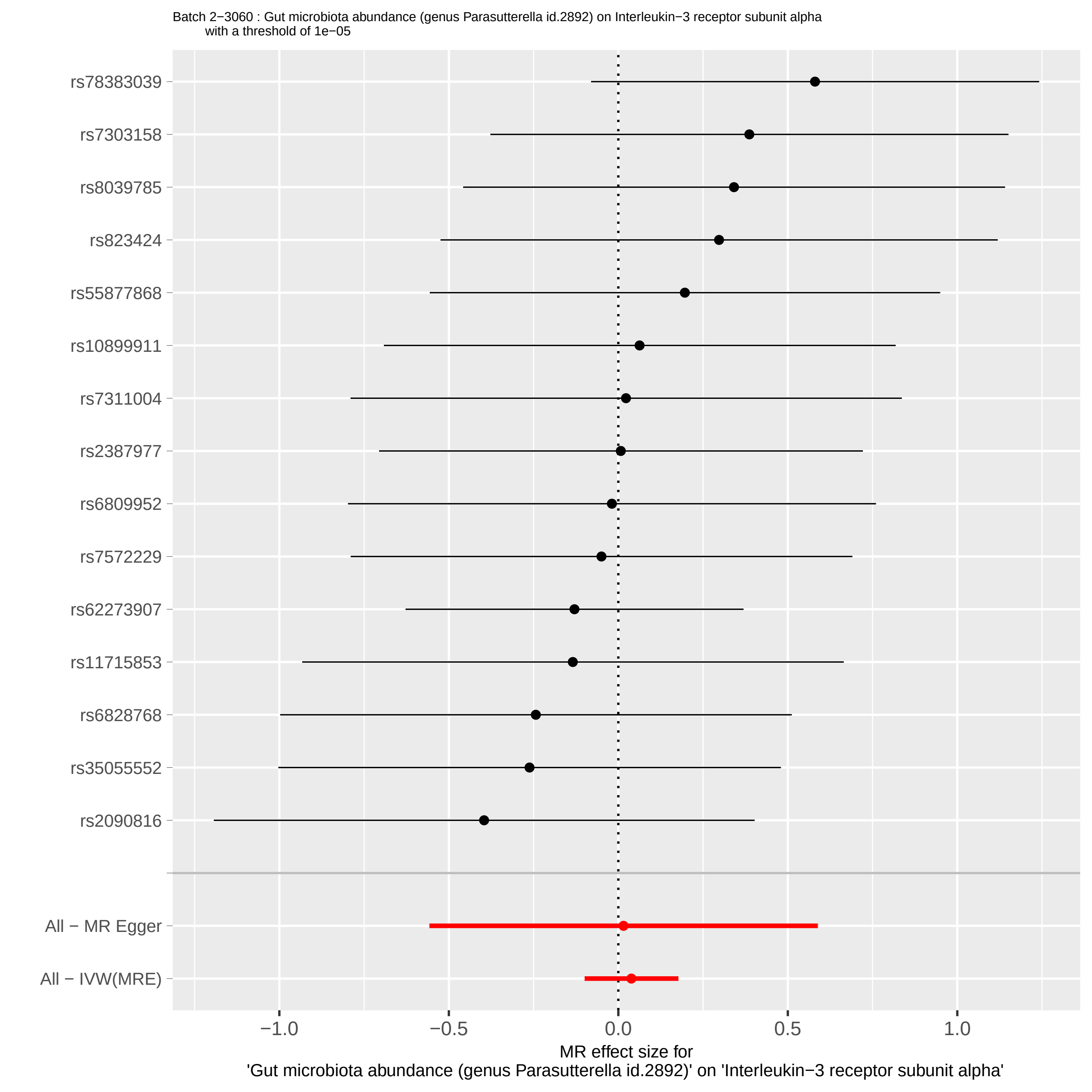

Batch 2−3060 : Gut microbiota abundance (genus Parasutterella id.2892) on Interleukin−3 receptor subunit alpha
with a threshold of 1e−05
rs78383039
rs7303158
rs8039785
rs823424
rs55877868
rs10899911
rs7311004
rs2387977
rs6809952
rs7572229
rs62273907
rs11715853
rs6828768
rs35055552
rs2090816
All − MR Egger
All − IVW(MRE)
−1.0
−0.5
0.0
0.5
1.0
MR effect size for
'Gut microbiota abundance (genus Parasutterella id.2892)' on 'Interleukin−3 receptor subunit alpha'

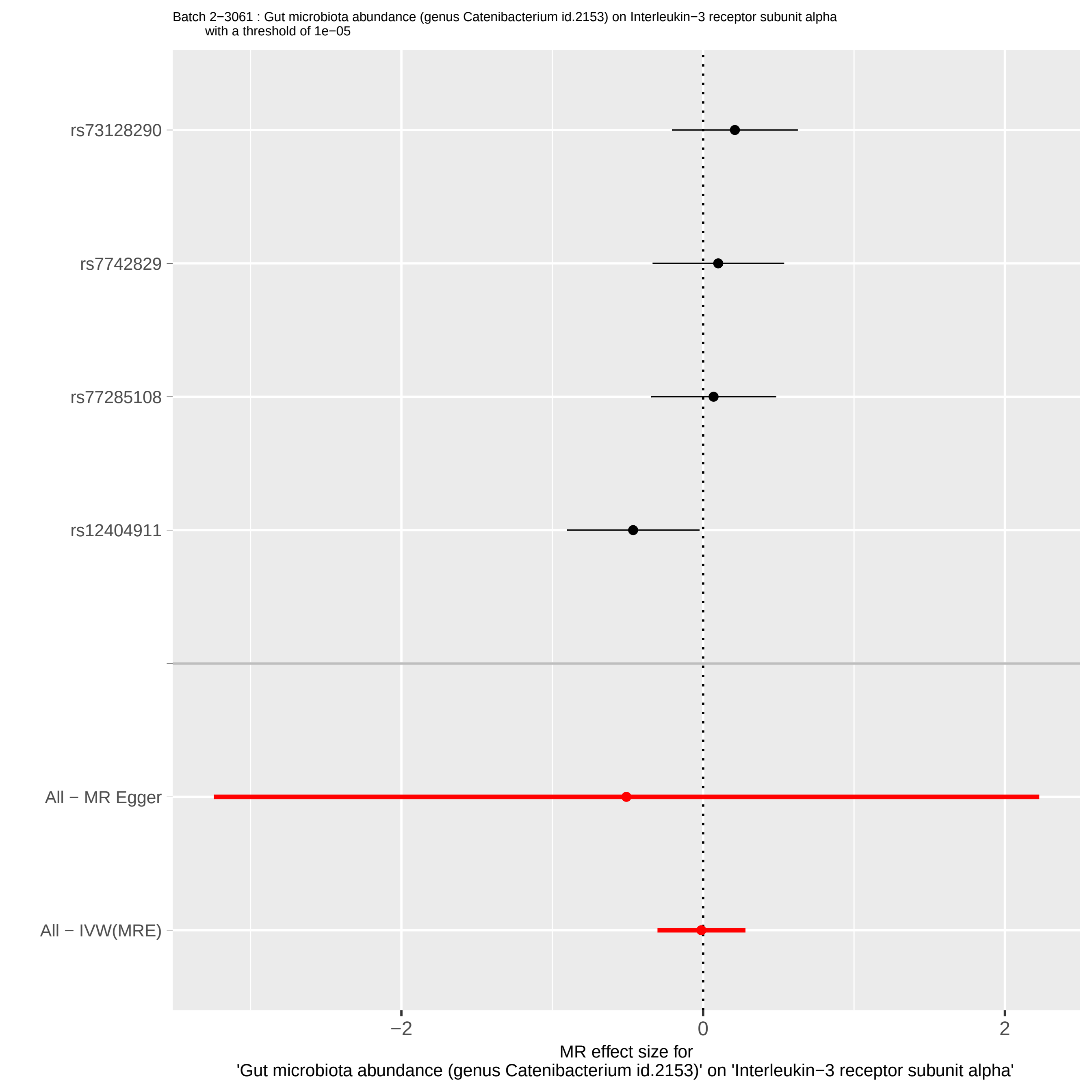

Batch 2−3061 : Gut microbiota abundance (genus Catenibacterium id.2153) on Interleukin−3 receptor subunit alpha
with a threshold of 1e−05
rs73128290
rs7742829
rs77285108
rs12404911
All − MR Egger
All − IVW(MRE)
−2
0
2
MR effect size for
'Gut microbiota abundance (genus Catenibacterium id.2153)' on 'Interleukin−3 receptor subunit alpha'

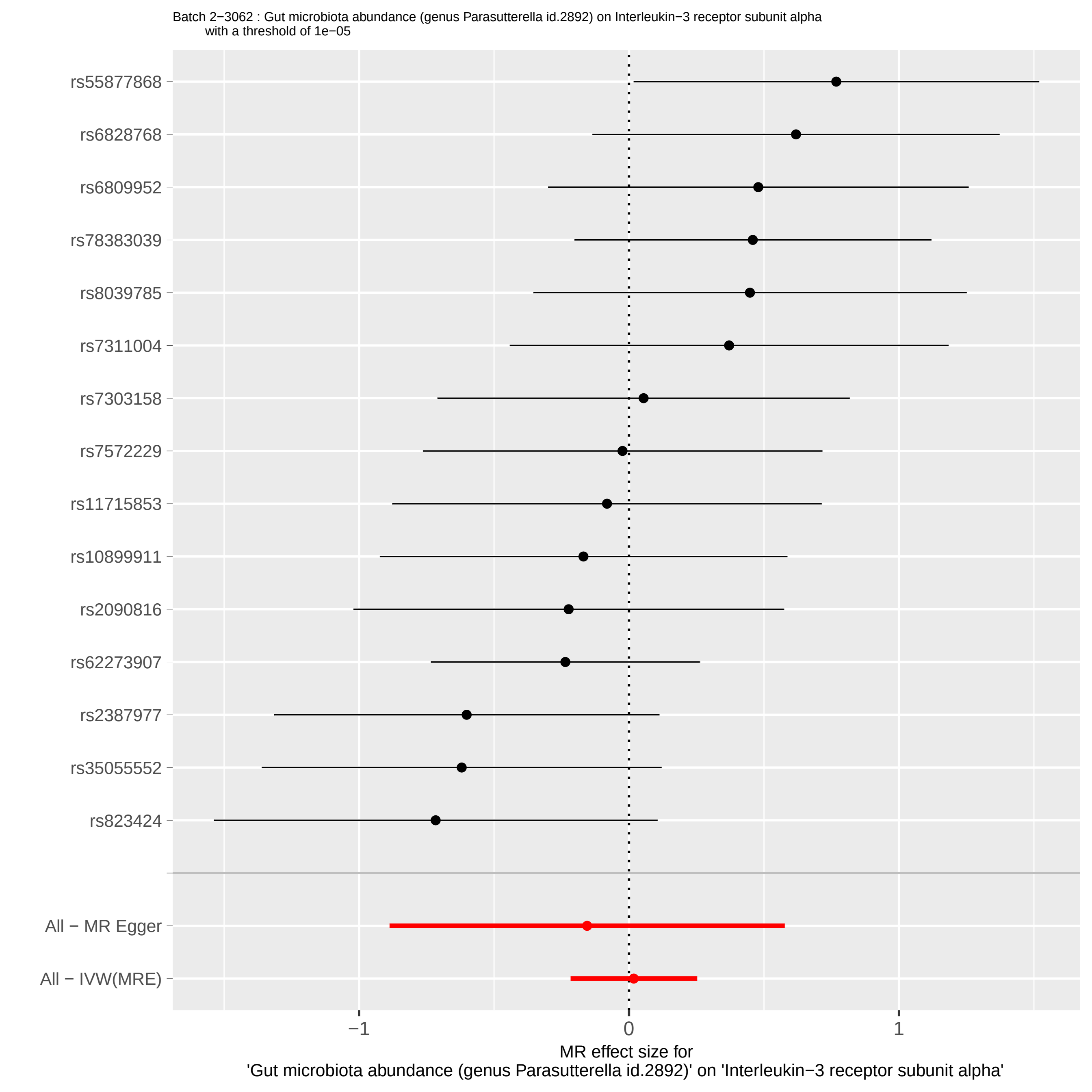

Batch 2−3062 : Gut microbiota abundance (genus Parasutterella id.2892) on Interleukin−3 receptor subunit alpha
with a threshold of 1e−05
rs55877868
rs6828768
rs6809952
rs78383039
rs8039785
rs7311004
rs7303158
rs7572229
rs11715853
rs10899911
rs2090816
rs62273907
rs2387977
rs35055552
rs823424
All − MR Egger
All − IVW(MRE)
−1
0
1
MR effect size for
'Gut microbiota abundance (genus Parasutterella id.2892)' on 'Interleukin−3 receptor subunit alpha'

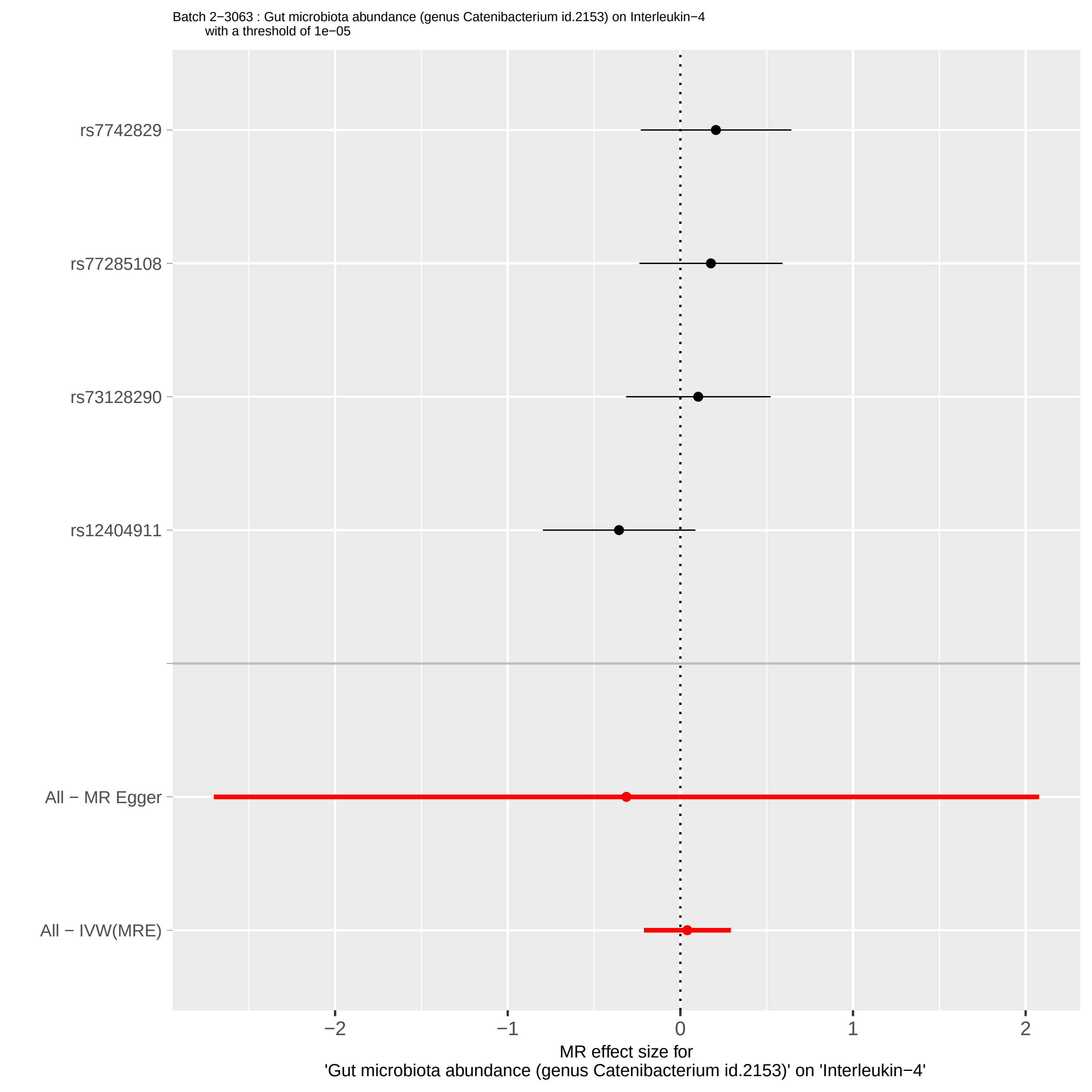

Batch 2−3063 : Gut microbiota abundance (genus Catenibacterium id.2153) on Interleukin−4
with a threshold of 1e−05
rs7742829
rs77285108
rs73128290
rs12404911
All − MR Egger
All − IVW(MRE)
−2
−1
0
1
2
MR effect size for
'Gut microbiota abundance (genus Catenibacterium id.2153)' on 'Interleukin−4'

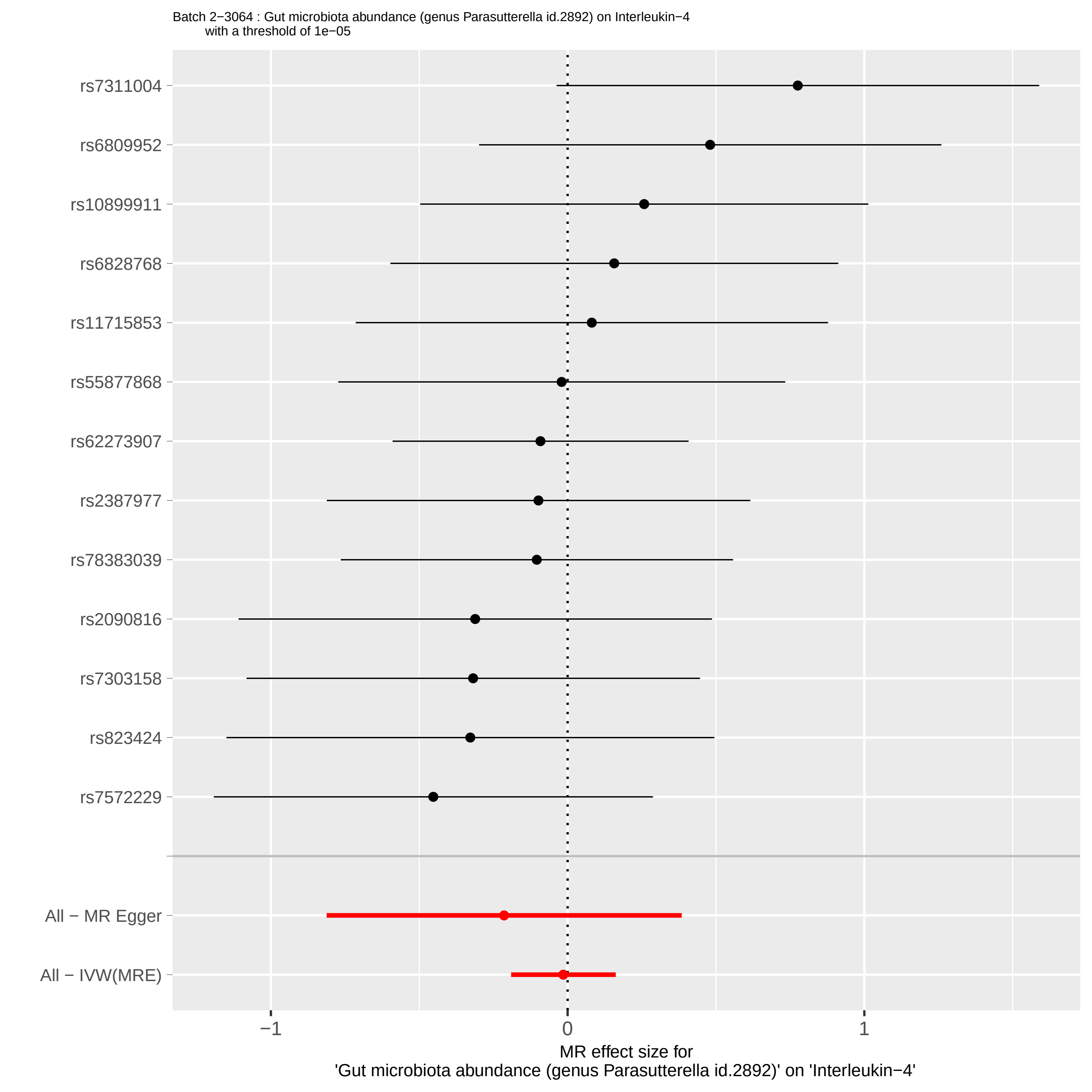

Batch 2−3064 : Gut microbiota abundance (genus Parasutterella id.2892) on Interleukin−4
with a threshold of 1e−05
rs7311004
rs6809952
rs10899911
rs6828768
rs11715853
rs55877868
rs62273907
rs2387977
rs78383039
rs2090816
rs7303158
rs823424
rs7572229
All − MR Egger
All − IVW(MRE)
−1
0
1
MR effect size for
'Gut microbiota abundance (genus Parasutterella id.2892)' on 'Interleukin−4'

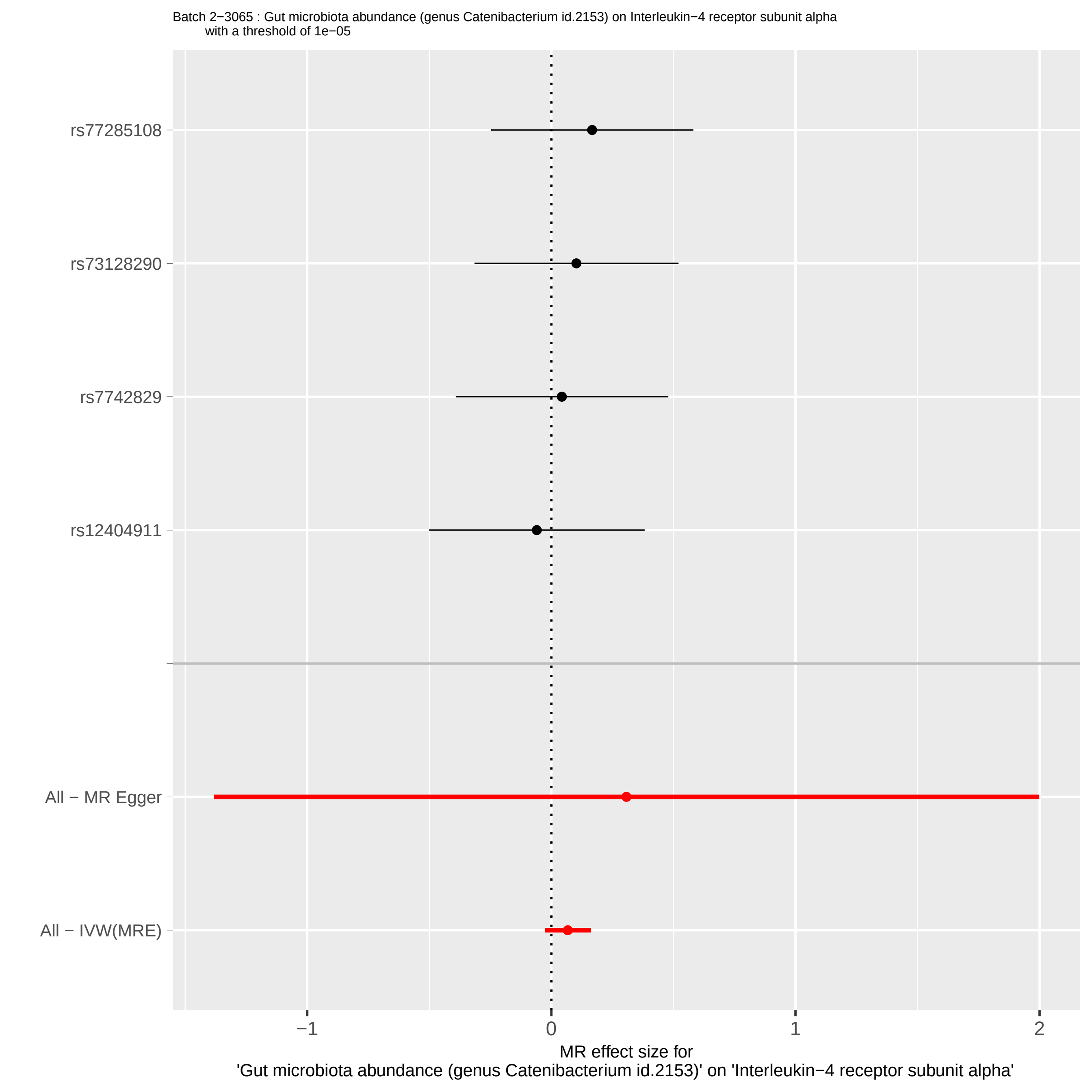

Batch 2−3065 : Gut microbiota abundance (genus Catenibacterium id.2153) on Interleukin−4 receptor subunit alpha
with a threshold of 1e−05
rs77285108
rs73128290
rs7742829
rs12404911
All − MR Egger
All − IVW(MRE)
−1
0
1
2
MR effect size for
'Gut microbiota abundance (genus Catenibacterium id.2153)' on 'Interleukin−4 receptor subunit alpha'

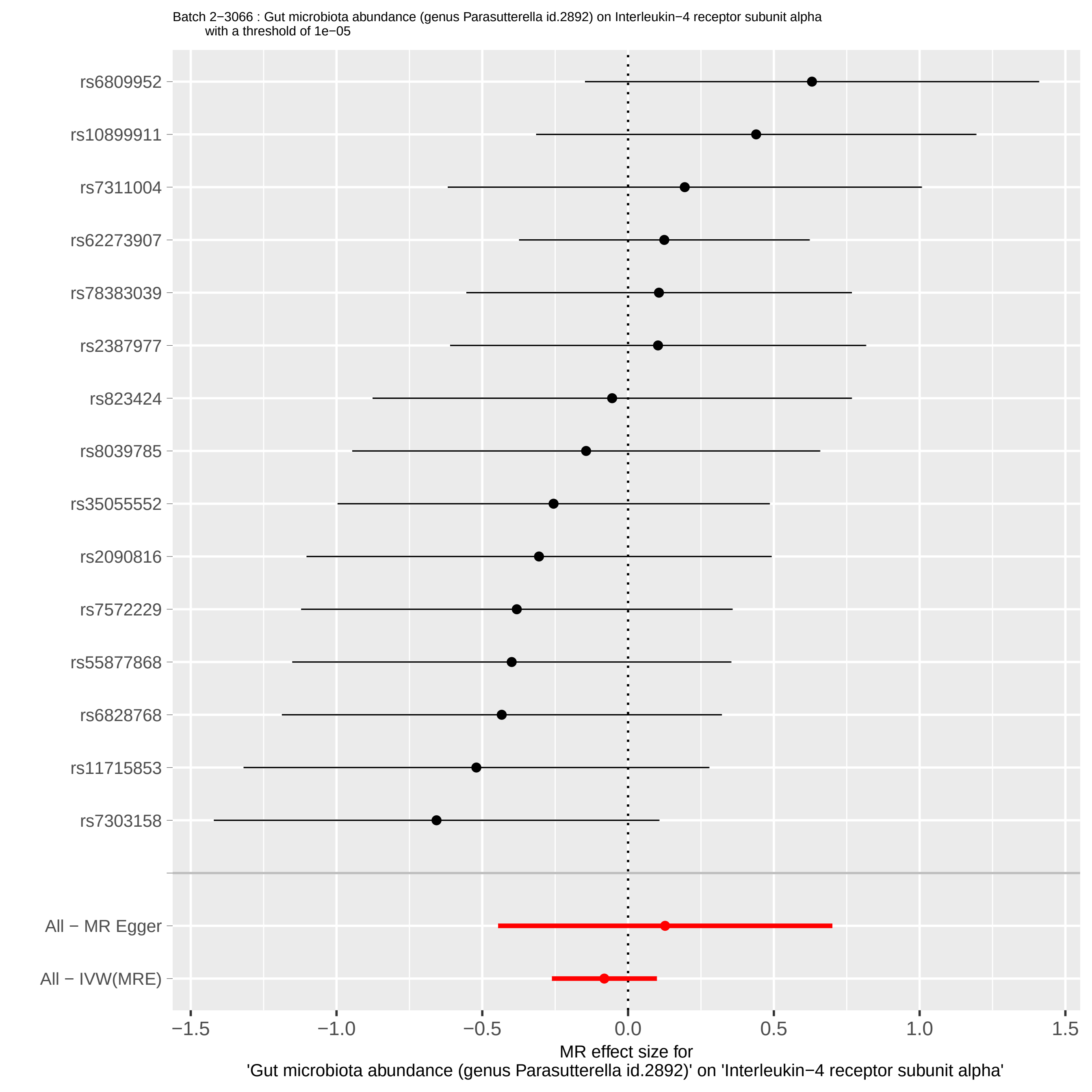
Batch 2−3066 : Gut microbiota abundance (genus Parasutterella id.2892) on Interleukin−4 receptor subunit alpha
with a threshold of 1e−05
rs6809952
rs10899911
rs7311004
rs62273907
rs78383039
rs2387977
rs823424
rs8039785
rs35055552
rs2090816
rs7572229
rs55877868
rs6828768
rs11715853
rs7303158
All − MR Egger
All − IVW(MRE)
−1.5
−1.0
−0.5
0.0
0.5
1.0
1.5
MR effect size for
'Gut microbiota abundance (genus Parasutterella id.2892)' on 'Interleukin−4 receptor subunit alpha'

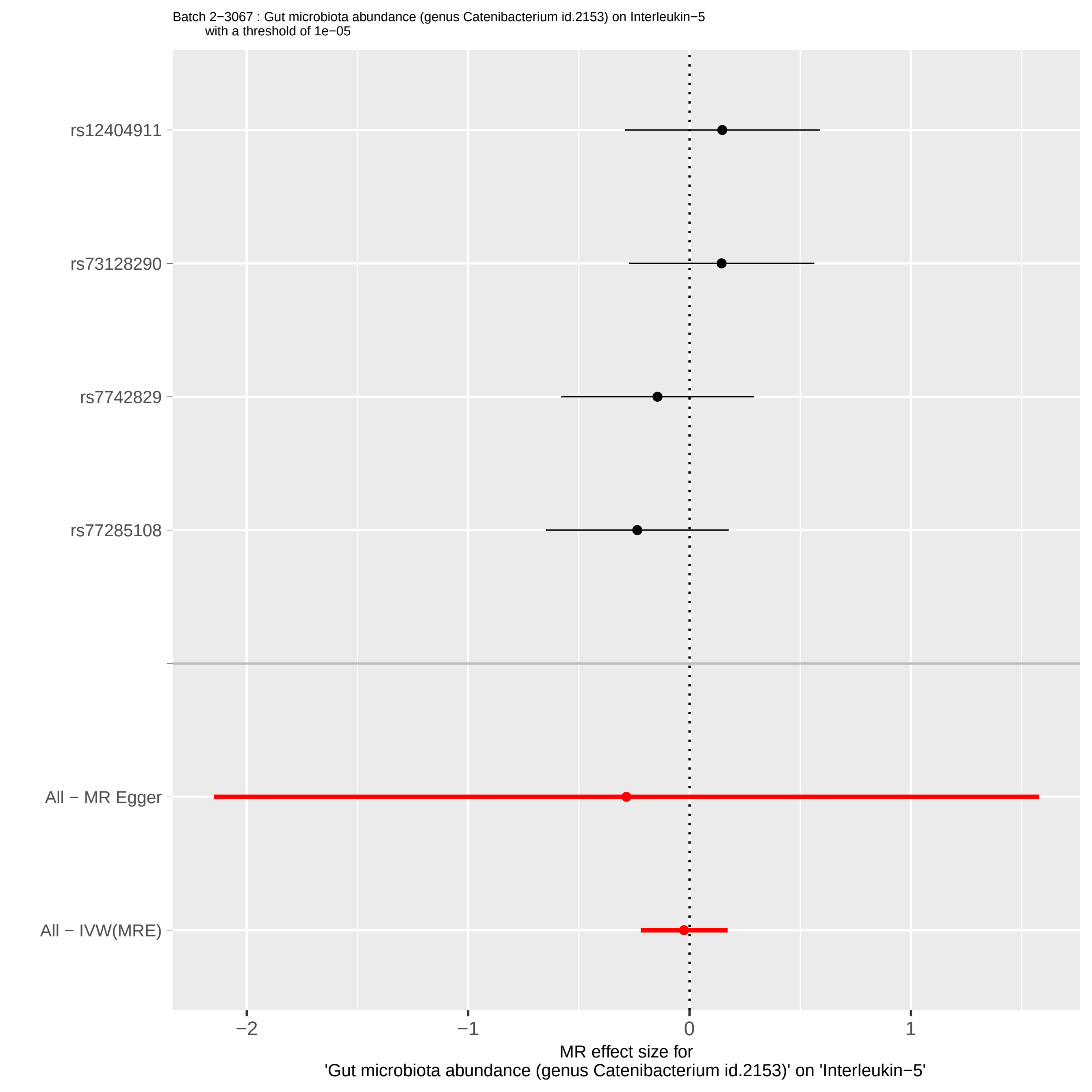
Batch 2−3067 : Gut microbiota abundance (genus Catenibacterium id.2153) on Interleukin−5
with a threshold of 1e−05
rs12404911
rs73128290
rs7742829
rs77285108
All − MR Egger
All − IVW(MRE)
−2
−1
0
1
MR effect size for
'Gut microbiota abundance (genus Catenibacterium id.2153)' on 'Interleukin−5'

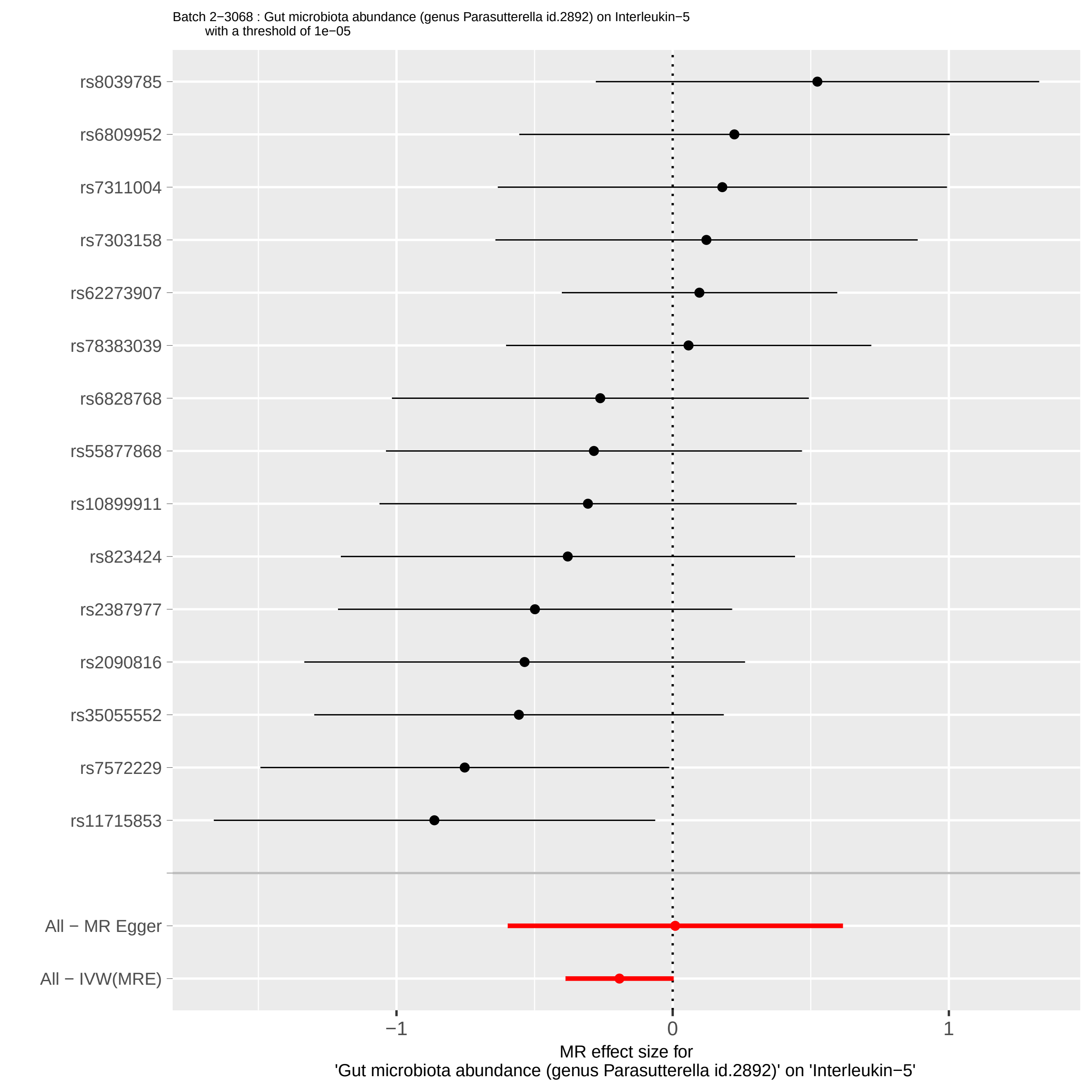
Batch 2−3068 : Gut microbiota abundance (genus Parasutterella id.2892) on Interleukin−5
with a threshold of 1e−05
rs8039785
rs6809952
rs7311004
rs7303158
rs62273907
rs78383039
rs6828768
rs55877868
rs10899911
rs823424
rs2387977
rs2090816
rs35055552
rs7572229
rs11715853
All − MR Egger
All − IVW(MRE)
−1
0
1
MR effect size for
'Gut microbiota abundance (genus Parasutterella id.2892)' on 'Interleukin−5'

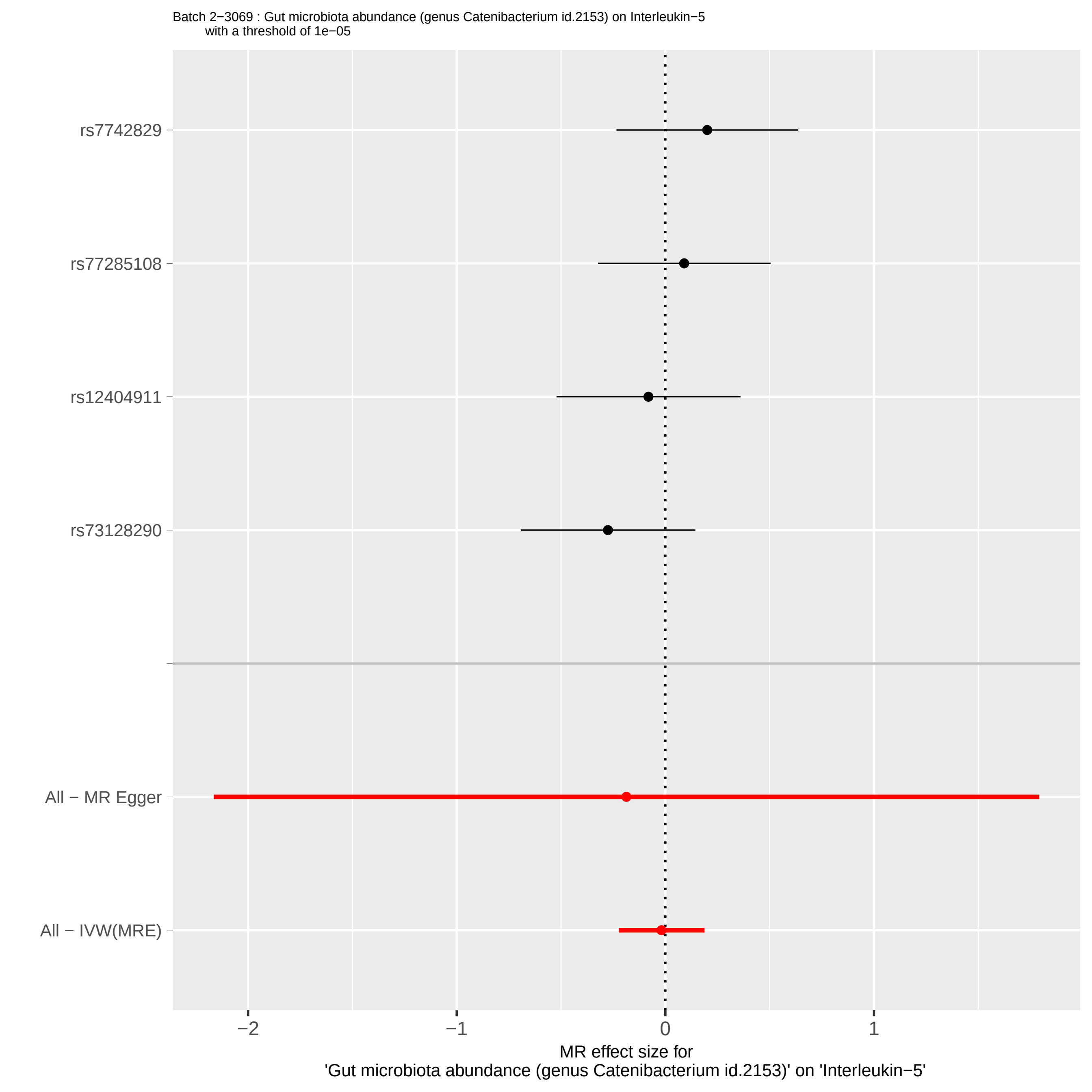
Batch 2−3069 : Gut microbiota abundance (genus Catenibacterium id.2153) on Interleukin−5
with a threshold of 1e−05
rs7742829
rs77285108
rs12404911
rs73128290
All − MR Egger
All − IVW(MRE)
−2
−1
0
1
MR effect size for
'Gut microbiota abundance (genus Catenibacterium id.2153)' on 'Interleukin−5'

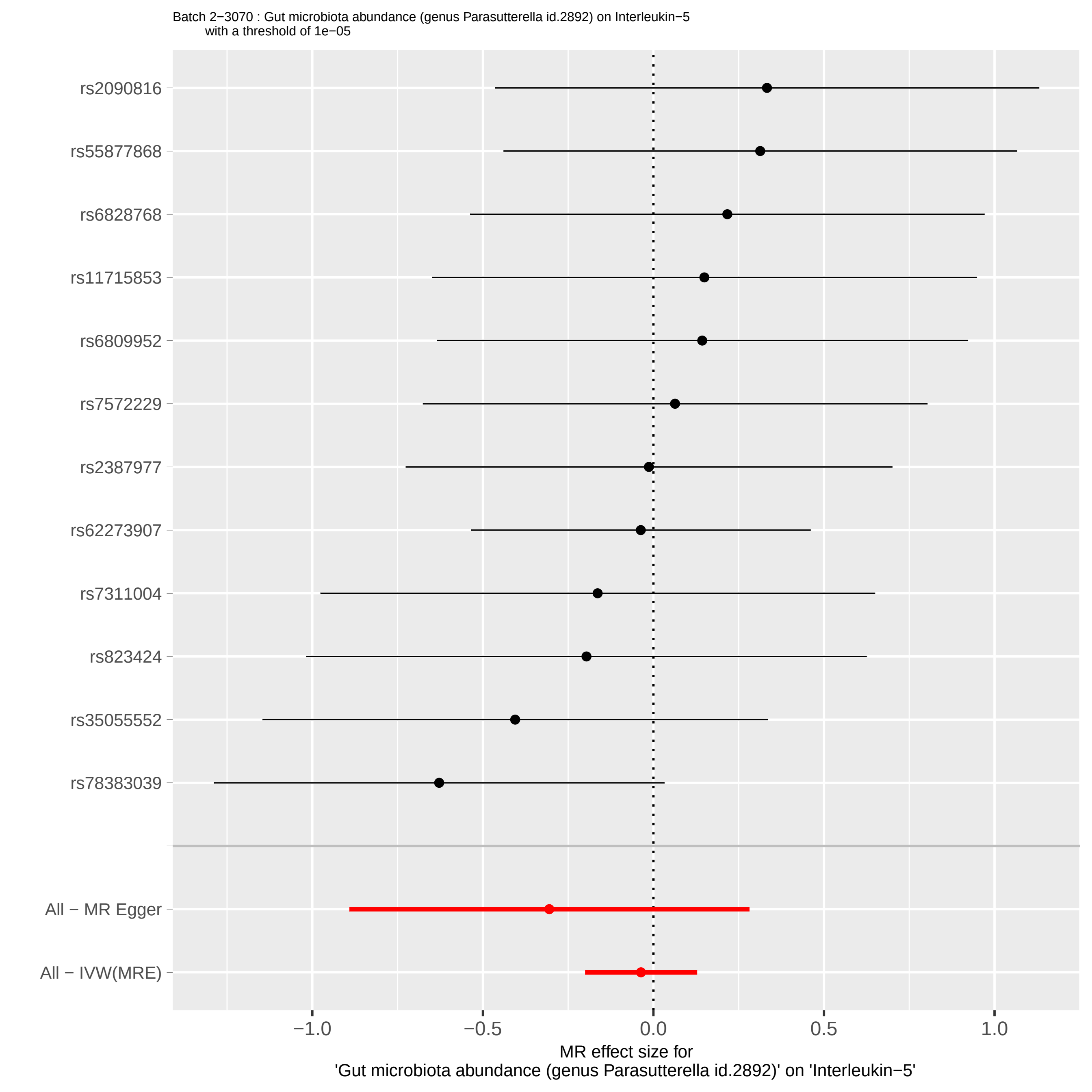

Batch 2−3070 : Gut microbiota abundance (genus Parasutterella id.2892) on Interleukin−5
with a threshold of 1e−05
rs2090816
rs55877868
rs6828768
rs11715853
rs6809952
rs7572229
rs2387977
rs62273907
rs7311004
rs823424
rs35055552
rs78383039
All − MR Egger
All − IVW(MRE)
−1.0
−0.5
0.0
0.5
1.0
MR effect size for
'Gut microbiota abundance (genus Parasutterella id.2892)' on 'Interleukin−5'

Batch 2−3071 : Gut microbiota abundance (genus Catenibacterium id.2153) on Interleukin−5 receptor subunit alpha
with a threshold of 1e−05

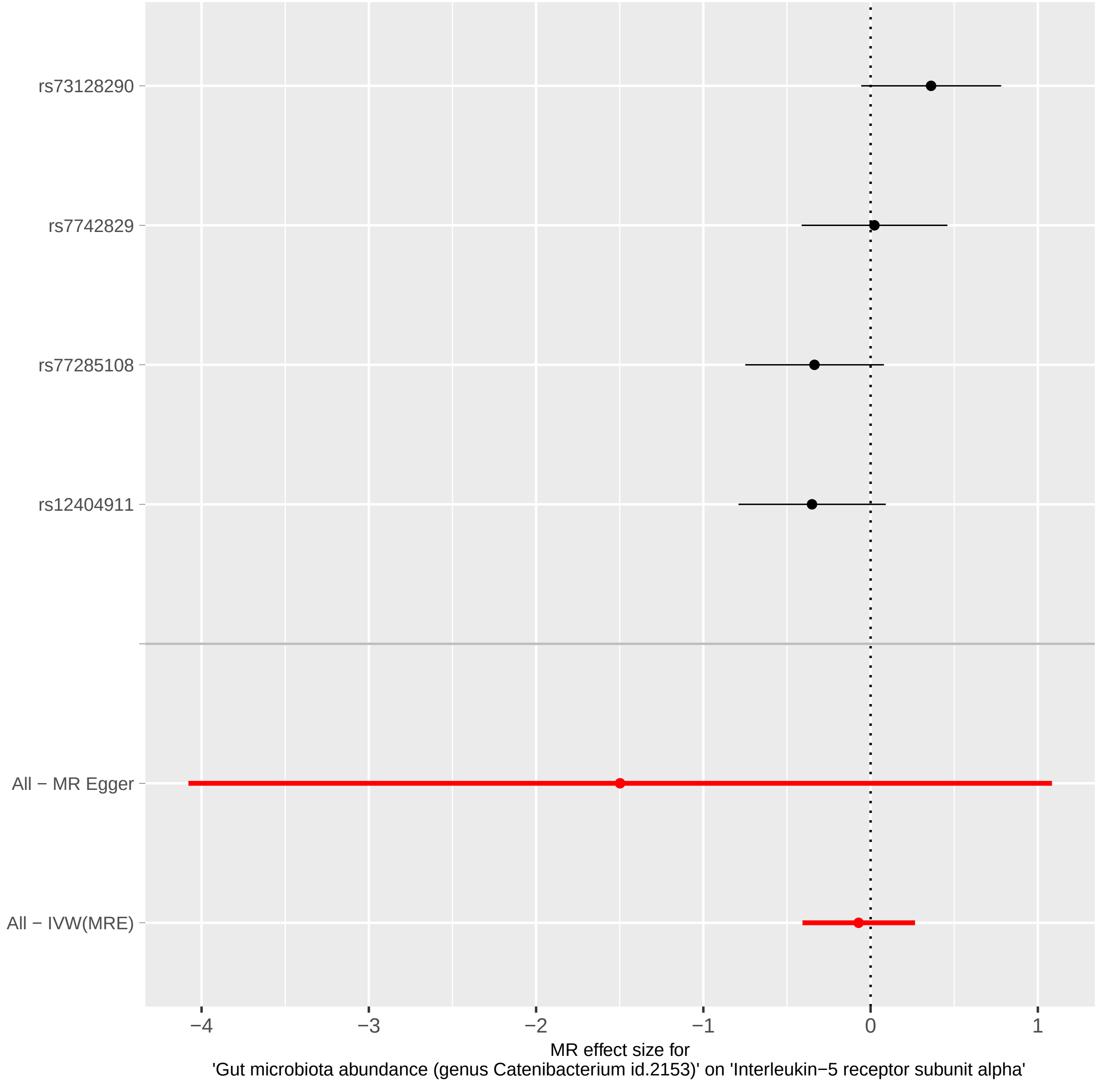

Batch 2−3072 : Gut microbiota abundance (genus Parasutterella id.2892) on Interleukin−5 receptor subunit alpha
with a threshold of 1e−05

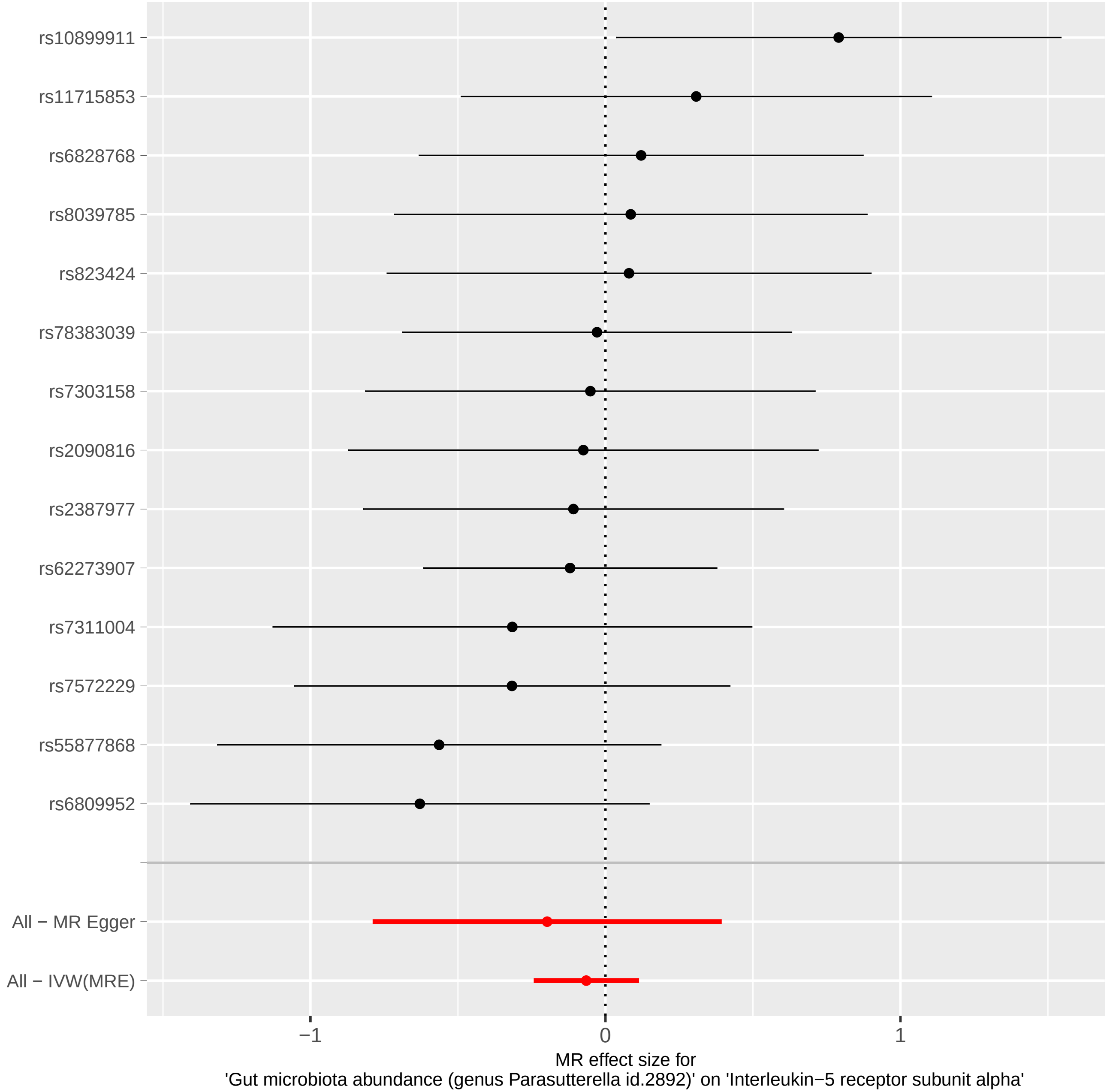

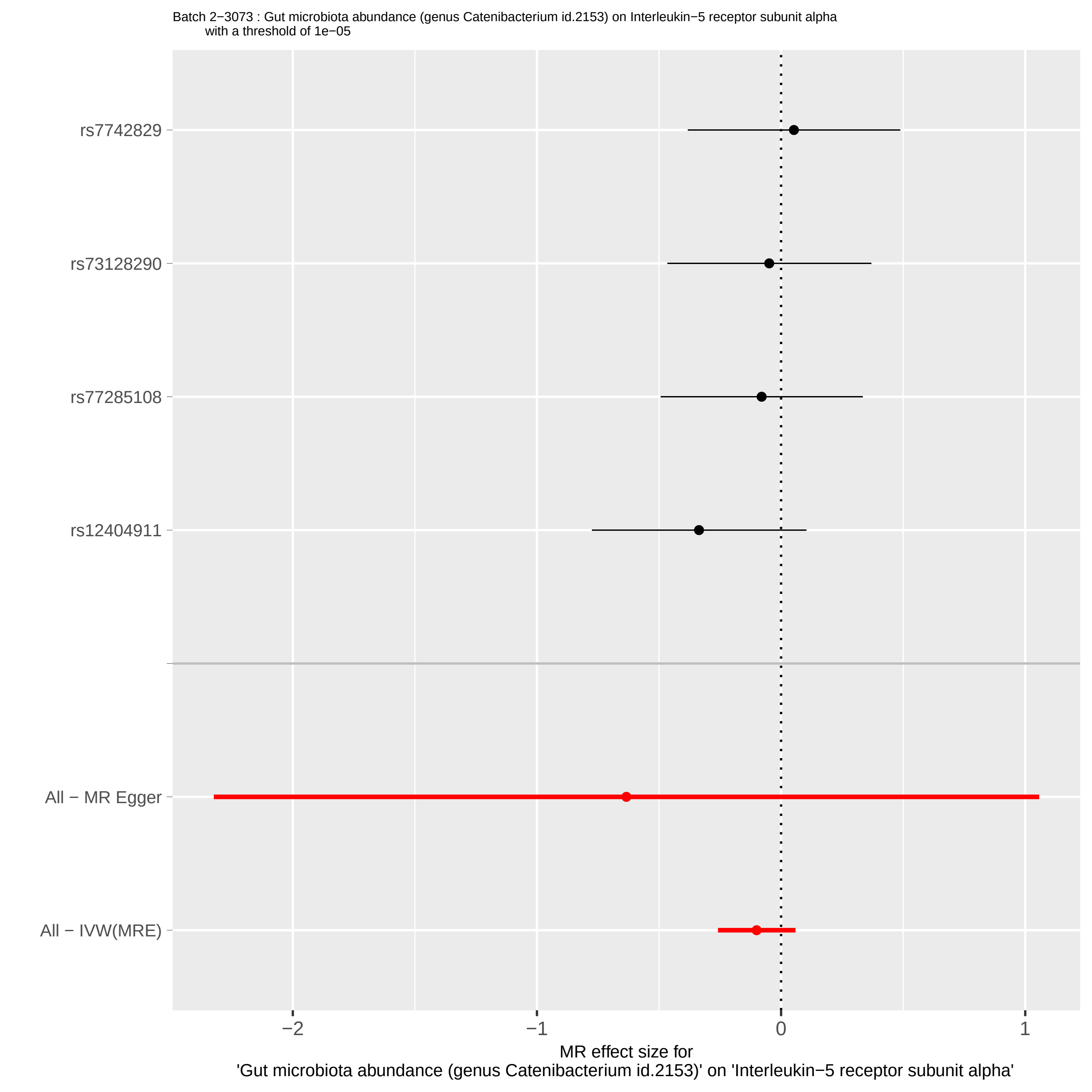

Batch 2−3073 : Gut microbiota abundance (genus Catenibacterium id.2153) on Interleukin−5 receptor subunit alpha
with a threshold of 1e−05
rs7742829
rs73128290
rs77285108
rs12404911
All − MR Egger
All − IVW(MRE)
−2
−1
0
1
MR effect size for
'Gut microbiota abundance (genus Catenibacterium id.2153)' on 'Interleukin−5 receptor subunit alpha'

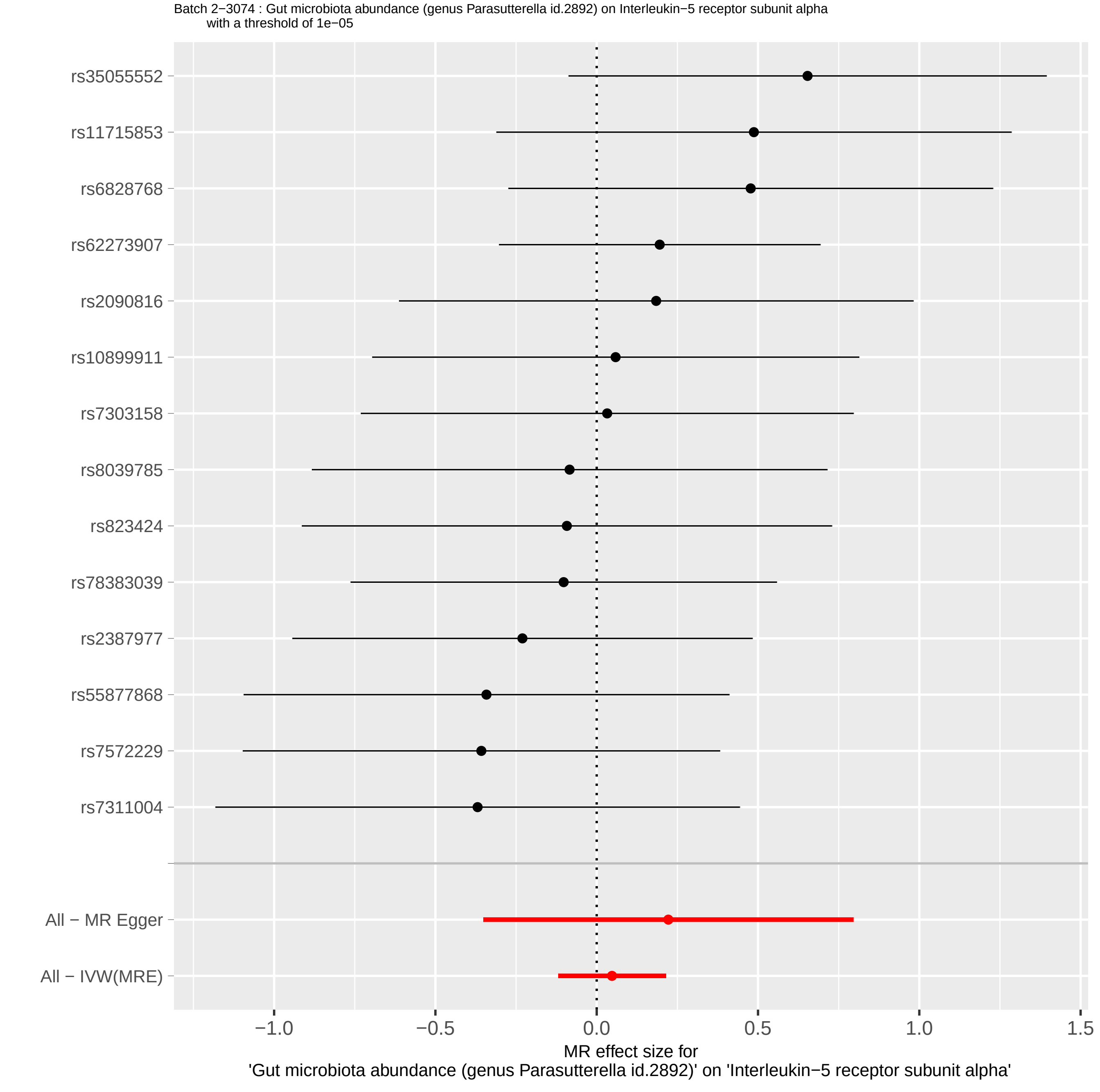

Batch 2−3074 : Gut microbiota abundance (genus Parasutterella id.2892) on Interleukin−5 receptor subunit alpha
with a threshold of 1e−05
rs35055552
rs11715853
rs6828768
rs62273907
rs2090816
rs10899911
rs7303158
rs8039785
rs823424
rs78383039
rs2387977
rs55877868
rs7572229
rs7311004
All − MR Egger
All − IVW(MRE)
−1.0
−0.5
0.0
0.5
1.0
1.5
MR effect size for
'Gut microbiota abundance (genus Parasutterella id.2892)' on 'Interleukin−5 receptor subunit alpha'

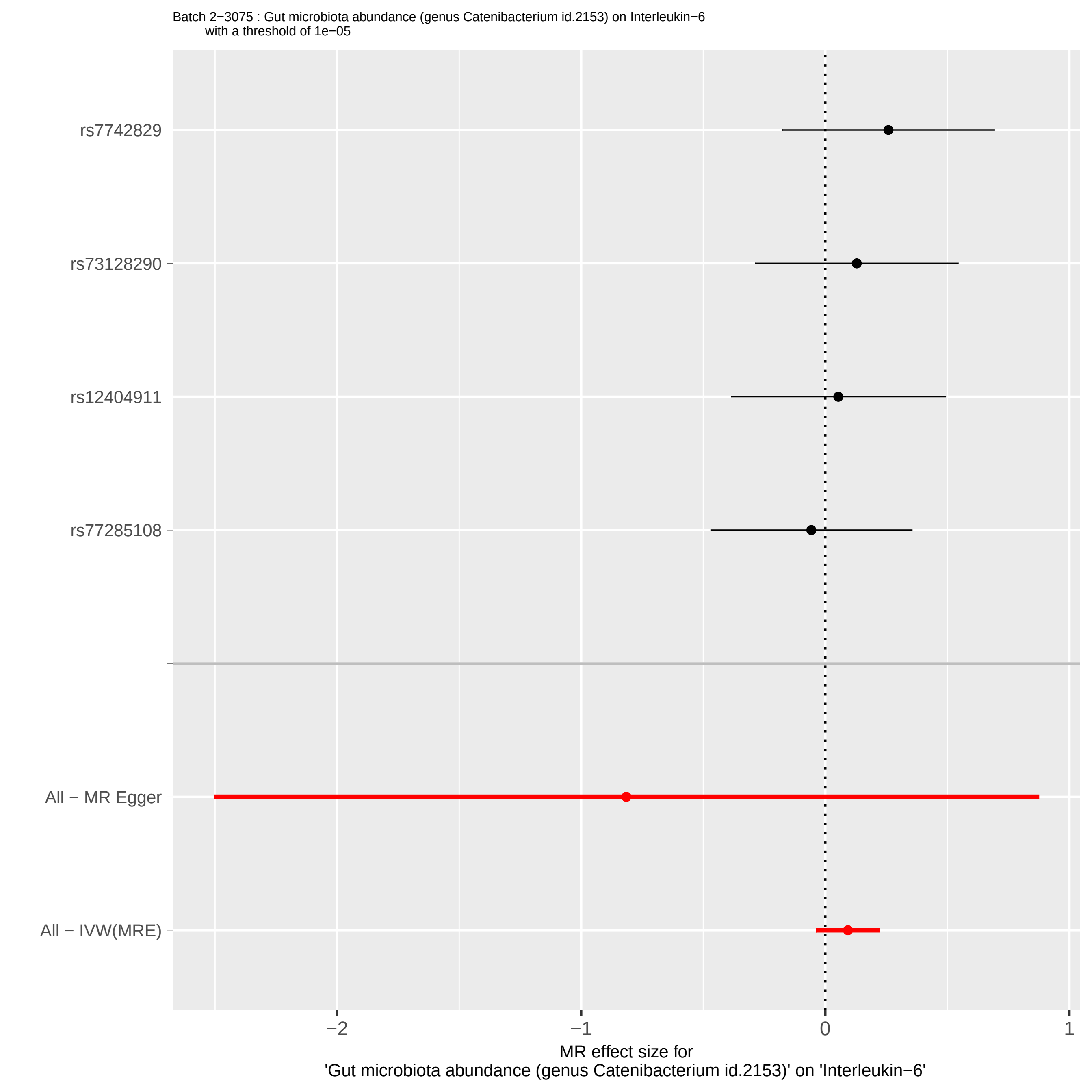
Batch 2−3075 : Gut microbiota abundance (genus Catenibacterium id.2153) on Interleukin−6
with a threshold of 1e−05
rs7742829
rs73128290
rs12404911
rs77285108
All − MR Egger
All − IVW(MRE)
−2
−1
0
1
MR effect size for
'Gut microbiota abundance (genus Catenibacterium id.2153)' on 'Interleukin−6'

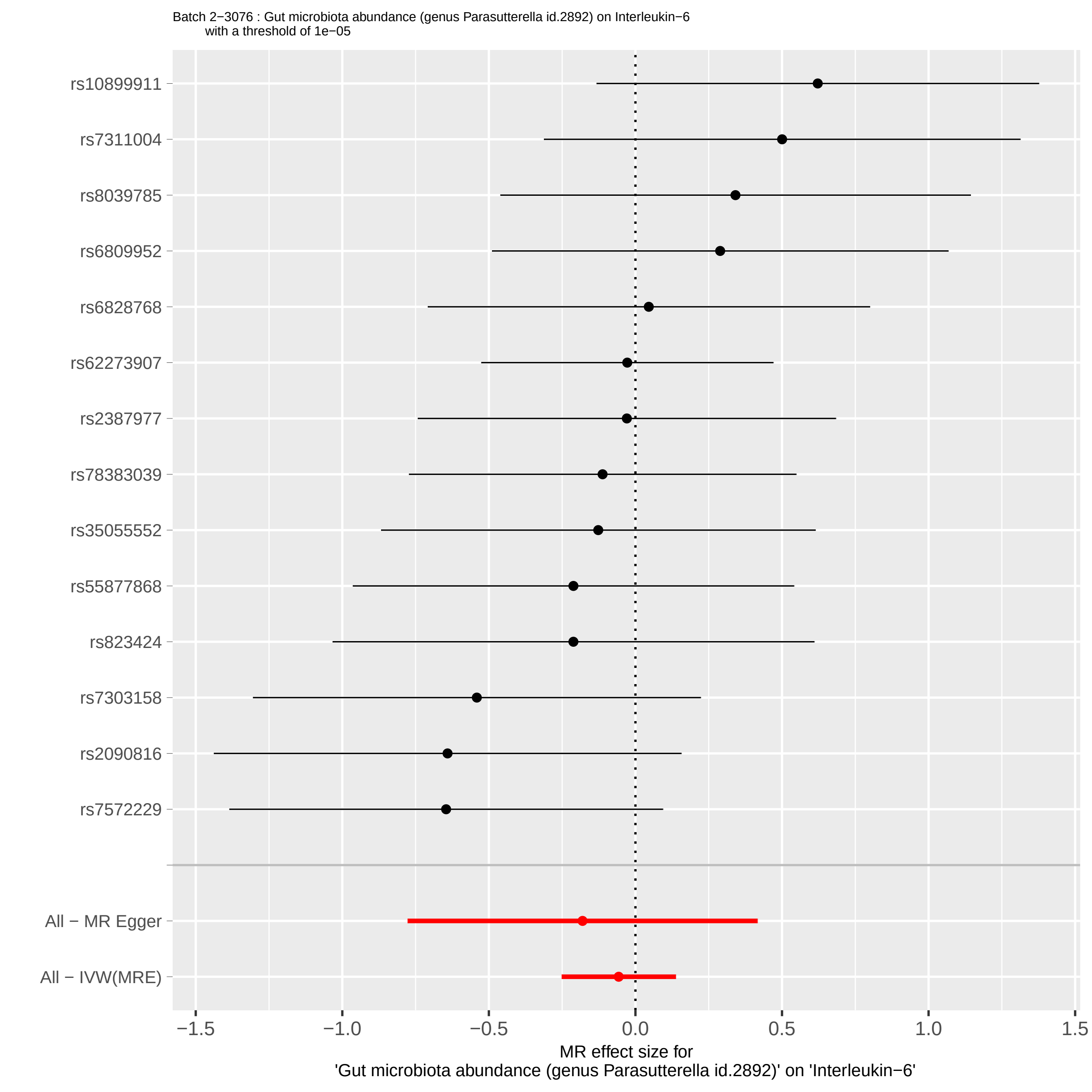

Batch 2−3076 : Gut microbiota abundance (genus Parasutterella id.2892) on Interleukin−6
with a threshold of 1e−05
rs10899911
rs7311004
rs8039785
rs6809952
rs6828768
rs62273907
rs2387977
rs78383039
rs35055552
rs55877868
rs823424
rs7303158
rs2090816
rs7572229
All − MR Egger
All − IVW(MRE)
−1.5
−1.0
−0.5
0.0
0.5
1.0
1.5
MR effect size for
'Gut microbiota abundance (genus Parasutterella id.2892)' on 'Interleukin−6'

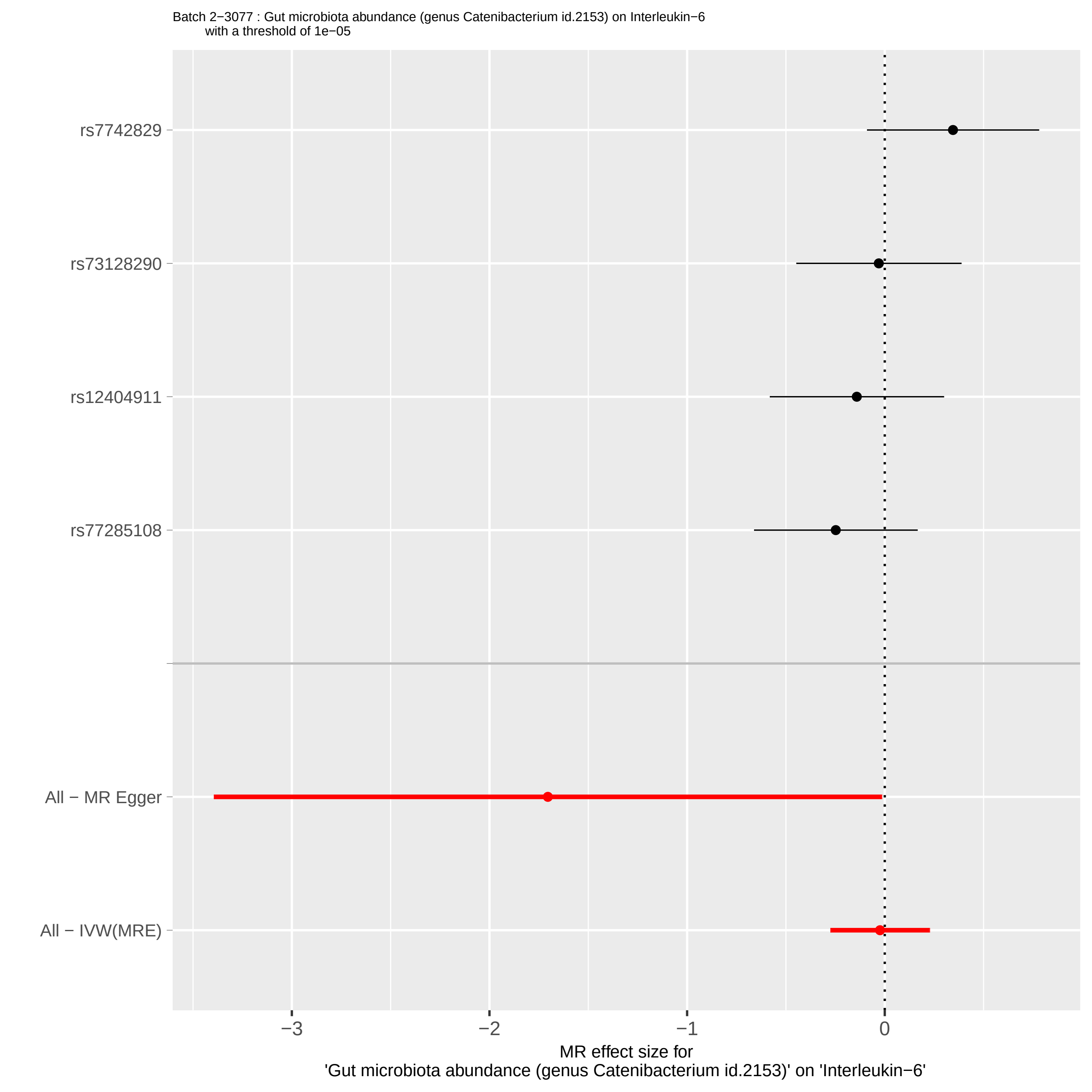

Batch 2−3077 : Gut microbiota abundance (genus Catenibacterium id.2153) on Interleukin−6
with a threshold of 1e−05
rs7742829
rs73128290
rs12404911
rs77285108
All − MR Egger
All − IVW(MRE)
−3
−2
−1
0
MR effect size for
'Gut microbiota abundance (genus Catenibacterium id.2153)' on 'Interleukin−6'

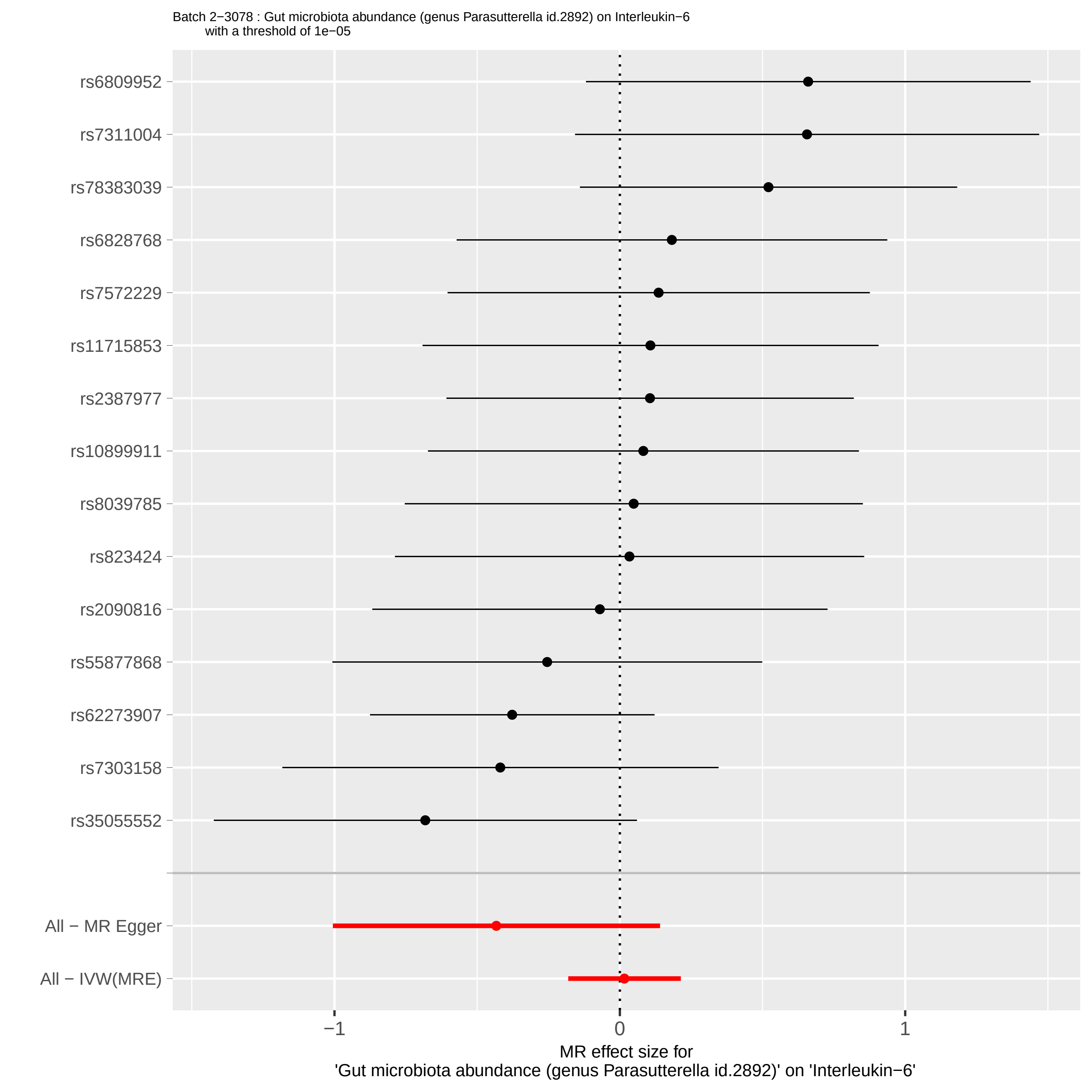

Batch 2−3078 : Gut microbiota abundance (genus Parasutterella id.2892) on Interleukin−6
with a threshold of 1e−05
rs6809952
rs7311004
rs78383039
rs6828768
rs7572229
rs11715853
rs2387977
rs10899911
rs8039785
rs823424
rs2090816
rs55877868
rs62273907
rs7303158
rs35055552
All − MR Egger
All − IVW(MRE)
−1
0
1
MR effect size for
'Gut microbiota abundance (genus Parasutterella id.2892)' on 'Interleukin−6'

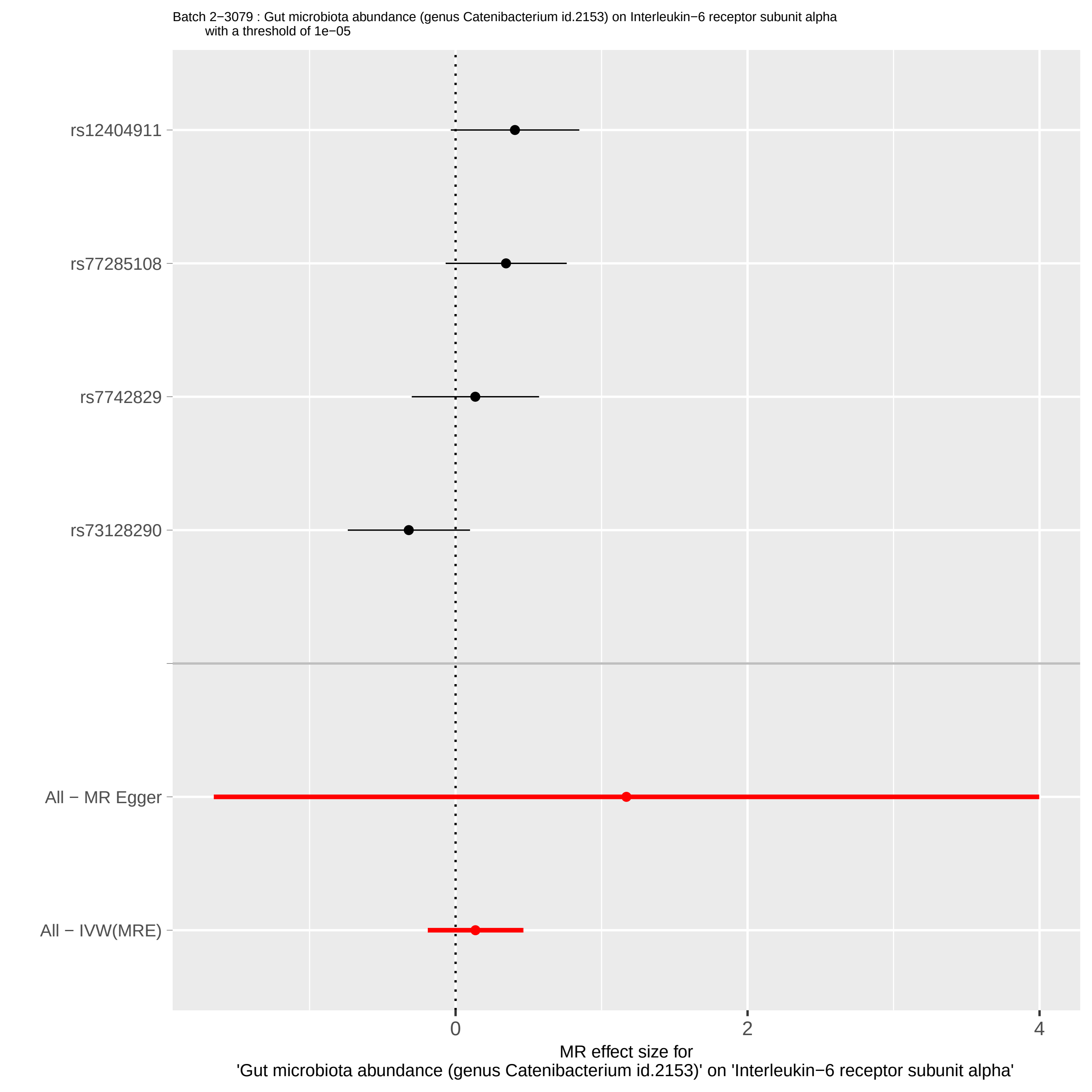

Batch 2−3079 : Gut microbiota abundance (genus Catenibacterium id.2153) on Interleukin−6 receptor subunit alpha
with a threshold of 1e−05
rs12404911
rs77285108
rs7742829
rs73128290
All − MR Egger
All − IVW(MRE)
0
2
4
MR effect size for
'Gut microbiota abundance (genus Catenibacterium id.2153)' on 'Interleukin−6 receptor subunit alpha'

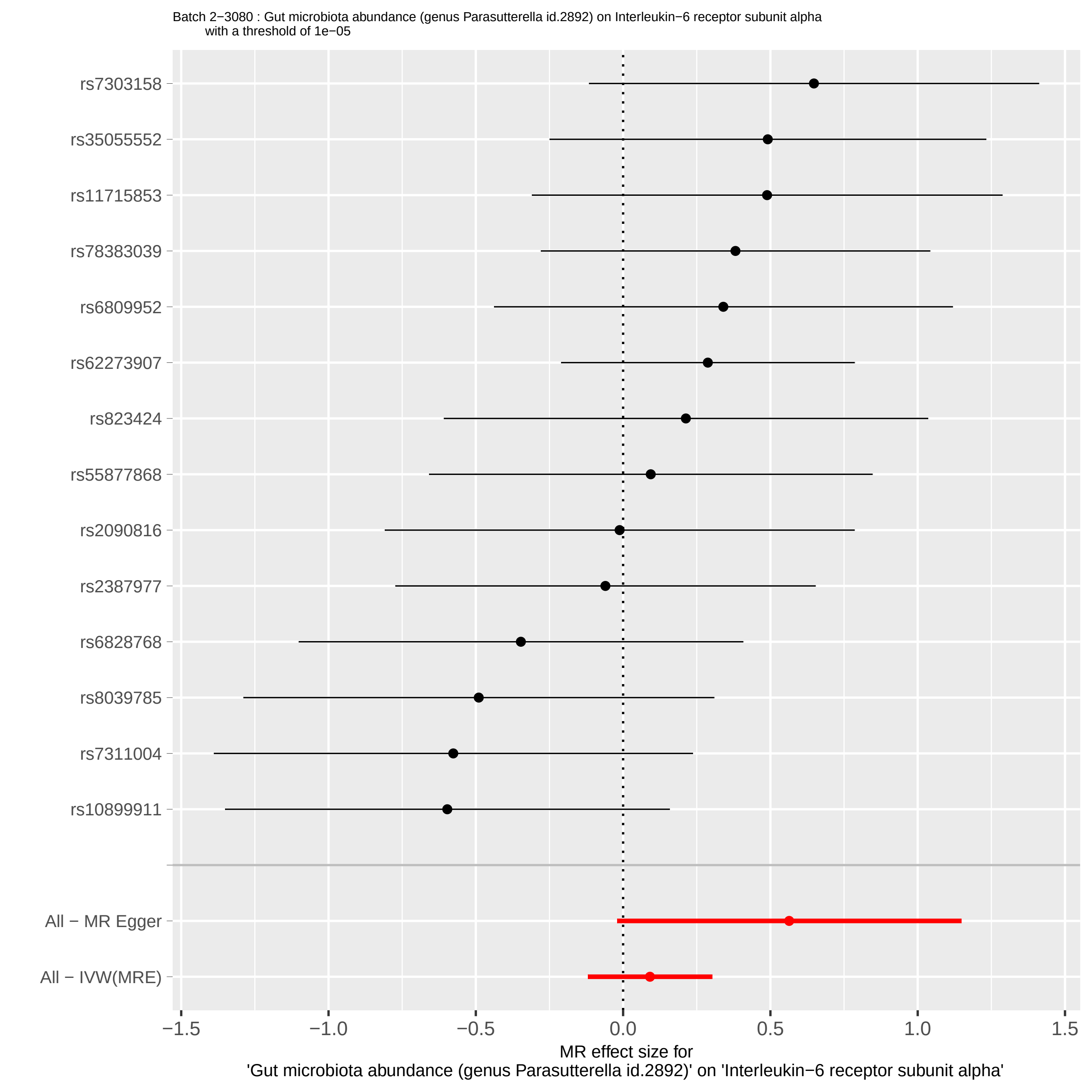

Batch 2−3080 : Gut microbiota abundance (genus Parasutterella id.2892) on Interleukin−6 receptor subunit alpha
with a threshold of 1e−05
rs7303158
rs35055552
rs11715853
rs78383039
rs6809952
rs62273907
rs823424
rs55877868
rs2090816
rs2387977
rs6828768
rs8039785
rs7311004
rs10899911
All − MR Egger
All − IVW(MRE)
−1.5
−1.0
−0.5
0.0
0.5
1.0
1.5
MR effect size for
'Gut microbiota abundance (genus Parasutterella id.2892)' on 'Interleukin−6 receptor subunit alpha'

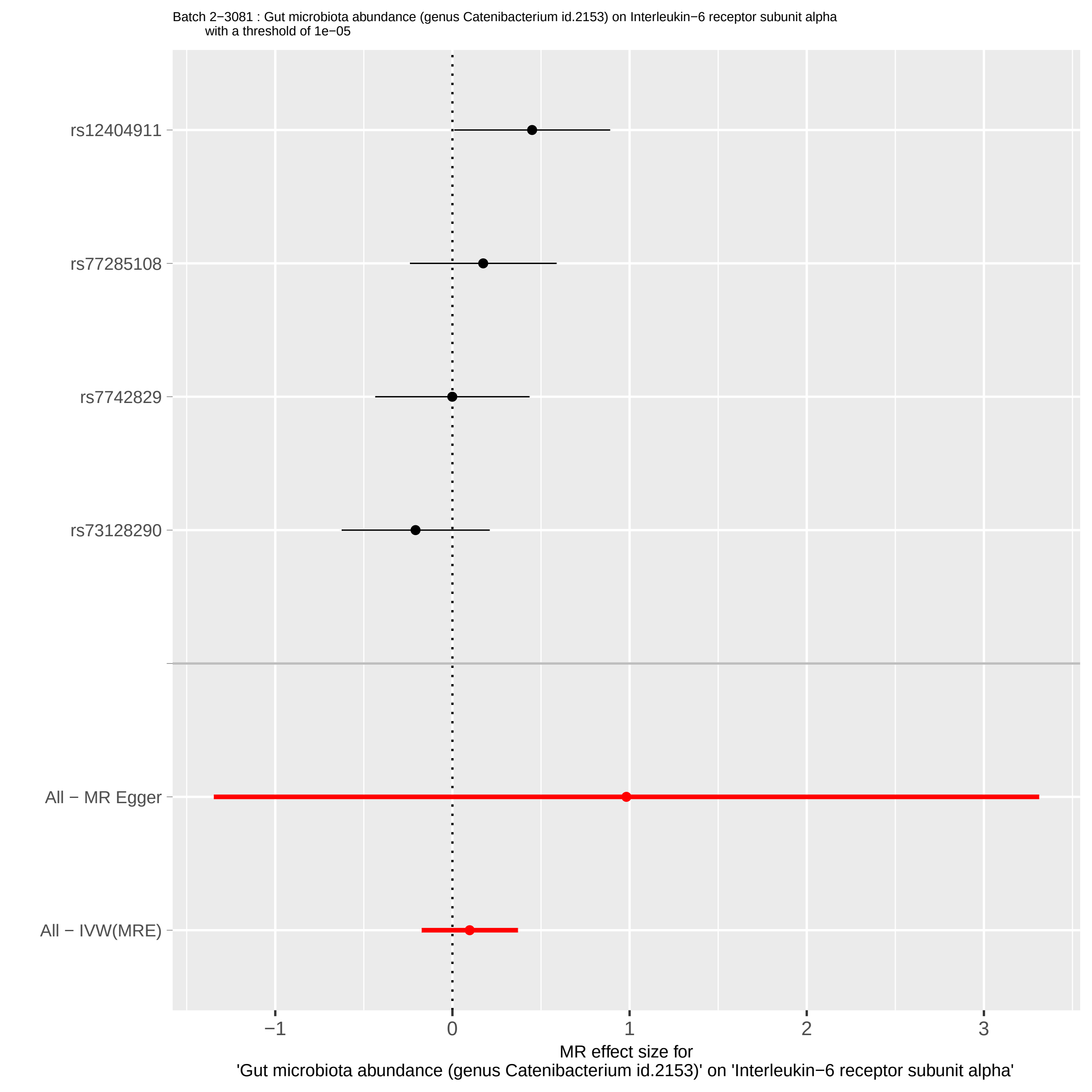
Batch 2−3081 : Gut microbiota abundance (genus Catenibacterium id.2153) on Interleukin−6 receptor subunit alpha
with a threshold of 1e−05
rs12404911
rs77285108
rs7742829
rs73128290
All − MR Egger
All − IVW(MRE)
−1
0
1
2
3
MR effect size for
'Gut microbiota abundance (genus Catenibacterium id.2153)' on 'Interleukin−6 receptor subunit alpha'

Batch 2−3082 : Gut microbiota abundance (genus Parasutterella id.2892) on Interleukin−6 receptor subunit alpha with a threshold of 1e−05

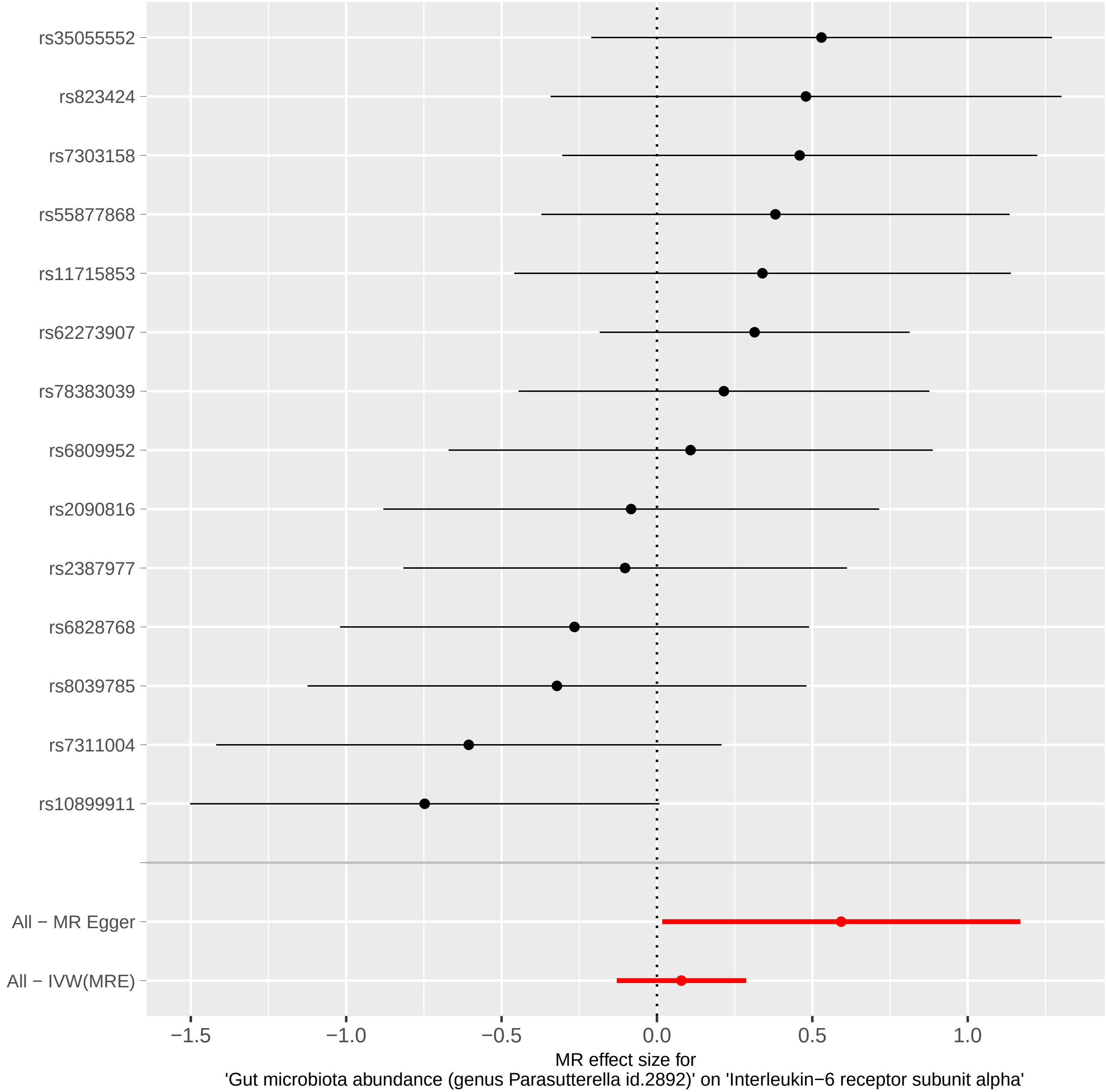

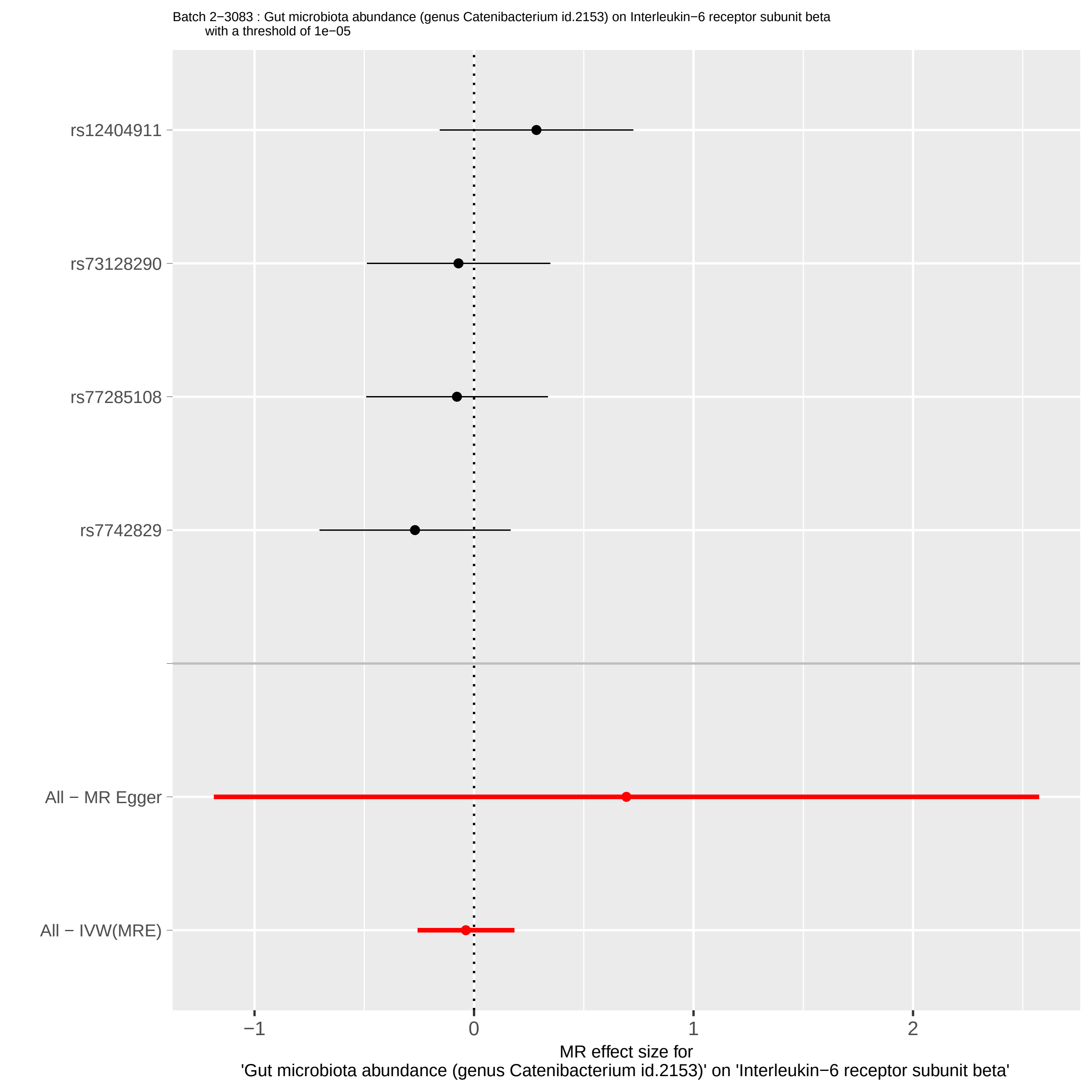

Batch 2−3083 : Gut microbiota abundance (genus Catenibacterium id.2153) on Interleukin−6 receptor subunit beta
with a threshold of 1e−05
rs12404911
rs73128290
rs77285108
rs7742829
All − MR Egger
All − IVW(MRE)
−1
0
1
2
MR effect size for
'Gut microbiota abundance (genus Catenibacterium id.2153)' on 'Interleukin−6 receptor subunit beta'

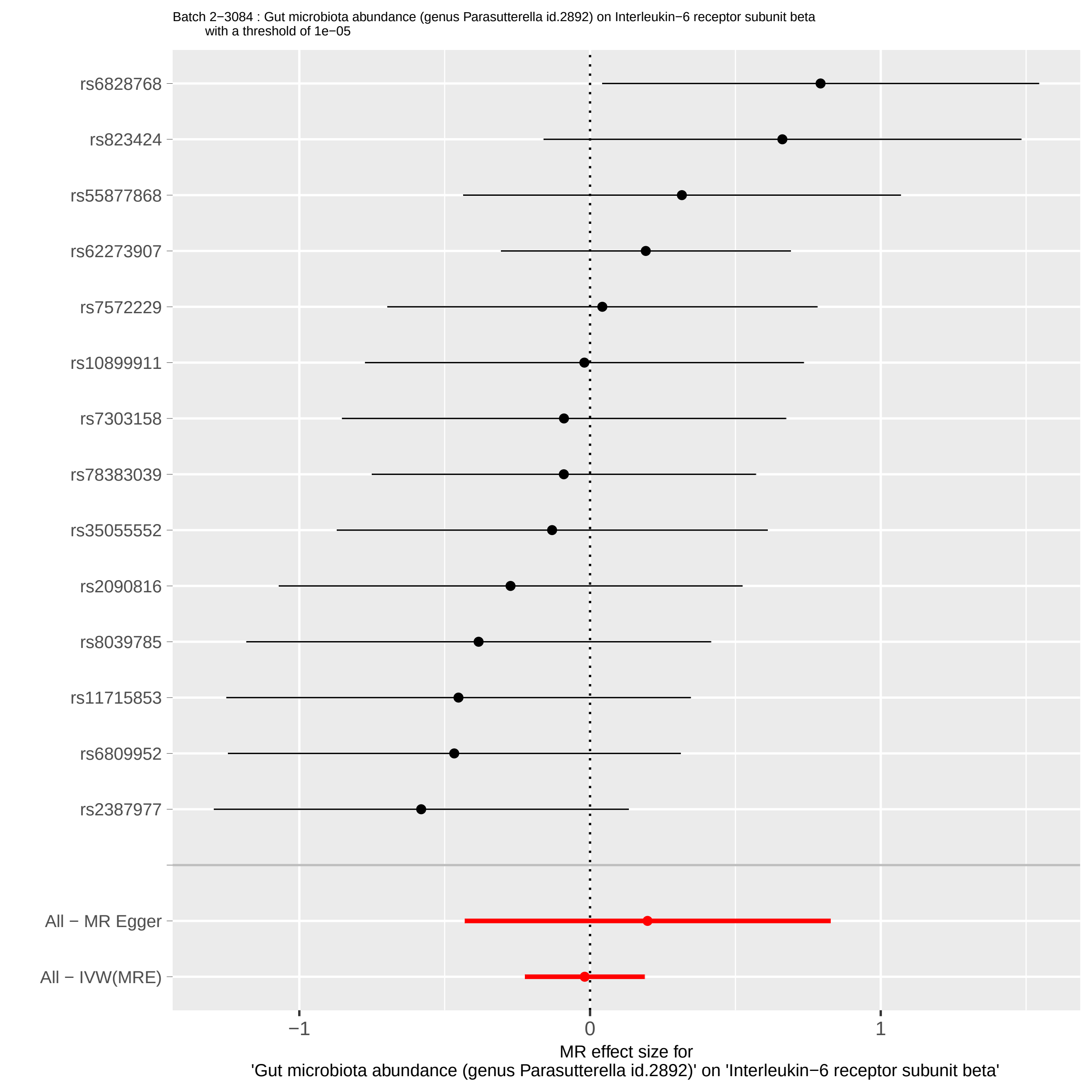
Batch 2−3084 : Gut microbiota abundance (genus Parasutterella id.2892) on Interleukin−6 receptor subunit beta
with a threshold of 1e−05
rs6828768
rs823424
rs55877868
rs62273907
rs7572229
rs10899911
rs7303158
rs78383039
rs35055552
rs2090816
rs8039785
rs11715853
rs6809952
rs2387977
All − MR Egger
All − IVW(MRE)
−1
0
1
MR effect size for
'Gut microbiota abundance (genus Parasutterella id.2892)' on 'Interleukin−6 receptor subunit beta'

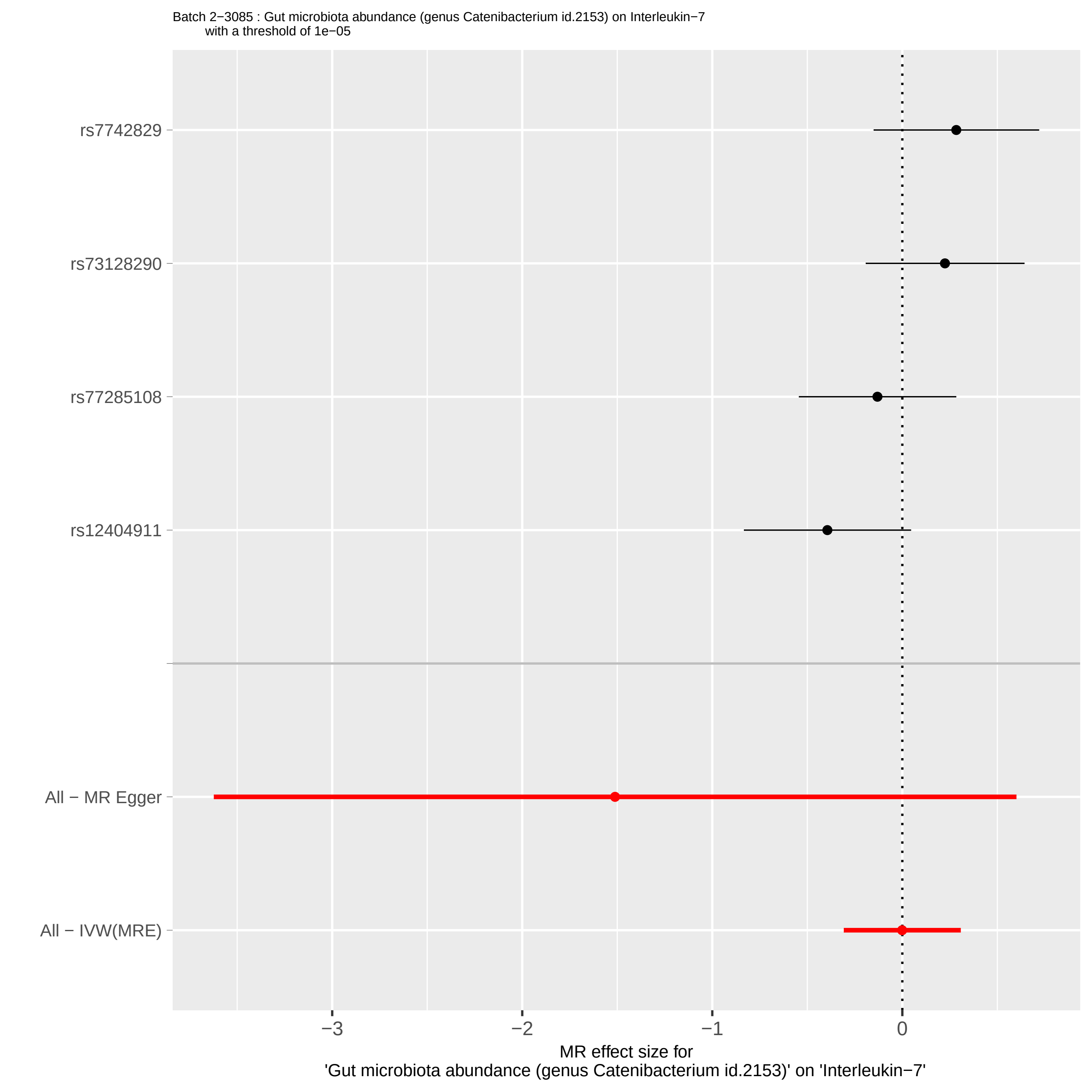
Batch 2−3085 : Gut microbiota abundance (genus Catenibacterium id.2153) on Interleukin−7
with a threshold of 1e−05
rs7742829
rs73128290
rs77285108
rs12404911
All − MR Egger
All − IVW(MRE)
−3
−2
−1
0
MR effect size for
'Gut microbiota abundance (genus Catenibacterium id.2153)' on 'Interleukin−7'

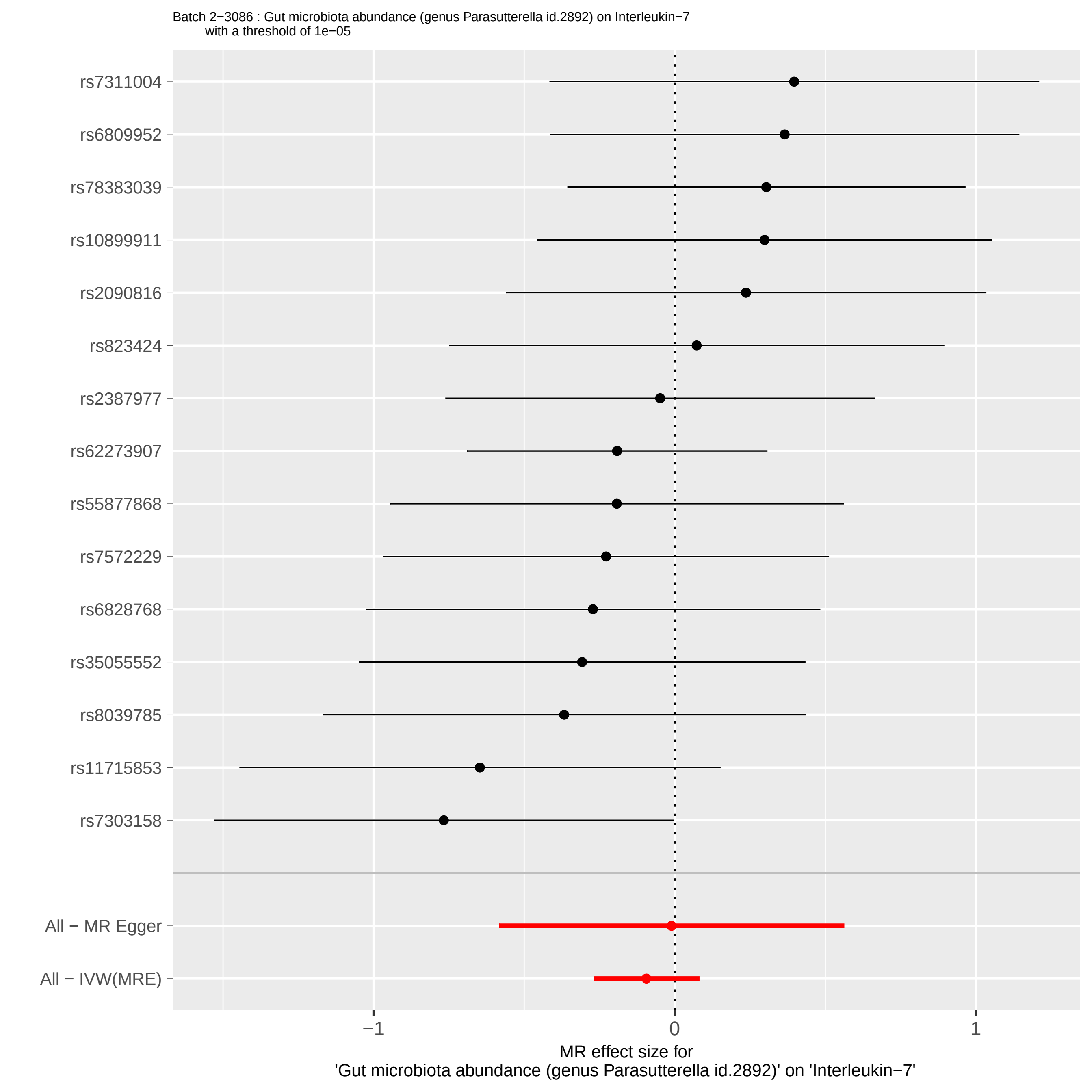
Batch 2−3086 : Gut microbiota abundance (genus Parasutterella id.2892) on Interleukin−7
with a threshold of 1e−05
rs7311004
rs6809952
rs78383039
rs10899911
rs2090816
rs823424
rs2387977
rs62273907
rs55877868
rs7572229
rs6828768
rs35055552
rs8039785
rs11715853
rs7303158
All − MR Egger
All − IVW(MRE)
−1
0
1
MR effect size for
'Gut microbiota abundance (genus Parasutterella id.2892)' on 'Interleukin−7'

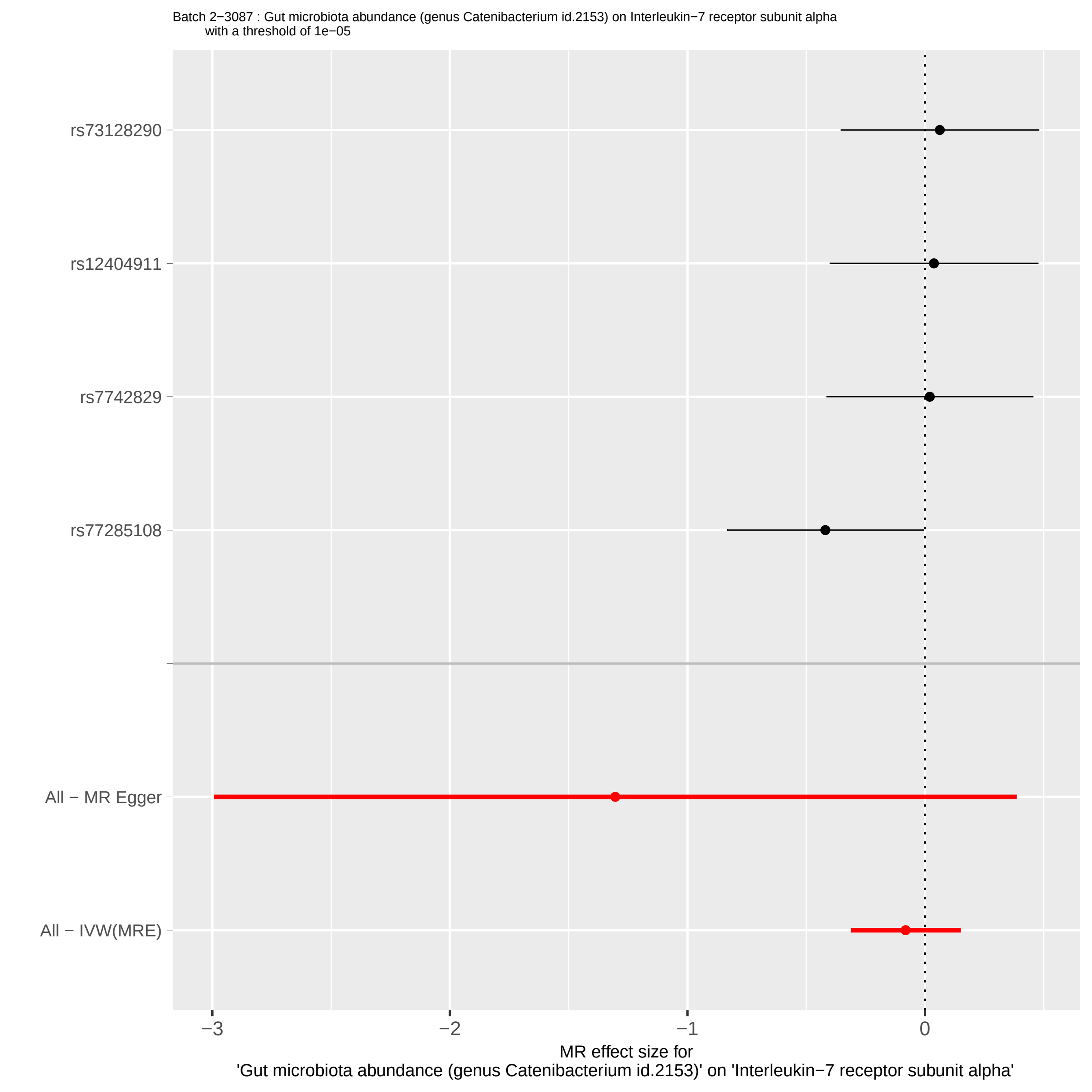

Batch 2−3087 : Gut microbiota abundance (genus Catenibacterium id.2153) on Interleukin−7 receptor subunit alpha
with a threshold of 1e−05
rs73128290
rs12404911
rs7742829
rs77285108
All − MR Egger
All − IVW(MRE)
−3
−2
−1
0
MR effect size for
'Gut microbiota abundance (genus Catenibacterium id.2153)' on 'Interleukin−7 receptor subunit alpha'

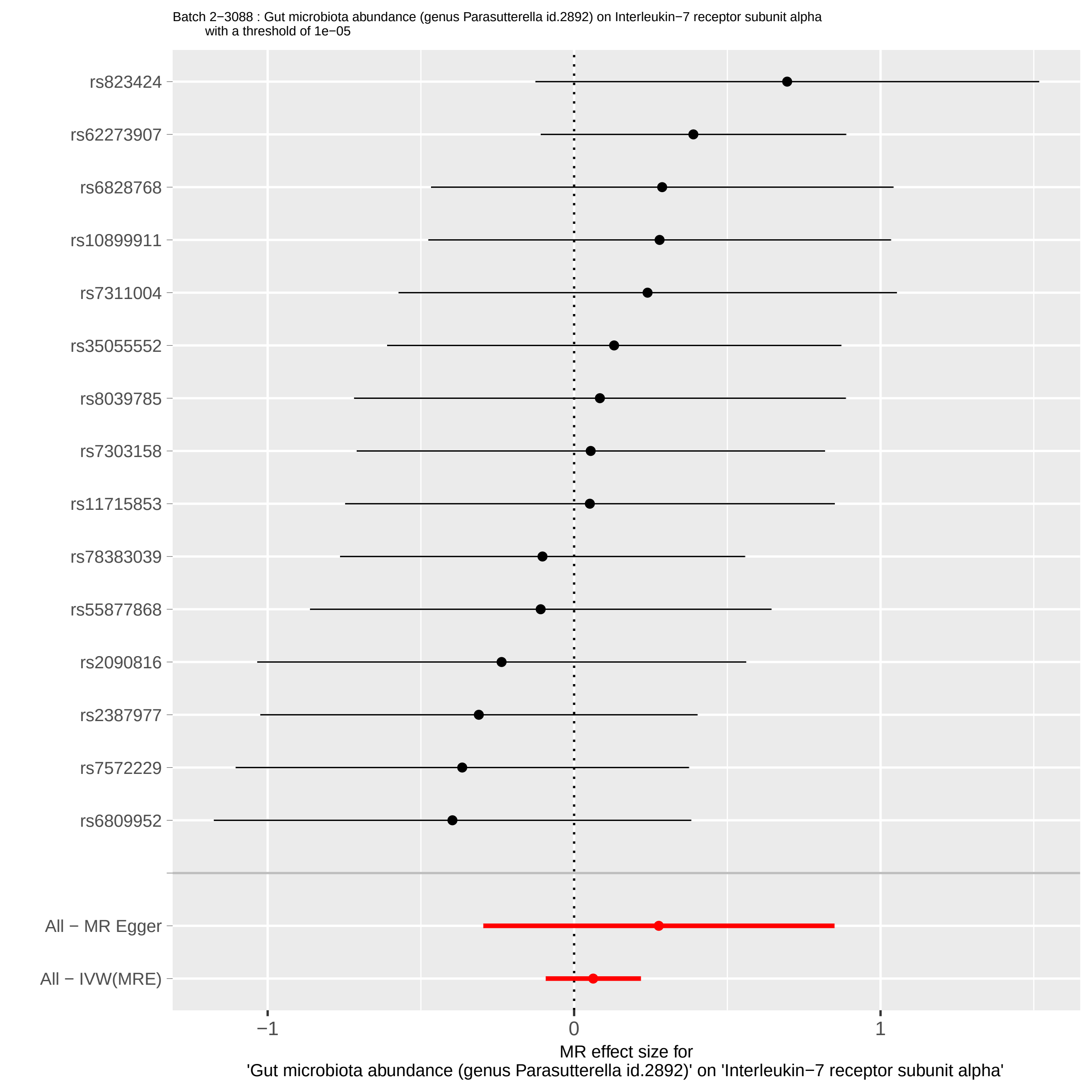
Batch 2−3088 : Gut microbiota abundance (genus Parasutterella id.2892) on Interleukin−7 receptor subunit alpha
with a threshold of 1e−05
rs823424
rs62273907
rs6828768
rs10899911
rs7311004
rs35055552
rs8039785
rs7303158
rs11715853
rs78383039
rs55877868
rs2090816
rs2387977
rs7572229
rs6809952
All − MR Egger
All − IVW(MRE)
−1
0
1
MR effect size for
'Gut microbiota abundance (genus Parasutterella id.2892)' on 'Interleukin−7 receptor subunit alpha'

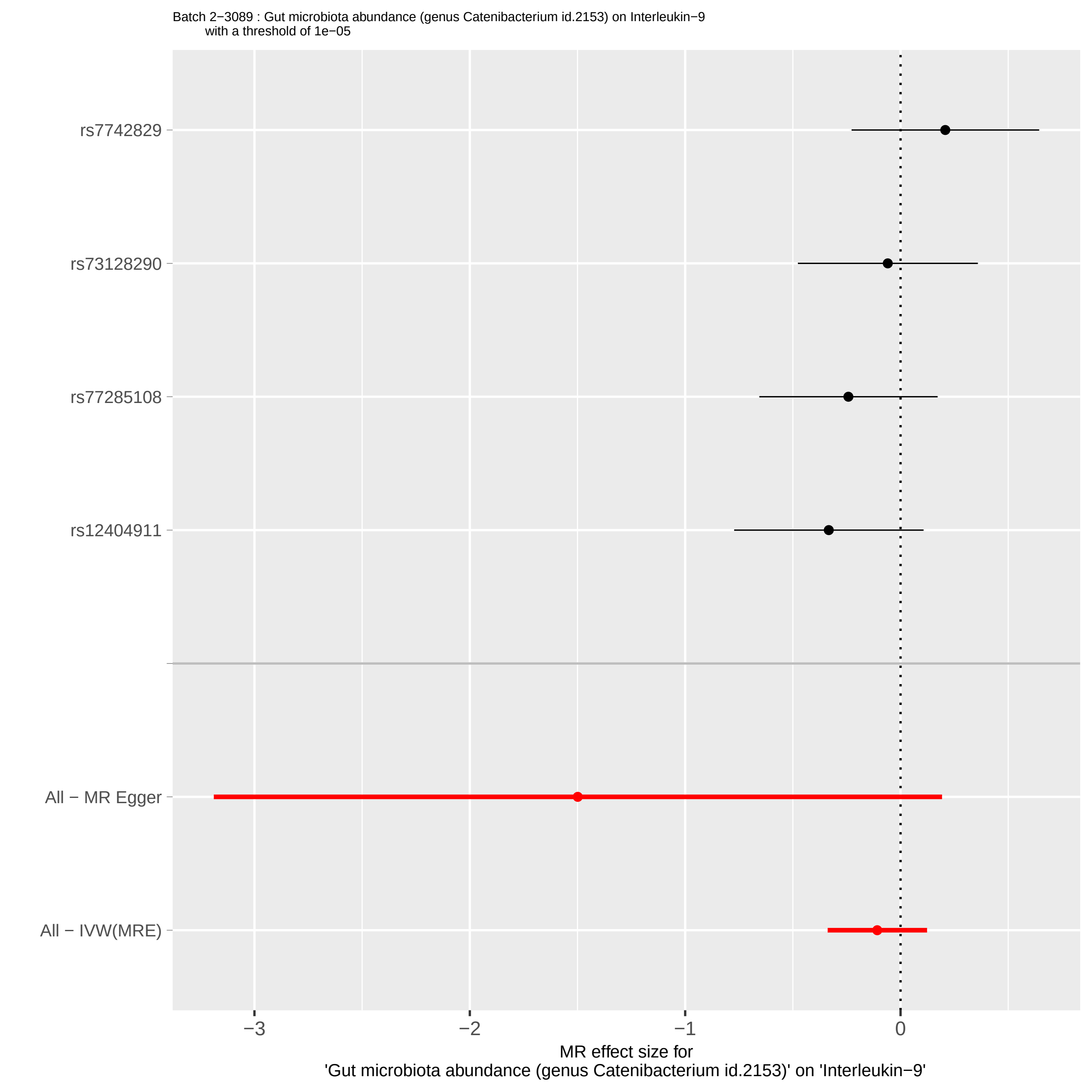

Batch 2−3089 : Gut microbiota abundance (genus Catenibacterium id.2153) on Interleukin−9
with a threshold of 1e−05
rs7742829
rs73128290
rs77285108
rs12404911
All − MR Egger
All − IVW(MRE)
−3
−2
−1
0
MR effect size for
'Gut microbiota abundance (genus Catenibacterium id.2153)' on 'Interleukin−9'

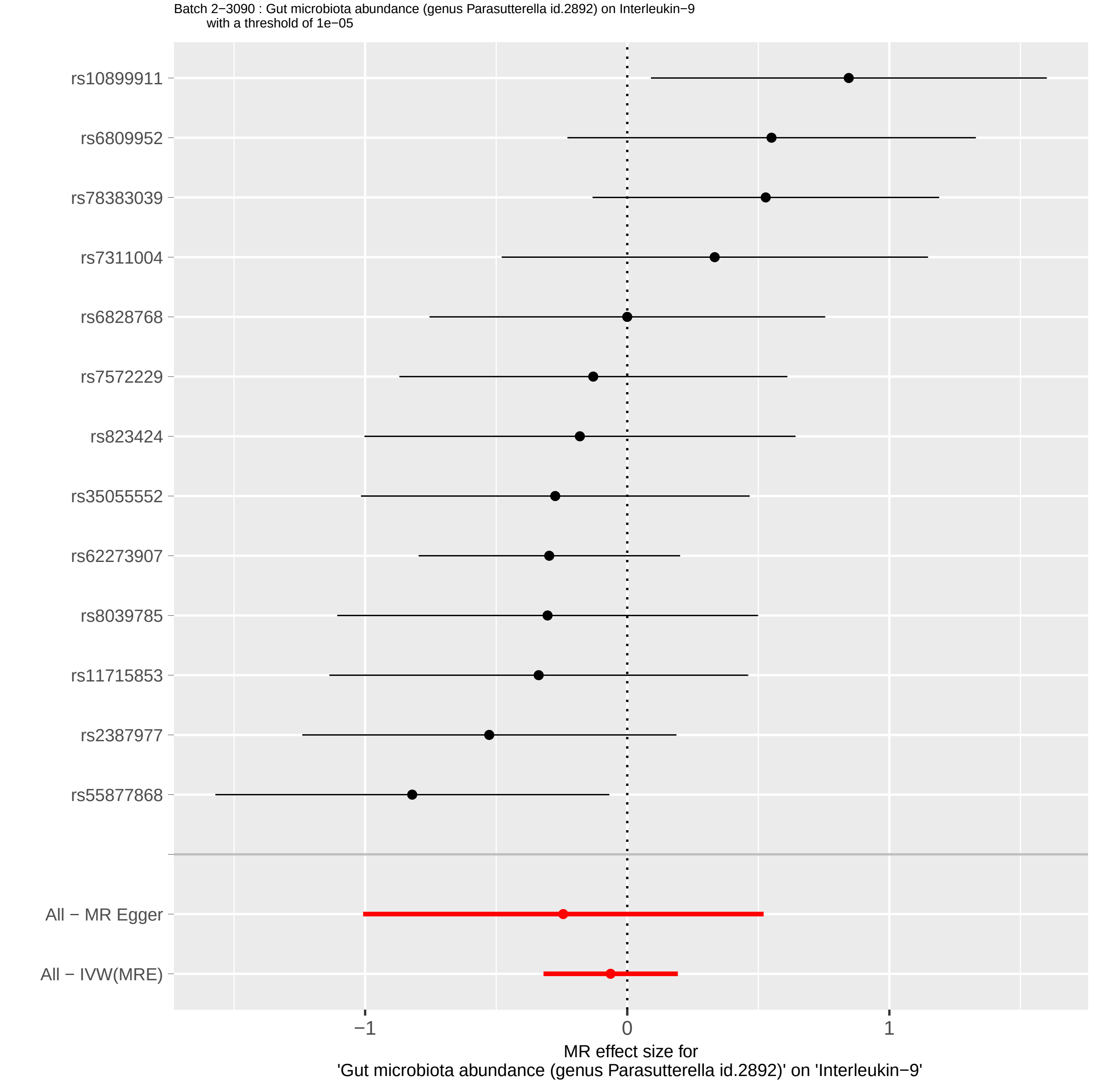

Batch 2−3090 : Gut microbiota abundance (genus Parasutterella id.2892) on Interleukin−9
with a threshold of 1e−05
rs10899911
rs6809952
rs78383039
rs7311004
rs6828768
rs7572229
rs823424
rs35055552
rs62273907
rs8039785
rs11715853
rs2387977
rs55877868
All − MR Egger
All − IVW(MRE)
−1
0
1
MR effect size for
'Gut microbiota abundance (genus Parasutterella id.2892)' on 'Interleukin−9'

Batch 2−3091 : Gut microbiota abundance (genus Catenibacterium id.2153) on Interleukin−9
with a threshold of 1e−05

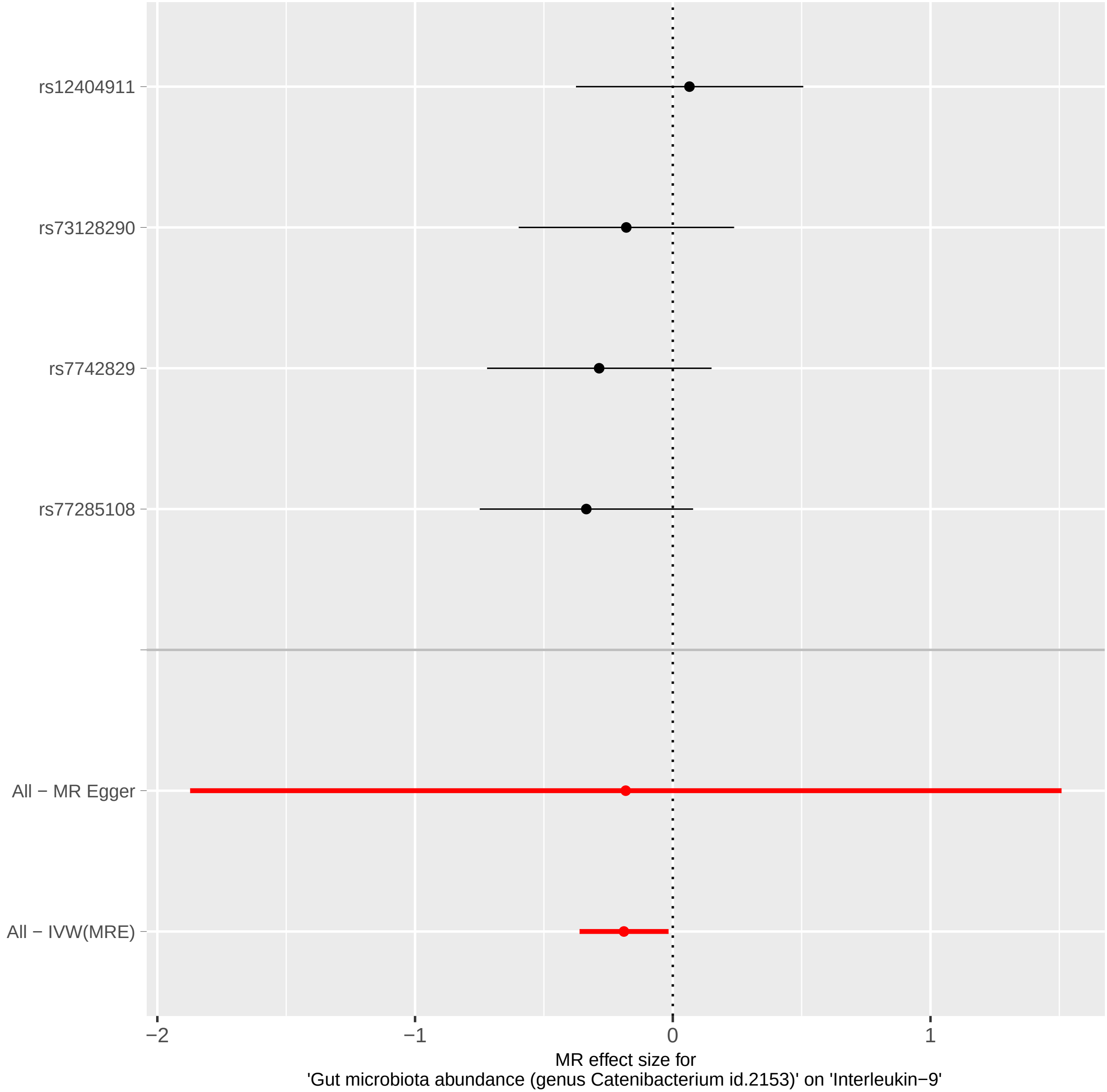

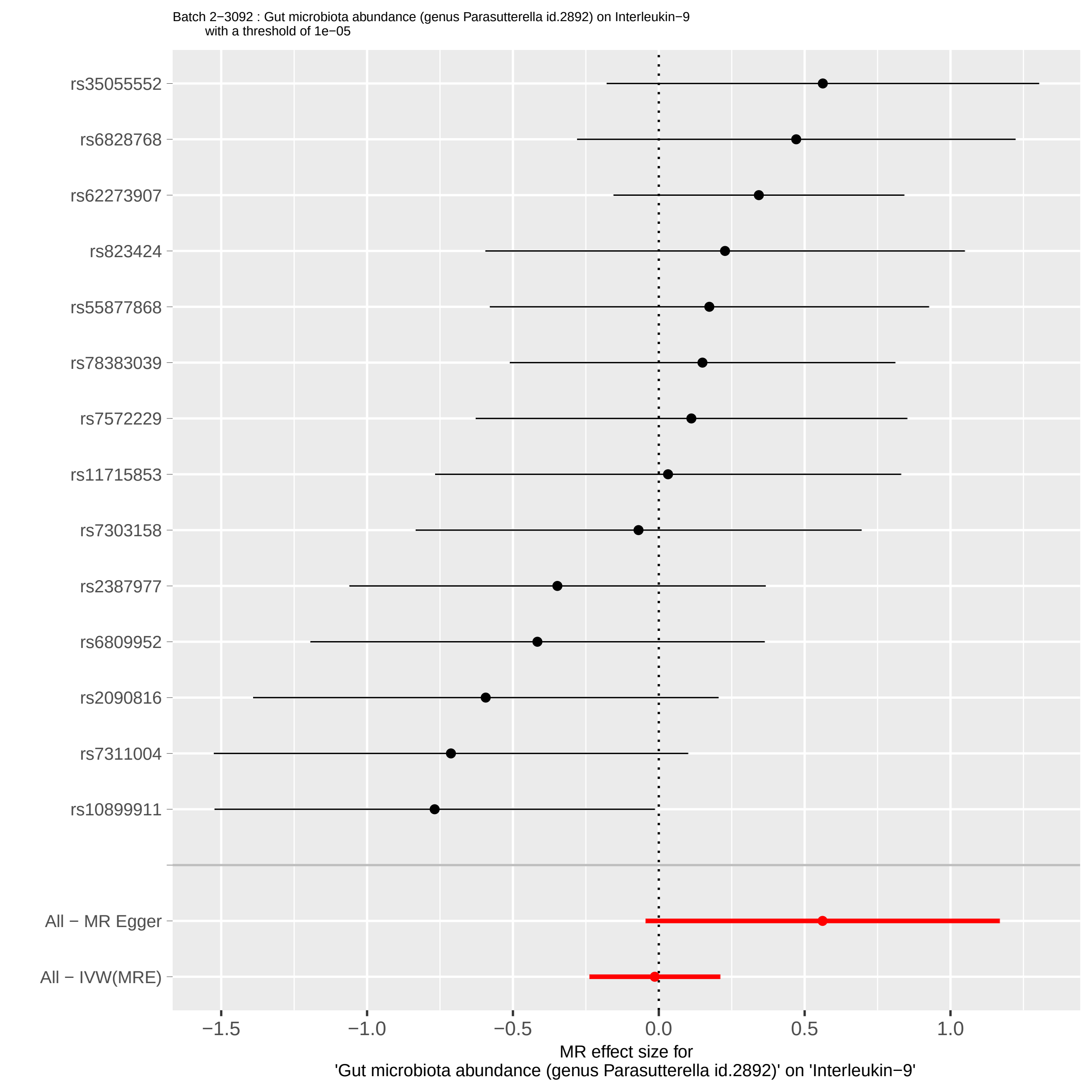

Batch 2−3092 : Gut microbiota abundance (genus Parasutterella id.2892) on Interleukin−9
with a threshold of 1e−05
rs35055552
rs6828768
rs62273907
rs823424
rs55877868
rs78383039
rs7572229
rs11715853
rs7303158
rs2387977
rs6809952
rs2090816
rs7311004
rs10899911
All − MR Egger
All − IVW(MRE)
−1.5
−1.0
−0.5
0.0
0.5
1.0
MR effect size for
'Gut microbiota abundance (genus Parasutterella id.2892)' on 'Interleukin−9'

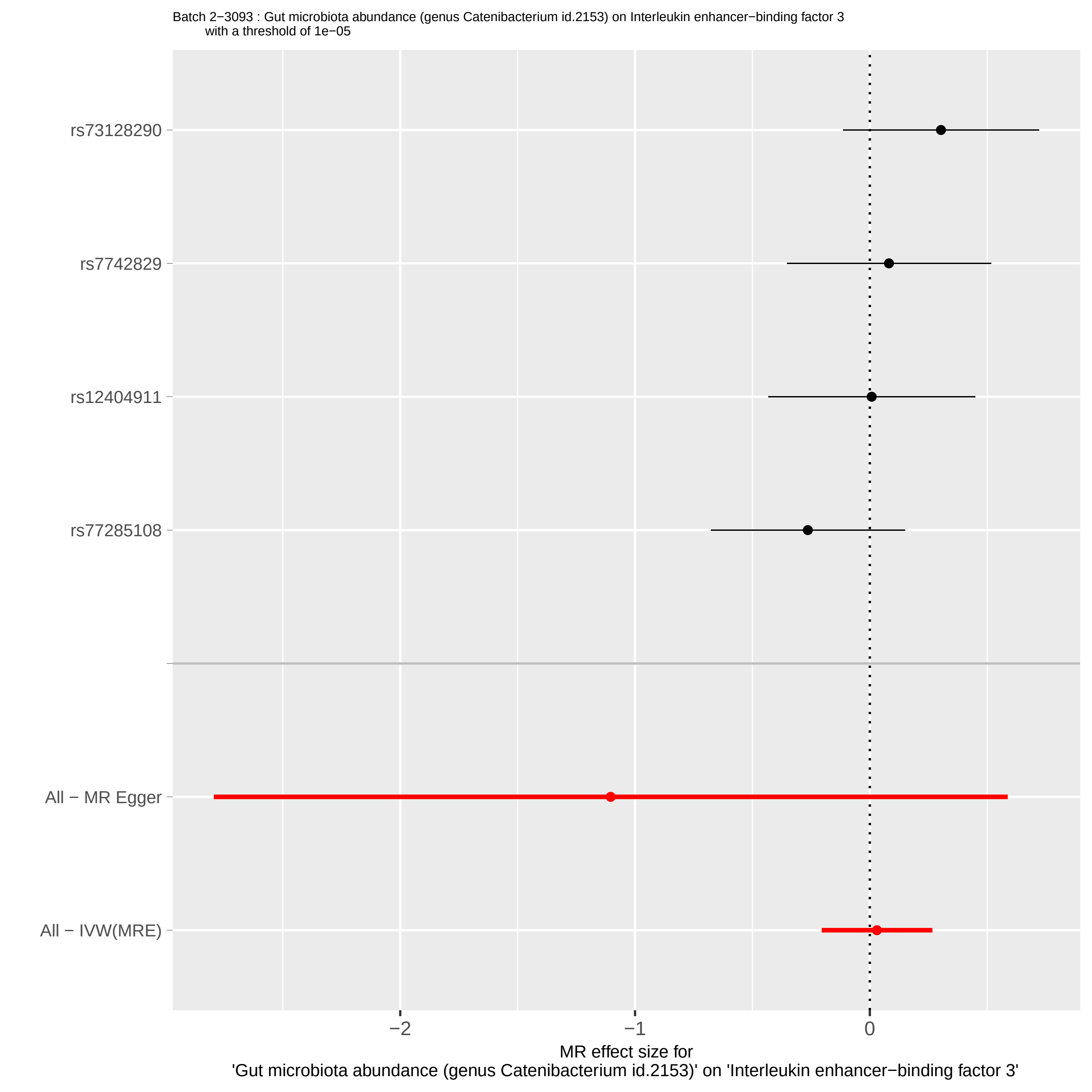
Batch 2−3093 : Gut microbiota abundance (genus Catenibacterium id.2153) on Interleukin enhancer−binding factor 3
with a threshold of 1e−05
rs73128290
rs7742829
rs12404911
rs77285108
All − MR Egger
All − IVW(MRE)
−2
−1
0
MR effect size for
'Gut microbiota abundance (genus Catenibacterium id.2153)' on 'Interleukin enhancer−binding factor 3'

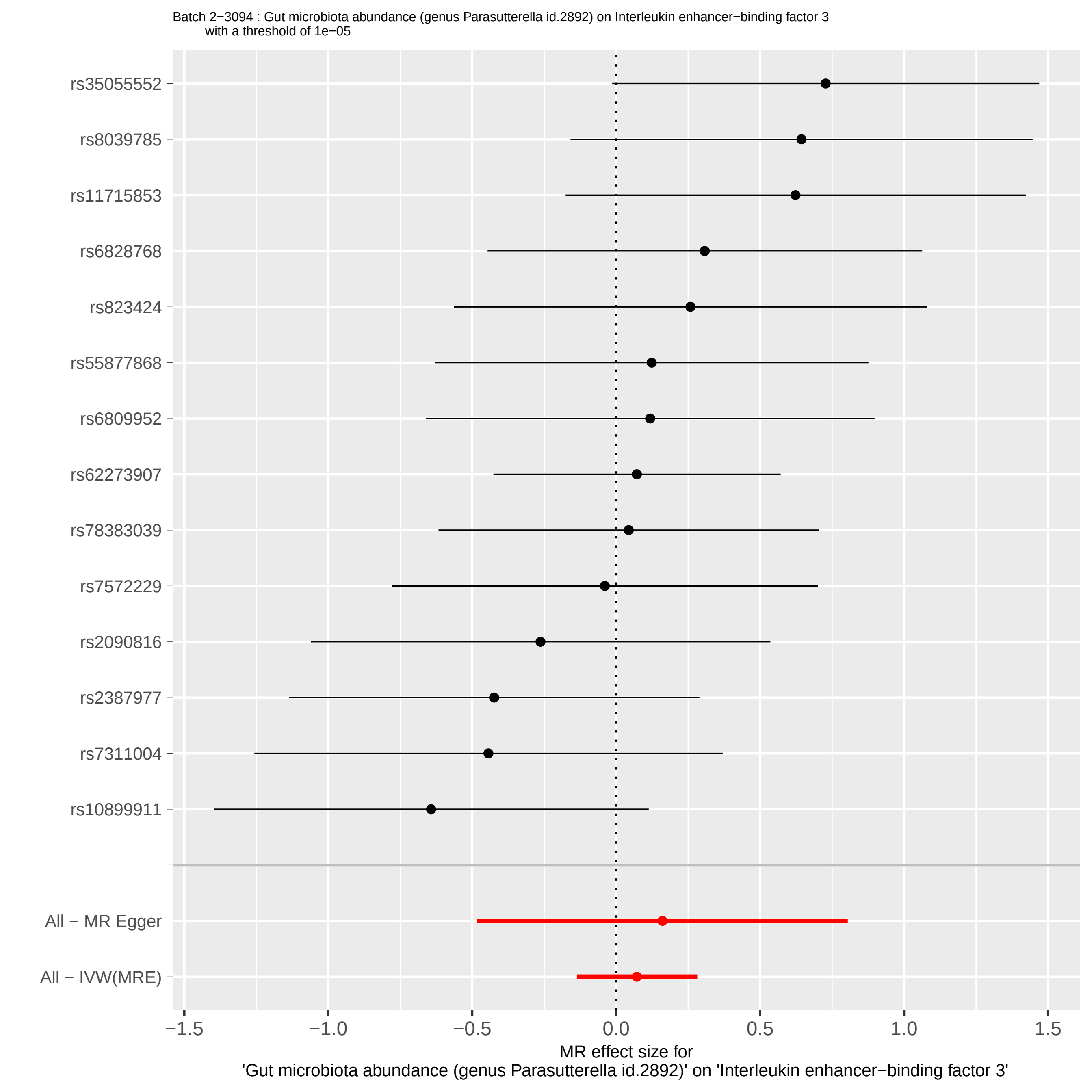
Batch 2−3094 : Gut microbiota abundance (genus Parasutterella id.2892) on Interleukin enhancer−binding factor 3
with a threshold of 1e−05
rs35055552
rs8039785
rs11715853
rs6828768
rs823424
rs55877868
rs6809952
rs62273907
rs78383039
rs7572229
rs2090816
rs2387977
rs7311004
rs10899911
All − MR Egger
All − IVW(MRE)
−1.5
−1.0
−0.5
0.0
0.5
1.0
1.5
MR effect size for
'Gut microbiota abundance (genus Parasutterella id.2892)' on 'Interleukin enhancer−binding factor 3'

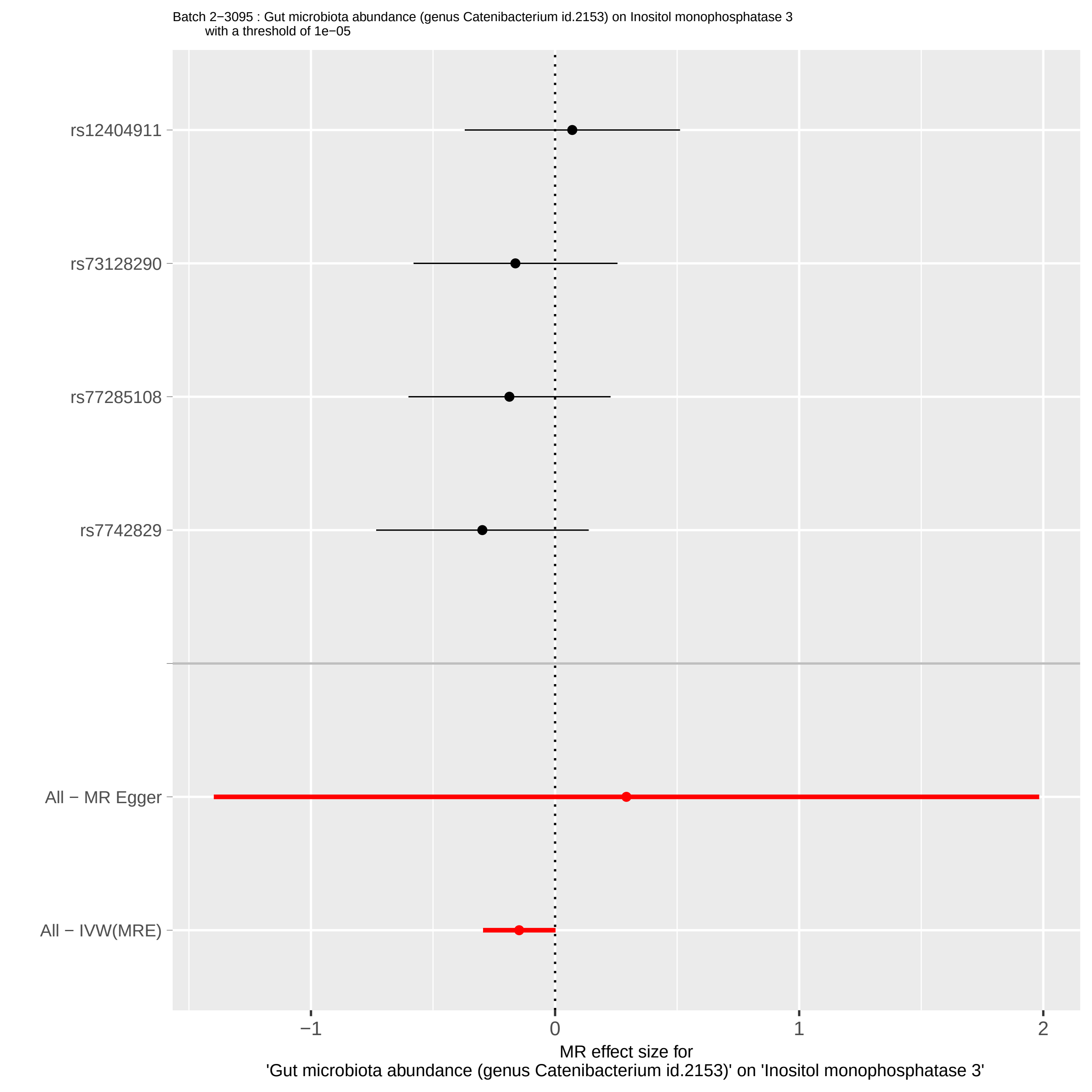
Batch 2−3095 : Gut microbiota abundance (genus Catenibacterium id.2153) on Inositol monophosphatase 3
with a threshold of 1e−05
rs12404911
rs73128290
rs77285108
rs7742829
All − MR Egger
All − IVW(MRE)
−1
0
1
2
MR effect size for
'Gut microbiota abundance (genus Catenibacterium id.2153)' on 'Inositol monophosphatase 3'

Batch 2−3096 : Gut microbiota abundance (genus Parasutterella id.2892) on Inositol monophosphatase 3
with a threshold of 1e−05

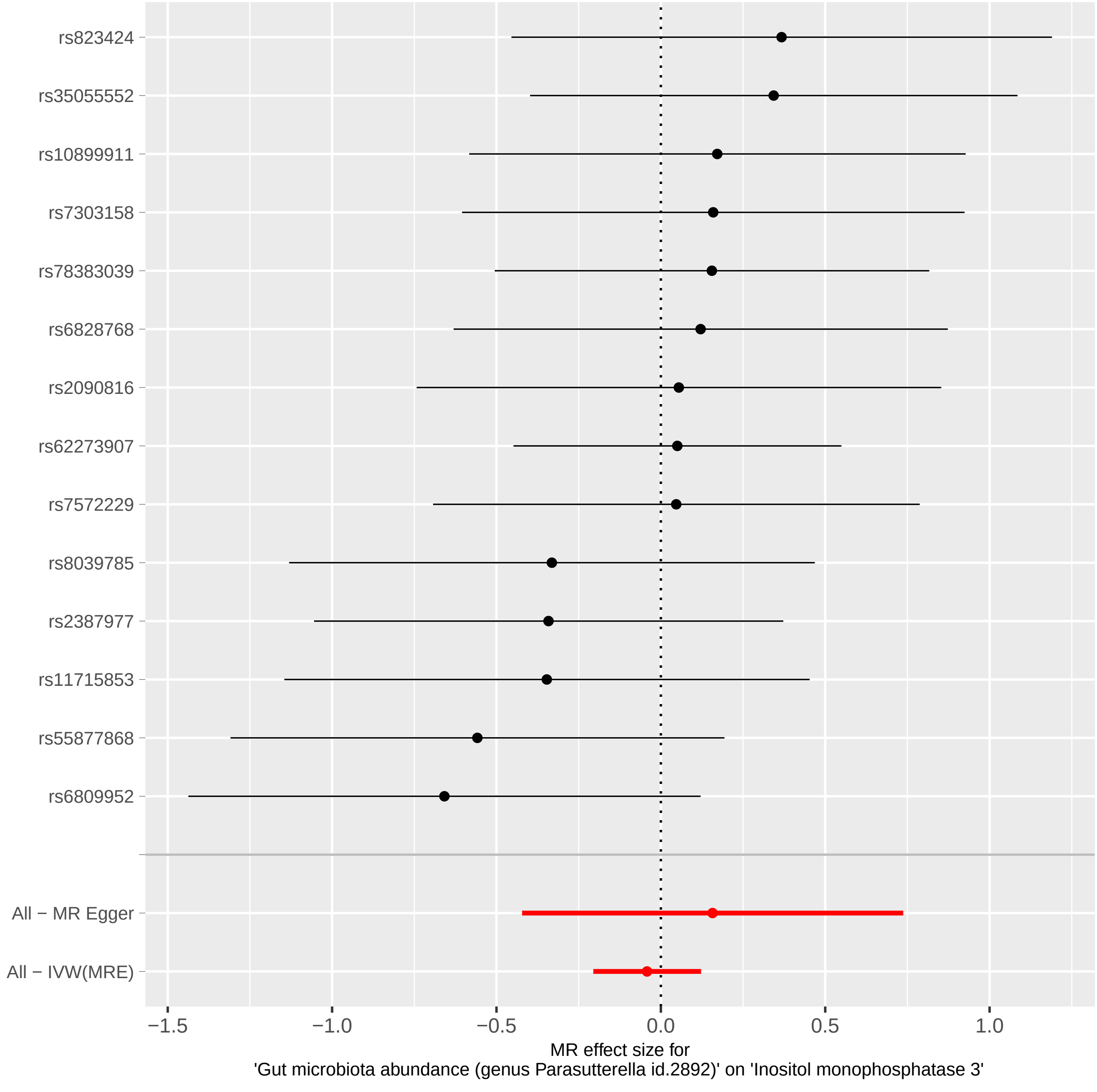

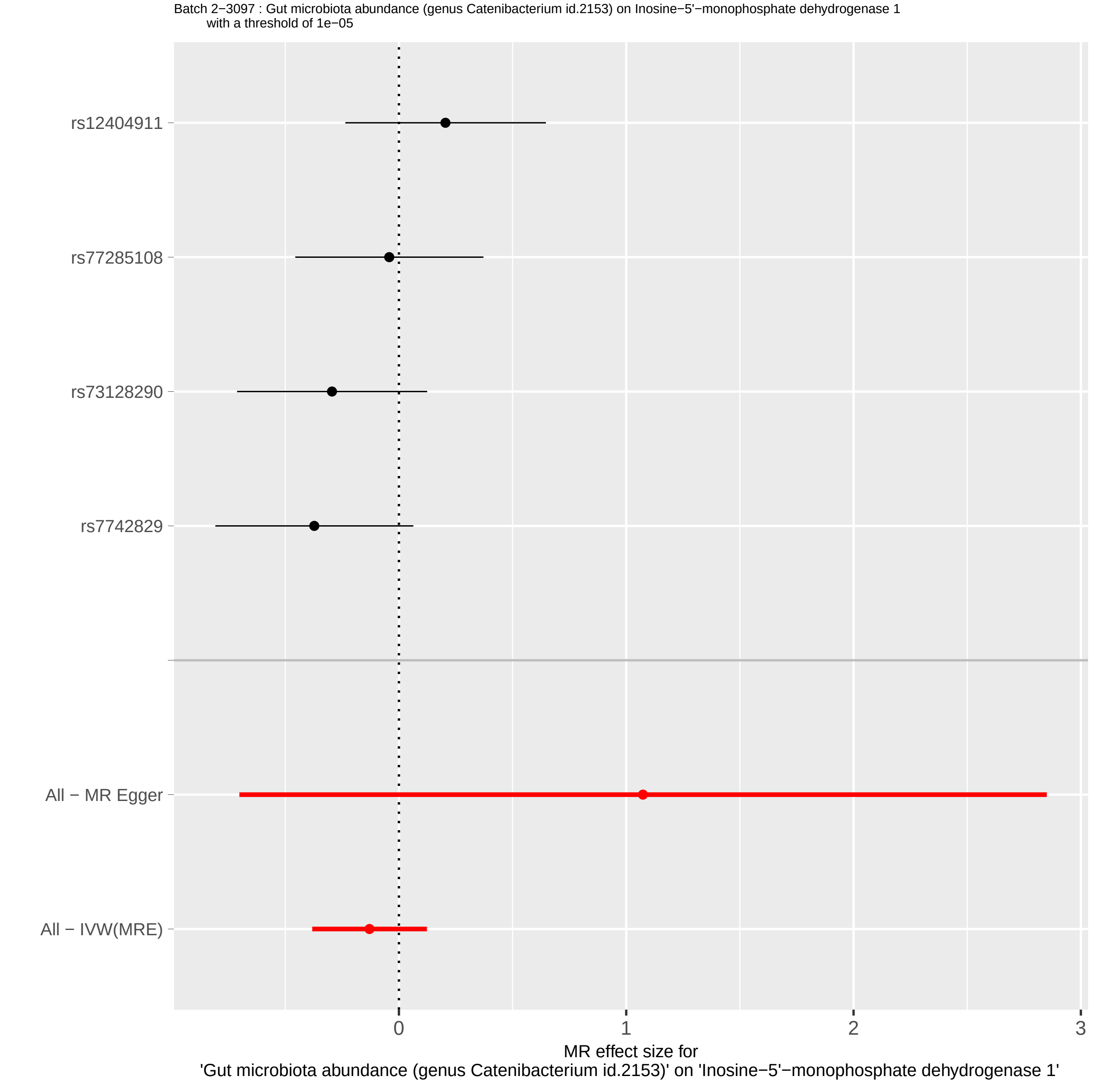

Batch 2−3097 : Gut microbiota abundance (genus Catenibacterium id.2153) on Inosine−5'−monophosphate dehydrogenase 1
with a threshold of 1e−05
rs12404911
rs77285108
rs73128290
rs7742829
All − MR Egger
All − IVW(MRE)
0
1
2
3
MR effect size for
'Gut microbiota abundance (genus Catenibacterium id.2153)' on 'Inosine−5'−monophosphate dehydrogenase 1'

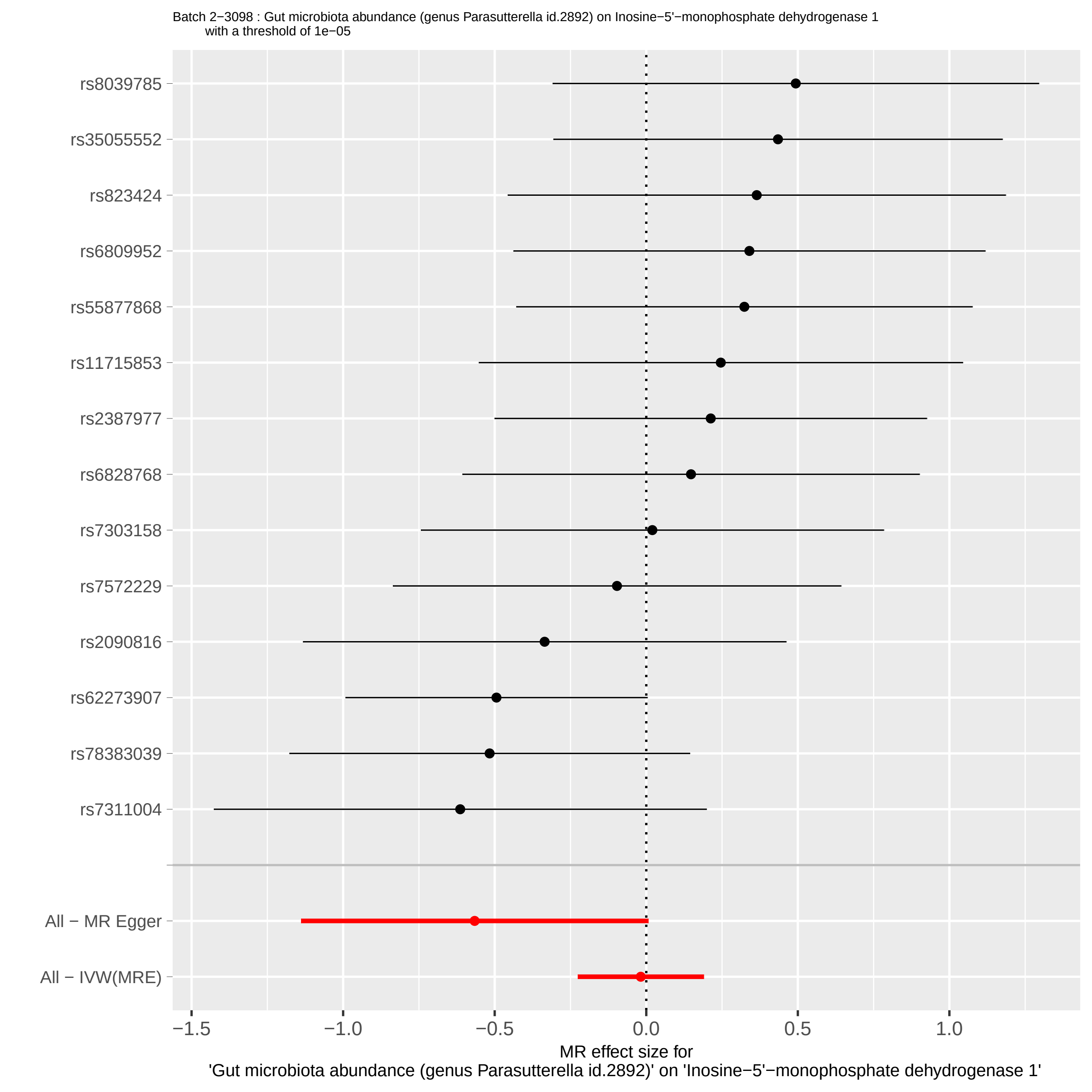

Batch 2−3098 : Gut microbiota abundance (genus Parasutterella id.2892) on Inosine−5'−monophosphate dehydrogenase 1
with a threshold of 1e−05
rs8039785
rs35055552
rs823424
rs6809952
rs55877868
rs11715853
rs2387977
rs6828768
rs7303158
rs7572229
rs2090816
rs62273907
rs78383039
rs7311004
All − MR Egger
All − IVW(MRE)
−1.5
−1.0
−0.5
0.0
0.5
1.0
MR effect size for
'Gut microbiota abundance (genus Parasutterella id.2892)' on 'Inosine−5'−monophosphate dehydrogenase 1'

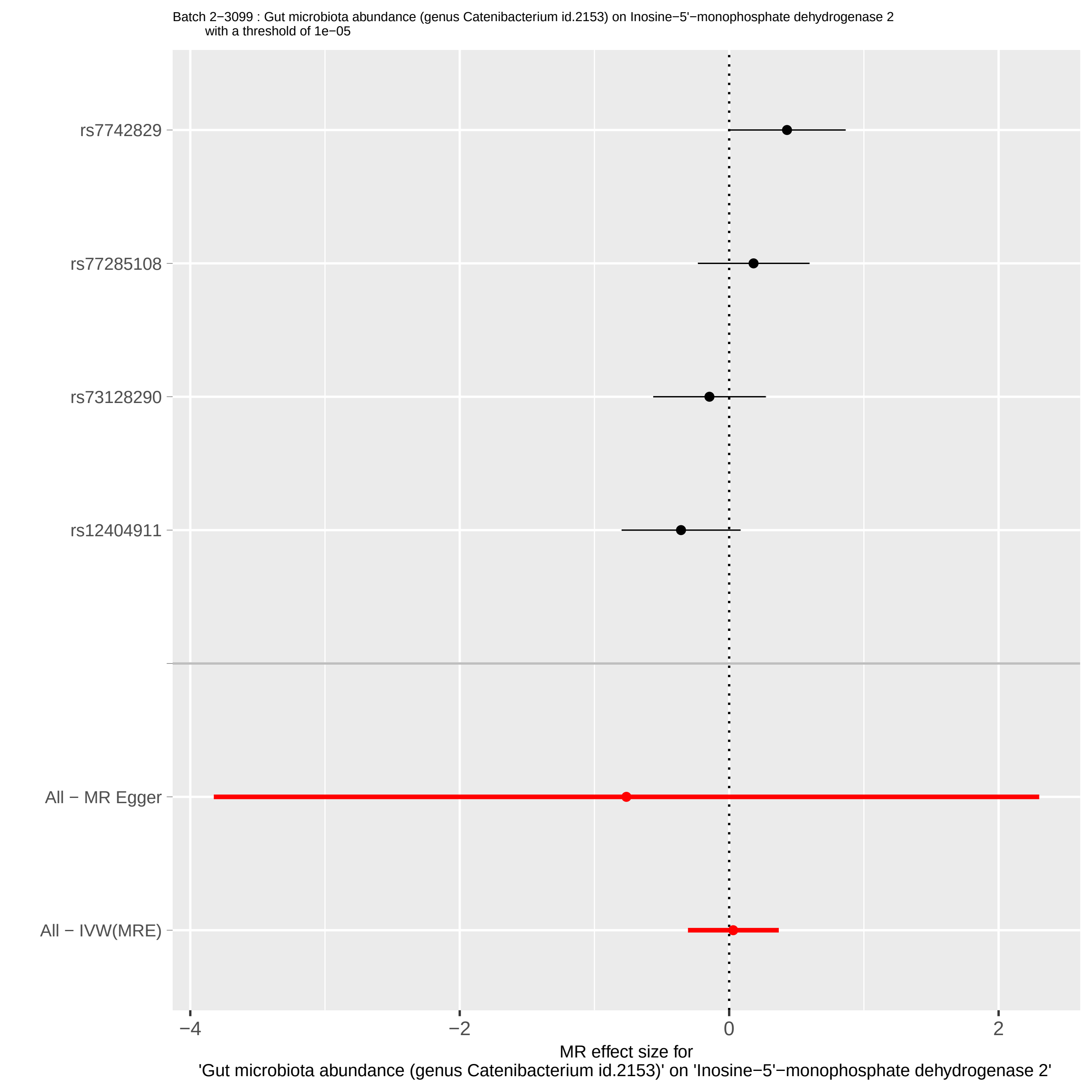
Batch 2−3099 : Gut microbiota abundance (genus Catenibacterium id.2153) on Inosine−5'−monophosphate dehydrogenase 2
with a threshold of 1e−05
rs7742829
rs77285108
rs73128290
rs12404911
All − MR Egger
All − IVW(MRE)
−4
−2
0
2
MR effect size for
'Gut microbiota abundance (genus Catenibacterium id.2153)' on 'Inosine−5'−monophosphate dehydrogenase 2'

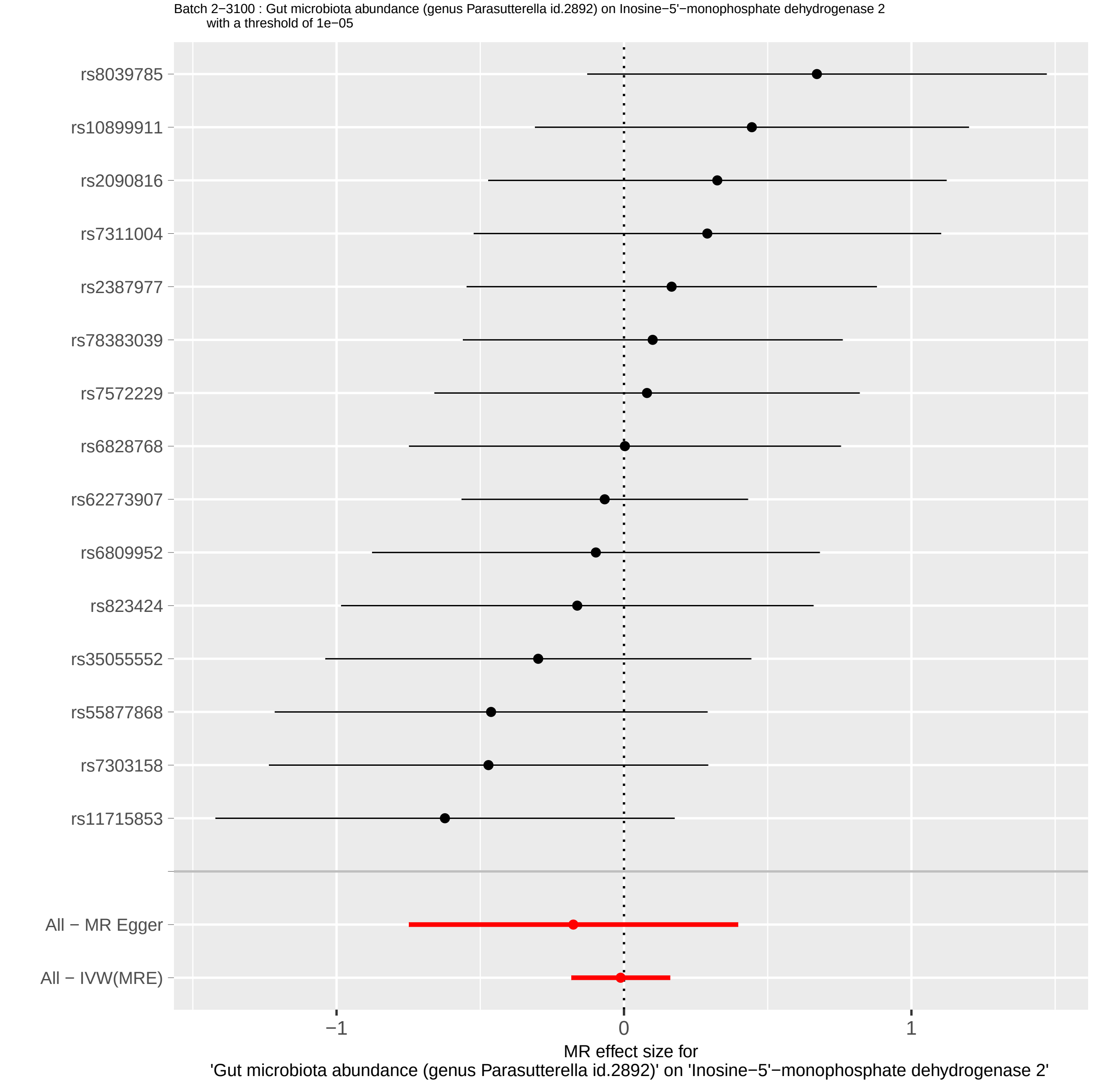

Batch 2−3100 : Gut microbiota abundance (genus Parasutterella id.2892) on Inosine−5'−monophosphate dehydrogenase 2
with a threshold of 1e−05
rs8039785
rs10899911
rs2090816
rs7311004
rs2387977
rs78383039
rs7572229
rs6828768
rs62273907
rs6809952
rs823424
rs35055552
rs55877868
rs7303158
rs11715853
All − MR Egger
All − IVW(MRE)
−1
0
1
MR effect size for
'Gut microbiota abundance (genus Parasutterella id.2892)' on 'Inosine−5'−monophosphate dehydrogenase 2'

Batch 2−3101 : Gut microbiota abundance (genus Catenibacterium id.2153) on Alpha−internexin
with a threshold of 1e−05

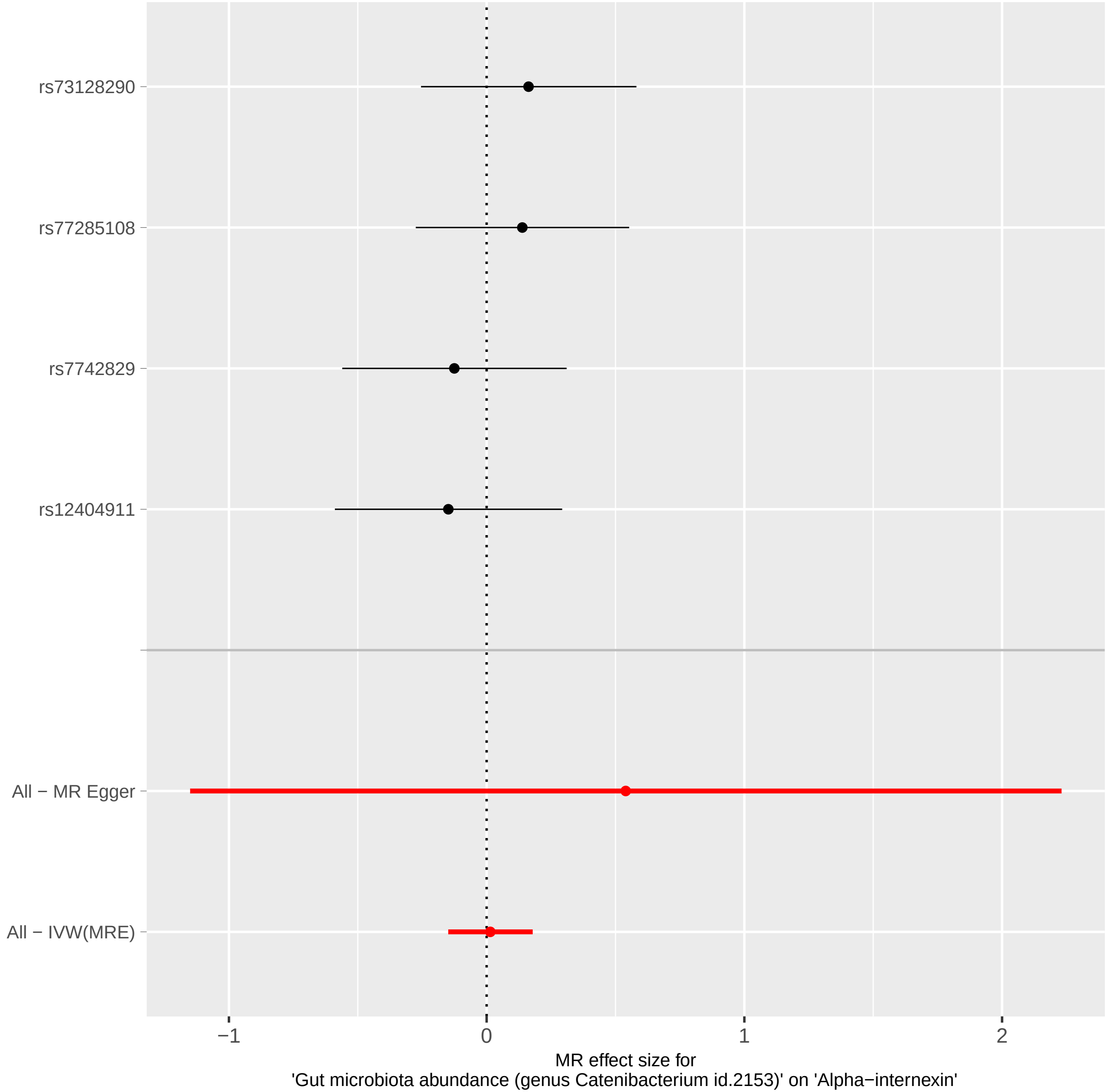

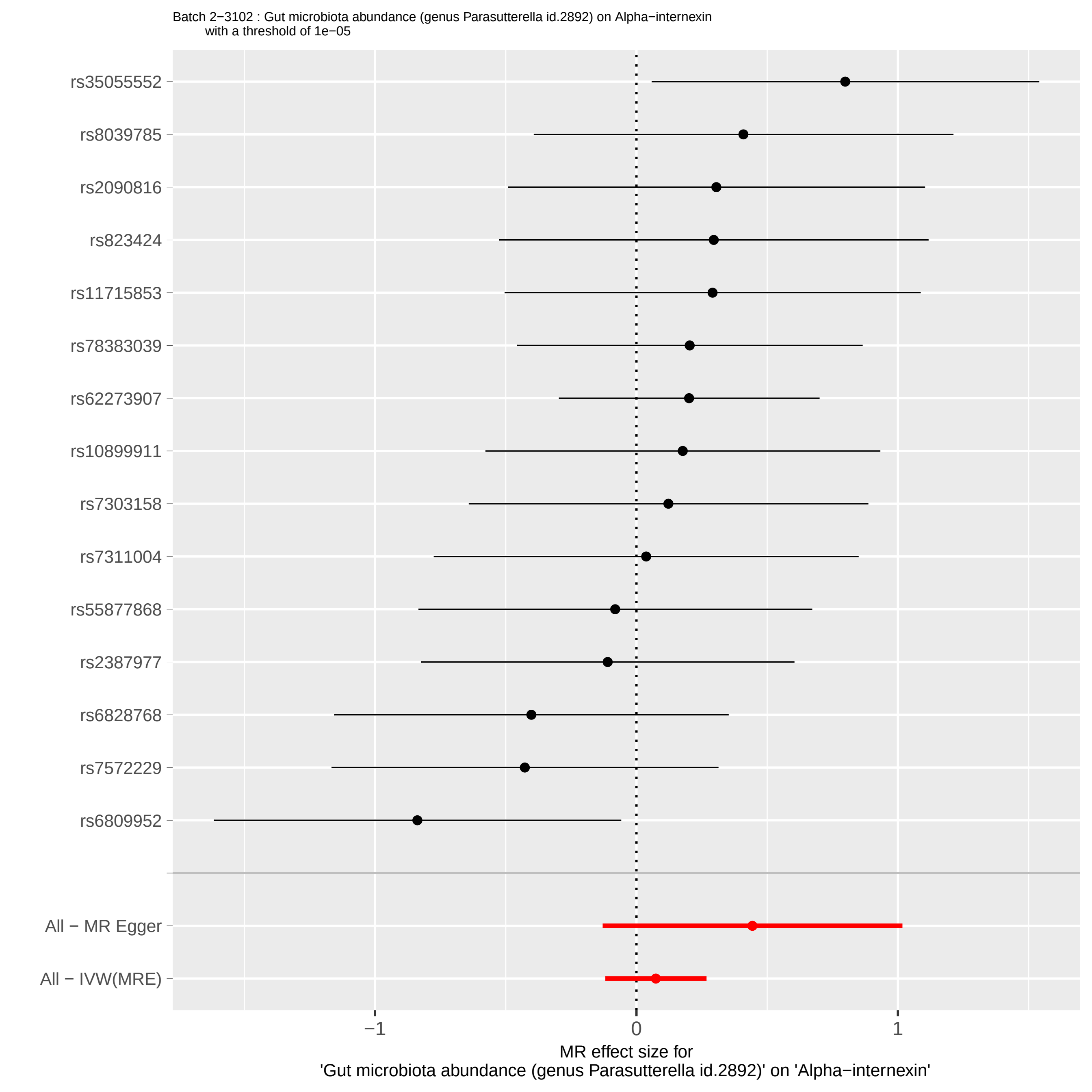

Batch 2−3102 : Gut microbiota abundance (genus Parasutterella id.2892) on Alpha−internexin
with a threshold of 1e−05
rs35055552
rs8039785
rs2090816
rs823424
rs11715853
rs78383039
rs62273907
rs10899911
rs7303158
rs7311004
rs55877868
rs2387977
rs6828768
rs7572229
rs6809952
All − MR Egger
All − IVW(MRE)
−1
0
1
MR effect size for
'Gut microbiota abundance (genus Parasutterella id.2892)' on 'Alpha−internexin'

Batch 2−3103 : Gut microbiota abundance (genus Catenibacterium id.2153) on Inhibitor of growth protein 1
with a threshold of 1e−05

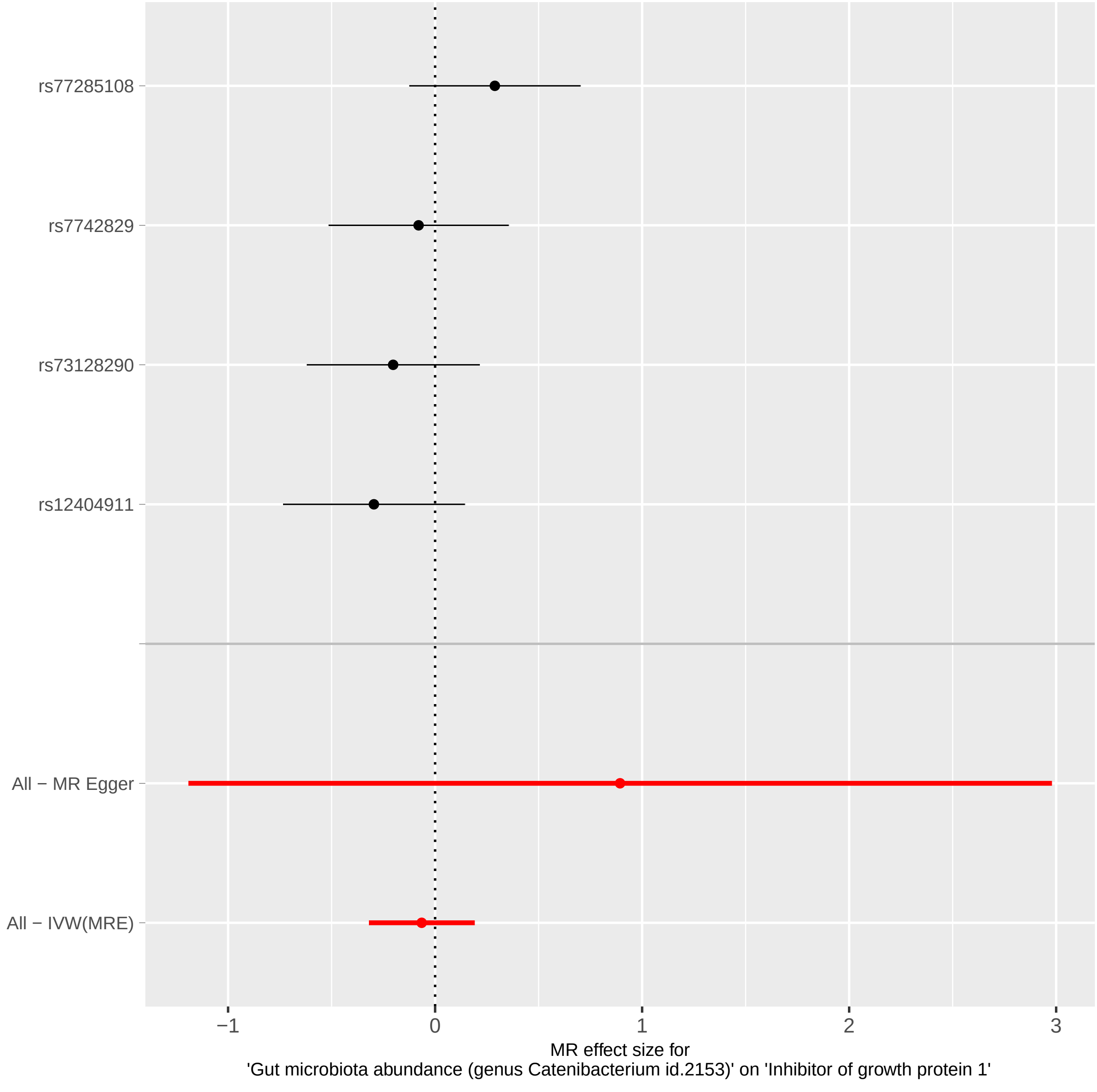

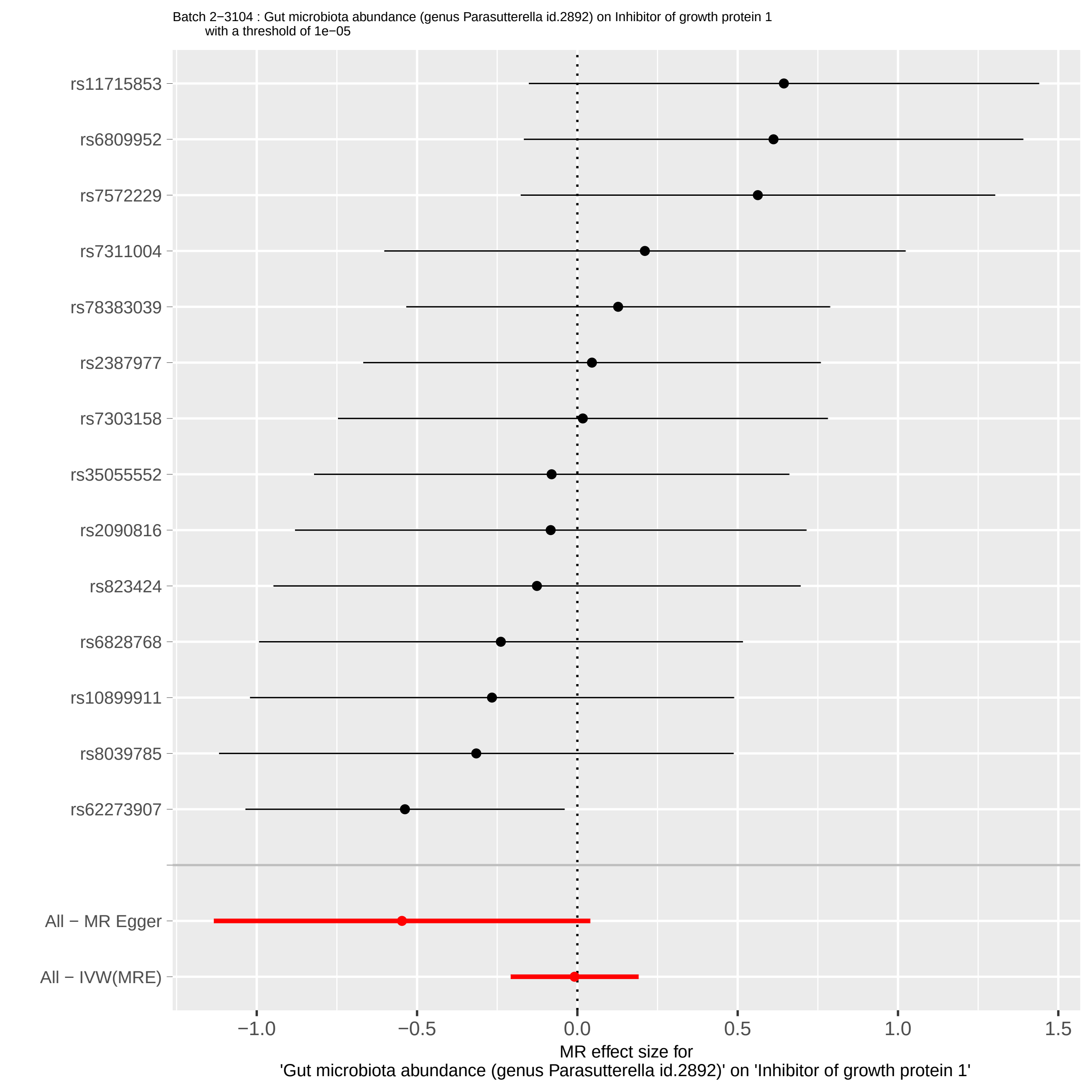
Batch 2−3104 : Gut microbiota abundance (genus Parasutterella id.2892) on Inhibitor of growth protein 1
with a threshold of 1e−05
rs11715853
rs6809952
rs7572229
rs7311004
rs78383039
rs2387977
rs7303158
rs35055552
rs2090816
rs823424
rs6828768
rs10899911
rs8039785
rs62273907
All − MR Egger
All − IVW(MRE)
−1.0
−0.5
0.0
0.5
1.0
1.5
MR effect size for
'Gut microbiota abundance (genus Parasutterella id.2892)' on 'Inhibitor of growth protein 1'

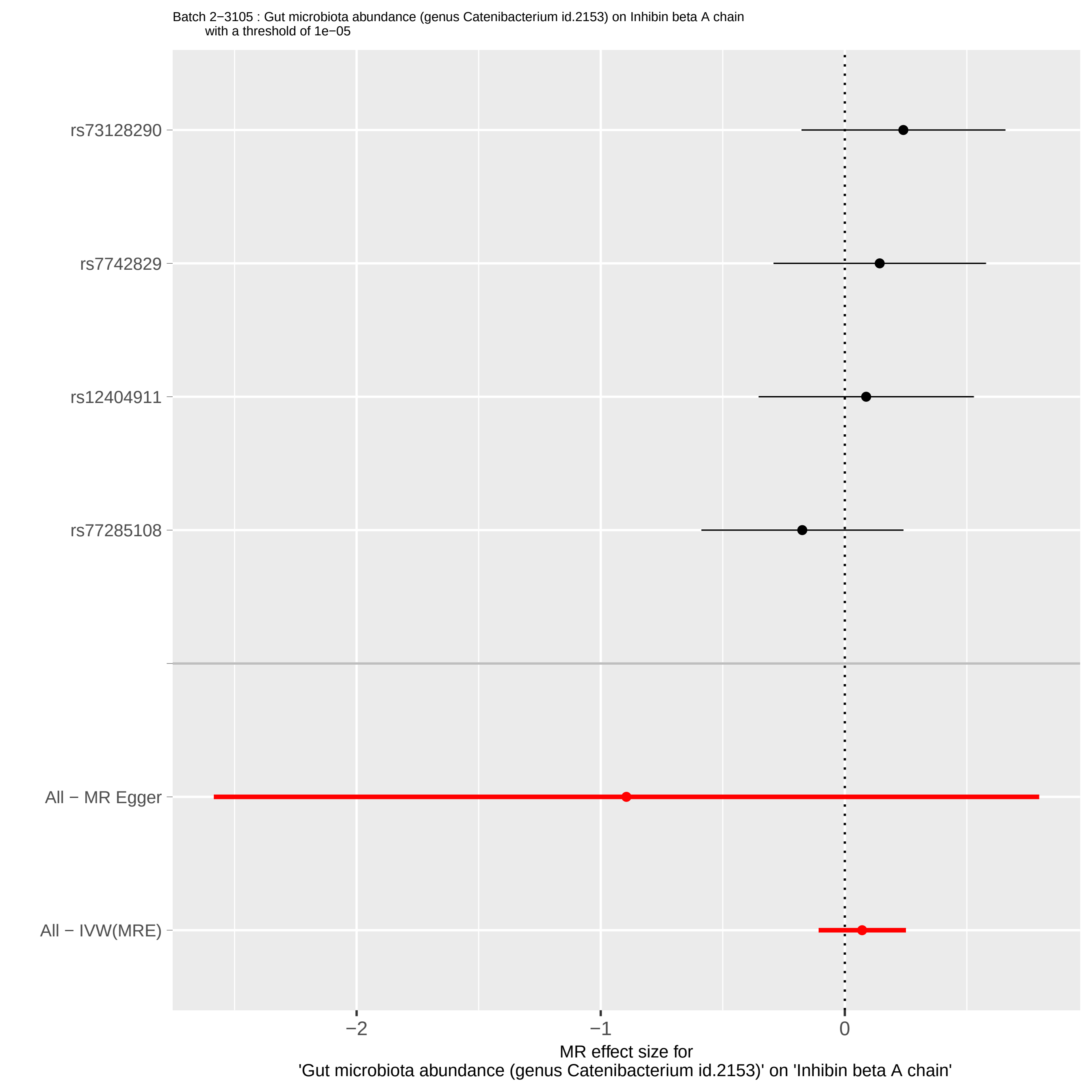
Batch 2−3105 : Gut microbiota abundance (genus Catenibacterium id.2153) on Inhibin beta A chain
with a threshold of 1e−05
rs73128290
rs7742829
rs12404911
rs77285108
All − MR Egger
All − IVW(MRE)
−2
−1
0
MR effect size for
'Gut microbiota abundance (genus Catenibacterium id.2153)' on 'Inhibin beta A chain'

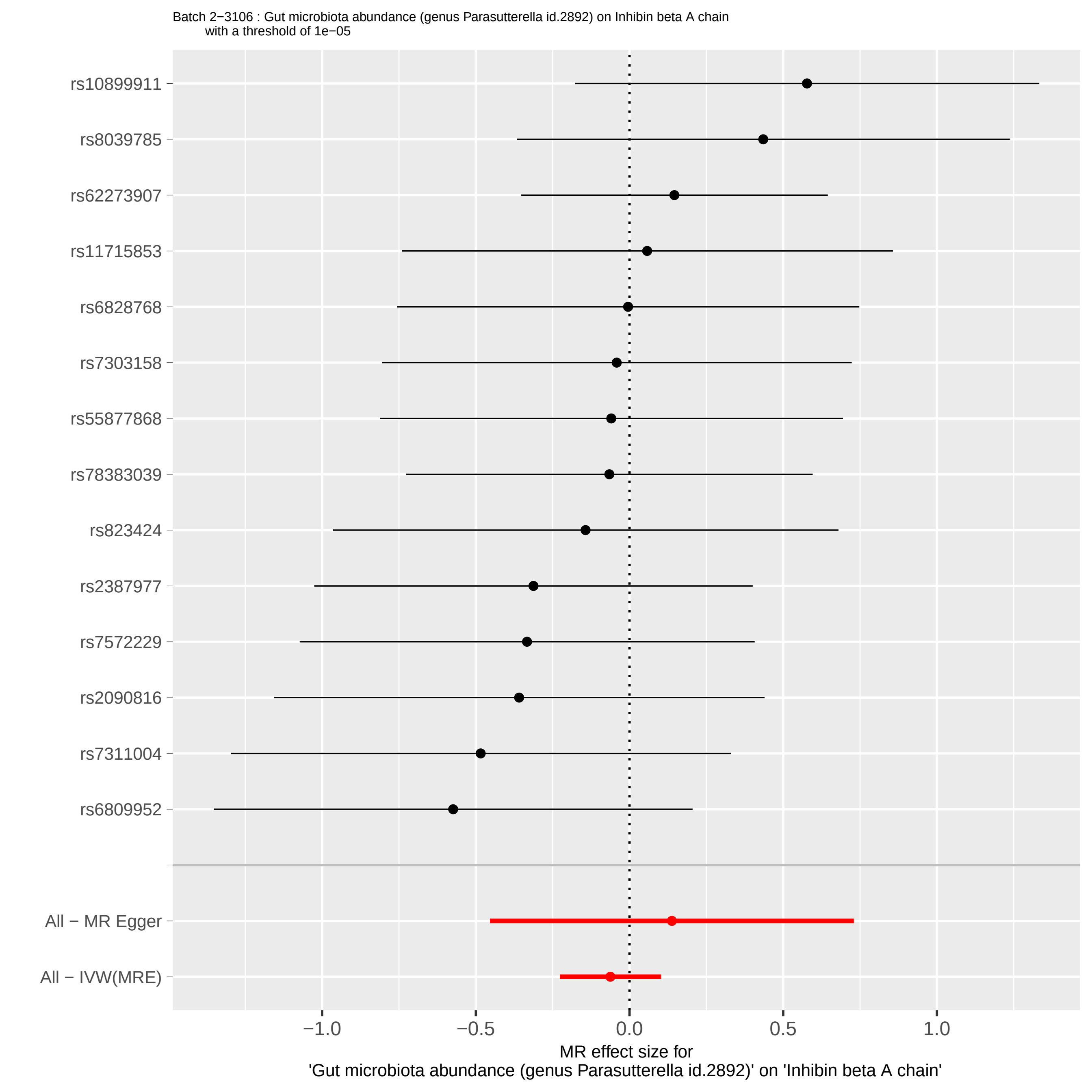

Batch 2−3106 : Gut microbiota abundance (genus Parasutterella id.2892) on Inhibin beta A chain
with a threshold of 1e−05
rs10899911
rs8039785
rs62273907
rs11715853
rs6828768
rs7303158
rs55877868
rs78383039
rs823424
rs2387977
rs7572229
rs2090816
rs7311004
rs6809952
All − MR Egger
All − IVW(MRE)
−1.0
−0.5
0.0
0.5
1.0
MR effect size for
'Gut microbiota abundance (genus Parasutterella id.2892)' on 'Inhibin beta A chain'

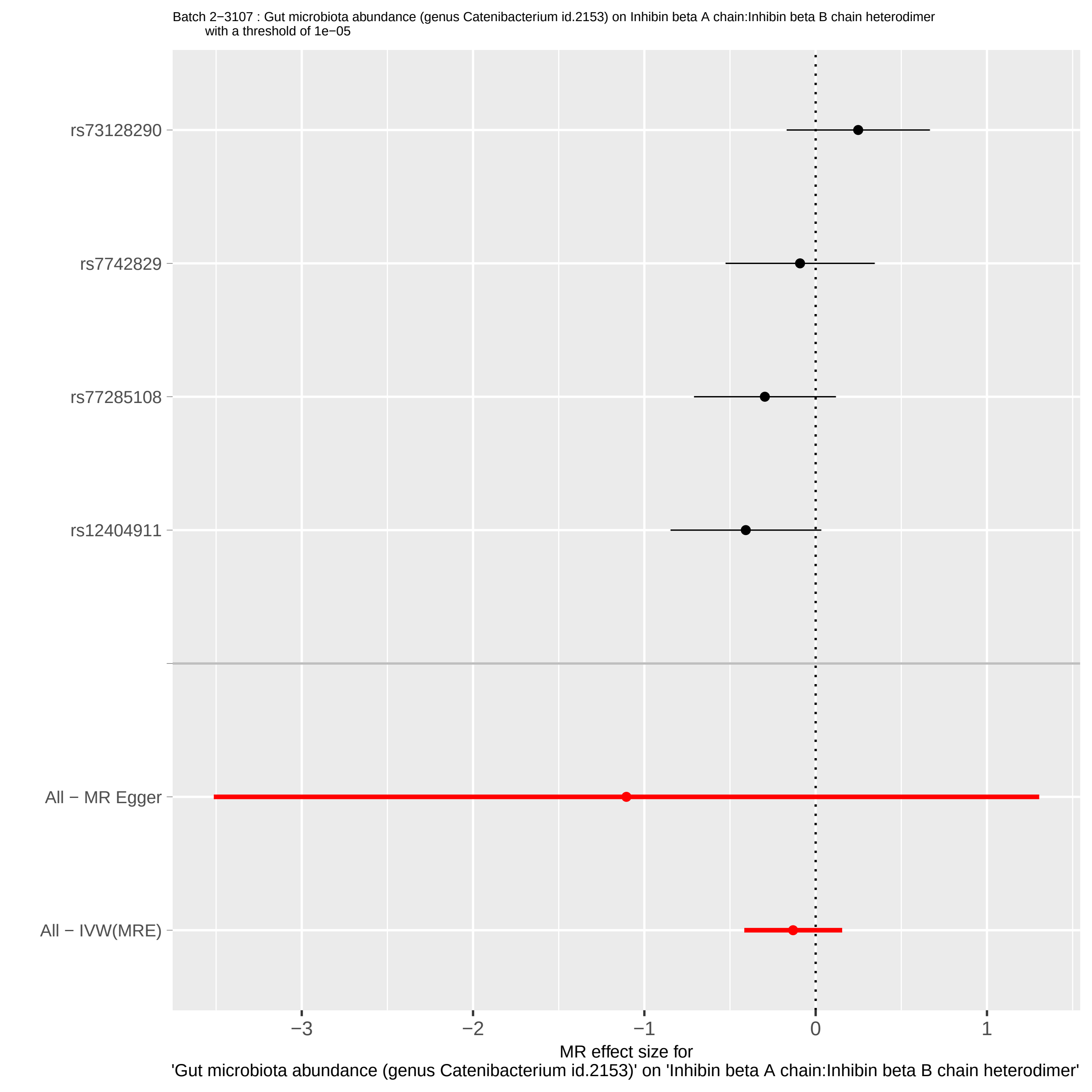

Batch 2−3107 : Gut microbiota abundance (genus Catenibacterium id.2153) on Inhibin beta A chain:Inhibin beta B chain heterodimer
with a threshold of 1e−05
rs73128290
rs7742829
rs77285108
rs12404911
All − MR Egger
All − IVW(MRE)
−3
−2
−1
0
1
MR effect size for
'Gut microbiota abundance (genus Catenibacterium id.2153)' on 'Inhibin beta A chain:Inhibin beta B chain heterodimer'

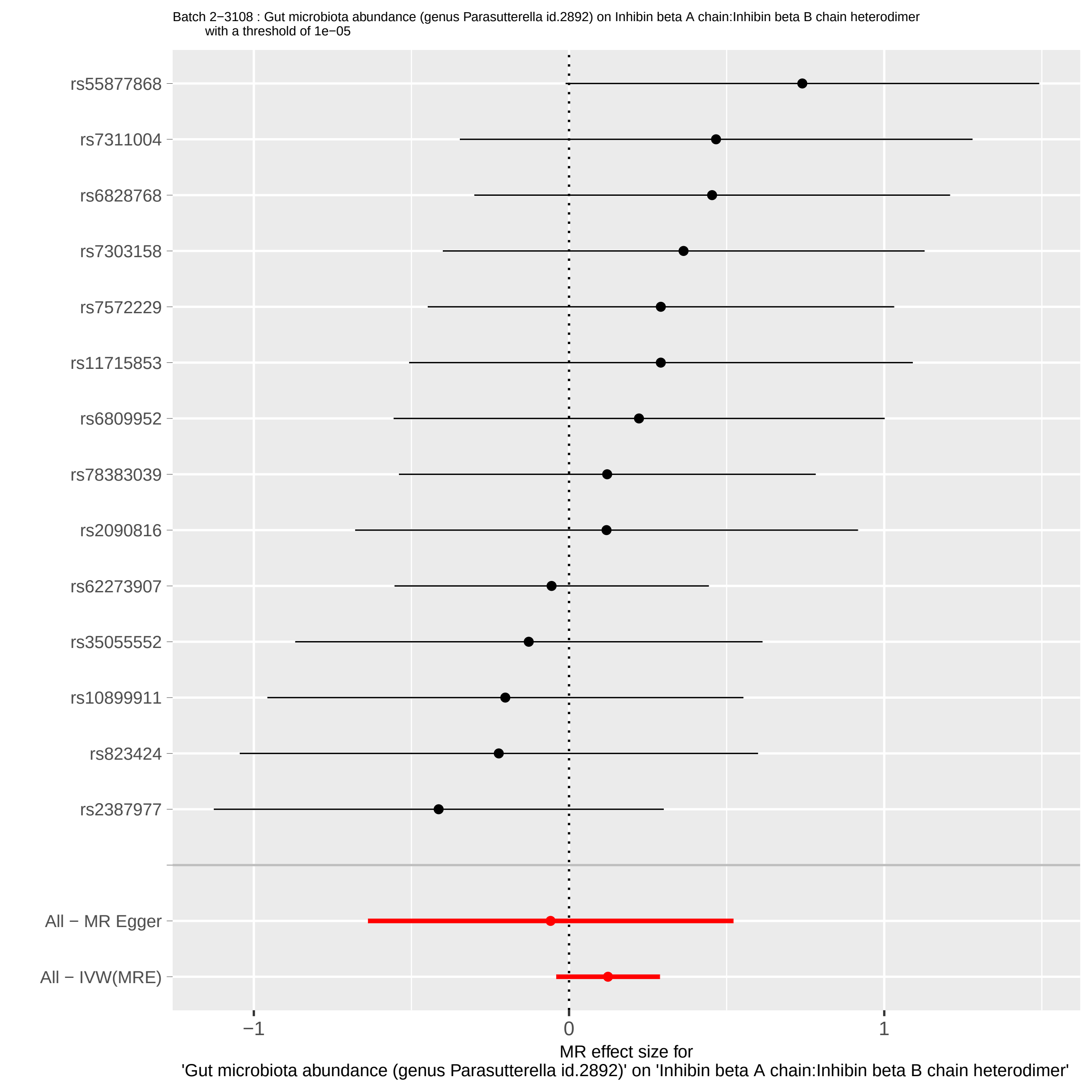
Batch 2−3108 : Gut microbiota abundance (genus Parasutterella id.2892) on Inhibin beta A chain:Inhibin beta B chain heterodimer
with a threshold of 1e−05
rs55877868
rs7311004
rs6828768
rs7303158
rs7572229
rs11715853
rs6809952
rs78383039
rs2090816
rs62273907
rs35055552
rs10899911
rs823424
rs2387977
All − MR Egger
All − IVW(MRE)
−1
0
1
MR effect size for
'Gut microbiota abundance (genus Parasutterella id.2892)' on 'Inhibin beta A chain:Inhibin beta B chain heterodimer'

Batch 2−3109 : Gut microbiota abundance (genus Catenibacterium id.2153) on Inhibin beta A chain:Inhibin beta B chain heterodimer
with a threshold of 1e−05

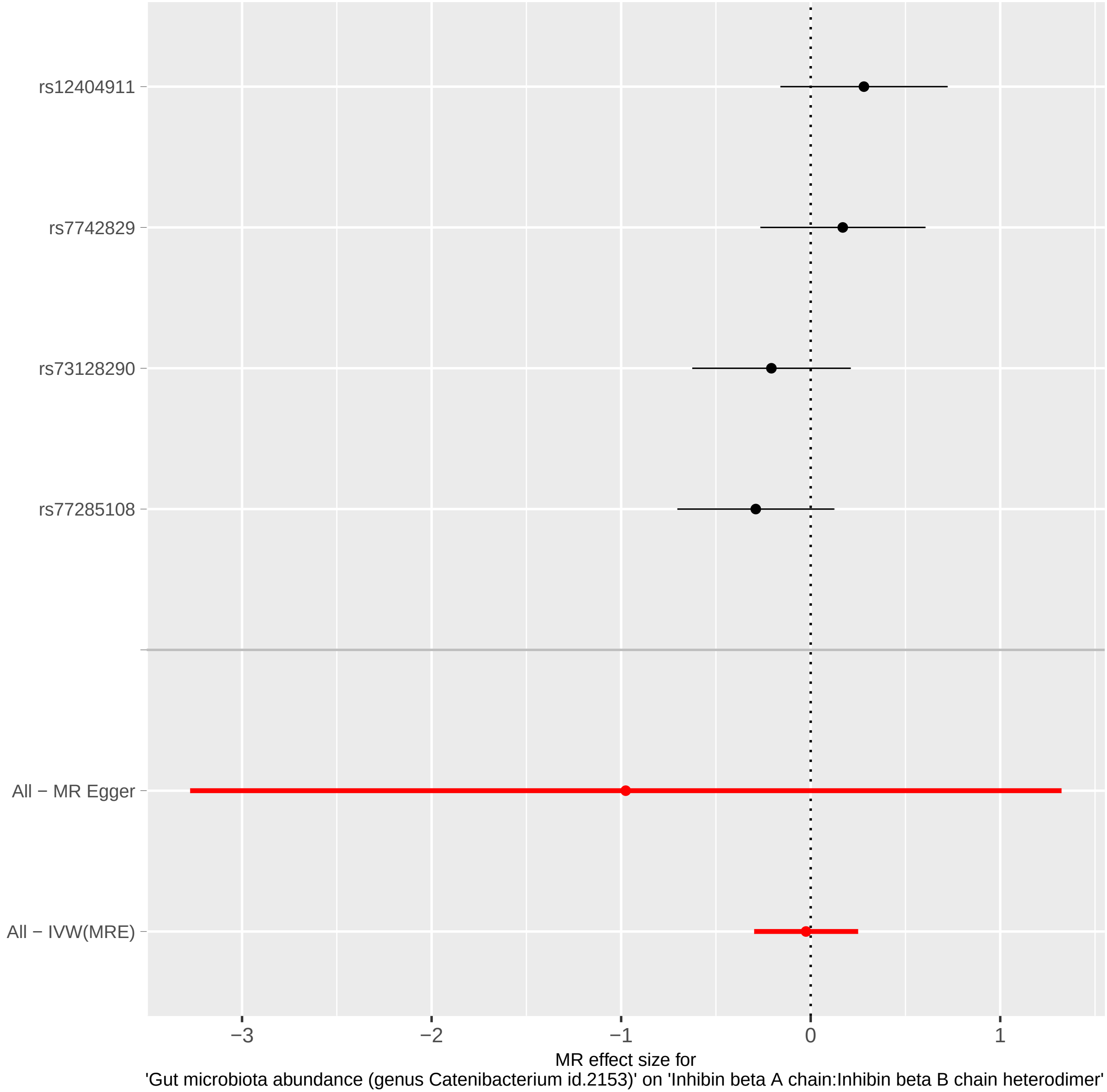

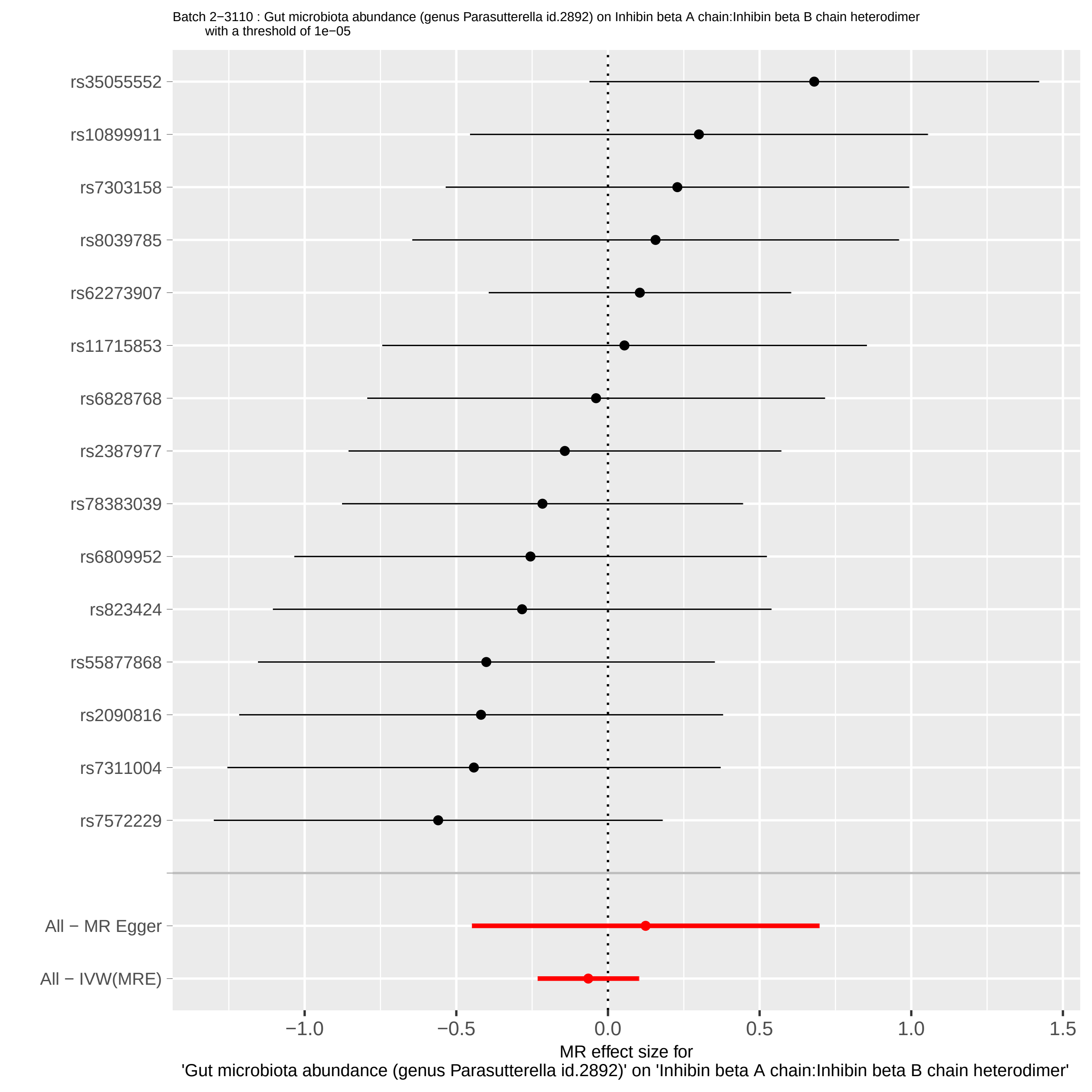

Batch 2−3110 : Gut microbiota abundance (genus Parasutterella id.2892) on Inhibin beta A chain:Inhibin beta B chain heterodimer
with a threshold of 1e−05
rs35055552
rs10899911
rs7303158
rs8039785
rs62273907
rs11715853
rs6828768
rs2387977
rs78383039
rs6809952
rs823424
rs55877868
rs2090816
rs7311004
rs7572229
All − MR Egger
All − IVW(MRE)
−1.0
−0.5
0.0
0.5
1.0
1.5
MR effect size for
'Gut microbiota abundance (genus Parasutterella id.2892)' on 'Inhibin beta A chain:Inhibin beta B chain heterodimer'

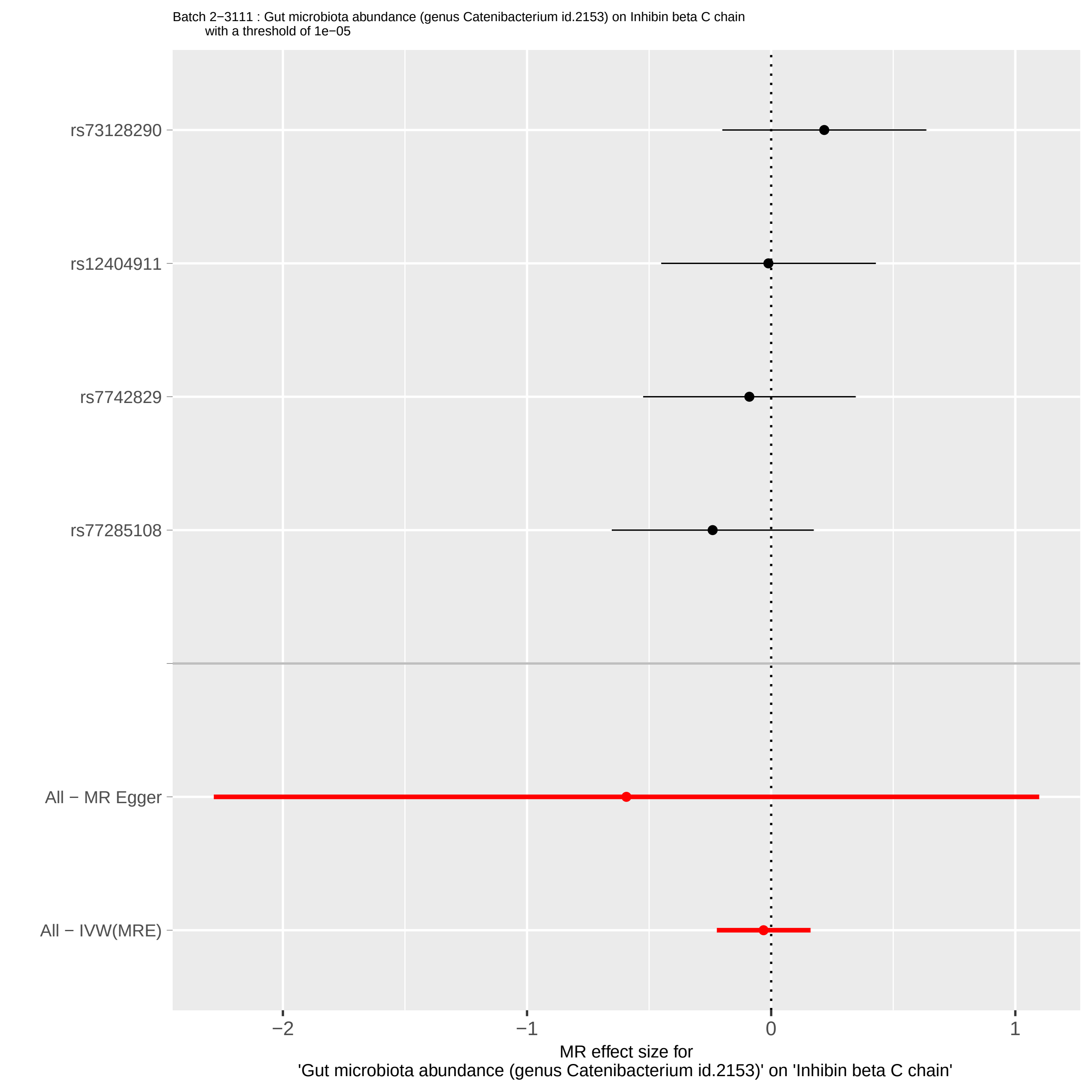
Batch 2−3111 : Gut microbiota abundance (genus Catenibacterium id.2153) on Inhibin beta C chain
with a threshold of 1e−05
rs73128290
rs12404911
rs7742829
rs77285108
All − MR Egger
All − IVW(MRE)
−2
−1
0
1
MR effect size for
'Gut microbiota abundance (genus Catenibacterium id.2153)' on 'Inhibin beta C chain'

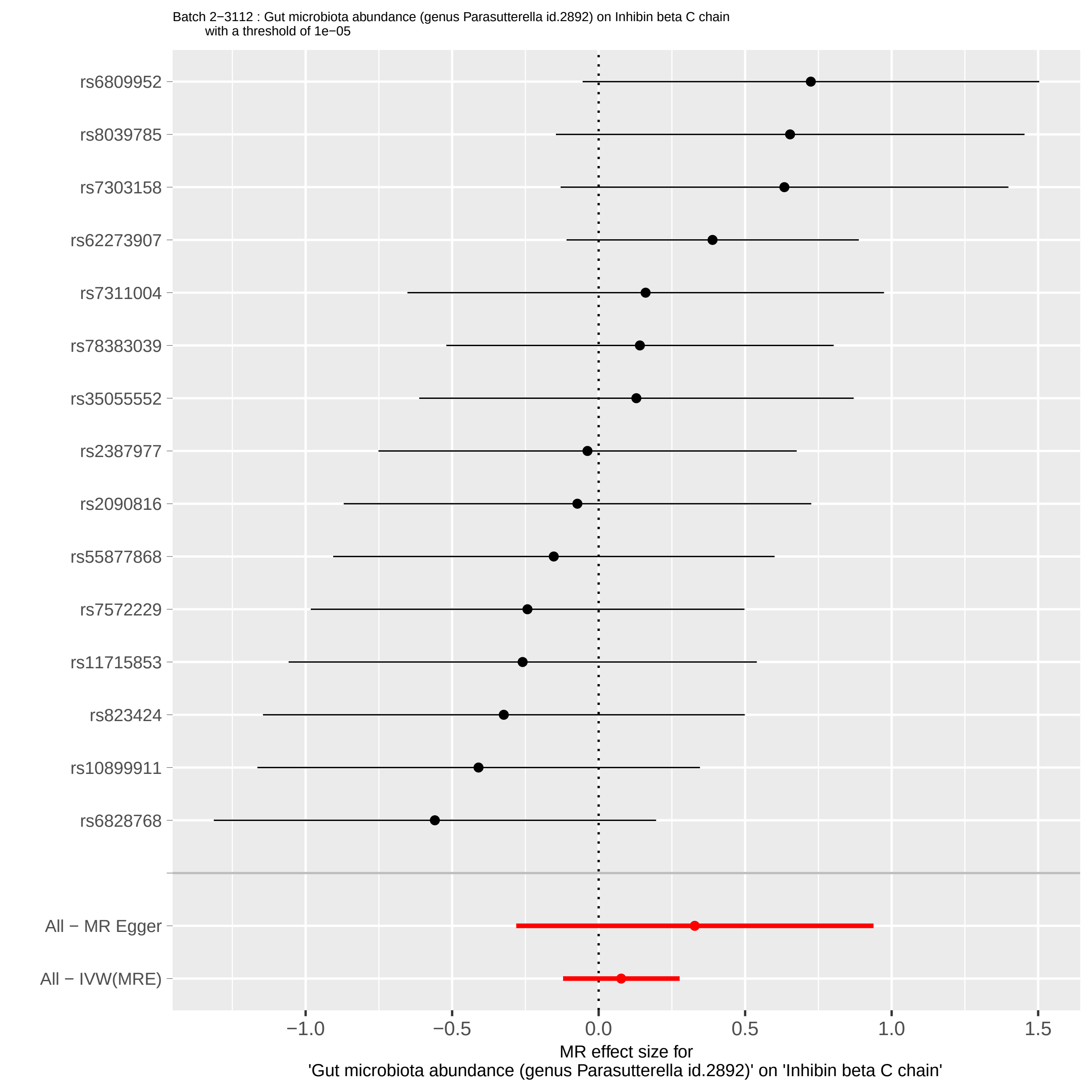
Batch 2−3112 : Gut microbiota abundance (genus Parasutterella id.2892) on Inhibin beta C chain
with a threshold of 1e−05
rs6809952
rs8039785
rs7303158
rs62273907
rs7311004
rs78383039
rs35055552
rs2387977
rs2090816
rs55877868
rs7572229
rs11715853
rs823424
rs10899911
rs6828768
All − MR Egger
All − IVW(MRE)
−1.0
−0.5
0.0
0.5
1.0
1.5
MR effect size for
'Gut microbiota abundance (genus Parasutterella id.2892)' on 'Inhibin beta C chain'

Batch 2−3113 : Gut microbiota abundance (genus Catenibacterium id.2153) on 72 kDa inositol polyphosphate 5−phosphatase with a threshold of 1e−05

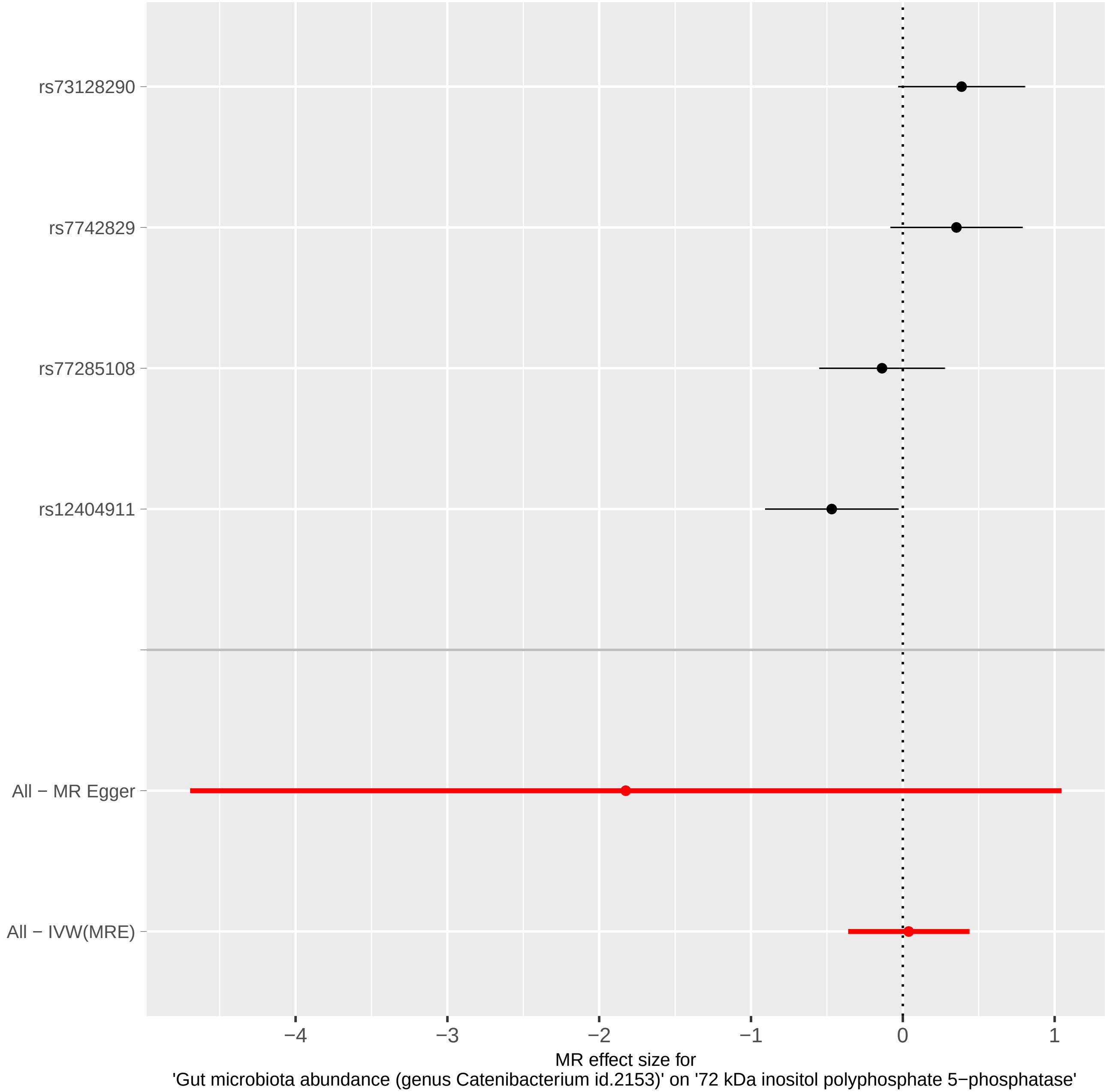

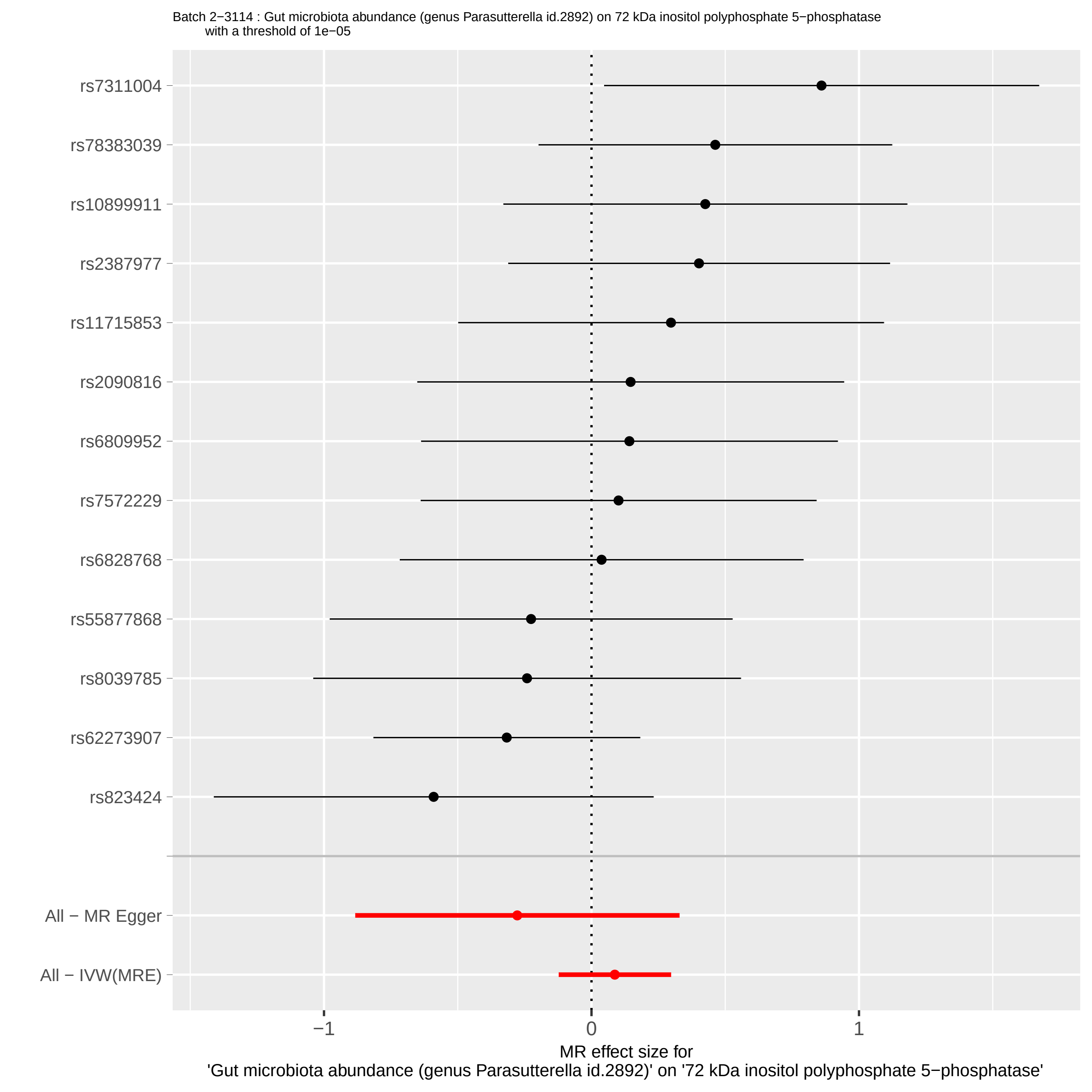
Batch 2−3114 : Gut microbiota abundance (genus Parasutterella id.2892) on 72 kDa inositol polyphosphate 5−phosphatase with a threshold of 1e−05
rs7311004
rs78383039
rs10899911
rs2387977
rs11715853
rs2090816
rs6809952
rs7572229
rs6828768
rs55877868
rs8039785
rs62273907
rs823424
All − MR Egger
All − IVW(MRE)
−1
0
1
MR effect size for
'Gut microbiota abundance (genus Parasutterella id.2892)' on '72 kDa inositol polyphosphate 5−phosphatase'

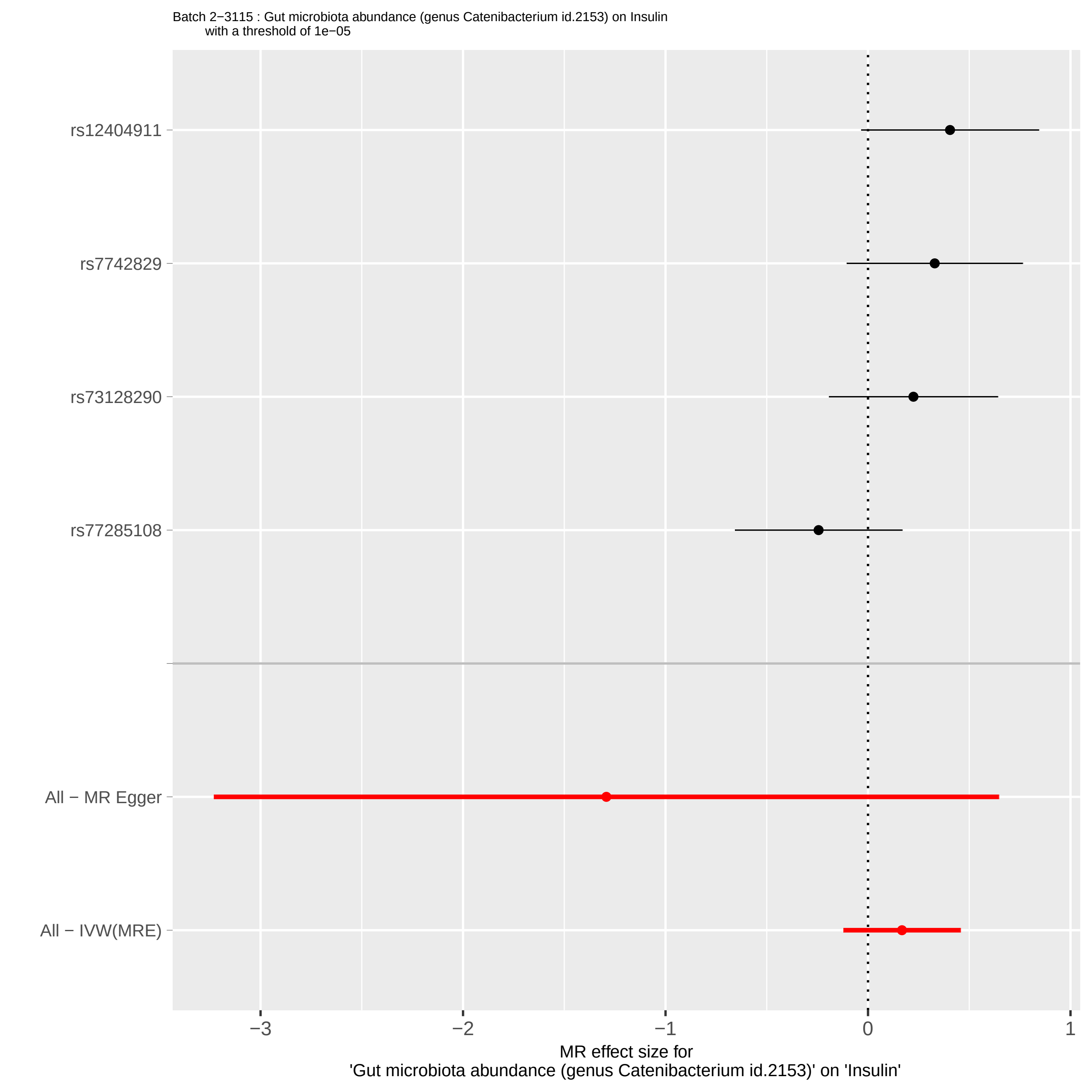

Batch 2−3115 : Gut microbiota abundance (genus Catenibacterium id.2153) on Insulin
with a threshold of 1e−05
rs12404911
rs7742829
rs73128290
rs77285108
All − MR Egger
All − IVW(MRE)
−3
−2
−1
0
1
MR effect size for
'Gut microbiota abundance (genus Catenibacterium id.2153)' on 'Insulin'

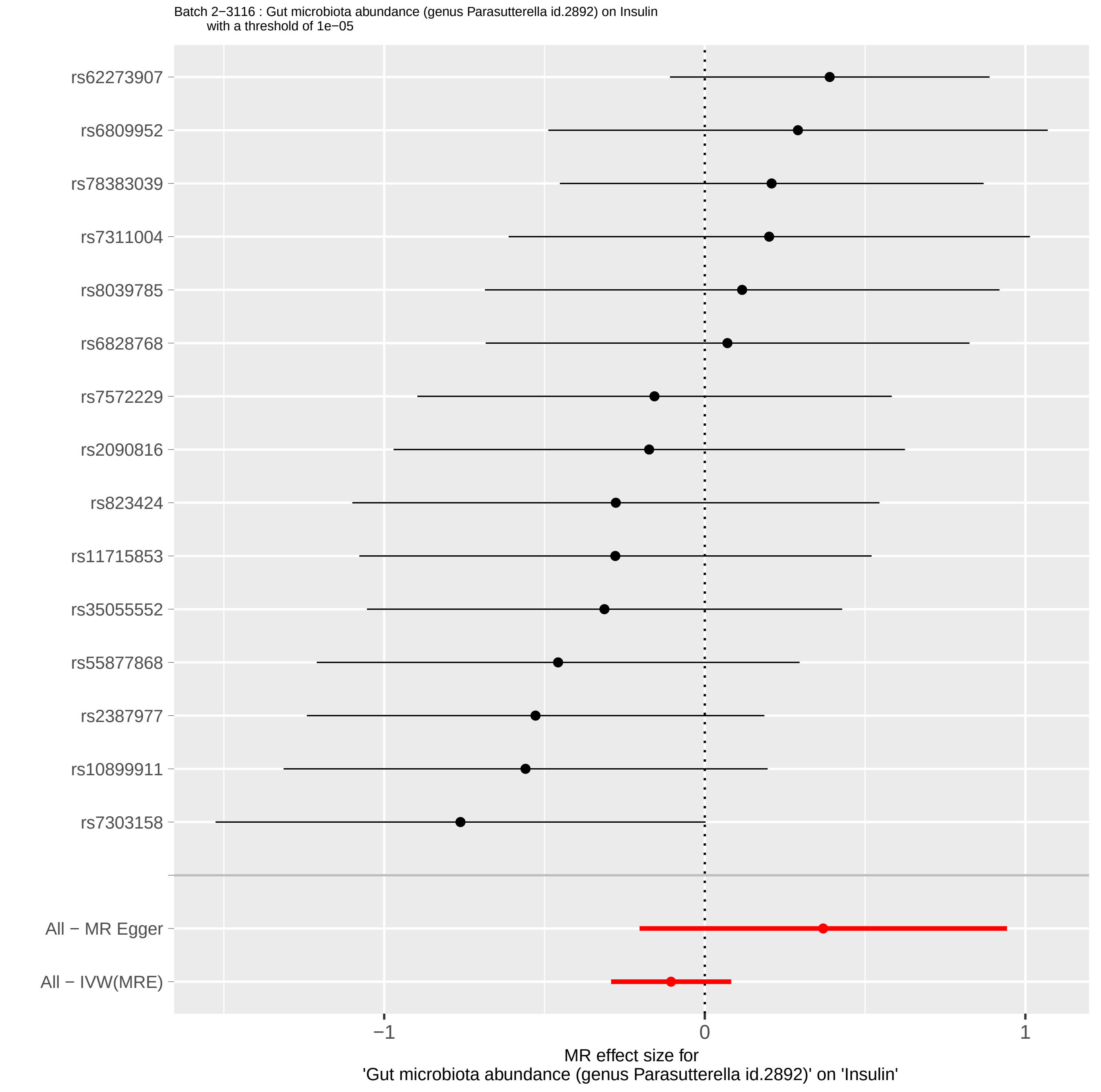

Batch 2−3116 : Gut microbiota abundance (genus Parasutterella id.2892) on Insulin
with a threshold of 1e−05
rs62273907
rs6809952
rs78383039
rs7311004
rs8039785
rs6828768
rs7572229
rs2090816
rs823424
rs11715853
rs35055552
rs55877868
rs2387977
rs10899911
rs7303158
All − MR Egger
All − IVW(MRE)
−1
0
1
MR effect size for
'Gut microbiota abundance (genus Parasutterella id.2892)' on 'Insulin'

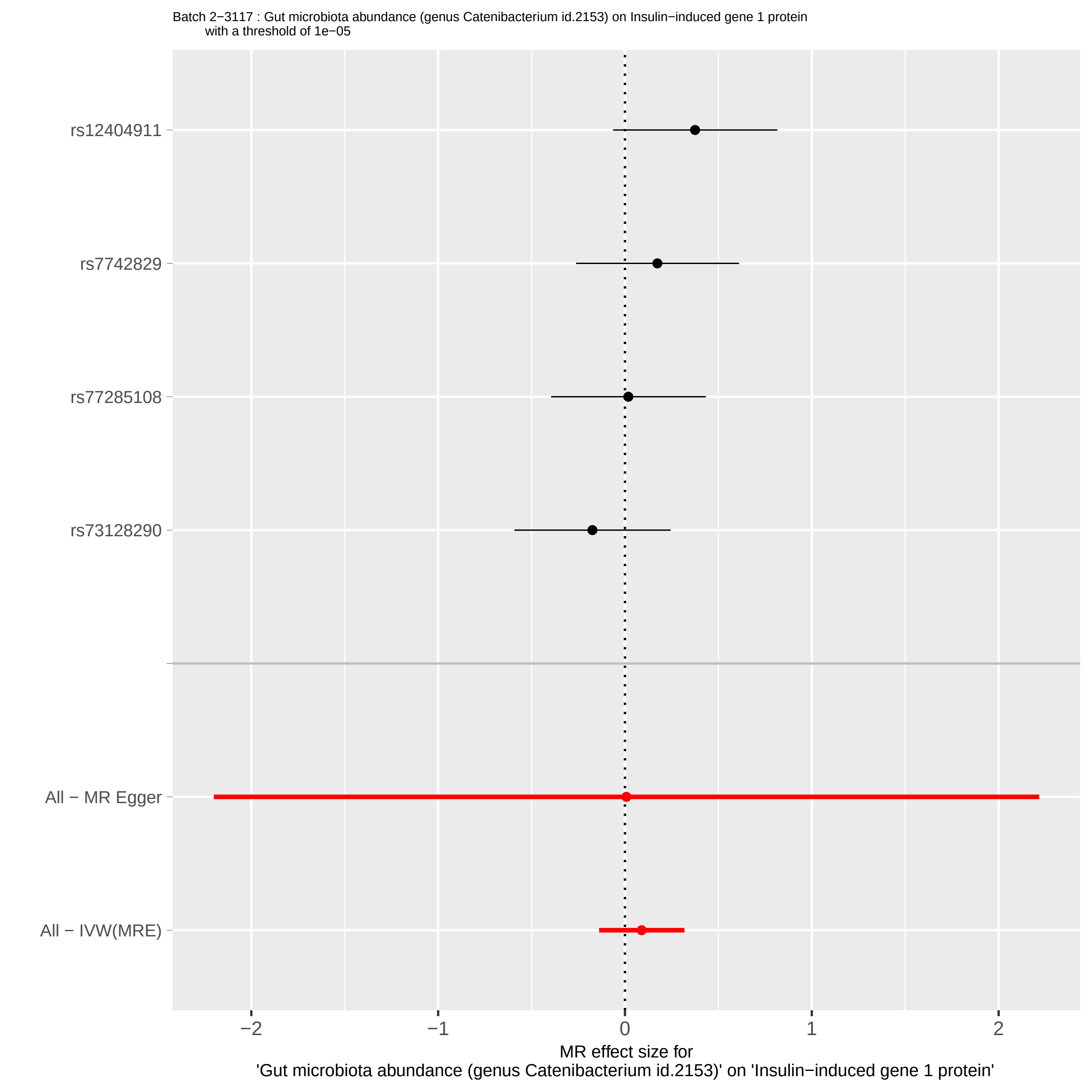

Batch 2−3117 : Gut microbiota abundance (genus Catenibacterium id.2153) on Insulin−induced gene 1 protein
with a threshold of 1e−05
rs12404911
rs7742829
rs77285108
rs73128290
All − MR Egger
All − IVW(MRE)
−2
−1
0
1
2
MR effect size for
'Gut microbiota abundance (genus Catenibacterium id.2153)' on 'Insulin−induced gene 1 protein'

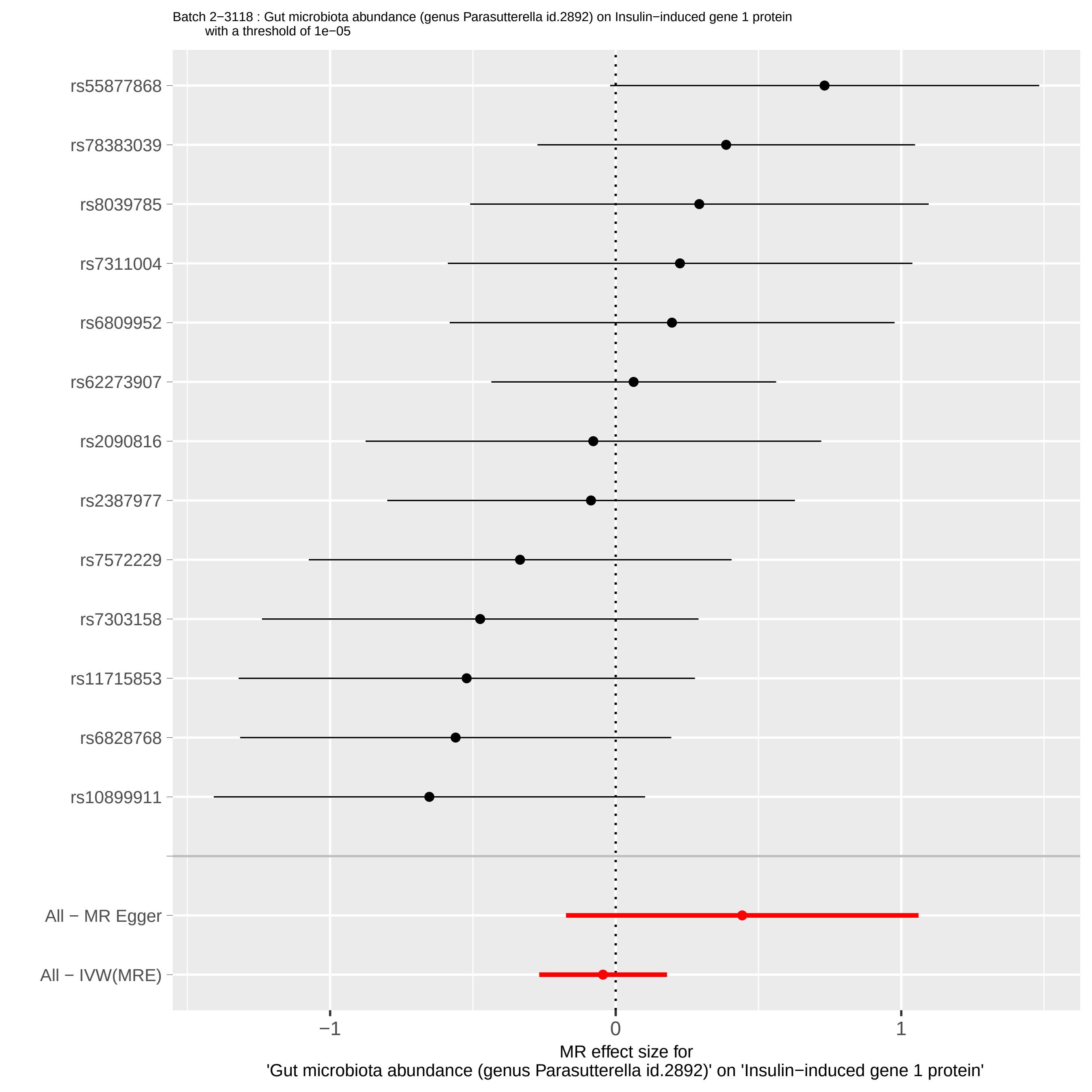

Batch 2−3118 : Gut microbiota abundance (genus Parasutterella id.2892) on Insulin−induced gene 1 protein
with a threshold of 1e−05
rs55877868
rs78383039
rs8039785
rs7311004
rs6809952
rs62273907
rs2090816
rs2387977
rs7572229
rs7303158
rs11715853
rs6828768
rs10899911
All − MR Egger
All − IVW(MRE)
−1
0
1
MR effect size for
'Gut microbiota abundance (genus Parasutterella id.2892)' on 'Insulin−induced gene 1 protein'

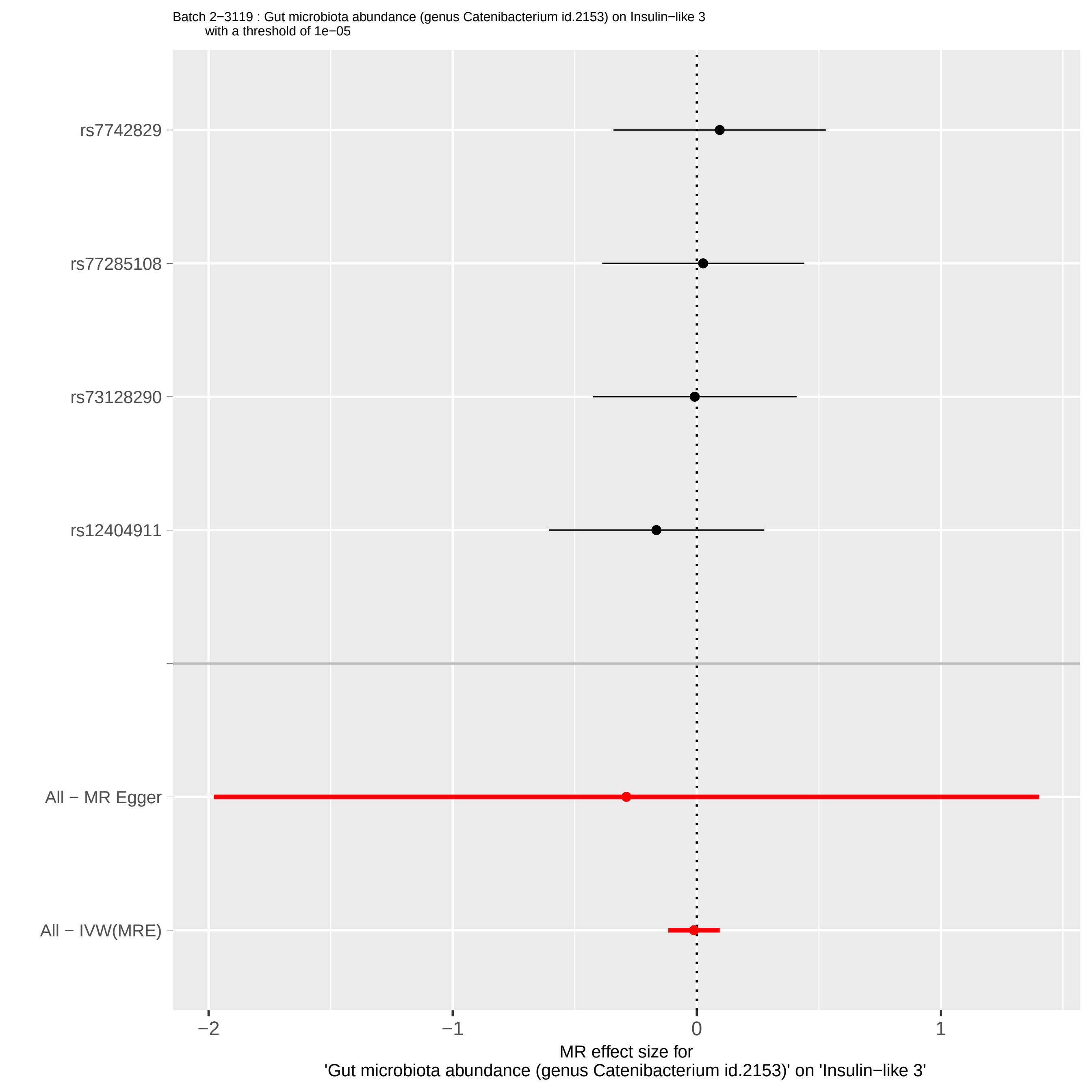
Batch 2−3119 : Gut microbiota abundance (genus Catenibacterium id.2153) on Insulin−like 3
with a threshold of 1e−05
rs7742829
rs77285108
rs73128290
rs12404911
All − MR Egger
All − IVW(MRE)
−2
−1
0
1
MR effect size for
'Gut microbiota abundance (genus Catenibacterium id.2153)' on 'Insulin−like 3'

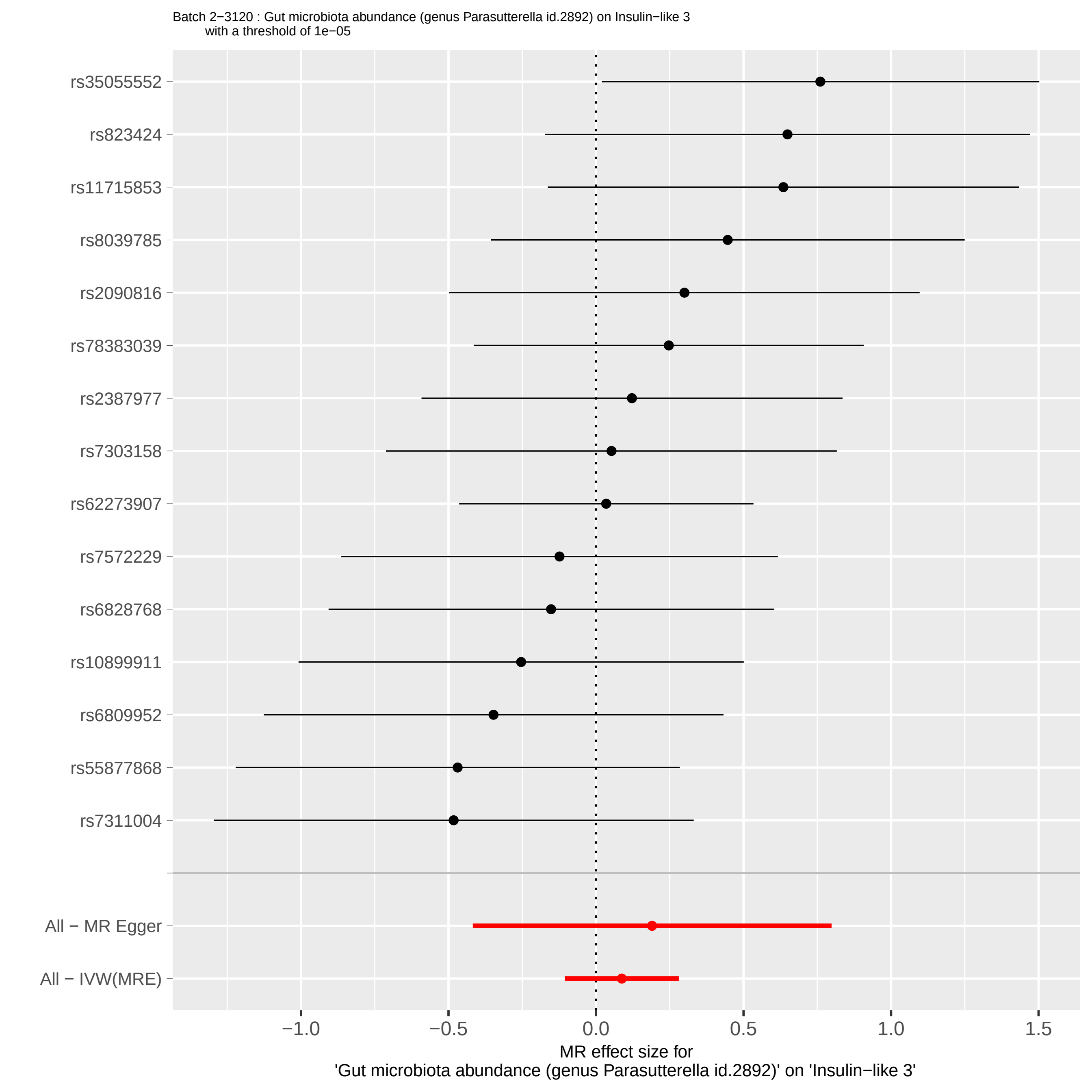

Batch 2−3120 : Gut microbiota abundance (genus Parasutterella id.2892) on Insulin−like 3
with a threshold of 1e−05
rs35055552
rs823424
rs11715853
rs8039785
rs2090816
rs78383039
rs2387977
rs7303158
rs62273907
rs7572229
rs6828768
rs10899911
rs6809952
rs55877868
rs7311004
All − MR Egger
All − IVW(MRE)
−1.0
−0.5
0.0
0.5
1.0
1.5
MR effect size for
'Gut microbiota abundance (genus Parasutterella id.2892)' on 'Insulin−like 3'

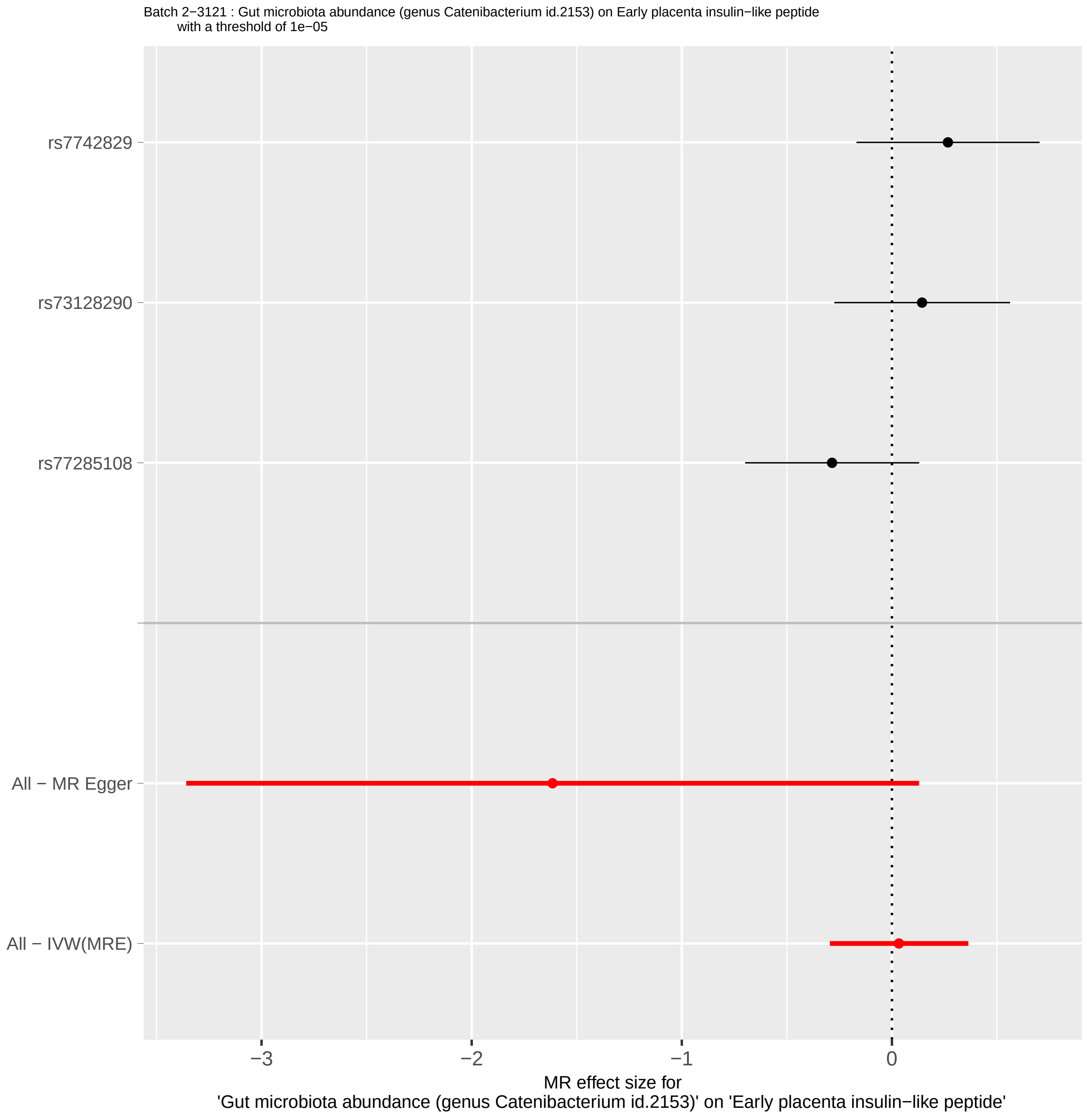
Batch 2−3121 : Gut microbiota abundance (genus Catenibacterium id.2153) on Early placenta insulin−like peptide
with a threshold of 1e−05
rs7742829
rs73128290
rs77285108
All − MR Egger
All − IVW(MRE)
−3
−2
−1
0
MR effect size for
'Gut microbiota abundance (genus Catenibacterium id.2153)' on 'Early placenta insulin−like peptide'

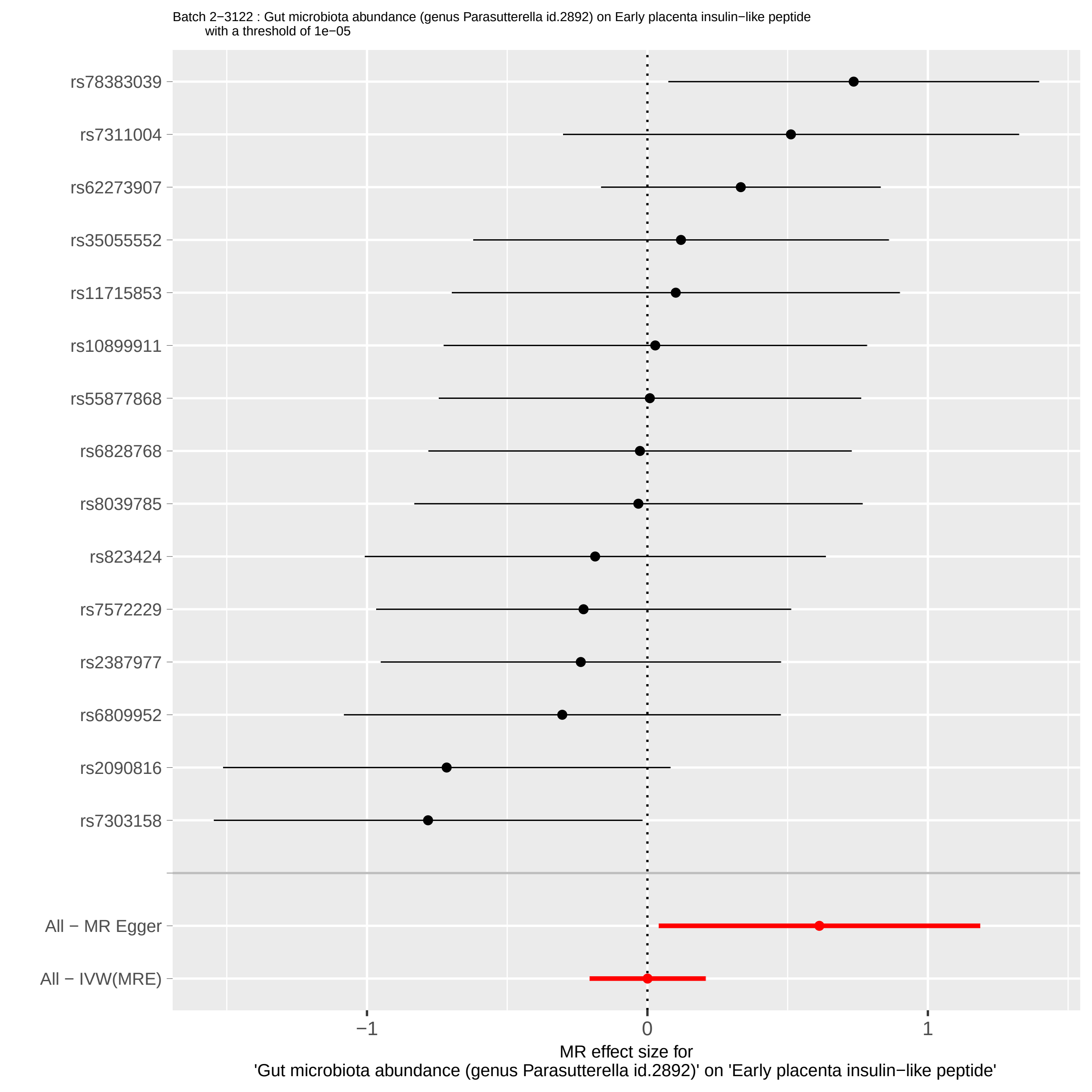

Batch 2−3122 : Gut microbiota abundance (genus Parasutterella id.2892) on Early placenta insulin−like peptide
with a threshold of 1e−05
rs78383039
rs7311004
rs62273907
rs35055552
rs11715853
rs10899911
rs55877868
rs6828768
rs8039785
rs823424
rs7572229
rs2387977
rs6809952
rs2090816
rs7303158
All − MR Egger
All − IVW(MRE)
−1
0
1
MR effect size for
'Gut microbiota abundance (genus Parasutterella id.2892)' on 'Early placenta insulin−like peptide'

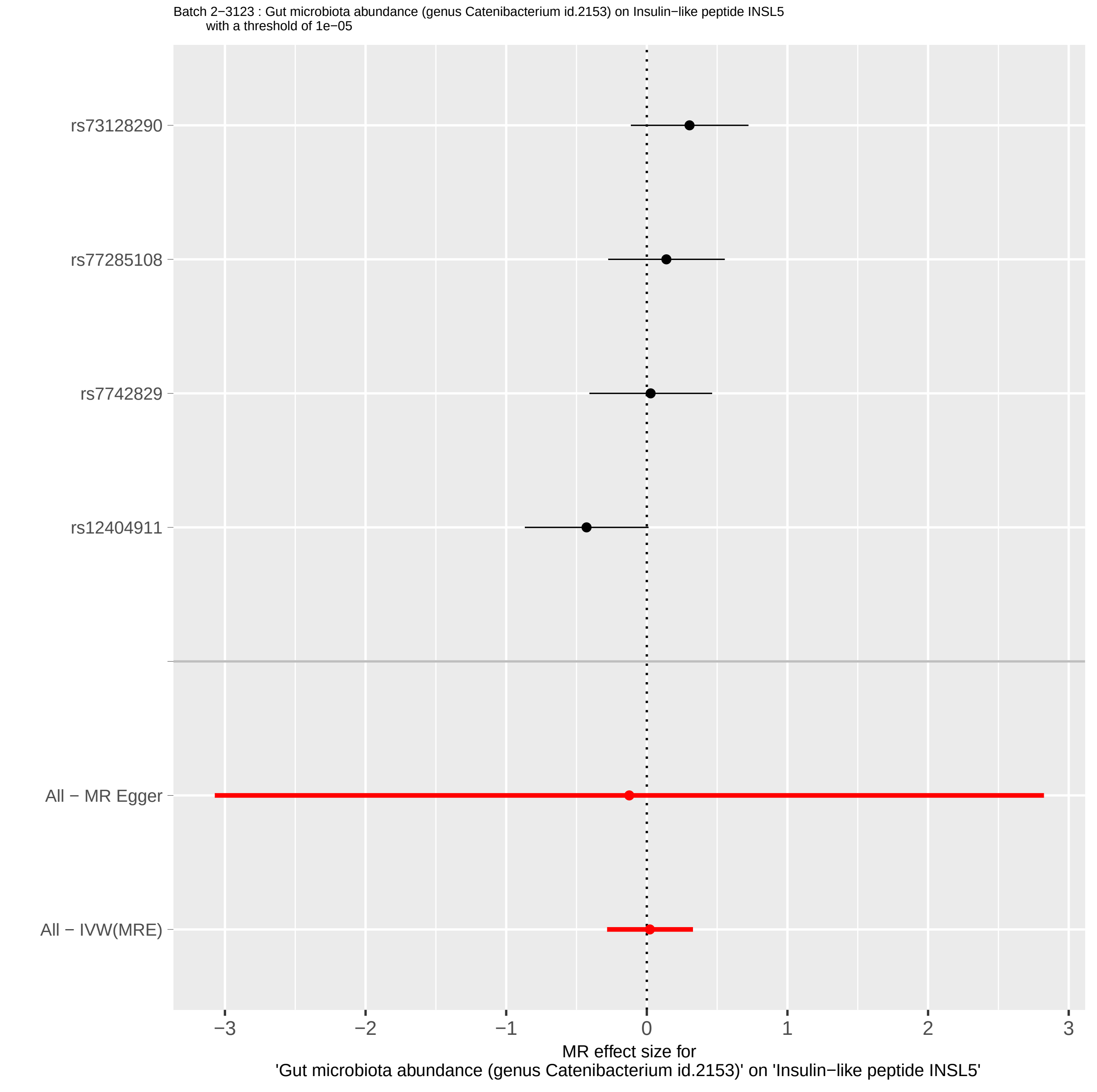

Batch 2−3123 : Gut microbiota abundance (genus Catenibacterium id.2153) on Insulin−like peptide INSL5
with a threshold of 1e−05
rs73128290
rs77285108
rs7742829
rs12404911
All − MR Egger
All − IVW(MRE)
−3
−2
−1
0
1
2
3
MR effect size for
'Gut microbiota abundance (genus Catenibacterium id.2153)' on 'Insulin−like peptide INSL5'

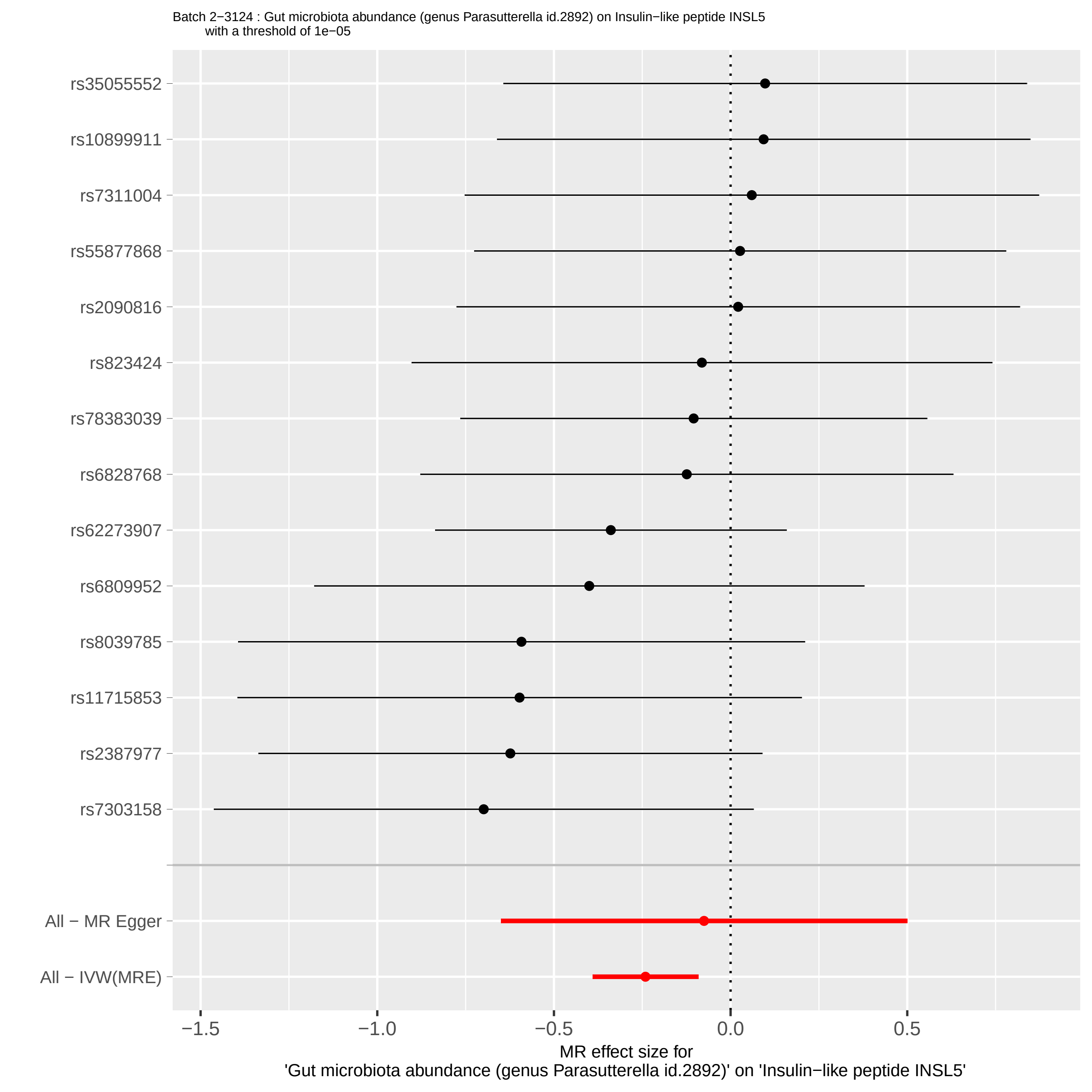

Batch 2−3124 : Gut microbiota abundance (genus Parasutterella id.2892) on Insulin−like peptide INSL5
with a threshold of 1e−05
rs35055552
rs10899911
rs7311004
rs55877868
rs2090816
rs823424
rs78383039
rs6828768
rs62273907
rs6809952
rs8039785
rs11715853
rs2387977
rs7303158
All − MR Egger
All − IVW(MRE)
−1.5
−1.0
−0.5
0.0
0.5
MR effect size for
'Gut microbiota abundance (genus Parasutterella id.2892)' on 'Insulin−like peptide INSL5'

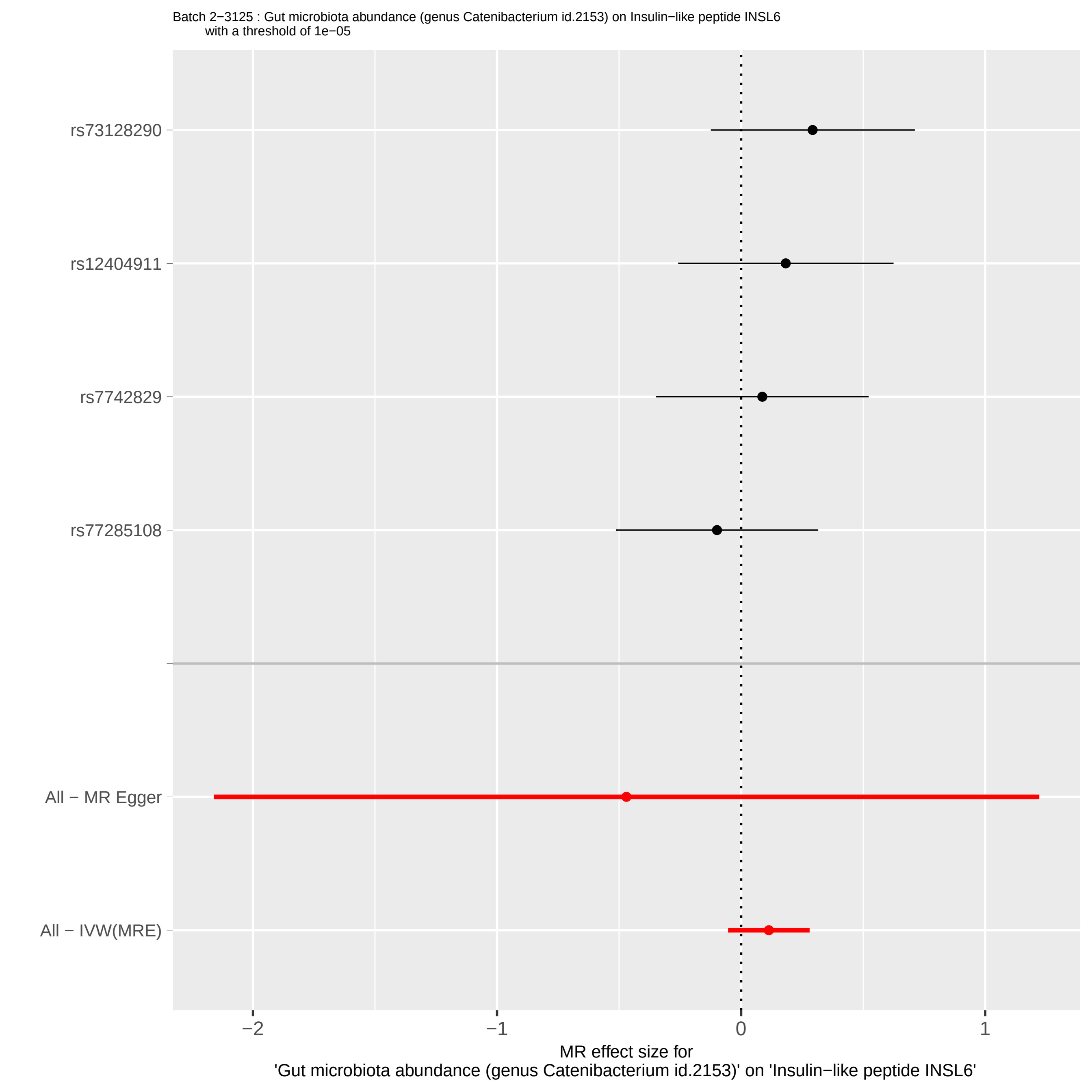
Batch 2−3125 : Gut microbiota abundance (genus Catenibacterium id.2153) on Insulin−like peptide INSL6
with a threshold of 1e−05
rs73128290
rs12404911
rs7742829
rs77285108
All − MR Egger
All − IVW(MRE)
−2
−1
0
1
MR effect size for
'Gut microbiota abundance (genus Catenibacterium id.2153)' on 'Insulin−like peptide INSL6'

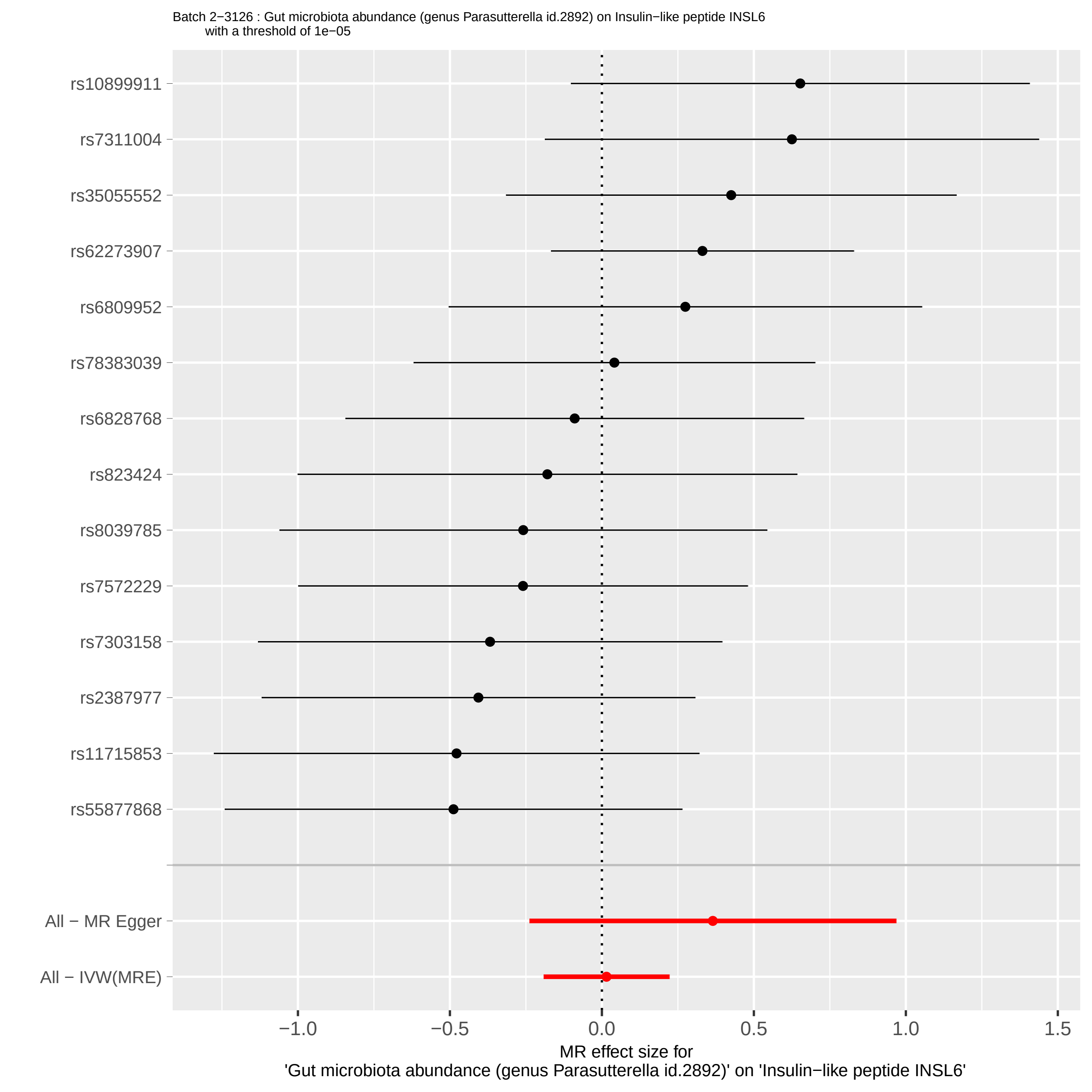

Batch 2−3126 : Gut microbiota abundance (genus Parasutterella id.2892) on Insulin−like peptide INSL6
with a threshold of 1e−05
rs10899911
rs7311004
rs35055552
rs62273907
rs6809952
rs78383039
rs6828768
rs823424
rs8039785
rs7572229
rs7303158
rs2387977
rs11715853
rs55877868
All − MR Egger
All − IVW(MRE)
−1.0
−0.5
0.0
0.5
1.0
1.5
MR effect size for
'Gut microbiota abundance (genus Parasutterella id.2892)' on 'Insulin−like peptide INSL6'

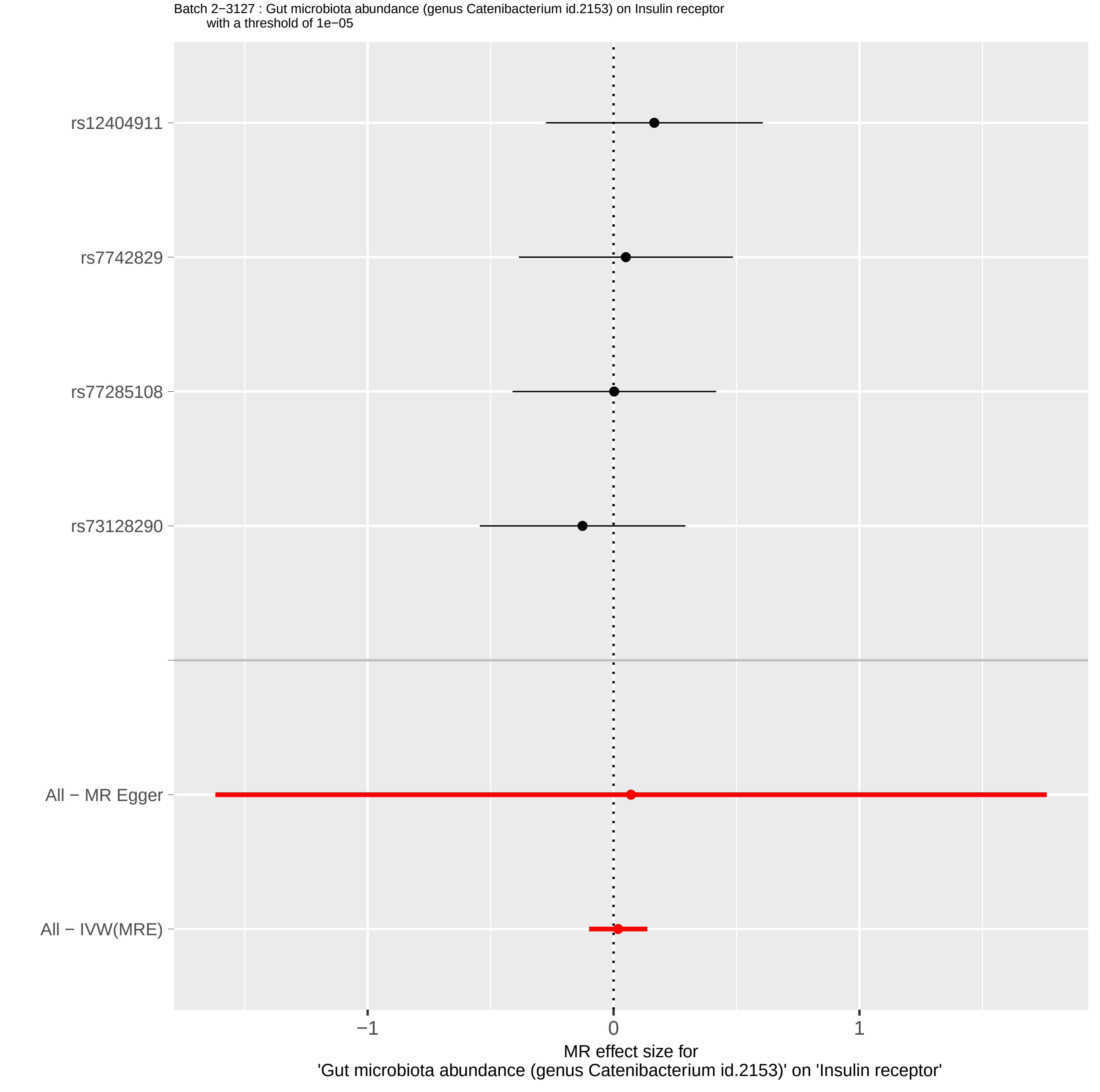

Batch 2−3127 : Gut microbiota abundance (genus Catenibacterium id.2153) on Insulin receptor
with a threshold of 1e−05
rs12404911
rs7742829
rs77285108
rs73128290
All − MR Egger
All − IVW(MRE)
−1
0
1
MR effect size for
'Gut microbiota abundance (genus Catenibacterium id.2153)' on 'Insulin receptor'

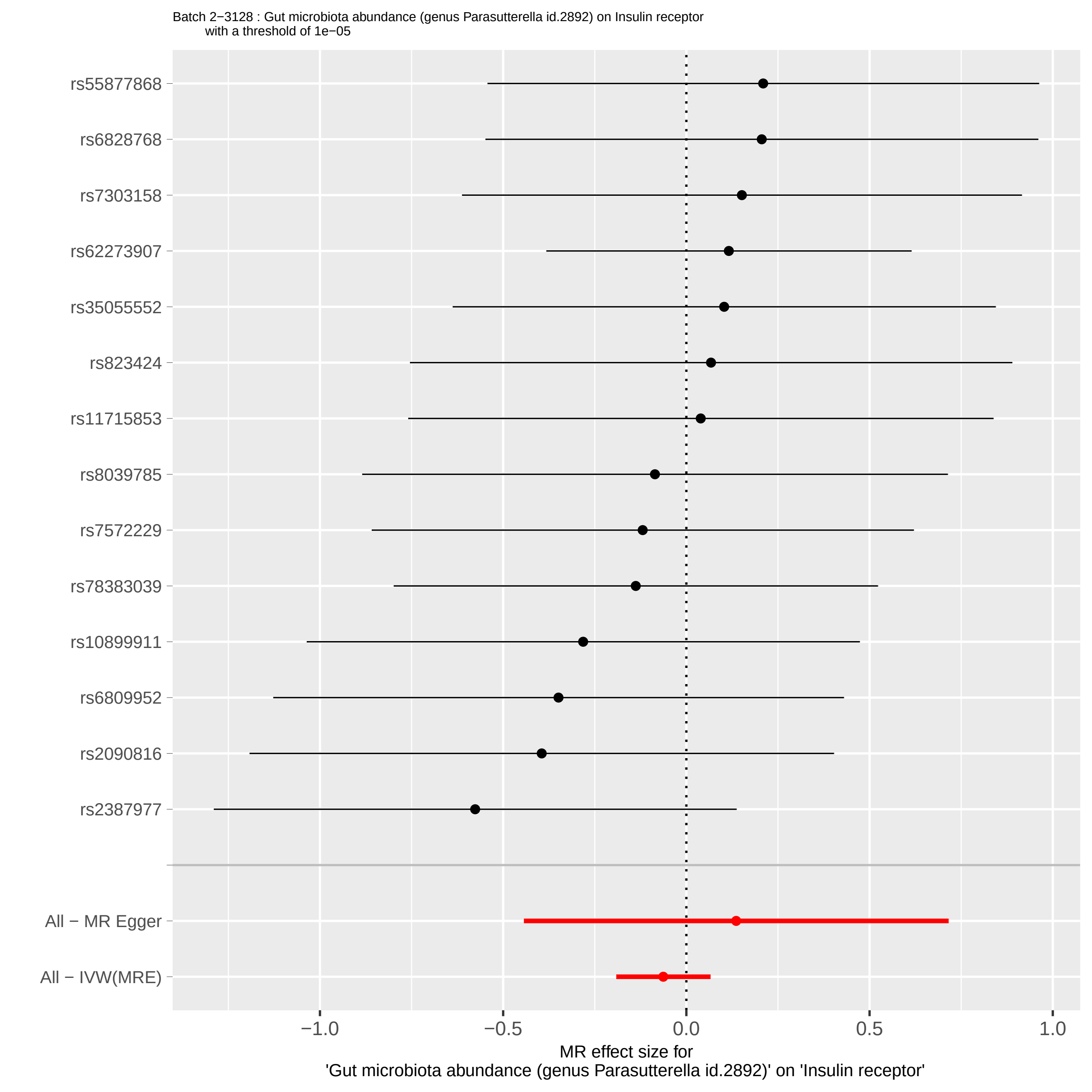

Batch 2−3128 : Gut microbiota abundance (genus Parasutterella id.2892) on Insulin receptor
with a threshold of 1e−05
rs55877868
rs6828768
rs7303158
rs62273907
rs35055552
rs823424
rs11715853
rs8039785
rs7572229
rs78383039
rs10899911
rs6809952
rs2090816
rs2387977
All − MR Egger
All − IVW(MRE)
−1.0
−0.5
0.0
0.5
1.0
MR effect size for
'Gut microbiota abundance (genus Parasutterella id.2892)' on 'Insulin receptor'

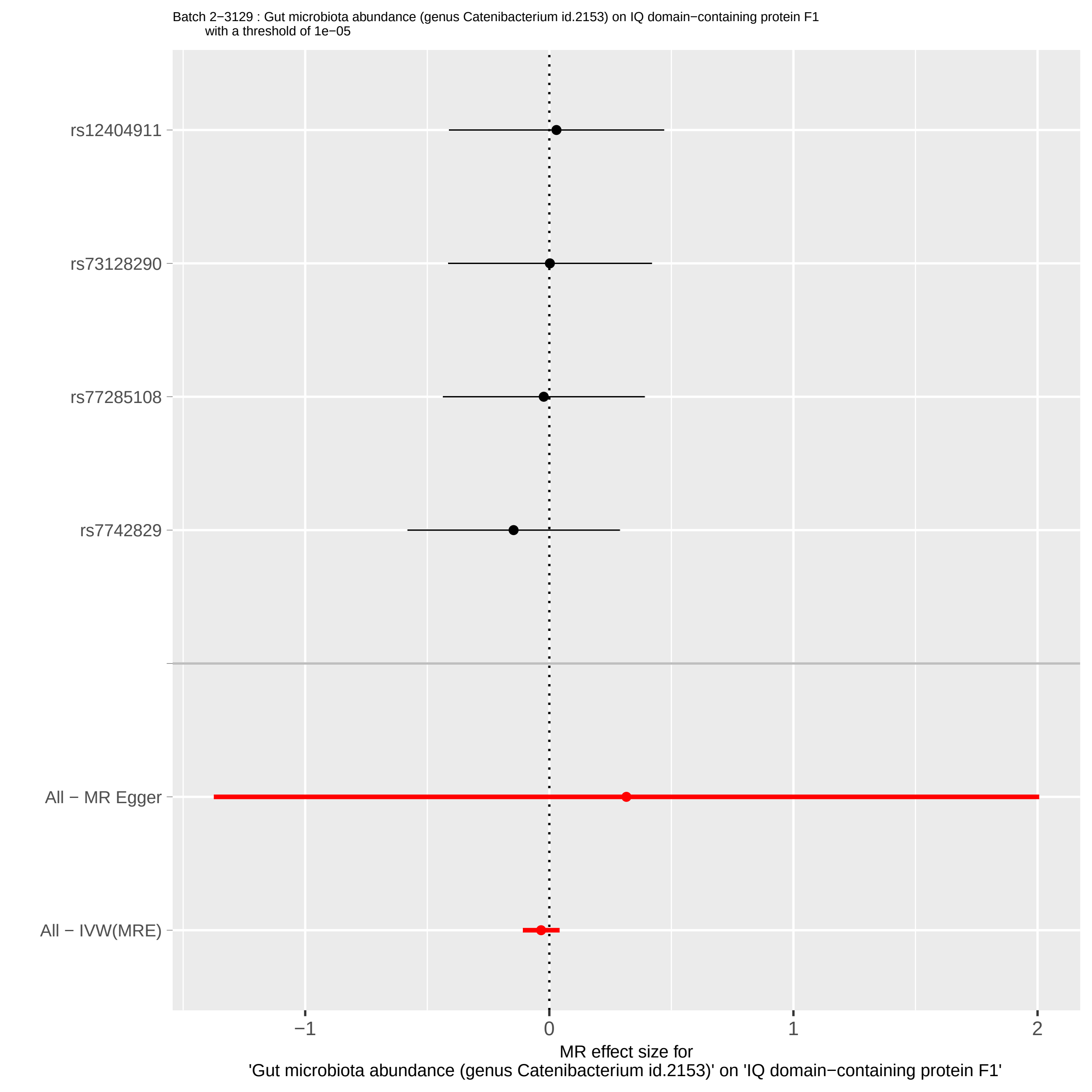
Batch 2−3129 : Gut microbiota abundance (genus Catenibacterium id.2153) on IQ domain−containing protein F1
with a threshold of 1e−05
rs12404911
rs73128290
rs77285108
rs7742829
All − MR Egger
All − IVW(MRE)
−1
0
1
2
MR effect size for
'Gut microbiota abundance (genus Catenibacterium id.2153)' on 'IQ domain−containing protein F1'

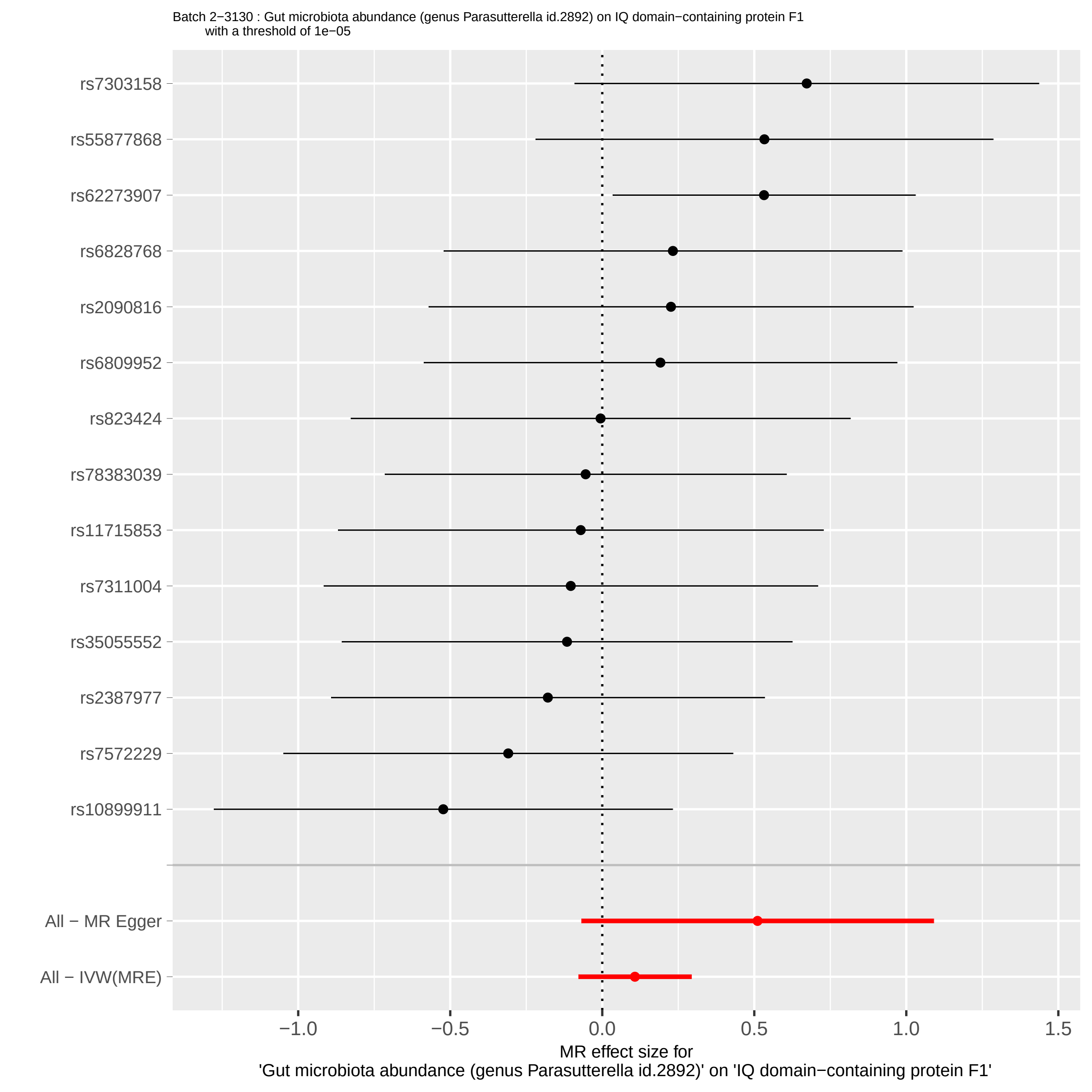
Batch 2−3130 : Gut microbiota abundance (genus Parasutterella id.2892) on IQ domain−containing protein F1
with a threshold of 1e−05
rs7303158
rs55877868
rs62273907
rs6828768
rs2090816
rs6809952
rs823424
rs78383039
rs11715853
rs7311004
rs35055552
rs2387977
rs7572229
rs10899911
All − MR Egger
All − IVW(MRE)
−1.0
−0.5
0.0
0.5
1.0
1.5
MR effect size for
'Gut microbiota abundance (genus Parasutterella id.2892)' on 'IQ domain−containing protein F1'

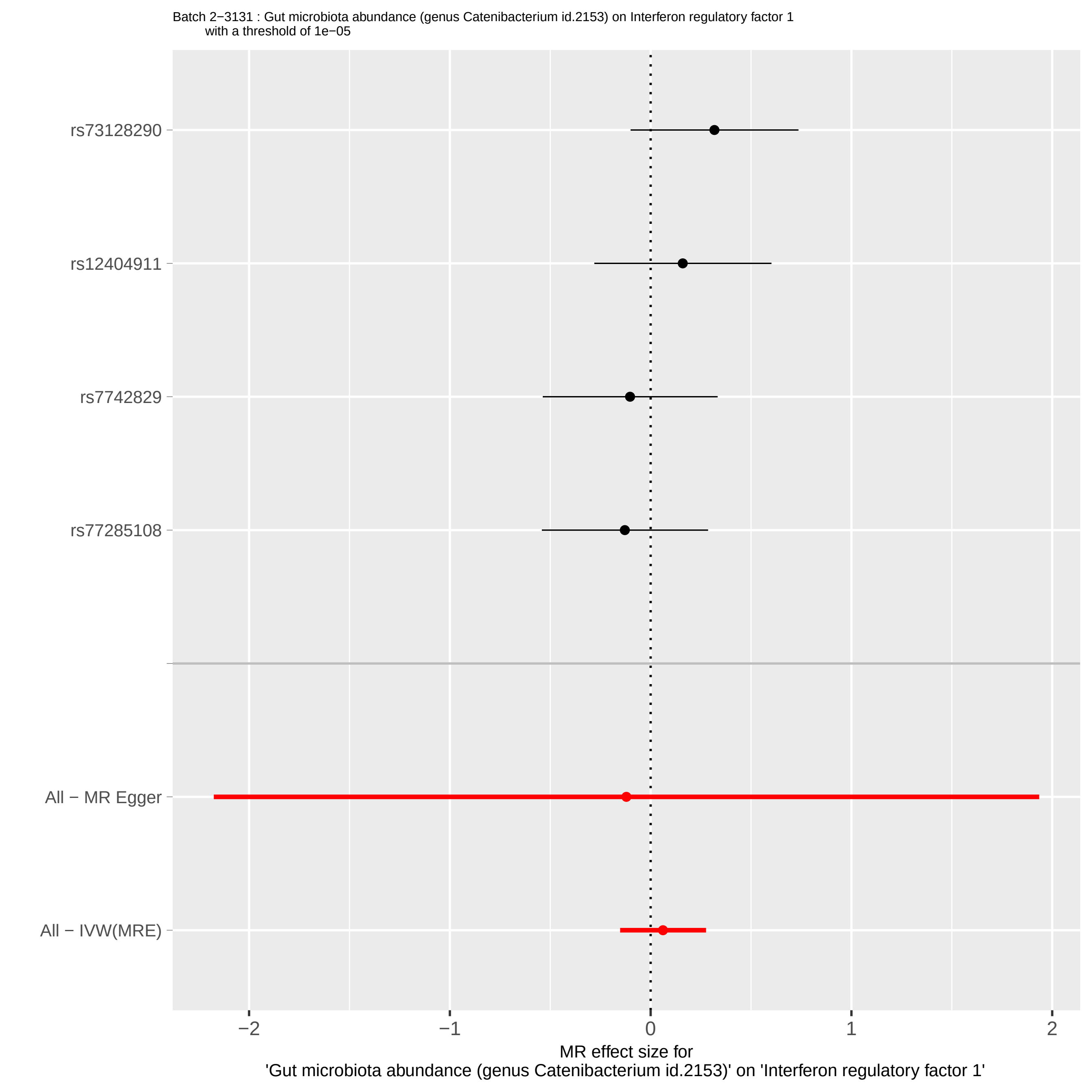
Batch 2−3131 : Gut microbiota abundance (genus Catenibacterium id.2153) on Interferon regulatory factor 1
with a threshold of 1e−05
rs73128290
rs12404911
rs7742829
rs77285108
All − MR Egger
All − IVW(MRE)
−2
−1
0
1
2
MR effect size for
'Gut microbiota abundance (genus Catenibacterium id.2153)' on 'Interferon regulatory factor 1'

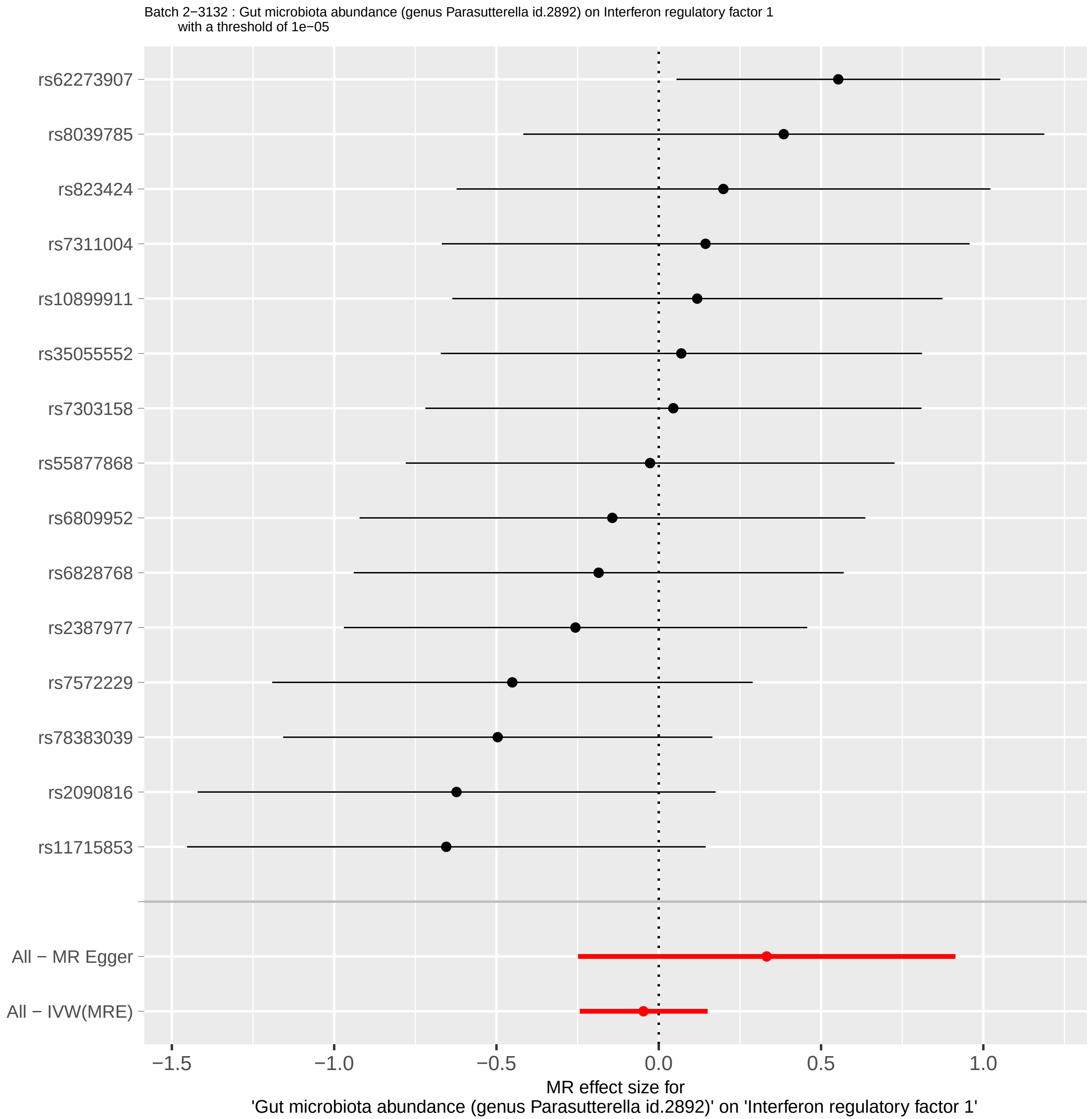
Batch 2−3132 : Gut microbiota abundance (genus Parasutterella id.2892) on Interferon regulatory factor 1
with a threshold of 1e−05
rs62273907
rs8039785
rs823424
rs7311004
rs10899911
rs35055552
rs7303158
rs55877868
rs6809952
rs6828768
rs2387977
rs7572229
rs78383039
rs2090816
rs11715853
All − MR Egger
All − IVW(MRE)
−1.5
−1.0
−0.5
0.0
0.5
1.0
MR effect size for
'Gut microbiota abundance (genus Parasutterella id.2892)' on 'Interferon regulatory factor 1'

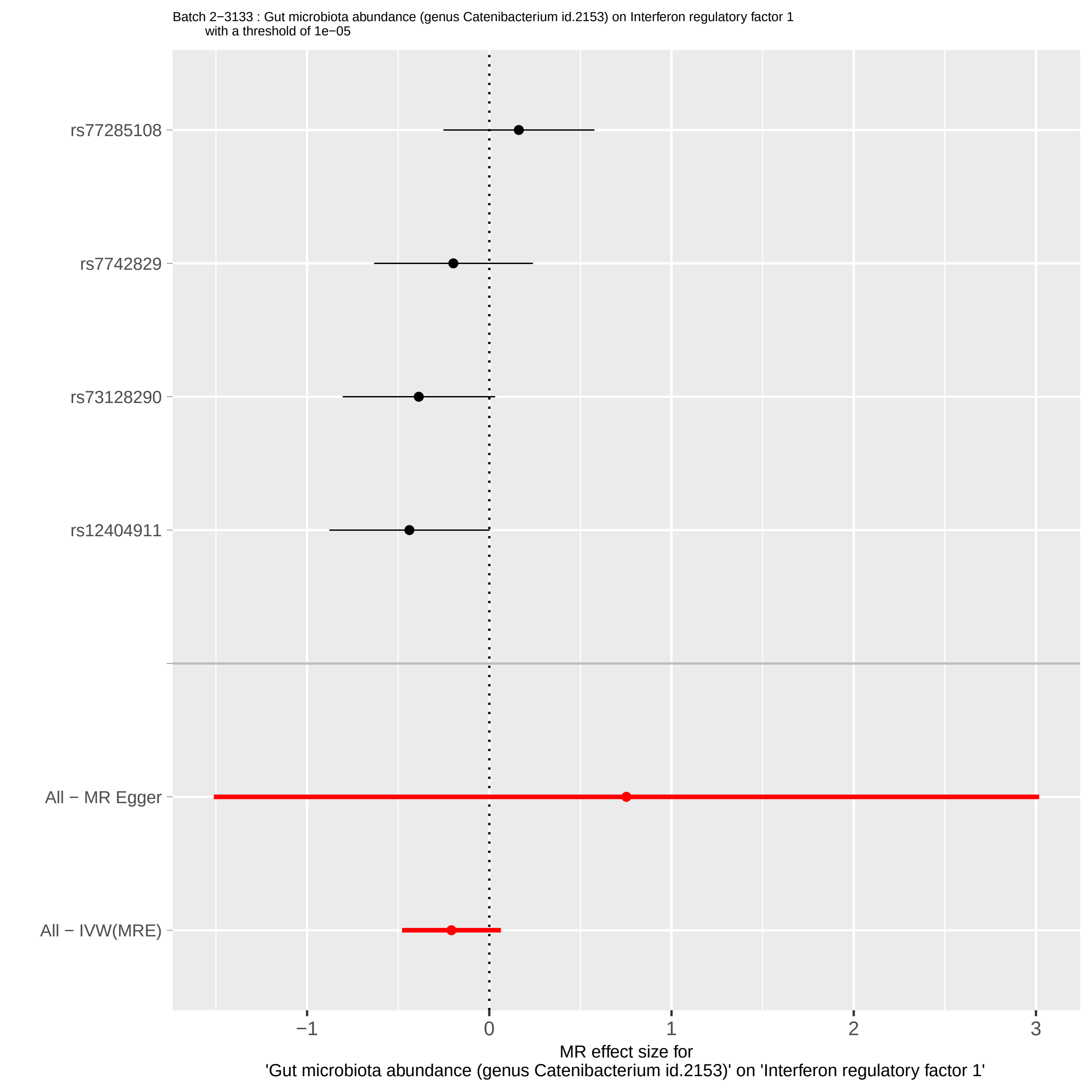
Batch 2−3133 : Gut microbiota abundance (genus Catenibacterium id.2153) on Interferon regulatory factor 1
with a threshold of 1e−05
rs77285108
rs7742829
rs73128290
rs12404911
All − MR Egger
All − IVW(MRE)
−1
0
1
2
3
MR effect size for
'Gut microbiota abundance (genus Catenibacterium id.2153)' on 'Interferon regulatory factor 1'

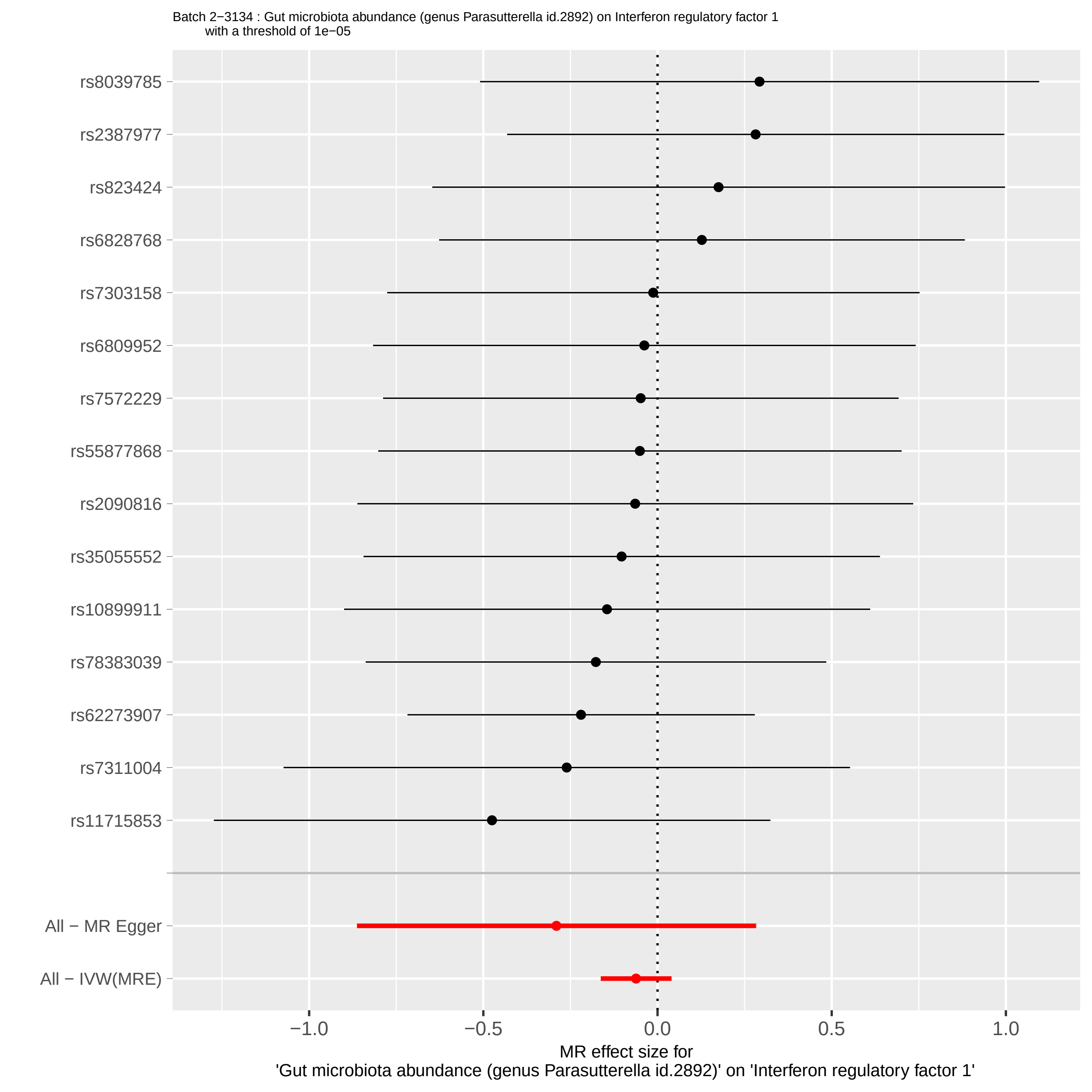

Batch 2−3134 : Gut microbiota abundance (genus Parasutterella id.2892) on Interferon regulatory factor 1
with a threshold of 1e−05
rs8039785
rs2387977
rs823424
rs6828768
rs7303158
rs6809952
rs7572229
rs55877868
rs2090816
rs35055552
rs10899911
rs78383039
rs62273907
rs7311004
rs11715853
All − MR Egger
All − IVW(MRE)
−1.0
−0.5
0.0
0.5
1.0
MR effect size for
'Gut microbiota abundance (genus Parasutterella id.2892)' on 'Interferon regulatory factor 1'

Batch 2−3135 : Gut microbiota abundance (genus Catenibacterium id.2153) on Interferon regulatory factor 2
with a threshold of 1e−05

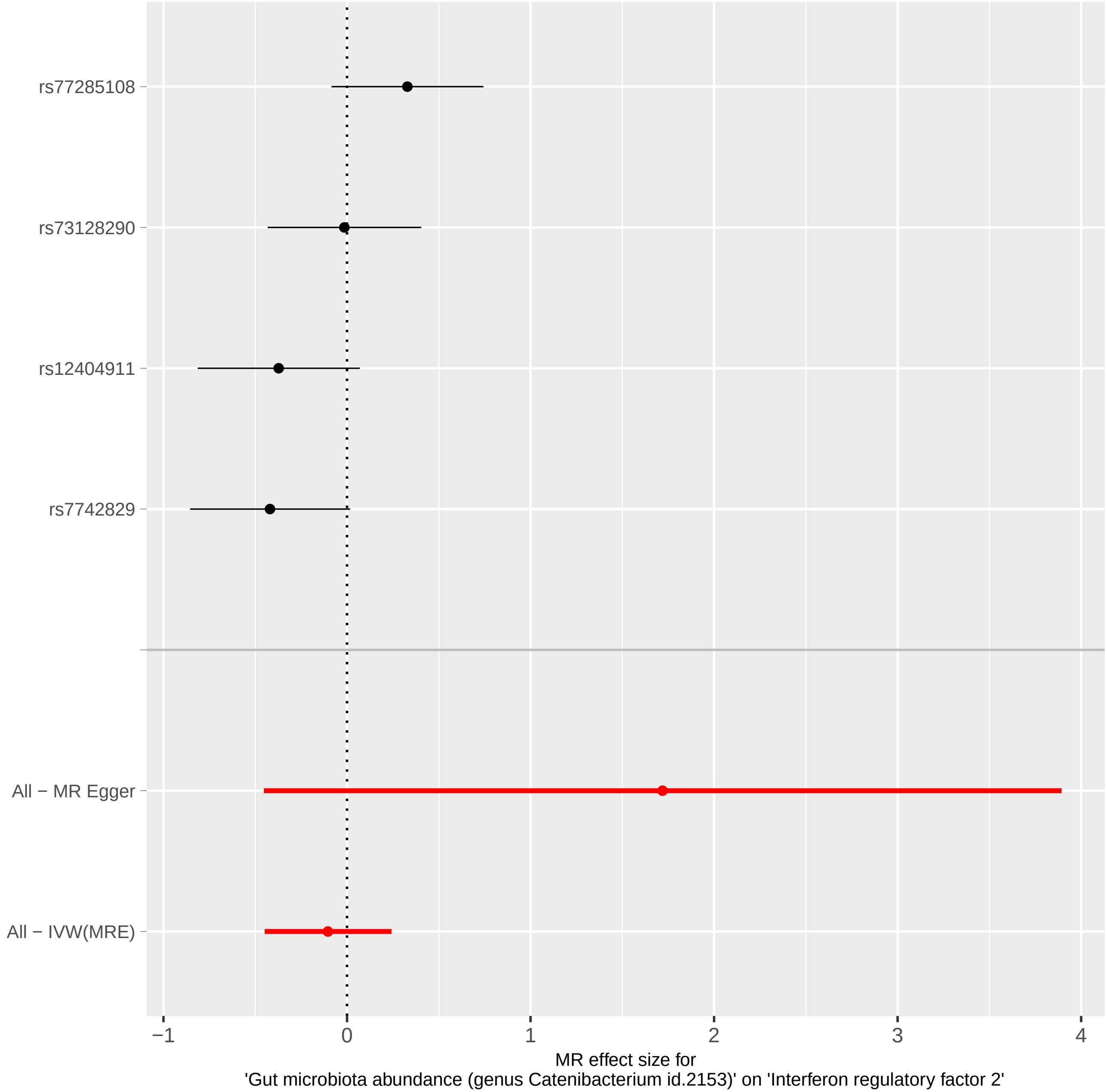

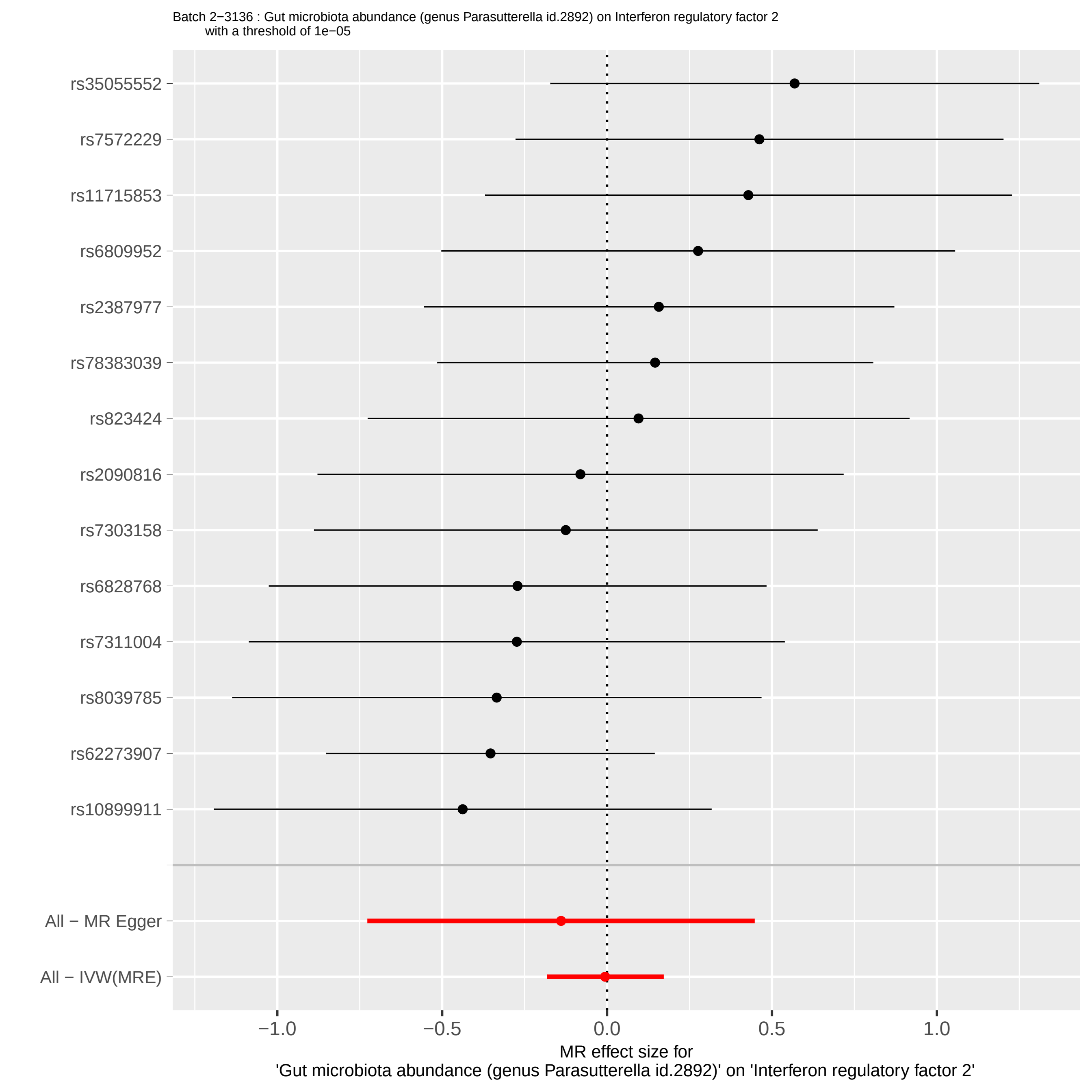
Batch 2−3136 : Gut microbiota abundance (genus Parasutterella id.2892) on Interferon regulatory factor 2
with a threshold of 1e−05
rs35055552
rs7572229
rs11715853
rs6809952
rs2387977
rs78383039
rs823424
rs2090816
rs7303158
rs6828768
rs7311004
rs8039785
rs62273907
rs10899911
All − MR Egger
All − IVW(MRE)
−1.0
−0.5
0.0
0.5
1.0
MR effect size for
'Gut microbiota abundance (genus Parasutterella id.2892)' on 'Interferon regulatory factor 2'

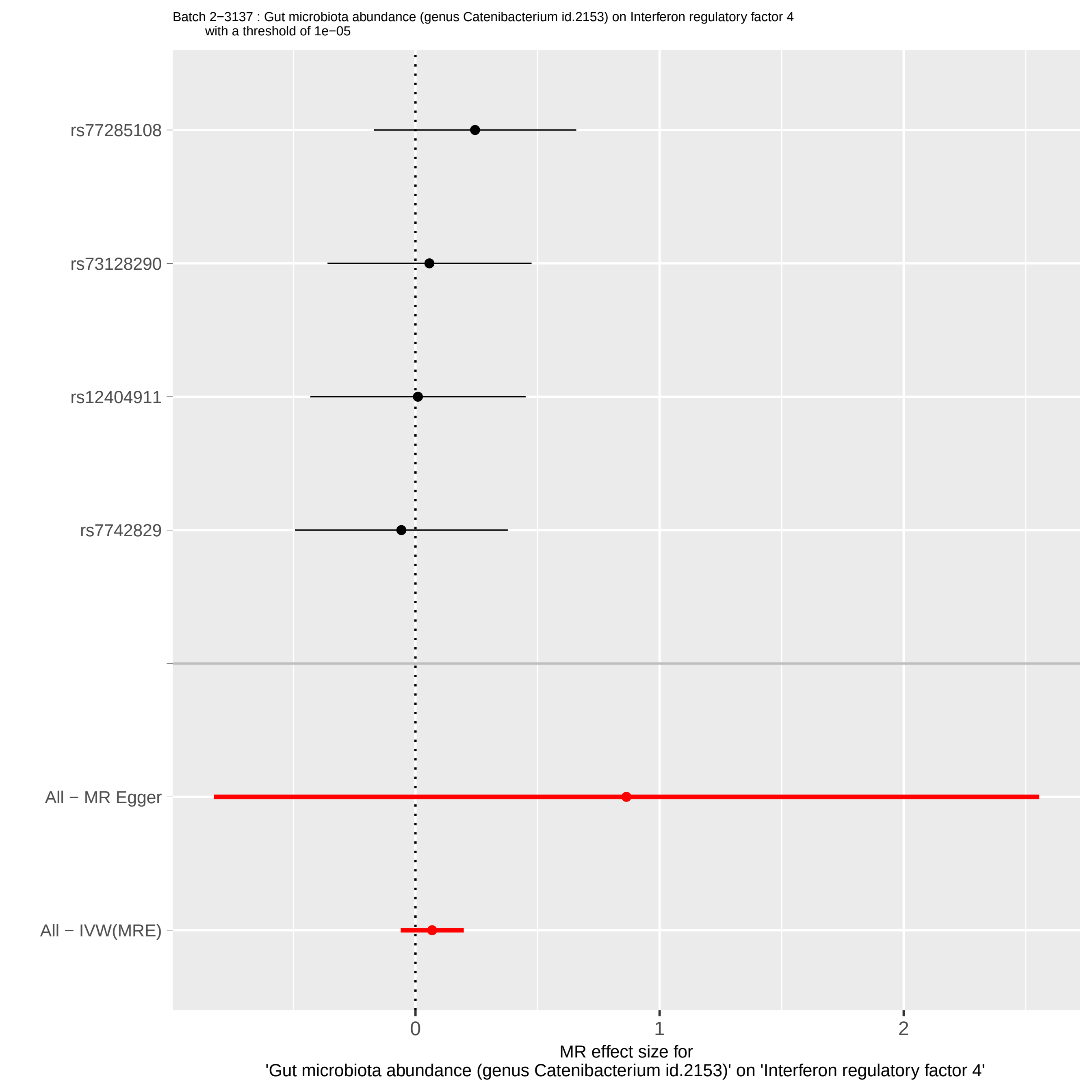

Batch 2−3137 : Gut microbiota abundance (genus Catenibacterium id.2153) on Interferon regulatory factor 4
with a threshold of 1e−05
rs77285108
rs73128290
rs12404911
rs7742829
All − MR Egger
All − IVW(MRE)
0
1
2
MR effect size for
'Gut microbiota abundance (genus Catenibacterium id.2153)' on 'Interferon regulatory factor 4'

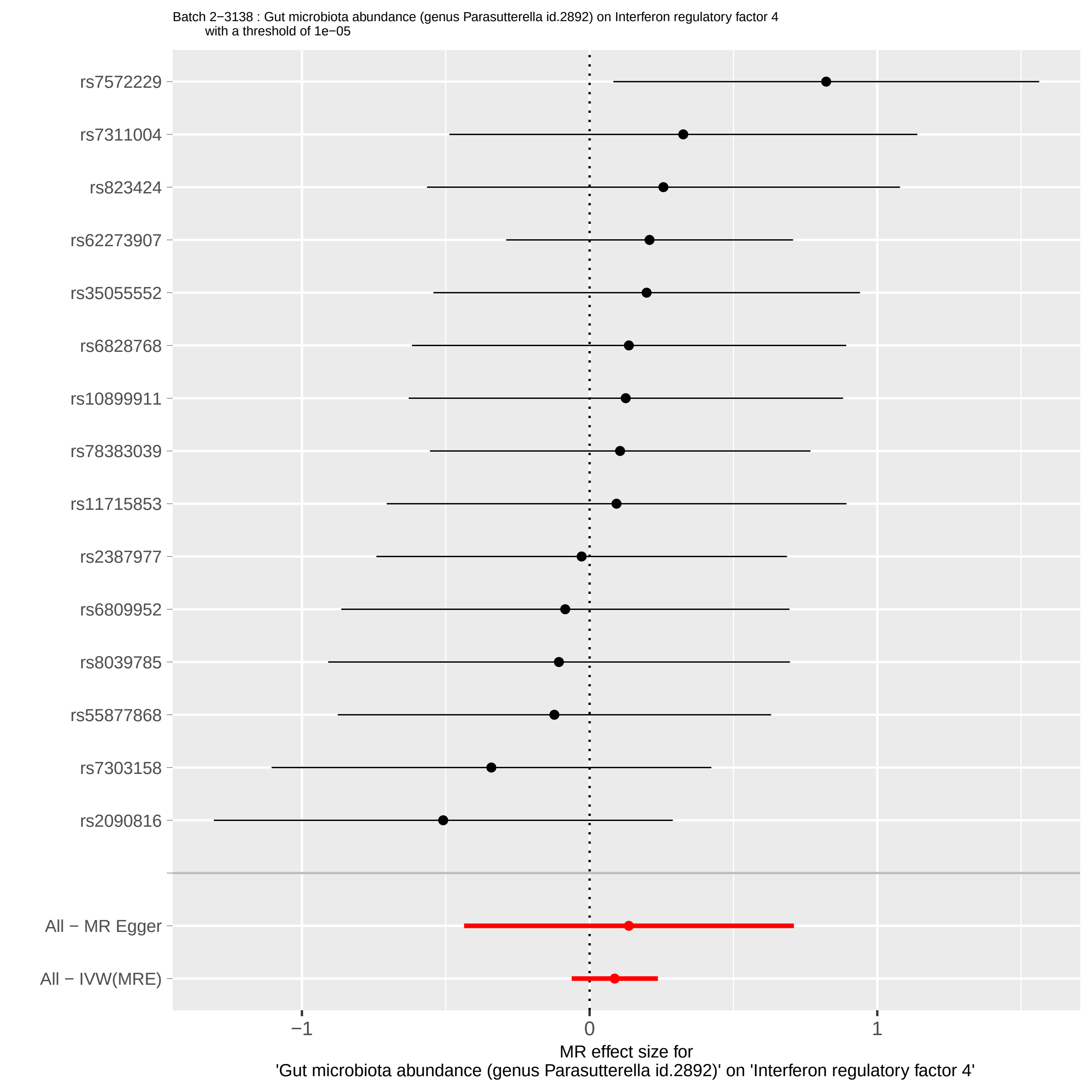

Batch 2−3138 : Gut microbiota abundance (genus Parasutterella id.2892) on Interferon regulatory factor 4
with a threshold of 1e−05
rs7572229
rs7311004
rs823424
rs62273907
rs35055552
rs6828768
rs10899911
rs78383039
rs11715853
rs2387977
rs6809952
rs8039785
rs55877868
rs7303158
rs2090816
All − MR Egger
All − IVW(MRE)
−1
0
1
MR effect size for
'Gut microbiota abundance (genus Parasutterella id.2892)' on 'Interferon regulatory factor 4'

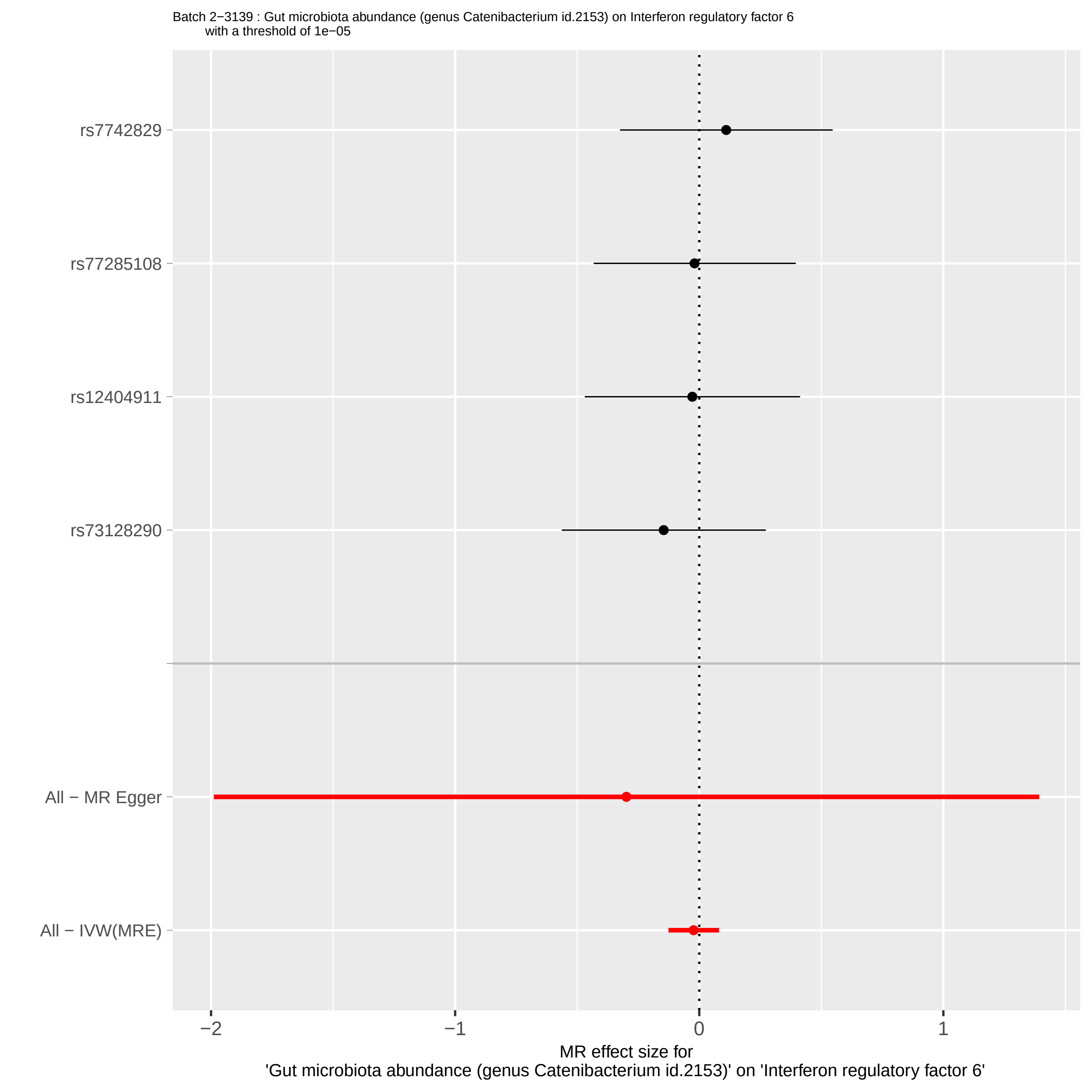
Batch 2−3139 : Gut microbiota abundance (genus Catenibacterium id.2153) on Interferon regulatory factor 6
with a threshold of 1e−05
rs7742829
rs77285108
rs12404911
rs73128290
All − MR Egger
All − IVW(MRE)
−2
−1
0
1
MR effect size for
'Gut microbiota abundance (genus Catenibacterium id.2153)' on 'Interferon regulatory factor 6'

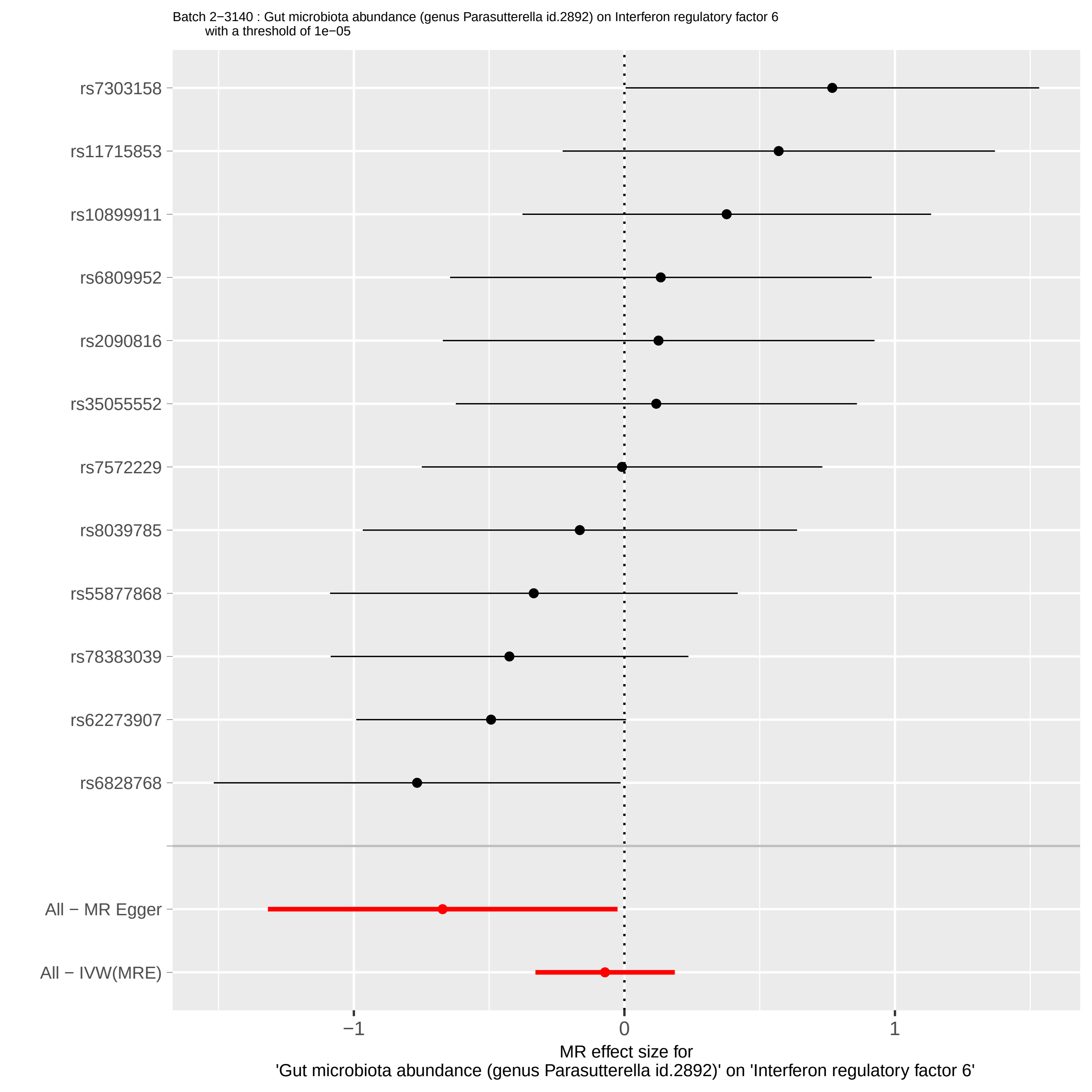

Batch 2−3140 : Gut microbiota abundance (genus Parasutterella id.2892) on Interferon regulatory factor 6
with a threshold of 1e−05
rs7303158
rs11715853
rs10899911
rs6809952
rs2090816
rs35055552
rs7572229
rs8039785
rs55877868
rs78383039
rs62273907
rs6828768
All − MR Egger
All − IVW(MRE)
−1
0
1
MR effect size for
'Gut microbiota abundance (genus Parasutterella id.2892)' on 'Interferon regulatory factor 6'

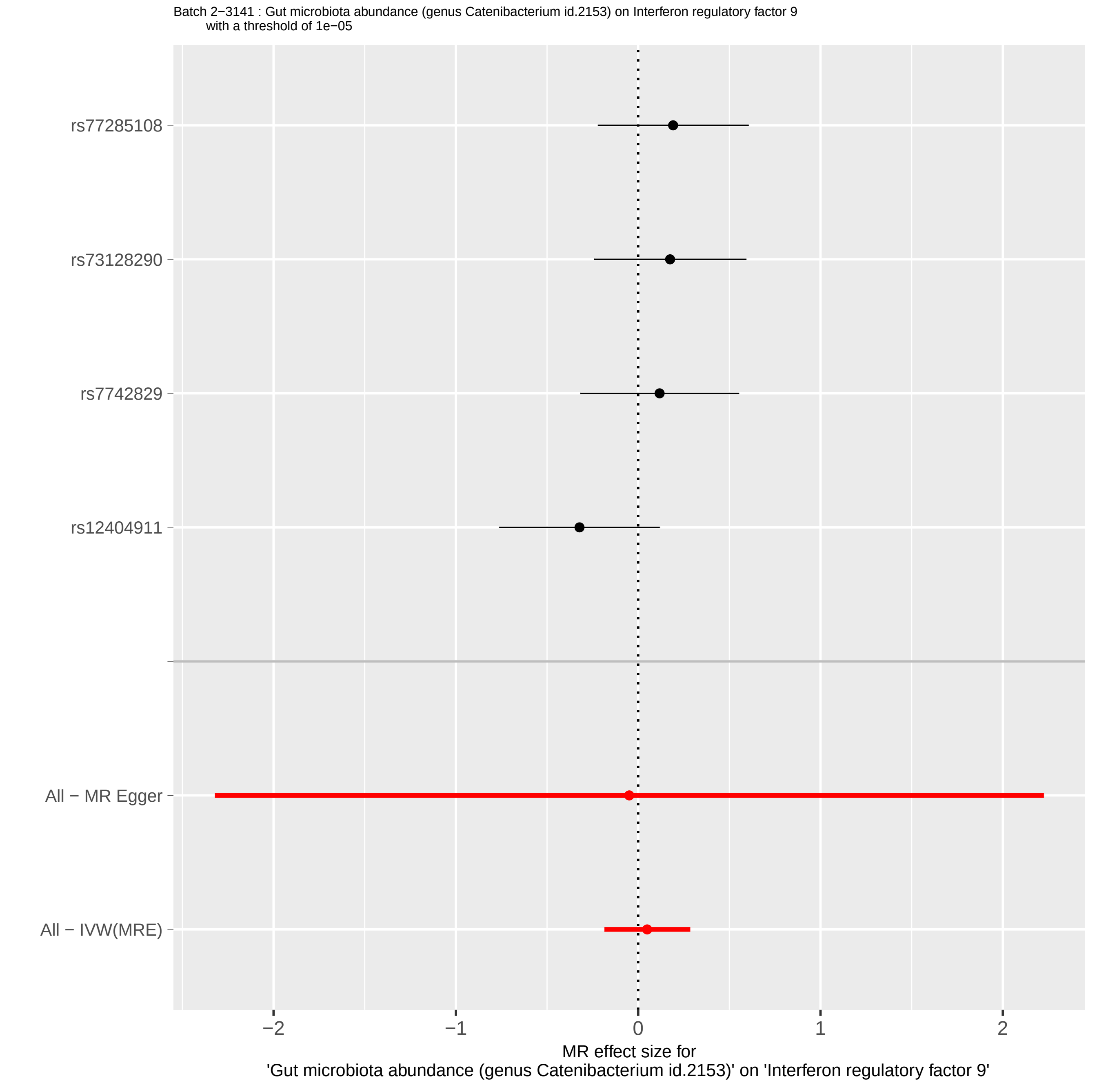
Batch 2−3141 : Gut microbiota abundance (genus Catenibacterium id.2153) on Interferon regulatory factor 9
with a threshold of 1e−05
rs77285108
rs73128290
rs7742829
rs12404911
All − MR Egger
All − IVW(MRE)
−2
−1
0
1
2
MR effect size for
'Gut microbiota abundance (genus Catenibacterium id.2153)' on 'Interferon regulatory factor 9'

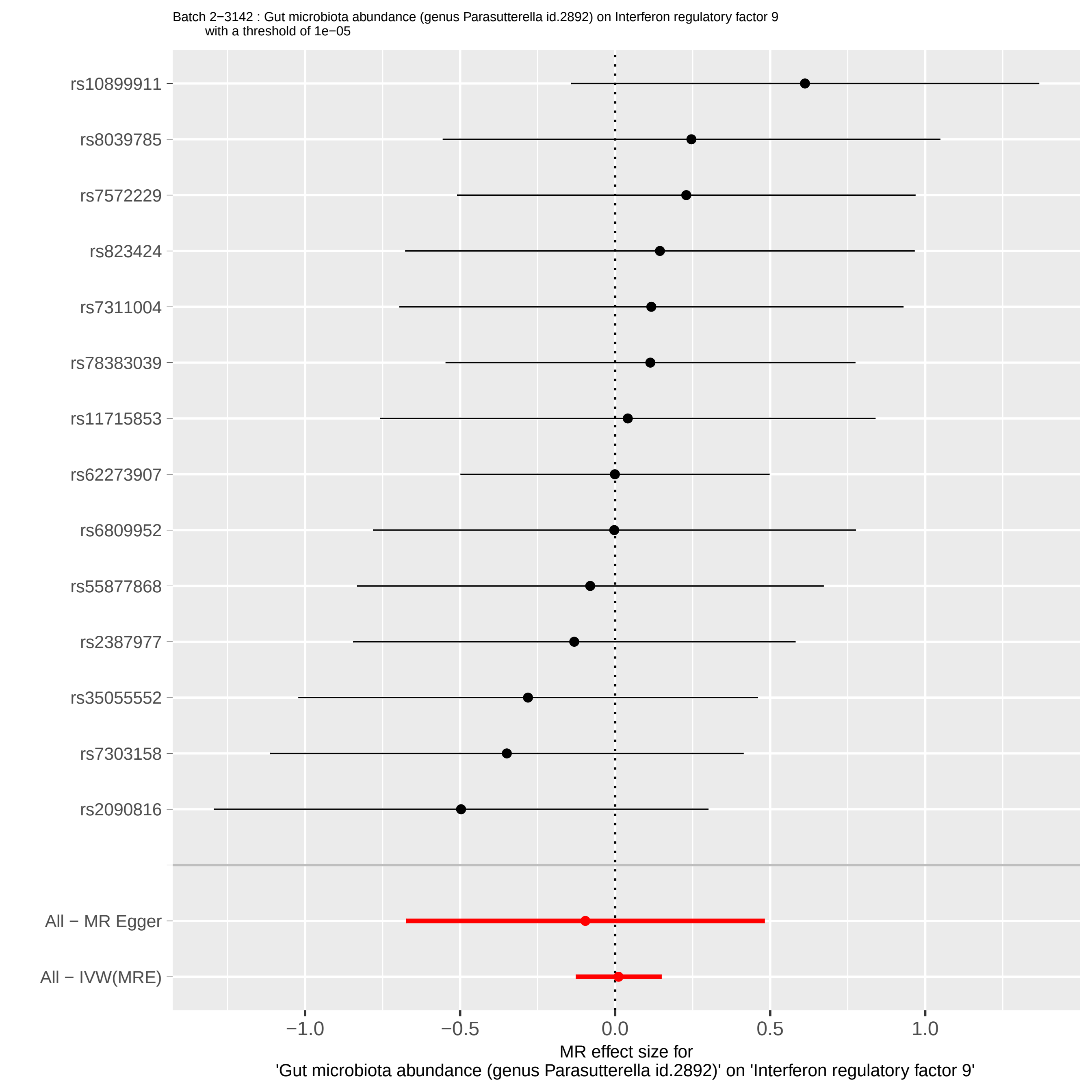
Batch 2−3142 : Gut microbiota abundance (genus Parasutterella id.2892) on Interferon regulatory factor 9
with a threshold of 1e−05
rs10899911
rs8039785
rs7572229
rs823424
rs7311004
rs78383039
rs11715853
rs62273907
rs6809952
rs55877868
rs2387977
rs35055552
rs7303158
rs2090816
All − MR Egger
All − IVW(MRE)
−1.0
−0.5
0.0
0.5
1.0
MR effect size for
'Gut microbiota abundance (genus Parasutterella id.2892)' on 'Interferon regulatory factor 9'

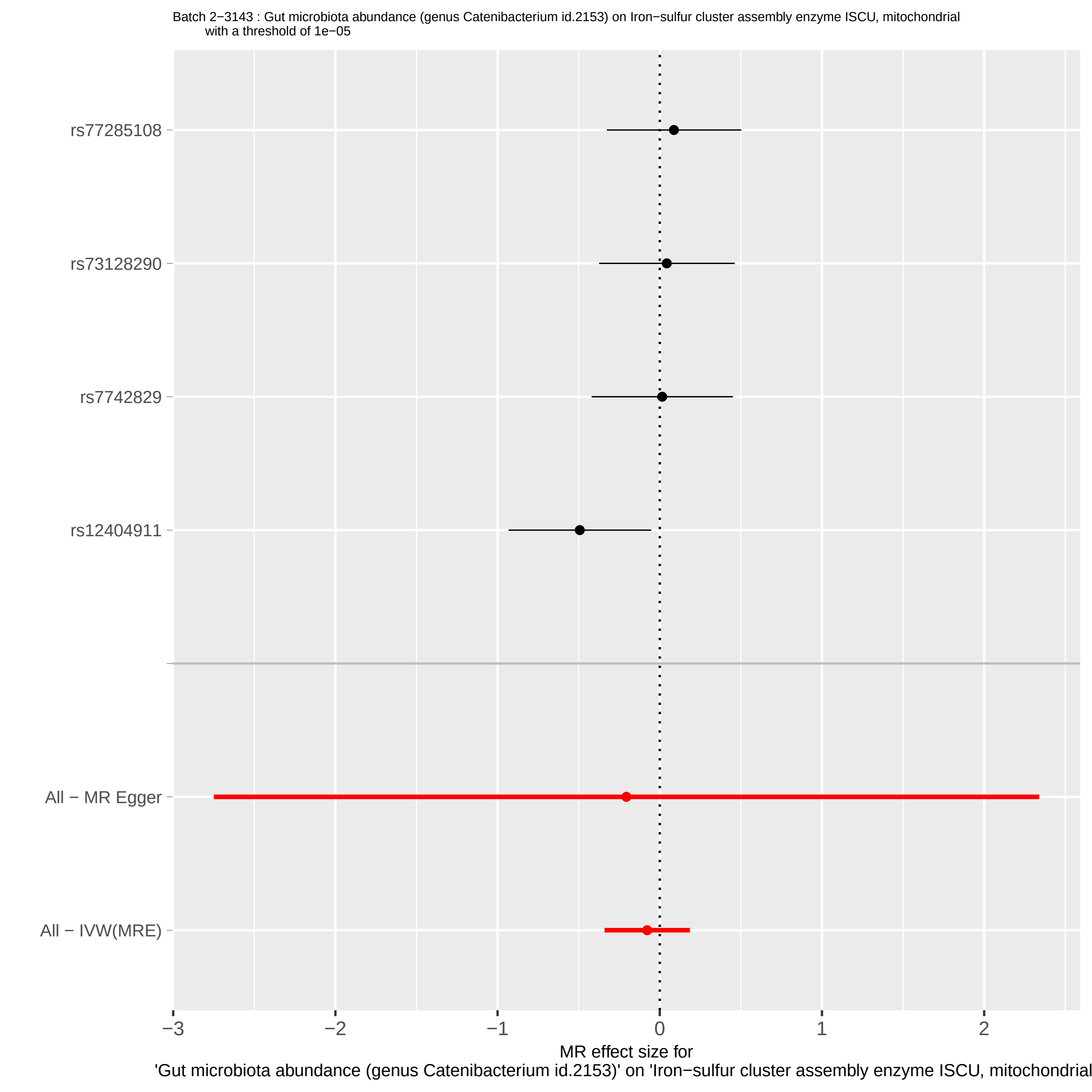
Batch 2−3143 : Gut microbiota abundance (genus Catenibacterium id.2153) on Iron−sulfur cluster assembly enzyme ISCU, mitochondrial
with a threshold of 1e−05
rs77285108
rs73128290
rs7742829
rs12404911
All − MR Egger
All − IVW(MRE)
−3
−2
−1
0
1
2
MR effect size for
'Gut microbiota abundance (genus Catenibacterium id.2153)' on 'Iron−sulfur cluster assembly enzyme ISCU, mitochondrial'

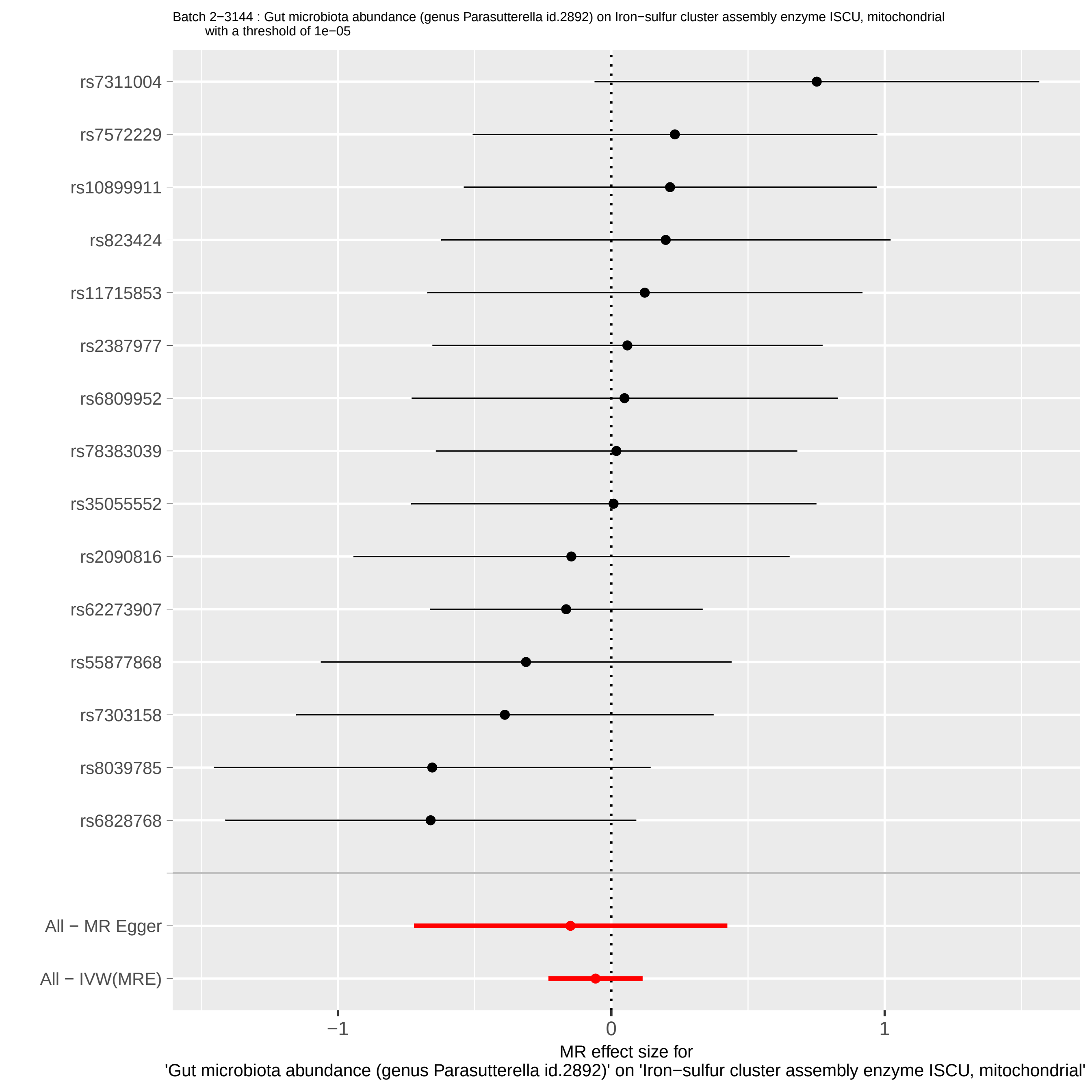

Batch 2−3144 : Gut microbiota abundance (genus Parasutterella id.2892) on Iron−sulfur cluster assembly enzyme ISCU, mitochondrial
with a threshold of 1e−05
rs7311004
rs7572229
rs10899911
rs823424
rs11715853
rs2387977
rs6809952
rs78383039
rs35055552
rs2090816
rs62273907
rs55877868
rs7303158
rs8039785
rs6828768
All − MR Egger
All − IVW(MRE)
−1
0
1
MR effect size for
'Gut microbiota abundance (genus Parasutterella id.2892)' on 'Iron−sulfur cluster assembly enzyme ISCU, mitochondrial'

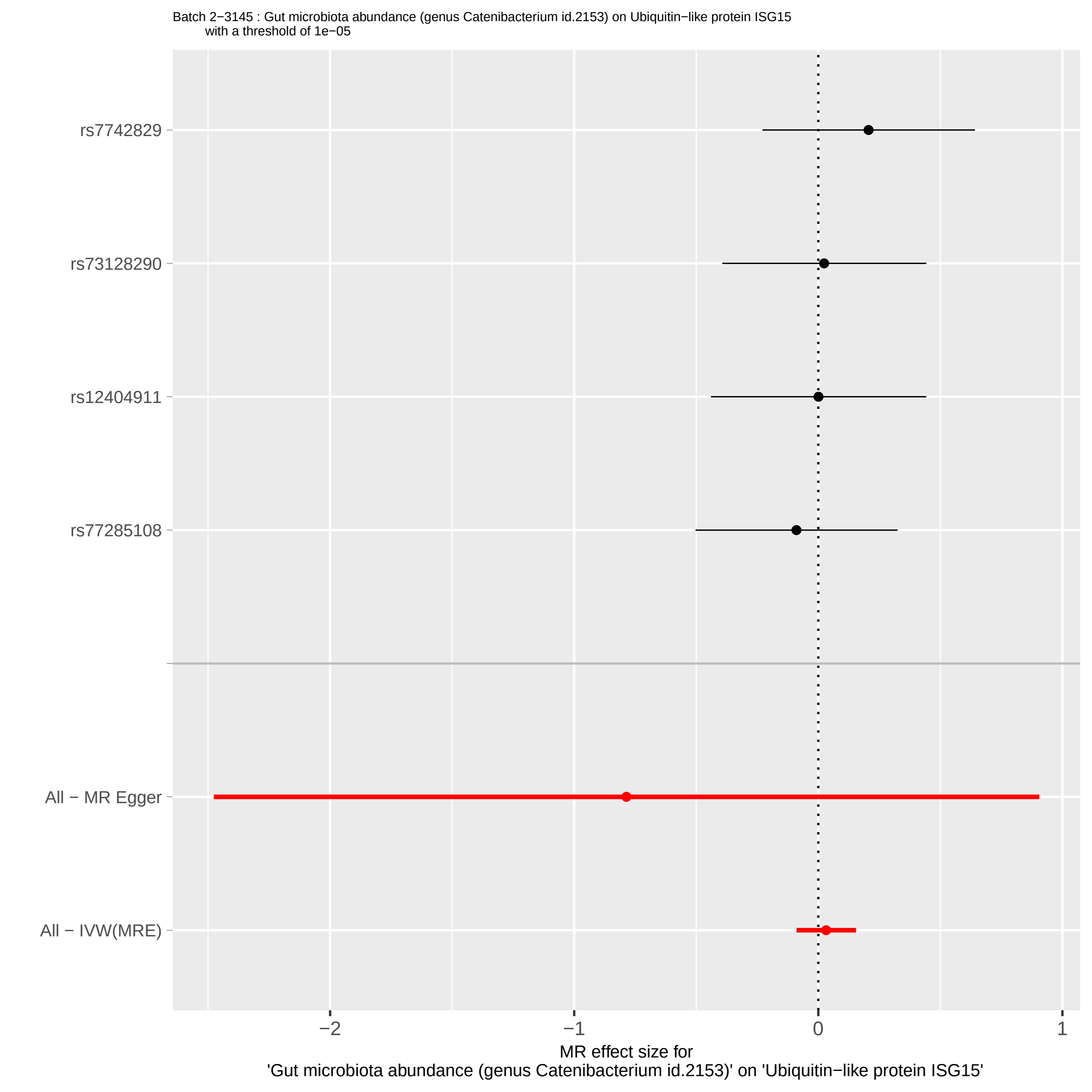
Batch 2−3145 : Gut microbiota abundance (genus Catenibacterium id.2153) on Ubiquitin−like protein ISG15
with a threshold of 1e−05
rs7742829
rs73128290
rs12404911
rs77285108
All − MR Egger
All − IVW(MRE)
−2
−1
0
1
MR effect size for
'Gut microbiota abundance (genus Catenibacterium id.2153)' on 'Ubiquitin−like protein ISG15'

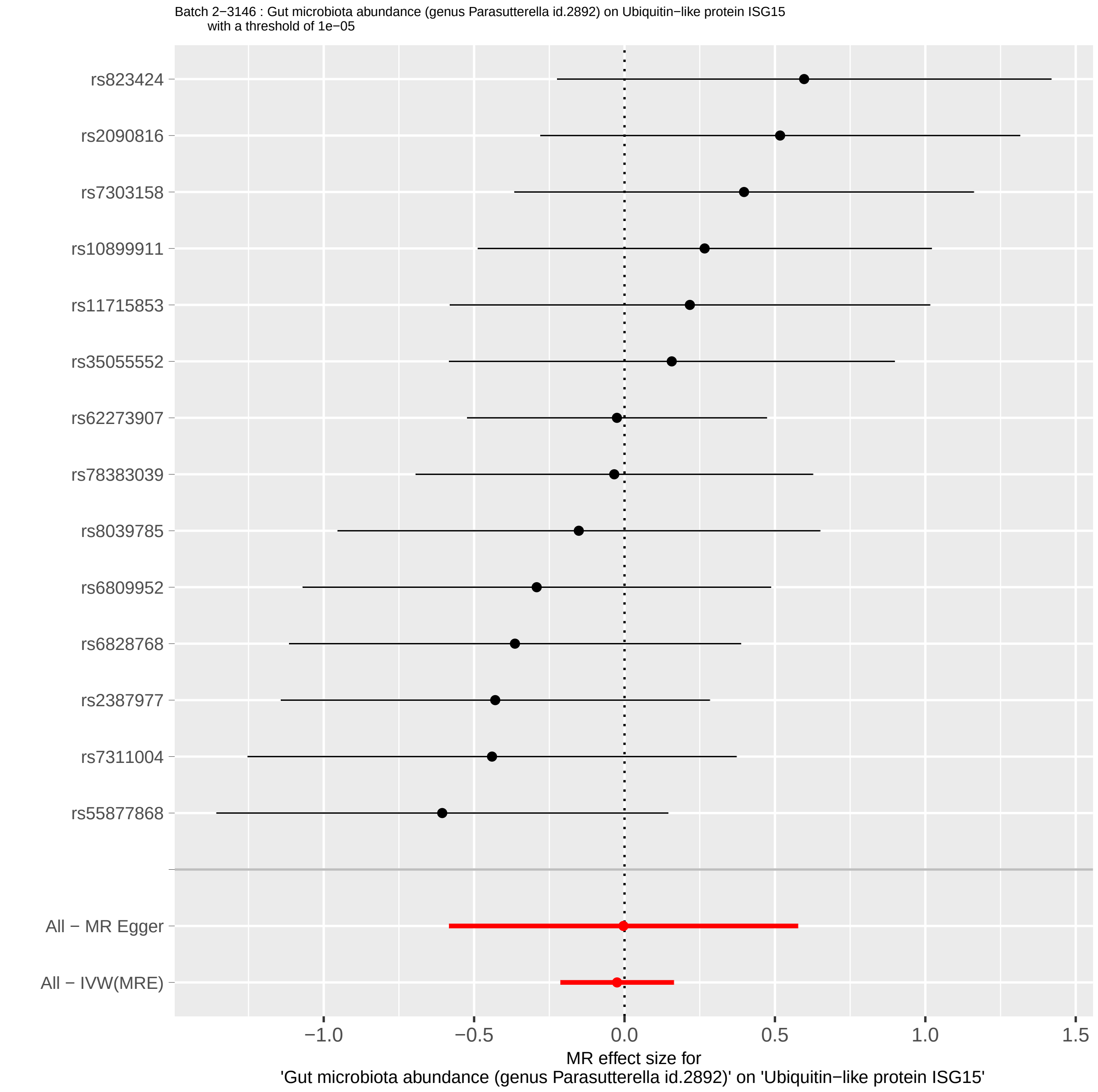
Batch 2−3146 : Gut microbiota abundance (genus Parasutterella id.2892) on Ubiquitin−like protein ISG15
with a threshold of 1e−05
rs823424
rs2090816
rs7303158
rs10899911
rs11715853
rs35055552
rs62273907
rs78383039
rs8039785
rs6809952
rs6828768
rs2387977
rs7311004
rs55877868
All − MR Egger
All − IVW(MRE)
−1.0
−0.5
0.0
0.5
1.0
1.5
MR effect size for
'Gut microbiota abundance (genus Parasutterella id.2892)' on 'Ubiquitin−like protein ISG15'

Batch 2−3147 : Gut microbiota abundance (genus Catenibacterium id.2153) on Ubiquitin−like protein ISG15
with a threshold of 1e−05

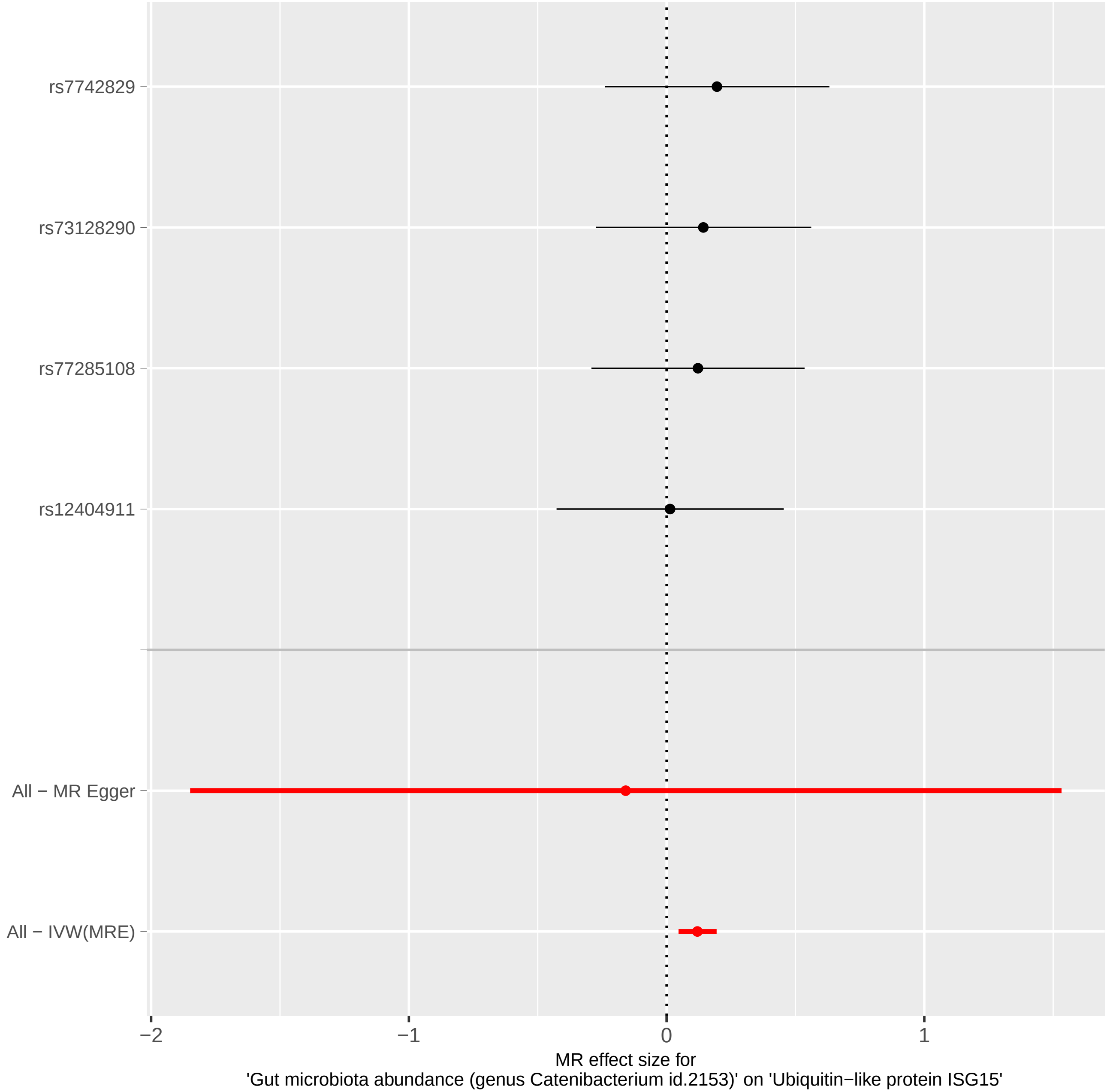

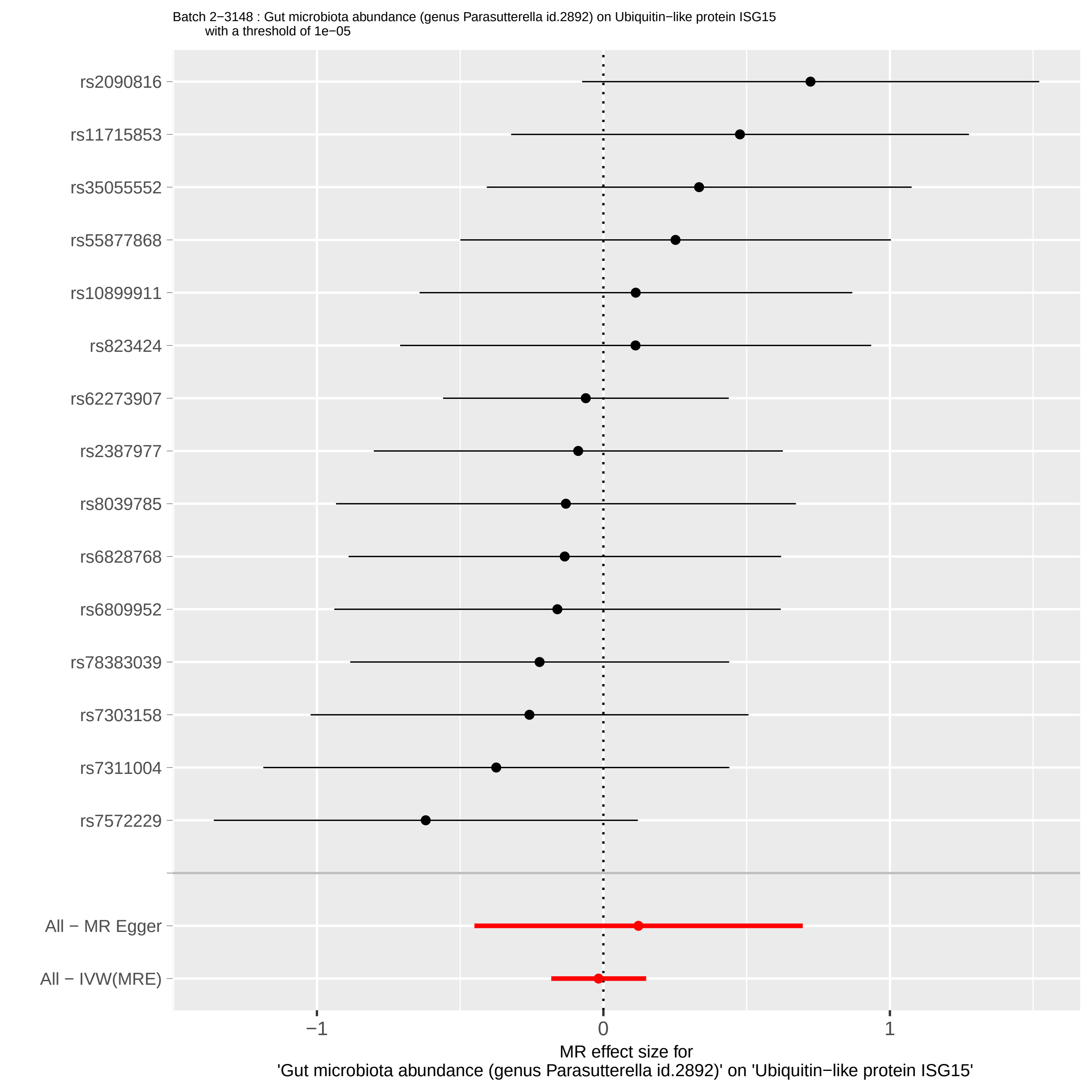

Batch 2−3148 : Gut microbiota abundance (genus Parasutterella id.2892) on Ubiquitin−like protein ISG15
with a threshold of 1e−05
rs2090816
rs11715853
rs35055552
rs55877868
rs10899911
rs823424
rs62273907
rs2387977
rs8039785
rs6828768
rs6809952
rs78383039
rs7303158
rs7311004
rs7572229
All − MR Egger
All − IVW(MRE)
−1
0
1
MR effect size for
'Gut microbiota abundance (genus Parasutterella id.2892)' on 'Ubiquitin−like protein ISG15'

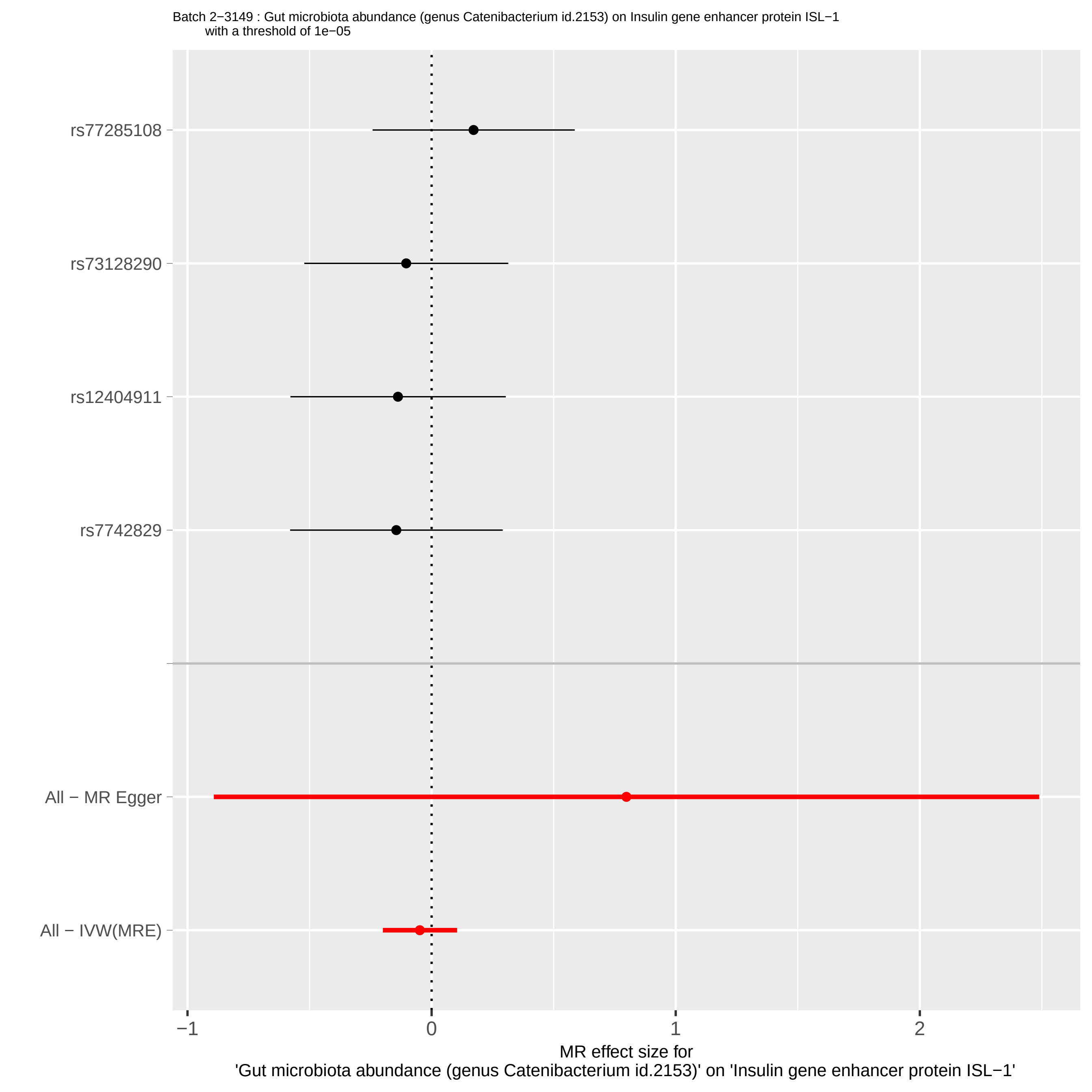

Batch 2−3149 : Gut microbiota abundance (genus Catenibacterium id.2153) on Insulin gene enhancer protein ISL−1
with a threshold of 1e−05
rs77285108
rs73128290
rs12404911
rs7742829
All − MR Egger
All − IVW(MRE)
−1
0
1
2
MR effect size for
'Gut microbiota abundance (genus Catenibacterium id.2153)' on 'Insulin gene enhancer protein ISL−1'

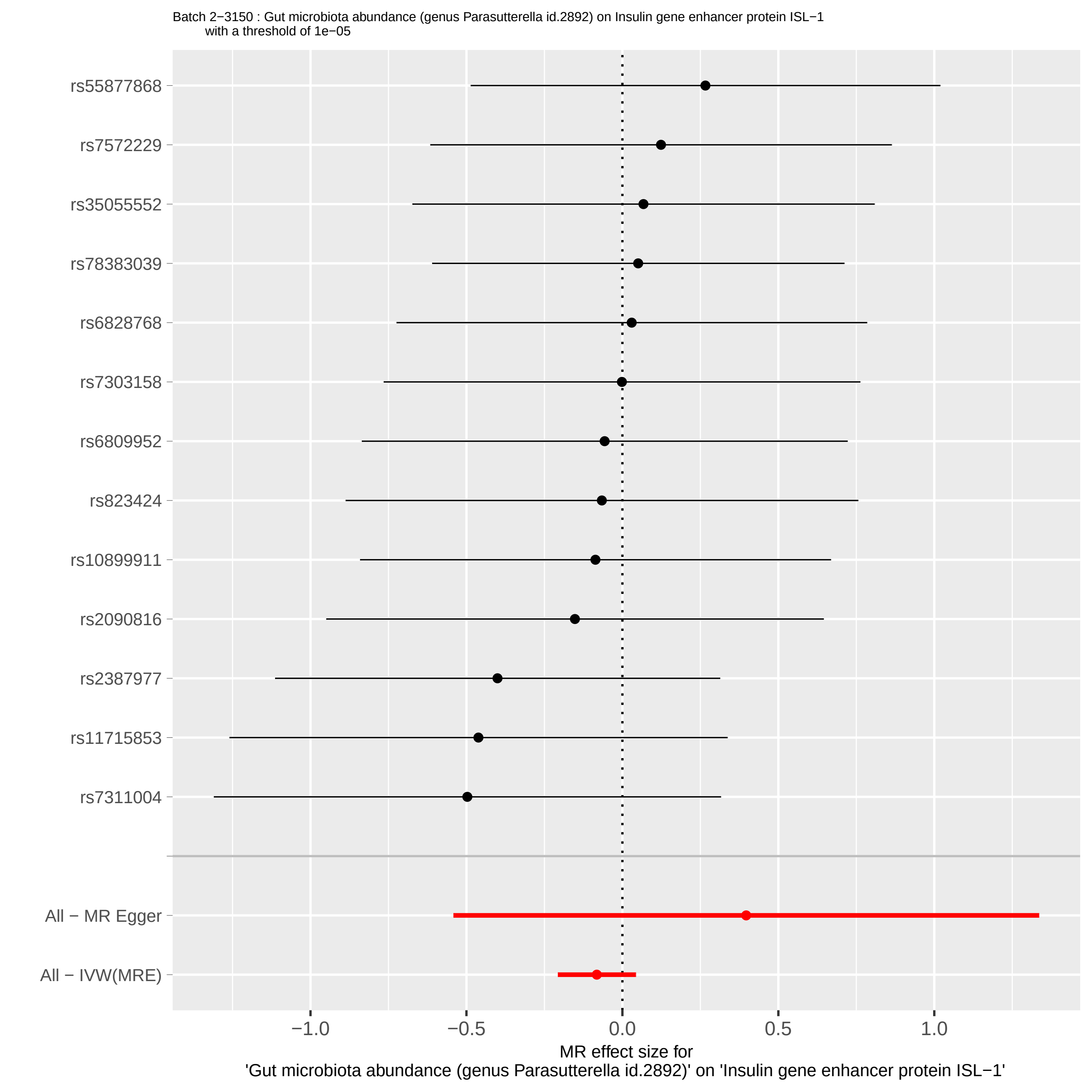

Batch 2−3150 : Gut microbiota abundance (genus Parasutterella id.2892) on Insulin gene enhancer protein ISL−1
with a threshold of 1e−05
rs55877868
rs7572229
rs35055552
rs78383039
rs6828768
rs7303158
rs6809952
rs823424
rs10899911
rs2090816
rs2387977
rs11715853
rs7311004
All − MR Egger
All − IVW(MRE)
−1.0
−0.5
0.0
0.5
1.0
MR effect size for
'Gut microbiota abundance (genus Parasutterella id.2892)' on 'Insulin gene enhancer protein ISL−1'

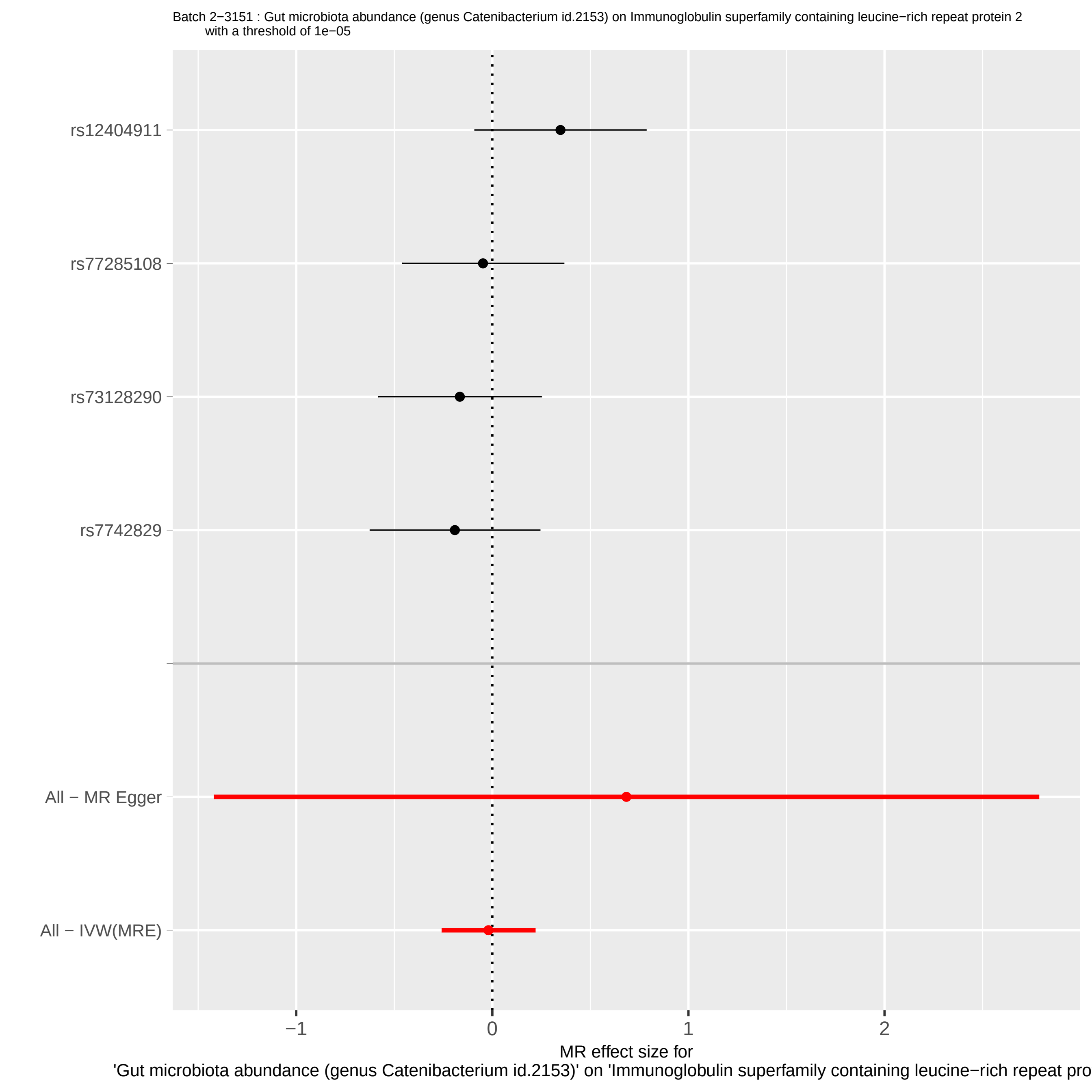
Batch 2−3151 : Gut microbiota abundance (genus Catenibacterium id.2153) on Immunoglobulin superfamily containing leucine−rich repeat protein 2 with a threshold of 1e−05
rs12404911
rs77285108
rs73128290
rs7742829
All − MR Egger
All − IVW(MRE)
−1
0
1
2
MR effect size for
'Gut microbiota abundance (genus Catenibacterium id.2153)' on 'Immunoglobulin superfamily containing leucine−rich repeat pro

Batch 2−3152 : Gut microbiota abundance (genus Parasutterella id.2892) on Immunoglobulin superfamily containing leucine−rich repeat protein 2
with a threshold of 1e−05

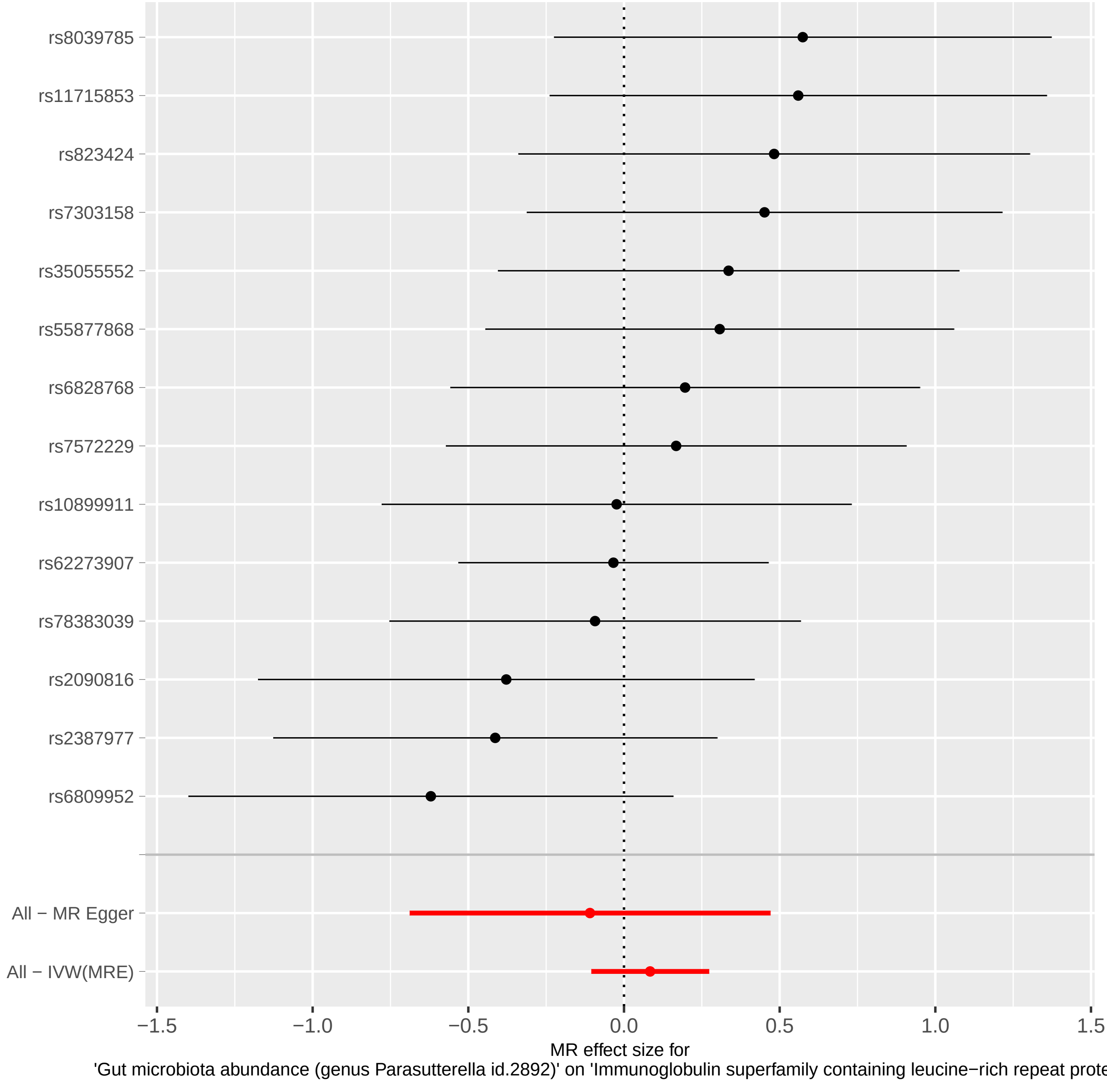

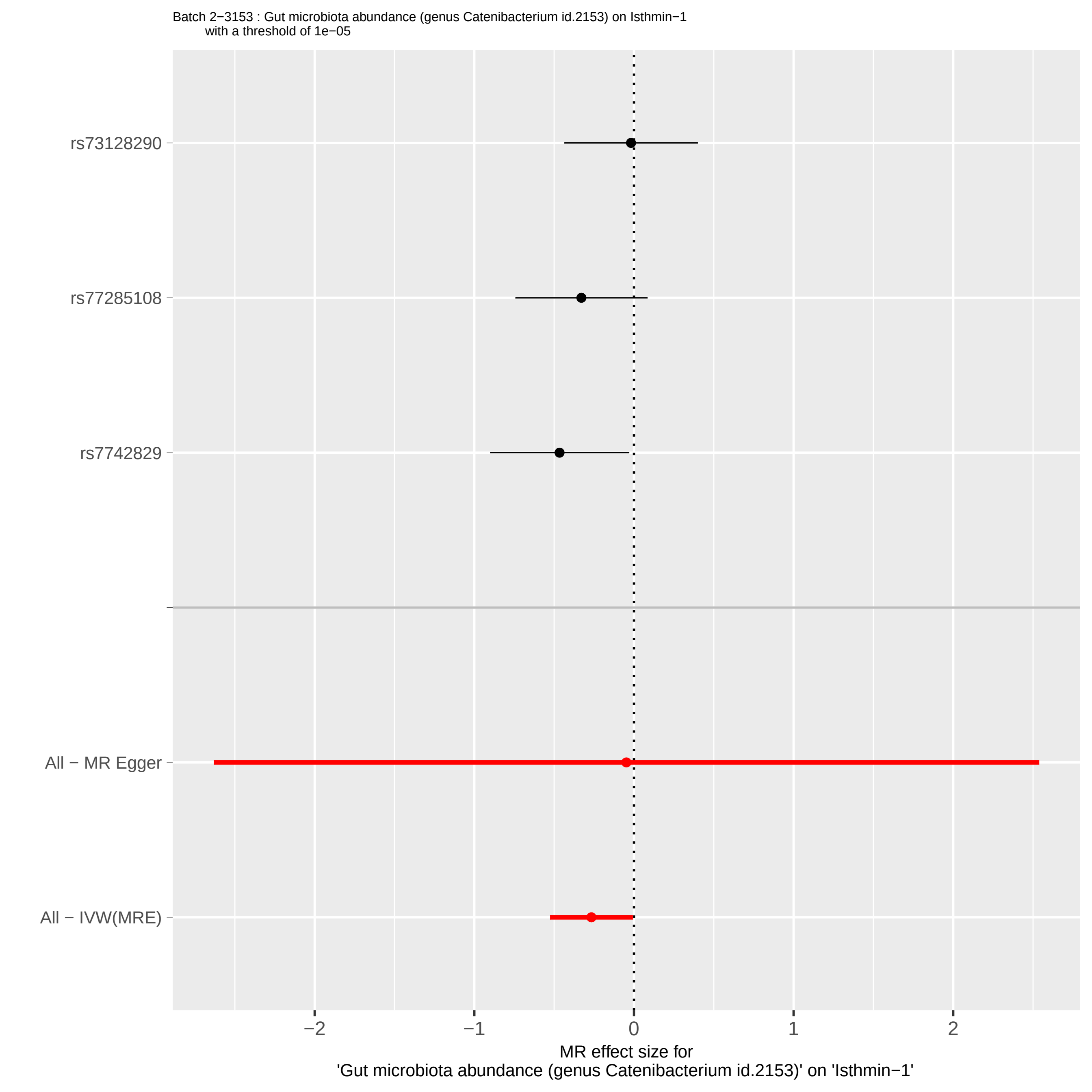
Batch 2−3153 : Gut microbiota abundance (genus Catenibacterium id.2153) on Isthmin−1
with a threshold of 1e−05
rs73128290
rs77285108
rs7742829
All − MR Egger
All − IVW(MRE)
−2
−1
0
1
2
MR effect size for
'Gut microbiota abundance (genus Catenibacterium id.2153)' on 'Isthmin−1'

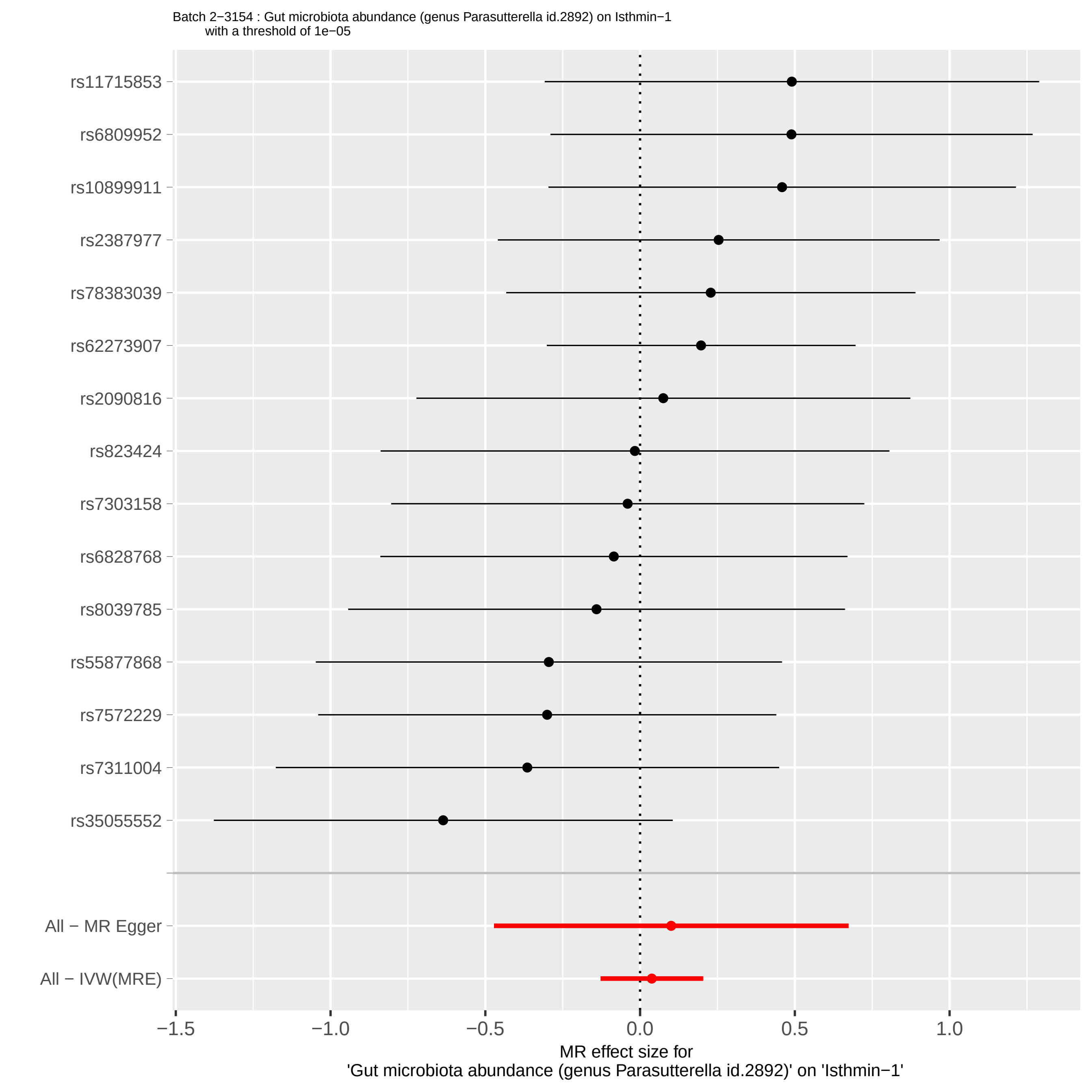
Batch 2−3154 : Gut microbiota abundance (genus Parasutterella id.2892) on Isthmin−1
with a threshold of 1e−05
rs11715853
rs6809952
rs10899911
rs2387977
rs78383039
rs62273907
rs2090816
rs823424
rs7303158
rs6828768
rs8039785
rs55877868
rs7572229
rs7311004
rs35055552
All − MR Egger
All − IVW(MRE)
−1.5
−1.0
−0.5
0.0
0.5
1.0
MR effect size for
'Gut microbiota abundance (genus Parasutterella id.2892)' on 'Isthmin−1'

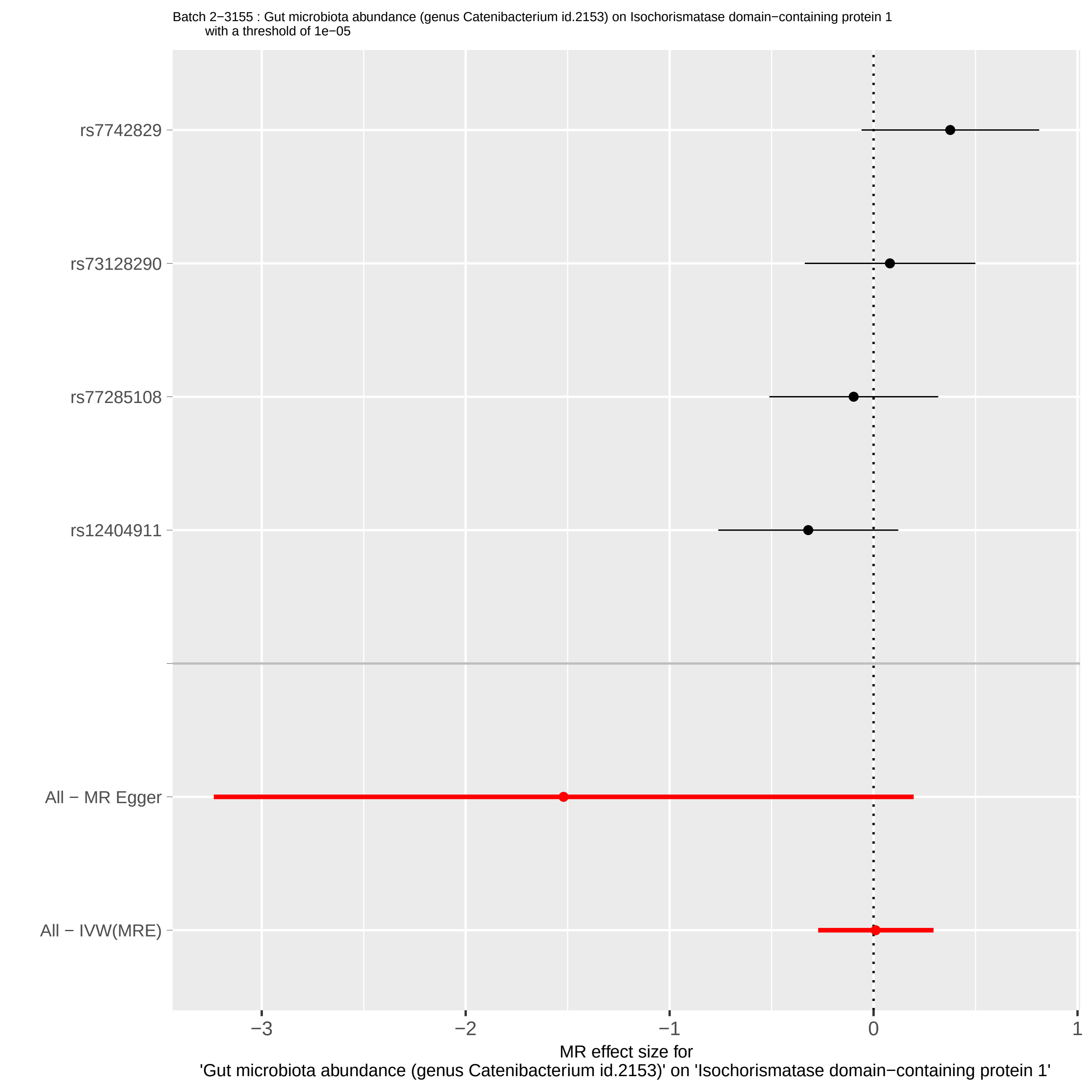

Batch 2−3155 : Gut microbiota abundance (genus Catenibacterium id.2153) on Isochorismatase domain−containing protein 1
with a threshold of 1e−05
rs7742829
rs73128290
rs77285108
rs12404911
All − MR Egger
All − IVW(MRE)
−3
−2
−1
0
1
MR effect size for
'Gut microbiota abundance (genus Catenibacterium id.2153)' on 'Isochorismatase domain−containing protein 1'

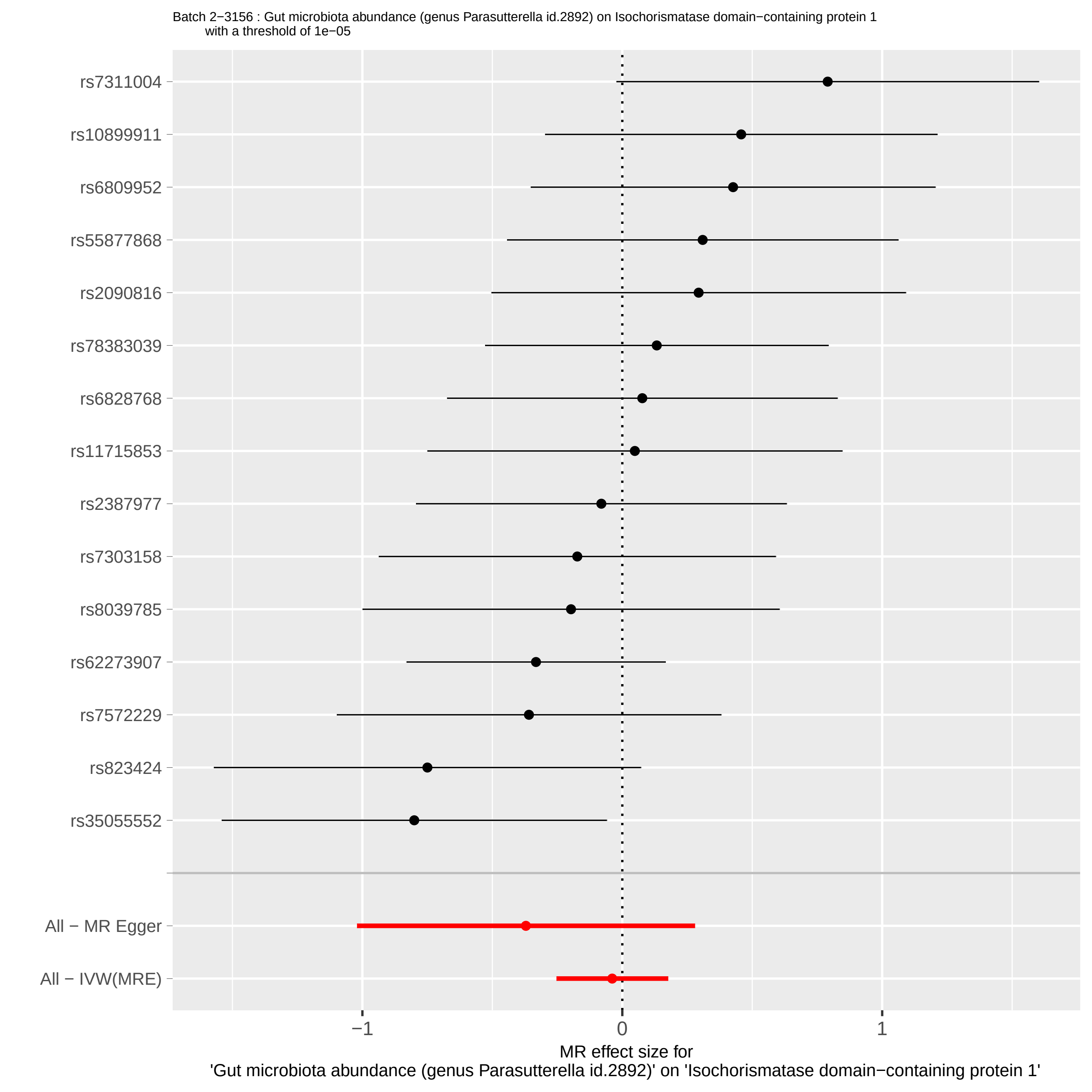

Batch 2−3156 : Gut microbiota abundance (genus Parasutterella id.2892) on Isochorismatase domain−containing protein 1
with a threshold of 1e−05
rs7311004
rs10899911
rs6809952
rs55877868
rs2090816
rs78383039
rs6828768
rs11715853
rs2387977
rs7303158
rs8039785
rs62273907
rs7572229
rs823424
rs35055552
All − MR Egger
All − IVW(MRE)
−1
0
1
MR effect size for
'Gut microbiota abundance (genus Parasutterella id.2892)' on 'Isochorismatase domain−containing protein 1'

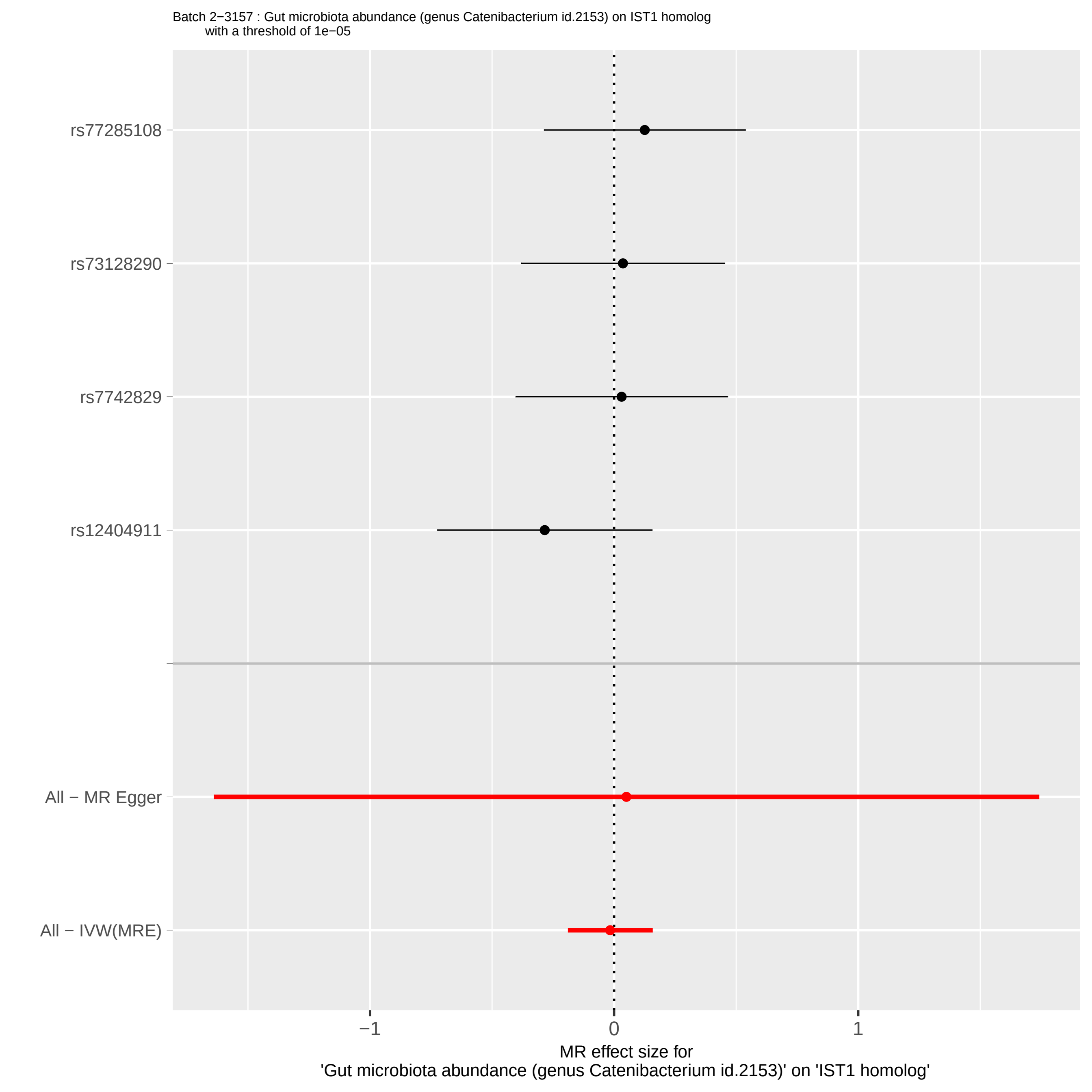
Batch 2−3157 : Gut microbiota abundance (genus Catenibacterium id.2153) on IST1 homolog
with a threshold of 1e−05
rs77285108
rs73128290
rs7742829
rs12404911
All − MR Egger
All − IVW(MRE)
−1
0
1
MR effect size for
'Gut microbiota abundance (genus Catenibacterium id.2153)' on 'IST1 homolog'

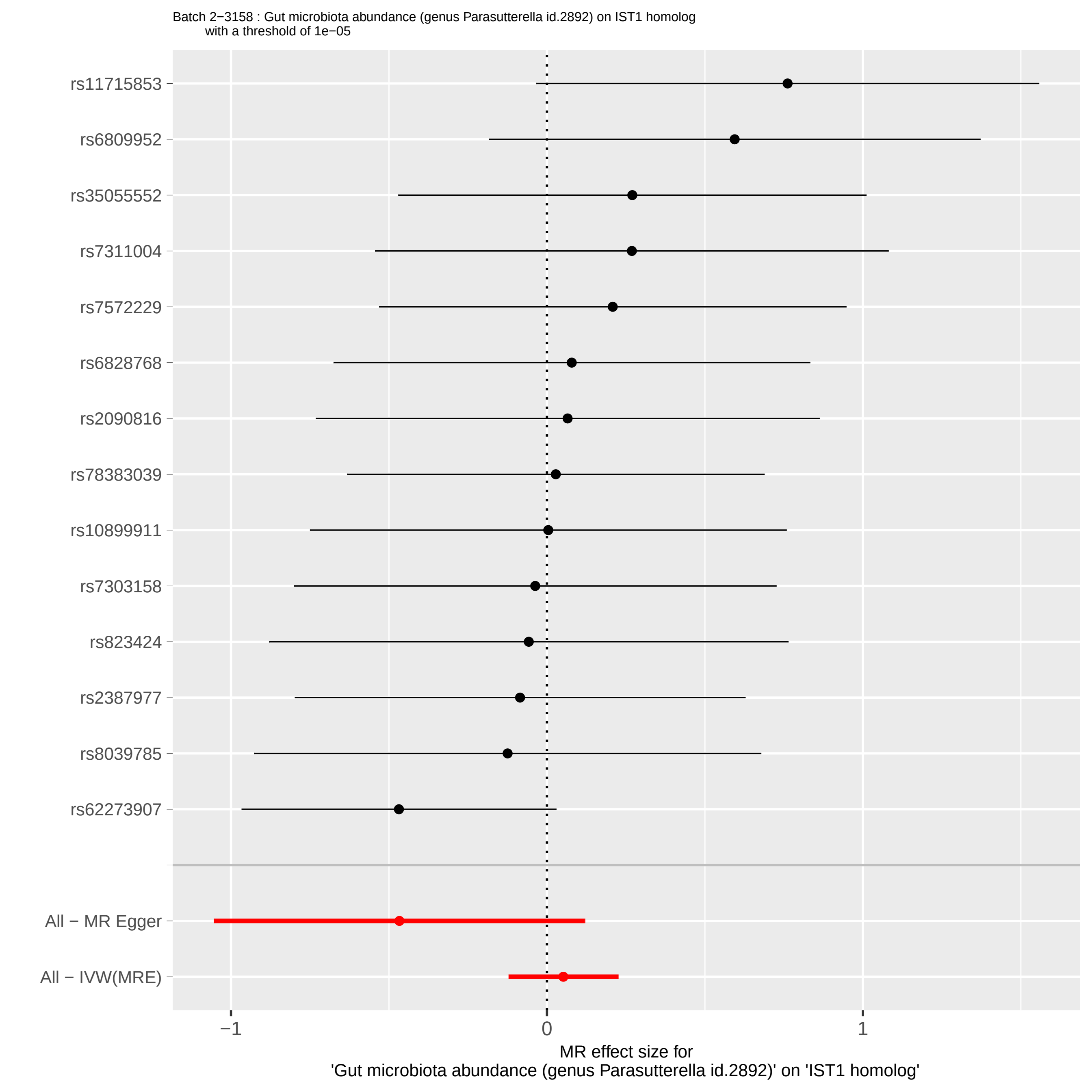

Batch 2−3158 : Gut microbiota abundance (genus Parasutterella id.2892) on IST1 homolog
with a threshold of 1e−05
rs11715853
rs6809952
rs35055552
rs7311004
rs7572229
rs6828768
rs2090816
rs78383039
rs10899911
rs7303158
rs823424
rs2387977
rs8039785
rs62273907
All − MR Egger
All − IVW(MRE)
−1
0
1
MR effect size for
'Gut microbiota abundance (genus Parasutterella id.2892)' on 'IST1 homolog'

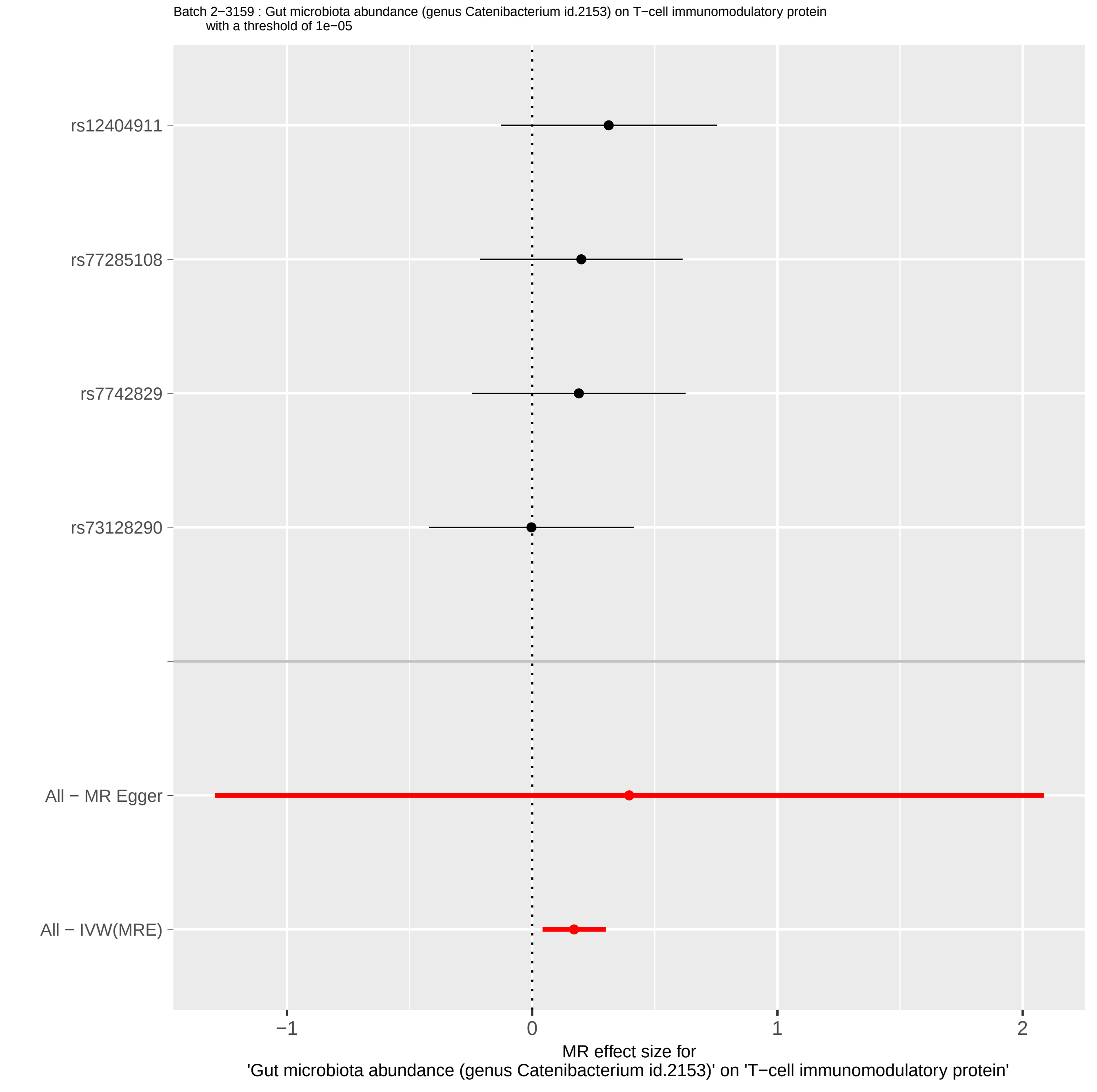

Batch 2−3159 : Gut microbiota abundance (genus Catenibacterium id.2153) on T−cell immunomodulatory protein
with a threshold of 1e−05
rs12404911
rs77285108
rs7742829
rs73128290
All − MR Egger
All − IVW(MRE)
−1
0
1
2
MR effect size for
'Gut microbiota abundance (genus Catenibacterium id.2153)' on 'T−cell immunomodulatory protein'

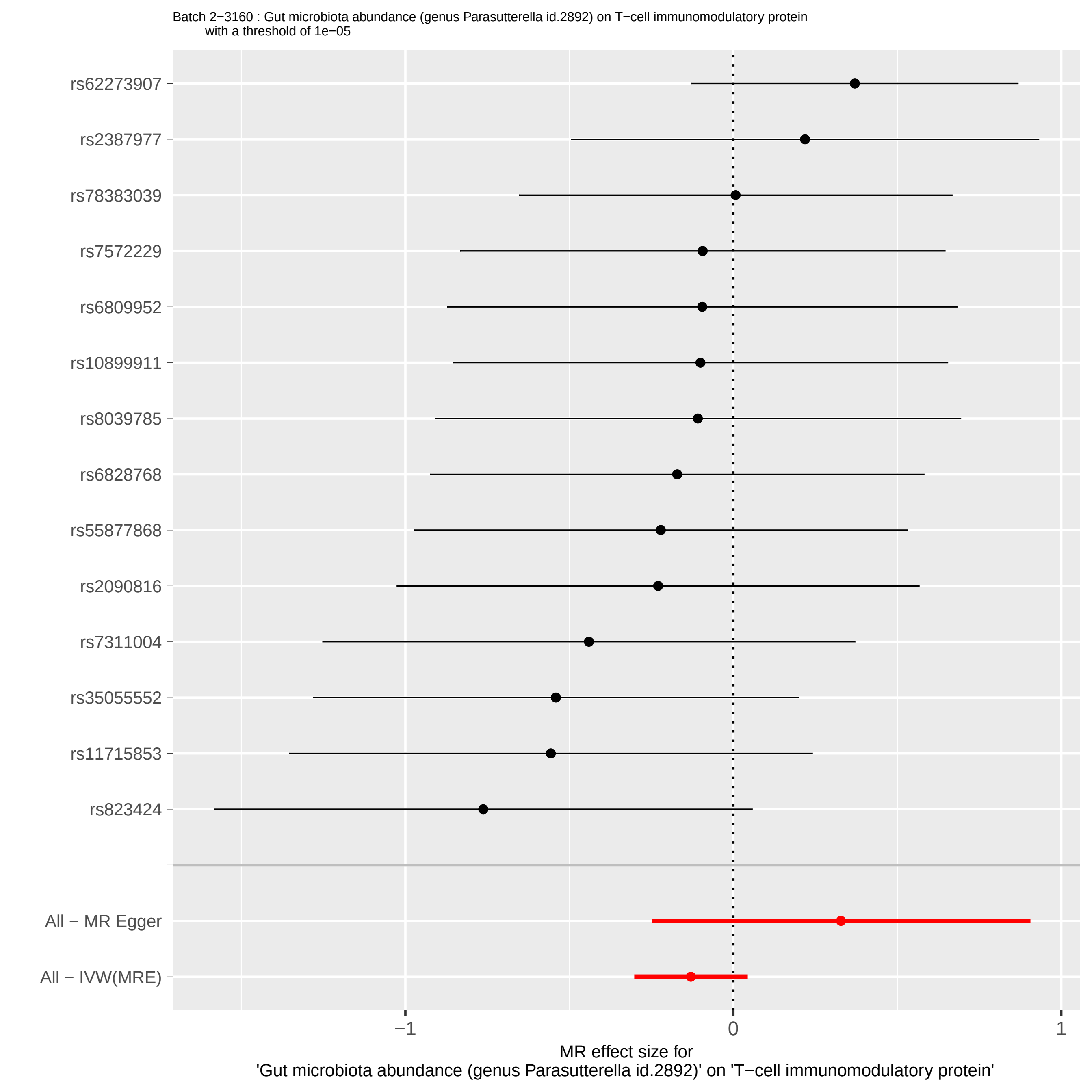

Batch 2−3160 : Gut microbiota abundance (genus Parasutterella id.2892) on T−cell immunomodulatory protein
with a threshold of 1e−05
rs62273907
rs2387977
rs78383039
rs7572229
rs6809952
rs10899911
rs8039785
rs6828768
rs55877868
rs2090816
rs7311004
rs35055552
rs11715853
rs823424
All − MR Egger
All − IVW(MRE)
−1
0
1
MR effect size for
'Gut microbiota abundance (genus Parasutterella id.2892)' on 'T−cell immunomodulatory protein'

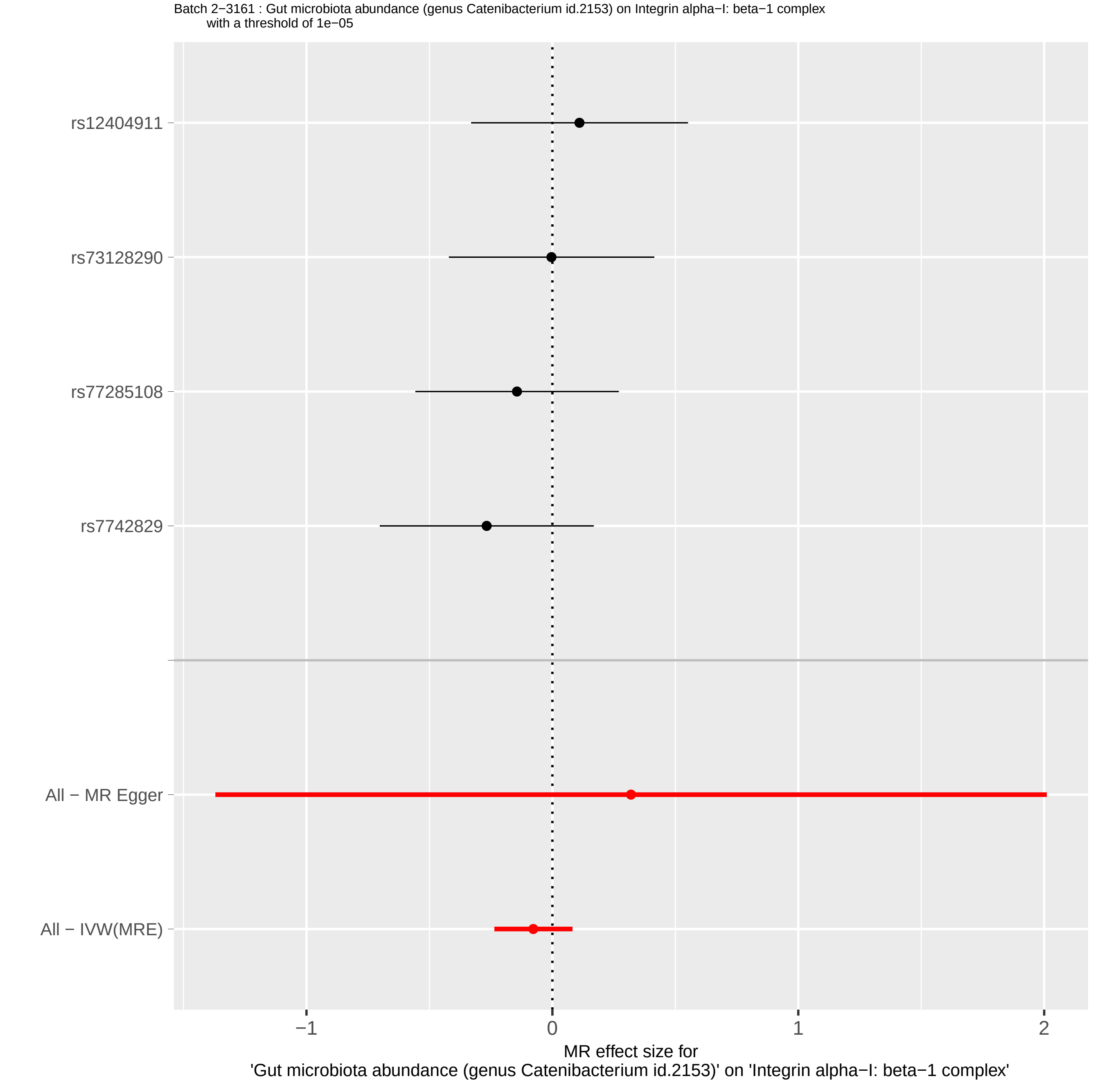
Batch 2−3161 : Gut microbiota abundance (genus Catenibacterium id.2153) on Integrin alpha−I: beta−1 complex
with a threshold of 1e−05
rs12404911
rs73128290
rs77285108
rs7742829
All − MR Egger
All − IVW(MRE)
−1
0
1
2
MR effect size for
'Gut microbiota abundance (genus Catenibacterium id.2153)' on 'Integrin alpha−I: beta−1 complex'

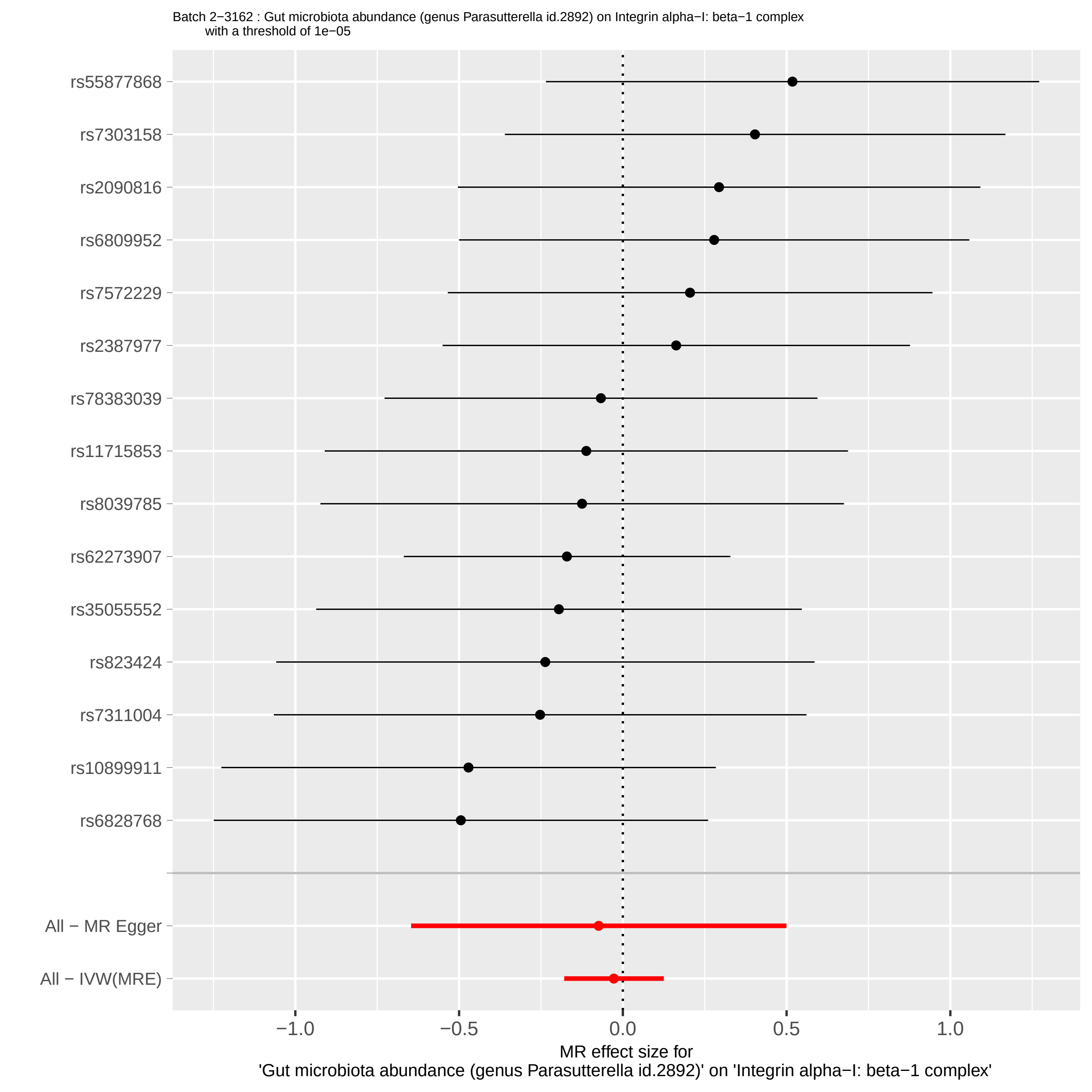

Batch 2−3162 : Gut microbiota abundance (genus Parasutterella id.2892) on Integrin alpha−I: beta−1 complex
with a threshold of 1e−05
rs55877868
rs7303158
rs2090816
rs6809952
rs7572229
rs2387977
rs78383039
rs11715853
rs8039785
rs62273907
rs35055552
rs823424
rs7311004
rs10899911
rs6828768
All − MR Egger
All − IVW(MRE)
−1.0
−0.5
0.0
0.5
1.0
MR effect size for
'Gut microbiota abundance (genus Parasutterella id.2892)' on 'Integrin alpha−I: beta−1 complex'

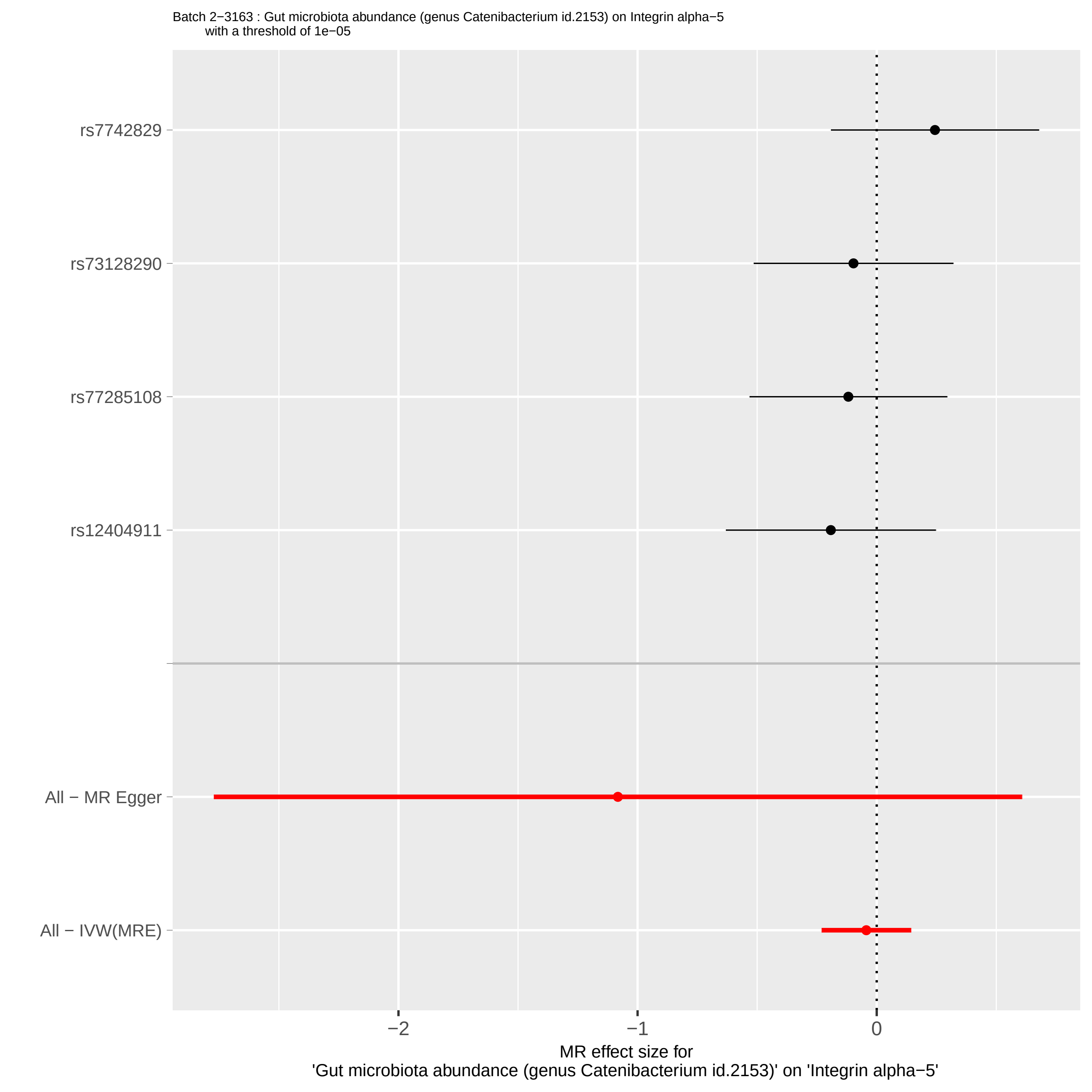

Batch 2−3163 : Gut microbiota abundance (genus Catenibacterium id.2153) on Integrin alpha−5
with a threshold of 1e−05
rs7742829
rs73128290
rs77285108
rs12404911
All − MR Egger
All − IVW(MRE)
−2
−1
0
MR effect size for
'Gut microbiota abundance (genus Catenibacterium id.2153)' on 'Integrin alpha−5'

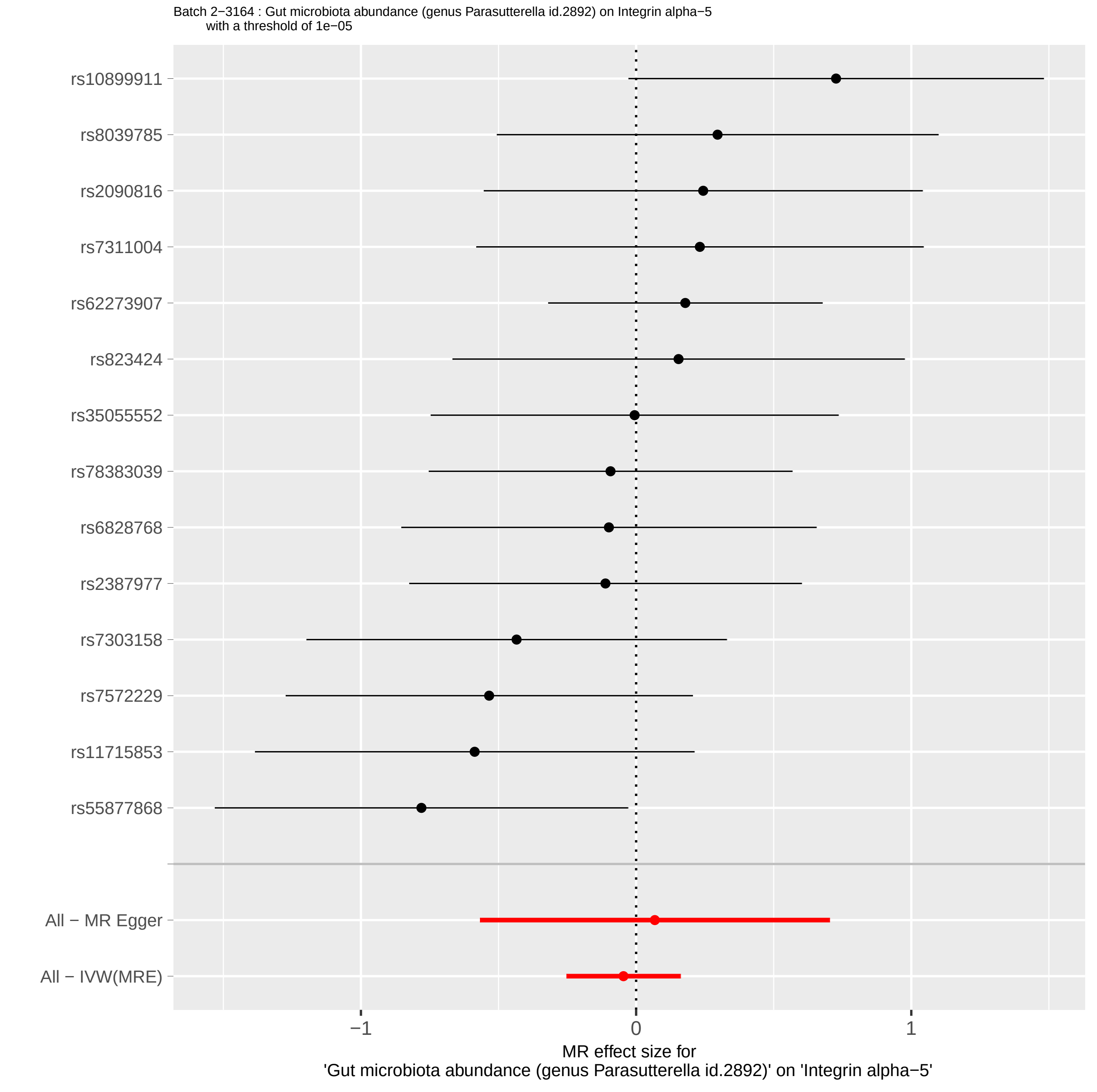

Batch 2−3164 : Gut microbiota abundance (genus Parasutterella id.2892) on Integrin alpha−5
with a threshold of 1e−05
rs10899911
rs8039785
rs2090816
rs7311004
rs62273907
rs823424
rs35055552
rs78383039
rs6828768
rs2387977
rs7303158
rs7572229
rs11715853
rs55877868
All − MR Egger
All − IVW(MRE)
−1
0
1
MR effect size for
'Gut microbiota abundance (genus Parasutterella id.2892)' on 'Integrin alpha−5'

Batch 2−3165 : Gut microbiota abundance (genus Catenibacterium id.2153) on Integrin alpha−L
with a threshold of 1e−05

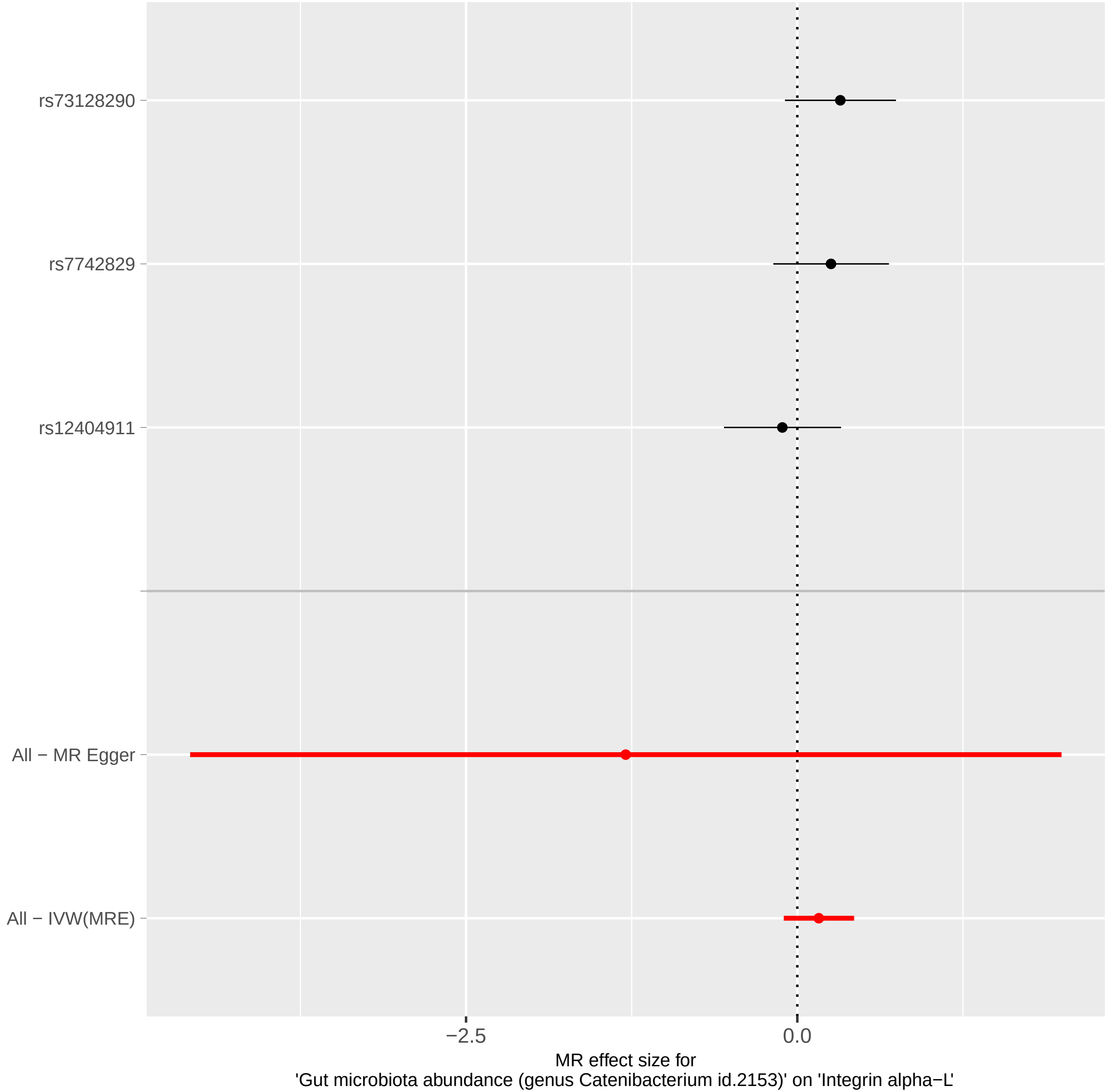

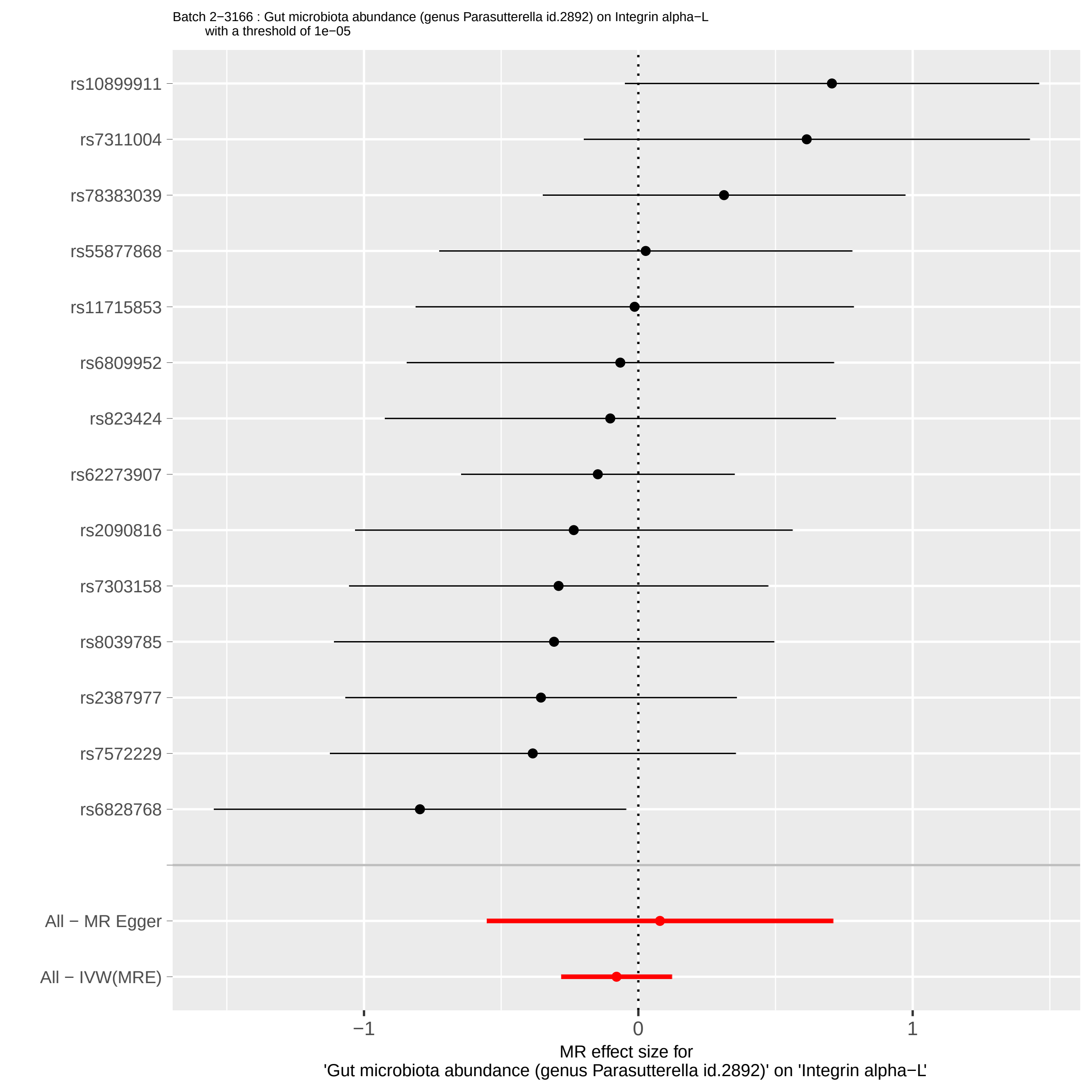

Batch 2−3166 : Gut microbiota abundance (genus Parasutterella id.2892) on Integrin alpha−L
with a threshold of 1e−05
rs10899911
rs7311004
rs78383039
rs55877868
rs11715853
rs6809952
rs823424
rs62273907
rs2090816
rs7303158
rs8039785
rs2387977
rs7572229
rs6828768
All − MR Egger
All − IVW(MRE)
−1
0
1
MR effect size for
'Gut microbiota abundance (genus Parasutterella id.2892)' on 'Integrin alpha−L'

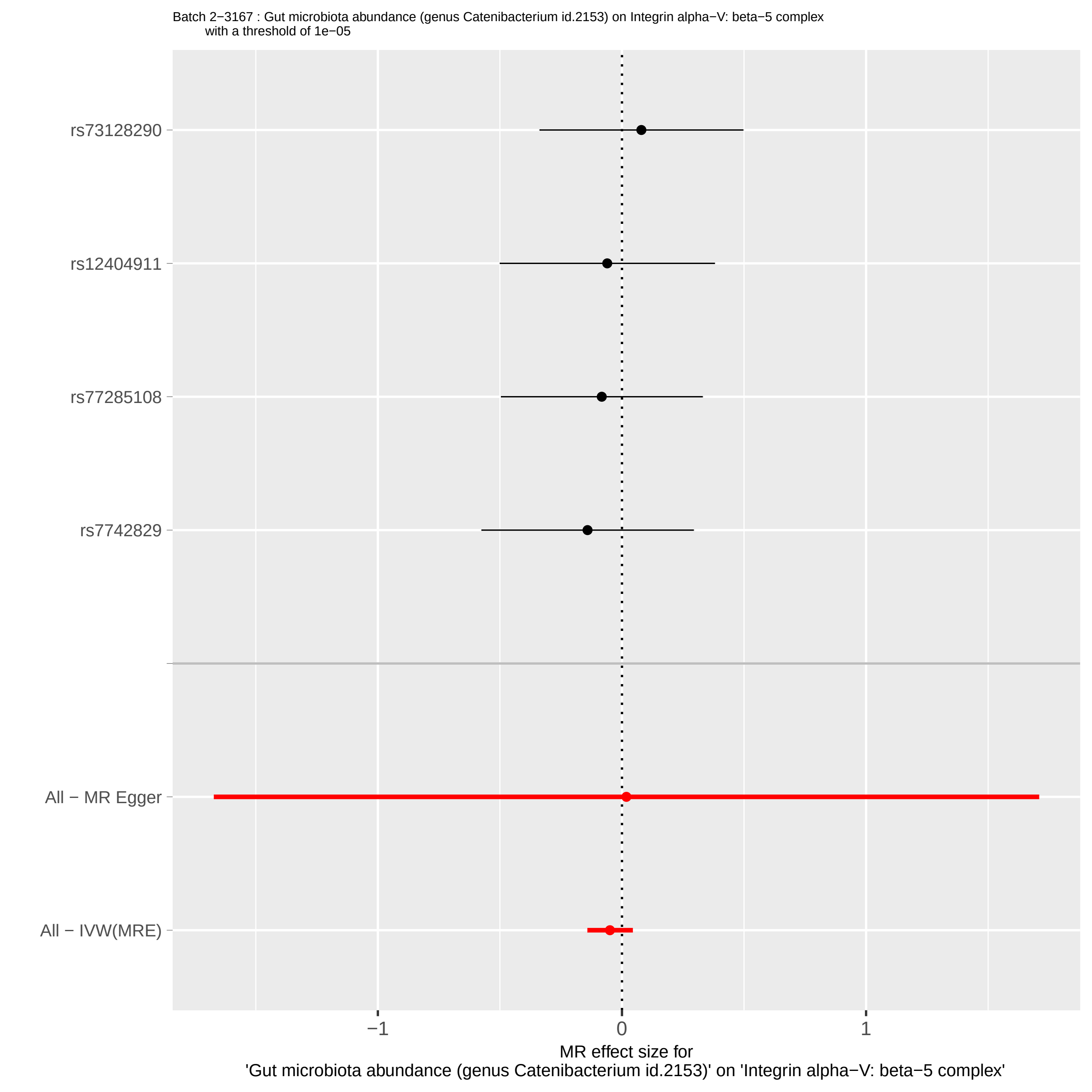
Batch 2−3167 : Gut microbiota abundance (genus Catenibacterium id.2153) on Integrin alpha−V: beta−5 complex
with a threshold of 1e−05
rs73128290
rs12404911
rs77285108
rs7742829
All − MR Egger
All − IVW(MRE)
−1
0
1
MR effect size for
'Gut microbiota abundance (genus Catenibacterium id.2153)' on 'Integrin alpha−V: beta−5 complex'

Batch 2−3168 : Gut microbiota abundance (genus Parasutterella id.2892) on Integrin alpha−V: beta−5 complex
with a threshold of 1e−05

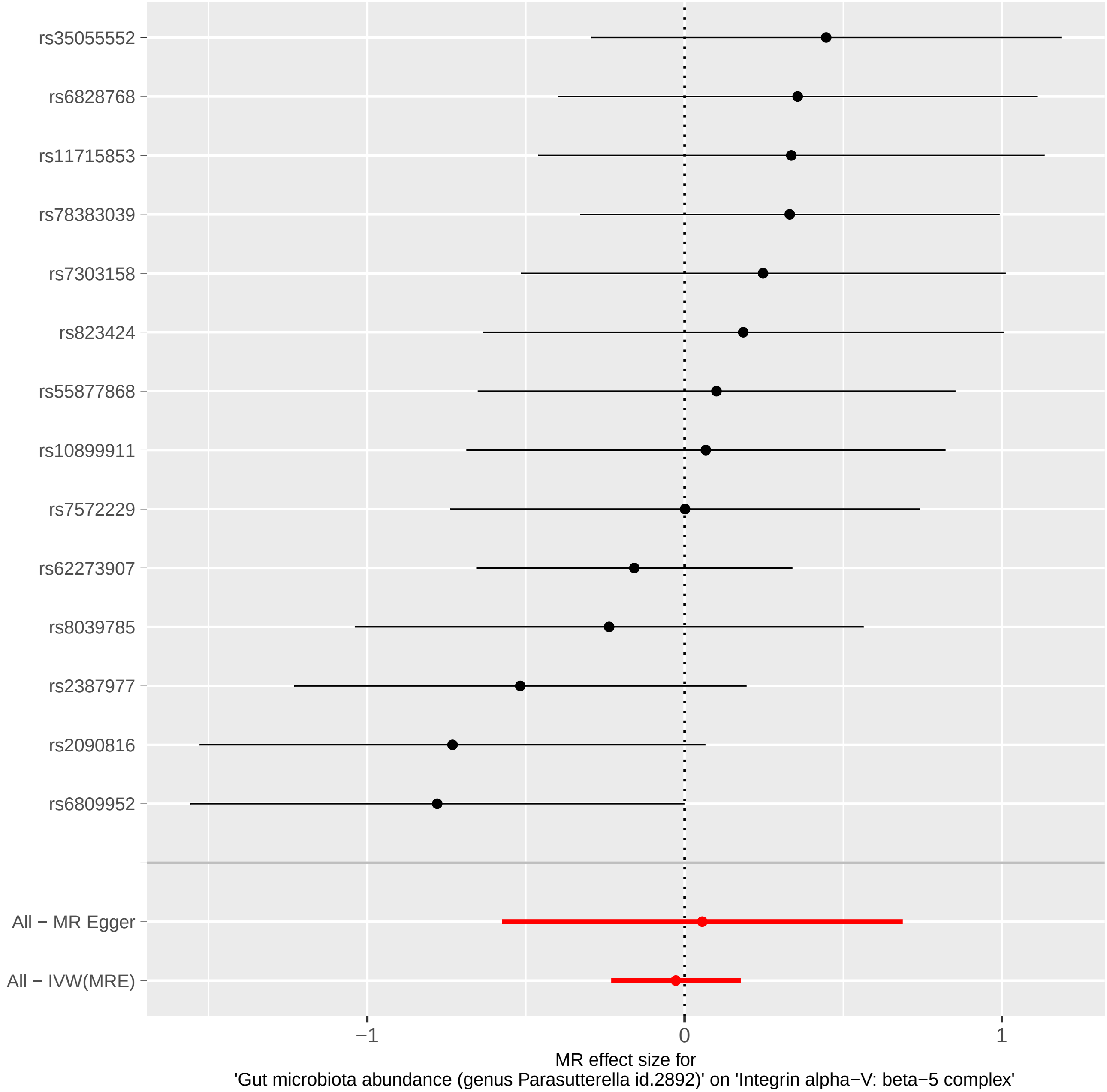

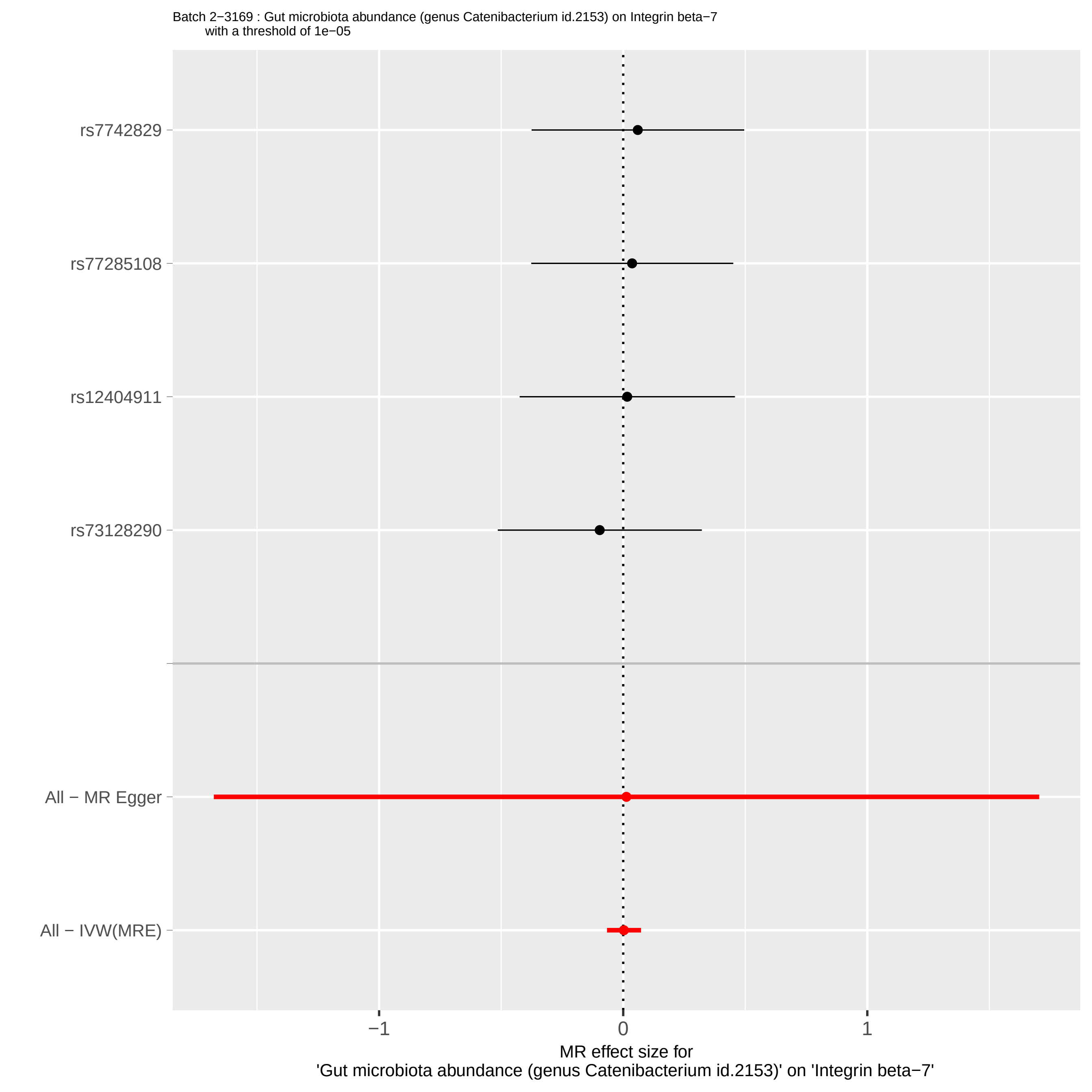
Batch 2−3169 : Gut microbiota abundance (genus Catenibacterium id.2153) on Integrin beta−7
with a threshold of 1e−05
rs7742829
rs77285108
rs12404911
rs73128290
All − MR Egger
All − IVW(MRE)
−1
0
1
MR effect size for
'Gut microbiota abundance (genus Catenibacterium id.2153)' on 'Integrin beta−7'

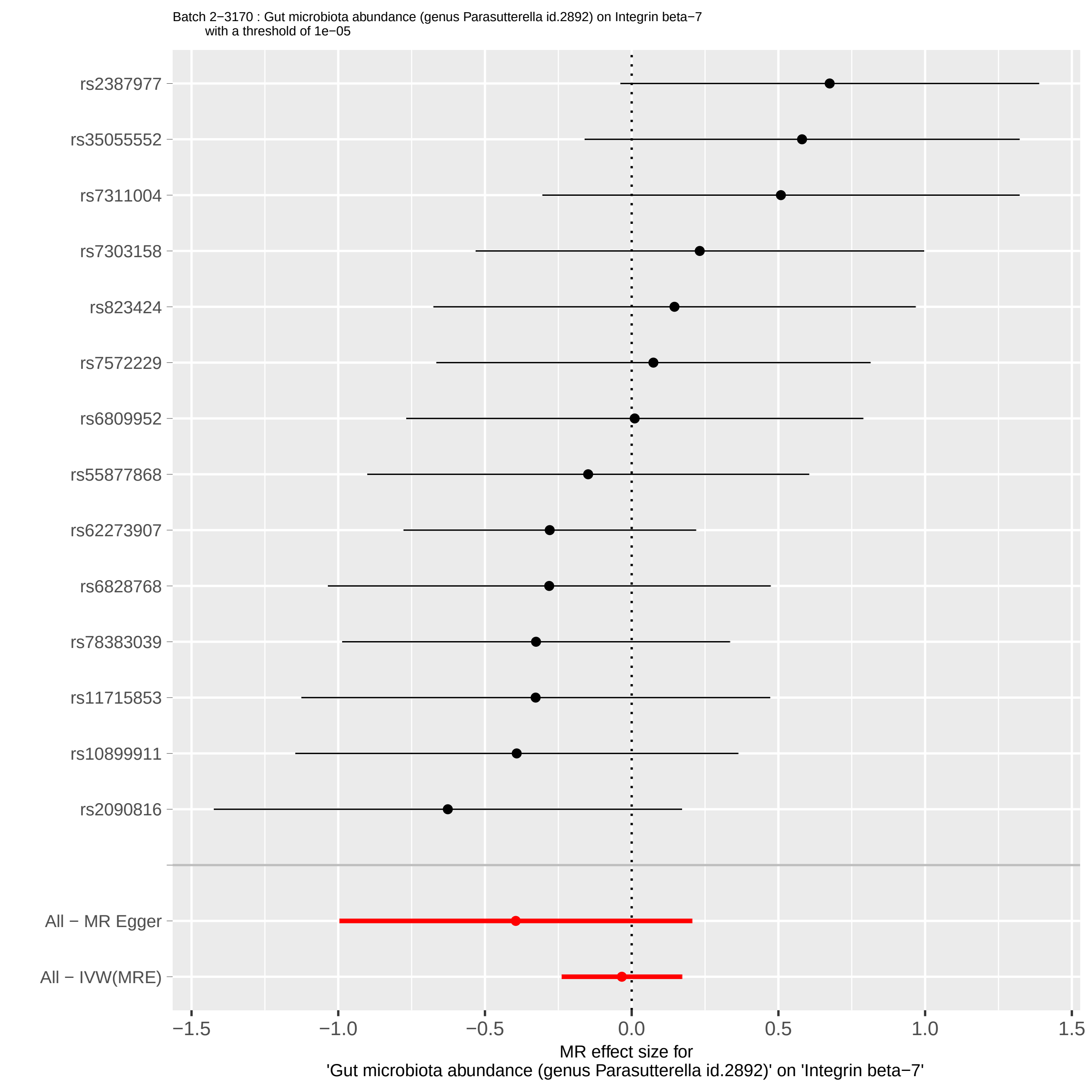
Batch 2−3170 : Gut microbiota abundance (genus Parasutterella id.2892) on Integrin beta−7
with a threshold of 1e−05
rs2387977
rs35055552
rs7311004
rs7303158
rs823424
rs7572229
rs6809952
rs55877868
rs62273907
rs6828768
rs78383039
rs11715853
rs10899911
rs2090816
All − MR Egger
All − IVW(MRE)
−1.5
−1.0
−0.5
0.0
0.5
1.0
1.5
MR effect size for
'Gut microbiota abundance (genus Parasutterella id.2892)' on 'Integrin beta−7'

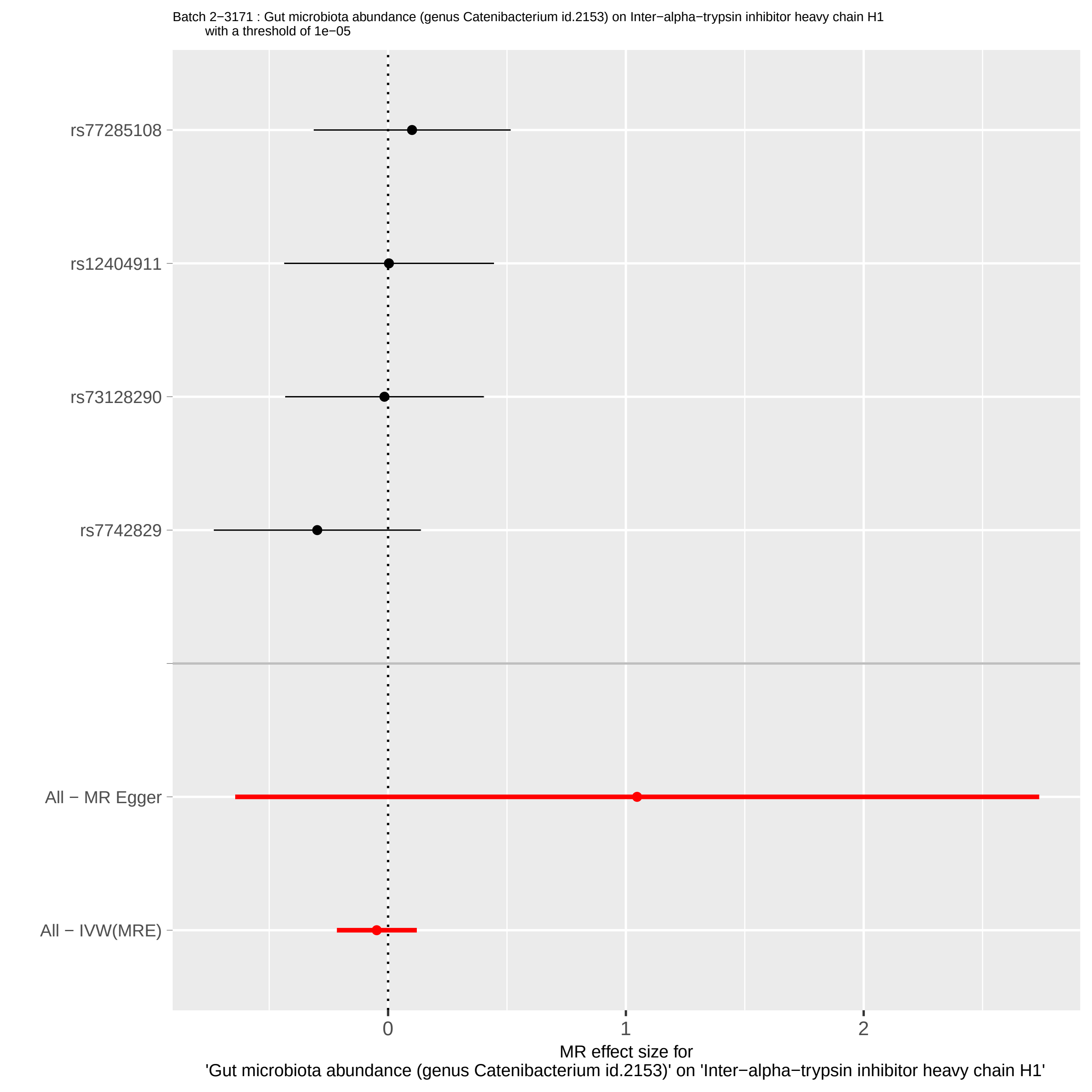

Batch 2−3171 : Gut microbiota abundance (genus Catenibacterium id.2153) on Inter−alpha−trypsin inhibitor heavy chain H1
with a threshold of 1e−05
rs77285108
rs12404911
rs73128290
rs7742829
All − MR Egger
All − IVW(MRE)
0
1
2
MR effect size for
'Gut microbiota abundance (genus Catenibacterium id.2153)' on 'Inter−alpha−trypsin inhibitor heavy chain H1'

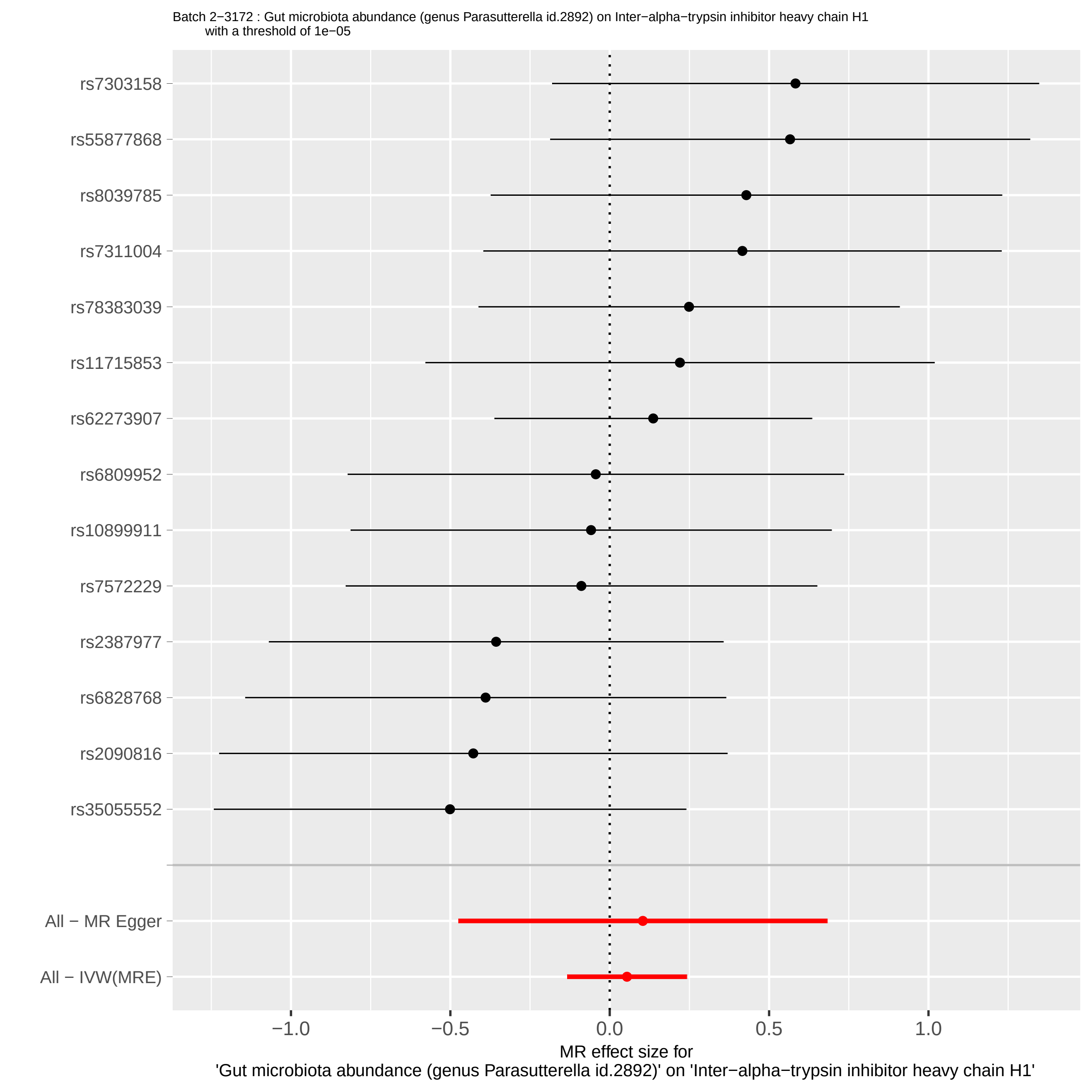

Batch 2−3172 : Gut microbiota abundance (genus Parasutterella id.2892) on Inter−alpha−trypsin inhibitor heavy chain H1
with a threshold of 1e−05
rs7303158
rs55877868
rs8039785
rs7311004
rs78383039
rs11715853
rs62273907
rs6809952
rs10899911
rs7572229
rs2387977
rs6828768
rs2090816
rs35055552
All − MR Egger
All − IVW(MRE)
−1.0
−0.5
0.0
0.5
1.0
MR effect size for
'Gut microbiota abundance (genus Parasutterella id.2892)' on 'Inter−alpha−trypsin inhibitor heavy chain H1'

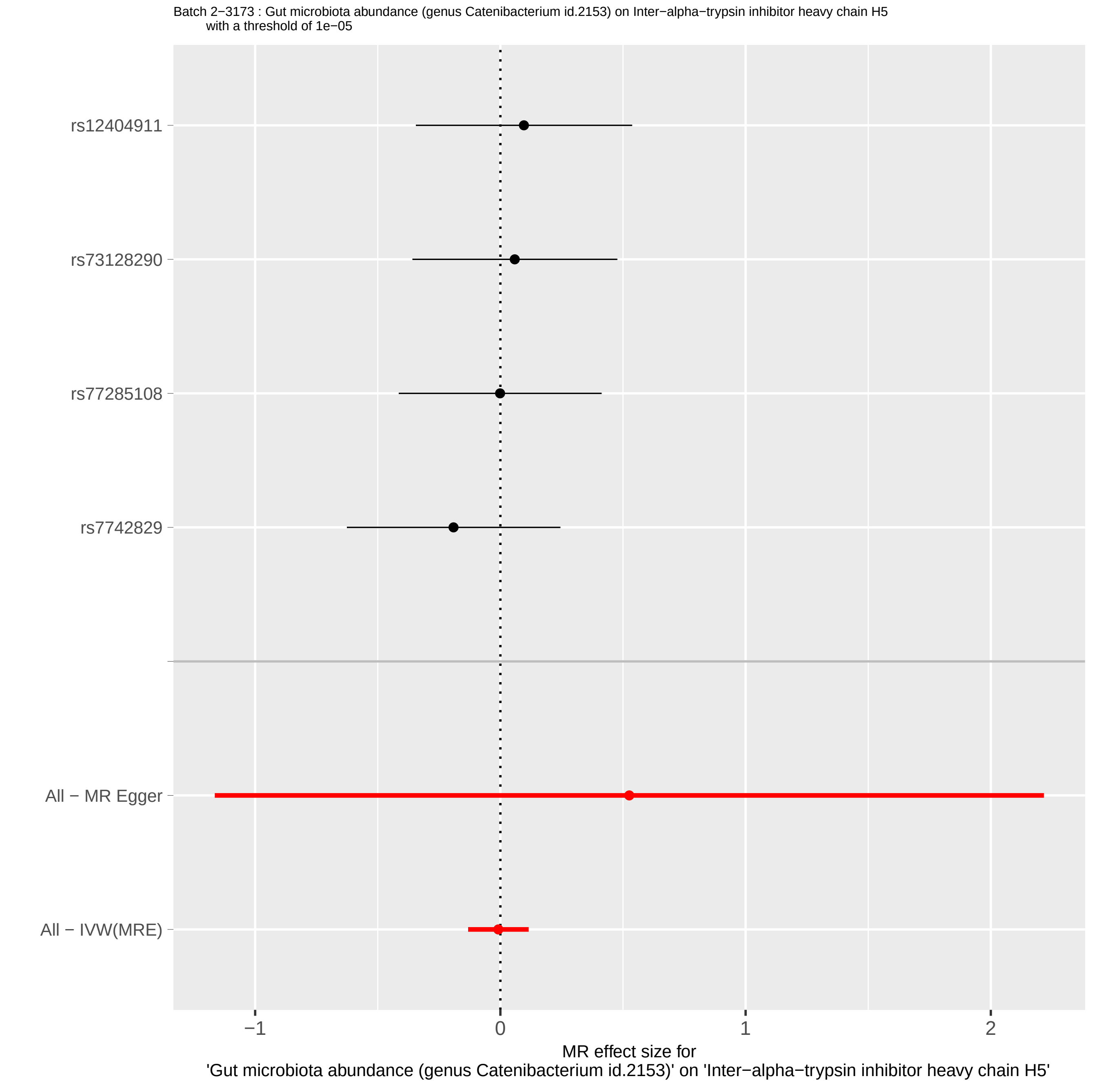
Batch 2−3173 : Gut microbiota abundance (genus Catenibacterium id.2153) on Inter−alpha−trypsin inhibitor heavy chain H5
with a threshold of 1e−05
rs12404911
rs73128290
rs77285108
rs7742829
All − MR Egger
All − IVW(MRE)
−1
0
1
2
MR effect size for
'Gut microbiota abundance (genus Catenibacterium id.2153)' on 'Inter−alpha−trypsin inhibitor heavy chain H5'

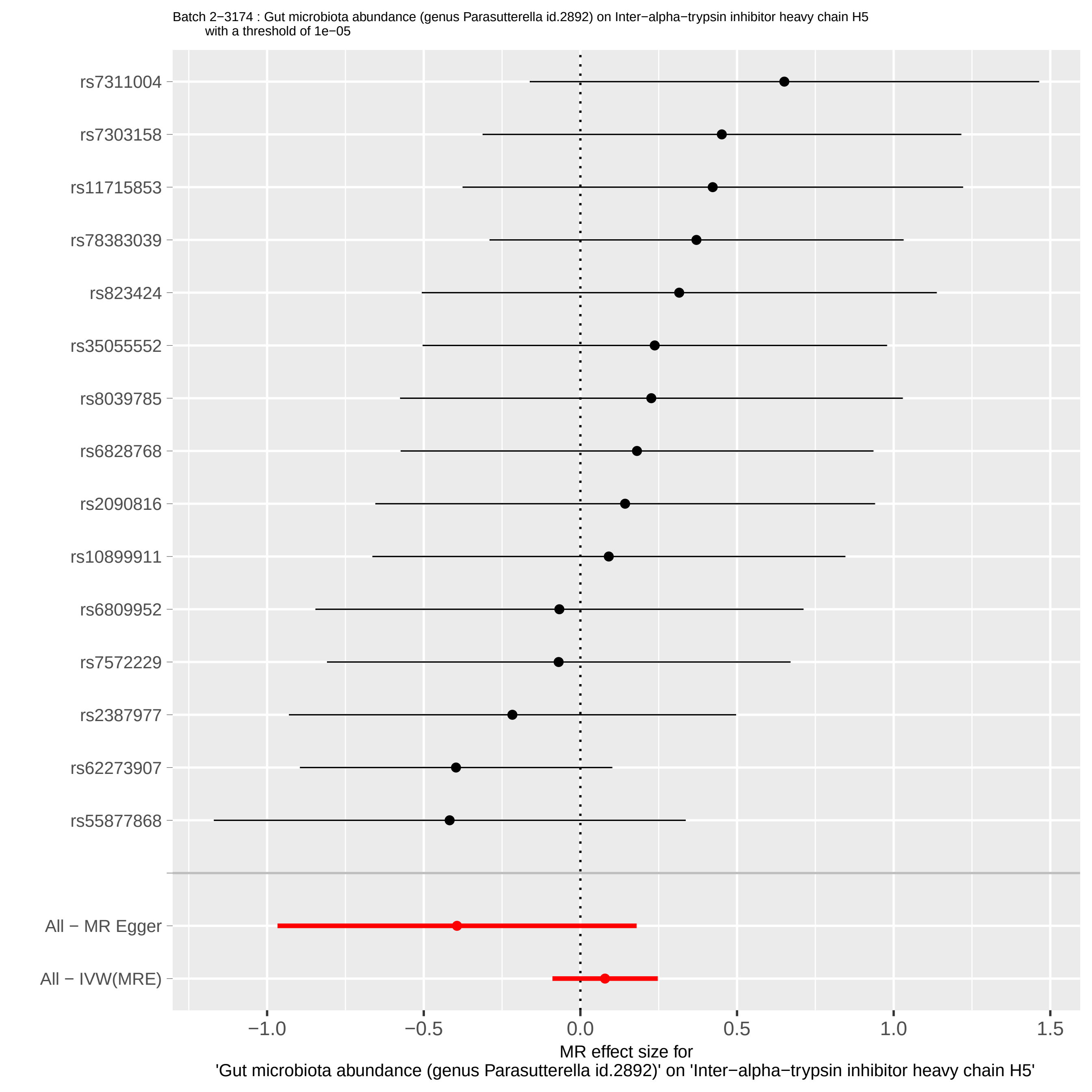

Batch 2−3174 : Gut microbiota abundance (genus Parasutterella id.2892) on Inter−alpha−trypsin inhibitor heavy chain H5
with a threshold of 1e−05
rs7311004
rs7303158
rs11715853
rs78383039
rs823424
rs35055552
rs8039785
rs6828768
rs2090816
rs10899911
rs6809952
rs7572229
rs2387977
rs62273907
rs55877868
All − MR Egger
All − IVW(MRE)
−1.0
−0.5
0.0
0.5
1.0
1.5
MR effect size for
'Gut microbiota abundance (genus Parasutterella id.2892)' on 'Inter−alpha−trypsin inhibitor heavy chain H5'

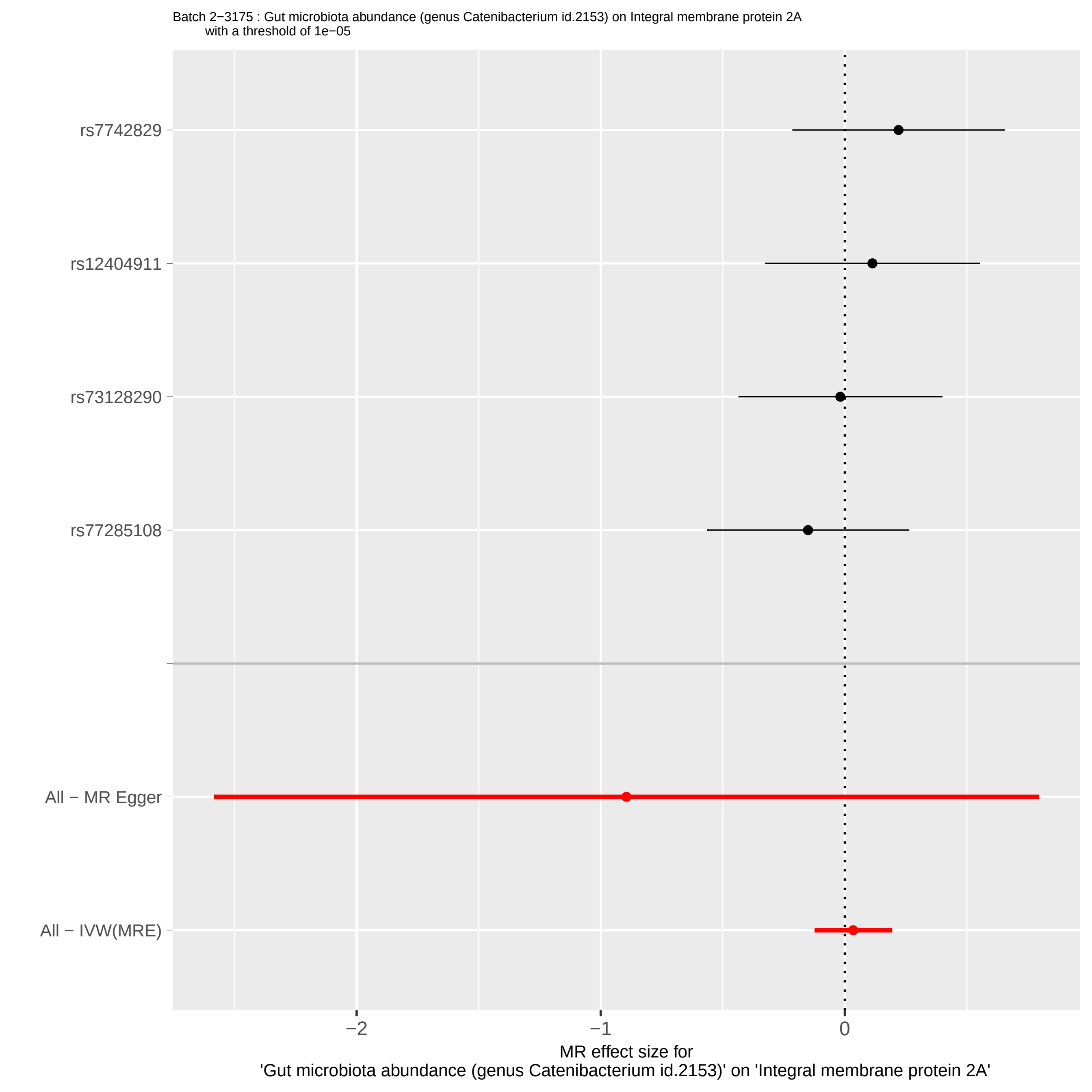
Batch 2−3175 : Gut microbiota abundance (genus Catenibacterium id.2153) on Integral membrane protein 2A
with a threshold of 1e−05
rs7742829
rs12404911
rs73128290
rs77285108
All − MR Egger
All − IVW(MRE)
−2
−1
0
MR effect size for
'Gut microbiota abundance (genus Catenibacterium id.2153)' on 'Integral membrane protein 2A'

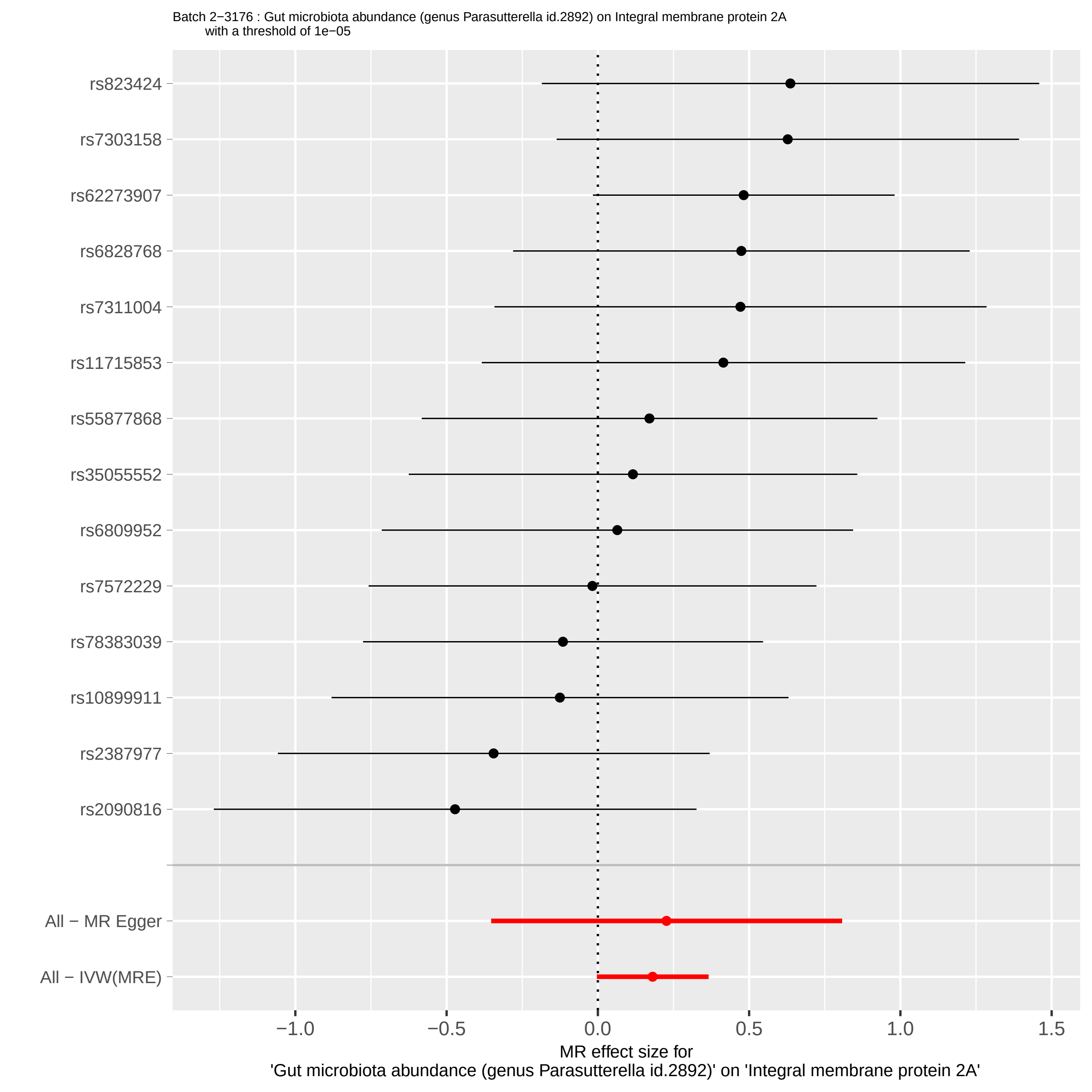

Batch 2−3176 : Gut microbiota abundance (genus Parasutterella id.2892) on Integral membrane protein 2A
with a threshold of 1e−05
rs823424
rs7303158
rs62273907
rs6828768
rs7311004
rs11715853
rs55877868
rs35055552
rs6809952
rs7572229
rs78383039
rs10899911
rs2387977
rs2090816
All − MR Egger
All − IVW(MRE)
−1.0
−0.5
0.0
0.5
1.0
1.5
MR effect size for
'Gut microbiota abundance (genus Parasutterella id.2892)' on 'Integral membrane protein 2A'

Batch 2−3177 : Gut microbiota abundance (genus Catenibacterium id.2153) on Integral membrane protein 2A
with a threshold of 1e−05

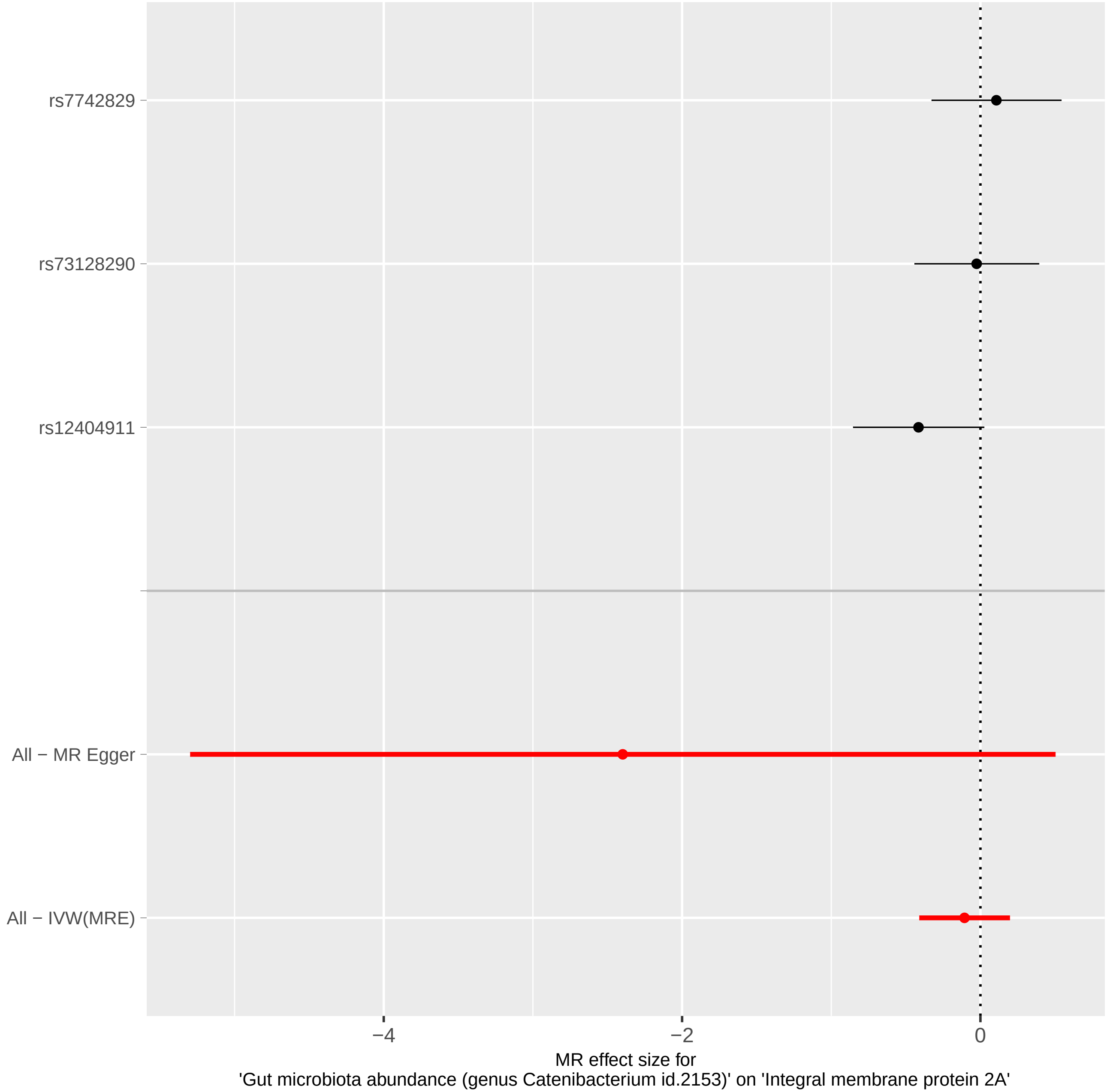

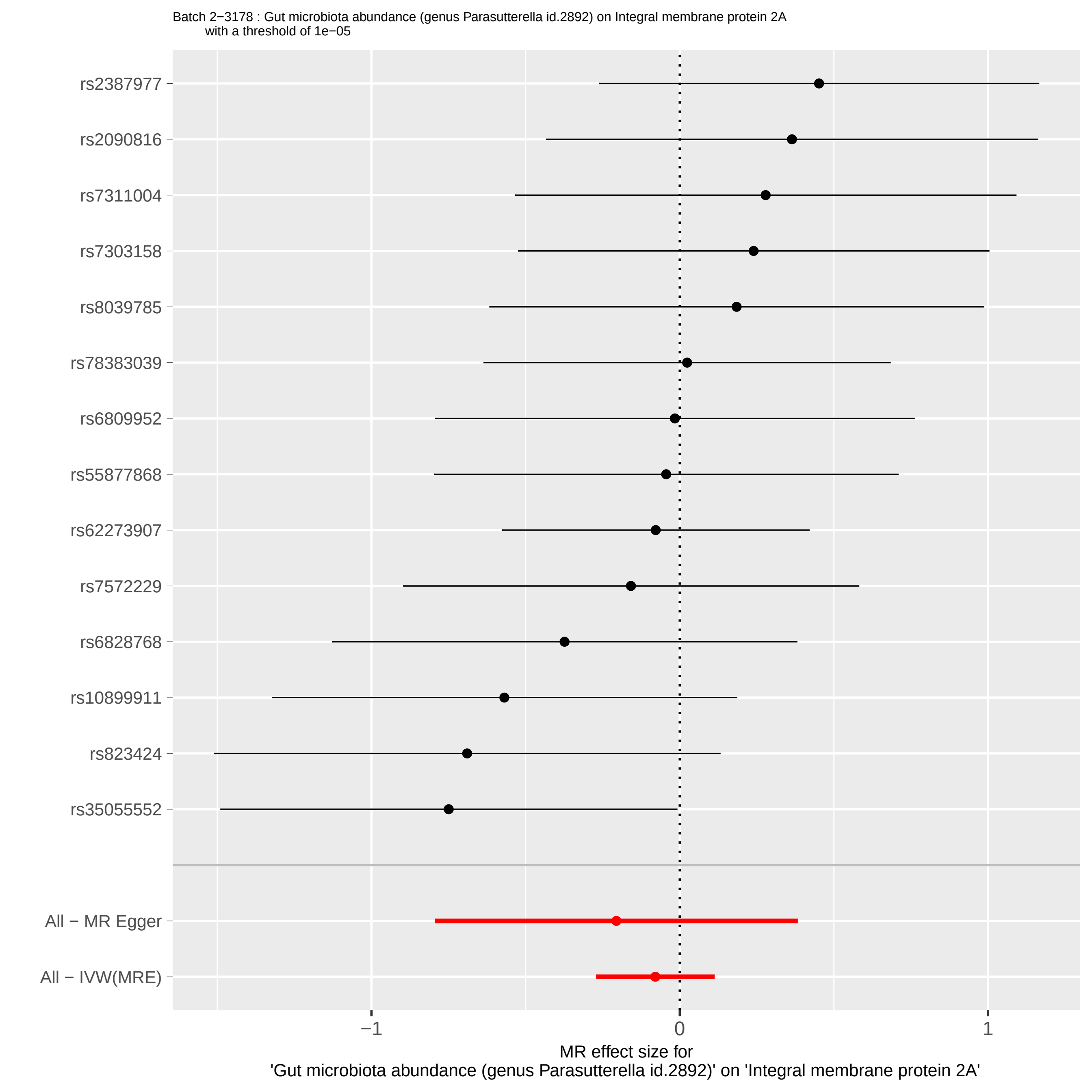
Batch 2−3178 : Gut microbiota abundance (genus Parasutterella id.2892) on Integral membrane protein 2A
with a threshold of 1e−05
rs2387977
rs2090816
rs7311004
rs7303158
rs8039785
rs78383039
rs6809952
rs55877868
rs62273907
rs7572229
rs6828768
rs10899911
rs823424
rs35055552
All − MR Egger
All − IVW(MRE)
−1
0
1
MR effect size for
'Gut microbiota abundance (genus Parasutterella id.2892)' on 'Integral membrane protein 2A'

Batch 2−3179 : Gut microbiota abundance (genus Catenibacterium id.2153) on Integral membrane protein 2B
with a threshold of 1e−05

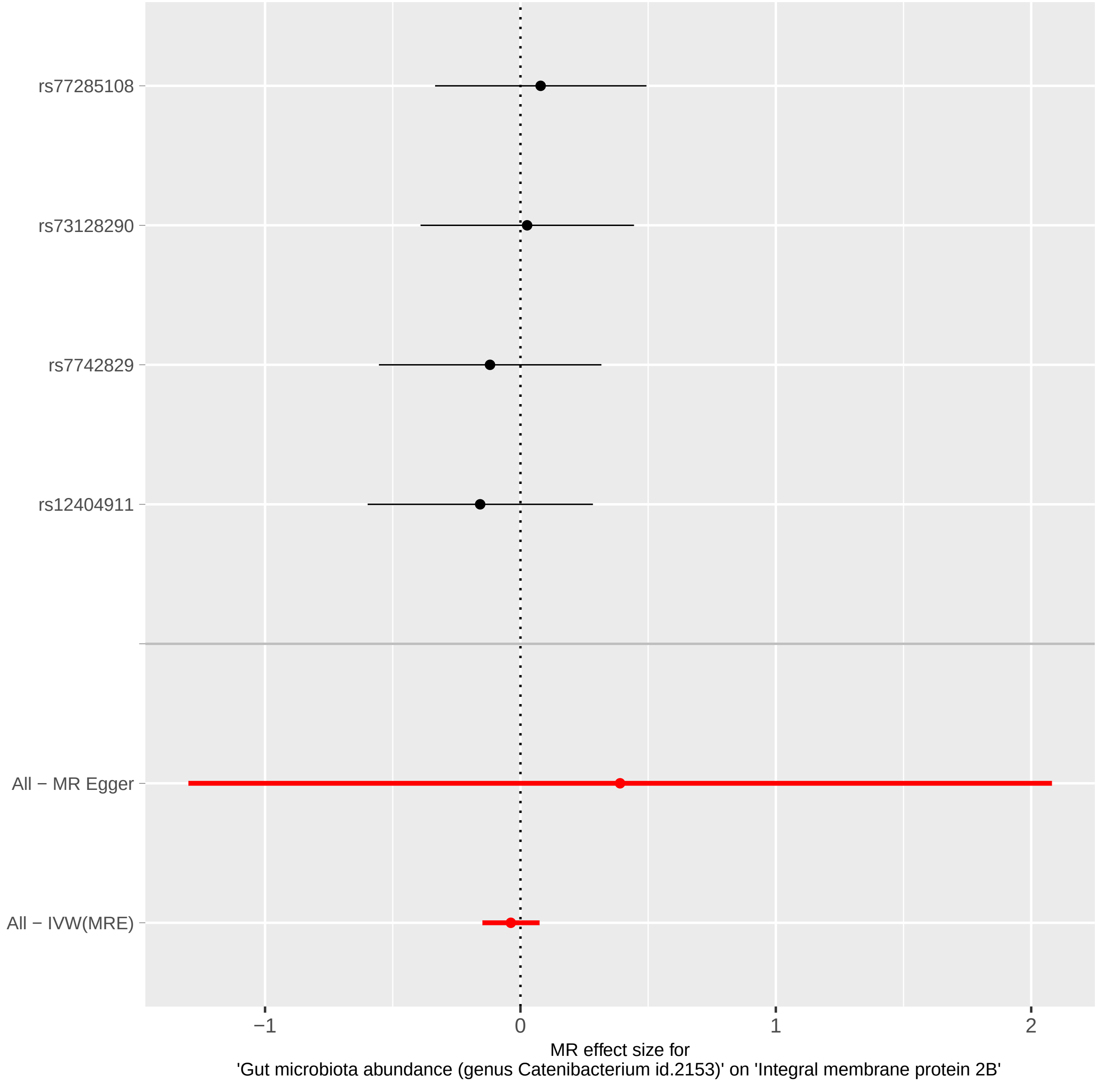

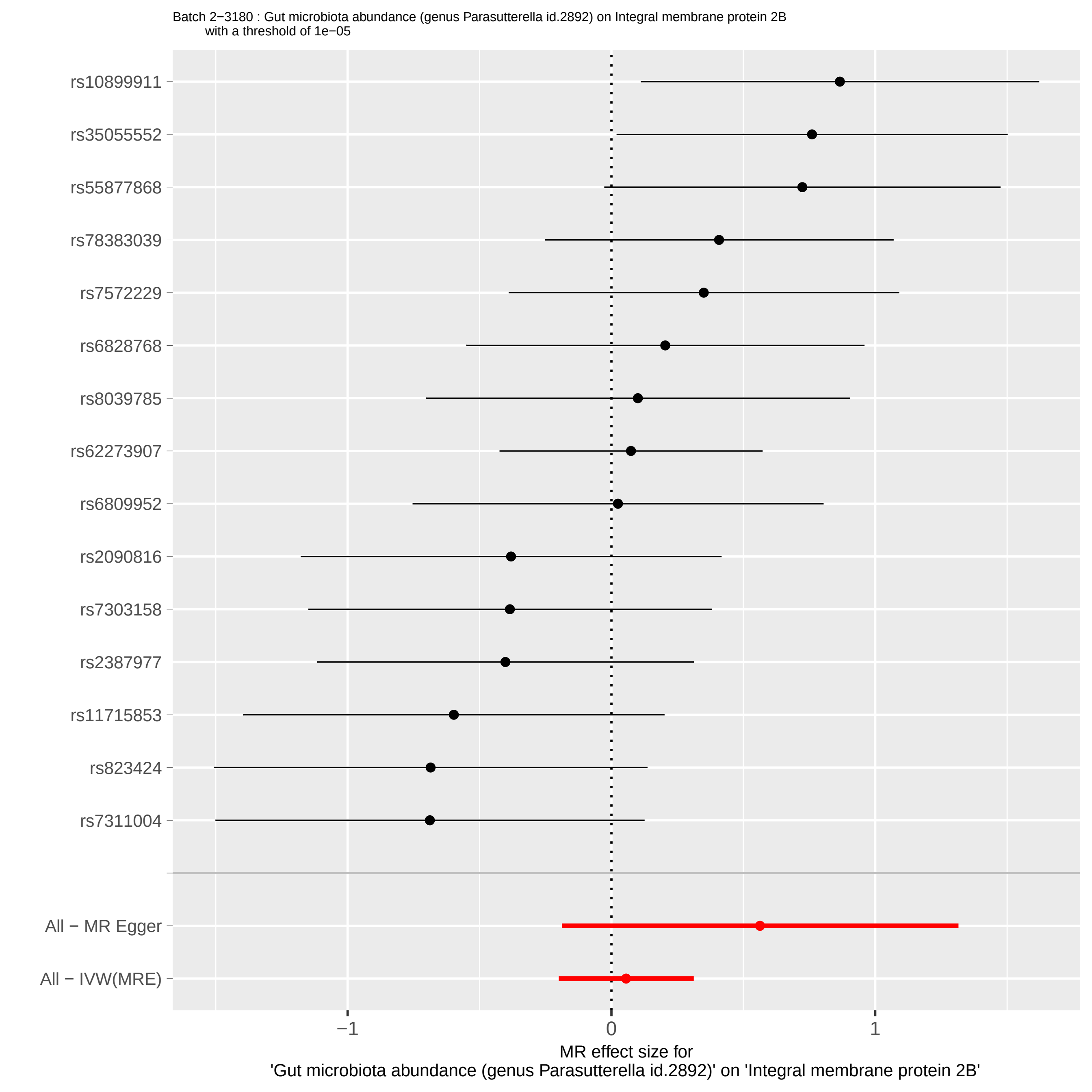

Batch 2−3180 : Gut microbiota abundance (genus Parasutterella id.2892) on Integral membrane protein 2B
with a threshold of 1e−05
rs10899911
rs35055552
rs55877868
rs78383039
rs7572229
rs6828768
rs8039785
rs62273907
rs6809952
rs2090816
rs7303158
rs2387977
rs11715853
rs823424
rs7311004
All − MR Egger
All − IVW(MRE)
−1
0
1
MR effect size for
'Gut microbiota abundance (genus Parasutterella id.2892)' on 'Integral membrane protein 2B'

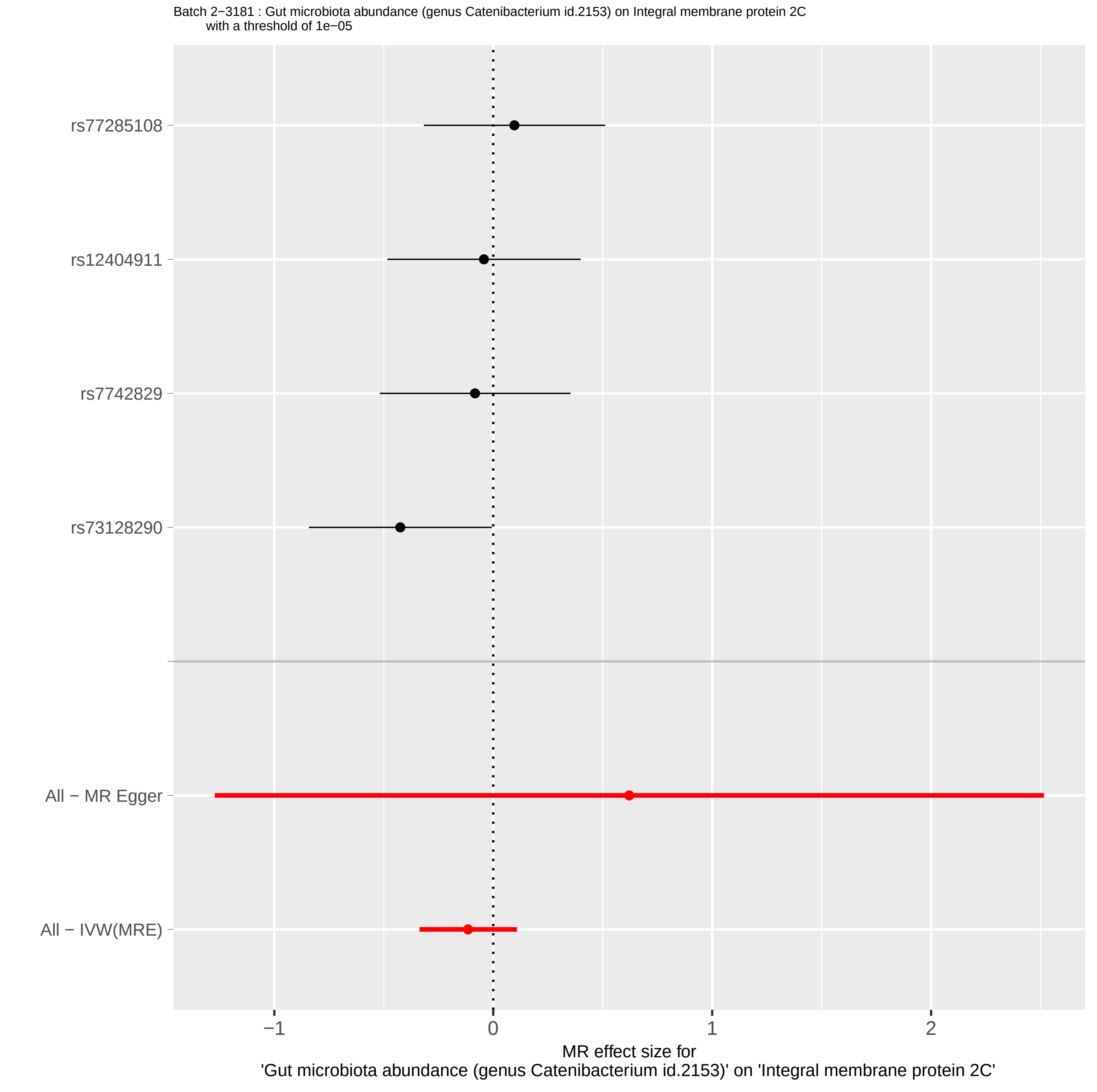
Batch 2−3181 : Gut microbiota abundance (genus Catenibacterium id.2153) on Integral membrane protein 2C
with a threshold of 1e−05
rs77285108
rs12404911
rs7742829
rs73128290
All − MR Egger
All − IVW(MRE)
−1
0
1
2
MR effect size for
'Gut microbiota abundance (genus Catenibacterium id.2153)' on 'Integral membrane protein 2C'

Batch 2−3182 : Gut microbiota abundance (genus Parasutterella id.2892) on Integral membrane protein 2C
with a threshold of 1e−05

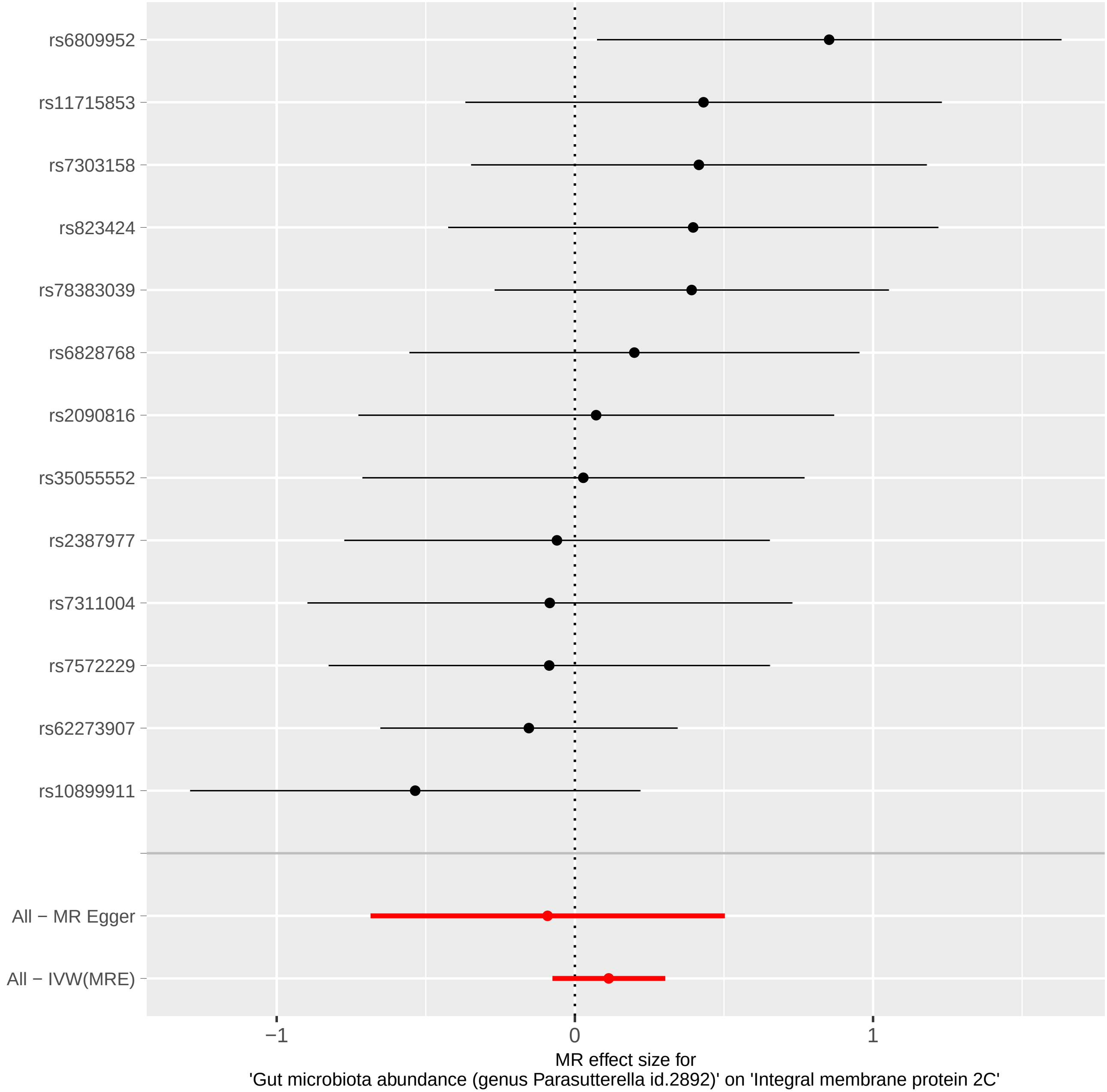

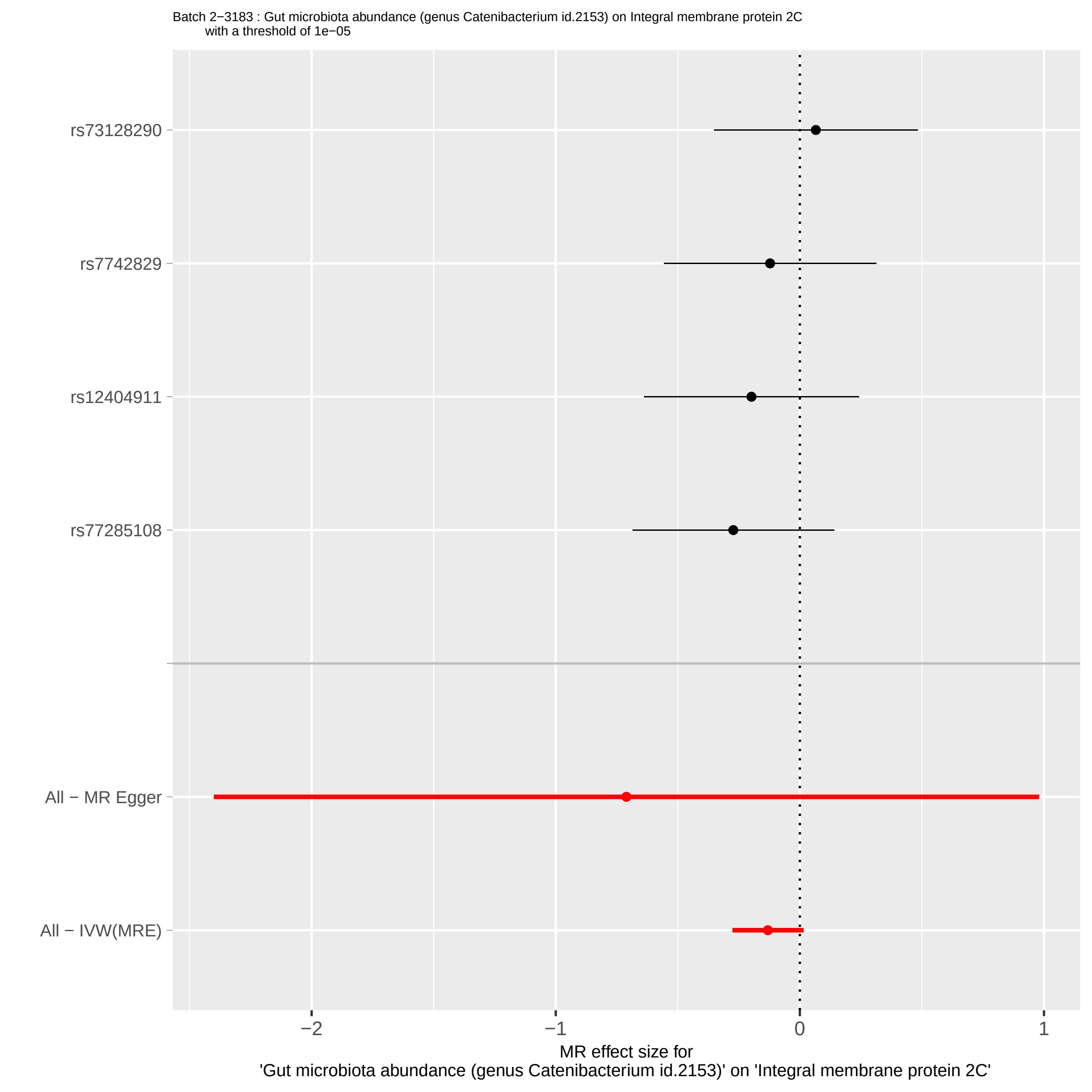

Batch 2−3183 : Gut microbiota abundance (genus Catenibacterium id.2153) on Integral membrane protein 2C
with a threshold of 1e−05
rs73128290
rs7742829
rs12404911
rs77285108
All − MR Egger
All − IVW(MRE)
−2
−1
0
1
MR effect size for
'Gut microbiota abundance (genus Catenibacterium id.2153)' on 'Integral membrane protein 2C'

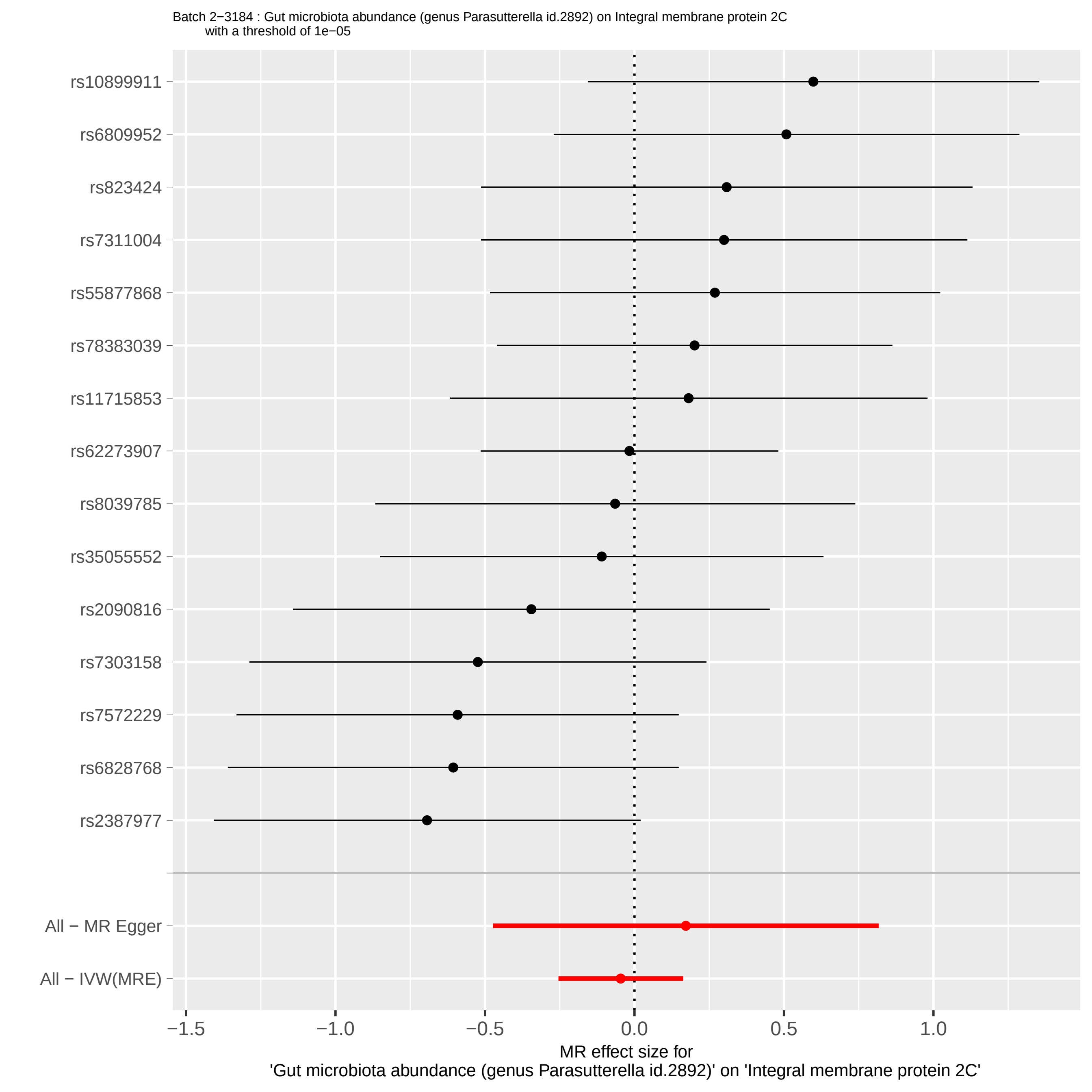
Batch 2−3184 : Gut microbiota abundance (genus Parasutterella id.2892) on Integral membrane protein 2C
with a threshold of 1e−05
rs10899911
rs6809952
rs823424
rs7311004
rs55877868
rs78383039
rs11715853
rs62273907
rs8039785
rs35055552
rs2090816
rs7303158
rs7572229
rs6828768
rs2387977
All − MR Egger
All − IVW(MRE)
−1.5
−1.0
−0.5
0.0
0.5
1.0
MR effect size for
'Gut microbiota abundance (genus Parasutterella id.2892)' on 'Integral membrane protein 2C'

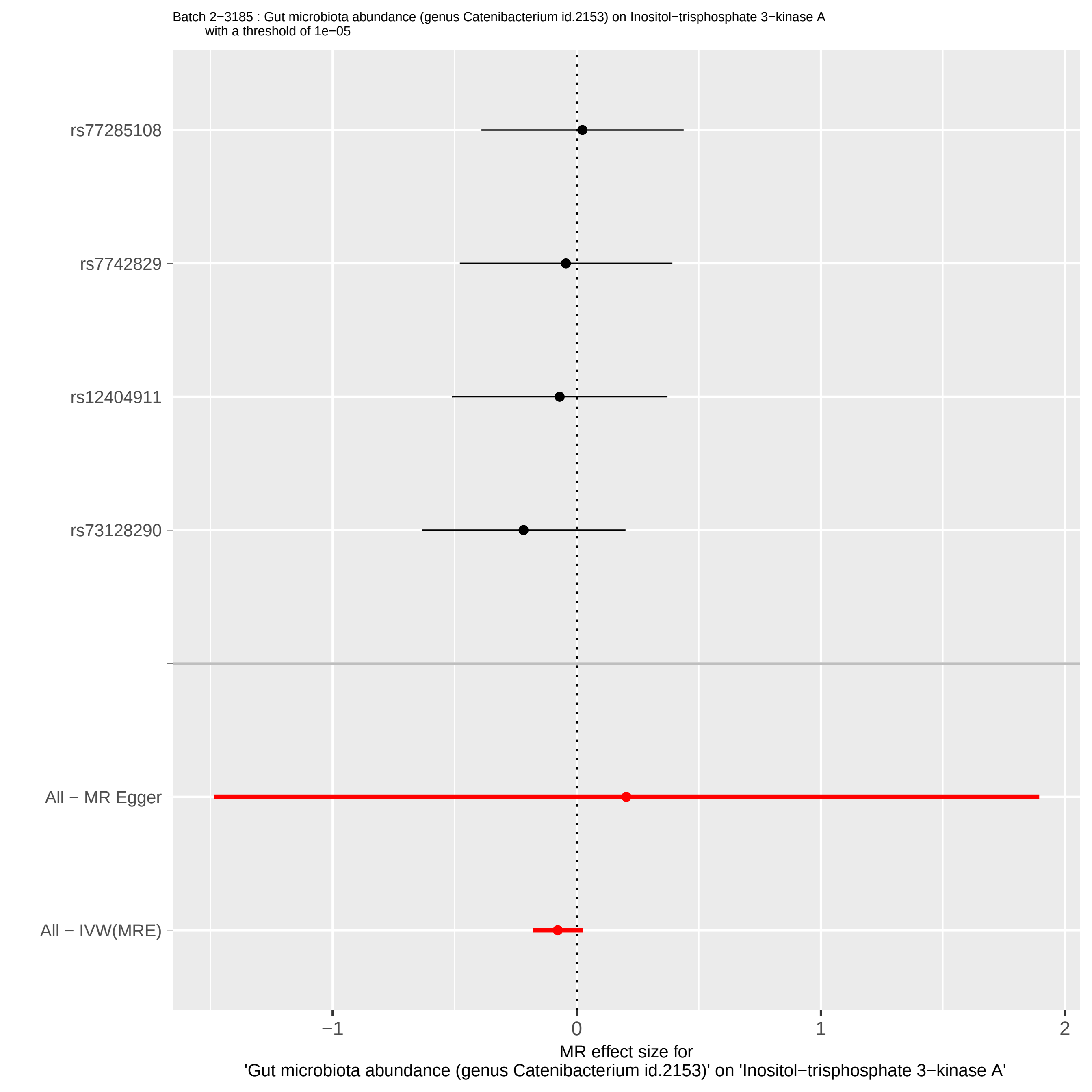

Batch 2−3185 : Gut microbiota abundance (genus Catenibacterium id.2153) on Inositol−trisphosphate 3−kinase A
with a threshold of 1e−05
rs77285108
rs7742829
rs12404911
rs73128290
All − MR Egger
All − IVW(MRE)
−1
0
1
2
MR effect size for
'Gut microbiota abundance (genus Catenibacterium id.2153)' on 'Inositol−trisphosphate 3−kinase A'

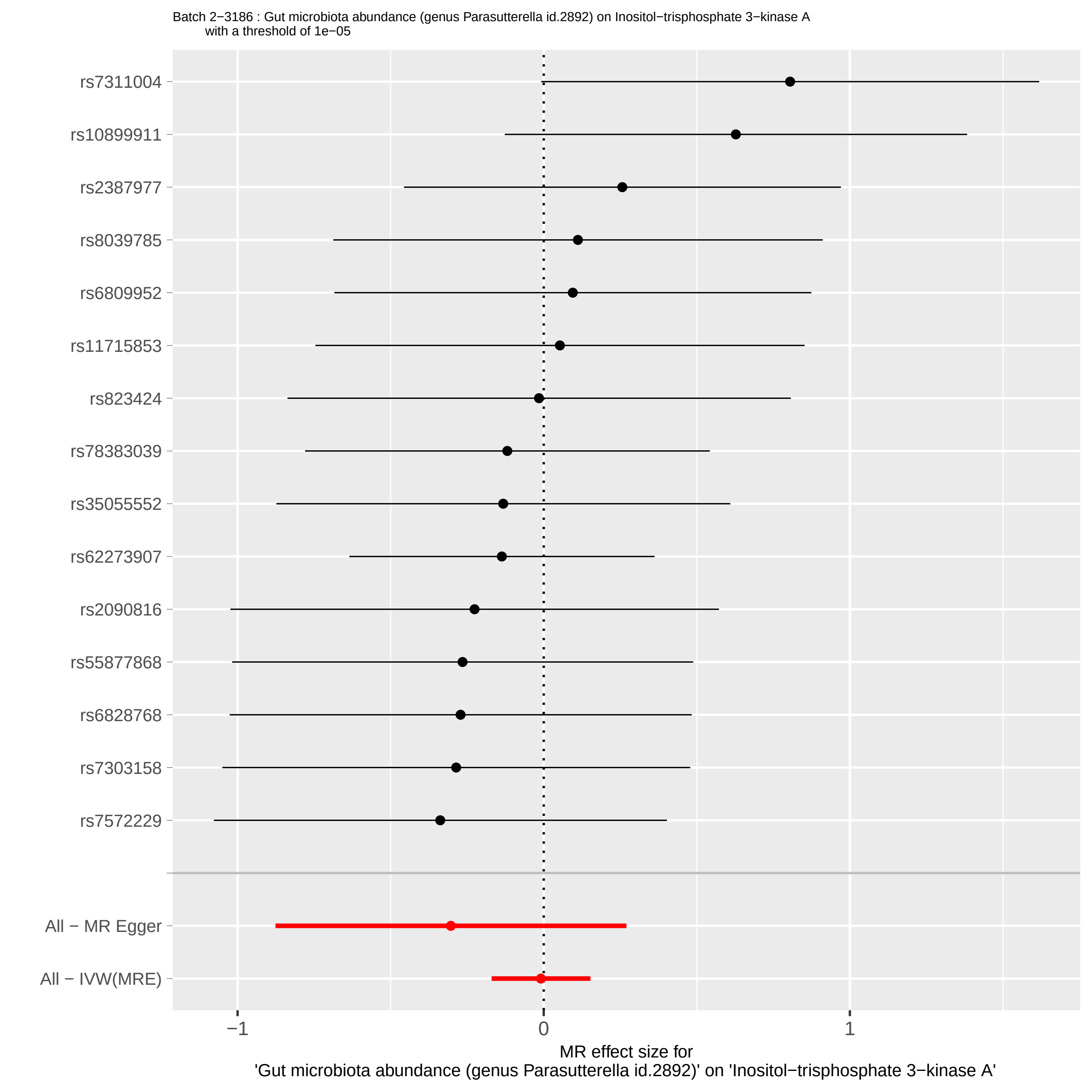

Batch 2−3186 : Gut microbiota abundance (genus Parasutterella id.2892) on Inositol−trisphosphate 3−kinase A
with a threshold of 1e−05
rs7311004
rs10899911
rs2387977
rs8039785
rs6809952
rs11715853
rs823424
rs78383039
rs35055552
rs62273907
rs2090816
rs55877868
rs6828768
rs7303158
rs7572229
All − MR Egger
All − IVW(MRE)
−1
0
1
MR effect size for
'Gut microbiota abundance (genus Parasutterella id.2892)' on 'Inositol−trisphosphate 3−kinase A'

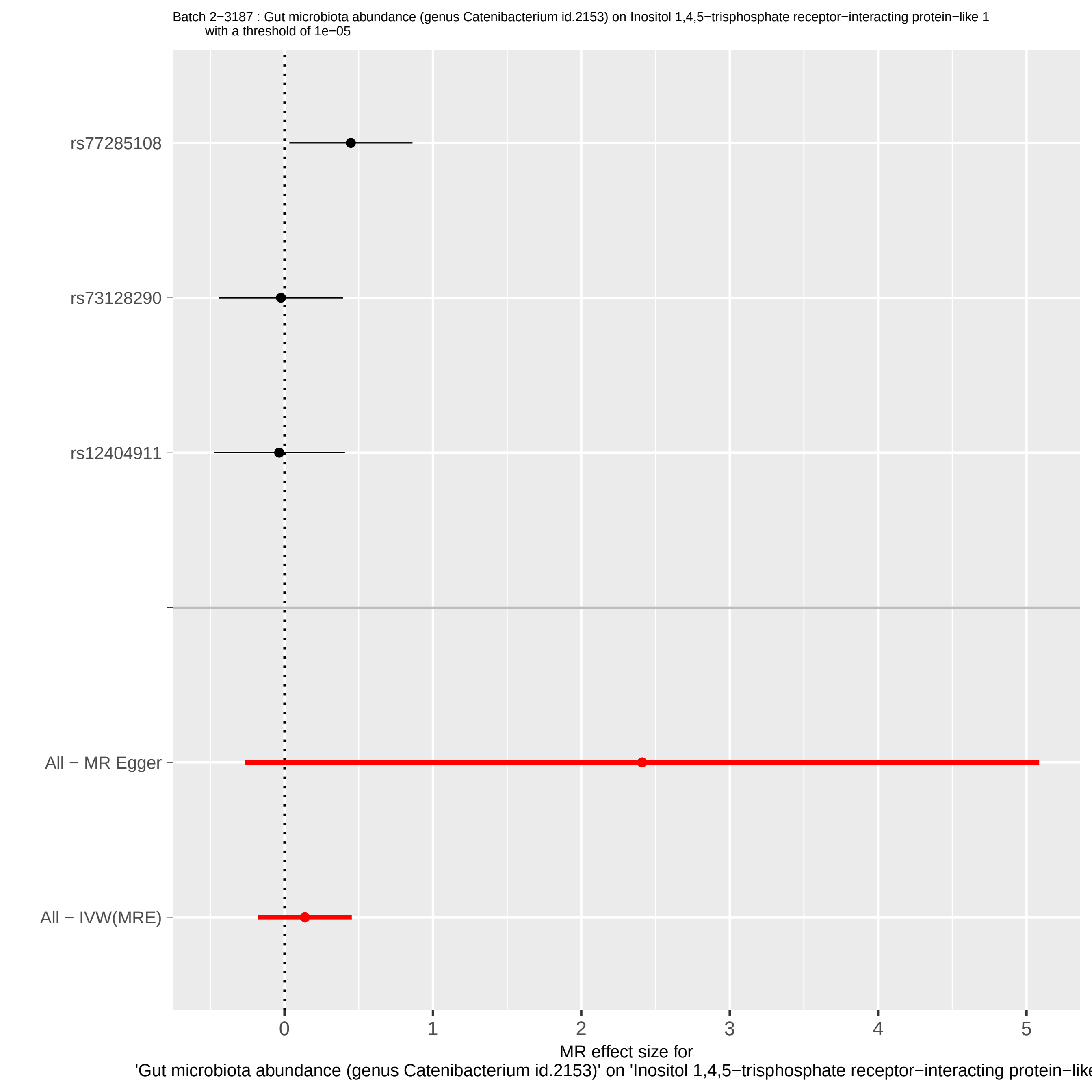

Batch 2−3187 : Gut microbiota abundance (genus Catenibacterium id.2153) on Inositol 1,4,5−trisphosphate receptor−interacting protein−like 1
with a threshold of 1e−05
rs77285108
rs73128290
rs12404911
All − MR Egger
All − IVW(MRE)
0
1
2
3
4
5
MR effect size for
'Gut microbiota abundance (genus Catenibacterium id.2153)' on 'Inositol 1,4,5−trisphosphate receptor−interacting protein−like

Batch 2−3188 : Gut microbiota abundance (genus Parasutterella id.2892) on Inositol 1,4,5−trisphosphate receptor−interacting protein−like 1 with a threshold of 1e−05

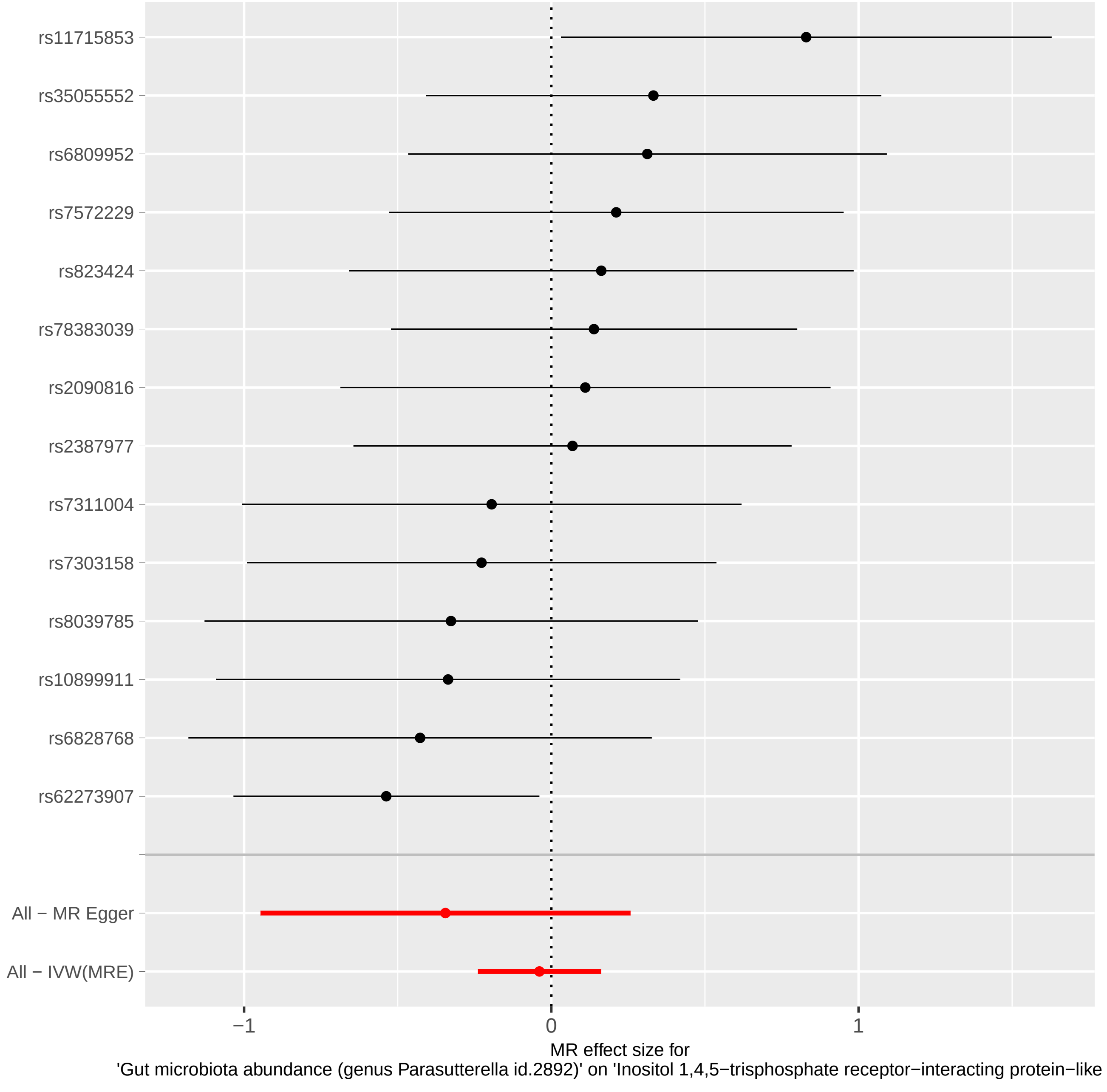

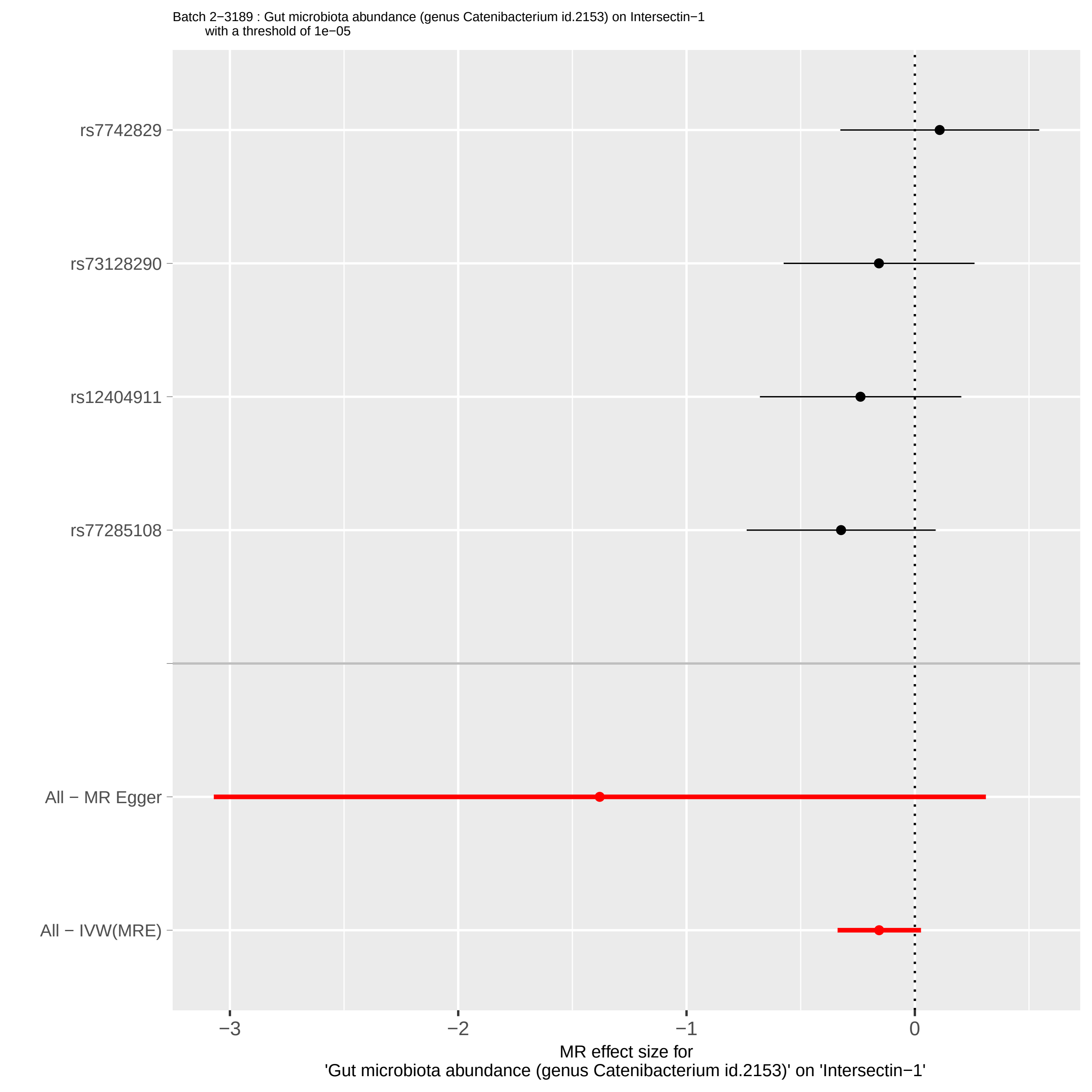
Batch 2−3189 : Gut microbiota abundance (genus Catenibacterium id.2153) on Intersectin−1
with a threshold of 1e−05
rs7742829
rs73128290
rs12404911
rs77285108
All − MR Egger
All − IVW(MRE)
−3
−2
−1
0
MR effect size for
'Gut microbiota abundance (genus Catenibacterium id.2153)' on 'Intersectin−1'

Batch 2−3190 : Gut microbiota abundance (genus Parasutterella id.2892) on Intersectin−1
with a threshold of 1e−05

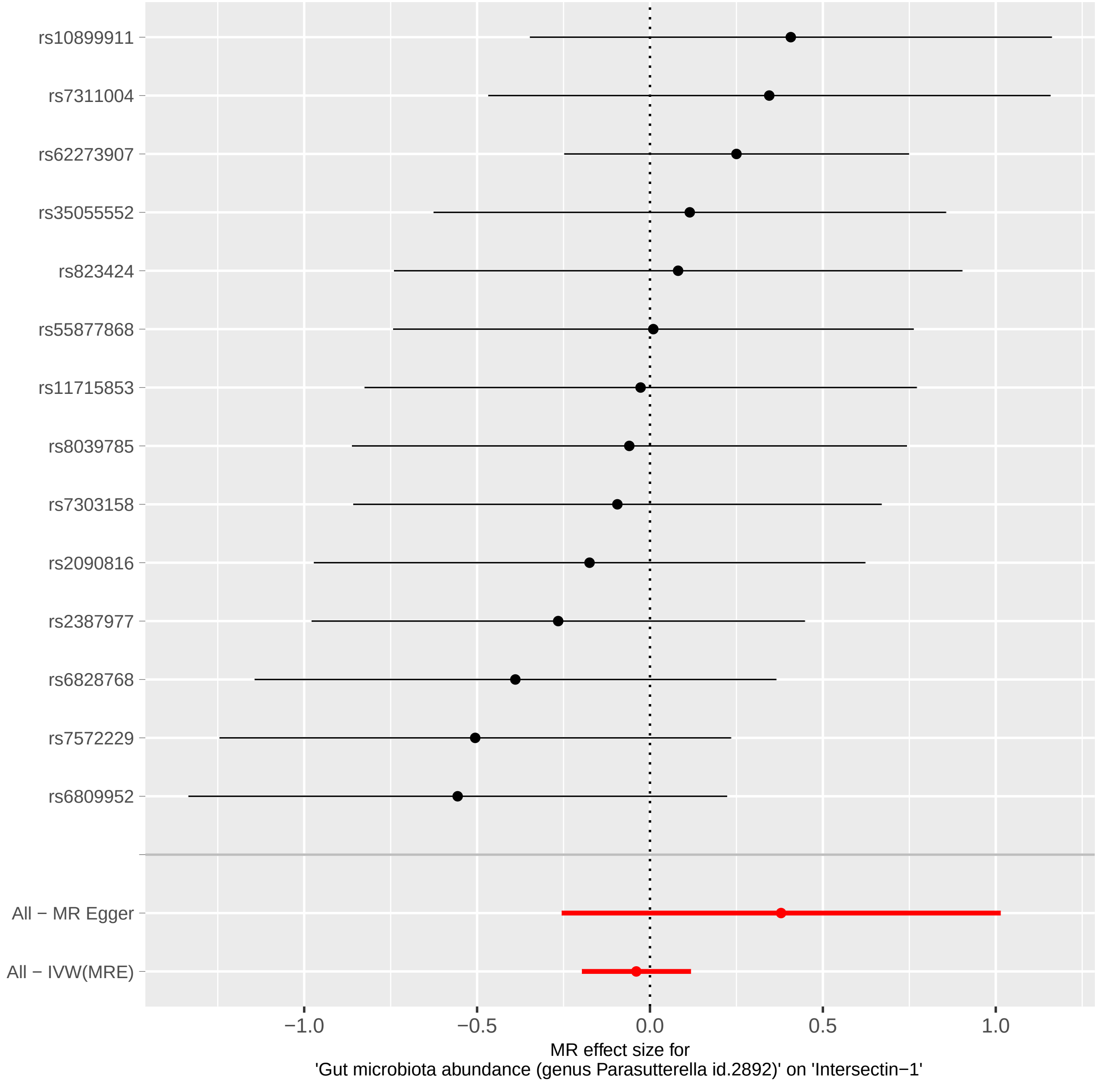

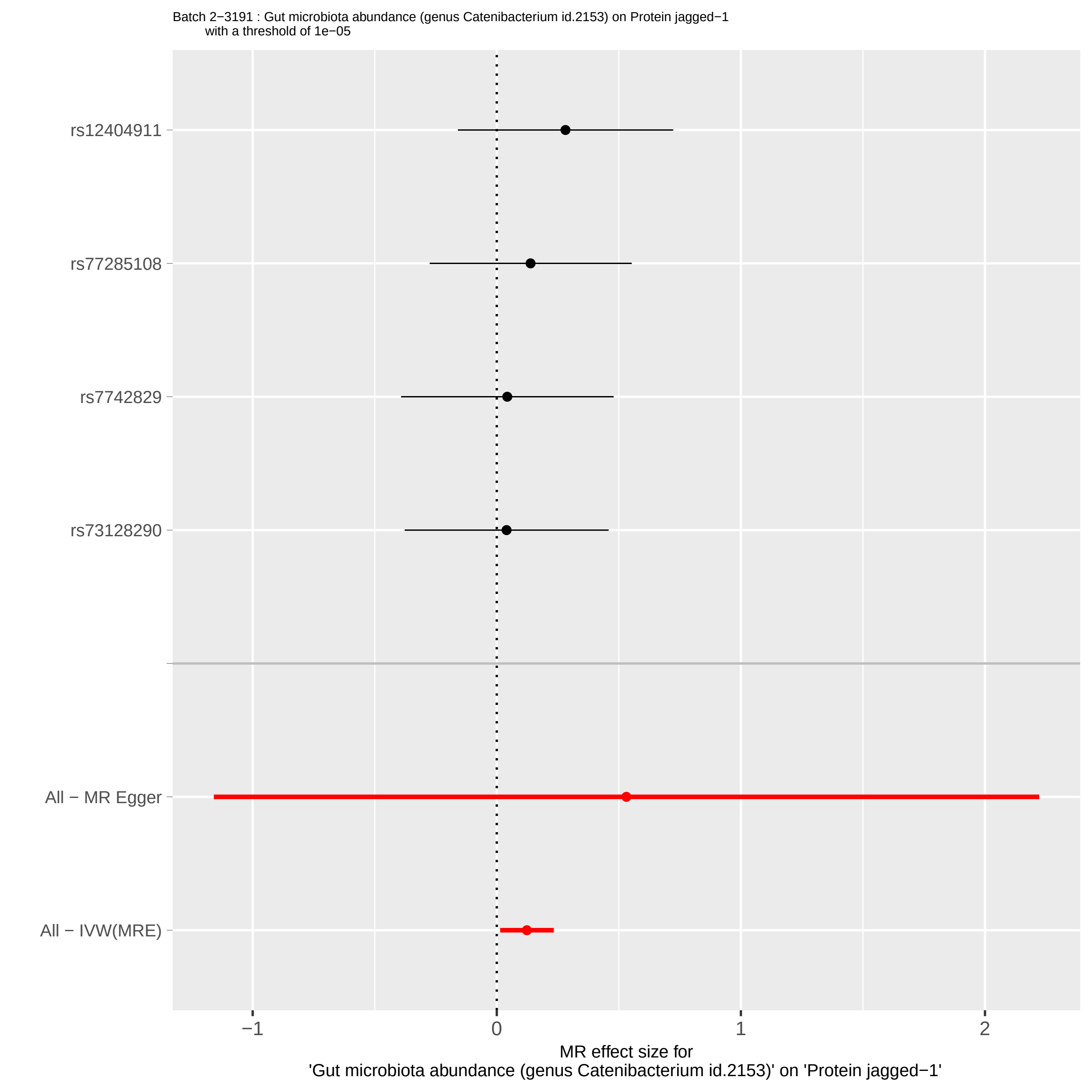

Batch 2−3191 : Gut microbiota abundance (genus Catenibacterium id.2153) on Protein jagged−1
with a threshold of 1e−05
rs12404911
rs77285108
rs7742829
rs73128290
All − MR Egger
All − IVW(MRE)
−1
0
1
2
MR effect size for
'Gut microbiota abundance (genus Catenibacterium id.2153)' on 'Protein jagged−1'

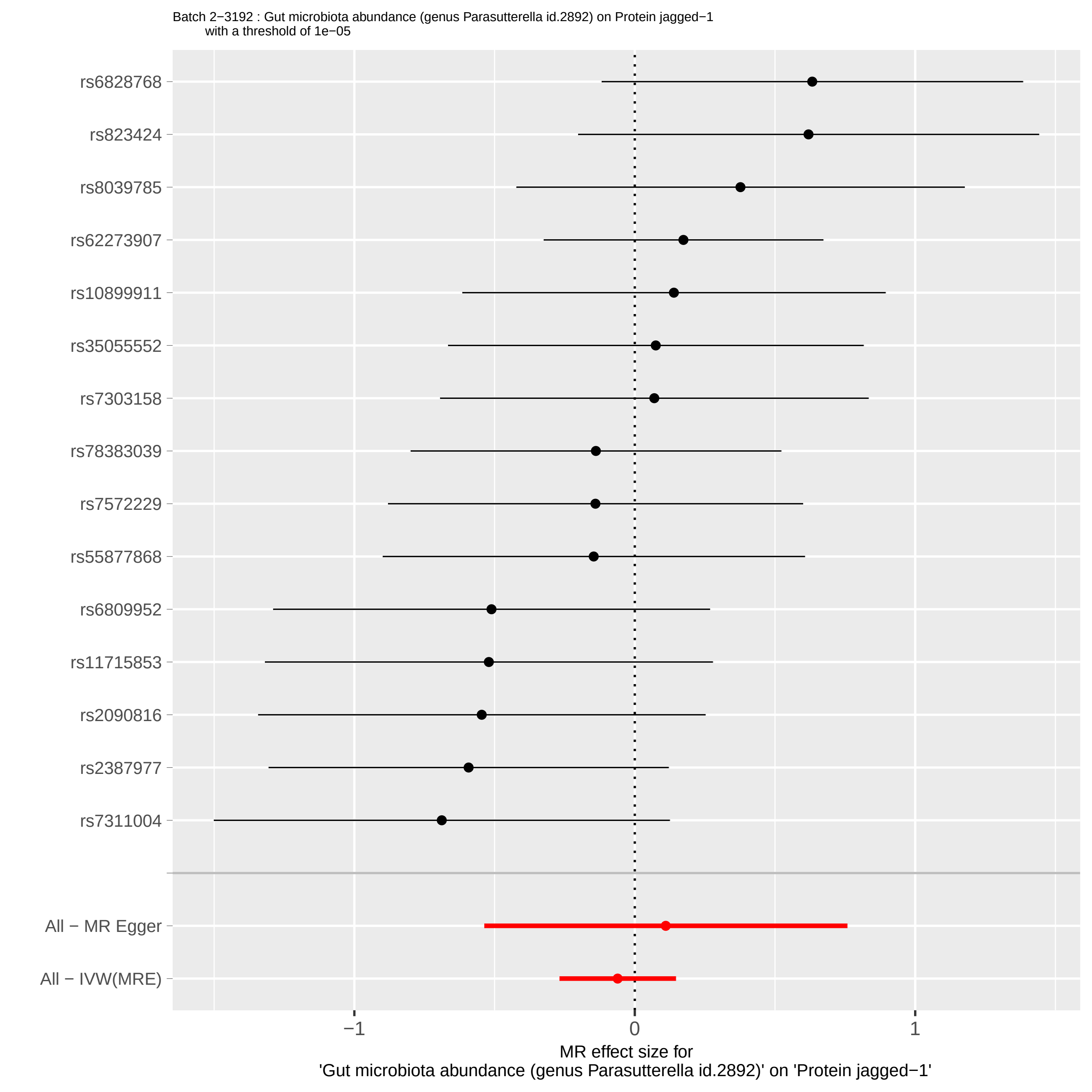
Batch 2−3192 : Gut microbiota abundance (genus Parasutterella id.2892) on Protein jagged−1
with a threshold of 1e−05
rs6828768
rs823424
rs8039785
rs62273907
rs10899911
rs35055552
rs7303158
rs78383039
rs7572229
rs55877868
rs6809952
rs11715853
rs2090816
rs2387977
rs7311004
All − MR Egger
All − IVW(MRE)
−1
0
1
MR effect size for
'Gut microbiota abundance (genus Parasutterella id.2892)' on 'Protein jagged−1'

Batch 2−3193 : Gut microbiota abundance (genus Catenibacterium id.2153) on Protein jagged−2
with a threshold of 1e−05

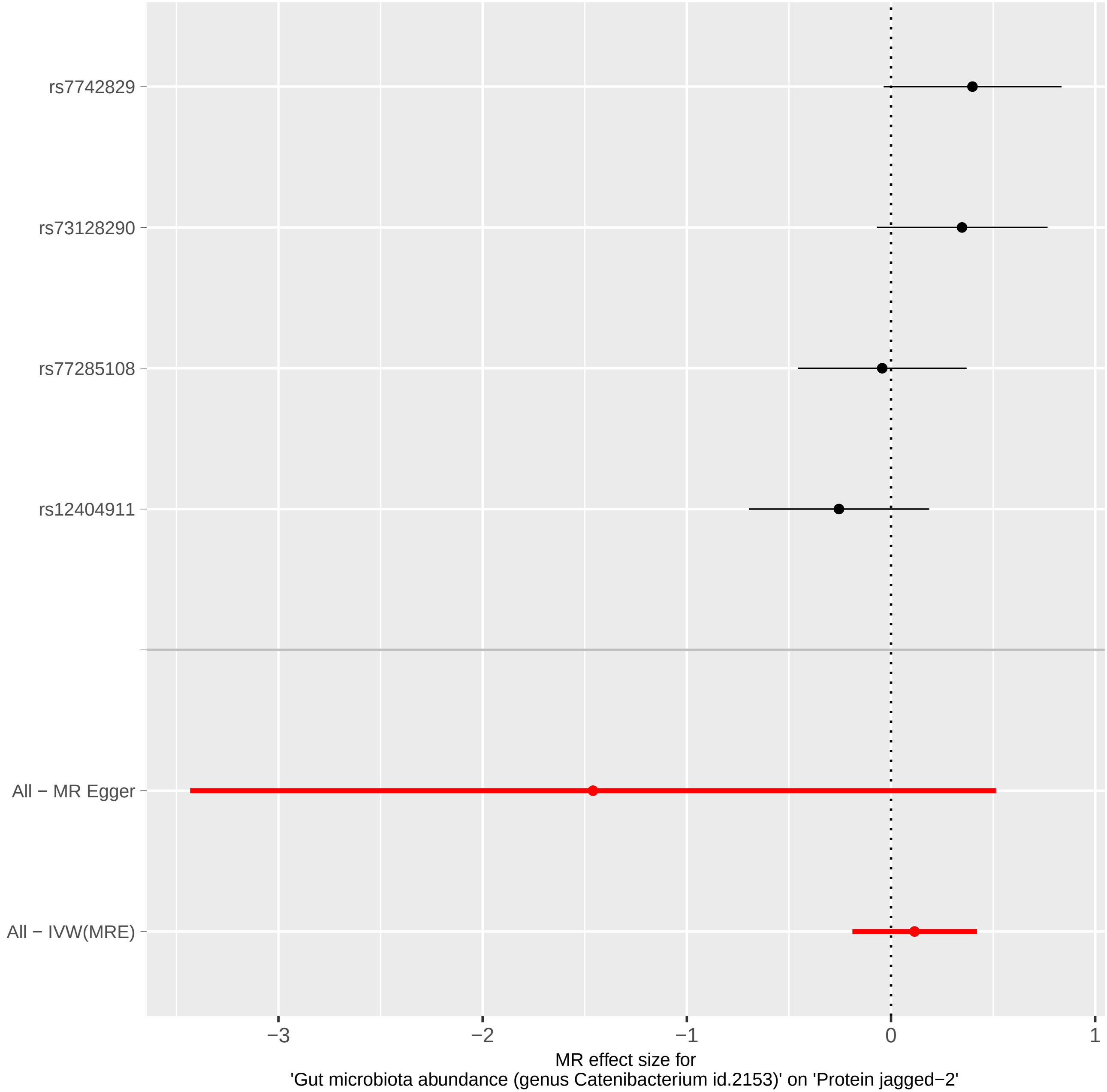

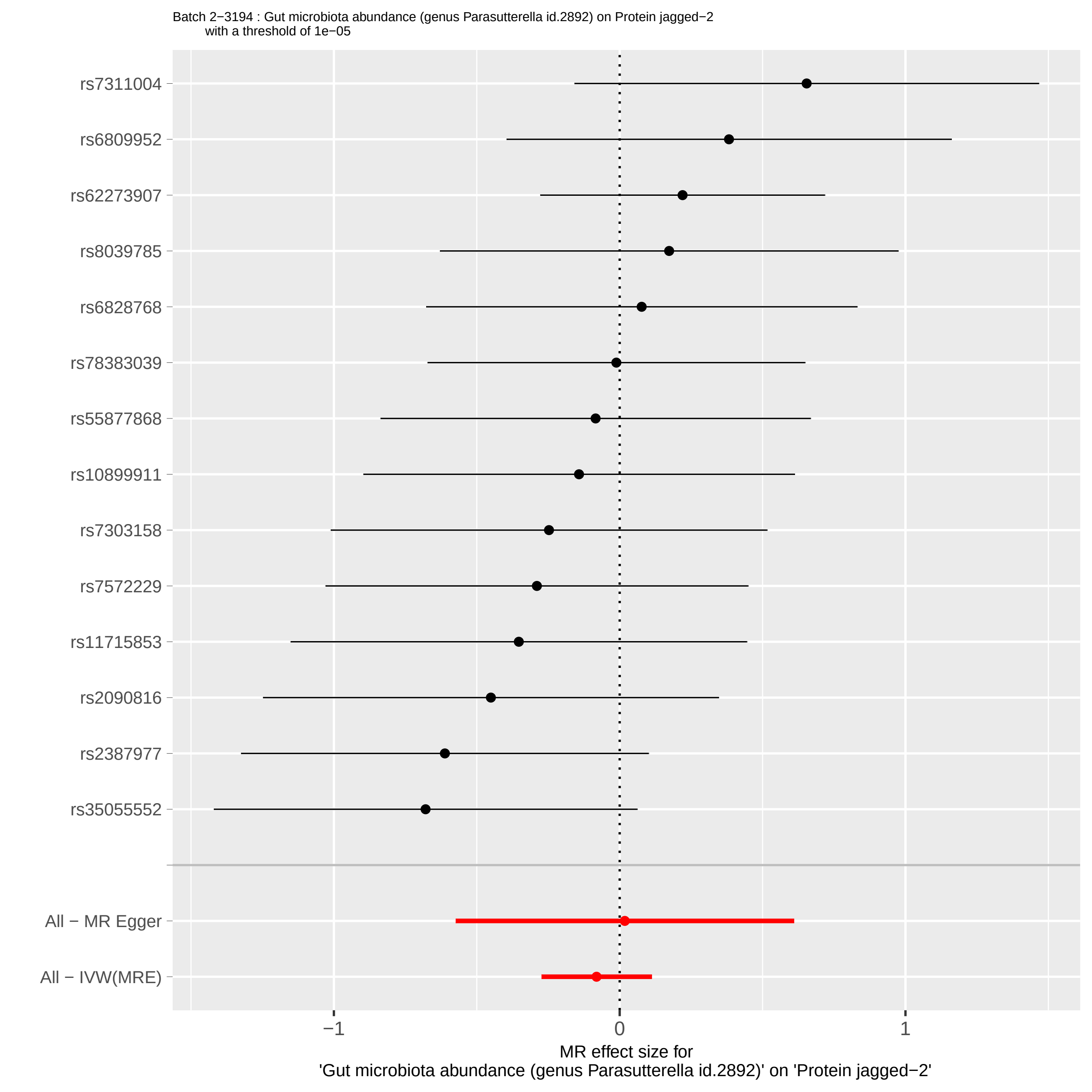
Batch 2−3194 : Gut microbiota abundance (genus Parasutterella id.2892) on Protein jagged−2
with a threshold of 1e−05
rs7311004
rs6809952
rs62273907
rs8039785
rs6828768
rs78383039
rs55877868
rs10899911
rs7303158
rs7572229
rs11715853
rs2090816
rs2387977
rs35055552
All − MR Egger
All − IVW(MRE)
−1
0
1
MR effect size for
'Gut microbiota abundance (genus Parasutterella id.2892)' on 'Protein jagged−2'

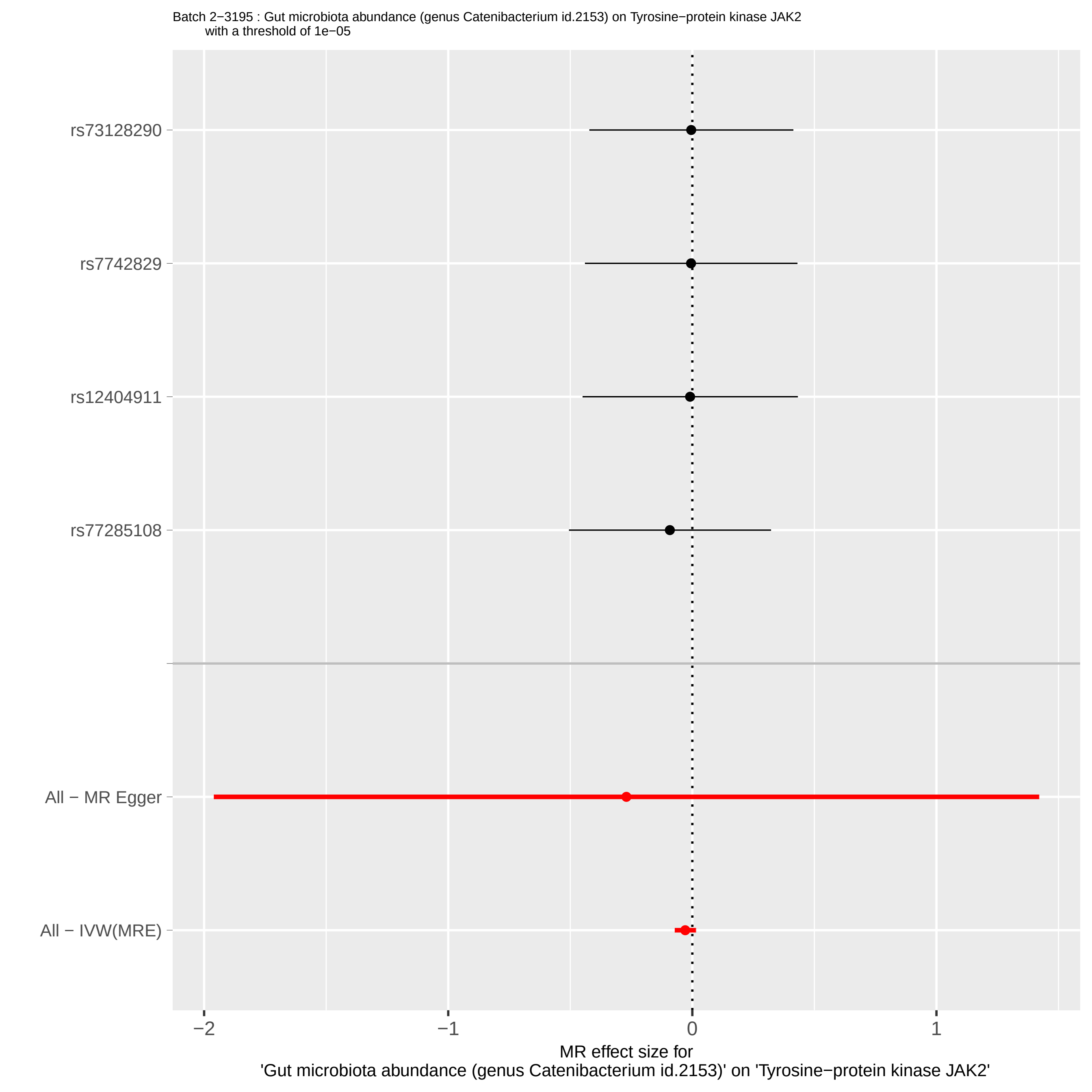
Batch 2−3195 : Gut microbiota abundance (genus Catenibacterium id.2153) on Tyrosine−protein kinase JAK2
with a threshold of 1e−05
rs73128290
rs7742829
rs12404911
rs77285108
All − MR Egger
All − IVW(MRE)
−2
−1
0
1
MR effect size for
'Gut microbiota abundance (genus Catenibacterium id.2153)' on 'Tyrosine−protein kinase JAK2'

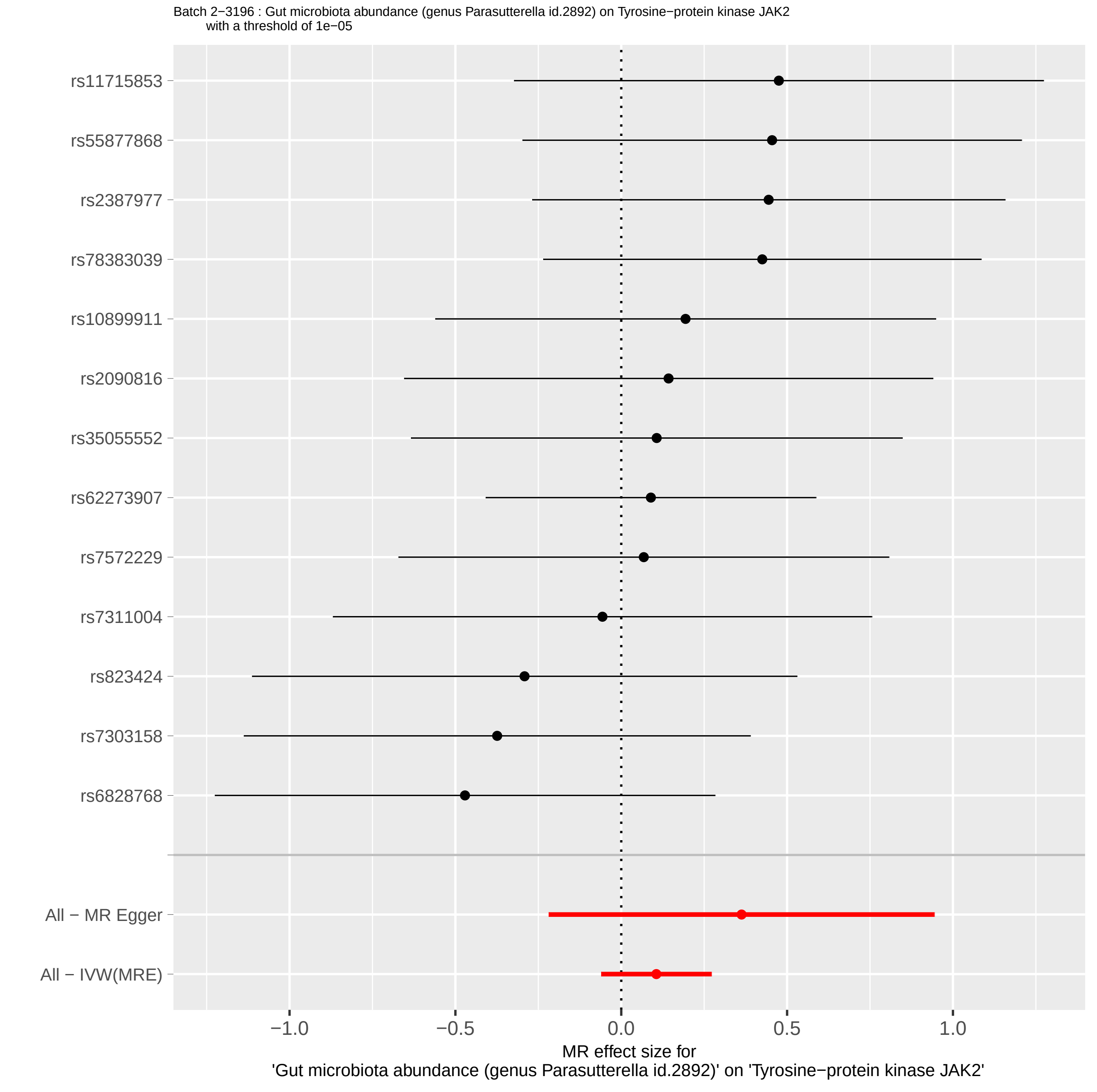

Batch 2−3196 : Gut microbiota abundance (genus Parasutterella id.2892) on Tyrosine−protein kinase JAK2
with a threshold of 1e−05
rs11715853
rs55877868
rs2387977
rs78383039
rs10899911
rs2090816
rs35055552
rs62273907
rs7572229
rs7311004
rs823424
rs7303158
rs6828768
All − MR Egger
All − IVW(MRE)
−1.0
−0.5
0.0
0.5
1.0
MR effect size for
'Gut microbiota abundance (genus Parasutterella id.2892)' on 'Tyrosine−protein kinase JAK2'

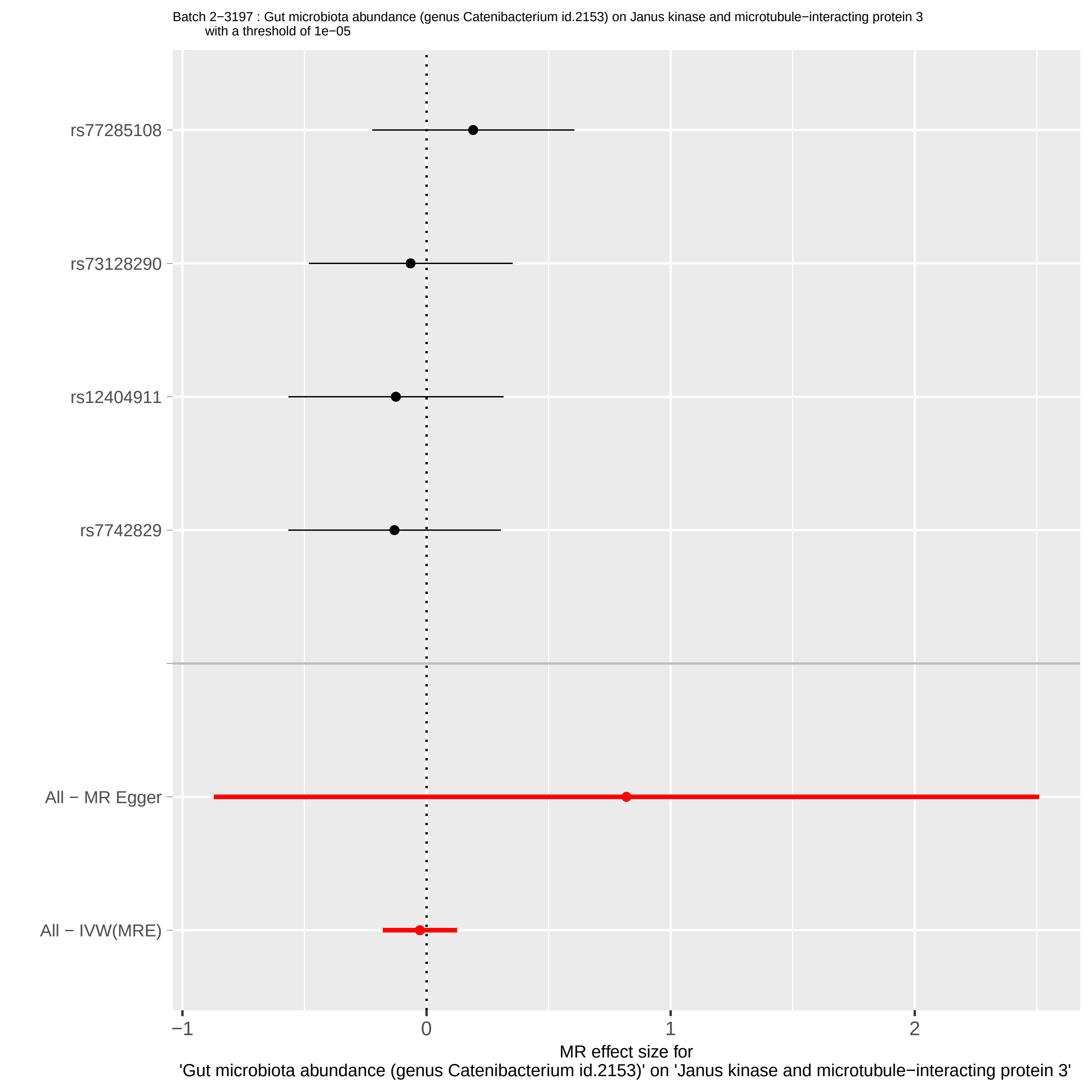
Batch 2−3197 : Gut microbiota abundance (genus Catenibacterium id.2153) on Janus kinase and microtubule−interacting protein 3
with a threshold of 1e−05
rs77285108
rs73128290
rs12404911
rs7742829
All − MR Egger
All − IVW(MRE)
−1
0
1
2
MR effect size for
'Gut microbiota abundance (genus Catenibacterium id.2153)' on 'Janus kinase and microtubule−interacting protein 3'

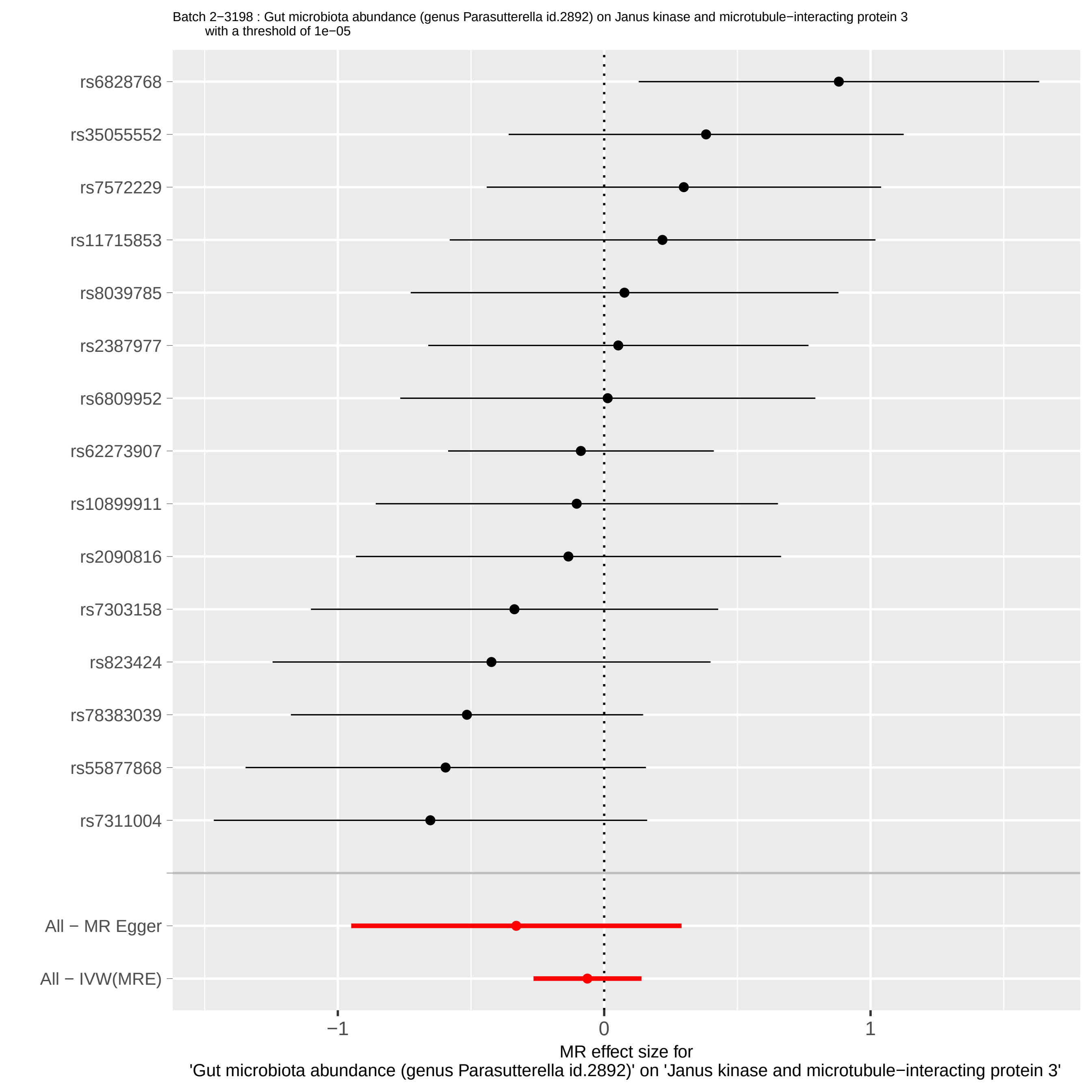
Batch 2−3198 : Gut microbiota abundance (genus Parasutterella id.2892) on Janus kinase and microtubule−interacting protein 3
with a threshold of 1e−05
rs6828768
rs35055552
rs7572229
rs11715853
rs8039785
rs2387977
rs6809952
rs62273907
rs10899911
rs2090816
rs7303158
rs823424
rs78383039
rs55877868
rs7311004
All − MR Egger
All − IVW(MRE)
−1
0
1
MR effect size for
'Gut microbiota abundance (genus Parasutterella id.2892)' on 'Janus kinase and microtubule−interacting protein 3'

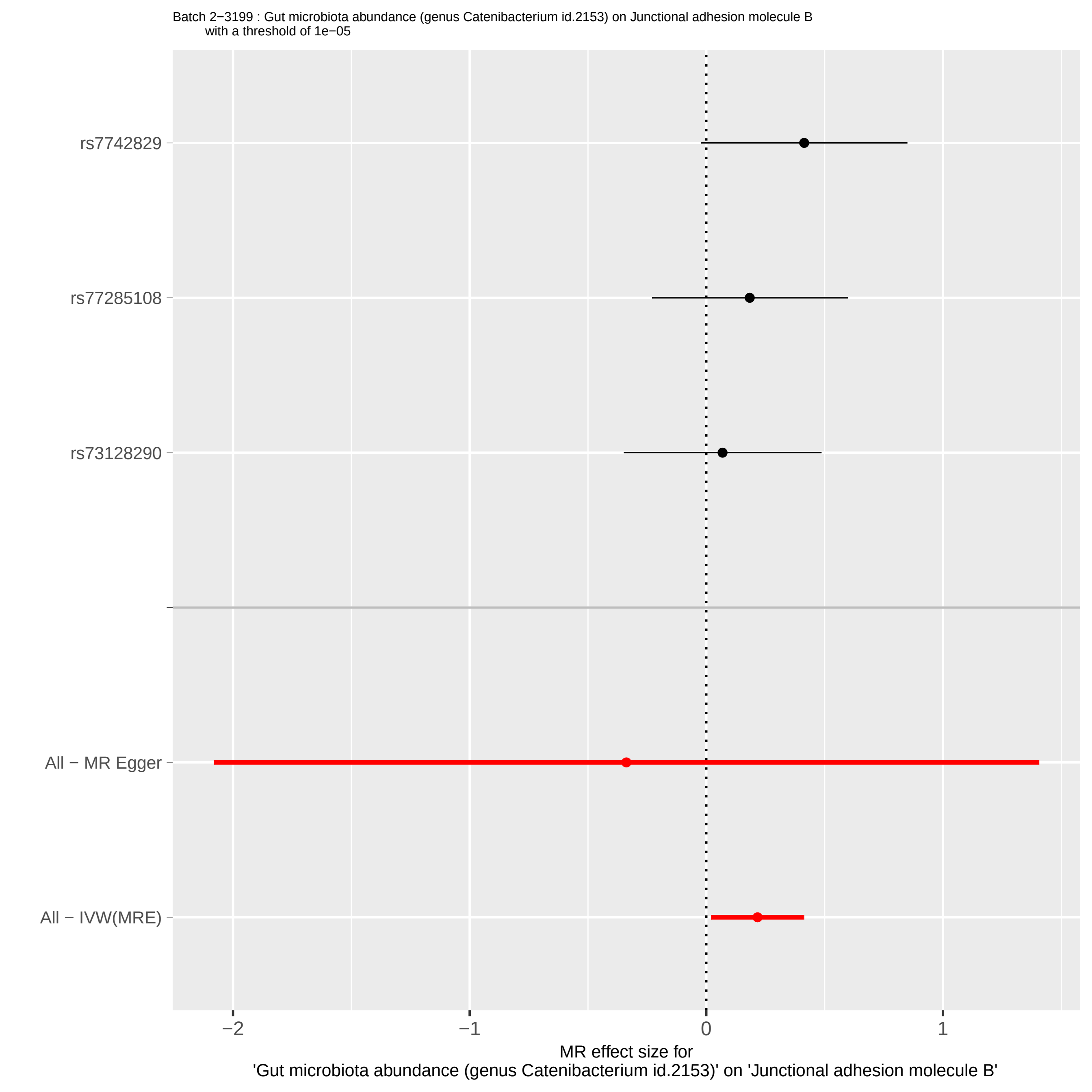

Batch 2−3199 : Gut microbiota abundance (genus Catenibacterium id.2153) on Junctional adhesion molecule B
with a threshold of 1e−05
rs7742829
rs77285108
rs73128290
All − MR Egger
All − IVW(MRE)
−2
−1
0
1
MR effect size for
'Gut microbiota abundance (genus Catenibacterium id.2153)' on 'Junctional adhesion molecule B'

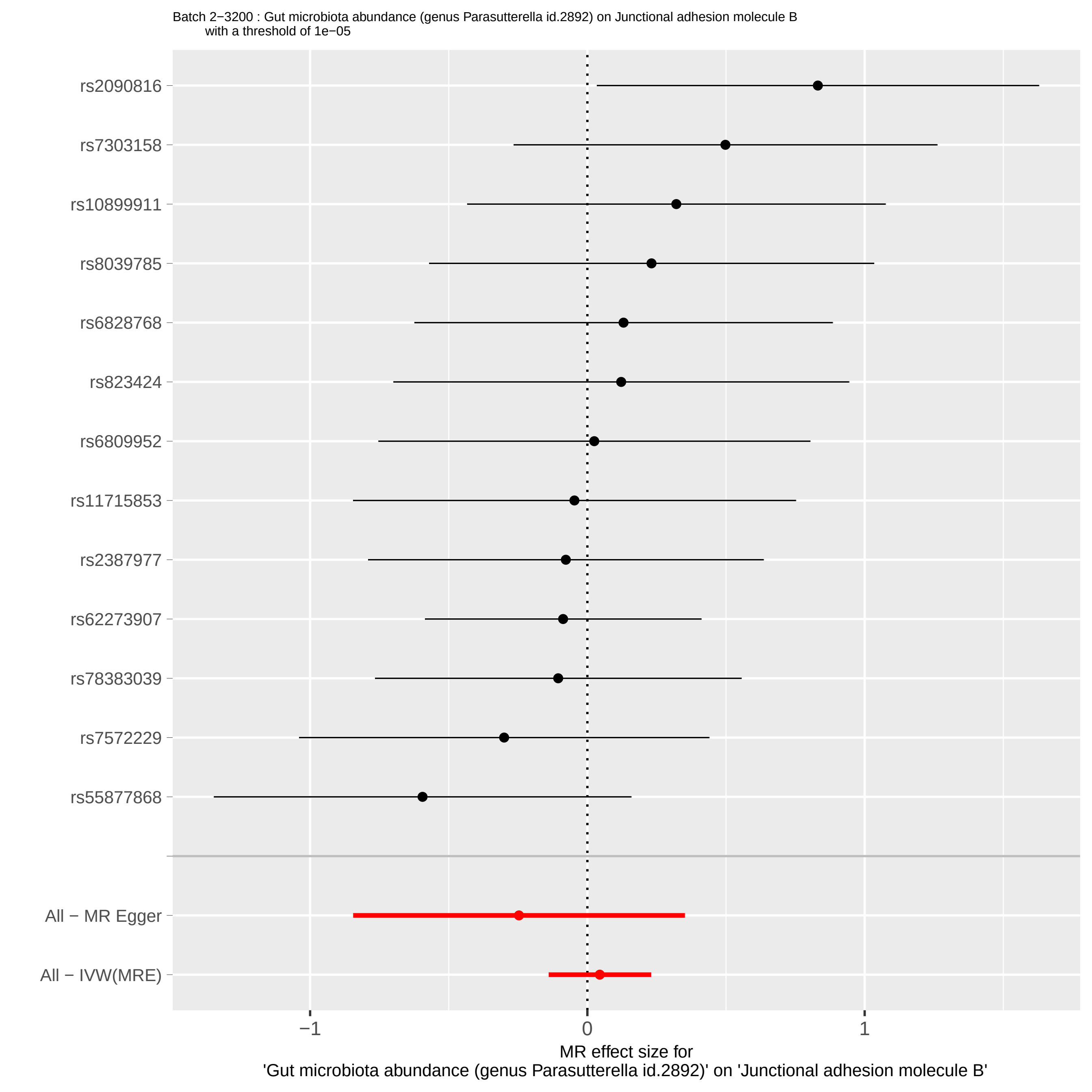
Batch 2−3200 : Gut microbiota abundance (genus Parasutterella id.2892) on Junctional adhesion molecule B
with a threshold of 1e−05
rs2090816
rs7303158
rs10899911
rs8039785
rs6828768
rs823424
rs6809952
rs11715853
rs2387977
rs62273907
rs78383039
rs7572229
rs55877868
All − MR Egger
All − IVW(MRE)
−1
0
1
MR effect size for
'Gut microbiota abundance (genus Parasutterella id.2892)' on 'Junctional adhesion molecule B'

Batch 2−3201 : Gut microbiota abundance (genus Catenibacterium id.2153) on Junctional adhesion molecule C
with a threshold of 1e−05

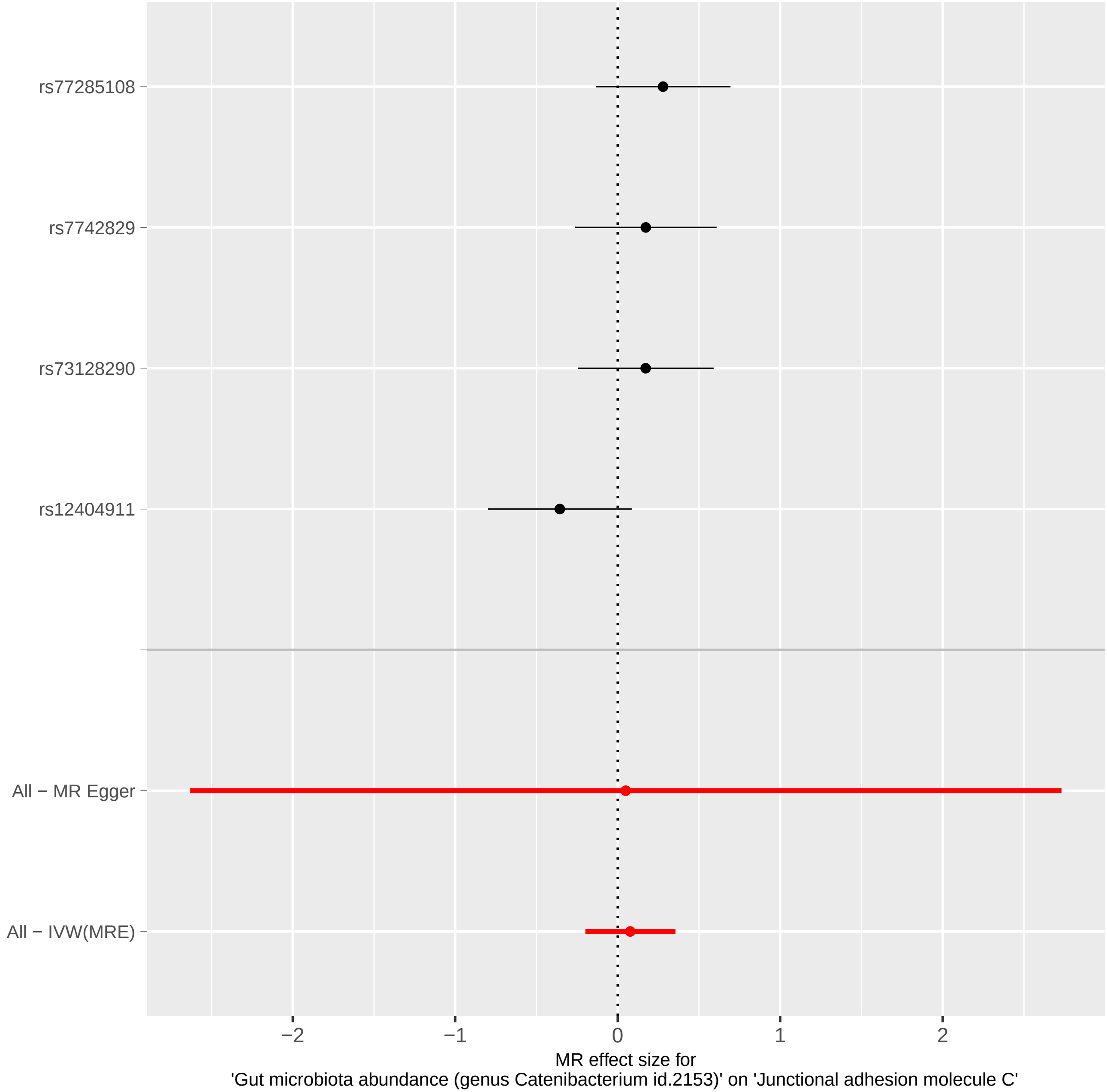

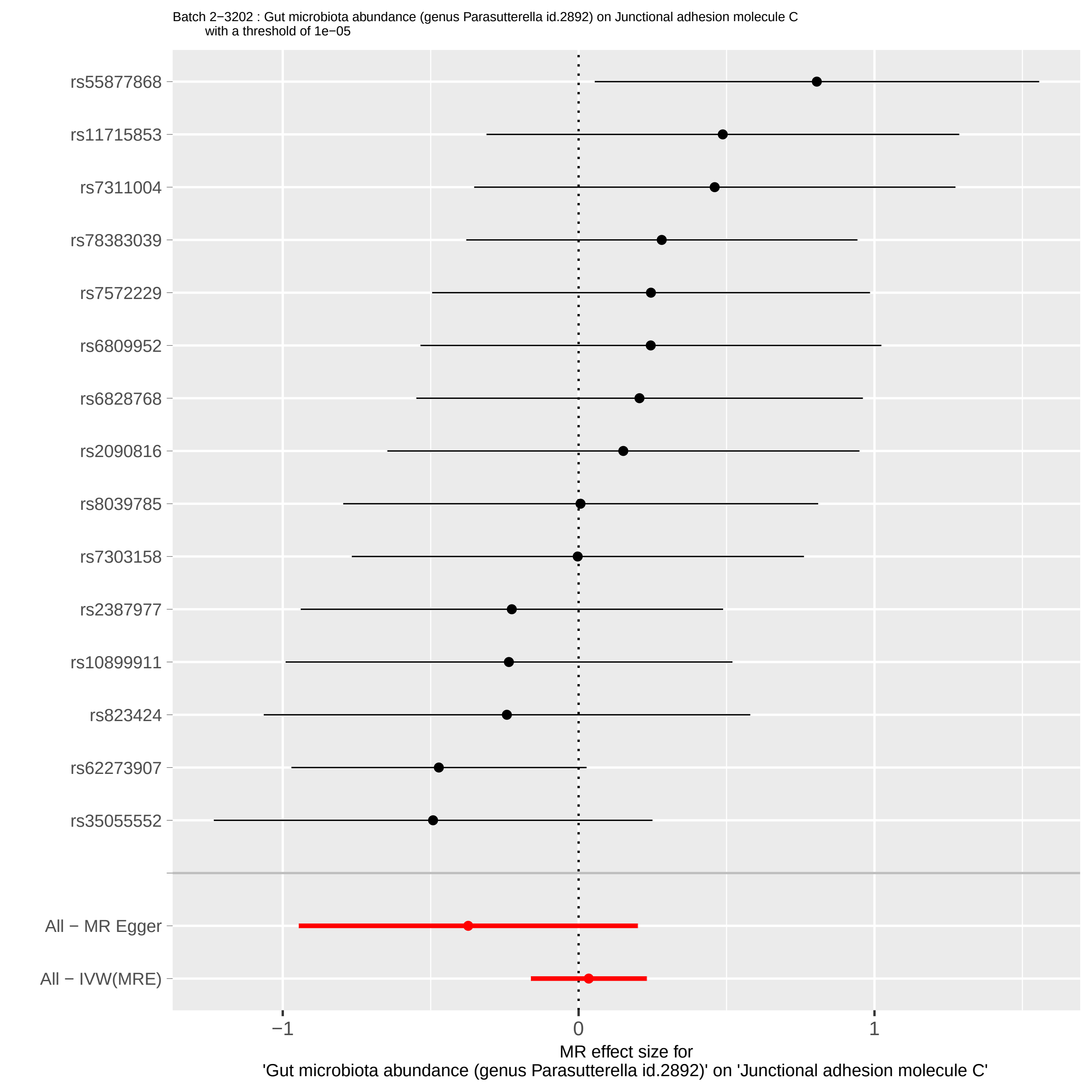
Batch 2−3202 : Gut microbiota abundance (genus Parasutterella id.2892) on Junctional adhesion molecule C
with a threshold of 1e−05
rs55877868
rs11715853
rs7311004
rs78383039
rs7572229
rs6809952
rs6828768
rs2090816
rs8039785
rs7303158
rs2387977
rs10899911
rs823424
rs62273907
rs35055552
All − MR Egger
All − IVW(MRE)
−1
0
1
MR effect size for
'Gut microbiota abundance (genus Parasutterella id.2892)' on 'Junctional adhesion molecule C'

Batch 2−3203 : Gut microbiota abundance (genus Catenibacterium id.2153) on Junctophilin−1
with a threshold of 1e−05

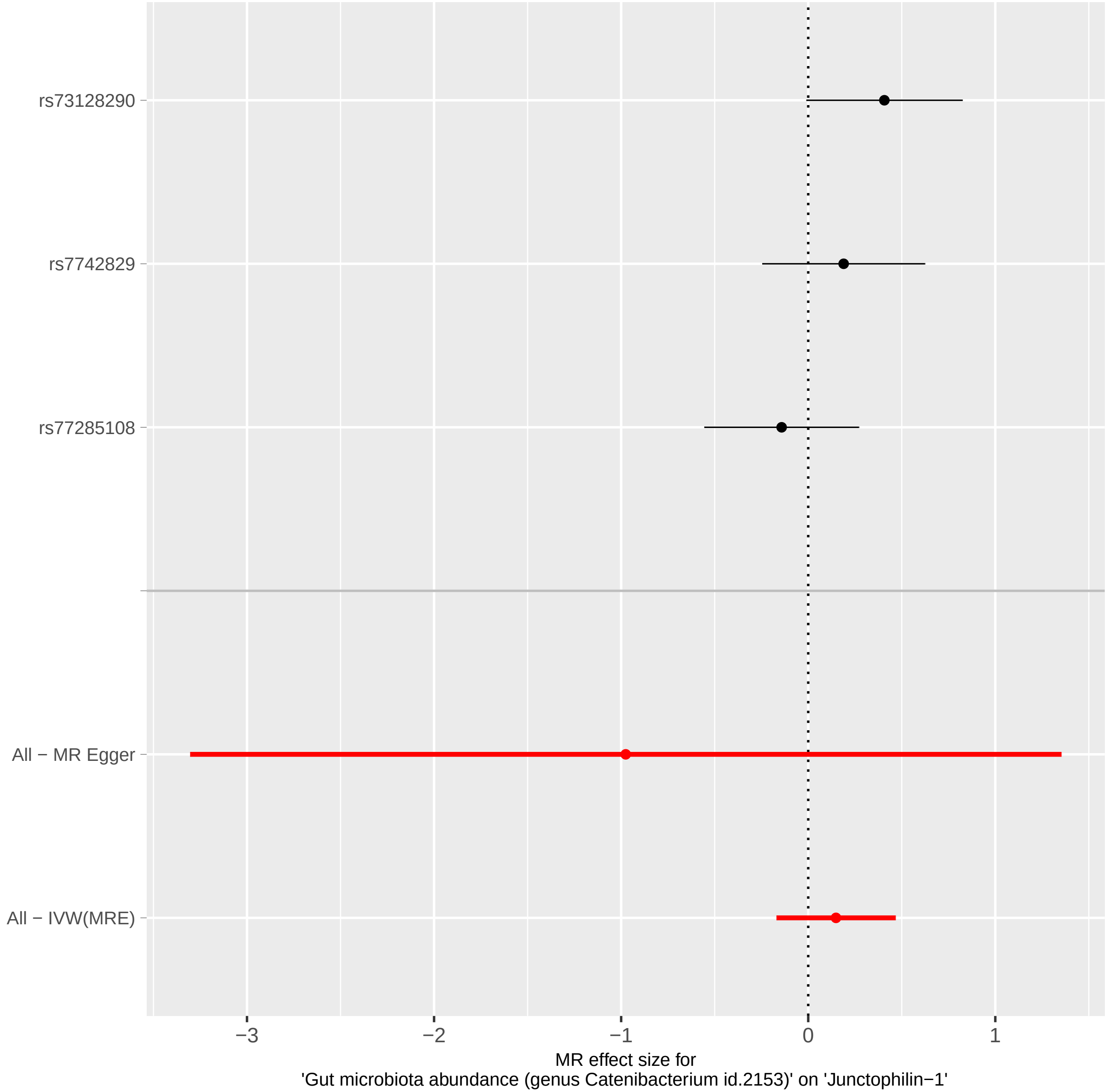

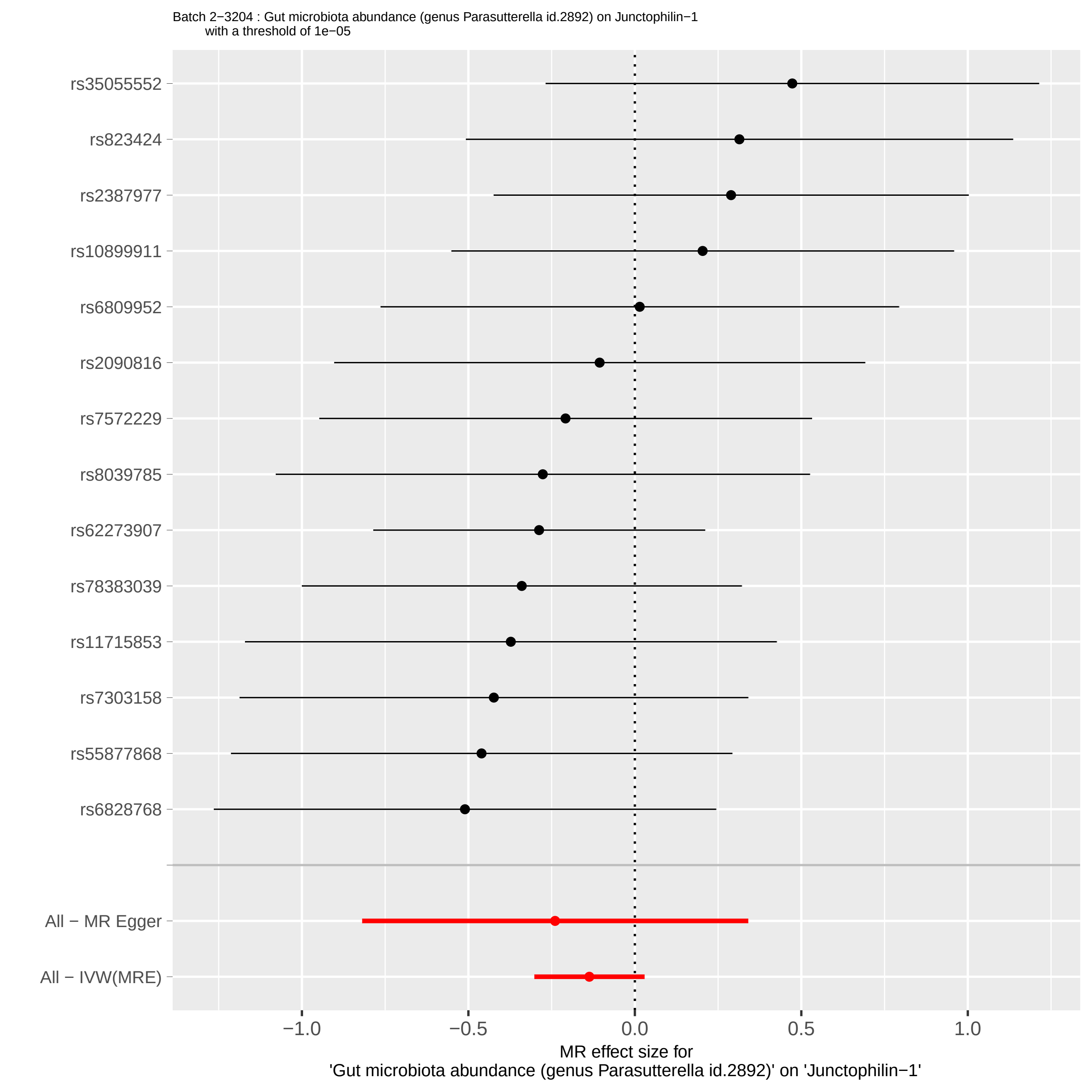

Batch 2−3204 : Gut microbiota abundance (genus Parasutterella id.2892) on Junctophilin−1
with a threshold of 1e−05
rs35055552
rs823424
rs2387977
rs10899911
rs6809952
rs2090816
rs7572229
rs8039785
rs62273907
rs78383039
rs11715853
rs7303158
rs55877868
rs6828768
All − MR Egger
All − IVW(MRE)
−1.0
−0.5
0.0
0.5
1.0
MR effect size for
'Gut microbiota abundance (genus Parasutterella id.2892)' on 'Junctophilin−1'

Batch 2−3205 : Gut microbiota abundance (genus Catenibacterium id.2153) on Junctophilin−3
with a threshold of 1e−05

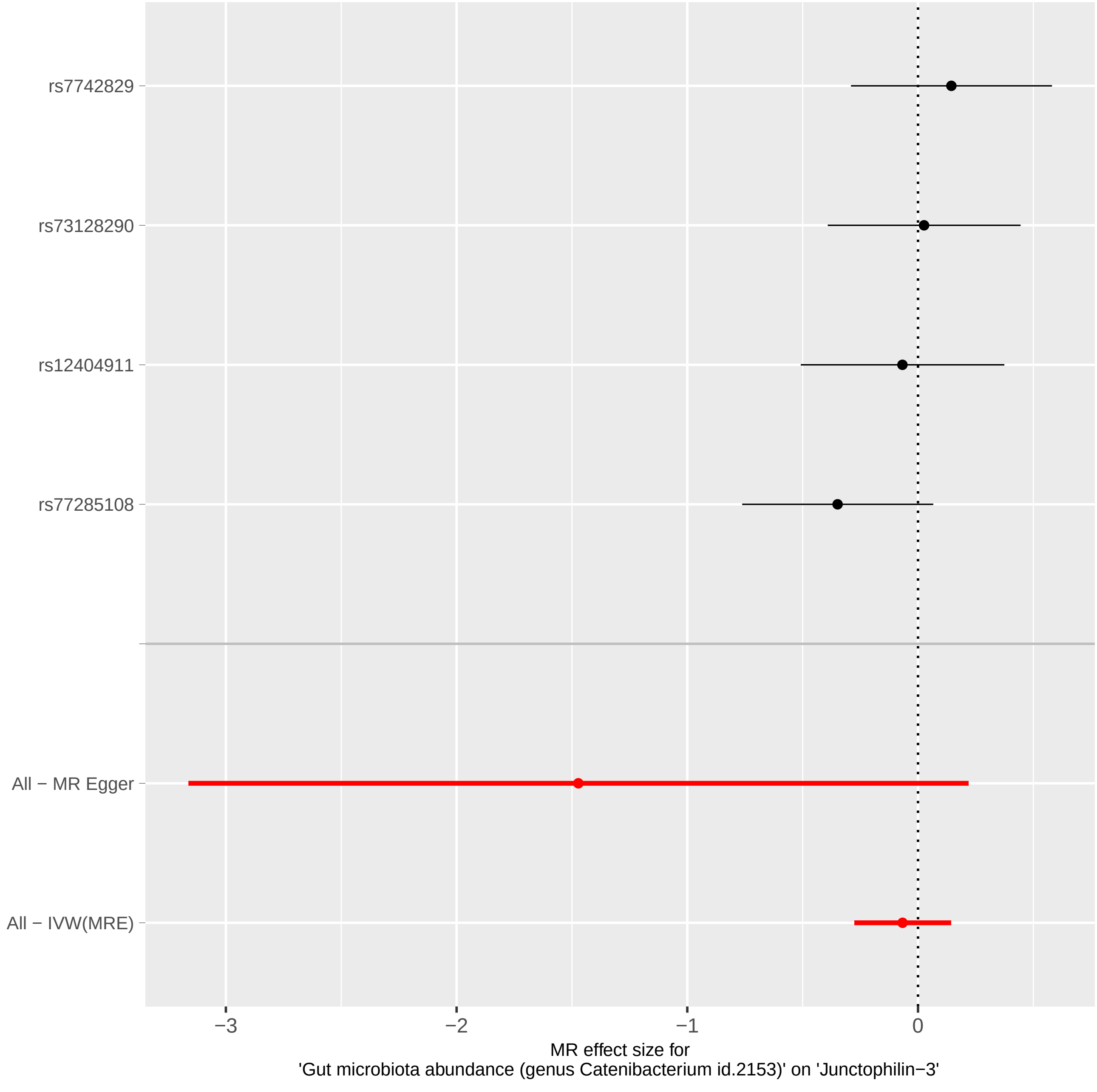

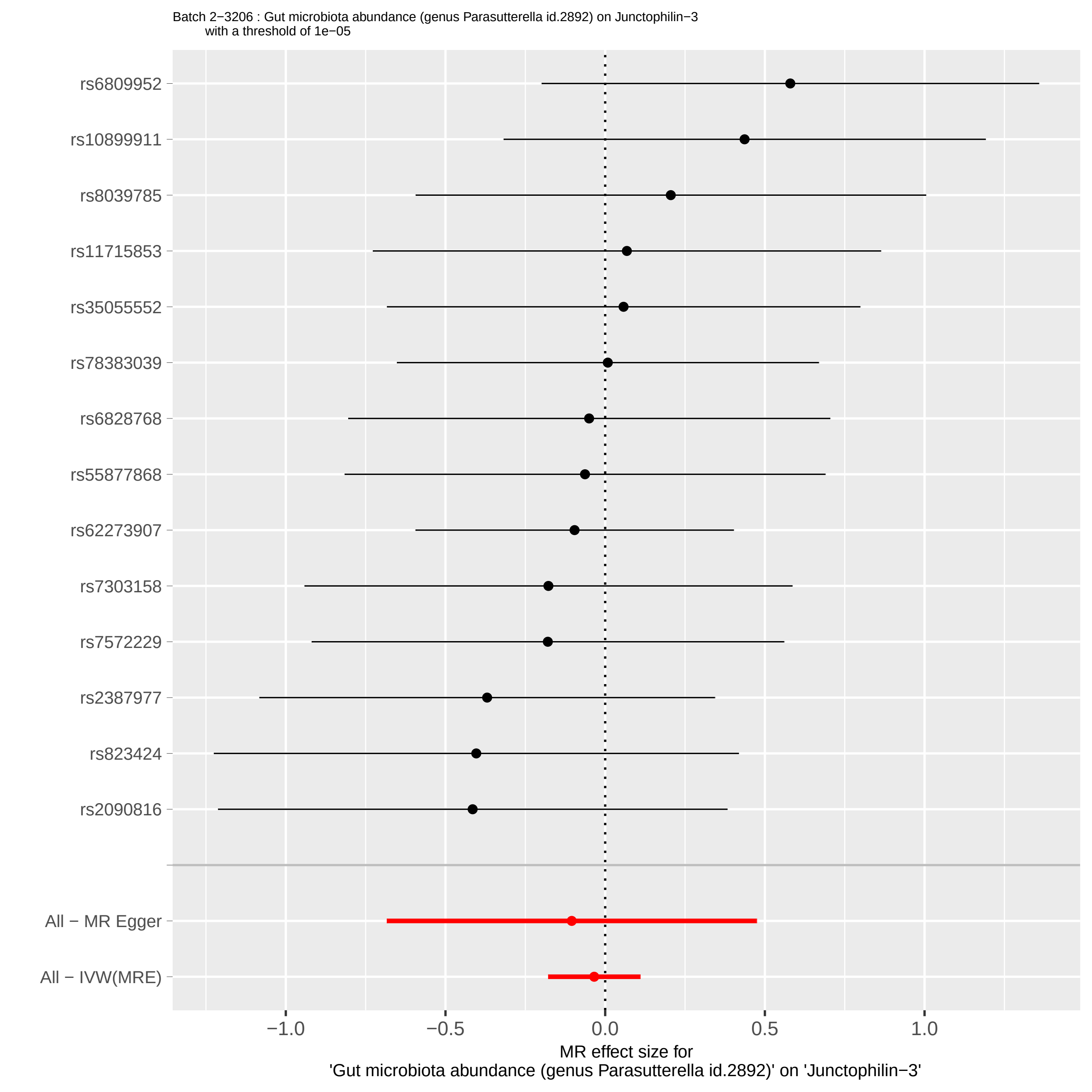

Batch 2−3206 : Gut microbiota abundance (genus Parasutterella id.2892) on Junctophilin−3
with a threshold of 1e−05
rs6809952
rs10899911
rs8039785
rs11715853
rs35055552
rs78383039
rs6828768
rs55877868
rs62273907
rs7303158
rs7572229
rs2387977
rs823424
rs2090816
All − MR Egger
All − IVW(MRE)
−1.0
−0.5
0.0
0.5
1.0
MR effect size for
'Gut microbiota abundance (genus Parasutterella id.2892)' on 'Junctophilin−3'

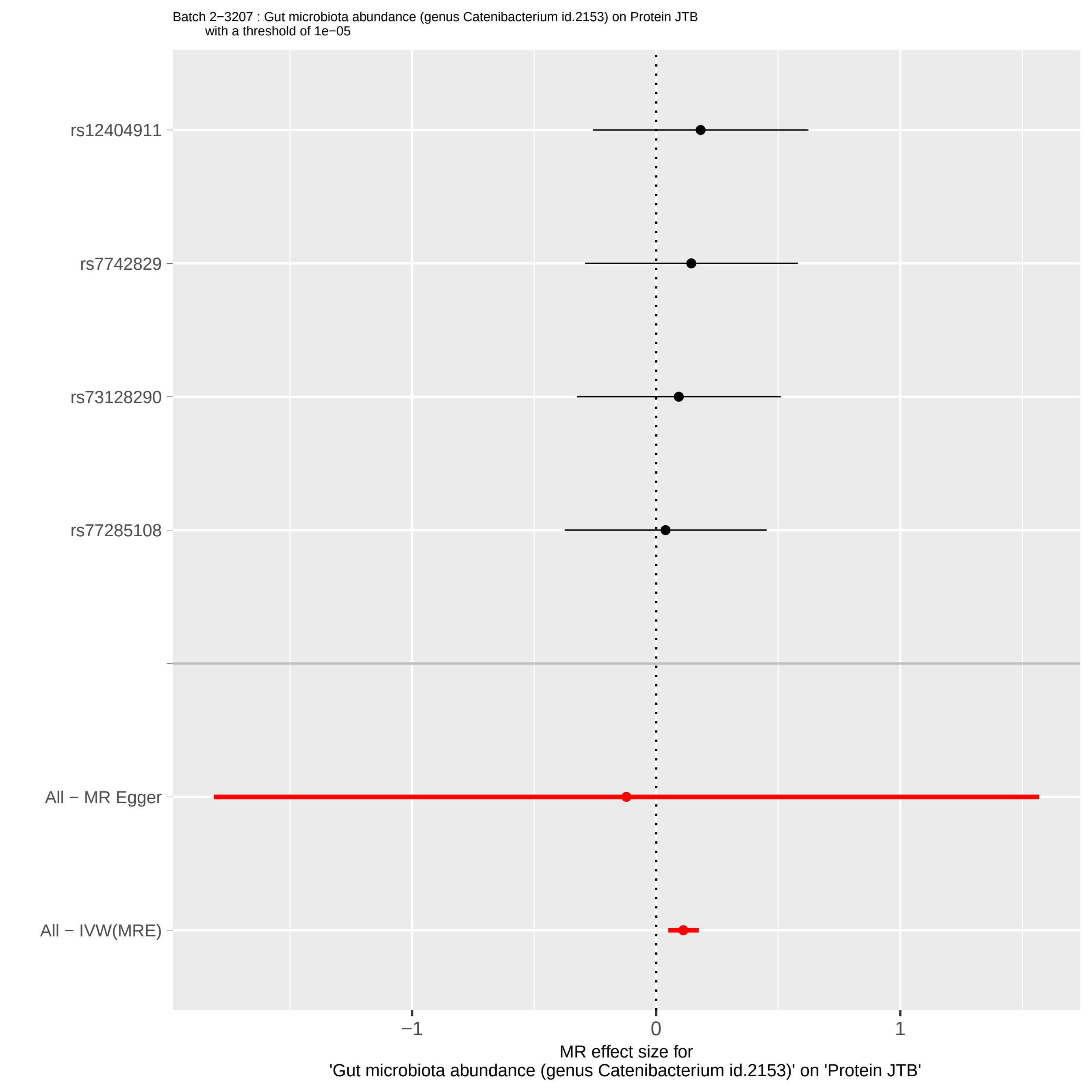

Batch 2−3207 : Gut microbiota abundance (genus Catenibacterium id.2153) on Protein JTB
with a threshold of 1e−05
rs12404911
rs7742829
rs73128290
rs77285108
All − MR Egger
All − IVW(MRE)
−1
0
1
MR effect size for
'Gut microbiota abundance (genus Catenibacterium id.2153)' on 'Protein JTB'

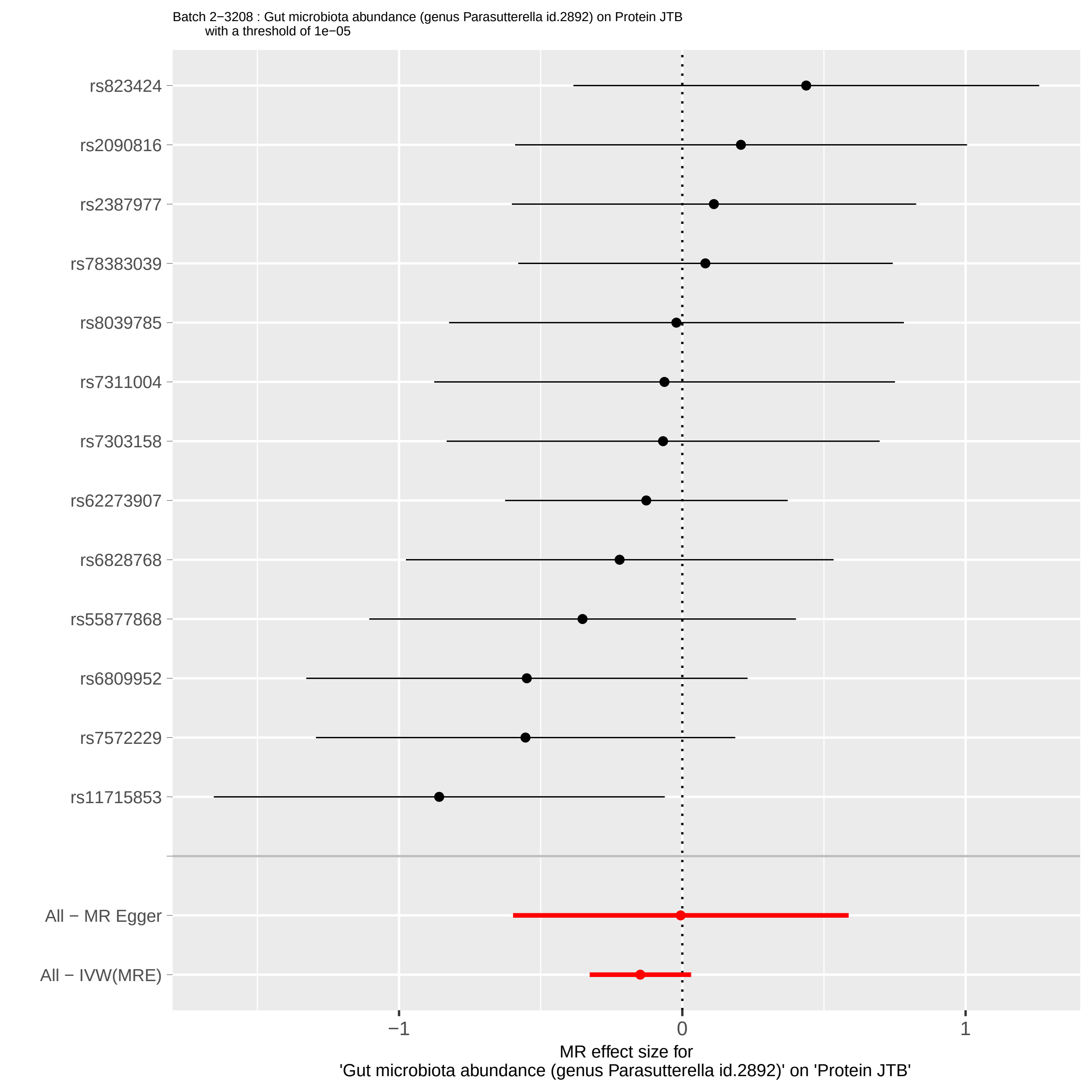

Batch 2−3208 : Gut microbiota abundance (genus Parasutterella id.2892) on Protein JTB
with a threshold of 1e−05
rs823424
rs2090816
rs2387977
rs78383039
rs8039785
rs7311004
rs7303158
rs62273907
rs6828768
rs55877868
rs6809952
rs7572229
rs11715853
All − MR Egger
All − IVW(MRE)
−1
0
1
MR effect size for
'Gut microbiota abundance (genus Parasutterella id.2892)' on 'Protein JTB'

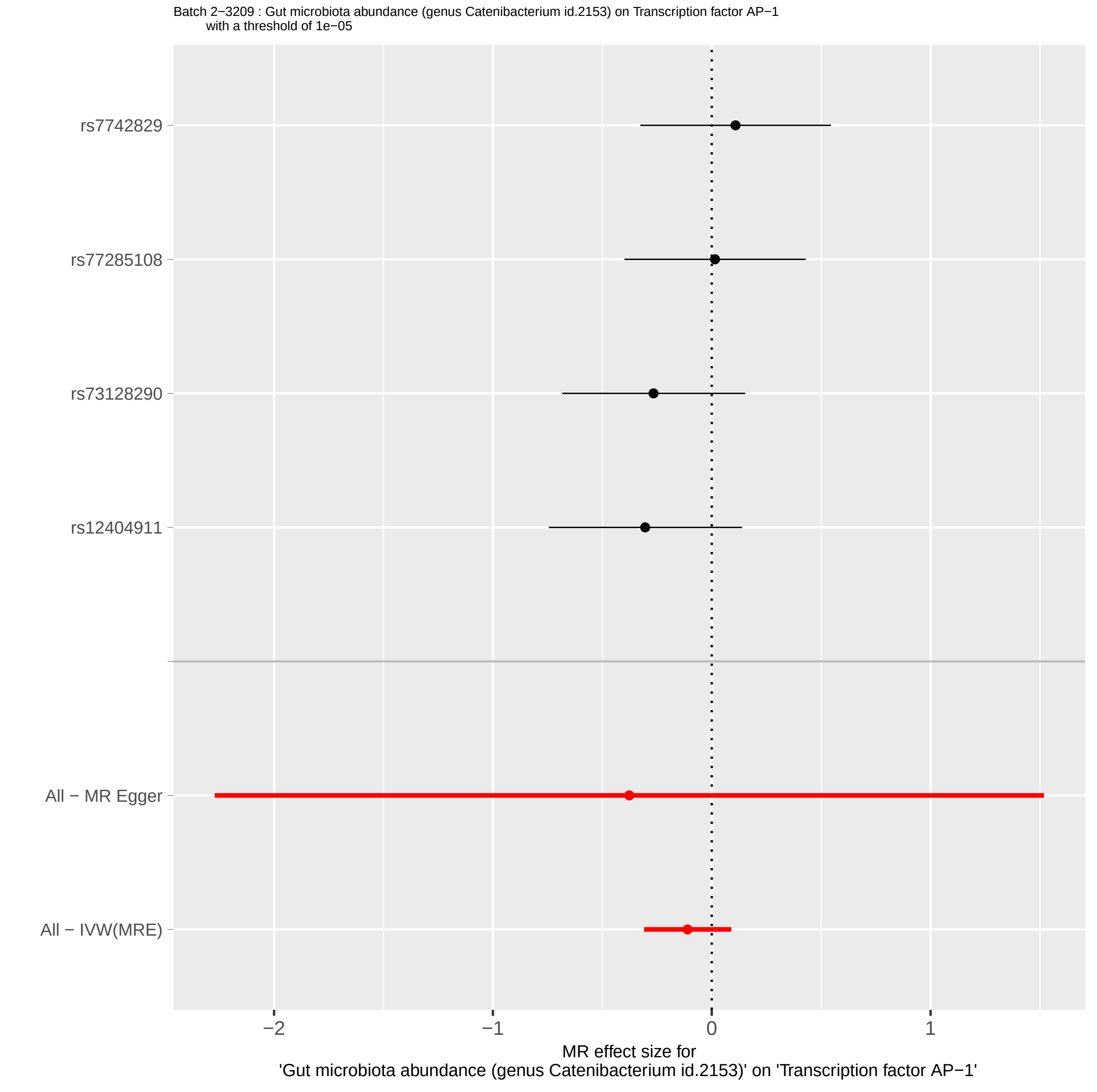

Batch 2−3209 : Gut microbiota abundance (genus Catenibacterium id.2153) on Transcription factor AP−1
with a threshold of 1e−05
rs7742829
rs77285108
rs73128290
rs12404911
All − MR Egger
All − IVW(MRE)
−2
−1
0
1
MR effect size for
'Gut microbiota abundance (genus Catenibacterium id.2153)' on 'Transcription factor AP−1'

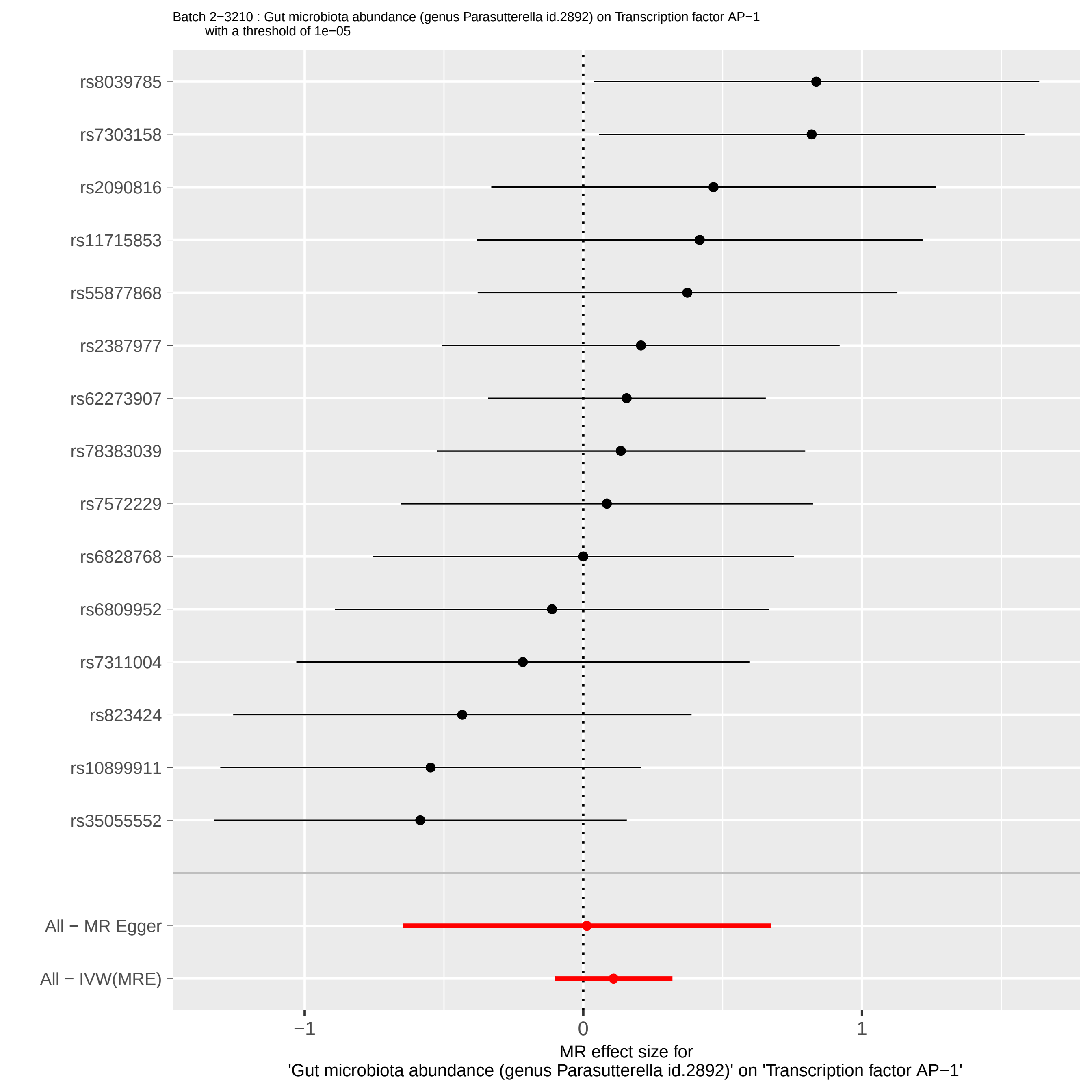

Batch 2−3210 : Gut microbiota abundance (genus Parasutterella id.2892) on Transcription factor AP−1
with a threshold of 1e−05
rs8039785
rs7303158
rs2090816
rs11715853
rs55877868
rs2387977
rs62273907
rs78383039
rs7572229
rs6828768
rs6809952
rs7311004
rs823424
rs10899911
rs35055552
All − MR Egger
All − IVW(MRE)
−1
0
1
MR effect size for
'Gut microbiota abundance (genus Parasutterella id.2892)' on 'Transcription factor AP−1'

Batch 2−3211 : Gut microbiota abundance (genus Catenibacterium id.2153) on Anosmin−1
with a threshold of 1e−05

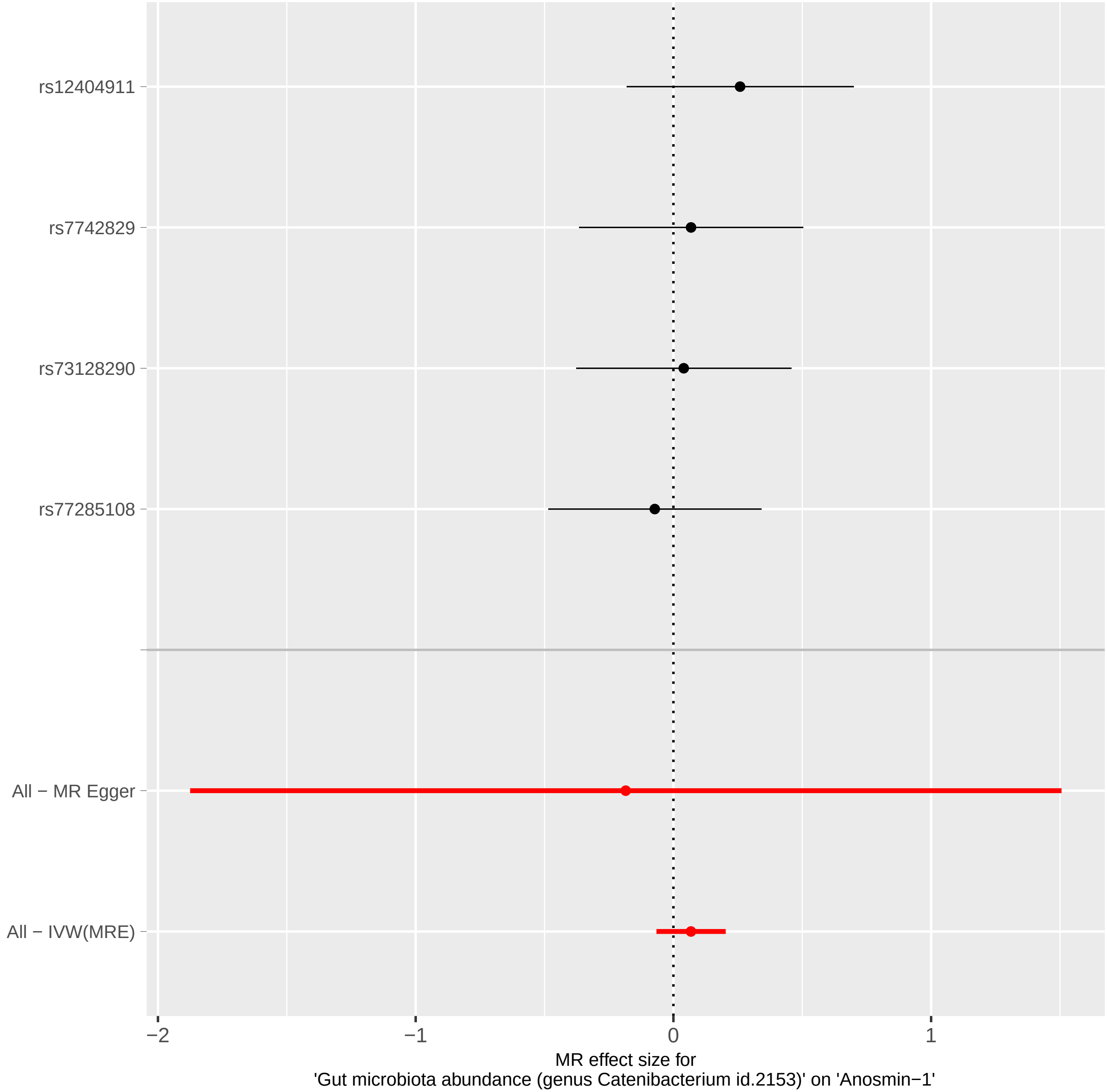

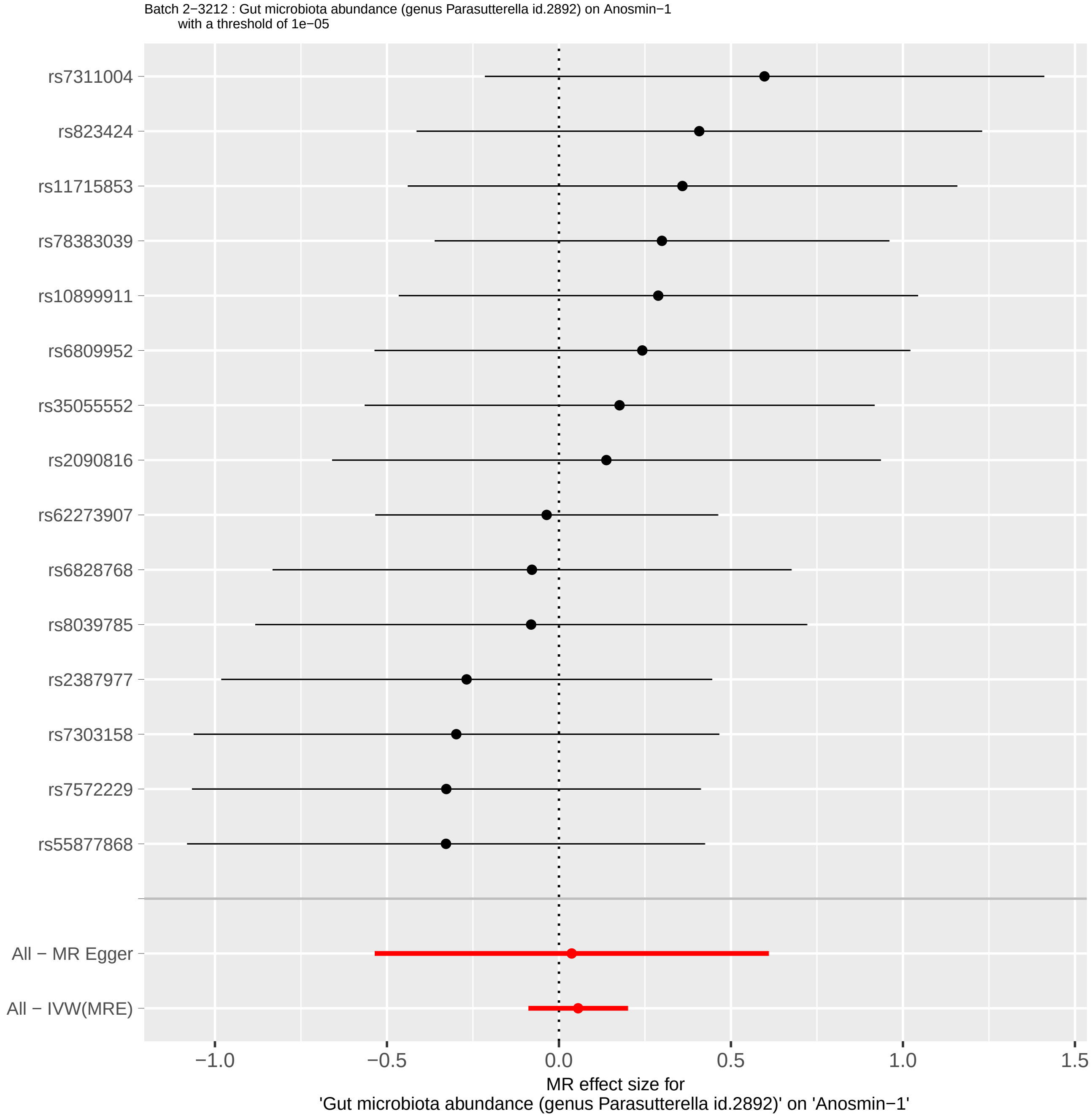
Batch 2−3212 : Gut microbiota abundance (genus Parasutterella id.2892) on Anosmin−1
with a threshold of 1e−05
rs7311004
rs823424
rs11715853
rs78383039
rs10899911
rs6809952
rs35055552
rs2090816
rs62273907
rs6828768
rs8039785
rs2387977
rs7303158
rs7572229
rs55877868
All − MR Egger
All − IVW(MRE)
−1.0
−0.5
0.0
0.5
1.0
1.5
MR effect size for
'Gut microbiota abundance (genus Parasutterella id.2892)' on 'Anosmin−1'

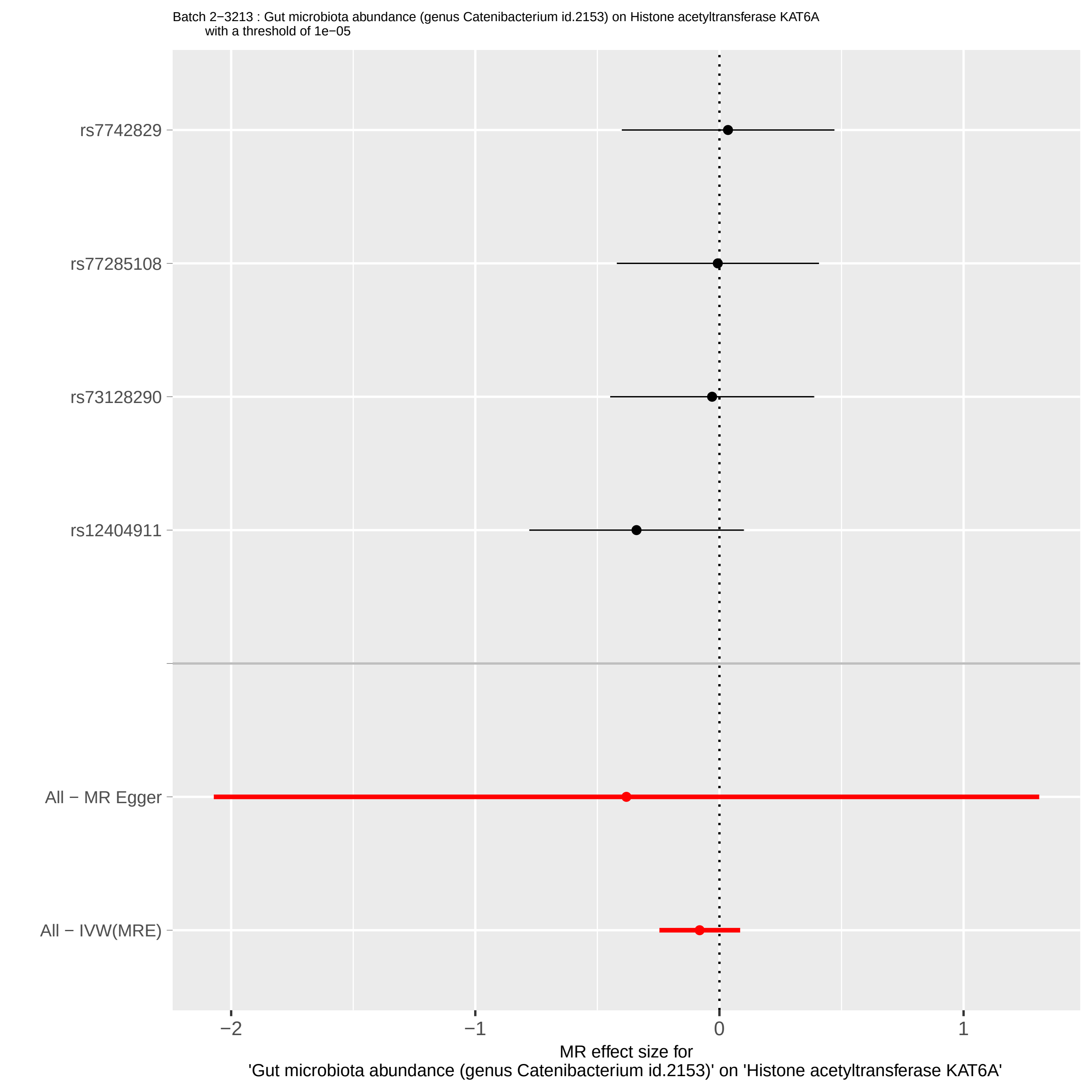

Batch 2−3213 : Gut microbiota abundance (genus Catenibacterium id.2153) on Histone acetyltransferase KAT6A
with a threshold of 1e−05
rs7742829
rs77285108
rs73128290
rs12404911
All − MR Egger
All − IVW(MRE)
−2
−1
0
1
MR effect size for
'Gut microbiota abundance (genus Catenibacterium id.2153)' on 'Histone acetyltransferase KAT6A'

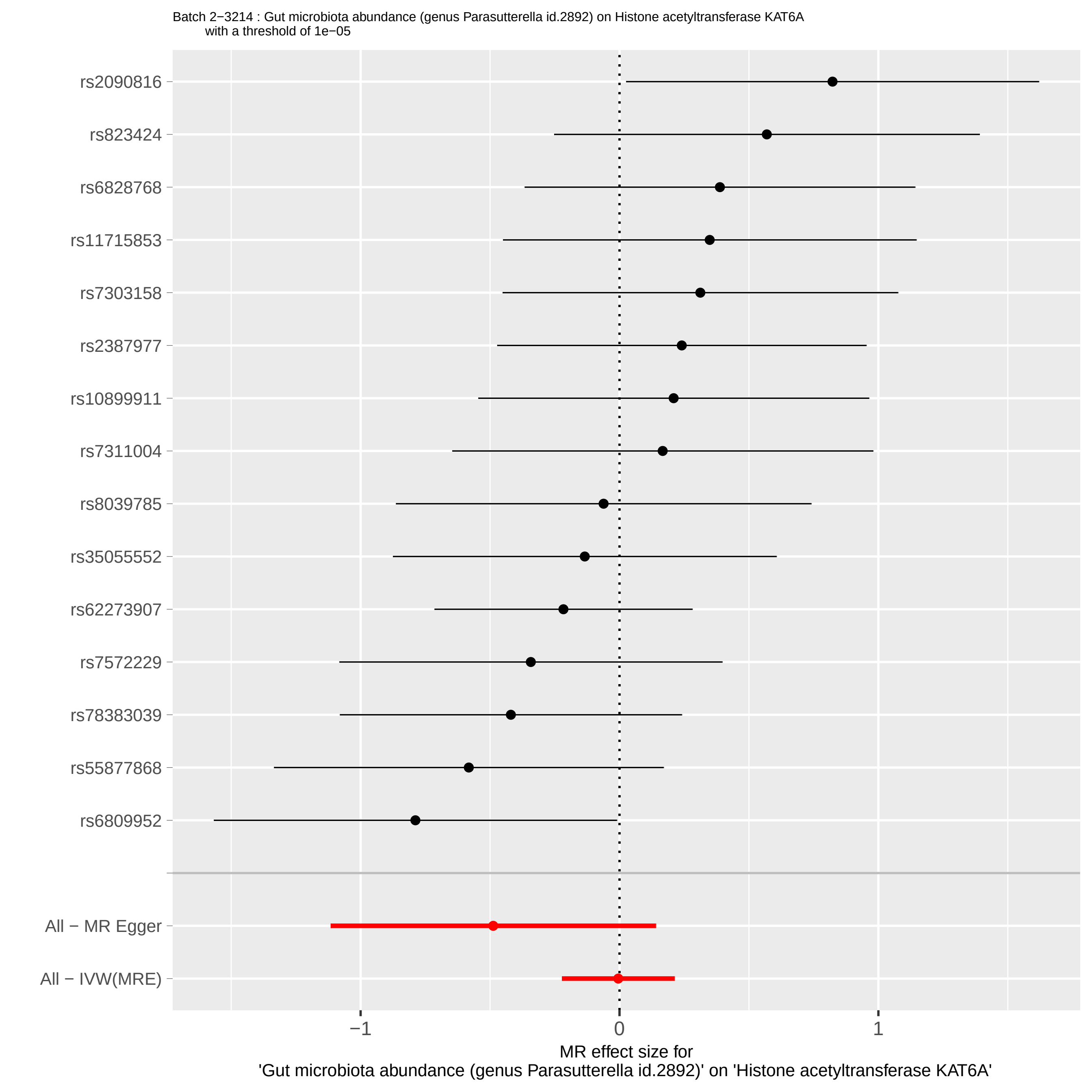
Batch 2−3214 : Gut microbiota abundance (genus Parasutterella id.2892) on Histone acetyltransferase KAT6A
with a threshold of 1e−05
rs2090816
rs823424
rs6828768
rs11715853
rs7303158
rs2387977
rs10899911
rs7311004
rs8039785
rs35055552
rs62273907
rs7572229
rs78383039
rs55877868
rs6809952
All − MR Egger
All − IVW(MRE)
−1
0
1
MR effect size for
'Gut microbiota abundance (genus Parasutterella id.2892)' on 'Histone acetyltransferase KAT6A'

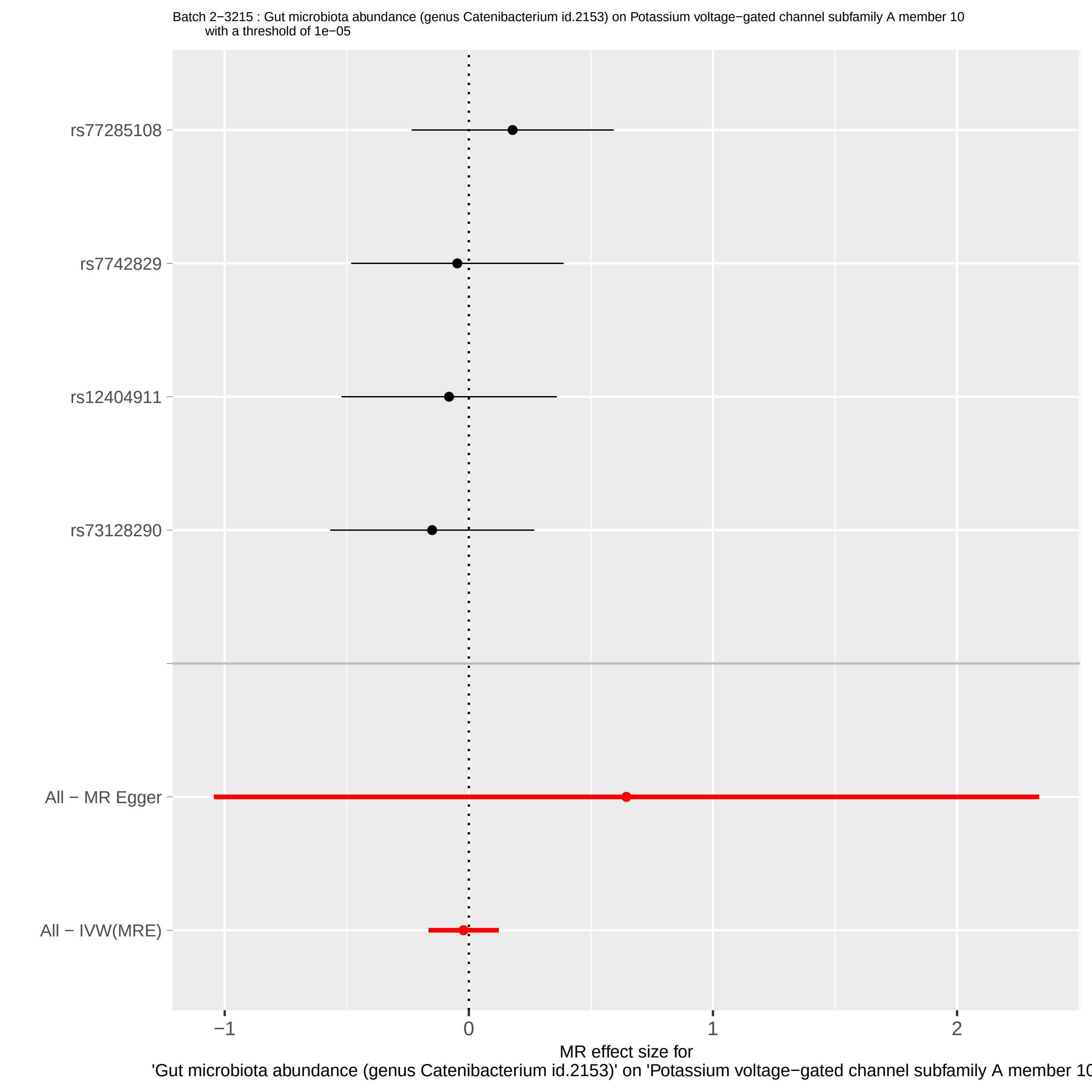
Batch 2−3215 : Gut microbiota abundance (genus Catenibacterium id.2153) on Potassium voltage−gated channel subfamily A member 10
with a threshold of 1e−05
rs77285108
rs7742829
rs12404911
rs73128290
All − MR Egger
All − IVW(MRE)
−1
0
1
2
MR effect size for
'Gut microbiota abundance (genus Catenibacterium id.2153)' on 'Potassium voltage−gated channel subfamily A member 10

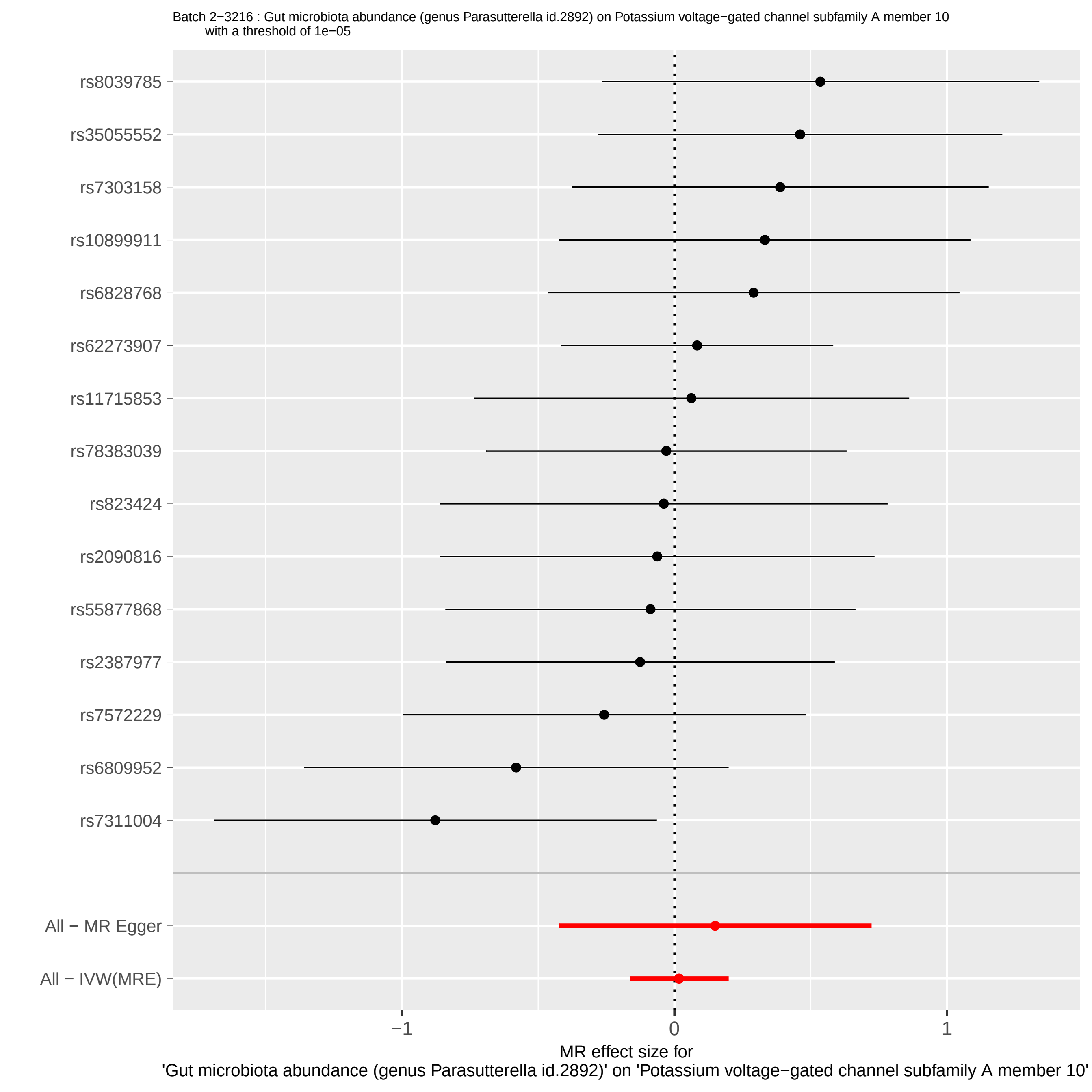

Batch 2−3216 : Gut microbiota abundance (genus Parasutterella id.2892) on Potassium voltage−gated channel subfamily A member 10 with a threshold of 1e−05
rs8039785
rs35055552
rs7303158
rs10899911
rs6828768
rs62273907
rs11715853
rs78383039
rs823424
rs2090816
rs55877868
rs2387977
rs7572229
rs6809952
rs7311004
All − MR Egger
All − IVW(MRE)
−1
0
1
MR effect size for
'Gut microbiota abundance (genus Parasutterella id.2892)' on 'Potassium voltage−gated channel subfamily A member 10'

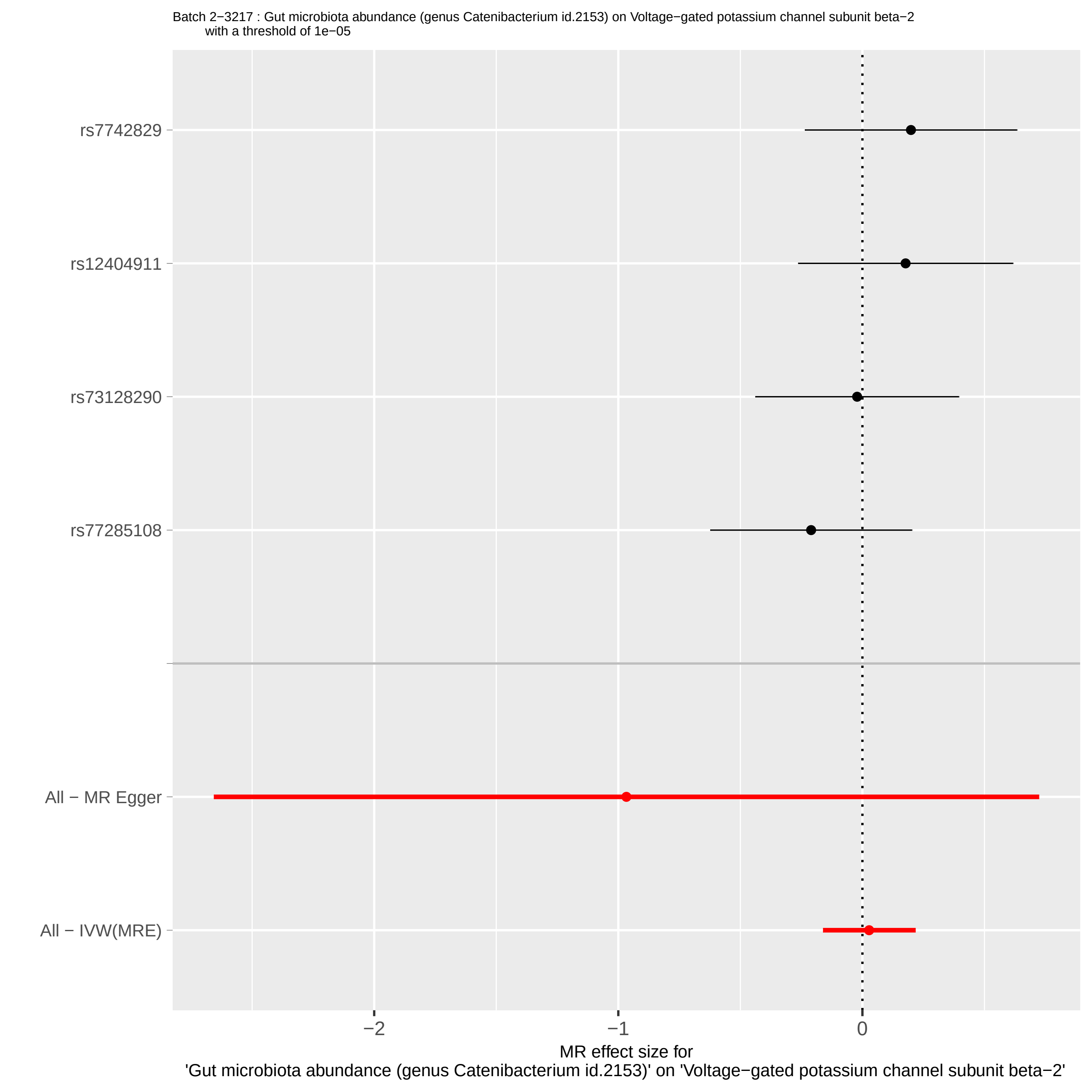

Batch 2−3217 : Gut microbiota abundance (genus Catenibacterium id.2153) on Voltage−gated potassium channel subunit beta−2
with a threshold of 1e−05
rs7742829
rs12404911
rs73128290
rs77285108
All − MR Egger
All − IVW(MRE)
−2
−1
0
MR effect size for
'Gut microbiota abundance (genus Catenibacterium id.2153)' on 'Voltage−gated potassium channel subunit beta−2'

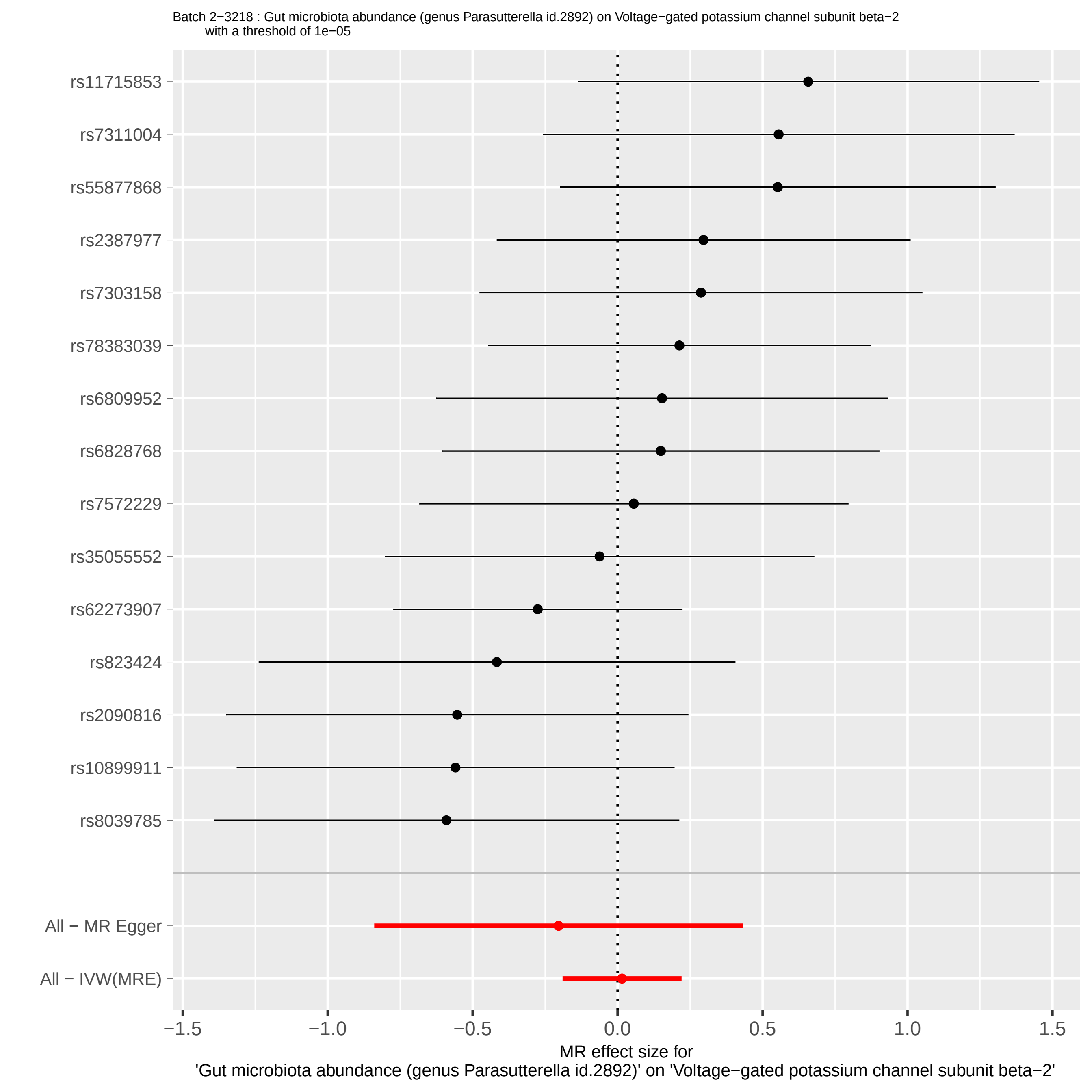

Batch 2−3218 : Gut microbiota abundance (genus Parasutterella id.2892) on Voltage−gated potassium channel subunit beta−2
with a threshold of 1e−05
rs11715853
rs7311004
rs55877868
rs2387977
rs7303158
rs78383039
rs6809952
rs6828768
rs7572229
rs35055552
rs62273907
rs823424
rs2090816
rs10899911
rs8039785
All − MR Egger
All − IVW(MRE)
−1.5
−1.0
−0.5
0.0
0.5
1.0
1.5
MR effect size for
'Gut microbiota abundance (genus Parasutterella id.2892)' on 'Voltage−gated potassium channel subunit beta−2'

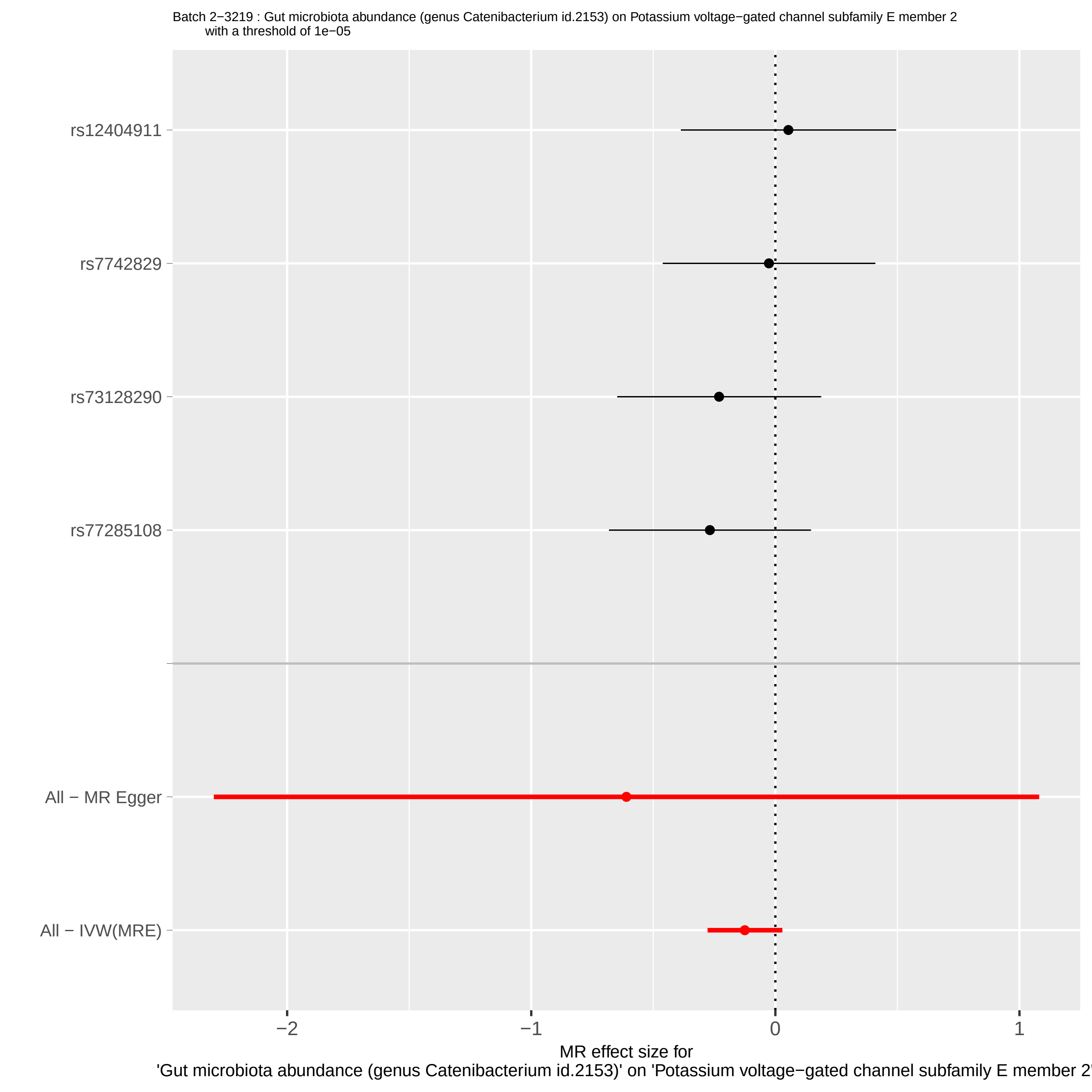

Batch 2−3219 : Gut microbiota abundance (genus Catenibacterium id.2153) on Potassium voltage−gated channel subfamily E member 2
with a threshold of 1e−05
rs12404911
rs7742829
rs73128290
rs77285108
All − MR Egger
All − IVW(MRE)
−2
−1
0
1
MR effect size for
'Gut microbiota abundance (genus Catenibacterium id.2153)' on 'Potassium voltage−gated channel subfamily E member 2'

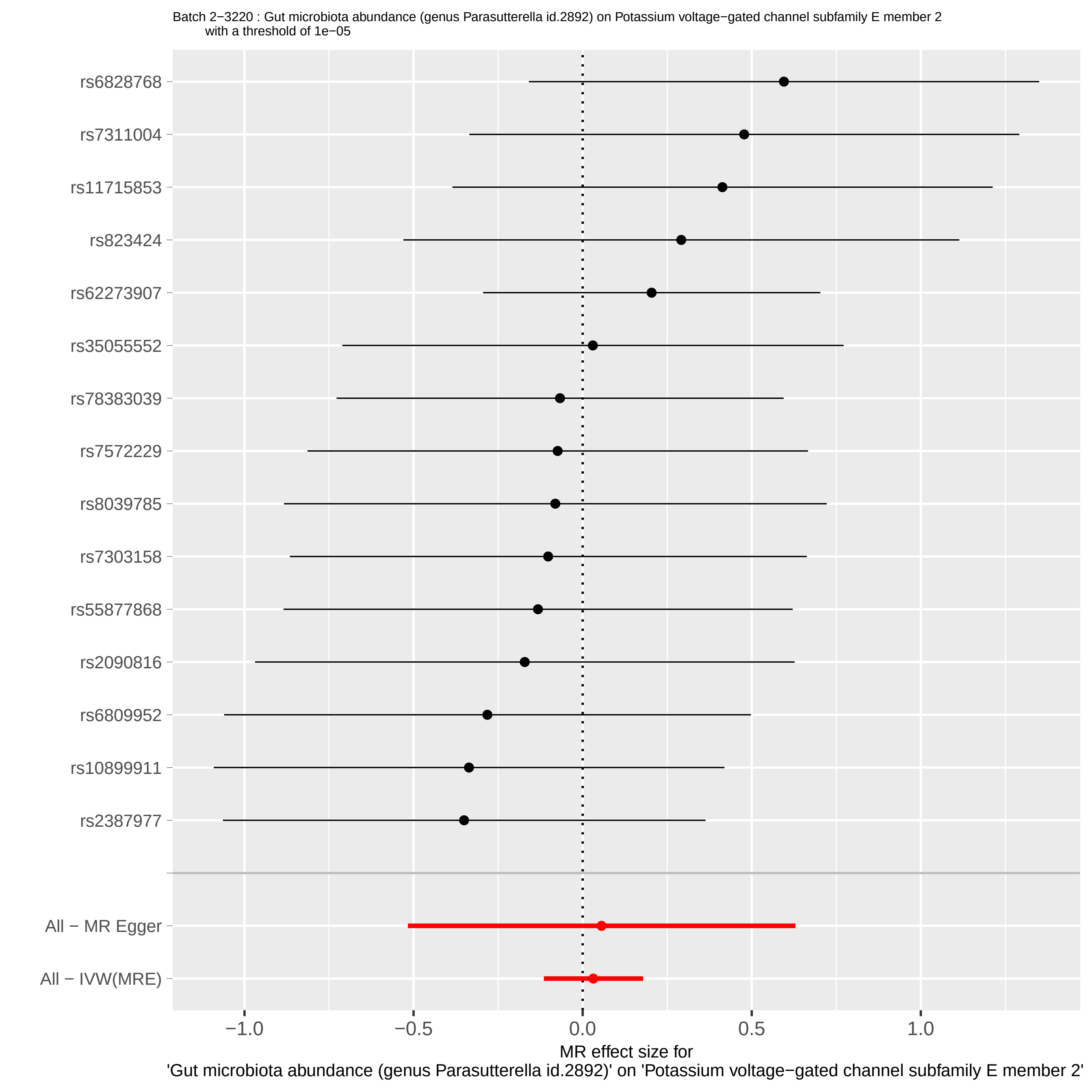

Batch 2−3220 : Gut microbiota abundance (genus Parasutterella id.2892) on Potassium voltage−gated channel subfamily E member 2
with a threshold of 1e−05
rs6828768
rs7311004
rs11715853
rs823424
rs62273907
rs35055552
rs78383039
rs7572229
rs8039785
rs7303158
rs55877868
rs2090816
rs6809952
rs10899911
rs2387977
All − MR Egger
All − IVW(MRE)
−1.0
−0.5
0.0
0.5
1.0
MR effect size for
'Gut microbiota abundance (genus Parasutterella id.2892)' on 'Potassium voltage−gated channel subfamily E member 2'

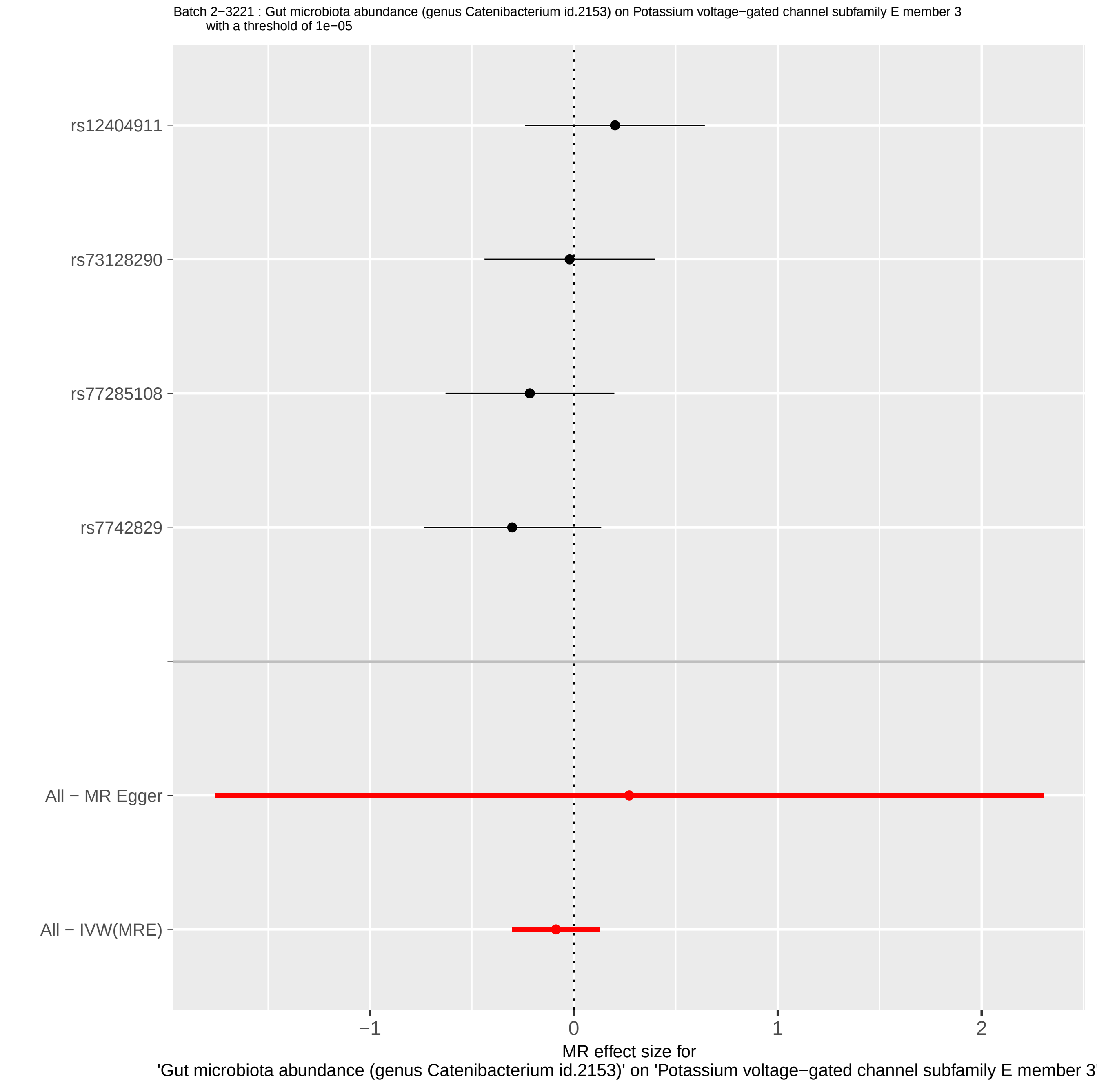

Batch 2−3221 : Gut microbiota abundance (genus Catenibacterium id.2153) on Potassium voltage−gated channel subfamily E member 3
with a threshold of 1e−05
rs12404911
rs73128290
rs77285108
rs7742829
All − MR Egger
All − IVW(MRE)
−1
0
1
2
MR effect size for
'Gut microbiota abundance (genus Catenibacterium id.2153)' on 'Potassium voltage−gated channel subfamily E member 3'

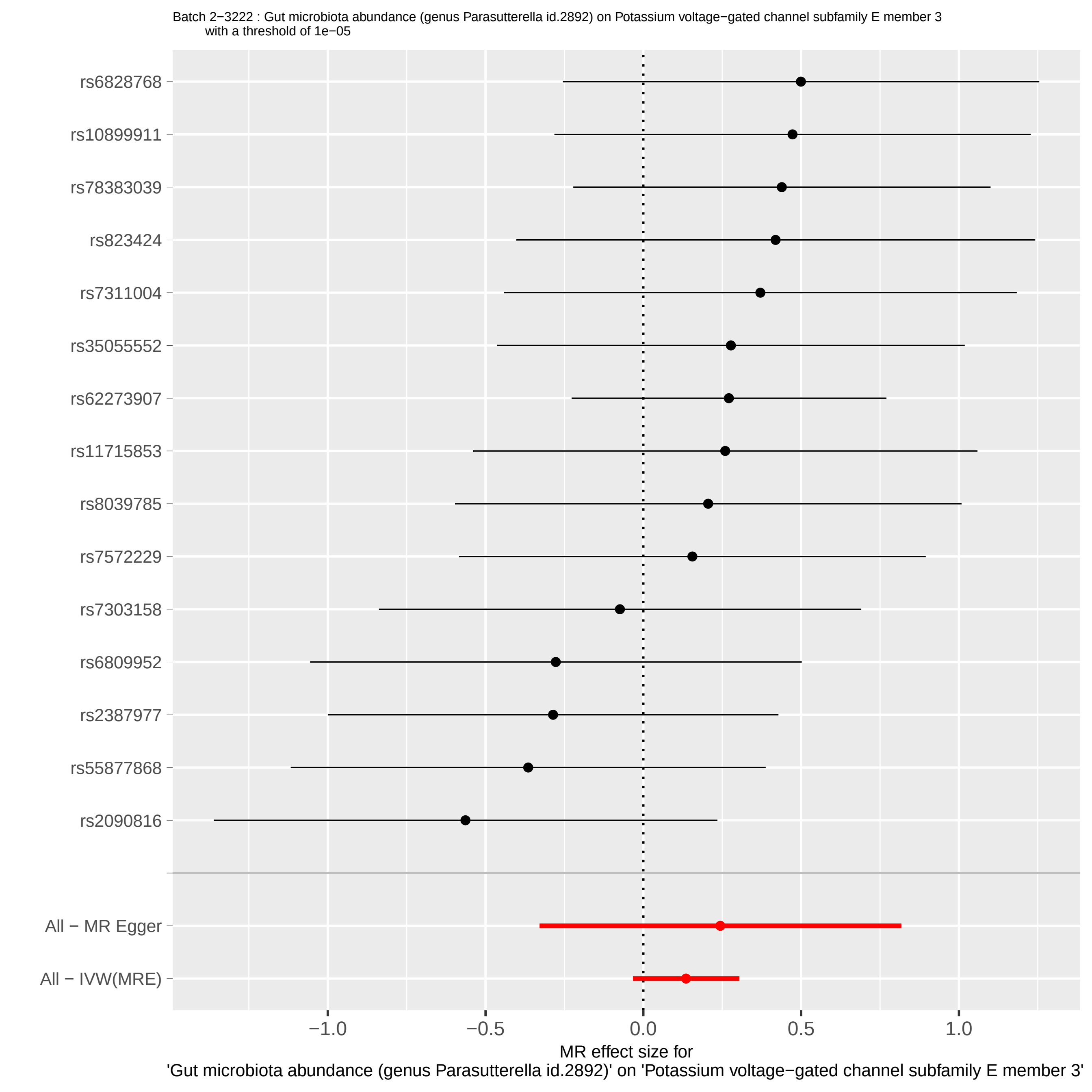
Batch 2−3222 : Gut microbiota abundance (genus Parasutterella id.2892) on Potassium voltage−gated channel subfamily E member 3
with a threshold of 1e−05
rs6828768
rs10899911
rs78383039
rs823424
rs7311004
rs35055552
rs62273907
rs11715853
rs8039785
rs7572229
rs7303158
rs6809952
rs2387977
rs55877868
rs2090816
All − MR Egger
All − IVW(MRE)
−1.0
−0.5
0.0
0.5
1.0
MR effect size for
'Gut microbiota abundance (genus Parasutterella id.2892)' on 'Potassium voltage−gated channel subfamily E member 3'

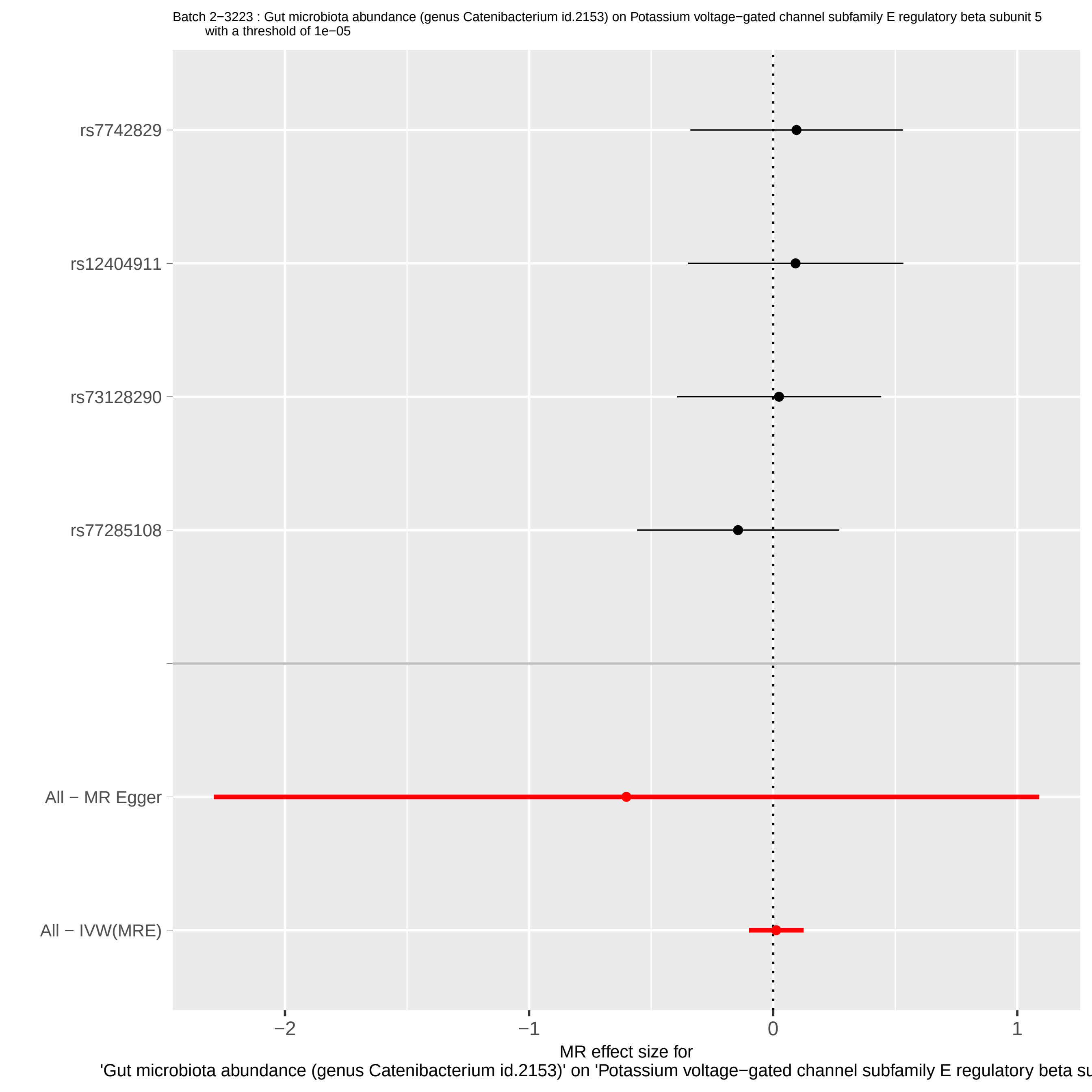
Batch 2−3223 : Gut microbiota abundance (genus Catenibacterium id.2153) on Potassium voltage−gated channel subfamily E regulatory beta subunit 5
with a threshold of 1e−05
rs7742829
rs12404911
rs73128290
rs77285108
All − MR Egger
All − IVW(MRE)
−2
−1
0
1
MR effect size for
'Gut microbiota abundance (genus Catenibacterium id.2153)' on 'Potassium voltage−gated channel subfamily E regulatory beta su

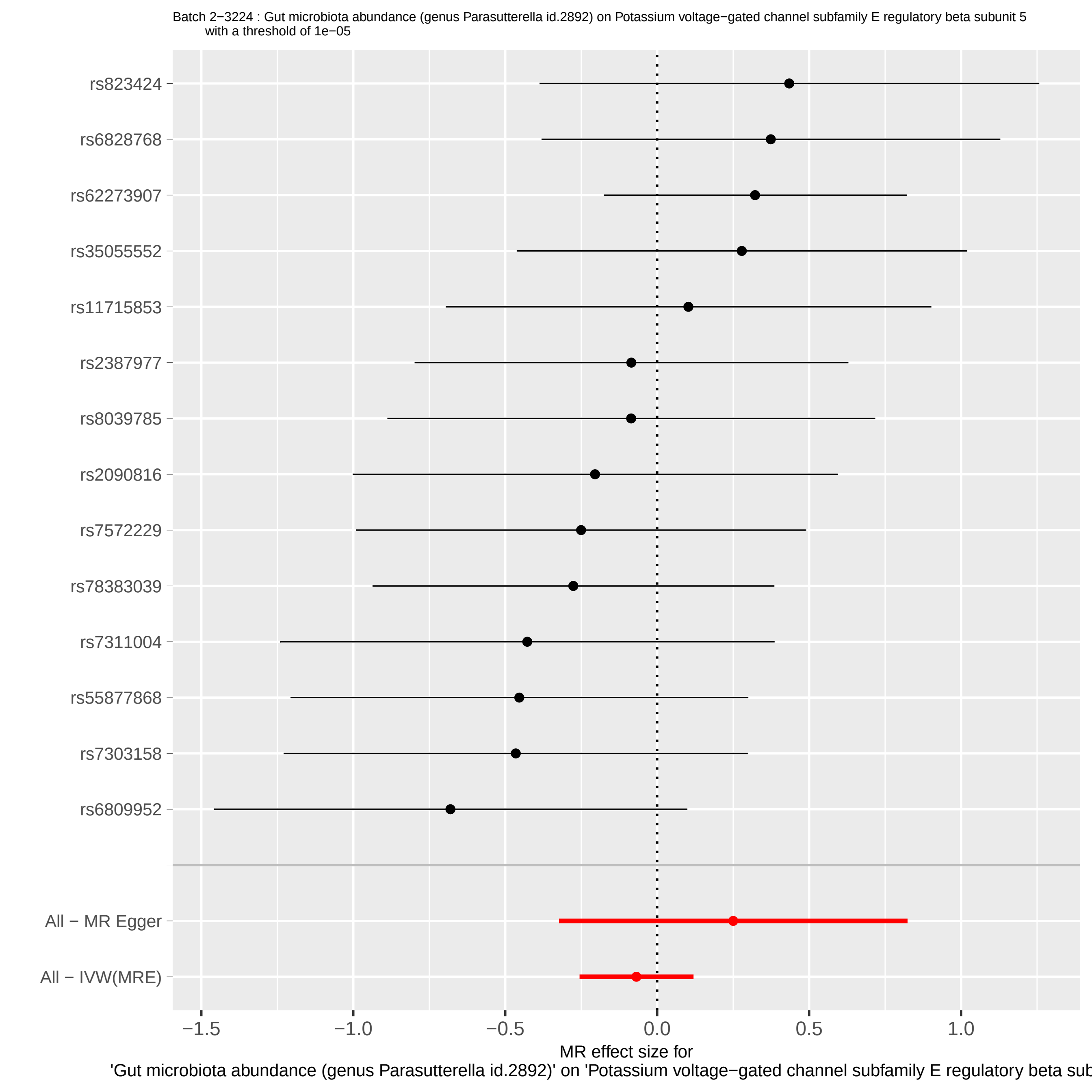

Batch 2−3224 : Gut microbiota abundance (genus Parasutterella id.2892) on Potassium voltage−gated channel subfamily E regulatory beta subunit 5
with a threshold of 1e−05
rs823424
rs6828768
rs62273907
rs35055552
rs11715853
rs2387977
rs8039785
rs2090816
rs7572229
rs78383039
rs7311004
rs55877868
rs7303158
rs6809952
All − MR Egger
All − IVW(MRE)
−1.5
−1.0
−0.5
0.0
0.5
1.0
MR effect size for
'Gut microbiota abundance (genus Parasutterella id.2892)' on 'Potassium voltage−gated channel subfamily E regulatory beta sub

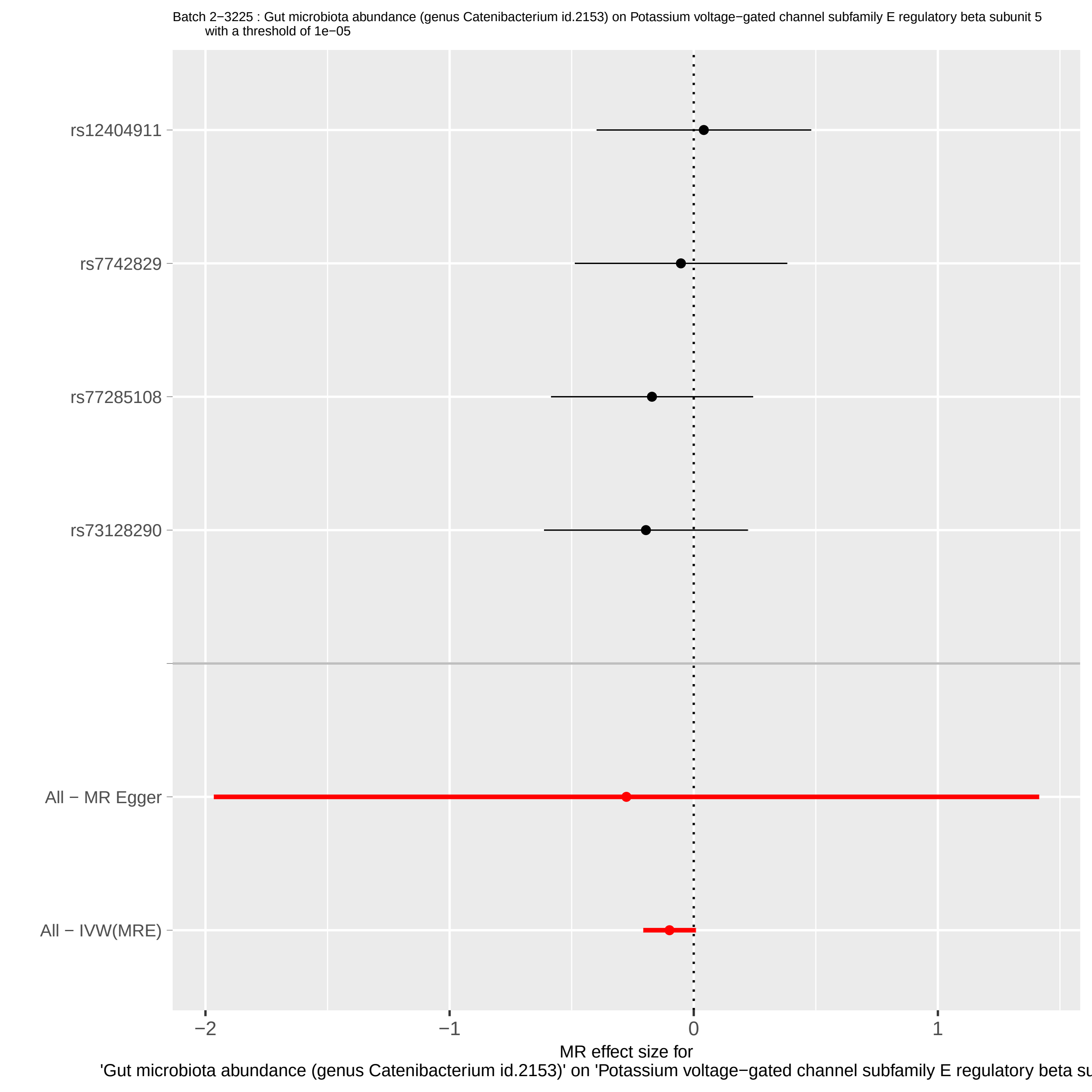

Batch 2−3225 : Gut microbiota abundance (genus Catenibacterium id.2153) on Potassium voltage−gated channel subfamily E regulatory beta subunit 5
with a threshold of 1e−05
rs12404911
rs7742829
rs77285108
rs73128290
All − MR Egger
All − IVW(MRE)
−2
−1
0
1
MR effect size for
'Gut microbiota abundance (genus Catenibacterium id.2153)' on 'Potassium voltage−gated channel subfamily E regulatory beta su

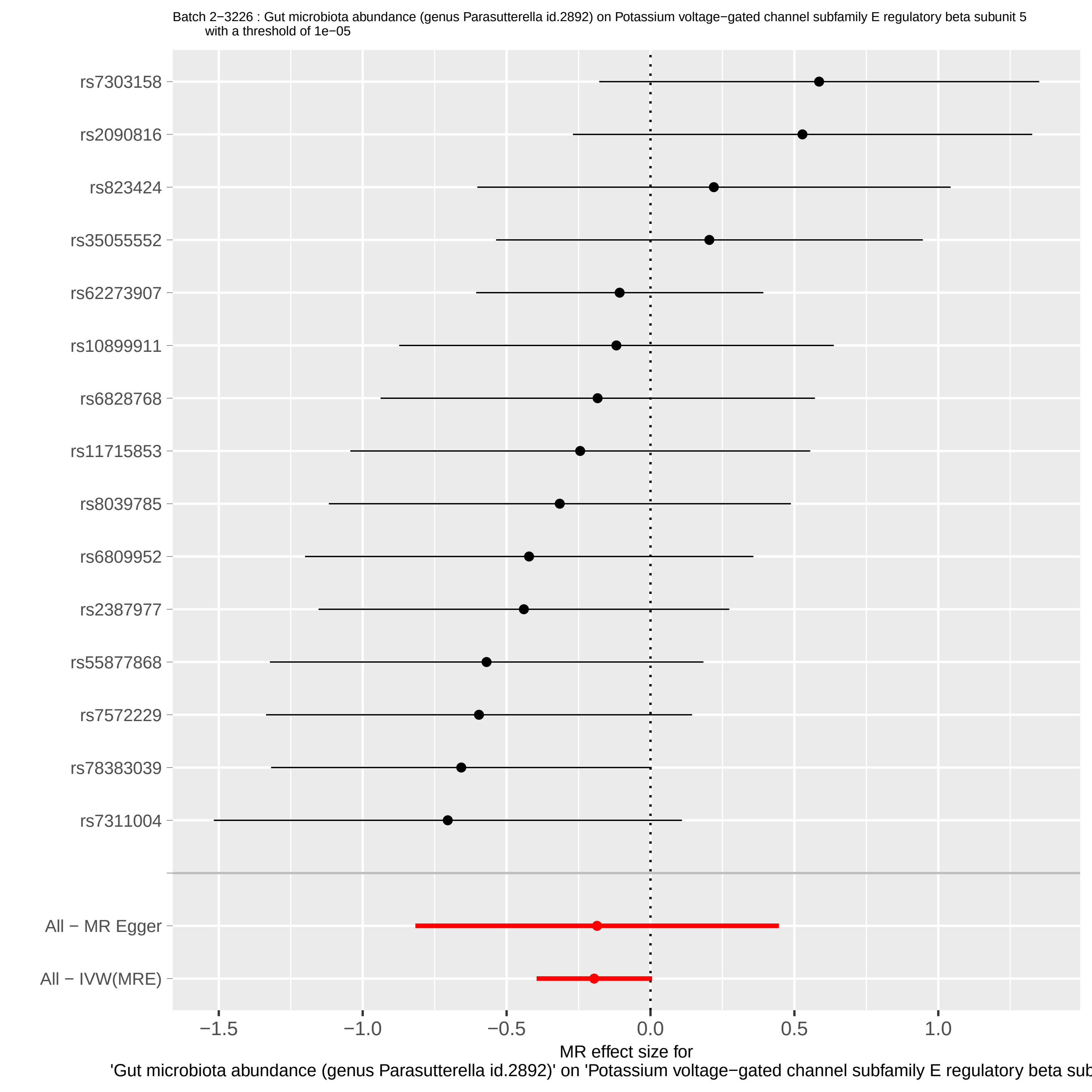

Batch 2−3226 : Gut microbiota abundance (genus Parasutterella id.2892) on Potassium voltage−gated channel subfamily E regulatory beta subunit 5
with a threshold of 1e−05
rs7303158
rs2090816
rs823424
rs35055552
rs62273907
rs10899911
rs6828768
rs11715853
rs8039785
rs6809952
rs2387977
rs55877868
rs7572229
rs78383039
rs7311004
All − MR Egger
All − IVW(MRE)
−1.5
−1.0
−0.5
0.0
0.5
1.0
MR effect size for
'Gut microbiota abundance (genus Parasutterella id.2892)' on 'Potassium voltage−gated channel subfamily E regulatory beta sub

Batch 2−3227 : Gut microbiota abundance (genus Catenibacterium id.2153) on Potassium voltage−gated channel subfamily F member 1 with a threshold of 1e−05

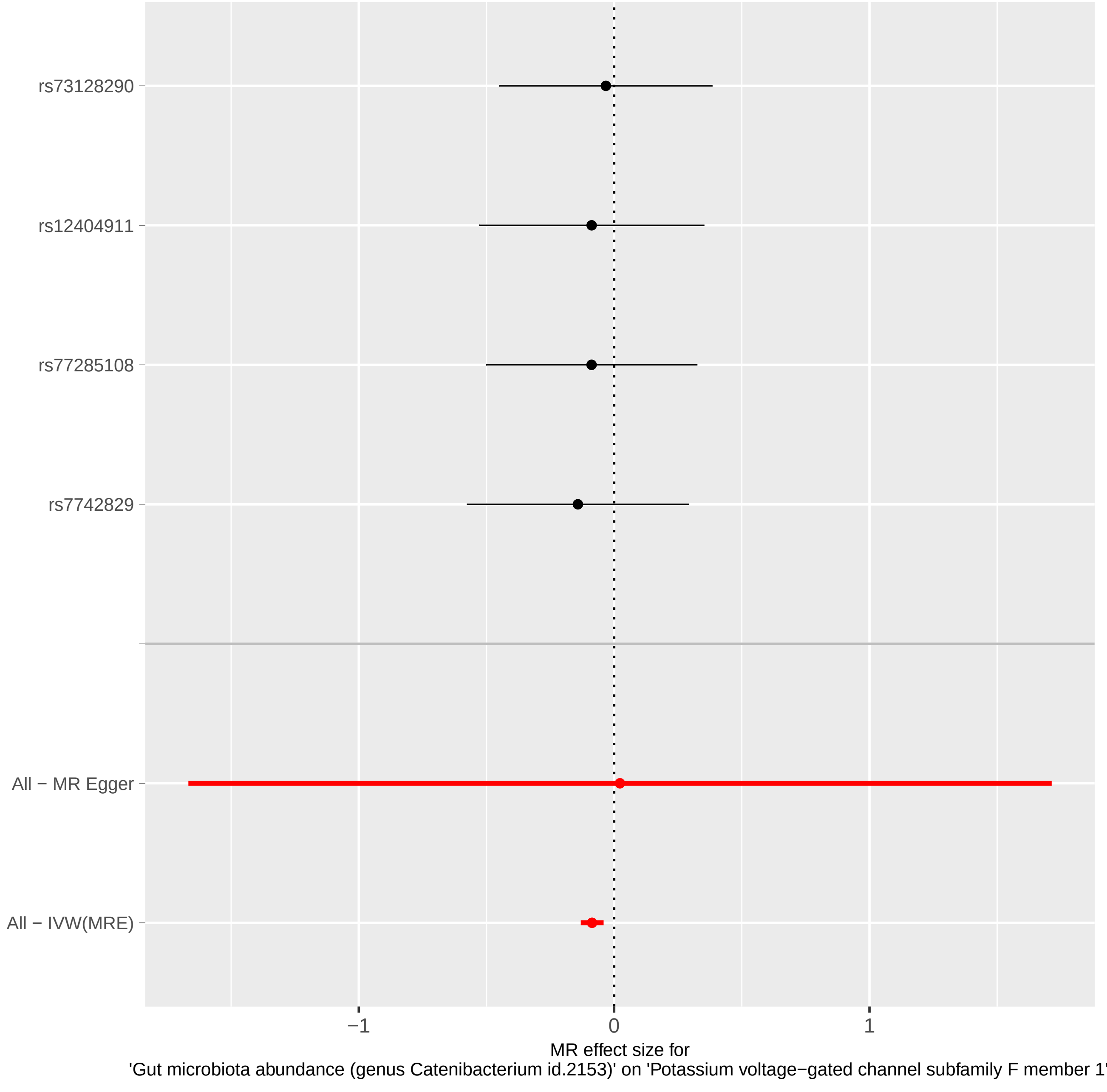

Batch 2−3228 : Gut microbiota abundance (genus Parasutterella id.2892) on Potassium voltage−gated channel subfamily F member 1 with a threshold of 1e−05

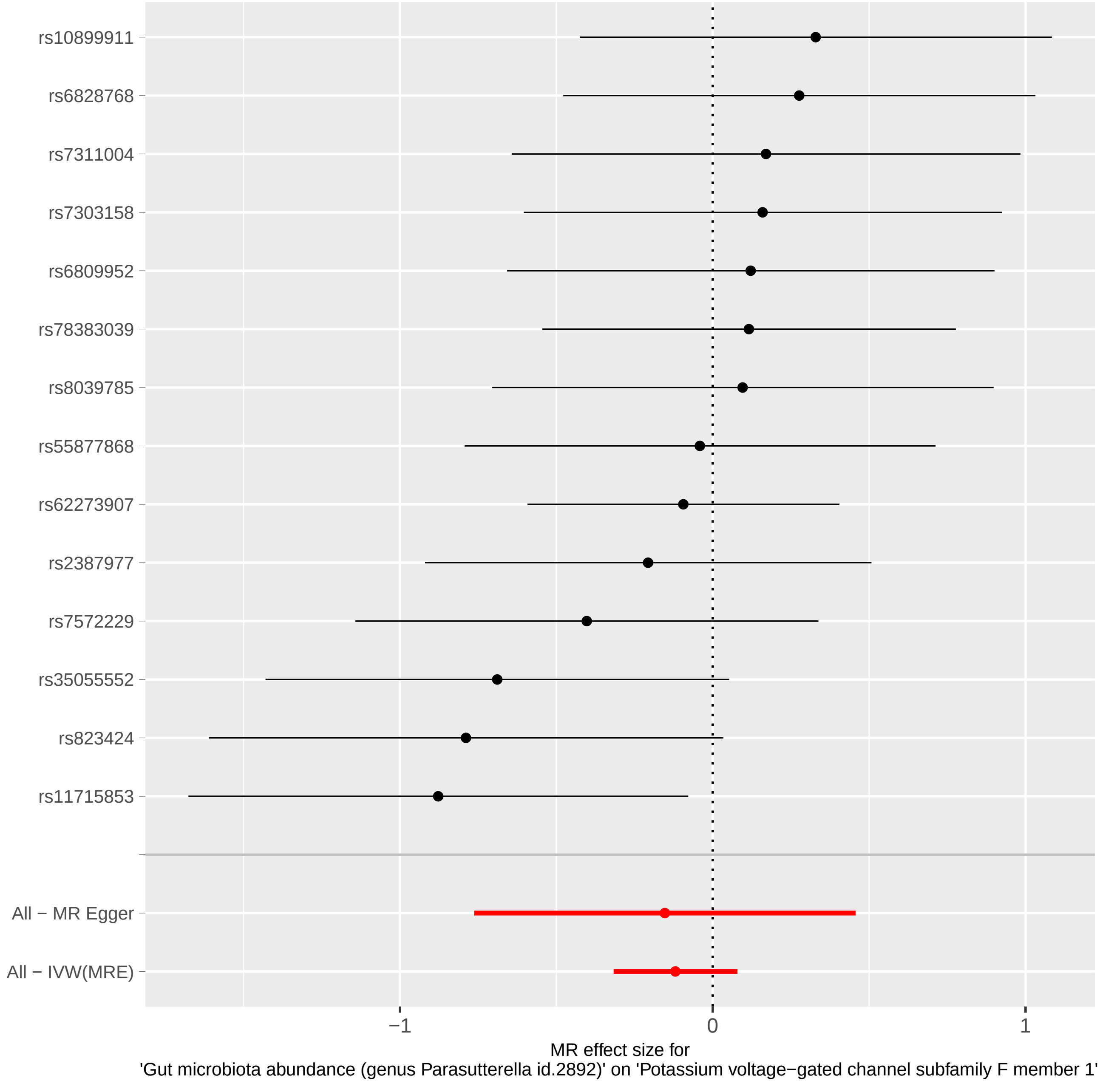

Batch 2−3229 : Gut microbiota abundance (genus Catenibacterium id.2153) on Potassium voltage−gated channel subfamily G member 4 with a threshold of 1e−05

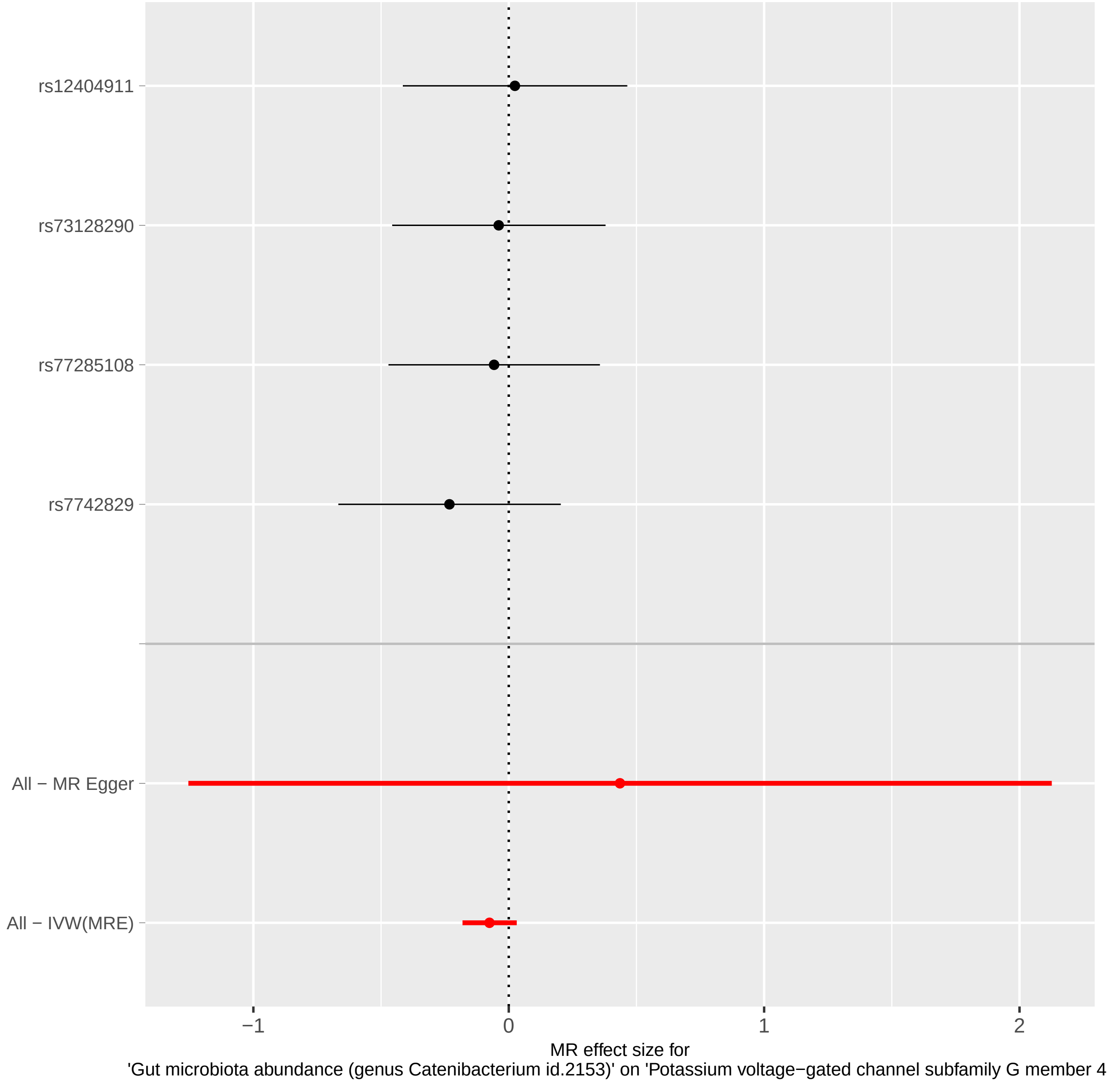

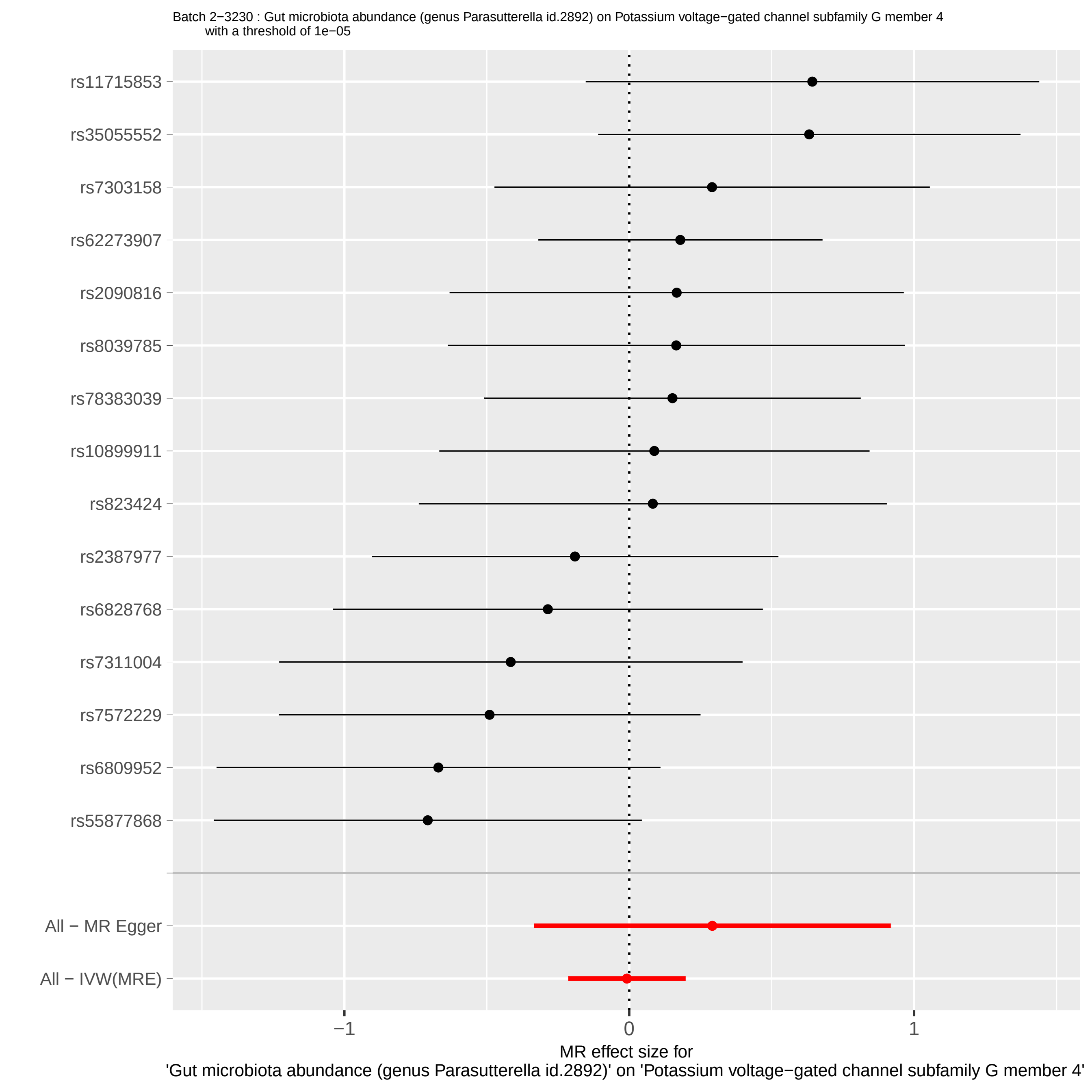

Batch 2−3230 : Gut microbiota abundance (genus Parasutterella id.2892) on Potassium voltage−gated channel subfamily G member 4
with a threshold of 1e−05
rs11715853
rs35055552
rs7303158
rs62273907
rs2090816
rs8039785
rs78383039
rs10899911
rs823424
rs2387977
rs6828768
rs7311004
rs7572229
rs6809952
rs55877868
All − MR Egger
All − IVW(MRE)
−1
0
1
MR effect size for
'Gut microbiota abundance (genus Parasutterella id.2892)' on 'Potassium voltage−gated channel subfamily G member 4'

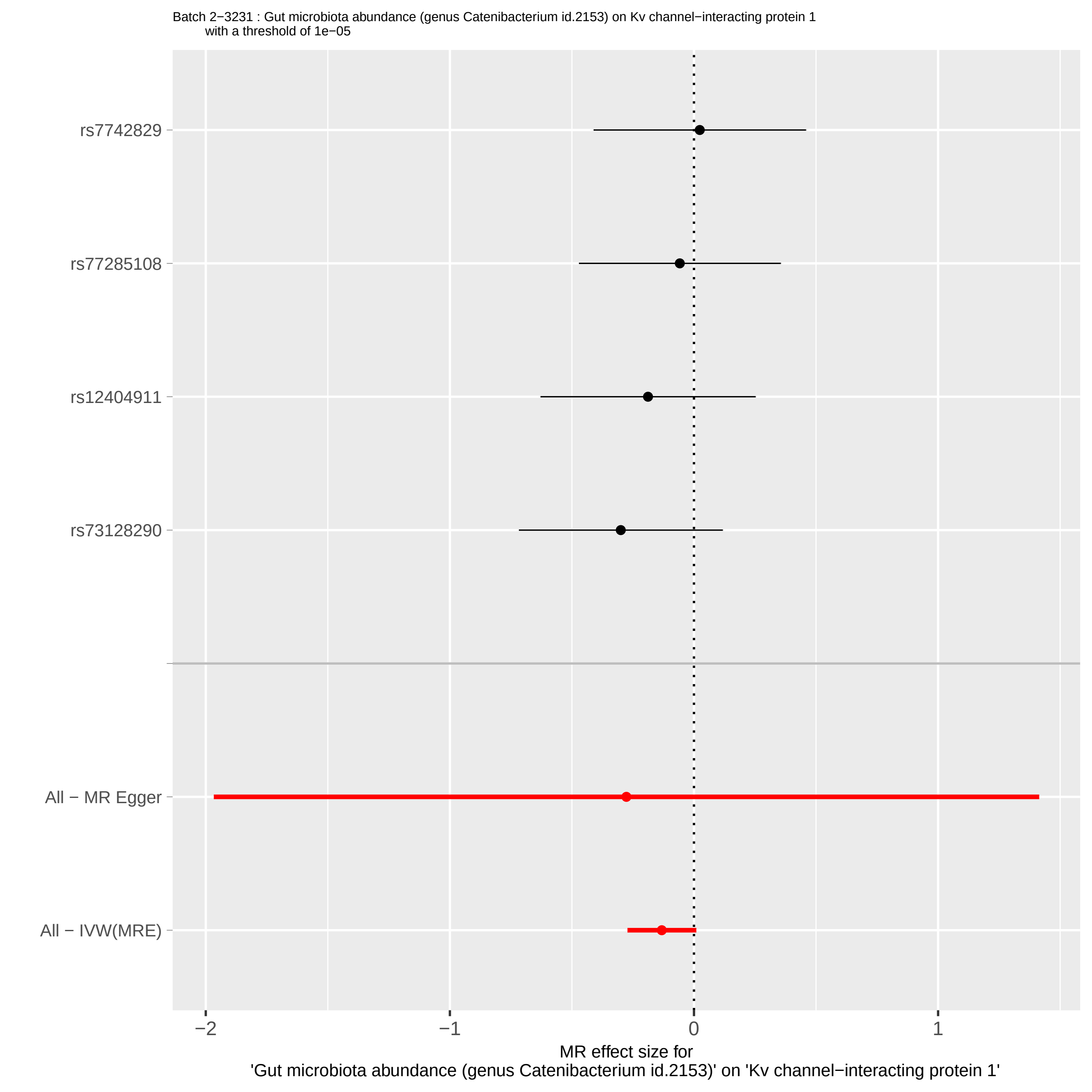

Batch 2−3231 : Gut microbiota abundance (genus Catenibacterium id.2153) on Kv channel−interacting protein 1
with a threshold of 1e−05
rs7742829
rs77285108
rs12404911
rs73128290
All − MR Egger
All − IVW(MRE)
−2
−1
0
1
MR effect size for
'Gut microbiota abundance (genus Catenibacterium id.2153)' on 'Kv channel−interacting protein 1'

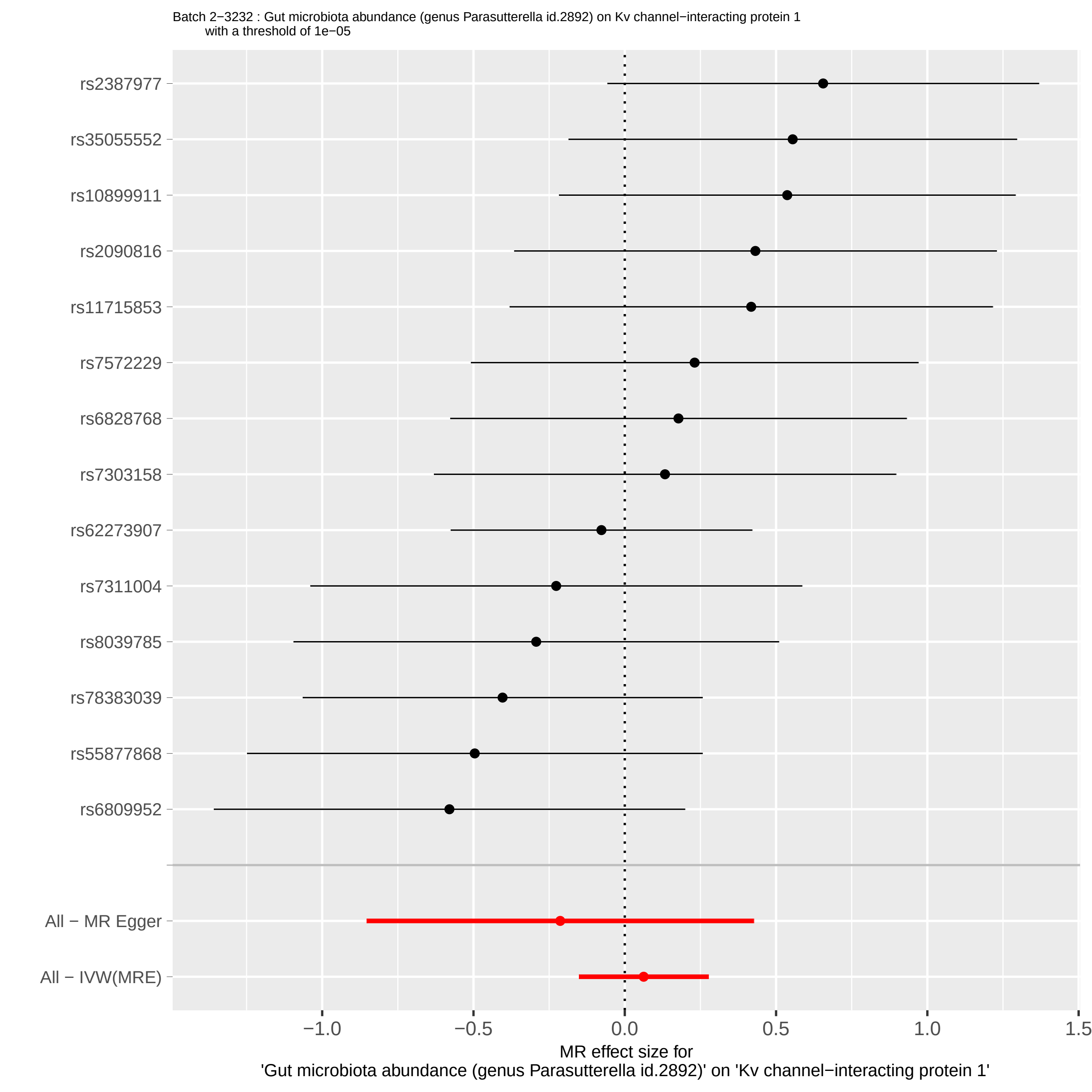

Batch 2−3232 : Gut microbiota abundance (genus Parasutterella id.2892) on Kv channel−interacting protein 1
with a threshold of 1e−05
rs2387977
rs35055552
rs10899911
rs2090816
rs11715853
rs7572229
rs6828768
rs7303158
rs62273907
rs7311004
rs8039785
rs78383039
rs55877868
rs6809952
All − MR Egger
All − IVW(MRE)
−1.0
−0.5
0.0
0.5
1.0
1.5
MR effect size for
'Gut microbiota abundance (genus Parasutterella id.2892)' on 'Kv channel−interacting protein 1'

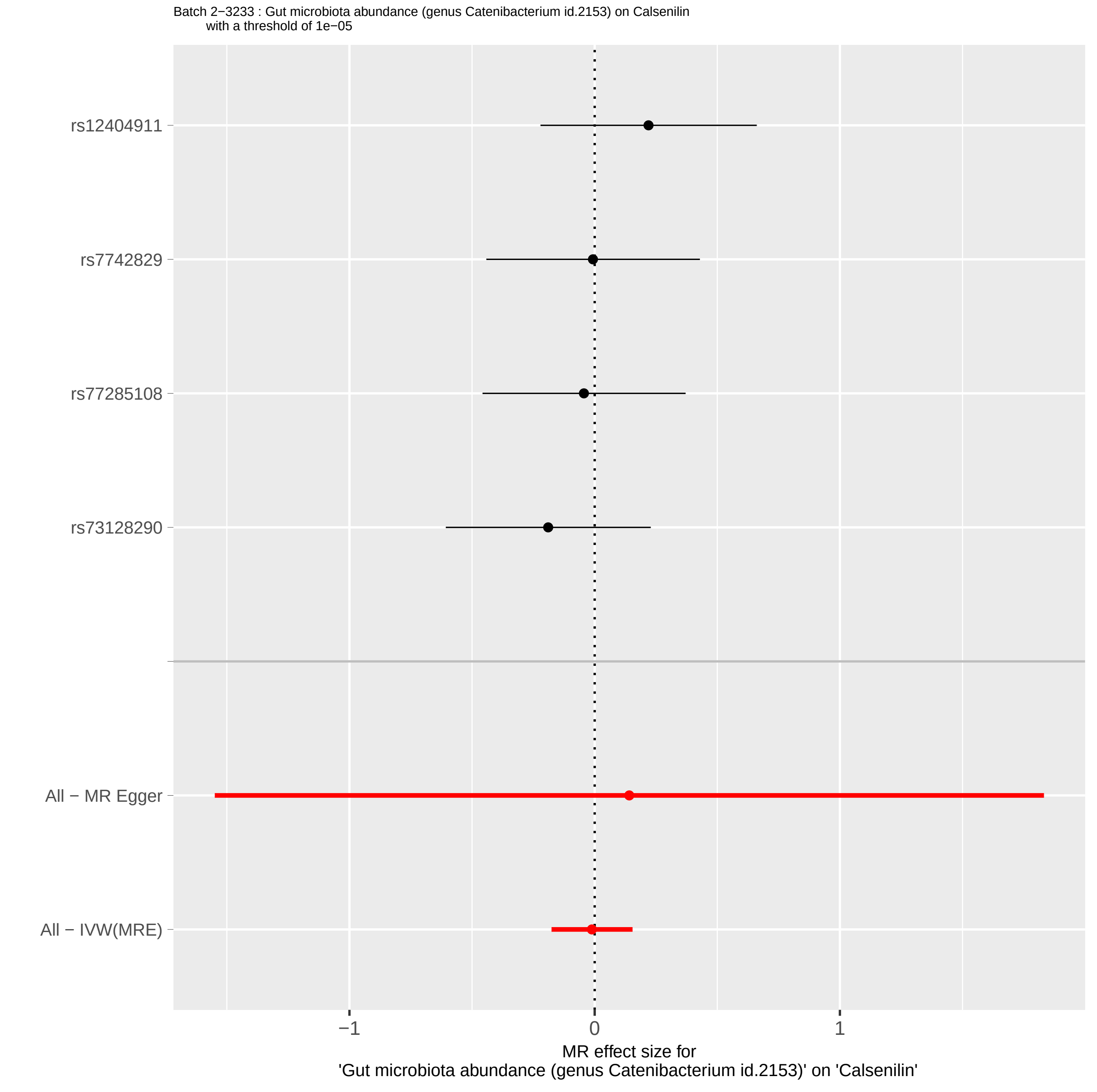

Batch 2−3233 : Gut microbiota abundance (genus Catenibacterium id.2153) on Calsenilin
with a threshold of 1e−05
rs12404911
rs7742829
rs77285108
rs73128290
All − MR Egger
All − IVW(MRE)
−1
0
1
MR effect size for
'Gut microbiota abundance (genus Catenibacterium id.2153)' on 'Calsenilin'

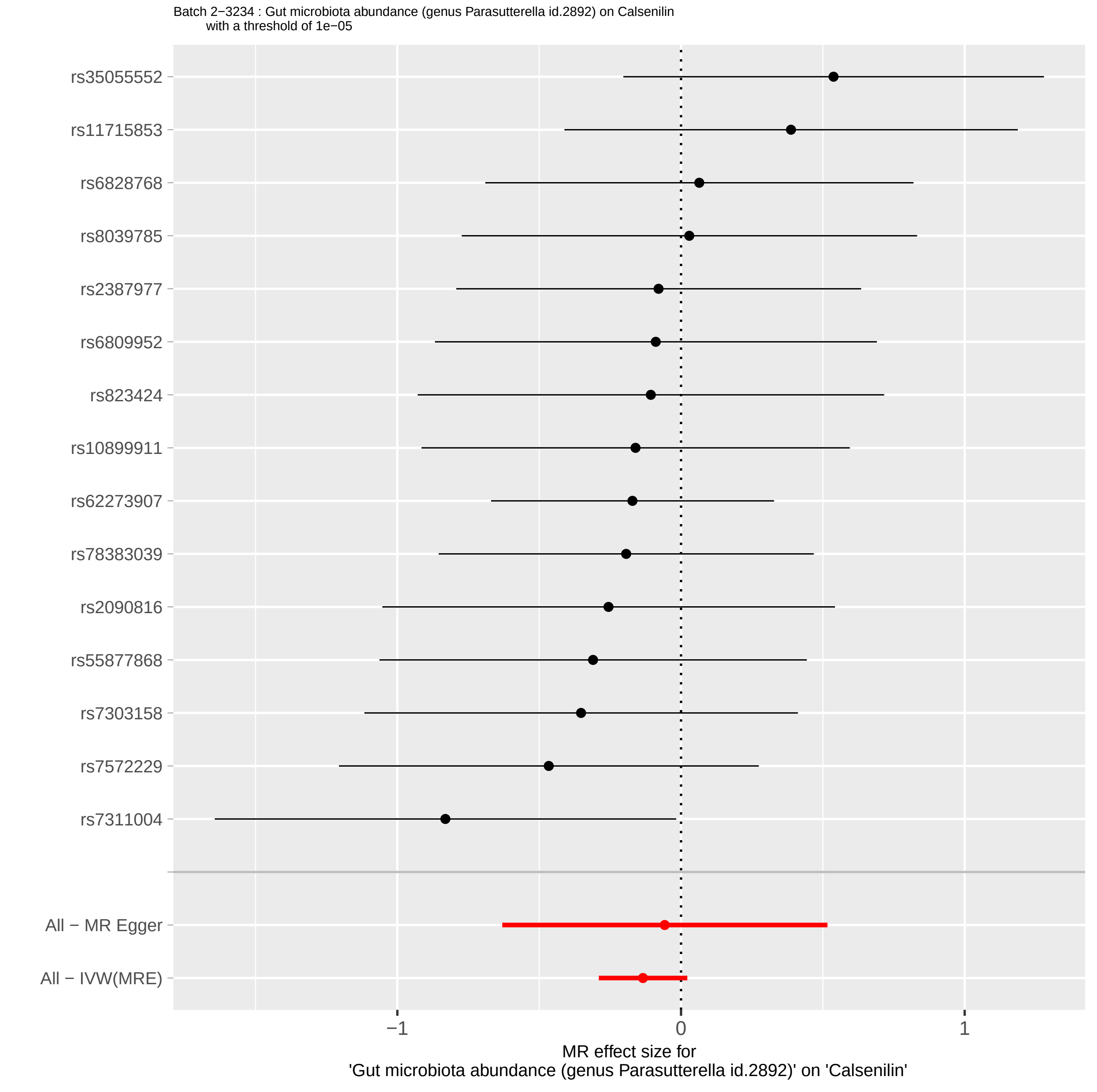

Batch 2−3234 : Gut microbiota abundance (genus Parasutterella id.2892) on Calsenilin
with a threshold of 1e−05
rs35055552
rs11715853
rs6828768
rs8039785
rs2387977
rs6809952
rs823424
rs10899911
rs62273907
rs78383039
rs2090816
rs55877868
rs7303158
rs7572229
rs7311004
All − MR Egger
All − IVW(MRE)
−1
0
1
MR effect size for
'Gut microbiota abundance (genus Parasutterella id.2892)' on 'Calsenilin'

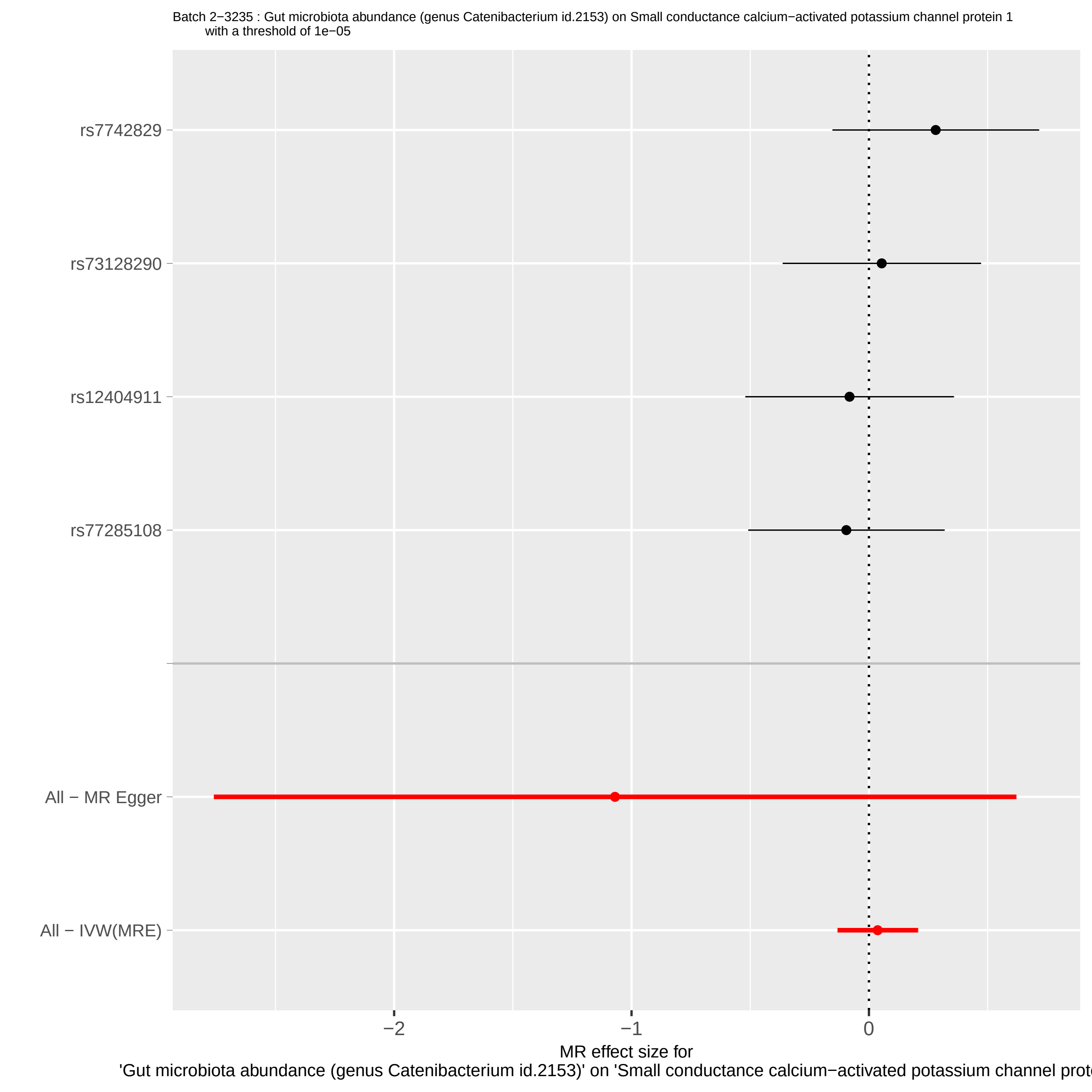

Batch 2−3235 : Gut microbiota abundance (genus Catenibacterium id.2153) on Small conductance calcium−activated potassium channel protein 1
with a threshold of 1e−05
rs7742829
rs73128290
rs12404911
rs77285108
All − MR Egger
All − IVW(MRE)
−2
−1
0
MR effect size for
'Gut microbiota abundance (genus Catenibacterium id.2153)' on 'Small conductance calcium−activated potassium channel prot

Batch 2−3236 : Gut microbiota abundance (genus Parasutterella id.2892) on Small conductance calcium−activated potassium channel protein 1
with a threshold of 1e−05

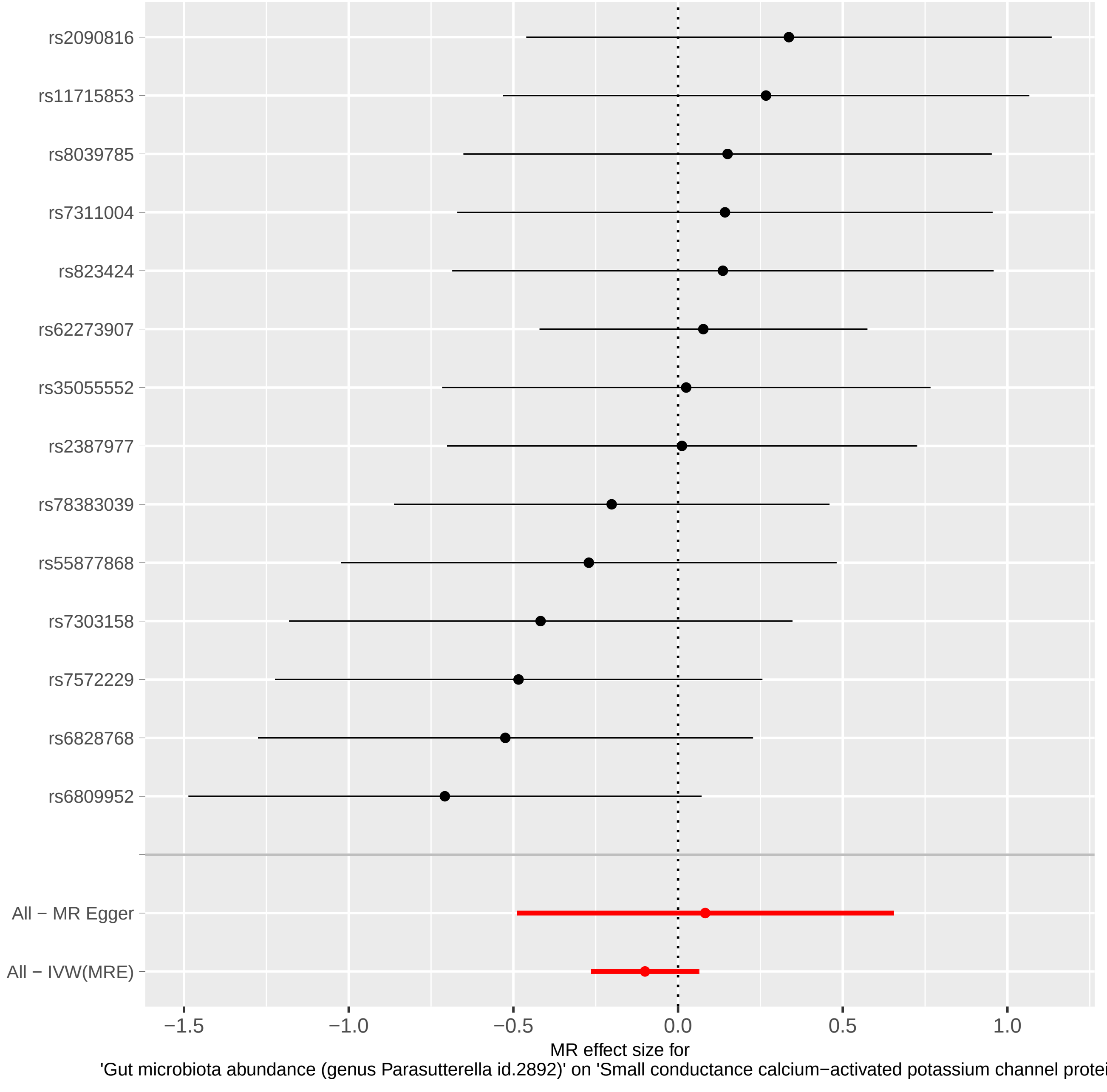

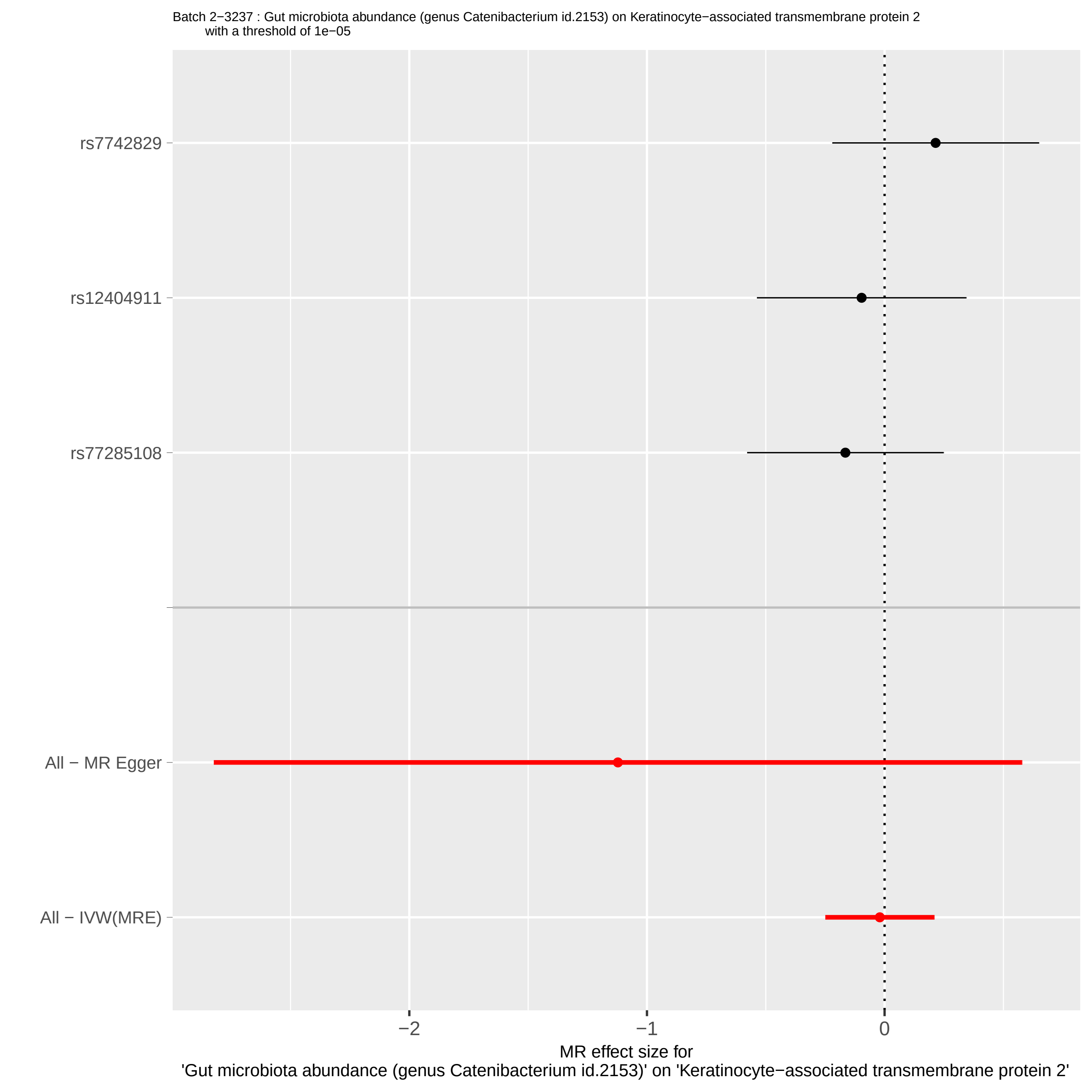

Batch 2−3237 : Gut microbiota abundance (genus Catenibacterium id.2153) on Keratinocyte−associated transmembrane protein 2
with a threshold of 1e−05
rs7742829
rs12404911
rs77285108
All − MR Egger
All − IVW(MRE)
−2
−1
0
MR effect size for
'Gut microbiota abundance (genus Catenibacterium id.2153)' on 'Keratinocyte−associated transmembrane protein 2'

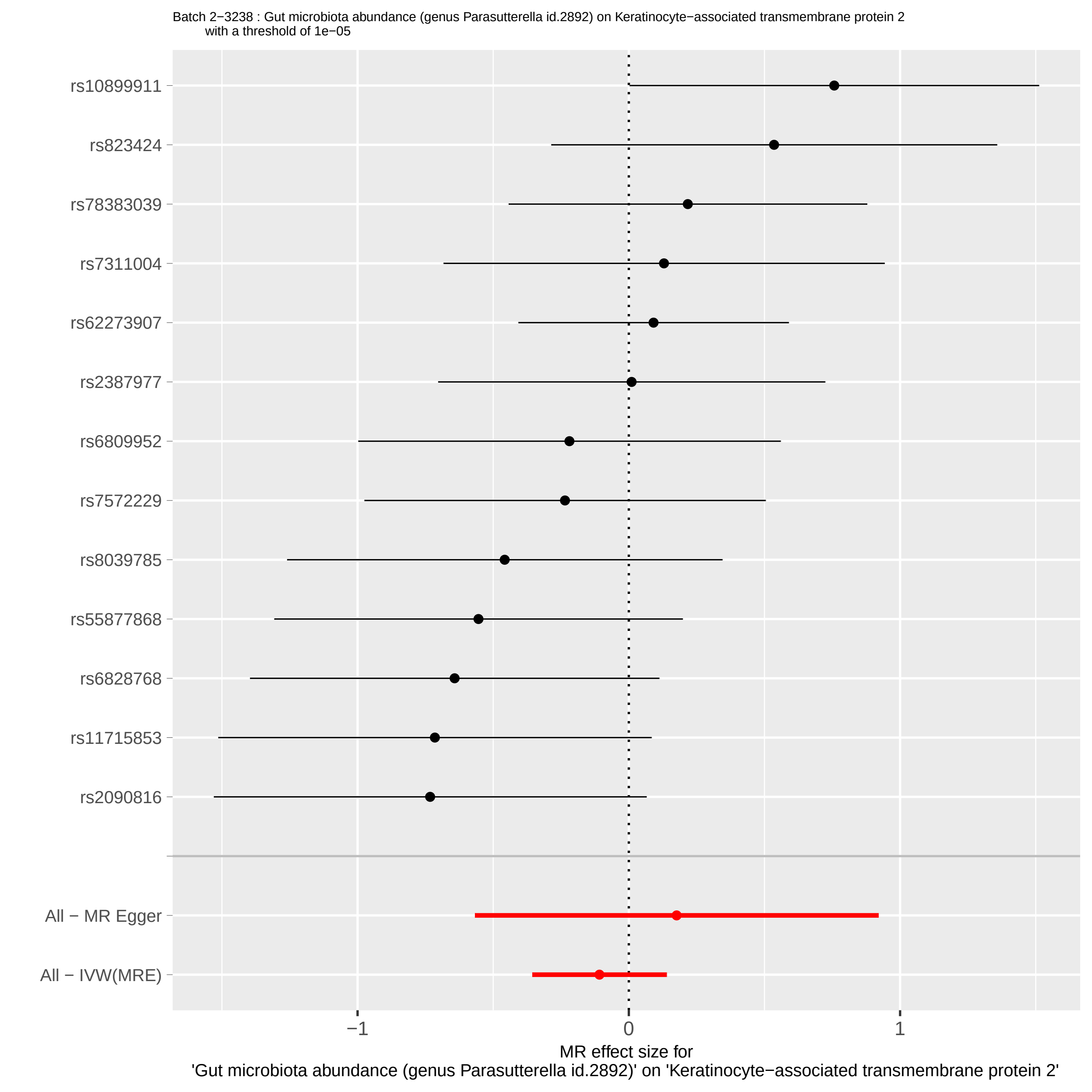
Batch 2−3238 : Gut microbiota abundance (genus Parasutterella id.2892) on Keratinocyte−associated transmembrane protein 2
with a threshold of 1e−05
rs10899911
rs823424
rs78383039
rs7311004
rs62273907
rs2387977
rs6809952
rs7572229
rs8039785
rs55877868
rs6828768
rs11715853
rs2090816
All − MR Egger
All − IVW(MRE)
−1
0
1
MR effect size for
'Gut microbiota abundance (genus Parasutterella id.2892)' on 'Keratinocyte−associated transmembrane protein 2'

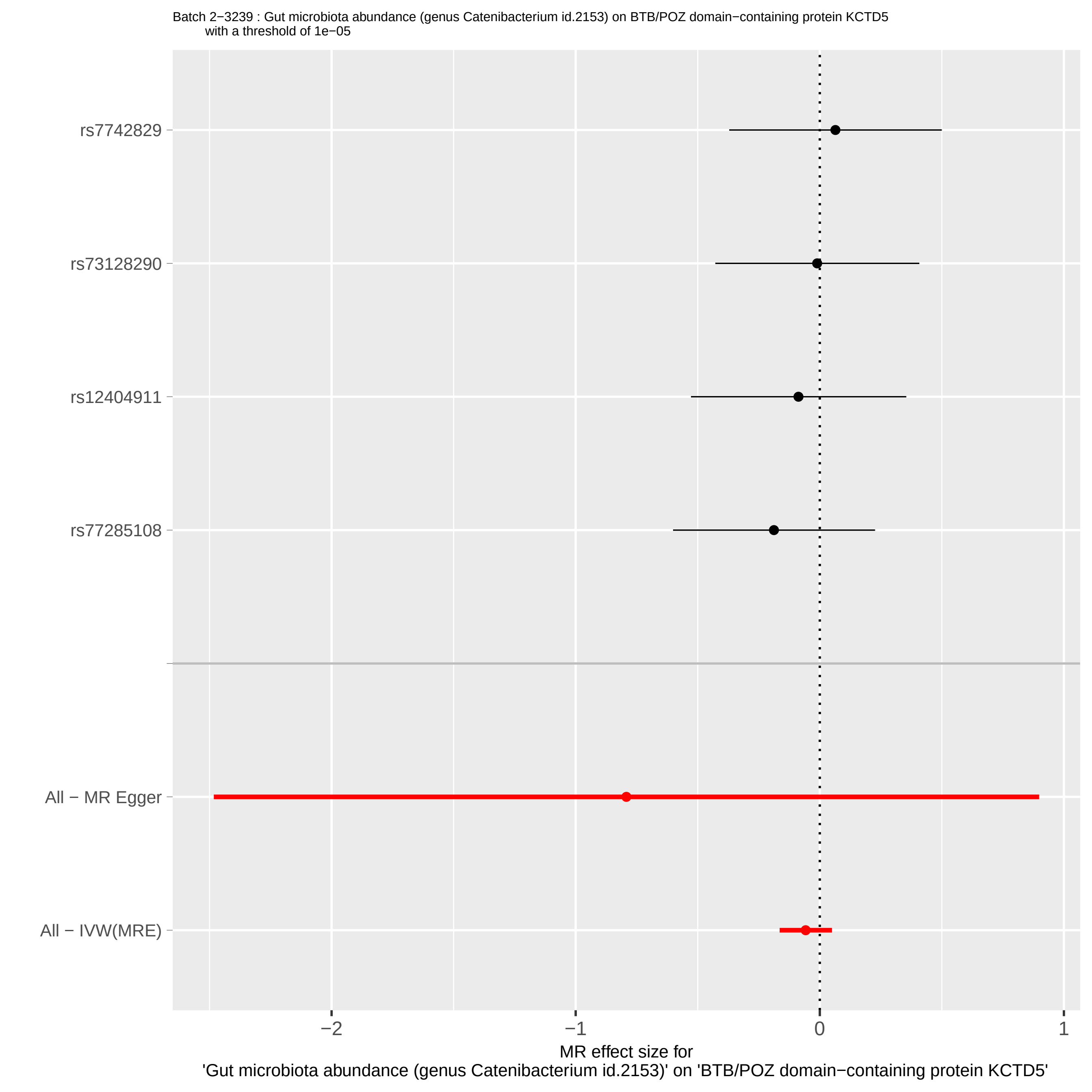

Batch 2−3239 : Gut microbiota abundance (genus Catenibacterium id.2153) on BTB/POZ domain−containing protein KCTD5
with a threshold of 1e−05
rs7742829
rs73128290
rs12404911
rs77285108
All − MR Egger
All − IVW(MRE)
−2
−1
0
1
MR effect size for
'Gut microbiota abundance (genus Catenibacterium id.2153)' on 'BTB/POZ domain−containing protein KCTD5'

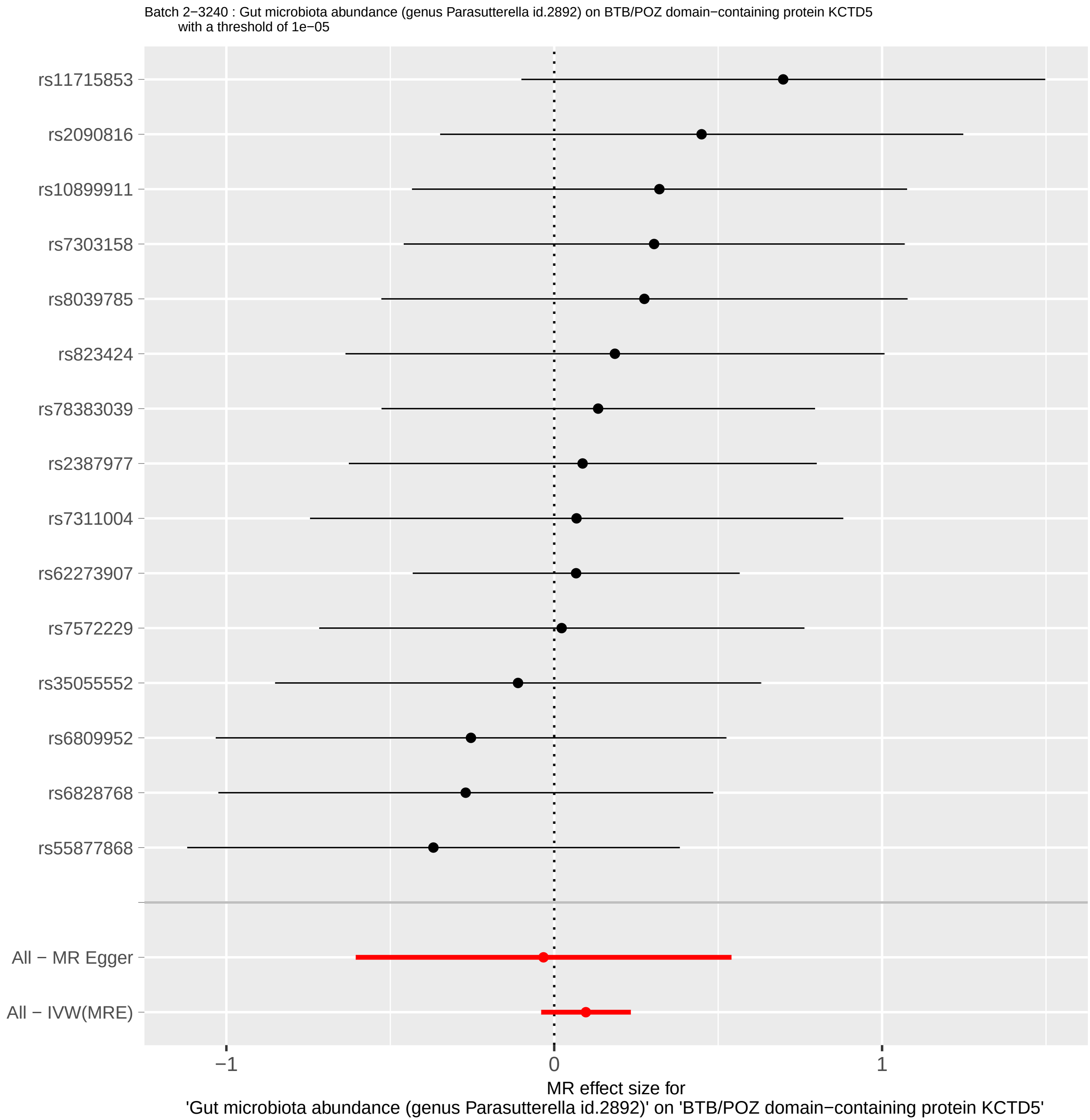
Batch 2−3240 : Gut microbiota abundance (genus Parasutterella id.2892) on BTB/POZ domain−containing protein KCTD5
with a threshold of 1e−05
rs11715853
rs2090816
rs10899911
rs7303158
rs8039785
rs823424
rs78383039
rs2387977
rs7311004
rs62273907
rs7572229
rs35055552
rs6809952
rs6828768
rs55877868
All − MR Egger
All − IVW(MRE)
−1
0
1
MR effect size for
'Gut microbiota abundance (genus Parasutterella id.2892)' on 'BTB/POZ domain−containing protein KCTD5'

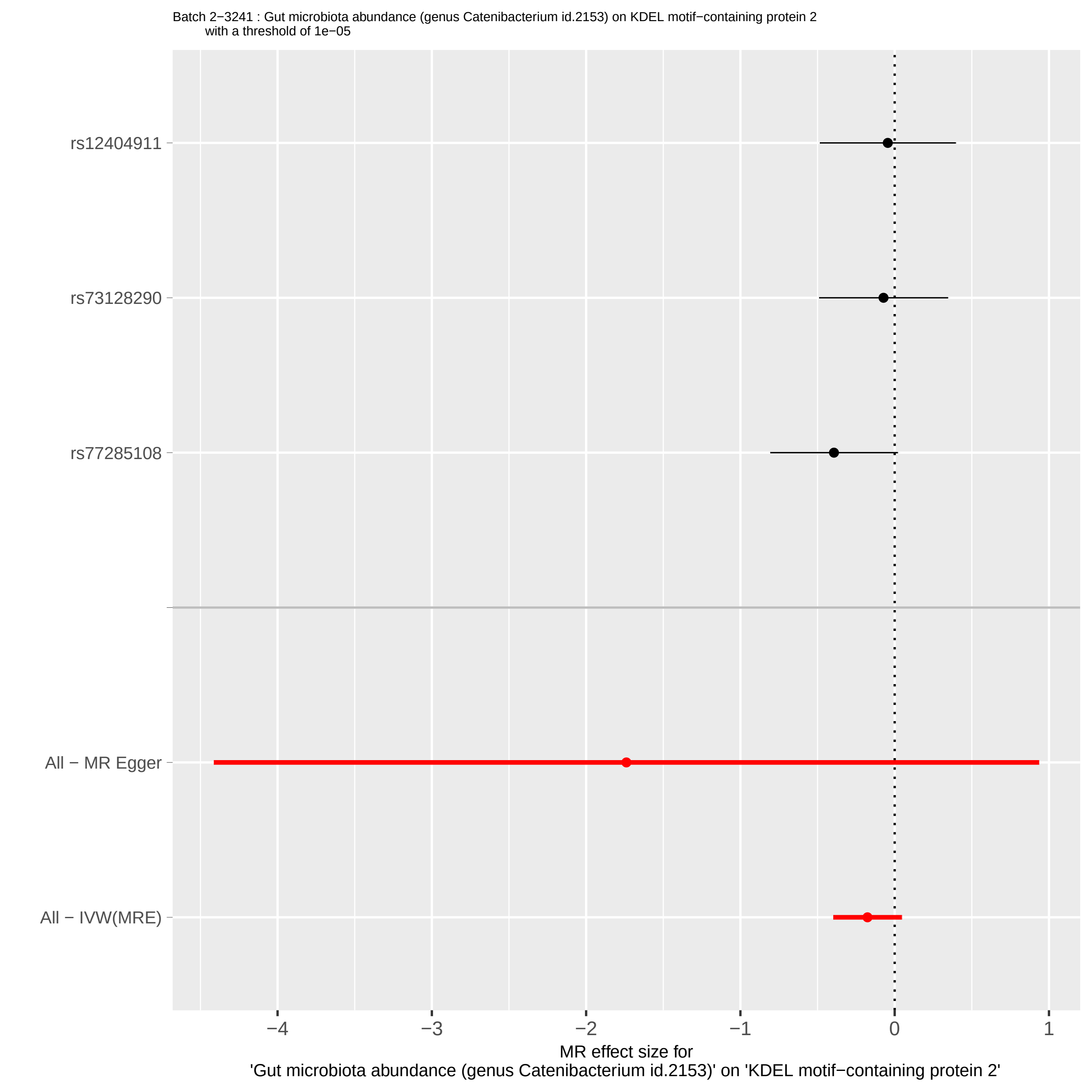
Batch 2−3241 : Gut microbiota abundance (genus Catenibacterium id.2153) on KDEL motif−containing protein 2
with a threshold of 1e−05
rs12404911
rs73128290
rs77285108
All − MR Egger
All − IVW(MRE)
−4
−3
−2
−1
0
1
MR effect size for
'Gut microbiota abundance (genus Catenibacterium id.2153)' on 'KDEL motif−containing protein 2'

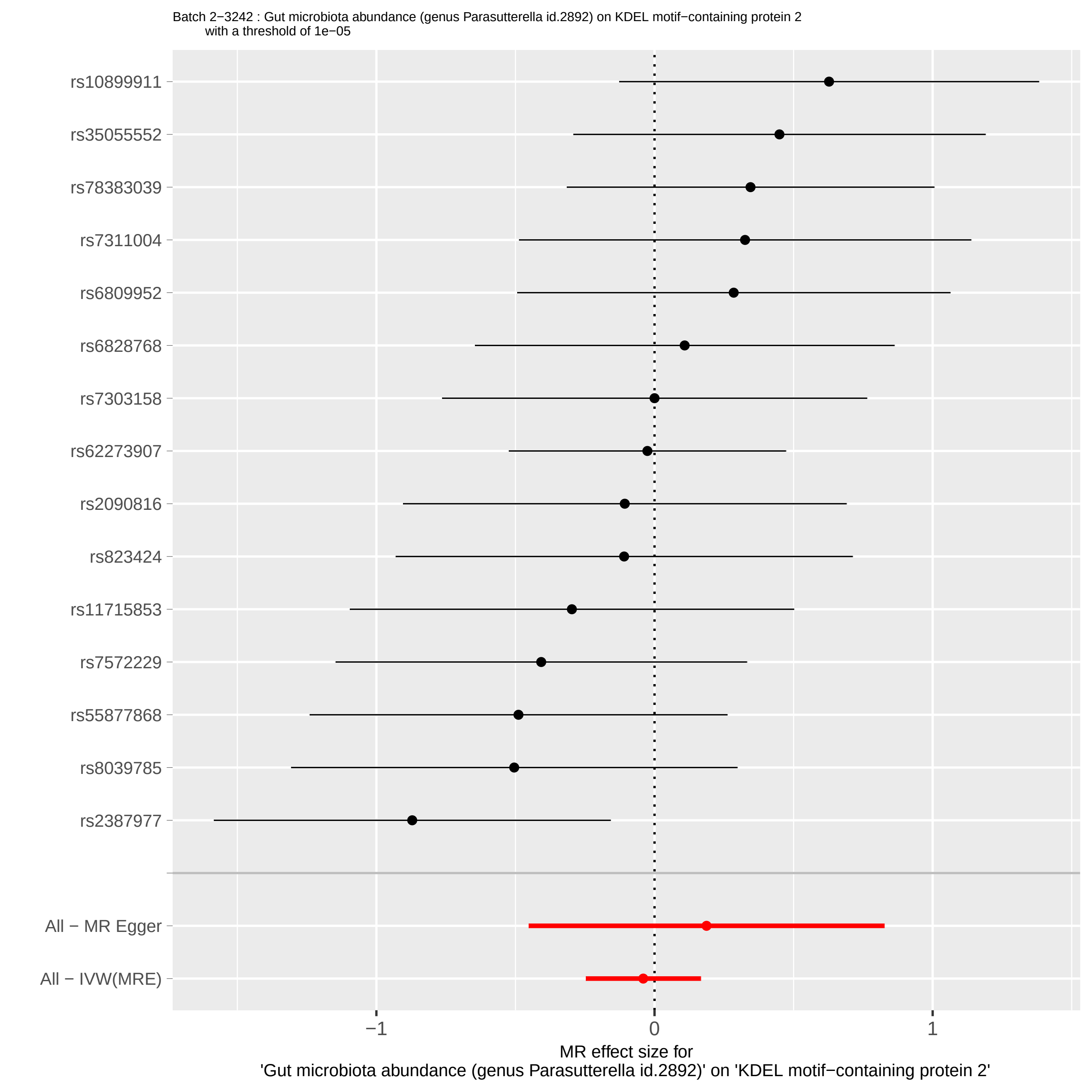

Batch 2−3242 : Gut microbiota abundance (genus Parasutterella id.2892) on KDEL motif−containing protein 2
with a threshold of 1e−05
rs10899911
rs35055552
rs78383039
rs7311004
rs6809952
rs6828768
rs7303158
rs62273907
rs2090816
rs823424
rs11715853
rs7572229
rs55877868
rs8039785
rs2387977
All − MR Egger
All − IVW(MRE)
−1
0
1
MR effect size for
'Gut microbiota abundance (genus Parasutterella id.2892)' on 'KDEL motif−containing protein 2'

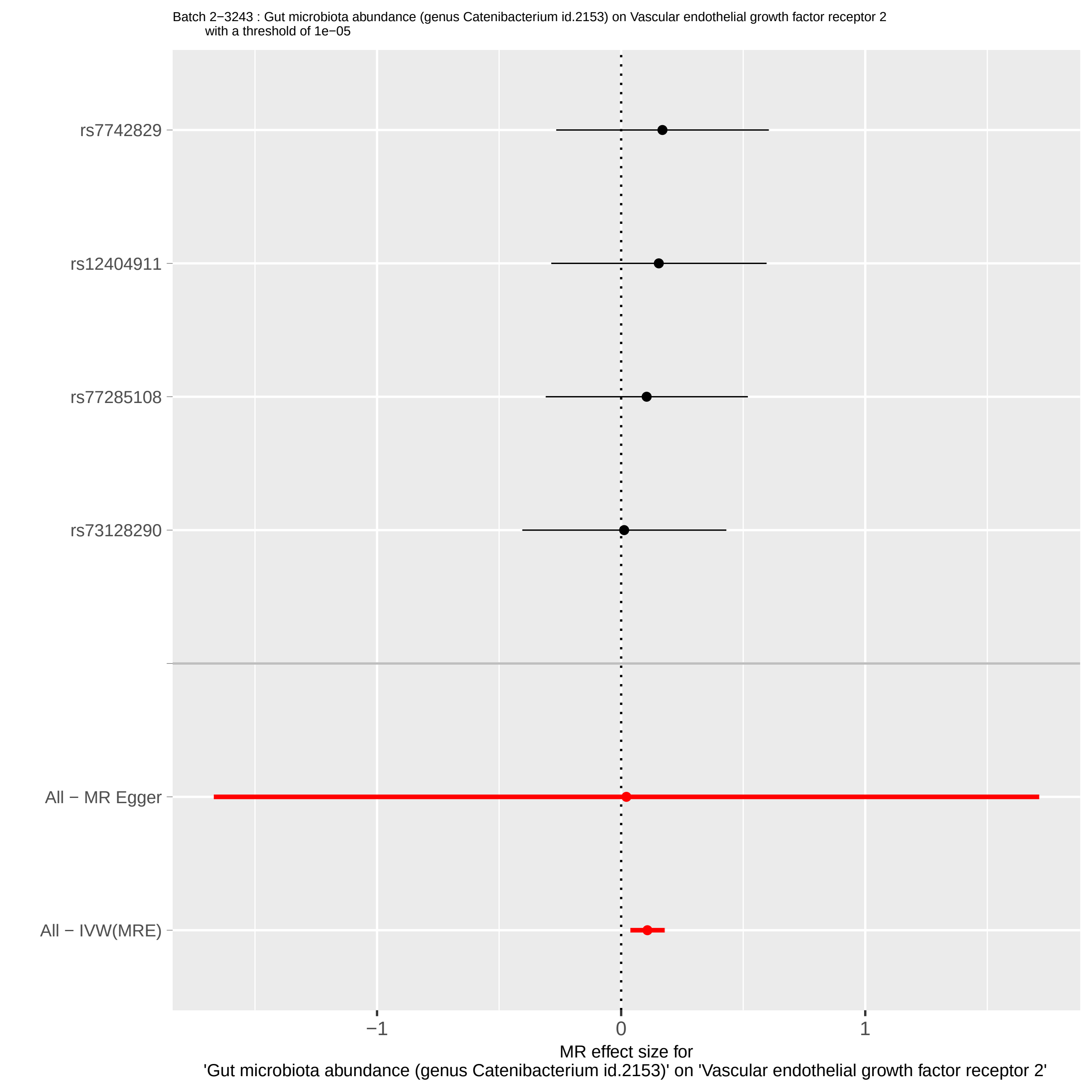

Batch 2−3243 : Gut microbiota abundance (genus Catenibacterium id.2153) on Vascular endothelial growth factor receptor 2
with a threshold of 1e−05
rs7742829
rs12404911
rs77285108
rs73128290
All − MR Egger
All − IVW(MRE)
−1
0
1
MR effect size for
'Gut microbiota abundance (genus Catenibacterium id.2153)' on 'Vascular endothelial growth factor receptor 2'

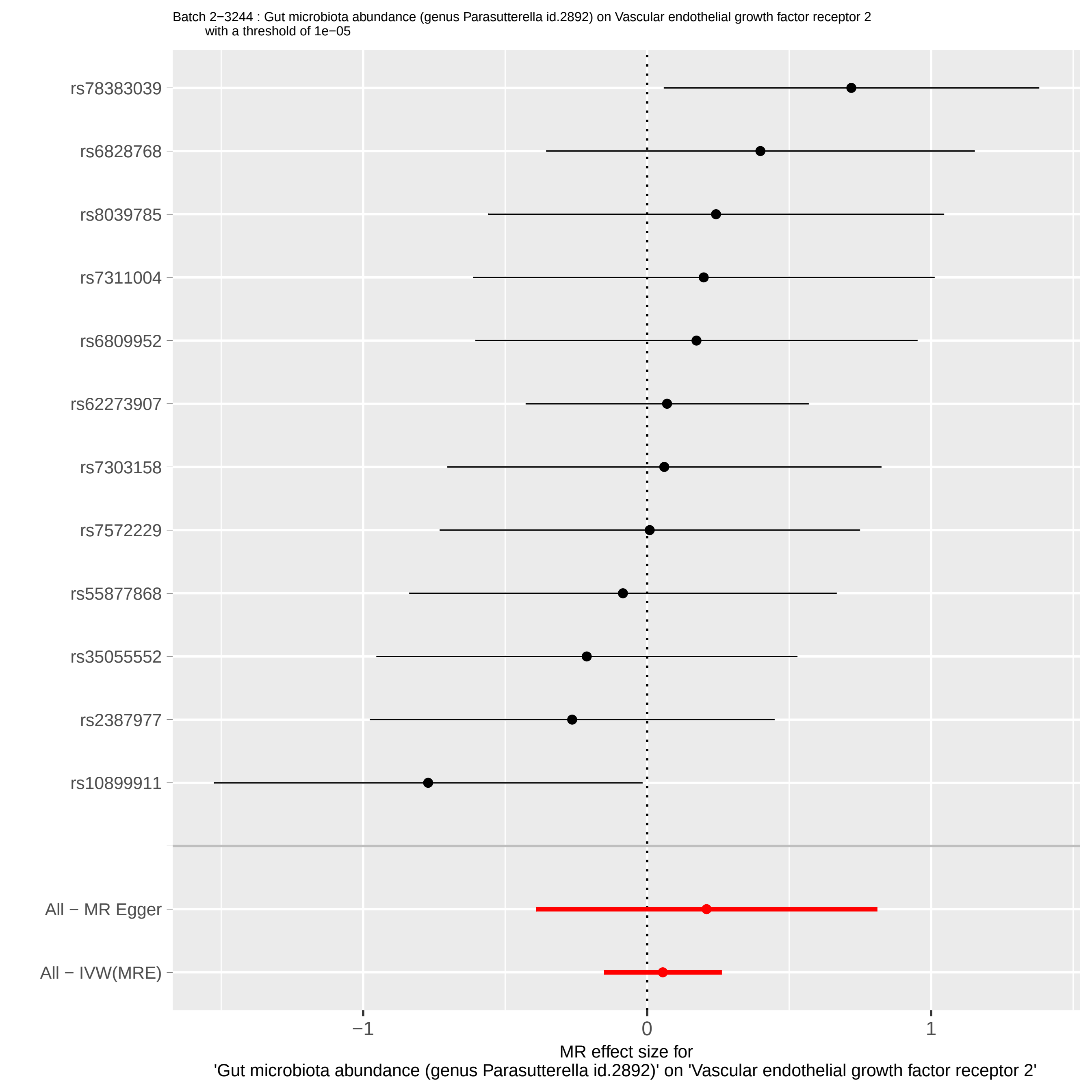

Batch 2−3244 : Gut microbiota abundance (genus Parasutterella id.2892) on Vascular endothelial growth factor receptor 2
with a threshold of 1e−05
rs78383039
rs6828768
rs8039785
rs7311004
rs6809952
rs62273907
rs7303158
rs7572229
rs55877868
rs35055552
rs2387977
rs10899911
All − MR Egger
All − IVW(MRE)
−1
0
1
MR effect size for
'Gut microbiota abundance (genus Parasutterella id.2892)' on 'Vascular endothelial growth factor receptor 2'

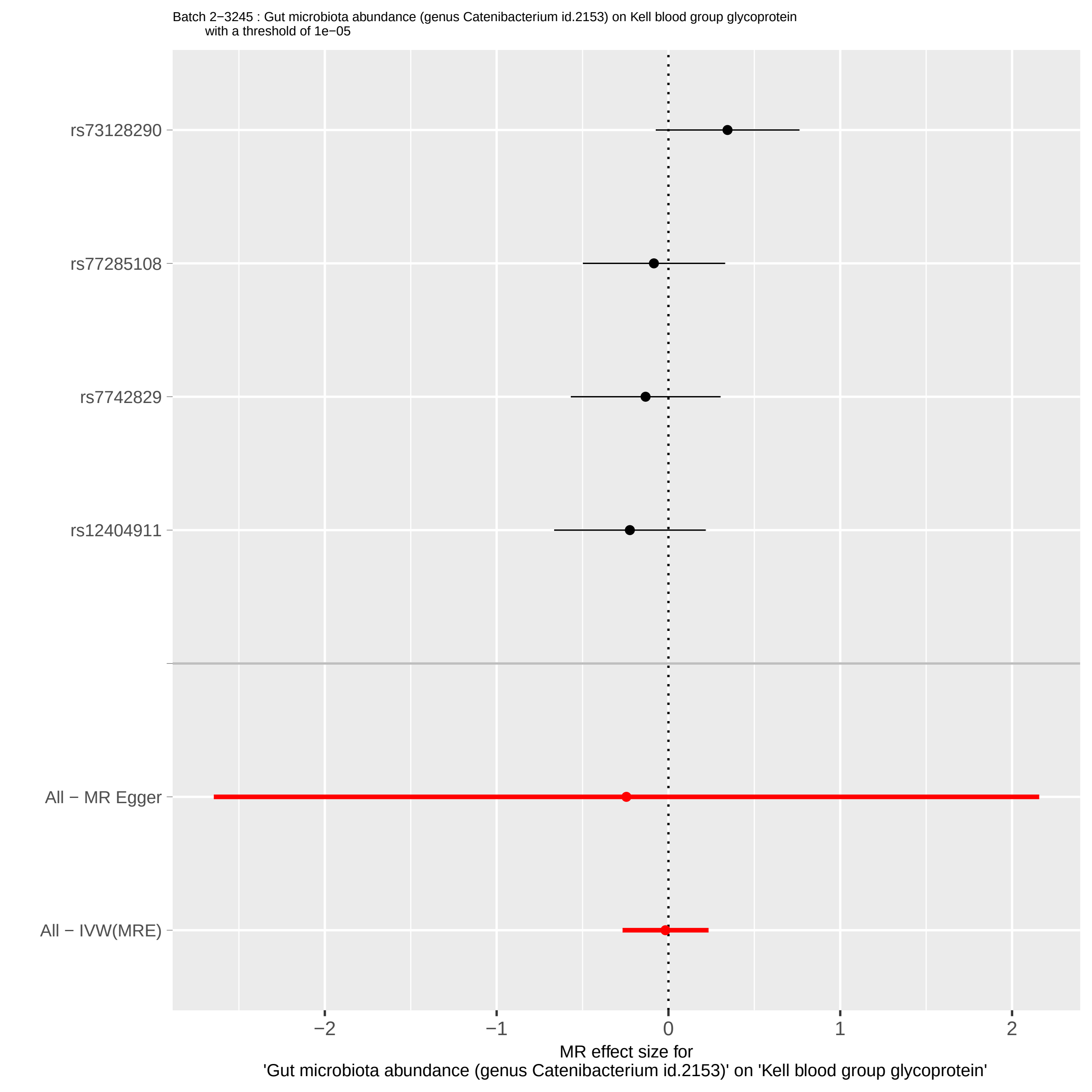
Batch 2−3245 : Gut microbiota abundance (genus Catenibacterium id.2153) on Kell blood group glycoprotein
with a threshold of 1e−05
rs73128290
rs77285108
rs7742829
rs12404911
All − MR Egger
All − IVW(MRE)
−2
−1
0
1
2
MR effect size for
'Gut microbiota abundance (genus Catenibacterium id.2153)' on 'Kell blood group glycoprotein'

Batch 2−3246 : Gut microbiota abundance (genus Parasutterella id.2892) on Kell blood group glycoprotein
with a threshold of 1e−05

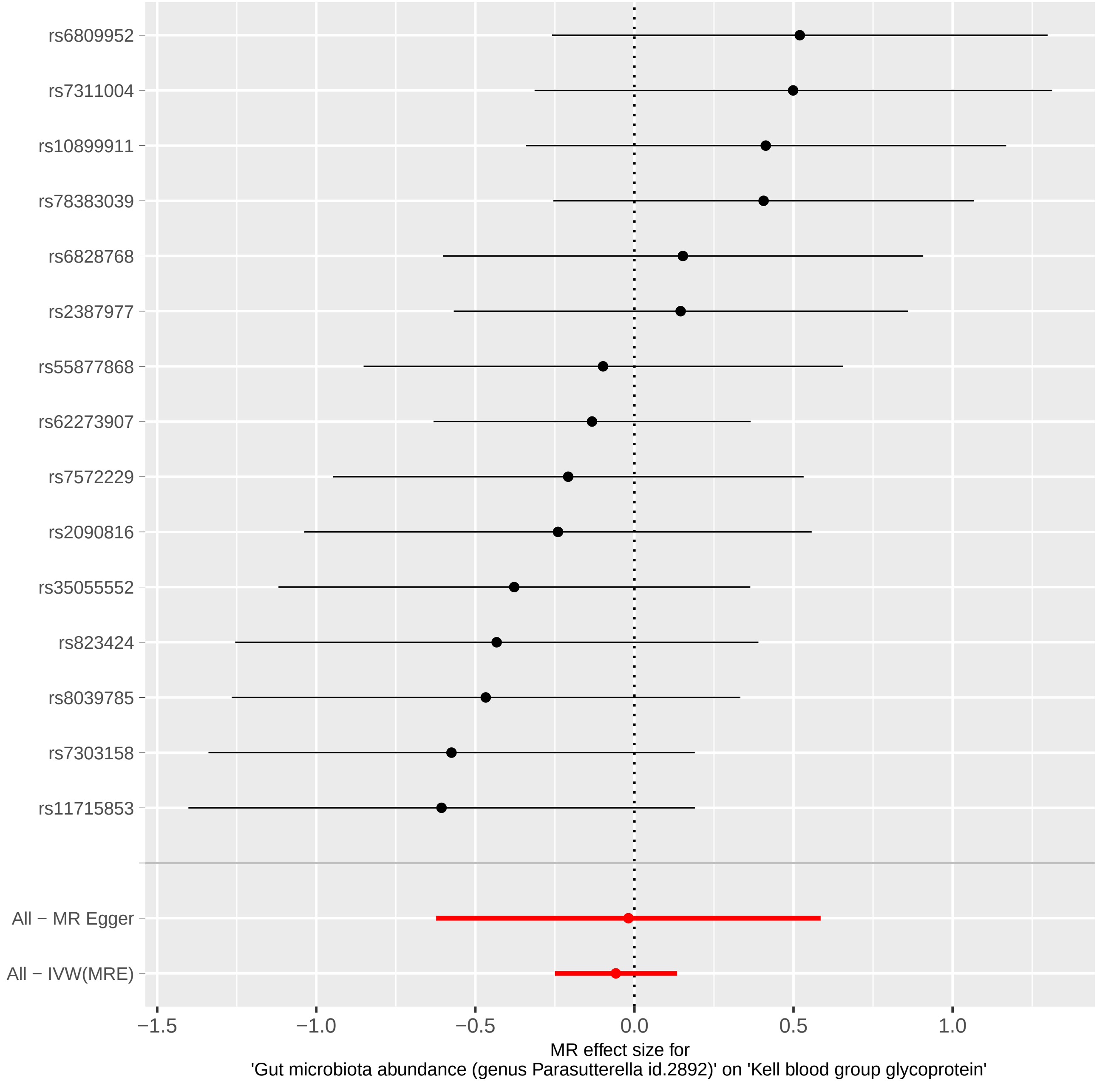

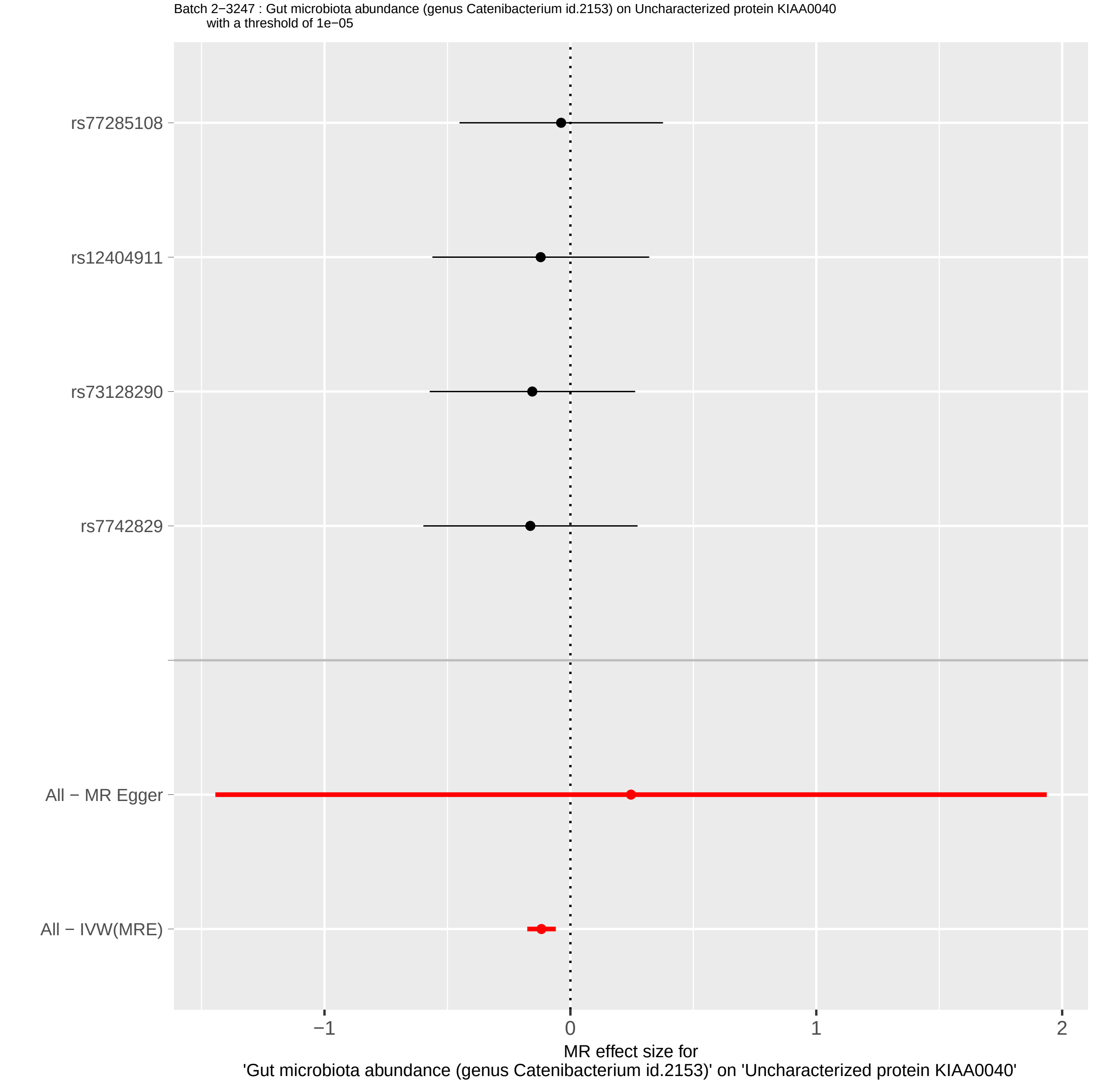

Batch 2−3247 : Gut microbiota abundance (genus Catenibacterium id.2153) on Uncharacterized protein KIAA0040
with a threshold of 1e−05
rs77285108
rs12404911
rs73128290
rs7742829
All − MR Egger
All − IVW(MRE)
−1
0
1
2
MR effect size for
'Gut microbiota abundance (genus Catenibacterium id.2153)' on 'Uncharacterized protein KIAA0040'

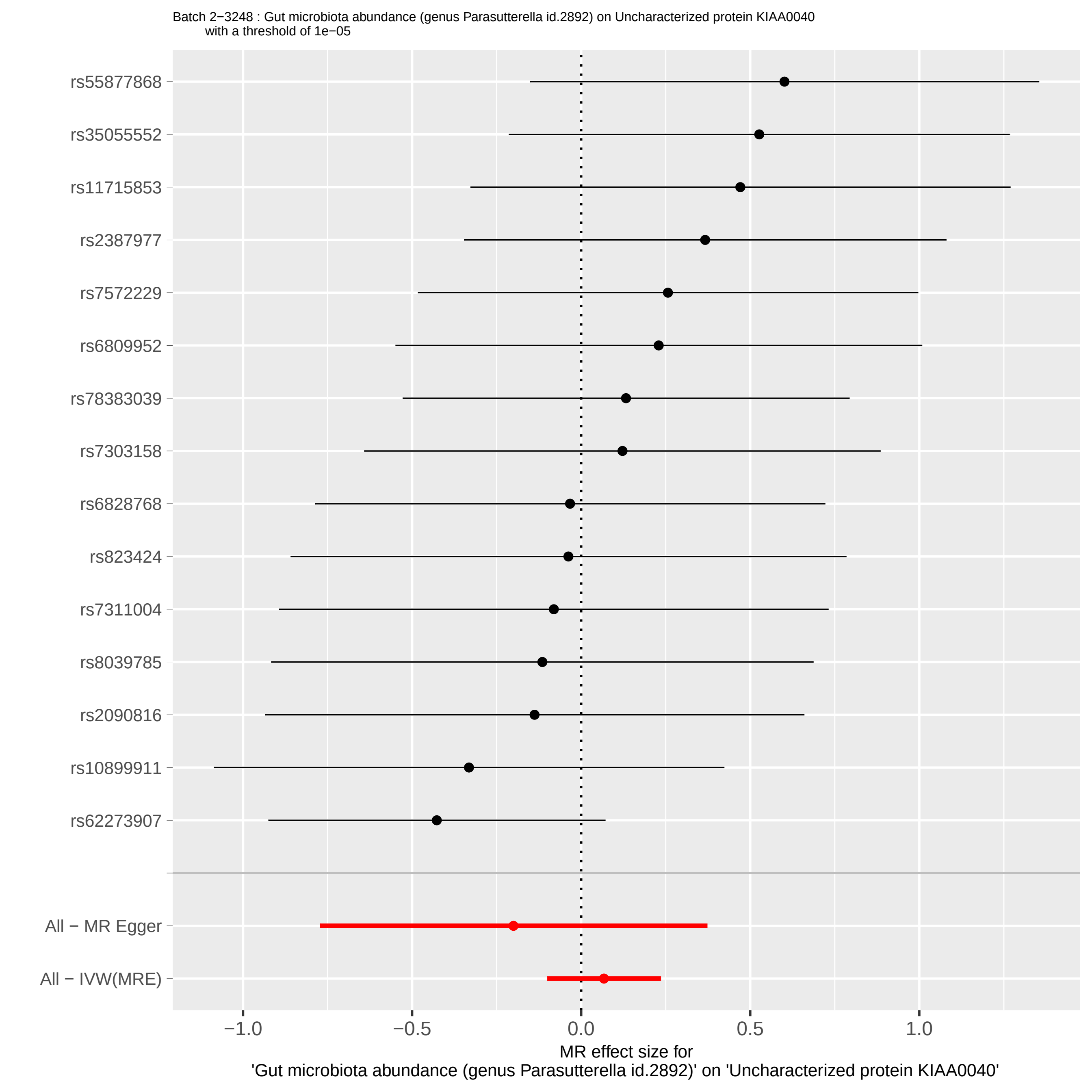

Batch 2−3248 : Gut microbiota abundance (genus Parasutterella id.2892) on Uncharacterized protein KIAA0040
with a threshold of 1e−05
rs55877868
rs35055552
rs11715853
rs2387977
rs7572229
rs6809952
rs78383039
rs7303158
rs6828768
rs823424
rs7311004
rs8039785
rs2090816
rs10899911
rs62273907
All − MR Egger
All − IVW(MRE)
−1.0
−0.5
0.0
0.5
1.0
MR effect size for
'Gut microbiota abundance (genus Parasutterella id.2892)' on 'Uncharacterized protein KIAA0040'

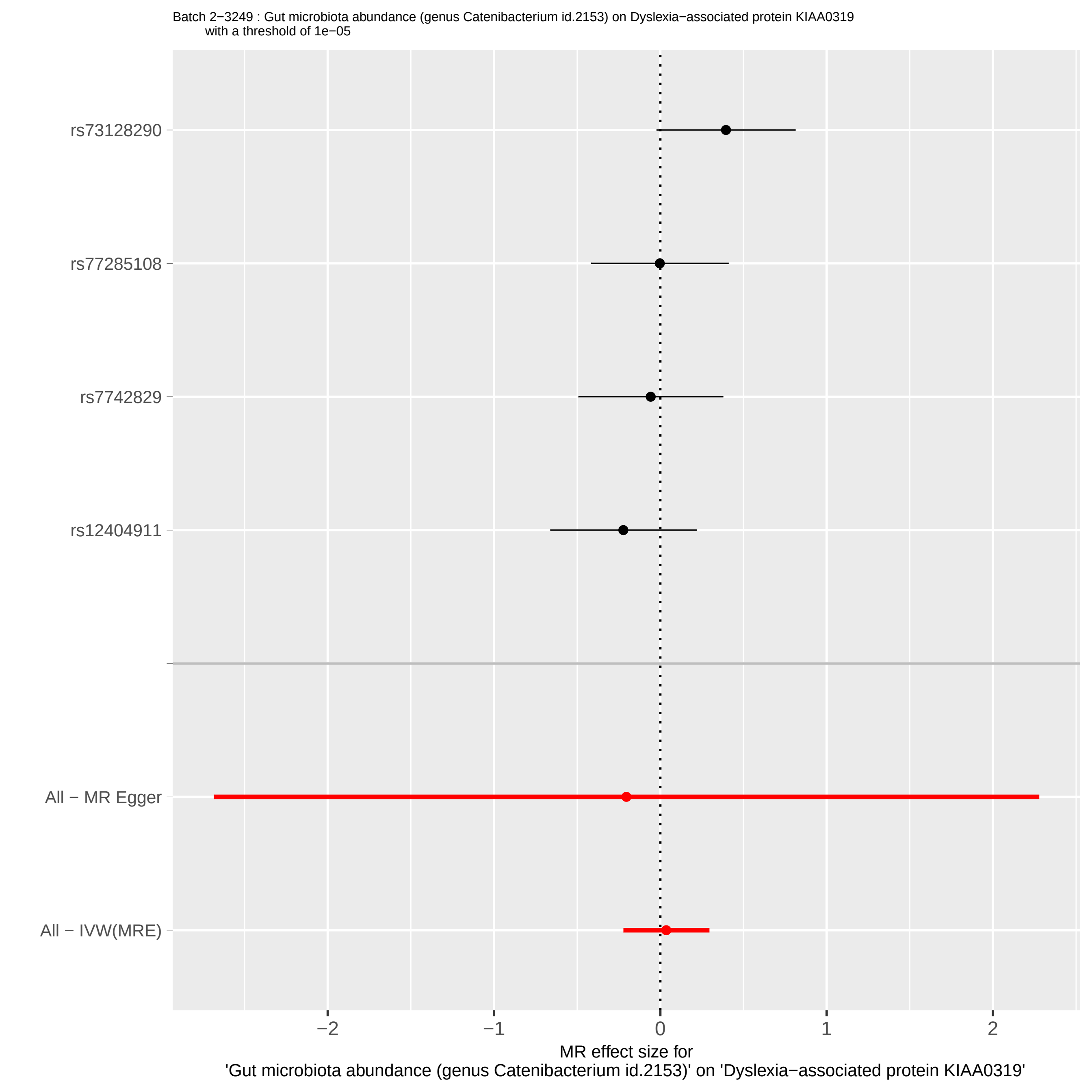
Batch 2−3249 : Gut microbiota abundance (genus Catenibacterium id.2153) on Dyslexia−associated protein KIAA0319
with a threshold of 1e−05
rs73128290
rs77285108
rs7742829
rs12404911
All − MR Egger
All − IVW(MRE)
−2
−1
0
1
2
MR effect size for
'Gut microbiota abundance (genus Catenibacterium id.2153)' on 'Dyslexia−associated protein KIAA0319'

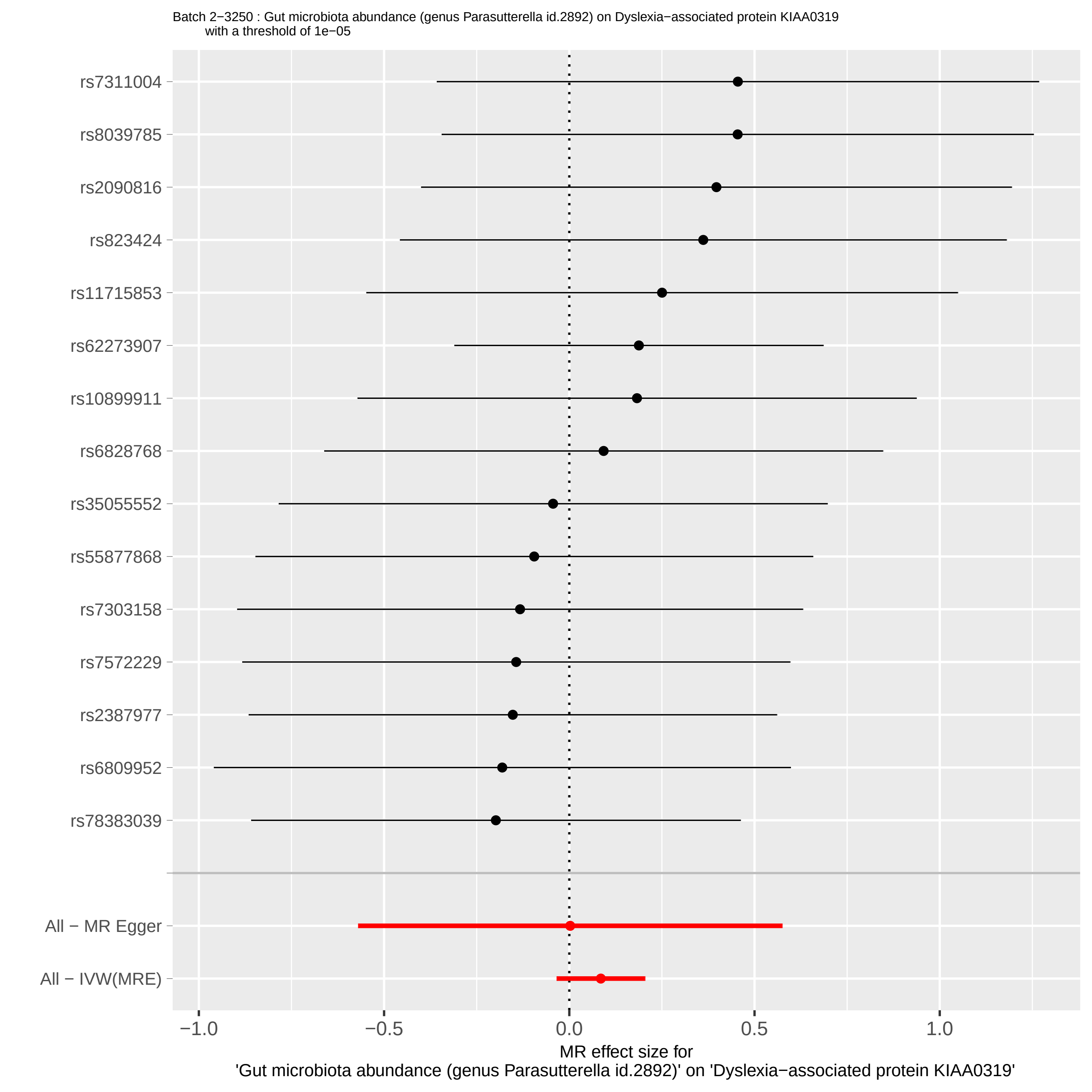
Batch 2−3250 : Gut microbiota abundance (genus Parasutterella id.2892) on Dyslexia−associated protein KIAA0319
with a threshold of 1e−05
rs7311004
rs8039785
rs2090816
rs823424
rs11715853
rs62273907
rs10899911
rs6828768
rs35055552
rs55877868
rs7303158
rs7572229
rs2387977
rs6809952
rs78383039
All − MR Egger
All − IVW(MRE)
−1.0
−0.5
0.0
0.5
1.0
MR effect size for
'Gut microbiota abundance (genus Parasutterella id.2892)' on 'Dyslexia−associated protein KIAA0319'

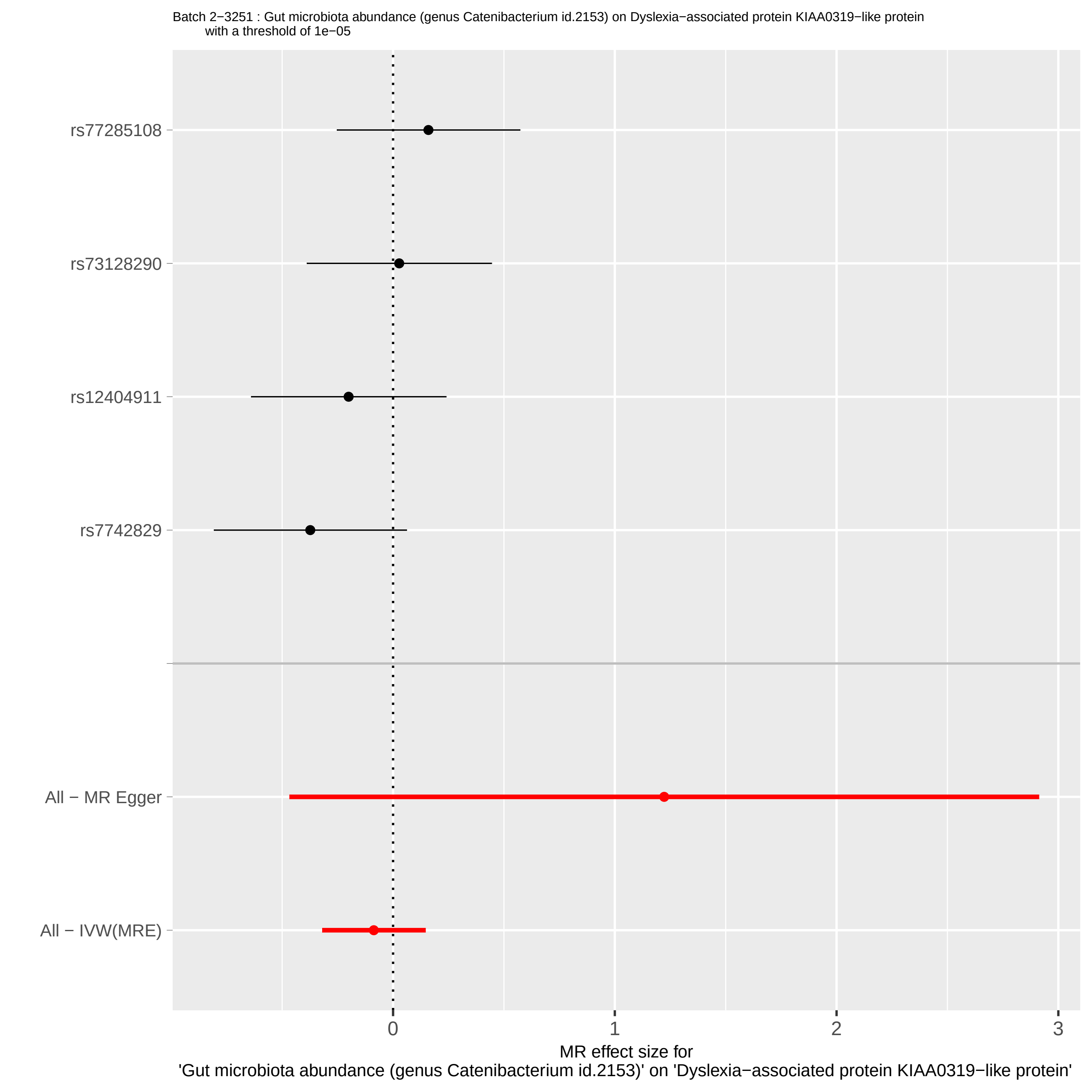

Batch 2−3251 : Gut microbiota abundance (genus Catenibacterium id.2153) on Dyslexia−associated protein KIAA0319−like protein
with a threshold of 1e−05
rs77285108
rs73128290
rs12404911
rs7742829
All − MR Egger
All − IVW(MRE)
0
1
2
3
MR effect size for
'Gut microbiota abundance (genus Catenibacterium id.2153)' on 'Dyslexia−associated protein KIAA0319−like protein'

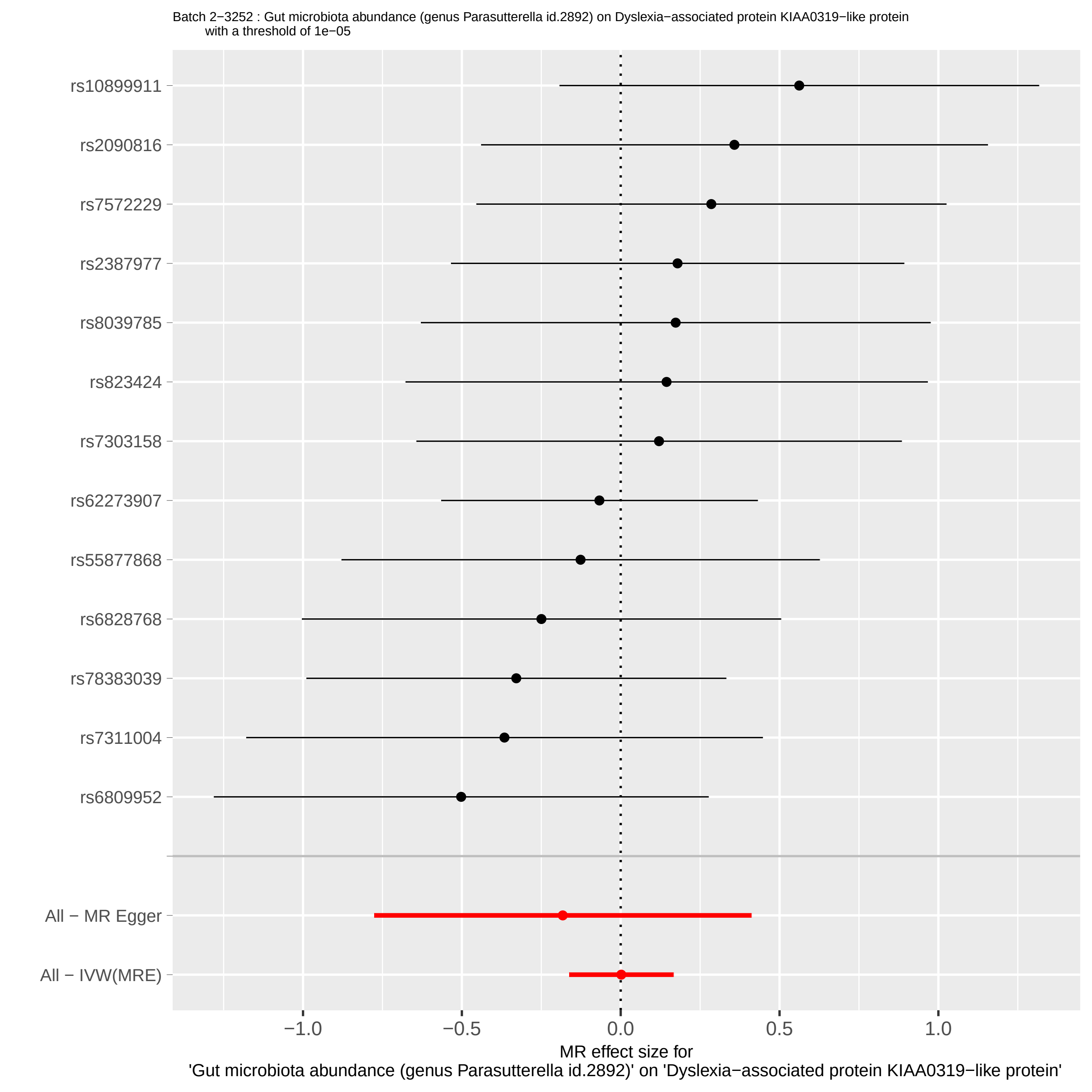

Batch 2−3252 : Gut microbiota abundance (genus Parasutterella id.2892) on Dyslexia−associated protein KIAA0319−like protein
with a threshold of 1e−05
rs10899911
rs2090816
rs7572229
rs2387977
rs8039785
rs823424
rs7303158
rs62273907
rs55877868
rs6828768
rs78383039
rs7311004
rs6809952
All − MR Egger
All − IVW(MRE)
−1.0
−0.5
0.0
0.5
1.0
MR effect size for
'Gut microbiota abundance (genus Parasutterella id.2892)' on 'Dyslexia−associated protein KIAA0319−like protein'

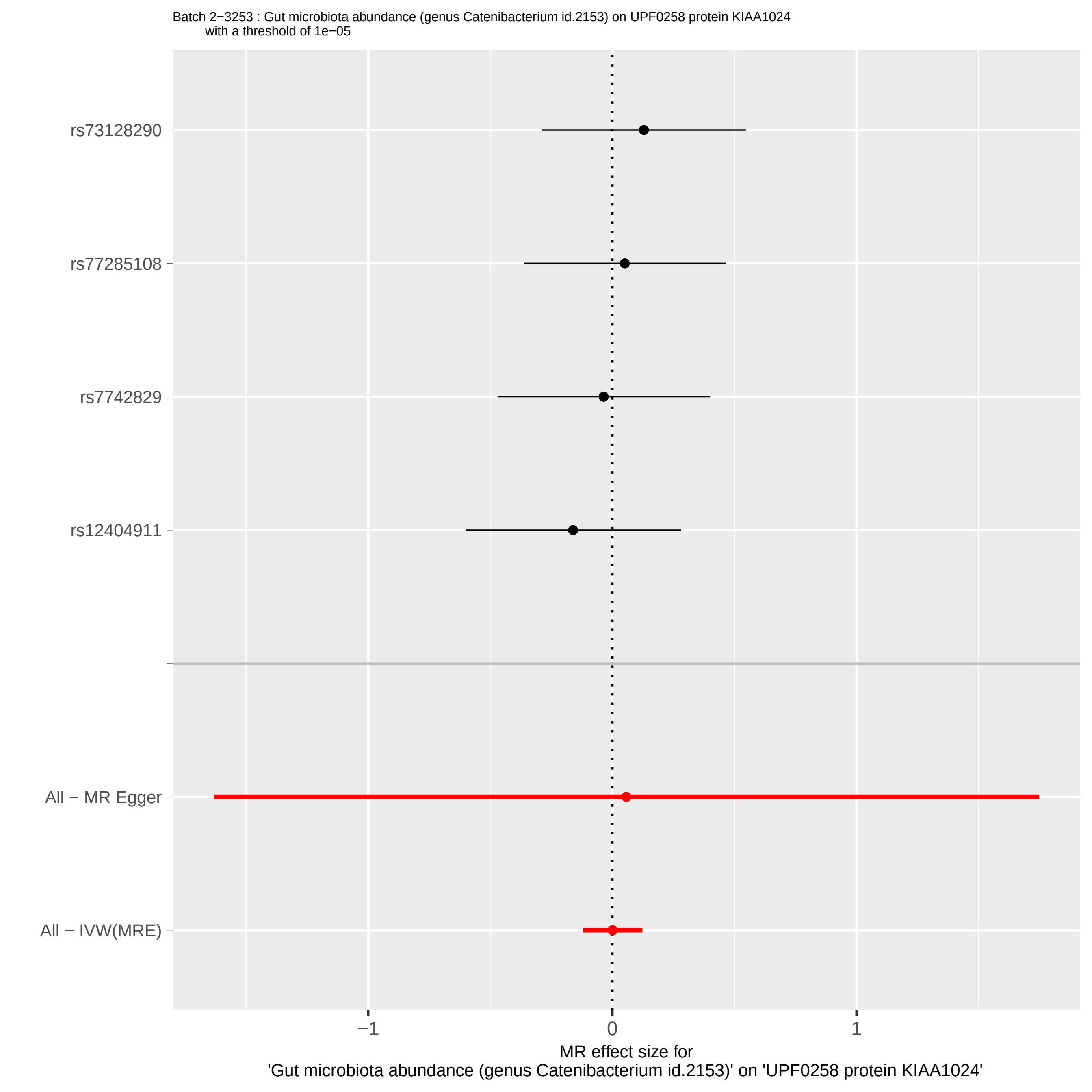
Batch 2−3253 : Gut microbiota abundance (genus Catenibacterium id.2153) on UPF0258 protein KIAA1024
with a threshold of 1e−05
rs73128290
rs77285108
rs7742829
rs12404911
All − MR Egger
All − IVW(MRE)
−1
0
1
MR effect size for
'Gut microbiota abundance (genus Catenibacterium id.2153)' on 'UPF0258 protein KIAA1024'

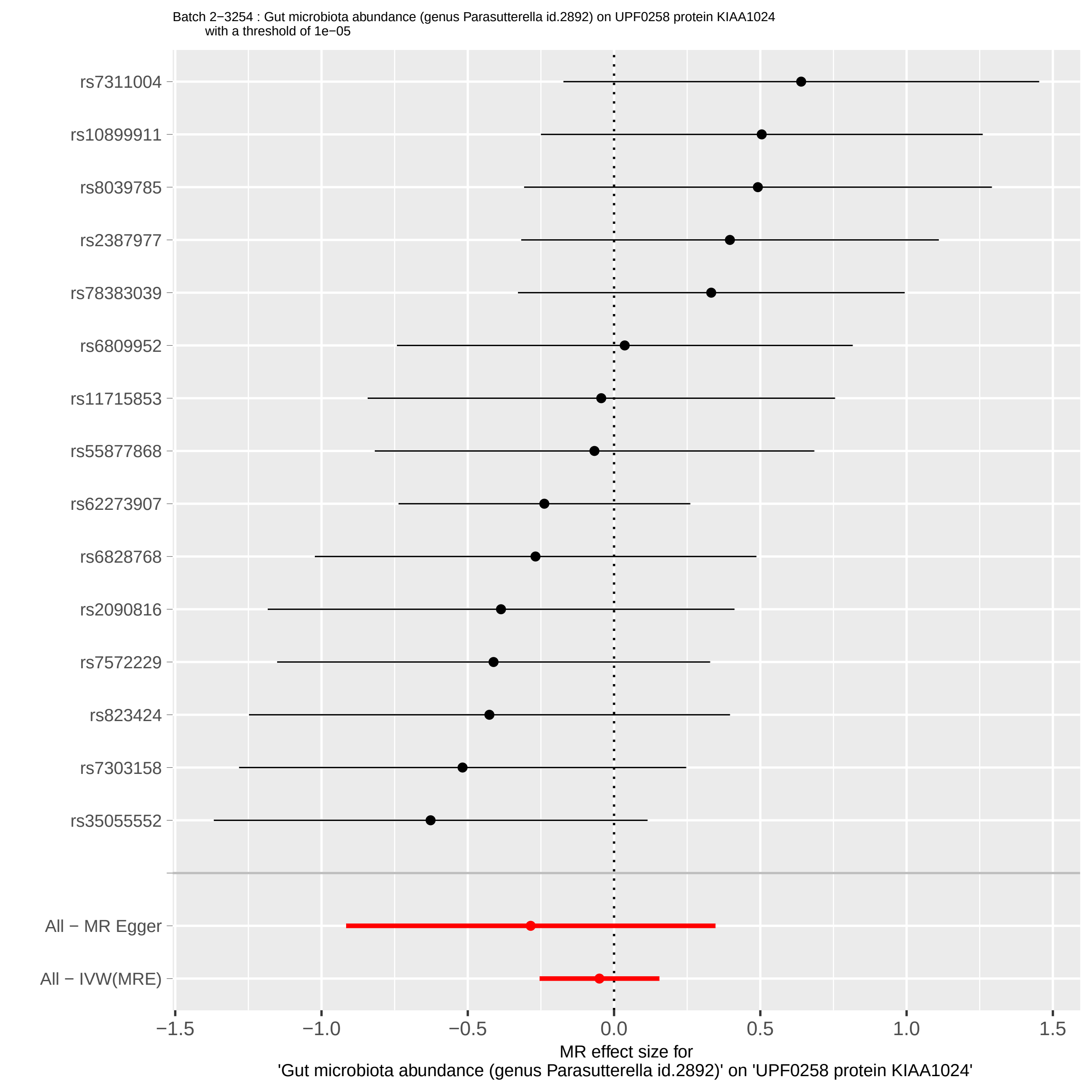
Batch 2−3254 : Gut microbiota abundance (genus Parasutterella id.2892) on UPF0258 protein KIAA1024
with a threshold of 1e−05
rs7311004
rs10899911
rs8039785
rs2387977
rs78383039
rs6809952
rs11715853
rs55877868
rs62273907
rs6828768
rs2090816
rs7572229
rs823424
rs7303158
rs35055552
All − MR Egger
All − IVW(MRE)
−1.5
−1.0
−0.5
0.0
0.5
1.0
1.5
MR effect size for
'Gut microbiota abundance (genus Parasutterella id.2892)' on 'UPF0258 protein KIAA1024'

Batch 2−3255 : Gut microbiota abundance (genus Catenibacterium id.2153) on Uncharacterized family 31 glucosidase KIAA1161
with a threshold of 1e−05

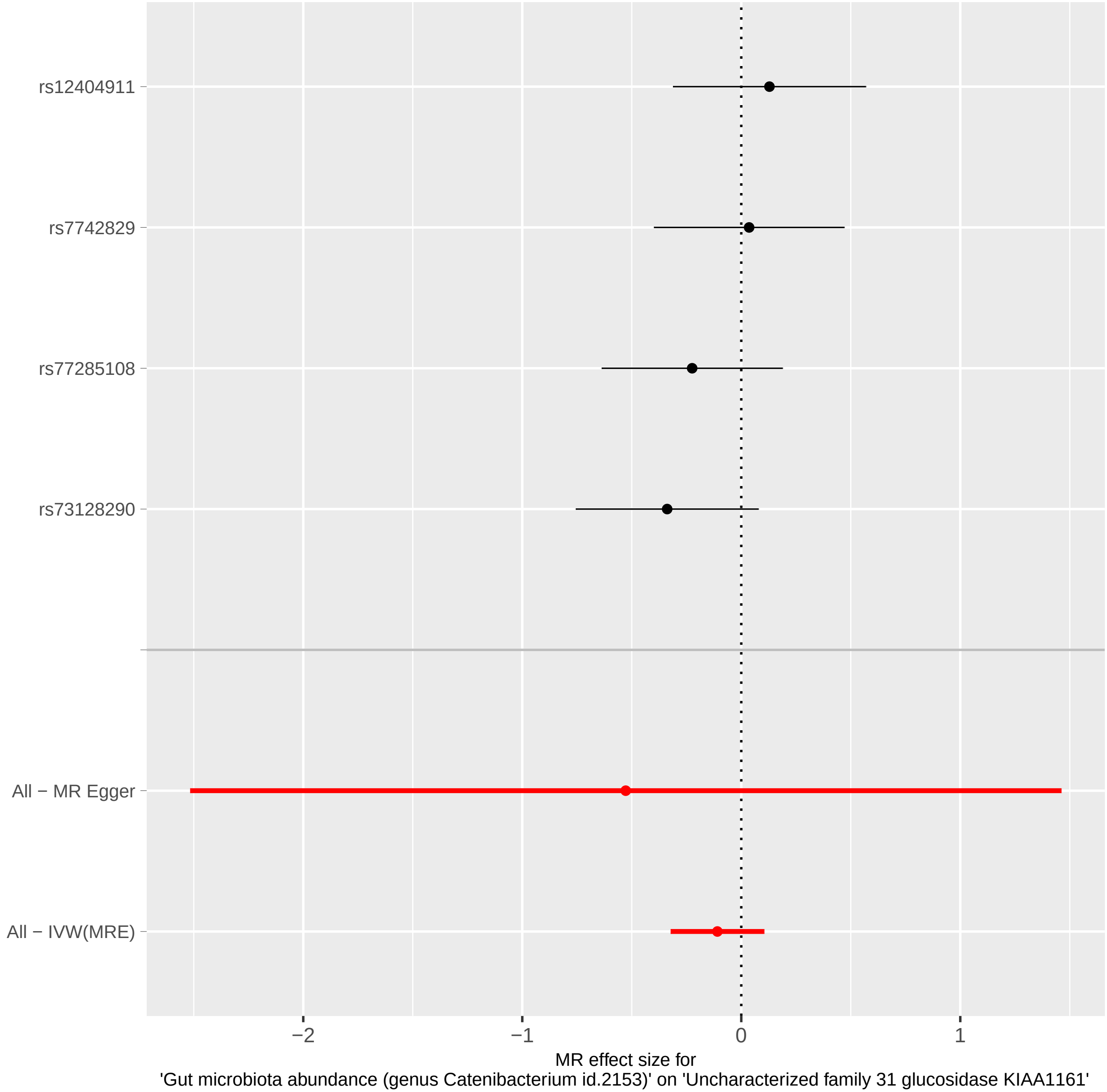

Batch 2−3256 : Gut microbiota abundance (genus Parasutterella id.2892) on Uncharacterized family 31 glucosidase KIAA1161
with a threshold of 1e−05

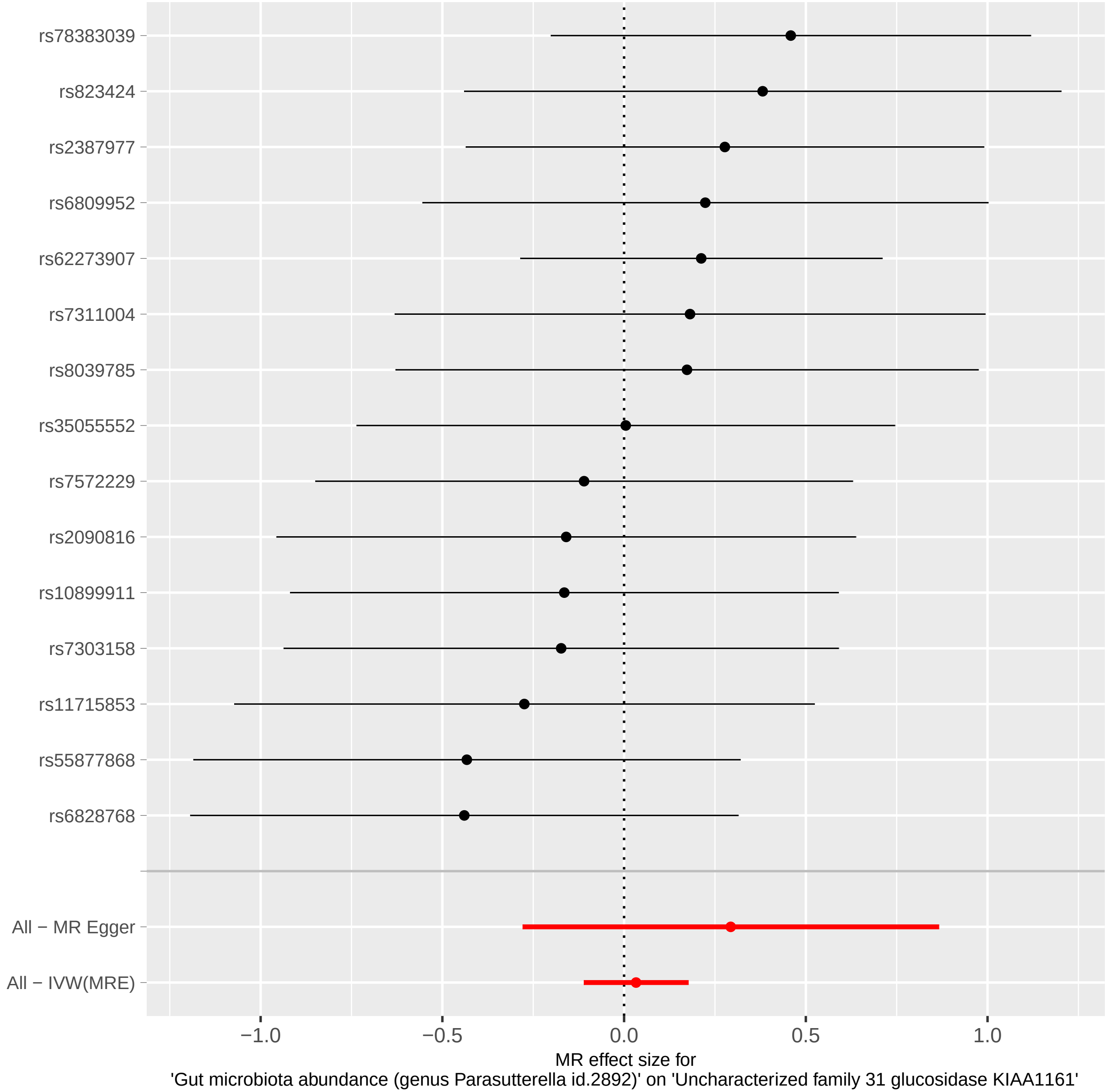

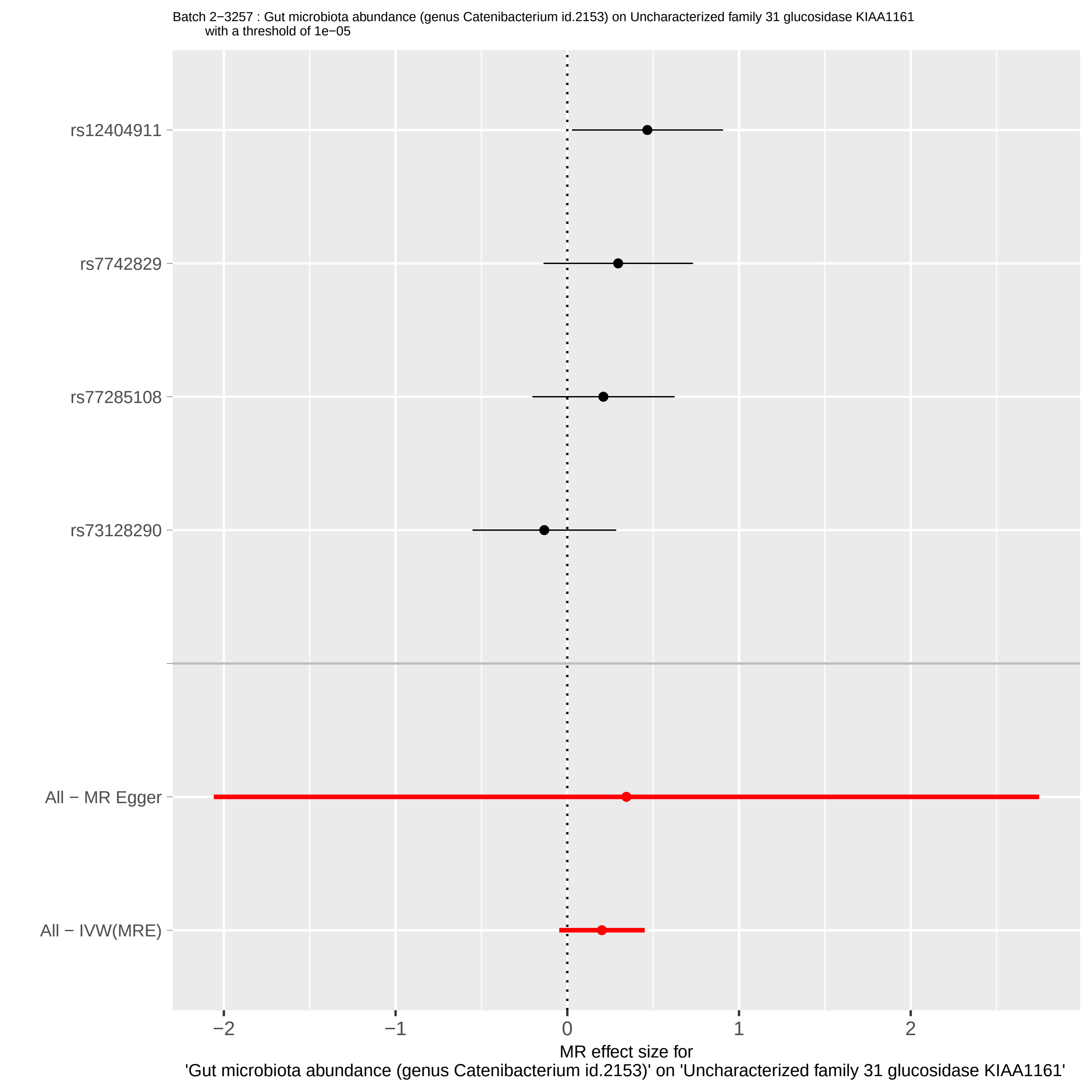

Batch 2−3257 : Gut microbiota abundance (genus Catenibacterium id.2153) on Uncharacterized family 31 glucosidase KIAA1161
with a threshold of 1e−05
rs12404911
rs7742829
rs77285108
rs73128290
All − MR Egger
All − IVW(MRE)
−2
−1
0
1
2
MR effect size for
'Gut microbiota abundance (genus Catenibacterium id.2153)' on 'Uncharacterized family 31 glucosidase KIAA1161'

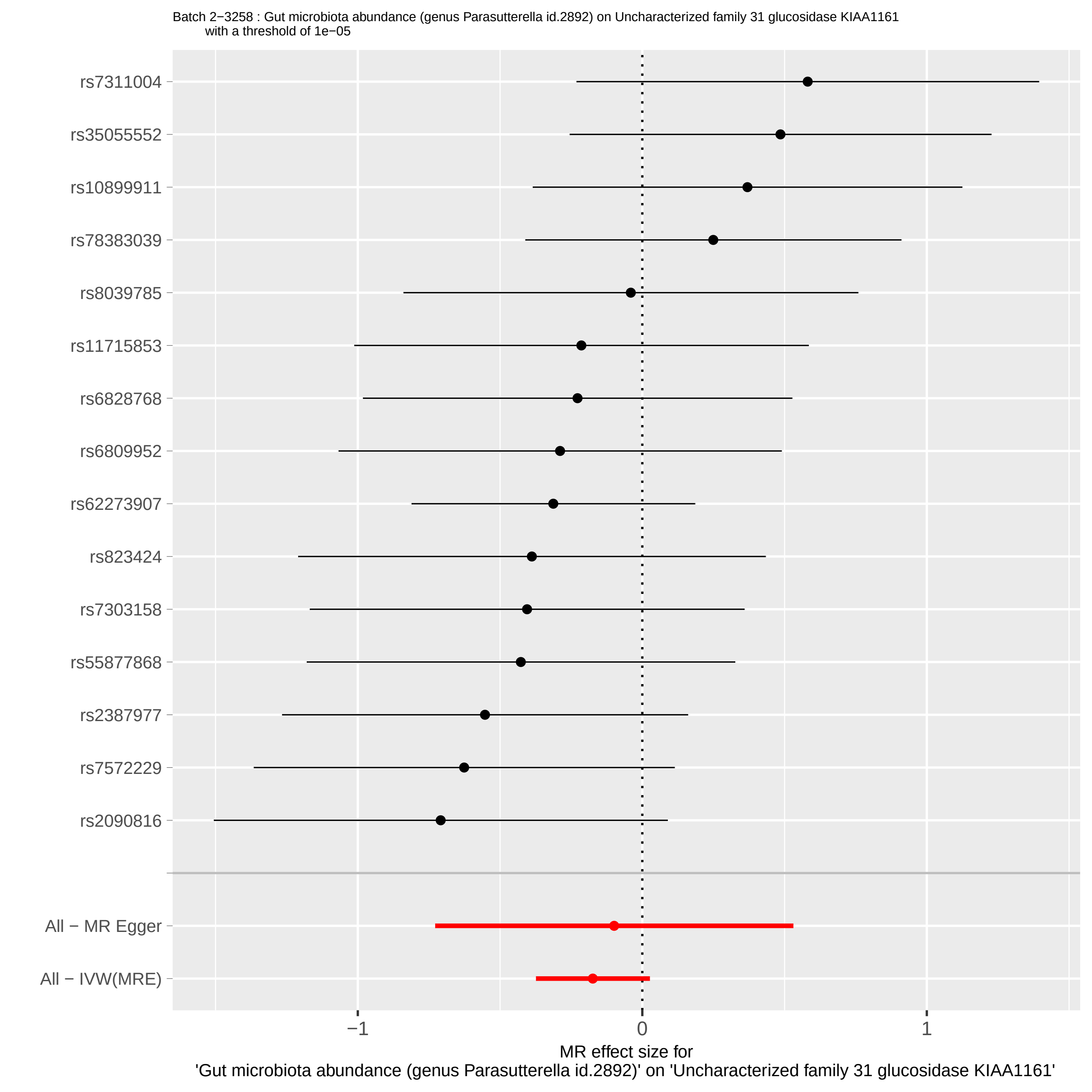

Batch 2−3258 : Gut microbiota abundance (genus Parasutterella id.2892) on Uncharacterized family 31 glucosidase KIAA1161
with a threshold of 1e−05
rs7311004
rs35055552
rs10899911
rs78383039
rs8039785
rs11715853
rs6828768
rs6809952
rs62273907
rs823424
rs7303158
rs55877868
rs2387977
rs7572229
rs2090816
All − MR Egger
All − IVW(MRE)
−1
0
1
MR effect size for
'Gut microbiota abundance (genus Parasutterella id.2892)' on 'Uncharacterized family 31 glucosidase KIAA1161'

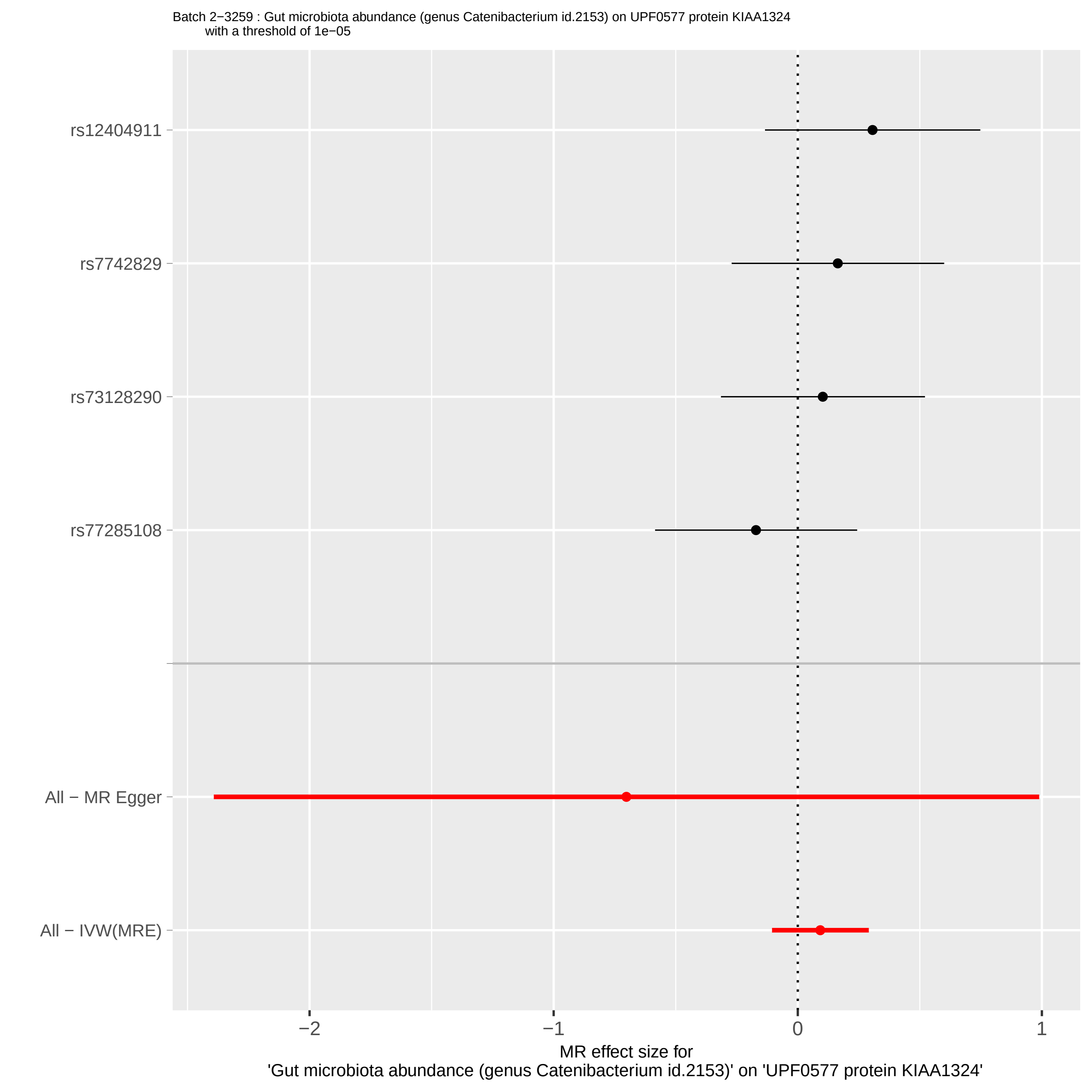
Batch 2−3259 : Gut microbiota abundance (genus Catenibacterium id.2153) on UPF0577 protein KIAA1324
with a threshold of 1e−05
rs12404911
rs7742829
rs73128290
rs77285108
All − MR Egger
All − IVW(MRE)
−2
−1
0
1
MR effect size for
'Gut microbiota abundance (genus Catenibacterium id.2153)' on 'UPF0577 protein KIAA1324'

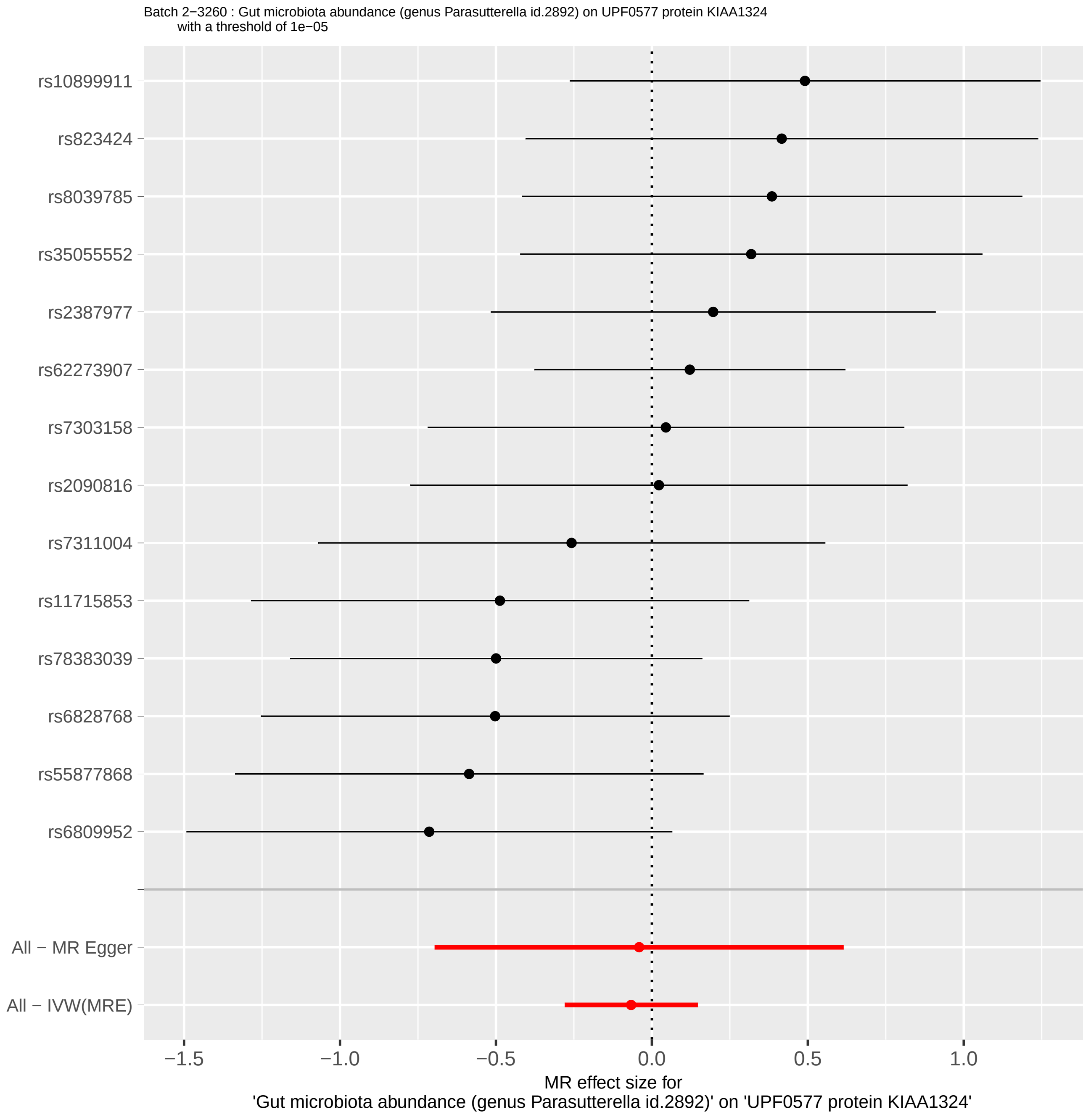

Batch 2−3260 : Gut microbiota abundance (genus Parasutterella id.2892) on UPF0577 protein KIAA1324
with a threshold of 1e−05
rs10899911
rs823424
rs8039785
rs35055552
rs2387977
rs62273907
rs7303158
rs2090816
rs7311004
rs11715853
rs78383039
rs6828768
rs55877868
rs6809952
All − MR Egger
All − IVW(MRE)
−1.5
−1.0
−0.5
0.0
0.5
1.0
MR effect size for
'Gut microbiota abundance (genus Parasutterella id.2892)' on 'UPF0577 protein KIAA1324'

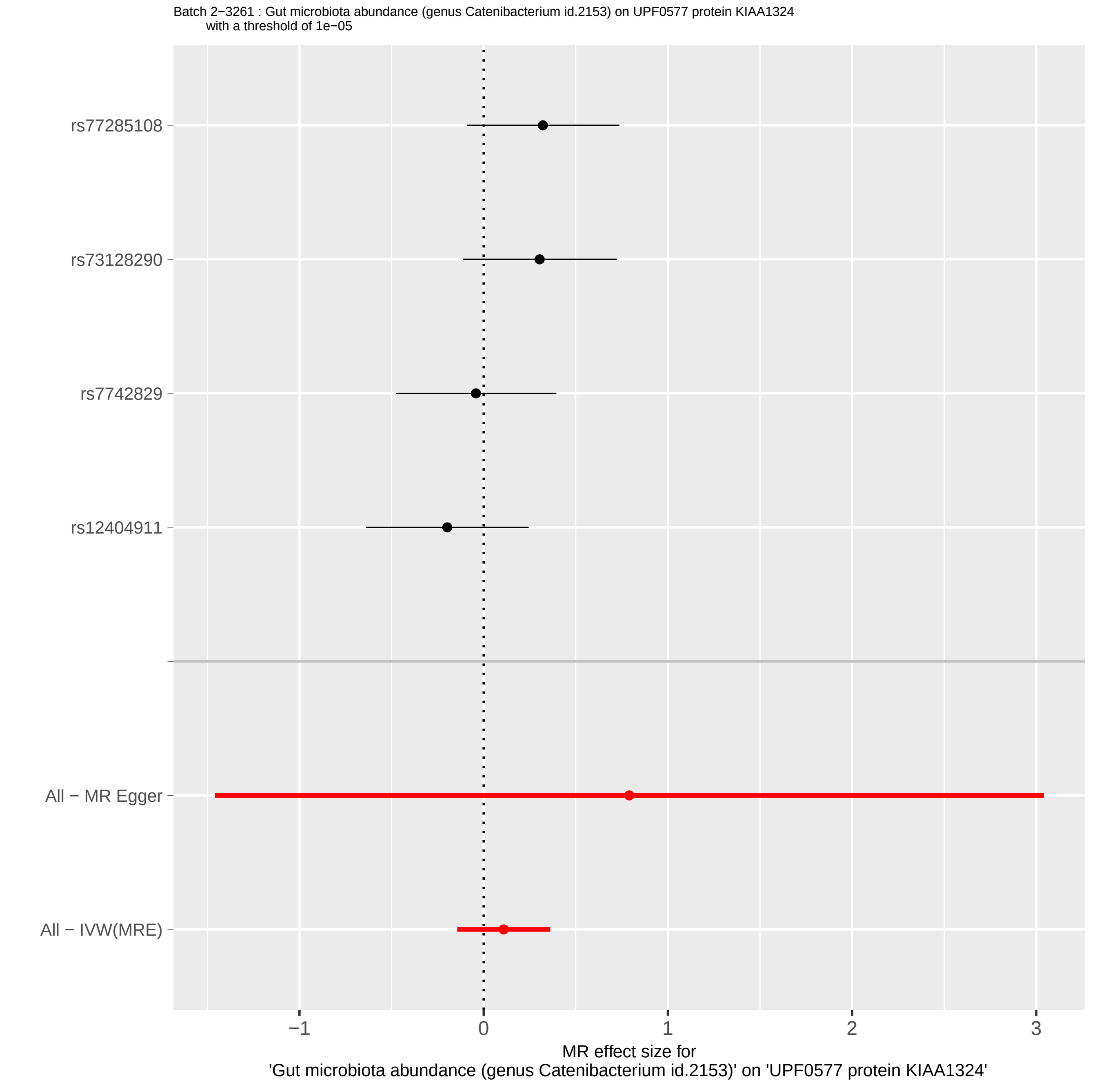
Batch 2−3261 : Gut microbiota abundance (genus Catenibacterium id.2153) on UPF0577 protein KIAA1324
with a threshold of 1e−05
rs77285108
rs73128290
rs7742829
rs12404911
All − MR Egger
All − IVW(MRE)
−1
0
1
2
3
MR effect size for
'Gut microbiota abundance (genus Catenibacterium id.2153)' on 'UPF0577 protein KIAA1324'

Batch 2−3262 : Gut microbiota abundance (genus Parasutterella id.2892) on UPF0577 protein KIAA1324
with a threshold of 1e−05

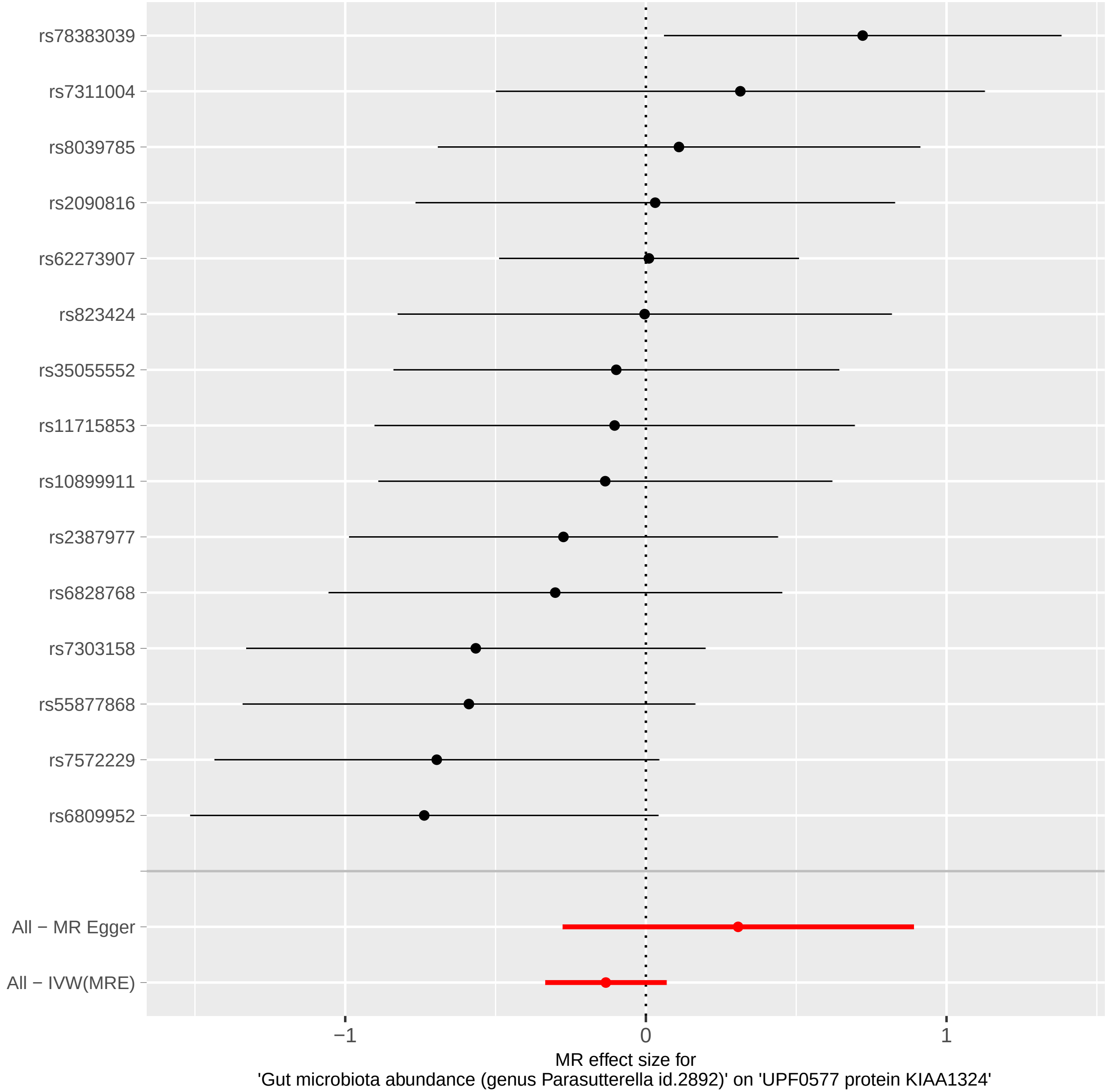

Batch 2−3263 : Gut microbiota abundance (genus Catenibacterium id.2153) on UPF0577 protein KIAA1324−like
with a threshold of 1e−05

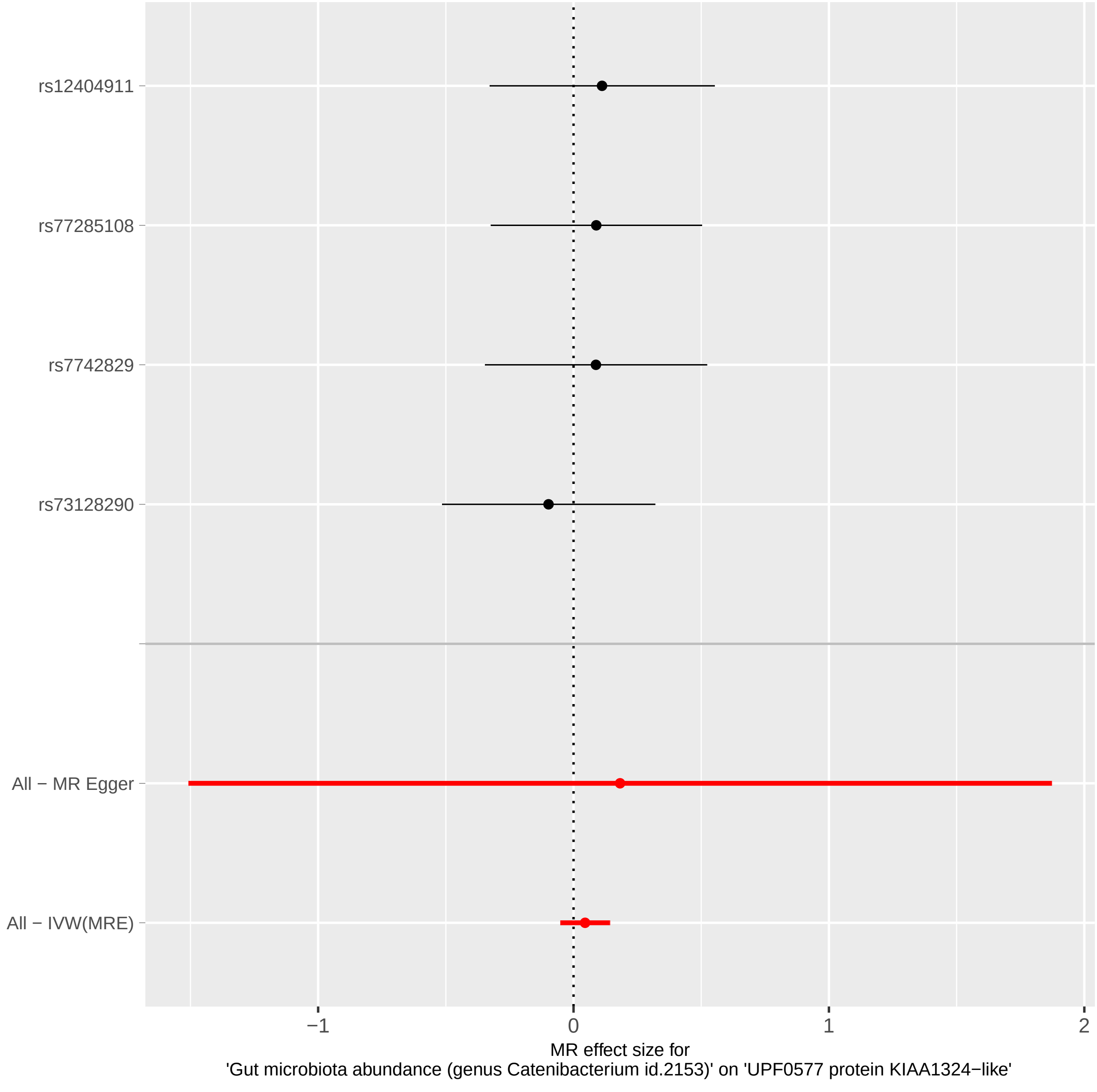

Batch 2−3264 : Gut microbiota abundance (genus Parasutterella id.2892) on UPF0577 protein KIAA1324−like
with a threshold of 1e−05

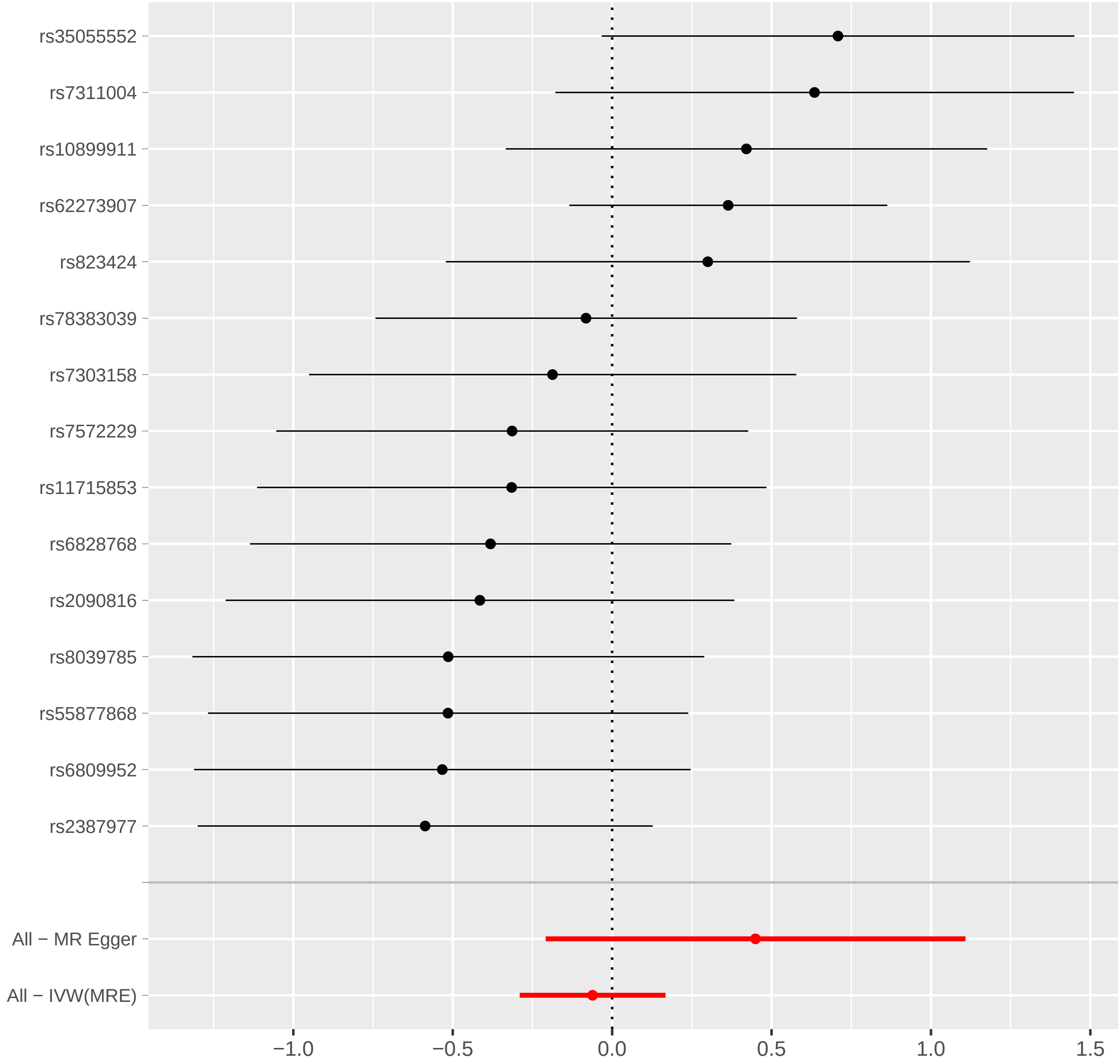

MR effect size for
'Gut microbiota abundance (genus Parasutterella id.2892)' on 'UPF0577 protein KIAA1324−like'

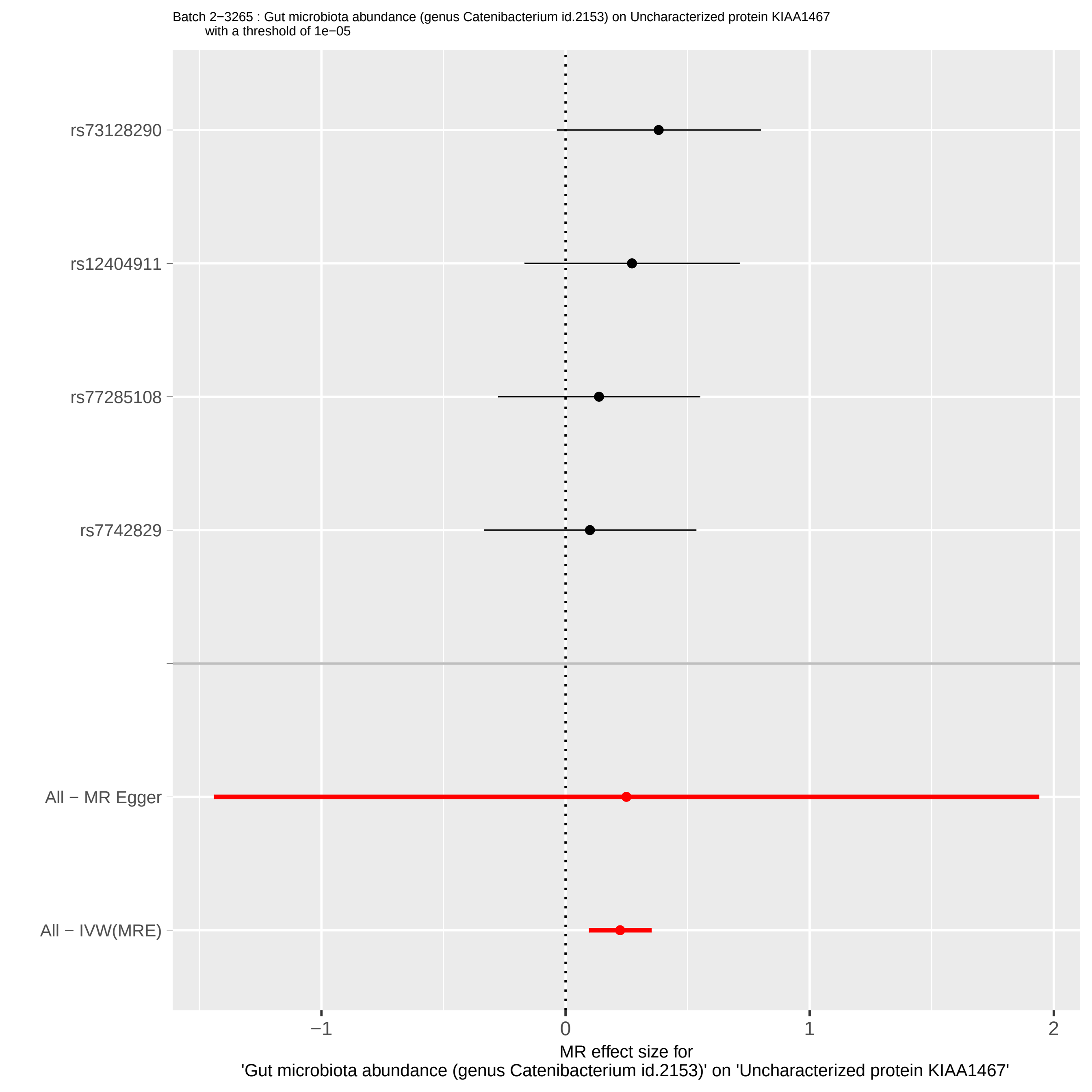
Batch 2−3265 : Gut microbiota abundance (genus Catenibacterium id.2153) on Uncharacterized protein KIAA1467
with a threshold of 1e−05
rs73128290
rs12404911
rs77285108
rs7742829
All − MR Egger
All − IVW(MRE)
−1
0
1
2
MR effect size for
'Gut microbiota abundance (genus Catenibacterium id.2153)' on 'Uncharacterized protein KIAA1467'

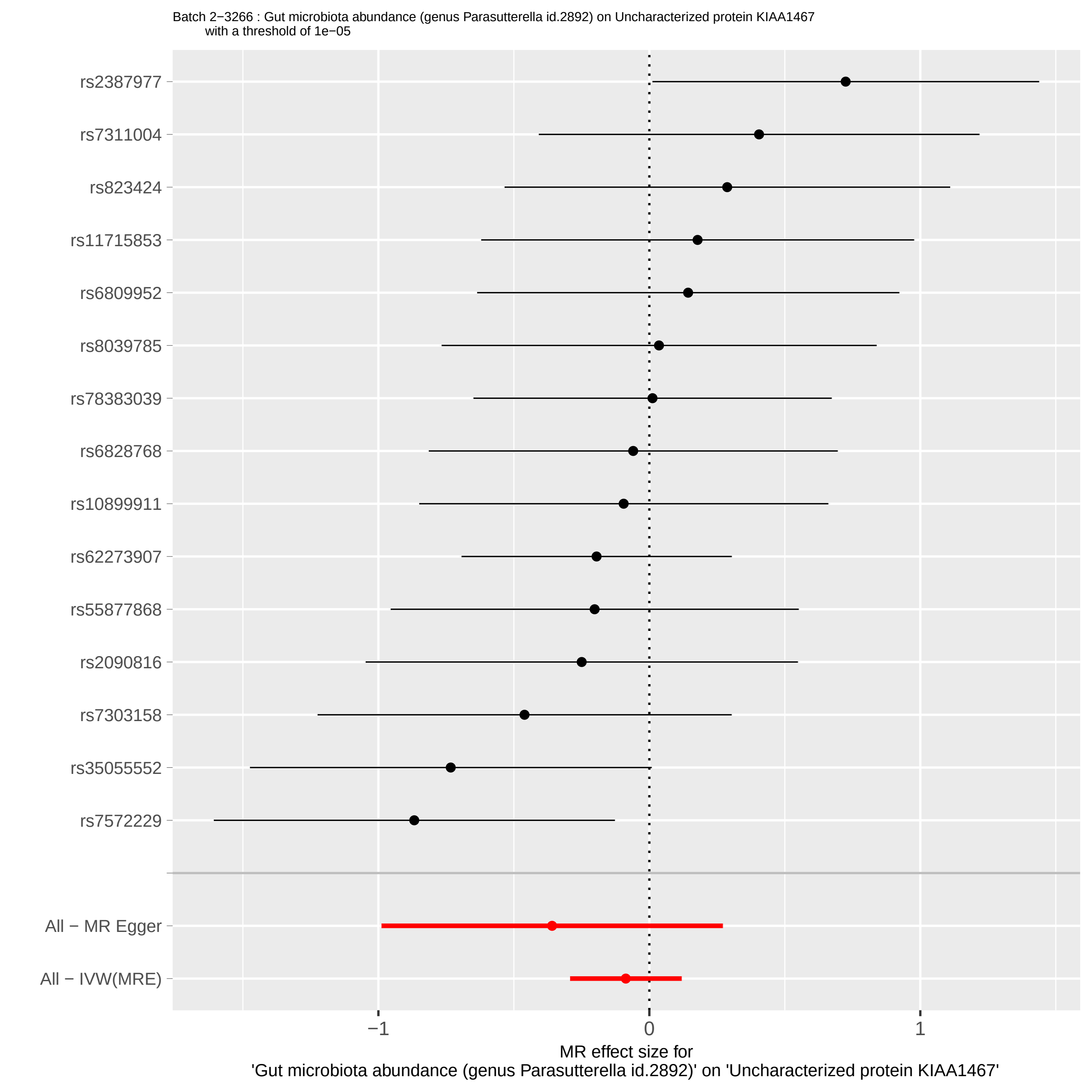

Batch 2−3266 : Gut microbiota abundance (genus Parasutterella id.2892) on Uncharacterized protein KIAA1467
with a threshold of 1e−05
rs2387977
rs7311004
rs823424
rs11715853
rs6809952
rs8039785
rs78383039
rs6828768
rs10899911
rs62273907
rs55877868
rs2090816
rs7303158
rs35055552
rs7572229
All − MR Egger
All − IVW(MRE)
−1
0
1
MR effect size for
'Gut microbiota abundance (genus Parasutterella id.2892)' on 'Uncharacterized protein KIAA1467'

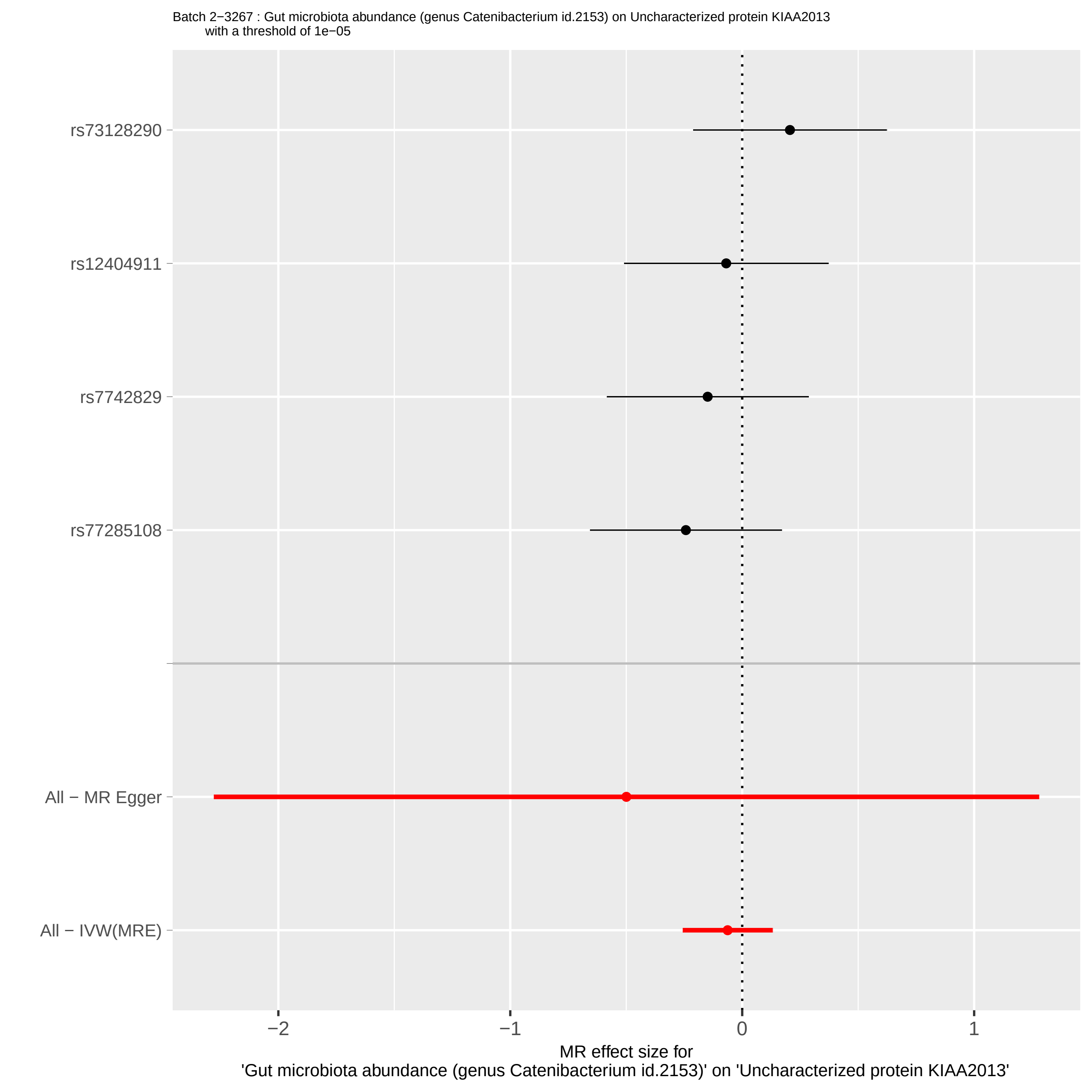

Batch 2−3267 : Gut microbiota abundance (genus Catenibacterium id.2153) on Uncharacterized protein KIAA2013
with a threshold of 1e−05
rs73128290
rs12404911
rs7742829
rs77285108
All − MR Egger
All − IVW(MRE)
−2
−1
0
1
MR effect size for
'Gut microbiota abundance (genus Catenibacterium id.2153)' on 'Uncharacterized protein KIAA2013'

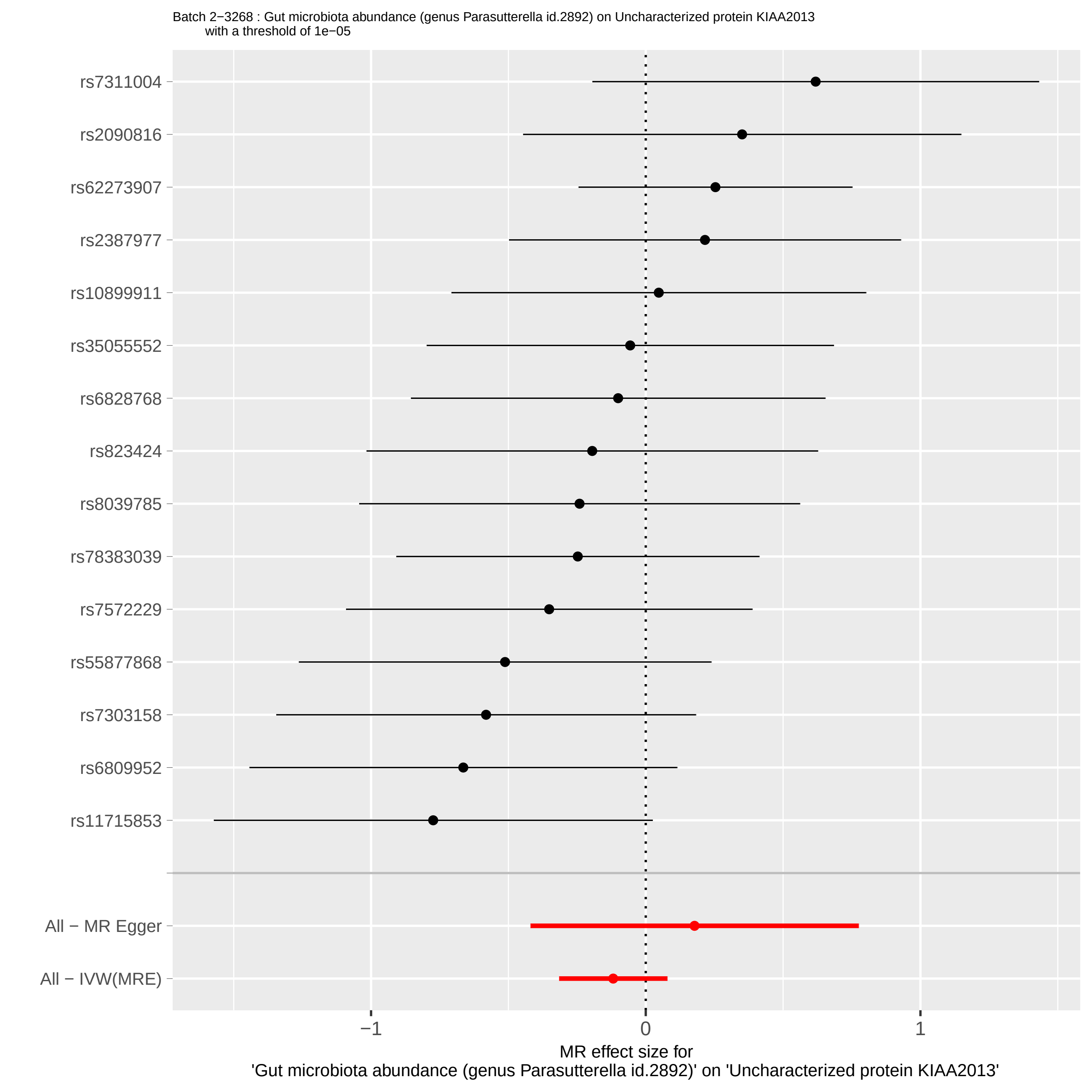
Batch 2−3268 : Gut microbiota abundance (genus Parasutterella id.2892) on Uncharacterized protein KIAA2013
with a threshold of 1e−05
rs7311004
rs2090816
rs62273907
rs2387977
rs10899911
rs35055552
rs6828768
rs823424
rs8039785
rs78383039
rs7572229
rs55877868
rs7303158
rs6809952
rs11715853
All − MR Egger
All − IVW(MRE)
−1
0
1
MR effect size for
'Gut microbiota abundance (genus Parasutterella id.2892)' on 'Uncharacterized protein KIAA2013'

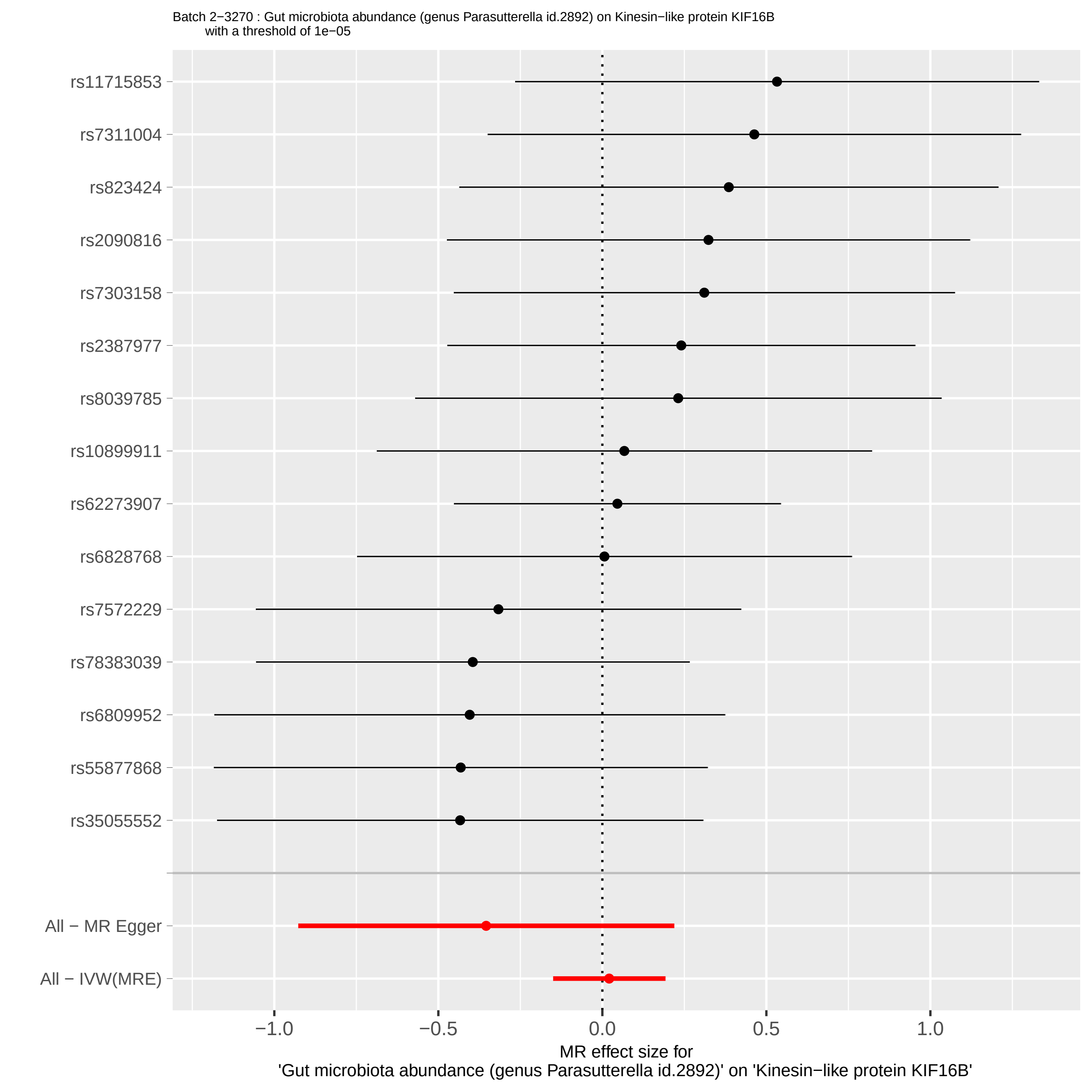

Batch 2−3270 : Gut microbiota abundance (genus Parasutterella id.2892) on Kinesin−like protein KIF16B
with a threshold of 1e−05
rs11715853
rs7311004
rs823424
rs2090816
rs7303158
rs2387977
rs8039785
rs10899911
rs62273907
rs6828768
rs7572229
rs78383039
rs6809952
rs55877868
rs35055552
All − MR Egger
All − IVW(MRE)
−1.0
−0.5
0.0
0.5
1.0
MR effect size for
'Gut microbiota abundance (genus Parasutterella id.2892)' on 'Kinesin−like protein KIF16B'

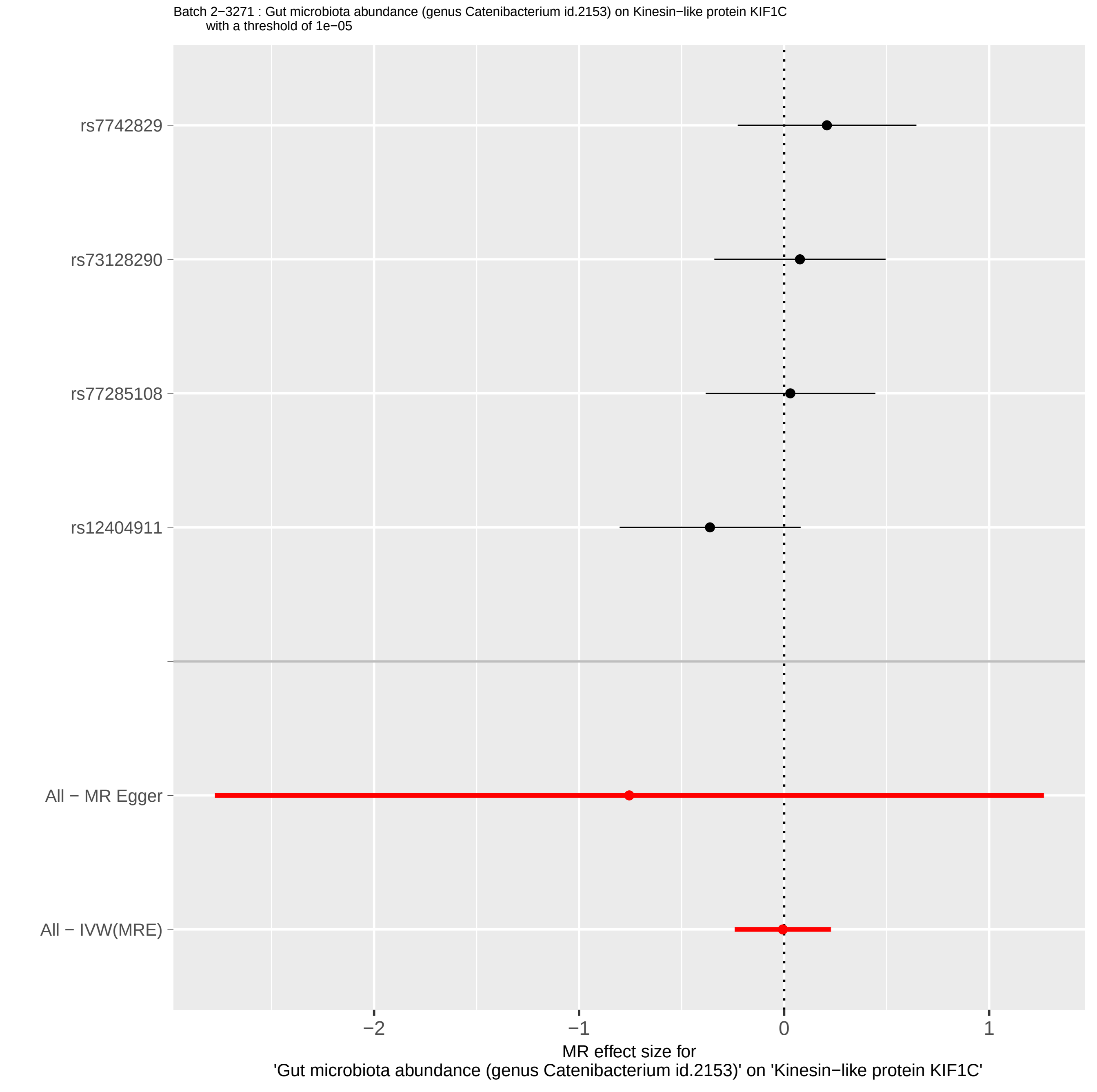

Batch 2−3271 : Gut microbiota abundance (genus Catenibacterium id.2153) on Kinesin−like protein KIF1C
with a threshold of 1e−05
rs7742829
rs73128290
rs77285108
rs12404911
All − MR Egger
All − IVW(MRE)
−2
−1
0
1
MR effect size for
'Gut microbiota abundance (genus Catenibacterium id.2153)' on 'Kinesin−like protein KIF1C'

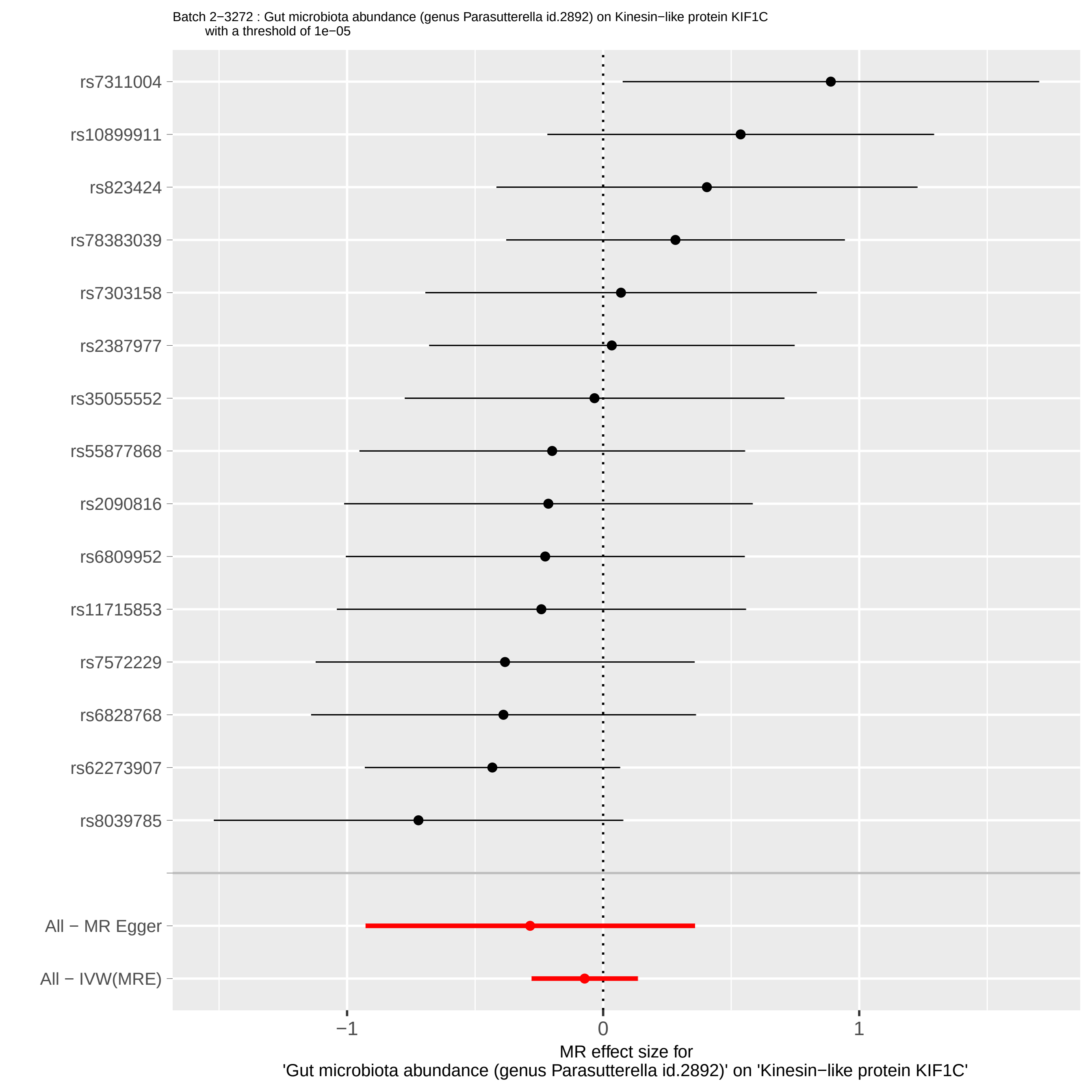

Batch 2−3272 : Gut microbiota abundance (genus Parasutterella id.2892) on Kinesin−like protein KIF1C
with a threshold of 1e−05
rs7311004
rs10899911
rs823424
rs78383039
rs7303158
rs2387977
rs35055552
rs55877868
rs2090816
rs6809952
rs11715853
rs7572229
rs6828768
rs62273907
rs8039785
All − MR Egger
All − IVW(MRE)
−1
0
1
MR effect size for
'Gut microbiota abundance (genus Parasutterella id.2892)' on 'Kinesin−like protein KIF1C'

Batch 2−3273 : Gut microbiota abundance (genus Catenibacterium id.2153) on Kinesin−like protein KIF23
with a threshold of 1e−05

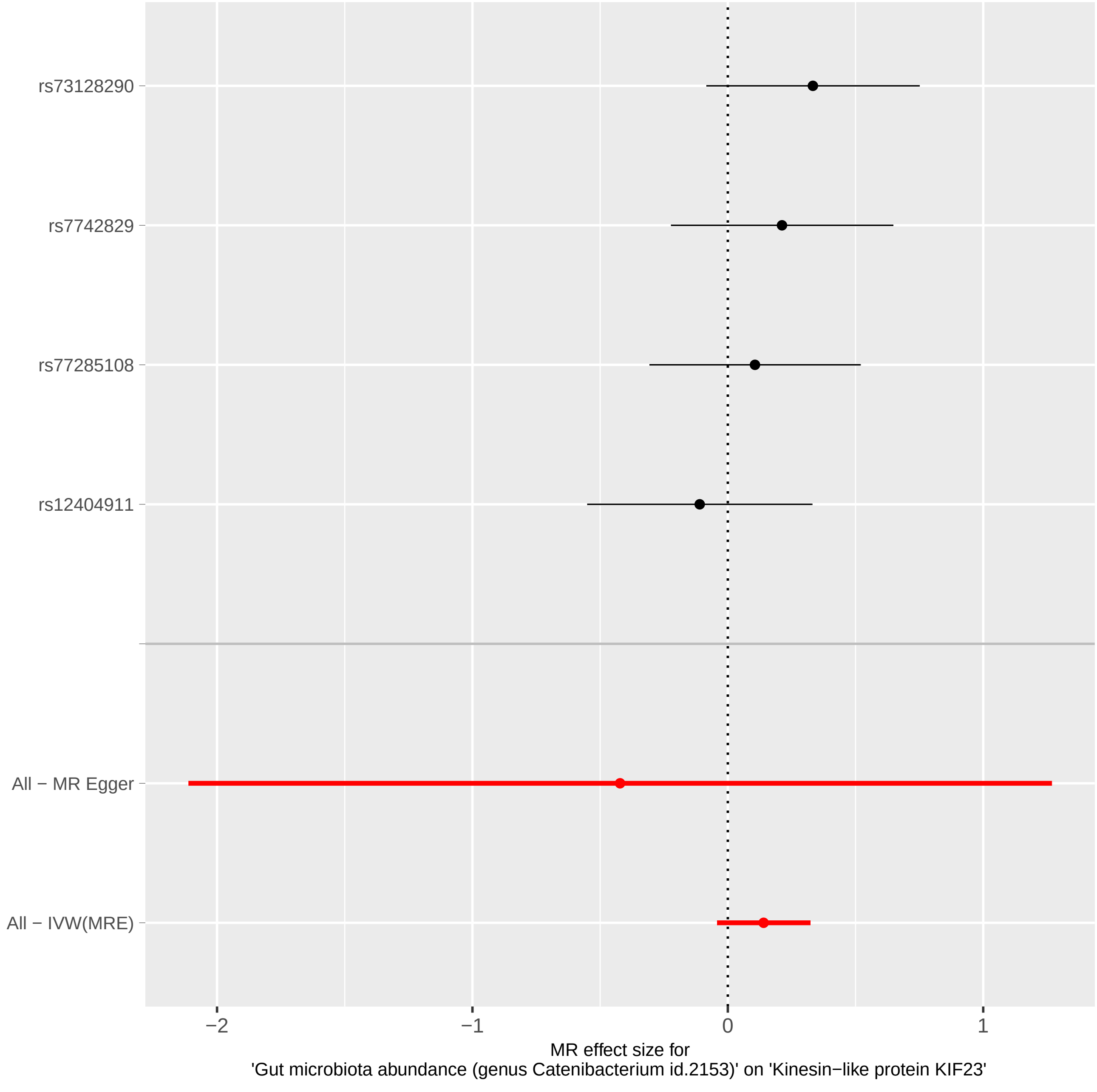

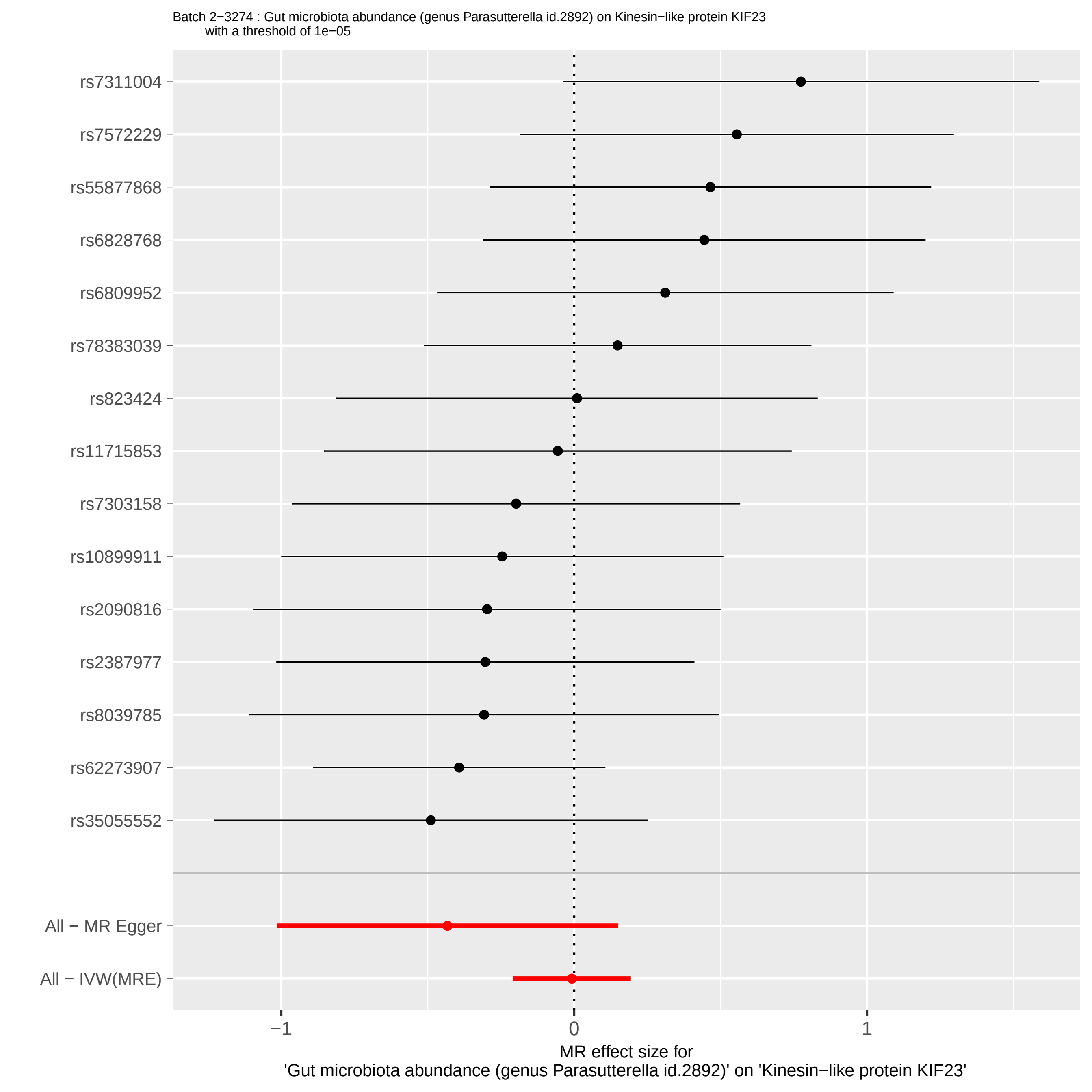

Batch 2−3274 : Gut microbiota abundance (genus Parasutterella id.2892) on Kinesin−like protein KIF23
with a threshold of 1e−05
rs7311004
rs7572229
rs55877868
rs6828768
rs6809952
rs78383039
rs823424
rs11715853
rs7303158
rs10899911
rs2090816
rs2387977
rs8039785
rs62273907
rs35055552
All − MR Egger
All − IVW(MRE)
−1
0
1
MR effect size for
'Gut microbiota abundance (genus Parasutterella id.2892)' on 'Kinesin−like protein KIF23'

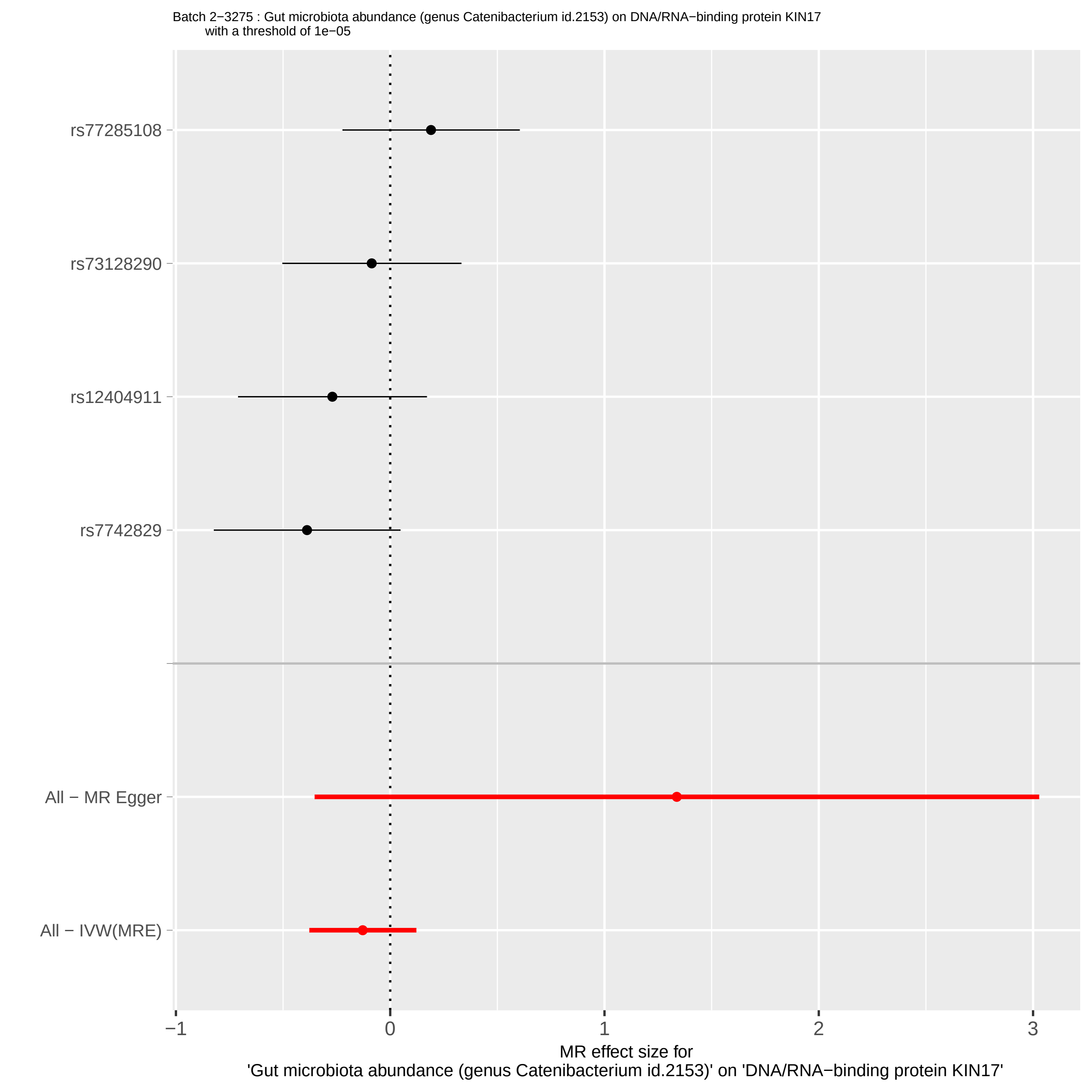

Batch 2−3275 : Gut microbiota abundance (genus Catenibacterium id.2153) on DNA/RNA−binding protein KIN17
with a threshold of 1e−05
rs77285108
rs73128290
rs12404911
rs7742829
All − MR Egger
All − IVW(MRE)
−1
0
1
2
3
MR effect size for
'Gut microbiota abundance (genus Catenibacterium id.2153)' on 'DNA/RNA−binding protein KIN17'

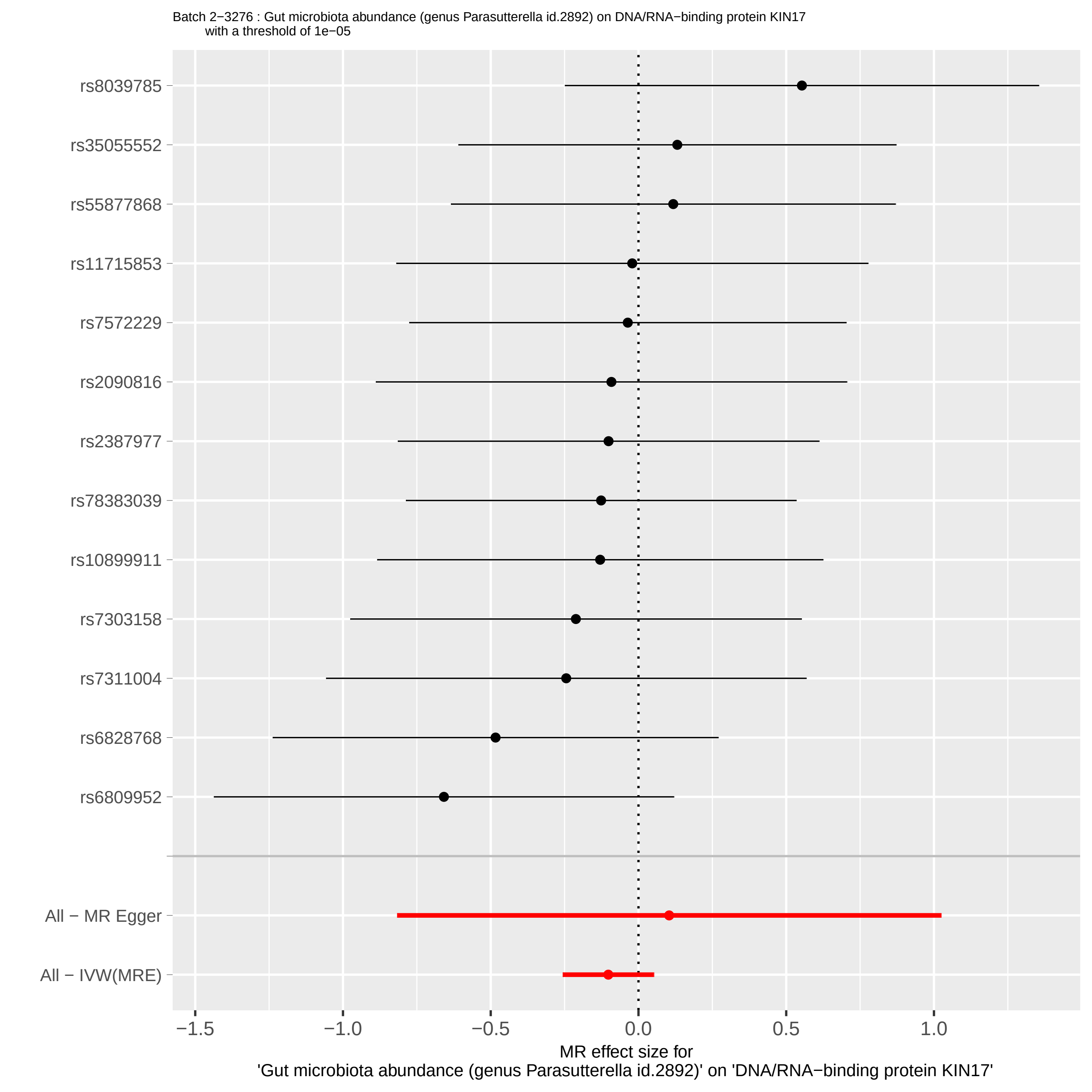

Batch 2−3276 : Gut microbiota abundance (genus Parasutterella id.2892) on DNA/RNA−binding protein KIN17
with a threshold of 1e−05
rs8039785
rs35055552
rs55877868
rs11715853
rs7572229
rs2090816
rs2387977
rs78383039
rs10899911
rs7303158
rs7311004
rs6828768
rs6809952
All − MR Egger
All − IVW(MRE)
−1.5
−1.0
−0.5
0.0
0.5
1.0
MR effect size for
'Gut microbiota abundance (genus Parasutterella id.2892)' on 'DNA/RNA−binding protein KIN17'

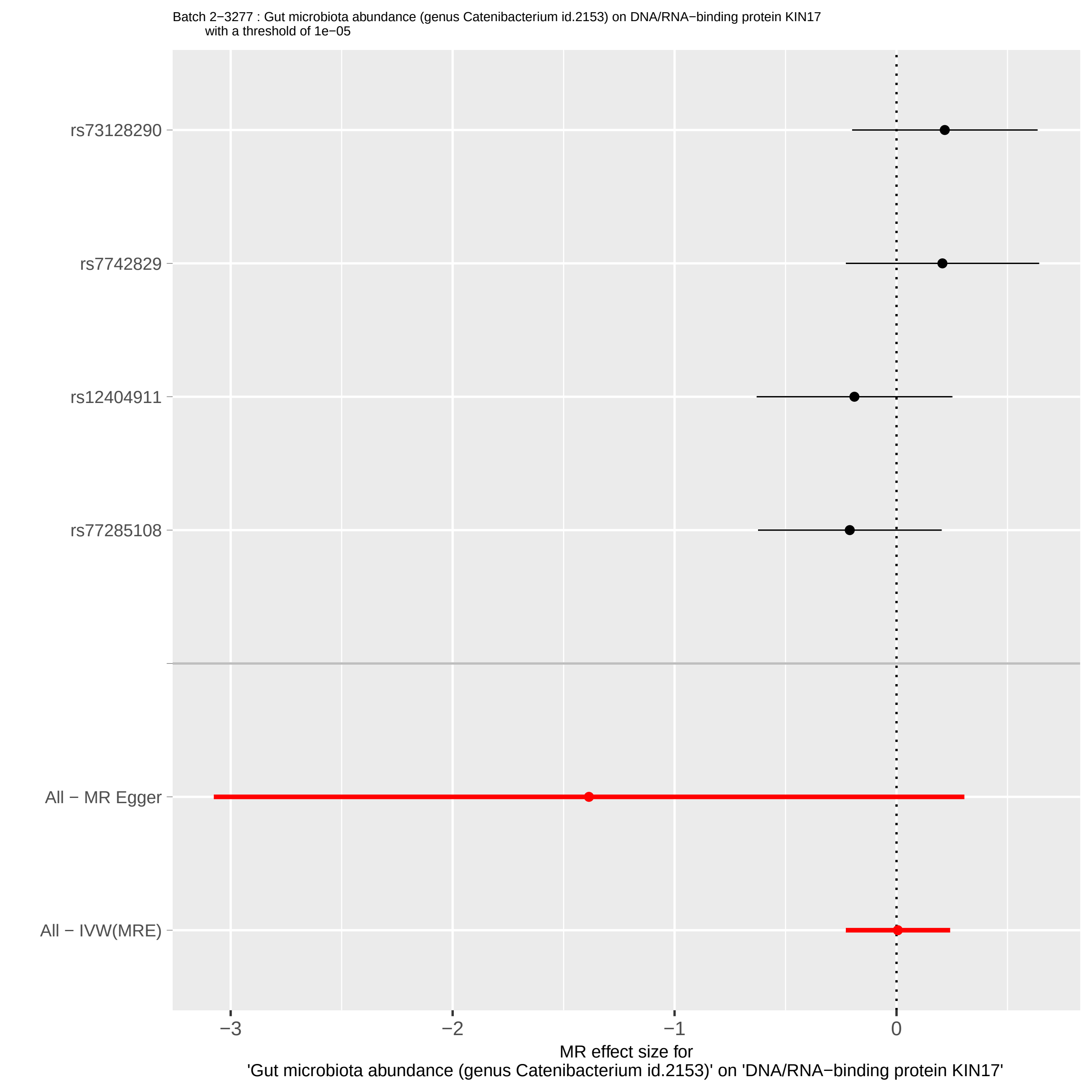

Batch 2−3277 : Gut microbiota abundance (genus Catenibacterium id.2153) on DNA/RNA−binding protein KIN17
with a threshold of 1e−05
rs73128290
rs7742829
rs12404911
rs77285108
All − MR Egger
All − IVW(MRE)
−3
−2
−1
0
MR effect size for
'Gut microbiota abundance (genus Catenibacterium id.2153)' on 'DNA/RNA−binding protein KIN17'

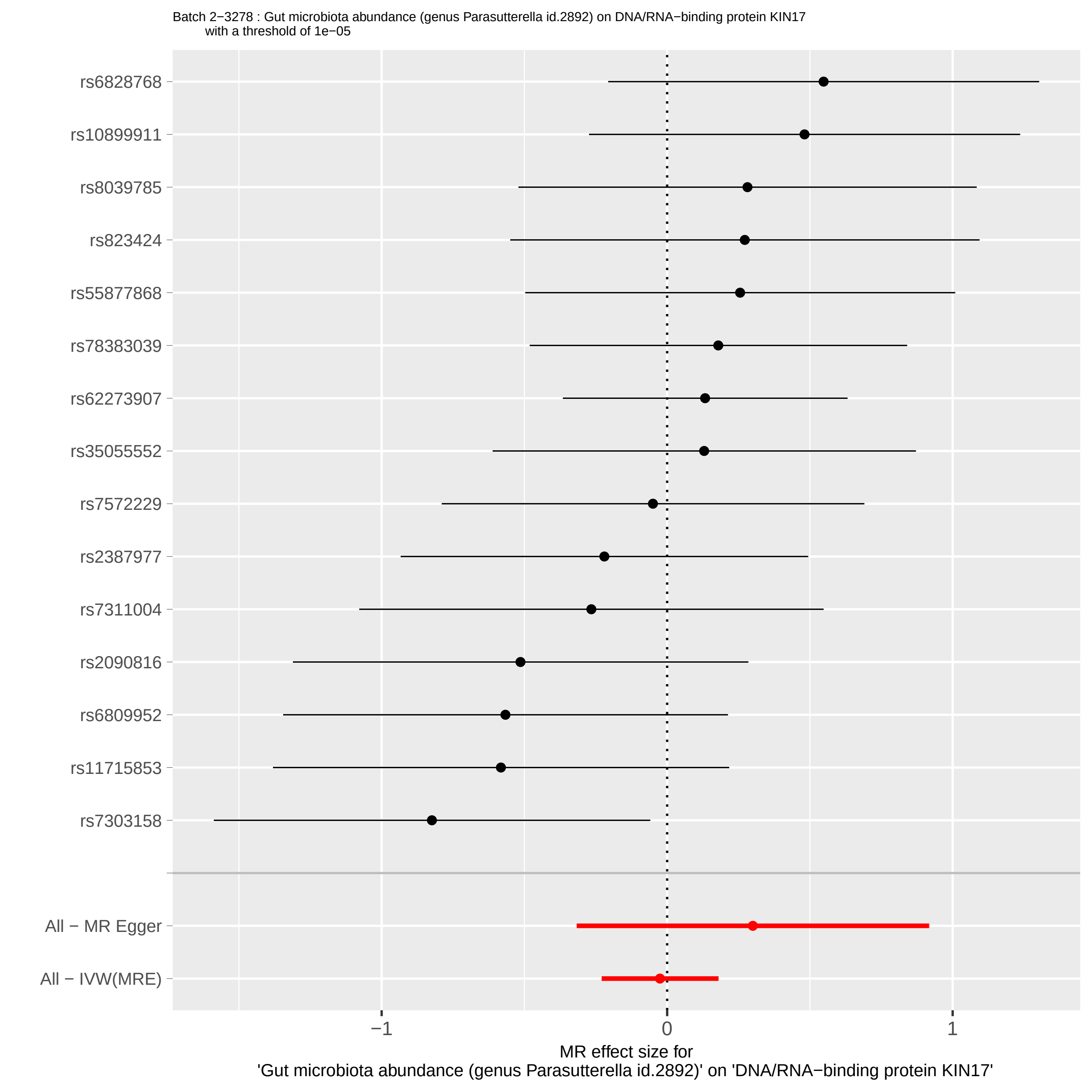

Batch 2−3278 : Gut microbiota abundance (genus Parasutterella id.2892) on DNA/RNA−binding protein KIN17
with a threshold of 1e−05
rs6828768
rs10899911
rs8039785
rs823424
rs55877868
rs78383039
rs62273907
rs35055552
rs7572229
rs2387977
rs7311004
rs2090816
rs6809952
rs11715853
rs7303158
All − MR Egger
All − IVW(MRE)
−1
0
1
MR effect size for
'Gut microbiota abundance (genus Parasutterella id.2892)' on 'DNA/RNA−binding protein KIN17'

Batch 2−3279 : Gut microbiota abundance (genus Catenibacterium id.2153) on Killer cell immunoglobulin−like receptor 2DL2
with a threshold of 1e−05

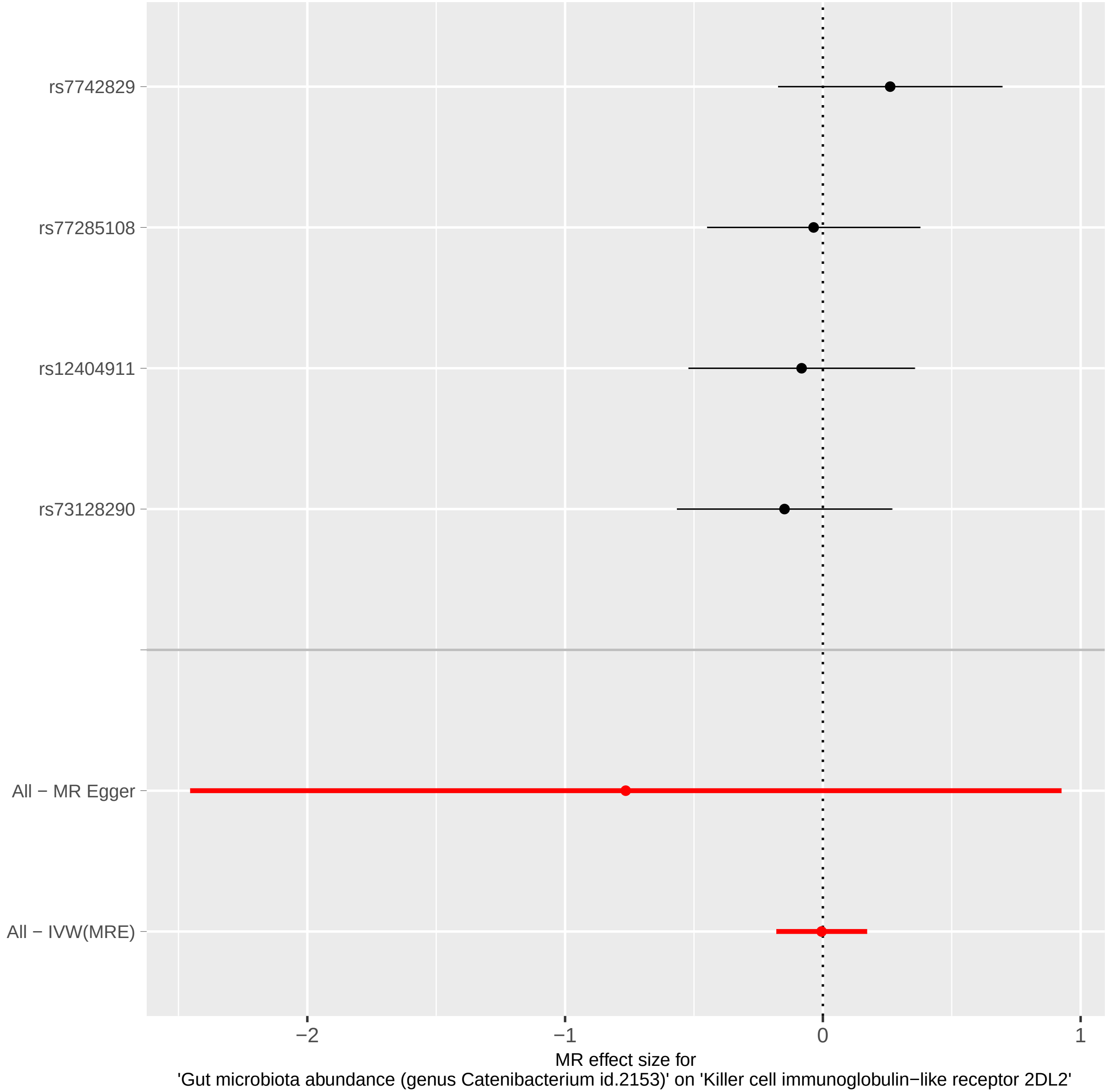

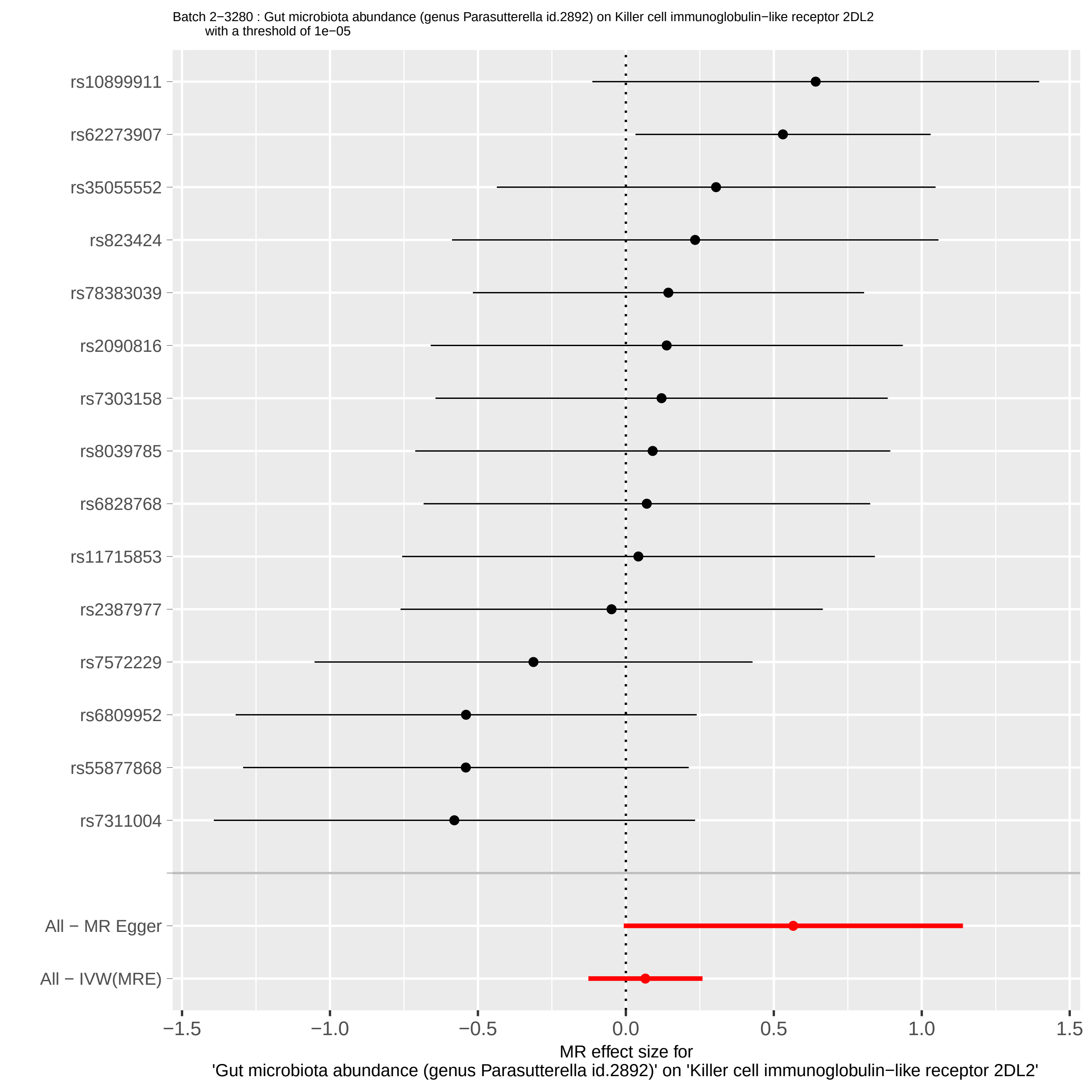

Batch 2−3280 : Gut microbiota abundance (genus Parasutterella id.2892) on Killer cell immunoglobulin−like receptor 2DL2
with a threshold of 1e−05
rs10899911
rs62273907
rs35055552
rs823424
rs78383039
rs2090816
rs7303158
rs8039785
rs6828768
rs11715853
rs2387977
rs7572229
rs6809952
rs55877868
rs7311004
All − MR Egger
All − IVW(MRE)
−1.5
−1.0
−0.5
0.0
0.5
1.0
1.5
MR effect size for
'Gut microbiota abundance (genus Parasutterella id.2892)' on 'Killer cell immunoglobulin−like receptor 2DL2'

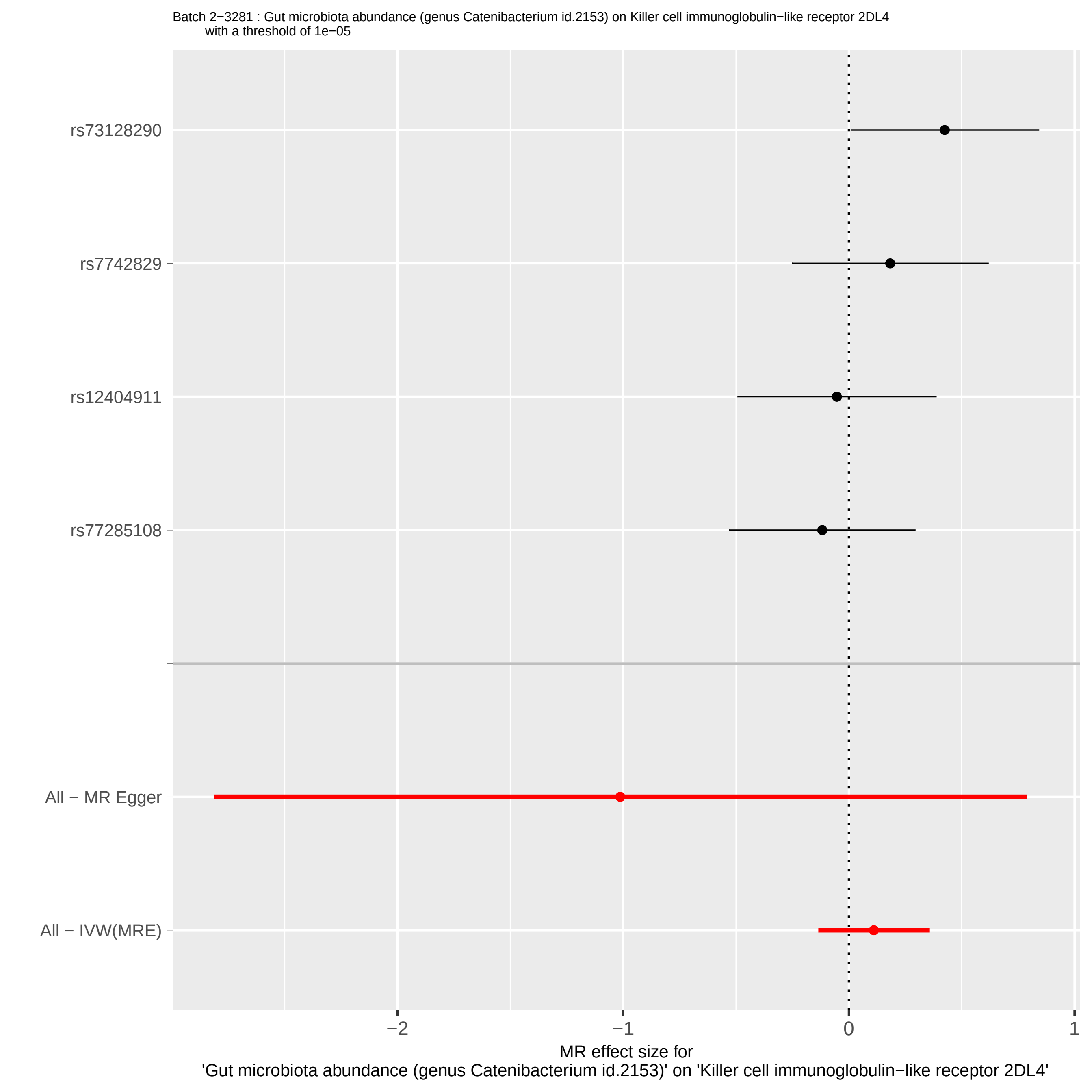

Batch 2−3281 : Gut microbiota abundance (genus Catenibacterium id.2153) on Killer cell immunoglobulin−like receptor 2DL4
with a threshold of 1e−05
rs73128290
rs7742829
rs12404911
rs77285108
All − MR Egger
All − IVW(MRE)
−2
−1
0
1
MR effect size for
'Gut microbiota abundance (genus Catenibacterium id.2153)' on 'Killer cell immunoglobulin−like receptor 2DL4'

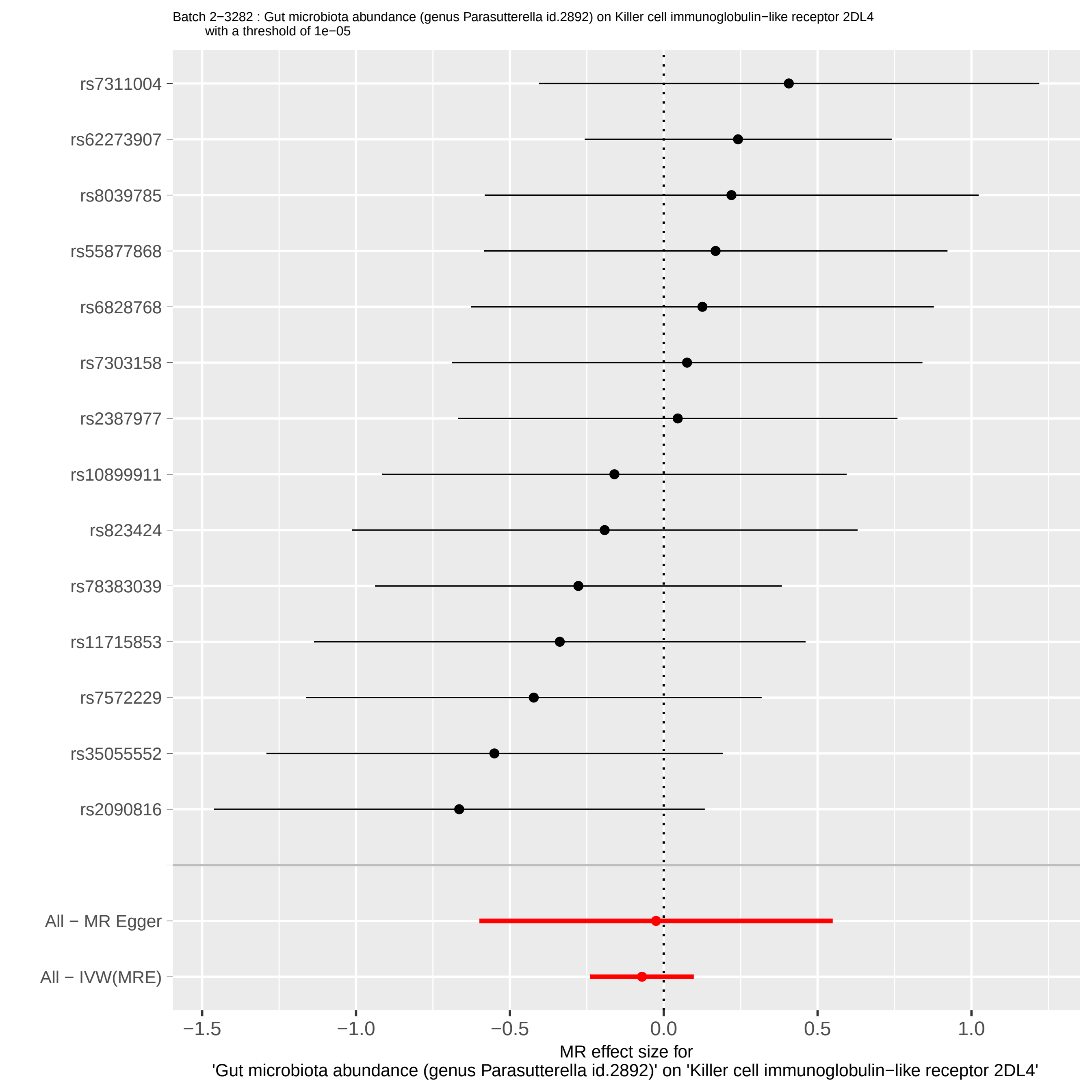
Batch 2−3282 : Gut microbiota abundance (genus Parasutterella id.2892) on Killer cell immunoglobulin−like receptor 2DL4
with a threshold of 1e−05
rs7311004
rs62273907
rs8039785
rs55877868
rs6828768
rs7303158
rs2387977
rs10899911
rs823424
rs78383039
rs11715853
rs7572229
rs35055552
rs2090816
All − MR Egger
All − IVW(MRE)
−1.5
−1.0
−0.5
0.0
0.5
1.0
MR effect size for
'Gut microbiota abundance (genus Parasutterella id.2892)' on 'Killer cell immunoglobulin−like receptor 2DL4'

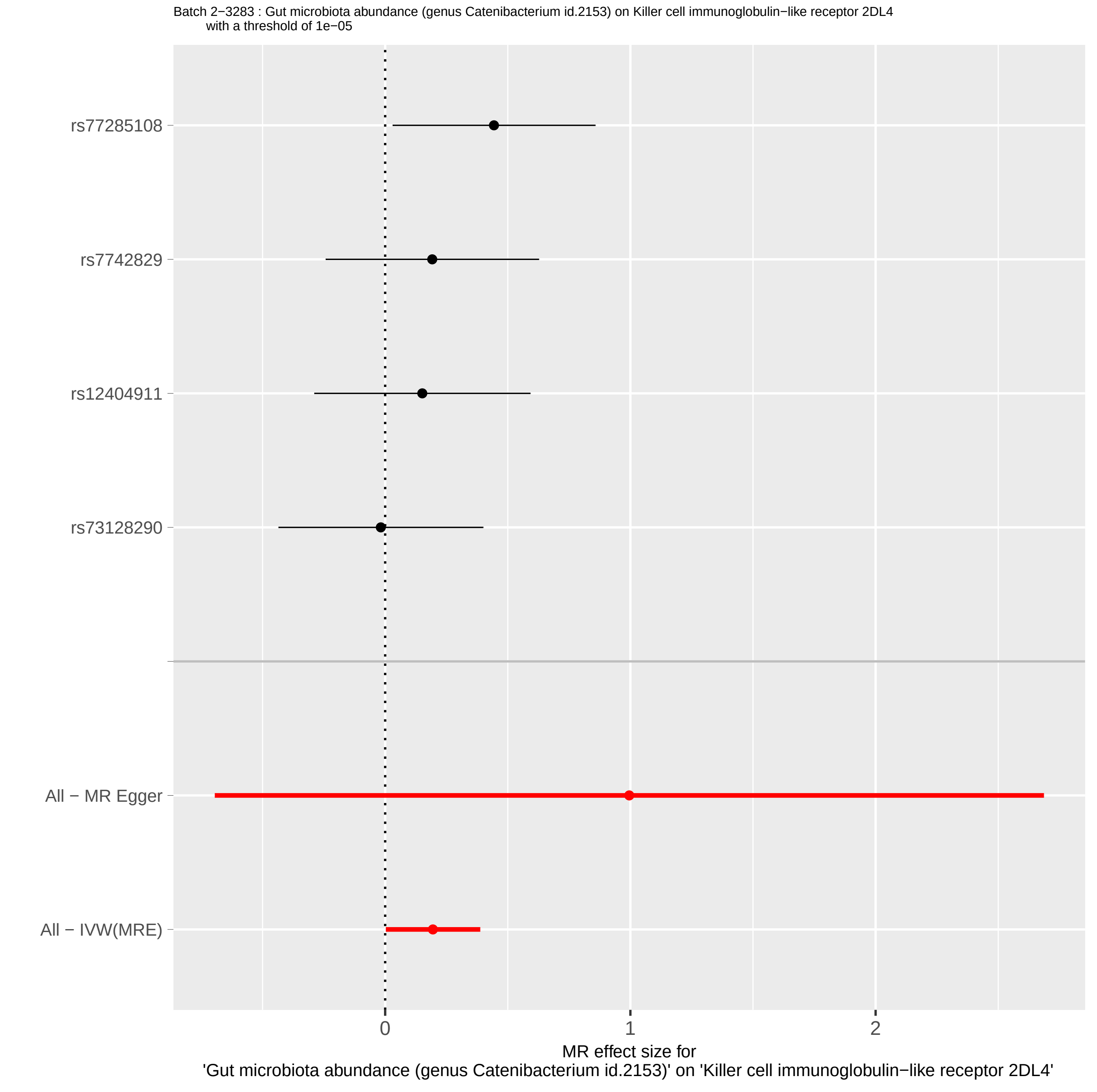
Batch 2−3283 : Gut microbiota abundance (genus Catenibacterium id.2153) on Killer cell immunoglobulin−like receptor 2DL4
with a threshold of 1e−05
rs77285108
rs7742829
rs12404911
rs73128290
All − MR Egger
All − IVW(MRE)
0
1
2
MR effect size for
'Gut microbiota abundance (genus Catenibacterium id.2153)' on 'Killer cell immunoglobulin−like receptor 2DL4'

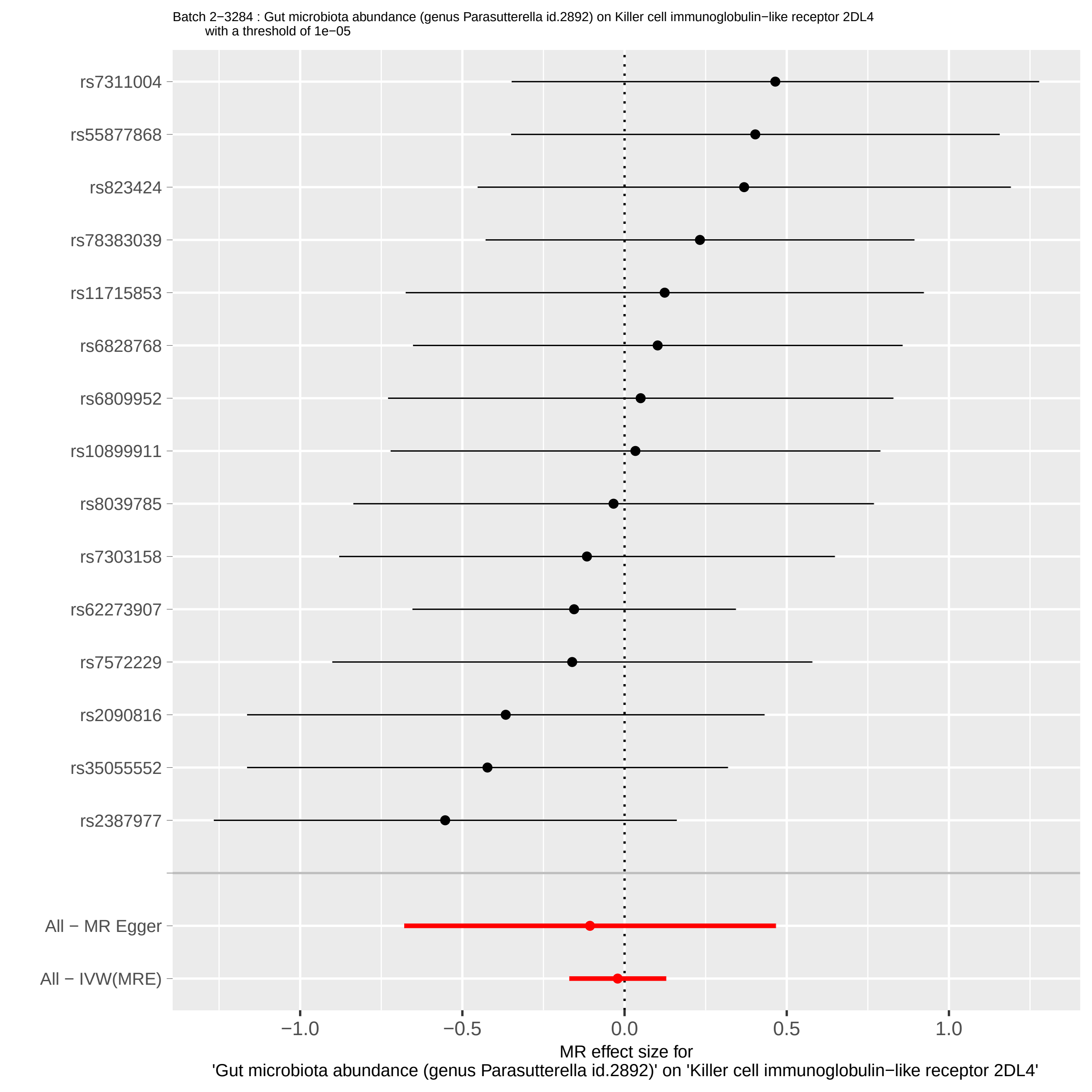
Batch 2−3284 : Gut microbiota abundance (genus Parasutterella id.2892) on Killer cell immunoglobulin−like receptor 2DL4
with a threshold of 1e−05
rs7311004
rs55877868
rs823424
rs78383039
rs11715853
rs6828768
rs6809952
rs10899911
rs8039785
rs7303158
rs62273907
rs7572229
rs2090816
rs35055552
rs2387977
All − MR Egger
All − IVW(MRE)
−1.0
−0.5
0.0
0.5
1.0
MR effect size for
'Gut microbiota abundance (genus Parasutterella id.2892)' on 'Killer cell immunoglobulin−like receptor 2DL4'

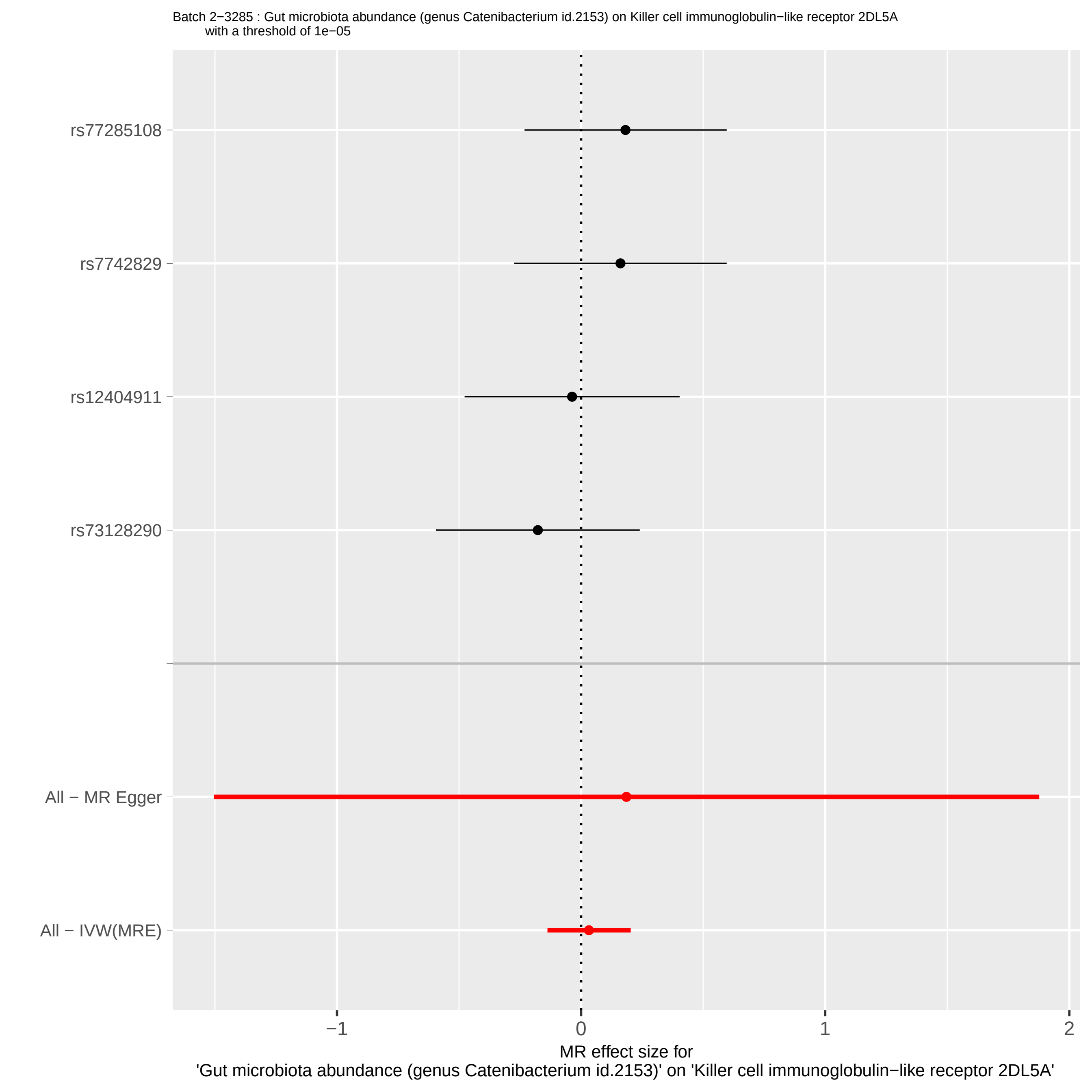

Batch 2−3285 : Gut microbiota abundance (genus Catenibacterium id.2153) on Killer cell immunoglobulin−like receptor 2DL5A
with a threshold of 1e−05
rs77285108
rs7742829
rs12404911
rs73128290
All − MR Egger
All − IVW(MRE)
−1
0
1
2
MR effect size for
'Gut microbiota abundance (genus Catenibacterium id.2153)' on 'Killer cell immunoglobulin−like receptor 2DL5A'

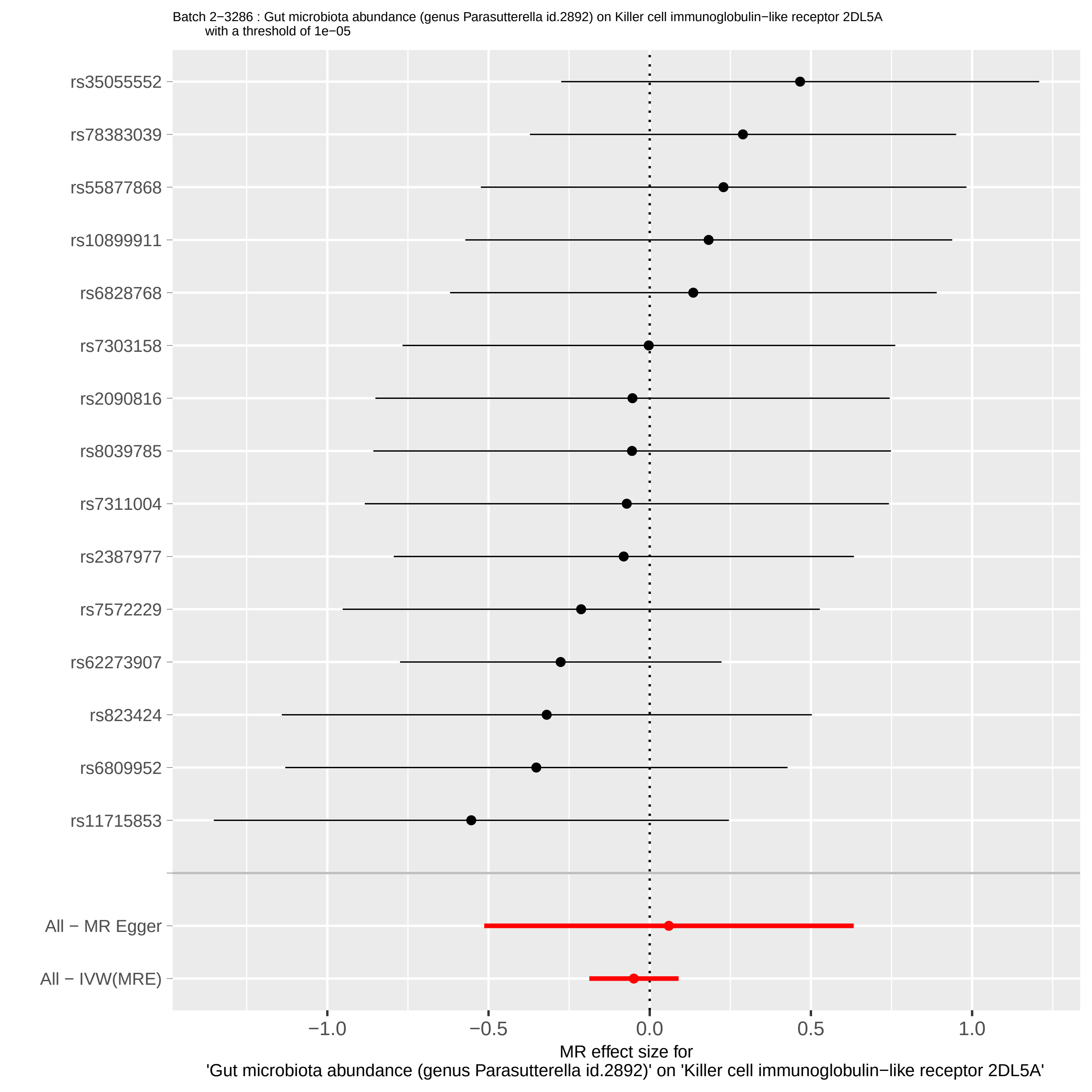

Batch 2−3286 : Gut microbiota abundance (genus Parasutterella id.2892) on Killer cell immunoglobulin−like receptor 2DL5A
with a threshold of 1e−05
rs35055552
rs78383039
rs55877868
rs10899911
rs6828768
rs7303158
rs2090816
rs8039785
rs7311004
rs2387977
rs7572229
rs62273907
rs823424
rs6809952
rs11715853
All − MR Egger
All − IVW(MRE)
−1.0
−0.5
0.0
0.5
1.0
MR effect size for
'Gut microbiota abundance (genus Parasutterella id.2892)' on 'Killer cell immunoglobulin−like receptor 2DL5A'

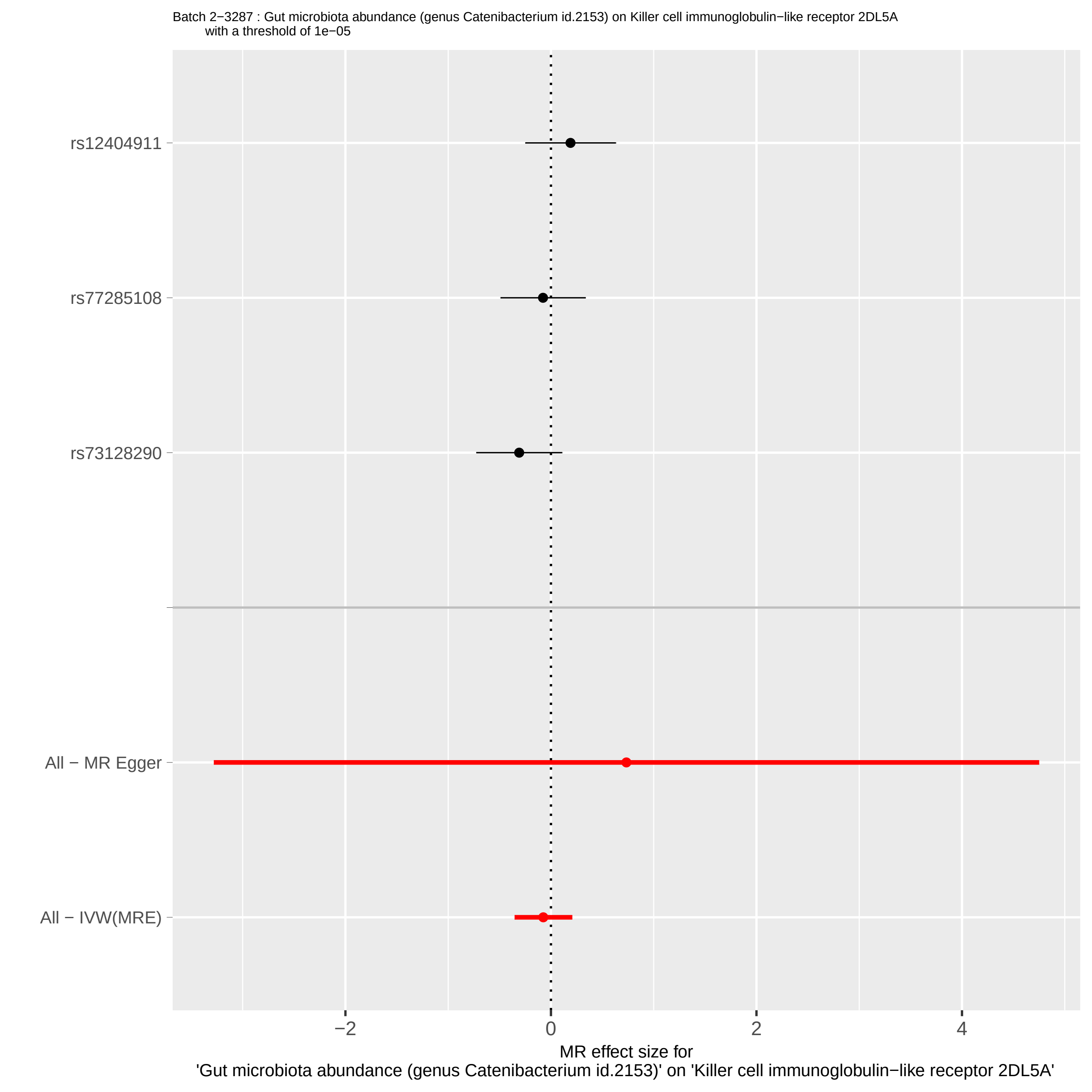

Batch 2−3287 : Gut microbiota abundance (genus Catenibacterium id.2153) on Killer cell immunoglobulin−like receptor 2DL5A
with a threshold of 1e−05
rs12404911
rs77285108
rs73128290
All − MR Egger
All − IVW(MRE)
−2
0
2
4
MR effect size for
'Gut microbiota abundance (genus Catenibacterium id.2153)' on 'Killer cell immunoglobulin−like receptor 2DL5A'

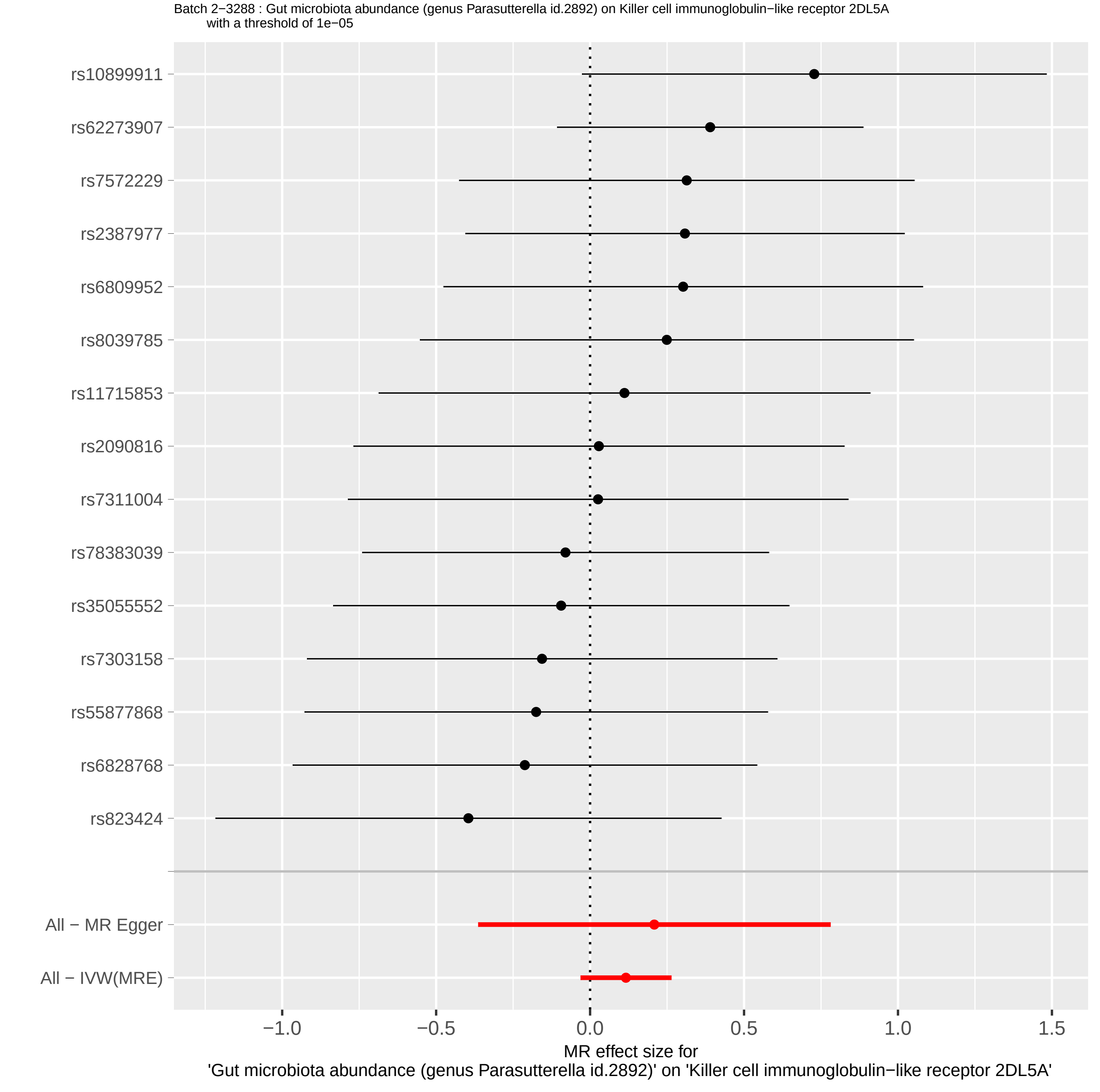

Batch 2−3288 : Gut microbiota abundance (genus Parasutterella id.2892) on Killer cell immunoglobulin−like receptor 2DL5A
with a threshold of 1e−05
rs10899911
rs62273907
rs7572229
rs2387977
rs6809952
rs8039785
rs11715853
rs2090816
rs7311004
rs78383039
rs35055552
rs7303158
rs55877868
rs6828768
rs823424
All − MR Egger
All − IVW(MRE)
−1.0
−0.5
0.0
0.5
1.0
1.5
MR effect size for
'Gut microbiota abundance (genus Parasutterella id.2892)' on 'Killer cell immunoglobulin−like receptor 2DL5A'

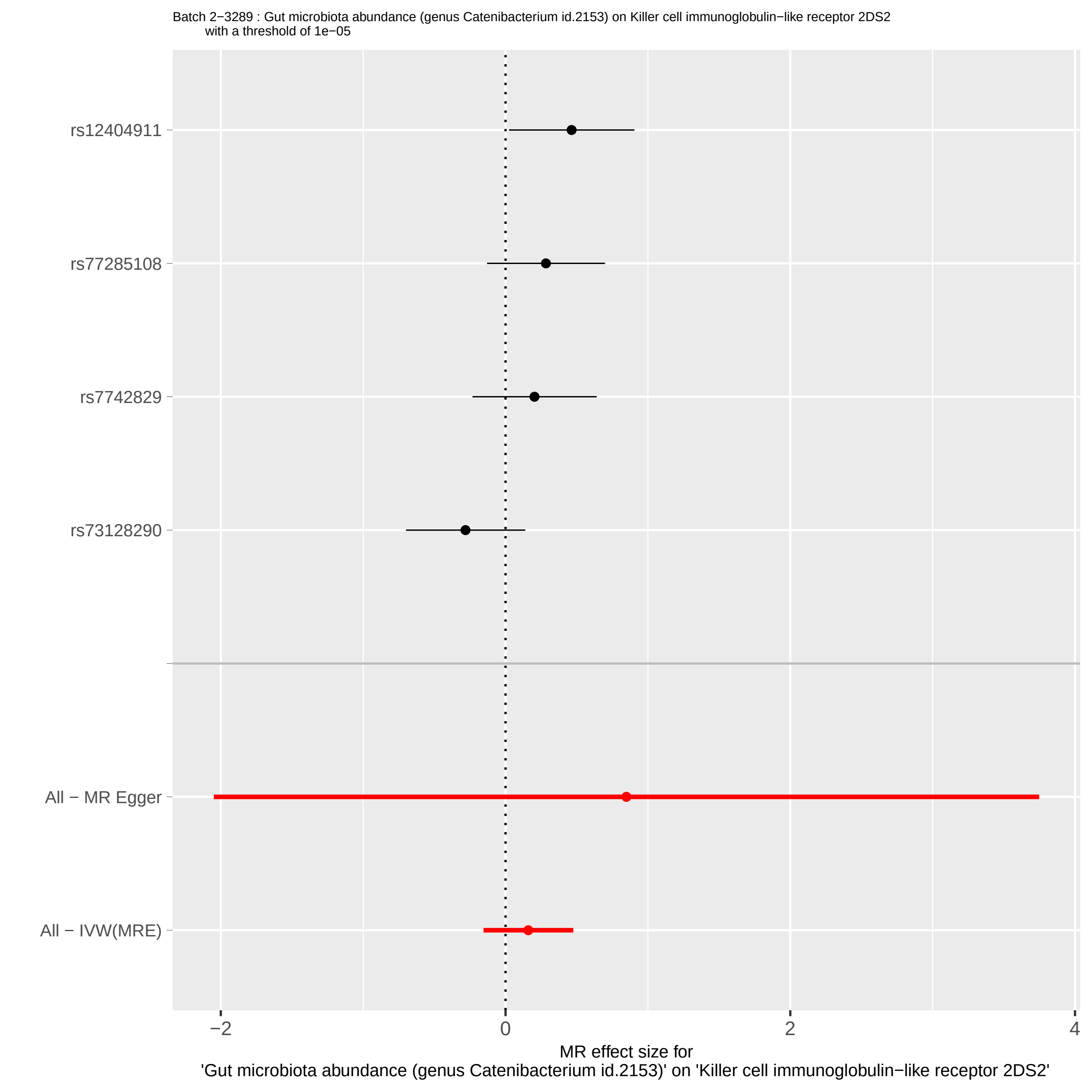
Batch 2−3289 : Gut microbiota abundance (genus Catenibacterium id.2153) on Killer cell immunoglobulin−like receptor 2DS2
with a threshold of 1e−05
rs12404911
rs77285108
rs7742829
rs73128290
All − MR Egger
All − IVW(MRE)
−2
0
2
4
MR effect size for
'Gut microbiota abundance (genus Catenibacterium id.2153)' on 'Killer cell immunoglobulin−like receptor 2DS2'

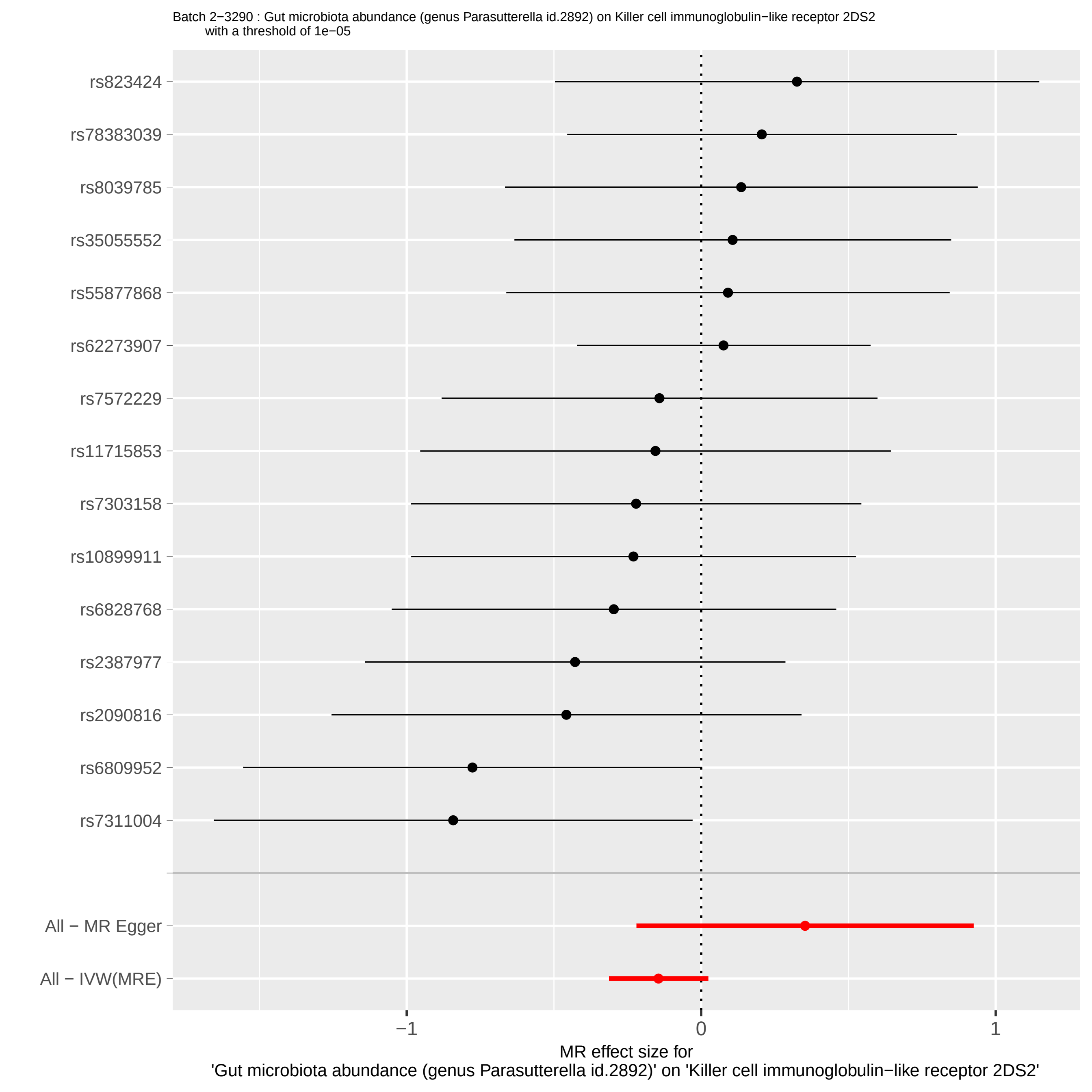

Batch 2−3290 : Gut microbiota abundance (genus Parasutterella id.2892) on Killer cell immunoglobulin−like receptor 2DS2
with a threshold of 1e−05
rs823424
rs78383039
rs8039785
rs35055552
rs55877868
rs62273907
rs7572229
rs11715853
rs7303158
rs10899911
rs6828768
rs2387977
rs2090816
rs6809952
rs7311004
All − MR Egger
All − IVW(MRE)
−1
0
1
MR effect size for
'Gut microbiota abundance (genus Parasutterella id.2892)' on 'Killer cell immunoglobulin−like receptor 2DS2'

Batch 2−3291 : Gut microbiota abundance (genus Catenibacterium id.2153) on Killer cell immunoglobulin−like receptor 3DL2
with a threshold of 1e−05

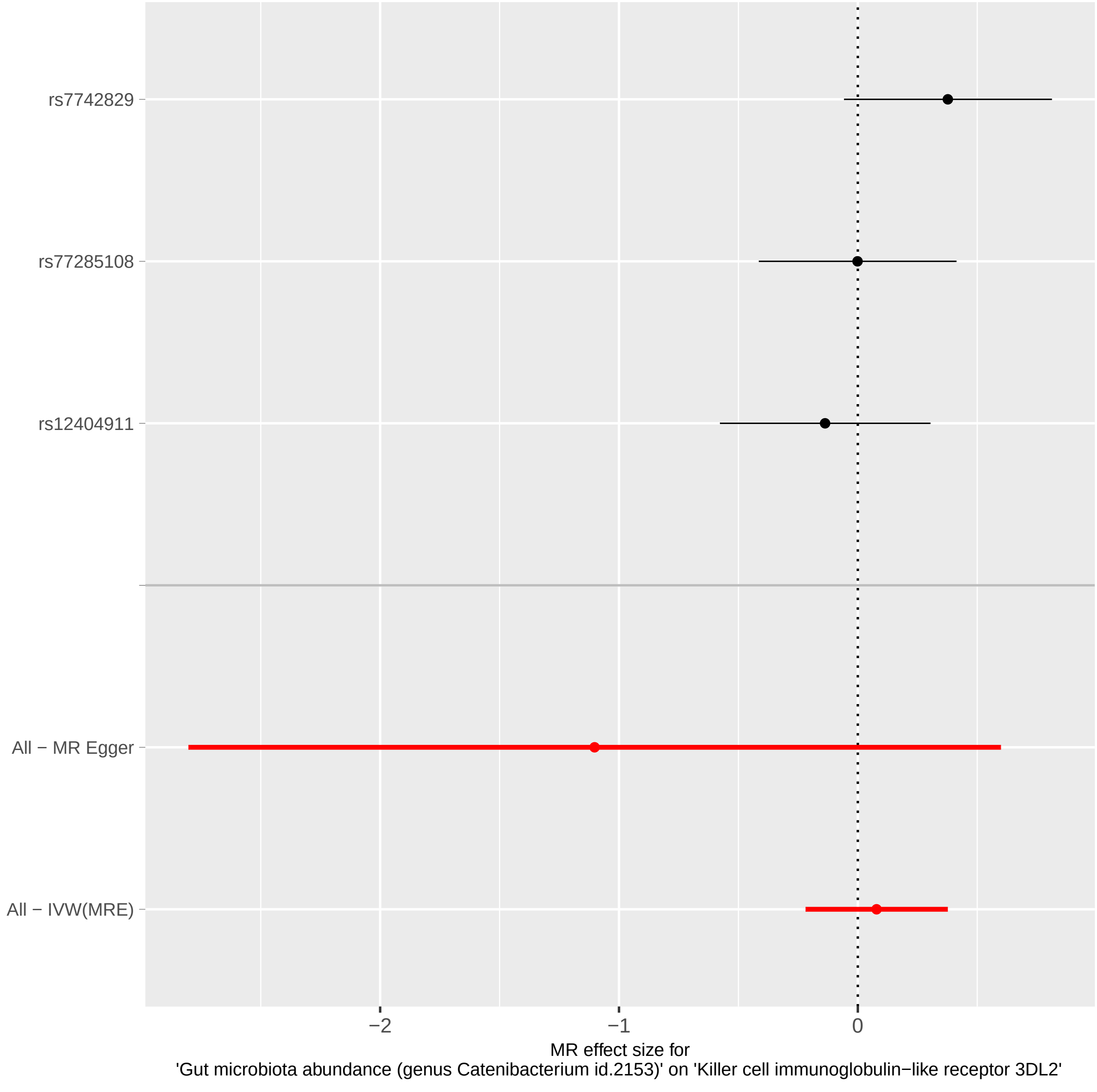

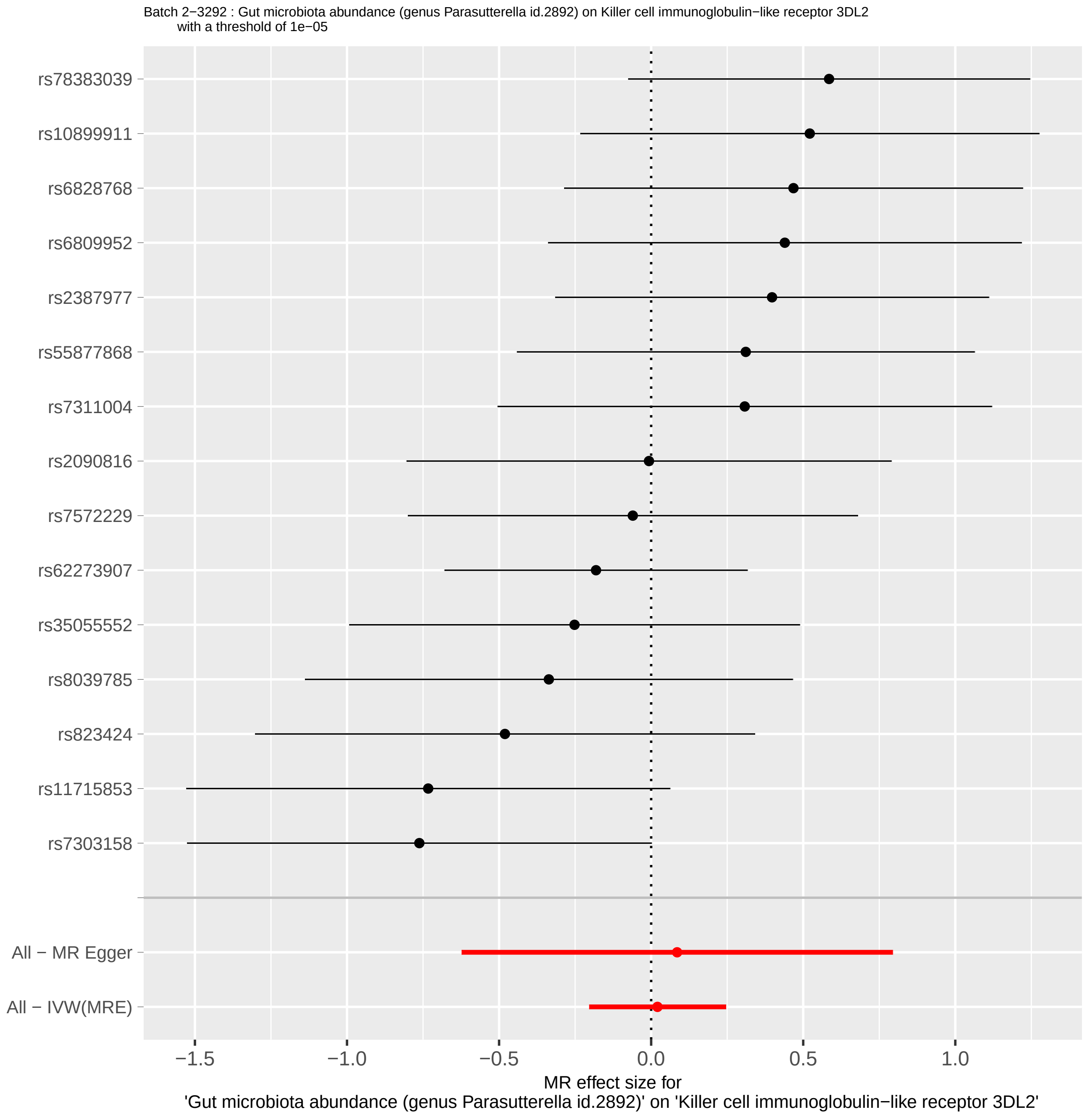

Batch 2−3292 : Gut microbiota abundance (genus Parasutterella id.2892) on Killer cell immunoglobulin−like receptor 3DL2
with a threshold of 1e−05
rs78383039
rs10899911
rs6828768
rs6809952
rs2387977
rs55877868
rs7311004
rs2090816
rs7572229
rs62273907
rs35055552
rs8039785
rs823424
rs11715853
rs7303158
All − MR Egger
All − IVW(MRE)
−1.5
−1.0
−0.5
0.0
0.5
1.0
MR effect size for
'Gut microbiota abundance (genus Parasutterella id.2892)' on 'Killer cell immunoglobulin−like receptor 3DL2'

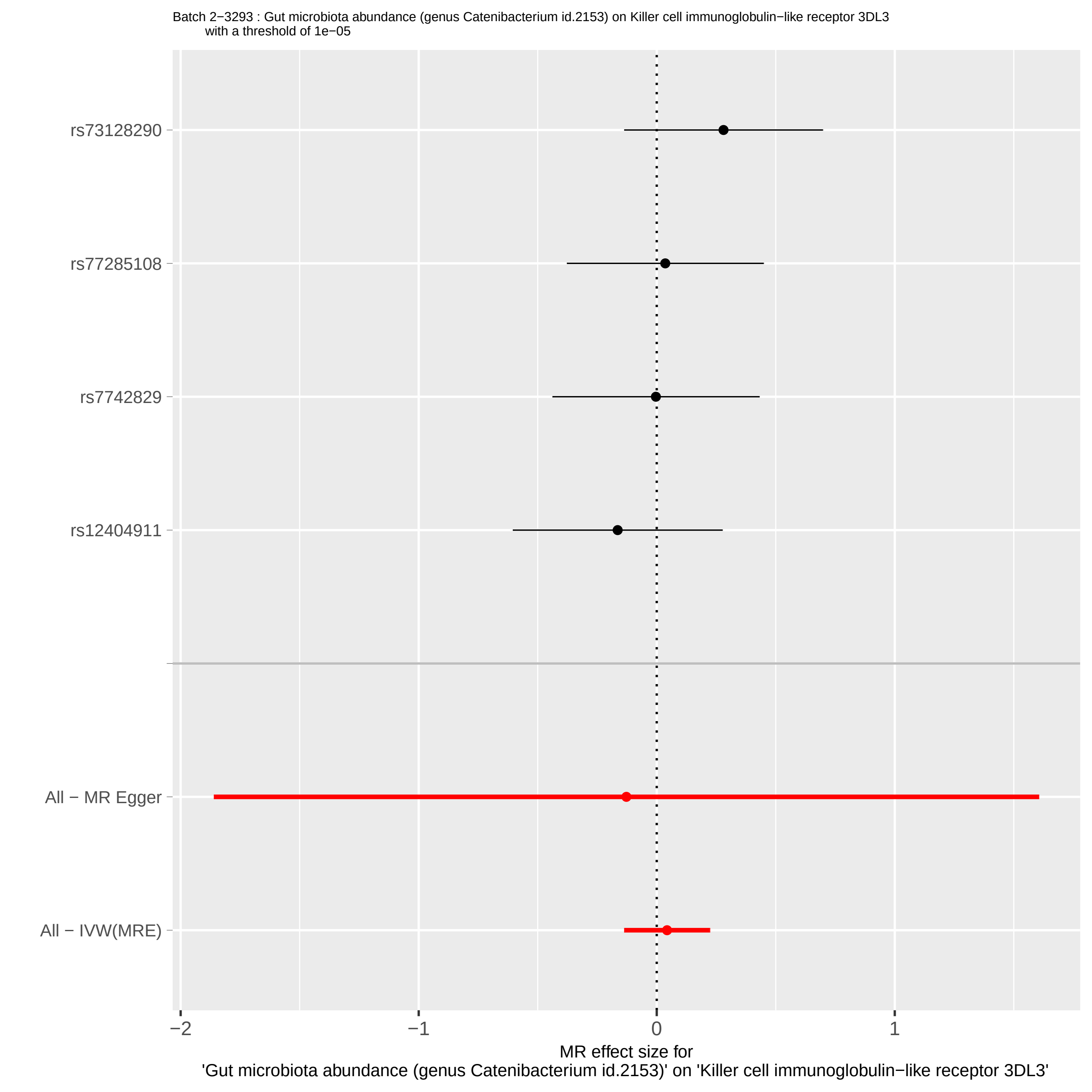

Batch 2−3293 : Gut microbiota abundance (genus Catenibacterium id.2153) on Killer cell immunoglobulin−like receptor 3DL3
with a threshold of 1e−05
rs73128290
rs77285108
rs7742829
rs12404911
All − MR Egger
All − IVW(MRE)
−2
−1
0
1
MR effect size for
'Gut microbiota abundance (genus Catenibacterium id.2153)' on 'Killer cell immunoglobulin−like receptor 3DL3'

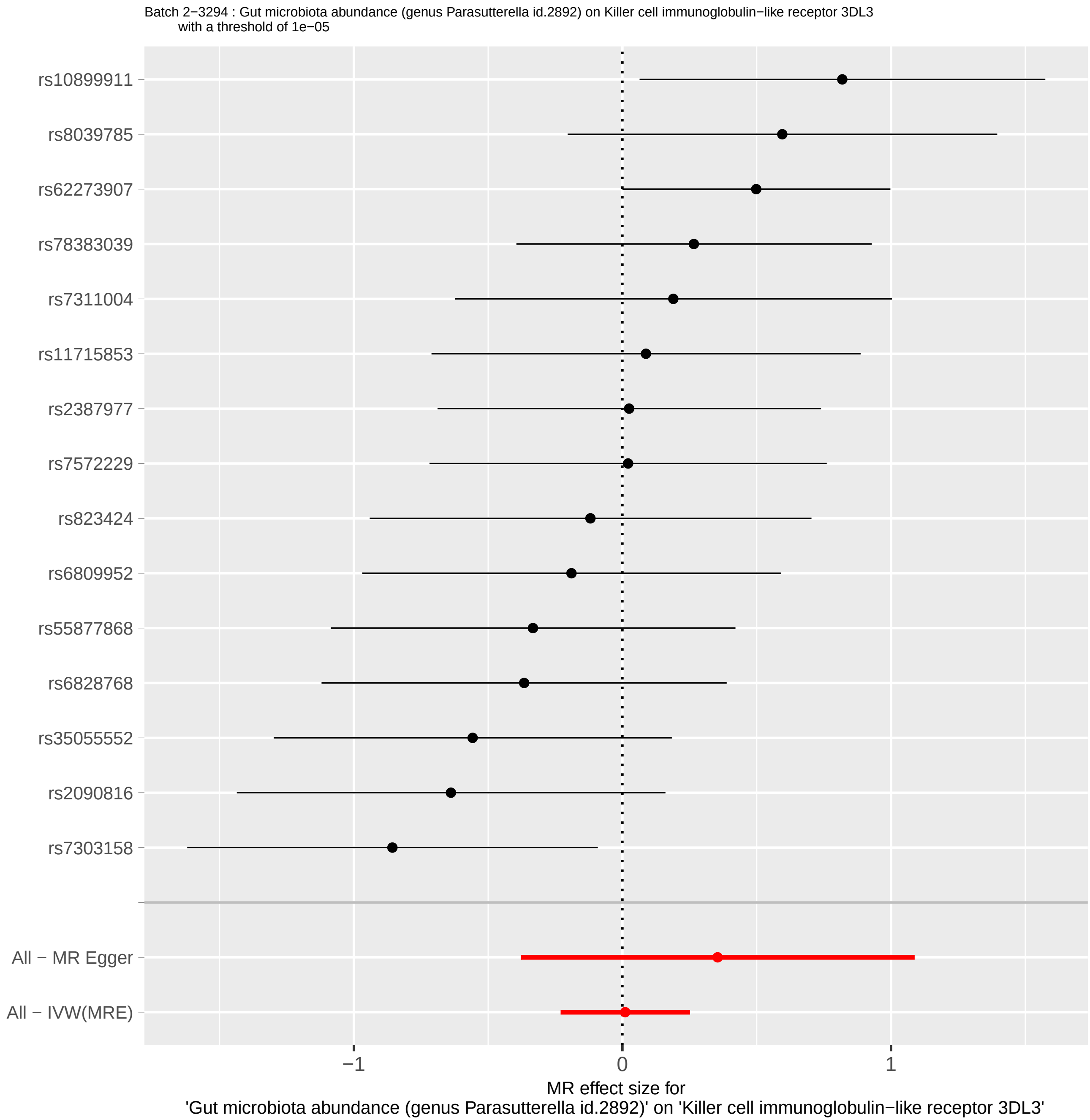

Batch 2−3294 : Gut microbiota abundance (genus Parasutterella id.2892) on Killer cell immunoglobulin−like receptor 3DL3
with a threshold of 1e−05
rs10899911
rs8039785
rs62273907
rs78383039
rs7311004
rs11715853
rs2387977
rs7572229
rs823424
rs6809952
rs55877868
rs6828768
rs35055552
rs2090816
rs7303158
All − MR Egger
All − IVW(MRE)
−1
0
1
MR effect size for
'Gut microbiota abundance (genus Parasutterella id.2892)' on 'Killer cell immunoglobulin−like receptor 3DL3'

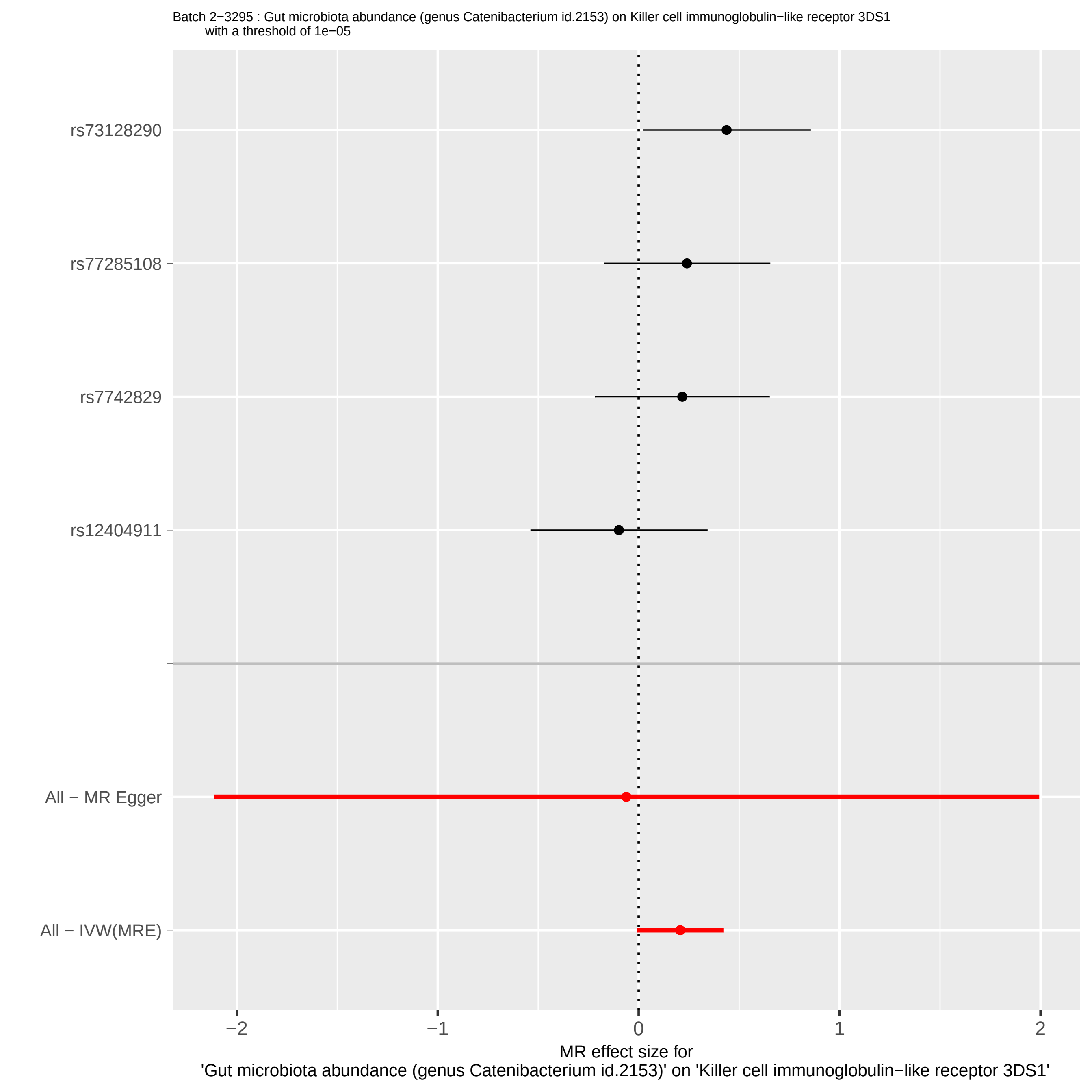
Batch 2−3295 : Gut microbiota abundance (genus Catenibacterium id.2153) on Killer cell immunoglobulin−like receptor 3DS1
with a threshold of 1e−05
rs73128290
rs77285108
rs7742829
rs12404911
All − MR Egger
All − IVW(MRE)
−2
−1
0
1
2
MR effect size for
'Gut microbiota abundance (genus Catenibacterium id.2153)' on 'Killer cell immunoglobulin−like receptor 3DS1'

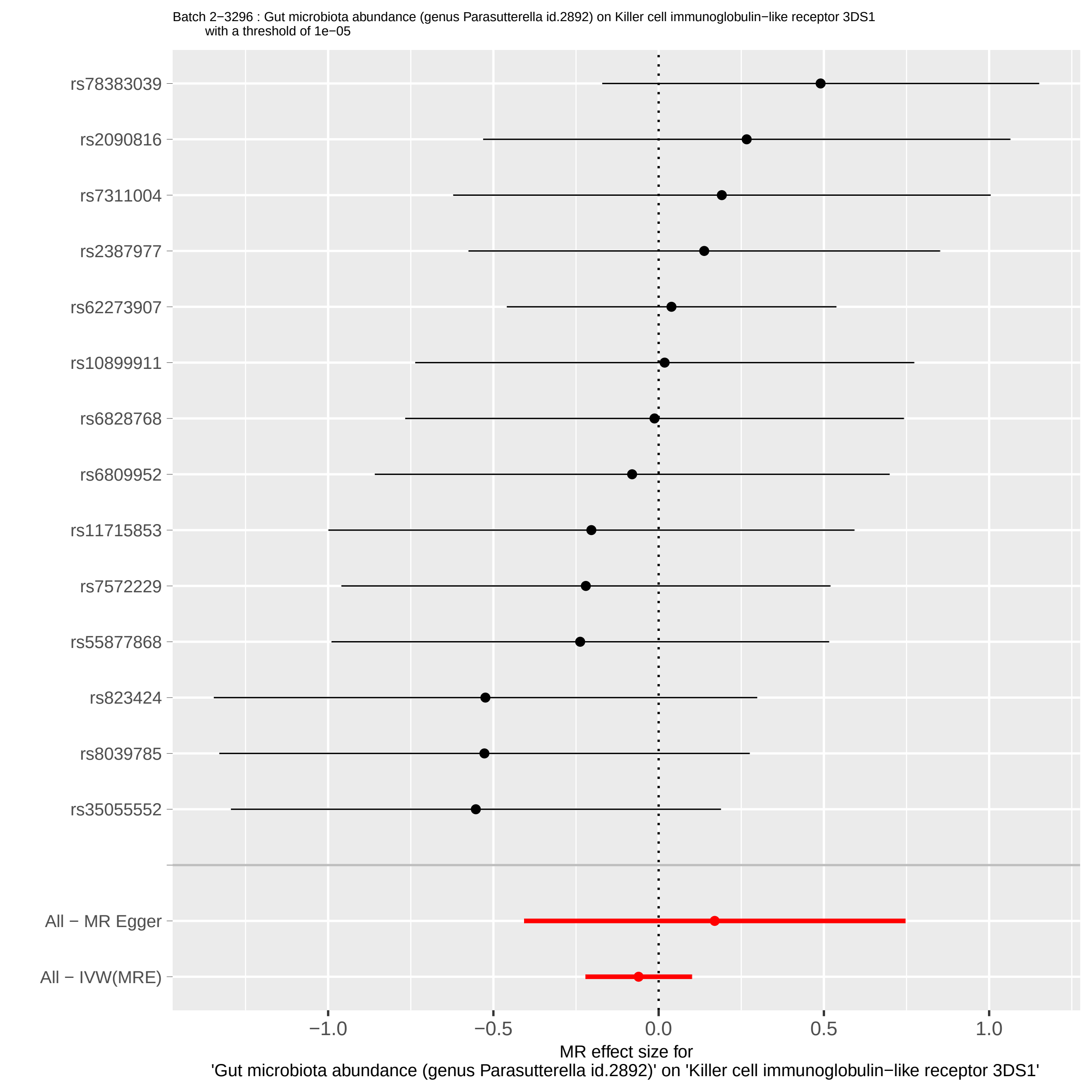

Batch 2−3296 : Gut microbiota abundance (genus Parasutterella id.2892) on Killer cell immunoglobulin−like receptor 3DS1
with a threshold of 1e−05
rs78383039
rs2090816
rs7311004
rs2387977
rs62273907
rs10899911
rs6828768
rs6809952
rs11715853
rs7572229
rs55877868
rs823424
rs8039785
rs35055552
All − MR Egger
All − IVW(MRE)
−1.0
−0.5
0.0
0.5
1.0
MR effect size for
'Gut microbiota abundance (genus Parasutterella id.2892)' on 'Killer cell immunoglobulin−like receptor 3DS1'

Batch 2−3297 : Gut microbiota abundance (genus Catenibacterium id.2153) on Kin of IRRE−like protein 2
with a threshold of 1e−05

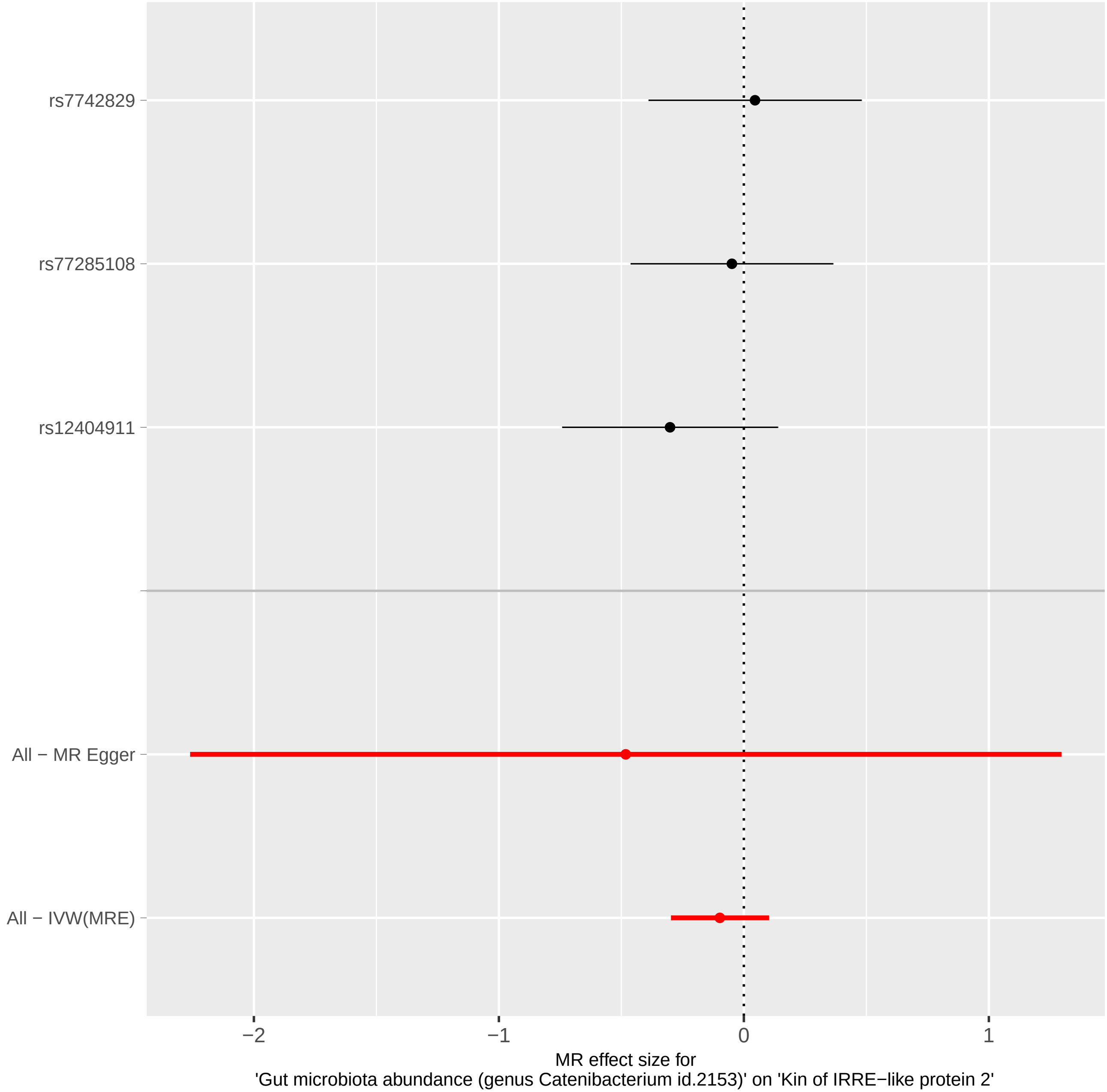

Batch 2−3298 : Gut microbiota abundance (genus Parasutterella id.2892) on Kin of IRRE−like protein 2
with a threshold of 1e−05

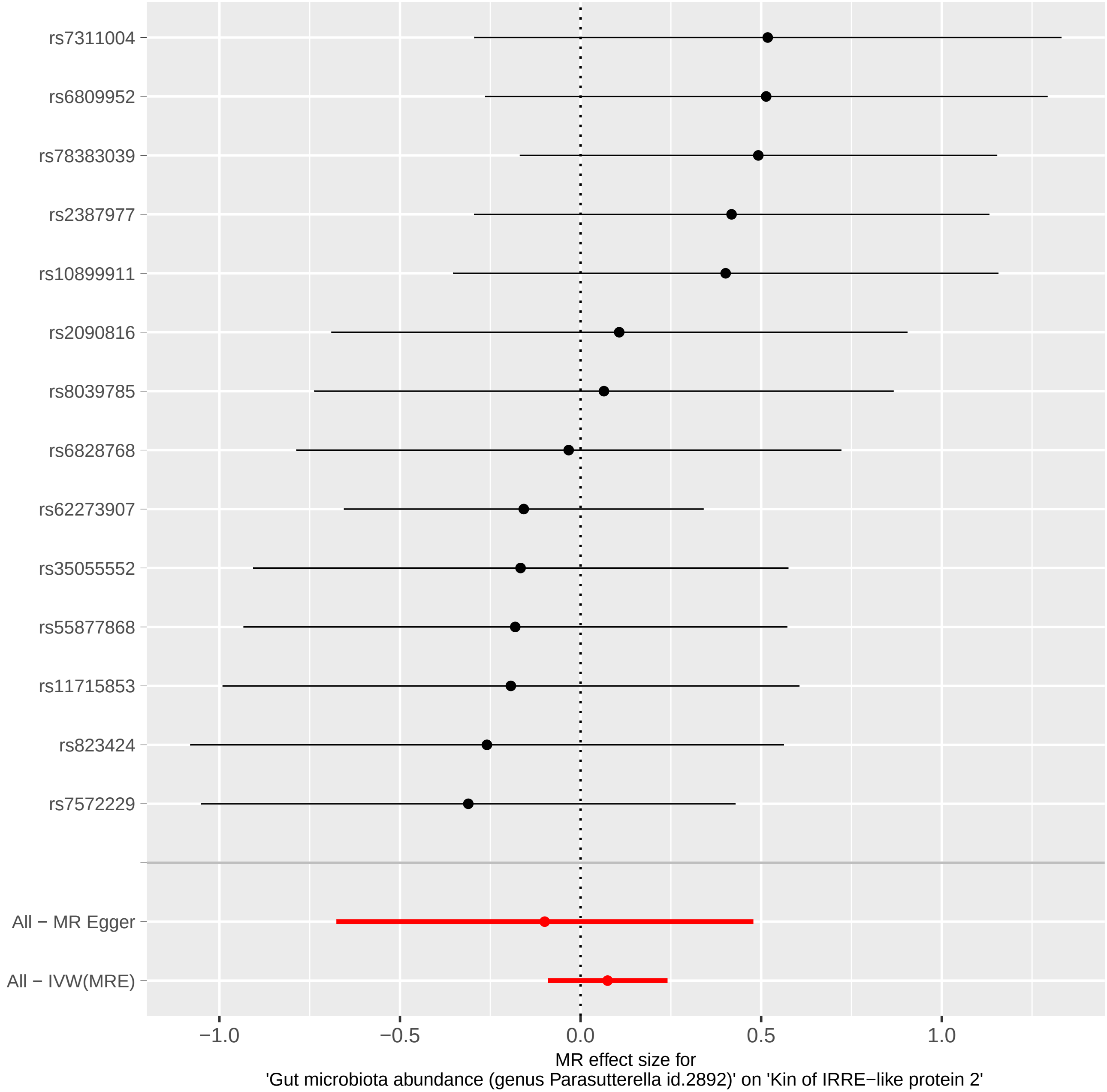

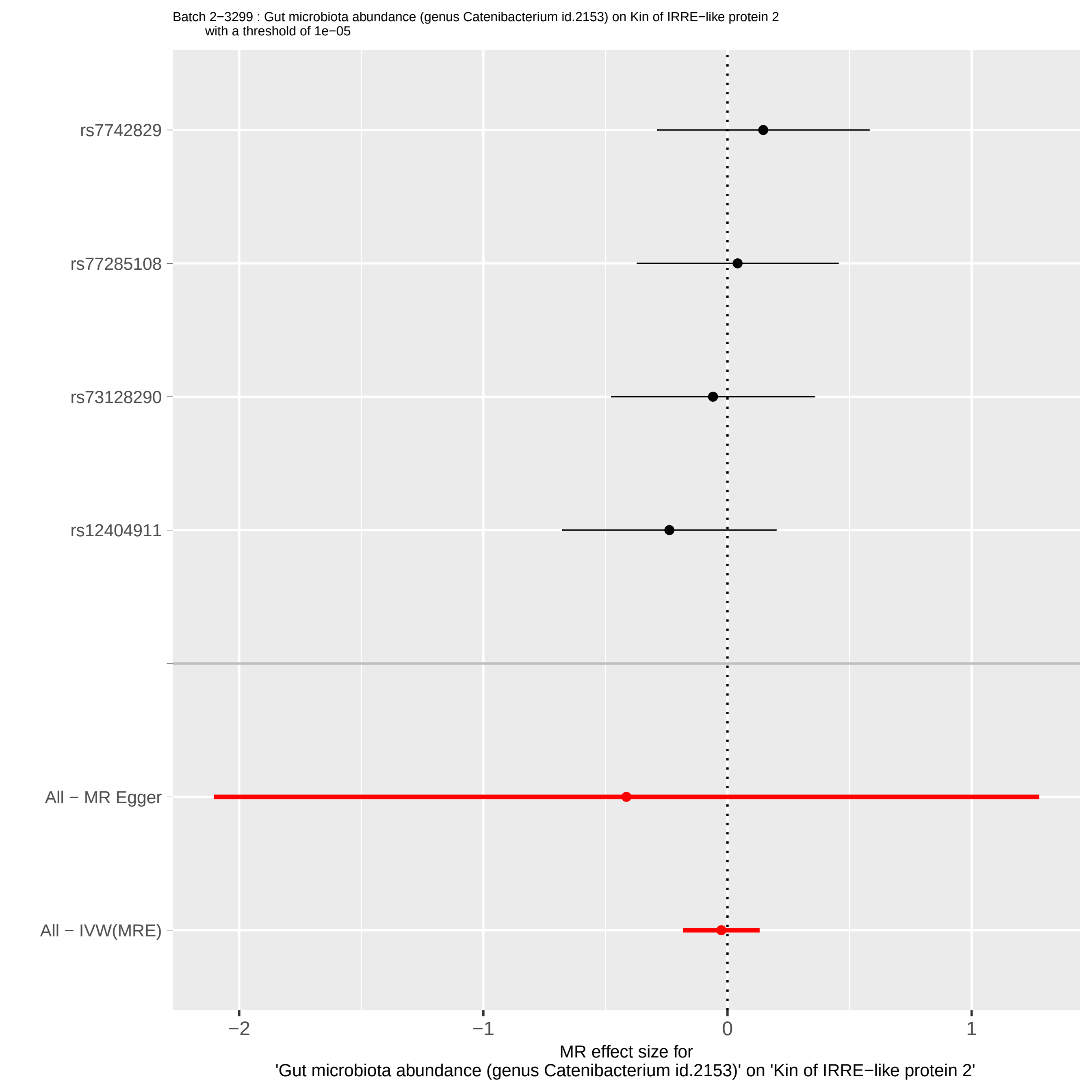

Batch 2−3299 : Gut microbiota abundance (genus Catenibacterium id.2153) on Kin of IRRE−like protein 2
with a threshold of 1e−05
rs7742829
rs77285108
rs73128290
rs12404911
All − MR Egger
All − IVW(MRE)
−2
−1
0
1
MR effect size for
'Gut microbiota abundance (genus Catenibacterium id.2153)' on 'Kin of IRRE−like protein 2'

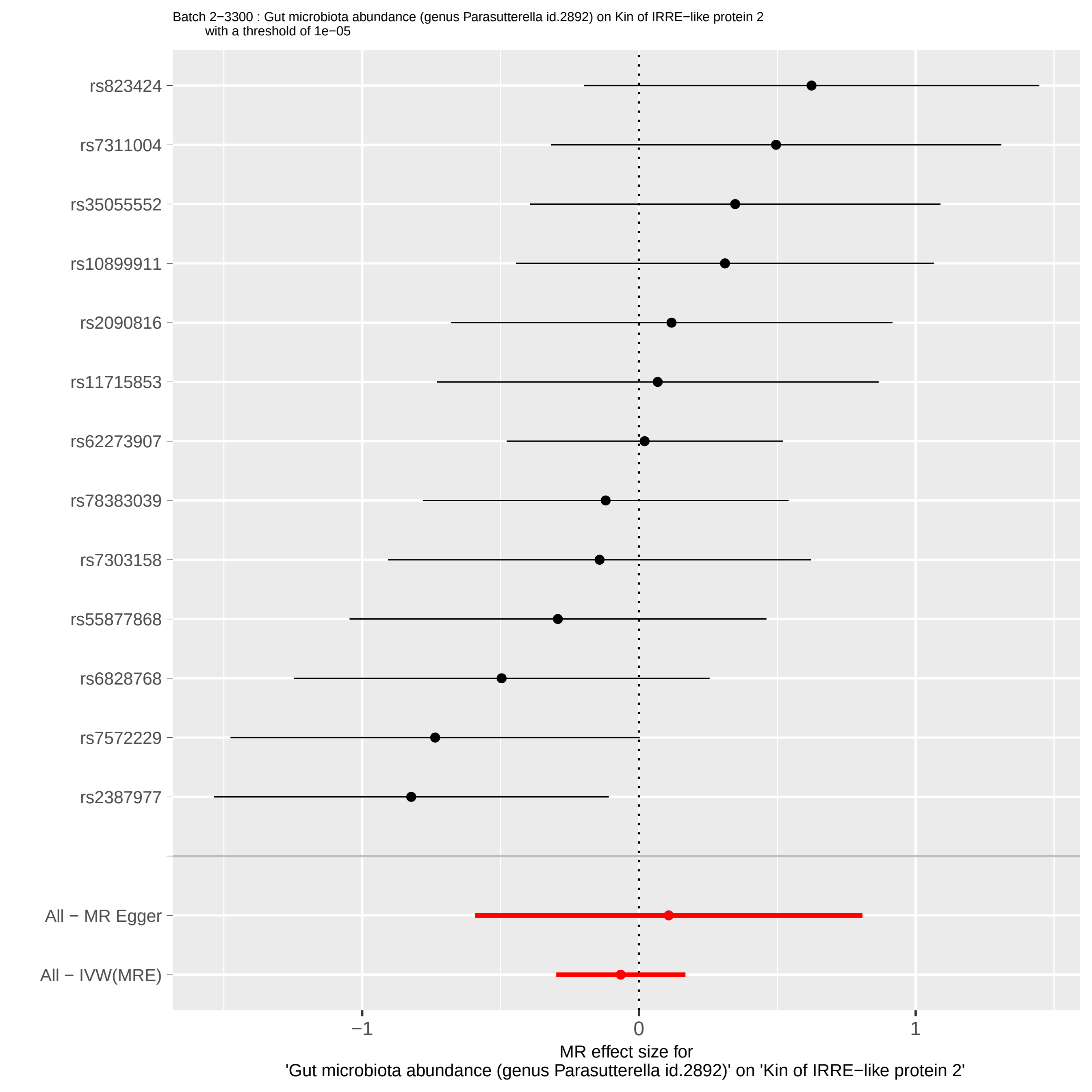

Batch 2−3300 : Gut microbiota abundance (genus Parasutterella id.2892) on Kin of IRRE−like protein 2
with a threshold of 1e−05
rs823424
rs7311004
rs35055552
rs10899911
rs2090816
rs11715853
rs62273907
rs78383039
rs7303158
rs55877868
rs6828768
rs7572229
rs2387977
All − MR Egger
All − IVW(MRE)
−1
0
1
MR effect size for
'Gut microbiota abundance (genus Parasutterella id.2892)' on 'Kin of IRRE−like protein 2'

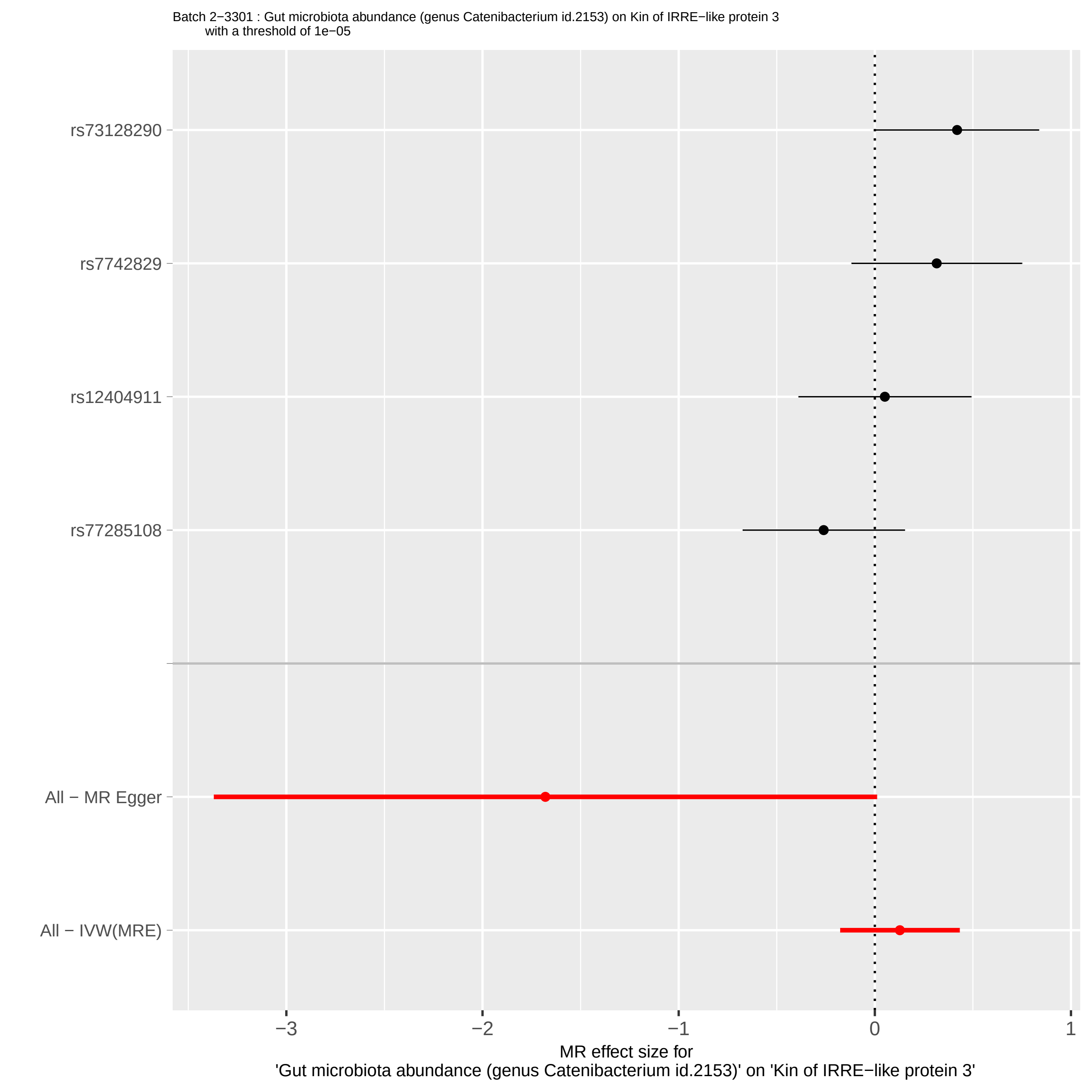
Batch 2−3301 : Gut microbiota abundance (genus Catenibacterium id.2153) on Kin of IRRE−like protein 3
with a threshold of 1e−05
rs73128290
rs7742829
rs12404911
rs77285108
All − MR Egger
All − IVW(MRE)
−3
−2
−1
0
1
MR effect size for
'Gut microbiota abundance (genus Catenibacterium id.2153)' on 'Kin of IRRE−like protein 3'

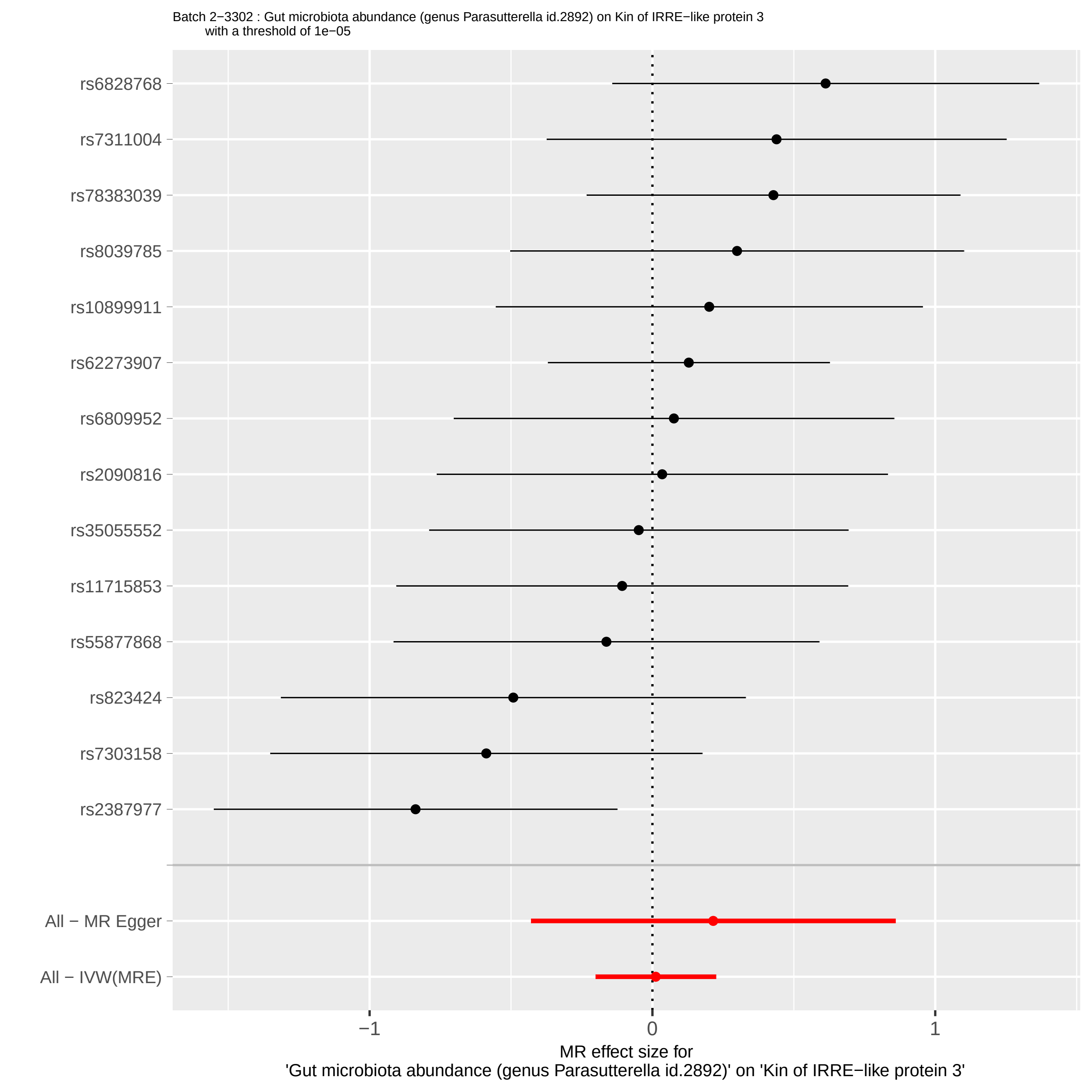
Batch 2−3302 : Gut microbiota abundance (genus Parasutterella id.2892) on Kin of IRRE−like protein 3
with a threshold of 1e−05
rs6828768
rs7311004
rs78383039
rs8039785
rs10899911
rs62273907
rs6809952
rs2090816
rs35055552
rs11715853
rs55877868
rs823424
rs7303158
rs2387977
All − MR Egger
All − IVW(MRE)
−1
0
1
MR effect size for
'Gut microbiota abundance (genus Parasutterella id.2892)' on 'Kin of IRRE−like protein 3'

Batch 2−3303 : Gut microbiota abundance (genus Catenibacterium id.2153) on Mast/stem cell growth factor receptor Kit
with a threshold of 1e−05

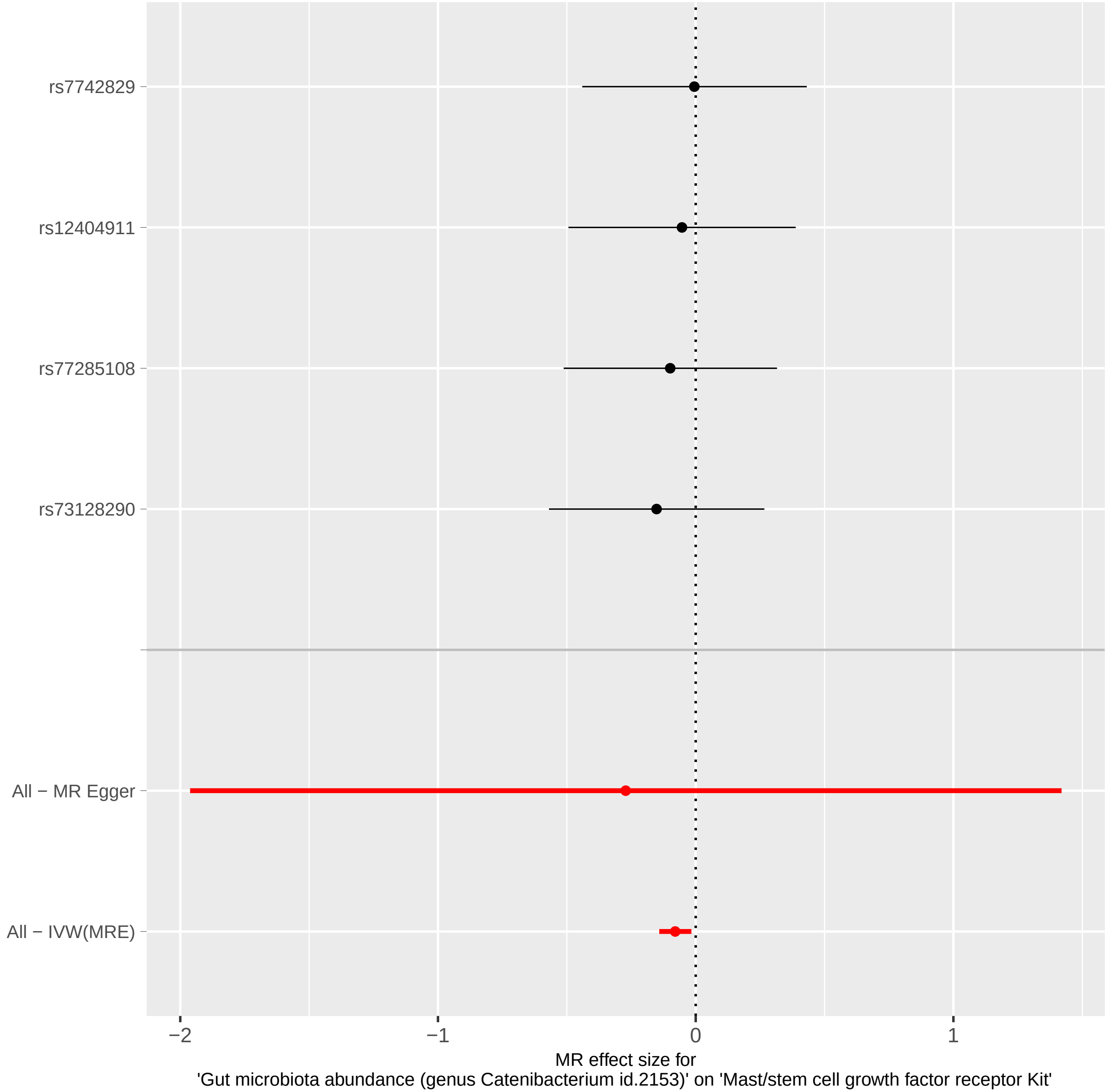

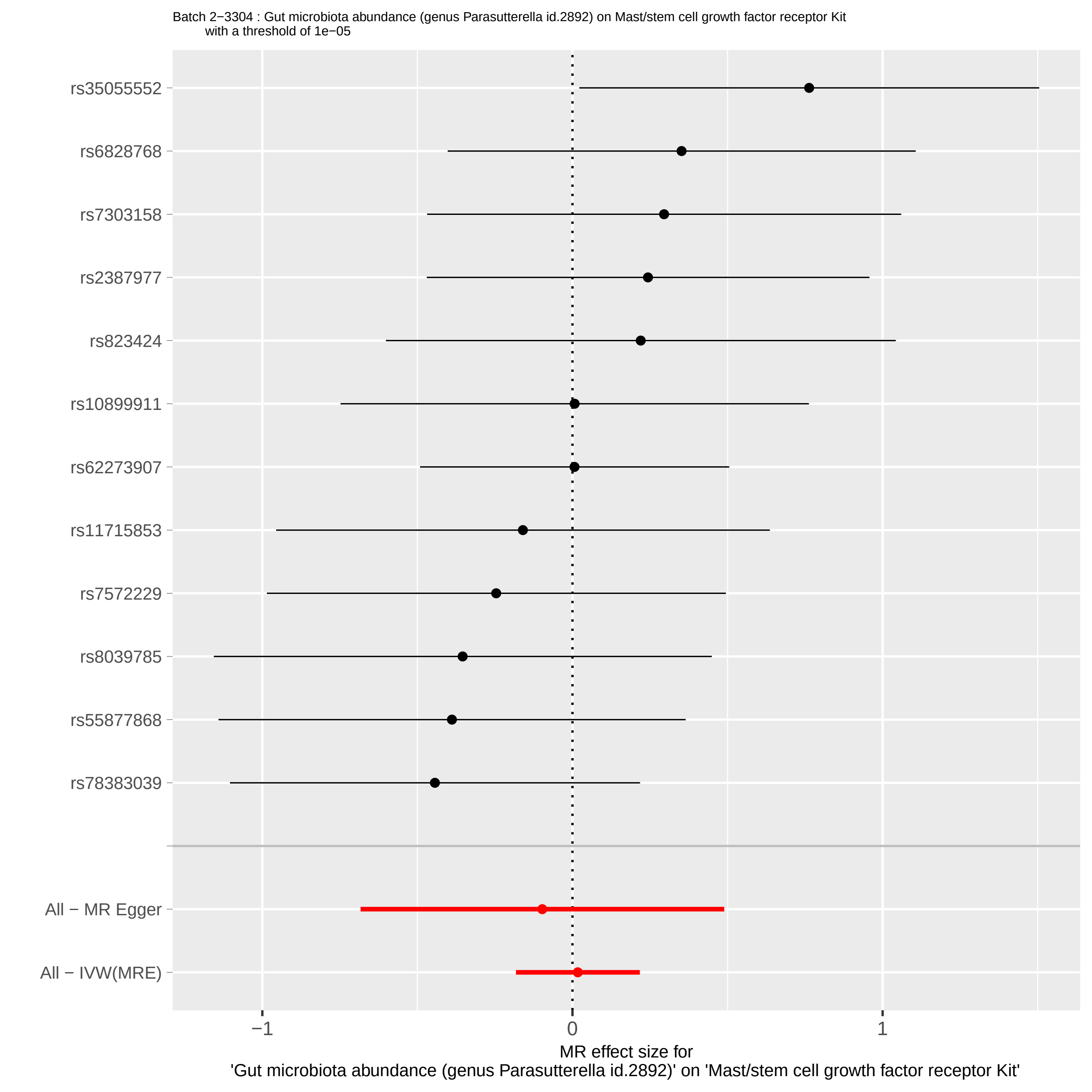
Batch 2−3304 : Gut microbiota abundance (genus Parasutterella id.2892) on Mast/stem cell growth factor receptor Kit
with a threshold of 1e−05
rs35055552
rs6828768
rs7303158
rs2387977
rs823424
rs10899911
rs62273907
rs11715853
rs7572229
rs8039785
rs55877868
rs78383039
All − MR Egger
All − IVW(MRE)
−1
0
1
MR effect size for
'Gut microbiota abundance (genus Parasutterella id.2892)' on 'Mast/stem cell growth factor receptor Kit'

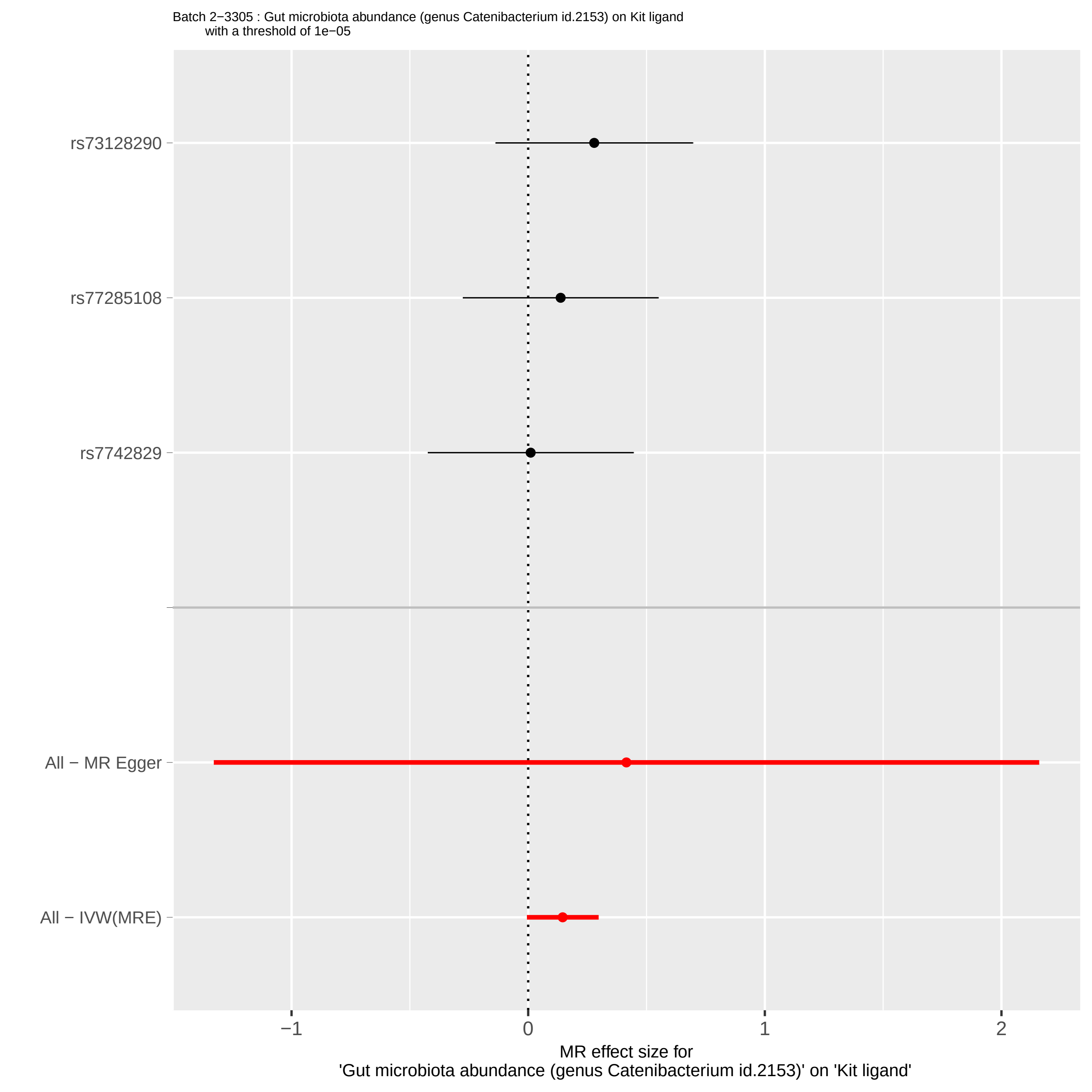
Batch 2−3305 : Gut microbiota abundance (genus Catenibacterium id.2153) on Kit ligand
with a threshold of 1e−05
rs73128290
rs77285108
rs7742829
All − MR Egger
All − IVW(MRE)
−1
0
1
2
MR effect size for
'Gut microbiota abundance (genus Catenibacterium id.2153)' on 'Kit ligand'

Batch 2−3306 : Gut microbiota abundance (genus Parasutterella id.2892) on Kit ligand
with a threshold of 1e−05

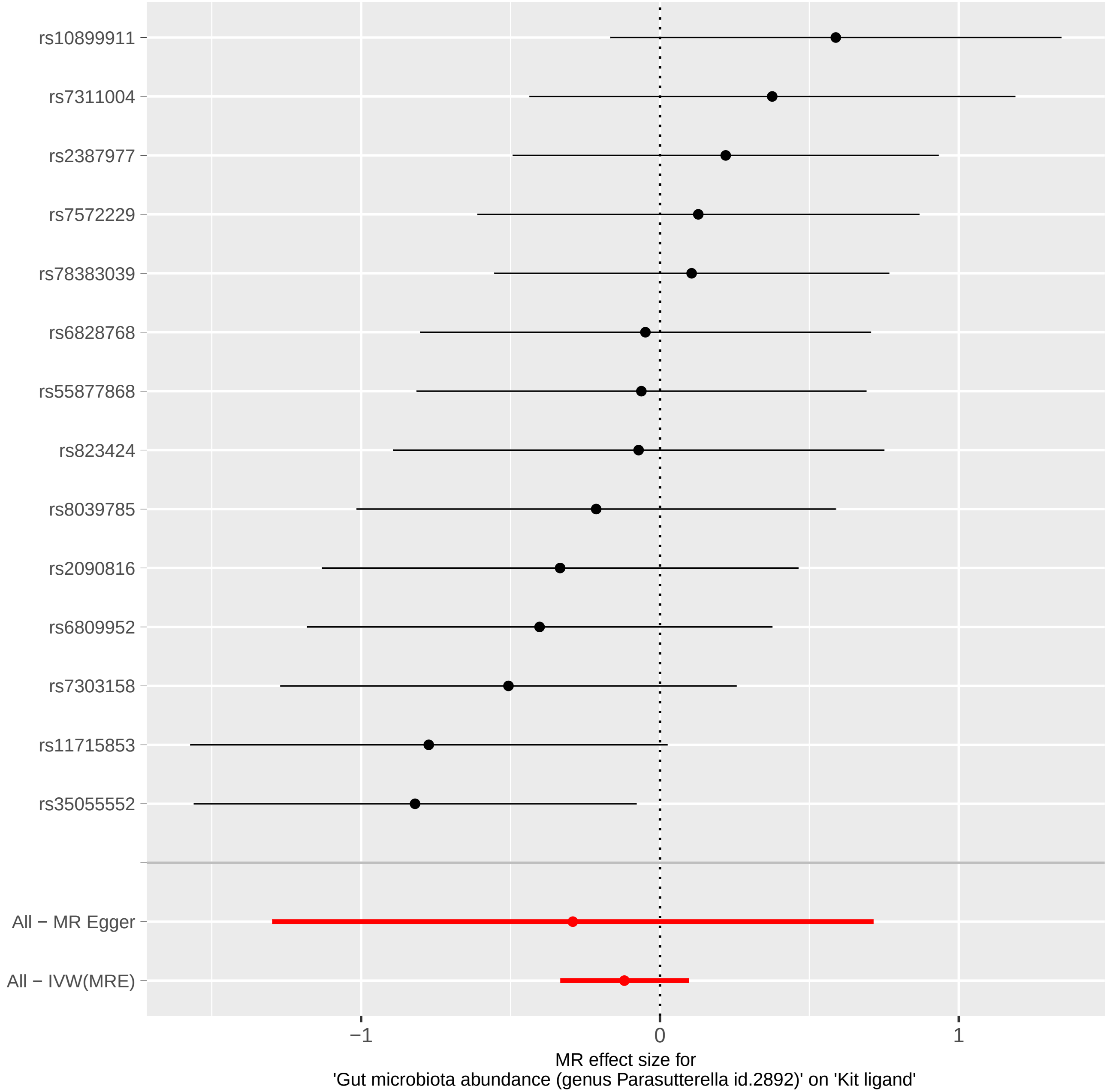

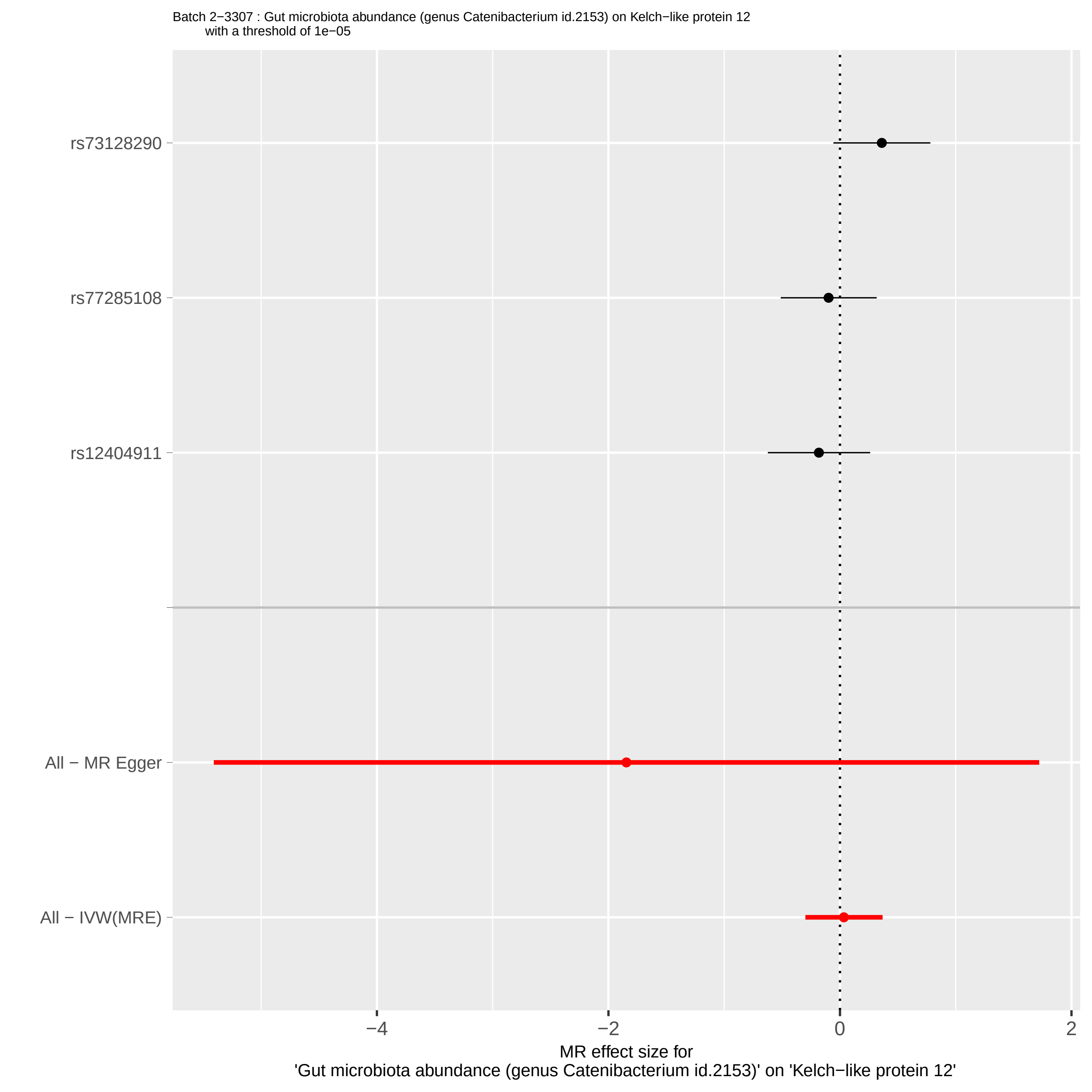

Batch 2−3307 : Gut microbiota abundance (genus Catenibacterium id.2153) on Kelch−like protein 12
with a threshold of 1e−05
rs73128290
rs77285108
rs12404911
All − MR Egger
All − IVW(MRE)
−4
−2
0
2
MR effect size for
'Gut microbiota abundance (genus Catenibacterium id.2153)' on 'Kelch−like protein 12'

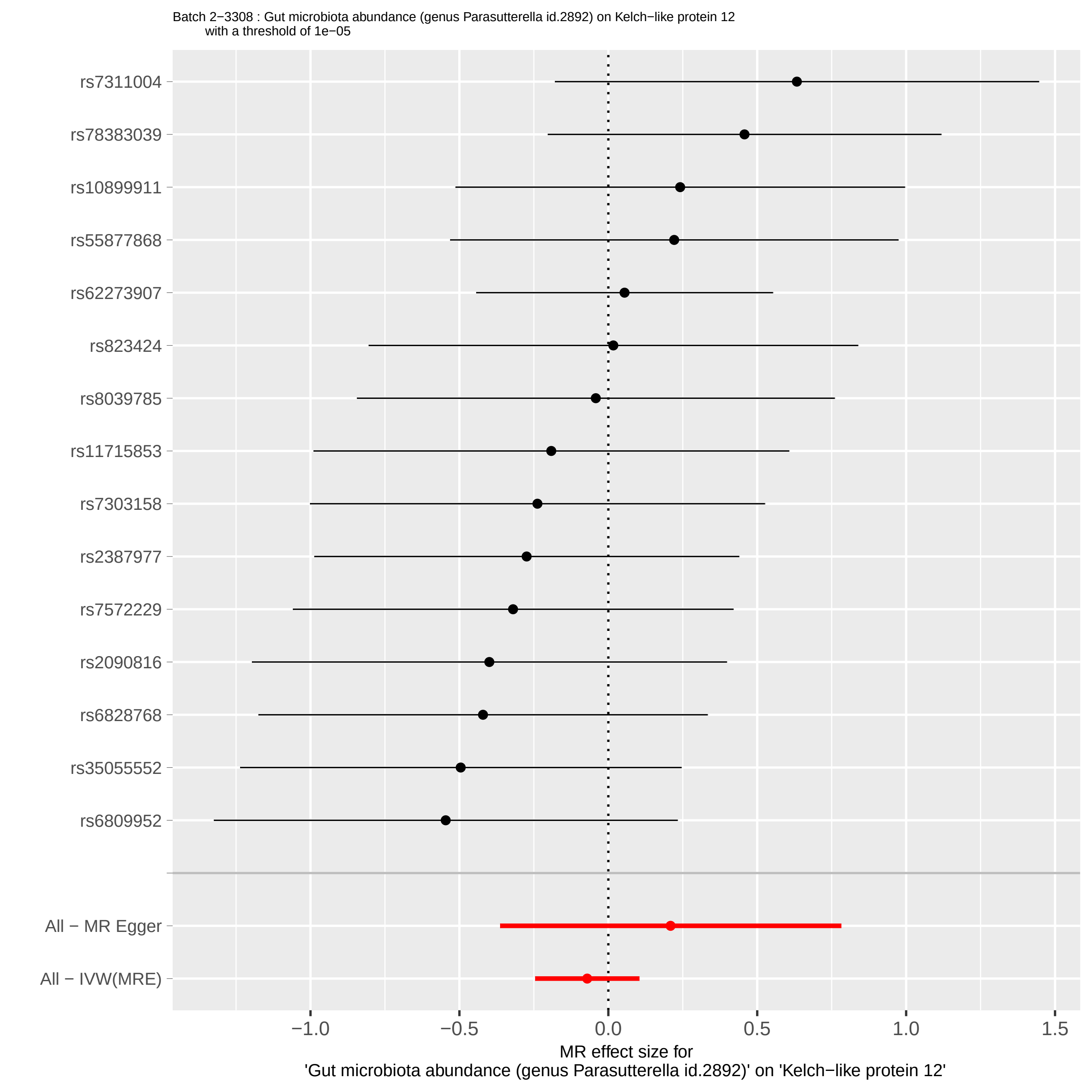
Batch 2−3308 : Gut microbiota abundance (genus Parasutterella id.2892) on Kelch−like protein 12
with a threshold of 1e−05
rs7311004
rs78383039
rs10899911
rs55877868
rs62273907
rs823424
rs8039785
rs11715853
rs7303158
rs2387977
rs7572229
rs2090816
rs6828768
rs35055552
rs6809952
All − MR Egger
All − IVW(MRE)
−1.0
−0.5
0.0
0.5
1.0
1.5
MR effect size for
'Gut microbiota abundance (genus Parasutterella id.2892)' on 'Kelch−like protein 12'

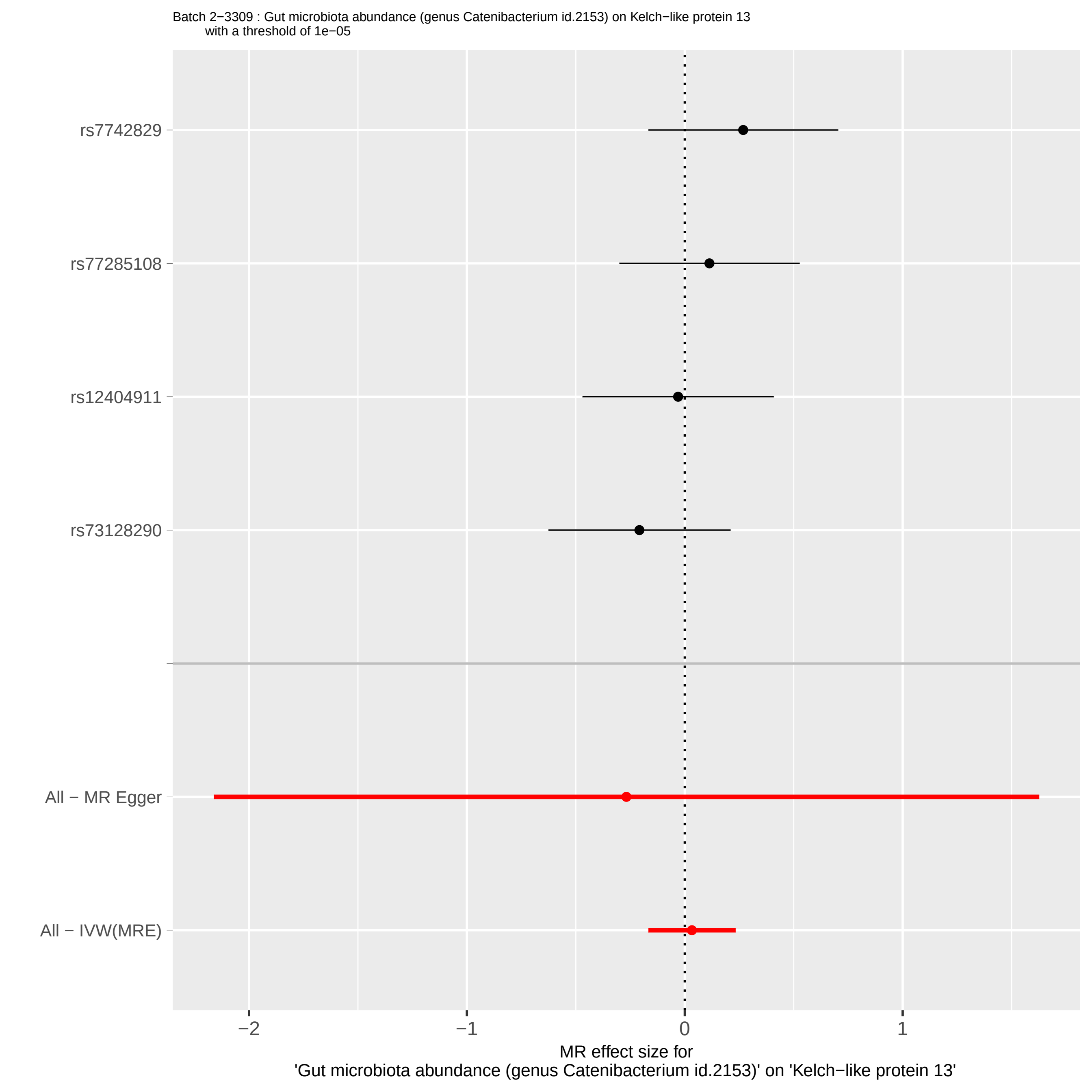
Batch 2−3309 : Gut microbiota abundance (genus Catenibacterium id.2153) on Kelch−like protein 13
with a threshold of 1e−05
rs7742829
rs77285108
rs12404911
rs73128290
All − MR Egger
All − IVW(MRE)
−2
−1
0
1
MR effect size for
'Gut microbiota abundance (genus Catenibacterium id.2153)' on 'Kelch−like protein 13'

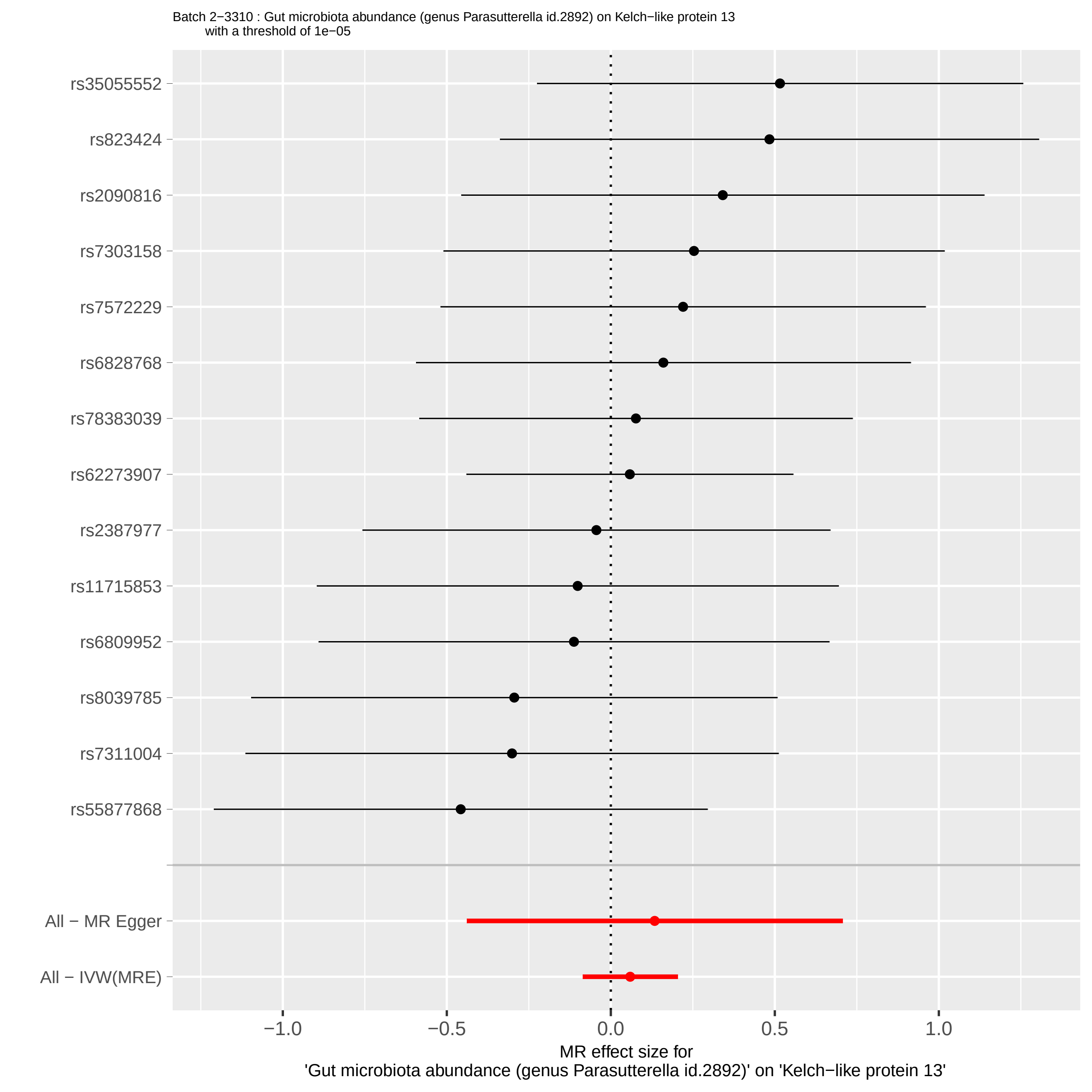

Batch 2−3310 : Gut microbiota abundance (genus Parasutterella id.2892) on Kelch−like protein 13
with a threshold of 1e−05
rs35055552
rs823424
rs2090816
rs7303158
rs7572229
rs6828768
rs78383039
rs62273907
rs2387977
rs11715853
rs6809952
rs8039785
rs7311004
rs55877868
All − MR Egger
All − IVW(MRE)
−1.0
−0.5
0.0
0.5
1.0
MR effect size for
'Gut microbiota abundance (genus Parasutterella id.2892)' on 'Kelch−like protein 13'

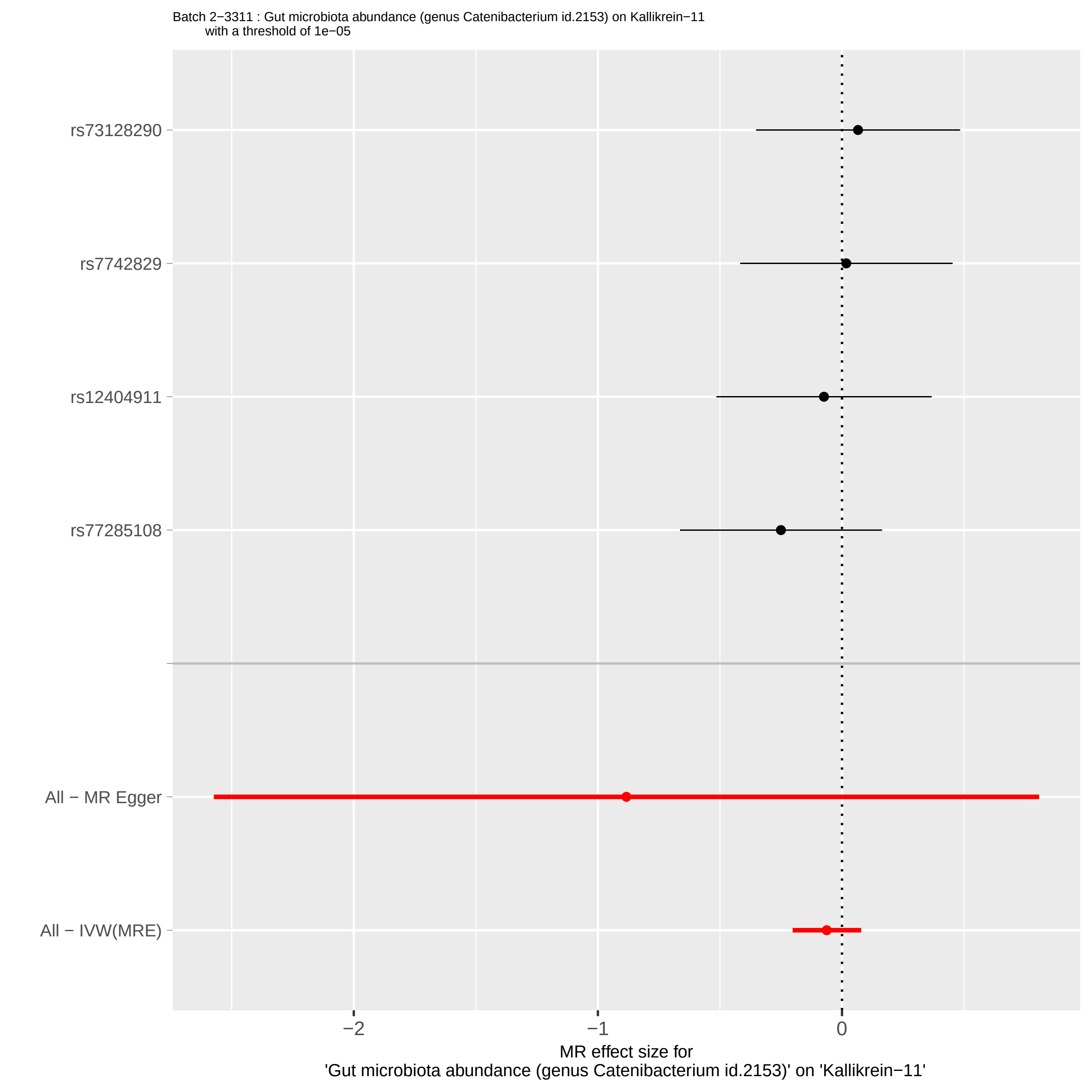
Batch 2−3311 : Gut microbiota abundance (genus Catenibacterium id.2153) on Kallikrein−11
with a threshold of 1e−05
rs73128290
rs7742829
rs12404911
rs77285108
All − MR Egger
All − IVW(MRE)
−2
−1
0
MR effect size for
'Gut microbiota abundance (genus Catenibacterium id.2153)' on 'Kallikrein−11'

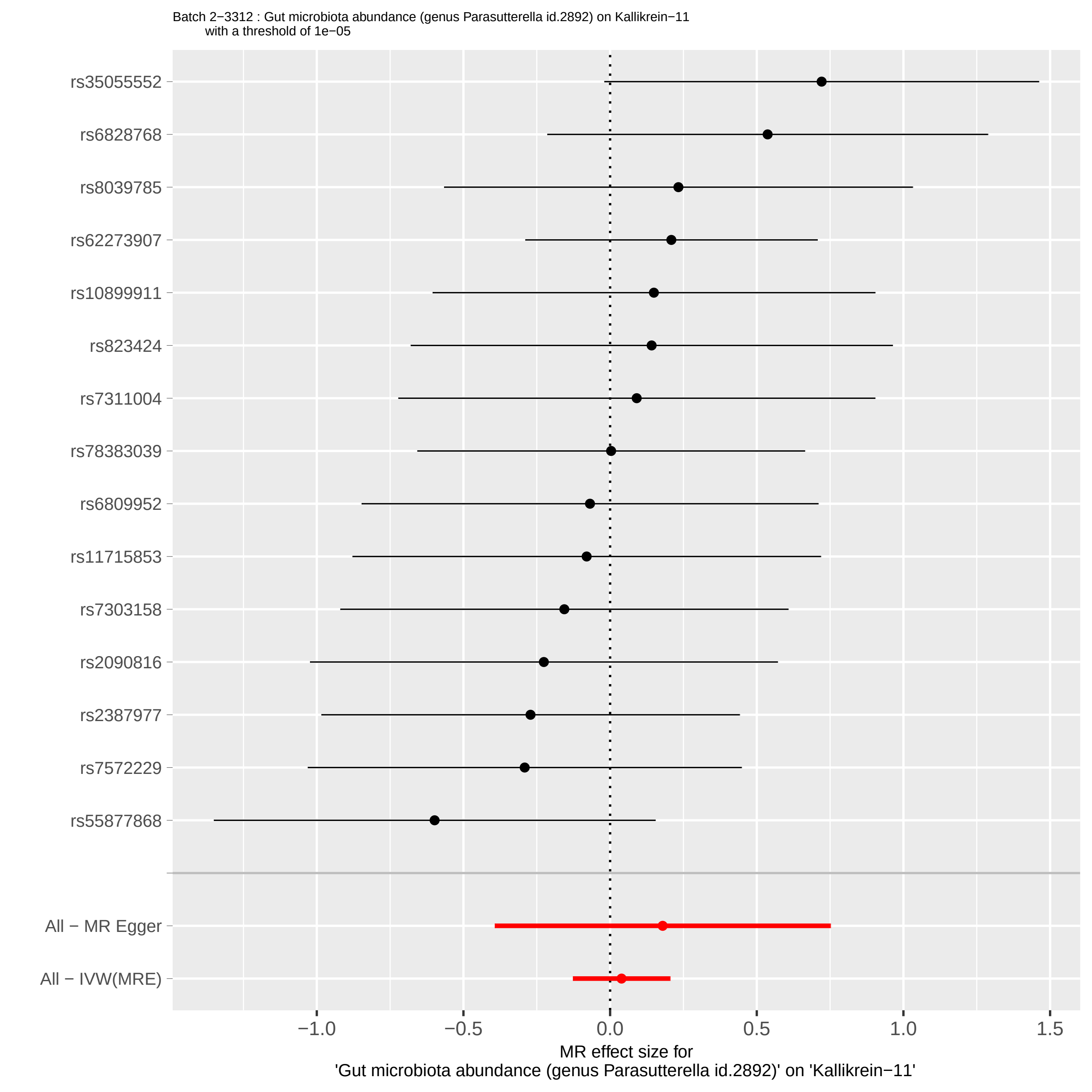

Batch 2−3312 : Gut microbiota abundance (genus Parasutterella id.2892) on Kallikrein−11
with a threshold of 1e−05
rs35055552
rs6828768
rs8039785
rs62273907
rs10899911
rs823424
rs7311004
rs78383039
rs6809952
rs11715853
rs7303158
rs2090816
rs2387977
rs7572229
rs55877868
All − MR Egger
All − IVW(MRE)
−1.0
−0.5
0.0
0.5
1.0
1.5
MR effect size for
'Gut microbiota abundance (genus Parasutterella id.2892)' on 'Kallikrein−11'

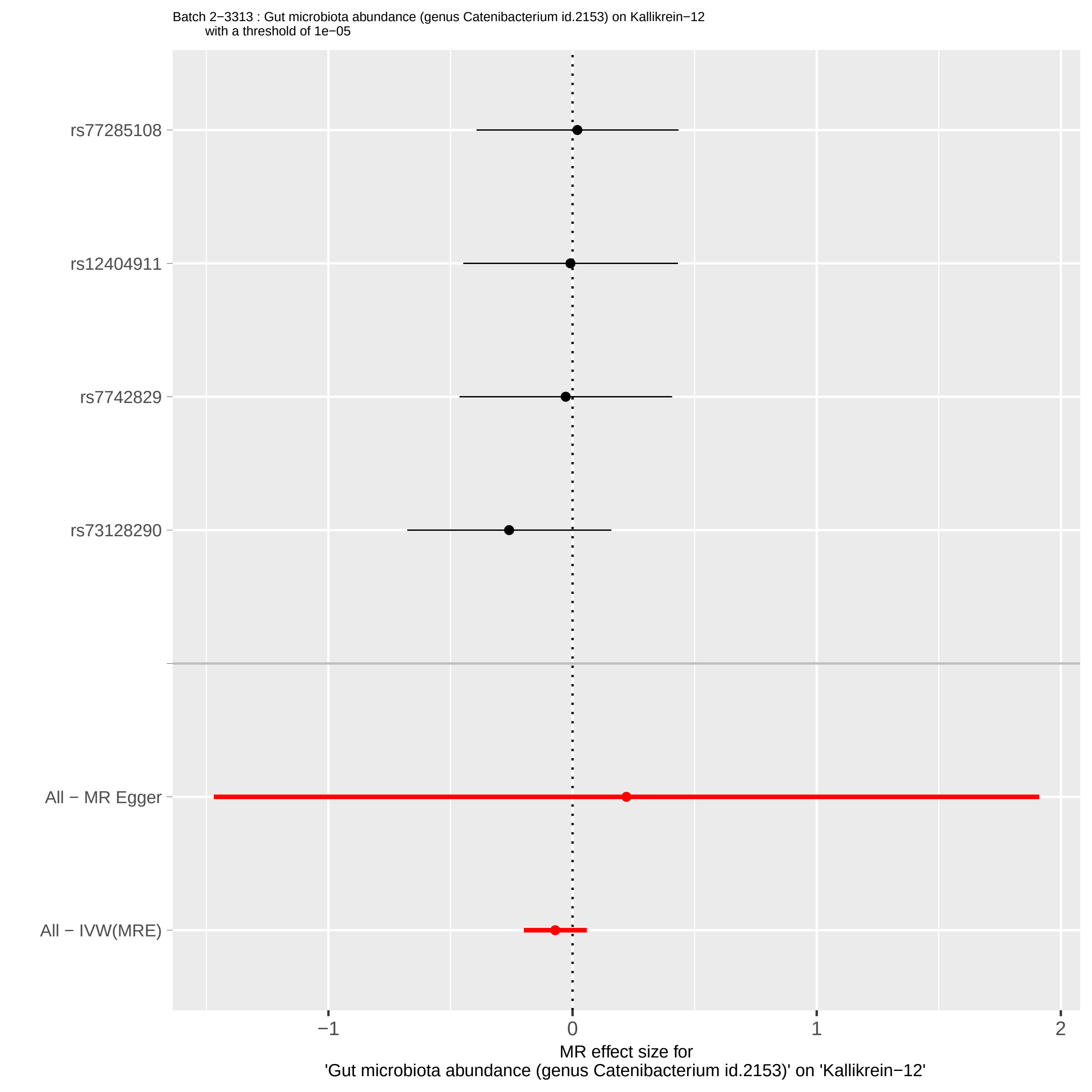

Batch 2−3313 : Gut microbiota abundance (genus Catenibacterium id.2153) on Kallikrein−12
with a threshold of 1e−05
rs77285108
rs12404911
rs7742829
rs73128290
All − MR Egger
All − IVW(MRE)
−1
0
1
2
MR effect size for
'Gut microbiota abundance (genus Catenibacterium id.2153)' on 'Kallikrein−12'

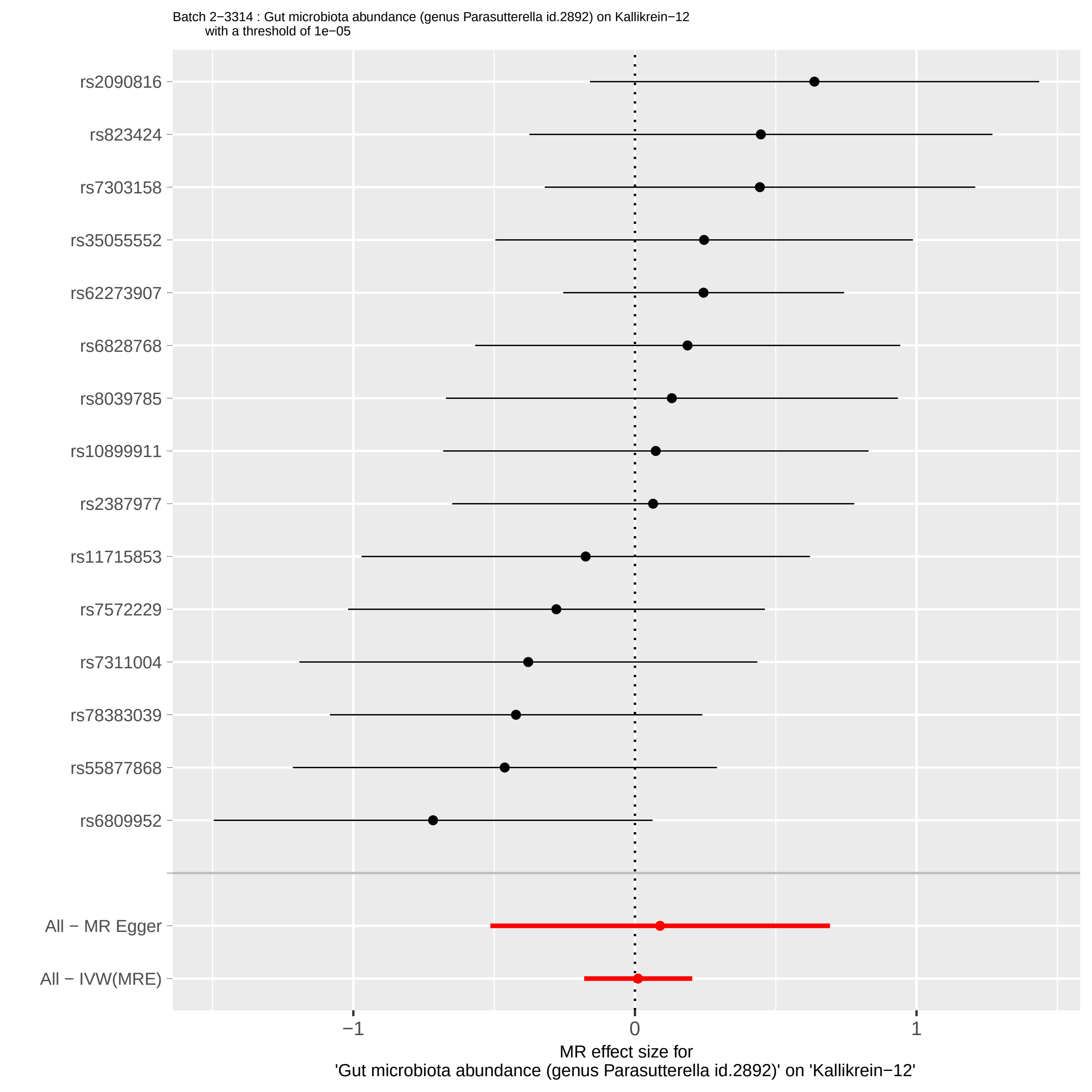

Batch 2−3314 : Gut microbiota abundance (genus Parasutterella id.2892) on Kallikrein−12
with a threshold of 1e−05
rs2090816
rs823424
rs7303158
rs35055552
rs62273907
rs6828768
rs8039785
rs10899911
rs2387977
rs11715853
rs7572229
rs7311004
rs78383039
rs55877868
rs6809952
All − MR Egger
All − IVW(MRE)
−1
0
1
MR effect size for
'Gut microbiota abundance (genus Parasutterella id.2892)' on 'Kallikrein−12'

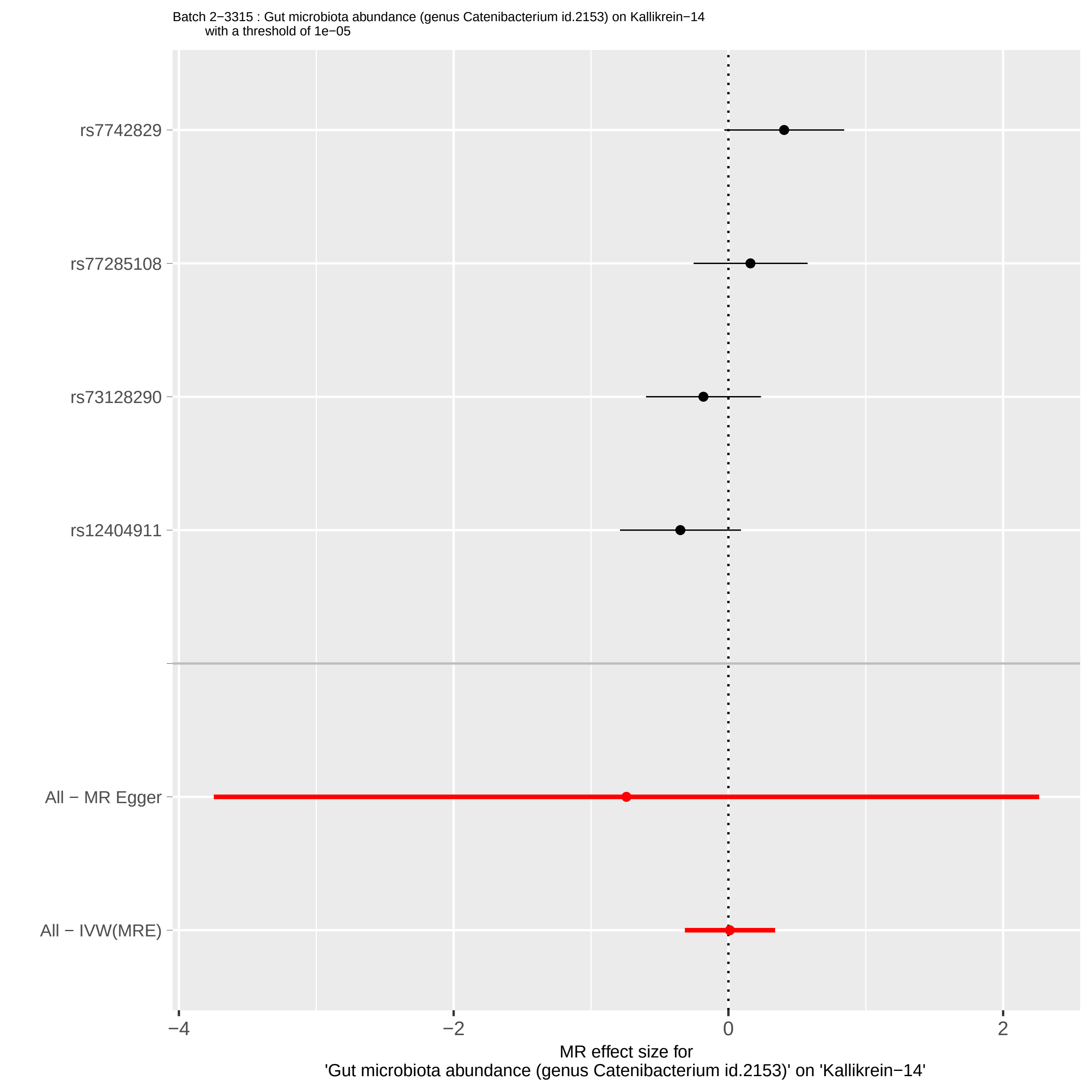
Batch 2−3315 : Gut microbiota abundance (genus Catenibacterium id.2153) on Kallikrein−14
with a threshold of 1e−05
rs7742829
rs77285108
rs73128290
rs12404911
All − MR Egger
All − IVW(MRE)
−4
−2
0
2
MR effect size for
'Gut microbiota abundance (genus Catenibacterium id.2153)' on 'Kallikrein−14'

Batch 2−3316 : Gut microbiota abundance (genus Parasutterella id.2892) on Kallikrein−14
with a threshold of 1e−05

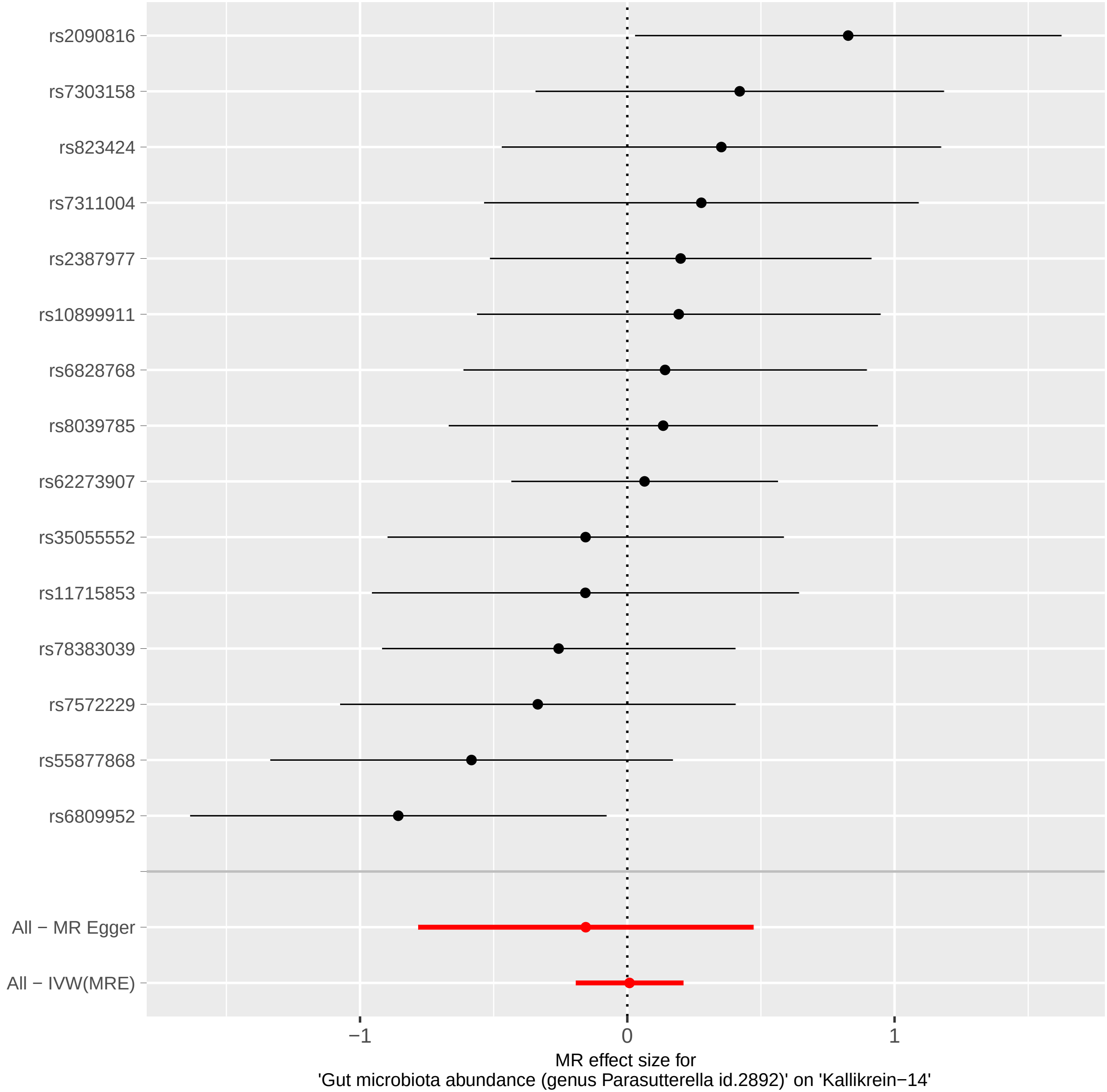

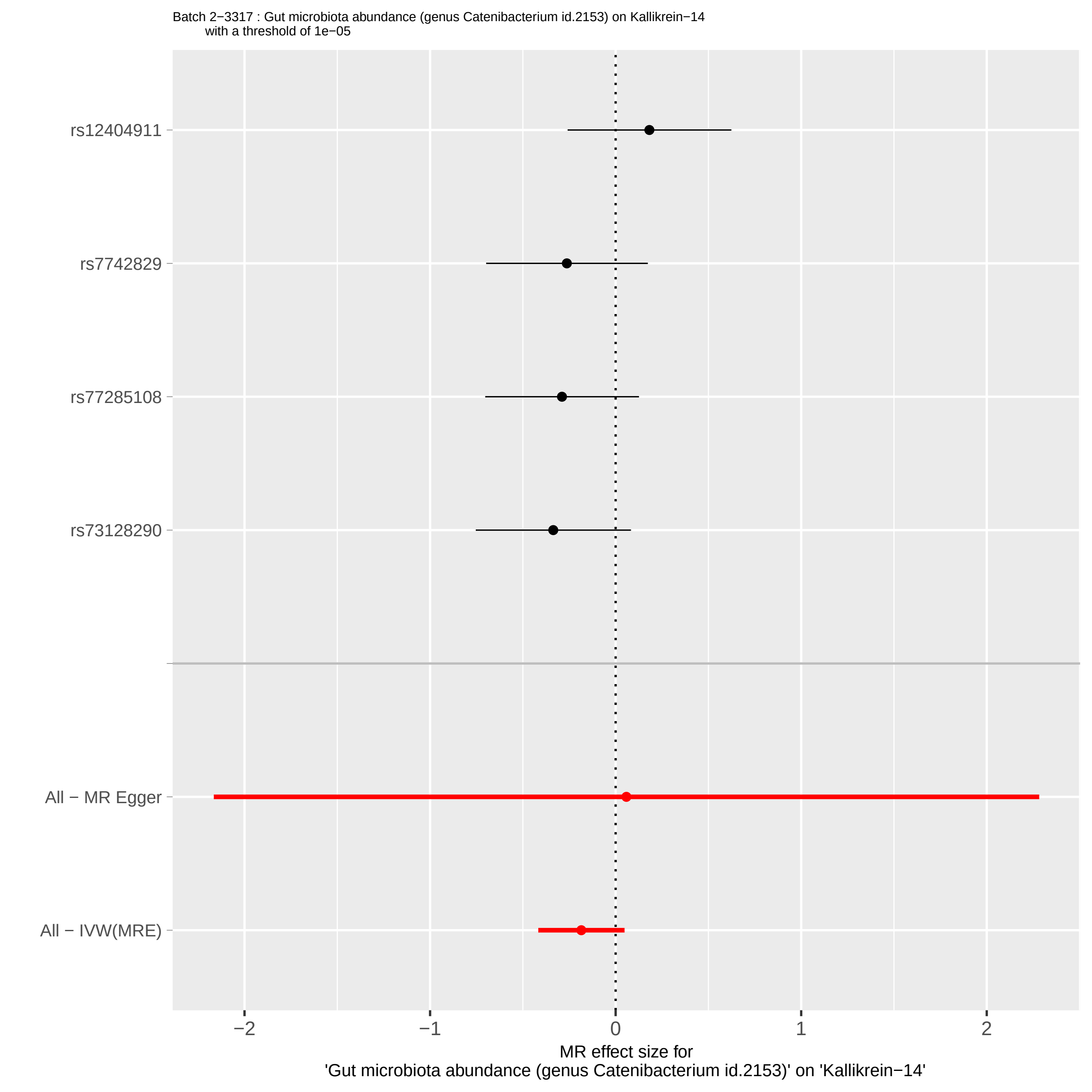
Batch 2−3317 : Gut microbiota abundance (genus Catenibacterium id.2153) on Kallikrein−14
with a threshold of 1e−05
rs12404911
rs7742829
rs77285108
rs73128290
All − MR Egger
All − IVW(MRE)
−2
−1
0
1
2
MR effect size for
'Gut microbiota abundance (genus Catenibacterium id.2153)' on 'Kallikrein−14'

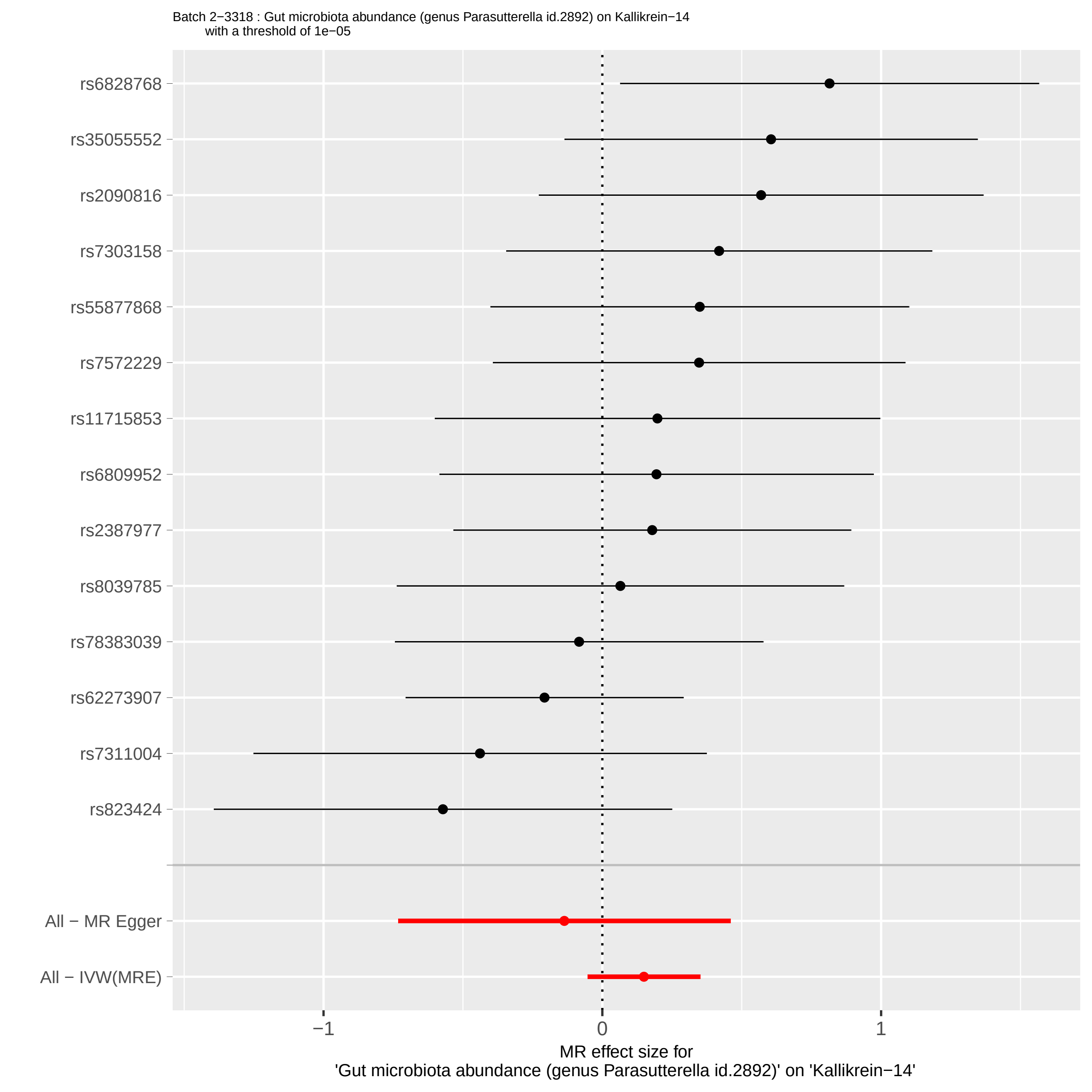

Batch 2−3318 : Gut microbiota abundance (genus Parasutterella id.2892) on Kallikrein−14
with a threshold of 1e−05
rs6828768
rs35055552
rs2090816
rs7303158
rs55877868
rs7572229
rs11715853
rs6809952
rs2387977
rs8039785
rs78383039
rs62273907
rs7311004
rs823424
All − MR Egger
All − IVW(MRE)
−1
0
1
MR effect size for
'Gut microbiota abundance (genus Parasutterella id.2892)' on 'Kallikrein−14'

Batch 2−3319 : Gut microbiota abundance (genus Catenibacterium id.2153) on Kallikrein−15
with a threshold of 1e−05

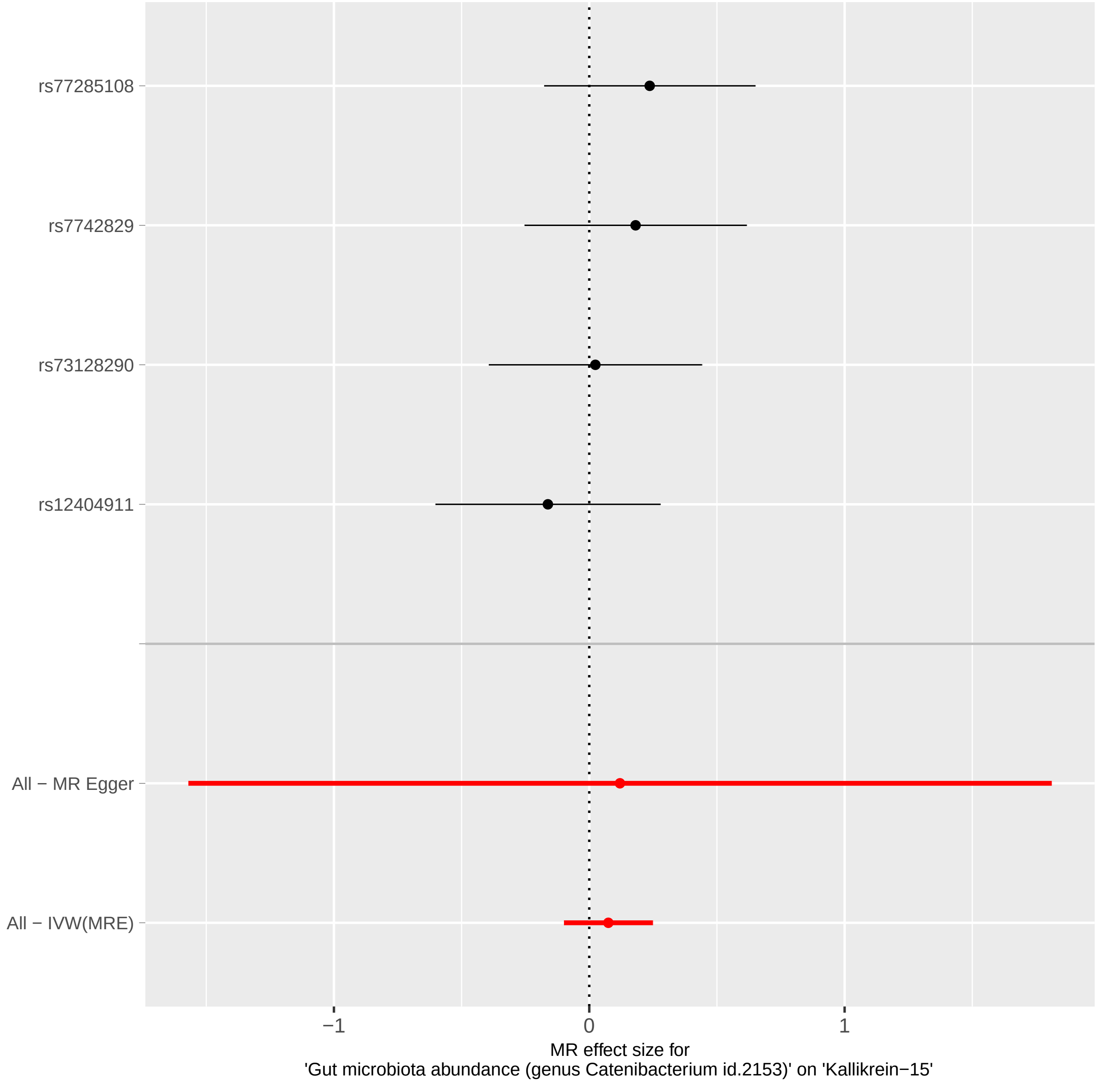

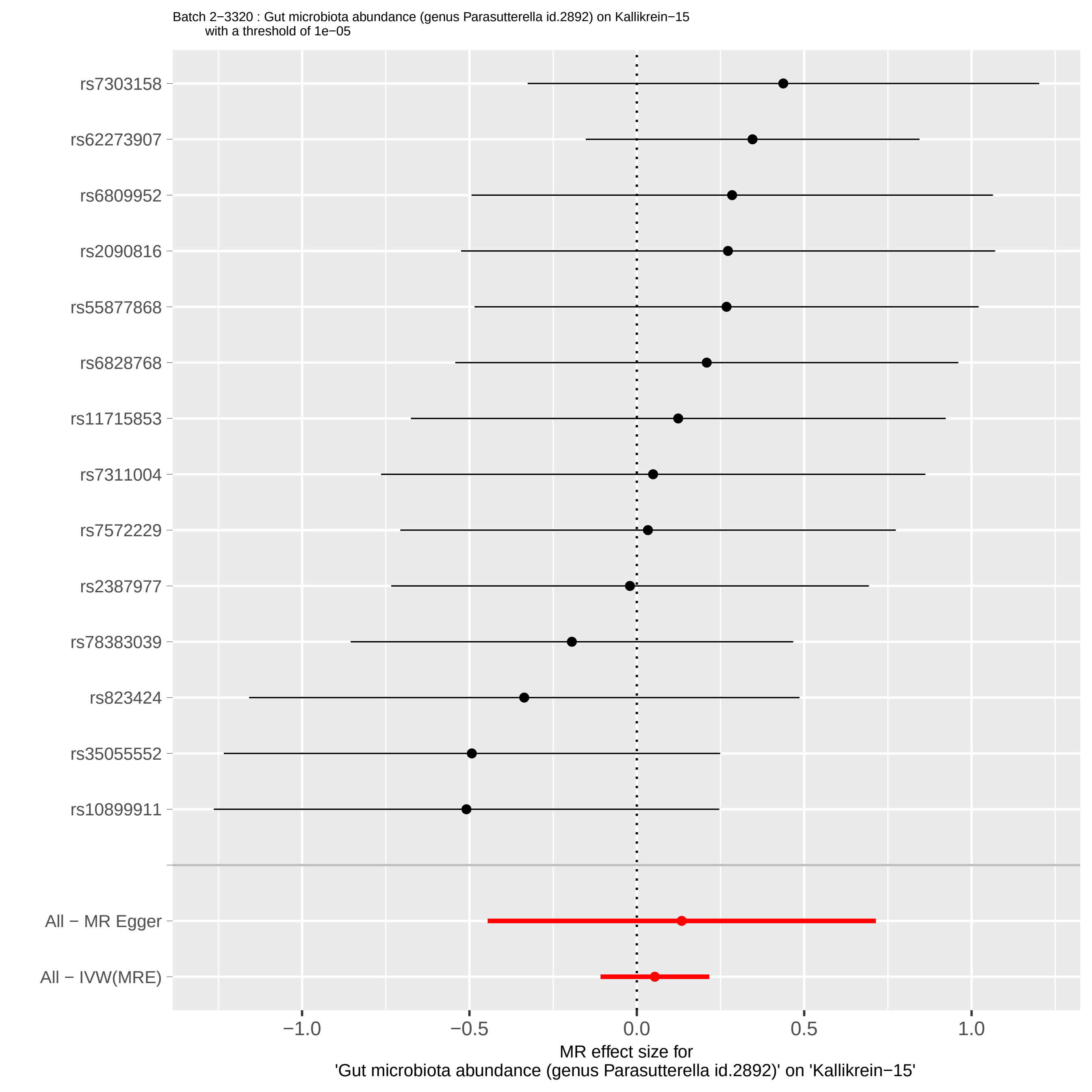

Batch 2−3320 : Gut microbiota abundance (genus Parasutterella id.2892) on Kallikrein−15
with a threshold of 1e−05
rs7303158
rs62273907
rs6809952
rs2090816
rs55877868
rs6828768
rs11715853
rs7311004
rs7572229
rs2387977
rs78383039
rs823424
rs35055552
rs10899911
All − MR Egger
All − IVW(MRE)
−1.0
−0.5
0.0
0.5
1.0
MR effect size for
'Gut microbiota abundance (genus Parasutterella id.2892)' on 'Kallikrein−15'

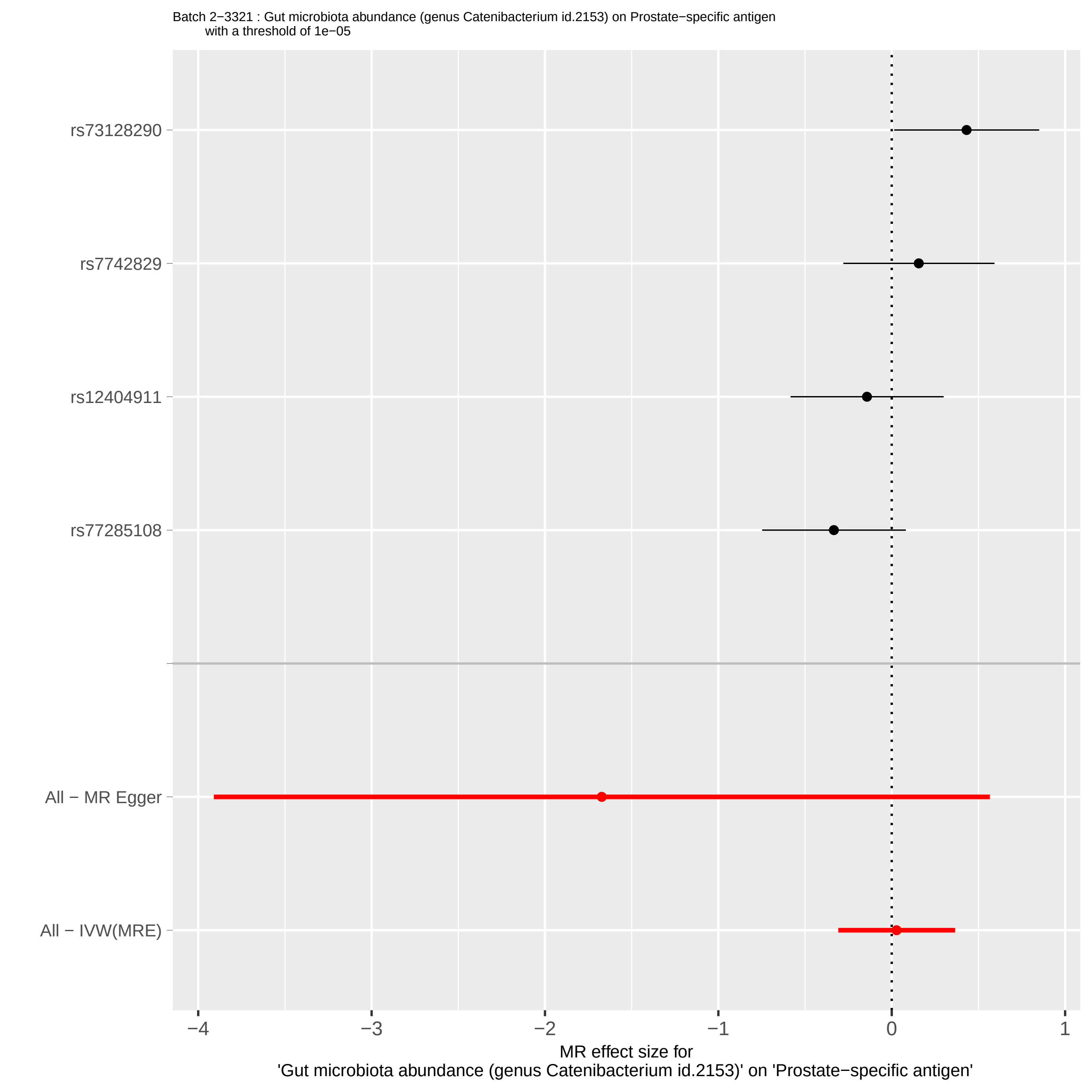

Batch 2−3321 : Gut microbiota abundance (genus Catenibacterium id.2153) on Prostate−specific antigen
with a threshold of 1e−05
rs73128290
rs7742829
rs12404911
rs77285108
All − MR Egger
All − IVW(MRE)
−4
−3
−2
−1
0
1
MR effect size for
'Gut microbiota abundance (genus Catenibacterium id.2153)' on 'Prostate−specific antigen'

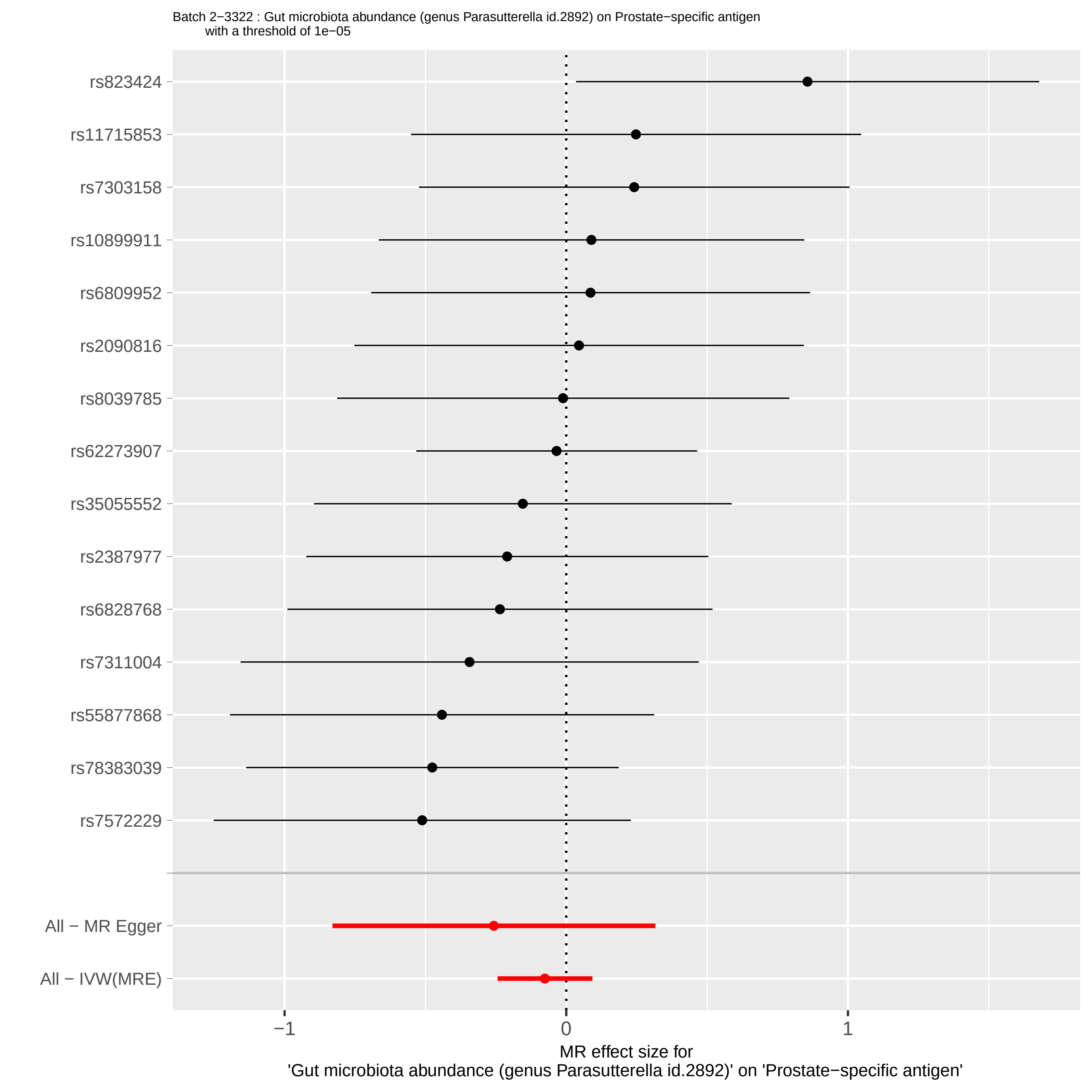

Batch 2−3322 : Gut microbiota abundance (genus Parasutterella id.2892) on Prostate−specific antigen
with a threshold of 1e−05
rs823424
rs11715853
rs7303158
rs10899911
rs6809952
rs2090816
rs8039785
rs62273907
rs35055552
rs2387977
rs6828768
rs7311004
rs55877868
rs78383039
rs7572229
All − MR Egger
All − IVW(MRE)
−1
0
1
MR effect size for
'Gut microbiota abundance (genus Parasutterella id.2892)' on 'Prostate−specific antigen'

Batch 2−3323 : Gut microbiota abundance (genus Catenibacterium id.2153) on Kallikrein−4
with a threshold of 1e−05

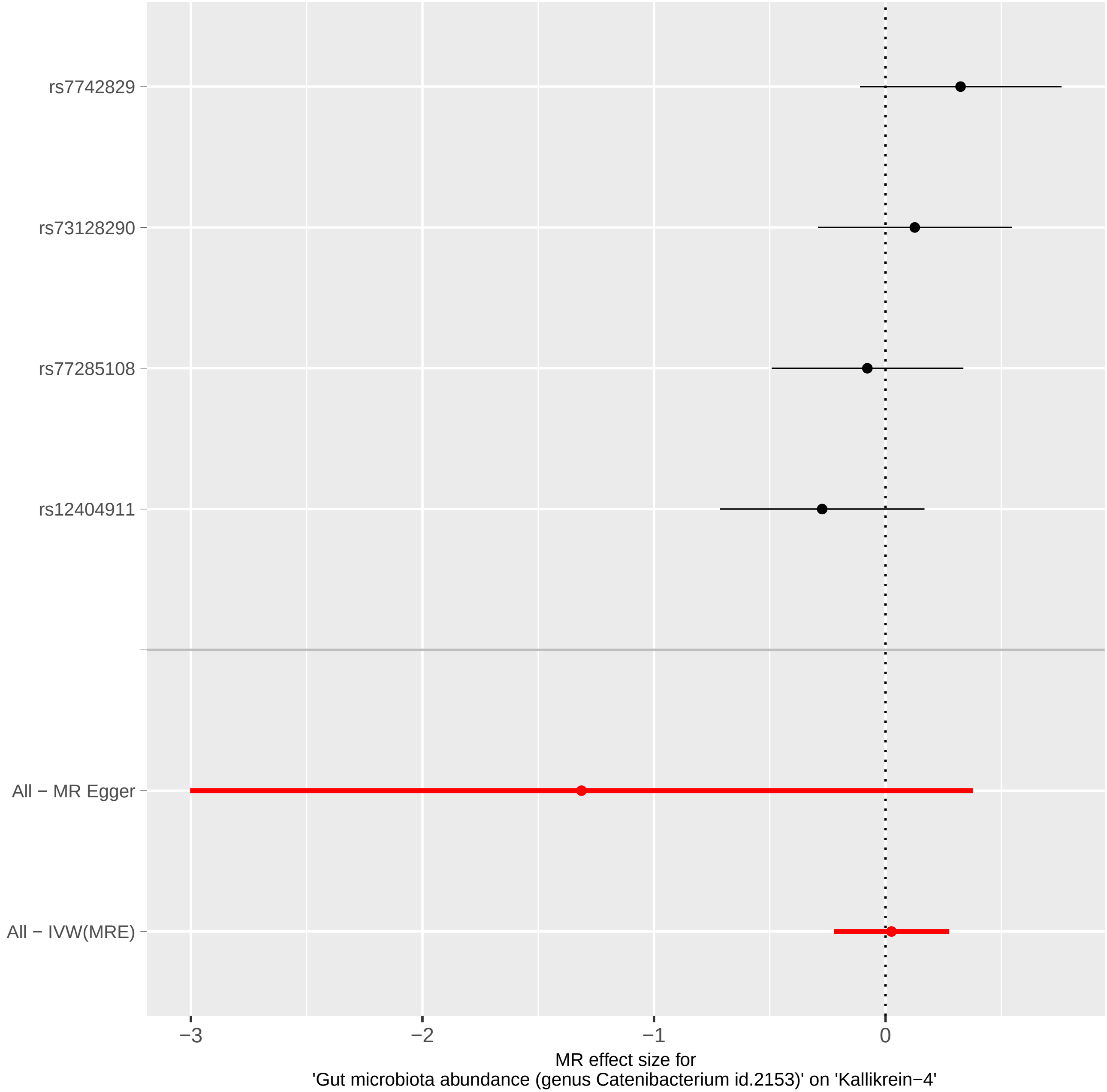

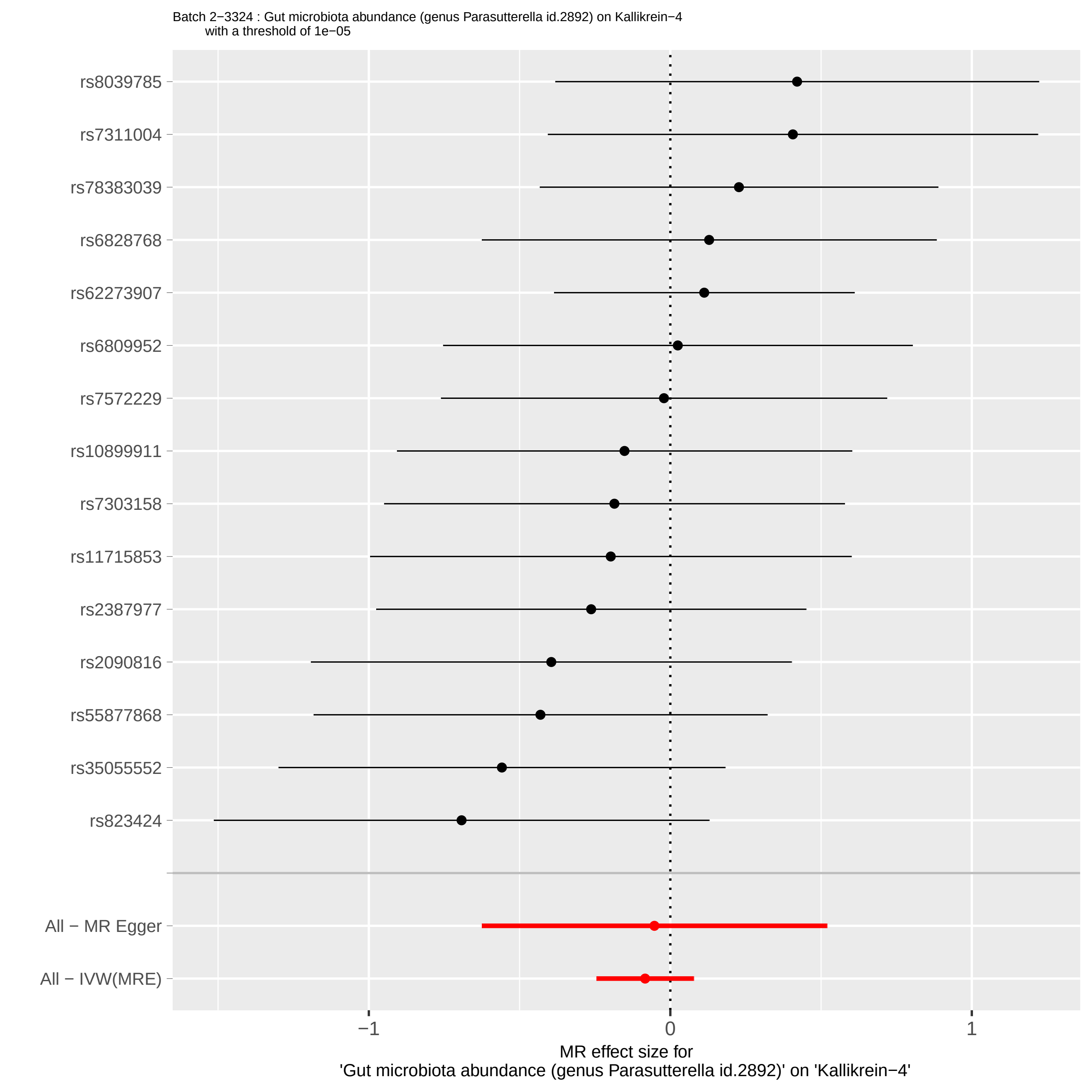

Batch 2−3324 : Gut microbiota abundance (genus Parasutterella id.2892) on Kallikrein−4
with a threshold of 1e−05
rs8039785
rs7311004
rs78383039
rs6828768
rs62273907
rs6809952
rs7572229
rs10899911
rs7303158
rs11715853
rs2387977
rs2090816
rs55877868
rs35055552
rs823424
All − MR Egger
All − IVW(MRE)
−1
0
1
MR effect size for
'Gut microbiota abundance (genus Parasutterella id.2892)' on 'Kallikrein−4'

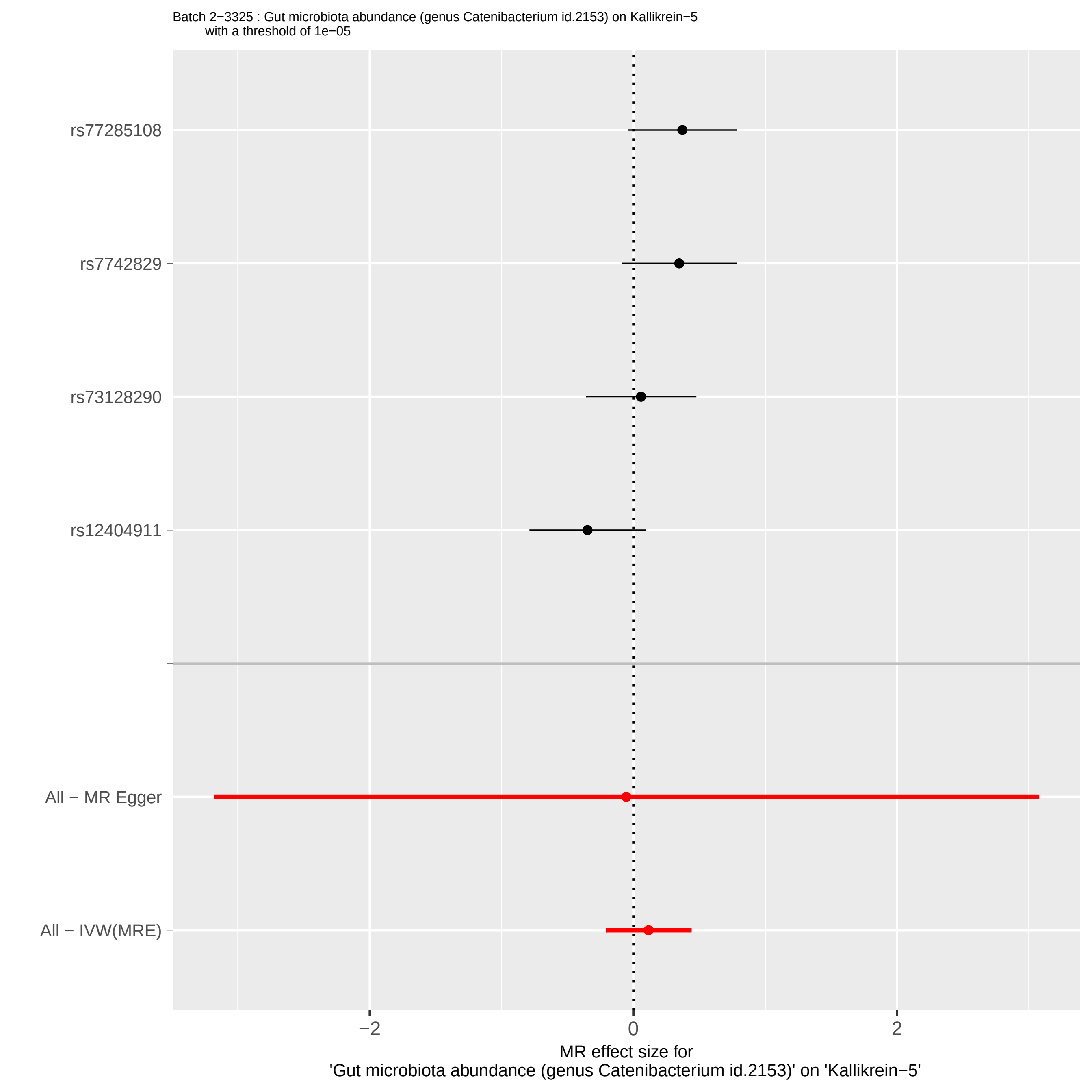

Batch 2−3325 : Gut microbiota abundance (genus Catenibacterium id.2153) on Kallikrein−5
with a threshold of 1e−05
rs77285108
rs7742829
rs73128290
rs12404911
All − MR Egger
All − IVW(MRE)
−2
0
2
MR effect size for
'Gut microbiota abundance (genus Catenibacterium id.2153)' on 'Kallikrein−5'

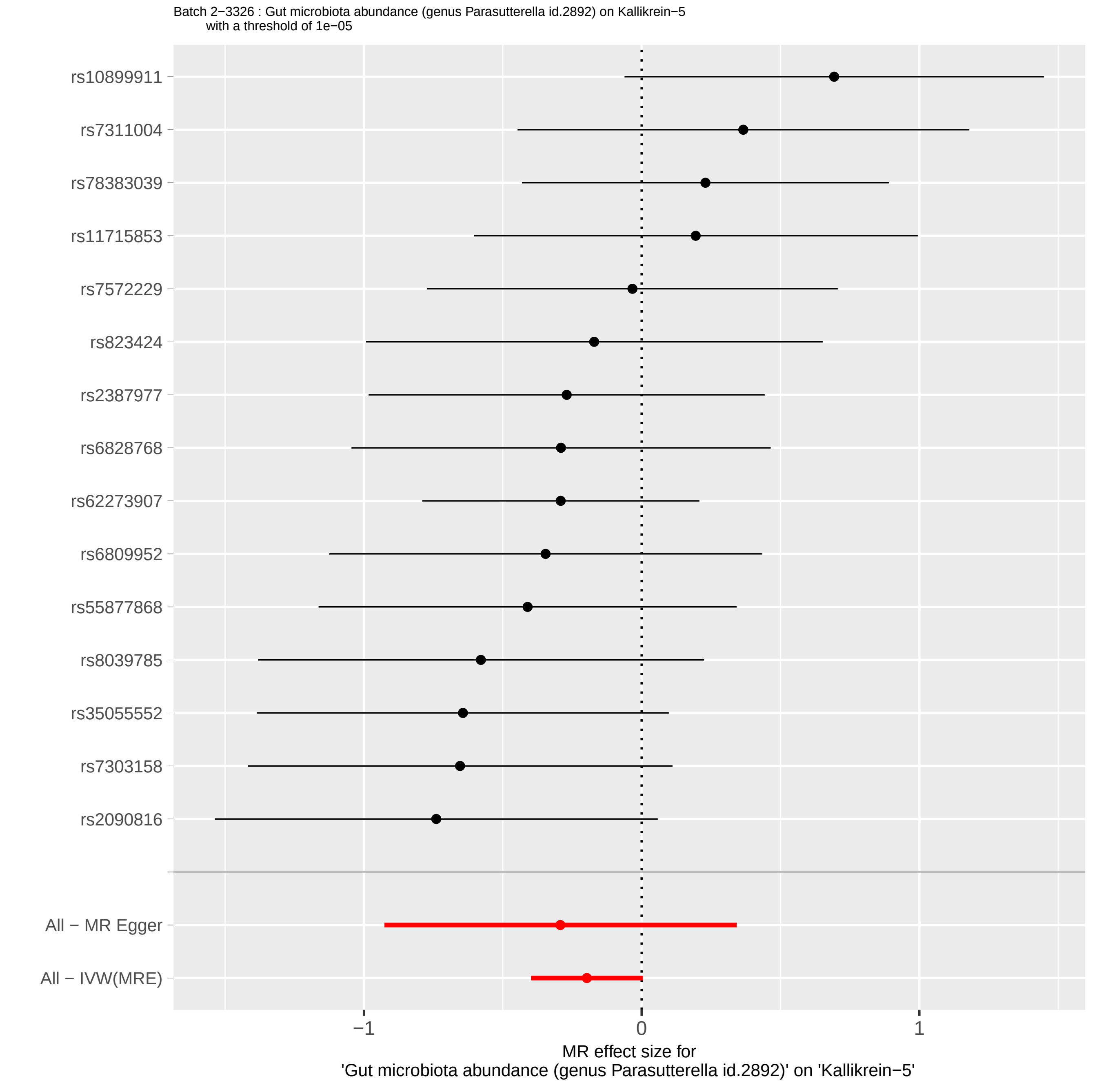
Batch 2−3326 : Gut microbiota abundance (genus Parasutterella id.2892) on Kallikrein−5
with a threshold of 1e−05
rs10899911
rs7311004
rs78383039
rs11715853
rs7572229
rs823424
rs2387977
rs6828768
rs62273907
rs6809952
rs55877868
rs8039785
rs35055552
rs7303158
rs2090816
All − MR Egger
All − IVW(MRE)
−1
0
1
MR effect size for
'Gut microbiota abundance (genus Parasutterella id.2892)' on 'Kallikrein−5'

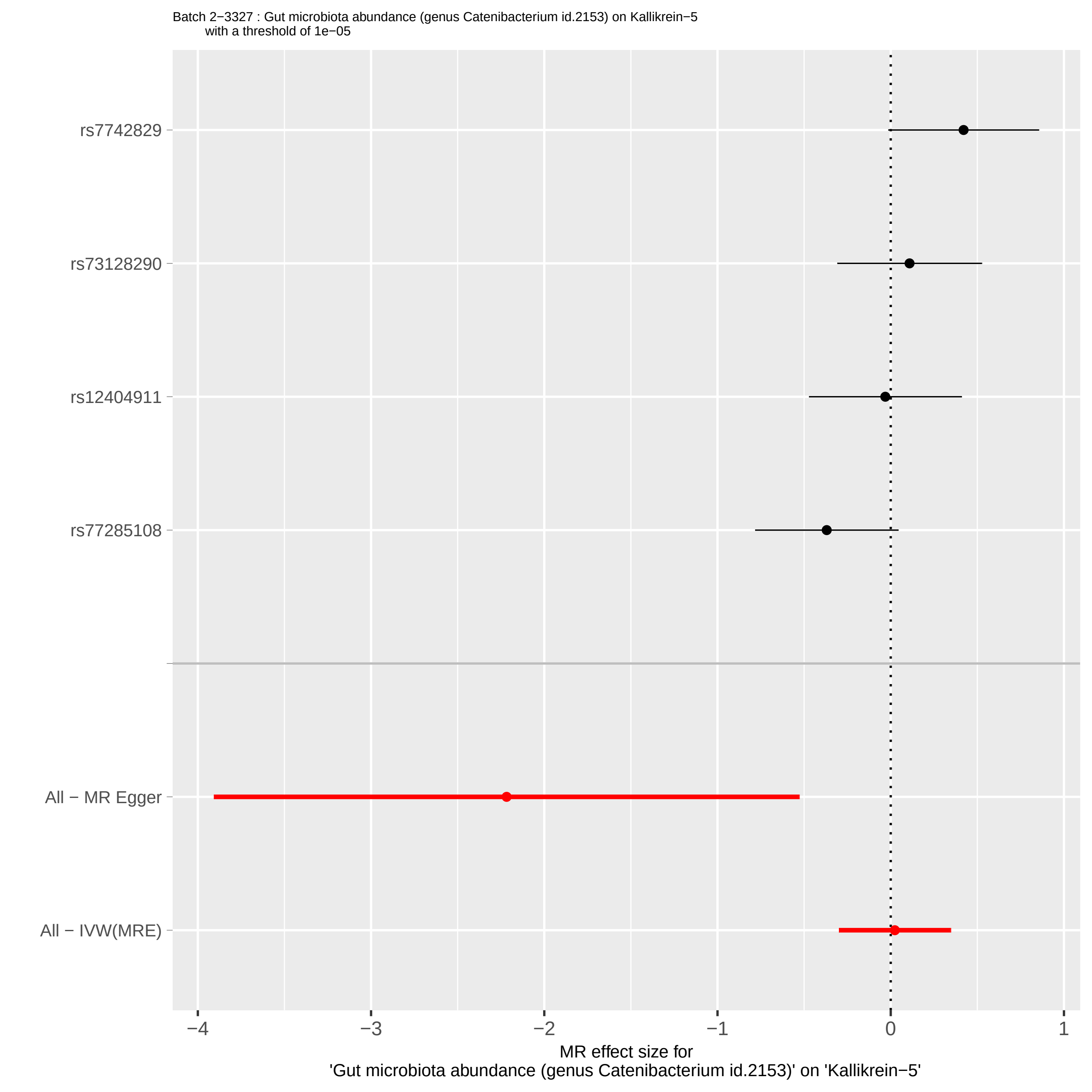

Batch 2−3327 : Gut microbiota abundance (genus Catenibacterium id.2153) on Kallikrein−5
with a threshold of 1e−05
rs7742829
rs73128290
rs12404911
rs77285108
All − MR Egger
All − IVW(MRE)
−4
−3
−2
−1
0
1
MR effect size for
'Gut microbiota abundance (genus Catenibacterium id.2153)' on 'Kallikrein−5'

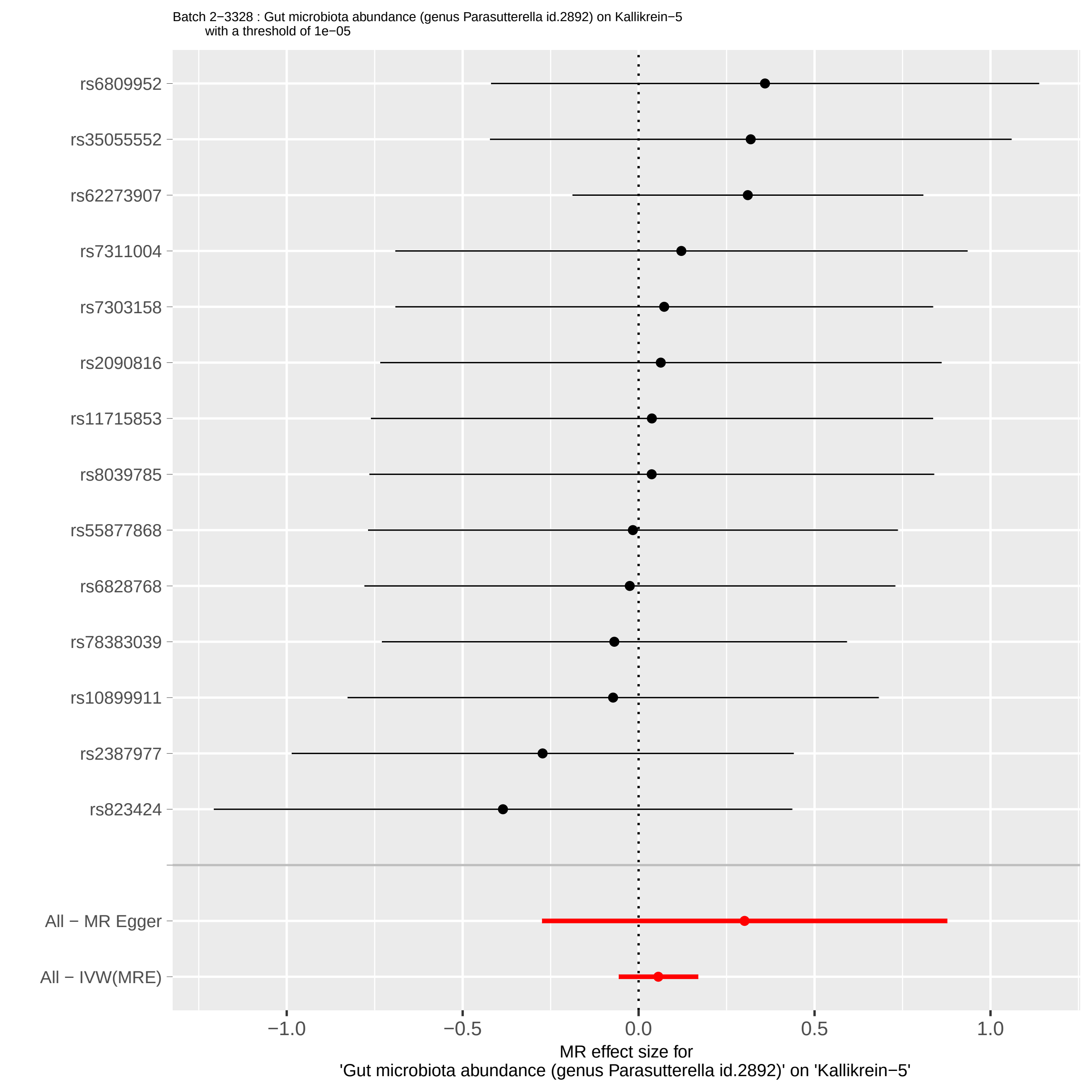

Batch 2−3328 : Gut microbiota abundance (genus Parasutterella id.2892) on Kallikrein−5
with a threshold of 1e−05
rs6809952
rs35055552
rs62273907
rs7311004
rs7303158
rs2090816
rs11715853
rs8039785
rs55877868
rs6828768
rs78383039
rs10899911
rs2387977
rs823424
All − MR Egger
All − IVW(MRE)
−1.0
−0.5
0.0
0.5
1.0
MR effect size for
'Gut microbiota abundance (genus Parasutterella id.2892)' on 'Kallikrein−5'

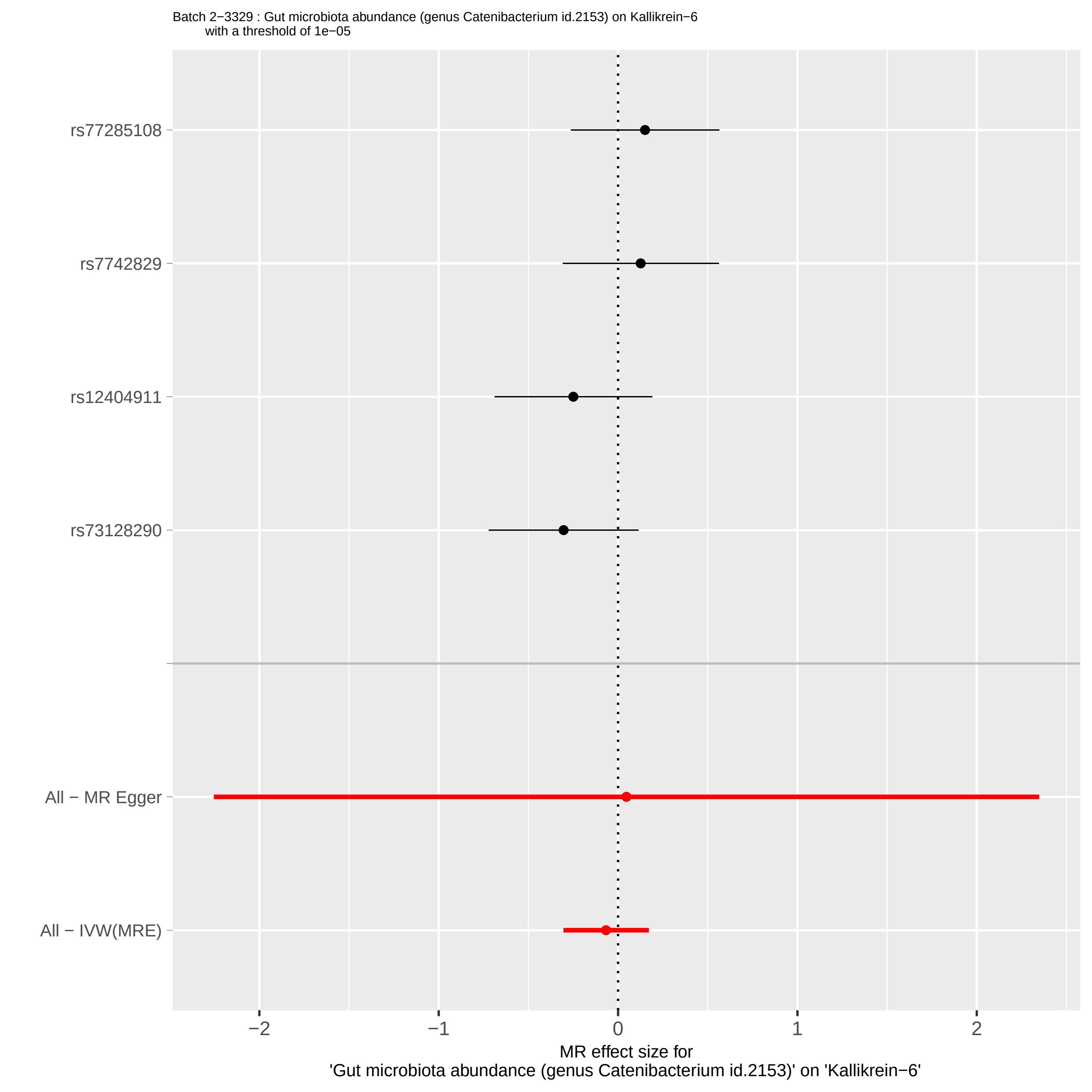

Batch 2−3329 : Gut microbiota abundance (genus Catenibacterium id.2153) on Kallikrein−6
with a threshold of 1e−05
rs77285108
rs7742829
rs12404911
rs73128290
All − MR Egger
All − IVW(MRE)
−2
−1
0
1
2
MR effect size for
'Gut microbiota abundance (genus Catenibacterium id.2153)' on 'Kallikrein−6'

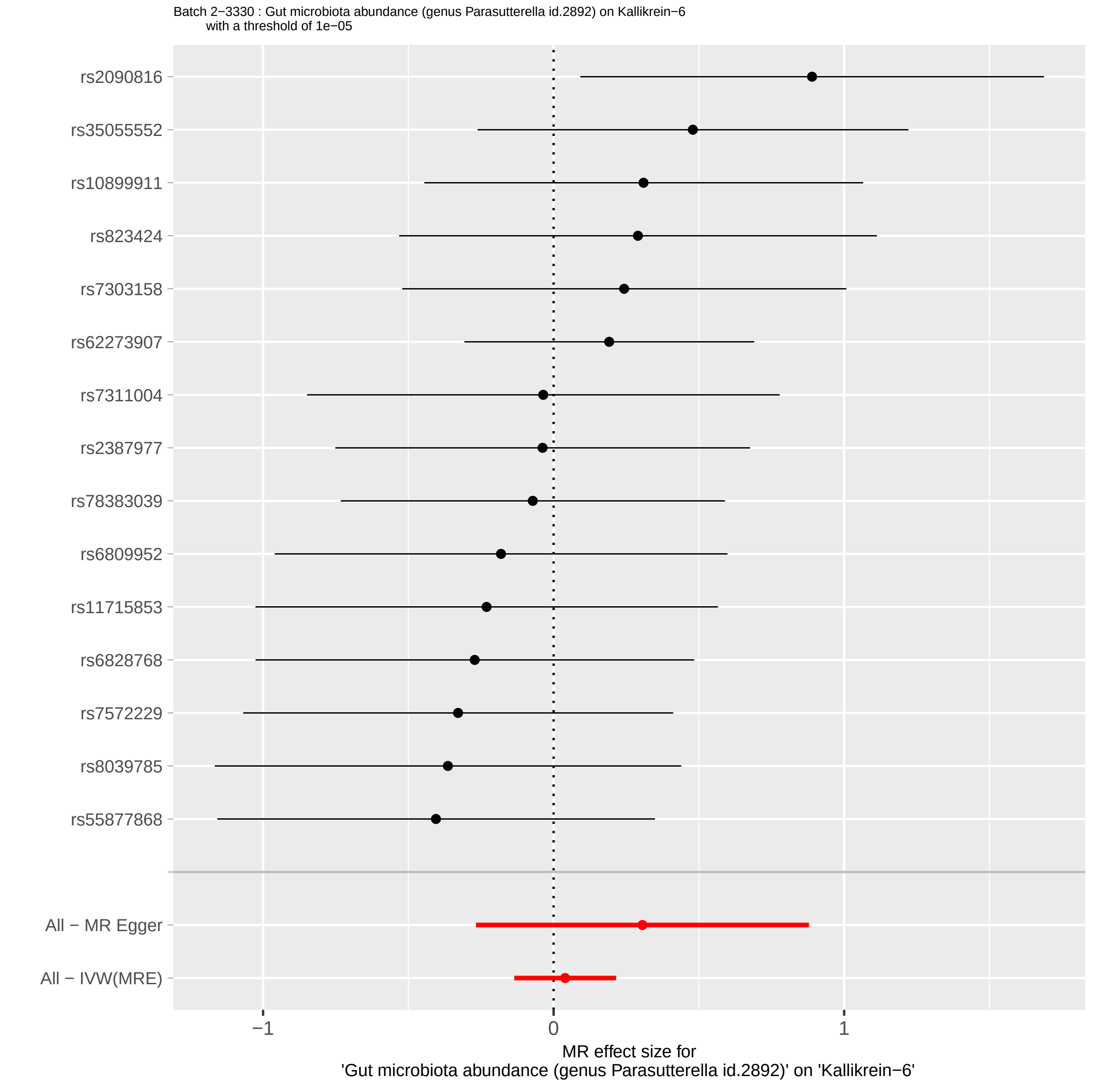

Batch 2−3330 : Gut microbiota abundance (genus Parasutterella id.2892) on Kallikrein−6
with a threshold of 1e−05
rs2090816
rs35055552
rs10899911
rs823424
rs7303158
rs62273907
rs7311004
rs2387977
rs78383039
rs6809952
rs11715853
rs6828768
rs7572229
rs8039785
rs55877868
All − MR Egger
All − IVW(MRE)
−1
0
1
MR effect size for
'Gut microbiota abundance (genus Parasutterella id.2892)' on 'Kallikrein−6'

Batch 2−3331 : Gut microbiota abundance (genus Catenibacterium id.2153) on Kallikrein−7
with a threshold of 1e−05

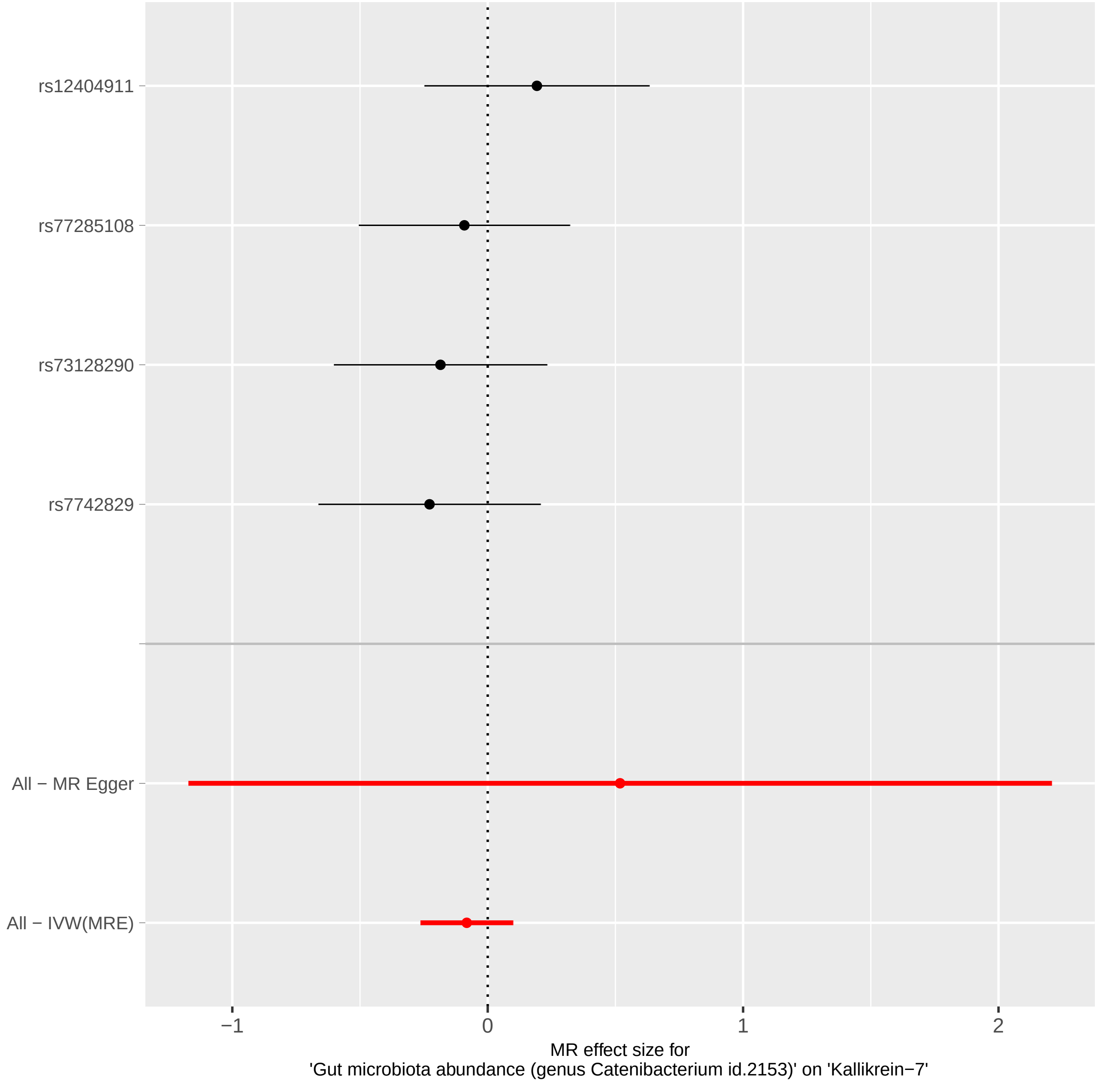

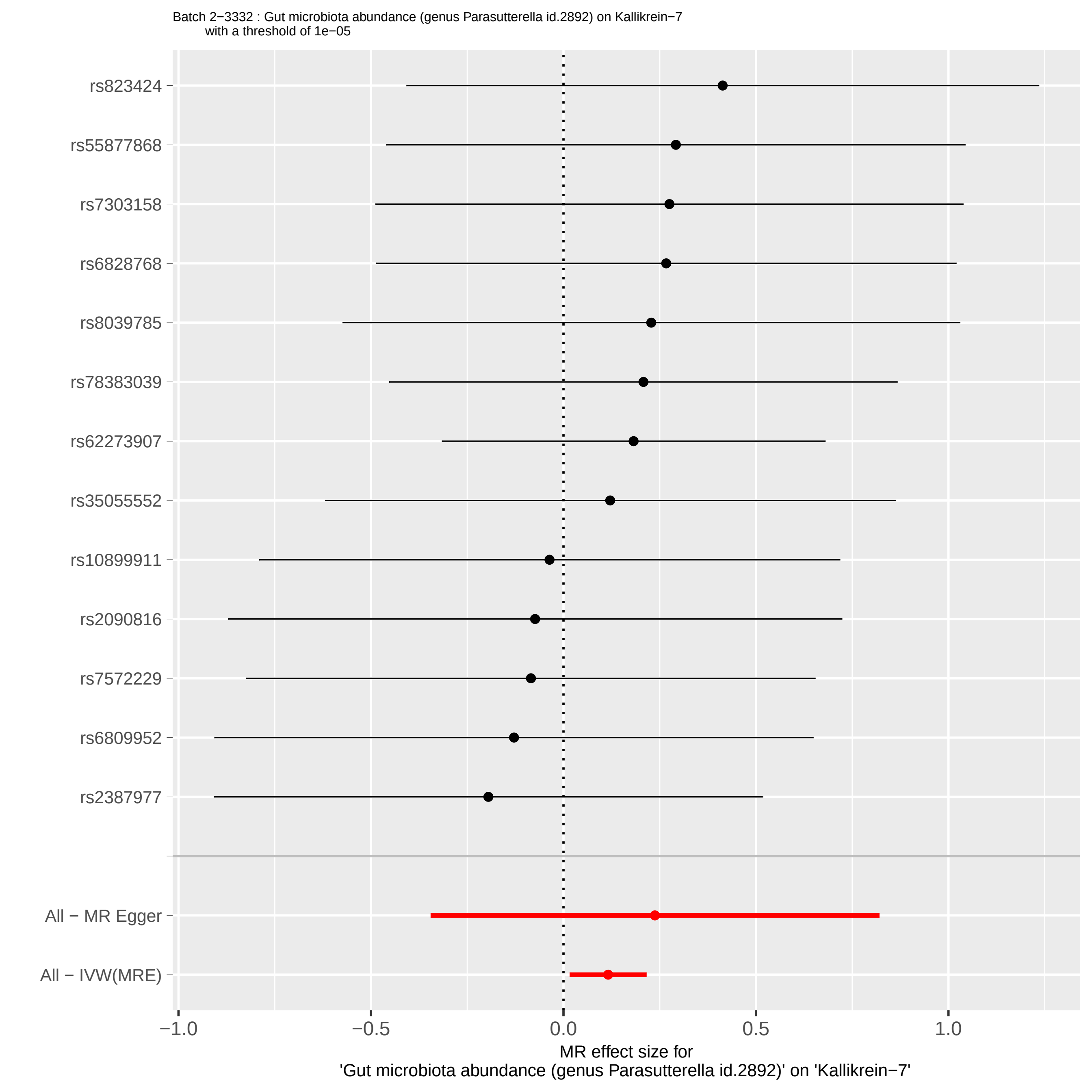

Batch 2−3332 : Gut microbiota abundance (genus Parasutterella id.2892) on Kallikrein−7
with a threshold of 1e−05
rs823424
rs55877868
rs7303158
rs6828768
rs8039785
rs78383039
rs62273907
rs35055552
rs10899911
rs2090816
rs7572229
rs6809952
rs2387977
All − MR Egger
All − IVW(MRE)
−1.0
−0.5
0.0
0.5
1.0
MR effect size for
'Gut microbiota abundance (genus Parasutterella id.2892)' on 'Kallikrein−7'

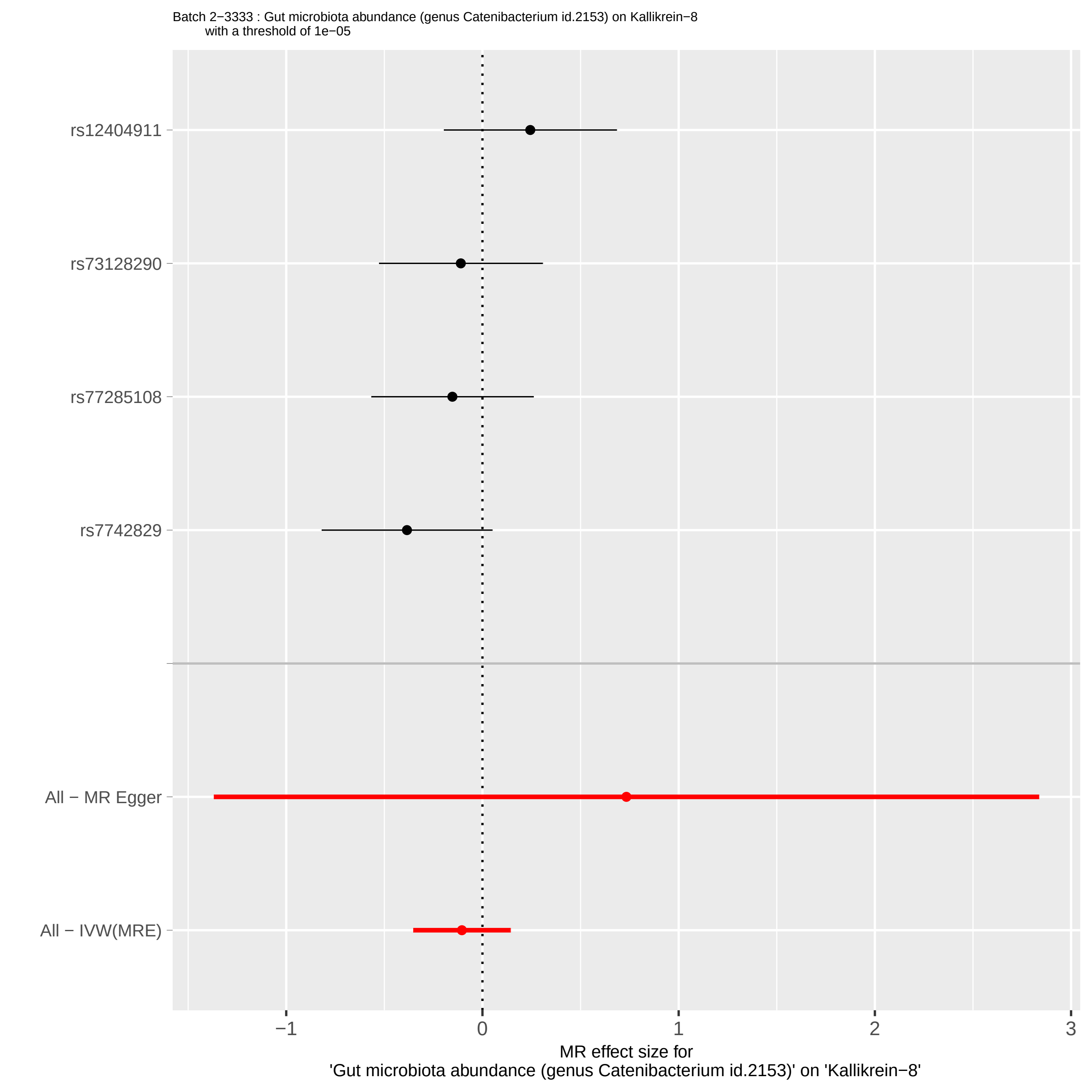

Batch 2−3333 : Gut microbiota abundance (genus Catenibacterium id.2153) on Kallikrein−8
with a threshold of 1e−05
rs12404911
rs73128290
rs77285108
rs7742829
All − MR Egger
All − IVW(MRE)
−1
0
1
2
3
MR effect size for
'Gut microbiota abundance (genus Catenibacterium id.2153)' on 'Kallikrein−8'

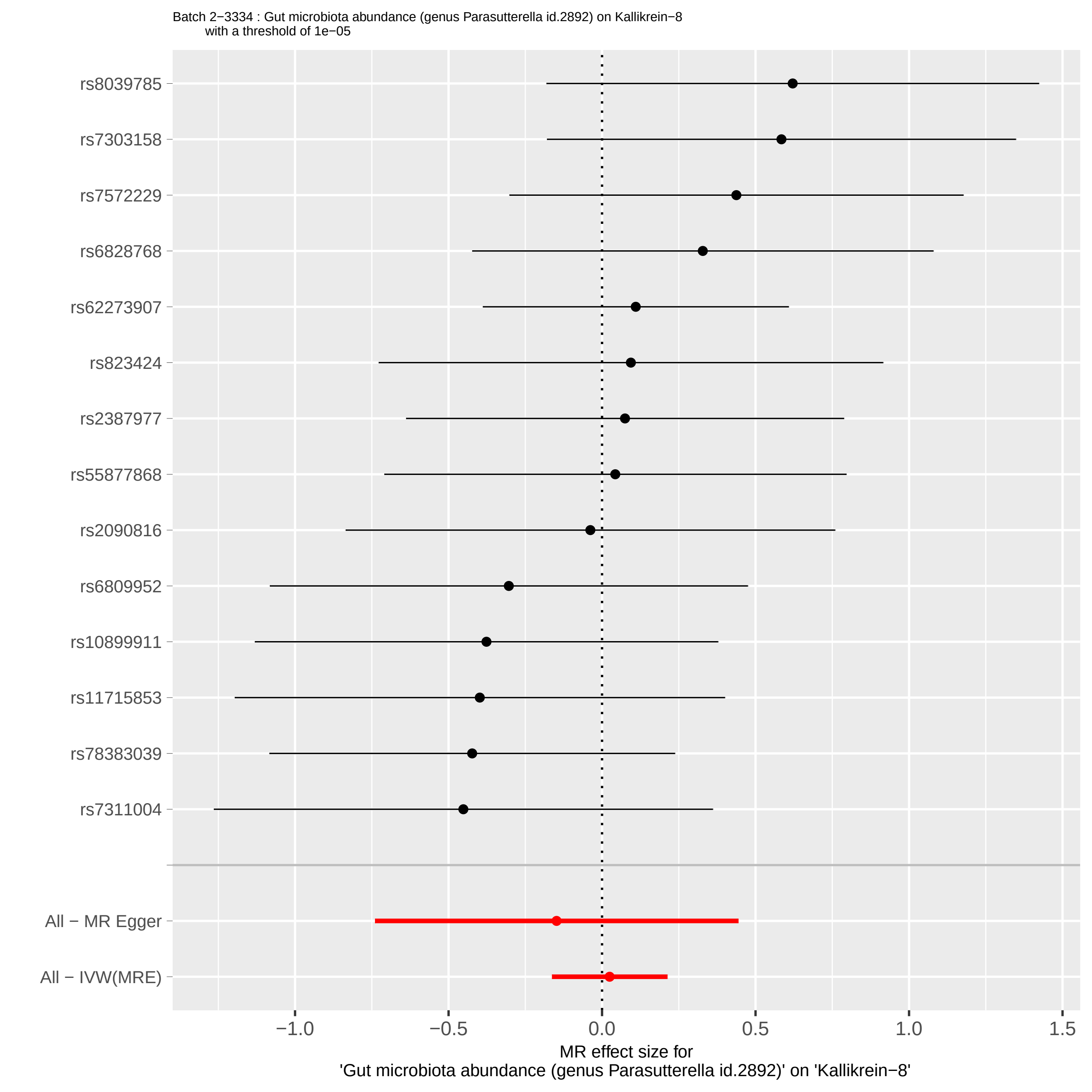
Batch 2−3334 : Gut microbiota abundance (genus Parasutterella id.2892) on Kallikrein−8
with a threshold of 1e−05
rs8039785
rs7303158
rs7572229
rs6828768
rs62273907
rs823424
rs2387977
rs55877868
rs2090816
rs6809952
rs10899911
rs11715853
rs78383039
rs7311004
All − MR Egger
All − IVW(MRE)
−1.0
−0.5
0.0
0.5
1.0
1.5
MR effect size for
'Gut microbiota abundance (genus Parasutterella id.2892)' on 'Kallikrein−8'

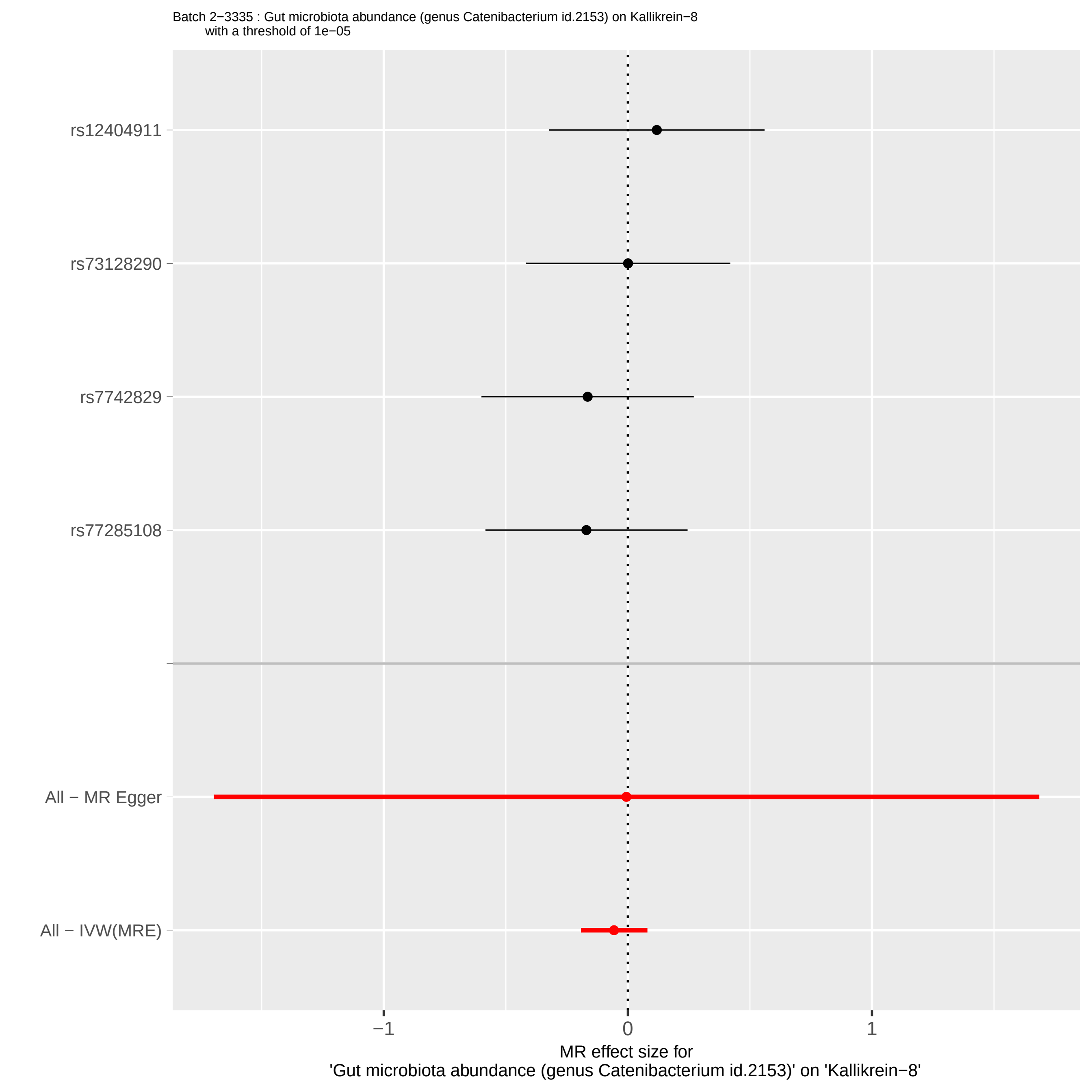
Batch 2−3335 : Gut microbiota abundance (genus Catenibacterium id.2153) on Kallikrein−8
with a threshold of 1e−05
rs12404911
rs73128290
rs7742829
rs77285108
All − MR Egger
All − IVW(MRE)
−1
0
1
MR effect size for
'Gut microbiota abundance (genus Catenibacterium id.2153)' on 'Kallikrein−8'

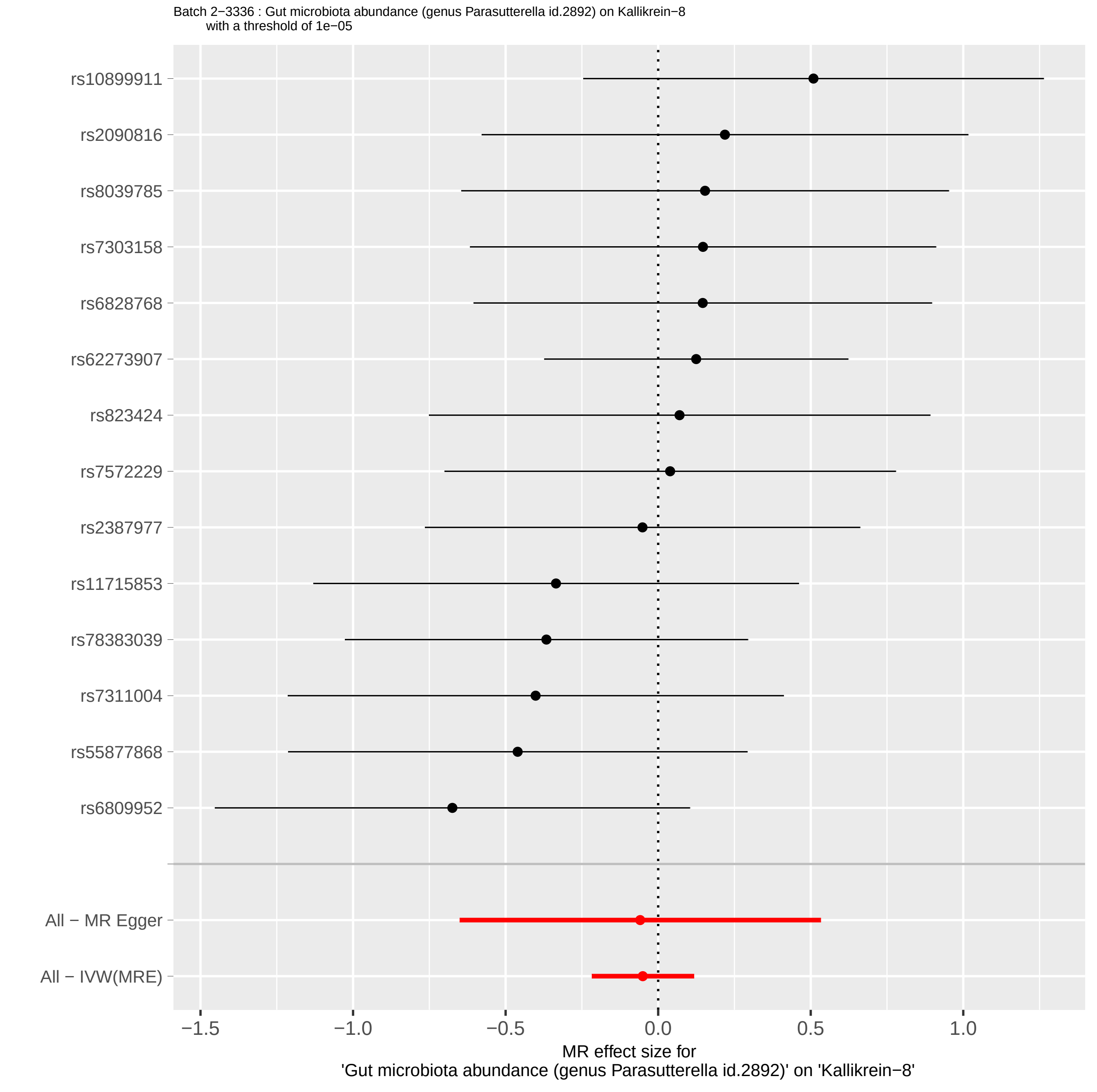
Batch 2−3336 : Gut microbiota abundance (genus Parasutterella id.2892) on Kallikrein−8
with a threshold of 1e−05
rs10899911
rs2090816
rs8039785
rs7303158
rs6828768
rs62273907
rs823424
rs7572229
rs2387977
rs11715853
rs78383039
rs7311004
rs55877868
rs6809952
All − MR Egger
All − IVW(MRE)
−1.5
−1.0
−0.5
0.0
0.5
1.0
MR effect size for
'Gut microbiota abundance (genus Parasutterella id.2892)' on 'Kallikrein−8'

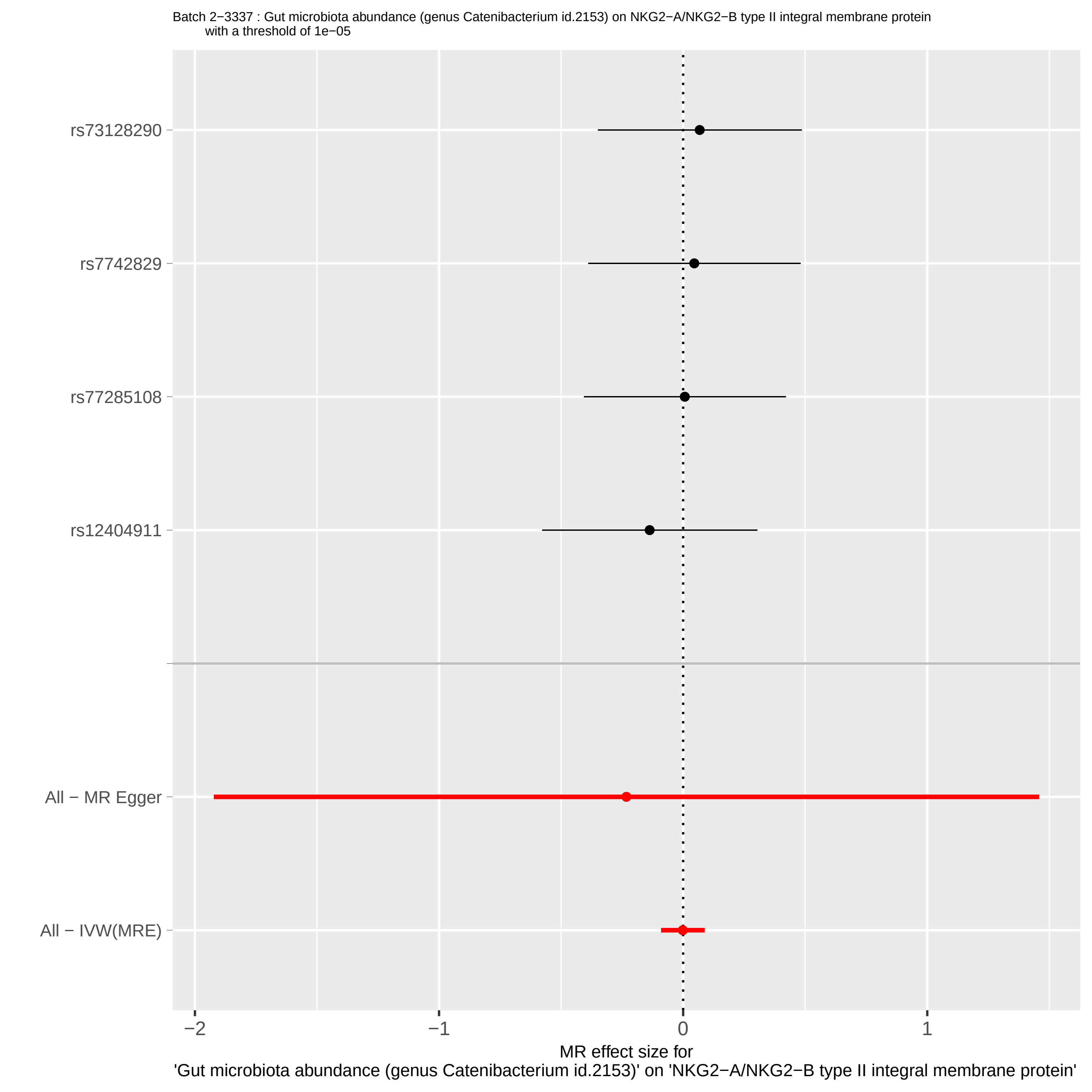

Batch 2−3337 : Gut microbiota abundance (genus Catenibacterium id.2153) on NKG2−A/NKG2−B type II integral membrane protein
with a threshold of 1e−05
rs73128290
rs7742829
rs77285108
rs12404911
All − MR Egger
All − IVW(MRE)
−2
−1
0
1
MR effect size for
'Gut microbiota abundance (genus Catenibacterium id.2153)' on 'NKG2−A/NKG2−B type II integral membrane protein'

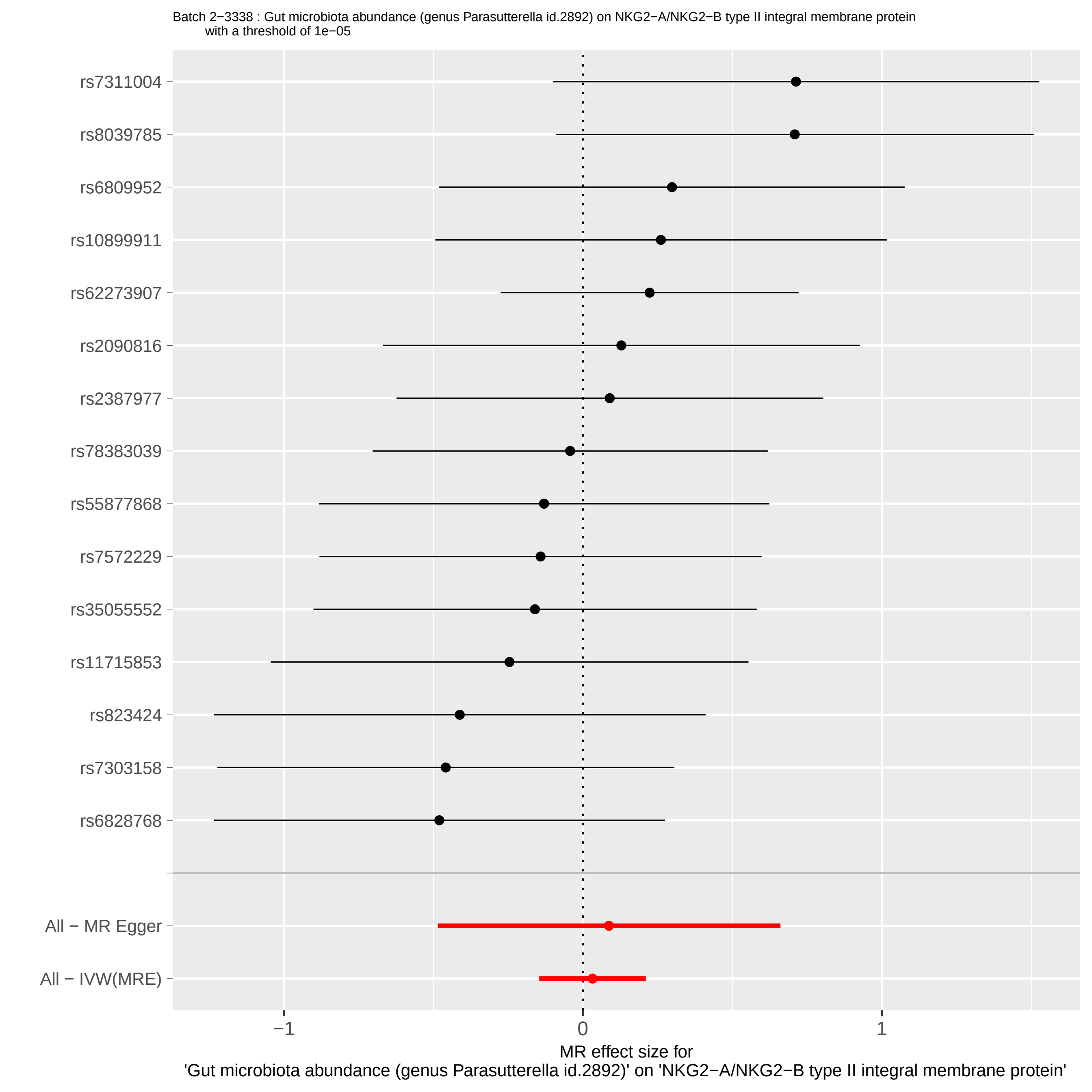

Batch 2−3338 : Gut microbiota abundance (genus Parasutterella id.2892) on NKG2−A/NKG2−B type II integral membrane protein
with a threshold of 1e−05
rs7311004
rs8039785
rs6809952
rs10899911
rs62273907
rs2090816
rs2387977
rs78383039
rs55877868
rs7572229
rs35055552
rs11715853
rs823424
rs7303158
rs6828768
All − MR Egger
All − IVW(MRE)
−1
0
1
MR effect size for
'Gut microbiota abundance (genus Parasutterella id.2892)' on 'NKG2−A/NKG2−B type II integral membrane protein'

Batch 2−3339 : Gut microbiota abundance (genus Catenibacterium id.2153) on NKG2−E type II integral membrane protein
with a threshold of 1e−05

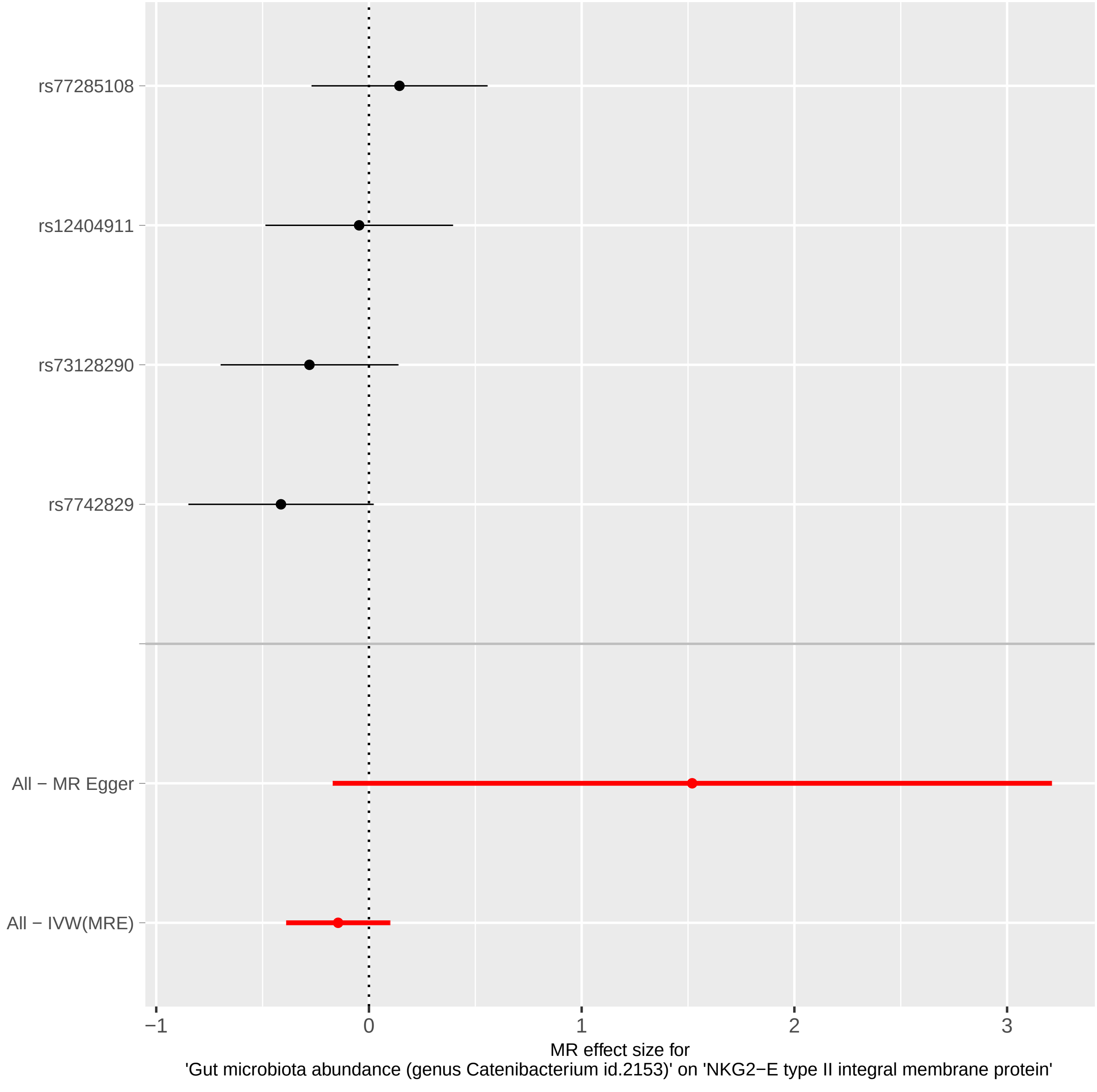

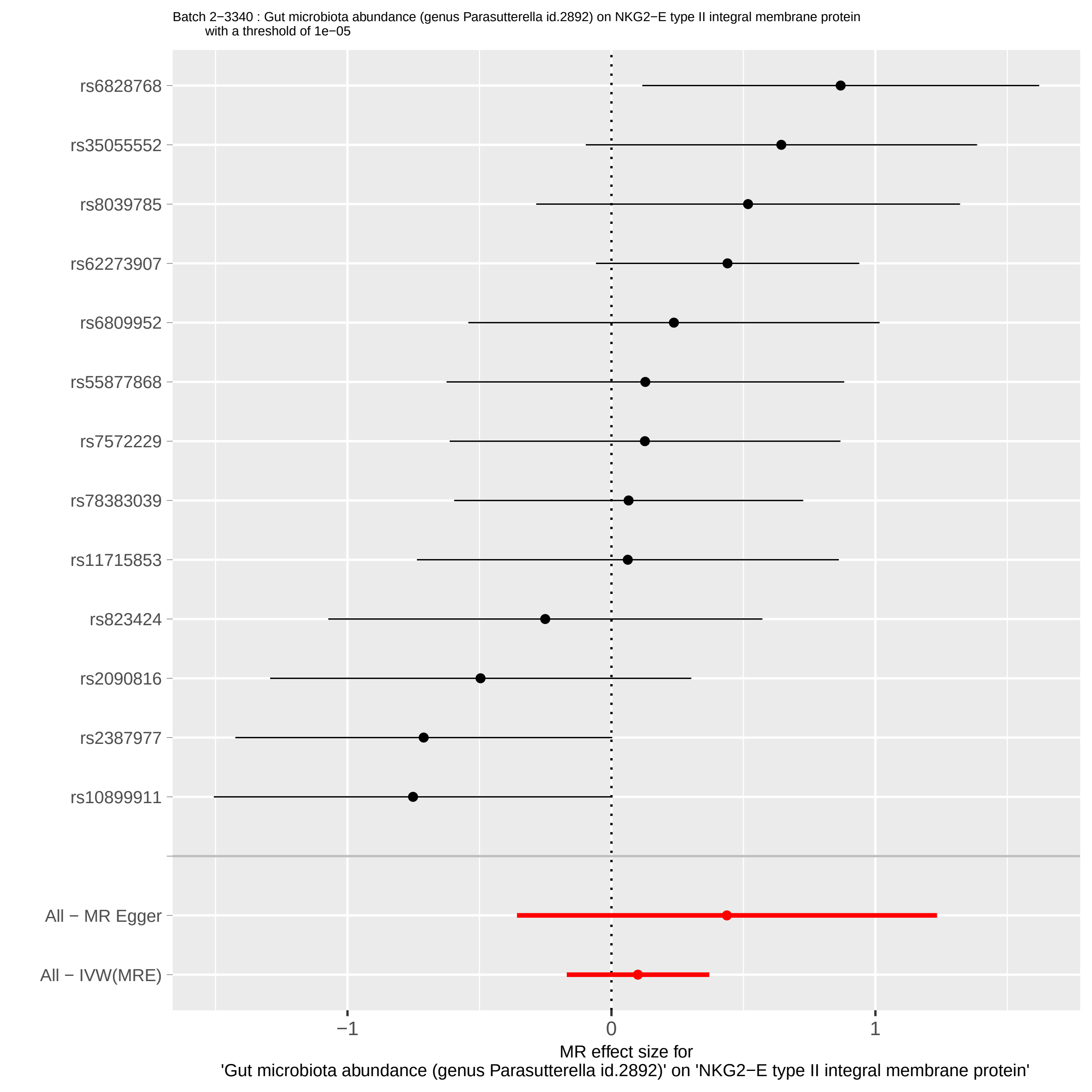

Batch 2−3340 : Gut microbiota abundance (genus Parasutterella id.2892) on NKG2−E type II integral membrane protein
with a threshold of 1e−05
rs6828768
rs35055552
rs8039785
rs62273907
rs6809952
rs55877868
rs7572229
rs78383039
rs11715853
rs823424
rs2090816
rs2387977
rs10899911
All − MR Egger
All − IVW(MRE)
−1
0
1
MR effect size for
'Gut microbiota abundance (genus Parasutterella id.2892)' on 'NKG2−E type II integral membrane protein'

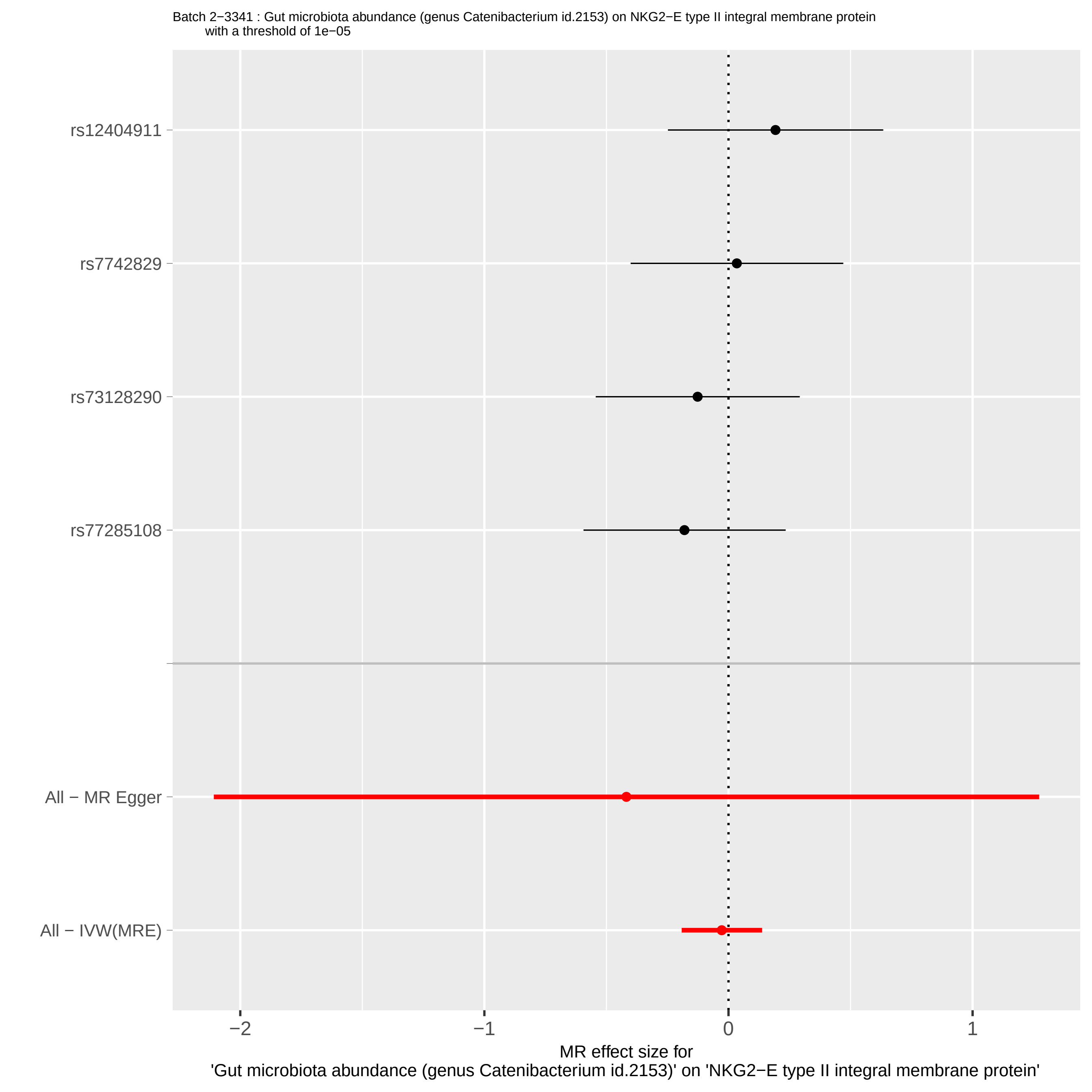
Batch 2−3341 : Gut microbiota abundance (genus Catenibacterium id.2153) on NKG2−E type II integral membrane protein
with a threshold of 1e−05
rs12404911
rs7742829
rs73128290
rs77285108
All − MR Egger
All − IVW(MRE)
−2
−1
0
1
MR effect size for
'Gut microbiota abundance (genus Catenibacterium id.2153)' on 'NKG2−E type II integral membrane protein'

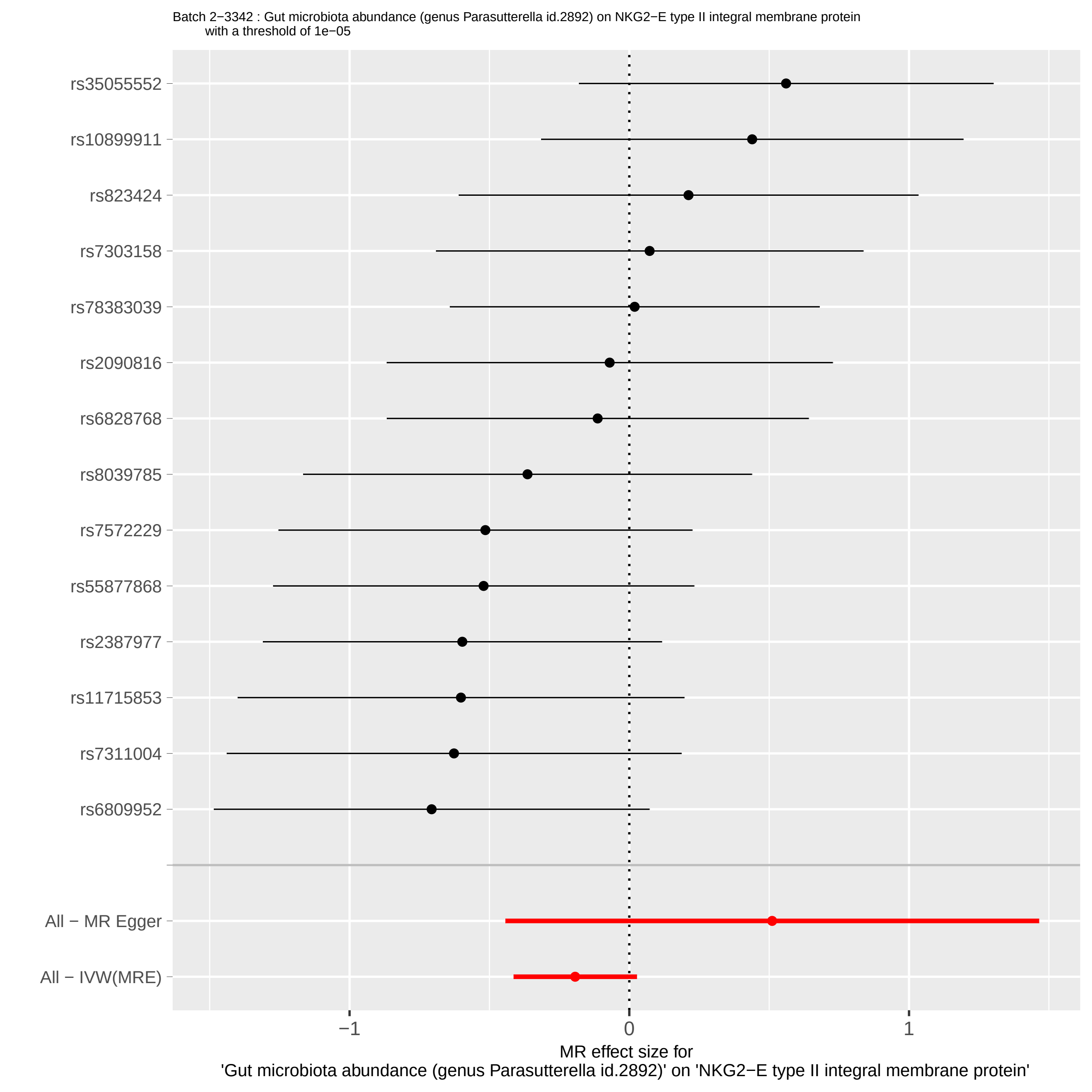
Batch 2−3342 : Gut microbiota abundance (genus Parasutterella id.2892) on NKG2−E type II integral membrane protein
with a threshold of 1e−05
rs35055552
rs10899911
rs823424
rs7303158
rs78383039
rs2090816
rs6828768
rs8039785
rs7572229
rs55877868
rs2387977
rs11715853
rs7311004
rs6809952
All − MR Egger
All − IVW(MRE)
−1
0
1
MR effect size for
'Gut microbiota abundance (genus Parasutterella id.2892)' on 'NKG2−E type II integral membrane protein'

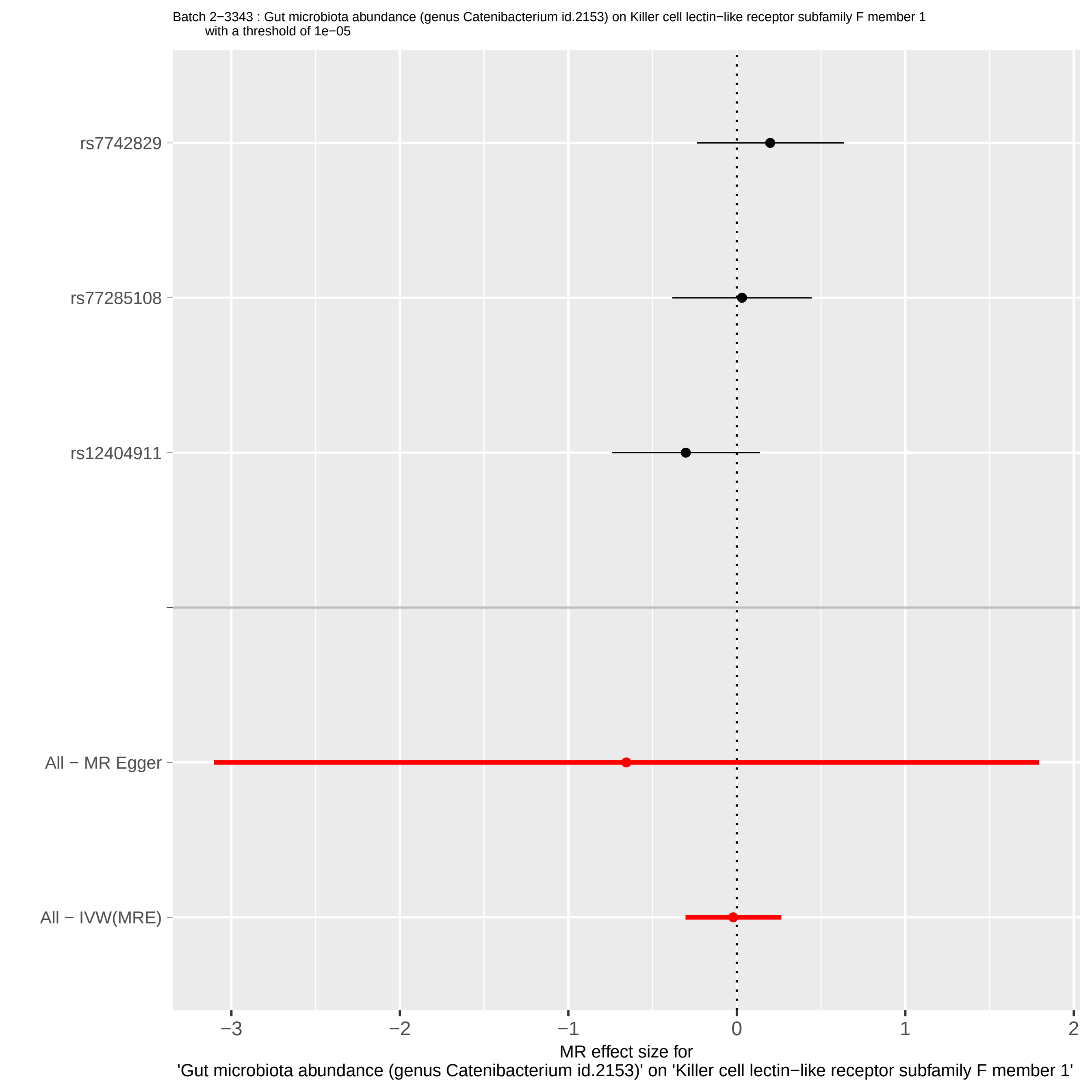
Batch 2−3343 : Gut microbiota abundance (genus Catenibacterium id.2153) on Killer cell lectin−like receptor subfamily F member 1
with a threshold of 1e−05
rs7742829
rs77285108
rs12404911
All − MR Egger
All − IVW(MRE)
−3
−2
−1
0
1
2
MR effect size for
'Gut microbiota abundance (genus Catenibacterium id.2153)' on 'Killer cell lectin−like receptor subfamily F member 1'

Batch 2−3344 : Gut microbiota abundance (genus Parasutterella id.2892) on Killer cell lectin−like receptor subfamily F member 1 with a threshold of 1e−05

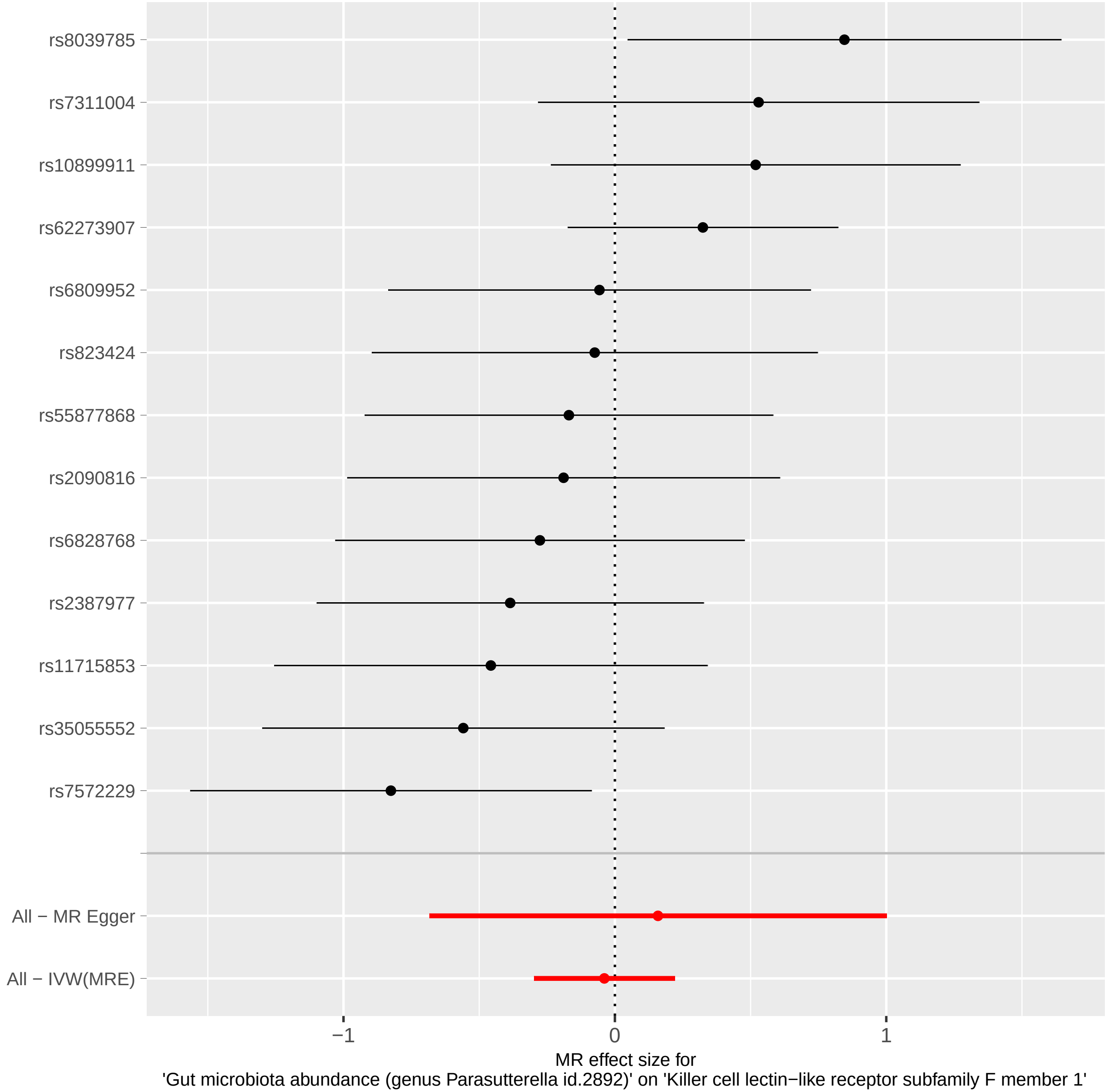

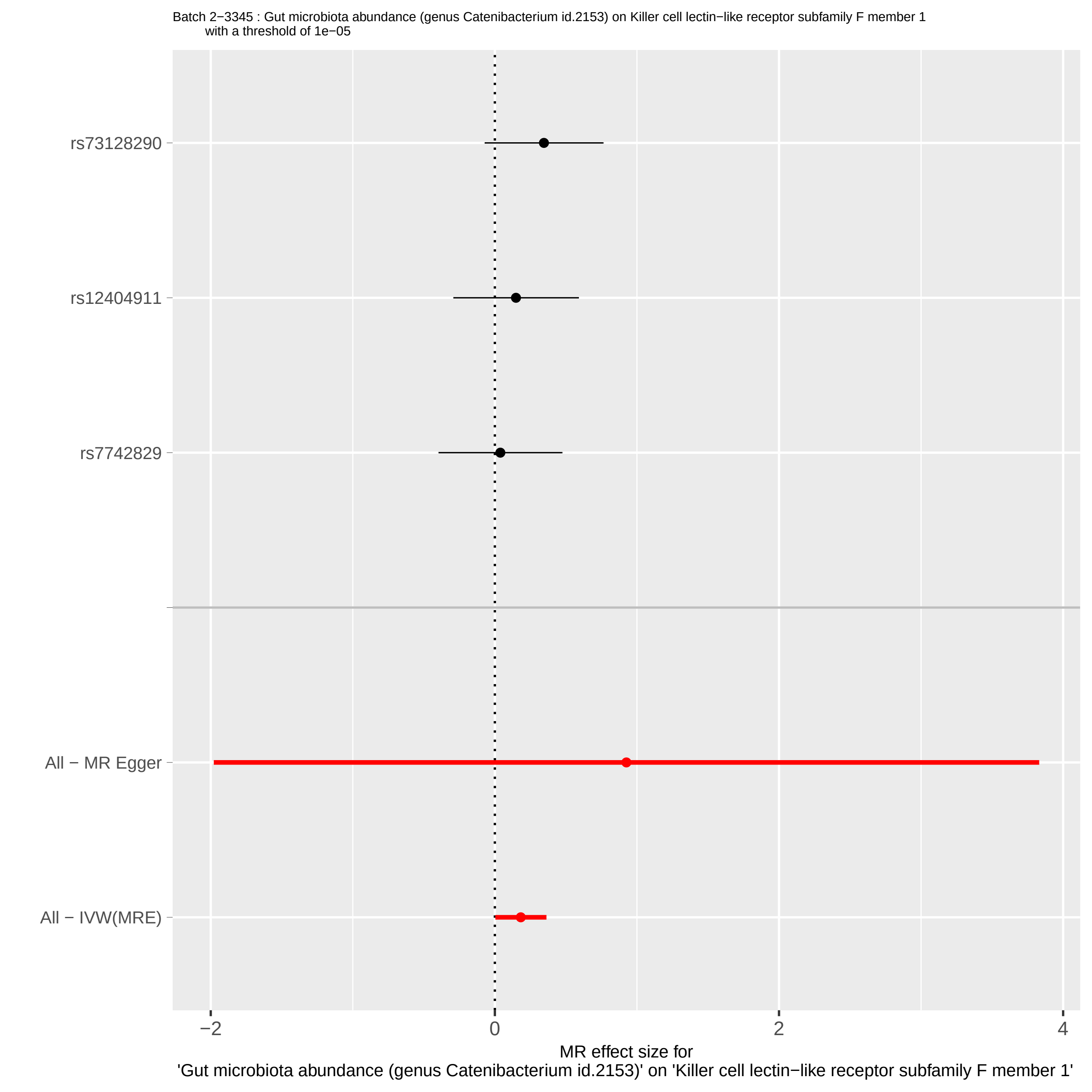

Batch 2−3345 : Gut microbiota abundance (genus Catenibacterium id.2153) on Killer cell lectin−like receptor subfamily F member 1
with a threshold of 1e−05
rs73128290
rs12404911
rs7742829
All − MR Egger
All − IVW(MRE)
−2
0
2
4
MR effect size for
'Gut microbiota abundance (genus Catenibacterium id.2153)' on 'Killer cell lectin−like receptor subfamily F member 1'

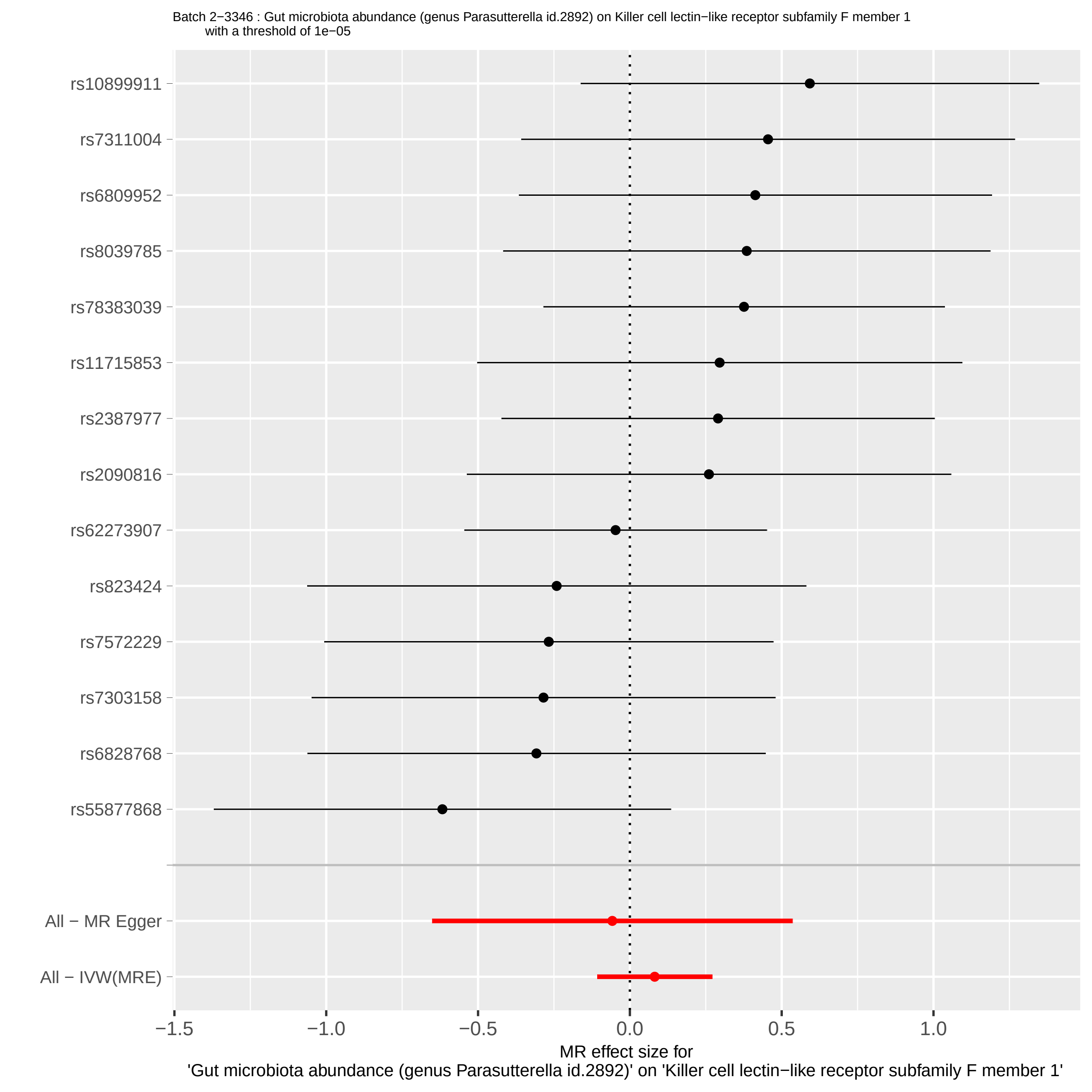
Batch 2−3346 : Gut microbiota abundance (genus Parasutterella id.2892) on Killer cell lectin−like receptor subfamily F member 1
with a threshold of 1e−05
rs10899911
rs7311004
rs6809952
rs8039785
rs78383039
rs11715853
rs2387977
rs2090816
rs62273907
rs823424
rs7572229
rs7303158
rs6828768
rs55877868
All − MR Egger
All − IVW(MRE)
−1.5
−1.0
−0.5
0.0
0.5
1.0
MR effect size for
'Gut microbiota abundance (genus Parasutterella id.2892)' on 'Killer cell lectin−like receptor subfamily F member 1'

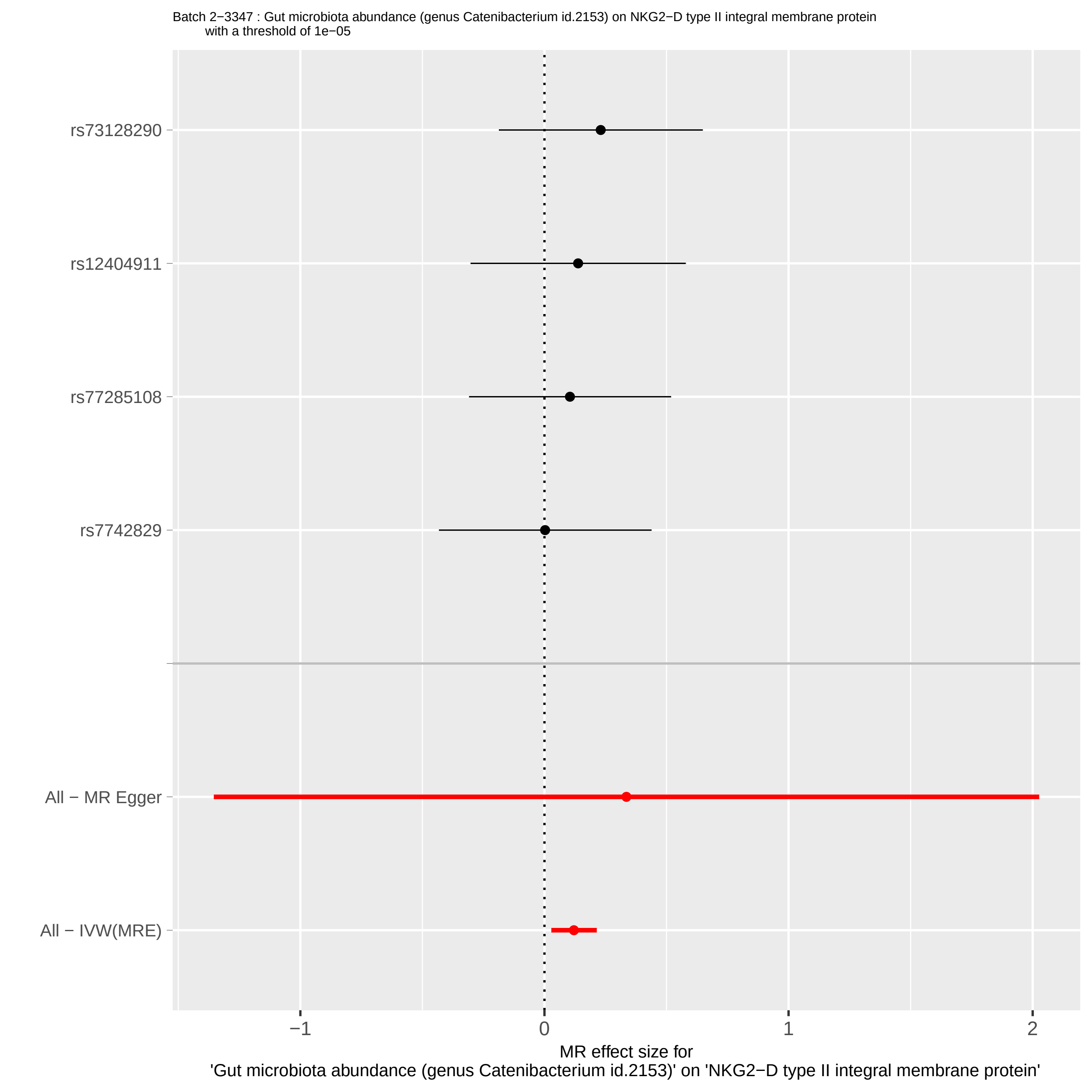

Batch 2−3347 : Gut microbiota abundance (genus Catenibacterium id.2153) on NKG2−D type II integral membrane protein
with a threshold of 1e−05
rs73128290
rs12404911
rs77285108
rs7742829
All − MR Egger
All − IVW(MRE)
−1
0
1
2
MR effect size for
'Gut microbiota abundance (genus Catenibacterium id.2153)' on 'NKG2−D type II integral membrane protein'

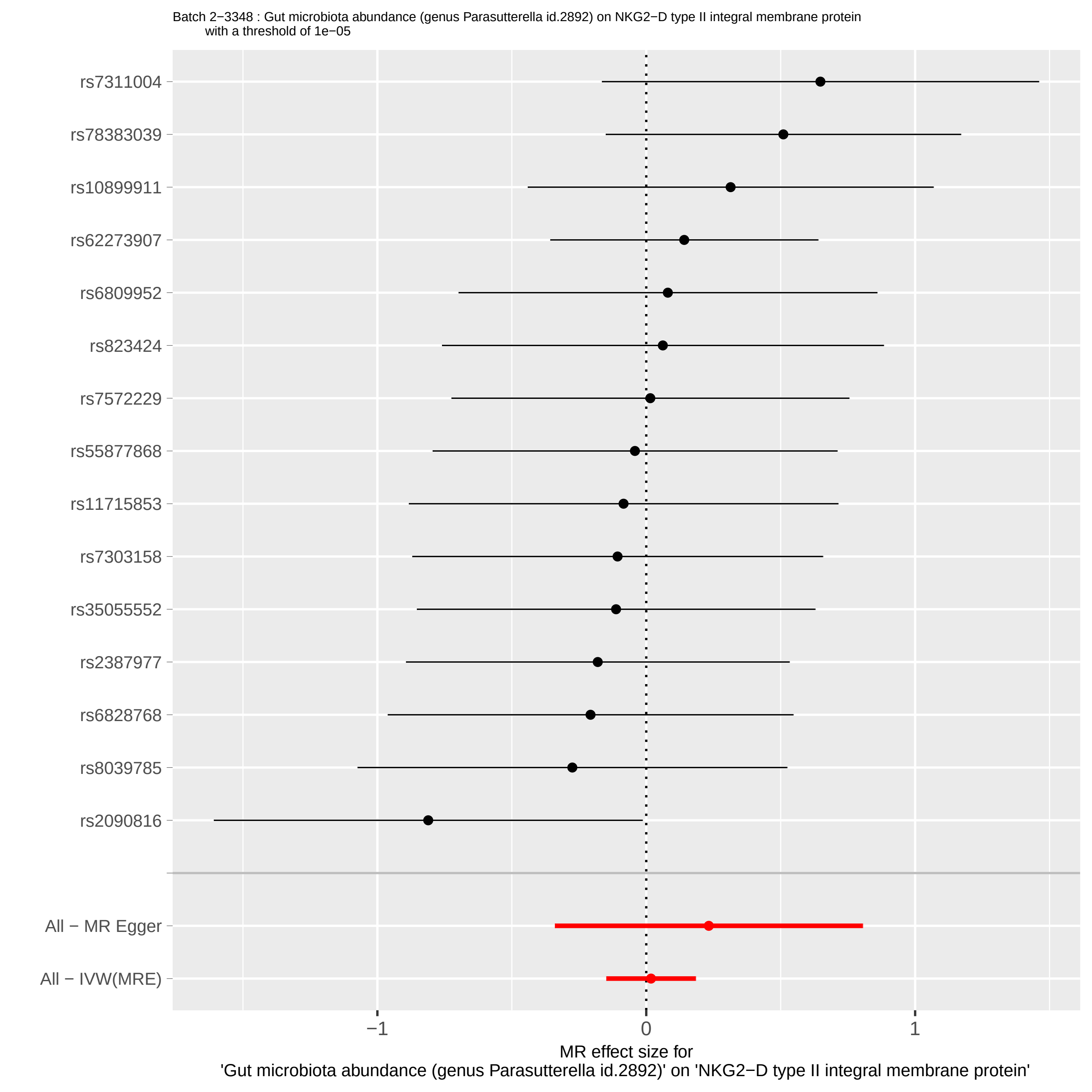

Batch 2−3348 : Gut microbiota abundance (genus Parasutterella id.2892) on NKG2−D type II integral membrane protein
with a threshold of 1e−05
rs7311004
rs78383039
rs10899911
rs62273907
rs6809952
rs823424
rs7572229
rs55877868
rs11715853
rs7303158
rs35055552
rs2387977
rs6828768
rs8039785
rs2090816
All − MR Egger
All − IVW(MRE)
−1
0
1
MR effect size for
'Gut microbiota abundance (genus Parasutterella id.2892)' on 'NKG2−D type II integral membrane protein'

Batch 2−3349 : Gut microbiota abundance (genus Catenibacterium id.2153) on NKG2−D type II integral membrane protein with a threshold of 1e−05

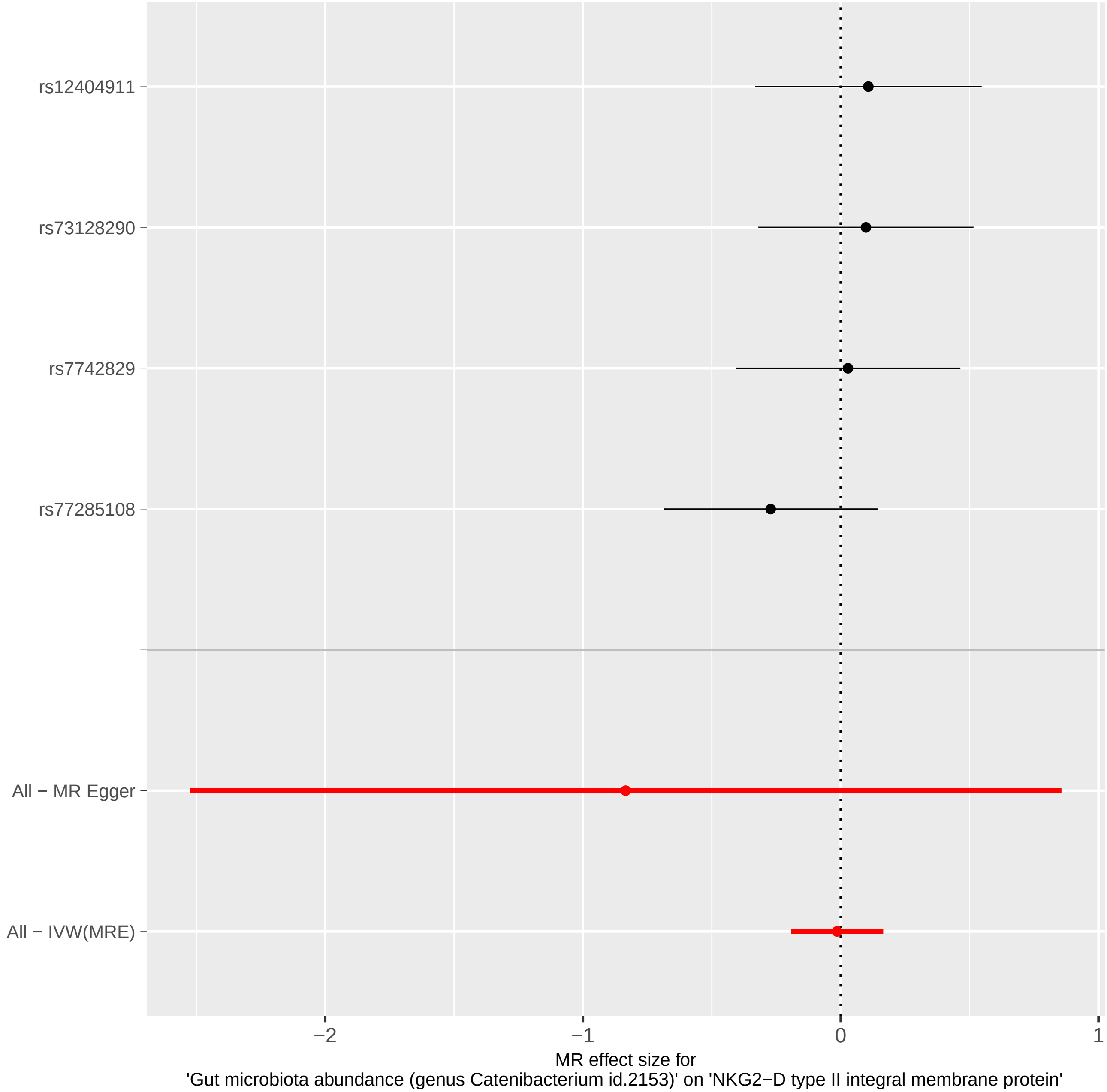

Batch 2−3350 : Gut microbiota abundance (genus Parasutterella id.2892) on NKG2−D type II integral membrane protein with a threshold of 1e−05

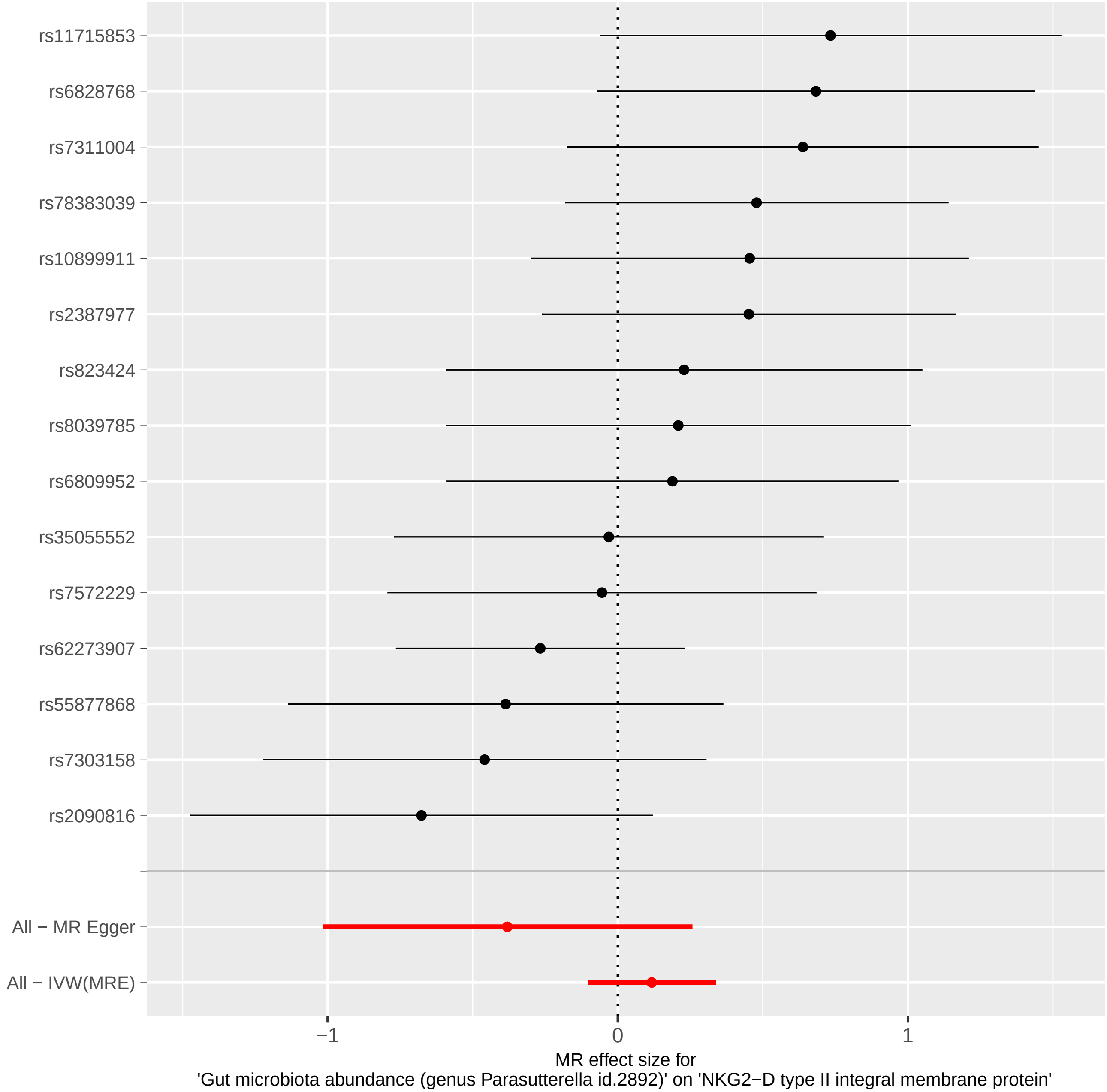

Batch 2−3351 : Gut microbiota abundance (genus Catenibacterium id.2153) on Histone−lysine N−methyltransferase 2C
with a threshold of 1e−05

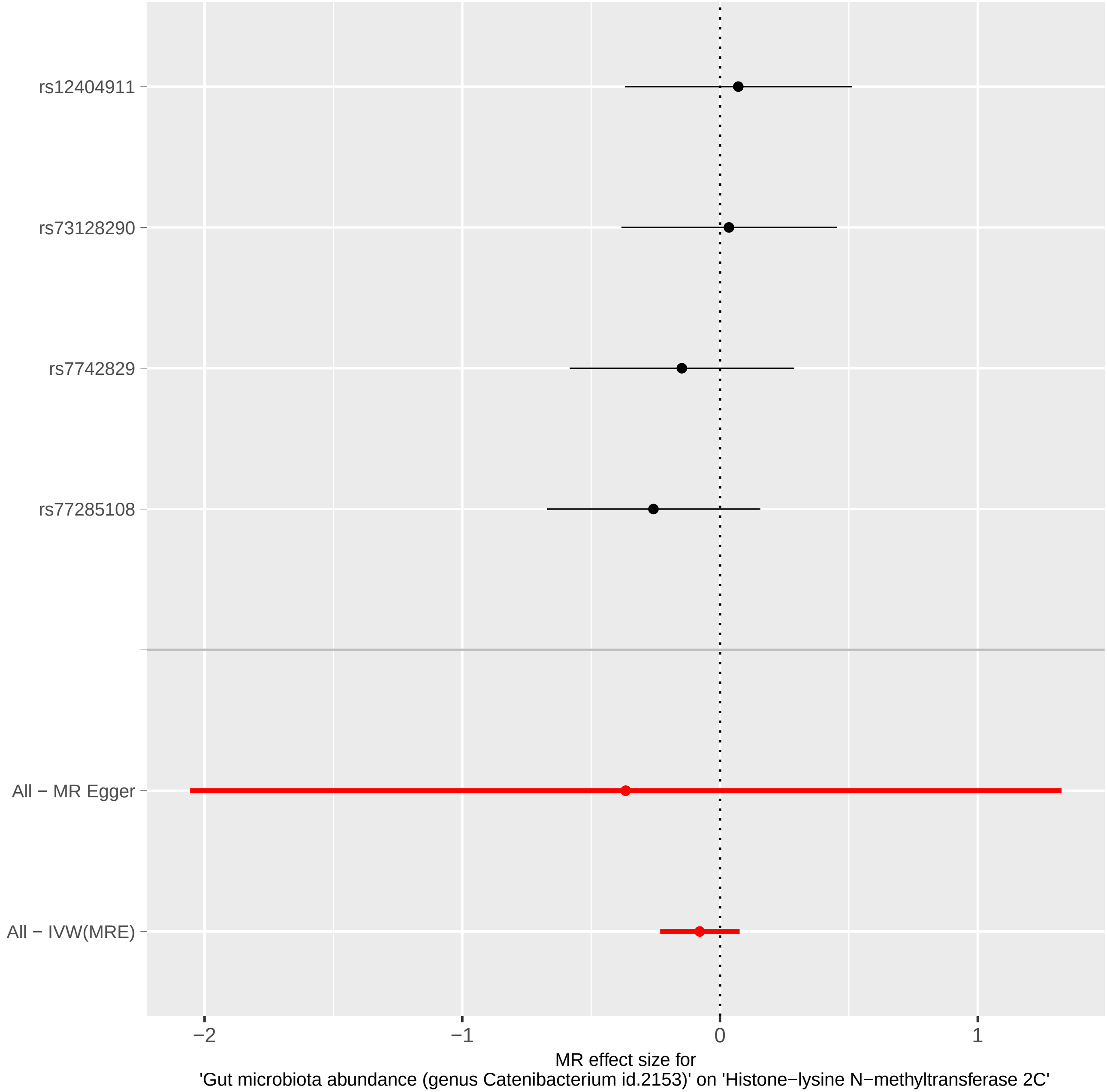

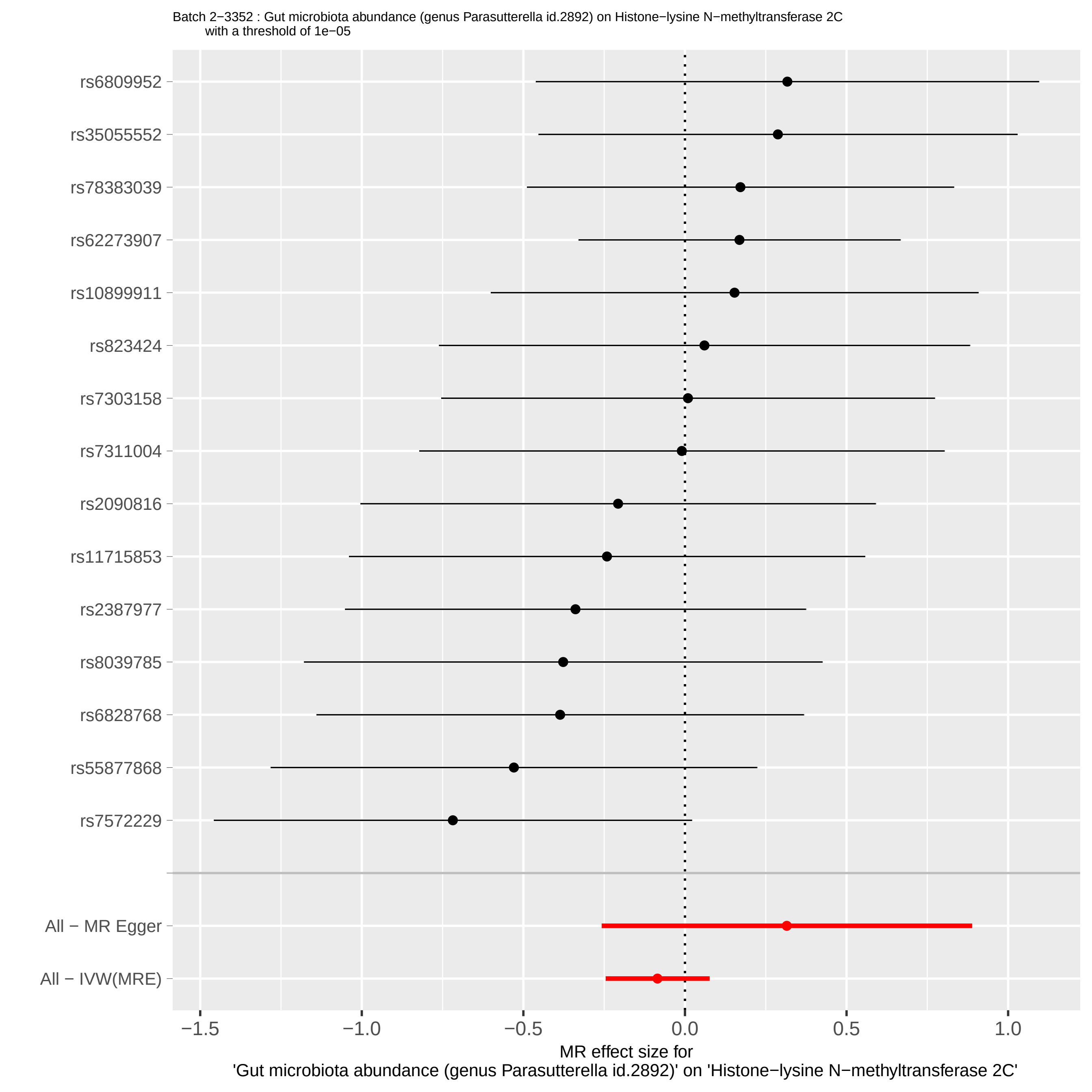
Batch 2−3352 : Gut microbiota abundance (genus Parasutterella id.2892) on Histone−lysine N−methyltransferase 2C
with a threshold of 1e−05
rs6809952
rs35055552
rs78383039
rs62273907
rs10899911
rs823424
rs7303158
rs7311004
rs2090816
rs11715853
rs2387977
rs8039785
rs6828768
rs55877868
rs7572229
All − MR Egger
All − IVW(MRE)
−1.5
−1.0
−0.5
0.0
0.5
1.0
MR effect size for
'Gut microbiota abundance (genus Parasutterella id.2892)' on 'Histone−lysine N−methyltransferase 2C'

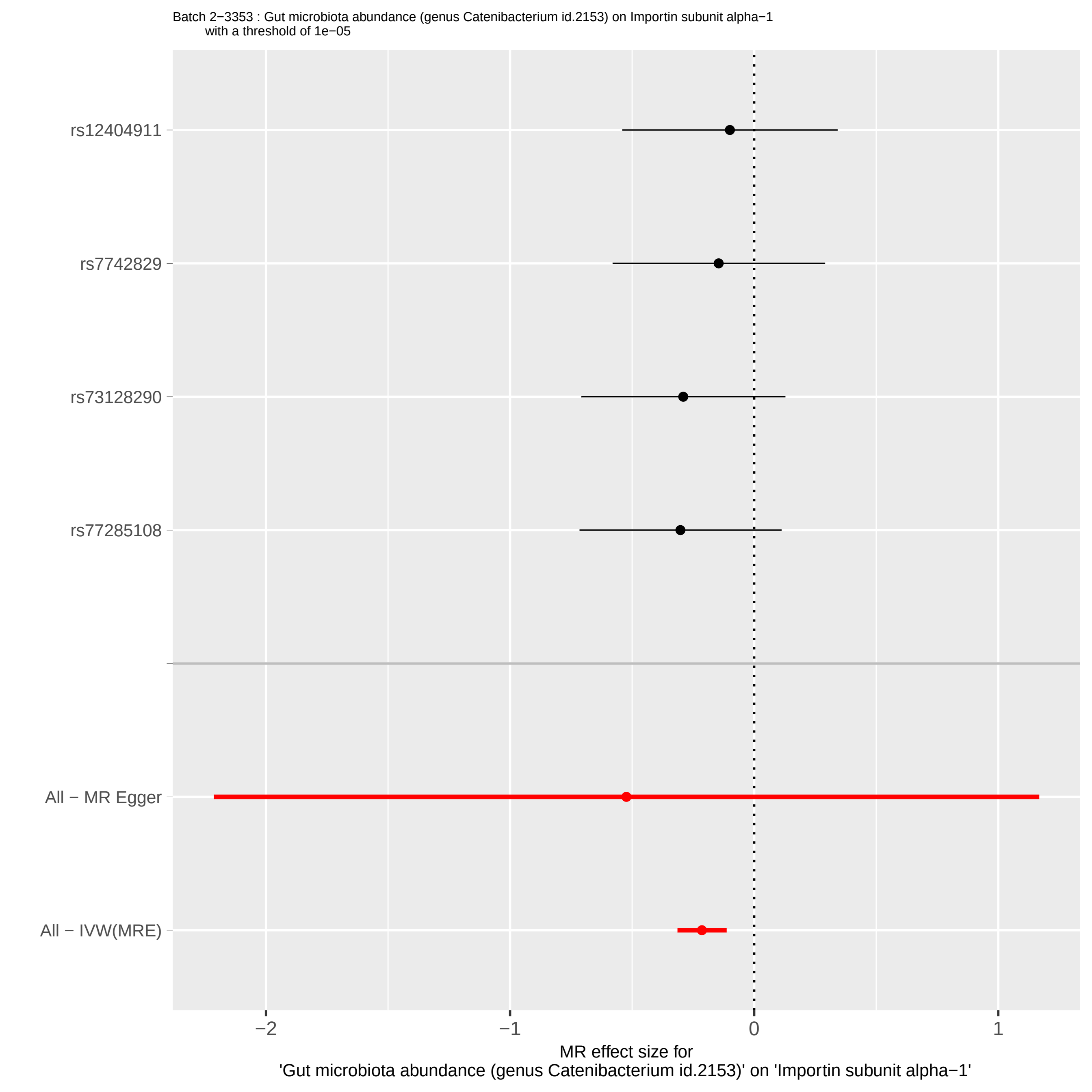

Batch 2−3353 : Gut microbiota abundance (genus Catenibacterium id.2153) on Importin subunit alpha−1
with a threshold of 1e−05
rs12404911
rs7742829
rs73128290
rs77285108
All − MR Egger
All − IVW(MRE)
−2
−1
0
1
MR effect size for
'Gut microbiota abundance (genus Catenibacterium id.2153)' on 'Importin subunit alpha−1'

Batch 2−3354 : Gut microbiota abundance (genus Parasutterella id.2892) on Importin subunit alpha−1
with a threshold of 1e−05

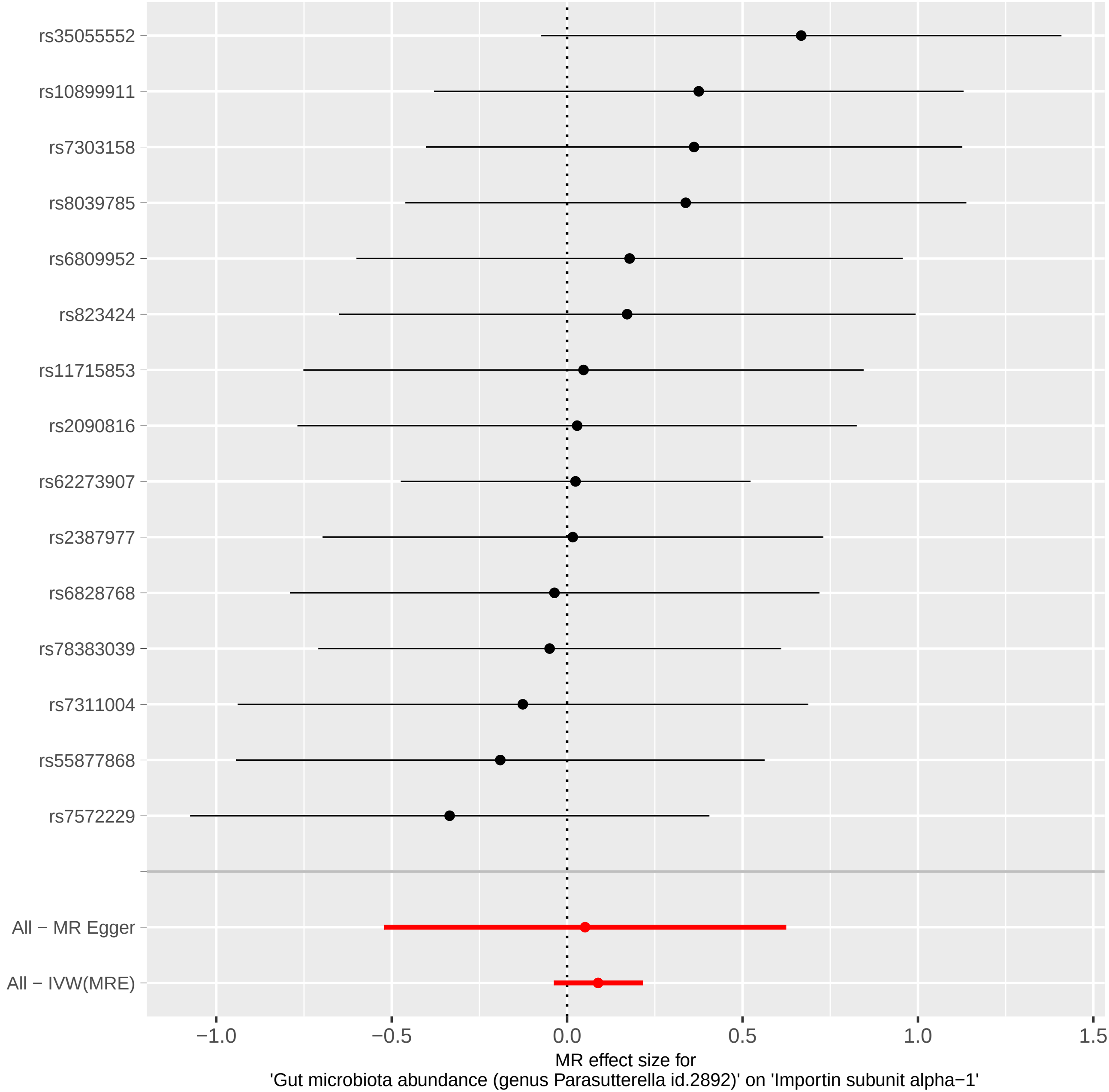

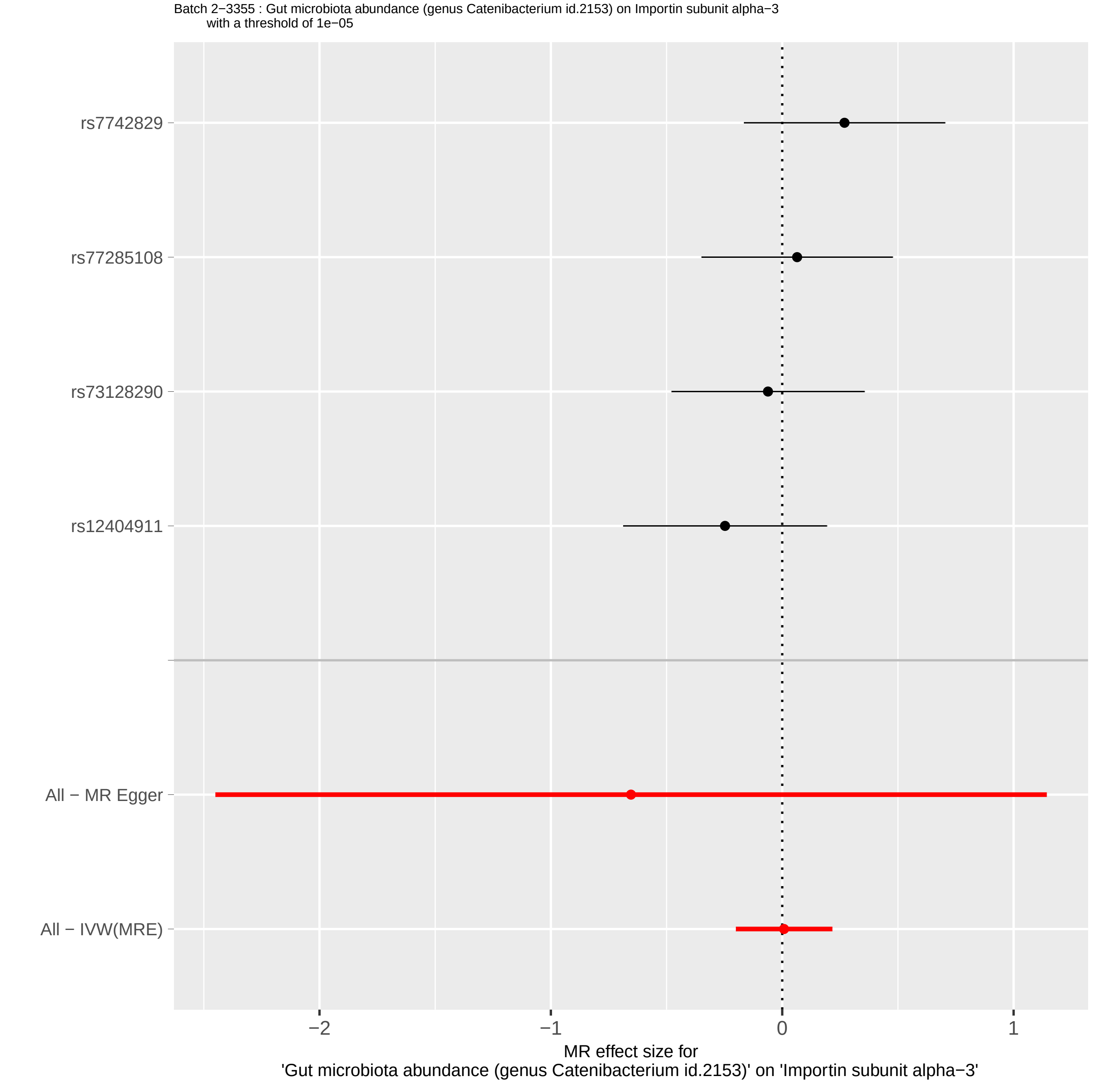
Batch 2−3355 : Gut microbiota abundance (genus Catenibacterium id.2153) on Importin subunit alpha−3
with a threshold of 1e−05
rs7742829
rs77285108
rs73128290
rs12404911
All − MR Egger
All − IVW(MRE)
−2
−1
0
1
MR effect size for
'Gut microbiota abundance (genus Catenibacterium id.2153)' on 'Importin subunit alpha−3'

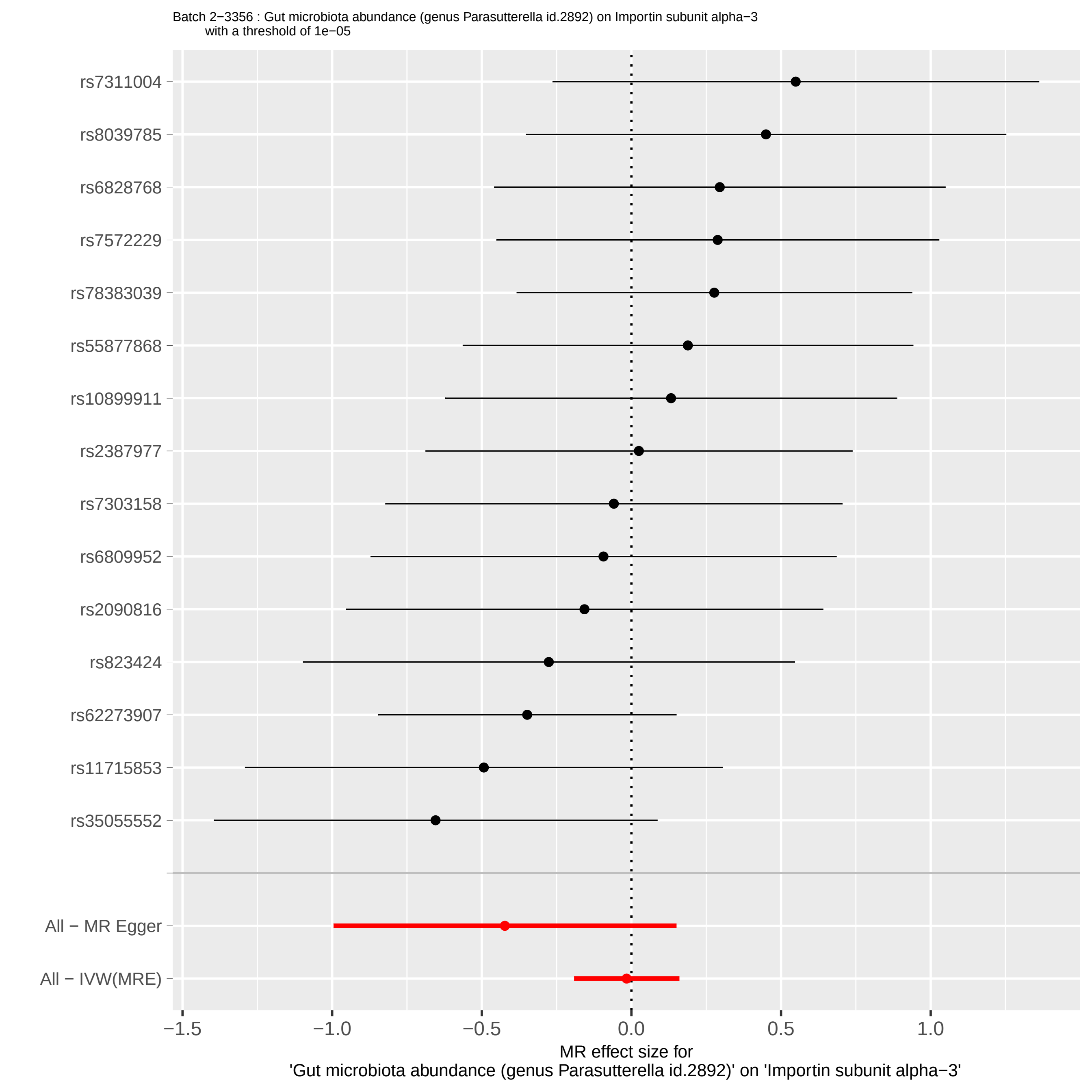
Batch 2−3356 : Gut microbiota abundance (genus Parasutterella id.2892) on Importin subunit alpha−3
with a threshold of 1e−05
rs7311004
rs8039785
rs6828768
rs7572229
rs78383039
rs55877868
rs10899911
rs2387977
rs7303158
rs6809952
rs2090816
rs823424
rs62273907
rs11715853
rs35055552
All − MR Egger
All − IVW(MRE)
−1.5
−1.0
−0.5
0.0
0.5
1.0
MR effect size for
'Gut microbiota abundance (genus Parasutterella id.2892)' on 'Importin subunit alpha−3'

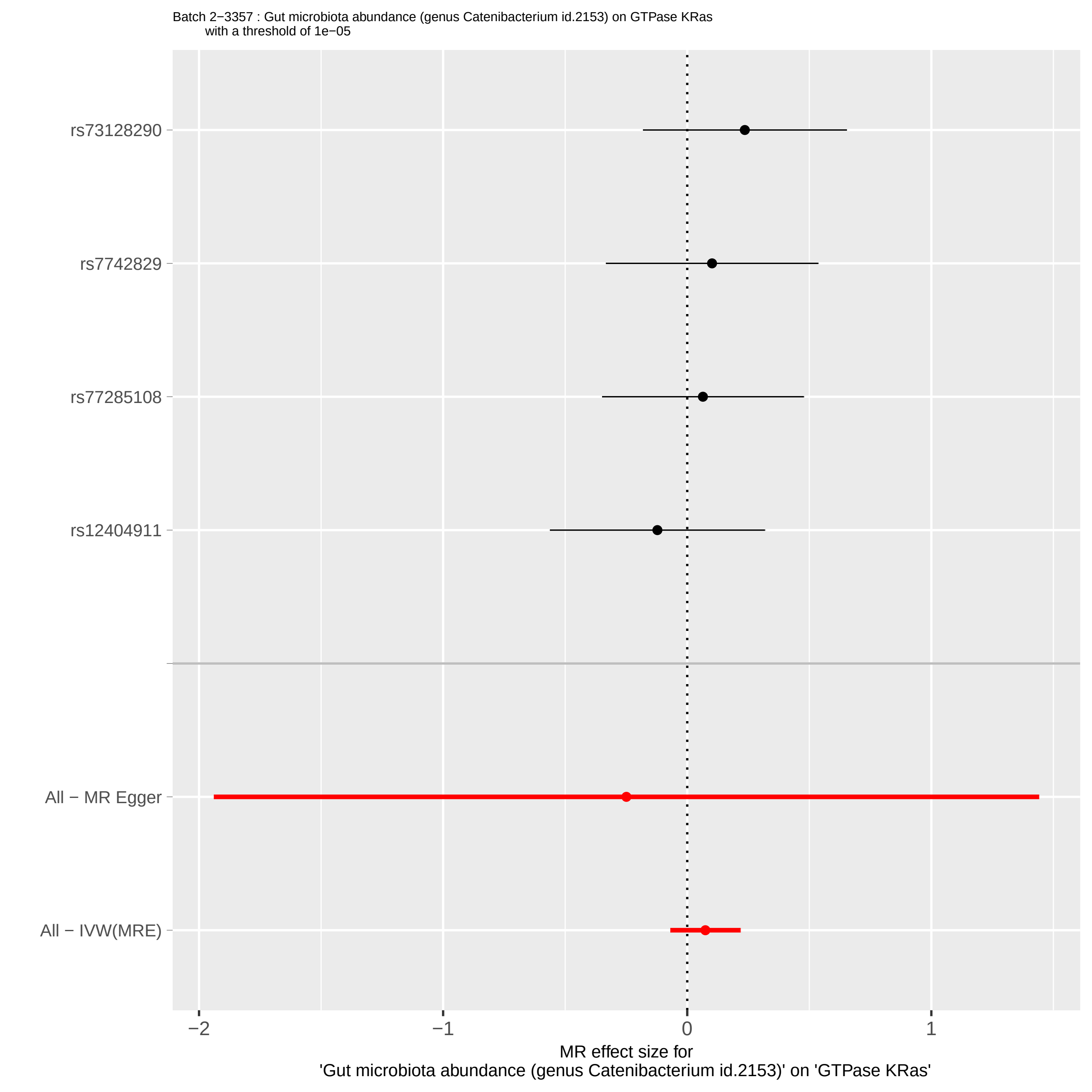

Batch 2−3357 : Gut microbiota abundance (genus Catenibacterium id.2153) on GTPase KRas
with a threshold of 1e−05
rs73128290
rs7742829
rs77285108
rs12404911
All − MR Egger
All − IVW(MRE)
−2
−1
0
1
MR effect size for
'Gut microbiota abundance (genus Catenibacterium id.2153)' on 'GTPase KRas'

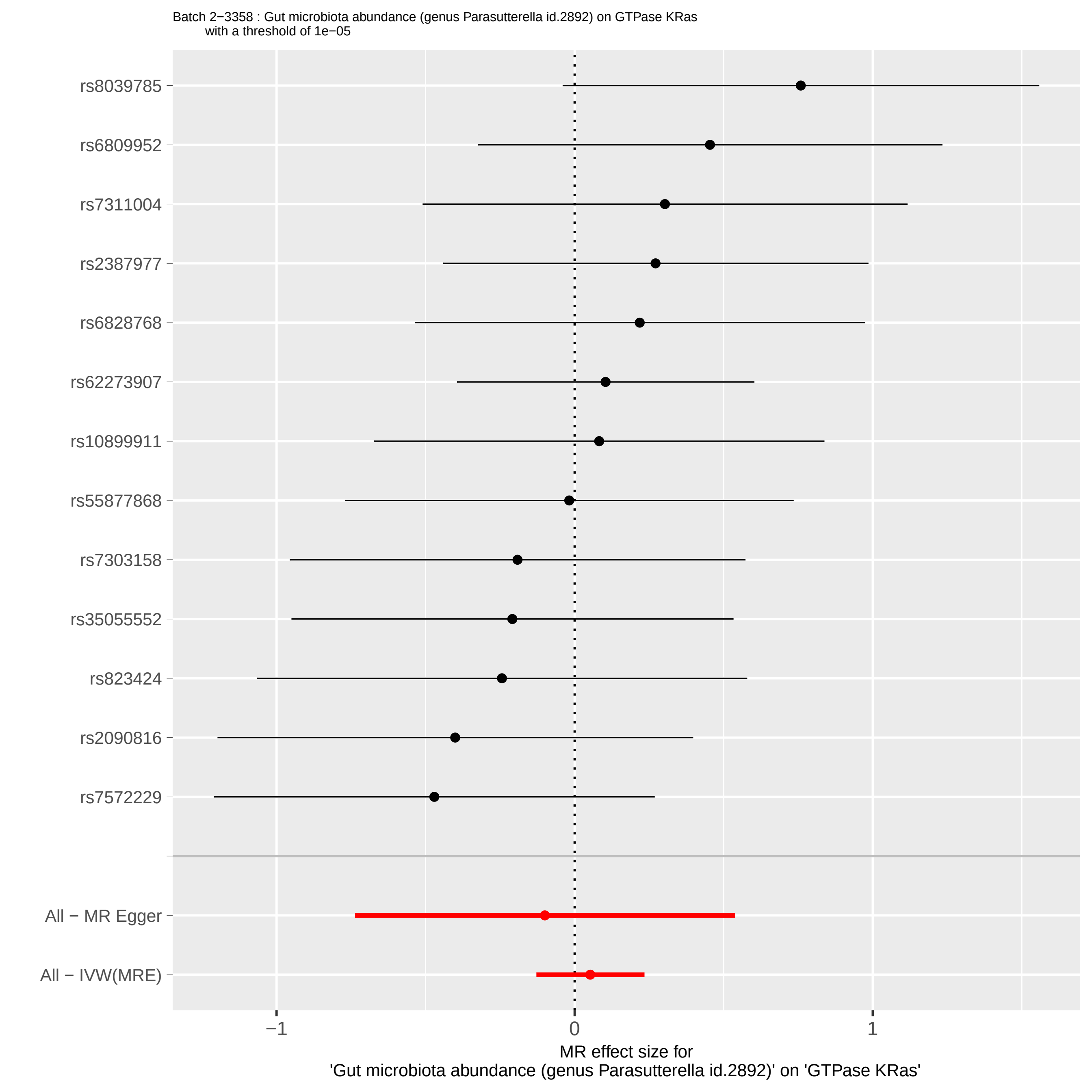

Batch 2−3358 : Gut microbiota abundance (genus Parasutterella id.2892) on GTPase KRas
with a threshold of 1e−05
rs8039785
rs6809952
rs7311004
rs2387977
rs6828768
rs62273907
rs10899911
rs55877868
rs7303158
rs35055552
rs823424
rs2090816
rs7572229
All − MR Egger
All − IVW(MRE)
−1
0
1
MR effect size for
'Gut microbiota abundance (genus Parasutterella id.2892)' on 'GTPase KRas'

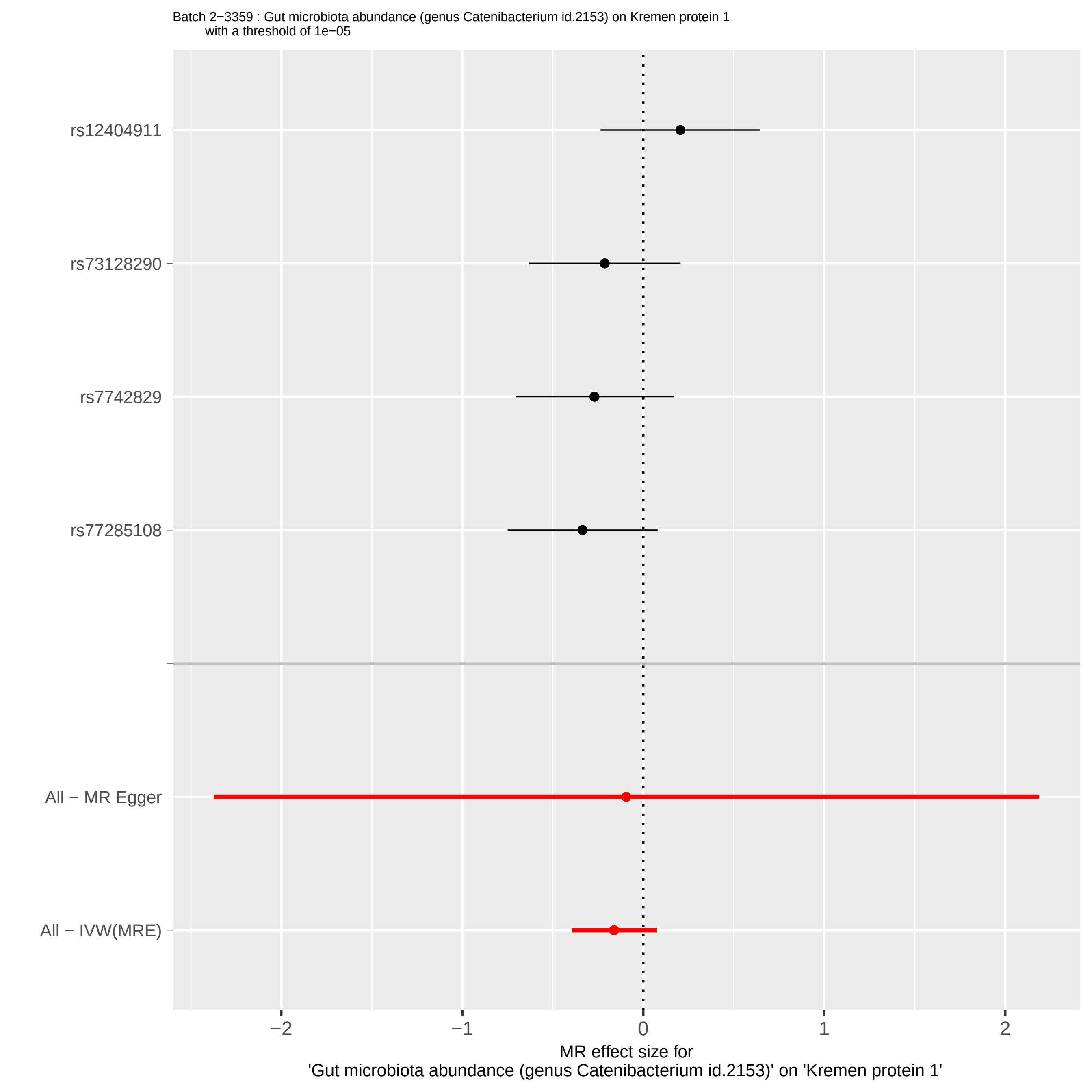
Batch 2−3359 : Gut microbiota abundance (genus Catenibacterium id.2153) on Kremen protein 1
with a threshold of 1e−05
rs12404911
rs73128290
rs7742829
rs77285108
All − MR Egger
All − IVW(MRE)
−2
−1
0
1
2
MR effect size for
'Gut microbiota abundance (genus Catenibacterium id.2153)' on 'Kremen protein 1'

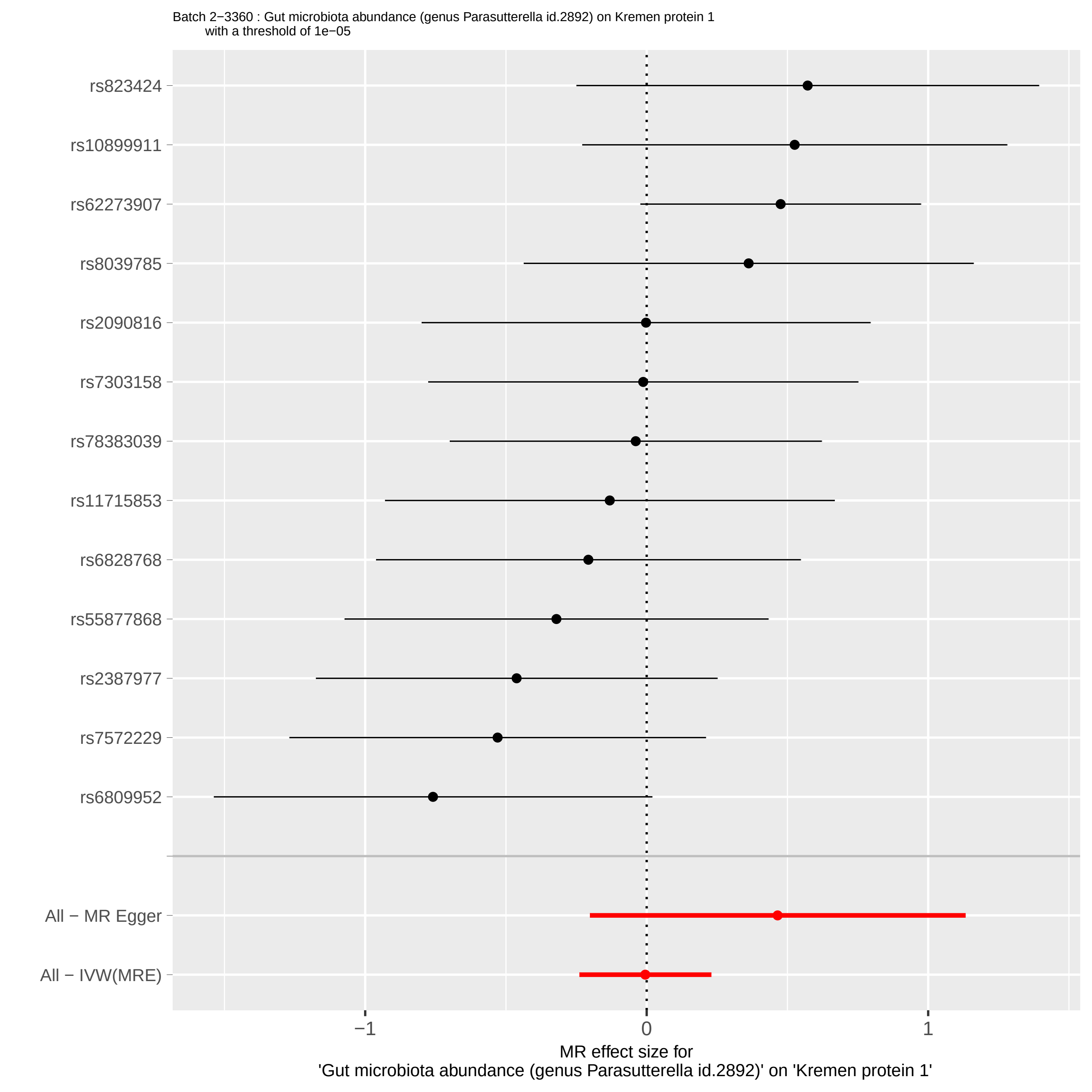

Batch 2−3360 : Gut microbiota abundance (genus Parasutterella id.2892) on Kremen protein 1
with a threshold of 1e−05
rs823424
rs10899911
rs62273907
rs8039785
rs2090816
rs7303158
rs78383039
rs11715853
rs6828768
rs55877868
rs2387977
rs7572229
rs6809952
All − MR Egger
All − IVW(MRE)
−1
0
1
MR effect size for
'Gut microbiota abundance (genus Parasutterella id.2892)' on 'Kremen protein 1'

Batch 2−3361 : Gut microbiota abundance (genus Catenibacterium id.2153) on Kremen protein 2
with a threshold of 1e−05

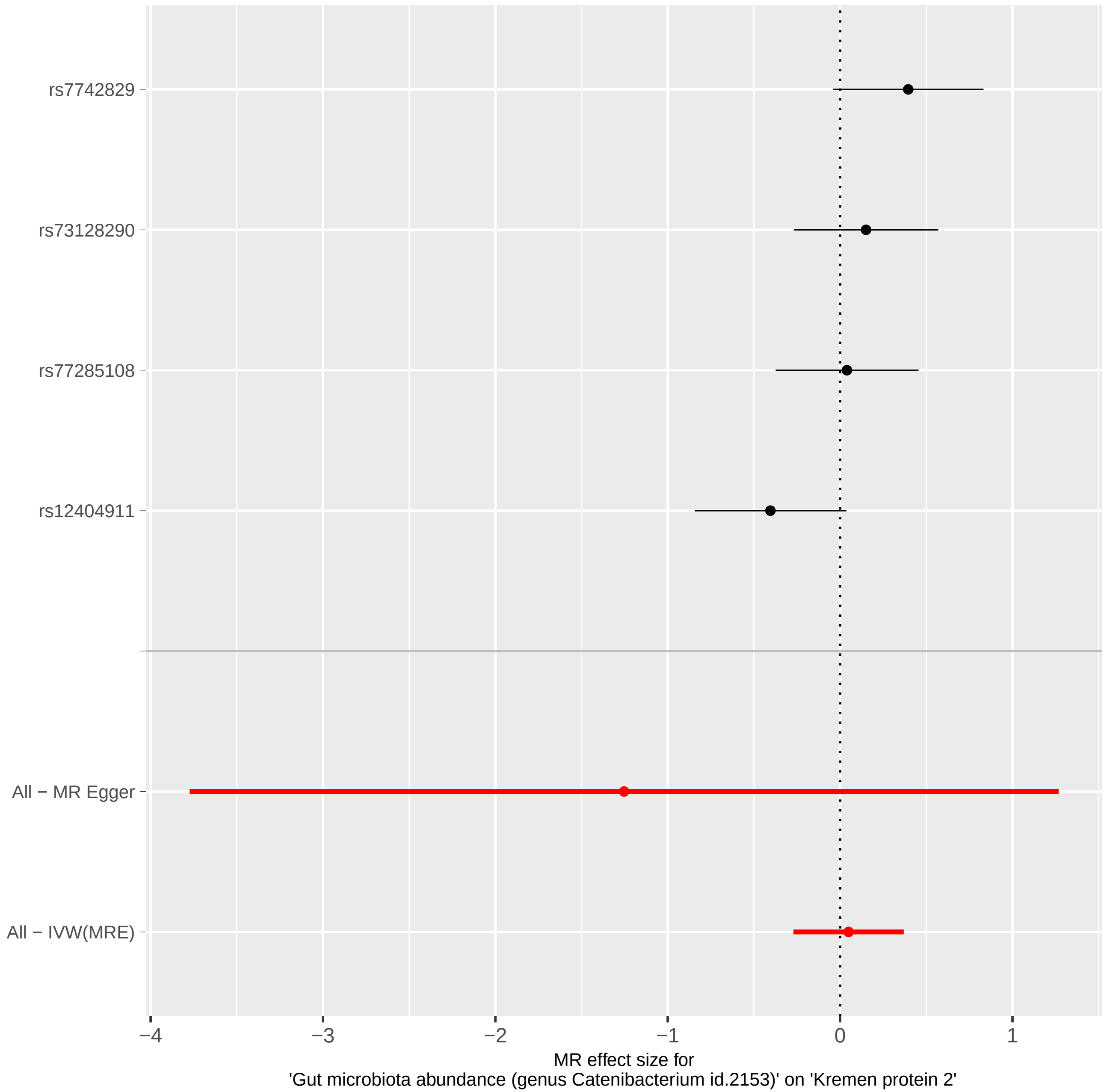

Batch 2−3362 : Gut microbiota abundance (genus Parasutterella id.2892) on Kremen protein 2
with a threshold of 1e−05

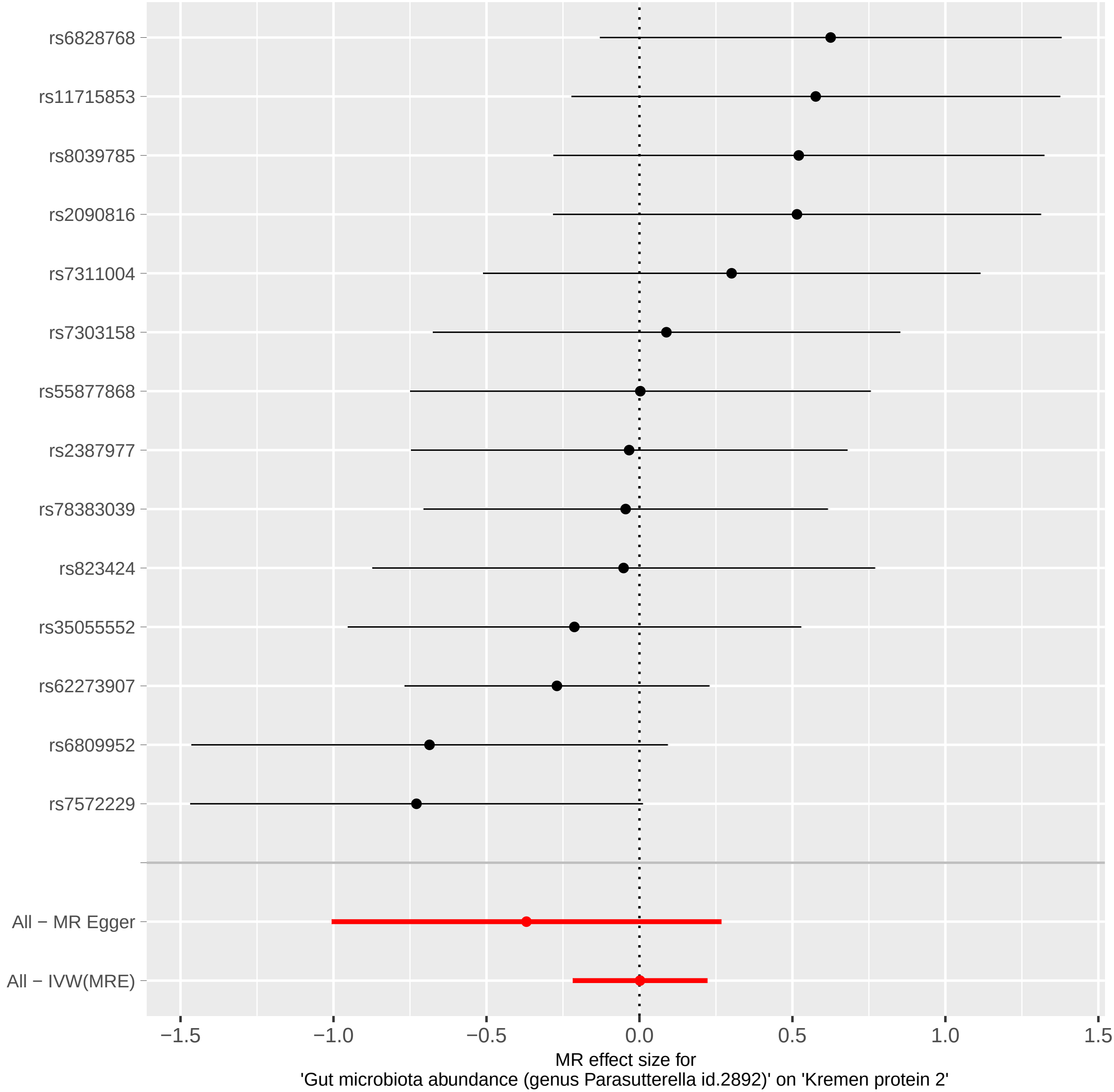

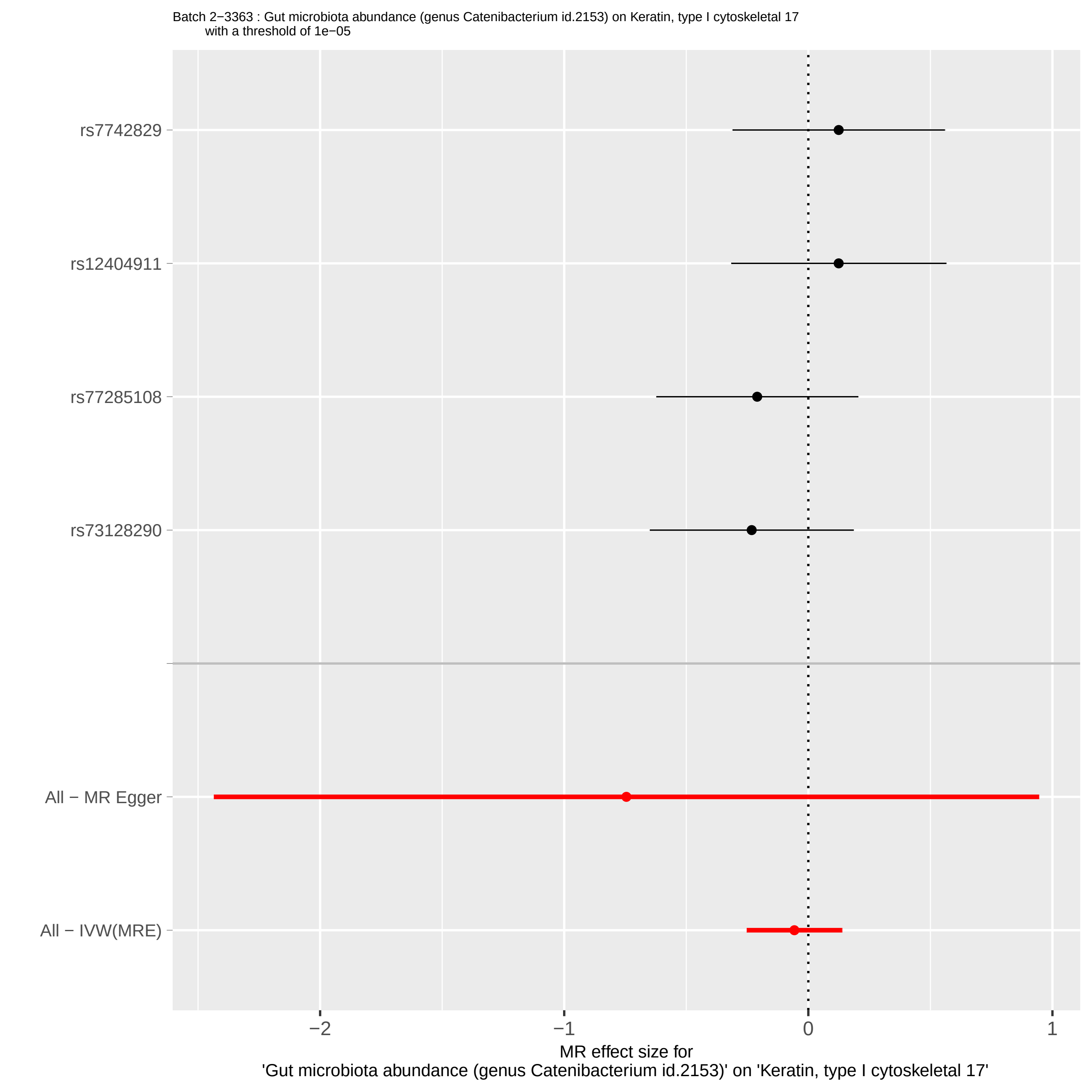
Batch 2−3363 : Gut microbiota abundance (genus Catenibacterium id.2153) on Keratin, type I cytoskeletal 17
with a threshold of 1e−05
rs7742829
rs12404911
rs77285108
rs73128290
All − MR Egger
All − IVW(MRE)
−2
−1
0
1
MR effect size for
'Gut microbiota abundance (genus Catenibacterium id.2153)' on 'Keratin, type I cytoskeletal 17'

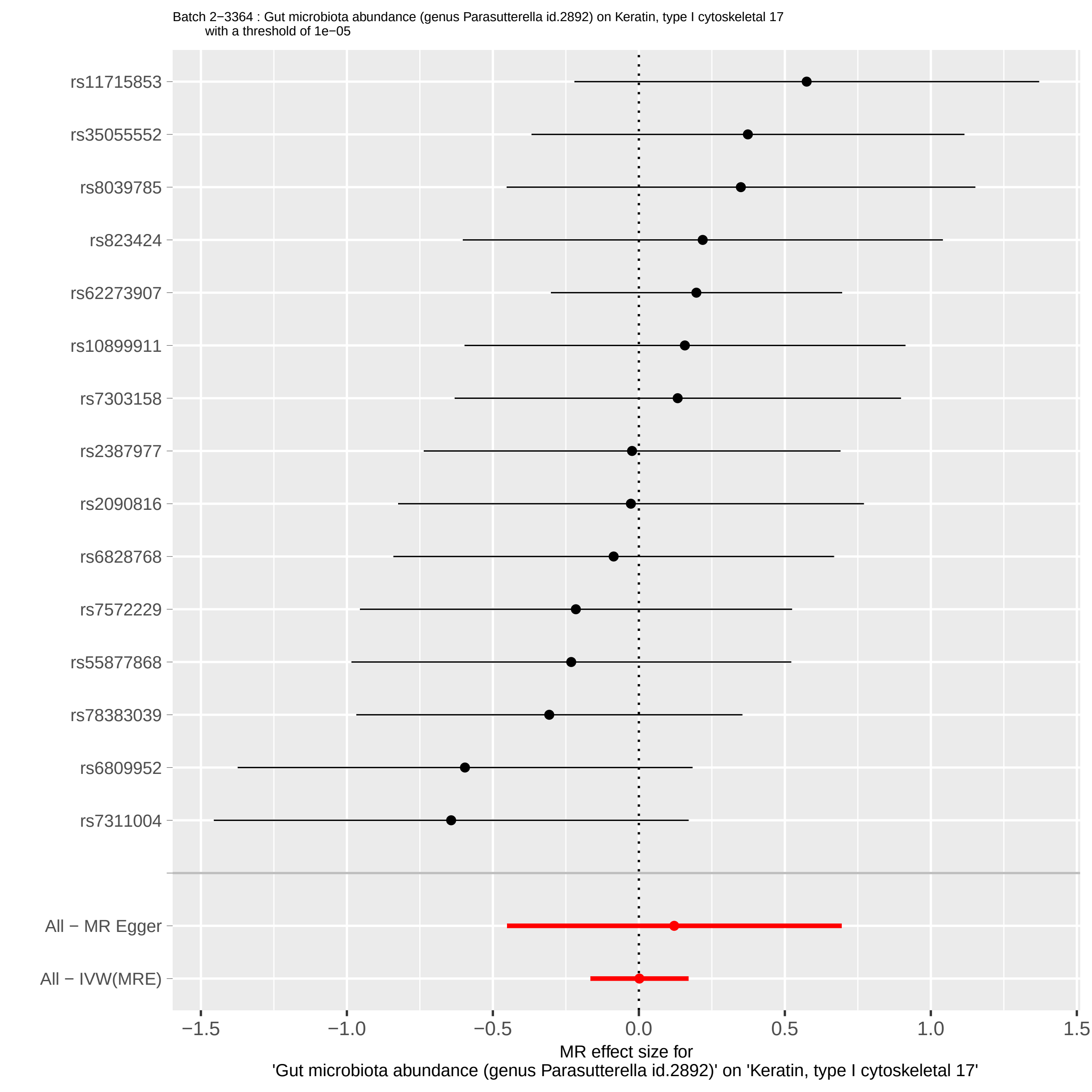

Batch 2−3364 : Gut microbiota abundance (genus Parasutterella id.2892) on Keratin, type I cytoskeletal 17
with a threshold of 1e−05
rs11715853
rs35055552
rs8039785
rs823424
rs62273907
rs10899911
rs7303158
rs2387977
rs2090816
rs6828768
rs7572229
rs55877868
rs78383039
rs6809952
rs7311004
All − MR Egger
All − IVW(MRE)
−1.5
−1.0
−0.5
0.0
0.5
1.0
1.5
MR effect size for
'Gut microbiota abundance (genus Parasutterella id.2892)' on 'Keratin, type I cytoskeletal 17'

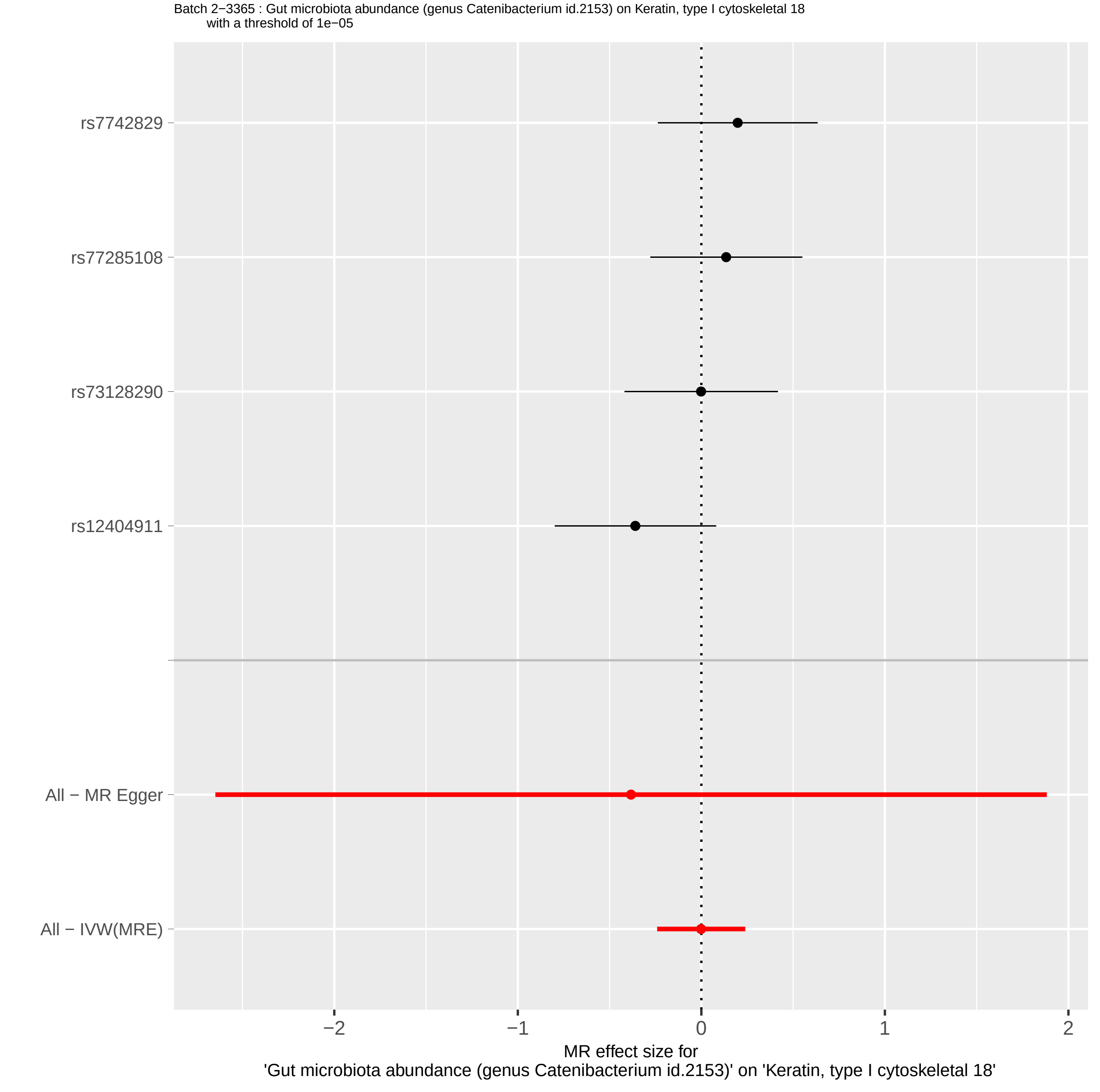

Batch 2−3365 : Gut microbiota abundance (genus Catenibacterium id.2153) on Keratin, type I cytoskeletal 18
with a threshold of 1e−05
rs7742829
rs77285108
rs73128290
rs12404911
All − MR Egger
All − IVW(MRE)
−2
−1
0
1
2
MR effect size for
'Gut microbiota abundance (genus Catenibacterium id.2153)' on 'Keratin, type I cytoskeletal 18'

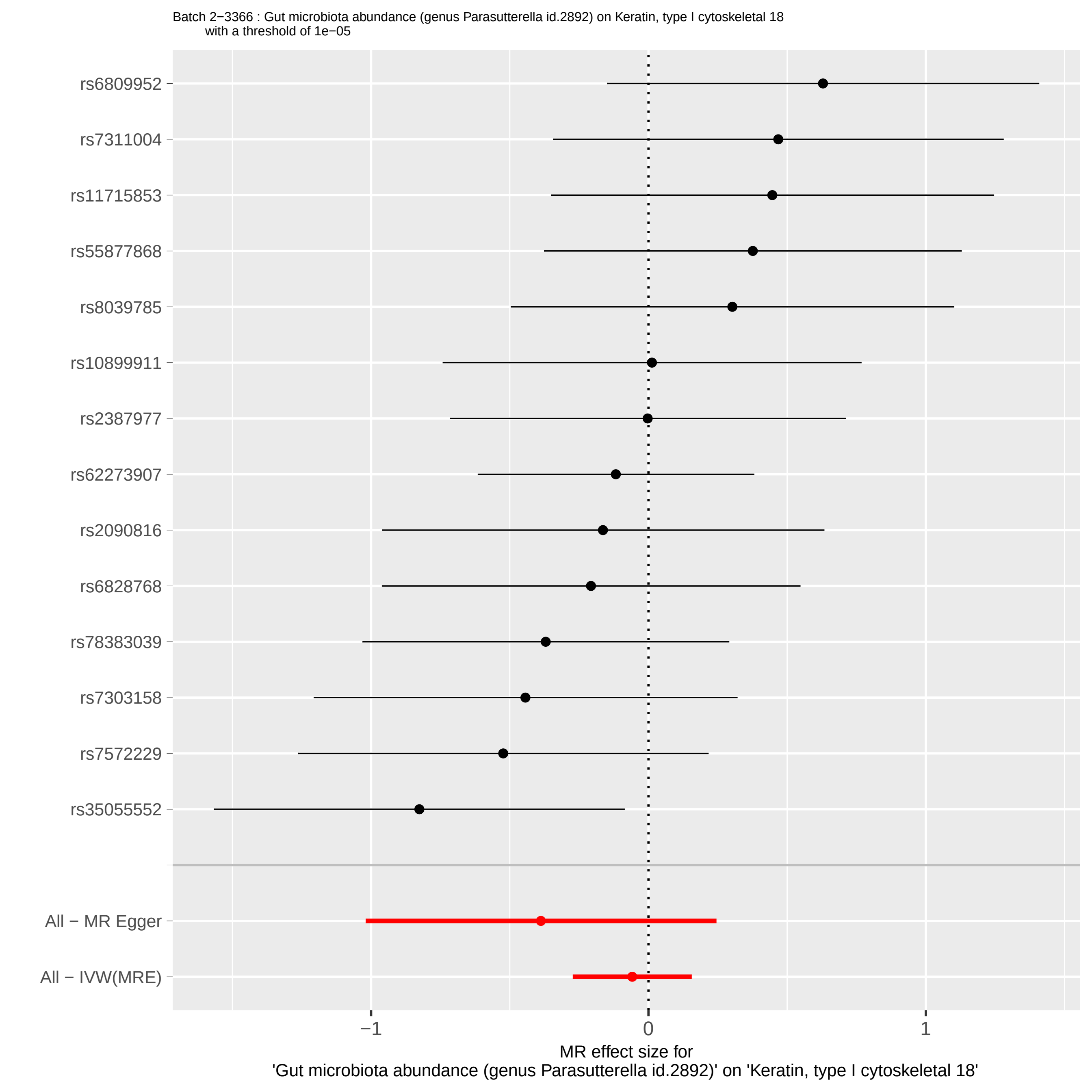
Batch 2−3366 : Gut microbiota abundance (genus Parasutterella id.2892) on Keratin, type I cytoskeletal 18
with a threshold of 1e−05
rs6809952
rs7311004
rs11715853
rs55877868
rs8039785
rs10899911
rs2387977
rs62273907
rs2090816
rs6828768
rs78383039
rs7303158
rs7572229
rs35055552
All − MR Egger
All − IVW(MRE)
−1
0
1
MR effect size for
'Gut microbiota abundance (genus Parasutterella id.2892)' on 'Keratin, type I cytoskeletal 18'

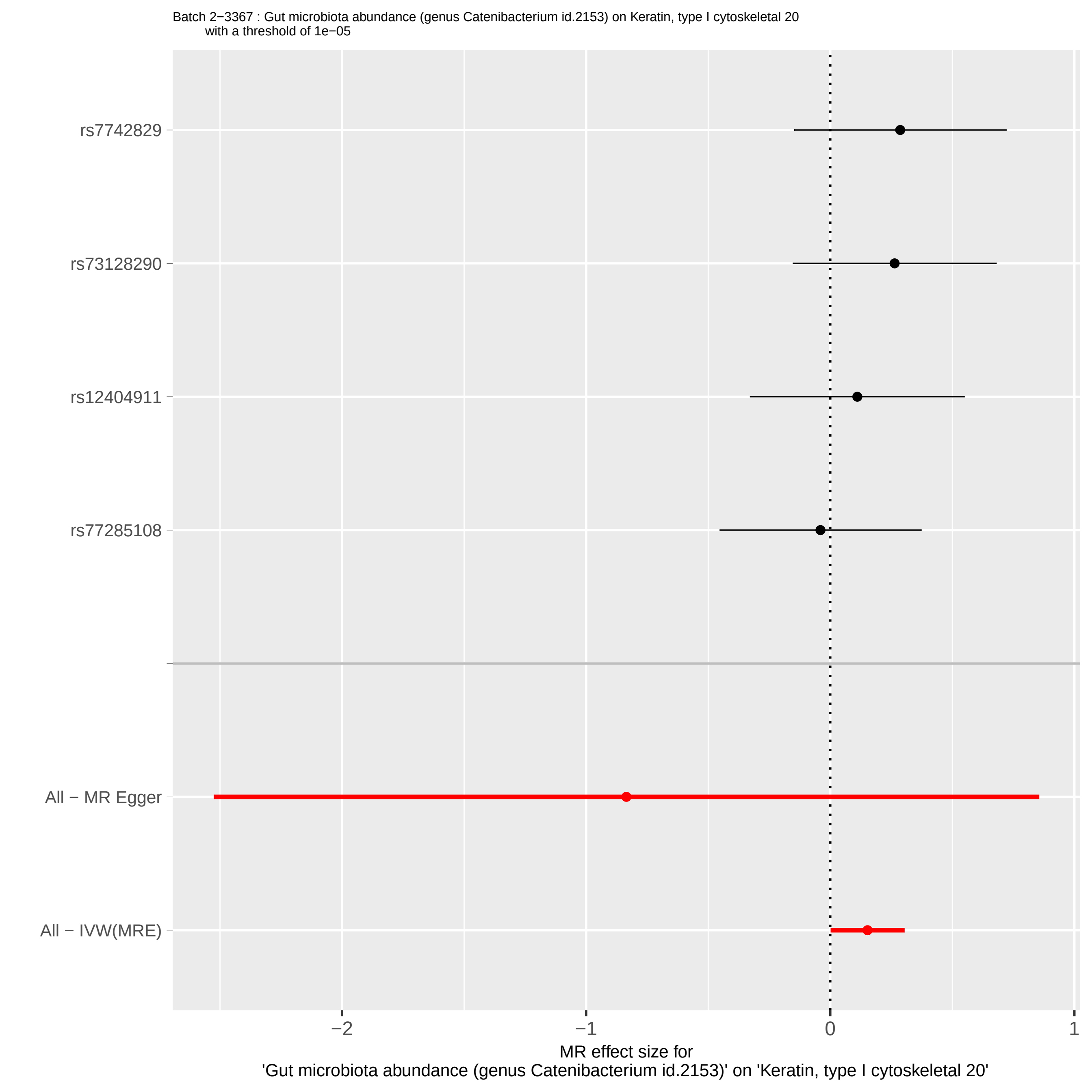

Batch 2−3367 : Gut microbiota abundance (genus Catenibacterium id.2153) on Keratin, type I cytoskeletal 20
with a threshold of 1e−05
rs7742829
rs73128290
rs12404911
rs77285108
All − MR Egger
All − IVW(MRE)
−2
−1
0
1
MR effect size for
'Gut microbiota abundance (genus Catenibacterium id.2153)' on 'Keratin, type I cytoskeletal 20'

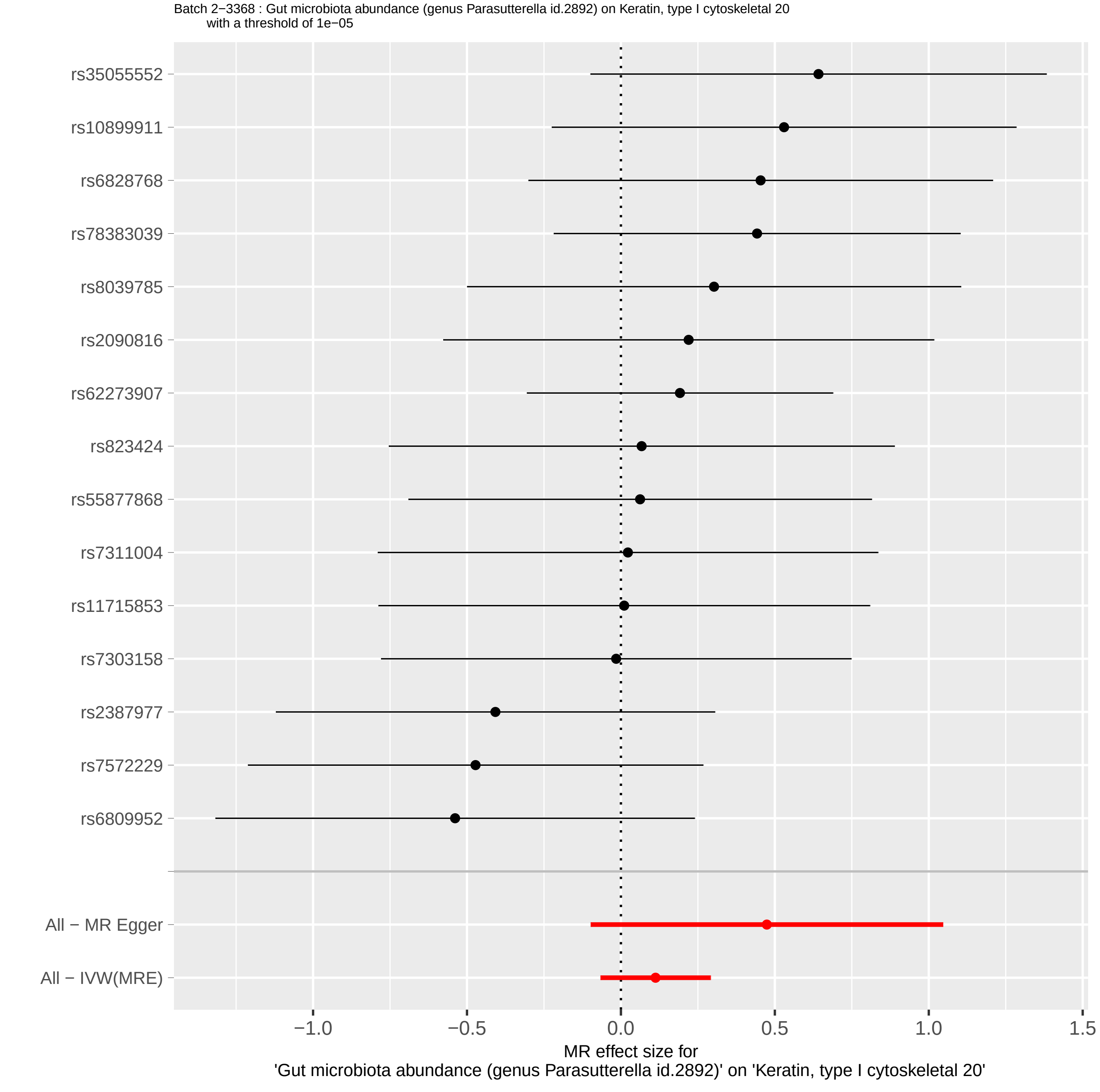

Batch 2−3368 : Gut microbiota abundance (genus Parasutterella id.2892) on Keratin, type I cytoskeletal 20
with a threshold of 1e−05
rs35055552
rs10899911
rs6828768
rs78383039
rs8039785
rs2090816
rs62273907
rs823424
rs55877868
rs7311004
rs11715853
rs7303158
rs2387977
rs7572229
rs6809952
All − MR Egger
All − IVW(MRE)
−1.0
−0.5
0.0
0.5
1.0
1.5
MR effect size for
'Gut microbiota abundance (genus Parasutterella id.2892)' on 'Keratin, type I cytoskeletal 20'

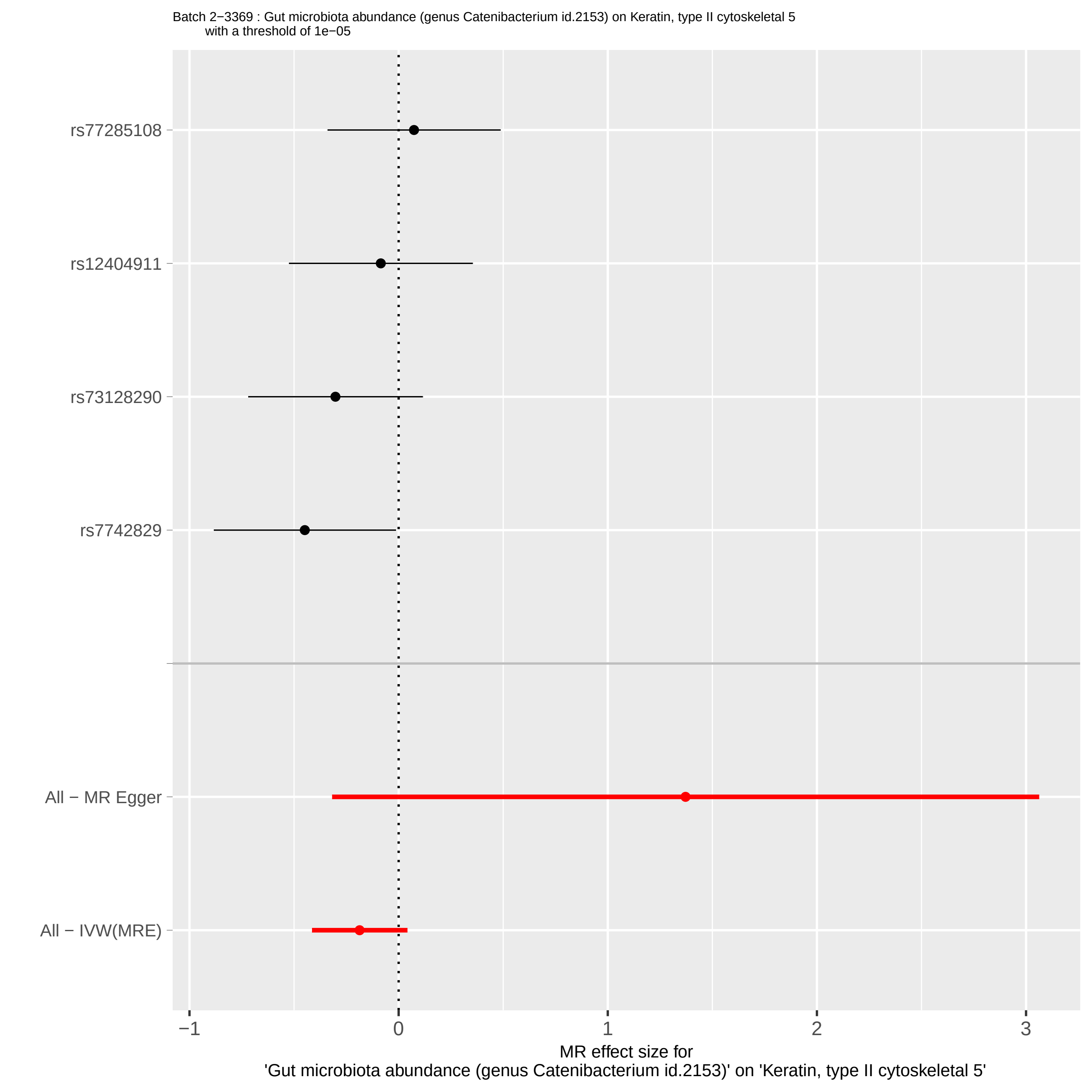
Batch 2−3369 : Gut microbiota abundance (genus Catenibacterium id.2153) on Keratin, type II cytoskeletal 5
with a threshold of 1e−05
rs77285108
rs12404911
rs73128290
rs7742829
All − MR Egger
All − IVW(MRE)
−1
0
1
2
3
MR effect size for
'Gut microbiota abundance (genus Catenibacterium id.2153)' on 'Keratin, type II cytoskeletal 5'

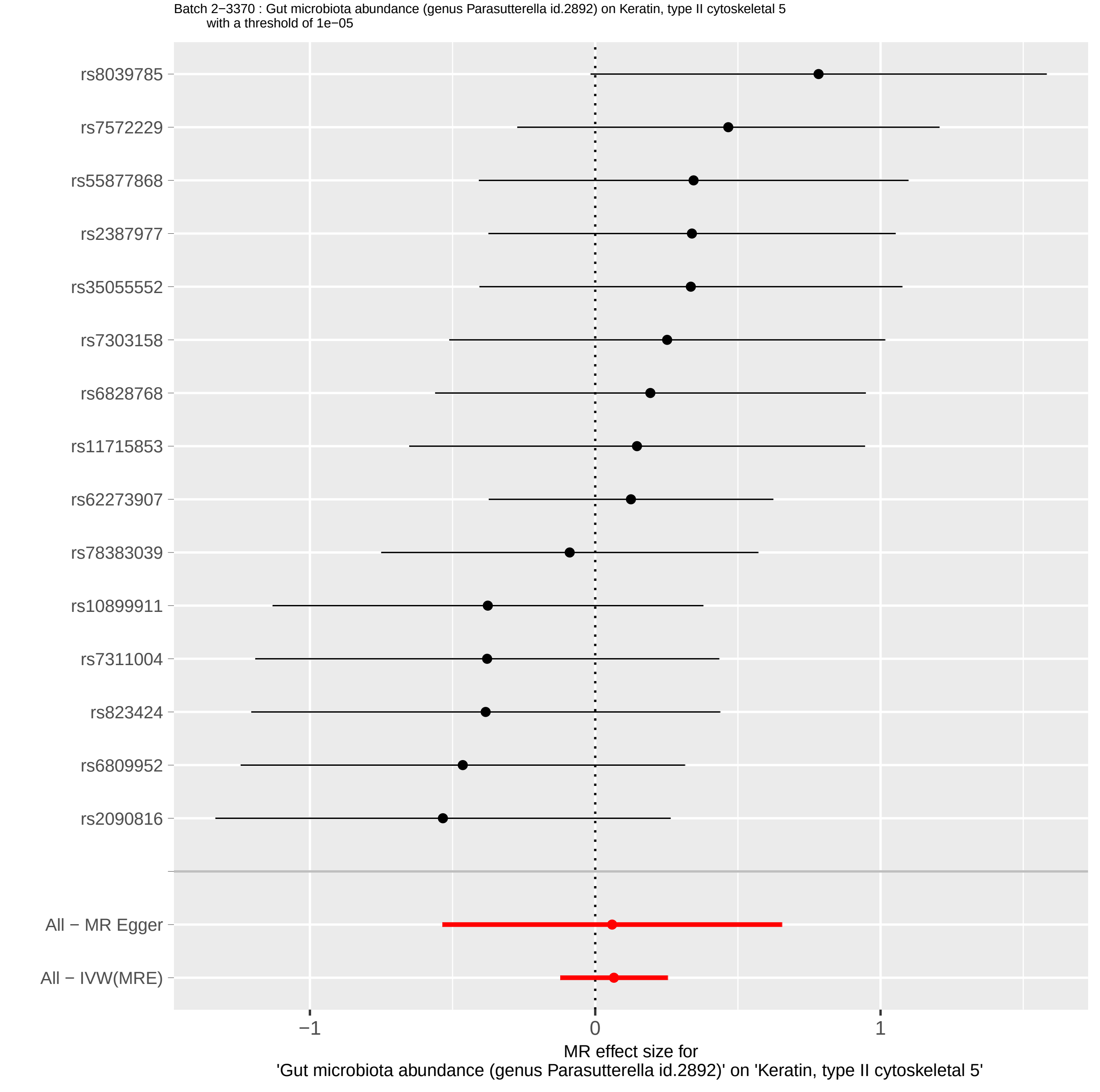
Batch 2−3370 : Gut microbiota abundance (genus Parasutterella id.2892) on Keratin, type II cytoskeletal 5
with a threshold of 1e−05
rs8039785
rs7572229
rs55877868
rs2387977
rs35055552
rs7303158
rs6828768
rs11715853
rs62273907
rs78383039
rs10899911
rs7311004
rs823424
rs6809952
rs2090816
All − MR Egger
All − IVW(MRE)
−1
0
1
MR effect size for
'Gut microbiota abundance (genus Parasutterella id.2892)' on 'Keratin, type II cytoskeletal 5'

Batch 2−3371 : Gut microbiota abundance (genus Catenibacterium id.2153) on Keratin, type II cytoskeletal 7
with a threshold of 1e−05

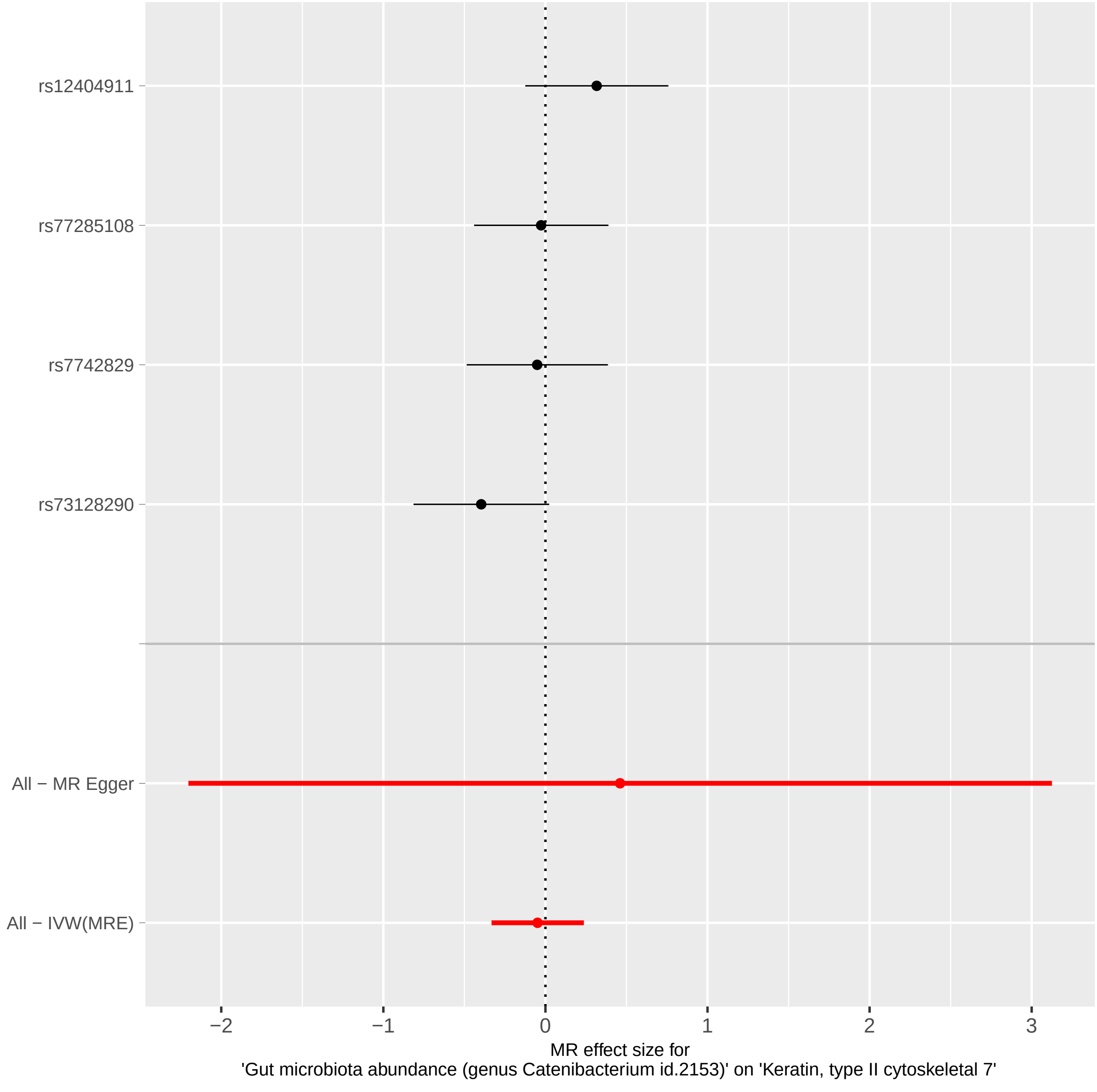

Batch 2−3372 : Gut microbiota abundance (genus Parasutterella id.2892) on Keratin, type II cytoskeletal 7
with a threshold of 1e−05

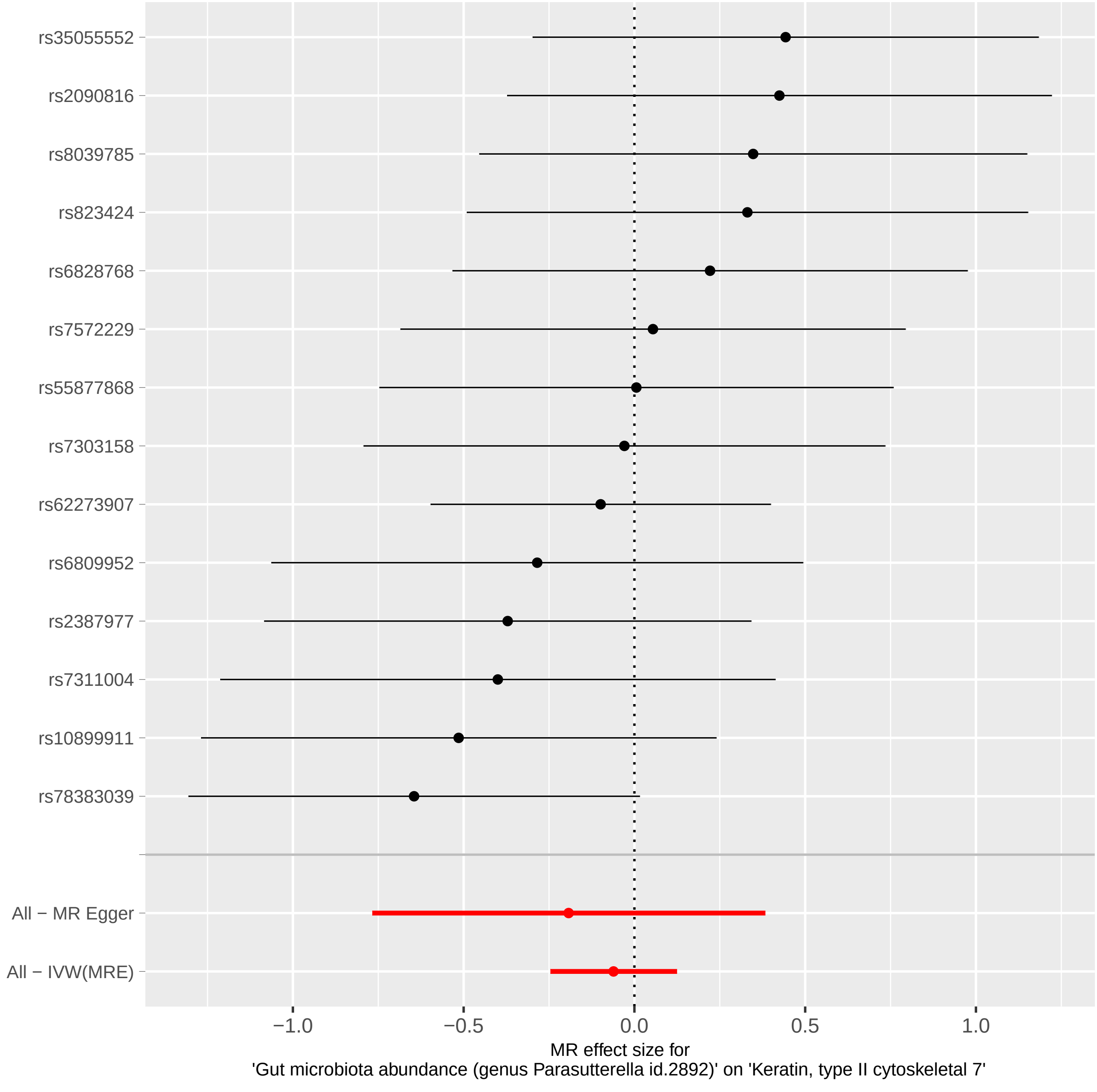

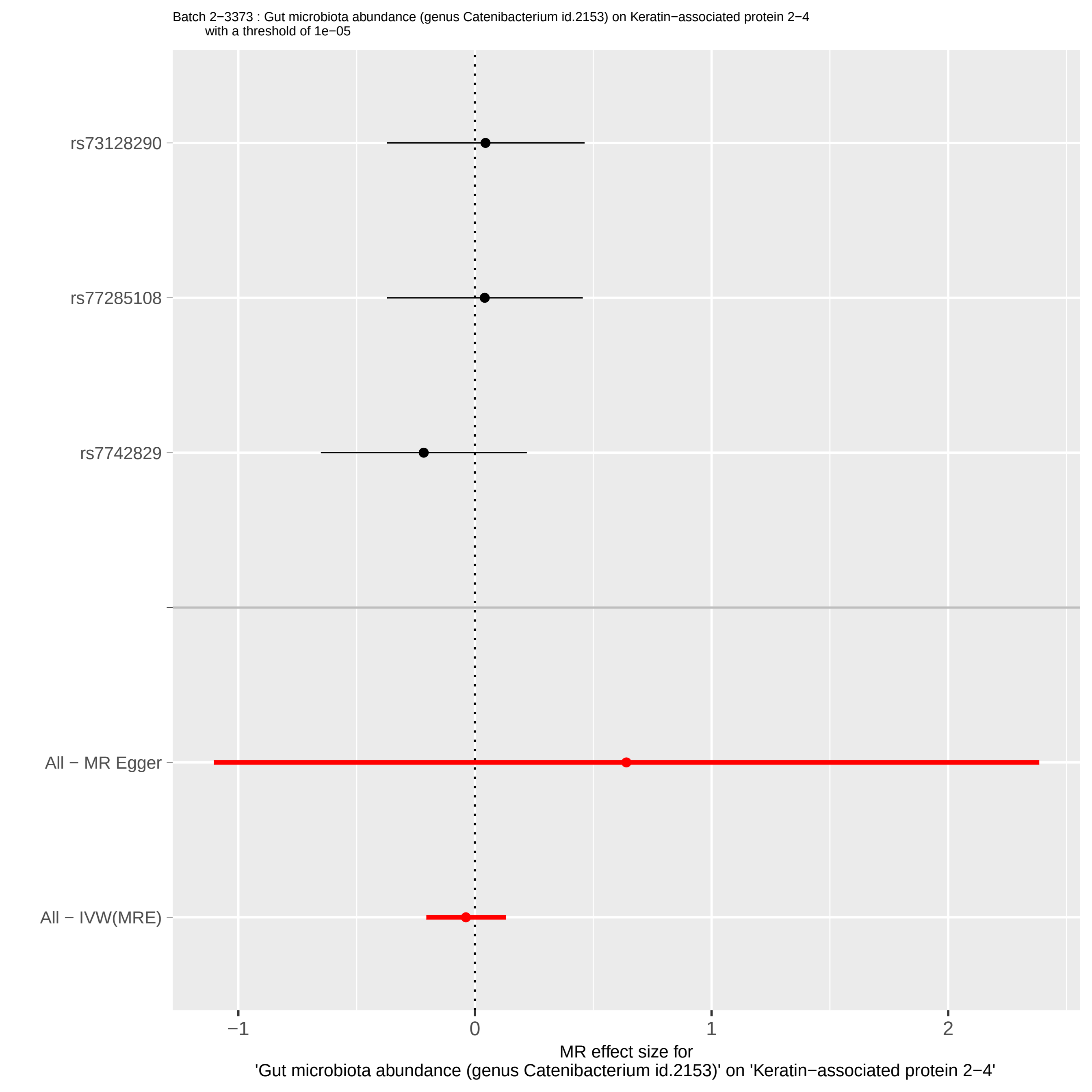

Batch 2−3373 : Gut microbiota abundance (genus Catenibacterium id.2153) on Keratin−associated protein 2−4
with a threshold of 1e−05
rs73128290
rs77285108
rs7742829
All − MR Egger
All − IVW(MRE)
−1
0
1
2
MR effect size for
'Gut microbiota abundance (genus Catenibacterium id.2153)' on 'Keratin−associated protein 2−4'

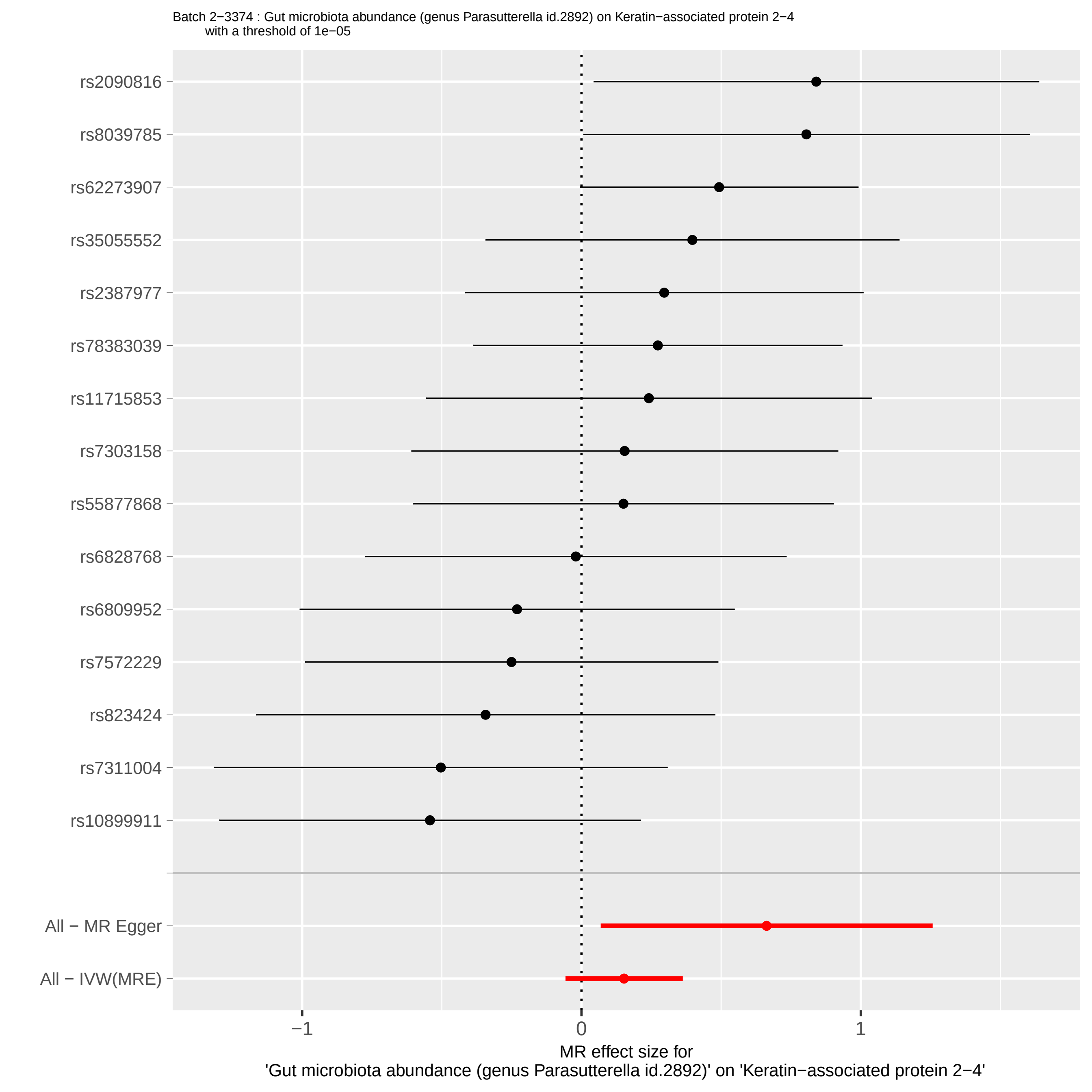
Batch 2−3374 : Gut microbiota abundance (genus Parasutterella id.2892) on Keratin−associated protein 2−4
with a threshold of 1e−05
rs2090816
rs8039785
rs62273907
rs35055552
rs2387977
rs78383039
rs11715853
rs7303158
rs55877868
rs6828768
rs6809952
rs7572229
rs823424
rs7311004
rs10899911
All − MR Egger
All − IVW(MRE)
−1
0
1
MR effect size for
'Gut microbiota abundance (genus Parasutterella id.2892)' on 'Keratin−associated protein 2−4'

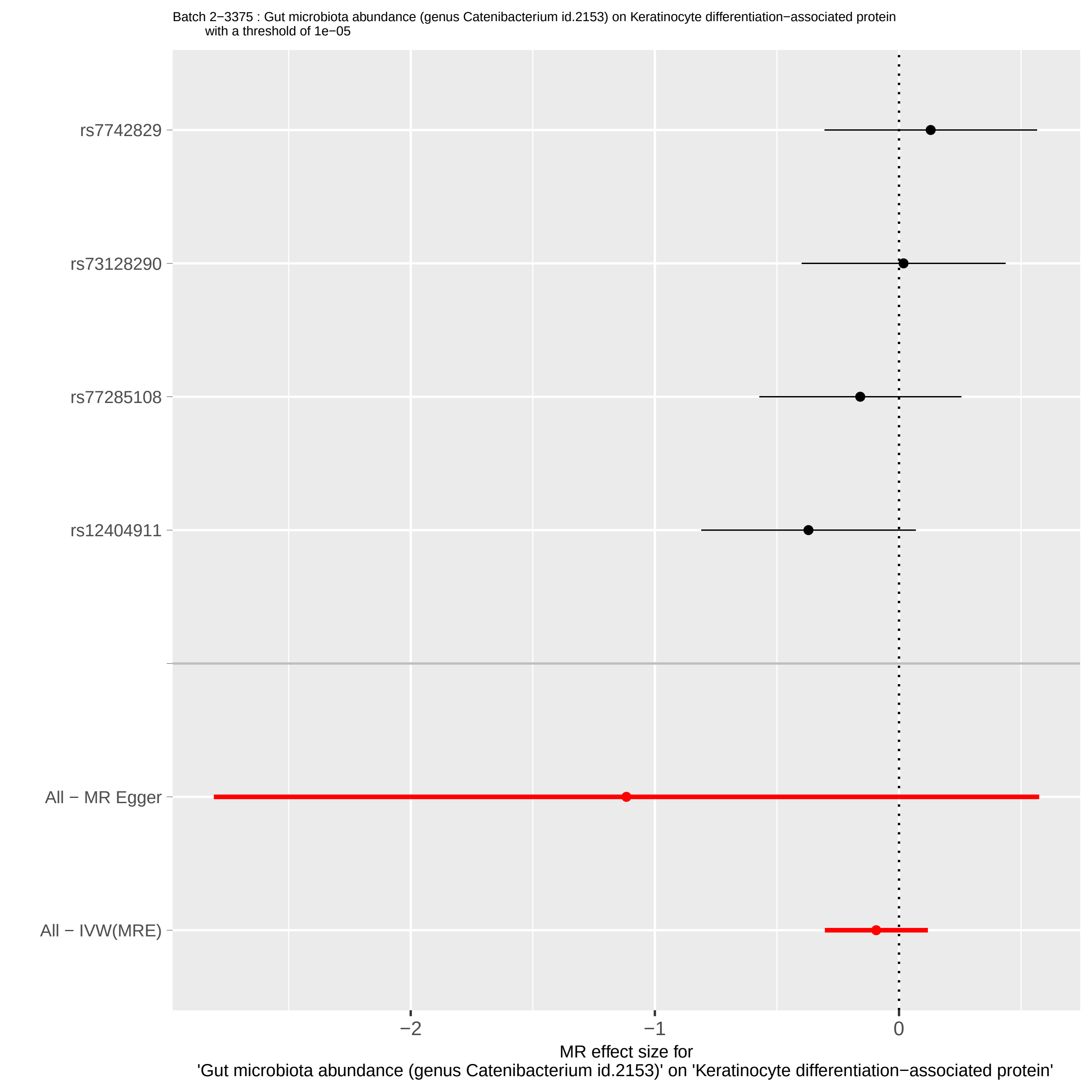
Batch 2−3375 : Gut microbiota abundance (genus Catenibacterium id.2153) on Keratinocyte differentiation−associated protein with a threshold of 1e−05
rs7742829
rs73128290
rs77285108
rs12404911
All − MR Egger
All − IVW(MRE)
−2
−1
0
MR effect size for
'Gut microbiota abundance (genus Catenibacterium id.2153)' on 'Keratinocyte differentiation−associated protein'

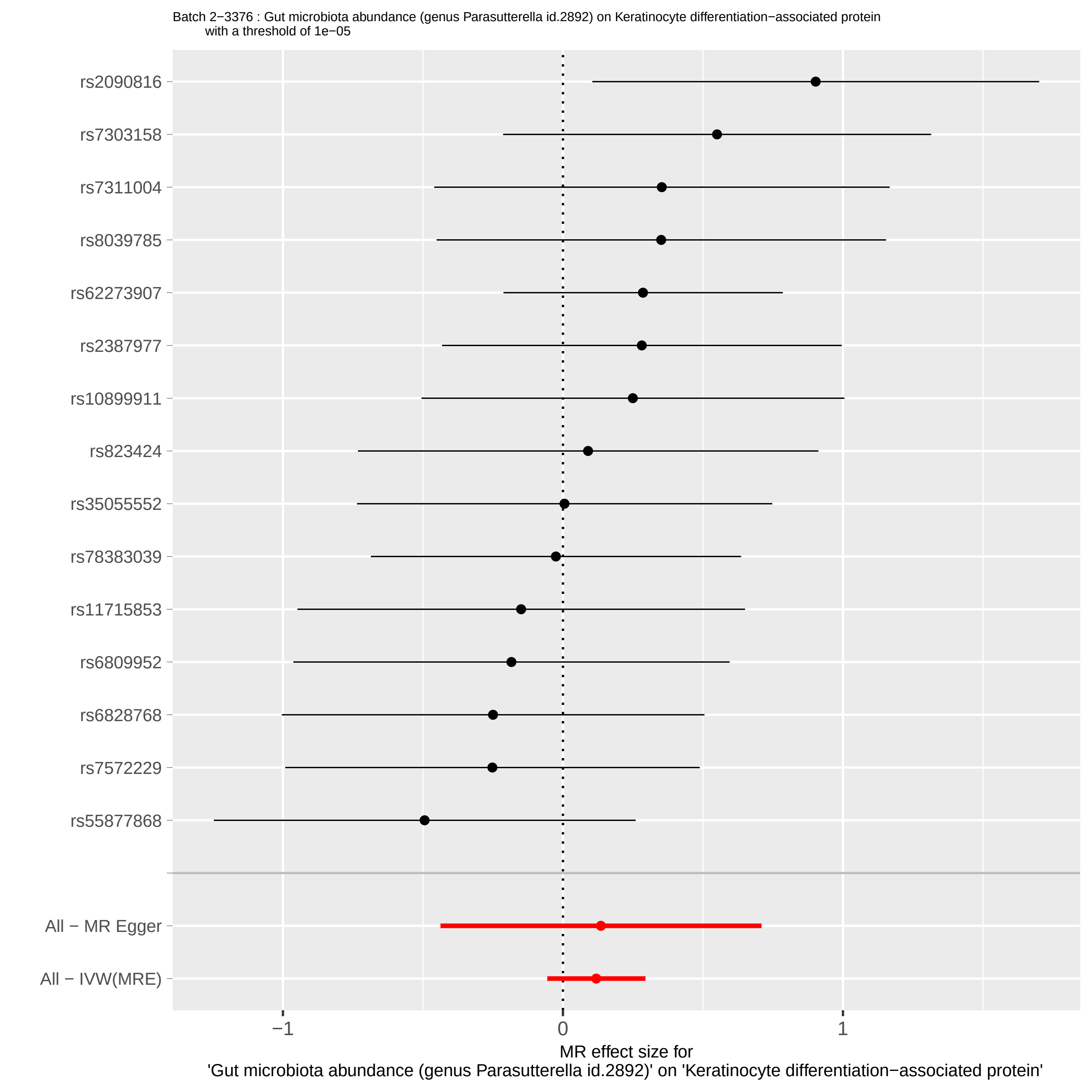

Batch 2−3376 : Gut microbiota abundance (genus Parasutterella id.2892) on Keratinocyte differentiation−associated protein
with a threshold of 1e−05
rs2090816
rs7303158
rs7311004
rs8039785
rs62273907
rs2387977
rs10899911
rs823424
rs35055552
rs78383039
rs11715853
rs6809952
rs6828768
rs7572229
rs55877868
All − MR Egger
All − IVW(MRE)
−1
0
1
MR effect size for
'Gut microbiota abundance (genus Parasutterella id.2892)' on 'Keratinocyte differentiation−associated protein'

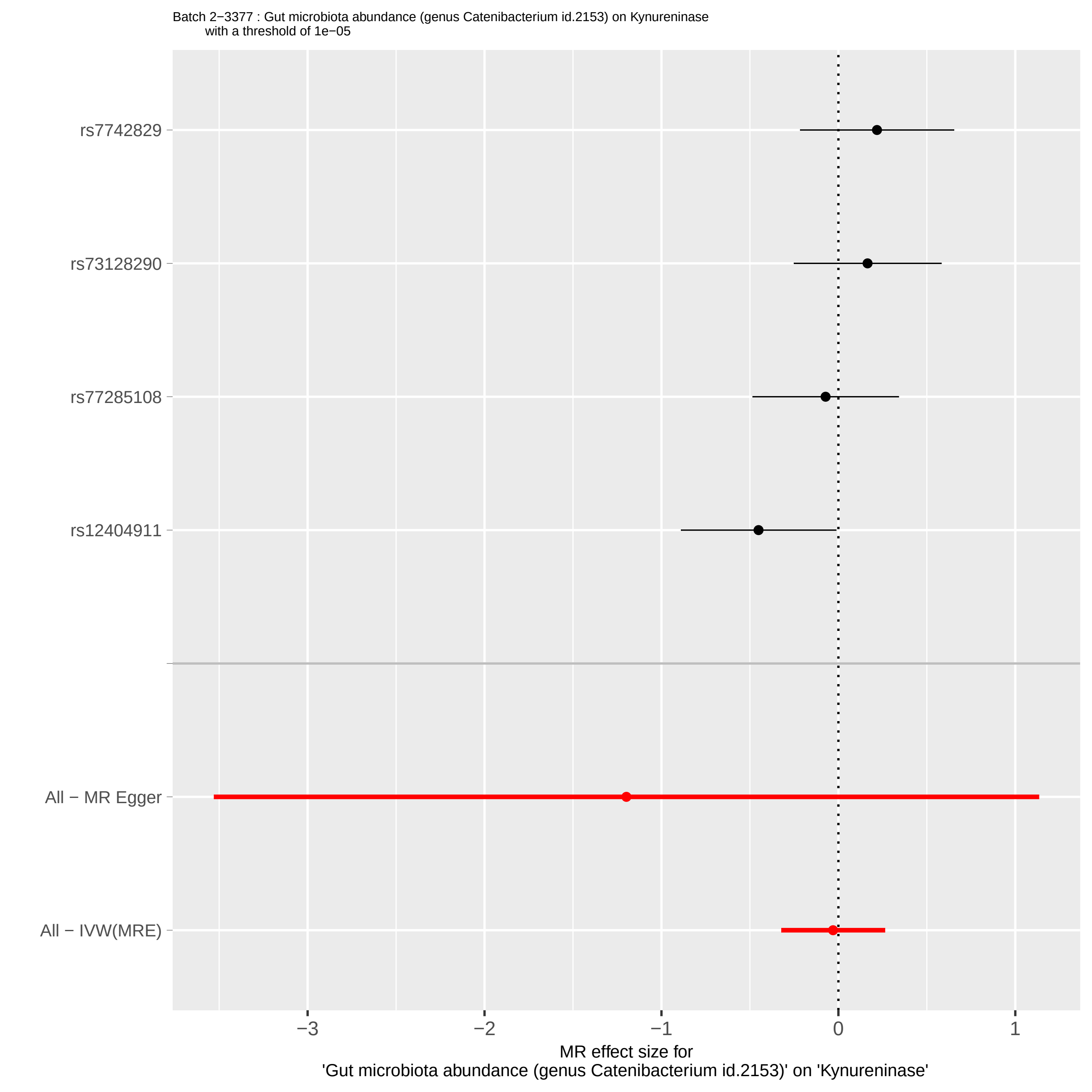

Batch 2−3377 : Gut microbiota abundance (genus Catenibacterium id.2153) on Kynureninase
with a threshold of 1e−05
rs7742829
rs73128290
rs77285108
rs12404911
All − MR Egger
All − IVW(MRE)
−3
−2
−1
0
1
MR effect size for
'Gut microbiota abundance (genus Catenibacterium id.2153)' on 'Kynureninase'

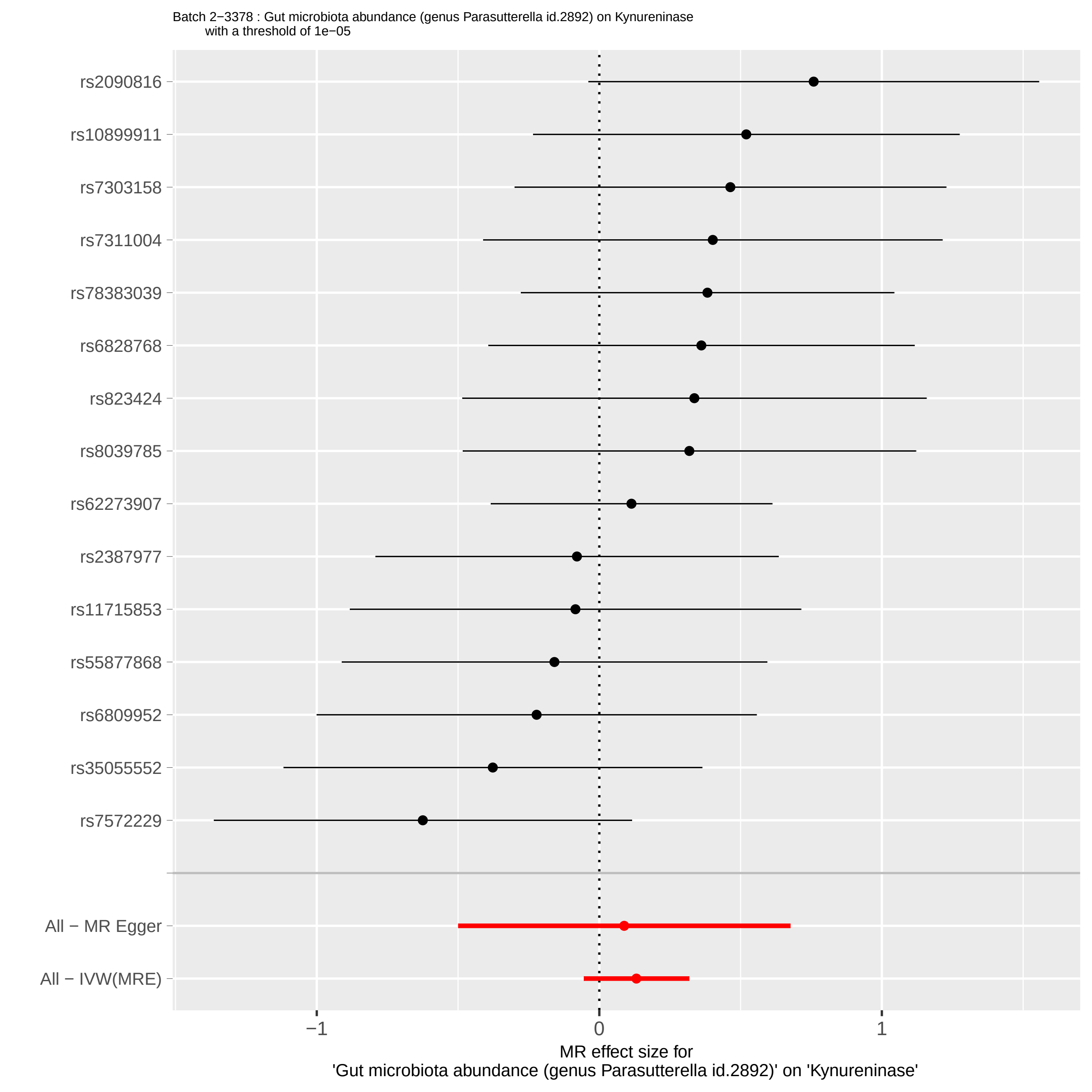
Batch 2−3378 : Gut microbiota abundance (genus Parasutterella id.2892) on Kynureninase
with a threshold of 1e−05
rs2090816
rs10899911
rs7303158
rs7311004
rs78383039
rs6828768
rs823424
rs8039785
rs62273907
rs2387977
rs11715853
rs55877868
rs6809952
rs35055552
rs7572229
All − MR Egger
All − IVW(MRE)
−1
0
1
MR effect size for
'Gut microbiota abundance (genus Parasutterella id.2892)' on 'Kynureninase'

Batch 2−3379 : Gut microbiota abundance (genus Catenibacterium id.2153) on Neural cell adhesion molecule L1
with a threshold of 1e−05

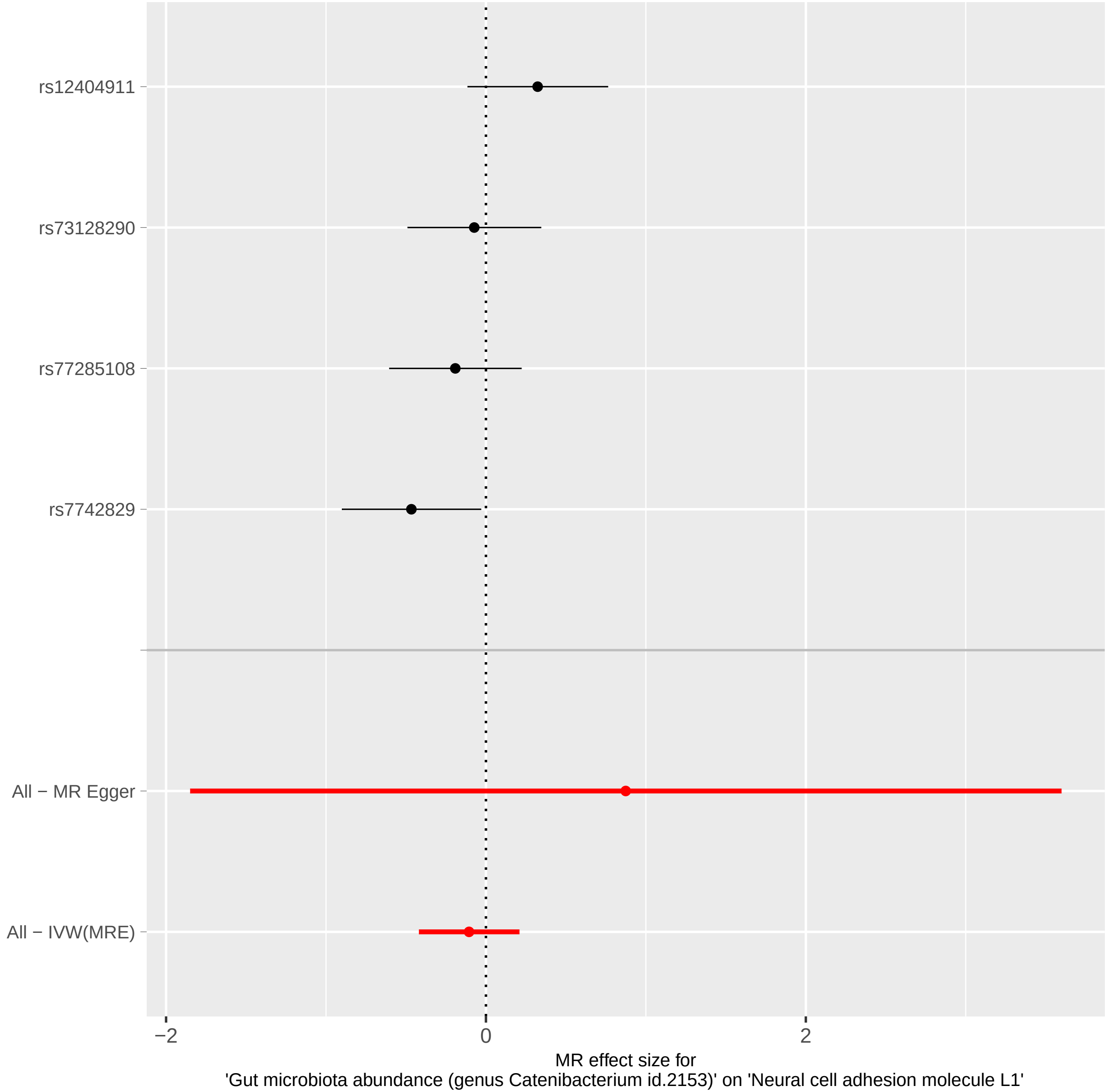

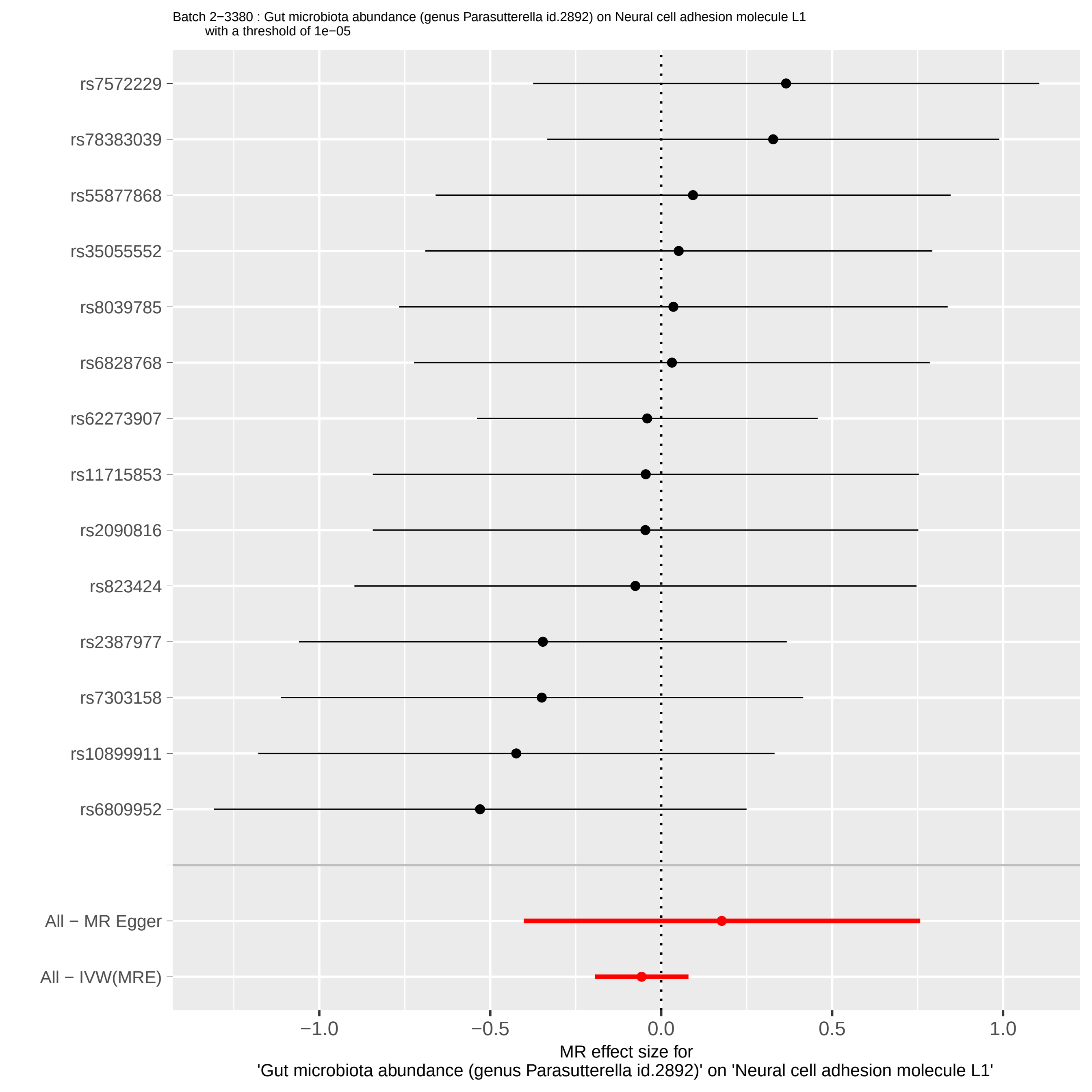

Batch 2−3380 : Gut microbiota abundance (genus Parasutterella id.2892) on Neural cell adhesion molecule L1
with a threshold of 1e−05
rs7572229
rs78383039
rs55877868
rs35055552
rs8039785
rs6828768
rs62273907
rs11715853
rs2090816
rs823424
rs2387977
rs7303158
rs10899911
rs6809952
All − MR Egger
All − IVW(MRE)
−1.0
−0.5
0.0
0.5
1.0
MR effect size for
'Gut microbiota abundance (genus Parasutterella id.2892)' on 'Neural cell adhesion molecule L1'

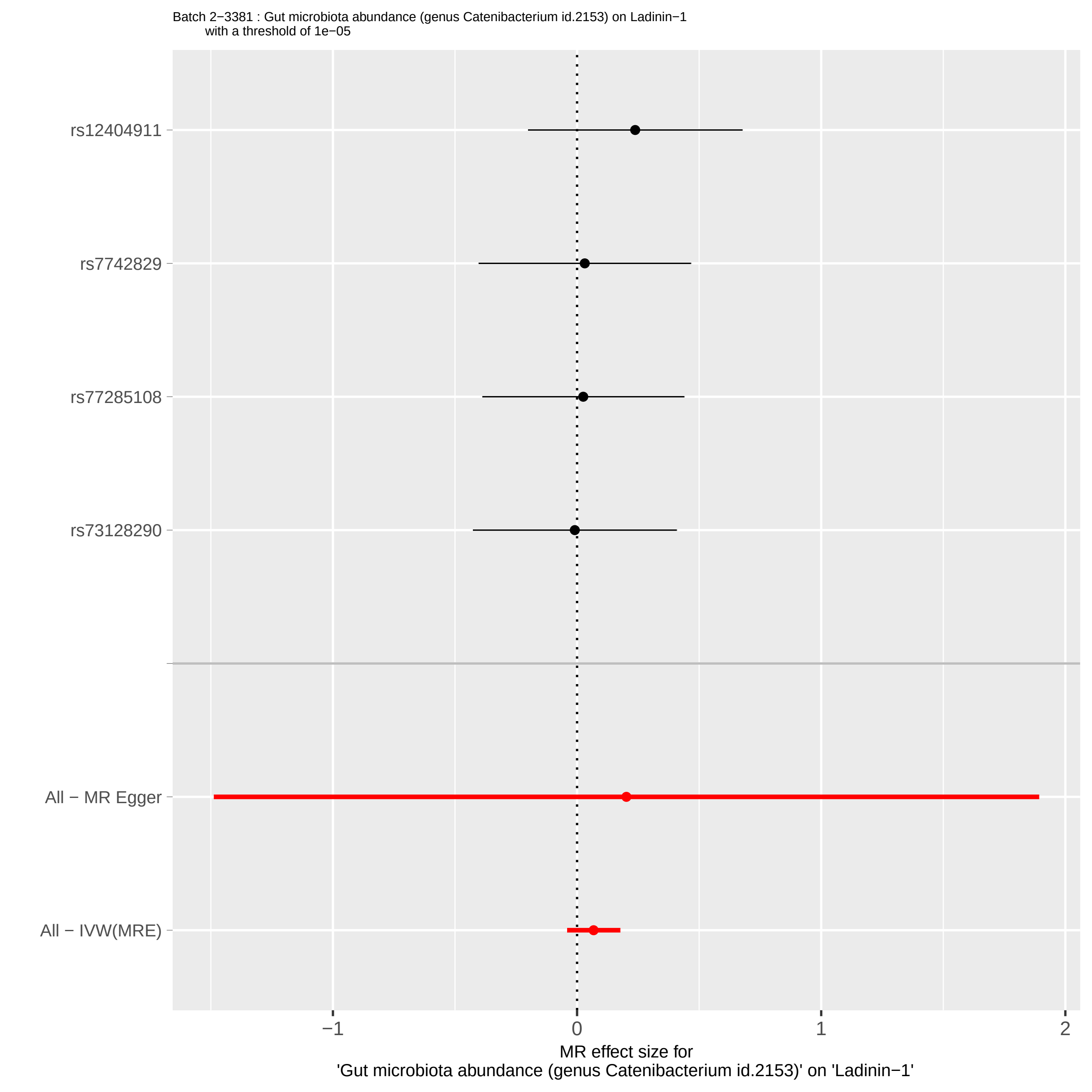

Batch 2−3381 : Gut microbiota abundance (genus Catenibacterium id.2153) on Ladinin−1
with a threshold of 1e−05
rs12404911
rs7742829
rs77285108
rs73128290
All − MR Egger
All − IVW(MRE)
−1
0
1
2
MR effect size for
'Gut microbiota abundance (genus Catenibacterium id.2153)' on 'Ladinin−1'

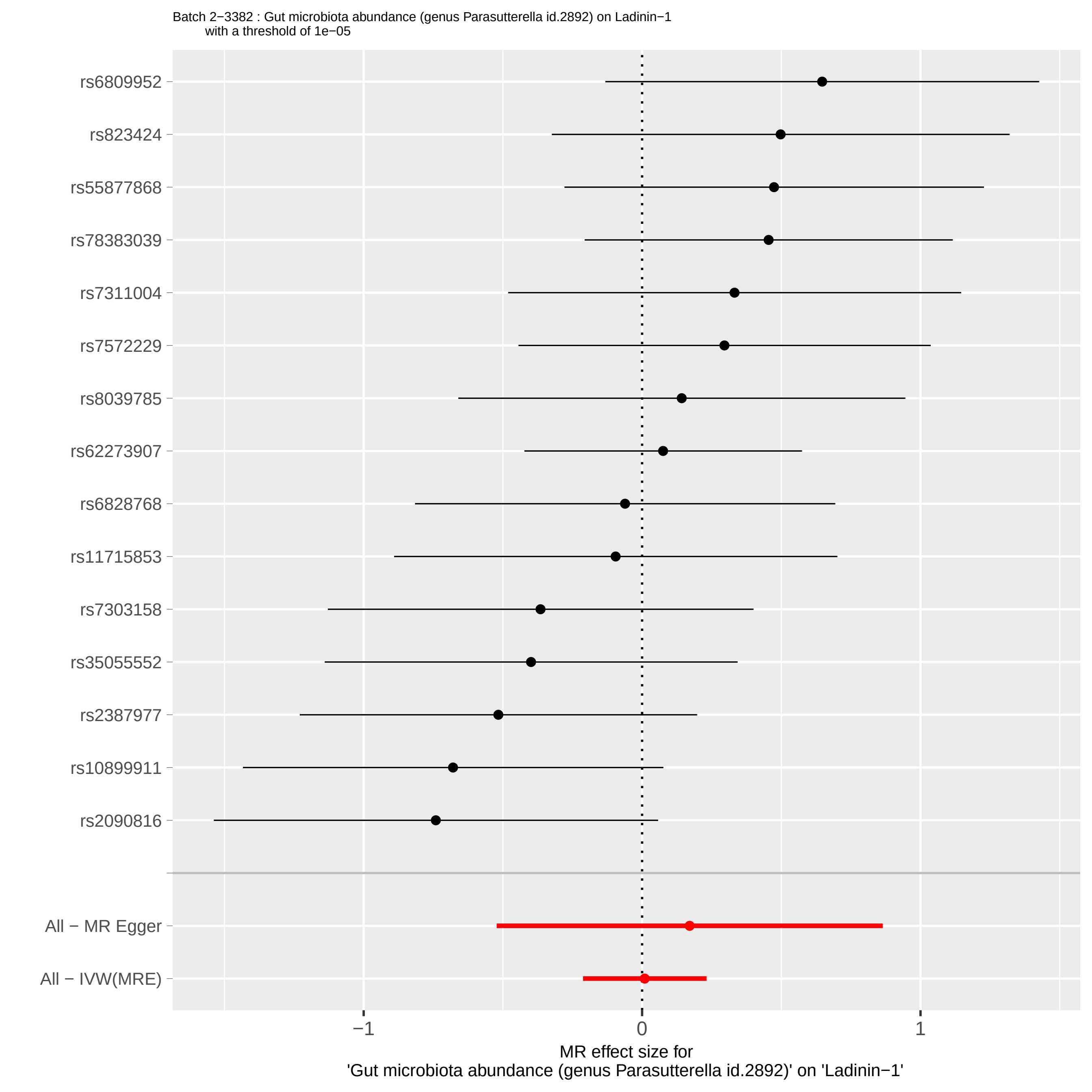
Batch 2−3382 : Gut microbiota abundance (genus Parasutterella id.2892) on Ladinin−1
with a threshold of 1e−05
rs6809952
rs823424
rs55877868
rs78383039
rs7311004
rs7572229
rs8039785
rs62273907
rs6828768
rs11715853
rs7303158
rs35055552
rs2387977
rs10899911
rs2090816
All − MR Egger
All − IVW(MRE)
−1
0
1
MR effect size for
'Gut microbiota abundance (genus Parasutterella id.2892)' on 'Ladinin−1'

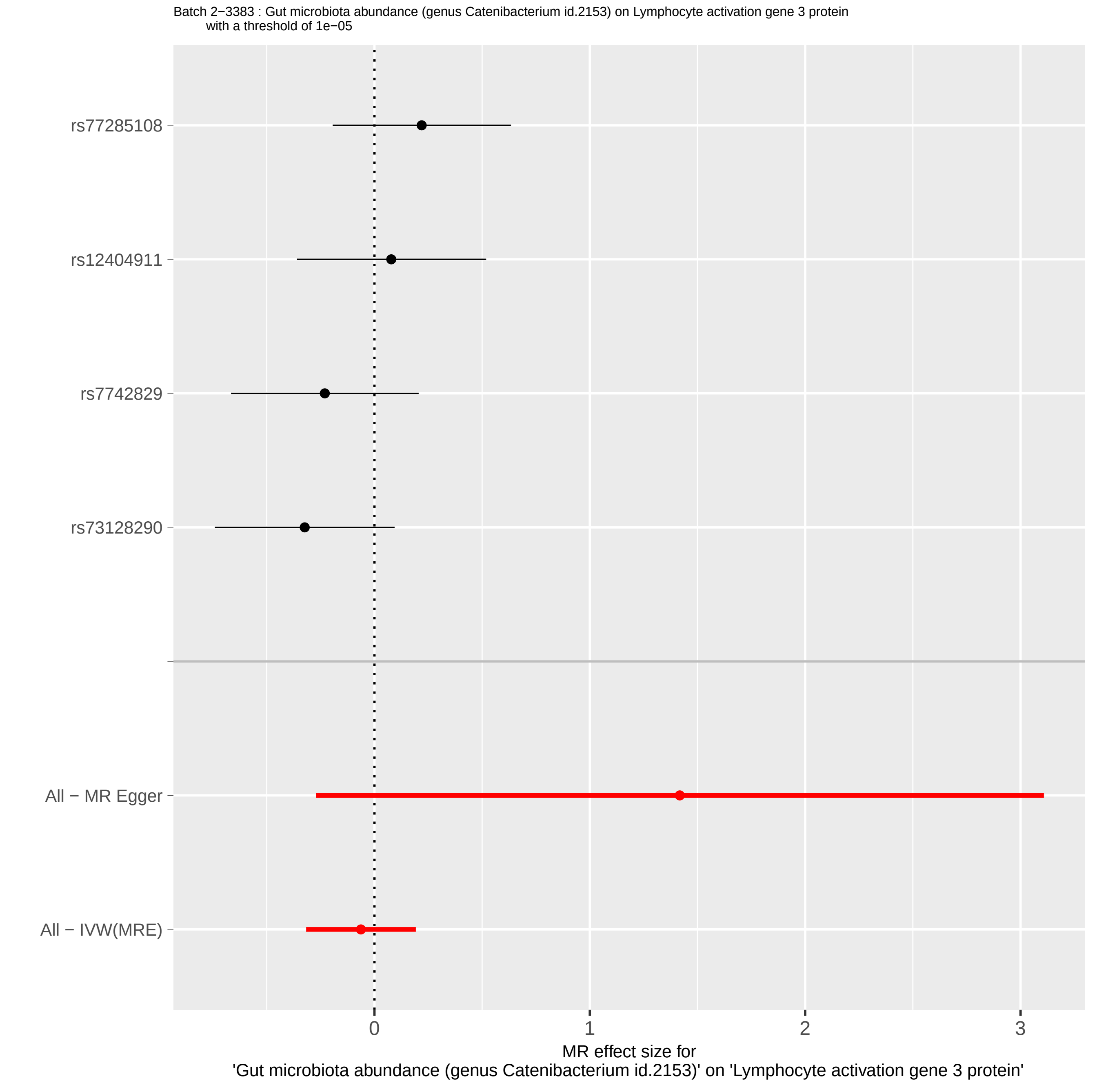

Batch 2−3383 : Gut microbiota abundance (genus Catenibacterium id.2153) on Lymphocyte activation gene 3 protein
with a threshold of 1e−05
rs77285108
rs12404911
rs7742829
rs73128290
All − MR Egger
All − IVW(MRE)
0
1
2
3
MR effect size for
'Gut microbiota abundance (genus Catenibacterium id.2153)' on 'Lymphocyte activation gene 3 protein'

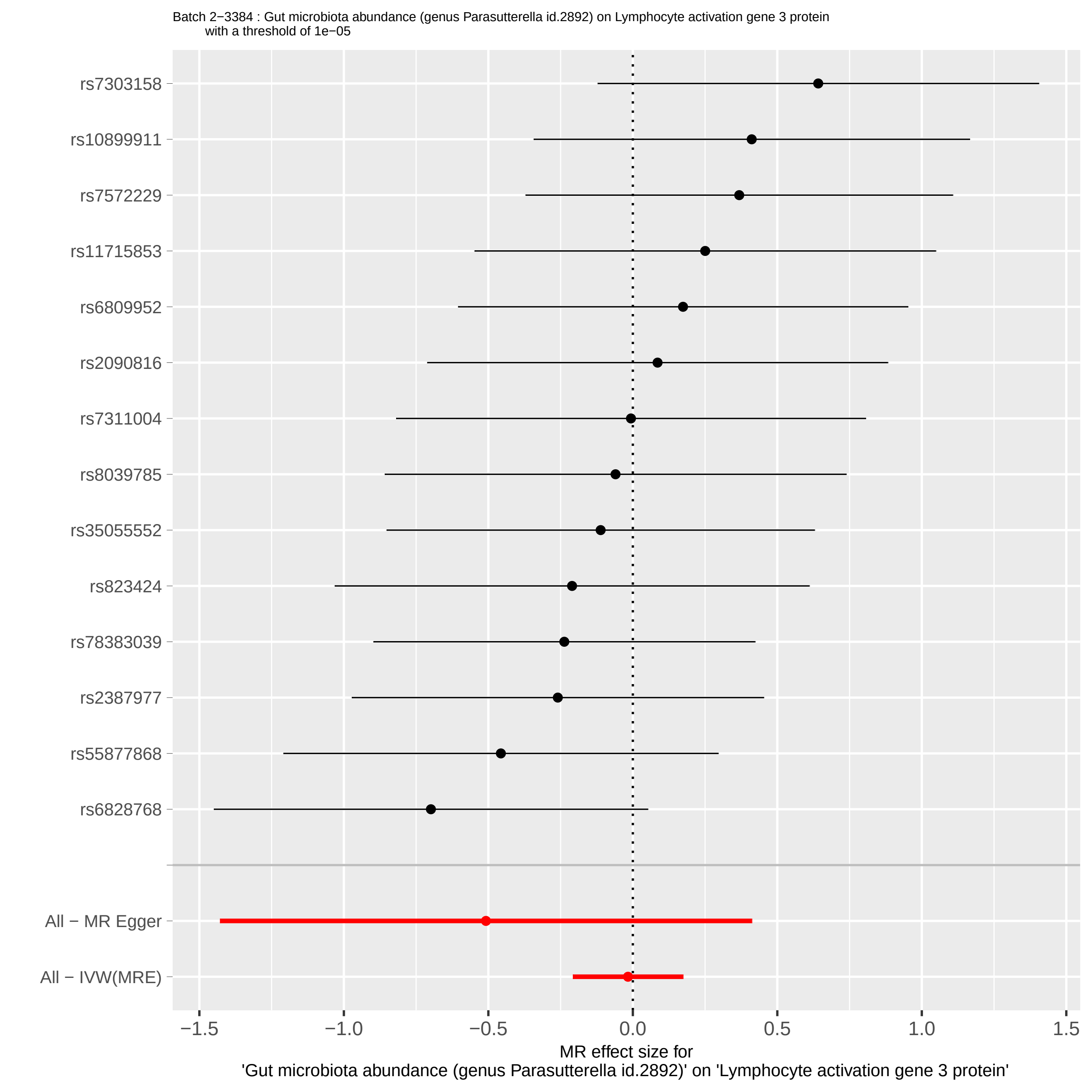

Batch 2−3384 : Gut microbiota abundance (genus Parasutterella id.2892) on Lymphocyte activation gene 3 protein
with a threshold of 1e−05
rs7303158
rs10899911
rs7572229
rs11715853
rs6809952
rs2090816
rs7311004
rs8039785
rs35055552
rs823424
rs78383039
rs2387977
rs55877868
rs6828768
All − MR Egger
All − IVW(MRE)
−1.5
−1.0
−0.5
0.0
0.5
1.0
1.5
MR effect size for
'Gut microbiota abundance (genus Parasutterella id.2892)' on 'Lymphocyte activation gene 3 protein'

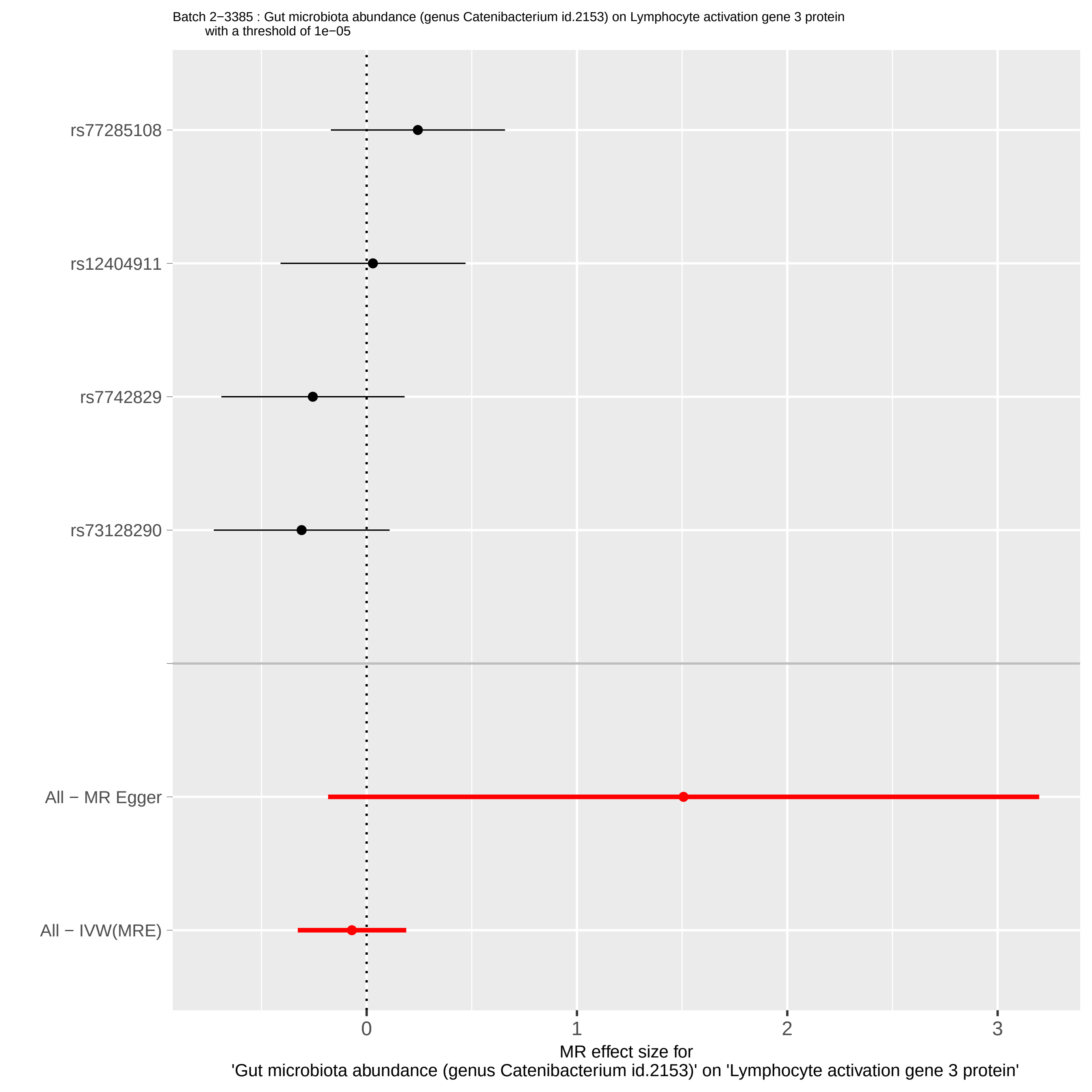
Batch 2−3385 : Gut microbiota abundance (genus Catenibacterium id.2153) on Lymphocyte activation gene 3 protein
with a threshold of 1e−05
rs77285108
rs12404911
rs7742829
rs73128290
All − MR Egger
All − IVW(MRE)
0
1
2
3
MR effect size for
'Gut microbiota abundance (genus Catenibacterium id.2153)' on 'Lymphocyte activation gene 3 protein'

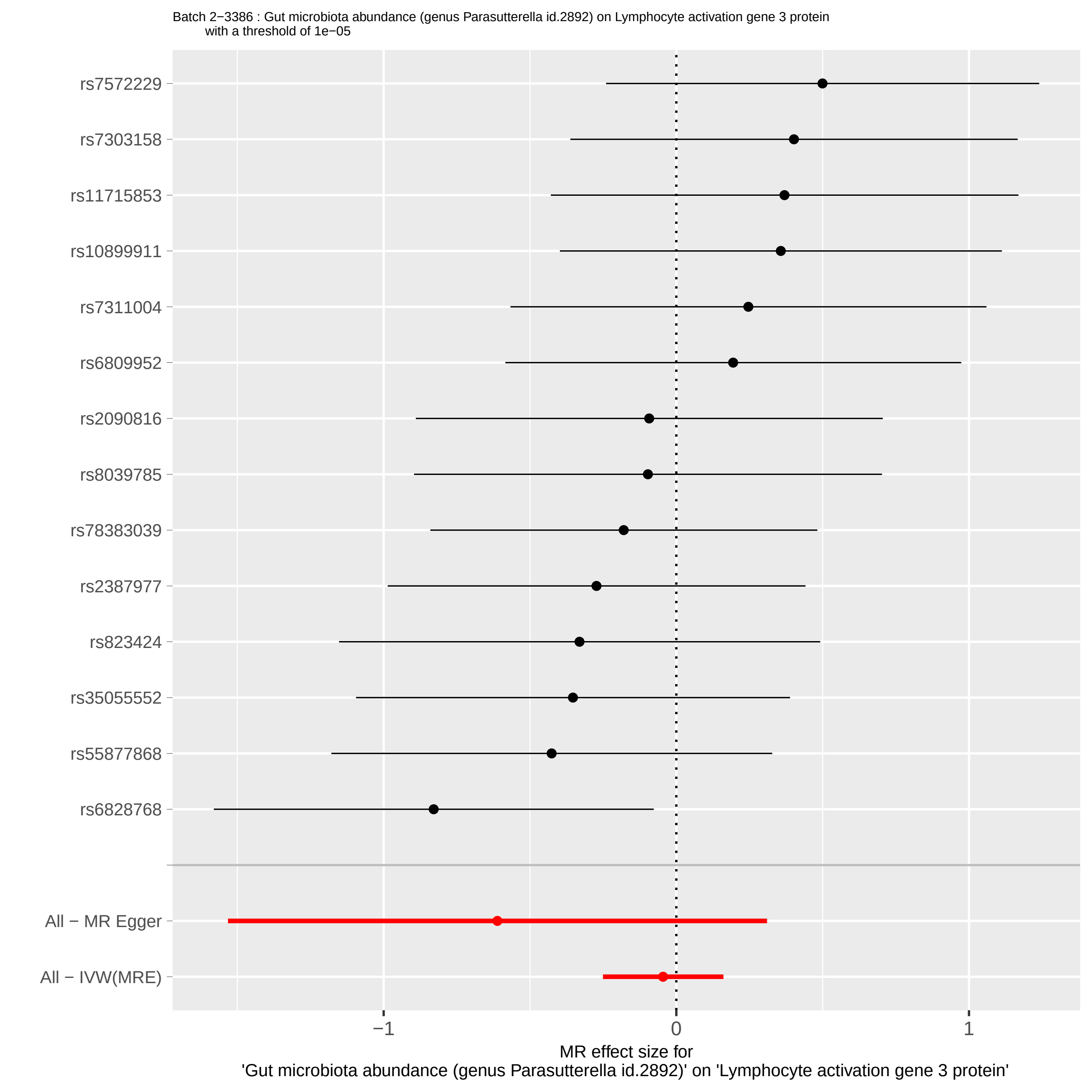

Batch 2−3386 : Gut microbiota abundance (genus Parasutterella id.2892) on Lymphocyte activation gene 3 protein
with a threshold of 1e−05
rs7572229
rs7303158
rs11715853
rs10899911
rs7311004
rs6809952
rs2090816
rs8039785
rs78383039
rs2387977
rs823424
rs35055552
rs55877868
rs6828768
All − MR Egger
All − IVW(MRE)
−1
0
1
MR effect size for
'Gut microbiota abundance (genus Parasutterella id.2892)' on 'Lymphocyte activation gene 3 protein'

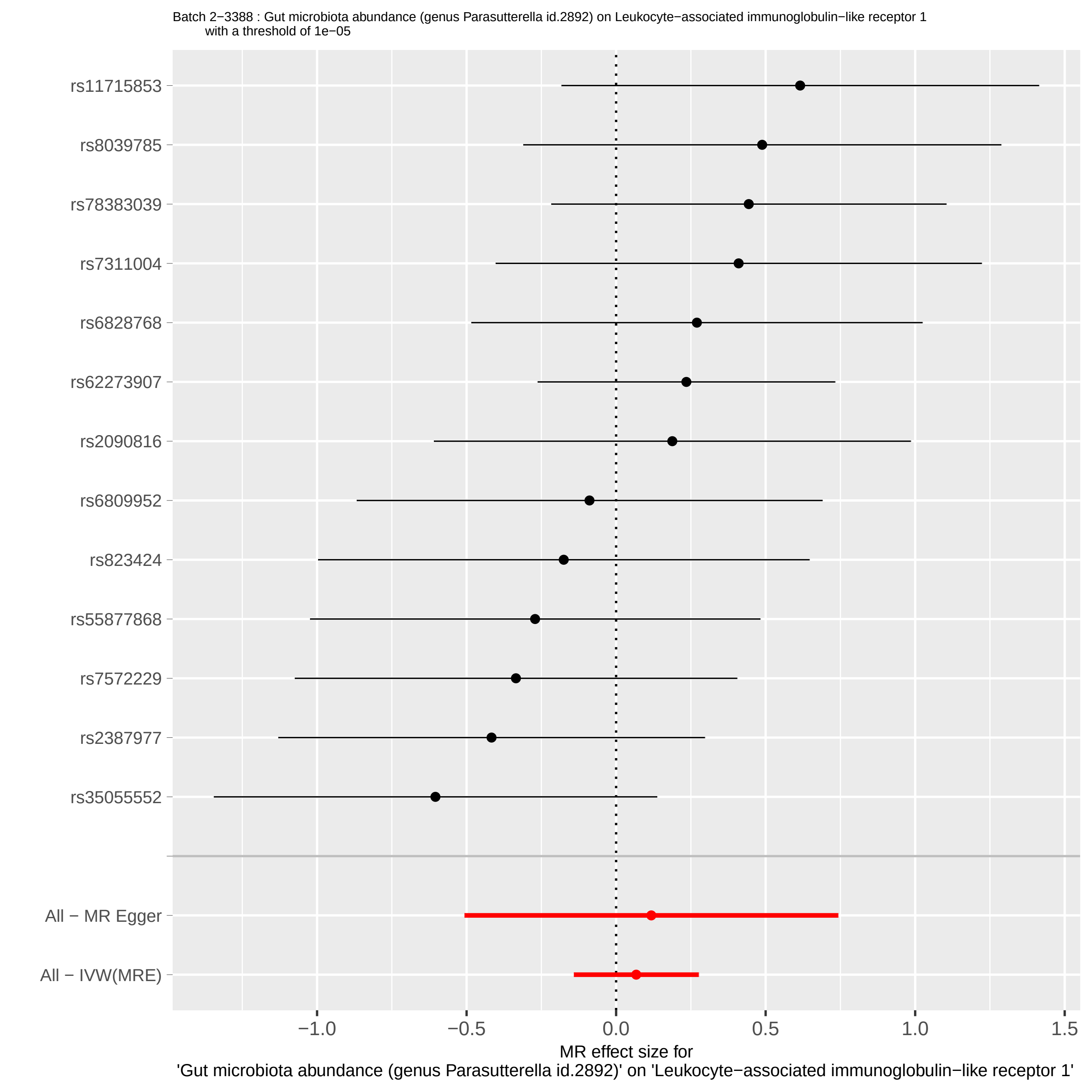
Batch 2−3388 : Gut microbiota abundance (genus Parasutterella id.2892) on Leukocyte−associated immunoglobulin−like receptor 1
with a threshold of 1e−05
rs11715853
rs8039785
rs78383039
rs7311004
rs6828768
rs62273907
rs2090816
rs6809952
rs823424
rs55877868
rs7572229
rs2387977
rs35055552
All − MR Egger
All − IVW(MRE)
−1.0
−0.5
0.0
0.5
1.0
1.5
MR effect size for
'Gut microbiota abundance (genus Parasutterella id.2892)' on 'Leukocyte−associated immunoglobulin−like receptor 1'

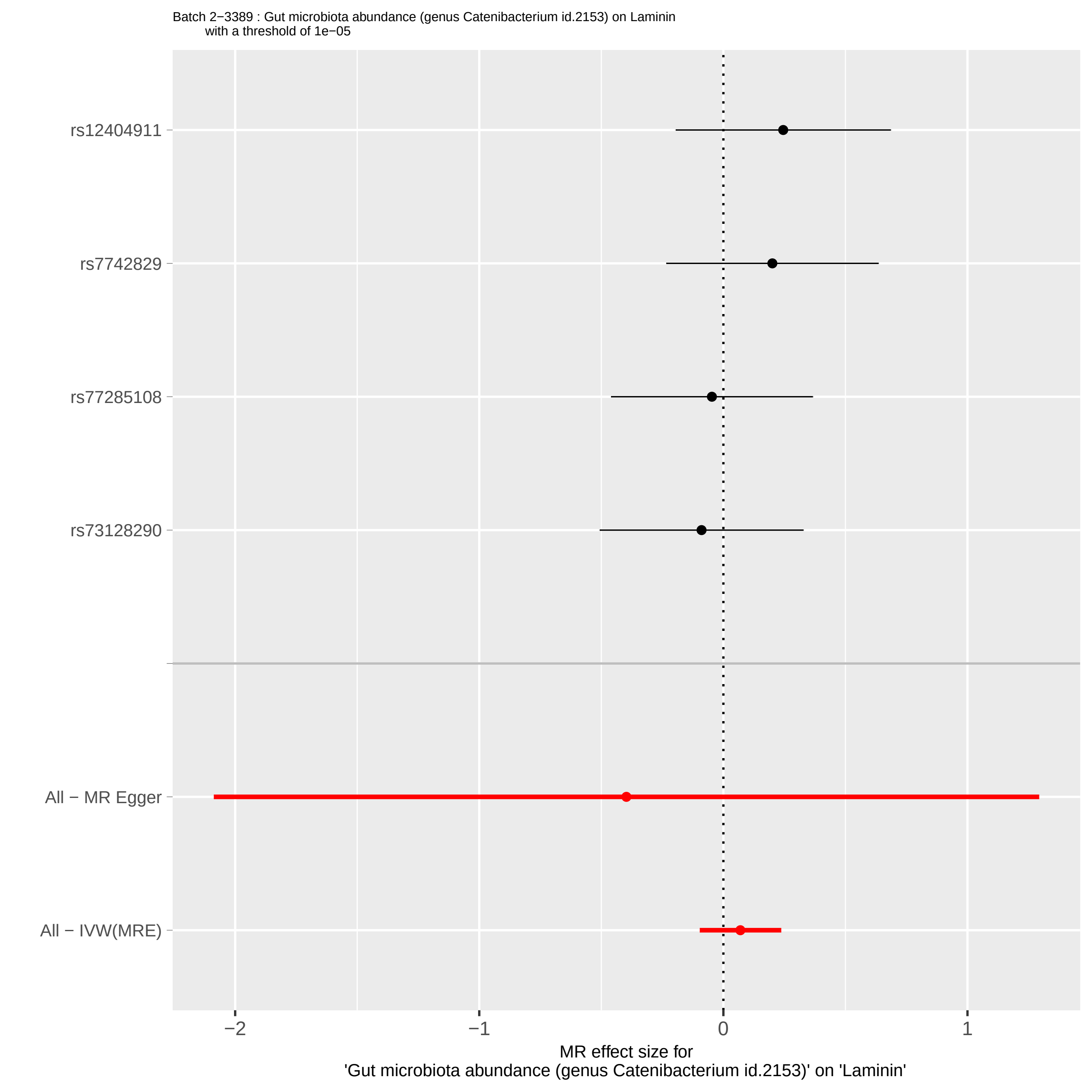
Batch 2−3389 : Gut microbiota abundance (genus Catenibacterium id.2153) on Laminin
with a threshold of 1e−05
rs12404911
rs7742829
rs77285108
rs73128290
All − MR Egger
All − IVW(MRE)
−2
−1
0
1
MR effect size for
'Gut microbiota abundance (genus Catenibacterium id.2153)' on 'Laminin'

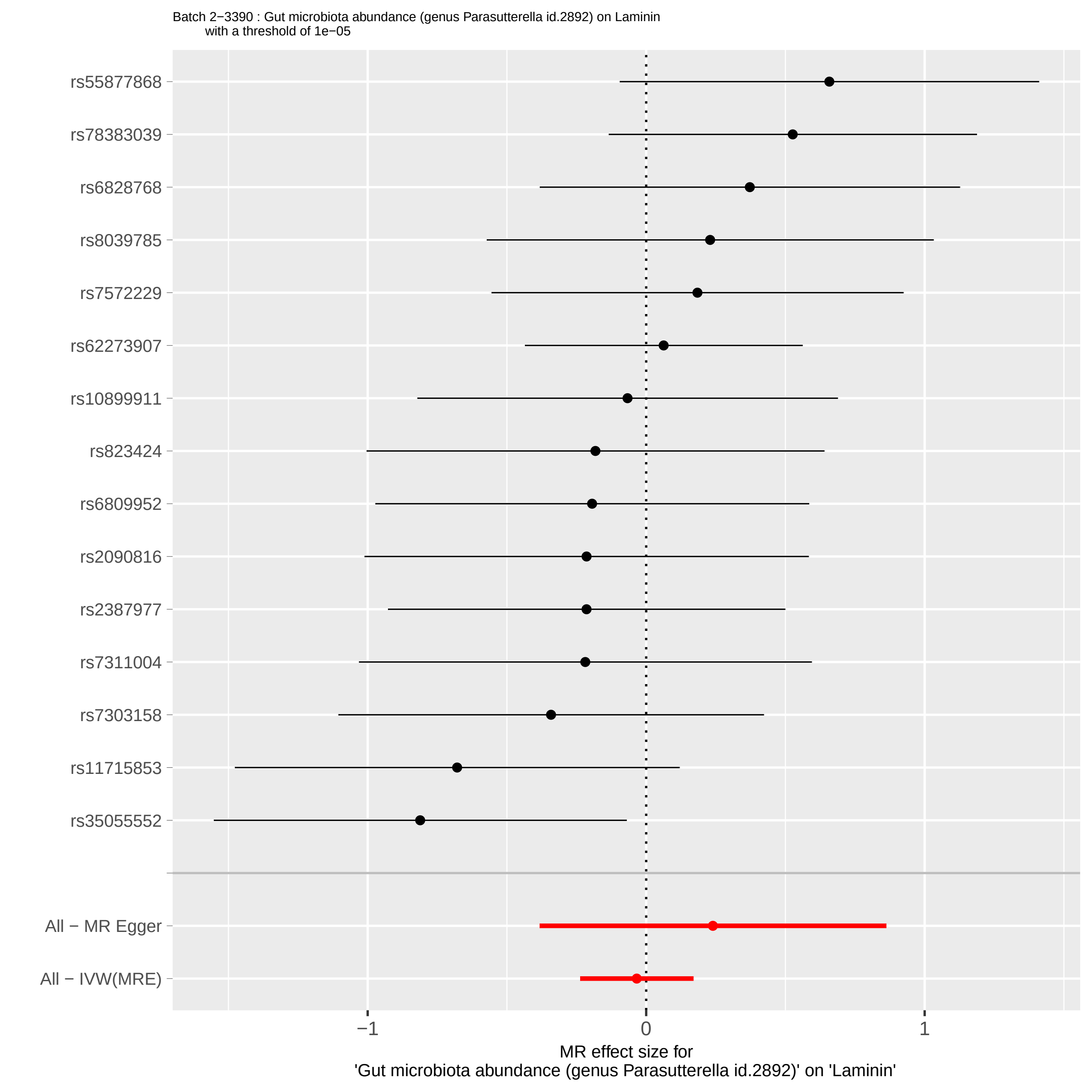
Batch 2−3390 : Gut microbiota abundance (genus Parasutterella id.2892) on Laminin
with a threshold of 1e−05
rs55877868
rs78383039
rs6828768
rs8039785
rs7572229
rs62273907
rs10899911
rs823424
rs6809952
rs2090816
rs2387977
rs7311004
rs7303158
rs11715853
rs35055552
All − MR Egger
All − IVW(MRE)
−1
0
1
MR effect size for
'Gut microbiota abundance (genus Parasutterella id.2892)' on 'Laminin'

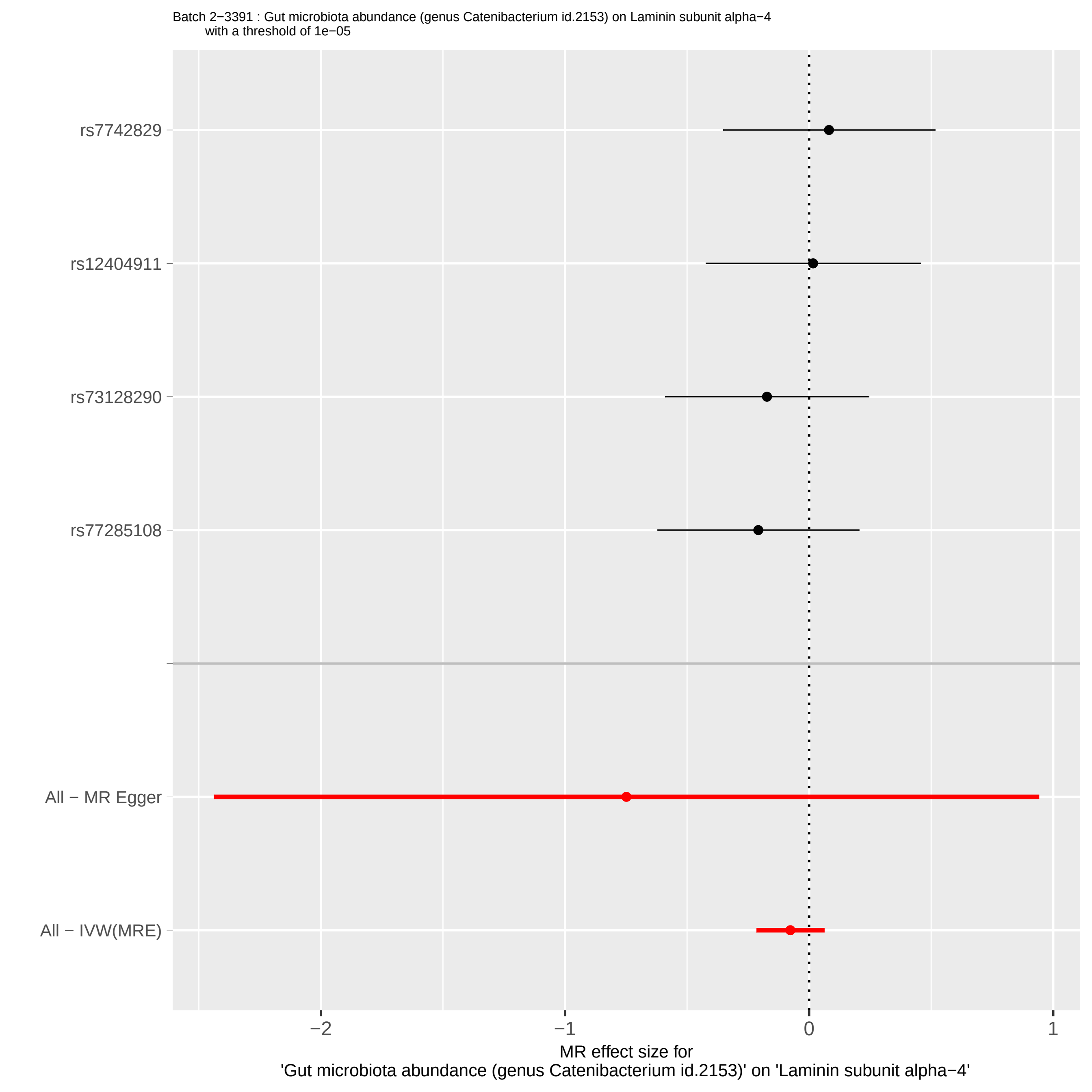
Batch 2−3391 : Gut microbiota abundance (genus Catenibacterium id.2153) on Laminin subunit alpha−4
with a threshold of 1e−05
rs7742829
rs12404911
rs73128290
rs77285108
All − MR Egger
All − IVW(MRE)
−2
−1
0
1
MR effect size for
'Gut microbiota abundance (genus Catenibacterium id.2153)' on 'Laminin subunit alpha−4'

Batch 2−3392 : Gut microbiota abundance (genus Parasutterella id.2892) on Laminin subunit alpha−4
with a threshold of 1e−05

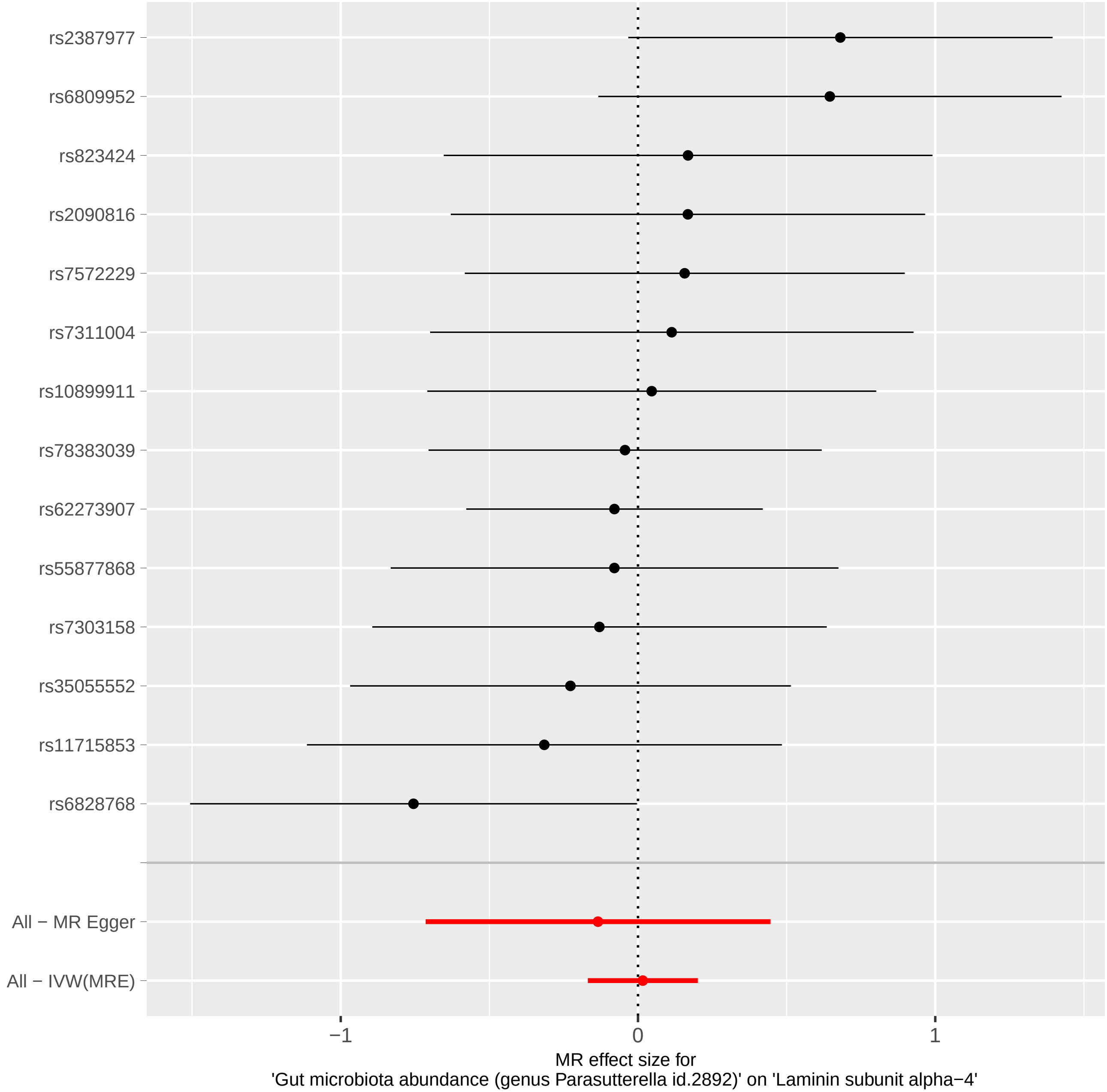

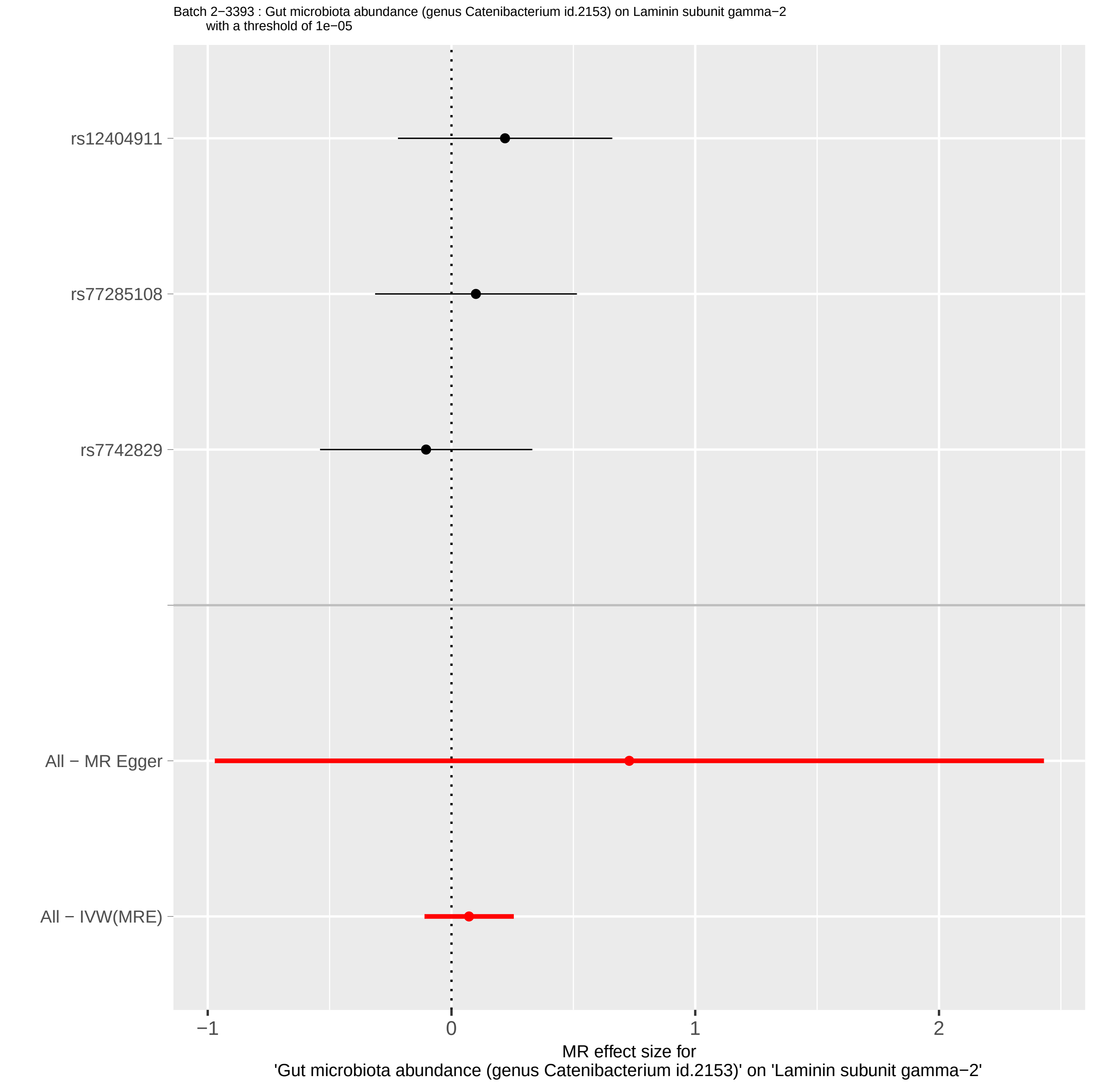

Batch 2−3393 : Gut microbiota abundance (genus Catenibacterium id.2153) on Laminin subunit gamma−2
with a threshold of 1e−05
rs12404911
rs77285108
rs7742829
All − MR Egger
All − IVW(MRE)
−1
0
1
2
MR effect size for
'Gut microbiota abundance (genus Catenibacterium id.2153)' on 'Laminin subunit gamma−2'

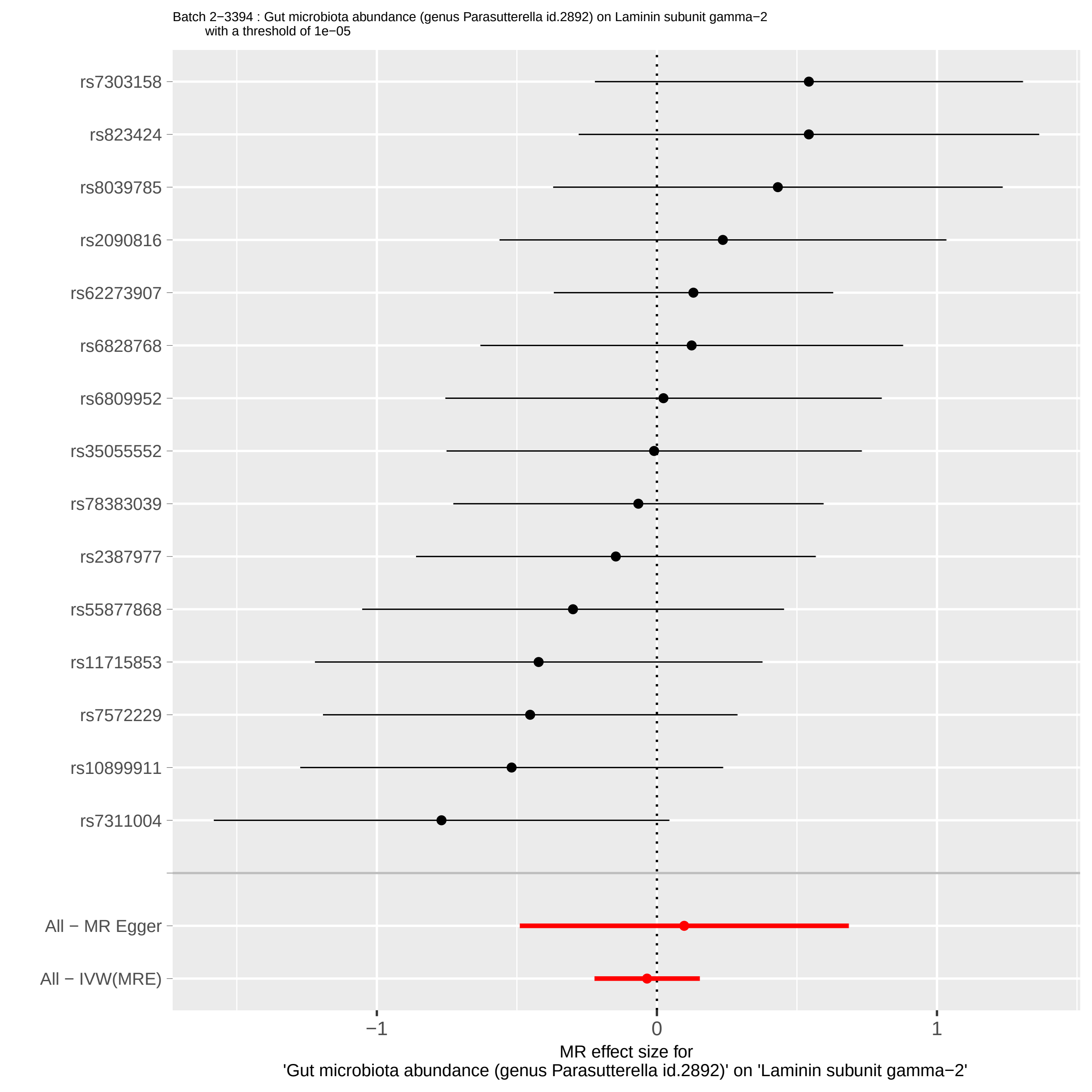

Batch 2−3394 : Gut microbiota abundance (genus Parasutterella id.2892) on Laminin subunit gamma−2
with a threshold of 1e−05
rs7303158
rs823424
rs8039785
rs2090816
rs62273907
rs6828768
rs6809952
rs35055552
rs78383039
rs2387977
rs55877868
rs11715853
rs7572229
rs10899911
rs7311004
All − MR Egger
All − IVW(MRE)
−1
0
1
MR effect size for
'Gut microbiota abundance (genus Parasutterella id.2892)' on 'Laminin subunit gamma−2'

Batch 2−3395 : Gut microbiota abundance (genus Catenibacterium id.2153) on Lysosome−associated membrane glycoprotein 3
with a threshold of 1e−05

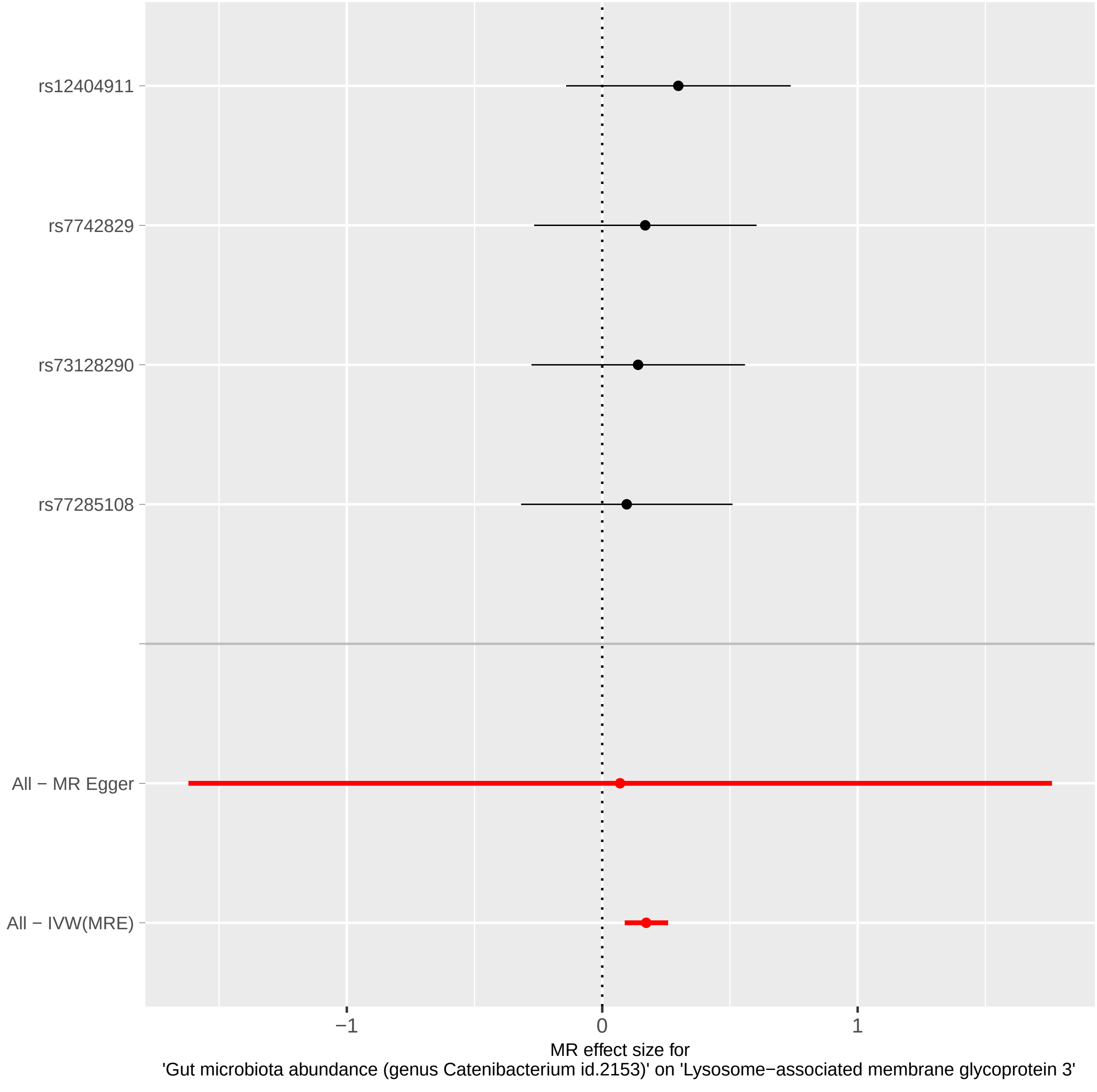

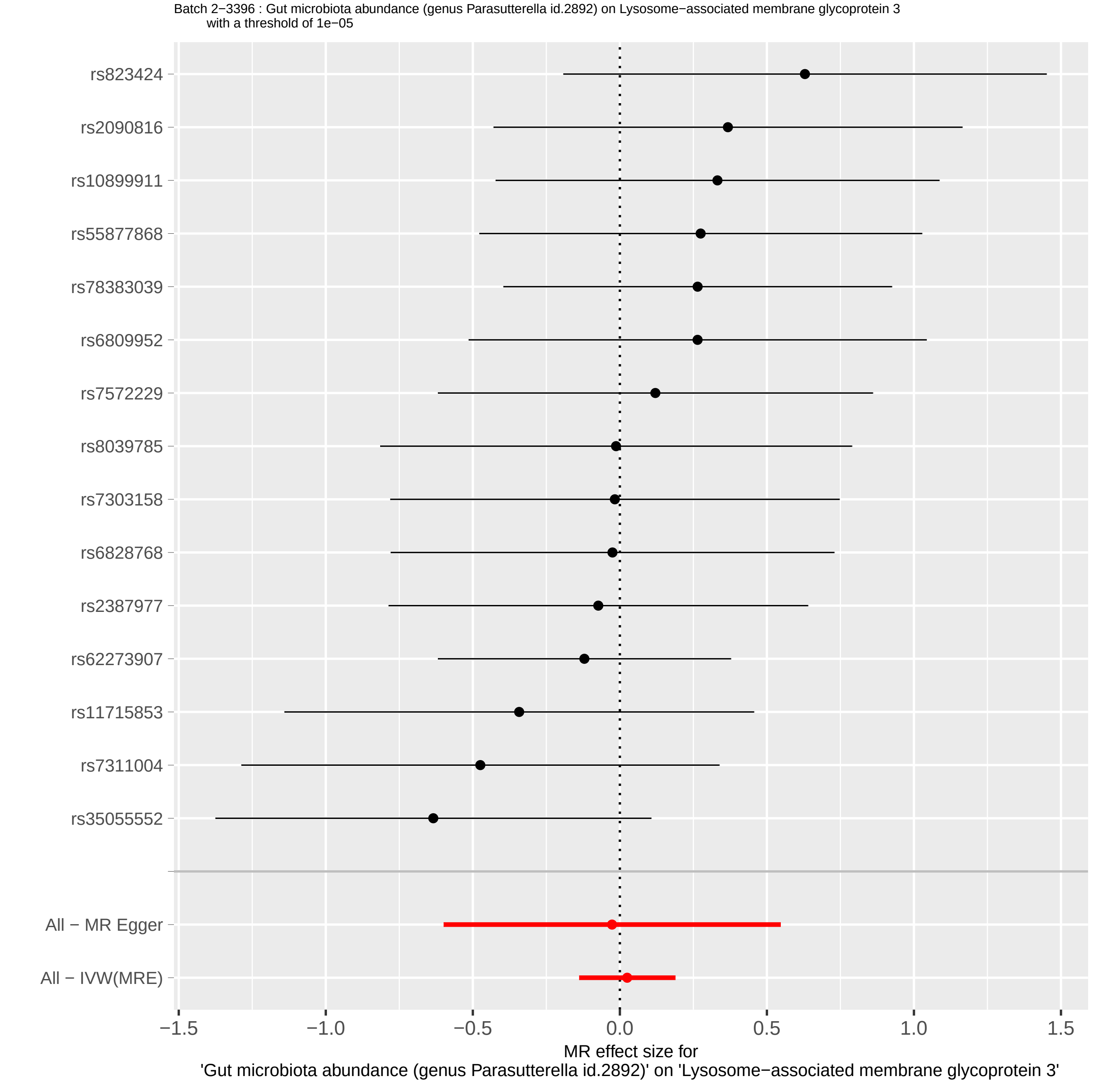

Batch 2−3396 : Gut microbiota abundance (genus Parasutterella id.2892) on Lysosome−associated membrane glycoprotein 3 with a threshold of 1e−05
rs823424
rs2090816
rs10899911
rs55877868
rs78383039
rs6809952
rs7572229
rs8039785
rs7303158
rs6828768
rs2387977
rs62273907
rs11715853
rs7311004
rs35055552
All − MR Egger
All − IVW(MRE)
−1.5
−1.0
−0.5
0.0
0.5
1.0
1.5
MR effect size for
'Gut microbiota abundance (genus Parasutterella id.2892)' on 'Lysosome−associated membrane glycoprotein 3'

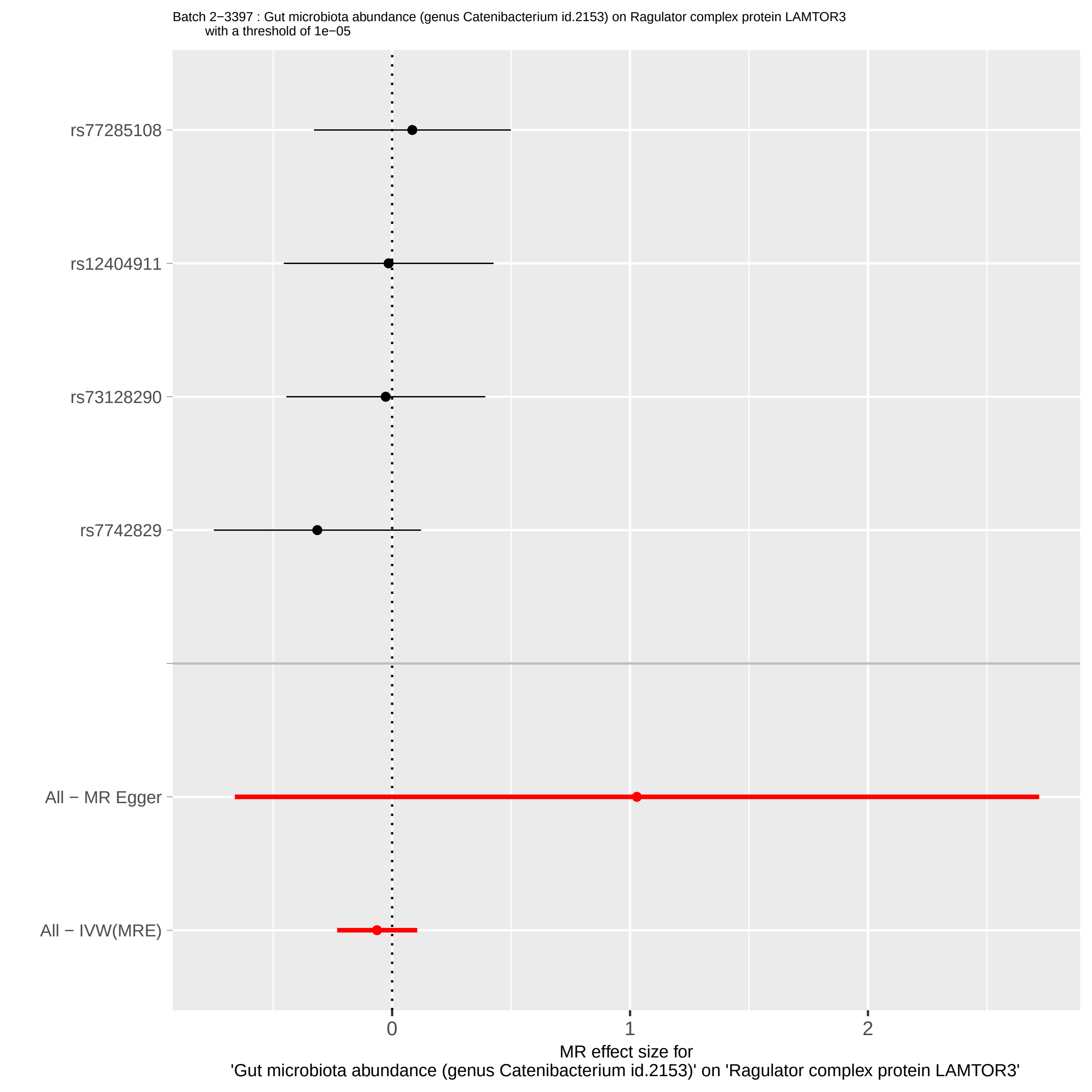

Batch 2−3397 : Gut microbiota abundance (genus Catenibacterium id.2153) on Ragulator complex protein LAMTOR3
with a threshold of 1e−05
rs77285108
rs12404911
rs73128290
rs7742829
All − MR Egger
All − IVW(MRE)
0
1
2
MR effect size for
'Gut microbiota abundance (genus Catenibacterium id.2153)' on 'Ragulator complex protein LAMTOR3'

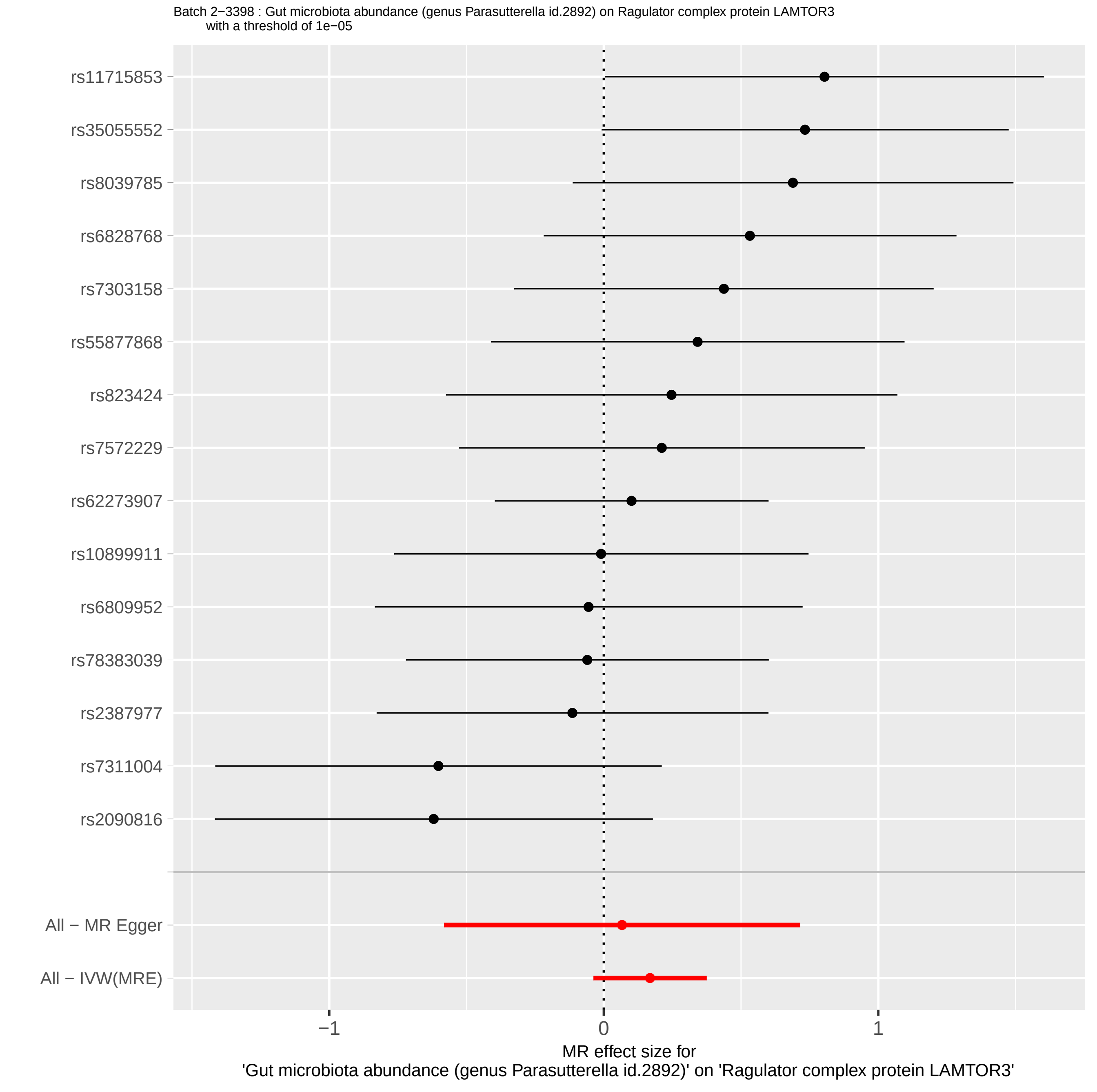

Batch 2−3398 : Gut microbiota abundance (genus Parasutterella id.2892) on Ragulator complex protein LAMTOR3
with a threshold of 1e−05
rs11715853
rs35055552
rs8039785
rs6828768
rs7303158
rs55877868
rs823424
rs7572229
rs62273907
rs10899911
rs6809952
rs78383039
rs2387977
rs7311004
rs2090816
All − MR Egger
All − IVW(MRE)
−1
0
1
MR effect size for
'Gut microbiota abundance (genus Parasutterella id.2892)' on 'Ragulator complex protein LAMTOR3'

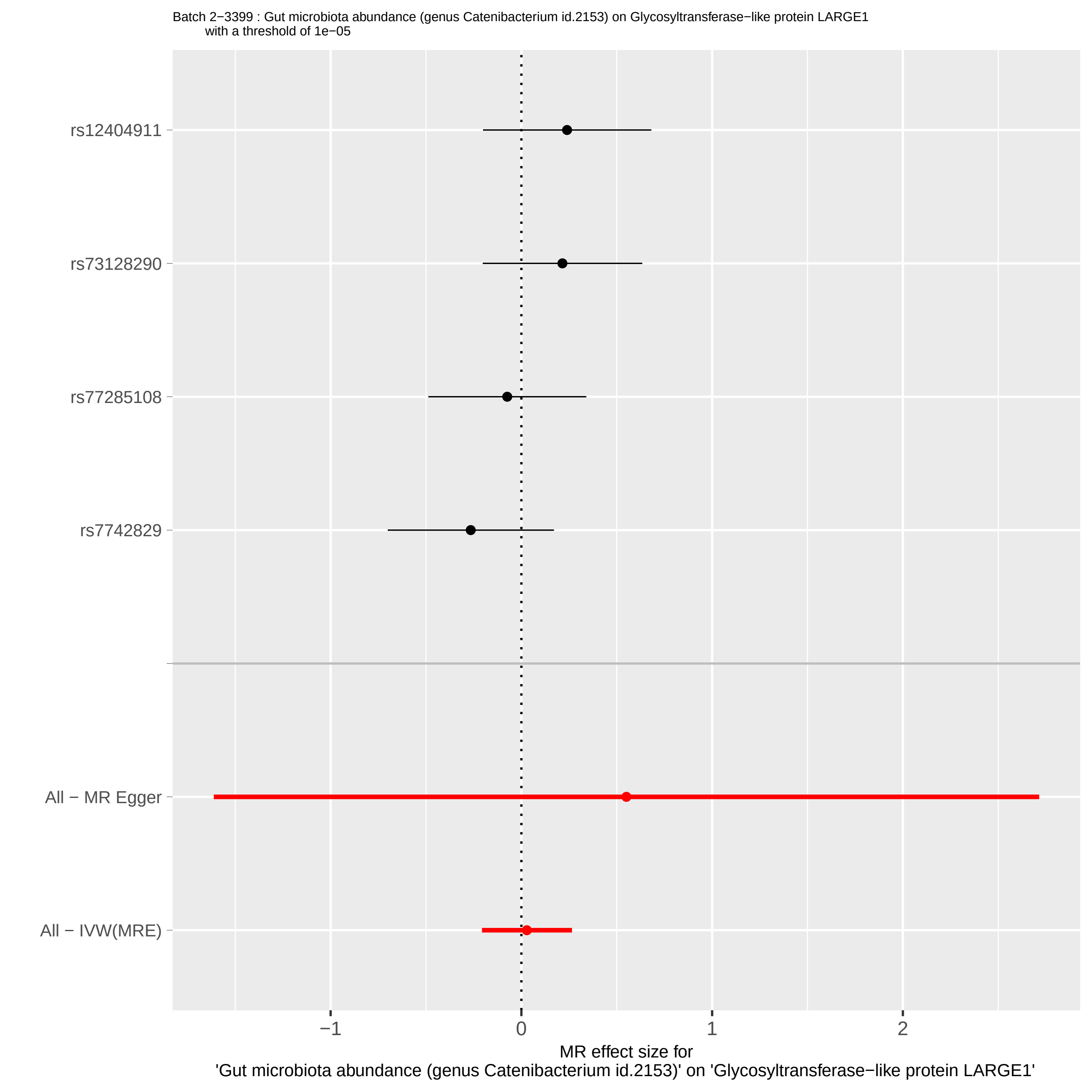
Batch 2−3399 : Gut microbiota abundance (genus Catenibacterium id.2153) on Glycosyltransferase−like protein LARGE1
with a threshold of 1e−05
rs12404911
rs73128290
rs77285108
rs7742829
All − MR Egger
All − IVW(MRE)
−1
0
1
2
MR effect size for
'Gut microbiota abundance (genus Catenibacterium id.2153)' on 'Glycosyltransferase−like protein LARGE1'

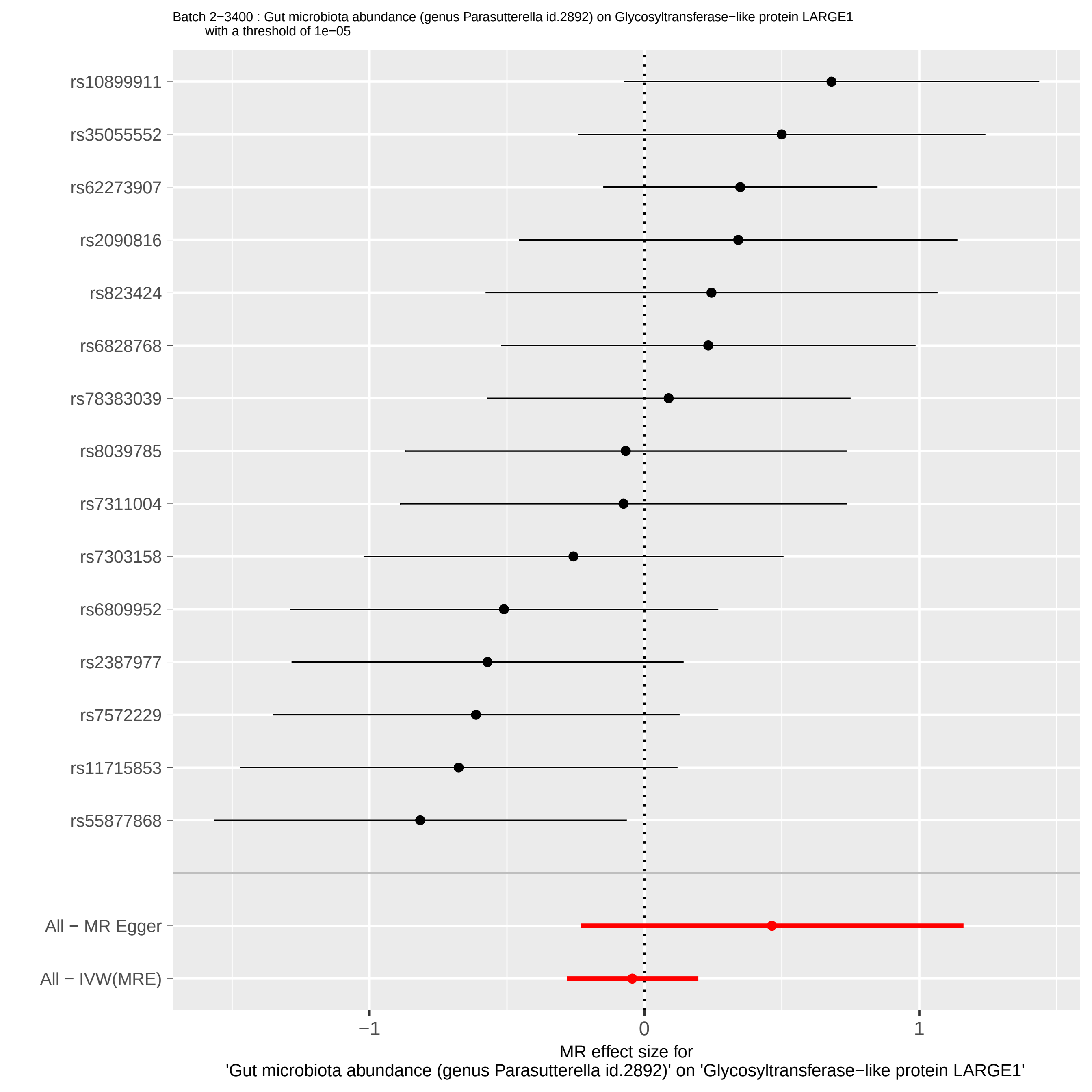

Batch 2−3400 : Gut microbiota abundance (genus Parasutterella id.2892) on Glycosyltransferase−like protein LARGE1
with a threshold of 1e−05
rs10899911
rs35055552
rs62273907
rs2090816
rs823424
rs6828768
rs78383039
rs8039785
rs7311004
rs7303158
rs6809952
rs2387977
rs7572229
rs11715853
rs55877868
All − MR Egger
All − IVW(MRE)
−1
0
1
MR effect size for
'Gut microbiota abundance (genus Parasutterella id.2892)' on 'Glycosyltransferase−like protein LARGE1'

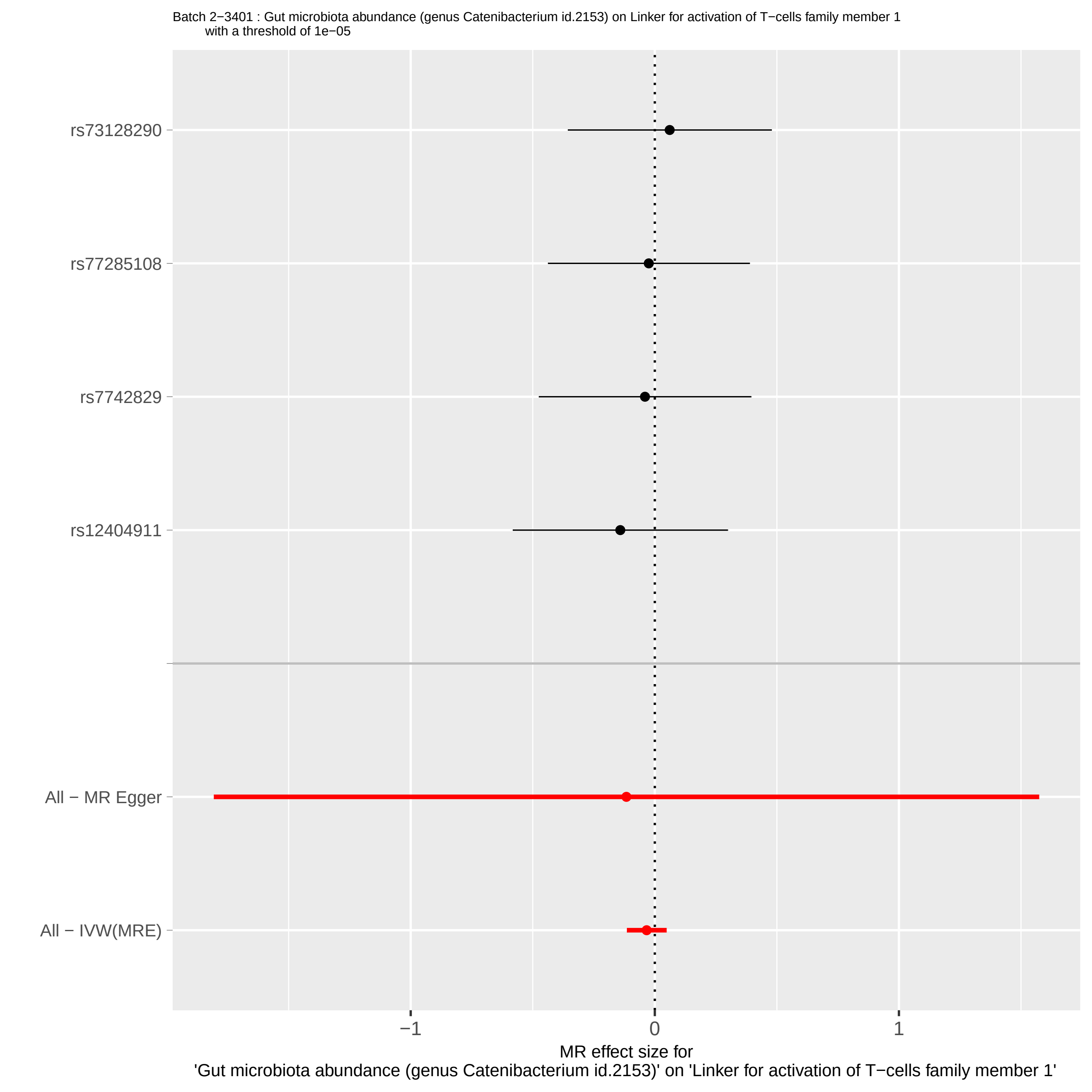
Batch 2−3401 : Gut microbiota abundance (genus Catenibacterium id.2153) on Linker for activation of T−cells family member 1
with a threshold of 1e−05
rs73128290
rs77285108
rs7742829
rs12404911
All − MR Egger
All − IVW(MRE)
−1
0
1
MR effect size for
'Gut microbiota abundance (genus Catenibacterium id.2153)' on 'Linker for activation of T−cells family member 1'

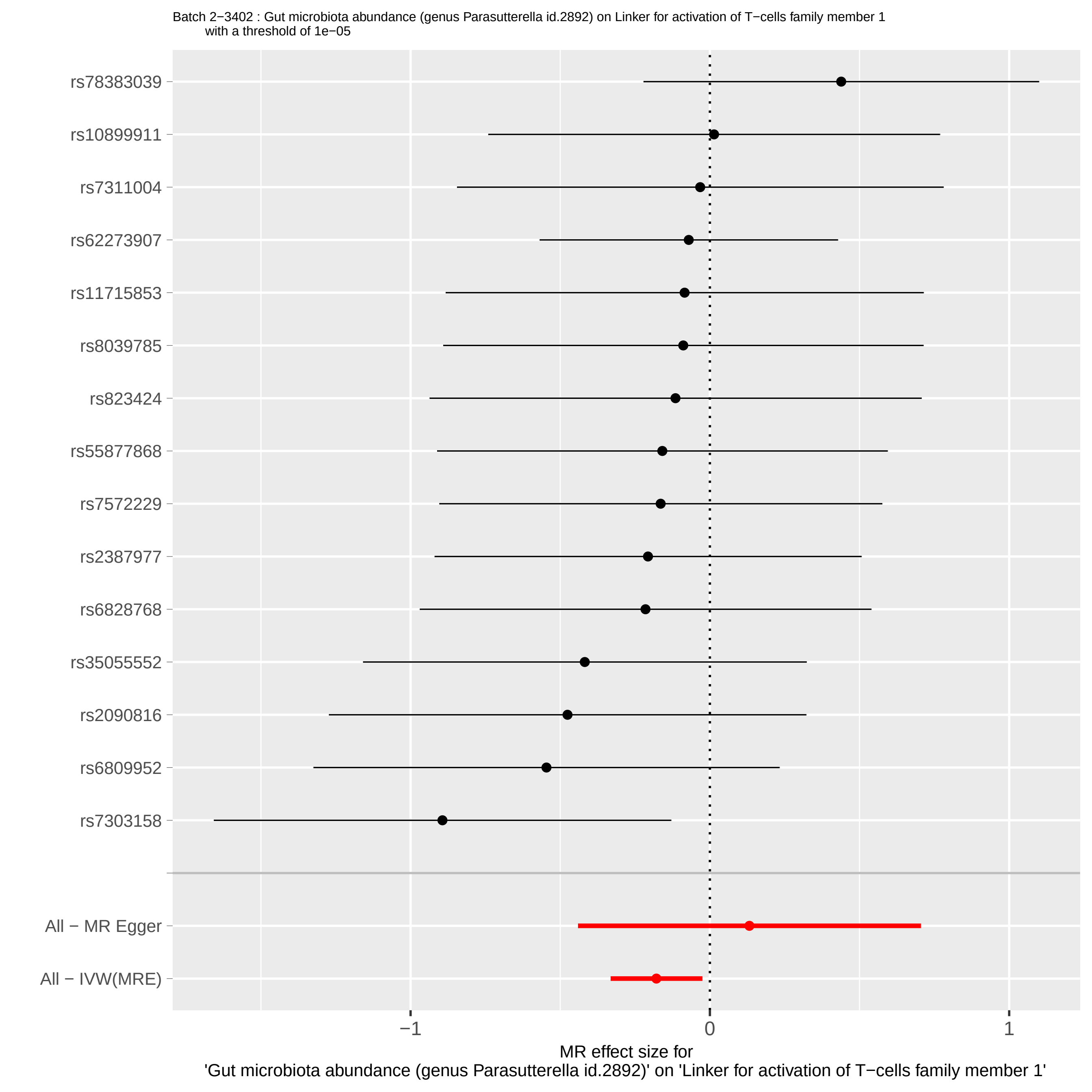
Batch 2−3402 : Gut microbiota abundance (genus Parasutterella id.2892) on Linker for activation of T−cells family member 1
with a threshold of 1e−05
rs78383039
rs10899911
rs7311004
rs62273907
rs11715853
rs8039785
rs823424
rs55877868
rs7572229
rs2387977
rs6828768
rs35055552
rs2090816
rs6809952
rs7303158
All − MR Egger
All − IVW(MRE)
−1
0
1
MR effect size for
'Gut microbiota abundance (genus Parasutterella id.2892)' on 'Linker for activation of T−cells family member 1'

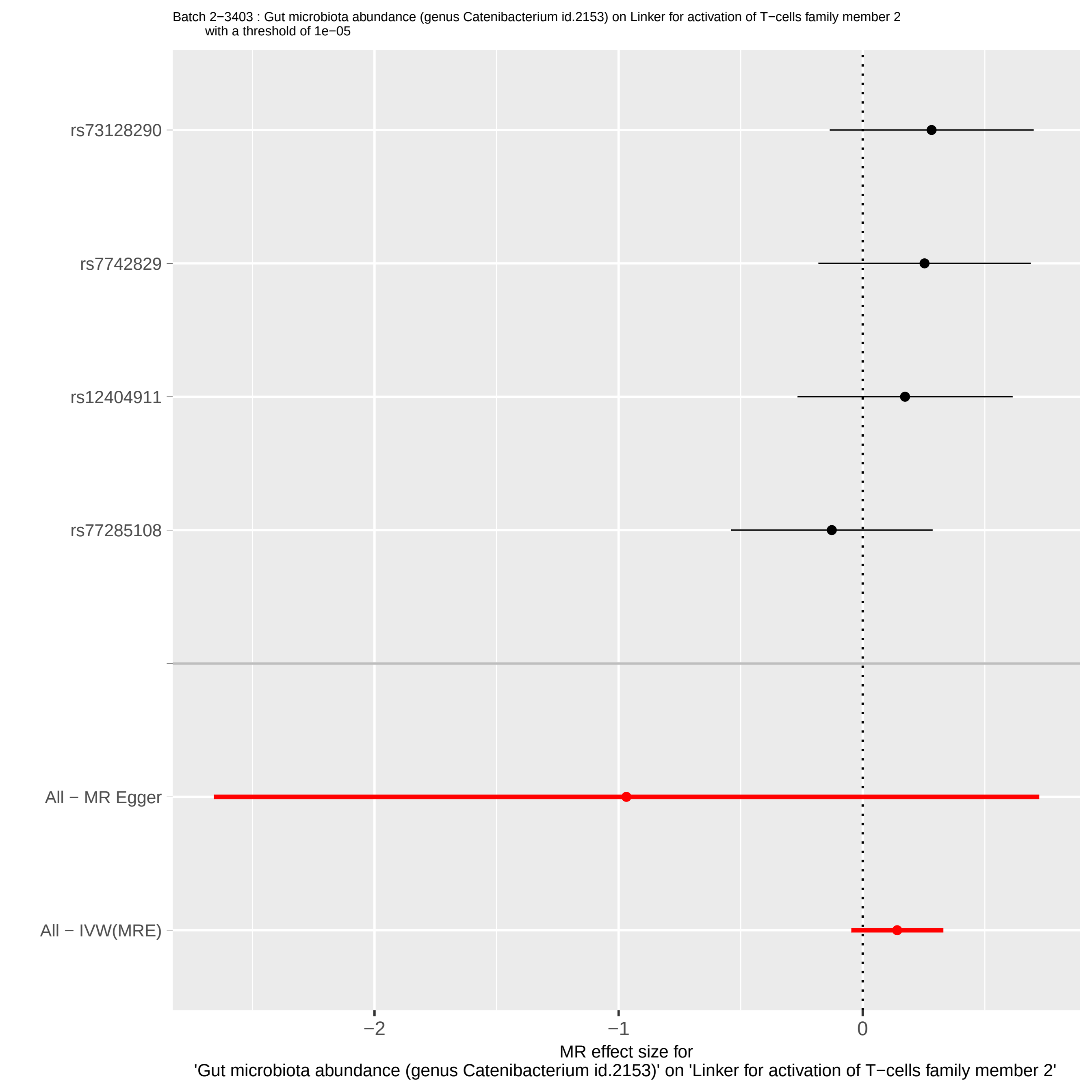
Batch 2−3403 : Gut microbiota abundance (genus Catenibacterium id.2153) on Linker for activation of T−cells family member 2
with a threshold of 1e−05
rs73128290
rs7742829
rs12404911
rs77285108
All − MR Egger
All − IVW(MRE)
−2
−1
0
MR effect size for
'Gut microbiota abundance (genus Catenibacterium id.2153)' on 'Linker for activation of T−cells family member 2'

Batch 2−3404 : Gut microbiota abundance (genus Parasutterella id.2892) on Linker for activation of T−cells family member 2
with a threshold of 1e−05

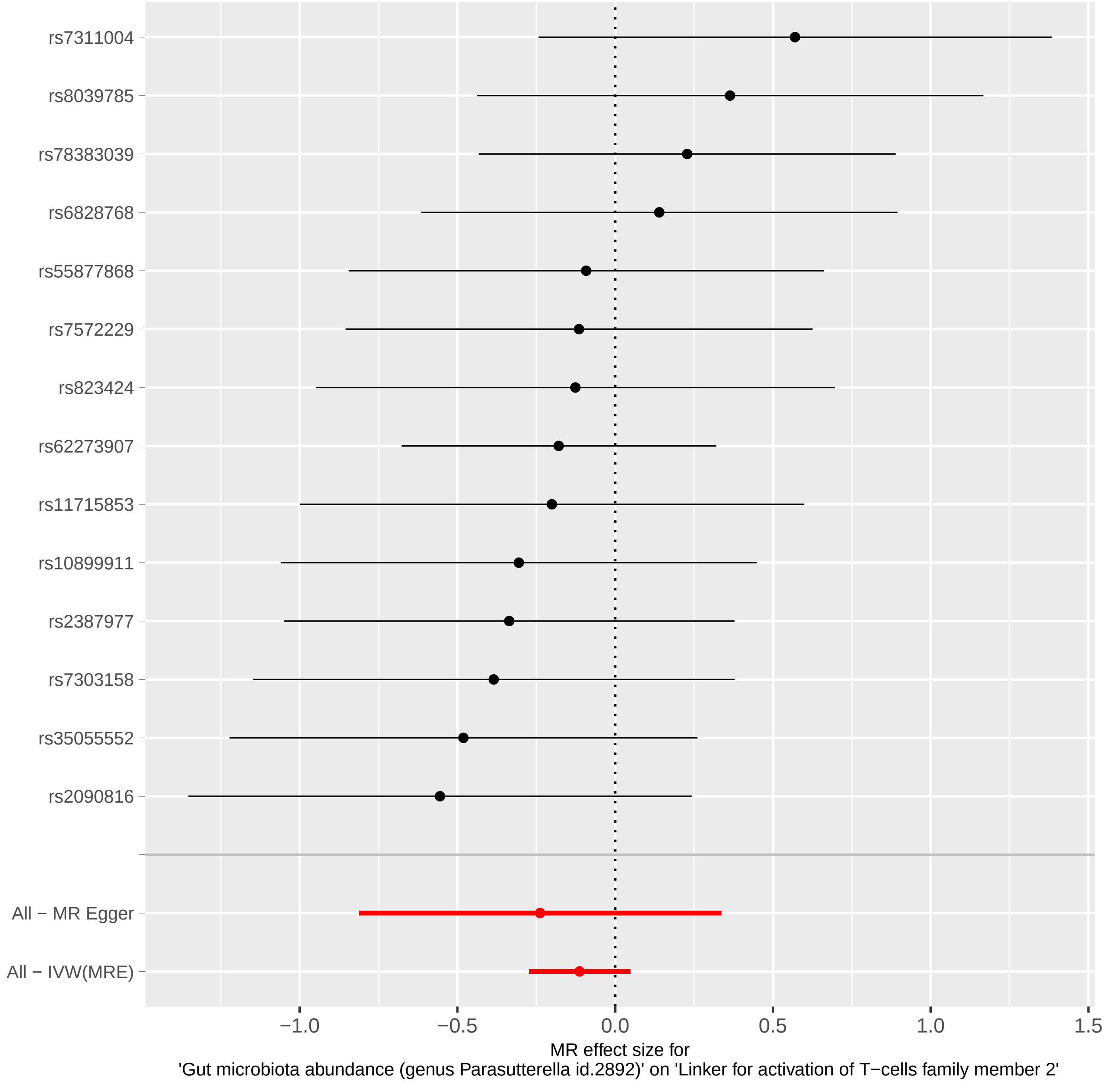

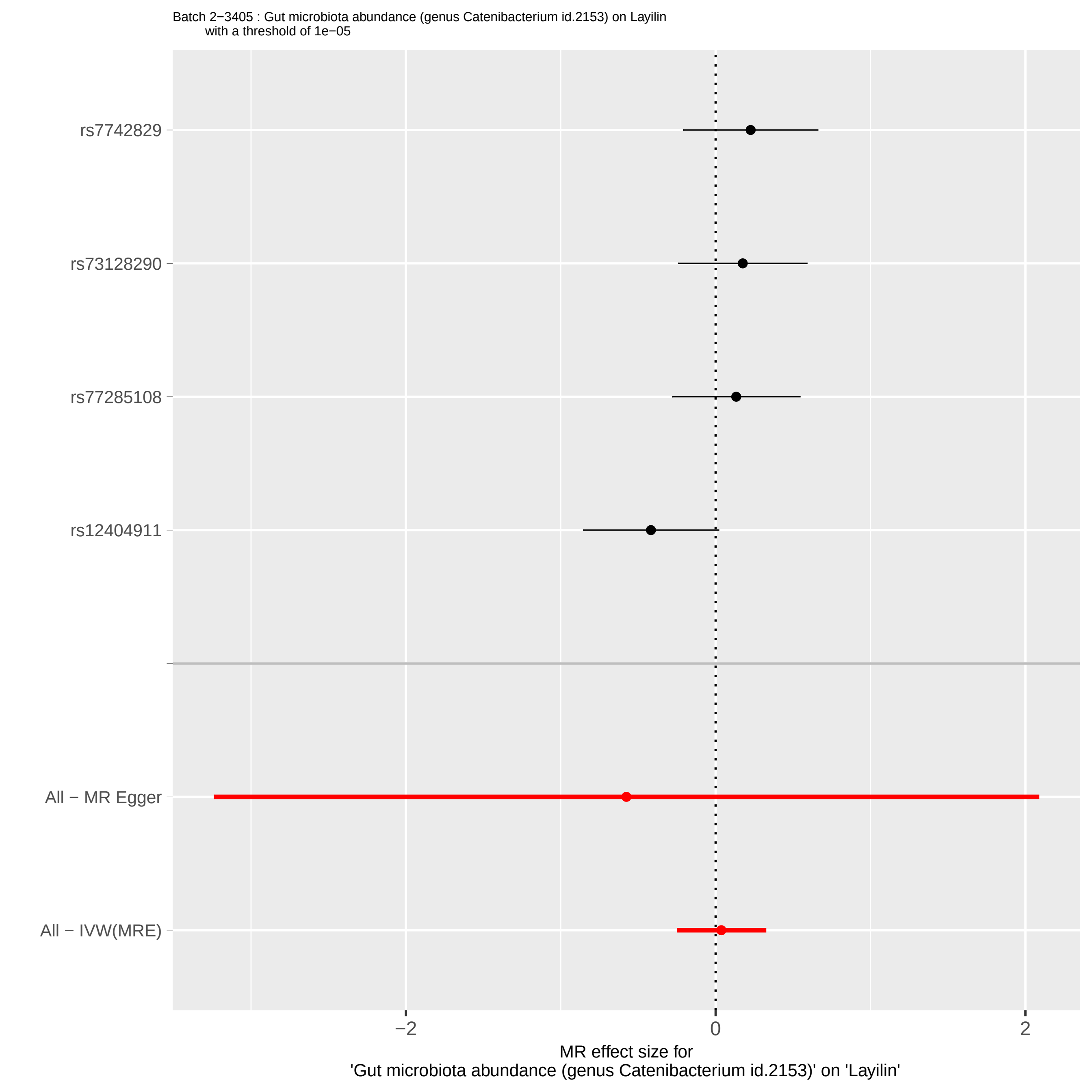
Batch 2−3405 : Gut microbiota abundance (genus Catenibacterium id.2153) on Layilin
with a threshold of 1e−05
rs7742829
rs73128290
rs77285108
rs12404911
All − MR Egger
All − IVW(MRE)
−2
0
2
MR effect size for
'Gut microbiota abundance (genus Catenibacterium id.2153)' on 'Layilin'

Batch 2−3406 : Gut microbiota abundance (genus Parasutterella id.2892) on Layilin
with a threshold of 1e−05

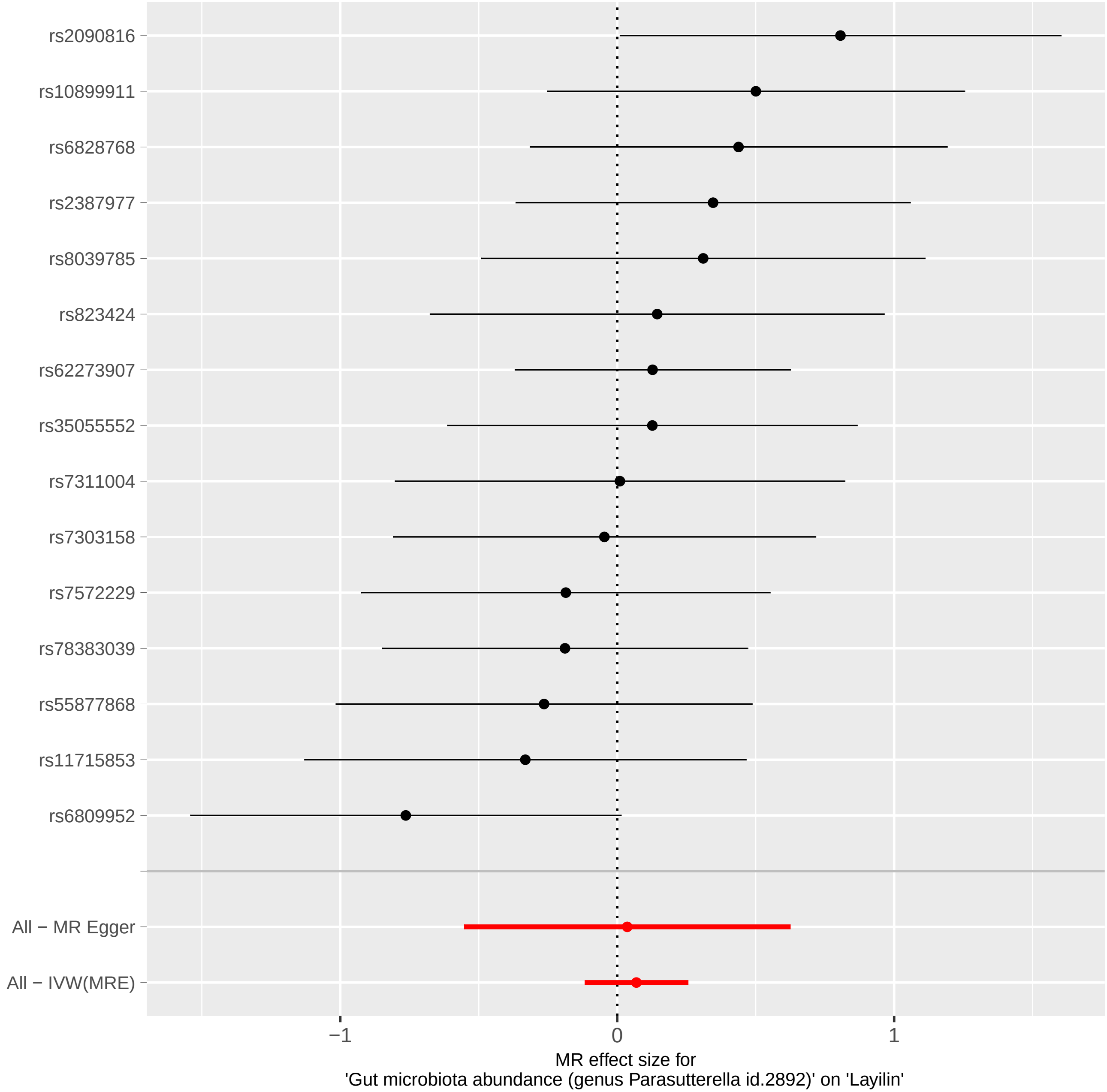

Batch 2−3407 : Gut microbiota abundance (genus Catenibacterium id.2153) on Tyrosine−protein kinase Lck
with a threshold of 1e−05

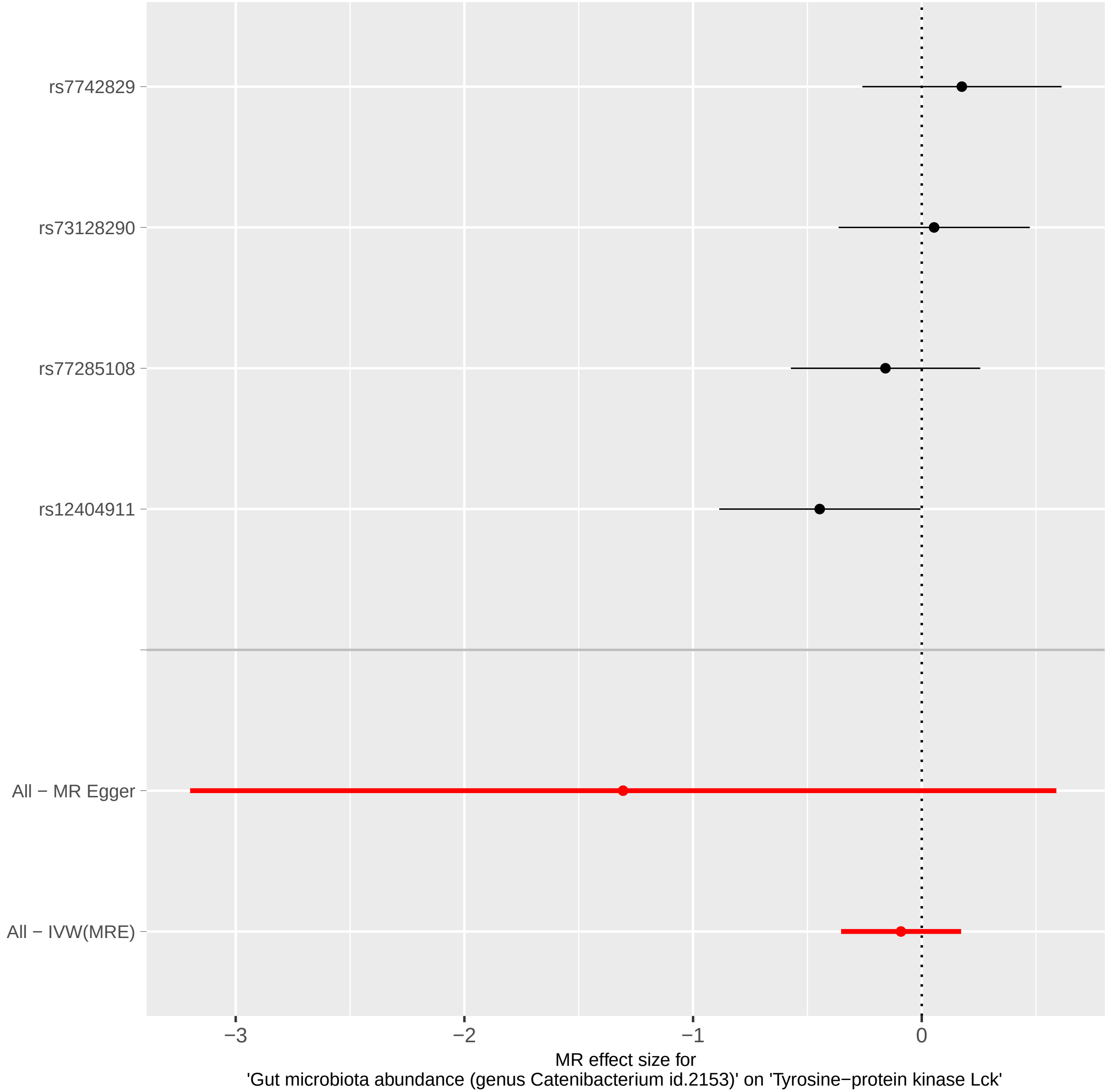

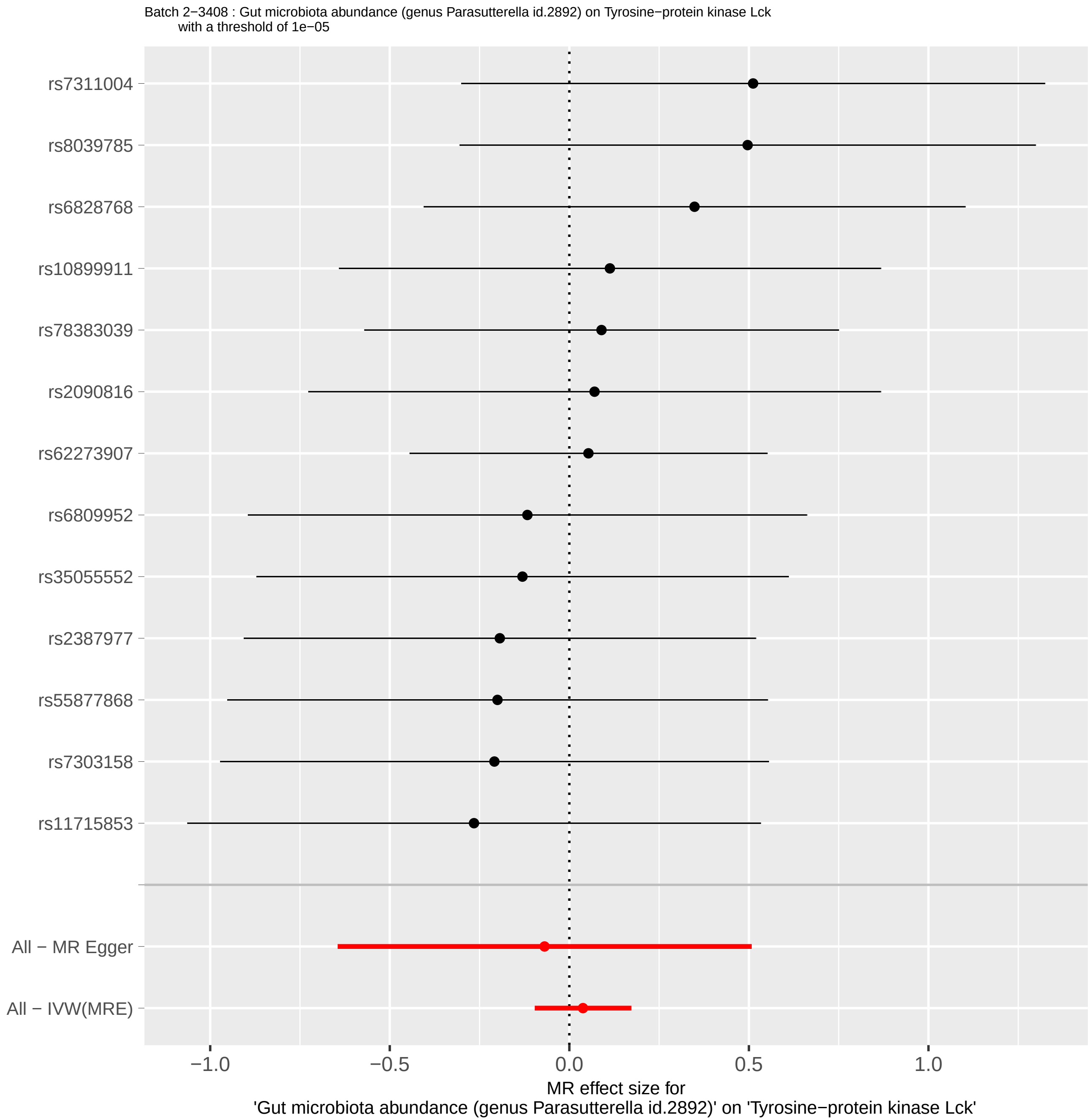

Batch 2−3408 : Gut microbiota abundance (genus Parasutterella id.2892) on Tyrosine−protein kinase Lck
with a threshold of 1e−05
rs7311004
rs8039785
rs6828768
rs10899911
rs78383039
rs2090816
rs62273907
rs6809952
rs35055552
rs2387977
rs55877868
rs7303158
rs11715853
All − MR Egger
All − IVW(MRE)
−1.0
−0.5
0.0
0.5
1.0
MR effect size for
'Gut microbiota abundance (genus Parasutterella id.2892)' on 'Tyrosine−protein kinase Lck'

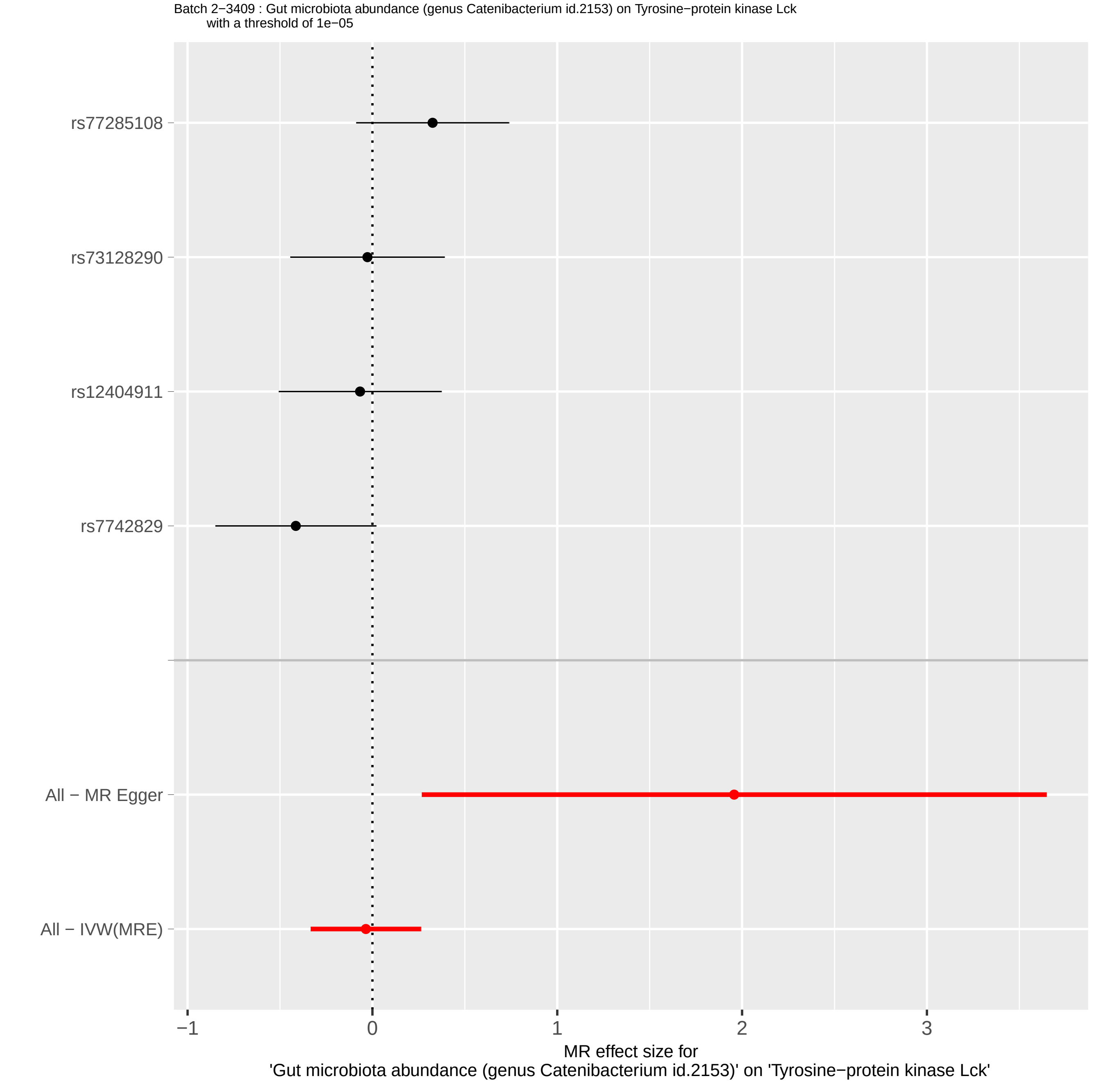

Batch 2−3409 : Gut microbiota abundance (genus Catenibacterium id.2153) on Tyrosine−protein kinase Lck
with a threshold of 1e−05
rs77285108
rs73128290
rs12404911
rs7742829
All − MR Egger
All − IVW(MRE)
−1
0
1
2
3
MR effect size for
'Gut microbiota abundance (genus Catenibacterium id.2153)' on 'Tyrosine−protein kinase Lck'

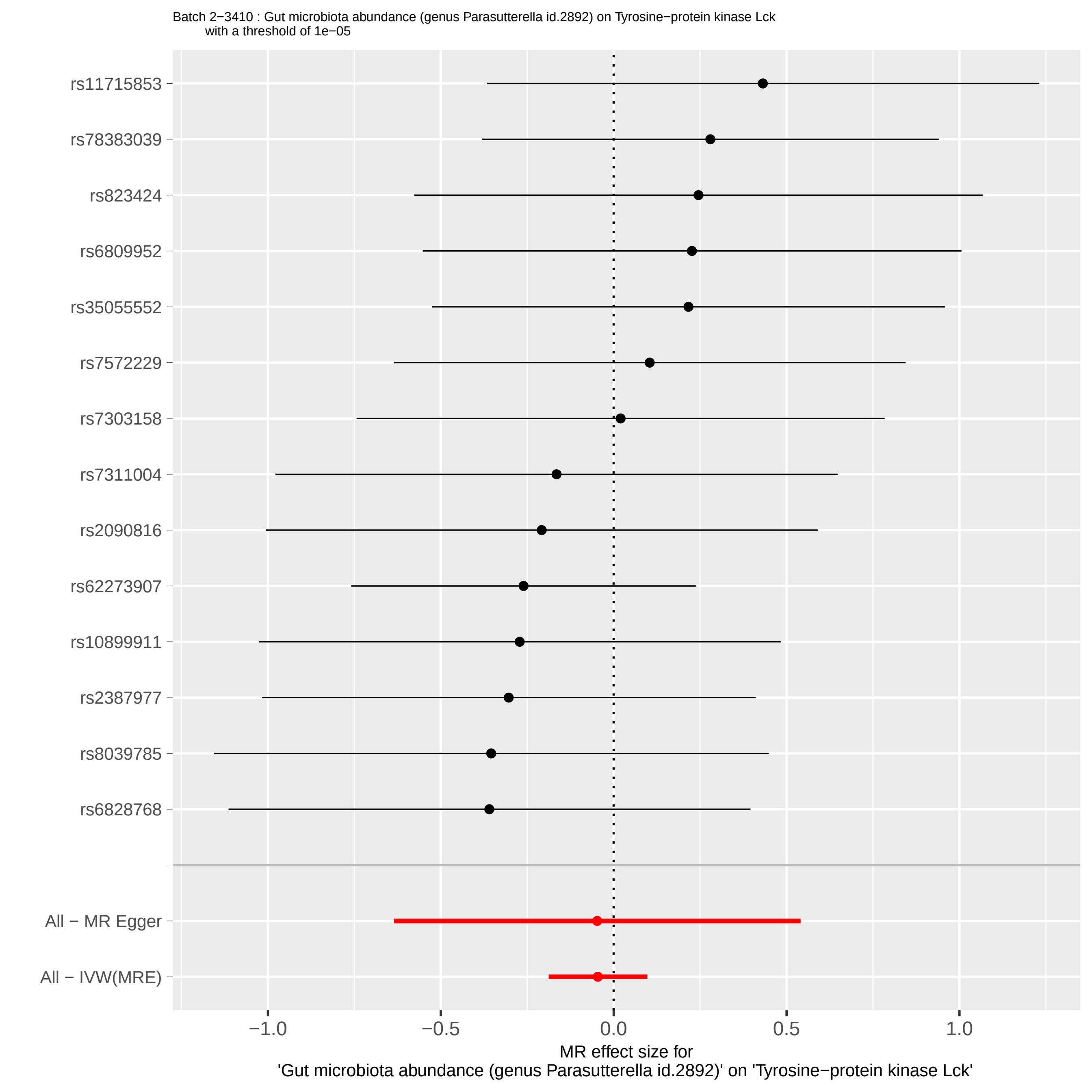
Batch 2−3410 : Gut microbiota abundance (genus Parasutterella id.2892) on Tyrosine−protein kinase Lck
with a threshold of 1e−05
rs11715853
rs78383039
rs823424
rs6809952
rs35055552
rs7572229
rs7303158
rs7311004
rs2090816
rs62273907
rs10899911
rs2387977
rs8039785
rs6828768
All − MR Egger
All − IVW(MRE)
−1.0
−0.5
0.0
0.5
1.0
MR effect size for
'Gut microbiota abundance (genus Parasutterella id.2892)' on 'Tyrosine−protein kinase Lck'

Batch 2−3411 : Gut microbiota abundance (genus Catenibacterium id.2153) on Leucine carboxyl methyltransferase 1
with a threshold of 1e−05

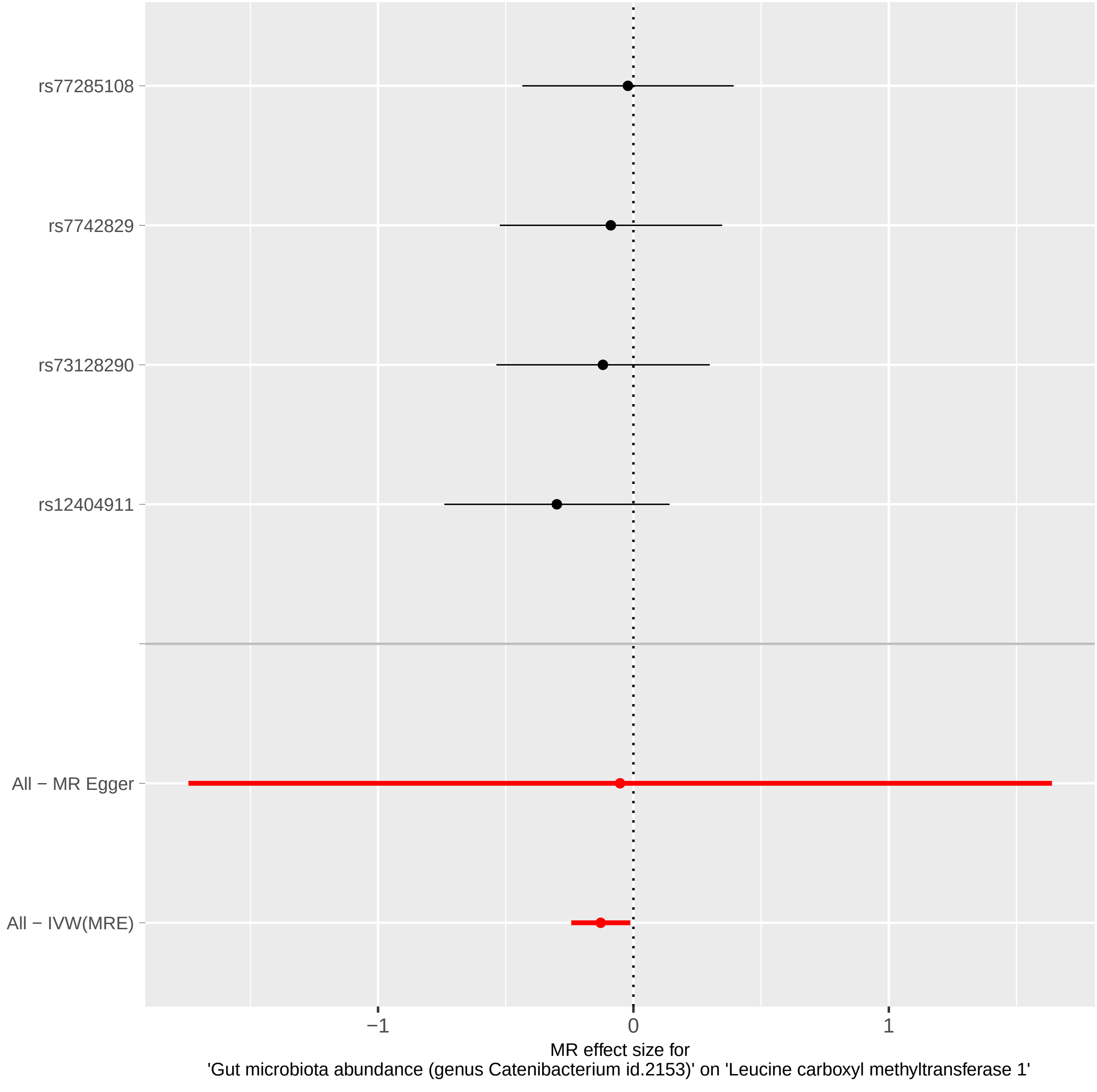

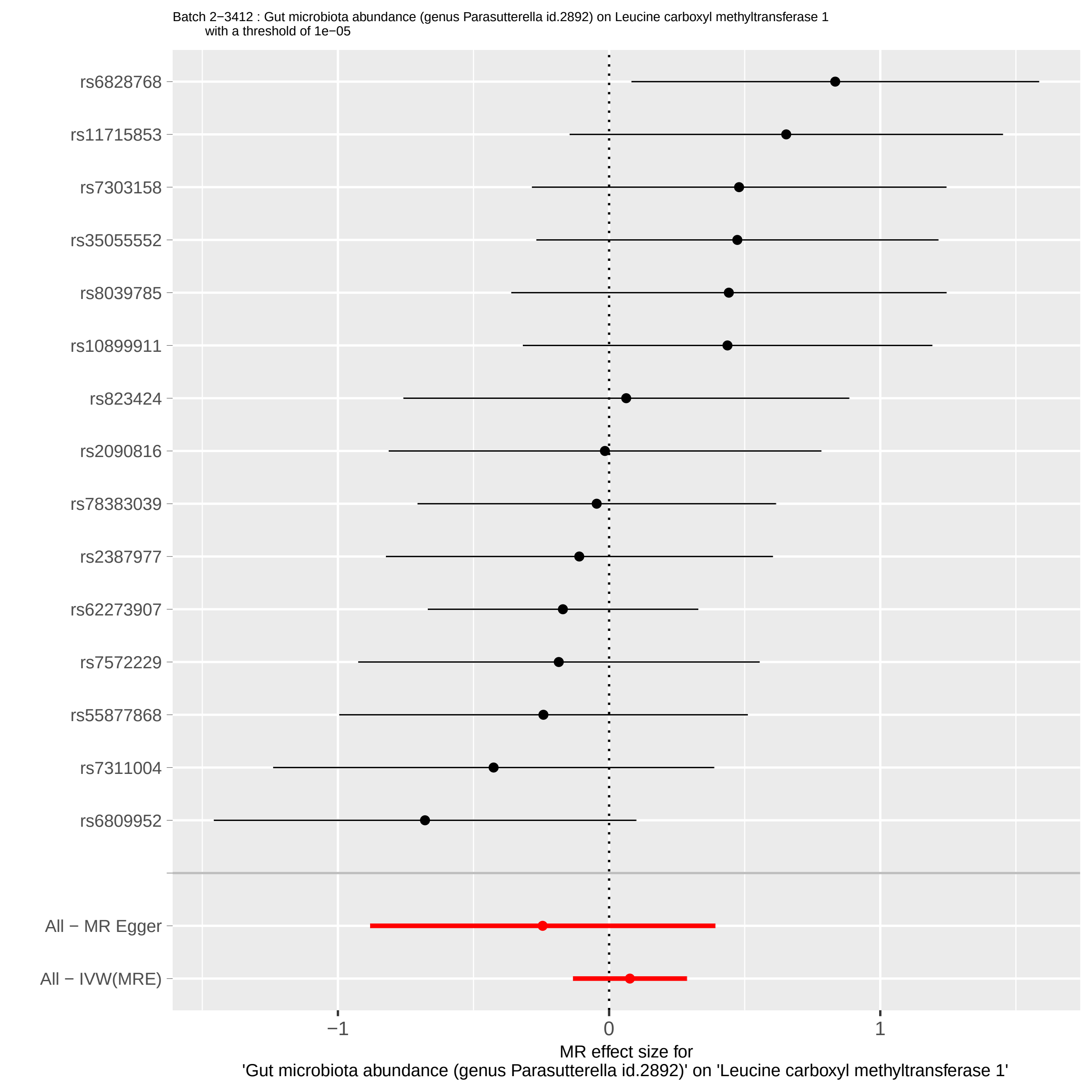
Batch 2−3412 : Gut microbiota abundance (genus Parasutterella id.2892) on Leucine carboxyl methyltransferase 1
with a threshold of 1e−05
rs6828768
rs11715853
rs7303158
rs35055552
rs8039785
rs10899911
rs823424
rs2090816
rs78383039
rs2387977
rs62273907
rs7572229
rs55877868
rs7311004
rs6809952
All − MR Egger
All − IVW(MRE)
−1
0
1
MR effect size for
'Gut microbiota abundance (genus Parasutterella id.2892)' on 'Leucine carboxyl methyltransferase 1'

Batch 2−3413 : Gut microbiota abundance (genus Catenibacterium id.2153) on Epididymal−specific lipocalin−10
with a threshold of 1e−05

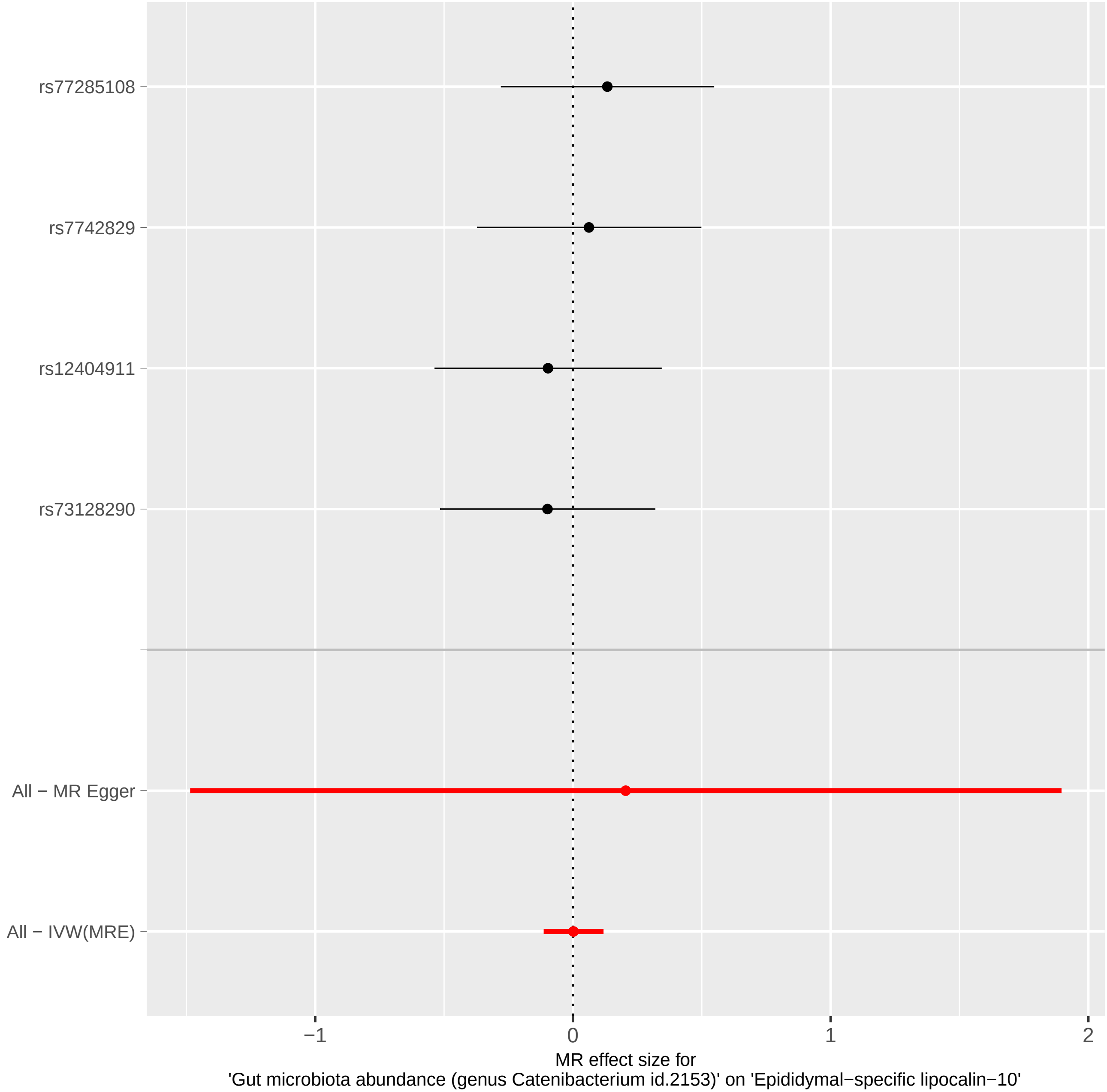

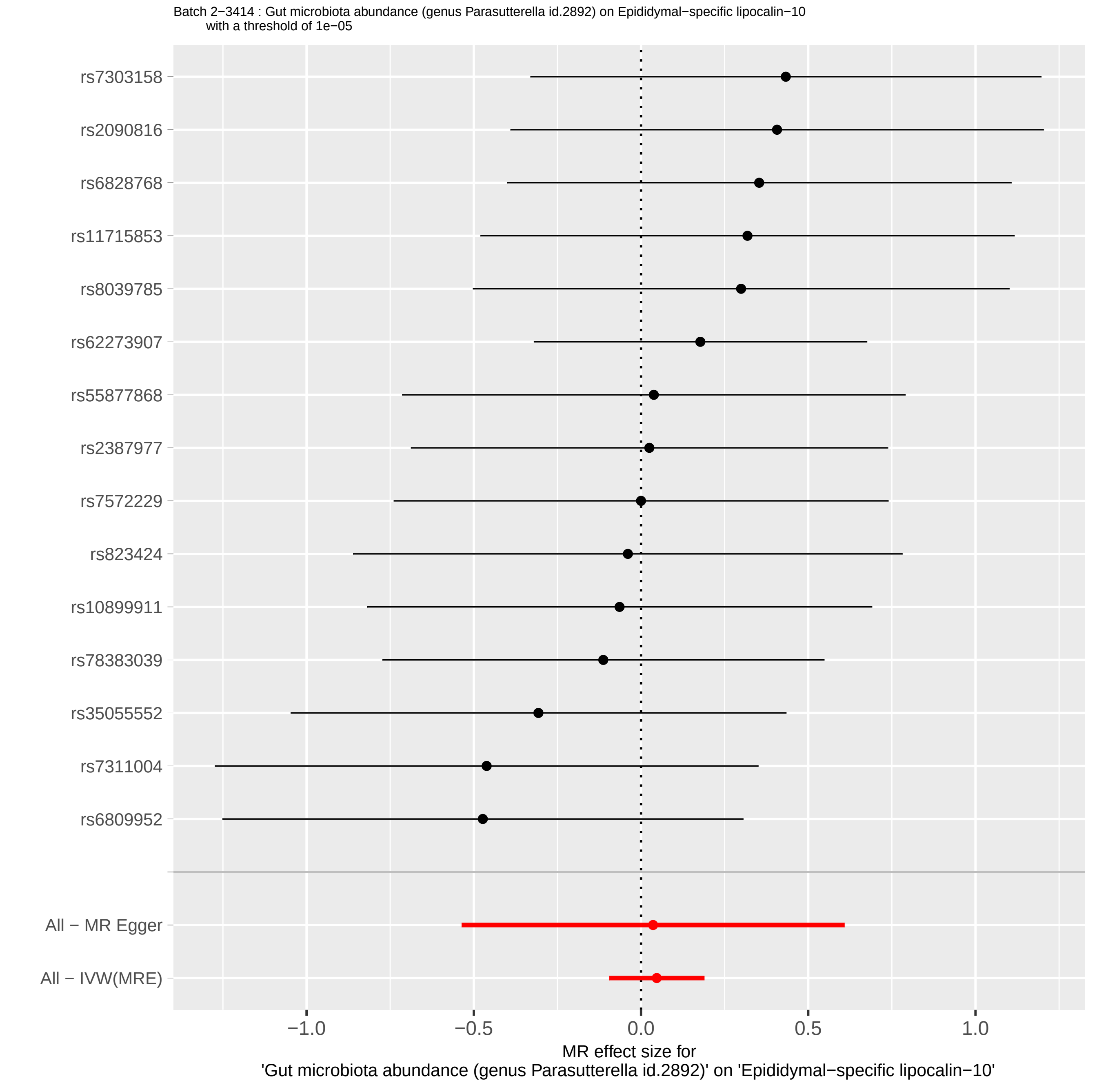
Batch 2−3414 : Gut microbiota abundance (genus Parasutterella id.2892) on Epididymal−specific lipocalin−10
with a threshold of 1e−05
rs7303158
rs2090816
rs6828768
rs11715853
rs8039785
rs62273907
rs55877868
rs2387977
rs7572229
rs823424
rs10899911
rs78383039
rs35055552
rs7311004
rs6809952
All − MR Egger
All − IVW(MRE)
−1.0
−0.5
0.0
0.5
1.0
MR effect size for
'Gut microbiota abundance (genus Parasutterella id.2892)' on 'Epididymal−specific lipocalin−10'

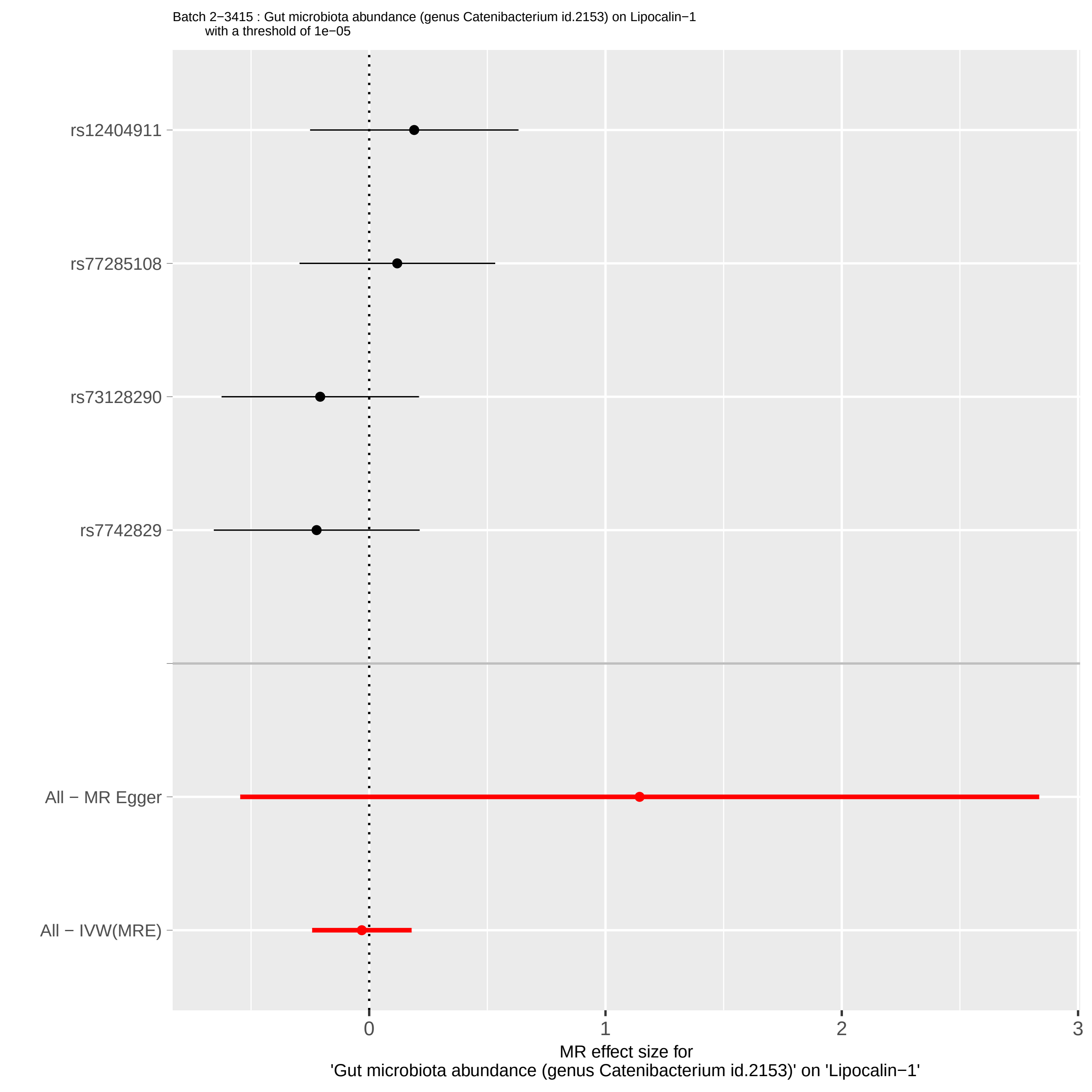
Batch 2−3415 : Gut microbiota abundance (genus Catenibacterium id.2153) on Lipocalin−1
with a threshold of 1e−05
rs12404911
rs77285108
rs73128290
rs7742829
All − MR Egger
All − IVW(MRE)
0
1
2
3
MR effect size for
'Gut microbiota abundance (genus Catenibacterium id.2153)' on 'Lipocalin−1'

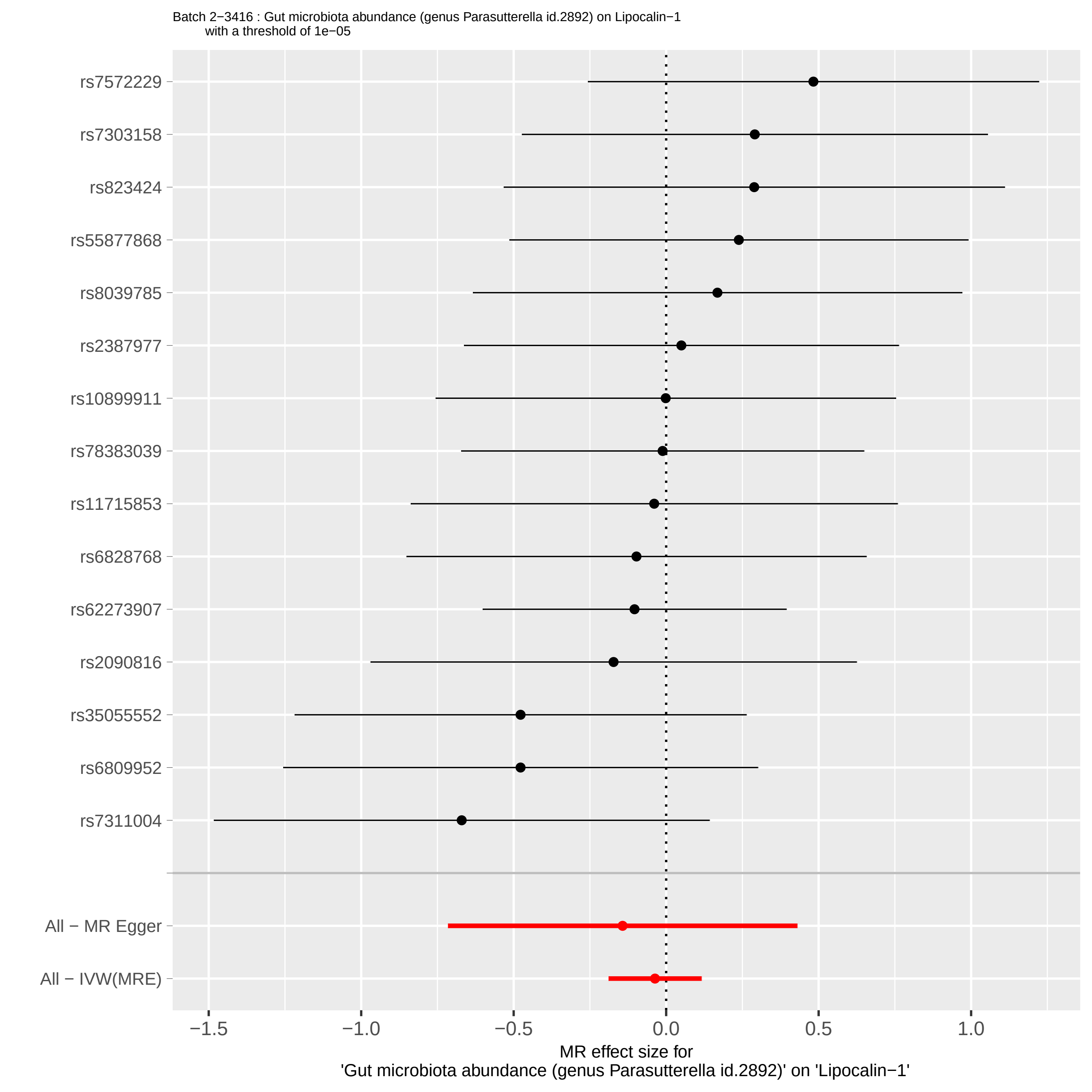

Batch 2−3416 : Gut microbiota abundance (genus Parasutterella id.2892) on Lipocalin−1
with a threshold of 1e−05
rs7572229
rs7303158
rs823424
rs55877868
rs8039785
rs2387977
rs10899911
rs78383039
rs11715853
rs6828768
rs62273907
rs2090816
rs35055552
rs6809952
rs7311004
All − MR Egger
All − IVW(MRE)
−1.5
−1.0
−0.5
0.0
0.5
1.0
MR effect size for
'Gut microbiota abundance (genus Parasutterella id.2892)' on 'Lipocalin−1'

Batch 2−3417 : Gut microbiota abundance (genus Catenibacterium id.2153) on Neutrophil gelatinase−associated lipocalin
with a threshold of 1e−05

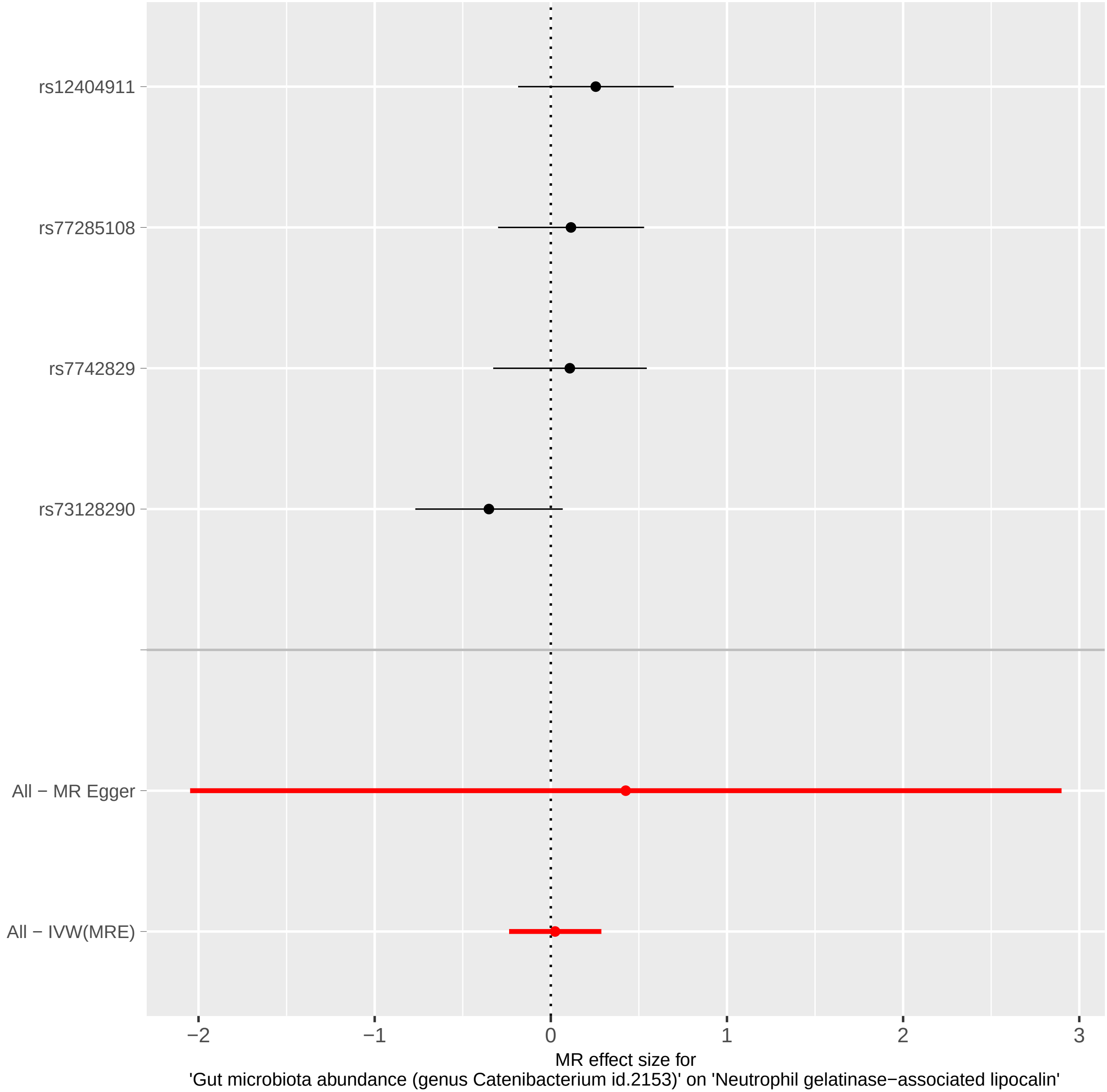

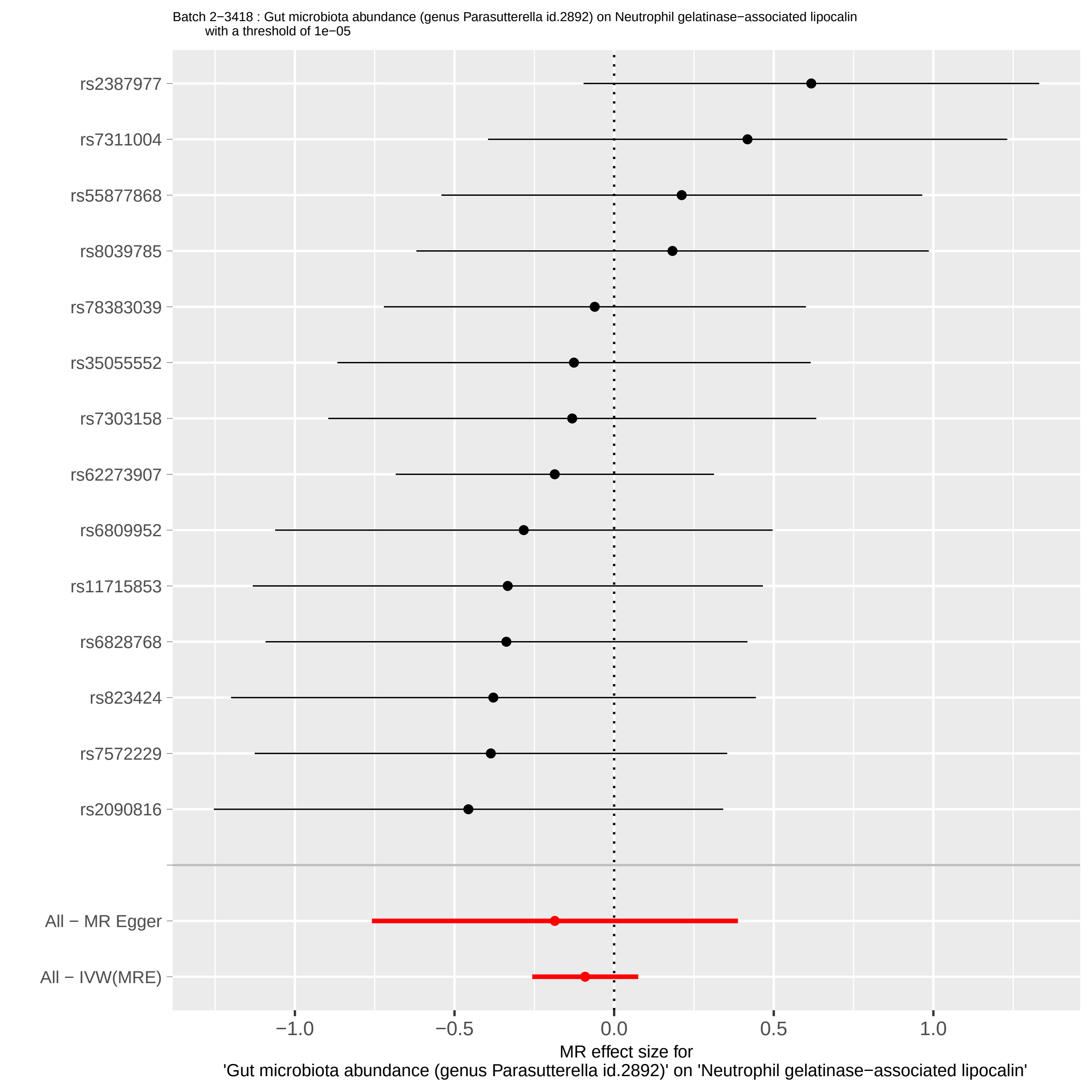

Batch 2−3418 : Gut microbiota abundance (genus Parasutterella id.2892) on Neutrophil gelatinase−associated lipocalin with a threshold of 1e−05
rs2387977
rs7311004
rs55877868
rs8039785
rs78383039
rs35055552
rs7303158
rs62273907
rs6809952
rs11715853
rs6828768
rs823424
rs7572229
rs2090816
All − MR Egger
All − IVW(MRE)
−1.0
−0.5
0.0
0.5
1.0
MR effect size for
'Gut microbiota abundance (genus Parasutterella id.2892)' on 'Neutrophil gelatinase−associated lipocalin'

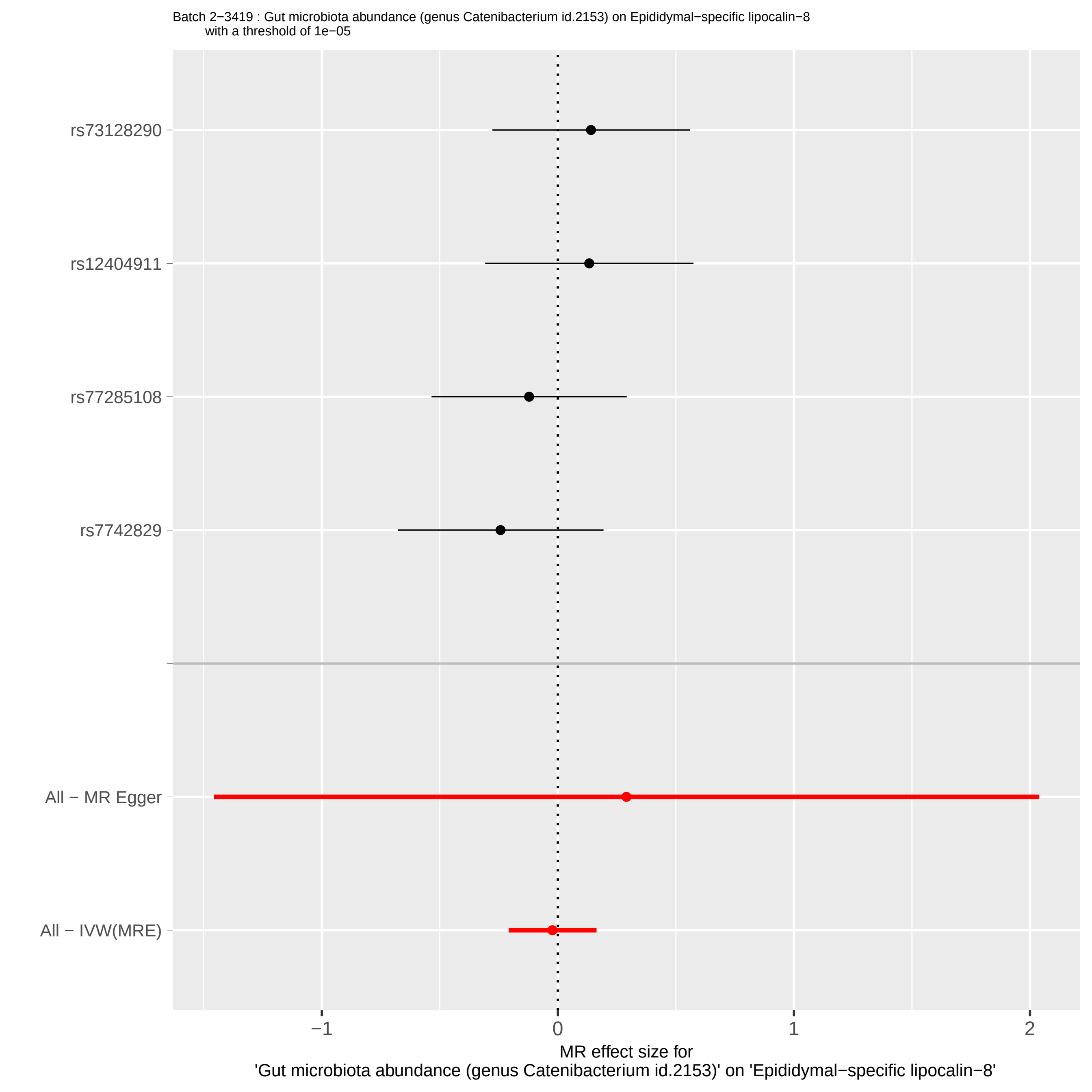

Batch 2−3419 : Gut microbiota abundance (genus Catenibacterium id.2153) on Epididymal−specific lipocalin−8
with a threshold of 1e−05
rs73128290
rs12404911
rs77285108
rs7742829
All − MR Egger
All − IVW(MRE)
−1
0
1
2
MR effect size for
'Gut microbiota abundance (genus Catenibacterium id.2153)' on 'Epididymal−specific lipocalin−8'

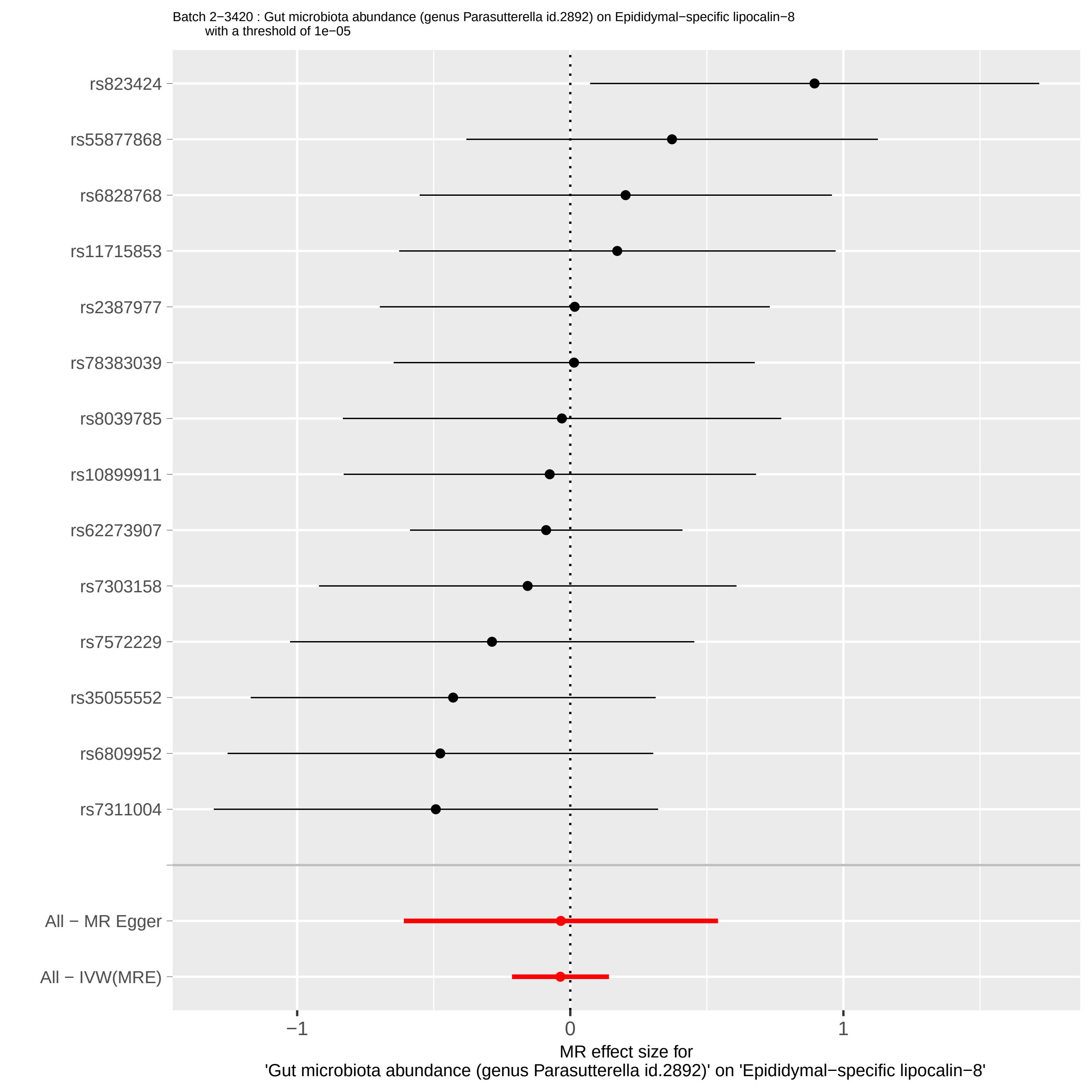

Batch 2−3420 : Gut microbiota abundance (genus Parasutterella id.2892) on Epididymal−specific lipocalin−8
with a threshold of 1e−05
rs823424
rs55877868
rs6828768
rs11715853
rs2387977
rs78383039
rs8039785
rs10899911
rs62273907
rs7303158
rs7572229
rs35055552
rs6809952
rs7311004
All − MR Egger
All − IVW(MRE)
−1
0
1
MR effect size for
'Gut microbiota abundance (genus Parasutterella id.2892)' on 'Epididymal−specific lipocalin−8'

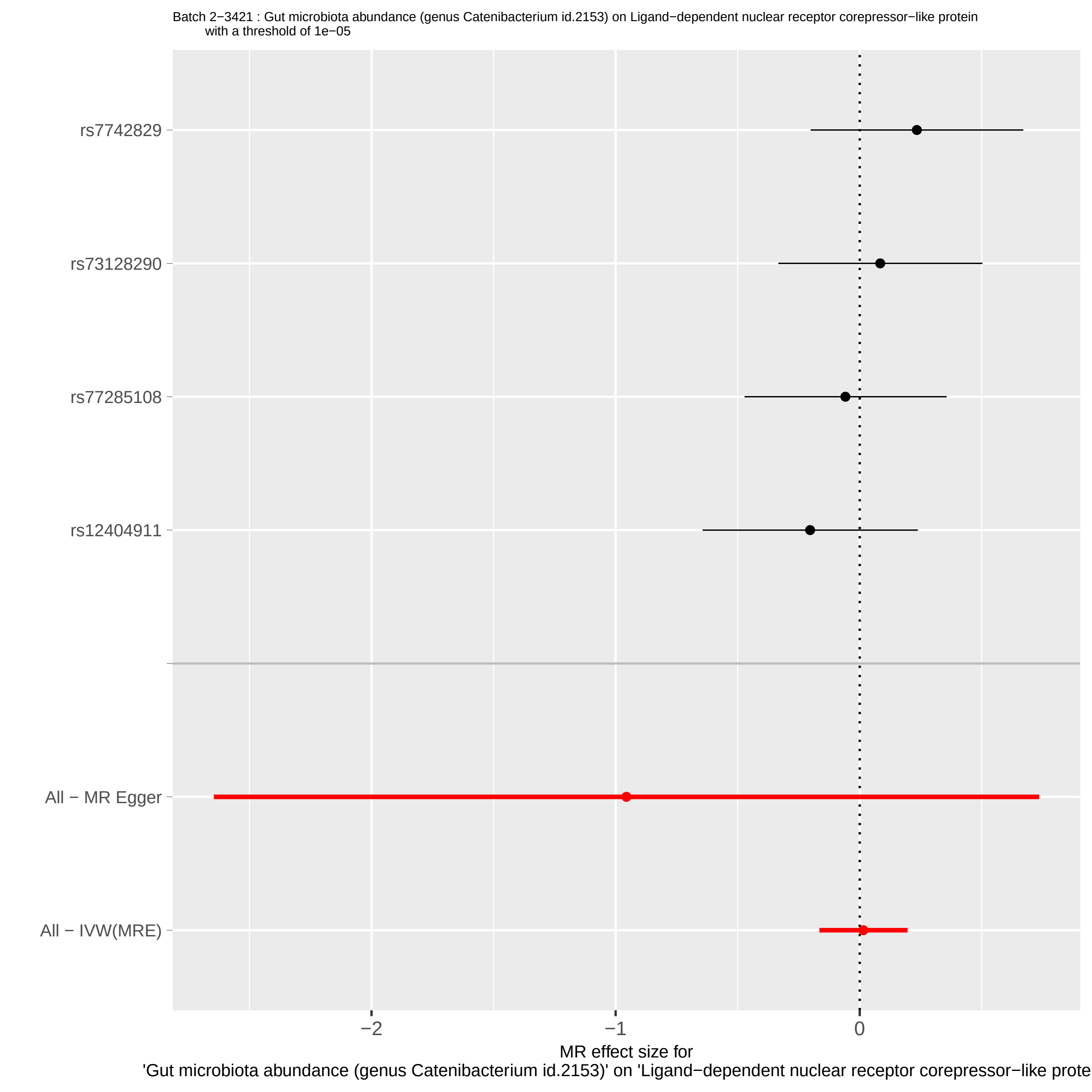

Batch 2−3421 : Gut microbiota abundance (genus Catenibacterium id.2153) on Ligand−dependent nuclear receptor corepressor−like protein
with a threshold of 1e−05
rs7742829
rs73128290
rs77285108
rs12404911
All − MR Egger
All − IVW(MRE)
−2
−1
0
MR effect size for
'Gut microbiota abundance (genus Catenibacterium id.2153)' on 'Ligand−dependent nuclear receptor corepressor−like prote

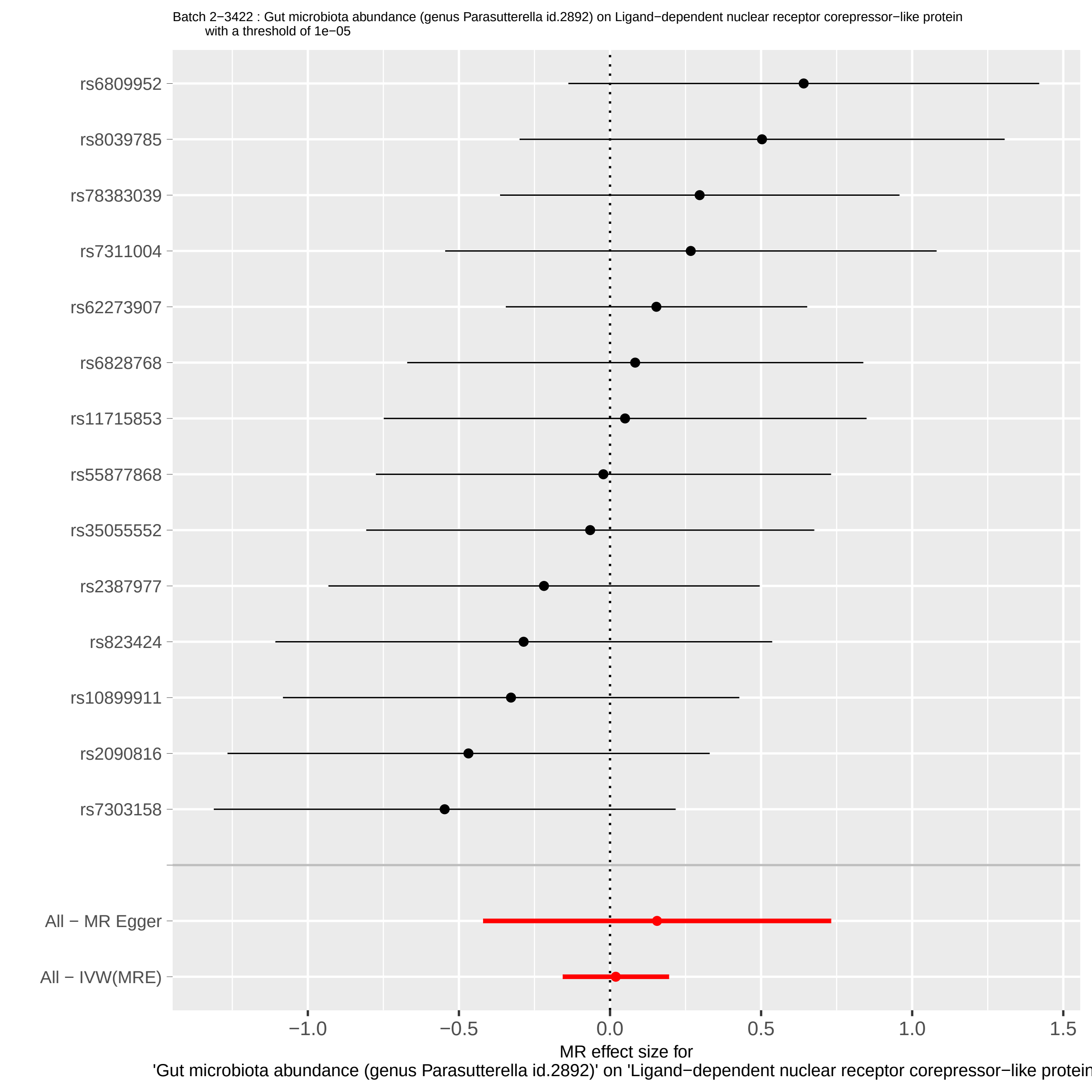

Batch 2−3422 : Gut microbiota abundance (genus Parasutterella id.2892) on Ligand−dependent nuclear receptor corepressor−like protein with a threshold of 1e−05
rs6809952
rs8039785
rs78383039
rs7311004
rs62273907
rs6828768
rs11715853
rs55877868
rs35055552
rs2387977
rs823424
rs10899911
rs2090816
rs7303158
All − MR Egger
All − IVW(MRE)
−1.0
−0.5
0.0
0.5
1.0
1.5
MR effect size for
'Gut microbiota abundance (genus Parasutterella id.2892)' on 'Ligand−dependent nuclear receptor corepressor−like protein

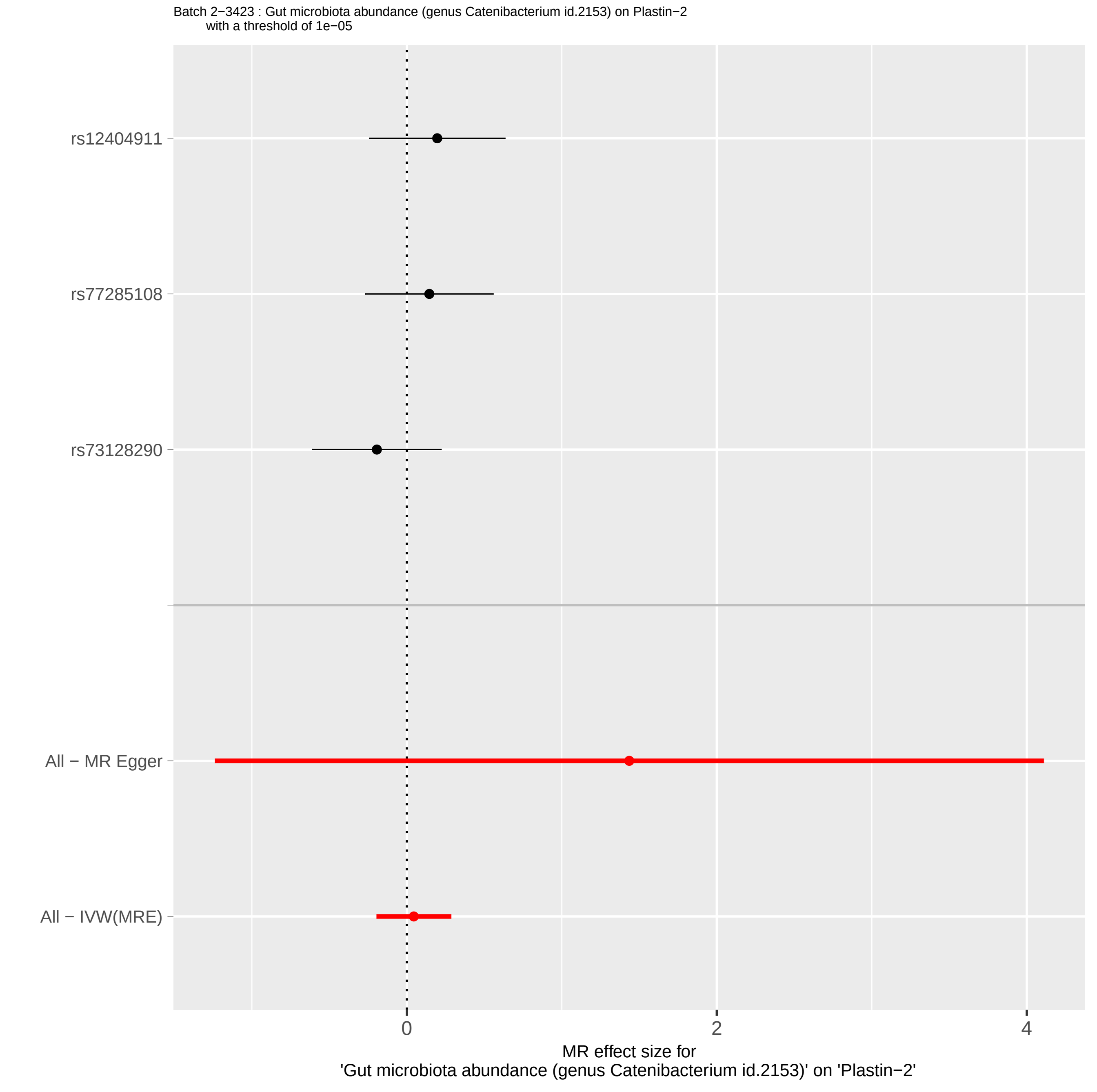
Batch 2−3423 : Gut microbiota abundance (genus Catenibacterium id.2153) on Plastin−2
with a threshold of 1e−05
rs12404911
rs77285108
rs73128290
All − MR Egger
All − IVW(MRE)
0
2
4
MR effect size for
'Gut microbiota abundance (genus Catenibacterium id.2153)' on 'Plastin−2'

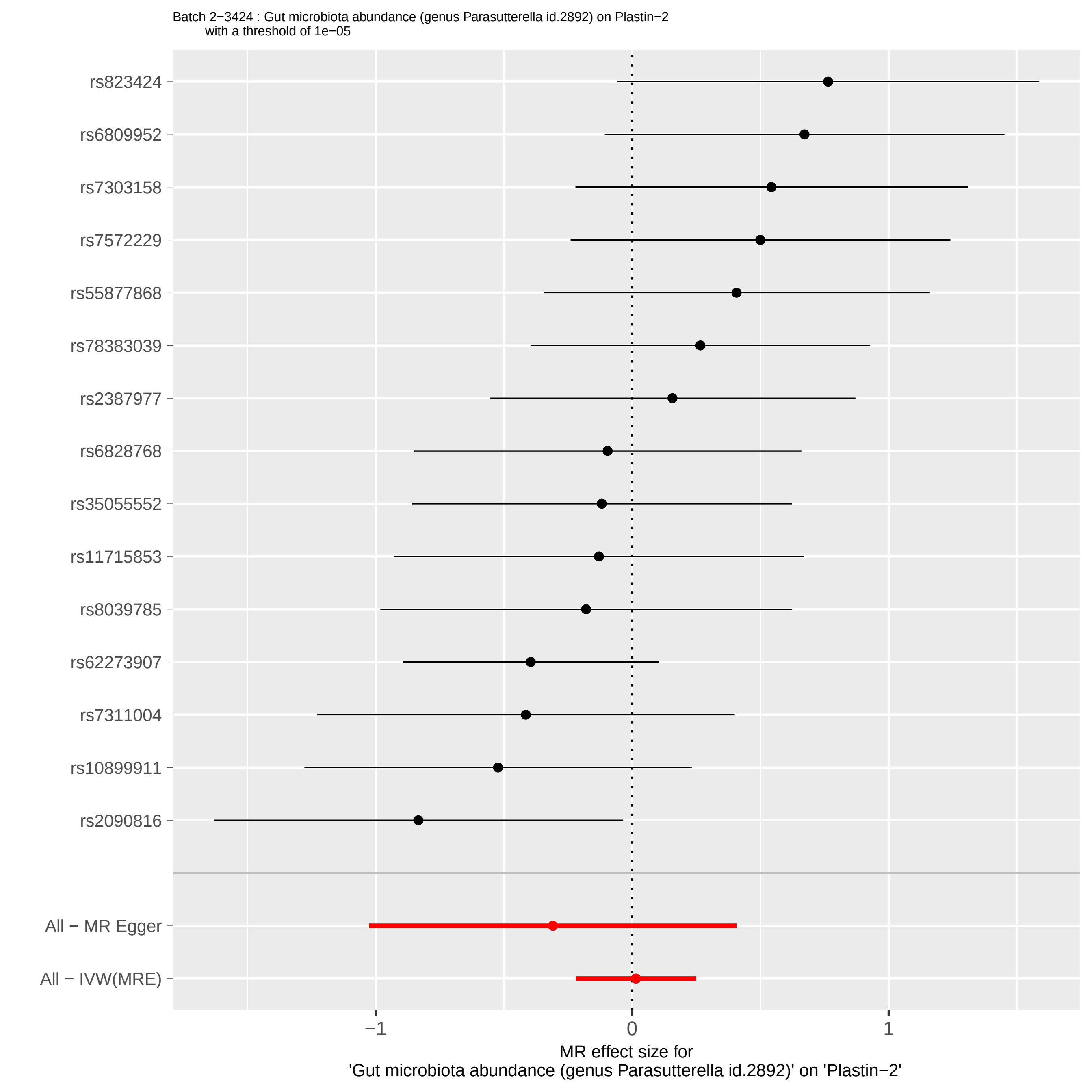
Batch 2−3424 : Gut microbiota abundance (genus Parasutterella id.2892) on Plastin−2
with a threshold of 1e−05
rs823424
rs6809952
rs7303158
rs7572229
rs55877868
rs78383039
rs2387977
rs6828768
rs35055552
rs11715853
rs8039785
rs62273907
rs7311004
rs10899911
rs2090816
All − MR Egger
All − IVW(MRE)
−1
0
1
MR effect size for
'Gut microbiota abundance (genus Parasutterella id.2892)' on 'Plastin−2'

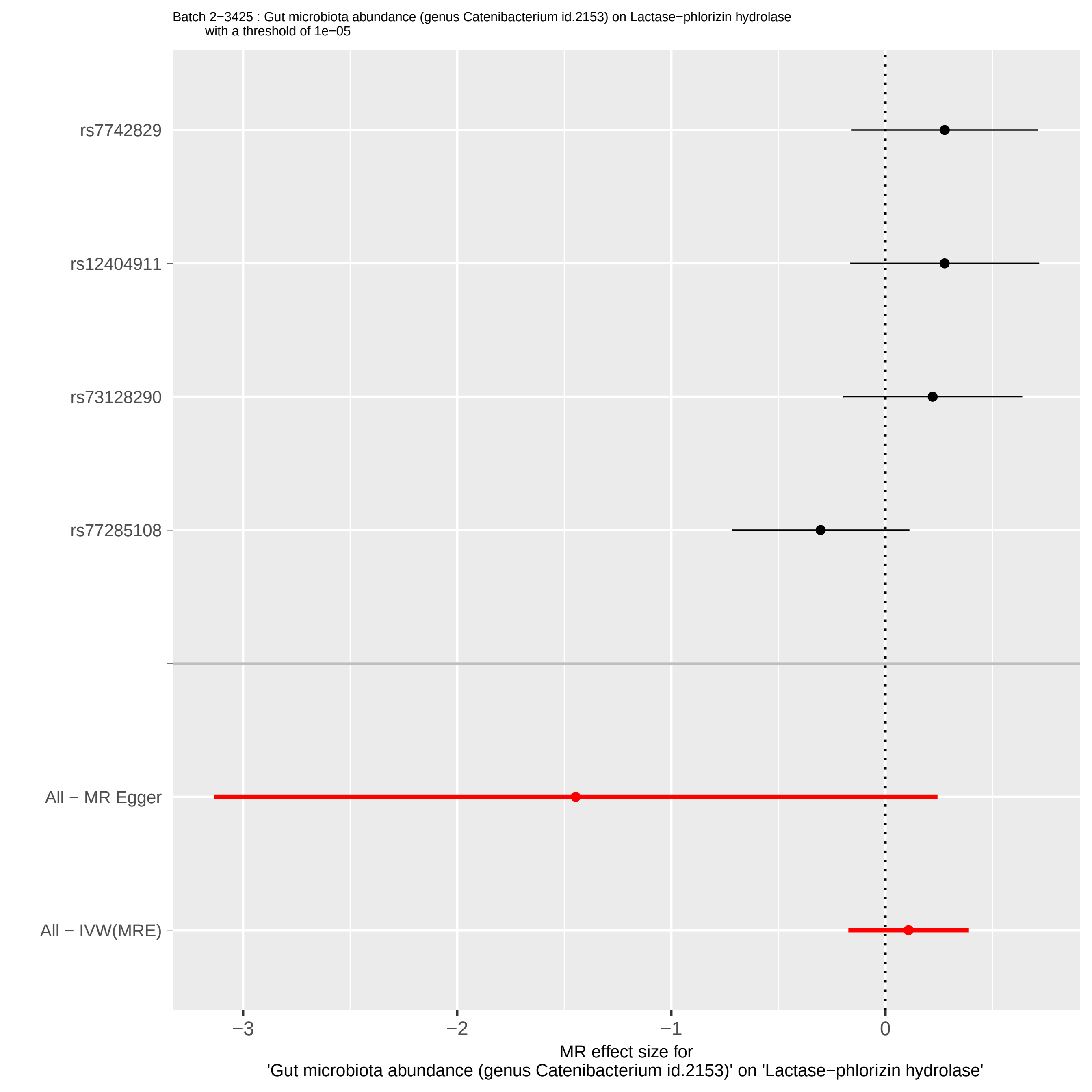

Batch 2−3425 : Gut microbiota abundance (genus Catenibacterium id.2153) on Lactase−phlorizin hydrolase
with a threshold of 1e−05
rs7742829
rs12404911
rs73128290
rs77285108
All − MR Egger
All − IVW(MRE)
−3
−2
−1
0
MR effect size for
'Gut microbiota abundance (genus Catenibacterium id.2153)' on 'Lactase−phlorizin hydrolase'

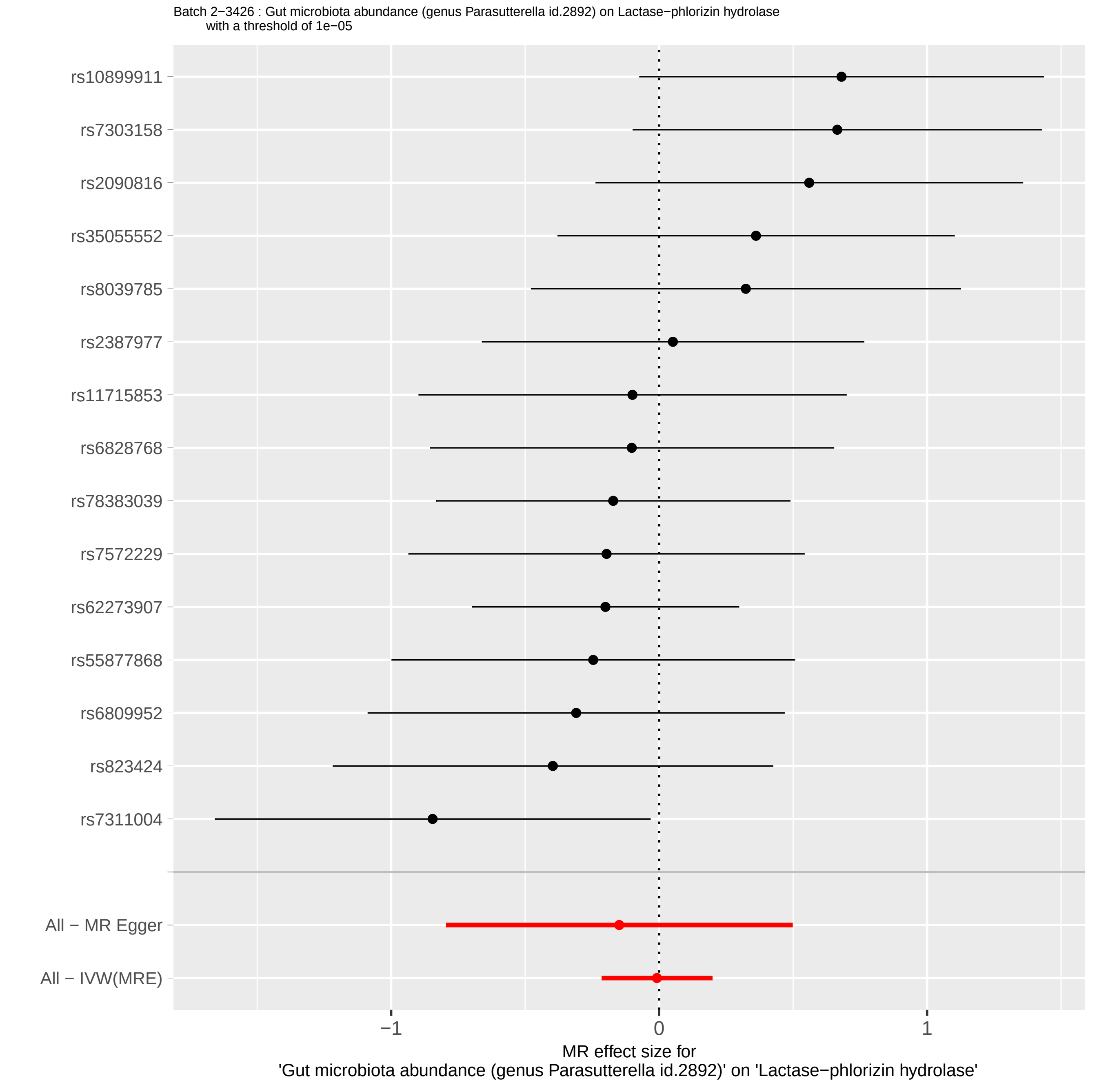

Batch 2−3426 : Gut microbiota abundance (genus Parasutterella id.2892) on Lactase−phlorizin hydrolase
with a threshold of 1e−05
rs10899911
rs7303158
rs2090816
rs35055552
rs8039785
rs2387977
rs11715853
rs6828768
rs78383039
rs7572229
rs62273907
rs55877868
rs6809952
rs823424
rs7311004
All − MR Egger
All − IVW(MRE)
−1
0
1
MR effect size for
'Gut microbiota abundance (genus Parasutterella id.2892)' on 'Lactase−phlorizin hydrolase'

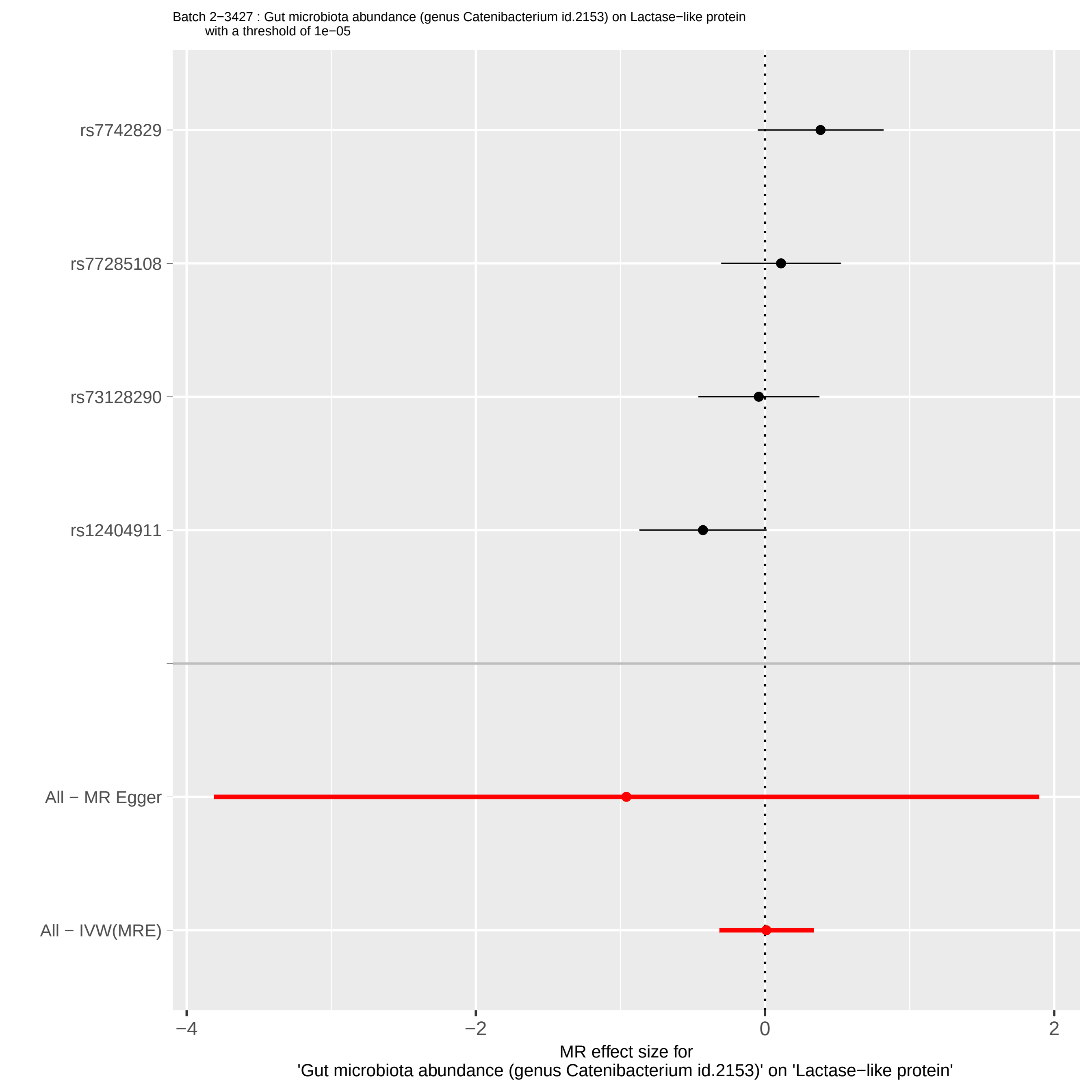

Batch 2−3427 : Gut microbiota abundance (genus Catenibacterium id.2153) on Lactase−like protein
with a threshold of 1e−05
rs7742829
rs77285108
rs73128290
rs12404911
All − MR Egger
All − IVW(MRE)
−4
−2
0
2
MR effect size for
'Gut microbiota abundance (genus Catenibacterium id.2153)' on 'Lactase−like protein'

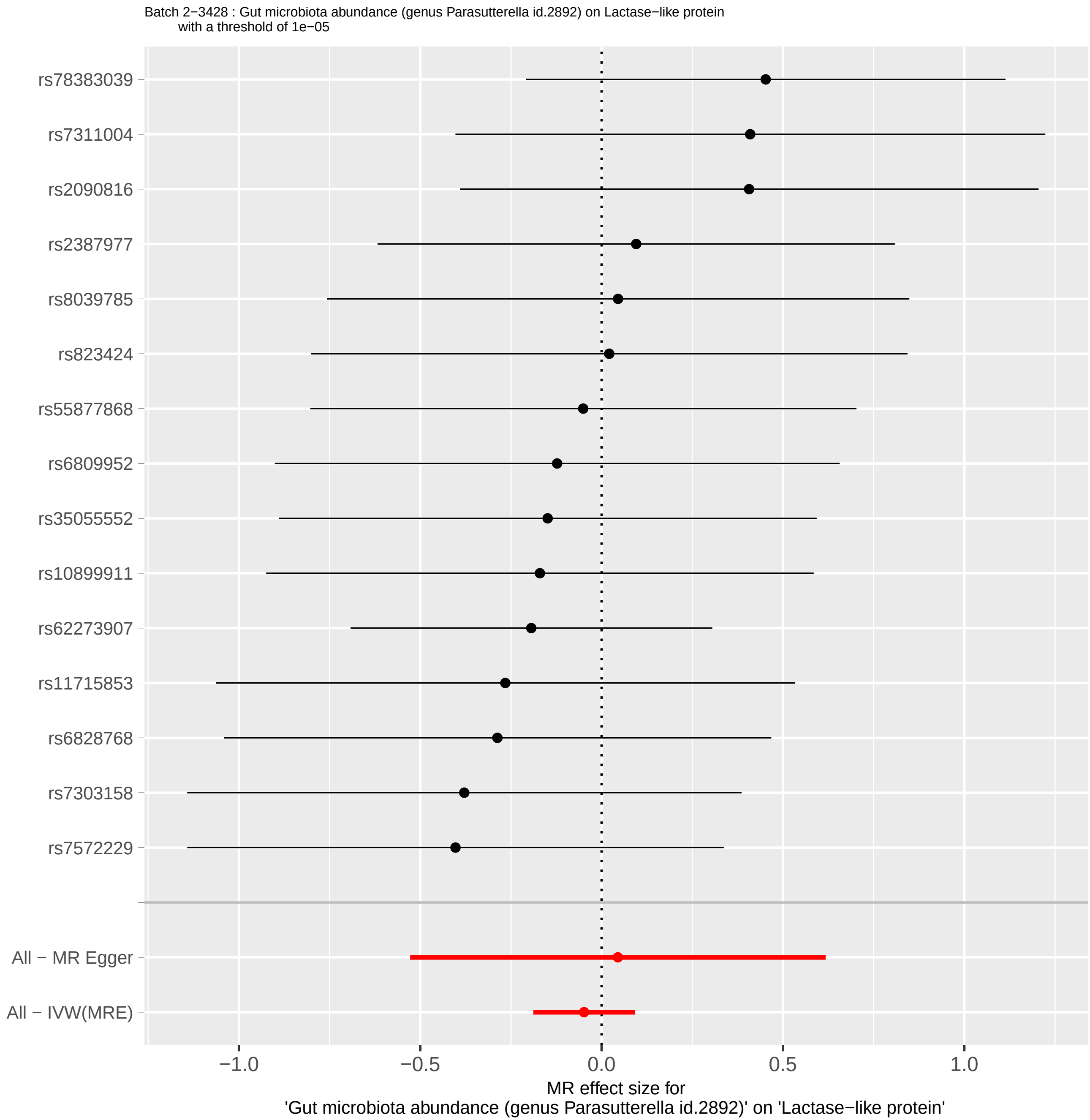
Batch 2−3428 : Gut microbiota abundance (genus Parasutterella id.2892) on Lactase−like protein
with a threshold of 1e−05
rs78383039
rs7311004
rs2090816
rs2387977
rs8039785
rs823424
rs55877868
rs6809952
rs35055552
rs10899911
rs62273907
rs11715853
rs6828768
rs7303158
rs7572229
All − MR Egger
All − IVW(MRE)
−1.0
−0.5
0.0
0.5
1.0
MR effect size for
'Gut microbiota abundance (genus Parasutterella id.2892)' on 'Lactase−like protein'

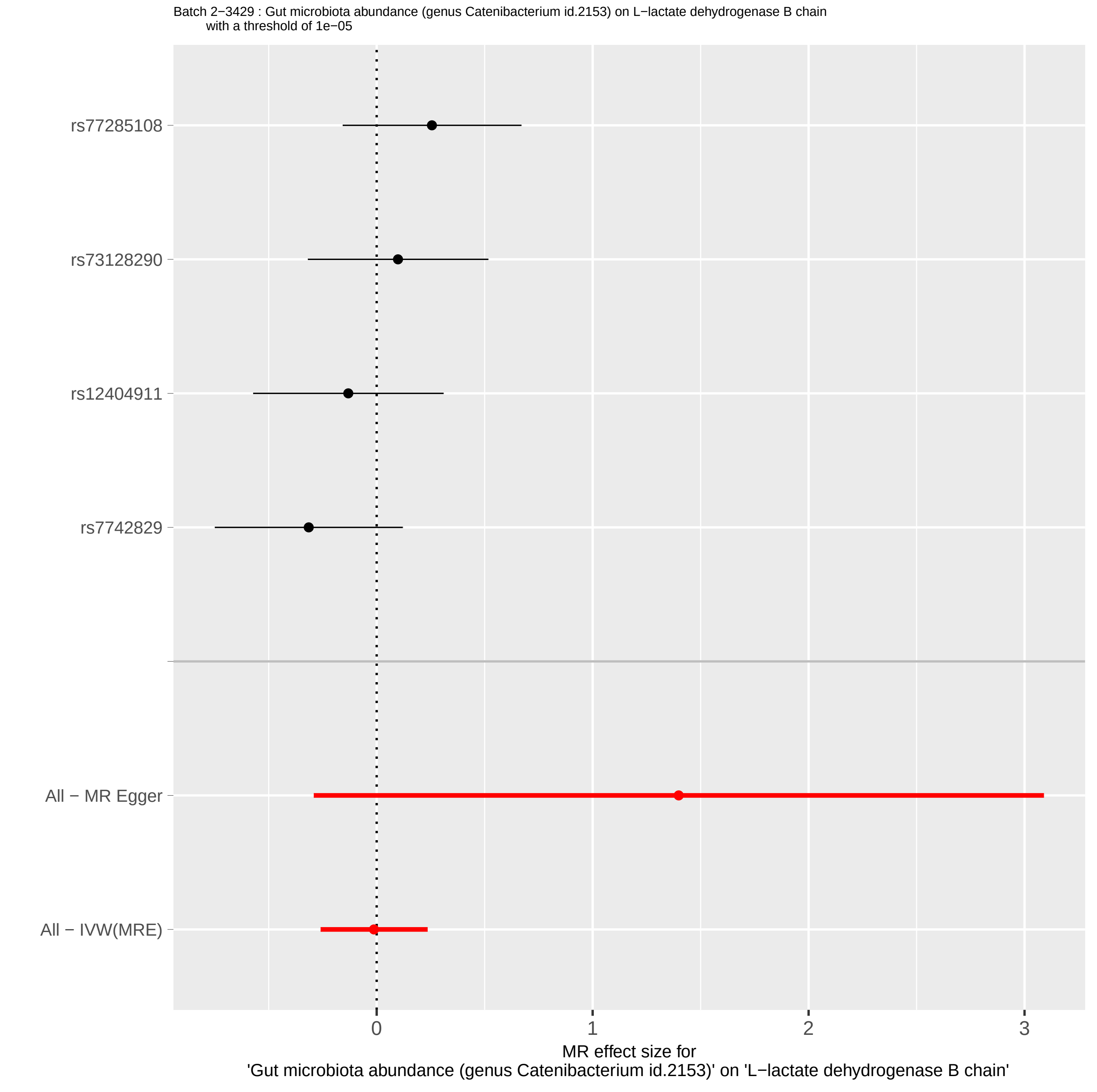

Batch 2−3429 : Gut microbiota abundance (genus Catenibacterium id.2153) on L−lactate dehydrogenase B chain
with a threshold of 1e−05
rs77285108
rs73128290
rs12404911
rs7742829
All − MR Egger
All − IVW(MRE)
0
1
2
3
MR effect size for
'Gut microbiota abundance (genus Catenibacterium id.2153)' on 'L−lactate dehydrogenase B chain'

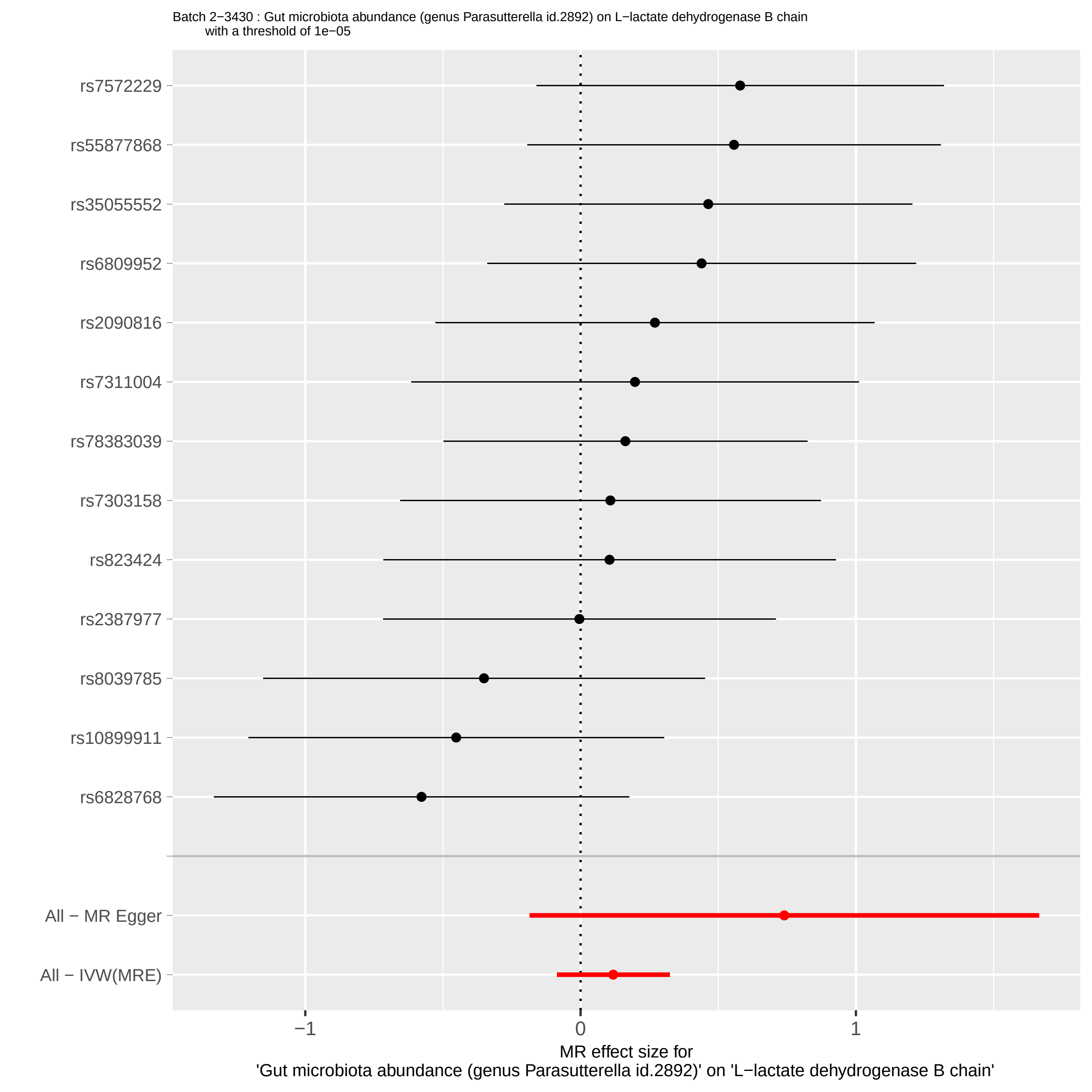

Batch 2−3430 : Gut microbiota abundance (genus Parasutterella id.2892) on L−lactate dehydrogenase B chain
with a threshold of 1e−05
rs7572229
rs55877868
rs35055552
rs6809952
rs2090816
rs7311004
rs78383039
rs7303158
rs823424
rs2387977
rs8039785
rs10899911
rs6828768
All − MR Egger
All − IVW(MRE)
−1
0
1
MR effect size for
'Gut microbiota abundance (genus Parasutterella id.2892)' on 'L−lactate dehydrogenase B chain'

Batch 2−3431 : Gut microbiota abundance (genus Catenibacterium id.2153) on L−lactate dehydrogenase C chain
with a threshold of 1e−05

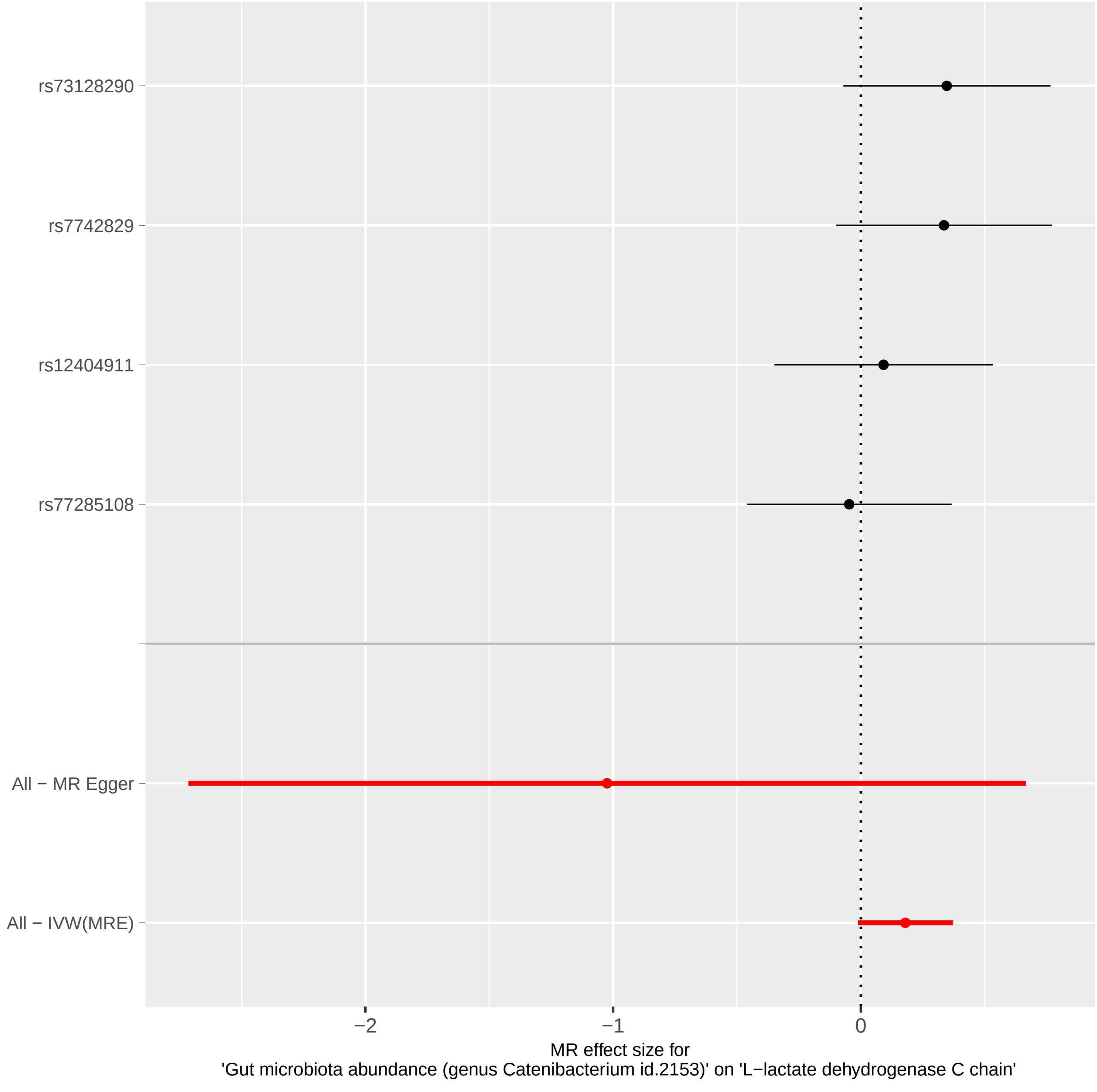

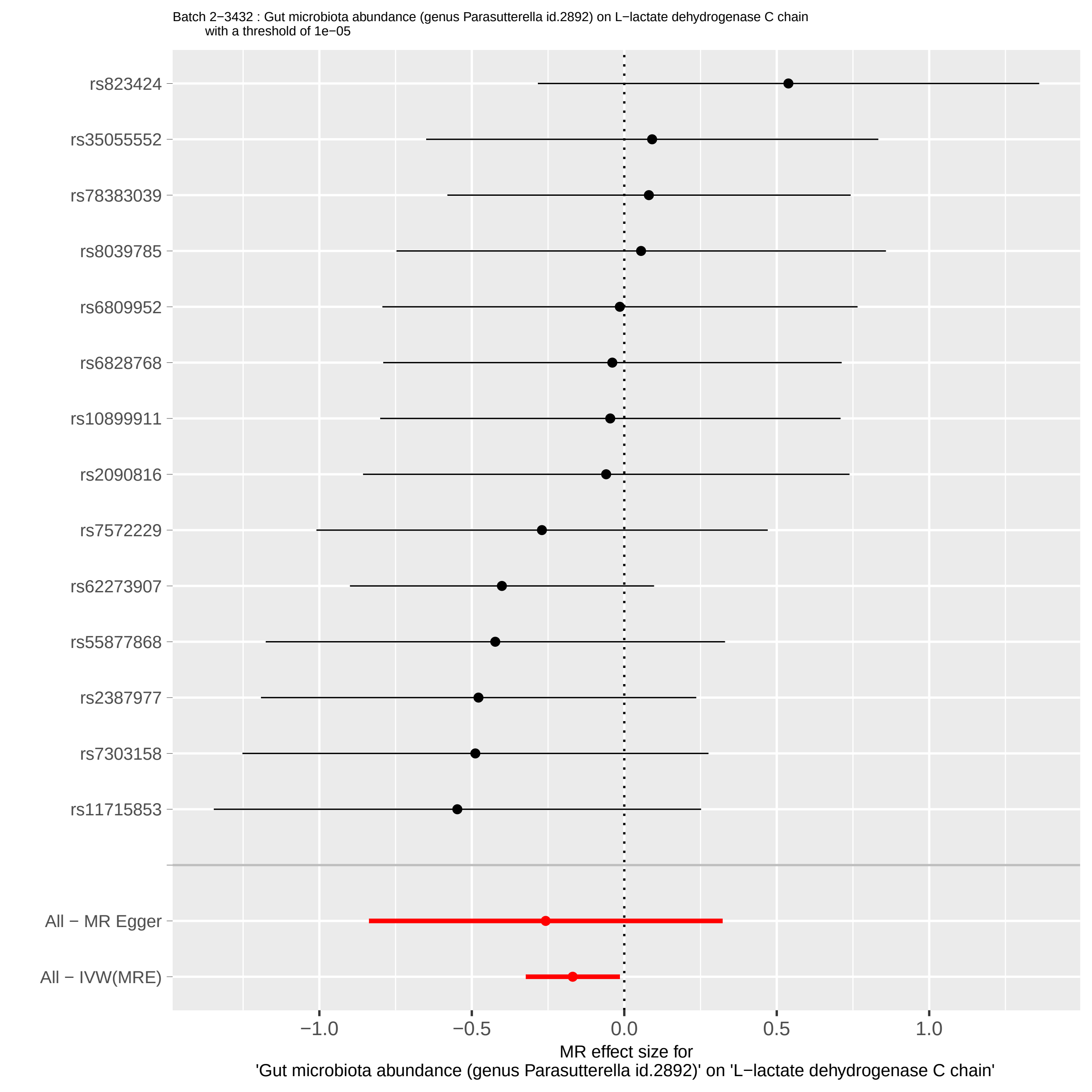

Batch 2−3432 : Gut microbiota abundance (genus Parasutterella id.2892) on L−lactate dehydrogenase C chain
with a threshold of 1e−05
rs823424
rs35055552
rs78383039
rs8039785
rs6809952
rs6828768
rs10899911
rs2090816
rs7572229
rs62273907
rs55877868
rs2387977
rs7303158
rs11715853
All − MR Egger
All − IVW(MRE)
−1.0
−0.5
0.0
0.5
1.0
MR effect size for
'Gut microbiota abundance (genus Parasutterella id.2892)' on 'L−lactate dehydrogenase C chain'

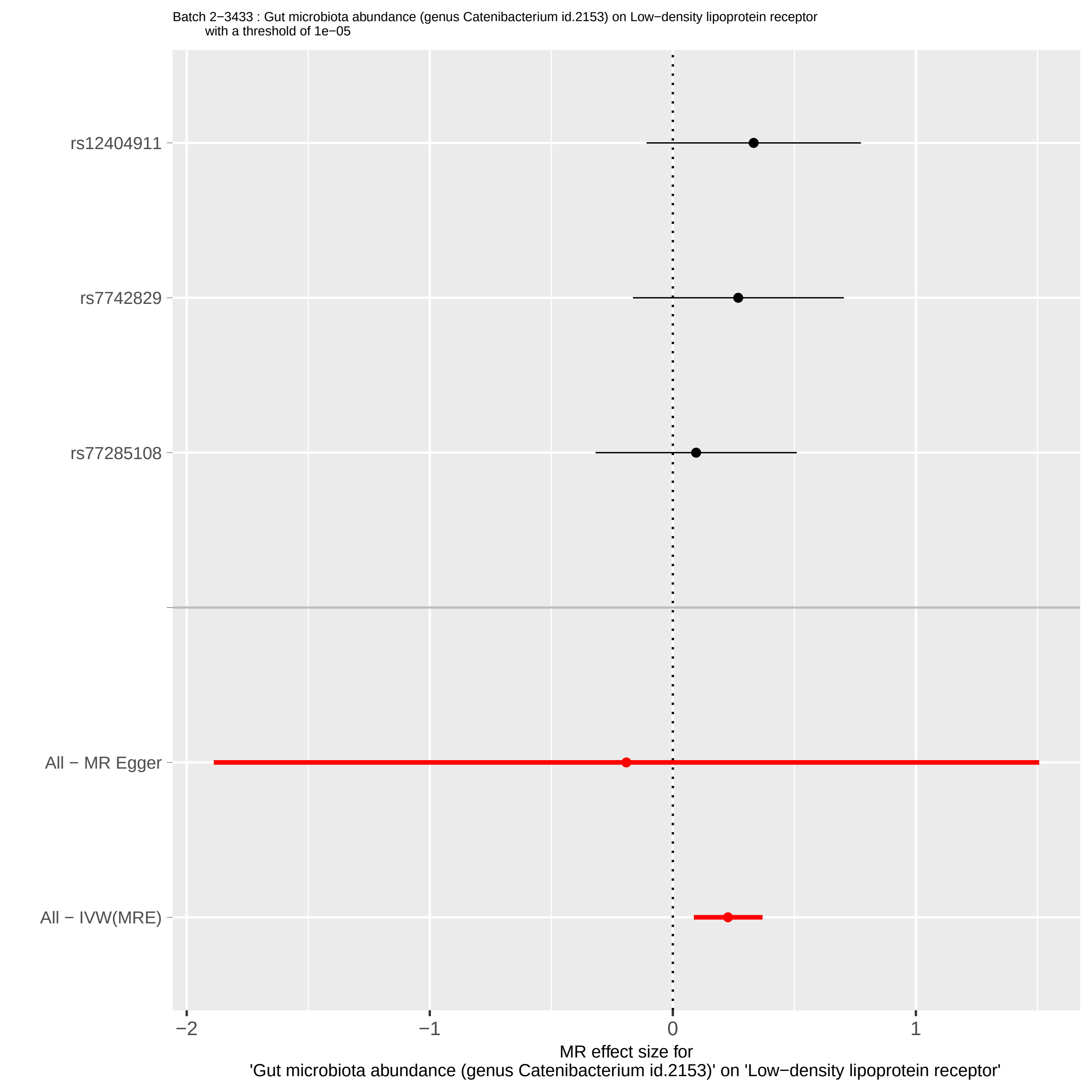

Batch 2−3433 : Gut microbiota abundance (genus Catenibacterium id.2153) on Low−density lipoprotein receptor
with a threshold of 1e−05
rs12404911
rs7742829
rs77285108
All − MR Egger
All − IVW(MRE)
−2
−1
0
1
MR effect size for
'Gut microbiota abundance (genus Catenibacterium id.2153)' on 'Low−density lipoprotein receptor'

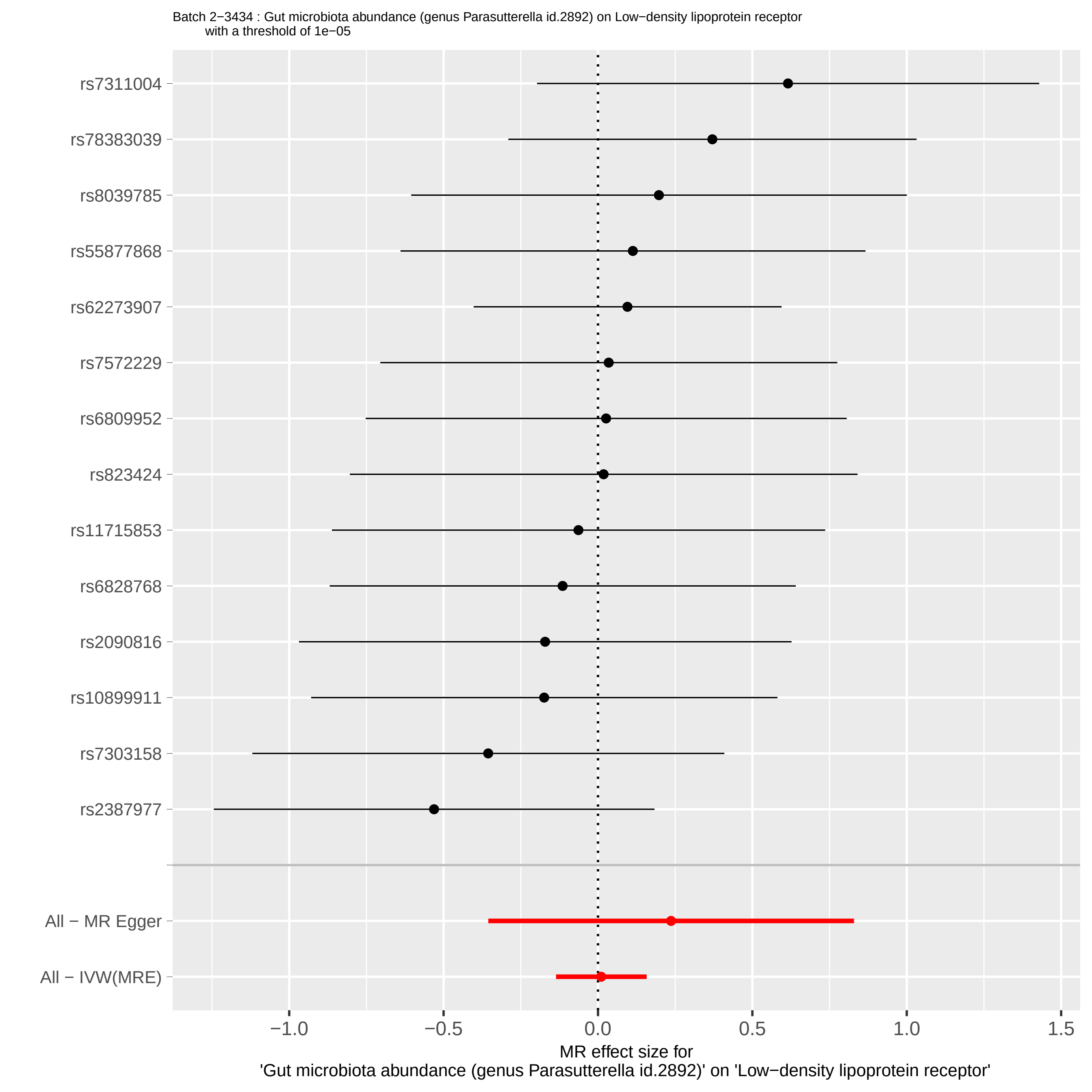

Batch 2−3434 : Gut microbiota abundance (genus Parasutterella id.2892) on Low−density lipoprotein receptor
with a threshold of 1e−05
rs7311004
rs78383039
rs8039785
rs55877868
rs62273907
rs7572229
rs6809952
rs823424
rs11715853
rs6828768
rs2090816
rs10899911
rs7303158
rs2387977
All − MR Egger
All − IVW(MRE)
−1.0
−0.5
0.0
0.5
1.0
1.5
MR effect size for
'Gut microbiota abundance (genus Parasutterella id.2892)' on 'Low−density lipoprotein receptor'

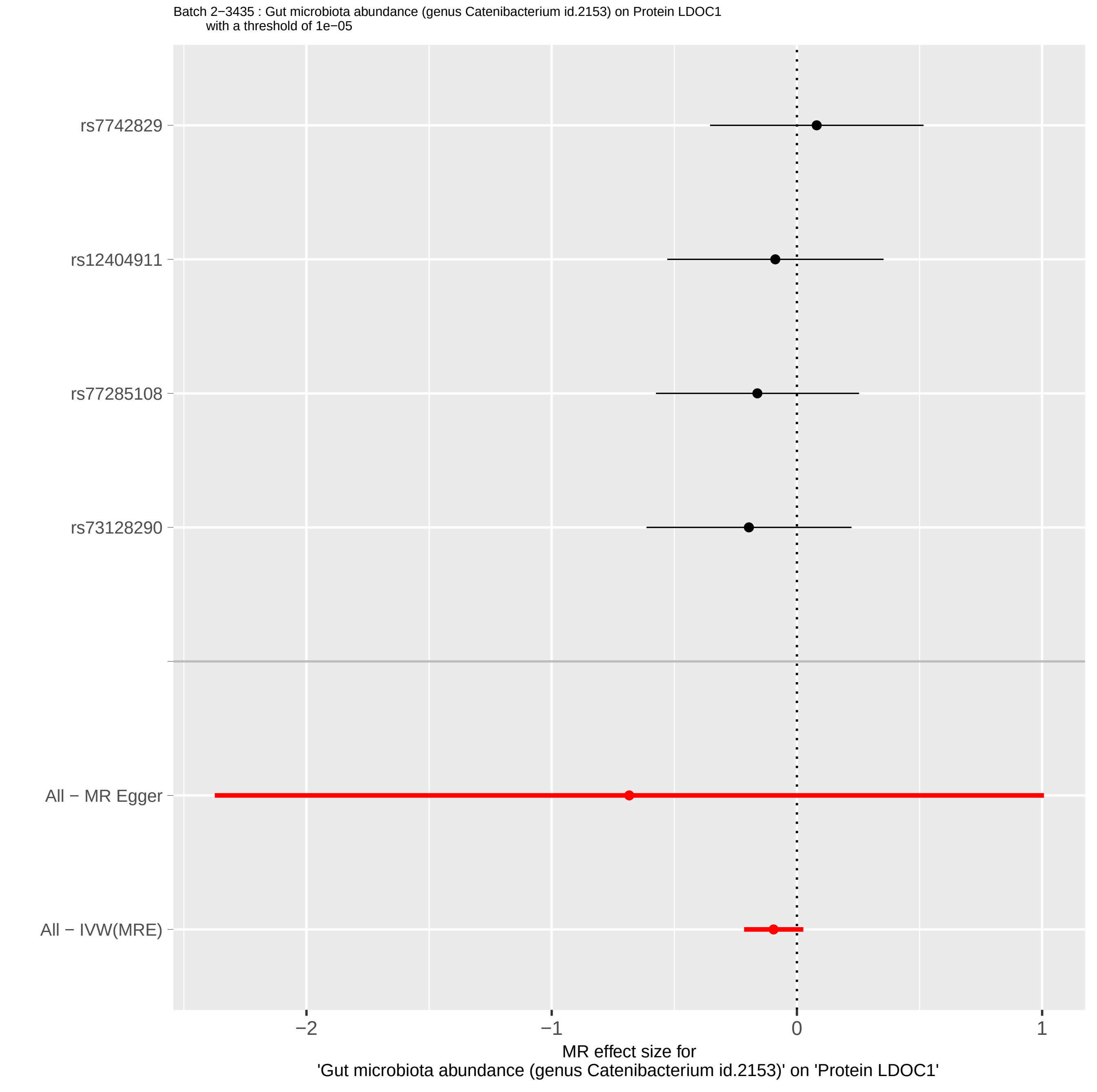

Batch 2−3435 : Gut microbiota abundance (genus Catenibacterium id.2153) on Protein LDOC1
with a threshold of 1e−05
rs7742829
rs12404911
rs77285108
rs73128290
All − MR Egger
All − IVW(MRE)
−2
−1
0
1
MR effect size for
'Gut microbiota abundance (genus Catenibacterium id.2153)' on 'Protein LDOC1'

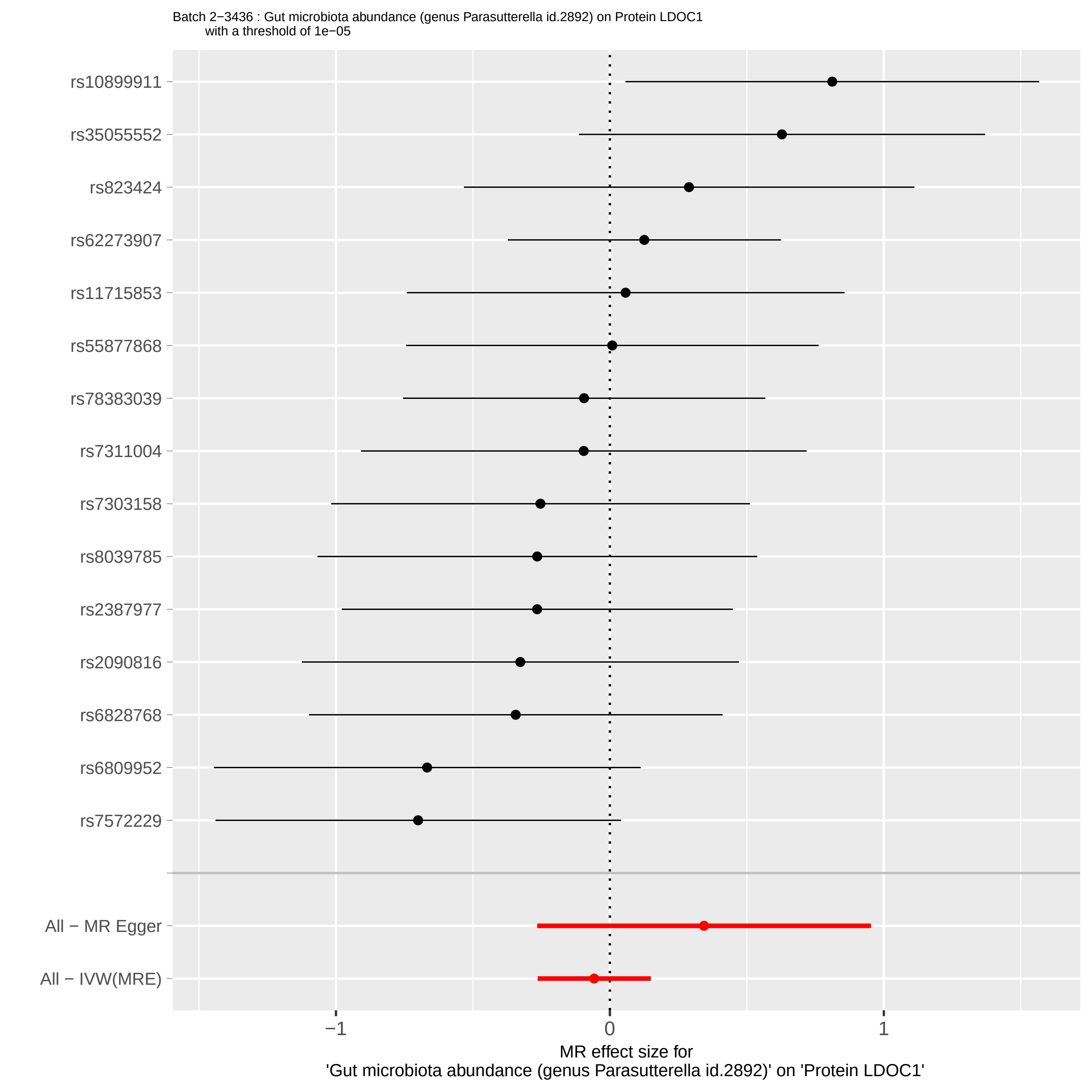

Batch 2−3436 : Gut microbiota abundance (genus Parasutterella id.2892) on Protein LDOC1
with a threshold of 1e−05
rs10899911
rs35055552
rs823424
rs62273907
rs11715853
rs55877868
rs78383039
rs7311004
rs7303158
rs8039785
rs2387977
rs2090816
rs6828768
rs6809952
rs7572229
All − MR Egger
All − IVW(MRE)
−1
0
1
MR effect size for
'Gut microbiota abundance (genus Parasutterella id.2892)' on 'Protein LDOC1'

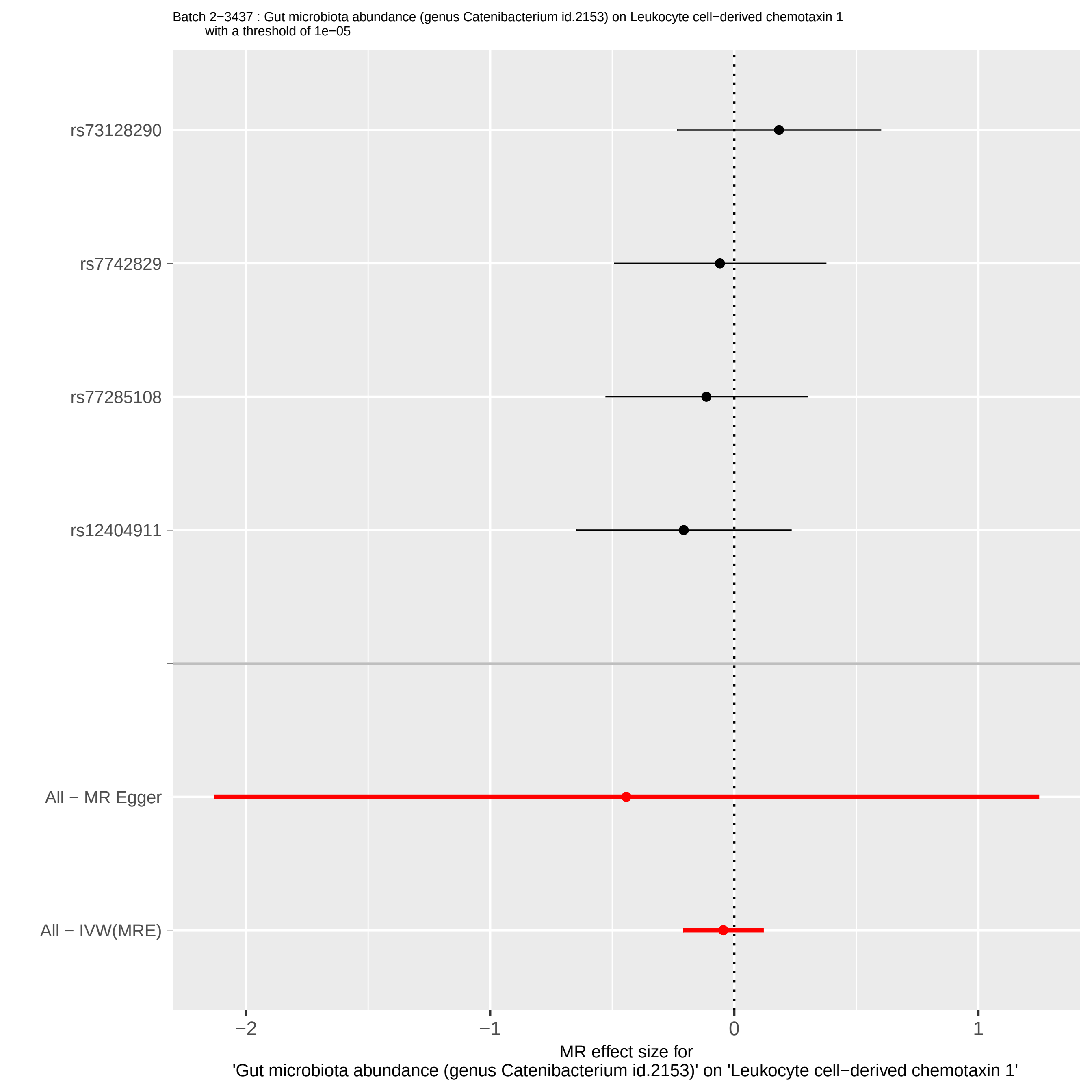
Batch 2−3437 : Gut microbiota abundance (genus Catenibacterium id.2153) on Leukocyte cell−derived chemotaxin 1
with a threshold of 1e−05
rs73128290
rs7742829
rs77285108
rs12404911
All − MR Egger
All − IVW(MRE)
−2
−1
0
1
MR effect size for
'Gut microbiota abundance (genus Catenibacterium id.2153)' on 'Leukocyte cell−derived chemotaxin 1'

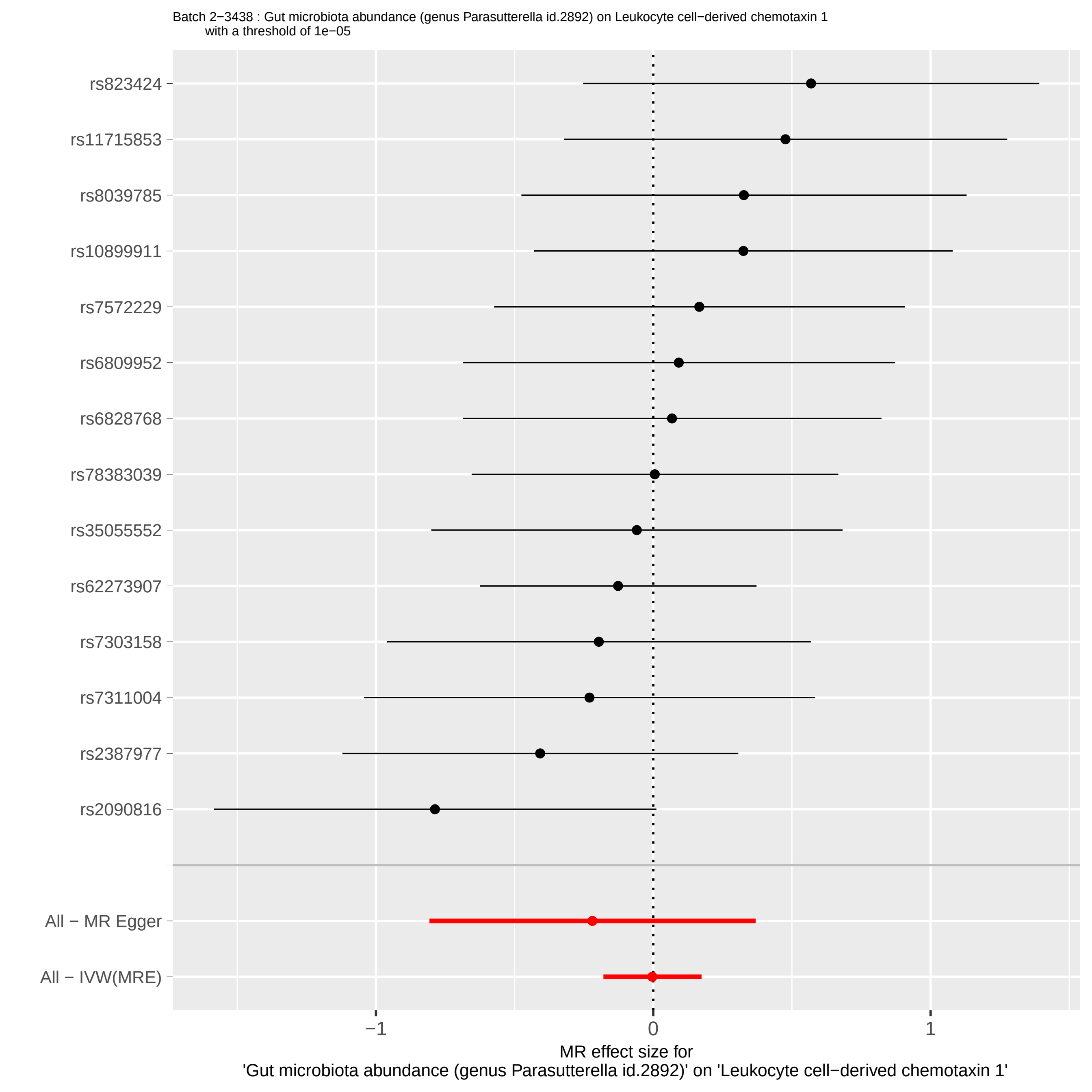

Batch 2−3438 : Gut microbiota abundance (genus Parasutterella id.2892) on Leukocyte cell−derived chemotaxin 1
with a threshold of 1e−05
rs823424
rs11715853
rs8039785
rs10899911
rs7572229
rs6809952
rs6828768
rs78383039
rs35055552
rs62273907
rs7303158
rs7311004
rs2387977
rs2090816
All − MR Egger
All − IVW(MRE)
−1
0
1
MR effect size for
'Gut microbiota abundance (genus Parasutterella id.2892)' on 'Leukocyte cell−derived chemotaxin 1'

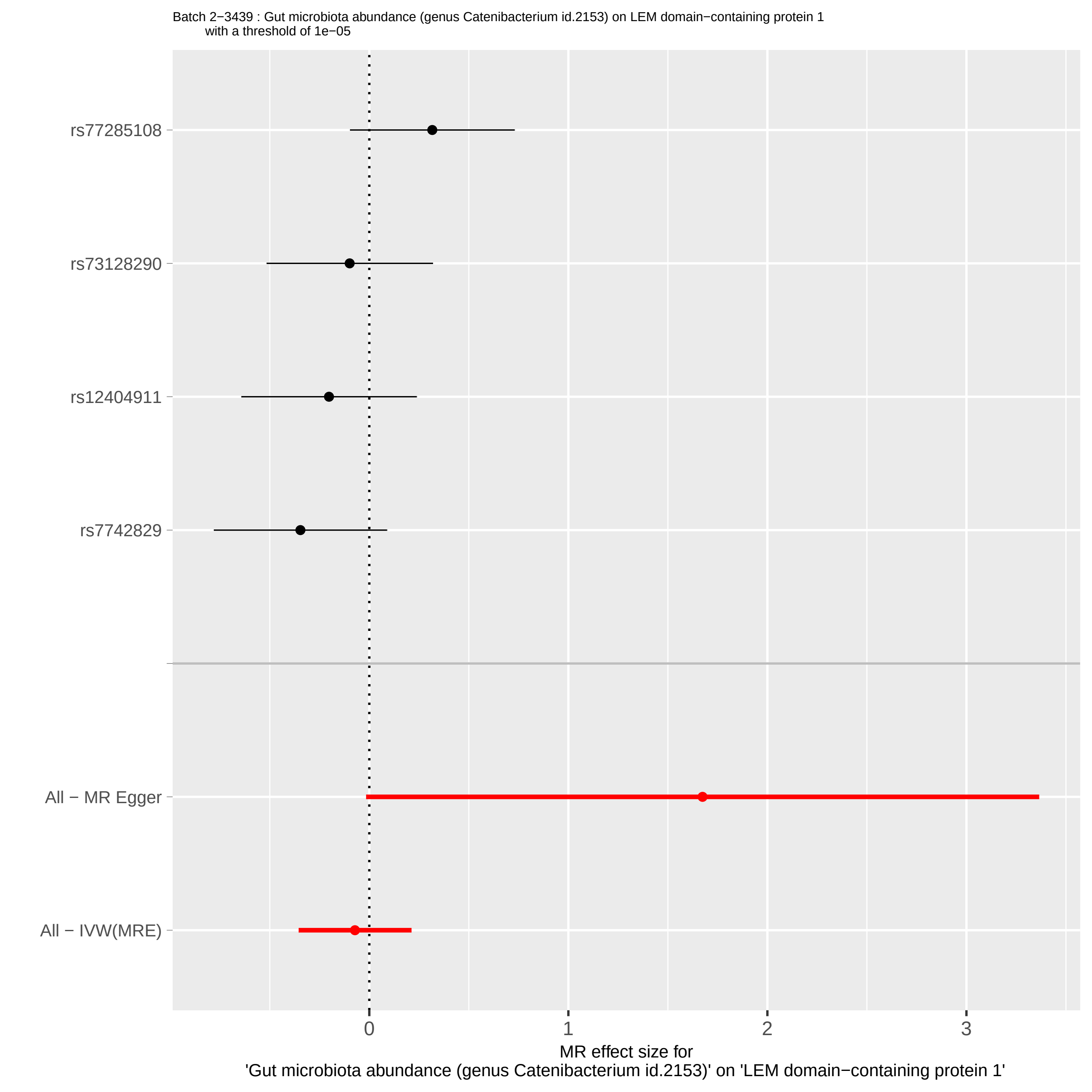

Batch 2−3439 : Gut microbiota abundance (genus Catenibacterium id.2153) on LEM domain−containing protein 1
with a threshold of 1e−05
rs77285108
rs73128290
rs12404911
rs7742829
All − MR Egger
All − IVW(MRE)
0
1
2
3
MR effect size for
'Gut microbiota abundance (genus Catenibacterium id.2153)' on 'LEM domain−containing protein 1'

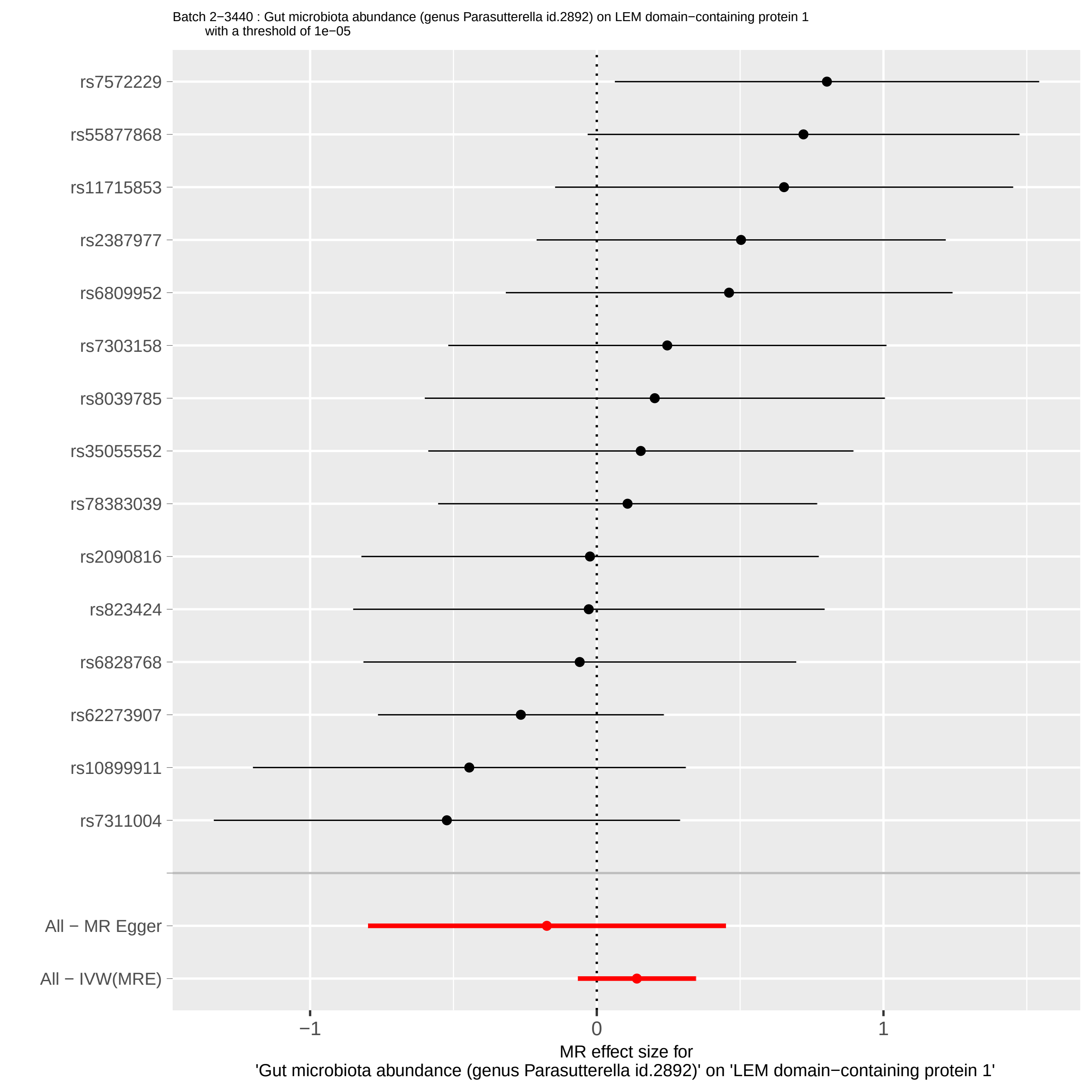

Batch 2−3440 : Gut microbiota abundance (genus Parasutterella id.2892) on LEM domain−containing protein 1
with a threshold of 1e−05
rs7572229
rs55877868
rs11715853
rs2387977
rs6809952
rs7303158
rs8039785
rs35055552
rs78383039
rs2090816
rs823424
rs6828768
rs62273907
rs10899911
rs7311004
All − MR Egger
All − IVW(MRE)
−1
0
1
MR effect size for
'Gut microbiota abundance (genus Parasutterella id.2892)' on 'LEM domain−containing protein 1'

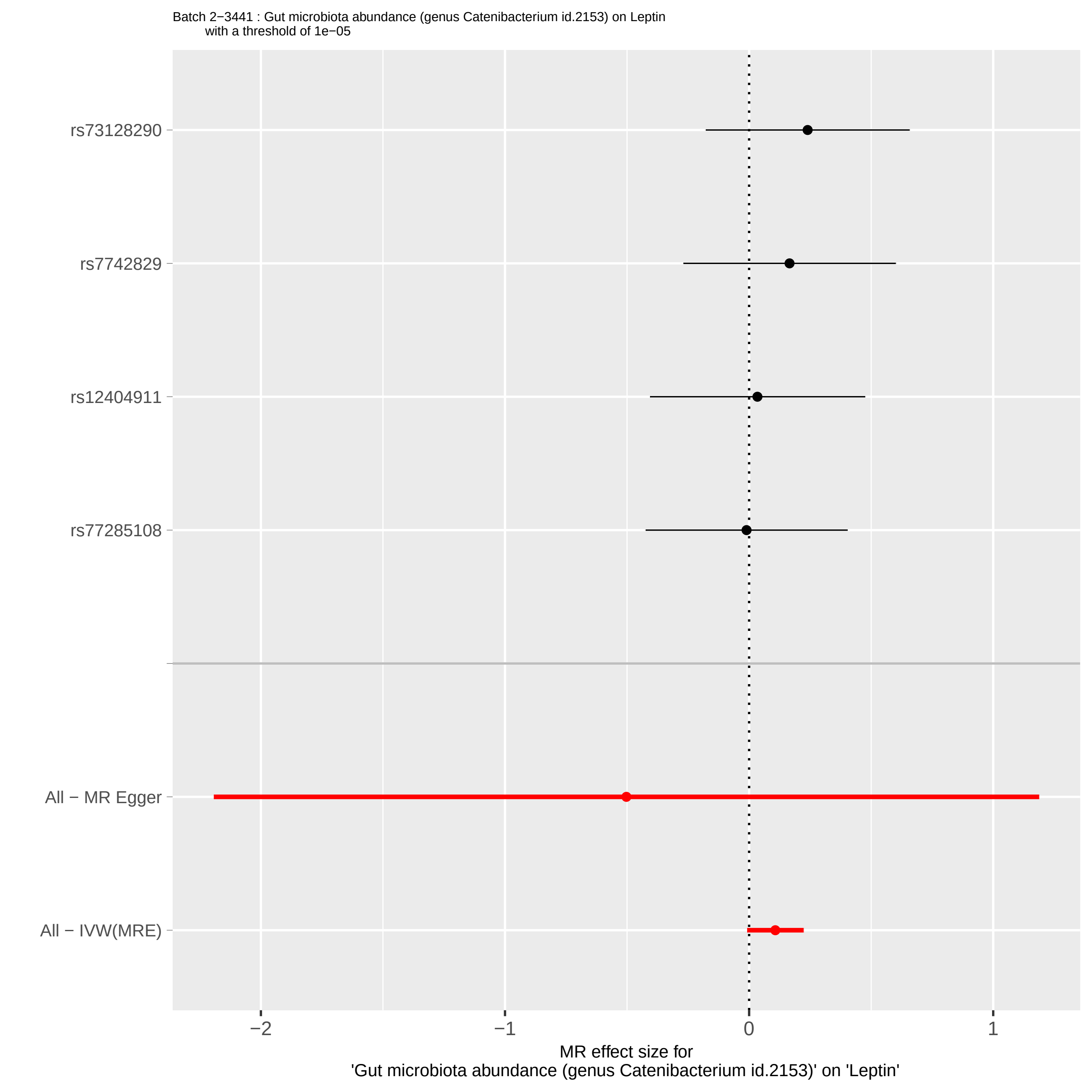

Batch 2−3441 : Gut microbiota abundance (genus Catenibacterium id.2153) on Leptin
with a threshold of 1e−05
rs73128290
rs7742829
rs12404911
rs77285108
All − MR Egger
All − IVW(MRE)
−2
−1
0
1
MR effect size for
'Gut microbiota abundance (genus Catenibacterium id.2153)' on 'Leptin'

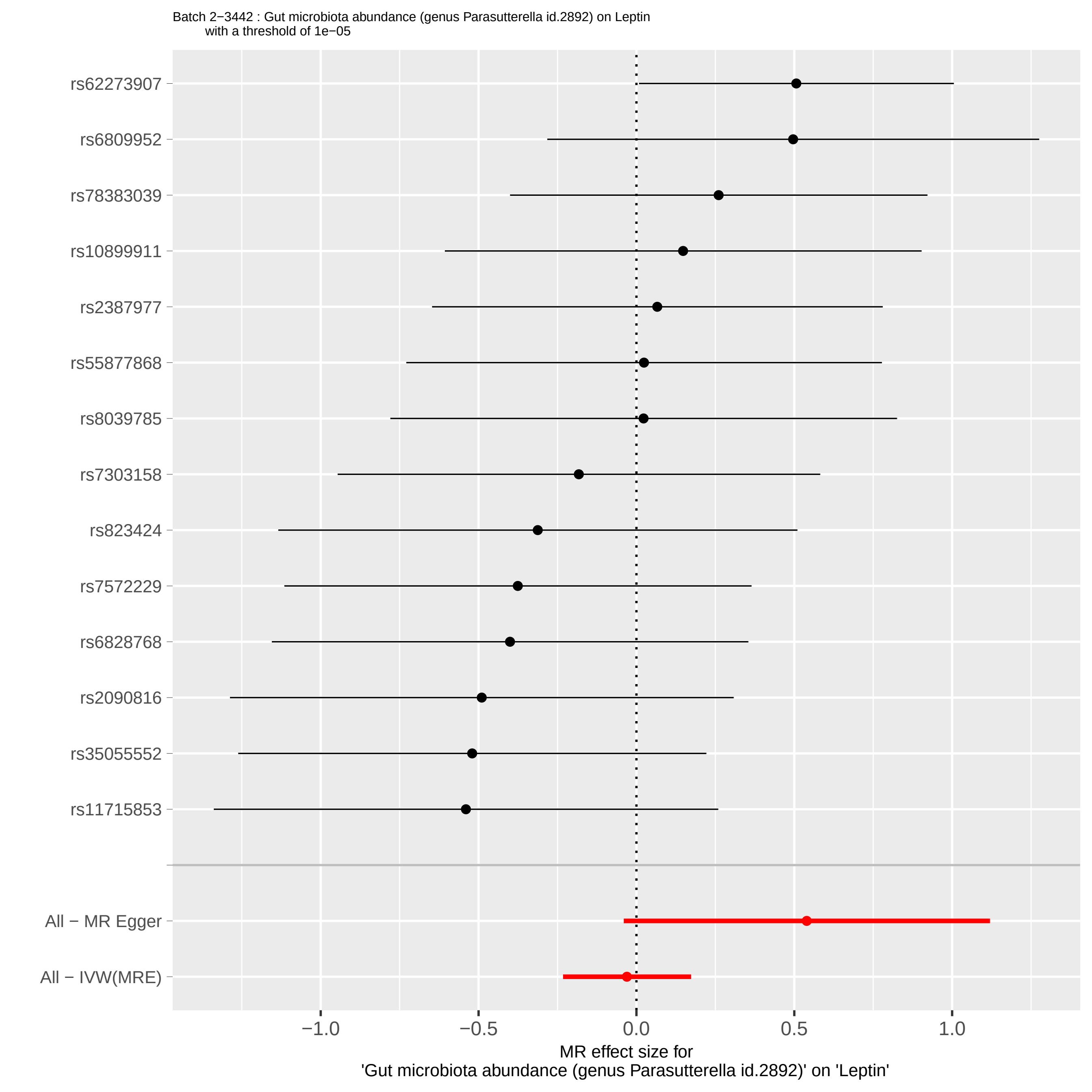

Batch 2−3442 : Gut microbiota abundance (genus Parasutterella id.2892) on Leptin
with a threshold of 1e−05
rs62273907
rs6809952
rs78383039
rs10899911
rs2387977
rs55877868
rs8039785
rs7303158
rs823424
rs7572229
rs6828768
rs2090816
rs35055552
rs11715853
All − MR Egger
All − IVW(MRE)
−1.0
−0.5
0.0
0.5
1.0
MR effect size for
'Gut microbiota abundance (genus Parasutterella id.2892)' on 'Leptin'

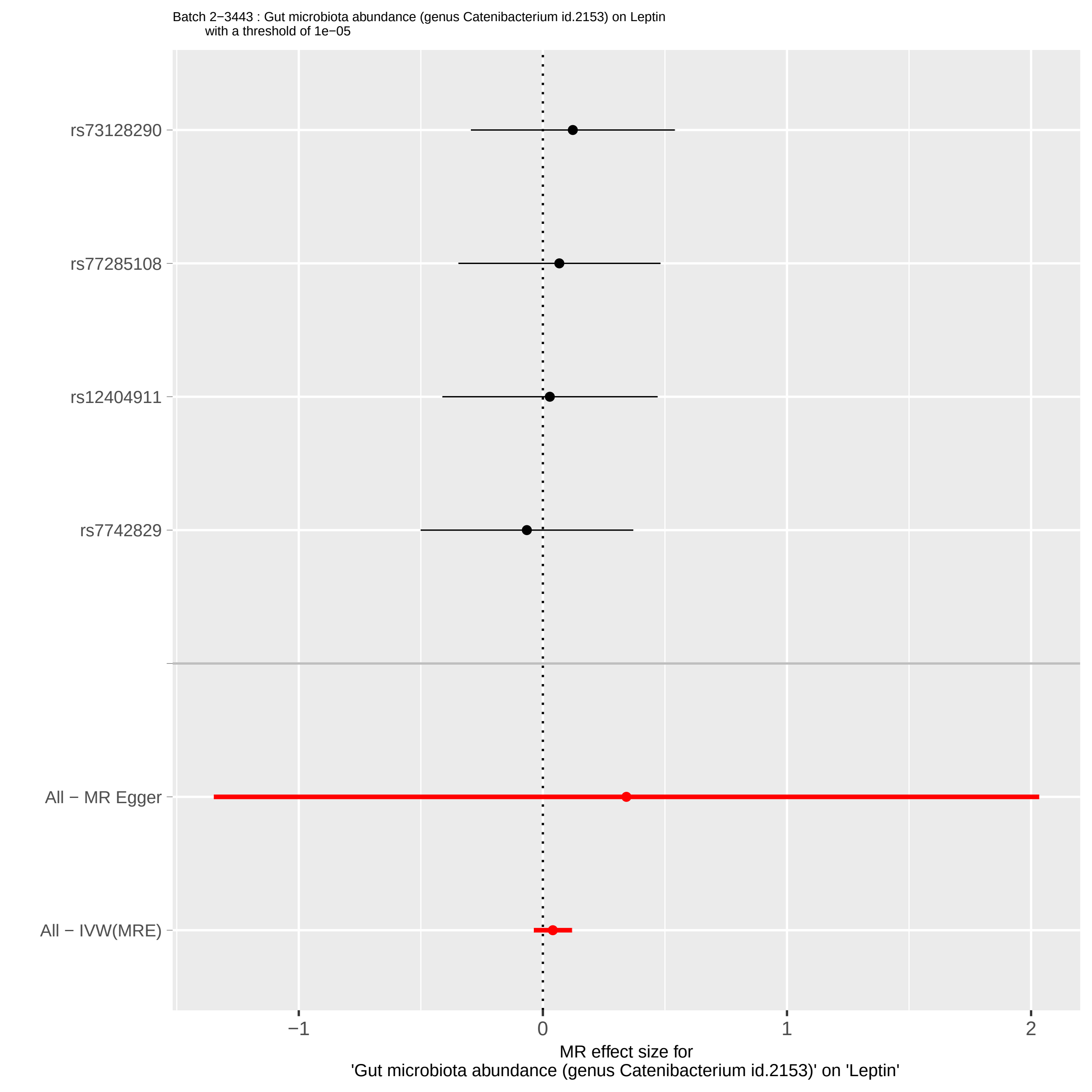

Batch 2−3443 : Gut microbiota abundance (genus Catenibacterium id.2153) on Leptin
with a threshold of 1e−05
rs73128290
rs77285108
rs12404911
rs7742829
All − MR Egger
All − IVW(MRE)
−1
0
1
2
MR effect size for
'Gut microbiota abundance (genus Catenibacterium id.2153)' on 'Leptin'

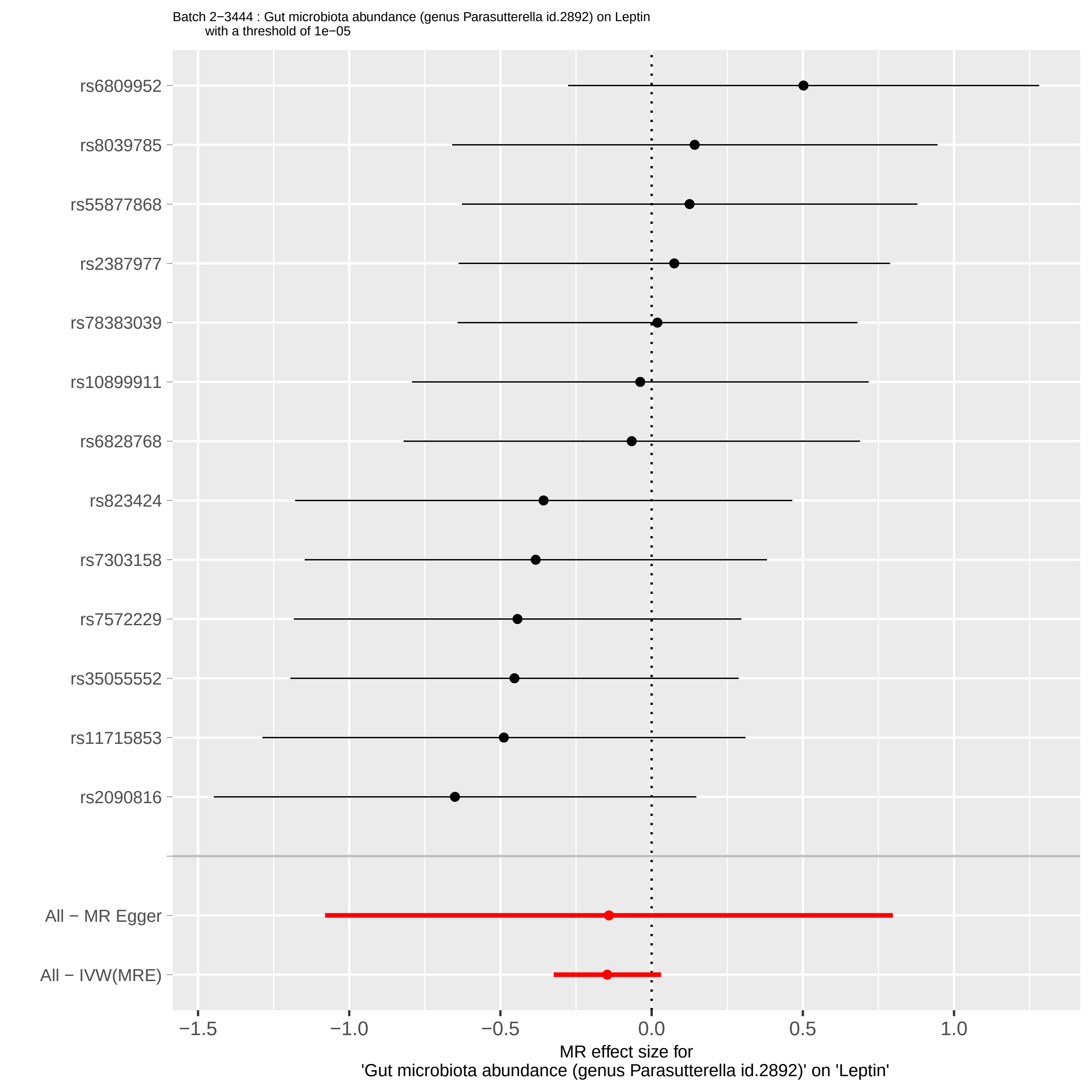
Batch 2−3444 : Gut microbiota abundance (genus Parasutterella id.2892) on Leptin
with a threshold of 1e−05
rs6809952
rs8039785
rs55877868
rs2387977
rs78383039
rs10899911
rs6828768
rs823424
rs7303158
rs7572229
rs35055552
rs11715853
rs2090816
All − MR Egger
All − IVW(MRE)
−1.5
−1.0
−0.5
0.0
0.5
1.0
MR effect size for
'Gut microbiota abundance (genus Parasutterella id.2892)' on 'Leptin'

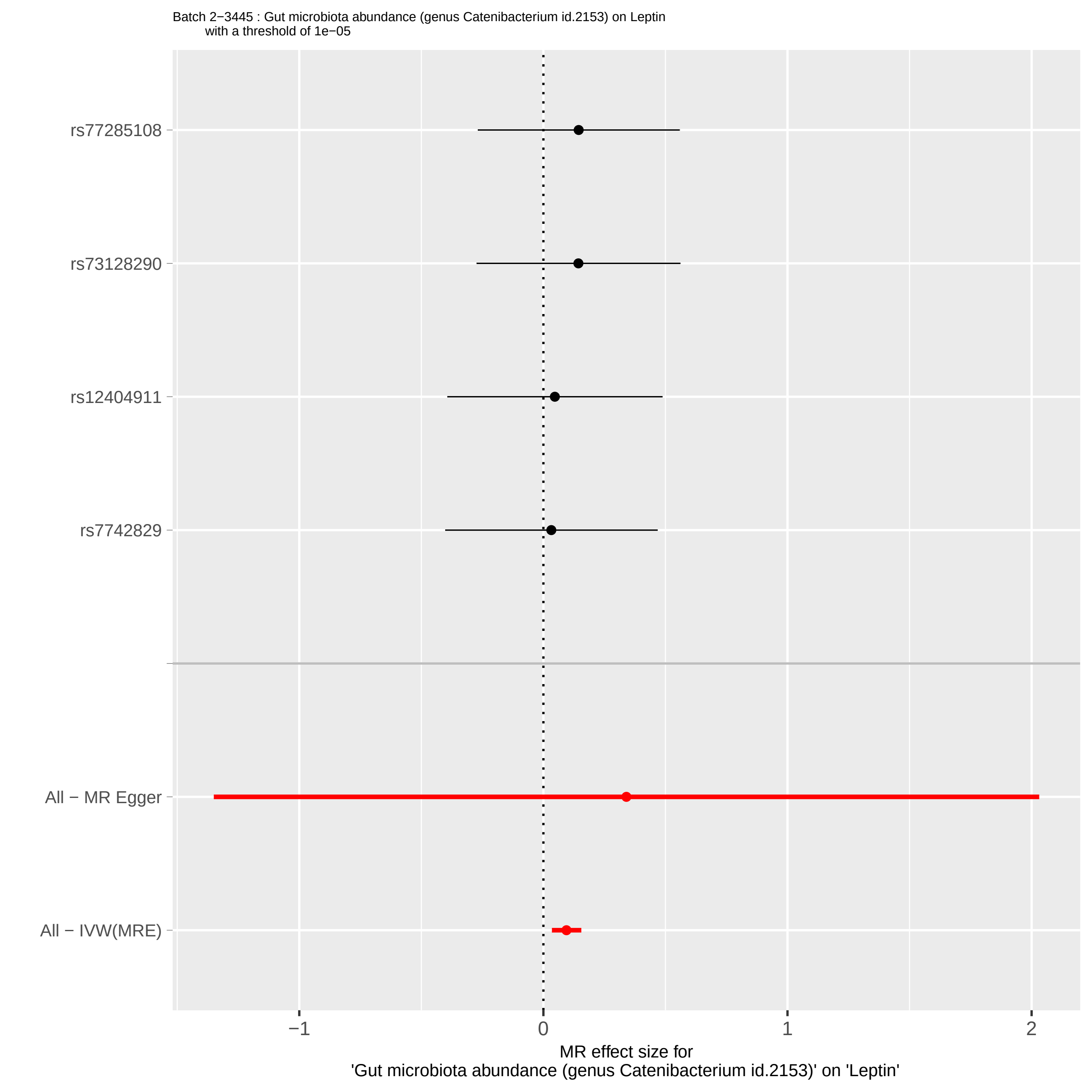
Batch 2−3445 : Gut microbiota abundance (genus Catenibacterium id.2153) on Leptin
with a threshold of 1e−05
rs77285108
rs73128290
rs12404911
rs7742829
All − MR Egger
All − IVW(MRE)
−1
0
1
2
MR effect size for
'Gut microbiota abundance (genus Catenibacterium id.2153)' on 'Leptin'

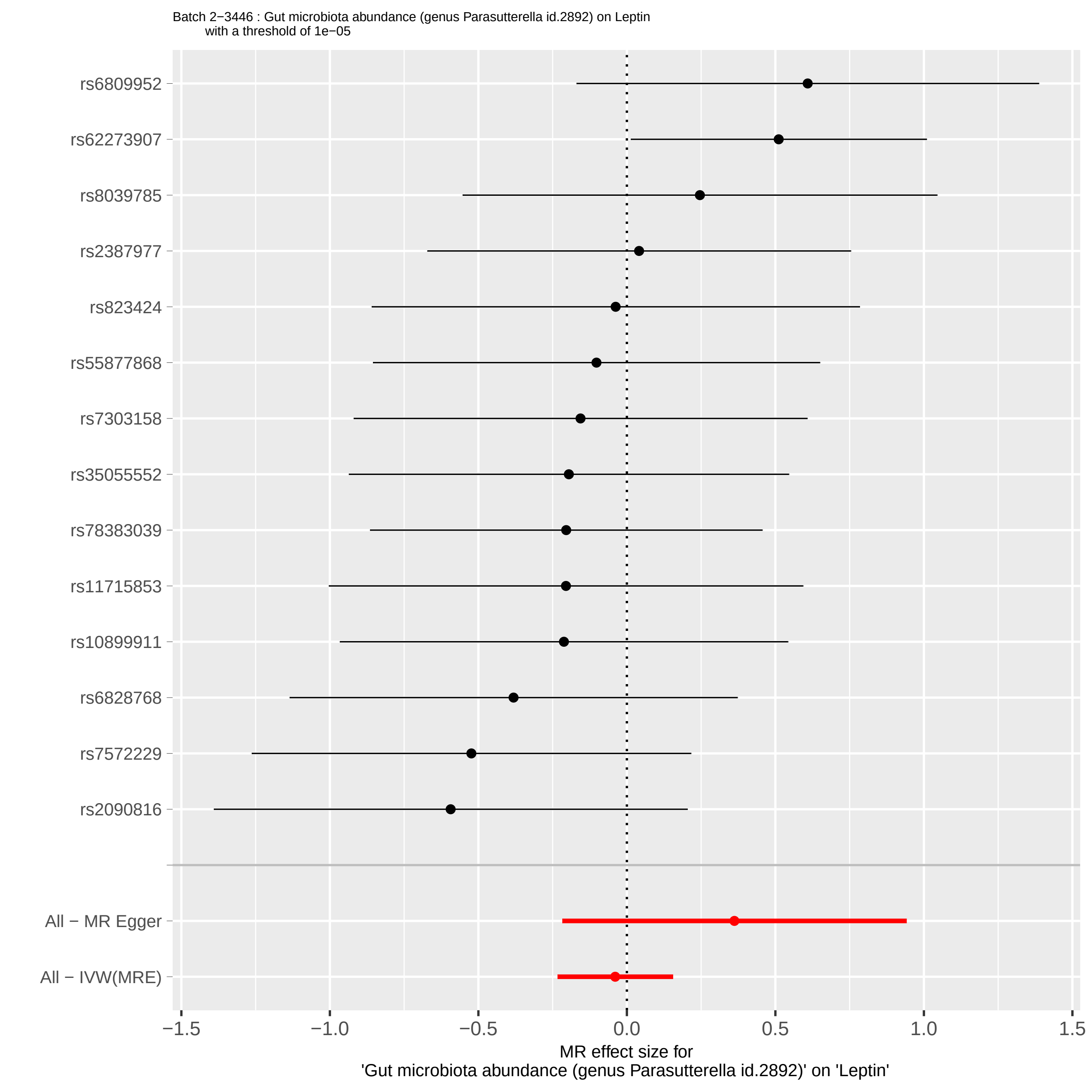
Batch 2−3446 : Gut microbiota abundance (genus Parasutterella id.2892) on Leptin
with a threshold of 1e−05
rs6809952
rs62273907
rs8039785
rs2387977
rs823424
rs55877868
rs7303158
rs35055552
rs78383039
rs11715853
rs10899911
rs6828768
rs7572229
rs2090816
All − MR Egger
All − IVW(MRE)
−1.5
−1.0
−0.5
0.0
0.5
1.0
1.5
MR effect size for
'Gut microbiota abundance (genus Parasutterella id.2892)' on 'Leptin'

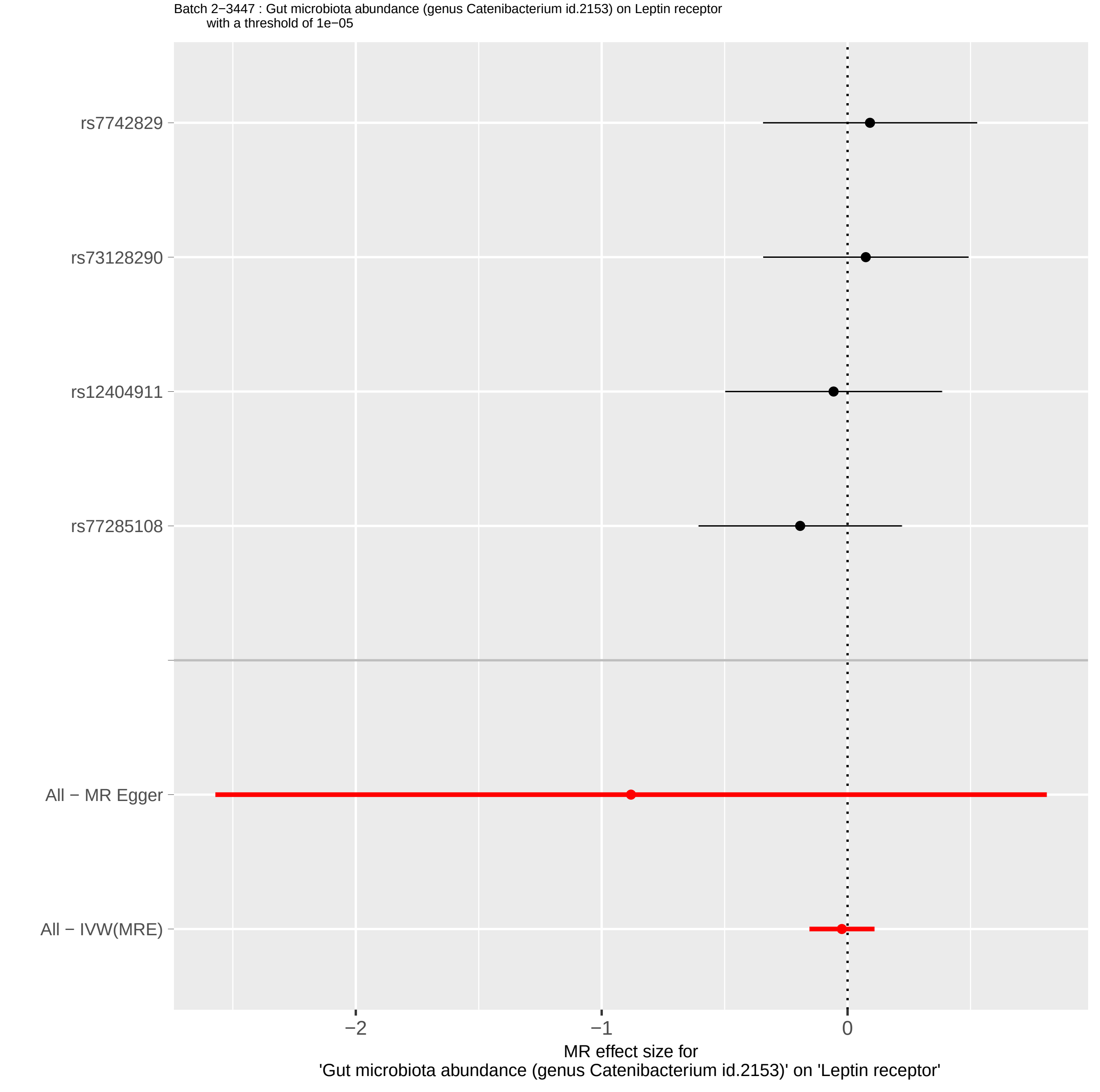

Batch 2−3447 : Gut microbiota abundance (genus Catenibacterium id.2153) on Leptin receptor
with a threshold of 1e−05
rs7742829
rs73128290
rs12404911
rs77285108
All − MR Egger
All − IVW(MRE)
−2
−1
0
MR effect size for
'Gut microbiota abundance (genus Catenibacterium id.2153)' on 'Leptin receptor'

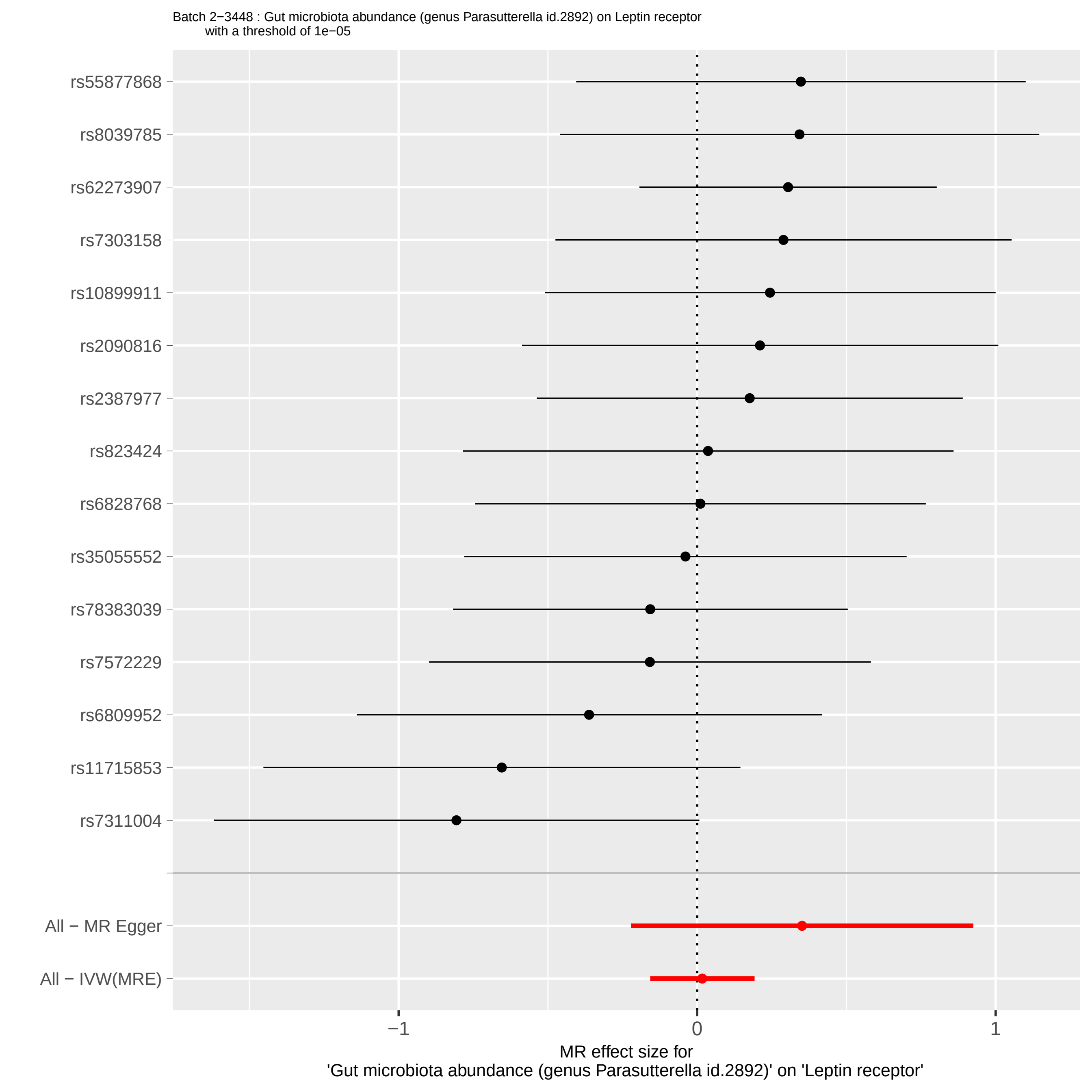

Batch 2−3448 : Gut microbiota abundance (genus Parasutterella id.2892) on Leptin receptor
with a threshold of 1e−05
rs55877868
rs8039785
rs62273907
rs7303158
rs10899911
rs2090816
rs2387977
rs823424
rs6828768
rs35055552
rs78383039
rs7572229
rs6809952
rs11715853
rs7311004
All − MR Egger
All − IVW(MRE)
−1
0
1
MR effect size for
'Gut microbiota abundance (genus Parasutterella id.2892)' on 'Leptin receptor'

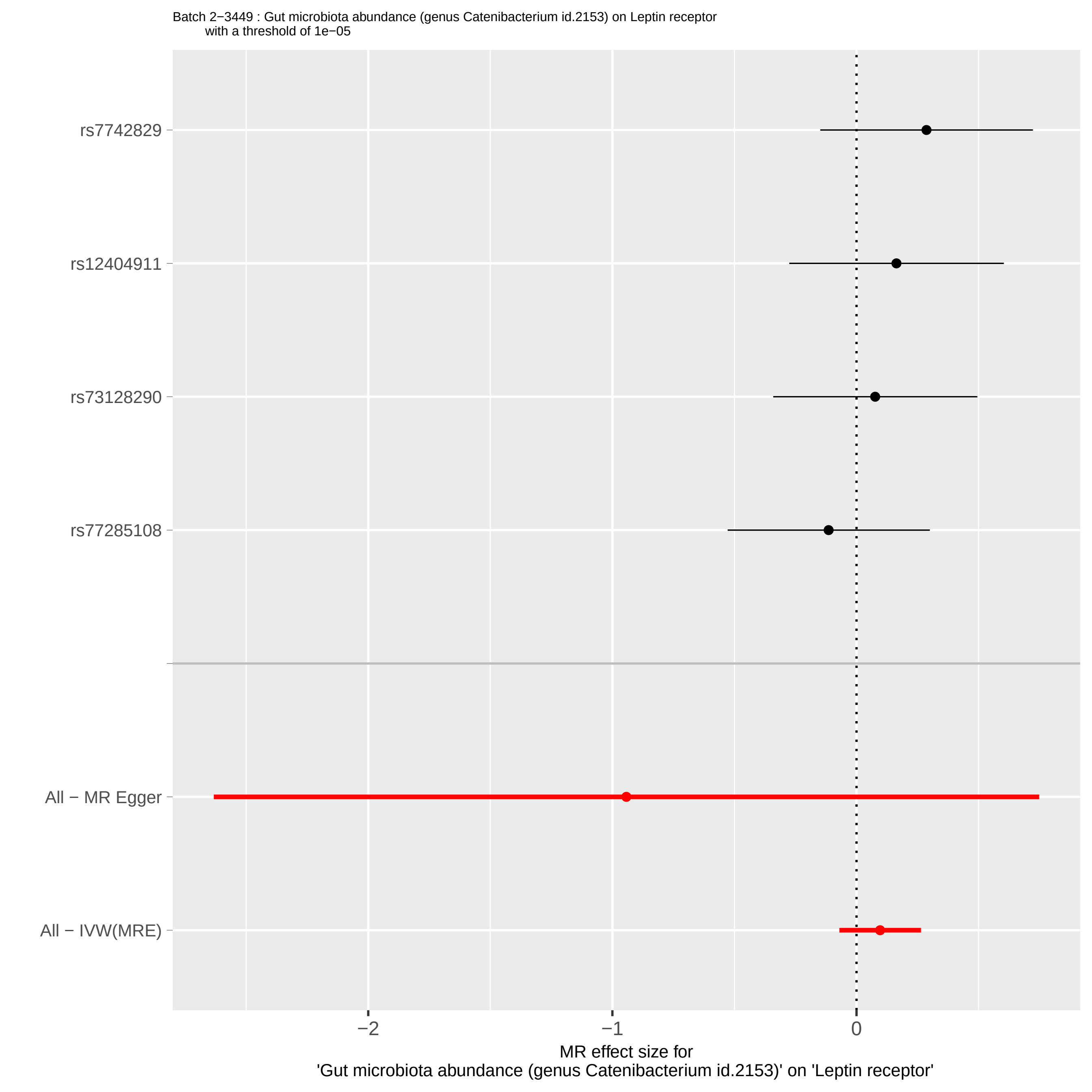
Batch 2−3449 : Gut microbiota abundance (genus Catenibacterium id.2153) on Leptin receptor
with a threshold of 1e−05
rs7742829
rs12404911
rs73128290
rs77285108
All − MR Egger
All − IVW(MRE)
−2
−1
0
MR effect size for
'Gut microbiota abundance (genus Catenibacterium id.2153)' on 'Leptin receptor'

Batch 2−3450 : Gut microbiota abundance (genus Parasutterella id.2892) on Leptin receptor
with a threshold of 1e−05

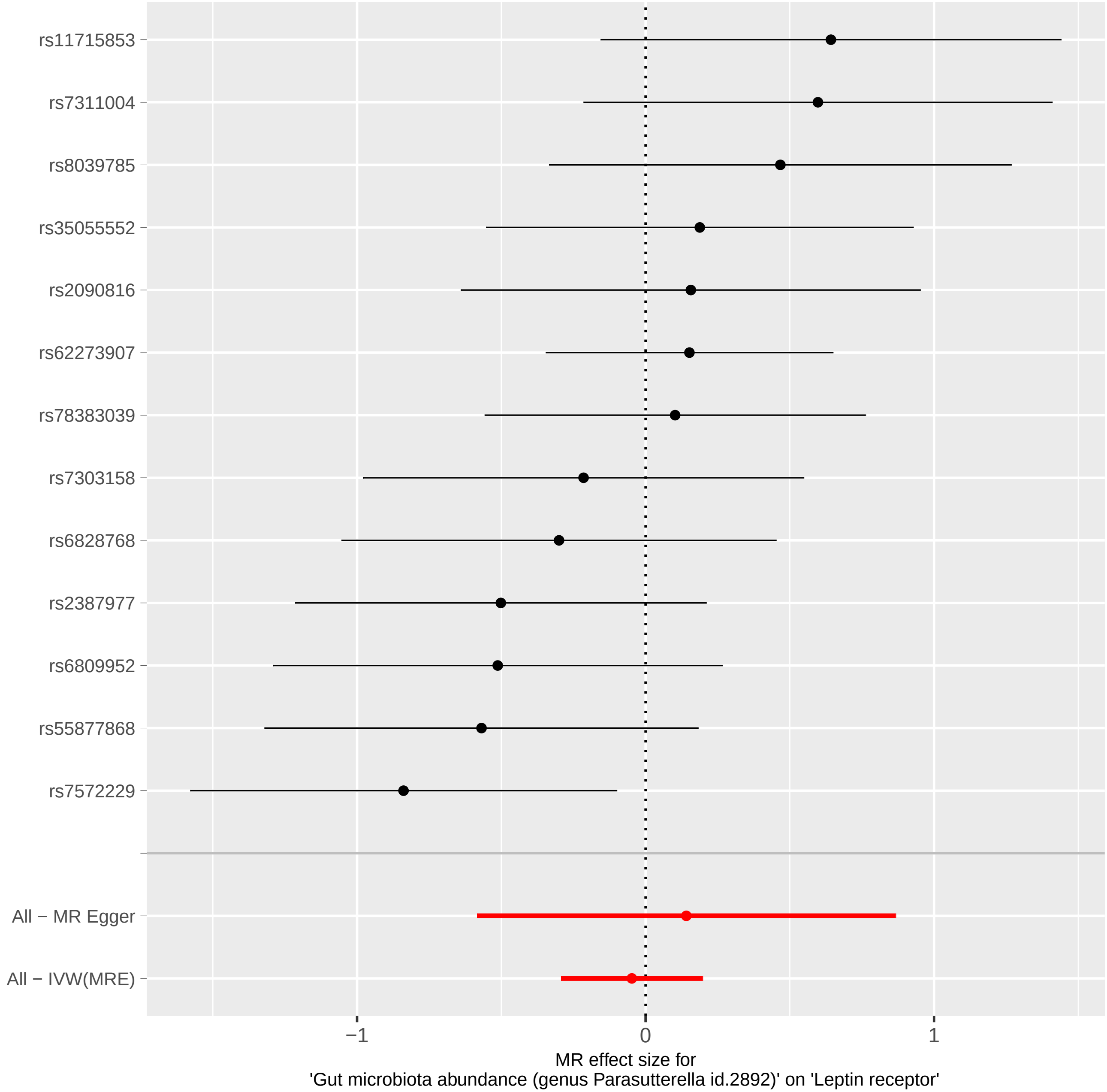

Batch 2−3451 : Gut microbiota abundance (genus Catenibacterium id.2153) on Galectin−1
with a threshold of 1e−05

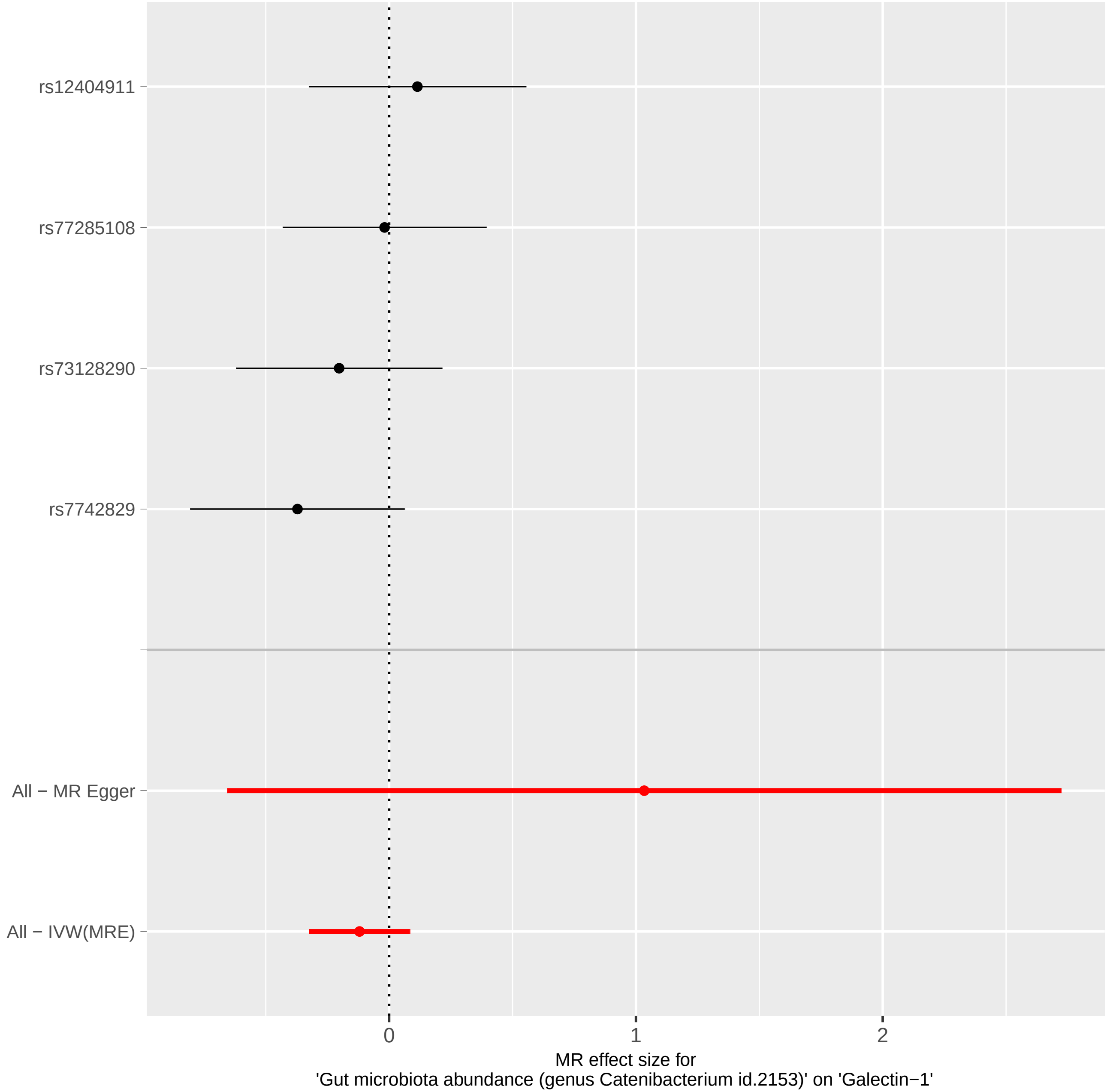

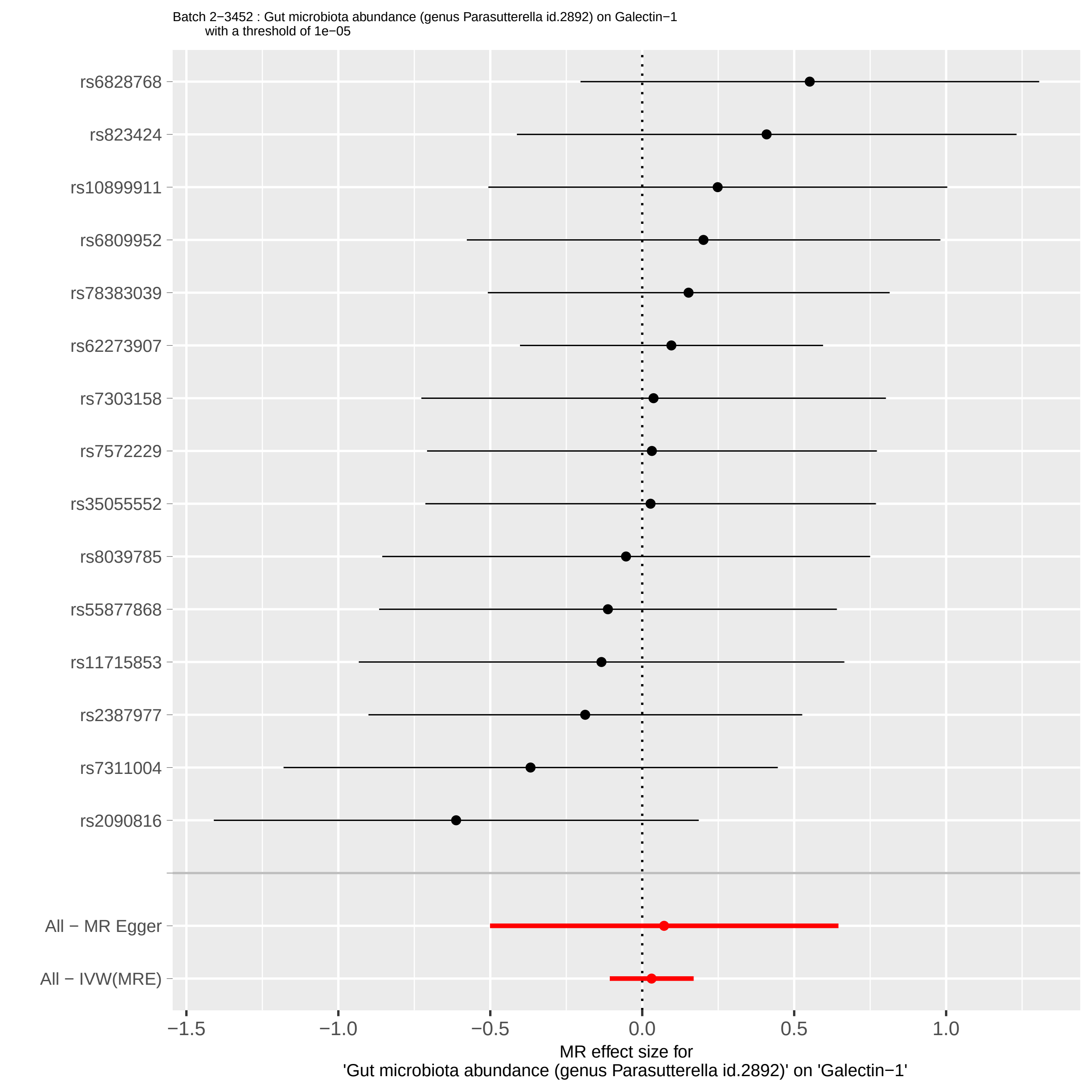

Batch 2−3452 : Gut microbiota abundance (genus Parasutterella id.2892) on Galectin−1
with a threshold of 1e−05
rs6828768
rs823424
rs10899911
rs6809952
rs78383039
rs62273907
rs7303158
rs7572229
rs35055552
rs8039785
rs55877868
rs11715853
rs2387977
rs7311004
rs2090816
All − MR Egger
All − IVW(MRE)
−1.5
−1.0
−0.5
0.0
0.5
1.0
MR effect size for
'Gut microbiota abundance (genus Parasutterella id.2892)' on 'Galectin−1'

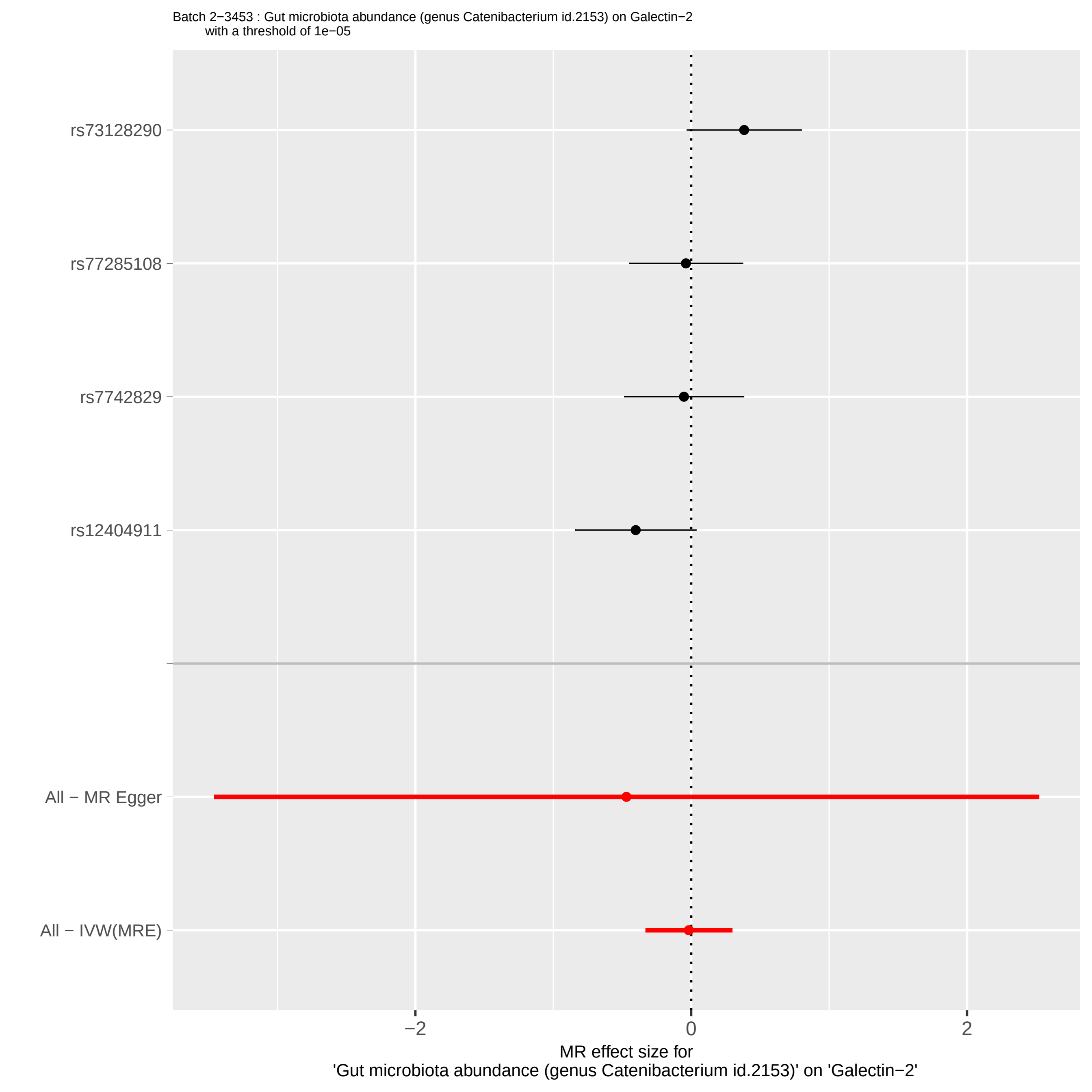
Batch 2−3453 : Gut microbiota abundance (genus Catenibacterium id.2153) on Galectin−2
with a threshold of 1e−05
rs73128290
rs77285108
rs7742829
rs12404911
All − MR Egger
All − IVW(MRE)
−2
0
2
MR effect size for
'Gut microbiota abundance (genus Catenibacterium id.2153)' on 'Galectin−2'

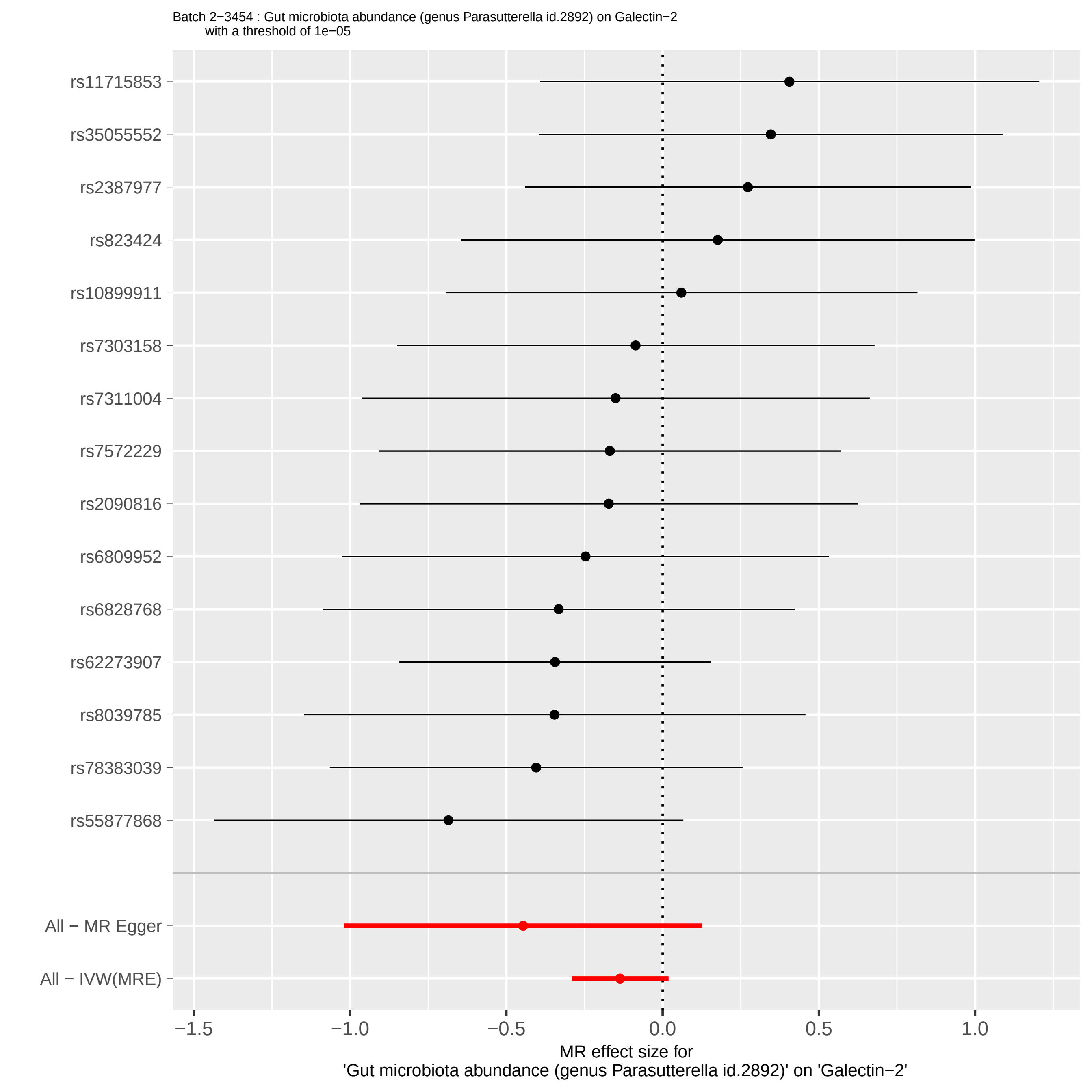

Batch 2−3454 : Gut microbiota abundance (genus Parasutterella id.2892) on Galectin−2
with a threshold of 1e−05
rs11715853
rs35055552
rs2387977
rs823424
rs10899911
rs7303158
rs7311004
rs7572229
rs2090816
rs6809952
rs6828768
rs62273907
rs8039785
rs78383039
rs55877868
All − MR Egger
All − IVW(MRE)
−1.5
−1.0
−0.5
0.0
0.5
1.0
MR effect size for
'Gut microbiota abundance (genus Parasutterella id.2892)' on 'Galectin−2'

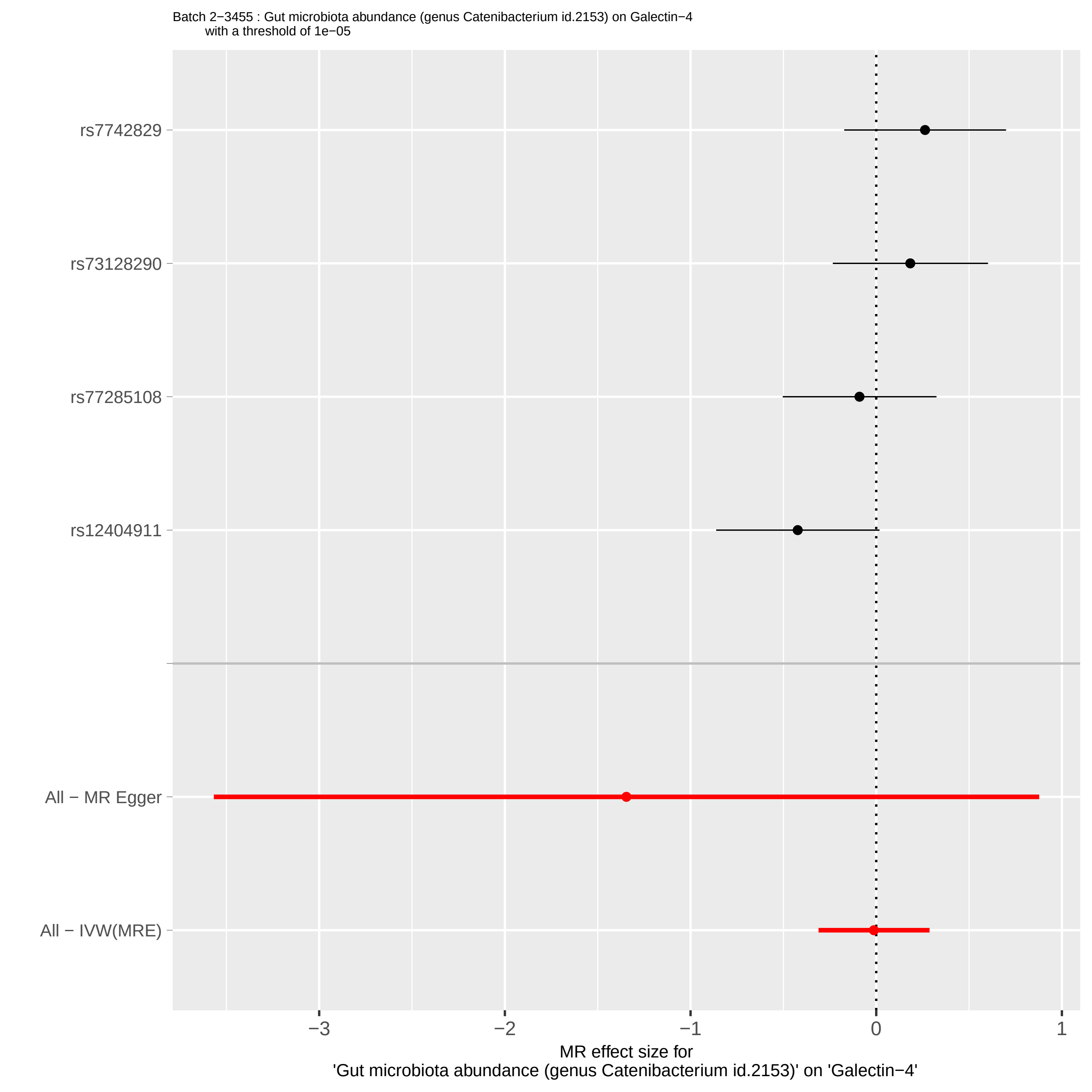

Batch 2−3455 : Gut microbiota abundance (genus Catenibacterium id.2153) on Galectin−4
with a threshold of 1e−05
rs7742829
rs73128290
rs77285108
rs12404911
All − MR Egger
All − IVW(MRE)
−3
−2
−1
0
1
MR effect size for
'Gut microbiota abundance (genus Catenibacterium id.2153)' on 'Galectin−4'

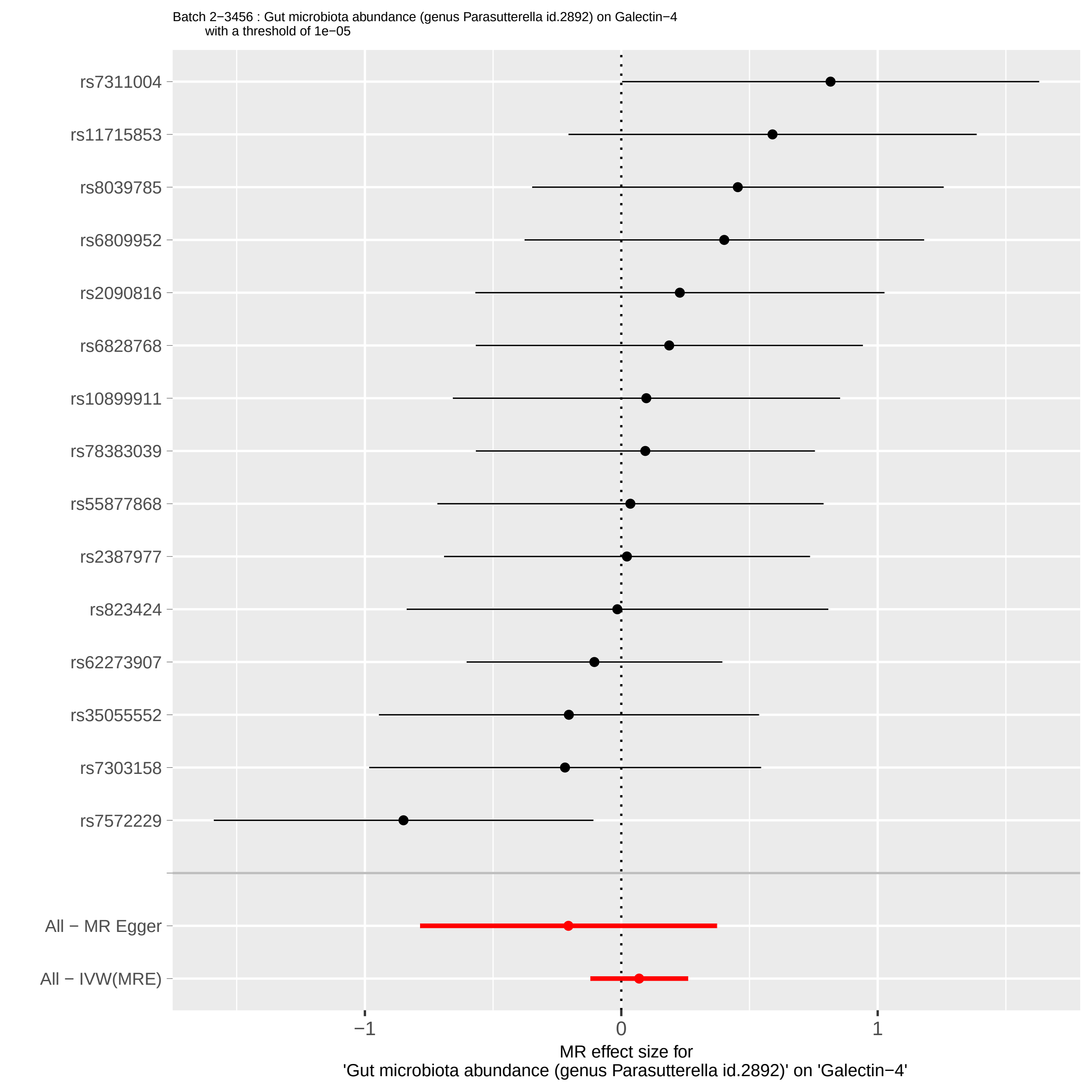

Batch 2−3456 : Gut microbiota abundance (genus Parasutterella id.2892) on Galectin−4
with a threshold of 1e−05
rs7311004
rs11715853
rs8039785
rs6809952
rs2090816
rs6828768
rs10899911
rs78383039
rs55877868
rs2387977
rs823424
rs62273907
rs35055552
rs7303158
rs7572229
All − MR Egger
All − IVW(MRE)
−1
0
1
MR effect size for
'Gut microbiota abundance (genus Parasutterella id.2892)' on 'Galectin−4'

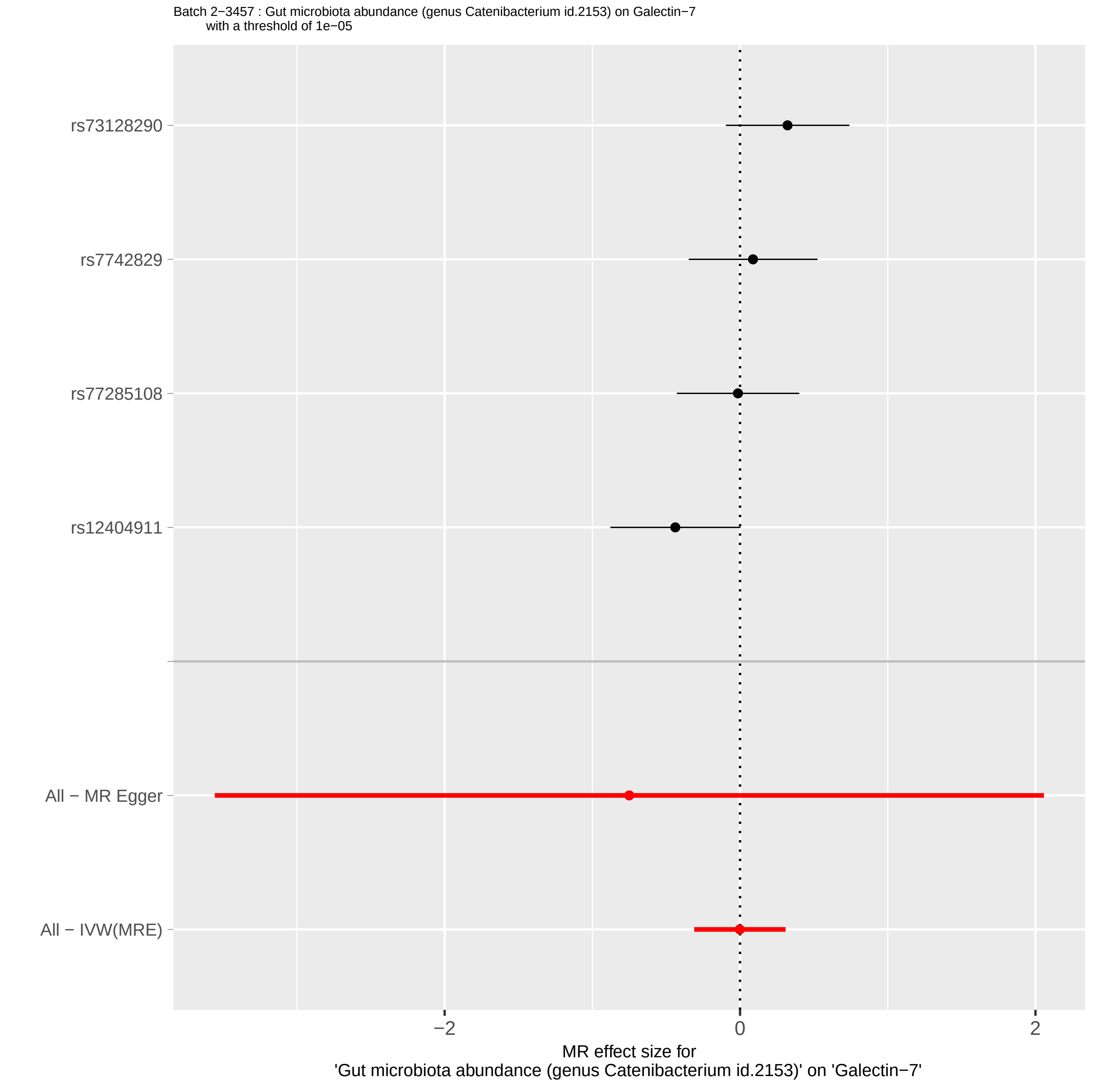
Batch 2−3457 : Gut microbiota abundance (genus Catenibacterium id.2153) on Galectin−7
with a threshold of 1e−05
rs73128290
rs7742829
rs77285108
rs12404911
All − MR Egger
All − IVW(MRE)
−2
0
2
MR effect size for
'Gut microbiota abundance (genus Catenibacterium id.2153)' on 'Galectin−7'

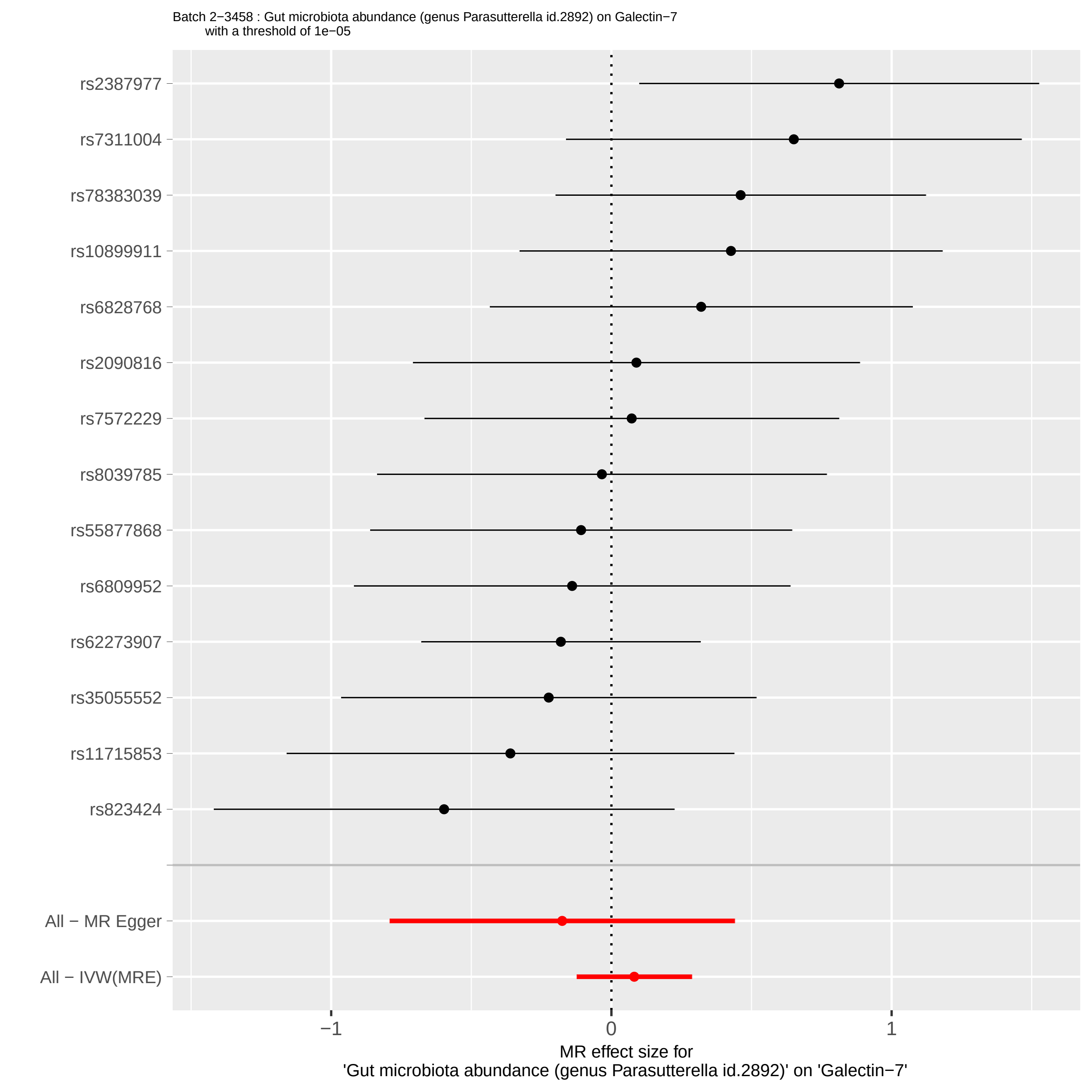

Batch 2−3458 : Gut microbiota abundance (genus Parasutterella id.2892) on Galectin−7
with a threshold of 1e−05
rs2387977
rs7311004
rs78383039
rs10899911
rs6828768
rs2090816
rs7572229
rs8039785
rs55877868
rs6809952
rs62273907
rs35055552
rs11715853
rs823424
All − MR Egger
All − IVW(MRE)
−1
0
1
MR effect size for
'Gut microbiota abundance (genus Parasutterella id.2892)' on 'Galectin−7'

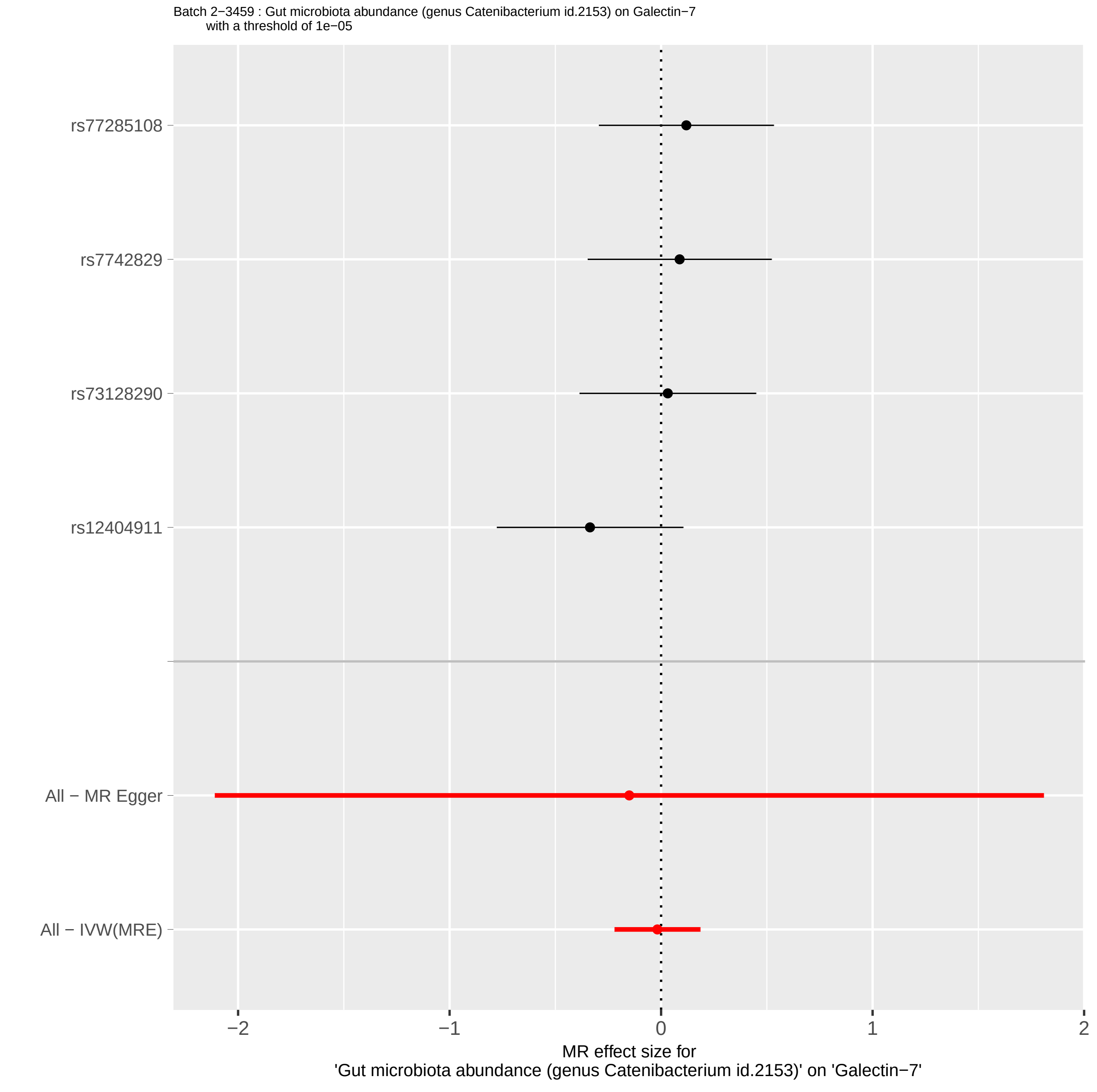
Batch 2−3459 : Gut microbiota abundance (genus Catenibacterium id.2153) on Galectin−7
with a threshold of 1e−05
rs77285108
rs7742829
rs73128290
rs12404911
All − MR Egger
All − IVW(MRE)
−2
−1
0
1
2
MR effect size for
'Gut microbiota abundance (genus Catenibacterium id.2153)' on 'Galectin−7'

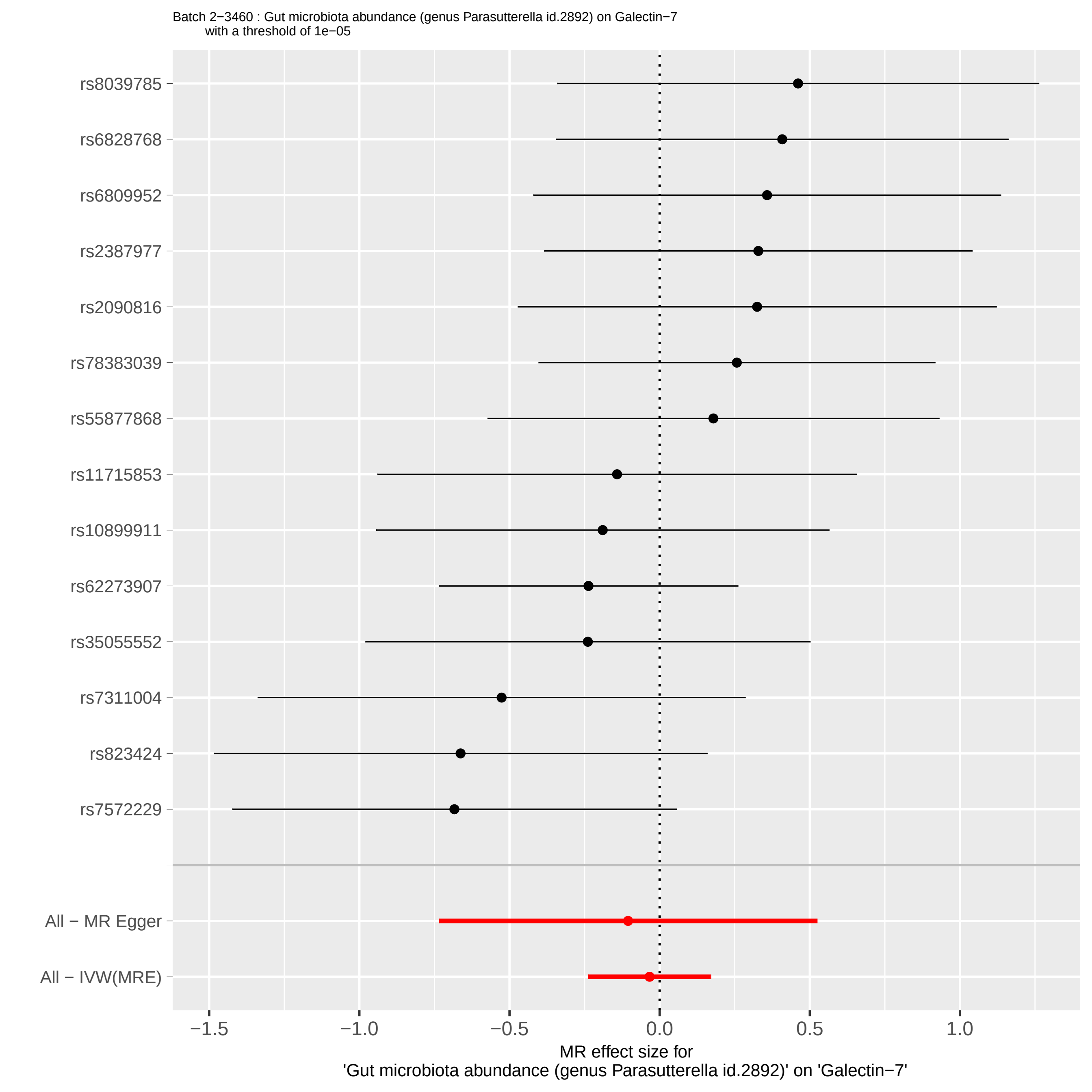
Batch 2−3460 : Gut microbiota abundance (genus Parasutterella id.2892) on Galectin−7
with a threshold of 1e−05
rs8039785
rs6828768
rs6809952
rs2387977
rs2090816
rs78383039
rs55877868
rs11715853
rs10899911
rs62273907
rs35055552
rs7311004
rs823424
rs7572229
All − MR Egger
All − IVW(MRE)
−1.5
−1.0
−0.5
0.0
0.5
1.0
MR effect size for
'Gut microbiota abundance (genus Parasutterella id.2892)' on 'Galectin−7'

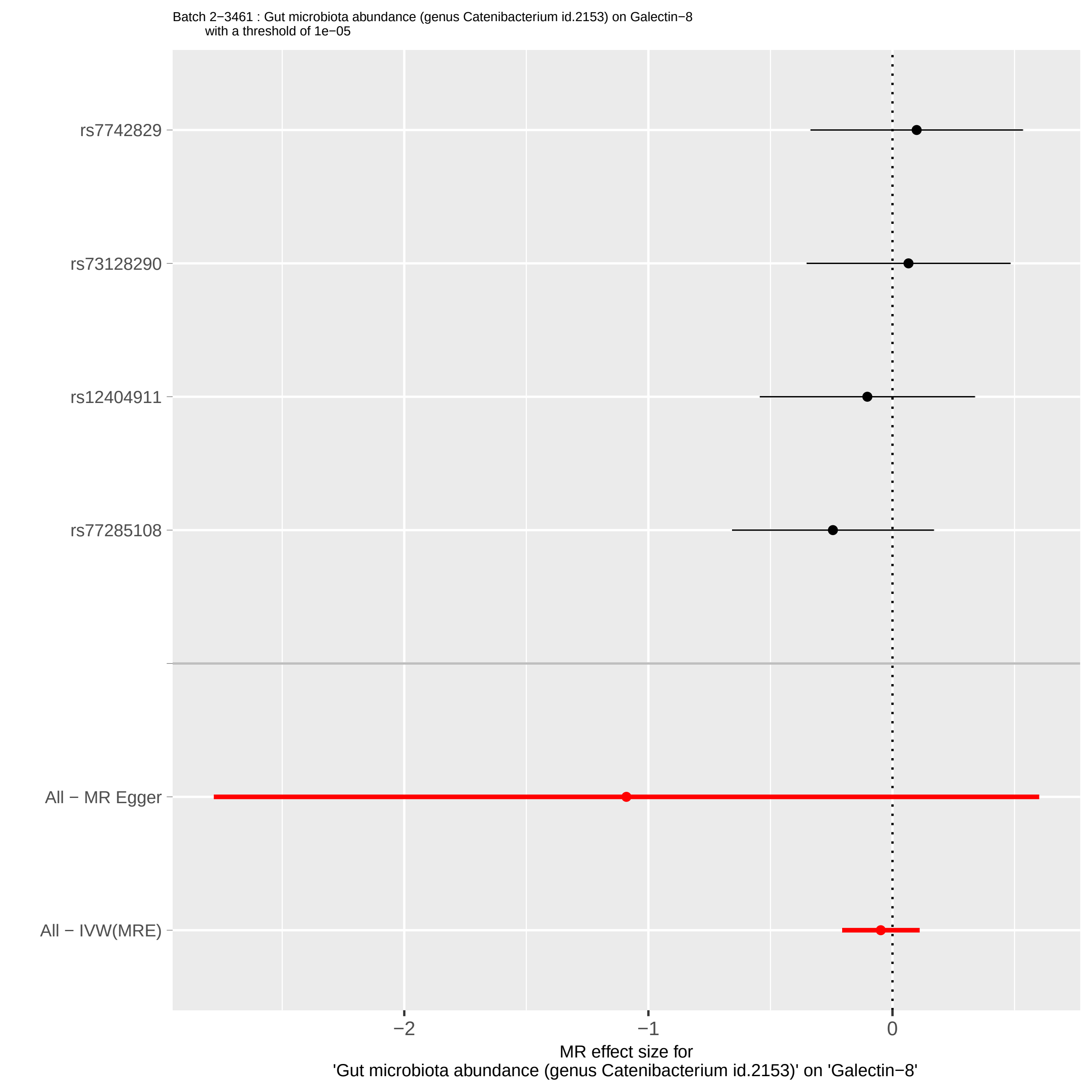
Batch 2−3461 : Gut microbiota abundance (genus Catenibacterium id.2153) on Galectin−8
with a threshold of 1e−05
rs7742829
rs73128290
rs12404911
rs77285108
All − MR Egger
All − IVW(MRE)
−2
−1
0
MR effect size for
'Gut microbiota abundance (genus Catenibacterium id.2153)' on 'Galectin−8'

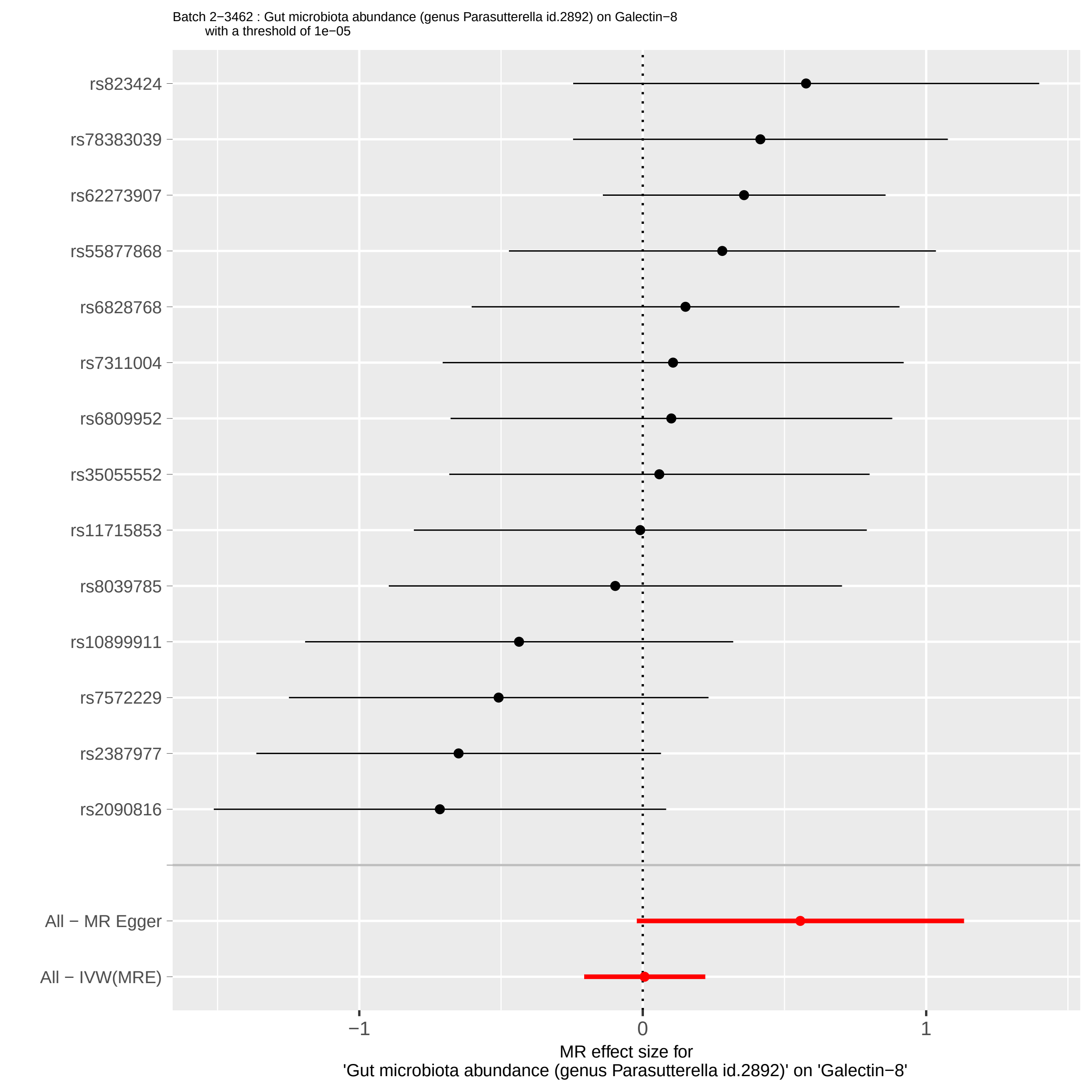

Batch 2−3462 : Gut microbiota abundance (genus Parasutterella id.2892) on Galectin−8
with a threshold of 1e−05
rs823424
rs78383039
rs62273907
rs55877868
rs6828768
rs7311004
rs6809952
rs35055552
rs11715853
rs8039785
rs10899911
rs7572229
rs2387977
rs2090816
All − MR Egger
All − IVW(MRE)
−1
0
1
MR effect size for
'Gut microbiota abundance (genus Parasutterella id.2892)' on 'Galectin−8'

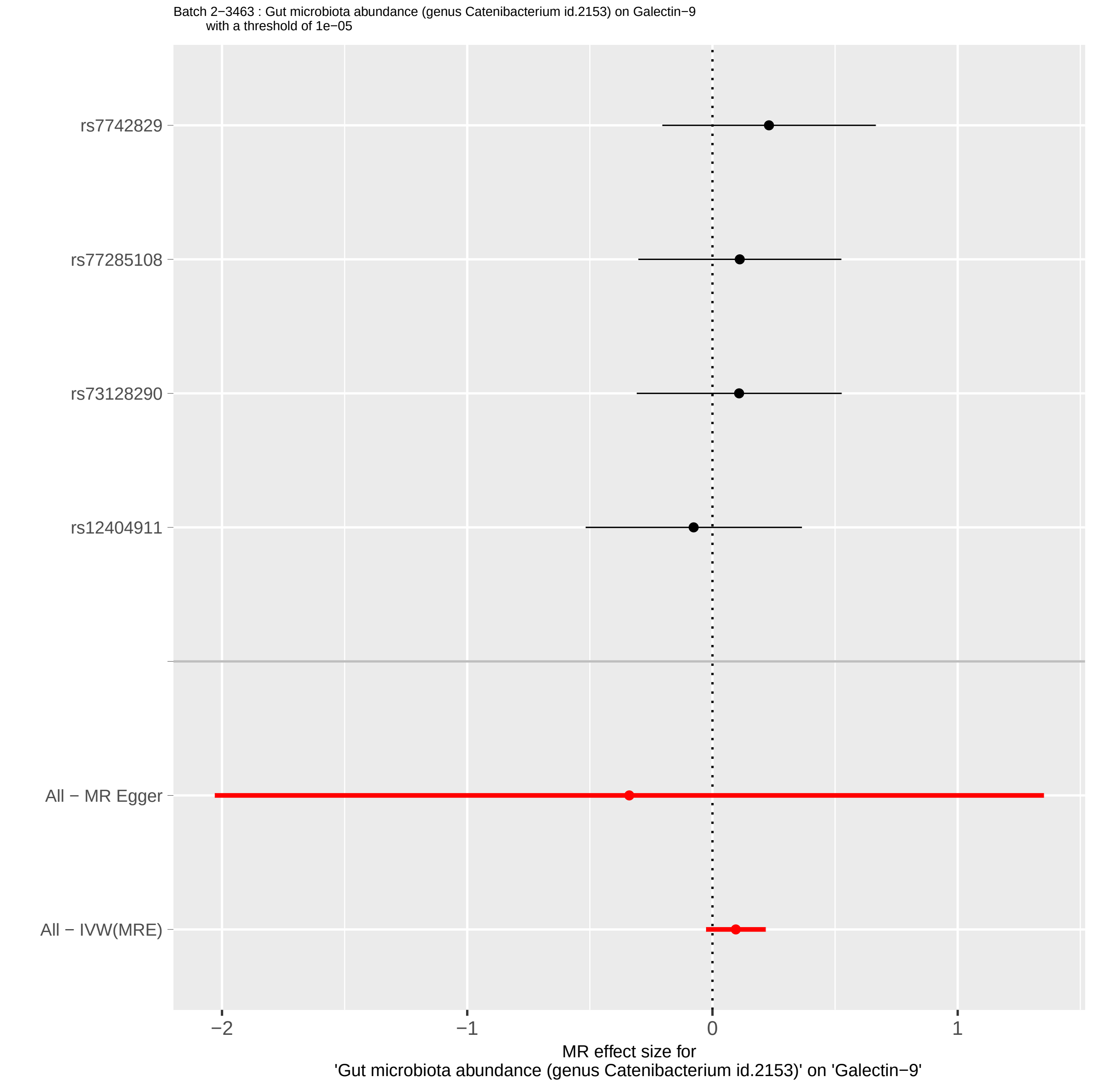
Batch 2−3463 : Gut microbiota abundance (genus Catenibacterium id.2153) on Galectin−9
with a threshold of 1e−05
rs7742829
rs77285108
rs73128290
rs12404911
All − MR Egger
All − IVW(MRE)
−2
−1
0
1
MR effect size for
'Gut microbiota abundance (genus Catenibacterium id.2153)' on 'Galectin−9'

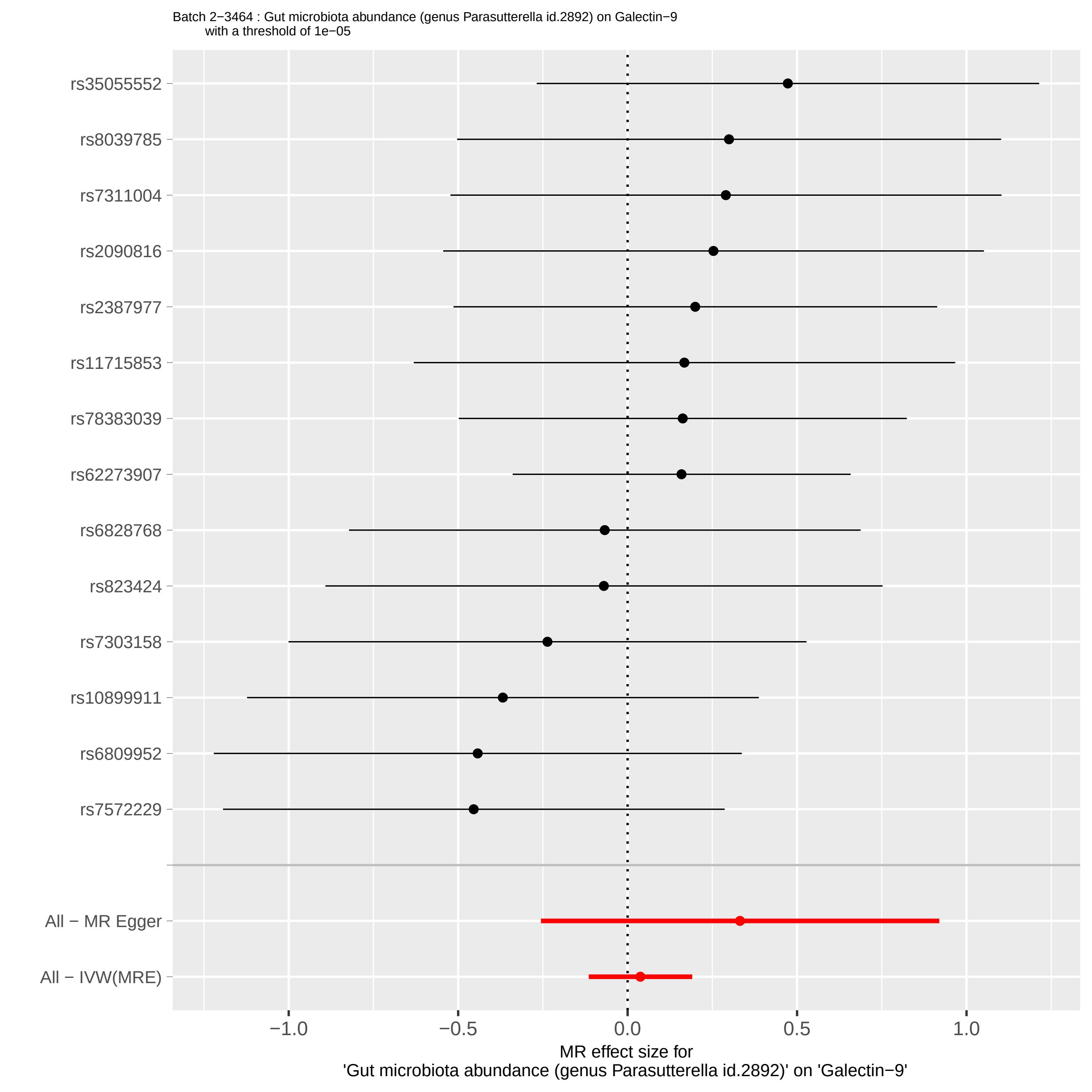

Batch 2−3464 : Gut microbiota abundance (genus Parasutterella id.2892) on Galectin−9
with a threshold of 1e−05
rs35055552
rs8039785
rs7311004
rs2090816
rs2387977
rs11715853
rs78383039
rs62273907
rs6828768
rs823424
rs7303158
rs10899911
rs6809952
rs7572229
All − MR Egger
All − IVW(MRE)
−1.0
−0.5
0.0
0.5
1.0
MR effect size for
'Gut microbiota abundance (genus Parasutterella id.2892)' on 'Galectin−9'

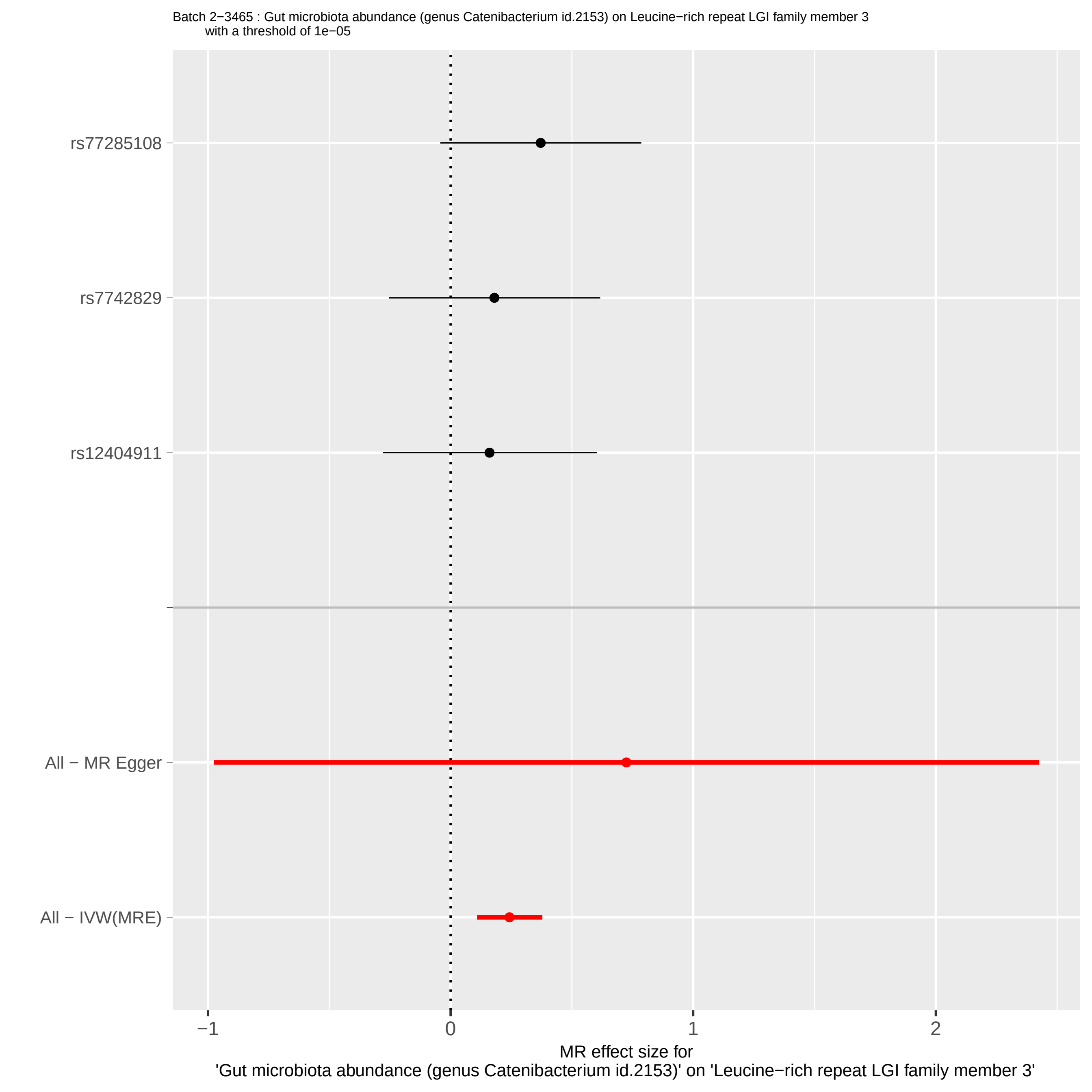

Batch 2−3465 : Gut microbiota abundance (genus Catenibacterium id.2153) on Leucine−rich repeat LGI family member 3
with a threshold of 1e−05
rs77285108
rs7742829
rs12404911
All − MR Egger
All − IVW(MRE)
−1
0
1
2
MR effect size for
'Gut microbiota abundance (genus Catenibacterium id.2153)' on 'Leucine−rich repeat LGI family member 3'

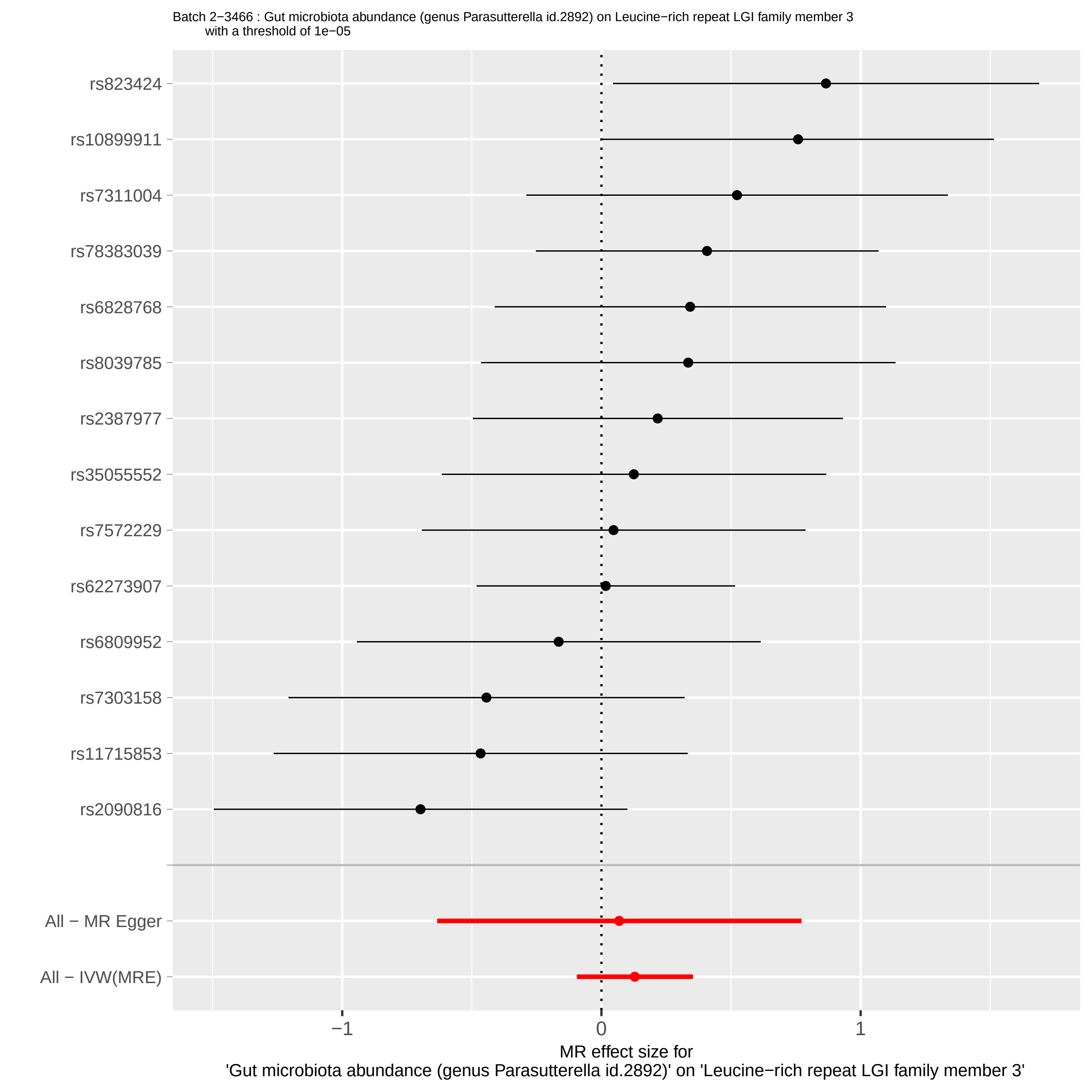
Batch 2−3466 : Gut microbiota abundance (genus Parasutterella id.2892) on Leucine−rich repeat LGI family member 3
with a threshold of 1e−05
rs823424
rs10899911
rs7311004
rs78383039
rs6828768
rs8039785
rs2387977
rs35055552
rs7572229
rs62273907
rs6809952
rs7303158
rs11715853
rs2090816
All − MR Egger
All − IVW(MRE)
−1
0
1
MR effect size for
'Gut microbiota abundance (genus Parasutterella id.2892)' on 'Leucine−rich repeat LGI family member 3'

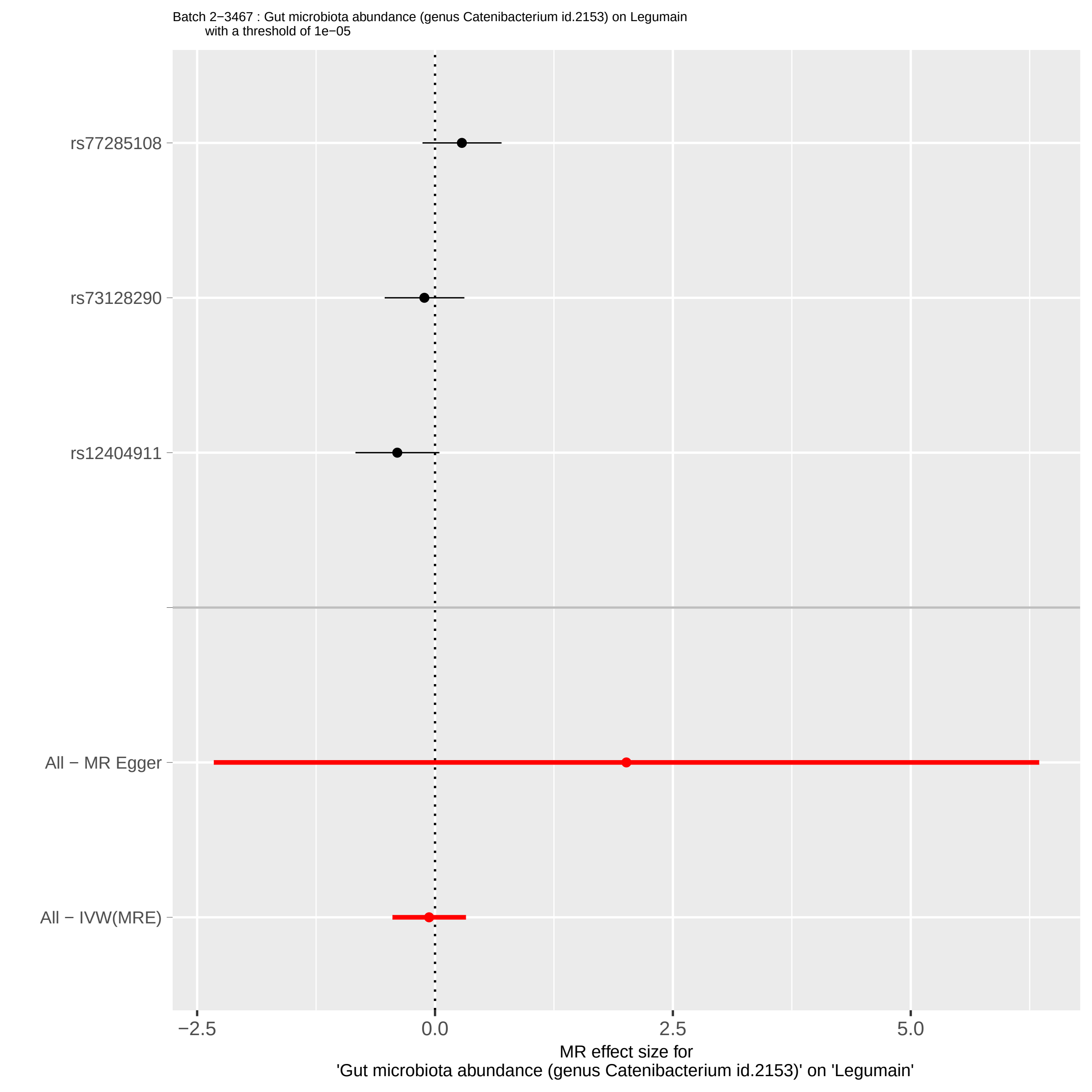
Batch 2−3467 : Gut microbiota abundance (genus Catenibacterium id.2153) on Legumain
with a threshold of 1e−05
rs77285108
rs73128290
rs12404911
All − MR Egger
All − IVW(MRE)
−2.5
0.0
2.5
5.0
MR effect size for
'Gut microbiota abundance (genus Catenibacterium id.2153)' on 'Legumain'

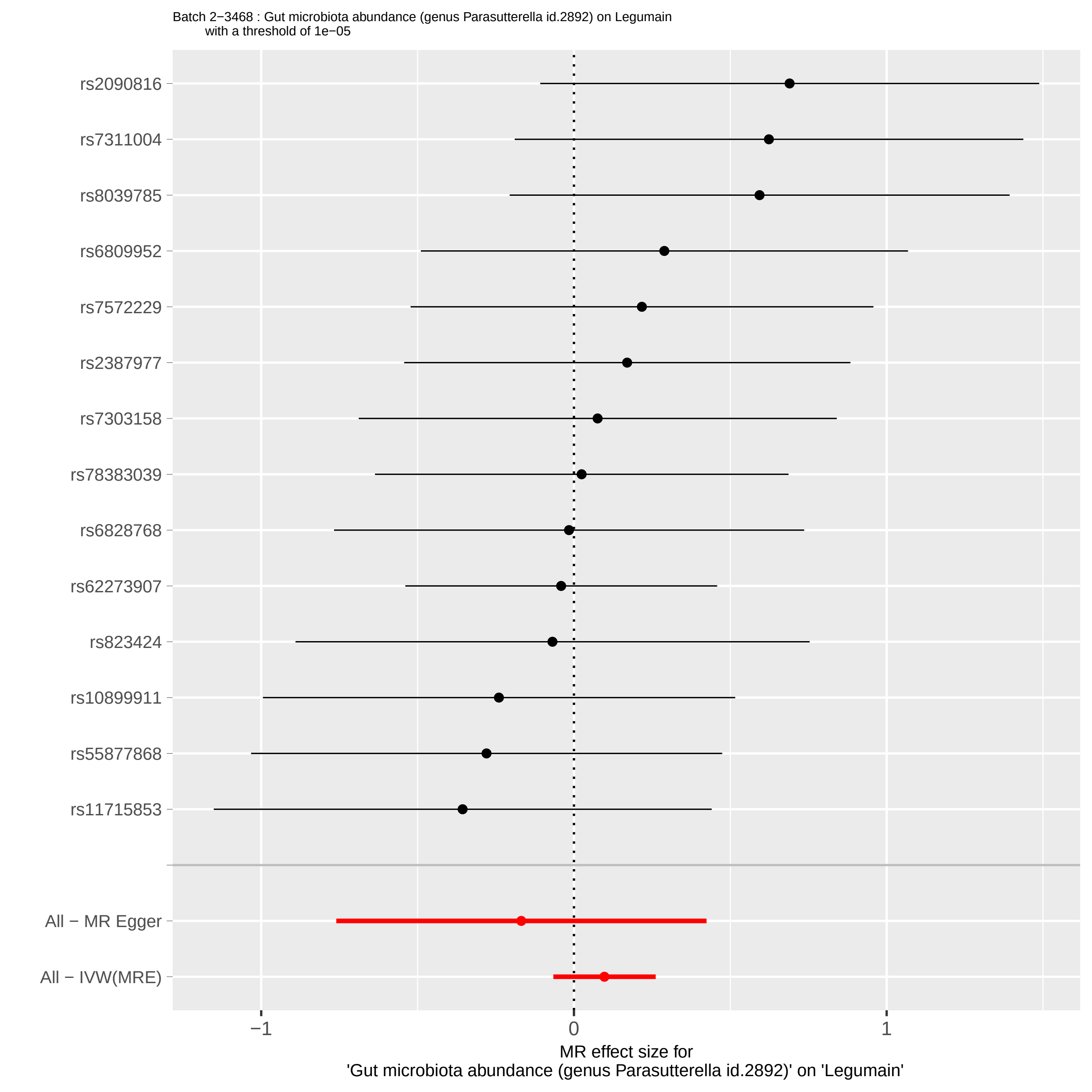

Batch 2−3468 : Gut microbiota abundance (genus Parasutterella id.2892) on Legumain
with a threshold of 1e−05
rs2090816
rs7311004
rs8039785
rs6809952
rs7572229
rs2387977
rs7303158
rs78383039
rs6828768
rs62273907
rs823424
rs10899911
rs55877868
rs11715853
All − MR Egger
All − IVW(MRE)
−1
0
1
MR effect size for
'Gut microbiota abundance (genus Parasutterella id.2892)' on 'Legumain'

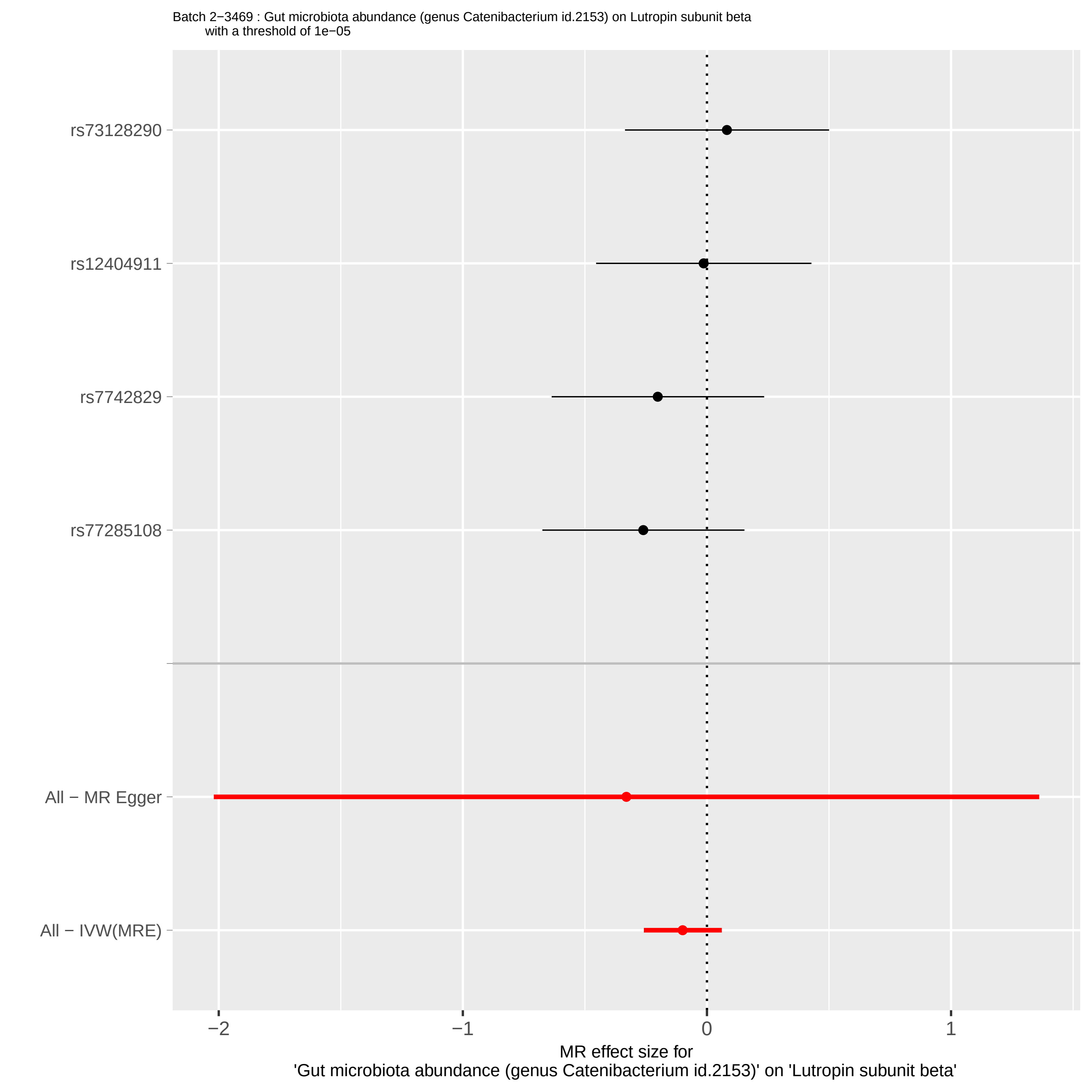
Batch 2−3469 : Gut microbiota abundance (genus Catenibacterium id.2153) on Lutropin subunit beta
with a threshold of 1e−05
rs73128290
rs12404911
rs7742829
rs77285108
All − MR Egger
All − IVW(MRE)
−2
−1
0
1
MR effect size for
'Gut microbiota abundance (genus Catenibacterium id.2153)' on 'Lutropin subunit beta'

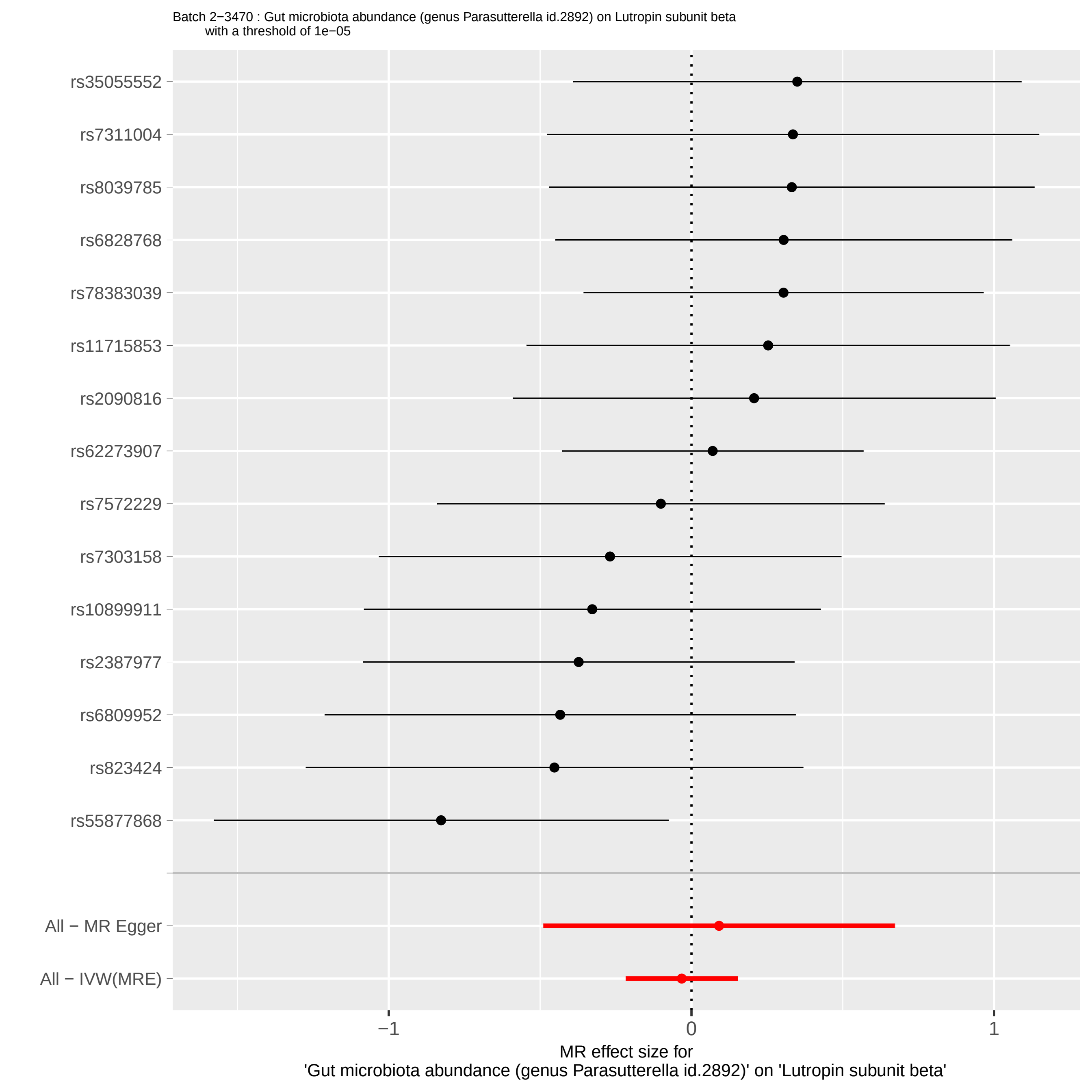

Batch 2−3470 : Gut microbiota abundance (genus Parasutterella id.2892) on Lutropin subunit beta
with a threshold of 1e−05
rs35055552
rs7311004
rs8039785
rs6828768
rs78383039
rs11715853
rs2090816
rs62273907
rs7572229
rs7303158
rs10899911
rs2387977
rs6809952
rs823424
rs55877868
All − MR Egger
All − IVW(MRE)
−1
0
1
MR effect size for
'Gut microbiota abundance (genus Parasutterella id.2892)' on 'Lutropin subunit beta'

Batch 2−3471 : Gut microbiota abundance (genus Catenibacterium id.2153) on Leukemia inhibitory factor
with a threshold of 1e−05

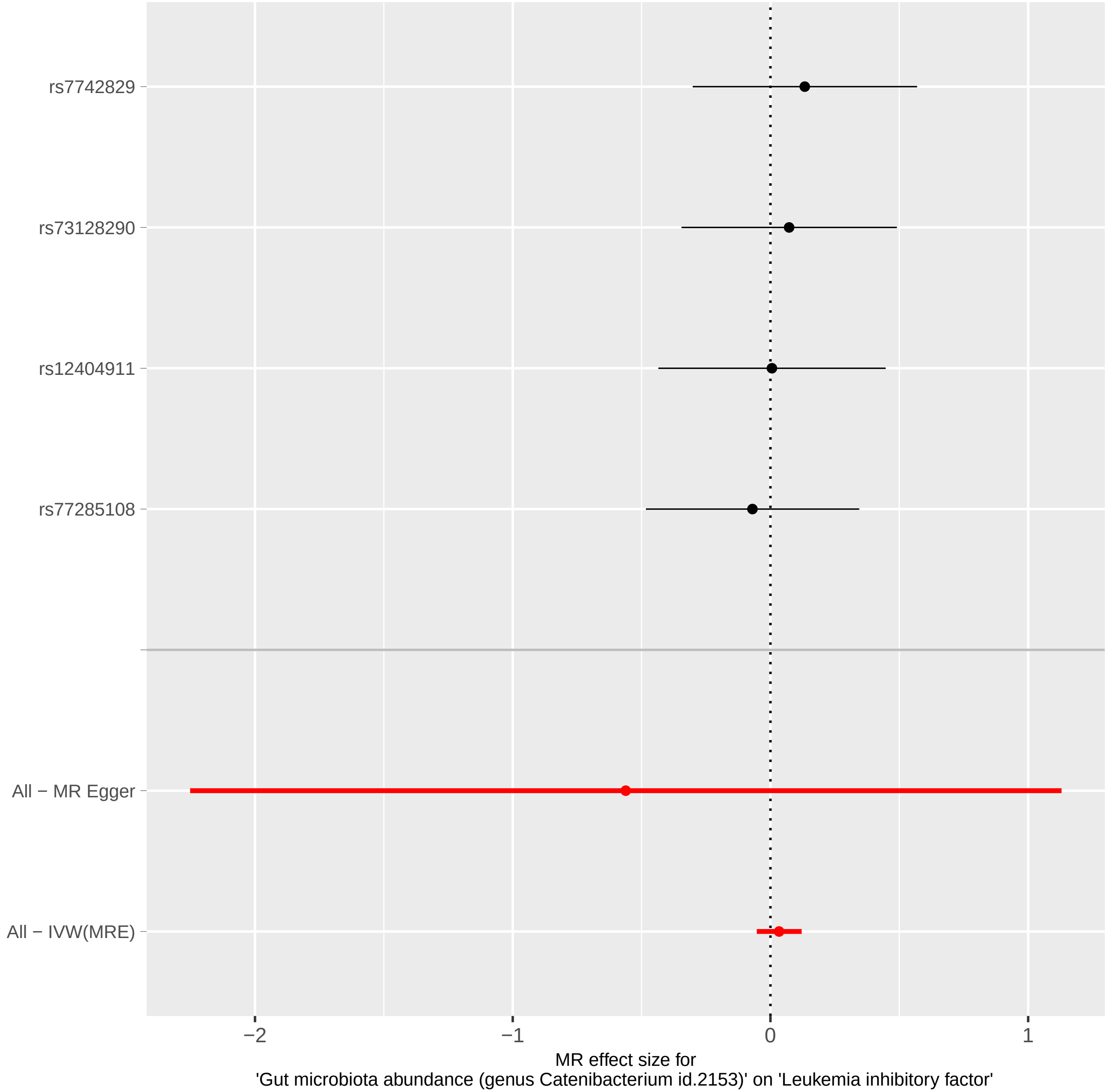

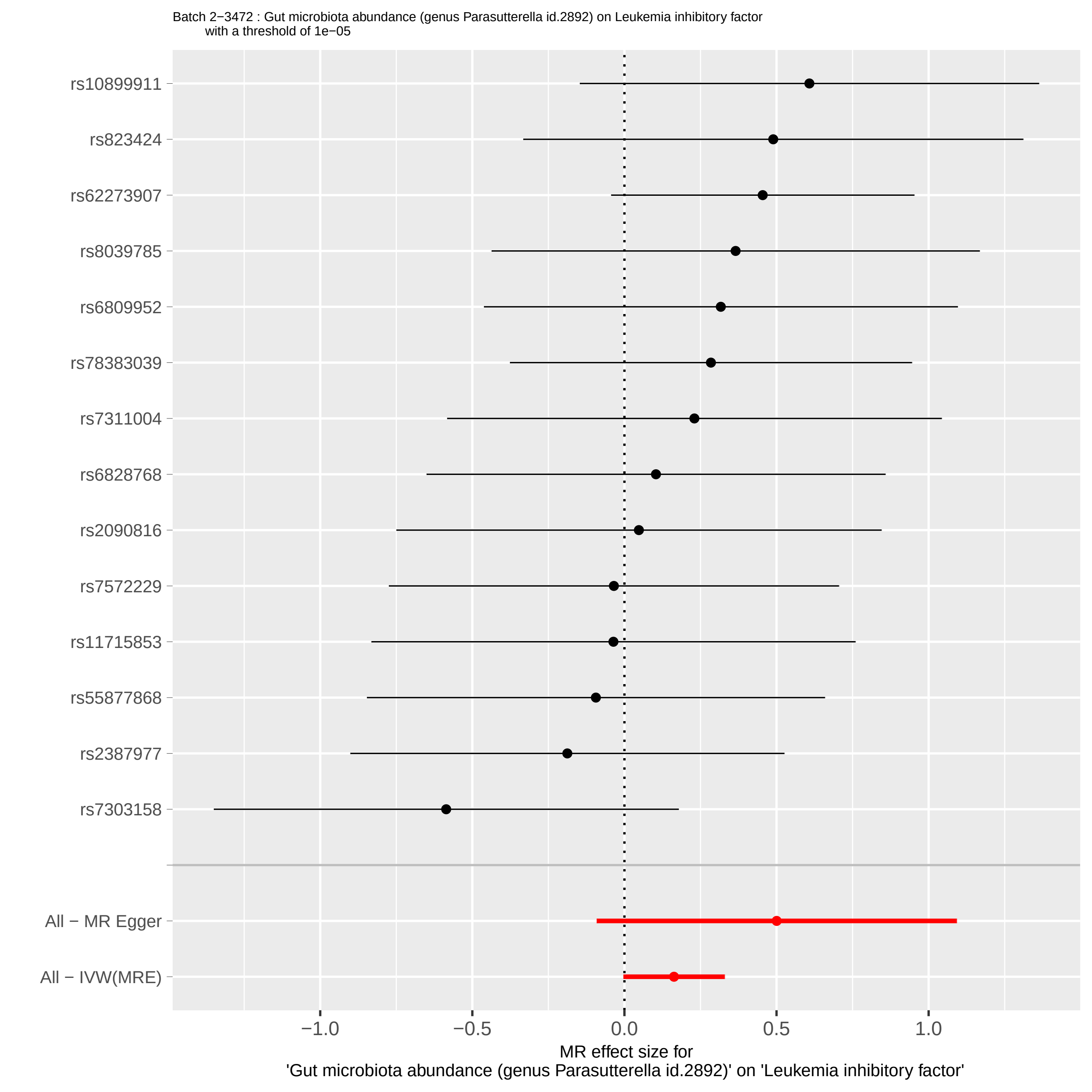

Batch 2−3472 : Gut microbiota abundance (genus Parasutterella id.2892) on Leukemia inhibitory factor
with a threshold of 1e−05
rs10899911
rs823424
rs62273907
rs8039785
rs6809952
rs78383039
rs7311004
rs6828768
rs2090816
rs7572229
rs11715853
rs55877868
rs2387977
rs7303158
All − MR Egger
All − IVW(MRE)
−1.0
−0.5
0.0
0.5
1.0
MR effect size for
'Gut microbiota abundance (genus Parasutterella id.2892)' on 'Leukemia inhibitory factor'

Batch 2−3473 : Gut microbiota abundance (genus Catenibacterium id.2153) on Leukemia inhibitory factor receptor with a threshold of 1e−05

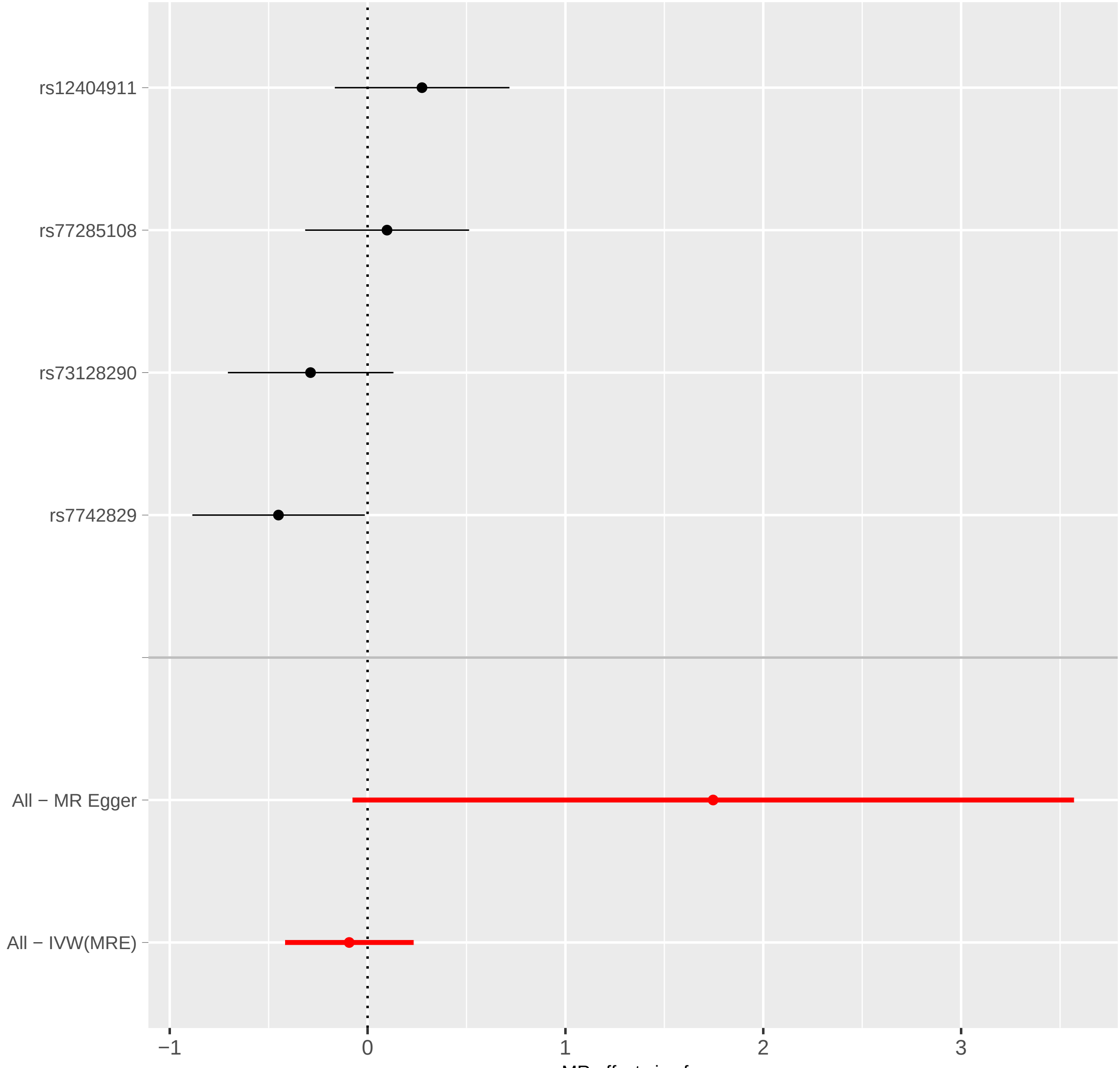

MR effect size for
'Gut microbiota abundance (genus Catenibacterium id.2153)' on 'Leukemia inhibitory factor receptor'

Batch 2−3474 : Gut microbiota abundance (genus Parasutterella id.2892) on Leukemia inhibitory factor receptor
with a threshold of 1e−05

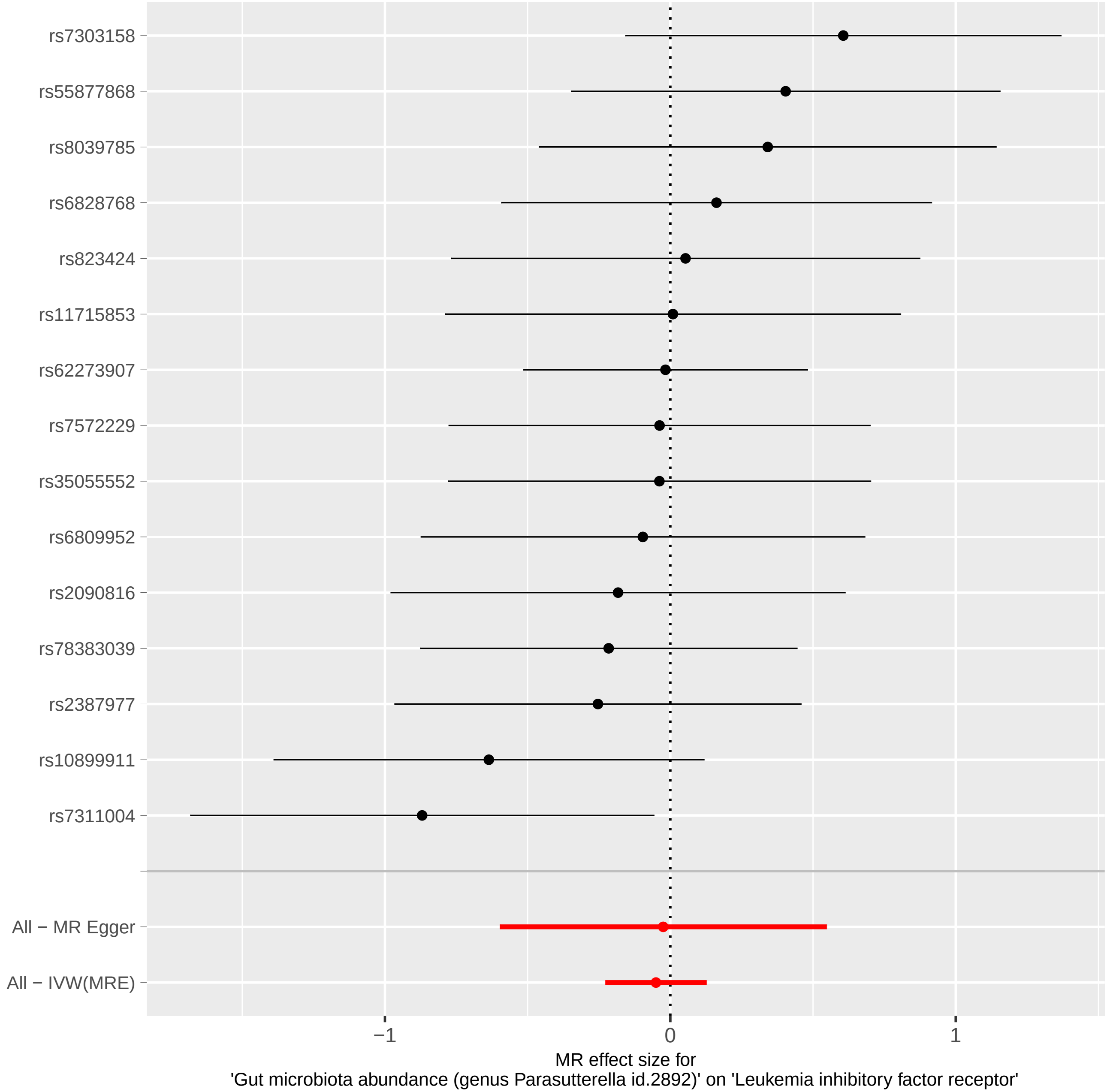

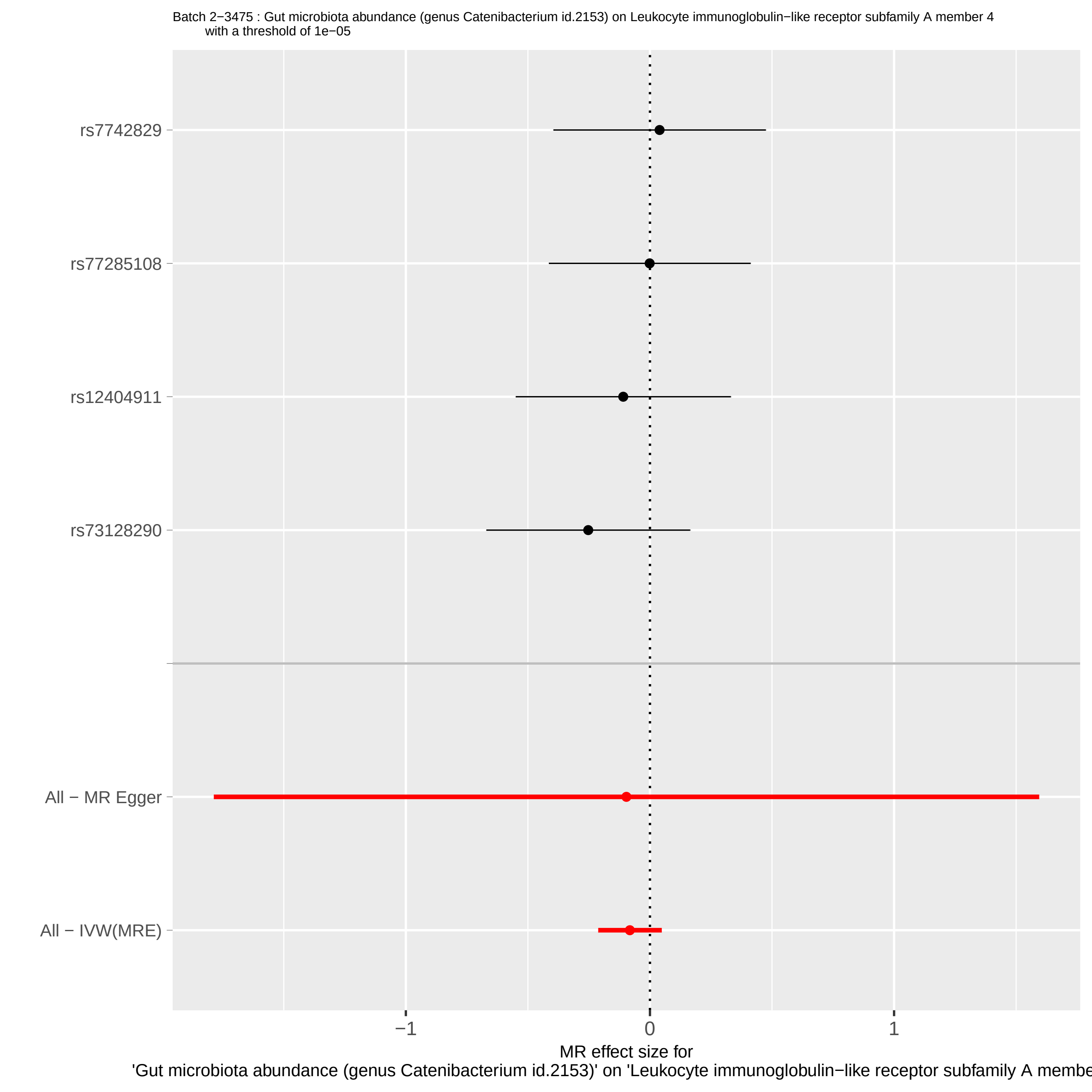
Batch 2−3475 : Gut microbiota abundance (genus Catenibacterium id.2153) on Leukocyte immunoglobulin−like receptor subfamily A member 4
with a threshold of 1e−05
rs7742829
rs77285108
rs12404911
rs73128290
All − MR Egger
All − IVW(MRE)
−1
0
1
MR effect size for
'Gut microbiota abundance (genus Catenibacterium id.2153)' on 'Leukocyte immunoglobulin−like receptor subfamily A membe

Batch 2−3476 : Gut microbiota abundance (genus Parasutterella id.2892) on Leukocyte immunoglobulin−like receptor subfamily A member 4
with a threshold of 1e−05

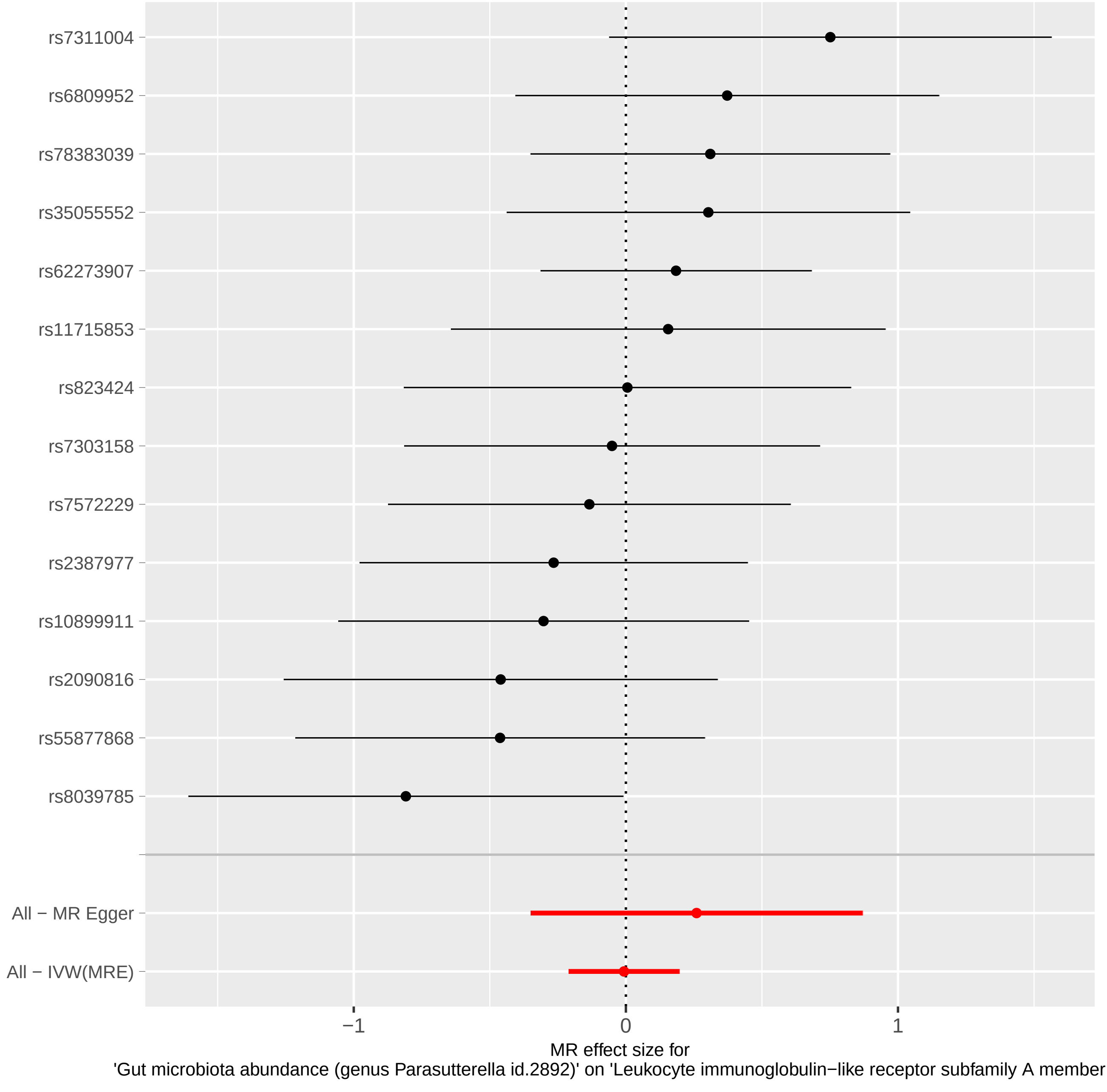

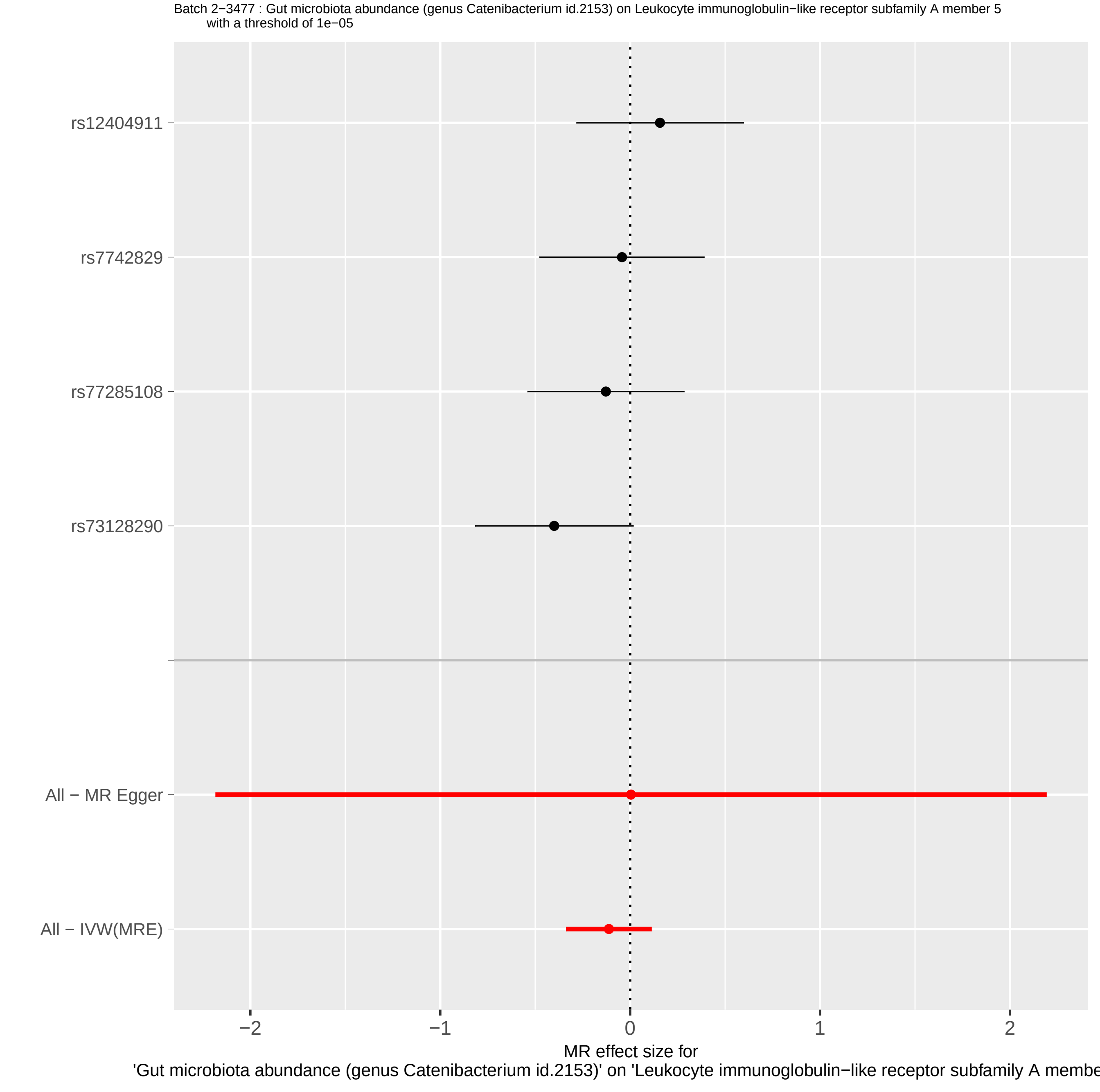
Batch 2−3477 : Gut microbiota abundance (genus Catenibacterium id.2153) on Leukocyte immunoglobulin−like receptor subfamily A member 5 with a threshold of 1e−05
rs12404911
rs7742829
rs77285108
rs73128290
All − MR Egger
All − IVW(MRE)
−2
−1
0
1
2
MR effect size for
'Gut microbiota abundance (genus Catenibacterium id.2153)' on 'Leukocyte immunoglobulin−like receptor subfamily A membe

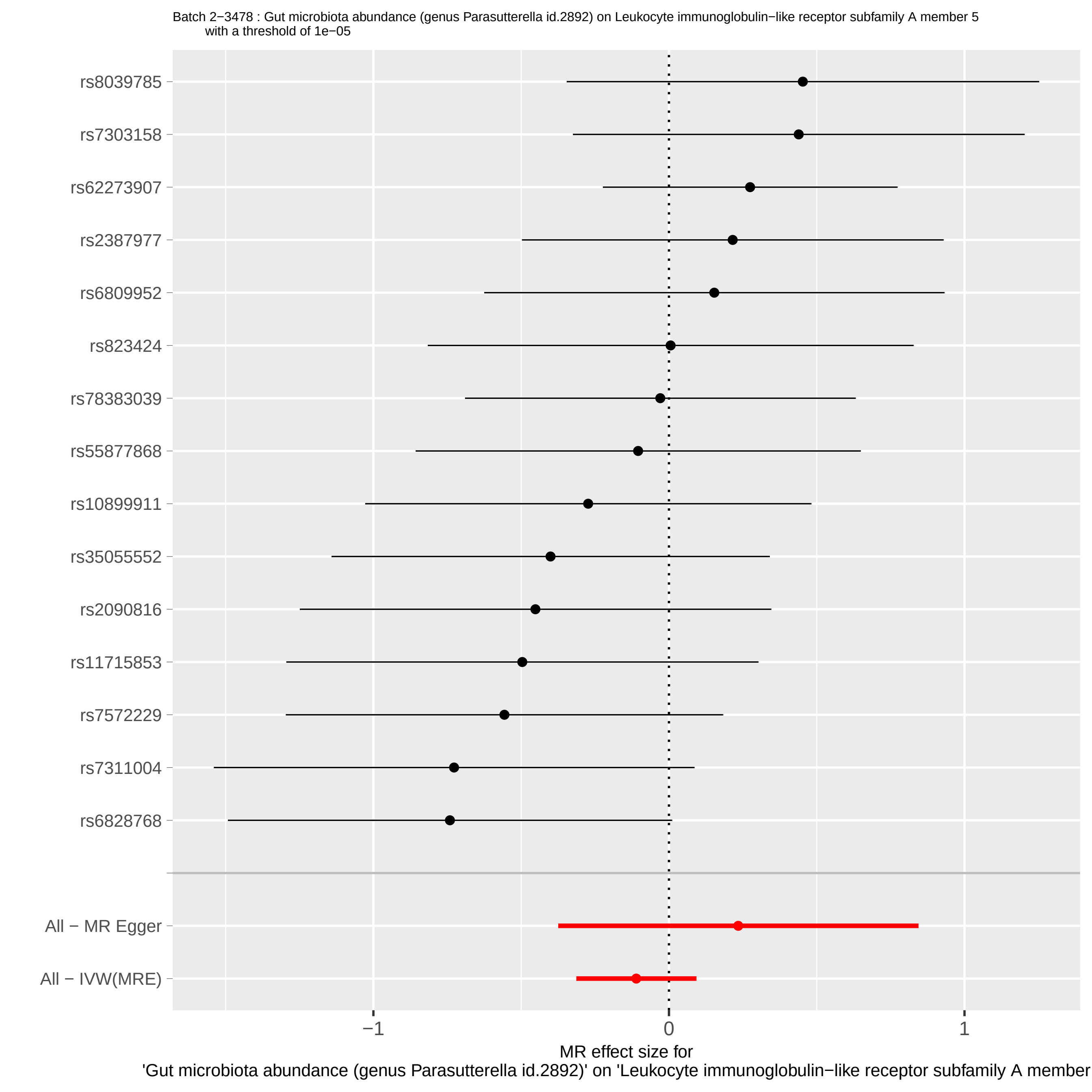
Batch 2−3478 : Gut microbiota abundance (genus Parasutterella id.2892) on Leukocyte immunoglobulin−like receptor subfamily A member 5
with a threshold of 1e−05
rs8039785
rs7303158
rs62273907
rs2387977
rs6809952
rs823424
rs78383039
rs55877868
rs10899911
rs35055552
rs2090816
rs11715853
rs7572229
rs7311004
rs6828768
All − MR Egger
All − IVW(MRE)
−1
0
1
MR effect size for
'Gut microbiota abundance (genus Parasutterella id.2892)' on 'Leukocyte immunoglobulin−like receptor subfamily A member

Batch 2−3479 : Gut microbiota abundance (genus Catenibacterium id.2153) on Leukocyte immunoglobulin−like receptor subfamily A member 5
with a threshold of 1e−05

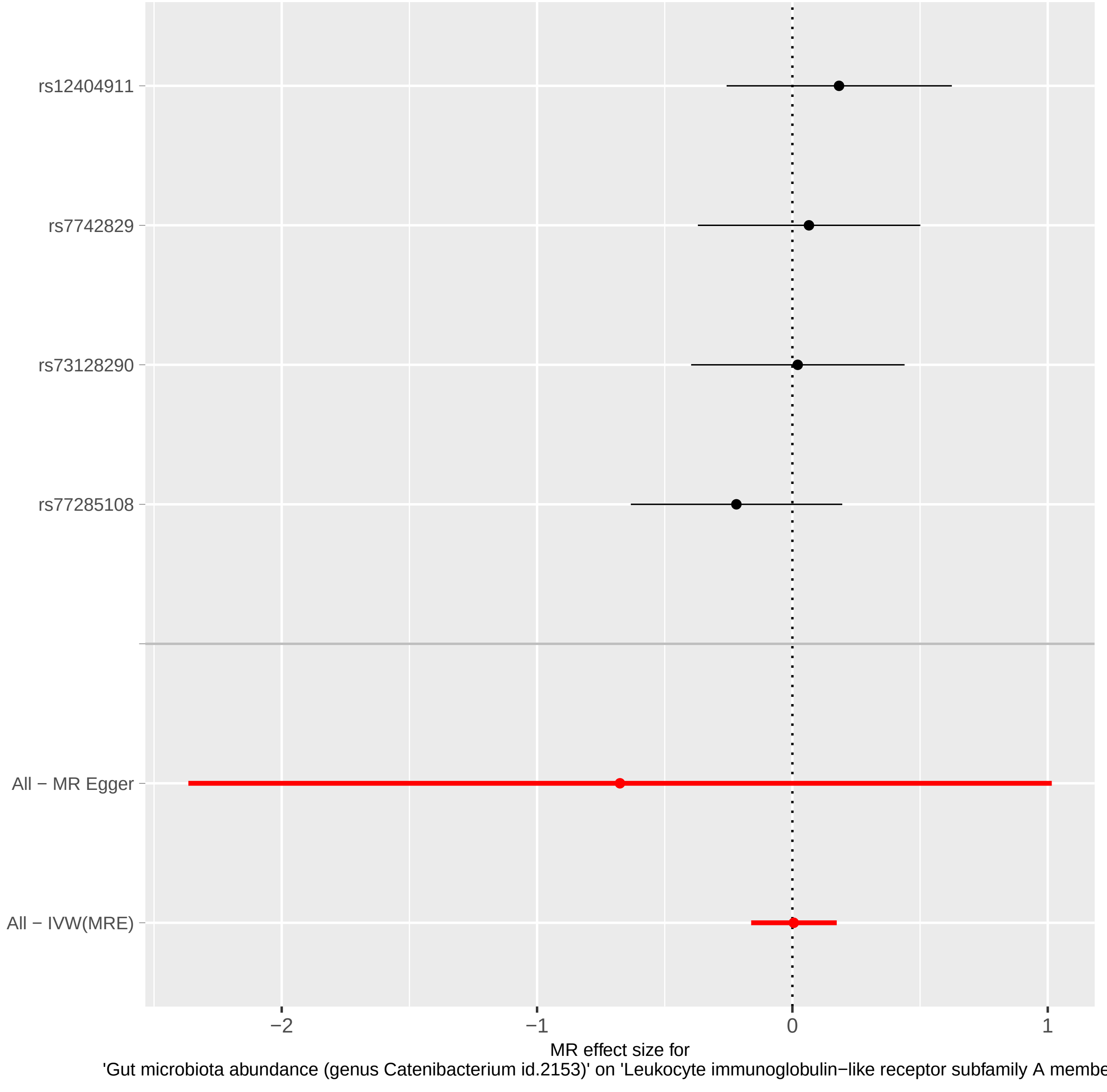

Batch 2−3480 : Gut microbiota abundance (genus Parasutterella id.2892) on Leukocyte immunoglobulin−like receptor subfamily A member 5 with a threshold of 1e−05

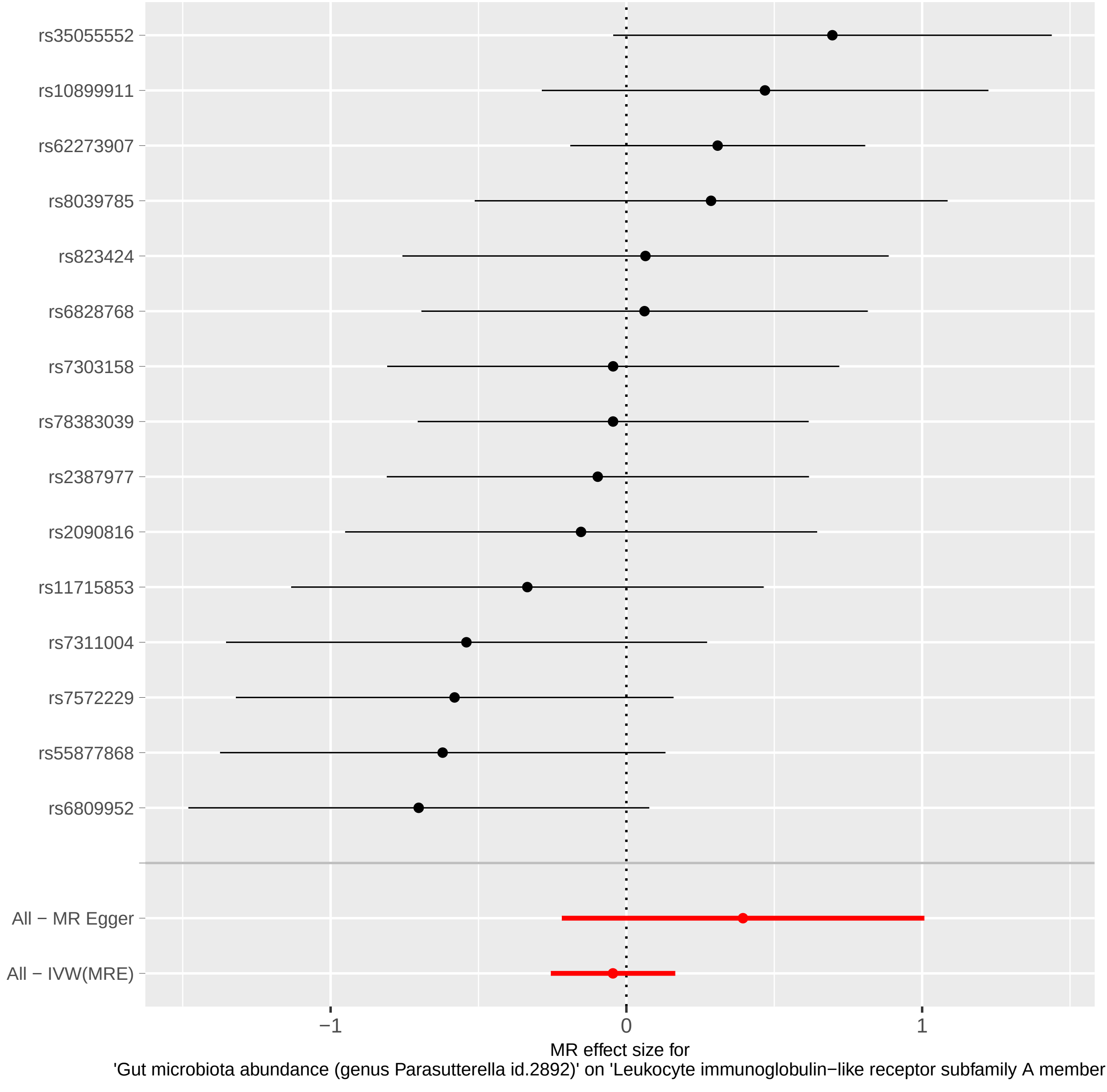

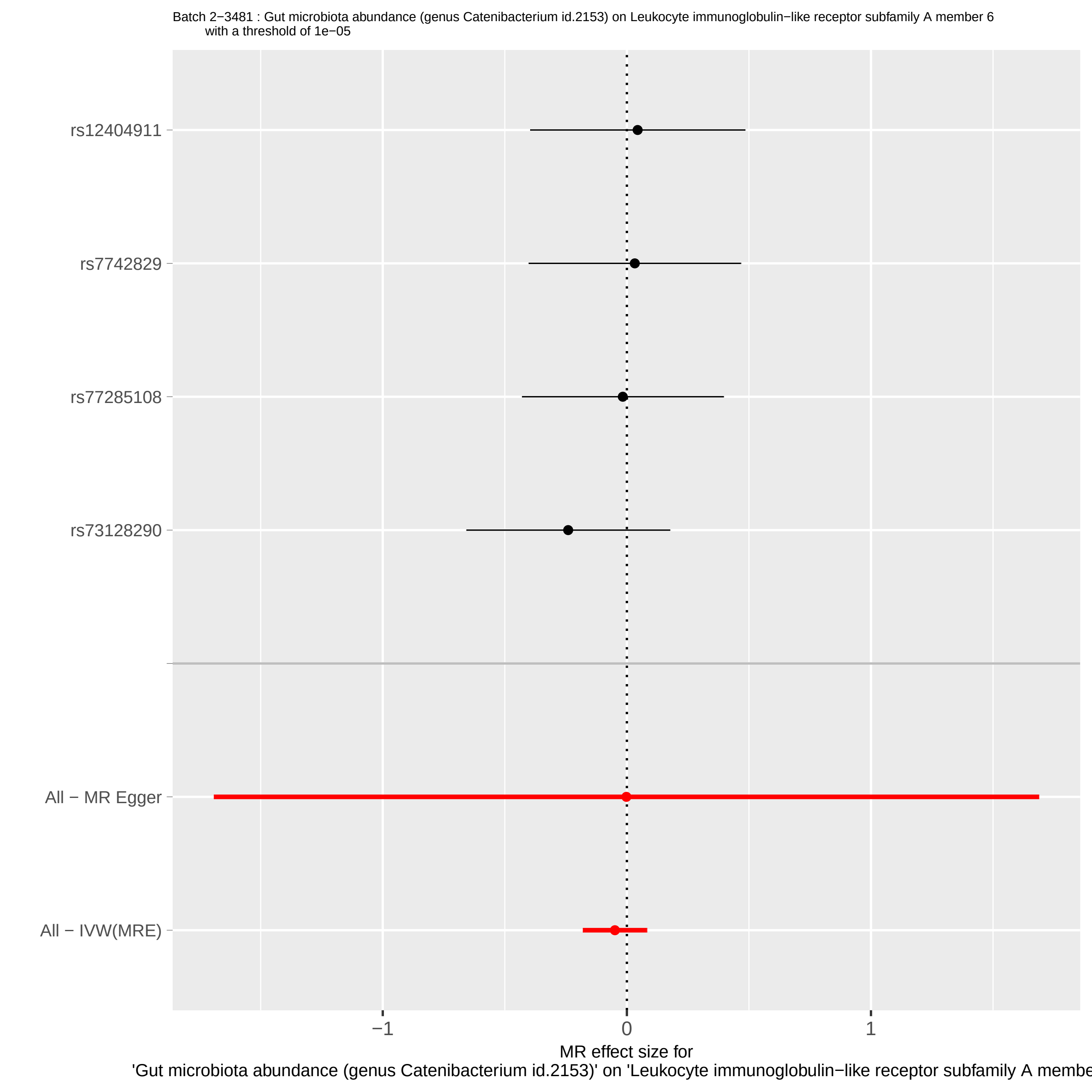

Batch 2−3481 : Gut microbiota abundance (genus Catenibacterium id.2153) on Leukocyte immunoglobulin−like receptor subfamily A member 6
with a threshold of 1e−05
rs12404911
rs7742829
rs77285108
rs73128290
All − MR Egger
All − IVW(MRE)
−1
0
1
MR effect size for
'Gut microbiota abundance (genus Catenibacterium id.2153)' on 'Leukocyte immunoglobulin−like receptor subfamily A membe

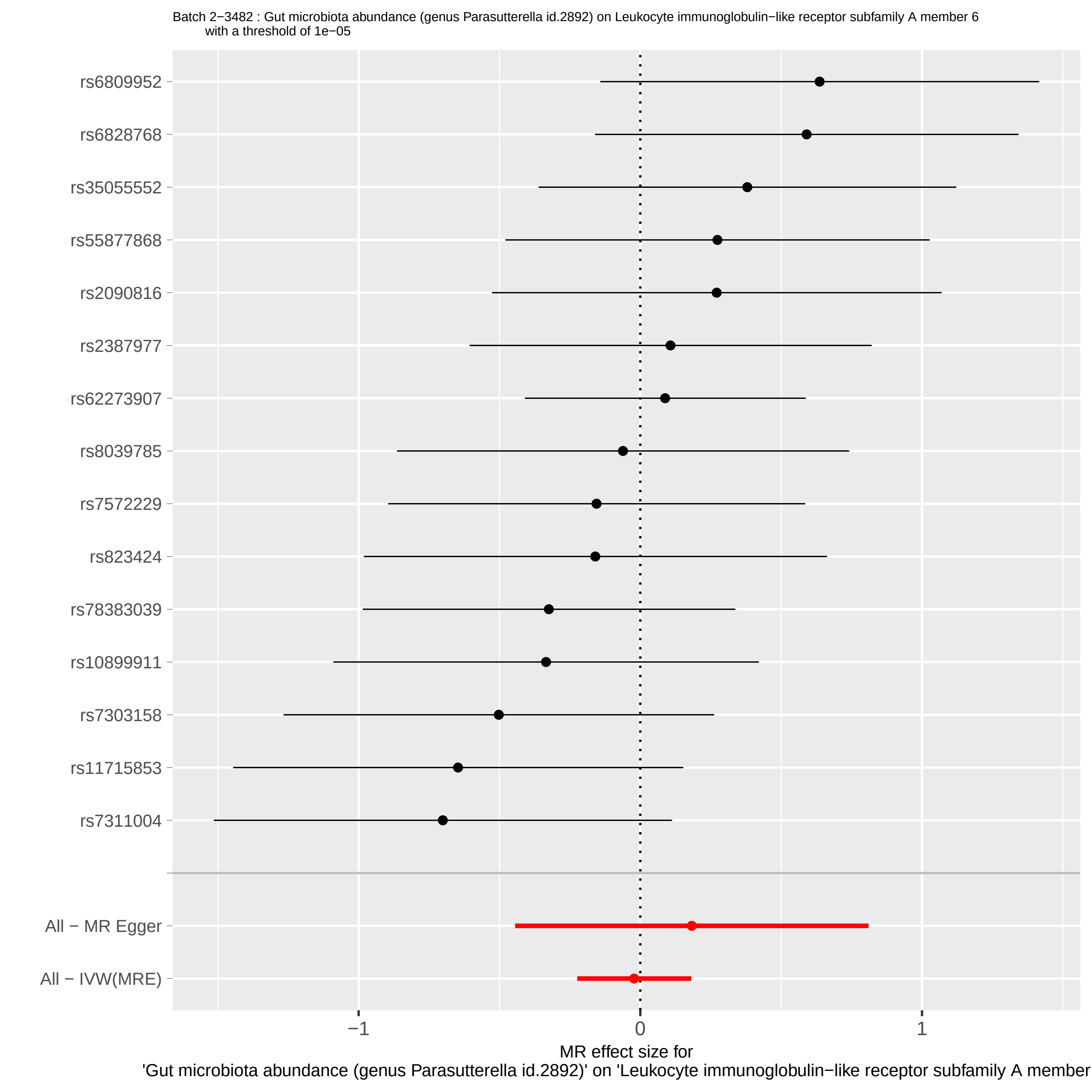
Batch 2−3482 : Gut microbiota abundance (genus Parasutterella id.2892) on Leukocyte immunoglobulin−like receptor subfamily A member 6
with a threshold of 1e−05
rs6809952
rs6828768
rs35055552
rs55877868
rs2090816
rs2387977
rs62273907
rs8039785
rs7572229
rs823424
rs78383039
rs10899911
rs7303158
rs11715853
rs7311004
All − MR Egger
All − IVW(MRE)
−1
0
1
MR effect size for
'Gut microbiota abundance (genus Parasutterella id.2892)' on 'Leukocyte immunoglobulin−like receptor subfamily A member

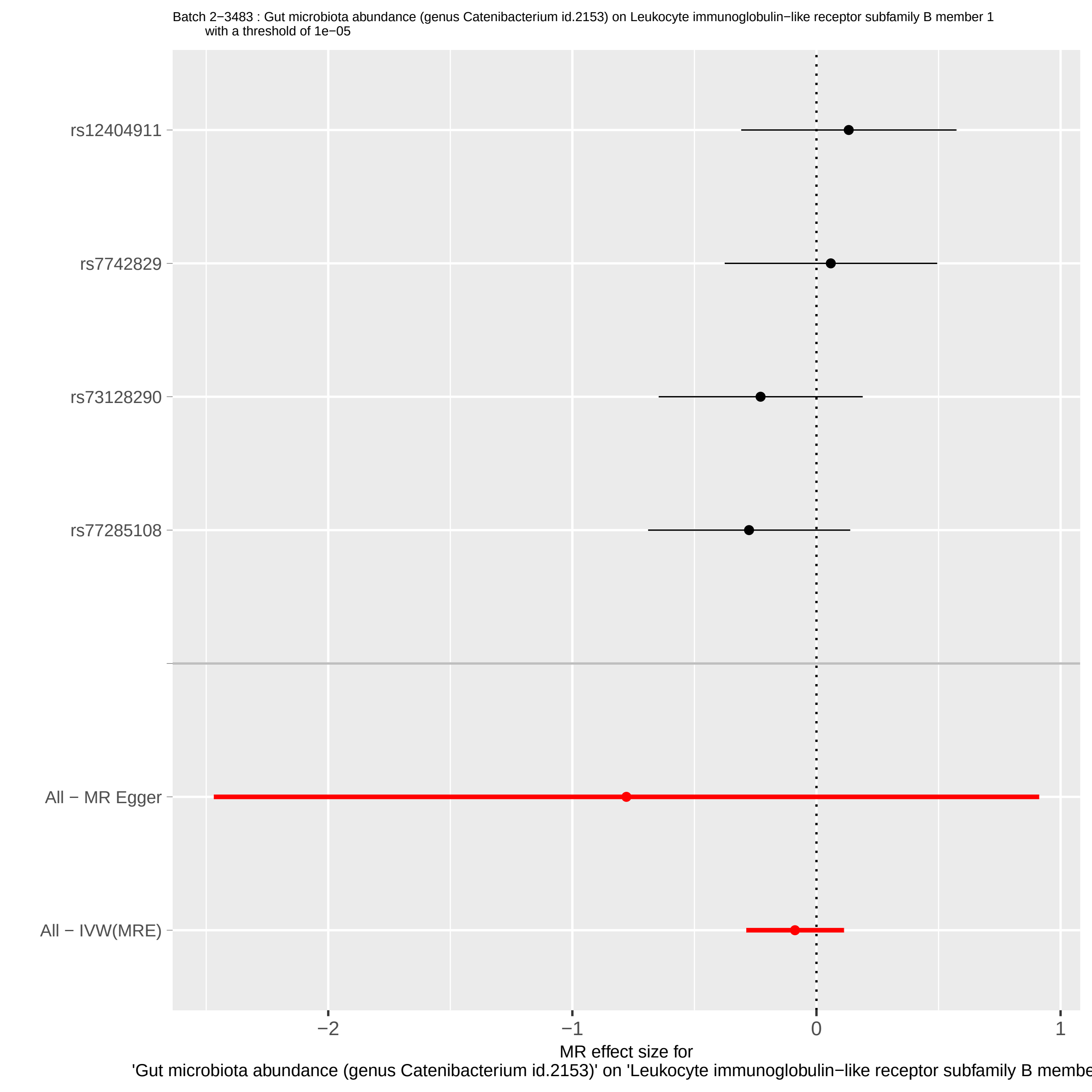
Batch 2−3483 : Gut microbiota abundance (genus Catenibacterium id.2153) on Leukocyte immunoglobulin−like receptor subfamily B member 1
with a threshold of 1e−05
rs12404911
rs7742829
rs73128290
rs77285108
All − MR Egger
All − IVW(MRE)
−2
−1
0
1
MR effect size for
'Gut microbiota abundance (genus Catenibacterium id.2153)' on 'Leukocyte immunoglobulin−like receptor subfamily B membe

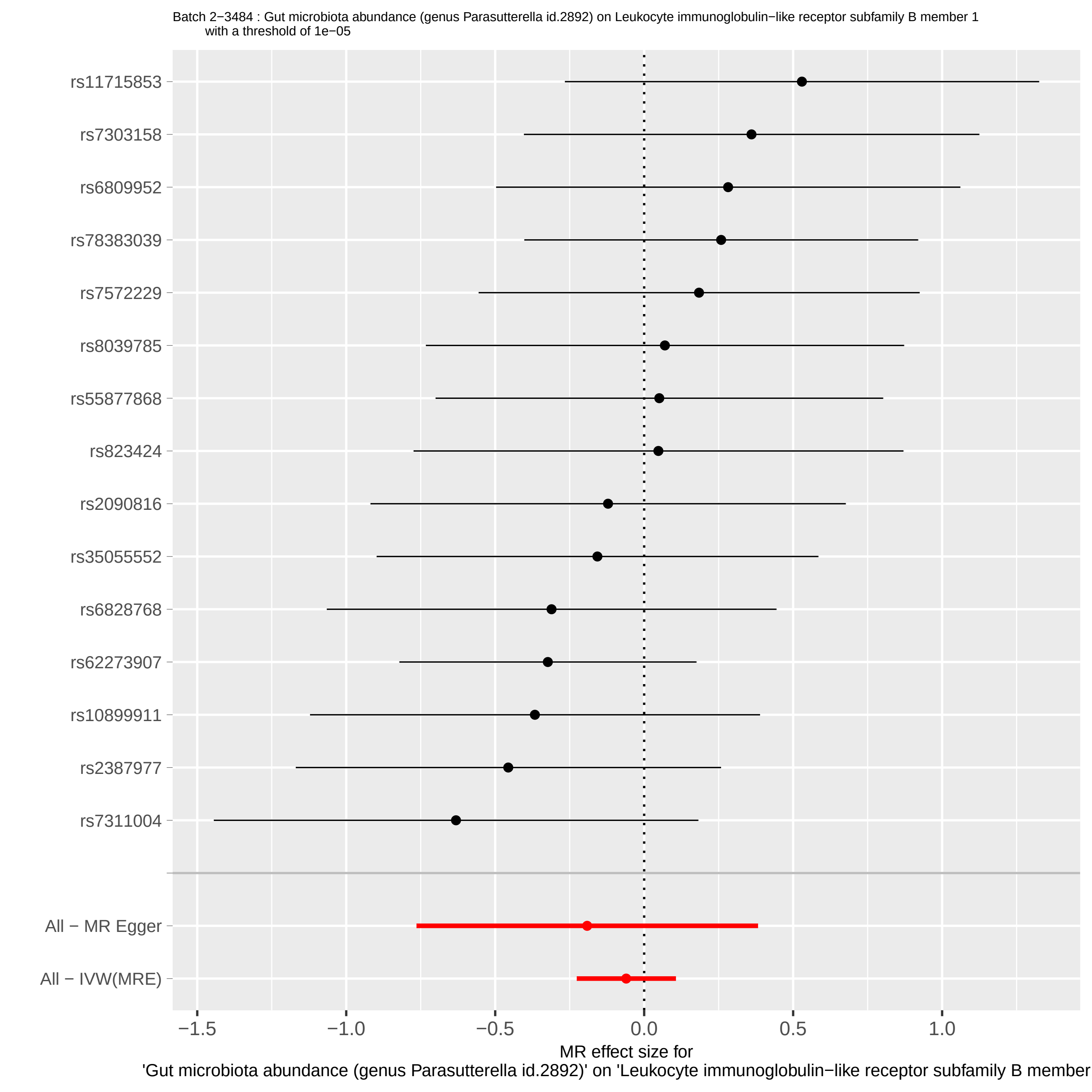

Batch 2−3484 : Gut microbiota abundance (genus Parasutterella id.2892) on Leukocyte immunoglobulin−like receptor subfamily B member 1 with a threshold of 1e−05
rs11715853
rs7303158
rs6809952
rs78383039
rs7572229
rs8039785
rs55877868
rs823424
rs2090816
rs35055552
rs6828768
rs62273907
rs10899911
rs2387977
rs7311004
All − MR Egger
All − IVW(MRE)
−1.5
−1.0
−0.5
0.0
0.5
1.0
MR effect size for
'Gut microbiota abundance (genus Parasutterella id.2892)' on 'Leukocyte immunoglobulin−like receptor subfamily B member

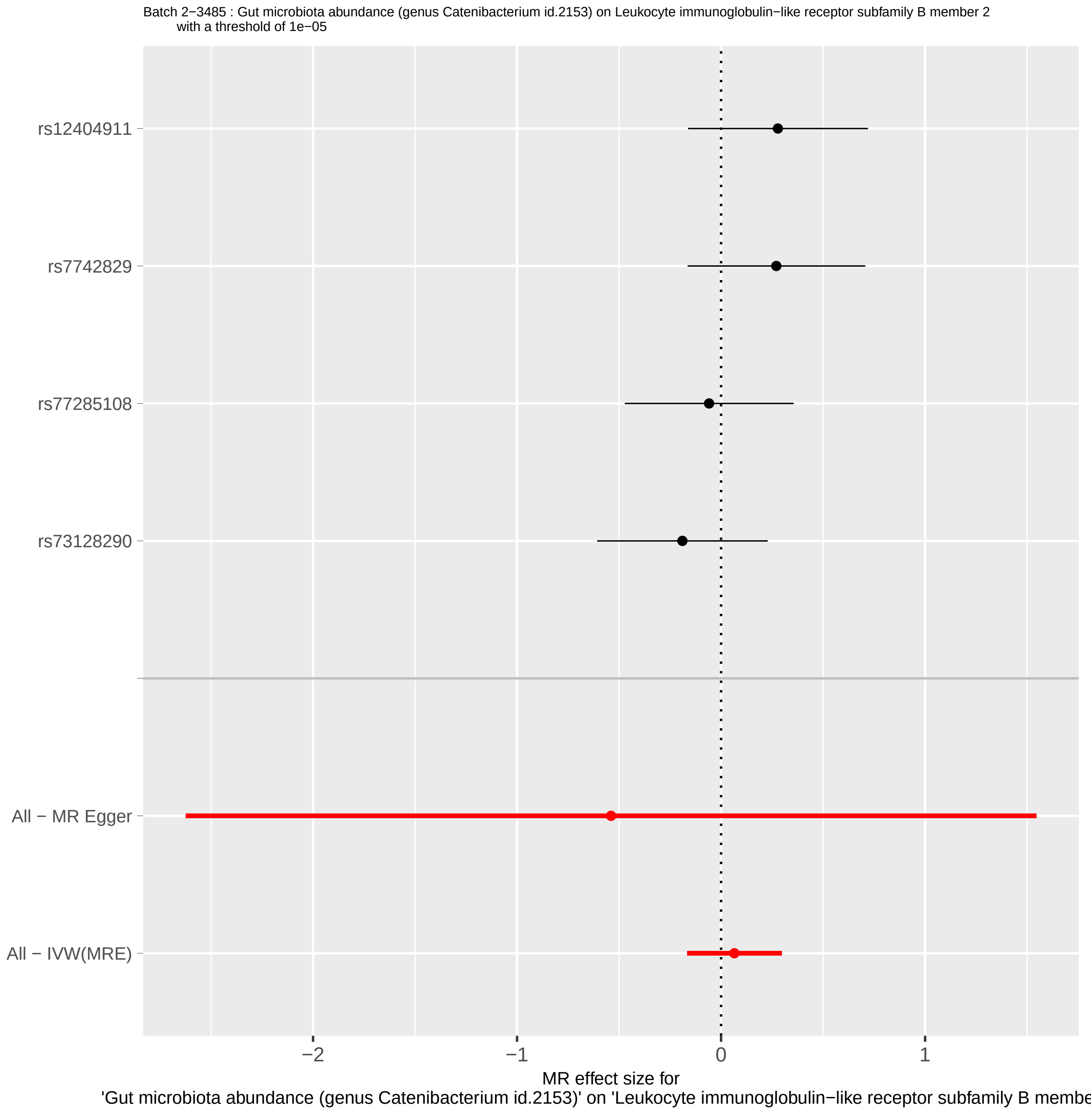
Batch 2−3485 : Gut microbiota abundance (genus Catenibacterium id.2153) on Leukocyte immunoglobulin−like receptor subfamily B member 2
with a threshold of 1e−05
rs12404911
rs7742829
rs77285108
rs73128290
All − MR Egger
All − IVW(MRE)
−2
−1
0
1
MR effect size for
'Gut microbiota abundance (genus Catenibacterium id.2153)' on 'Leukocyte immunoglobulin−like receptor subfamily B membe

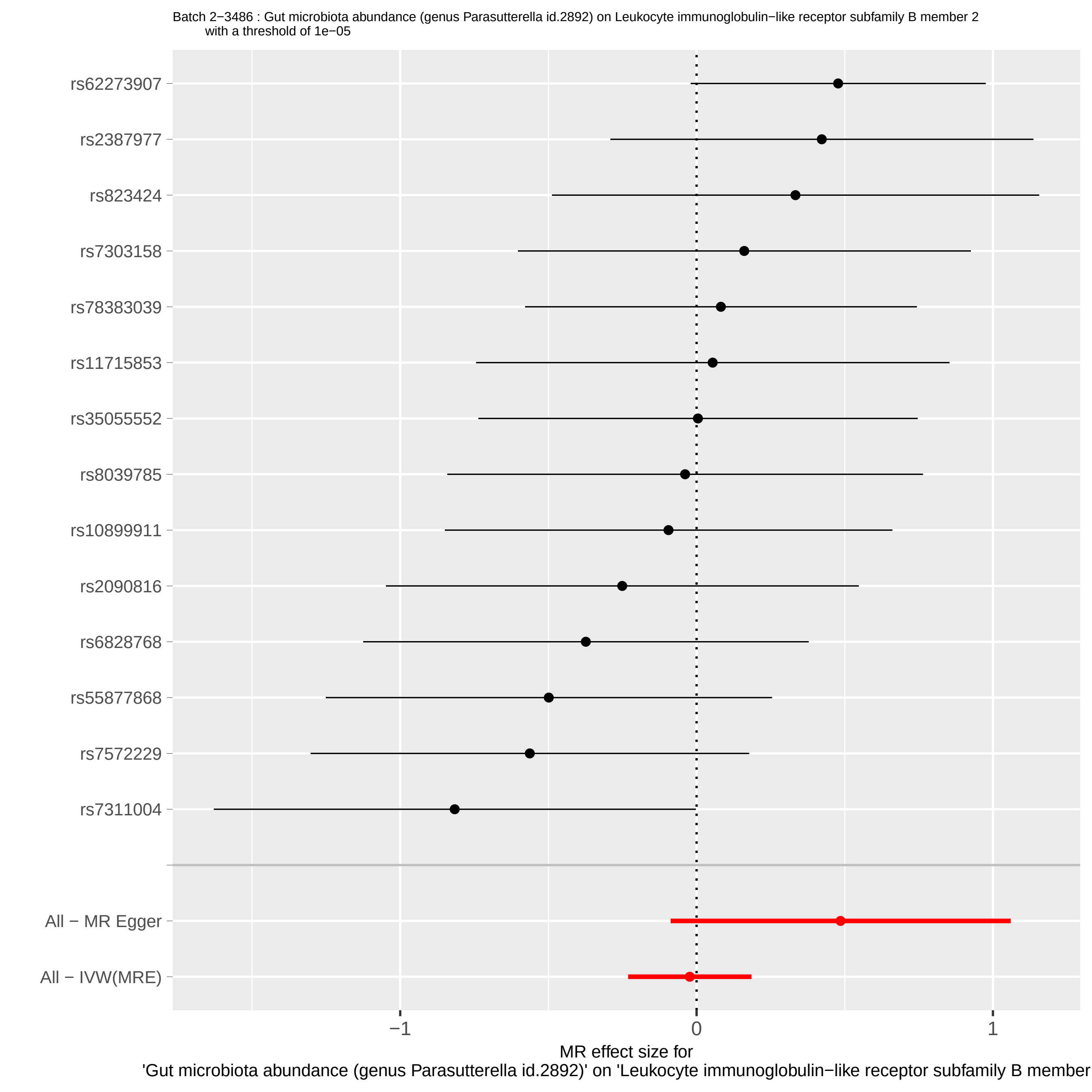

Batch 2−3486 : Gut microbiota abundance (genus Parasutterella id.2892) on Leukocyte immunoglobulin−like receptor subfamily B member 2
with a threshold of 1e−05
rs62273907
rs2387977
rs823424
rs7303158
rs78383039
rs11715853
rs35055552
rs8039785
rs10899911
rs2090816
rs6828768
rs55877868
rs7572229
rs7311004
All − MR Egger
All − IVW(MRE)
−1
0
1
MR effect size for
'Gut microbiota abundance (genus Parasutterella id.2892)' on 'Leukocyte immunoglobulin−like receptor subfamily B member

Batch 2−3487 : Gut microbiota abundance (genus Catenibacterium id.2153) on Leukocyte immunoglobulin−like receptor subfamily B member 2
with a threshold of 1e−05

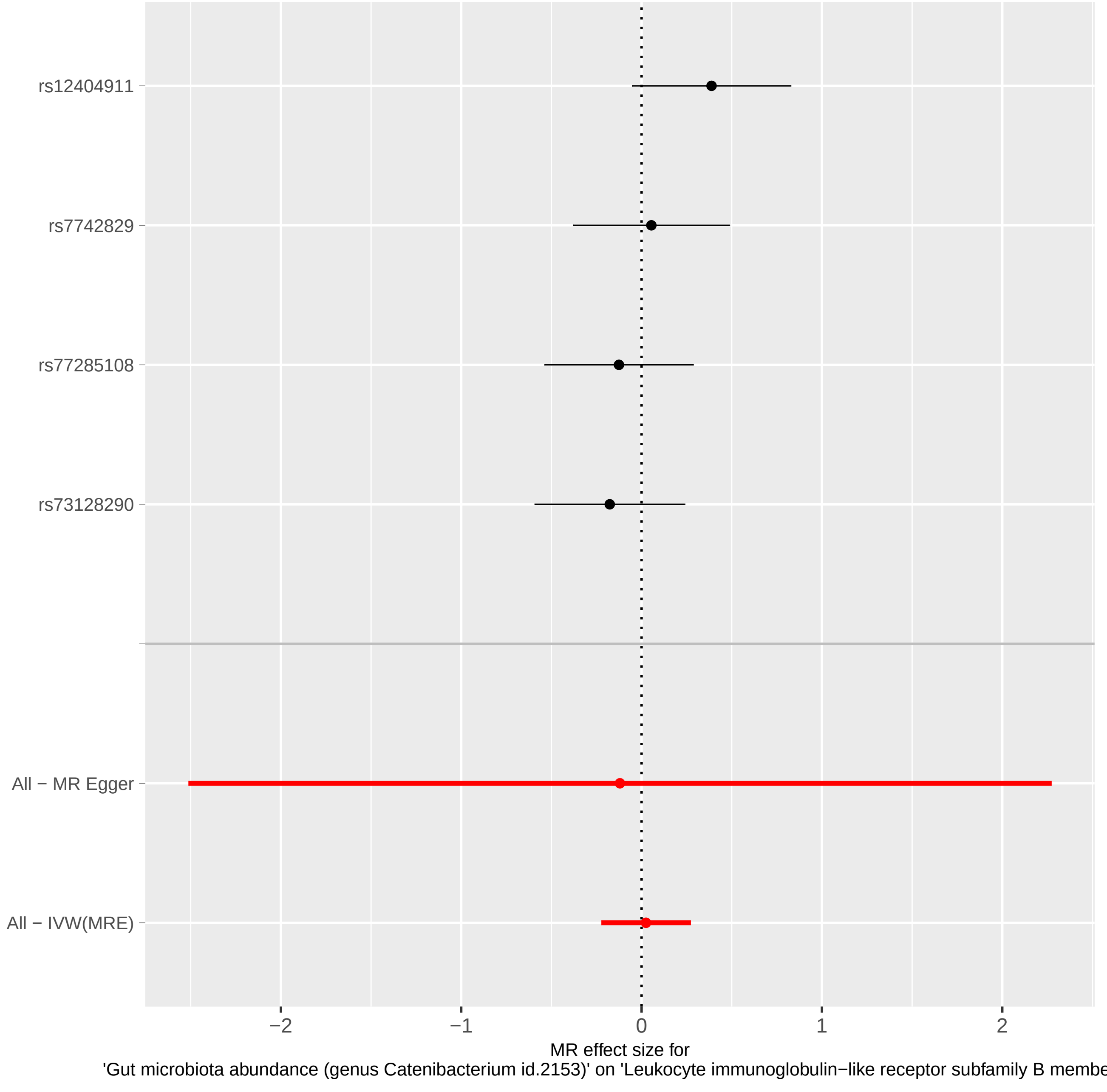

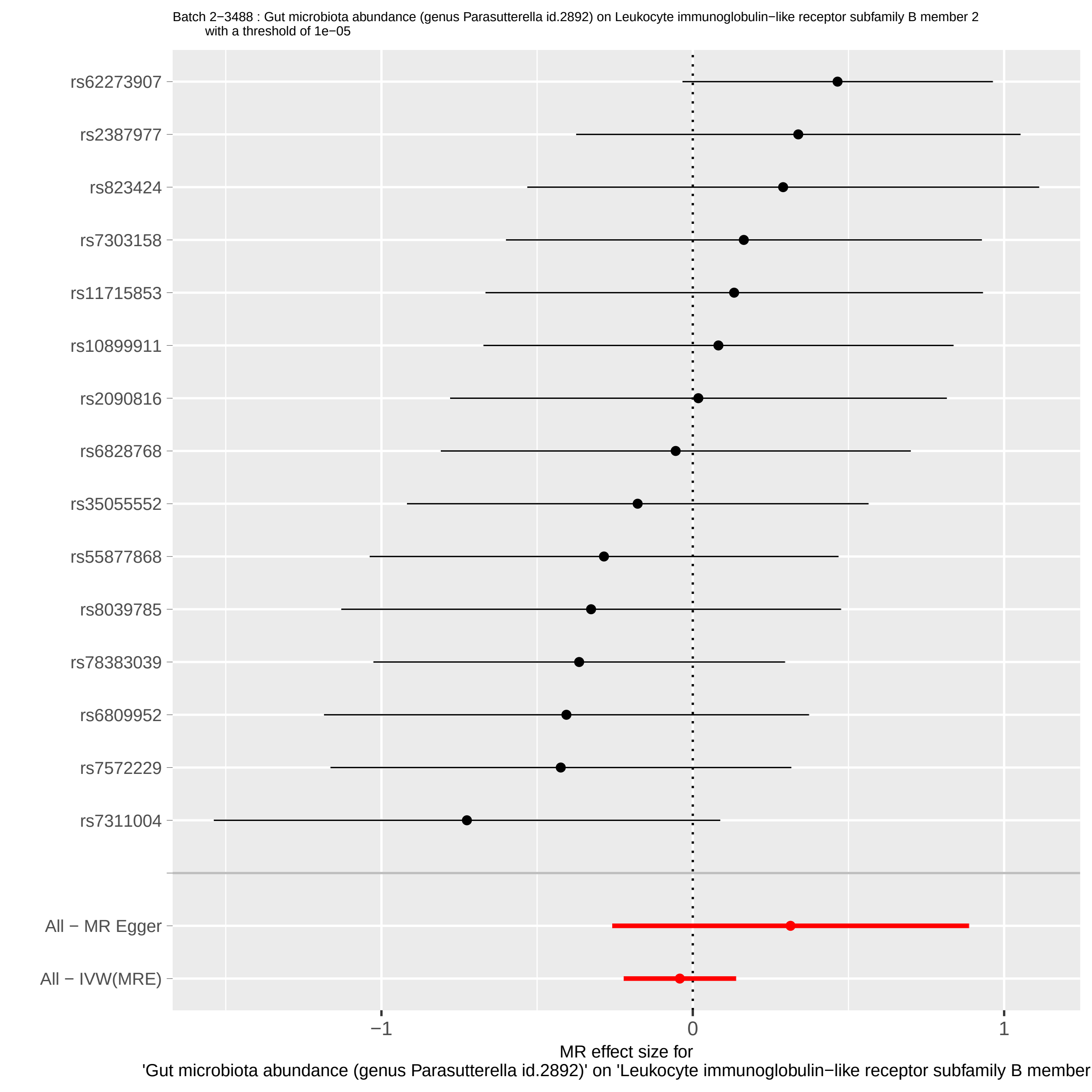

Batch 2−3488 : Gut microbiota abundance (genus Parasutterella id.2892) on Leukocyte immunoglobulin−like receptor subfamily B member 2
with a threshold of 1e−05
rs62273907
rs2387977
rs823424
rs7303158
rs11715853
rs10899911
rs2090816
rs6828768
rs35055552
rs55877868
rs8039785
rs78383039
rs6809952
rs7572229
rs7311004
All − MR Egger
All − IVW(MRE)
−1
0
1
MR effect size for
'Gut microbiota abundance (genus Parasutterella id.2892)' on 'Leukocyte immunoglobulin−like receptor subfamily B member

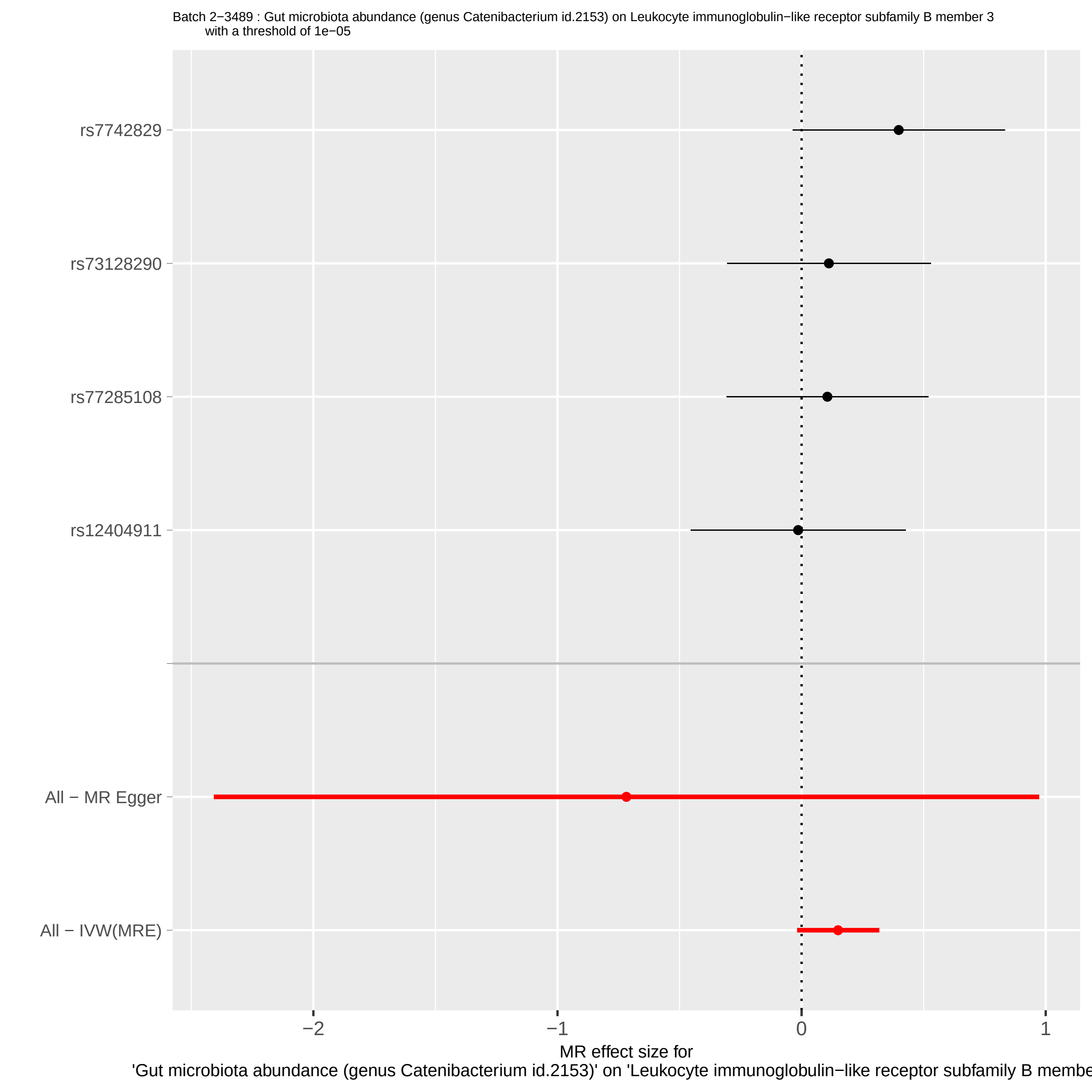

Batch 2−3489 : Gut microbiota abundance (genus Catenibacterium id.2153) on Leukocyte immunoglobulin−like receptor subfamily B member 3
with a threshold of 1e−05
rs7742829
rs73128290
rs77285108
rs12404911
All − MR Egger
All − IVW(MRE)
−2
−1
0
1
MR effect size for
'Gut microbiota abundance (genus Catenibacterium id.2153)' on 'Leukocyte immunoglobulin−like receptor subfamily B membe

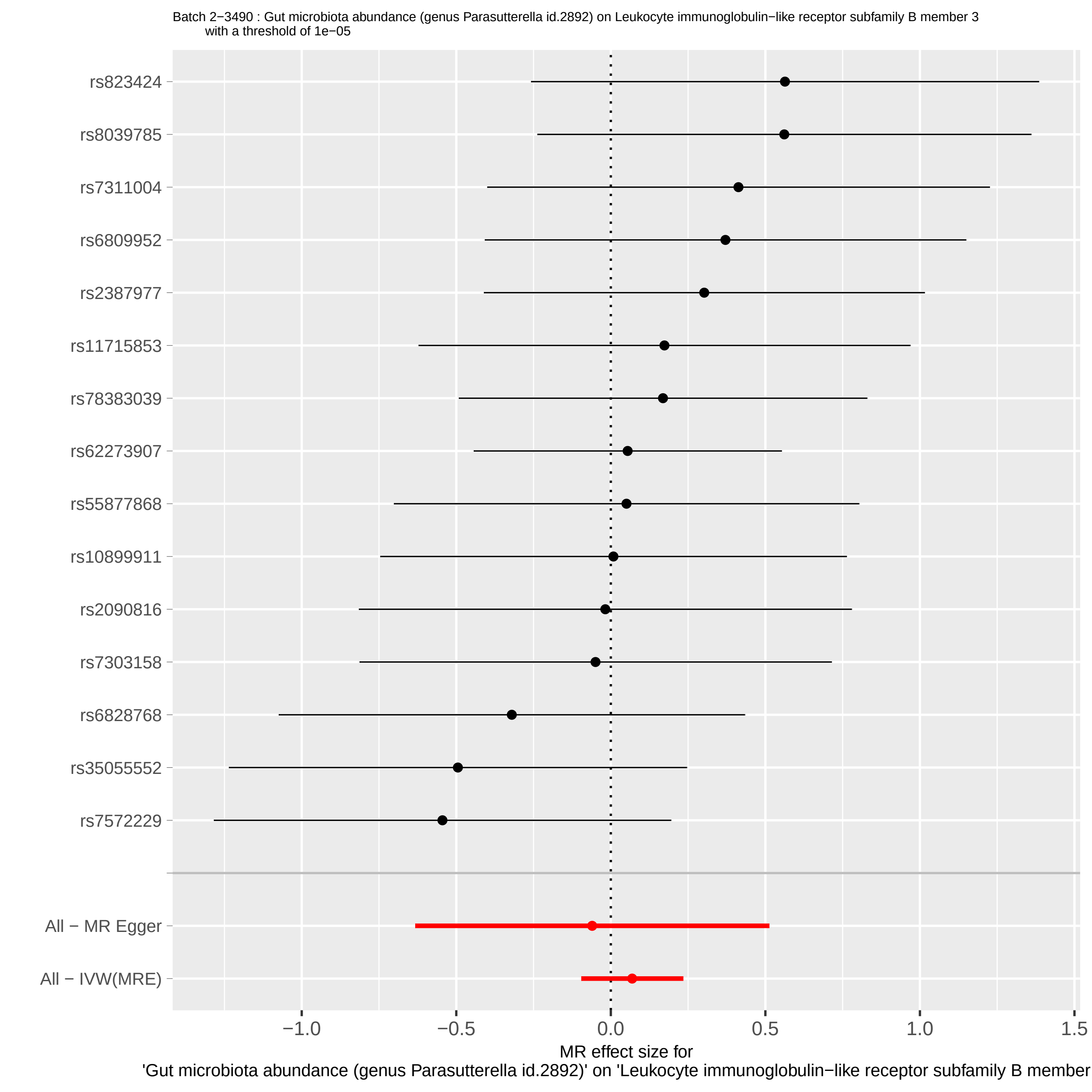

Batch 2−3490 : Gut microbiota abundance (genus Parasutterella id.2892) on Leukocyte immunoglobulin−like receptor subfamily B member 3
with a threshold of 1e−05
rs823424
rs8039785
rs7311004
rs6809952
rs2387977
rs11715853
rs78383039
rs62273907
rs55877868
rs10899911
rs2090816
rs7303158
rs6828768
rs35055552
rs7572229
All − MR Egger
All − IVW(MRE)
−1.0
−0.5
0.0
0.5
1.0
1.5
MR effect size for
'Gut microbiota abundance (genus Parasutterella id.2892)' on 'Leukocyte immunoglobulin−like receptor subfamily B member

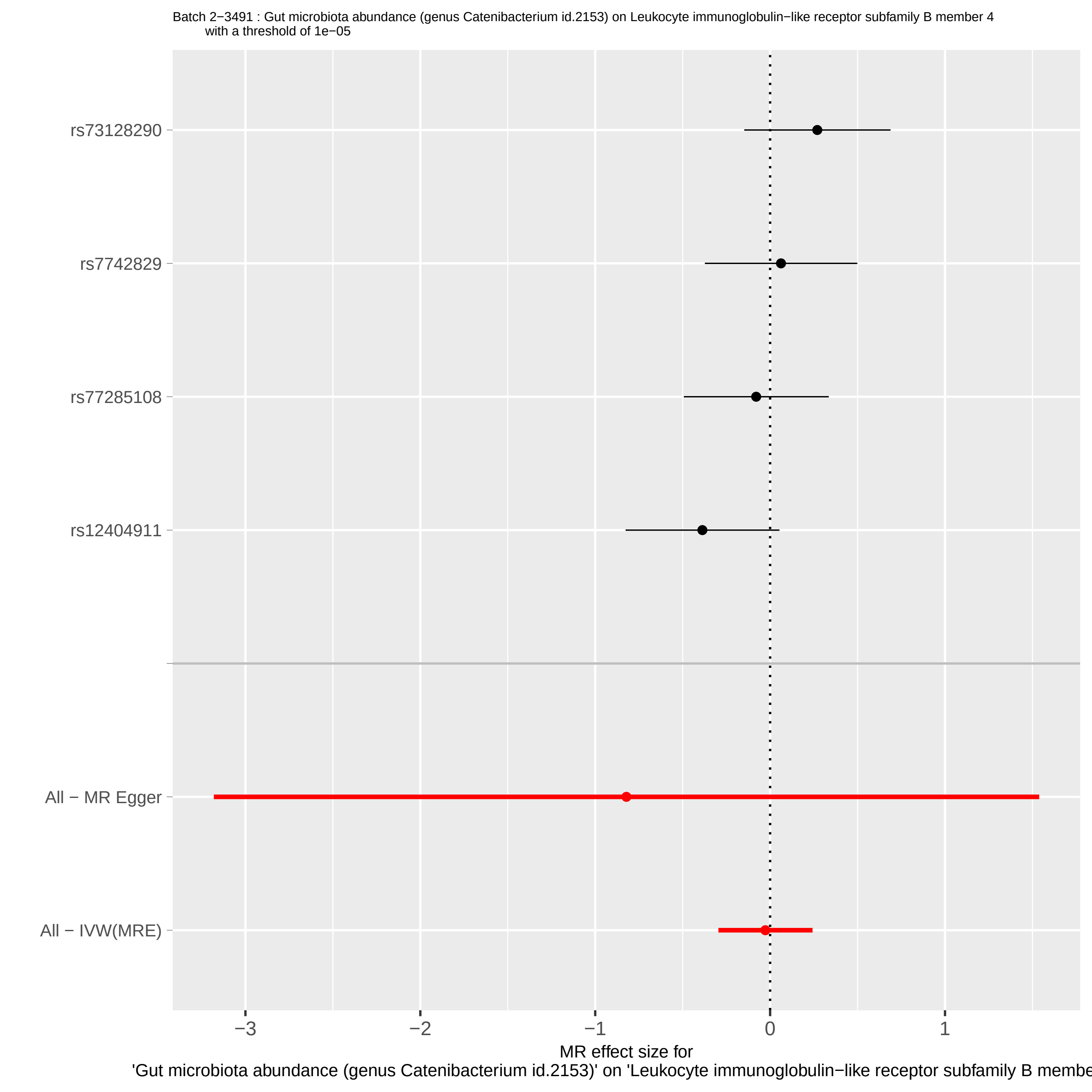
Batch 2−3491 : Gut microbiota abundance (genus Catenibacterium id.2153) on Leukocyte immunoglobulin−like receptor subfamily B member 4
with a threshold of 1e−05
rs73128290
rs7742829
rs77285108
rs12404911
All − MR Egger
All − IVW(MRE)
−3
−2
−1
0
1
MR effect size for
'Gut microbiota abundance (genus Catenibacterium id.2153)' on 'Leukocyte immunoglobulin−like receptor subfamily B membe

Batch 2−3492 : Gut microbiota abundance (genus Parasutterella id.2892) on Leukocyte immunoglobulin−like receptor subfamily B member 4 with a threshold of 1e−05

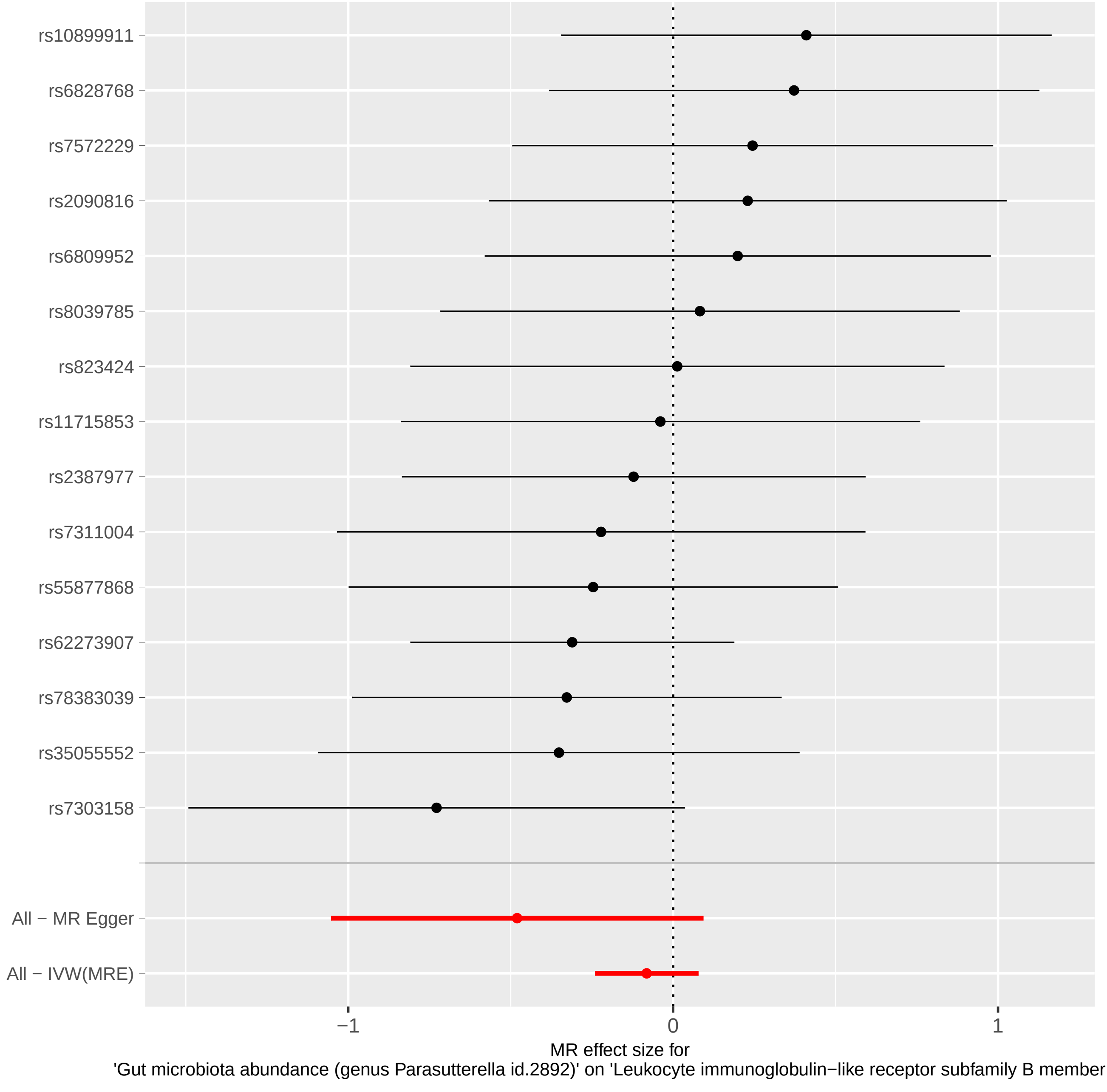

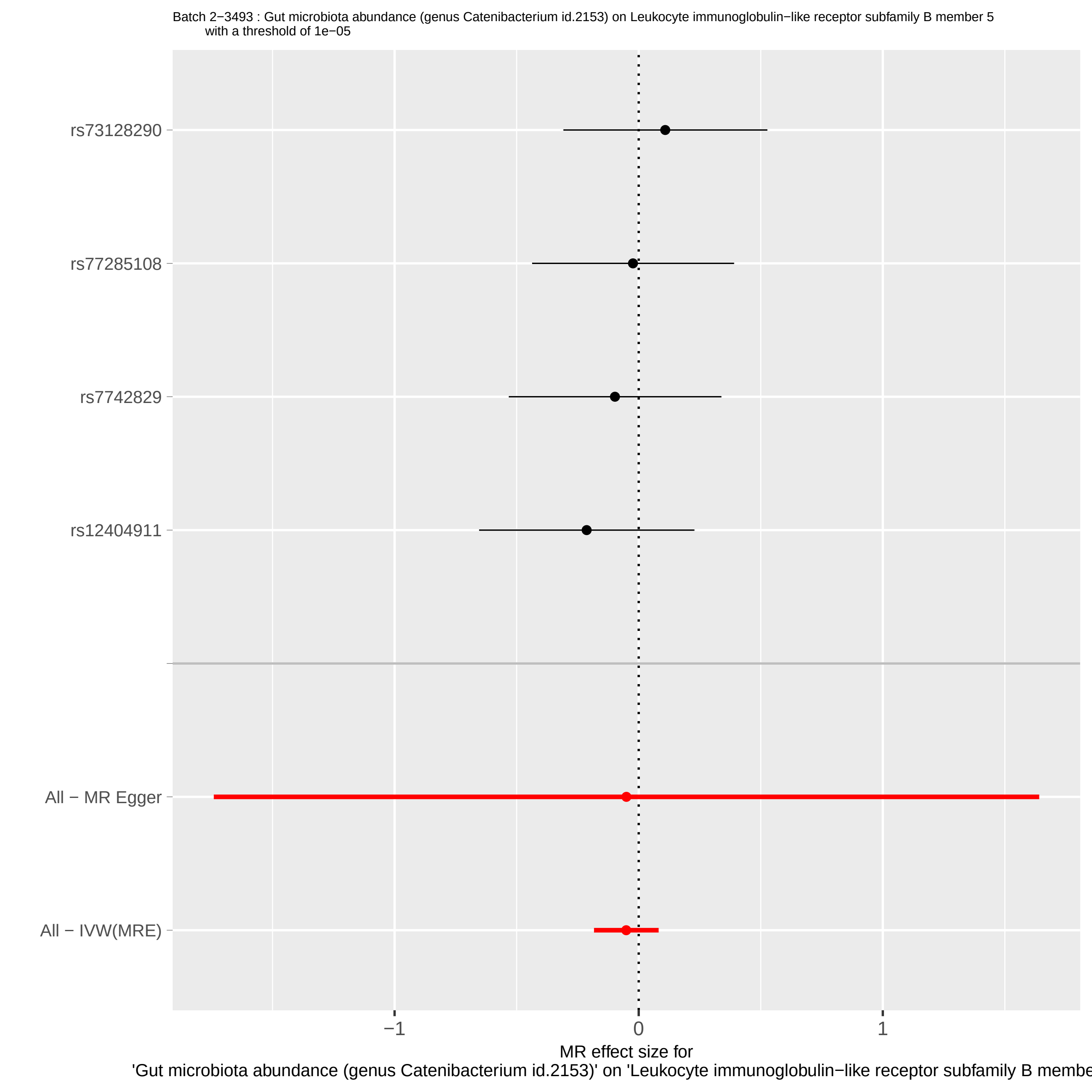

Batch 2−3493 : Gut microbiota abundance (genus Catenibacterium id.2153) on Leukocyte immunoglobulin−like receptor subfamily B member 5
with a threshold of 1e−05
rs73128290
rs77285108
rs7742829
rs12404911
All − MR Egger
All − IVW(MRE)
−1
0
1
MR effect size for
'Gut microbiota abundance (genus Catenibacterium id.2153)' on 'Leukocyte immunoglobulin−like receptor subfamily B membe

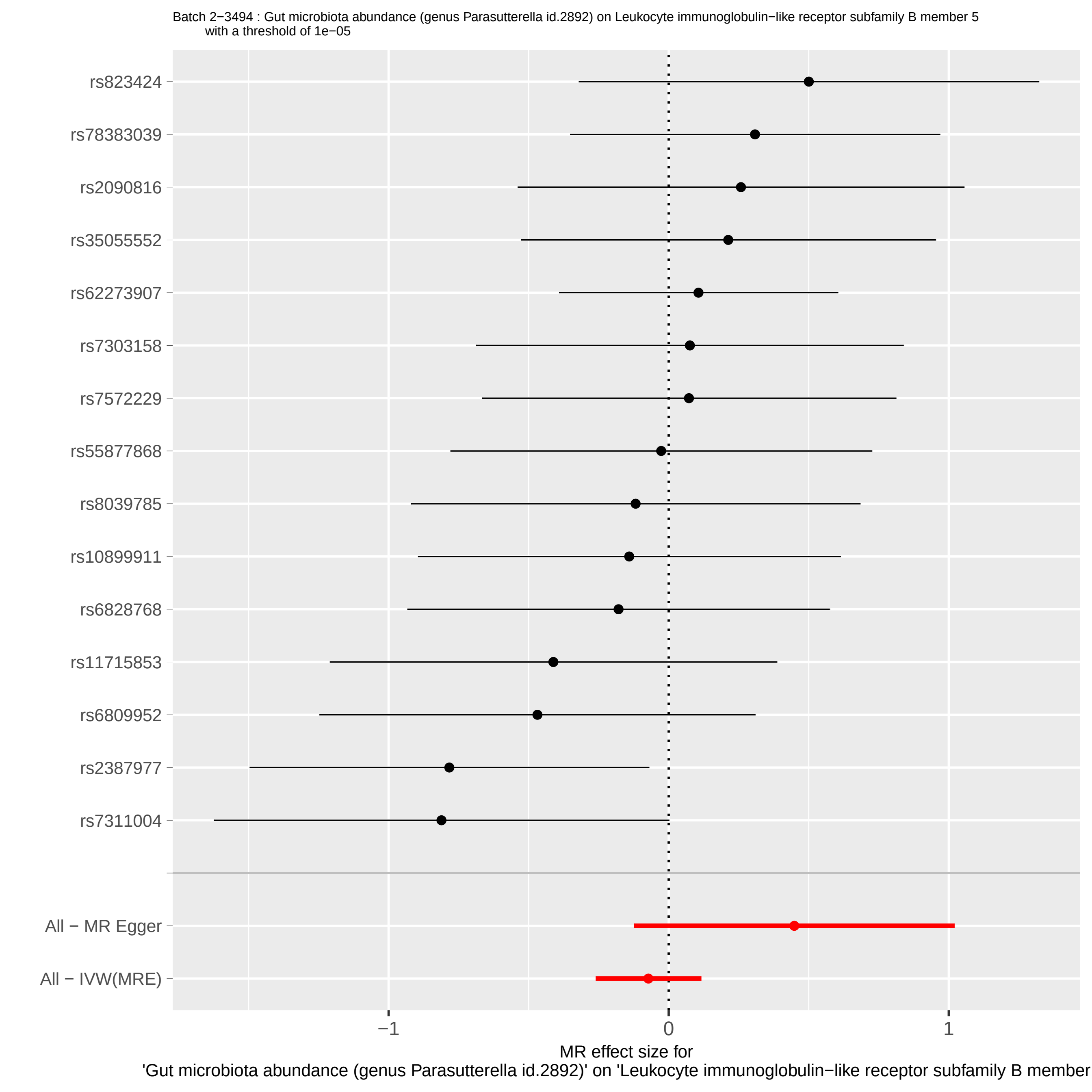

Batch 2−3494 : Gut microbiota abundance (genus Parasutterella id.2892) on Leukocyte immunoglobulin−like receptor subfamily B member 5
with a threshold of 1e−05
rs823424
rs78383039
rs2090816
rs35055552
rs62273907
rs7303158
rs7572229
rs55877868
rs8039785
rs10899911
rs6828768
rs11715853
rs6809952
rs2387977
rs7311004
All − MR Egger
All − IVW(MRE)
−1
0
1
MR effect size for
'Gut microbiota abundance (genus Parasutterella id.2892)' on 'Leukocyte immunoglobulin−like receptor subfamily B member

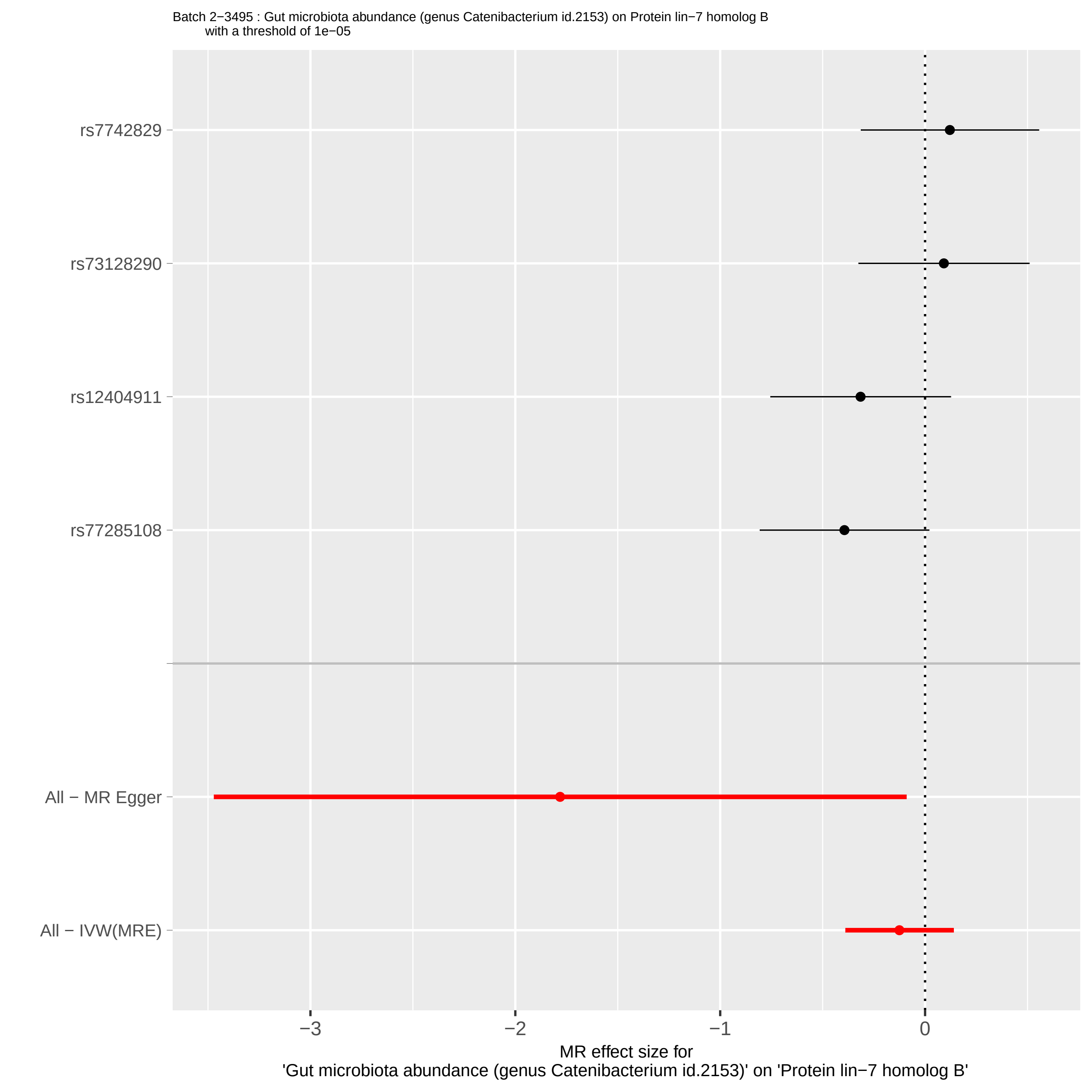

Batch 2−3495 : Gut microbiota abundance (genus Catenibacterium id.2153) on Protein lin−7 homolog B
with a threshold of 1e−05
rs7742829
rs73128290
rs12404911
rs77285108
All − MR Egger
All − IVW(MRE)
−3
−2
−1
0
MR effect size for
'Gut microbiota abundance (genus Catenibacterium id.2153)' on 'Protein lin−7 homolog B'

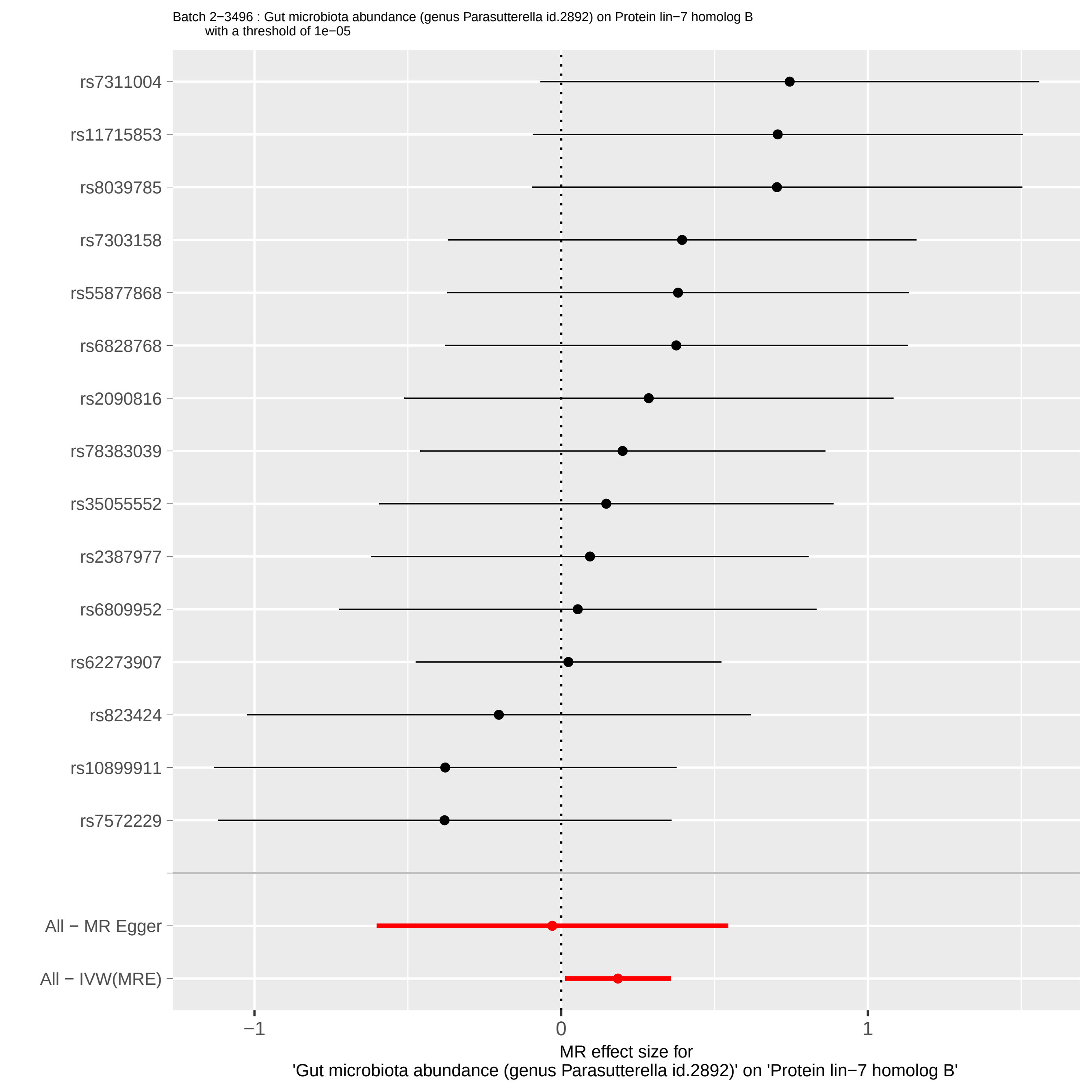

Batch 2−3496 : Gut microbiota abundance (genus Parasutterella id.2892) on Protein lin−7 homolog B
with a threshold of 1e−05
rs7311004
rs11715853
rs8039785
rs7303158
rs55877868
rs6828768
rs2090816
rs78383039
rs35055552
rs2387977
rs6809952
rs62273907
rs823424
rs10899911
rs7572229
All − MR Egger
All − IVW(MRE)
−1
0
1
MR effect size for
'Gut microbiota abundance (genus Parasutterella id.2892)' on 'Protein lin−7 homolog B'

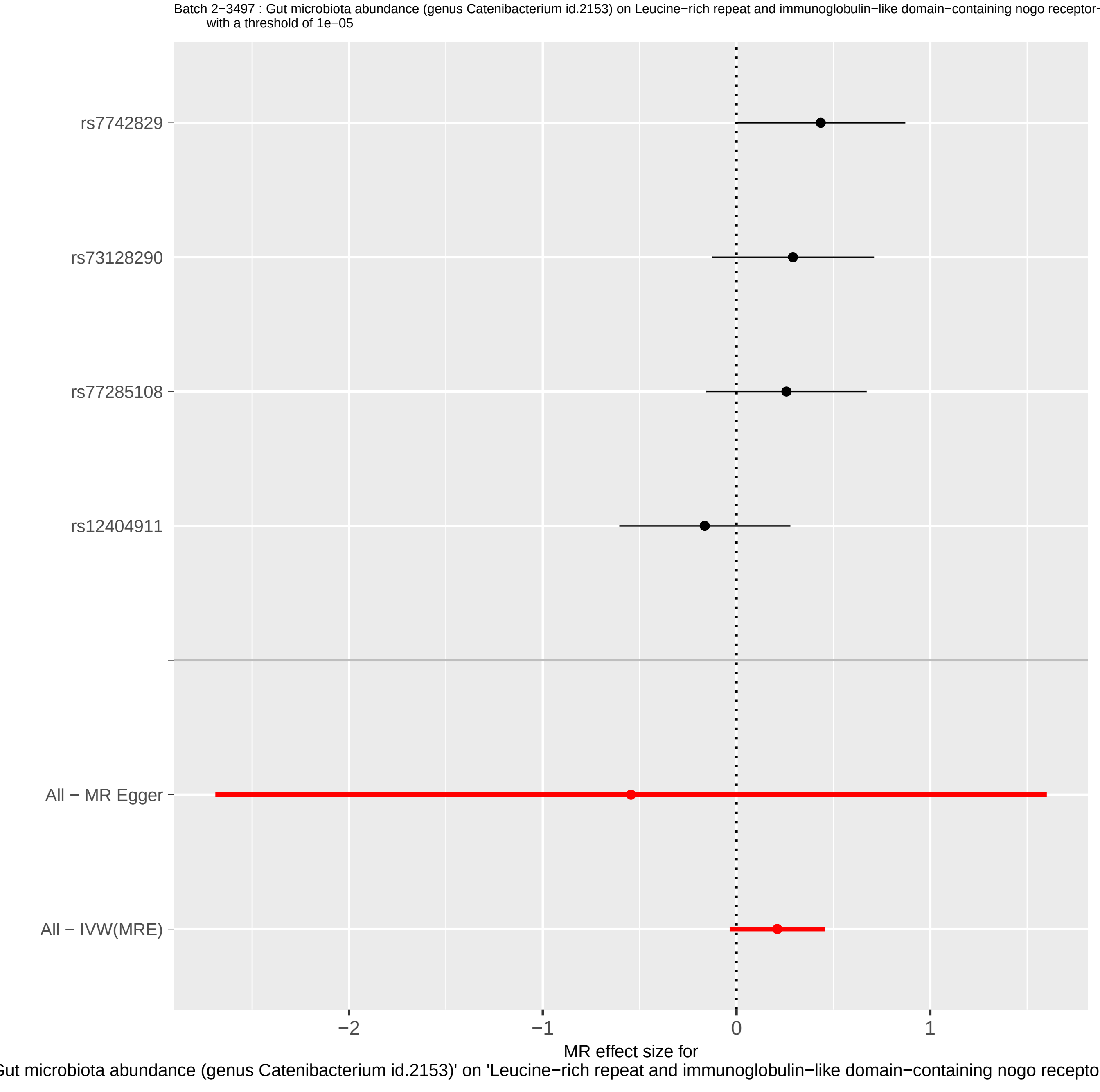

Batch 2−3497 : Gut microbiota abundance (genus Catenibacterium id.2153) on Leucine−rich repeat and immunoglobulin−like domain−containing nogo receptor−
with a threshold of 1e−05
rs7742829
rs73128290
rs77285108
rs12404911
All − MR Egger
All − IVW(MRE)
−2
−1
0
1
MR effect size for
Gut microbiota abundance (genus Catenibacterium id.2153)' on 'Leucine−rich repeat and immunoglobulin−like domain−containing nogo recepto

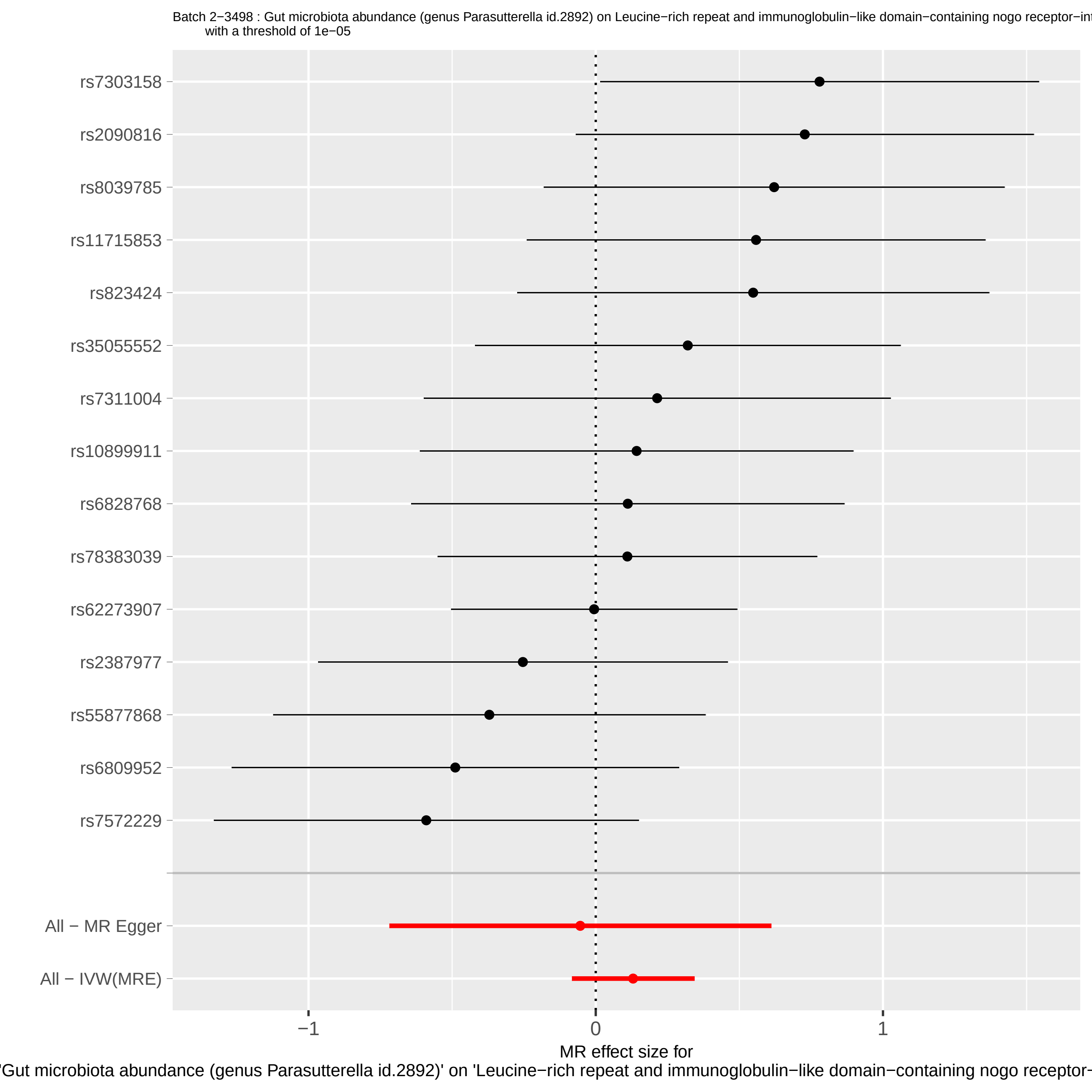
Batch 2−3498 : Gut microbiota abundance (genus Parasutterella id.2892) on Leucine−rich repeat and immunoglobulin−like domain−containing nogo receptor−in
with a threshold of 1e−05
rs7303158
rs2090816
rs8039785
rs11715853
rs823424
rs35055552
rs7311004
rs10899911
rs6828768
rs78383039
rs62273907
rs2387977
rs55877868
rs6809952
rs7572229
All − MR Egger
All − IVW(MRE)
−1
0
1
MR effect size for
'Gut microbiota abundance (genus Parasutterella id.2892)' on 'Leucine−rich repeat and immunoglobulin−like domain−containing nogo receptor−

Batch 2−3499 : Gut microbiota abundance (genus Catenibacterium id.2153) on Leucine−rich repeat and immunoglobulin−like domain−containing nogo receptor−
with a threshold of 1e−05

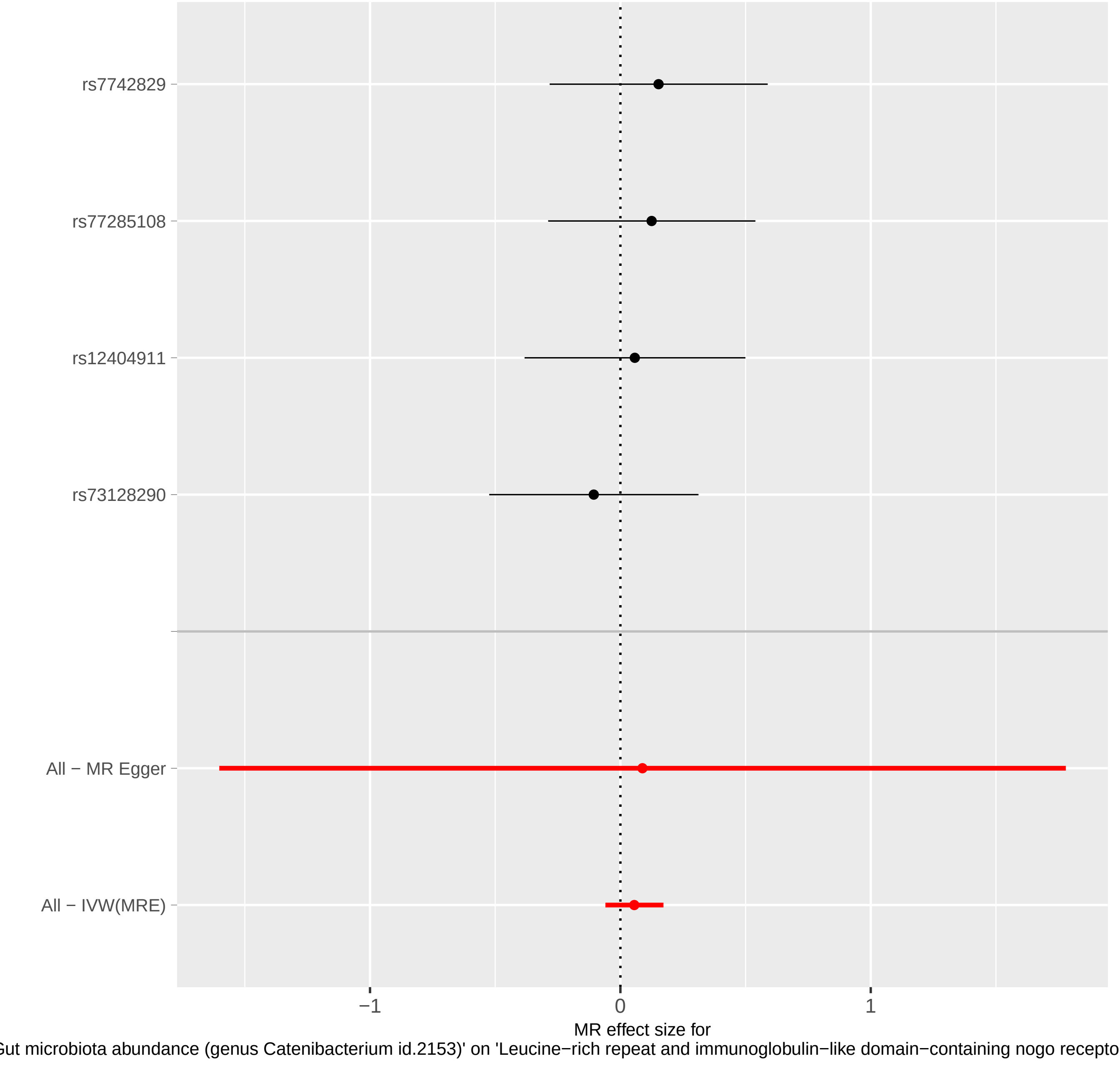

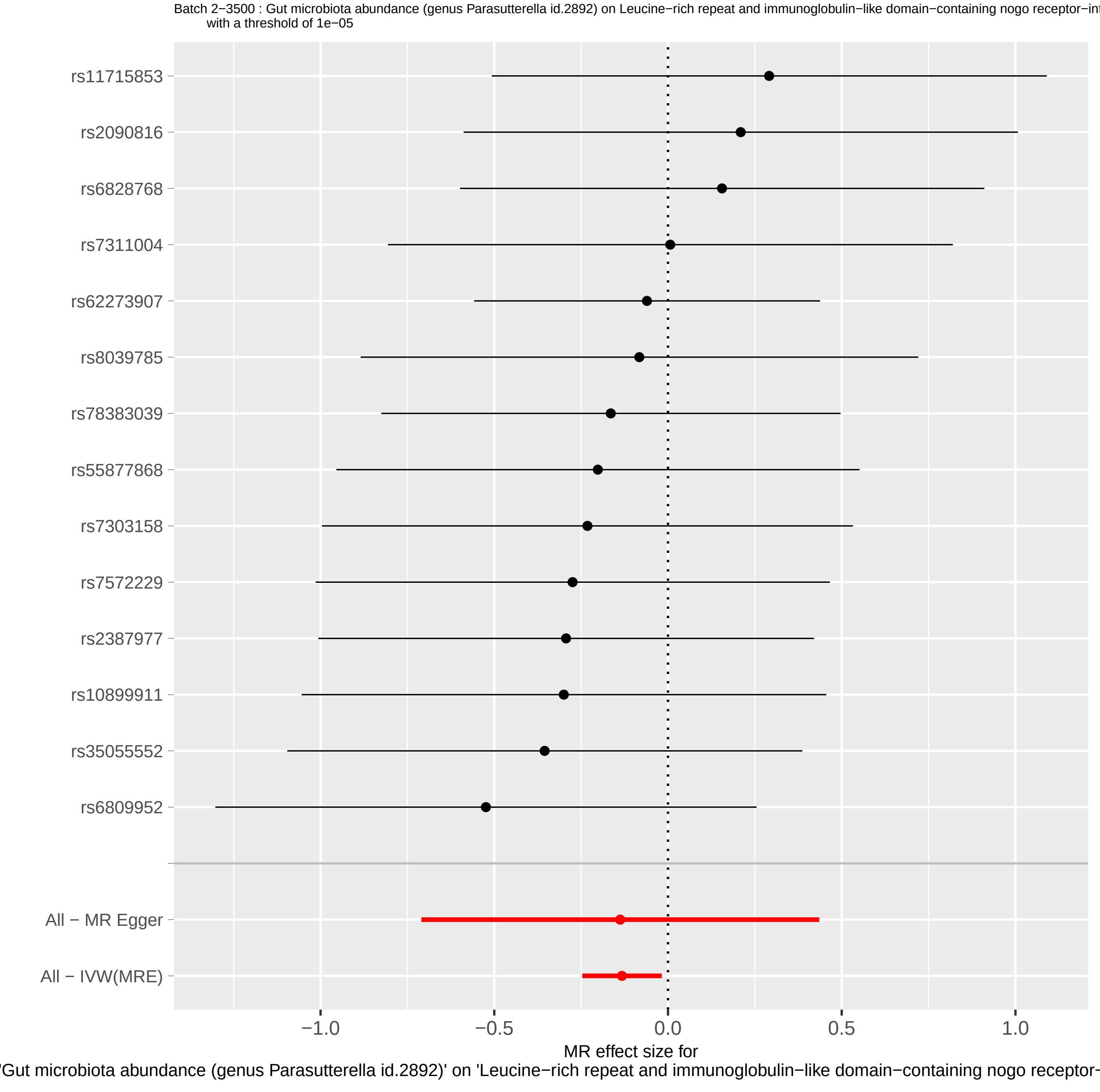

Batch 2−3500 : Gut microbiota abundance (genus Parasutterella id.2892) on Leucine−rich repeat and immunoglobulin−like domain−containing nogo receptor−in
with a threshold of 1e−05
rs11715853
rs2090816
rs6828768
rs7311004
rs62273907
rs8039785
rs78383039
rs55877868
rs7303158
rs7572229
rs2387977
rs10899911
rs35055552
rs6809952
All − MR Egger
All − IVW(MRE)
−1.0
−0.5
0.0
0.5
1.0
MR effect size for
'Gut microbiota abundance (genus Parasutterella id.2892)' on 'Leucine−rich repeat and immunoglobulin−like domain−containing nogo receptor−

Batch 2−3501 : Gut microbiota abundance (genus Catenibacterium id.2153) on Gastric triacylglycerol lipase
with a threshold of 1e−05

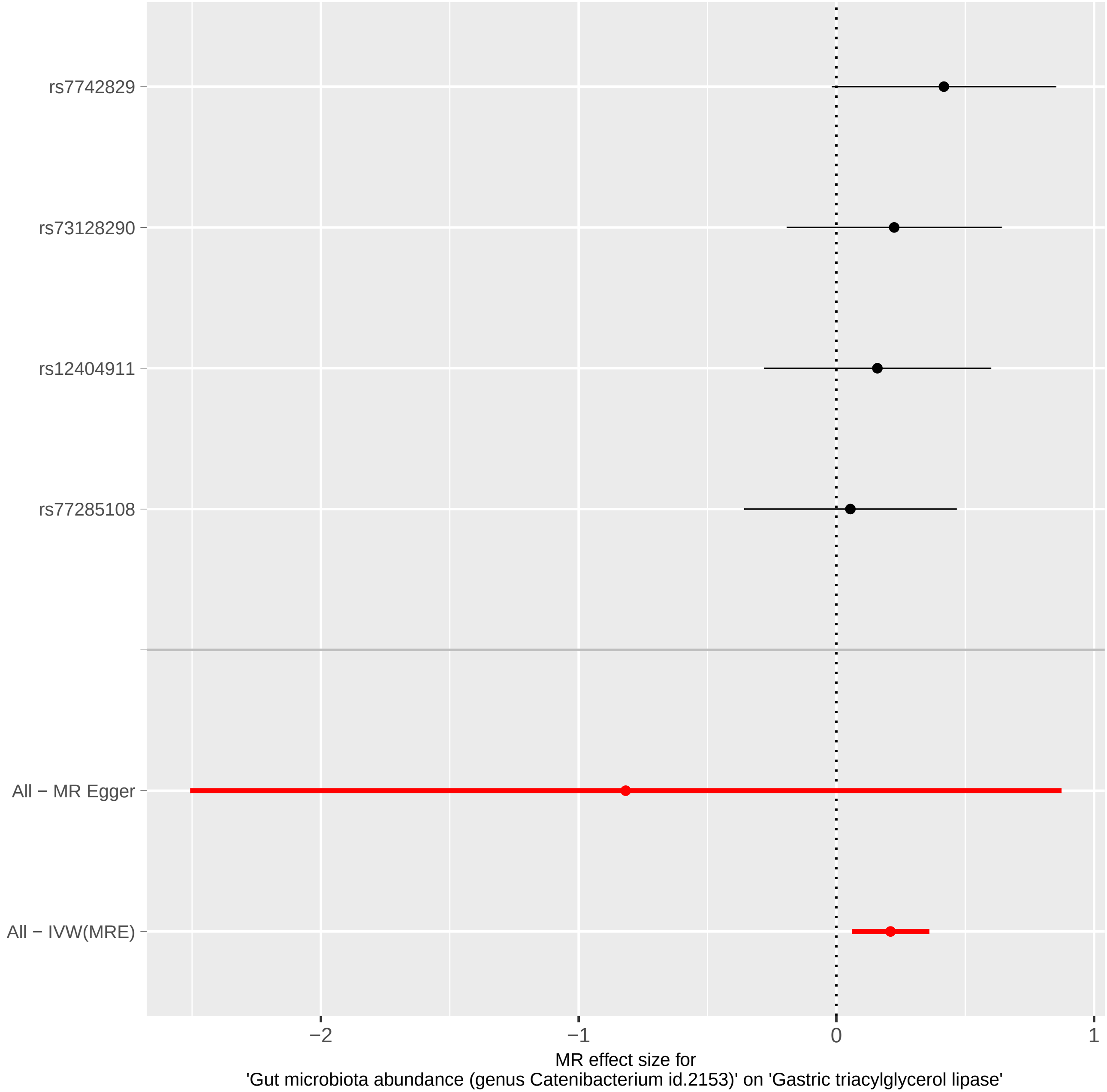

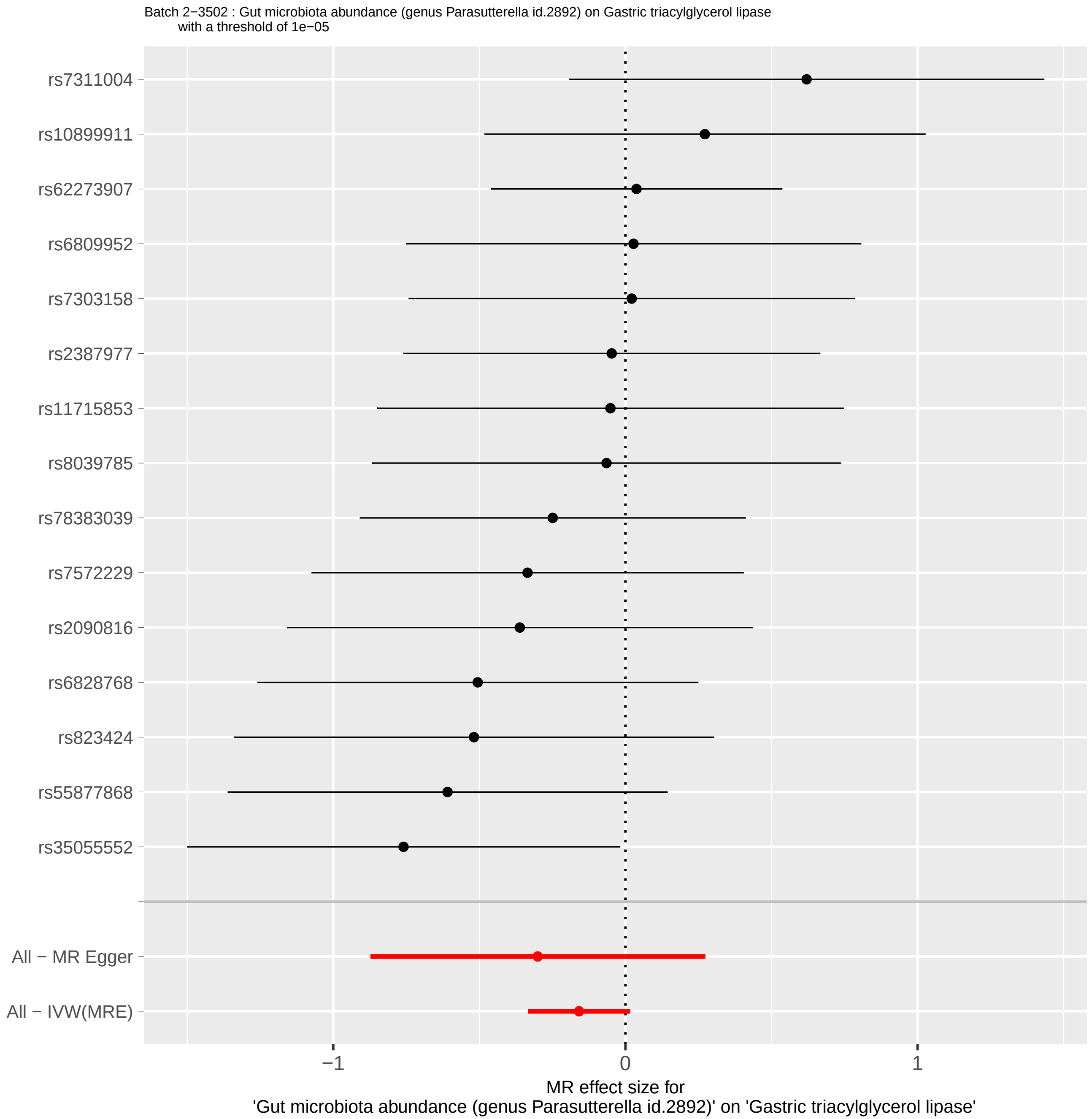

Batch 2−3502 : Gut microbiota abundance (genus Parasutterella id.2892) on Gastric triacylglycerol lipase
with a threshold of 1e−05
rs7311004
rs10899911
rs62273907
rs6809952
rs7303158
rs2387977
rs11715853
rs8039785
rs78383039
rs7572229
rs2090816
rs6828768
rs823424
rs55877868
rs35055552
All − MR Egger
All − IVW(MRE)
−1
0
1
MR effect size for
'Gut microbiota abundance (genus Parasutterella id.2892)' on 'Gastric triacylglycerol lipase'

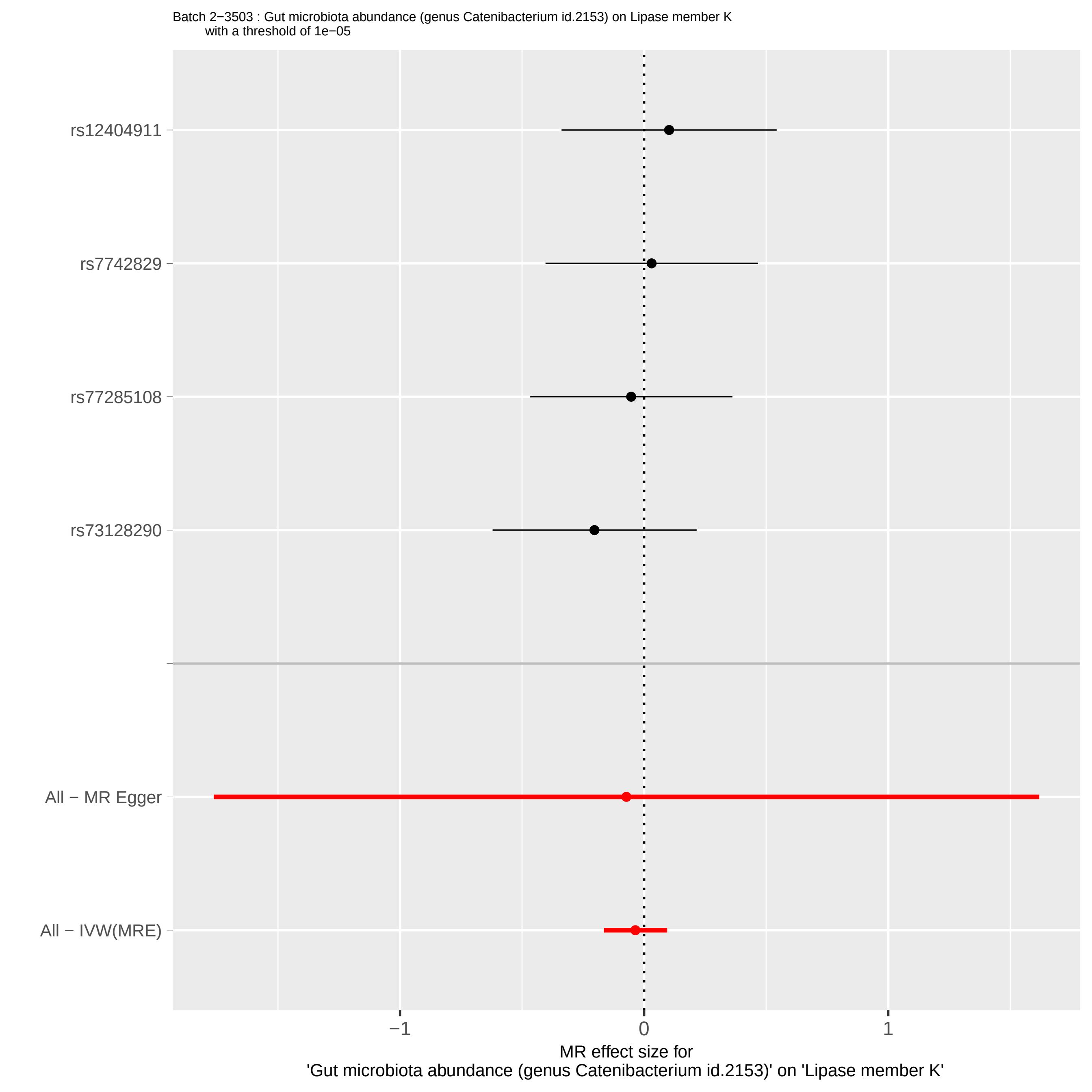
Batch 2−3503 : Gut microbiota abundance (genus Catenibacterium id.2153) on Lipase member K
with a threshold of 1e−05
rs12404911
rs7742829
rs77285108
rs73128290
All − MR Egger
All − IVW(MRE)
−1
0
1
MR effect size for
'Gut microbiota abundance (genus Catenibacterium id.2153)' on 'Lipase member K'

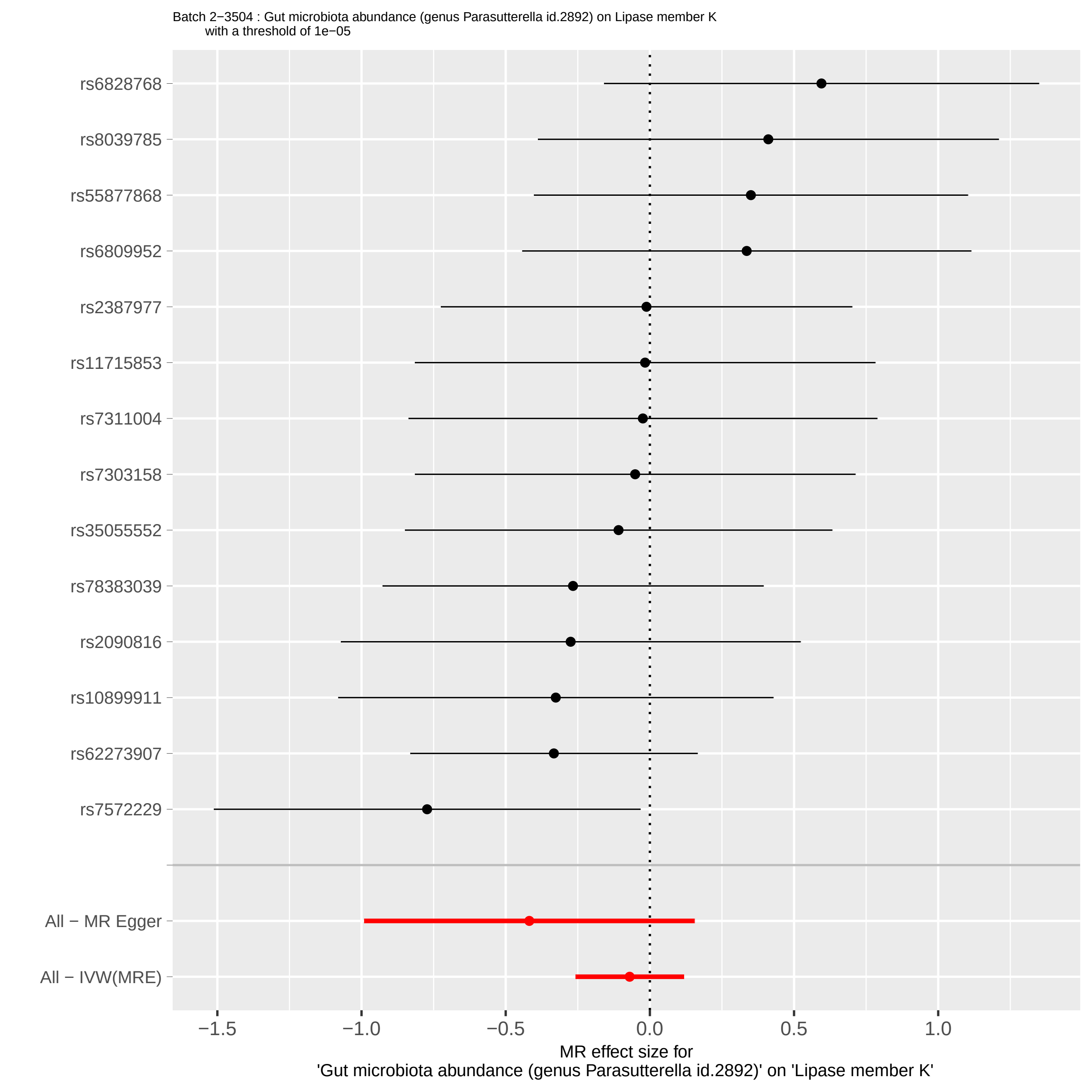
Batch 2−3504 : Gut microbiota abundance (genus Parasutterella id.2892) on Lipase member K
with a threshold of 1e−05
rs6828768
rs8039785
rs55877868
rs6809952
rs2387977
rs11715853
rs7311004
rs7303158
rs35055552
rs78383039
rs2090816
rs10899911
rs62273907
rs7572229
All − MR Egger
All − IVW(MRE)
−1.5
−1.0
−0.5
0.0
0.5
1.0
MR effect size for
'Gut microbiota abundance (genus Parasutterella id.2892)' on 'Lipase member K'

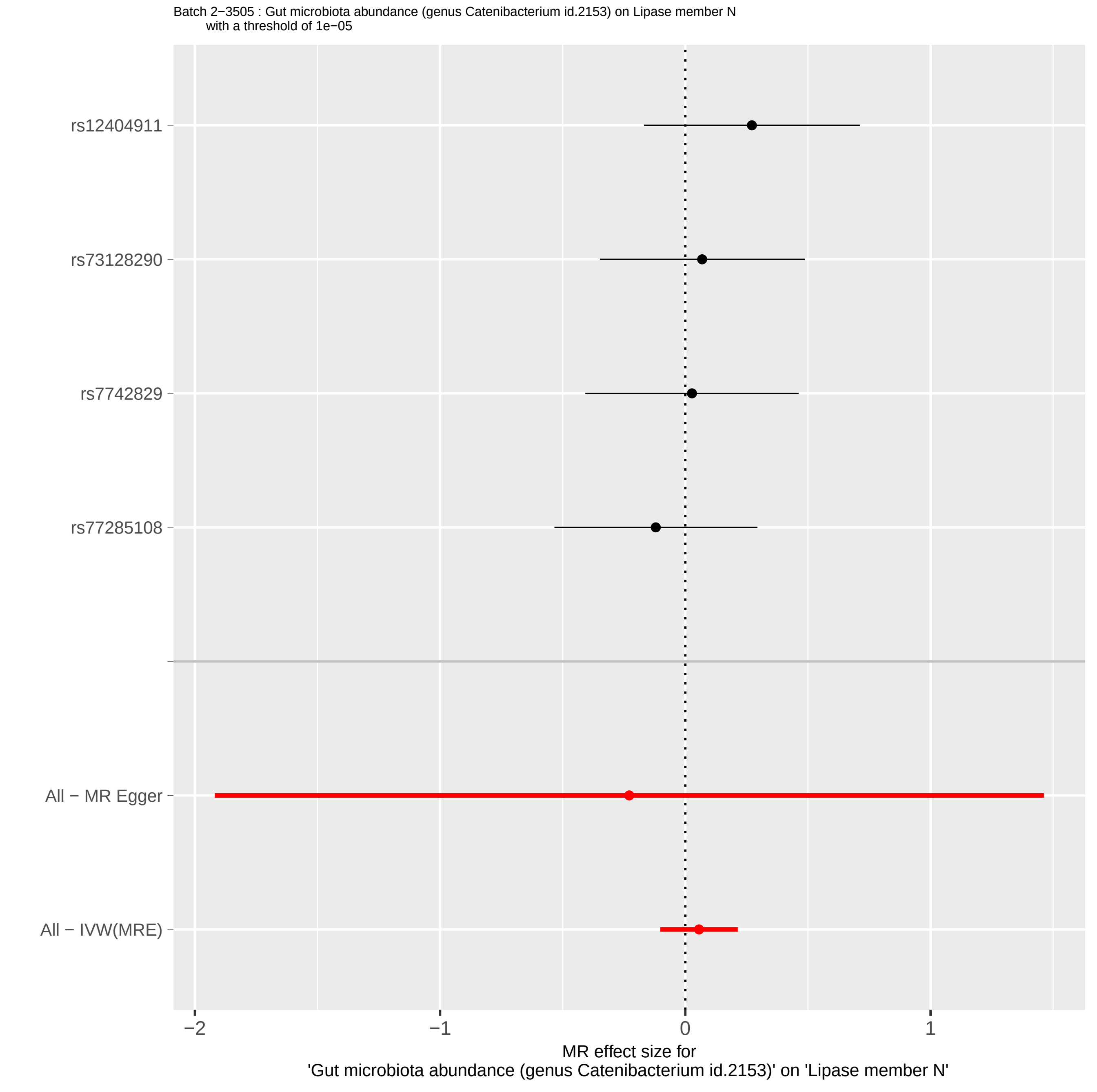
Batch 2−3505 : Gut microbiota abundance (genus Catenibacterium id.2153) on Lipase member N
with a threshold of 1e−05
rs12404911
rs73128290
rs7742829
rs77285108
All − MR Egger
All − IVW(MRE)
−2
−1
0
1
MR effect size for
'Gut microbiota abundance (genus Catenibacterium id.2153)' on 'Lipase member N'

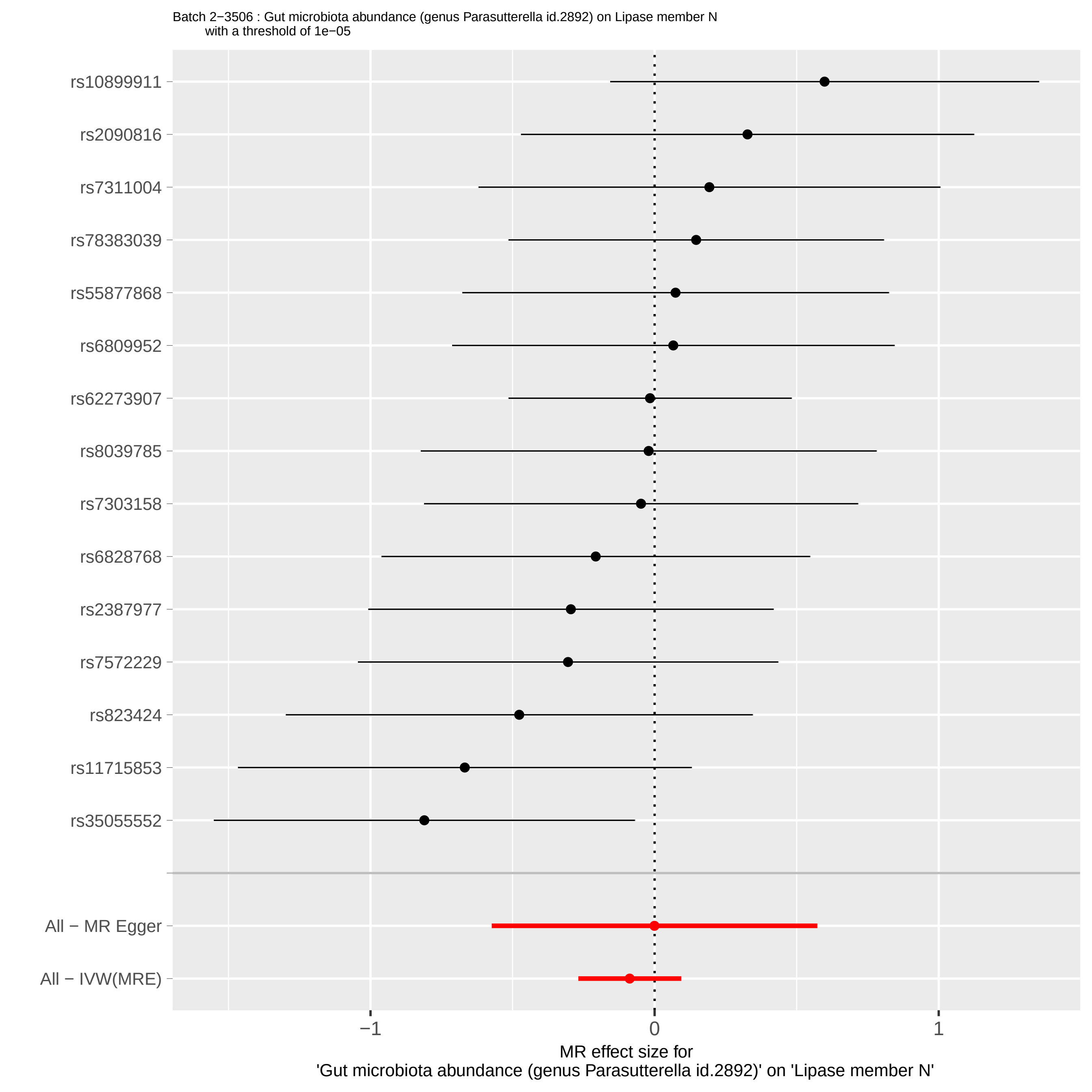

Batch 2−3506 : Gut microbiota abundance (genus Parasutterella id.2892) on Lipase member N
with a threshold of 1e−05
rs10899911
rs2090816
rs7311004
rs78383039
rs55877868
rs6809952
rs62273907
rs8039785
rs7303158
rs6828768
rs2387977
rs7572229
rs823424
rs11715853
rs35055552
All − MR Egger
All − IVW(MRE)
−1
0
1
MR effect size for
'Gut microbiota abundance (genus Parasutterella id.2892)' on 'Lipase member N'

Batch 2−3507 : Gut microbiota abundance (genus Catenibacterium id.2153) on Vesicular integral−membrane protein VIP36
with a threshold of 1e−05

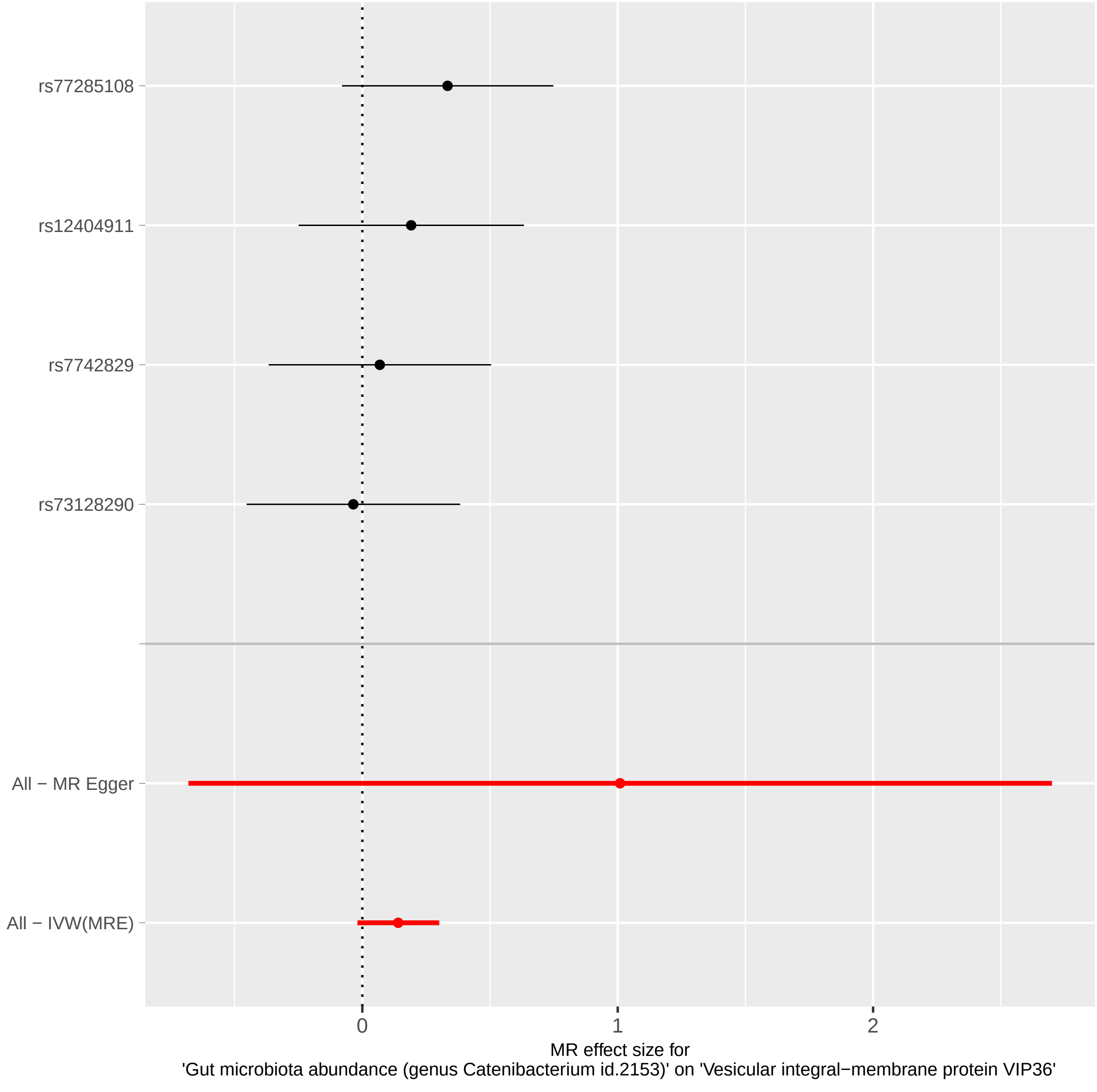

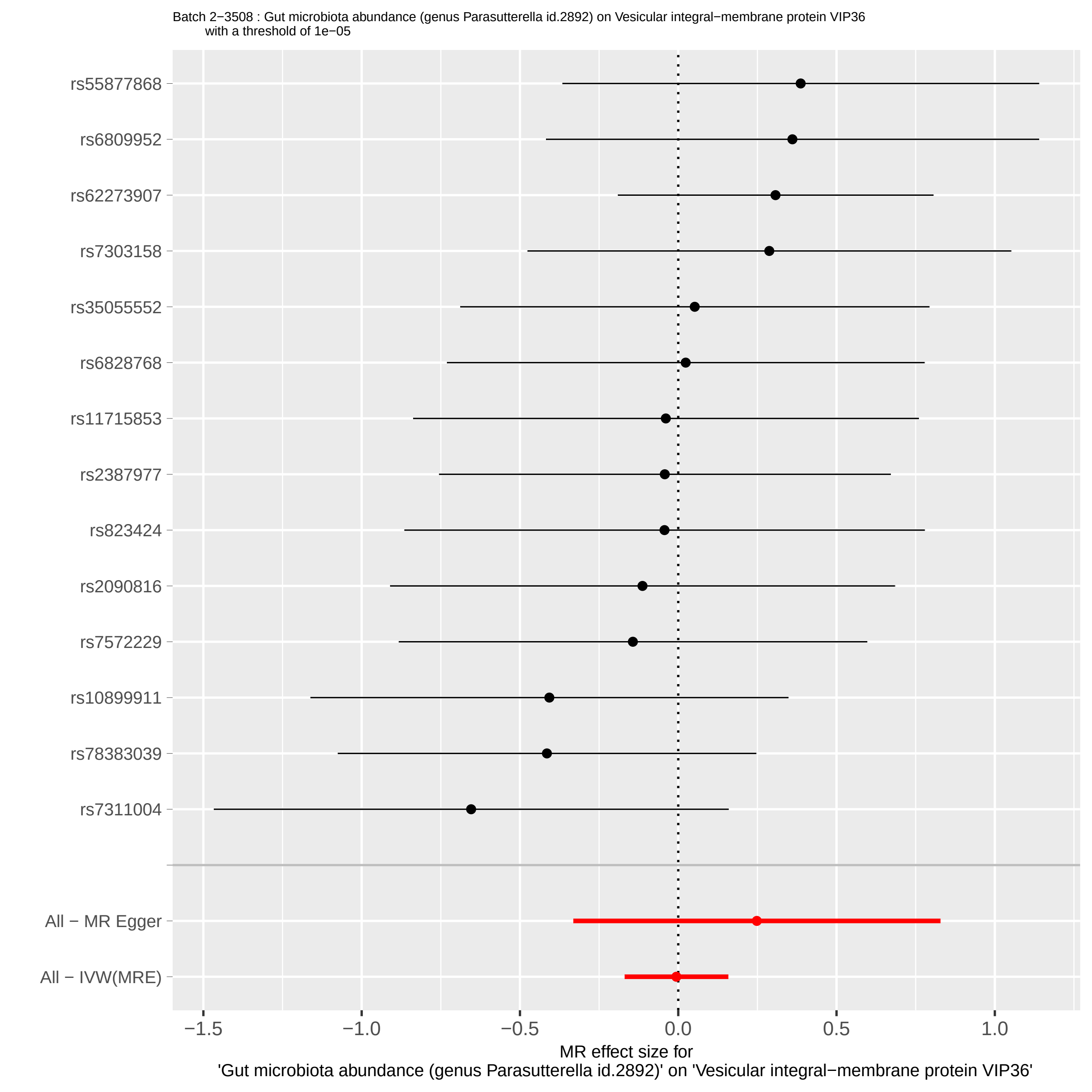

Batch 2−3508 : Gut microbiota abundance (genus Parasutterella id.2892) on Vesicular integral−membrane protein VIP36
with a threshold of 1e−05
rs55877868
rs6809952
rs62273907
rs7303158
rs35055552
rs6828768
rs11715853
rs2387977
rs823424
rs2090816
rs7572229
rs10899911
rs78383039
rs7311004
All − MR Egger
All − IVW(MRE)
−1.5
−1.0
−0.5
0.0
0.5
1.0
MR effect size for
'Gut microbiota abundance (genus Parasutterella id.2892)' on 'Vesicular integral−membrane protein VIP36'

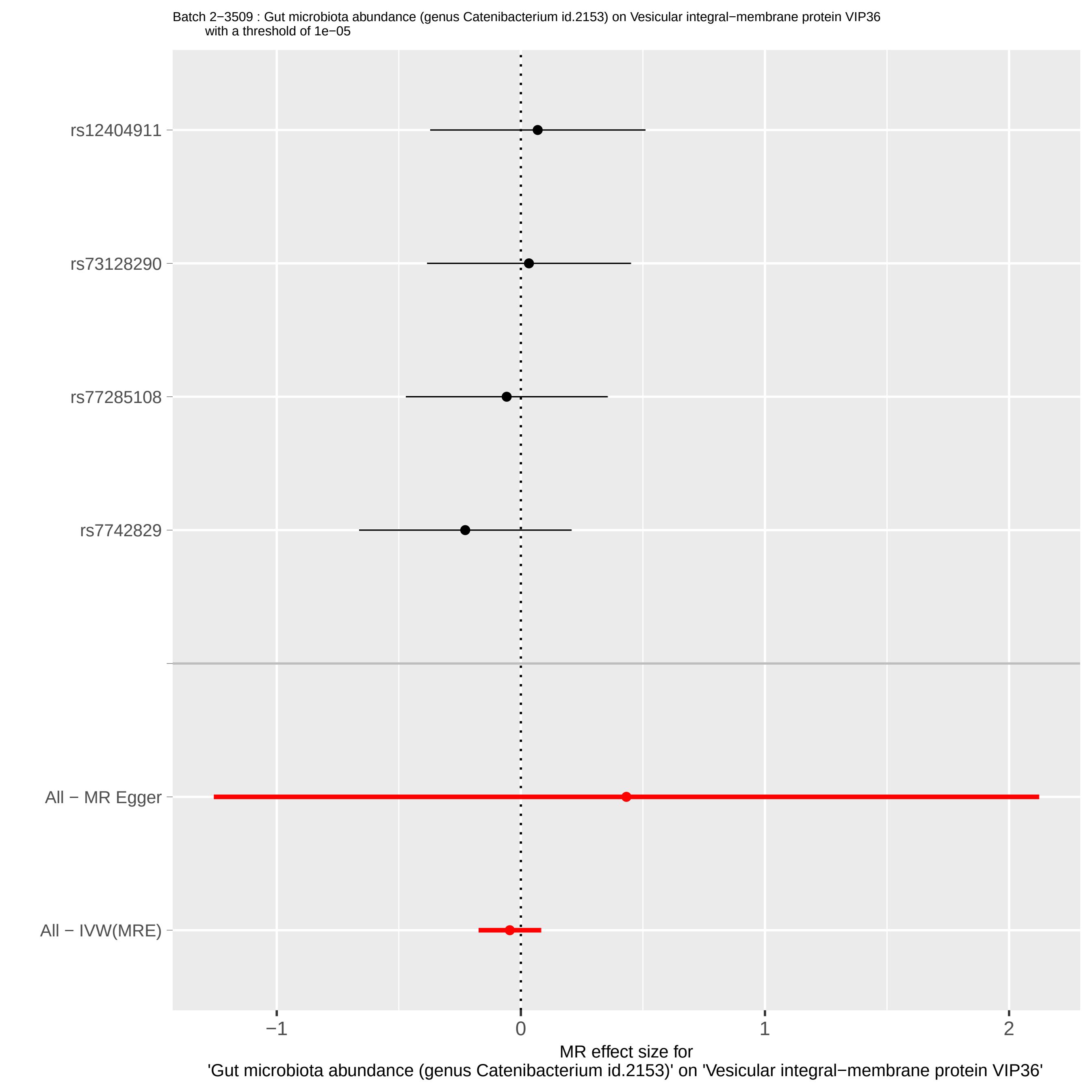
Batch 2−3509 : Gut microbiota abundance (genus Catenibacterium id.2153) on Vesicular integral−membrane protein VIP36
with a threshold of 1e−05
rs12404911
rs73128290
rs77285108
rs7742829
All − MR Egger
All − IVW(MRE)
−1
0
1
2
MR effect size for
'Gut microbiota abundance (genus Catenibacterium id.2153)' on 'Vesicular integral−membrane protein VIP36'

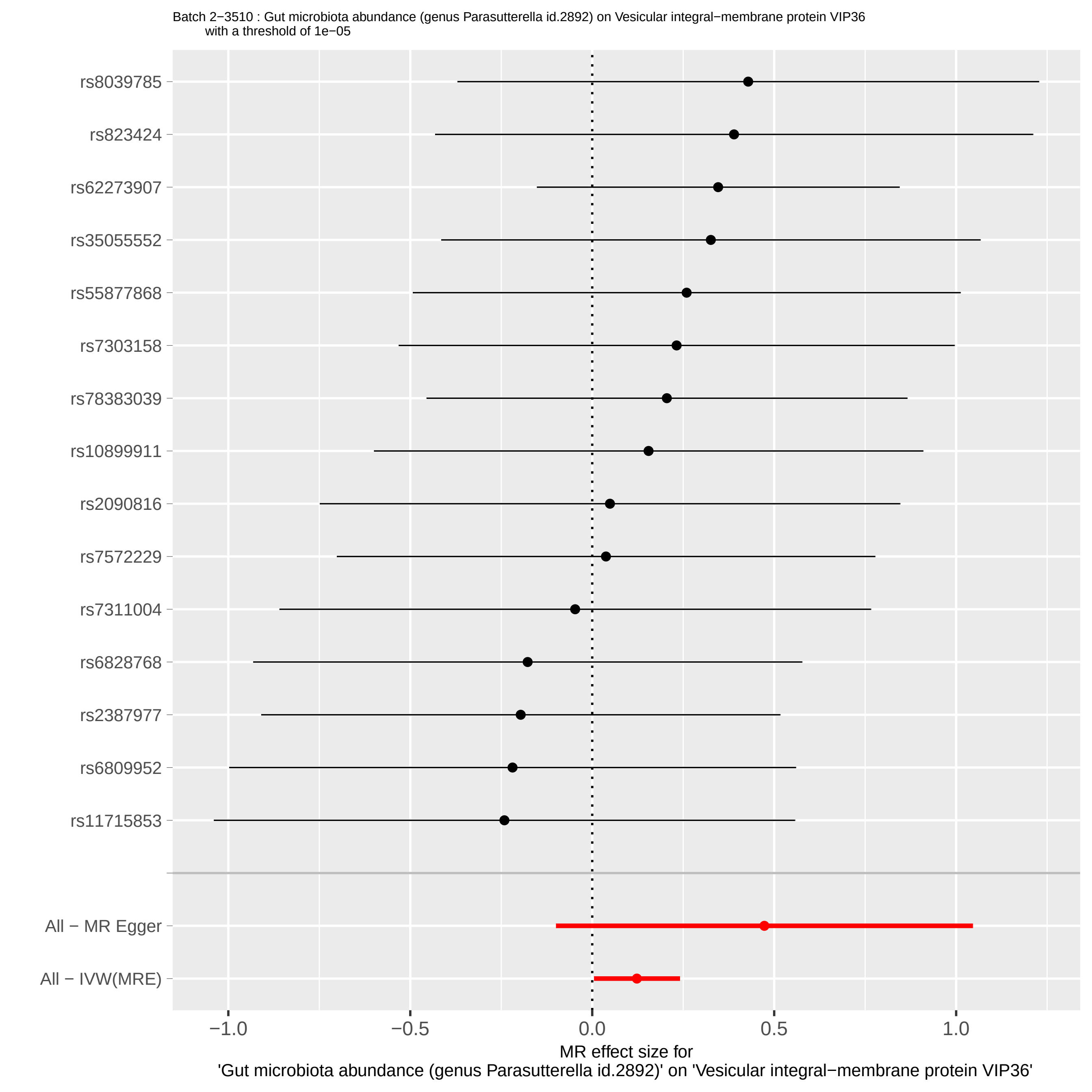
Batch 2−3510 : Gut microbiota abundance (genus Parasutterella id.2892) on Vesicular integral−membrane protein VIP36
with a threshold of 1e−05
rs8039785
rs823424
rs62273907
rs35055552
rs55877868
rs7303158
rs78383039
rs10899911
rs2090816
rs7572229
rs7311004
rs6828768
rs2387977
rs6809952
rs11715853
All − MR Egger
All − IVW(MRE)
−1.0
−0.5
0.0
0.5
1.0
MR effect size for
'Gut microbiota abundance (genus Parasutterella id.2892)' on 'Vesicular integral−membrane protein VIP36'

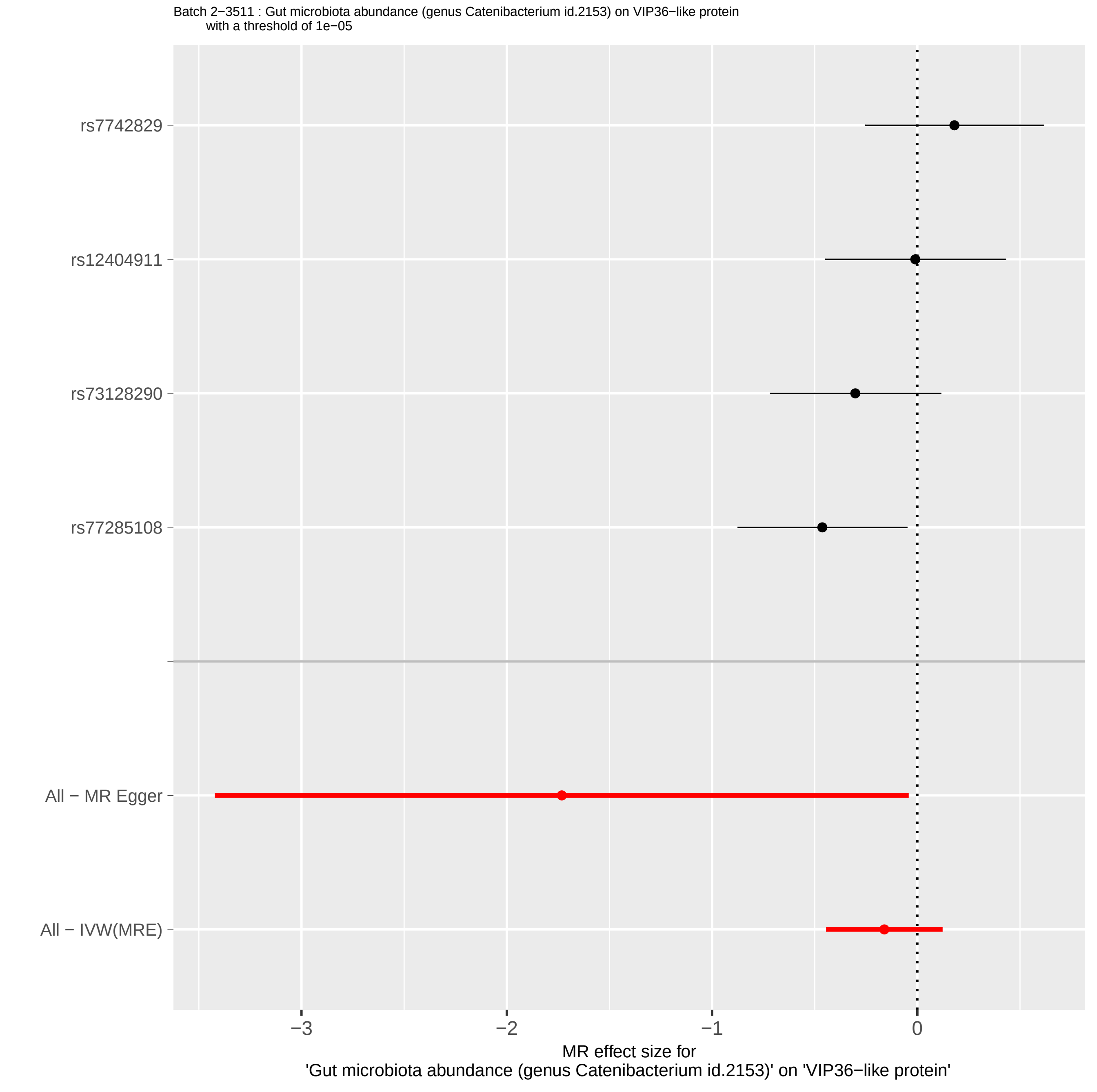

Batch 2−3511 : Gut microbiota abundance (genus Catenibacterium id.2153) on VIP36−like protein
with a threshold of 1e−05
rs7742829
rs12404911
rs73128290
rs77285108
All − MR Egger
All − IVW(MRE)
−3
−2
−1
0
MR effect size for
'Gut microbiota abundance (genus Catenibacterium id.2153)' on 'VIP36−like protein'

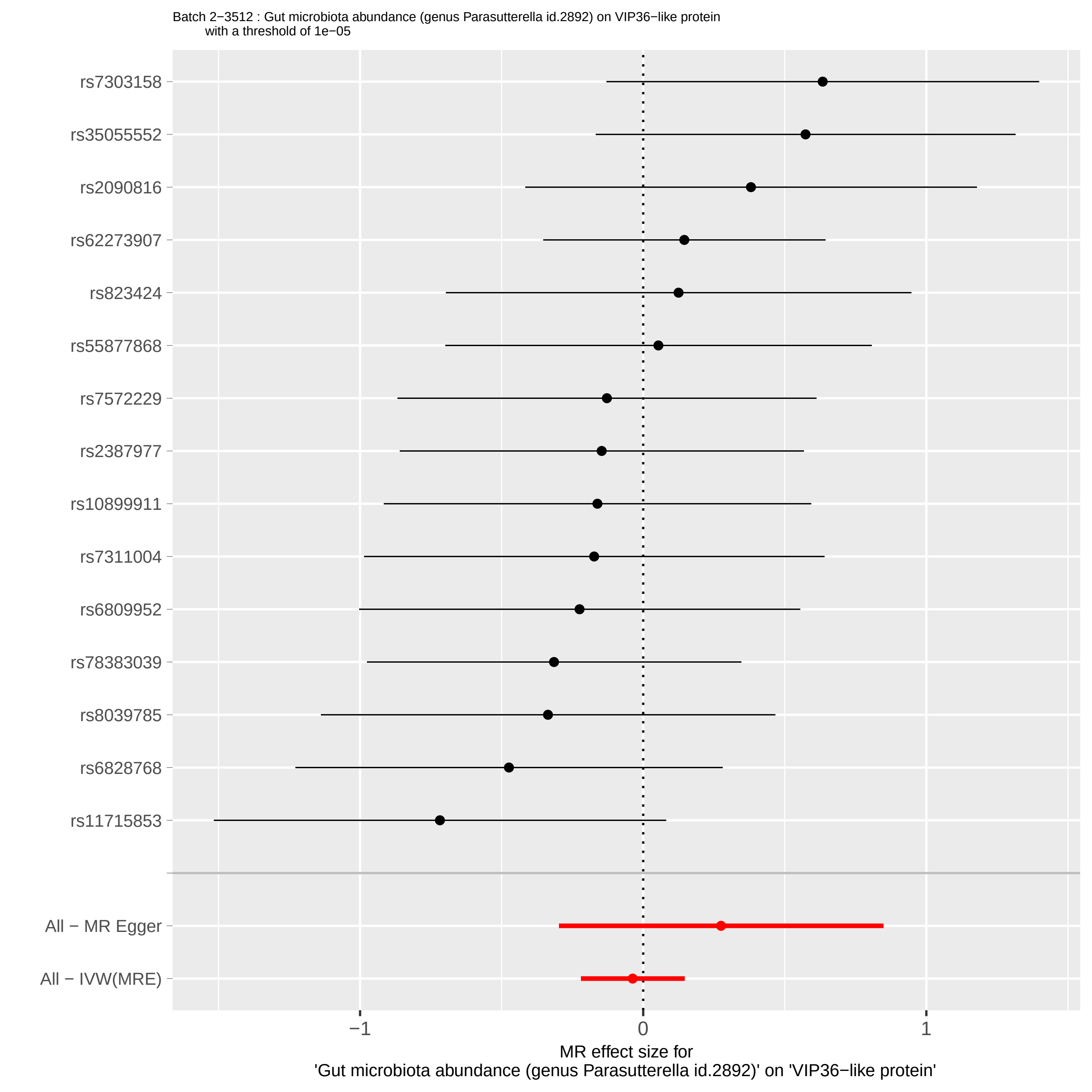
Batch 2−3512 : Gut microbiota abundance (genus Parasutterella id.2892) on VIP36−like protein
with a threshold of 1e−05
rs7303158
rs35055552
rs2090816
rs62273907
rs823424
rs55877868
rs7572229
rs2387977
rs10899911
rs7311004
rs6809952
rs78383039
rs8039785
rs6828768
rs11715853
All − MR Egger
All − IVW(MRE)
−1
0
1
MR effect size for
'Gut microbiota abundance (genus Parasutterella id.2892)' on 'VIP36−like protein'

Batch 2−3513 : Gut microbiota abundance (genus Catenibacterium id.2153) on LIM and cysteine−rich domains protein 1
with a threshold of 1e−05

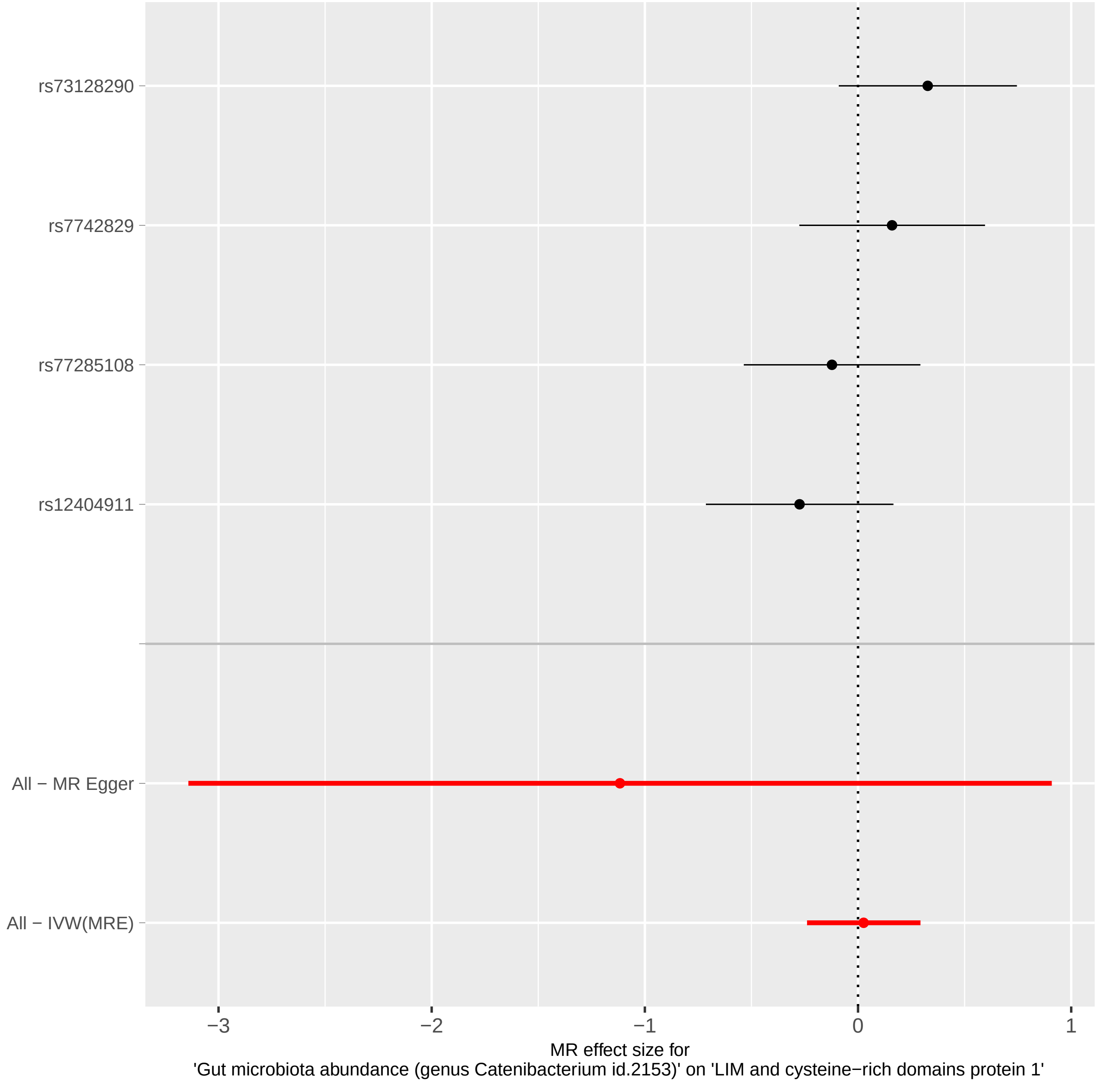

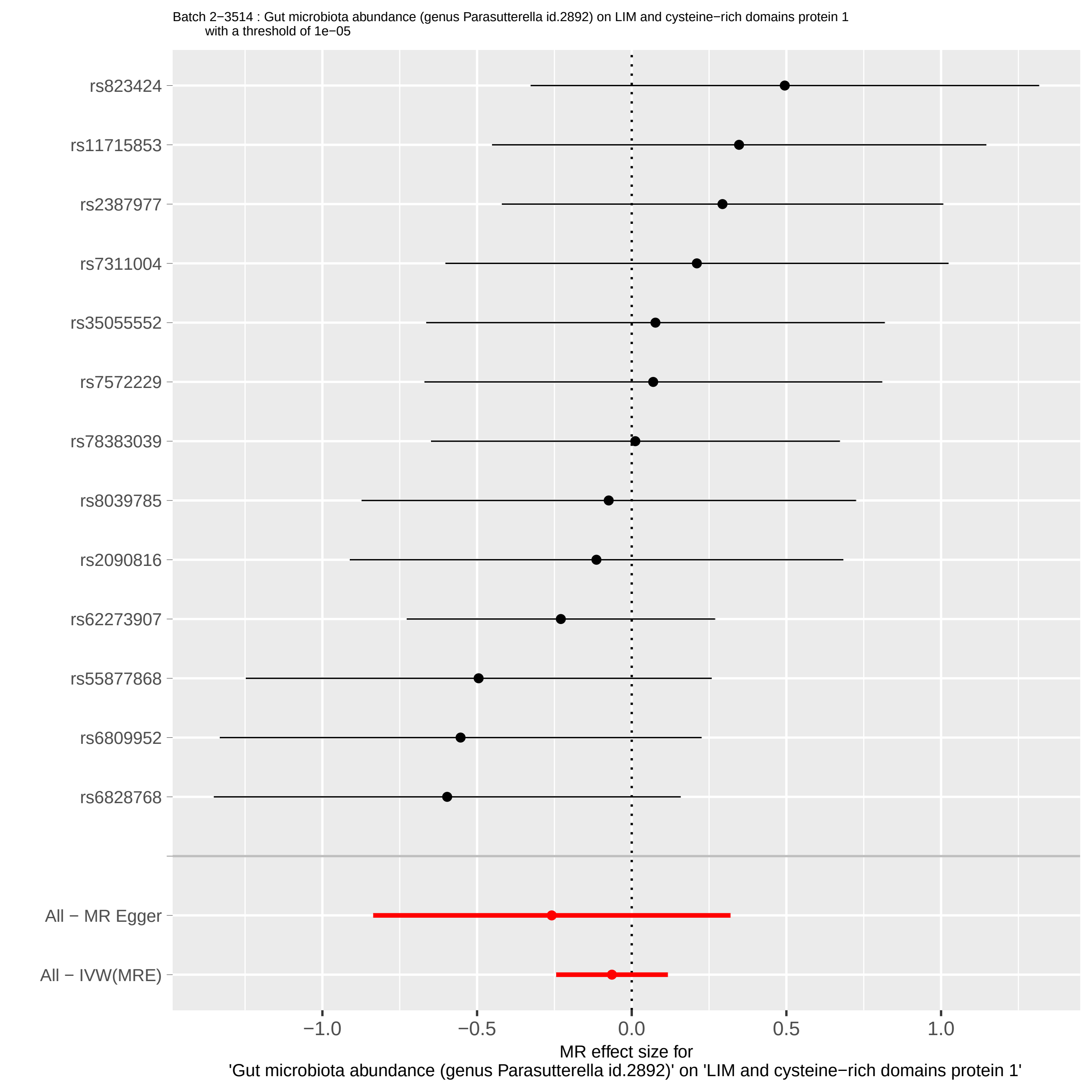
Batch 2−3514 : Gut microbiota abundance (genus Parasutterella id.2892) on LIM and cysteine−rich domains protein 1
with a threshold of 1e−05
rs823424
rs11715853
rs2387977
rs7311004
rs35055552
rs7572229
rs78383039
rs8039785
rs2090816
rs62273907
rs55877868
rs6809952
rs6828768
All − MR Egger
All − IVW(MRE)
−1.0
−0.5
0.0
0.5
1.0
MR effect size for
'Gut microbiota abundance (genus Parasutterella id.2892)' on 'LIM and cysteine−rich domains protein 1'

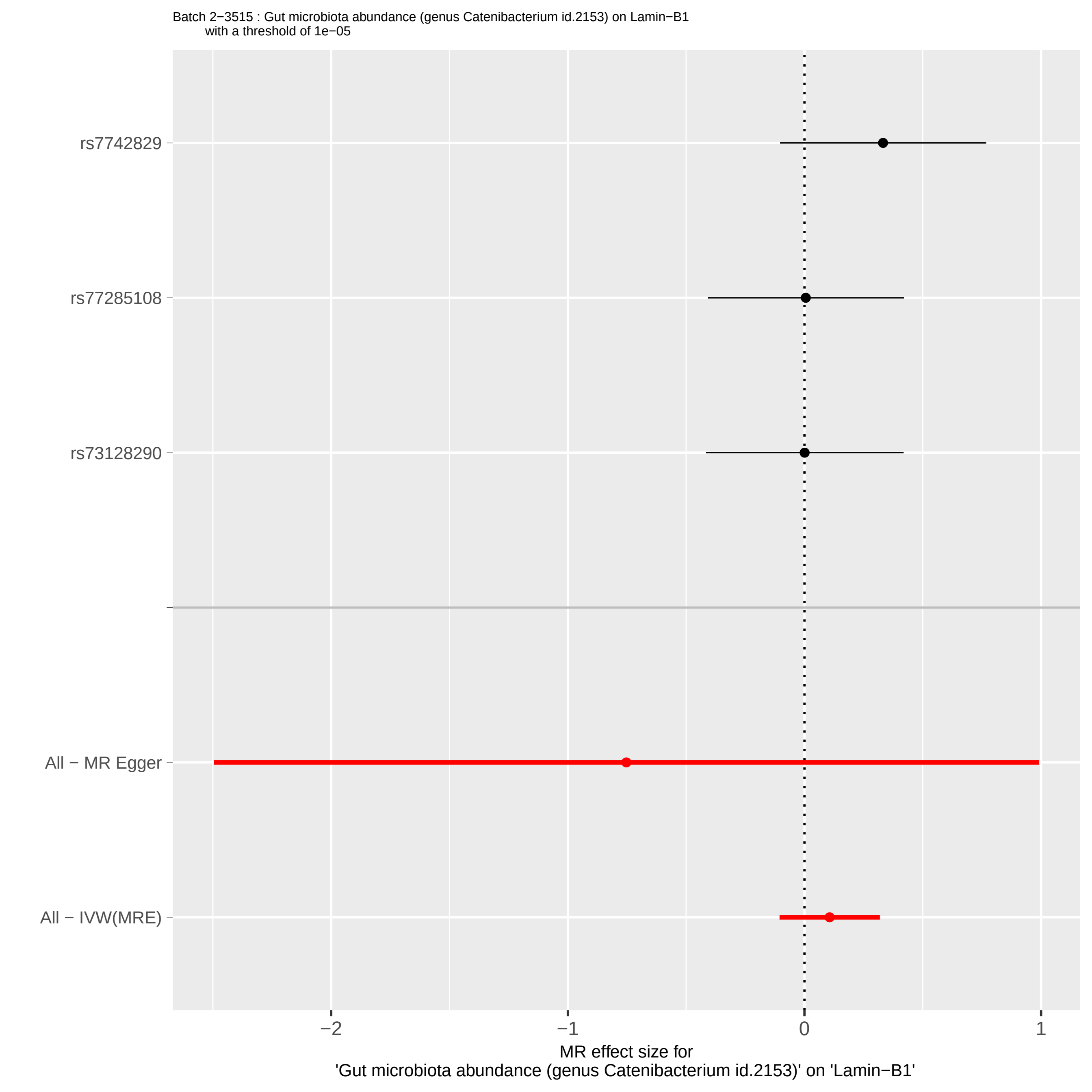

Batch 2−3515 : Gut microbiota abundance (genus Catenibacterium id.2153) on Lamin−B1
with a threshold of 1e−05
rs7742829
rs77285108
rs73128290
All − MR Egger
All − IVW(MRE)
−2
−1
0
1
MR effect size for
'Gut microbiota abundance (genus Catenibacterium id.2153)' on 'Lamin−B1'

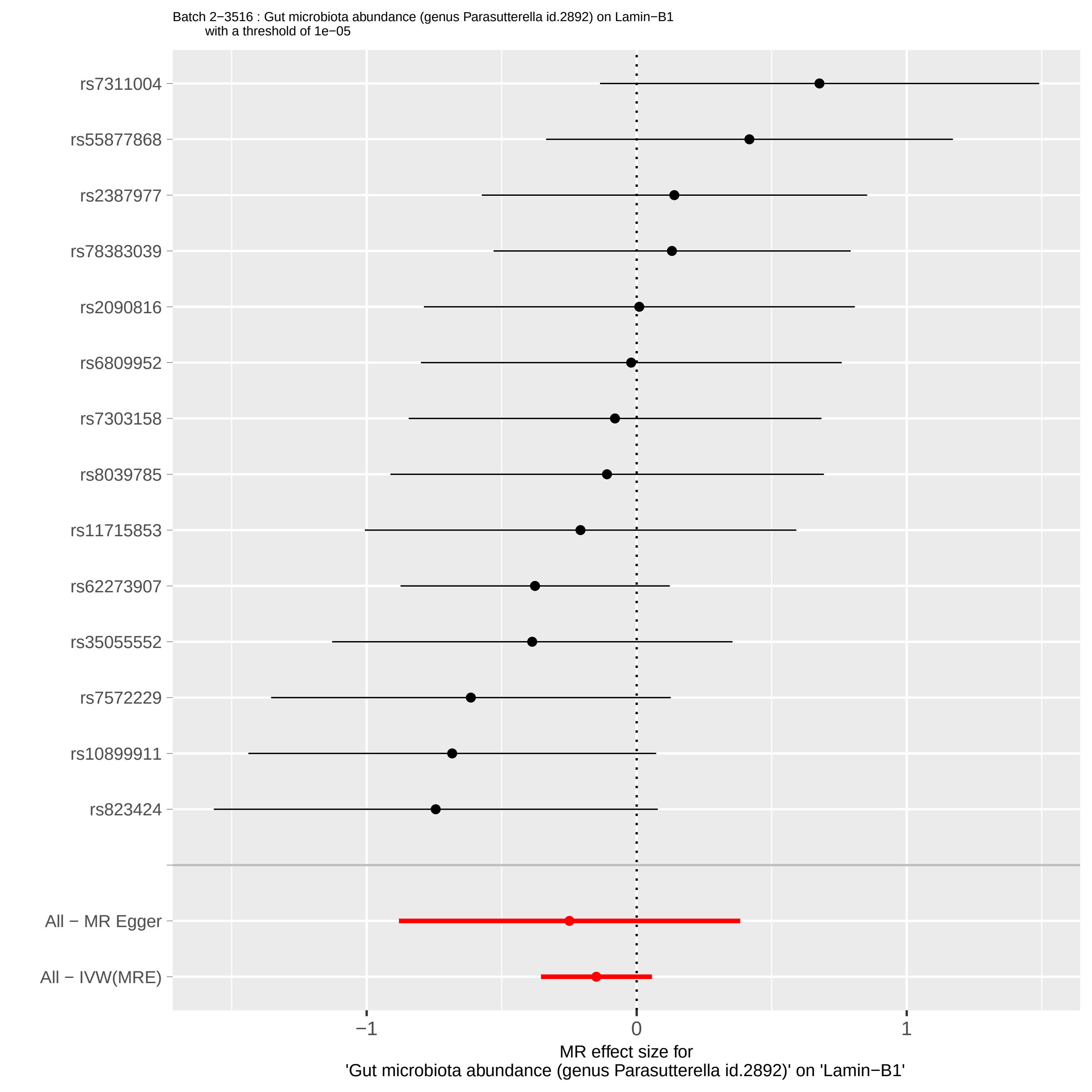

Batch 2−3516 : Gut microbiota abundance (genus Parasutterella id.2892) on Lamin−B1
with a threshold of 1e−05
rs7311004
rs55877868
rs2387977
rs78383039
rs2090816
rs6809952
rs7303158
rs8039785
rs11715853
rs62273907
rs35055552
rs7572229
rs10899911
rs823424
All − MR Egger
All − IVW(MRE)
−1
0
1
MR effect size for
'Gut microbiota abundance (genus Parasutterella id.2892)' on 'Lamin−B1'

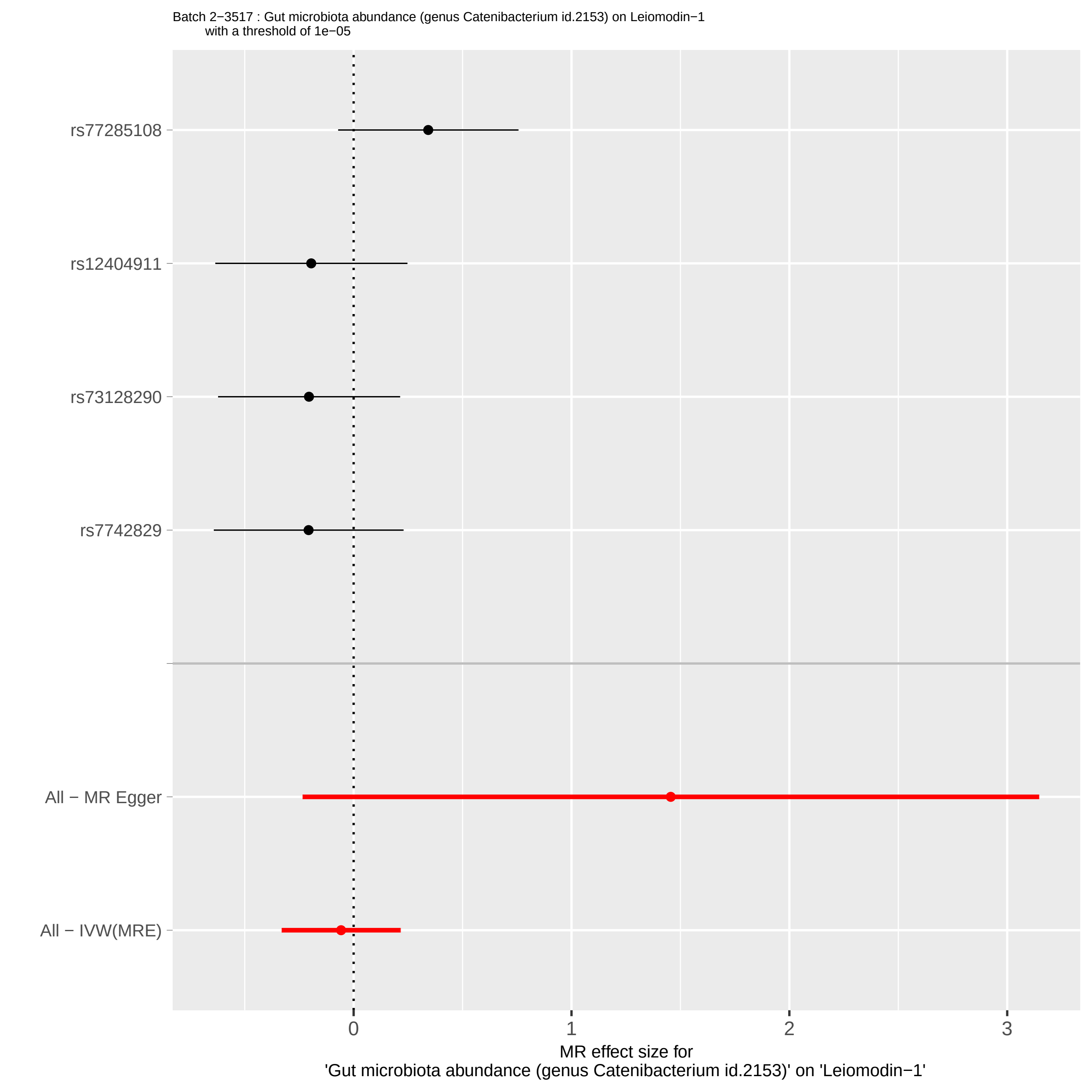
Batch 2−3517 : Gut microbiota abundance (genus Catenibacterium id.2153) on Leiomodin−1
with a threshold of 1e−05
rs77285108
rs12404911
rs73128290
rs7742829
All − MR Egger
All − IVW(MRE)
0
1
2
3
MR effect size for
'Gut microbiota abundance (genus Catenibacterium id.2153)' on 'Leiomodin−1'

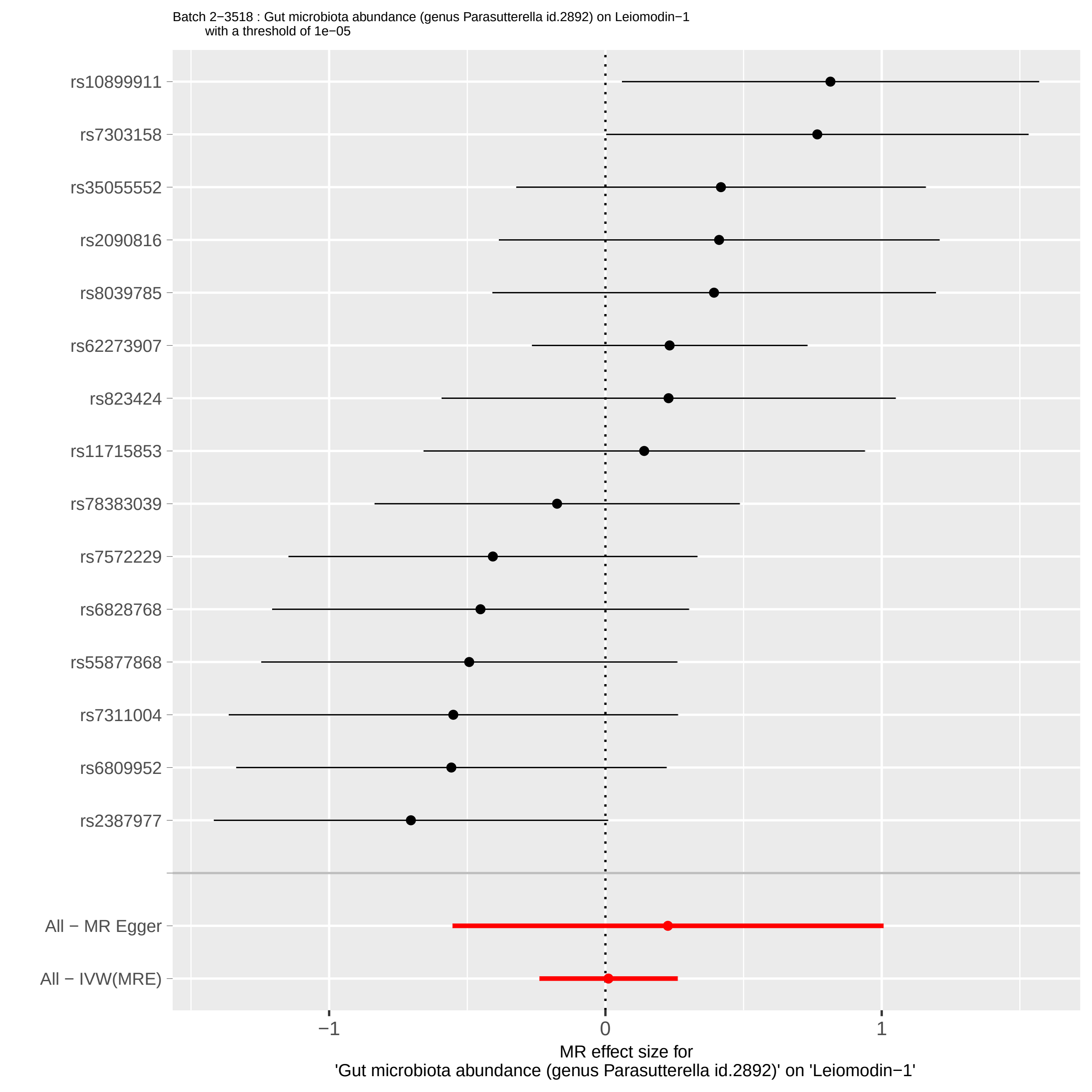
Batch 2−3518 : Gut microbiota abundance (genus Parasutterella id.2892) on Leiomodin−1
with a threshold of 1e−05
rs10899911
rs7303158
rs35055552
rs2090816
rs8039785
rs62273907
rs823424
rs11715853
rs78383039
rs7572229
rs6828768
rs55877868
rs7311004
rs6809952
rs2387977
All − MR Egger
All − IVW(MRE)
−1
0
1
MR effect size for
'Gut microbiota abundance (genus Parasutterella id.2892)' on 'Leiomodin−1'

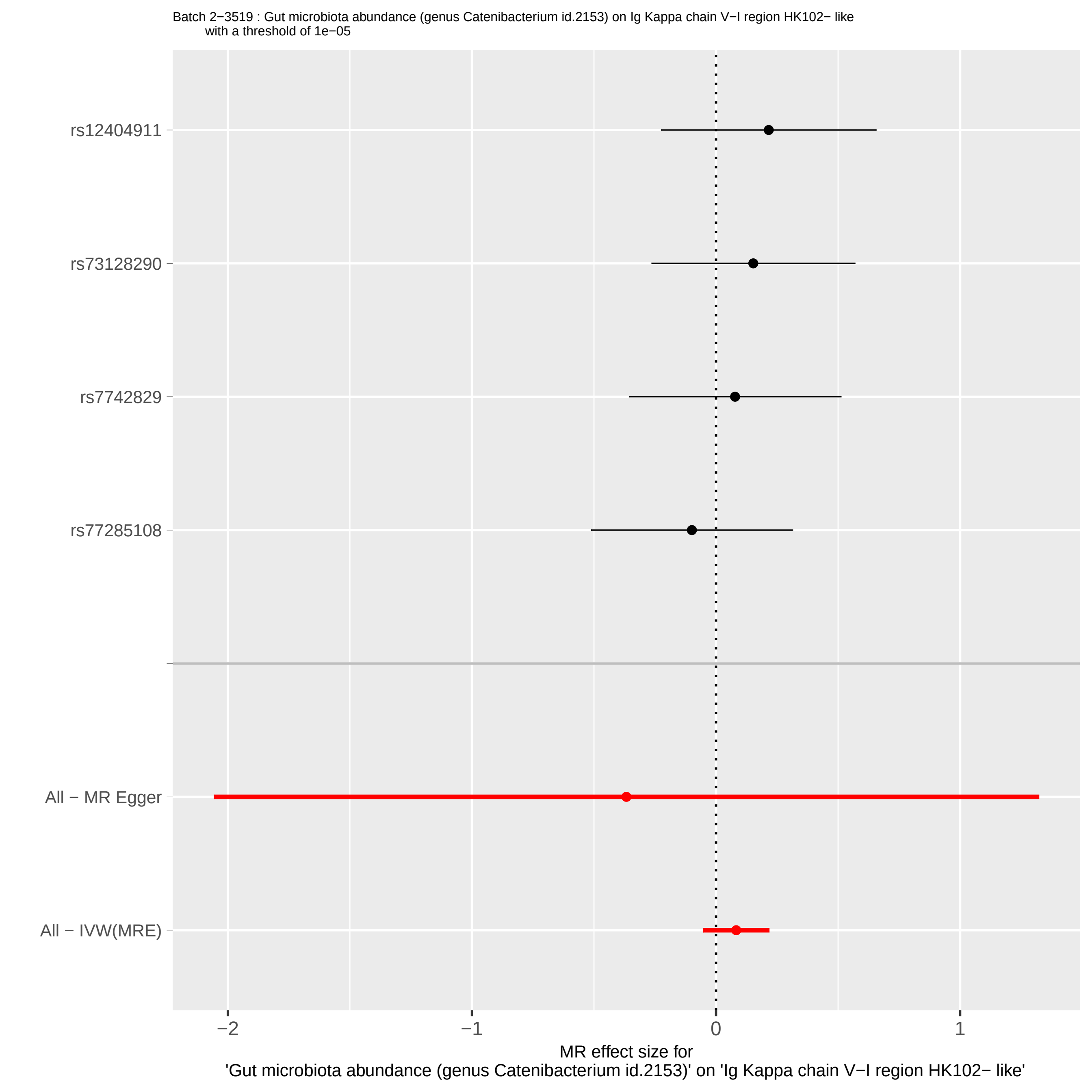
Batch 2−3519 : Gut microbiota abundance (genus Catenibacterium id.2153) on Ig Kappa chain V−I region HK102− like
with a threshold of 1e−05
rs12404911
rs73128290
rs7742829
rs77285108
All − MR Egger
All − IVW(MRE)
−2
−1
0
1
MR effect size for
'Gut microbiota abundance (genus Catenibacterium id.2153)' on 'Ig Kappa chain V−I region HK102− like'

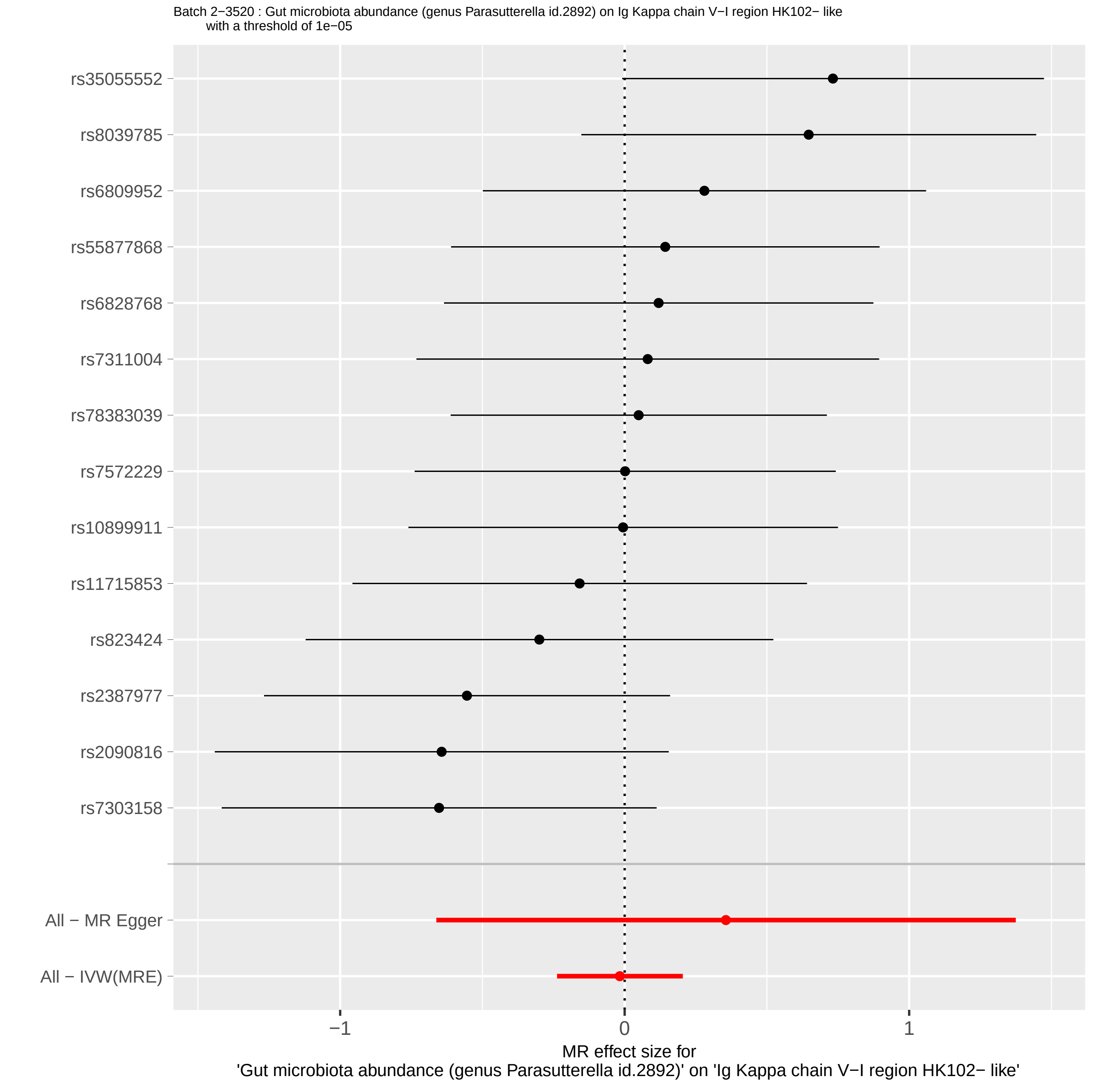

Batch 2−3520 : Gut microbiota abundance (genus Parasutterella id.2892) on Ig Kappa chain V−I region HK102− like
with a threshold of 1e−05
rs35055552
rs8039785
rs6809952
rs55877868
rs6828768
rs7311004
rs78383039
rs7572229
rs10899911
rs11715853
rs823424
rs2387977
rs2090816
rs7303158
All − MR Egger
All − IVW(MRE)
−1
0
1
MR effect size for
'Gut microbiota abundance (genus Parasutterella id.2892)' on 'Ig Kappa chain V−I region HK102− like'

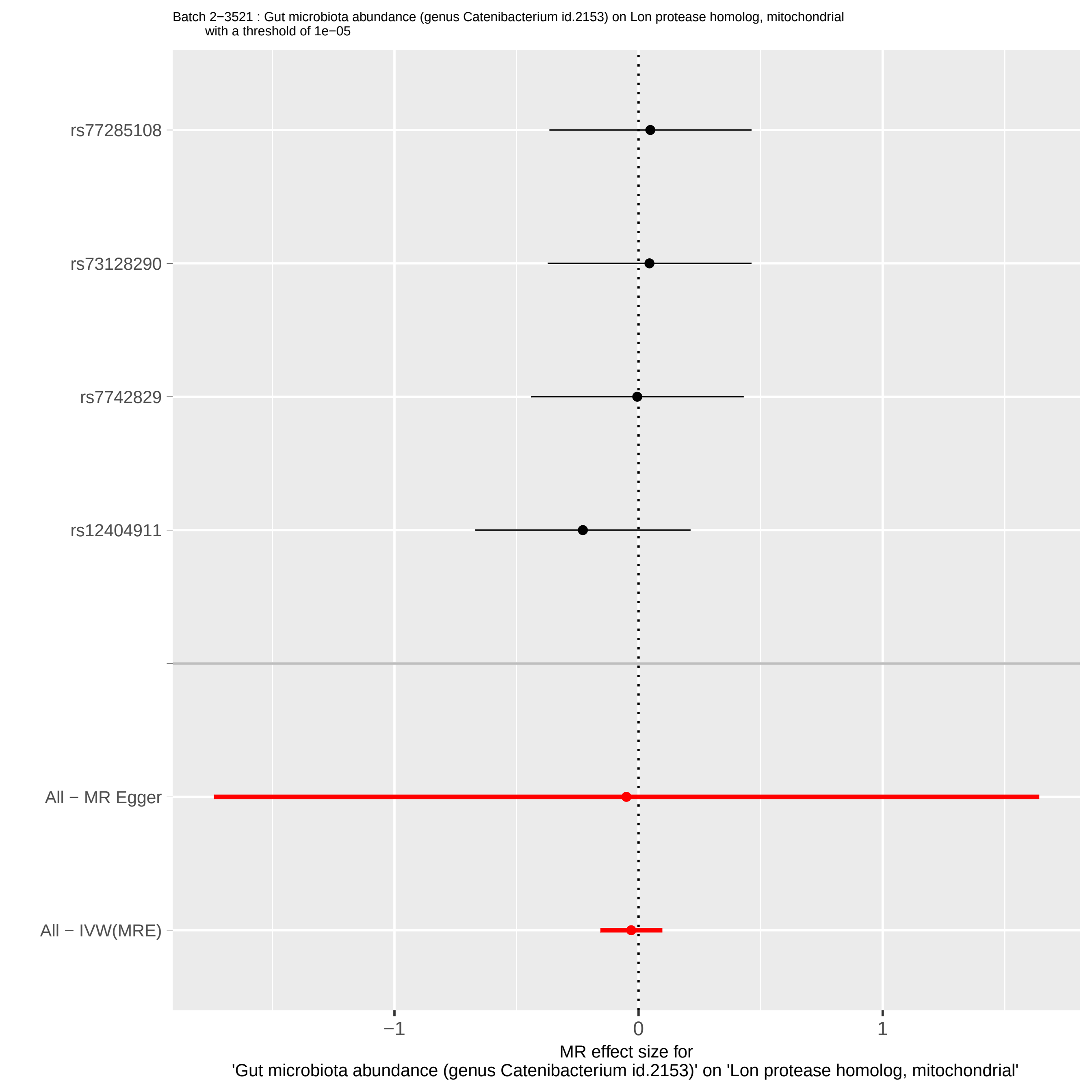

Batch 2−3521 : Gut microbiota abundance (genus Catenibacterium id.2153) on Lon protease homolog, mitochondrial
with a threshold of 1e−05
rs77285108
rs73128290
rs7742829
rs12404911
All − MR Egger
All − IVW(MRE)
−1
0
1
MR effect size for
'Gut microbiota abundance (genus Catenibacterium id.2153)' on 'Lon protease homolog, mitochondrial'

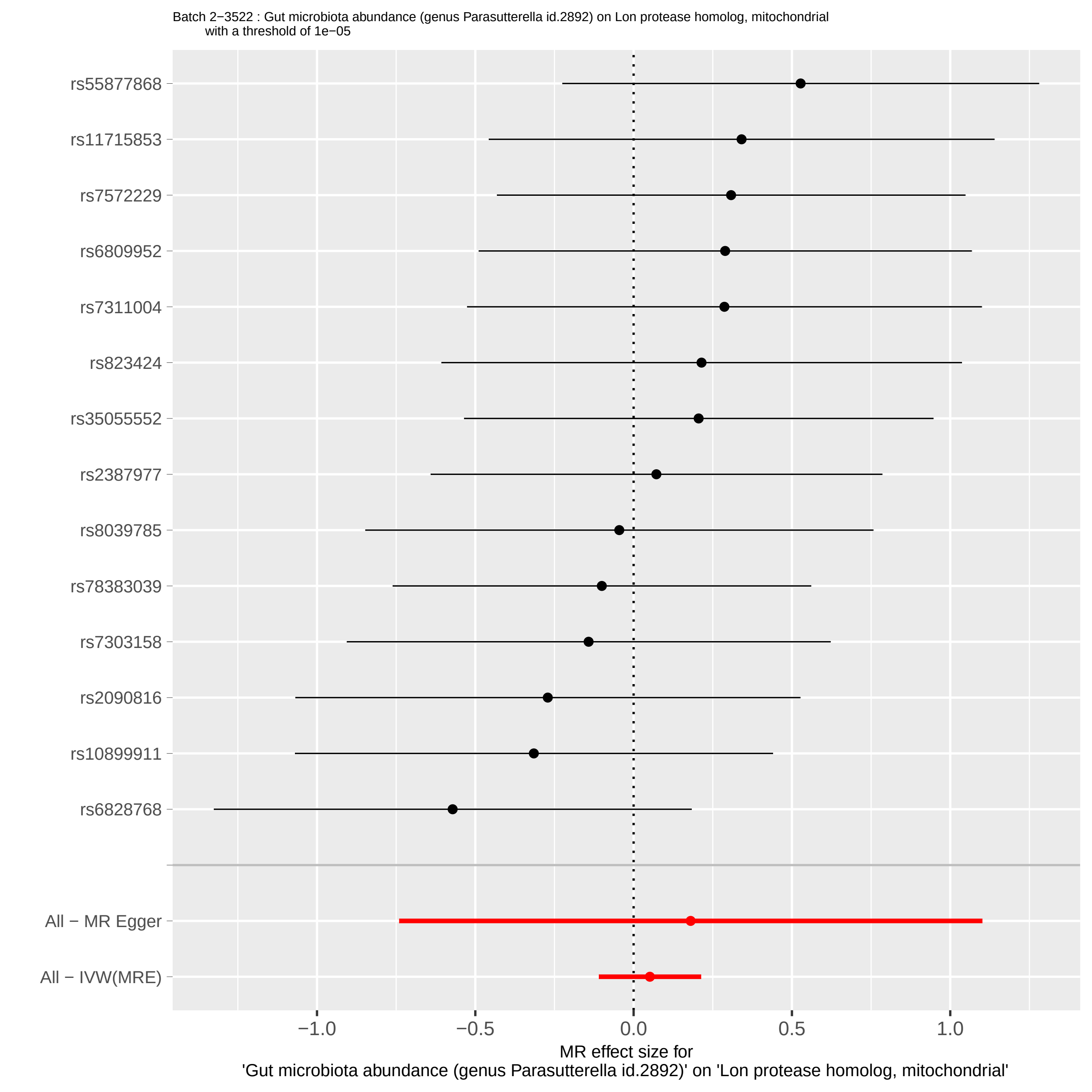

Batch 2−3522 : Gut microbiota abundance (genus Parasutterella id.2892) on Lon protease homolog, mitochondrial
with a threshold of 1e−05
rs55877868
rs11715853
rs7572229
rs6809952
rs7311004
rs823424
rs35055552
rs2387977
rs8039785
rs78383039
rs7303158
rs2090816
rs10899911
rs6828768
All − MR Egger
All − IVW(MRE)
−1.0
−0.5
0.0
0.5
1.0
MR effect size for
'Gut microbiota abundance (genus Parasutterella id.2892)' on 'Lon protease homolog, mitochondrial'

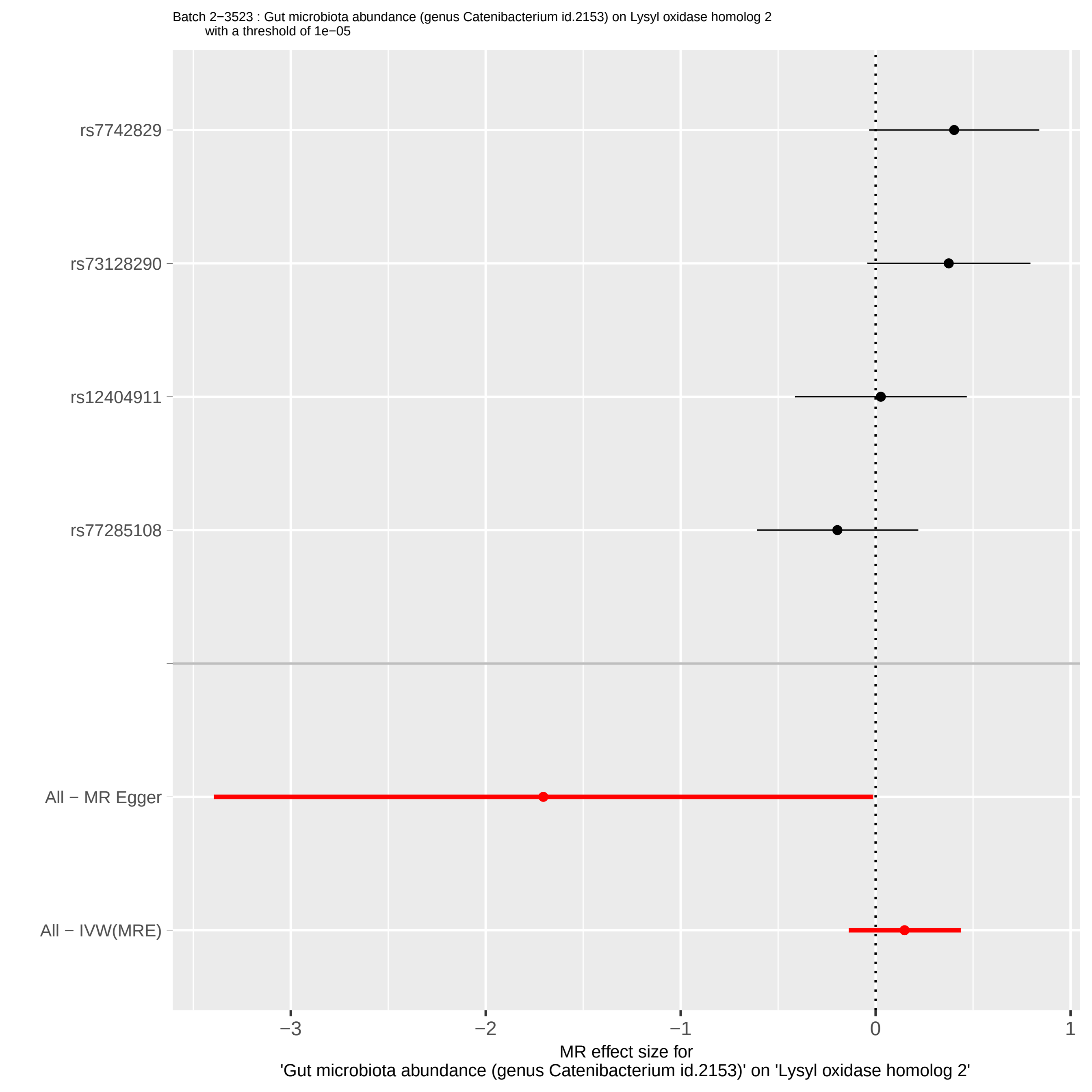

Batch 2−3523 : Gut microbiota abundance (genus Catenibacterium id.2153) on Lysyl oxidase homolog 2
with a threshold of 1e−05
rs7742829
rs73128290
rs12404911
rs77285108
All − MR Egger
All − IVW(MRE)
−3
−2
−1
0
1
MR effect size for
'Gut microbiota abundance (genus Catenibacterium id.2153)' on 'Lysyl oxidase homolog 2'

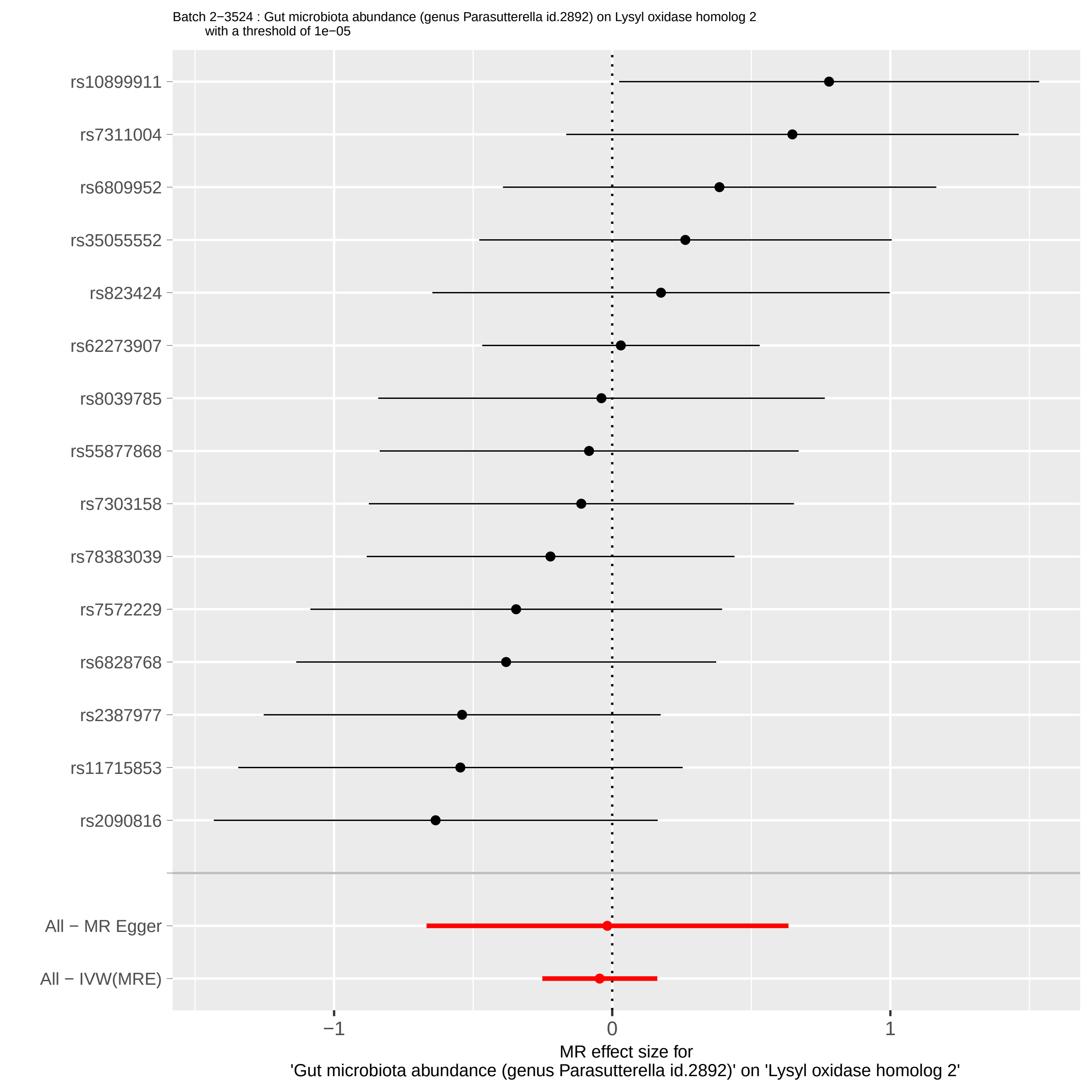
Batch 2−3524 : Gut microbiota abundance (genus Parasutterella id.2892) on Lysyl oxidase homolog 2
with a threshold of 1e−05
rs10899911
rs7311004
rs6809952
rs35055552
rs823424
rs62273907
rs8039785
rs55877868
rs7303158
rs78383039
rs7572229
rs6828768
rs2387977
rs11715853
rs2090816
All − MR Egger
All − IVW(MRE)
−1
0
1
MR effect size for
'Gut microbiota abundance (genus Parasutterella id.2892)' on 'Lysyl oxidase homolog 2'

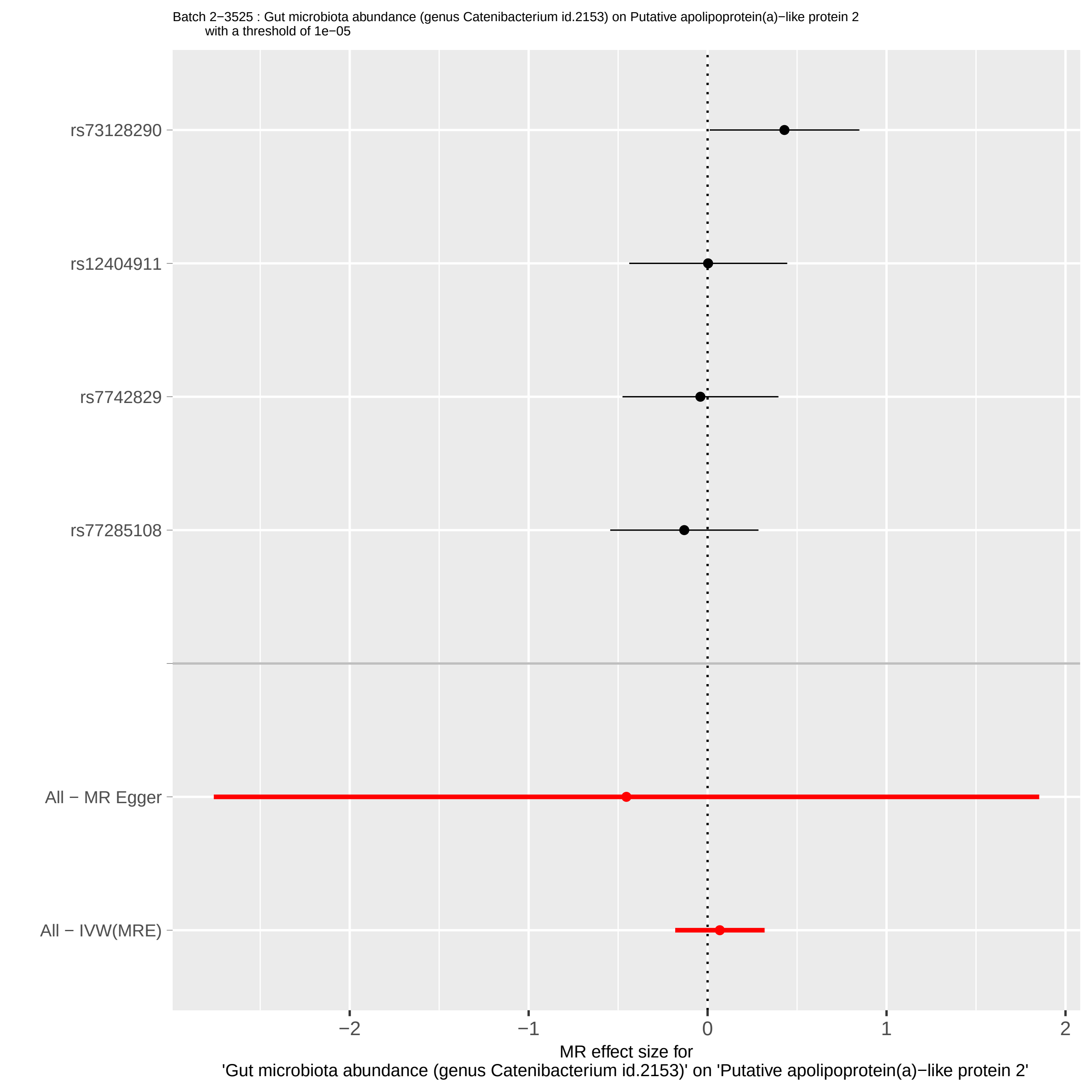

Batch 2−3525 : Gut microbiota abundance (genus Catenibacterium id.2153) on Putative apolipoprotein(a)−like protein 2
with a threshold of 1e−05
rs73128290
rs12404911
rs7742829
rs77285108
All − MR Egger
All − IVW(MRE)
−2
−1
0
1
2
MR effect size for
'Gut microbiota abundance (genus Catenibacterium id.2153)' on 'Putative apolipoprotein(a)−like protein 2'

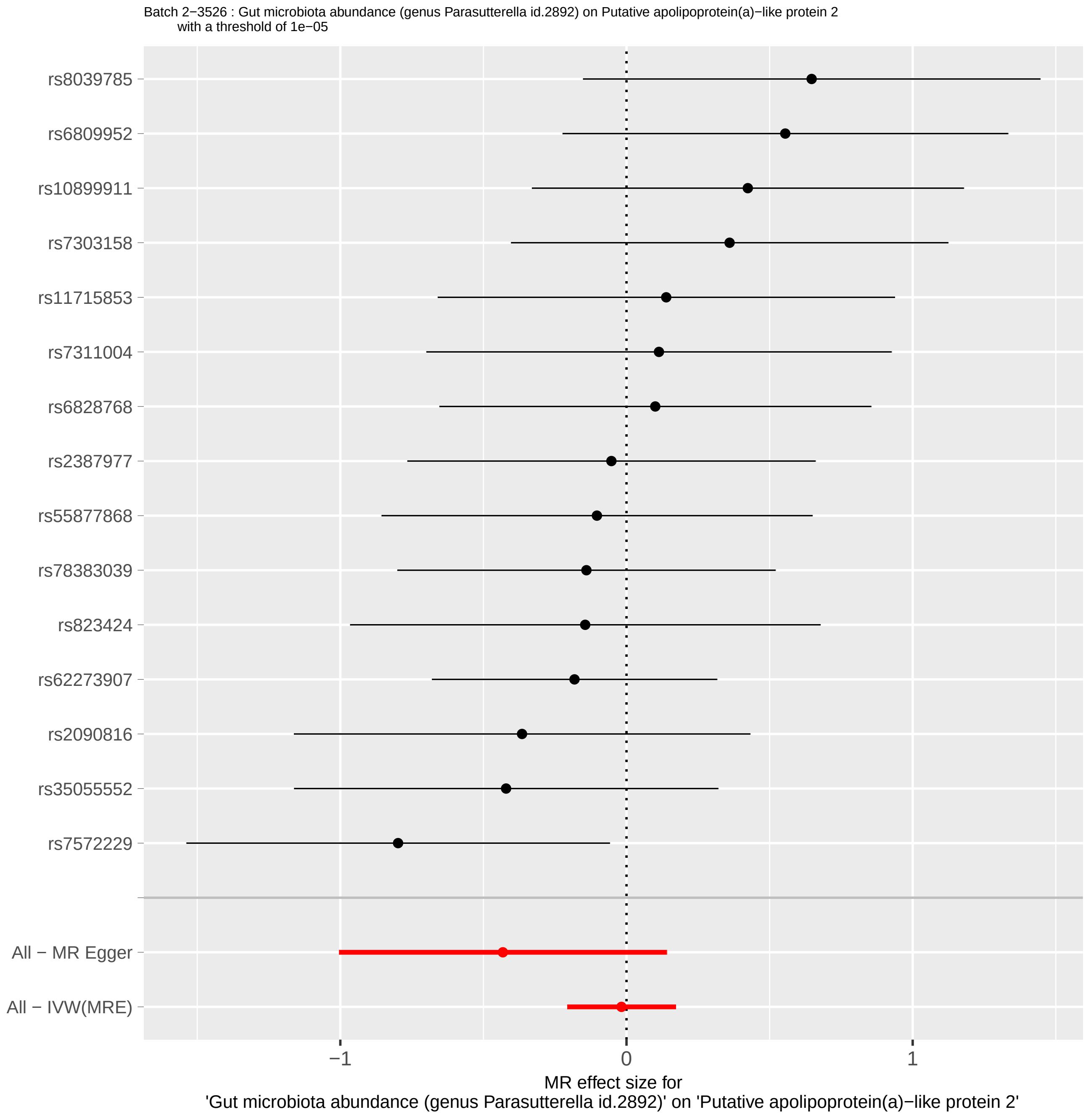

Batch 2−3526 : Gut microbiota abundance (genus Parasutterella id.2892) on Putative apolipoprotein(a)−like protein 2
with a threshold of 1e−05
rs8039785
rs6809952
rs10899911
rs7303158
rs11715853
rs7311004
rs6828768
rs2387977
rs55877868
rs78383039
rs823424
rs62273907
rs2090816
rs35055552
rs7572229
All − MR Egger
All − IVW(MRE)
−1
0
1
MR effect size for
'Gut microbiota abundance (genus Parasutterella id.2892)' on 'Putative apolipoprotein(a)−like protein 2'

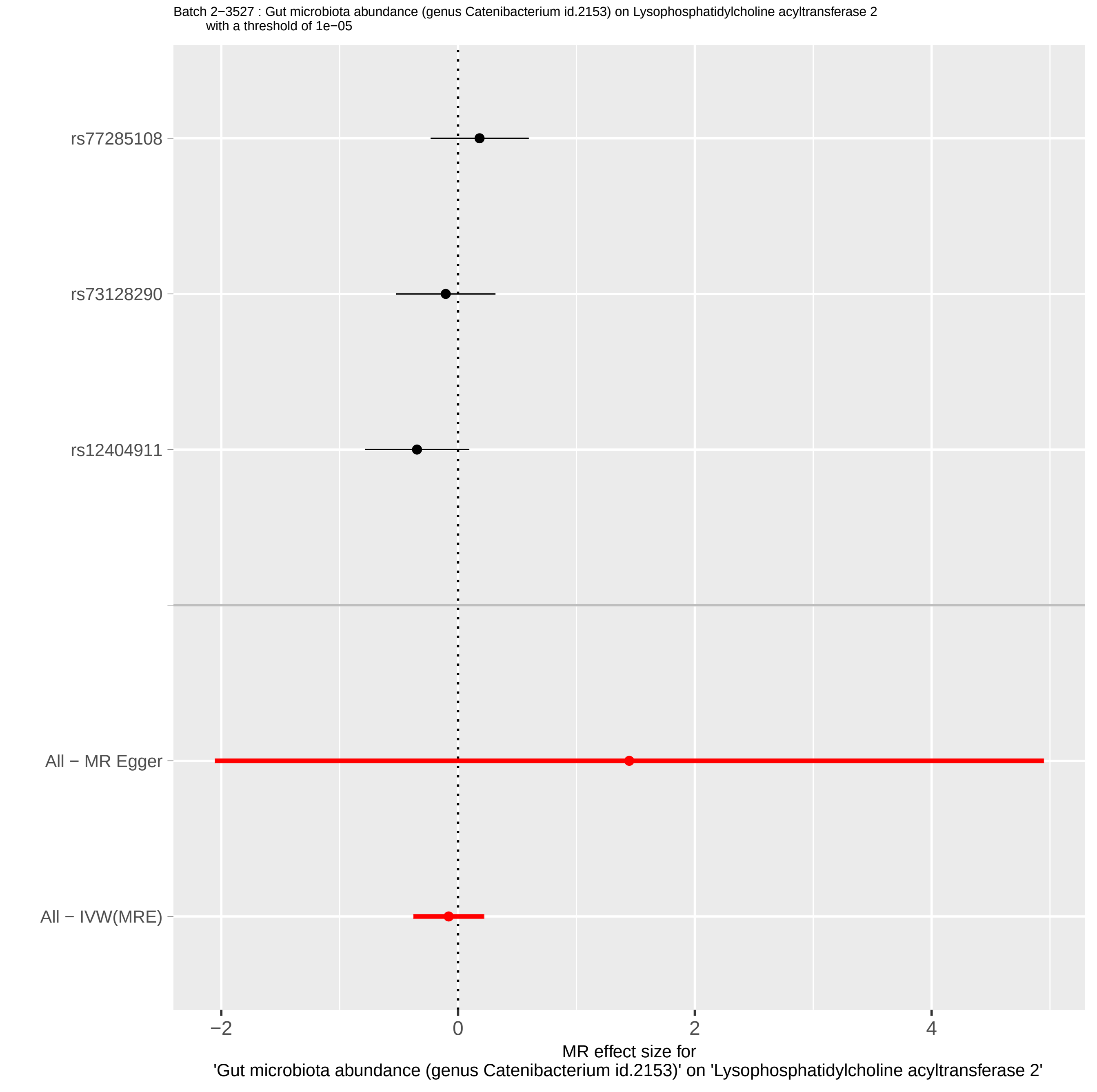

Batch 2−3527 : Gut microbiota abundance (genus Catenibacterium id.2153) on Lysophosphatidylcholine acyltransferase 2
with a threshold of 1e−05
rs77285108
rs73128290
rs12404911
All − MR Egger
All − IVW(MRE)
−2
0
2
4
MR effect size for
'Gut microbiota abundance (genus Catenibacterium id.2153)' on 'Lysophosphatidylcholine acyltransferase 2'

Batch 2−3528 : Gut microbiota abundance (genus Parasutterella id.2892) on Lysophosphatidylcholine acyltransferase 2
with a threshold of 1e−05

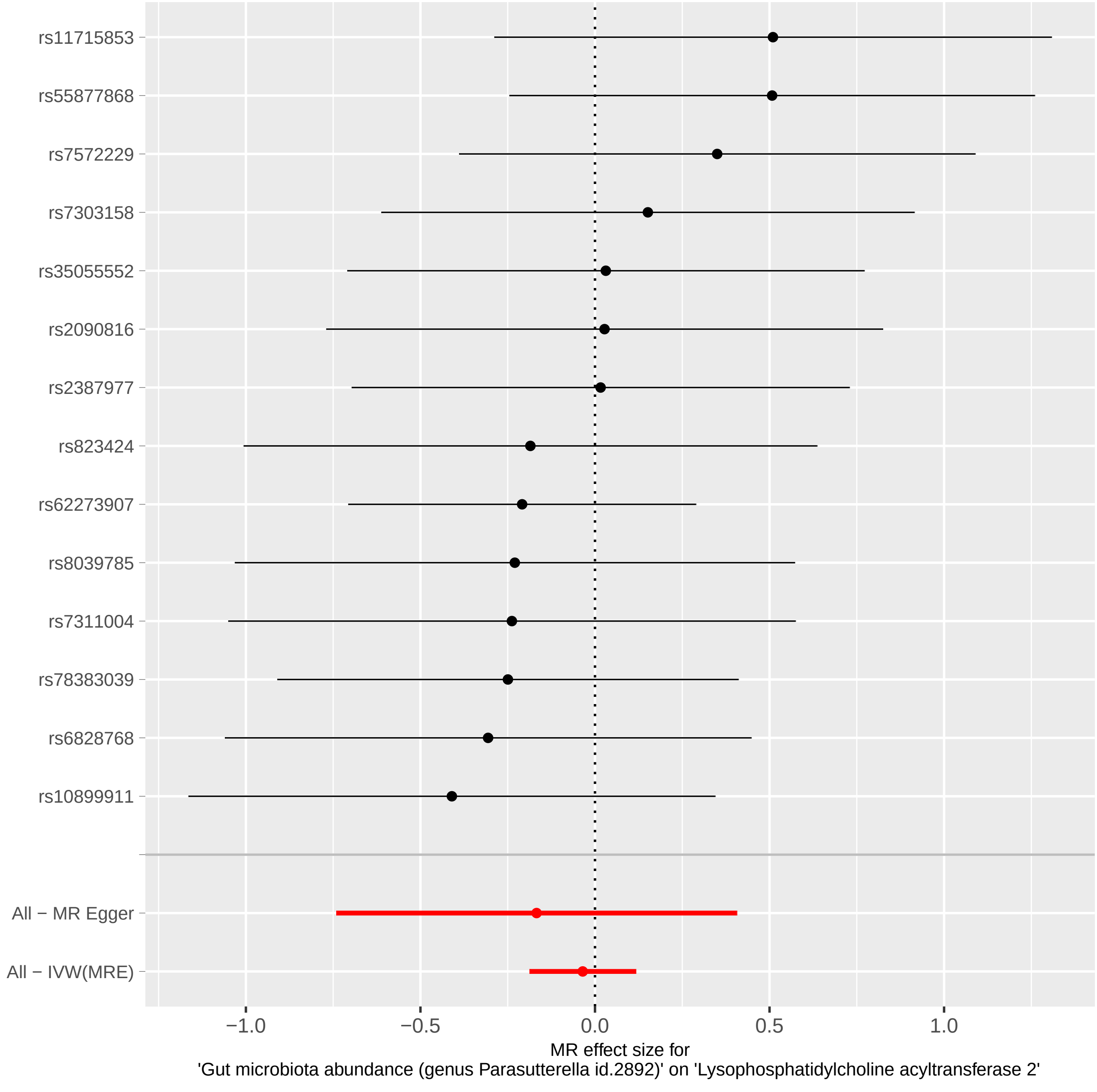

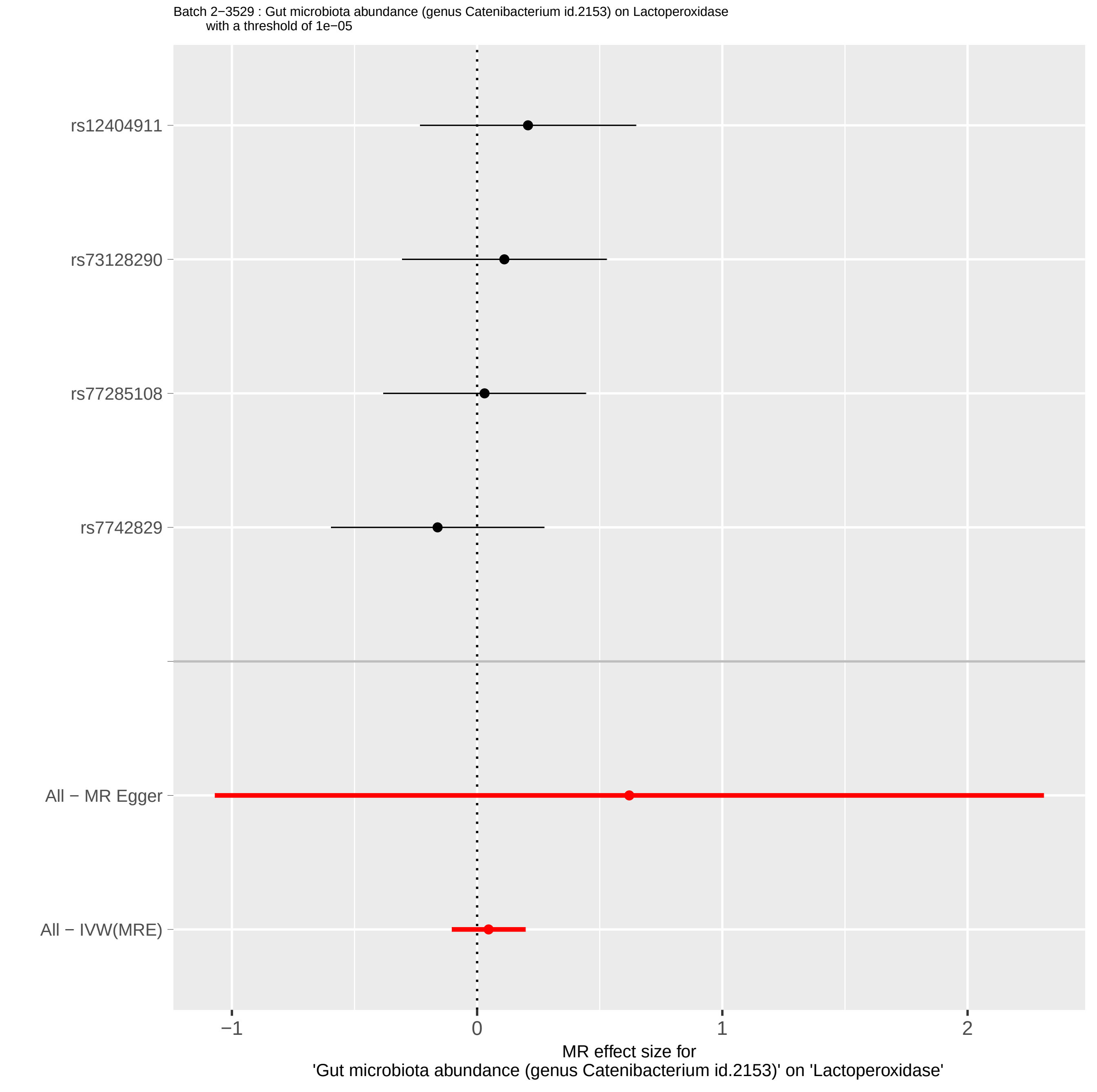

Batch 2−3529 : Gut microbiota abundance (genus Catenibacterium id.2153) on Lactoperoxidase
with a threshold of 1e−05
rs12404911
rs73128290
rs77285108
rs7742829
All − MR Egger
All − IVW(MRE)
−1
0
1
2
MR effect size for
'Gut microbiota abundance (genus Catenibacterium id.2153)' on 'Lactoperoxidase'

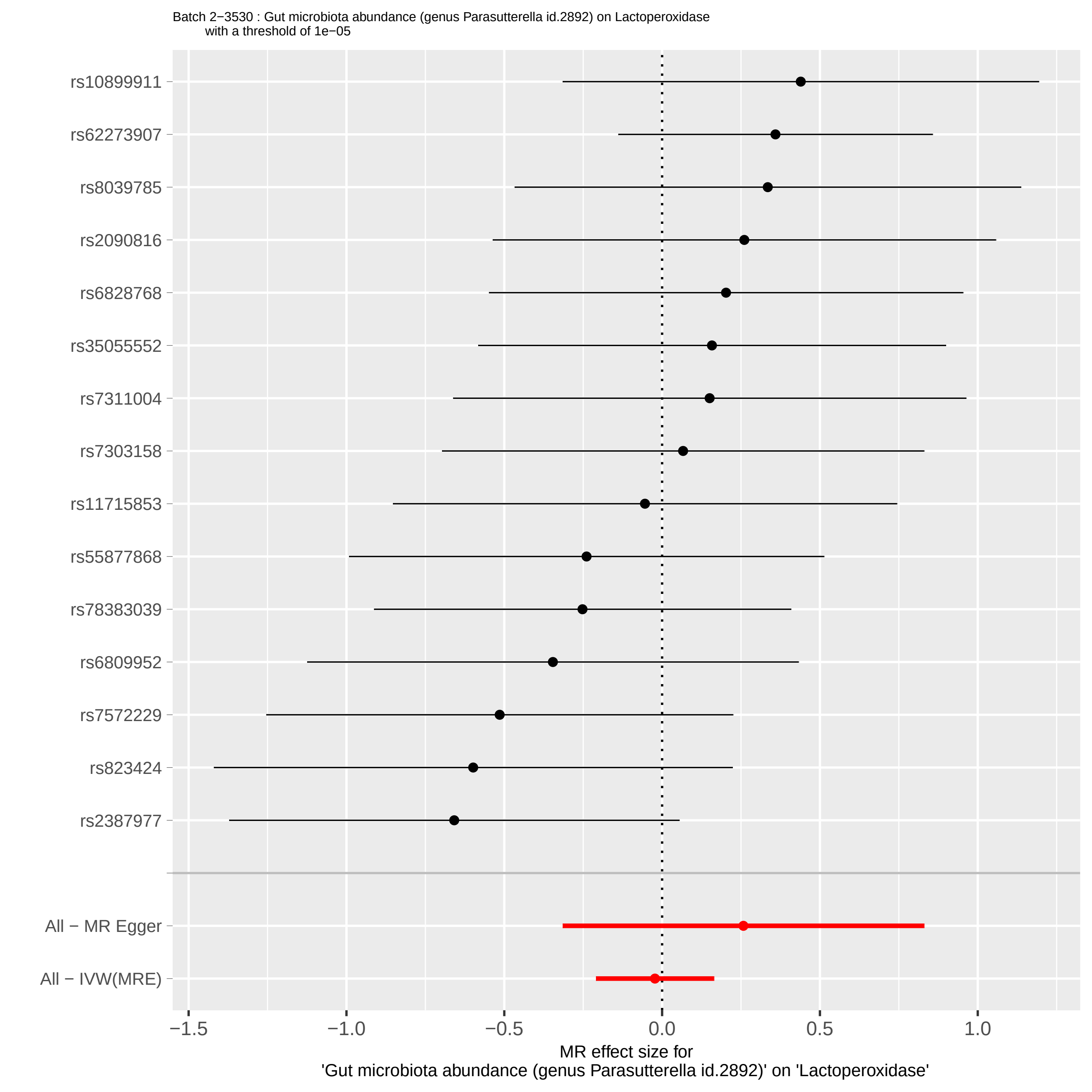
Batch 2−3530 : Gut microbiota abundance (genus Parasutterella id.2892) on Lactoperoxidase
with a threshold of 1e−05
rs10899911
rs62273907
rs8039785
rs2090816
rs6828768
rs35055552
rs7311004
rs7303158
rs11715853
rs55877868
rs78383039
rs6809952
rs7572229
rs823424
rs2387977
All − MR Egger
All − IVW(MRE)
−1.5
−1.0
−0.5
0.0
0.5
1.0
MR effect size for
'Gut microbiota abundance (genus Parasutterella id.2892)' on 'Lactoperoxidase'

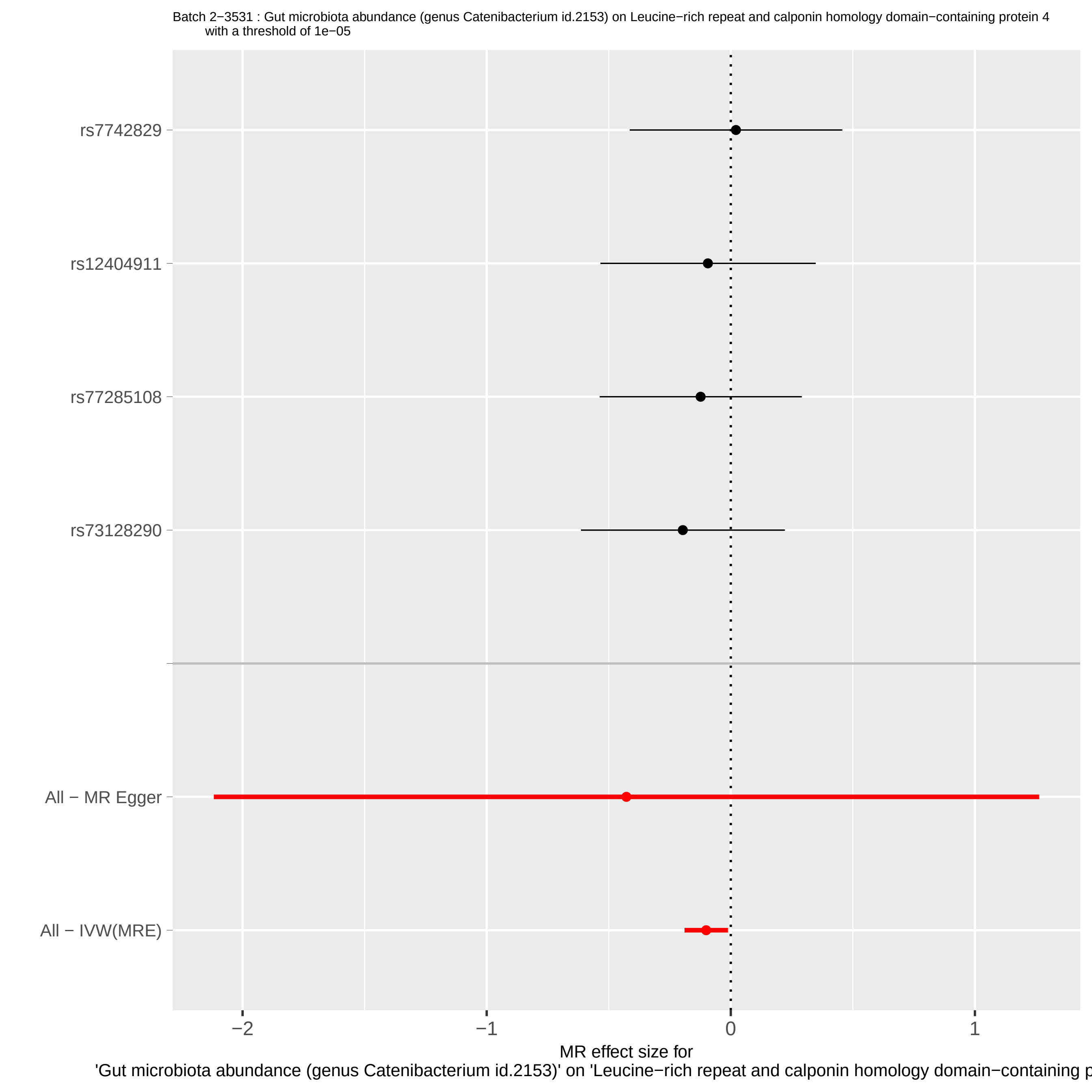

Batch 2−3531 : Gut microbiota abundance (genus Catenibacterium id.2153) on Leucine−rich repeat and calponin homology domain−containing protein 4
with a threshold of 1e−05
rs7742829
rs12404911
rs77285108
rs73128290
All − MR Egger
All − IVW(MRE)
−2
−1
0
1
MR effect size for
'Gut microbiota abundance (genus Catenibacterium id.2153)' on 'Leucine−rich repeat and calponin homology domain−containing p

Batch 2−3532 : Gut microbiota abundance (genus Parasutterella id.2892) on Leucine−rich repeat and calponin homology domain−containing protein 4 with a threshold of 1e−05

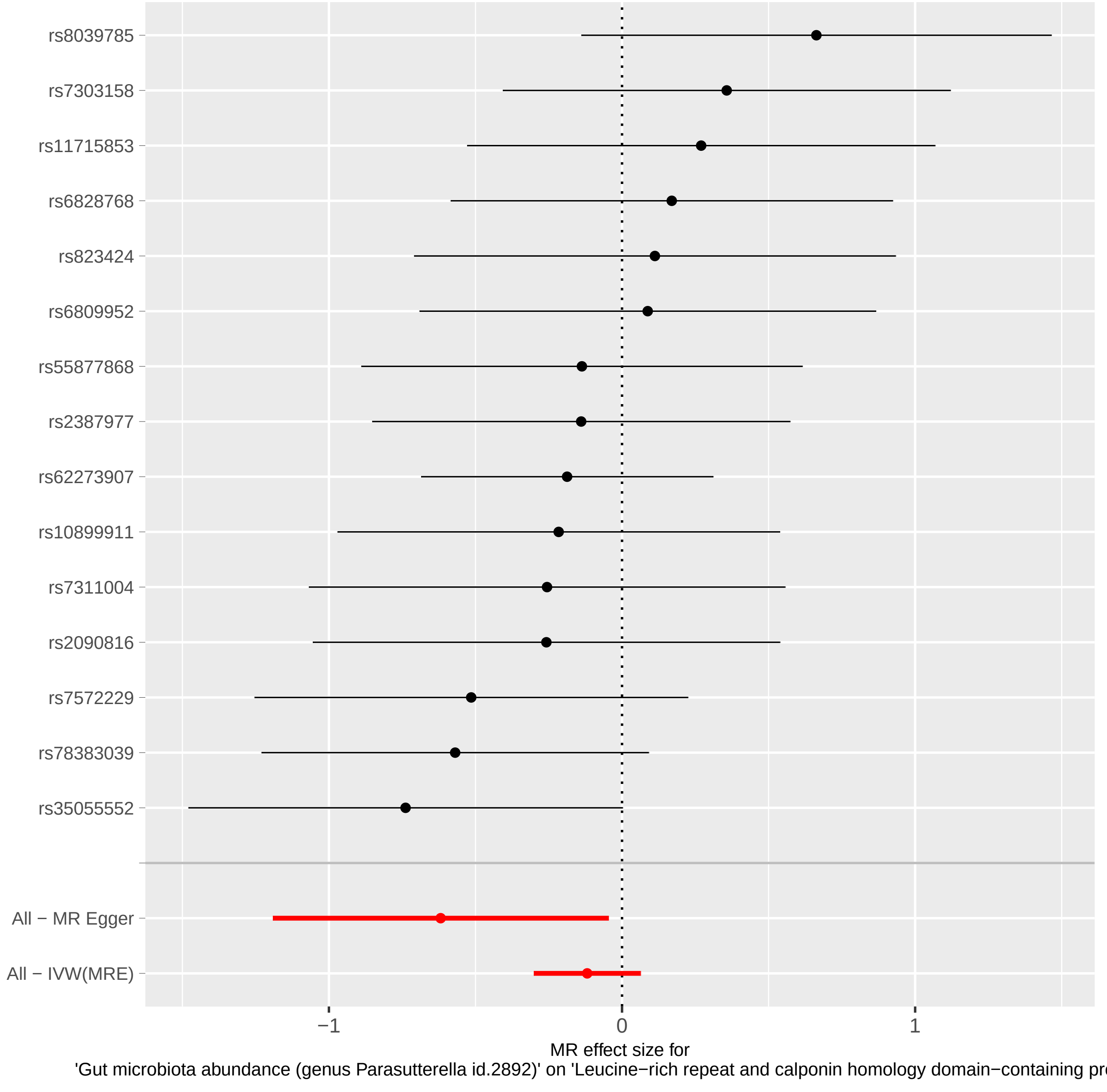

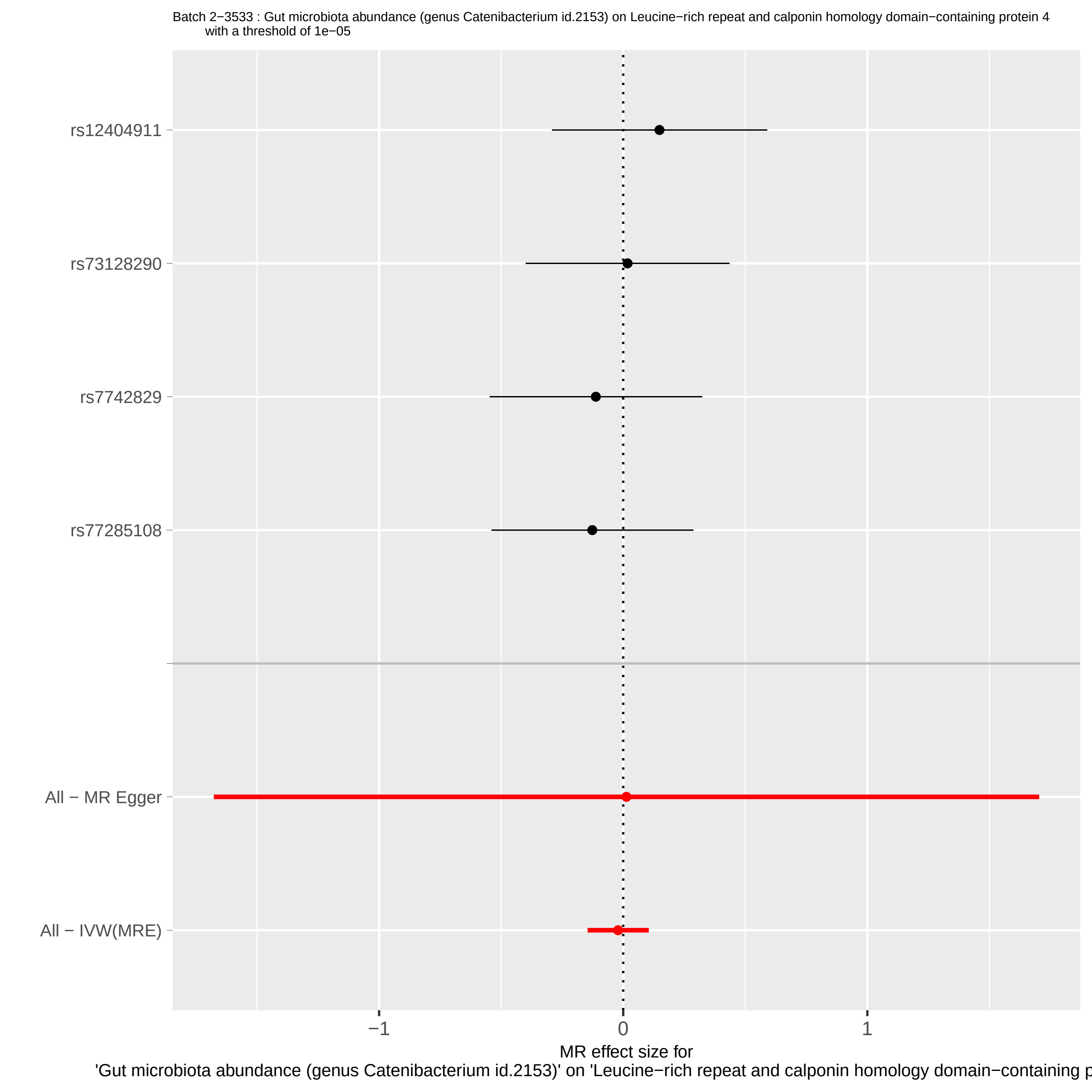

Batch 2−3533 : Gut microbiota abundance (genus Catenibacterium id.2153) on Leucine−rich repeat and calponin homology domain−containing protein 4
with a threshold of 1e−05
rs12404911
rs73128290
rs7742829
rs77285108
All − MR Egger
All − IVW(MRE)
−1
0
1
MR effect size for
'Gut microbiota abundance (genus Catenibacterium id.2153)' on 'Leucine−rich repeat and calponin homology domain−containing p

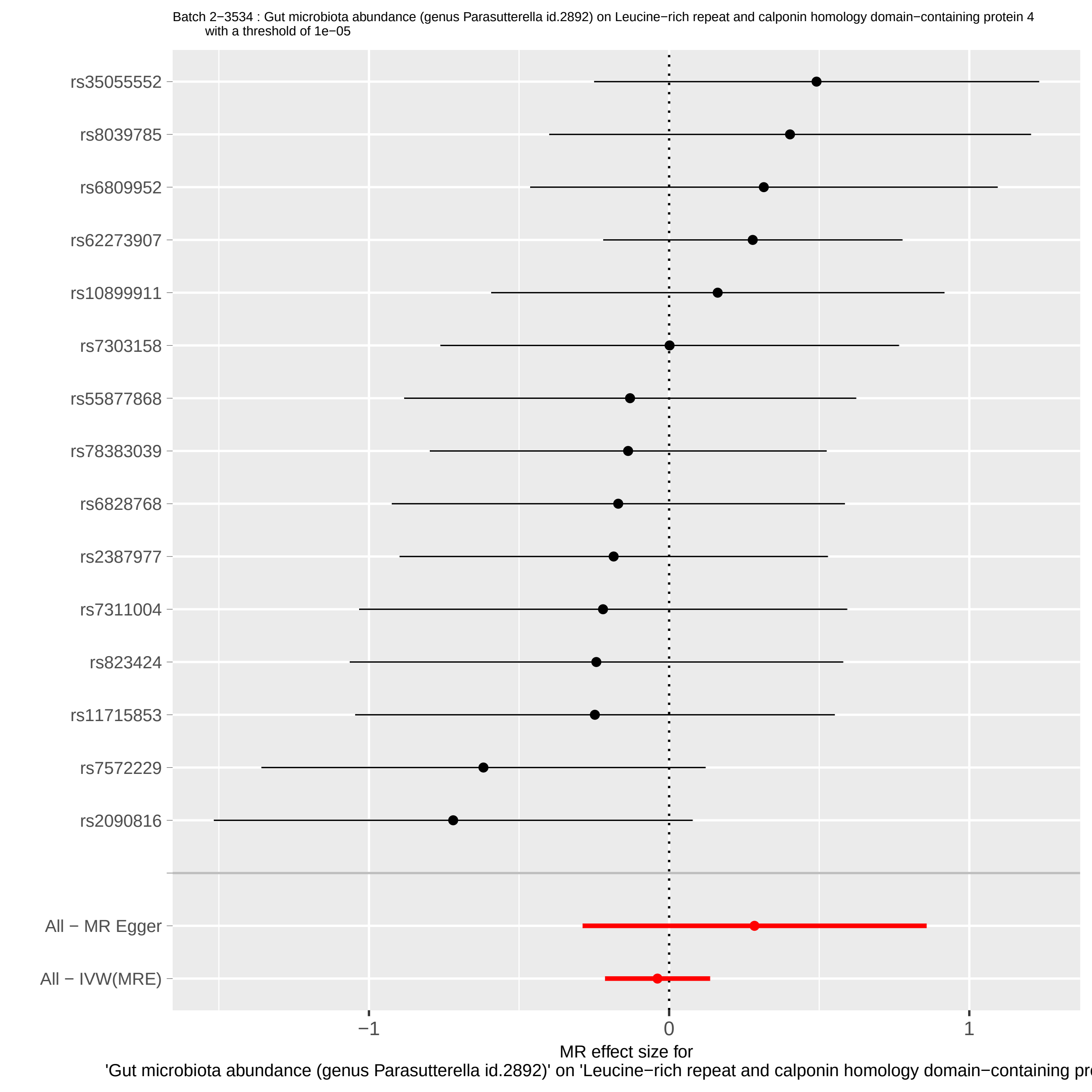

Batch 2−3534 : Gut microbiota abundance (genus Parasutterella id.2892) on Leucine−rich repeat and calponin homology domain−containing protein 4
with a threshold of 1e−05
rs35055552
rs8039785
rs6809952
rs62273907
rs10899911
rs7303158
rs55877868
rs78383039
rs6828768
rs2387977
rs7311004
rs823424
rs11715853
rs7572229
rs2090816
All − MR Egger
All − IVW(MRE)
−1
0
1
MR effect size for
'Gut microbiota abundance (genus Parasutterella id.2892)' on 'Leucine−rich repeat and calponin homology domain−containing pr

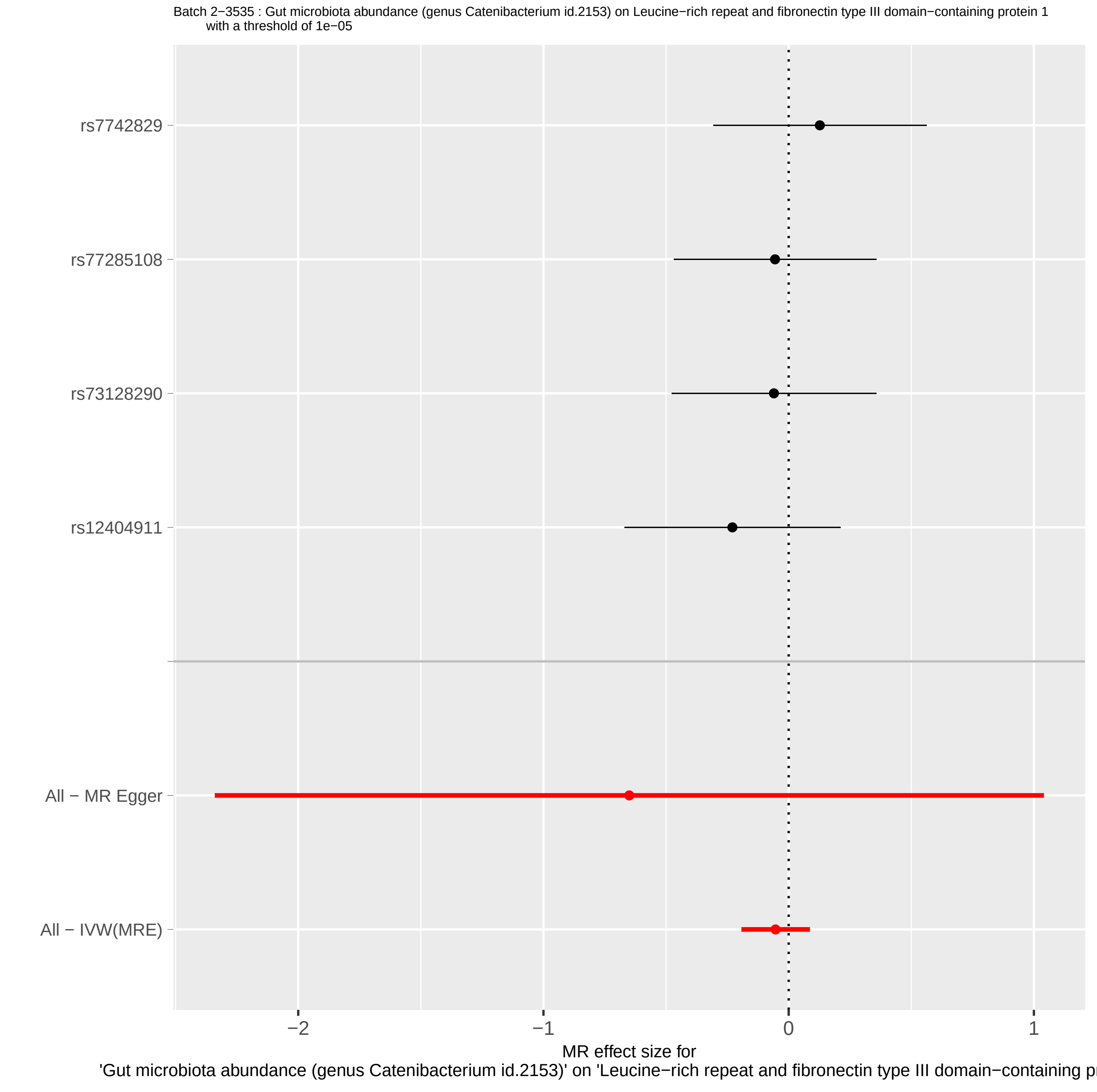
Batch 2−3535 : Gut microbiota abundance (genus Catenibacterium id.2153) on Leucine−rich repeat and fibronectin type III domain−containing protein 1
with a threshold of 1e−05
rs7742829
rs77285108
rs73128290
rs12404911
All − MR Egger
All − IVW(MRE)
−2
−1
0
1
MR effect size for
'Gut microbiota abundance (genus Catenibacterium id.2153)' on 'Leucine−rich repeat and fibronectin type III domain−containing p

Batch 2−3536 : Gut microbiota abundance (genus Parasutterella id.2892) on Leucine−rich repeat and fibronectin type III domain−containing protein 1
with a threshold of 1e−05

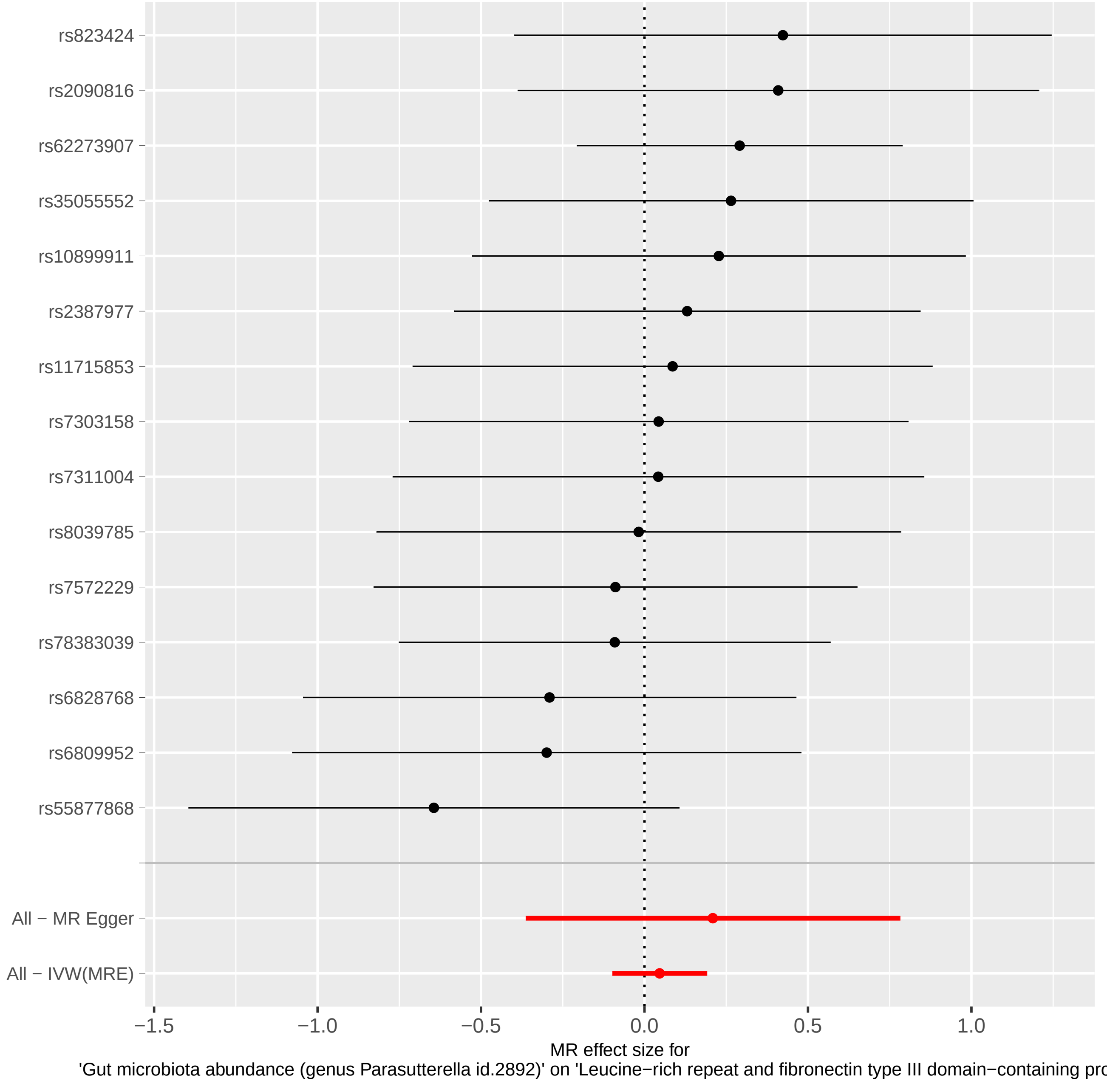

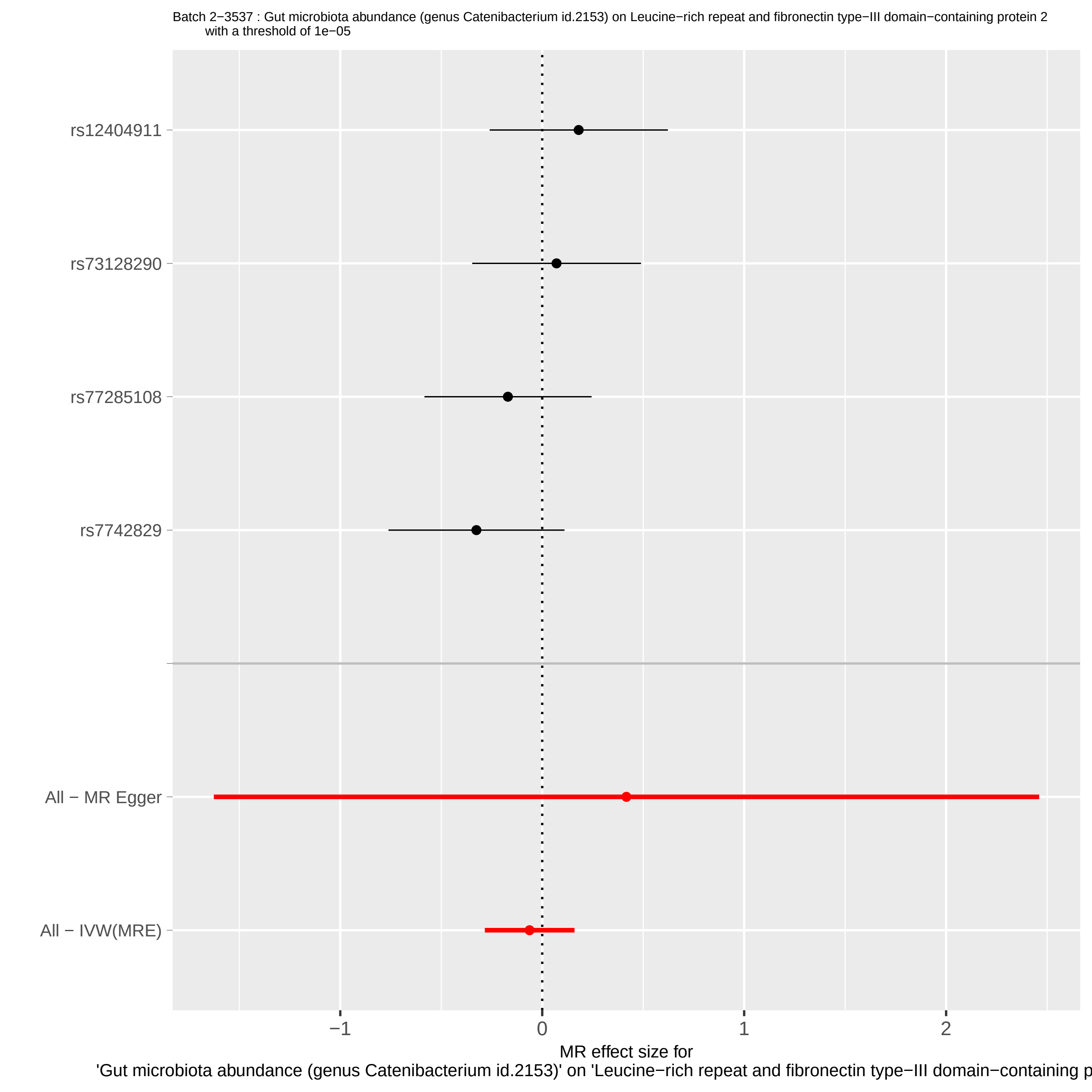

Batch 2−3537 : Gut microbiota abundance (genus Catenibacterium id.2153) on Leucine−rich repeat and fibronectin type−III domain−containing protein 2
with a threshold of 1e−05
rs12404911
rs73128290
rs77285108
rs7742829
All − MR Egger
All − IVW(MRE)
−1
0
1
2
MR effect size for
'Gut microbiota abundance (genus Catenibacterium id.2153)' on 'Leucine−rich repeat and fibronectin type−III domain−containing p

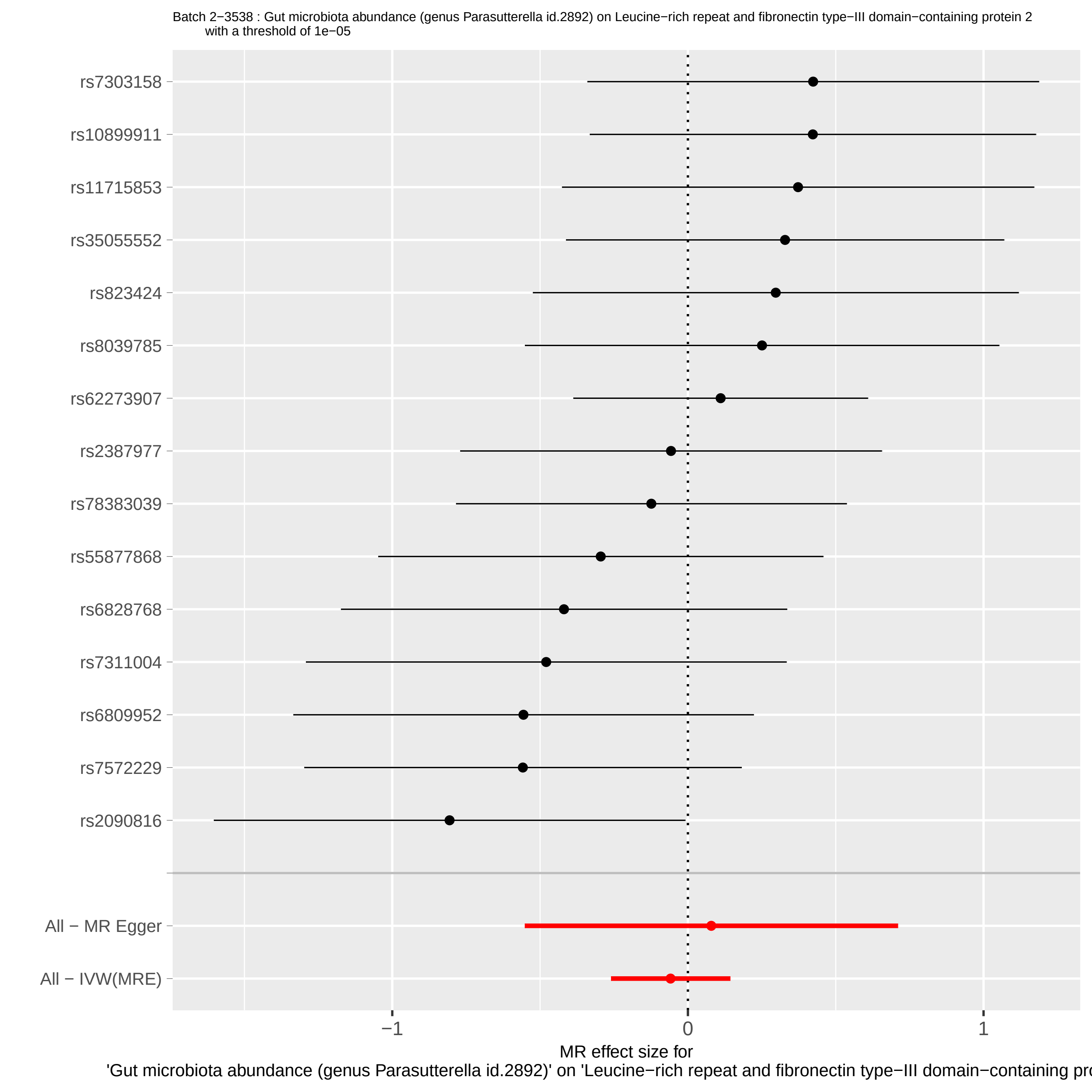

Batch 2−3538 : Gut microbiota abundance (genus Parasutterella id.2892) on Leucine−rich repeat and fibronectin type−III domain−containing protein 2
with a threshold of 1e−05
rs7303158
rs10899911
rs11715853
rs35055552
rs823424
rs8039785
rs62273907
rs2387977
rs78383039
rs55877868
rs6828768
rs7311004
rs6809952
rs7572229
rs2090816
All − MR Egger
All − IVW(MRE)
−1
0
1
MR effect size for
'Gut microbiota abundance (genus Parasutterella id.2892)' on 'Leucine−rich repeat and fibronectin type−III domain−containing pro

Batch 2−3539 : Gut microbiota abundance (genus Catenibacterium id.2153) on Leucine−rich repeats and immunoglobulin−like domains protein 3
with a threshold of 1e−05

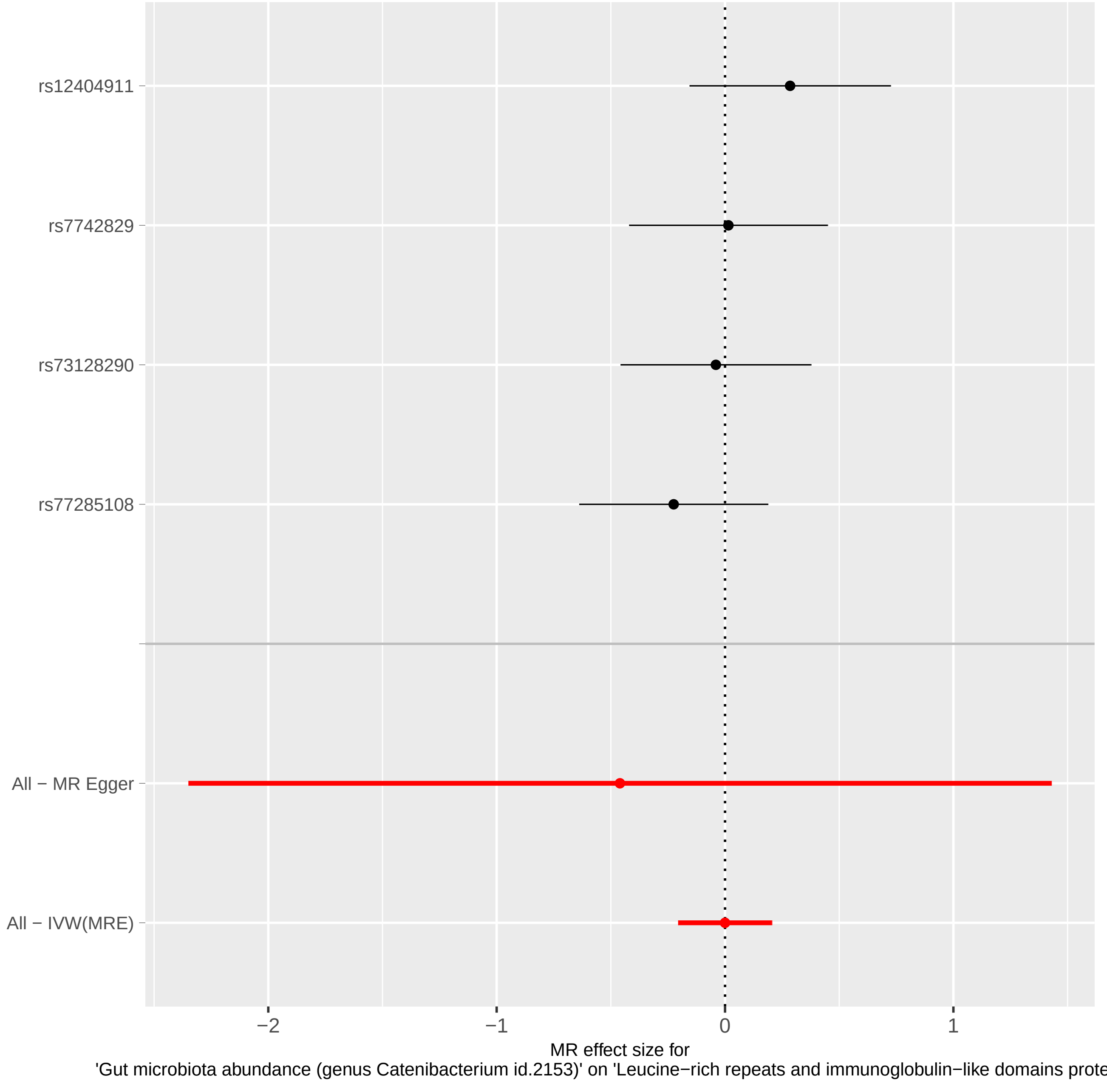

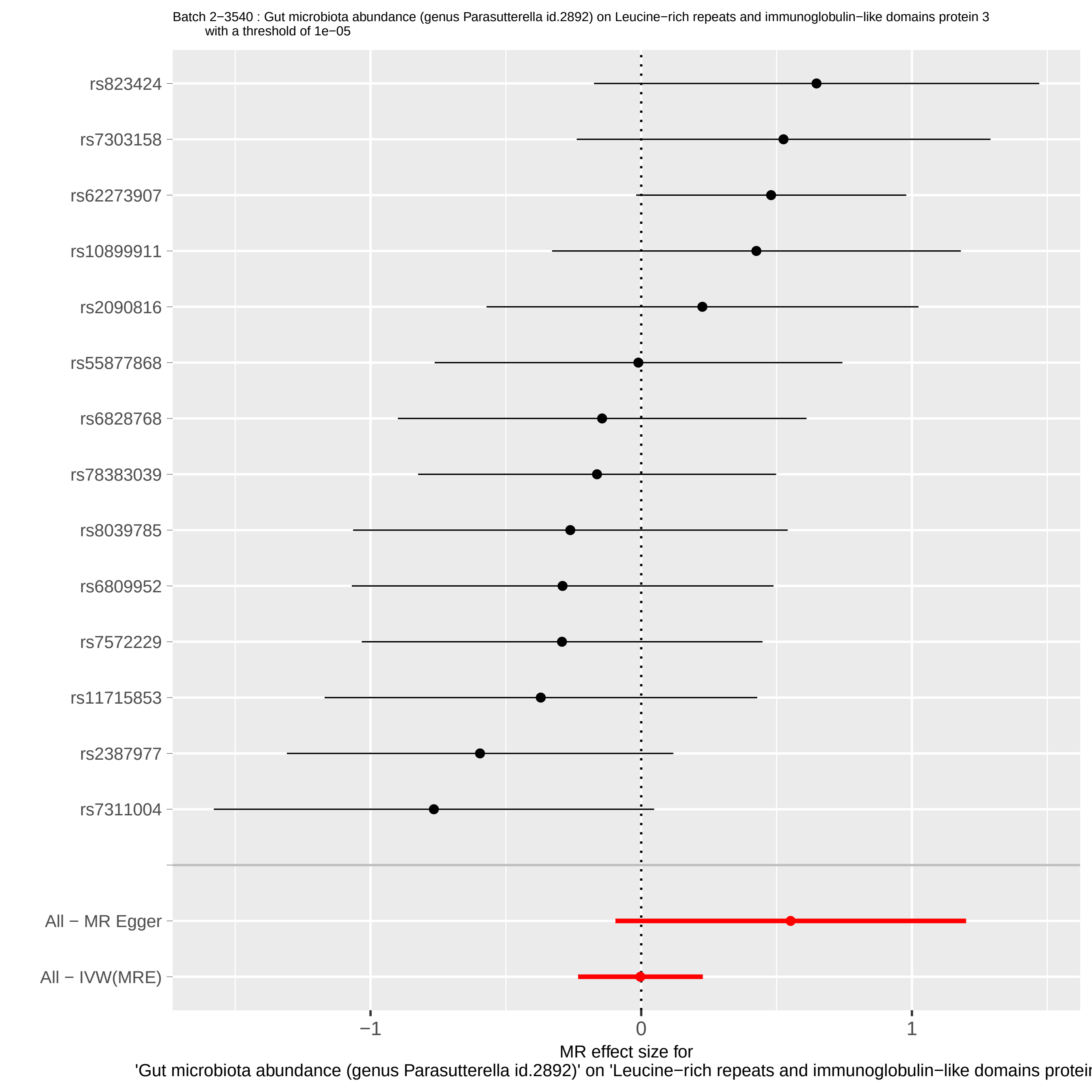
Batch 2−3540 : Gut microbiota abundance (genus Parasutterella id.2892) on Leucine−rich repeats and immunoglobulin−like domains protein 3
with a threshold of 1e−05
rs823424
rs7303158
rs62273907
rs10899911
rs2090816
rs55877868
rs6828768
rs78383039
rs8039785
rs6809952
rs7572229
rs11715853
rs2387977
rs7311004
All − MR Egger
All − IVW(MRE)
−1
0
1
MR effect size for
'Gut microbiota abundance (genus Parasutterella id.2892)' on 'Leucine−rich repeats and immunoglobulin−like domains protei

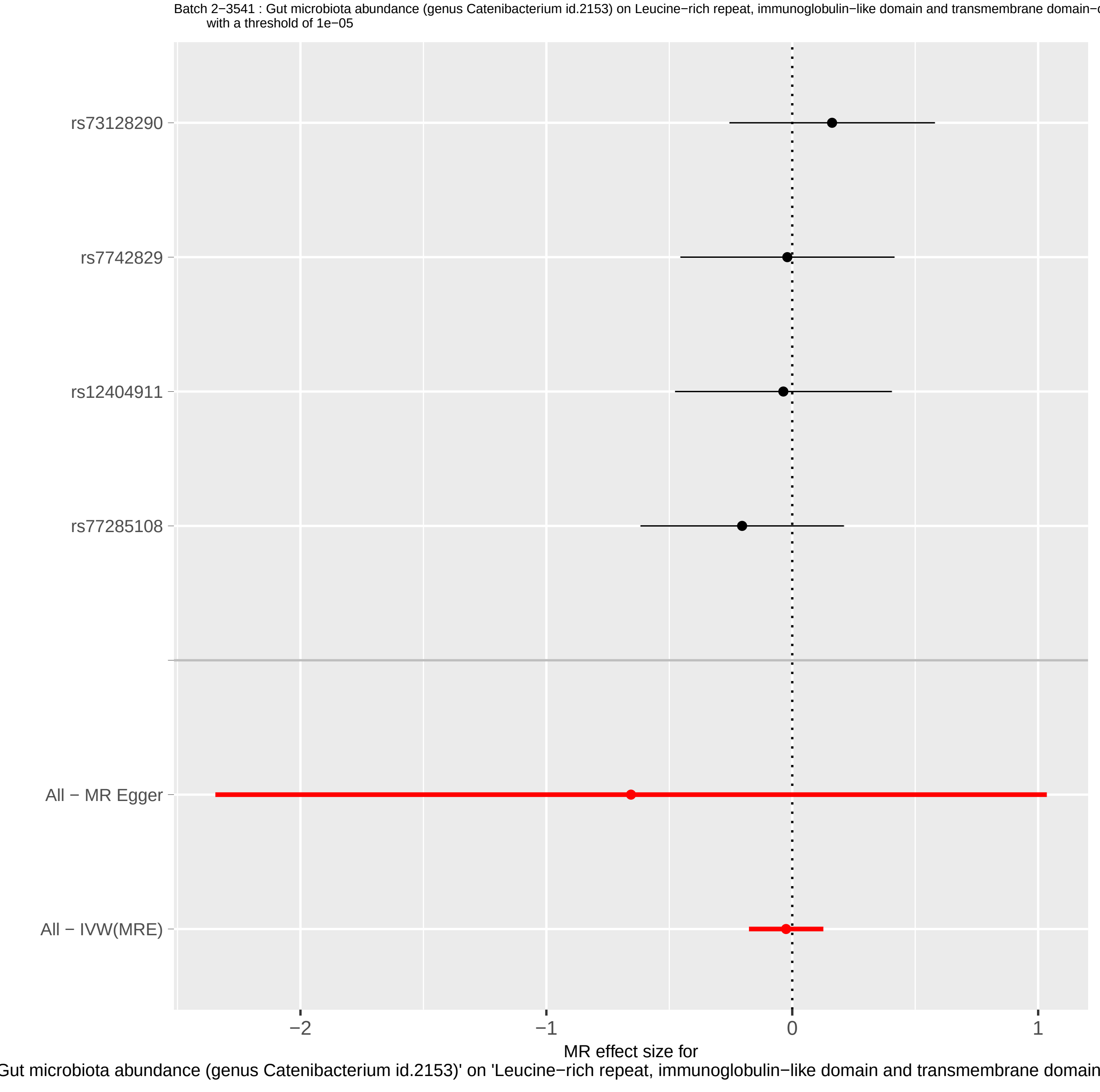

Batch 2−3541 : Gut microbiota abundance (genus Catenibacterium id.2153) on Leucine−rich repeat, immunoglobulin−like domain and transmembrane domain−c
with a threshold of 1e−05
rs73128290
rs7742829
rs12404911
rs77285108
All − MR Egger
All − IVW(MRE)
−2
−1
0
1
MR effect size for
Gut microbiota abundance (genus Catenibacterium id.2153)' on 'Leucine−rich repeat, immunoglobulin−like domain and transmembrane domain

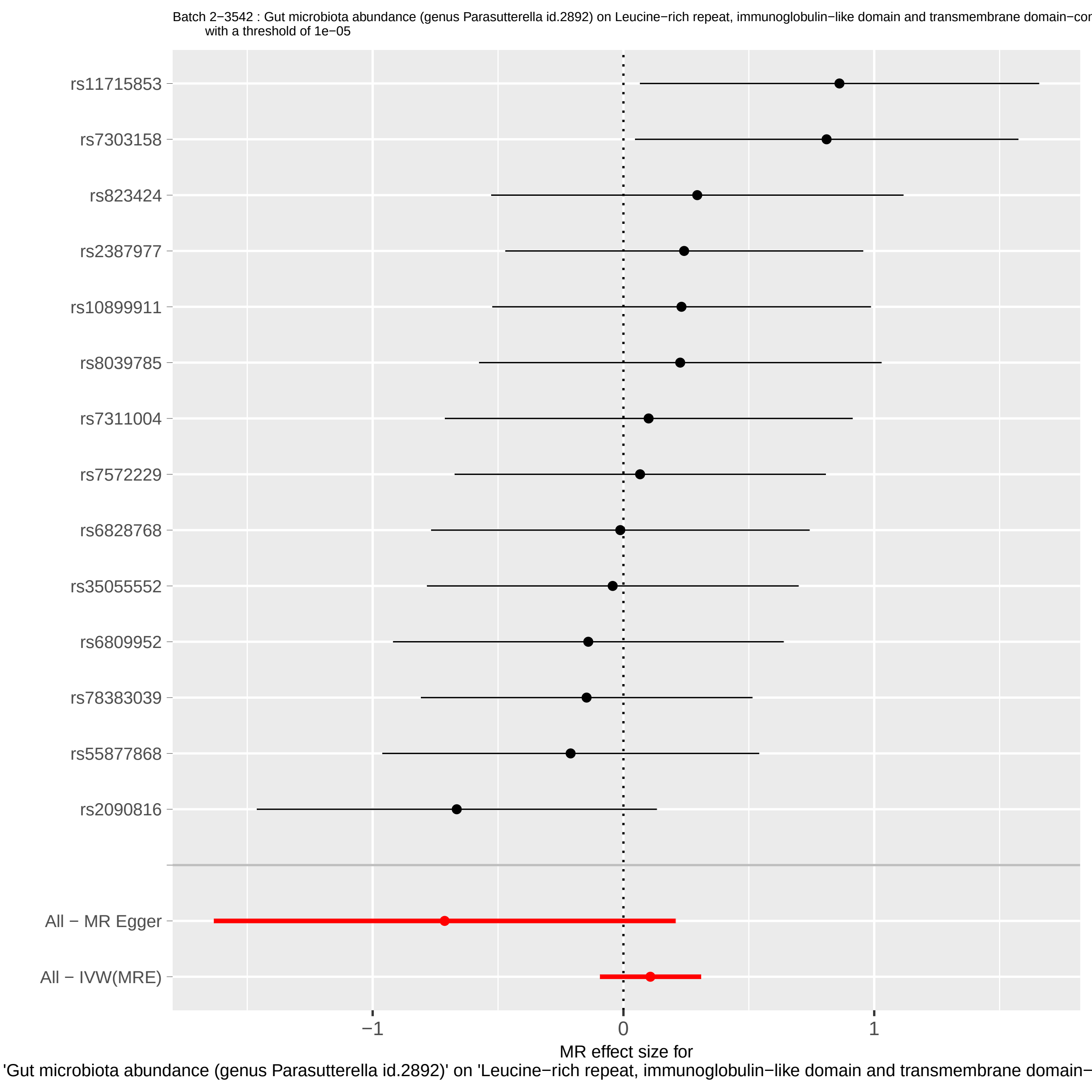
Batch 2−3542 : Gut microbiota abundance (genus Parasutterella id.2892) on Leucine−rich repeat, immunoglobulin−like domain and transmembrane domain−co
with a threshold of 1e−05
rs11715853
rs7303158
rs823424
rs2387977
rs10899911
rs8039785
rs7311004
rs7572229
rs6828768
rs35055552
rs6809952
rs78383039
rs55877868
rs2090816
All − MR Egger
All − IVW(MRE)
−1
0
1
MR effect size for
'Gut microbiota abundance (genus Parasutterella id.2892)' on 'Leucine−rich repeat, immunoglobulin−like domain and transmembrane domain−

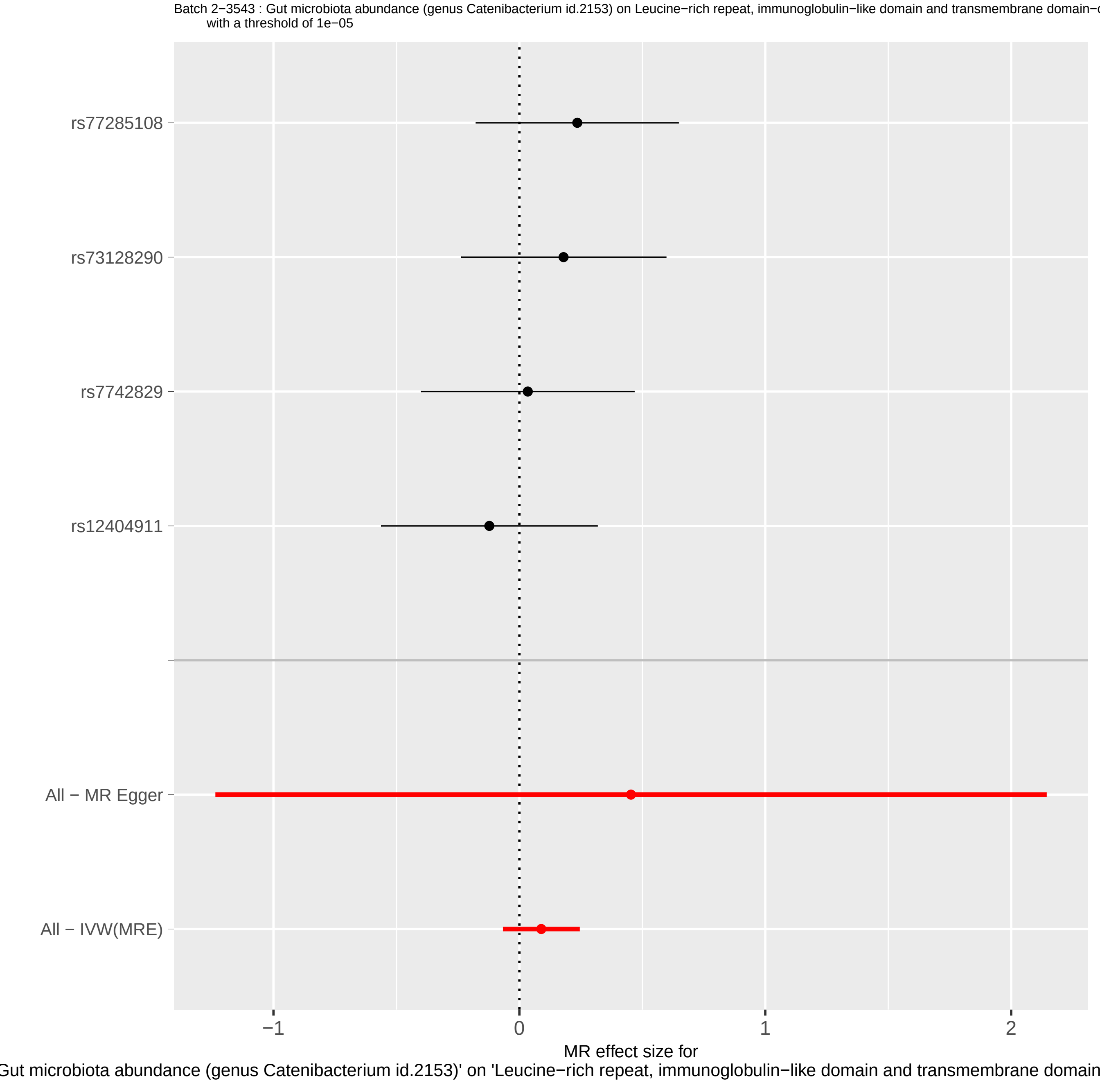
Batch 2−3543 : Gut microbiota abundance (genus Catenibacterium id.2153) on Leucine−rich repeat, immunoglobulin−like domain and transmembrane domain−c
with a threshold of 1e−05
rs77285108
rs73128290
rs7742829
rs12404911
All − MR Egger
All − IVW(MRE)
−1
0
1
2
MR effect size for
Gut microbiota abundance (genus Catenibacterium id.2153)' on 'Leucine−rich repeat, immunoglobulin−like domain and transmembrane domain

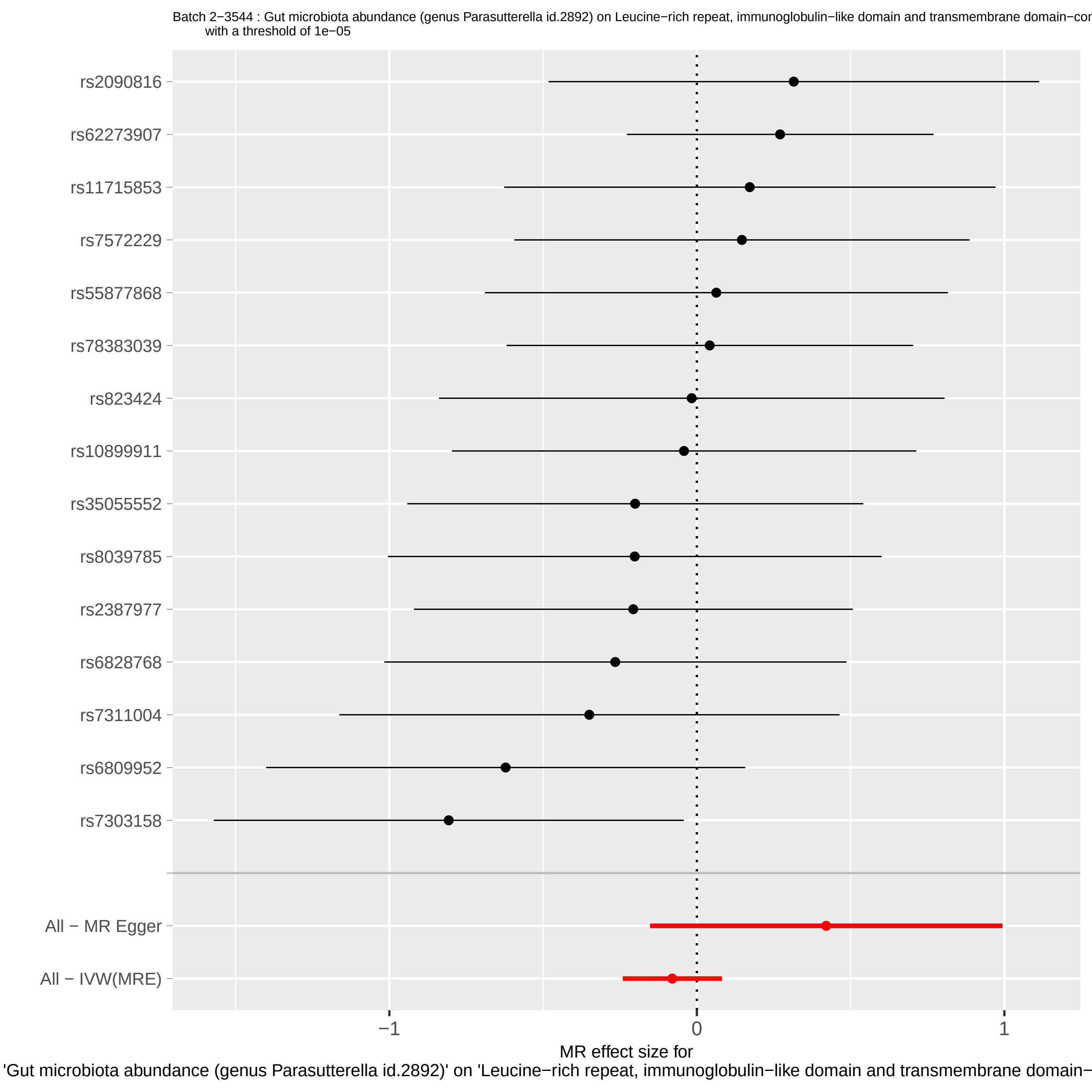

Batch 2−3544 : Gut microbiota abundance (genus Parasutterella id.2892) on Leucine−rich repeat, immunoglobulin−like domain and transmembrane domain−cor
with a threshold of 1e−05
rs2090816
rs62273907
rs11715853
rs7572229
rs55877868
rs78383039
rs823424
rs10899911
rs35055552
rs8039785
rs2387977
rs6828768
rs7311004
rs6809952
rs7303158
All − MR Egger
All − IVW(MRE)
−1
0
1
MR effect size for
'Gut microbiota abundance (genus Parasutterella id.2892)' on 'Leucine−rich repeat, immunoglobulin−like domain and transmembrane domain−

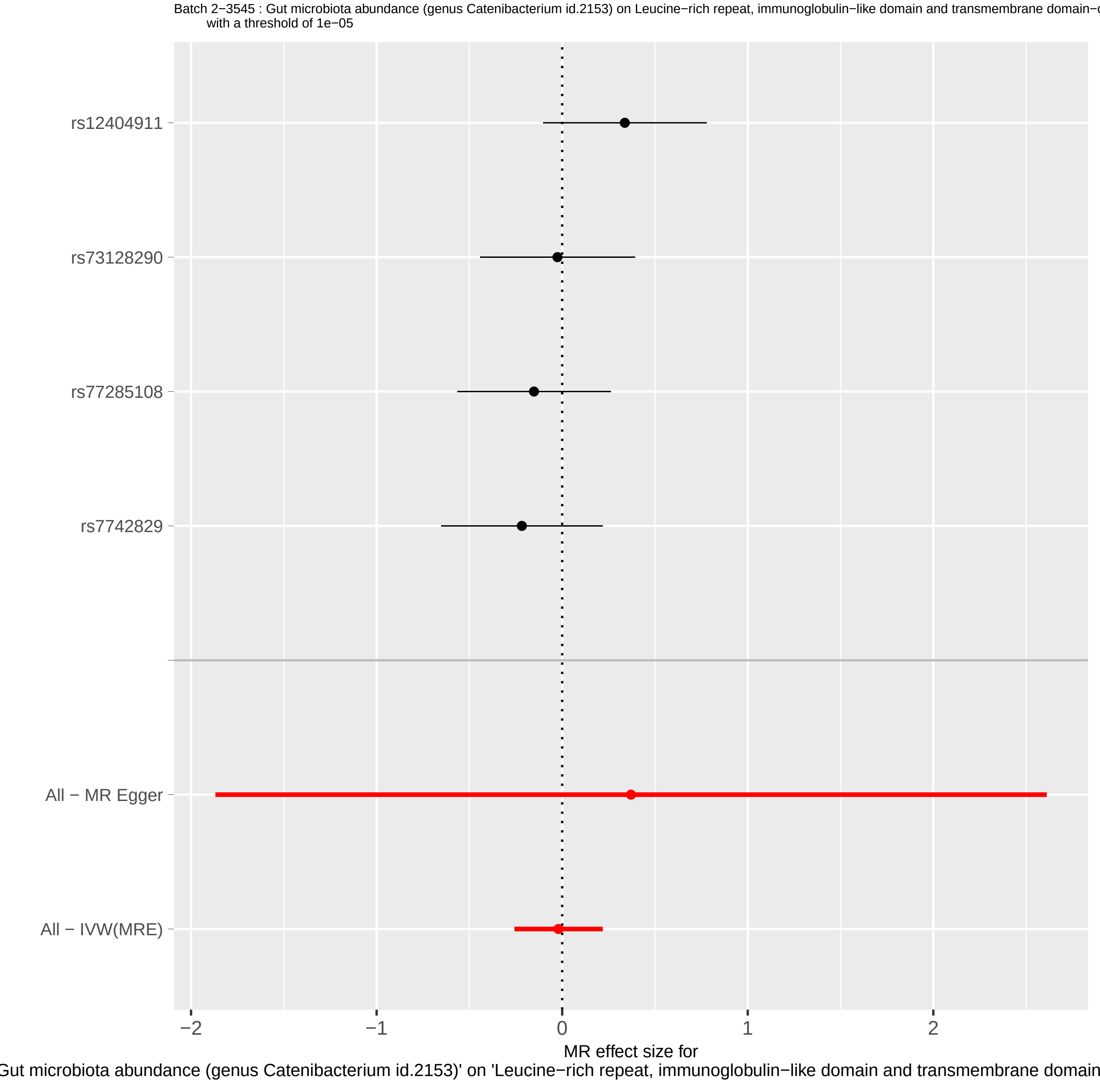

Batch 2−3545 : Gut microbiota abundance (genus Catenibacterium id.2153) on Leucine−rich repeat, immunoglobulin−like domain and transmembrane domain−c
with a threshold of 1e−05
rs12404911
rs73128290
rs77285108
rs7742829
All − MR Egger
All − IVW(MRE)
−2
−1
0
1
2
MR effect size for
'Gut microbiota abundance (genus Catenibacterium id.2153)' on 'Leucine−rich repeat, immunoglobulin−like domain and transmembrane domain

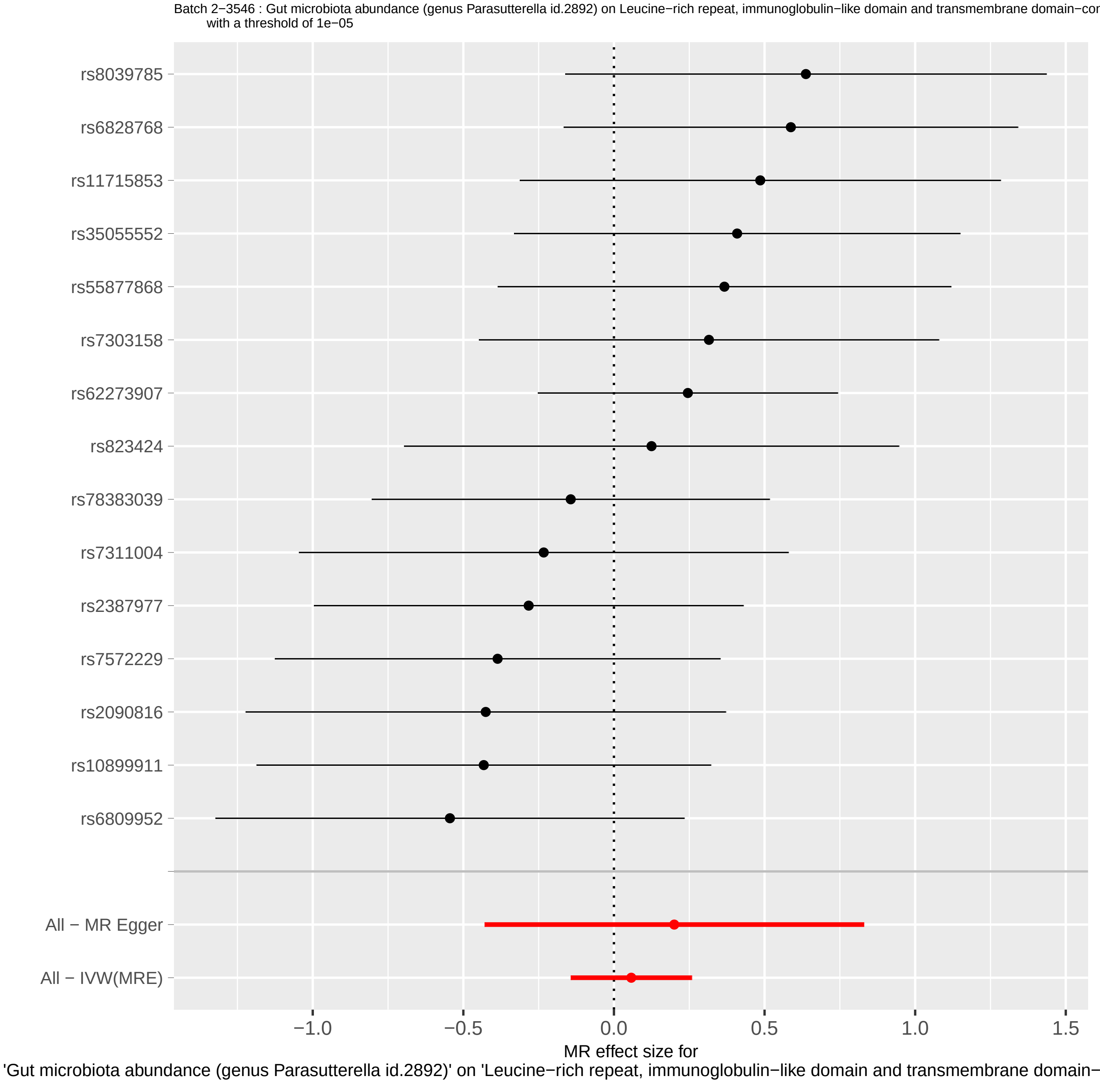

Batch 2−3546 : Gut microbiota abundance (genus Parasutterella id.2892) on Leucine−rich repeat, immunoglobulin−like domain and transmembrane domain−cor
with a threshold of 1e−05
rs8039785
rs6828768
rs11715853
rs35055552
rs55877868
rs7303158
rs62273907
rs823424
rs78383039
rs7311004
rs2387977
rs7572229
rs2090816
rs10899911
rs6809952
All − MR Egger
All − IVW(MRE)
−1.0
−0.5
0.0
0.5
1.0
1.5
MR effect size for
'Gut microbiota abundance (genus Parasutterella id.2892)' on 'Leucine−rich repeat, immunoglobulin−like domain and transmembrane domain−

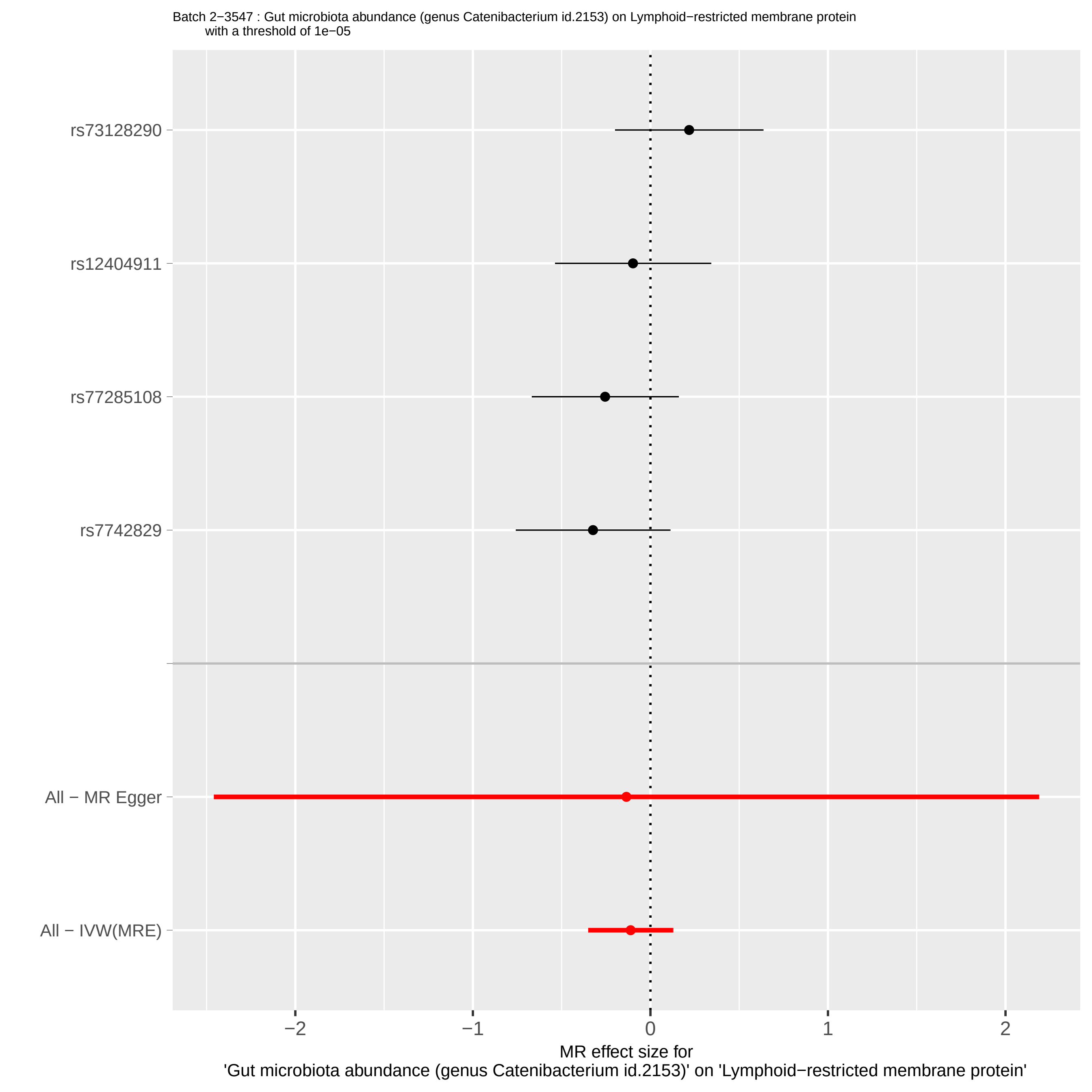
Batch 2−3547 : Gut microbiota abundance (genus Catenibacterium id.2153) on Lymphoid−restricted membrane protein
with a threshold of 1e−05
rs73128290
rs12404911
rs77285108
rs7742829
All − MR Egger
All − IVW(MRE)
−2
−1
0
1
2
MR effect size for
'Gut microbiota abundance (genus Catenibacterium id.2153)' on 'Lymphoid−restricted membrane protein'

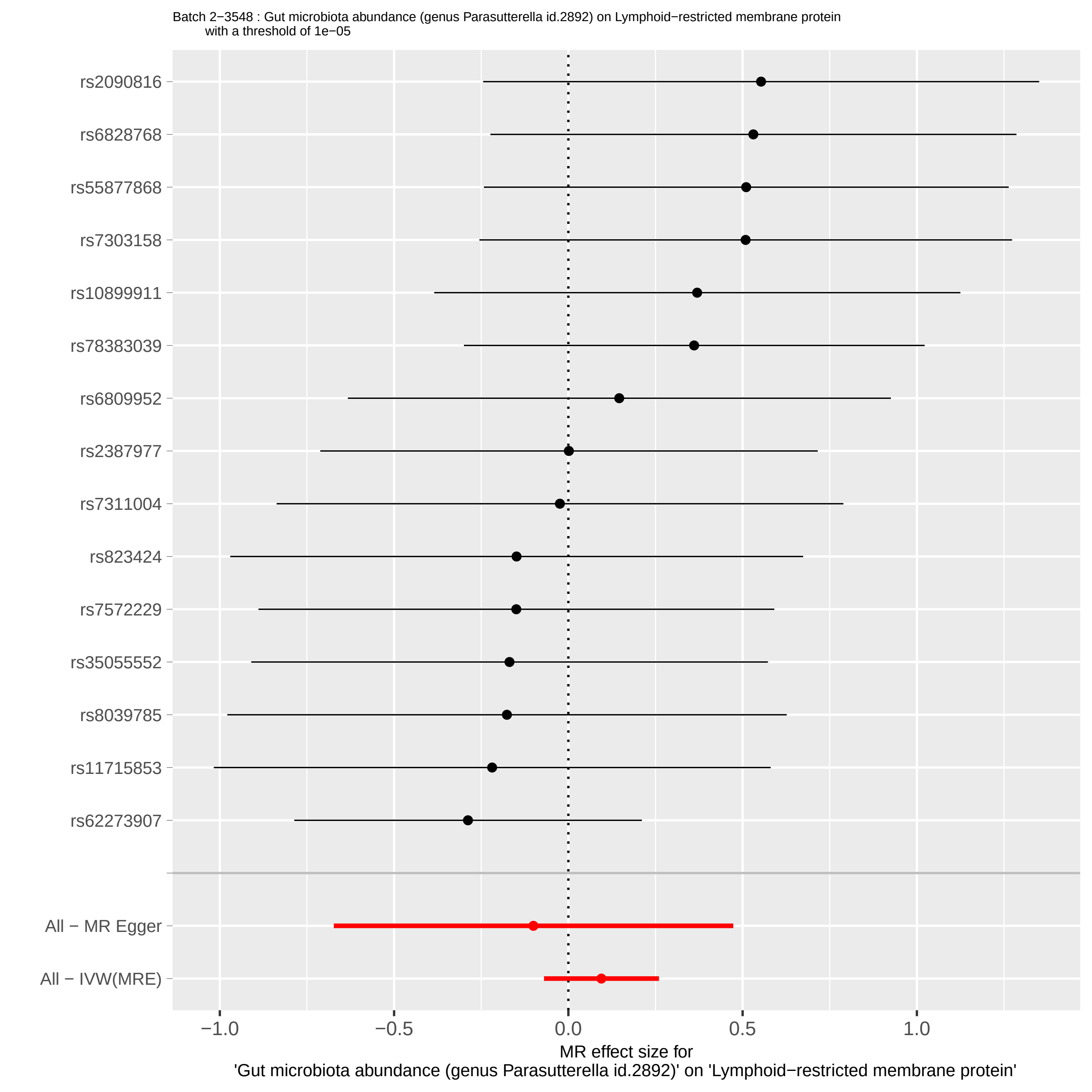
Batch 2−3548 : Gut microbiota abundance (genus Parasutterella id.2892) on Lymphoid−restricted membrane protein
with a threshold of 1e−05
rs2090816
rs6828768
rs55877868
rs7303158
rs10899911
rs78383039
rs6809952
rs2387977
rs7311004
rs823424
rs7572229
rs35055552
rs8039785
rs11715853
rs62273907
All − MR Egger
All − IVW(MRE)
−1.0
−0.5
0.0
0.5
1.0
MR effect size for
'Gut microbiota abundance (genus Parasutterella id.2892)' on 'Lymphoid−restricted membrane protein'

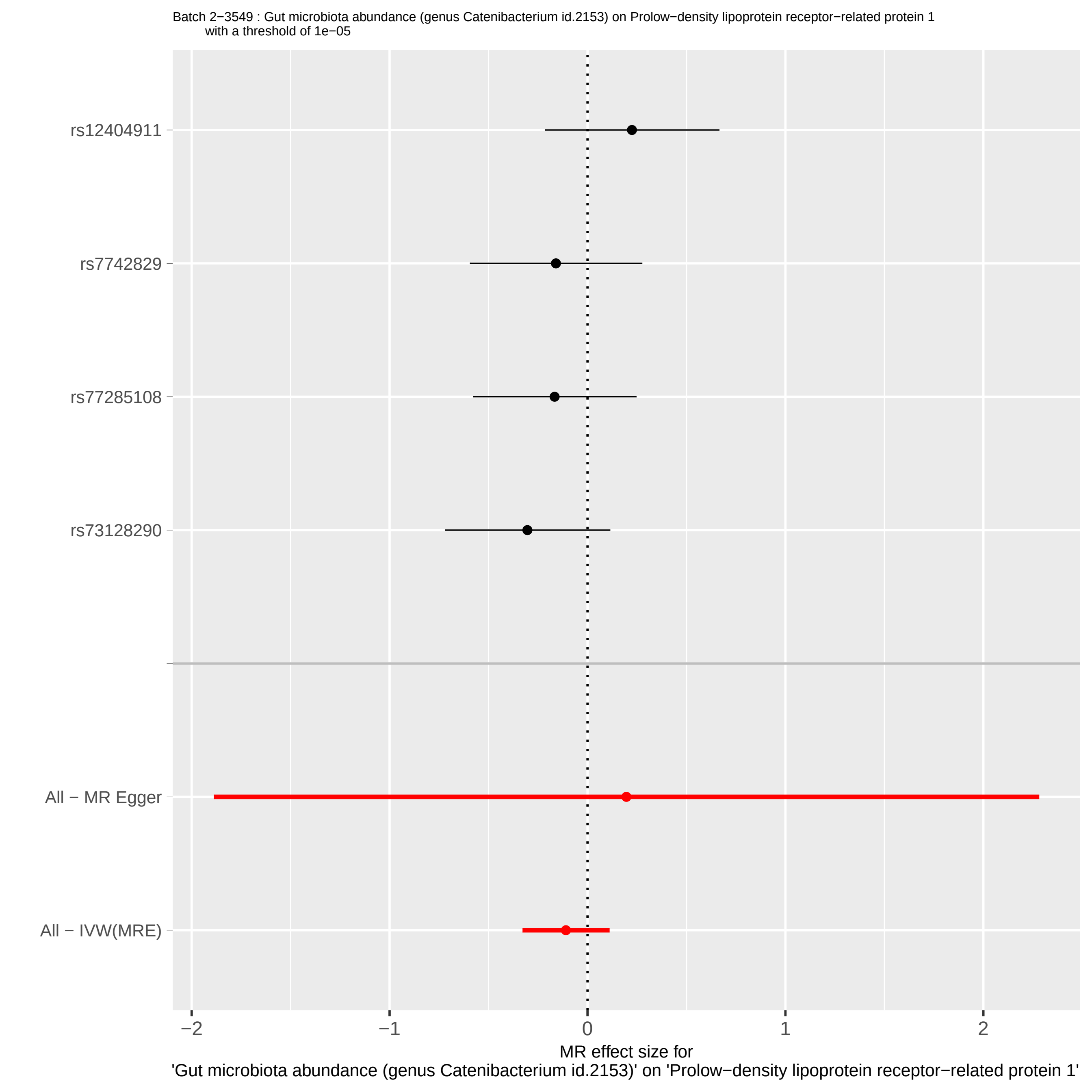
Batch 2−3549 : Gut microbiota abundance (genus Catenibacterium id.2153) on Prolow−density lipoprotein receptor−related protein 1
with a threshold of 1e−05
rs12404911
rs7742829
rs77285108
rs73128290
All − MR Egger
All − IVW(MRE)
−2
−1
0
1
2
MR effect size for
'Gut microbiota abundance (genus Catenibacterium id.2153)' on 'Prolow−density lipoprotein receptor−related protein 1'

Batch 2−3550 : Gut microbiota abundance (genus Parasutterella id.2892) on Prolow−density lipoprotein receptor−related protein 1 with a threshold of 1e−05

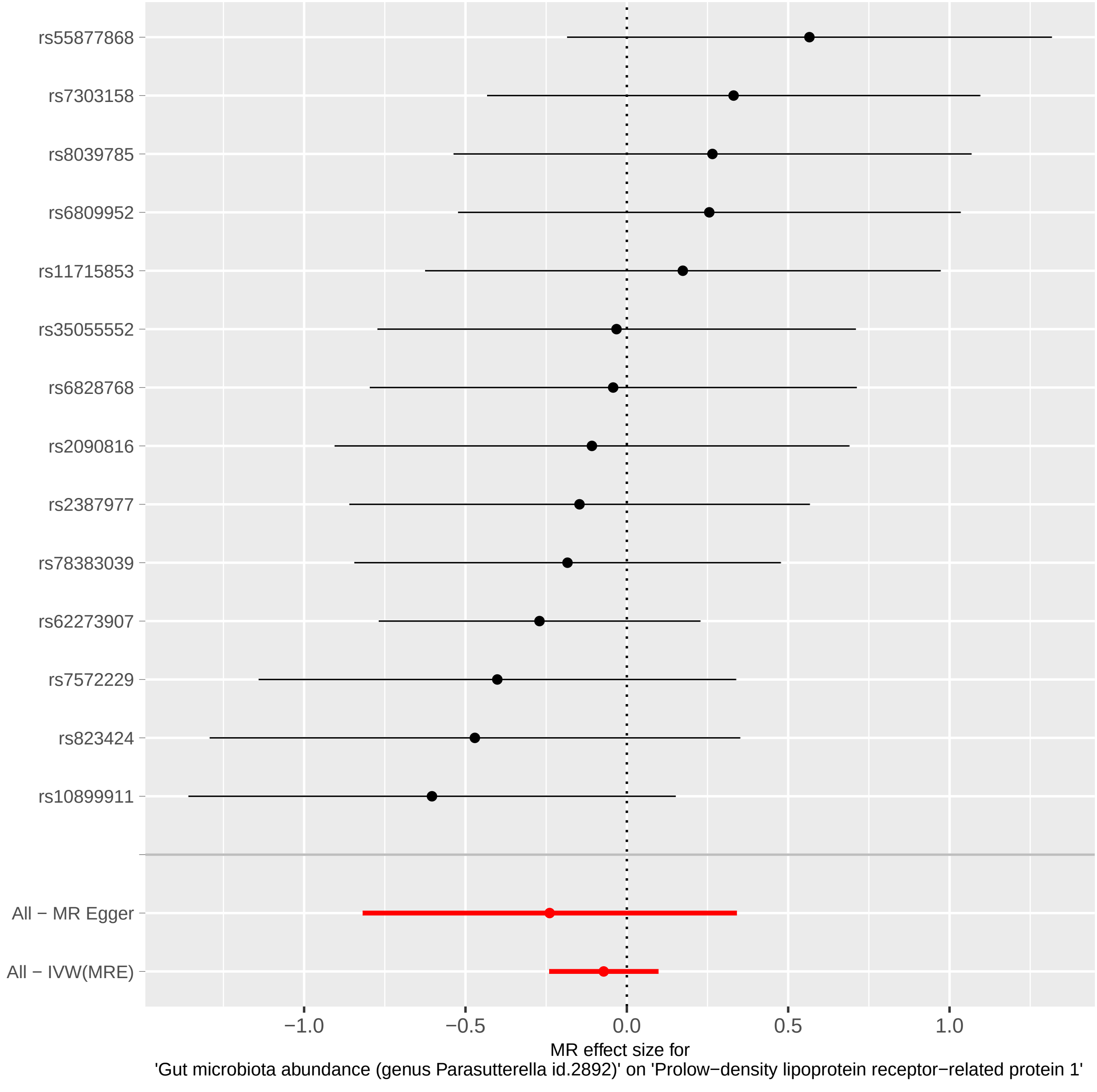

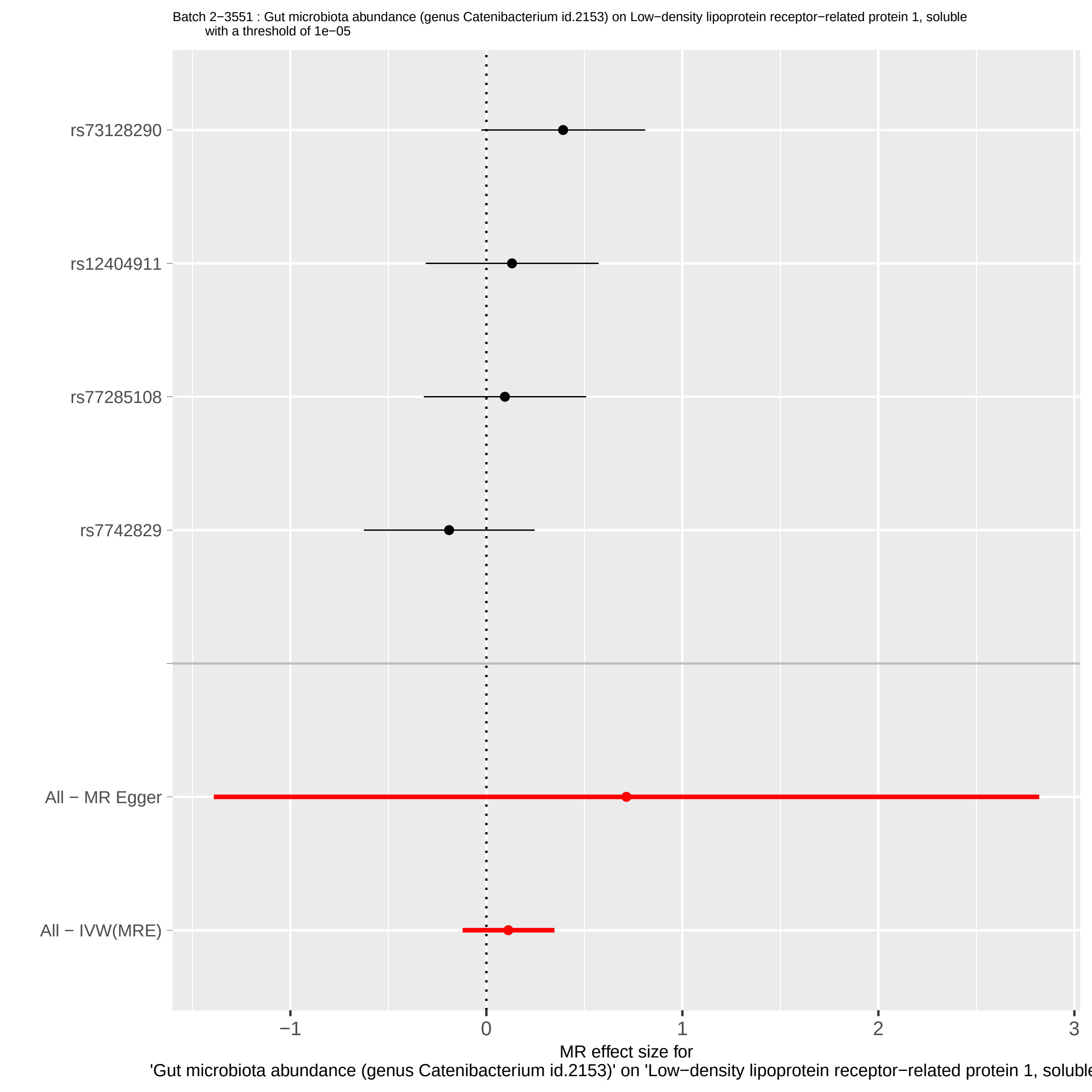

Batch 2−3551 : Gut microbiota abundance (genus Catenibacterium id.2153) on Low−density lipoprotein receptor−related protein 1, soluble
with a threshold of 1e−05
rs73128290
rs12404911
rs77285108
rs7742829
All − MR Egger
All − IVW(MRE)
−1
0
1
2
3
MR effect size for
'Gut microbiota abundance (genus Catenibacterium id.2153)' on 'Low−density lipoprotein receptor−related protein 1, soluble

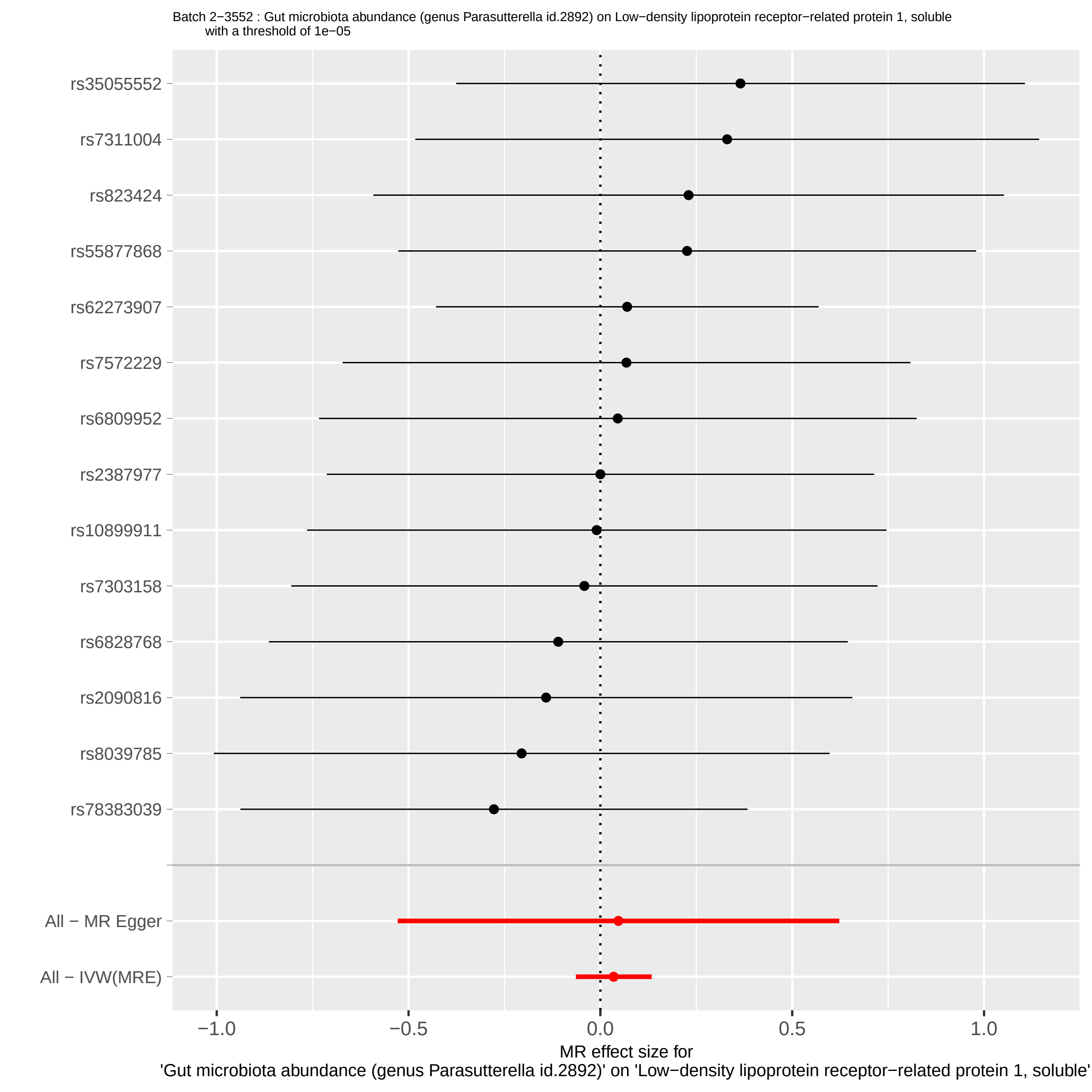
Batch 2−3552 : Gut microbiota abundance (genus Parasutterella id.2892) on Low−density lipoprotein receptor−related protein 1, soluble
with a threshold of 1e−05
rs35055552
rs7311004
rs823424
rs55877868
rs62273907
rs7572229
rs6809952
rs2387977
rs10899911
rs7303158
rs6828768
rs2090816
rs8039785
rs78383039
All − MR Egger
All − IVW(MRE)
−1.0
−0.5
0.0
0.5
1.0
MR effect size for
'Gut microbiota abundance (genus Parasutterella id.2892)' on 'Low−density lipoprotein receptor−related protein 1, soluble'

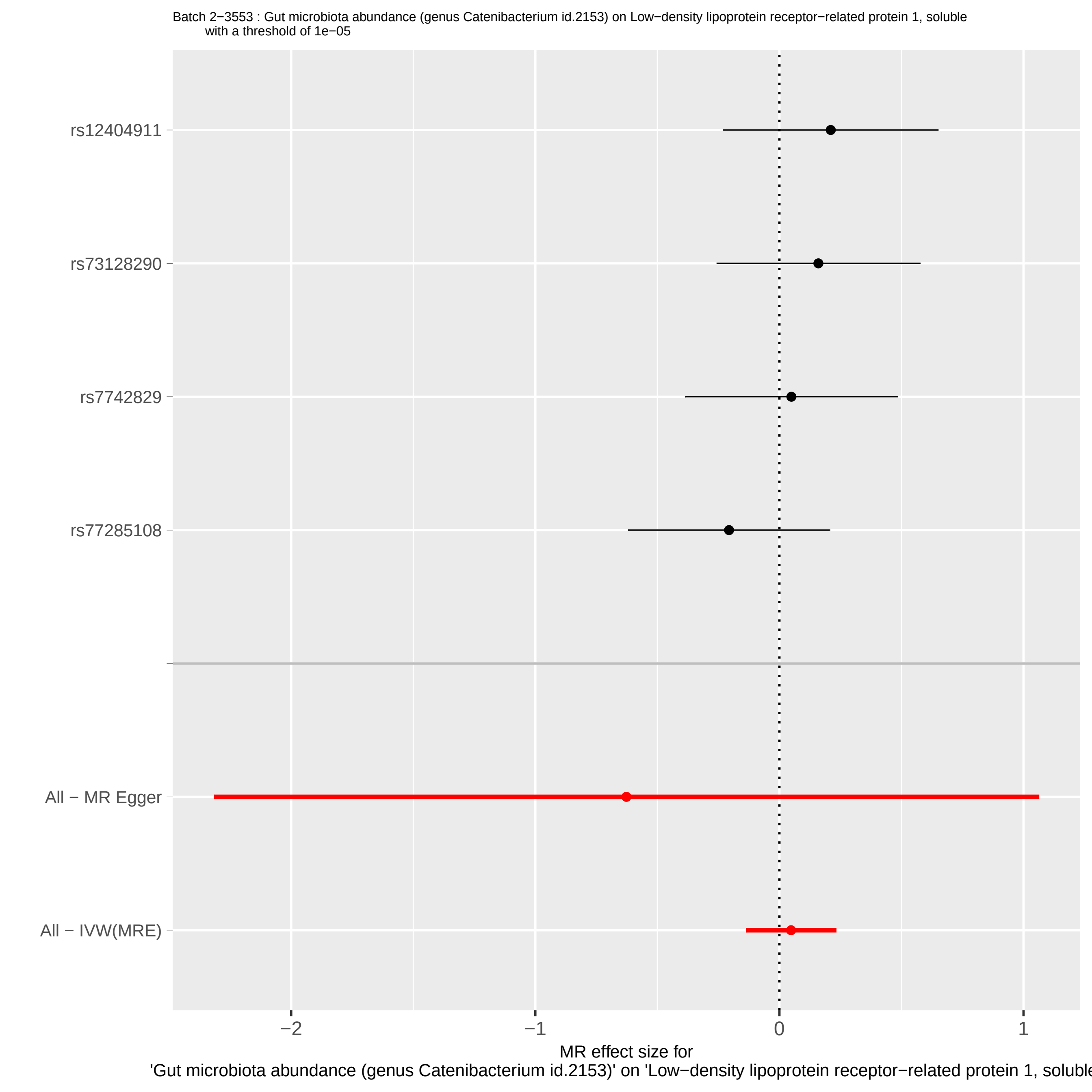

Batch 2−3553 : Gut microbiota abundance (genus Catenibacterium id.2153) on Low−density lipoprotein receptor−related protein 1, soluble
with a threshold of 1e−05
rs12404911
rs73128290
rs7742829
rs77285108
All − MR Egger
All − IVW(MRE)
−2
−1
0
1
MR effect size for
'Gut microbiota abundance (genus Catenibacterium id.2153)' on 'Low−density lipoprotein receptor−related protein 1, soluble

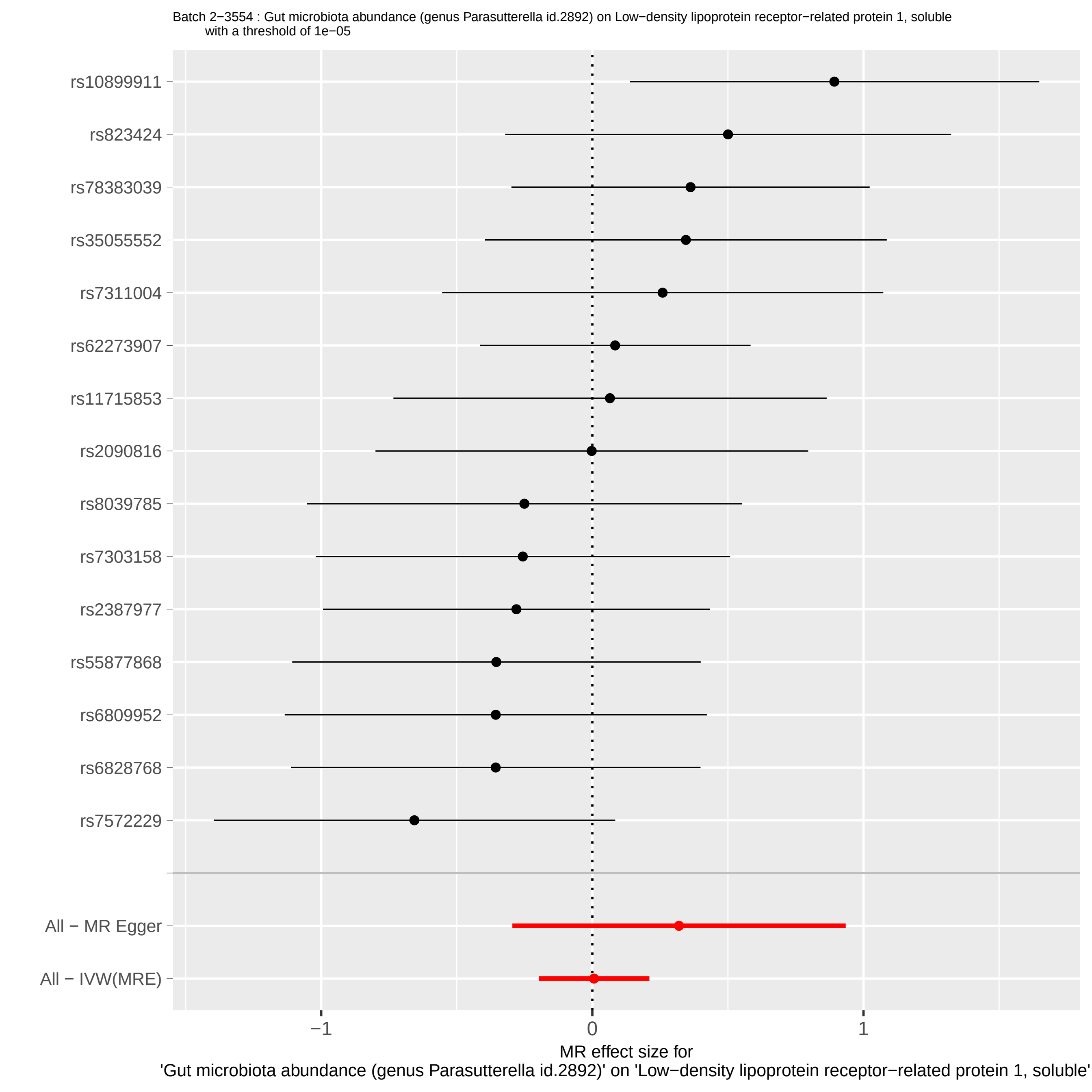
Batch 2−3554 : Gut microbiota abundance (genus Parasutterella id.2892) on Low−density lipoprotein receptor−related protein 1, soluble
with a threshold of 1e−05
rs10899911
rs823424
rs78383039
rs35055552
rs7311004
rs62273907
rs11715853
rs2090816
rs8039785
rs7303158
rs2387977
rs55877868
rs6809952
rs6828768
rs7572229
All − MR Egger
All − IVW(MRE)
−1
0
1
MR effect size for
'Gut microbiota abundance (genus Parasutterella id.2892)' on 'Low−density lipoprotein receptor−related protein 1, soluble'

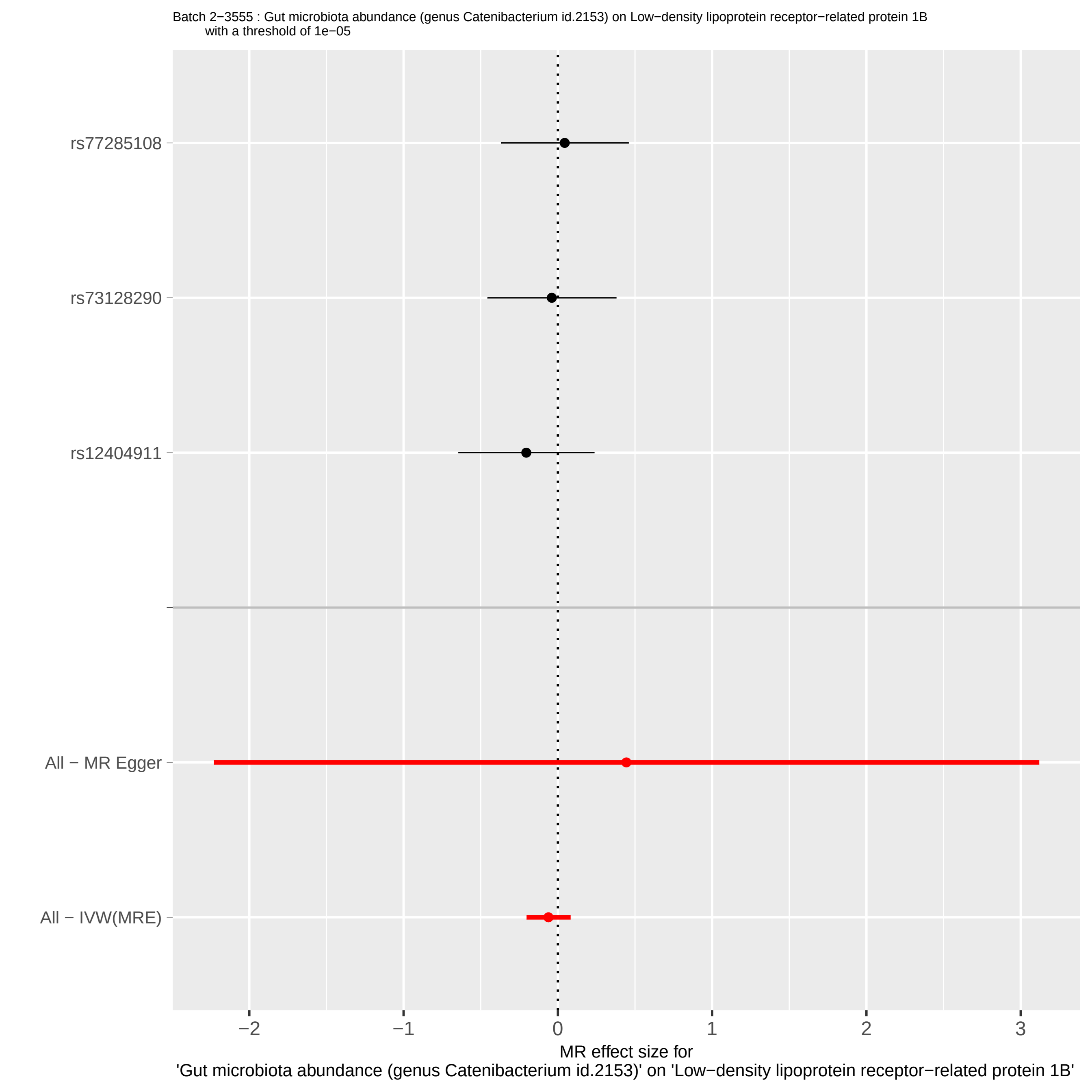

Batch 2−3555 : Gut microbiota abundance (genus Catenibacterium id.2153) on Low−density lipoprotein receptor−related protein 1B
with a threshold of 1e−05
rs77285108
rs73128290
rs12404911
All − MR Egger
All − IVW(MRE)
−2
−1
0
1
2
3
MR effect size for
'Gut microbiota abundance (genus Catenibacterium id.2153)' on 'Low−density lipoprotein receptor−related protein 1B'

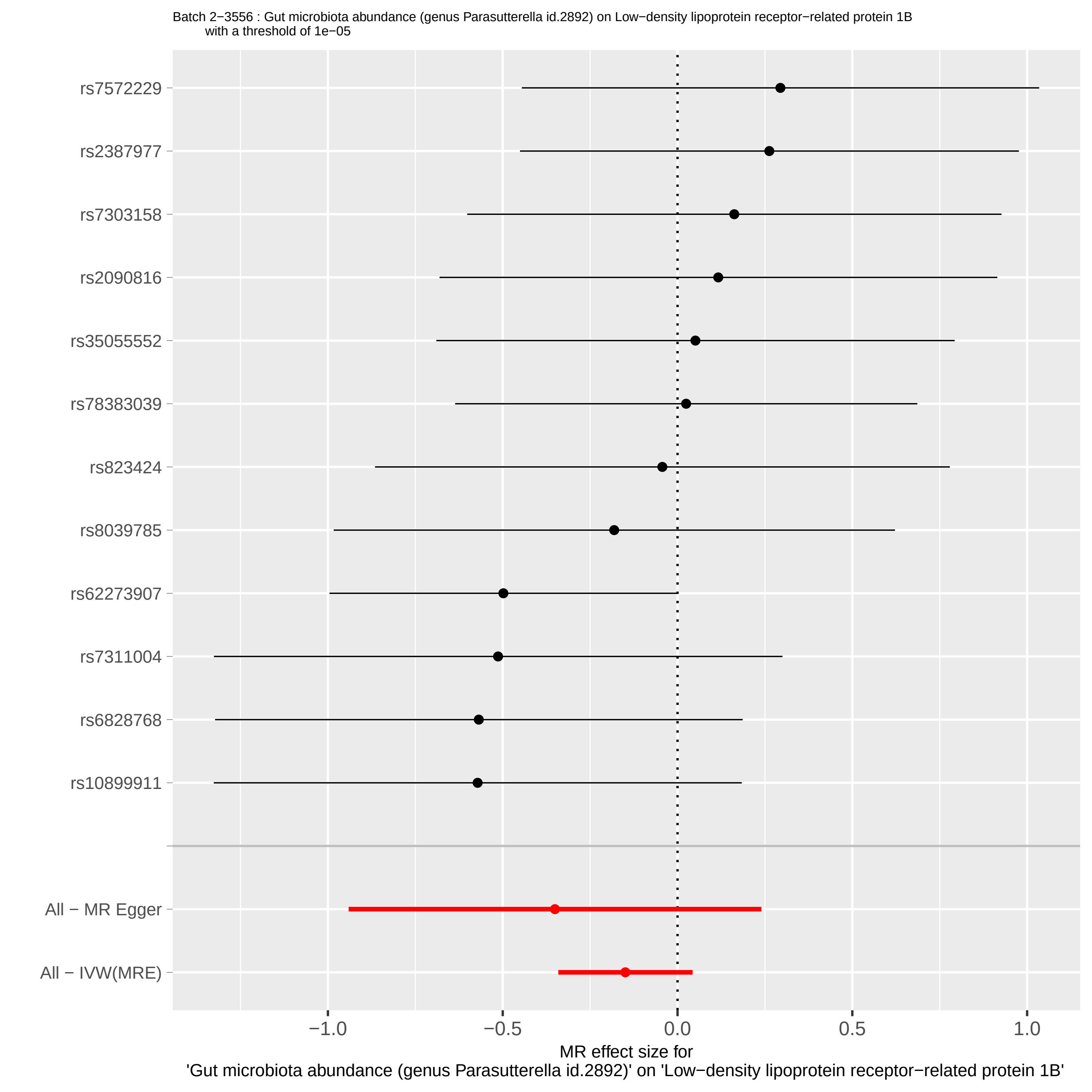

Batch 2−3556 : Gut microbiota abundance (genus Parasutterella id.2892) on Low−density lipoprotein receptor−related protein 1B
with a threshold of 1e−05
rs7572229
rs2387977
rs7303158
rs2090816
rs35055552
rs78383039
rs823424
rs8039785
rs62273907
rs7311004
rs6828768
rs10899911
All − MR Egger
All − IVW(MRE)
−1.0
−0.5
0.0
0.5
1.0
MR effect size for
'Gut microbiota abundance (genus Parasutterella id.2892)' on 'Low−density lipoprotein receptor−related protein 1B'

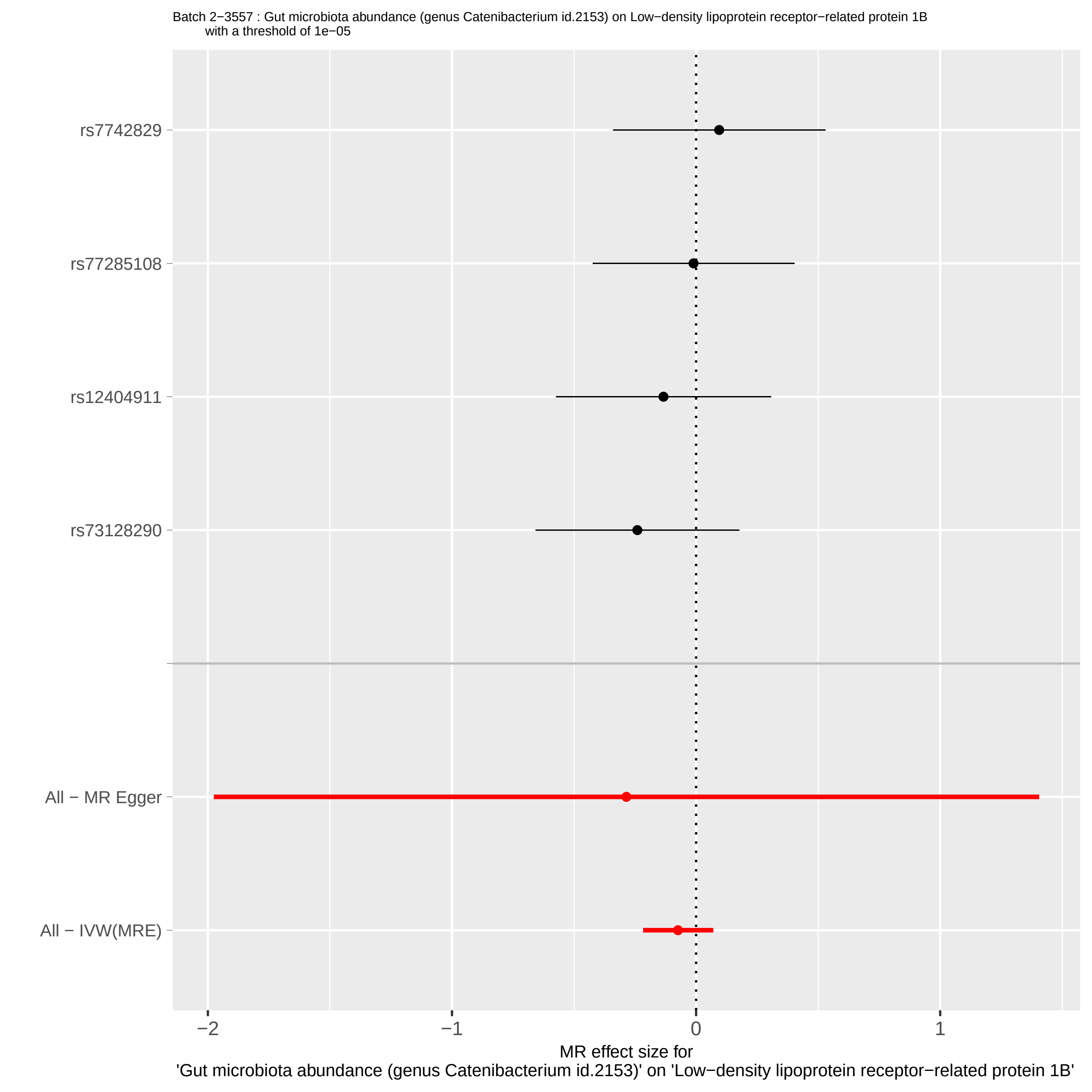

Batch 2−3557 : Gut microbiota abundance (genus Catenibacterium id.2153) on Low−density lipoprotein receptor−related protein 1B
with a threshold of 1e−05
rs7742829
rs77285108
rs12404911
rs73128290
All − MR Egger
All − IVW(MRE)
−2
−1
0
1
MR effect size for
'Gut microbiota abundance (genus Catenibacterium id.2153)' on 'Low−density lipoprotein receptor−related protein 1B'

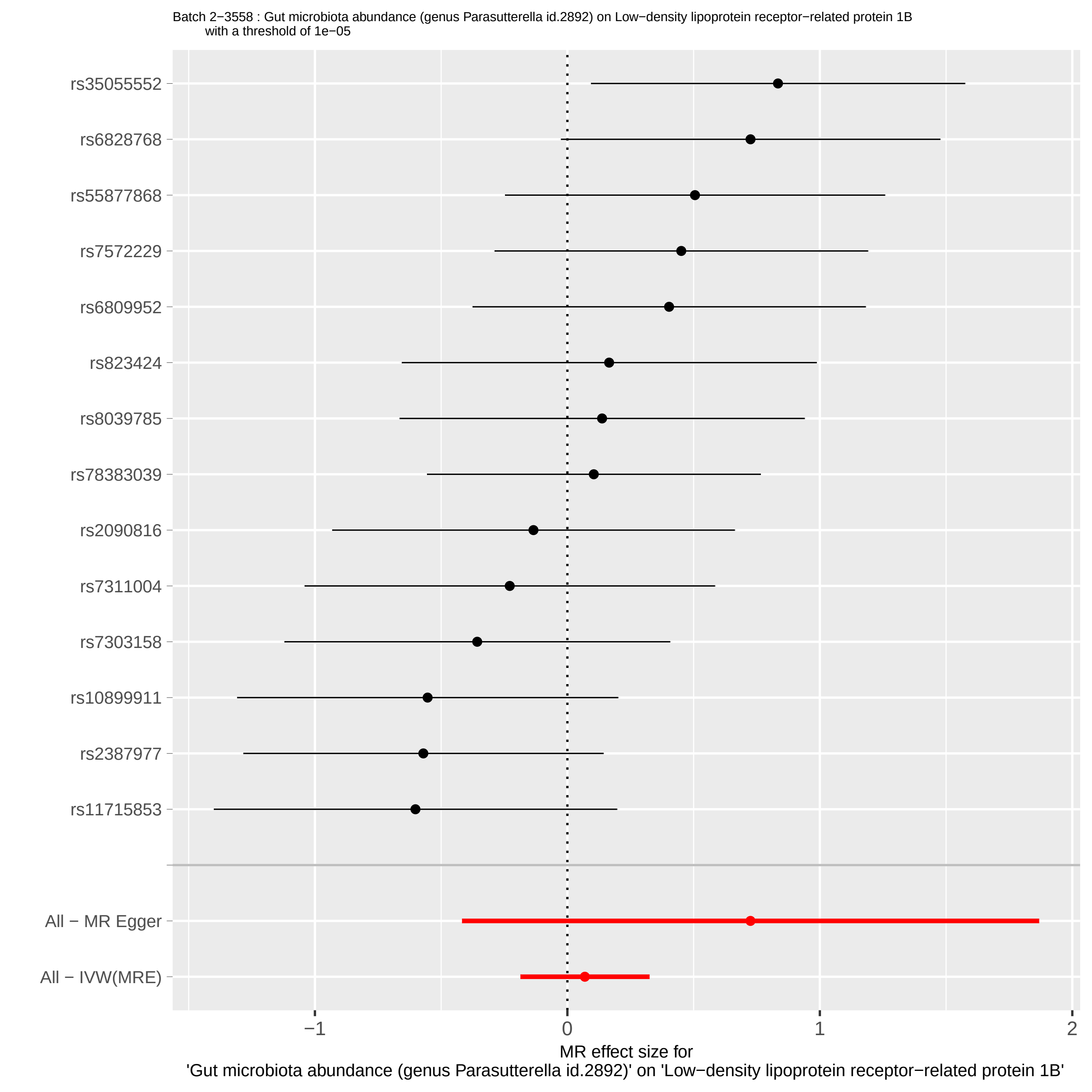

Batch 2−3558 : Gut microbiota abundance (genus Parasutterella id.2892) on Low−density lipoprotein receptor−related protein 1B with a threshold of 1e−05
rs35055552
rs6828768
rs55877868
rs7572229
rs6809952
rs823424
rs8039785
rs78383039
rs2090816
rs7311004
rs7303158
rs10899911
rs2387977
rs11715853
All − MR Egger
All − IVW(MRE)
−1
0
1
2
MR effect size for
'Gut microbiota abundance (genus Parasutterella id.2892)' on 'Low−density lipoprotein receptor−related protein 1B'

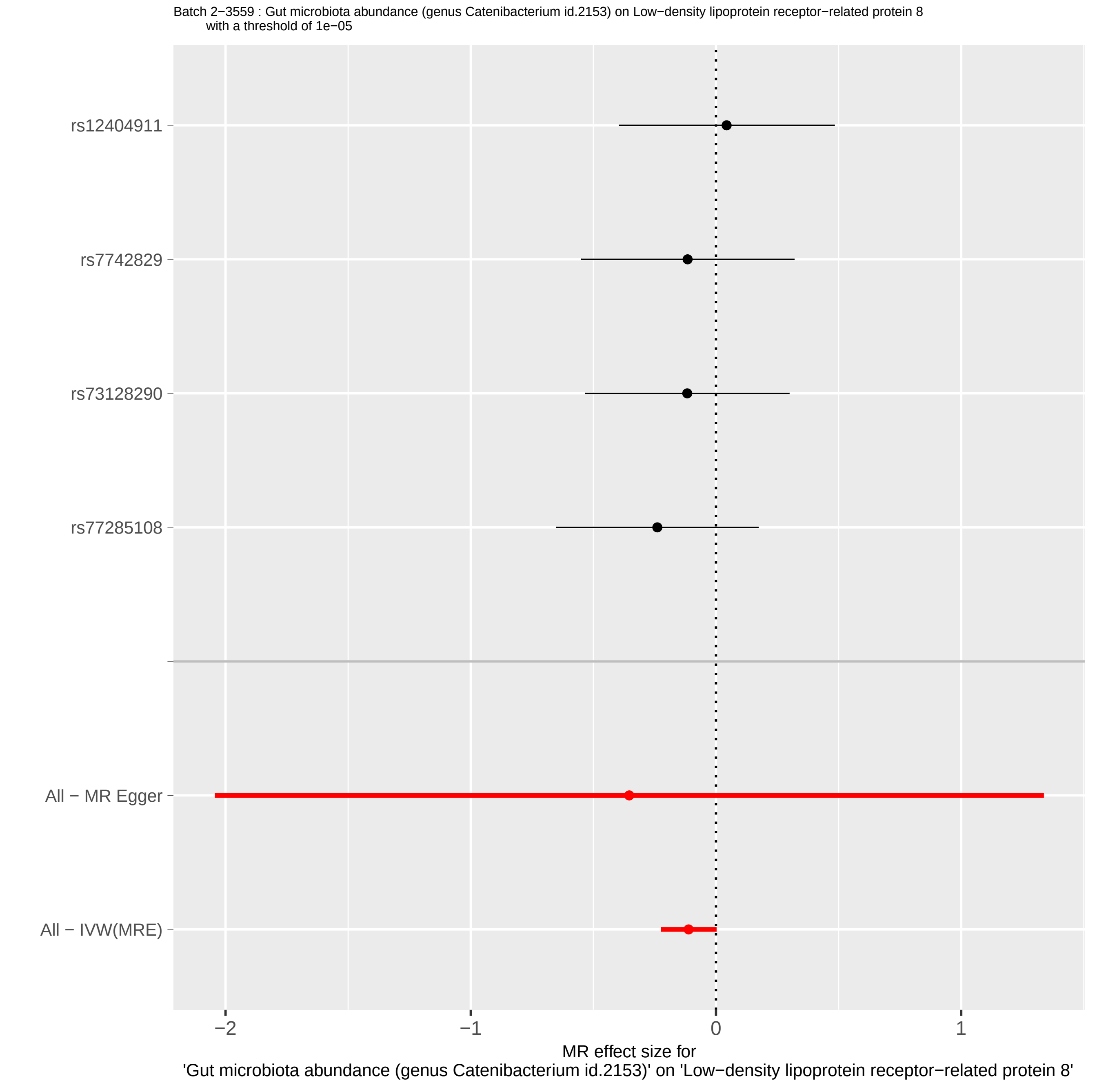
Batch 2−3559 : Gut microbiota abundance (genus Catenibacterium id.2153) on Low−density lipoprotein receptor−related protein 8
with a threshold of 1e−05
rs12404911
rs7742829
rs73128290
rs77285108
All − MR Egger
All − IVW(MRE)
−2
−1
0
1
MR effect size for
'Gut microbiota abundance (genus Catenibacterium id.2153)' on 'Low−density lipoprotein receptor−related protein 8'

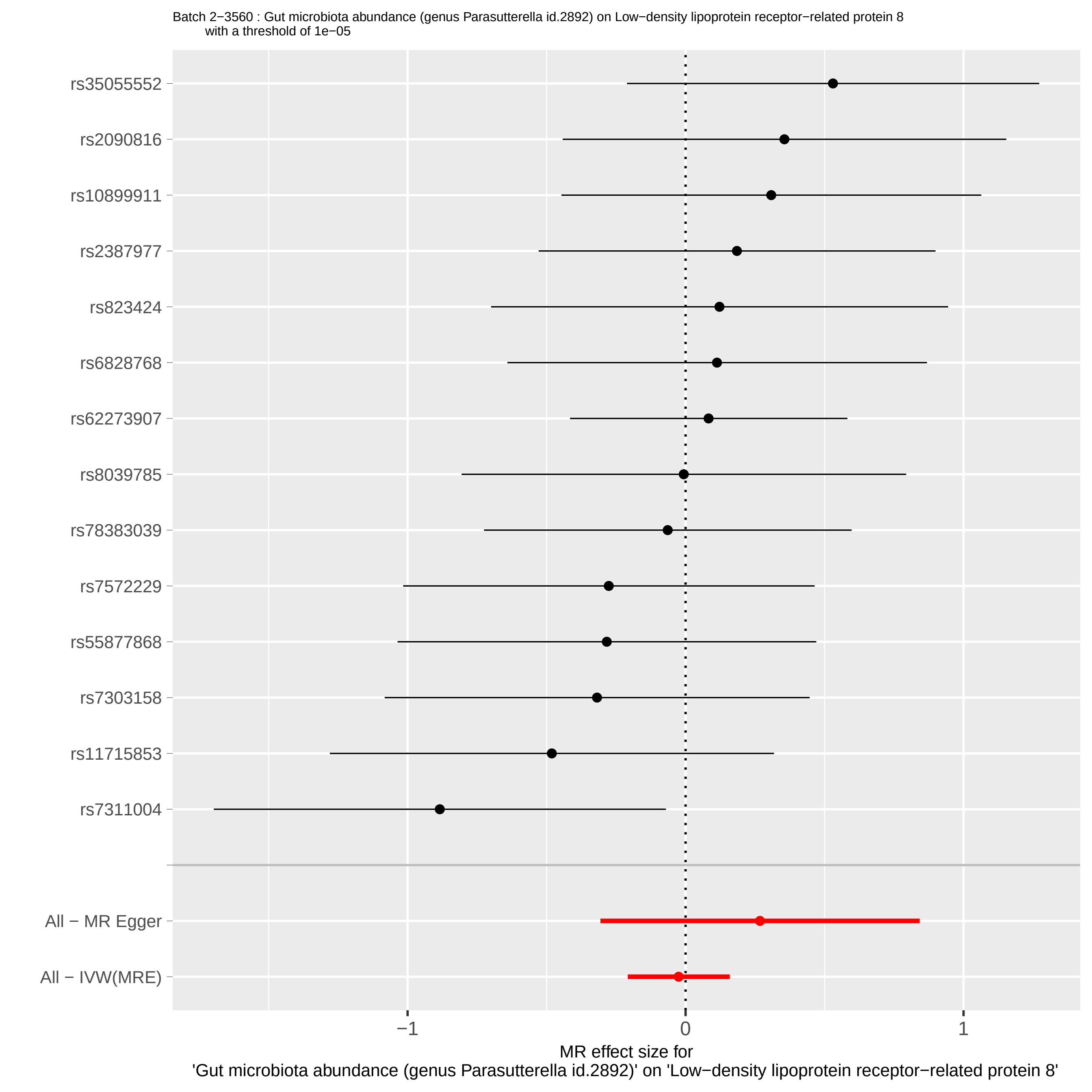
Batch 2−3560 : Gut microbiota abundance (genus Parasutterella id.2892) on Low−density lipoprotein receptor−related protein 8
with a threshold of 1e−05
rs35055552
rs2090816
rs10899911
rs2387977
rs823424
rs6828768
rs62273907
rs8039785
rs78383039
rs7572229
rs55877868
rs7303158
rs11715853
rs7311004
All − MR Egger
All − IVW(MRE)
−1
0
1
MR effect size for
'Gut microbiota abundance (genus Parasutterella id.2892)' on 'Low−density lipoprotein receptor−related protein 8'

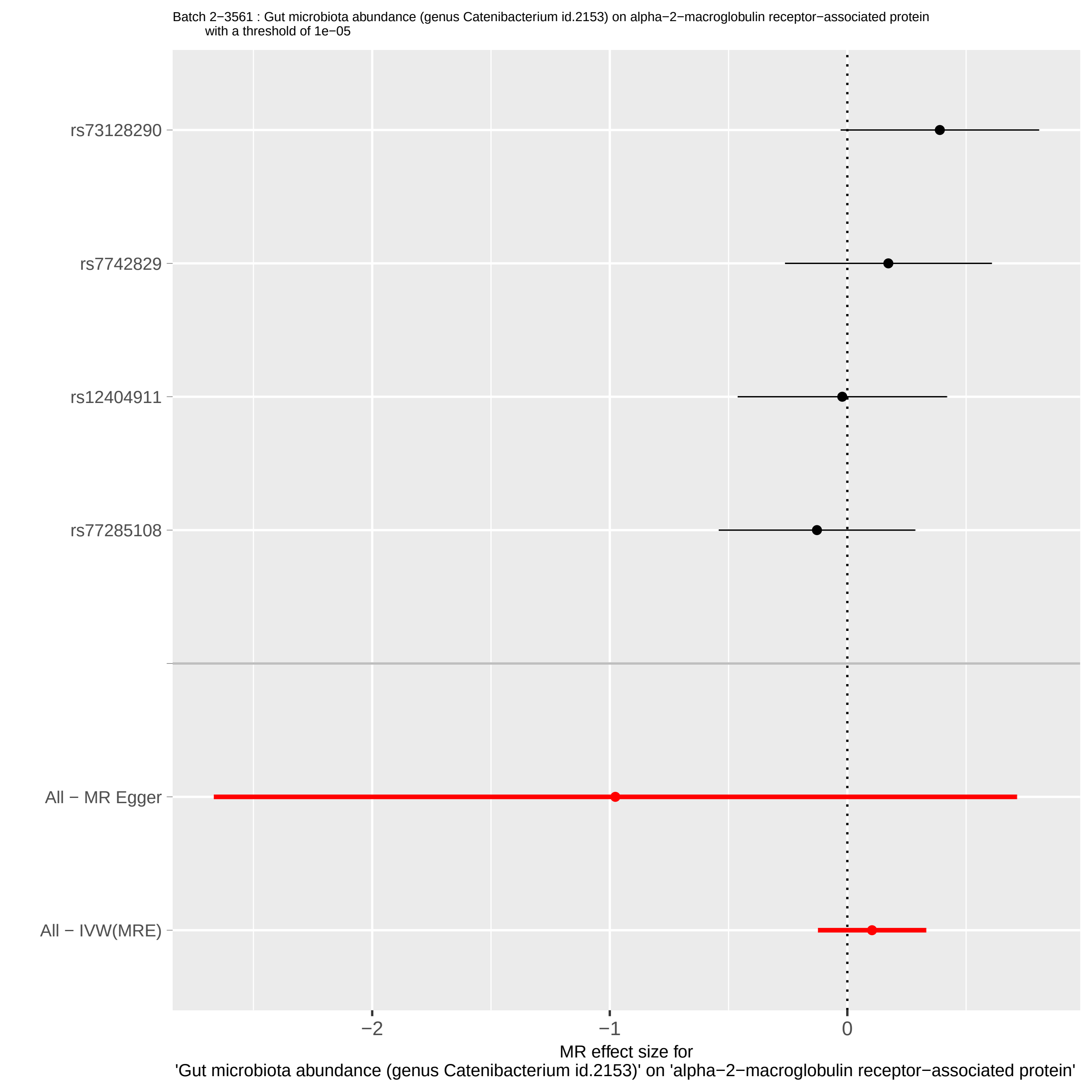

Batch 2−3561 : Gut microbiota abundance (genus Catenibacterium id.2153) on alpha−2−macroglobulin receptor−associated protein
with a threshold of 1e−05
rs73128290
rs7742829
rs12404911
rs77285108
All − MR Egger
All − IVW(MRE)
−2
−1
0
MR effect size for
'Gut microbiota abundance (genus Catenibacterium id.2153)' on 'alpha−2−macroglobulin receptor−associated protein'

Batch 2−3562 : Gut microbiota abundance (genus Parasutterella id.2892) on alpha−2−macroglobulin receptor−associated protein
with a threshold of 1e−05

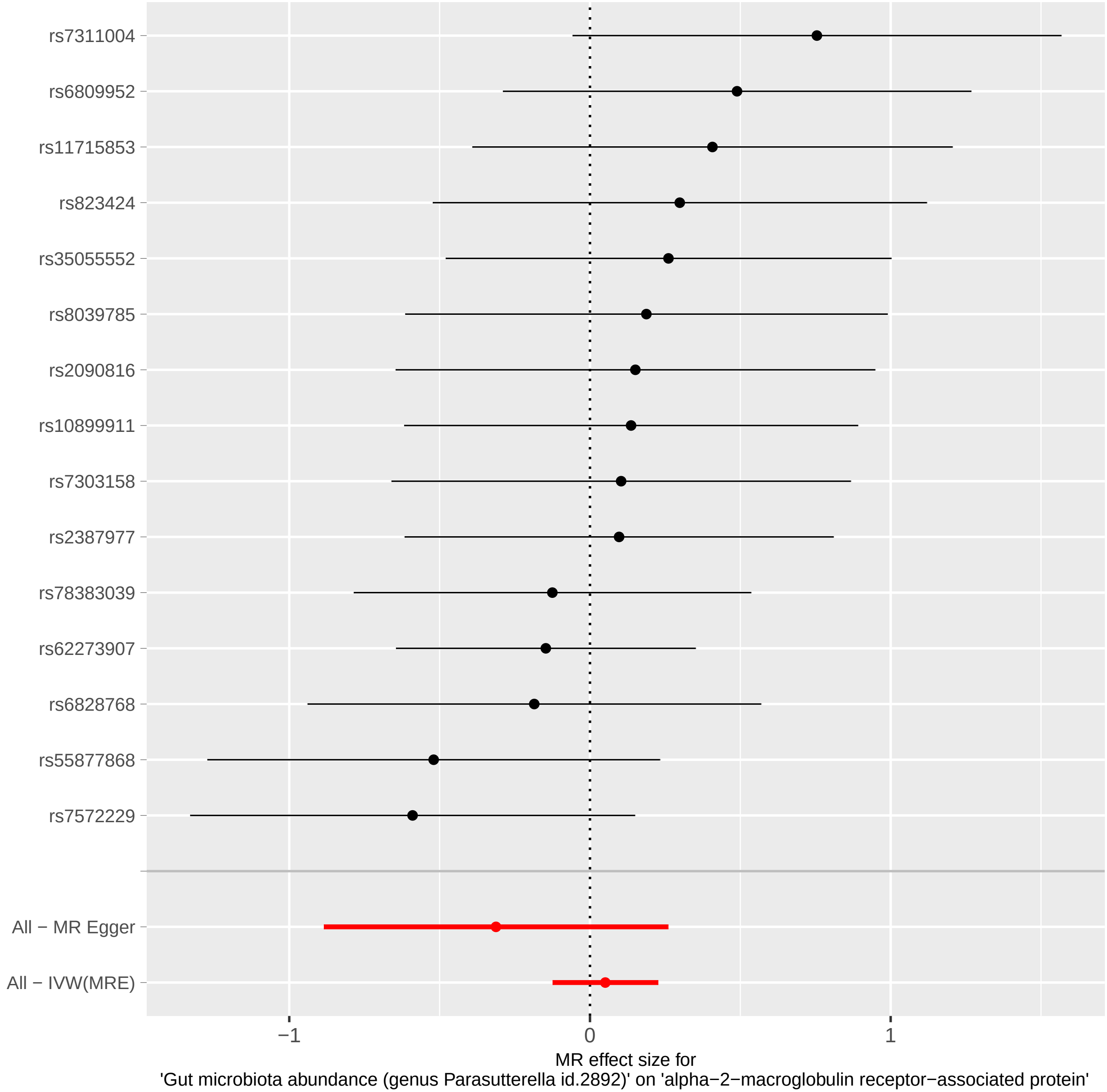

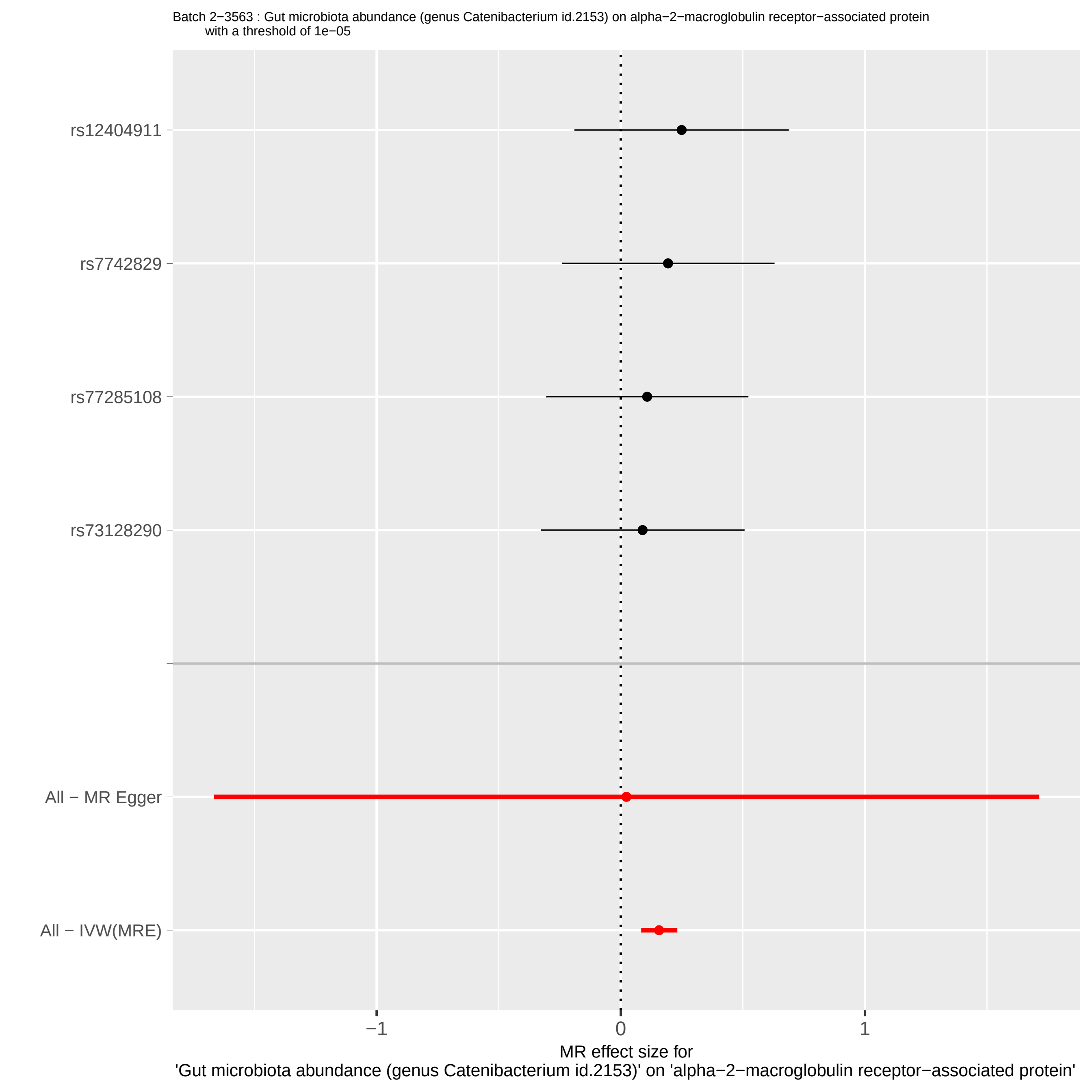
Batch 2−3563 : Gut microbiota abundance (genus Catenibacterium id.2153) on alpha−2−macroglobulin receptor−associated protein
with a threshold of 1e−05
rs12404911
rs7742829
rs77285108
rs73128290
All − MR Egger
All − IVW(MRE)
−1
0
1
MR effect size for
'Gut microbiota abundance (genus Catenibacterium id.2153)' on 'alpha−2−macroglobulin receptor−associated protein'

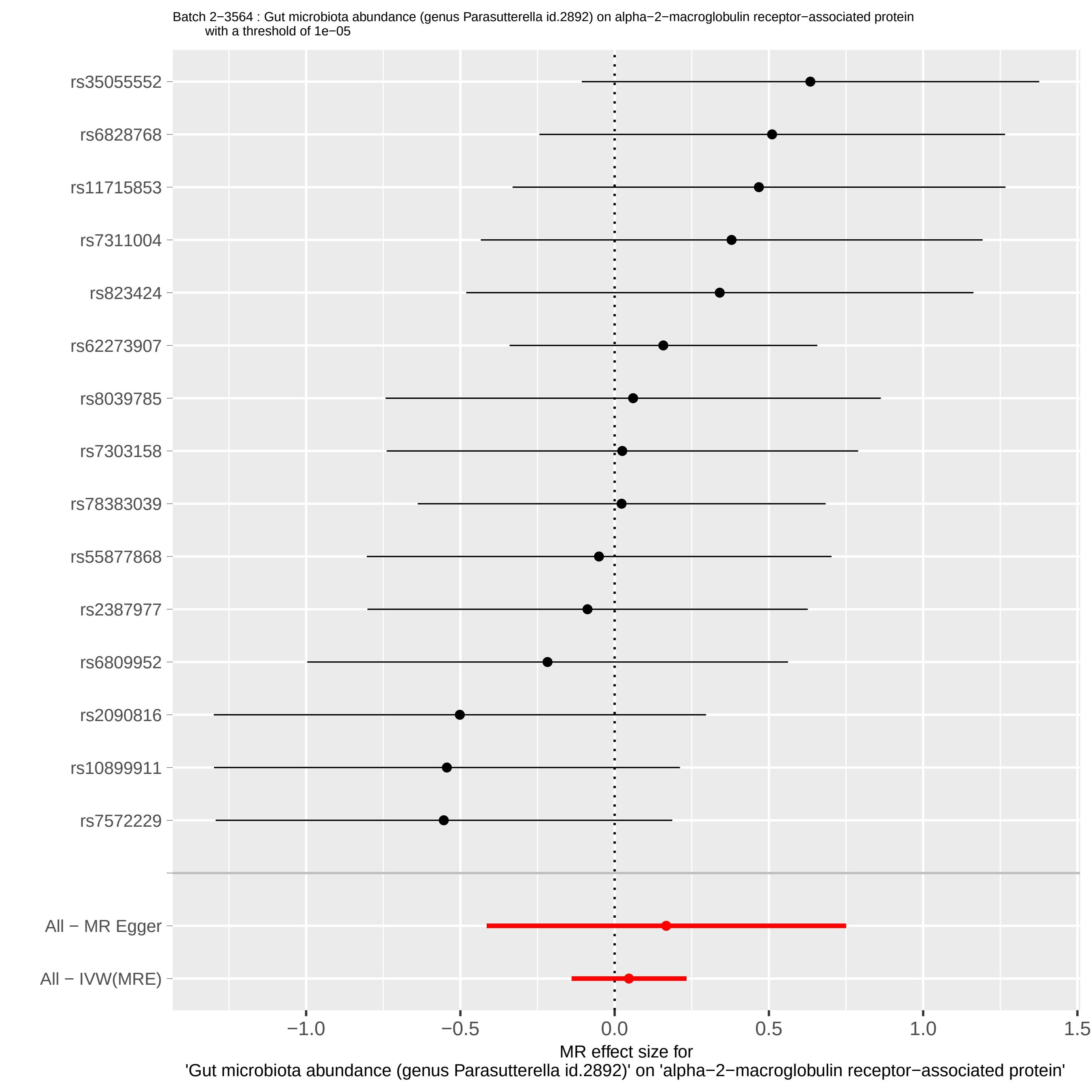

Batch 2−3564 : Gut microbiota abundance (genus Parasutterella id.2892) on alpha−2−macroglobulin receptor−associated protein with a threshold of 1e−05
rs35055552
rs6828768
rs11715853
rs7311004
rs823424
rs62273907
rs8039785
rs7303158
rs78383039
rs55877868
rs2387977
rs6809952
rs2090816
rs10899911
rs7572229
All − MR Egger
All − IVW(MRE)
−1.0
−0.5
0.0
0.5
1.0
1.5
MR effect size for
'Gut microbiota abundance (genus Parasutterella id.2892)' on 'alpha−2−macroglobulin receptor−associated protein'

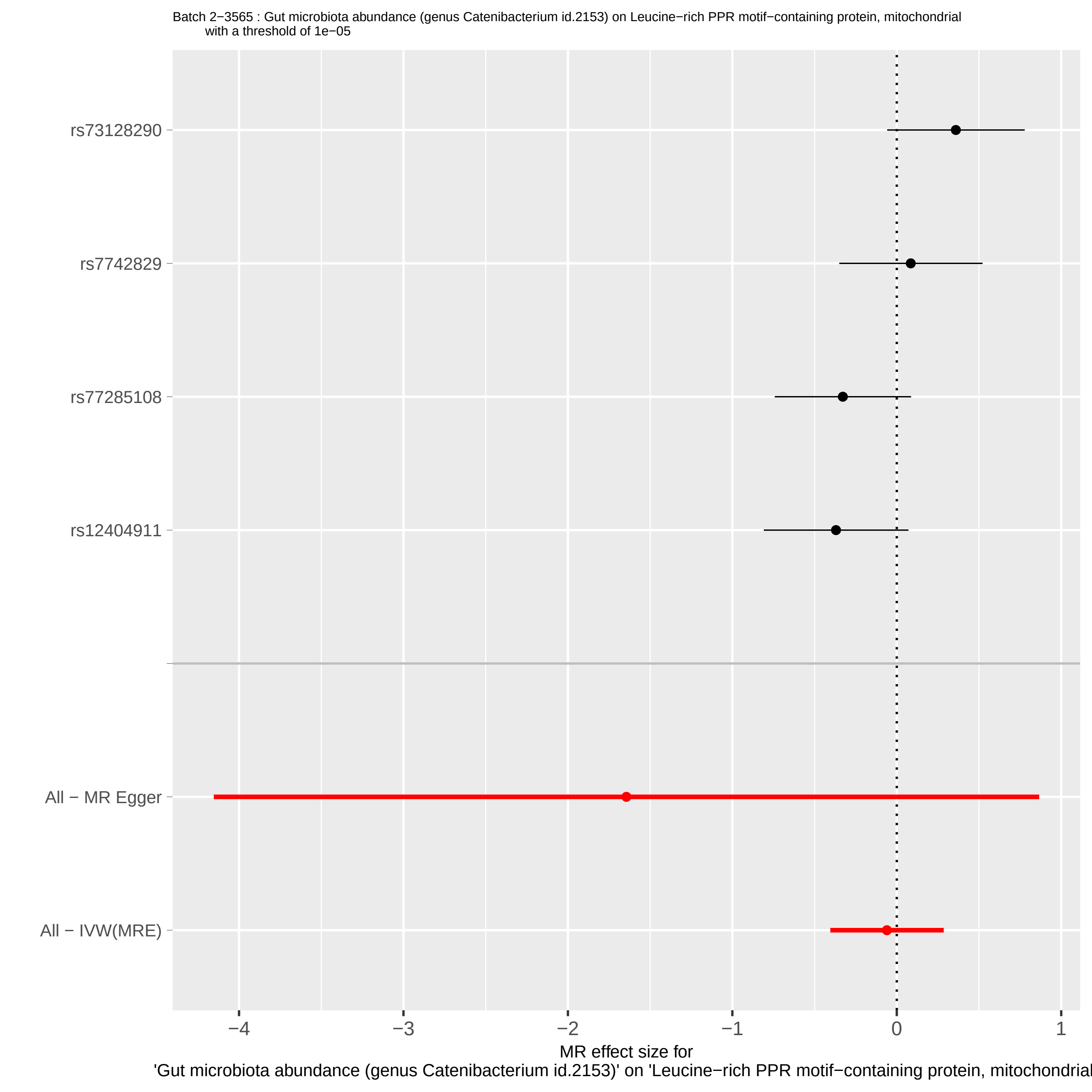

Batch 2−3565 : Gut microbiota abundance (genus Catenibacterium id.2153) on Leucine−rich PPR motif−containing protein, mitochondrial
with a threshold of 1e−05
rs73128290
rs7742829
rs77285108
rs12404911
All − MR Egger
All − IVW(MRE)
−4
−3
−2
−1
0
1
MR effect size for
'Gut microbiota abundance (genus Catenibacterium id.2153)' on 'Leucine−rich PPR motif−containing protein, mitochondrial'

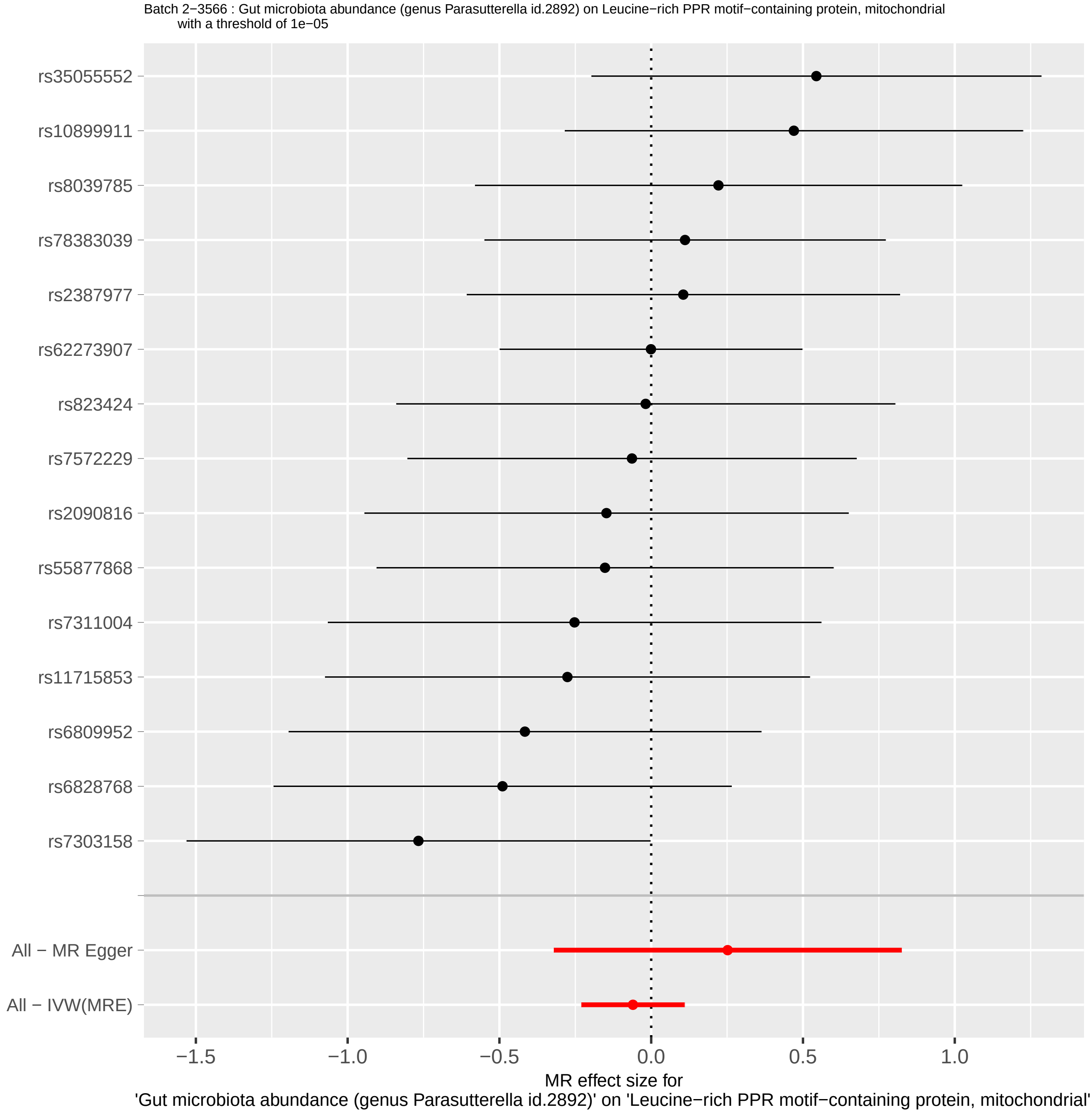

Batch 2−3566 : Gut microbiota abundance (genus Parasutterella id.2892) on Leucine−rich PPR motif−containing protein, mitochondrial
with a threshold of 1e−05
rs35055552
rs10899911
rs8039785
rs78383039
rs2387977
rs62273907
rs823424
rs7572229
rs2090816
rs55877868
rs7311004
rs11715853
rs6809952
rs6828768
rs7303158
All − MR Egger
All − IVW(MRE)
−1.5
−1.0
−0.5
0.0
0.5
1.0
MR effect size for
'Gut microbiota abundance (genus Parasutterella id.2892)' on 'Leucine−rich PPR motif−containing protein, mitochondrial'

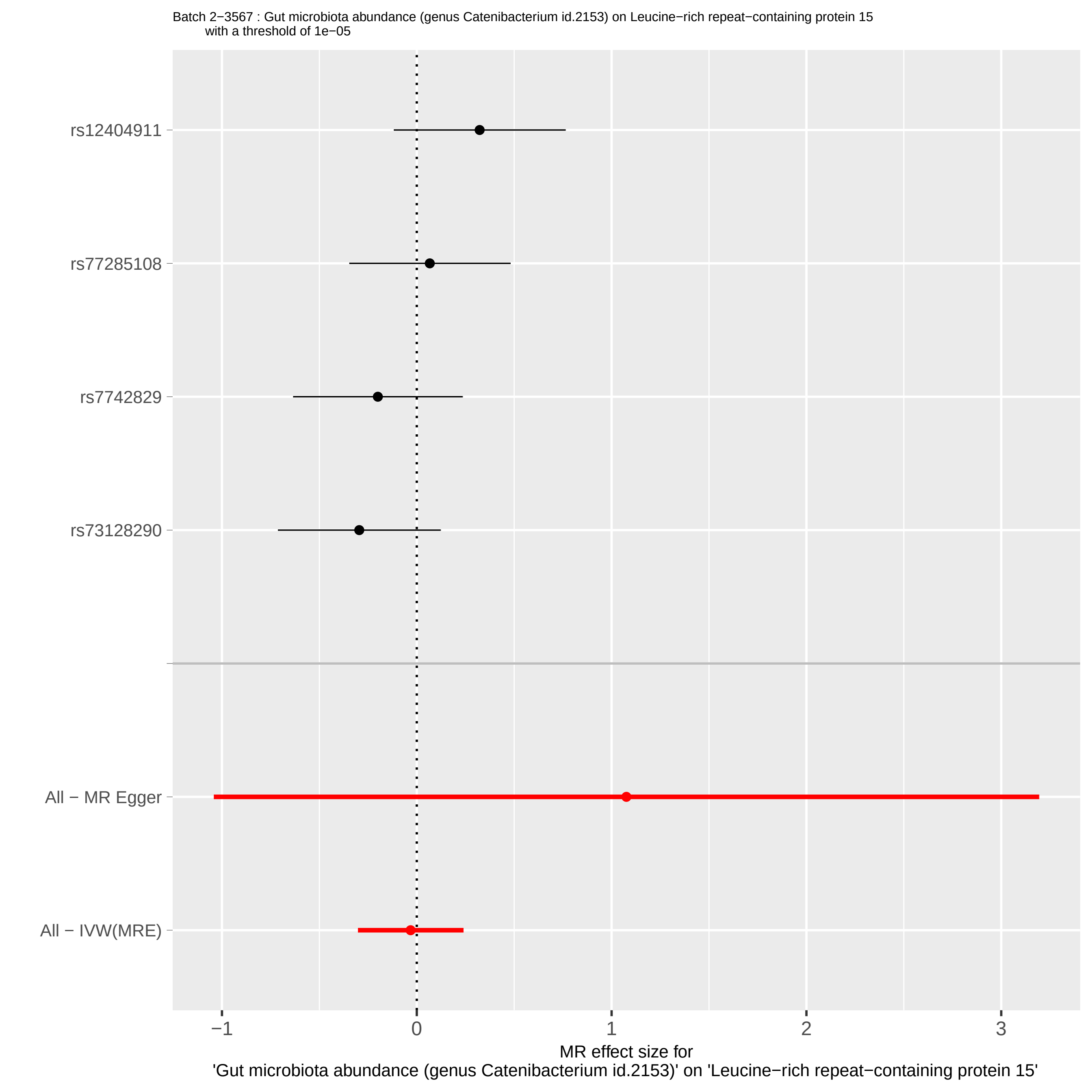
Batch 2−3567 : Gut microbiota abundance (genus Catenibacterium id.2153) on Leucine−rich repeat−containing protein 15
with a threshold of 1e−05
rs12404911
rs77285108
rs7742829
rs73128290
All − MR Egger
All − IVW(MRE)
−1
0
1
2
3
MR effect size for
'Gut microbiota abundance (genus Catenibacterium id.2153)' on 'Leucine−rich repeat−containing protein 15'

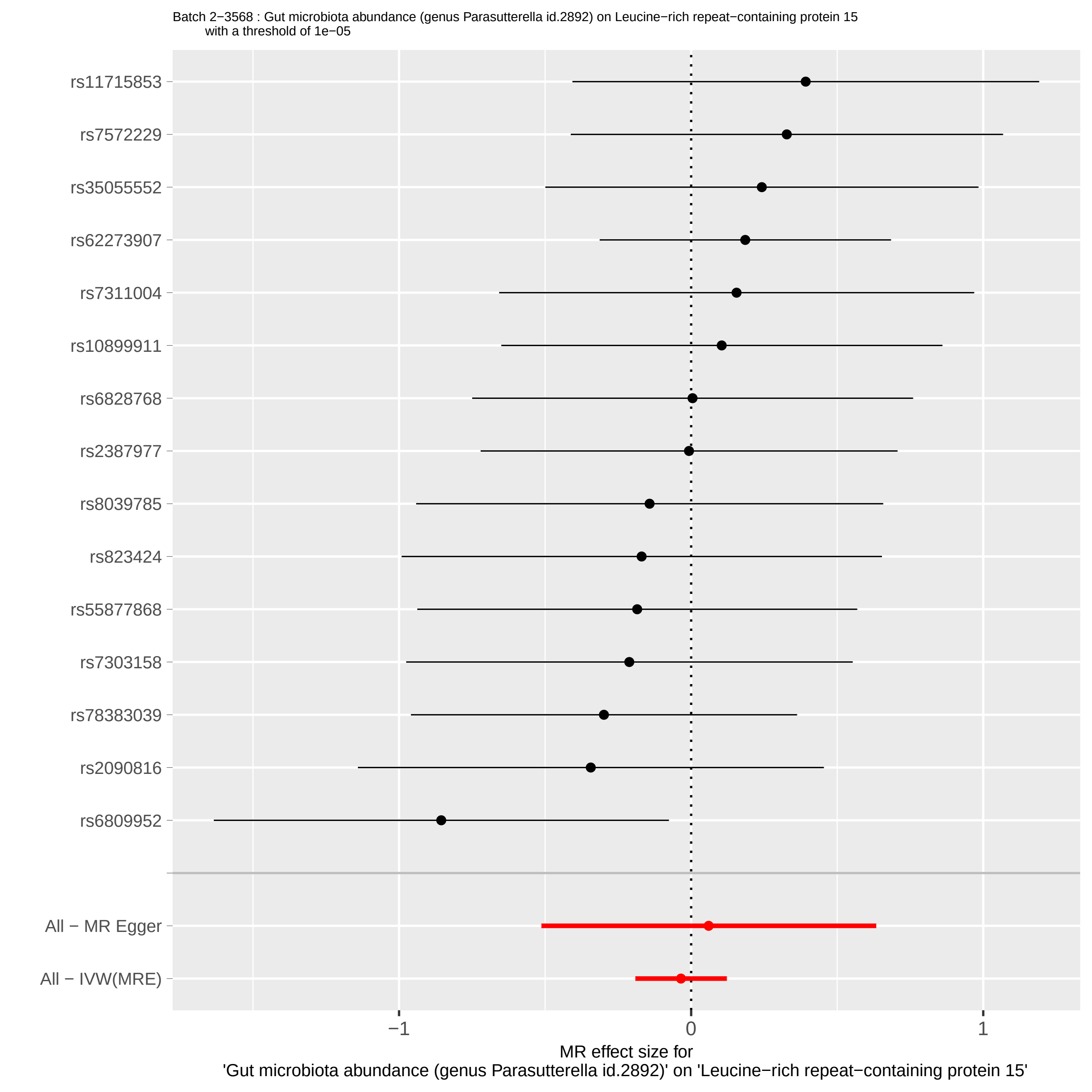

Batch 2−3568 : Gut microbiota abundance (genus Parasutterella id.2892) on Leucine−rich repeat−containing protein 15
with a threshold of 1e−05
rs11715853
rs7572229
rs35055552
rs62273907
rs7311004
rs10899911
rs6828768
rs2387977
rs8039785
rs823424
rs55877868
rs7303158
rs78383039
rs2090816
rs6809952
All − MR Egger
All − IVW(MRE)
−1
0
1
MR effect size for
'Gut microbiota abundance (genus Parasutterella id.2892)' on 'Leucine−rich repeat−containing protein 15'

Batch 2−3569 : Gut microbiota abundance (genus Catenibacterium id.2153) on Leucine−rich repeat−containing protein 19
with a threshold of 1e−05

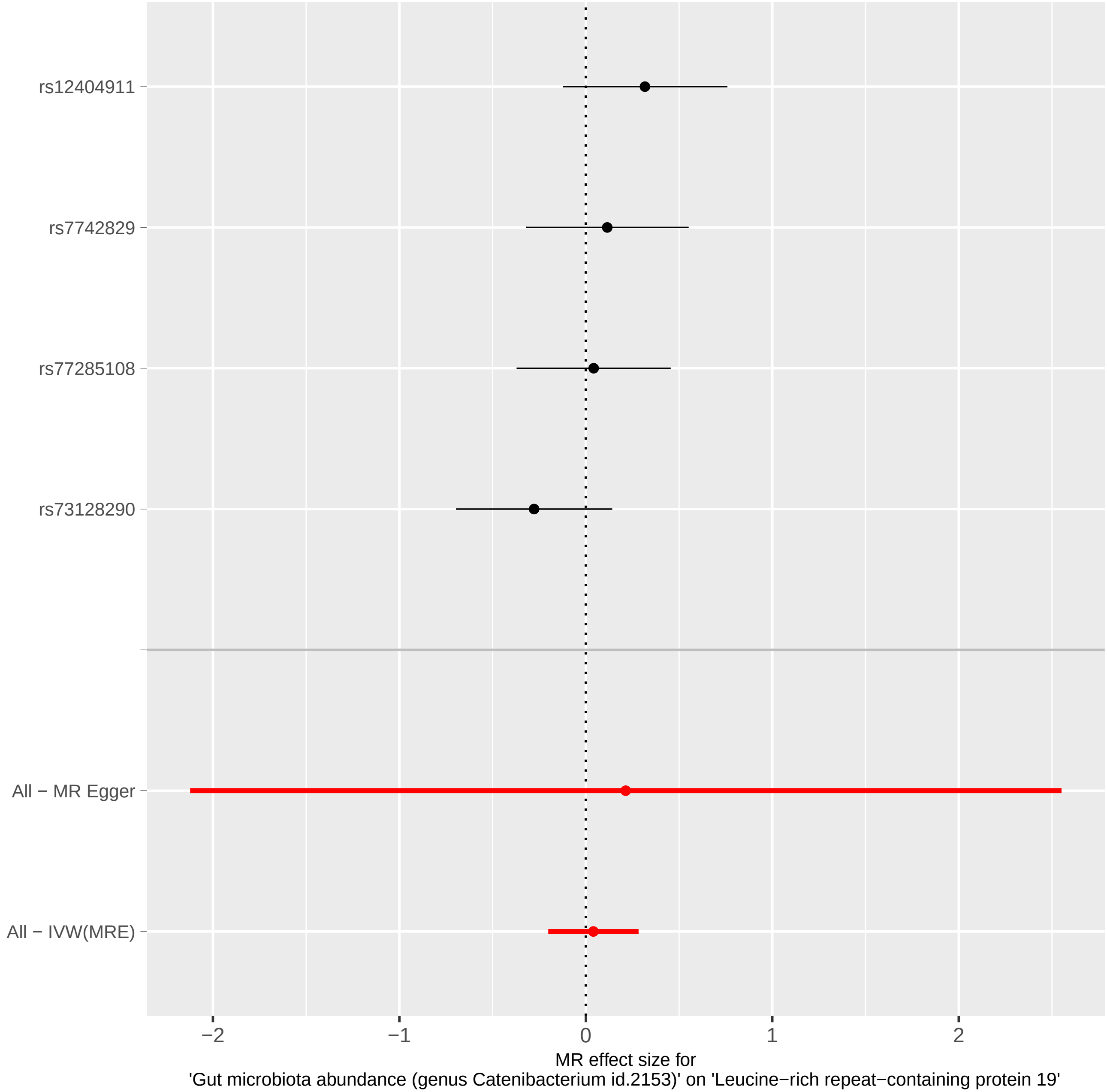

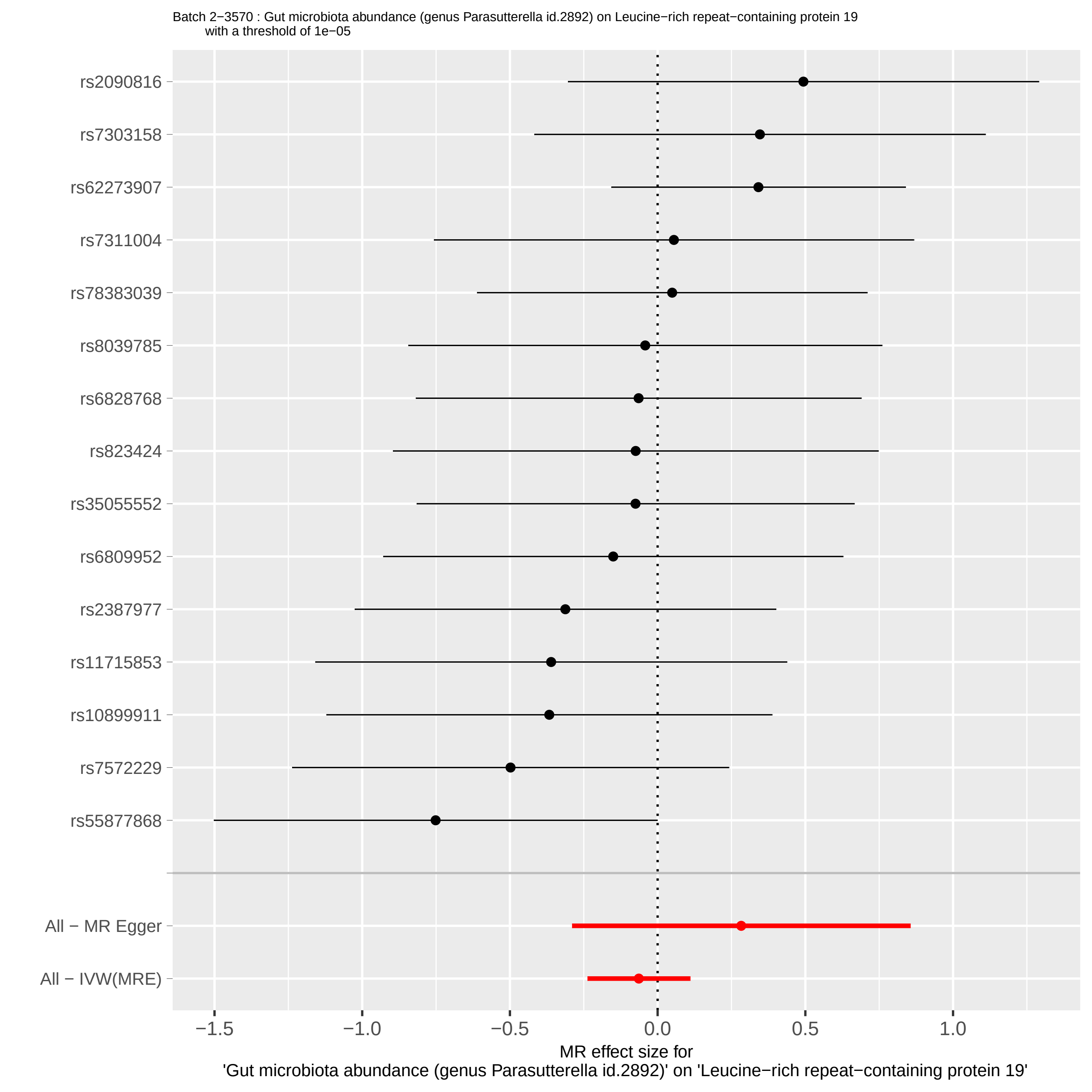

Batch 2−3570 : Gut microbiota abundance (genus Parasutterella id.2892) on Leucine−rich repeat−containing protein 19
with a threshold of 1e−05
rs2090816
rs7303158
rs62273907
rs7311004
rs78383039
rs8039785
rs6828768
rs823424
rs35055552
rs6809952
rs2387977
rs11715853
rs10899911
rs7572229
rs55877868
All − MR Egger
All − IVW(MRE)
−1.5
−1.0
−0.5
0.0
0.5
1.0
MR effect size for
'Gut microbiota abundance (genus Parasutterella id.2892)' on 'Leucine−rich repeat−containing protein 19'

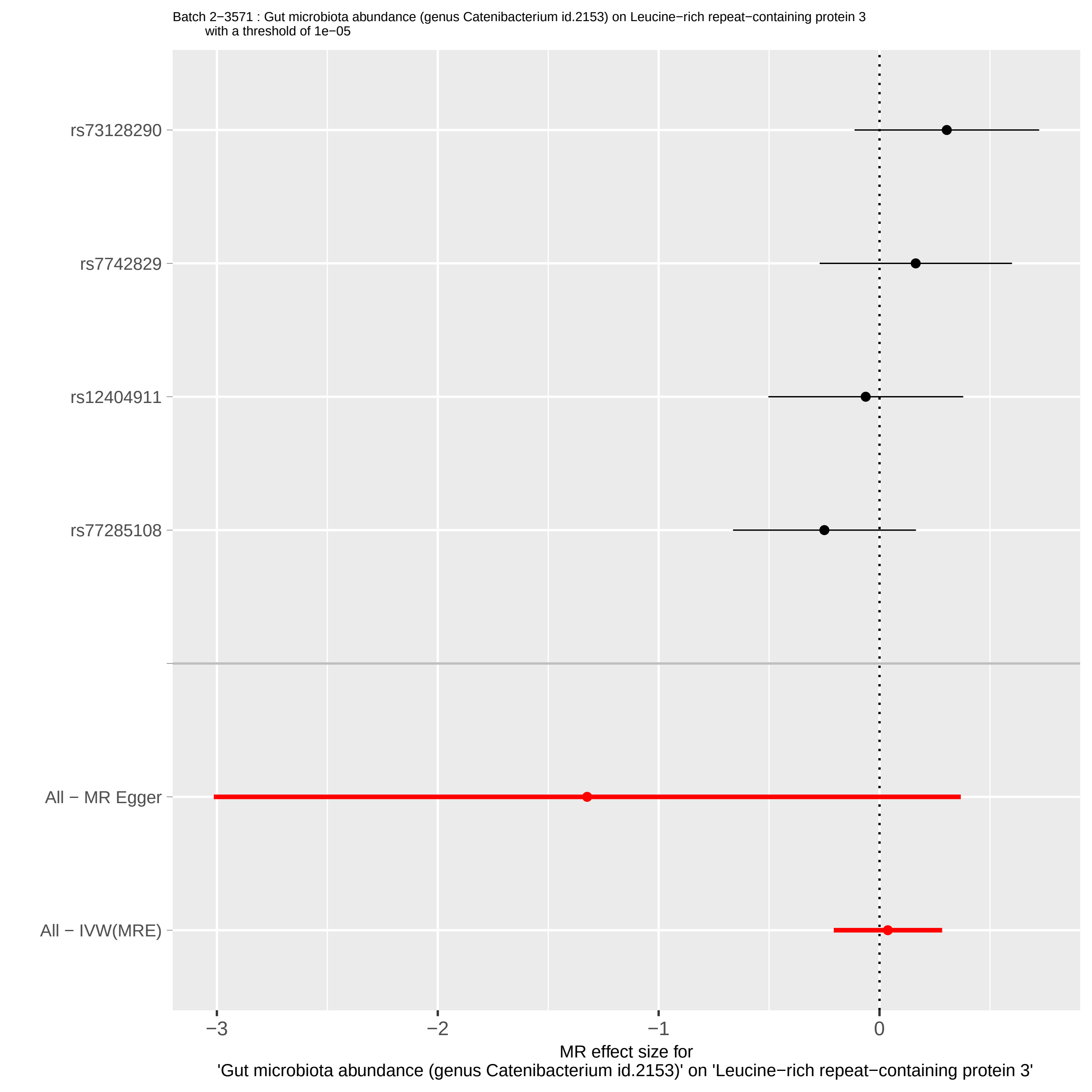

Batch 2−3571 : Gut microbiota abundance (genus Catenibacterium id.2153) on Leucine−rich repeat−containing protein 3
with a threshold of 1e−05
rs73128290
rs7742829
rs12404911
rs77285108
All − MR Egger
All − IVW(MRE)
−3
−2
−1
0
MR effect size for
'Gut microbiota abundance (genus Catenibacterium id.2153)' on 'Leucine−rich repeat−containing protein 3'

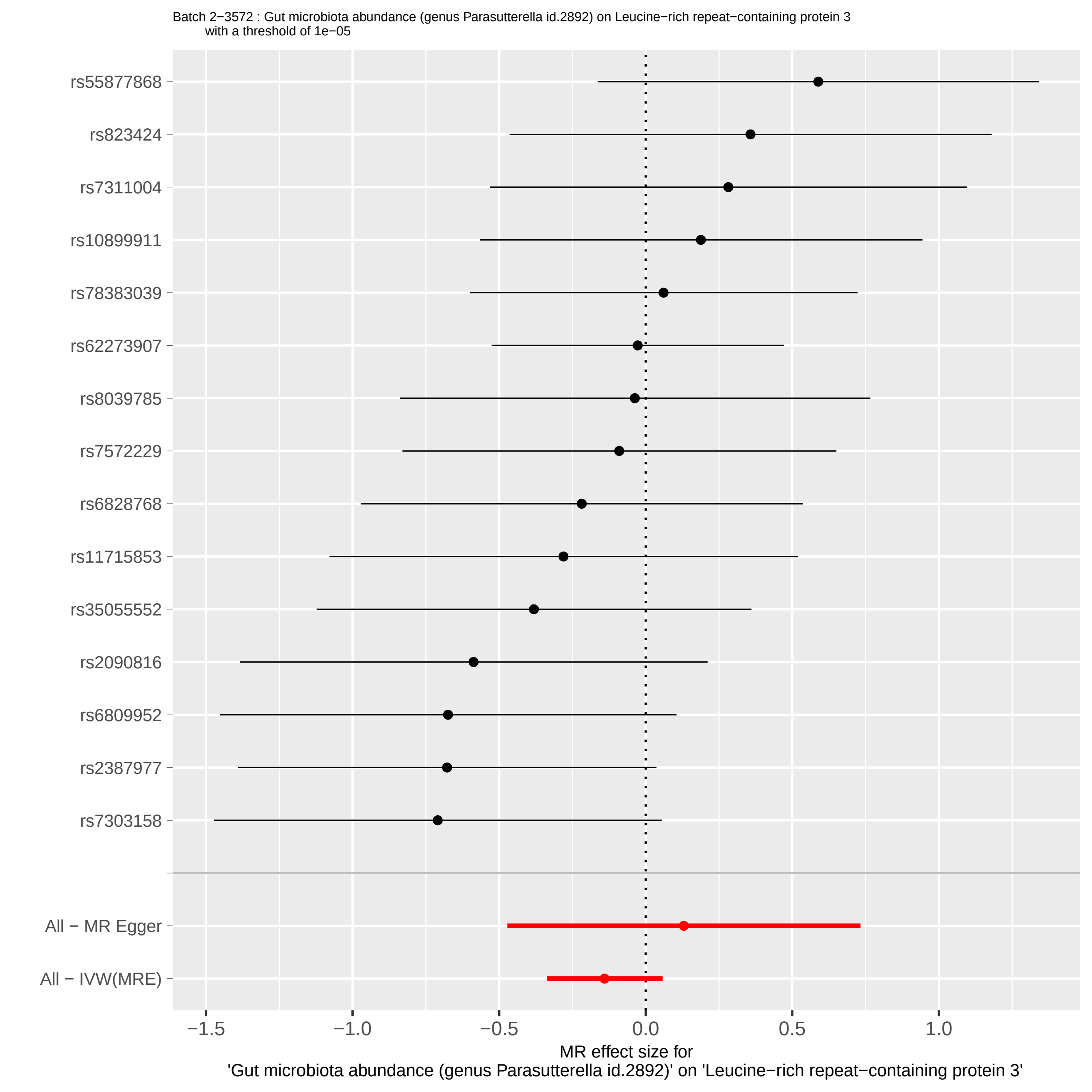

Batch 2−3572 : Gut microbiota abundance (genus Parasutterella id.2892) on Leucine−rich repeat−containing protein 3
with a threshold of 1e−05
rs55877868
rs823424
rs7311004
rs10899911
rs78383039
rs62273907
rs8039785
rs7572229
rs6828768
rs11715853
rs35055552
rs2090816
rs6809952
rs2387977
rs7303158
All − MR Egger
All − IVW(MRE)
−1.5
−1.0
−0.5
0.0
0.5
1.0
MR effect size for
'Gut microbiota abundance (genus Parasutterella id.2892)' on 'Leucine−rich repeat−containing protein 3'

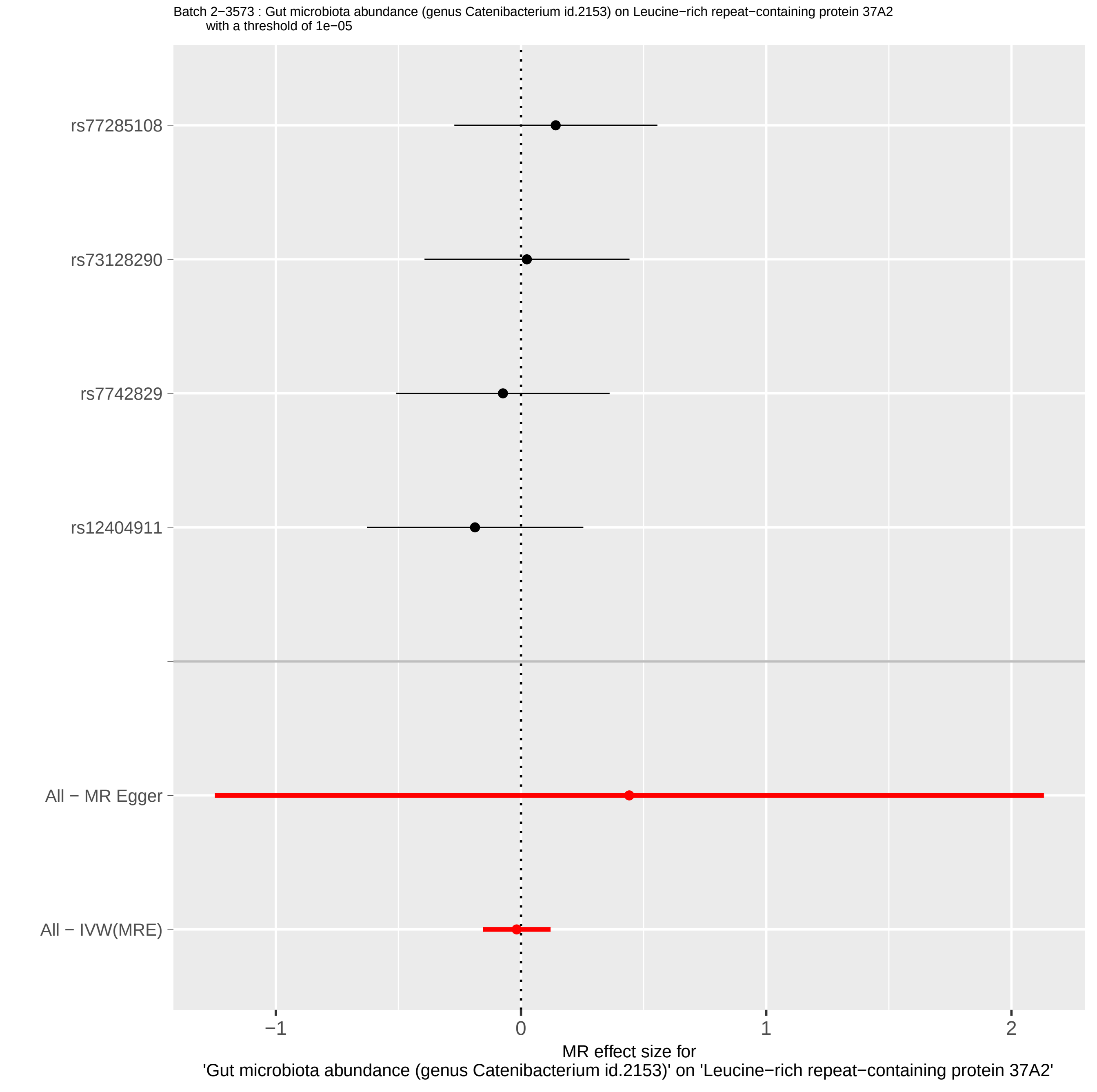

Batch 2−3573 : Gut microbiota abundance (genus Catenibacterium id.2153) on Leucine−rich repeat−containing protein 37A2
with a threshold of 1e−05
rs77285108
rs73128290
rs7742829
rs12404911
All − MR Egger
All − IVW(MRE)
−1
0
1
2
MR effect size for
'Gut microbiota abundance (genus Catenibacterium id.2153)' on 'Leucine−rich repeat−containing protein 37A2'

Batch 2−3574 : Gut microbiota abundance (genus Parasutterella id.2892) on Leucine−rich repeat−containing protein 37A2
with a threshold of 1e−05

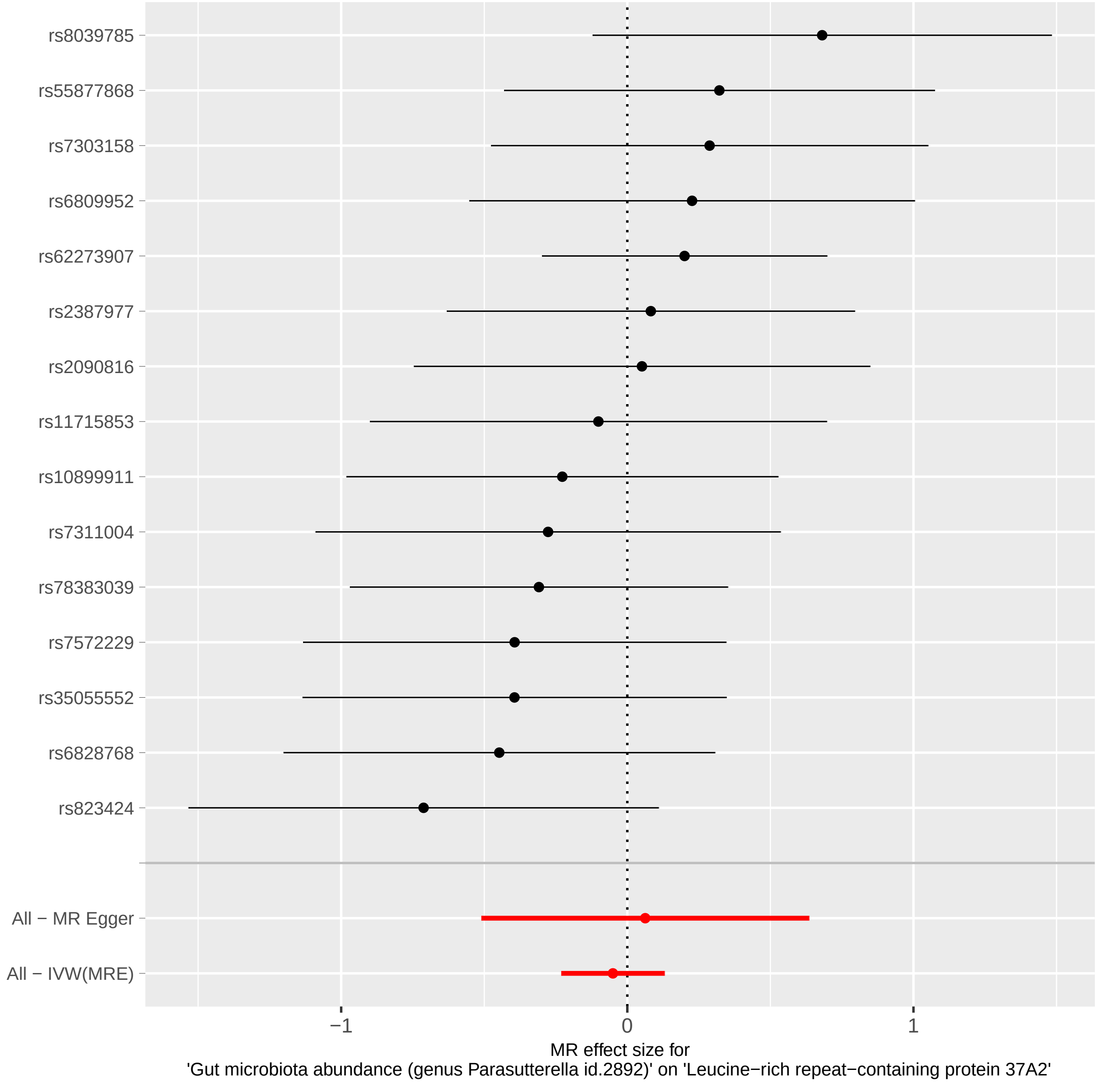

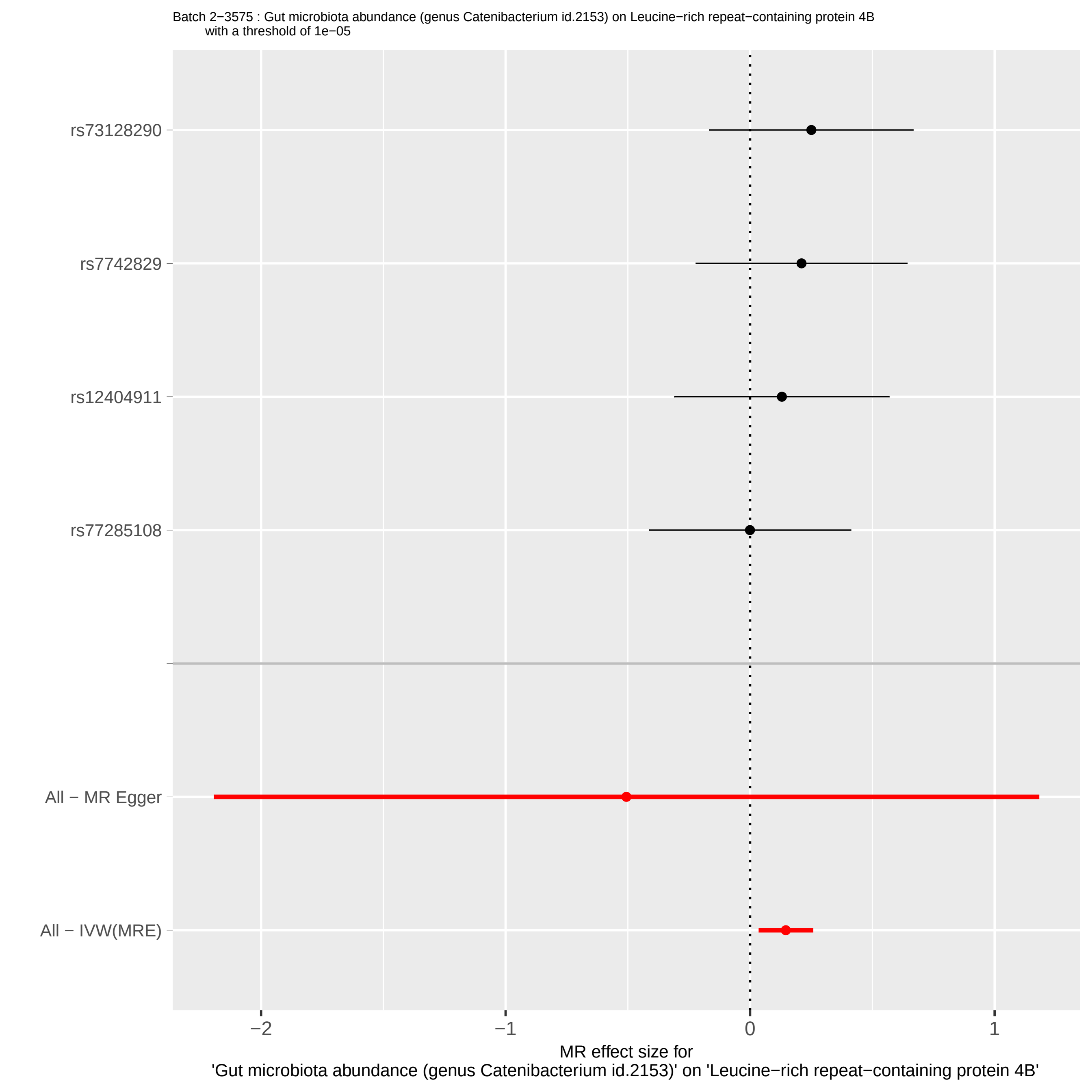
Batch 2−3575 : Gut microbiota abundance (genus Catenibacterium id.2153) on Leucine−rich repeat−containing protein 4B
with a threshold of 1e−05
rs73128290
rs7742829
rs12404911
rs77285108
All − MR Egger
All − IVW(MRE)
−2
−1
0
1
MR effect size for
'Gut microbiota abundance (genus Catenibacterium id.2153)' on 'Leucine−rich repeat−containing protein 4B'

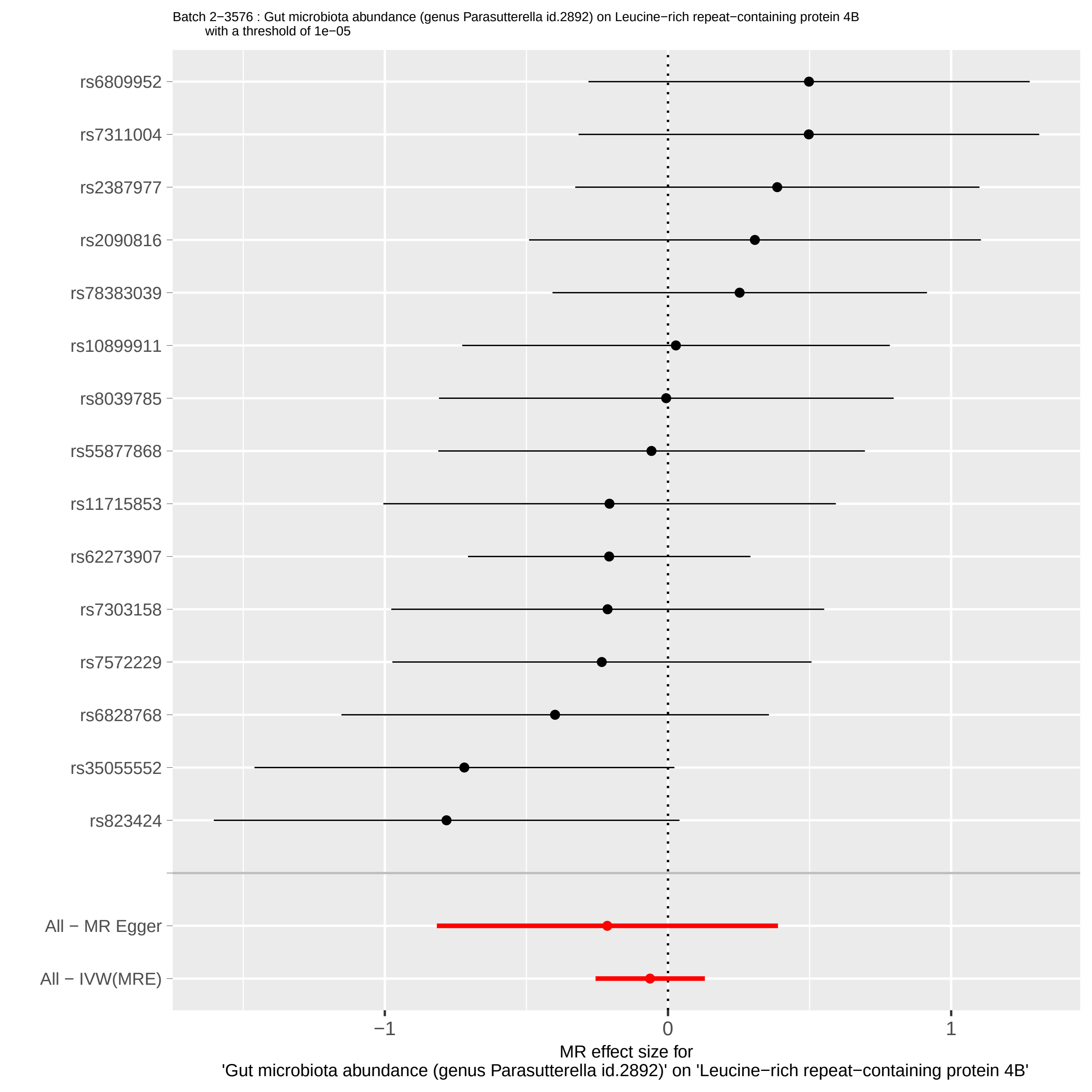
Batch 2−3576 : Gut microbiota abundance (genus Parasutterella id.2892) on Leucine−rich repeat−containing protein 4B with a threshold of 1e−05
rs6809952
rs7311004
rs2387977
rs2090816
rs78383039
rs10899911
rs8039785
rs55877868
rs11715853
rs62273907
rs7303158
rs7572229
rs6828768
rs35055552
rs823424
All − MR Egger
All − IVW(MRE)
−1
0
1
MR effect size for
'Gut microbiota abundance (genus Parasutterella id.2892)' on 'Leucine−rich repeat−containing protein 4B'

Batch 2−3577 : Gut microbiota abundance (genus Catenibacterium id.2153) on Leucine−rich repeat−containing protein 4C
with a threshold of 1e−05

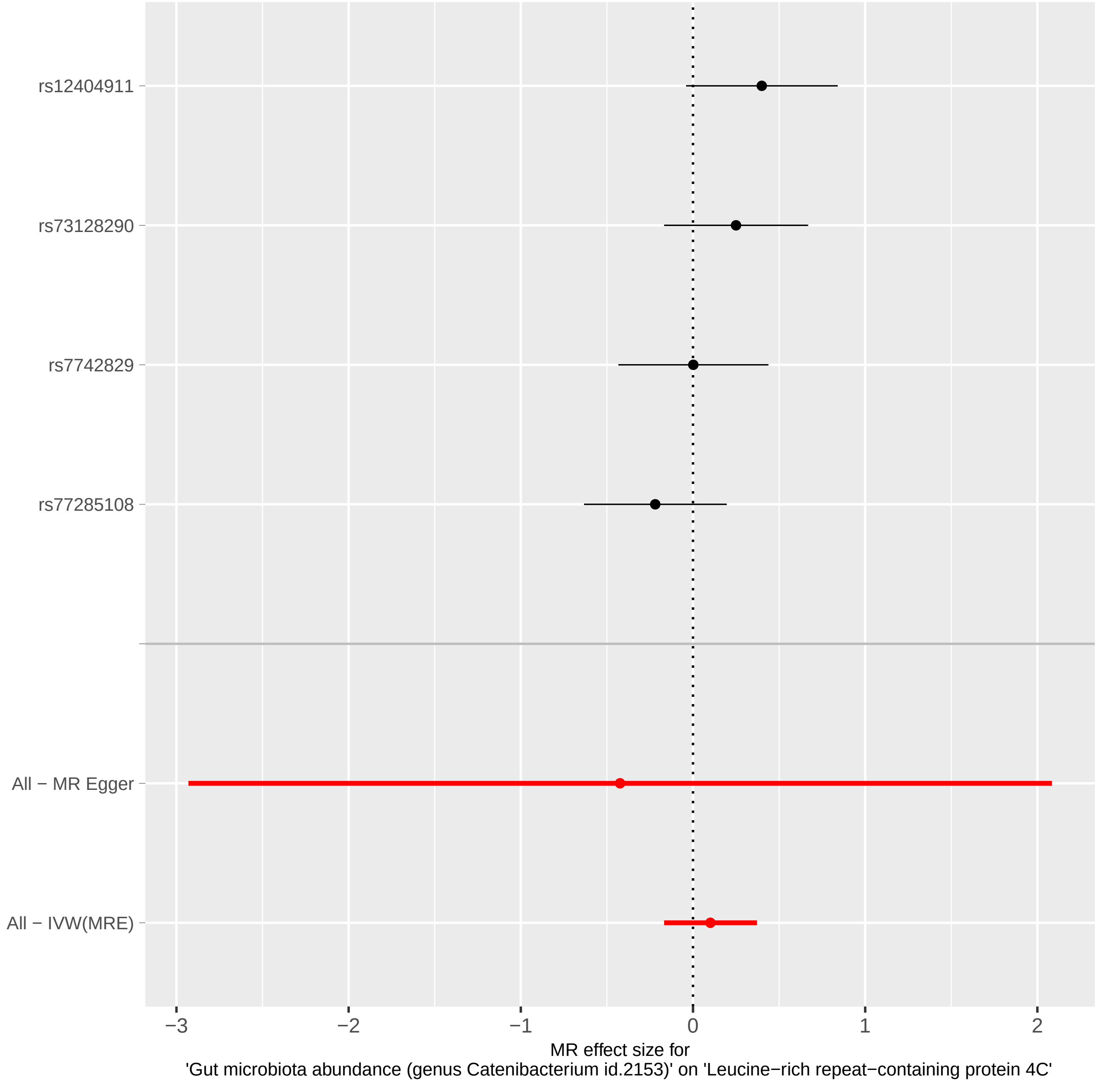

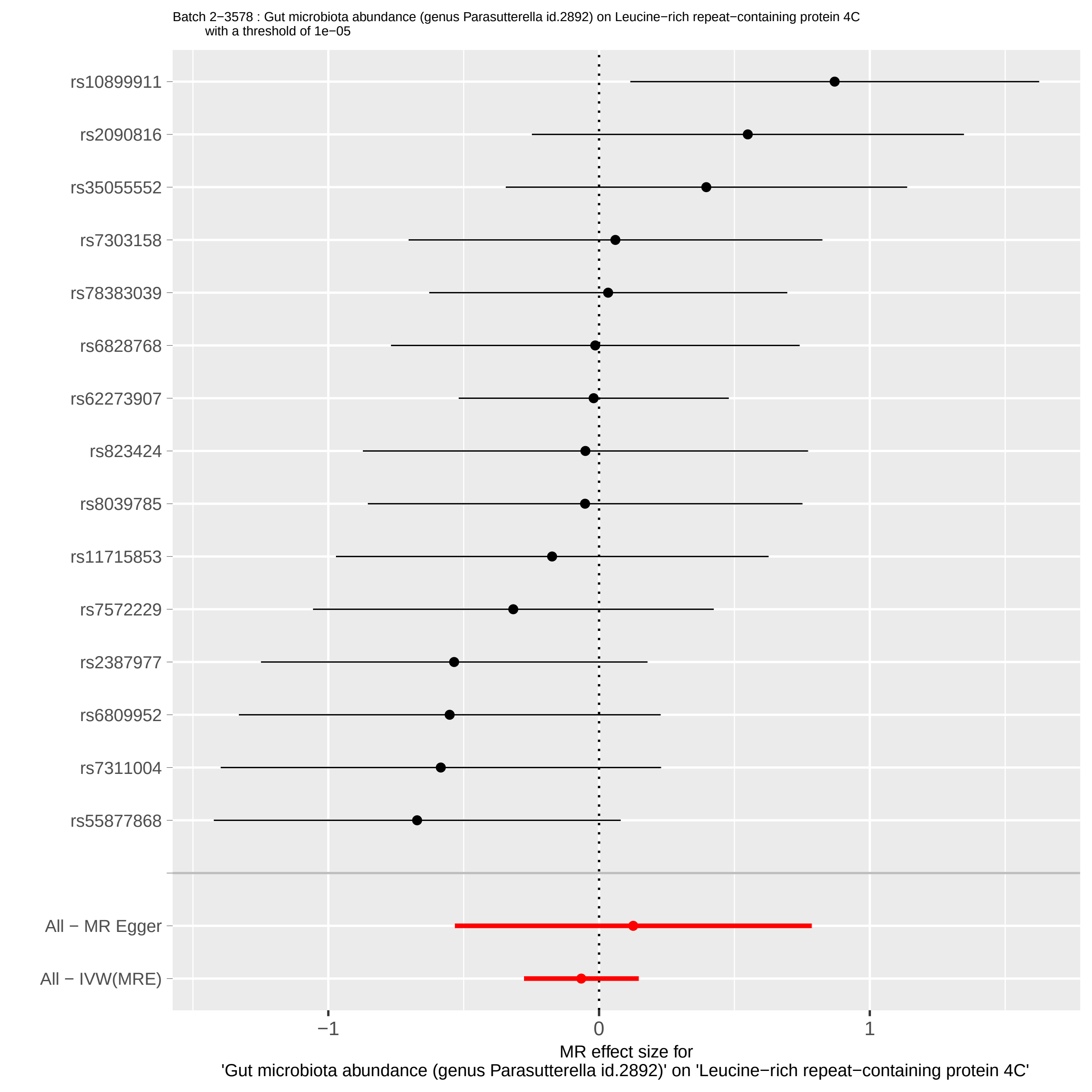
Batch 2−3578 : Gut microbiota abundance (genus Parasutterella id.2892) on Leucine−rich repeat−containing protein 4C
with a threshold of 1e−05
rs10899911
rs2090816
rs35055552
rs7303158
rs78383039
rs6828768
rs62273907
rs823424
rs8039785
rs11715853
rs7572229
rs2387977
rs6809952
rs7311004
rs55877868
All − MR Egger
All − IVW(MRE)
−1
0
1
MR effect size for
'Gut microbiota abundance (genus Parasutterella id.2892)' on 'Leucine−rich repeat−containing protein 4C'

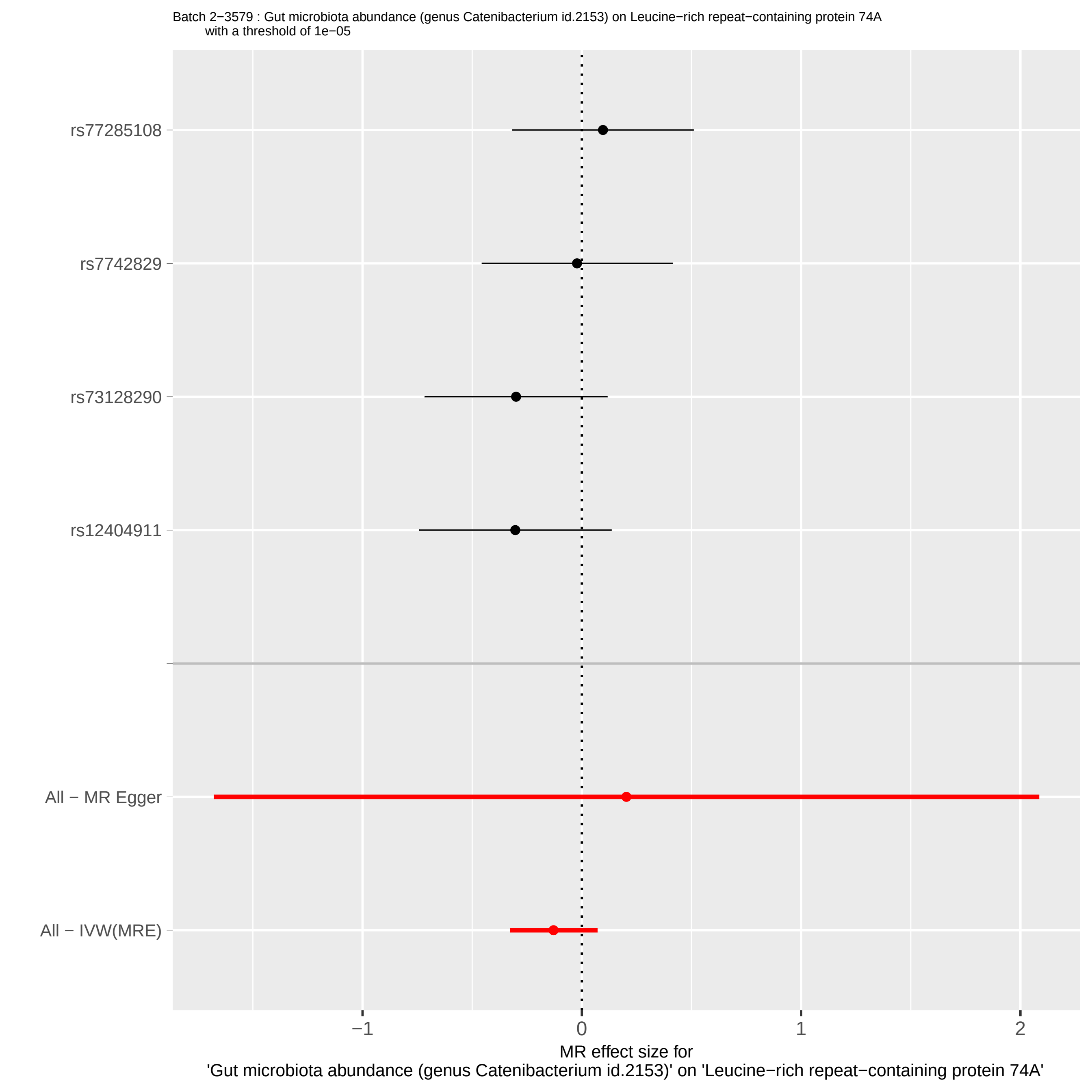

Batch 2−3579 : Gut microbiota abundance (genus Catenibacterium id.2153) on Leucine−rich repeat−containing protein 74A
with a threshold of 1e−05
rs77285108
rs7742829
rs73128290
rs12404911
All − MR Egger
All − IVW(MRE)
−1
0
1
2
MR effect size for
'Gut microbiota abundance (genus Catenibacterium id.2153)' on 'Leucine−rich repeat−containing protein 74A'

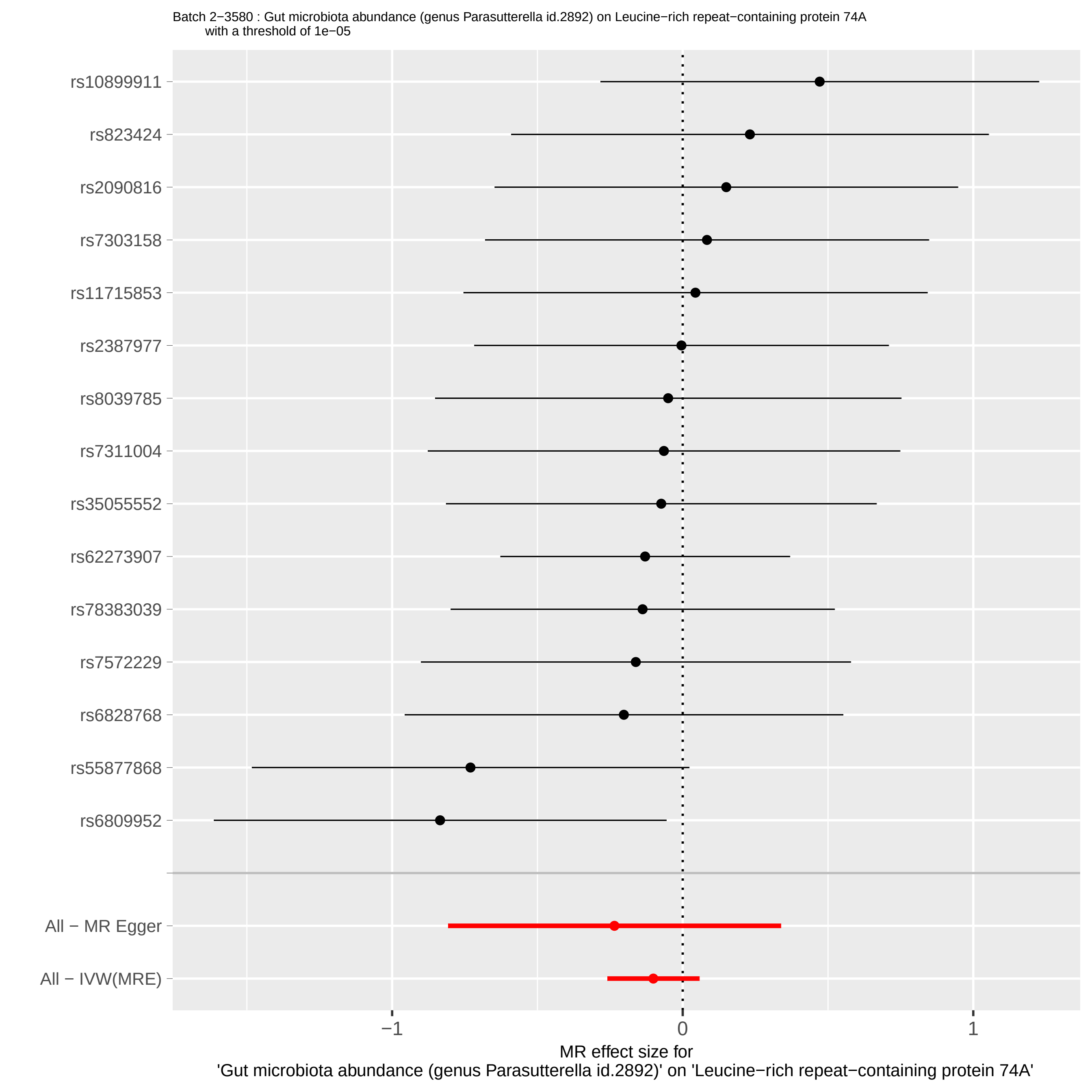
Batch 2−3580 : Gut microbiota abundance (genus Parasutterella id.2892) on Leucine−rich repeat−containing protein 74A
with a threshold of 1e−05
rs10899911
rs823424
rs2090816
rs7303158
rs11715853
rs2387977
rs8039785
rs7311004
rs35055552
rs62273907
rs78383039
rs7572229
rs6828768
rs55877868
rs6809952
All − MR Egger
All − IVW(MRE)
−1
0
1
MR effect size for
'Gut microbiota abundance (genus Parasutterella id.2892)' on 'Leucine−rich repeat−containing protein 74A'

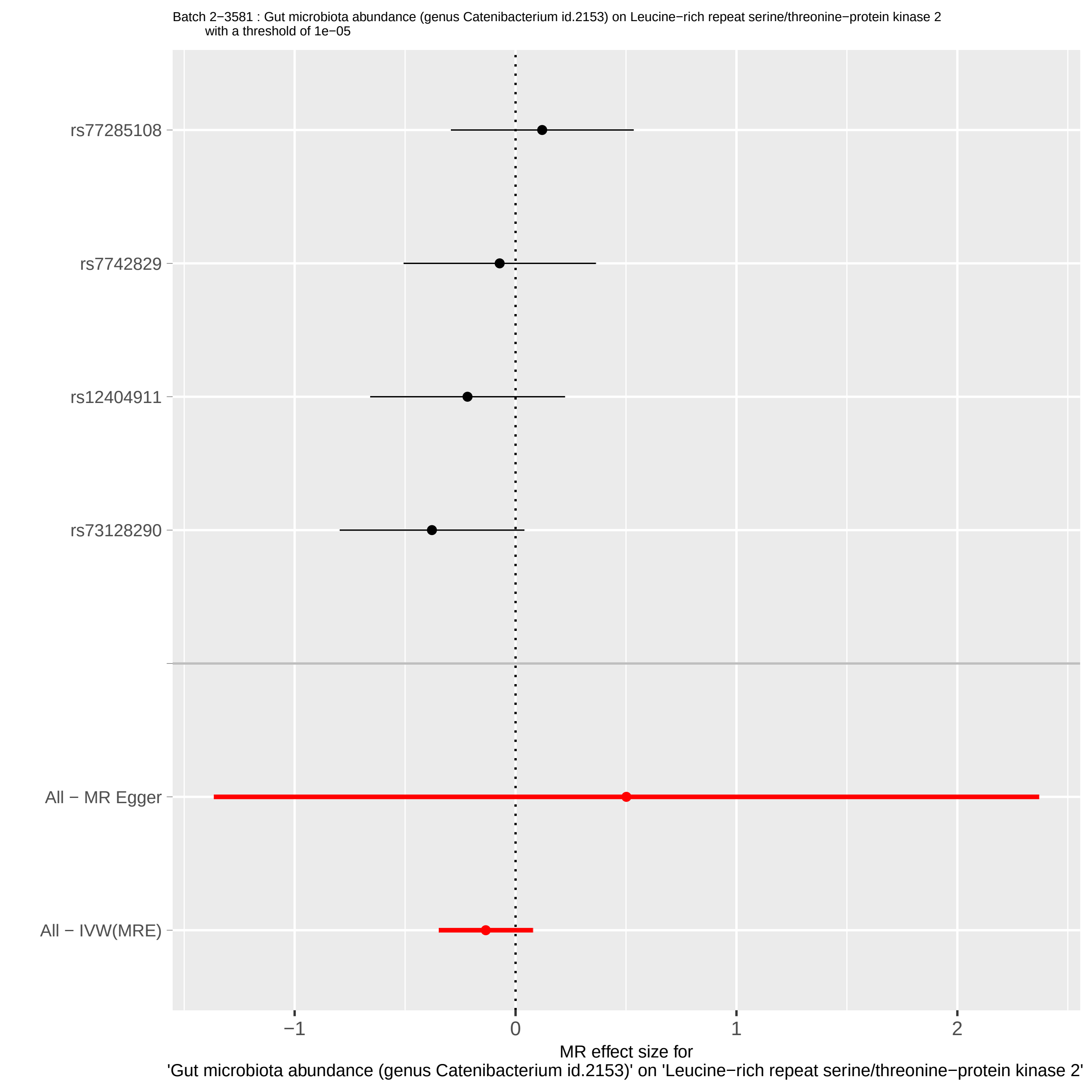

Batch 2−3581 : Gut microbiota abundance (genus Catenibacterium id.2153) on Leucine−rich repeat serine/threonine−protein kinase 2
with a threshold of 1e−05
rs77285108
rs7742829
rs12404911
rs73128290
All − MR Egger
All − IVW(MRE)
−1
0
1
2
MR effect size for
'Gut microbiota abundance (genus Catenibacterium id.2153)' on 'Leucine−rich repeat serine/threonine−protein kinase 2'

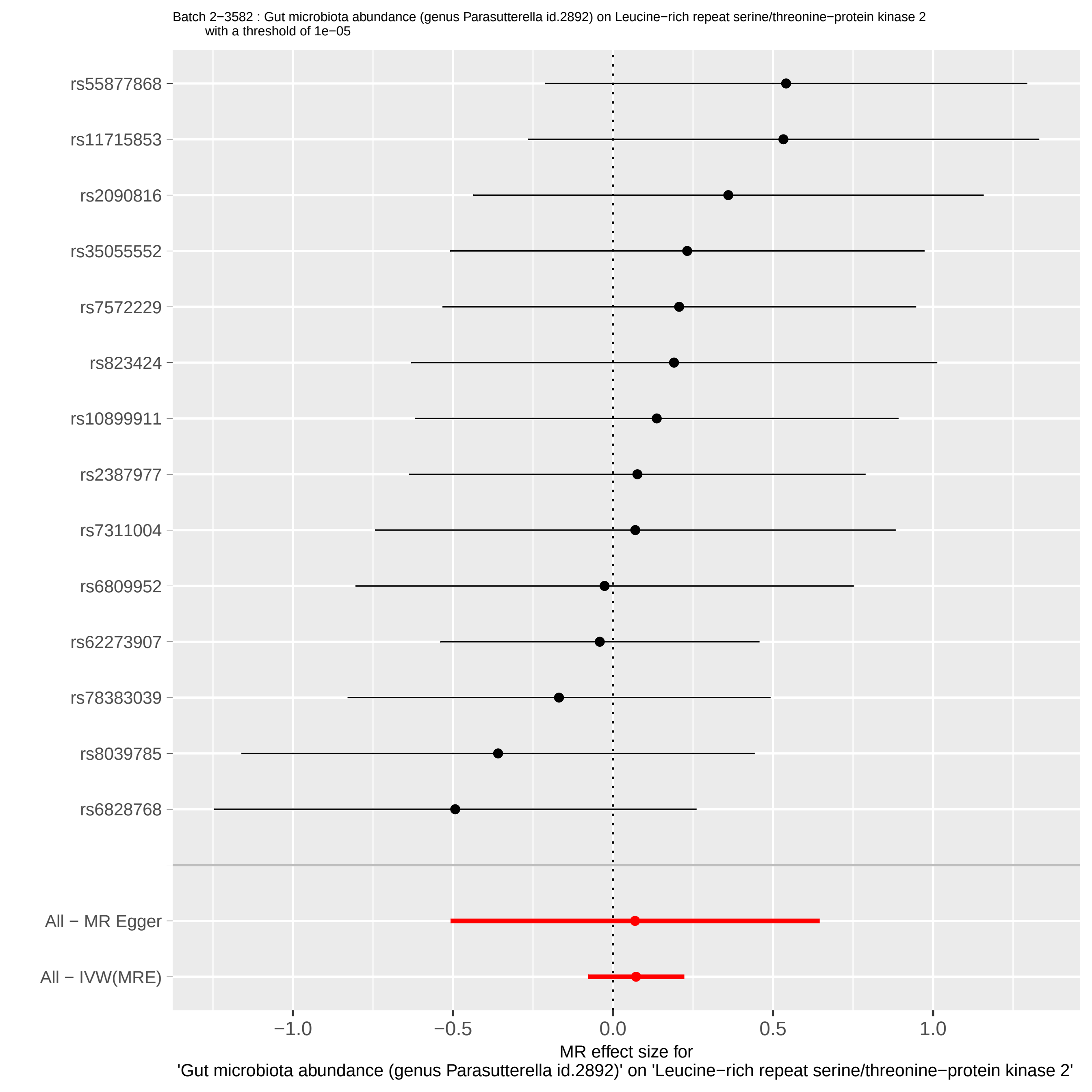
Batch 2−3582 : Gut microbiota abundance (genus Parasutterella id.2892) on Leucine−rich repeat serine/threonine−protein kinase 2
with a threshold of 1e−05
rs55877868
rs11715853
rs2090816
rs35055552
rs7572229
rs823424
rs10899911
rs2387977
rs7311004
rs6809952
rs62273907
rs78383039
rs8039785
rs6828768
All − MR Egger
All − IVW(MRE)
−1.0
−0.5
0.0
0.5
1.0
MR effect size for
'Gut microbiota abundance (genus Parasutterella id.2892)' on 'Leucine−rich repeat serine/threonine−protein kinase 2'

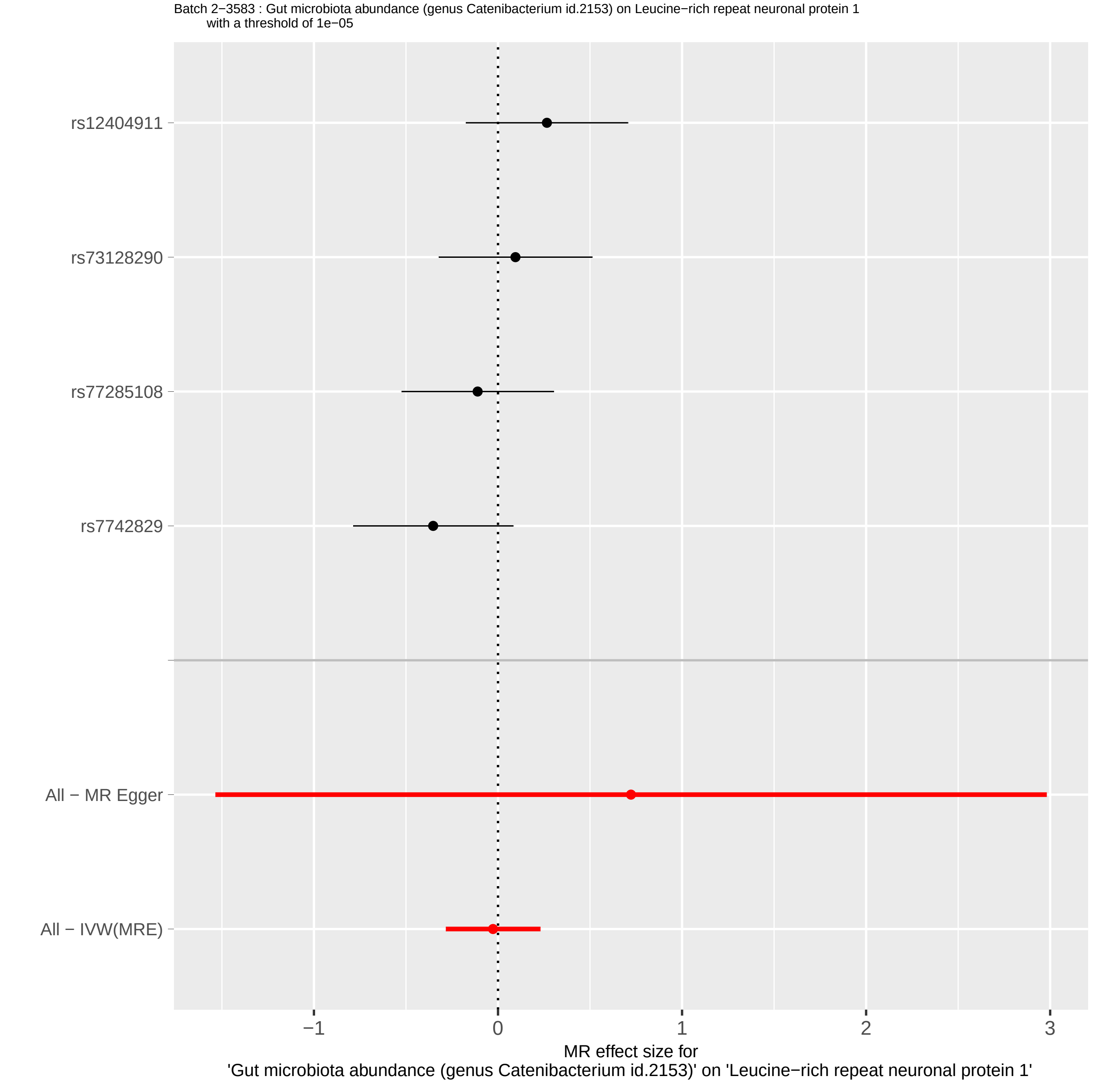
Batch 2−3583 : Gut microbiota abundance (genus Catenibacterium id.2153) on Leucine−rich repeat neuronal protein 1
with a threshold of 1e−05
rs12404911
rs73128290
rs77285108
rs7742829
All − MR Egger
All − IVW(MRE)
−1
0
1
2
3
MR effect size for
'Gut microbiota abundance (genus Catenibacterium id.2153)' on 'Leucine−rich repeat neuronal protein 1'

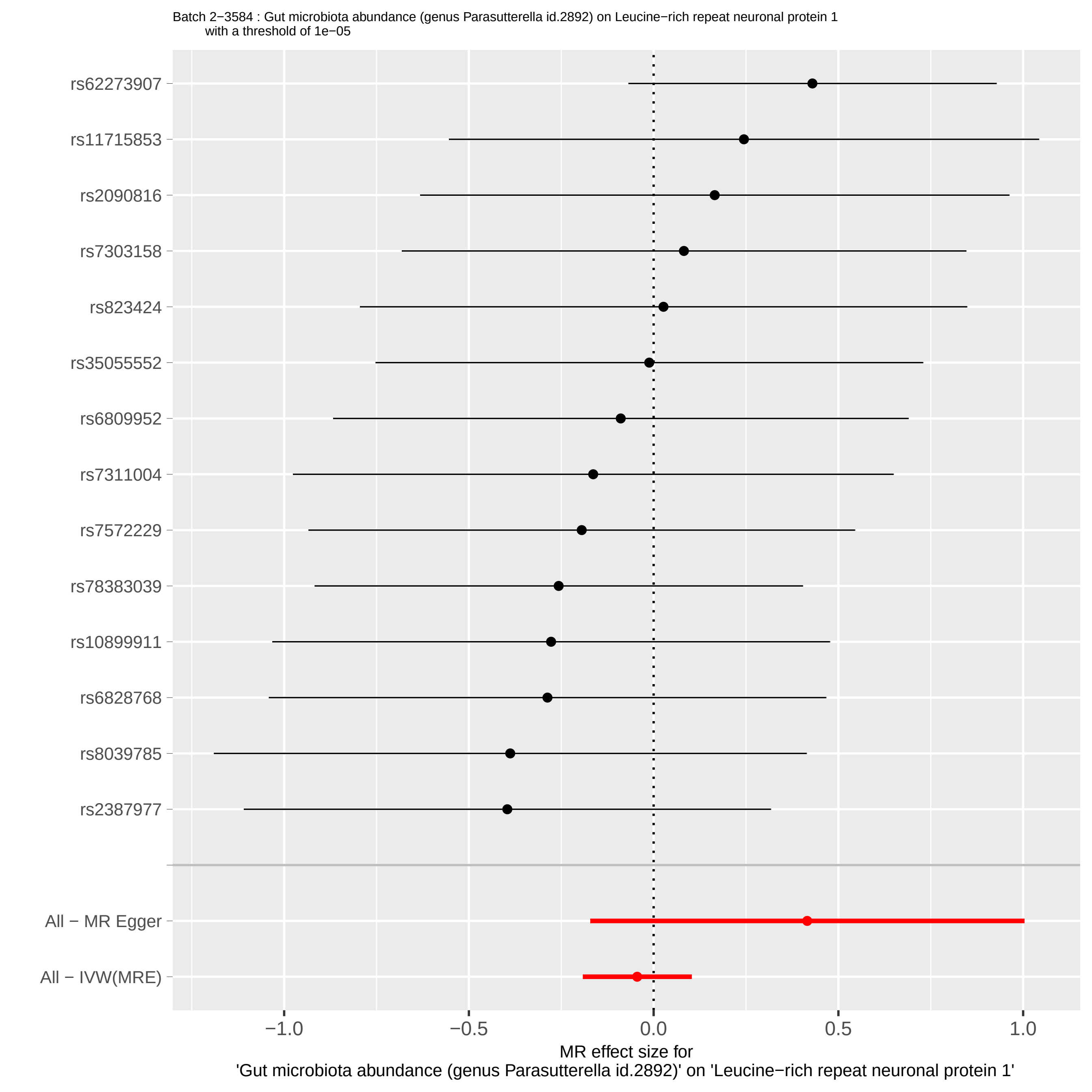
Batch 2−3584 : Gut microbiota abundance (genus Parasutterella id.2892) on Leucine−rich repeat neuronal protein 1
with a threshold of 1e−05
rs62273907
rs11715853
rs2090816
rs7303158
rs823424
rs35055552
rs6809952
rs7311004
rs7572229
rs78383039
rs10899911
rs6828768
rs8039785
rs2387977
All − MR Egger
All − IVW(MRE)
−1.0
−0.5
0.0
0.5
1.0
MR effect size for
'Gut microbiota abundance (genus Parasutterella id.2892)' on 'Leucine−rich repeat neuronal protein 1'

Batch 2−3585 : Gut microbiota abundance (genus Catenibacterium id.2153) on Leucine−rich repeat neuronal protein 1
with a threshold of 1e−05

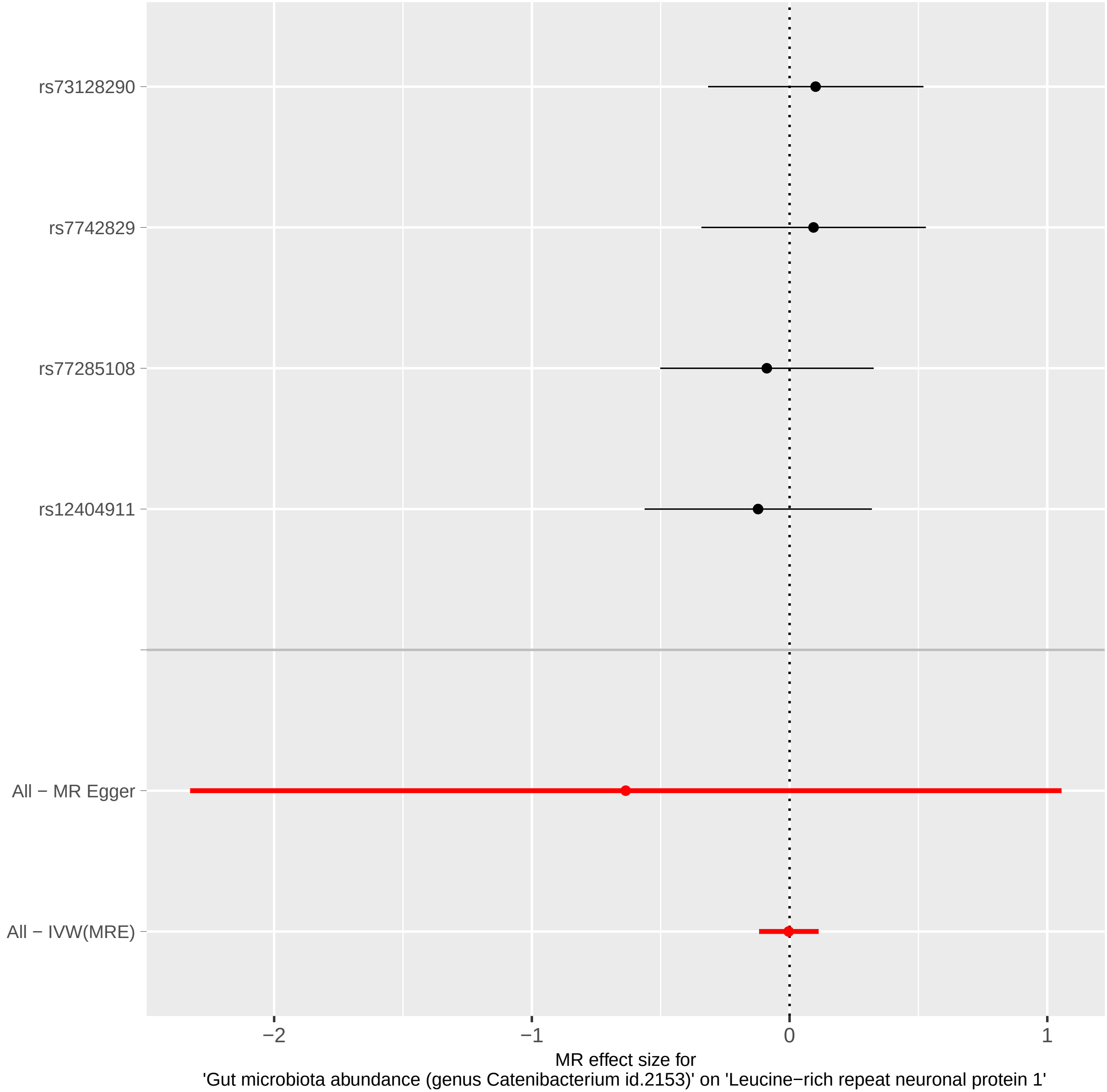

Batch 2−3586 : Gut microbiota abundance (genus Parasutterella id.2892) on Leucine−rich repeat neuronal protein 1
with a threshold of 1e−05

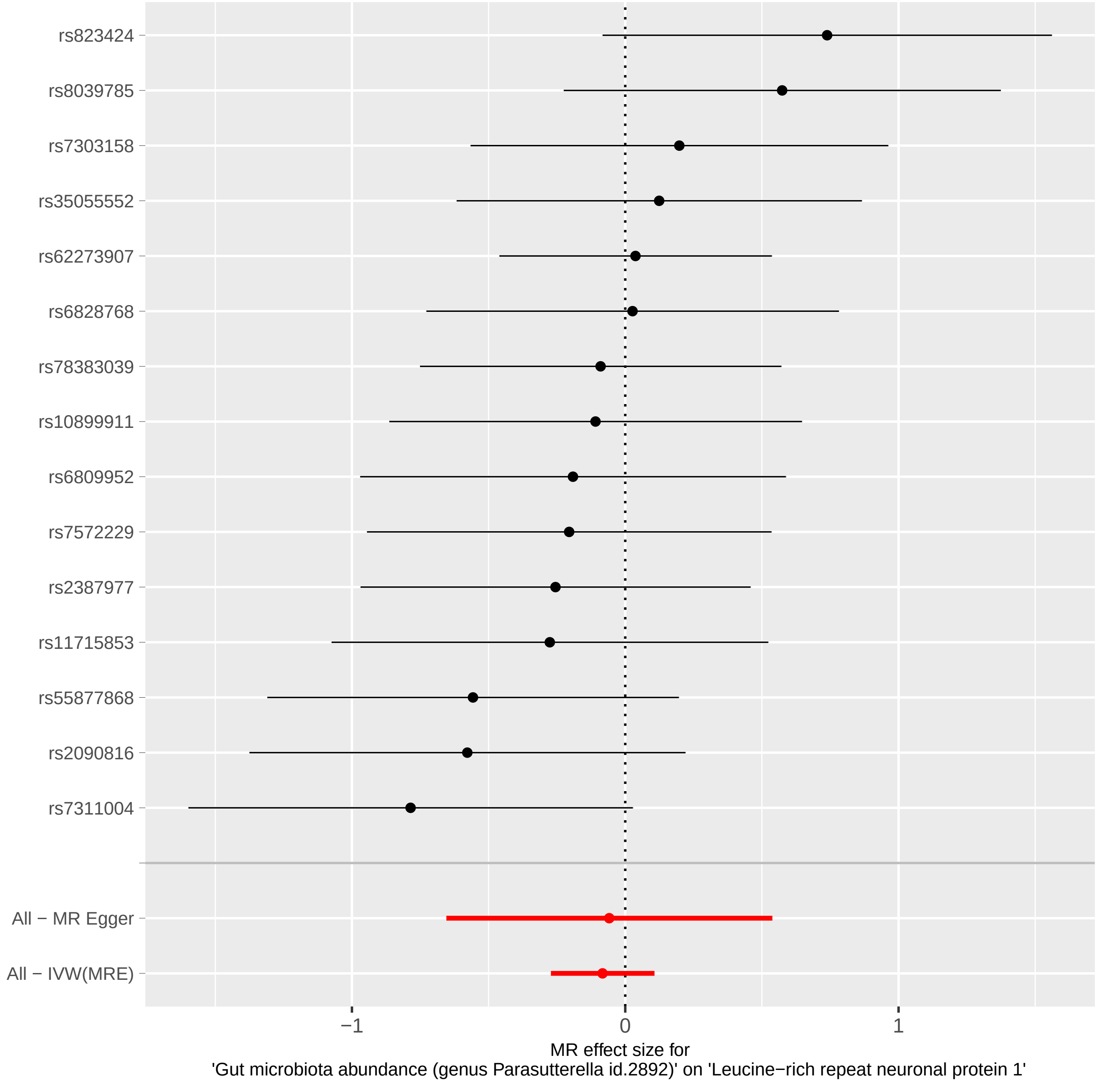

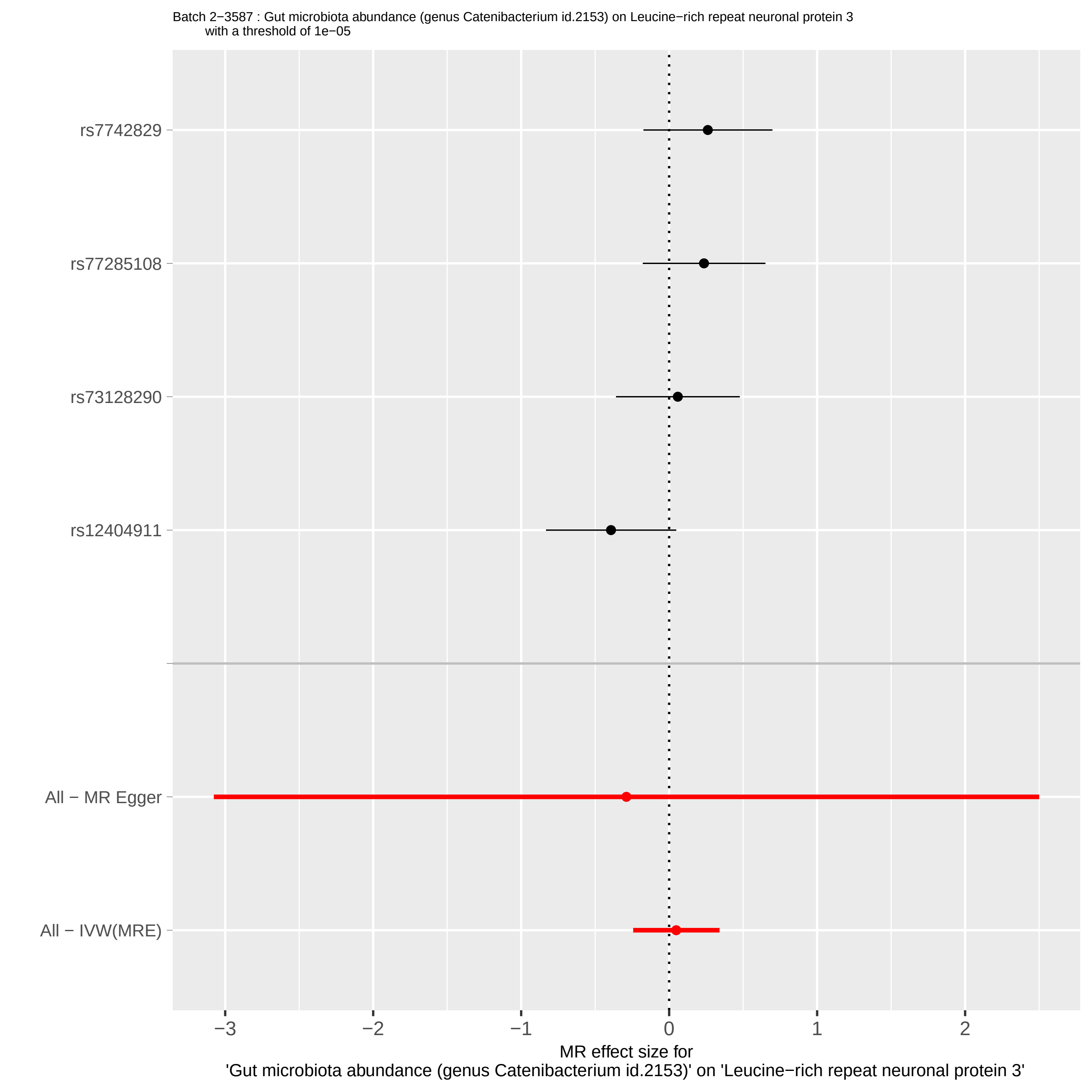

Batch 2−3587 : Gut microbiota abundance (genus Catenibacterium id.2153) on Leucine−rich repeat neuronal protein 3
with a threshold of 1e−05
rs7742829
rs77285108
rs73128290
rs12404911
All − MR Egger
All − IVW(MRE)
−3
−2
−1
0
1
2
MR effect size for
'Gut microbiota abundance (genus Catenibacterium id.2153)' on 'Leucine−rich repeat neuronal protein 3'

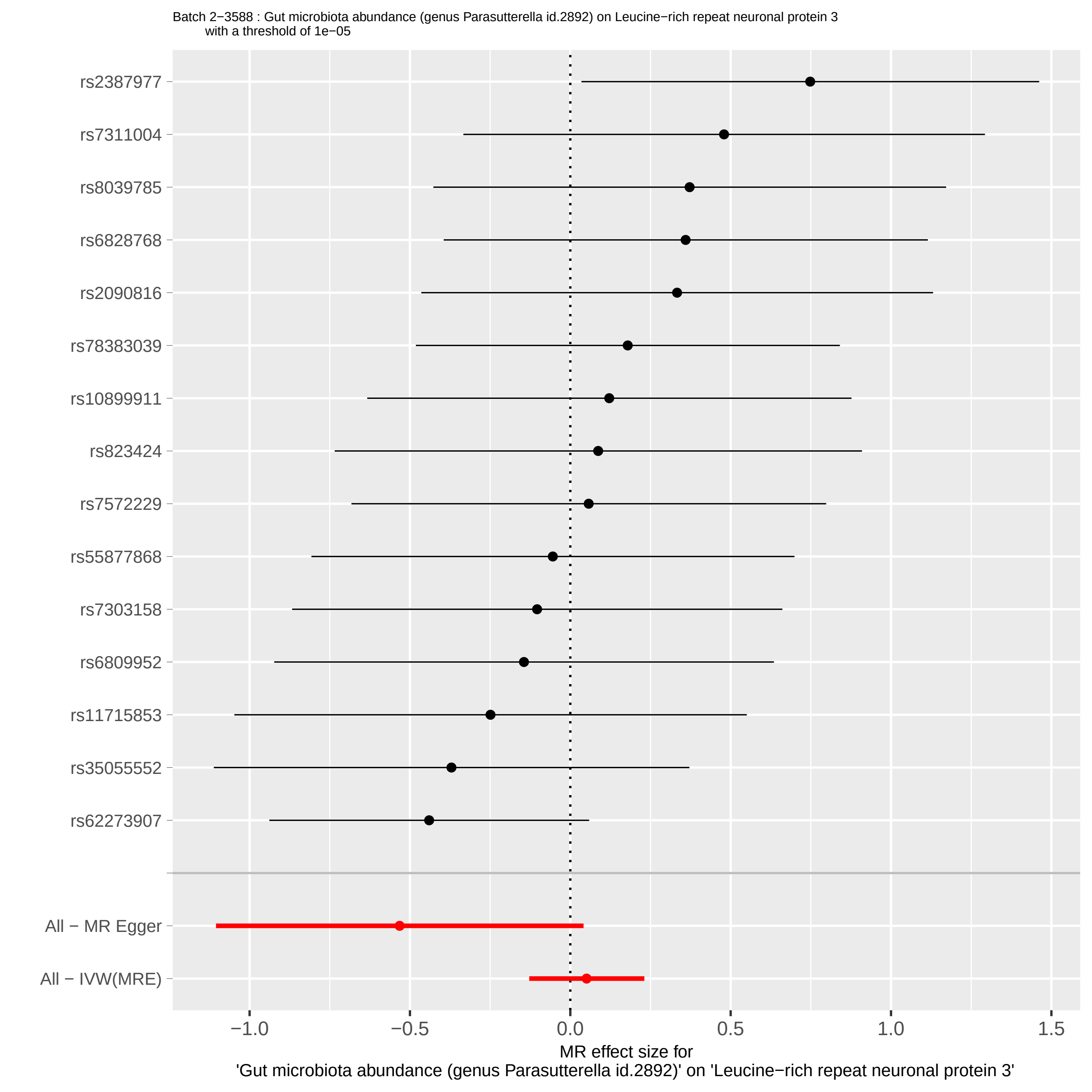

Batch 2−3588 : Gut microbiota abundance (genus Parasutterella id.2892) on Leucine−rich repeat neuronal protein 3
with a threshold of 1e−05
rs2387977
rs7311004
rs8039785
rs6828768
rs2090816
rs78383039
rs10899911
rs823424
rs7572229
rs55877868
rs7303158
rs6809952
rs11715853
rs35055552
rs62273907
All − MR Egger
All − IVW(MRE)
−1.0
−0.5
0.0
0.5
1.0
1.5
MR effect size for
'Gut microbiota abundance (genus Parasutterella id.2892)' on 'Leucine−rich repeat neuronal protein 3'

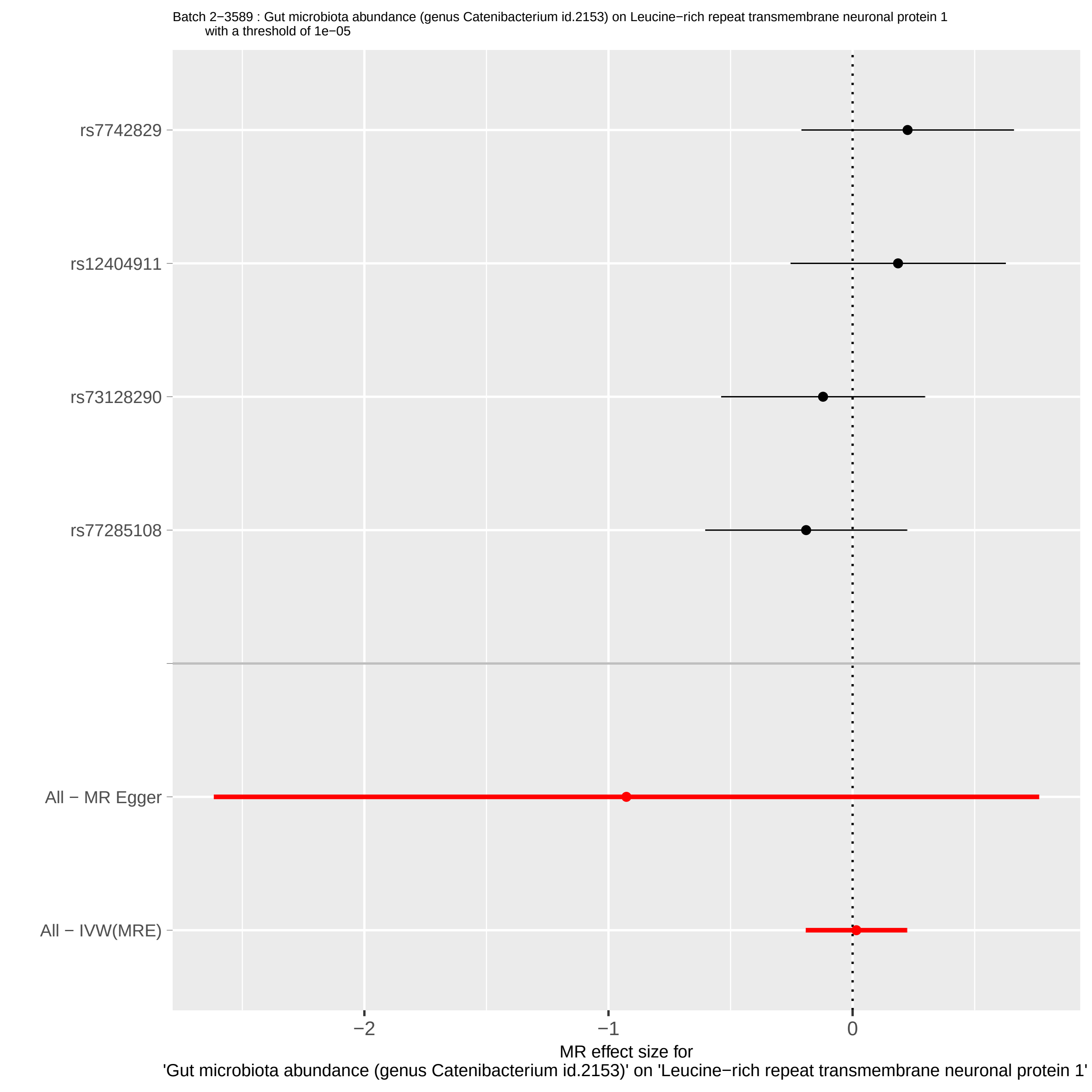
Batch 2−3589 : Gut microbiota abundance (genus Catenibacterium id.2153) on Leucine−rich repeat transmembrane neuronal protein 1
with a threshold of 1e−05
rs7742829
rs12404911
rs73128290
rs77285108
All − MR Egger
All − IVW(MRE)
−2
−1
0
MR effect size for
'Gut microbiota abundance (genus Catenibacterium id.2153)' on 'Leucine−rich repeat transmembrane neuronal protein 1'

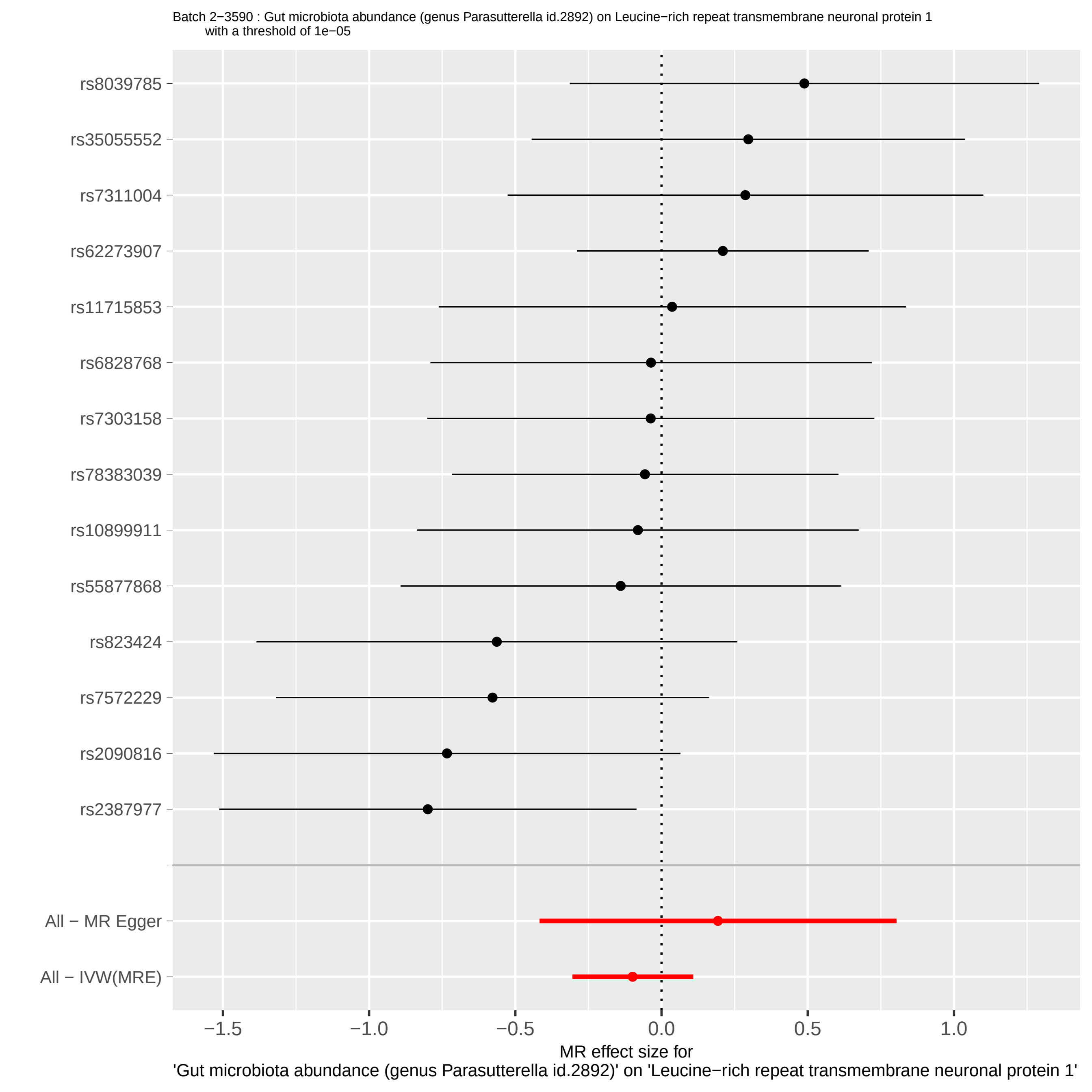

Batch 2−3590 : Gut microbiota abundance (genus Parasutterella id.2892) on Leucine−rich repeat transmembrane neuronal protein 1
with a threshold of 1e−05
rs8039785
rs35055552
rs7311004
rs62273907
rs11715853
rs6828768
rs7303158
rs78383039
rs10899911
rs55877868
rs823424
rs7572229
rs2090816
rs2387977
All − MR Egger
All − IVW(MRE)
−1.5
−1.0
−0.5
0.0
0.5
1.0
MR effect size for
'Gut microbiota abundance (genus Parasutterella id.2892)' on 'Leucine−rich repeat transmembrane neuronal protein 1'

Batch 2−3591 : Gut microbiota abundance (genus Catenibacterium id.2153) on Leucine−rich repeat transmembrane neuronal protein 2
with a threshold of 1e−05

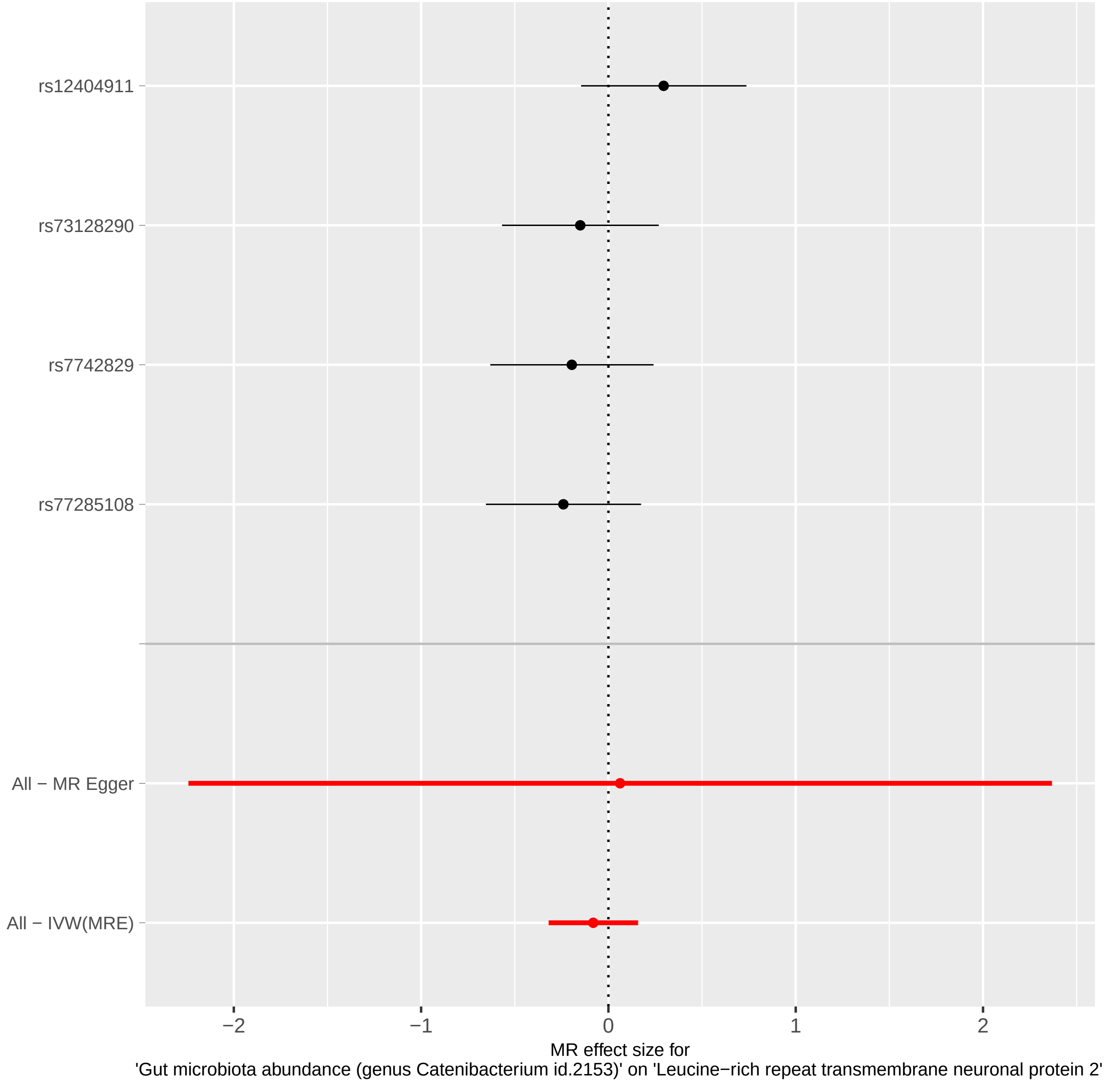

Batch 2−3592 : Gut microbiota abundance (genus Parasutterella id.2892) on Leucine−rich repeat transmembrane neuronal protein 2
with a threshold of 1e−05

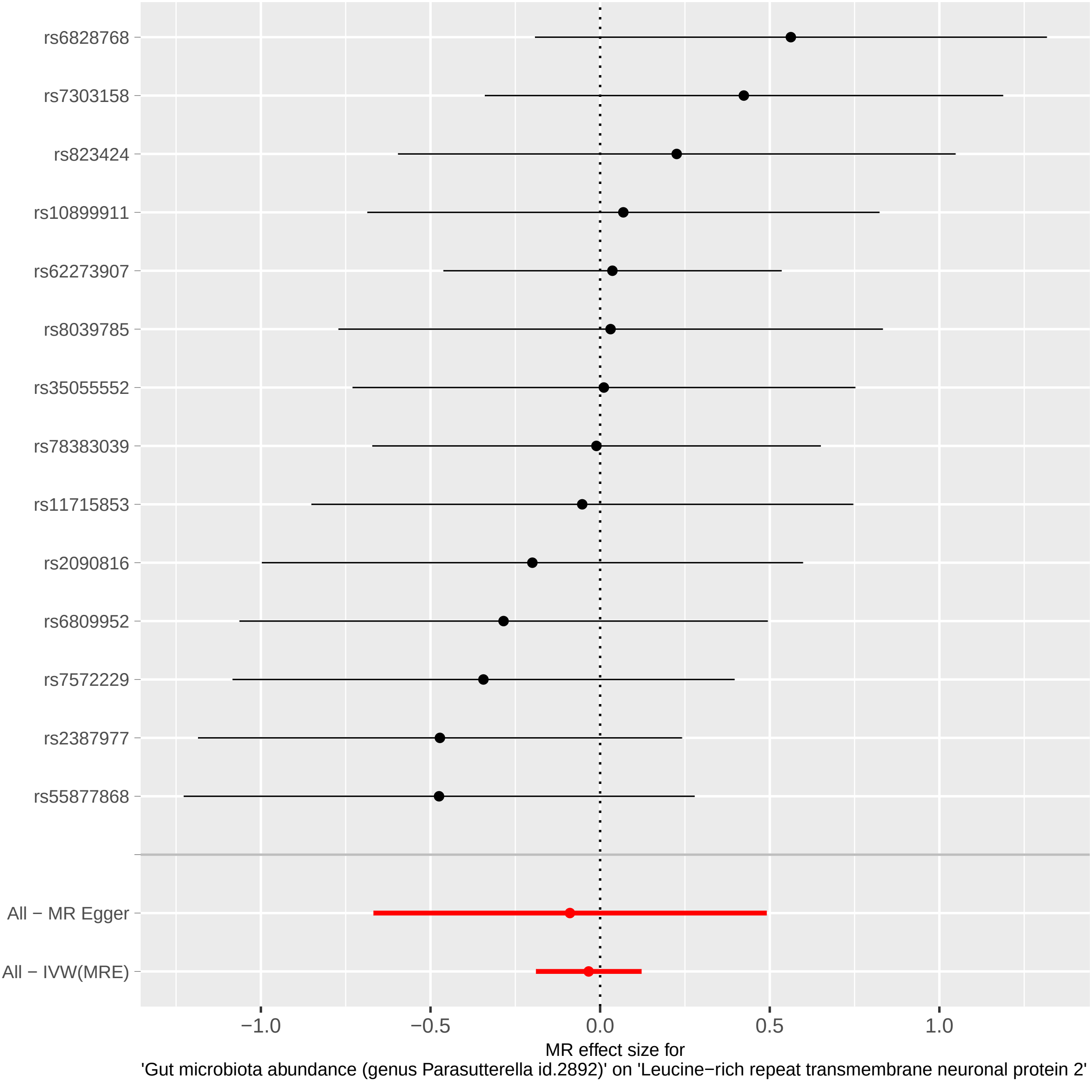

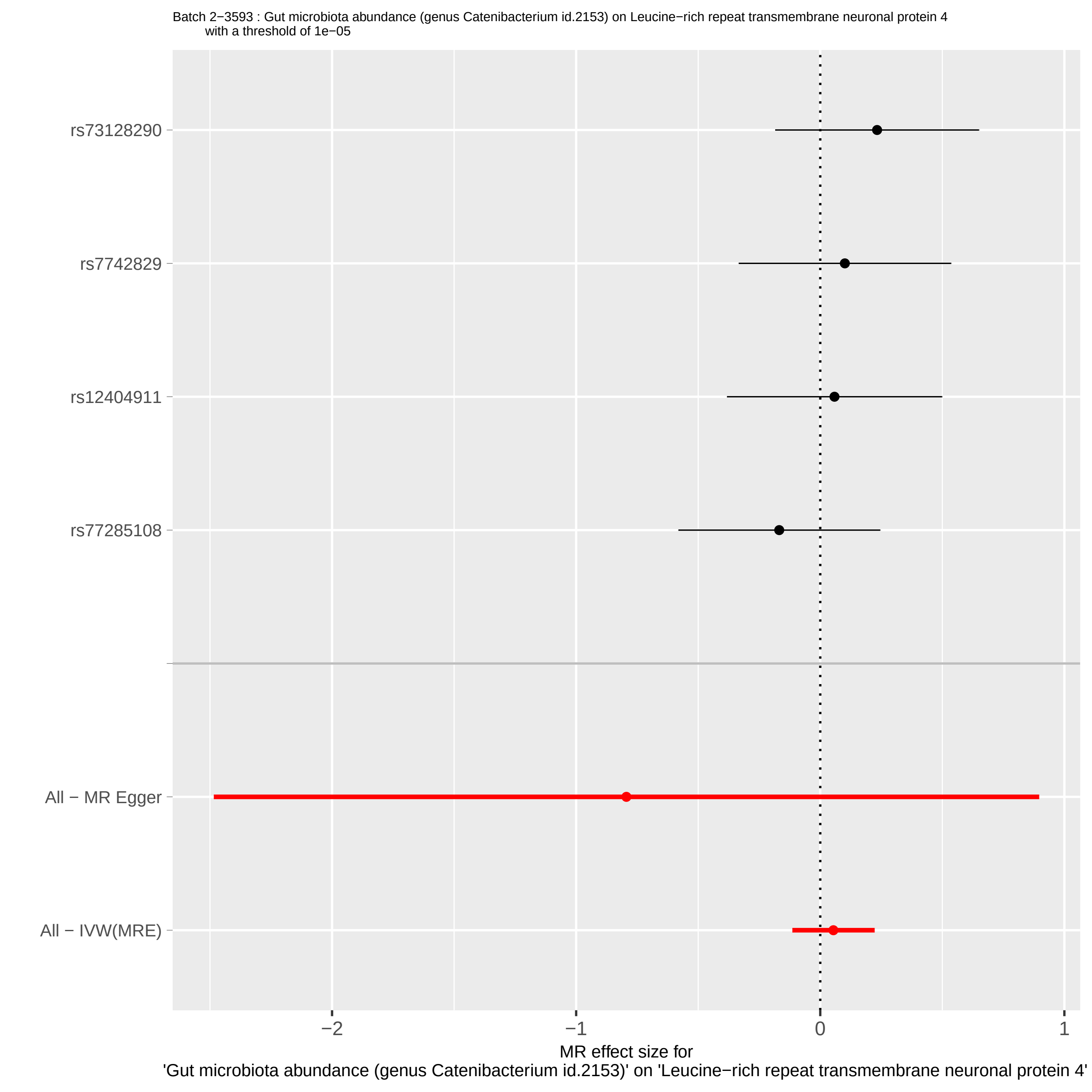
Batch 2−3593 : Gut microbiota abundance (genus Catenibacterium id.2153) on Leucine−rich repeat transmembrane neuronal protein 4
with a threshold of 1e−05
rs73128290
rs7742829
rs12404911
rs77285108
All − MR Egger
All − IVW(MRE)
−2
−1
0
1
MR effect size for
'Gut microbiota abundance (genus Catenibacterium id.2153)' on 'Leucine−rich repeat transmembrane neuronal protein 4'

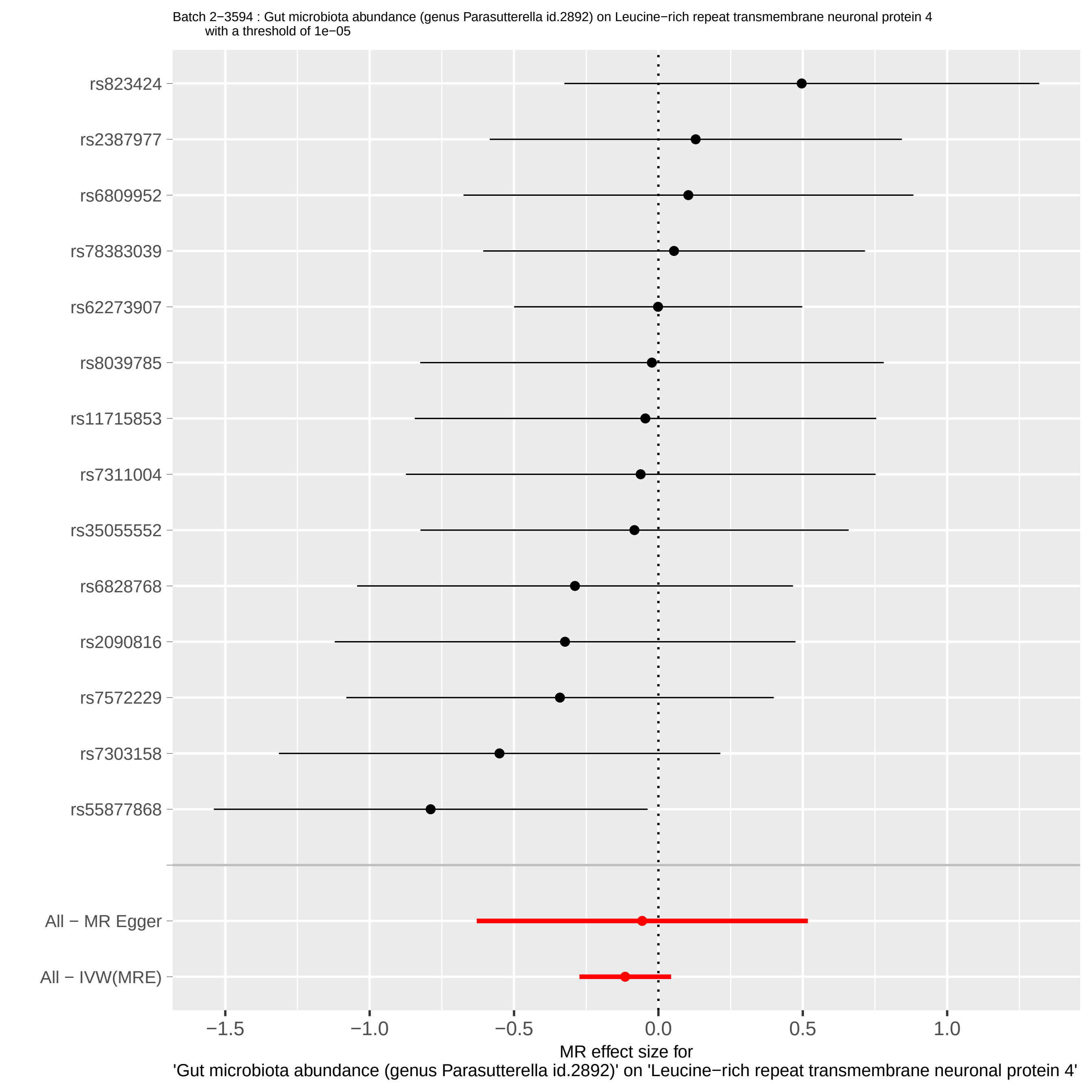
Batch 2−3594 : Gut microbiota abundance (genus Parasutterella id.2892) on Leucine−rich repeat transmembrane neuronal protein 4
with a threshold of 1e−05
rs823424
rs2387977
rs6809952
rs78383039
rs62273907
rs8039785
rs11715853
rs7311004
rs35055552
rs6828768
rs2090816
rs7572229
rs7303158
rs55877868
All − MR Egger
All − IVW(MRE)
−1.5
−1.0
−0.5
0.0
0.5
1.0
MR effect size for
'Gut microbiota abundance (genus Parasutterella id.2892)' on 'Leucine−rich repeat transmembrane neuronal protein 4'

Batch 2−3595 : Gut microbiota abundance (genus Catenibacterium id.2153) on Leucine−rich repeat transmembrane neuronal protein 4
with a threshold of 1e−05

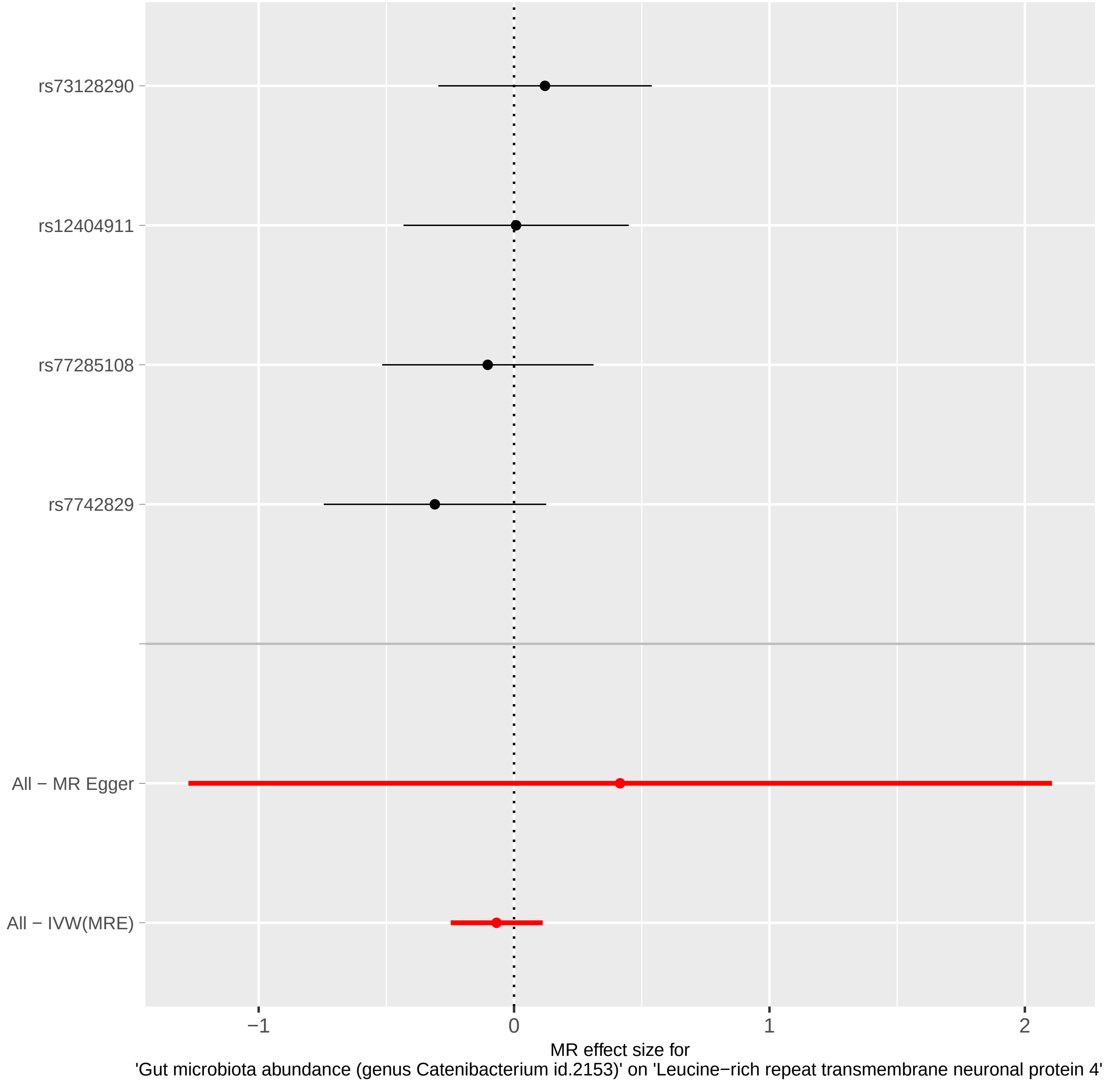

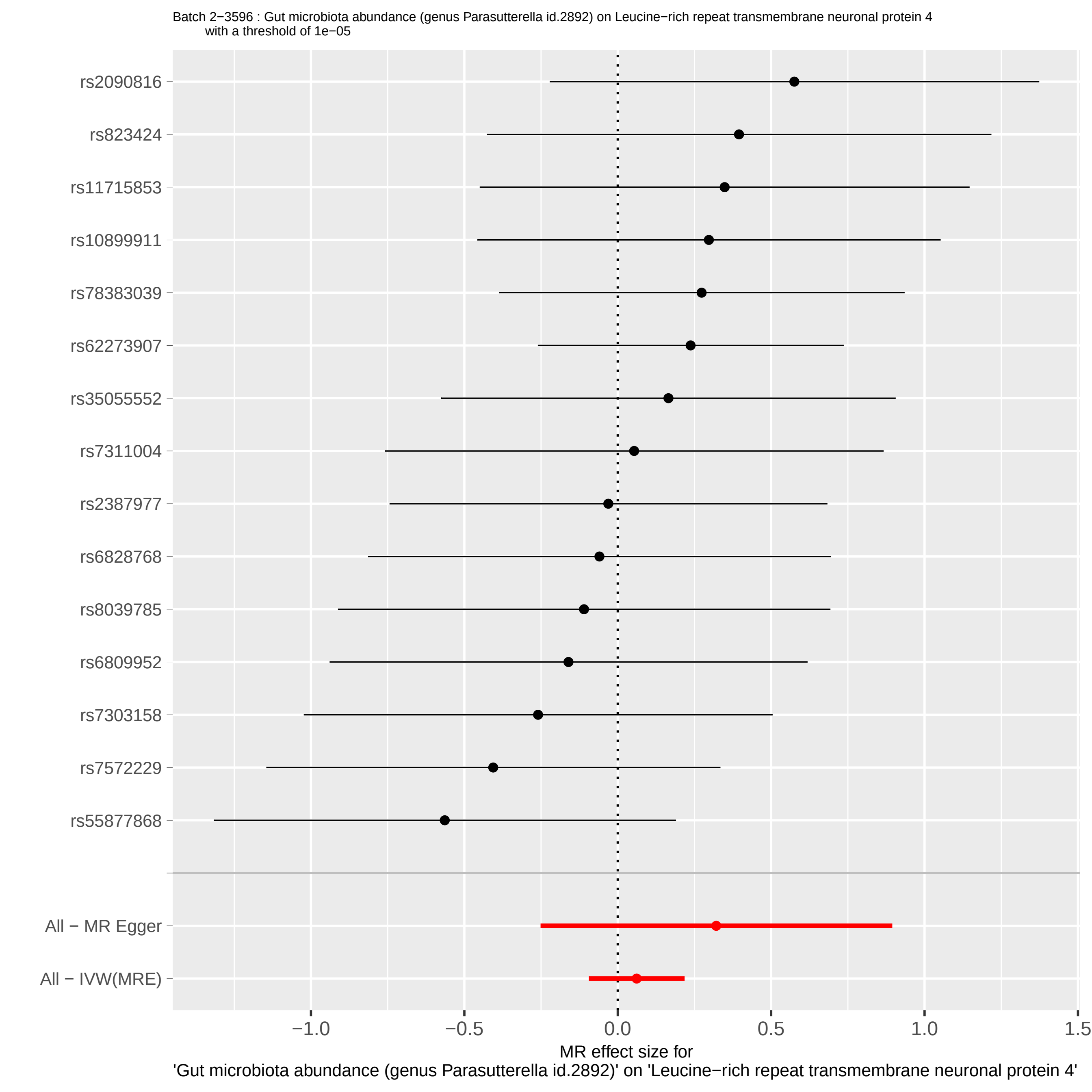
Batch 2−3596 : Gut microbiota abundance (genus Parasutterella id.2892) on Leucine−rich repeat transmembrane neuronal protein 4
with a threshold of 1e−05
rs2090816
rs823424
rs11715853
rs10899911
rs78383039
rs62273907
rs35055552
rs7311004
rs2387977
rs6828768
rs8039785
rs6809952
rs7303158
rs7572229
rs55877868
All − MR Egger
All − IVW(MRE)
−1.0
−0.5
0.0
0.5
1.0
1.5
MR effect size for
'Gut microbiota abundance (genus Parasutterella id.2892)' on 'Leucine−rich repeat transmembrane neuronal protein 4'

Batch 2−3597 : Gut microbiota abundance (genus Catenibacterium id.2153) on Leucine−rich repeat and transmembrane domain−containing protein 1 with a threshold of 1e−05

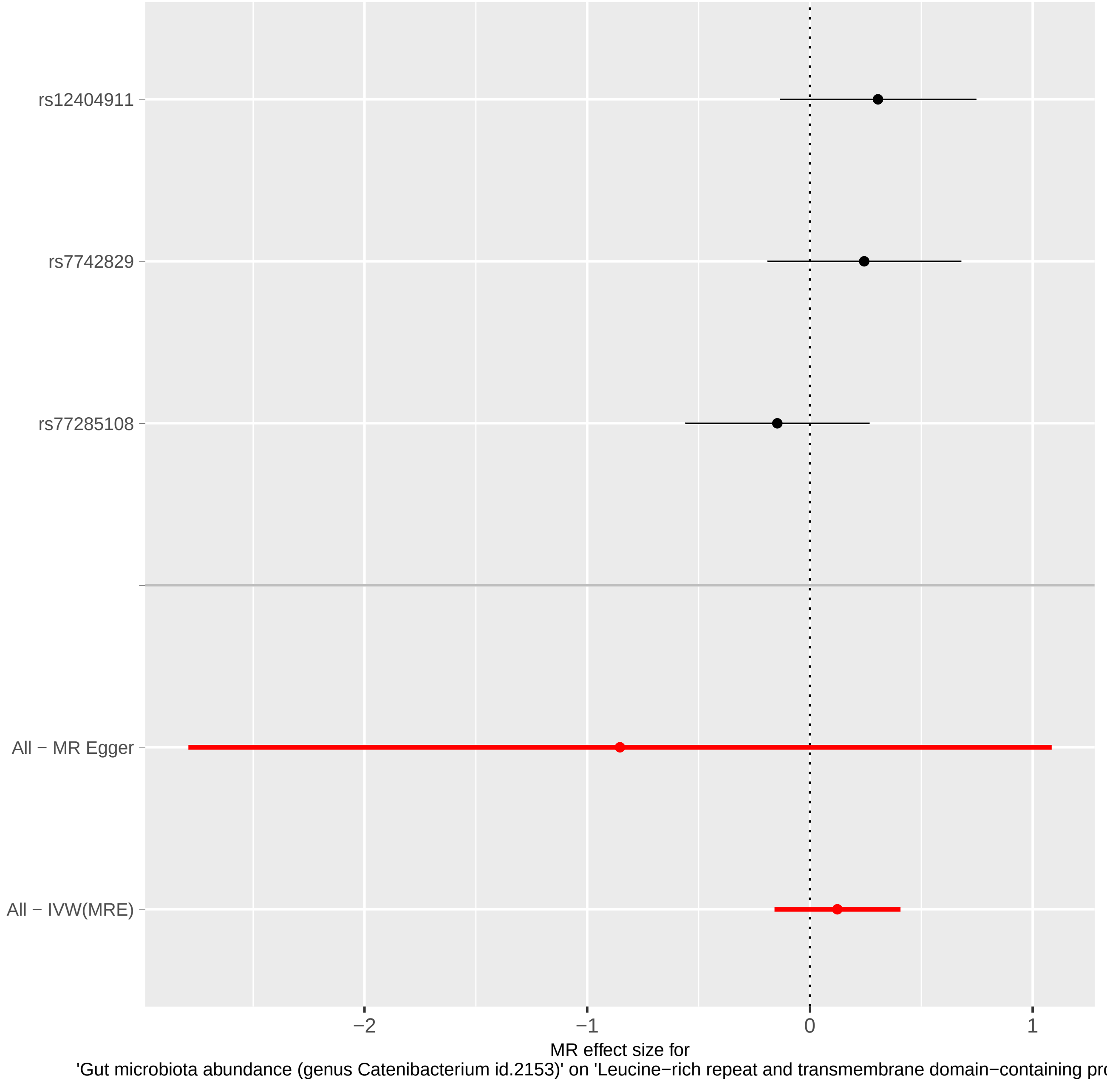

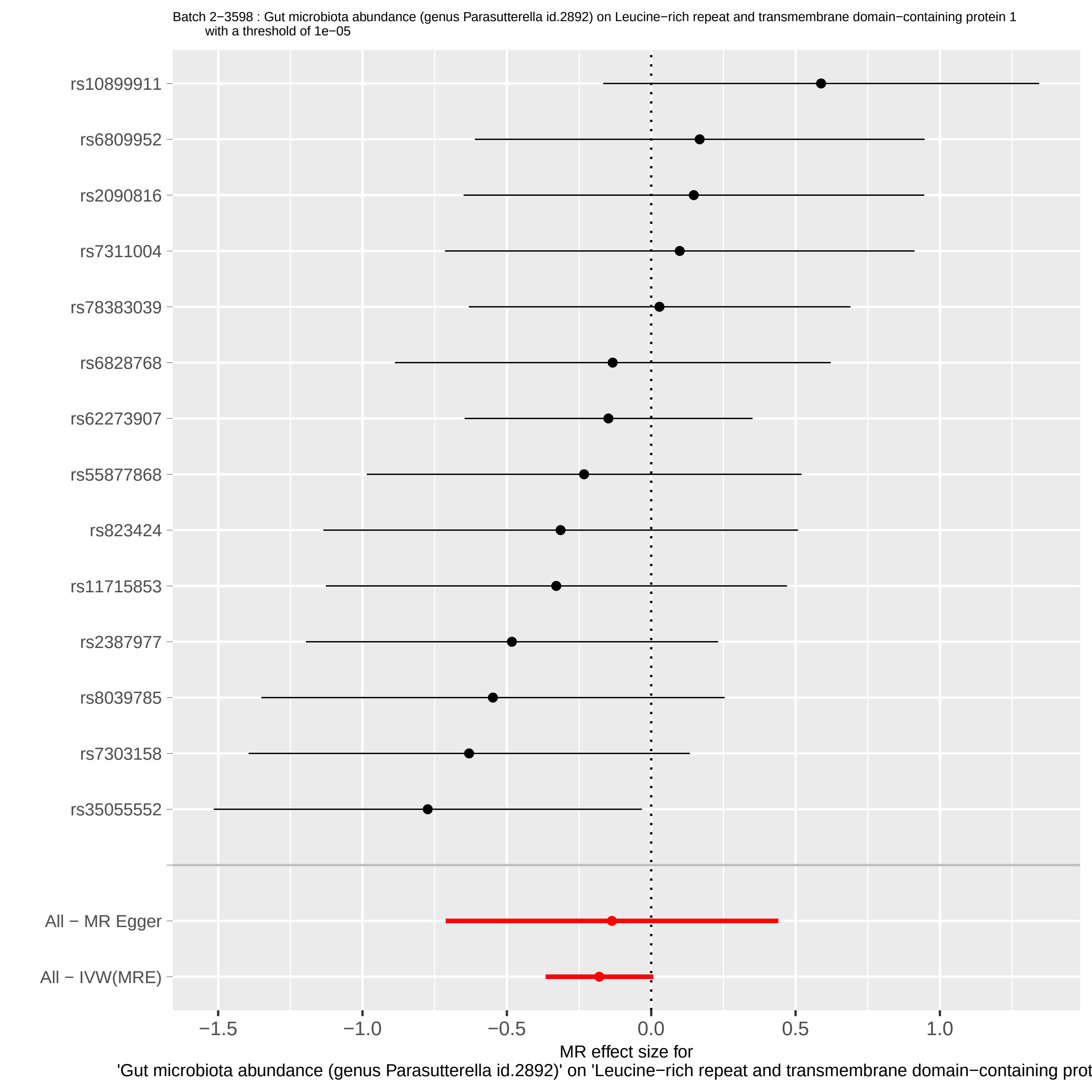

Batch 2−3598 : Gut microbiota abundance (genus Parasutterella id.2892) on Leucine−rich repeat and transmembrane domain−containing protein 1
with a threshold of 1e−05
rs10899911
rs6809952
rs2090816
rs7311004
rs78383039
rs6828768
rs62273907
rs55877868
rs823424
rs11715853
rs2387977
rs8039785
rs7303158
rs35055552
All − MR Egger
All − IVW(MRE)
−1.5
−1.0
−0.5
0.0
0.5
1.0
MR effect size for
'Gut microbiota abundance (genus Parasutterella id.2892)' on 'Leucine−rich repeat and transmembrane domain−containing prot

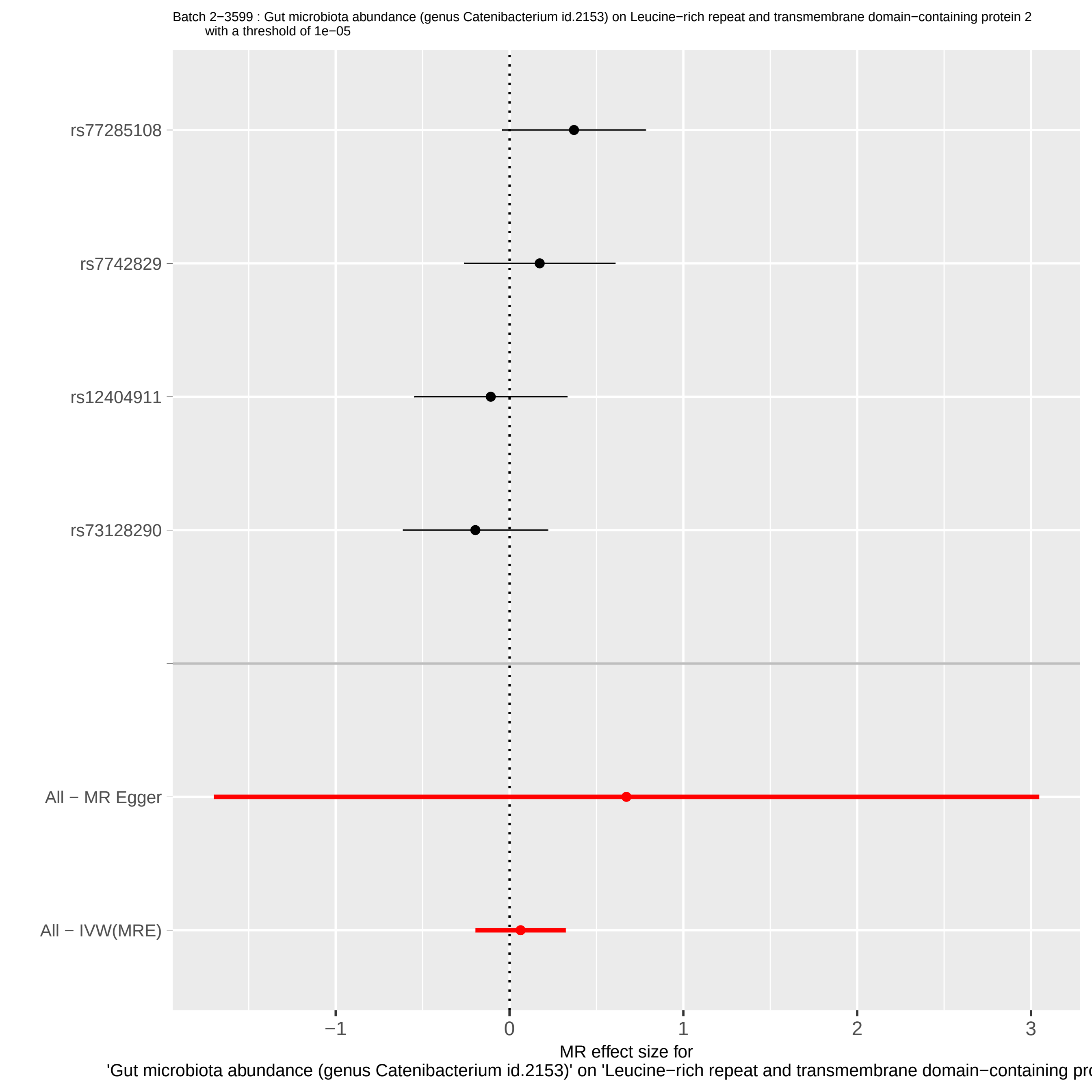

Batch 2−3599 : Gut microbiota abundance (genus Catenibacterium id.2153) on Leucine−rich repeat and transmembrane domain−containing protein 2
with a threshold of 1e−05
rs77285108
rs7742829
rs12404911
rs73128290
All − MR Egger
All − IVW(MRE)
−1
0
1
2
3
MR effect size for
'Gut microbiota abundance (genus Catenibacterium id.2153)' on 'Leucine−rich repeat and transmembrane domain−containing pro

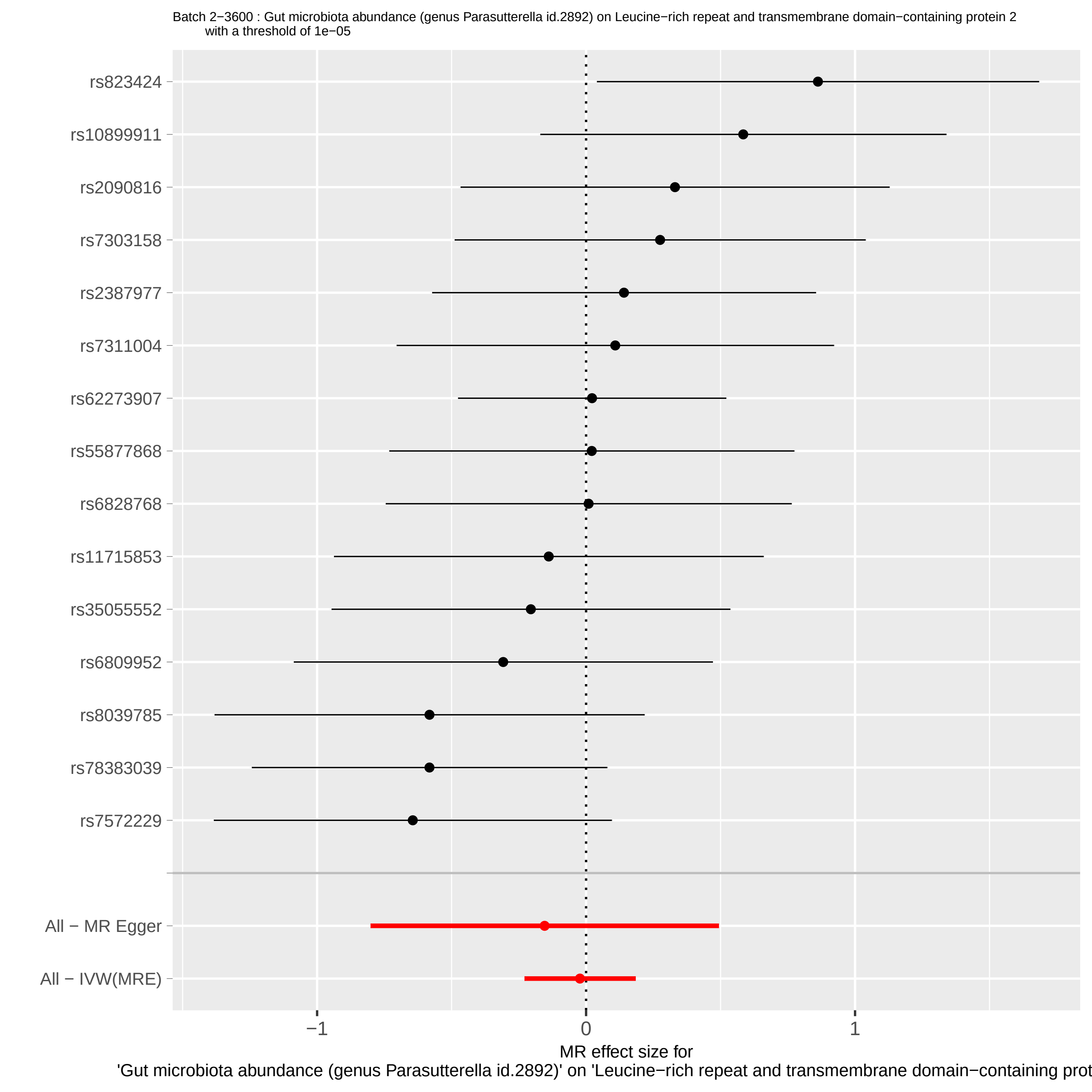
Batch 2−3600 : Gut microbiota abundance (genus Parasutterella id.2892) on Leucine−rich repeat and transmembrane domain−containing protein 2
with a threshold of 1e−05
rs823424
rs10899911
rs2090816
rs7303158
rs2387977
rs7311004
rs62273907
rs55877868
rs6828768
rs11715853
rs35055552
rs6809952
rs8039785
rs78383039
rs7572229
All − MR Egger
All − IVW(MRE)
−1
0
1
MR effect size for
'Gut microbiota abundance (genus Parasutterella id.2892)' on 'Leucine−rich repeat and transmembrane domain−containing prot

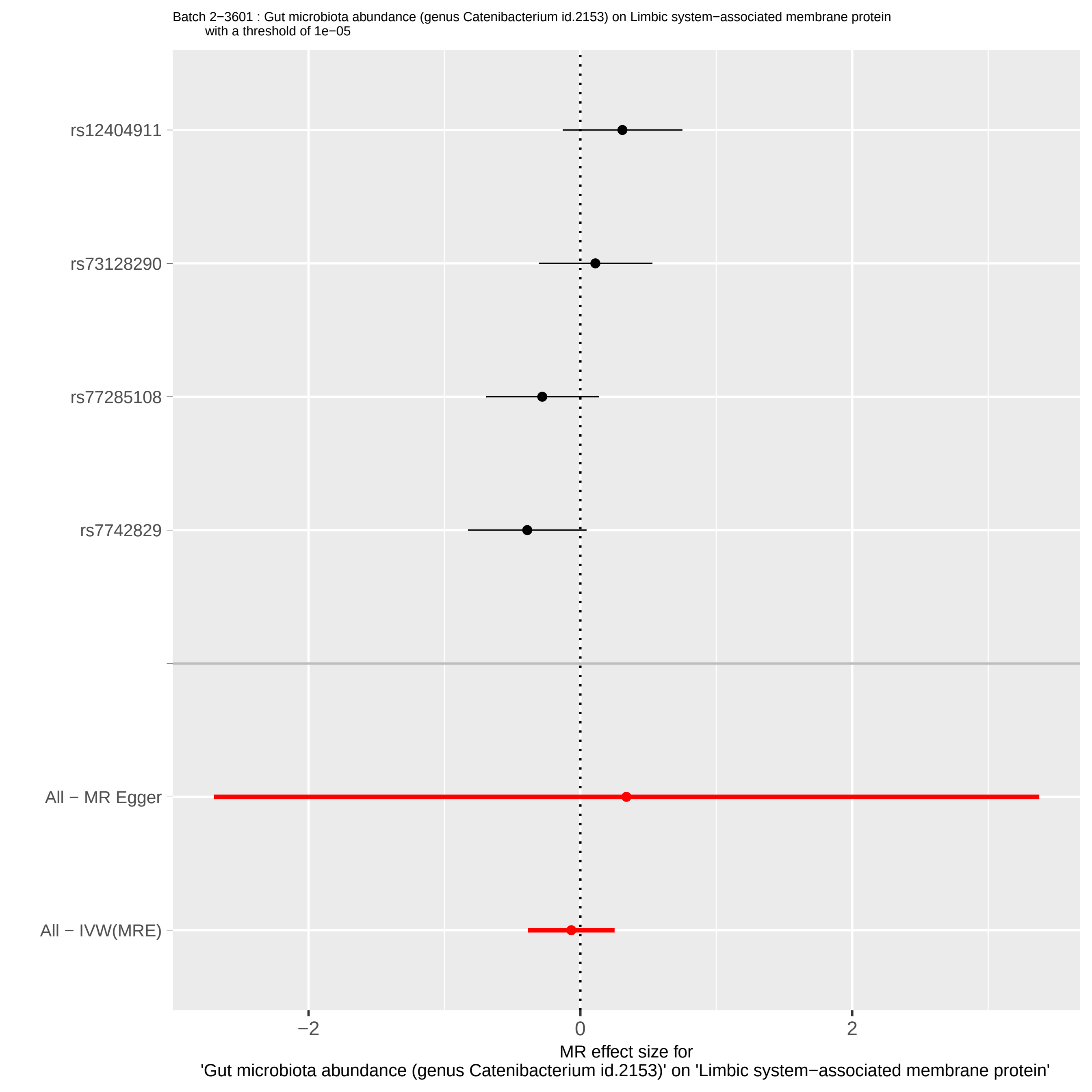

Batch 2−3601 : Gut microbiota abundance (genus Catenibacterium id.2153) on Limbic system−associated membrane protein
with a threshold of 1e−05
rs12404911
rs73128290
rs77285108
rs7742829
All − MR Egger
All − IVW(MRE)
−2
0
2
MR effect size for
'Gut microbiota abundance (genus Catenibacterium id.2153)' on 'Limbic system−associated membrane protein'

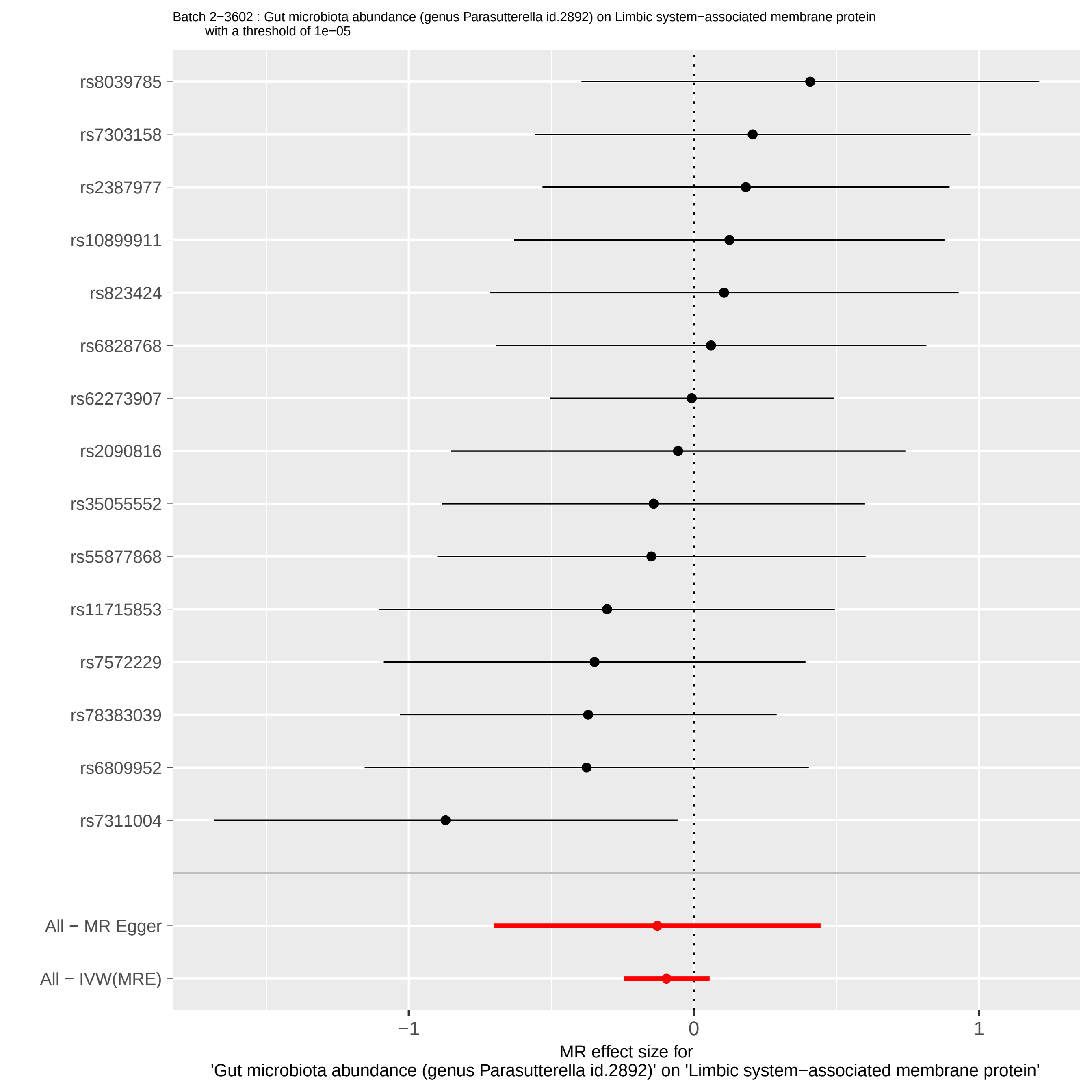

Batch 2−3602 : Gut microbiota abundance (genus Parasutterella id.2892) on Limbic system−associated membrane protein
with a threshold of 1e−05
rs8039785
rs7303158
rs2387977
rs10899911
rs823424
rs6828768
rs62273907
rs2090816
rs35055552
rs55877868
rs11715853
rs7572229
rs78383039
rs6809952
rs7311004
All − MR Egger
All − IVW(MRE)
−1
0
1
MR effect size for
'Gut microbiota abundance (genus Parasutterella id.2892)' on 'Limbic system−associated membrane protein'

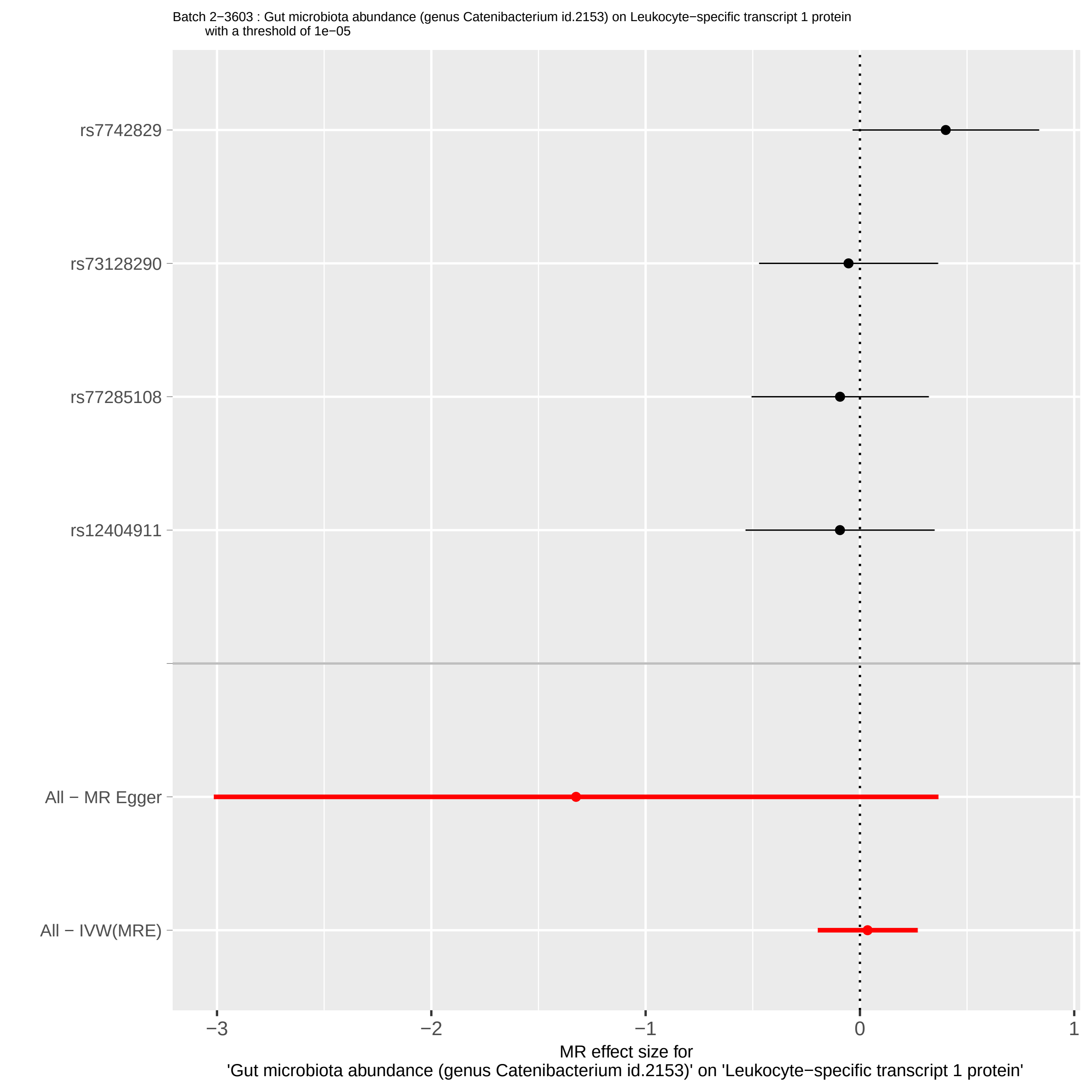

Batch 2−3603 : Gut microbiota abundance (genus Catenibacterium id.2153) on Leukocyte−specific transcript 1 protein
with a threshold of 1e−05
rs7742829
rs73128290
rs77285108
rs12404911
All − MR Egger
All − IVW(MRE)
−3
−2
−1
0
1
MR effect size for
'Gut microbiota abundance (genus Catenibacterium id.2153)' on 'Leukocyte−specific transcript 1 protein'

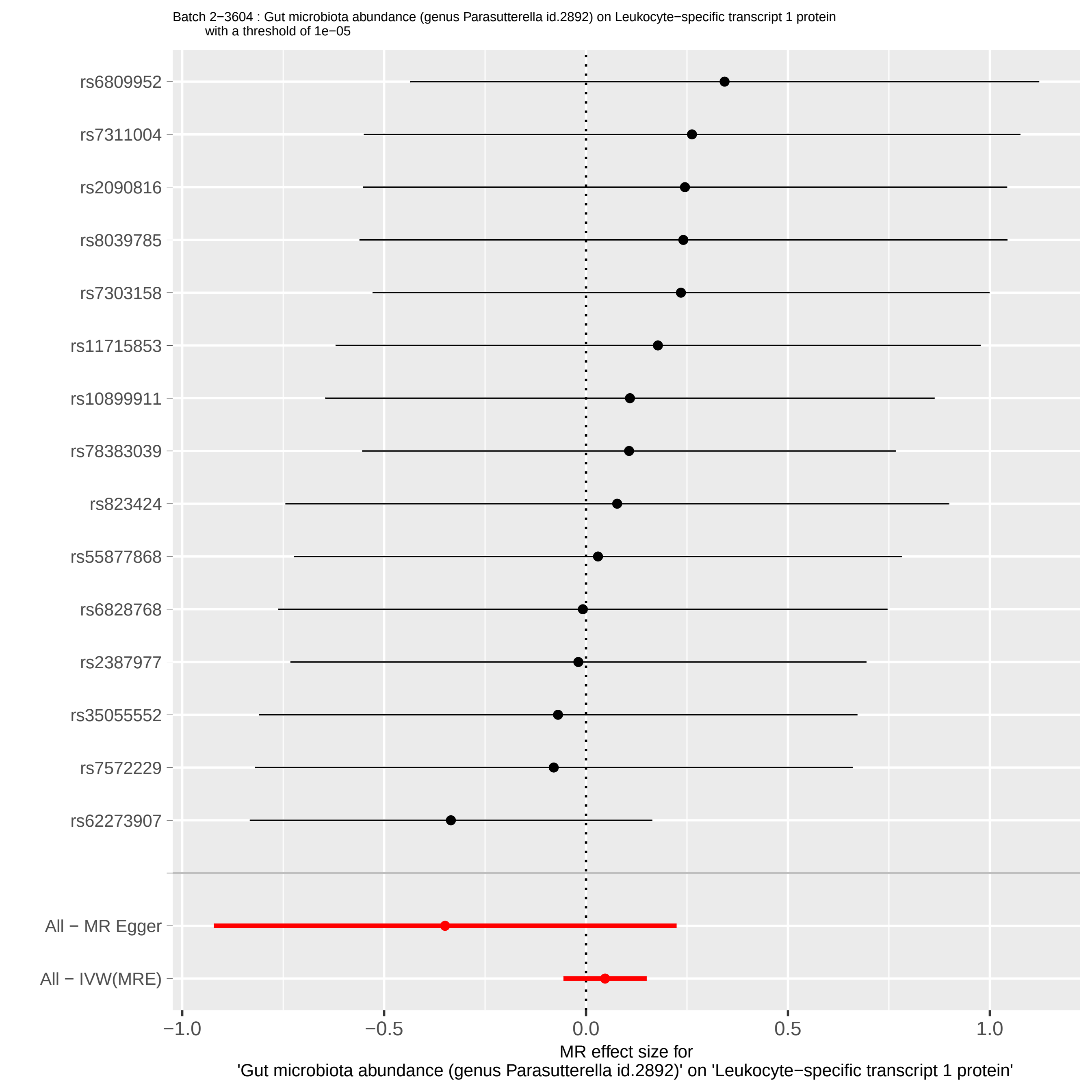
Batch 2−3604 : Gut microbiota abundance (genus Parasutterella id.2892) on Leukocyte−specific transcript 1 protein
with a threshold of 1e−05
rs6809952
rs7311004
rs2090816
rs8039785
rs7303158
rs11715853
rs10899911
rs78383039
rs823424
rs55877868
rs6828768
rs2387977
rs35055552
rs7572229
rs62273907
All − MR Egger
All − IVW(MRE)
−1.0
−0.5
0.0
0.5
1.0
MR effect size for
'Gut microbiota abundance (genus Parasutterella id.2892)' on 'Leukocyte−specific transcript 1 protein'

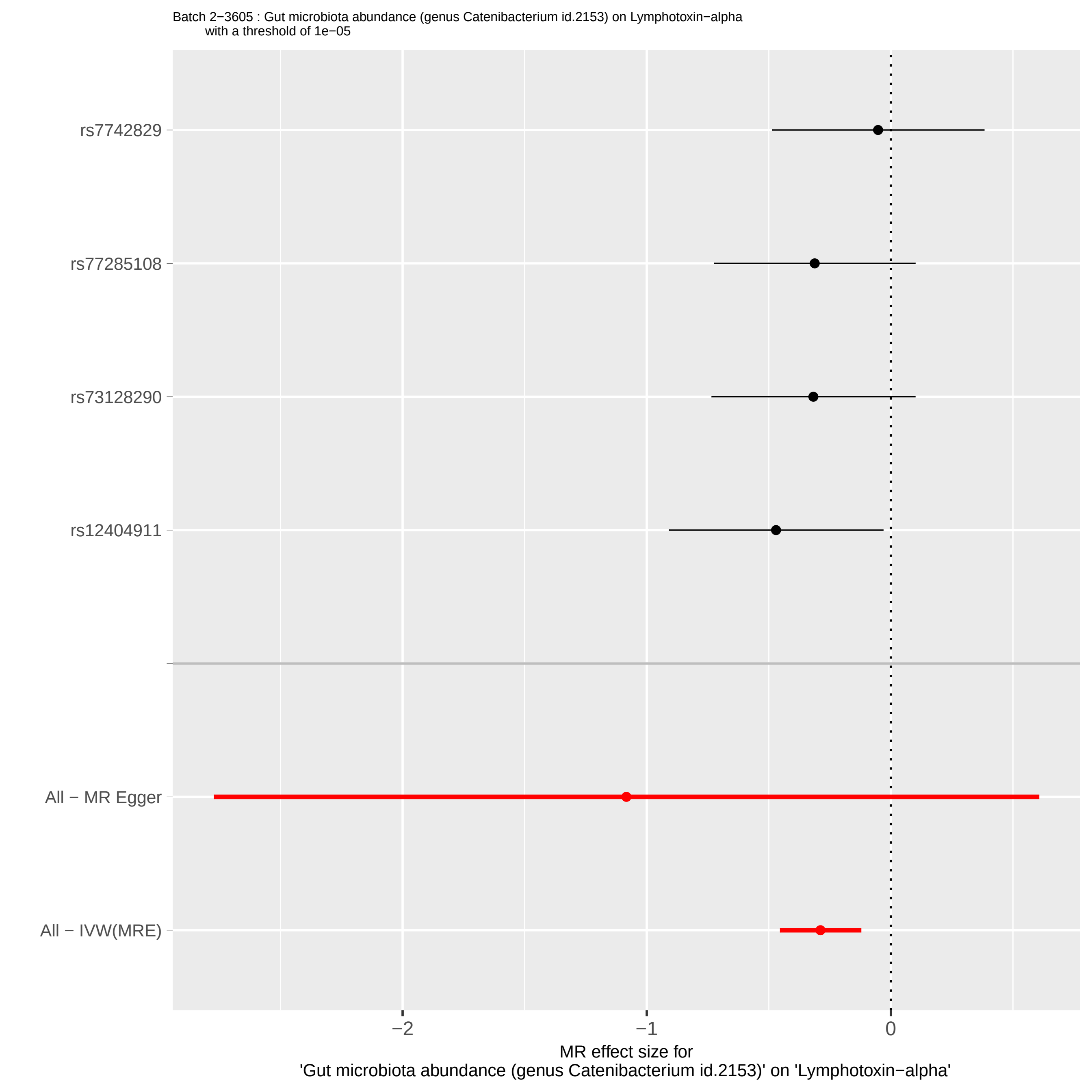

Batch 2−3605 : Gut microbiota abundance (genus Catenibacterium id.2153) on Lymphotoxin−alpha
with a threshold of 1e−05
rs7742829
rs77285108
rs73128290
rs12404911
All − MR Egger
All − IVW(MRE)
−2
−1
0
MR effect size for
'Gut microbiota abundance (genus Catenibacterium id.2153)' on 'Lymphotoxin−alpha'

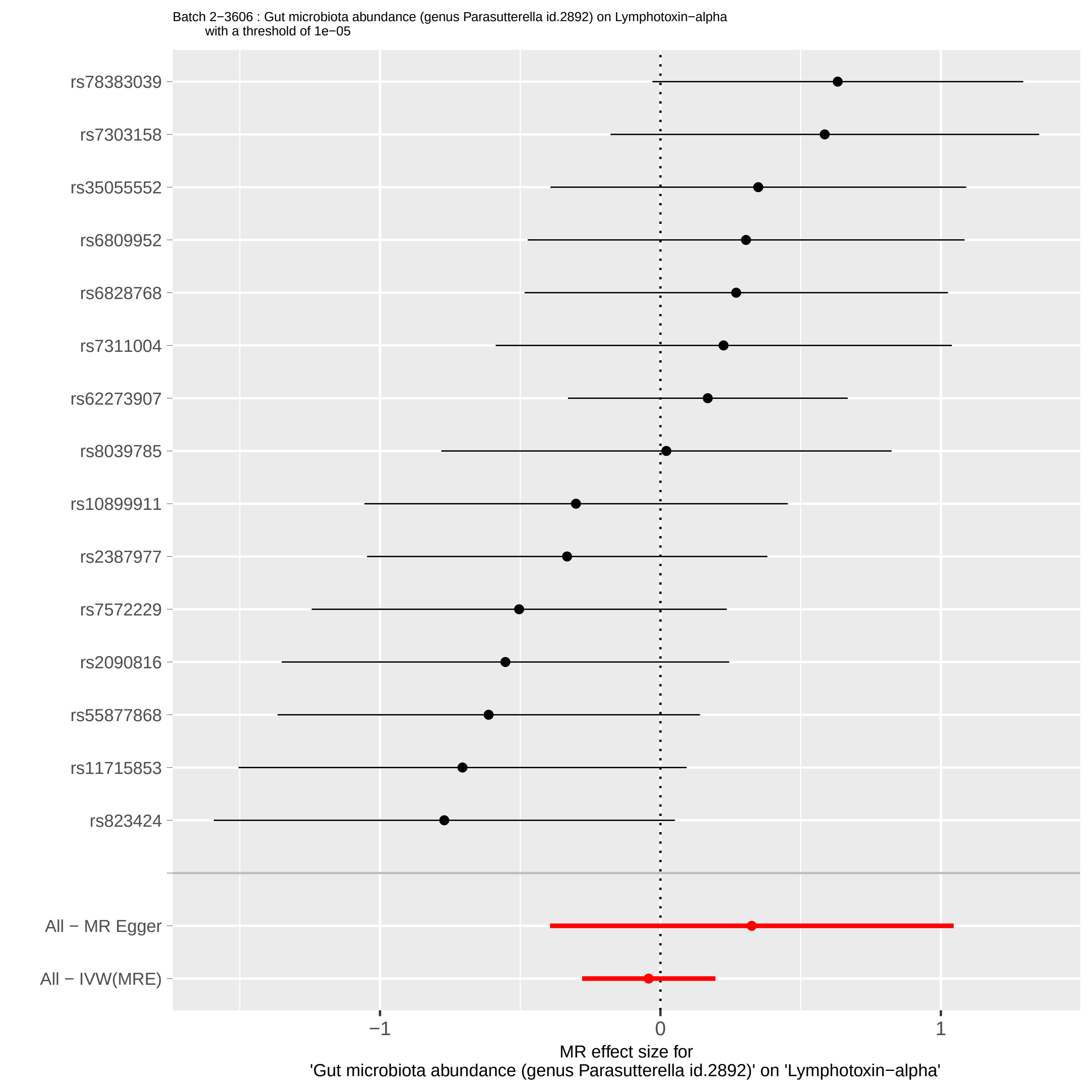

Batch 2−3606 : Gut microbiota abundance (genus Parasutterella id.2892) on Lymphotoxin−alpha
with a threshold of 1e−05
rs78383039
rs7303158
rs35055552
rs6809952
rs6828768
rs7311004
rs62273907
rs8039785
rs10899911
rs2387977
rs7572229
rs2090816
rs55877868
rs11715853
rs823424
All − MR Egger
All − IVW(MRE)
−1
0
1
MR effect size for
'Gut microbiota abundance (genus Parasutterella id.2892)' on 'Lymphotoxin−alpha'

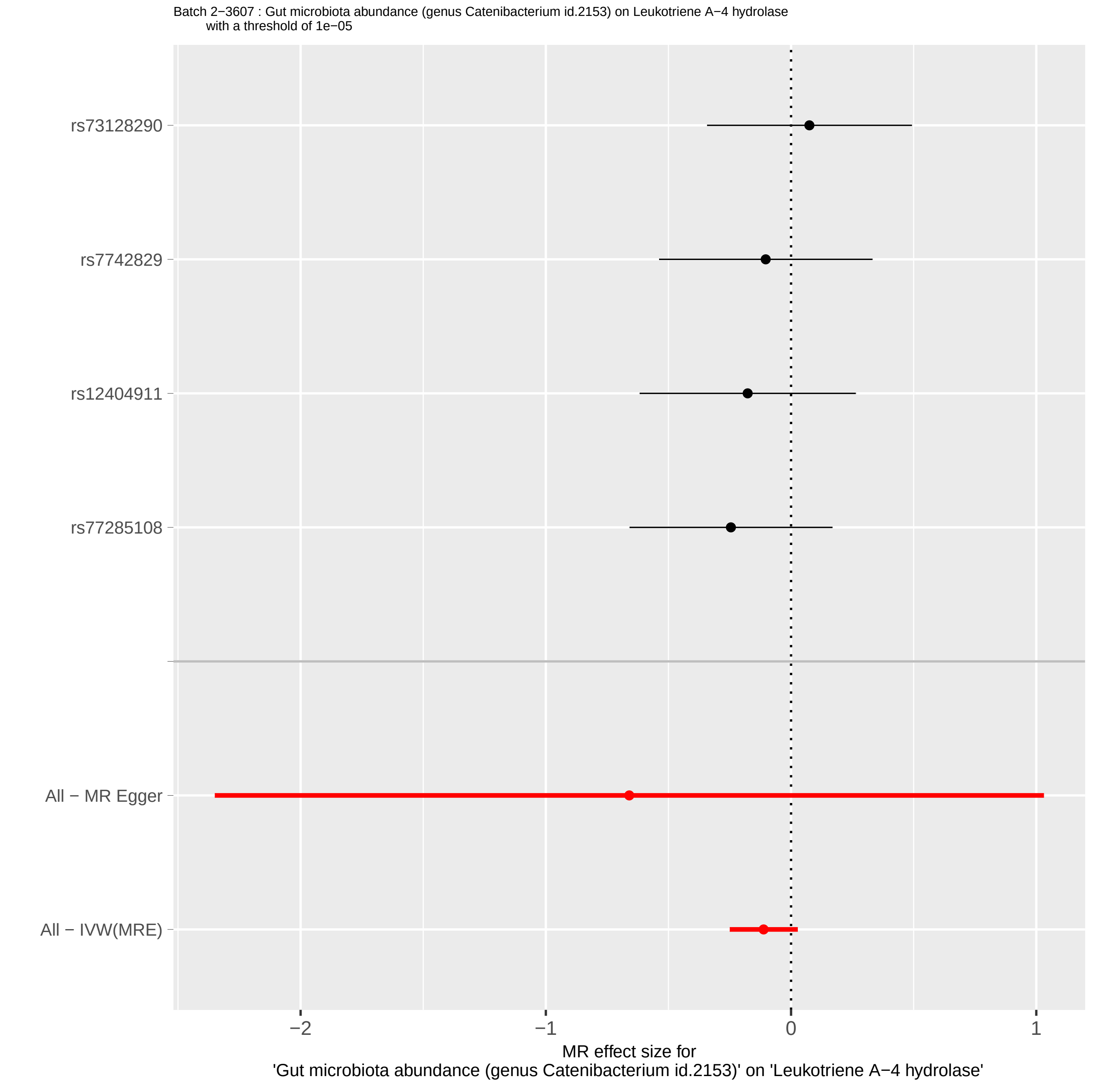

Batch 2−3607 : Gut microbiota abundance (genus Catenibacterium id.2153) on Leukotriene A−4 hydrolase
with a threshold of 1e−05
rs73128290
rs7742829
rs12404911
rs77285108
All − MR Egger
All − IVW(MRE)
−2
−1
0
1
MR effect size for
'Gut microbiota abundance (genus Catenibacterium id.2153)' on 'Leukotriene A−4 hydrolase'

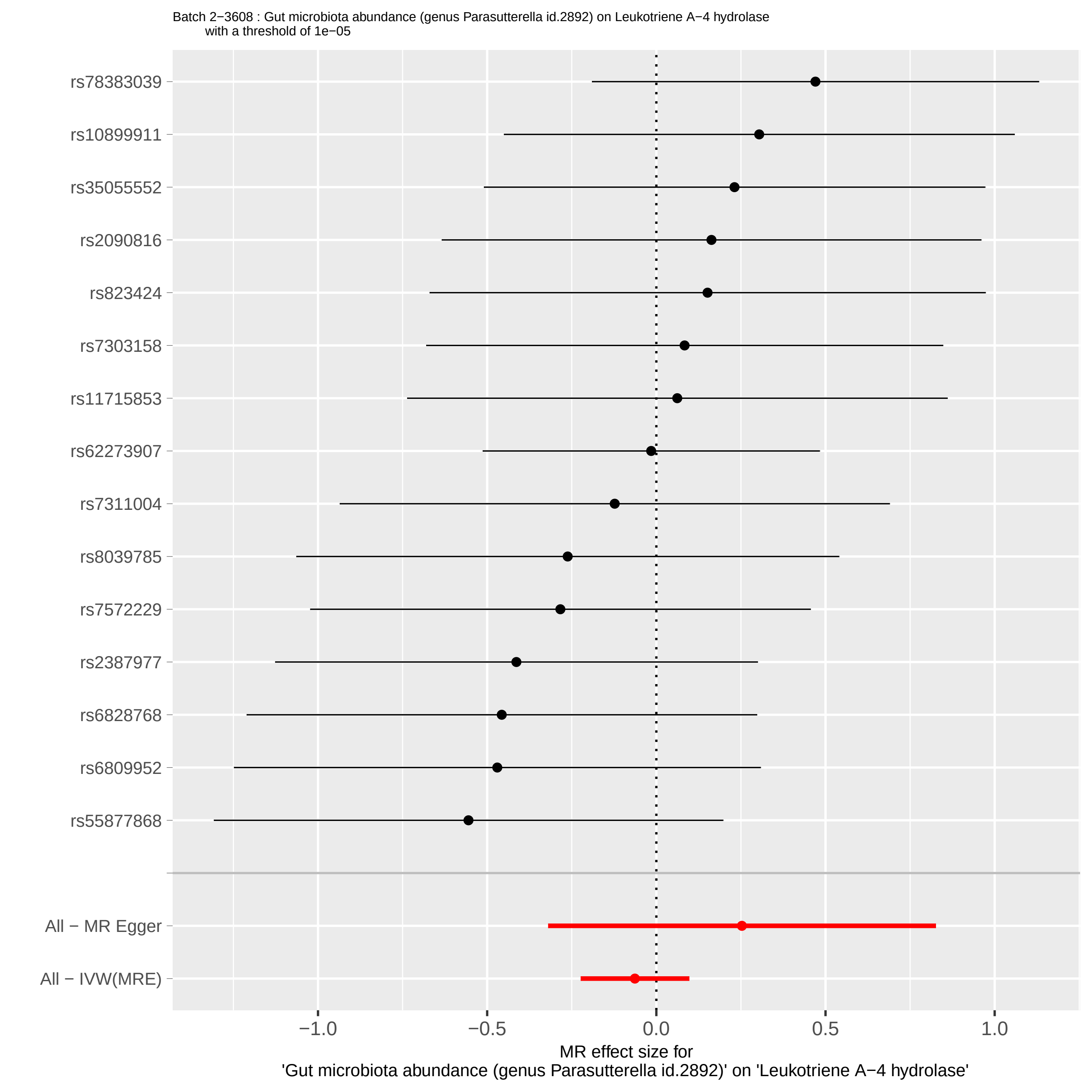

Batch 2−3608 : Gut microbiota abundance (genus Parasutterella id.2892) on Leukotriene A−4 hydrolase
with a threshold of 1e−05
rs78383039
rs10899911
rs35055552
rs2090816
rs823424
rs7303158
rs11715853
rs62273907
rs7311004
rs8039785
rs7572229
rs2387977
rs6828768
rs6809952
rs55877868
All − MR Egger
All − IVW(MRE)
−1.0
−0.5
0.0
0.5
1.0
MR effect size for
'Gut microbiota abundance (genus Parasutterella id.2892)' on 'Leukotriene A−4 hydrolase'

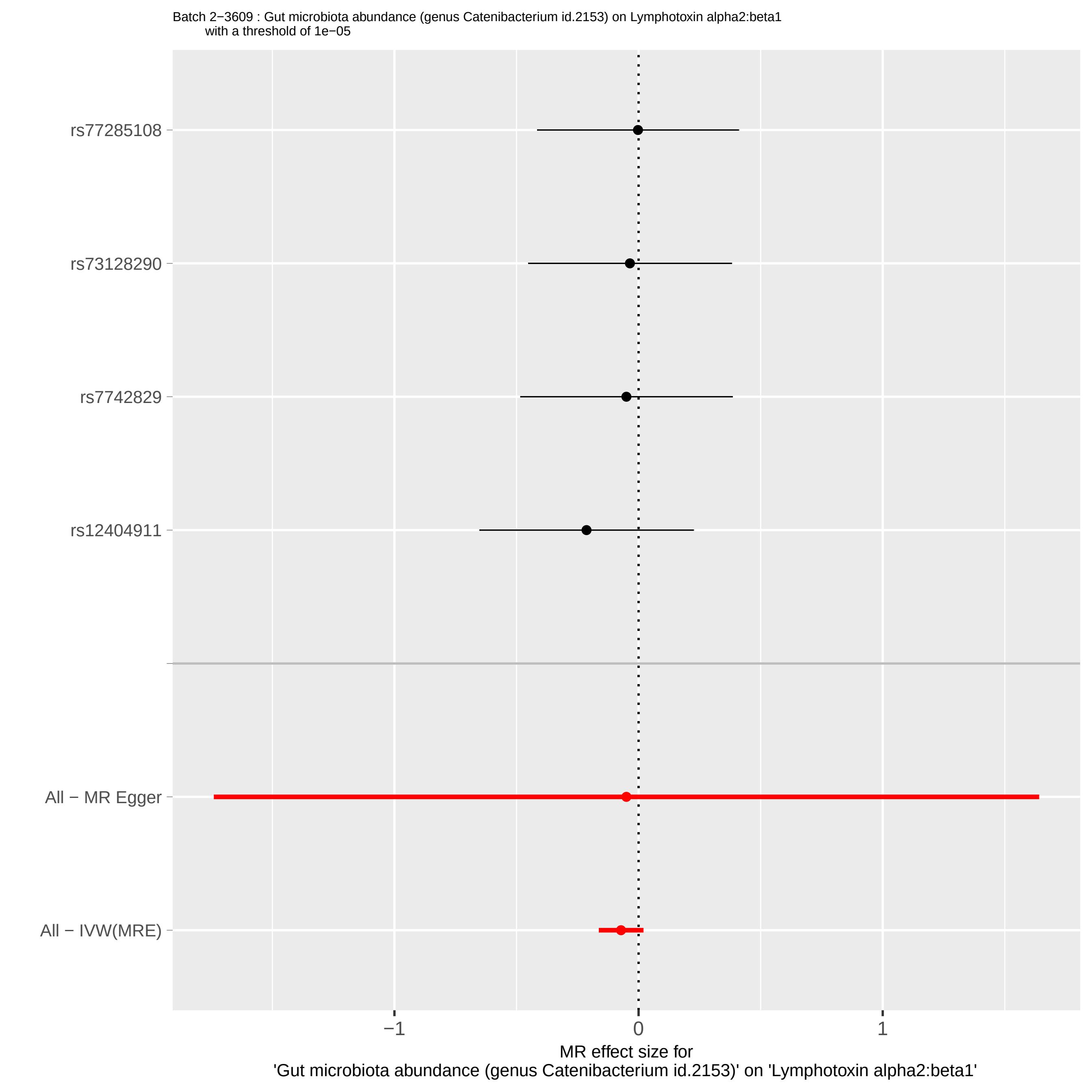

Batch 2−3609 : Gut microbiota abundance (genus Catenibacterium id.2153) on Lymphotoxin alpha2:beta1
with a threshold of 1e−05
rs77285108
rs73128290
rs7742829
rs12404911
All − MR Egger
All − IVW(MRE)
−1
0
1
MR effect size for
'Gut microbiota abundance (genus Catenibacterium id.2153)' on 'Lymphotoxin alpha2:beta1'

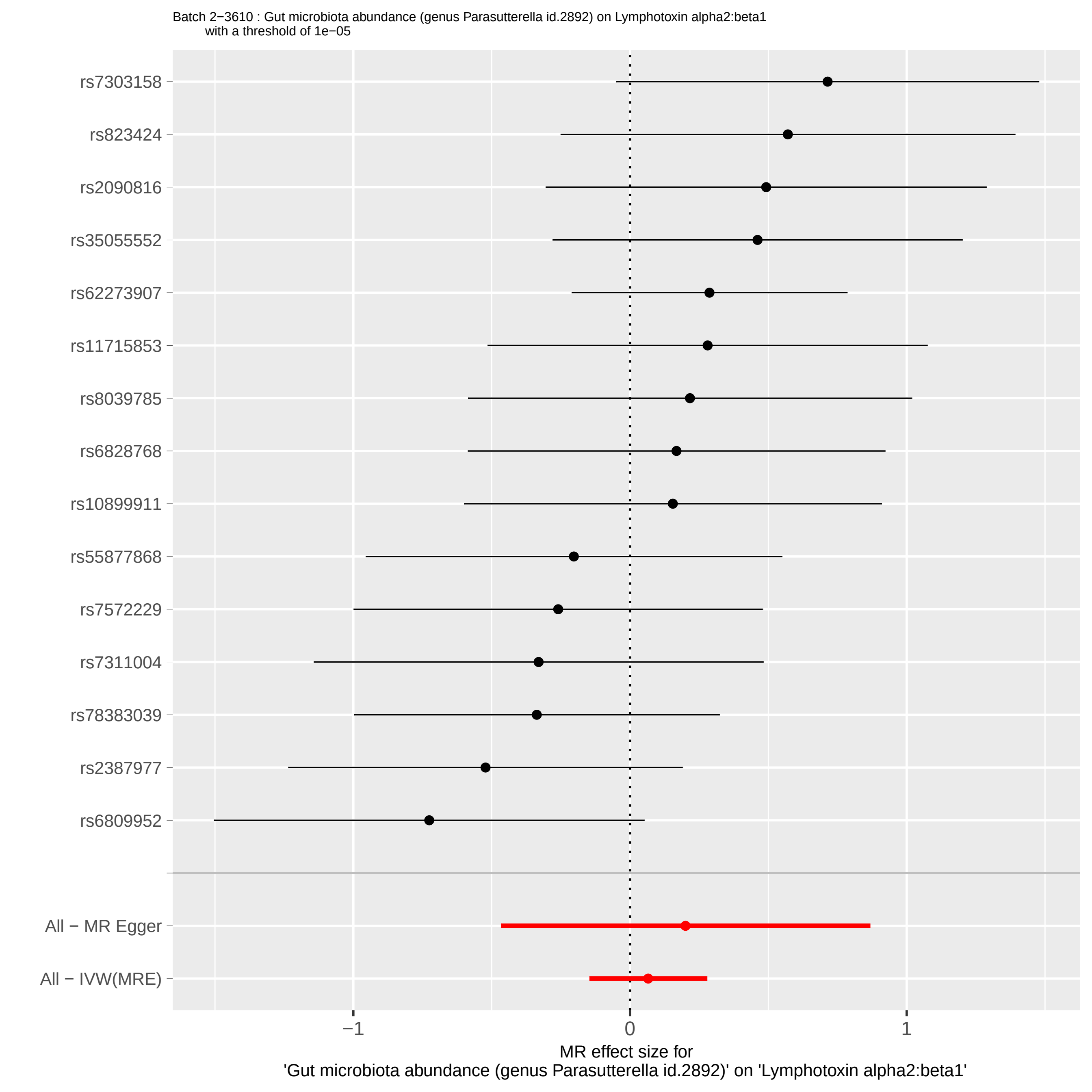

Batch 2−3610 : Gut microbiota abundance (genus Parasutterella id.2892) on Lymphotoxin alpha2:beta1
with a threshold of 1e−05
rs7303158
rs823424
rs2090816
rs35055552
rs62273907
rs11715853
rs8039785
rs6828768
rs10899911
rs55877868
rs7572229
rs7311004
rs78383039
rs2387977
rs6809952
All − MR Egger
All − IVW(MRE)
−1
0
1
MR effect size for
'Gut microbiota abundance (genus Parasutterella id.2892)' on 'Lymphotoxin alpha2:beta1'

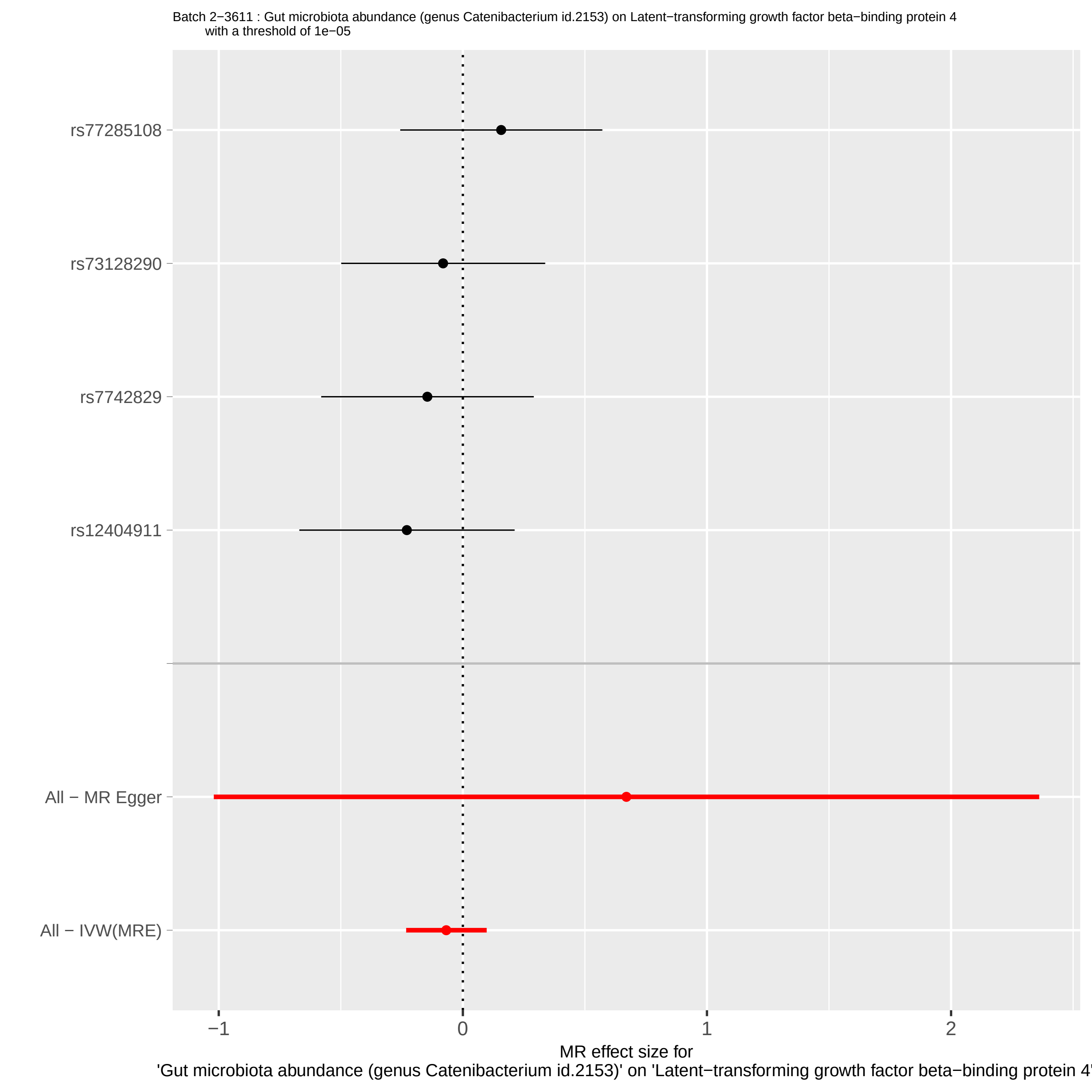

Batch 2−3611 : Gut microbiota abundance (genus Catenibacterium id.2153) on Latent−transforming growth factor beta−binding protein 4
with a threshold of 1e−05
rs77285108
rs73128290
rs7742829
rs12404911
All − MR Egger
All − IVW(MRE)
−1
0
1
2
MR effect size for
'Gut microbiota abundance (genus Catenibacterium id.2153)' on 'Latent−transforming growth factor beta−binding protein 4'

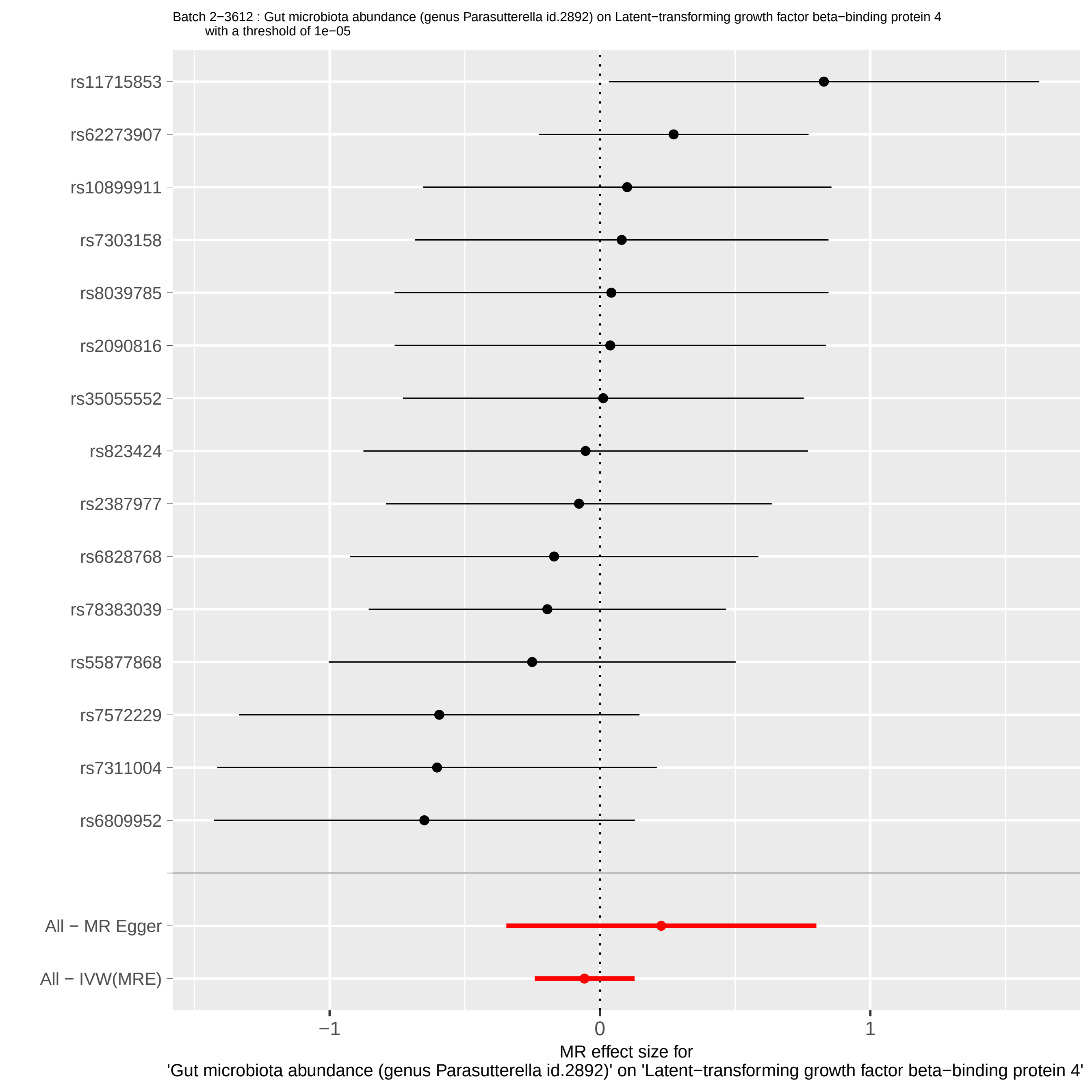
Batch 2−3612 : Gut microbiota abundance (genus Parasutterella id.2892) on Latent−transforming growth factor beta−binding protein 4
with a threshold of 1e−05
rs11715853
rs62273907
rs10899911
rs7303158
rs8039785
rs2090816
rs35055552
rs823424
rs2387977
rs6828768
rs78383039
rs55877868
rs7572229
rs7311004
rs6809952
All − MR Egger
All − IVW(MRE)
−1
0
1
MR effect size for
'Gut microbiota abundance (genus Parasutterella id.2892)' on 'Latent−transforming growth factor beta−binding protein 4'

Batch 2−3613 : Gut microbiota abundance (genus Catenibacterium id.2153) on Tumor necrosis factor receptor superfamily member 3
with a threshold of 1e−05

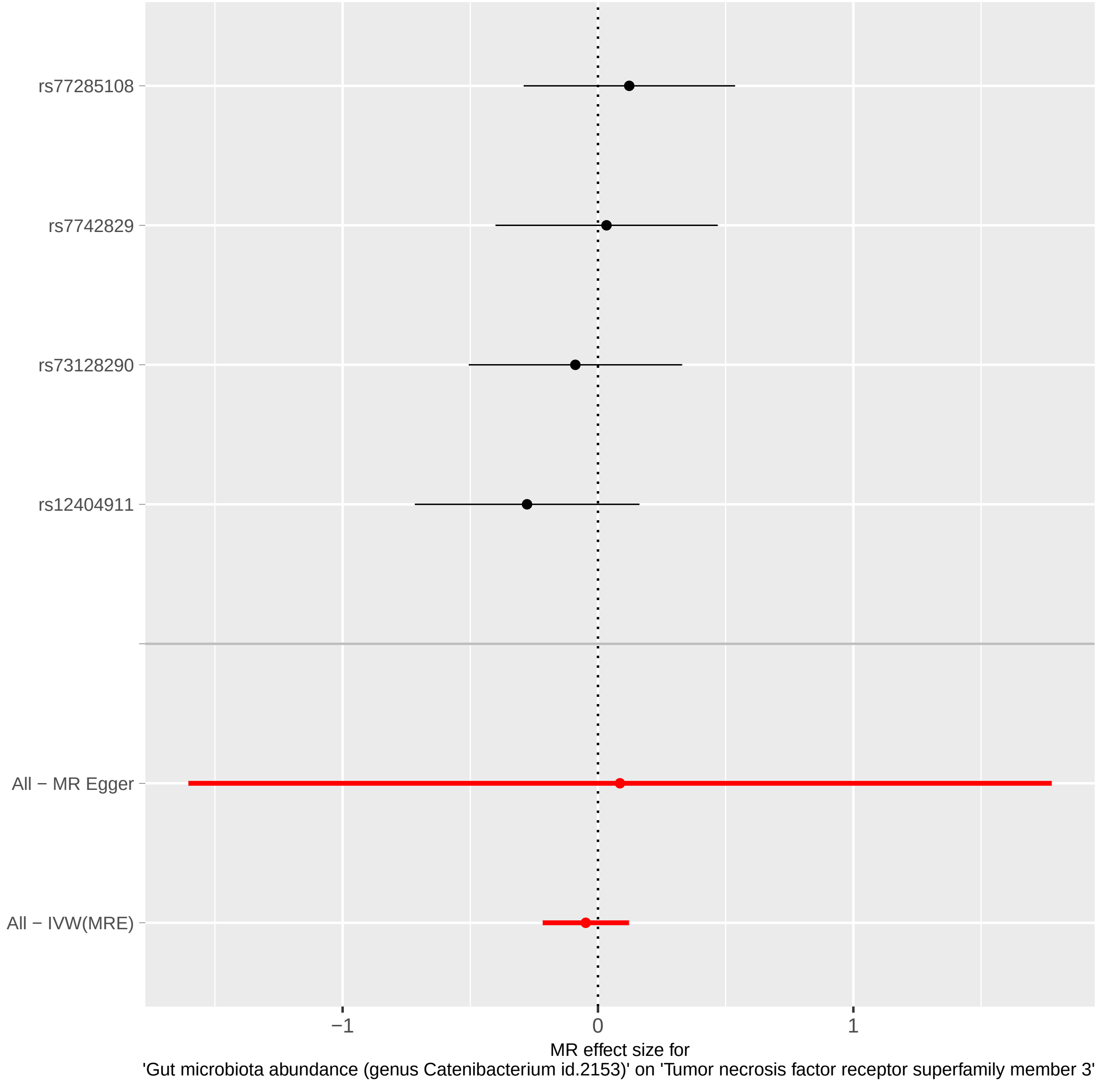

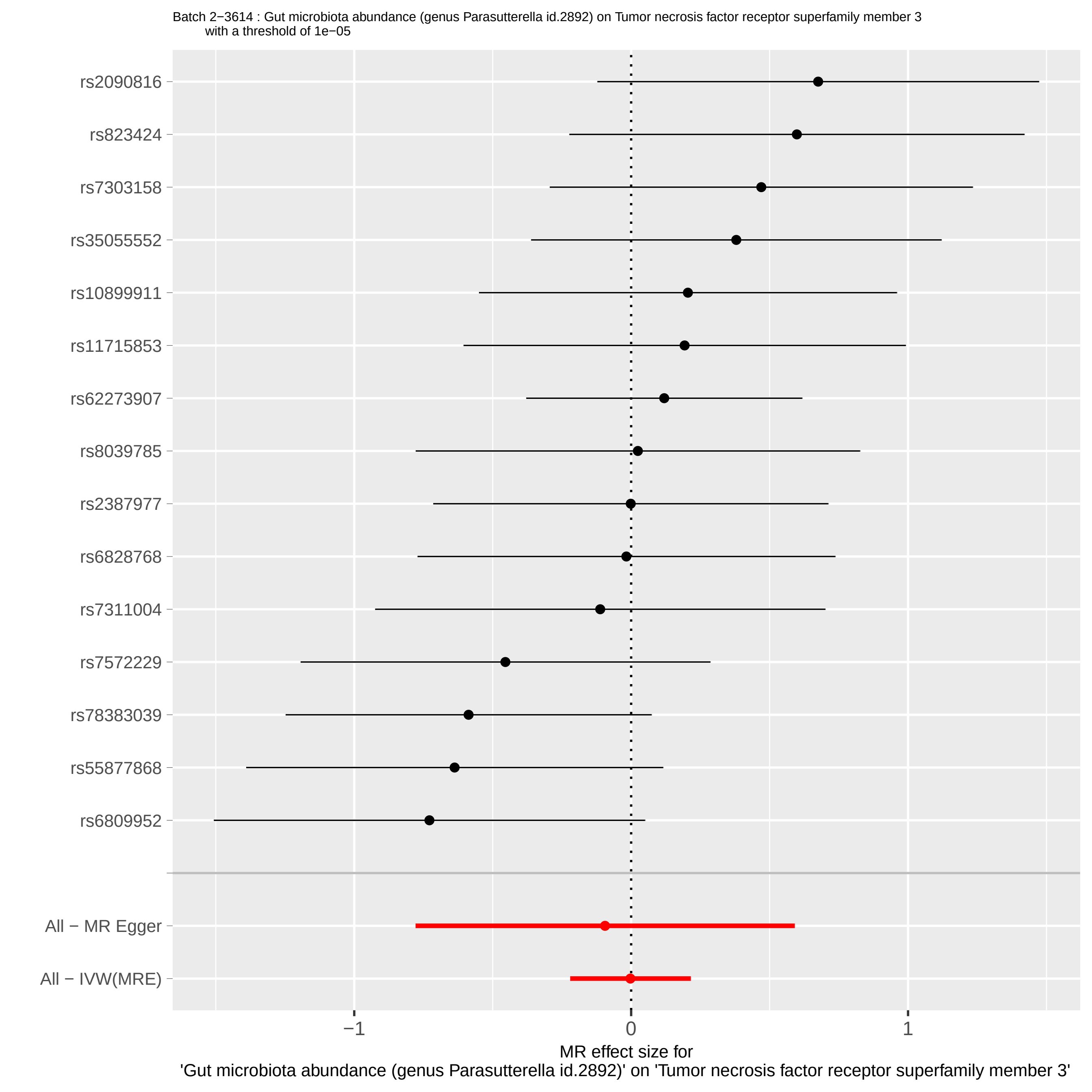

Batch 2−3614 : Gut microbiota abundance (genus Parasutterella id.2892) on Tumor necrosis factor receptor superfamily member 3
with a threshold of 1e−05
rs2090816
rs823424
rs7303158
rs35055552
rs10899911
rs11715853
rs62273907
rs8039785
rs2387977
rs6828768
rs7311004
rs7572229
rs78383039
rs55877868
rs6809952
All − MR Egger
All − IVW(MRE)
−1
0
1
MR effect size for
'Gut microbiota abundance (genus Parasutterella id.2892)' on 'Tumor necrosis factor receptor superfamily member 3'

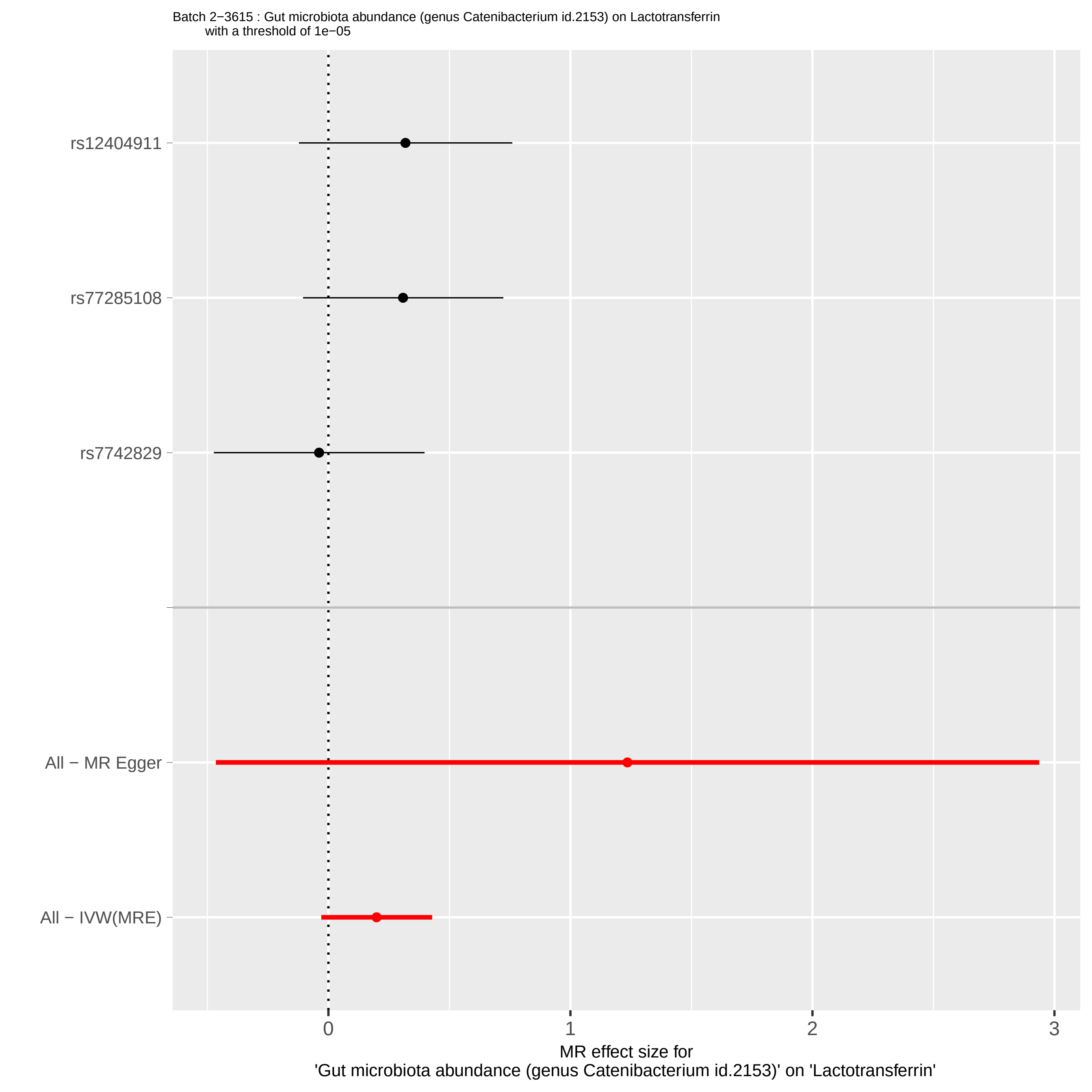
Batch 2−3615 : Gut microbiota abundance (genus Catenibacterium id.2153) on Lactotransferrin
with a threshold of 1e−05
rs12404911
rs77285108
rs7742829
All − MR Egger
All − IVW(MRE)
0
1
2
3
MR effect size for
'Gut microbiota abundance (genus Catenibacterium id.2153)' on 'Lactotransferrin'

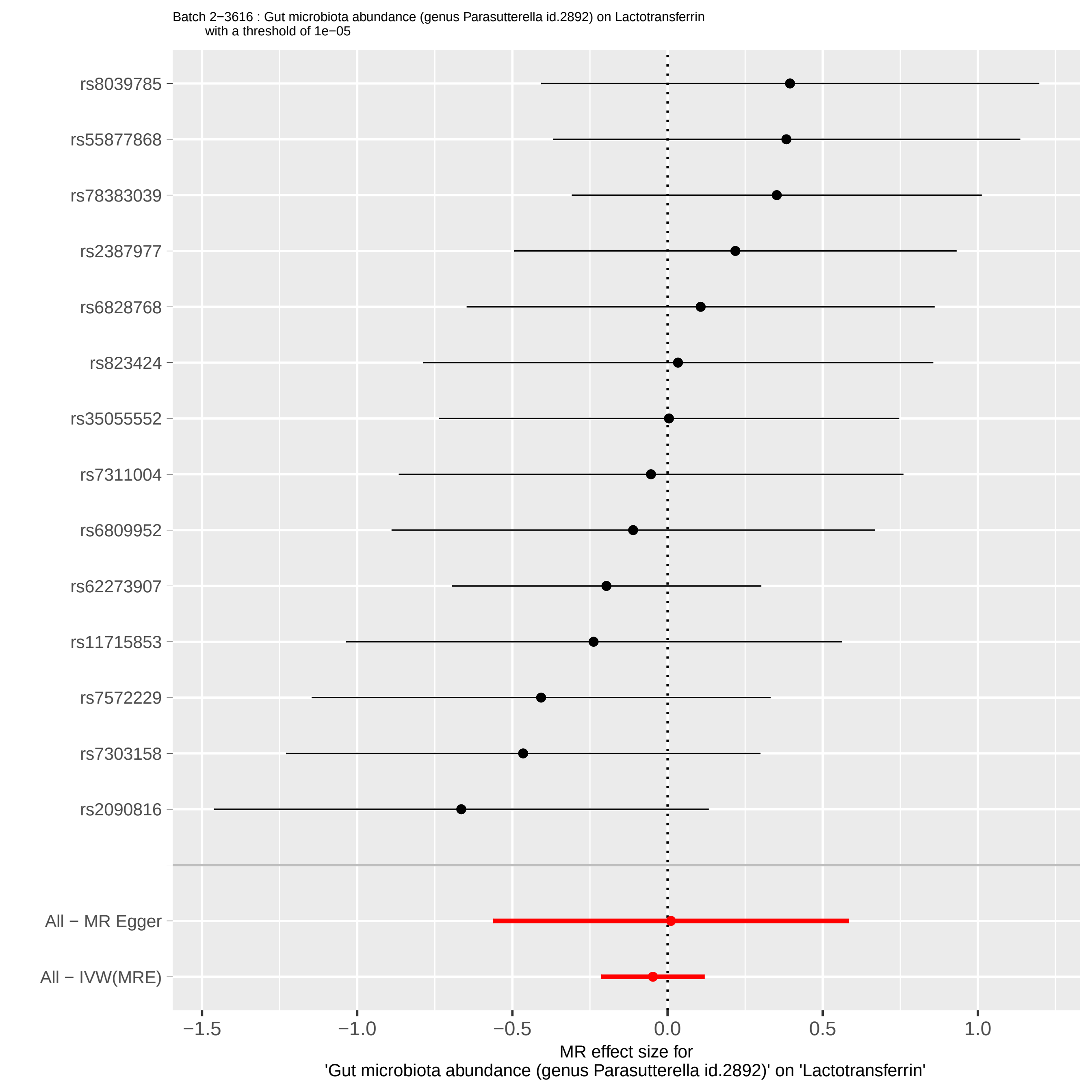
Batch 2−3616 : Gut microbiota abundance (genus Parasutterella id.2892) on Lactotransferrin
with a threshold of 1e−05
rs8039785
rs55877868
rs78383039
rs2387977
rs6828768
rs823424
rs35055552
rs7311004
rs6809952
rs62273907
rs11715853
rs7572229
rs7303158
rs2090816
All − MR Egger
All − IVW(MRE)
−1.5
−1.0
−0.5
0.0
0.5
1.0
MR effect size for
'Gut microbiota abundance (genus Parasutterella id.2892)' on 'Lactotransferrin'

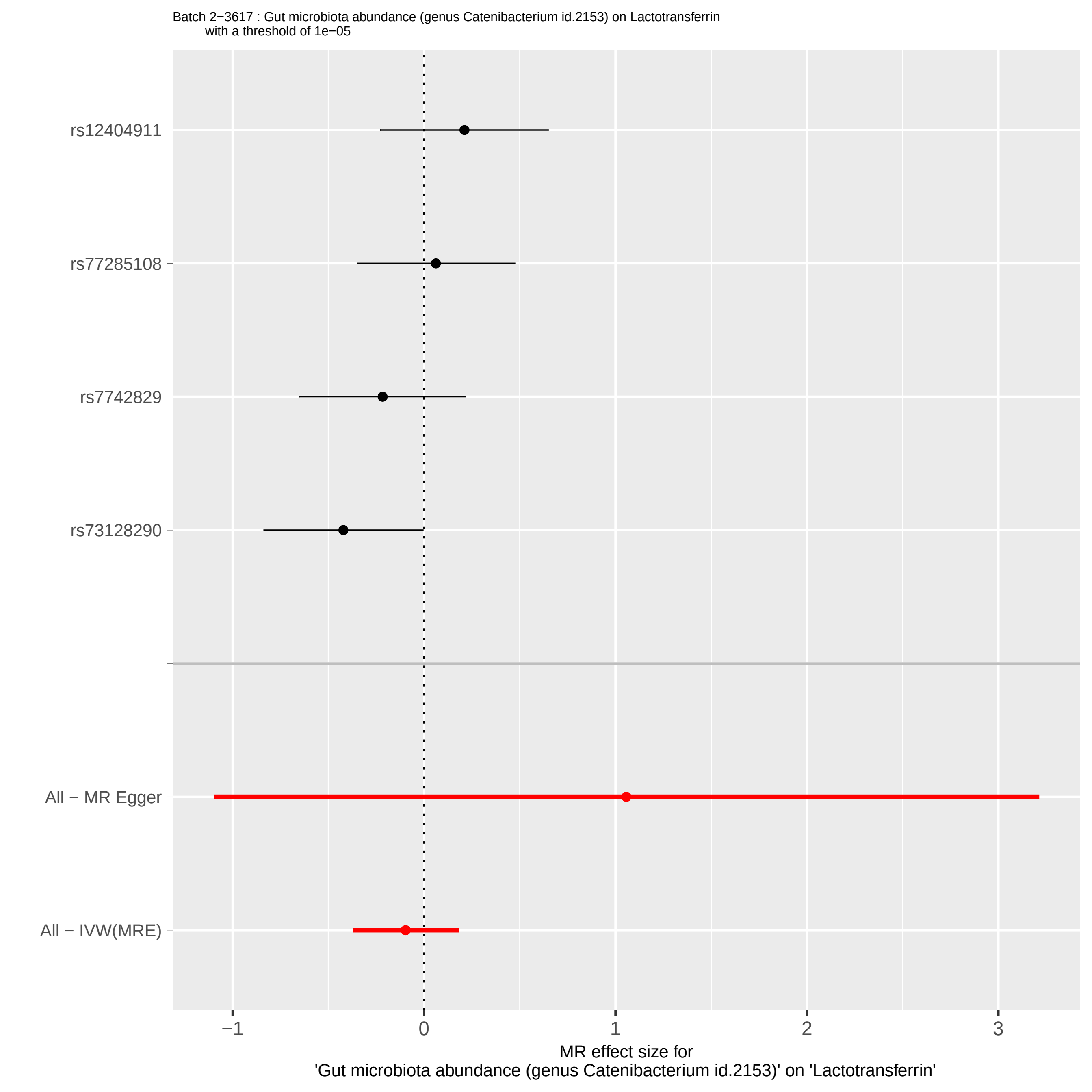
Batch 2−3617 : Gut microbiota abundance (genus Catenibacterium id.2153) on Lactotransferrin
with a threshold of 1e−05
rs12404911
rs77285108
rs7742829
rs73128290
All − MR Egger
All − IVW(MRE)
−1
0
1
2
3
MR effect size for
'Gut microbiota abundance (genus Catenibacterium id.2153)' on 'Lactotransferrin'

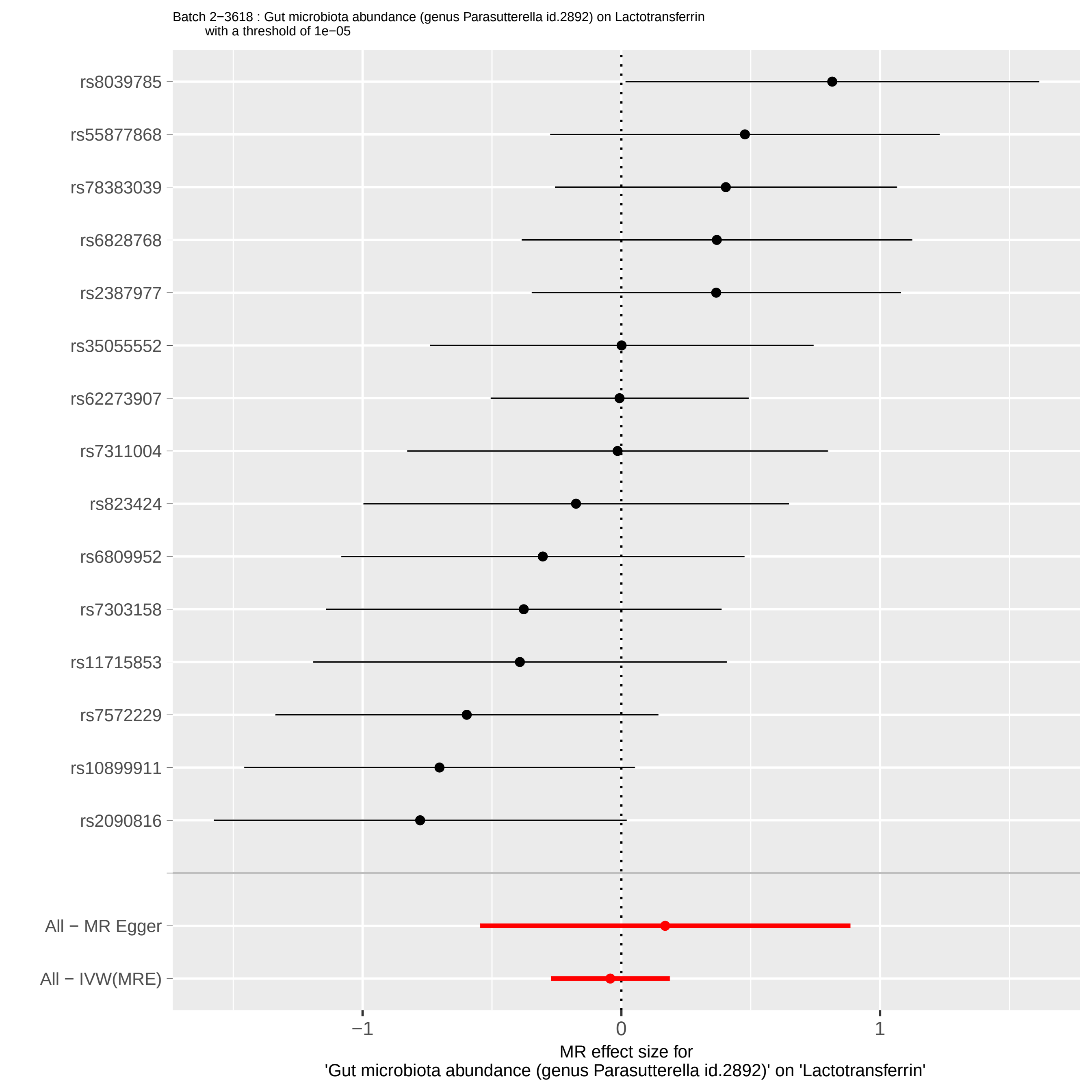

Batch 2−3618 : Gut microbiota abundance (genus Parasutterella id.2892) on Lactotransferrin
with a threshold of 1e−05
rs8039785
rs55877868
rs78383039
rs6828768
rs2387977
rs35055552
rs62273907
rs7311004
rs823424
rs6809952
rs7303158
rs11715853
rs7572229
rs10899911
rs2090816
All − MR Egger
All − IVW(MRE)
−1
0
1
MR effect size for
'Gut microbiota abundance (genus Parasutterella id.2892)' on 'Lactotransferrin'

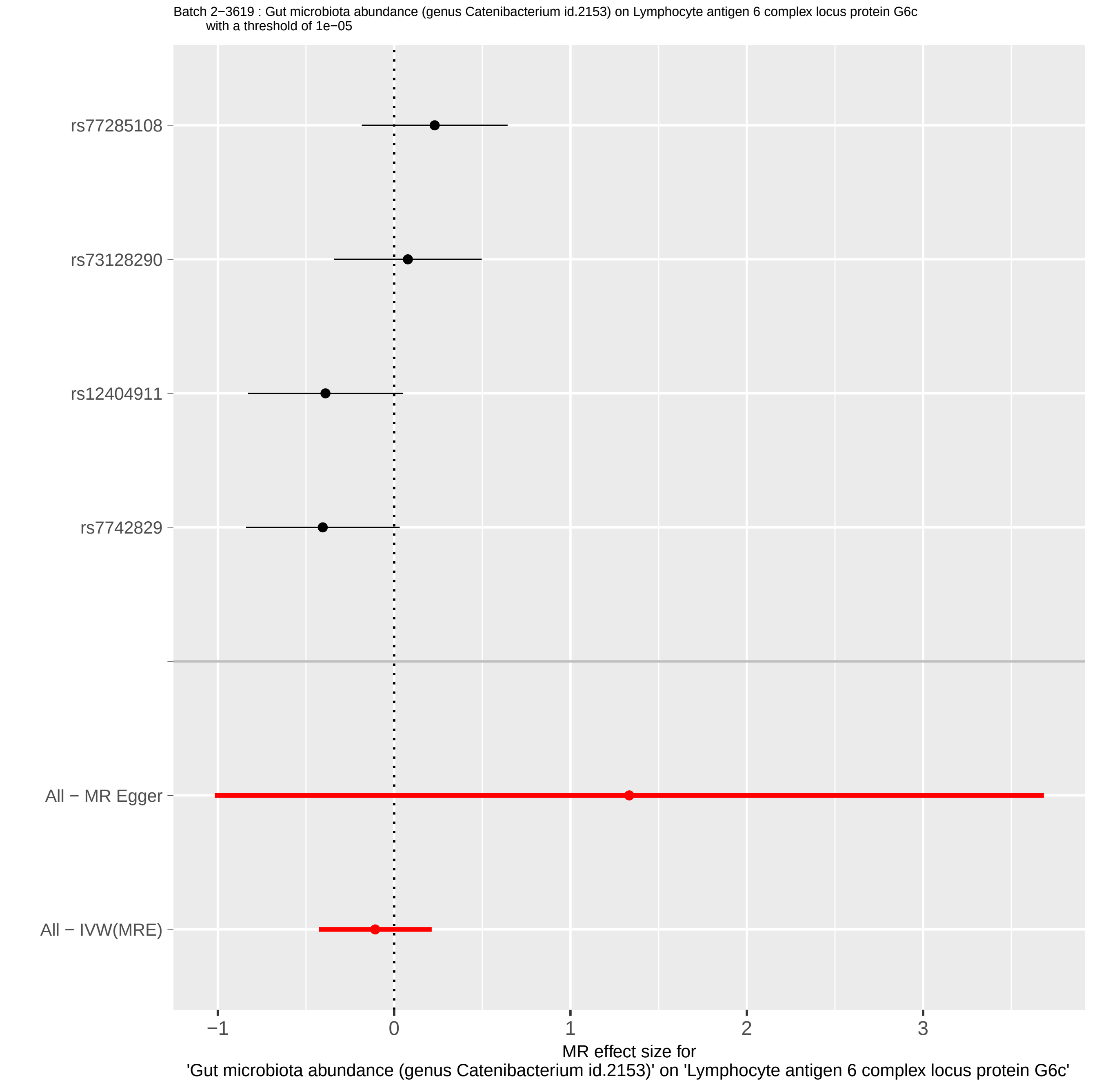

Batch 2−3619 : Gut microbiota abundance (genus Catenibacterium id.2153) on Lymphocyte antigen 6 complex locus protein G6c
with a threshold of 1e−05
rs77285108
rs73128290
rs12404911
rs7742829
All − MR Egger
All − IVW(MRE)
−1
0
1
2
3
MR effect size for
'Gut microbiota abundance (genus Catenibacterium id.2153)' on 'Lymphocyte antigen 6 complex locus protein G6c'

Batch 2−3620 : Gut microbiota abundance (genus Parasutterella id.2892) on Lymphocyte antigen 6 complex locus protein G6c
with a threshold of 1e−05

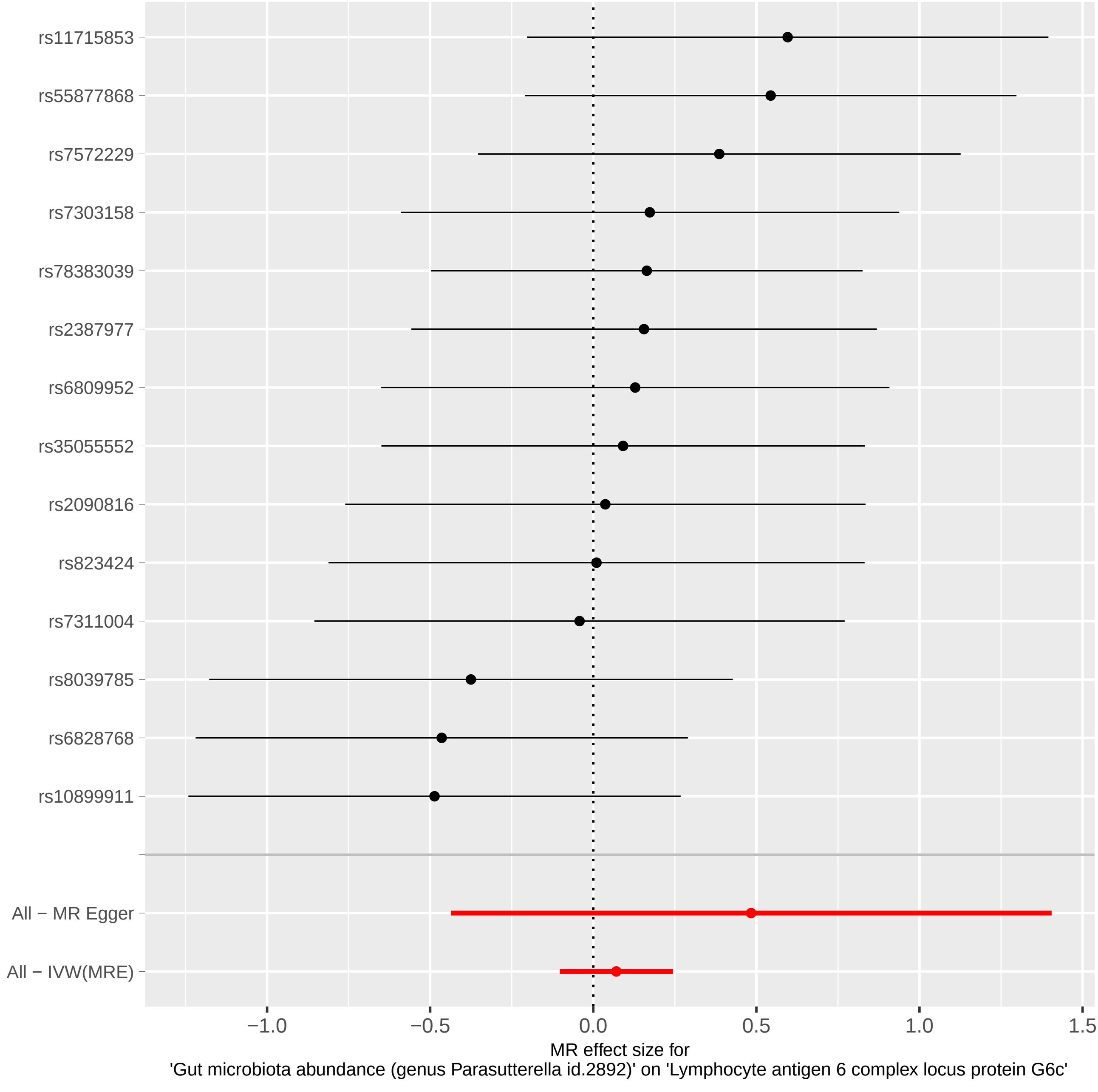

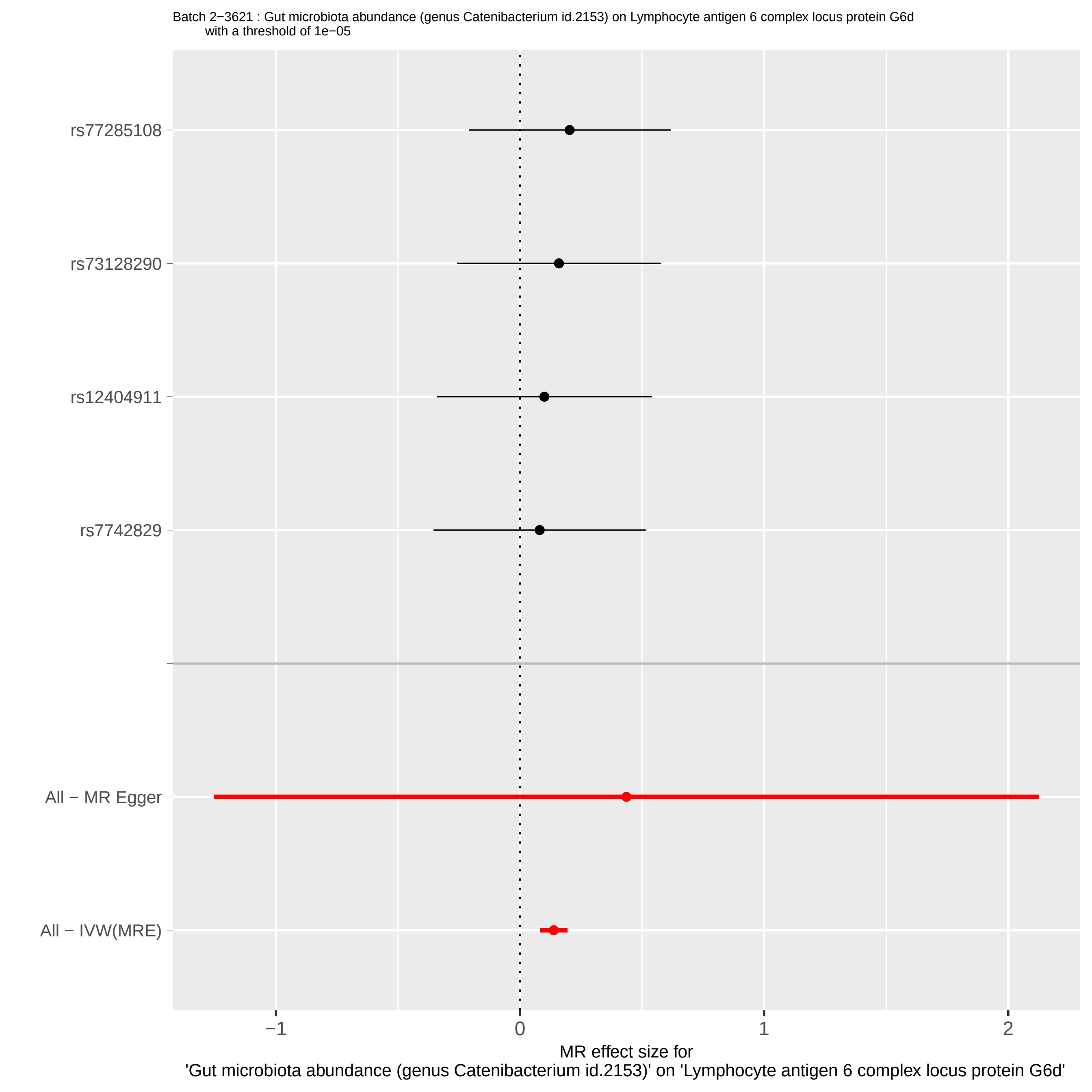
Batch 2−3621 : Gut microbiota abundance (genus Catenibacterium id.2153) on Lymphocyte antigen 6 complex locus protein G6d
with a threshold of 1e−05
rs77285108
rs73128290
rs12404911
rs7742829
All − MR Egger
All − IVW(MRE)
−1
0
1
2
MR effect size for
'Gut microbiota abundance (genus Catenibacterium id.2153)' on 'Lymphocyte antigen 6 complex locus protein G6d'

Batch 2−3622 : Gut microbiota abundance (genus Parasutterella id.2892) on Lymphocyte antigen 6 complex locus protein G6d
with a threshold of 1e−05

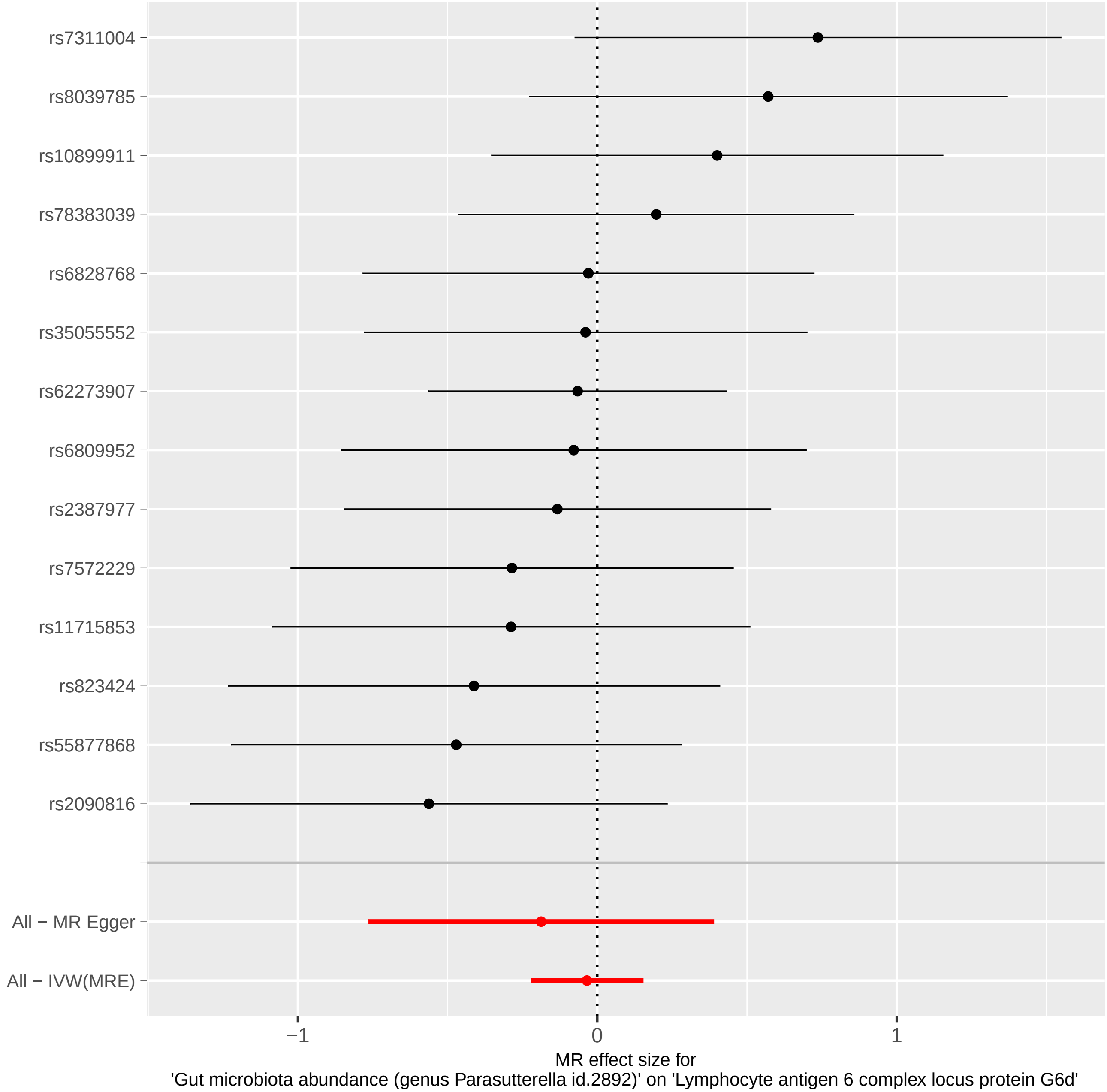

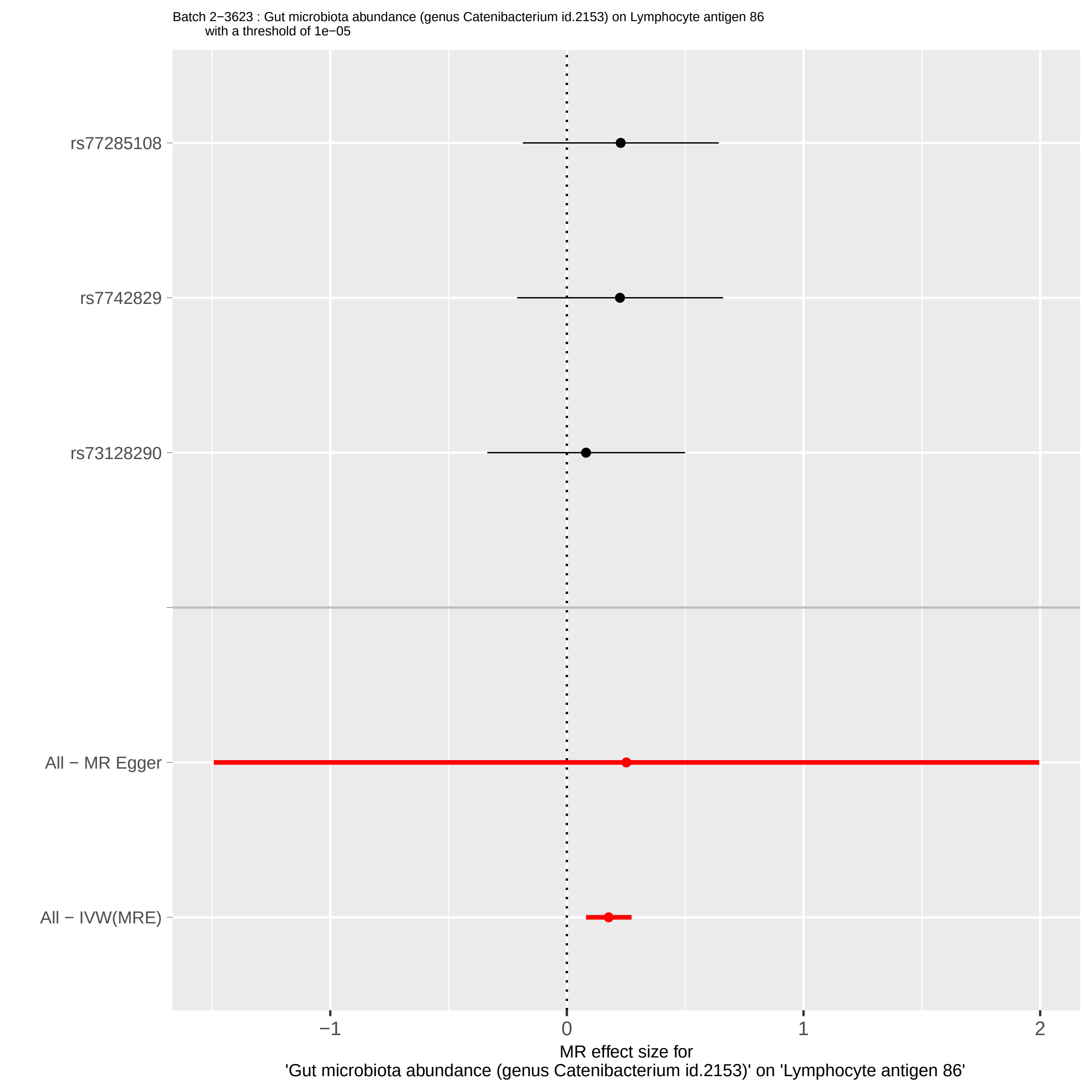
Batch 2−3623 : Gut microbiota abundance (genus Catenibacterium id.2153) on Lymphocyte antigen 86
with a threshold of 1e−05
rs77285108
rs7742829
rs73128290
All − MR Egger
All − IVW(MRE)
−1
0
1
2
MR effect size for
'Gut microbiota abundance (genus Catenibacterium id.2153)' on 'Lymphocyte antigen 86'

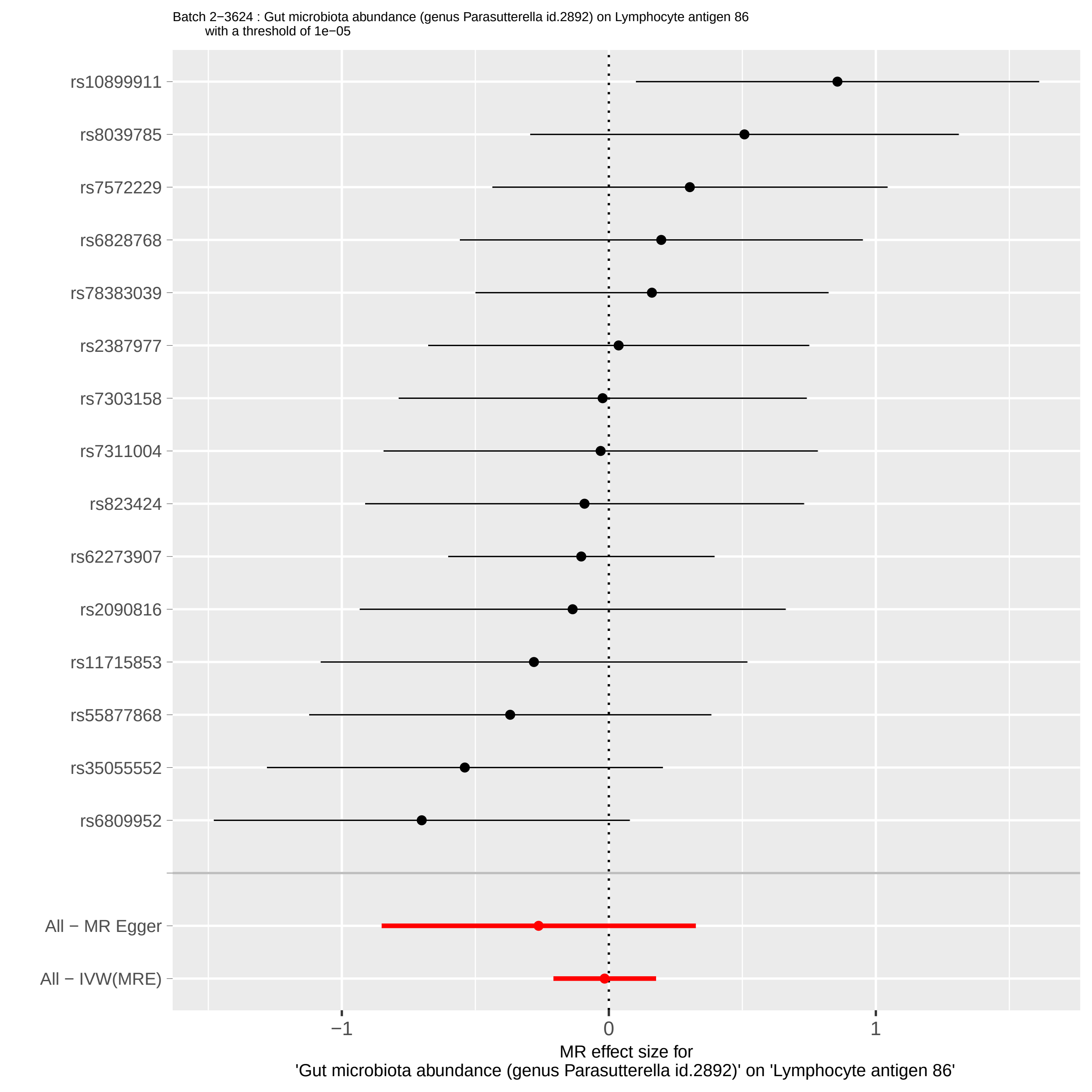
Batch 2−3624 : Gut microbiota abundance (genus Parasutterella id.2892) on Lymphocyte antigen 86
with a threshold of 1e−05
rs10899911
rs8039785
rs7572229
rs6828768
rs78383039
rs2387977
rs7303158
rs7311004
rs823424
rs62273907
rs2090816
rs11715853
rs55877868
rs35055552
rs6809952
All − MR Egger
All − IVW(MRE)
−1
0
1
MR effect size for
'Gut microbiota abundance (genus Parasutterella id.2892)' on 'Lymphocyte antigen 86'

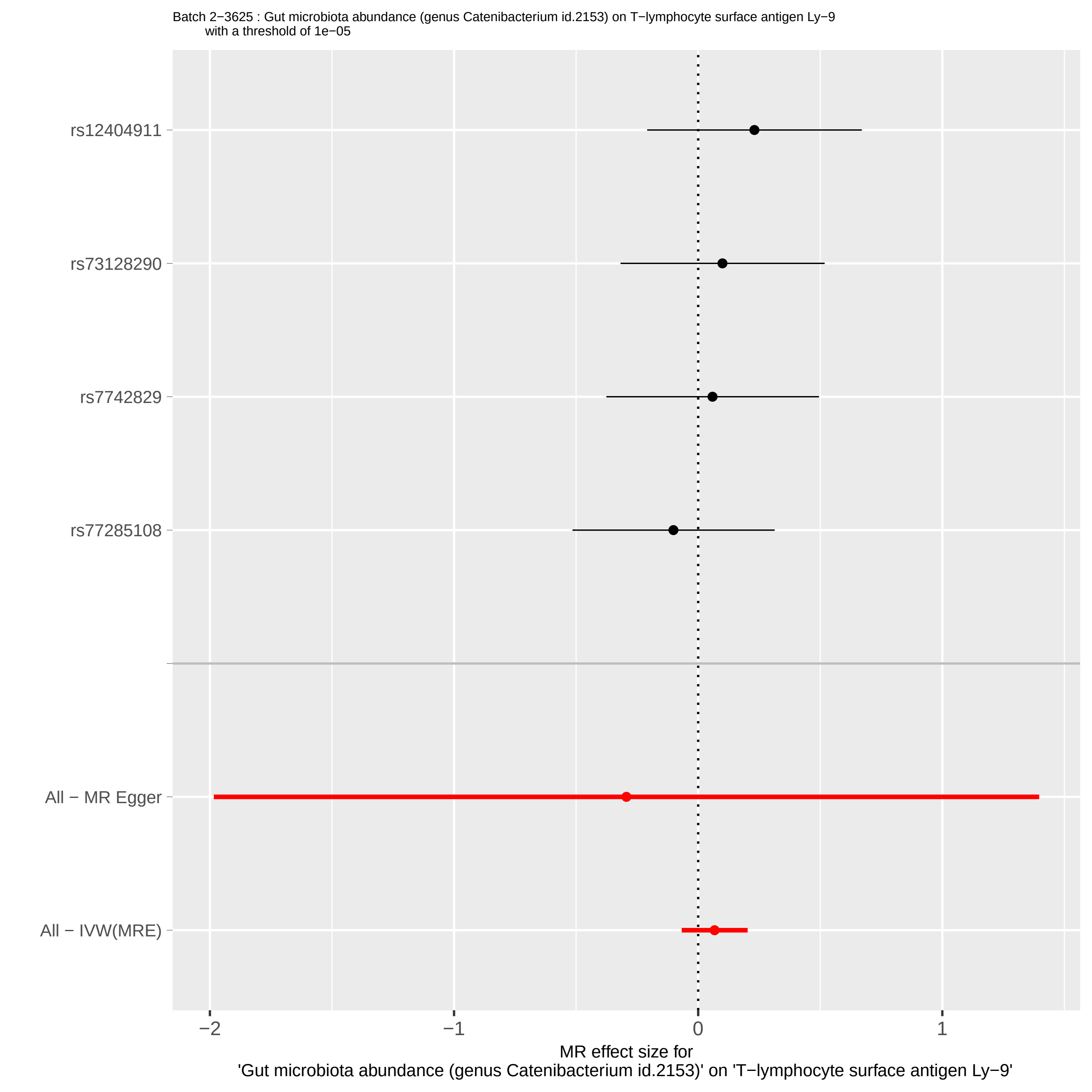
Batch 2−3625 : Gut microbiota abundance (genus Catenibacterium id.2153) on T−lymphocyte surface antigen Ly−9
with a threshold of 1e−05
rs12404911
rs73128290
rs7742829
rs77285108
All − MR Egger
All − IVW(MRE)
−2
−1
0
1
MR effect size for
'Gut microbiota abundance (genus Catenibacterium id.2153)' on 'T−lymphocyte surface antigen Ly−9'

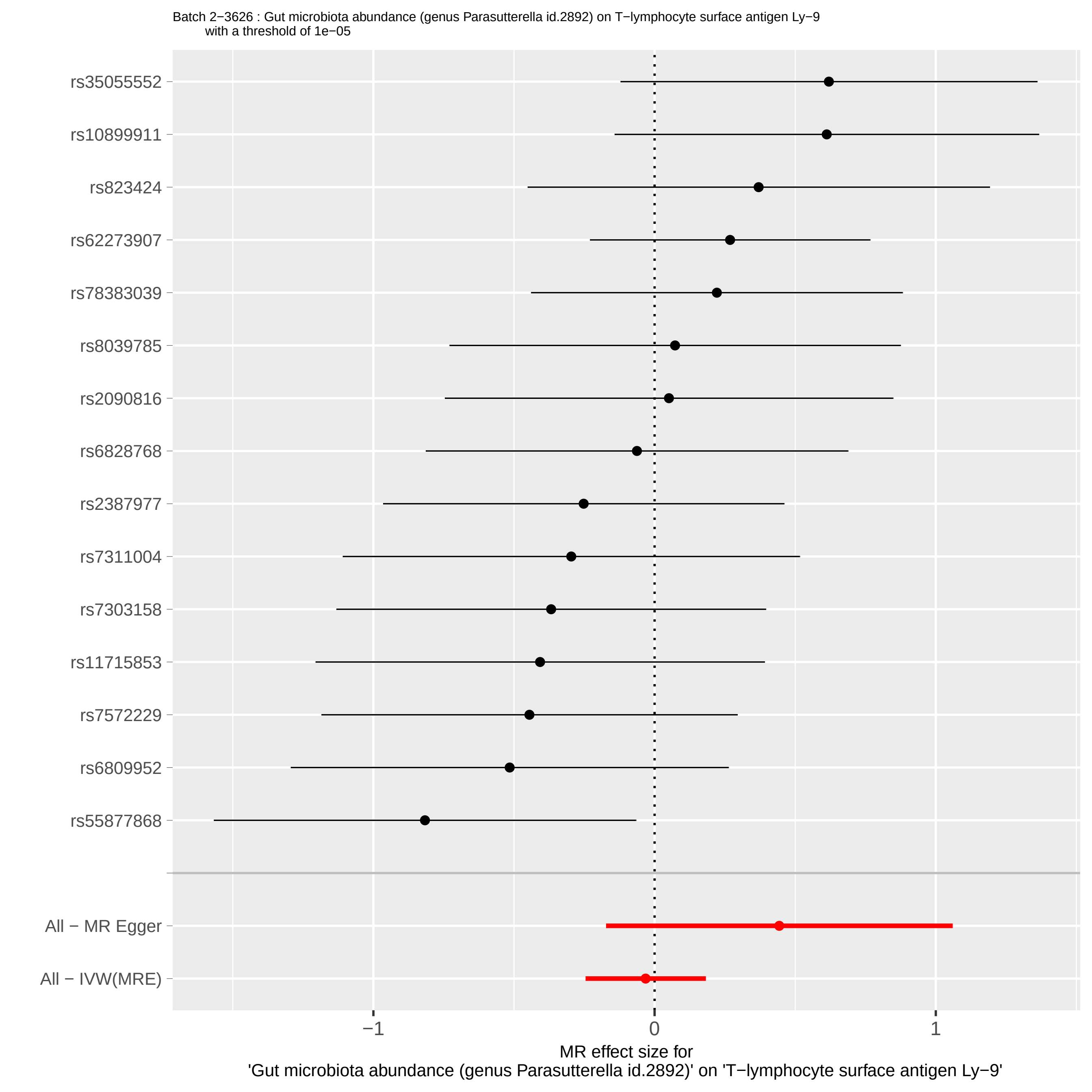
Batch 2−3626 : Gut microbiota abundance (genus Parasutterella id.2892) on T−lymphocyte surface antigen Ly−9
with a threshold of 1e−05
rs35055552
rs10899911
rs823424
rs62273907
rs78383039
rs8039785
rs2090816
rs6828768
rs2387977
rs7311004
rs7303158
rs11715853
rs7572229
rs6809952
rs55877868
All − MR Egger
All − IVW(MRE)
−1
0
1
MR effect size for
'Gut microbiota abundance (genus Parasutterella id.2892)' on 'T−lymphocyte surface antigen Ly−9'

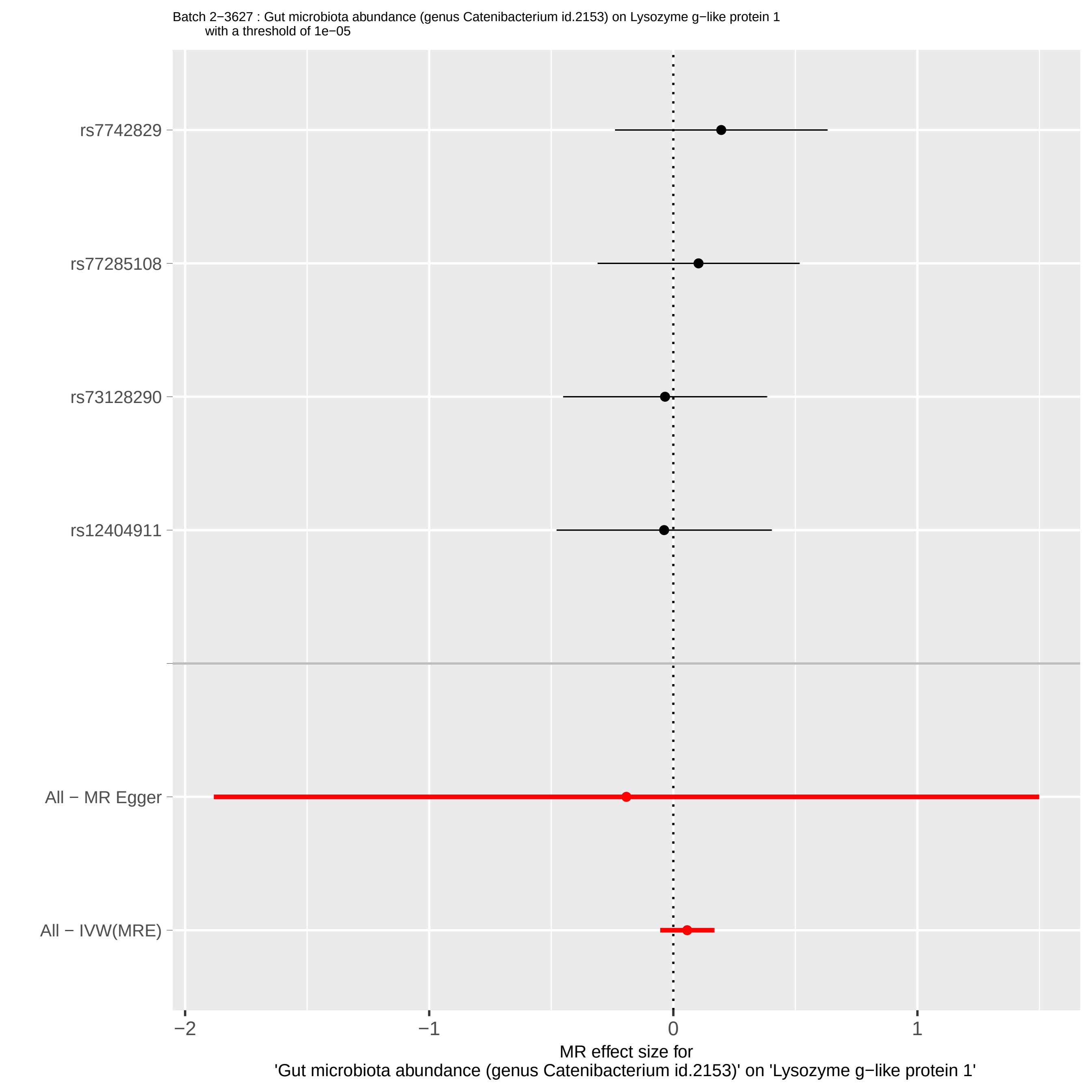

Batch 2−3627 : Gut microbiota abundance (genus Catenibacterium id.2153) on Lysozyme g−like protein 1
with a threshold of 1e−05
rs7742829
rs77285108
rs73128290
rs12404911
All − MR Egger
All − IVW(MRE)
−2
−1
0
1
MR effect size for
'Gut microbiota abundance (genus Catenibacterium id.2153)' on 'Lysozyme g−like protein 1'

Batch 2−3628 : Gut microbiota abundance (genus Parasutterella id.2892) on Lysozyme g−like protein 1
with a threshold of 1e−05

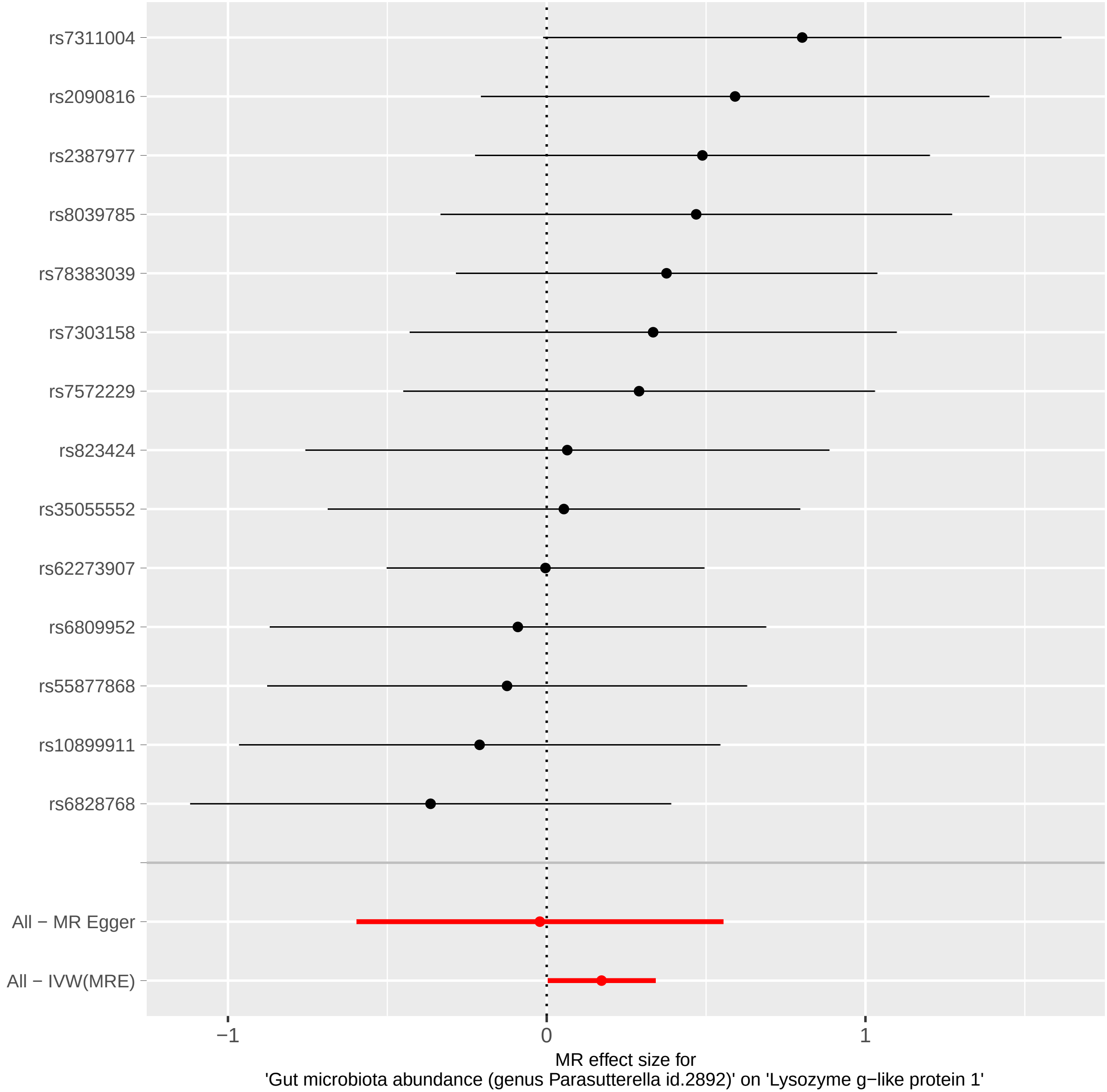

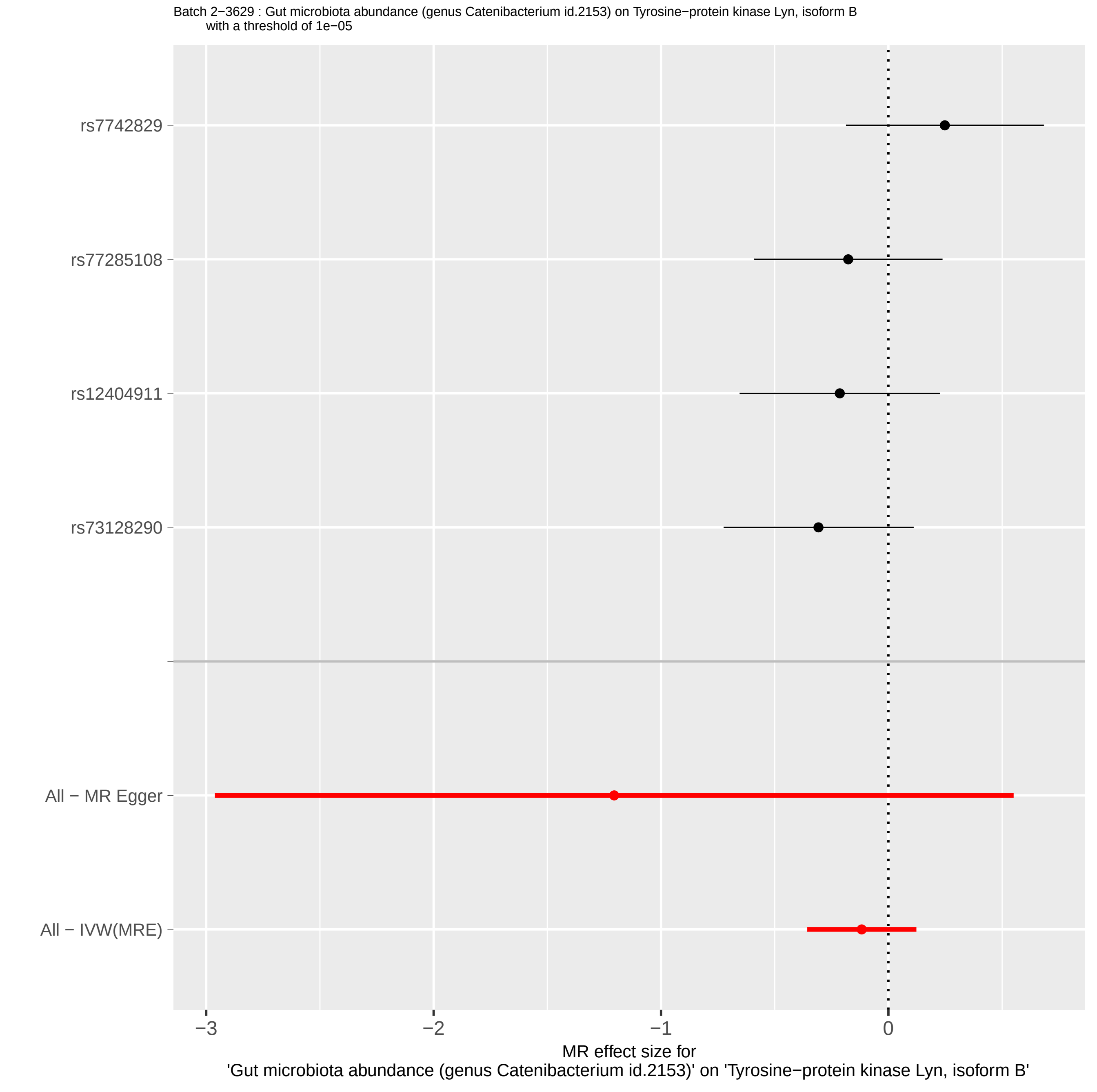

Batch 2−3629 : Gut microbiota abundance (genus Catenibacterium id.2153) on Tyrosine−protein kinase Lyn, isoform B
with a threshold of 1e−05
rs7742829
rs77285108
rs12404911
rs73128290
All − MR Egger
All − IVW(MRE)
−3
−2
−1
0
MR effect size for
'Gut microbiota abundance (genus Catenibacterium id.2153)' on 'Tyrosine−protein kinase Lyn, isoform B'

Batch 2−3630 : Gut microbiota abundance (genus Parasutterella id.2892) on Tyrosine−protein kinase Lyn, isoform B
with a threshold of 1e−05

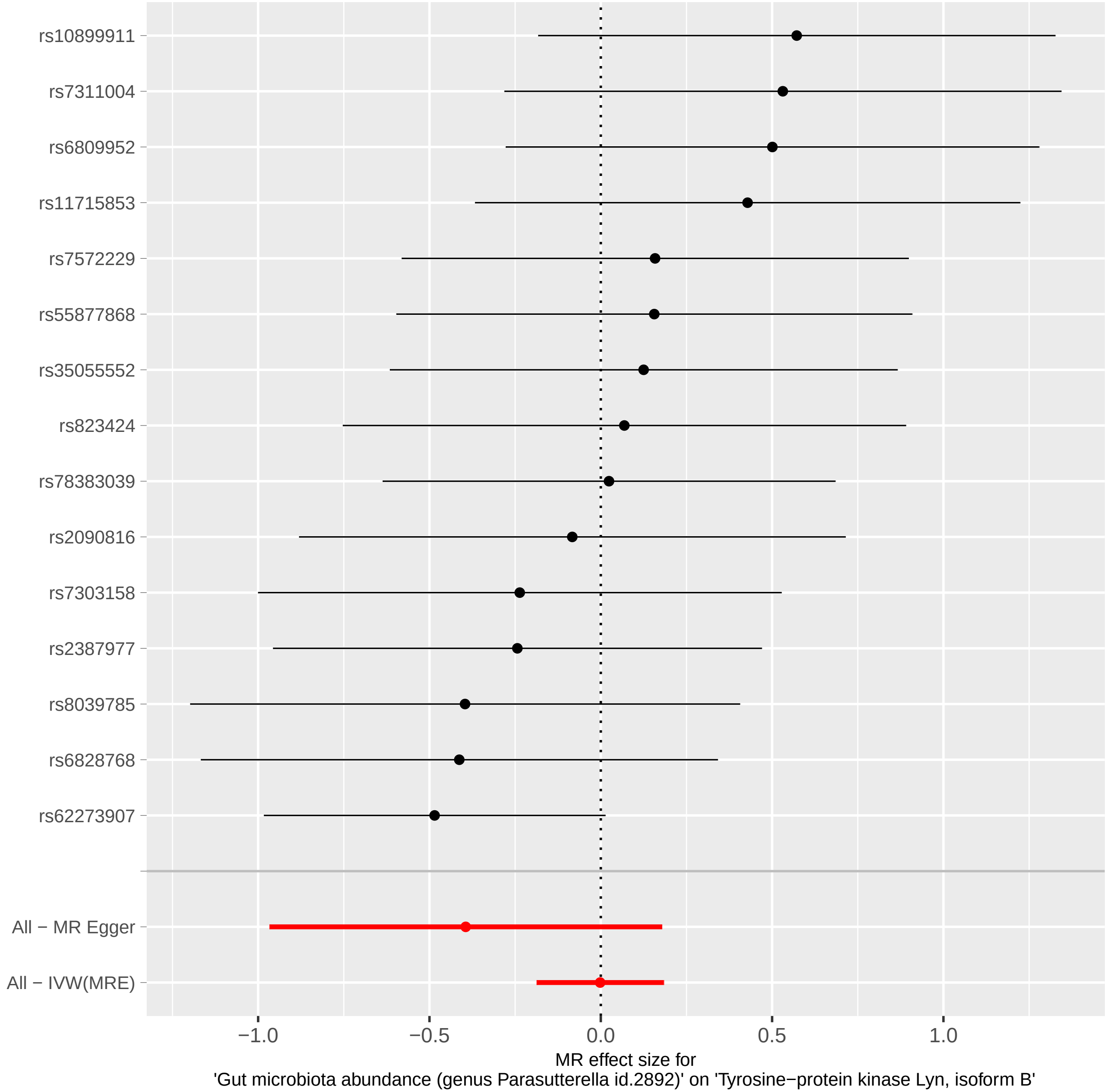

Batch 2−3631 : Gut microbiota abundance (genus Catenibacterium id.2153) on Tyrosine−protein kinase Lyn
with a threshold of 1e−05

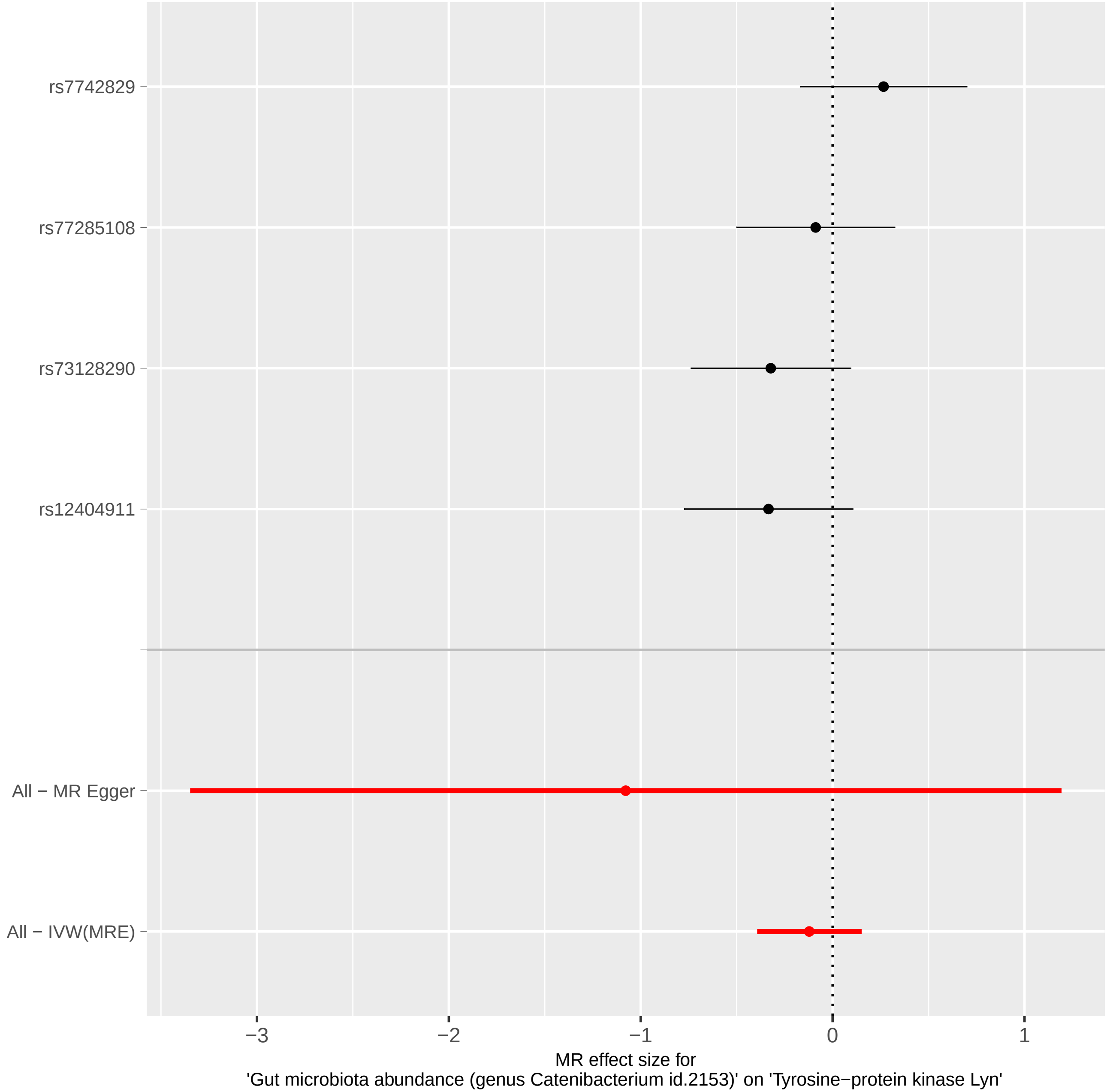

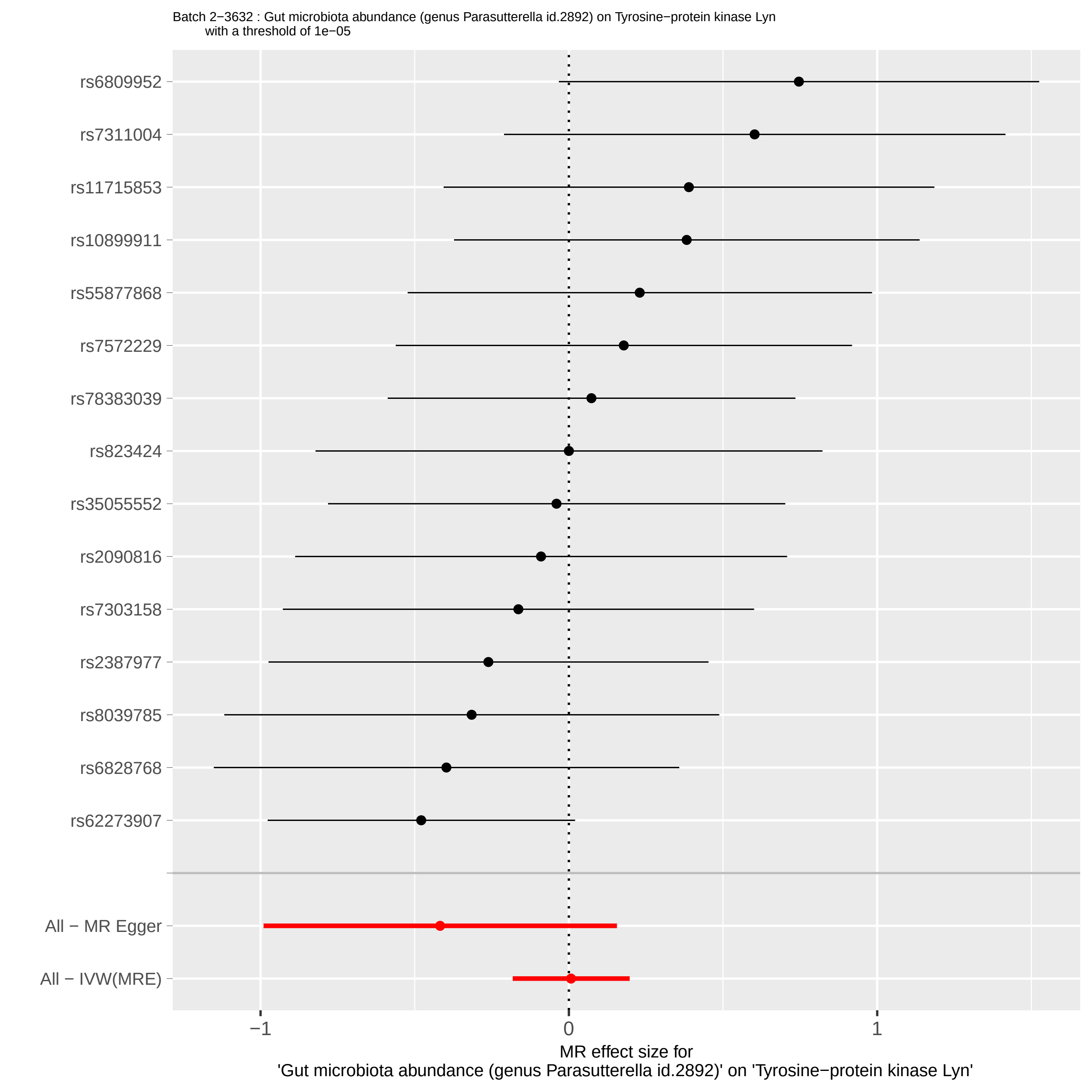
Batch 2−3632 : Gut microbiota abundance (genus Parasutterella id.2892) on Tyrosine−protein kinase Lyn
with a threshold of 1e−05
rs6809952
rs7311004
rs11715853
rs10899911
rs55877868
rs7572229
rs78383039
rs823424
rs35055552
rs2090816
rs7303158
rs2387977
rs8039785
rs6828768
rs62273907
All − MR Egger
All − IVW(MRE)
−1
0
1
MR effect size for
'Gut microbiota abundance (genus Parasutterella id.2892)' on 'Tyrosine−protein kinase Lyn'

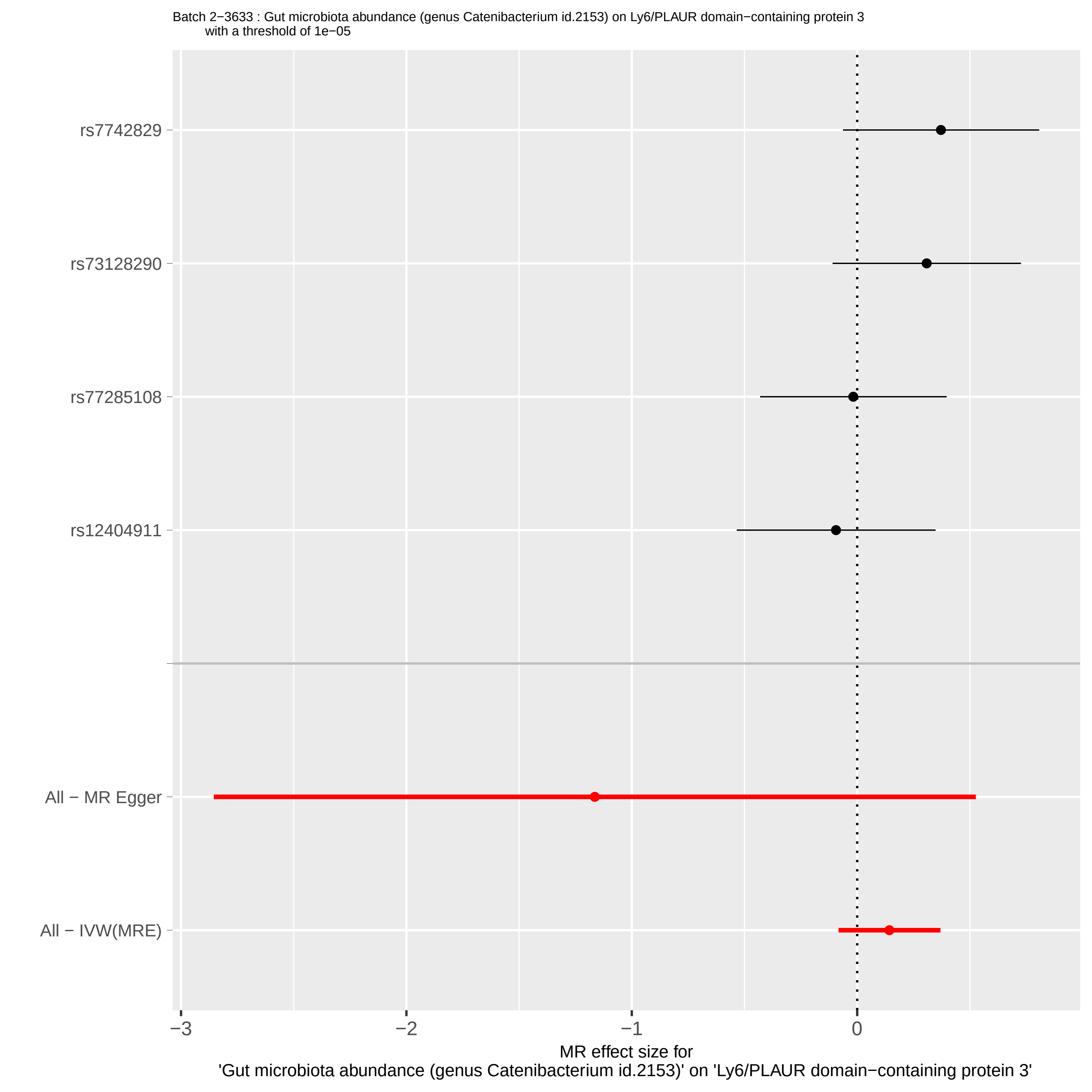

Batch 2−3633 : Gut microbiota abundance (genus Catenibacterium id.2153) on Ly6/PLAUR domain−containing protein 3
with a threshold of 1e−05
rs7742829
rs73128290
rs77285108
rs12404911
All − MR Egger
All − IVW(MRE)
−3
−2
−1
0
MR effect size for
'Gut microbiota abundance (genus Catenibacterium id.2153)' on 'Ly6/PLAUR domain−containing protein 3'

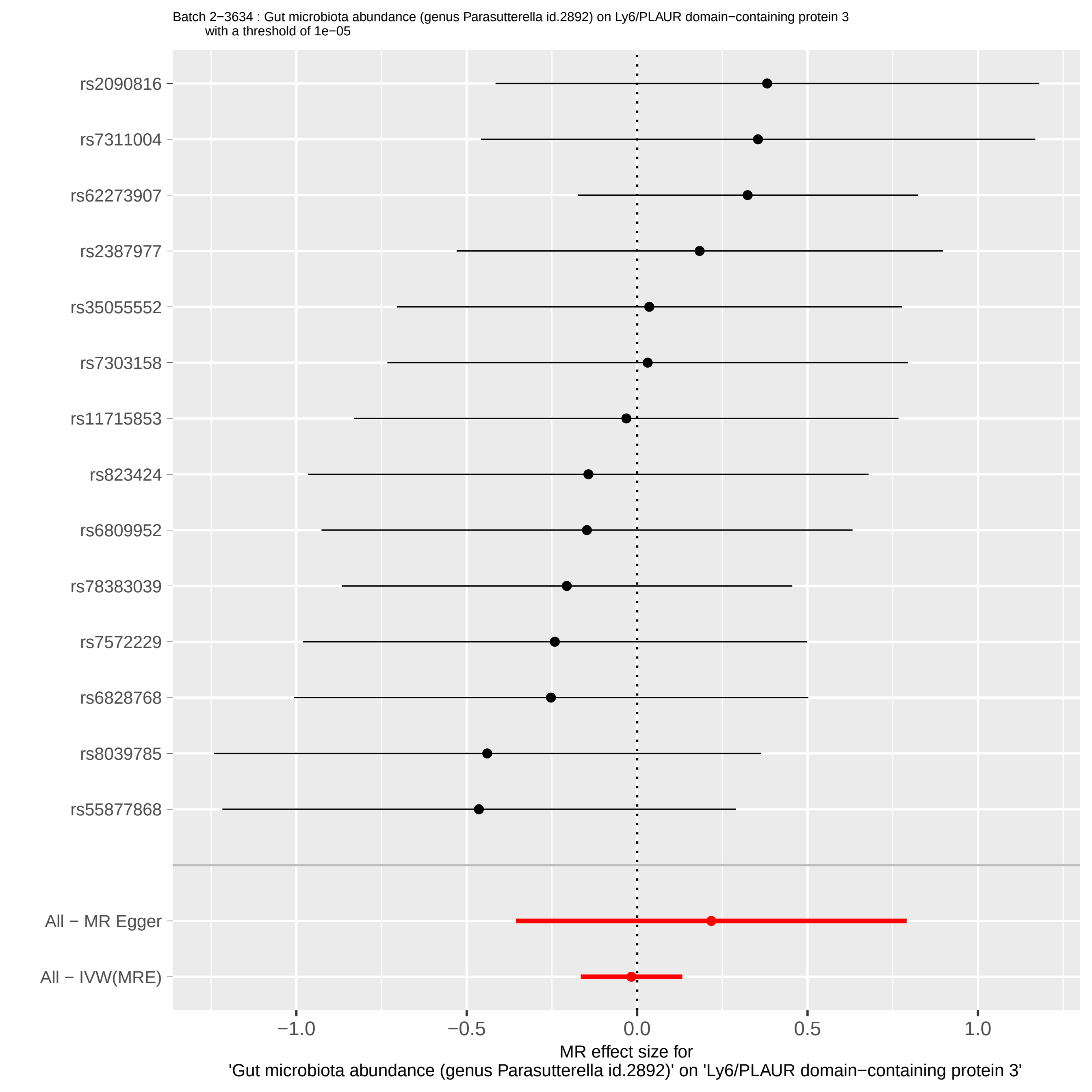
Batch 2−3634 : Gut microbiota abundance (genus Parasutterella id.2892) on Ly6/PLAUR domain−containing protein 3
with a threshold of 1e−05
rs2090816
rs7311004
rs62273907
rs2387977
rs35055552
rs7303158
rs11715853
rs823424
rs6809952
rs78383039
rs7572229
rs6828768
rs8039785
rs55877868
All − MR Egger
All − IVW(MRE)
−1.0
−0.5
0.0
0.5
1.0
MR effect size for
'Gut microbiota abundance (genus Parasutterella id.2892)' on 'Ly6/PLAUR domain−containing protein 3'

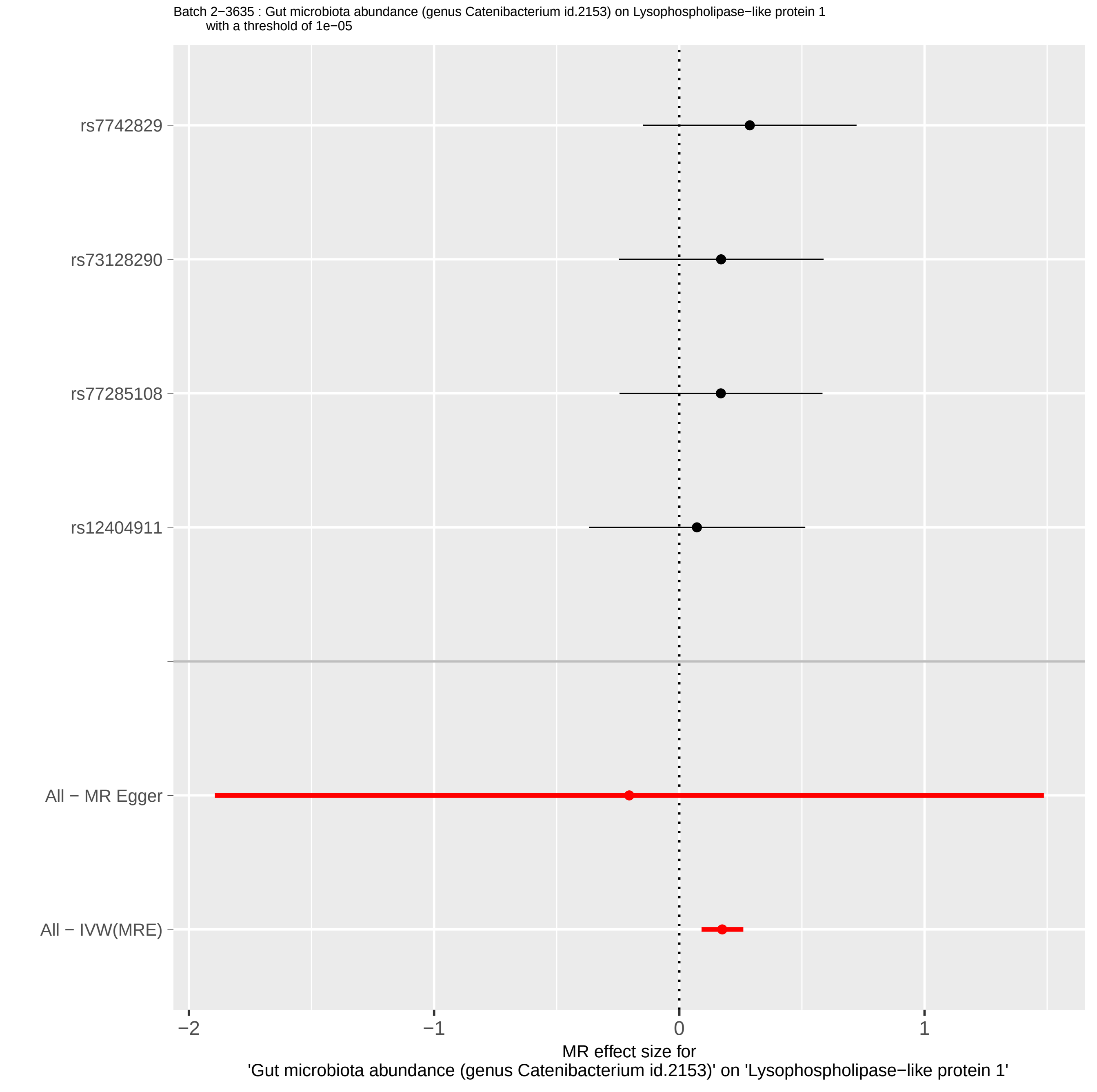

Batch 2−3635 : Gut microbiota abundance (genus Catenibacterium id.2153) on Lysophospholipase−like protein 1
with a threshold of 1e−05
rs7742829
rs73128290
rs77285108
rs12404911
All − MR Egger
All − IVW(MRE)
−2
−1
0
1
MR effect size for
'Gut microbiota abundance (genus Catenibacterium id.2153)' on 'Lysophospholipase−like protein 1'

Batch 2−3636 : Gut microbiota abundance (genus Parasutterella id.2892) on Lysophospholipase−like protein 1
with a threshold of 1e−05

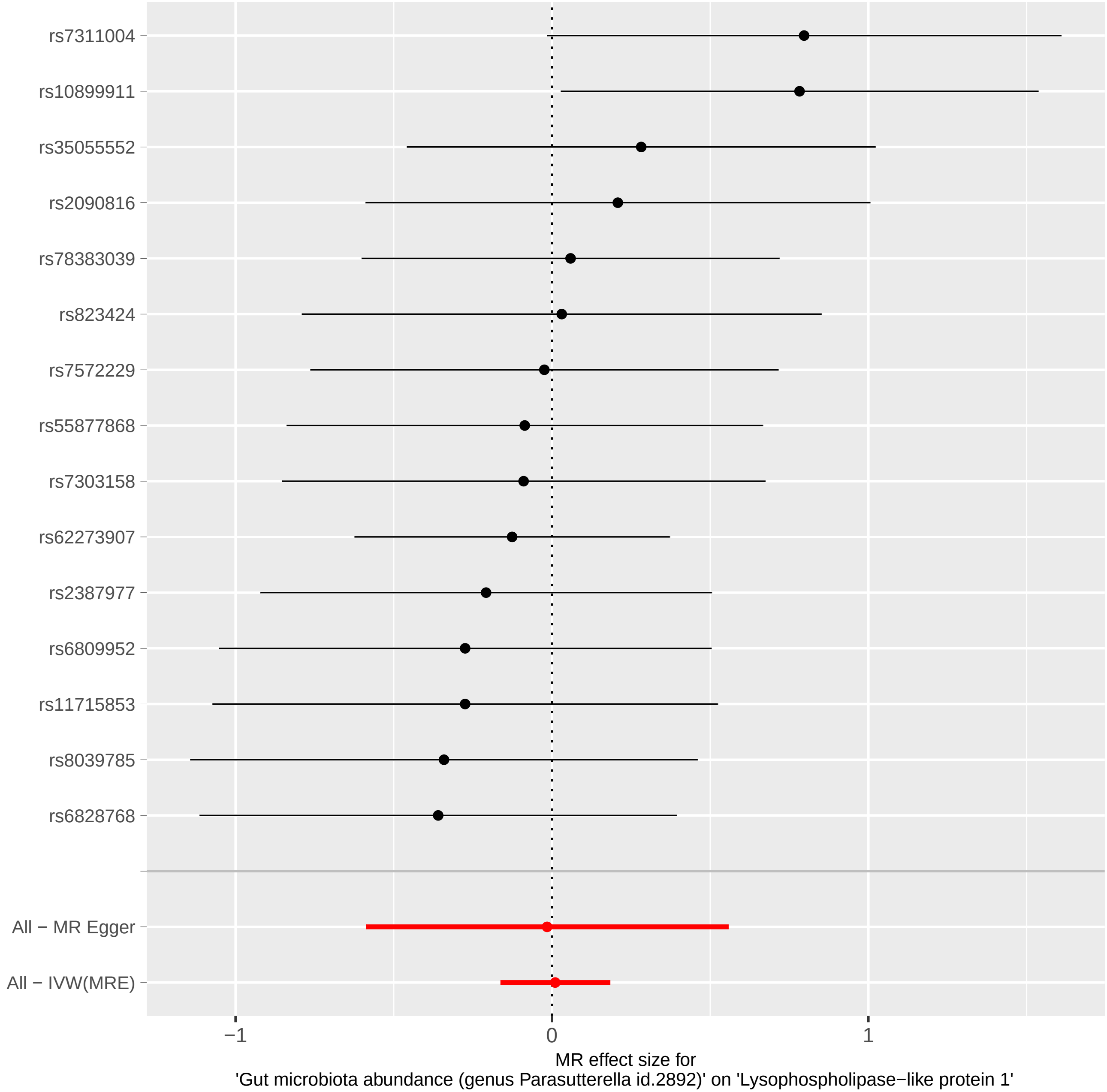

Batch 2−3637 : Gut microbiota abundance (genus Catenibacterium id.2153) on LysM and putative peptidoglycan−binding domain−containing protein 3
with a threshold of 1e−05

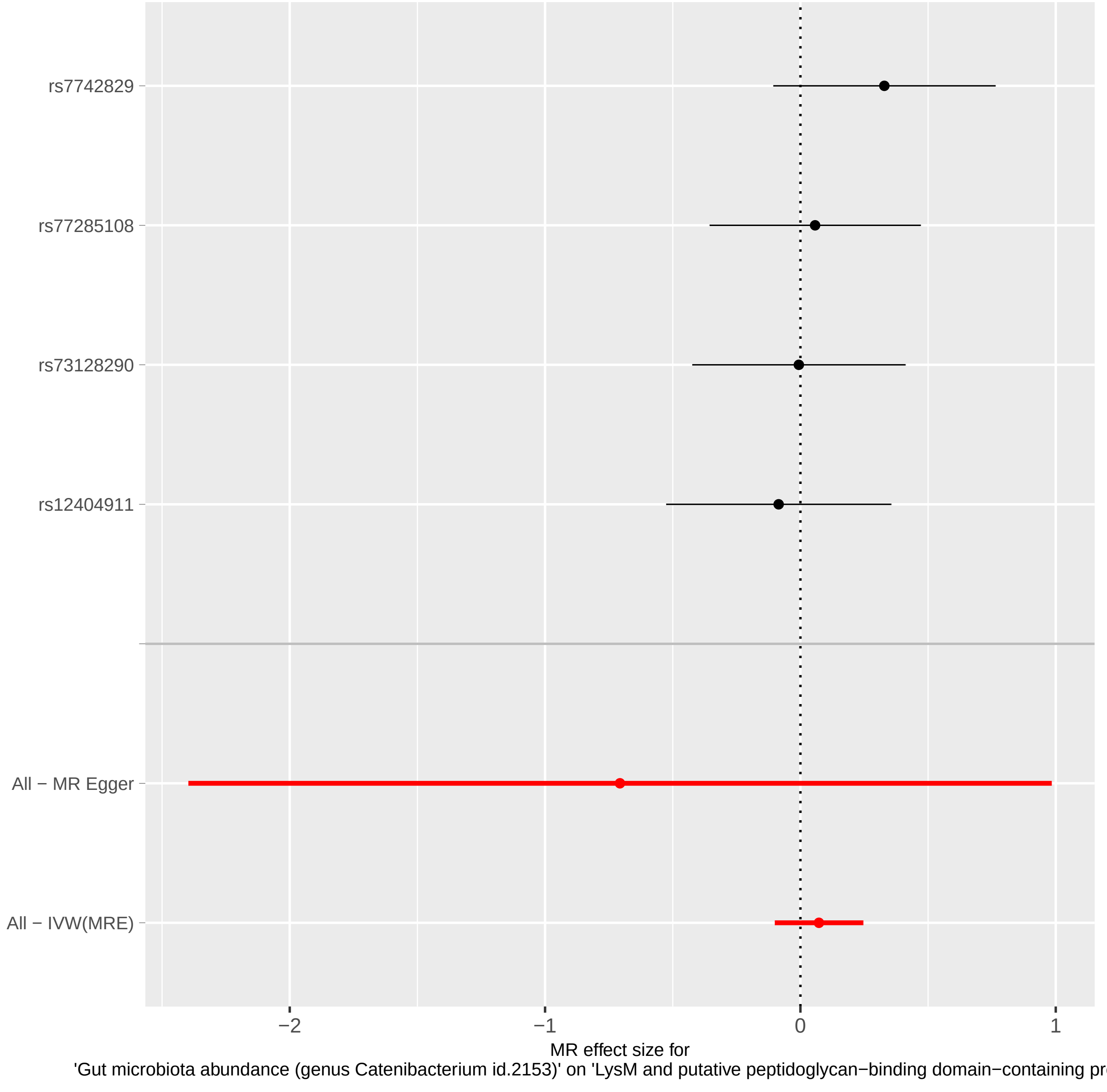

Batch 2−3638 : Gut microbiota abundance (genus Parasutterella id.2892) on LysM and putative peptidoglycan−binding domain−containing protein 3 with a threshold of 1e−05

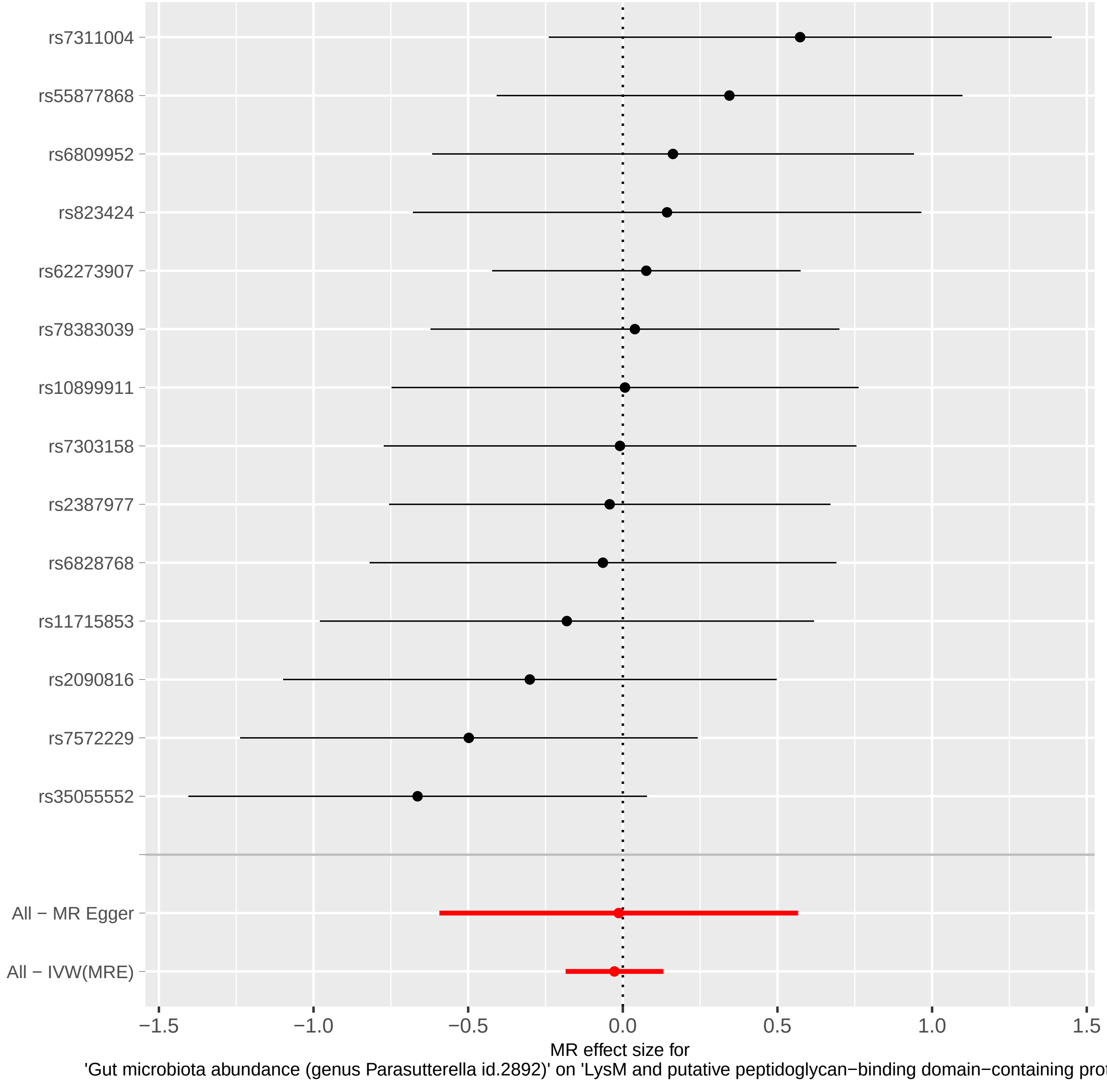

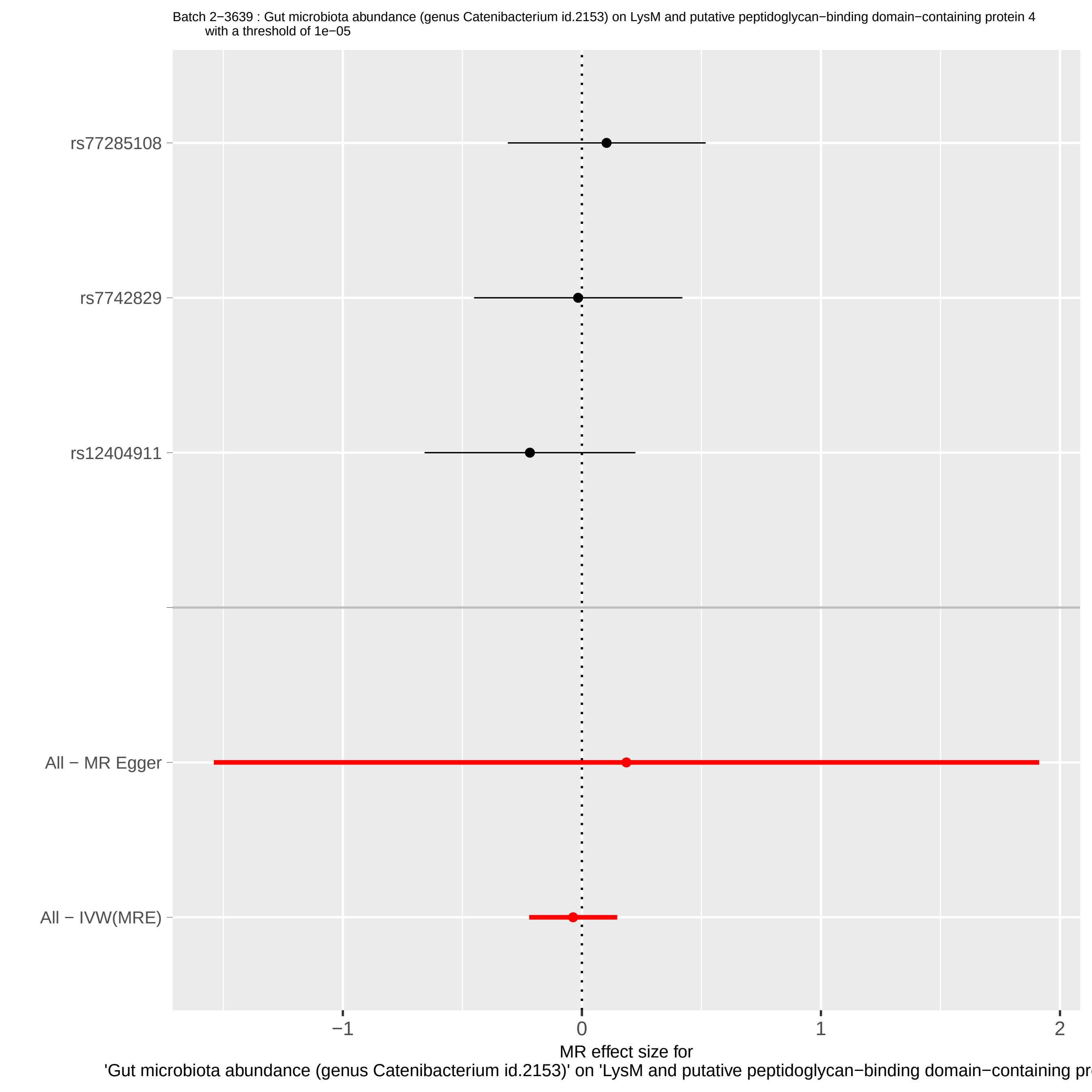
Batch 2−3639 : Gut microbiota abundance (genus Catenibacterium id.2153) on LysM and putative peptidoglycan−binding domain−containing protein 4
with a threshold of 1e−05
rs77285108
rs7742829
rs12404911
All − MR Egger
All − IVW(MRE)
−1
0
1
2
MR effect size for
'Gut microbiota abundance (genus Catenibacterium id.2153)' on 'LysM and putative peptidoglycan−binding domain−containing pr

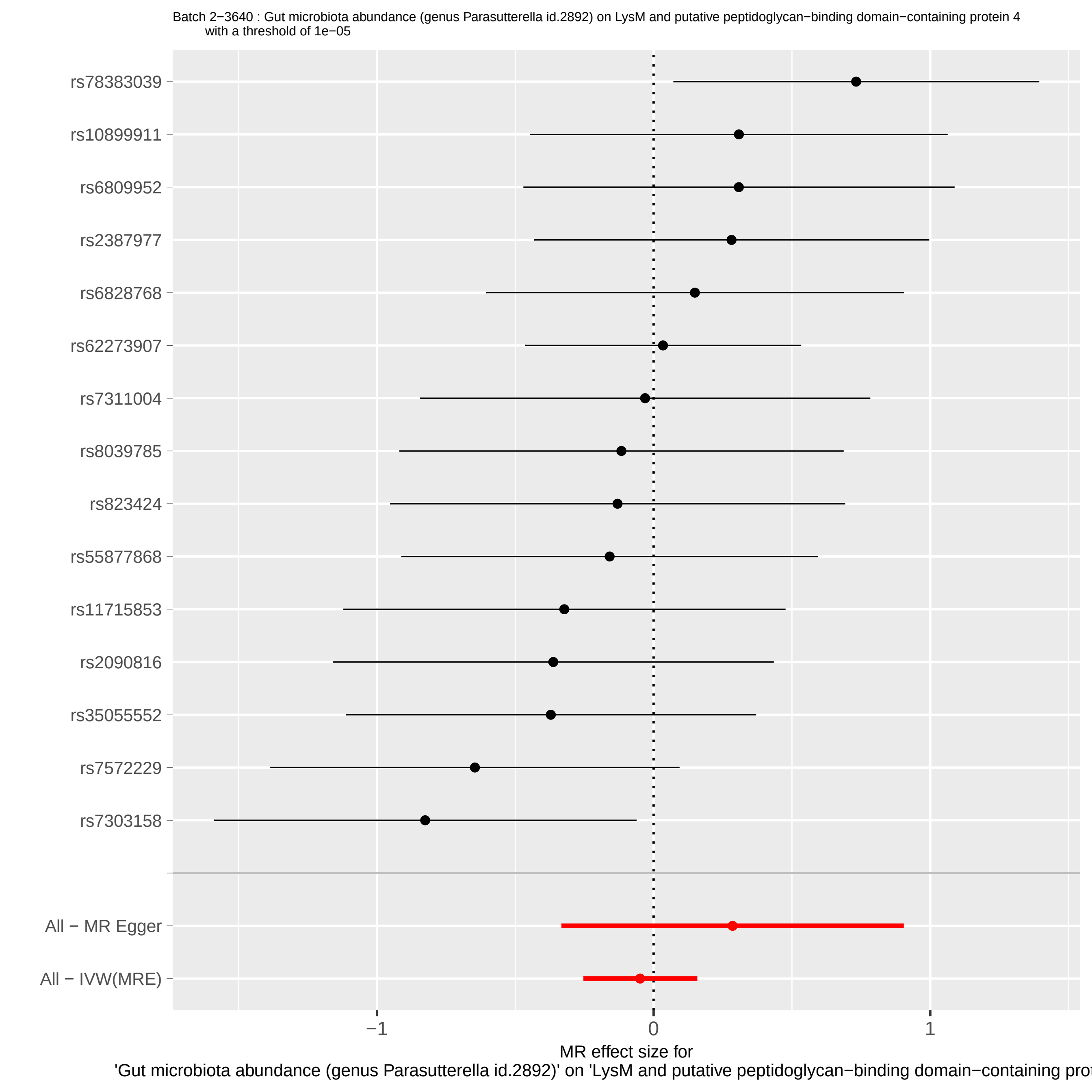

Batch 2−3640 : Gut microbiota abundance (genus Parasutterella id.2892) on LysM and putative peptidoglycan−binding domain−containing protein 4
with a threshold of 1e−05
rs78383039
rs10899911
rs6809952
rs2387977
rs6828768
rs62273907
rs7311004
rs8039785
rs823424
rs55877868
rs11715853
rs2090816
rs35055552
rs7572229
rs7303158
All − MR Egger
All − IVW(MRE)
−1
0
1
MR effect size for
'Gut microbiota abundance (genus Parasutterella id.2892)' on 'LysM and putative peptidoglycan−binding domain−containing pro

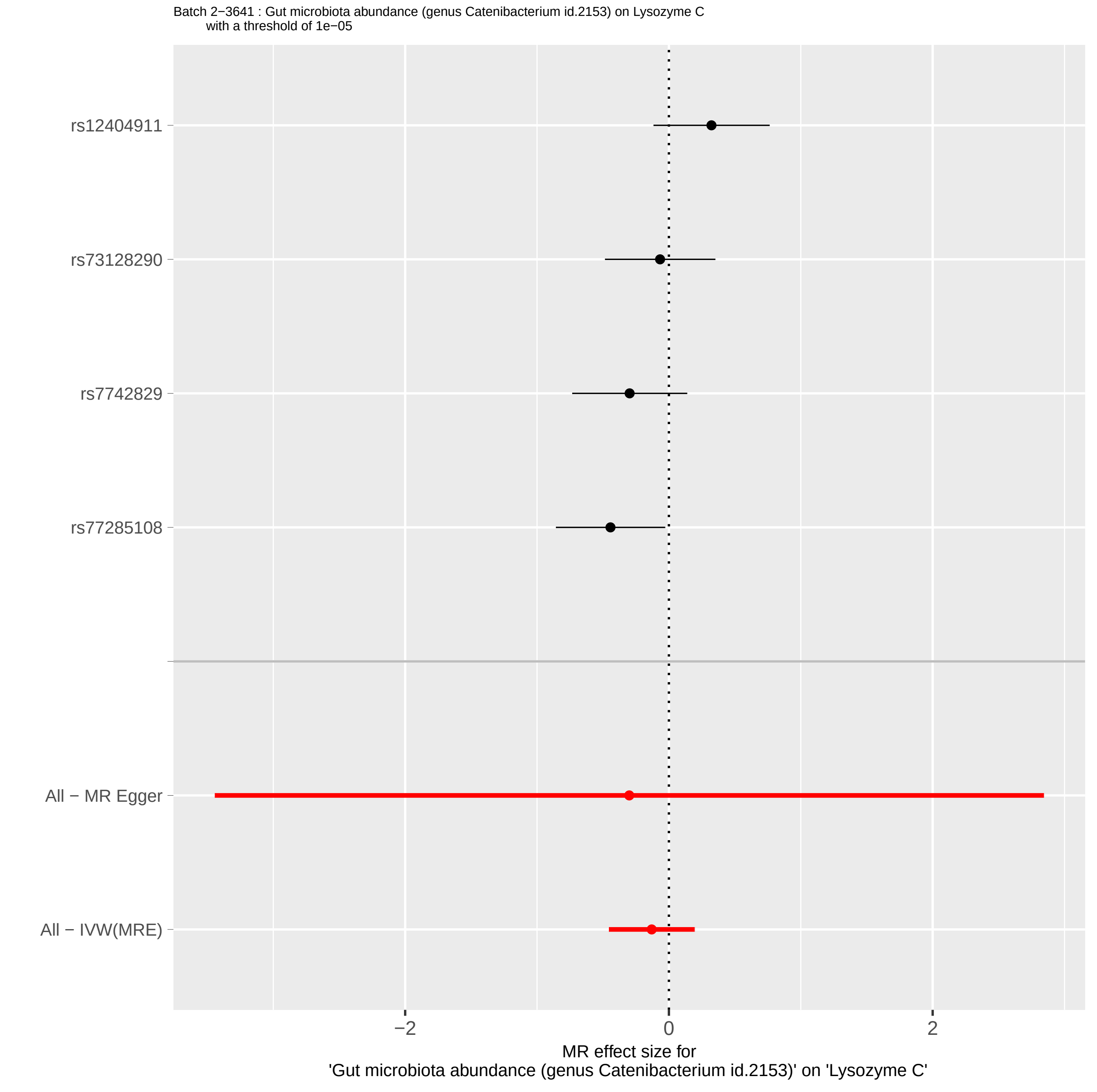

Batch 2−3641 : Gut microbiota abundance (genus Catenibacterium id.2153) on Lysozyme C
with a threshold of 1e−05
rs12404911
rs73128290
rs7742829
rs77285108
All − MR Egger
All − IVW(MRE)
−2
0
2
MR effect size for
'Gut microbiota abundance (genus Catenibacterium id.2153)' on 'Lysozyme C'

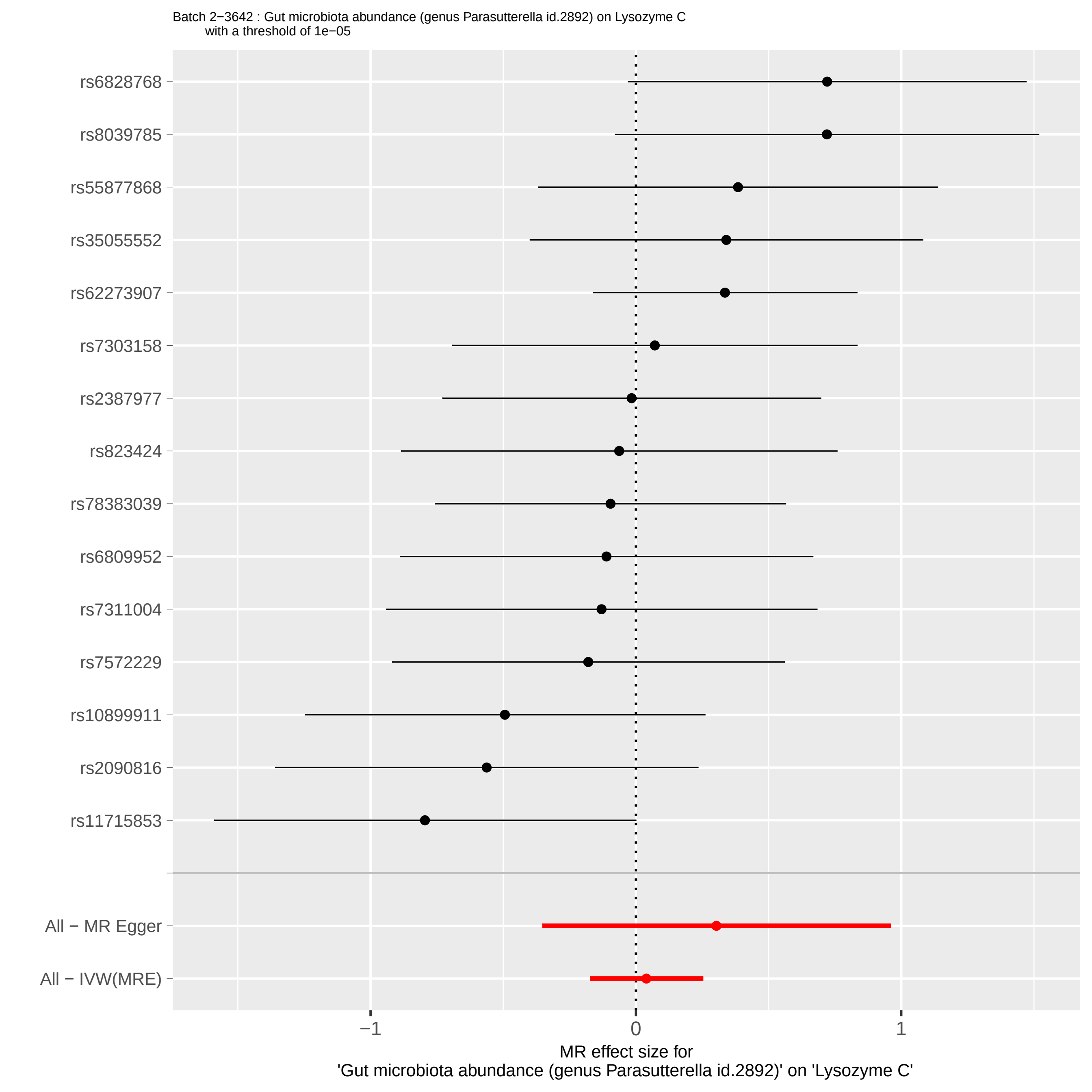
Batch 2−3642 : Gut microbiota abundance (genus Parasutterella id.2892) on Lysozyme C
with a threshold of 1e−05
rs6828768
rs8039785
rs55877868
rs35055552
rs62273907
rs7303158
rs2387977
rs823424
rs78383039
rs6809952
rs7311004
rs7572229
rs10899911
rs2090816
rs11715853
All − MR Egger
All − IVW(MRE)
−1
0
1
MR effect size for
'Gut microbiota abundance (genus Parasutterella id.2892)' on 'Lysozyme C'

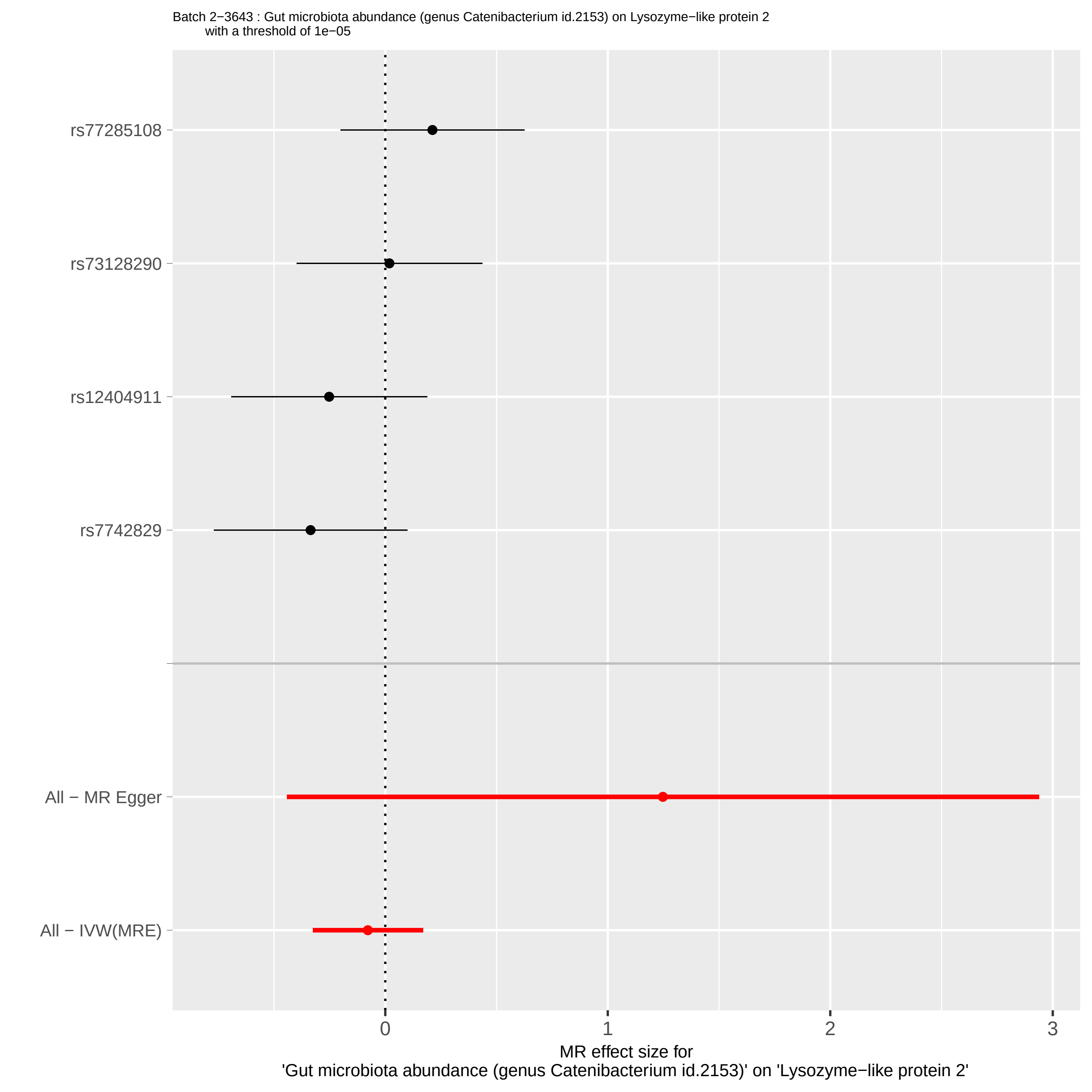

Batch 2−3643 : Gut microbiota abundance (genus Catenibacterium id.2153) on Lysozyme−like protein 2
with a threshold of 1e−05
rs77285108
rs73128290
rs12404911
rs7742829
All − MR Egger
All − IVW(MRE)
0
1
2
3
MR effect size for
'Gut microbiota abundance (genus Catenibacterium id.2153)' on 'Lysozyme−like protein 2'

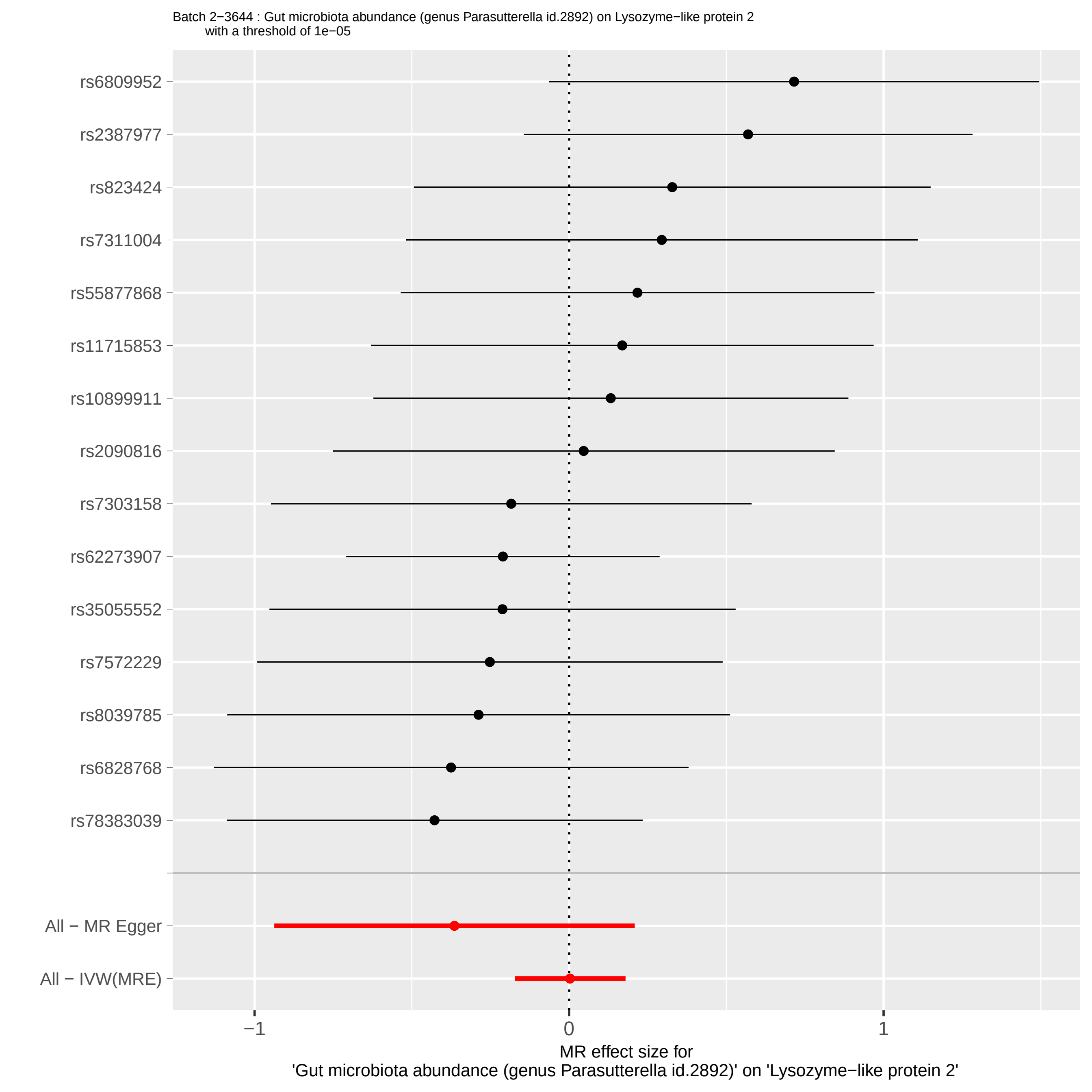

Batch 2−3644 : Gut microbiota abundance (genus Parasutterella id.2892) on Lysozyme−like protein 2
with a threshold of 1e−05
rs6809952
rs2387977
rs823424
rs7311004
rs55877868
rs11715853
rs10899911
rs2090816
rs7303158
rs62273907
rs35055552
rs7572229
rs8039785
rs6828768
rs78383039
All − MR Egger
All − IVW(MRE)
−1
0
1
MR effect size for
'Gut microbiota abundance (genus Parasutterella id.2892)' on 'Lysozyme−like protein 2'

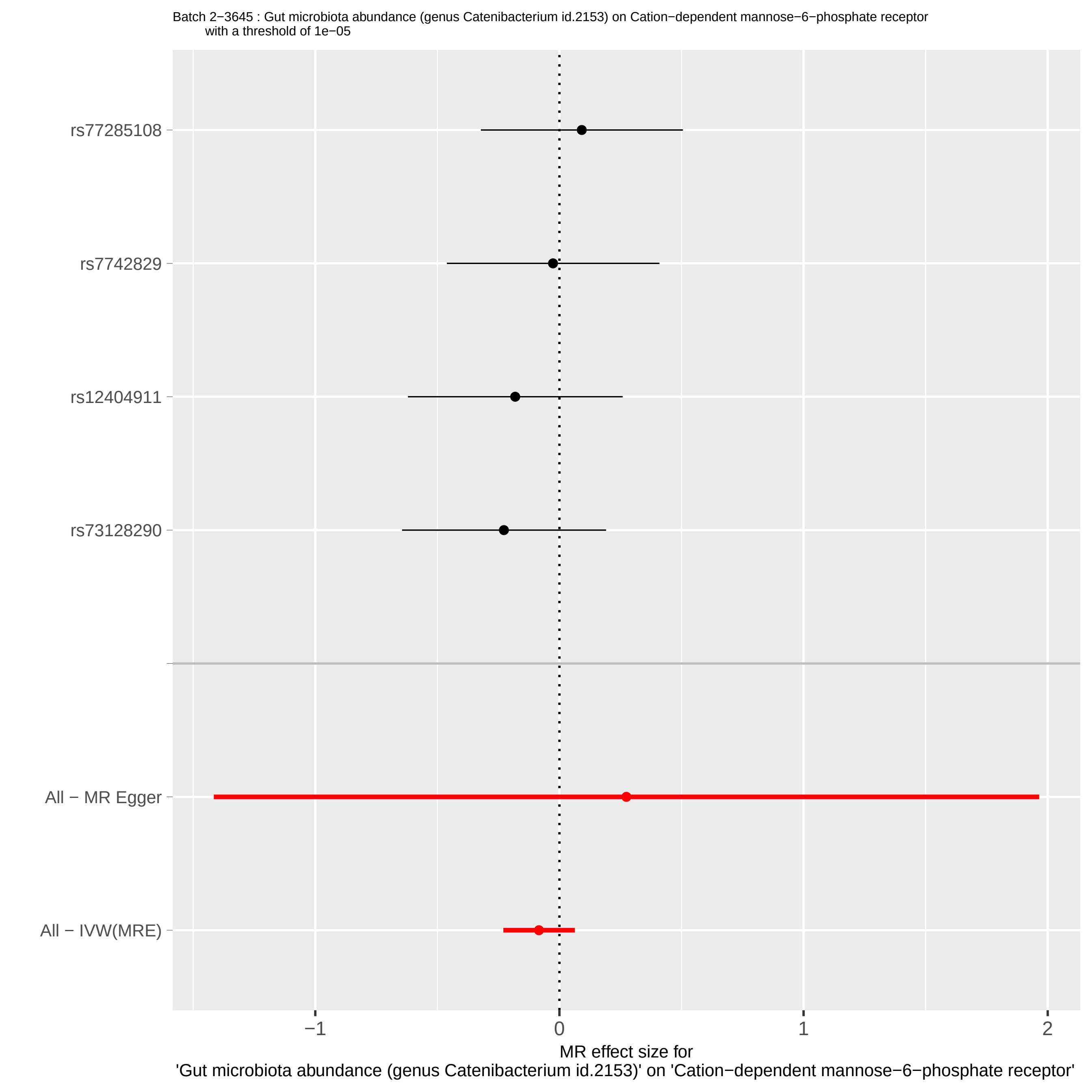
Batch 2−3645 : Gut microbiota abundance (genus Catenibacterium id.2153) on Cation−dependent mannose−6−phosphate receptor
with a threshold of 1e−05
rs77285108
rs7742829
rs12404911
rs73128290
All − MR Egger
All − IVW(MRE)
−1
0
1
2
MR effect size for
'Gut microbiota abundance (genus Catenibacterium id.2153)' on 'Cation−dependent mannose−6−phosphate receptor'

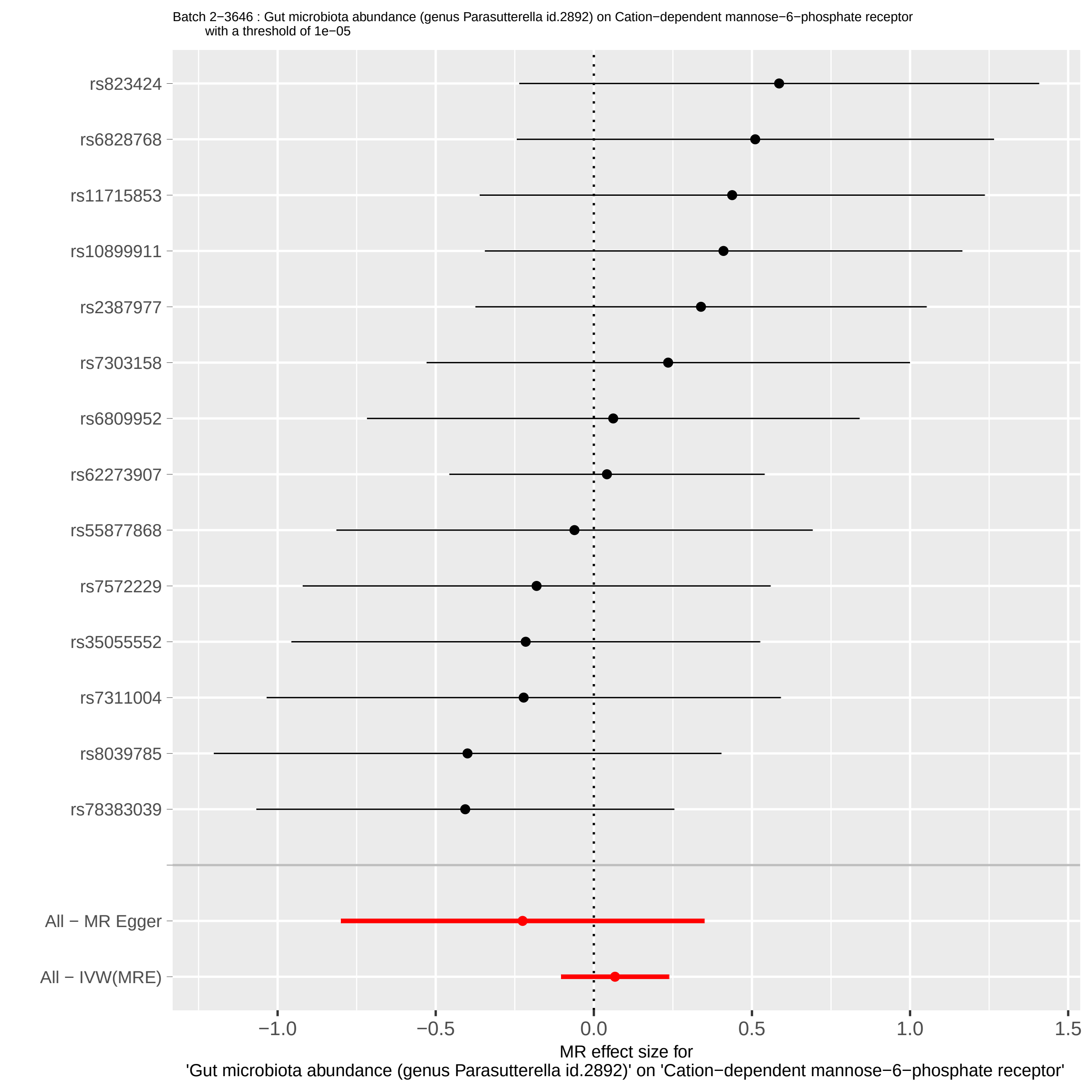
Batch 2−3646 : Gut microbiota abundance (genus Parasutterella id.2892) on Cation−dependent mannose−6−phosphate receptor
with a threshold of 1e−05
rs823424
rs6828768
rs11715853
rs10899911
rs2387977
rs7303158
rs6809952
rs62273907
rs55877868
rs7572229
rs35055552
rs7311004
rs8039785
rs78383039
All − MR Egger
All − IVW(MRE)
−1.0
−0.5
0.0
0.5
1.0
1.5
MR effect size for
'Gut microbiota abundance (genus Parasutterella id.2892)' on 'Cation−dependent mannose−6−phosphate receptor'

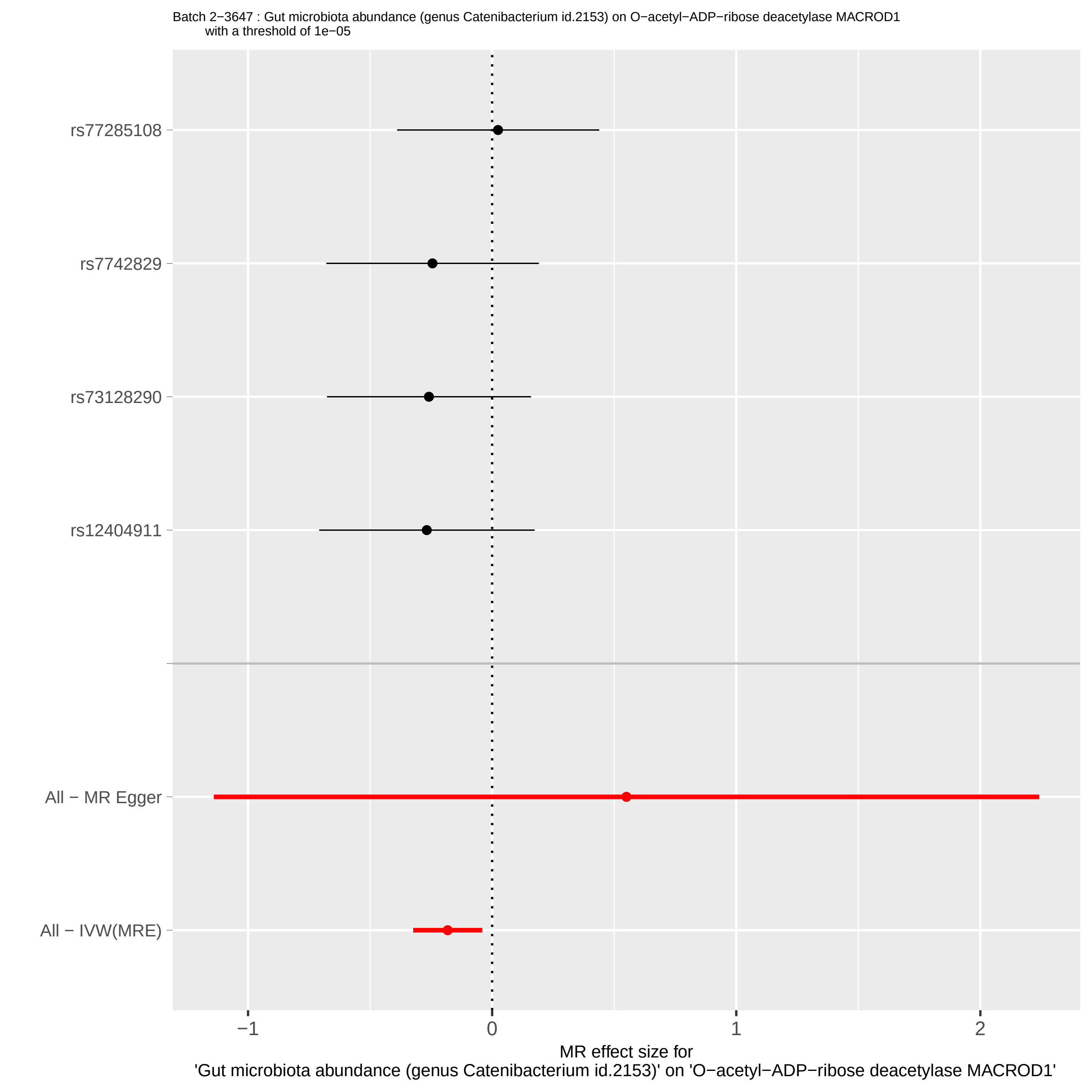

Batch 2−3647 : Gut microbiota abundance (genus Catenibacterium id.2153) on O−acetyl−ADP−ribose deacetylase MACROD1
with a threshold of 1e−05
rs77285108
rs7742829
rs73128290
rs12404911
All − MR Egger
All − IVW(MRE)
−1
0
1
2
MR effect size for
'Gut microbiota abundance (genus Catenibacterium id.2153)' on 'O−acetyl−ADP−ribose deacetylase MACROD1'

Batch 2−3648 : Gut microbiota abundance (genus Parasutterella id.2892) on O−acetyl−ADP−ribose deacetylase MACROD1
with a threshold of 1e−05

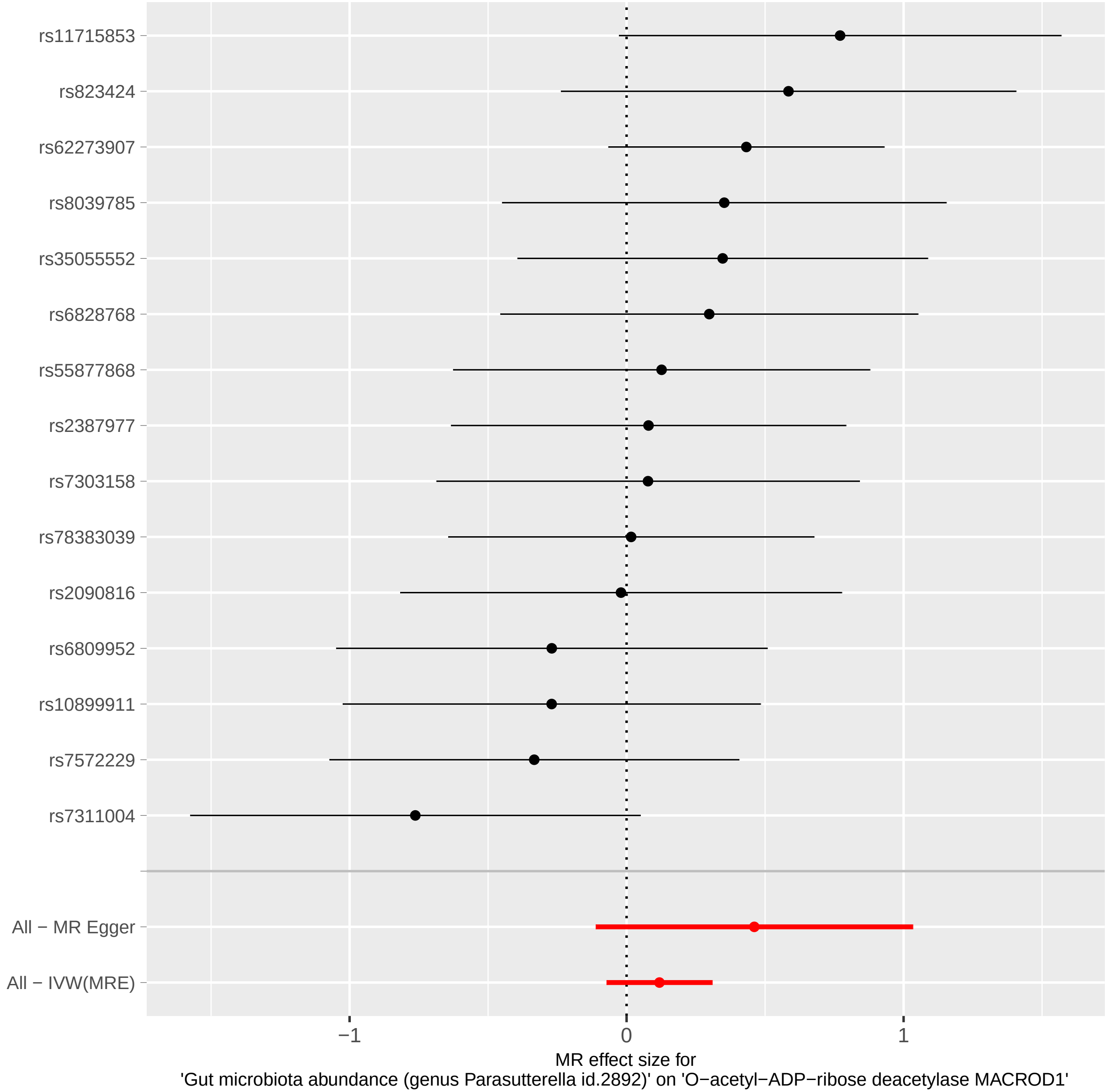

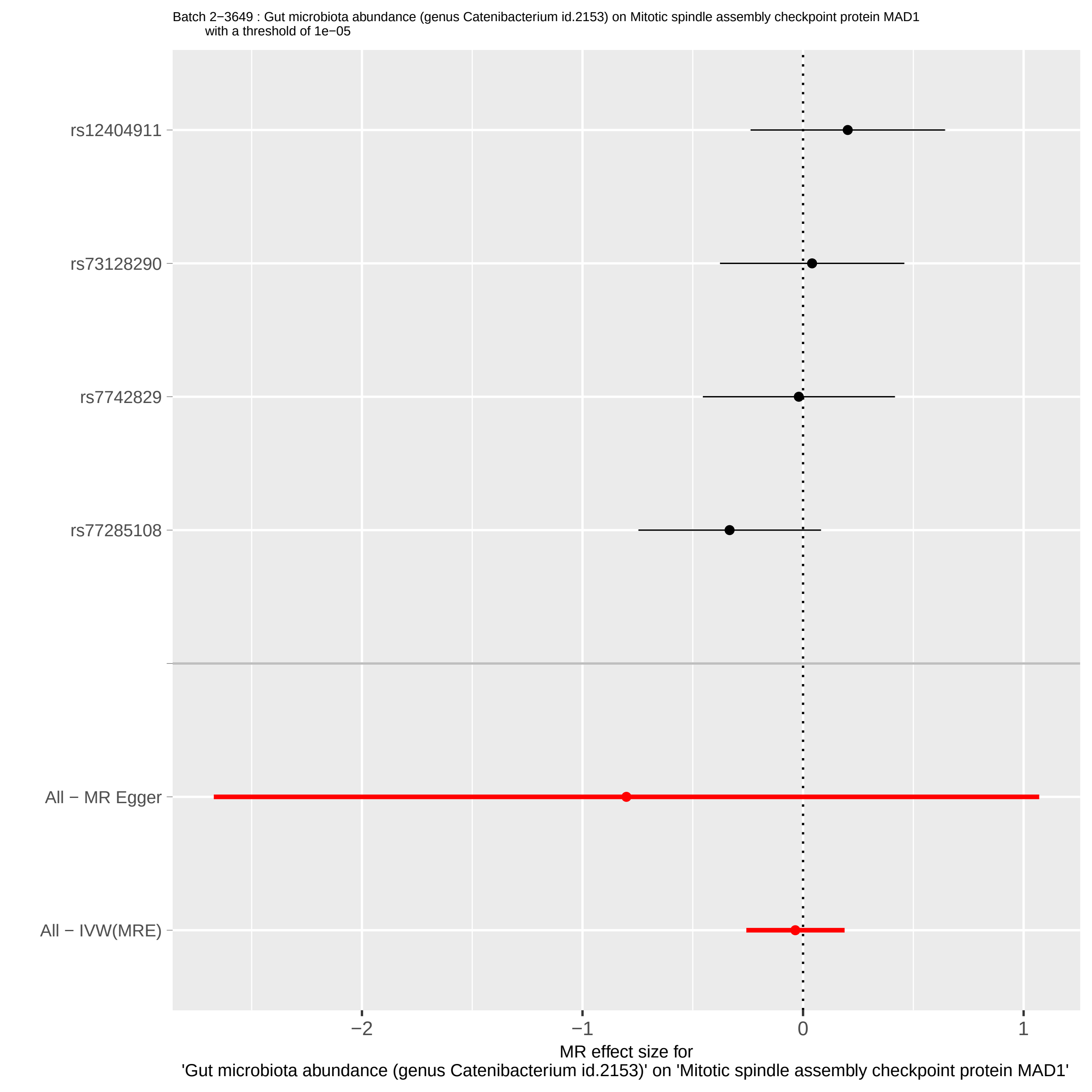

Batch 2−3649 : Gut microbiota abundance (genus Catenibacterium id.2153) on Mitotic spindle assembly checkpoint protein MAD1
with a threshold of 1e−05
rs12404911
rs73128290
rs7742829
rs77285108
All − MR Egger
All − IVW(MRE)
−2
−1
0
1
MR effect size for
'Gut microbiota abundance (genus Catenibacterium id.2153)' on 'Mitotic spindle assembly checkpoint protein MAD1'

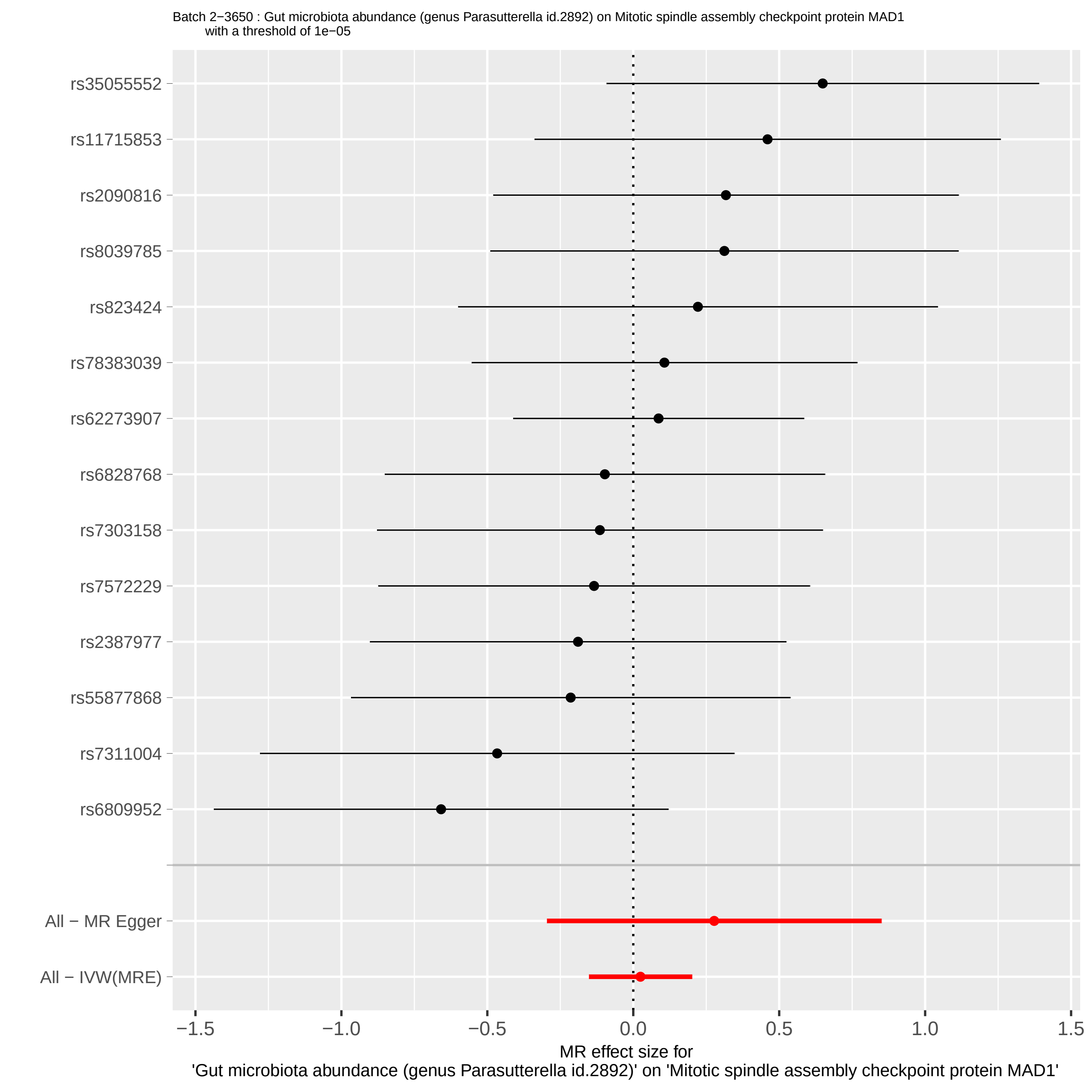
Batch 2−3650 : Gut microbiota abundance (genus Parasutterella id.2892) on Mitotic spindle assembly checkpoint protein MAD1
with a threshold of 1e−05
rs35055552
rs11715853
rs2090816
rs8039785
rs823424
rs78383039
rs62273907
rs6828768
rs7303158
rs7572229
rs2387977
rs55877868
rs7311004
rs6809952
All − MR Egger
All − IVW(MRE)
−1.5
−1.0
−0.5
0.0
0.5
1.0
1.5
MR effect size for
'Gut microbiota abundance (genus Parasutterella id.2892)' on 'Mitotic spindle assembly checkpoint protein MAD1'

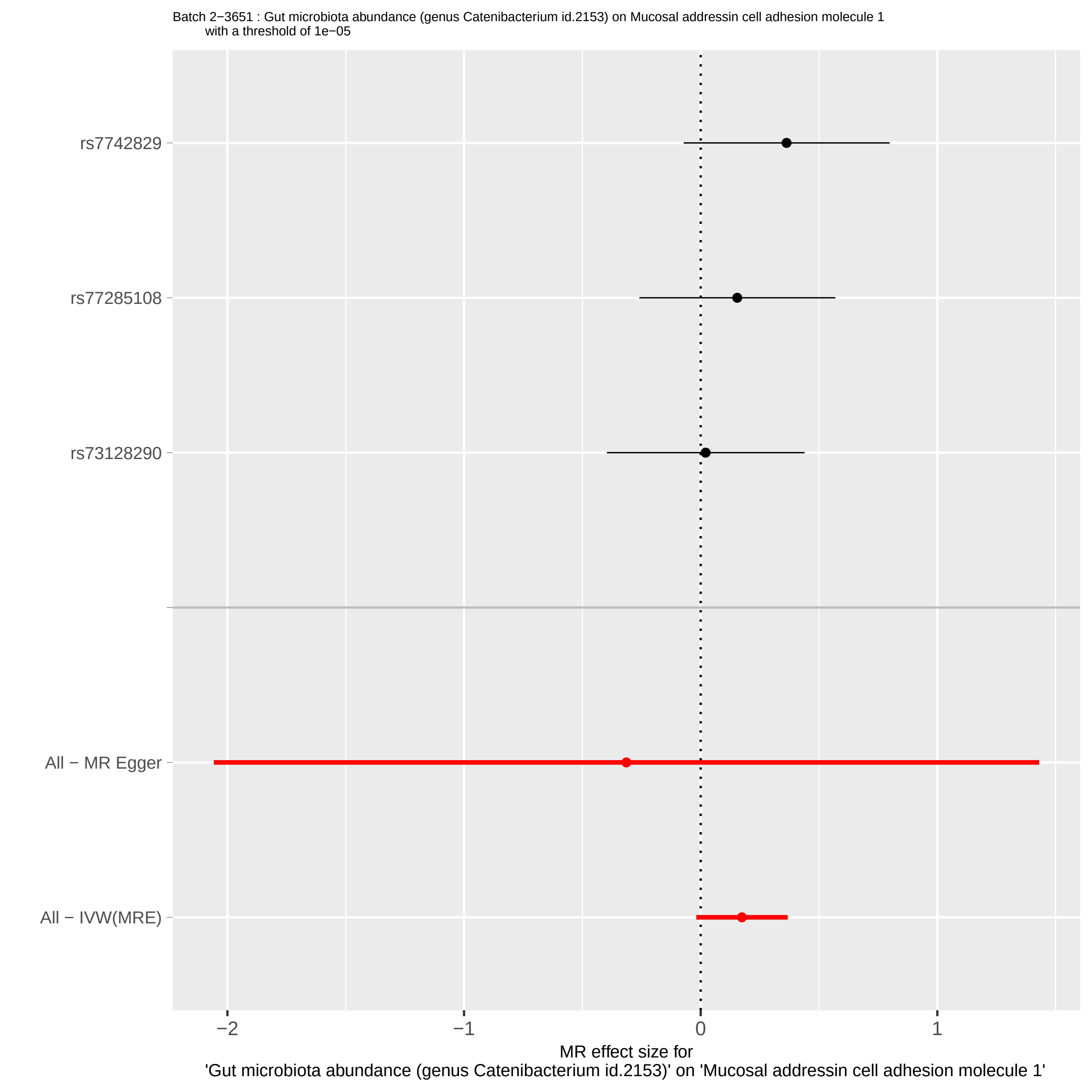

Batch 2−3651 : Gut microbiota abundance (genus Catenibacterium id.2153) on Mucosal addressin cell adhesion molecule 1
with a threshold of 1e−05
rs7742829
rs77285108
rs73128290
All − MR Egger
All − IVW(MRE)
−2
−1
0
1
MR effect size for
'Gut microbiota abundance (genus Catenibacterium id.2153)' on 'Mucosal addressin cell adhesion molecule 1'

Batch 2−3652 : Gut microbiota abundance (genus Parasutterella id.2892) on Mucosal addressin cell adhesion molecule 1
with a threshold of 1e−05

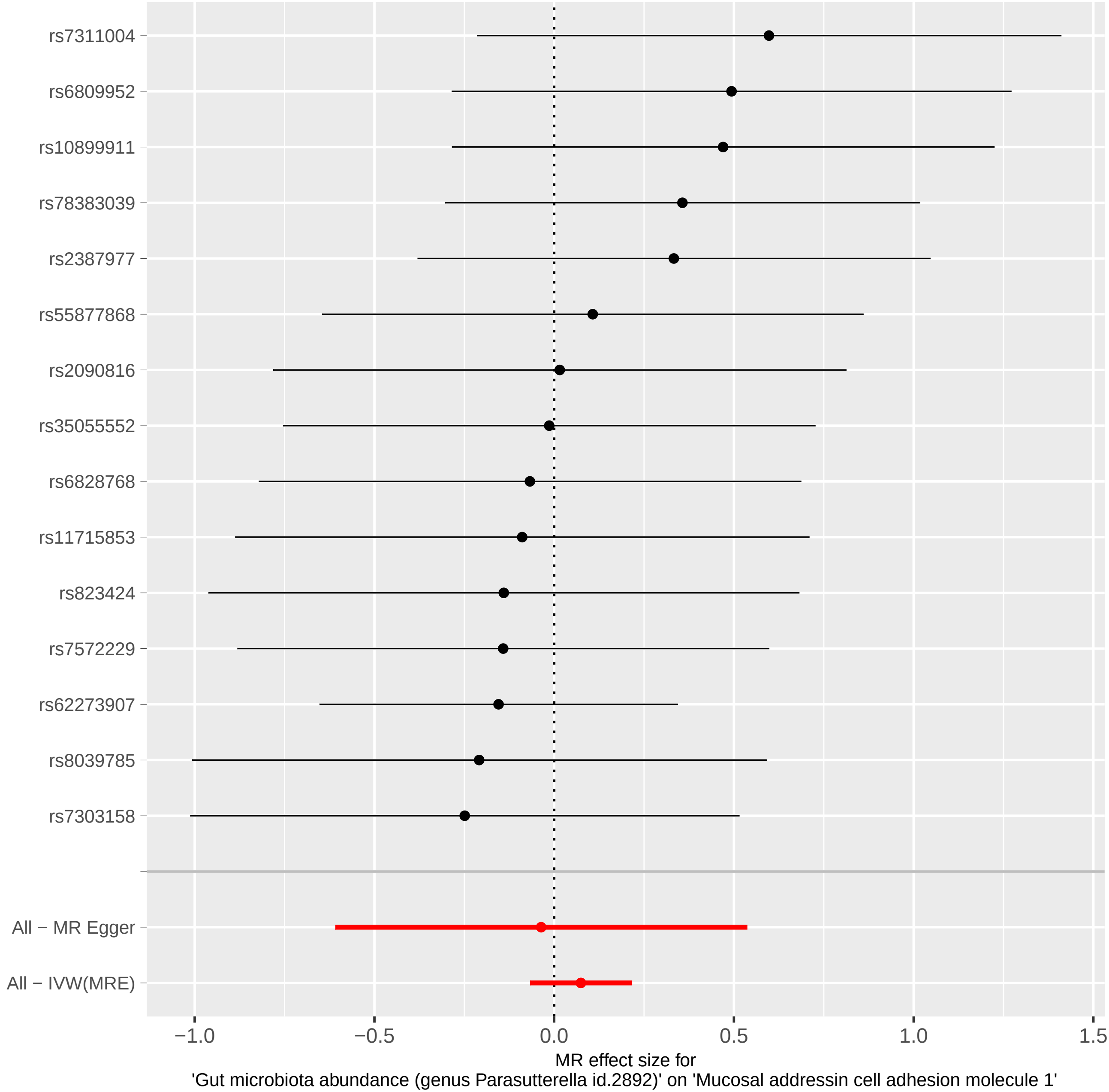

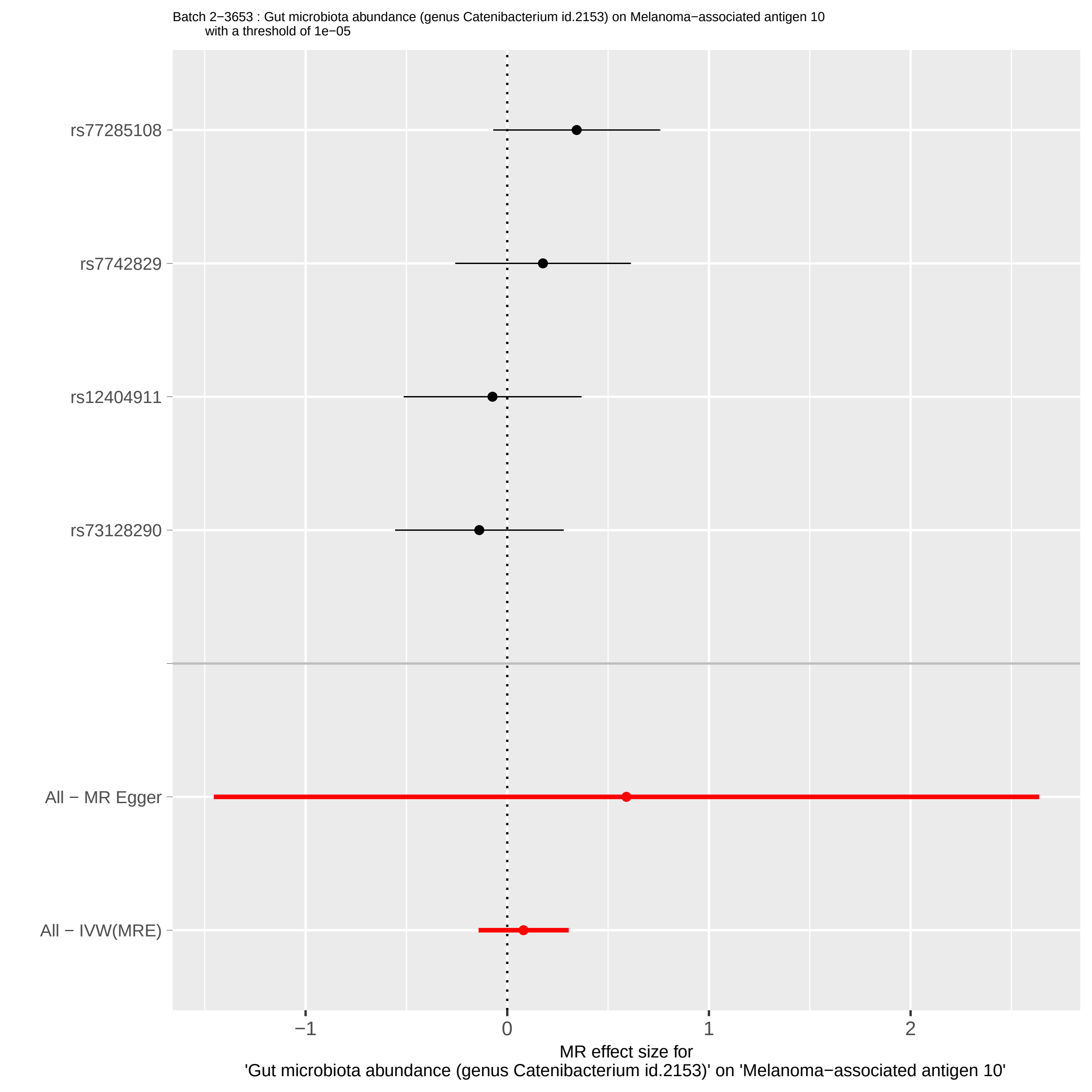
Batch 2−3653 : Gut microbiota abundance (genus Catenibacterium id.2153) on Melanoma−associated antigen 10
with a threshold of 1e−05
rs77285108
rs7742829
rs12404911
rs73128290
All − MR Egger
All − IVW(MRE)
−1
0
1
2
MR effect size for
'Gut microbiota abundance (genus Catenibacterium id.2153)' on 'Melanoma−associated antigen 10'

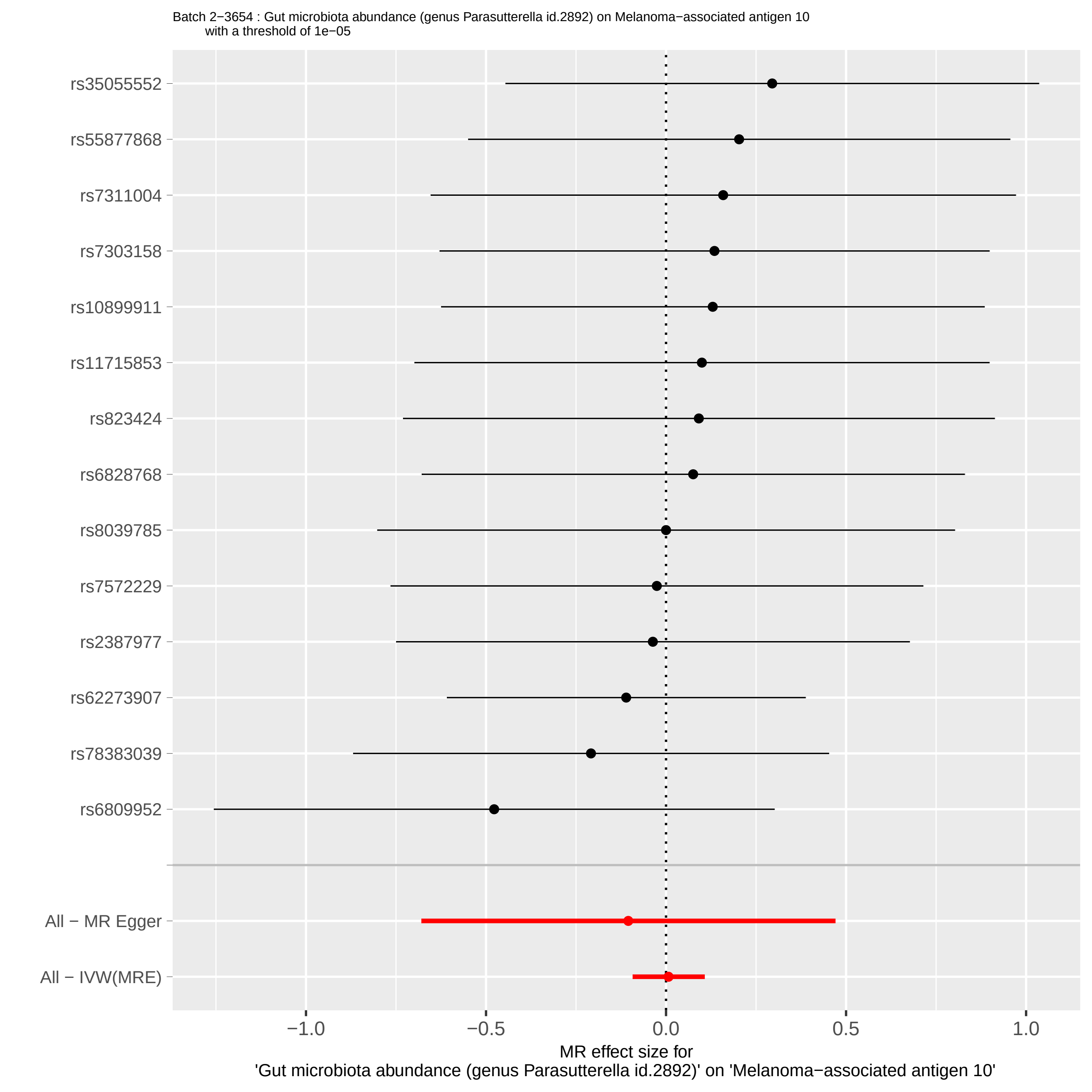
Batch 2−3654 : Gut microbiota abundance (genus Parasutterella id.2892) on Melanoma−associated antigen 10
with a threshold of 1e−05
rs35055552
rs55877868
rs7311004
rs7303158
rs10899911
rs11715853
rs823424
rs6828768
rs8039785
rs7572229
rs2387977
rs62273907
rs78383039
rs6809952
All − MR Egger
All − IVW(MRE)
−1.0
−0.5
0.0
0.5
1.0
MR effect size for
'Gut microbiota abundance (genus Parasutterella id.2892)' on 'Melanoma−associated antigen 10'

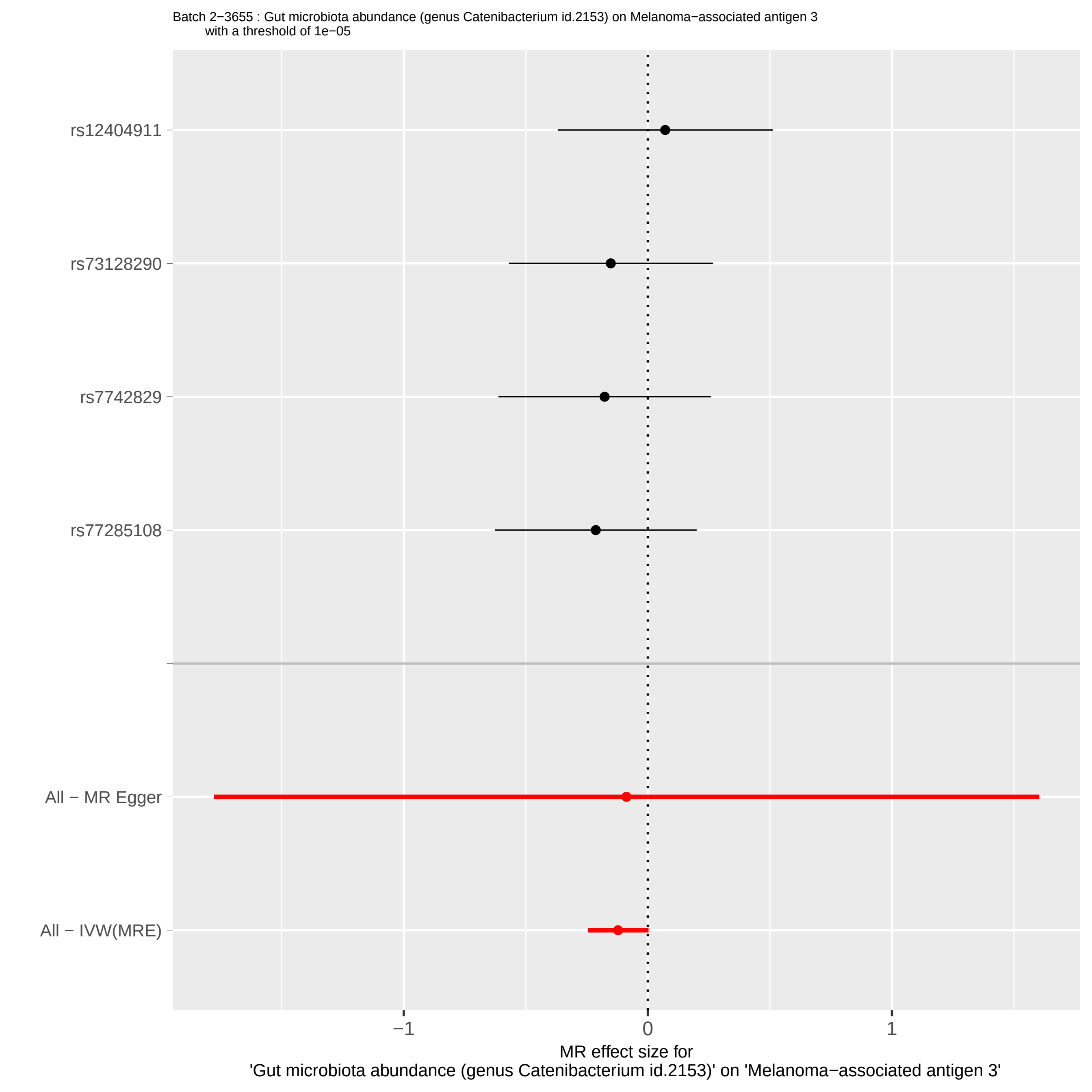

Batch 2−3655 : Gut microbiota abundance (genus Catenibacterium id.2153) on Melanoma−associated antigen 3
with a threshold of 1e−05
rs12404911
rs73128290
rs7742829
rs77285108
All − MR Egger
All − IVW(MRE)
−1
0
1
MR effect size for
'Gut microbiota abundance (genus Catenibacterium id.2153)' on 'Melanoma−associated antigen 3'

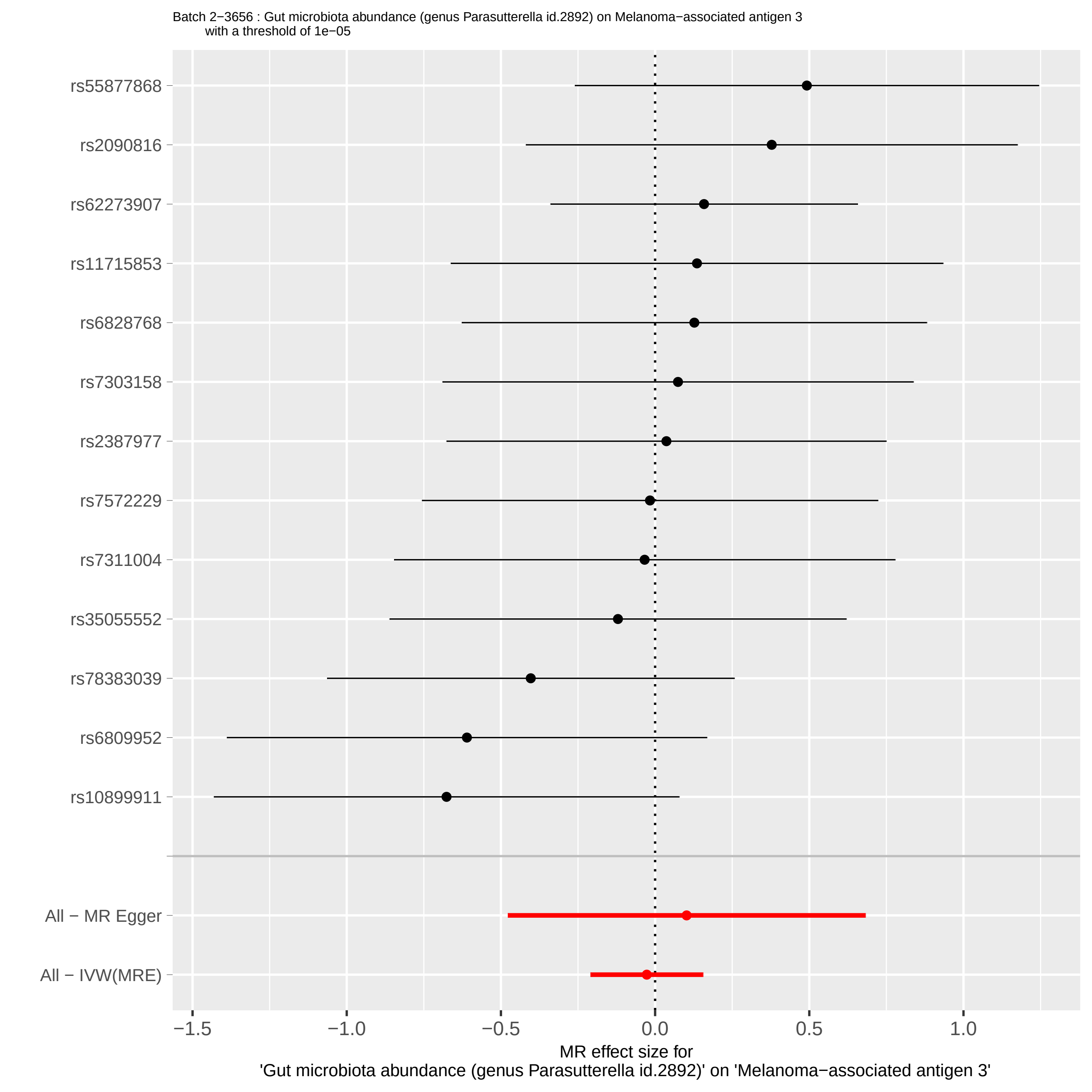

Batch 2−3656 : Gut microbiota abundance (genus Parasutterella id.2892) on Melanoma−associated antigen 3
with a threshold of 1e−05
rs55877868
rs2090816
rs62273907
rs11715853
rs6828768
rs7303158
rs2387977
rs7572229
rs7311004
rs35055552
rs78383039
rs6809952
rs10899911
All − MR Egger
All − IVW(MRE)
−1.5
−1.0
−0.5
0.0
0.5
1.0
MR effect size for
'Gut microbiota abundance (genus Parasutterella id.2892)' on 'Melanoma−associated antigen 3'

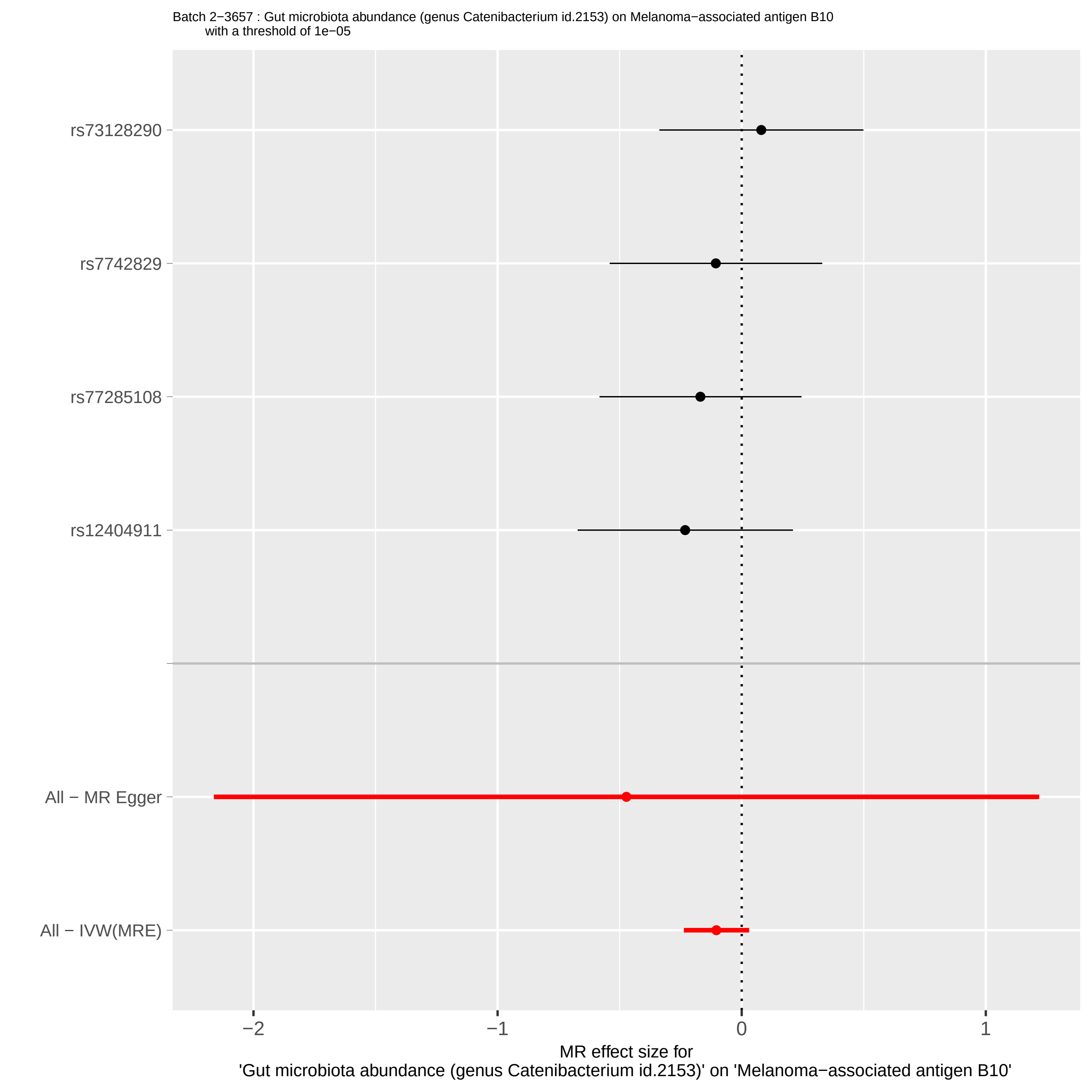
Batch 2−3657 : Gut microbiota abundance (genus Catenibacterium id.2153) on Melanoma−associated antigen B10
with a threshold of 1e−05
rs73128290
rs7742829
rs77285108
rs12404911
All − MR Egger
All − IVW(MRE)
−2
−1
0
1
MR effect size for
'Gut microbiota abundance (genus Catenibacterium id.2153)' on 'Melanoma−associated antigen B10'

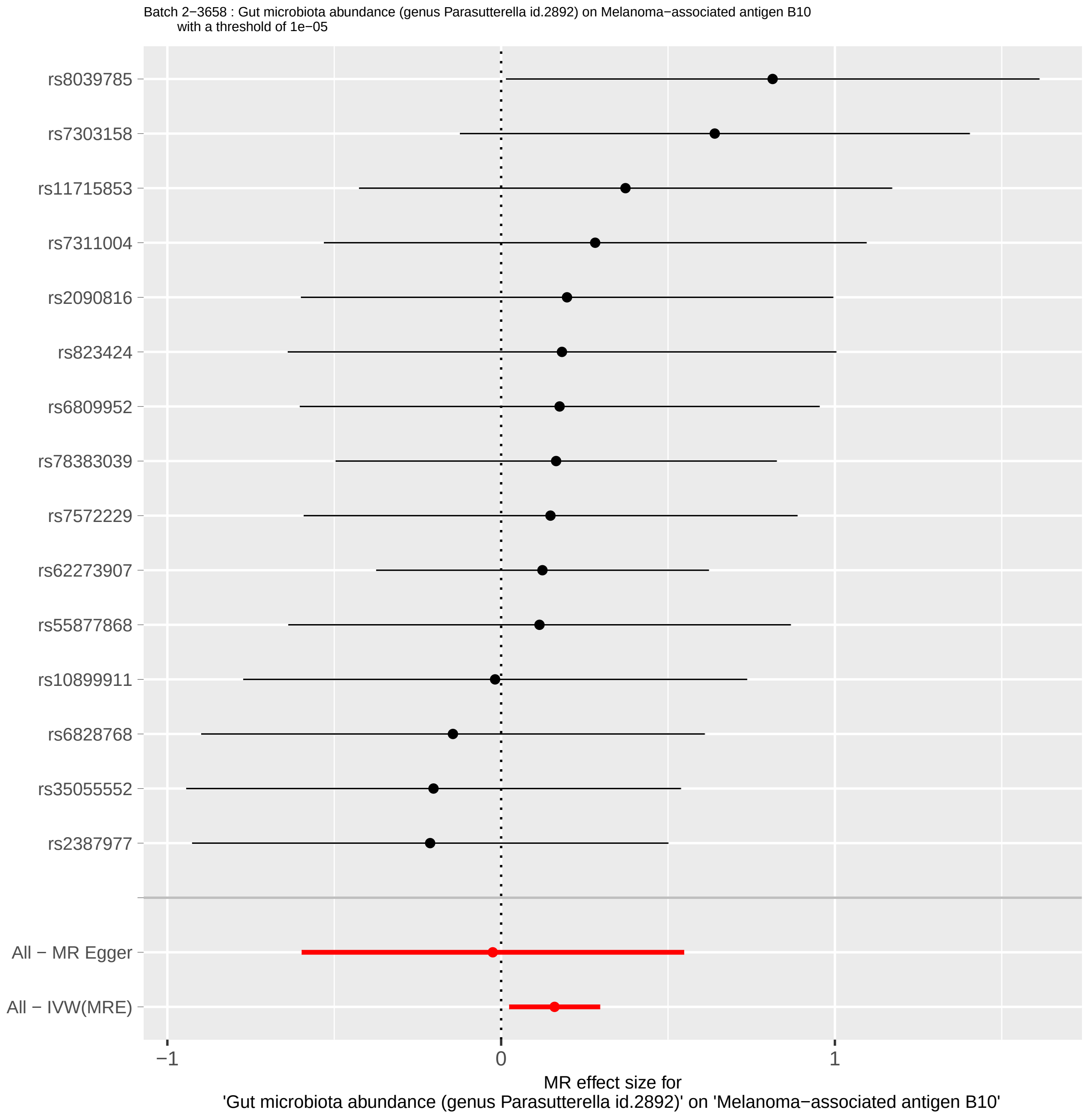

Batch 2−3658 : Gut microbiota abundance (genus Parasutterella id.2892) on Melanoma−associated antigen B10
with a threshold of 1e−05
rs8039785
rs7303158
rs11715853
rs7311004
rs2090816
rs823424
rs6809952
rs78383039
rs7572229
rs62273907
rs55877868
rs10899911
rs6828768
rs35055552
rs2387977
All − MR Egger
All − IVW(MRE)
−1
0
1
MR effect size for
'Gut microbiota abundance (genus Parasutterella id.2892)' on 'Melanoma−associated antigen B10'

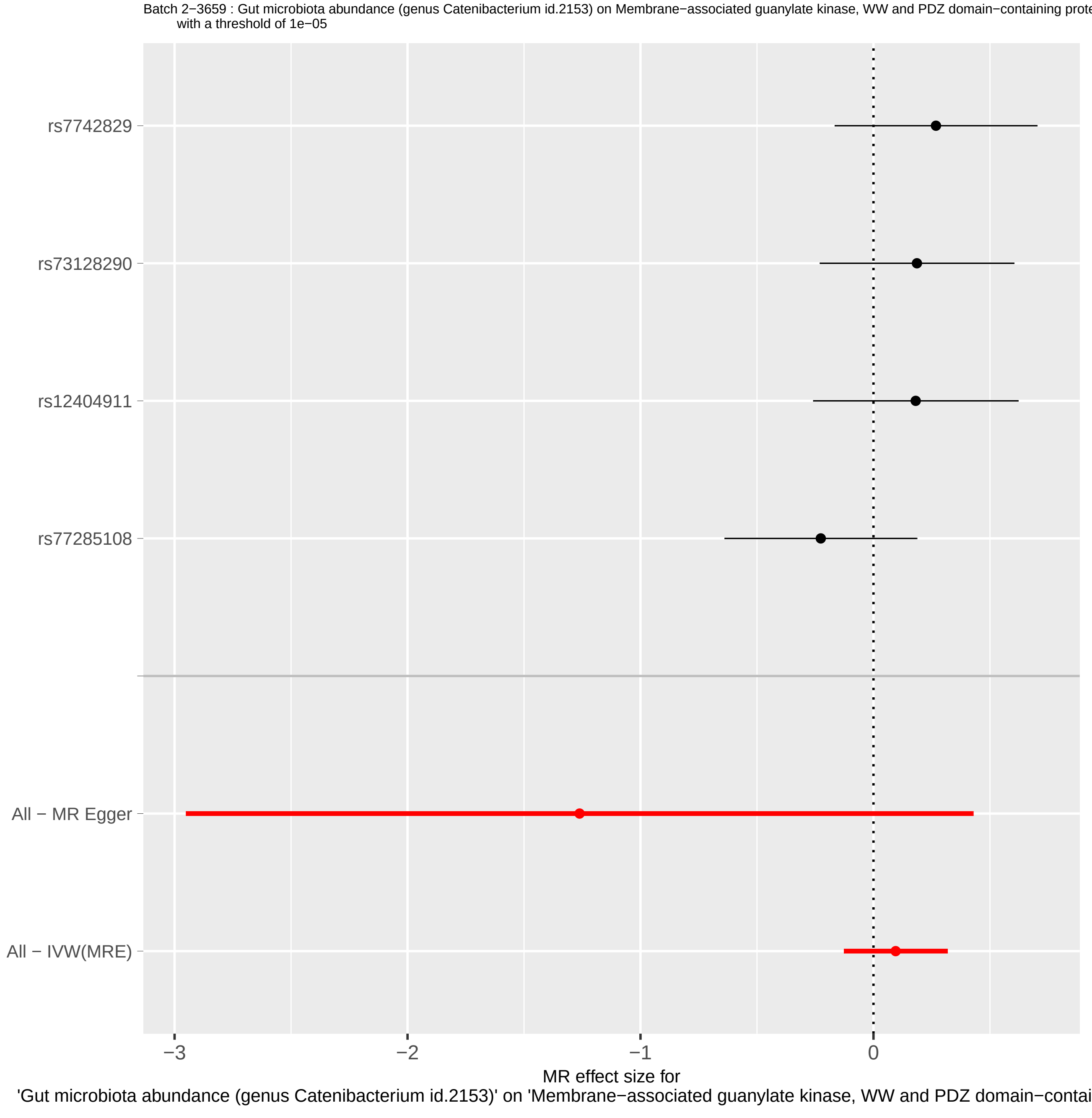

Batch 2−3659 : Gut microbiota abundance (genus Catenibacterium id.2153) on Membrane−associated guanylate kinase, WW and PDZ domain−containing prote
with a threshold of 1e−05
rs7742829
rs73128290
rs12404911
rs77285108
All − MR Egger
All − IVW(MRE)
−3
−2
−1
0
MR effect size for
'Gut microbiota abundance (genus Catenibacterium id.2153)' on 'Membrane−associated guanylate kinase, WW and PDZ domain−contai

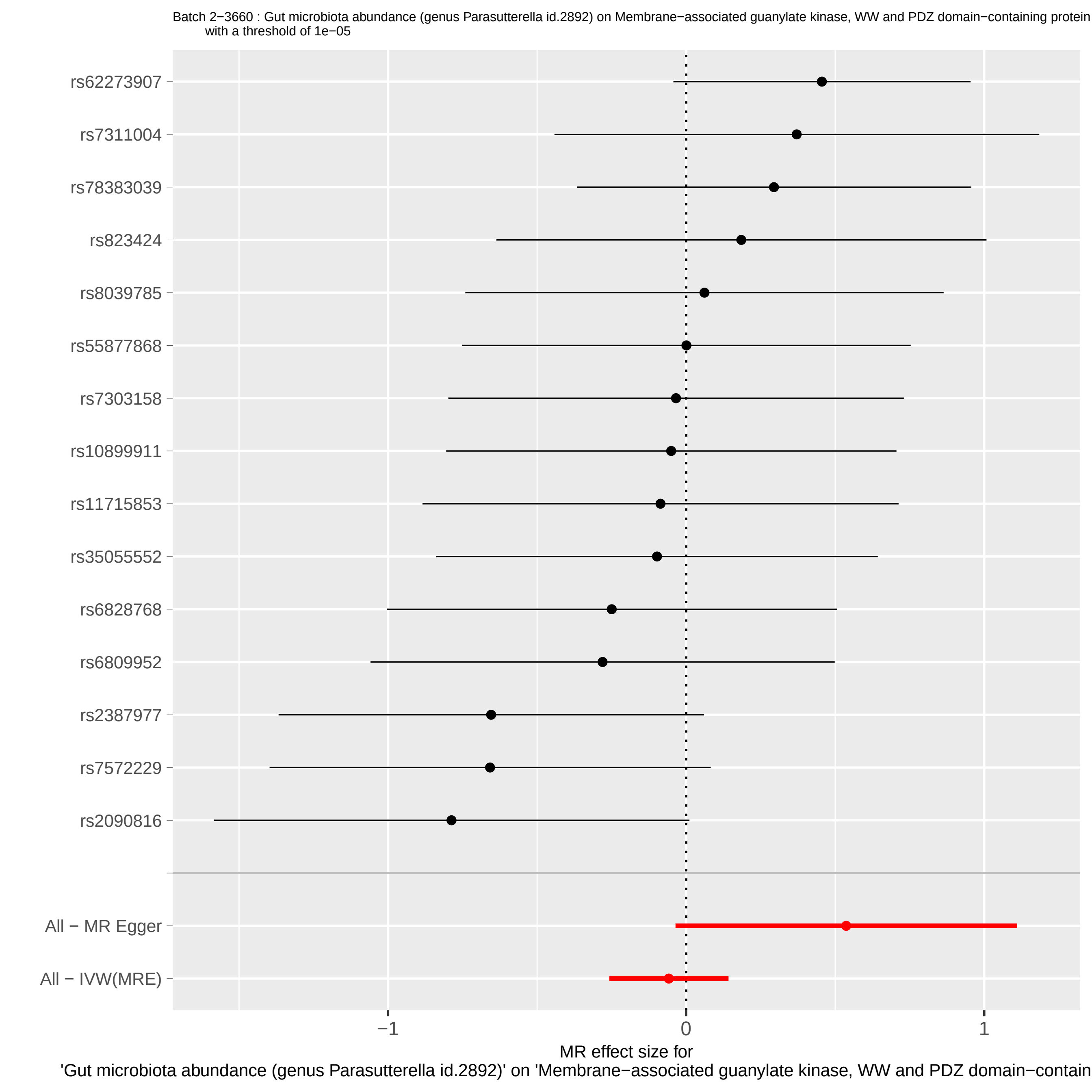

Batch 2−3660 : Gut microbiota abundance (genus Parasutterella id.2892) on Membrane−associated guanylate kinase, WW and PDZ domain−containing protein
with a threshold of 1e−05
rs62273907
rs7311004
rs78383039
rs823424
rs8039785
rs55877868
rs7303158
rs10899911
rs11715853
rs35055552
rs6828768
rs6809952
rs2387977
rs7572229
rs2090816
All − MR Egger
All − IVW(MRE)
−1
0
1
MR effect size for
'Gut microbiota abundance (genus Parasutterella id.2892)' on 'Membrane−associated guanylate kinase, WW and PDZ domain−contain

Batch 2−3661 : Gut microbiota abundance (genus Catenibacterium id.2153) on MAM domain−containing protein 2
with a threshold of 1e−05

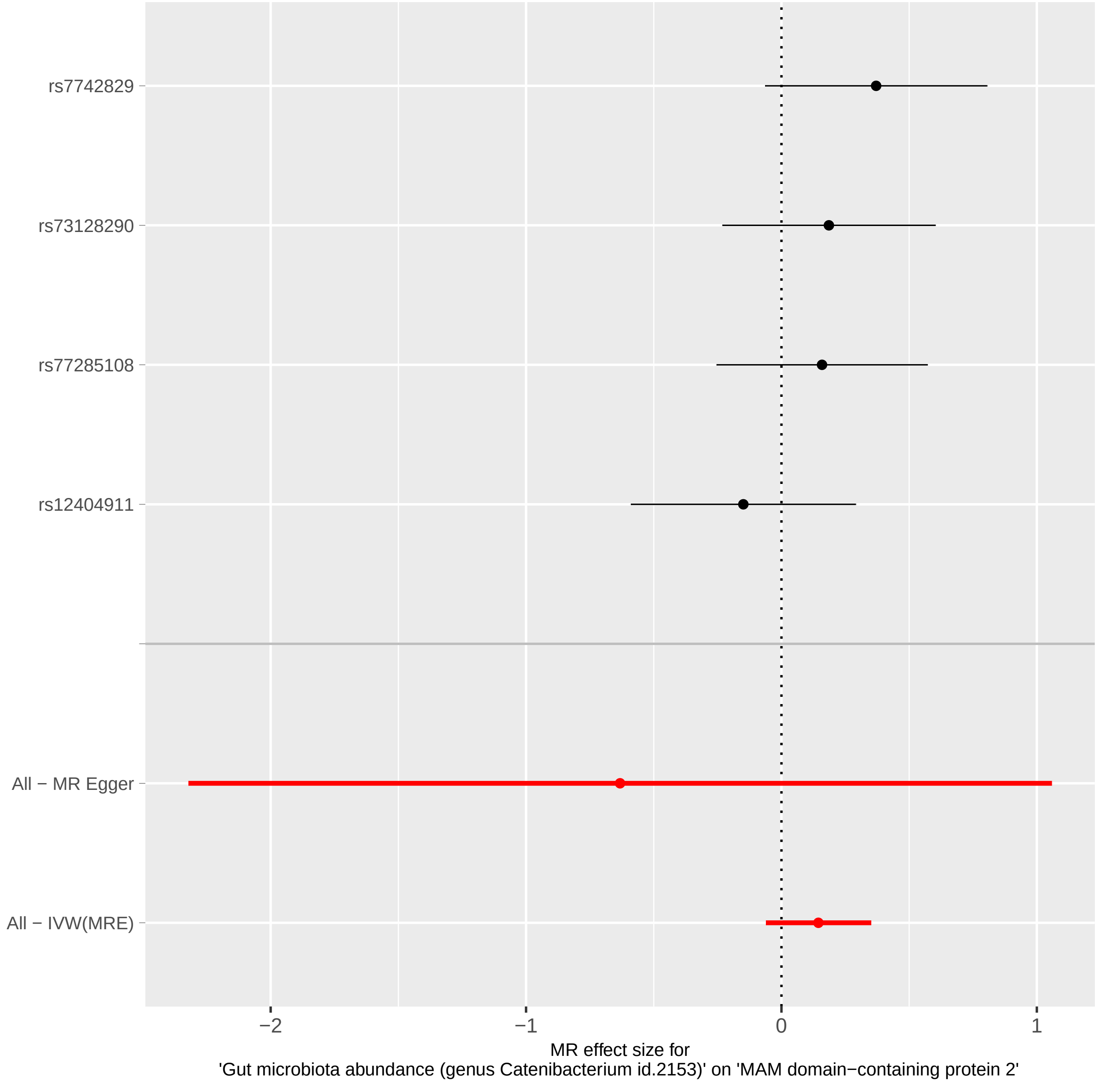

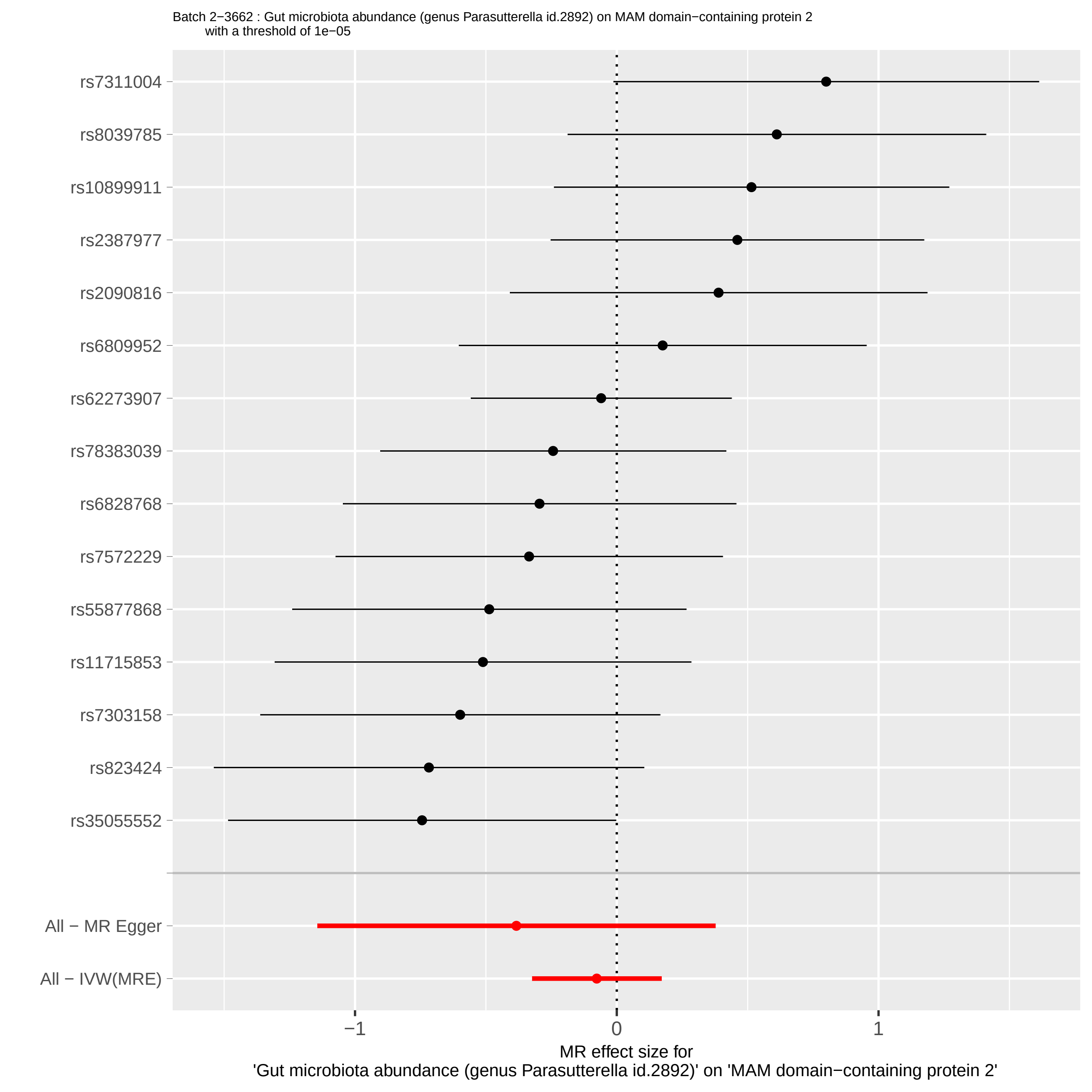

Batch 2−3662 : Gut microbiota abundance (genus Parasutterella id.2892) on MAM domain−containing protein 2
with a threshold of 1e−05
rs7311004
rs8039785
rs10899911
rs2387977
rs2090816
rs6809952
rs62273907
rs78383039
rs6828768
rs7572229
rs55877868
rs11715853
rs7303158
rs823424
rs35055552
All − MR Egger
All − IVW(MRE)
−1
0
1
MR effect size for
'Gut microbiota abundance (genus Parasutterella id.2892)' on 'MAM domain−containing protein 2'

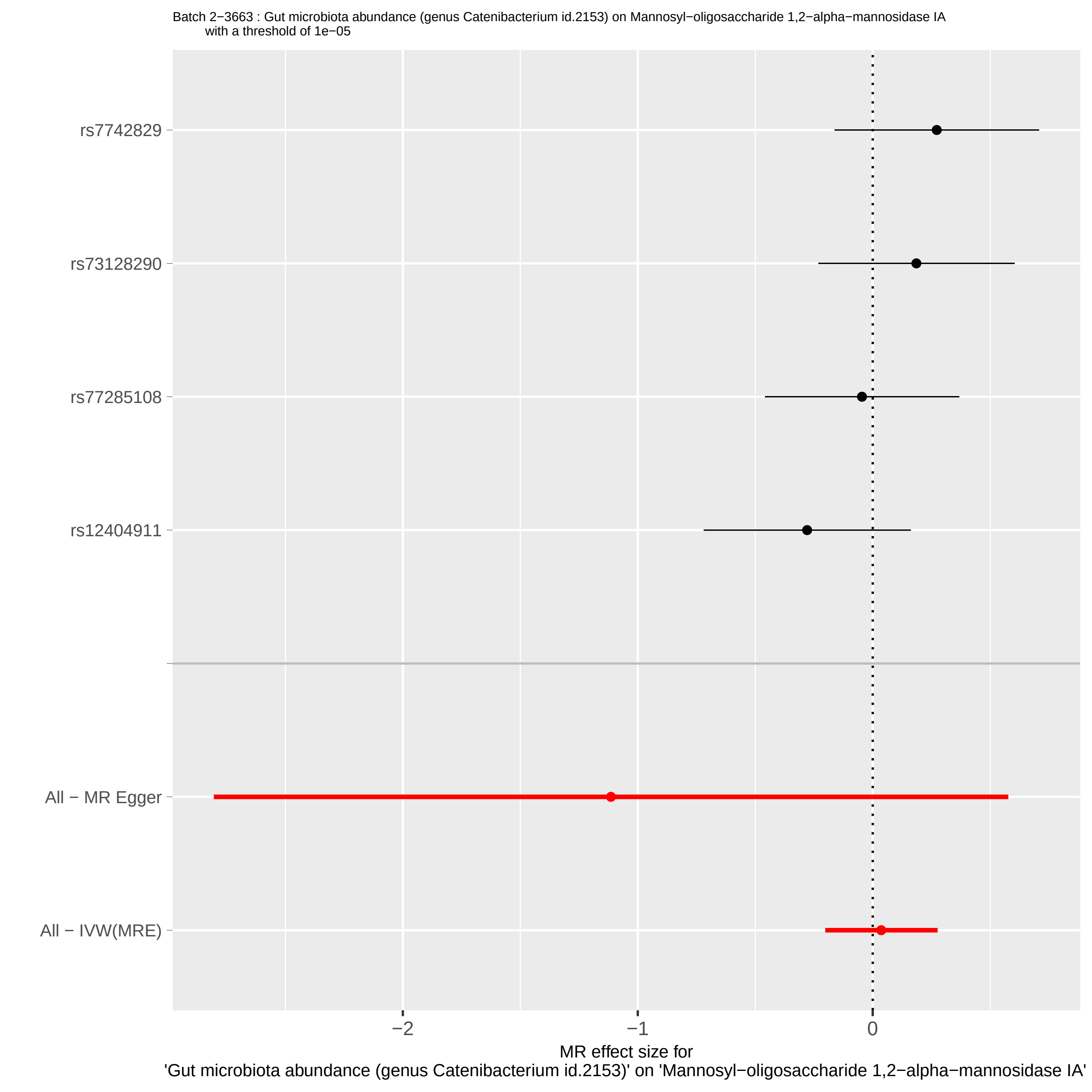

Batch 2−3663 : Gut microbiota abundance (genus Catenibacterium id.2153) on Mannosyl−oligosaccharide 1,2−alpha−mannosidase IA
with a threshold of 1e−05
rs7742829
rs73128290
rs77285108
rs12404911
All − MR Egger
All − IVW(MRE)
−2
−1
0
MR effect size for
'Gut microbiota abundance (genus Catenibacterium id.2153)' on 'Mannosyl−oligosaccharide 1,2−alpha−mannosidase IA'

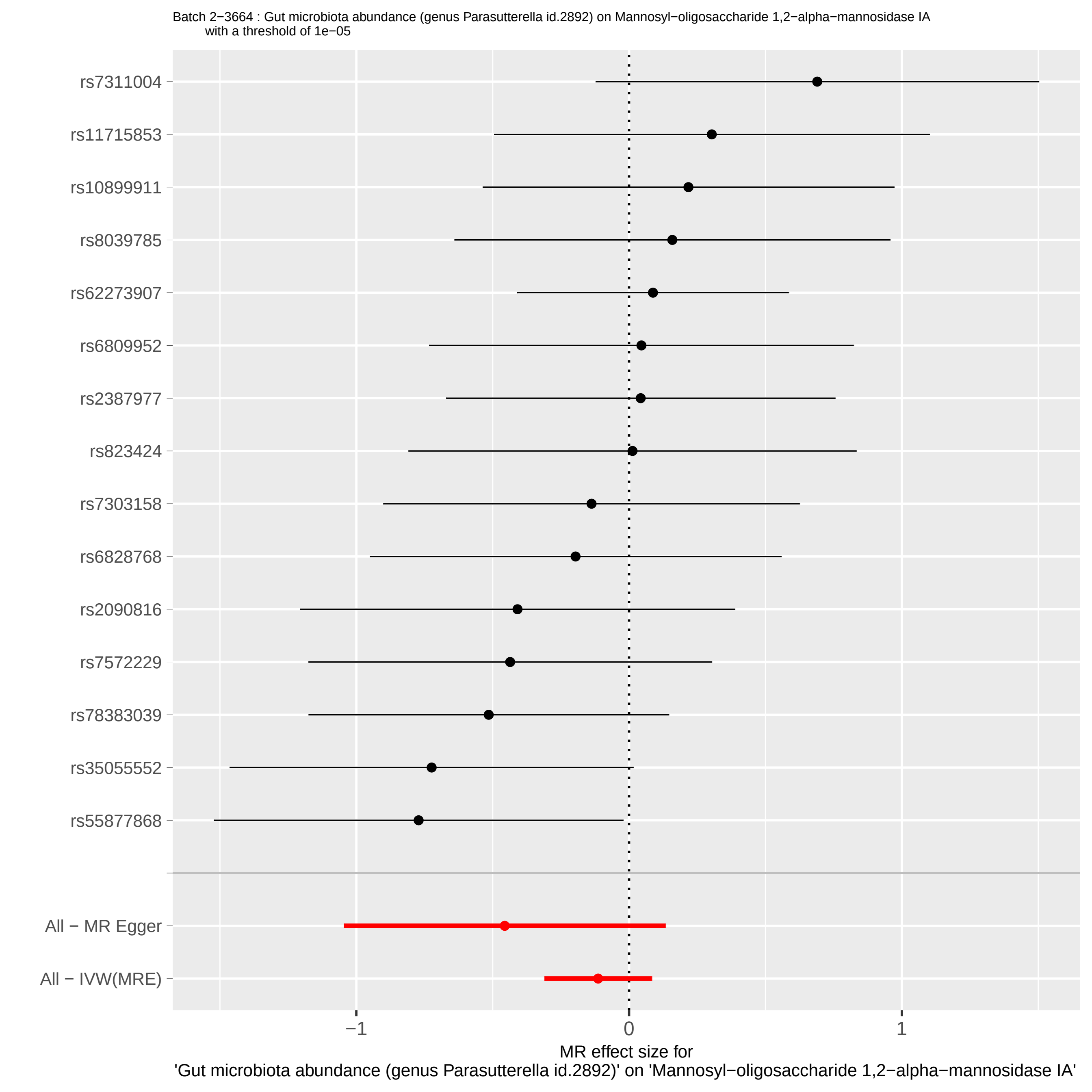

Batch 2−3664 : Gut microbiota abundance (genus Parasutterella id.2892) on Mannosyl−oligosaccharide 1,2−alpha−mannosidase IA
with a threshold of 1e−05
rs7311004
rs11715853
rs10899911
rs8039785
rs62273907
rs6809952
rs2387977
rs823424
rs7303158
rs6828768
rs2090816
rs7572229
rs78383039
rs35055552
rs55877868
All − MR Egger
All − IVW(MRE)
−1
0
1
MR effect size for
'Gut microbiota abundance (genus Parasutterella id.2892)' on 'Mannosyl−oligosaccharide 1,2−alpha−mannosidase IA'

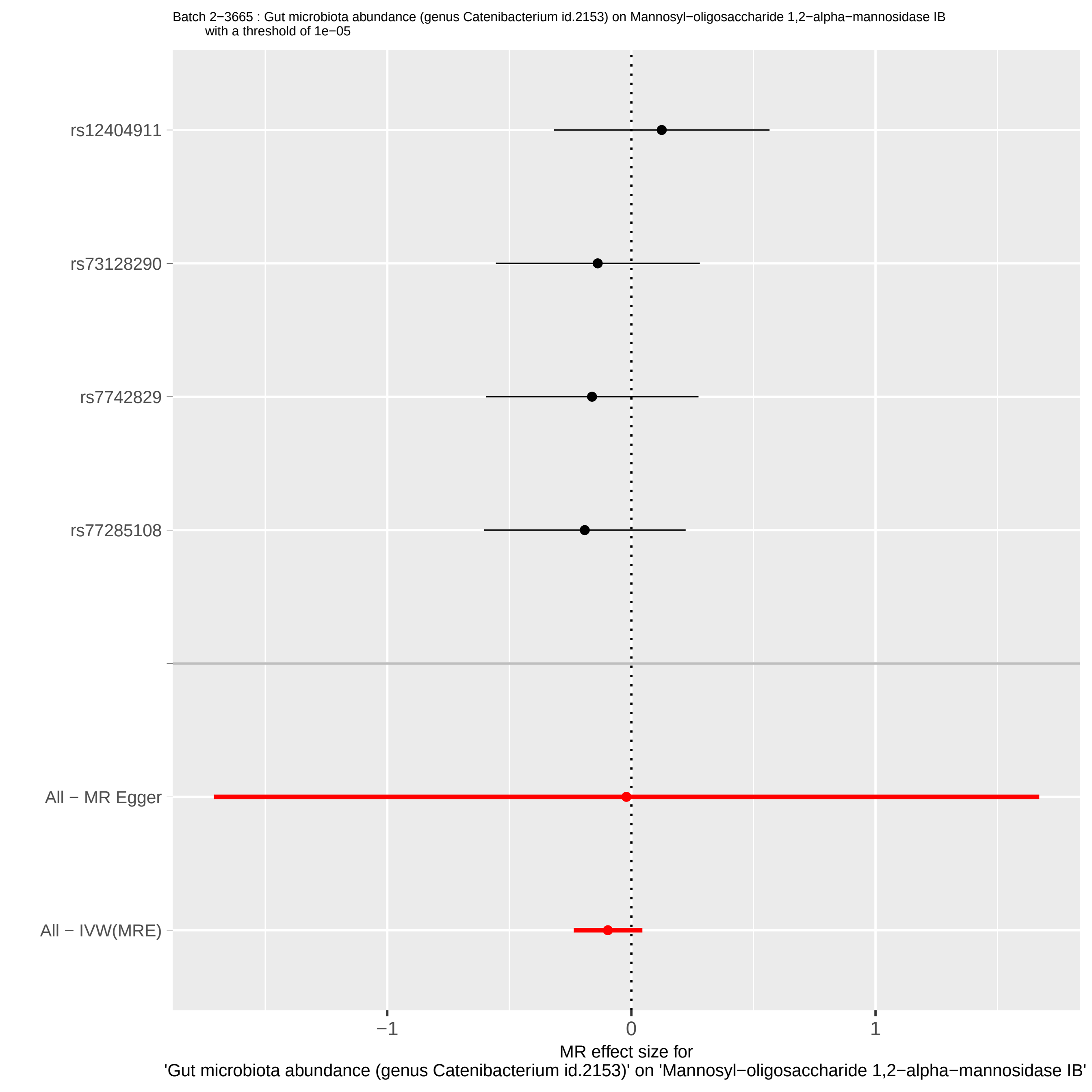
Batch 2−3665 : Gut microbiota abundance (genus Catenibacterium id.2153) on Mannosyl−oligosaccharide 1,2−alpha−mannosidase IB
with a threshold of 1e−05
rs12404911
rs73128290
rs7742829
rs77285108
All − MR Egger
All − IVW(MRE)
−1
0
1
MR effect size for
'Gut microbiota abundance (genus Catenibacterium id.2153)' on 'Mannosyl−oligosaccharide 1,2−alpha−mannosidase IB'

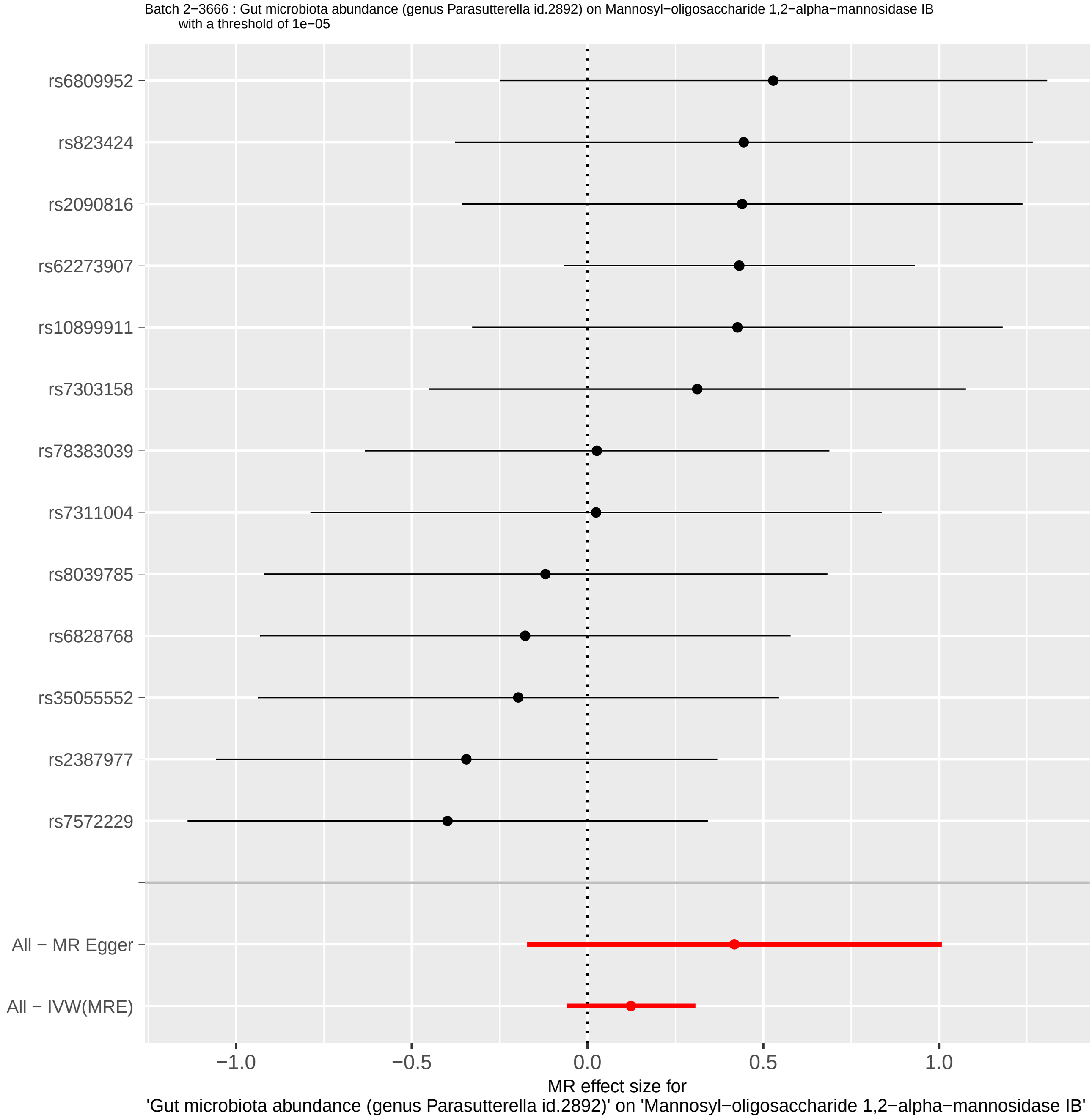
Batch 2−3666 : Gut microbiota abundance (genus Parasutterella id.2892) on Mannosyl−oligosaccharide 1,2−alpha−mannosidase IB with a threshold of 1e−05
rs6809952
rs823424
rs2090816
rs62273907
rs10899911
rs7303158
rs78383039
rs7311004
rs8039785
rs6828768
rs35055552
rs2387977
rs7572229
All − MR Egger
All − IVW(MRE)
−1.0
−0.5
0.0
0.5
1.0
MR effect size for
'Gut microbiota abundance (genus Parasutterella id.2892)' on 'Mannosyl−oligosaccharide 1,2−alpha−mannosidase IB'

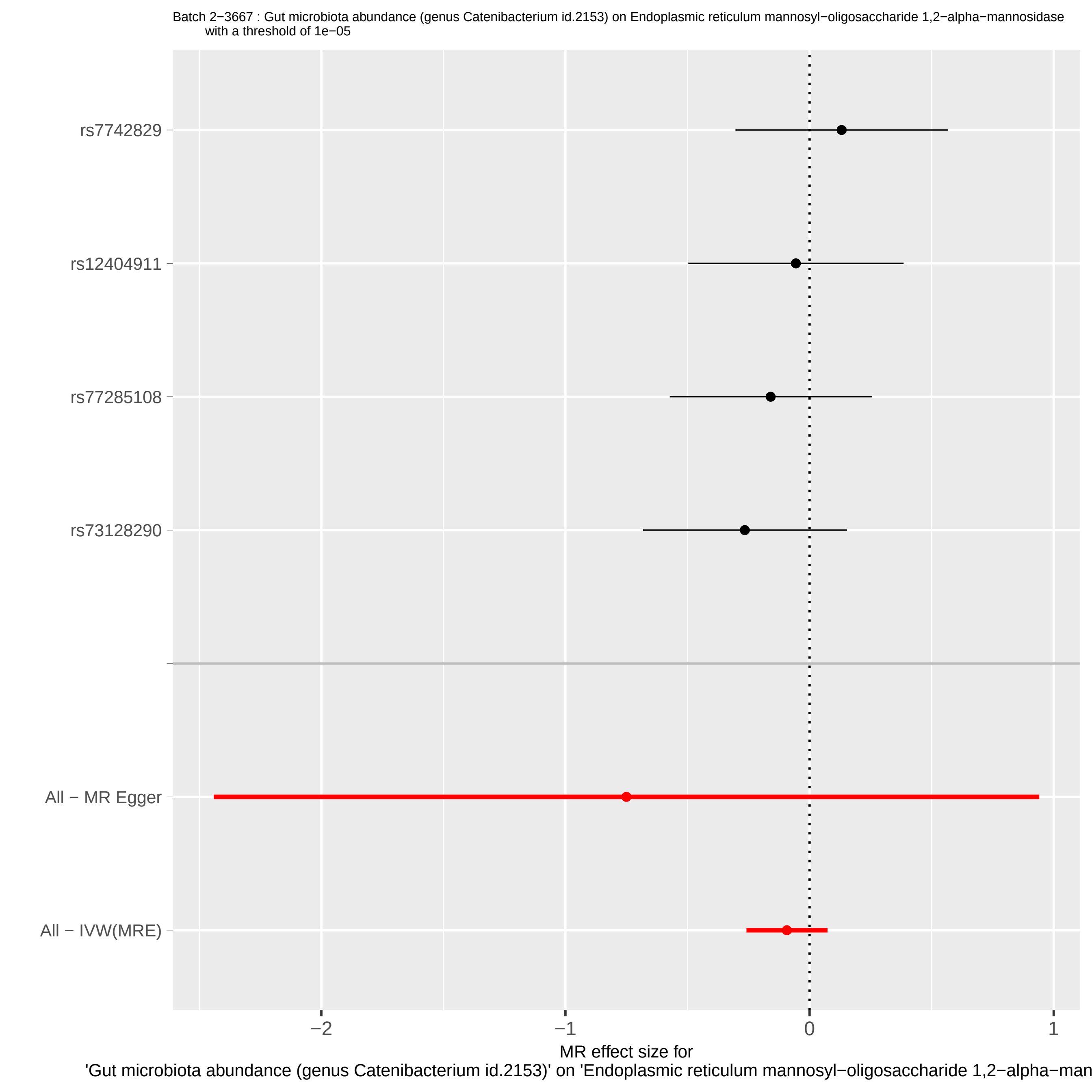

Batch 2−3667 : Gut microbiota abundance (genus Catenibacterium id.2153) on Endoplasmic reticulum mannosyl−oligosaccharide 1,2−alpha−mannosidase with a threshold of 1e−05
rs7742829
rs12404911
rs77285108
rs73128290
All − MR Egger
All − IVW(MRE)
−2
−1
0
1
MR effect size for
'Gut microbiota abundance (genus Catenibacterium id.2153)' on 'Endoplasmic reticulum mannosyl−oligosaccharide 1,2−alpha−man

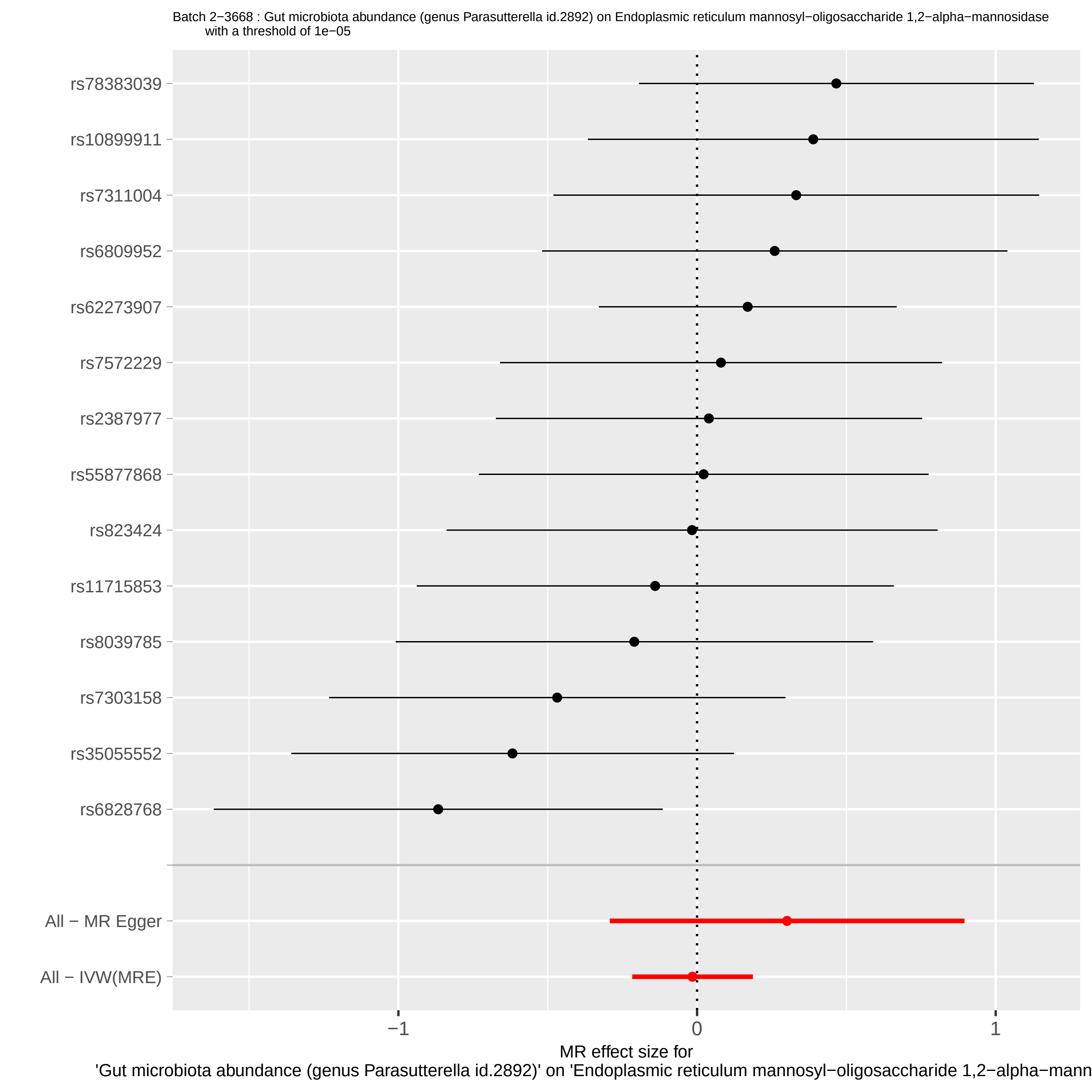

Batch 2−3668 : Gut microbiota abundance (genus Parasutterella id.2892) on Endoplasmic reticulum mannosyl−oligosaccharide 1,2−alpha−mannosidase with a threshold of 1e−05
rs78383039
rs10899911
rs7311004
rs6809952
rs62273907
rs7572229
rs2387977
rs55877868
rs823424
rs11715853
rs8039785
rs7303158
rs35055552
rs6828768
All − MR Egger
All − IVW(MRE)
−1
0
1
MR effect size for
'Gut microbiota abundance (genus Parasutterella id.2892)' on 'Endoplasmic reticulum mannosyl−oligosaccharide 1,2−alpha−mann

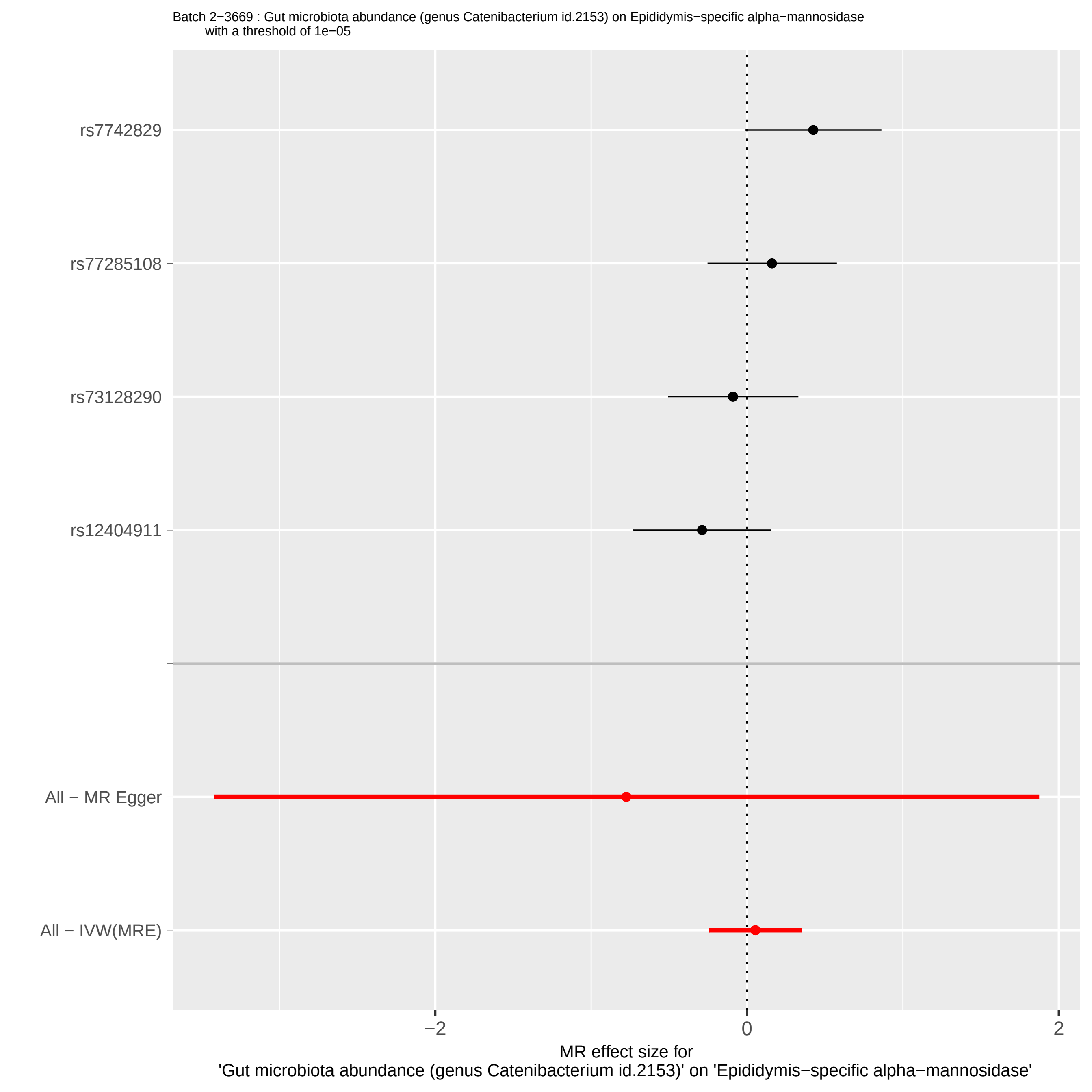

Batch 2−3669 : Gut microbiota abundance (genus Catenibacterium id.2153) on Epididymis−specific alpha−mannosidase
with a threshold of 1e−05
rs7742829
rs77285108
rs73128290
rs12404911
All − MR Egger
All − IVW(MRE)
−2
0
2
MR effect size for
'Gut microbiota abundance (genus Catenibacterium id.2153)' on 'Epididymis−specific alpha−mannosidase'

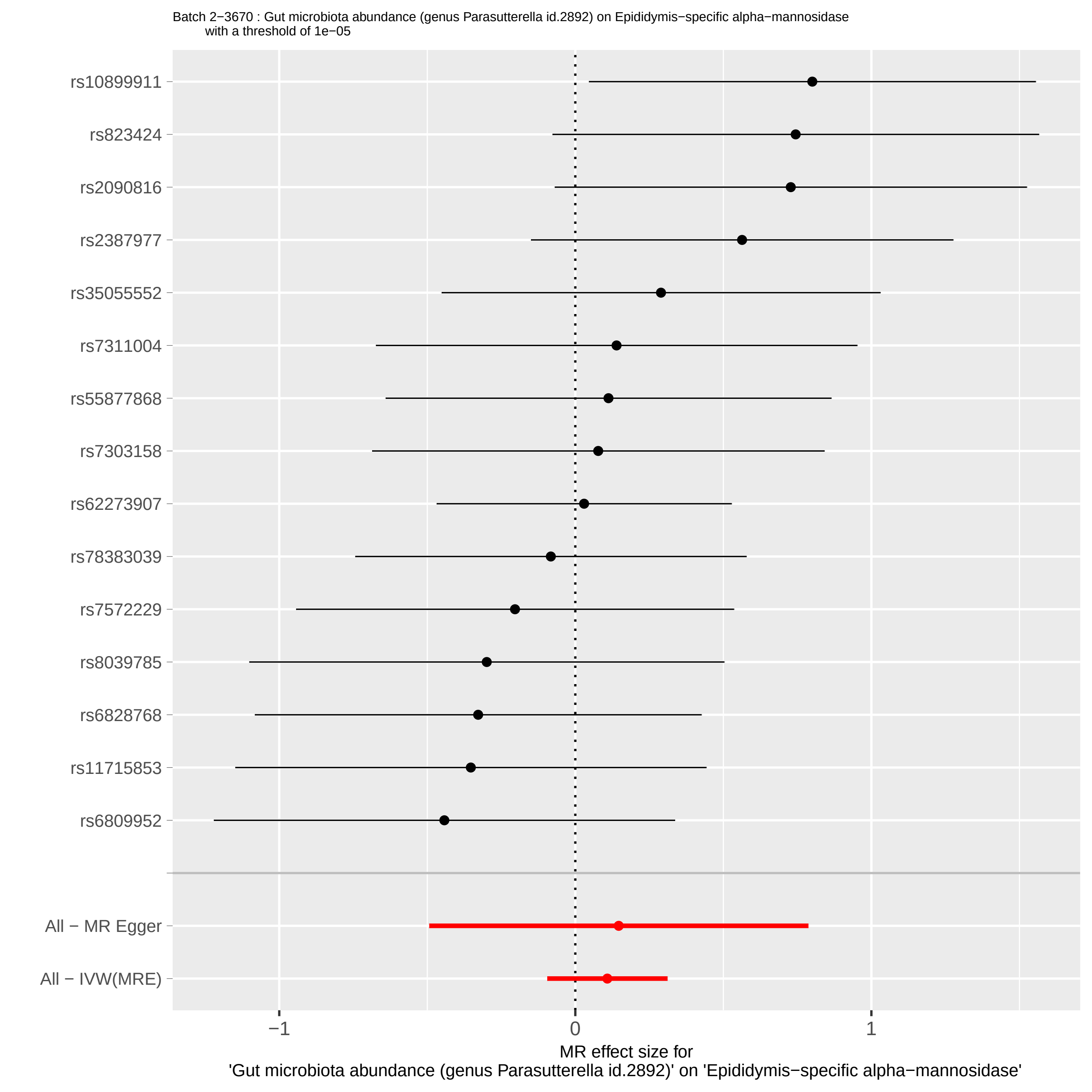

Batch 2−3670 : Gut microbiota abundance (genus Parasutterella id.2892) on Epididymis−specific alpha−mannosidase
with a threshold of 1e−05
rs10899911
rs823424
rs2090816
rs2387977
rs35055552
rs7311004
rs55877868
rs7303158
rs62273907
rs78383039
rs7572229
rs8039785
rs6828768
rs11715853
rs6809952
All − MR Egger
All − IVW(MRE)
−1
0
1
MR effect size for
'Gut microbiota abundance (genus Parasutterella id.2892)' on 'Epididymis−specific alpha−mannosidase'

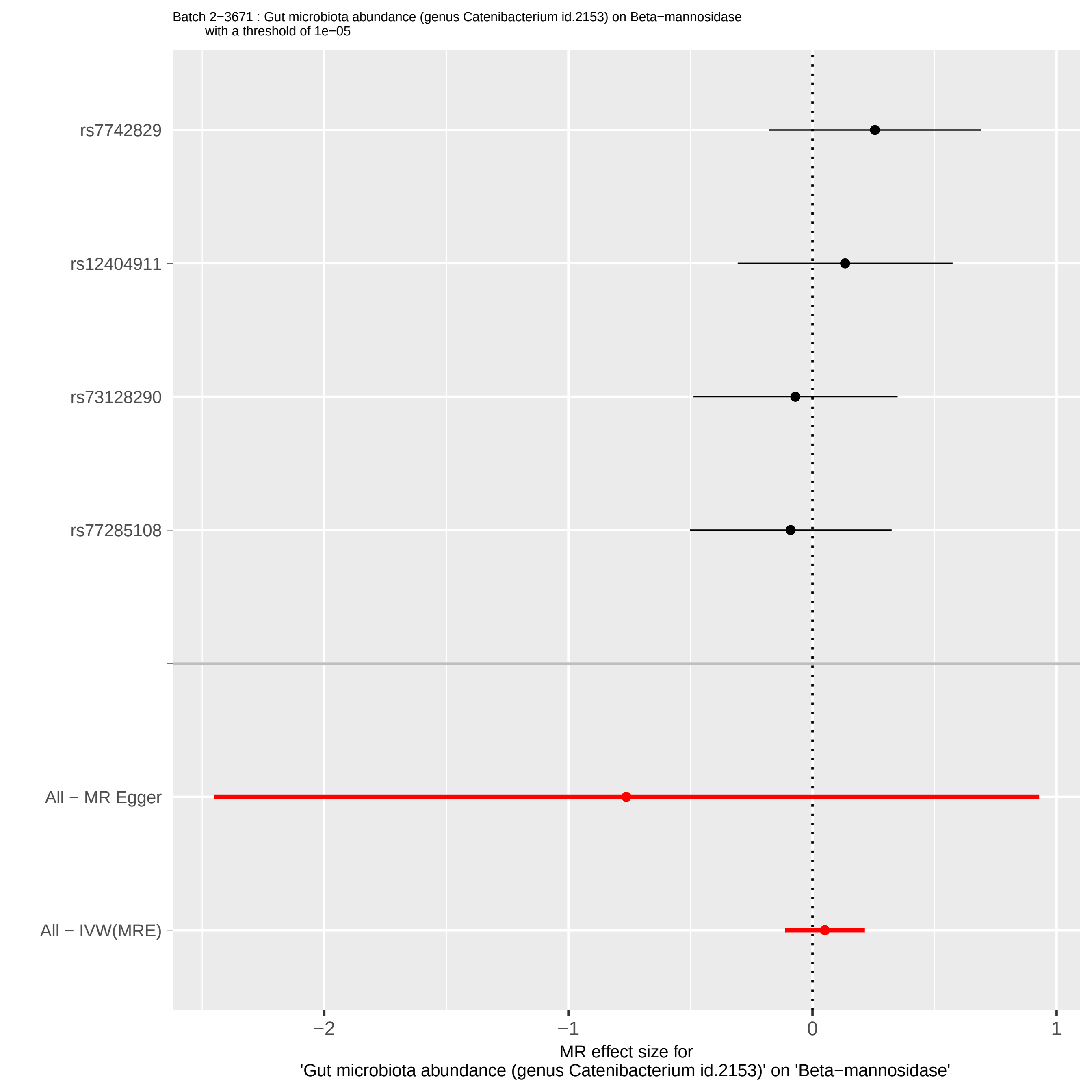
Batch 2−3671 : Gut microbiota abundance (genus Catenibacterium id.2153) on Beta−mannosidase
with a threshold of 1e−05
rs7742829
rs12404911
rs73128290
rs77285108
All − MR Egger
All − IVW(MRE)
−2
−1
0
1
MR effect size for
'Gut microbiota abundance (genus Catenibacterium id.2153)' on 'Beta−mannosidase'

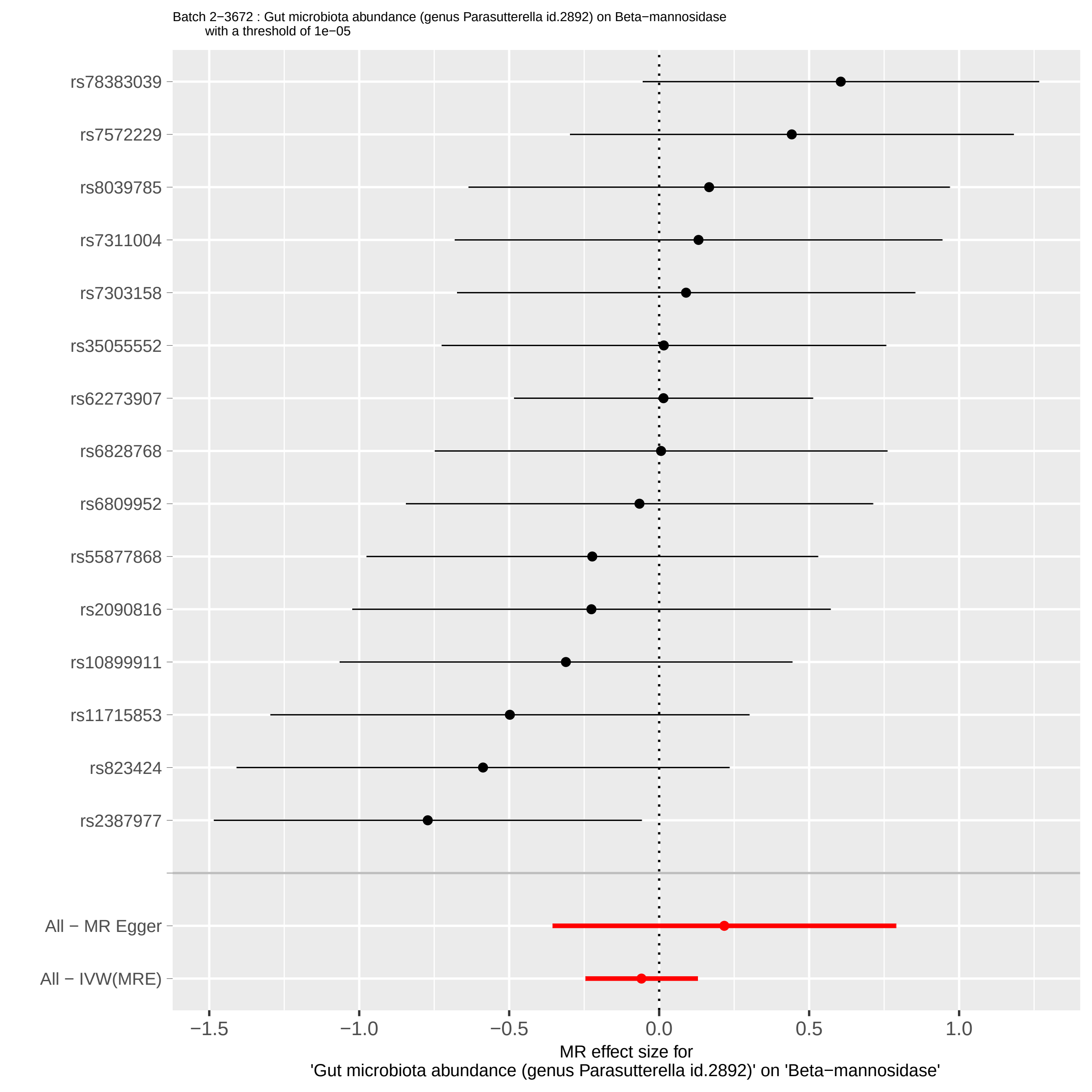

Batch 2−3672 : Gut microbiota abundance (genus Parasutterella id.2892) on Beta−mannosidase
with a threshold of 1e−05
rs78383039
rs7572229
rs8039785
rs7311004
rs7303158
rs35055552
rs62273907
rs6828768
rs6809952
rs55877868
rs2090816
rs10899911
rs11715853
rs823424
rs2387977
All − MR Egger
All − IVW(MRE)
−1.5
−1.0
−0.5
0.0
0.5
1.0
MR effect size for
'Gut microbiota abundance (genus Parasutterella id.2892)' on 'Beta−mannosidase'

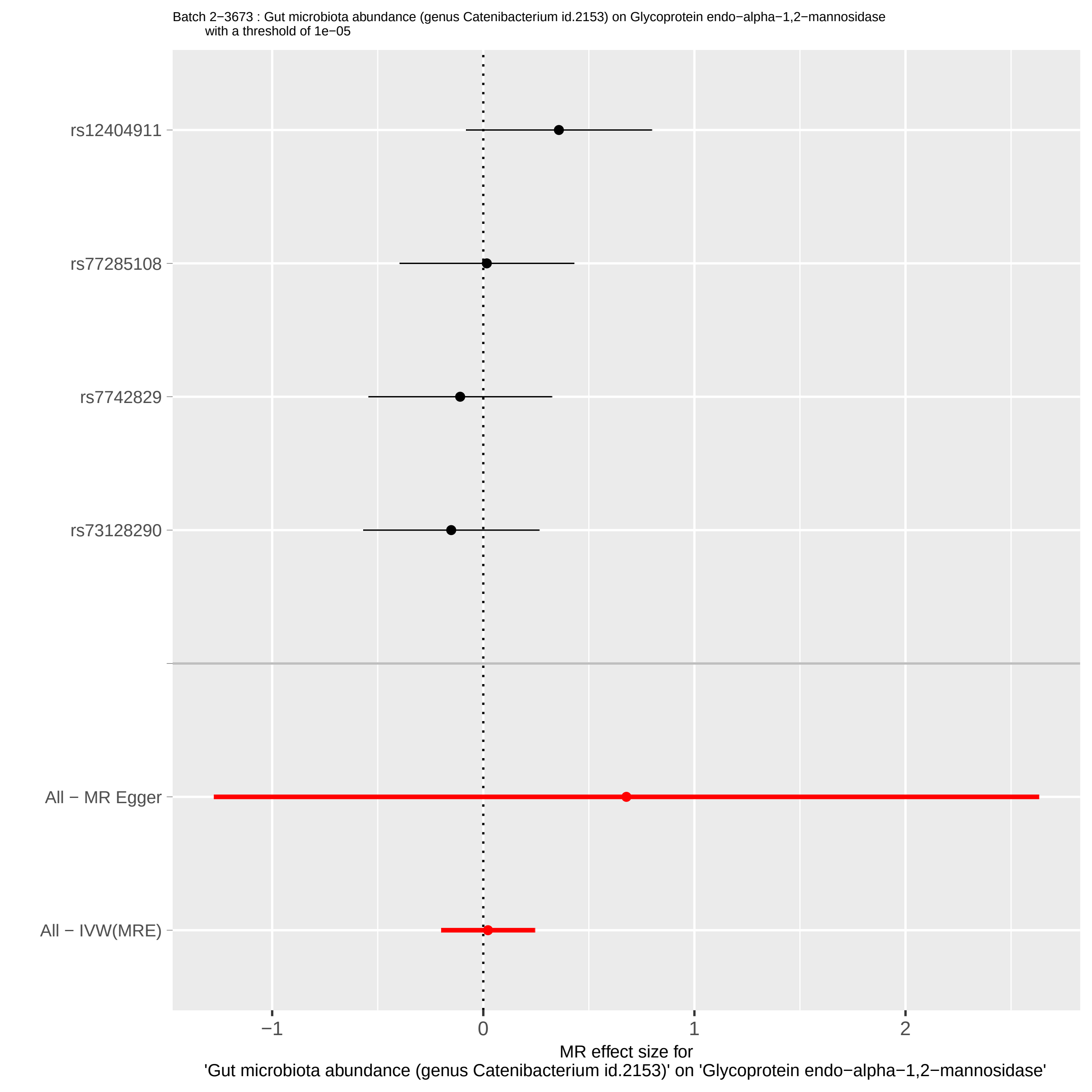
Batch 2−3673 : Gut microbiota abundance (genus Catenibacterium id.2153) on Glycoprotein endo−alpha−1,2−mannosidase with a threshold of 1e−05
rs12404911
rs77285108
rs7742829
rs73128290
All − MR Egger
All − IVW(MRE)
−1
0
1
2
MR effect size for
'Gut microbiota abundance (genus Catenibacterium id.2153)' on 'Glycoprotein endo−alpha−1,2−mannosidase'

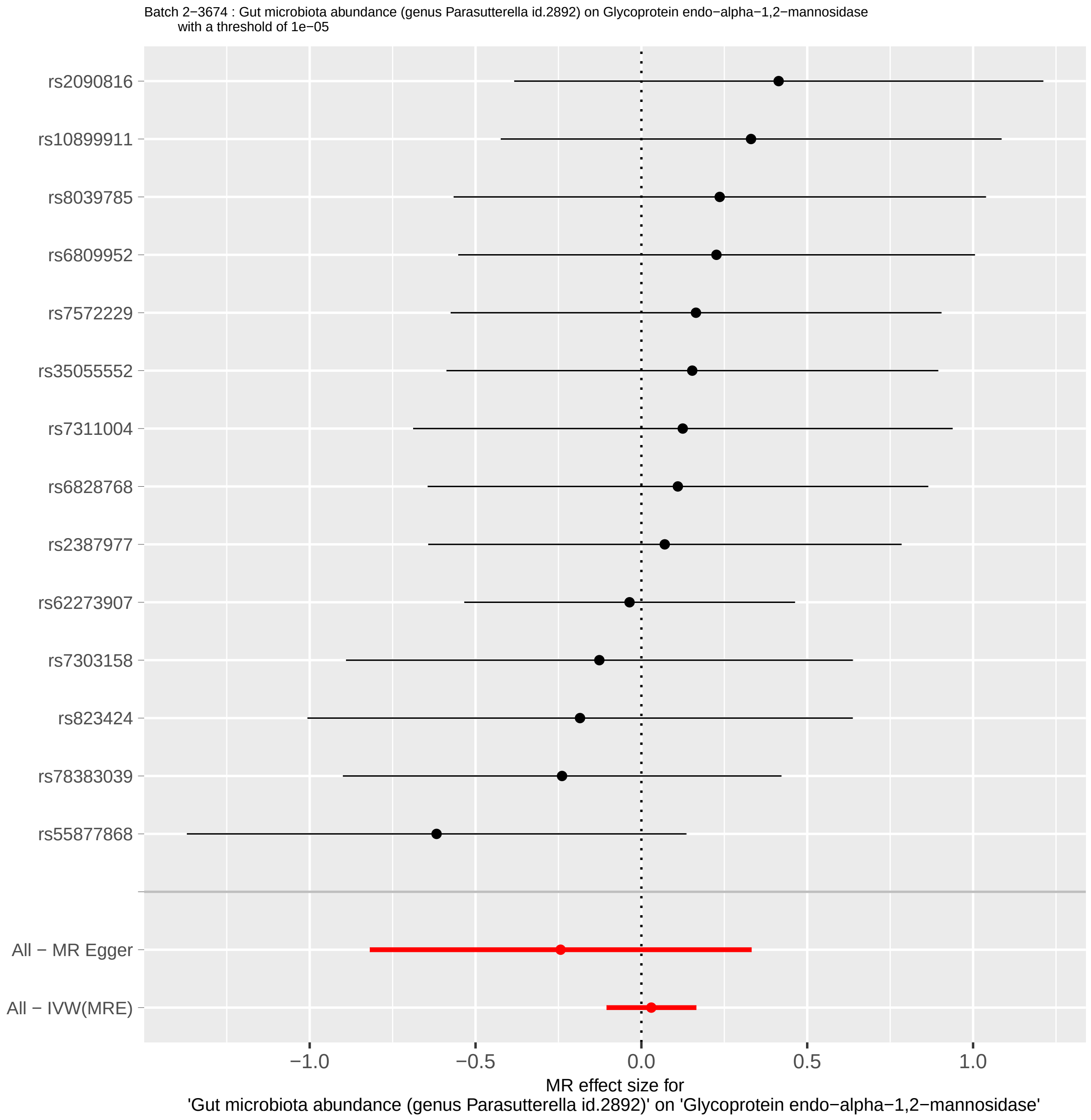
Batch 2−3674 : Gut microbiota abundance (genus Parasutterella id.2892) on Glycoprotein endo−alpha−1,2−mannosidase
with a threshold of 1e−05
rs2090816
rs10899911
rs8039785
rs6809952
rs7572229
rs35055552
rs7311004
rs6828768
rs2387977
rs62273907
rs7303158
rs823424
rs78383039
rs55877868
All − MR Egger
All − IVW(MRE)
−1.0
−0.5
0.0
0.5
1.0
MR effect size for
'Gut microbiota abundance (genus Parasutterella id.2892)' on 'Glycoprotein endo−alpha−1,2−mannosidase'

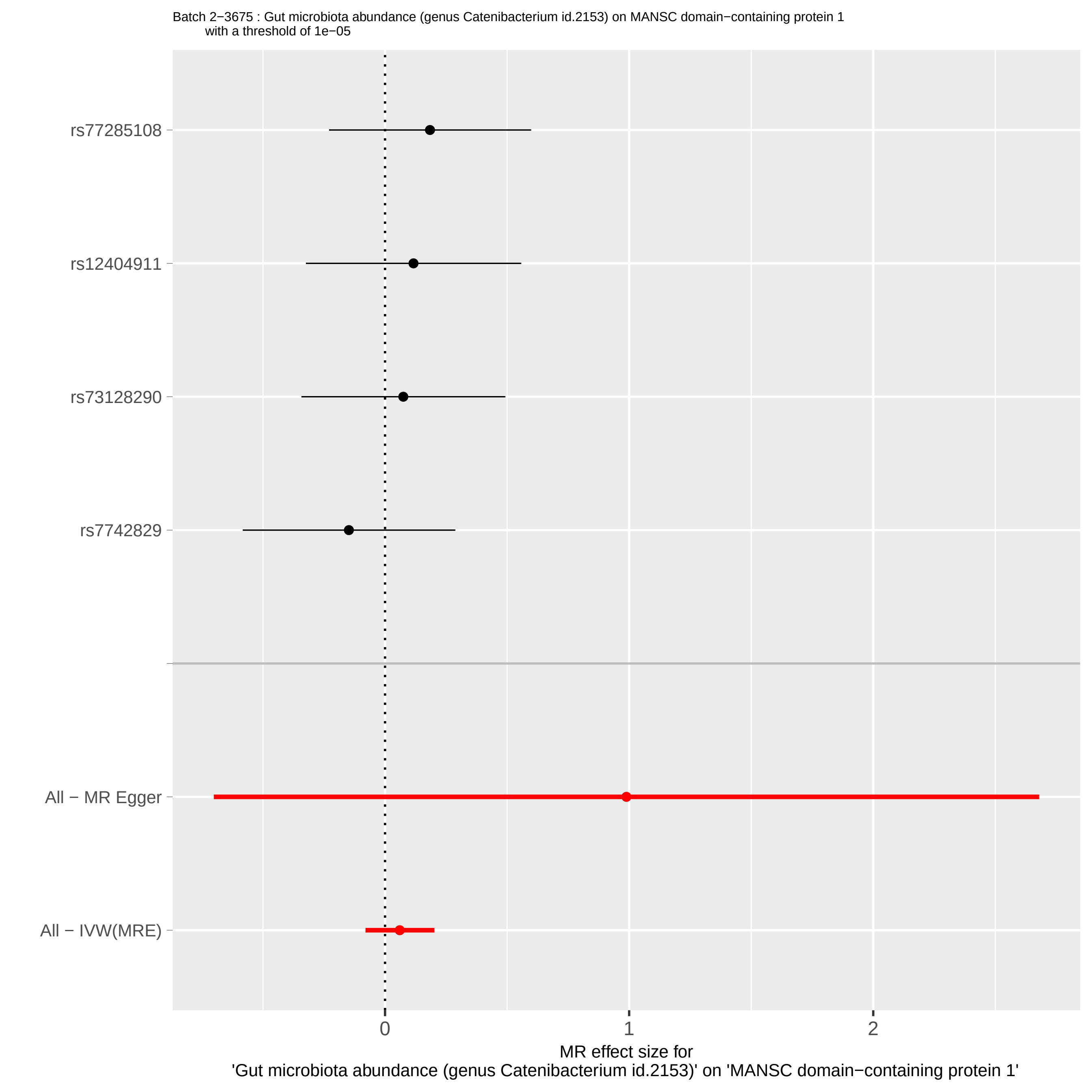

Batch 2−3675 : Gut microbiota abundance (genus Catenibacterium id.2153) on MANSC domain−containing protein 1
with a threshold of 1e−05
rs77285108
rs12404911
rs73128290
rs7742829
All − MR Egger
All − IVW(MRE)
0
1
2
MR effect size for
'Gut microbiota abundance (genus Catenibacterium id.2153)' on 'MANSC domain−containing protein 1'

Batch 2−3676 : Gut microbiota abundance (genus Parasutterella id.2892) on MANSC domain−containing protein 1
with a threshold of 1e−05

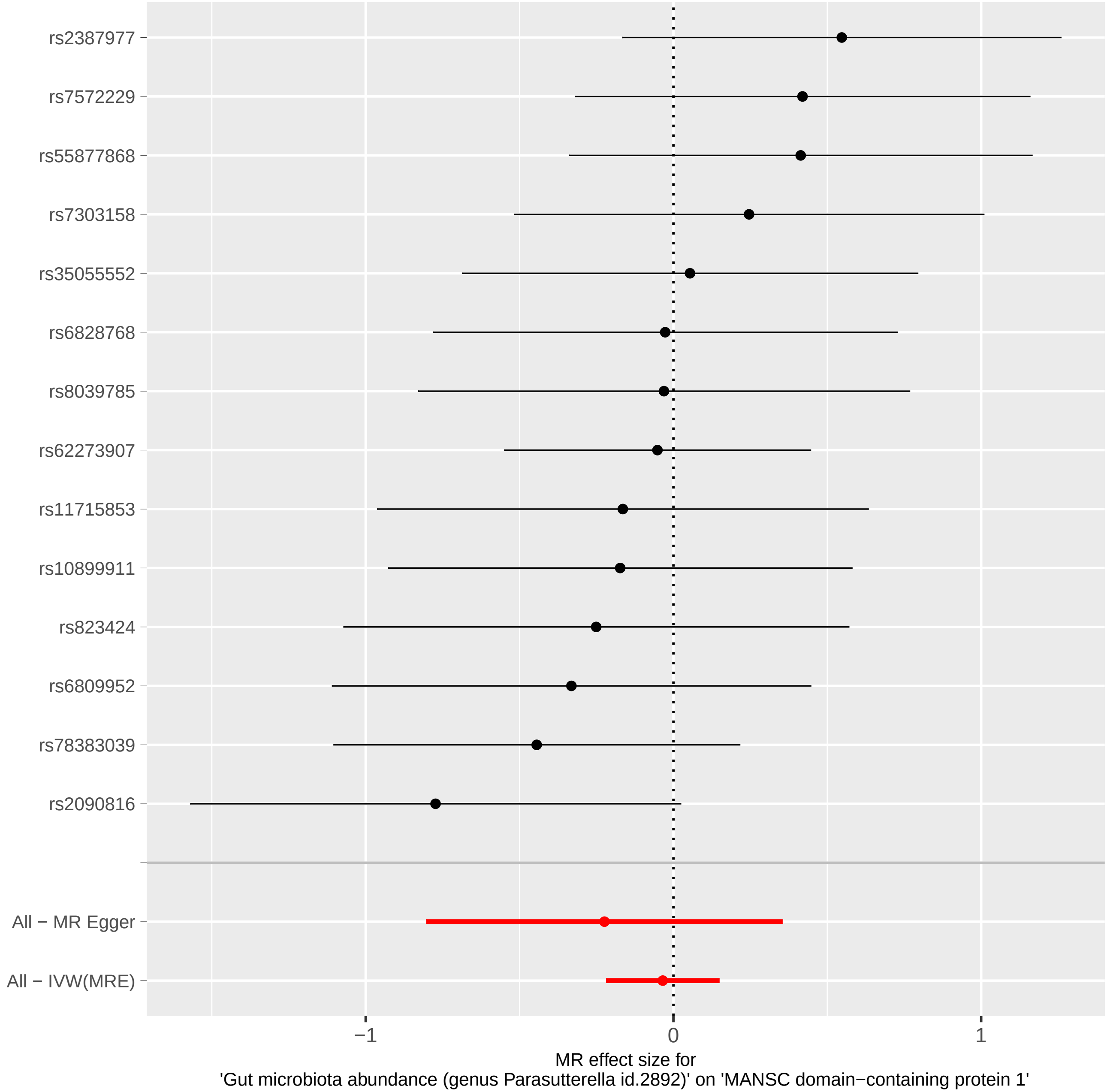

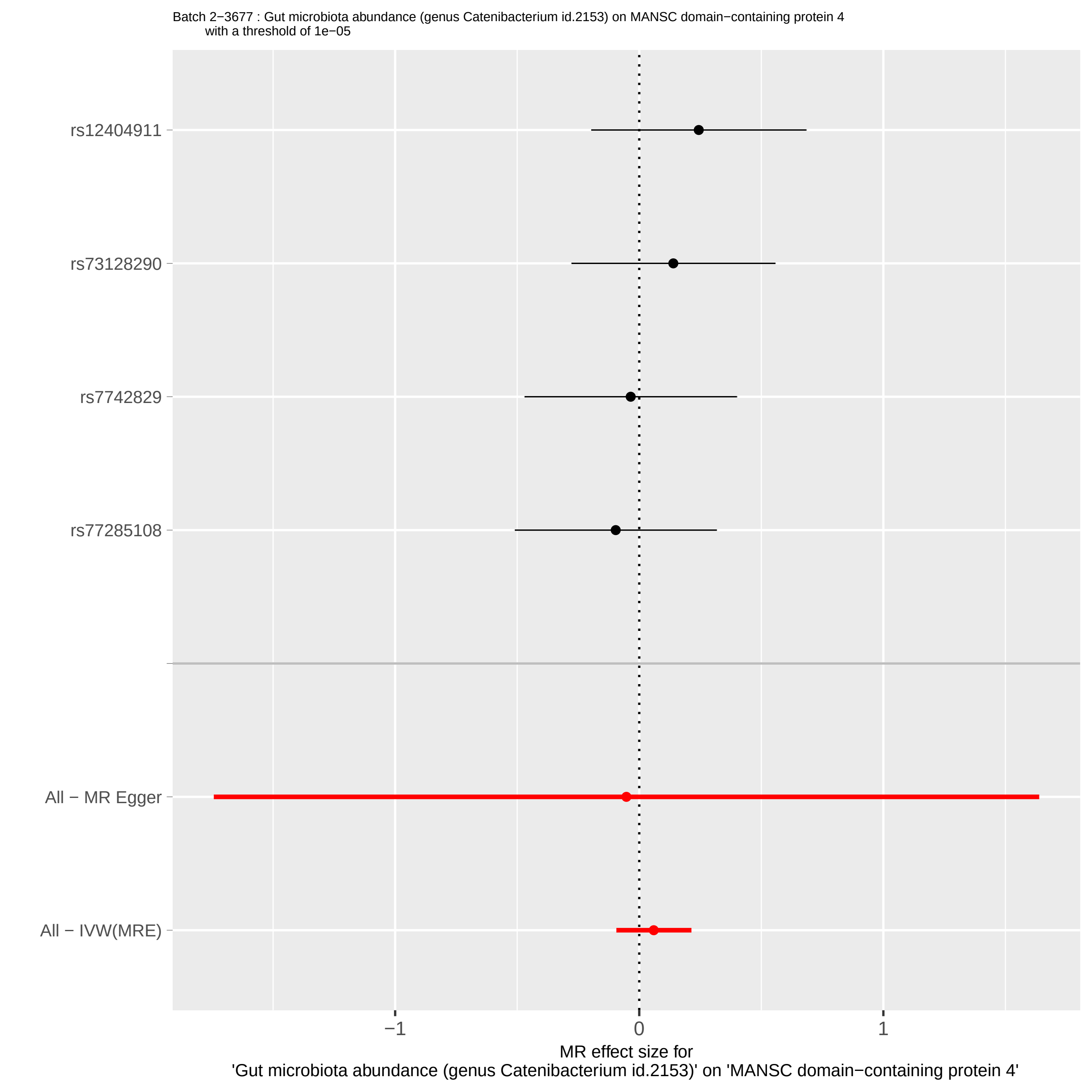

Batch 2−3677 : Gut microbiota abundance (genus Catenibacterium id.2153) on MANSC domain−containing protein 4
with a threshold of 1e−05
rs12404911
rs73128290
rs7742829
rs77285108
All − MR Egger
All − IVW(MRE)
−1
0
1
MR effect size for
'Gut microbiota abundance (genus Catenibacterium id.2153)' on 'MANSC domain−containing protein 4'

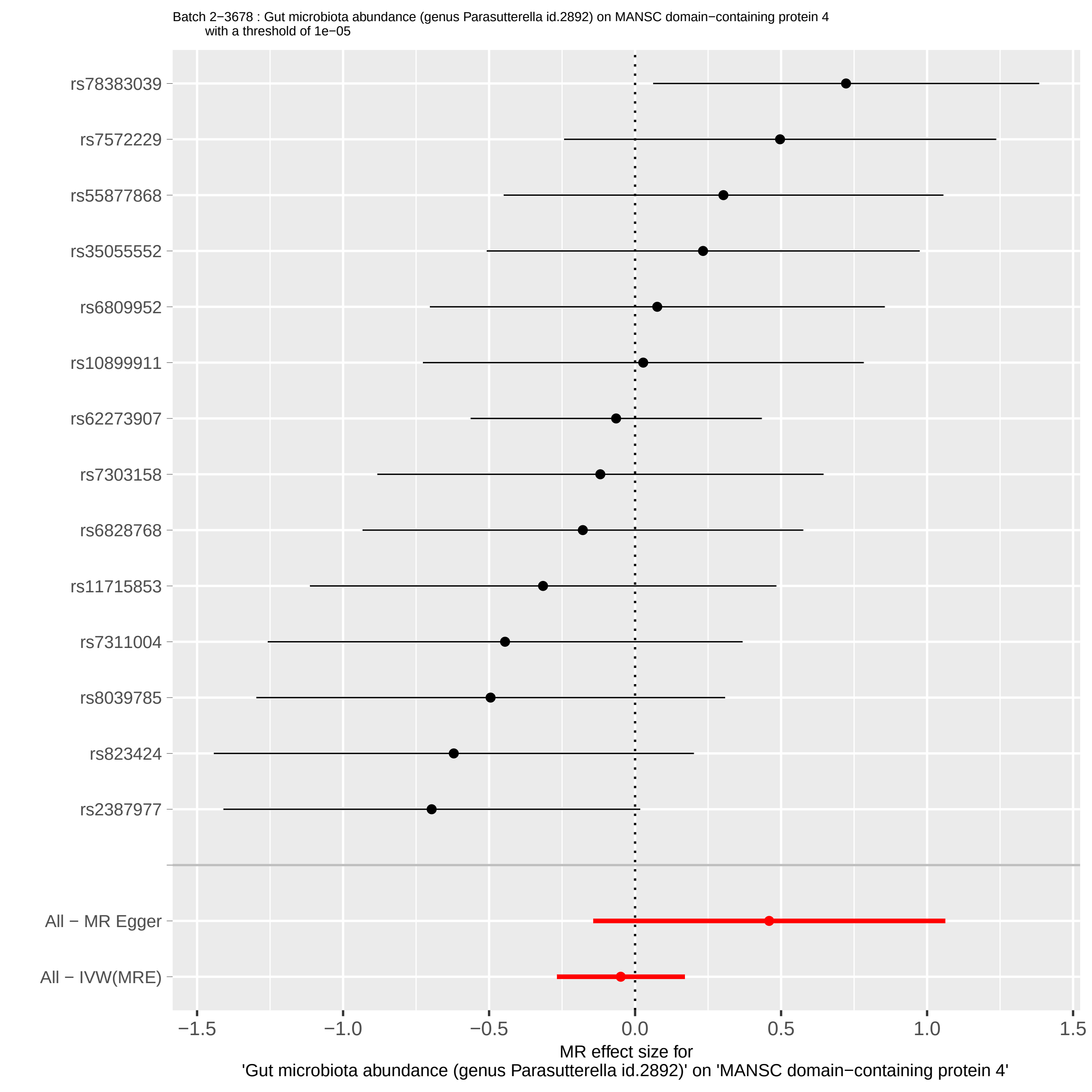

Batch 2−3678 : Gut microbiota abundance (genus Parasutterella id.2892) on MANSC domain−containing protein 4
with a threshold of 1e−05
rs78383039
rs7572229
rs55877868
rs35055552
rs6809952
rs10899911
rs62273907
rs7303158
rs6828768
rs11715853
rs7311004
rs8039785
rs823424
rs2387977
All − MR Egger
All − IVW(MRE)
−1.5
−1.0
−0.5
0.0
0.5
1.0
1.5
MR effect size for
'Gut microbiota abundance (genus Parasutterella id.2892)' on 'MANSC domain−containing protein 4'

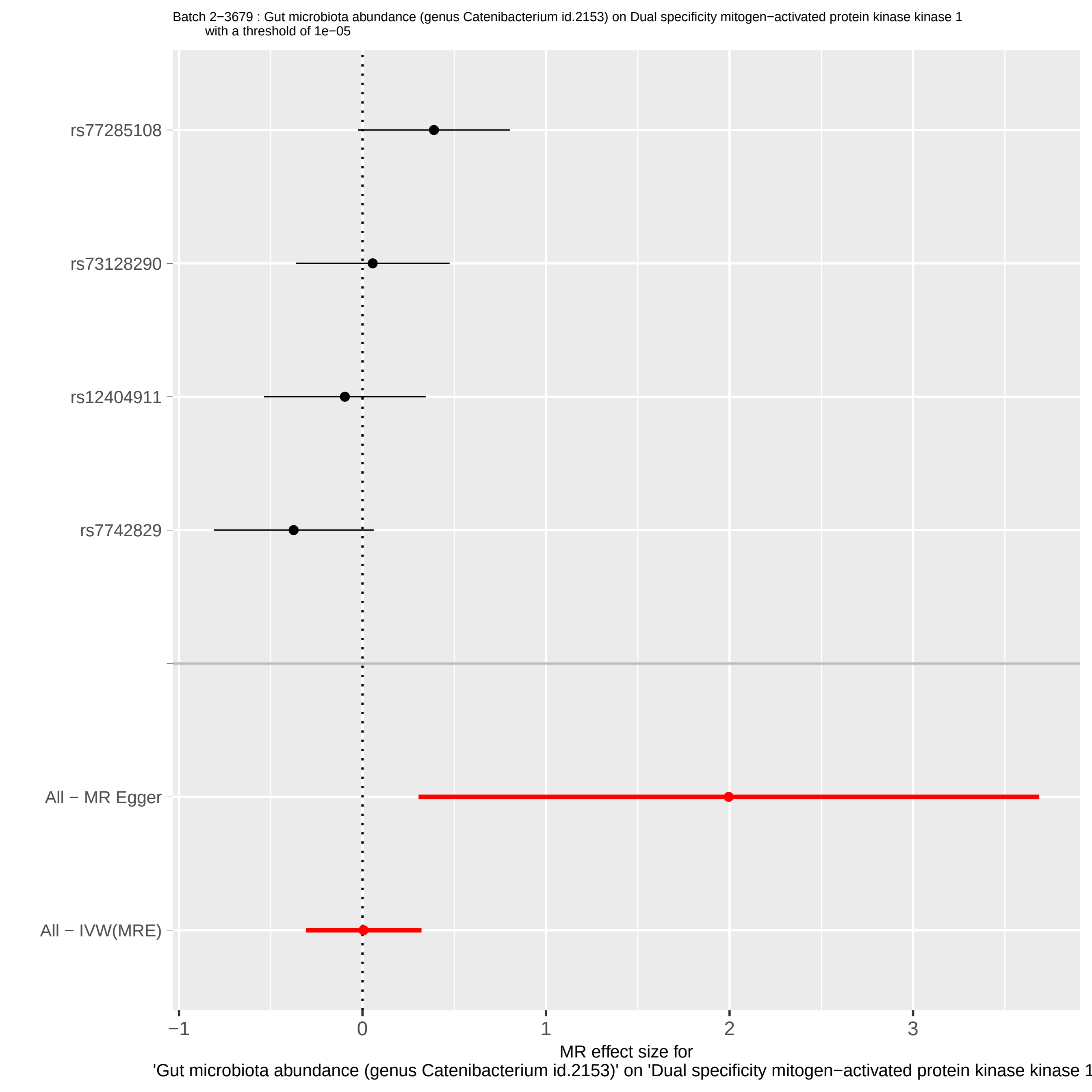
Batch 2−3679 : Gut microbiota abundance (genus Catenibacterium id.2153) on Dual specificity mitogen−activated protein kinase kinase 1
with a threshold of 1e−05
rs77285108
rs73128290
rs12404911
rs7742829
All − MR Egger
All − IVW(MRE)
−1
0
1
2
3
MR effect size for
'Gut microbiota abundance (genus Catenibacterium id.2153)' on 'Dual specificity mitogen−activated protein kinase kinase 1

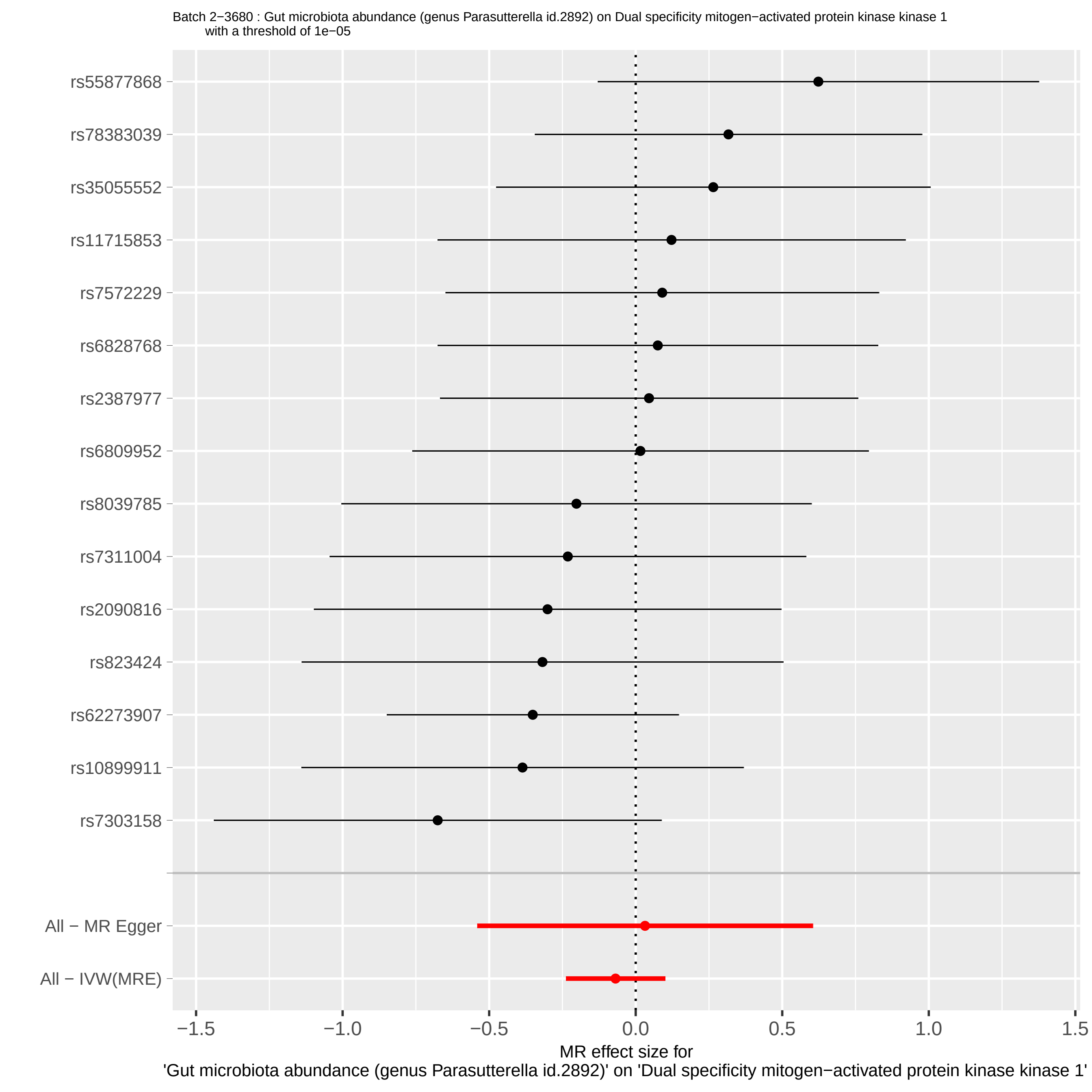

Batch 2−3680 : Gut microbiota abundance (genus Parasutterella id.2892) on Dual specificity mitogen−activated protein kinase kinase 1 with a threshold of 1e−05
rs55877868
rs78383039
rs35055552
rs11715853
rs7572229
rs6828768
rs2387977
rs6809952
rs8039785
rs7311004
rs2090816
rs823424
rs62273907
rs10899911
rs7303158
All − MR Egger
All − IVW(MRE)
−1.5
−1.0
−0.5
0.0
0.5
1.0
1.5
MR effect size for
'Gut microbiota abundance (genus Parasutterella id.2892)' on 'Dual specificity mitogen−activated protein kinase kinase 1'

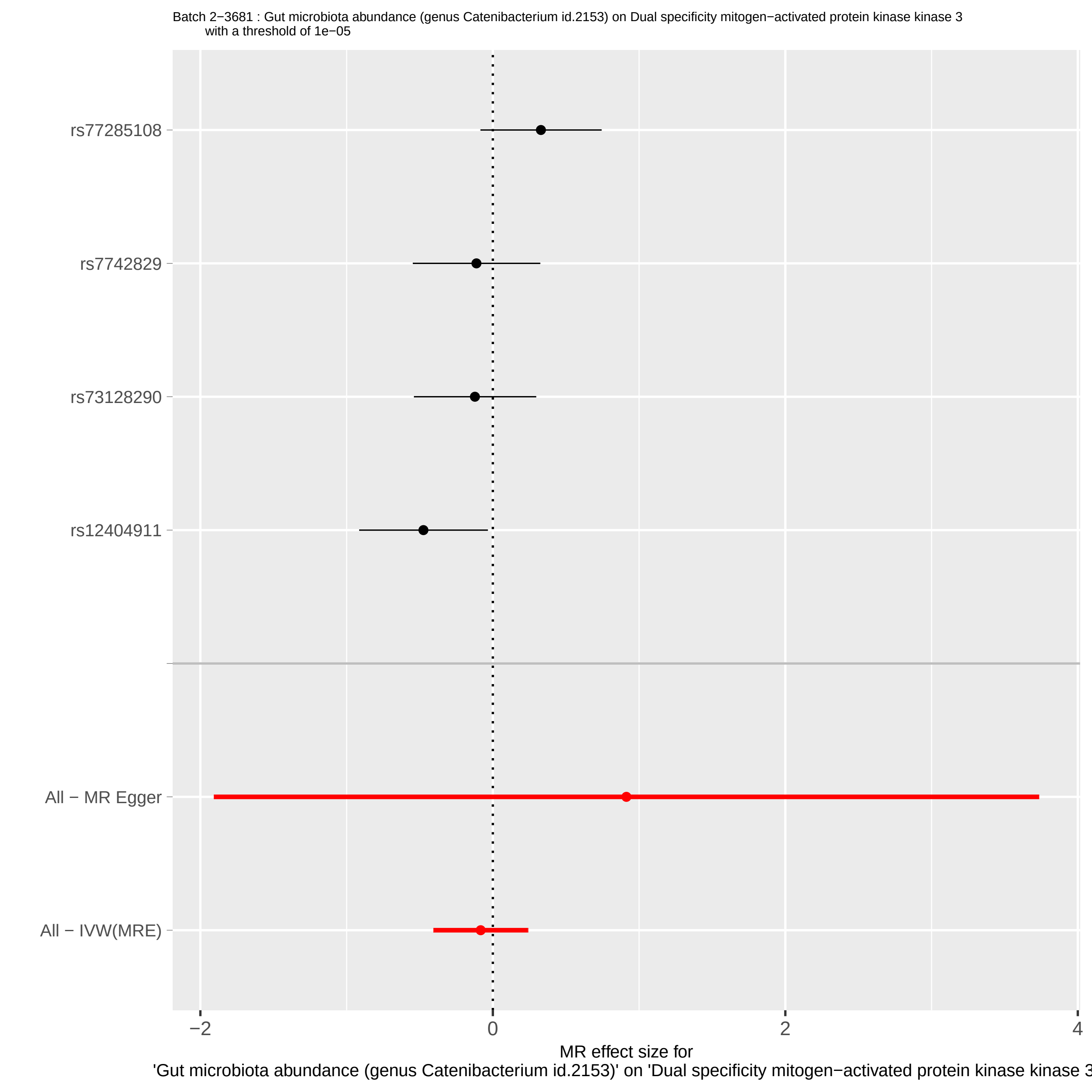
Batch 2−3681 : Gut microbiota abundance (genus Catenibacterium id.2153) on Dual specificity mitogen−activated protein kinase kinase 3
with a threshold of 1e−05
rs77285108
rs7742829
rs73128290
rs12404911
All − MR Egger
All − IVW(MRE)
−2
0
2
4
MR effect size for
'Gut microbiota abundance (genus Catenibacterium id.2153)' on 'Dual specificity mitogen−activated protein kinase kinase 3

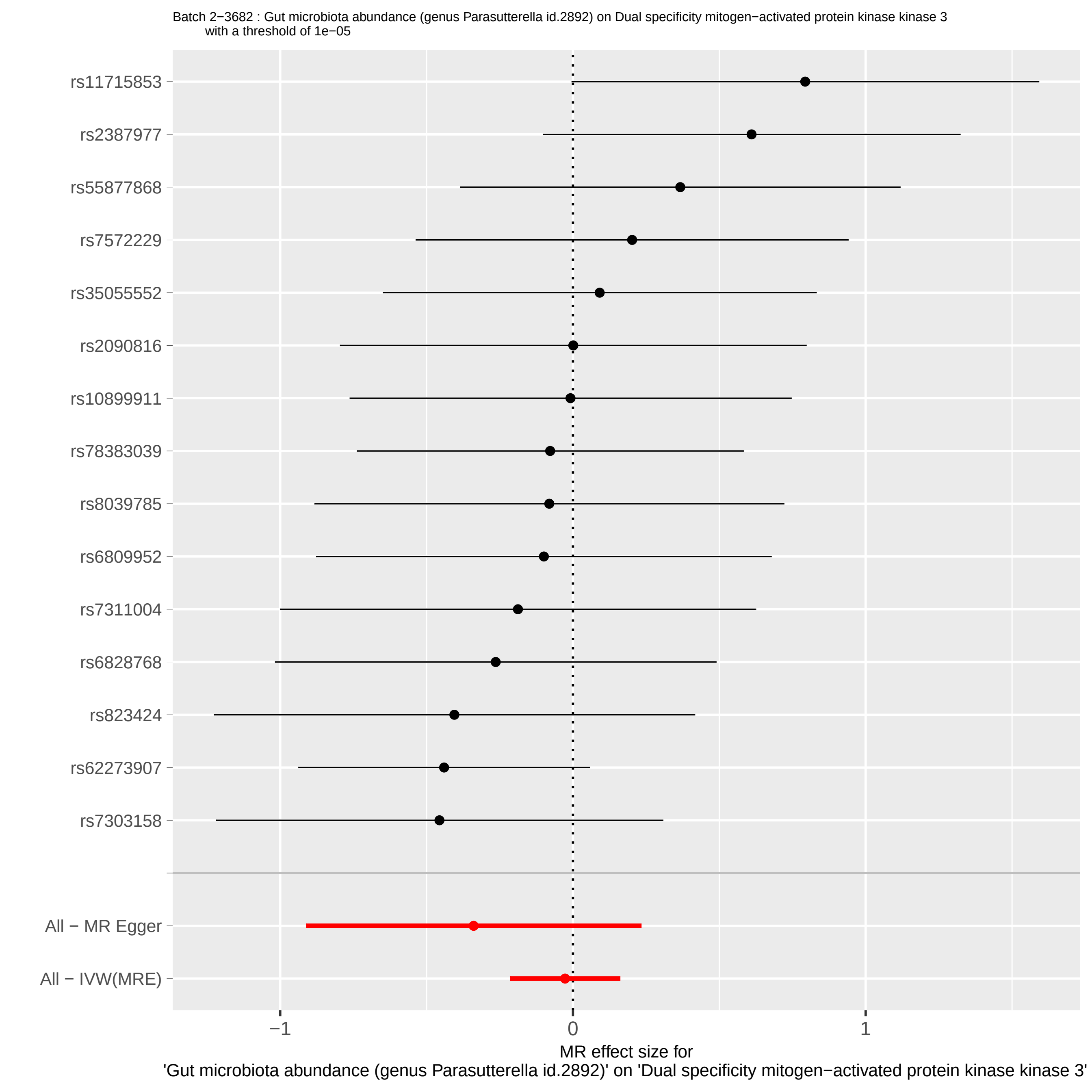
Batch 2−3682 : Gut microbiota abundance (genus Parasutterella id.2892) on Dual specificity mitogen−activated protein kinase kinase 3
with a threshold of 1e−05
rs11715853
rs2387977
rs55877868
rs7572229
rs35055552
rs2090816
rs10899911
rs78383039
rs8039785
rs6809952
rs7311004
rs6828768
rs823424
rs62273907
rs7303158
All − MR Egger
All − IVW(MRE)
−1
0
1
MR effect size for
'Gut microbiota abundance (genus Parasutterella id.2892)' on 'Dual specificity mitogen−activated protein kinase kinase 3'

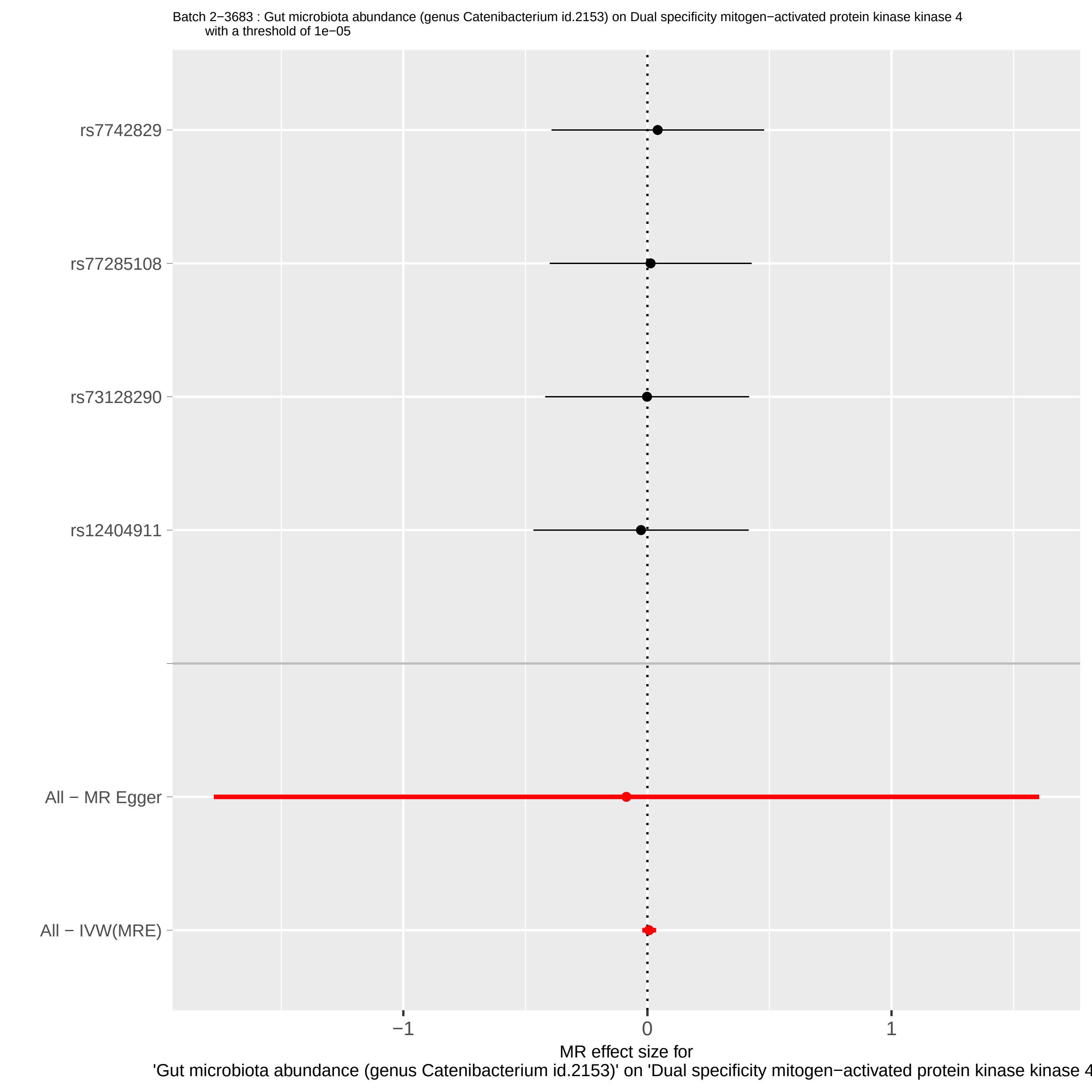
Batch 2−3683 : Gut microbiota abundance (genus Catenibacterium id.2153) on Dual specificity mitogen−activated protein kinase kinase 4
with a threshold of 1e−05
rs7742829
rs77285108
rs73128290
rs12404911
All − MR Egger
All − IVW(MRE)
−1
0
1
MR effect size for
'Gut microbiota abundance (genus Catenibacterium id.2153)' on 'Dual specificity mitogen−activated protein kinase kinase 4

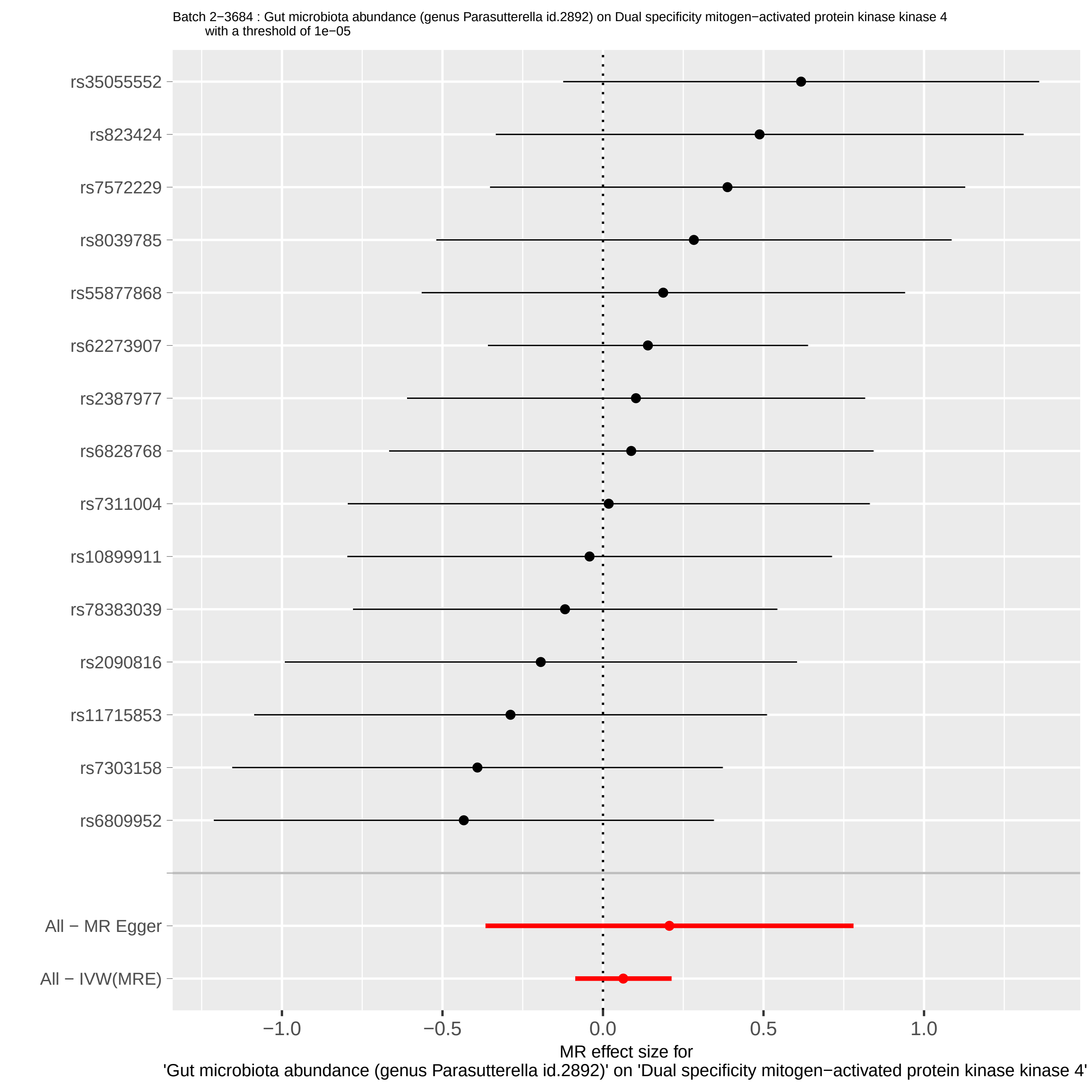

Batch 2−3684 : Gut microbiota abundance (genus Parasutterella id.2892) on Dual specificity mitogen−activated protein kinase kinase 4
with a threshold of 1e−05
rs35055552
rs823424
rs7572229
rs8039785
rs55877868
rs62273907
rs2387977
rs6828768
rs7311004
rs10899911
rs78383039
rs2090816
rs11715853
rs7303158
rs6809952
All − MR Egger
All − IVW(MRE)
−1.0
−0.5
0.0
0.5
1.0
MR effect size for
'Gut microbiota abundance (genus Parasutterella id.2892)' on 'Dual specificity mitogen−activated protein kinase kinase 4'

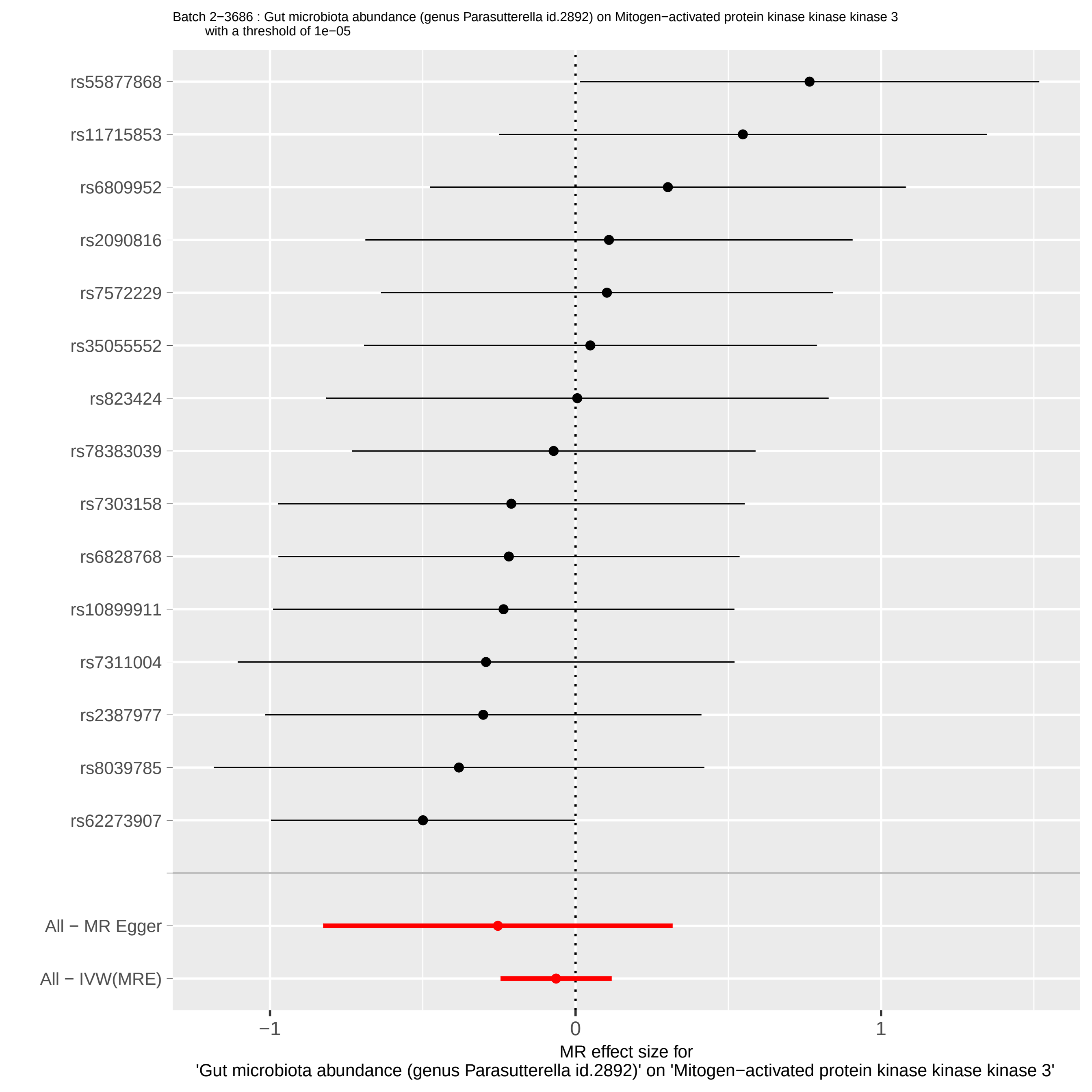

Batch 2−3686 : Gut microbiota abundance (genus Parasutterella id.2892) on Mitogen−activated protein kinase kinase kinase 3
with a threshold of 1e−05
rs55877868
rs11715853
rs6809952
rs2090816
rs7572229
rs35055552
rs823424
rs78383039
rs7303158
rs6828768
rs10899911
rs7311004
rs2387977
rs8039785
rs62273907
All − MR Egger
All − IVW(MRE)
−1
0
1
MR effect size for
'Gut microbiota abundance (genus Parasutterella id.2892)' on 'Mitogen−activated protein kinase kinase kinase 3'

Batch 2−3687 : Gut microbiota abundance (genus Catenibacterium id.2153) on Mitogen−activated protein kinase kinase kinase 7:TGF−beta−activated kinase 1 a
with a threshold of 1e−05

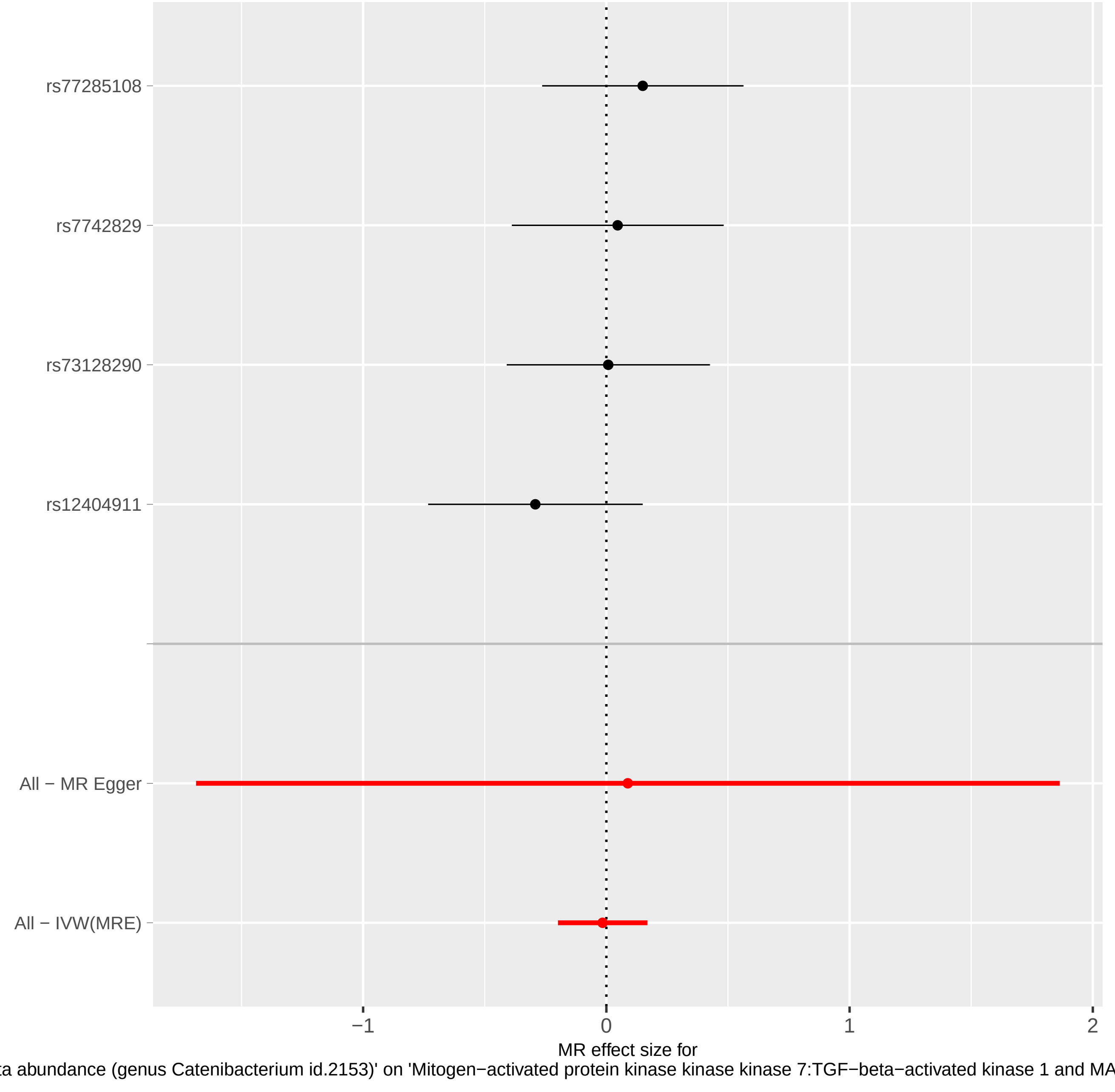

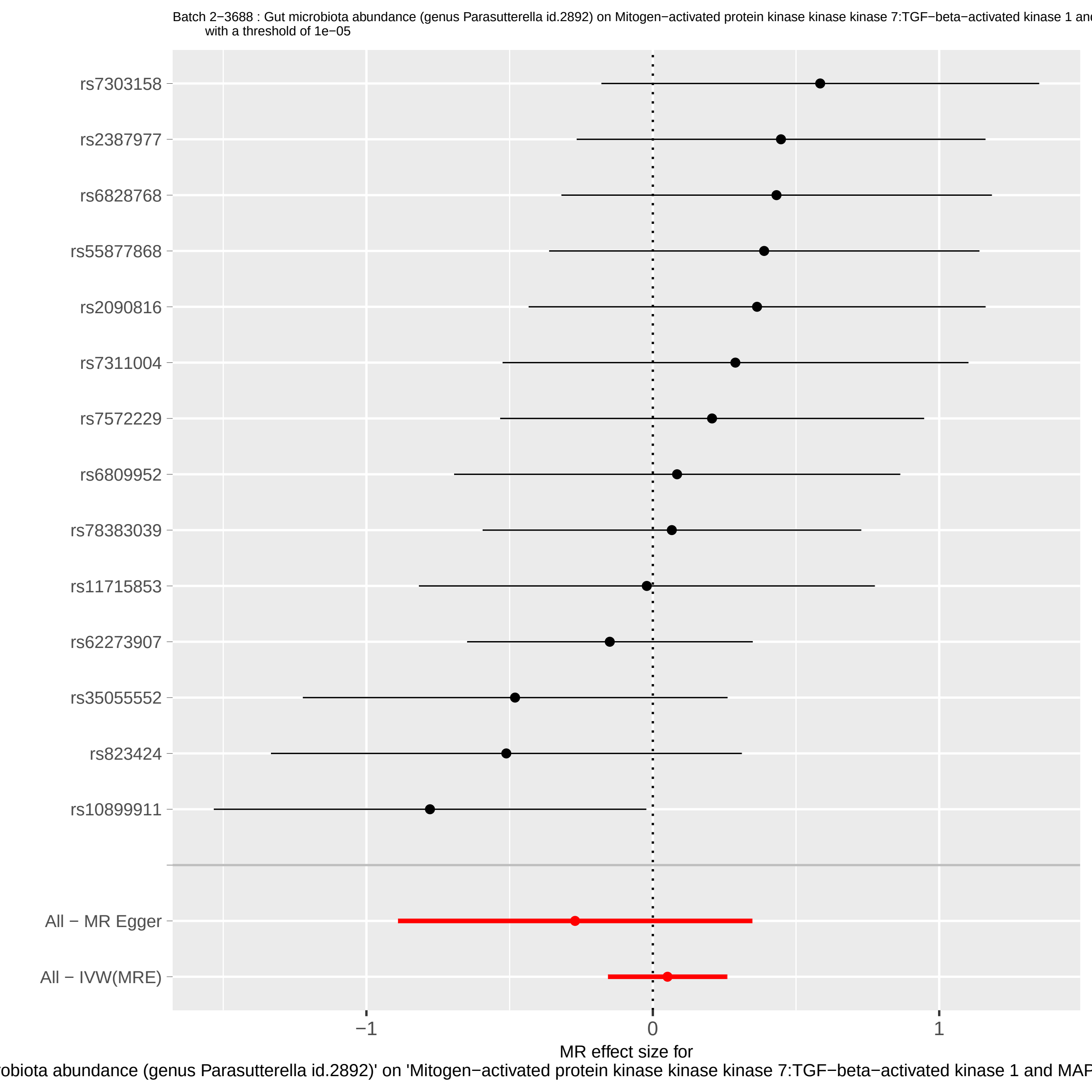

Batch 2−3688 : Gut microbiota abundance (genus Parasutterella id.2892) on Mitogen−activated protein kinase kinase kinase 7:TGF−beta−activated kinase 1 and
with a threshold of 1e−05
rs7303158
rs2387977
rs6828768
rs55877868
rs2090816
rs7311004
rs7572229
rs6809952
rs78383039
rs11715853
rs62273907
rs35055552
rs823424
rs10899911
All − MR Egger
All − IVW(MRE)
−1
0
1
MR effect size for
robiota abundance (genus Parasutterella id.2892)' on 'Mitogen−activated protein kinase kinase kinase 7:TGF−beta−activated kinase 1 and MAP

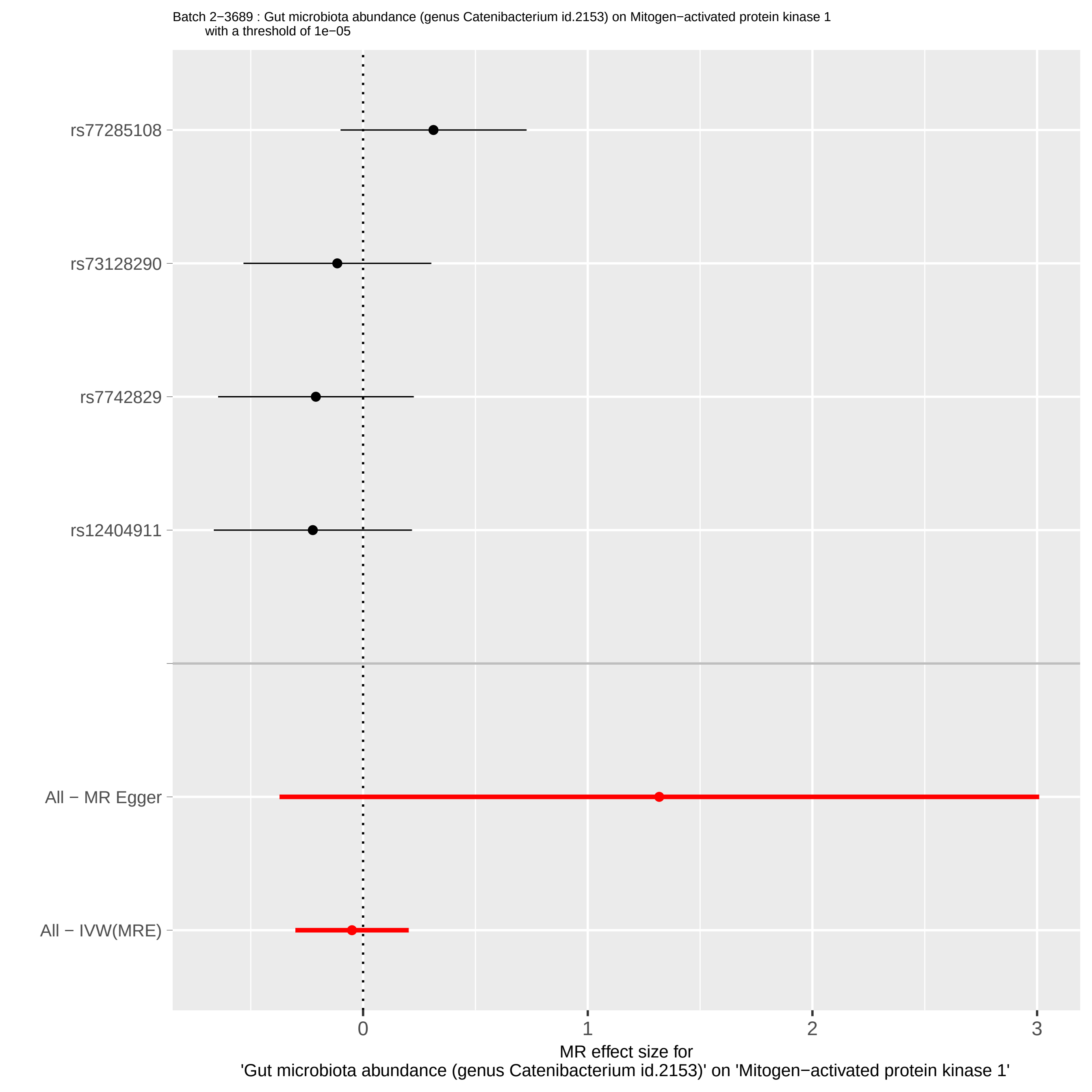

Batch 2−3689 : Gut microbiota abundance (genus Catenibacterium id.2153) on Mitogen−activated protein kinase 1
with a threshold of 1e−05
rs77285108
rs73128290
rs7742829
rs12404911
All − MR Egger
All − IVW(MRE)
0
1
2
3
MR effect size for
'Gut microbiota abundance (genus Catenibacterium id.2153)' on 'Mitogen−activated protein kinase 1'

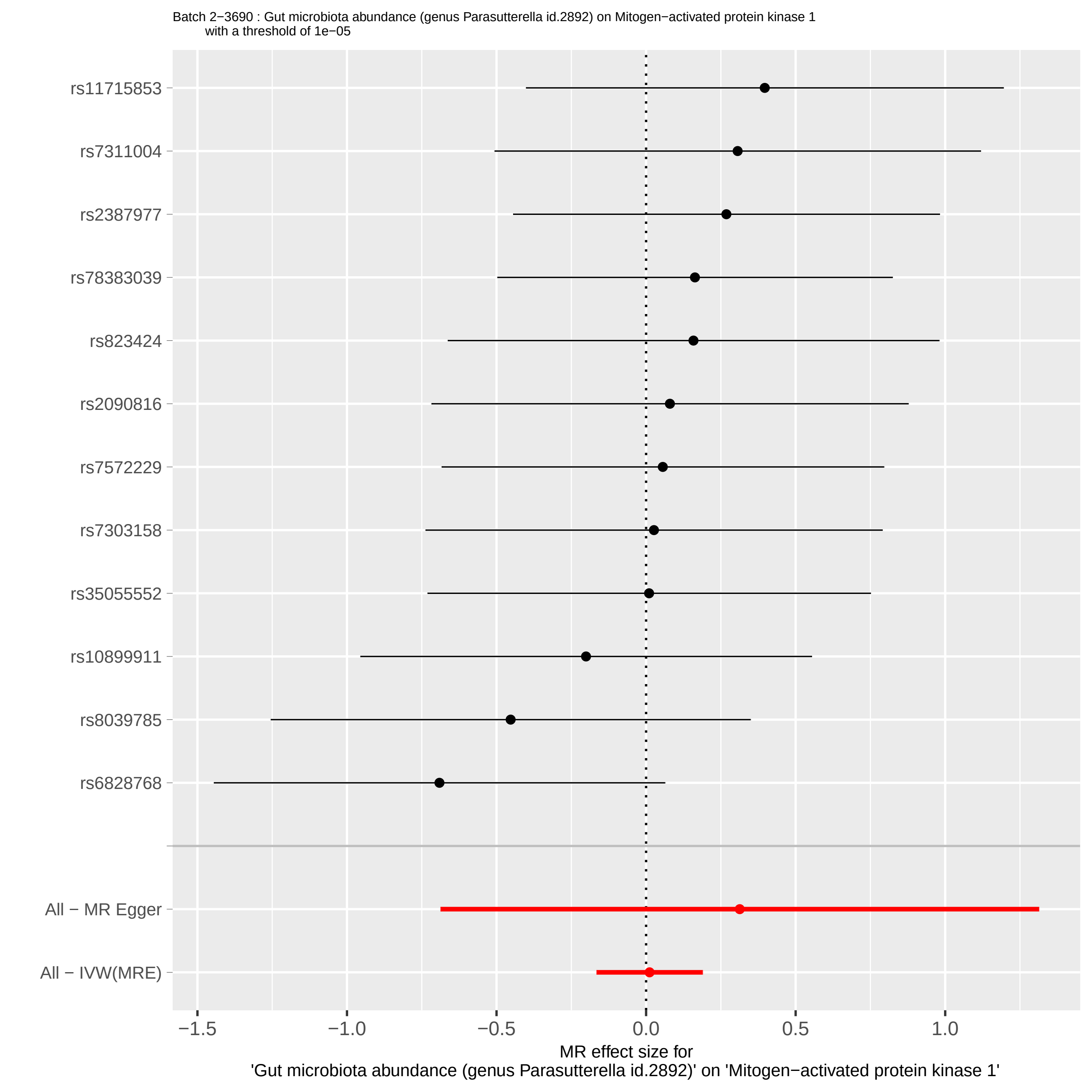

Batch 2−3690 : Gut microbiota abundance (genus Parasutterella id.2892) on Mitogen−activated protein kinase 1
with a threshold of 1e−05
rs11715853
rs7311004
rs2387977
rs78383039
rs823424
rs2090816
rs7572229
rs7303158
rs35055552
rs10899911
rs8039785
rs6828768
All − MR Egger
All − IVW(MRE)
−1.5
−1.0
−0.5
0.0
0.5
1.0
MR effect size for
'Gut microbiota abundance (genus Parasutterella id.2892)' on 'Mitogen−activated protein kinase 1'

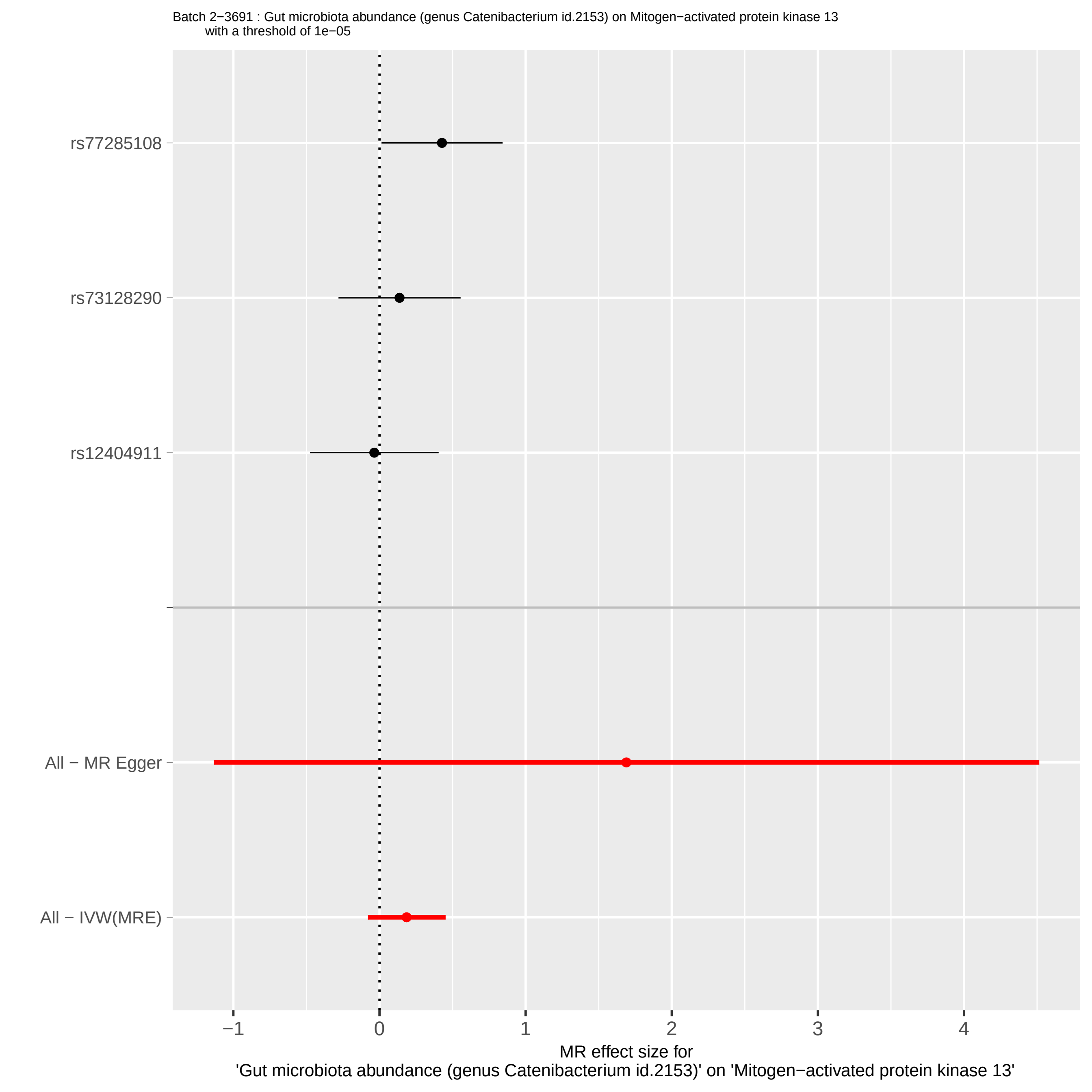

Batch 2−3691 : Gut microbiota abundance (genus Catenibacterium id.2153) on Mitogen−activated protein kinase 13
with a threshold of 1e−05
rs77285108
rs73128290
rs12404911
All − MR Egger
All − IVW(MRE)
−1
0
1
2
3
4
MR effect size for
'Gut microbiota abundance (genus Catenibacterium id.2153)' on 'Mitogen−activated protein kinase 13'

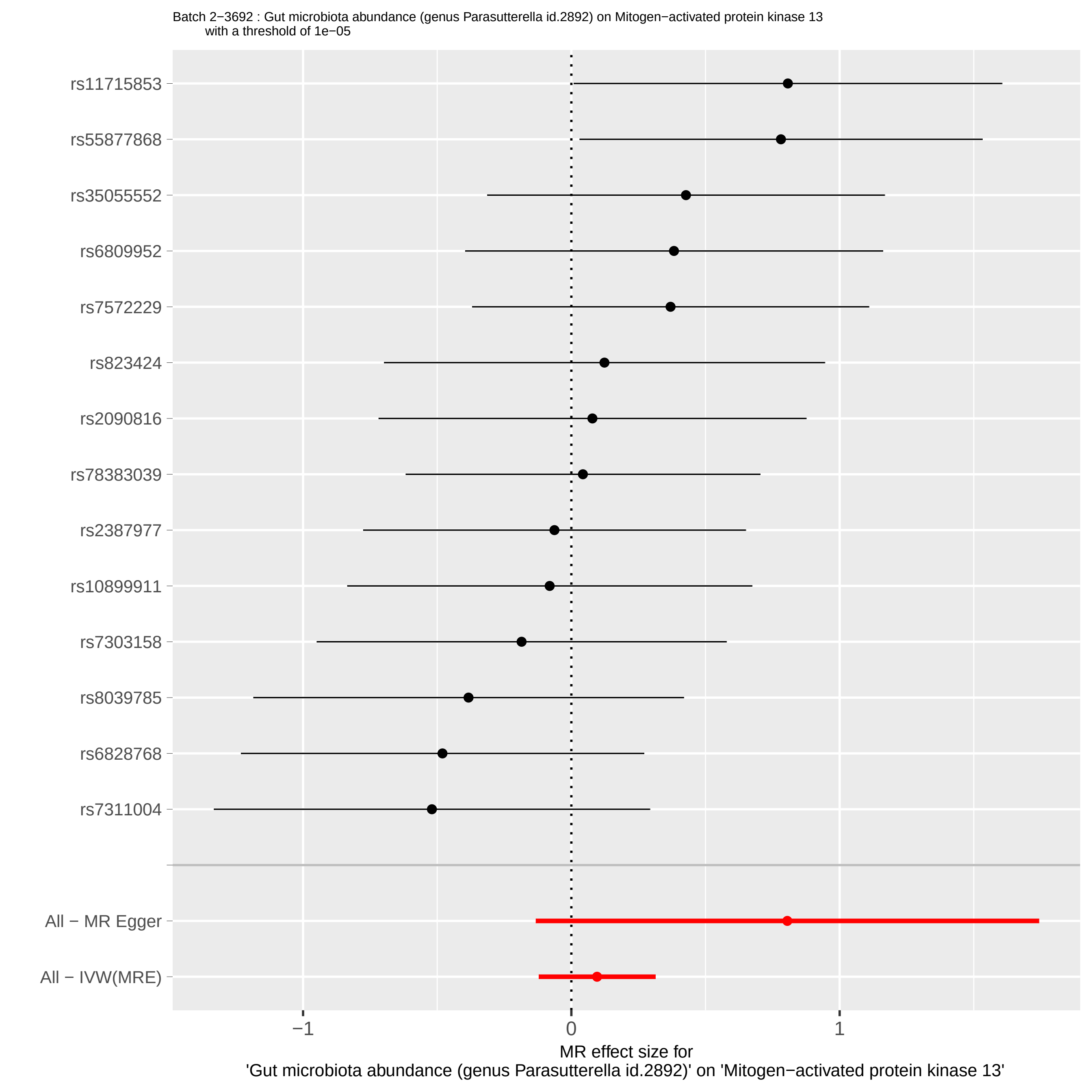

Batch 2−3692 : Gut microbiota abundance (genus Parasutterella id.2892) on Mitogen−activated protein kinase 13
with a threshold of 1e−05
rs11715853
rs55877868
rs35055552
rs6809952
rs7572229
rs823424
rs2090816
rs78383039
rs2387977
rs10899911
rs7303158
rs8039785
rs6828768
rs7311004
All − MR Egger
All − IVW(MRE)
−1
0
1
MR effect size for
'Gut microbiota abundance (genus Parasutterella id.2892)' on 'Mitogen−activated protein kinase 13'

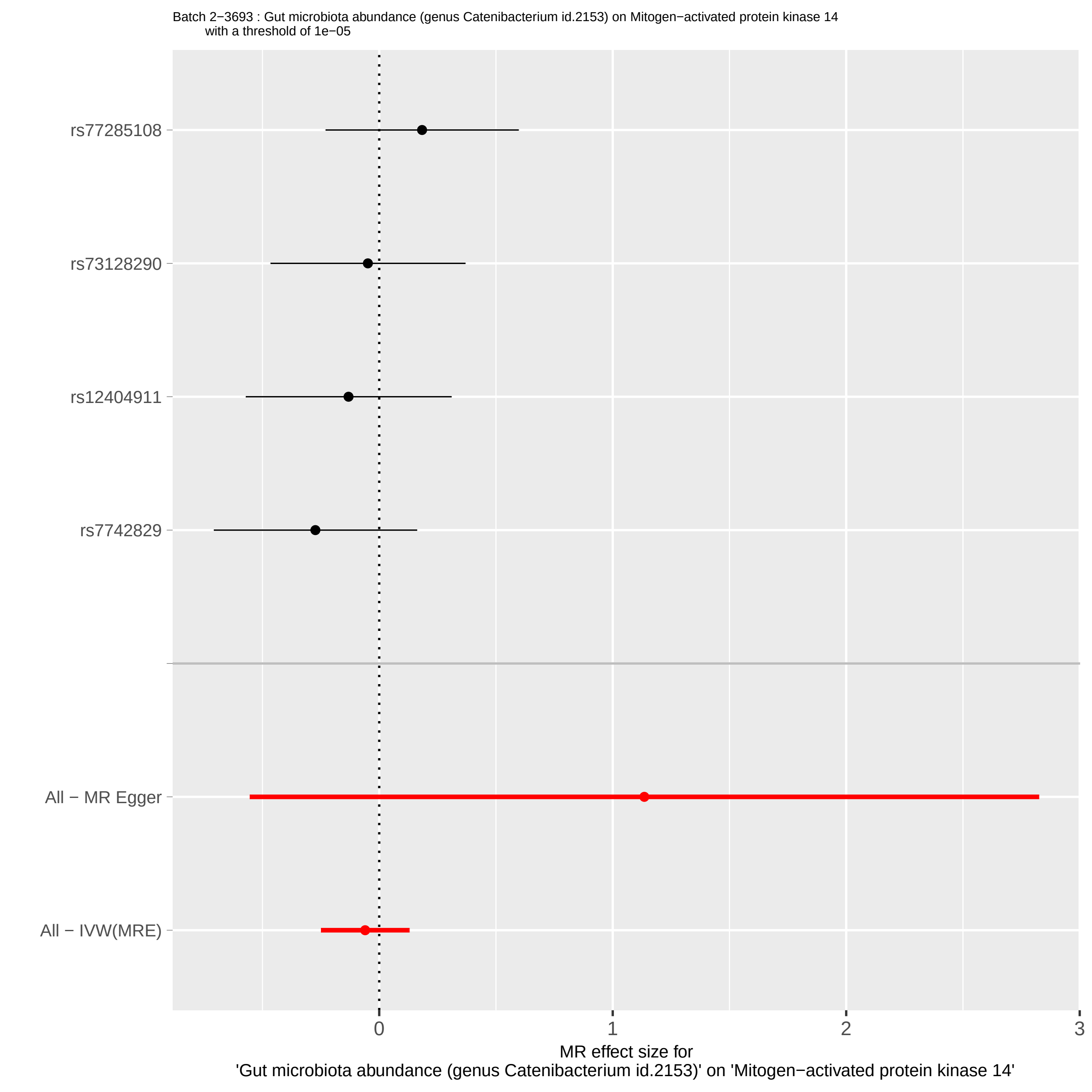

Batch 2−3693 : Gut microbiota abundance (genus Catenibacterium id.2153) on Mitogen−activated protein kinase 14
with a threshold of 1e−05
rs77285108
rs73128290
rs12404911
rs7742829
All − MR Egger
All − IVW(MRE)
0
1
2
3
MR effect size for
'Gut microbiota abundance (genus Catenibacterium id.2153)' on 'Mitogen−activated protein kinase 14'

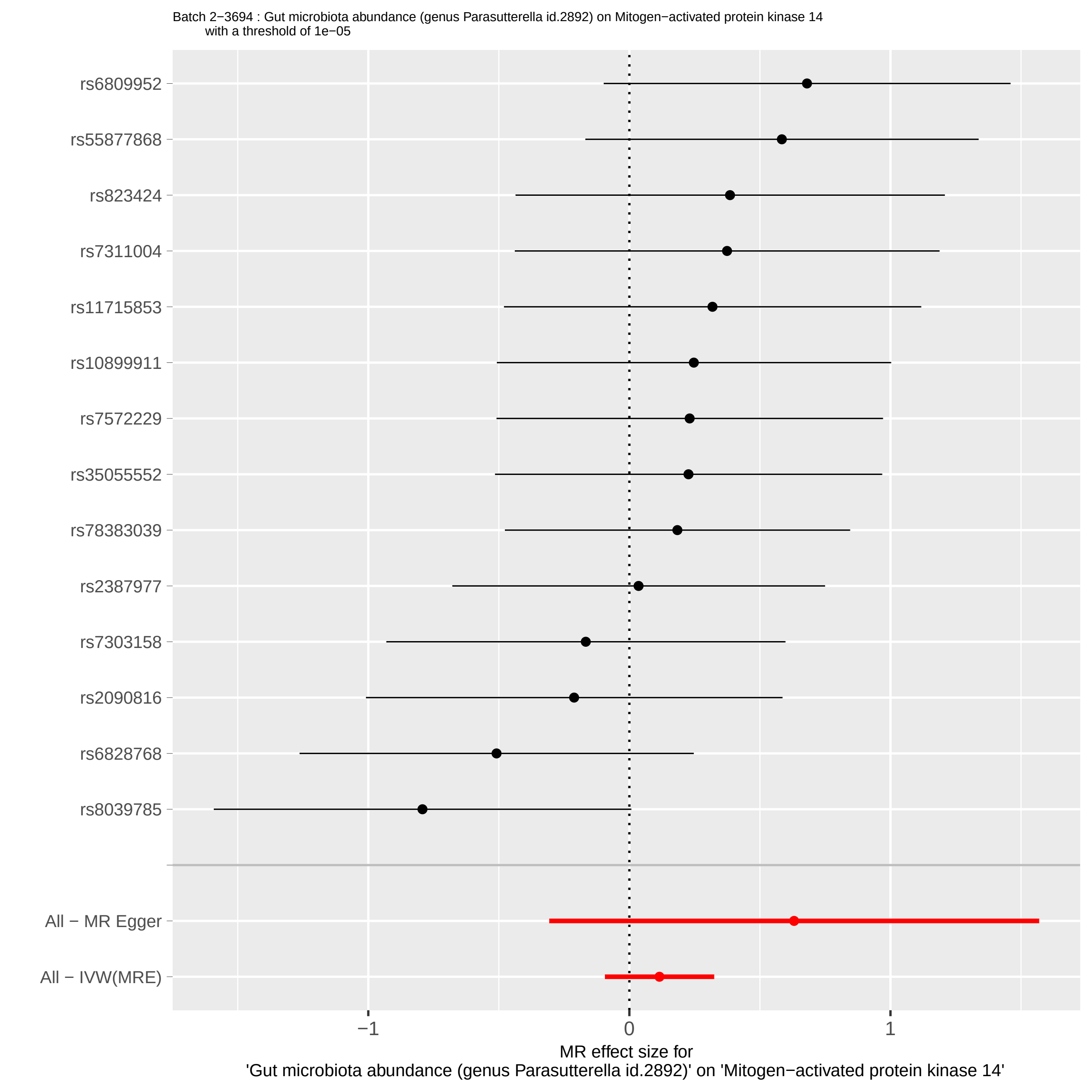

Batch 2−3694 : Gut microbiota abundance (genus Parasutterella id.2892) on Mitogen−activated protein kinase 14
with a threshold of 1e−05
rs6809952
rs55877868
rs823424
rs7311004
rs11715853
rs10899911
rs7572229
rs35055552
rs78383039
rs2387977
rs7303158
rs2090816
rs6828768
rs8039785
All − MR Egger
All − IVW(MRE)
−1
0
1
MR effect size for
'Gut microbiota abundance (genus Parasutterella id.2892)' on 'Mitogen−activated protein kinase 14'

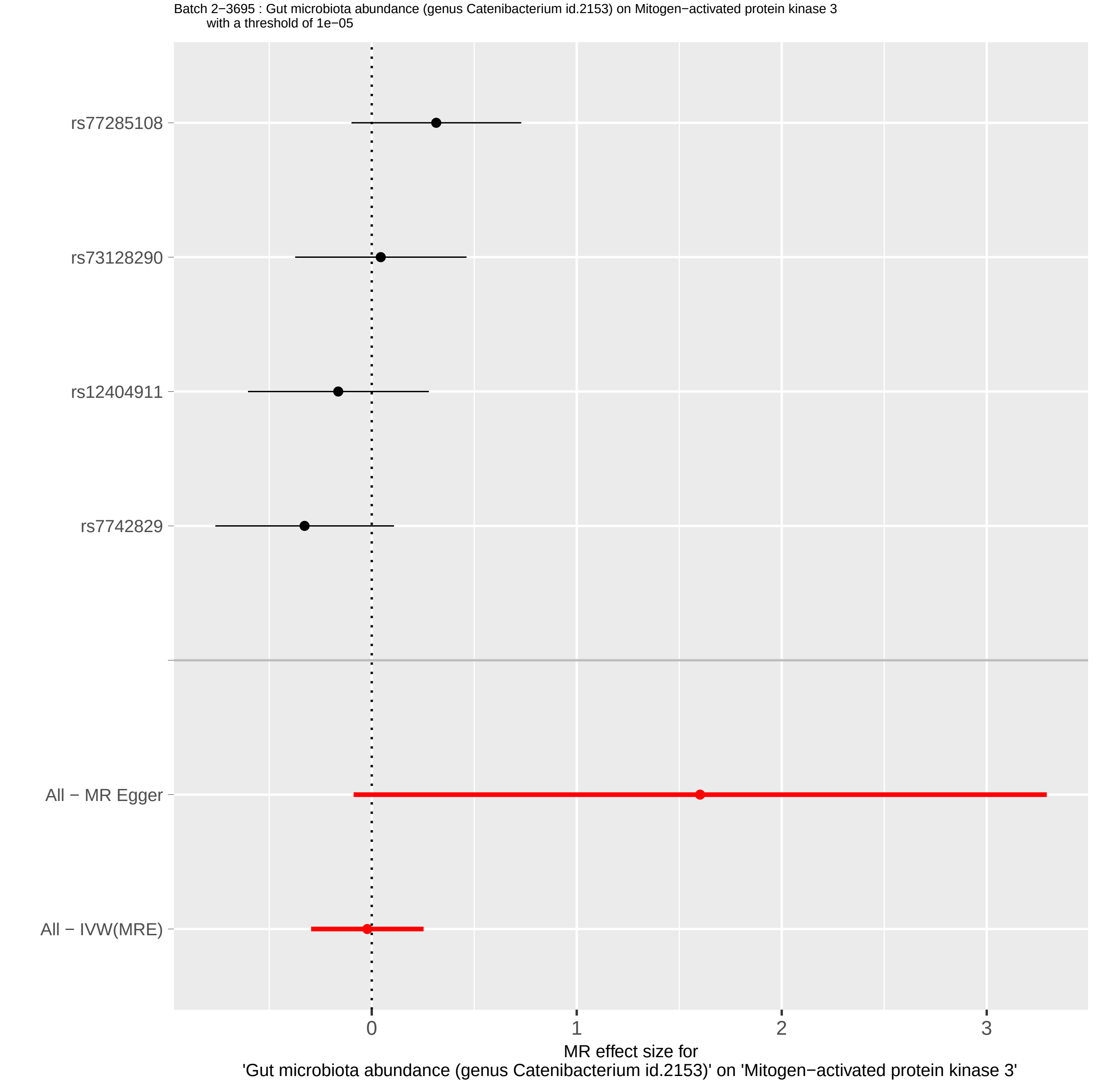

Batch 2−3695 : Gut microbiota abundance (genus Catenibacterium id.2153) on Mitogen−activated protein kinase 3
with a threshold of 1e−05
rs77285108
rs73128290
rs12404911
rs7742829
All − MR Egger
All − IVW(MRE)
0
1
2
3
MR effect size for
'Gut microbiota abundance (genus Catenibacterium id.2153)' on 'Mitogen−activated protein kinase 3'

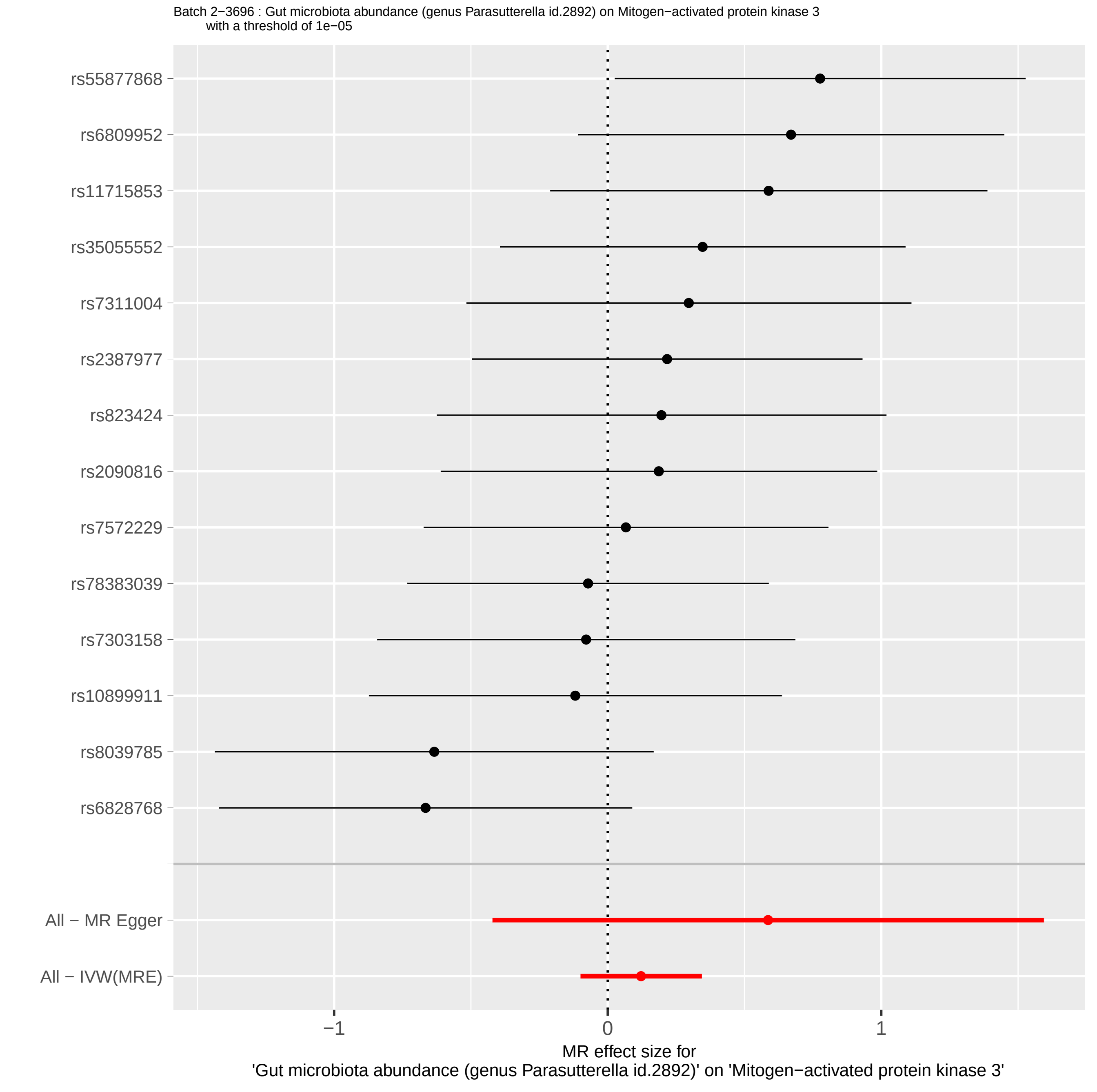

Batch 2−3696 : Gut microbiota abundance (genus Parasutterella id.2892) on Mitogen−activated protein kinase 3
with a threshold of 1e−05
rs55877868
rs6809952
rs11715853
rs35055552
rs7311004
rs2387977
rs823424
rs2090816
rs7572229
rs78383039
rs7303158
rs10899911
rs8039785
rs6828768
All − MR Egger
All − IVW(MRE)
−1
0
1
MR effect size for
'Gut microbiota abundance (genus Parasutterella id.2892)' on 'Mitogen−activated protein kinase 3'

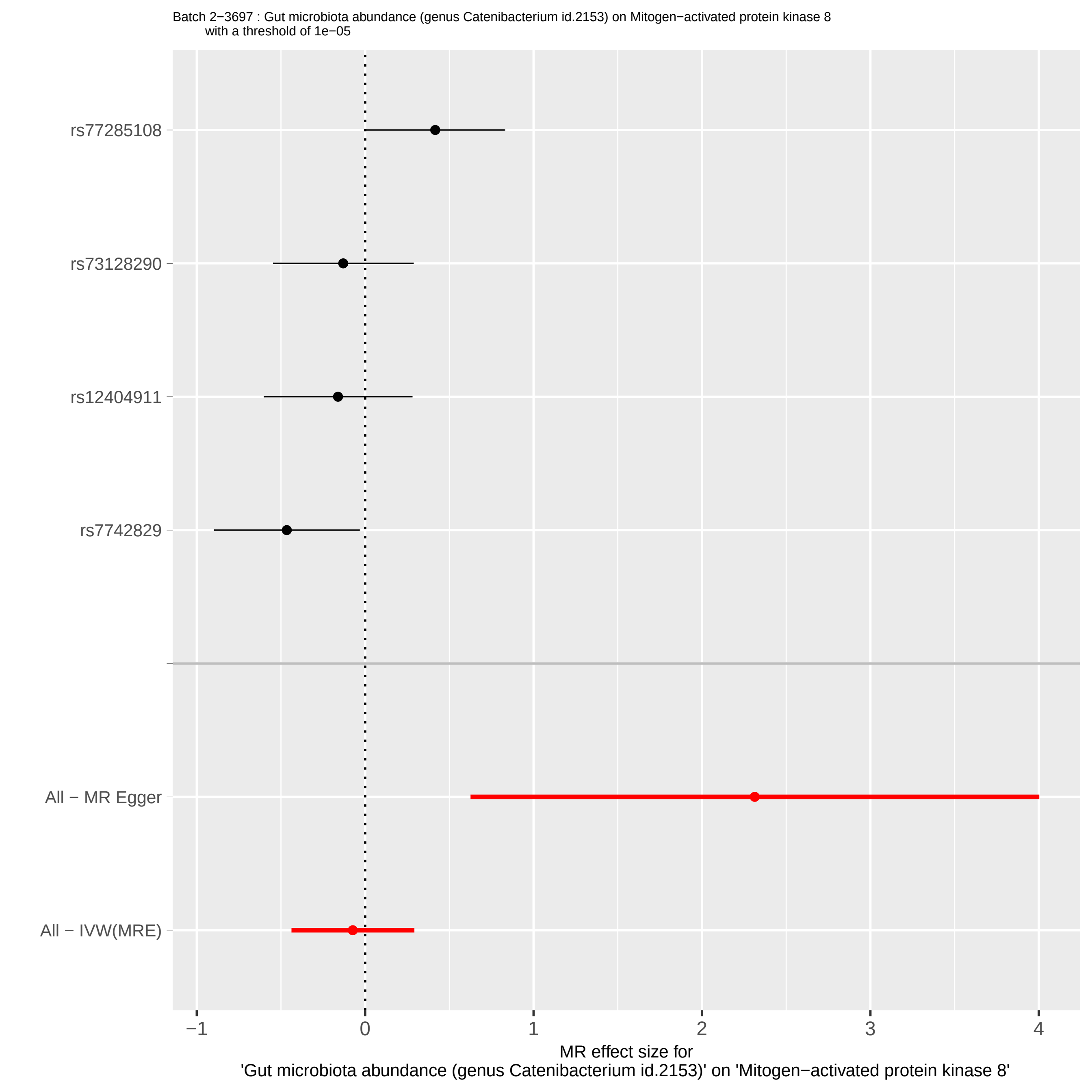

Batch 2−3697 : Gut microbiota abundance (genus Catenibacterium id.2153) on Mitogen−activated protein kinase 8
with a threshold of 1e−05
rs77285108
rs73128290
rs12404911
rs7742829
All − MR Egger
All − IVW(MRE)
−1
0
1
2
3
4
MR effect size for
'Gut microbiota abundance (genus Catenibacterium id.2153)' on 'Mitogen−activated protein kinase 8'

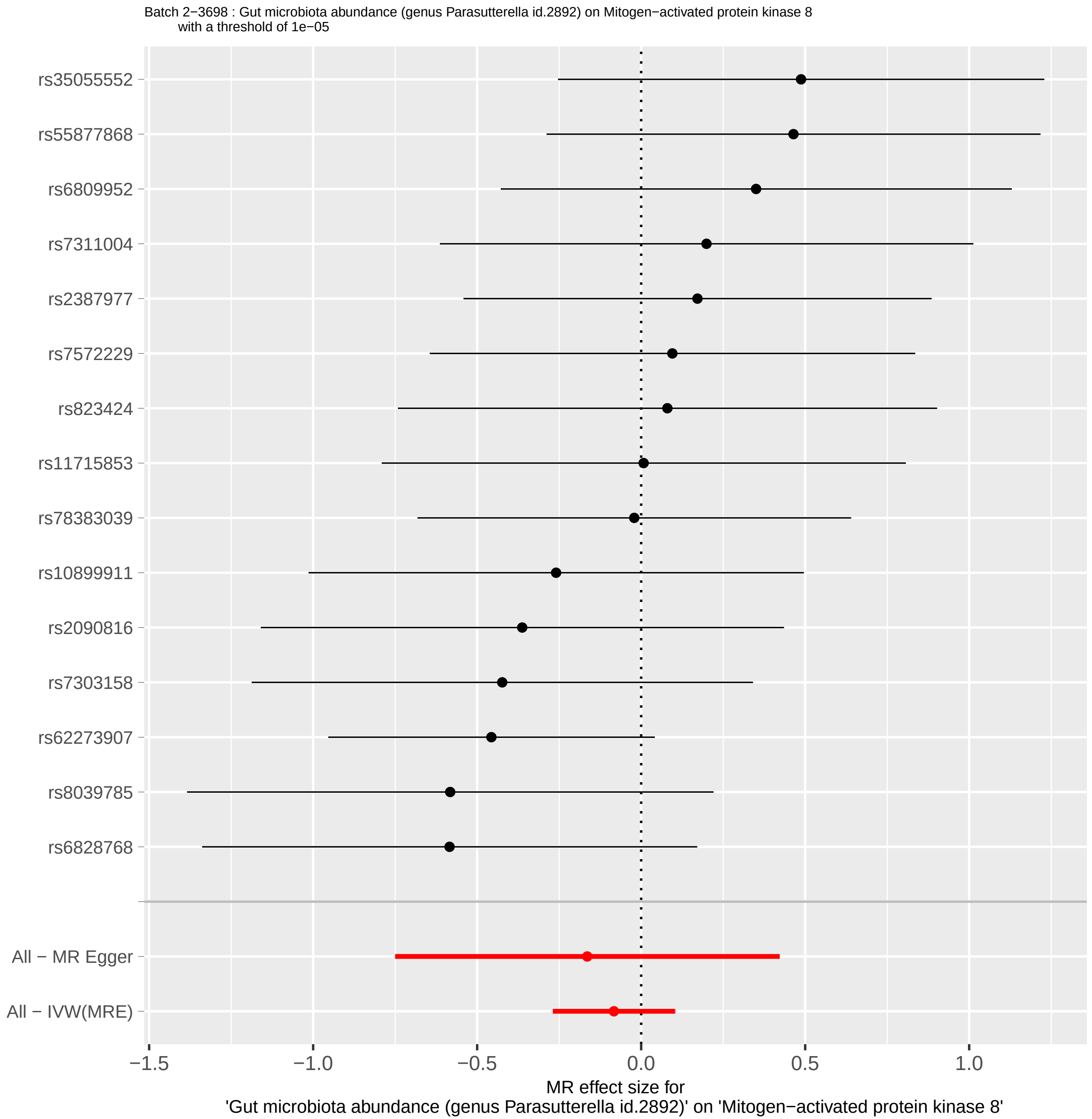

Batch 2−3698 : Gut microbiota abundance (genus Parasutterella id.2892) on Mitogen−activated protein kinase 8
with a threshold of 1e−05
rs35055552
rs55877868
rs6809952
rs7311004
rs2387977
rs7572229
rs823424
rs11715853
rs78383039
rs10899911
rs2090816
rs7303158
rs62273907
rs8039785
rs6828768
All − MR Egger
All − IVW(MRE)
−1.5
−1.0
−0.5
0.0
0.5
1.0
MR effect size for
'Gut microbiota abundance (genus Parasutterella id.2892)' on 'Mitogen−activated protein kinase 8'

Batch 2−3699 : Gut microbiota abundance (genus Catenibacterium id.2153) on Mitogen−activated protein kinase 9
with a threshold of 1e−05

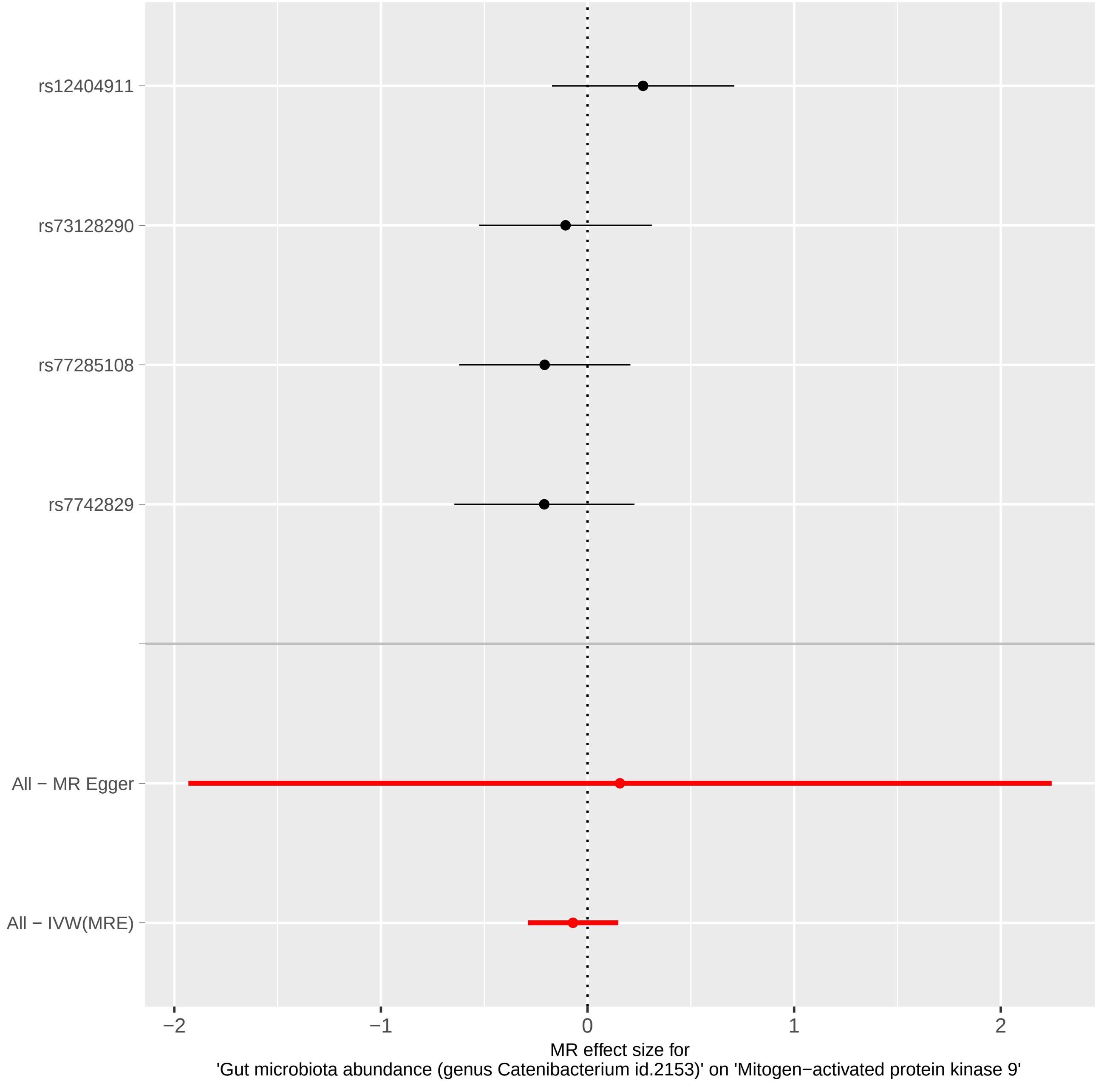

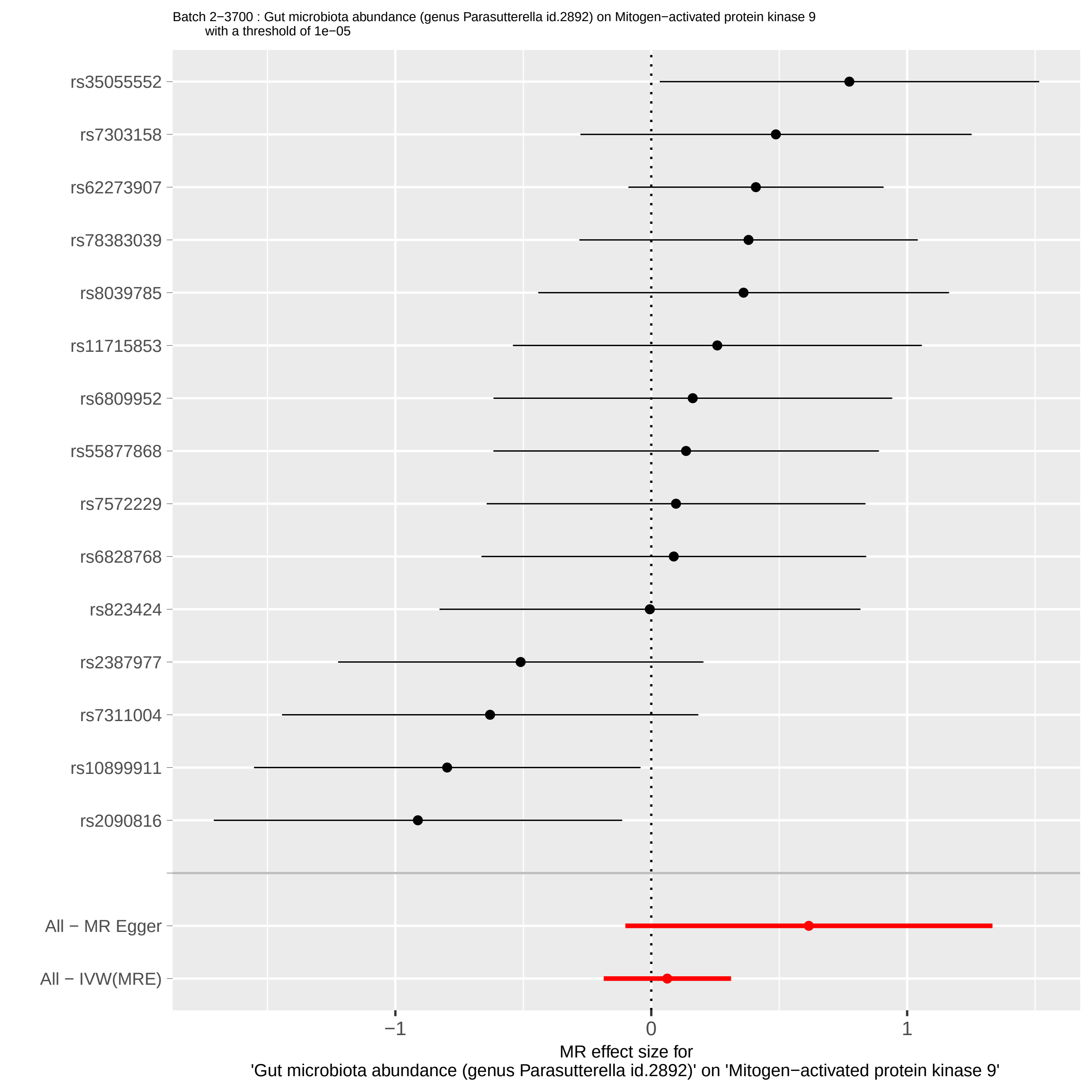

Batch 2−3700 : Gut microbiota abundance (genus Parasutterella id.2892) on Mitogen−activated protein kinase 9
with a threshold of 1e−05
rs35055552
rs7303158
rs62273907
rs78383039
rs8039785
rs11715853
rs6809952
rs55877868
rs7572229
rs6828768
rs823424
rs2387977
rs7311004
rs10899911
rs2090816
All − MR Egger
All − IVW(MRE)
−1
0
1
MR effect size for
'Gut microbiota abundance (genus Parasutterella id.2892)' on 'Mitogen−activated protein kinase 9'

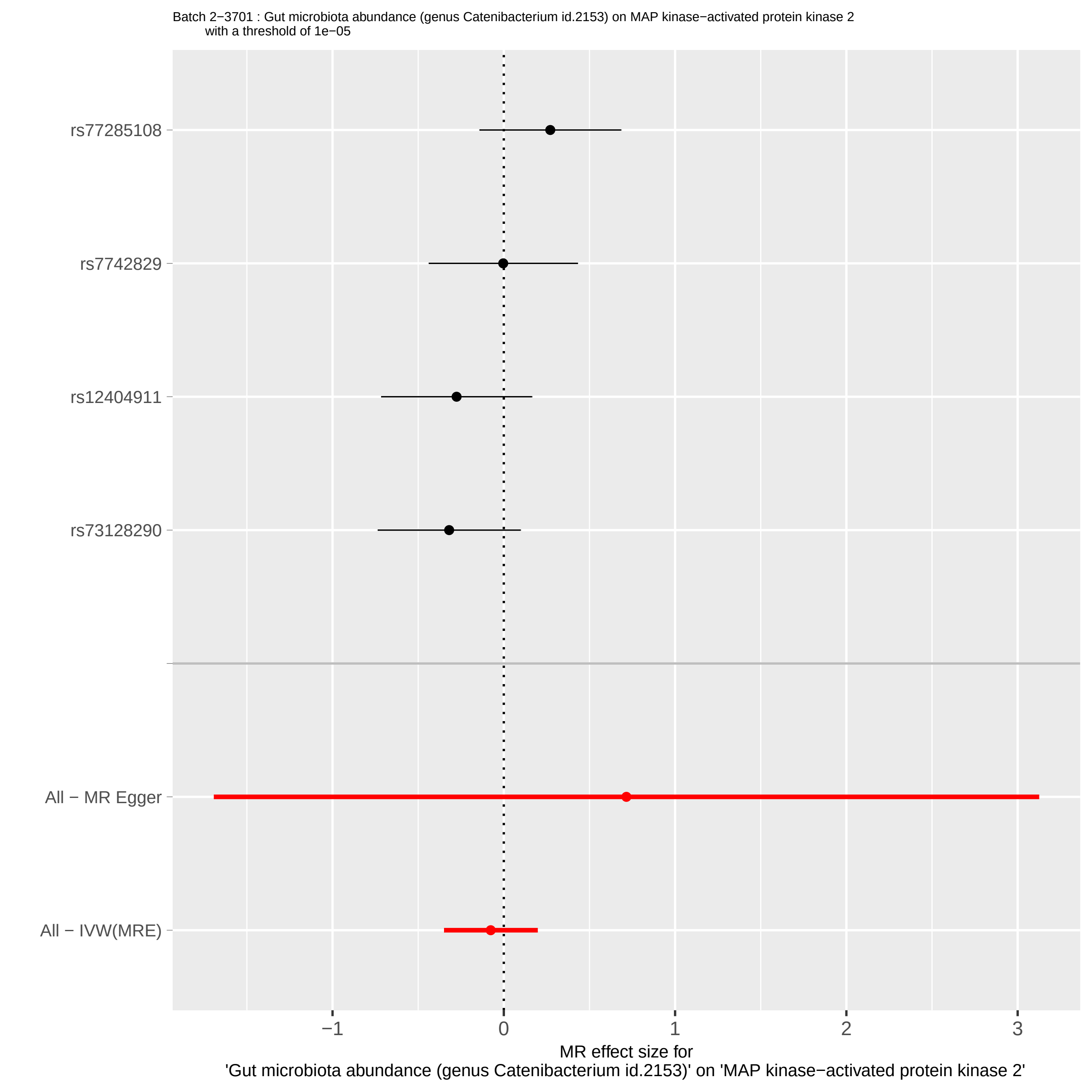

Batch 2−3701 : Gut microbiota abundance (genus Catenibacterium id.2153) on MAP kinase−activated protein kinase 2
with a threshold of 1e−05
rs77285108
rs7742829
rs12404911
rs73128290
All − MR Egger
All − IVW(MRE)
−1
0
1
2
3
MR effect size for
'Gut microbiota abundance (genus Catenibacterium id.2153)' on 'MAP kinase−activated protein kinase 2'

Batch 2−3702 : Gut microbiota abundance (genus Parasutterella id.2892) on MAP kinase−activated protein kinase 2
with a threshold of 1e−05

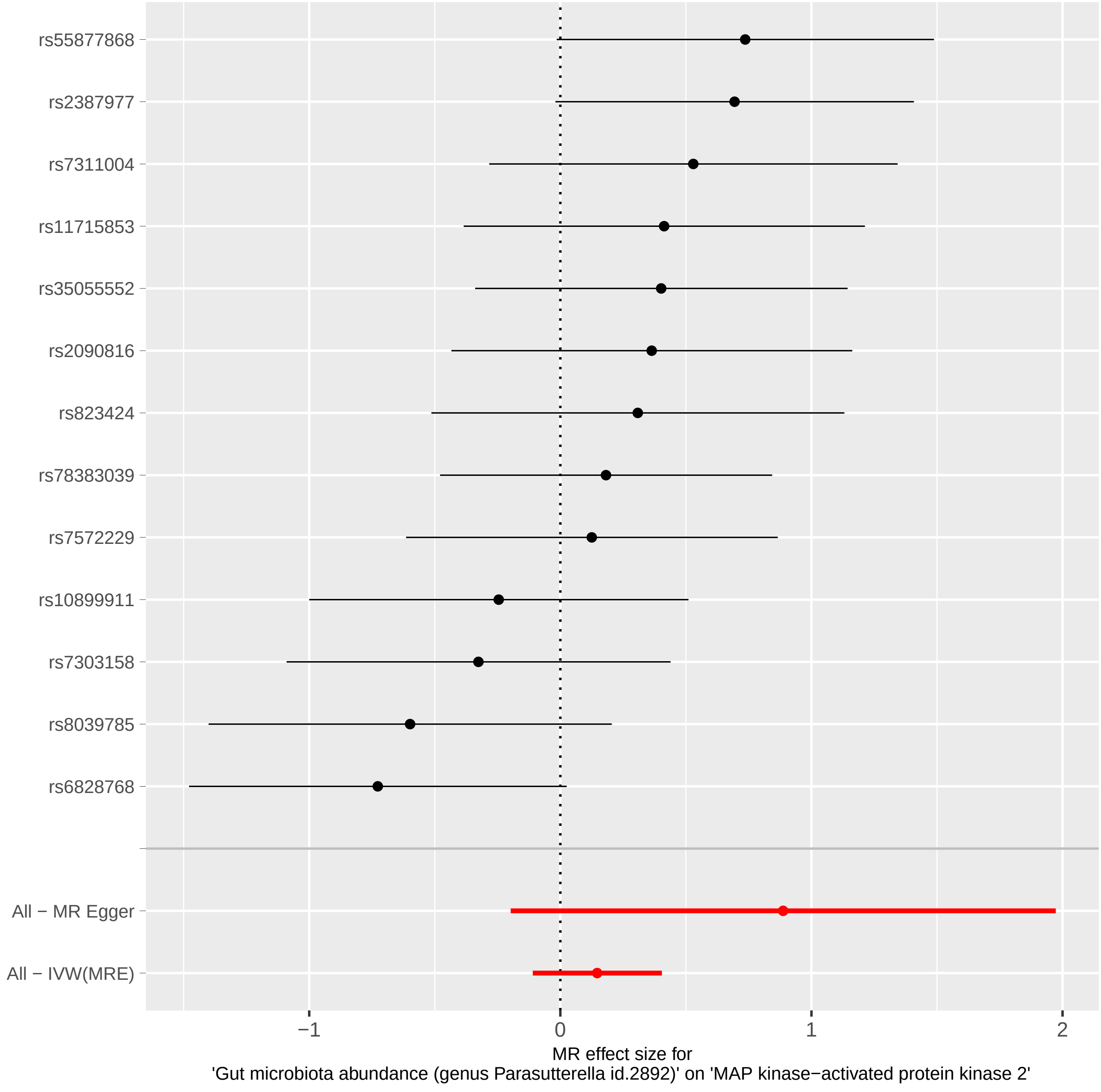

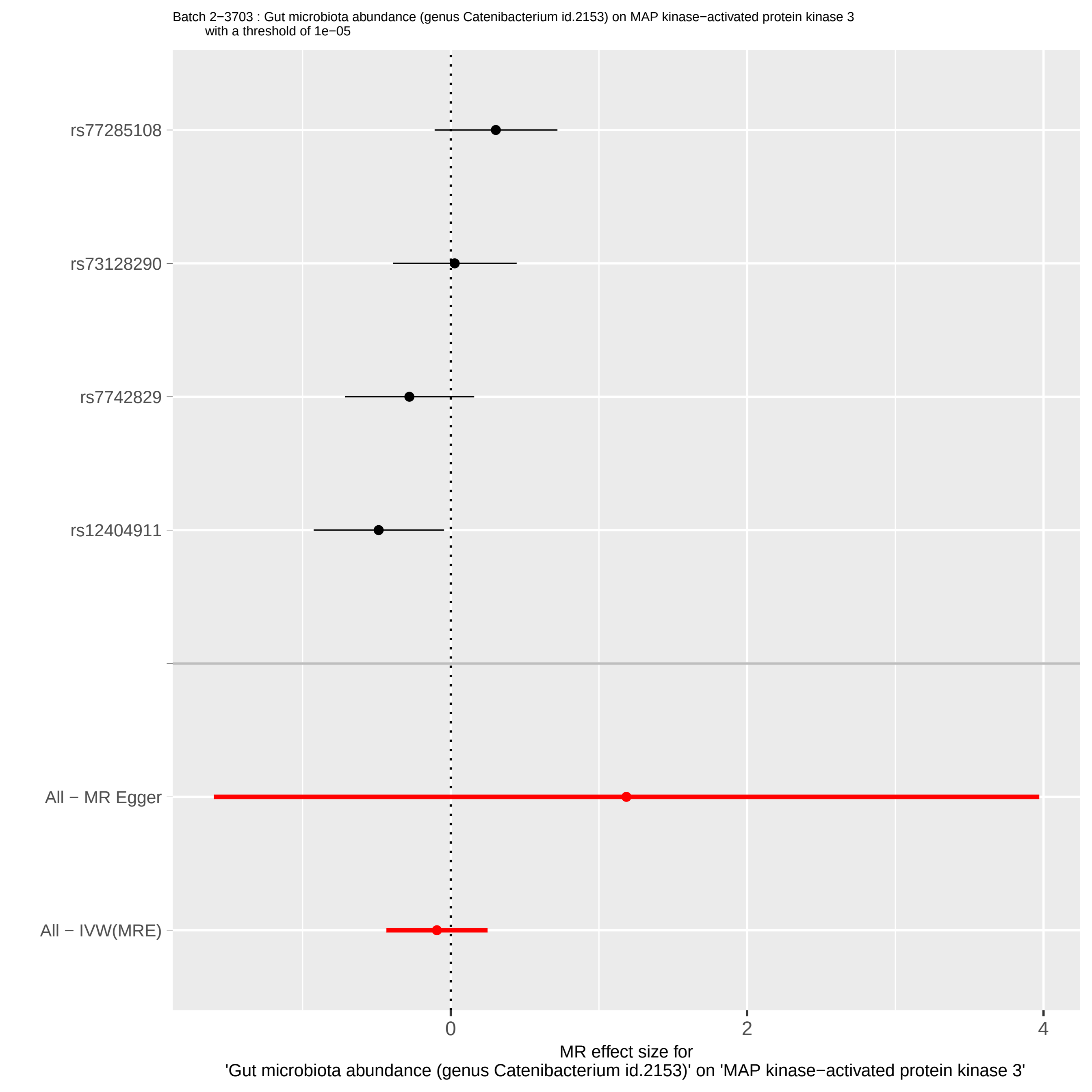
Batch 2−3703 : Gut microbiota abundance (genus Catenibacterium id.2153) on MAP kinase−activated protein kinase 3
with a threshold of 1e−05
rs77285108
rs73128290
rs7742829
rs12404911
All − MR Egger
All − IVW(MRE)
0
2
4
MR effect size for
'Gut microbiota abundance (genus Catenibacterium id.2153)' on 'MAP kinase−activated protein kinase 3'

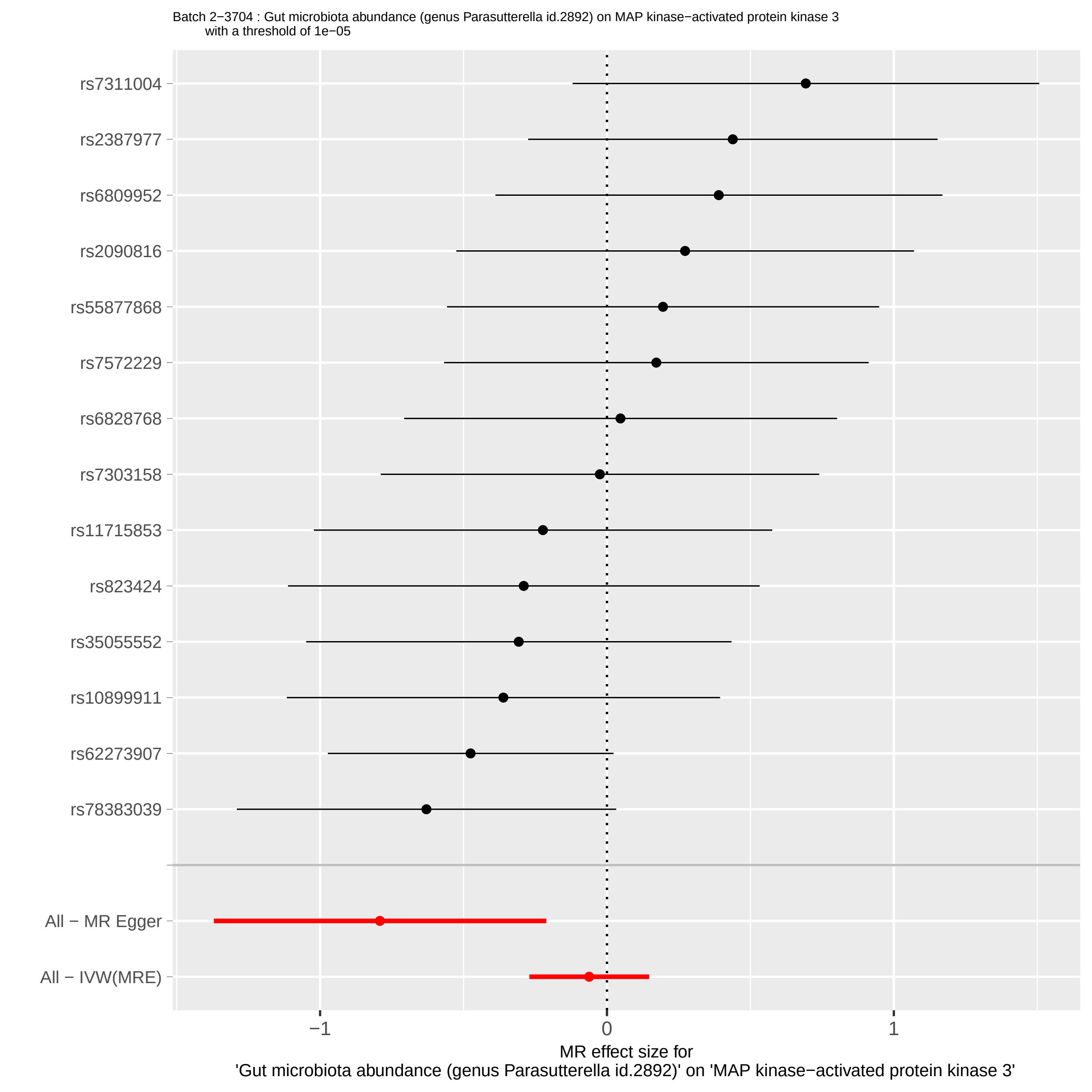

Batch 2−3704 : Gut microbiota abundance (genus Parasutterella id.2892) on MAP kinase−activated protein kinase 3
with a threshold of 1e−05
rs7311004
rs2387977
rs6809952
rs2090816
rs55877868
rs7572229
rs6828768
rs7303158
rs11715853
rs823424
rs35055552
rs10899911
rs62273907
rs78383039
All − MR Egger
All − IVW(MRE)
−1
0
1
MR effect size for
'Gut microbiota abundance (genus Parasutterella id.2892)' on 'MAP kinase−activated protein kinase 3'

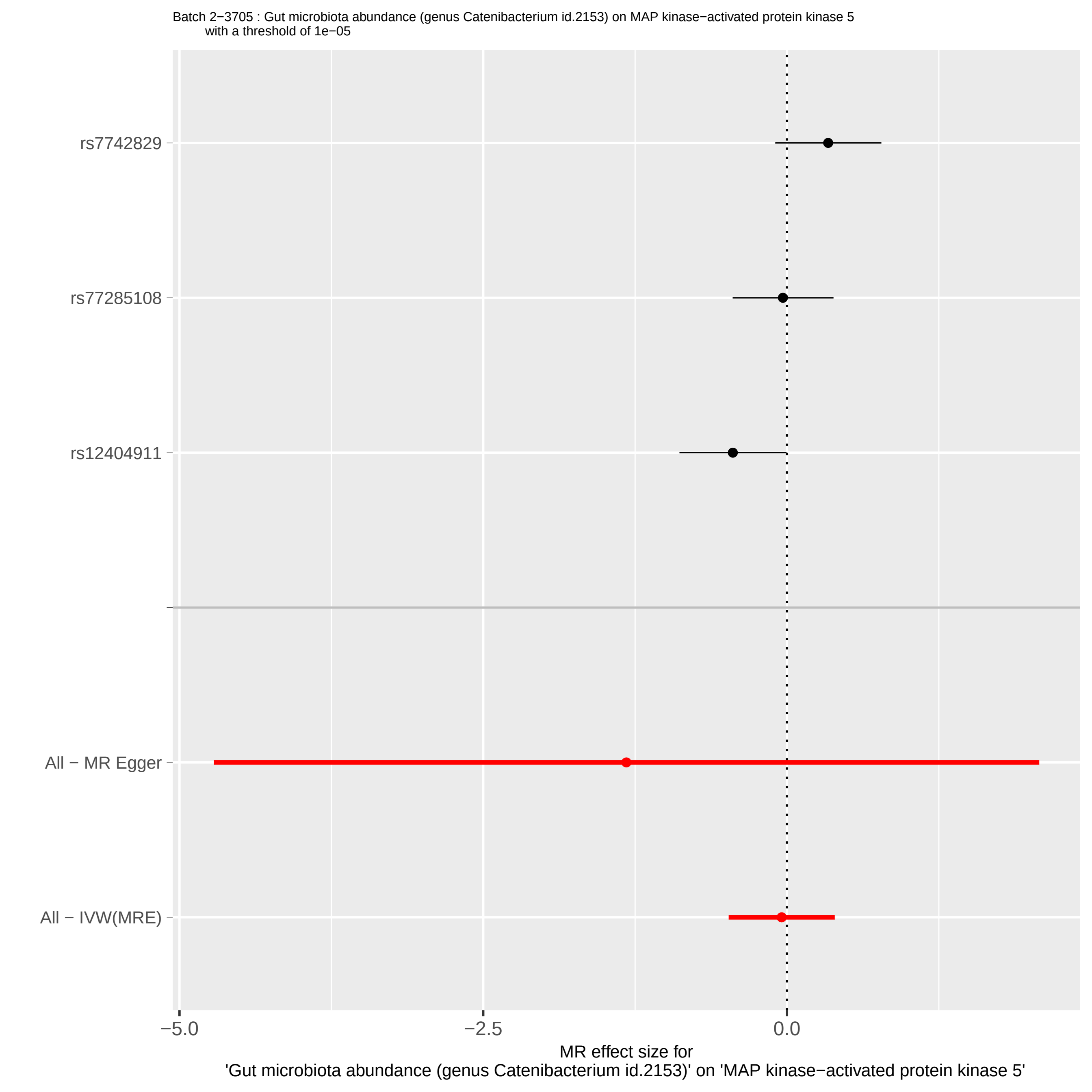

Batch 2−3705 : Gut microbiota abundance (genus Catenibacterium id.2153) on MAP kinase−activated protein kinase 5
with a threshold of 1e−05
rs7742829
rs77285108
rs12404911
All − MR Egger
All − IVW(MRE)
−5.0
−2.5
0.0
MR effect size for
'Gut microbiota abundance (genus Catenibacterium id.2153)' on 'MAP kinase−activated protein kinase 5'

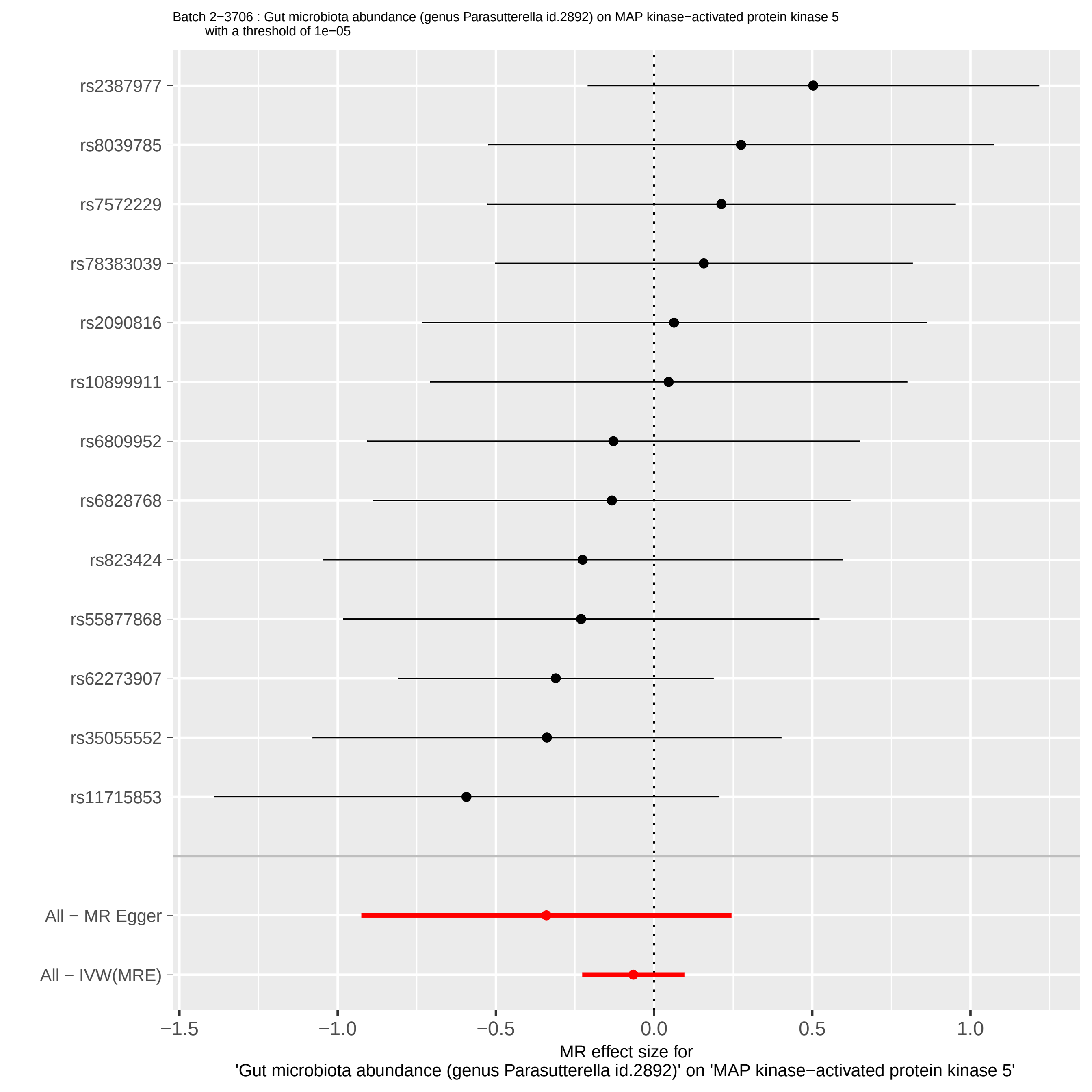

Batch 2−3706 : Gut microbiota abundance (genus Parasutterella id.2892) on MAP kinase−activated protein kinase 5
with a threshold of 1e−05
rs2387977
rs8039785
rs7572229
rs78383039
rs2090816
rs10899911
rs6809952
rs6828768
rs823424
rs55877868
rs62273907
rs35055552
rs11715853
All − MR Egger
All − IVW(MRE)
−1.5
−1.0
−0.5
0.0
0.5
1.0
MR effect size for
'Gut microbiota abundance (genus Parasutterella id.2892)' on 'MAP kinase−activated protein kinase 5'

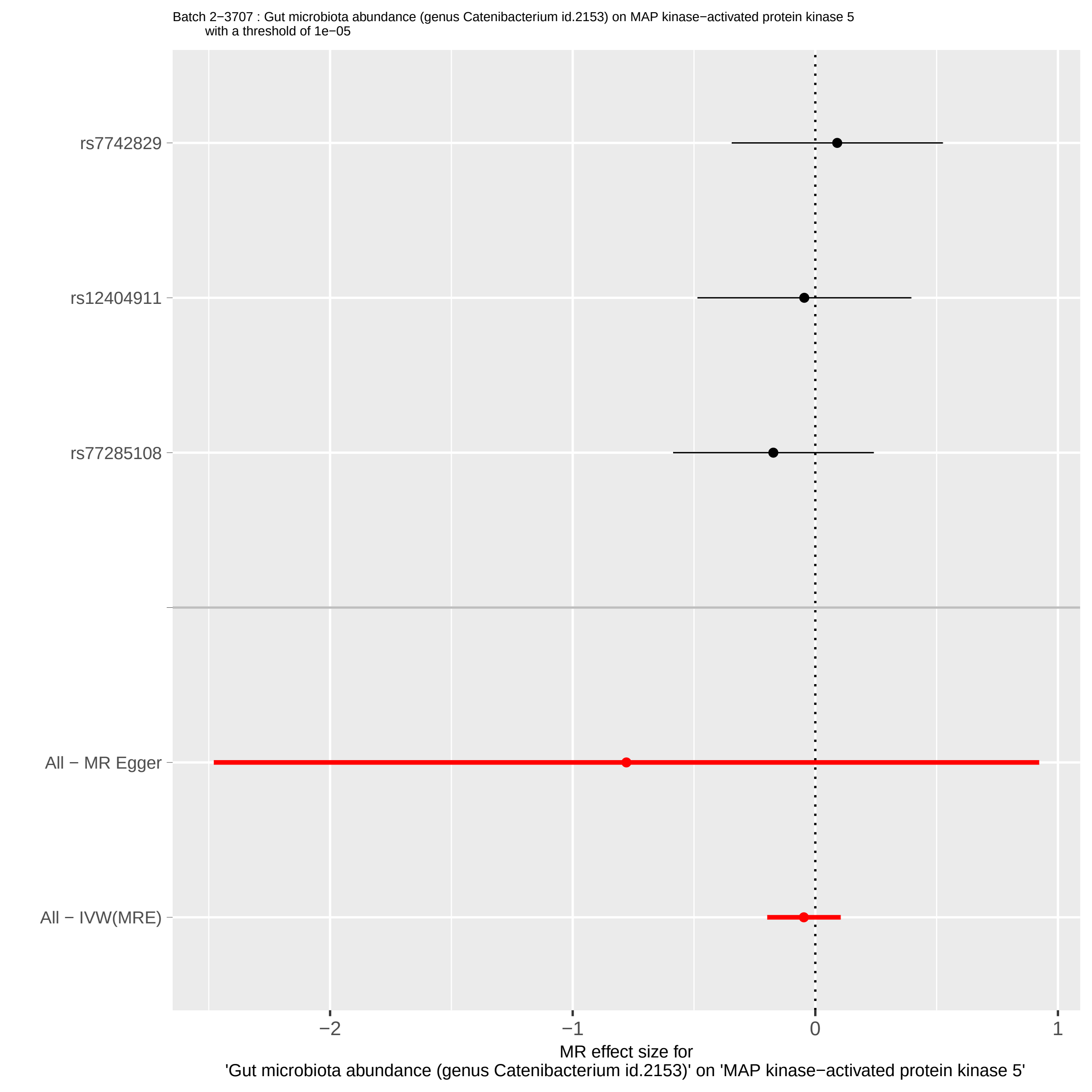

Batch 2−3707 : Gut microbiota abundance (genus Catenibacterium id.2153) on MAP kinase−activated protein kinase 5
with a threshold of 1e−05
rs7742829
rs12404911
rs77285108
All − MR Egger
All − IVW(MRE)
−2
−1
0
1
MR effect size for
'Gut microbiota abundance (genus Catenibacterium id.2153)' on 'MAP kinase−activated protein kinase 5'

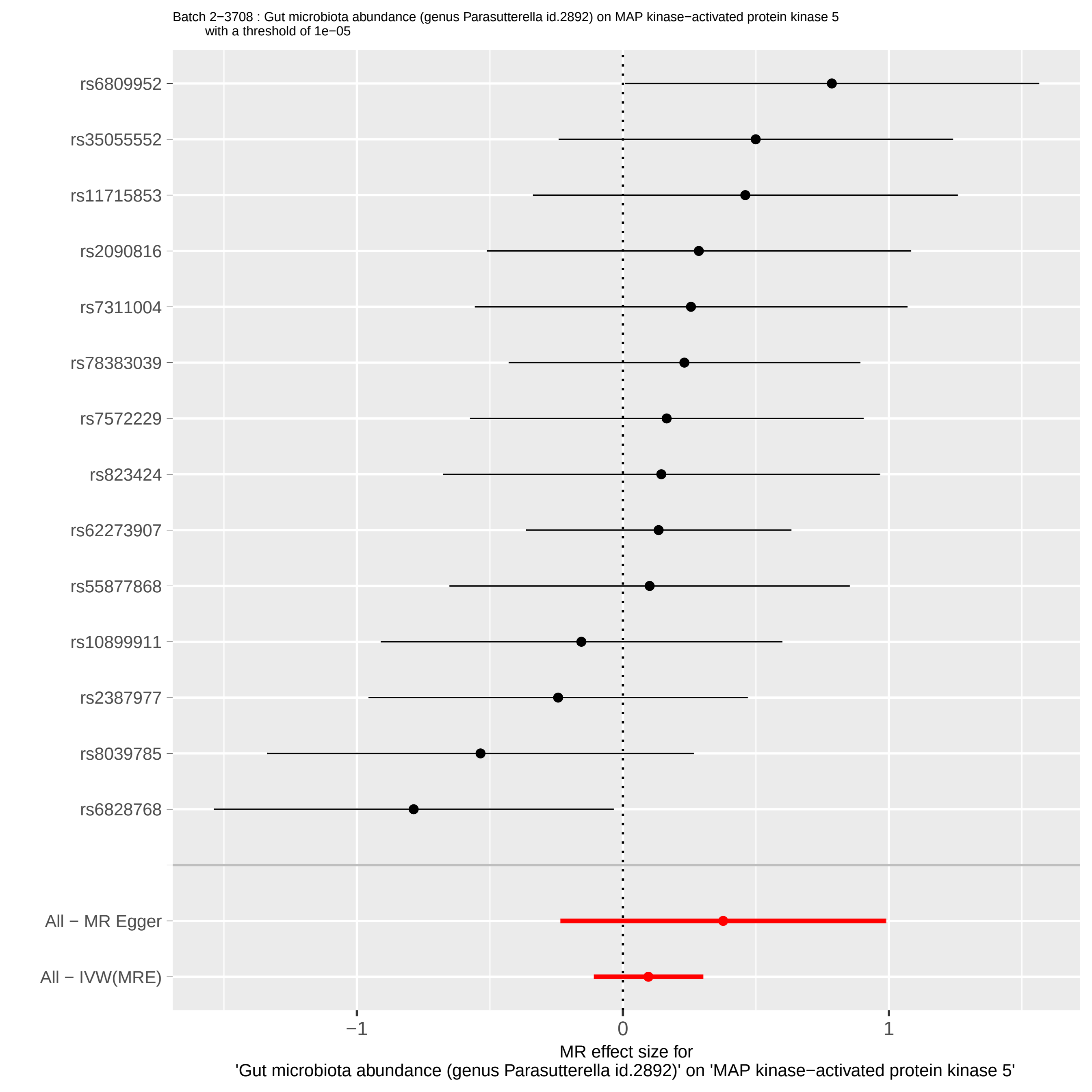

Batch 2−3708 : Gut microbiota abundance (genus Parasutterella id.2892) on MAP kinase−activated protein kinase 5
with a threshold of 1e−05
rs6809952
rs35055552
rs11715853
rs2090816
rs7311004
rs78383039
rs7572229
rs823424
rs62273907
rs55877868
rs10899911
rs2387977
rs8039785
rs6828768
All − MR Egger
All − IVW(MRE)
−1
0
1
MR effect size for
'Gut microbiota abundance (genus Parasutterella id.2892)' on 'MAP kinase−activated protein kinase 5'

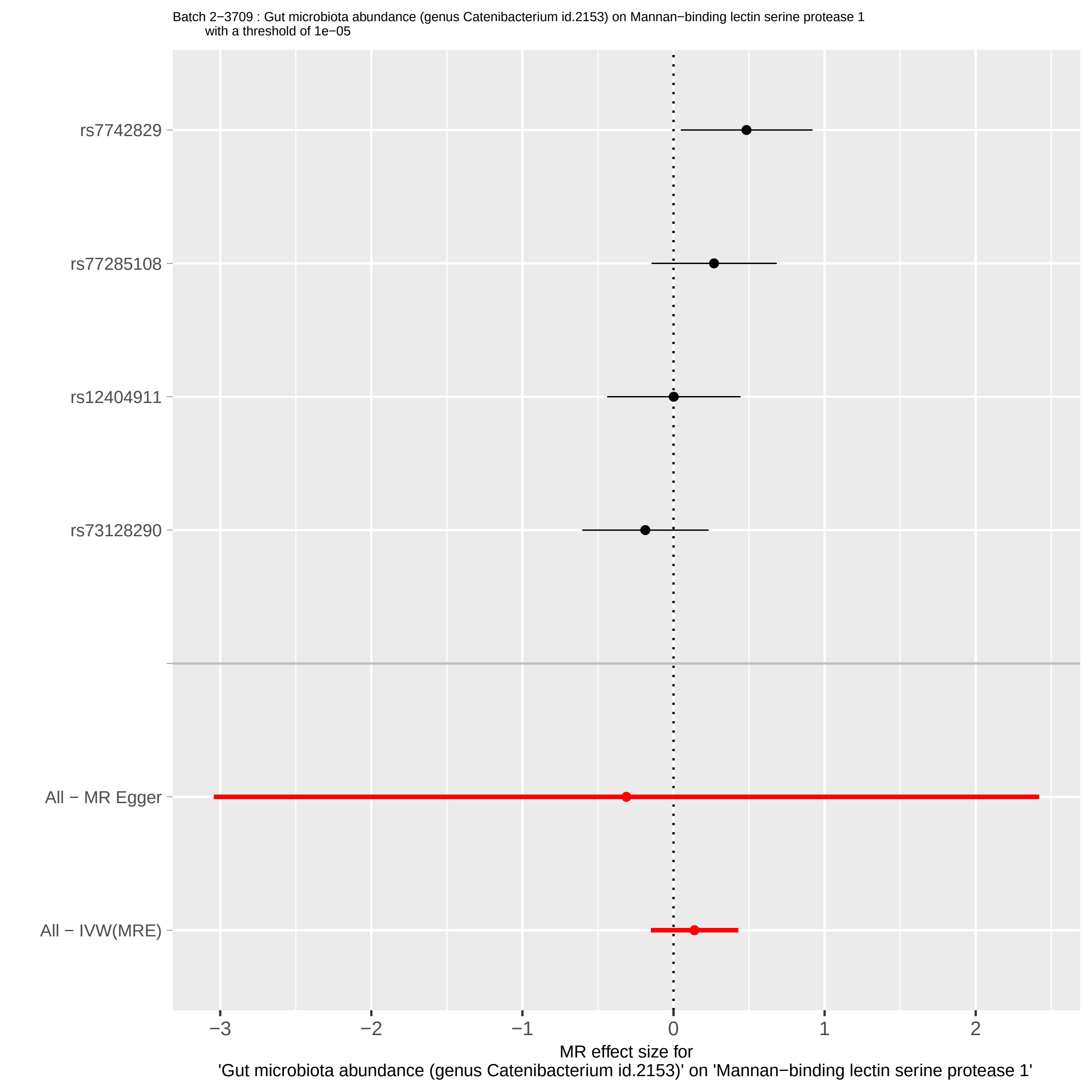
Batch 2−3709 : Gut microbiota abundance (genus Catenibacterium id.2153) on Mannan−binding lectin serine protease 1
with a threshold of 1e−05
rs7742829
rs77285108
rs12404911
rs73128290
All − MR Egger
All − IVW(MRE)
−3
−2
−1
0
1
2
MR effect size for
'Gut microbiota abundance (genus Catenibacterium id.2153)' on 'Mannan−binding lectin serine protease 1'

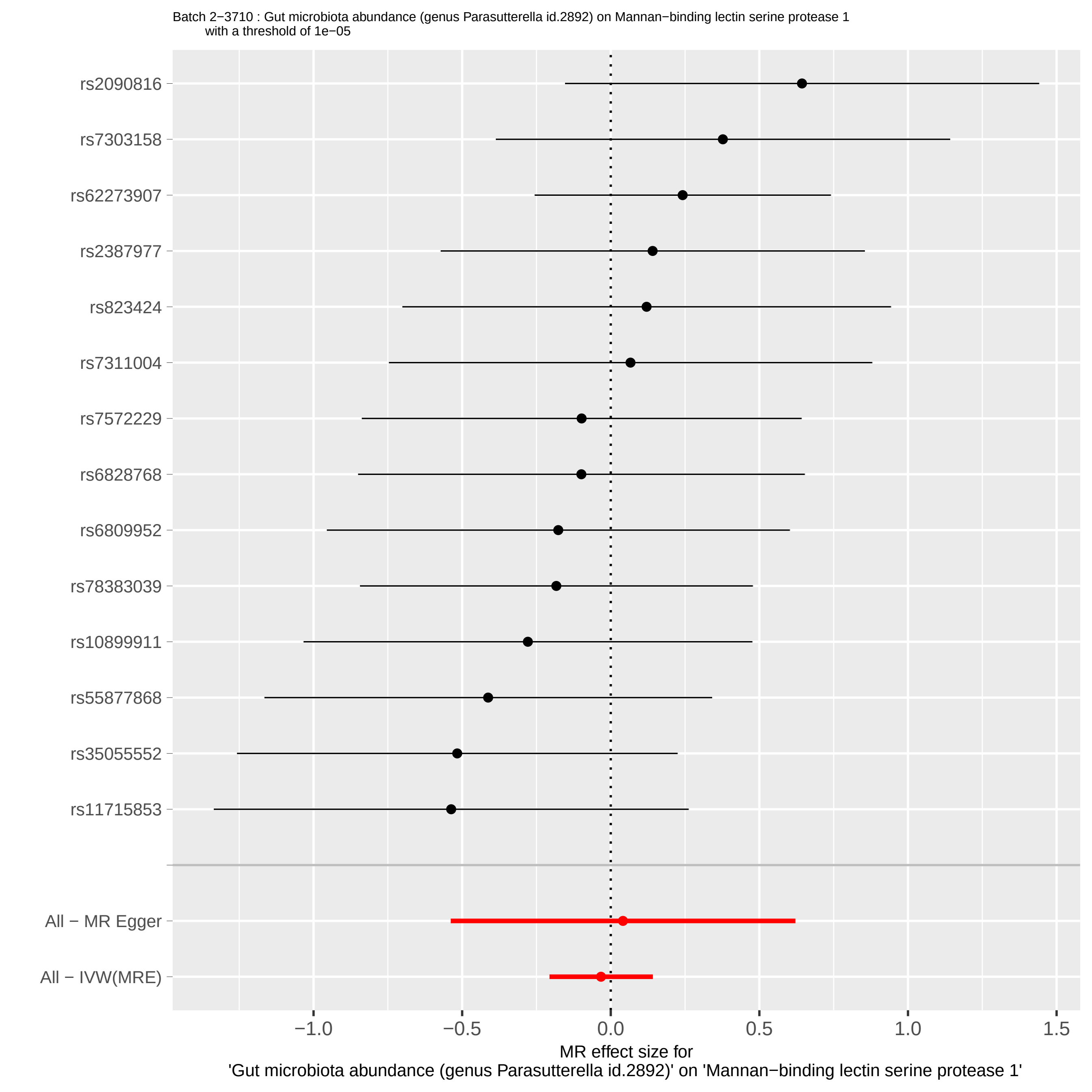
Batch 2−3710 : Gut microbiota abundance (genus Parasutterella id.2892) on Mannan−binding lectin serine protease 1
with a threshold of 1e−05
rs2090816
rs7303158
rs62273907
rs2387977
rs823424
rs7311004
rs7572229
rs6828768
rs6809952
rs78383039
rs10899911
rs55877868
rs35055552
rs11715853
All − MR Egger
All − IVW(MRE)
−1.0
−0.5
0.0
0.5
1.0
1.5
MR effect size for
'Gut microbiota abundance (genus Parasutterella id.2892)' on 'Mannan−binding lectin serine protease 1'

Batch 2−3711 : Gut microbiota abundance (genus Catenibacterium id.2153) on Megakaryocyte−associated tyrosine−protein kinase
with a threshold of 1e−05

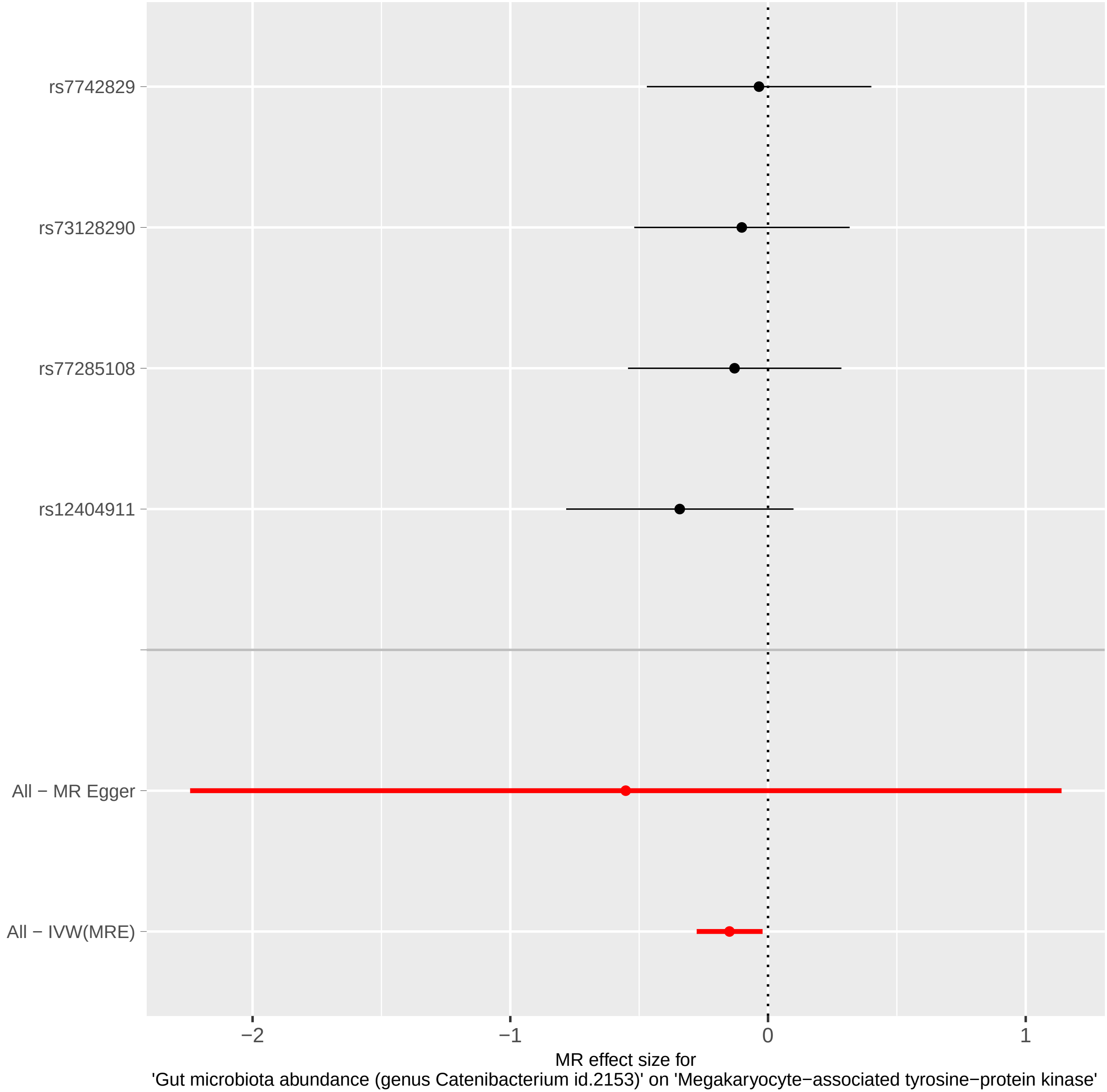

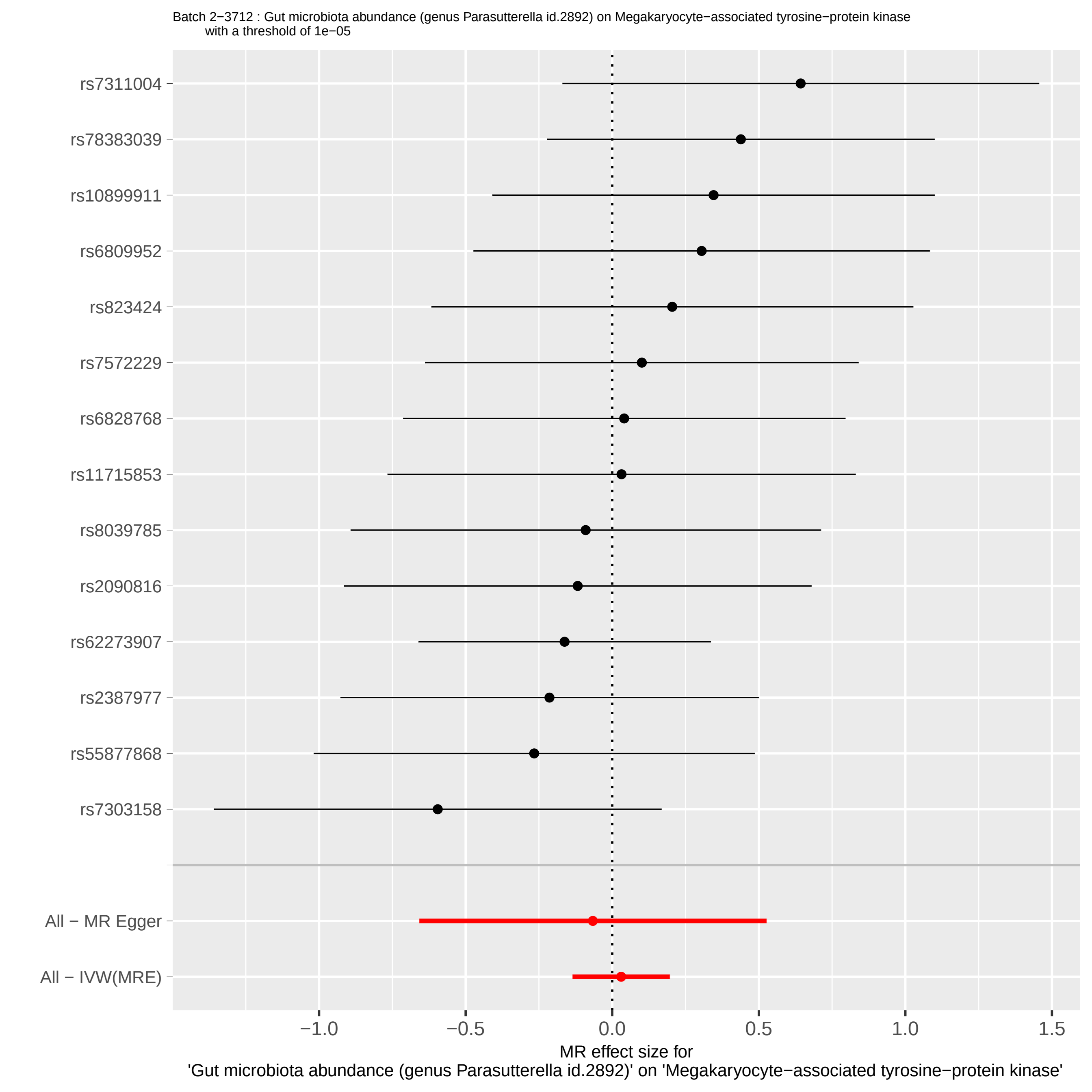
Batch 2−3712 : Gut microbiota abundance (genus Parasutterella id.2892) on Megakaryocyte−associated tyrosine−protein kinase
with a threshold of 1e−05
rs7311004
rs78383039
rs10899911
rs6809952
rs823424
rs7572229
rs6828768
rs11715853
rs8039785
rs2090816
rs62273907
rs2387977
rs55877868
rs7303158
All − MR Egger
All − IVW(MRE)
−1.0
−0.5
0.0
0.5
1.0
1.5
MR effect size for
'Gut microbiota abundance (genus Parasutterella id.2892)' on 'Megakaryocyte−associated tyrosine−protein kinase'

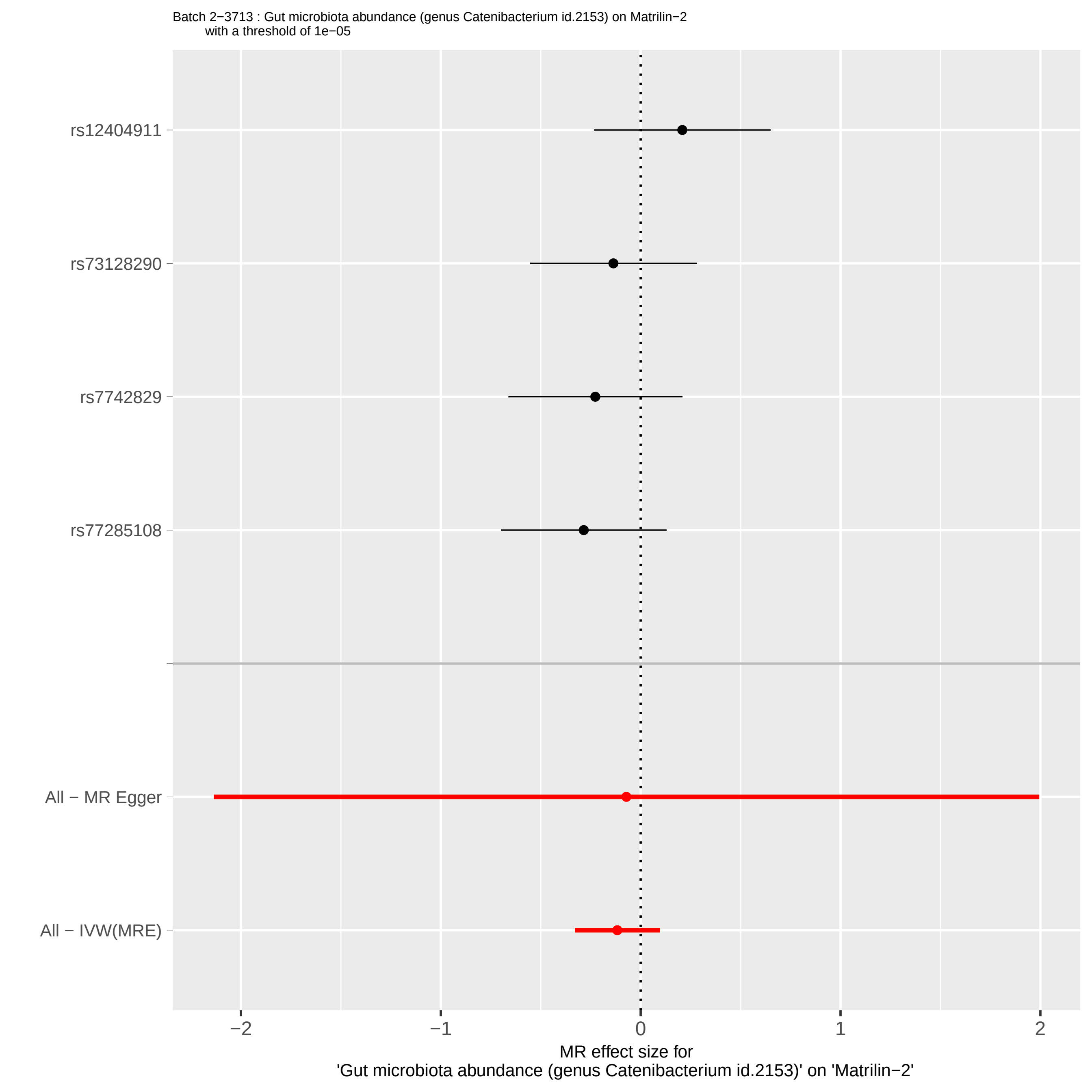

Batch 2−3713 : Gut microbiota abundance (genus Catenibacterium id.2153) on Matrilin−2
with a threshold of 1e−05
rs12404911
rs73128290
rs7742829
rs77285108
All − MR Egger
All − IVW(MRE)
−2
−1
0
1
2
MR effect size for
'Gut microbiota abundance (genus Catenibacterium id.2153)' on 'Matrilin−2'

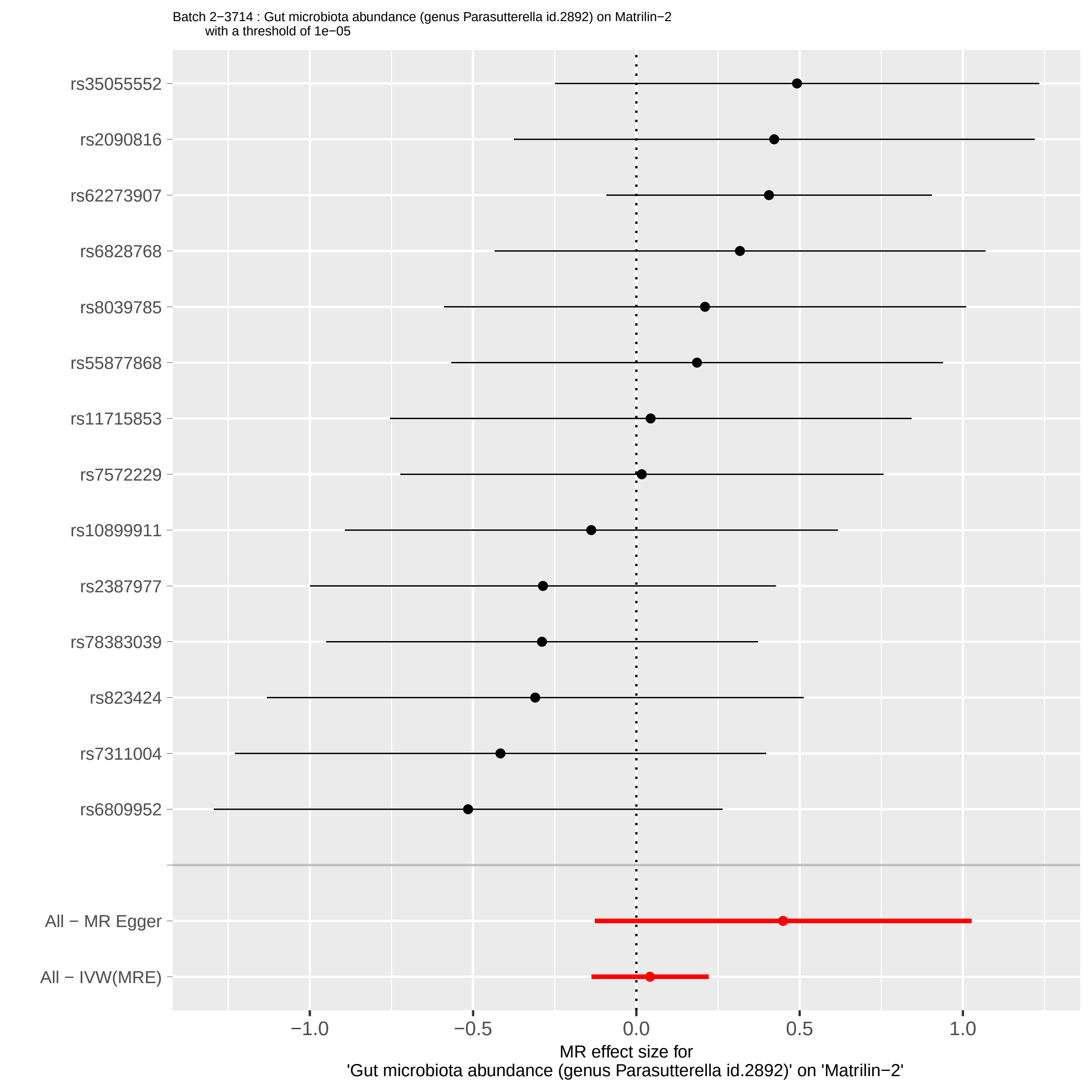

Batch 2−3714 : Gut microbiota abundance (genus Parasutterella id.2892) on Matrilin−2
with a threshold of 1e−05
rs35055552
rs2090816
rs62273907
rs6828768
rs8039785
rs55877868
rs11715853
rs7572229
rs10899911
rs2387977
rs78383039
rs823424
rs7311004
rs6809952
All − MR Egger
All − IVW(MRE)
−1.0
−0.5
0.0
0.5
1.0
MR effect size for
'Gut microbiota abundance (genus Parasutterella id.2892)' on 'Matrilin−2'

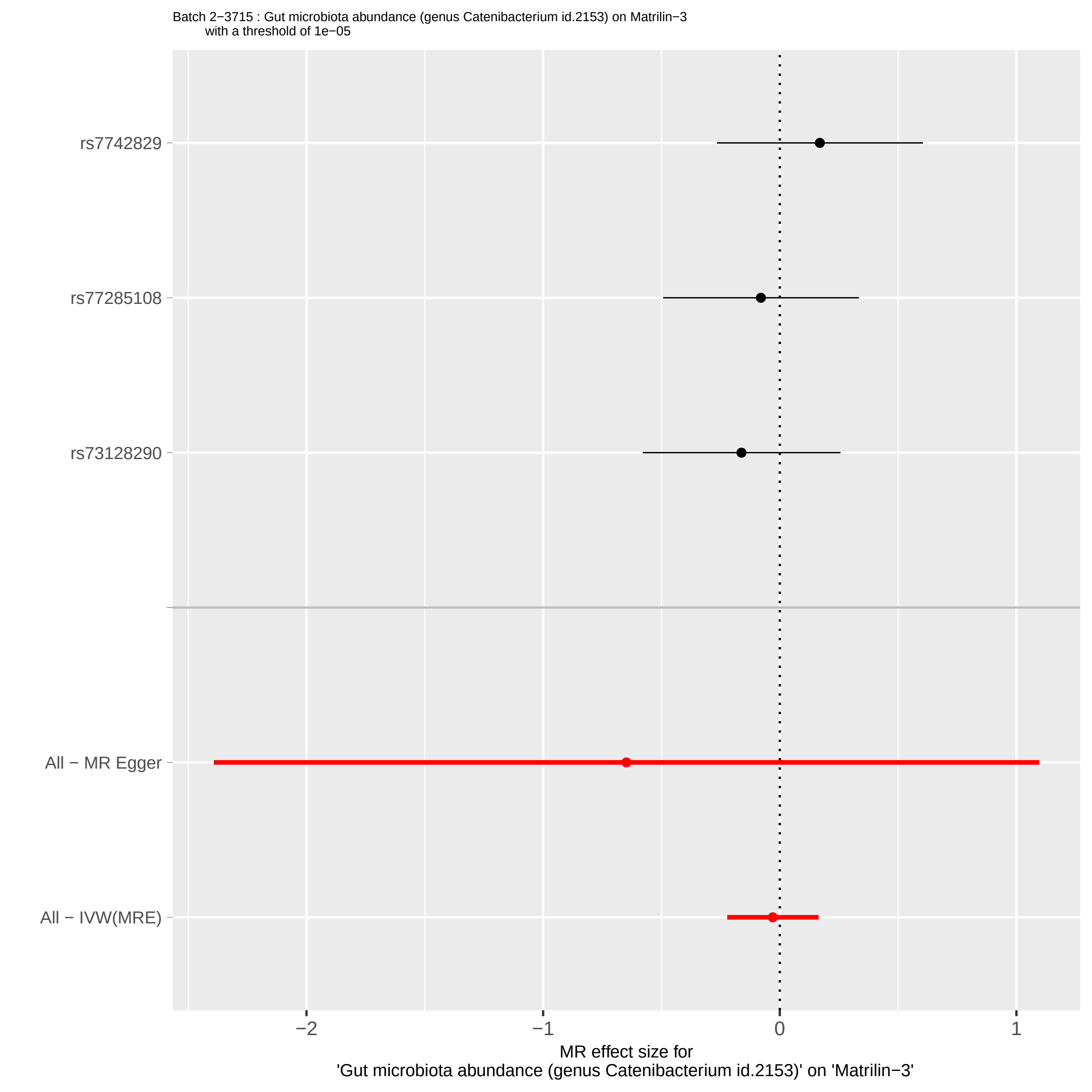

Batch 2−3715 : Gut microbiota abundance (genus Catenibacterium id.2153) on Matrilin−3
with a threshold of 1e−05
rs7742829
rs77285108
rs73128290
All − MR Egger
All − IVW(MRE)
−2
−1
0
1
MR effect size for
'Gut microbiota abundance (genus Catenibacterium id.2153)' on 'Matrilin−3'

Batch 2−3716 : Gut microbiota abundance (genus Parasutterella id.2892) on Matrilin−3
with a threshold of 1e−05

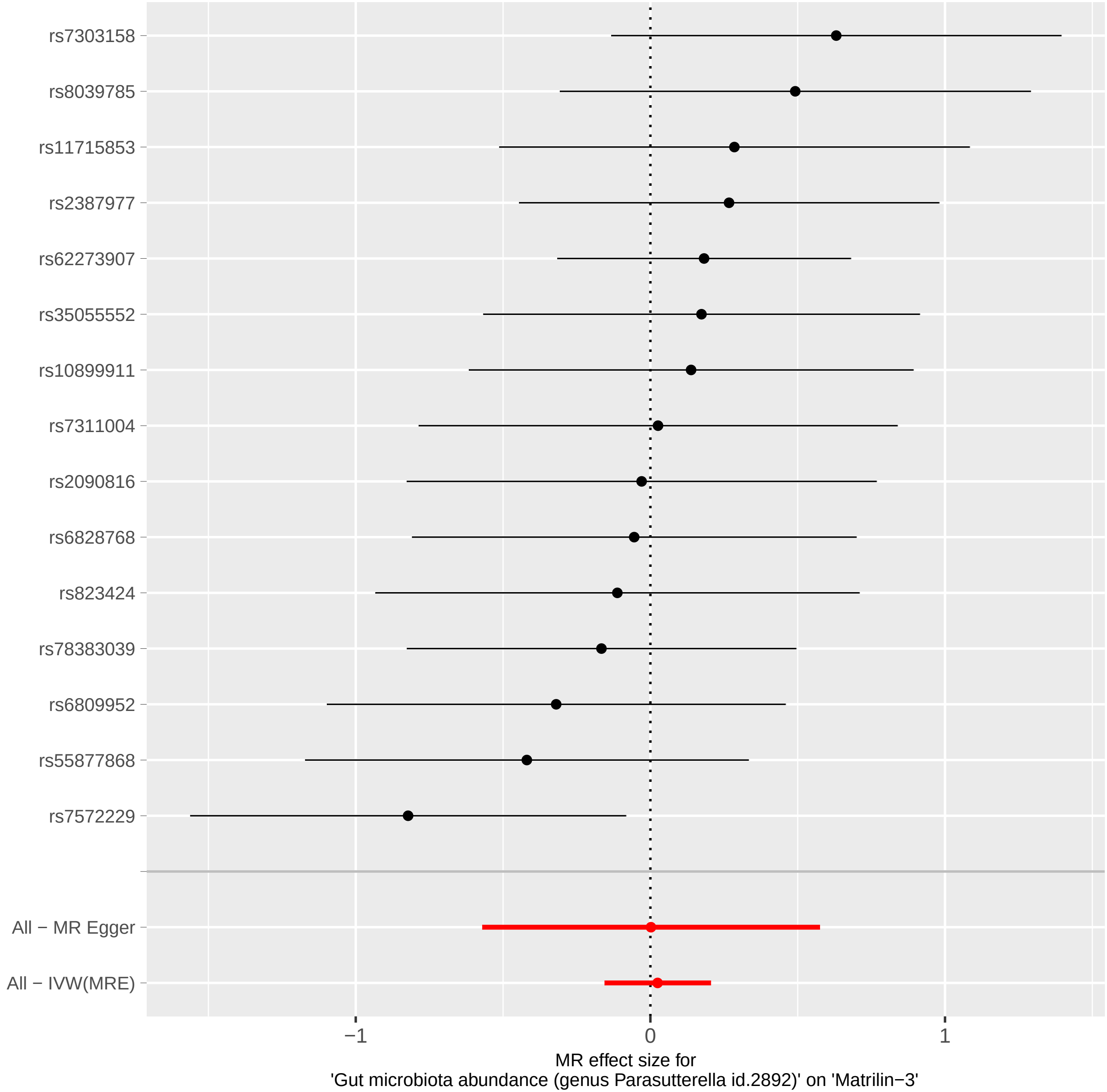

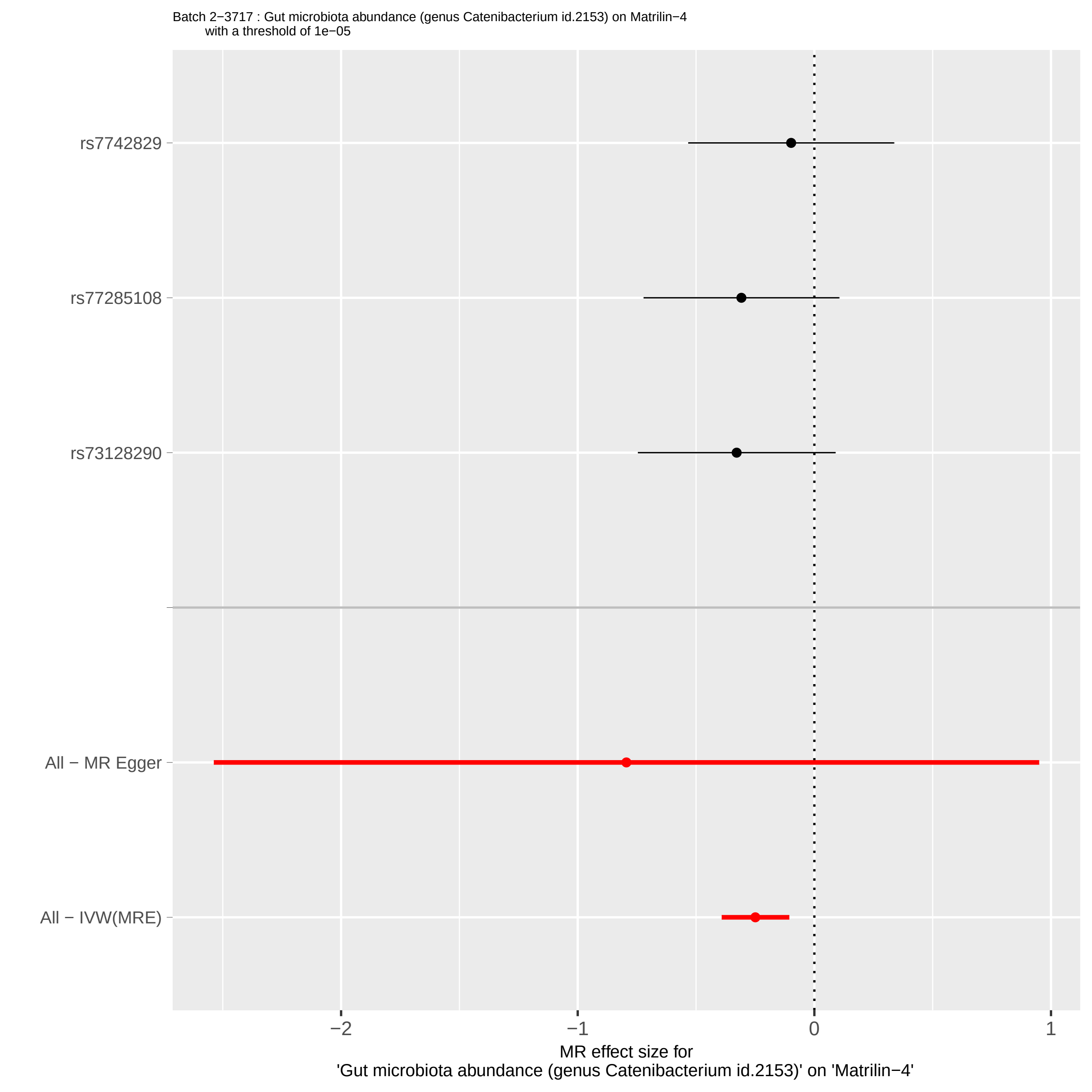
Batch 2−3717 : Gut microbiota abundance (genus Catenibacterium id.2153) on Matrilin−4
with a threshold of 1e−05
rs7742829
rs77285108
rs73128290
All − MR Egger
All − IVW(MRE)
−2
−1
0
1
MR effect size for
'Gut microbiota abundance (genus Catenibacterium id.2153)' on 'Matrilin−4'

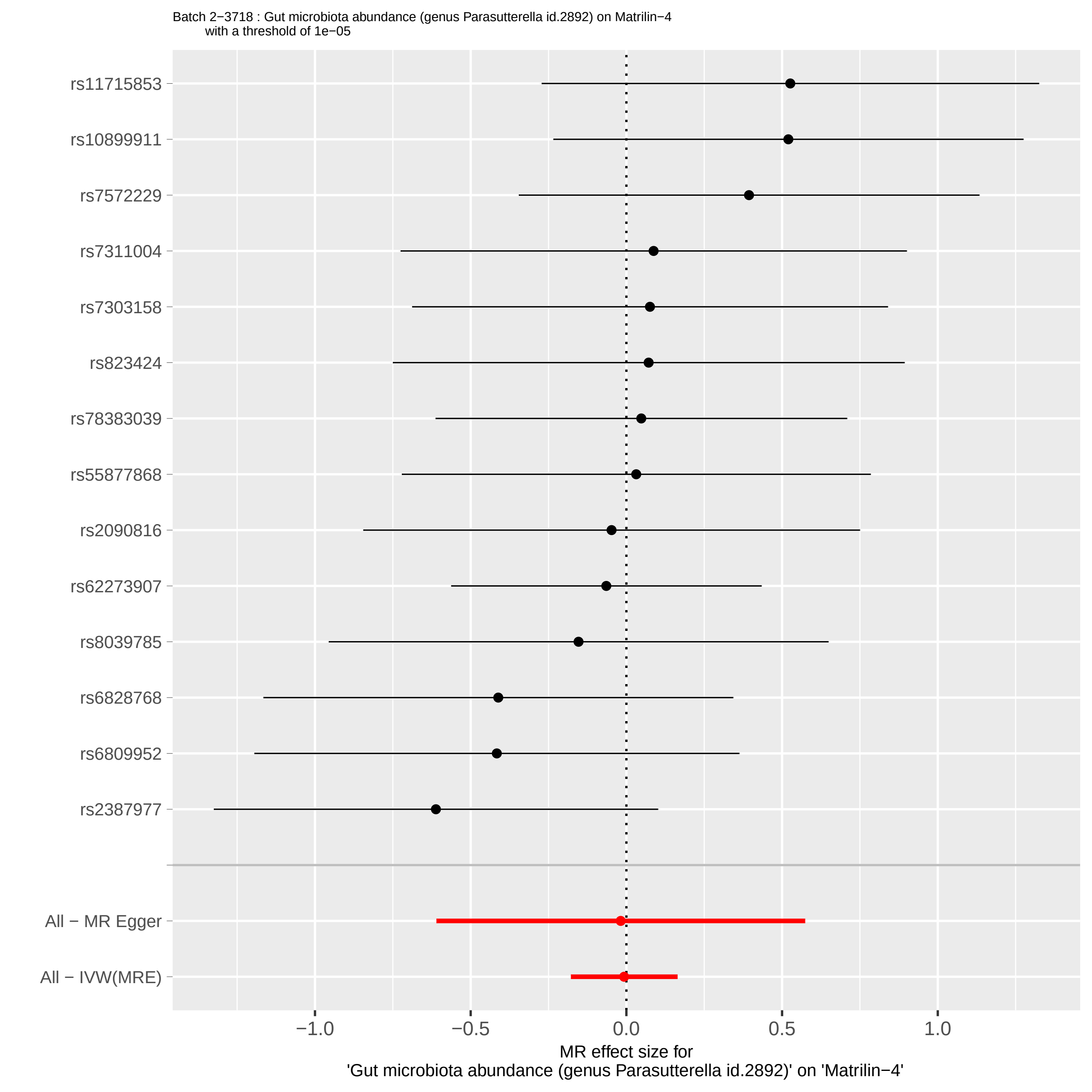
Batch 2−3718 : Gut microbiota abundance (genus Parasutterella id.2892) on Matrilin−4
with a threshold of 1e−05
rs11715853
rs10899911
rs7572229
rs7311004
rs7303158
rs823424
rs78383039
rs55877868
rs2090816
rs62273907
rs8039785
rs6828768
rs6809952
rs2387977
All − MR Egger
All − IVW(MRE)
−1.0
−0.5
0.0
0.5
1.0
MR effect size for
'Gut microbiota abundance (genus Parasutterella id.2892)' on 'Matrilin−4'

Batch 2−3719 : Gut microbiota abundance (genus Catenibacterium id.2153) on Myc−associated zinc finger protein
with a threshold of 1e−05

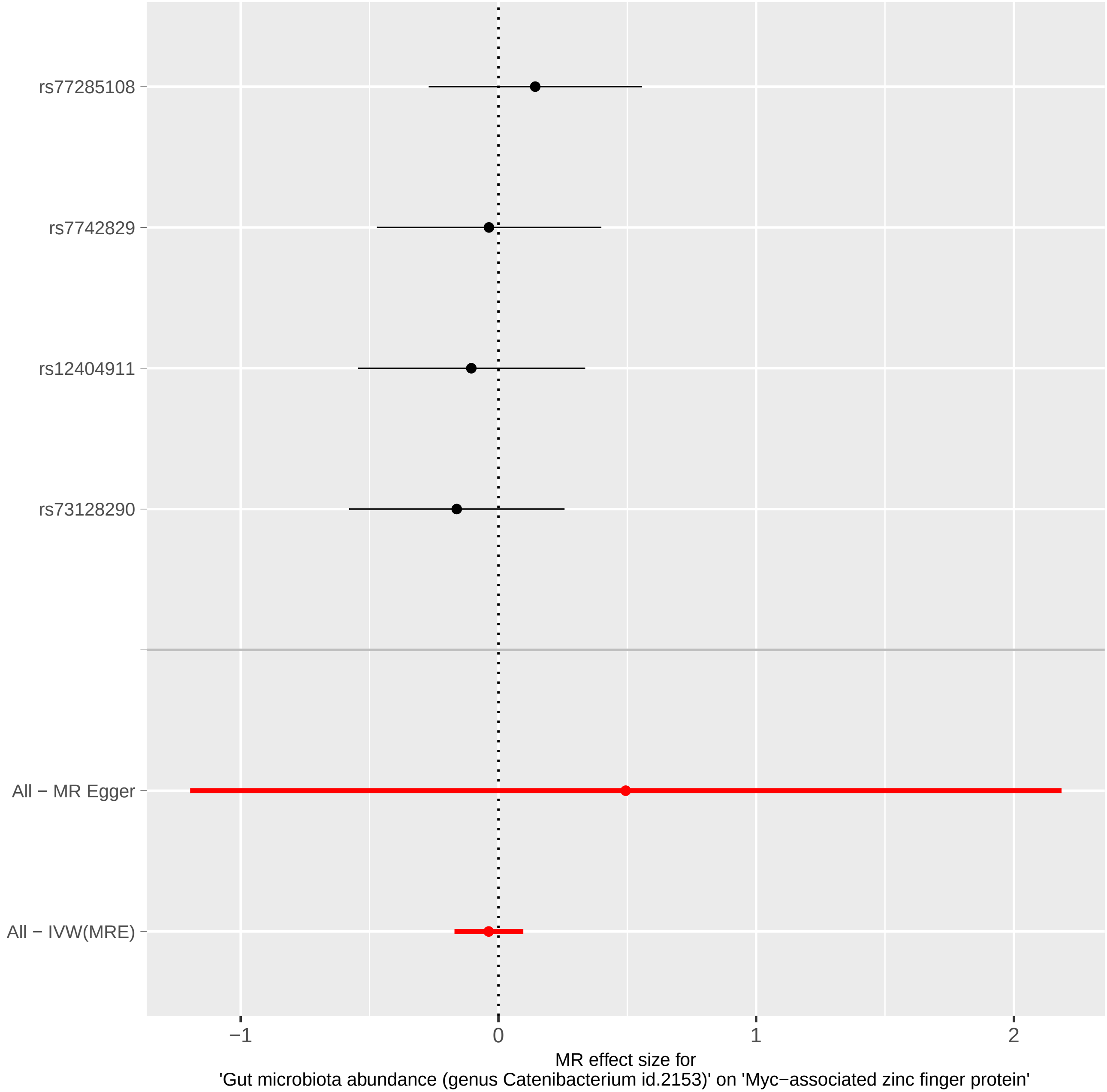

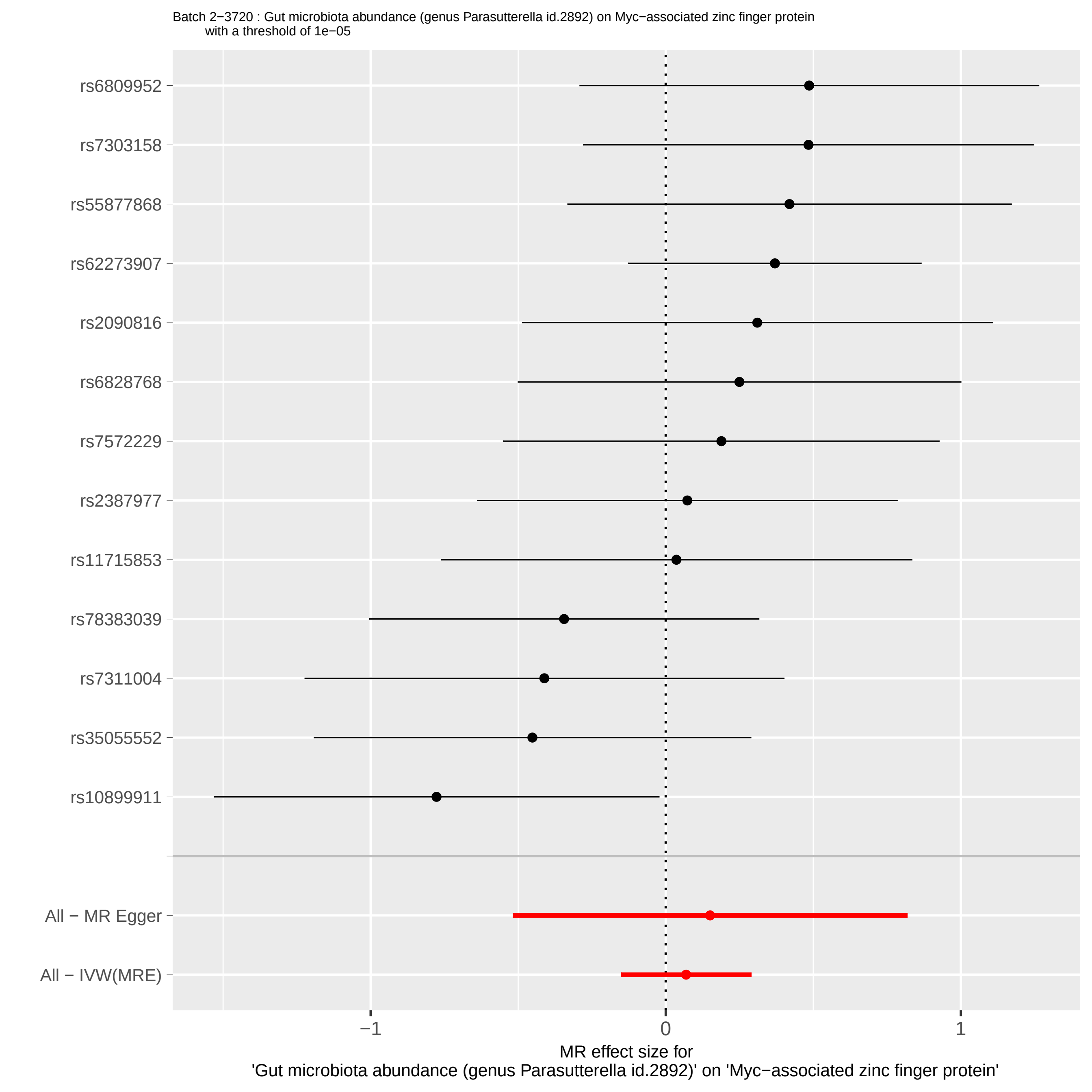

Batch 2−3720 : Gut microbiota abundance (genus Parasutterella id.2892) on Myc−associated zinc finger protein
with a threshold of 1e−05
rs6809952
rs7303158
rs55877868
rs62273907
rs2090816
rs6828768
rs7572229
rs2387977
rs11715853
rs78383039
rs7311004
rs35055552
rs10899911
All − MR Egger
All − IVW(MRE)
−1
0
1
MR effect size for
'Gut microbiota abundance (genus Parasutterella id.2892)' on 'Myc−associated zinc finger protein'

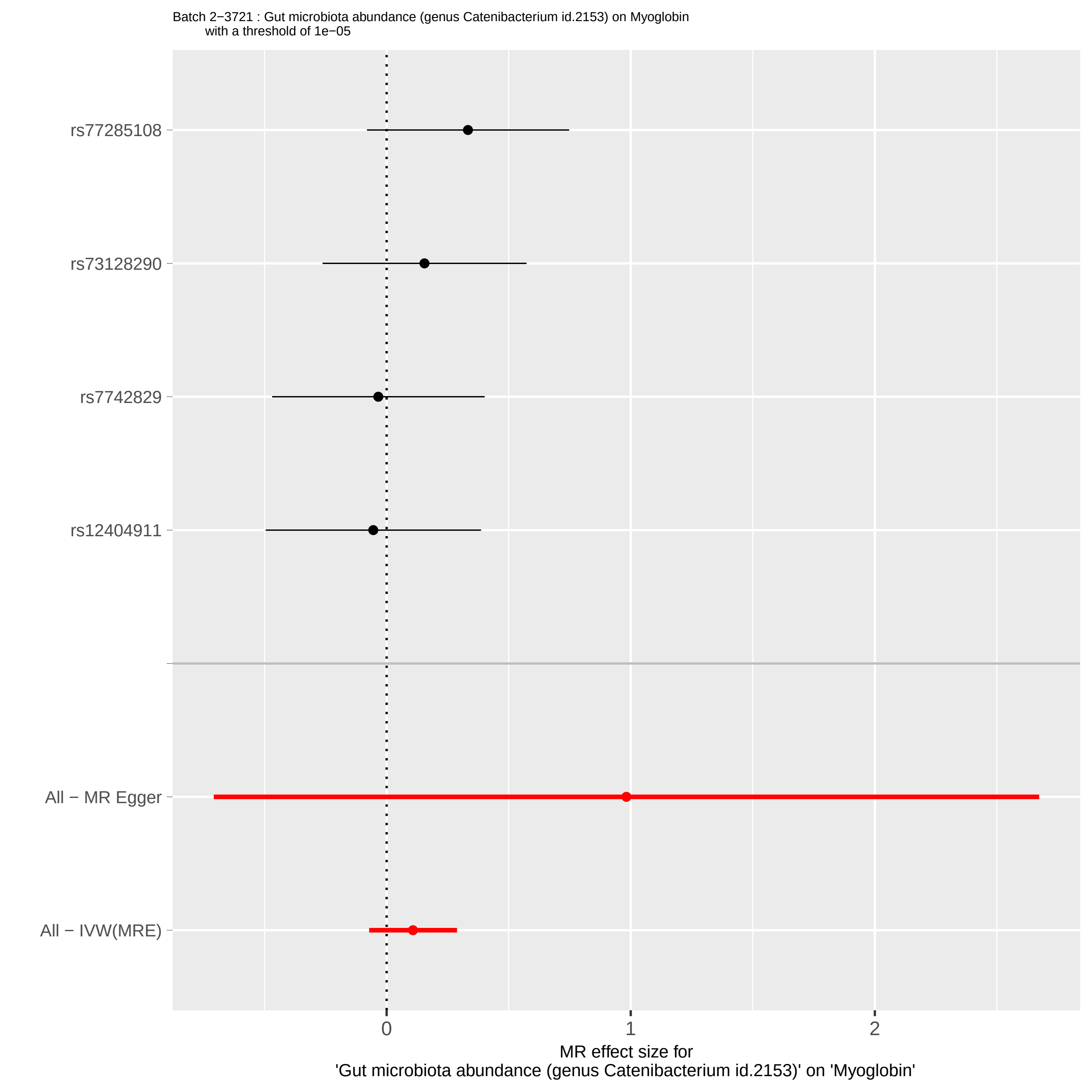
Batch 2−3721 : Gut microbiota abundance (genus Catenibacterium id.2153) on Myoglobin
with a threshold of 1e−05
rs77285108
rs73128290
rs7742829
rs12404911
All − MR Egger
All − IVW(MRE)
0
1
2
MR effect size for
'Gut microbiota abundance (genus Catenibacterium id.2153)' on 'Myoglobin'

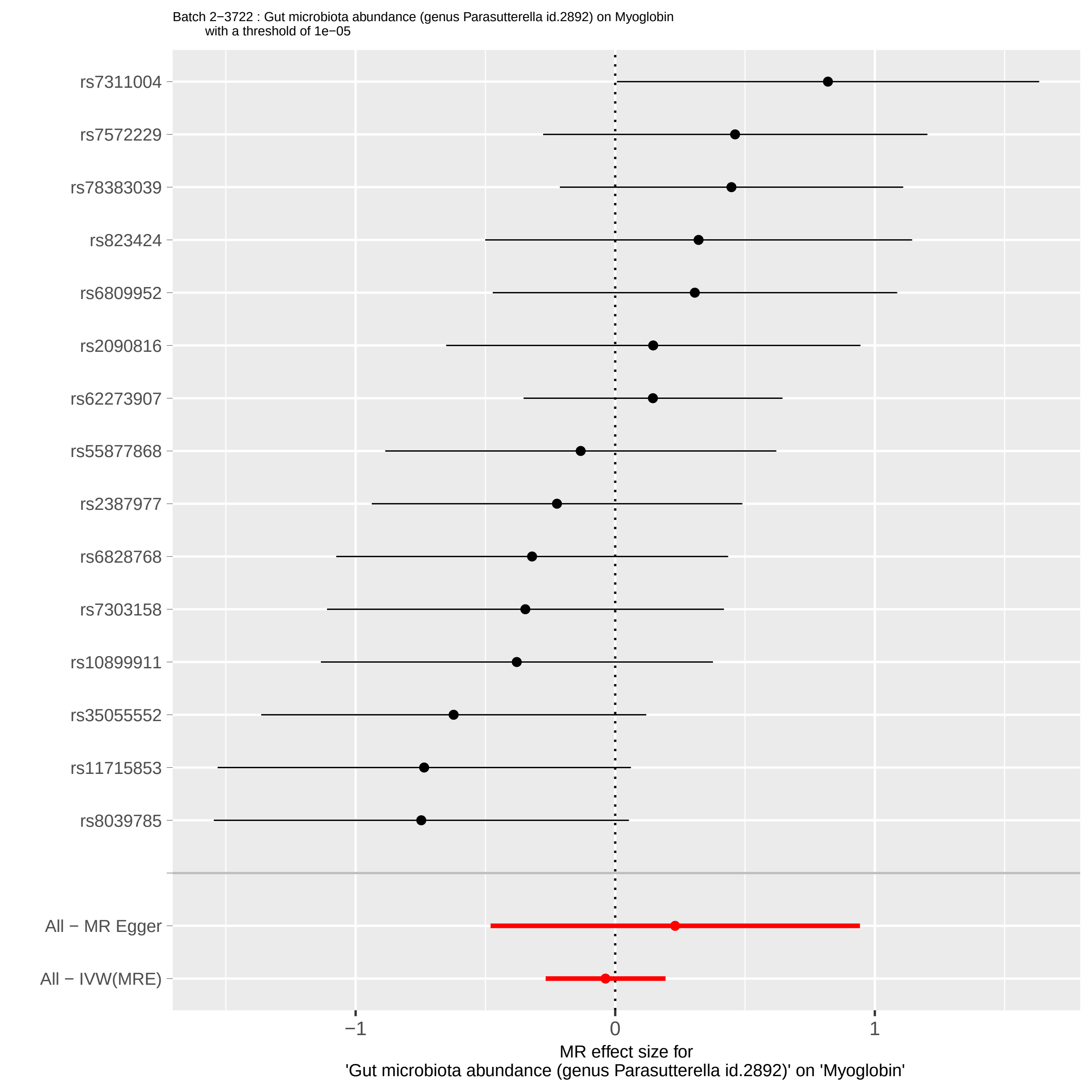

Batch 2−3722 : Gut microbiota abundance (genus Parasutterella id.2892) on Myoglobin
with a threshold of 1e−05
rs7311004
rs7572229
rs78383039
rs823424
rs6809952
rs2090816
rs62273907
rs55877868
rs2387977
rs6828768
rs7303158
rs10899911
rs35055552
rs11715853
rs8039785
All − MR Egger
All − IVW(MRE)
−1
0
1
MR effect size for
'Gut microbiota abundance (genus Parasutterella id.2892)' on 'Myoglobin'

Batch 2−3723 : Gut microbiota abundance (genus Catenibacterium id.2153) on Methyl−CpG−binding domain protein 4
with a threshold of 1e−05

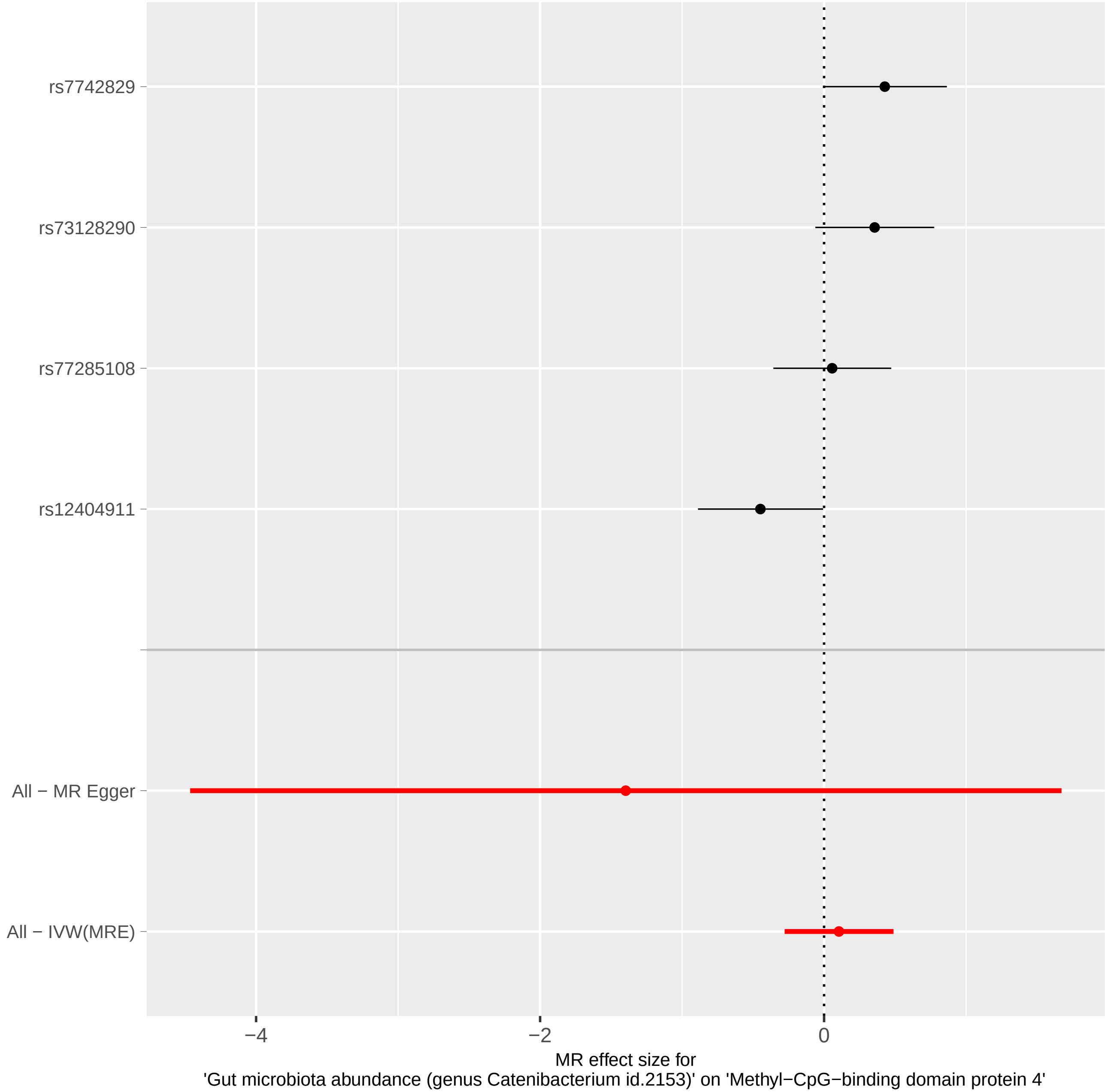

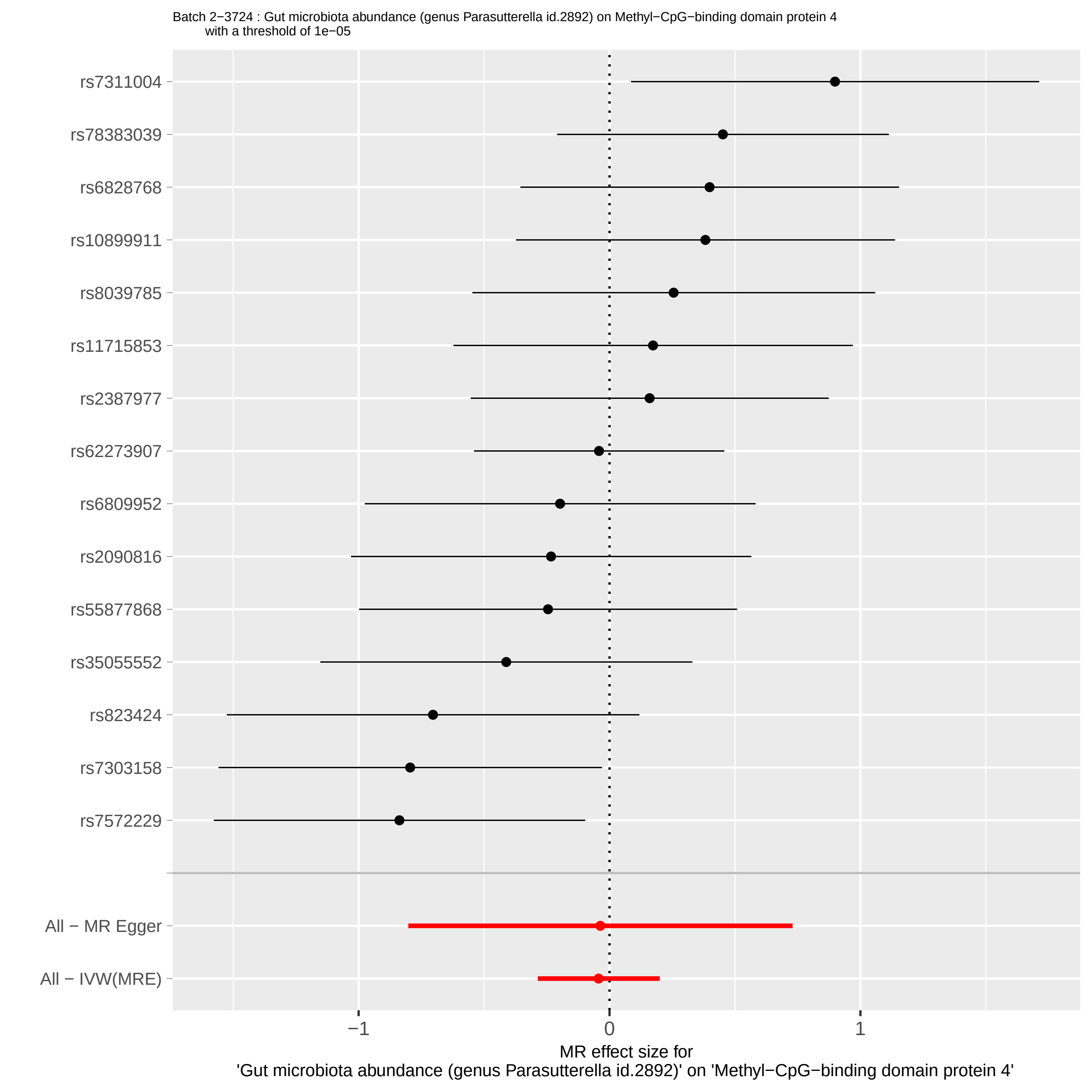

Batch 2−3724 : Gut microbiota abundance (genus Parasutterella id.2892) on Methyl−CpG−binding domain protein 4
with a threshold of 1e−05
rs7311004
rs78383039
rs6828768
rs10899911
rs8039785
rs11715853
rs2387977
rs62273907
rs6809952
rs2090816
rs55877868
rs35055552
rs823424
rs7303158
rs7572229
All − MR Egger
All − IVW(MRE)
−1
0
1
MR effect size for
'Gut microbiota abundance (genus Parasutterella id.2892)' on 'Methyl−CpG−binding domain protein 4'

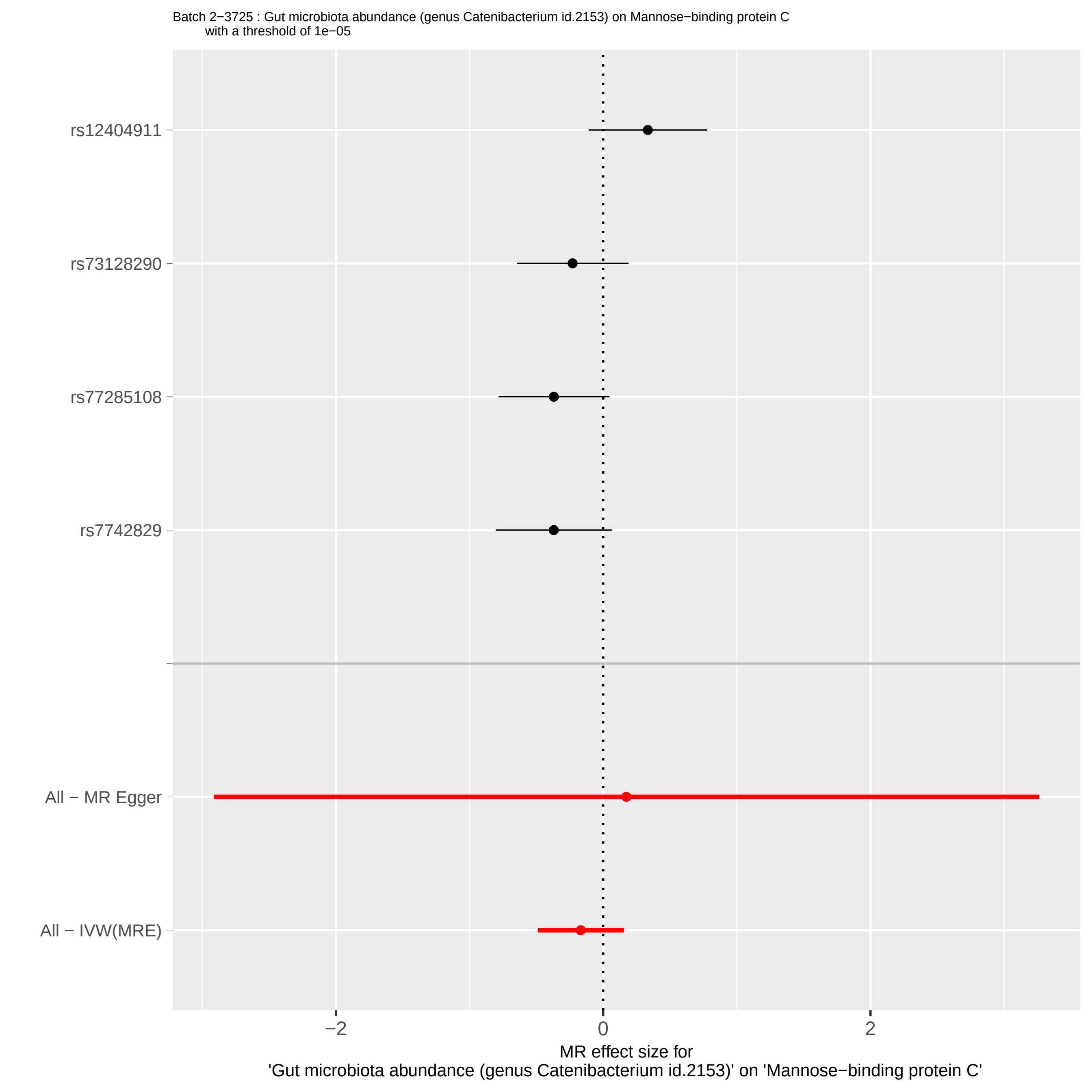
Batch 2−3725 : Gut microbiota abundance (genus Catenibacterium id.2153) on Mannose−binding protein C
with a threshold of 1e−05
rs12404911
rs73128290
rs77285108
rs7742829
All − MR Egger
All − IVW(MRE)
−2
0
2
MR effect size for
'Gut microbiota abundance (genus Catenibacterium id.2153)' on 'Mannose−binding protein C'

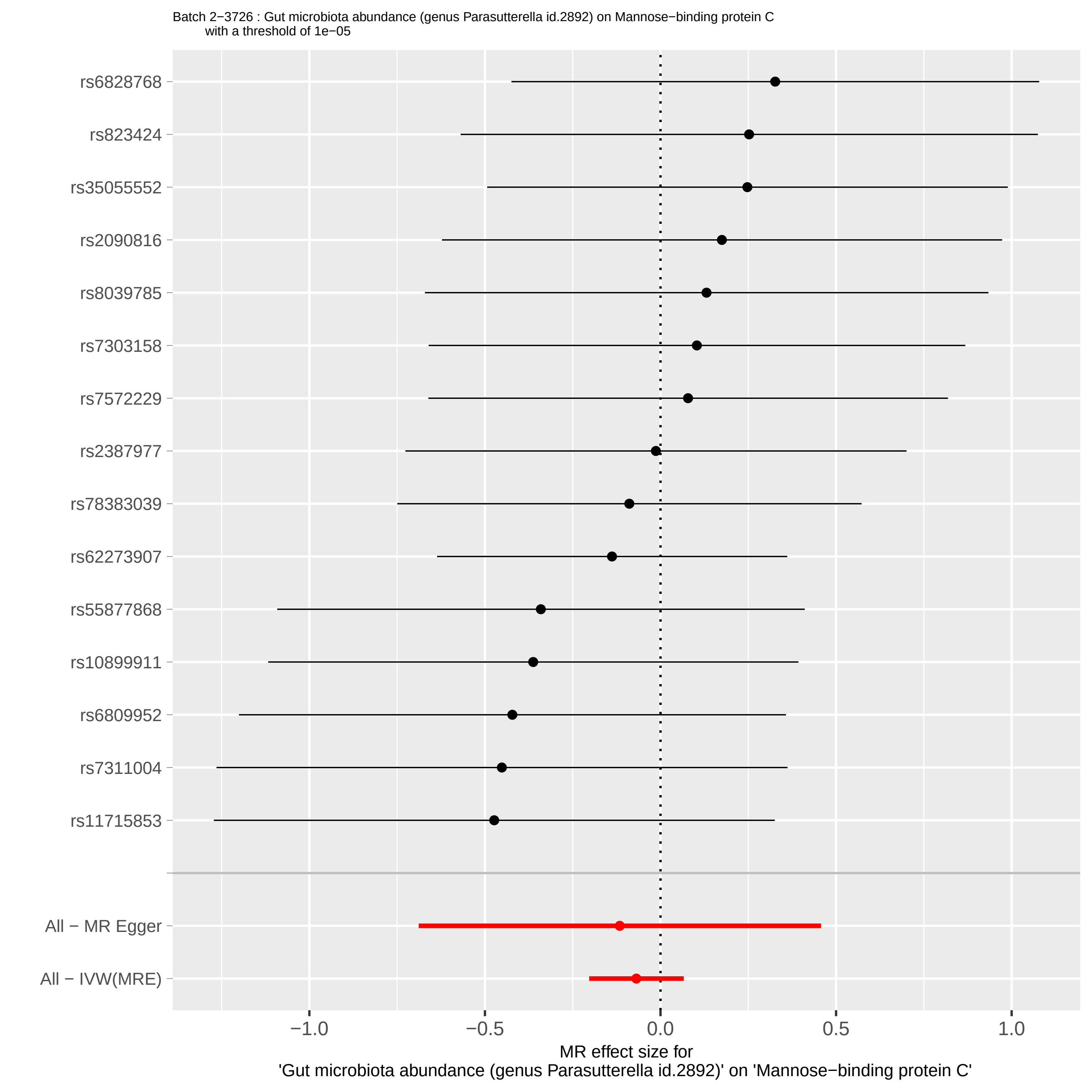

Batch 2−3726 : Gut microbiota abundance (genus Parasutterella id.2892) on Mannose−binding protein C
with a threshold of 1e−05
rs6828768
rs823424
rs35055552
rs2090816
rs8039785
rs7303158
rs7572229
rs2387977
rs78383039
rs62273907
rs55877868
rs10899911
rs6809952
rs7311004
rs11715853
All − MR Egger
All − IVW(MRE)
−1.0
−0.5
0.0
0.5
1.0
MR effect size for
'Gut microbiota abundance (genus Parasutterella id.2892)' on 'Mannose−binding protein C'

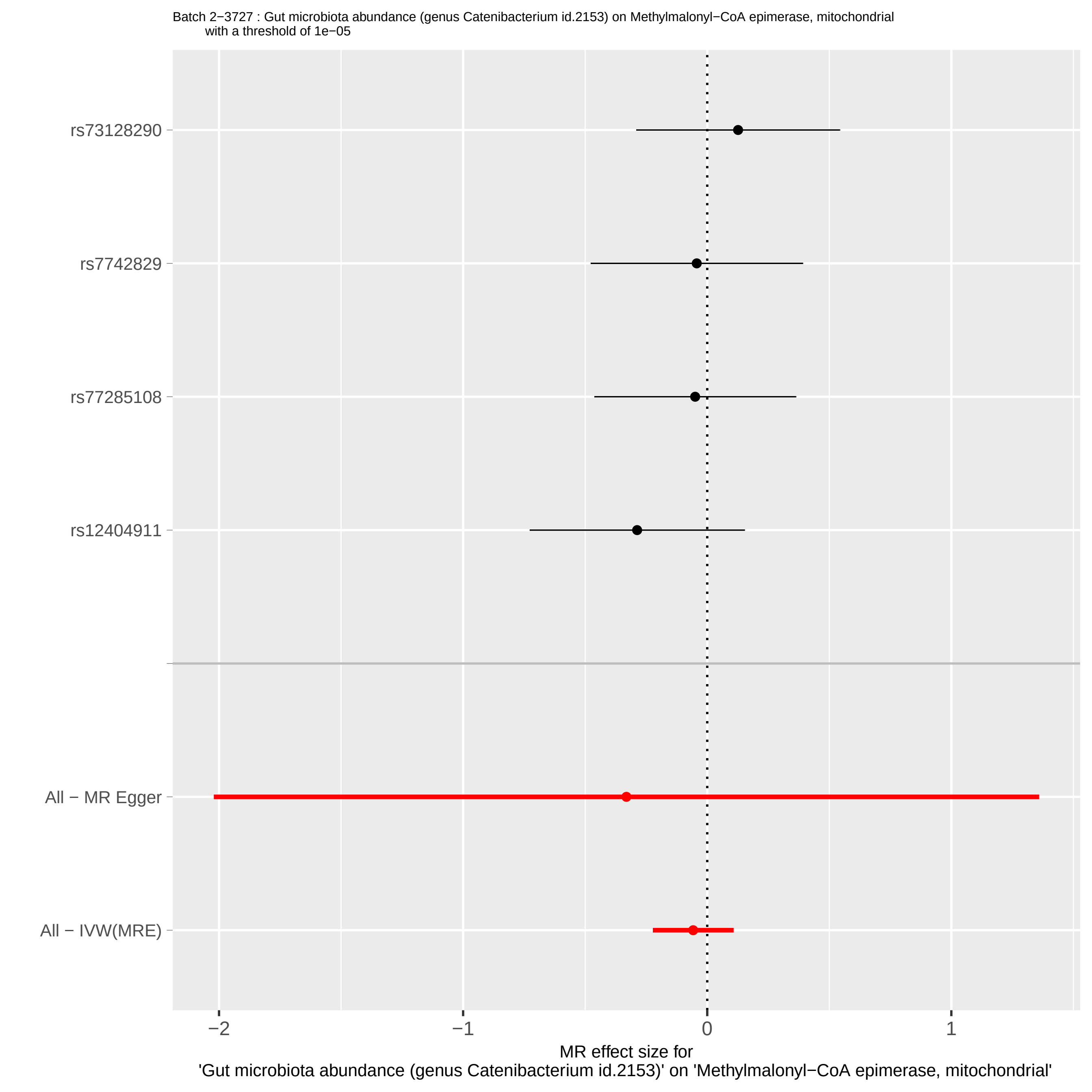
Batch 2−3727 : Gut microbiota abundance (genus Catenibacterium id.2153) on Methylmalonyl−CoA epimerase, mitochondrial
with a threshold of 1e−05
rs73128290
rs7742829
rs77285108
rs12404911
All − MR Egger
All − IVW(MRE)
−2
−1
0
1
MR effect size for
'Gut microbiota abundance (genus Catenibacterium id.2153)' on 'Methylmalonyl−CoA epimerase, mitochondrial'

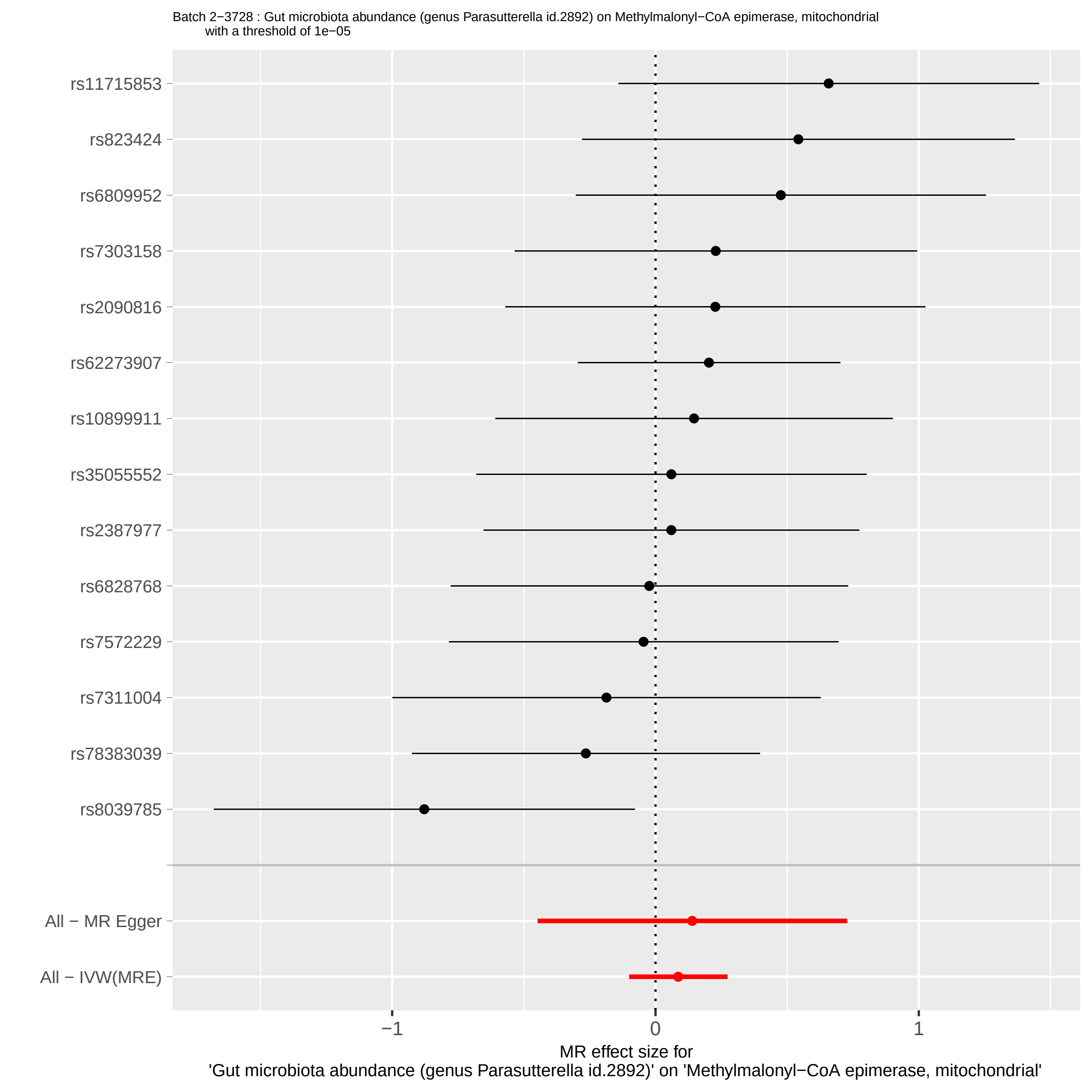

Batch 2−3728 : Gut microbiota abundance (genus Parasutterella id.2892) on Methylmalonyl−CoA epimerase, mitochondrial
with a threshold of 1e−05
rs11715853
rs823424
rs6809952
rs7303158
rs2090816
rs62273907
rs10899911
rs35055552
rs2387977
rs6828768
rs7572229
rs7311004
rs78383039
rs8039785
All − MR Egger
All − IVW(MRE)
−1
0
1
MR effect size for
'Gut microbiota abundance (genus Parasutterella id.2892)' on 'Methylmalonyl−CoA epimerase, mitochondrial'

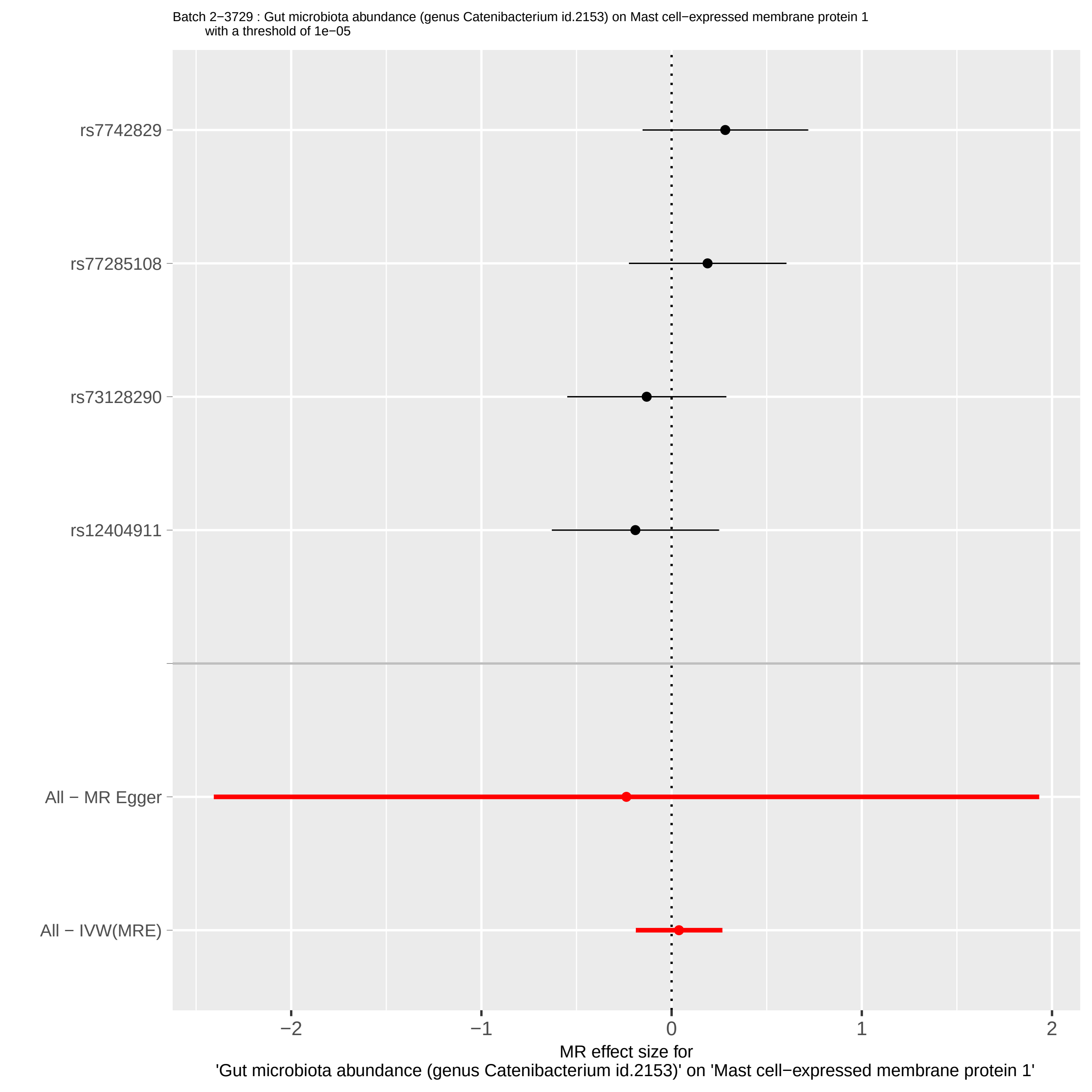
Batch 2−3729 : Gut microbiota abundance (genus Catenibacterium id.2153) on Mast cell−expressed membrane protein 1
with a threshold of 1e−05
rs7742829
rs77285108
rs73128290
rs12404911
All − MR Egger
All − IVW(MRE)
−2
−1
0
1
2
MR effect size for
'Gut microbiota abundance (genus Catenibacterium id.2153)' on 'Mast cell−expressed membrane protein 1'

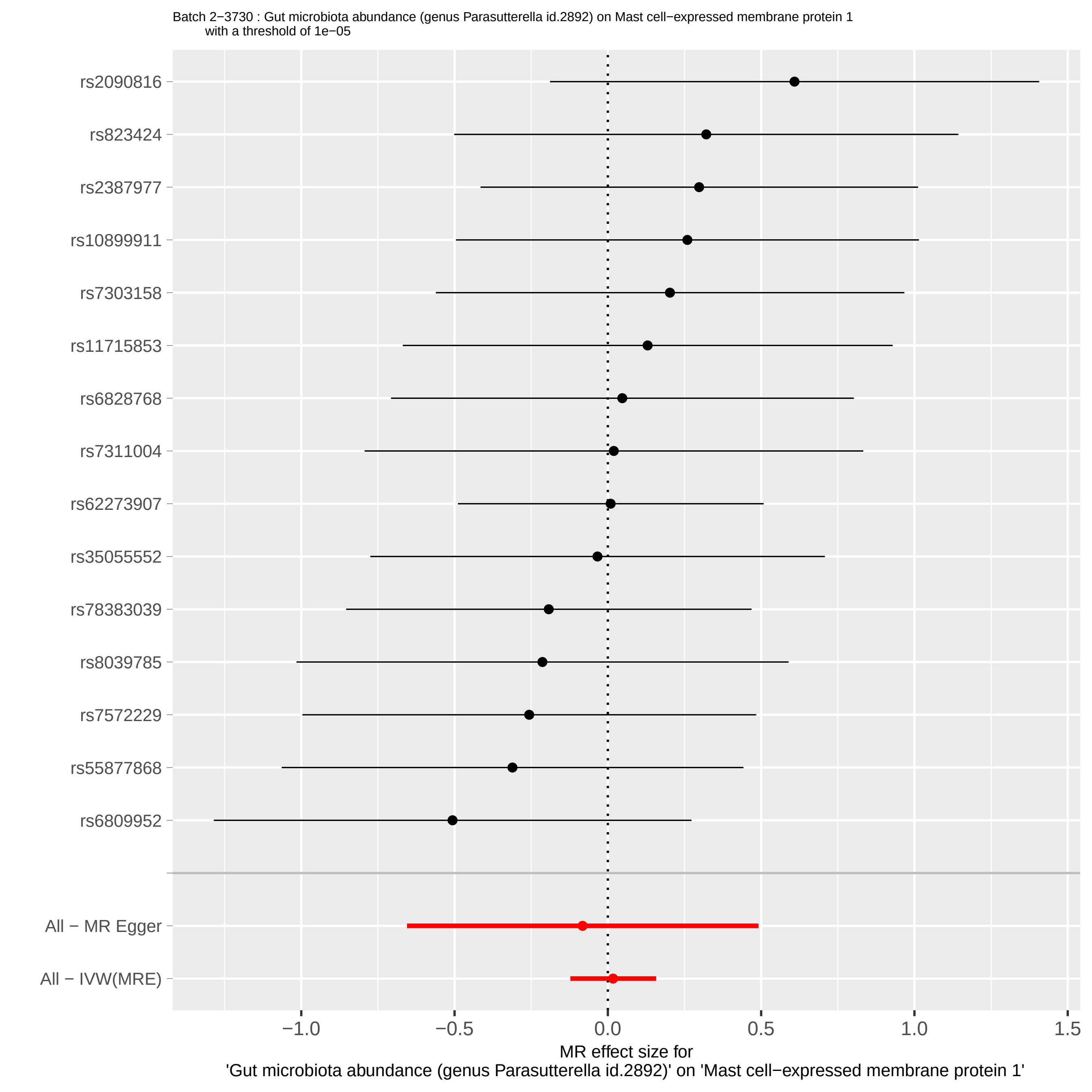

Batch 2−3730 : Gut microbiota abundance (genus Parasutterella id.2892) on Mast cell−expressed membrane protein 1
with a threshold of 1e−05
rs2090816
rs823424
rs2387977
rs10899911
rs7303158
rs11715853
rs6828768
rs7311004
rs62273907
rs35055552
rs78383039
rs8039785
rs7572229
rs55877868
rs6809952
All − MR Egger
All − IVW(MRE)
−1.0
−0.5
0.0
0.5
1.0
1.5
MR effect size for
'Gut microbiota abundance (genus Parasutterella id.2892)' on 'Mast cell−expressed membrane protein 1'

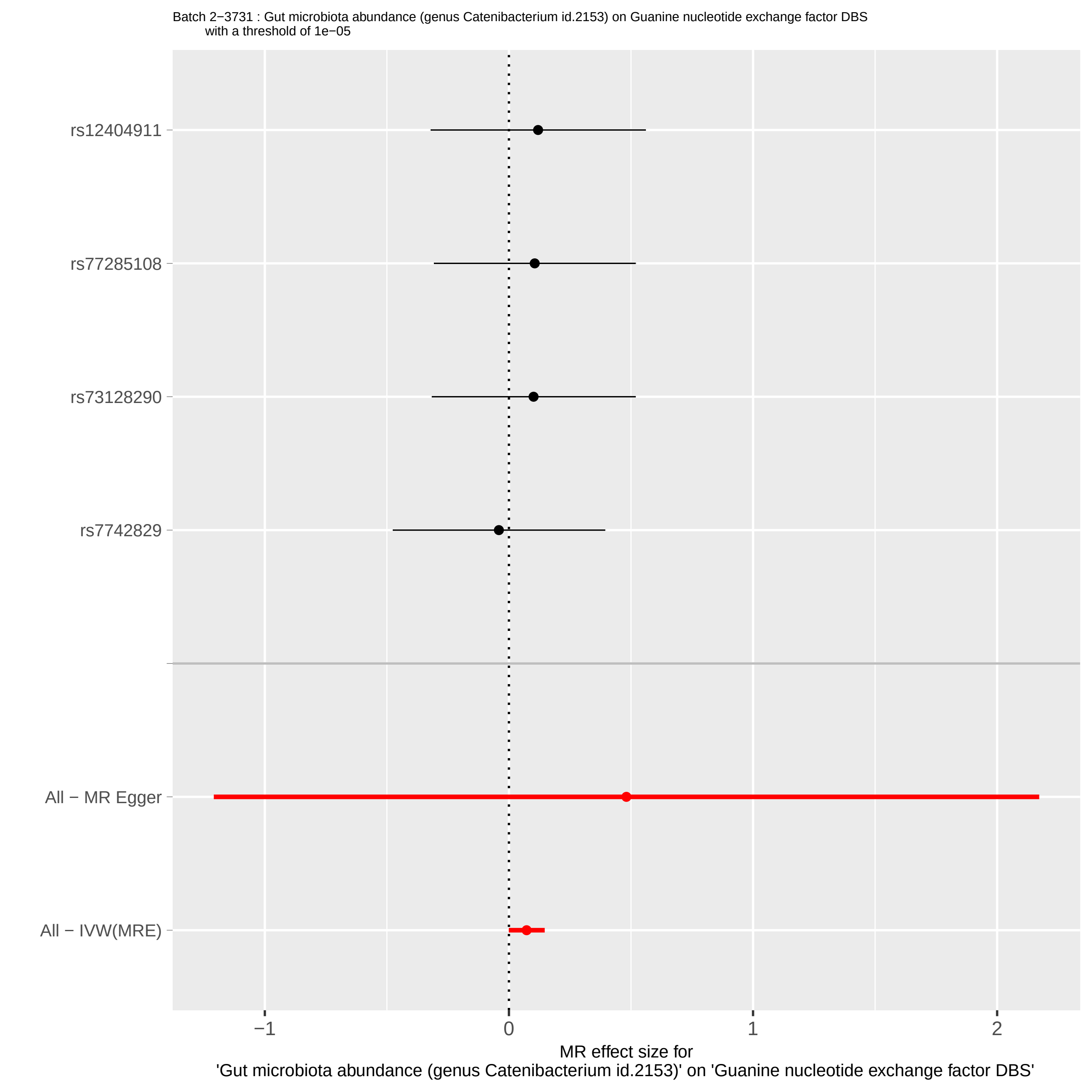
Batch 2−3731 : Gut microbiota abundance (genus Catenibacterium id.2153) on Guanine nucleotide exchange factor DBS
with a threshold of 1e−05
rs12404911
rs77285108
rs73128290
rs7742829
All − MR Egger
All − IVW(MRE)
−1
0
1
2
MR effect size for
'Gut microbiota abundance (genus Catenibacterium id.2153)' on 'Guanine nucleotide exchange factor DBS'

Batch 2−3732 : Gut microbiota abundance (genus Parasutterella id.2892) on Guanine nucleotide exchange factor DBS
with a threshold of 1e−05

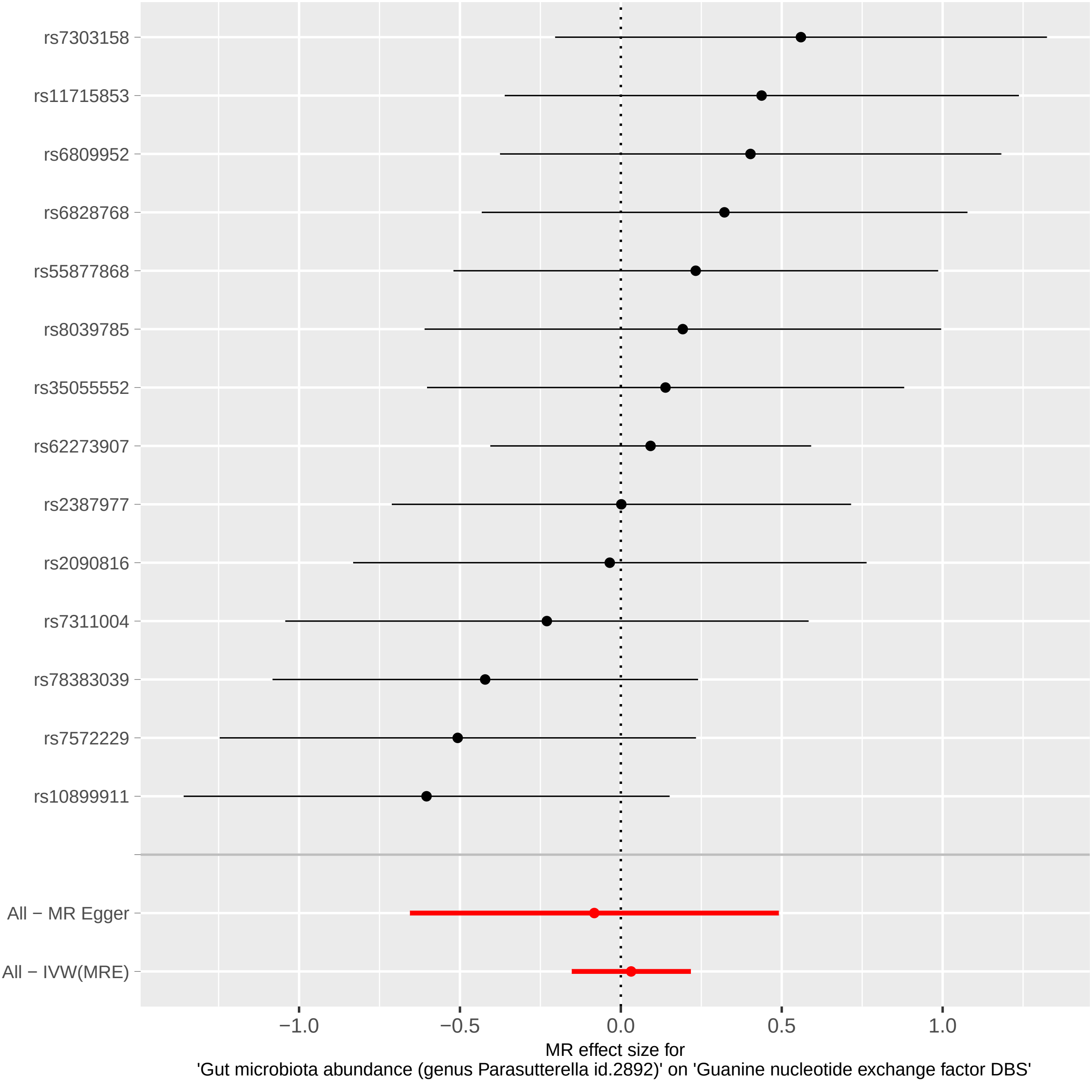

Batch 2−3733 : Gut microbiota abundance (genus Catenibacterium id.2153) on Multiple coagulation factor deficiency protein 2
with a threshold of 1e−05

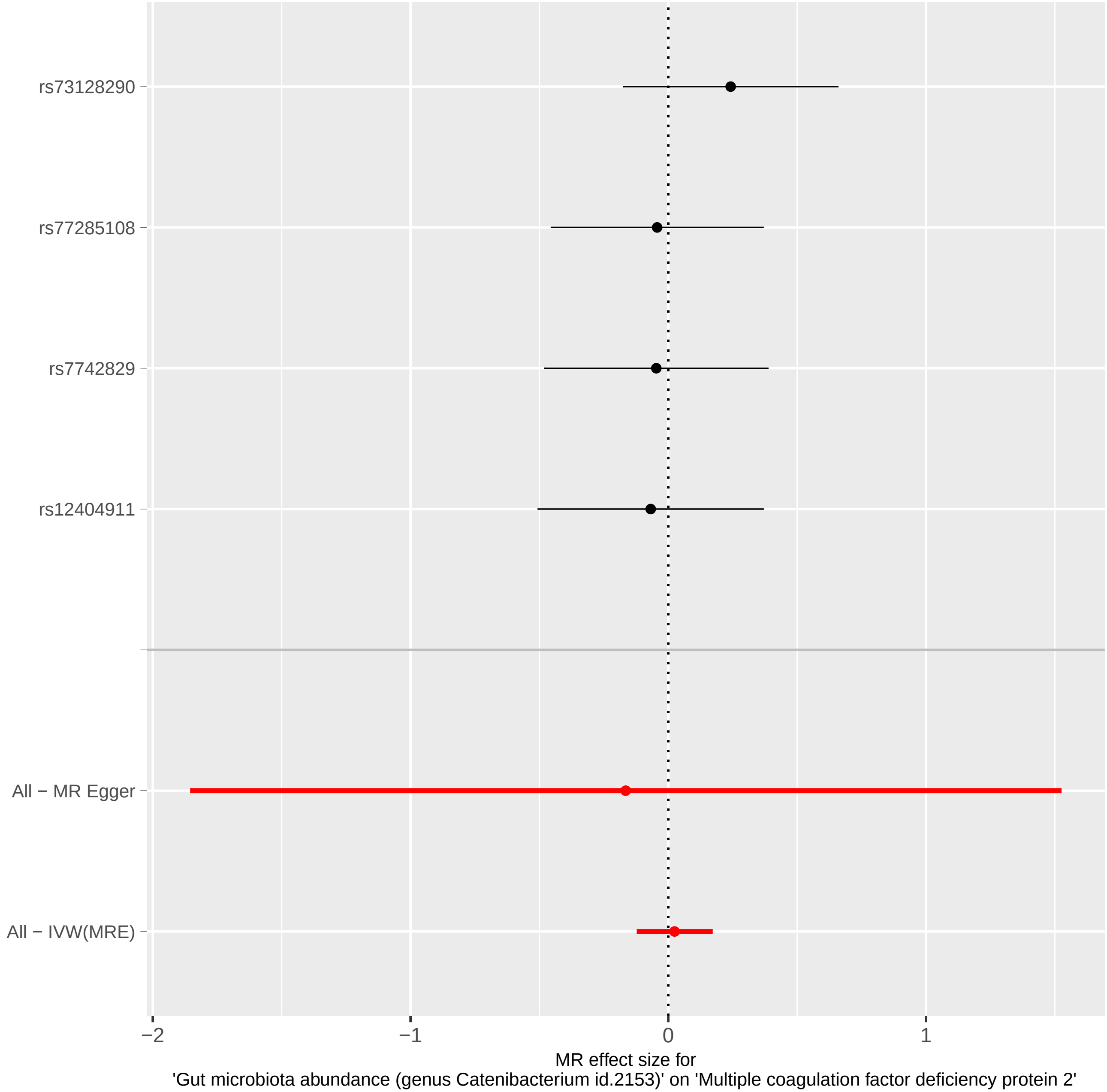

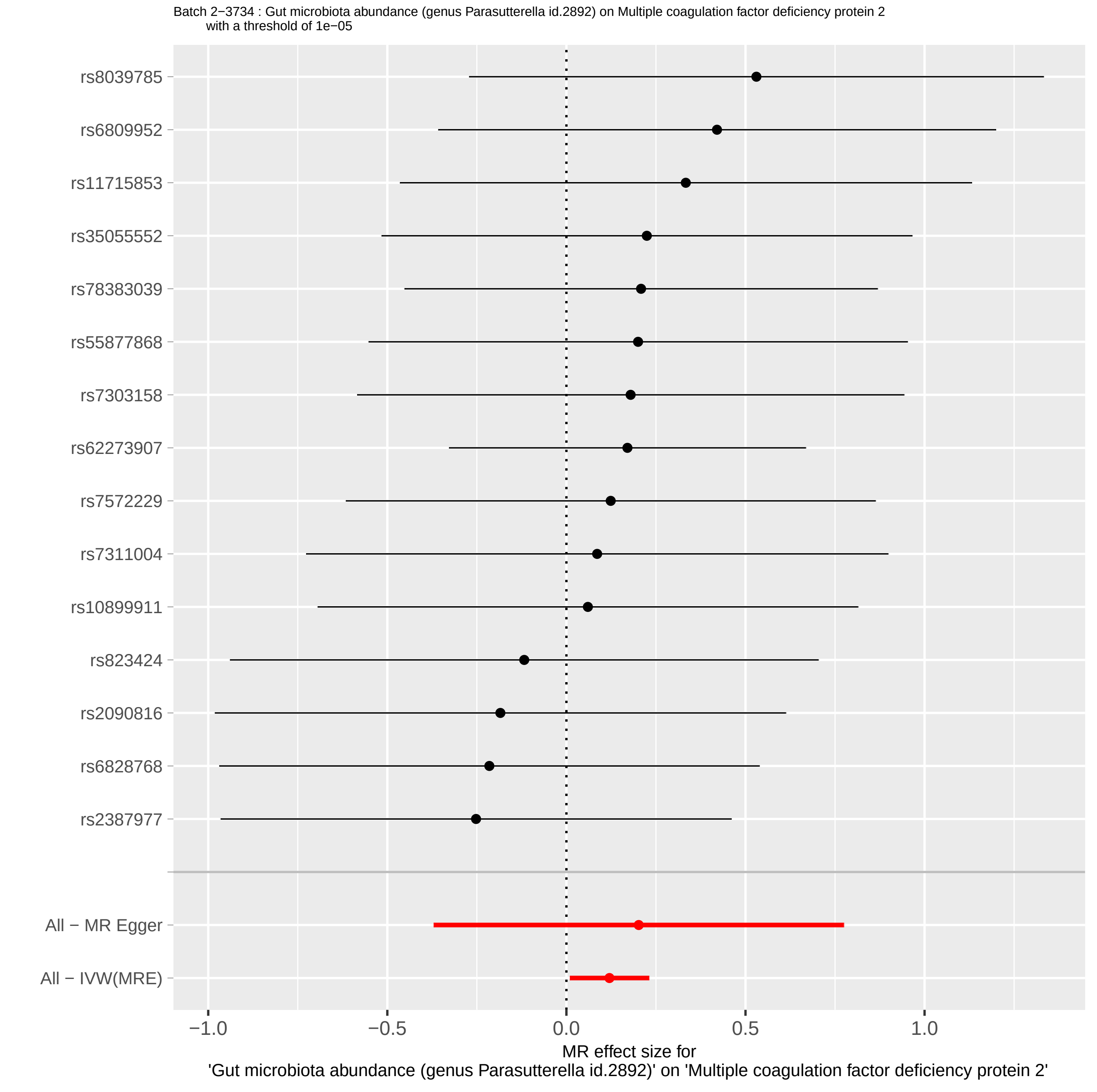

Batch 2−3734 : Gut microbiota abundance (genus Parasutterella id.2892) on Multiple coagulation factor deficiency protein 2
with a threshold of 1e−05
rs8039785
rs6809952
rs11715853
rs35055552
rs78383039
rs55877868
rs7303158
rs62273907
rs7572229
rs7311004
rs10899911
rs823424
rs2090816
rs6828768
rs2387977
All − MR Egger
All − IVW(MRE)
−1.0
−0.5
0.0
0.5
1.0
MR effect size for
'Gut microbiota abundance (genus Parasutterella id.2892)' on 'Multiple coagulation factor deficiency protein 2'

Batch 2−3735 : Gut microbiota abundance (genus Catenibacterium id.2153) on Induced myeloid leukemia cell differentiation protein Mcl−1 with a threshold of 1e−05

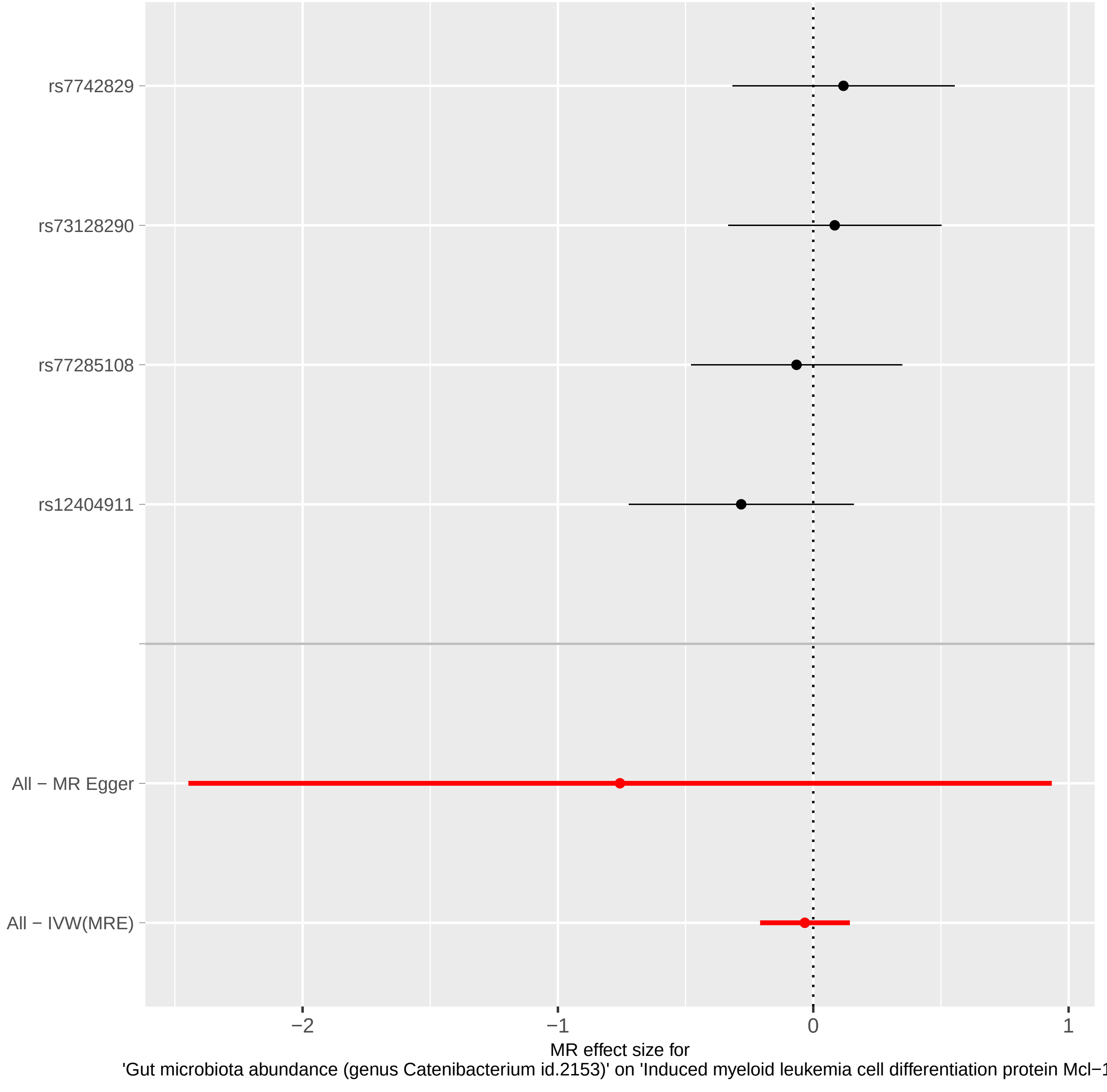

Batch 2−3736 : Gut microbiota abundance (genus Parasutterella id.2892) on Induced myeloid leukemia cell differentiation protein Mcl−1 with a threshold of 1e−05

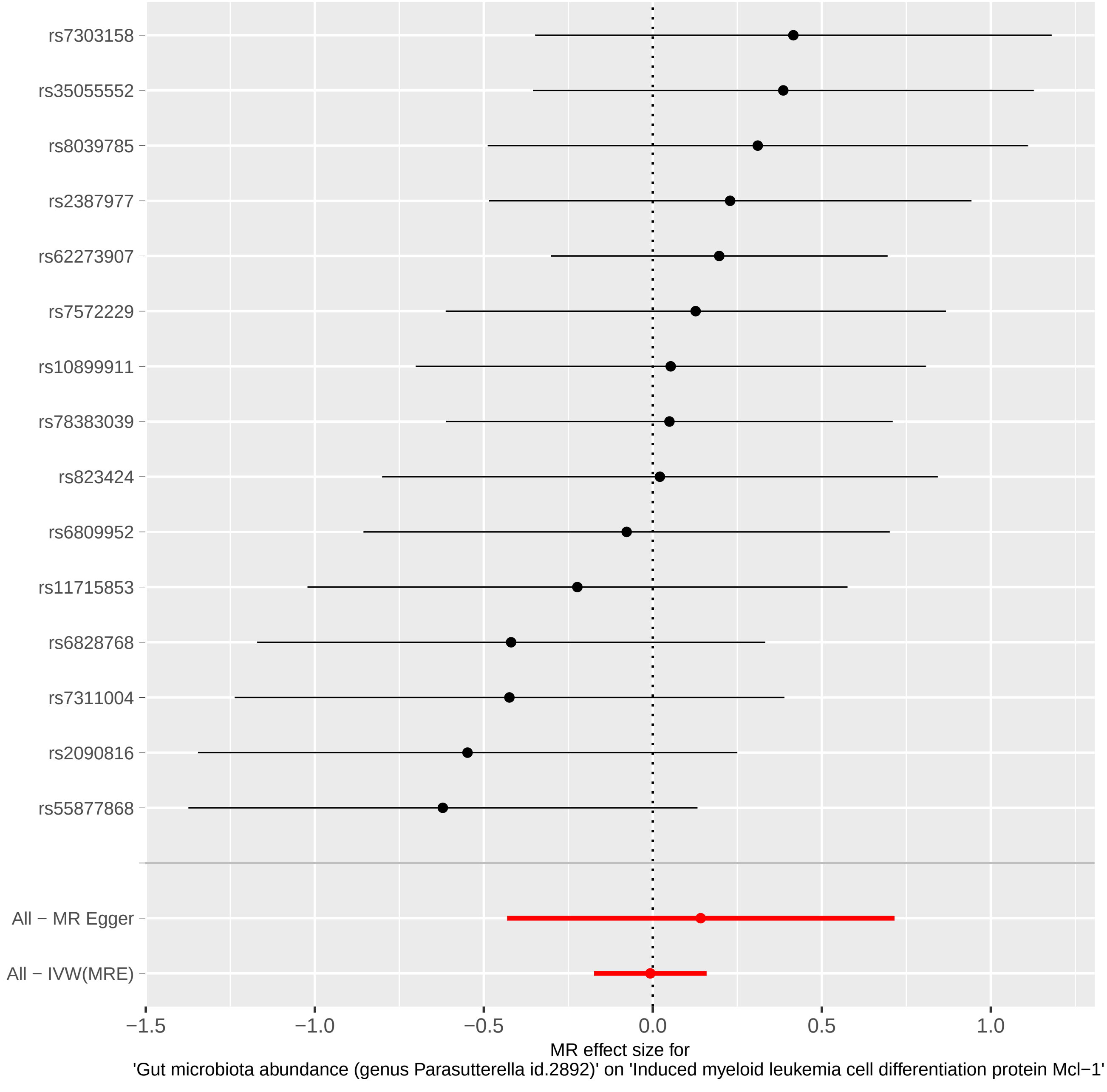

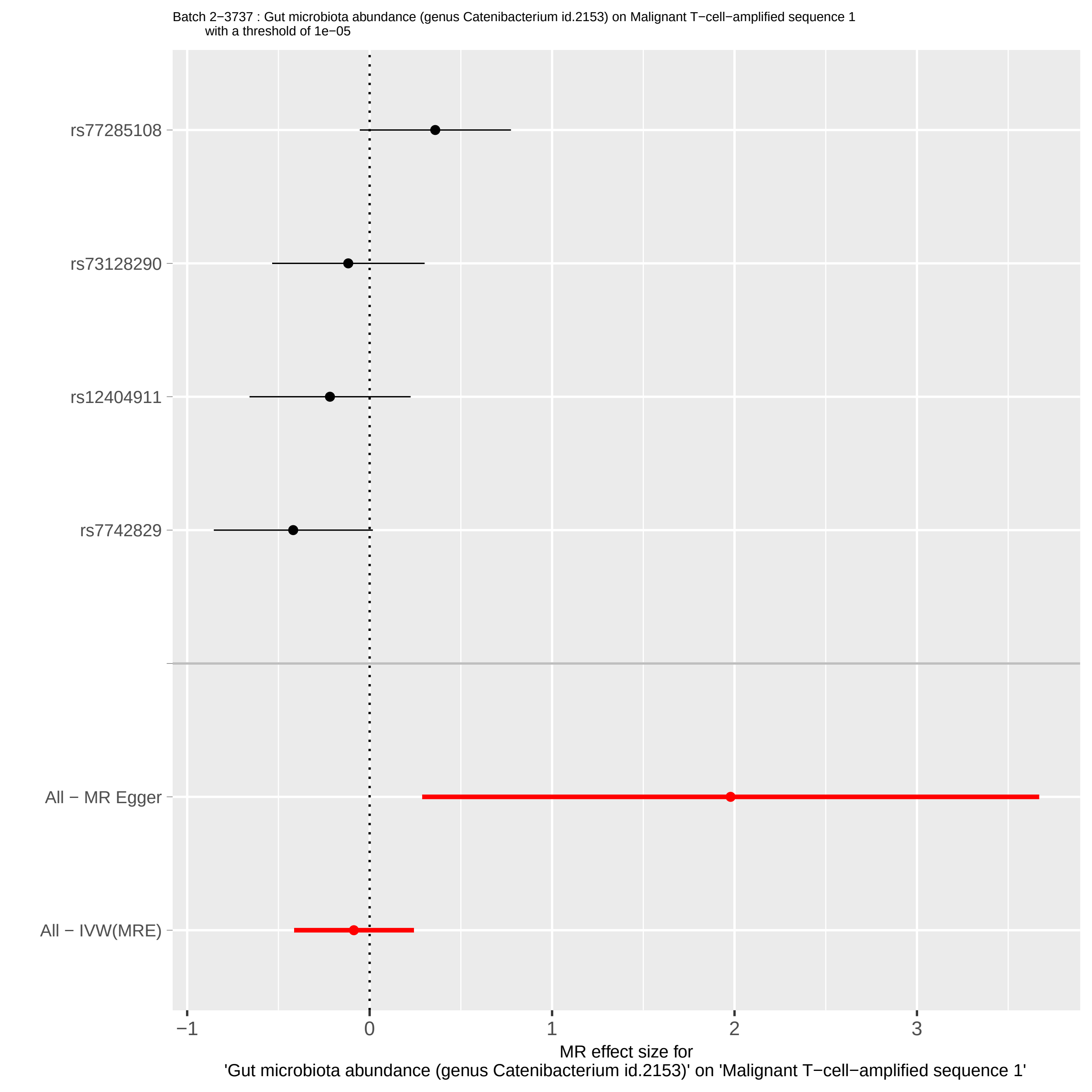
Batch 2−3737 : Gut microbiota abundance (genus Catenibacterium id.2153) on Malignant T−cell−amplified sequence 1
with a threshold of 1e−05
rs77285108
rs73128290
rs12404911
rs7742829
All − MR Egger
All − IVW(MRE)
−1
0
1
2
3
MR effect size for
'Gut microbiota abundance (genus Catenibacterium id.2153)' on 'Malignant T−cell−amplified sequence 1'

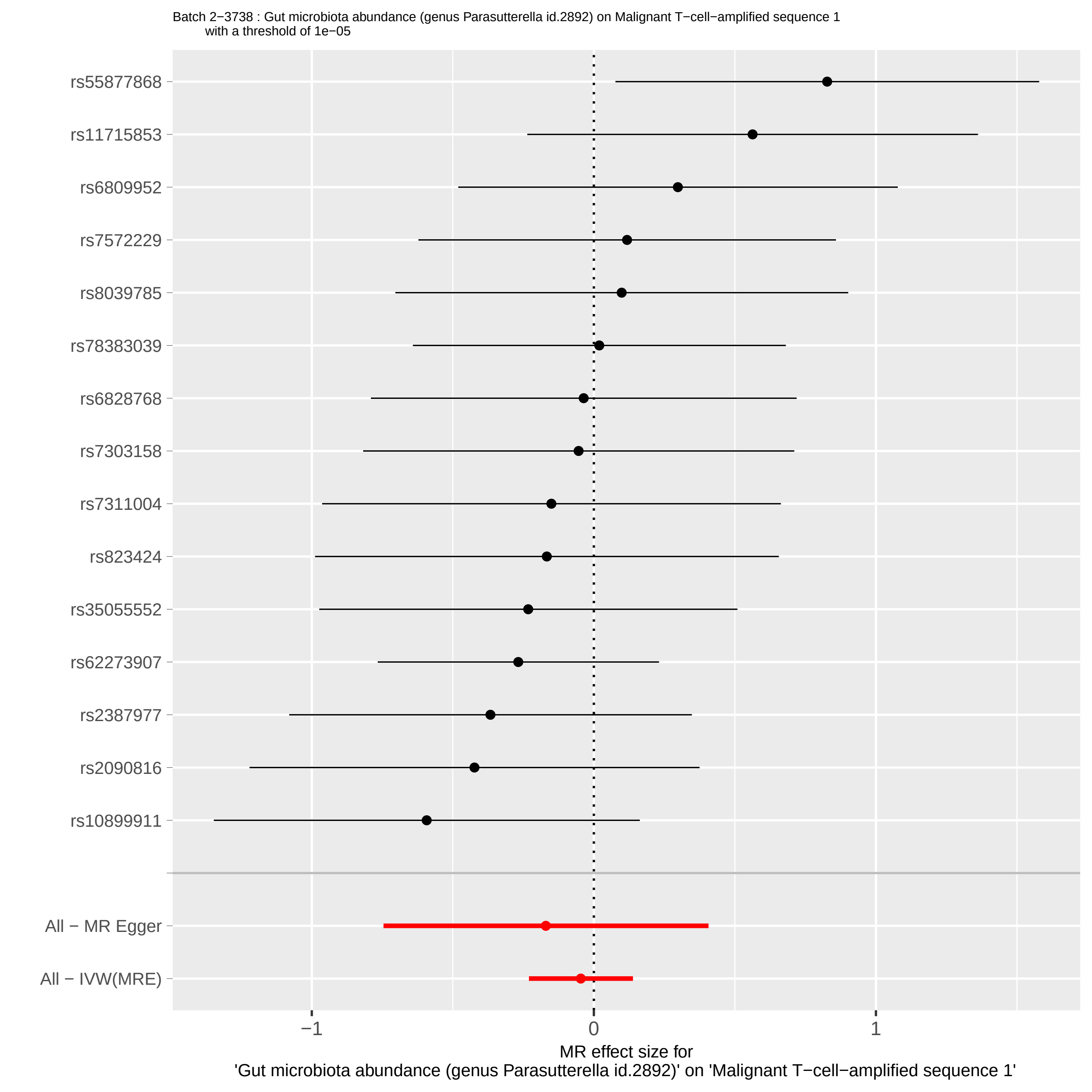

Batch 2−3738 : Gut microbiota abundance (genus Parasutterella id.2892) on Malignant T−cell−amplified sequence 1
with a threshold of 1e−05
rs55877868
rs11715853
rs6809952
rs7572229
rs8039785
rs78383039
rs6828768
rs7303158
rs7311004
rs823424
rs35055552
rs62273907
rs2387977
rs2090816
rs10899911
All − MR Egger
All − IVW(MRE)
−1
0
1
MR effect size for
'Gut microbiota abundance (genus Parasutterella id.2892)' on 'Malignant T−cell−amplified sequence 1'

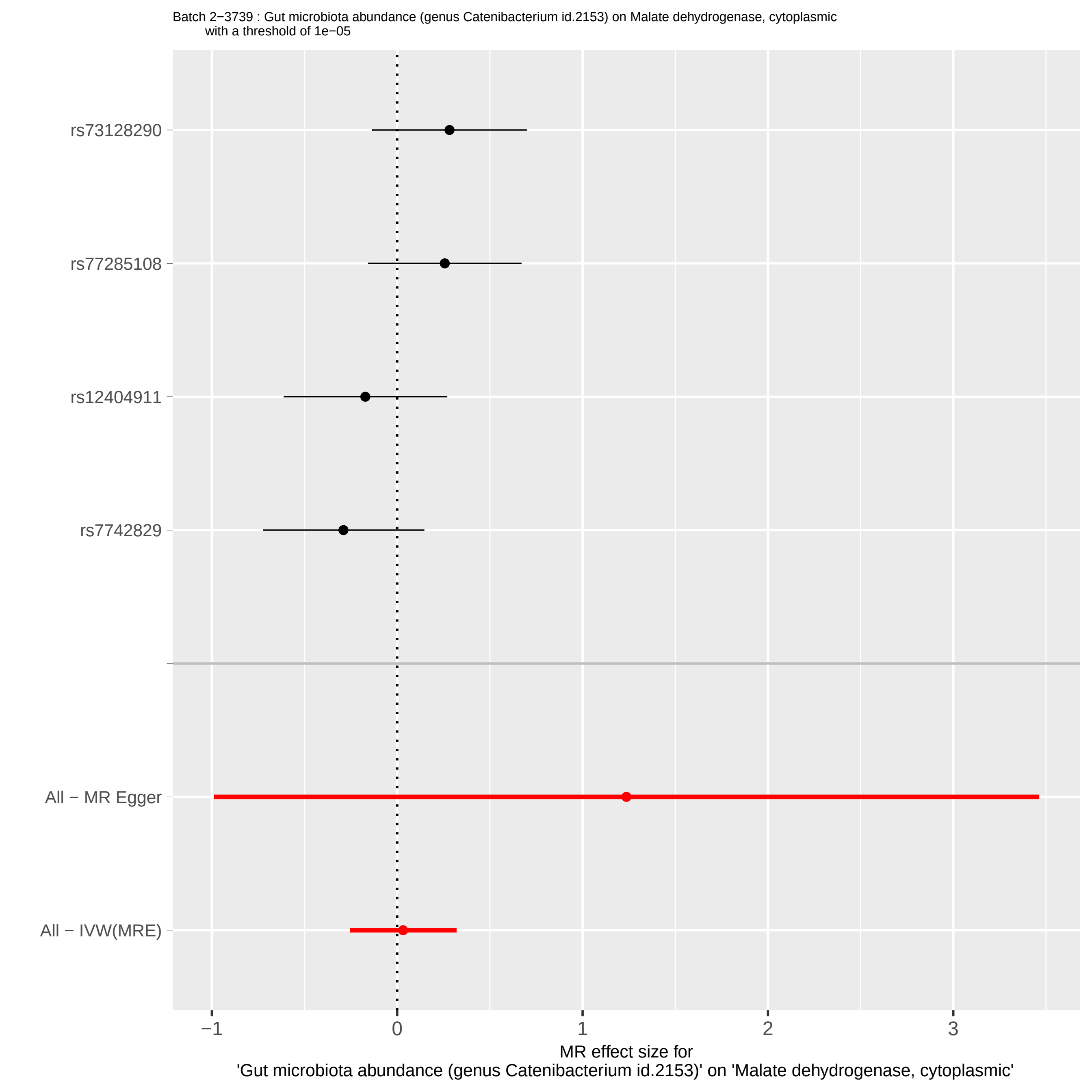
Batch 2−3739 : Gut microbiota abundance (genus Catenibacterium id.2153) on Malate dehydrogenase, cytoplasmic
with a threshold of 1e−05
rs73128290
rs77285108
rs12404911
rs7742829
All − MR Egger
All − IVW(MRE)
−1
0
1
2
3
MR effect size for
'Gut microbiota abundance (genus Catenibacterium id.2153)' on 'Malate dehydrogenase, cytoplasmic'

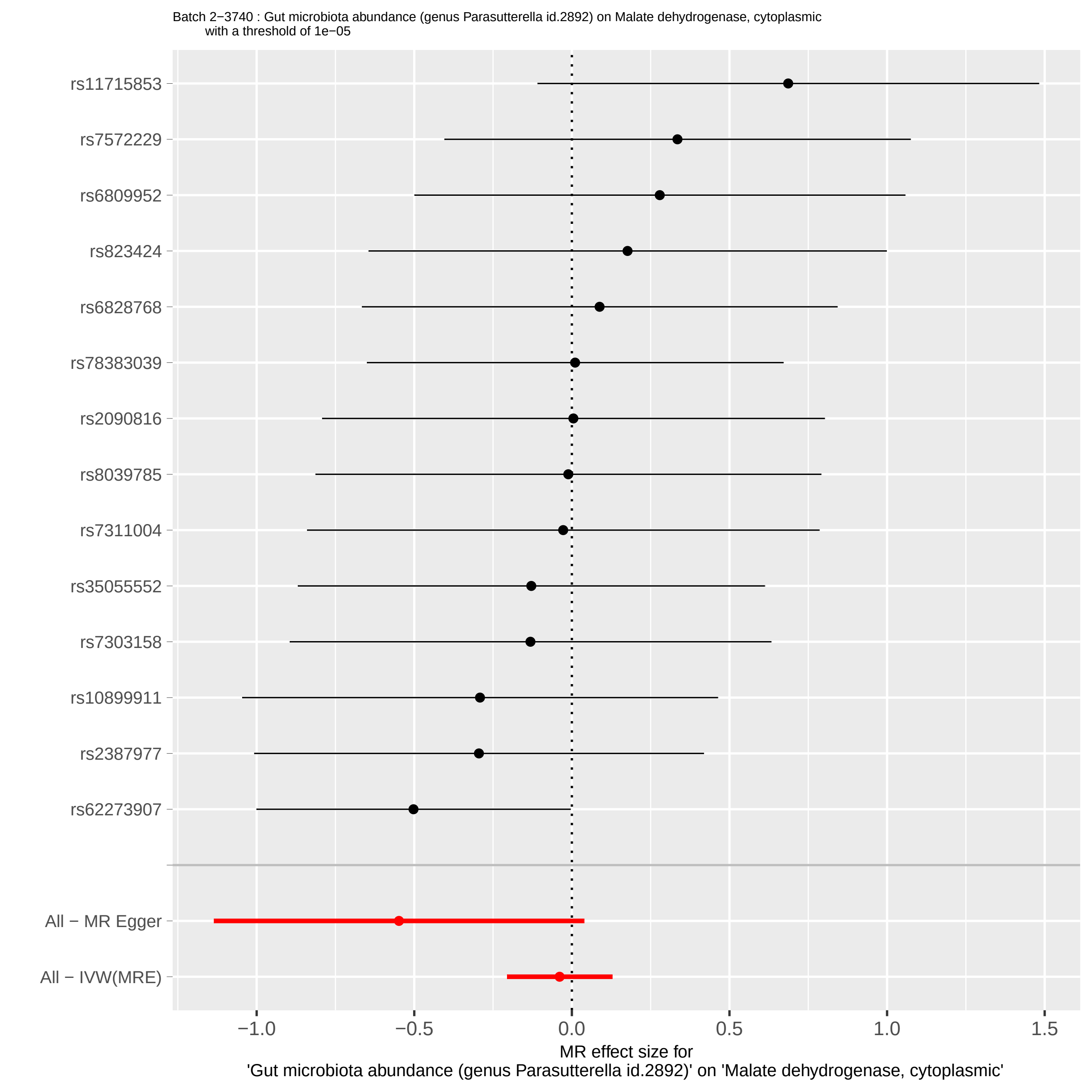

Batch 2−3740 : Gut microbiota abundance (genus Parasutterella id.2892) on Malate dehydrogenase, cytoplasmic
with a threshold of 1e−05
rs11715853
rs7572229
rs6809952
rs823424
rs6828768
rs78383039
rs2090816
rs8039785
rs7311004
rs35055552
rs7303158
rs10899911
rs2387977
rs62273907
All − MR Egger
All − IVW(MRE)
−1.0
−0.5
0.0
0.5
1.0
1.5
MR effect size for
'Gut microbiota abundance (genus Parasutterella id.2892)' on 'Malate dehydrogenase, cytoplasmic'

Batch 2−3741 : Gut microbiota abundance (genus Catenibacterium id.2153) on Midkine
with a threshold of 1e−05

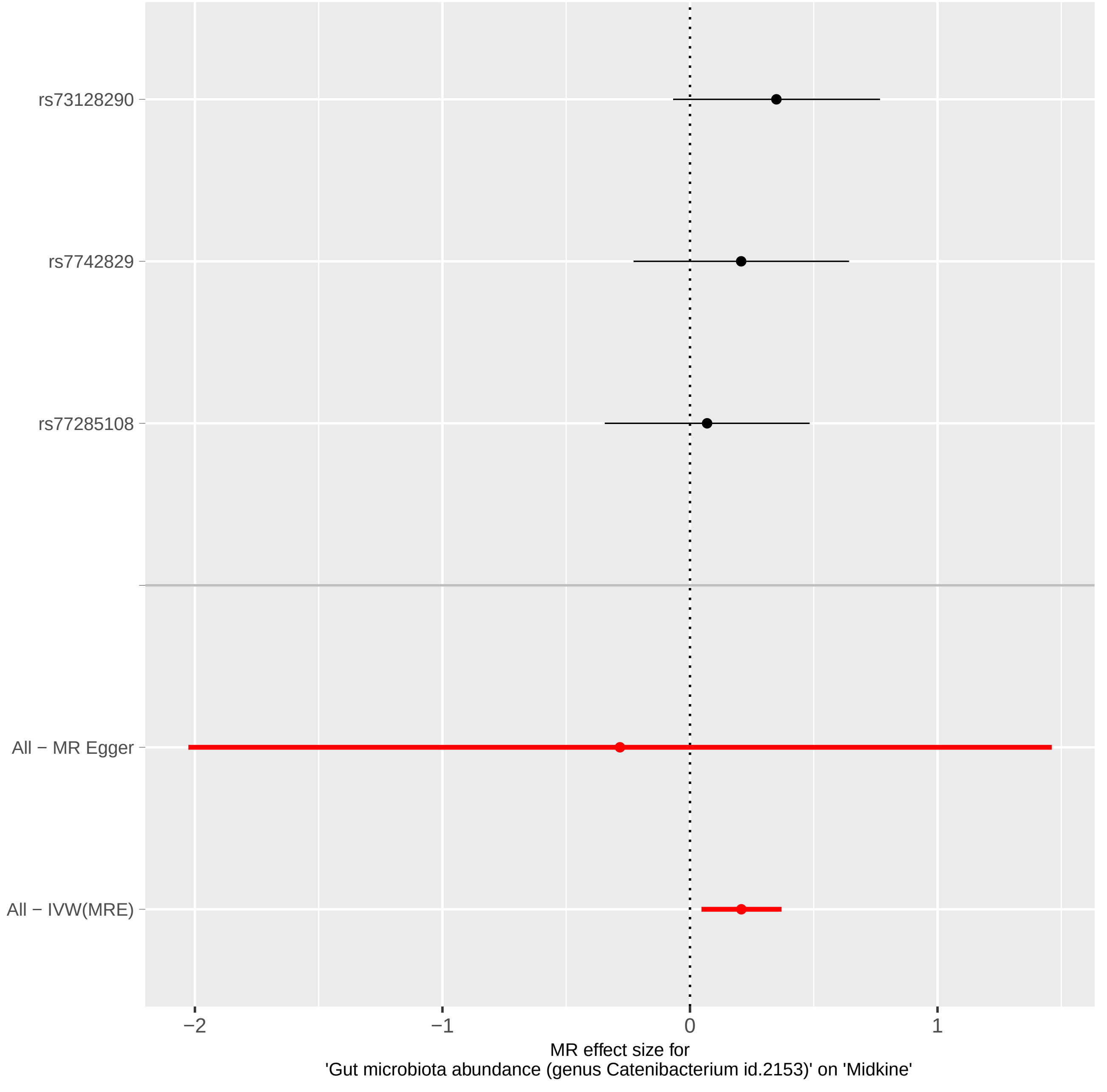

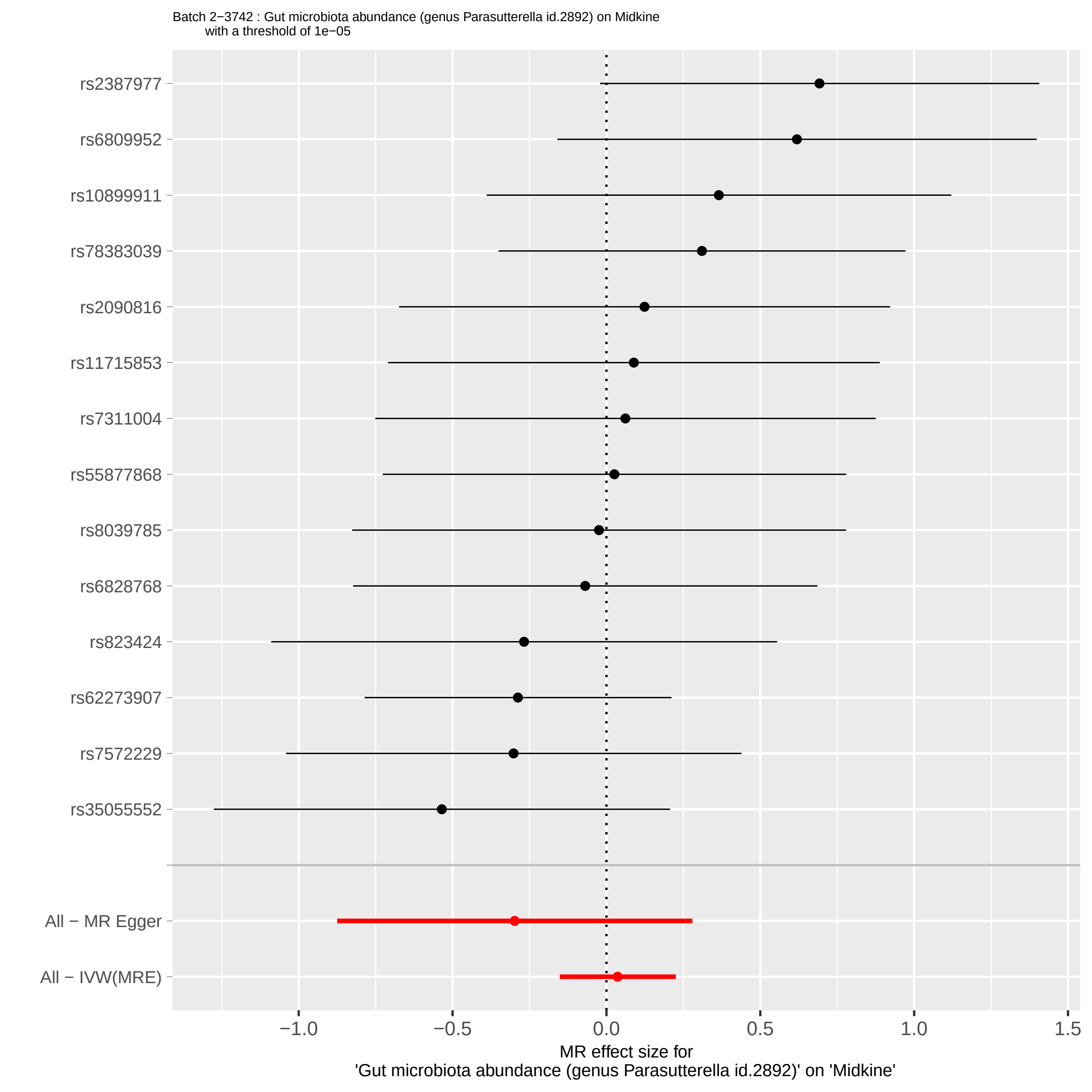
Batch 2−3742 : Gut microbiota abundance (genus Parasutterella id.2892) on Midkine
with a threshold of 1e−05
rs2387977
rs6809952
rs10899911
rs78383039
rs2090816
rs11715853
rs7311004
rs55877868
rs8039785
rs6828768
rs823424
rs62273907
rs7572229
rs35055552
All − MR Egger
All − IVW(MRE)
−1.0
−0.5
0.0
0.5
1.0
1.5
MR effect size for
'Gut microbiota abundance (genus Parasutterella id.2892)' on 'Midkine'

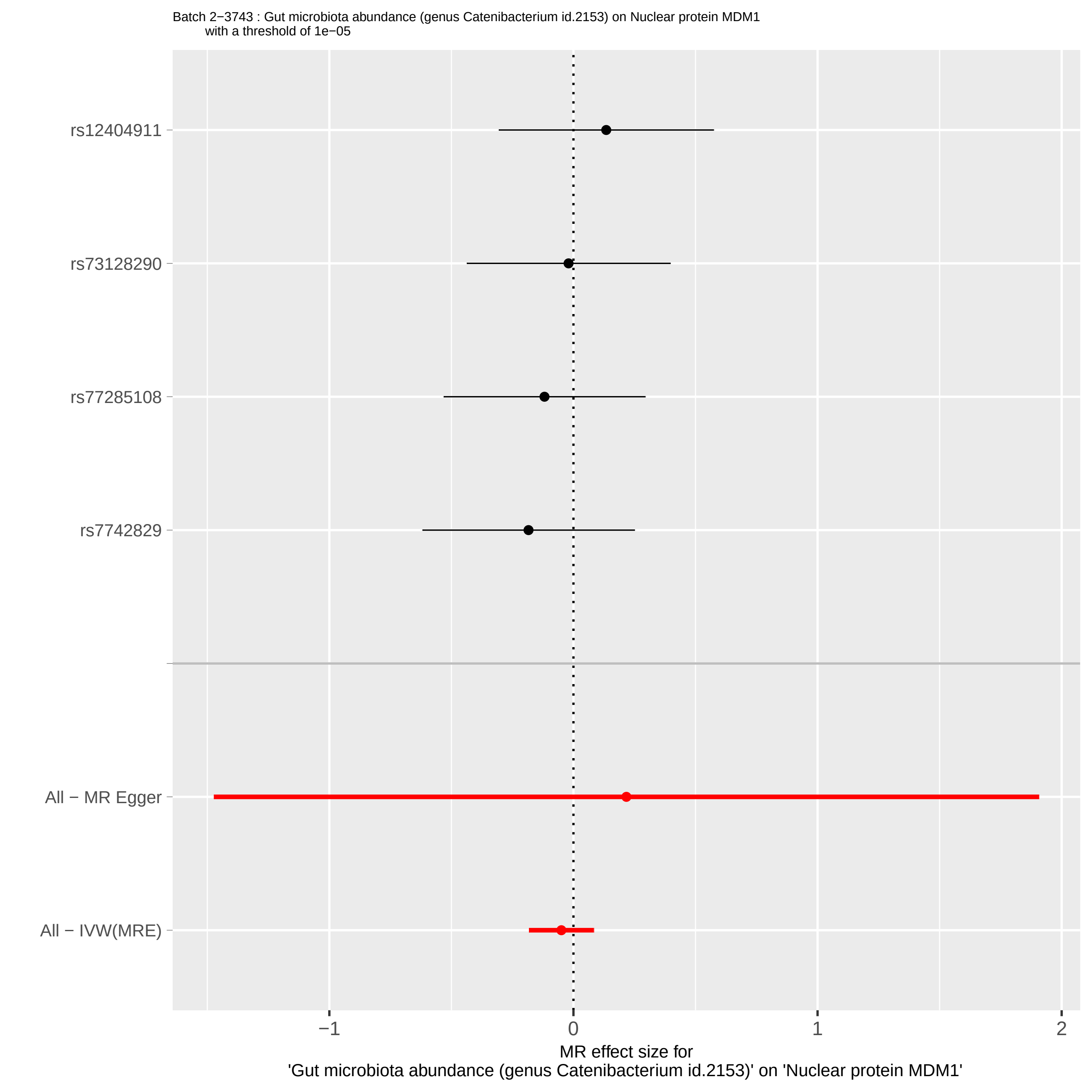
Batch 2−3743 : Gut microbiota abundance (genus Catenibacterium id.2153) on Nuclear protein MDM1
with a threshold of 1e−05
rs12404911
rs73128290
rs77285108
rs7742829
All − MR Egger
All − IVW(MRE)
−1
0
1
2
MR effect size for
'Gut microbiota abundance (genus Catenibacterium id.2153)' on 'Nuclear protein MDM1'

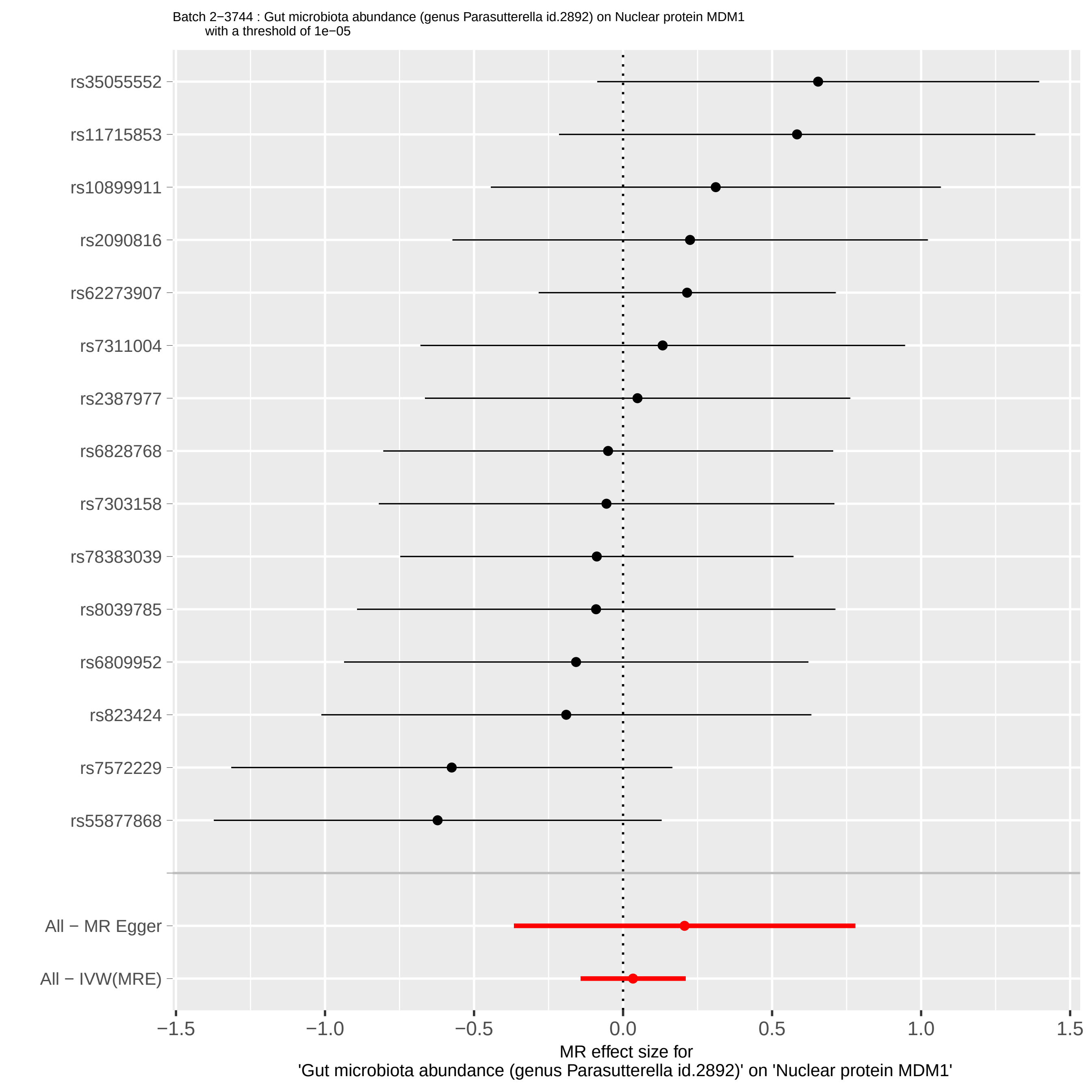

Batch 2−3744 : Gut microbiota abundance (genus Parasutterella id.2892) on Nuclear protein MDM1
with a threshold of 1e−05
rs35055552
rs11715853
rs10899911
rs2090816
rs62273907
rs7311004
rs2387977
rs6828768
rs7303158
rs78383039
rs8039785
rs6809952
rs823424
rs7572229
rs55877868
All − MR Egger
All − IVW(MRE)
−1.5
−1.0
−0.5
0.0
0.5
1.0
1.5
MR effect size for
'Gut microbiota abundance (genus Parasutterella id.2892)' on 'Nuclear protein MDM1'

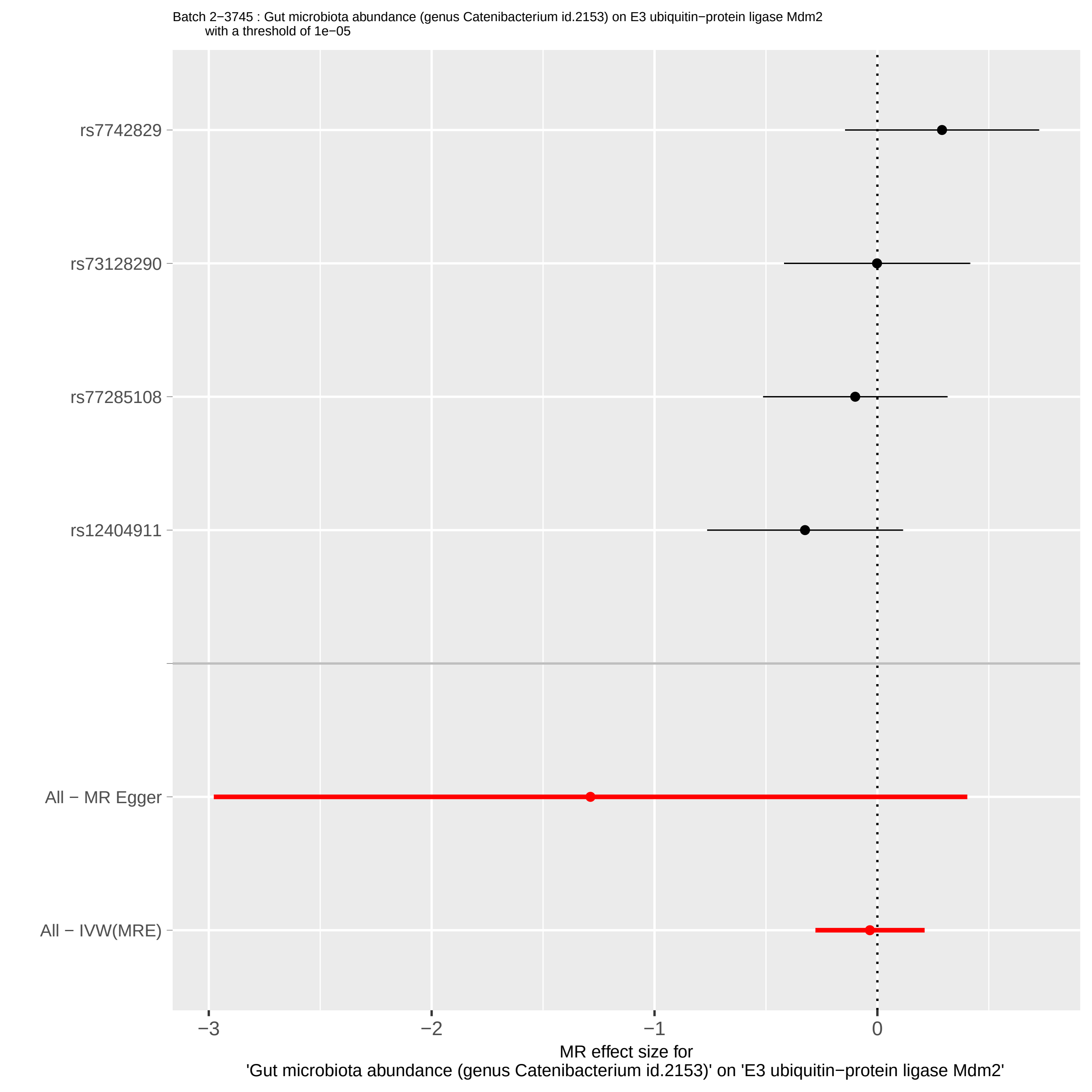
Batch 2−3745 : Gut microbiota abundance (genus Catenibacterium id.2153) on E3 ubiquitin−protein ligase Mdm2
with a threshold of 1e−05
rs7742829
rs73128290
rs77285108
rs12404911
All − MR Egger
All − IVW(MRE)
−3
−2
−1
0
MR effect size for
'Gut microbiota abundance (genus Catenibacterium id.2153)' on 'E3 ubiquitin−protein ligase Mdm2'

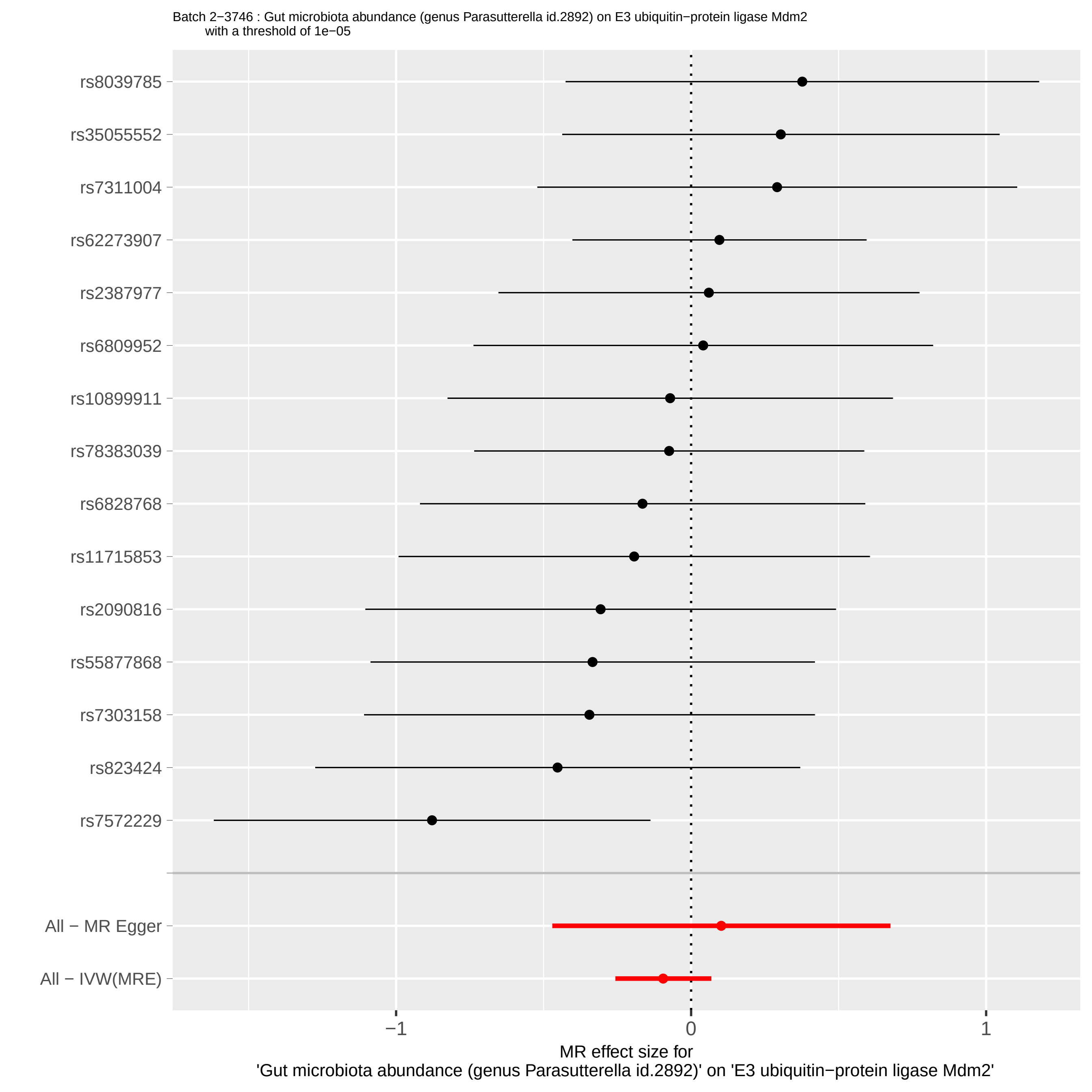
Batch 2−3746 : Gut microbiota abundance (genus Parasutterella id.2892) on E3 ubiquitin−protein ligase Mdm2
with a threshold of 1e−05
rs8039785
rs35055552
rs7311004
rs62273907
rs2387977
rs6809952
rs10899911
rs78383039
rs6828768
rs11715853
rs2090816
rs55877868
rs7303158
rs823424
rs7572229
All − MR Egger
All − IVW(MRE)
−1
0
1
MR effect size for
'Gut microbiota abundance (genus Parasutterella id.2892)' on 'E3 ubiquitin−protein ligase Mdm2'

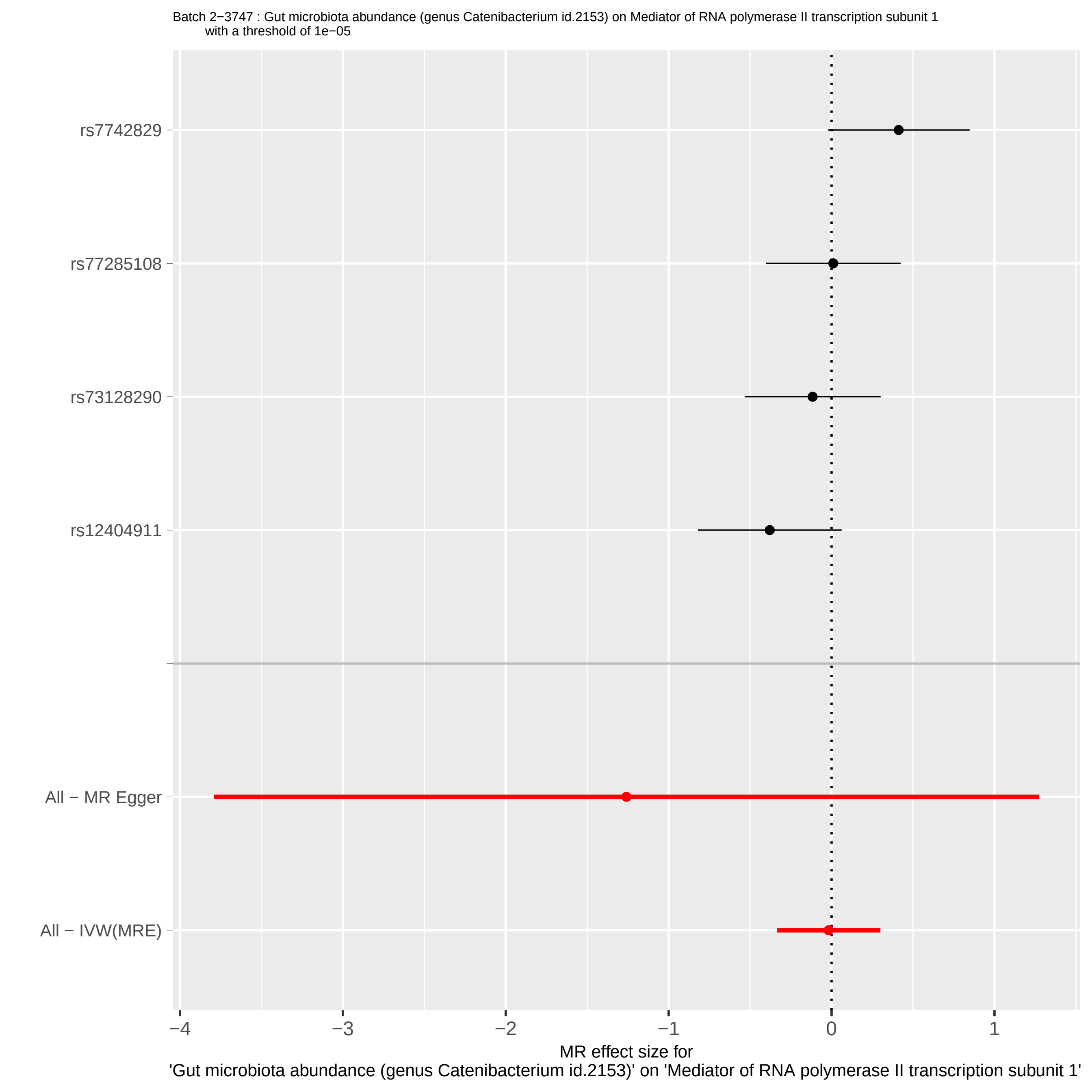

Batch 2−3747 : Gut microbiota abundance (genus Catenibacterium id.2153) on Mediator of RNA polymerase II transcription subunit 1
with a threshold of 1e−05
rs7742829
rs77285108
rs73128290
rs12404911
All − MR Egger
All − IVW(MRE)
−4
−3
−2
−1
0
1
MR effect size for
'Gut microbiota abundance (genus Catenibacterium id.2153)' on 'Mediator of RNA polymerase II transcription subunit 1'

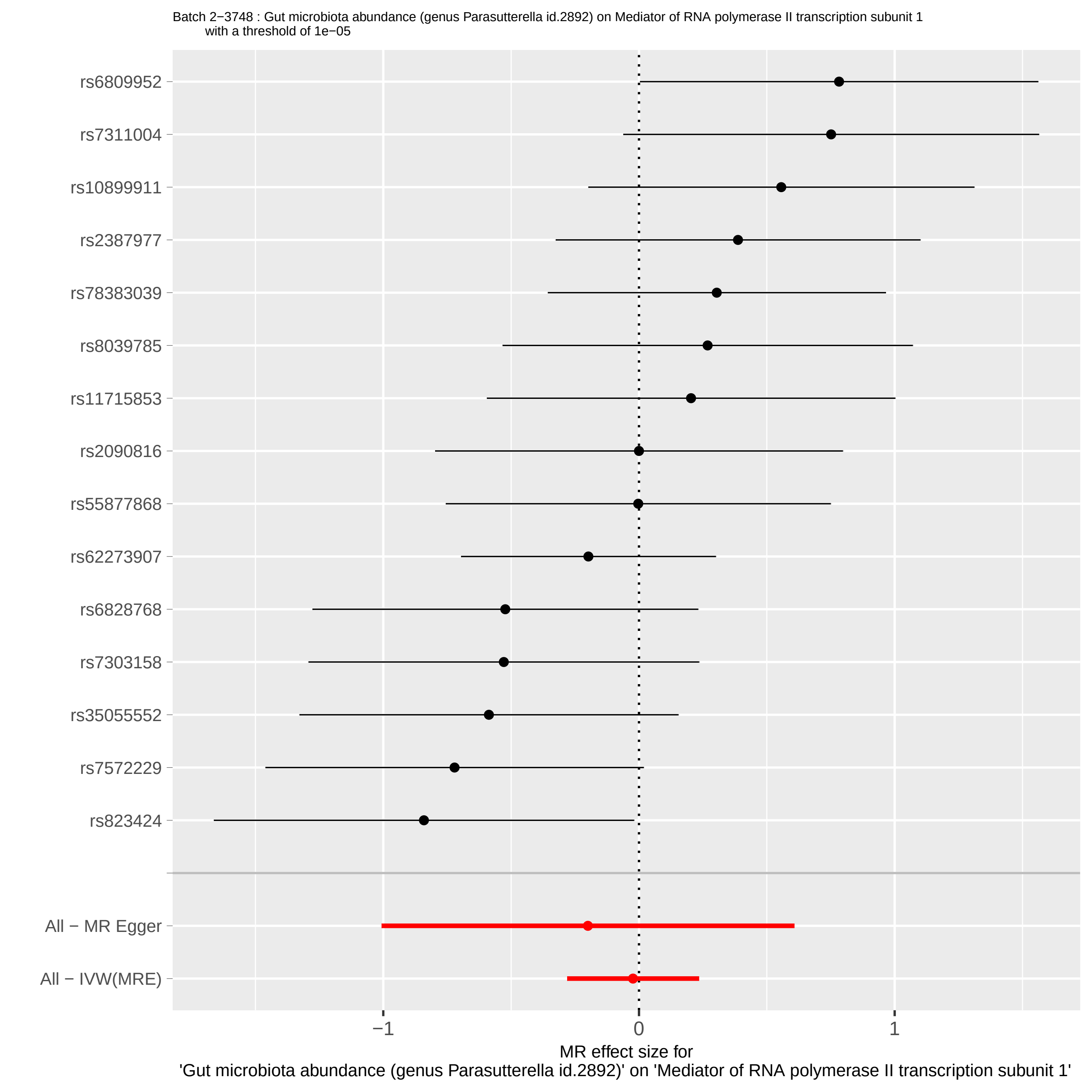

Batch 2−3748 : Gut microbiota abundance (genus Parasutterella id.2892) on Mediator of RNA polymerase II transcription subunit 1
with a threshold of 1e−05
rs6809952
rs7311004
rs10899911
rs2387977
rs78383039
rs8039785
rs11715853
rs2090816
rs55877868
rs62273907
rs6828768
rs7303158
rs35055552
rs7572229
rs823424
All − MR Egger
All − IVW(MRE)
−1
0
1
MR effect size for
'Gut microbiota abundance (genus Parasutterella id.2892)' on 'Mediator of RNA polymerase II transcription subunit 1'

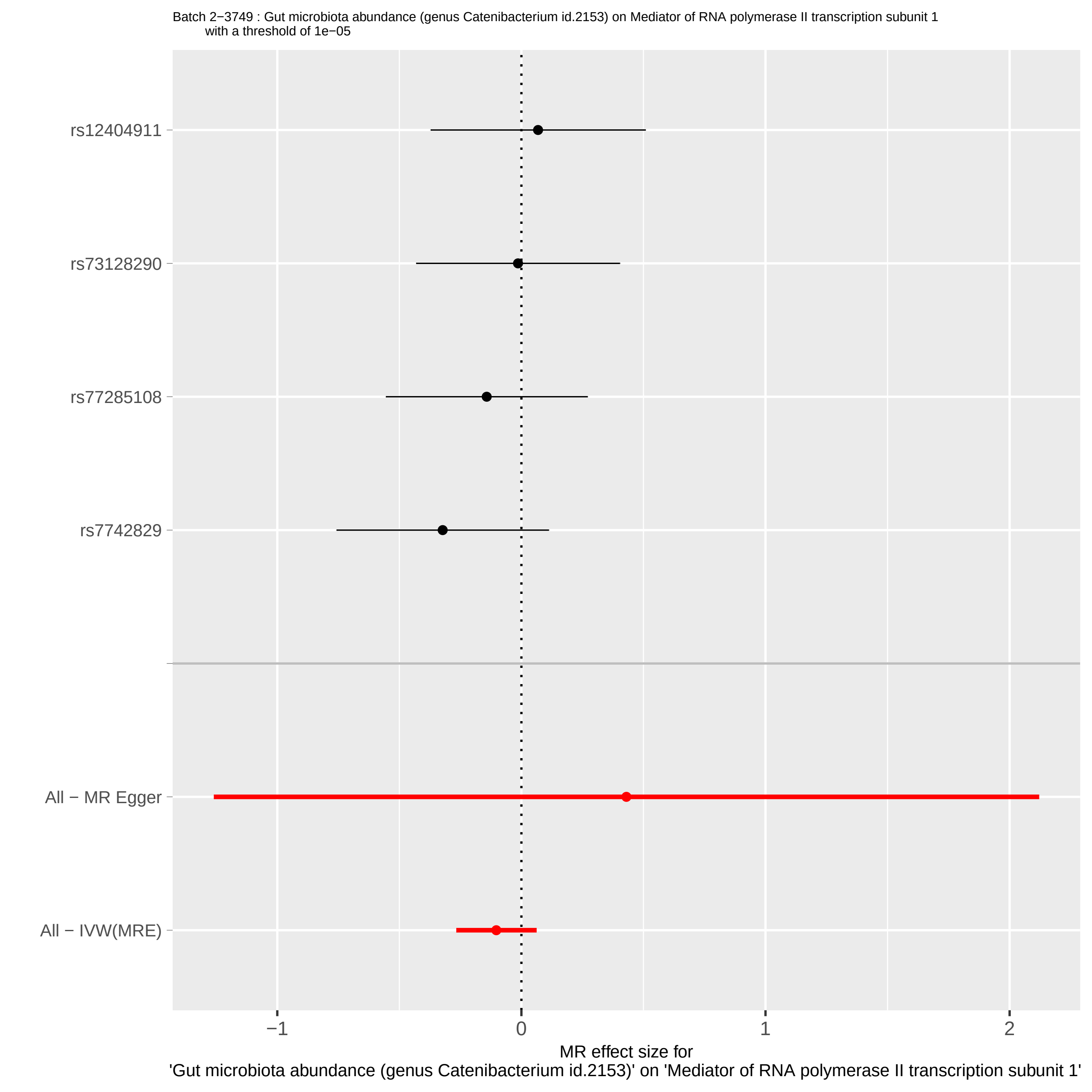

Batch 2−3749 : Gut microbiota abundance (genus Catenibacterium id.2153) on Mediator of RNA polymerase II transcription subunit 1
with a threshold of 1e−05
rs12404911
rs73128290
rs77285108
rs7742829
All − MR Egger
All − IVW(MRE)
−1
0
1
2
MR effect size for
'Gut microbiota abundance (genus Catenibacterium id.2153)' on 'Mediator of RNA polymerase II transcription subunit 1'

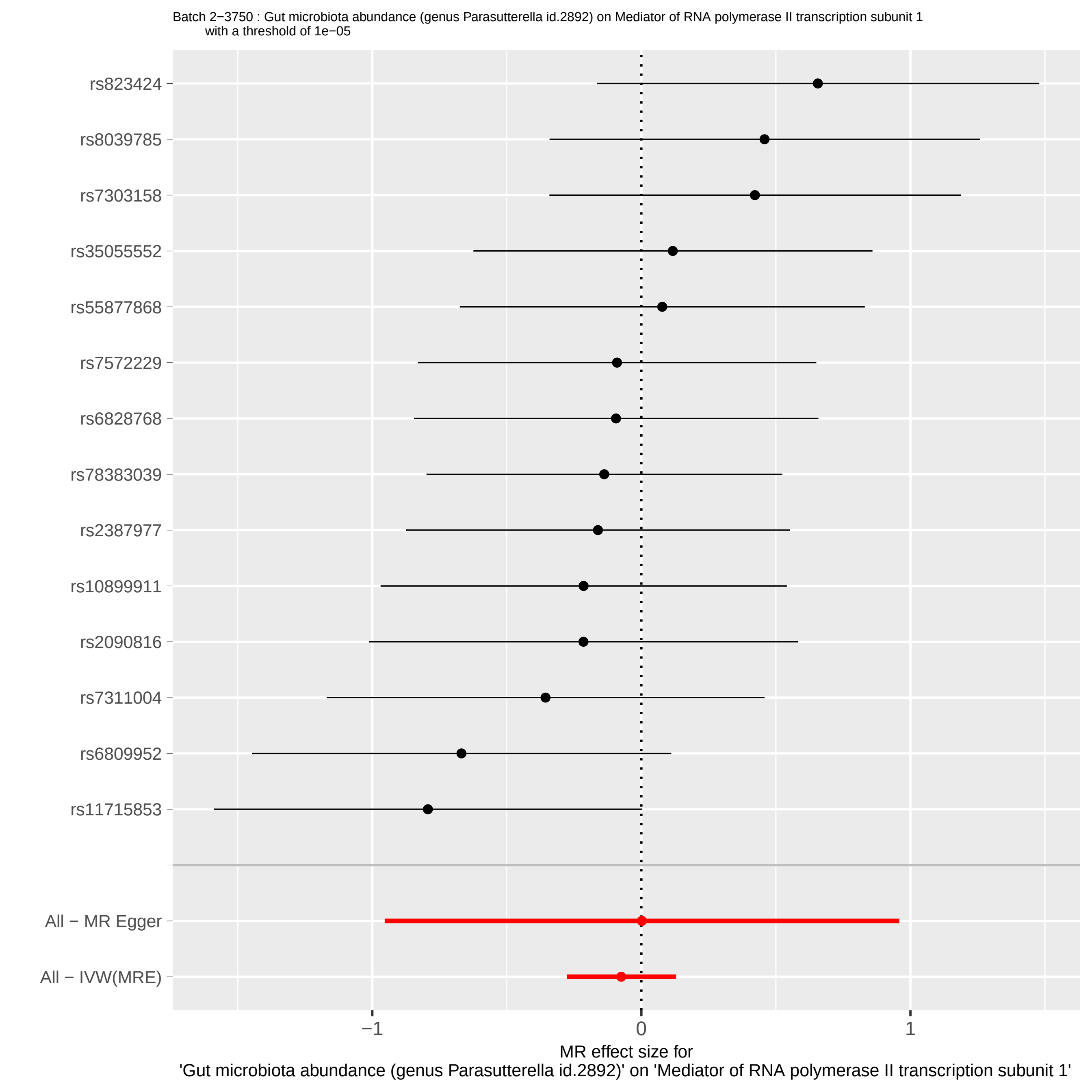

Batch 2−3750 : Gut microbiota abundance (genus Parasutterella id.2892) on Mediator of RNA polymerase II transcription subunit 1
with a threshold of 1e−05
rs823424
rs8039785
rs7303158
rs35055552
rs55877868
rs7572229
rs6828768
rs78383039
rs2387977
rs10899911
rs2090816
rs7311004
rs6809952
rs11715853
All − MR Egger
All − IVW(MRE)
−1
0
1
MR effect size for
'Gut microbiota abundance (genus Parasutterella id.2892)' on 'Mediator of RNA polymerase II transcription subunit 1'

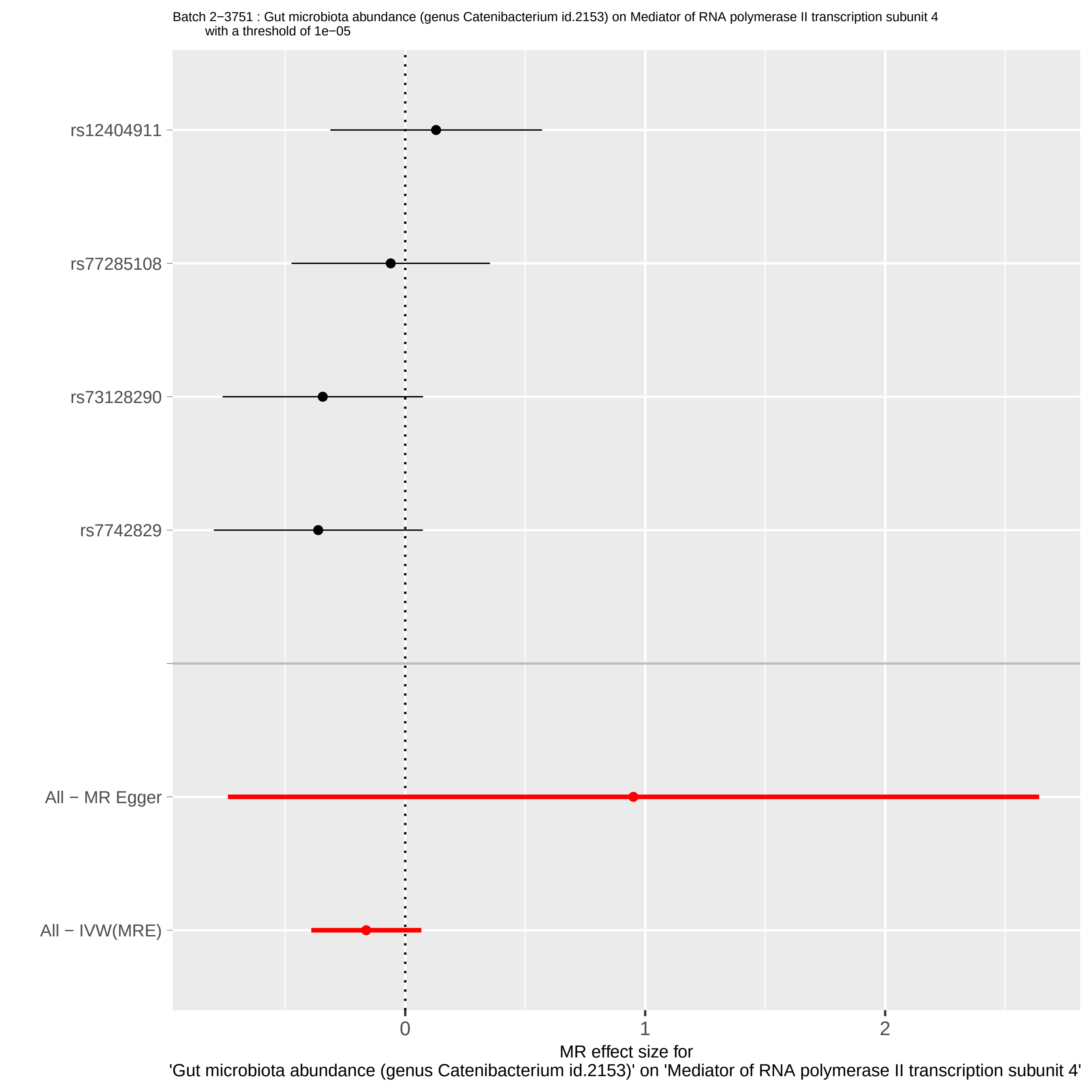

Batch 2−3751 : Gut microbiota abundance (genus Catenibacterium id.2153) on Mediator of RNA polymerase II transcription subunit 4
with a threshold of 1e−05
rs12404911
rs77285108
rs73128290
rs7742829
All − MR Egger
All − IVW(MRE)
0
1
2
MR effect size for
'Gut microbiota abundance (genus Catenibacterium id.2153)' on 'Mediator of RNA polymerase II transcription subunit 4'

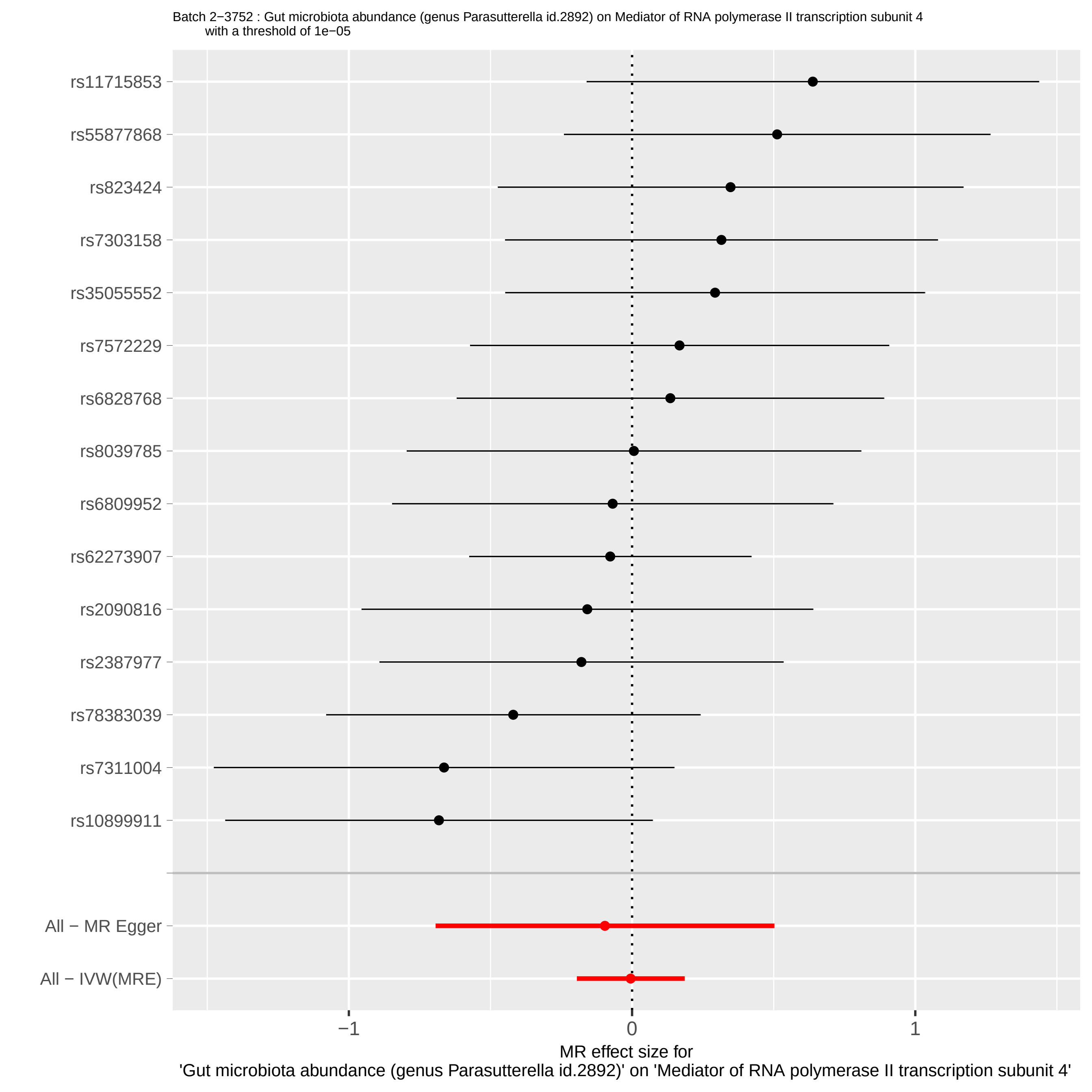

Batch 2−3752 : Gut microbiota abundance (genus Parasutterella id.2892) on Mediator of RNA polymerase II transcription subunit 4
with a threshold of 1e−05
rs11715853
rs55877868
rs823424
rs7303158
rs35055552
rs7572229
rs6828768
rs8039785
rs6809952
rs62273907
rs2090816
rs2387977
rs78383039
rs7311004
rs10899911
All − MR Egger
All − IVW(MRE)
−1
0
1
MR effect size for
'Gut microbiota abundance (genus Parasutterella id.2892)' on 'Mediator of RNA polymerase II transcription subunit 4'

Batch 2−3753 : Gut microbiota abundance (genus Catenibacterium id.2153) on Myocyte−specific enhancer factor 2C
with a threshold of 1e−05

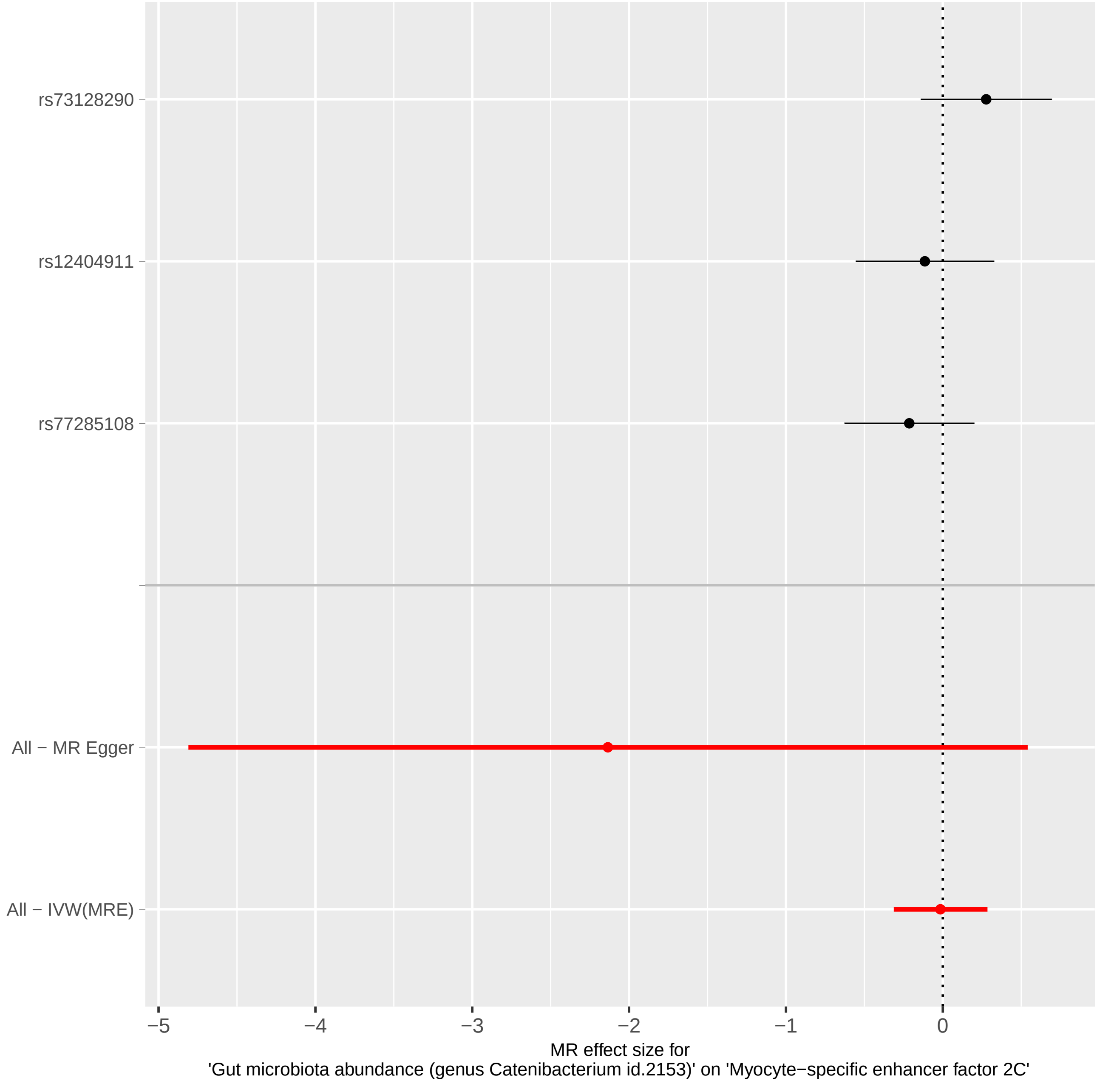

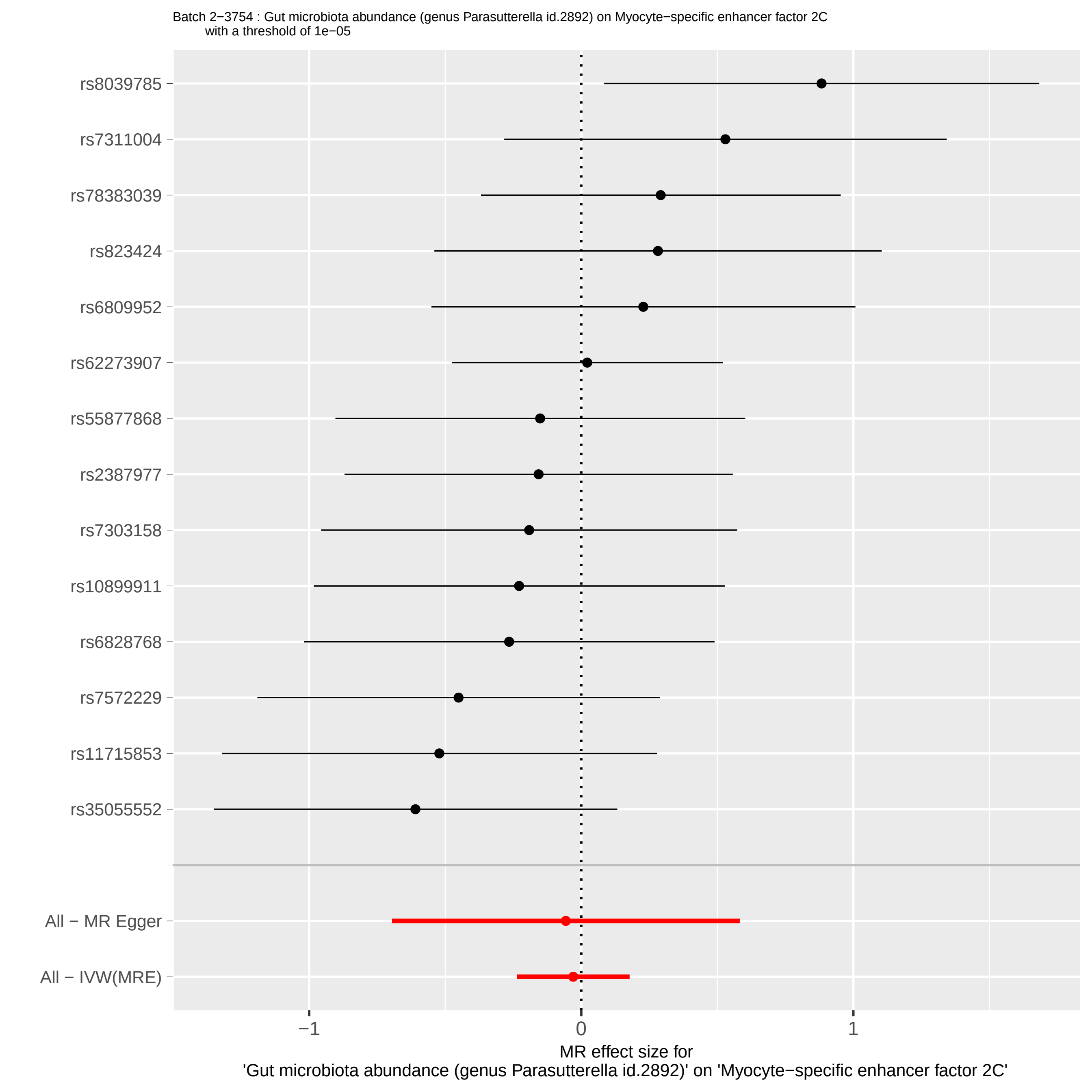

Batch 2−3754 : Gut microbiota abundance (genus Parasutterella id.2892) on Myocyte−specific enhancer factor 2C
with a threshold of 1e−05
rs8039785
rs7311004
rs78383039
rs823424
rs6809952
rs62273907
rs55877868
rs2387977
rs7303158
rs10899911
rs6828768
rs7572229
rs11715853
rs35055552
All − MR Egger
All − IVW(MRE)
−1
0
1
MR effect size for
'Gut microbiota abundance (genus Parasutterella id.2892)' on 'Myocyte−specific enhancer factor 2C'

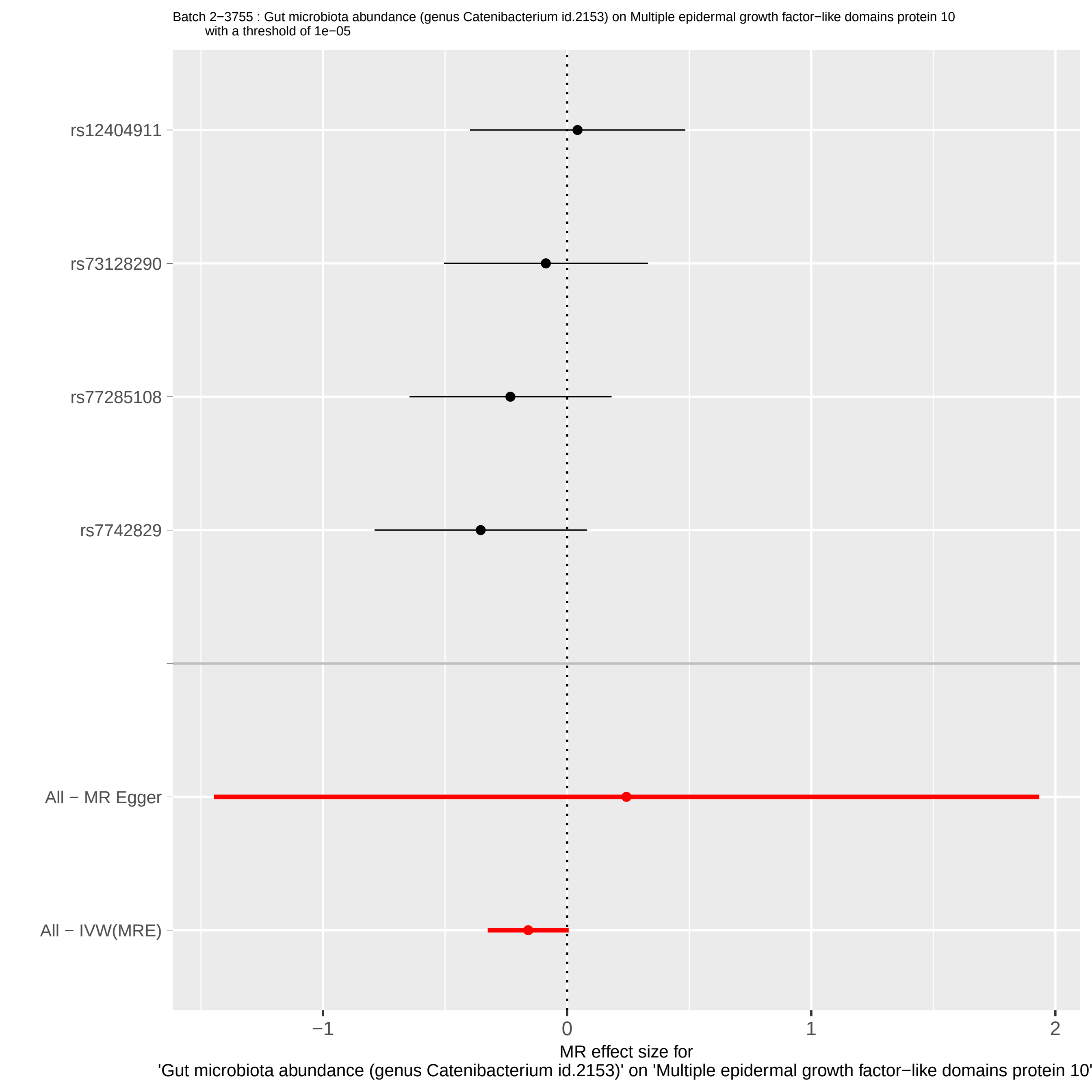
Batch 2−3755 : Gut microbiota abundance (genus Catenibacterium id.2153) on Multiple epidermal growth factor−like domains protein 10
with a threshold of 1e−05
rs12404911
rs73128290
rs77285108
rs7742829
All − MR Egger
All − IVW(MRE)
−1
0
1
2
MR effect size for
'Gut microbiota abundance (genus Catenibacterium id.2153)' on 'Multiple epidermal growth factor−like domains protein 10'

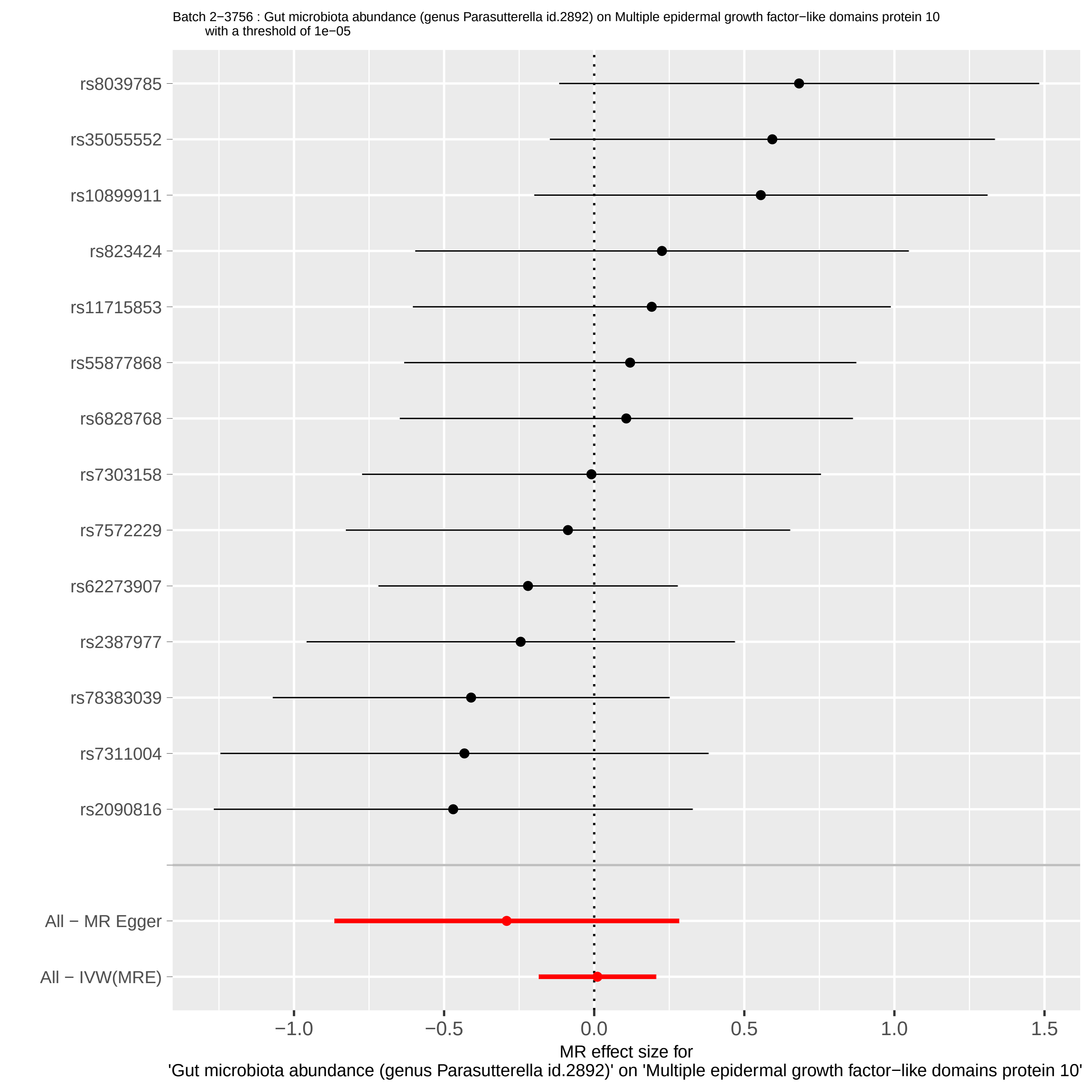

Batch 2−3756 : Gut microbiota abundance (genus Parasutterella id.2892) on Multiple epidermal growth factor−like domains protein 10
with a threshold of 1e−05
rs8039785
rs35055552
rs10899911
rs823424
rs11715853
rs55877868
rs6828768
rs7303158
rs7572229
rs62273907
rs2387977
rs78383039
rs7311004
rs2090816
All − MR Egger
All − IVW(MRE)
−1.0
−0.5
0.0
0.5
1.0
1.5
MR effect size for
'Gut microbiota abundance (genus Parasutterella id.2892)' on 'Multiple epidermal growth factor−like domains protein 10'

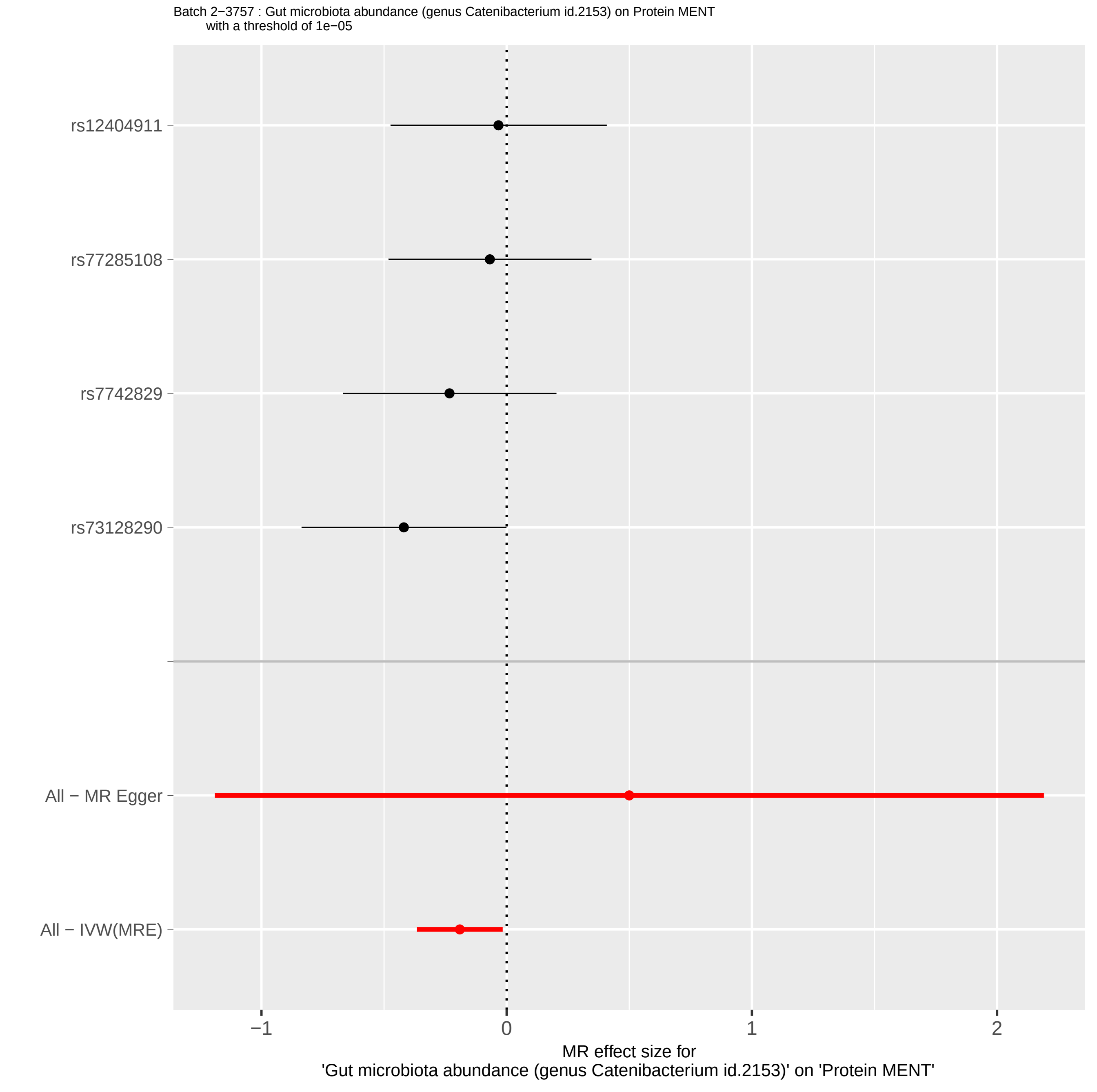
Batch 2−3757 : Gut microbiota abundance (genus Catenibacterium id.2153) on Protein MENT
with a threshold of 1e−05
rs12404911
rs77285108
rs7742829
rs73128290
All − MR Egger
All − IVW(MRE)
−1
0
1
2
MR effect size for
'Gut microbiota abundance (genus Catenibacterium id.2153)' on 'Protein MENT'

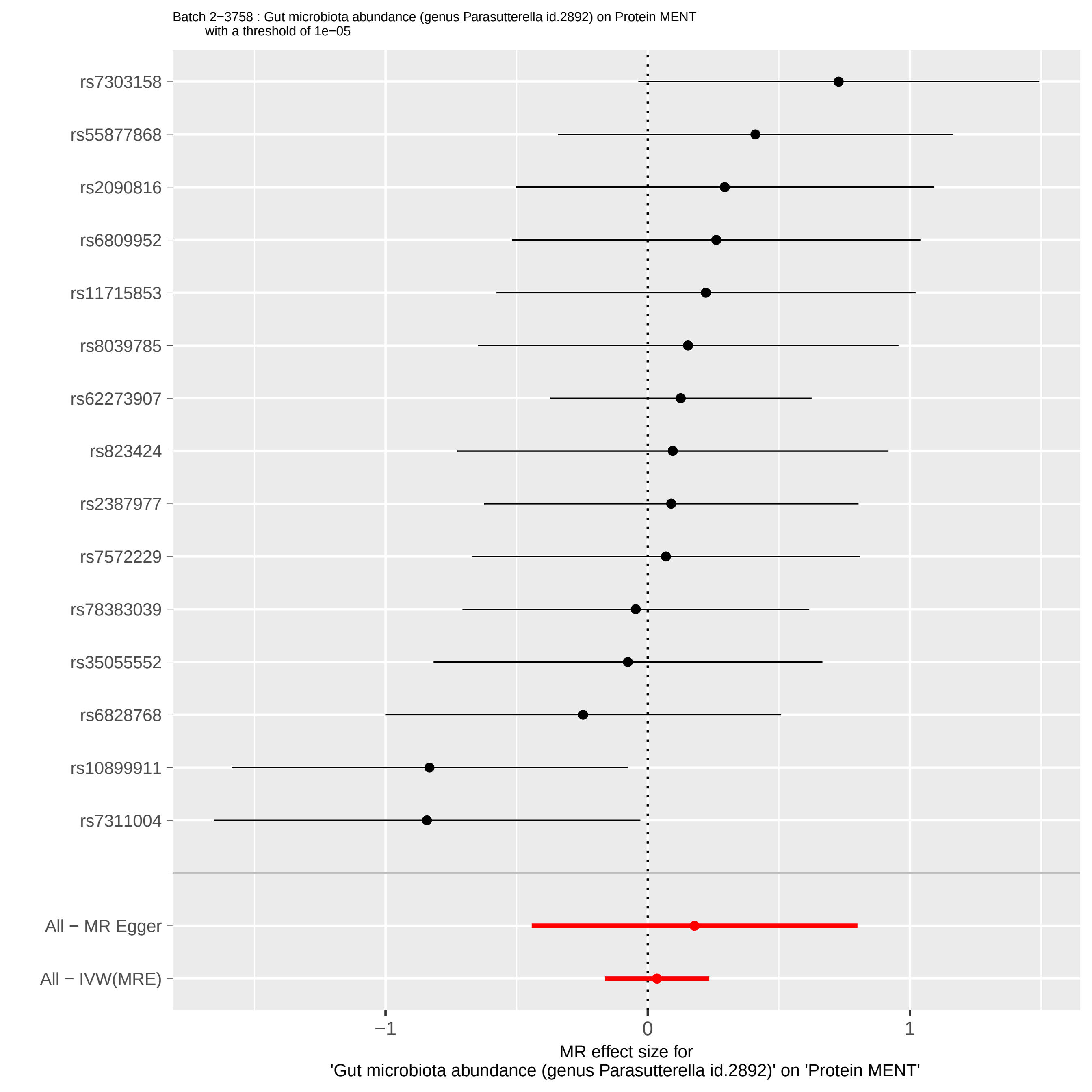

Batch 2−3758 : Gut microbiota abundance (genus Parasutterella id.2892) on Protein MENT
with a threshold of 1e−05
rs7303158
rs55877868
rs2090816
rs6809952
rs11715853
rs8039785
rs62273907
rs823424
rs2387977
rs7572229
rs78383039
rs35055552
rs6828768
rs10899911
rs7311004
All − MR Egger
All − IVW(MRE)
−1
0
1
MR effect size for
'Gut microbiota abundance (genus Parasutterella id.2892)' on 'Protein MENT'

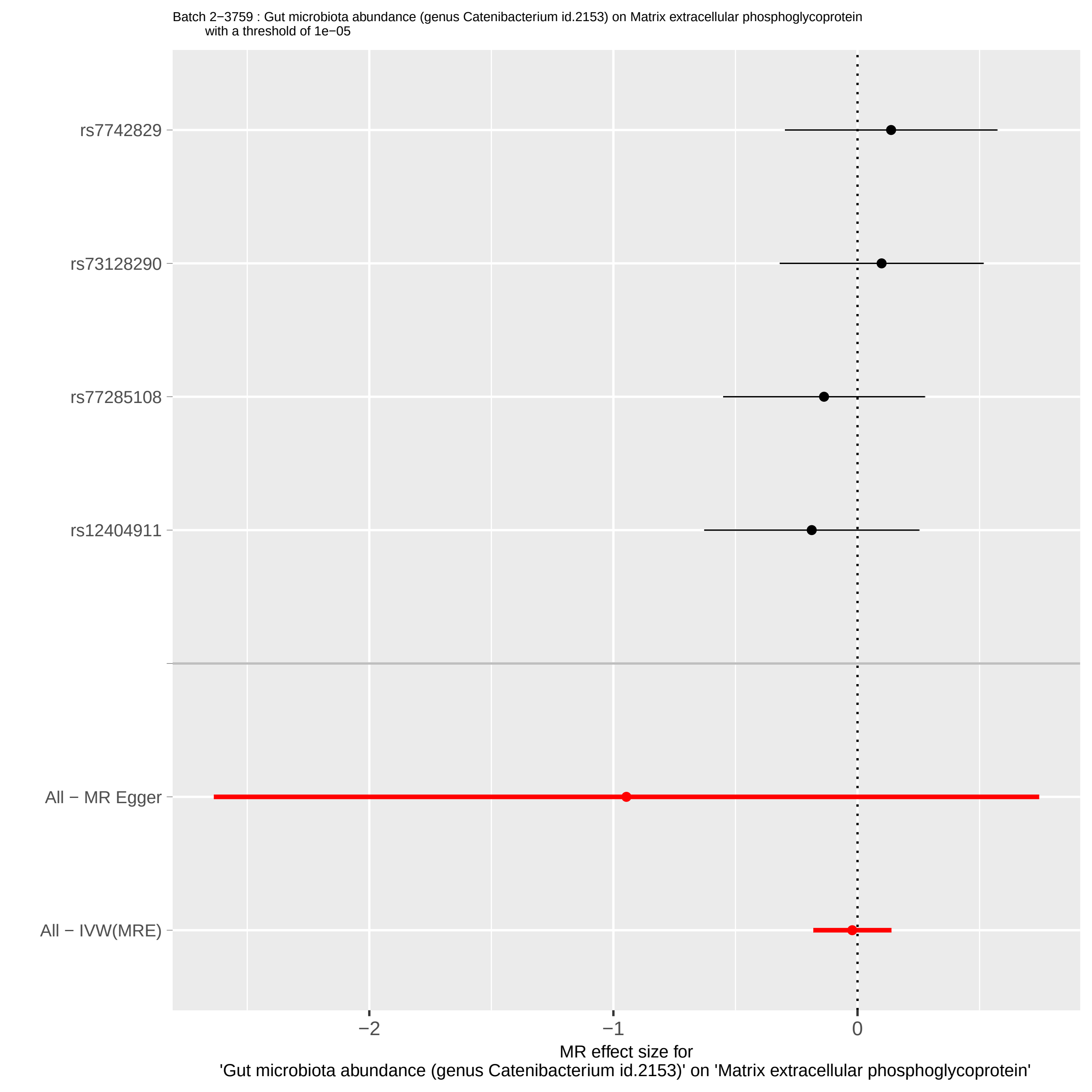

Batch 2−3759 : Gut microbiota abundance (genus Catenibacterium id.2153) on Matrix extracellular phosphoglycoprotein
with a threshold of 1e−05
rs7742829
rs73128290
rs77285108
rs12404911
All − MR Egger
All − IVW(MRE)
−2
−1
0
MR effect size for
'Gut microbiota abundance (genus Catenibacterium id.2153)' on 'Matrix extracellular phosphoglycoprotein'

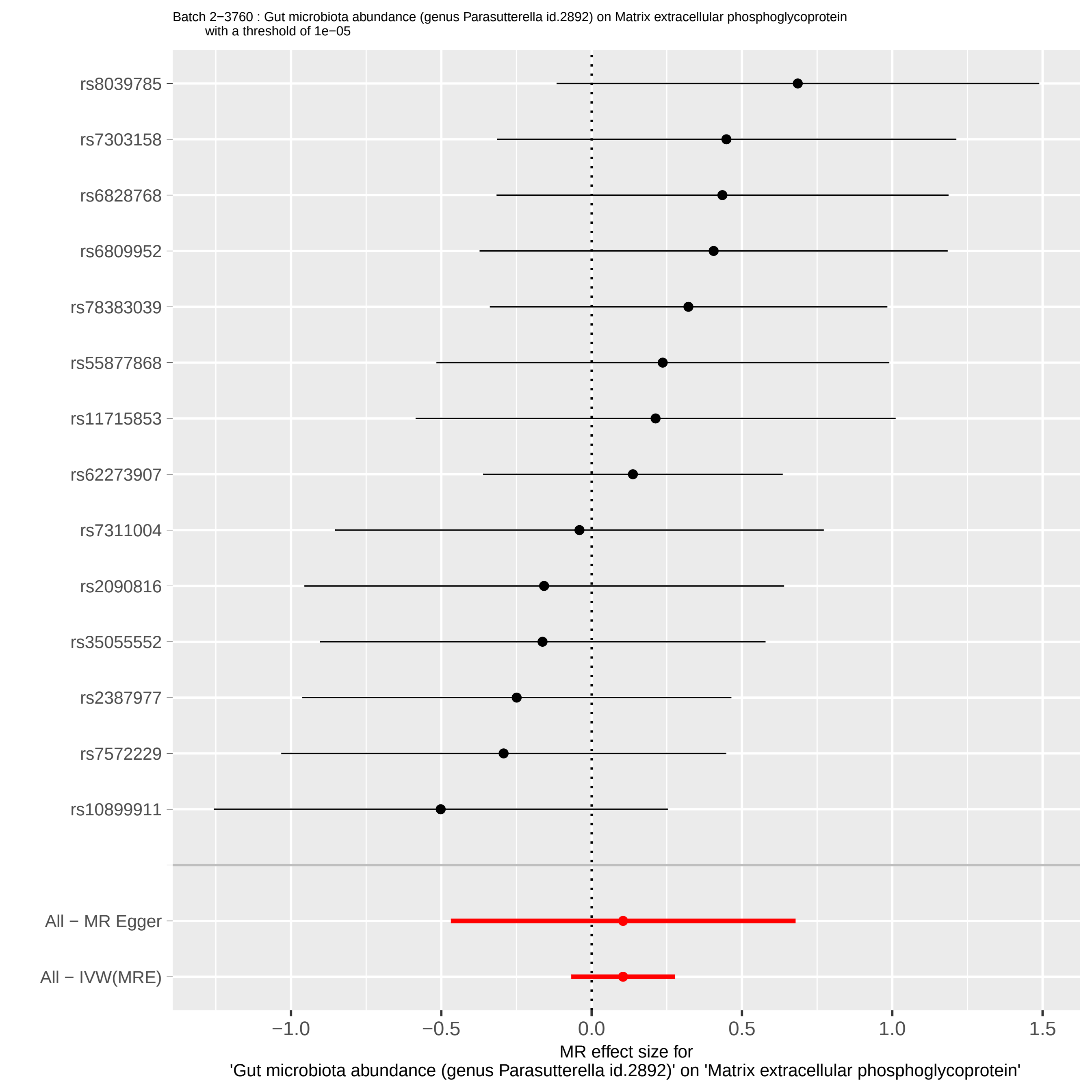
Batch 2−3760 : Gut microbiota abundance (genus Parasutterella id.2892) on Matrix extracellular phosphoglycoprotein
with a threshold of 1e−05
rs8039785
rs7303158
rs6828768
rs6809952
rs78383039
rs55877868
rs11715853
rs62273907
rs7311004
rs2090816
rs35055552
rs2387977
rs7572229
rs10899911
All − MR Egger
All − IVW(MRE)
−1.0
−0.5
0.0
0.5
1.0
1.5
MR effect size for
'Gut microbiota abundance (genus Parasutterella id.2892)' on 'Matrix extracellular phosphoglycoprotein'

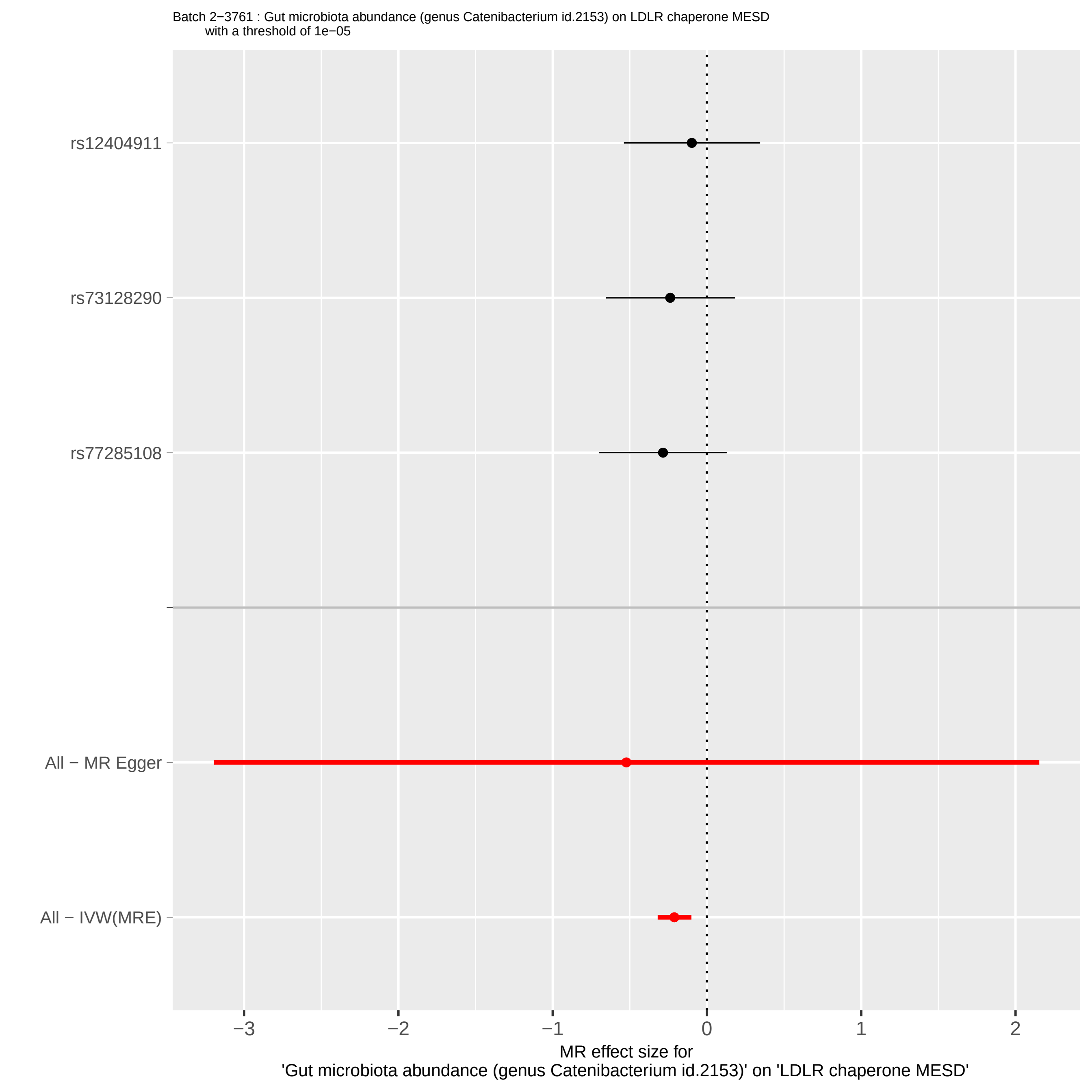

Batch 2−3761 : Gut microbiota abundance (genus Catenibacterium id.2153) on LDLR chaperone MESD
with a threshold of 1e−05
rs12404911
rs73128290
rs77285108
All − MR Egger
All − IVW(MRE)
−3
−2
−1
0
1
2
MR effect size for
'Gut microbiota abundance (genus Catenibacterium id.2153)' on 'LDLR chaperone MESD'

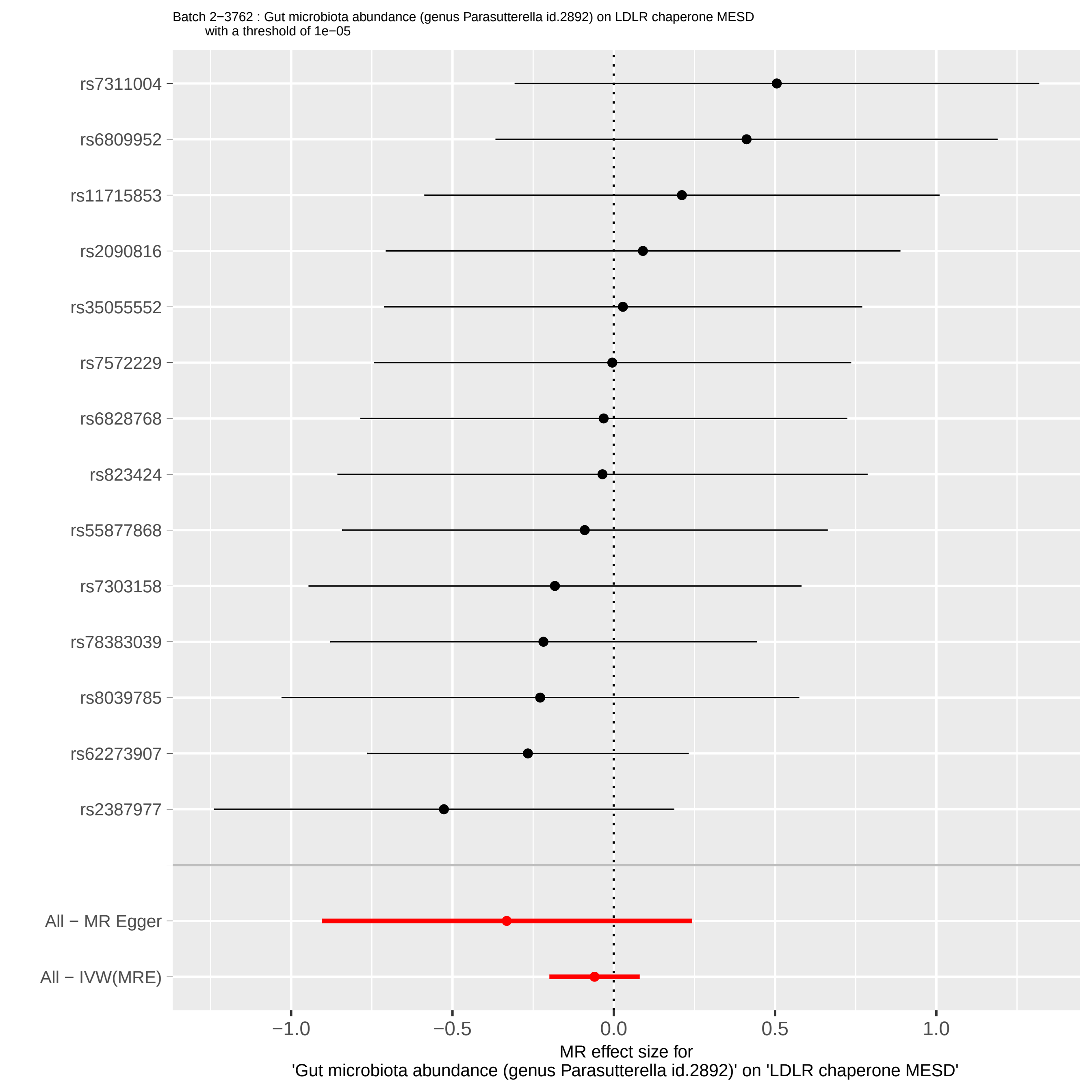
Batch 2−3762 : Gut microbiota abundance (genus Parasutterella id.2892) on LDLR chaperone MESD
with a threshold of 1e−05
rs7311004
rs6809952
rs11715853
rs2090816
rs35055552
rs7572229
rs6828768
rs823424
rs55877868
rs7303158
rs78383039
rs8039785
rs62273907
rs2387977
All − MR Egger
All − IVW(MRE)
−1.0
−0.5
0.0
0.5
1.0
MR effect size for
'Gut microbiota abundance (genus Parasutterella id.2892)' on 'LDLR chaperone MESD'

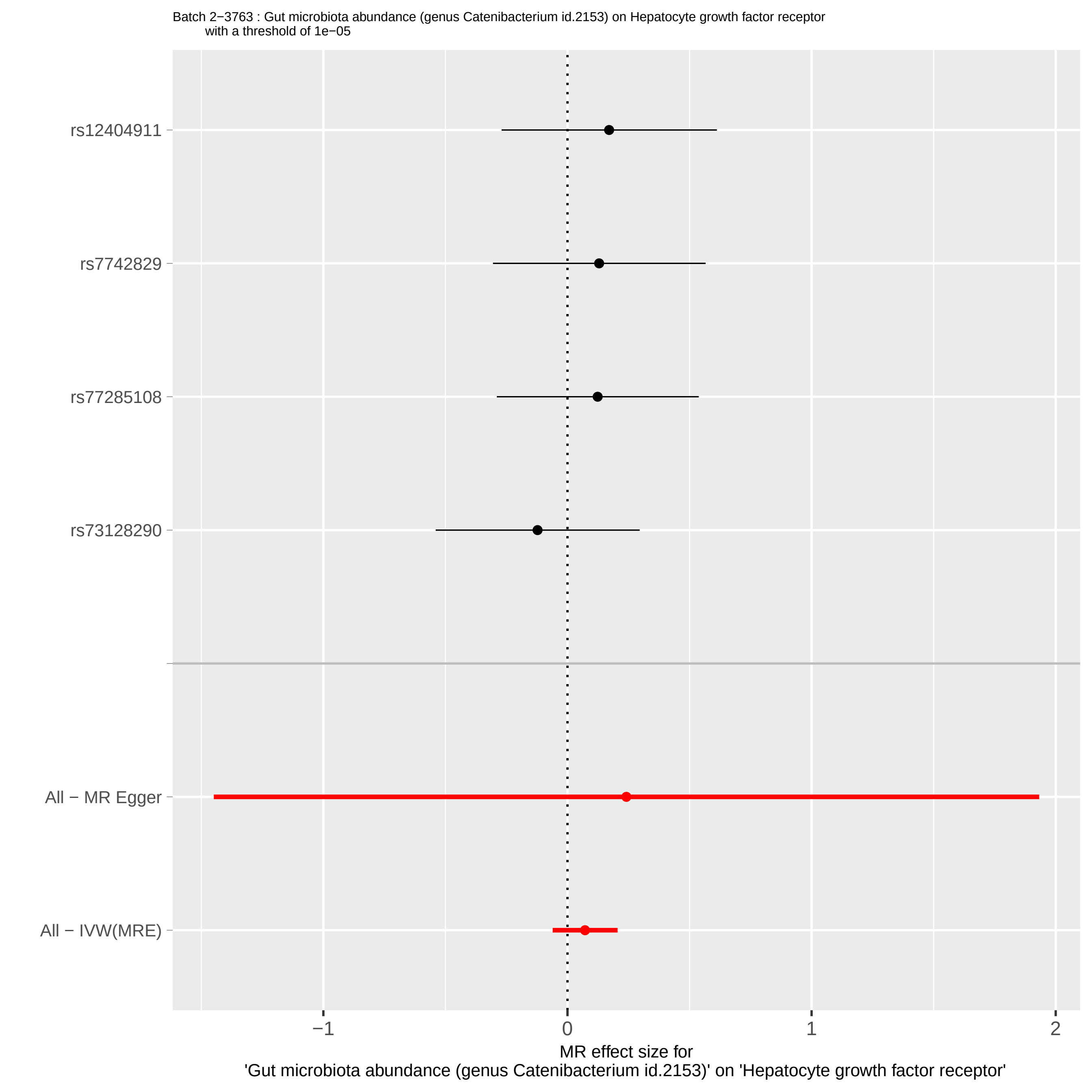

Batch 2−3763 : Gut microbiota abundance (genus Catenibacterium id.2153) on Hepatocyte growth factor receptor
with a threshold of 1e−05
rs12404911
rs7742829
rs77285108
rs73128290
All − MR Egger
All − IVW(MRE)
−1
0
1
2
MR effect size for
'Gut microbiota abundance (genus Catenibacterium id.2153)' on 'Hepatocyte growth factor receptor'

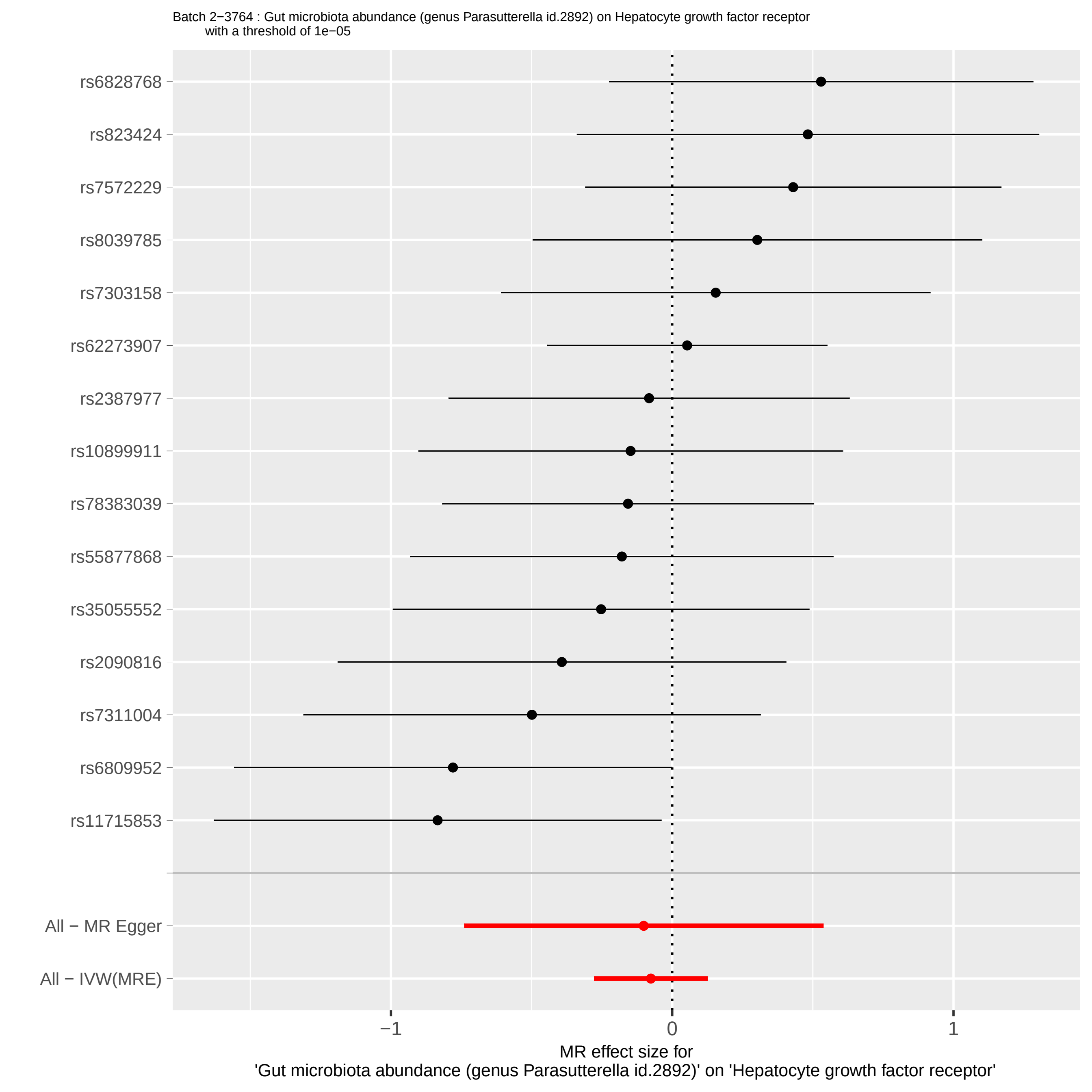
Batch 2−3764 : Gut microbiota abundance (genus Parasutterella id.2892) on Hepatocyte growth factor receptor
with a threshold of 1e−05
rs6828768
rs823424
rs7572229
rs8039785
rs7303158
rs62273907
rs2387977
rs10899911
rs78383039
rs55877868
rs35055552
rs2090816
rs7311004
rs6809952
rs11715853
All − MR Egger
All − IVW(MRE)
−1
0
1
MR effect size for
'Gut microbiota abundance (genus Parasutterella id.2892)' on 'Hepatocyte growth factor receptor'

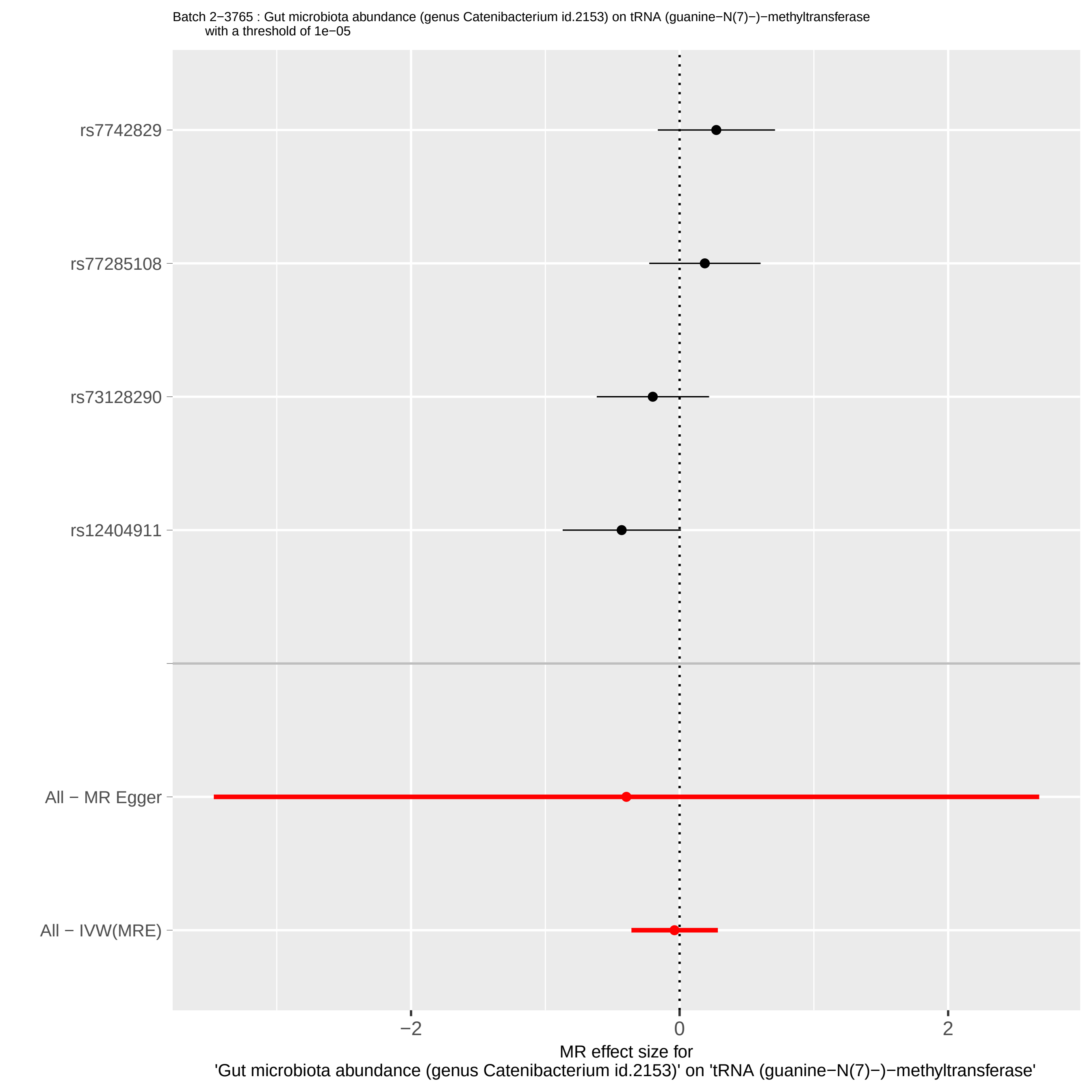
Batch 2−3765 : Gut microbiota abundance (genus Catenibacterium id.2153) on tRNA (guanine−N(7)−)−methyltransferase
with a threshold of 1e−05
rs7742829
rs77285108
rs73128290
rs12404911
All − MR Egger
All − IVW(MRE)
−2
0
2
MR effect size for
'Gut microbiota abundance (genus Catenibacterium id.2153)' on 'tRNA (guanine−N(7)−)−methyltransferase'

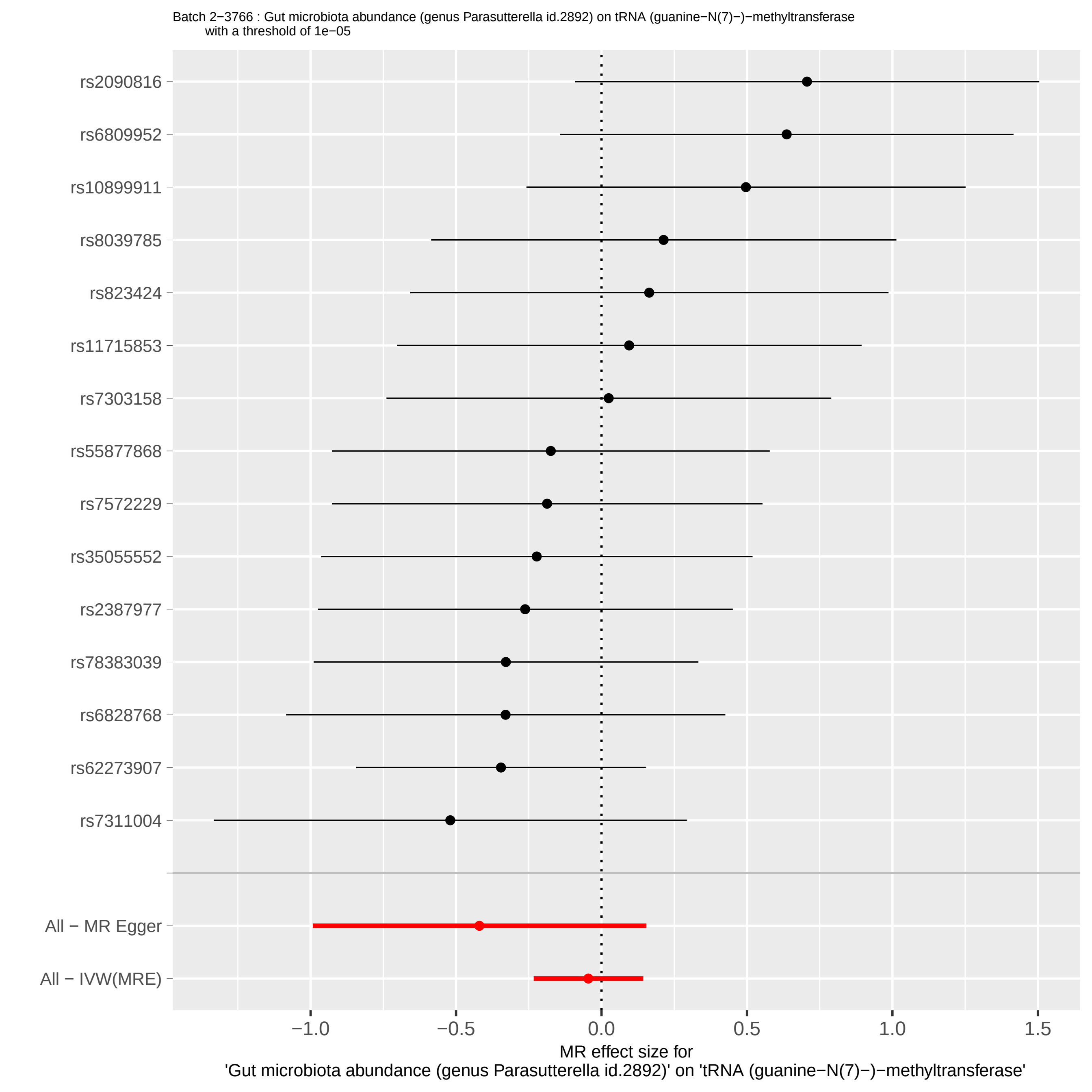

Batch 2−3766 : Gut microbiota abundance (genus Parasutterella id.2892) on tRNA (guanine−N(7)−)−methyltransferase
with a threshold of 1e−05
rs2090816
rs6809952
rs10899911
rs8039785
rs823424
rs11715853
rs7303158
rs55877868
rs7572229
rs35055552
rs2387977
rs78383039
rs6828768
rs62273907
rs7311004
All − MR Egger
All − IVW(MRE)
−1.0
−0.5
0.0
0.5
1.0
1.5
MR effect size for
'Gut microbiota abundance (genus Parasutterella id.2892)' on 'tRNA (guanine−N(7)−)−methyltransferase'

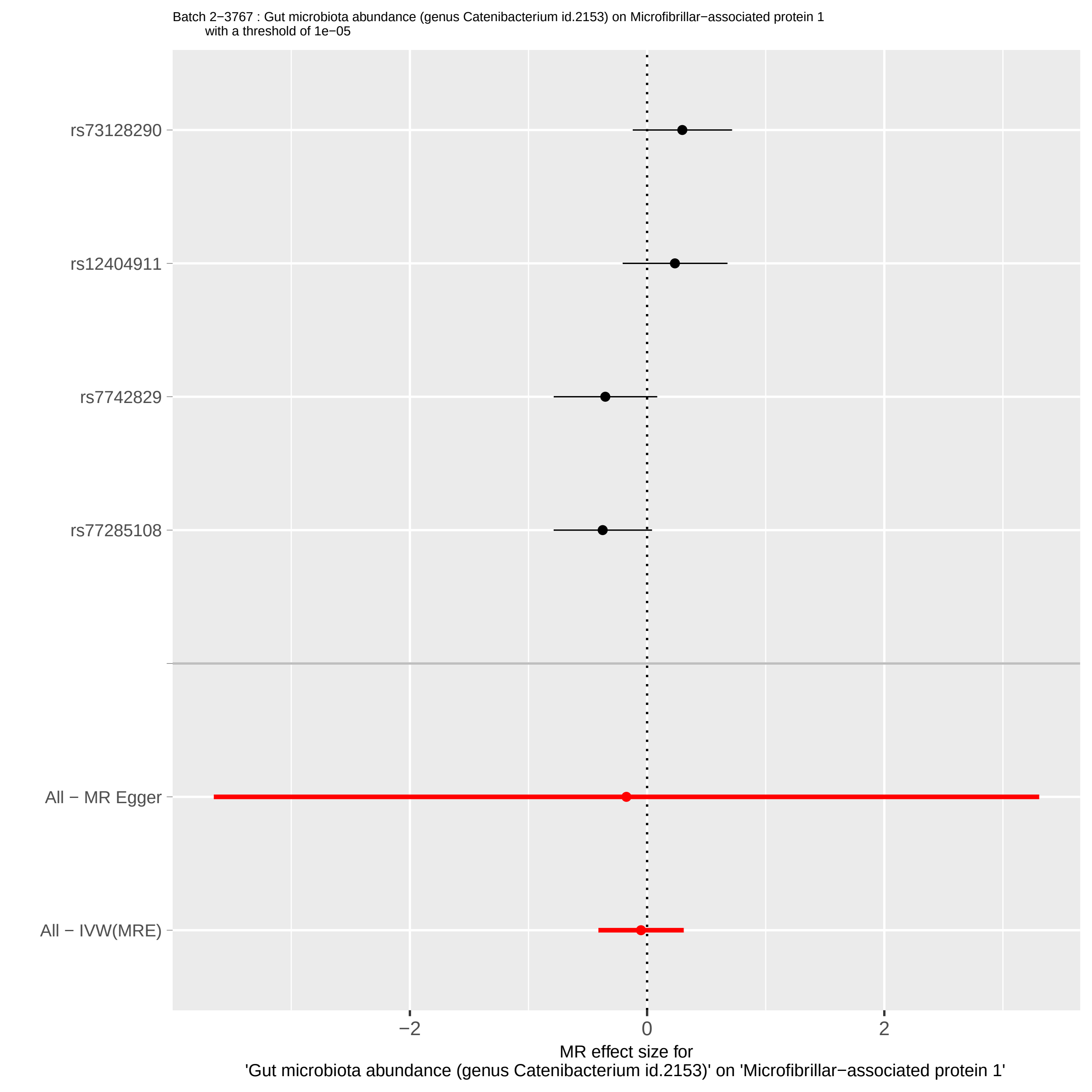

Batch 2−3767 : Gut microbiota abundance (genus Catenibacterium id.2153) on Microfibrillar−associated protein 1
with a threshold of 1e−05
rs73128290
rs12404911
rs7742829
rs77285108
All − MR Egger
All − IVW(MRE)
−2
0
2
MR effect size for
'Gut microbiota abundance (genus Catenibacterium id.2153)' on 'Microfibrillar−associated protein 1'

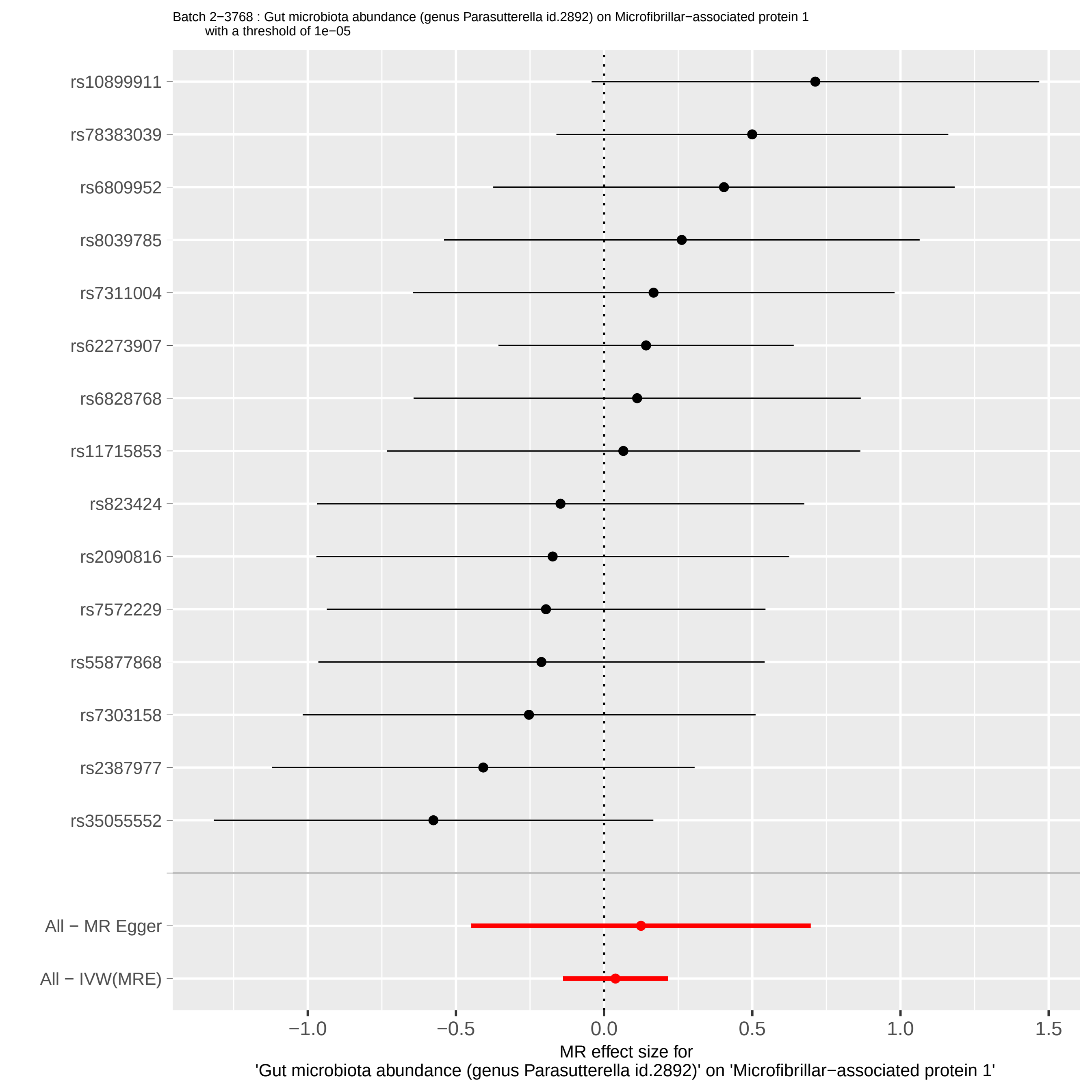

Batch 2−3768 : Gut microbiota abundance (genus Parasutterella id.2892) on Microfibrillar−associated protein 1
with a threshold of 1e−05
rs10899911
rs78383039
rs6809952
rs8039785
rs7311004
rs62273907
rs6828768
rs11715853
rs823424
rs2090816
rs7572229
rs55877868
rs7303158
rs2387977
rs35055552
All − MR Egger
All − IVW(MRE)
−1.0
−0.5
0.0
0.5
1.0
1.5
MR effect size for
'Gut microbiota abundance (genus Parasutterella id.2892)' on 'Microfibrillar−associated protein 1'

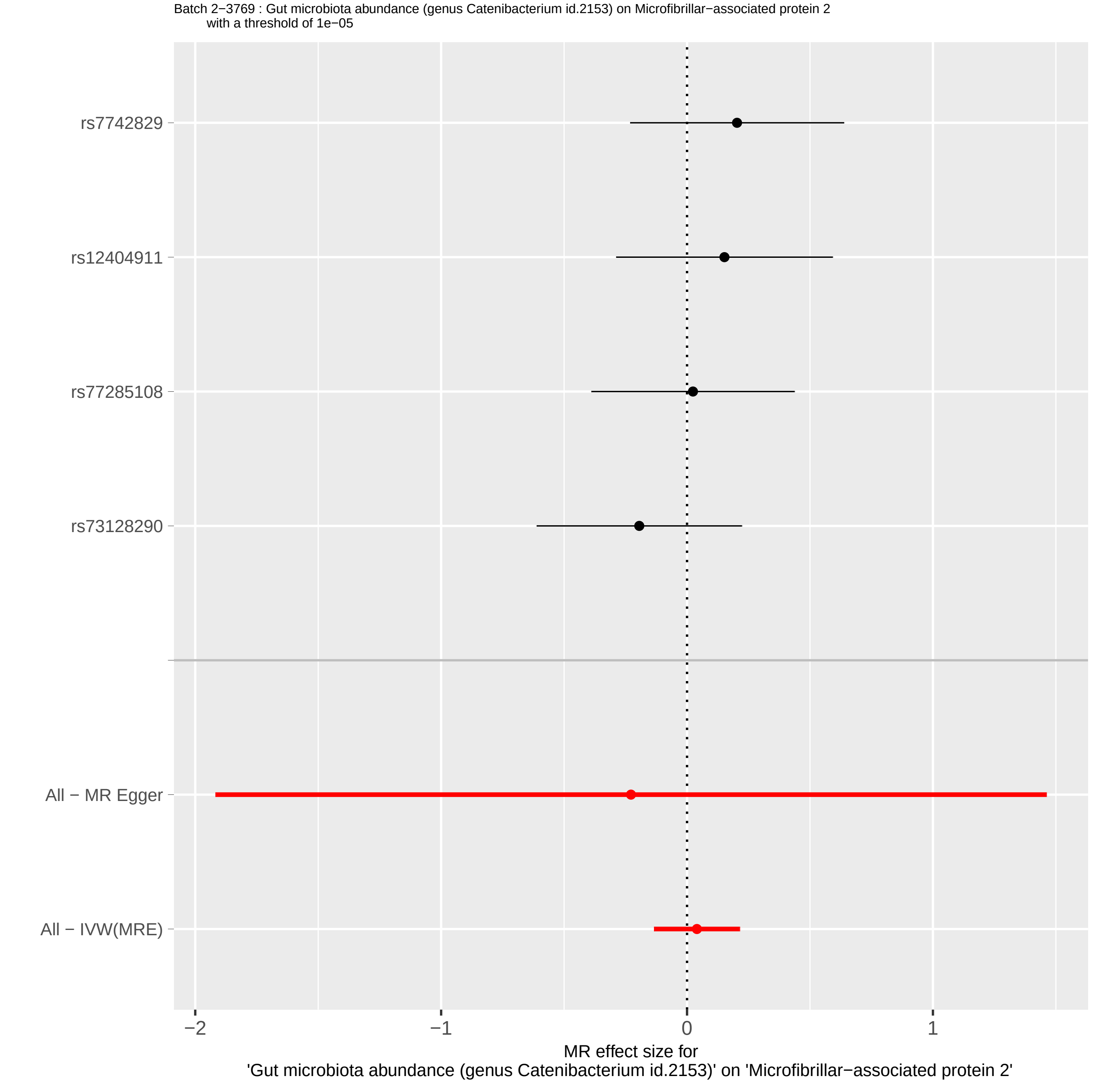
Batch 2−3769 : Gut microbiota abundance (genus Catenibacterium id.2153) on Microfibrillar−associated protein 2
with a threshold of 1e−05
rs7742829
rs12404911
rs77285108
rs73128290
All − MR Egger
All − IVW(MRE)
−2
−1
0
1
MR effect size for
'Gut microbiota abundance (genus Catenibacterium id.2153)' on 'Microfibrillar−associated protein 2'

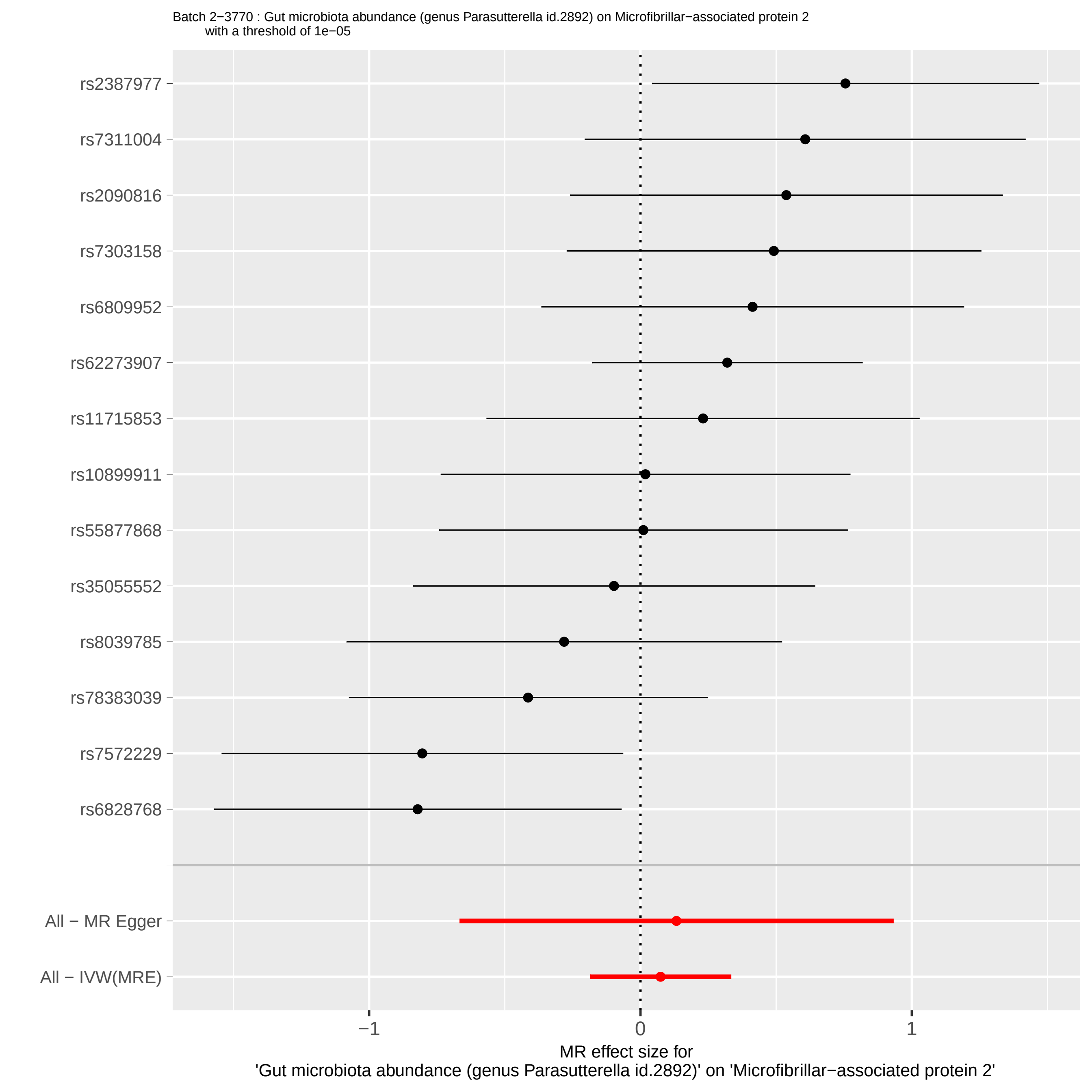
Batch 2−3770 : Gut microbiota abundance (genus Parasutterella id.2892) on Microfibrillar−associated protein 2
with a threshold of 1e−05
rs2387977
rs7311004
rs2090816
rs7303158
rs6809952
rs62273907
rs11715853
rs10899911
rs55877868
rs35055552
rs8039785
rs78383039
rs7572229
rs6828768
All − MR Egger
All − IVW(MRE)
−1
0
1
MR effect size for
'Gut microbiota abundance (genus Parasutterella id.2892)' on 'Microfibrillar−associated protein 2'

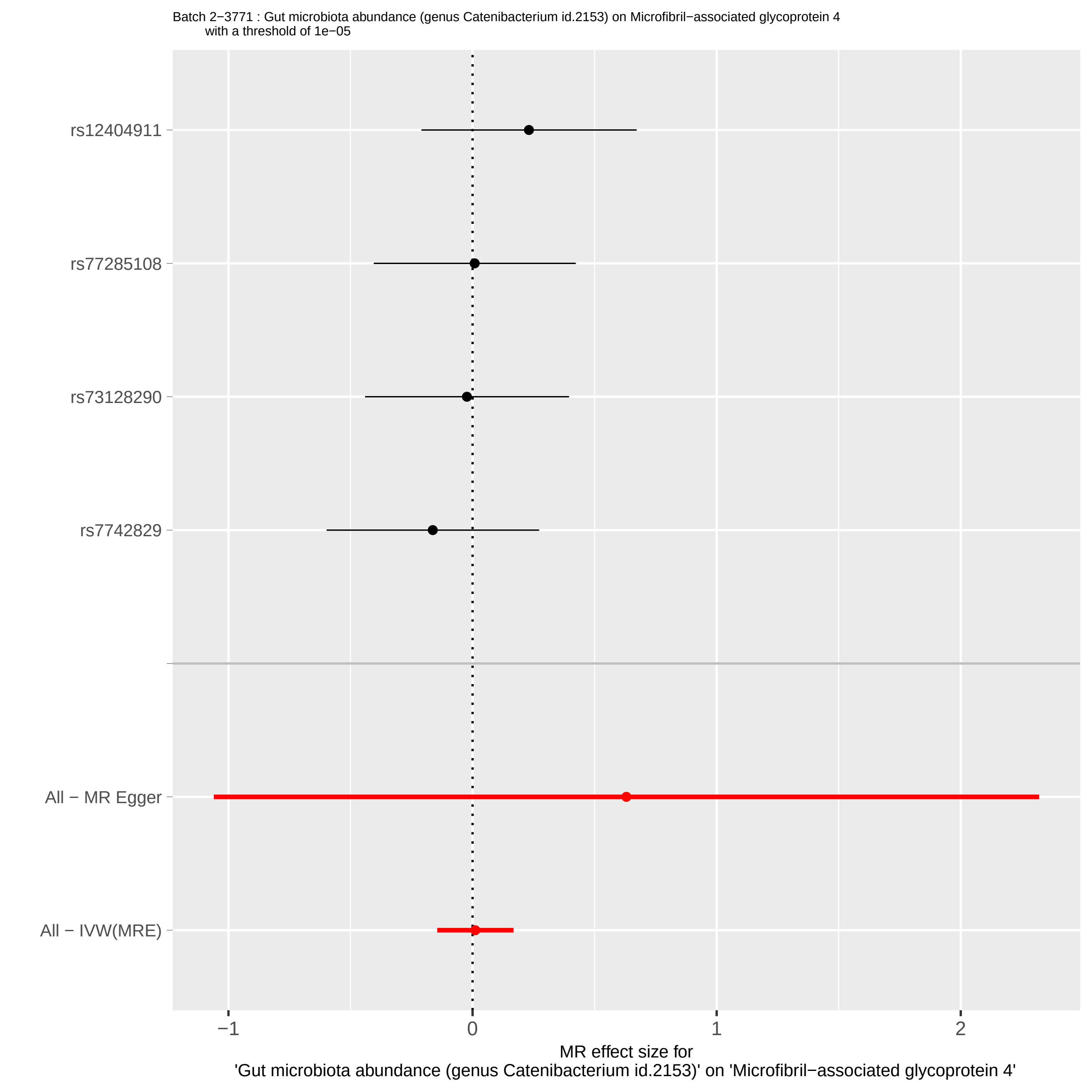

Batch 2−3771 : Gut microbiota abundance (genus Catenibacterium id.2153) on Microfibril−associated glycoprotein 4
with a threshold of 1e−05
rs12404911
rs77285108
rs73128290
rs7742829
All − MR Egger
All − IVW(MRE)
−1
0
1
2
MR effect size for
'Gut microbiota abundance (genus Catenibacterium id.2153)' on 'Microfibril−associated glycoprotein 4'

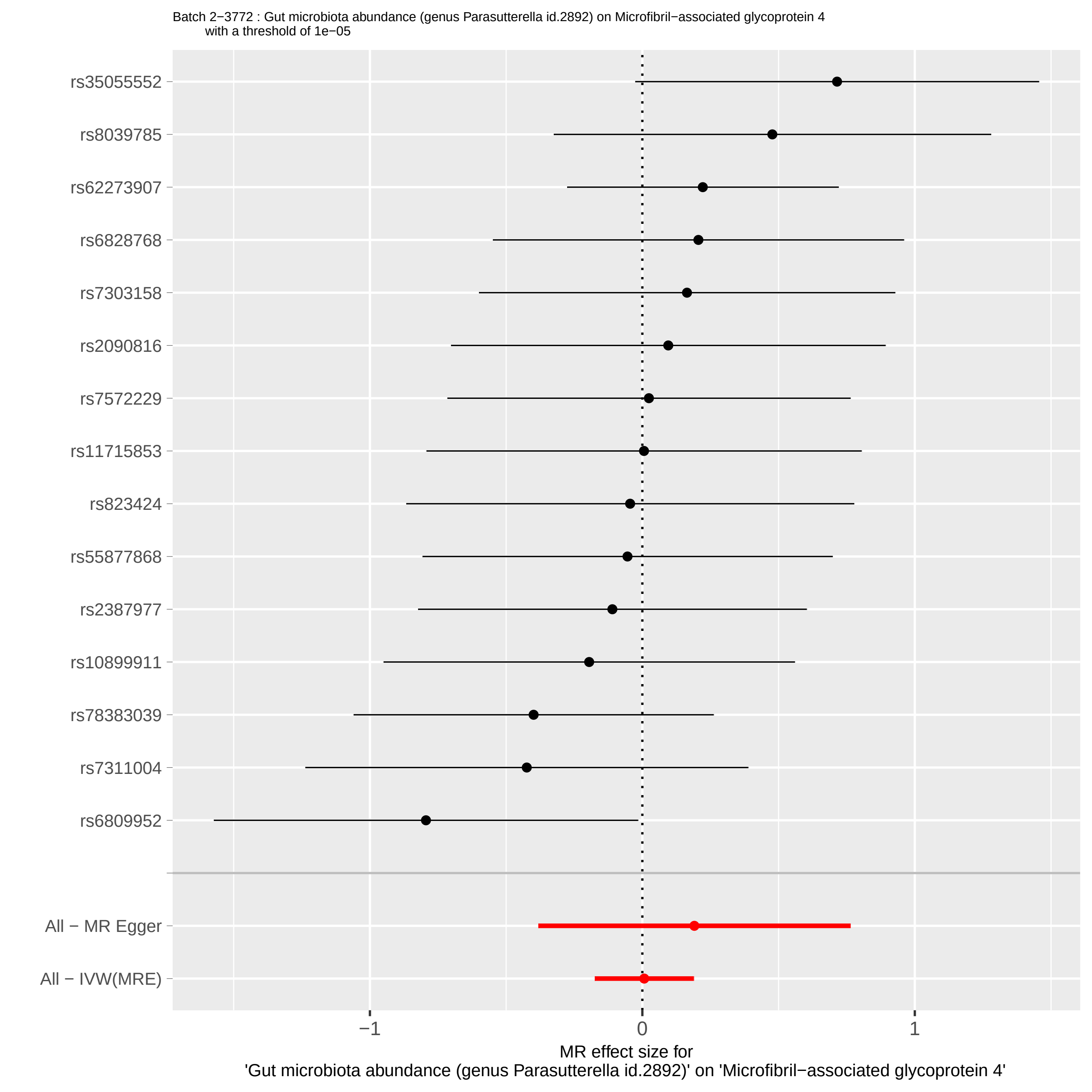
Batch 2−3772 : Gut microbiota abundance (genus Parasutterella id.2892) on Microfibril−associated glycoprotein 4
with a threshold of 1e−05
rs35055552
rs8039785
rs62273907
rs6828768
rs7303158
rs2090816
rs7572229
rs11715853
rs823424
rs55877868
rs2387977
rs10899911
rs78383039
rs7311004
rs6809952
All − MR Egger
All − IVW(MRE)
−1
0
1
MR effect size for
'Gut microbiota abundance (genus Parasutterella id.2892)' on 'Microfibril−associated glycoprotein 4'

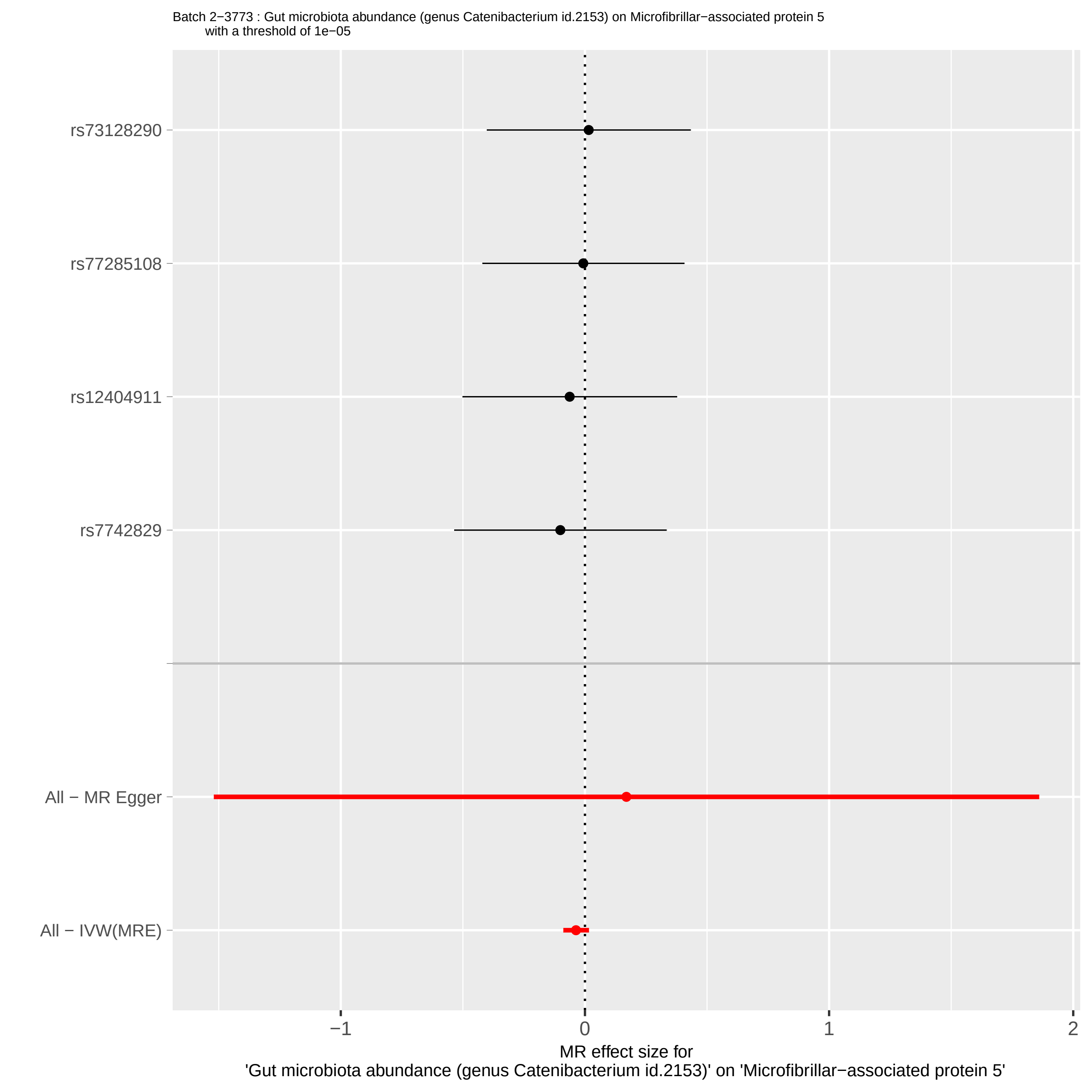

Batch 2−3773 : Gut microbiota abundance (genus Catenibacterium id.2153) on Microfibrillar−associated protein 5
with a threshold of 1e−05
rs73128290
rs77285108
rs12404911
rs7742829
All − MR Egger
All − IVW(MRE)
−1
0
1
2
MR effect size for
'Gut microbiota abundance (genus Catenibacterium id.2153)' on 'Microfibrillar−associated protein 5'

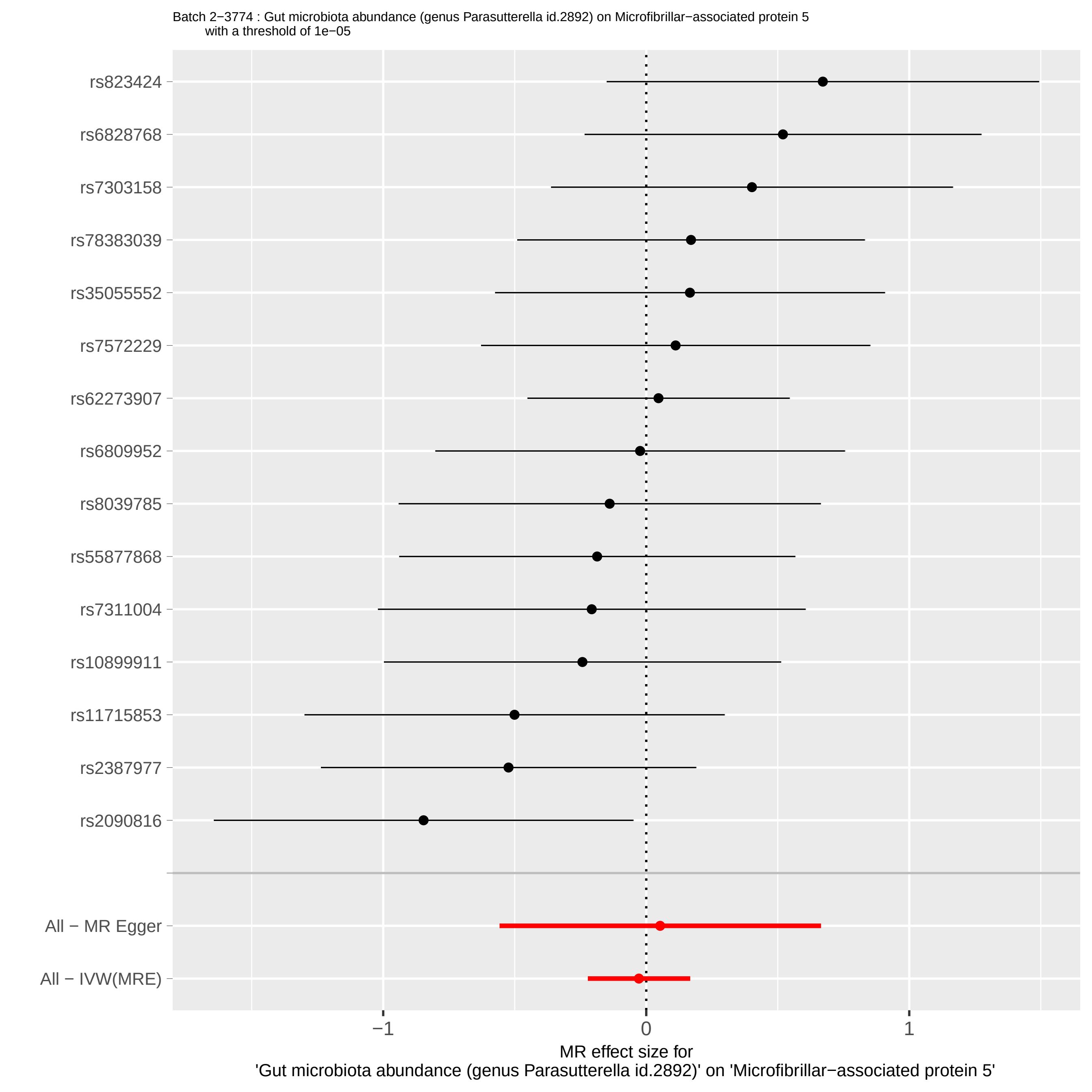

Batch 2−3774 : Gut microbiota abundance (genus Parasutterella id.2892) on Microfibrillar−associated protein 5
with a threshold of 1e−05
rs823424
rs6828768
rs7303158
rs78383039
rs35055552
rs7572229
rs62273907
rs6809952
rs8039785
rs55877868
rs7311004
rs10899911
rs11715853
rs2387977
rs2090816
All − MR Egger
All − IVW(MRE)
−1
0
1
MR effect size for
'Gut microbiota abundance (genus Parasutterella id.2892)' on 'Microfibrillar−associated protein 5'

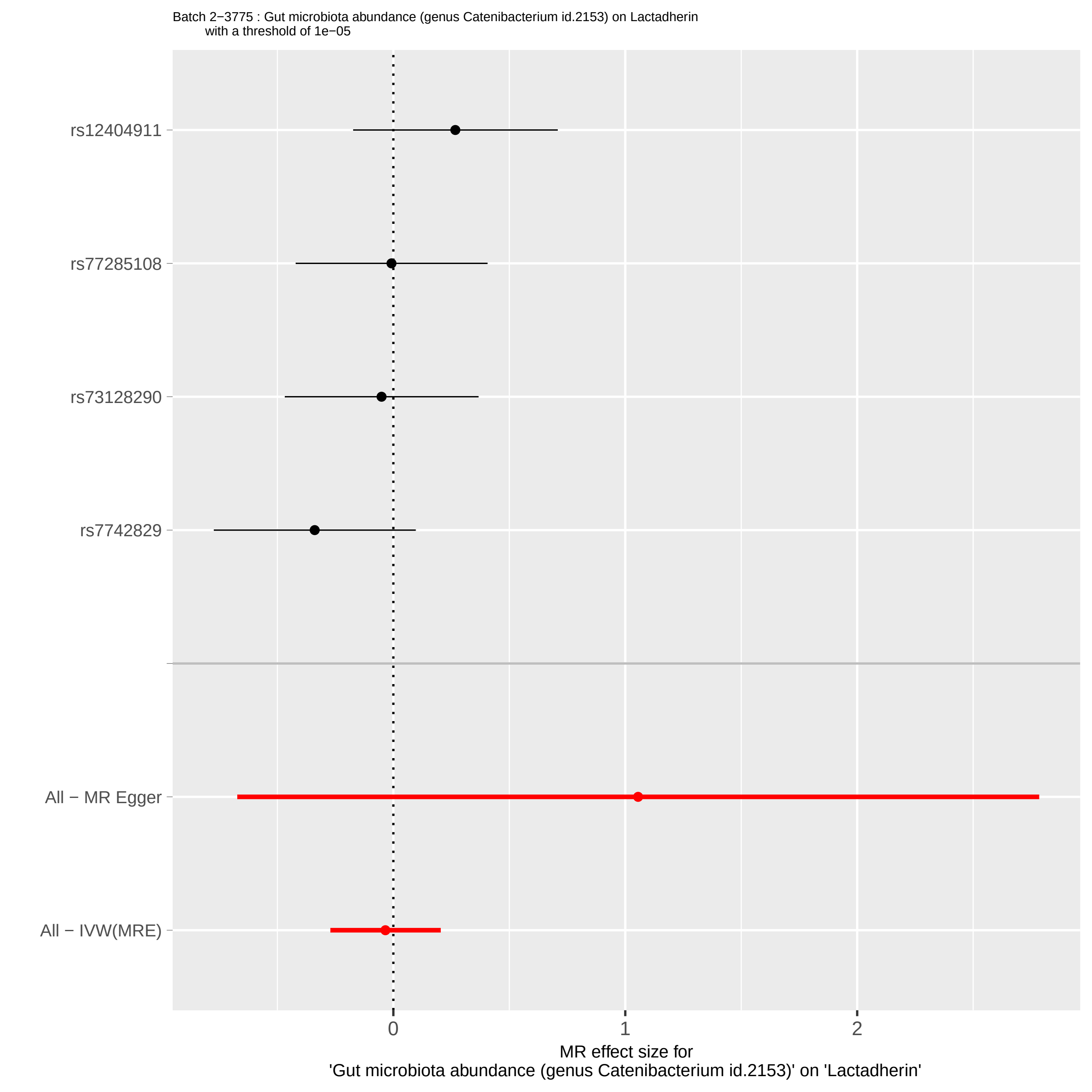

Batch 2−3775 : Gut microbiota abundance (genus Catenibacterium id.2153) on Lactadherin
with a threshold of 1e−05
rs12404911
rs77285108
rs73128290
rs7742829
All − MR Egger
All − IVW(MRE)
0
1
2
MR effect size for
'Gut microbiota abundance (genus Catenibacterium id.2153)' on 'Lactadherin'

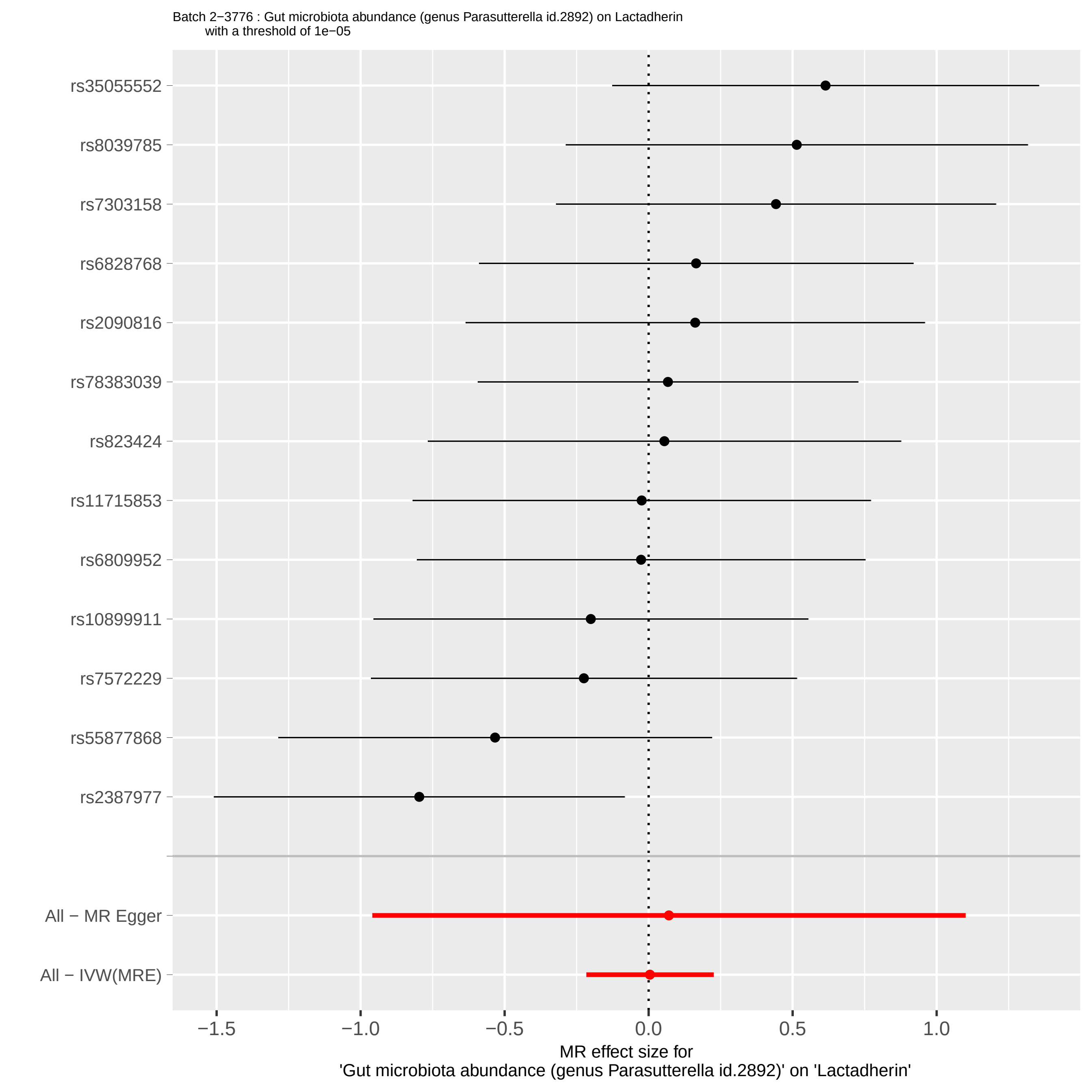
Batch 2−3776 : Gut microbiota abundance (genus Parasutterella id.2892) on Lactadherin
with a threshold of 1e−05
rs35055552
rs8039785
rs7303158
rs6828768
rs2090816
rs78383039
rs823424
rs11715853
rs6809952
rs10899911
rs7572229
rs55877868
rs2387977
All − MR Egger
All − IVW(MRE)
−1.5
−1.0
−0.5
0.0
0.5
1.0
MR effect size for
'Gut microbiota abundance (genus Parasutterella id.2892)' on 'Lactadherin'

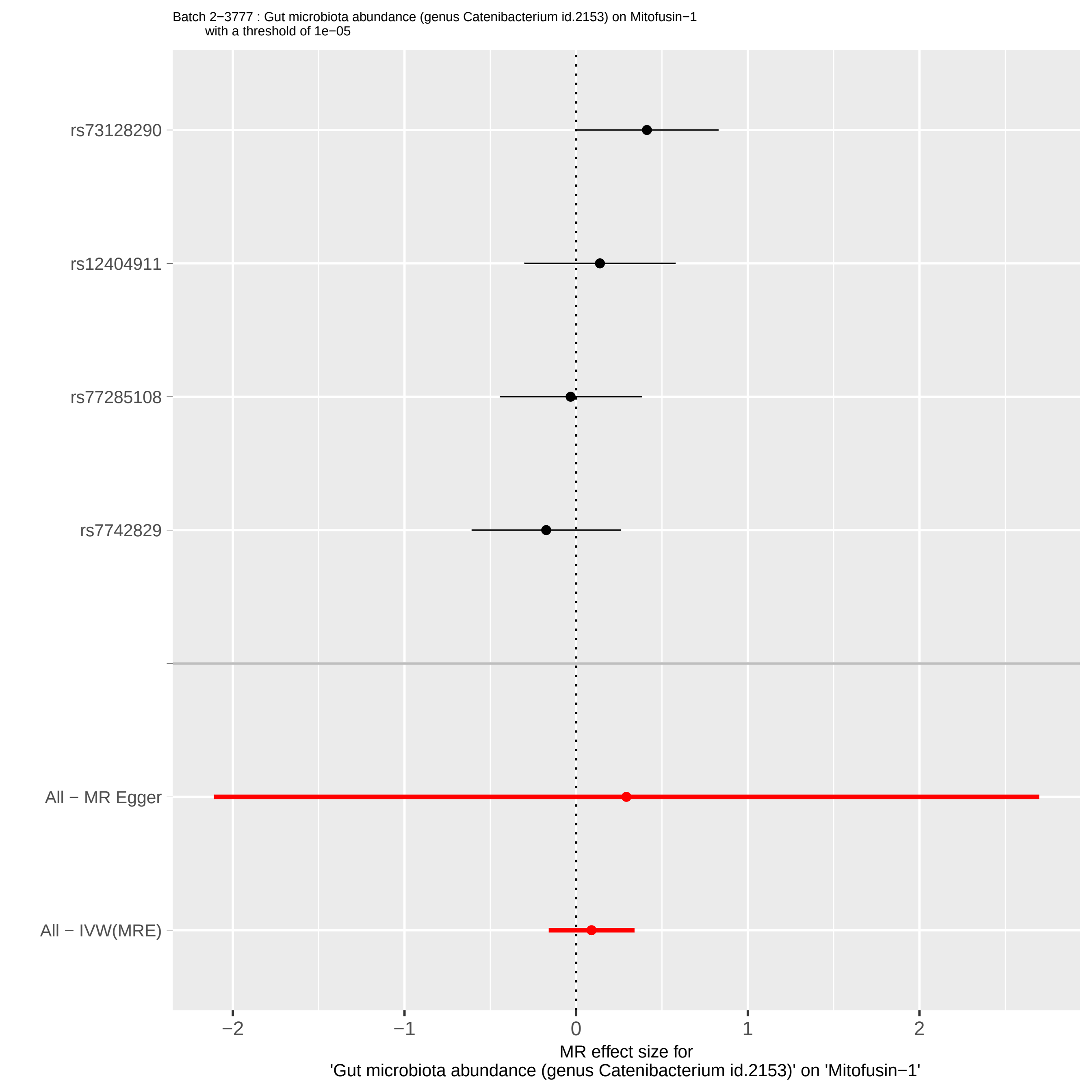
Batch 2−3777 : Gut microbiota abundance (genus Catenibacterium id.2153) on Mitofusin−1
with a threshold of 1e−05
rs73128290
rs12404911
rs77285108
rs7742829
All − MR Egger
All − IVW(MRE)
−2
−1
0
1
2
MR effect size for
'Gut microbiota abundance (genus Catenibacterium id.2153)' on 'Mitofusin−1'

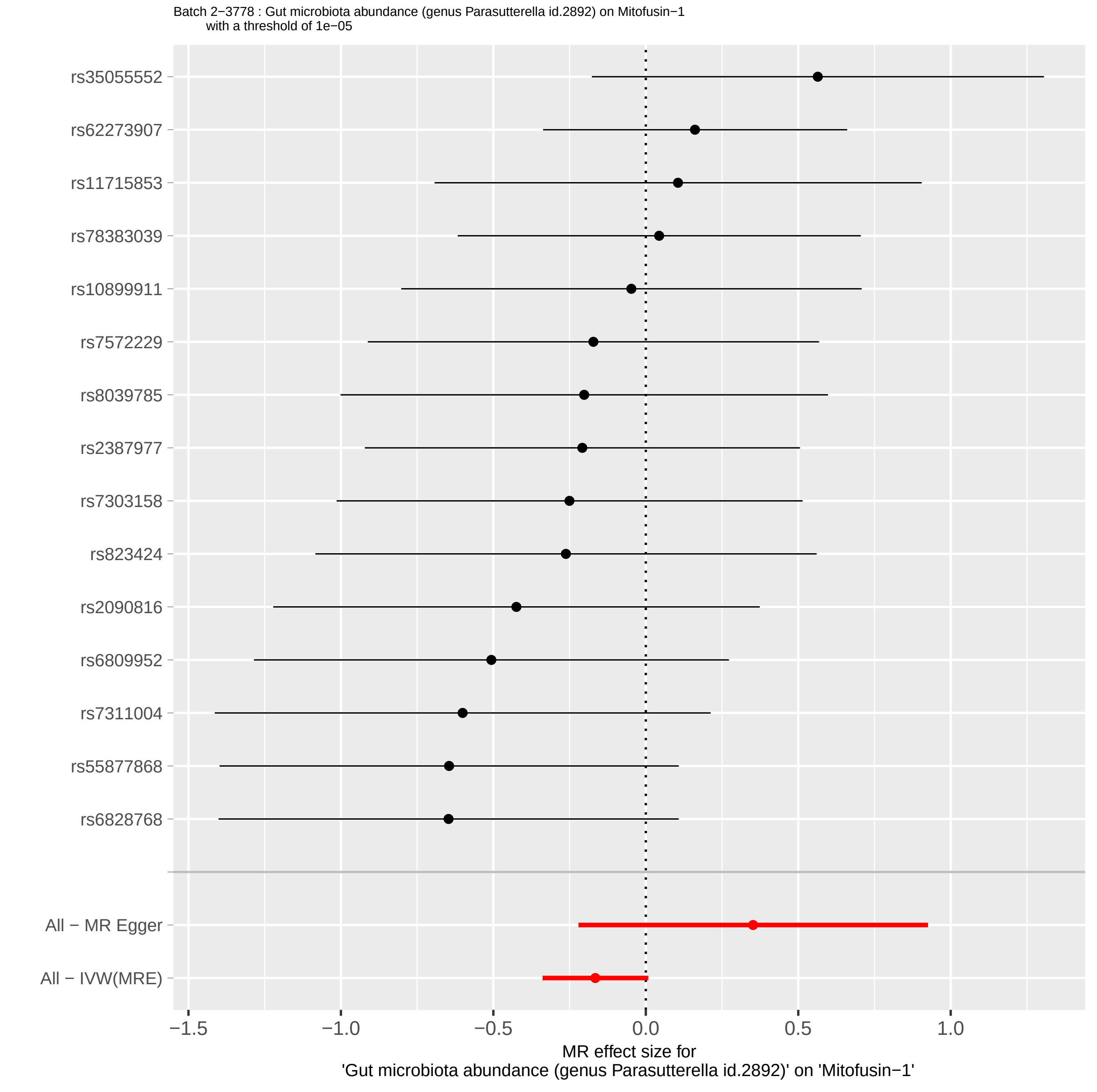

Batch 2−3778 : Gut microbiota abundance (genus Parasutterella id.2892) on Mitofusin−1
with a threshold of 1e−05
rs35055552
rs62273907
rs11715853
rs78383039
rs10899911
rs7572229
rs8039785
rs2387977
rs7303158
rs823424
rs2090816
rs6809952
rs7311004
rs55877868
rs6828768
All − MR Egger
All − IVW(MRE)
−1.5
−1.0
−0.5
0.0
0.5
1.0
MR effect size for
'Gut microbiota abundance (genus Parasutterella id.2892)' on 'Mitofusin−1'

Batch 2−3779 : Gut microbiota abundance (genus Catenibacterium id.2153) on Beta−1,3−N−acetylglucosaminyltransferase manic fringe with a threshold of 1e−05

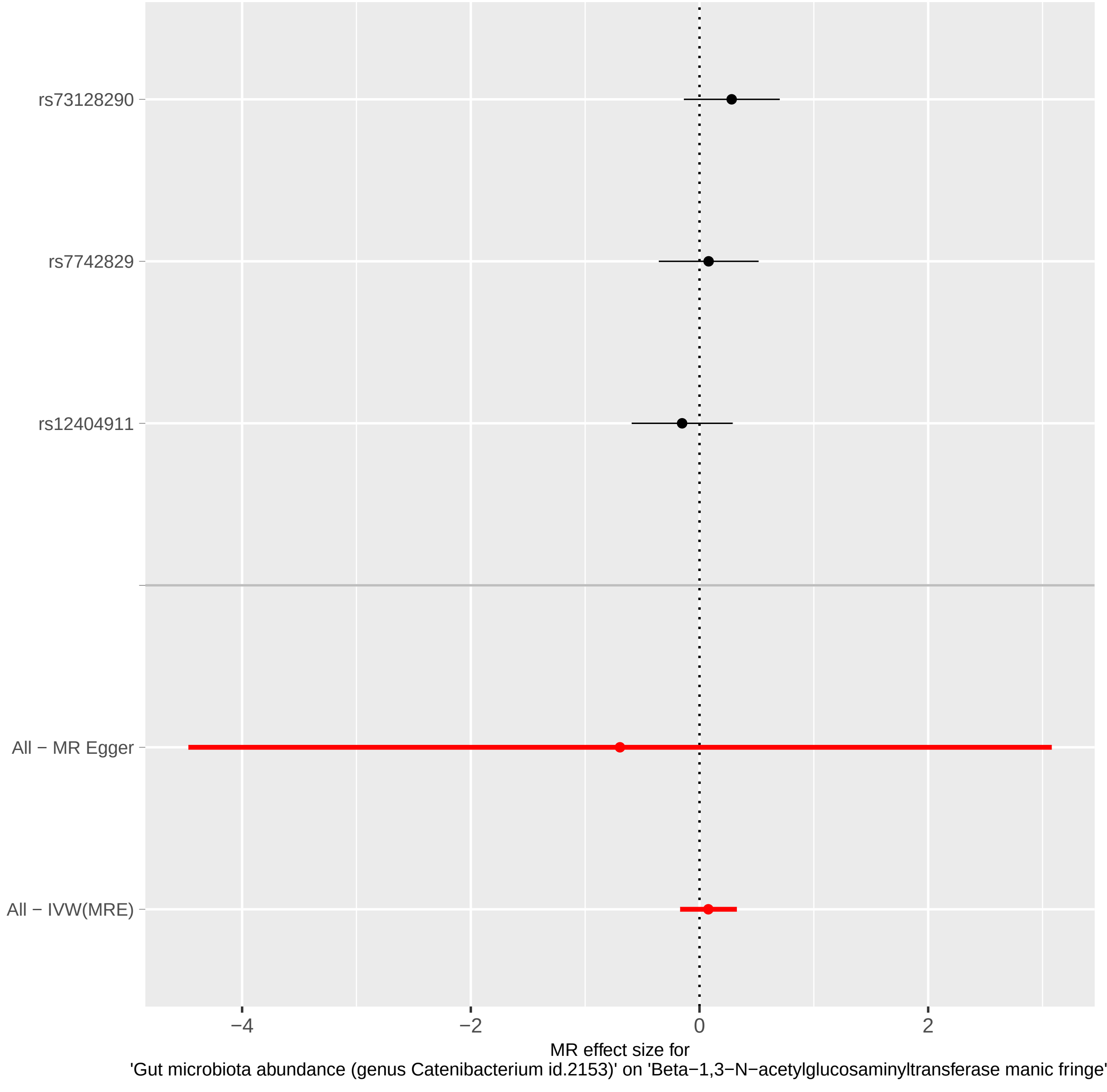

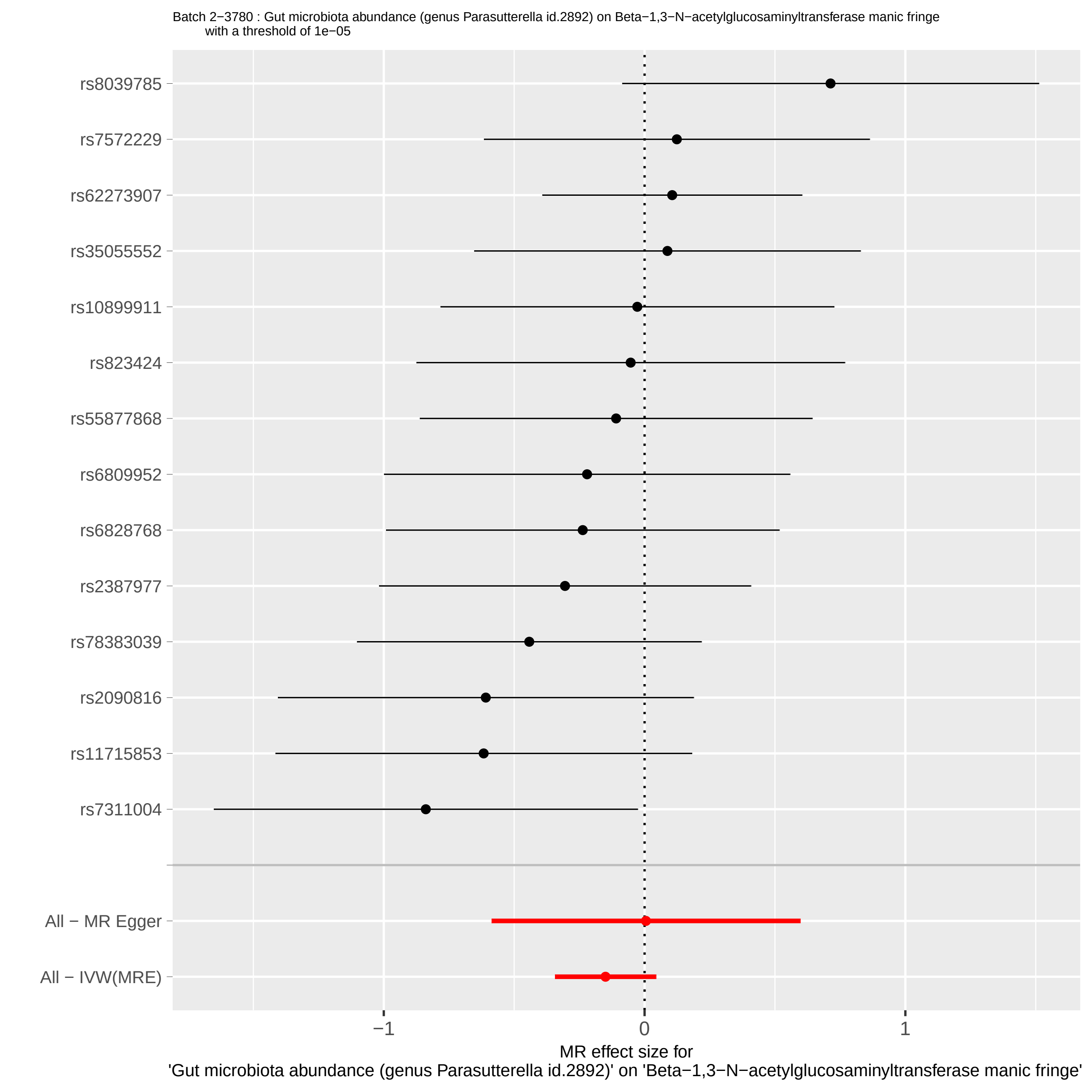
Batch 2−3780 : Gut microbiota abundance (genus Parasutterella id.2892) on Beta−1,3−N−acetylglucosaminyltransferase manic fringe with a threshold of 1e−05
rs8039785
rs7572229
rs62273907
rs35055552
rs10899911
rs823424
rs55877868
rs6809952
rs6828768
rs2387977
rs78383039
rs2090816
rs11715853
rs7311004
All − MR Egger
All − IVW(MRE)
−1
0
1
MR effect size for
'Gut microbiota abundance (genus Parasutterella id.2892)' on 'Beta−1,3−N−acetylglucosaminyltransferase manic fringe'

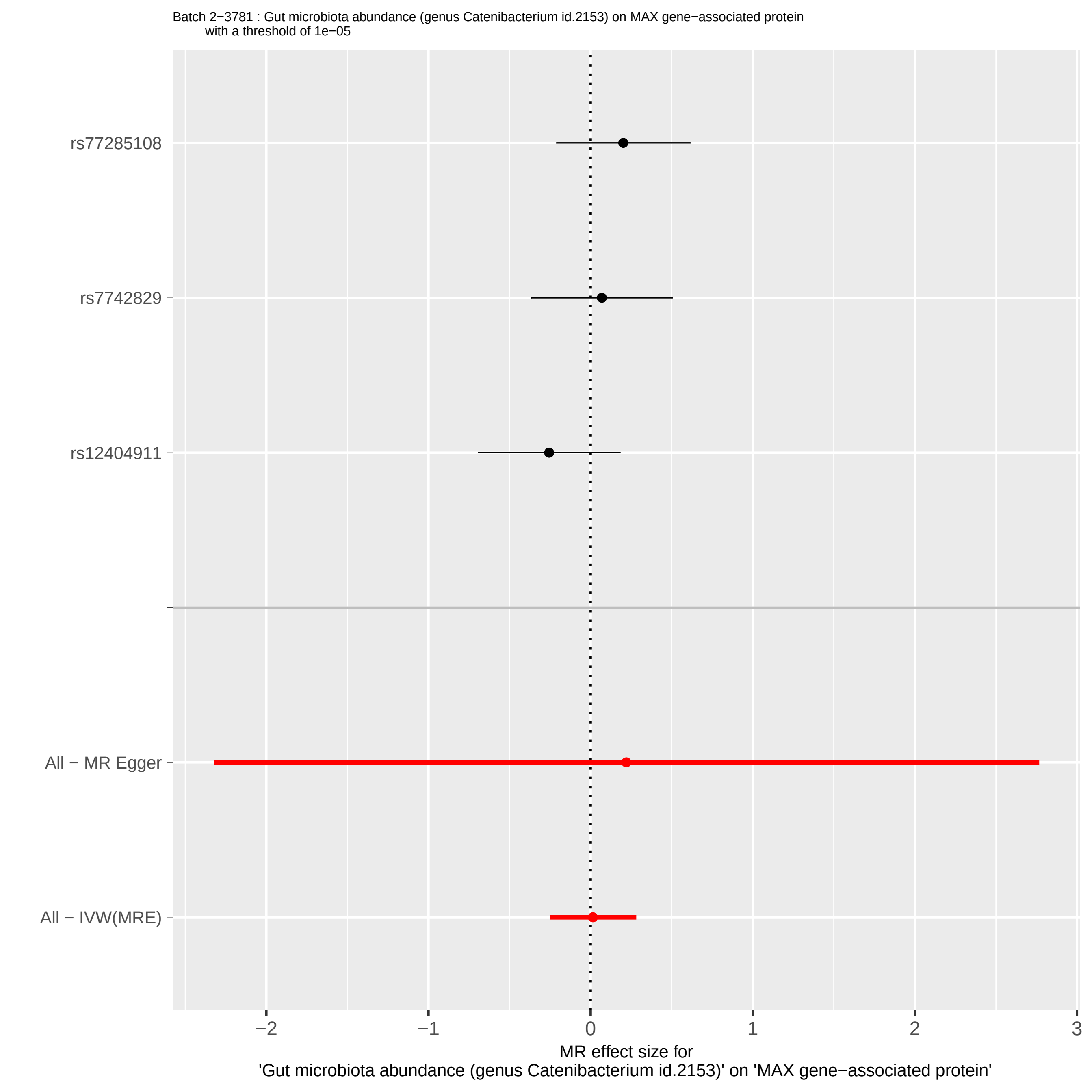

Batch 2−3781 : Gut microbiota abundance (genus Catenibacterium id.2153) on MAX gene−associated protein
with a threshold of 1e−05
rs77285108
rs7742829
rs12404911
All − MR Egger
All − IVW(MRE)
−2
−1
0
1
2
3
MR effect size for
'Gut microbiota abundance (genus Catenibacterium id.2153)' on 'MAX gene−associated protein'

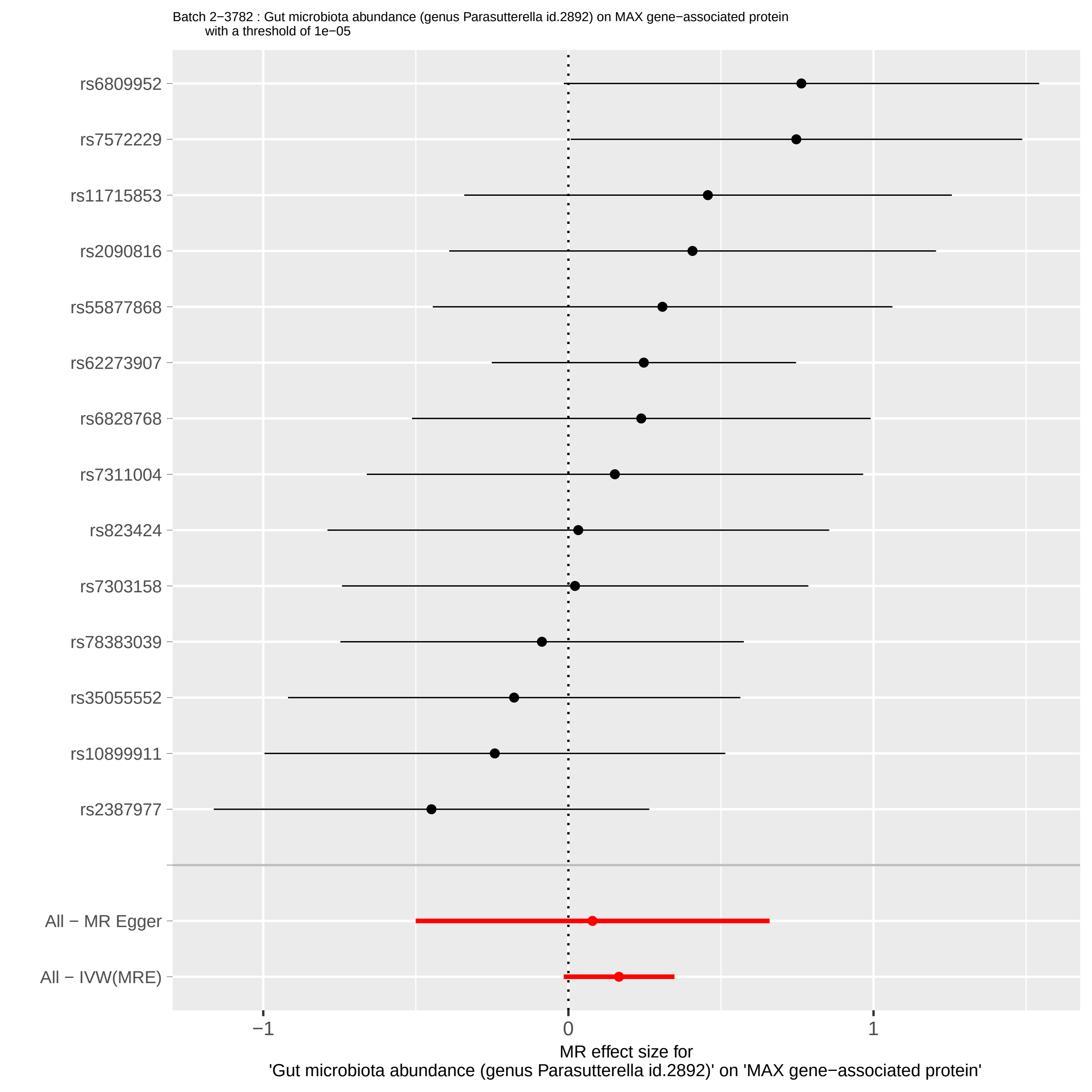

Batch 2−3782 : Gut microbiota abundance (genus Parasutterella id.2892) on MAX gene−associated protein
with a threshold of 1e−05
rs6809952
rs7572229
rs11715853
rs2090816
rs55877868
rs62273907
rs6828768
rs7311004
rs823424
rs7303158
rs78383039
rs35055552
rs10899911
rs2387977
All − MR Egger
All − IVW(MRE)
−1
0
1
MR effect size for
'Gut microbiota abundance (genus Parasutterella id.2892)' on 'MAX gene−associated protein'

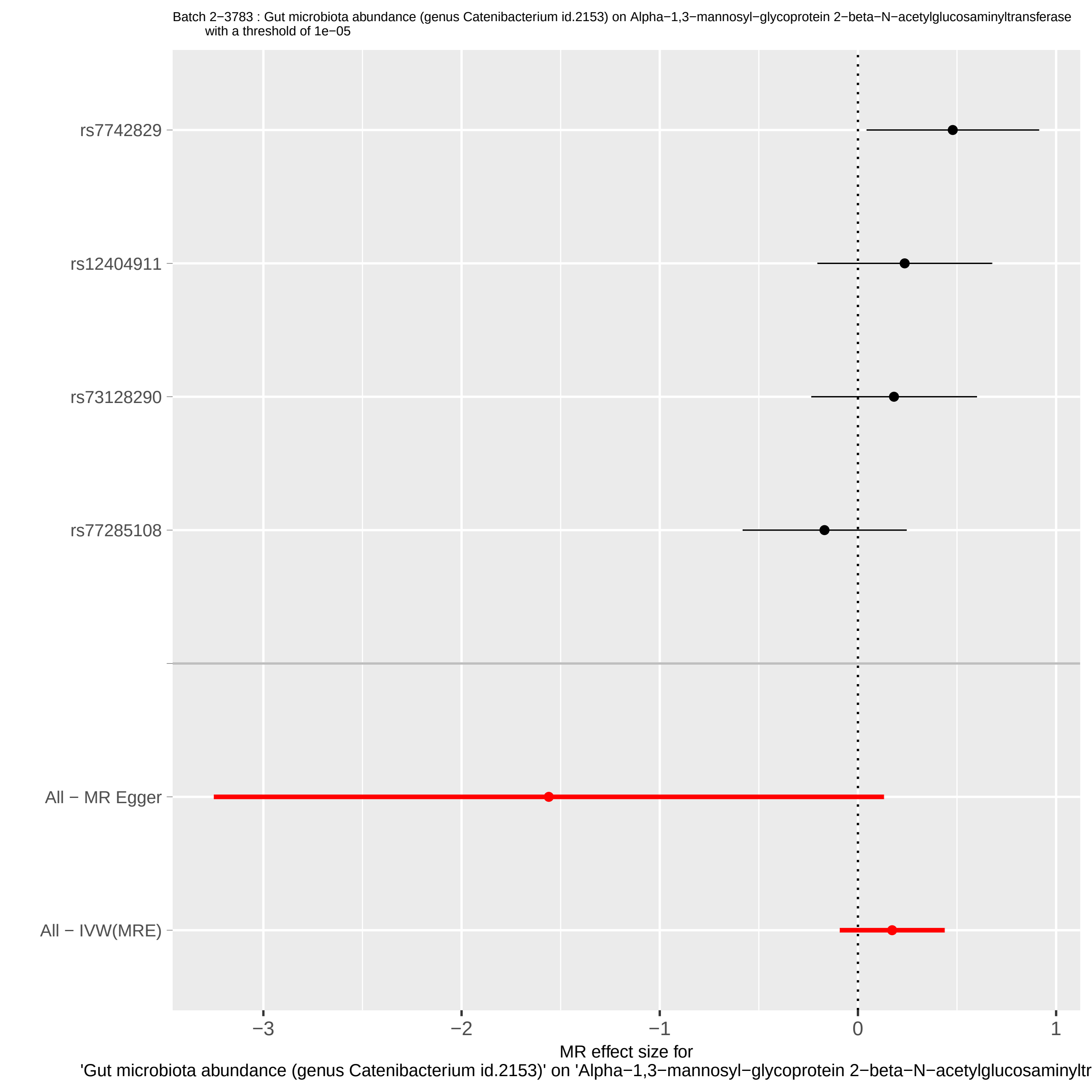

Batch 2−3783 : Gut microbiota abundance (genus Catenibacterium id.2153) on Alpha−1,3−mannosyl−glycoprotein 2−beta−N−acetylglucosaminyltransferase with a threshold of 1e−05
rs7742829
rs12404911
rs73128290
rs77285108
All − MR Egger
All − IVW(MRE)
−3
−2
−1
0
1
MR effect size for
'Gut microbiota abundance (genus Catenibacterium id.2153)' on 'Alpha−1,3−mannosyl−glycoprotein 2−beta−N−acetylglucosaminyltr

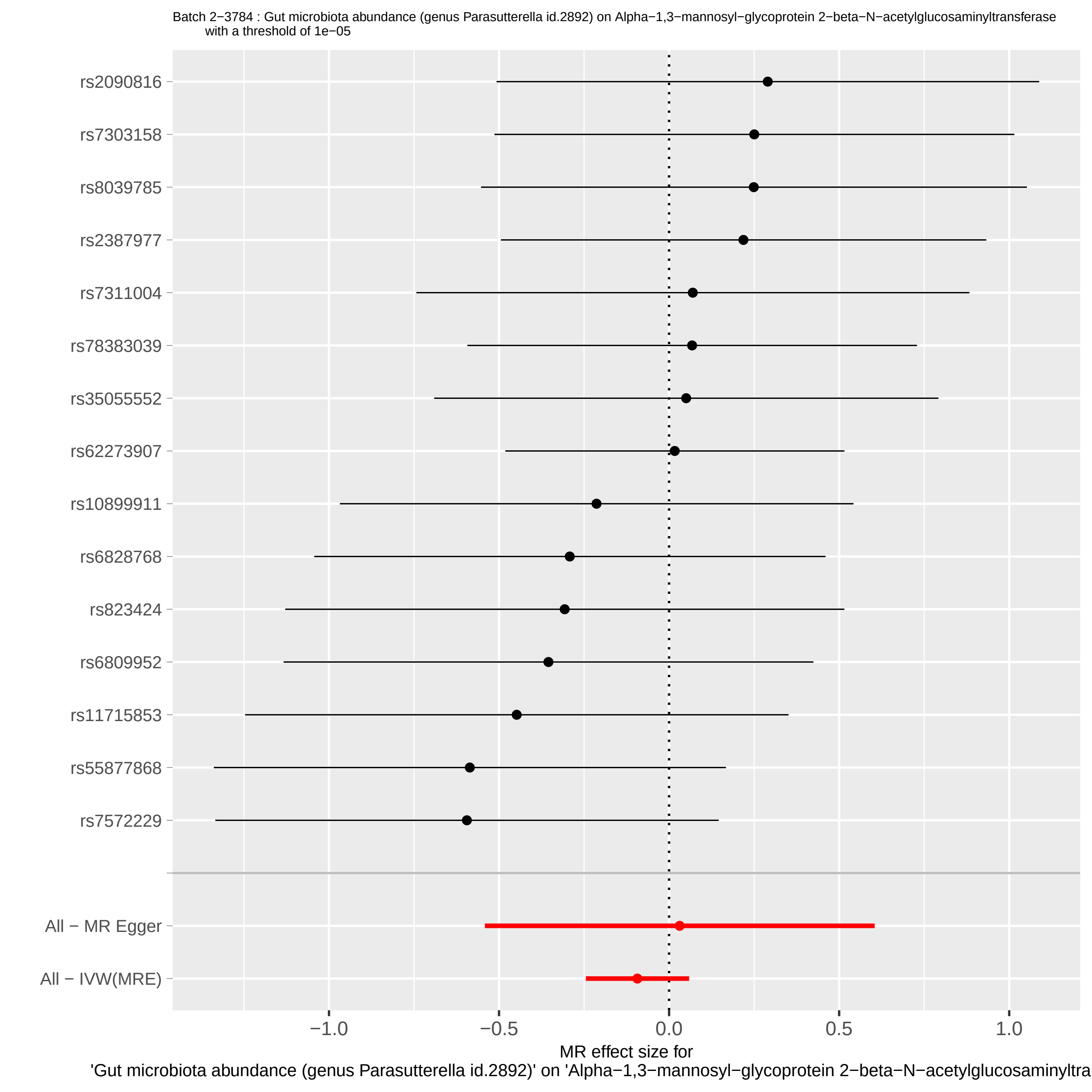
Batch 2−3784 : Gut microbiota abundance (genus Parasutterella id.2892) on Alpha−1,3−mannosyl−glycoprotein 2−beta−N−acetylglucosaminyltransferase
with a threshold of 1e−05
rs2090816
rs7303158
rs8039785
rs2387977
rs7311004
rs78383039
rs35055552
rs62273907
rs10899911
rs6828768
rs823424
rs6809952
rs11715853
rs55877868
rs7572229
All − MR Egger
All − IVW(MRE)
−1.0
−0.5
0.0
0.5
1.0
MR effect size for
'Gut microbiota abundance (genus Parasutterella id.2892)' on 'Alpha−1,3−mannosyl−glycoprotein 2−beta−N−acetylglucosaminyltra

Batch 2−3785 : Gut microbiota abundance (genus Catenibacterium id.2153) on Alpha−1,6−mannosyl−glycoprotein 2−beta−N−acetylglucosaminyltransferase with a threshold of 1e−05

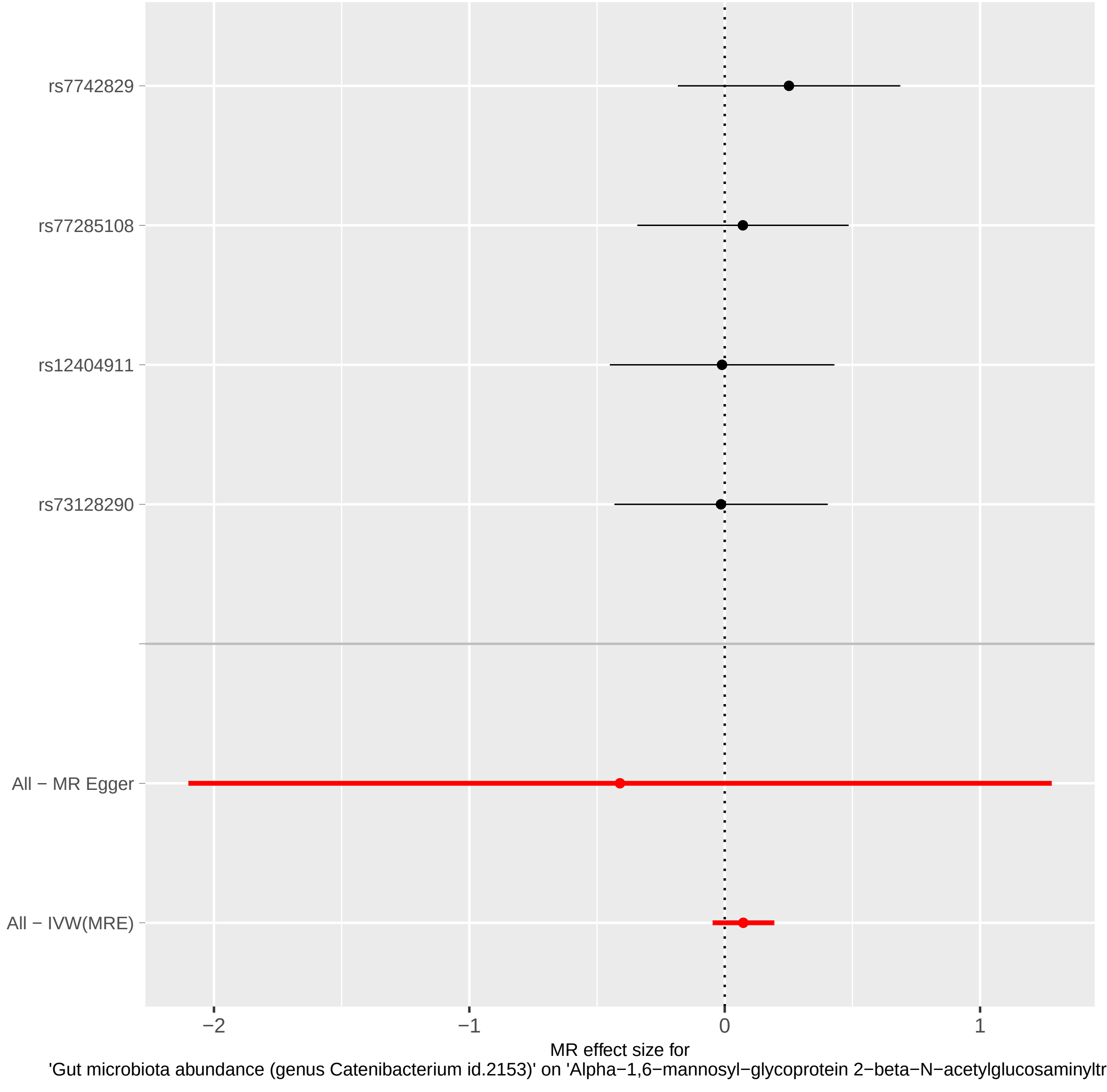

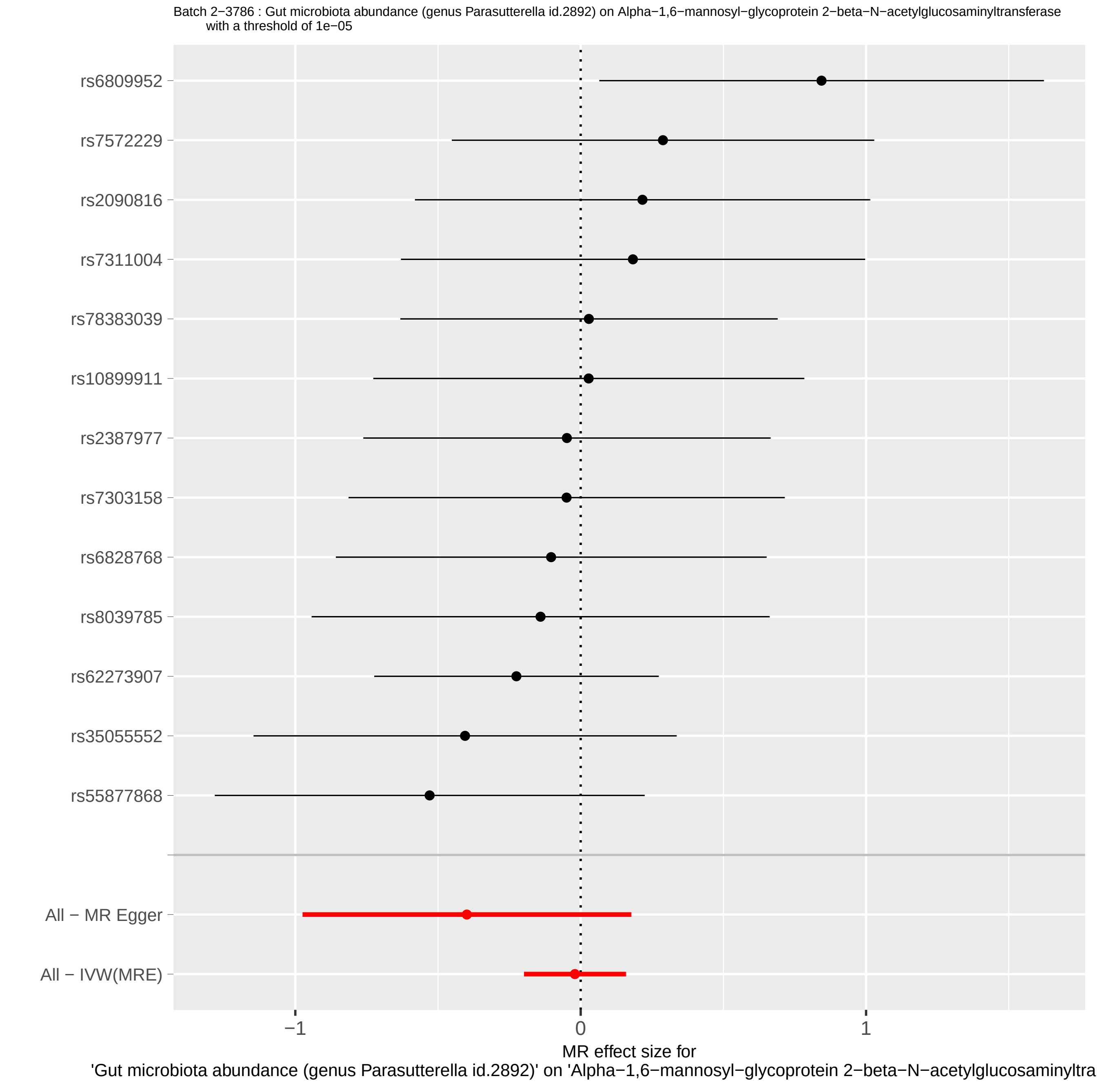

Batch 2−3786 : Gut microbiota abundance (genus Parasutterella id.2892) on Alpha−1,6−mannosyl−glycoprotein 2−beta−N−acetylglucosaminyltransferase with a threshold of 1e−05
rs6809952
rs7572229
rs2090816
rs7311004
rs78383039
rs10899911
rs2387977
rs7303158
rs6828768
rs8039785
rs62273907
rs35055552
rs55877868
All − MR Egger
All − IVW(MRE)
−1
0
1
MR effect size for
'Gut microbiota abundance (genus Parasutterella id.2892)' on 'Alpha−1,6−mannosyl−glycoprotein 2−beta−N−acetylglucosaminyltra

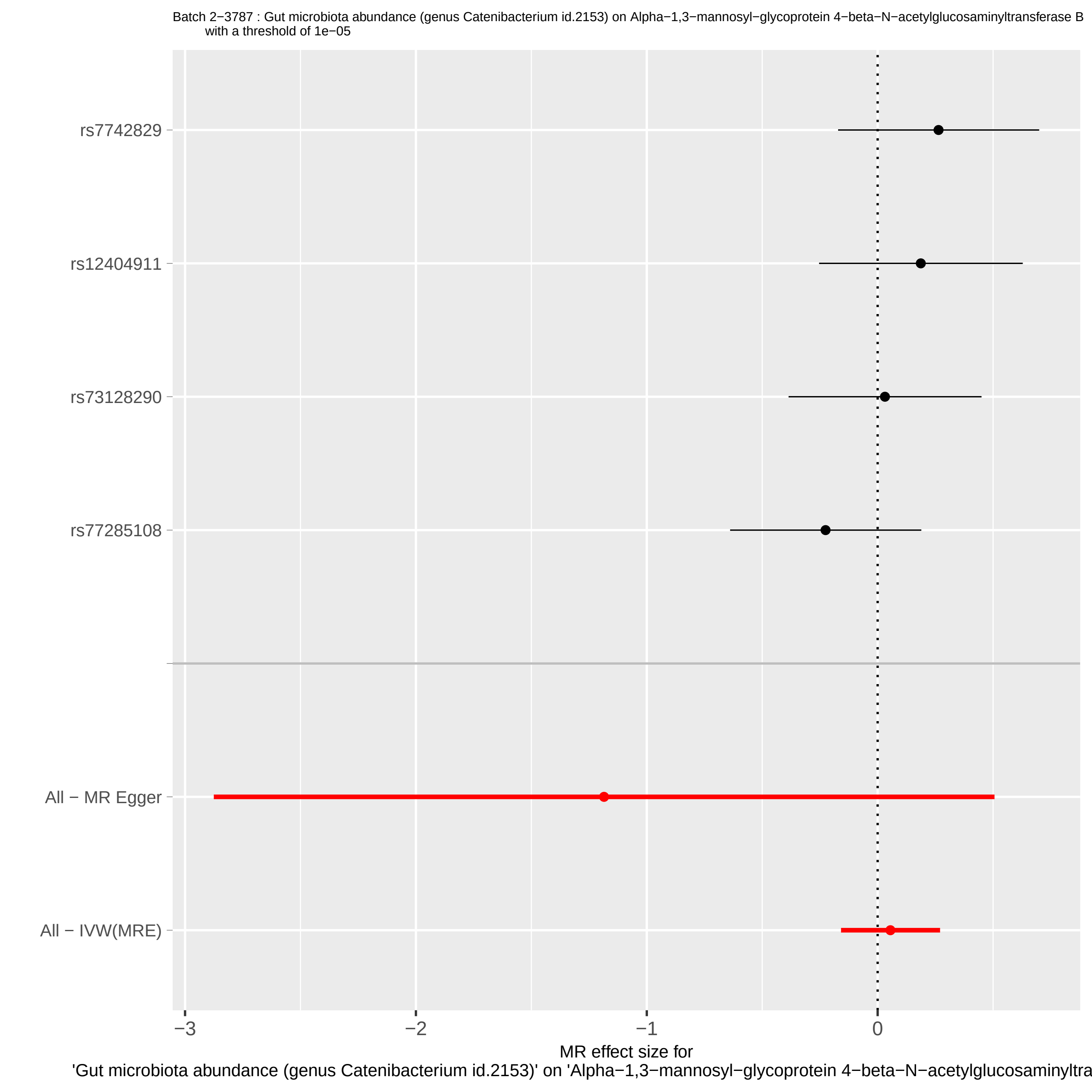

Batch 2−3787 : Gut microbiota abundance (genus Catenibacterium id.2153) on Alpha−1,3−mannosyl−glycoprotein 4−beta−N−acetylglucosaminyltransferase B
with a threshold of 1e−05
rs7742829
rs12404911
rs73128290
rs77285108
All − MR Egger
All − IVW(MRE)
−3
−2
−1
0
MR effect size for
'Gut microbiota abundance (genus Catenibacterium id.2153)' on 'Alpha−1,3−mannosyl−glycoprotein 4−beta−N−acetylglucosaminyltra

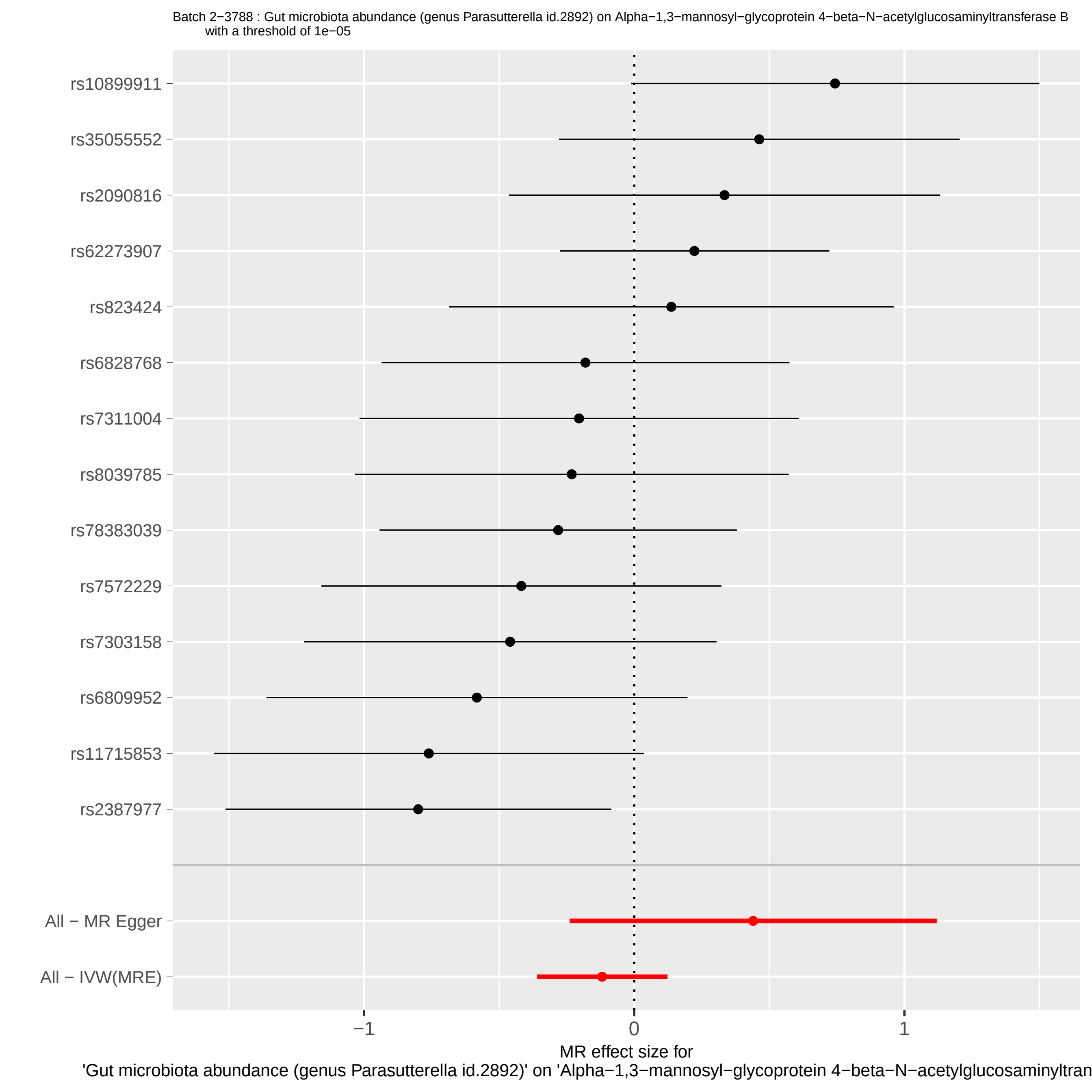
Batch 2−3788 : Gut microbiota abundance (genus Parasutterella id.2892) on Alpha−1,3−mannosyl−glycoprotein 4−beta−N−acetylglucosaminyltransferase B
with a threshold of 1e−05
rs10899911
rs35055552
rs2090816
rs62273907
rs823424
rs6828768
rs7311004
rs8039785
rs78383039
rs7572229
rs7303158
rs6809952
rs11715853
rs2387977
All − MR Egger
All − IVW(MRE)
−1
0
1
MR effect size for
'Gut microbiota abundance (genus Parasutterella id.2892)' on 'Alpha−1,3−mannosyl−glycoprotein 4−beta−N−acetylglucosaminyltran

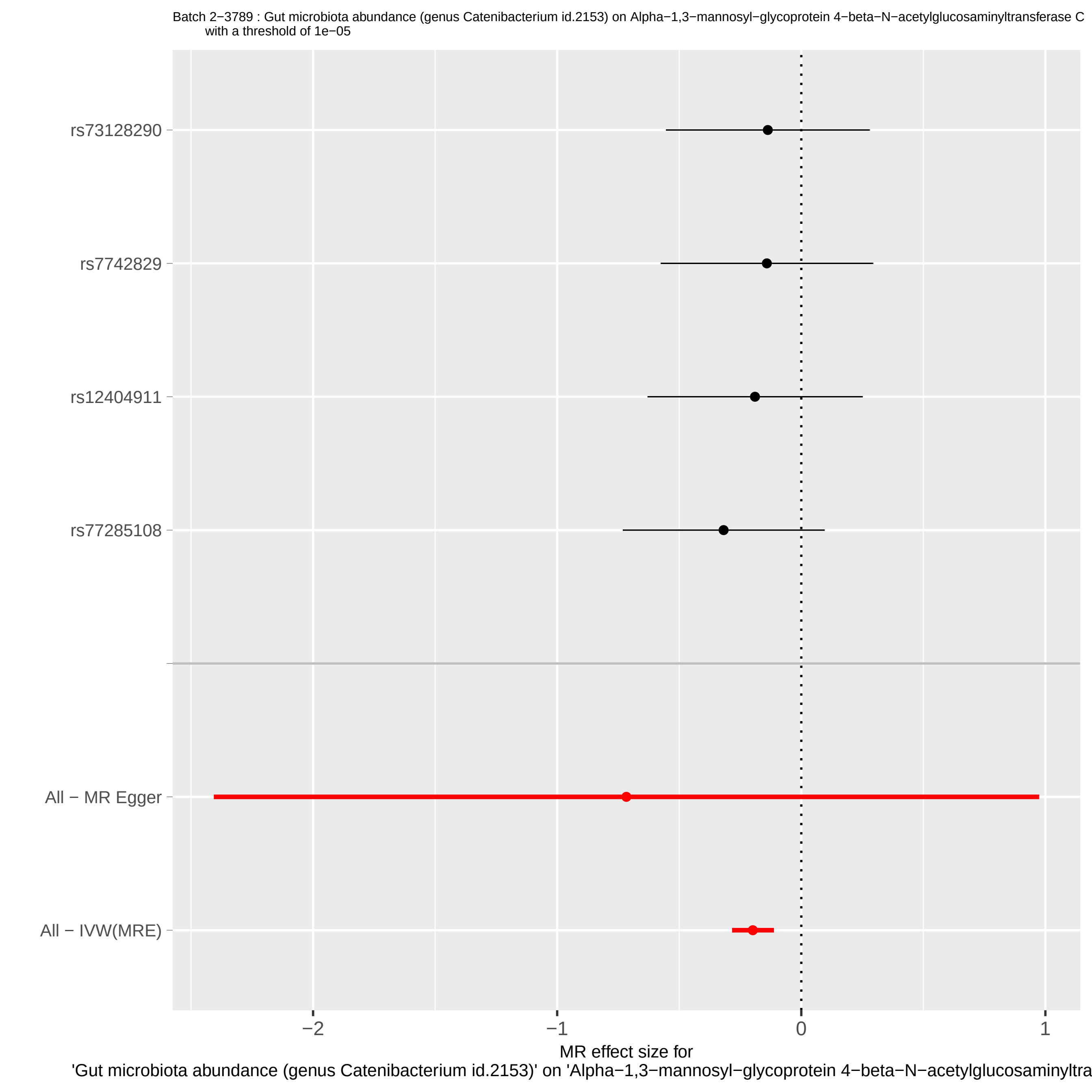

Batch 2−3789 : Gut microbiota abundance (genus Catenibacterium id.2153) on Alpha−1,3−mannosyl−glycoprotein 4−beta−N−acetylglucosaminyltransferase C
with a threshold of 1e−05
rs73128290
rs7742829
rs12404911
rs77285108
All − MR Egger
All − IVW(MRE)
−2
−1
0
1
MR effect size for
'Gut microbiota abundance (genus Catenibacterium id.2153)' on 'Alpha−1,3−mannosyl−glycoprotein 4−beta−N−acetylglucosaminyltra

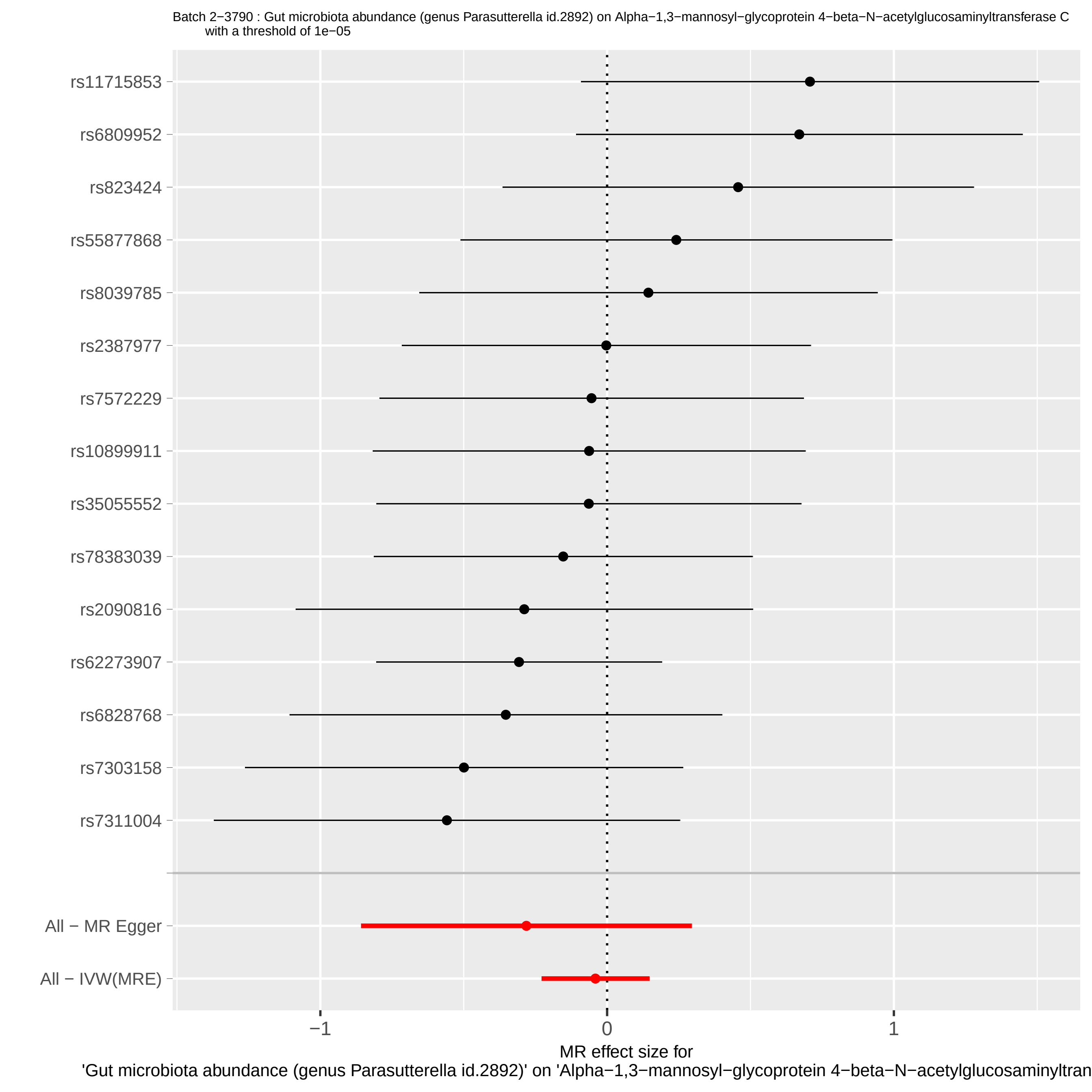

Batch 2−3790 : Gut microbiota abundance (genus Parasutterella id.2892) on Alpha−1,3−mannosyl−glycoprotein 4−beta−N−acetylglucosaminyltransferase C
with a threshold of 1e−05
rs11715853
rs6809952
rs823424
rs55877868
rs8039785
rs2387977
rs7572229
rs10899911
rs35055552
rs78383039
rs2090816
rs62273907
rs6828768
rs7303158
rs7311004
All − MR Egger
All − IVW(MRE)
−1
0
1
MR effect size for
'Gut microbiota abundance (genus Parasutterella id.2892)' on 'Alpha−1,3−mannosyl−glycoprotein 4−beta−N−acetylglucosaminyltran

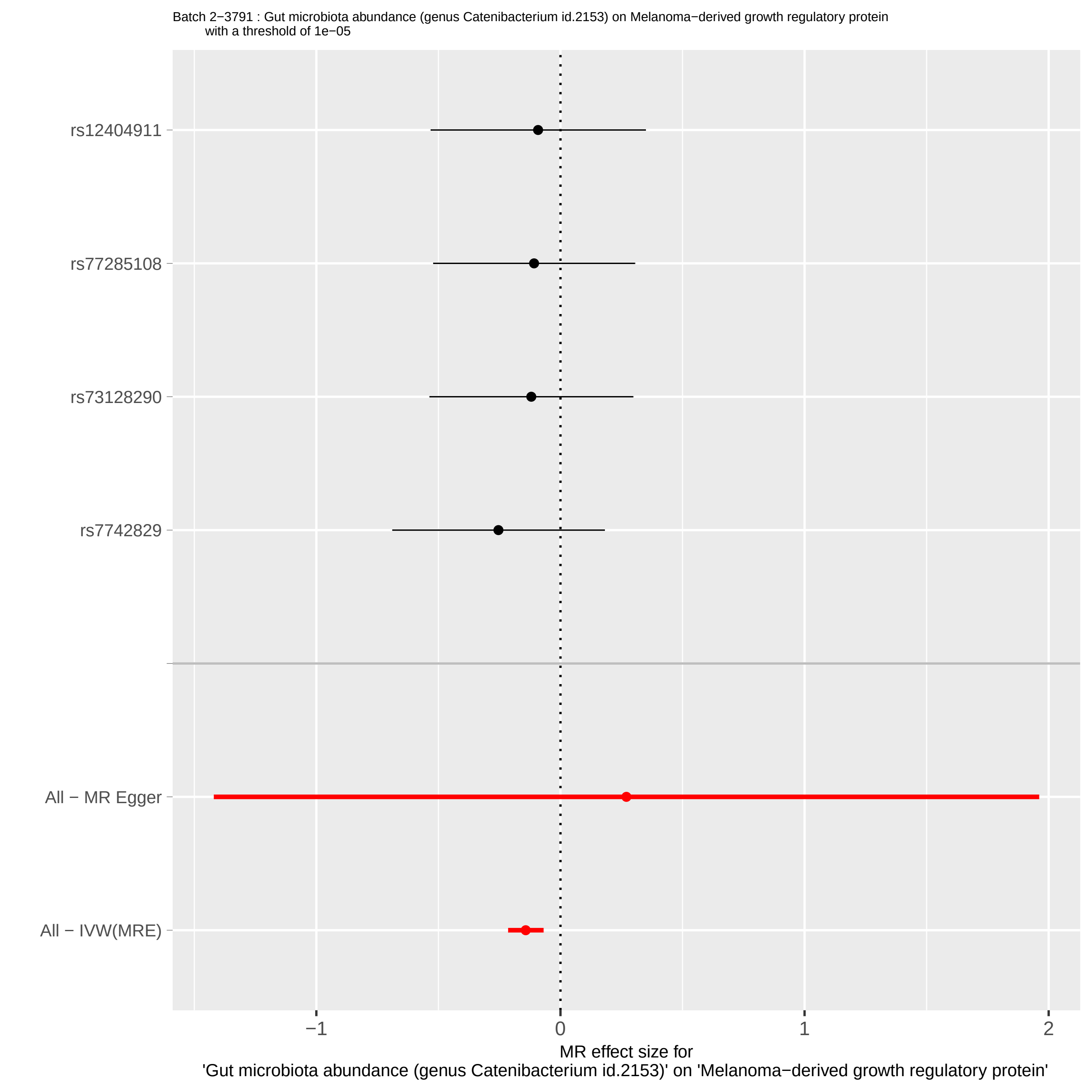
Batch 2−3791 : Gut microbiota abundance (genus Catenibacterium id.2153) on Melanoma−derived growth regulatory protein
with a threshold of 1e−05
rs12404911
rs77285108
rs73128290
rs7742829
All − MR Egger
All − IVW(MRE)
−1
0
1
2
MR effect size for
'Gut microbiota abundance (genus Catenibacterium id.2153)' on 'Melanoma−derived growth regulatory protein'

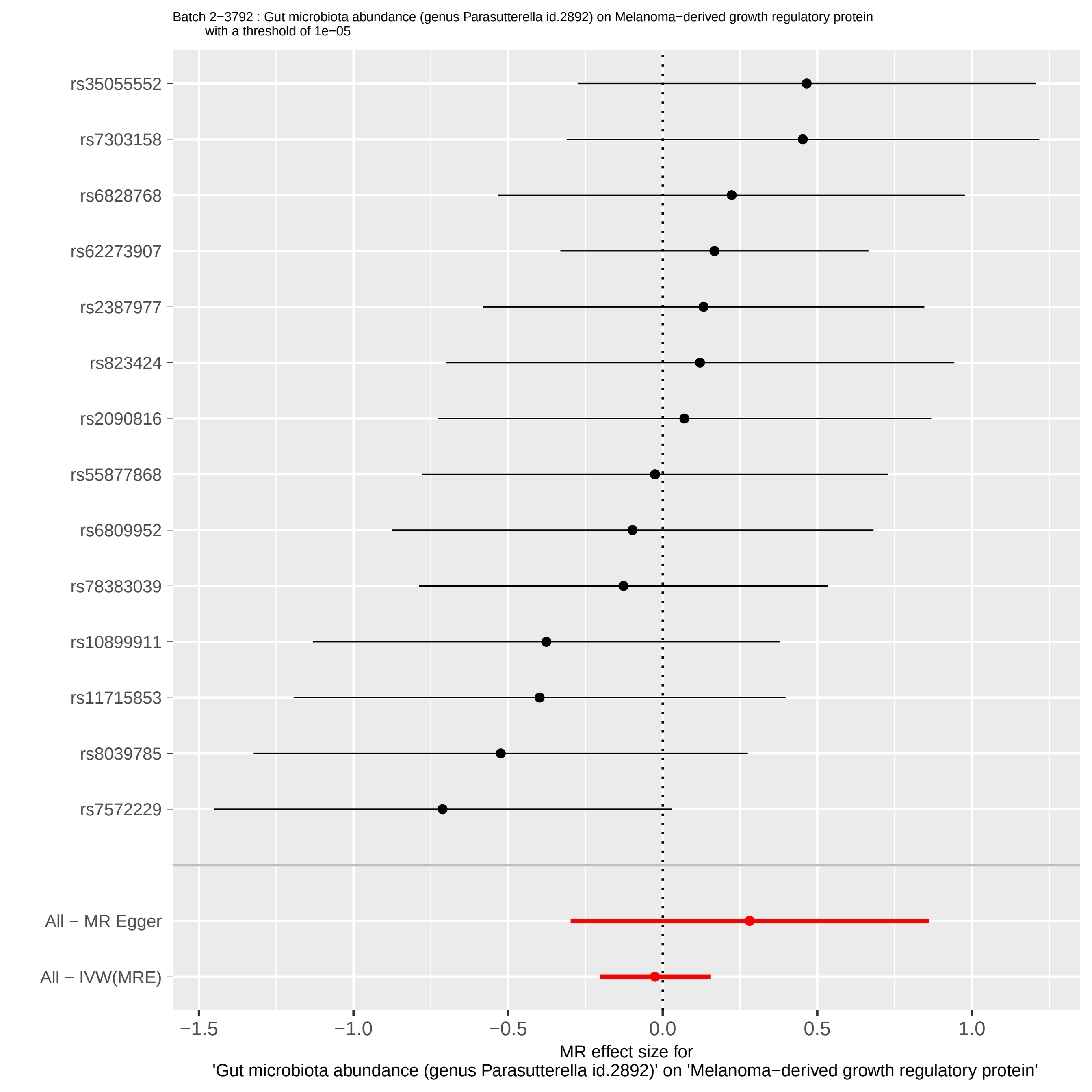
Batch 2−3792 : Gut microbiota abundance (genus Parasutterella id.2892) on Melanoma−derived growth regulatory protein
with a threshold of 1e−05
rs35055552
rs7303158
rs6828768
rs62273907
rs2387977
rs823424
rs2090816
rs55877868
rs6809952
rs78383039
rs10899911
rs11715853
rs8039785
rs7572229
All − MR Egger
All − IVW(MRE)
−1.5
−1.0
−0.5
0.0
0.5
1.0
MR effect size for
'Gut microbiota abundance (genus Parasutterella id.2892)' on 'Melanoma−derived growth regulatory protein'

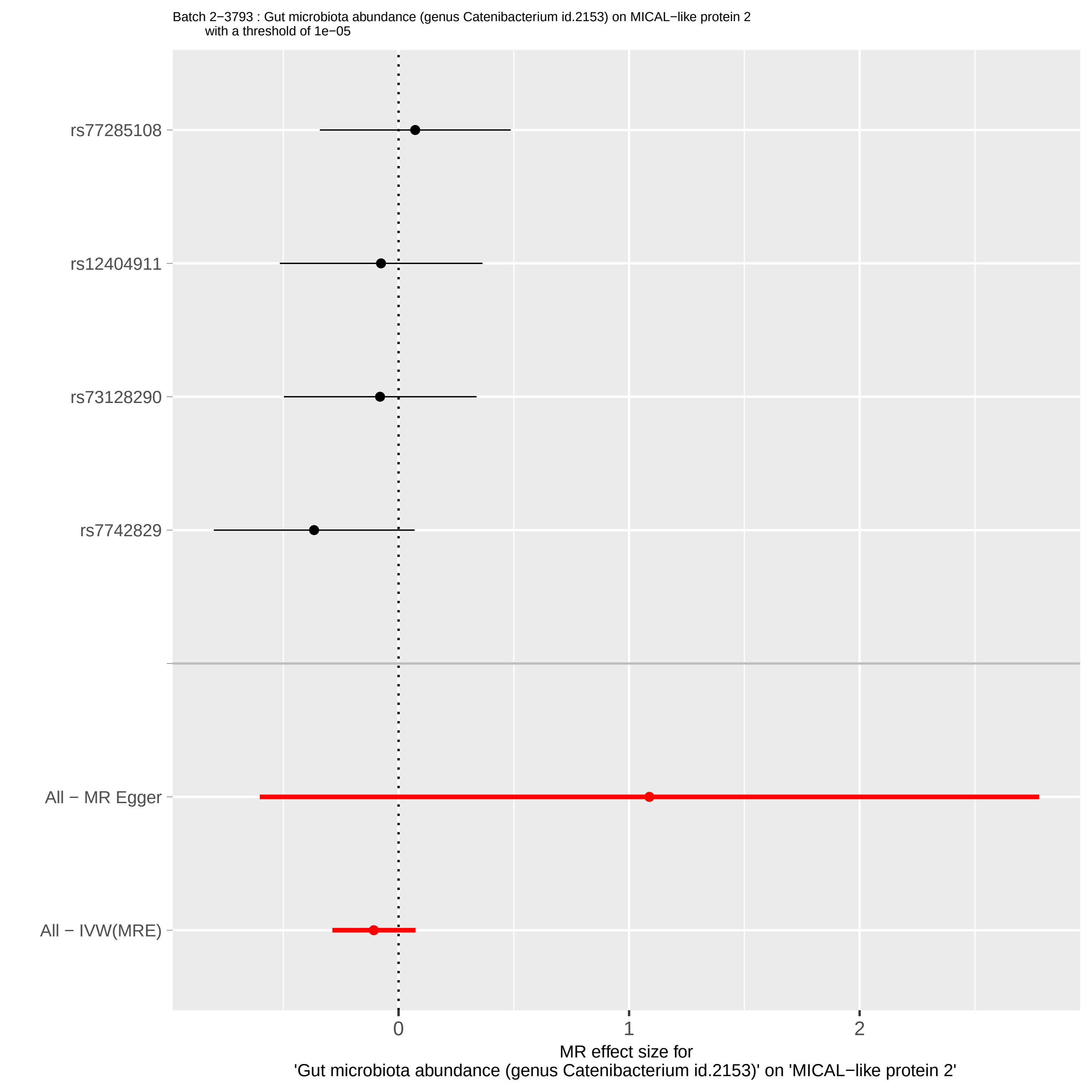

Batch 2−3793 : Gut microbiota abundance (genus Catenibacterium id.2153) on MICAL−like protein 2
with a threshold of 1e−05
rs77285108
rs12404911
rs73128290
rs7742829
All − MR Egger
All − IVW(MRE)
0
1
2
MR effect size for
'Gut microbiota abundance (genus Catenibacterium id.2153)' on 'MICAL−like protein 2'

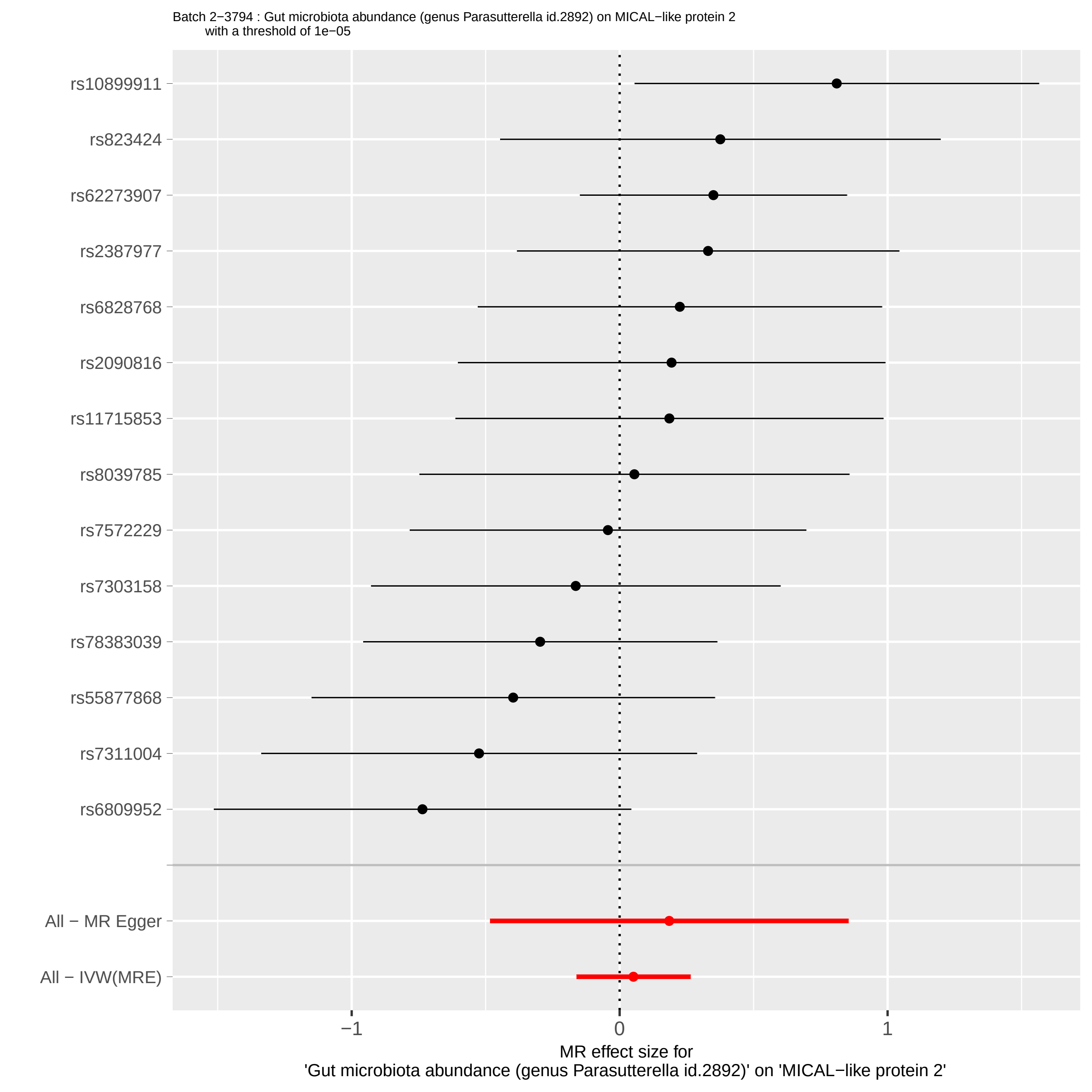

Batch 2−3794 : Gut microbiota abundance (genus Parasutterella id.2892) on MICAL−like protein 2
with a threshold of 1e−05
rs10899911
rs823424
rs62273907
rs2387977
rs6828768
rs2090816
rs11715853
rs8039785
rs7572229
rs7303158
rs78383039
rs55877868
rs7311004
rs6809952
All − MR Egger
All − IVW(MRE)
−1
0
1
MR effect size for
'Gut microbiota abundance (genus Parasutterella id.2892)' on 'MICAL−like protein 2'

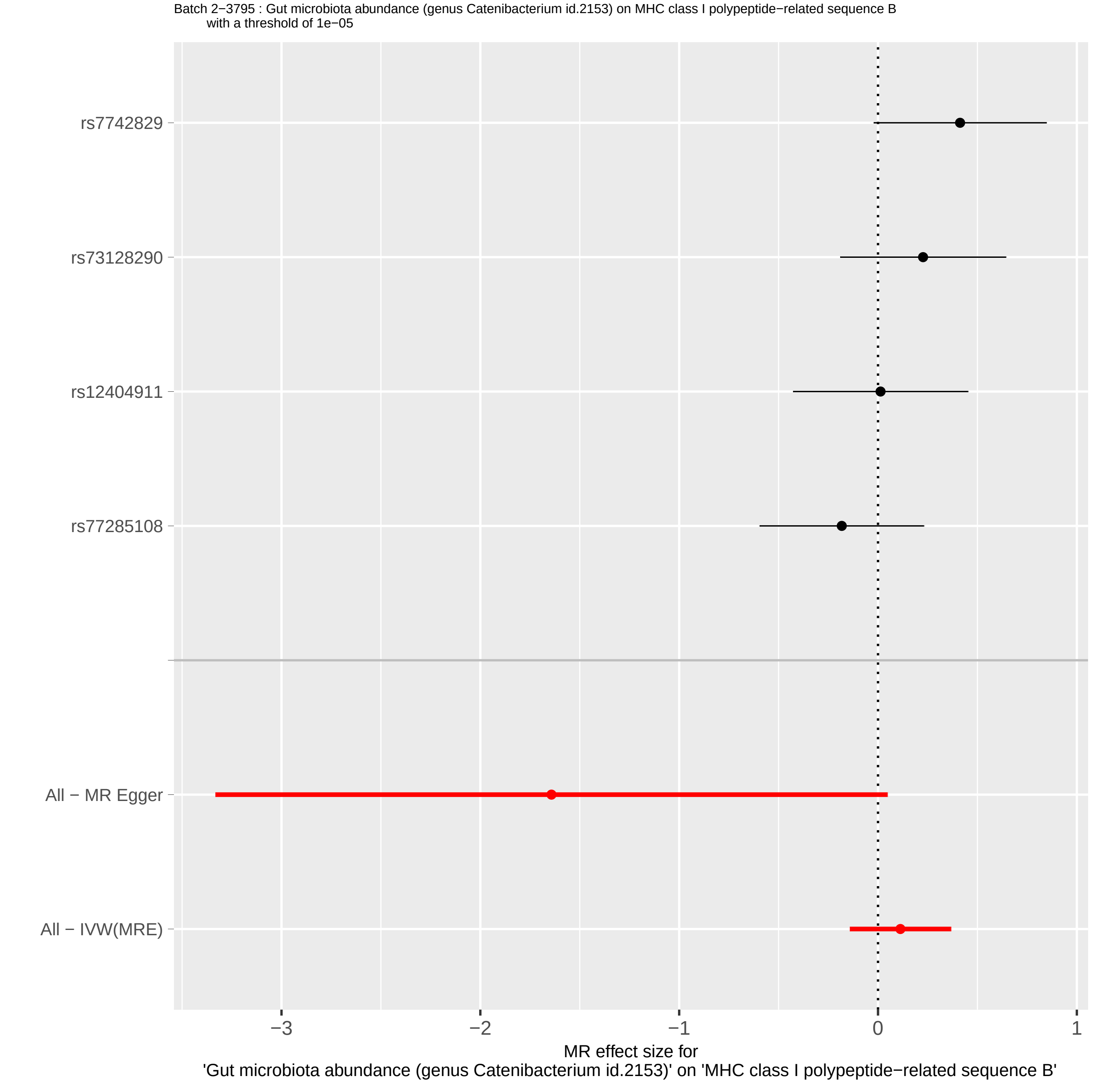

Batch 2−3795 : Gut microbiota abundance (genus Catenibacterium id.2153) on MHC class I polypeptide−related sequence B with a threshold of 1e−05
rs7742829
rs73128290
rs12404911
rs77285108
All − MR Egger
All − IVW(MRE)
−3
−2
−1
0
1
MR effect size for
'Gut microbiota abundance (genus Catenibacterium id.2153)' on 'MHC class I polypeptide−related sequence B'

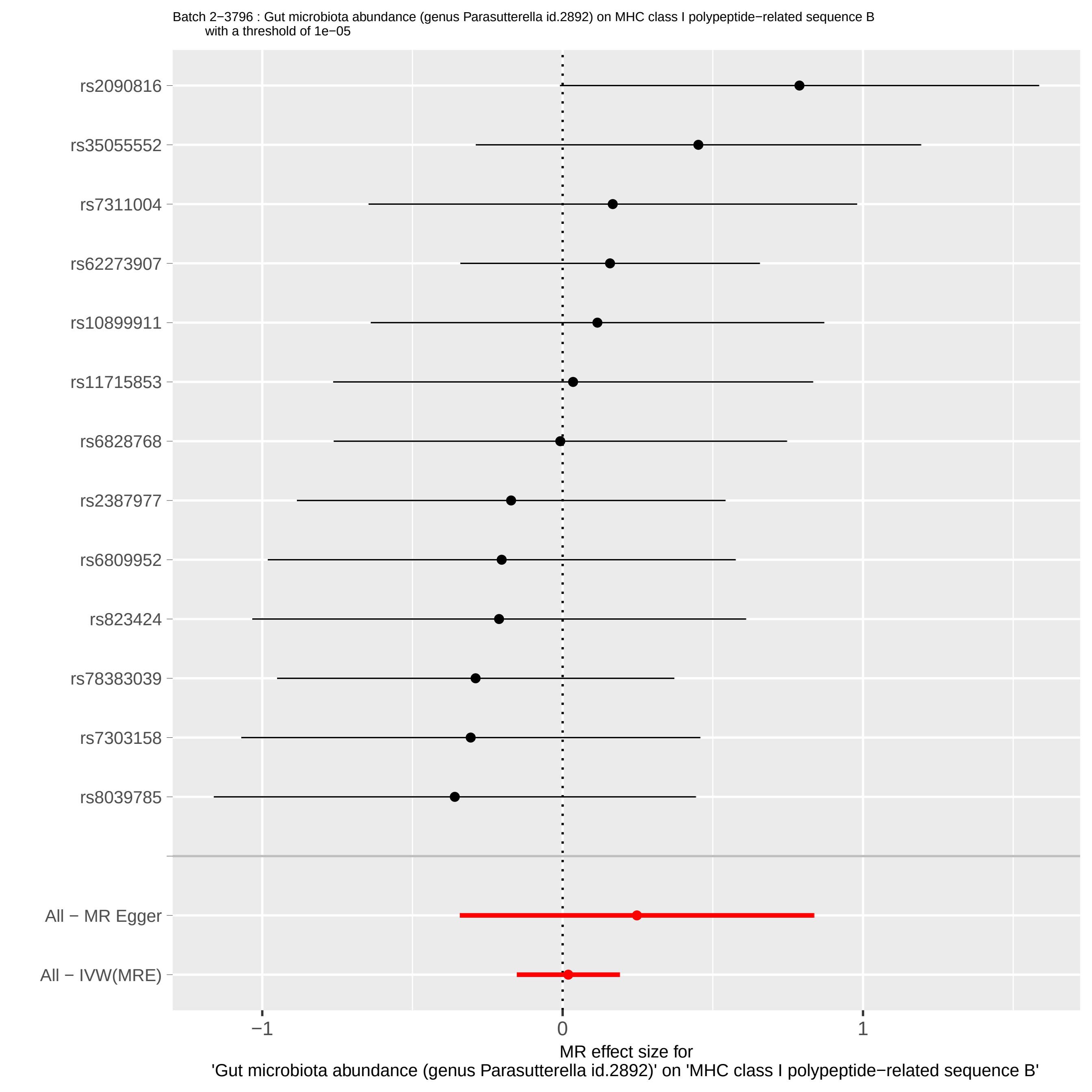
Batch 2−3796 : Gut microbiota abundance (genus Parasutterella id.2892) on MHC class I polypeptide−related sequence B
with a threshold of 1e−05
rs2090816
rs35055552
rs7311004
rs62273907
rs10899911
rs11715853
rs6828768
rs2387977
rs6809952
rs823424
rs78383039
rs7303158
rs8039785
All − MR Egger
All − IVW(MRE)
−1
0
1
MR effect size for
'Gut microbiota abundance (genus Parasutterella id.2892)' on 'MHC class I polypeptide−related sequence B'

Batch 2−3797 : Gut microbiota abundance (genus Catenibacterium id.2153) on Probable E3 ubiquitin−protein ligase MID2
with a threshold of 1e−05

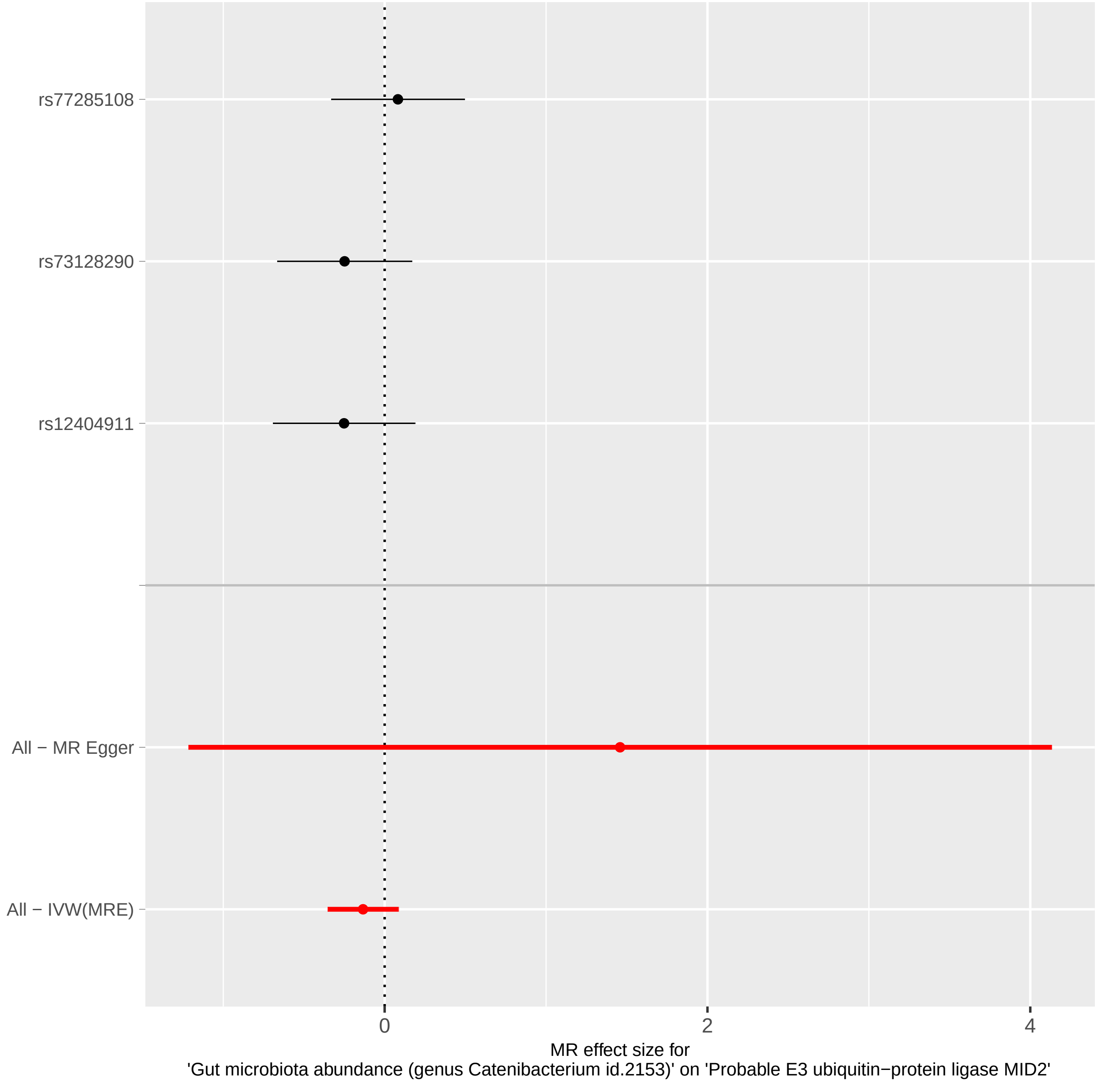

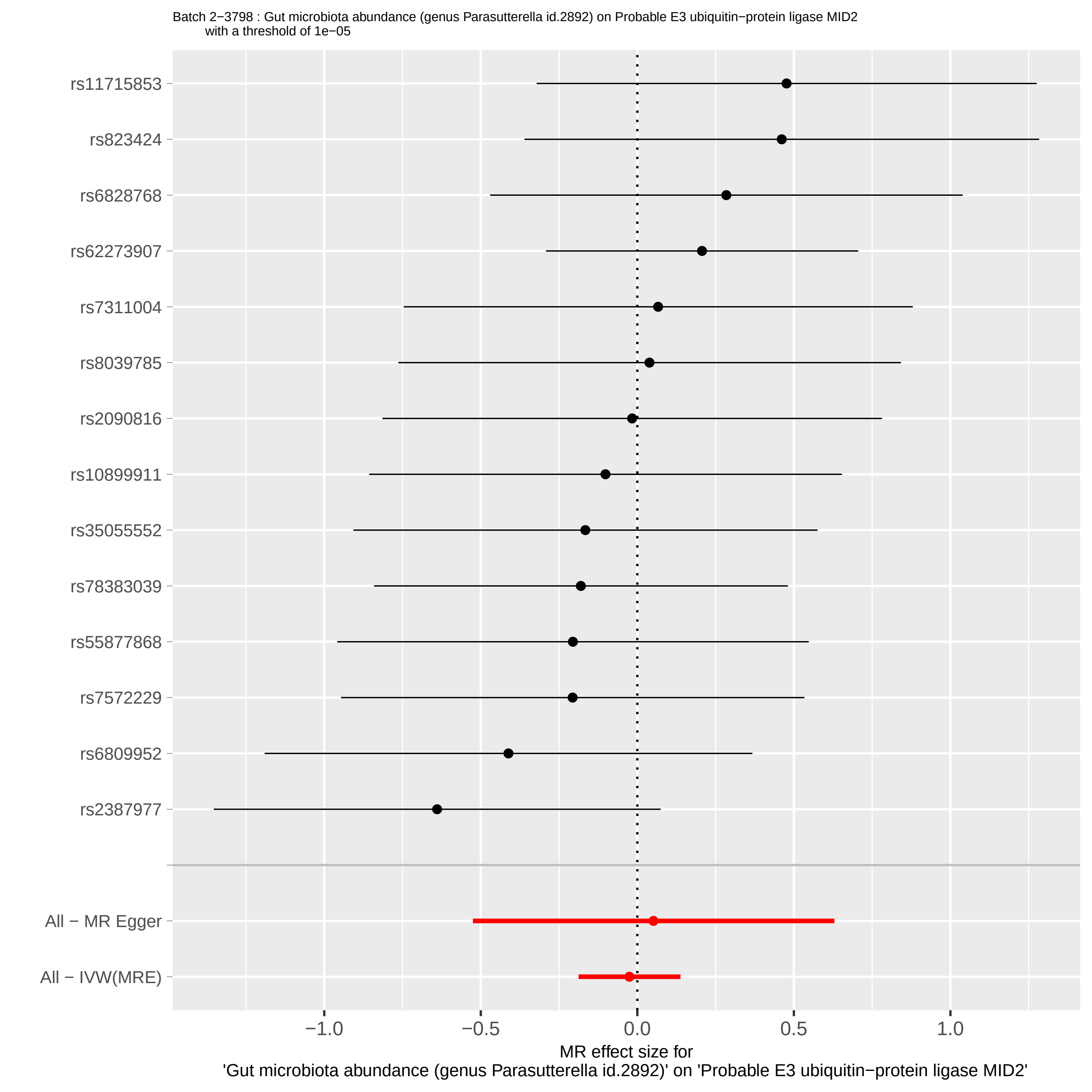

Batch 2−3798 : Gut microbiota abundance (genus Parasutterella id.2892) on Probable E3 ubiquitin−protein ligase MID2
with a threshold of 1e−05
rs11715853
rs823424
rs6828768
rs62273907
rs7311004
rs8039785
rs2090816
rs10899911
rs35055552
rs78383039
rs55877868
rs7572229
rs6809952
rs2387977
All − MR Egger
All − IVW(MRE)
−1.0
−0.5
0.0
0.5
1.0
MR effect size for
'Gut microbiota abundance (genus Parasutterella id.2892)' on 'Probable E3 ubiquitin−protein ligase MID2'

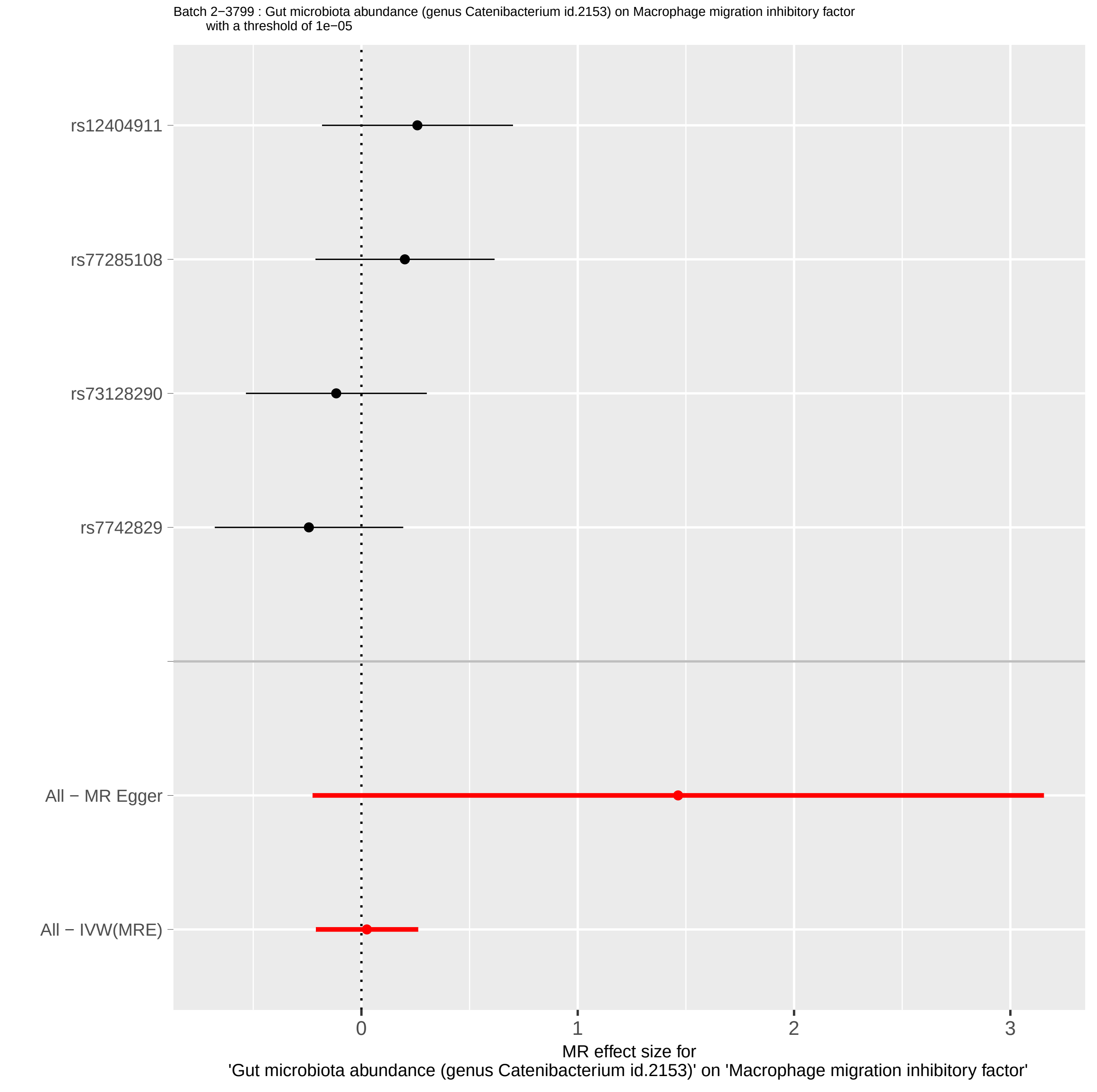
Batch 2−3799 : Gut microbiota abundance (genus Catenibacterium id.2153) on Macrophage migration inhibitory factor
with a threshold of 1e−05
rs12404911
rs77285108
rs73128290
rs7742829
All − MR Egger
All − IVW(MRE)
0
1
2
3
MR effect size for
'Gut microbiota abundance (genus Catenibacterium id.2153)' on 'Macrophage migration inhibitory factor'

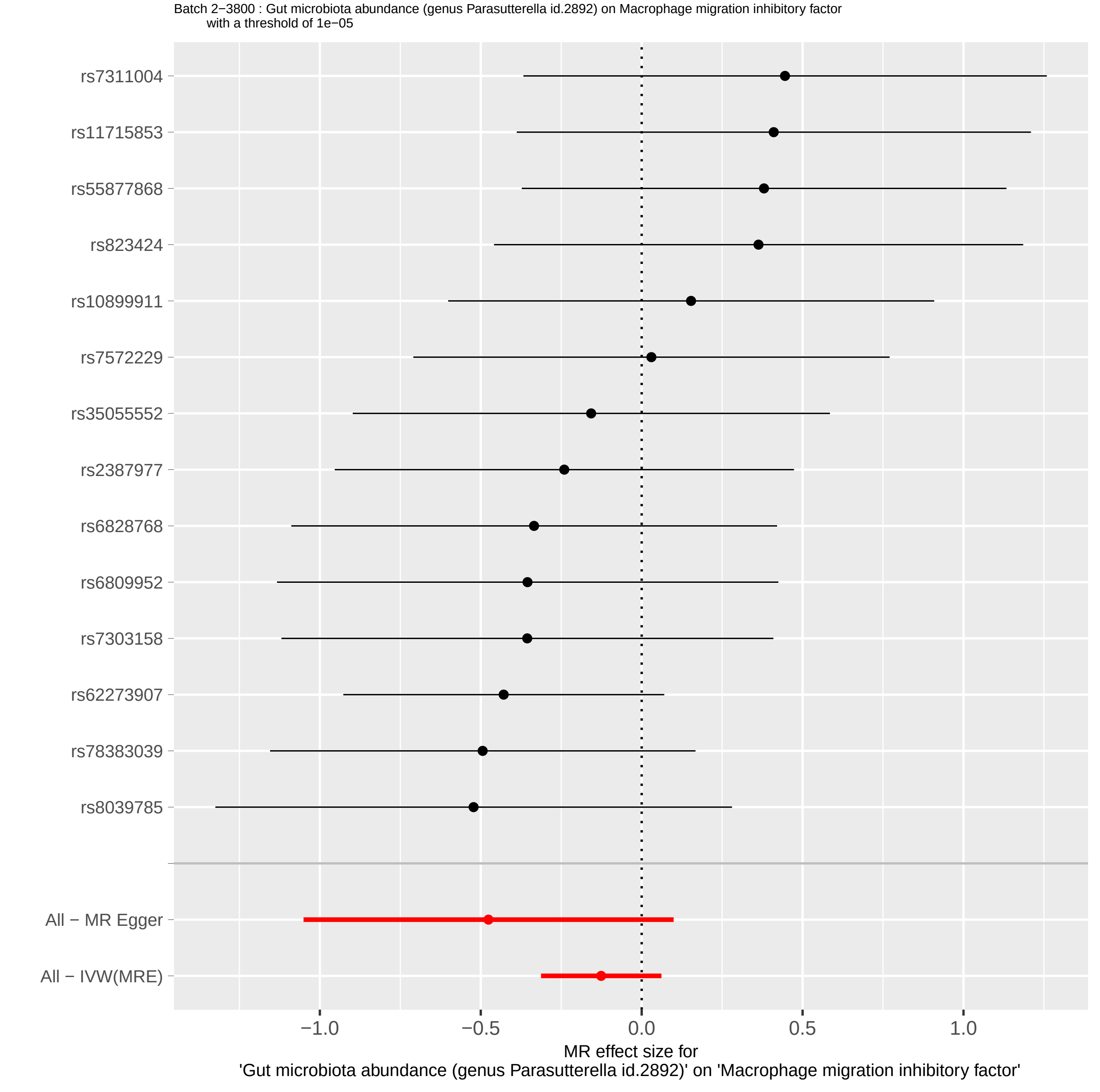
Batch 2−3800 : Gut microbiota abundance (genus Parasutterella id.2892) on Macrophage migration inhibitory factor
with a threshold of 1e−05
rs7311004
rs11715853
rs55877868
rs823424
rs10899911
rs7572229
rs35055552
rs2387977
rs6828768
rs6809952
rs7303158
rs62273907
rs78383039
rs8039785
All − MR Egger
All − IVW(MRE)
−1.0
−0.5
0.0
0.5
1.0
MR effect size for
'Gut microbiota abundance (genus Parasutterella id.2892)' on 'Macrophage migration inhibitory factor'

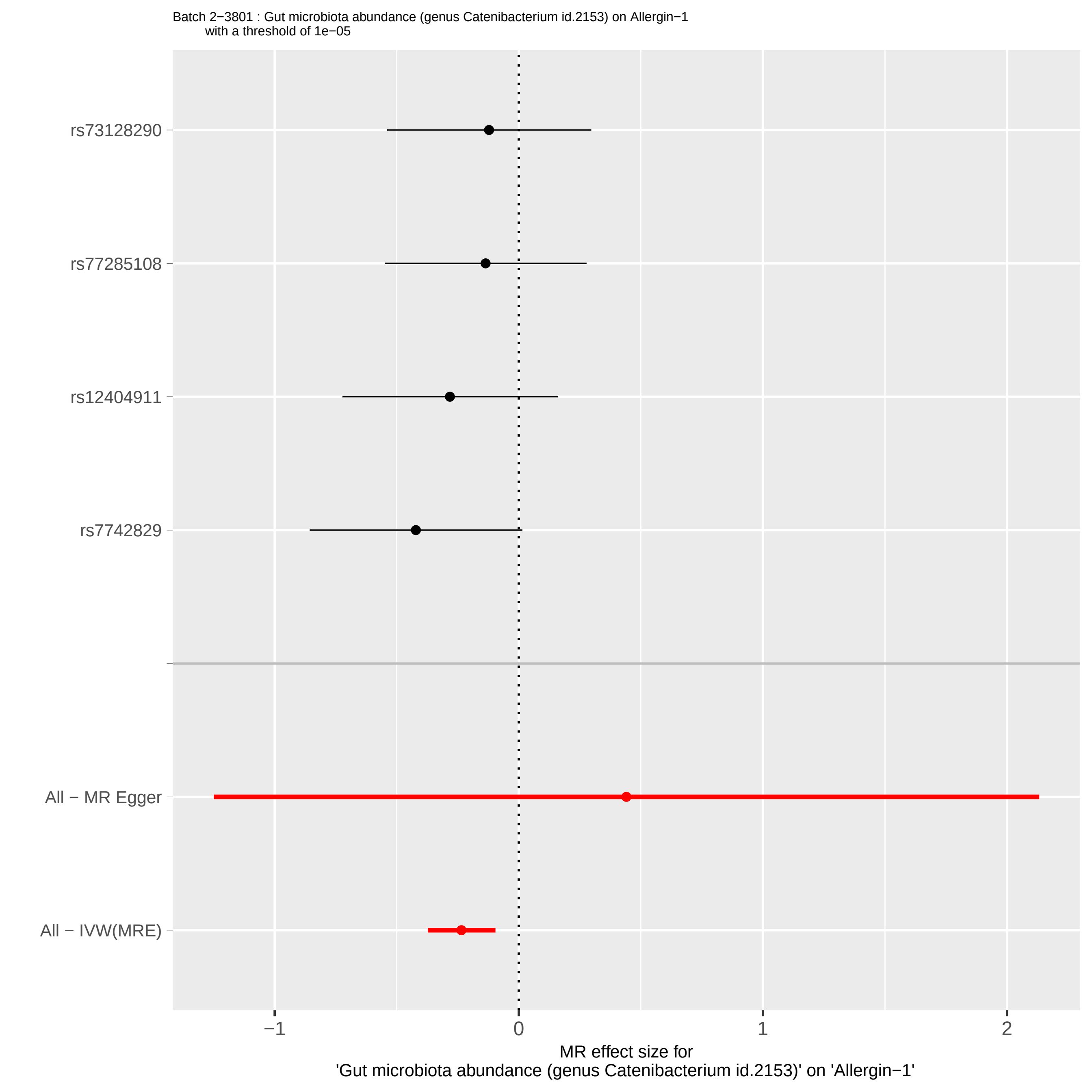
Batch 2−3801 : Gut microbiota abundance (genus Catenibacterium id.2153) on Allergin−1
with a threshold of 1e−05
rs73128290
rs77285108
rs12404911
rs7742829
All − MR Egger
All − IVW(MRE)
−1
0
1
2
MR effect size for
'Gut microbiota abundance (genus Catenibacterium id.2153)' on 'Allergin−1'

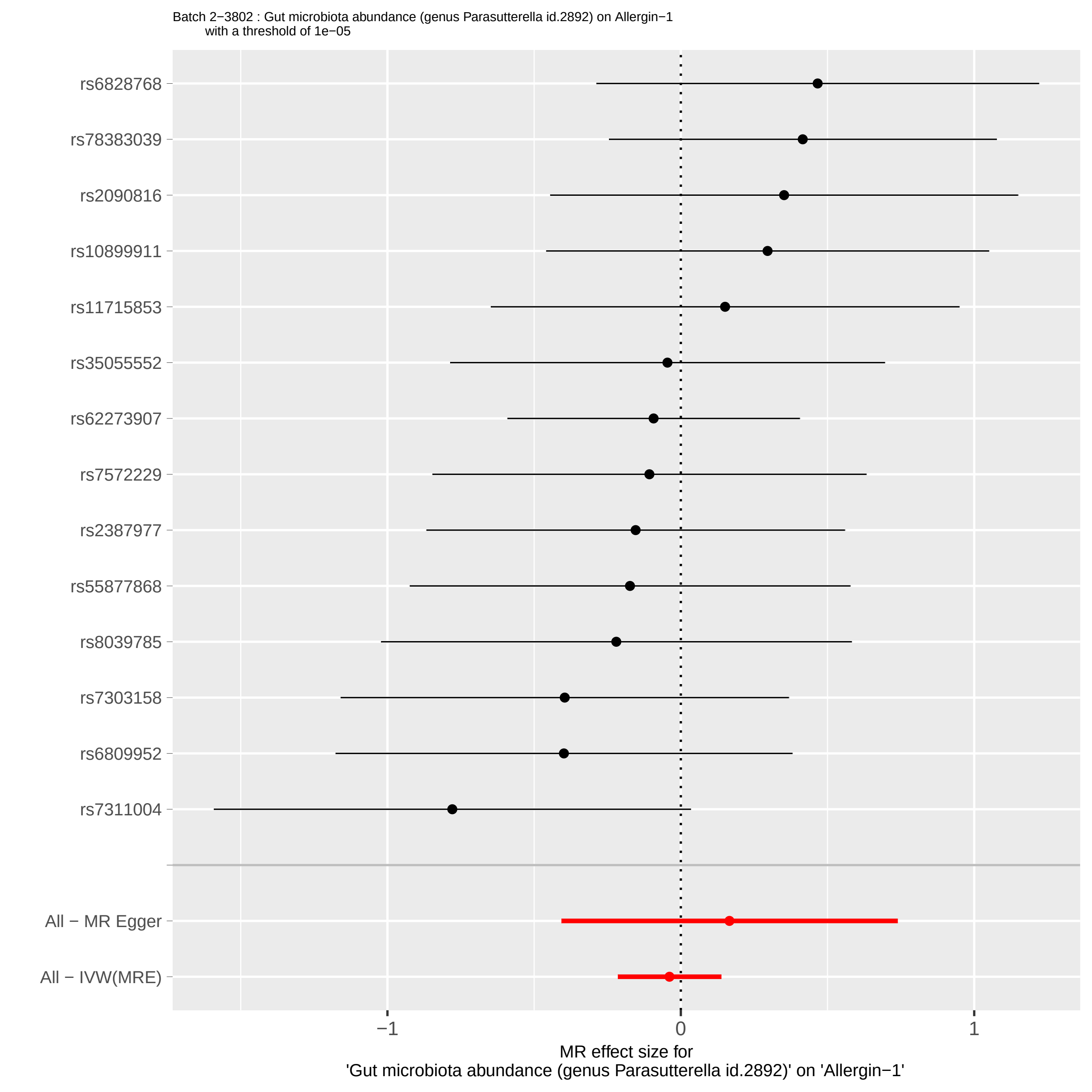

Batch 2−3802 : Gut microbiota abundance (genus Parasutterella id.2892) on Allergin−1
with a threshold of 1e−05
rs6828768
rs78383039
rs2090816
rs10899911
rs11715853
rs35055552
rs62273907
rs7572229
rs2387977
rs55877868
rs8039785
rs7303158
rs6809952
rs7311004
All − MR Egger
All − IVW(MRE)
−1
0
1
MR effect size for
'Gut microbiota abundance (genus Parasutterella id.2892)' on 'Allergin−1'

Batch 2−3803 : Gut microbiota abundance (genus Catenibacterium id.2153) on MICOS complex subunit MIC10
with a threshold of 1e−05

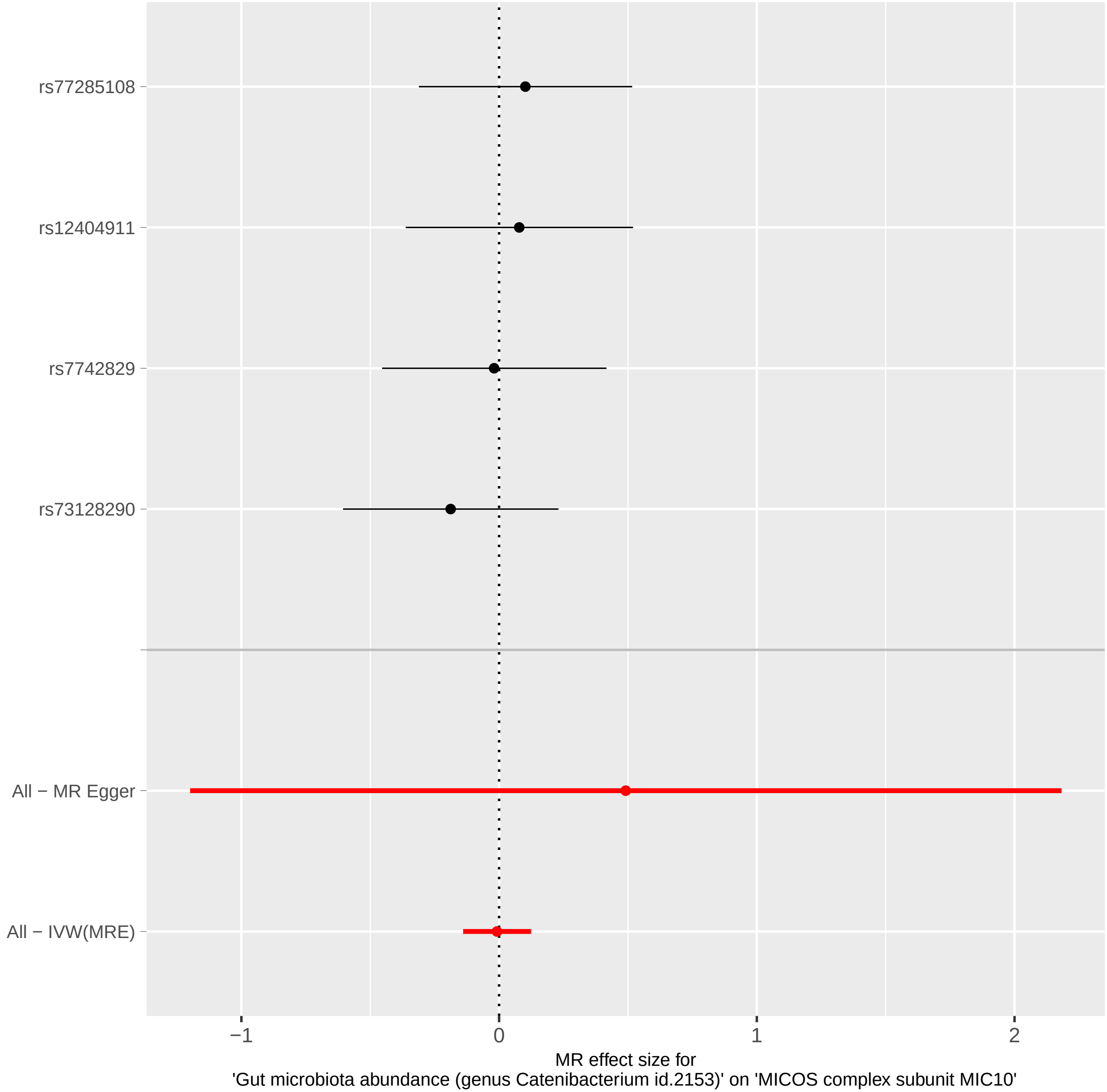

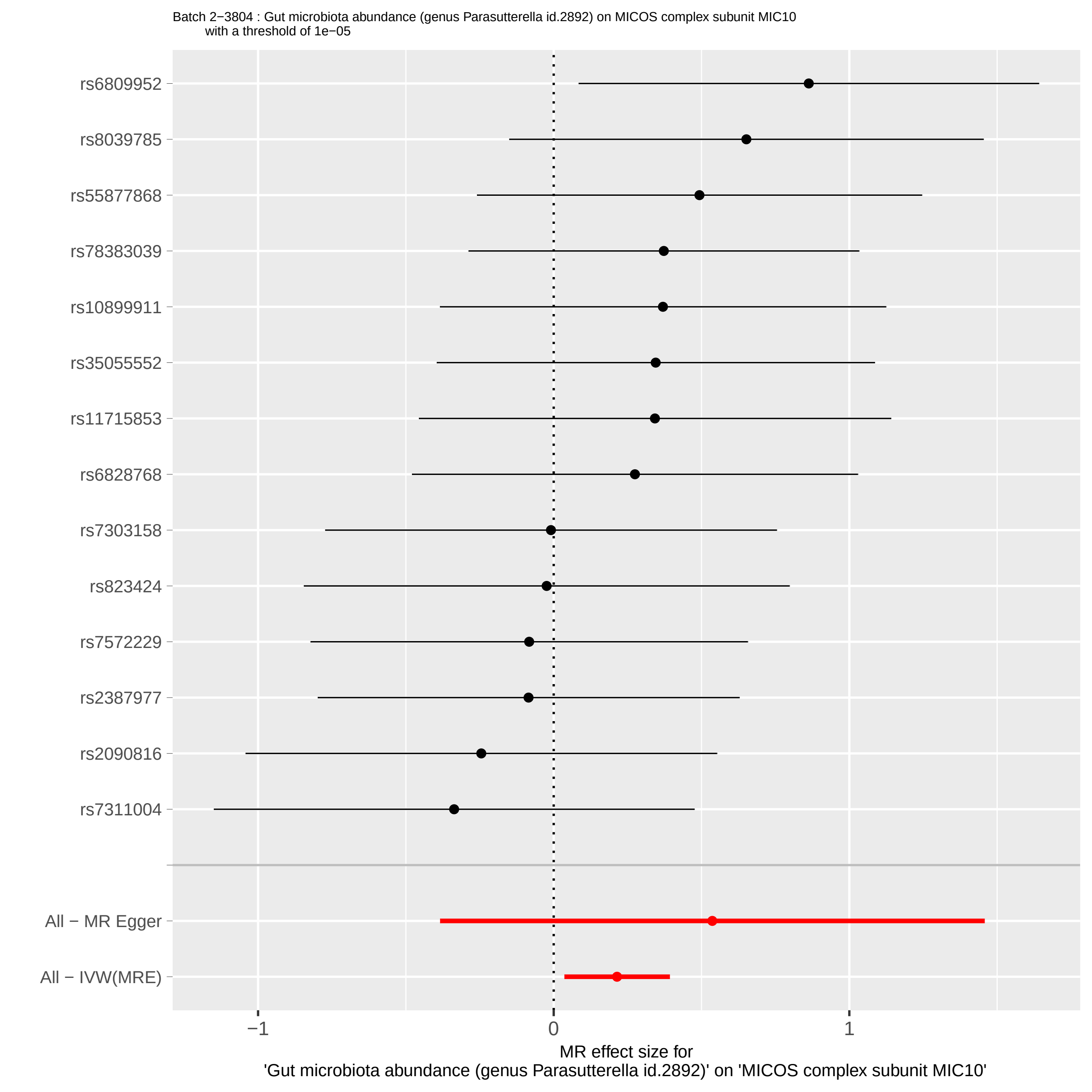

Batch 2−3804 : Gut microbiota abundance (genus Parasutterella id.2892) on MICOS complex subunit MIC10
with a threshold of 1e−05
rs6809952
rs8039785
rs55877868
rs78383039
rs10899911
rs35055552
rs11715853
rs6828768
rs7303158
rs823424
rs7572229
rs2387977
rs2090816
rs7311004
All − MR Egger
All − IVW(MRE)
−1
0
1
MR effect size for
'Gut microbiota abundance (genus Parasutterella id.2892)' on 'MICOS complex subunit MIC10'

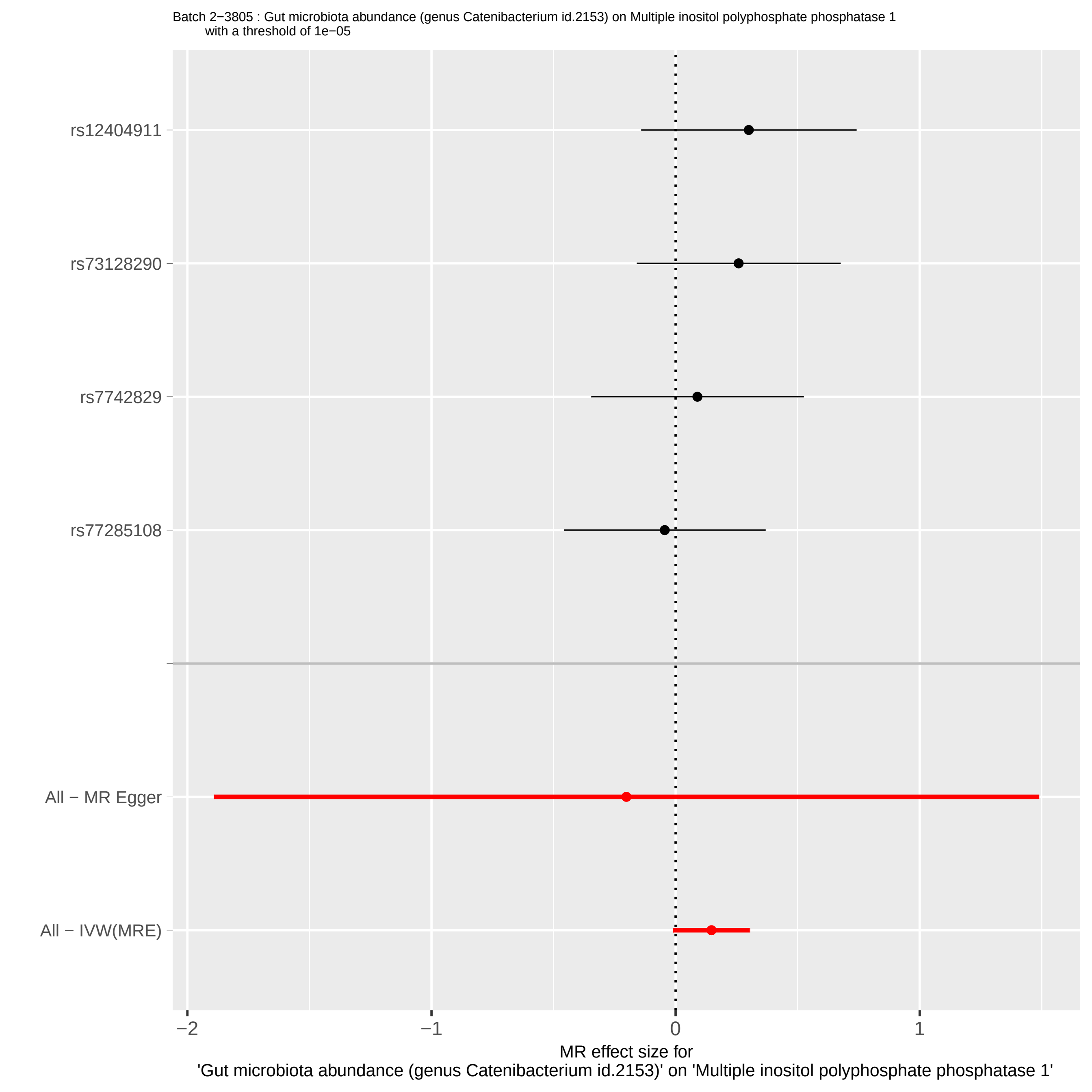
Batch 2−3805 : Gut microbiota abundance (genus Catenibacterium id.2153) on Multiple inositol polyphosphate phosphatase 1
with a threshold of 1e−05
rs12404911
rs73128290
rs7742829
rs77285108
All − MR Egger
All − IVW(MRE)
−2
−1
0
1
MR effect size for
'Gut microbiota abundance (genus Catenibacterium id.2153)' on 'Multiple inositol polyphosphate phosphatase 1'

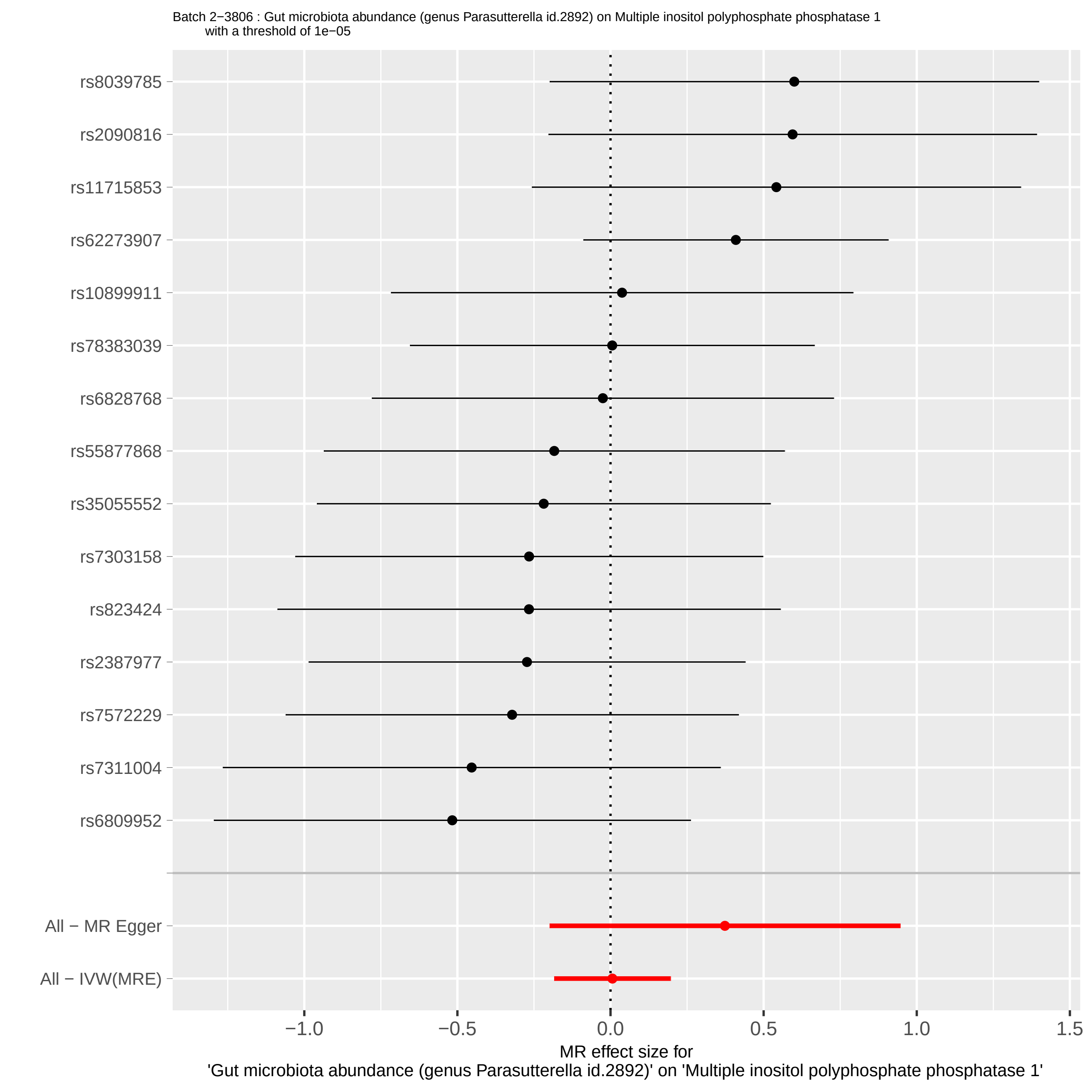

Batch 2−3806 : Gut microbiota abundance (genus Parasutterella id.2892) on Multiple inositol polyphosphate phosphatase 1
with a threshold of 1e−05
rs8039785
rs2090816
rs11715853
rs62273907
rs10899911
rs78383039
rs6828768
rs55877868
rs35055552
rs7303158
rs823424
rs2387977
rs7572229
rs7311004
rs6809952
All − MR Egger
All − IVW(MRE)
−1.0
−0.5
0.0
0.5
1.0
1.5
MR effect size for
'Gut microbiota abundance (genus Parasutterella id.2892)' on 'Multiple inositol polyphosphate phosphatase 1'

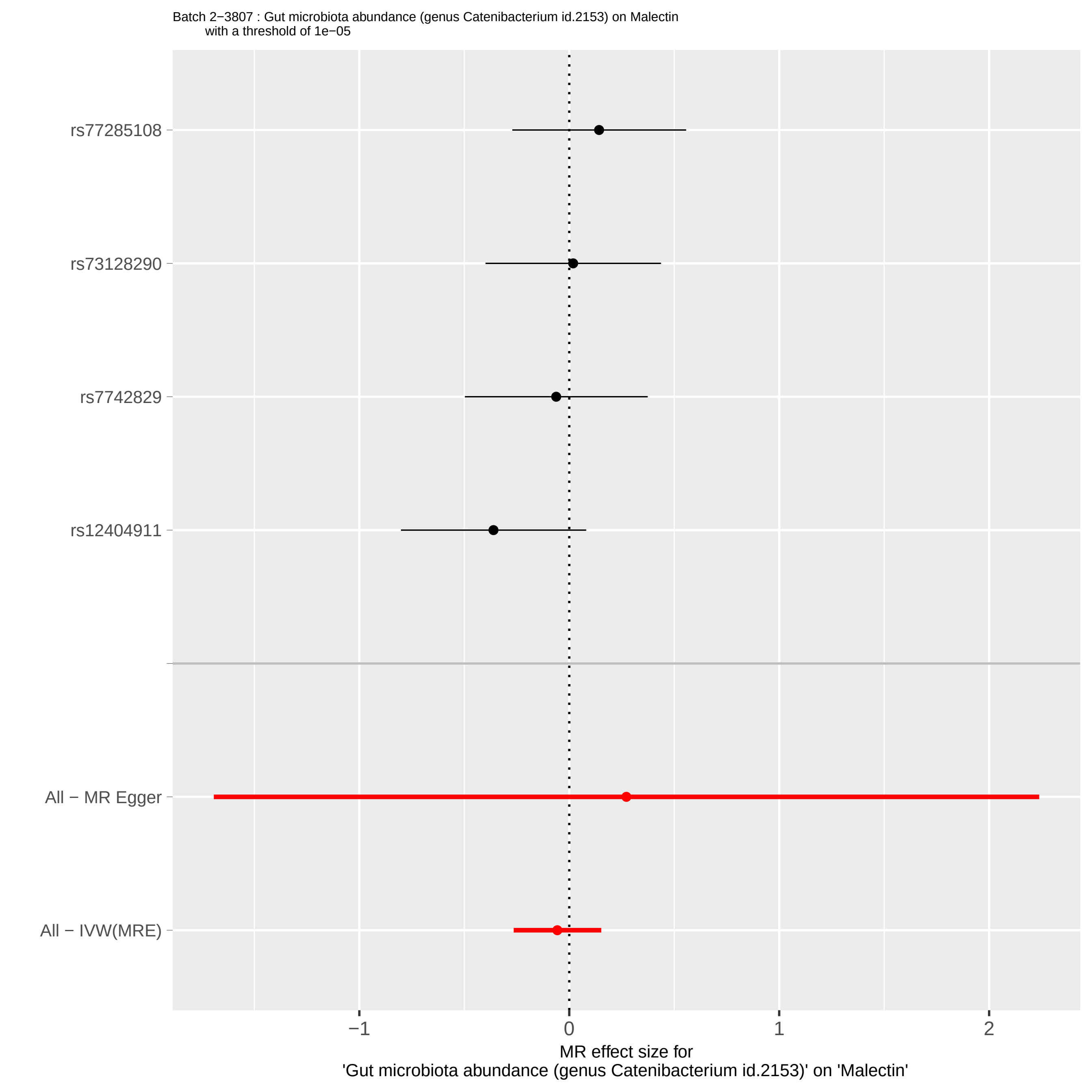
Batch 2−3807 : Gut microbiota abundance (genus Catenibacterium id.2153) on Malectin
with a threshold of 1e−05
rs77285108
rs73128290
rs7742829
rs12404911
All − MR Egger
All − IVW(MRE)
−1
0
1
2
MR effect size for
'Gut microbiota abundance (genus Catenibacterium id.2153)' on 'Malectin'

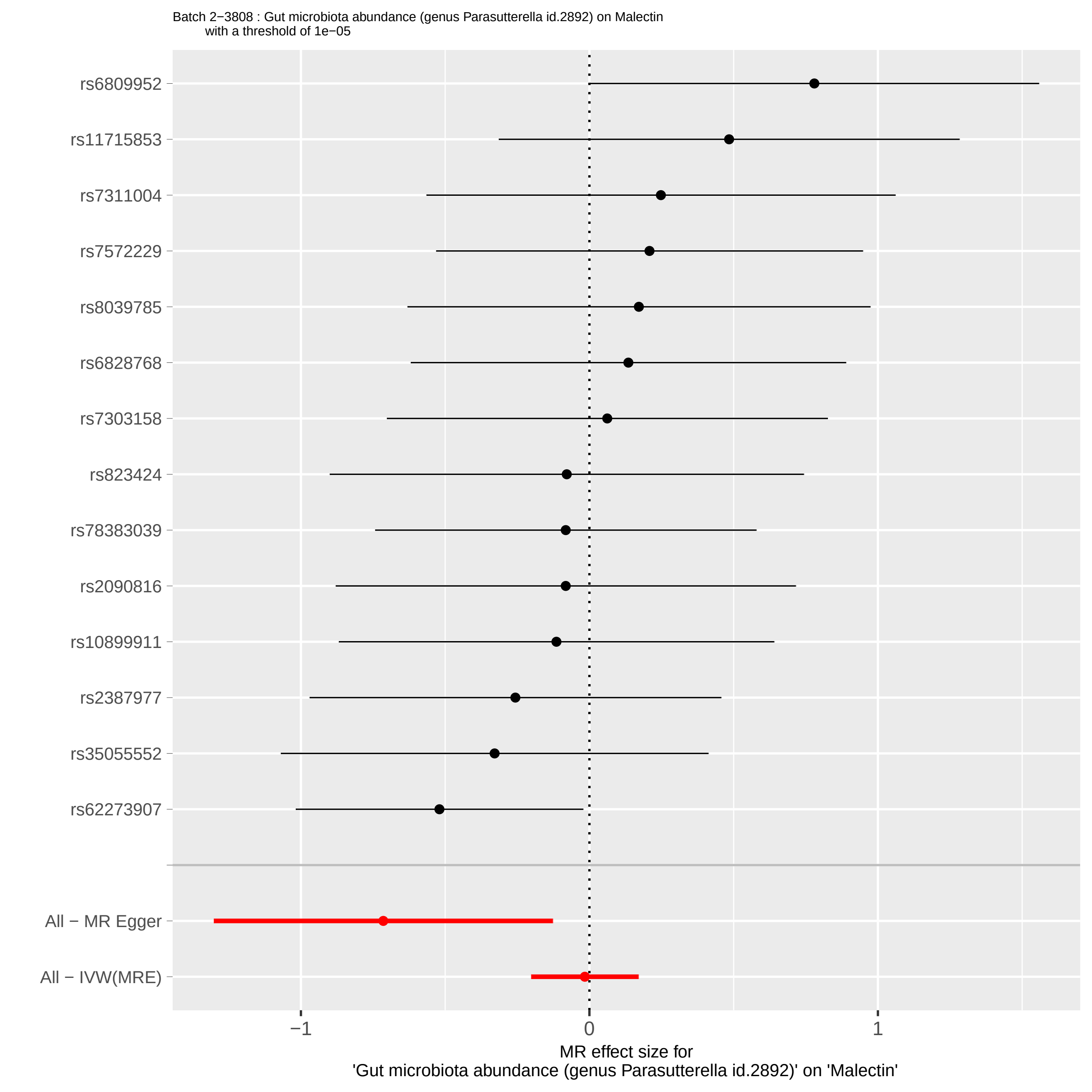
Batch 2−3808 : Gut microbiota abundance (genus Parasutterella id.2892) on Malectin
with a threshold of 1e−05
rs6809952
rs11715853
rs7311004
rs7572229
rs8039785
rs6828768
rs7303158
rs823424
rs78383039
rs2090816
rs10899911
rs2387977
rs35055552
rs62273907
All − MR Egger
All − IVW(MRE)
−1
0
1
MR effect size for
'Gut microbiota abundance (genus Parasutterella id.2892)' on 'Malectin'

Batch 2−3809 : Gut microbiota abundance (genus Catenibacterium id.2153) on Histone−lysine N−methyltransferase 2D
with a threshold of 1e−05

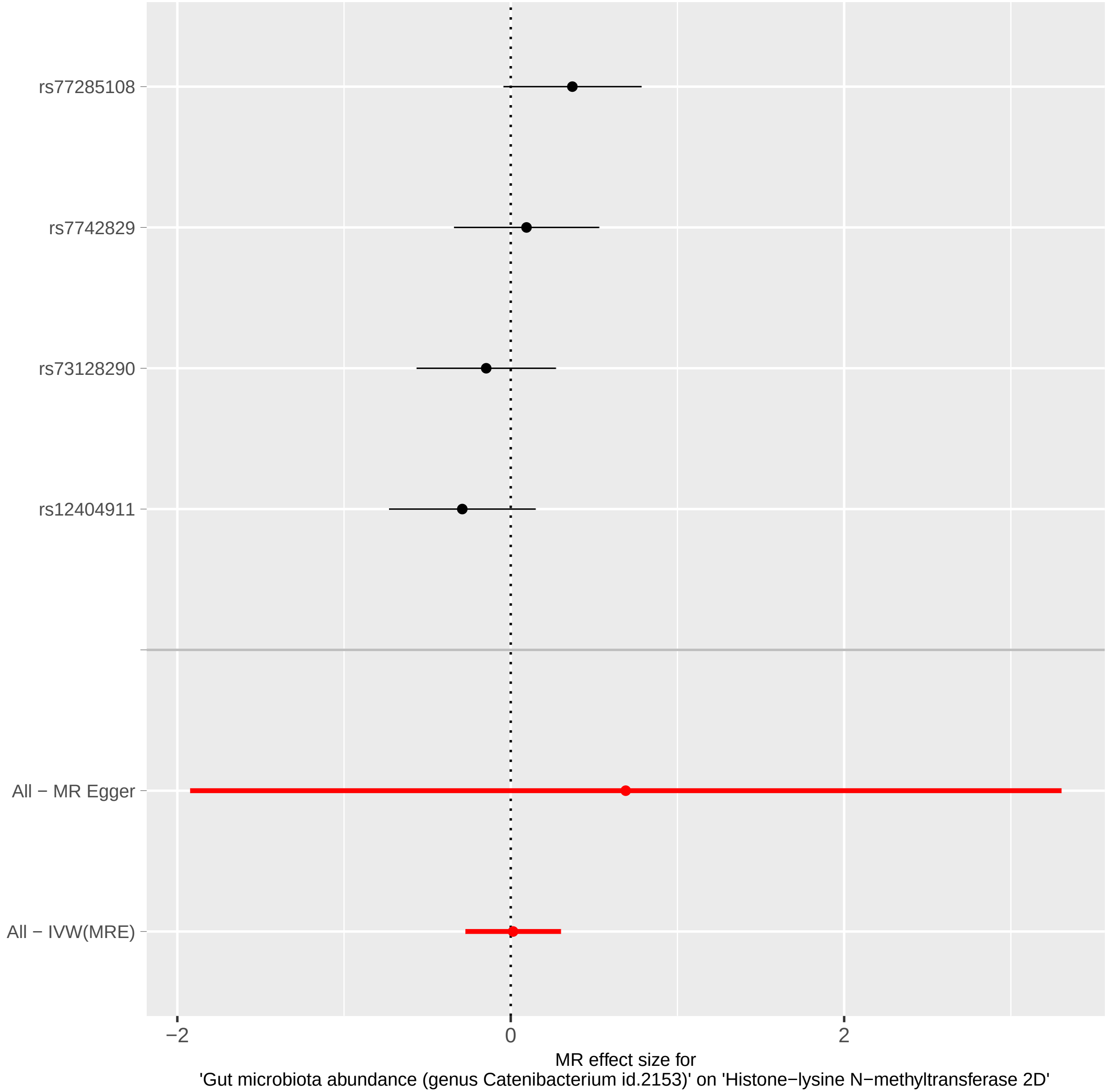

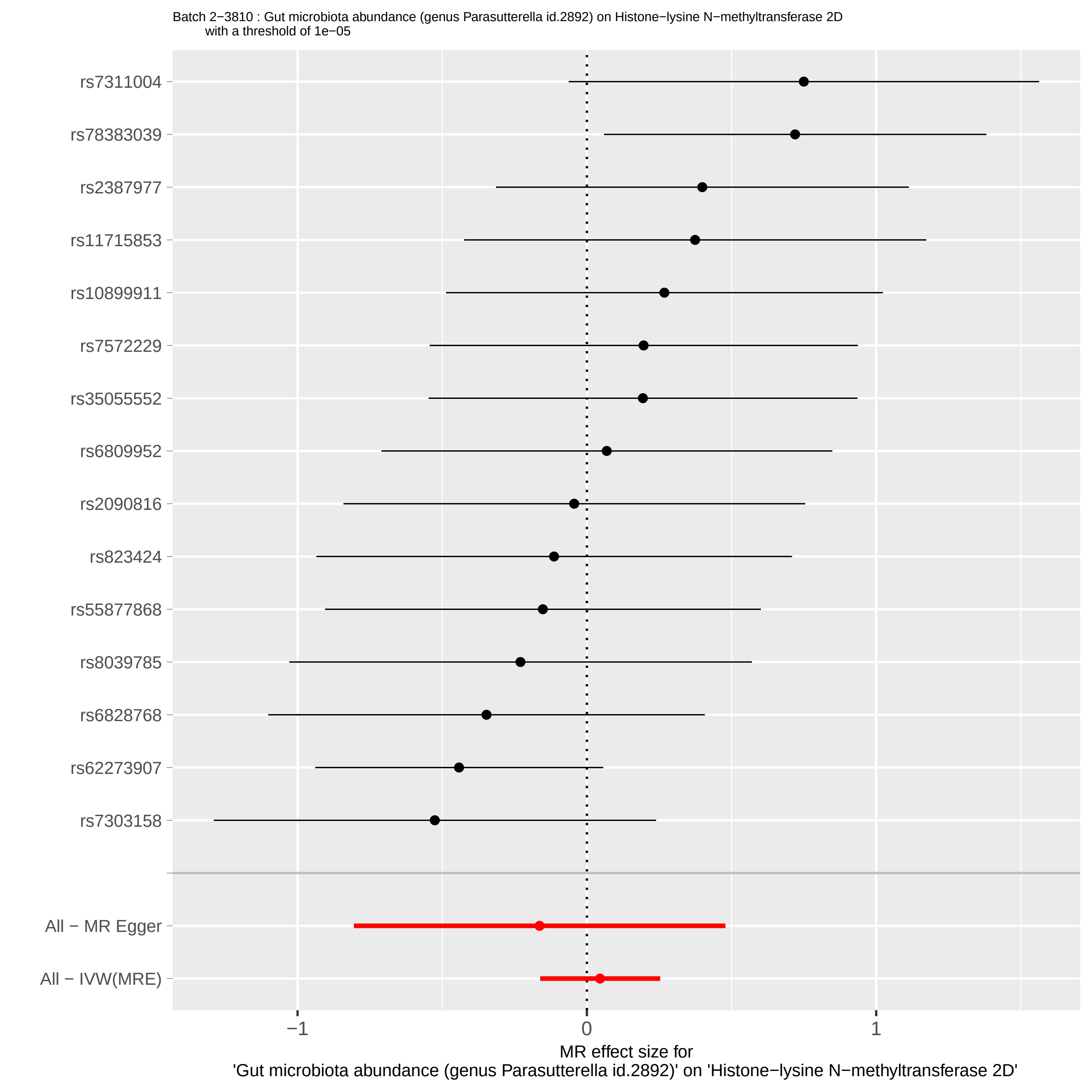
Batch 2−3810 : Gut microbiota abundance (genus Parasutterella id.2892) on Histone−lysine N−methyltransferase 2D
with a threshold of 1e−05
rs7311004
rs78383039
rs2387977
rs11715853
rs10899911
rs7572229
rs35055552
rs6809952
rs2090816
rs823424
rs55877868
rs8039785
rs6828768
rs62273907
rs7303158
All − MR Egger
All − IVW(MRE)
−1
0
1
MR effect size for
'Gut microbiota abundance (genus Parasutterella id.2892)' on 'Histone−lysine N−methyltransferase 2D'

Batch 2−3811 : Gut microbiota abundance (genus Catenibacterium id.2153) on Promotilin
with a threshold of 1e−05

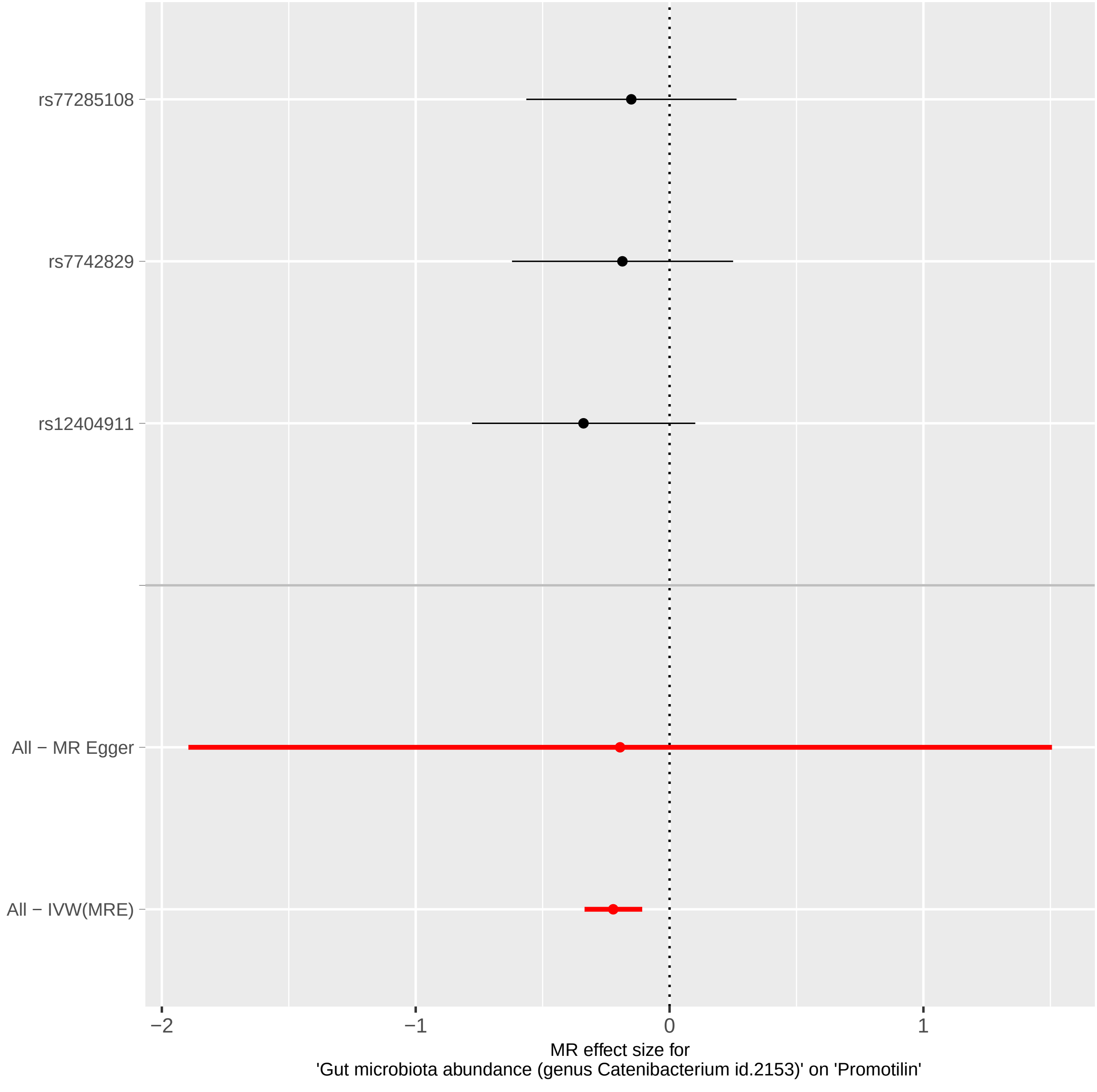

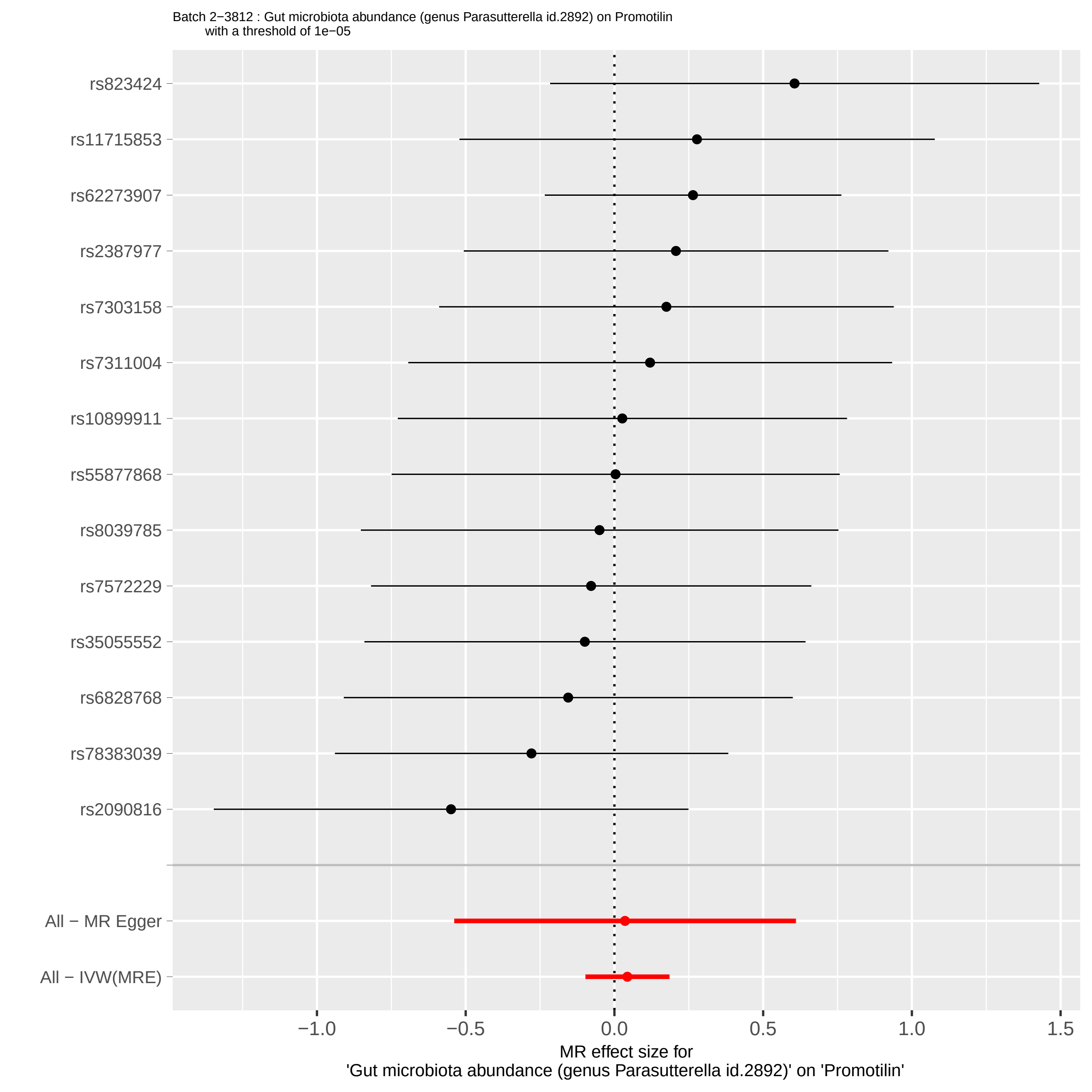
Batch 2−3812 : Gut microbiota abundance (genus Parasutterella id.2892) on Promotilin
with a threshold of 1e−05
rs823424
rs11715853
rs62273907
rs2387977
rs7303158
rs7311004
rs10899911
rs55877868
rs8039785
rs7572229
rs35055552
rs6828768
rs78383039
rs2090816
All − MR Egger
All − IVW(MRE)
−1.0
−0.5
0.0
0.5
1.0
1.5
MR effect size for
'Gut microbiota abundance (genus Parasutterella id.2892)' on 'Promotilin'

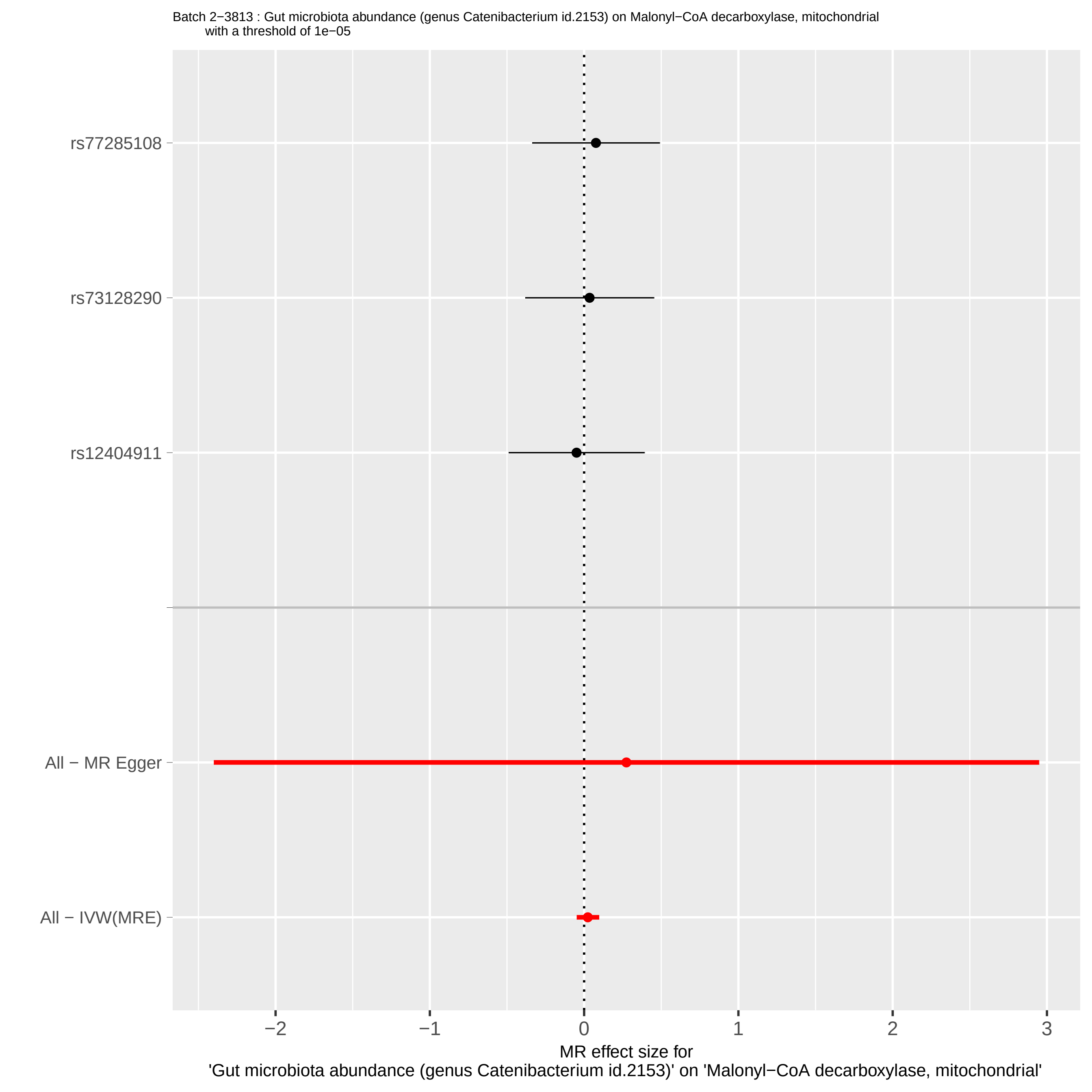

Batch 2−3813 : Gut microbiota abundance (genus Catenibacterium id.2153) on Malonyl−CoA decarboxylase, mitochondrial
with a threshold of 1e−05
rs77285108
rs73128290
rs12404911
All − MR Egger
All − IVW(MRE)
−2
−1
0
1
2
3
MR effect size for
'Gut microbiota abundance (genus Catenibacterium id.2153)' on 'Malonyl−CoA decarboxylase, mitochondrial'

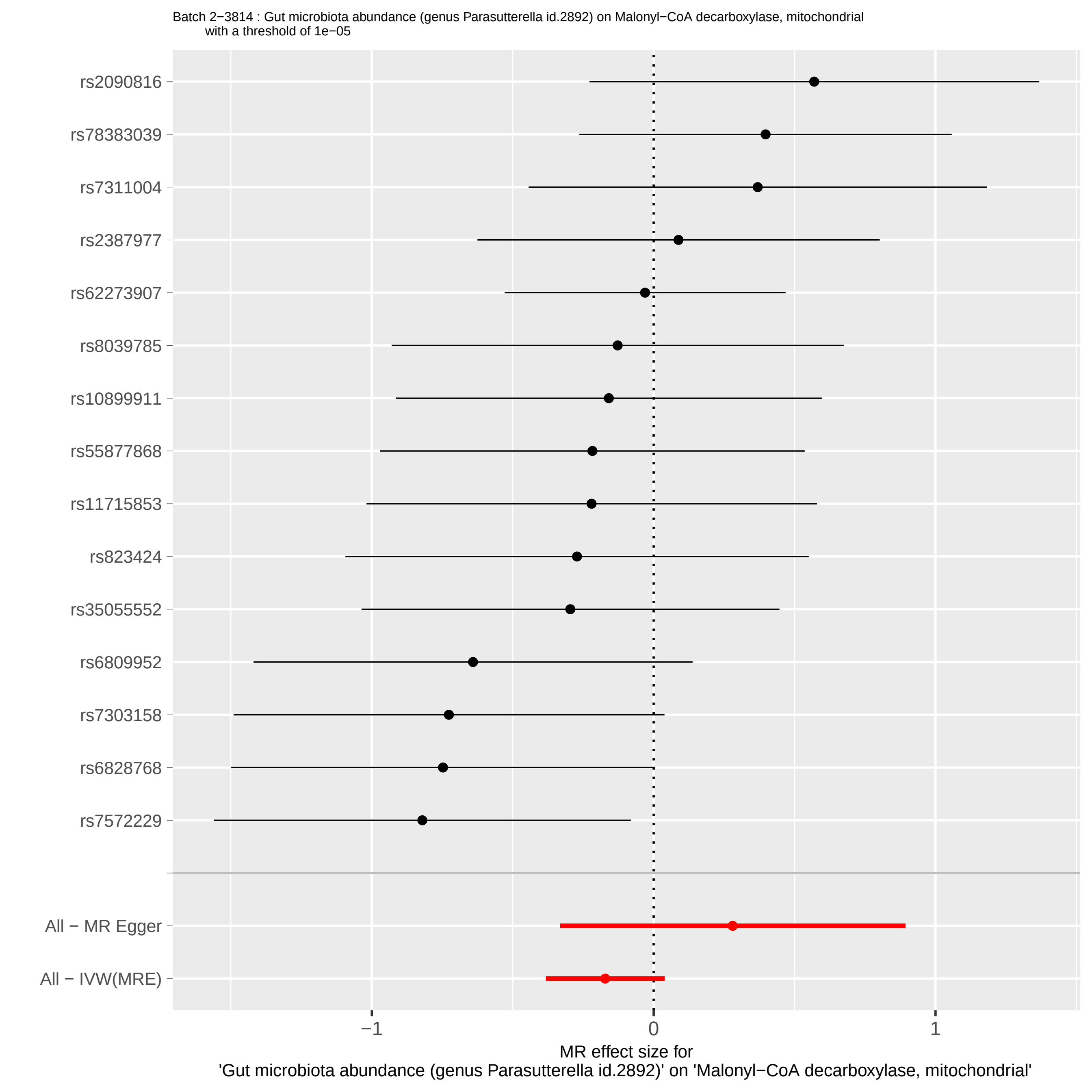
Batch 2−3814 : Gut microbiota abundance (genus Parasutterella id.2892) on Malonyl−CoA decarboxylase, mitochondrial
with a threshold of 1e−05
rs2090816
rs78383039
rs7311004
rs2387977
rs62273907
rs8039785
rs10899911
rs55877868
rs11715853
rs823424
rs35055552
rs6809952
rs7303158
rs6828768
rs7572229
All − MR Egger
All − IVW(MRE)
−1
0
1
MR effect size for
'Gut microbiota abundance (genus Parasutterella id.2892)' on 'Malonyl−CoA decarboxylase, mitochondrial'

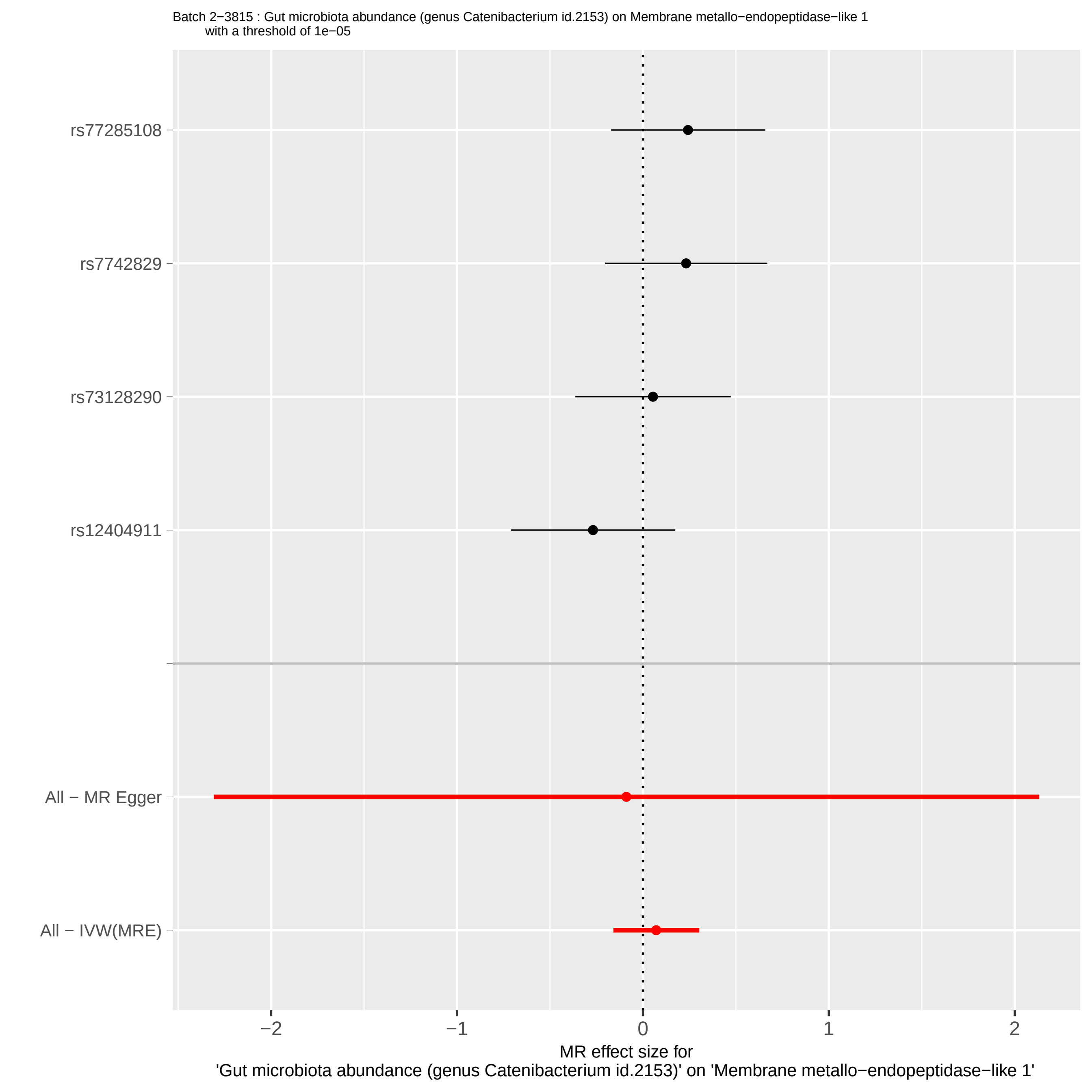

Batch 2−3815 : Gut microbiota abundance (genus Catenibacterium id.2153) on Membrane metallo−endopeptidase−like 1
with a threshold of 1e−05
rs77285108
rs7742829
rs73128290
rs12404911
All − MR Egger
All − IVW(MRE)
−2
−1
0
1
2
MR effect size for
'Gut microbiota abundance (genus Catenibacterium id.2153)' on 'Membrane metallo−endopeptidase−like 1'

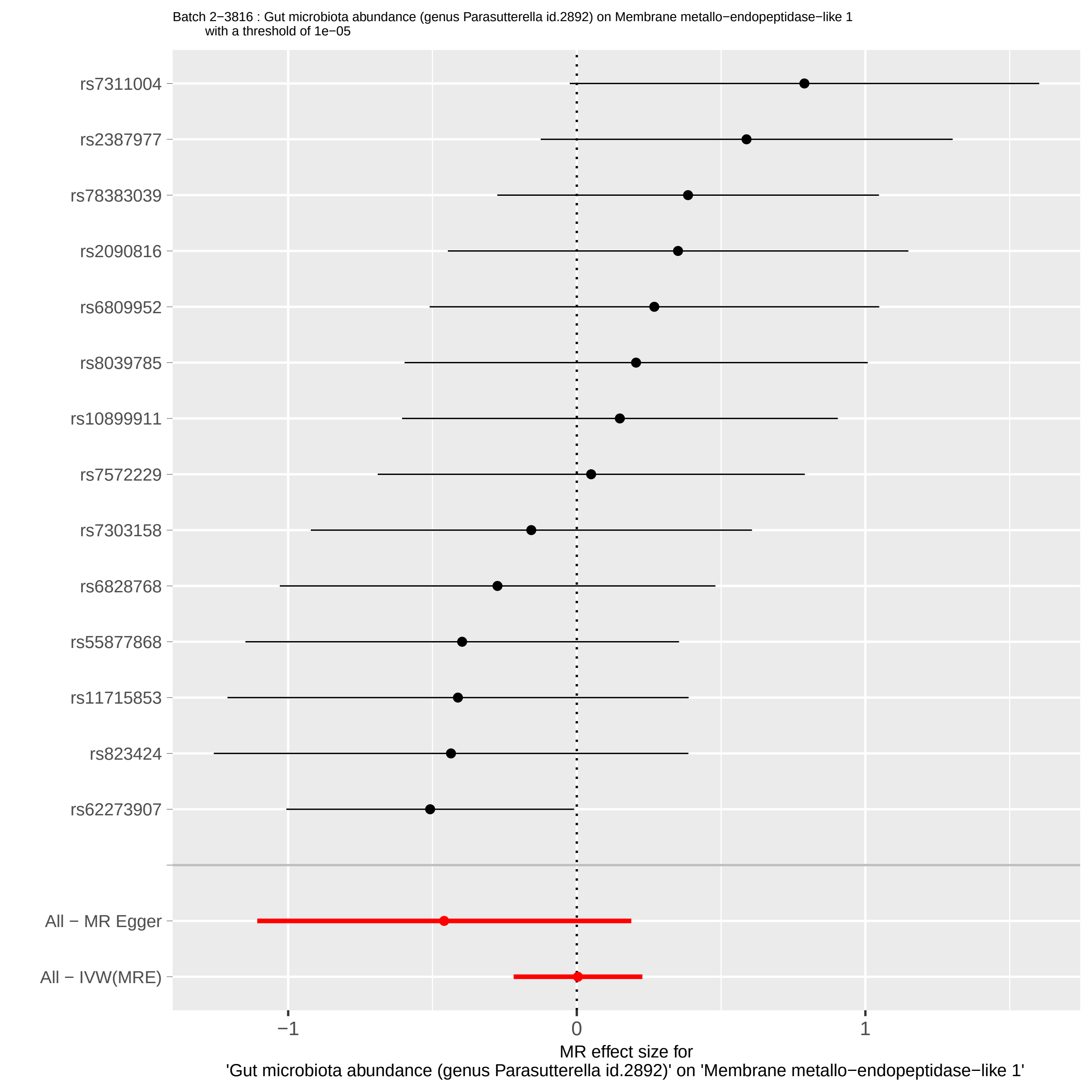
Batch 2−3816 : Gut microbiota abundance (genus Parasutterella id.2892) on Membrane metallo−endopeptidase−like 1
with a threshold of 1e−05
rs7311004
rs2387977
rs78383039
rs2090816
rs6809952
rs8039785
rs10899911
rs7572229
rs7303158
rs6828768
rs55877868
rs11715853
rs823424
rs62273907
All − MR Egger
All − IVW(MRE)
−1
0
1
MR effect size for
'Gut microbiota abundance (genus Parasutterella id.2892)' on 'Membrane metallo−endopeptidase−like 1'

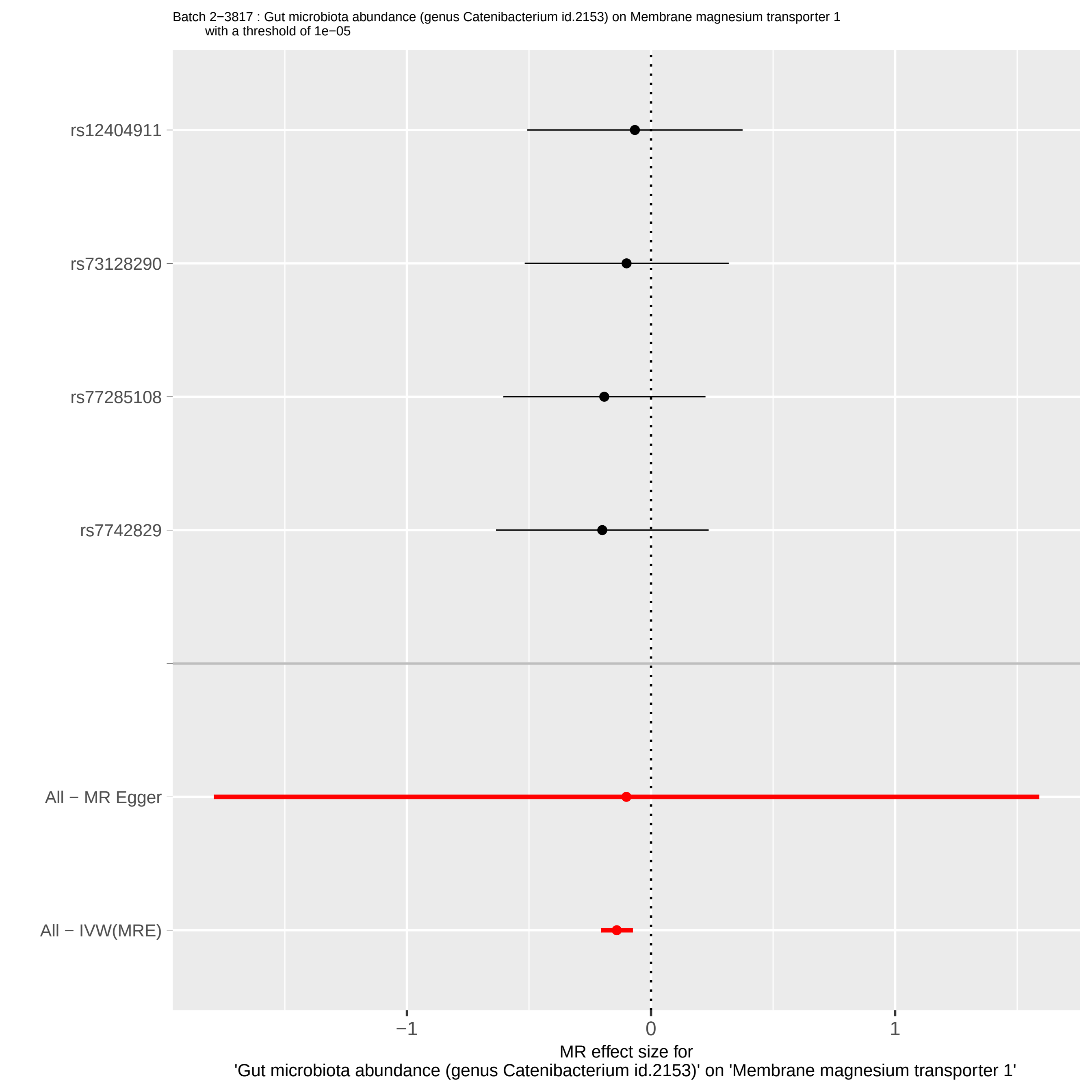

Batch 2−3817 : Gut microbiota abundance (genus Catenibacterium id.2153) on Membrane magnesium transporter 1
with a threshold of 1e−05
rs12404911
rs73128290
rs77285108
rs7742829
All − MR Egger
All − IVW(MRE)
−1
0
1
MR effect size for
'Gut microbiota abundance (genus Catenibacterium id.2153)' on 'Membrane magnesium transporter 1'

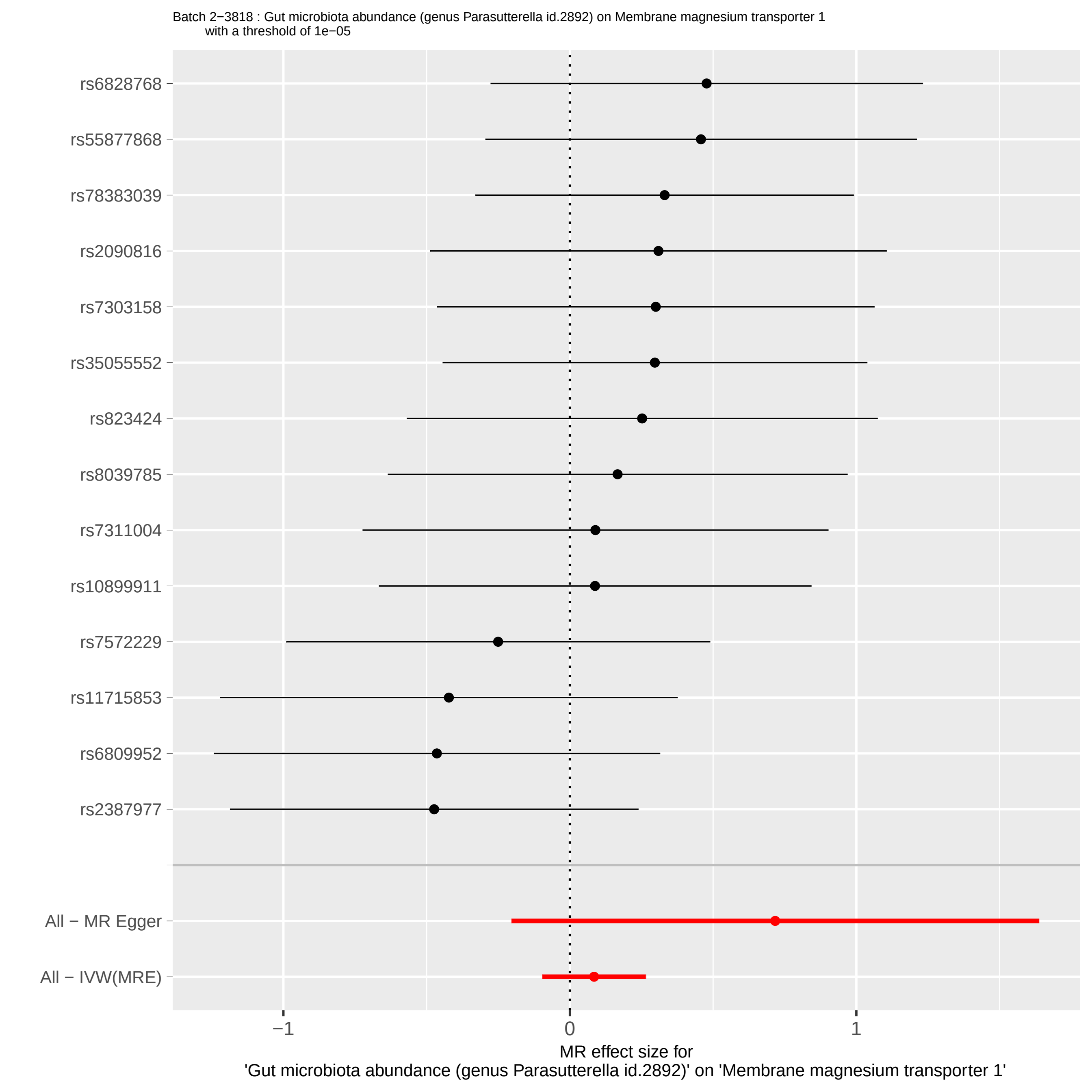
Batch 2−3818 : Gut microbiota abundance (genus Parasutterella id.2892) on Membrane magnesium transporter 1
with a threshold of 1e−05
rs6828768
rs55877868
rs78383039
rs2090816
rs7303158
rs35055552
rs823424
rs8039785
rs7311004
rs10899911
rs7572229
rs11715853
rs6809952
rs2387977
All − MR Egger
All − IVW(MRE)
−1
0
1
MR effect size for
'Gut microbiota abundance (genus Parasutterella id.2892)' on 'Membrane magnesium transporter 1'

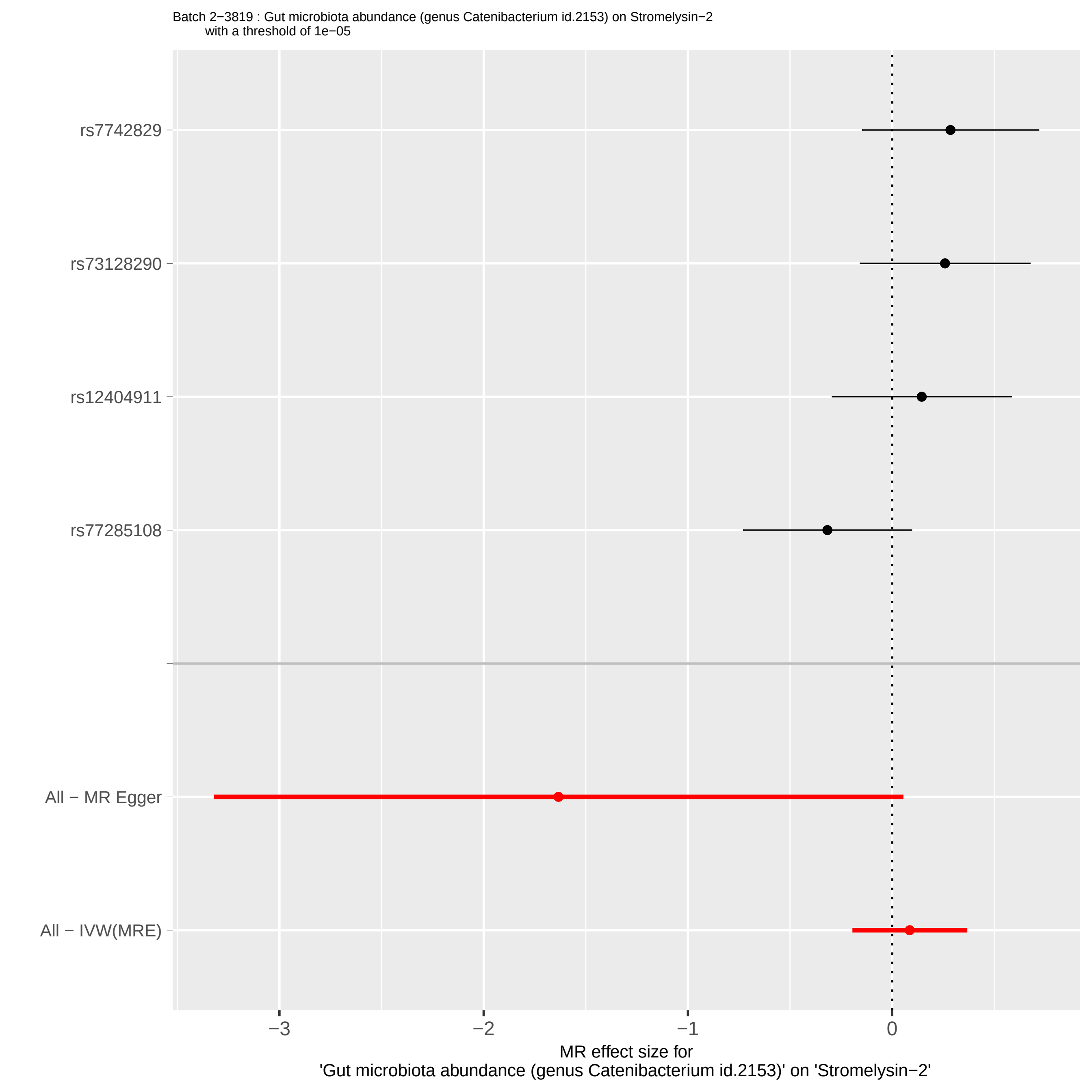

Batch 2−3819 : Gut microbiota abundance (genus Catenibacterium id.2153) on Stromelysin−2
with a threshold of 1e−05
rs7742829
rs73128290
rs12404911
rs77285108
All − MR Egger
All − IVW(MRE)
−3
−2
−1
0
MR effect size for
'Gut microbiota abundance (genus Catenibacterium id.2153)' on 'Stromelysin−2'

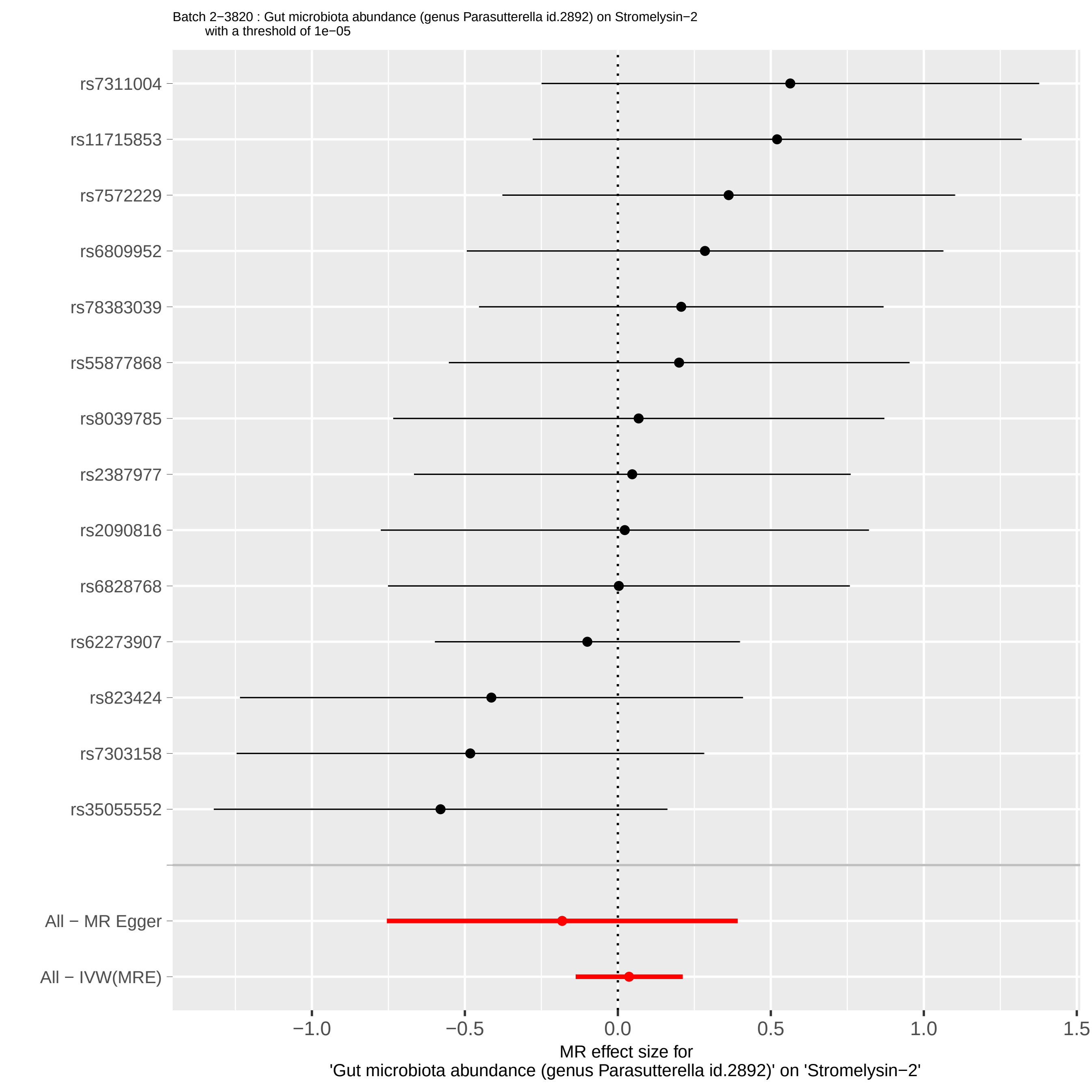
Batch 2−3820 : Gut microbiota abundance (genus Parasutterella id.2892) on Stromelysin−2
with a threshold of 1e−05
rs7311004
rs11715853
rs7572229
rs6809952
rs78383039
rs55877868
rs8039785
rs2387977
rs2090816
rs6828768
rs62273907
rs823424
rs7303158
rs35055552
All − MR Egger
All − IVW(MRE)
−1.0
−0.5
0.0
0.5
1.0
1.5
MR effect size for
'Gut microbiota abundance (genus Parasutterella id.2892)' on 'Stromelysin−2'

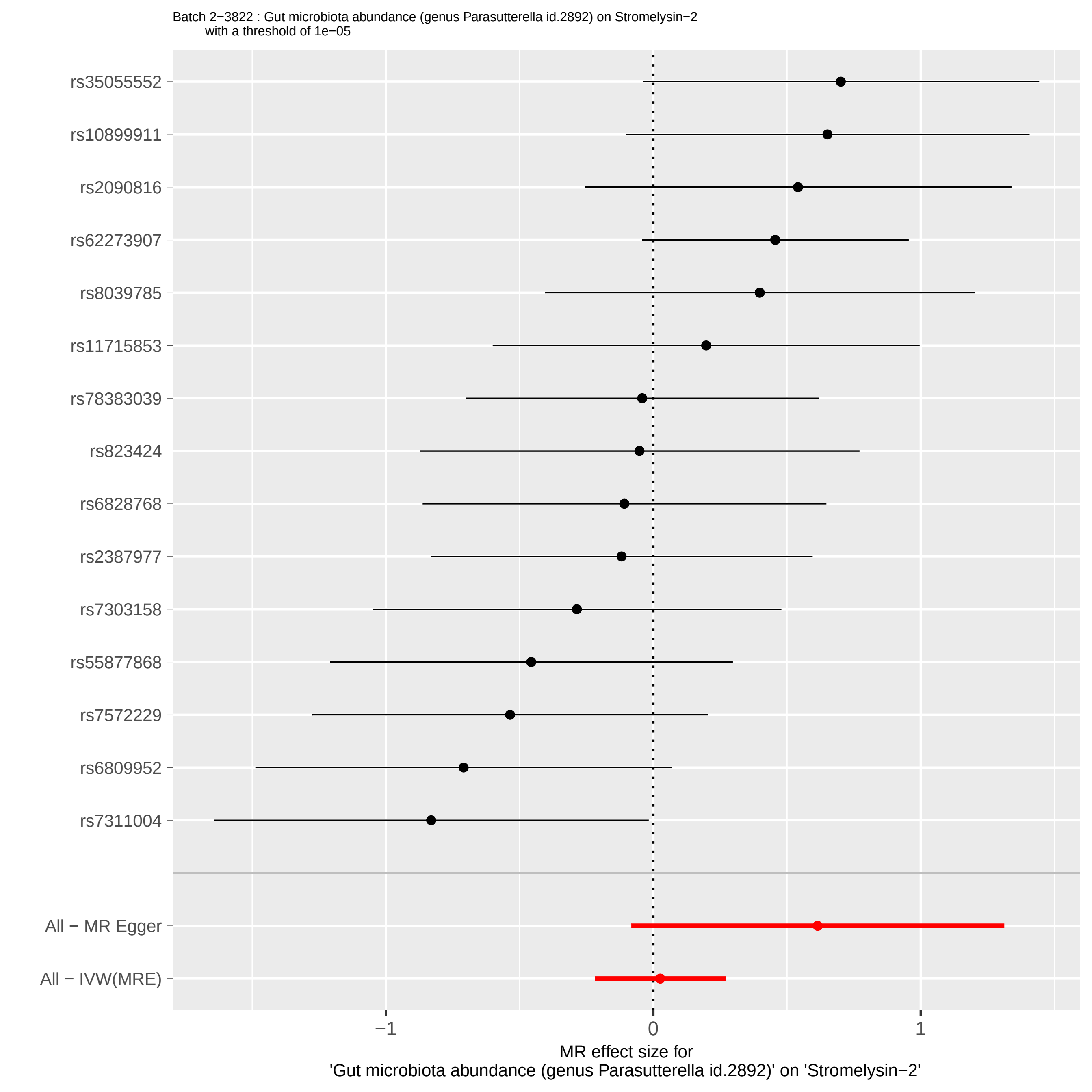

Batch 2−3822 : Gut microbiota abundance (genus Parasutterella id.2892) on Stromelysin−2
with a threshold of 1e−05
rs35055552
rs10899911
rs2090816
rs62273907
rs8039785
rs11715853
rs78383039
rs823424
rs6828768
rs2387977
rs7303158
rs55877868
rs7572229
rs6809952
rs7311004
All − MR Egger
All − IVW(MRE)
−1
0
1
MR effect size for
'Gut microbiota abundance (genus Parasutterella id.2892)' on 'Stromelysin−2'

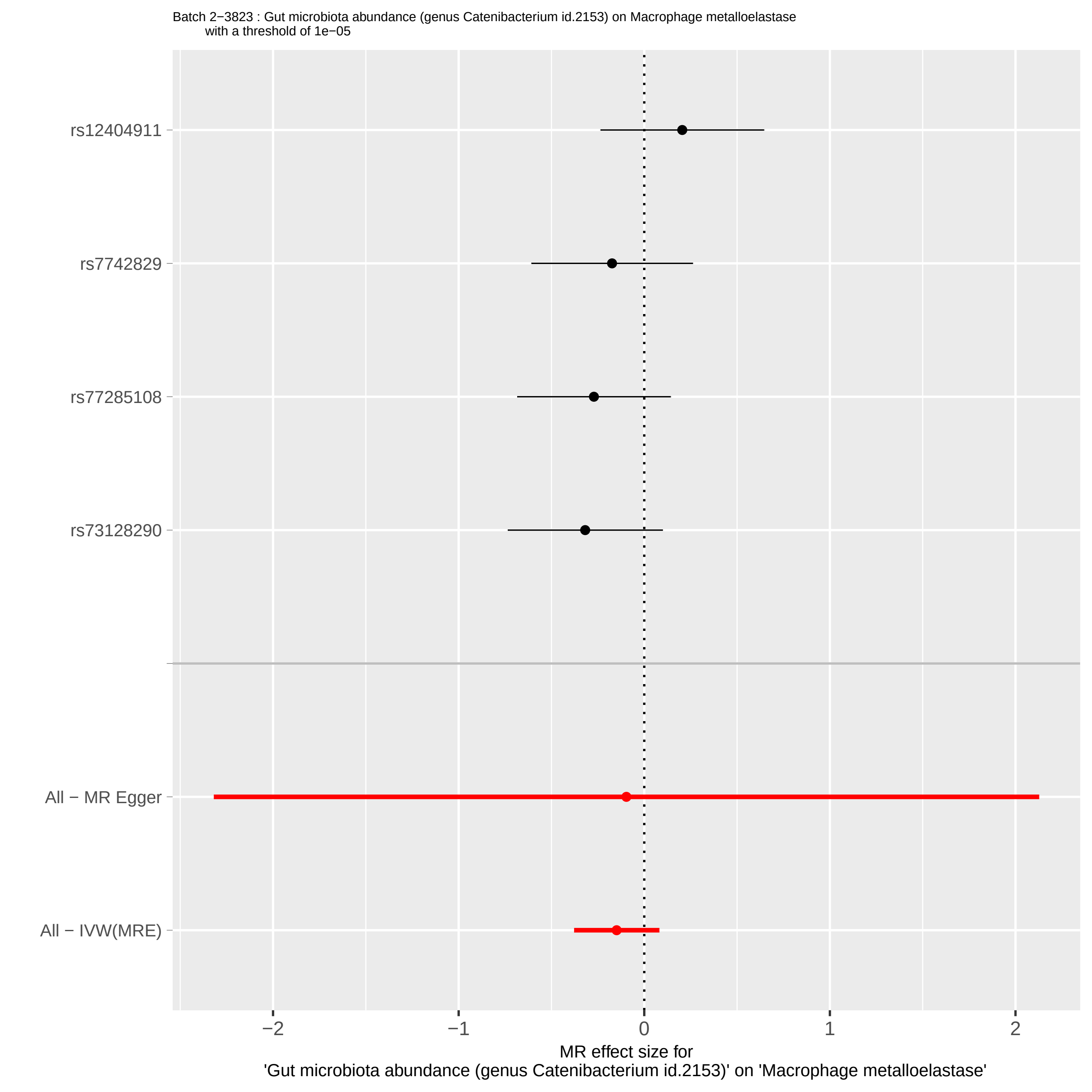
Batch 2−3823 : Gut microbiota abundance (genus Catenibacterium id.2153) on Macrophage metalloelastase
with a threshold of 1e−05
rs12404911
rs7742829
rs77285108
rs73128290
All − MR Egger
All − IVW(MRE)
−2
−1
0
1
2
MR effect size for
'Gut microbiota abundance (genus Catenibacterium id.2153)' on 'Macrophage metalloelastase'

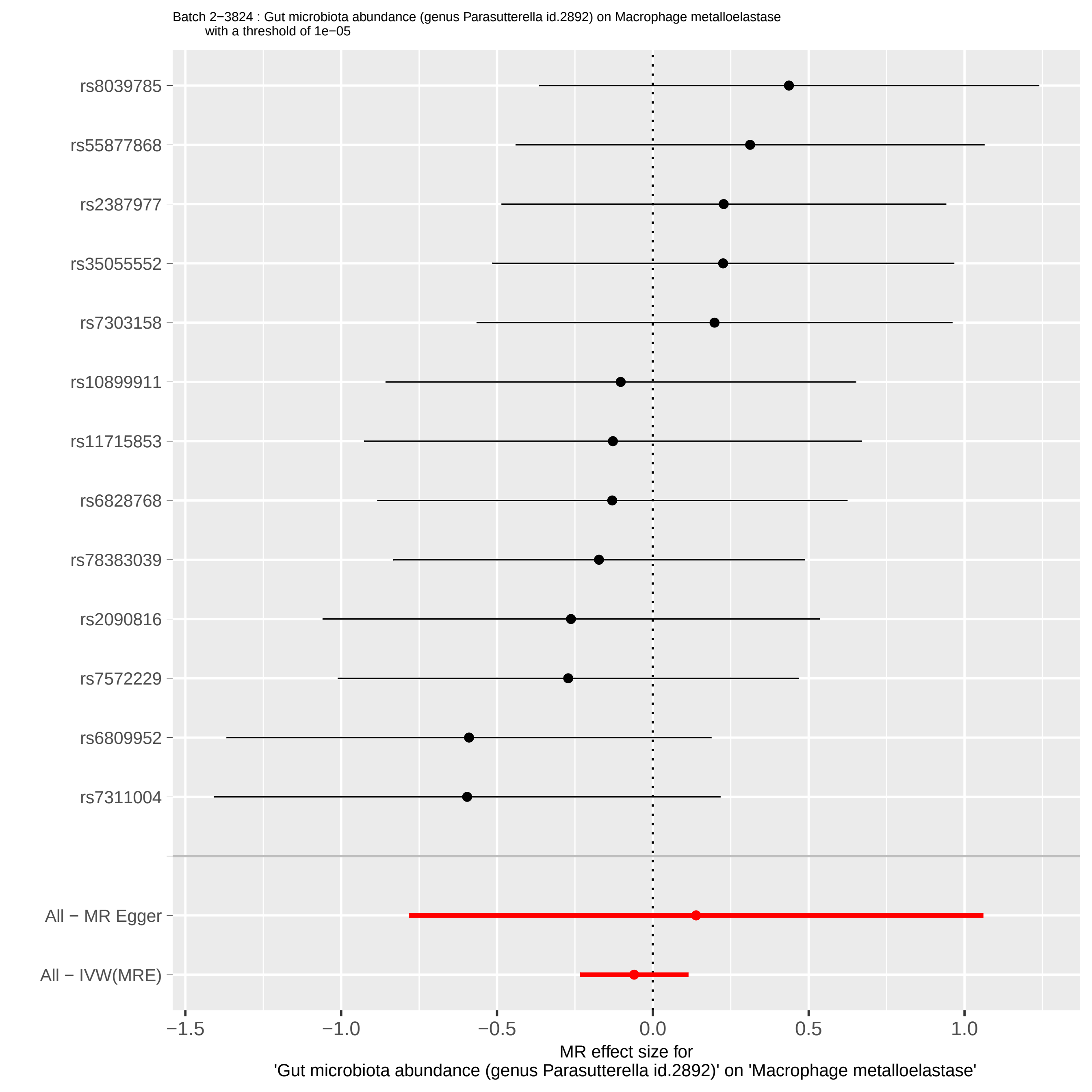

Batch 2−3824 : Gut microbiota abundance (genus Parasutterella id.2892) on Macrophage metalloelastase
with a threshold of 1e−05
rs8039785
rs55877868
rs2387977
rs35055552
rs7303158
rs10899911
rs11715853
rs6828768
rs78383039
rs2090816
rs7572229
rs6809952
rs7311004
All − MR Egger
All − IVW(MRE)
−1.5
−1.0
−0.5
0.0
0.5
1.0
MR effect size for
'Gut microbiota abundance (genus Parasutterella id.2892)' on 'Macrophage metalloelastase'

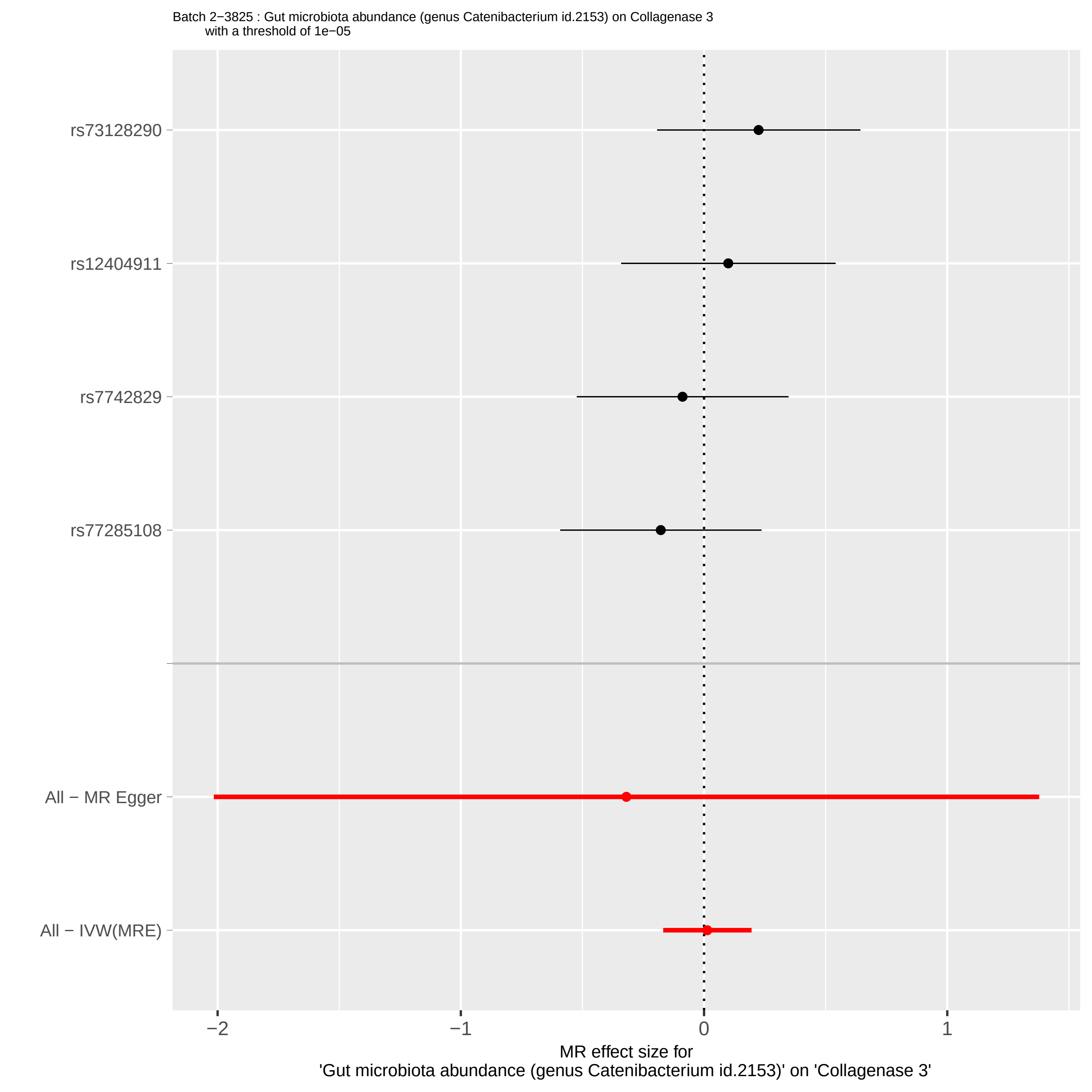
Batch 2−3825 : Gut microbiota abundance (genus Catenibacterium id.2153) on Collagenase 3
with a threshold of 1e−05
rs73128290
rs12404911
rs7742829
rs77285108
All − MR Egger
All − IVW(MRE)
−2
−1
0
1
MR effect size for
'Gut microbiota abundance (genus Catenibacterium id.2153)' on 'Collagenase 3'

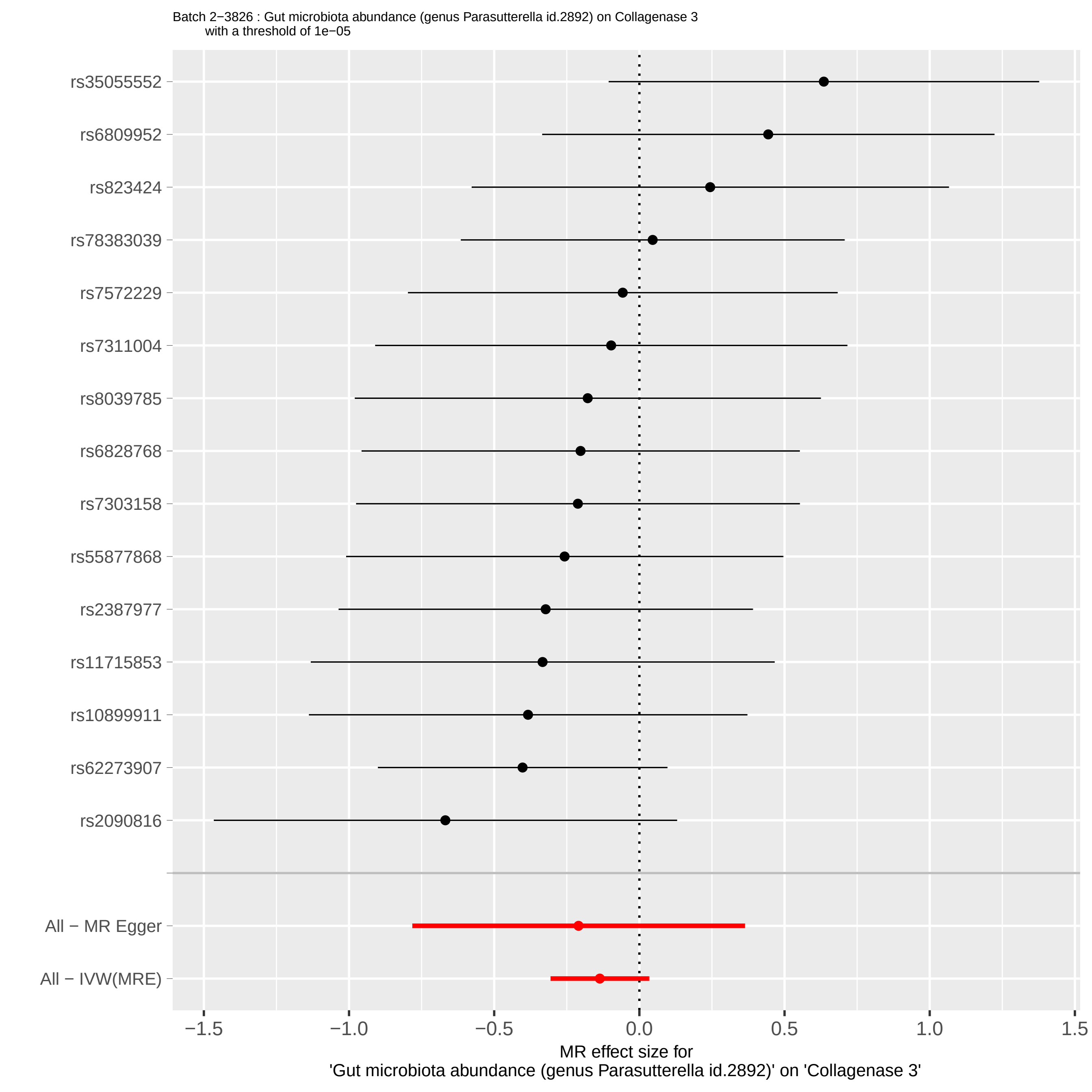
Batch 2−3826 : Gut microbiota abundance (genus Parasutterella id.2892) on Collagenase 3
with a threshold of 1e−05
rs35055552
rs6809952
rs823424
rs78383039
rs7572229
rs7311004
rs8039785
rs6828768
rs7303158
rs55877868
rs2387977
rs11715853
rs10899911
rs62273907
rs2090816
All − MR Egger
All − IVW(MRE)
−1.5
−1.0
−0.5
0.0
0.5
1.0
1.5
MR effect size for
'Gut microbiota abundance (genus Parasutterella id.2892)' on 'Collagenase 3'

Batch 2−3827 : Gut microbiota abundance (genus Catenibacterium id.2153) on Matrix metalloproteinase−14
with a threshold of 1e−05

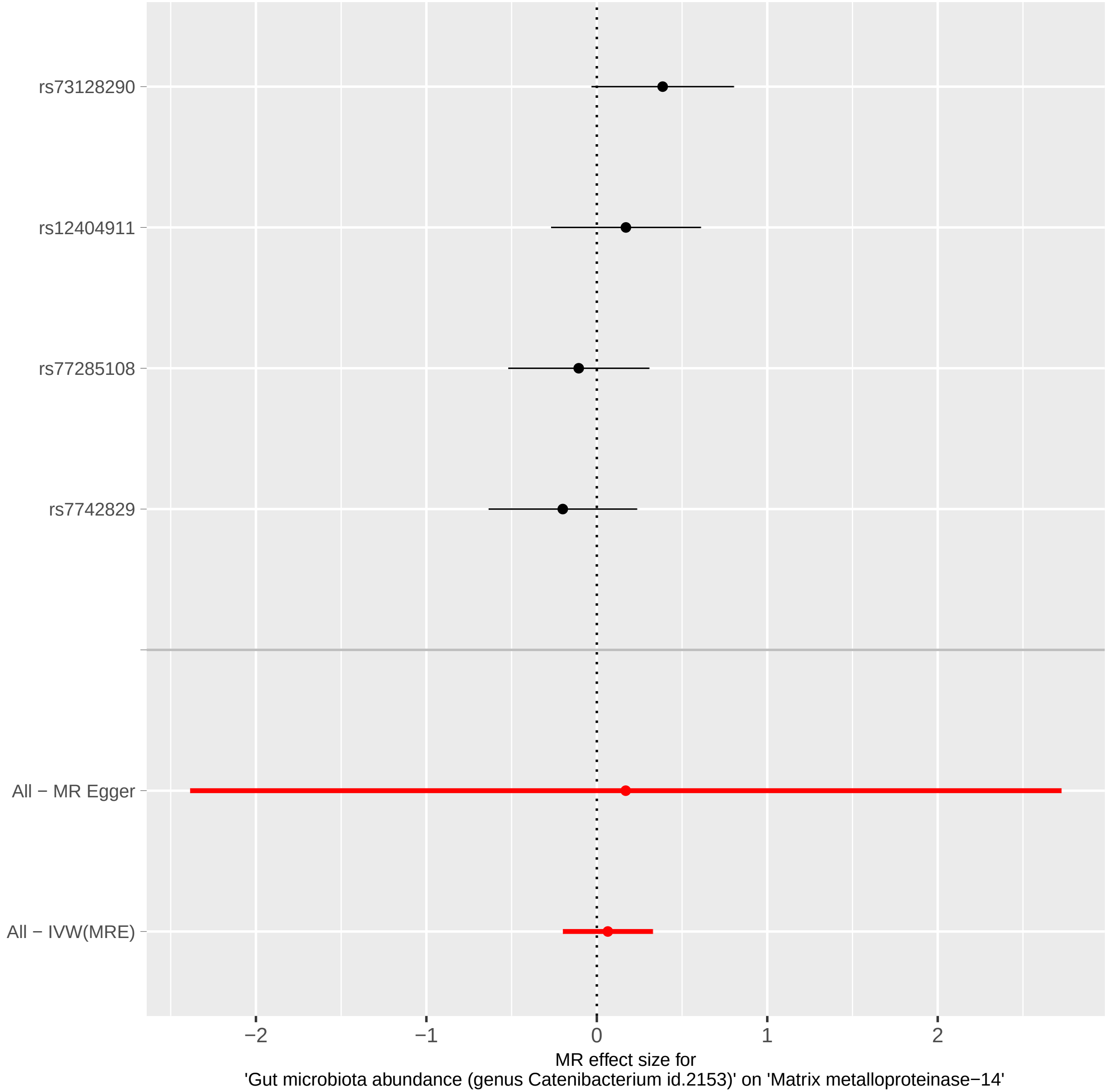

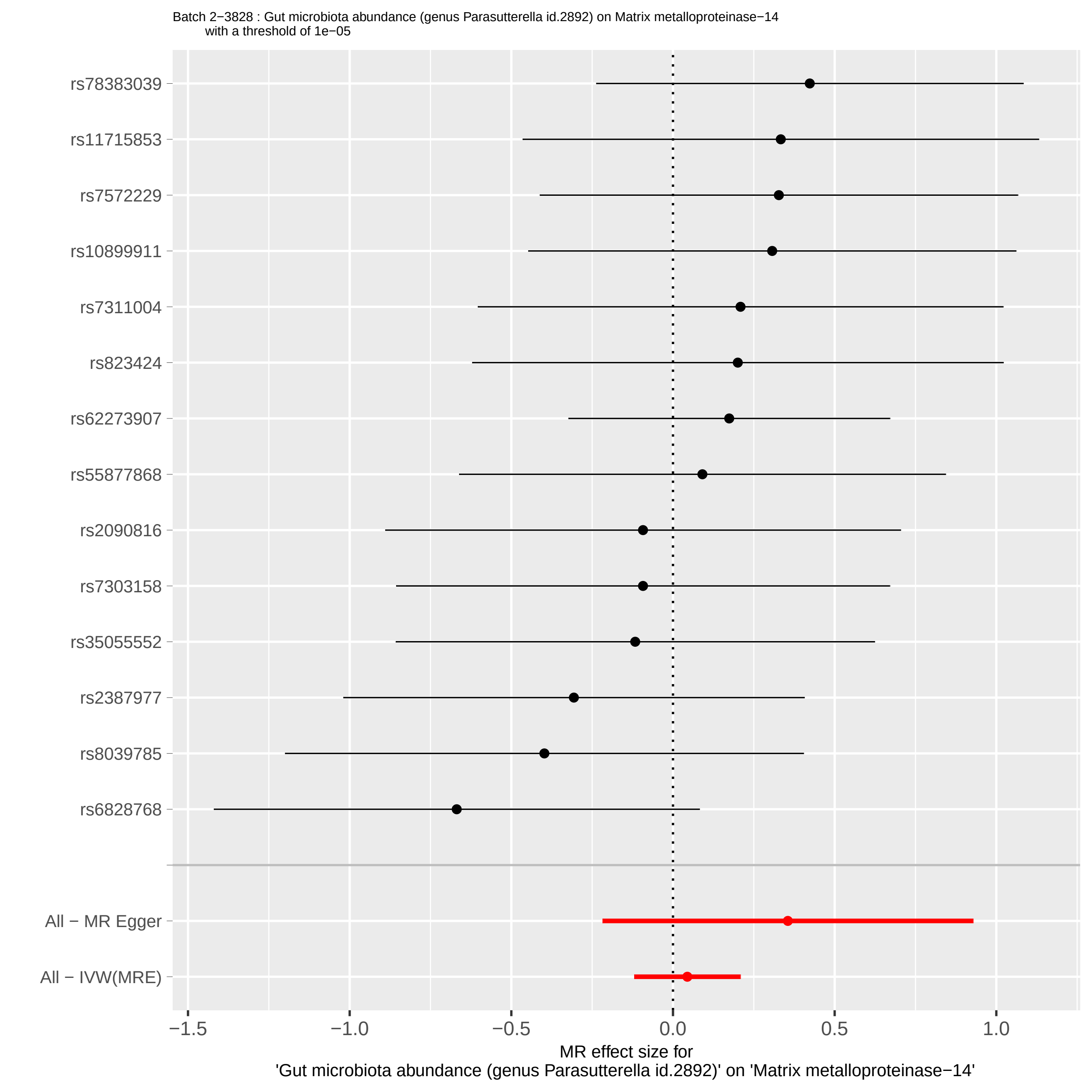

Batch 2−3828 : Gut microbiota abundance (genus Parasutterella id.2892) on Matrix metalloproteinase−14
with a threshold of 1e−05
rs78383039
rs11715853
rs7572229
rs10899911
rs7311004
rs823424
rs62273907
rs55877868
rs2090816
rs7303158
rs35055552
rs2387977
rs8039785
rs6828768
All − MR Egger
All − IVW(MRE)
−1.5
−1.0
−0.5
0.0
0.5
1.0
MR effect size for
'Gut microbiota abundance (genus Parasutterella id.2892)' on 'Matrix metalloproteinase−14'

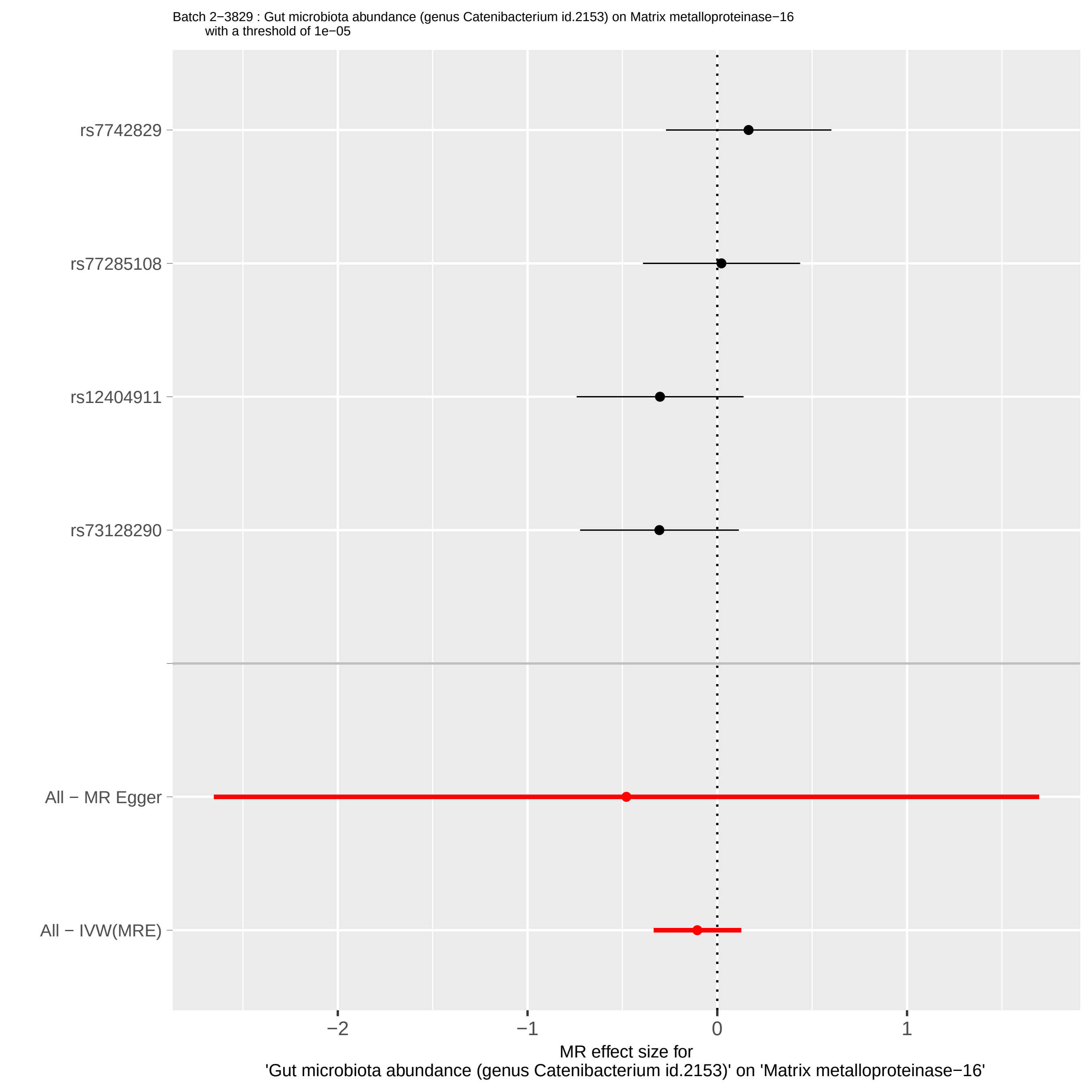

Batch 2−3829 : Gut microbiota abundance (genus Catenibacterium id.2153) on Matrix metalloproteinase−16
with a threshold of 1e−05
rs7742829
rs77285108
rs12404911
rs73128290
All − MR Egger
All − IVW(MRE)
−2
−1
0
1
MR effect size for
'Gut microbiota abundance (genus Catenibacterium id.2153)' on 'Matrix metalloproteinase−16'

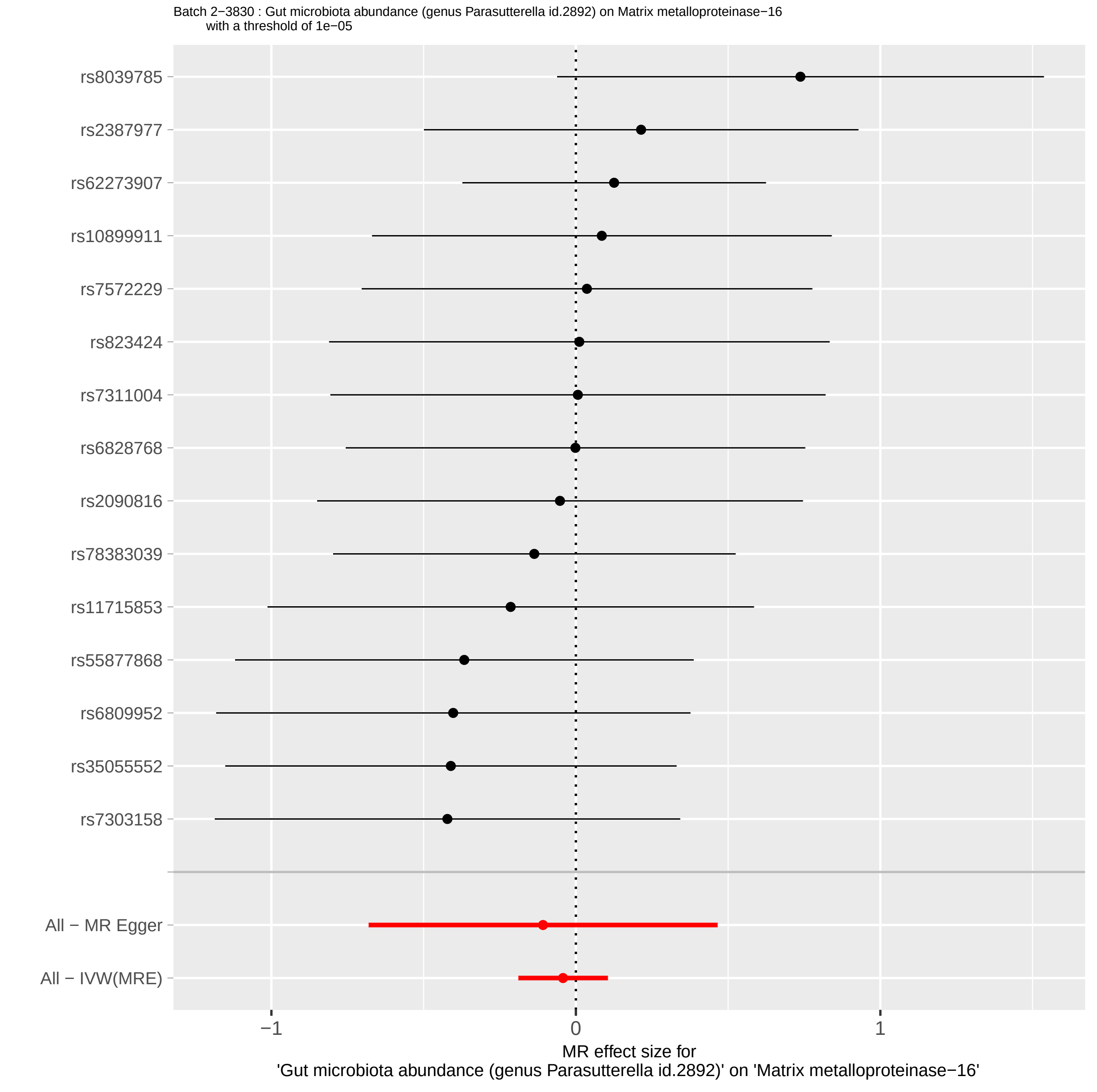

Batch 2−3830 : Gut microbiota abundance (genus Parasutterella id.2892) on Matrix metalloproteinase−16
with a threshold of 1e−05
rs8039785
rs2387977
rs62273907
rs10899911
rs7572229
rs823424
rs7311004
rs6828768
rs2090816
rs78383039
rs11715853
rs55877868
rs6809952
rs35055552
rs7303158
All − MR Egger
All − IVW(MRE)
−1
0
1
MR effect size for
'Gut microbiota abundance (genus Parasutterella id.2892)' on 'Matrix metalloproteinase−16'

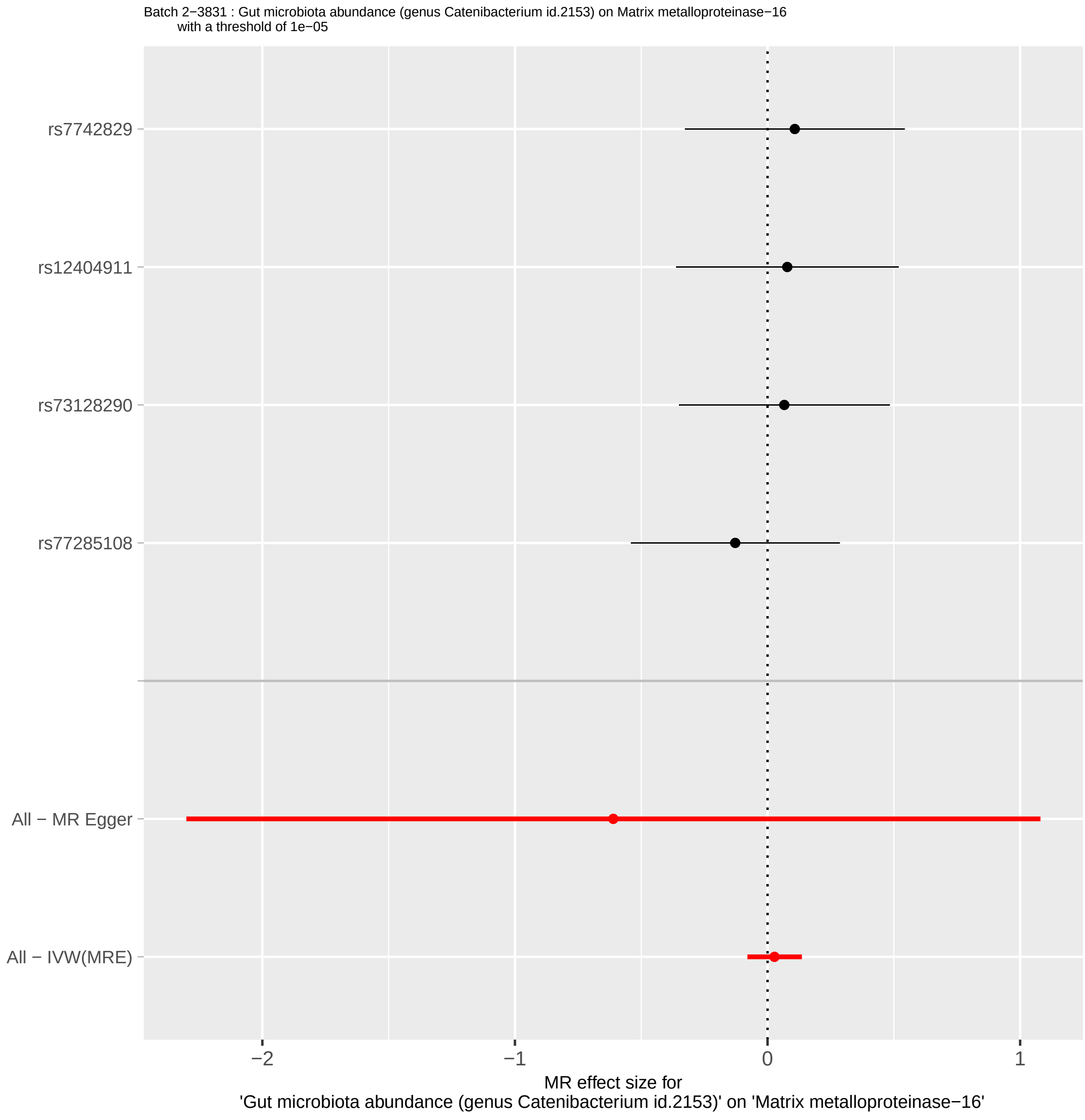
Batch 2−3831 : Gut microbiota abundance (genus Catenibacterium id.2153) on Matrix metalloproteinase−16
with a threshold of 1e−05
rs7742829
rs12404911
rs73128290
rs77285108
All − MR Egger
All − IVW(MRE)
−2
−1
0
1
MR effect size for
'Gut microbiota abundance (genus Catenibacterium id.2153)' on 'Matrix metalloproteinase−16'

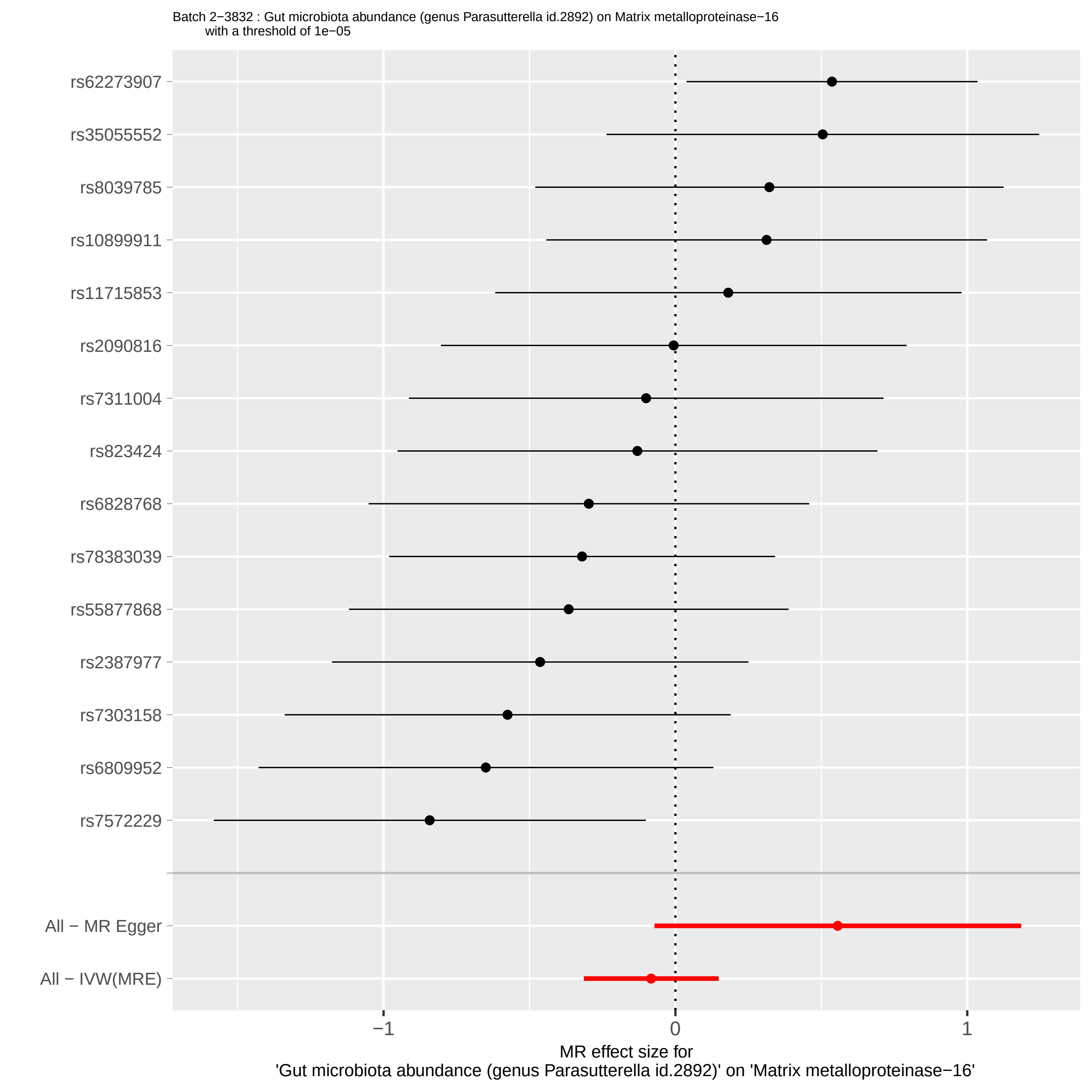
Batch 2−3832 : Gut microbiota abundance (genus Parasutterella id.2892) on Matrix metalloproteinase−16
with a threshold of 1e−05
rs62273907
rs35055552
rs8039785
rs10899911
rs11715853
rs2090816
rs7311004
rs823424
rs6828768
rs78383039
rs55877868
rs2387977
rs7303158
rs6809952
rs7572229
All − MR Egger
All − IVW(MRE)
−1
0
1
MR effect size for
'Gut microbiota abundance (genus Parasutterella id.2892)' on 'Matrix metalloproteinase−16'

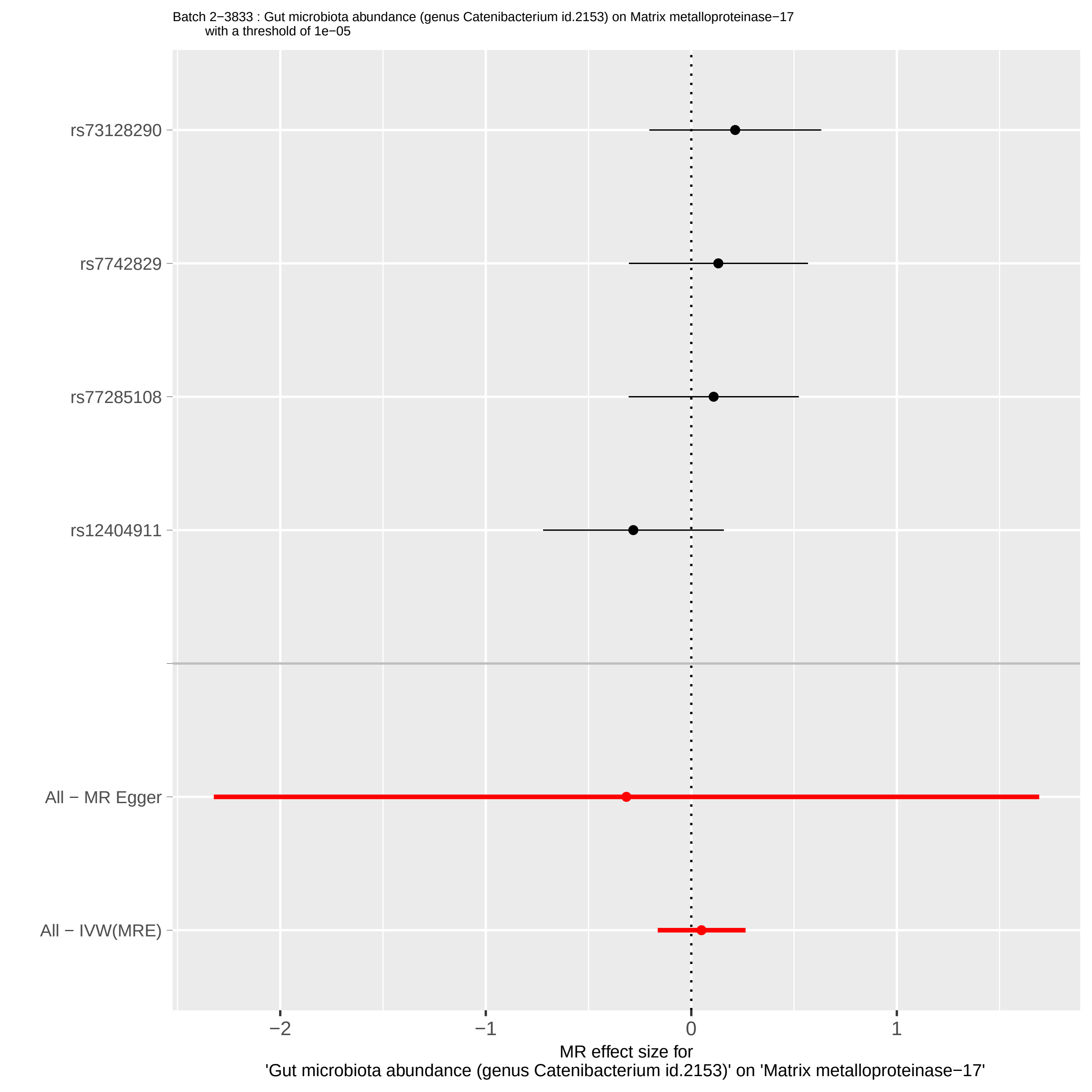

Batch 2−3833 : Gut microbiota abundance (genus Catenibacterium id.2153) on Matrix metalloproteinase−17
with a threshold of 1e−05
rs73128290
rs7742829
rs77285108
rs12404911
All − MR Egger
All − IVW(MRE)
−2
−1
0
1
MR effect size for
'Gut microbiota abundance (genus Catenibacterium id.2153)' on 'Matrix metalloproteinase−17'

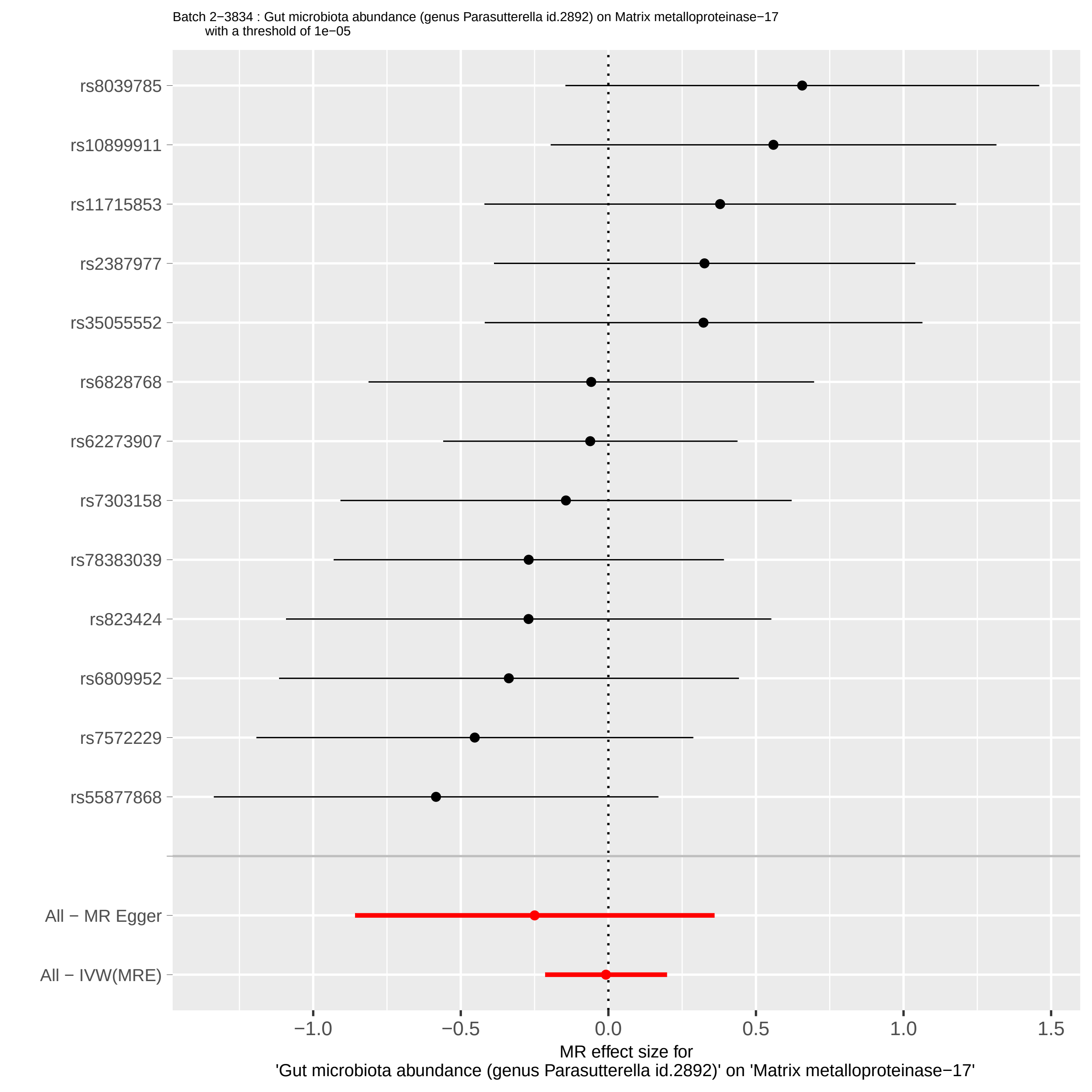
Batch 2−3834 : Gut microbiota abundance (genus Parasutterella id.2892) on Matrix metalloproteinase−17
with a threshold of 1e−05
rs8039785
rs10899911
rs11715853
rs2387977
rs35055552
rs6828768
rs62273907
rs7303158
rs78383039
rs823424
rs6809952
rs7572229
rs55877868
All − MR Egger
All − IVW(MRE)
−1.0
−0.5
0.0
0.5
1.0
1.5
MR effect size for
'Gut microbiota abundance (genus Parasutterella id.2892)' on 'Matrix metalloproteinase−17'

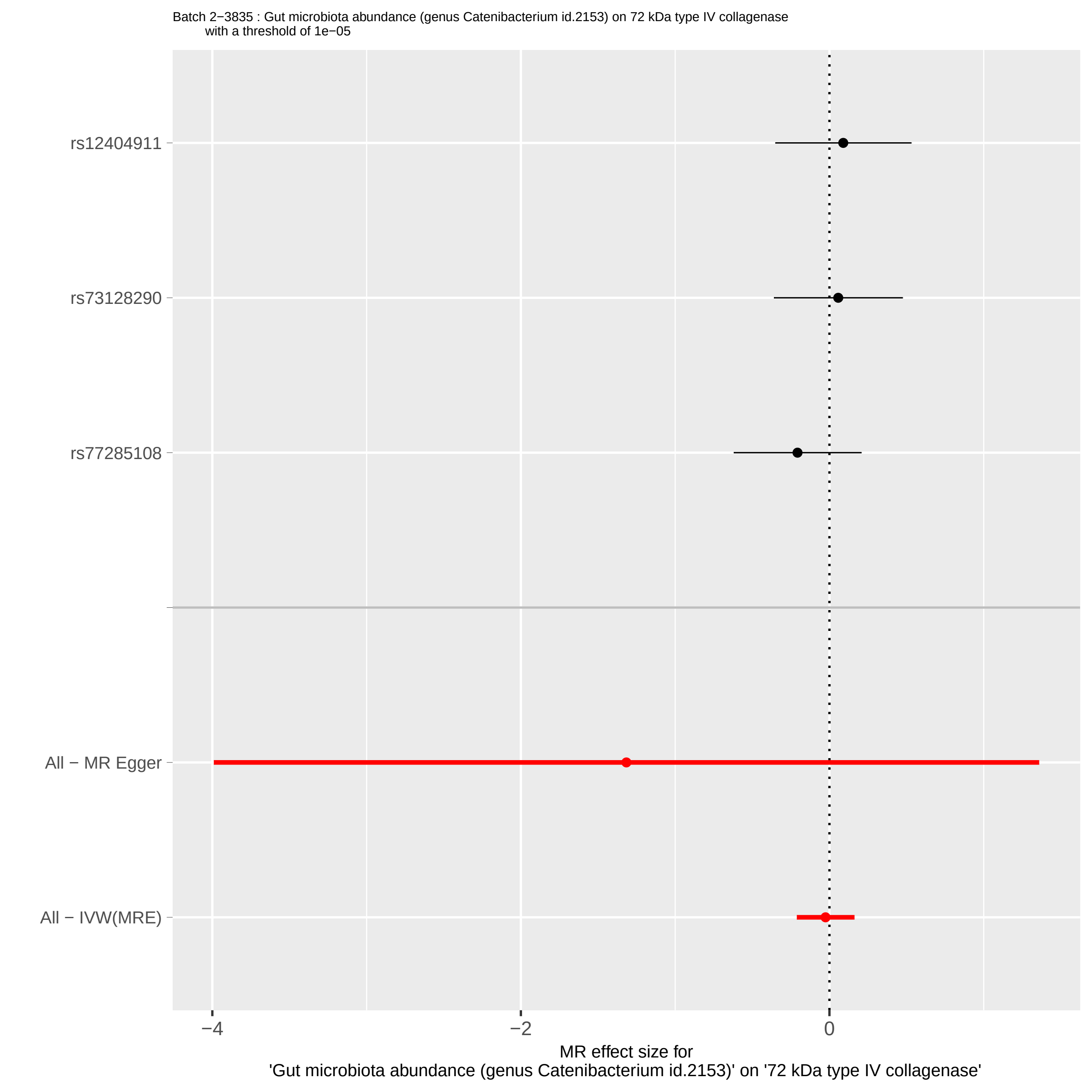

Batch 2−3835 : Gut microbiota abundance (genus Catenibacterium id.2153) on 72 kDa type IV collagenase
with a threshold of 1e−05
rs12404911
rs73128290
rs77285108
All − MR Egger
All − IVW(MRE)
−4
−2
0
MR effect size for
'Gut microbiota abundance (genus Catenibacterium id.2153)' on '72 kDa type IV collagenase'

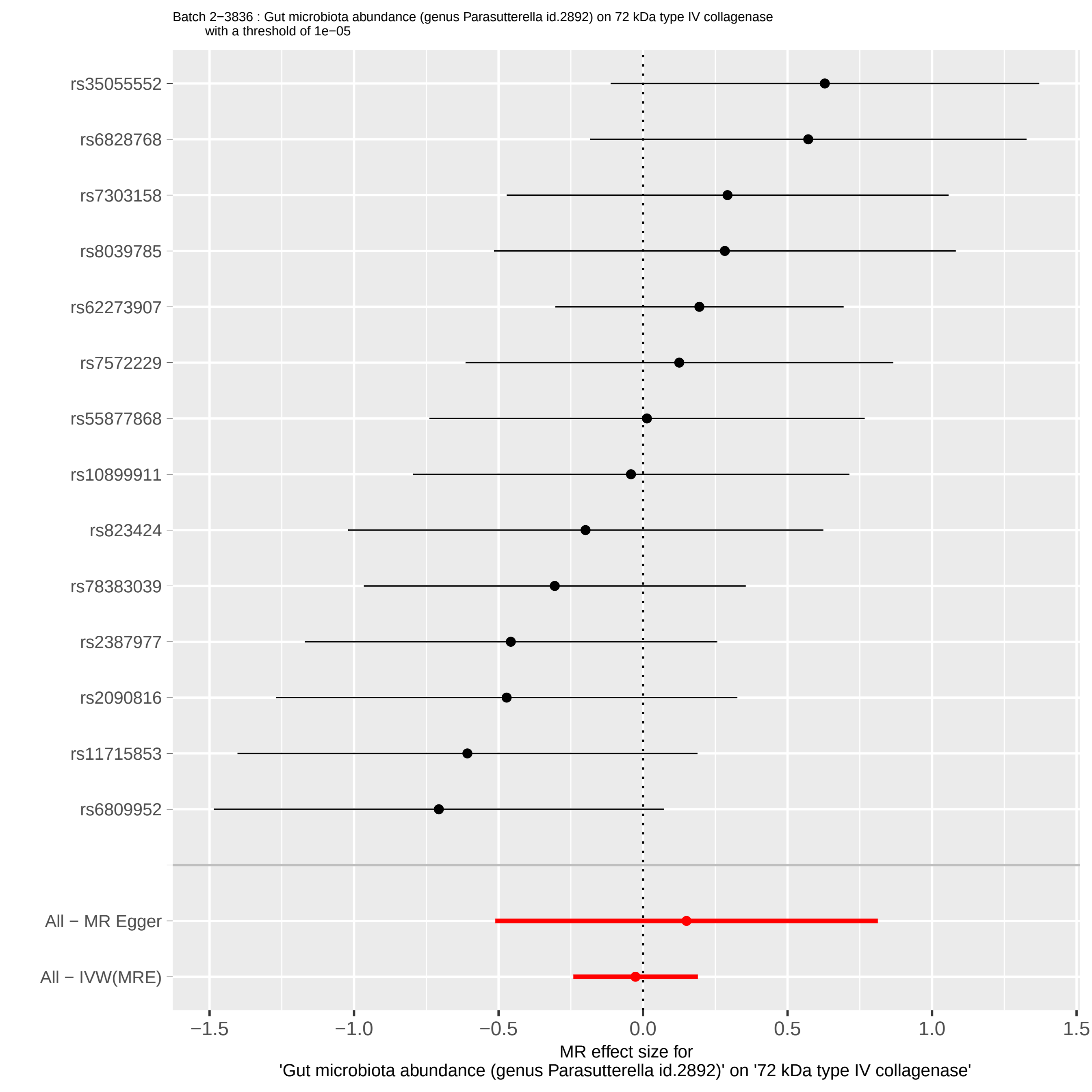
Batch 2−3836 : Gut microbiota abundance (genus Parasutterella id.2892) on 72 kDa type IV collagenase
with a threshold of 1e−05
rs35055552
rs6828768
rs7303158
rs8039785
rs62273907
rs7572229
rs55877868
rs10899911
rs823424
rs78383039
rs2387977
rs2090816
rs11715853
rs6809952
All − MR Egger
All − IVW(MRE)
−1.5
−1.0
−0.5
0.0
0.5
1.0
1.5
MR effect size for
'Gut microbiota abundance (genus Parasutterella id.2892)' on '72 kDa type IV collagenase'

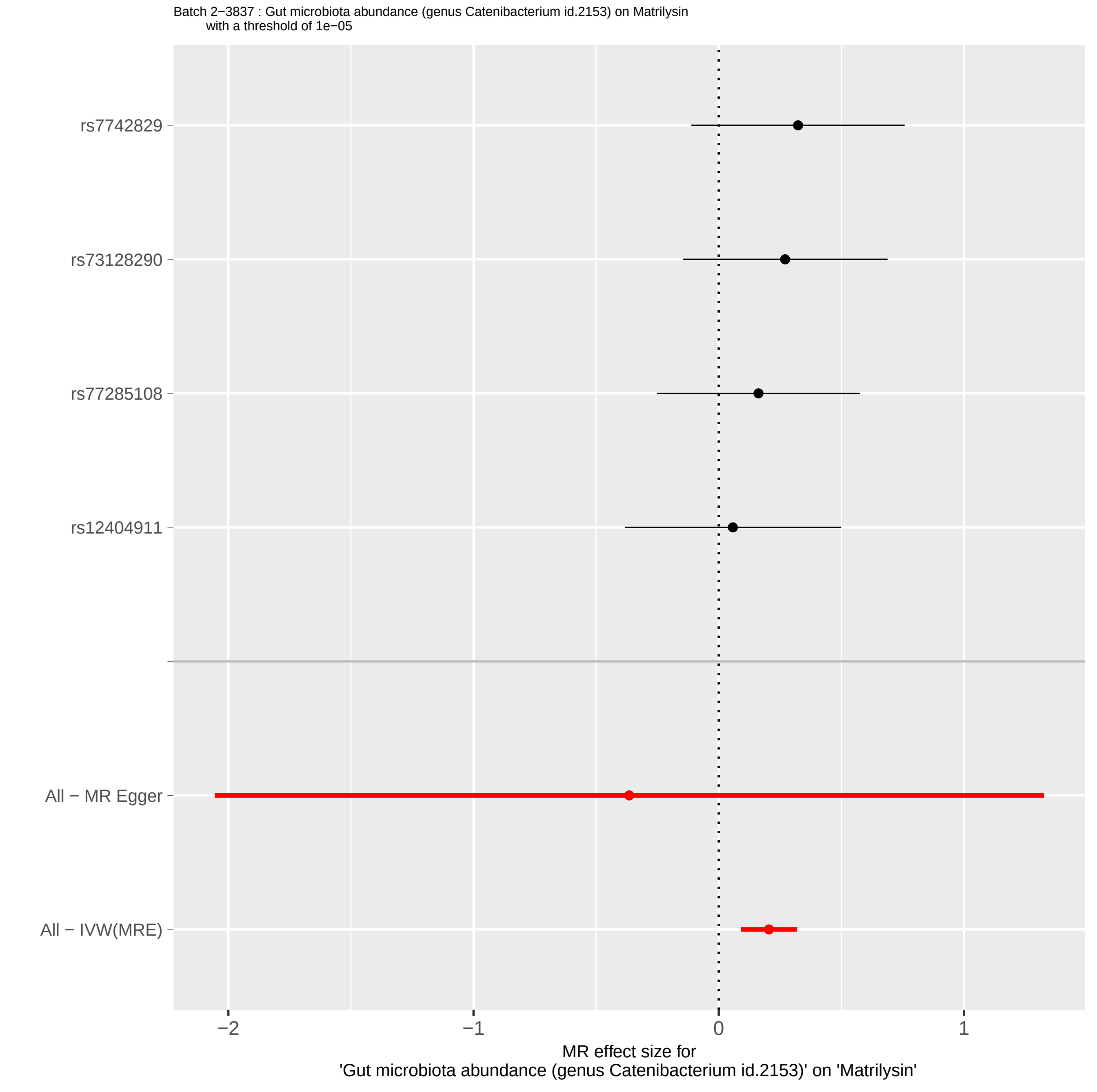
Batch 2−3837 : Gut microbiota abundance (genus Catenibacterium id.2153) on Matrilysin
with a threshold of 1e−05
rs7742829
rs73128290
rs77285108
rs12404911
All − MR Egger
All − IVW(MRE)
−2
−1
0
1
MR effect size for
'Gut microbiota abundance (genus Catenibacterium id.2153)' on 'Matrilysin'

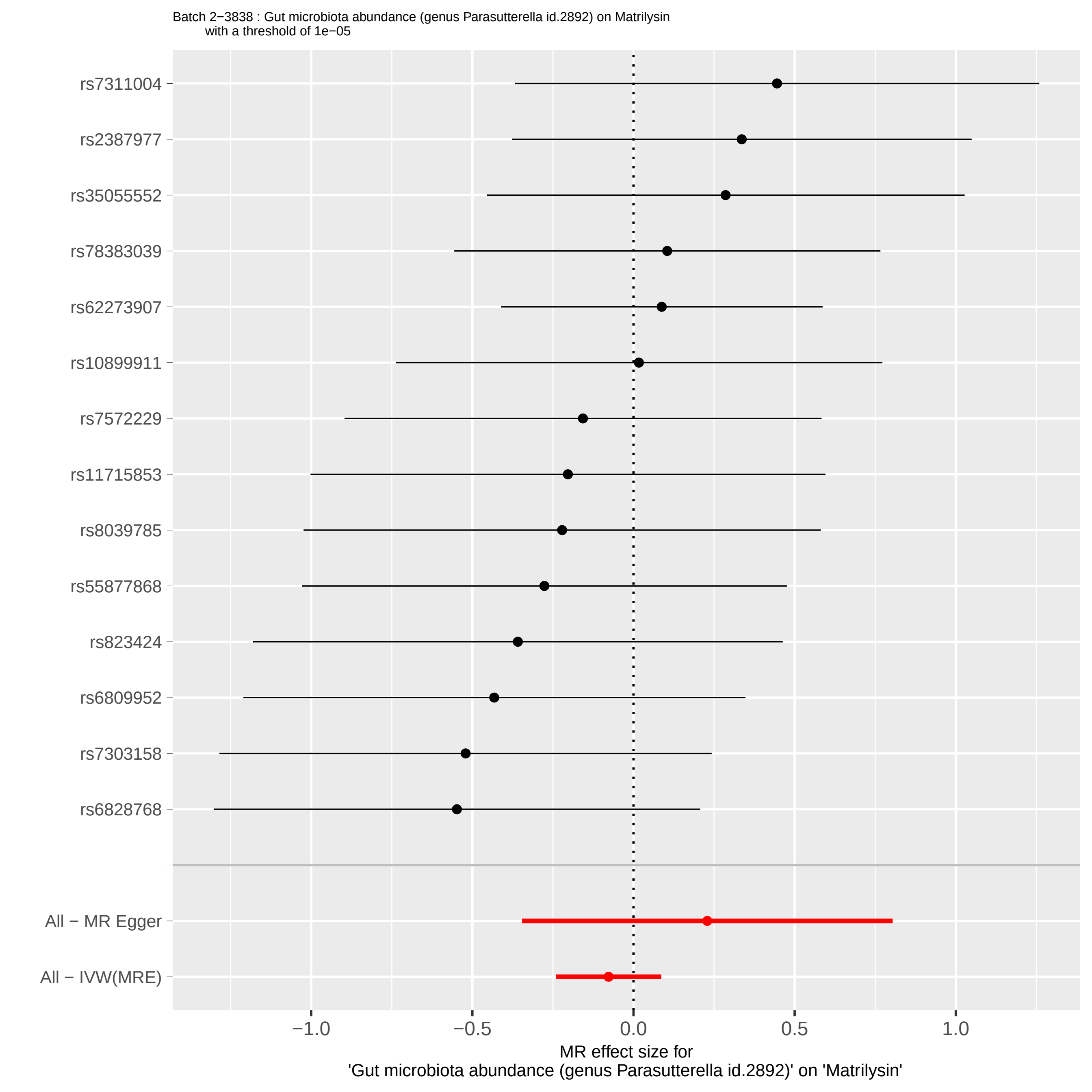
Batch 2−3838 : Gut microbiota abundance (genus Parasutterella id.2892) on Matrilysin
with a threshold of 1e−05
rs7311004
rs2387977
rs35055552
rs78383039
rs62273907
rs10899911
rs7572229
rs11715853
rs8039785
rs55877868
rs823424
rs6809952
rs7303158
rs6828768
All − MR Egger
All − IVW(MRE)
−1.0
−0.5
0.0
0.5
1.0
MR effect size for
'Gut microbiota abundance (genus Parasutterella id.2892)' on 'Matrilysin'

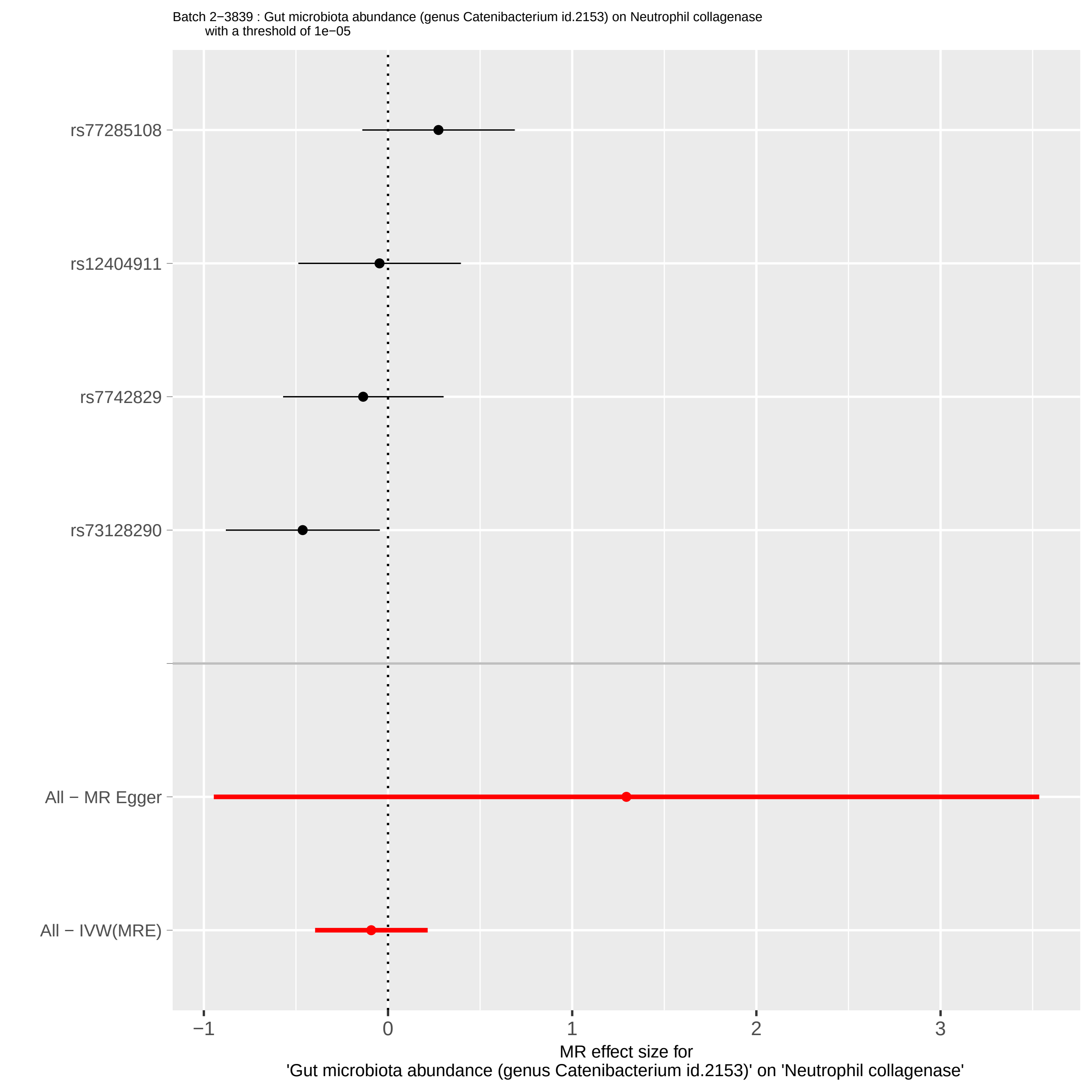
Batch 2−3839 : Gut microbiota abundance (genus Catenibacterium id.2153) on Neutrophil collagenase
with a threshold of 1e−05
rs77285108
rs12404911
rs7742829
rs73128290
All − MR Egger
All − IVW(MRE)
−1
0
1
2
3
MR effect size for
'Gut microbiota abundance (genus Catenibacterium id.2153)' on 'Neutrophil collagenase'

Batch 2−3840 : Gut microbiota abundance (genus Parasutterella id.2892) on Neutrophil collagenase
with a threshold of 1e−05

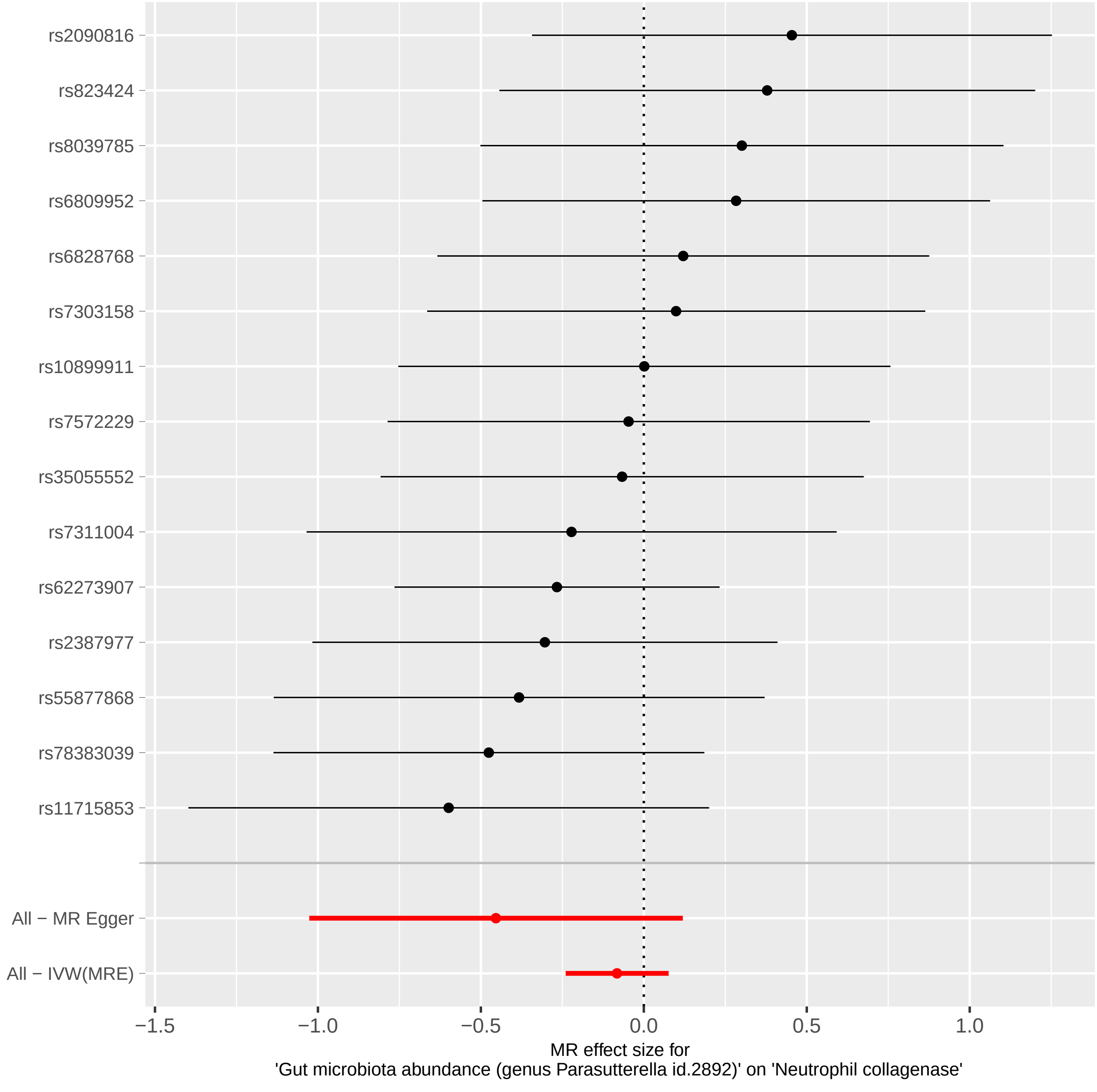

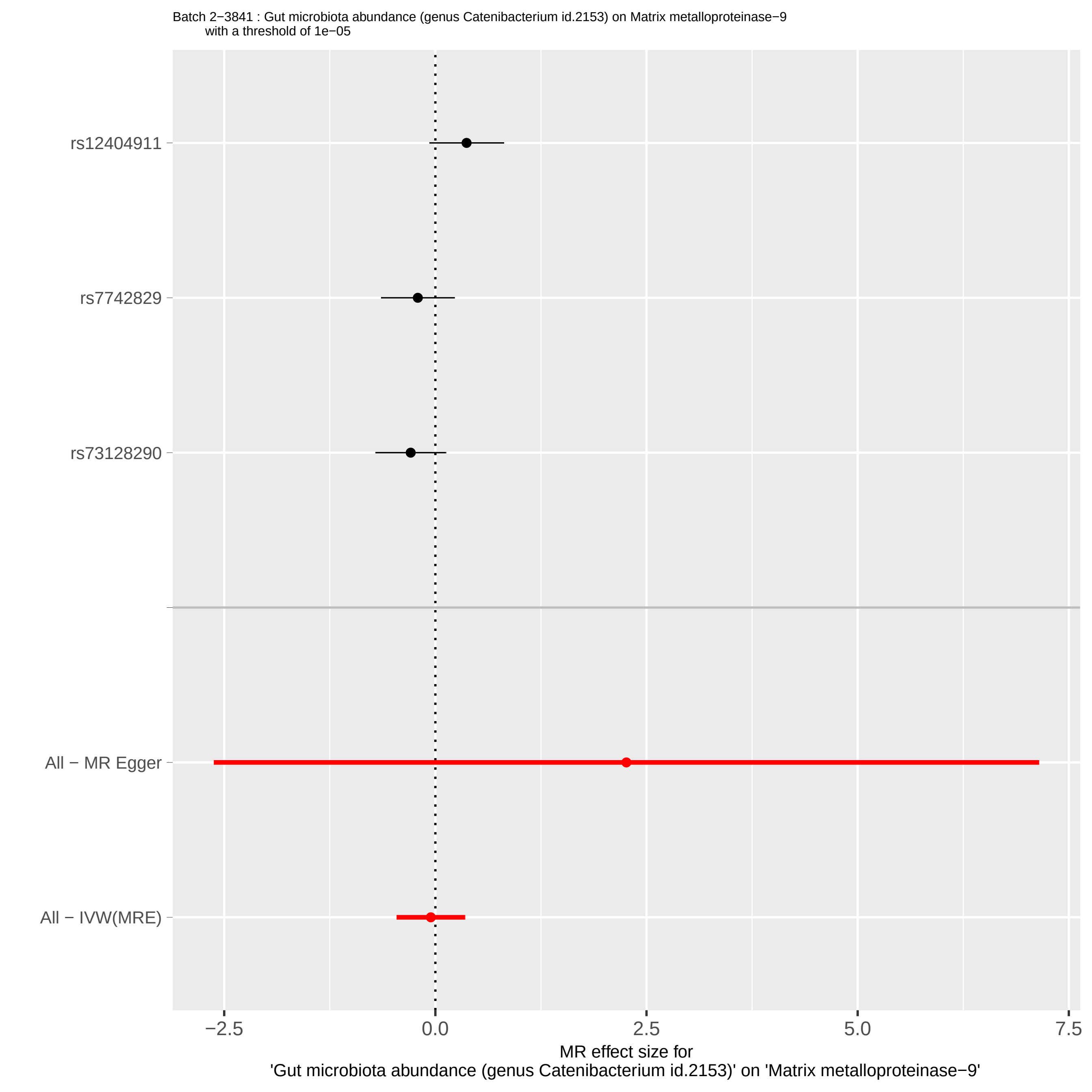

Batch 2−3841 : Gut microbiota abundance (genus Catenibacterium id.2153) on Matrix metalloproteinase−9
with a threshold of 1e−05
rs12404911
rs7742829
rs73128290
All − MR Egger
All − IVW(MRE)
−2.5
0.0
2.5
5.0
7.5
MR effect size for
'Gut microbiota abundance (genus Catenibacterium id.2153)' on 'Matrix metalloproteinase−9'

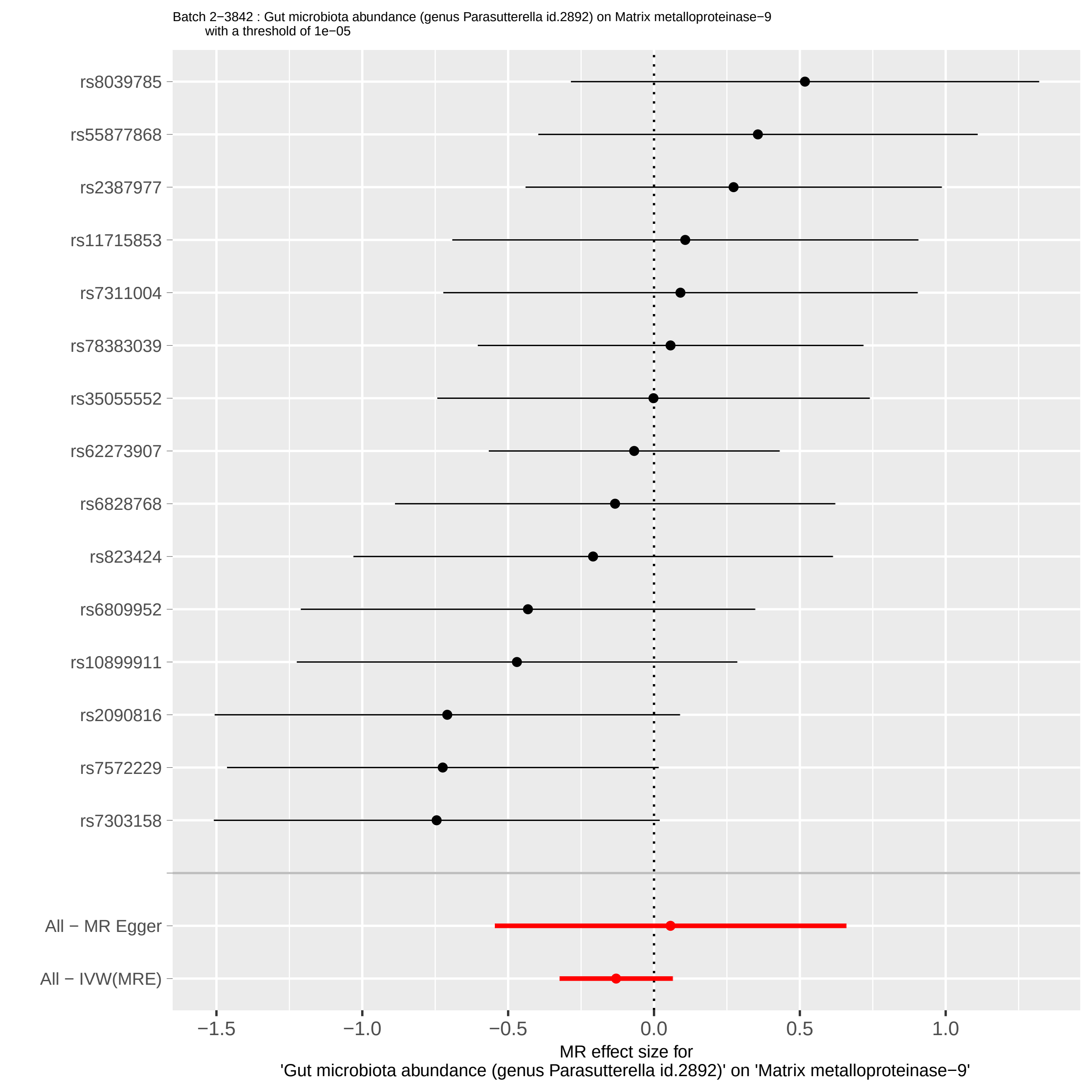
Batch 2−3842 : Gut microbiota abundance (genus Parasutterella id.2892) on Matrix metalloproteinase−9
with a threshold of 1e−05
rs8039785
rs55877868
rs2387977
rs11715853
rs7311004
rs78383039
rs35055552
rs62273907
rs6828768
rs823424
rs6809952
rs10899911
rs2090816
rs7572229
rs7303158
All − MR Egger
All − IVW(MRE)
−1.5
−1.0
−0.5
0.0
0.5
1.0
MR effect size for
'Gut microbiota abundance (genus Parasutterella id.2892)' on 'Matrix metalloproteinase−9'

Batch 2−3843 : Gut microbiota abundance (genus Catenibacterium id.2153) on Multimerin−2
with a threshold of 1e−05

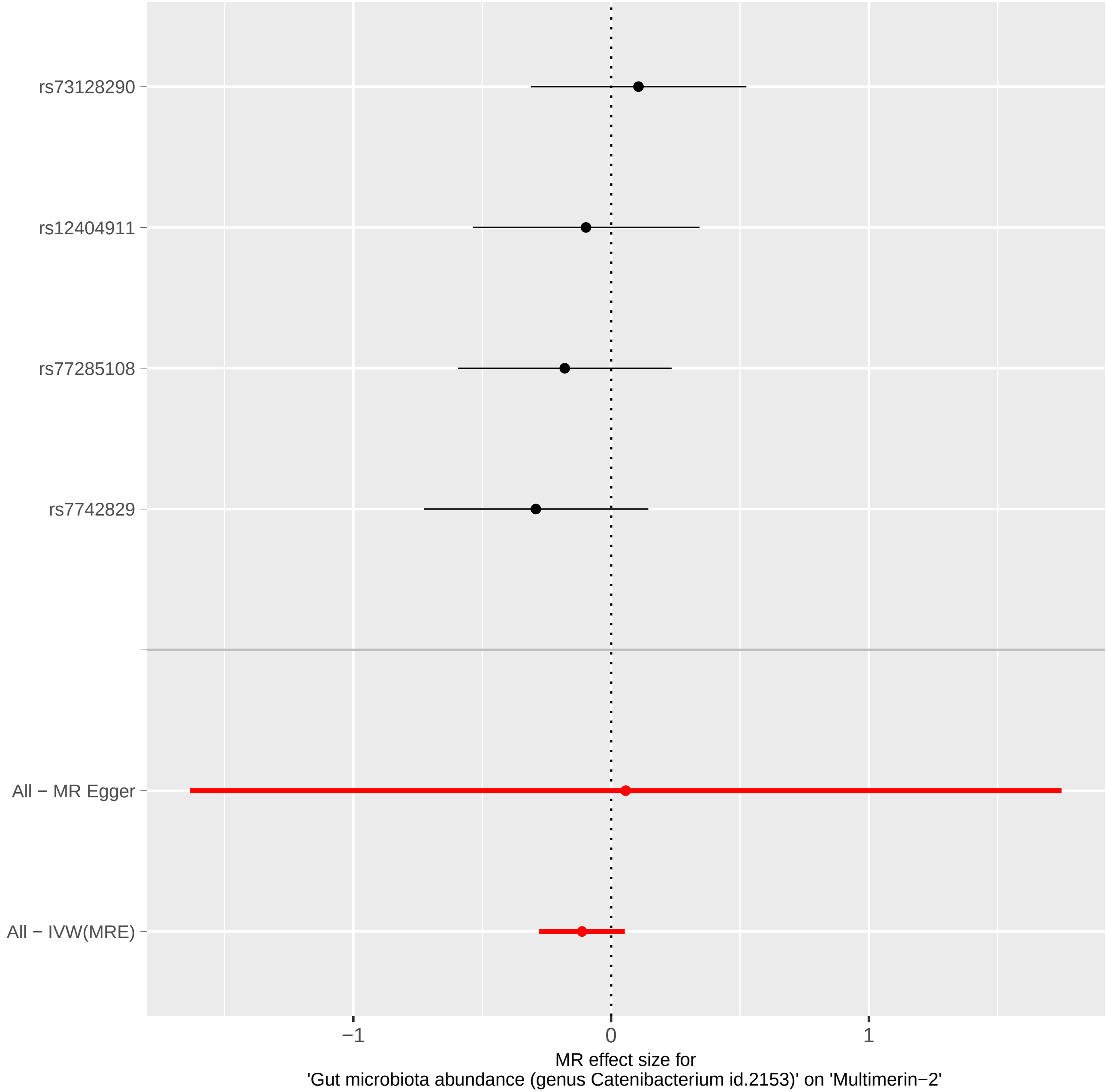

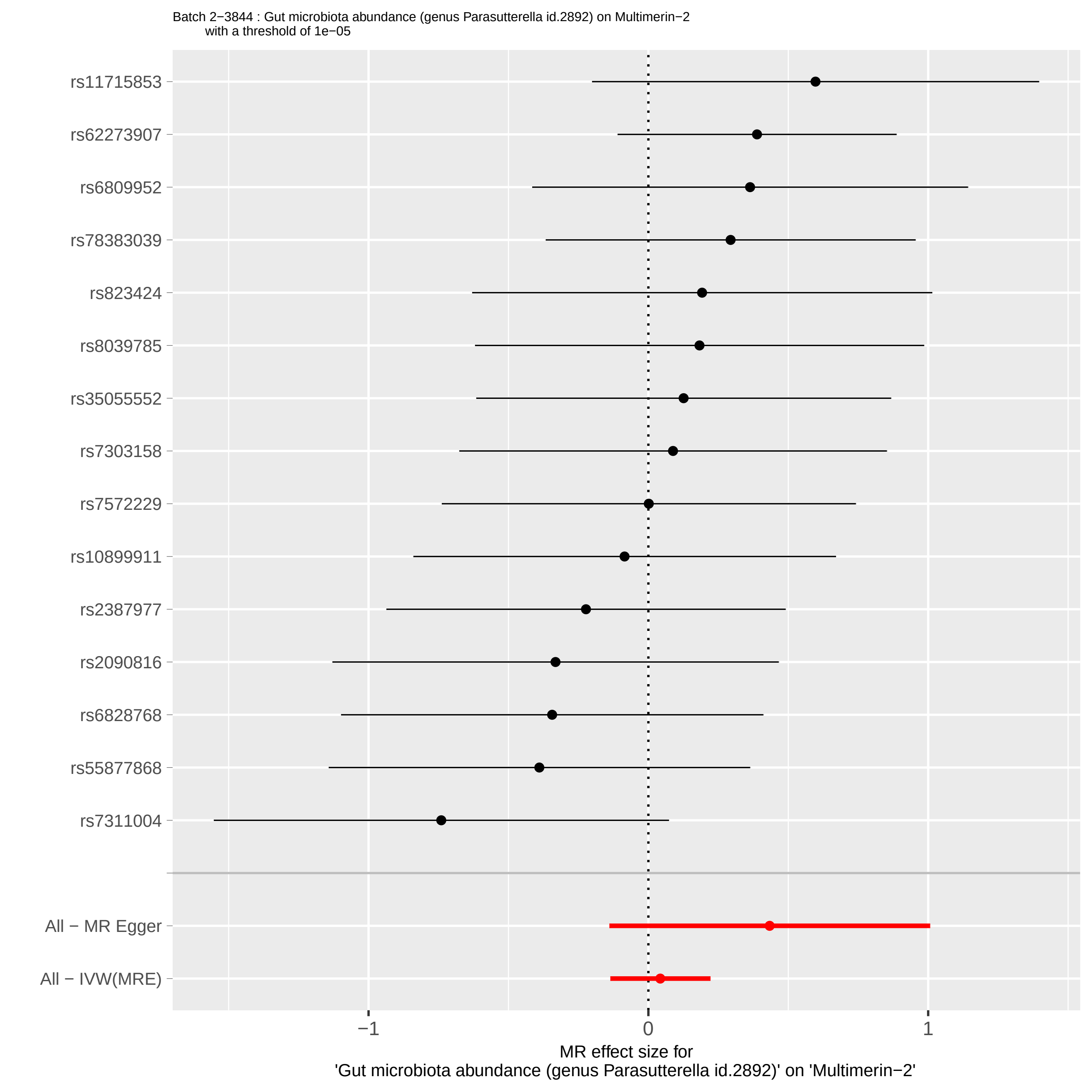

Batch 2−3844 : Gut microbiota abundance (genus Parasutterella id.2892) on Multimerin−2
with a threshold of 1e−05
rs11715853
rs62273907
rs6809952
rs78383039
rs823424
rs8039785
rs35055552
rs7303158
rs7572229
rs10899911
rs2387977
rs2090816
rs6828768
rs55877868
rs7311004
All − MR Egger
All − IVW(MRE)
−1
0
1
MR effect size for
'Gut microbiota abundance (genus Parasutterella id.2892)' on 'Multimerin−2'

Batch 2−3845 : Gut microbiota abundance (genus Catenibacterium id.2153) on Multimerin−2
with a threshold of 1e−05

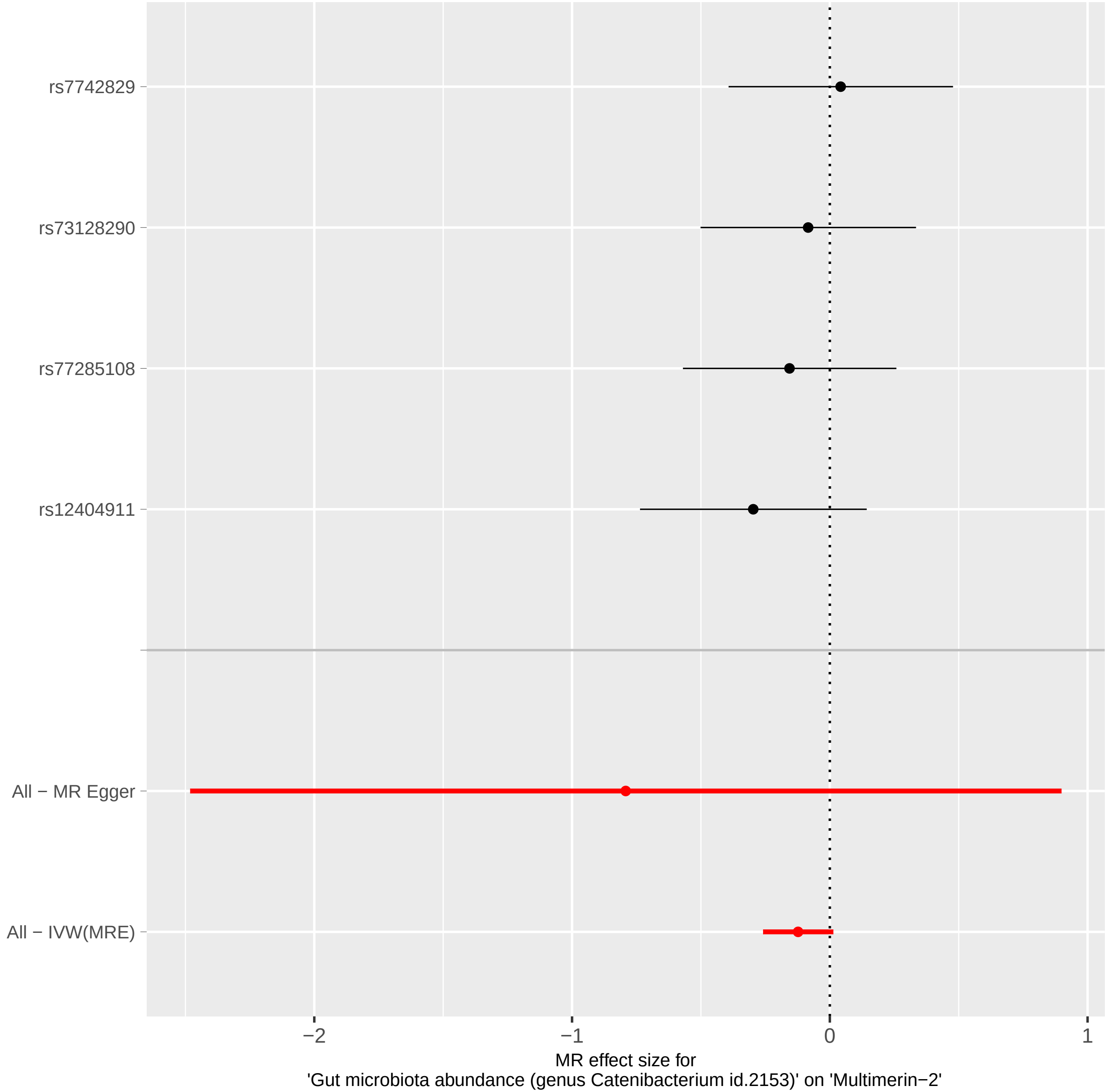

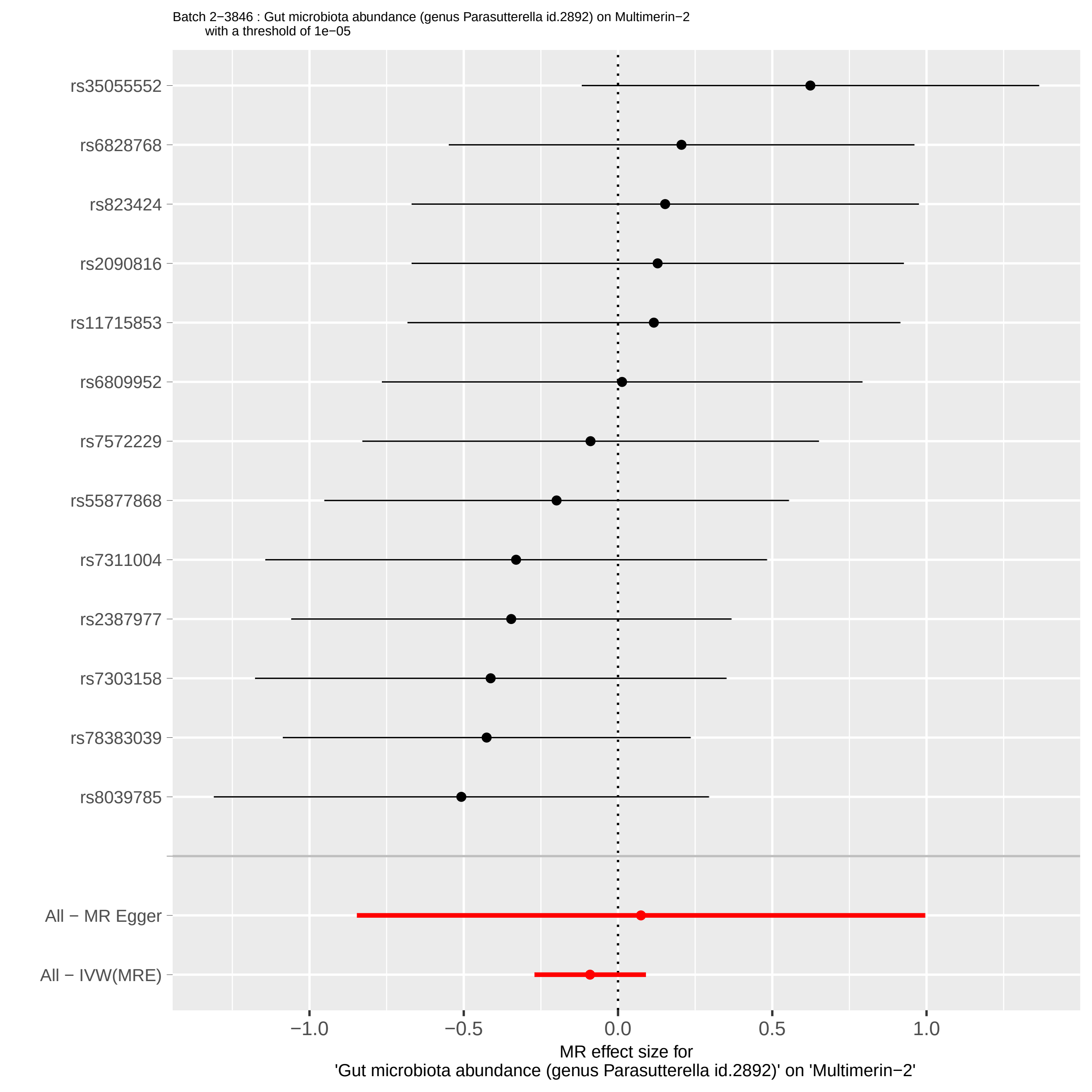
Batch 2−3846 : Gut microbiota abundance (genus Parasutterella id.2892) on Multimerin−2
with a threshold of 1e−05
rs35055552
rs6828768
rs823424
rs2090816
rs11715853
rs6809952
rs7572229
rs55877868
rs7311004
rs2387977
rs7303158
rs78383039
rs8039785
All − MR Egger
All − IVW(MRE)
−1.0
−0.5
0.0
0.5
1.0
MR effect size for
'Gut microbiota abundance (genus Parasutterella id.2892)' on 'Multimerin−2'

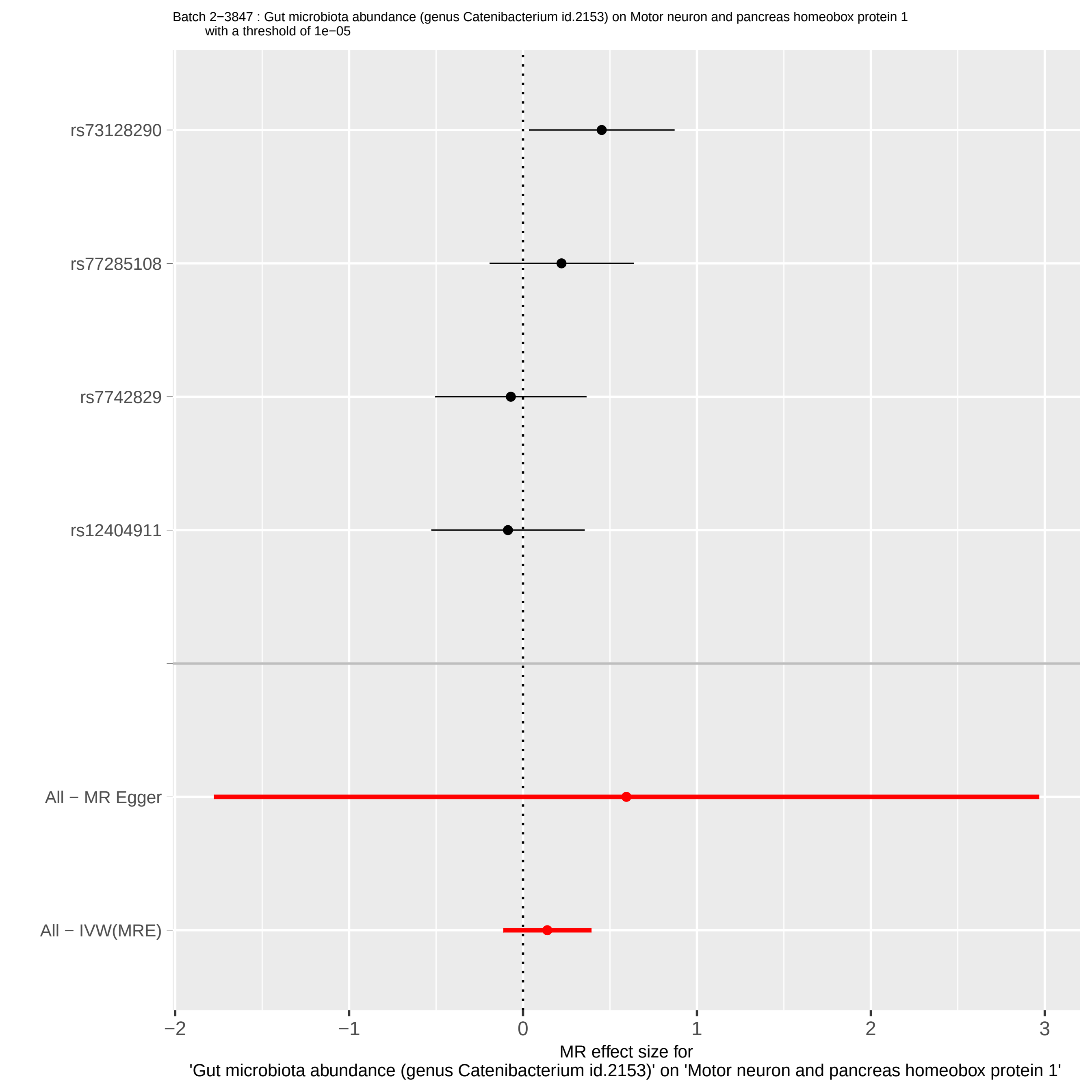
Batch 2−3847 : Gut microbiota abundance (genus Catenibacterium id.2153) on Motor neuron and pancreas homeobox protein 1
with a threshold of 1e−05
rs73128290
rs77285108
rs7742829
rs12404911
All − MR Egger
All − IVW(MRE)
−2
−1
0
1
2
3
MR effect size for
'Gut microbiota abundance (genus Catenibacterium id.2153)' on 'Motor neuron and pancreas homeobox protein 1'

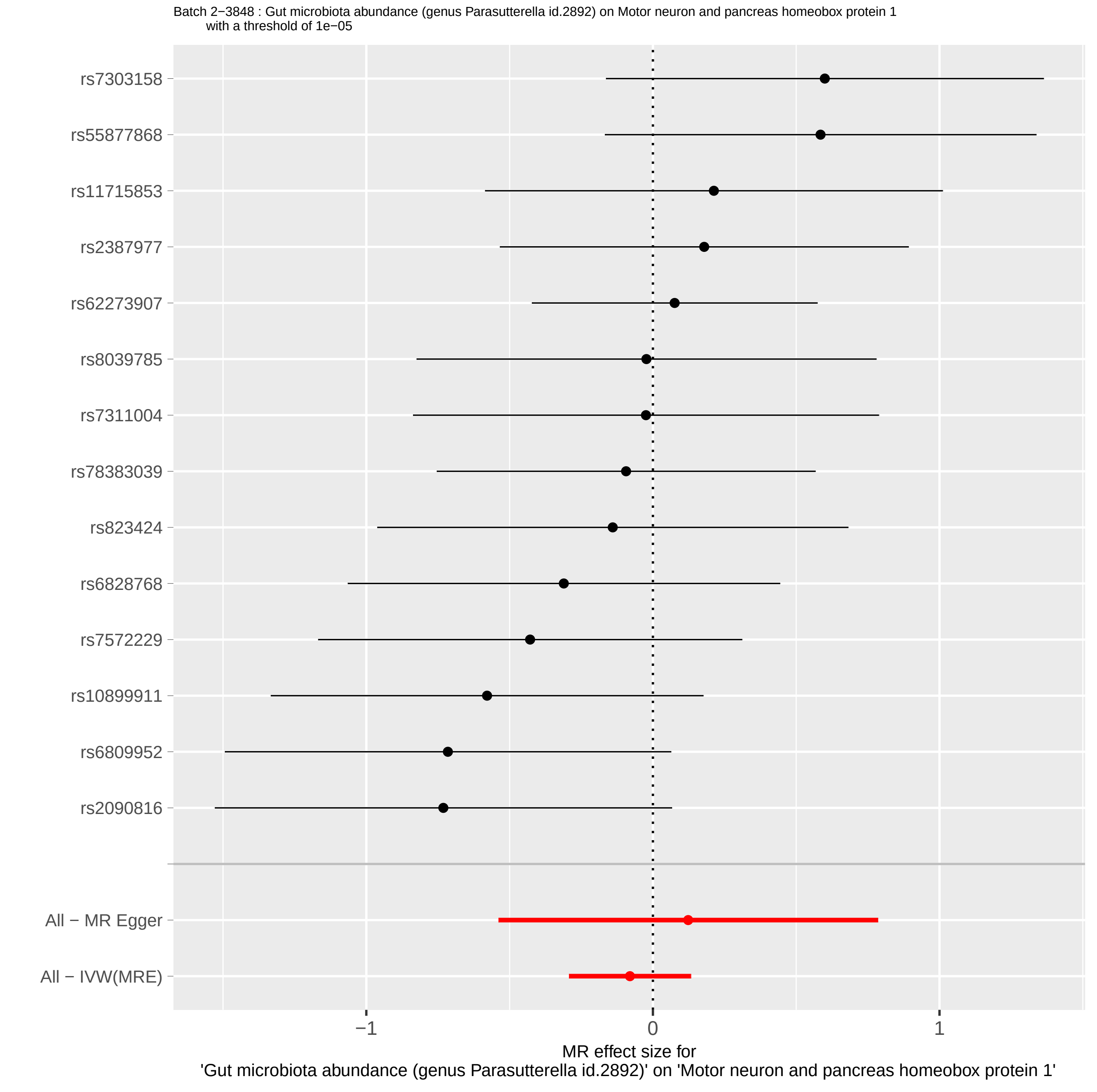

Batch 2−3848 : Gut microbiota abundance (genus Parasutterella id.2892) on Motor neuron and pancreas homeobox protein 1
with a threshold of 1e−05
rs7303158
rs55877868
rs11715853
rs2387977
rs62273907
rs8039785
rs7311004
rs78383039
rs823424
rs6828768
rs7572229
rs10899911
rs6809952
rs2090816
All − MR Egger
All − IVW(MRE)
−1
0
1
MR effect size for
'Gut microbiota abundance (genus Parasutterella id.2892)' on 'Motor neuron and pancreas homeobox protein 1'

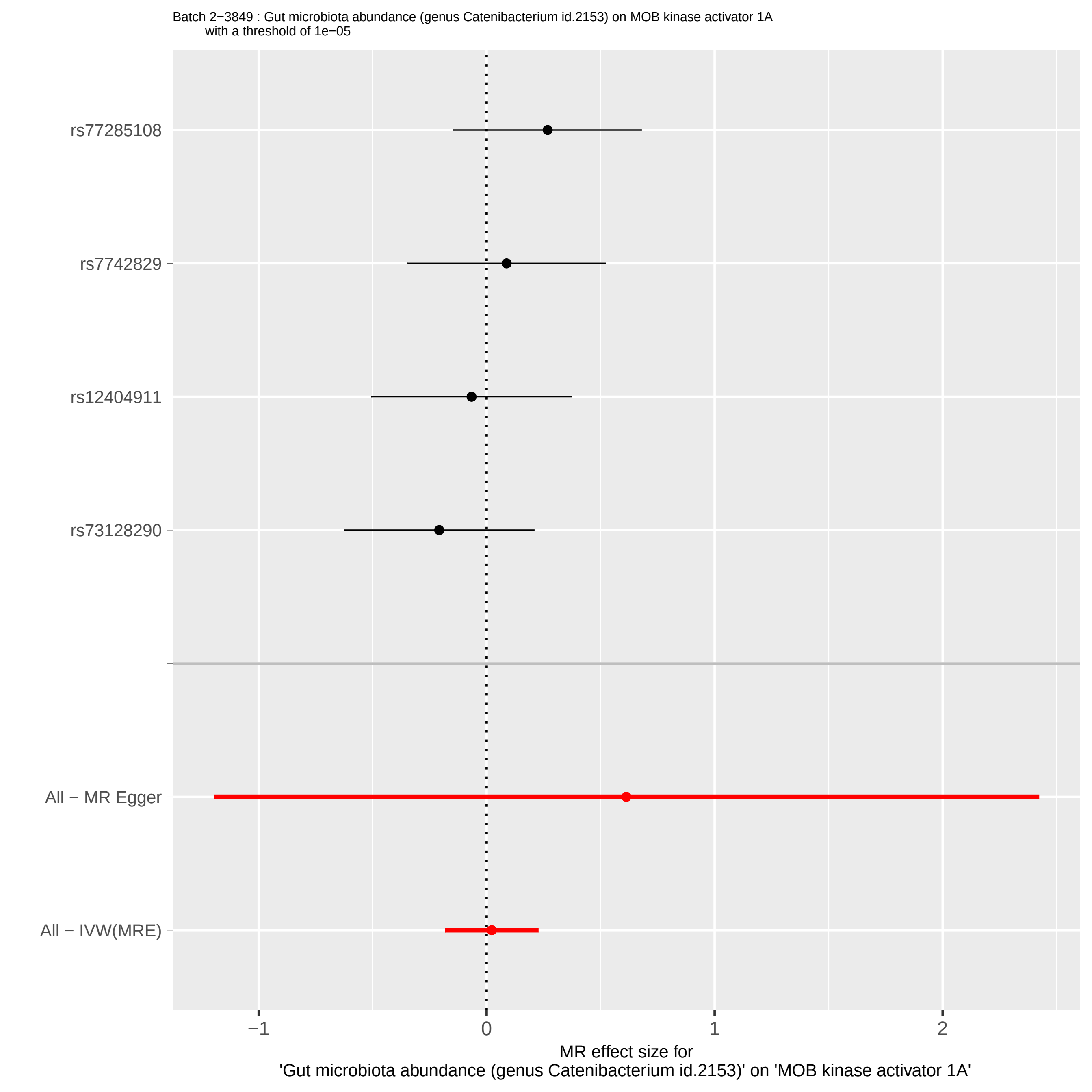
Batch 2−3849 : Gut microbiota abundance (genus Catenibacterium id.2153) on MOB kinase activator 1A
with a threshold of 1e−05
rs77285108
rs7742829
rs12404911
rs73128290
All − MR Egger
All − IVW(MRE)
−1
0
1
2
MR effect size for
'Gut microbiota abundance (genus Catenibacterium id.2153)' on 'MOB kinase activator 1A'

Batch 2−3850 : Gut microbiota abundance (genus Parasutterella id.2892) on MOB kinase activator 1A
with a threshold of 1e−05

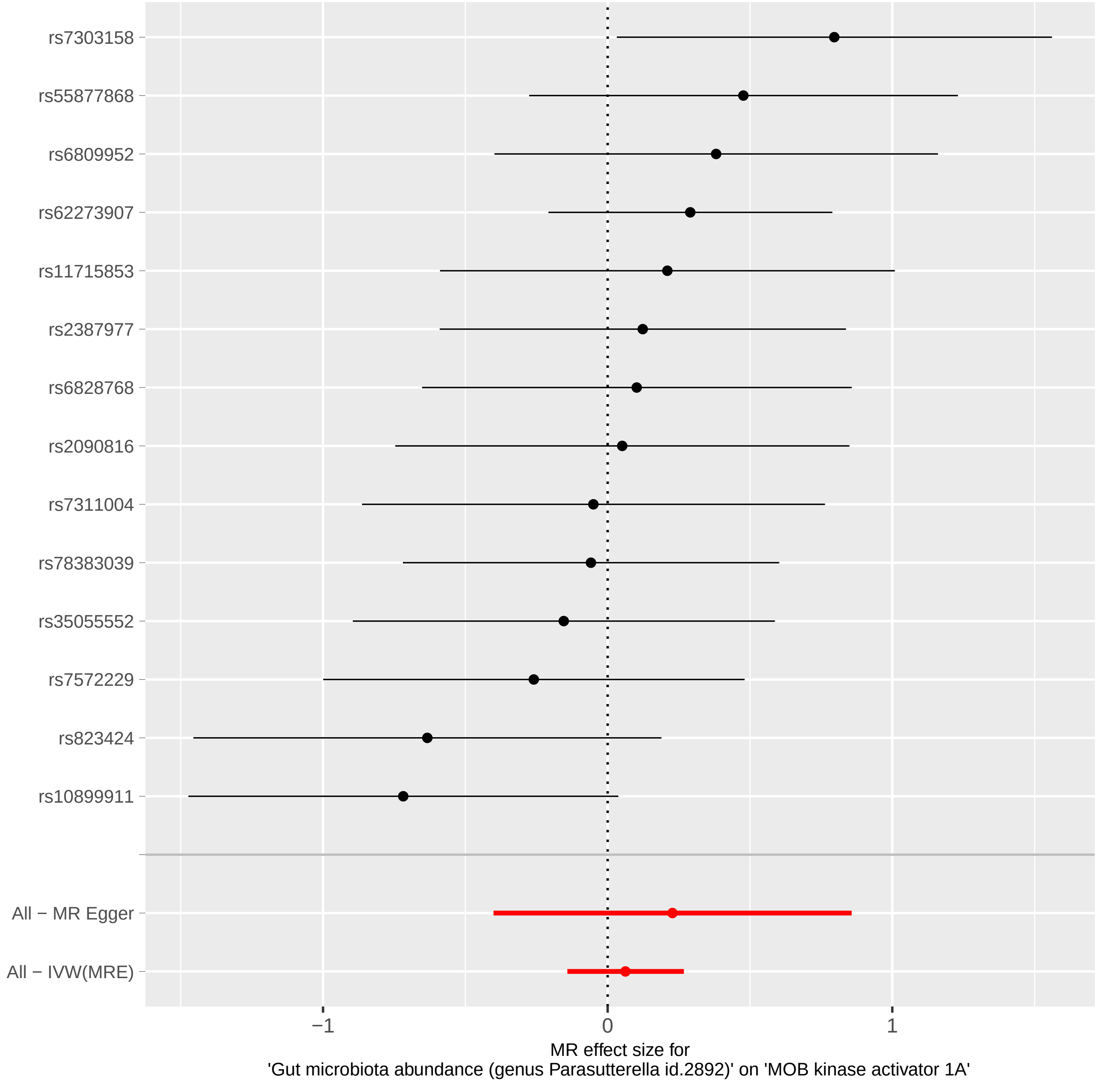

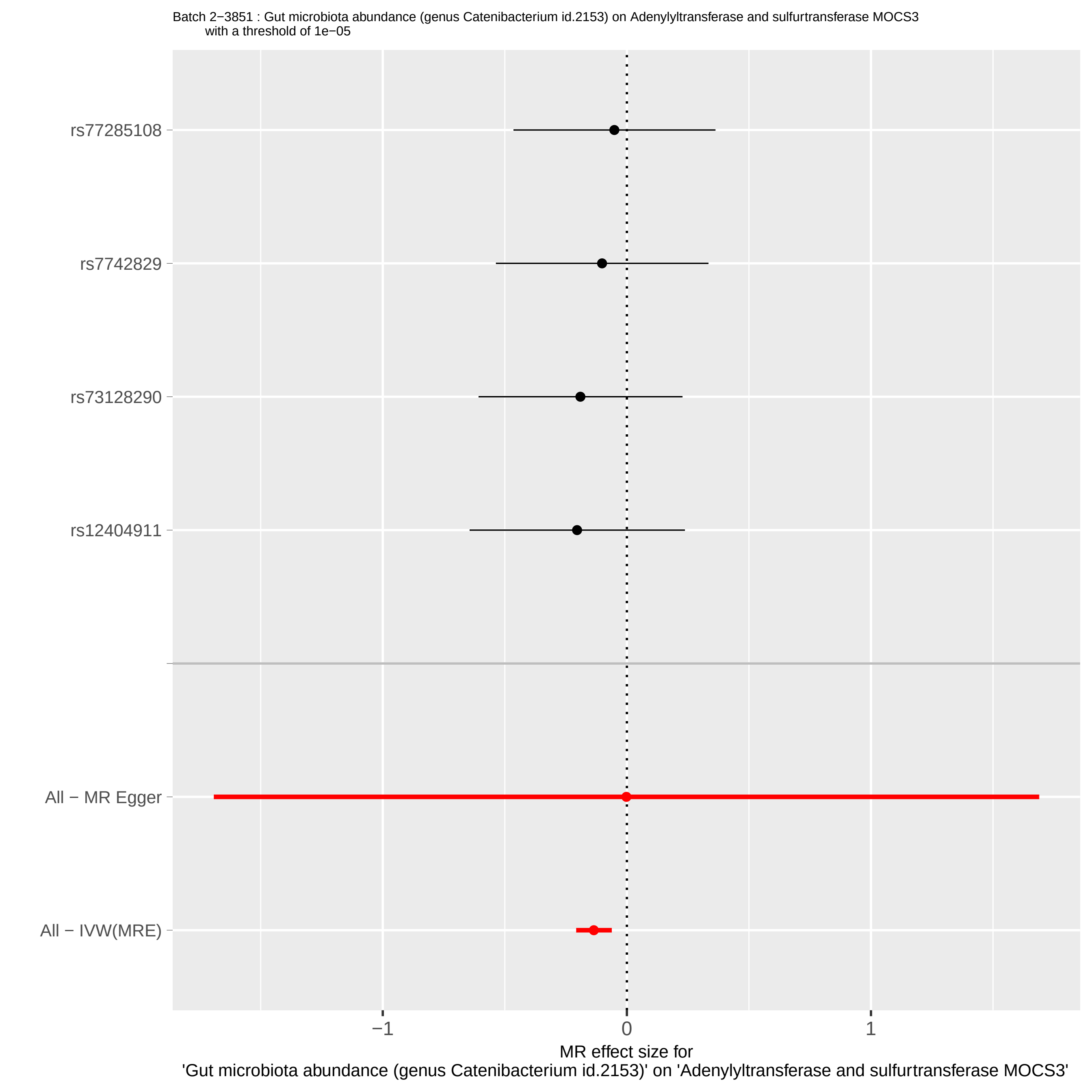
Batch 2−3851 : Gut microbiota abundance (genus Catenibacterium id.2153) on Adenylyltransferase and sulfurtransferase MOCS3
with a threshold of 1e−05
rs77285108
rs7742829
rs73128290
rs12404911
All − MR Egger
All − IVW(MRE)
−1
0
1
MR effect size for
'Gut microbiota abundance (genus Catenibacterium id.2153)' on 'Adenylyltransferase and sulfurtransferase MOCS3'

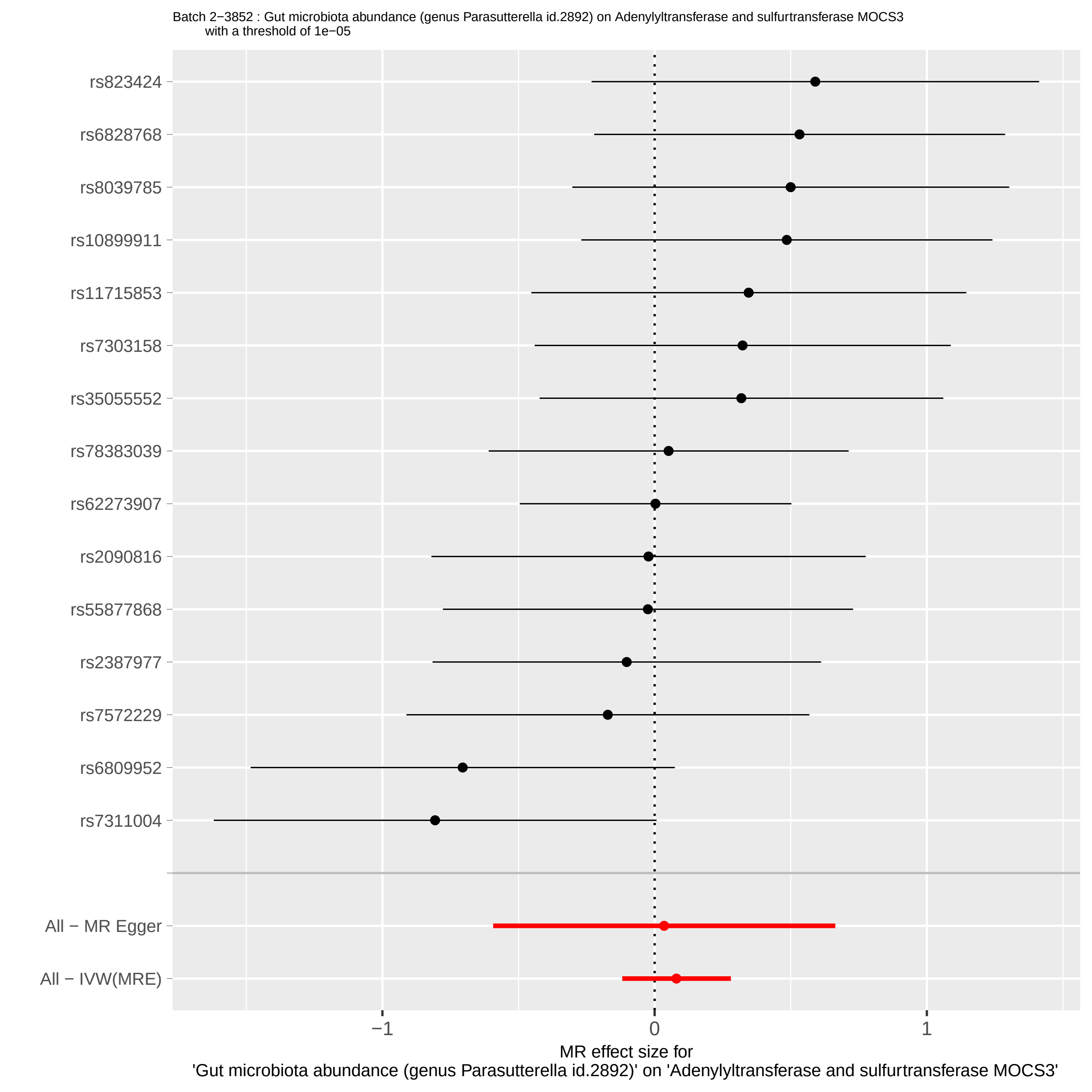

Batch 2−3852 : Gut microbiota abundance (genus Parasutterella id.2892) on Adenylyltransferase and sulfurtransferase MOCS3
with a threshold of 1e−05
rs823424
rs6828768
rs8039785
rs10899911
rs11715853
rs7303158
rs35055552
rs78383039
rs62273907
rs2090816
rs55877868
rs2387977
rs7572229
rs6809952
rs7311004
All − MR Egger
All − IVW(MRE)
−1
0
1
MR effect size for
'Gut microbiota abundance (genus Parasutterella id.2892)' on 'Adenylyltransferase and sulfurtransferase MOCS3'

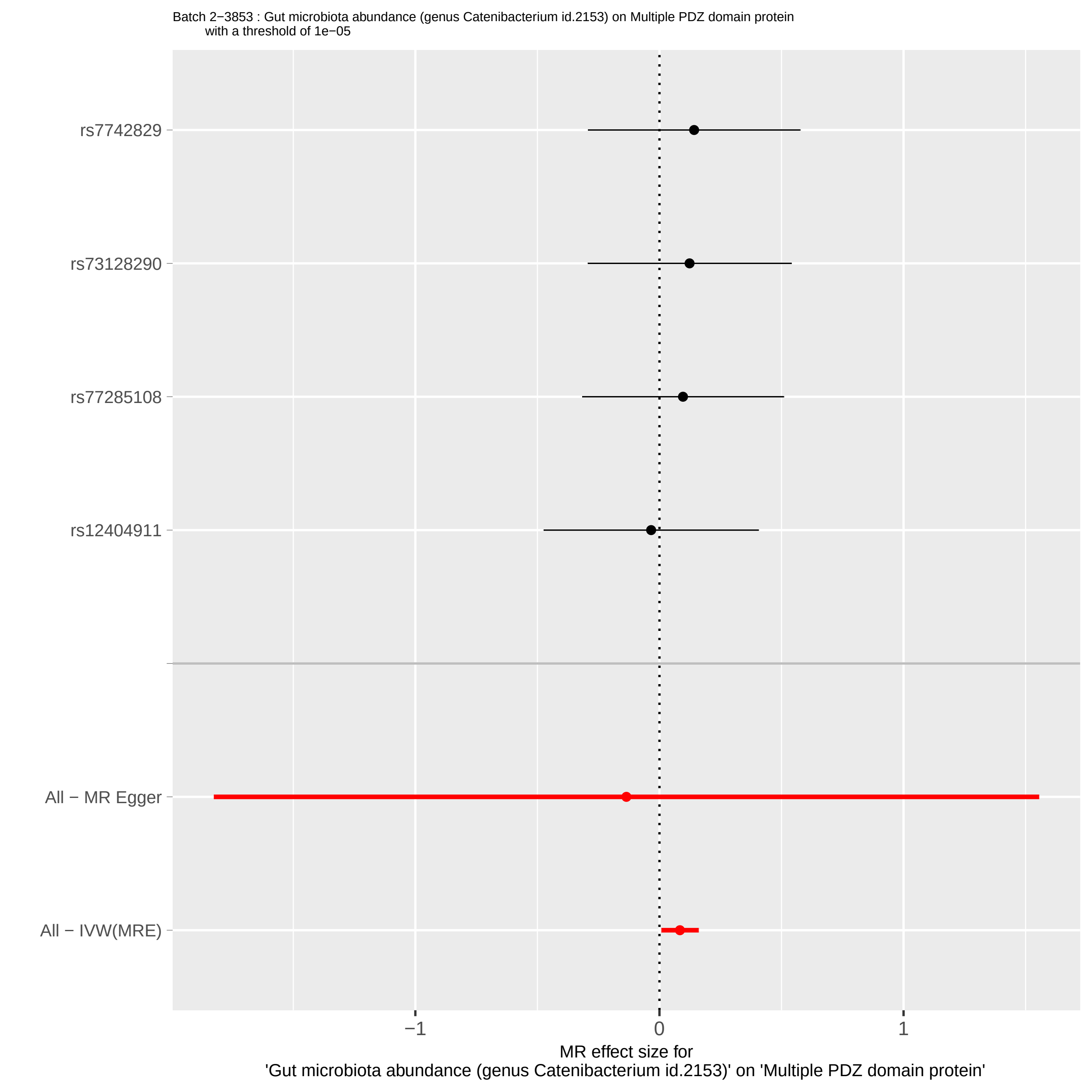

Batch 2−3853 : Gut microbiota abundance (genus Catenibacterium id.2153) on Multiple PDZ domain protein
with a threshold of 1e−05
rs7742829
rs73128290
rs77285108
rs12404911
All − MR Egger
All − IVW(MRE)
−1
0
1
MR effect size for
'Gut microbiota abundance (genus Catenibacterium id.2153)' on 'Multiple PDZ domain protein'

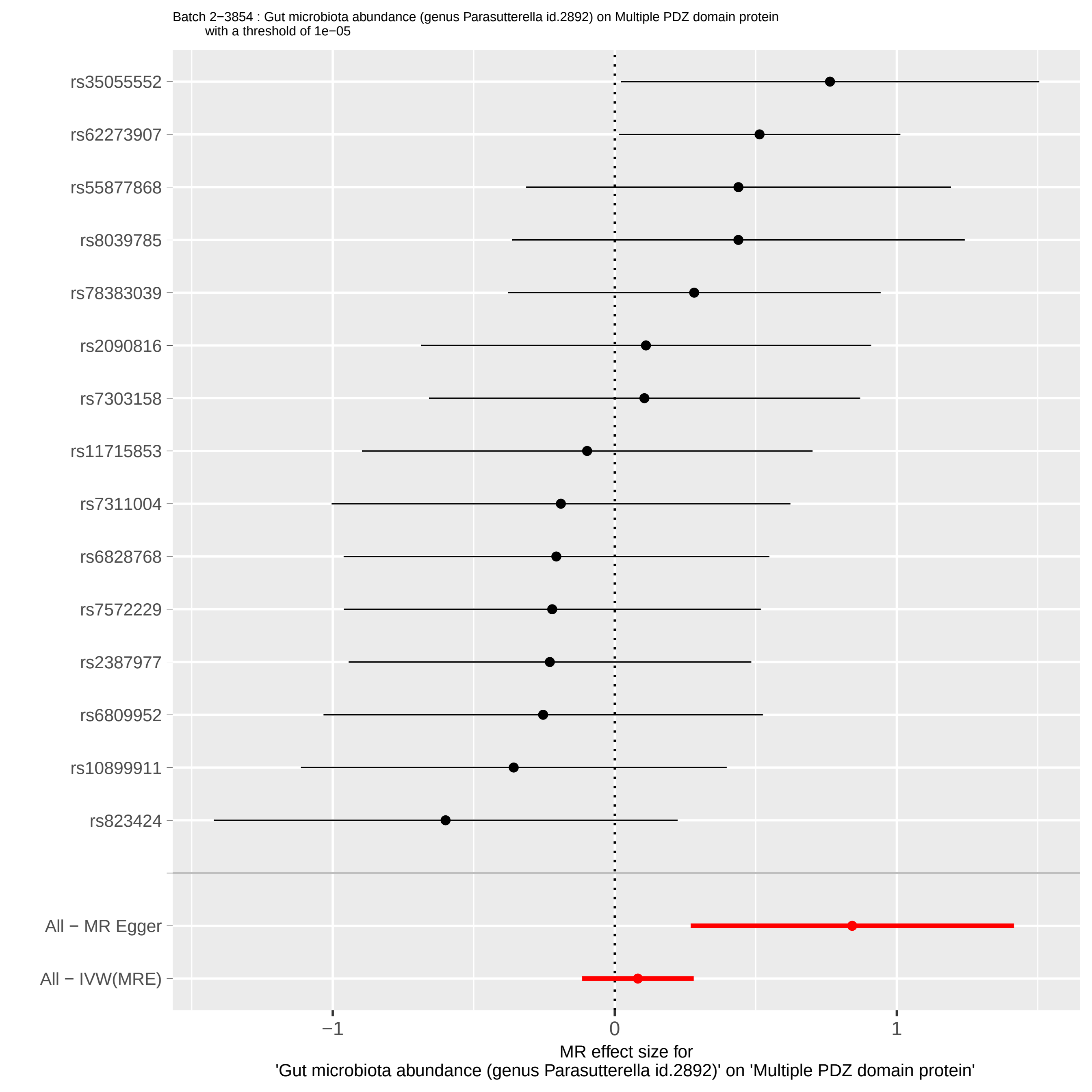

Batch 2−3854 : Gut microbiota abundance (genus Parasutterella id.2892) on Multiple PDZ domain protein
with a threshold of 1e−05
rs35055552
rs62273907
rs55877868
rs8039785
rs78383039
rs2090816
rs7303158
rs11715853
rs7311004
rs6828768
rs7572229
rs2387977
rs6809952
rs10899911
rs823424
All − MR Egger
All − IVW(MRE)
−1
0
1
MR effect size for
'Gut microbiota abundance (genus Parasutterella id.2892)' on 'Multiple PDZ domain protein'

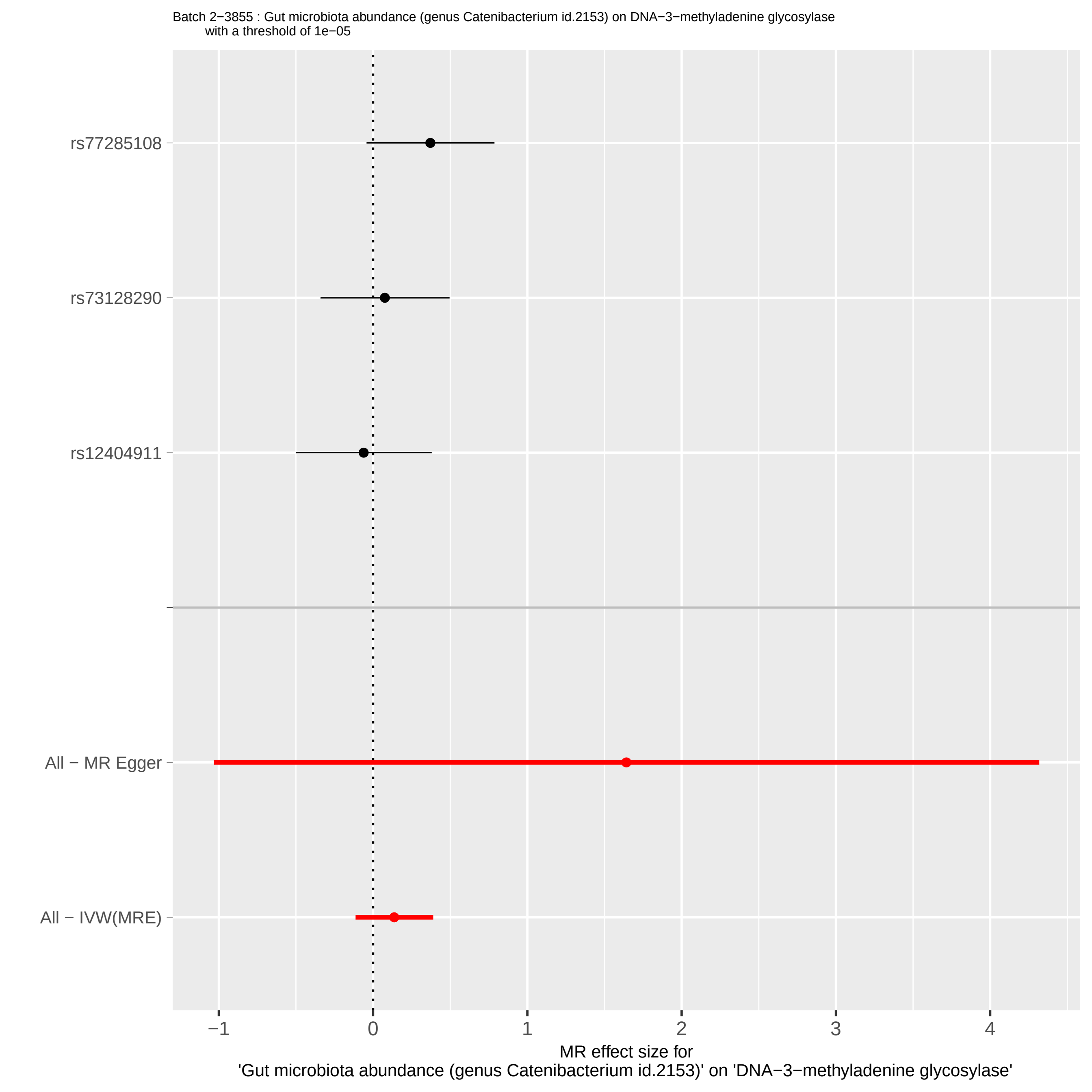

Batch 2−3855 : Gut microbiota abundance (genus Catenibacterium id.2153) on DNA−3−methyladenine glycosylase
with a threshold of 1e−05
rs77285108
rs73128290
rs12404911
All − MR Egger
All − IVW(MRE)
−1
0
1
2
3
4
MR effect size for
'Gut microbiota abundance (genus Catenibacterium id.2153)' on 'DNA−3−methyladenine glycosylase'

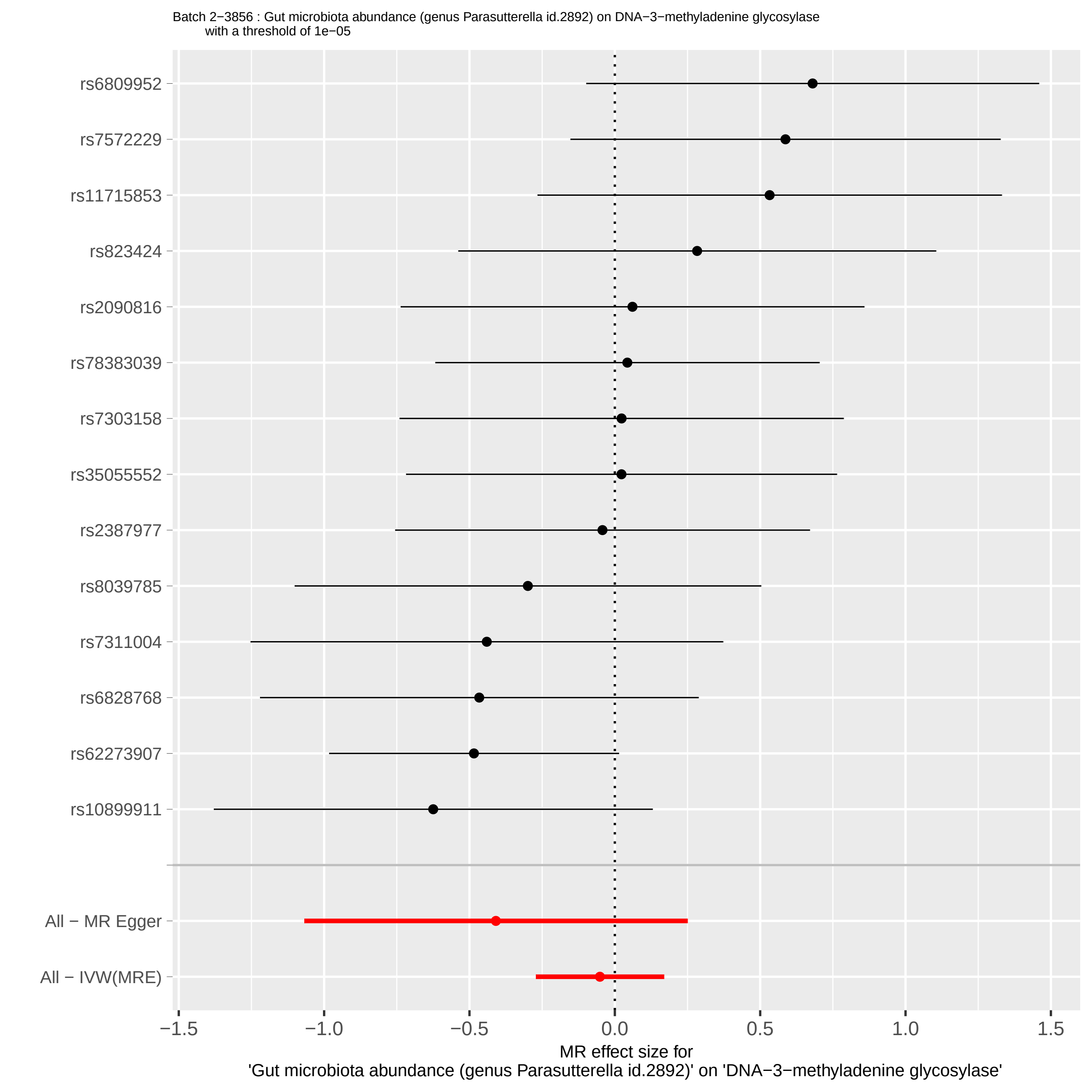

Batch 2−3856 : Gut microbiota abundance (genus Parasutterella id.2892) on DNA−3−methyladenine glycosylase
with a threshold of 1e−05
rs6809952
rs7572229
rs11715853
rs823424
rs2090816
rs78383039
rs7303158
rs35055552
rs2387977
rs8039785
rs7311004
rs6828768
rs62273907
rs10899911
All − MR Egger
All − IVW(MRE)
−1.5
−1.0
−0.5
0.0
0.5
1.0
1.5
MR effect size for
'Gut microbiota abundance (genus Parasutterella id.2892)' on 'DNA−3−methyladenine glycosylase'

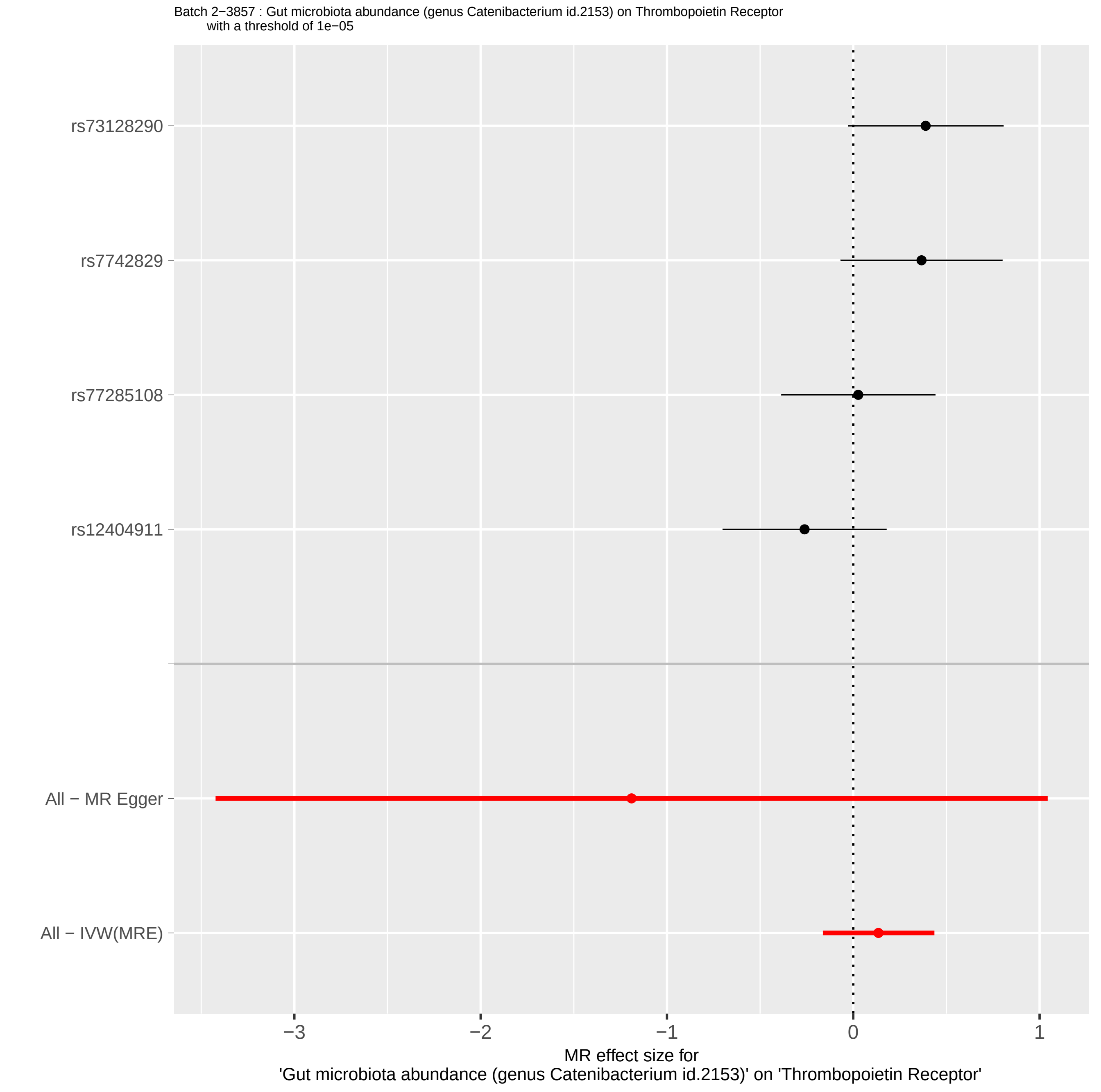
Batch 2−3857 : Gut microbiota abundance (genus Catenibacterium id.2153) on Thrombopoietin Receptor
with a threshold of 1e−05
rs73128290
rs7742829
rs77285108
rs12404911
All − MR Egger
All − IVW(MRE)
−3
−2
−1
0
1
MR effect size for
'Gut microbiota abundance (genus Catenibacterium id.2153)' on 'Thrombopoietin Receptor'

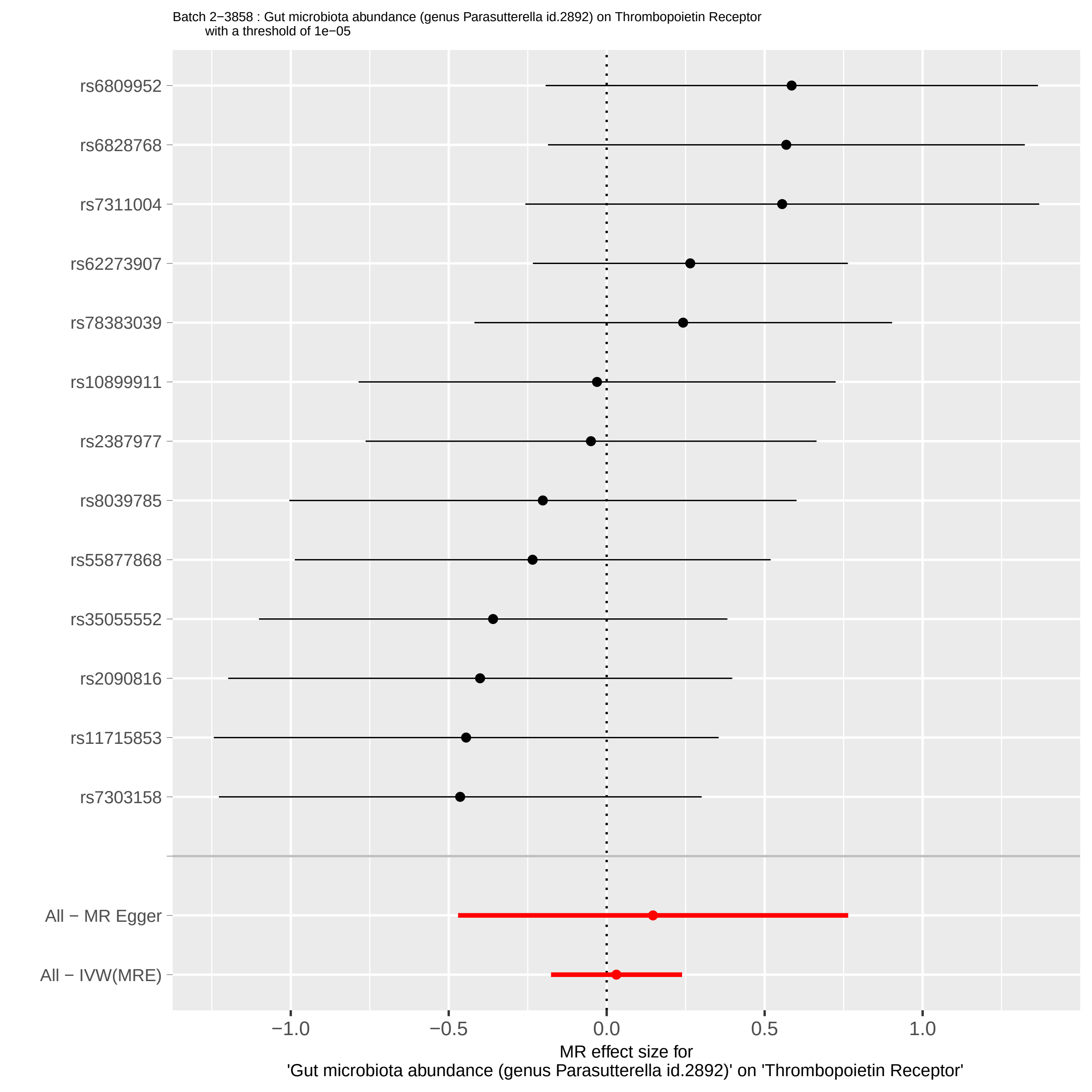

Batch 2−3858 : Gut microbiota abundance (genus Parasutterella id.2892) on Thrombopoietin Receptor
with a threshold of 1e−05
rs6809952
rs6828768
rs7311004
rs62273907
rs78383039
rs10899911
rs2387977
rs8039785
rs55877868
rs35055552
rs2090816
rs11715853
rs7303158
All − MR Egger
All − IVW(MRE)
−1.0
−0.5
0.0
0.5
1.0
MR effect size for
'Gut microbiota abundance (genus Parasutterella id.2892)' on 'Thrombopoietin Receptor'

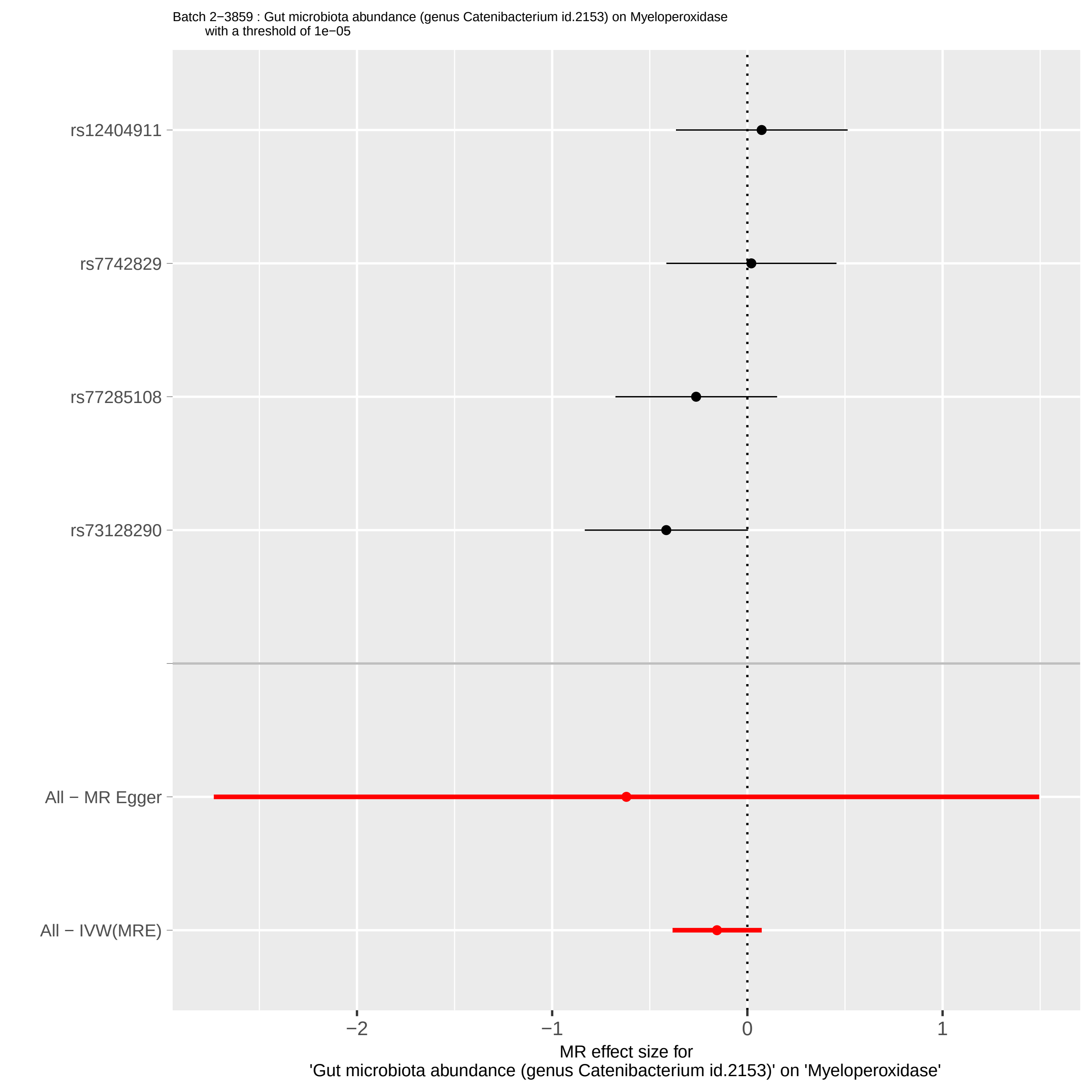
Batch 2−3859 : Gut microbiota abundance (genus Catenibacterium id.2153) on Myeloperoxidase
with a threshold of 1e−05
rs12404911
rs7742829
rs77285108
rs73128290
All − MR Egger
All − IVW(MRE)
−2
−1
0
1
MR effect size for
'Gut microbiota abundance (genus Catenibacterium id.2153)' on 'Myeloperoxidase'

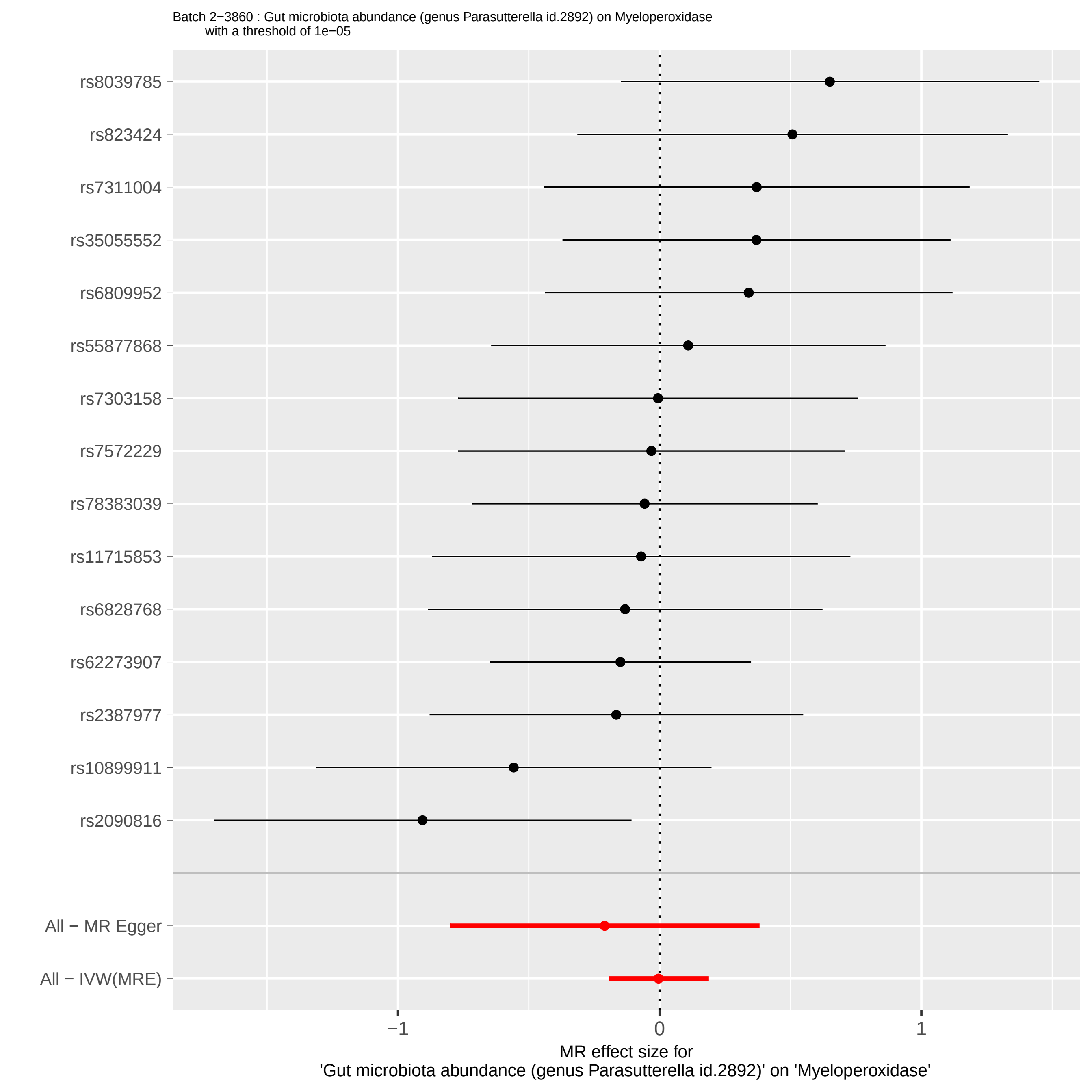

Batch 2−3860 : Gut microbiota abundance (genus Parasutterella id.2892) on Myeloperoxidase
with a threshold of 1e−05
rs8039785
rs823424
rs7311004
rs35055552
rs6809952
rs55877868
rs7303158
rs7572229
rs78383039
rs11715853
rs6828768
rs62273907
rs2387977
rs10899911
rs2090816
All − MR Egger
All − IVW(MRE)
−1
0
1
MR effect size for
'Gut microbiota abundance (genus Parasutterella id.2892)' on 'Myeloperoxidase'

Batch 2−3861 : Gut microbiota abundance (genus Catenibacterium id.2153) on MAGUK p55 subfamily member 6
with a threshold of 1e−05

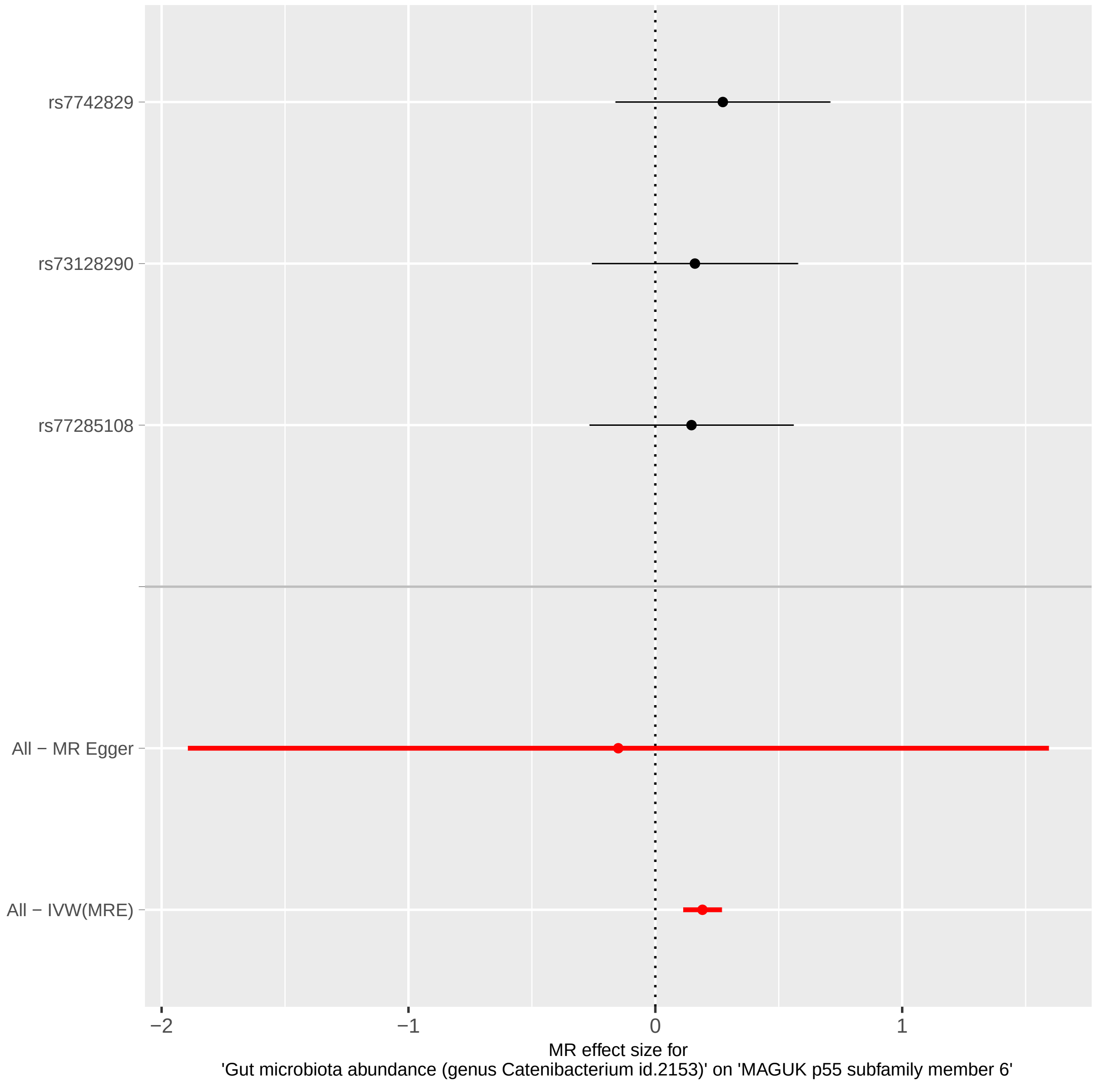

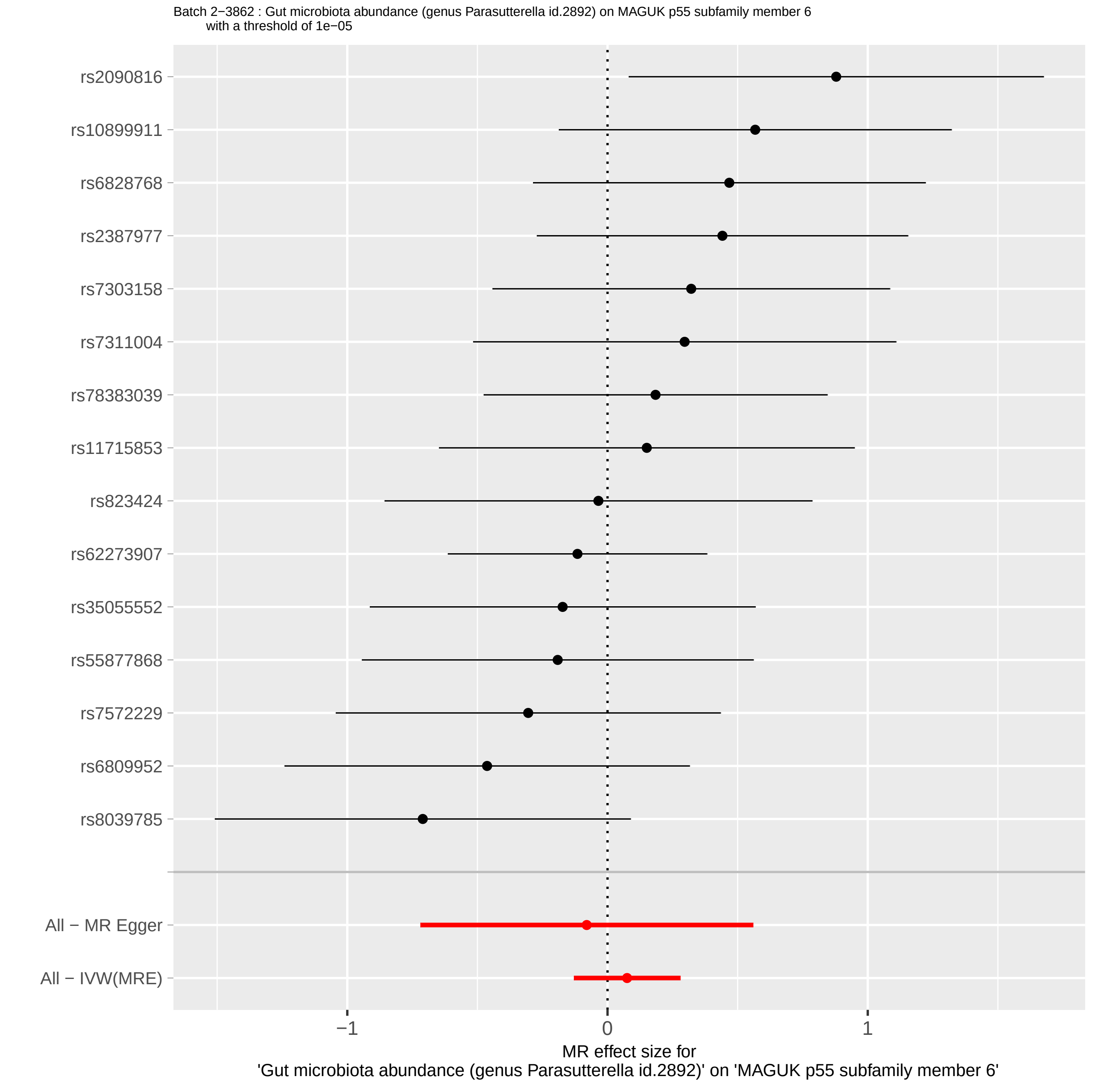

Batch 2−3862 : Gut microbiota abundance (genus Parasutterella id.2892) on MAGUK p55 subfamily member 6
with a threshold of 1e−05
rs2090816
rs10899911
rs6828768
rs2387977
rs7303158
rs7311004
rs78383039
rs11715853
rs823424
rs62273907
rs35055552
rs55877868
rs7572229
rs6809952
rs8039785
All − MR Egger
All − IVW(MRE)
−1
0
1
MR effect size for
'Gut microbiota abundance (genus Parasutterella id.2892)' on 'MAGUK p55 subfamily member 6'

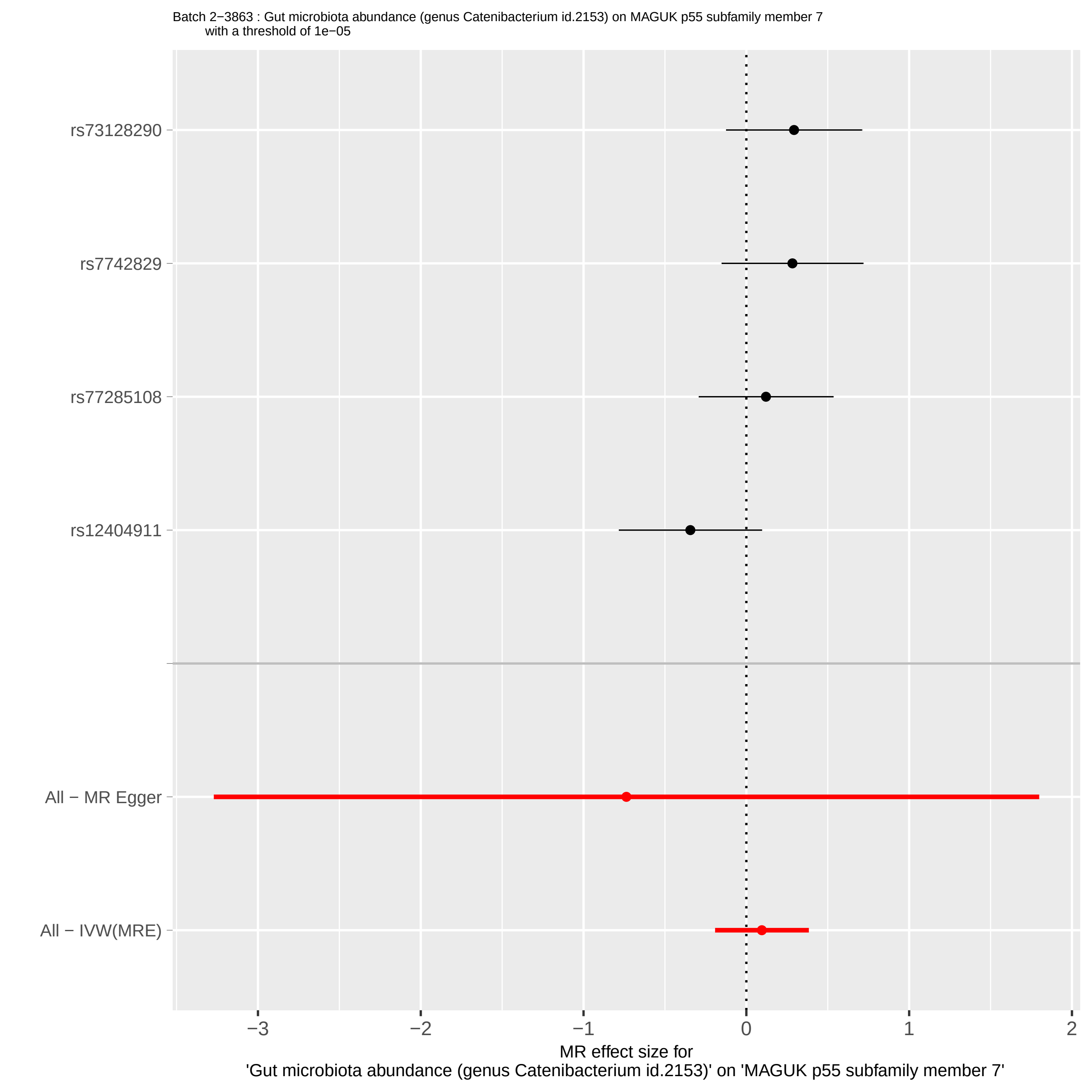

Batch 2−3863 : Gut microbiota abundance (genus Catenibacterium id.2153) on MAGUK p55 subfamily member 7
with a threshold of 1e−05
rs73128290
rs7742829
rs77285108
rs12404911
All − MR Egger
All − IVW(MRE)
−3
−2
−1
0
1
2
MR effect size for
'Gut microbiota abundance (genus Catenibacterium id.2153)' on 'MAGUK p55 subfamily member 7'

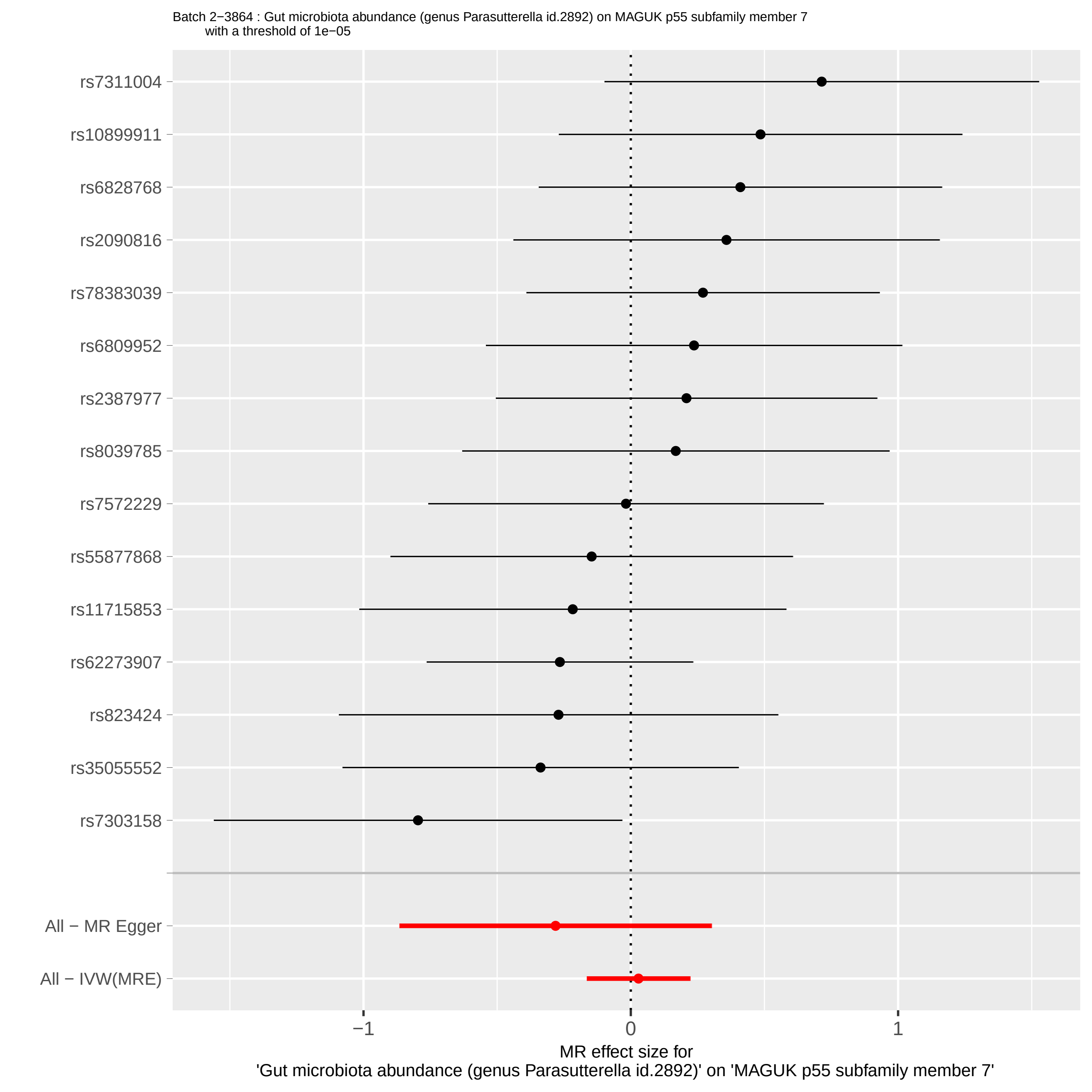
Batch 2−3864 : Gut microbiota abundance (genus Parasutterella id.2892) on MAGUK p55 subfamily member 7
with a threshold of 1e−05
rs7311004
rs10899911
rs6828768
rs2090816
rs78383039
rs6809952
rs2387977
rs8039785
rs7572229
rs55877868
rs11715853
rs62273907
rs823424
rs35055552
rs7303158
All − MR Egger
All − IVW(MRE)
−1
0
1
MR effect size for
'Gut microbiota abundance (genus Parasutterella id.2892)' on 'MAGUK p55 subfamily member 7'

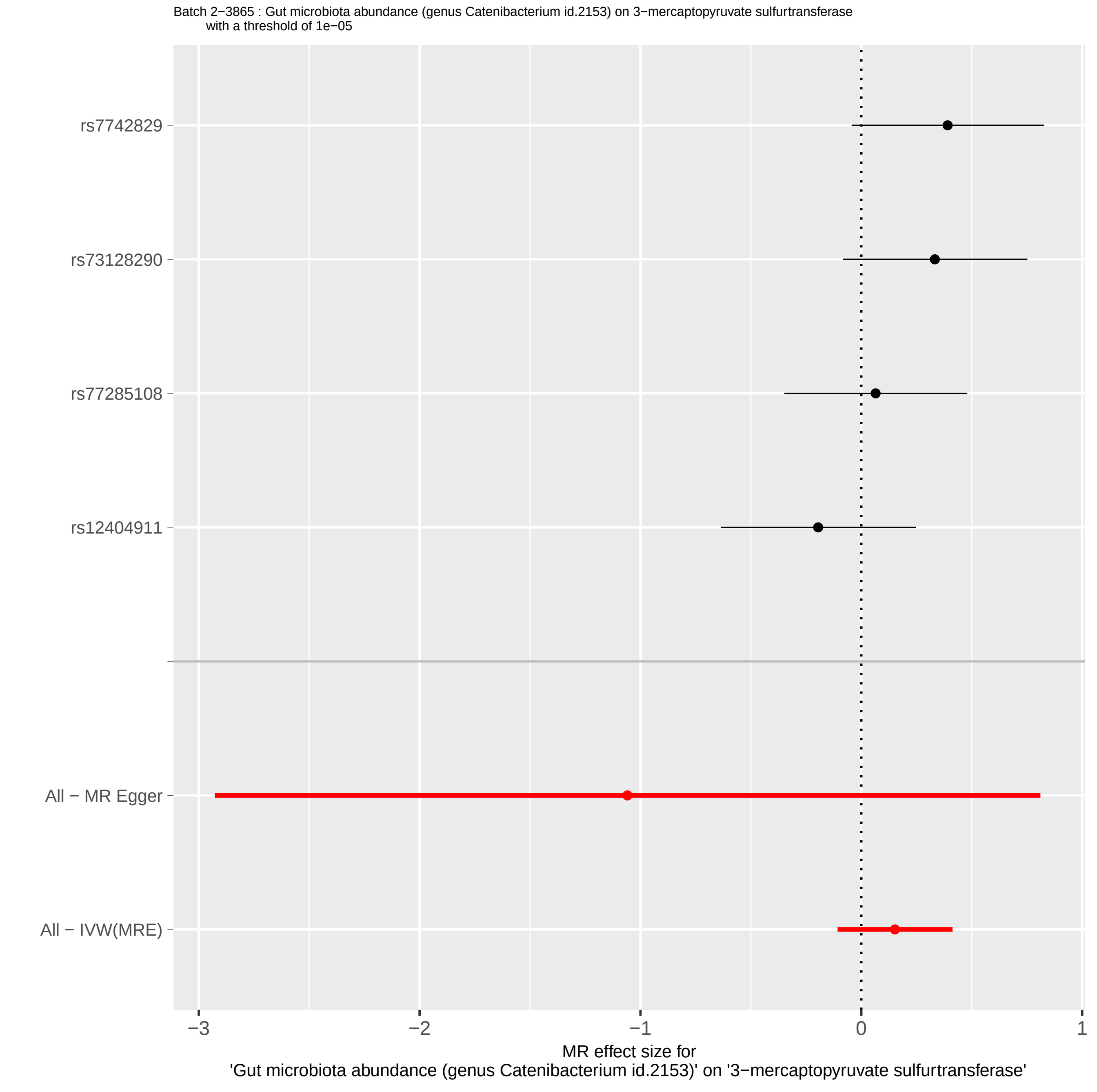

Batch 2−3865 : Gut microbiota abundance (genus Catenibacterium id.2153) on 3−mercaptopyruvate sulfurtransferase
with a threshold of 1e−05
rs7742829
rs73128290
rs77285108
rs12404911
All − MR Egger
All − IVW(MRE)
−3
−2
−1
0
1
MR effect size for
'Gut microbiota abundance (genus Catenibacterium id.2153)' on '3−mercaptopyruvate sulfurtransferase'

Batch 2−3866 : Gut microbiota abundance (genus Parasutterella id.2892) on 3−mercaptopyruvate sulfurtransferase with a threshold of 1e−05

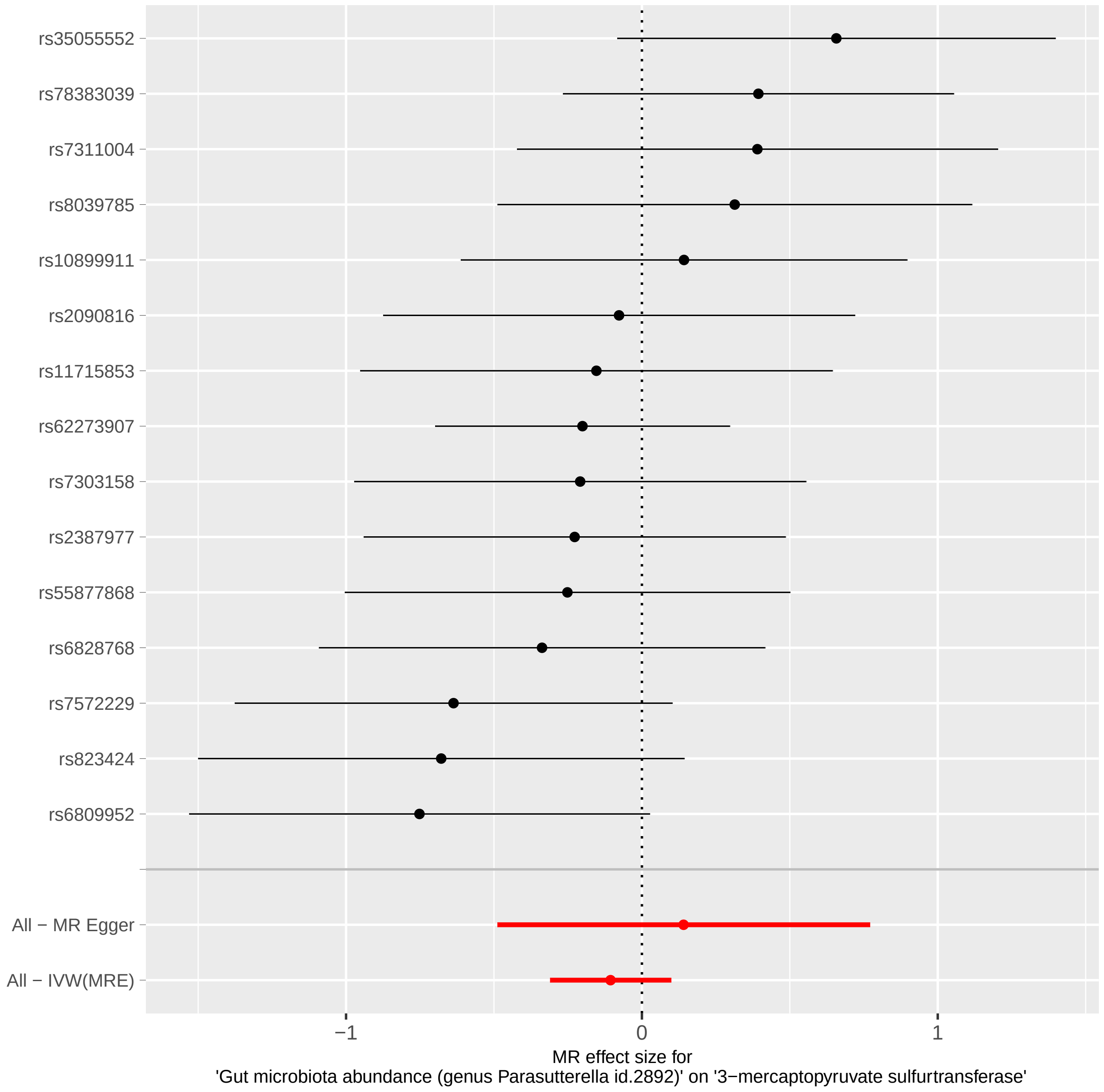

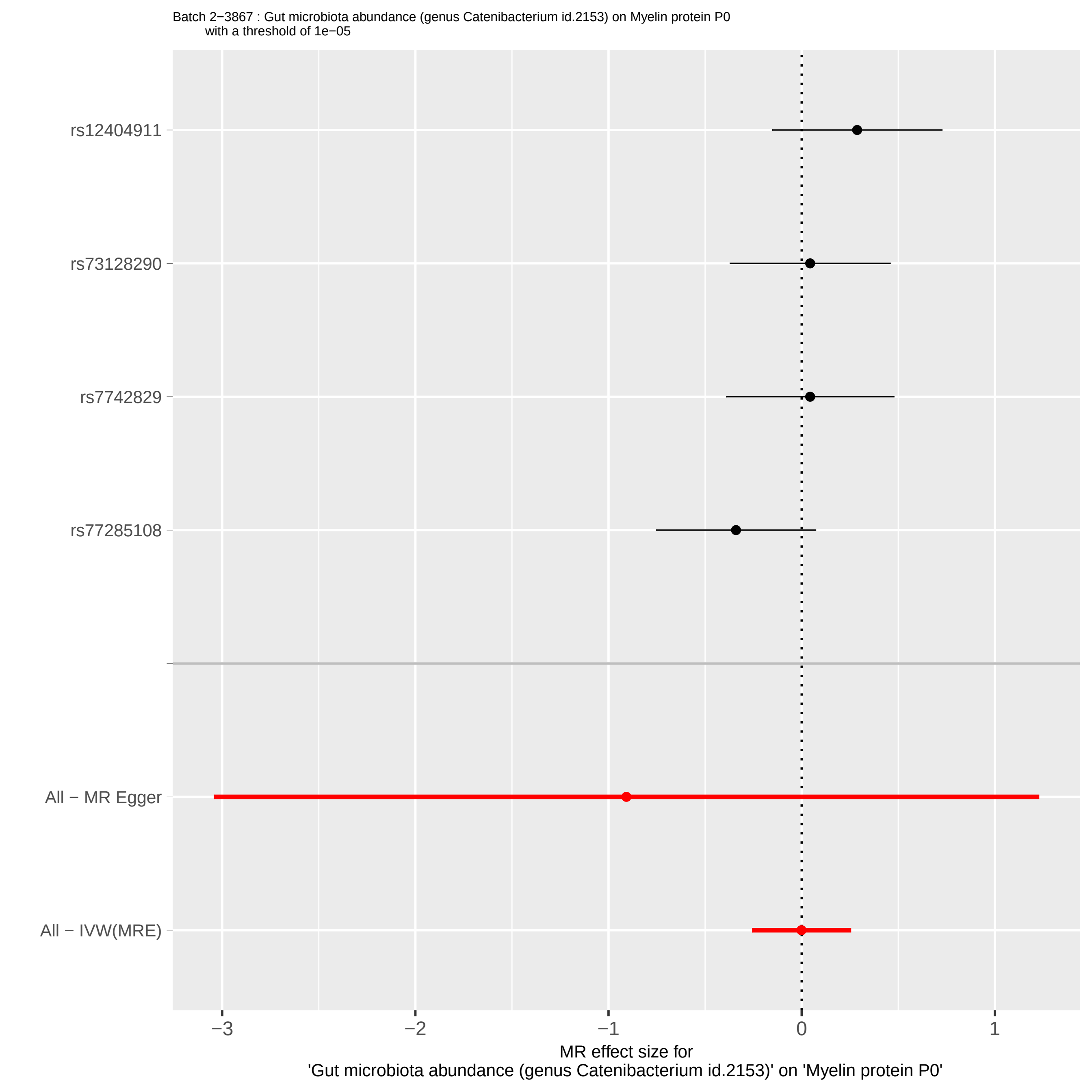
Batch 2−3867 : Gut microbiota abundance (genus Catenibacterium id.2153) on Myelin protein P0
with a threshold of 1e−05
rs12404911
rs73128290
rs7742829
rs77285108
All − MR Egger
All − IVW(MRE)
−3
−2
−1
0
1
MR effect size for
'Gut microbiota abundance (genus Catenibacterium id.2153)' on 'Myelin protein P0'

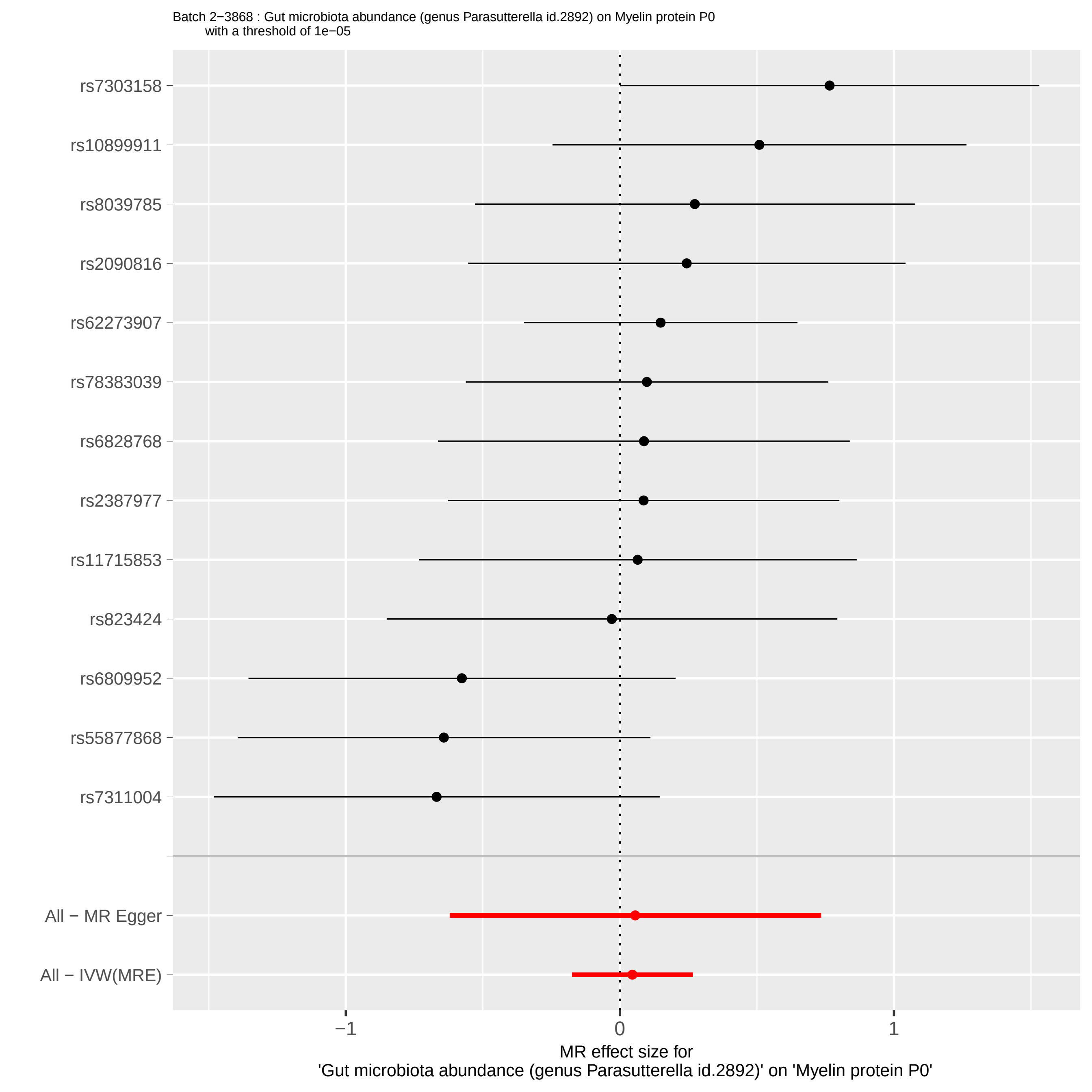
Batch 2−3868 : Gut microbiota abundance (genus Parasutterella id.2892) on Myelin protein P0
with a threshold of 1e−05
rs7303158
rs10899911
rs8039785
rs2090816
rs62273907
rs78383039
rs6828768
rs2387977
rs11715853
rs823424
rs6809952
rs55877868
rs7311004
All − MR Egger
All − IVW(MRE)
−1
0
1
MR effect size for
'Gut microbiota abundance (genus Parasutterella id.2892)' on 'Myelin protein P0'

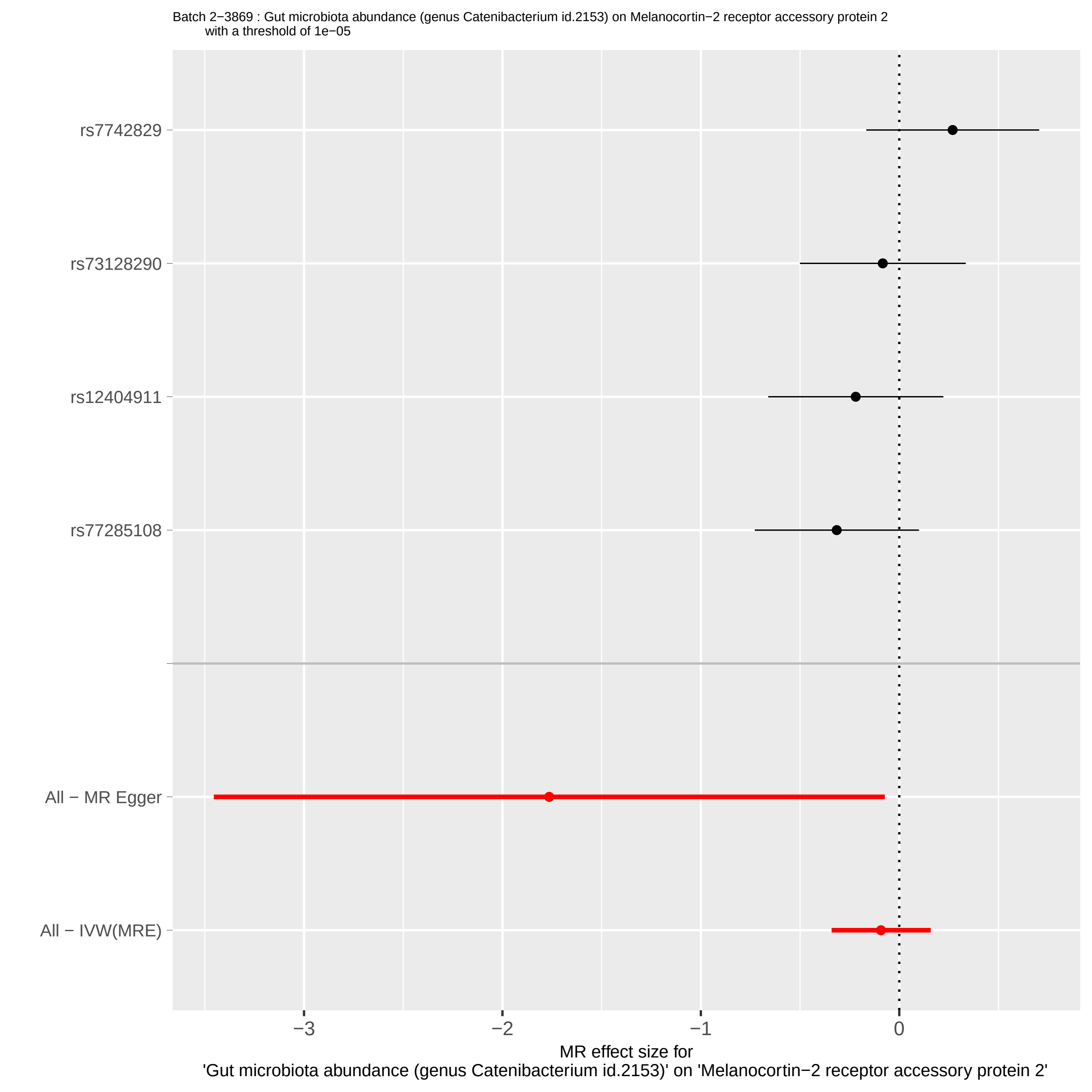
Batch 2−3869 : Gut microbiota abundance (genus Catenibacterium id.2153) on Melanocortin−2 receptor accessory protein 2
with a threshold of 1e−05
rs7742829
rs73128290
rs12404911
rs77285108
All − MR Egger
All − IVW(MRE)
−3
−2
−1
0
MR effect size for
'Gut microbiota abundance (genus Catenibacterium id.2153)' on 'Melanocortin−2 receptor accessory protein 2'

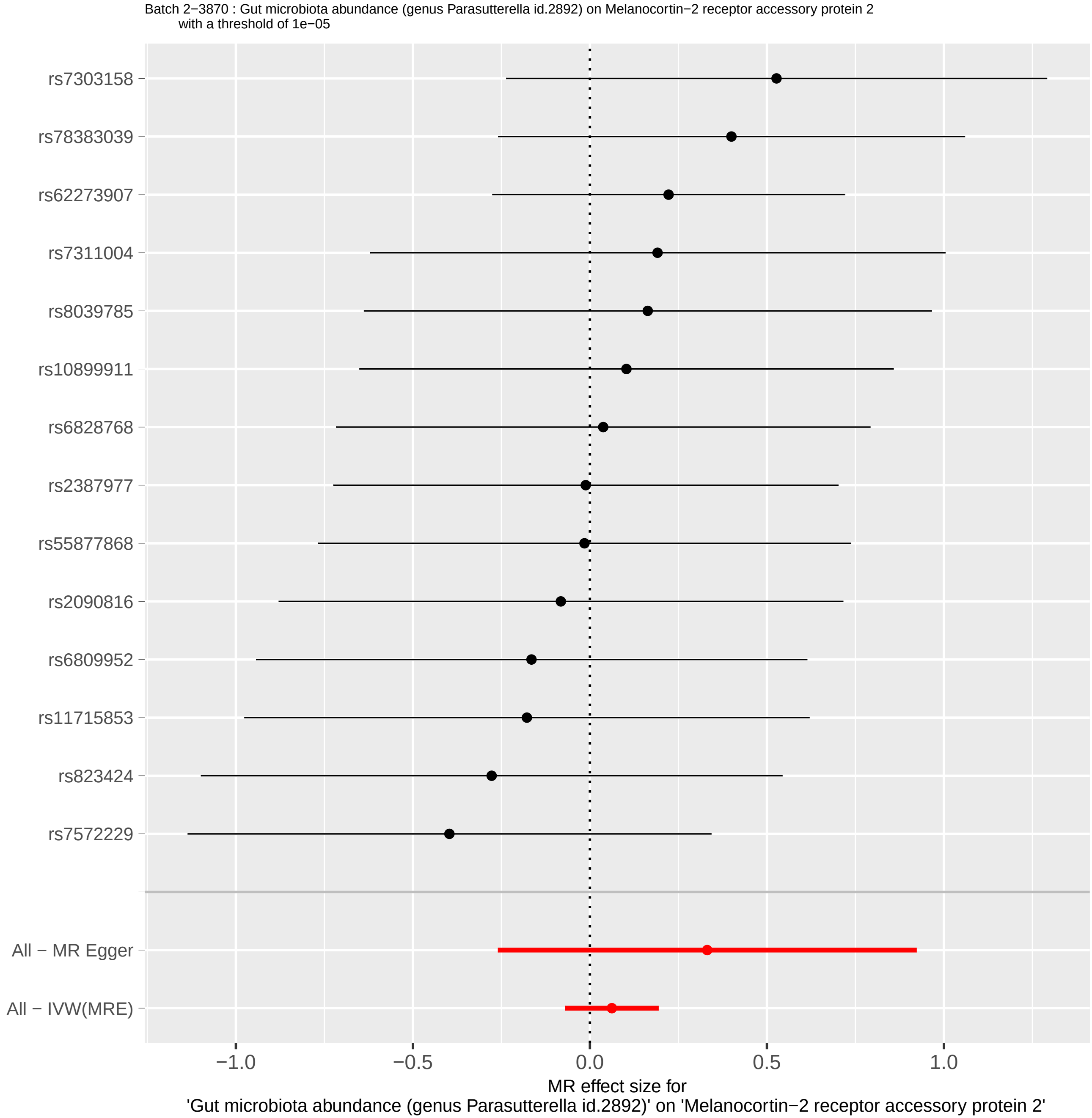
Batch 2−3870 : Gut microbiota abundance (genus Parasutterella id.2892) on Melanocortin−2 receptor accessory protein 2
with a threshold of 1e−05
rs7303158
rs78383039
rs62273907
rs7311004
rs8039785
rs10899911
rs6828768
rs2387977
rs55877868
rs2090816
rs6809952
rs11715853
rs823424
rs7572229
All − MR Egger
All − IVW(MRE)
−1.0
−0.5
0.0
0.5
1.0
MR effect size for
'Gut microbiota abundance (genus Parasutterella id.2892)' on 'Melanocortin−2 receptor accessory protein 2'

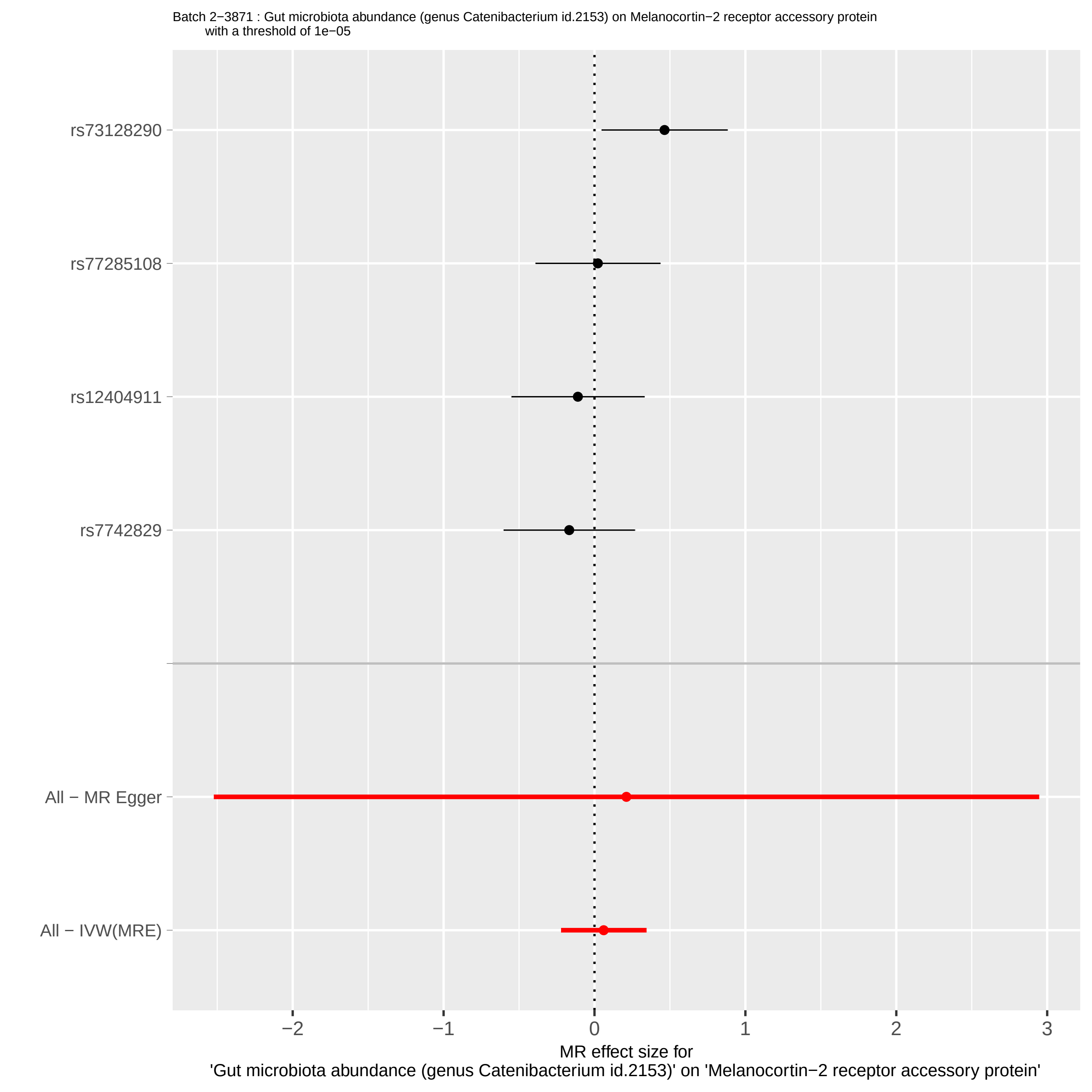
Batch 2−3871 : Gut microbiota abundance (genus Catenibacterium id.2153) on Melanocortin−2 receptor accessory protein
with a threshold of 1e−05
rs73128290
rs77285108
rs12404911
rs7742829
All − MR Egger
All − IVW(MRE)
−2
−1
0
1
2
3
MR effect size for
'Gut microbiota abundance (genus Catenibacterium id.2153)' on 'Melanocortin−2 receptor accessory protein'

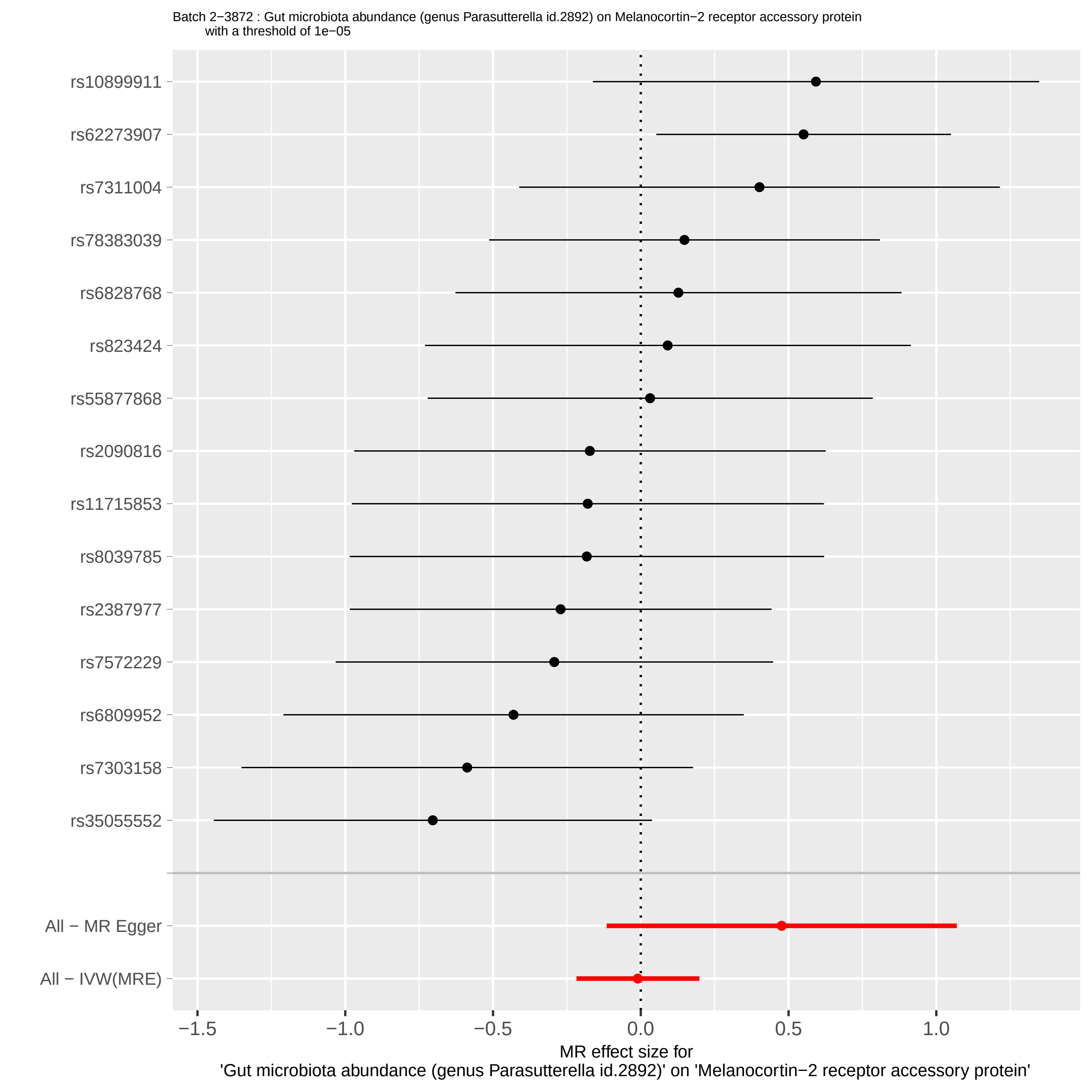

Batch 2−3872 : Gut microbiota abundance (genus Parasutterella id.2892) on Melanocortin−2 receptor accessory protein
with a threshold of 1e−05
rs10899911
rs62273907
rs7311004
rs78383039
rs6828768
rs823424
rs55877868
rs2090816
rs11715853
rs8039785
rs2387977
rs7572229
rs6809952
rs7303158
rs35055552
All − MR Egger
All − IVW(MRE)
−1.5
−1.0
−0.5
0.0
0.5
1.0
MR effect size for
'Gut microbiota abundance (genus Parasutterella id.2892)' on 'Melanocortin−2 receptor accessory protein'

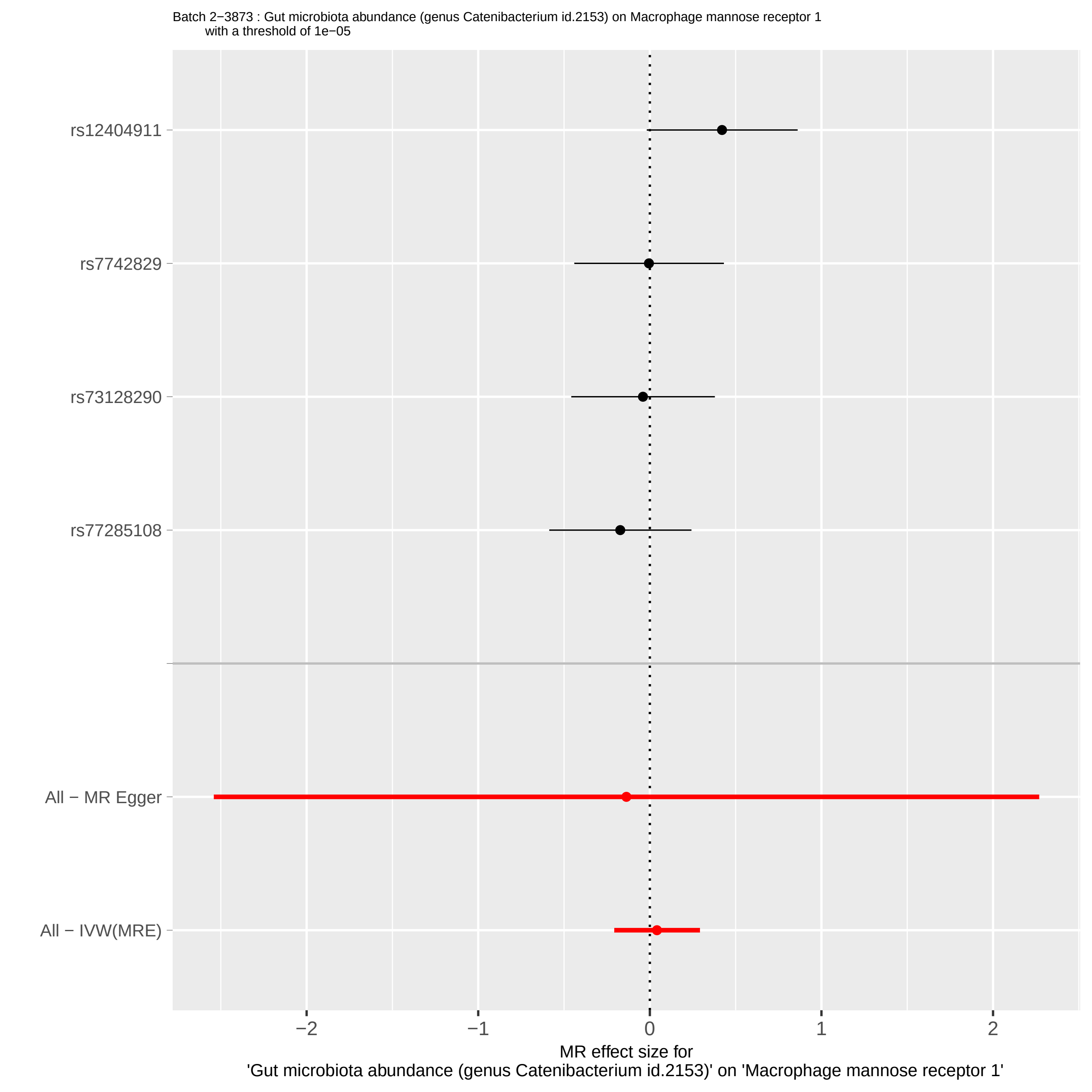

Batch 2−3873 : Gut microbiota abundance (genus Catenibacterium id.2153) on Macrophage mannose receptor 1
with a threshold of 1e−05
rs12404911
rs7742829
rs73128290
rs77285108
All − MR Egger
All − IVW(MRE)
−2
−1
0
1
2
MR effect size for
'Gut microbiota abundance (genus Catenibacterium id.2153)' on 'Macrophage mannose receptor 1'

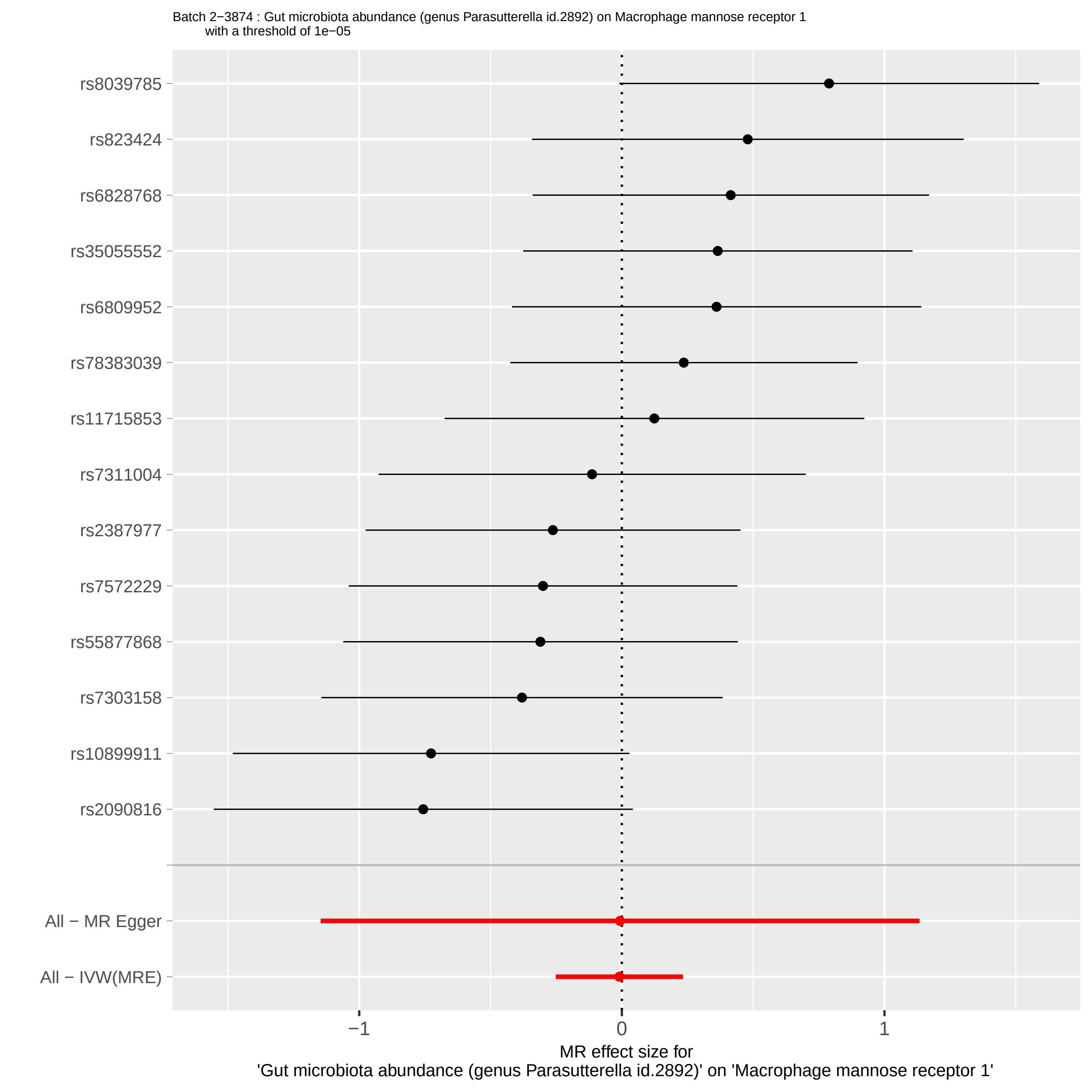

Batch 2−3874 : Gut microbiota abundance (genus Parasutterella id.2892) on Macrophage mannose receptor 1
with a threshold of 1e−05
rs8039785
rs823424
rs6828768
rs35055552
rs6809952
rs78383039
rs11715853
rs7311004
rs2387977
rs7572229
rs55877868
rs7303158
rs10899911
rs2090816
All − MR Egger
All − IVW(MRE)
−1
0
1
MR effect size for
'Gut microbiota abundance (genus Parasutterella id.2892)' on 'Macrophage mannose receptor 1'

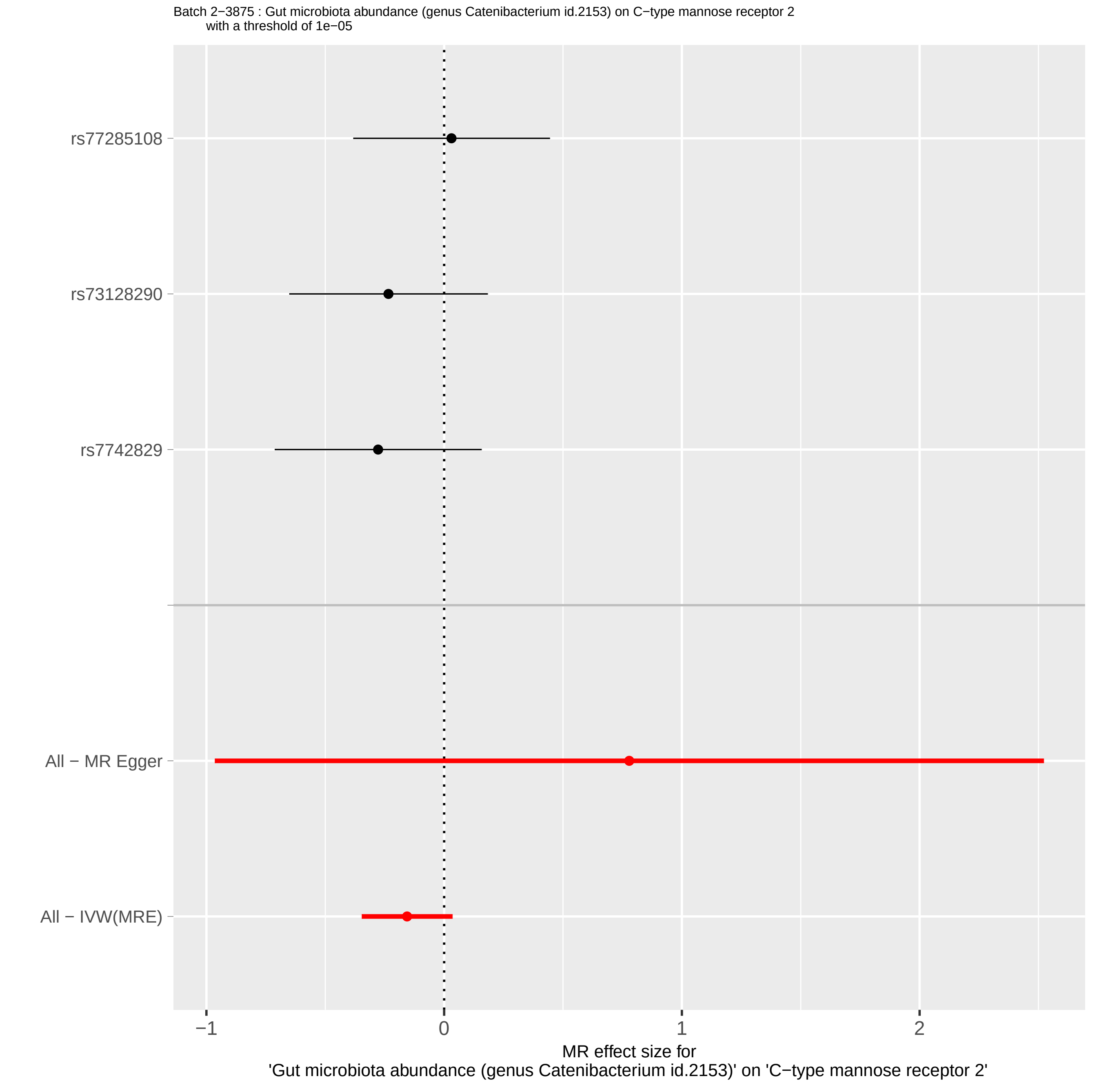

Batch 2−3875 : Gut microbiota abundance (genus Catenibacterium id.2153) on C−type mannose receptor 2
with a threshold of 1e−05
rs77285108
rs73128290
rs7742829
All − MR Egger
All − IVW(MRE)
−1
0
1
2
MR effect size for
'Gut microbiota abundance (genus Catenibacterium id.2153)' on 'C−type mannose receptor 2'

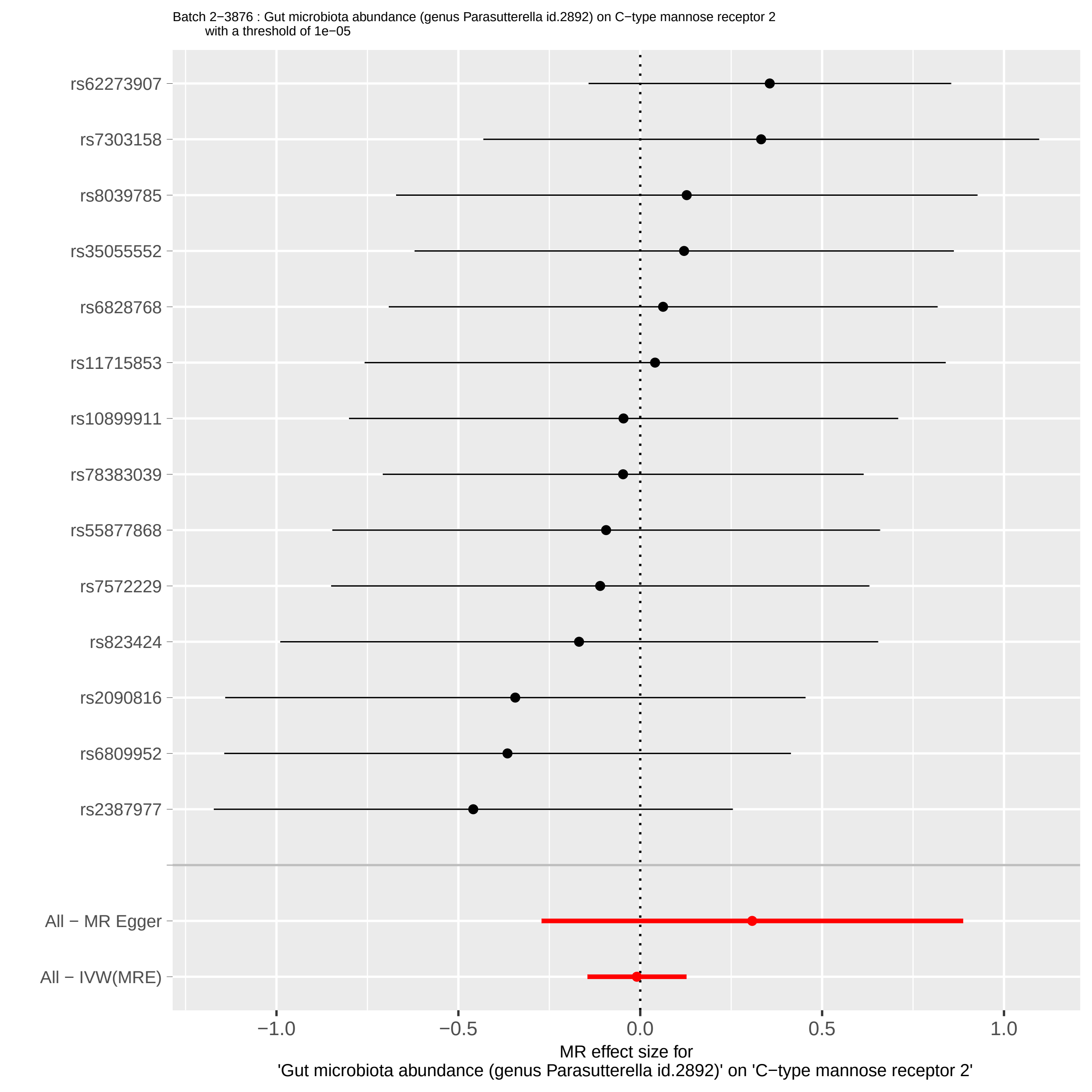

Batch 2−3876 : Gut microbiota abundance (genus Parasutterella id.2892) on C−type mannose receptor 2
with a threshold of 1e−05
rs62273907
rs7303158
rs8039785
rs35055552
rs6828768
rs11715853
rs10899911
rs78383039
rs55877868
rs7572229
rs823424
rs2090816
rs6809952
rs2387977
All − MR Egger
All − IVW(MRE)
−1.0
−0.5
0.0
0.5
1.0
MR effect size for
'Gut microbiota abundance (genus Parasutterella id.2892)' on 'C−type mannose receptor 2'

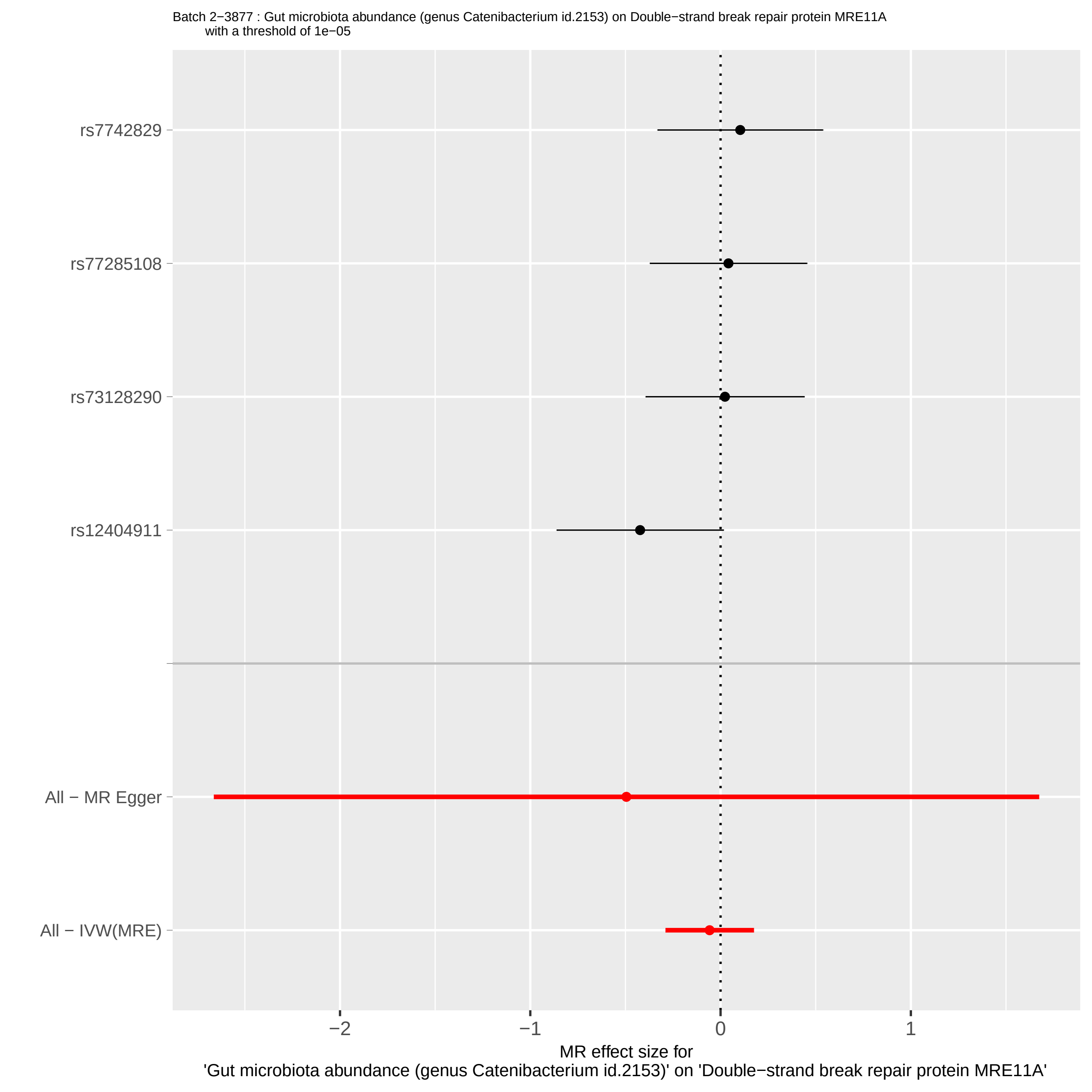

Batch 2−3877 : Gut microbiota abundance (genus Catenibacterium id.2153) on Double−strand break repair protein MRE11A
with a threshold of 1e−05
rs7742829
rs77285108
rs73128290
rs12404911
All − MR Egger
All − IVW(MRE)
−2
−1
0
1
MR effect size for
'Gut microbiota abundance (genus Catenibacterium id.2153)' on 'Double−strand break repair protein MRE11A'

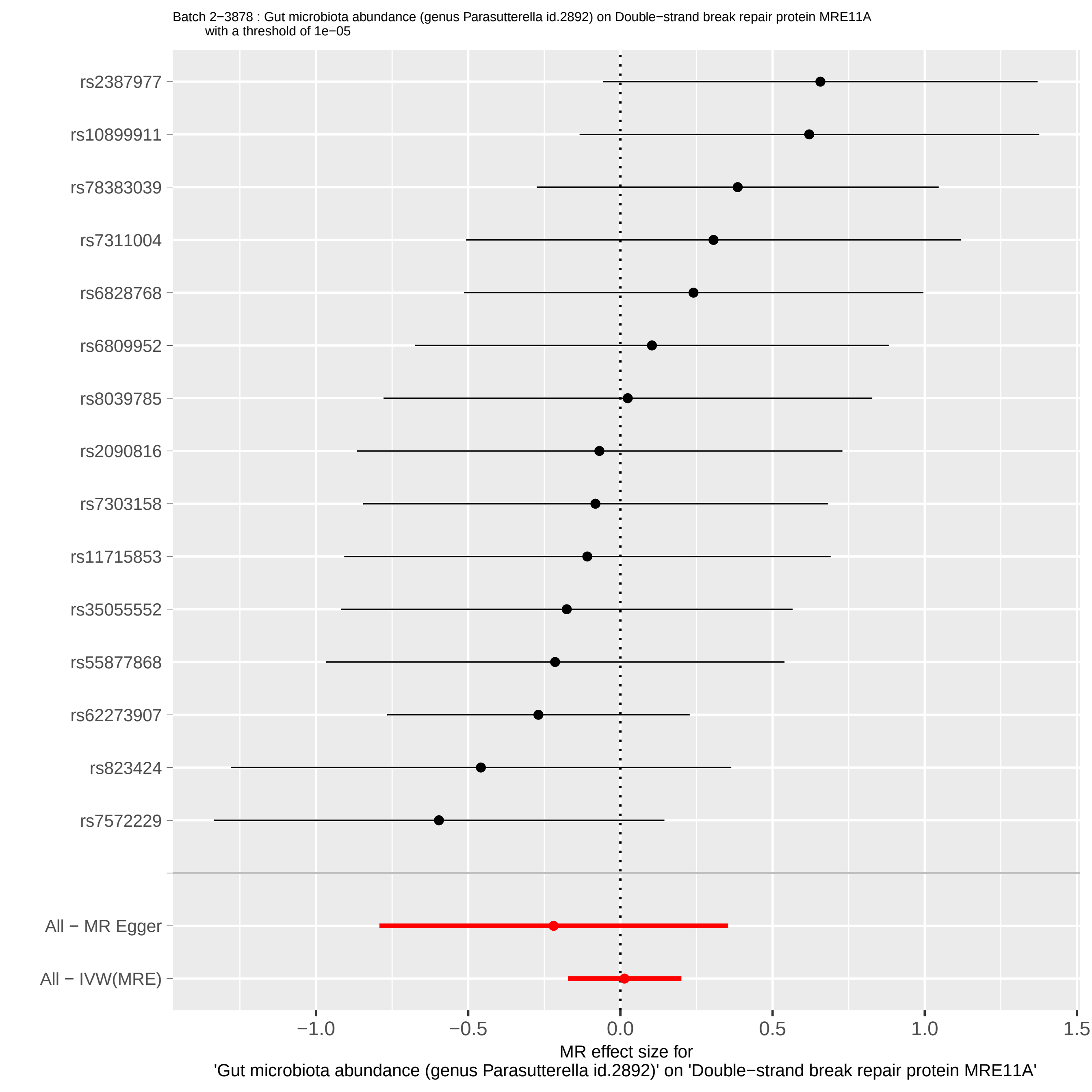
Batch 2−3878 : Gut microbiota abundance (genus Parasutterella id.2892) on Double−strand break repair protein MRE11A
with a threshold of 1e−05
rs2387977
rs10899911
rs78383039
rs7311004
rs6828768
rs6809952
rs8039785
rs2090816
rs7303158
rs11715853
rs35055552
rs55877868
rs62273907
rs823424
rs7572229
All − MR Egger
All − IVW(MRE)
−1.0
−0.5
0.0
0.5
1.0
1.5
MR effect size for
'Gut microbiota abundance (genus Parasutterella id.2892)' on 'Double−strand break repair protein MRE11A'

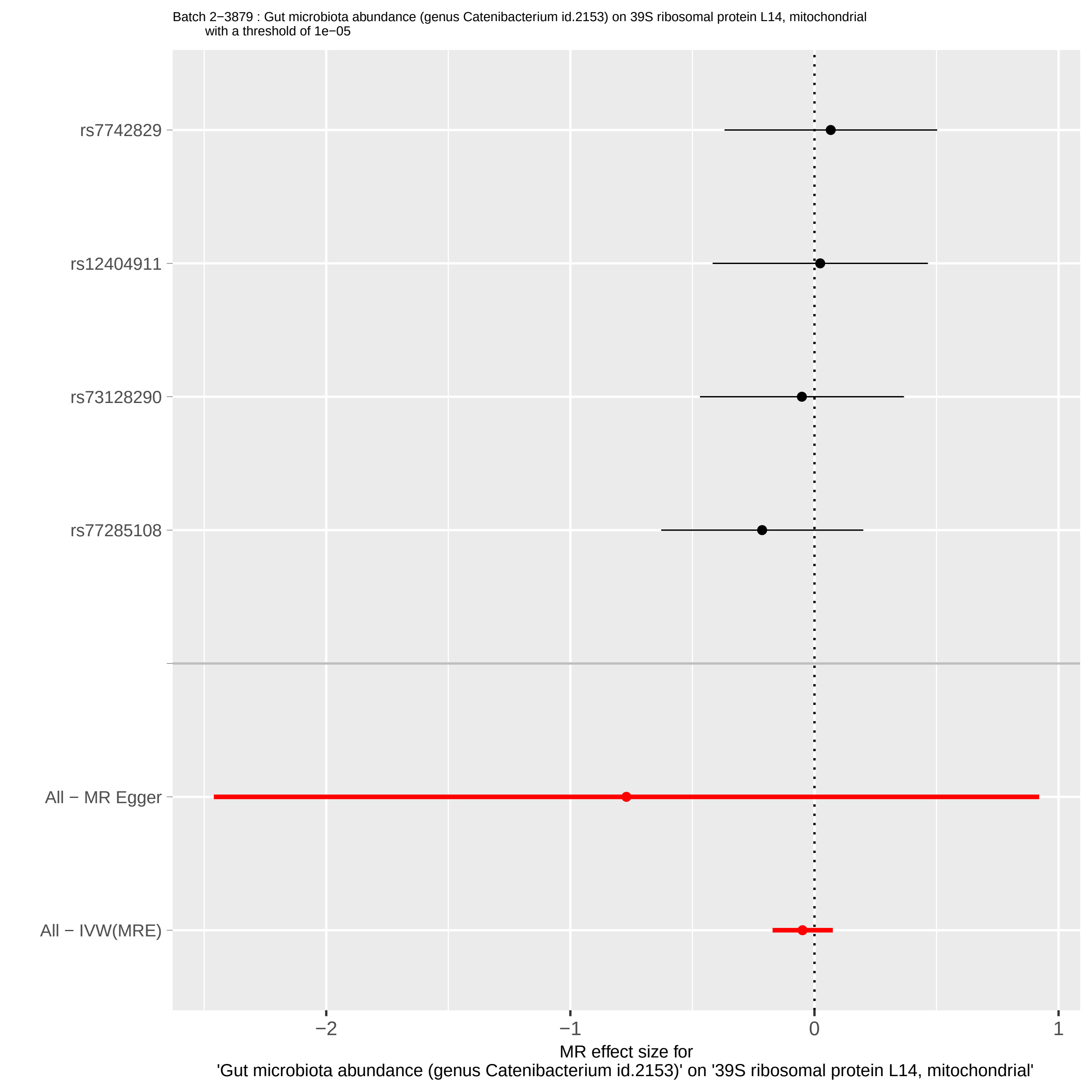

Batch 2−3879 : Gut microbiota abundance (genus Catenibacterium id.2153) on 39S ribosomal protein L14, mitochondrial
with a threshold of 1e−05
rs7742829
rs12404911
rs73128290
rs77285108
All − MR Egger
All − IVW(MRE)
−2
−1
0
1
MR effect size for
'Gut microbiota abundance (genus Catenibacterium id.2153)' on '39S ribosomal protein L14, mitochondrial'

Batch 2−3880 : Gut microbiota abundance (genus Parasutterella id.2892) on 39S ribosomal protein L14, mitochondrial
with a threshold of 1e−05

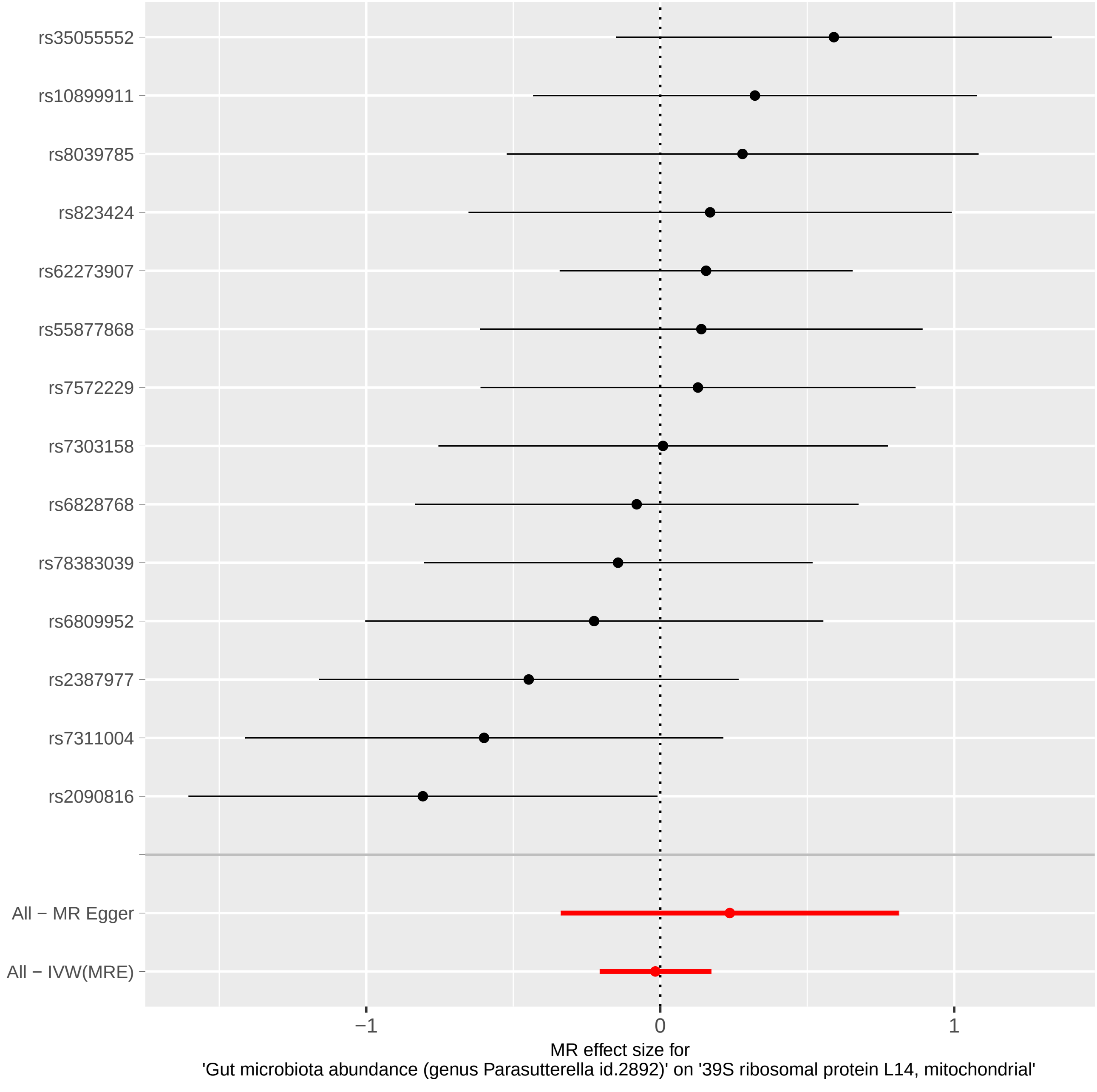

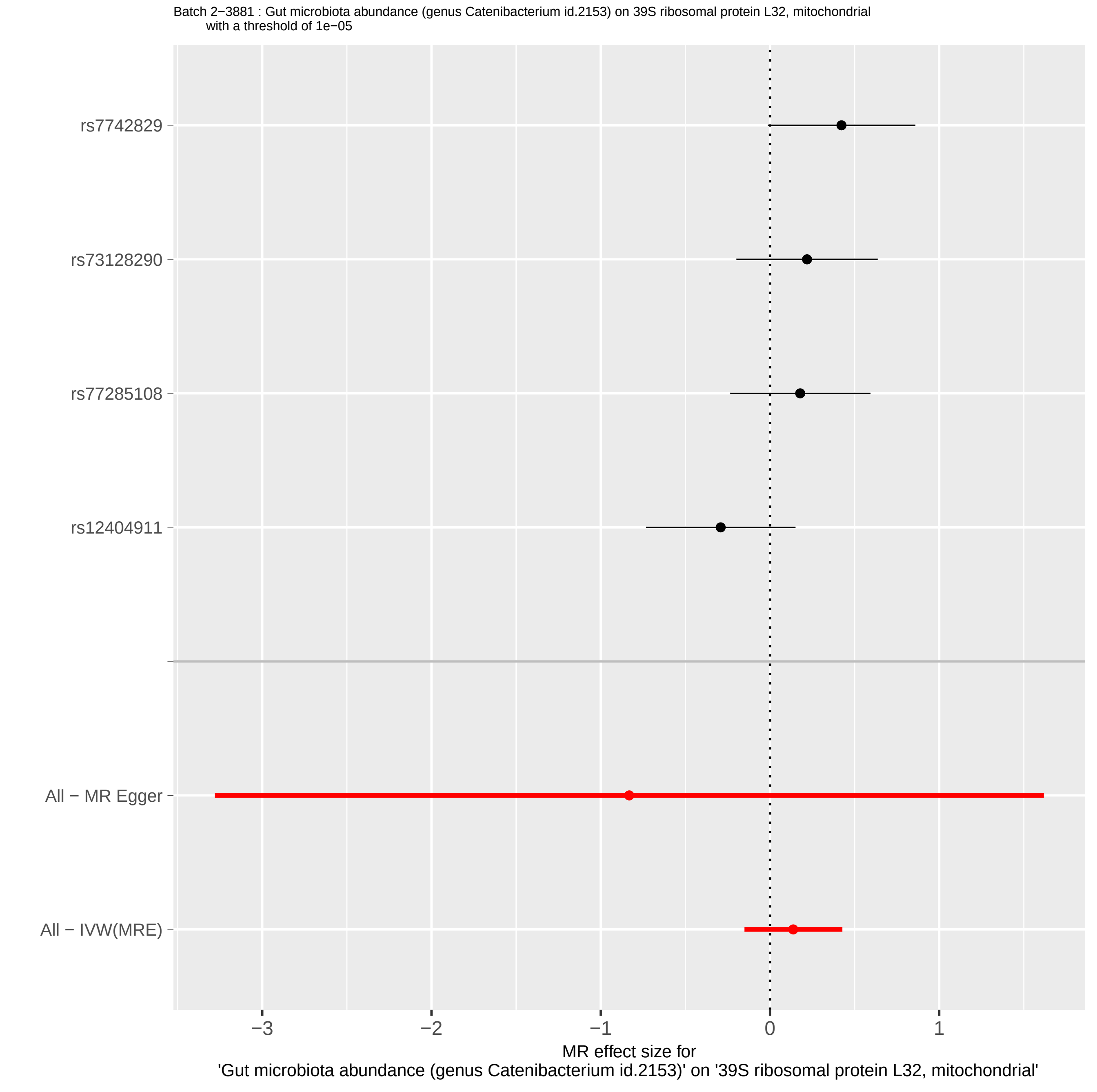

Batch 2−3881 : Gut microbiota abundance (genus Catenibacterium id.2153) on 39S ribosomal protein L32, mitochondrial
with a threshold of 1e−05
rs7742829
rs73128290
rs77285108
rs12404911
All − MR Egger
All − IVW(MRE)
−3
−2
−1
0
1
MR effect size for
'Gut microbiota abundance (genus Catenibacterium id.2153)' on '39S ribosomal protein L32, mitochondrial'

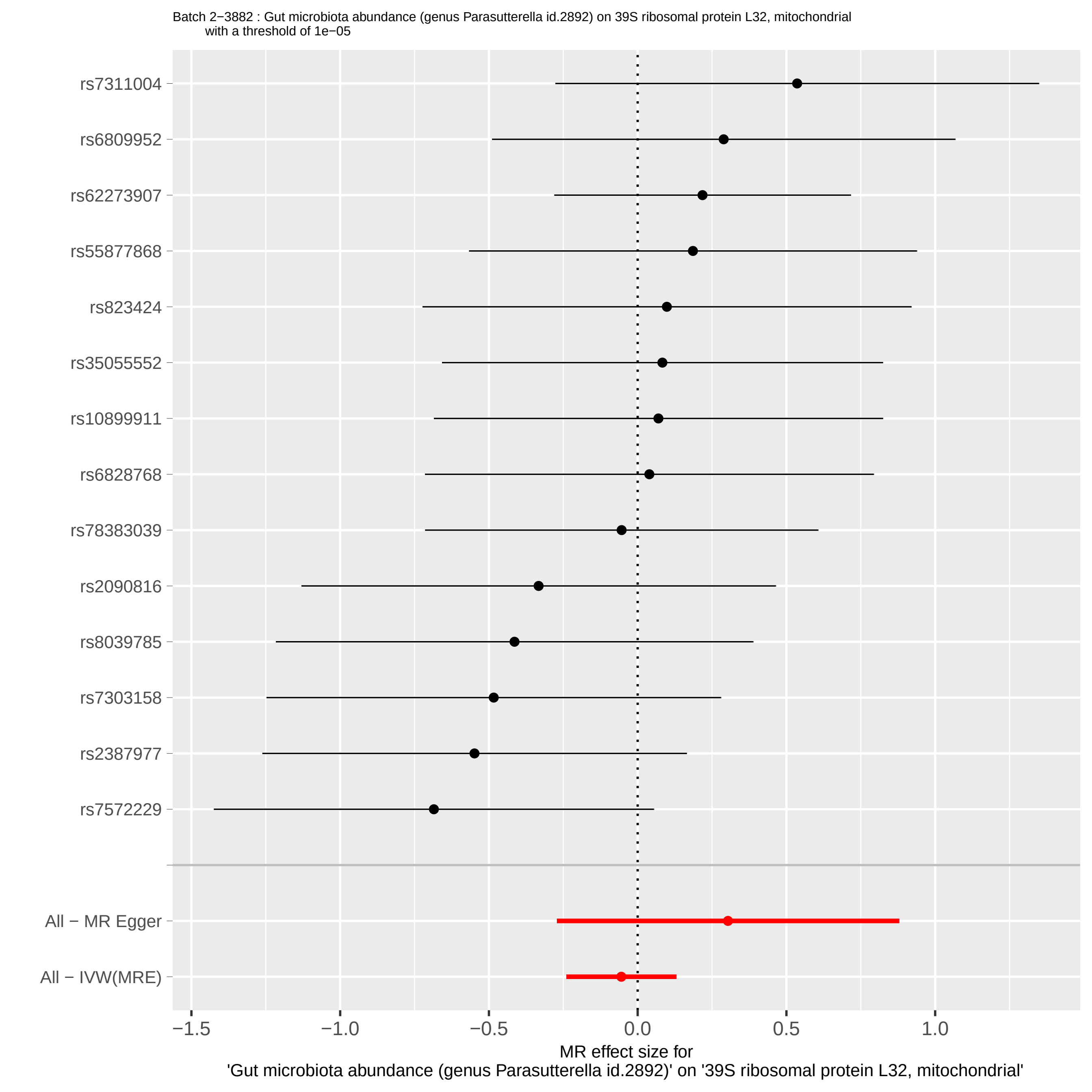

Batch 2−3882 : Gut microbiota abundance (genus Parasutterella id.2892) on 39S ribosomal protein L32, mitochondrial
with a threshold of 1e−05
rs7311004
rs6809952
rs62273907
rs55877868
rs823424
rs35055552
rs10899911
rs6828768
rs78383039
rs2090816
rs8039785
rs7303158
rs2387977
rs7572229
All − MR Egger
All − IVW(MRE)
−1.5
−1.0
−0.5
0.0
0.5
1.0
MR effect size for
'Gut microbiota abundance (genus Parasutterella id.2892)' on '39S ribosomal protein L32, mitochondrial'

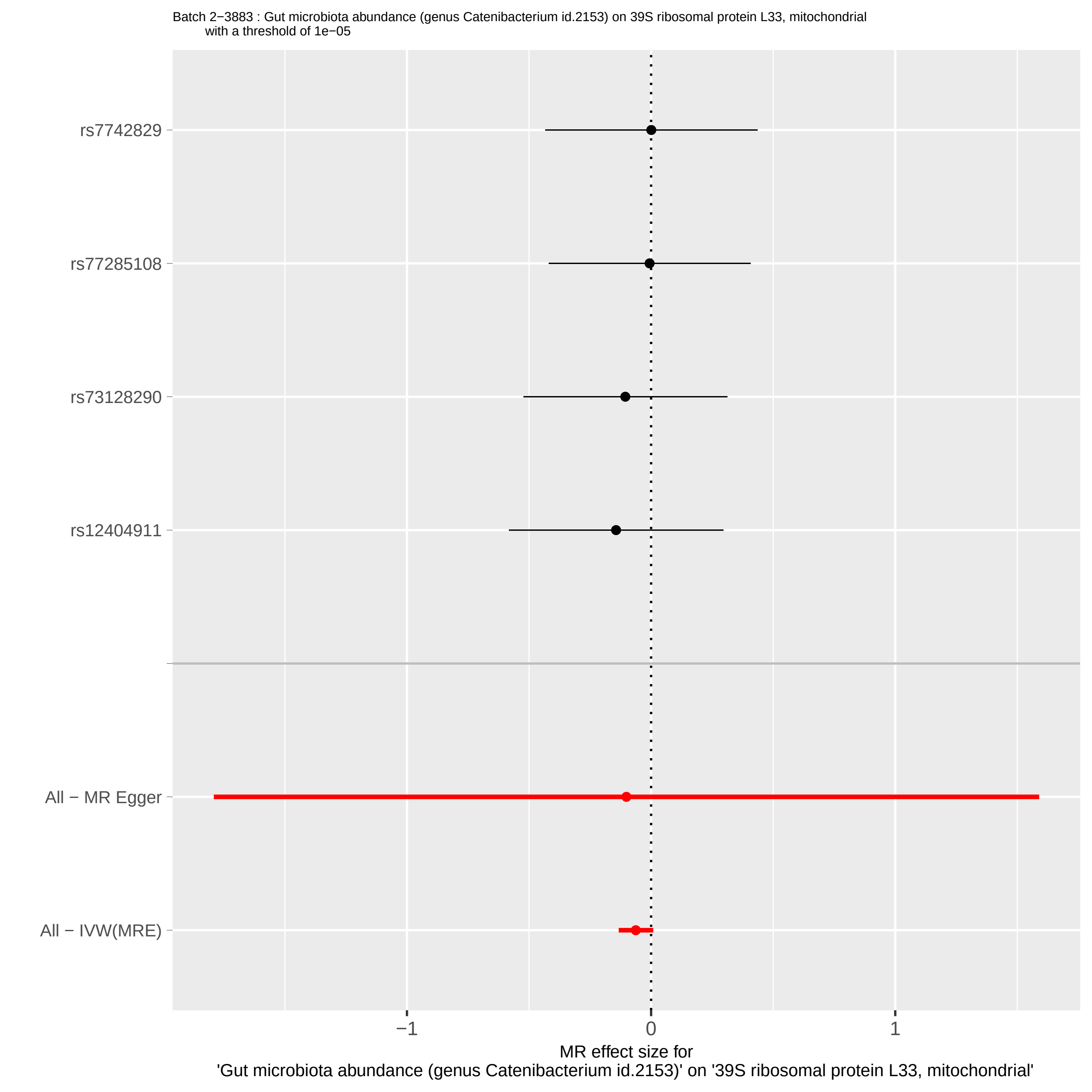

Batch 2−3883 : Gut microbiota abundance (genus Catenibacterium id.2153) on 39S ribosomal protein L33, mitochondrial
with a threshold of 1e−05
rs7742829
rs77285108
rs73128290
rs12404911
All − MR Egger
All − IVW(MRE)
−1
0
1
MR effect size for
'Gut microbiota abundance (genus Catenibacterium id.2153)' on '39S ribosomal protein L33, mitochondrial'

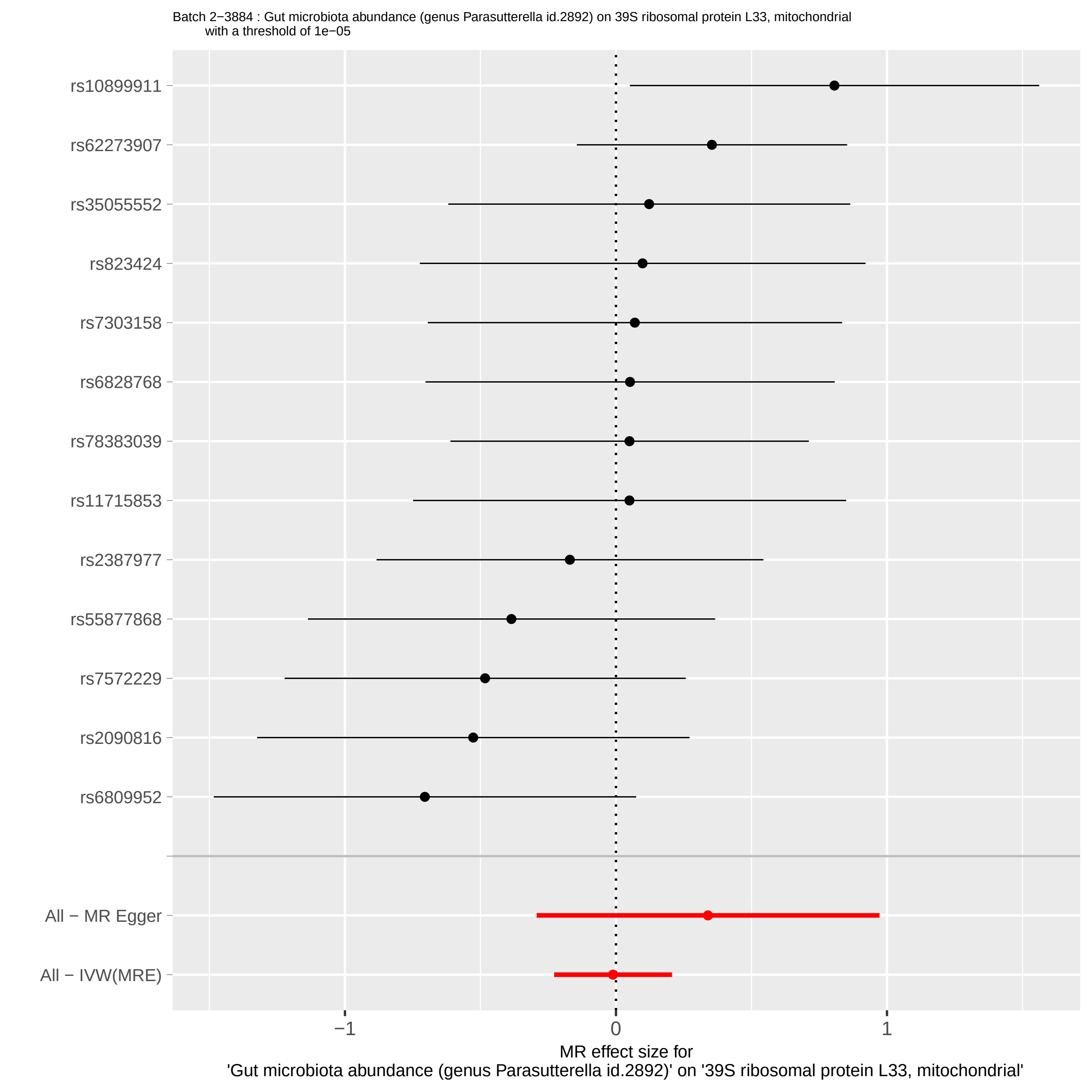
Batch 2−3884 : Gut microbiota abundance (genus Parasutterella id.2892) on 39S ribosomal protein L33, mitochondrial
with a threshold of 1e−05
rs10899911
rs62273907
rs35055552
rs823424
rs7303158
rs6828768
rs78383039
rs11715853
rs2387977
rs55877868
rs7572229
rs2090816
rs6809952
All − MR Egger
All − IVW(MRE)
−1
0
1
MR effect size for
'Gut microbiota abundance (genus Parasutterella id.2892)' on '39S ribosomal protein L33, mitochondrial'

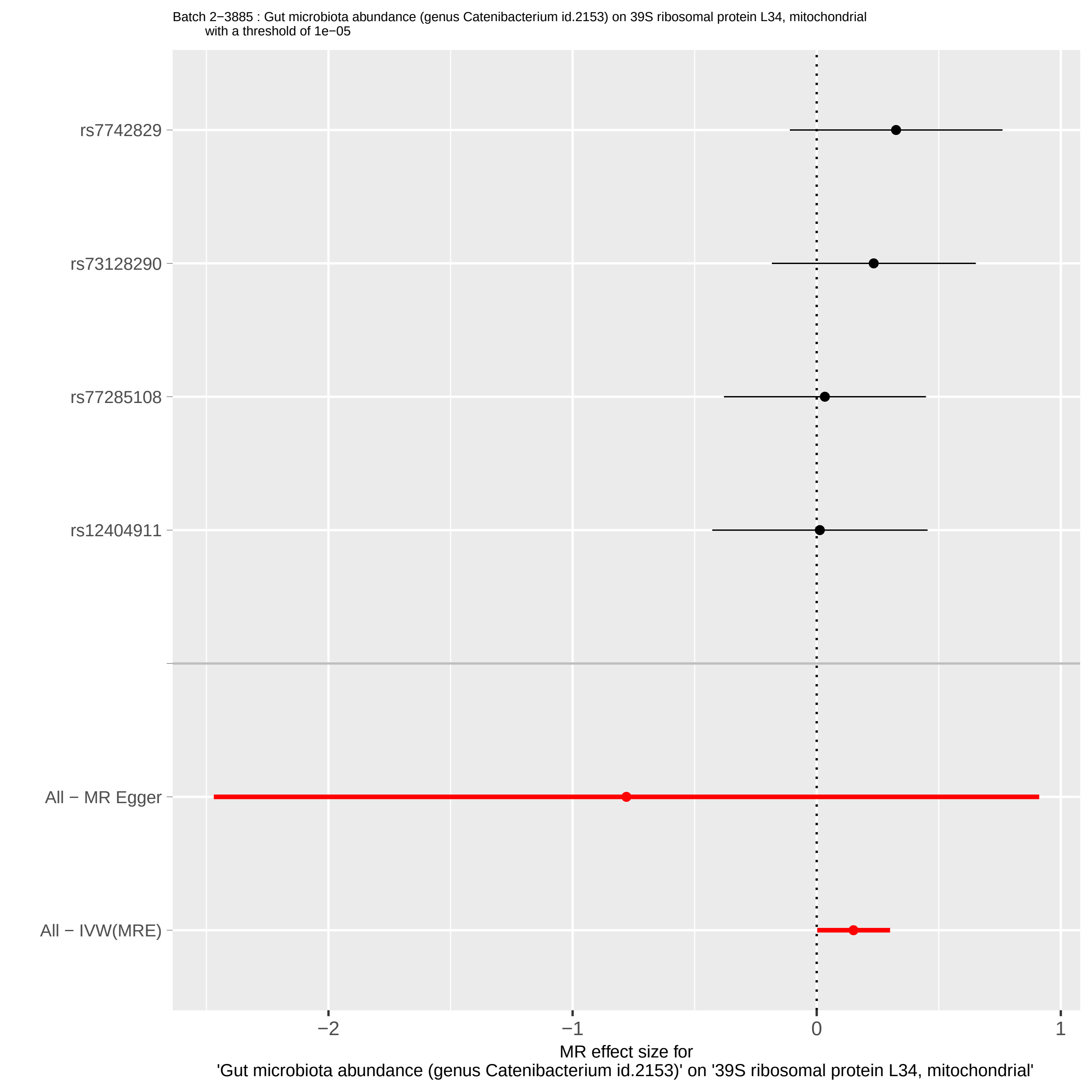

Batch 2−3885 : Gut microbiota abundance (genus Catenibacterium id.2153) on 39S ribosomal protein L34, mitochondrial
with a threshold of 1e−05
rs7742829
rs73128290
rs77285108
rs12404911
All − MR Egger
All − IVW(MRE)
−2
−1
0
1
MR effect size for
'Gut microbiota abundance (genus Catenibacterium id.2153)' on '39S ribosomal protein L34, mitochondrial'

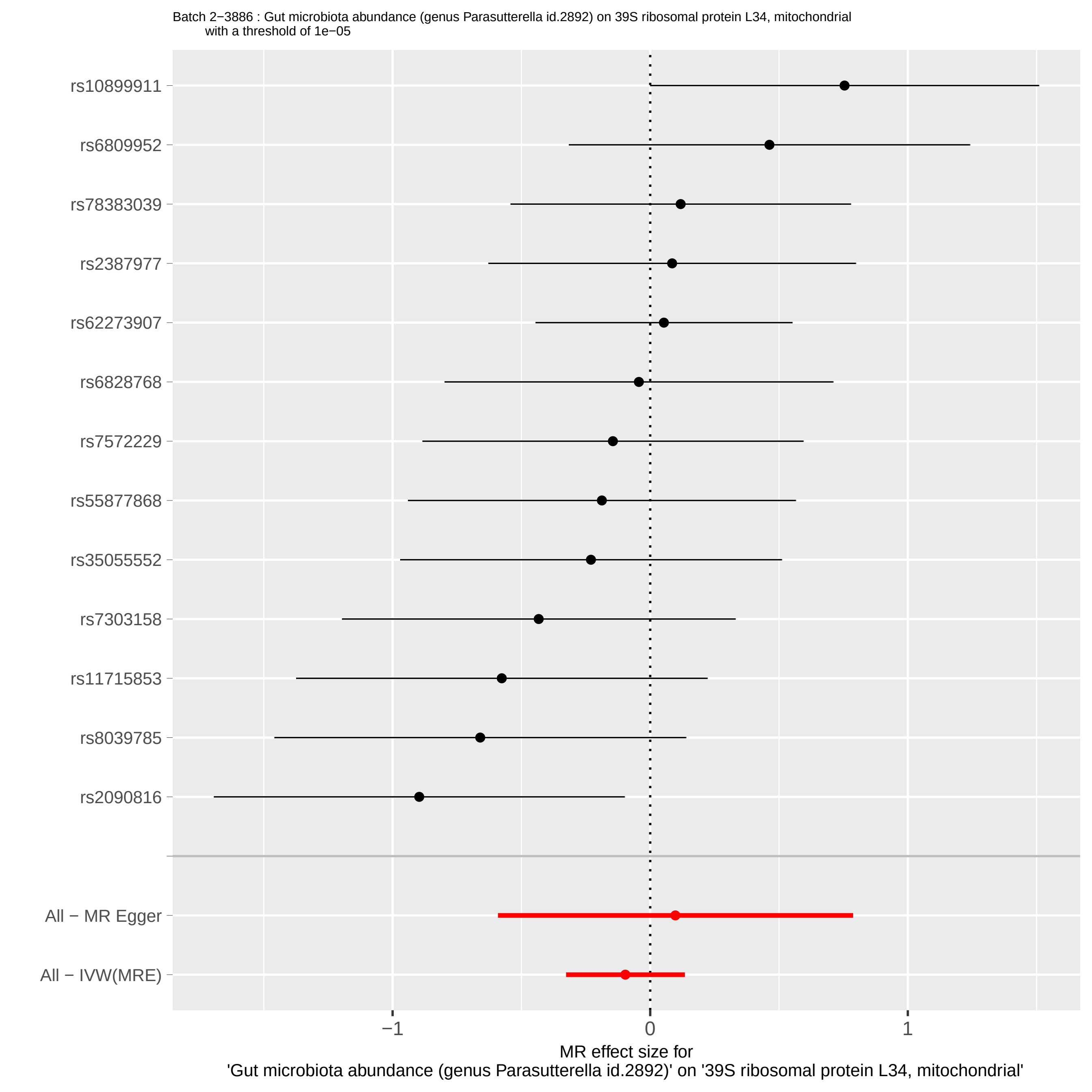
Batch 2−3886 : Gut microbiota abundance (genus Parasutterella id.2892) on 39S ribosomal protein L34, mitochondrial
with a threshold of 1e−05
rs10899911
rs6809952
rs78383039
rs2387977
rs62273907
rs6828768
rs7572229
rs55877868
rs35055552
rs7303158
rs11715853
rs8039785
rs2090816
All − MR Egger
All − IVW(MRE)
−1
0
1
MR effect size for
'Gut microbiota abundance (genus Parasutterella id.2892)' on '39S ribosomal protein L34, mitochondrial'

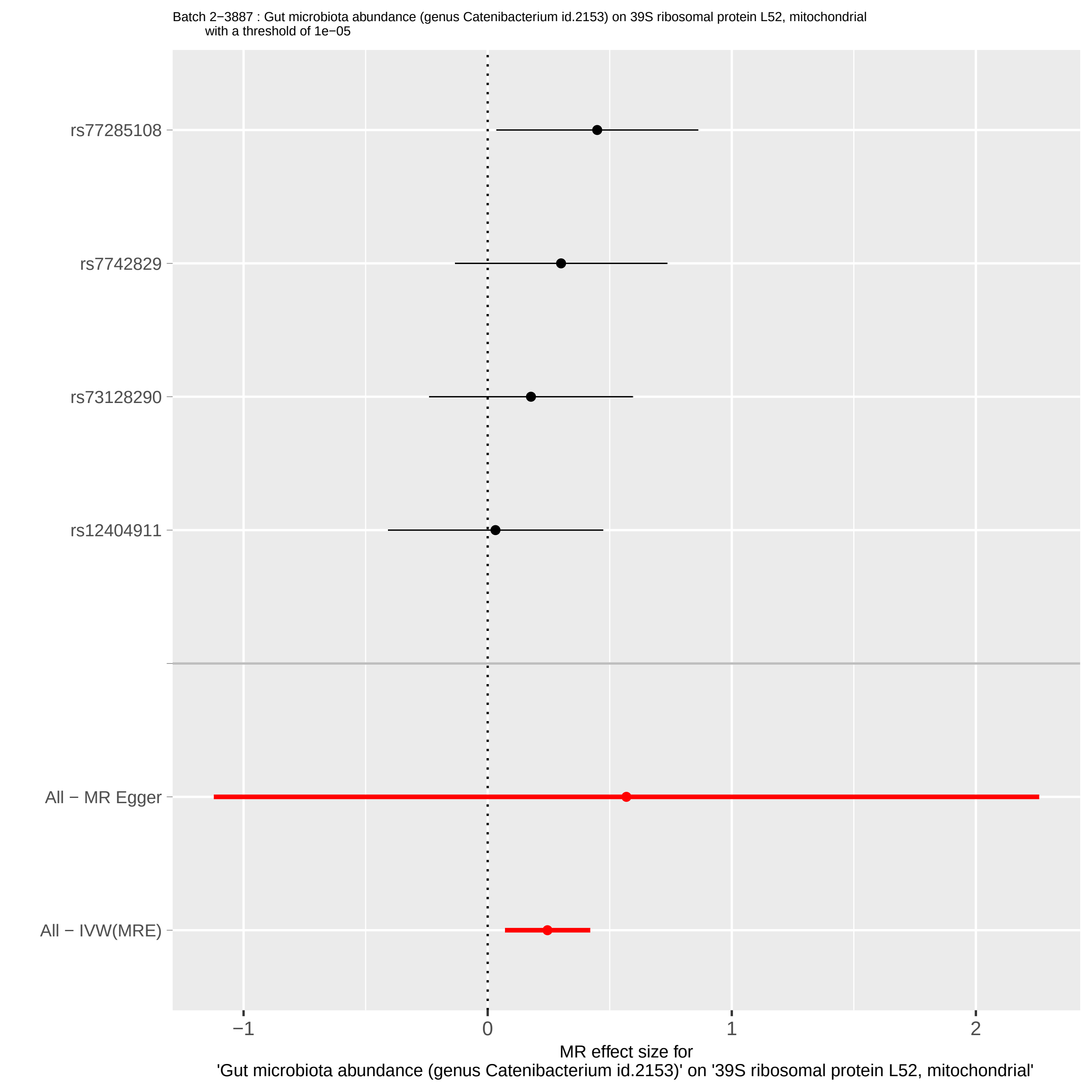
Batch 2−3887 : Gut microbiota abundance (genus Catenibacterium id.2153) on 39S ribosomal protein L52, mitochondrial
with a threshold of 1e−05
rs77285108
rs7742829
rs73128290
rs12404911
All − MR Egger
All − IVW(MRE)
−1
0
1
2
MR effect size for
'Gut microbiota abundance (genus Catenibacterium id.2153)' on '39S ribosomal protein L52, mitochondrial'

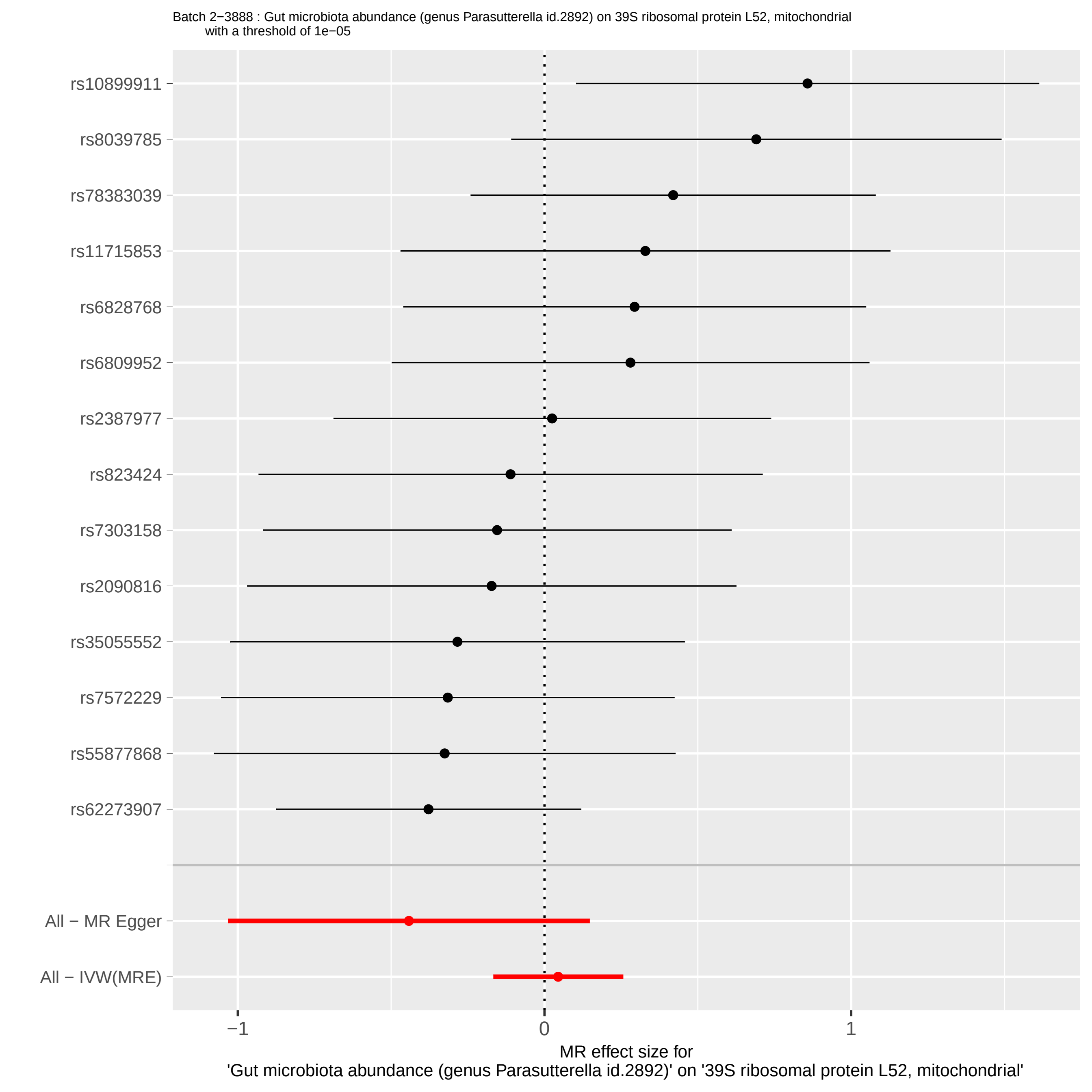

Batch 2−3888 : Gut microbiota abundance (genus Parasutterella id.2892) on 39S ribosomal protein L52, mitochondrial
with a threshold of 1e−05
rs10899911
rs8039785
rs78383039
rs11715853
rs6828768
rs6809952
rs2387977
rs823424
rs7303158
rs2090816
rs35055552
rs7572229
rs55877868
rs62273907
All − MR Egger
All − IVW(MRE)
−1
0
1
MR effect size for
'Gut microbiota abundance (genus Parasutterella id.2892)' on '39S ribosomal protein L52, mitochondrial'

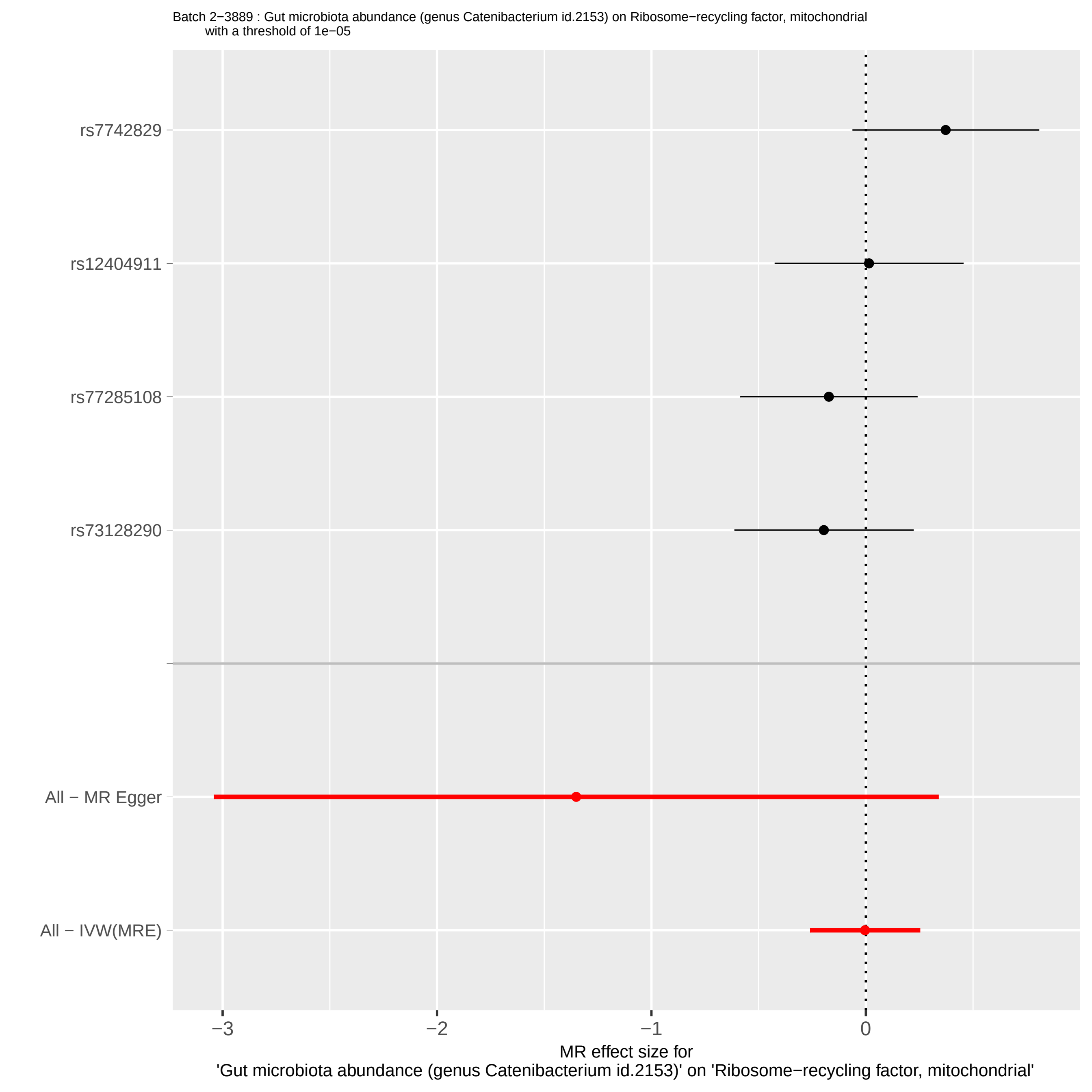

Batch 2−3889 : Gut microbiota abundance (genus Catenibacterium id.2153) on Ribosome−recycling factor, mitochondrial
with a threshold of 1e−05
rs7742829
rs12404911
rs77285108
rs73128290
All − MR Egger
All − IVW(MRE)
−3
−2
−1
0
MR effect size for
'Gut microbiota abundance (genus Catenibacterium id.2153)' on 'Ribosome−recycling factor, mitochondrial'

Batch 2−3890 : Gut microbiota abundance (genus Parasutterella id.2892) on Ribosome−recycling factor, mitochondrial
with a threshold of 1e−05

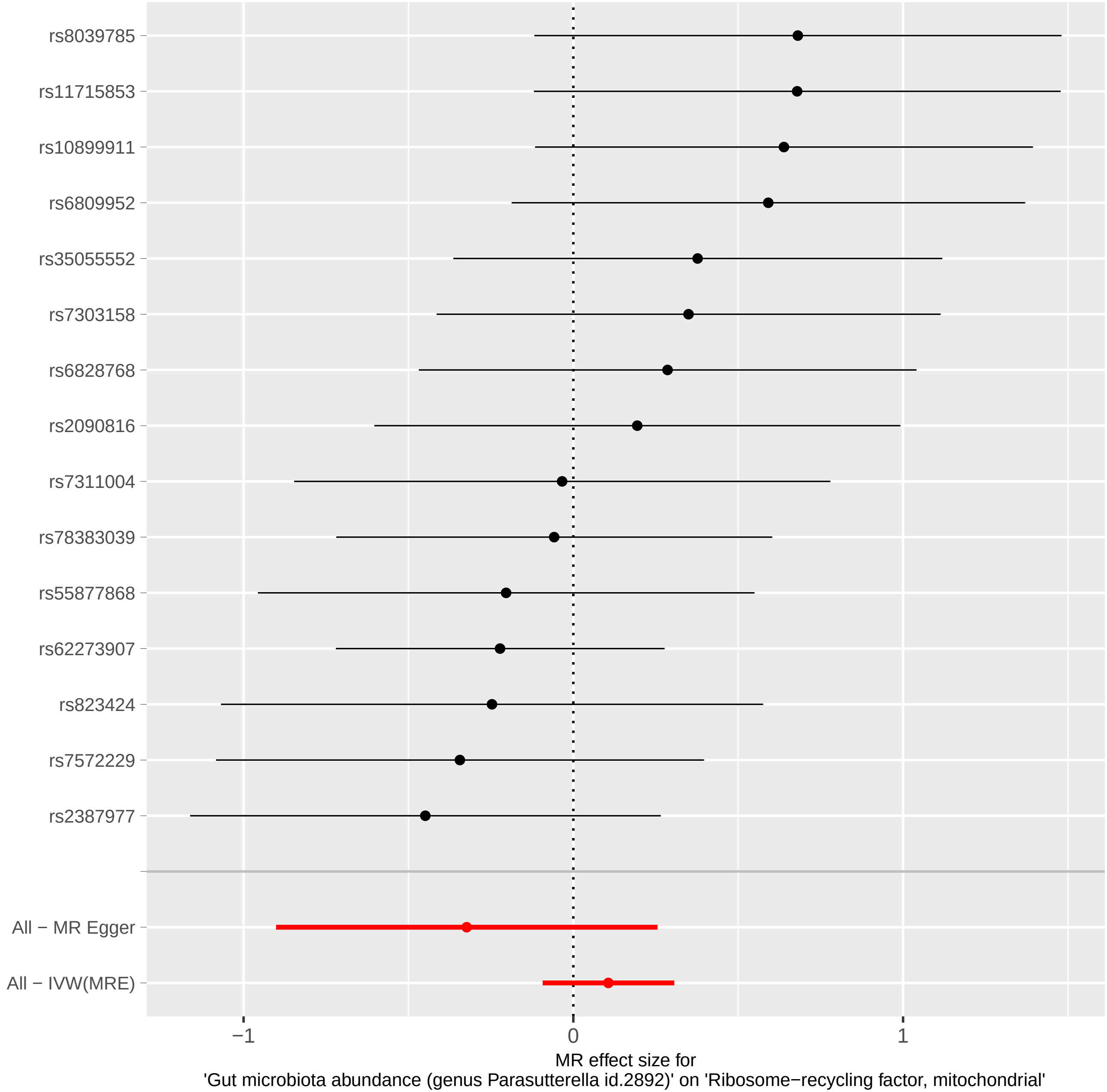

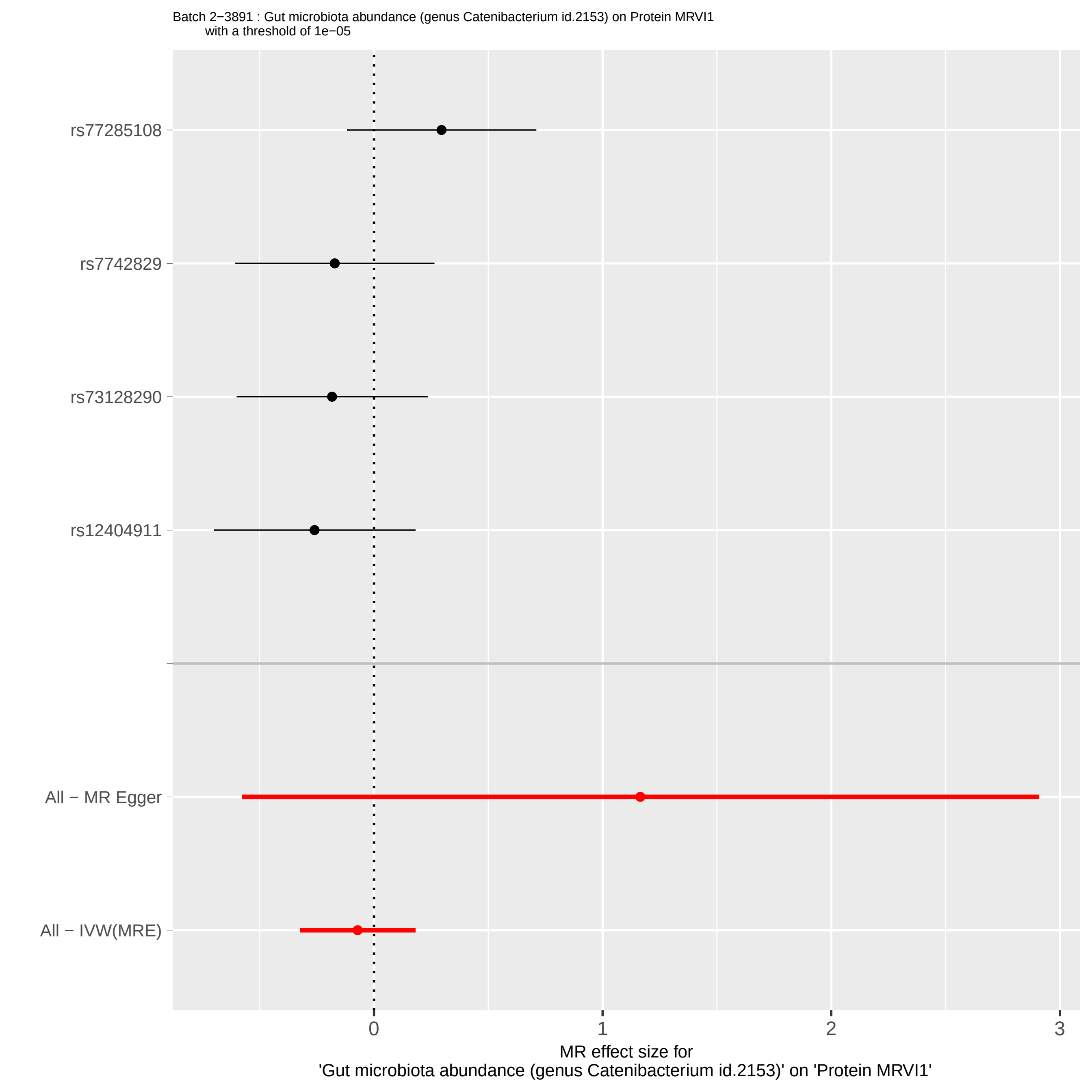

Batch 2−3891 : Gut microbiota abundance (genus Catenibacterium id.2153) on Protein MRVI1
with a threshold of 1e−05
rs77285108
rs7742829
rs73128290
rs12404911
All − MR Egger
All − IVW(MRE)
0
1
2
3
MR effect size for
'Gut microbiota abundance (genus Catenibacterium id.2153)' on 'Protein MRVI1'

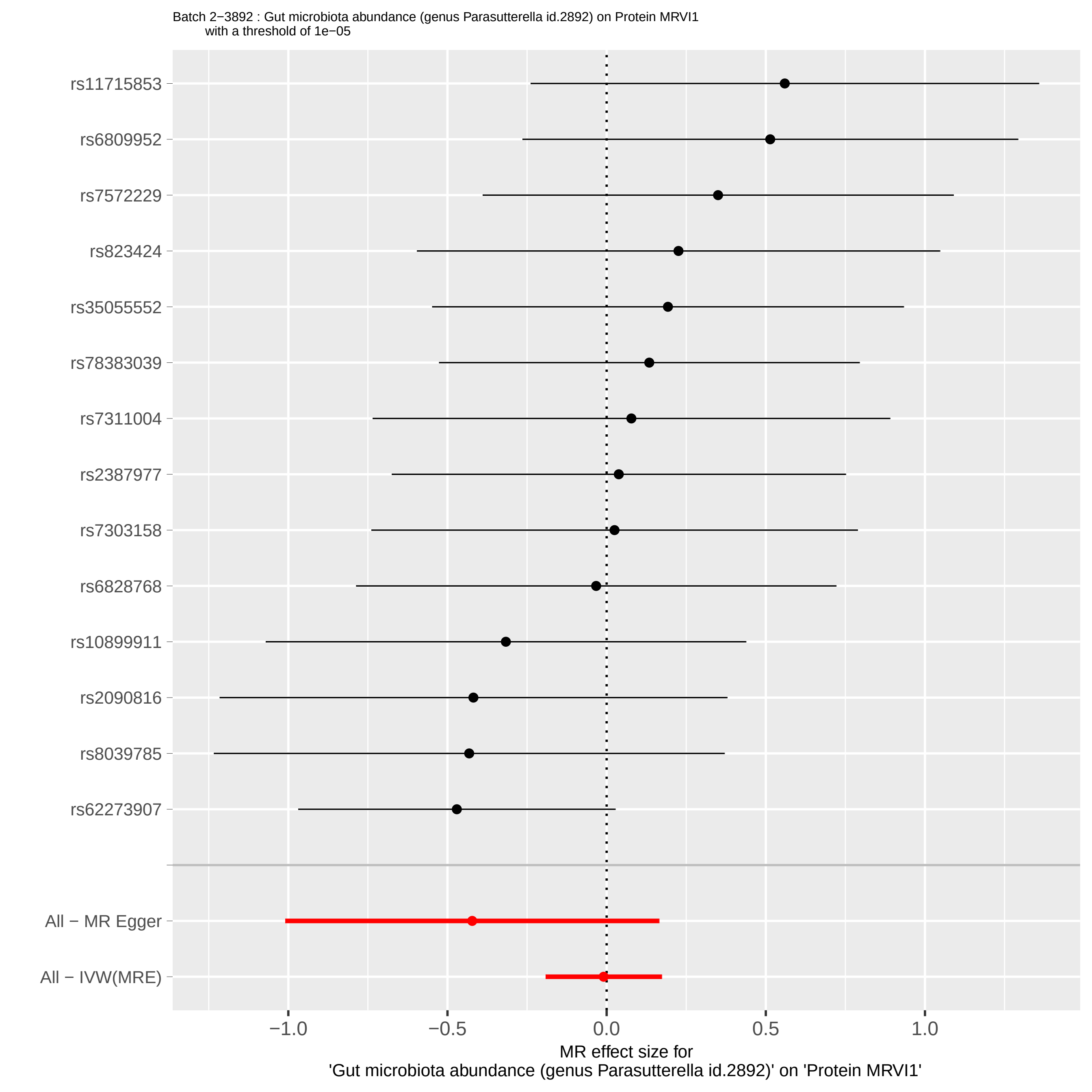

Batch 2−3892 : Gut microbiota abundance (genus Parasutterella id.2892) on Protein MRVI1
with a threshold of 1e−05
rs11715853
rs6809952
rs7572229
rs823424
rs35055552
rs78383039
rs7311004
rs2387977
rs7303158
rs6828768
rs10899911
rs2090816
rs8039785
rs62273907
All − MR Egger
All − IVW(MRE)
−1.0
−0.5
0.0
0.5
1.0
MR effect size for
'Gut microbiota abundance (genus Parasutterella id.2892)' on 'Protein MRVI1'

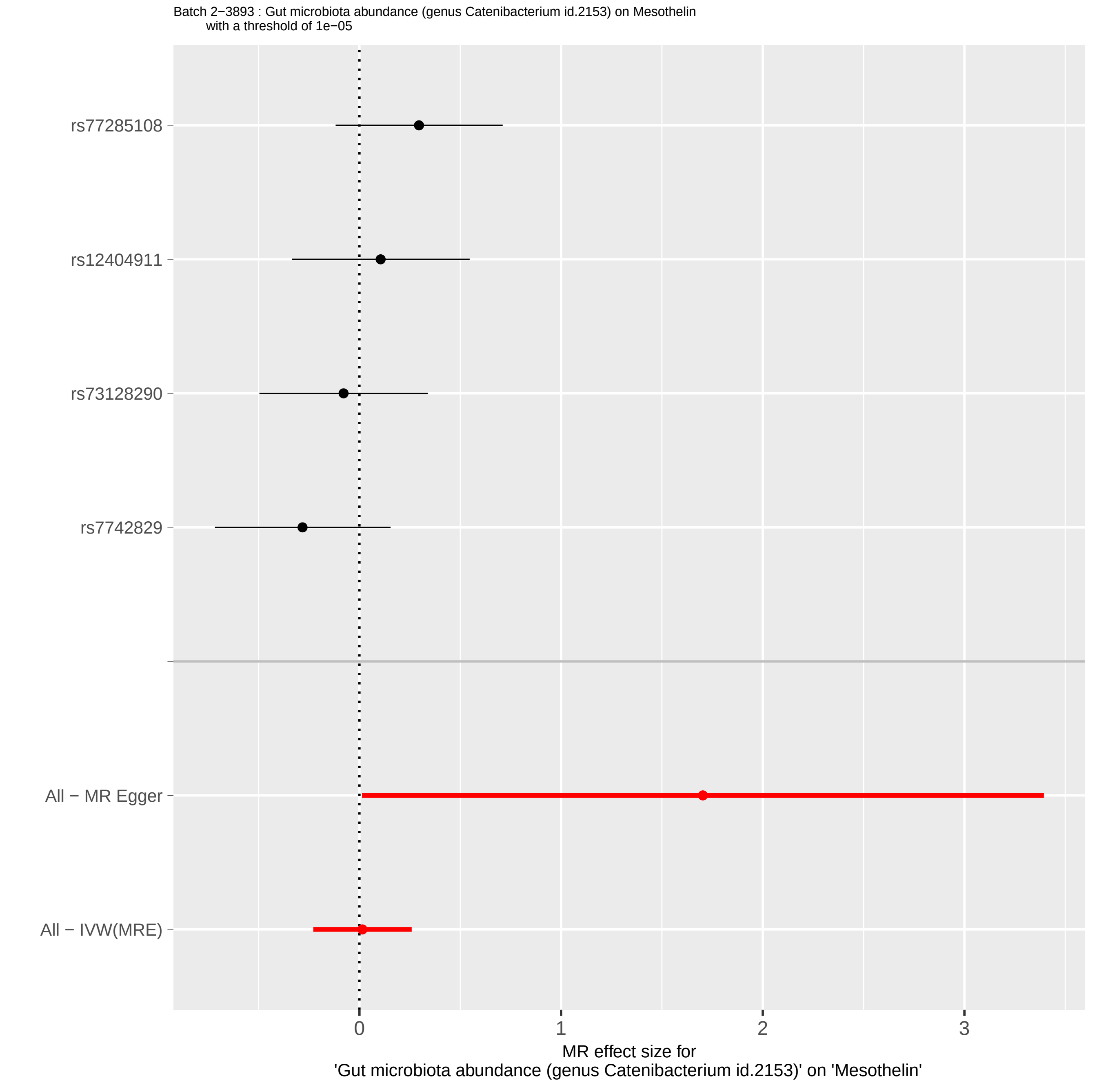

Batch 2−3893 : Gut microbiota abundance (genus Catenibacterium id.2153) on Mesothelin
with a threshold of 1e−05
rs77285108
rs12404911
rs73128290
rs7742829
All − MR Egger
All − IVW(MRE)
0
1
2
3
MR effect size for
'Gut microbiota abundance (genus Catenibacterium id.2153)' on 'Mesothelin'

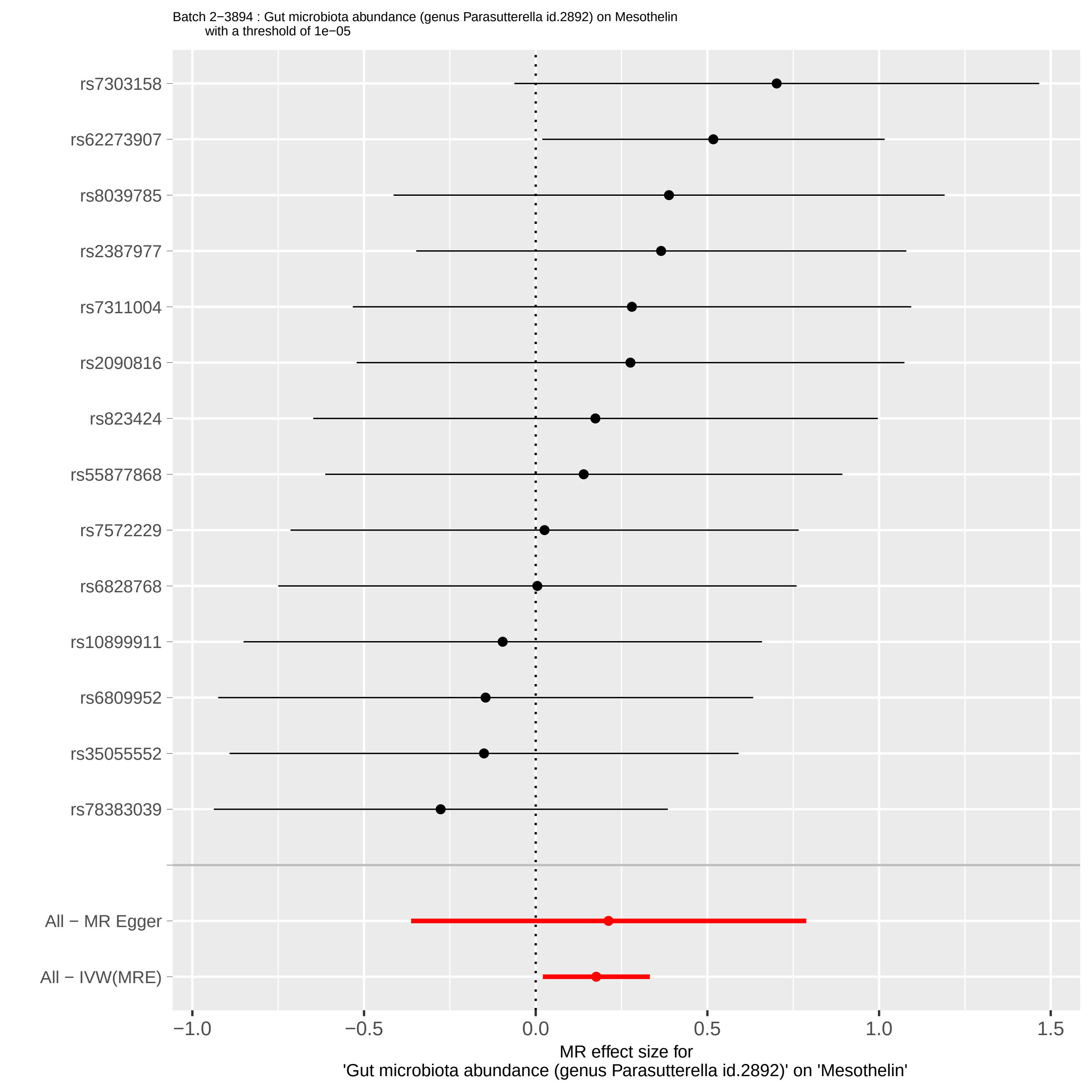
Batch 2−3894 : Gut microbiota abundance (genus Parasutterella id.2892) on Mesothelin
with a threshold of 1e−05
rs7303158
rs62273907
rs8039785
rs2387977
rs7311004
rs2090816
rs823424
rs55877868
rs7572229
rs6828768
rs10899911
rs6809952
rs35055552
rs78383039
All − MR Egger
All − IVW(MRE)
−1.0
−0.5
0.0
0.5
1.0
1.5
MR effect size for
'Gut microbiota abundance (genus Parasutterella id.2892)' on 'Mesothelin'

Batch 2−3895 : Gut microbiota abundance (genus Catenibacterium id.2153) on Mesothelin
with a threshold of 1e−05

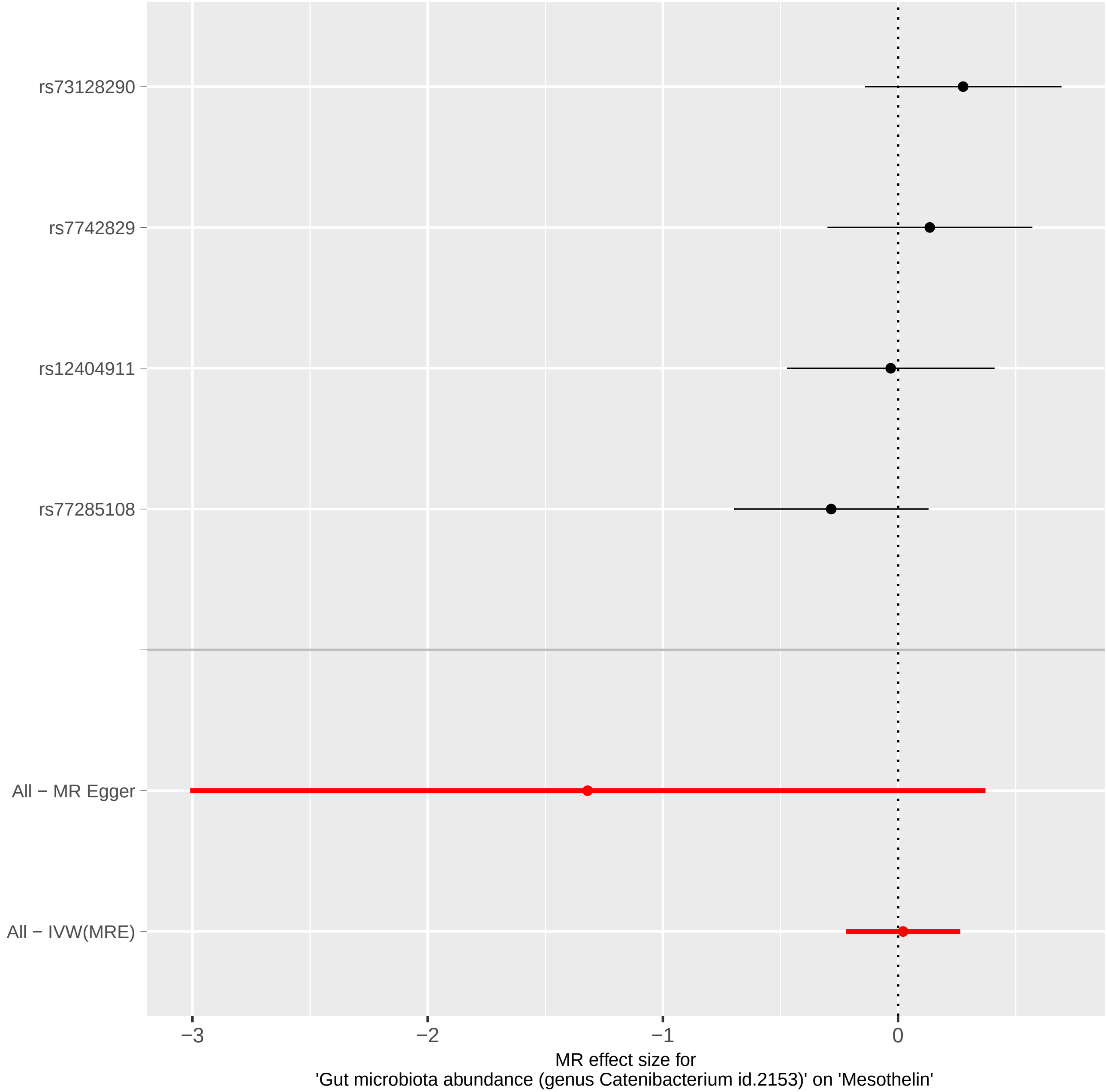

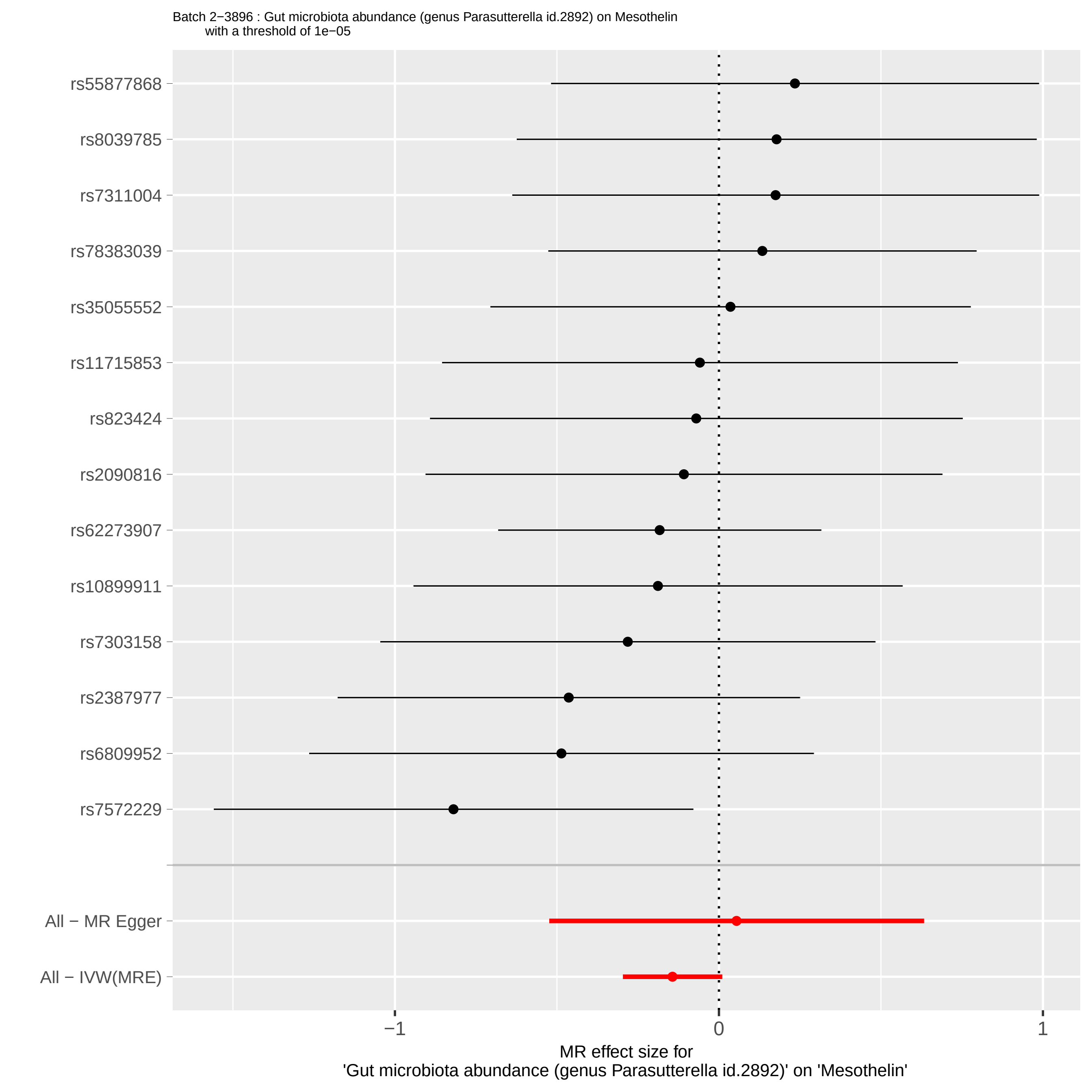

Batch 2−3896 : Gut microbiota abundance (genus Parasutterella id.2892) on Mesothelin
with a threshold of 1e−05
rs55877868
rs8039785
rs7311004
rs78383039
rs35055552
rs11715853
rs823424
rs2090816
rs62273907
rs10899911
rs7303158
rs2387977
rs6809952
rs7572229
All − MR Egger
All − IVW(MRE)
−1
0
1
MR effect size for
'Gut microbiota abundance (genus Parasutterella id.2892)' on 'Mesothelin'

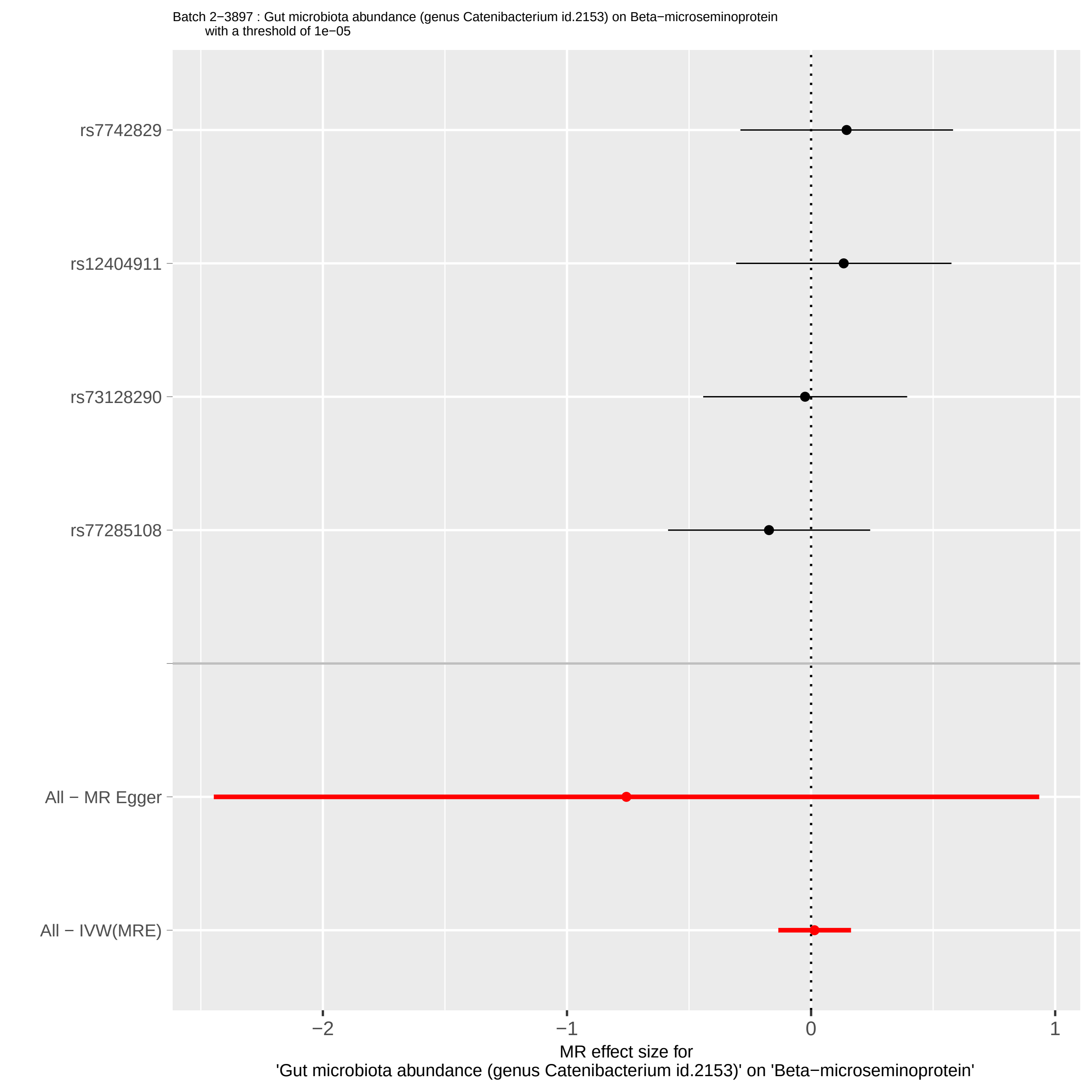
Batch 2−3897 : Gut microbiota abundance (genus Catenibacterium id.2153) on Beta−microseminoprotein
with a threshold of 1e−05
rs7742829
rs12404911
rs73128290
rs77285108
All − MR Egger
All − IVW(MRE)
−2
−1
0
1
MR effect size for
'Gut microbiota abundance (genus Catenibacterium id.2153)' on 'Beta−microseminoprotein'

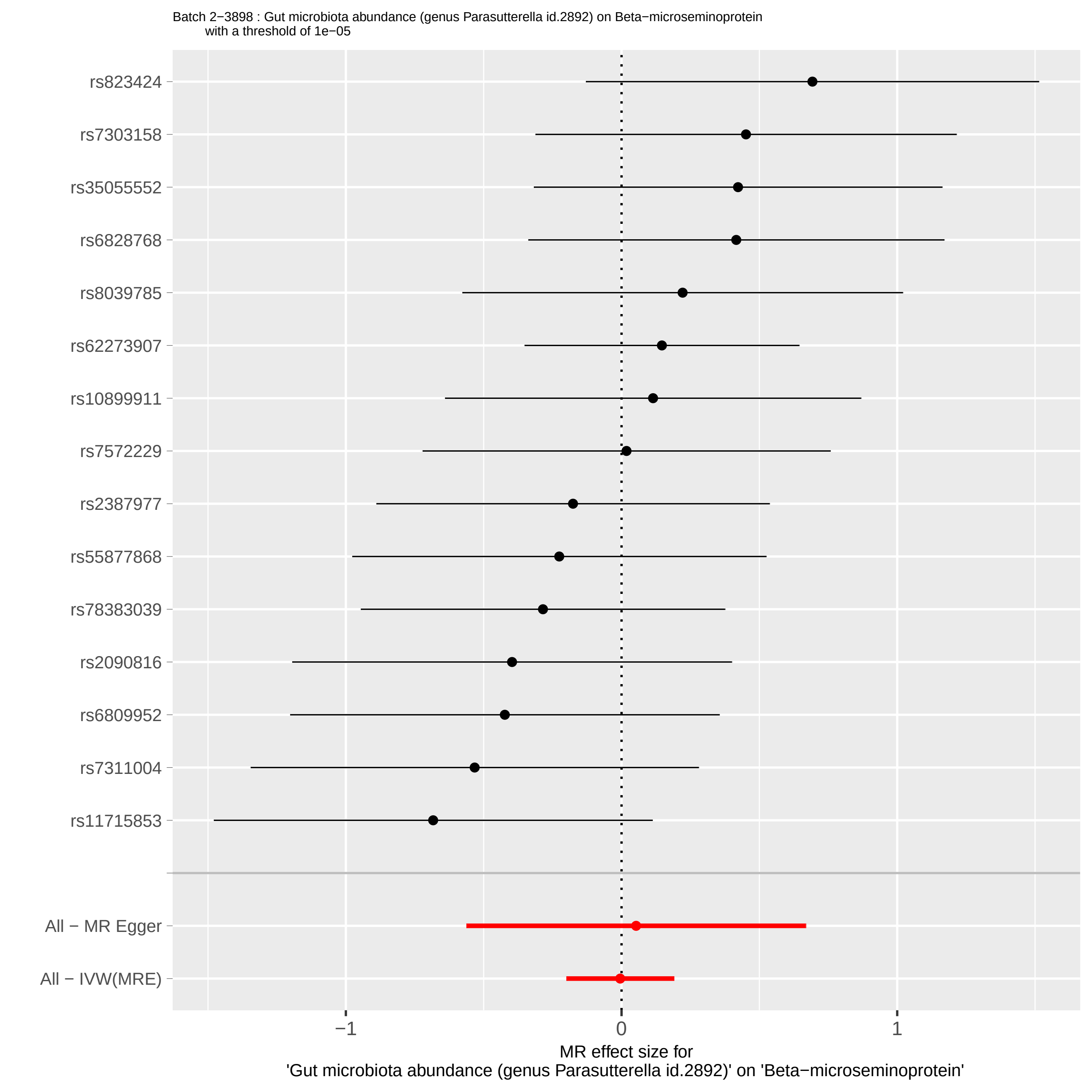

Batch 2−3898 : Gut microbiota abundance (genus Parasutterella id.2892) on Beta−microseminoprotein
with a threshold of 1e−05
rs823424
rs7303158
rs35055552
rs6828768
rs8039785
rs62273907
rs10899911
rs7572229
rs2387977
rs55877868
rs78383039
rs2090816
rs6809952
rs7311004
rs11715853
All − MR Egger
All − IVW(MRE)
−1
0
1
MR effect size for
'Gut microbiota abundance (genus Parasutterella id.2892)' on 'Beta−microseminoprotein'

Batch 2−3899 : Gut microbiota abundance (genus Catenibacterium id.2153) on Prostate−associated microseminoprotein
with a threshold of 1e−05

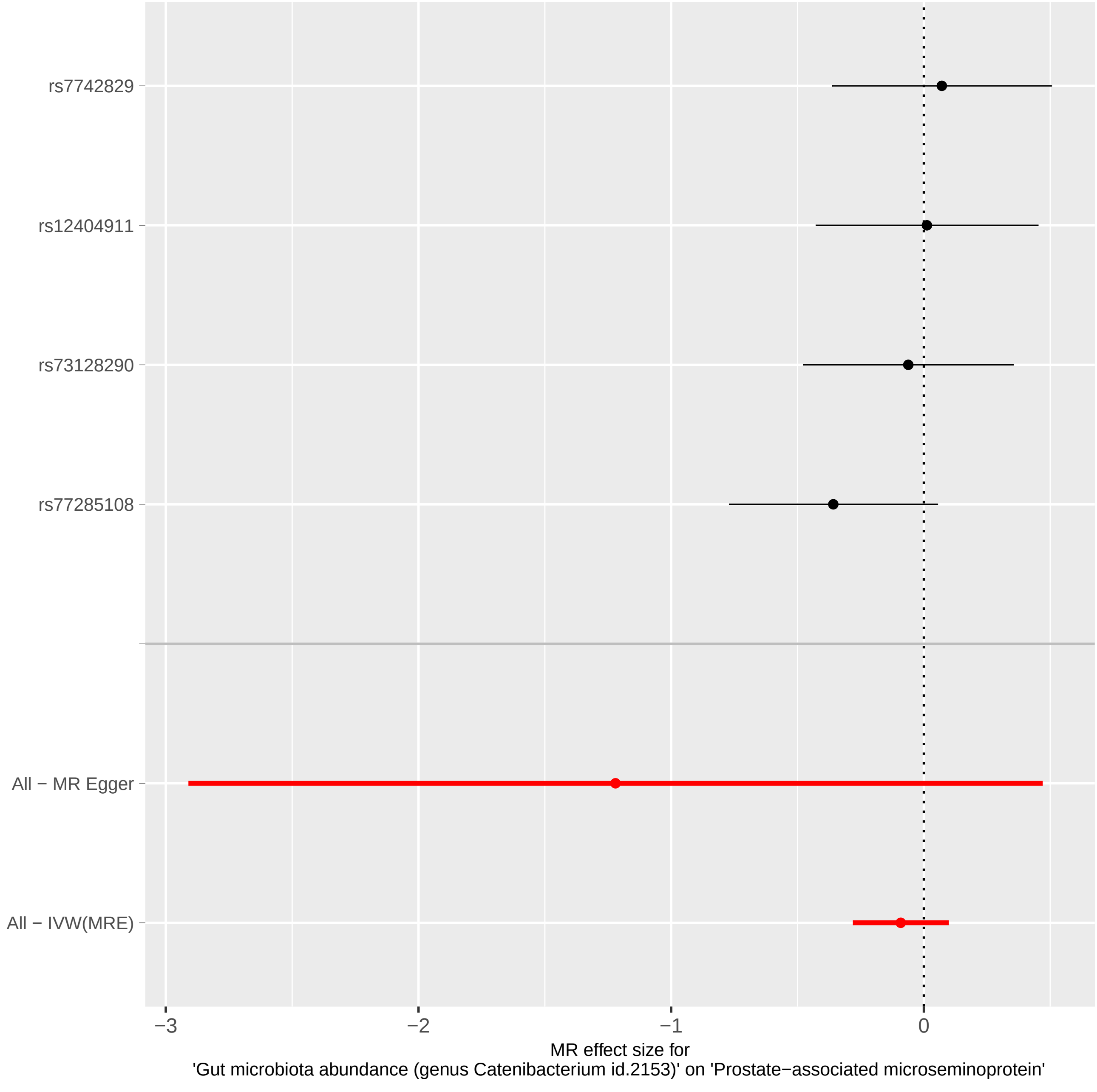

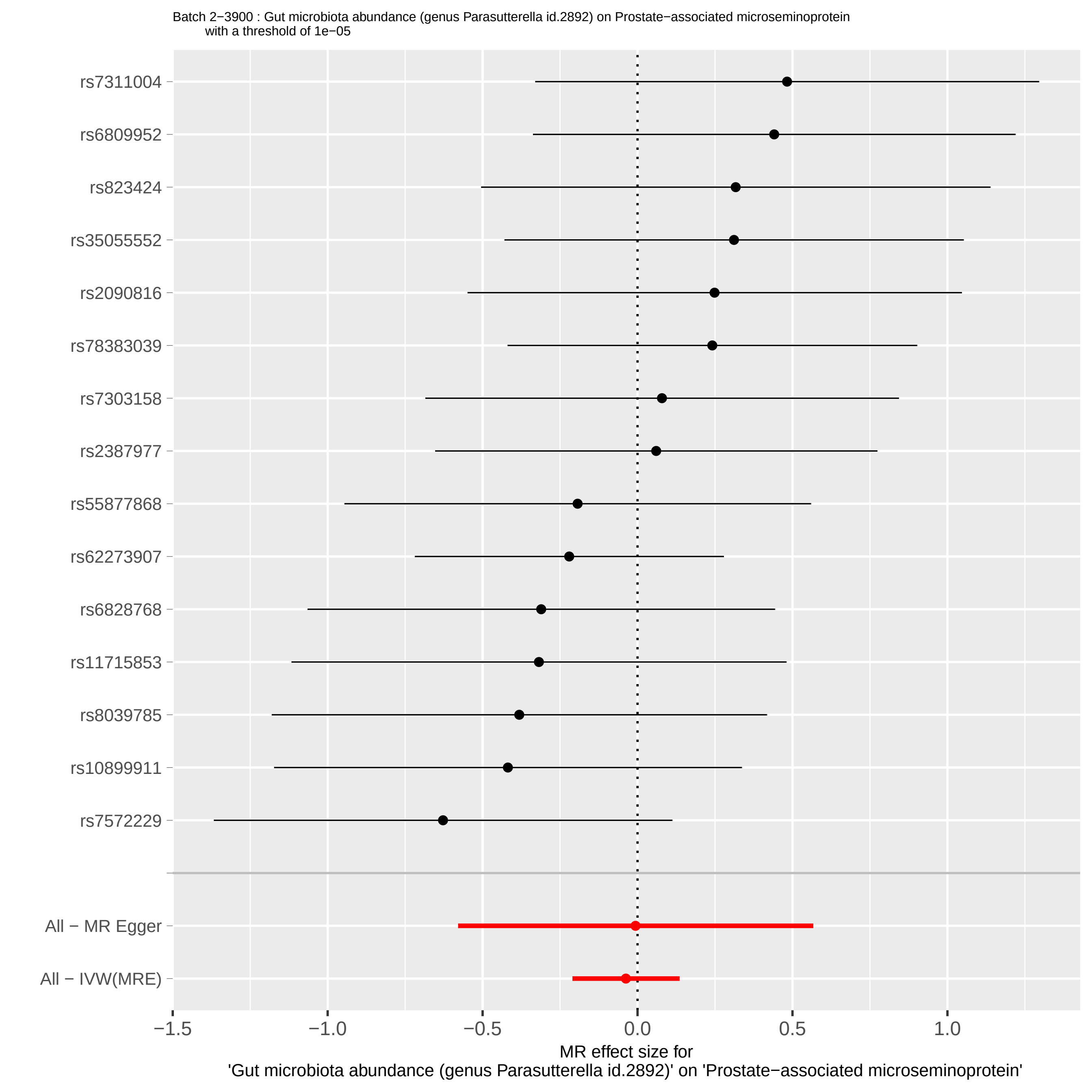
Batch 2−3900 : Gut microbiota abundance (genus Parasutterella id.2892) on Prostate−associated microseminoprotein
with a threshold of 1e−05
rs7311004
rs6809952
rs823424
rs35055552
rs2090816
rs78383039
rs7303158
rs2387977
rs55877868
rs62273907
rs6828768
rs11715853
rs8039785
rs10899911
rs7572229
All − MR Egger
All − IVW(MRE)
−1.5
−1.0
−0.5
0.0
0.5
1.0
MR effect size for
'Gut microbiota abundance (genus Parasutterella id.2892)' on 'Prostate−associated microseminoprotein'

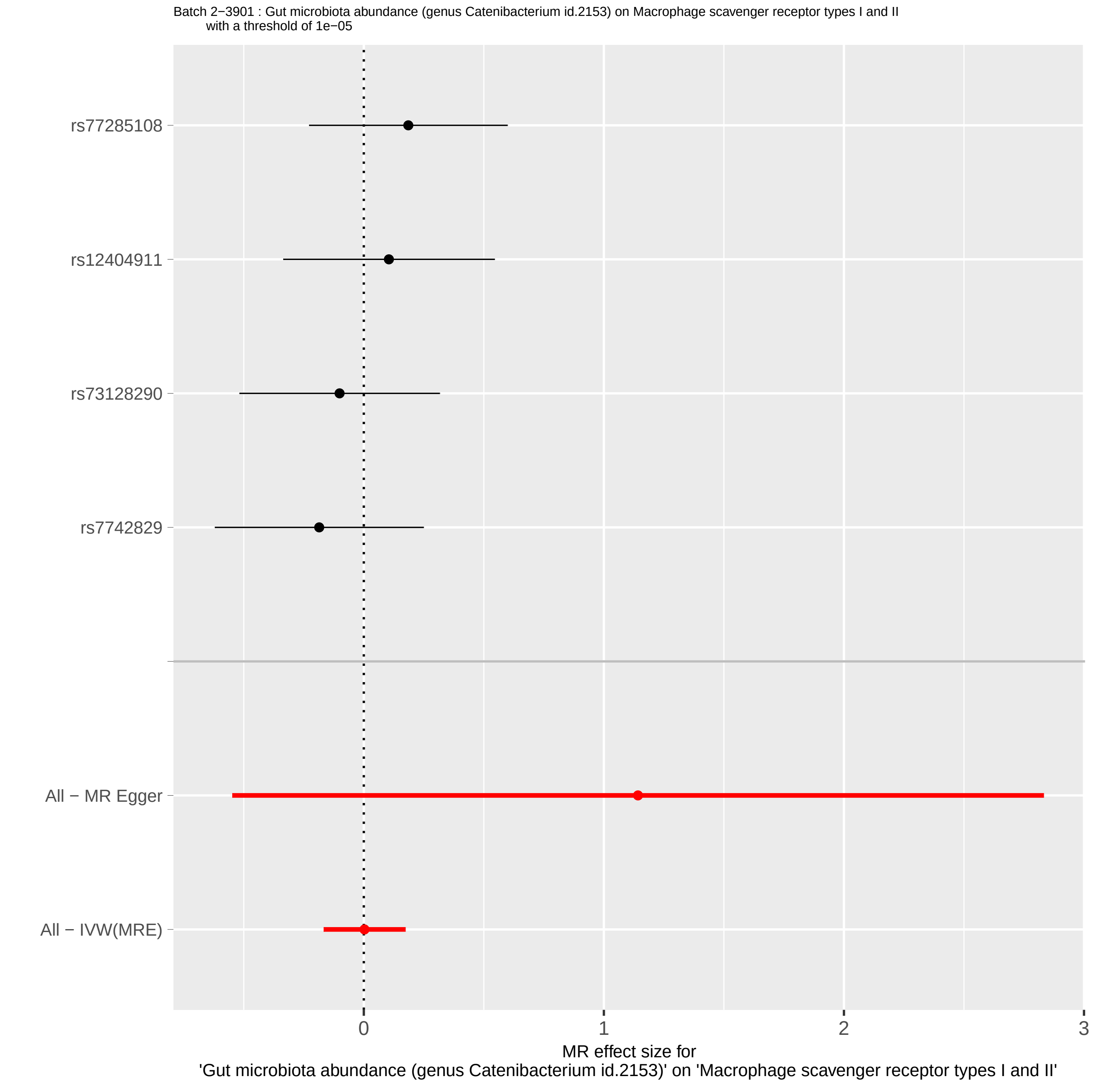

Batch 2−3901 : Gut microbiota abundance (genus Catenibacterium id.2153) on Macrophage scavenger receptor types I and II with a threshold of 1e−05
rs77285108
rs12404911
rs73128290
rs7742829
All − MR Egger
All − IVW(MRE)
0
1
2
3
MR effect size for
'Gut microbiota abundance (genus Catenibacterium id.2153)' on 'Macrophage scavenger receptor types I and II'

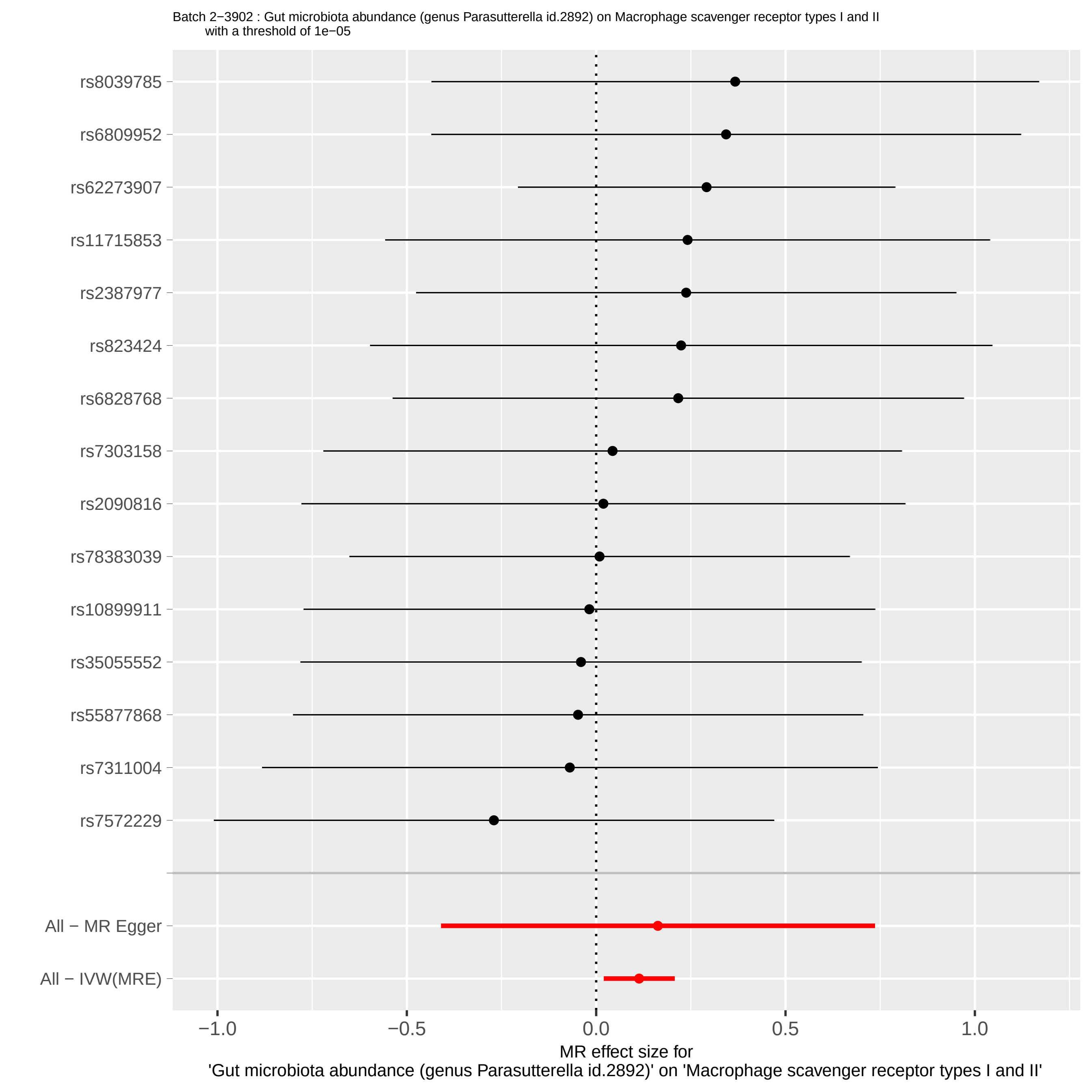

Batch 2−3902 : Gut microbiota abundance (genus Parasutterella id.2892) on Macrophage scavenger receptor types I and II
with a threshold of 1e−05
rs8039785
rs6809952
rs62273907
rs11715853
rs2387977
rs823424
rs6828768
rs7303158
rs2090816
rs78383039
rs10899911
rs35055552
rs55877868
rs7311004
rs7572229
All − MR Egger
All − IVW(MRE)
−1.0
−0.5
0.0
0.5
1.0
MR effect size for
'Gut microbiota abundance (genus Parasutterella id.2892)' on 'Macrophage scavenger receptor types I and II'

Batch 2−3903 : Gut microbiota abundance (genus Catenibacterium id.2153) on Macrophage scavenger receptor types I and II with a threshold of 1e−05

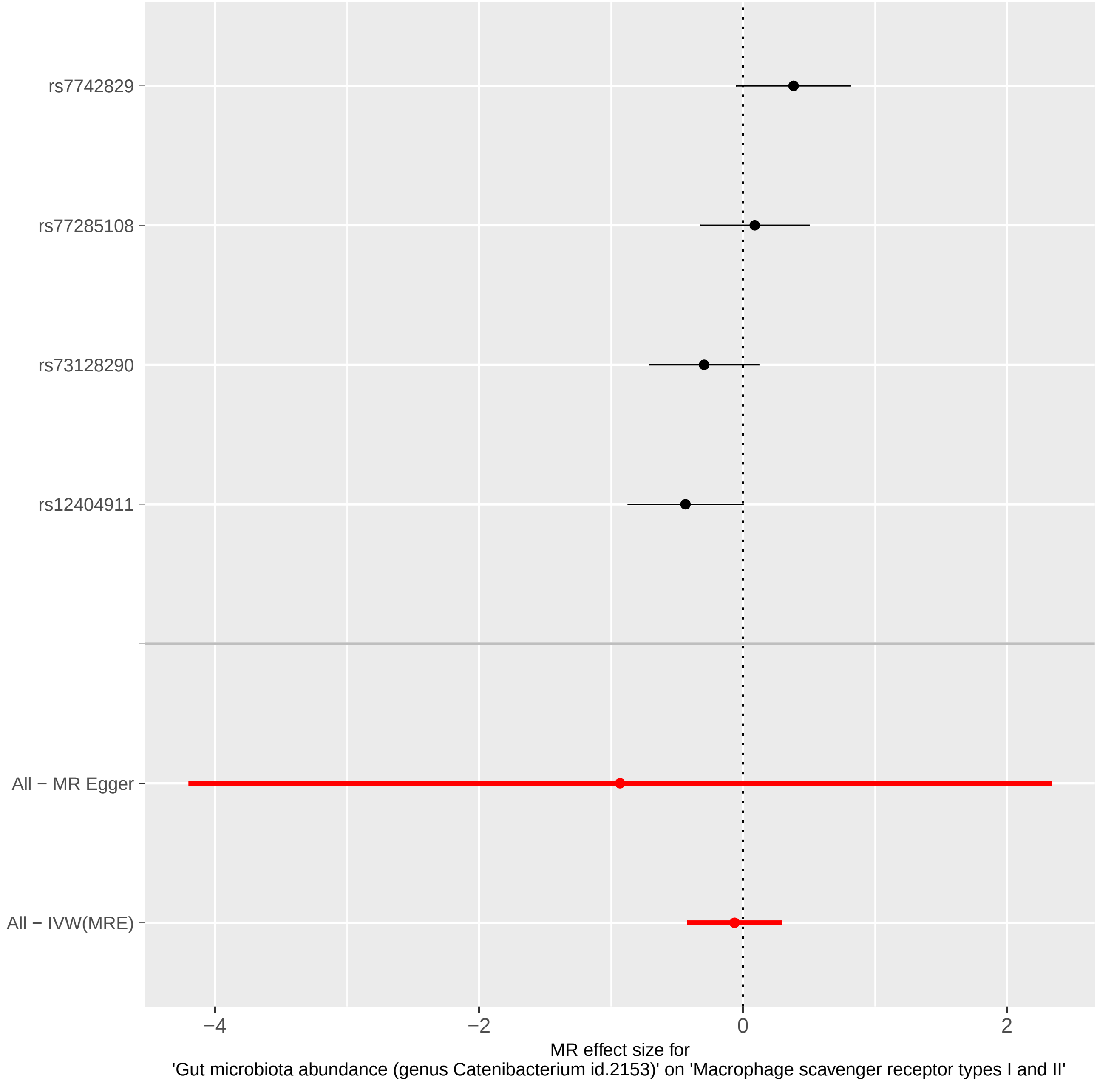

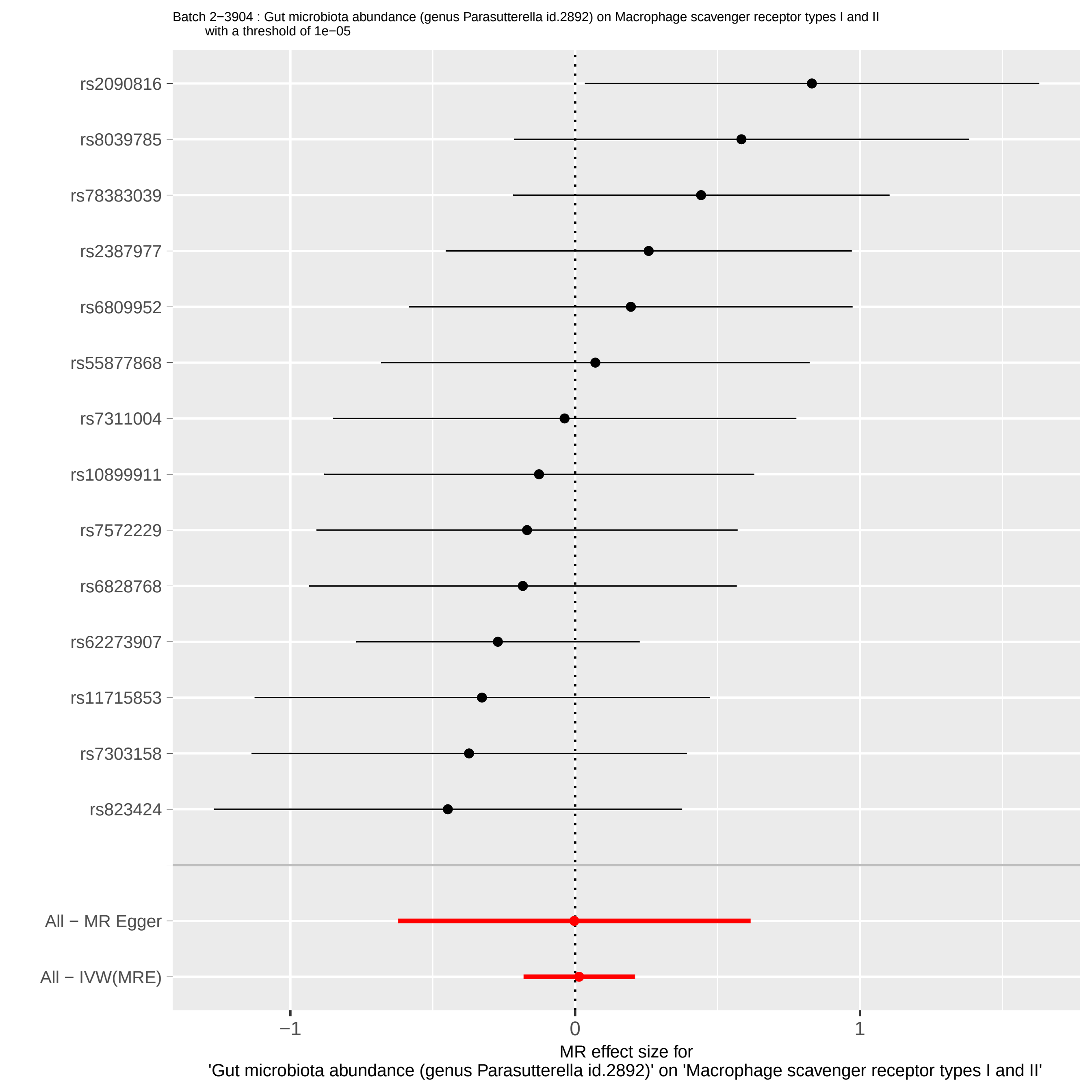
Batch 2−3904 : Gut microbiota abundance (genus Parasutterella id.2892) on Macrophage scavenger receptor types I and II
with a threshold of 1e−05
rs2090816
rs8039785
rs78383039
rs2387977
rs6809952
rs55877868
rs7311004
rs10899911
rs7572229
rs6828768
rs62273907
rs11715853
rs7303158
rs823424
All − MR Egger
All − IVW(MRE)
−1
0
1
MR effect size for
'Gut microbiota abundance (genus Parasutterella id.2892)' on 'Macrophage scavenger receptor types I and II'

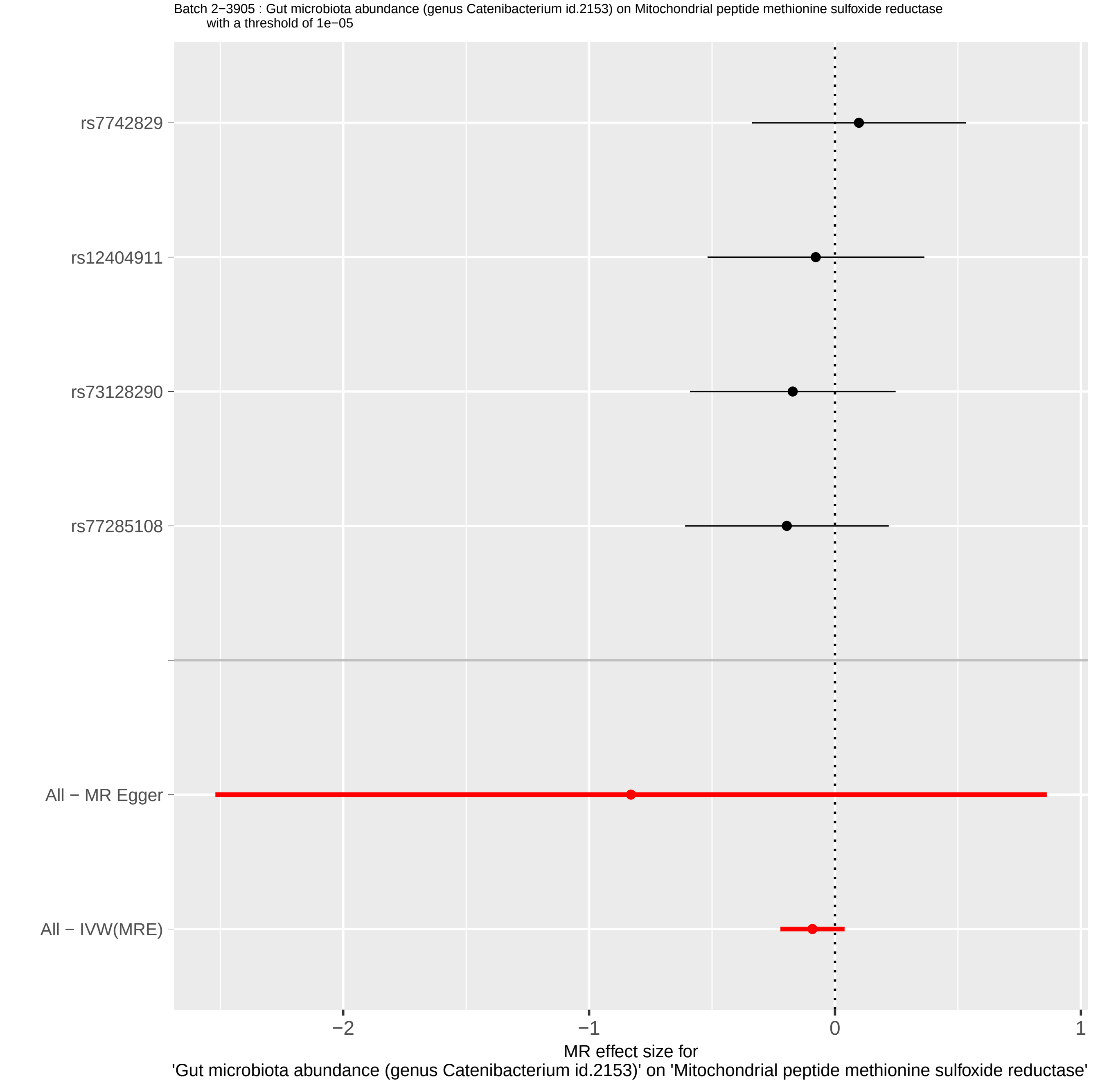
Batch 2−3905 : Gut microbiota abundance (genus Catenibacterium id.2153) on Mitochondrial peptide methionine sulfoxide reductase
with a threshold of 1e−05
rs7742829
rs12404911
rs73128290
rs77285108
All − MR Egger
All − IVW(MRE)
−2
−1
0
1
MR effect size for
'Gut microbiota abundance (genus Catenibacterium id.2153)' on 'Mitochondrial peptide methionine sulfoxide reductase'

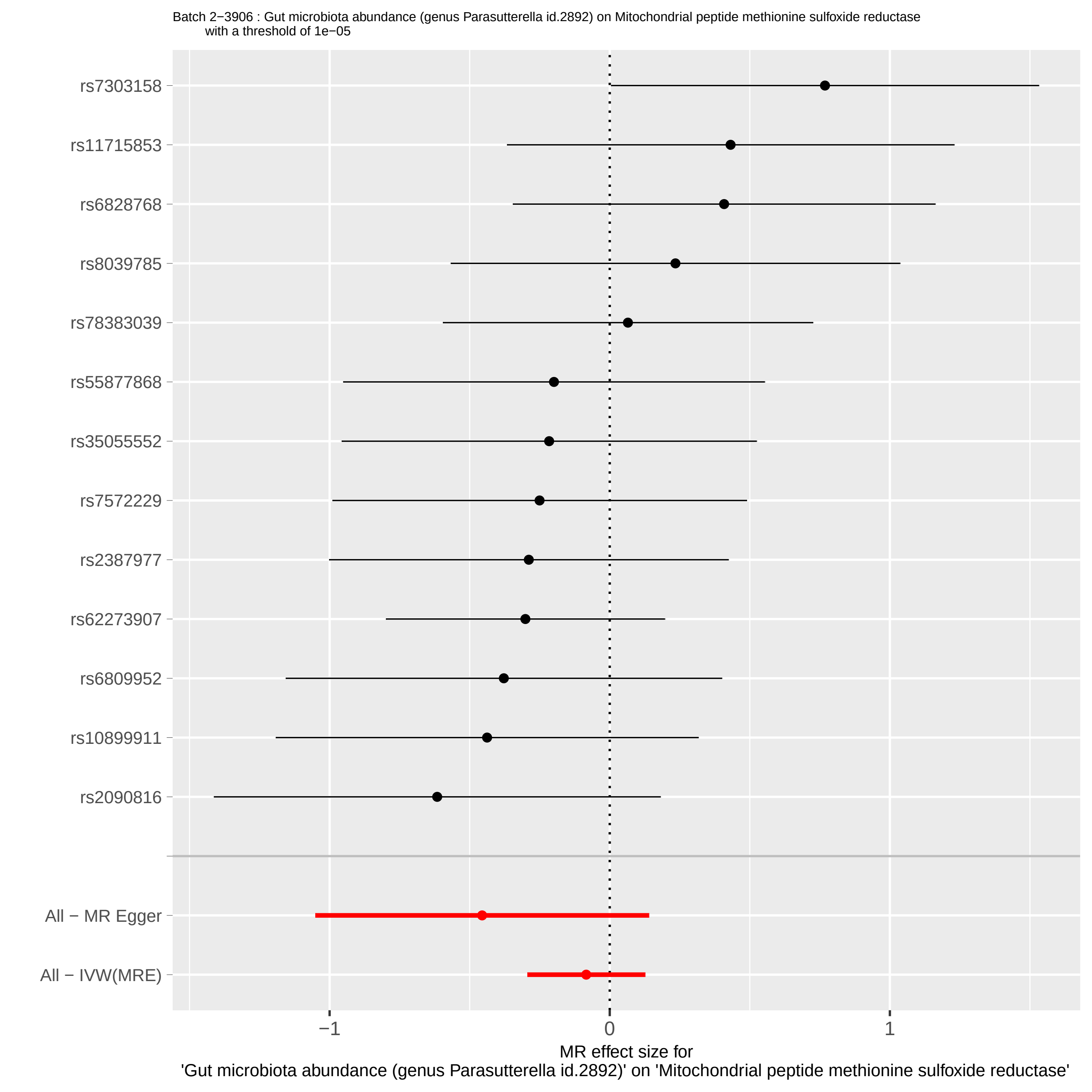
Batch 2−3906 : Gut microbiota abundance (genus Parasutterella id.2892) on Mitochondrial peptide methionine sulfoxide reductase
with a threshold of 1e−05
rs7303158
rs11715853
rs6828768
rs8039785
rs78383039
rs55877868
rs35055552
rs7572229
rs2387977
rs62273907
rs6809952
rs10899911
rs2090816
All − MR Egger
All − IVW(MRE)
−1
0
1
MR effect size for
'Gut microbiota abundance (genus Parasutterella id.2892)' on 'Mitochondrial peptide methionine sulfoxide reductase'

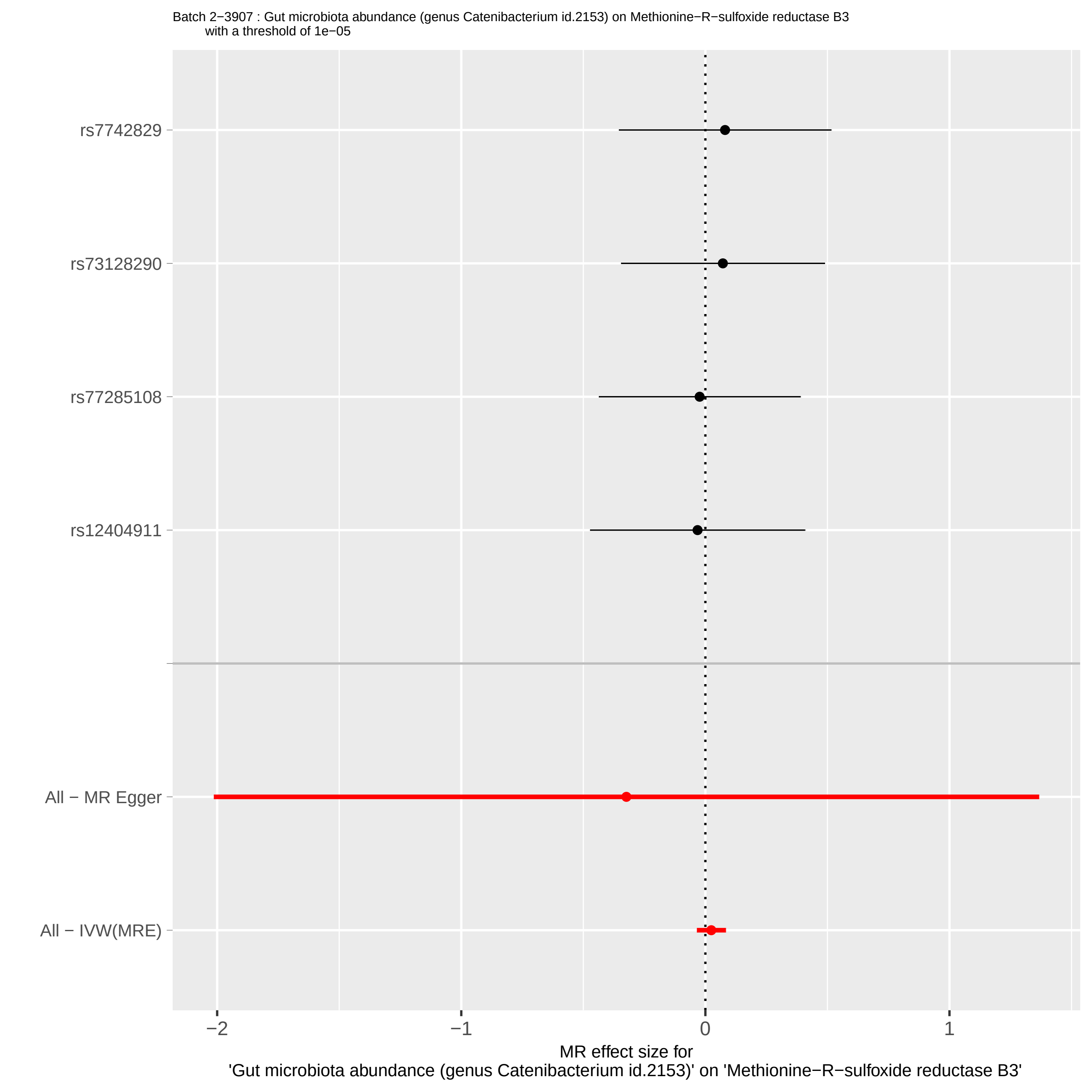
Batch 2−3907 : Gut microbiota abundance (genus Catenibacterium id.2153) on Methionine−R−sulfoxide reductase B3
with a threshold of 1e−05
rs7742829
rs73128290
rs77285108
rs12404911
All − MR Egger
All − IVW(MRE)
−2
−1
0
1
MR effect size for
'Gut microbiota abundance (genus Catenibacterium id.2153)' on 'Methionine−R−sulfoxide reductase B3'

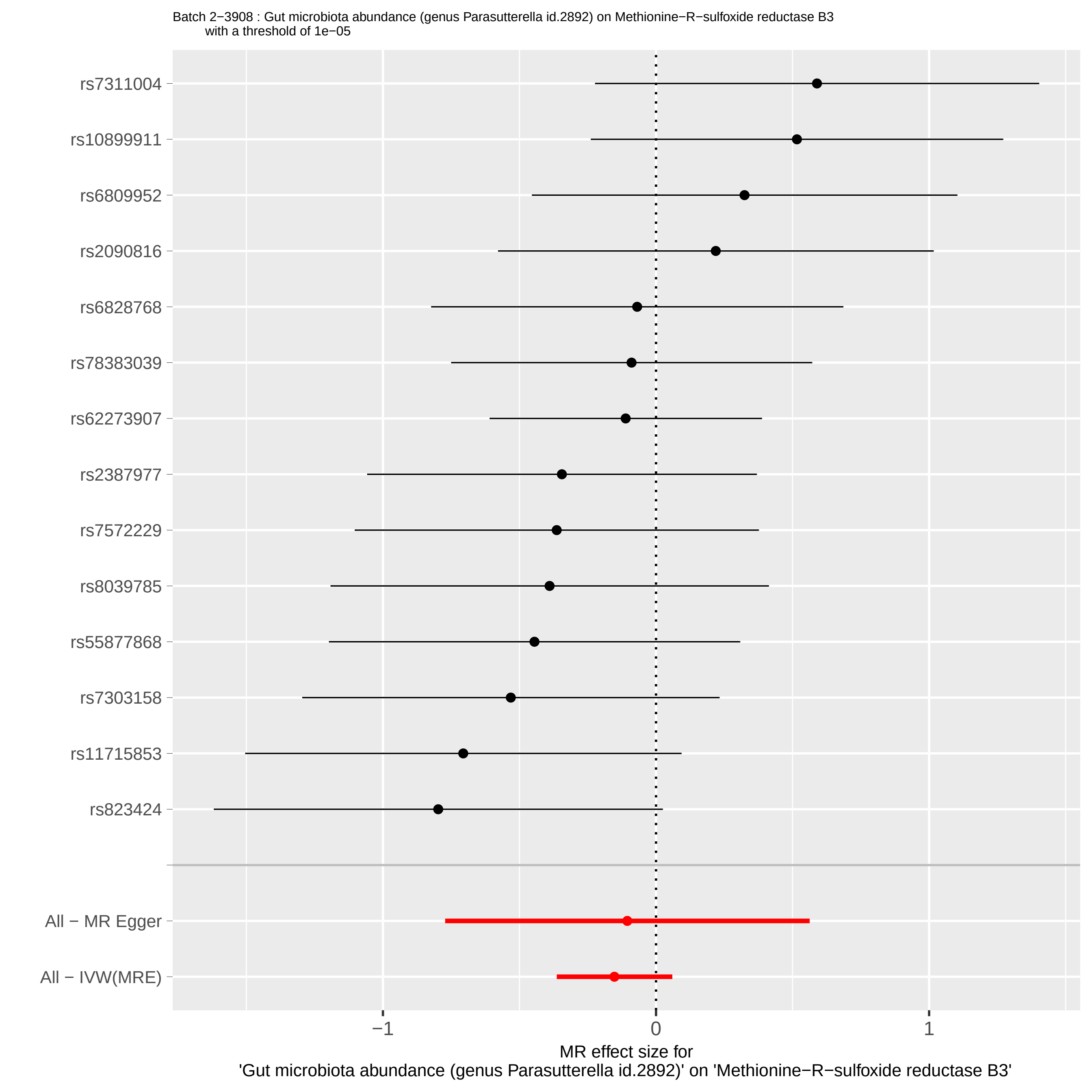

Batch 2−3908 : Gut microbiota abundance (genus Parasutterella id.2892) on Methionine−R−sulfoxide reductase B3
with a threshold of 1e−05
rs7311004
rs10899911
rs6809952
rs2090816
rs6828768
rs78383039
rs62273907
rs2387977
rs7572229
rs8039785
rs55877868
rs7303158
rs11715853
rs823424
All − MR Egger
All − IVW(MRE)
−1
0
1
MR effect size for
'Gut microbiota abundance (genus Parasutterella id.2892)' on 'Methionine−R−sulfoxide reductase B3'

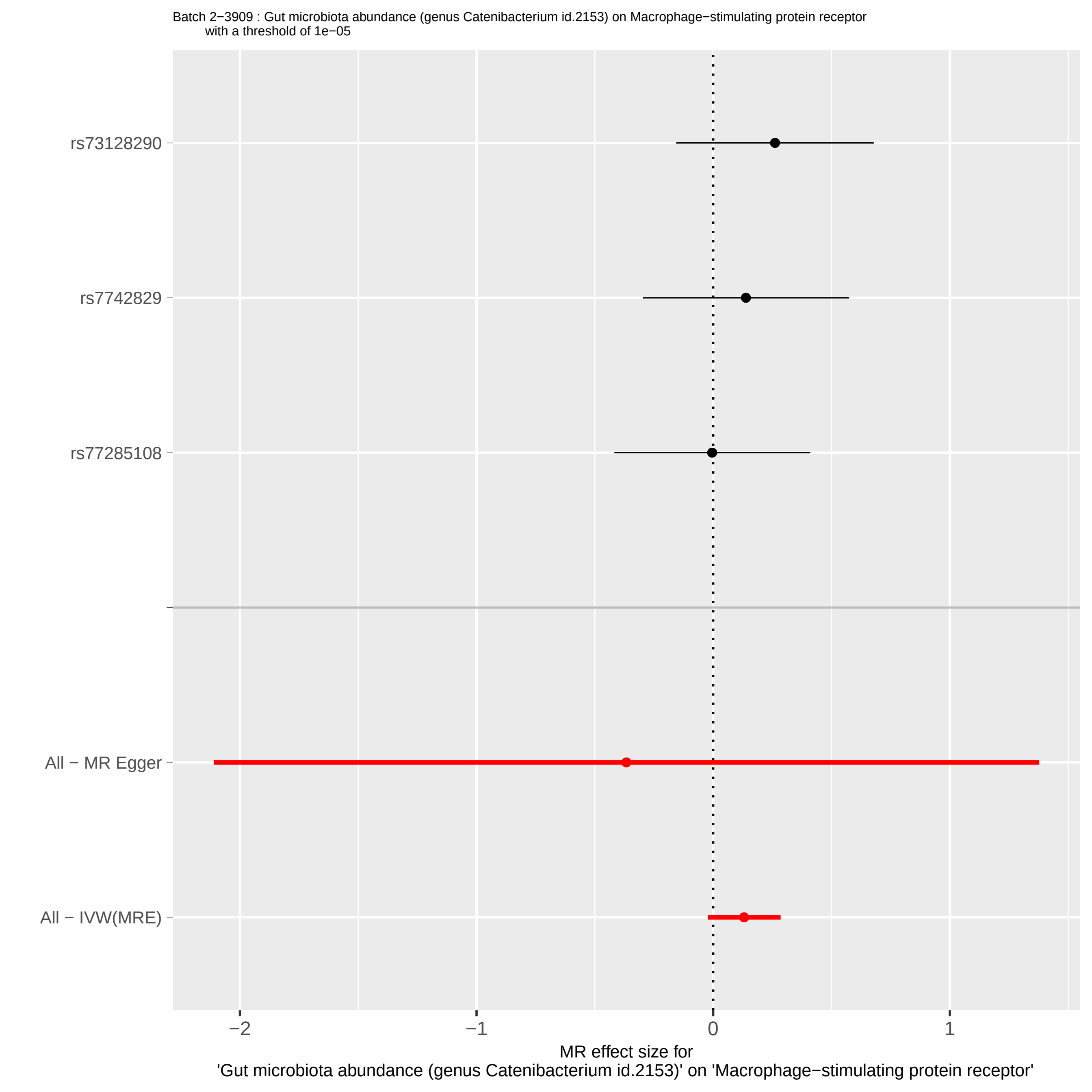

Batch 2−3909 : Gut microbiota abundance (genus Catenibacterium id.2153) on Macrophage−stimulating protein receptor
with a threshold of 1e−05
rs73128290
rs7742829
rs77285108
All − MR Egger
All − IVW(MRE)
−2
−1
0
1
MR effect size for
'Gut microbiota abundance (genus Catenibacterium id.2153)' on 'Macrophage−stimulating protein receptor'

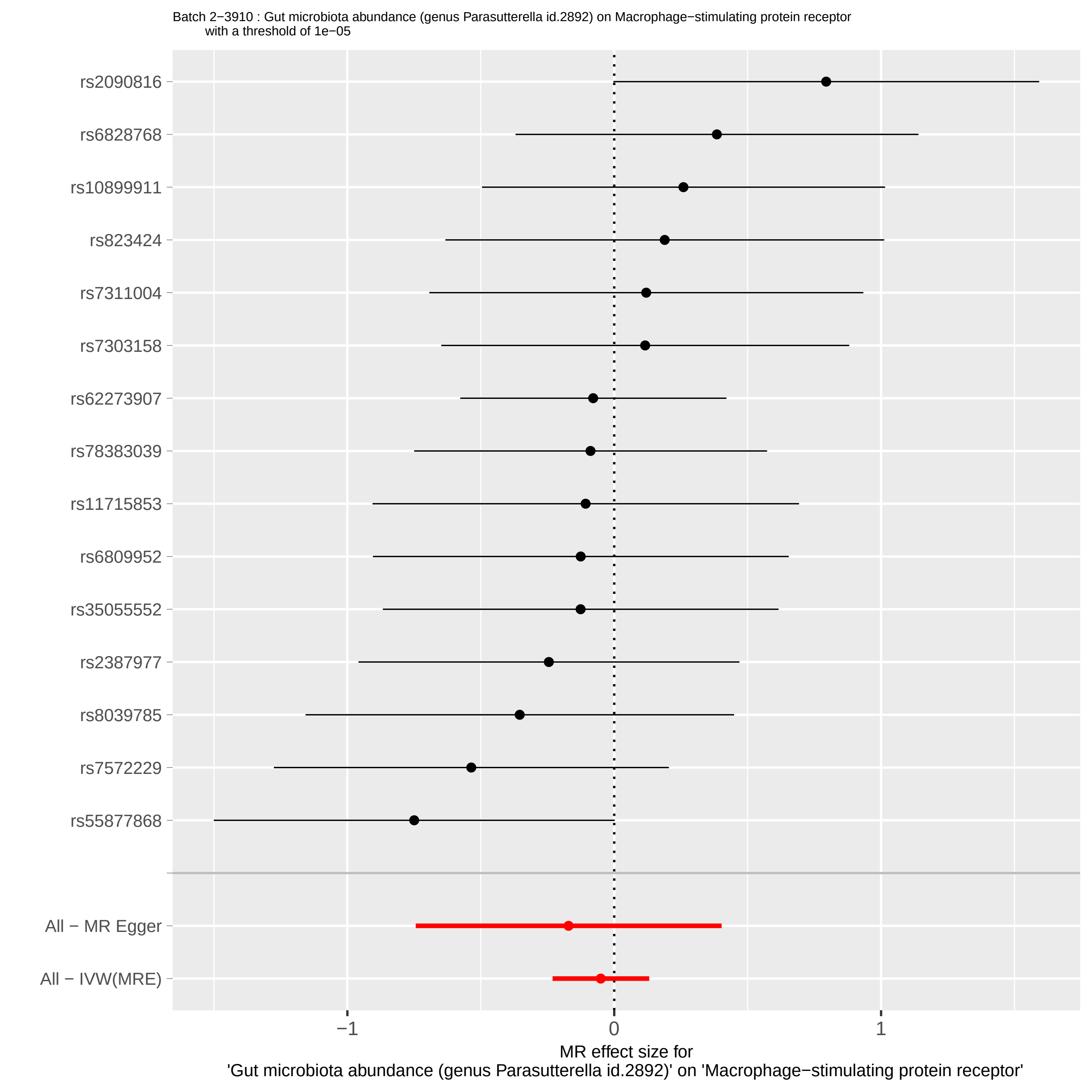
Batch 2−3910 : Gut microbiota abundance (genus Parasutterella id.2892) on Macrophage−stimulating protein receptor
with a threshold of 1e−05
rs2090816
rs6828768
rs10899911
rs823424
rs7311004
rs7303158
rs62273907
rs78383039
rs11715853
rs6809952
rs35055552
rs2387977
rs8039785
rs7572229
rs55877868
All − MR Egger
All − IVW(MRE)
−1
0
1
MR effect size for
'Gut microbiota abundance (genus Parasutterella id.2892)' on 'Macrophage−stimulating protein receptor'

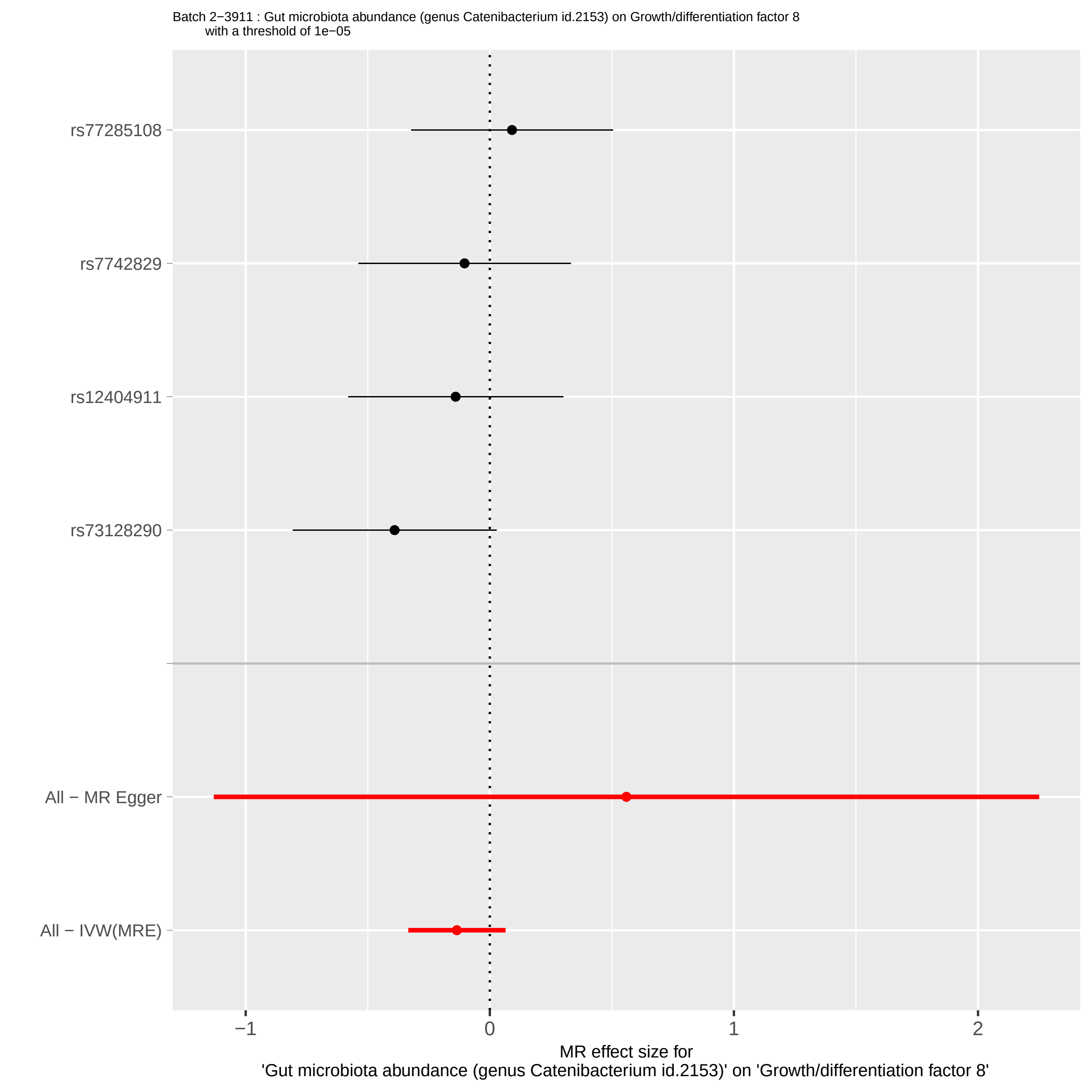

Batch 2−3911 : Gut microbiota abundance (genus Catenibacterium id.2153) on Growth/differentiation factor 8
with a threshold of 1e−05
rs77285108
rs7742829
rs12404911
rs73128290
All − MR Egger
All − IVW(MRE)
−1
0
1
2
MR effect size for
'Gut microbiota abundance (genus Catenibacterium id.2153)' on 'Growth/differentiation factor 8'

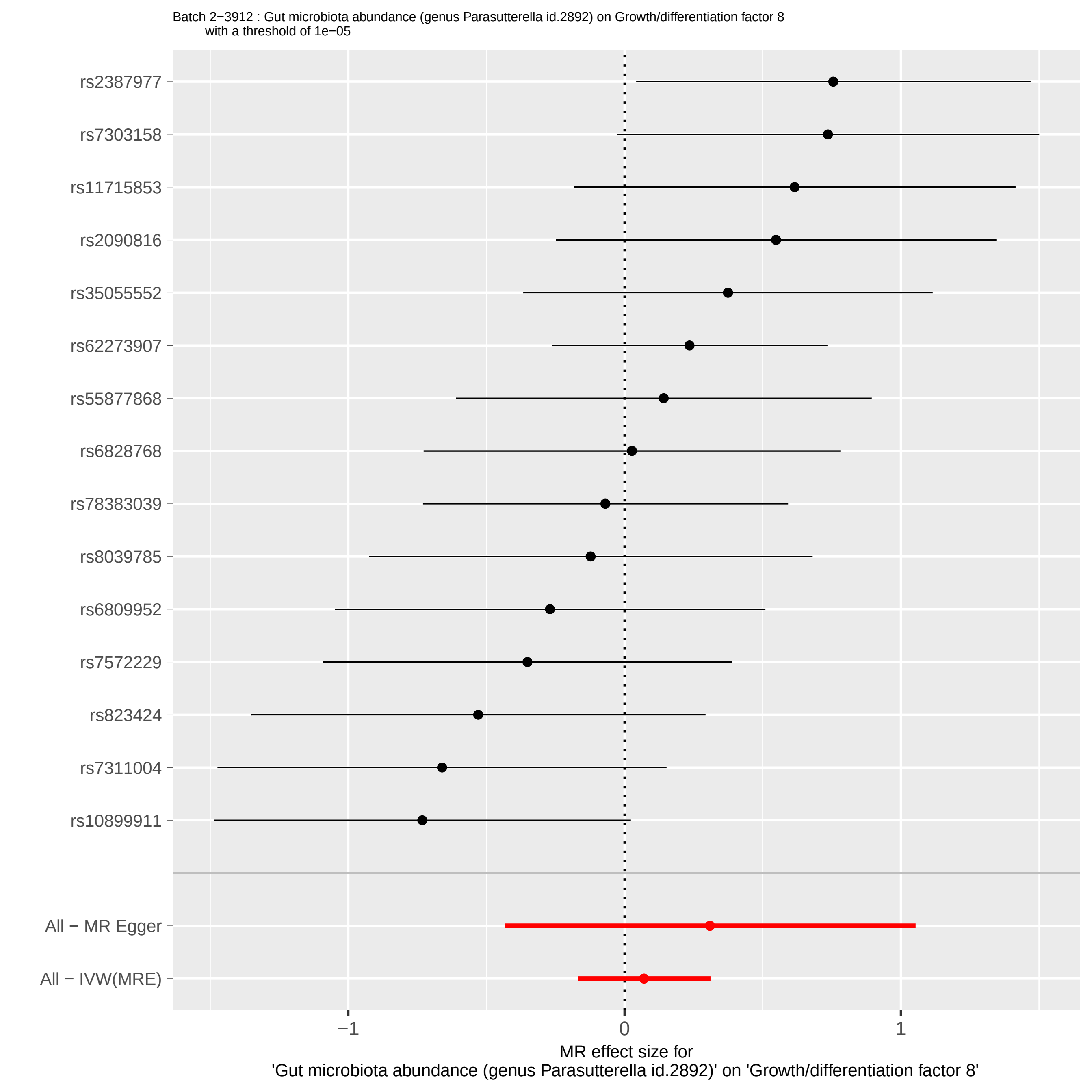

Batch 2−3912 : Gut microbiota abundance (genus Parasutterella id.2892) on Growth/differentiation factor 8
with a threshold of 1e−05
rs2387977
rs7303158
rs11715853
rs2090816
rs35055552
rs62273907
rs55877868
rs6828768
rs78383039
rs8039785
rs6809952
rs7572229
rs823424
rs7311004
rs10899911
All − MR Egger
All − IVW(MRE)
−1
0
1
MR effect size for
'Gut microbiota abundance (genus Parasutterella id.2892)' on 'Growth/differentiation factor 8'

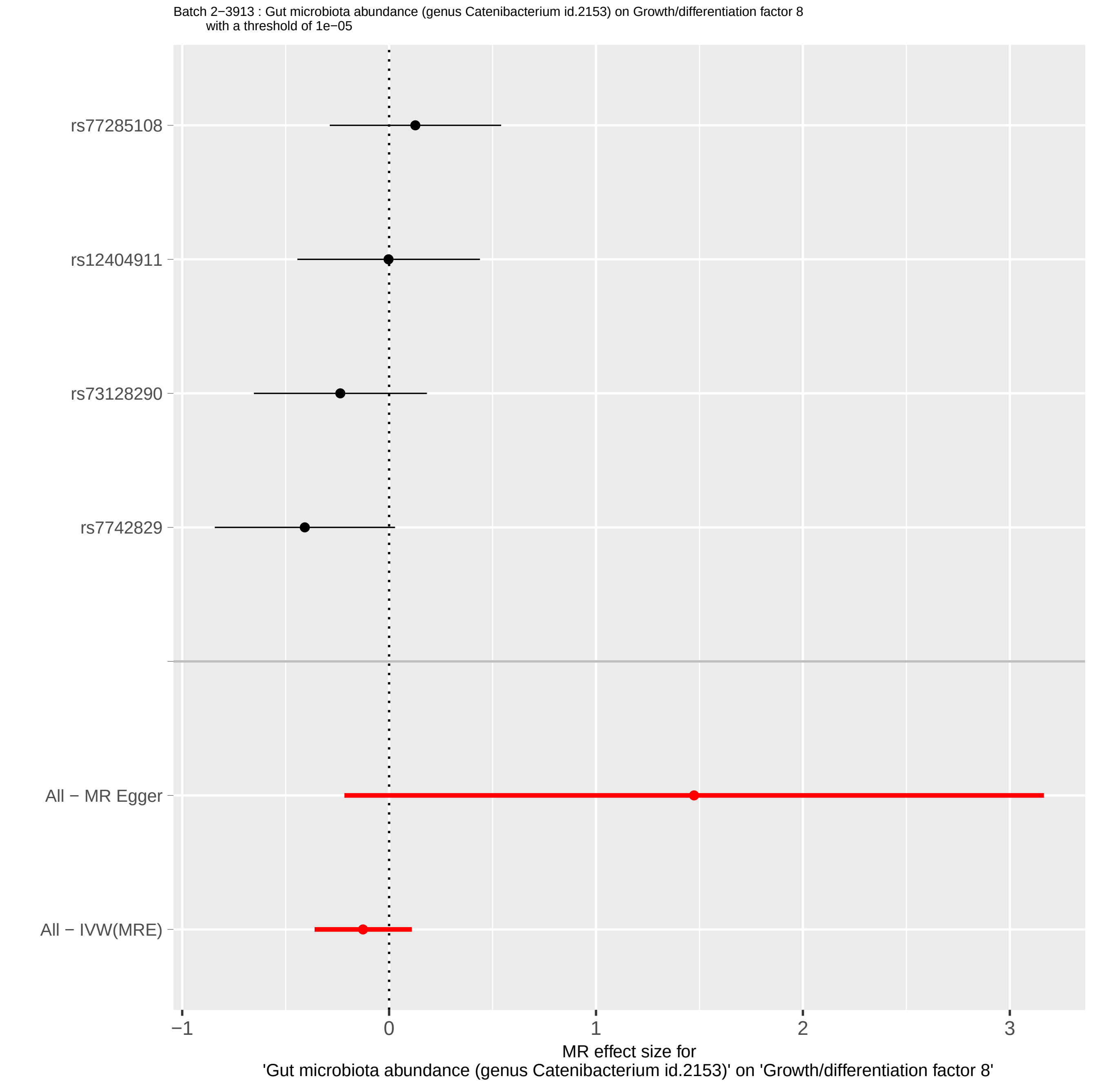
Batch 2−3913 : Gut microbiota abundance (genus Catenibacterium id.2153) on Growth/differentiation factor 8
with a threshold of 1e−05
rs77285108
rs12404911
rs73128290
rs7742829
All − MR Egger
All − IVW(MRE)
−1
0
1
2
3
MR effect size for
'Gut microbiota abundance (genus Catenibacterium id.2153)' on 'Growth/differentiation factor 8'

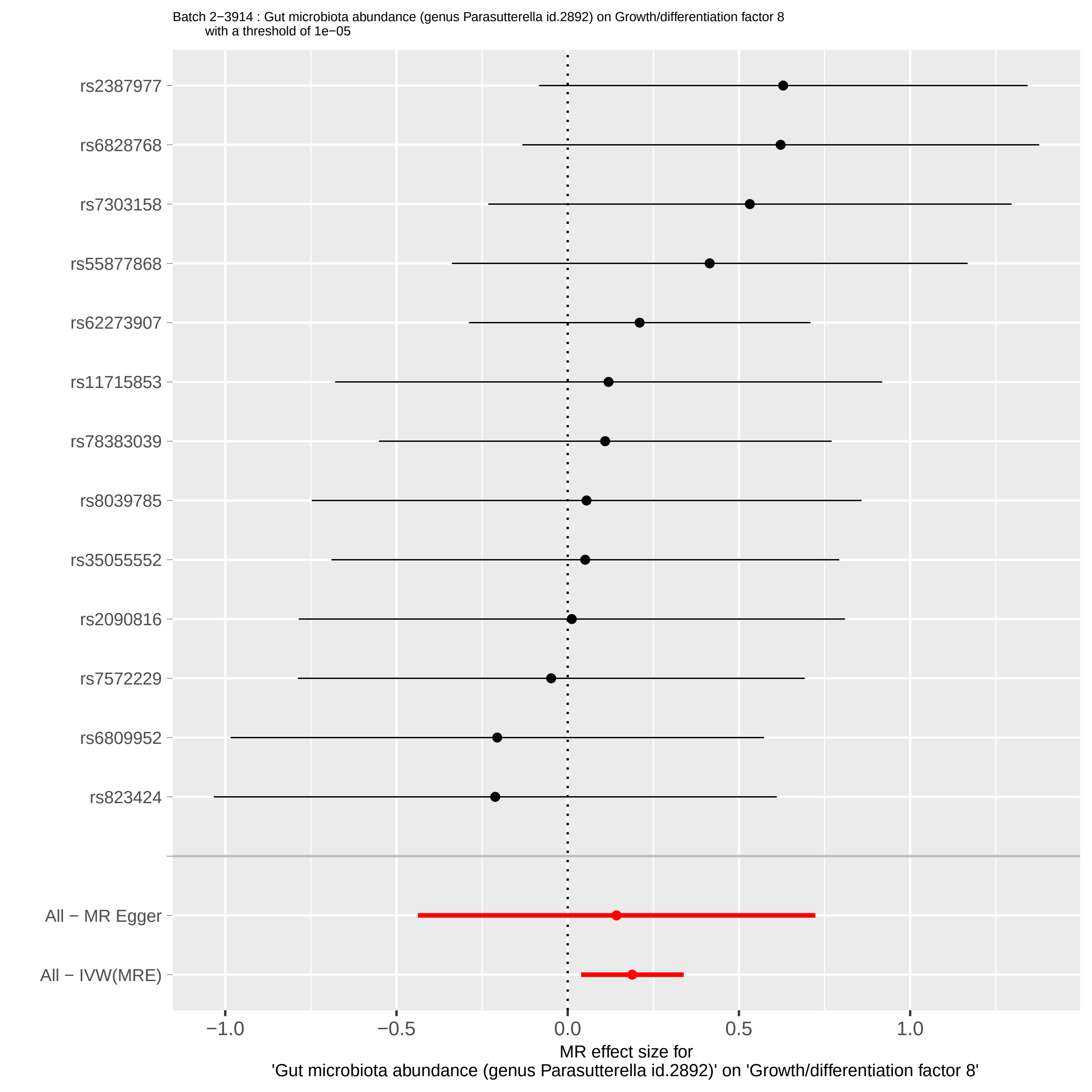

Batch 2−3914 : Gut microbiota abundance (genus Parasutterella id.2892) on Growth/differentiation factor 8
with a threshold of 1e−05
rs2387977
rs6828768
rs7303158
rs55877868
rs62273907
rs11715853
rs78383039
rs8039785
rs35055552
rs2090816
rs7572229
rs6809952
rs823424
All − MR Egger
All − IVW(MRE)
−1.0
−0.5
0.0
0.5
1.0
MR effect size for
'Gut microbiota abundance (genus Parasutterella id.2892)' on 'Growth/differentiation factor 8'

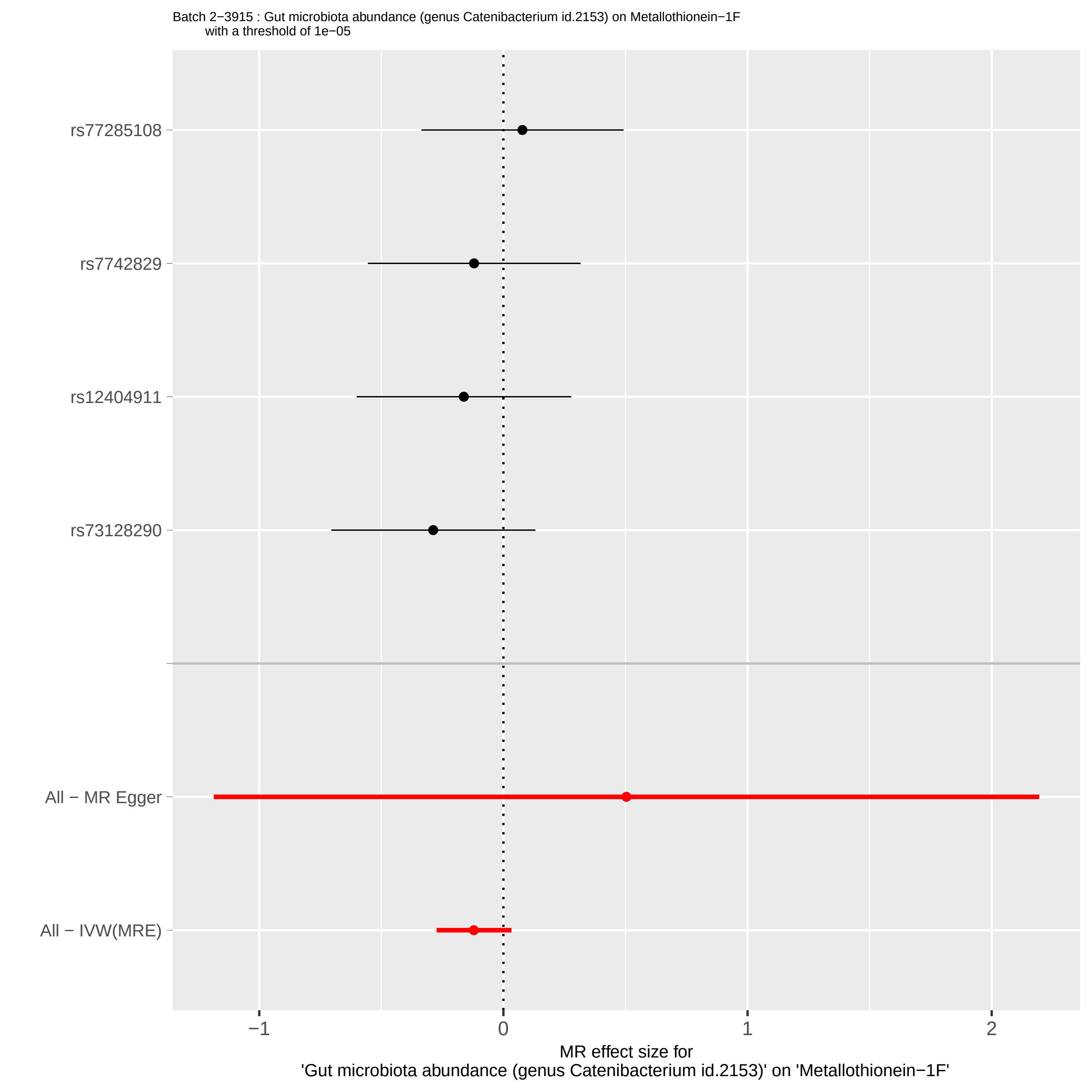
Batch 2−3915 : Gut microbiota abundance (genus Catenibacterium id.2153) on Metallothionein−1F
with a threshold of 1e−05
rs77285108
rs7742829
rs12404911
rs73128290
All − MR Egger
All − IVW(MRE)
−1
0
1
2
MR effect size for
'Gut microbiota abundance (genus Catenibacterium id.2153)' on 'Metallothionein−1F'

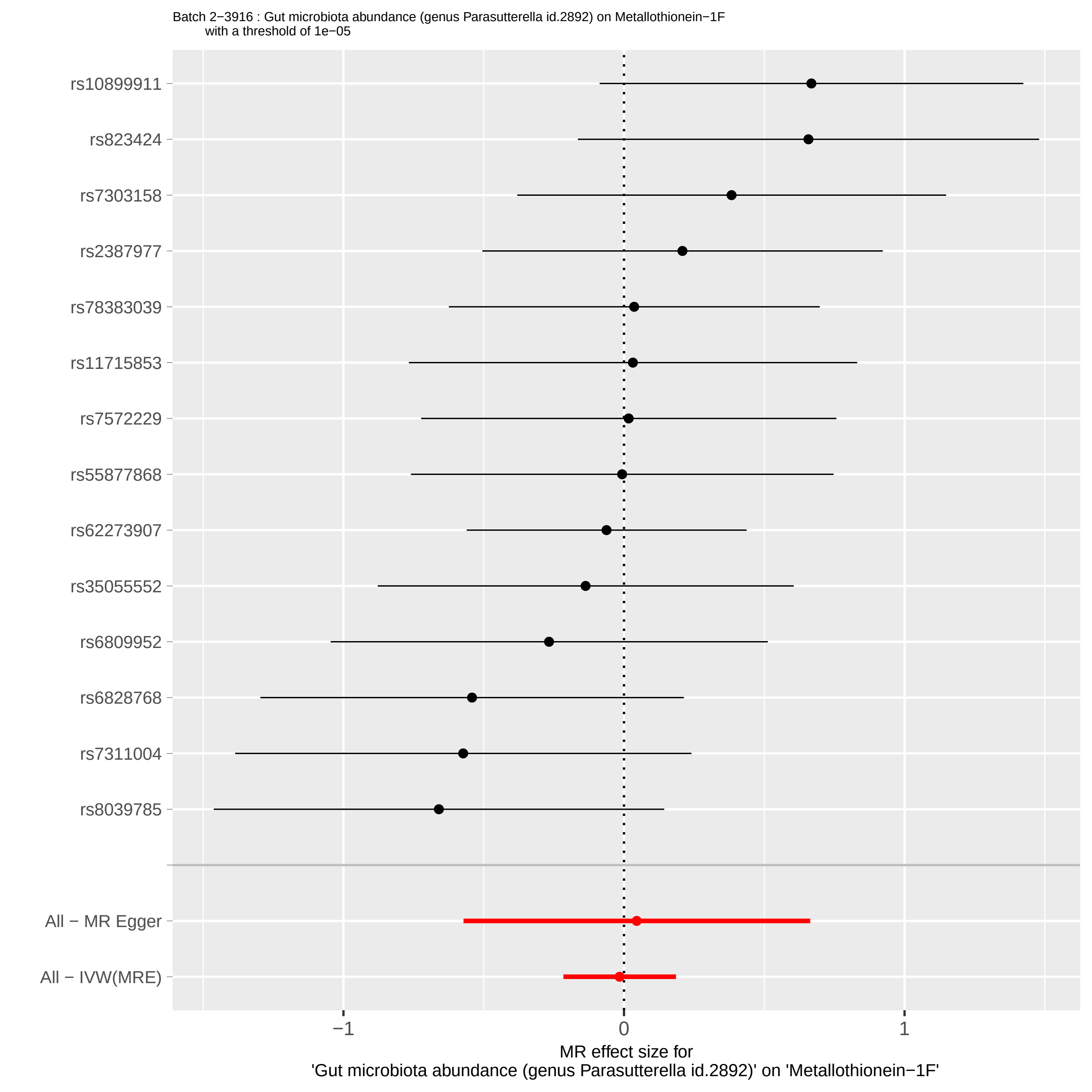

Batch 2−3916 : Gut microbiota abundance (genus Parasutterella id.2892) on Metallothionein−1F
with a threshold of 1e−05
rs10899911
rs823424
rs7303158
rs2387977
rs78383039
rs11715853
rs7572229
rs55877868
rs62273907
rs35055552
rs6809952
rs6828768
rs7311004
rs8039785
All − MR Egger
All − IVW(MRE)
−1
0
1
MR effect size for
'Gut microbiota abundance (genus Parasutterella id.2892)' on 'Metallothionein−1F'

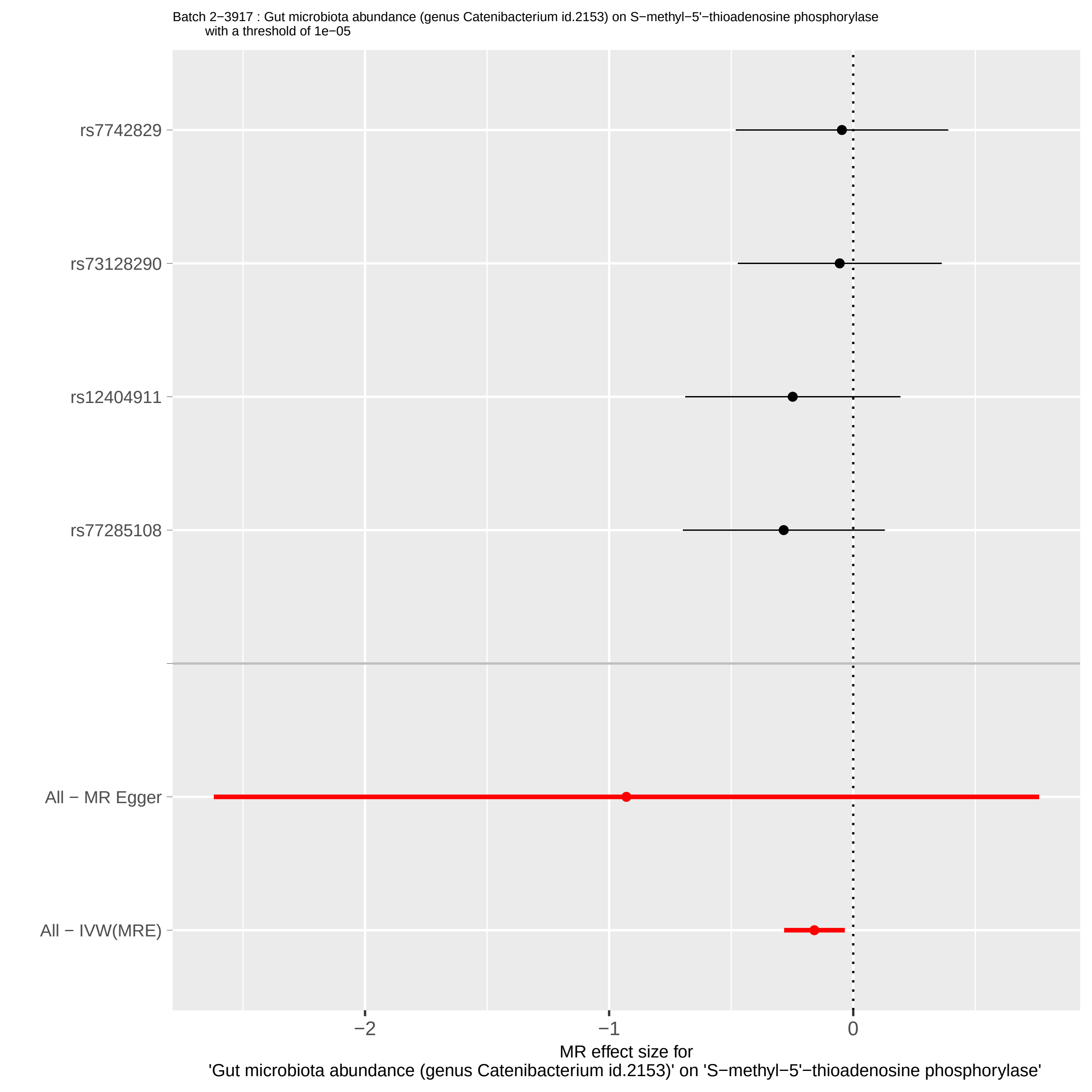

Batch 2−3917 : Gut microbiota abundance (genus Catenibacterium id.2153) on S−methyl−5'−thioadenosine phosphorylase
with a threshold of 1e−05
rs7742829
rs73128290
rs12404911
rs77285108
All − MR Egger
All − IVW(MRE)
−2
−1
0
MR effect size for
'Gut microbiota abundance (genus Catenibacterium id.2153)' on 'S−methyl−5'−thioadenosine phosphorylase'

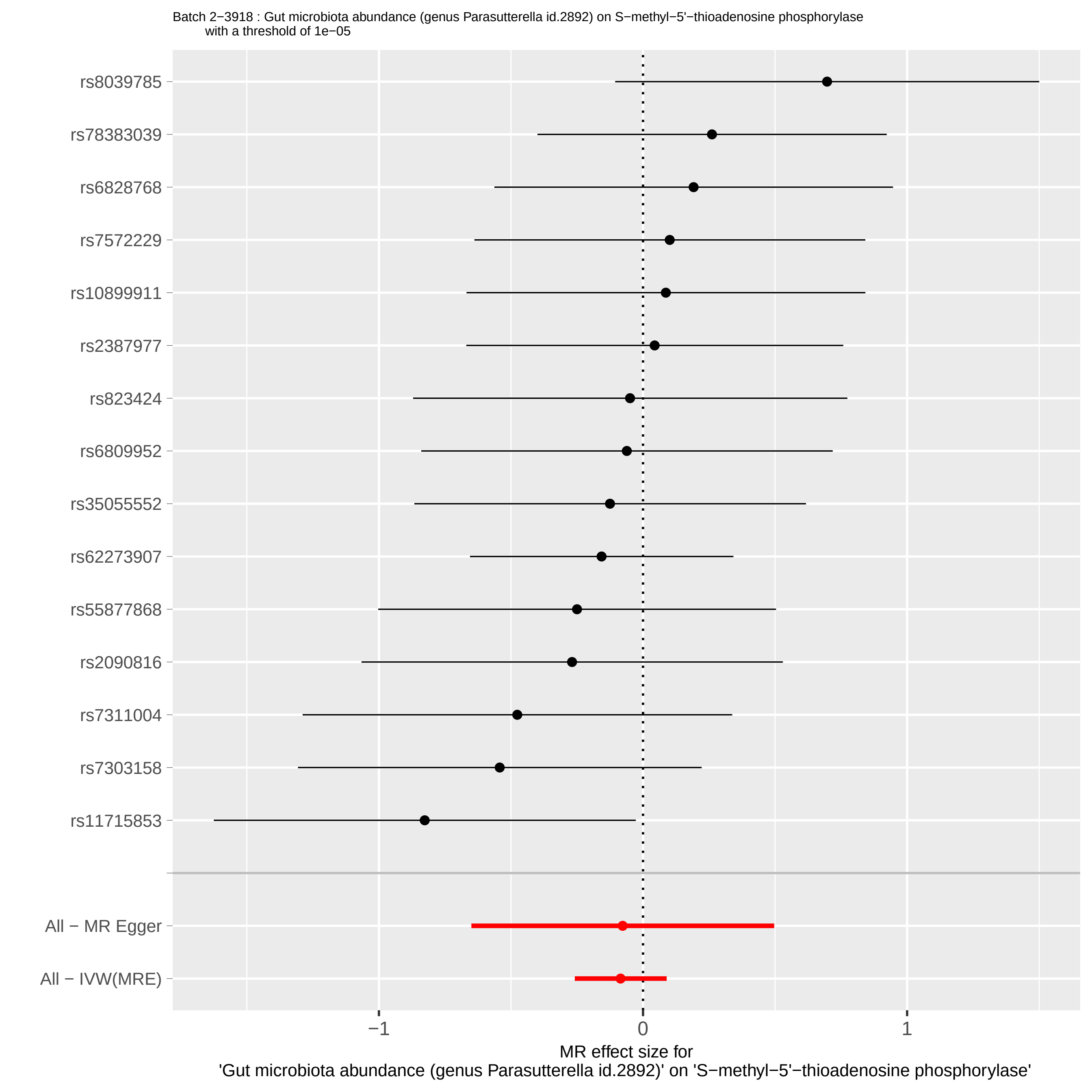

Batch 2−3918 : Gut microbiota abundance (genus Parasutterella id.2892) on S−methyl−5'−thioadenosine phosphorylase
with a threshold of 1e−05
rs8039785
rs78383039
rs6828768
rs7572229
rs10899911
rs2387977
rs823424
rs6809952
rs35055552
rs62273907
rs55877868
rs2090816
rs7311004
rs7303158
rs11715853
All − MR Egger
All − IVW(MRE)
−1
0
1
MR effect size for
'Gut microbiota abundance (genus Parasutterella id.2892)' on 'S−methyl−5'−thioadenosine phosphorylase'

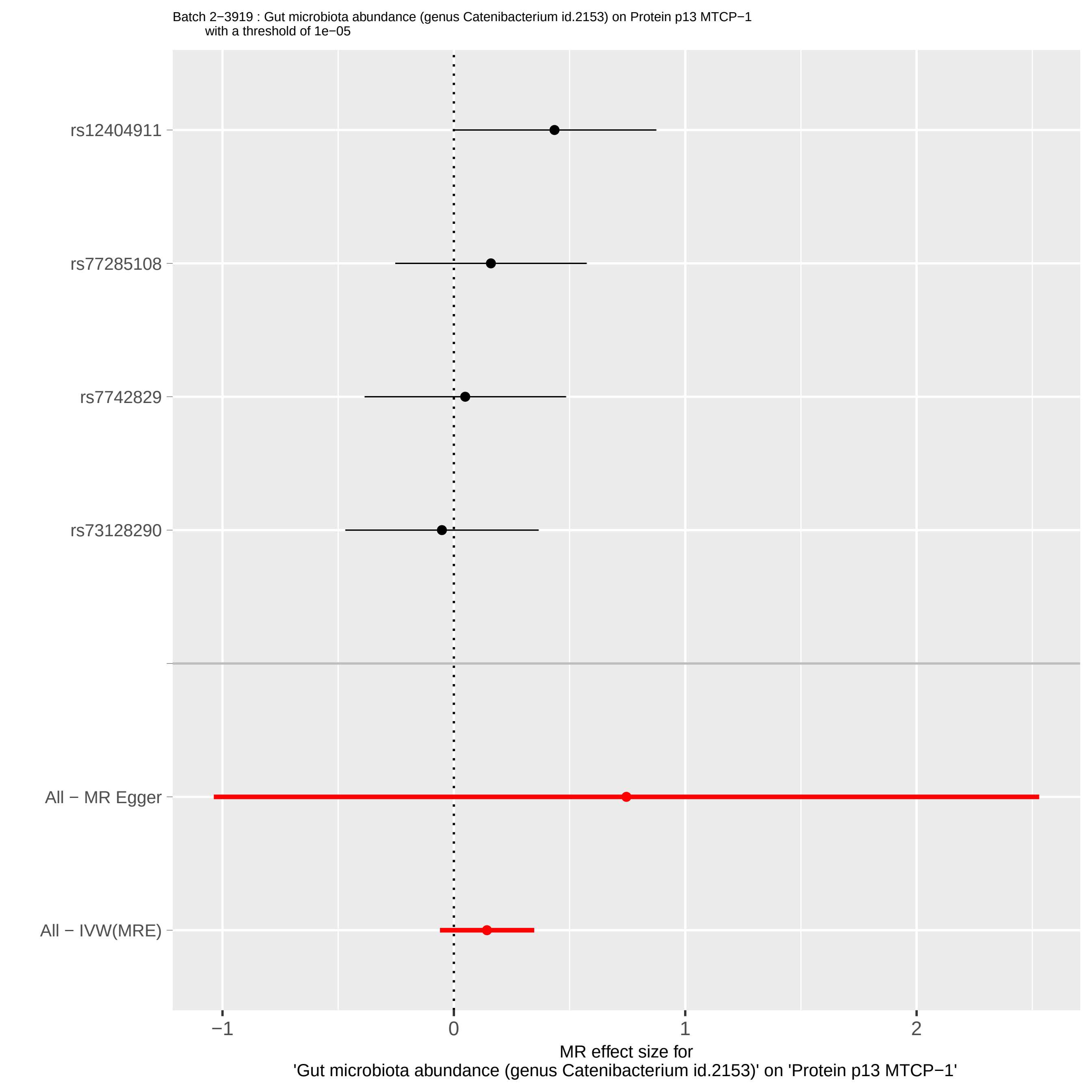
Batch 2−3919 : Gut microbiota abundance (genus Catenibacterium id.2153) on Protein p13 MTCP−1
with a threshold of 1e−05
rs12404911
rs77285108
rs7742829
rs73128290
All − MR Egger
All − IVW(MRE)
−1
0
1
2
MR effect size for
'Gut microbiota abundance (genus Catenibacterium id.2153)' on 'Protein p13 MTCP−1'

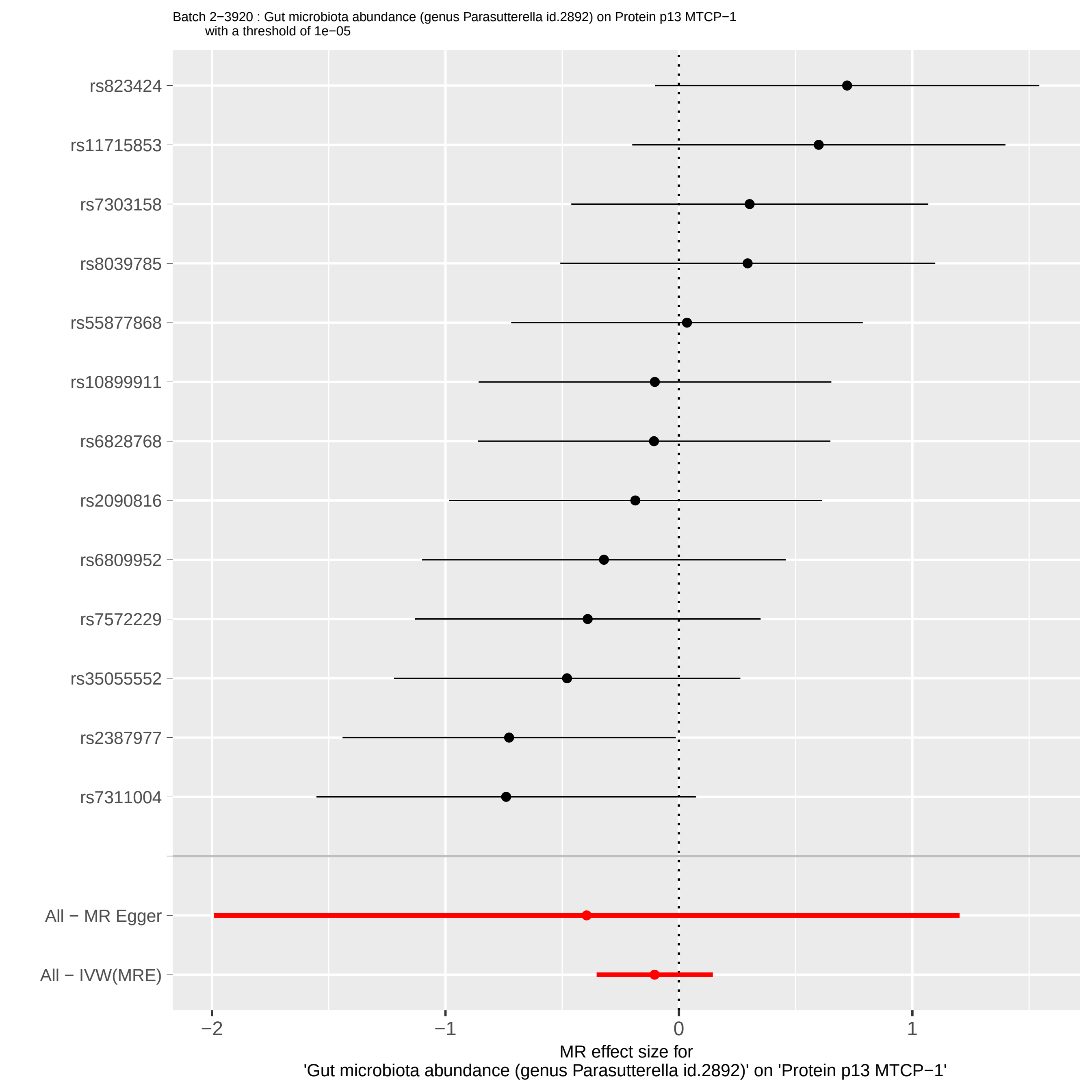

Batch 2−3920 : Gut microbiota abundance (genus Parasutterella id.2892) on Protein p13 MTCP−1
with a threshold of 1e−05
rs823424
rs11715853
rs7303158
rs8039785
rs55877868
rs10899911
rs6828768
rs2090816
rs6809952
rs7572229
rs35055552
rs2387977
rs7311004
All − MR Egger
All − IVW(MRE)
−2
−1
0
1
MR effect size for
'Gut microbiota abundance (genus Parasutterella id.2892)' on 'Protein p13 MTCP−1'

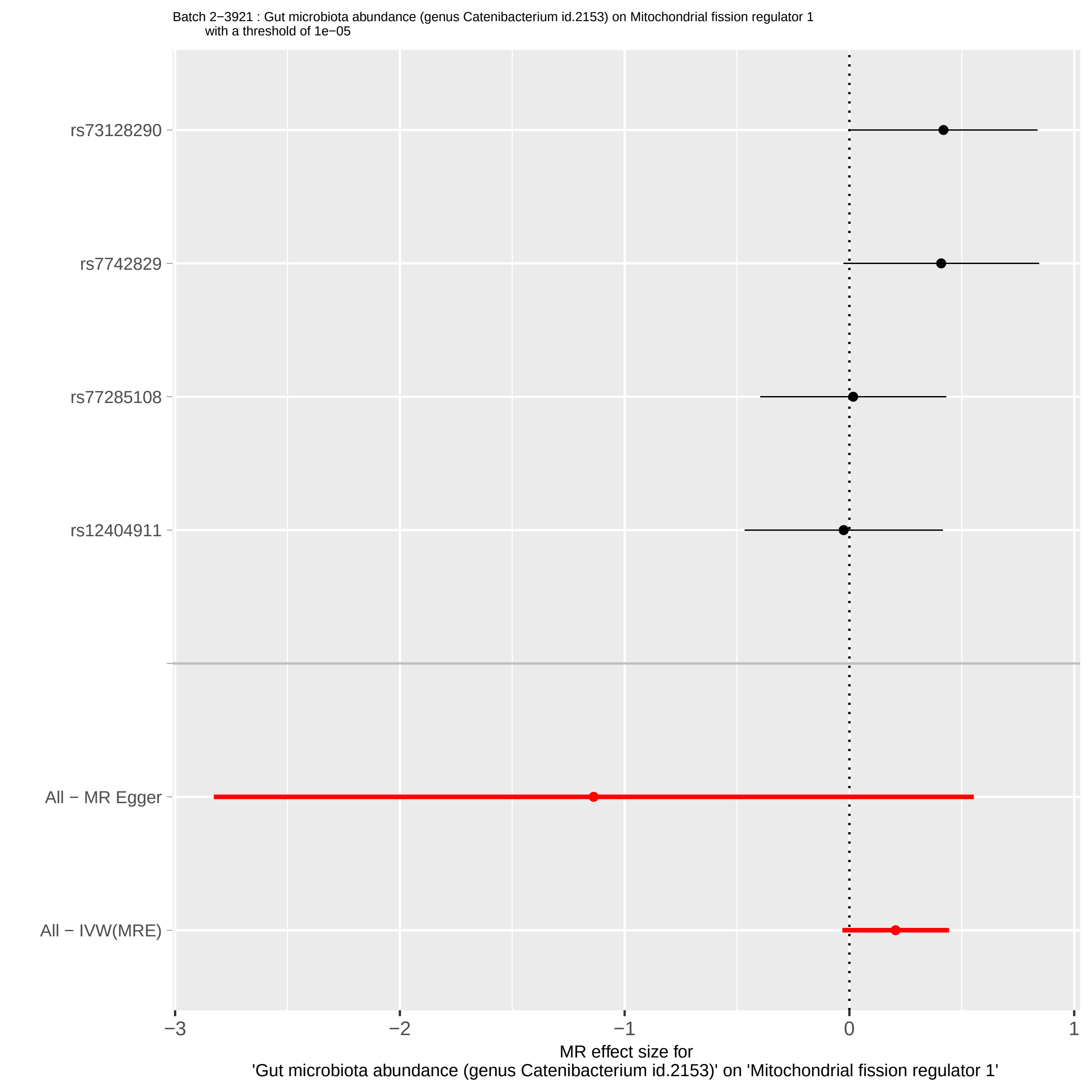

Batch 2−3921 : Gut microbiota abundance (genus Catenibacterium id.2153) on Mitochondrial fission regulator 1
with a threshold of 1e−05
rs73128290
rs7742829
rs77285108
rs12404911
All − MR Egger
All − IVW(MRE)
−3
−2
−1
0
1
MR effect size for
'Gut microbiota abundance (genus Catenibacterium id.2153)' on 'Mitochondrial fission regulator 1'

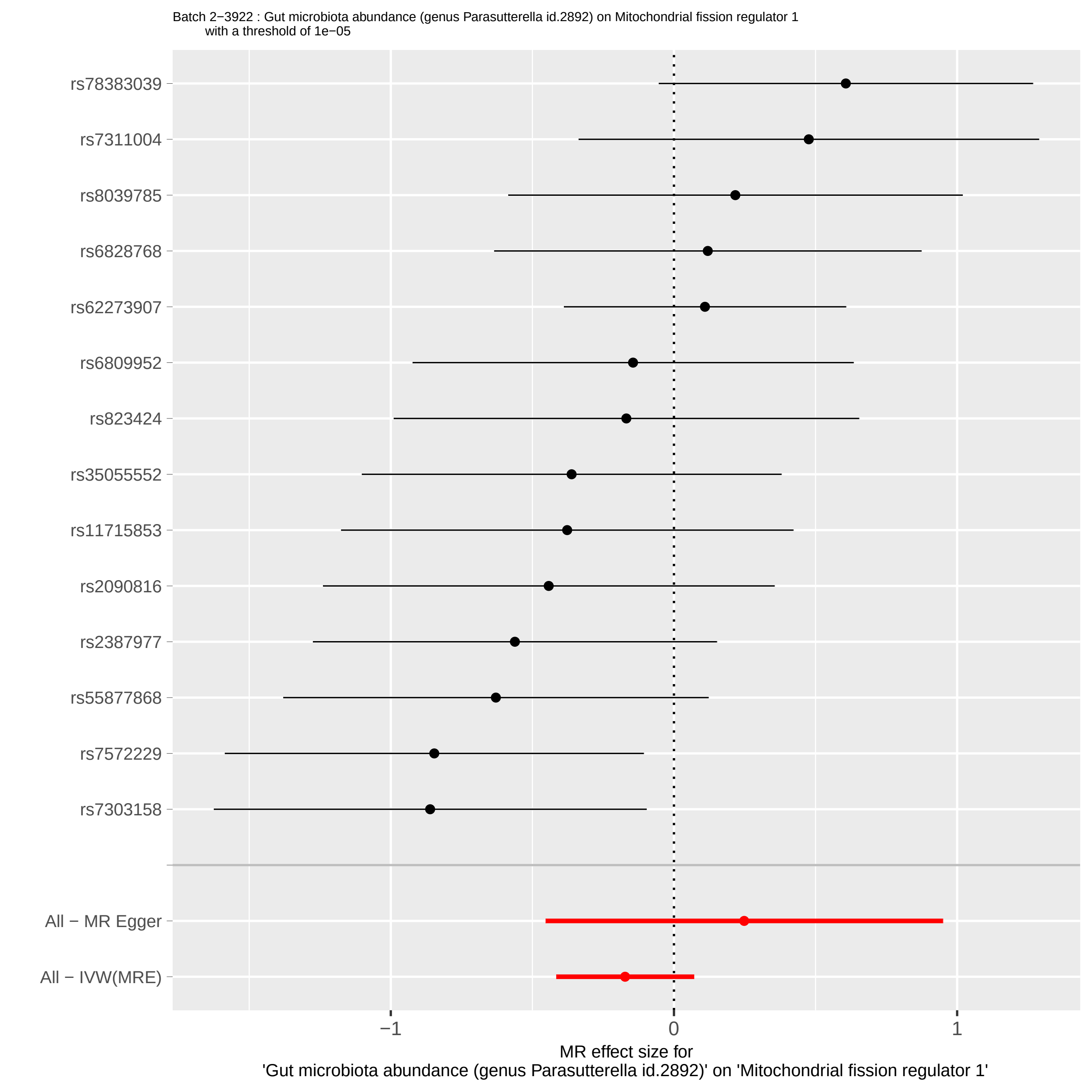

Batch 2−3922 : Gut microbiota abundance (genus Parasutterella id.2892) on Mitochondrial fission regulator 1
with a threshold of 1e−05
rs78383039
rs7311004
rs8039785
rs6828768
rs62273907
rs6809952
rs823424
rs35055552
rs11715853
rs2090816
rs2387977
rs55877868
rs7572229
rs7303158
All − MR Egger
All − IVW(MRE)
−1
0
1
MR effect size for
'Gut microbiota abundance (genus Parasutterella id.2892)' on 'Mitochondrial fission regulator 1'

Batch 2−3923 : Gut microbiota abundance (genus Catenibacterium id.2153) on 5−formyltetrahydrofolate cyclo−ligase
with a threshold of 1e−05

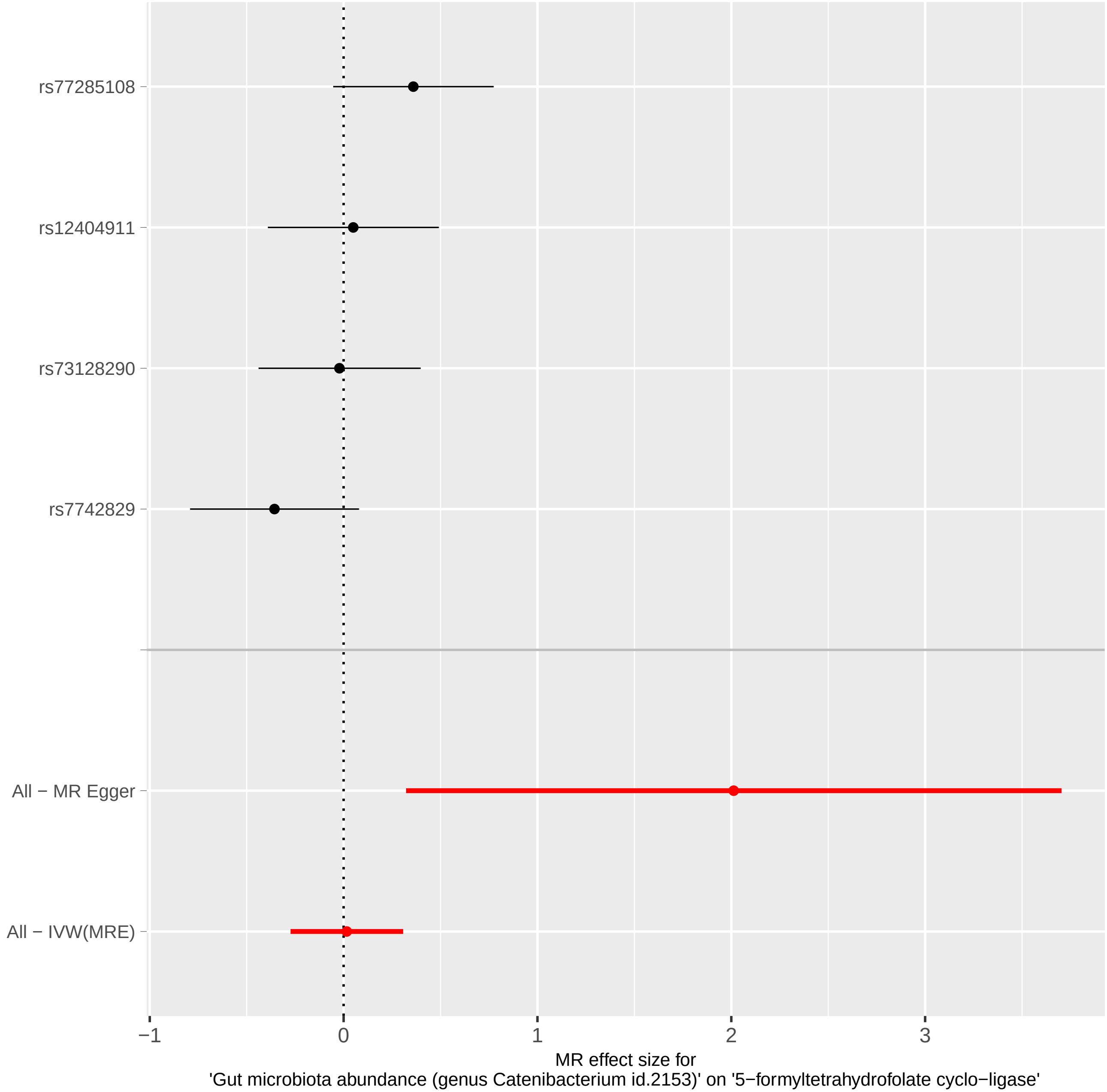

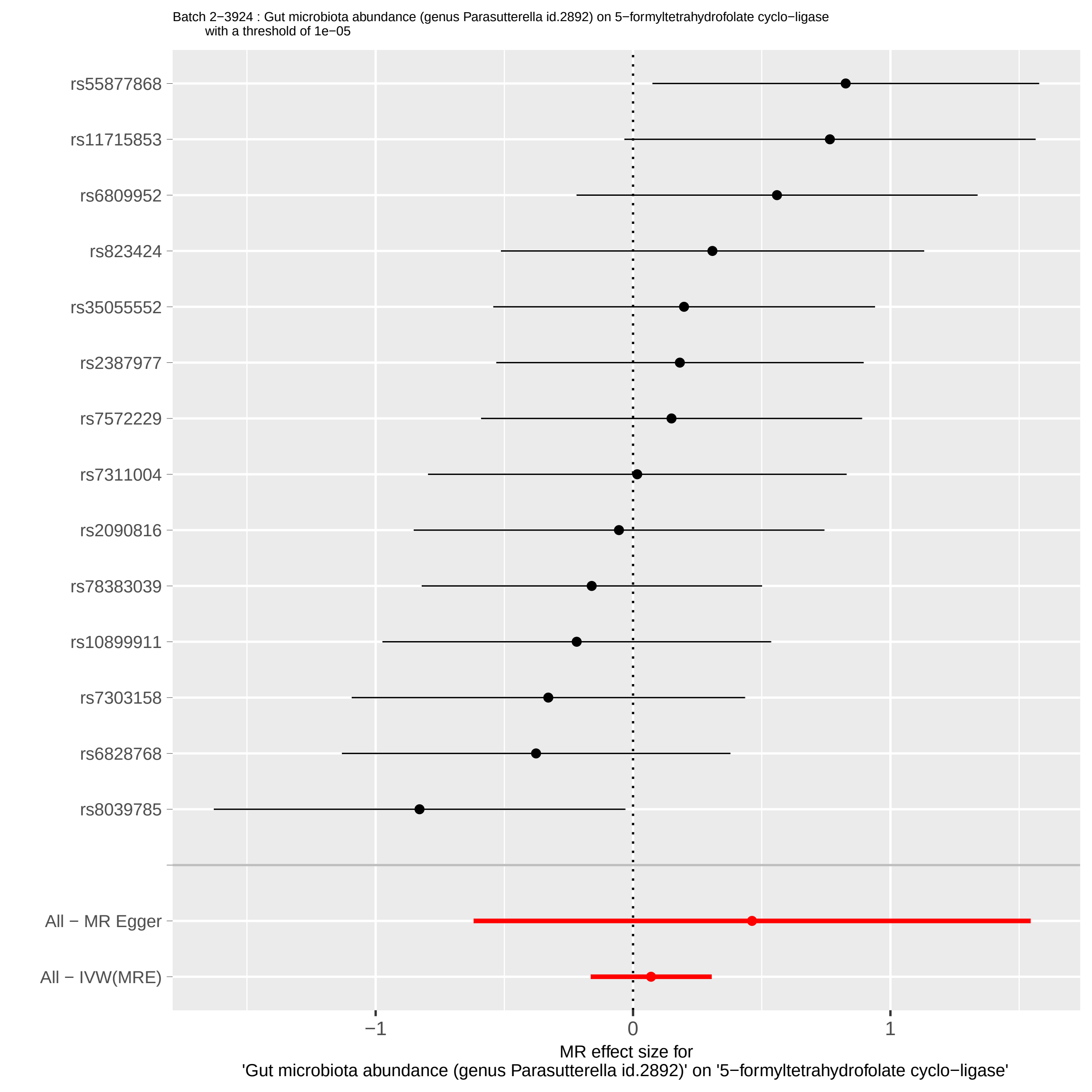

Batch 2−3924 : Gut microbiota abundance (genus Parasutterella id.2892) on 5−formyltetrahydrofolate cyclo−ligase
with a threshold of 1e−05
rs55877868
rs11715853
rs6809952
rs823424
rs35055552
rs2387977
rs7572229
rs7311004
rs2090816
rs78383039
rs10899911
rs7303158
rs6828768
rs8039785
All − MR Egger
All − IVW(MRE)
−1
0
1
MR effect size for
'Gut microbiota abundance (genus Parasutterella id.2892)' on '5−formyltetrahydrofolate cyclo−ligase'

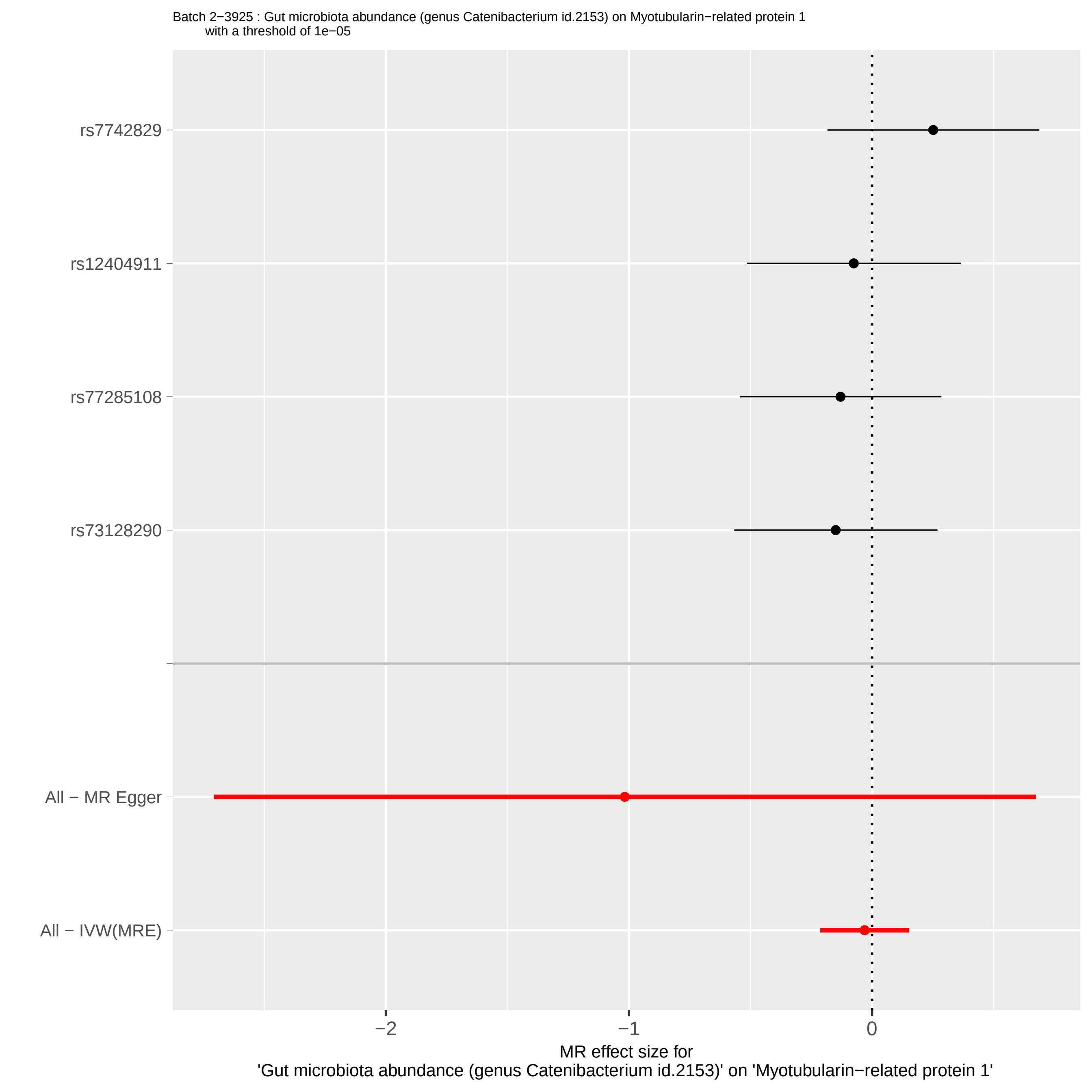

Batch 2−3925 : Gut microbiota abundance (genus Catenibacterium id.2153) on Myotubularin−related protein 1
with a threshold of 1e−05
rs7742829
rs12404911
rs77285108
rs73128290
All − MR Egger
All − IVW(MRE)
−2
−1
0
MR effect size for
'Gut microbiota abundance (genus Catenibacterium id.2153)' on 'Myotubularin−related protein 1'

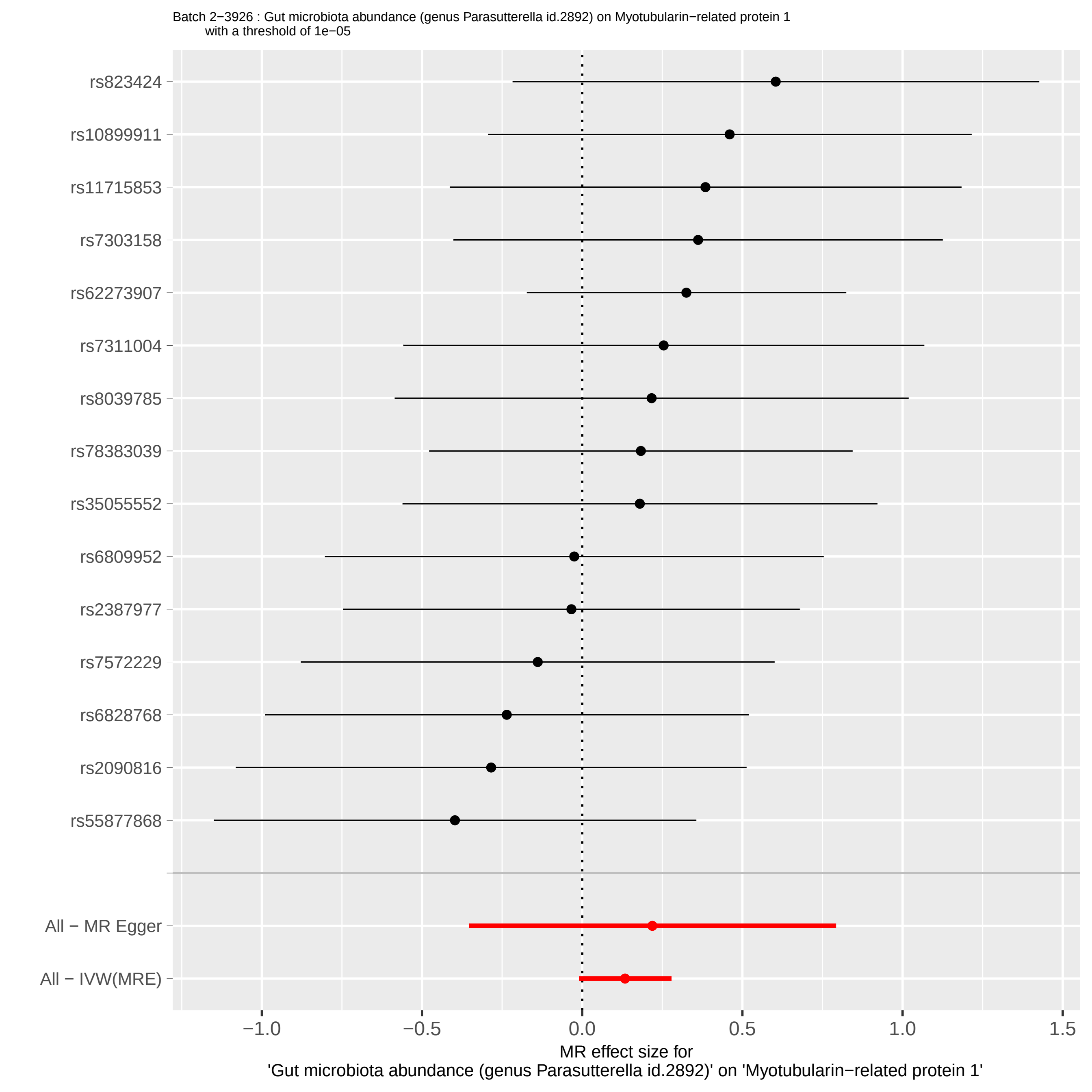

Batch 2−3926 : Gut microbiota abundance (genus Parasutterella id.2892) on Myotubularin−related protein 1
with a threshold of 1e−05
rs823424
rs10899911
rs11715853
rs7303158
rs62273907
rs7311004
rs8039785
rs78383039
rs35055552
rs6809952
rs2387977
rs7572229
rs6828768
rs2090816
rs55877868
All − MR Egger
All − IVW(MRE)
−1.0
−0.5
0.0
0.5
1.0
1.5
MR effect size for
'Gut microbiota abundance (genus Parasutterella id.2892)' on 'Myotubularin−related protein 1'

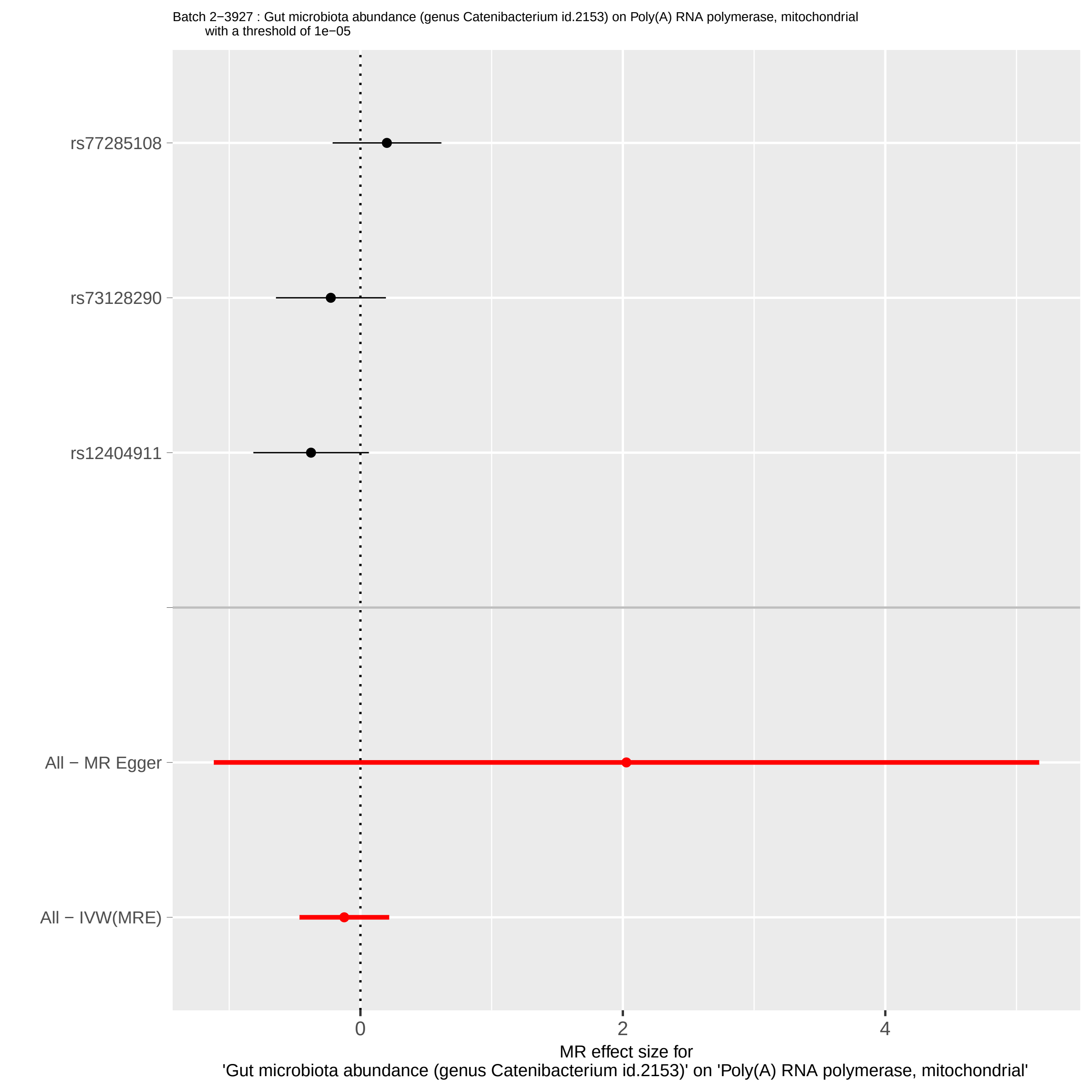

Batch 2−3927 : Gut microbiota abundance (genus Catenibacterium id.2153) on Poly(A) RNA polymerase, mitochondrial
with a threshold of 1e−05
rs77285108
rs73128290
rs12404911
All − MR Egger
All − IVW(MRE)
0
2
4
MR effect size for
'Gut microbiota abundance (genus Catenibacterium id.2153)' on 'Poly(A) RNA polymerase, mitochondrial'

Batch 2−3928 : Gut microbiota abundance (genus Parasutterella id.2892) on Poly(A) RNA polymerase, mitochondrial
with a threshold of 1e−05

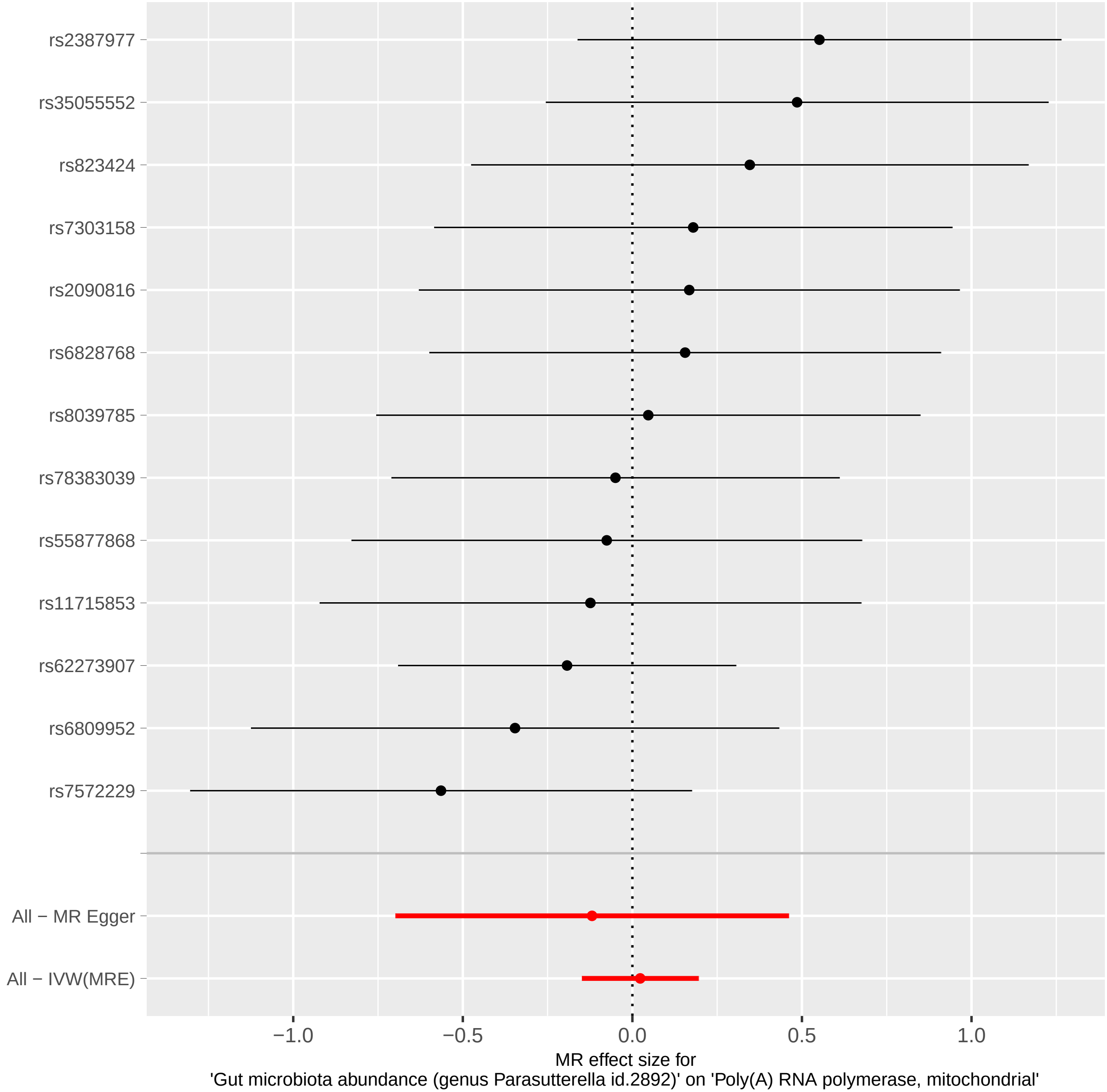

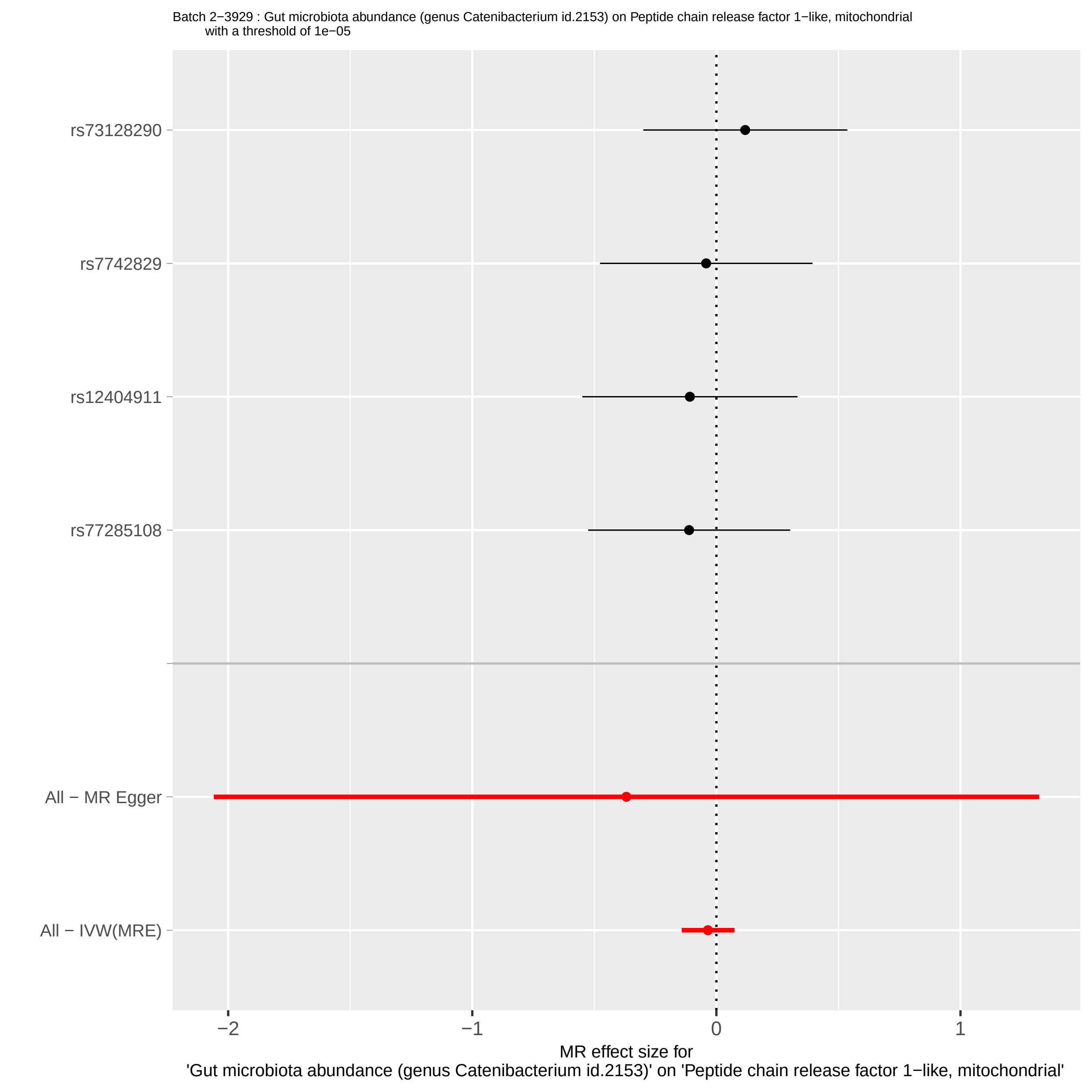
Batch 2−3929 : Gut microbiota abundance (genus Catenibacterium id.2153) on Peptide chain release factor 1−like, mitochondrial
with a threshold of 1e−05
rs73128290
rs7742829
rs12404911
rs77285108
All − MR Egger
All − IVW(MRE)
−2
−1
0
1
MR effect size for
'Gut microbiota abundance (genus Catenibacterium id.2153)' on 'Peptide chain release factor 1−like, mitochondrial'

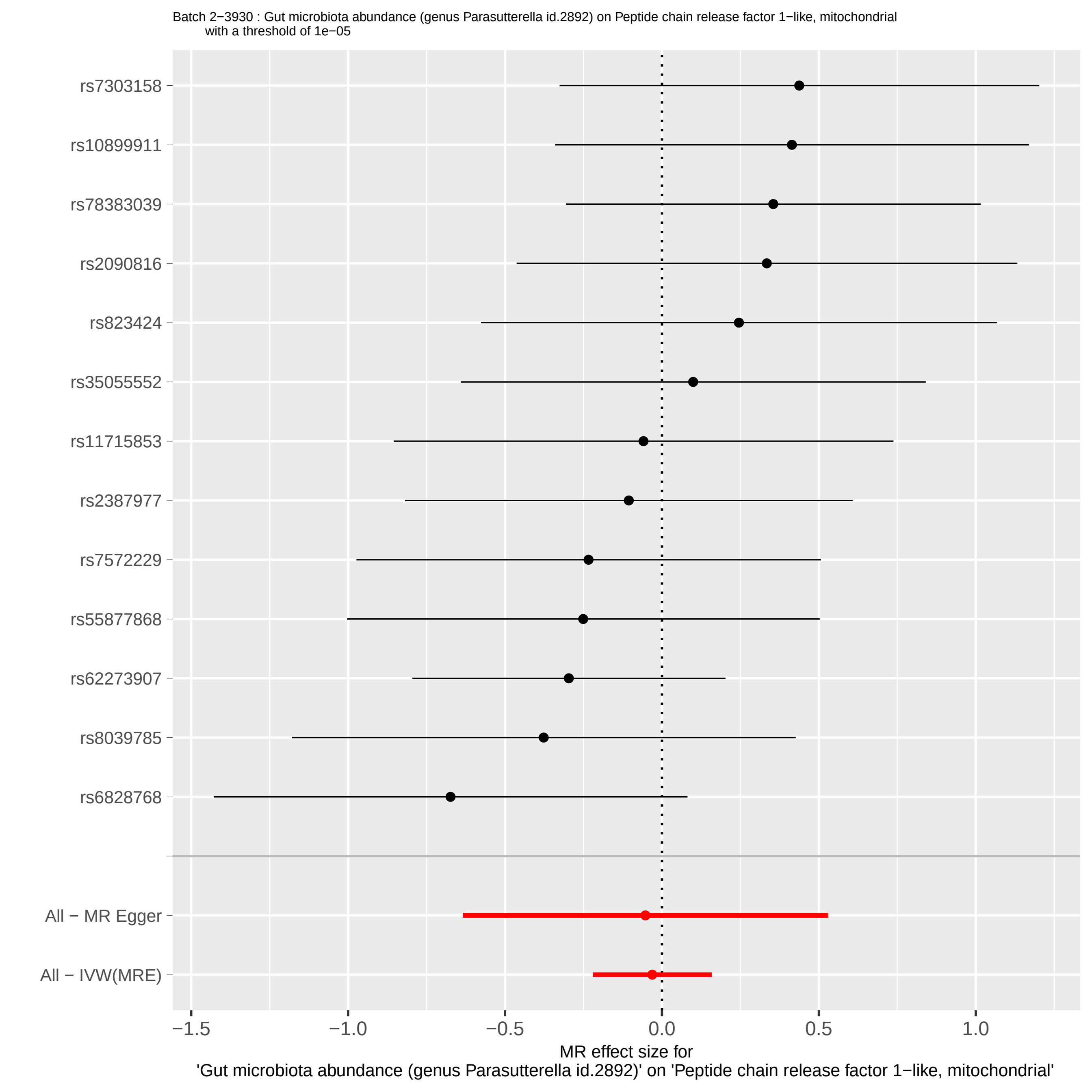

Batch 2−3930 : Gut microbiota abundance (genus Parasutterella id.2892) on Peptide chain release factor 1−like, mitochondrial
with a threshold of 1e−05
rs7303158
rs10899911
rs78383039
rs2090816
rs823424
rs35055552
rs11715853
rs2387977
rs7572229
rs55877868
rs62273907
rs8039785
rs6828768
All − MR Egger
All − IVW(MRE)
−1.5
−1.0
−0.5
0.0
0.5
1.0
MR effect size for
'Gut microbiota abundance (genus Parasutterella id.2892)' on 'Peptide chain release factor 1−like, mitochondrial'

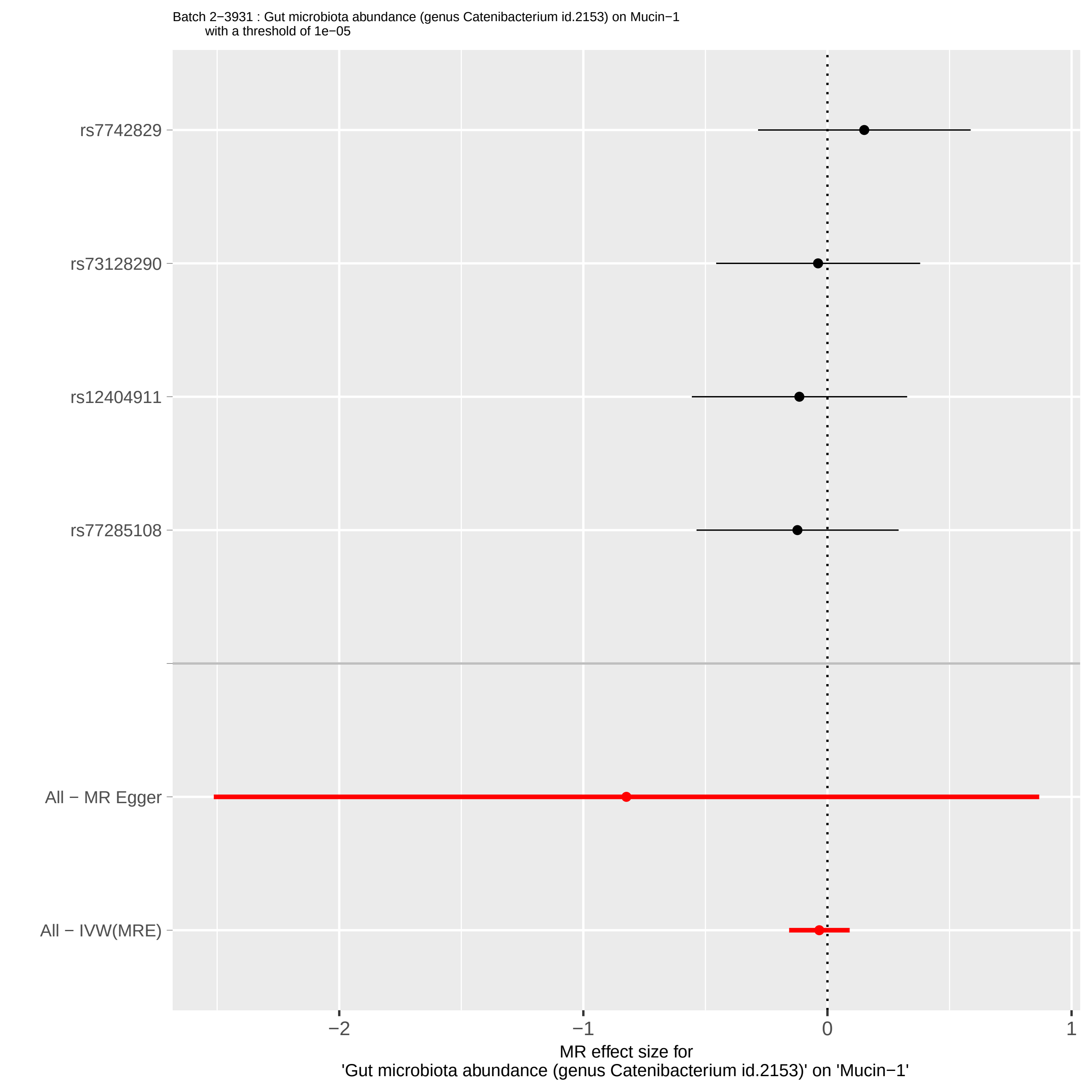

Batch 2−3931 : Gut microbiota abundance (genus Catenibacterium id.2153) on Mucin−1
with a threshold of 1e−05
rs7742829
rs73128290
rs12404911
rs77285108
All − MR Egger
All − IVW(MRE)
−2
−1
0
1
MR effect size for
'Gut microbiota abundance (genus Catenibacterium id.2153)' on 'Mucin−1'

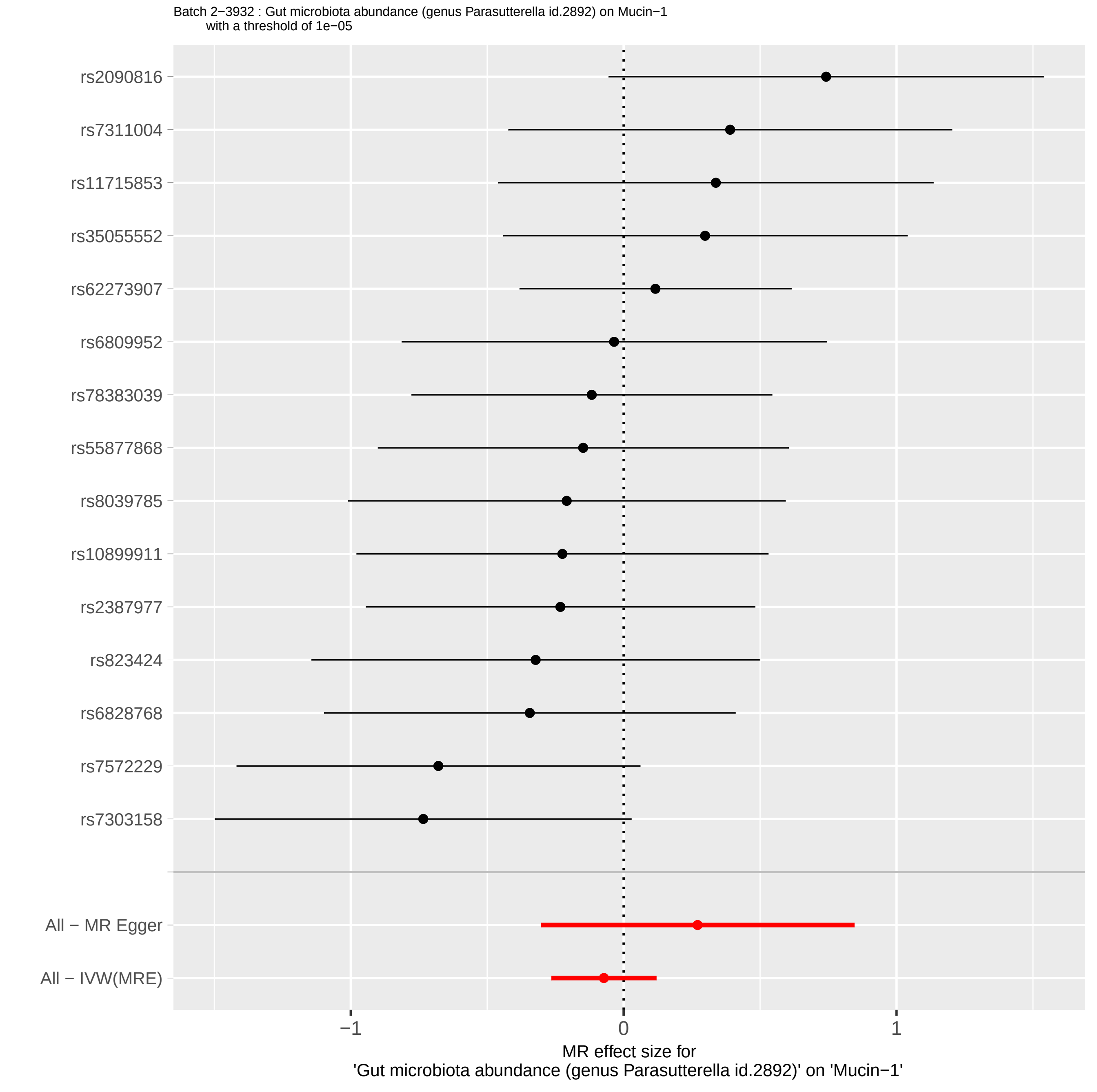

Batch 2−3932 : Gut microbiota abundance (genus Parasutterella id.2892) on Mucin−1
with a threshold of 1e−05
rs2090816
rs7311004
rs11715853
rs35055552
rs62273907
rs6809952
rs78383039
rs55877868
rs8039785
rs10899911
rs2387977
rs823424
rs6828768
rs7572229
rs7303158
All − MR Egger
All − IVW(MRE)
−1
0
1
MR effect size for
'Gut microbiota abundance (genus Parasutterella id.2892)' on 'Mucin−1'

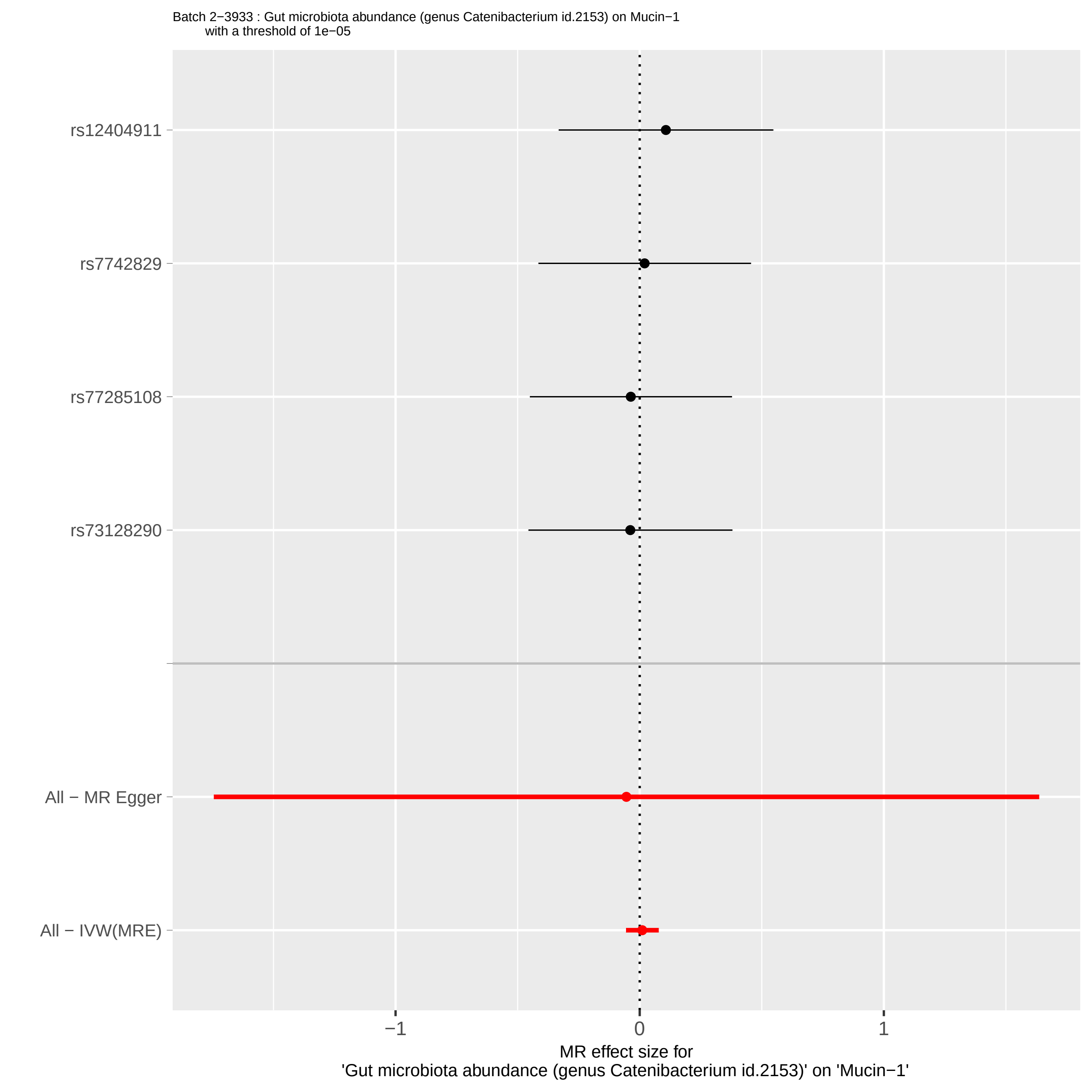

Batch 2−3933 : Gut microbiota abundance (genus Catenibacterium id.2153) on Mucin−1
with a threshold of 1e−05
rs12404911
rs7742829
rs77285108
rs73128290
All − MR Egger
All − IVW(MRE)
−1
0
1
MR effect size for
'Gut microbiota abundance (genus Catenibacterium id.2153)' on 'Mucin−1'

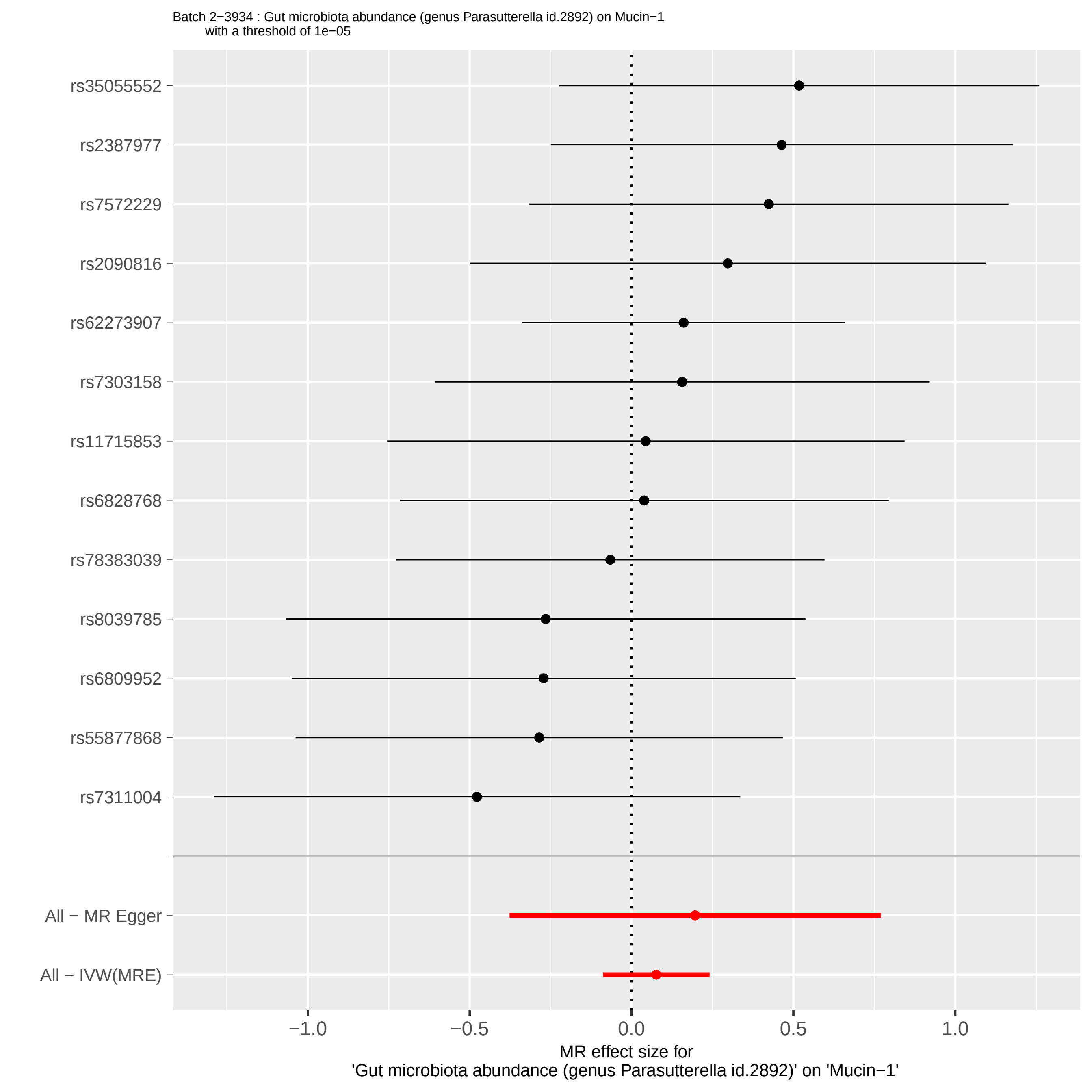

Batch 2−3934 : Gut microbiota abundance (genus Parasutterella id.2892) on Mucin−1
with a threshold of 1e−05
rs35055552
rs2387977
rs7572229
rs2090816
rs62273907
rs7303158
rs11715853
rs6828768
rs78383039
rs8039785
rs6809952
rs55877868
rs7311004
All − MR Egger
All − IVW(MRE)
−1.0
−0.5
0.0
0.5
1.0
MR effect size for
'Gut microbiota abundance (genus Parasutterella id.2892)' on 'Mucin−1'

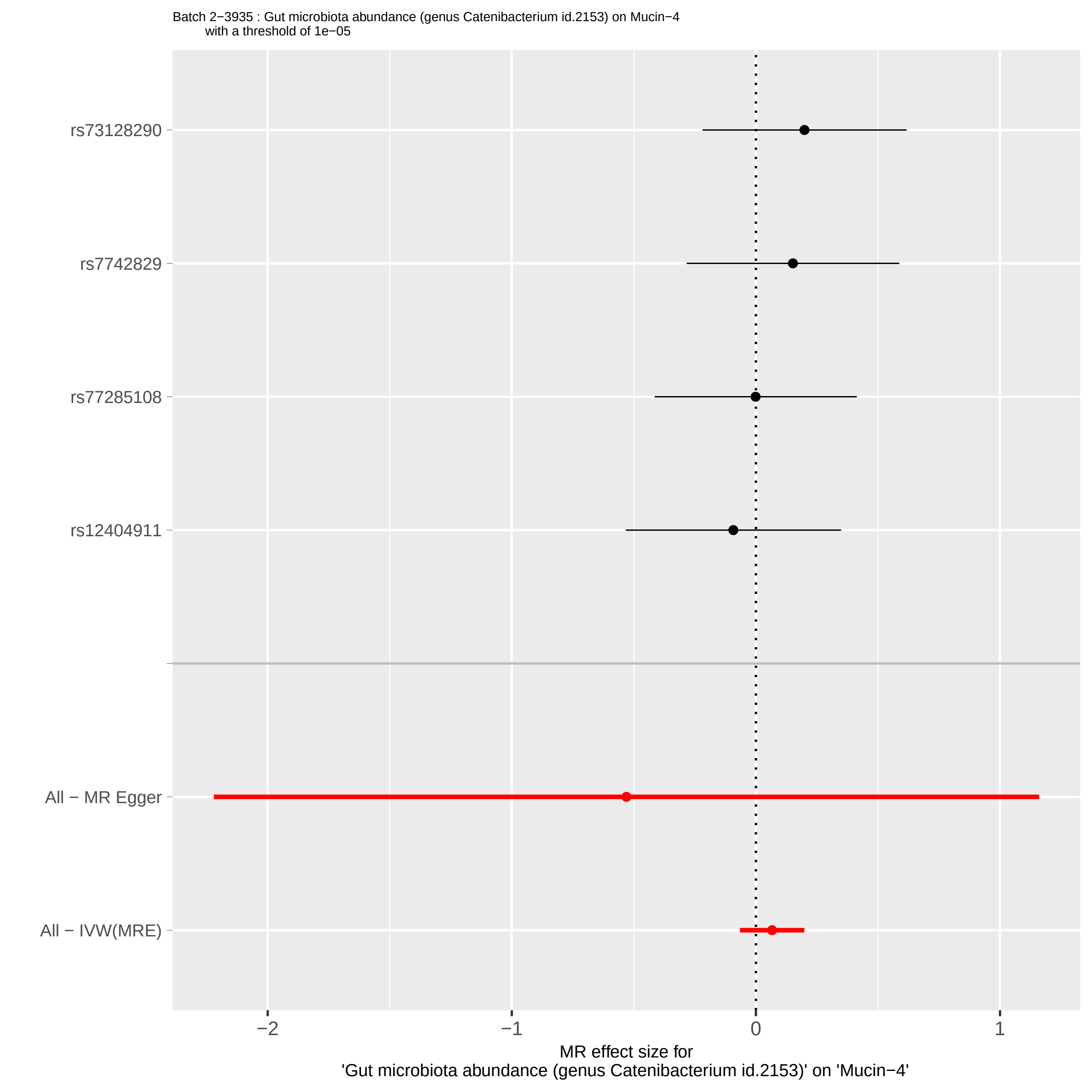

Batch 2−3935 : Gut microbiota abundance (genus Catenibacterium id.2153) on Mucin−4
with a threshold of 1e−05
rs73128290
rs7742829
rs77285108
rs12404911
All − MR Egger
All − IVW(MRE)
−2
−1
0
1
MR effect size for
'Gut microbiota abundance (genus Catenibacterium id.2153)' on 'Mucin−4'

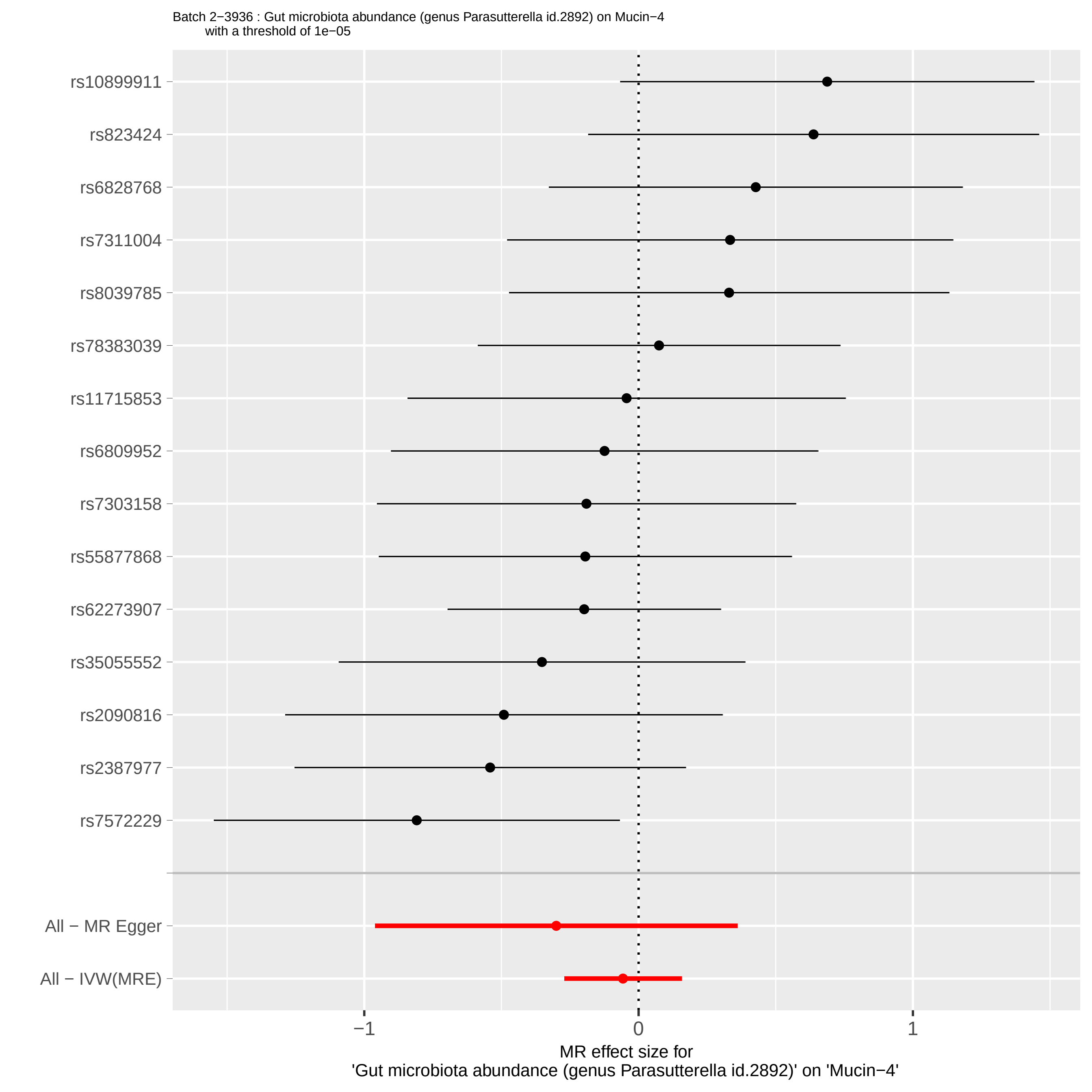

Batch 2−3936 : Gut microbiota abundance (genus Parasutterella id.2892) on Mucin−4
with a threshold of 1e−05
rs10899911
rs823424
rs6828768
rs7311004
rs8039785
rs78383039
rs11715853
rs6809952
rs7303158
rs55877868
rs62273907
rs35055552
rs2090816
rs2387977
rs7572229
All − MR Egger
All − IVW(MRE)
−1
0
1
MR effect size for
'Gut microbiota abundance (genus Parasutterella id.2892)' on 'Mucin−4'

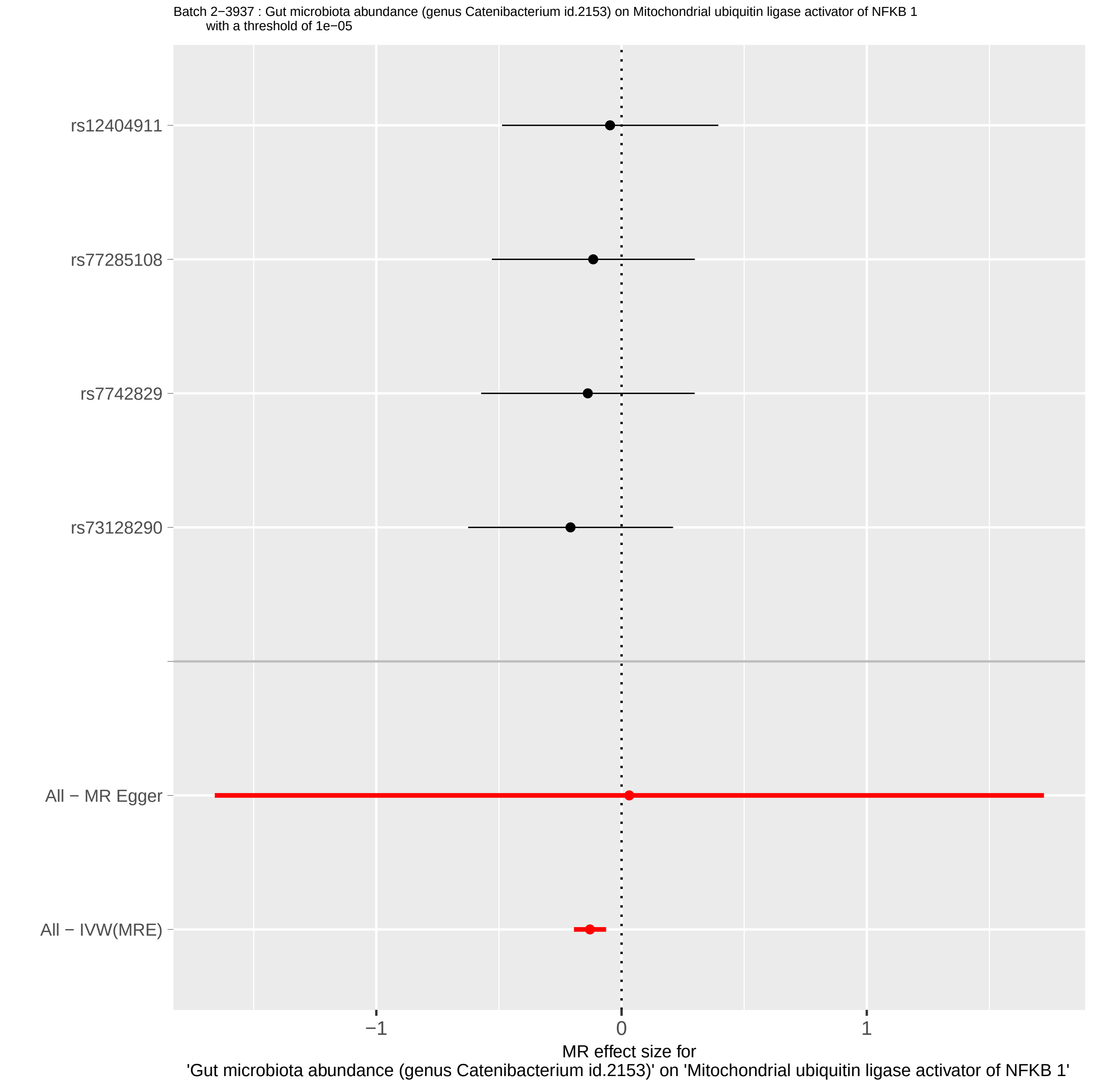

Batch 2−3937 : Gut microbiota abundance (genus Catenibacterium id.2153) on Mitochondrial ubiquitin ligase activator of NFKB 1
with a threshold of 1e−05
rs12404911
rs77285108
rs7742829
rs73128290
All − MR Egger
All − IVW(MRE)
−1
0
1
MR effect size for
'Gut microbiota abundance (genus Catenibacterium id.2153)' on 'Mitochondrial ubiquitin ligase activator of NFKB 1'

Batch 2−3938 : Gut microbiota abundance (genus Parasutterella id.2892) on Mitochondrial ubiquitin ligase activator of NFKB 1
with a threshold of 1e−05

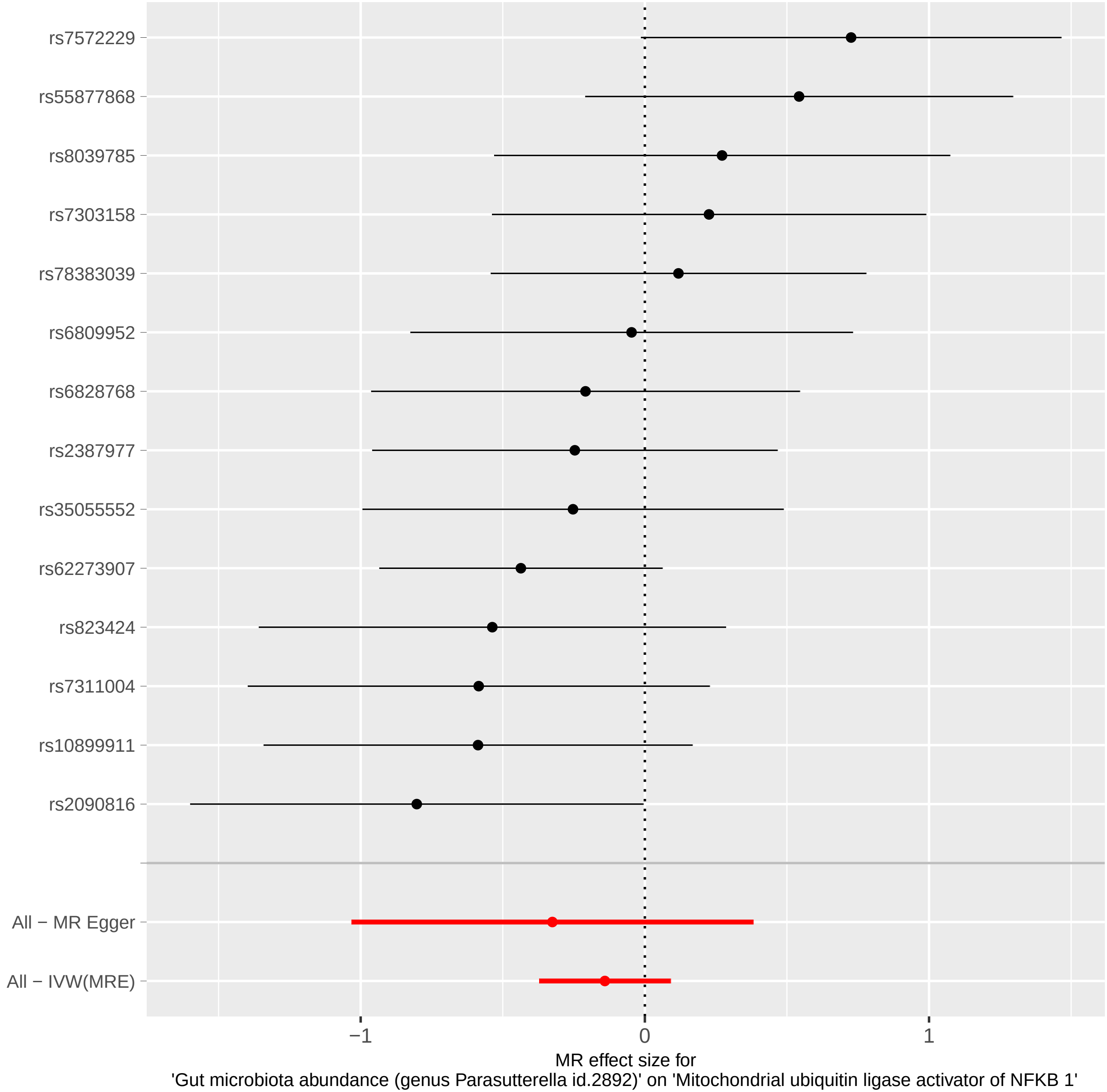

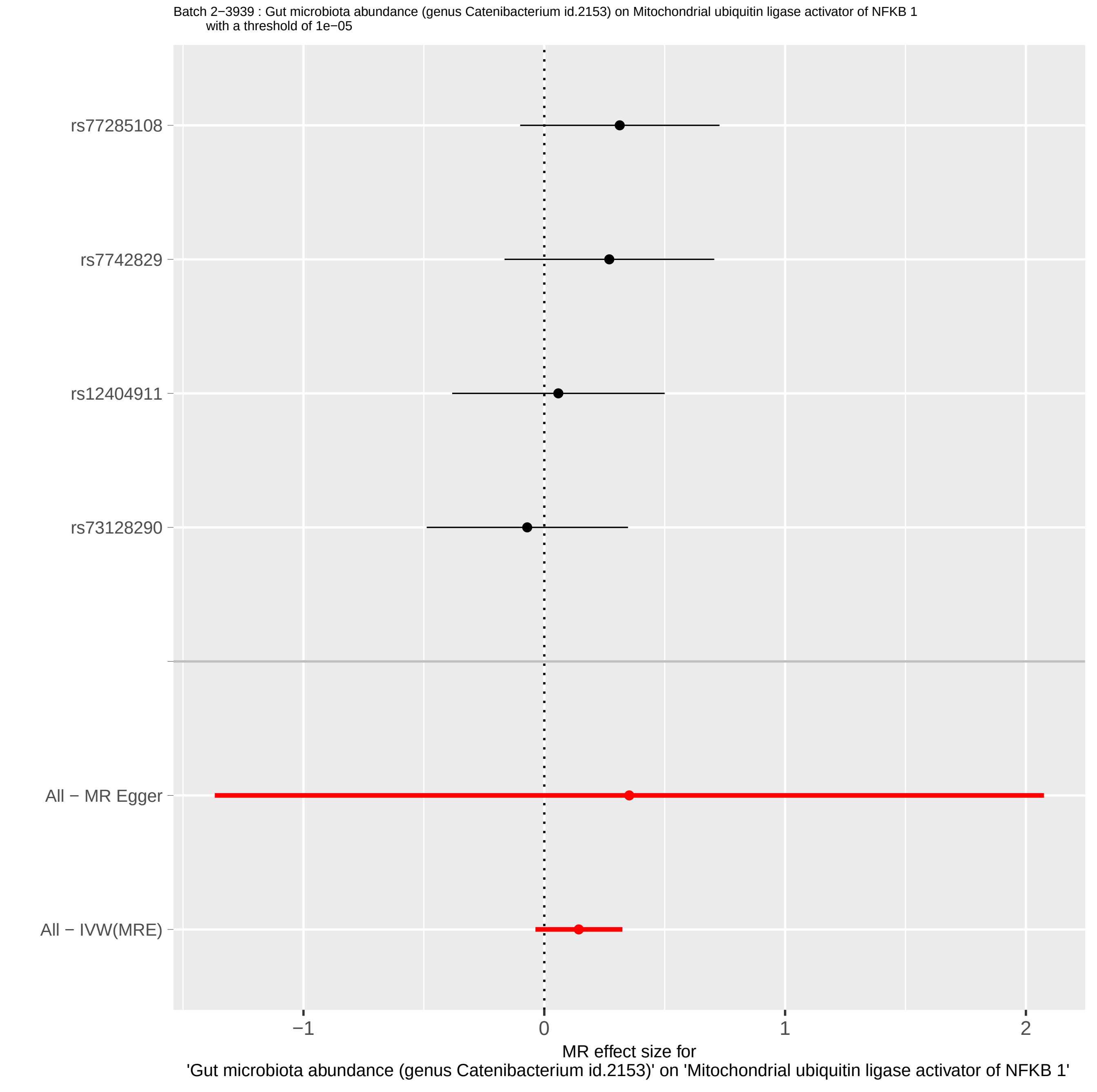

Batch 2−3939 : Gut microbiota abundance (genus Catenibacterium id.2153) on Mitochondrial ubiquitin ligase activator of NFKB 1
with a threshold of 1e−05
rs77285108
rs7742829
rs12404911
rs73128290
All − MR Egger
All − IVW(MRE)
−1
0
1
2
MR effect size for
'Gut microbiota abundance (genus Catenibacterium id.2153)' on 'Mitochondrial ubiquitin ligase activator of NFKB 1'

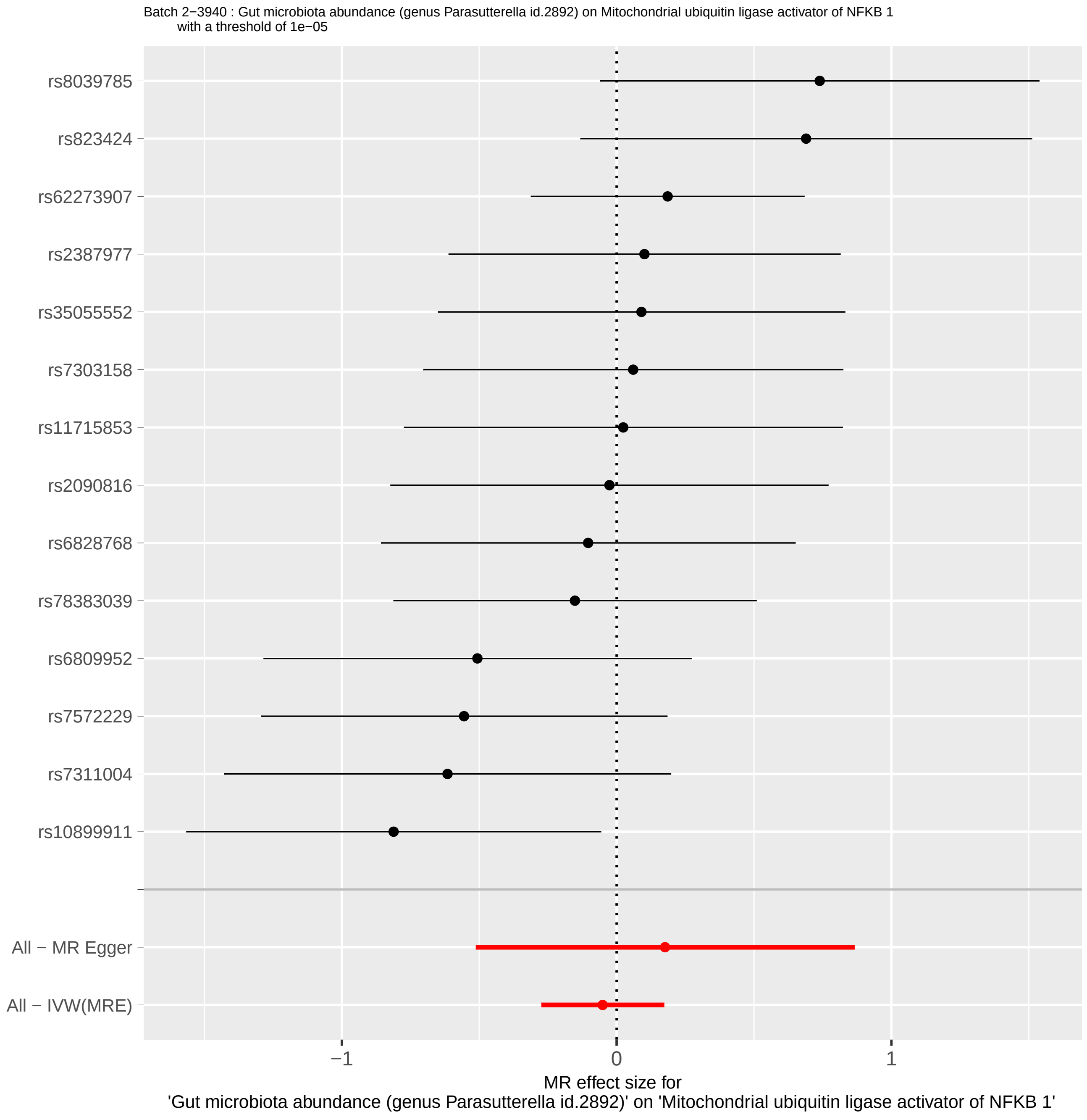
Batch 2−3940 : Gut microbiota abundance (genus Parasutterella id.2892) on Mitochondrial ubiquitin ligase activator of NFKB 1
with a threshold of 1e−05
rs8039785
rs823424
rs62273907
rs2387977
rs35055552
rs7303158
rs11715853
rs2090816
rs6828768
rs78383039
rs6809952
rs7572229
rs7311004
rs10899911
All − MR Egger
All − IVW(MRE)
−1
0
1
MR effect size for
'Gut microbiota abundance (genus Parasutterella id.2892)' on 'Mitochondrial ubiquitin ligase activator of NFKB 1'

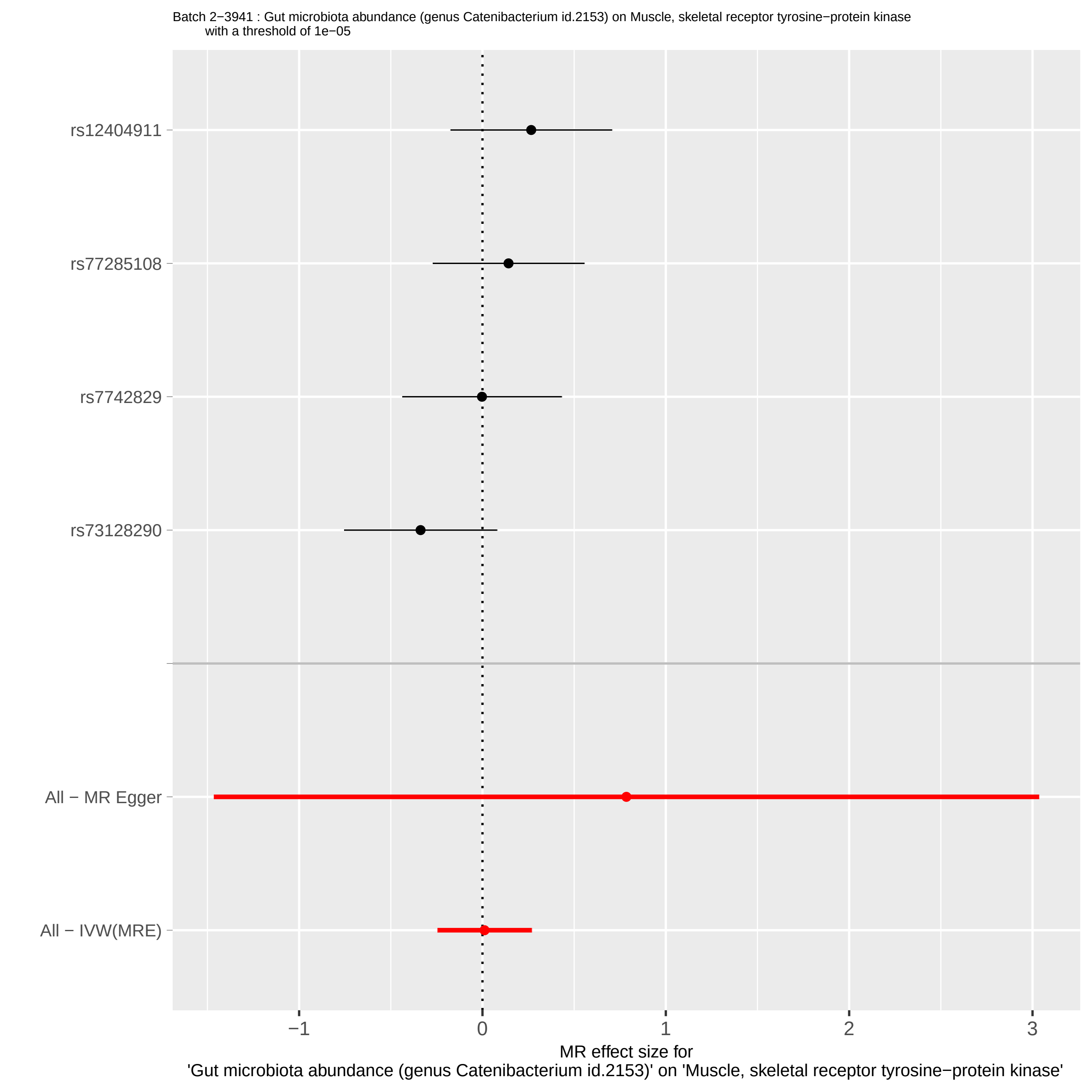

Batch 2−3941 : Gut microbiota abundance (genus Catenibacterium id.2153) on Muscle, skeletal receptor tyrosine−protein kinase
with a threshold of 1e−05
rs12404911
rs77285108
rs7742829
rs73128290
All − MR Egger
All − IVW(MRE)
−1
0
1
2
3
MR effect size for
'Gut microbiota abundance (genus Catenibacterium id.2153)' on 'Muscle, skeletal receptor tyrosine−protein kinase'

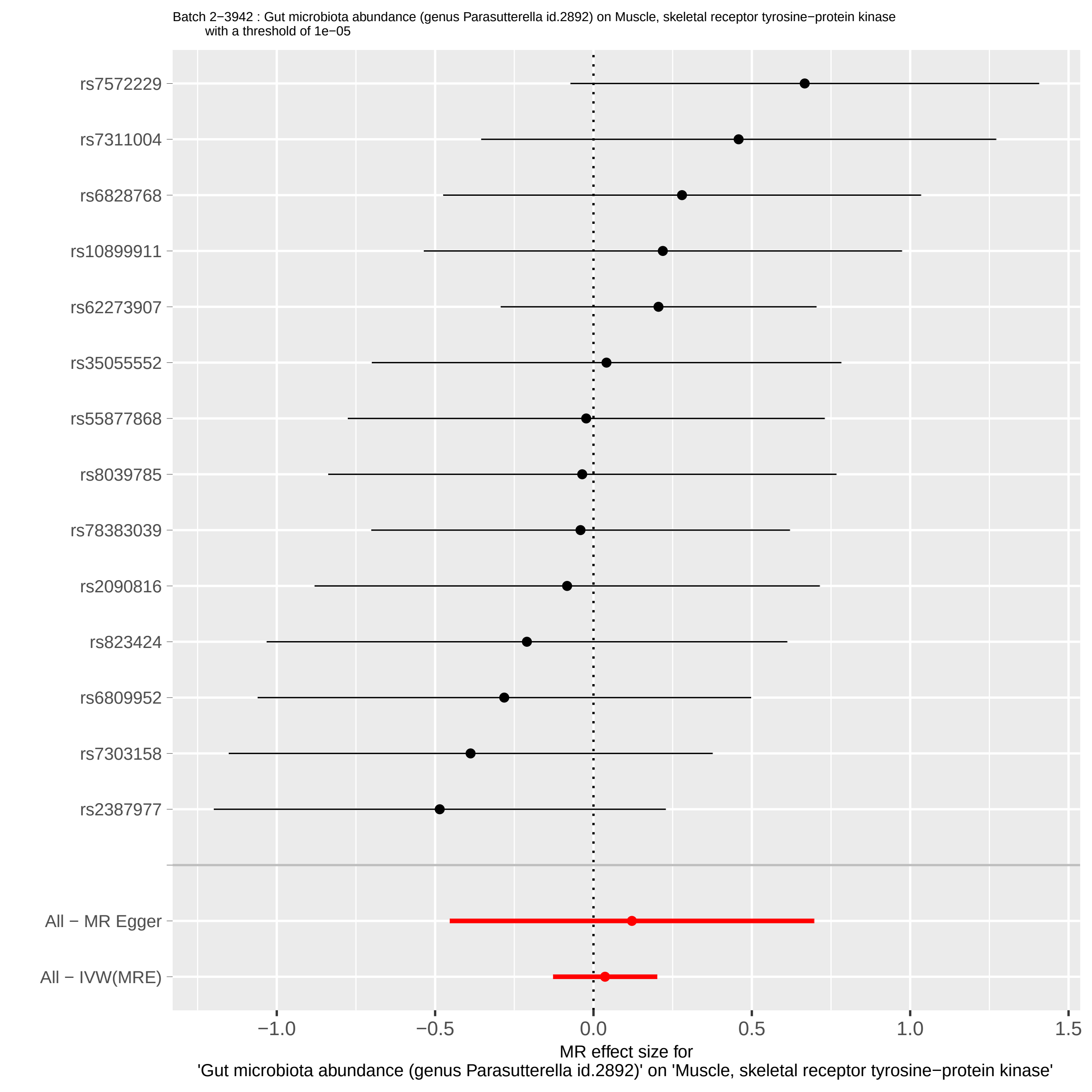
Batch 2−3942 : Gut microbiota abundance (genus Parasutterella id.2892) on Muscle, skeletal receptor tyrosine−protein kinase
with a threshold of 1e−05
rs7572229
rs7311004
rs6828768
rs10899911
rs62273907
rs35055552
rs55877868
rs8039785
rs78383039
rs2090816
rs823424
rs6809952
rs7303158
rs2387977
All − MR Egger
All − IVW(MRE)
−1.0
−0.5
0.0
0.5
1.0
1.5
MR effect size for
'Gut microbiota abundance (genus Parasutterella id.2892)' on 'Muscle, skeletal receptor tyrosine−protein kinase'

Batch 2−3943 : Gut microbiota abundance (genus Catenibacterium id.2153) on A/G−specific adenine DNA glycosylase
with a threshold of 1e−05

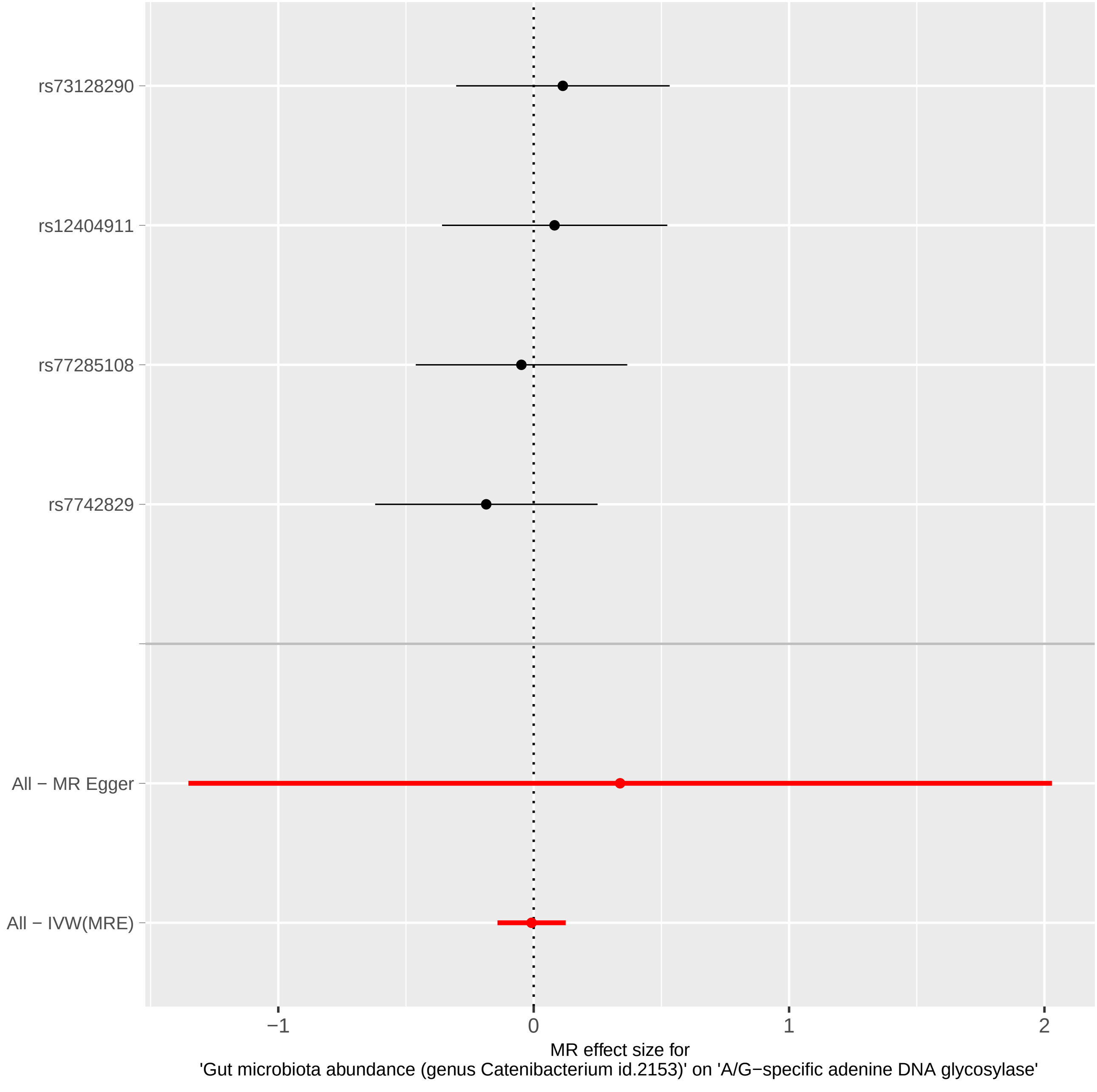

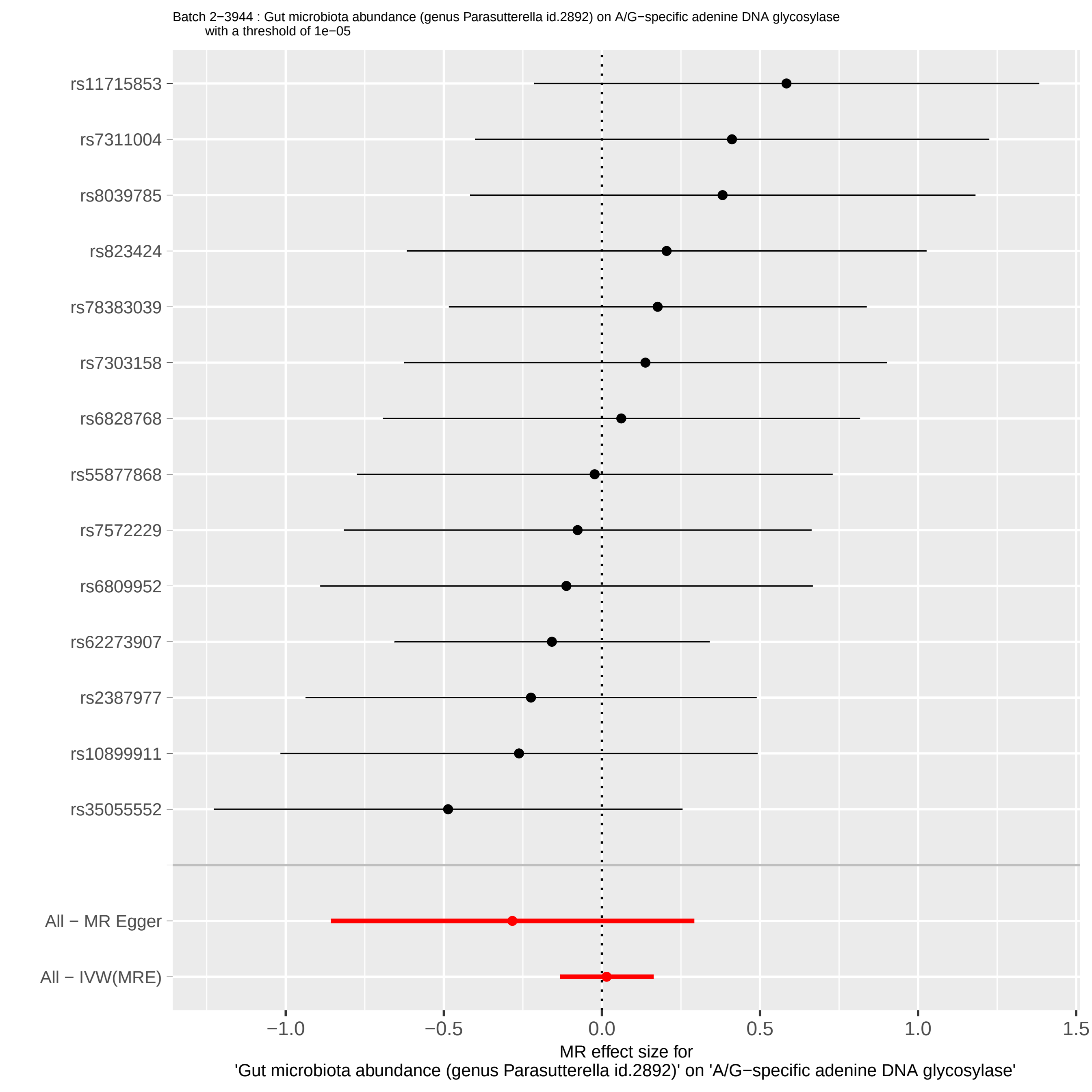

Batch 2−3944 : Gut microbiota abundance (genus Parasutterella id.2892) on A/G−specific adenine DNA glycosylase
with a threshold of 1e−05
rs11715853
rs7311004
rs8039785
rs823424
rs78383039
rs7303158
rs6828768
rs55877868
rs7572229
rs6809952
rs62273907
rs2387977
rs10899911
rs35055552
All − MR Egger
All − IVW(MRE)
−1.0
−0.5
0.0
0.5
1.0
1.5
MR effect size for
'Gut microbiota abundance (genus Parasutterella id.2892)' on 'A/G−specific adenine DNA glycosylase'

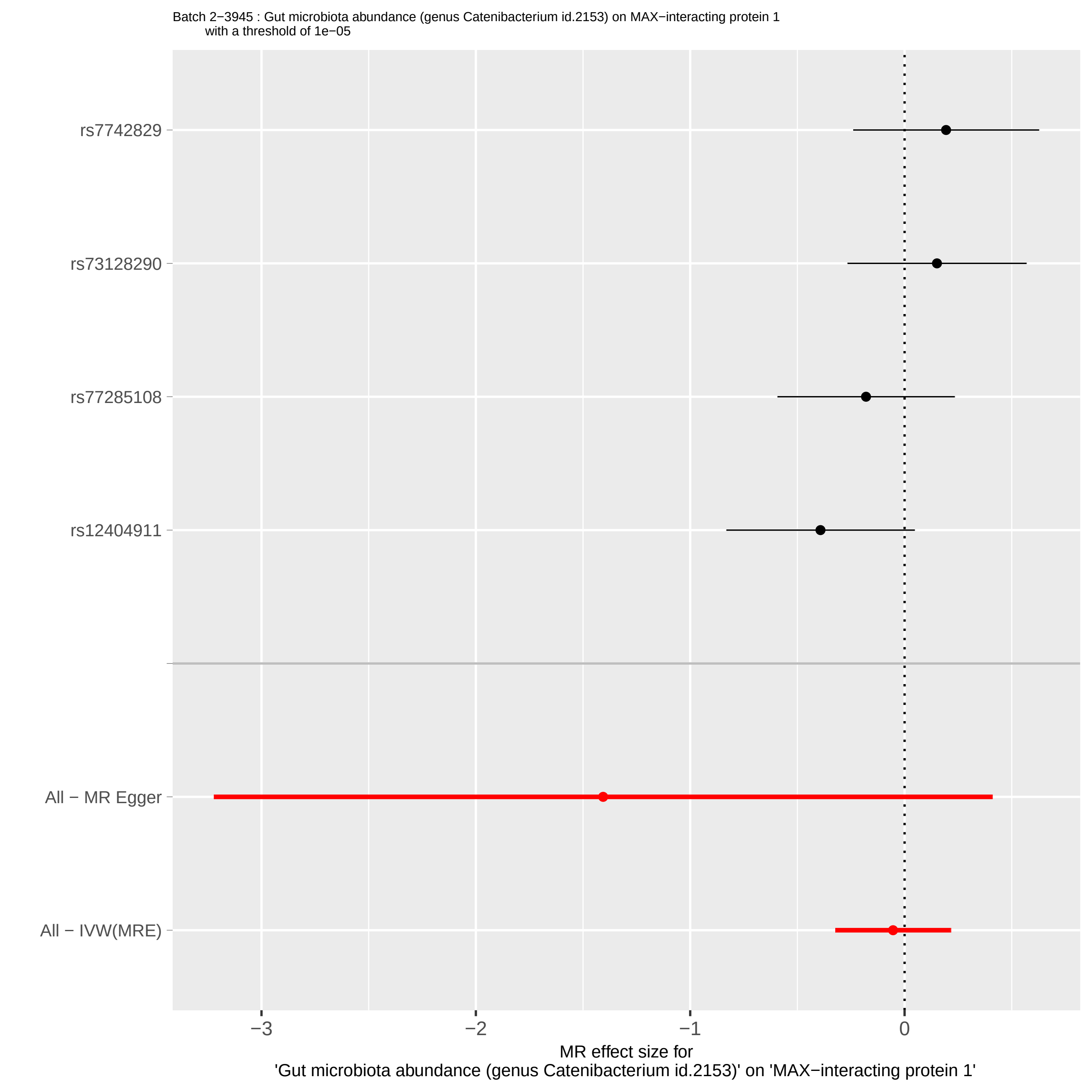

Batch 2−3945 : Gut microbiota abundance (genus Catenibacterium id.2153) on MAX−interacting protein 1
with a threshold of 1e−05
rs7742829
rs73128290
rs77285108
rs12404911
All − MR Egger
All − IVW(MRE)
−3
−2
−1
0
MR effect size for
'Gut microbiota abundance (genus Catenibacterium id.2153)' on 'MAX−interacting protein 1'

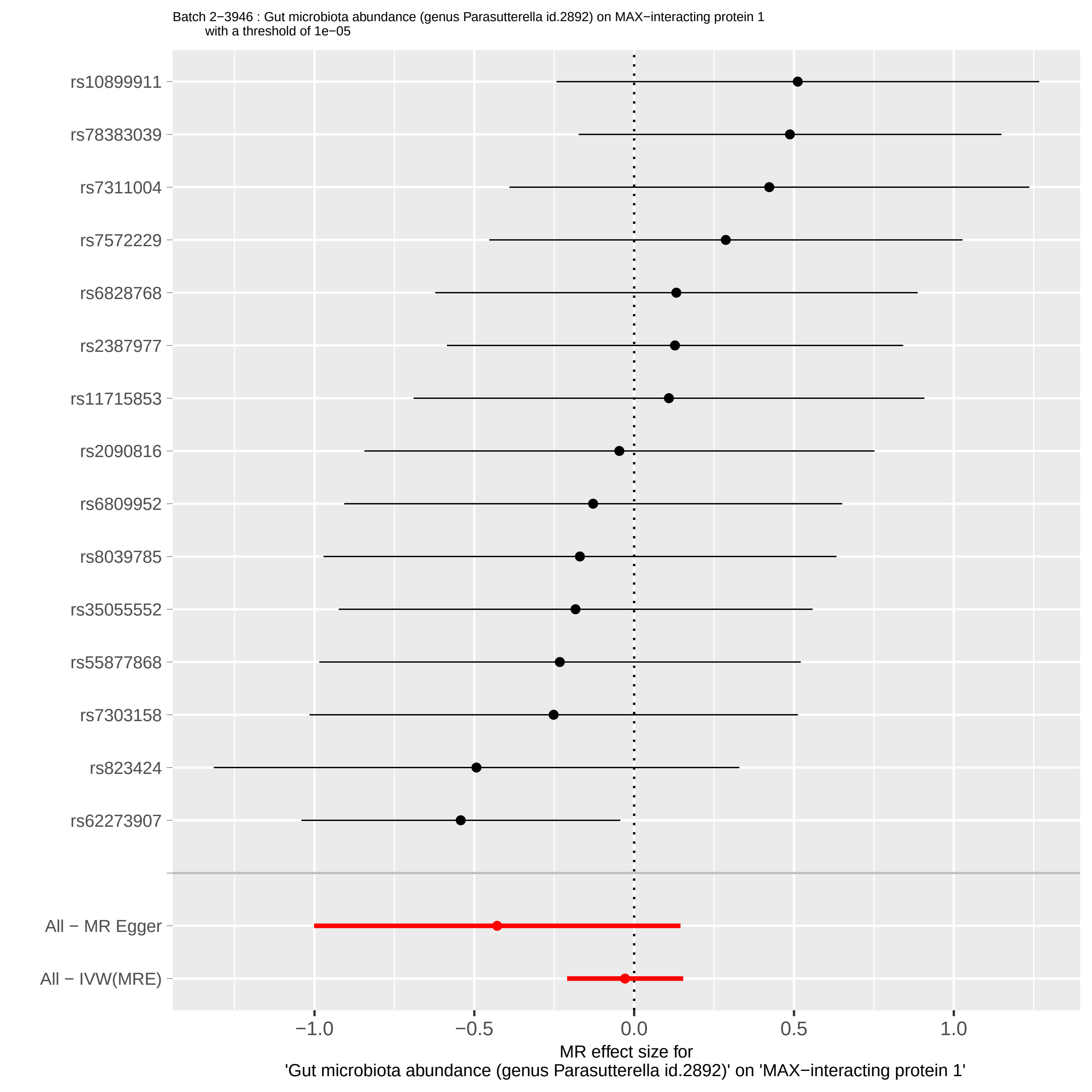
Batch 2−3946 : Gut microbiota abundance (genus Parasutterella id.2892) on MAX−interacting protein 1
with a threshold of 1e−05
rs10899911
rs78383039
rs7311004
rs7572229
rs6828768
rs2387977
rs11715853
rs2090816
rs6809952
rs8039785
rs35055552
rs55877868
rs7303158
rs823424
rs62273907
All − MR Egger
All − IVW(MRE)
−1.0
−0.5
0.0
0.5
1.0
MR effect size for
'Gut microbiota abundance (genus Parasutterella id.2892)' on 'MAX−interacting protein 1'

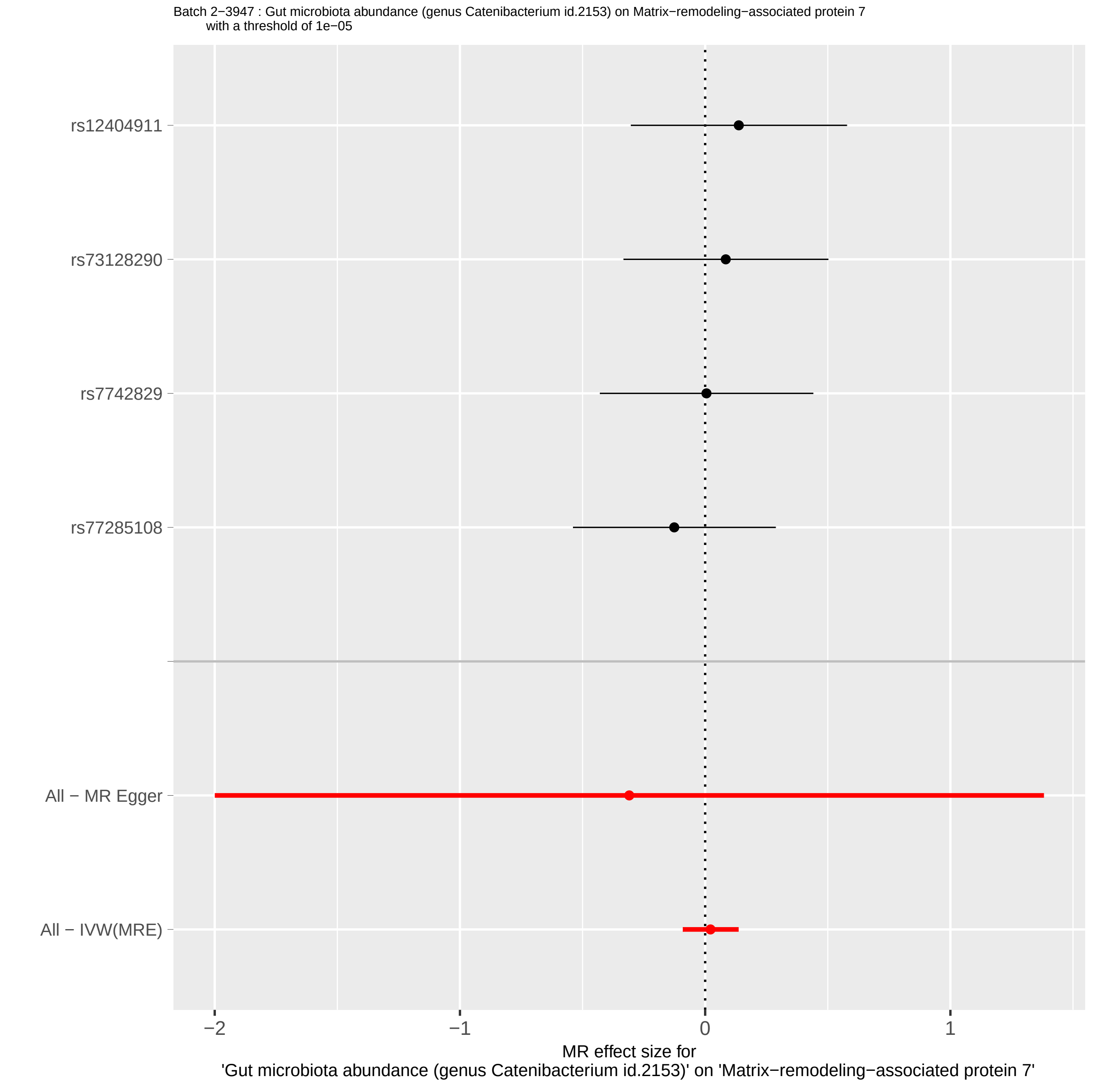

Batch 2−3947 : Gut microbiota abundance (genus Catenibacterium id.2153) on Matrix−remodeling−associated protein 7
with a threshold of 1e−05
rs12404911
rs73128290
rs7742829
rs77285108
All − MR Egger
All − IVW(MRE)
−2
−1
0
1
MR effect size for
'Gut microbiota abundance (genus Catenibacterium id.2153)' on 'Matrix−remodeling−associated protein 7'

Batch 2−3948 : Gut microbiota abundance (genus Parasutterella id.2892) on Matrix−remodeling−associated protein 7
with a threshold of 1e−05

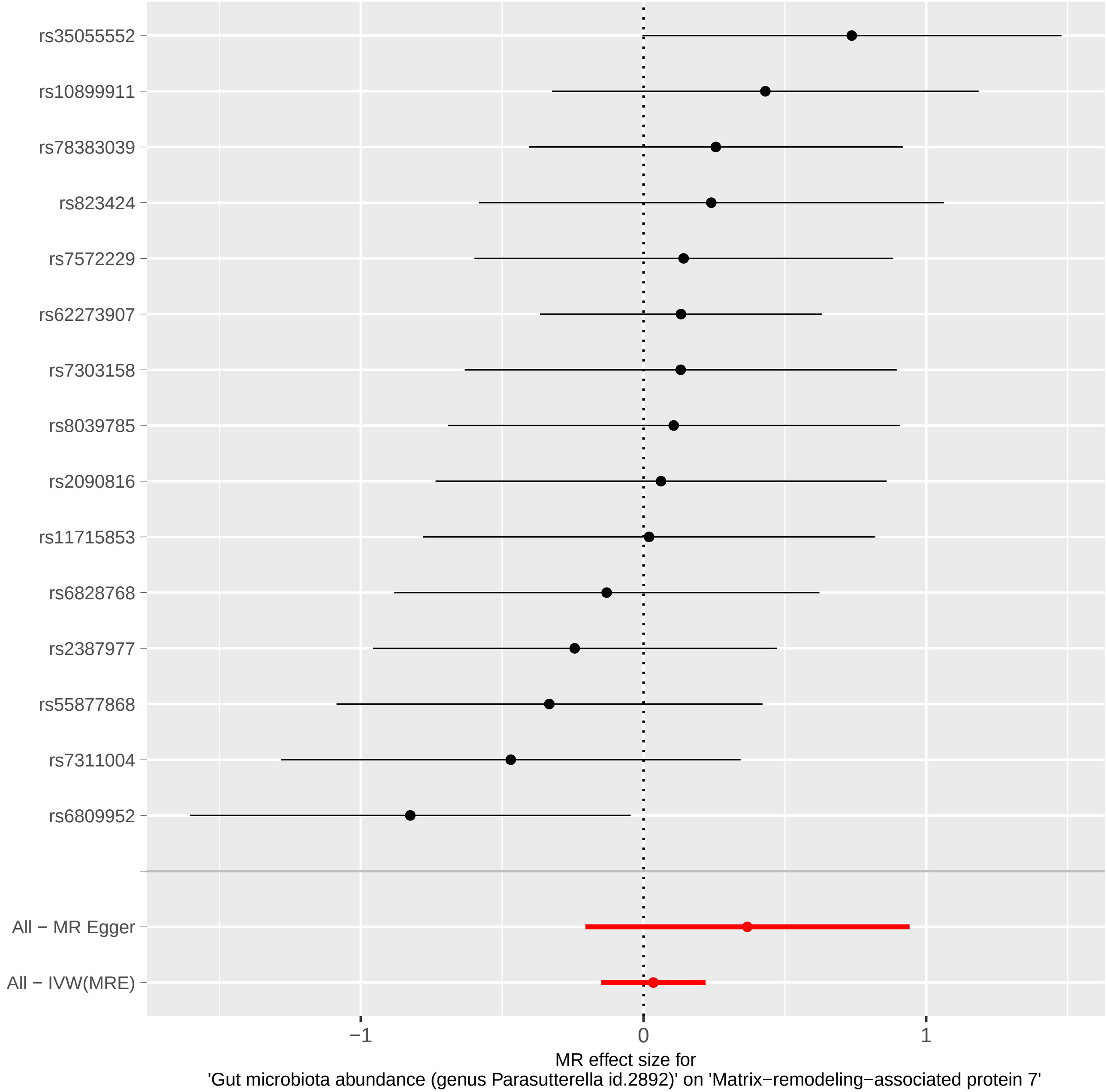

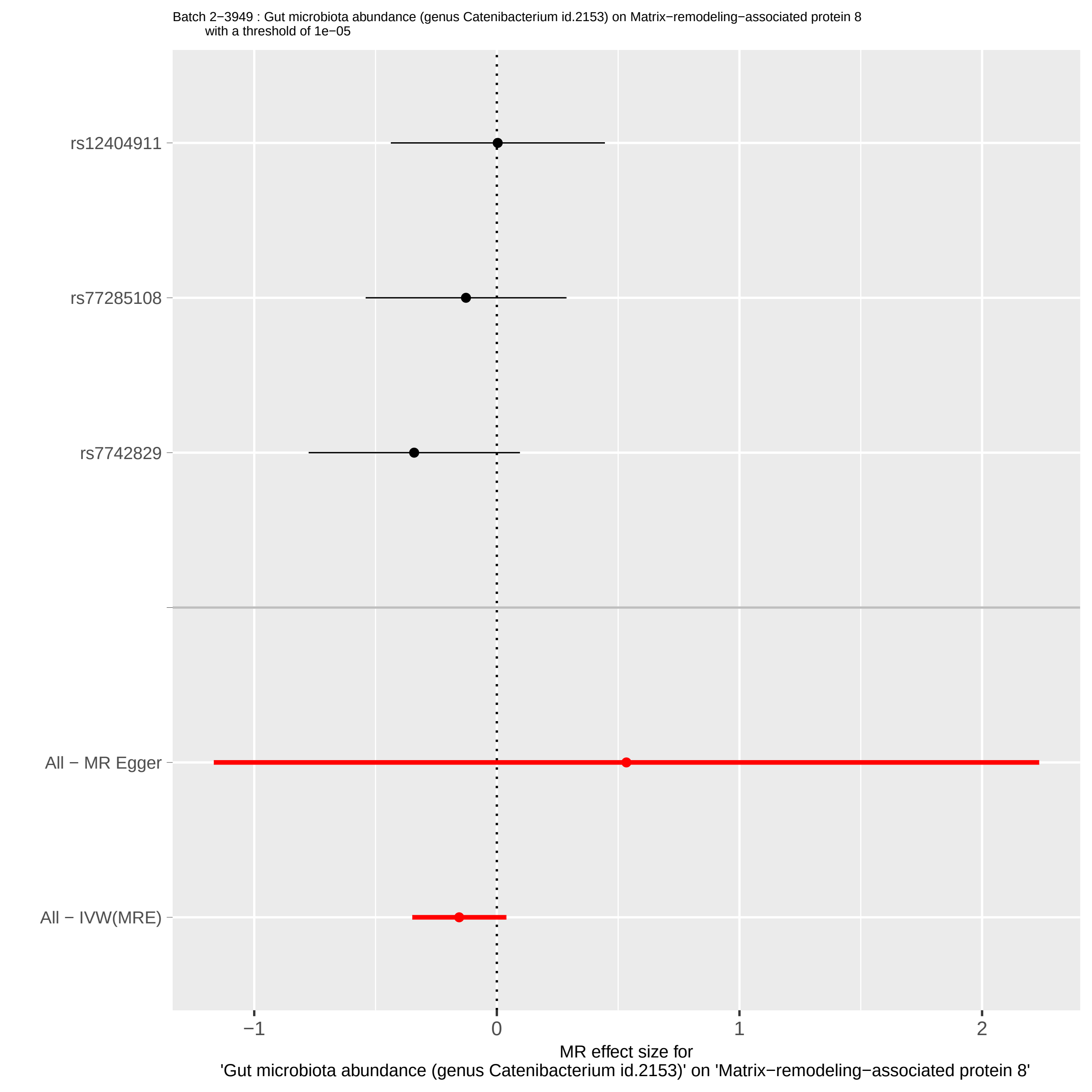
Batch 2−3949 : Gut microbiota abundance (genus Catenibacterium id.2153) on Matrix−remodeling−associated protein 8
with a threshold of 1e−05
rs12404911
rs77285108
rs7742829
All − MR Egger
All − IVW(MRE)
−1
0
1
2
MR effect size for
'Gut microbiota abundance (genus Catenibacterium id.2153)' on 'Matrix−remodeling−associated protein 8'

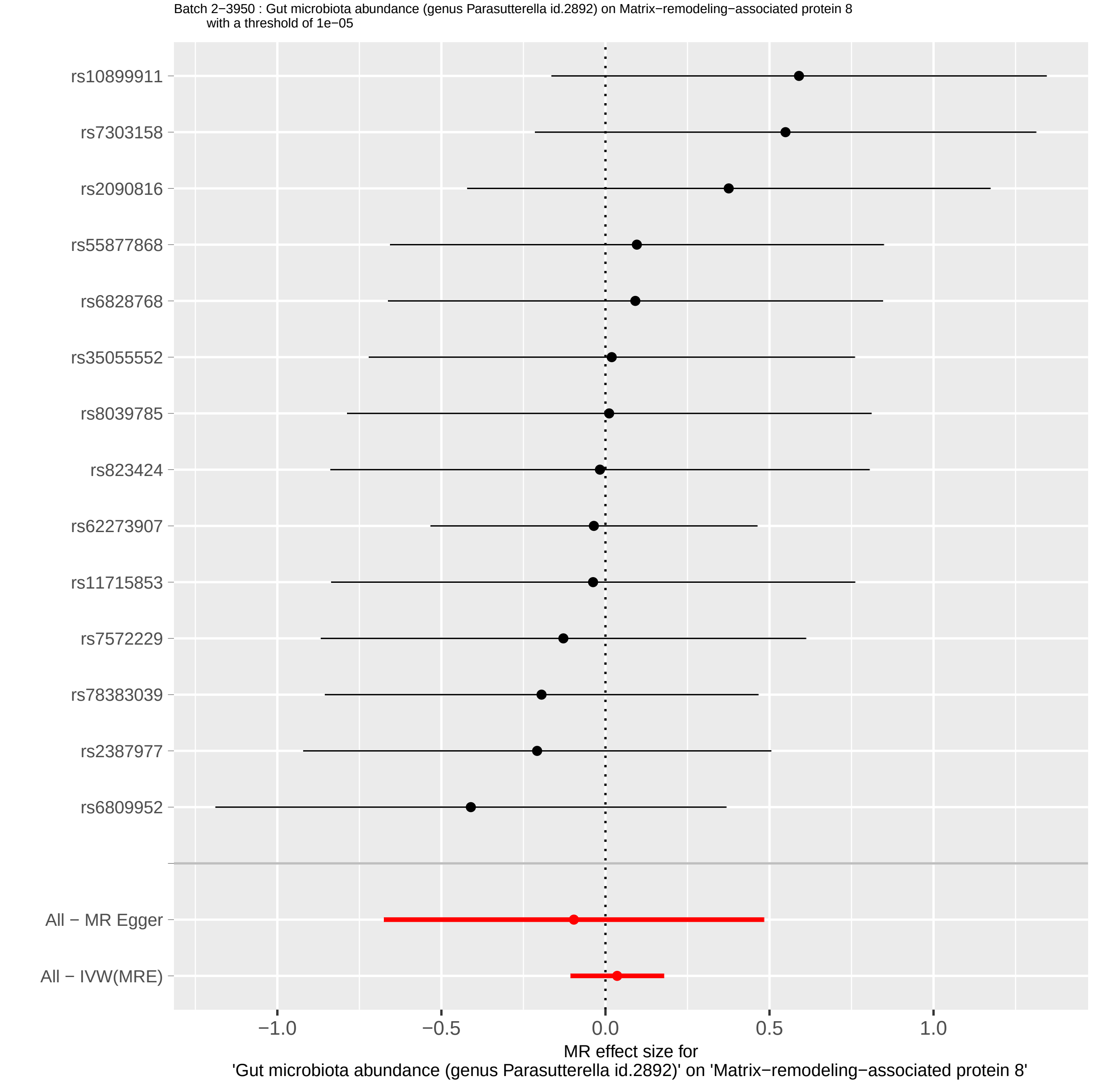

Batch 2−3950 : Gut microbiota abundance (genus Parasutterella id.2892) on Matrix−remodeling−associated protein 8
with a threshold of 1e−05
rs10899911
rs7303158
rs2090816
rs55877868
rs6828768
rs35055552
rs8039785
rs823424
rs62273907
rs11715853
rs7572229
rs78383039
rs2387977
rs6809952
All − MR Egger
All − IVW(MRE)
−1.0
−0.5
0.0
0.5
1.0
MR effect size for
'Gut microbiota abundance (genus Parasutterella id.2892)' on 'Matrix−remodeling−associated protein 8'

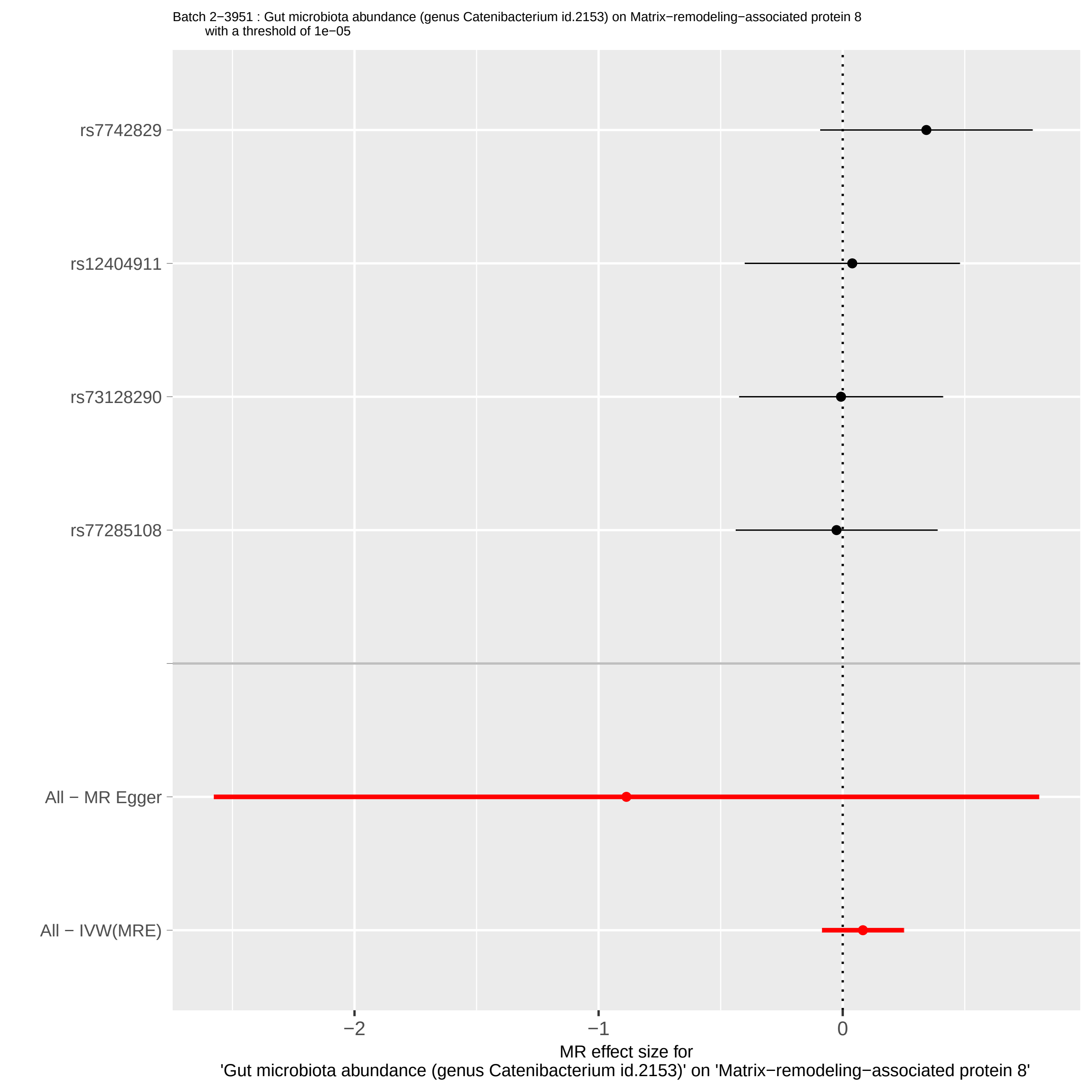

Batch 2−3951 : Gut microbiota abundance (genus Catenibacterium id.2153) on Matrix−remodeling−associated protein 8
with a threshold of 1e−05
rs7742829
rs12404911
rs73128290
rs77285108
All − MR Egger
All − IVW(MRE)
−2
−1
0
MR effect size for
'Gut microbiota abundance (genus Catenibacterium id.2153)' on 'Matrix−remodeling−associated protein 8'

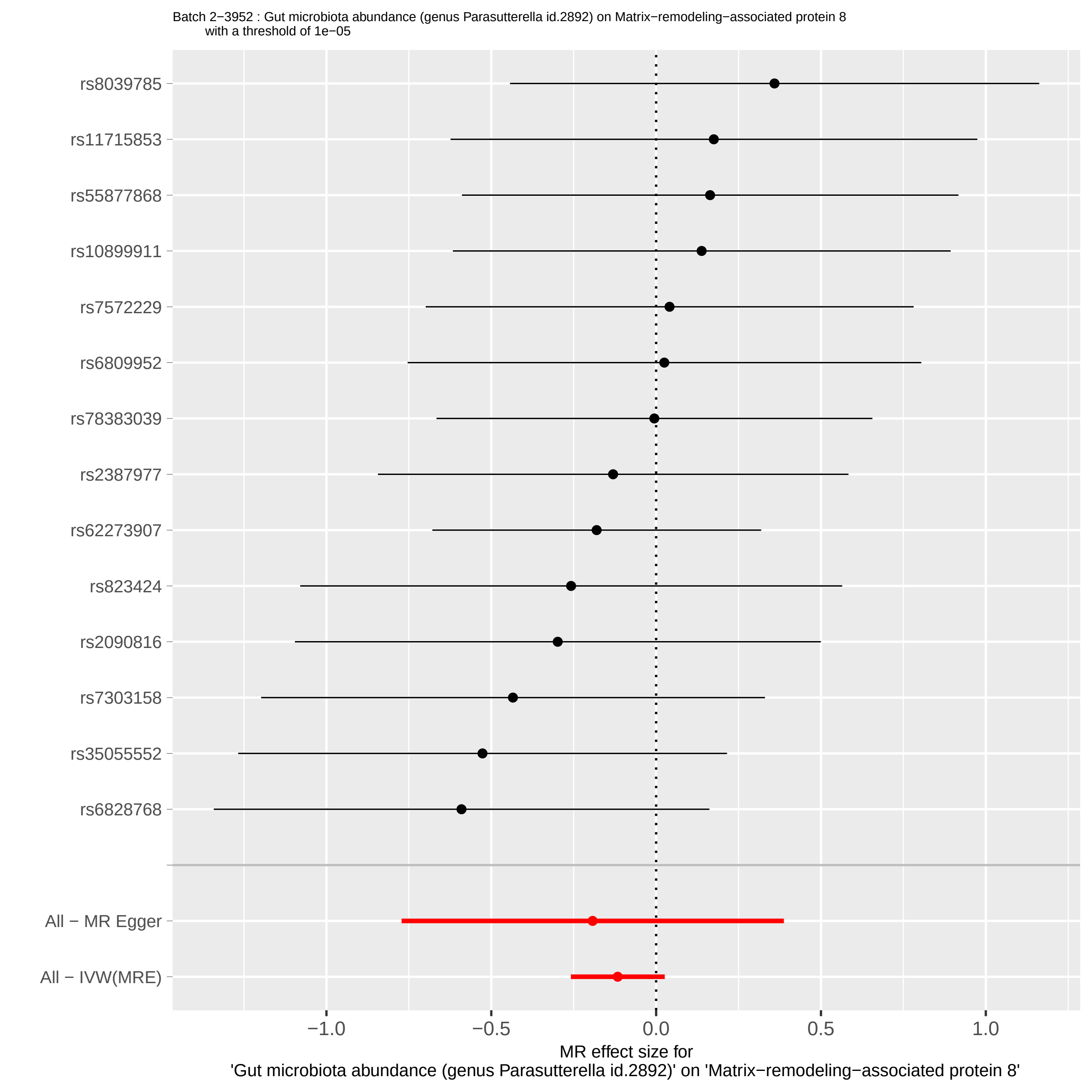

Batch 2−3952 : Gut microbiota abundance (genus Parasutterella id.2892) on Matrix−remodeling−associated protein 8
with a threshold of 1e−05
rs8039785
rs11715853
rs55877868
rs10899911
rs7572229
rs6809952
rs78383039
rs2387977
rs62273907
rs823424
rs2090816
rs7303158
rs35055552
rs6828768
All − MR Egger
All − IVW(MRE)
−1.0
−0.5
0.0
0.5
1.0
MR effect size for
'Gut microbiota abundance (genus Parasutterella id.2892)' on 'Matrix−remodeling−associated protein 8'

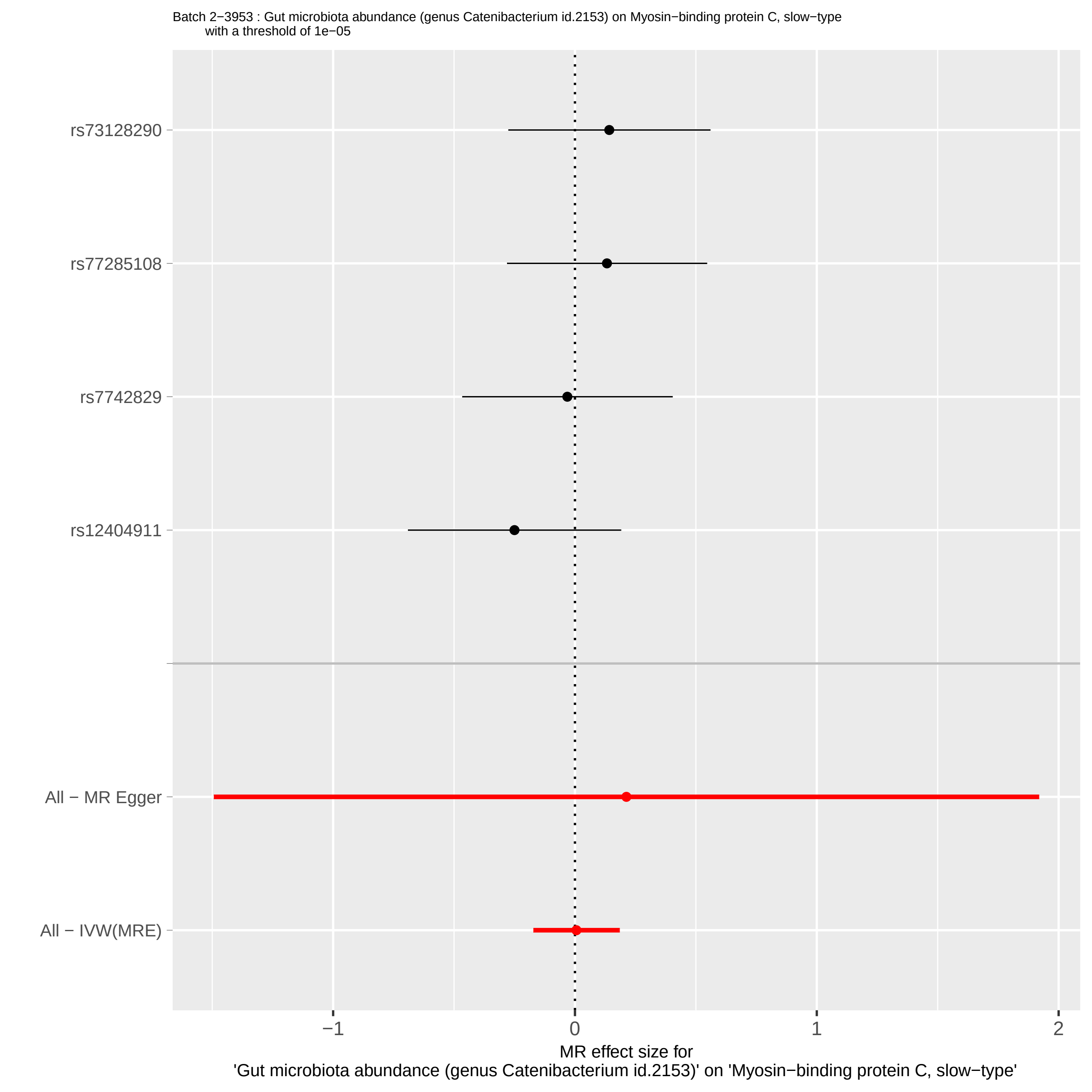
Batch 2−3953 : Gut microbiota abundance (genus Catenibacterium id.2153) on Myosin−binding protein C, slow−type
with a threshold of 1e−05
rs73128290
rs77285108
rs7742829
rs12404911
All − MR Egger
All − IVW(MRE)
−1
0
1
2
MR effect size for
'Gut microbiota abundance (genus Catenibacterium id.2153)' on 'Myosin−binding protein C, slow−type'

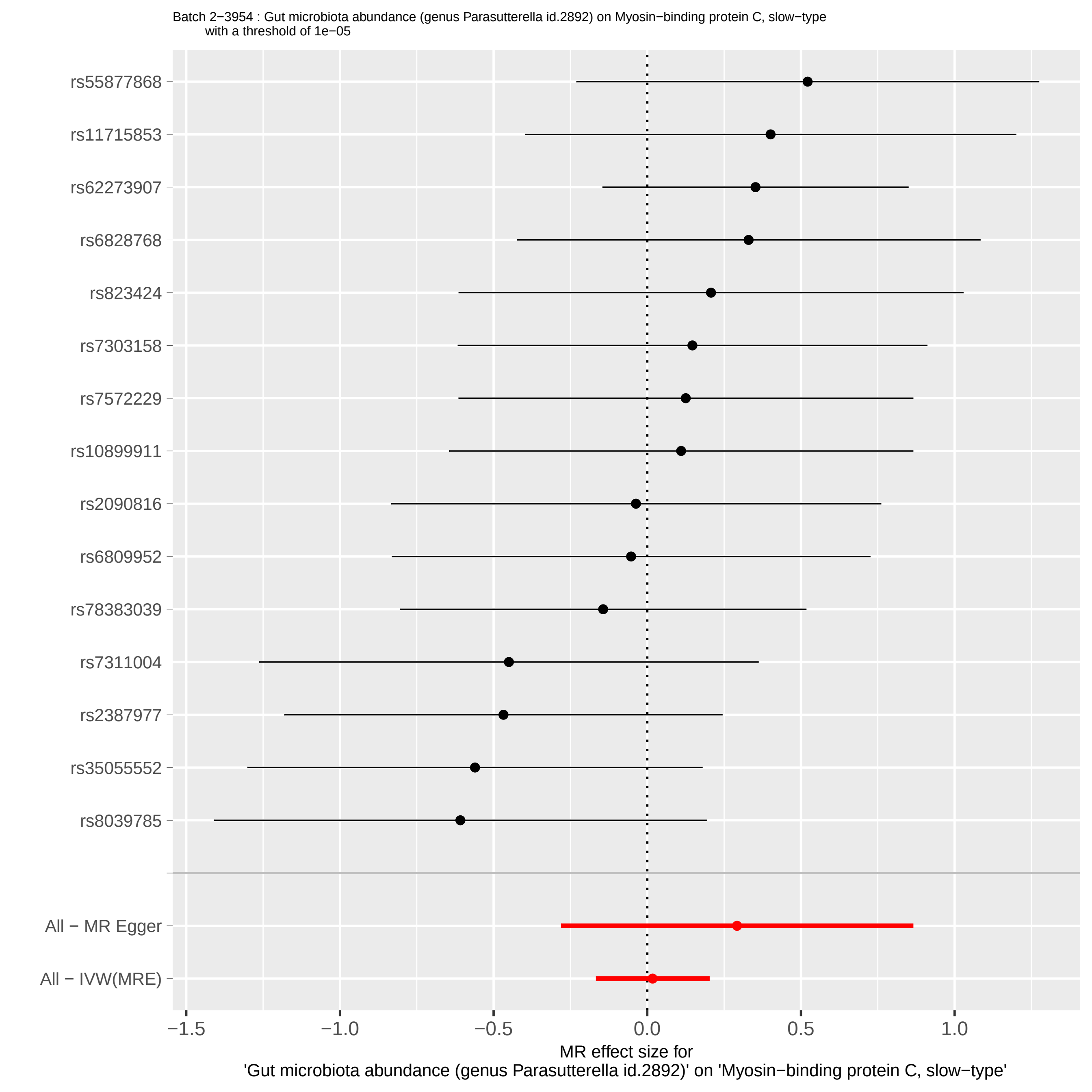
Batch 2−3954 : Gut microbiota abundance (genus Parasutterella id.2892) on Myosin−binding protein C, slow−type
with a threshold of 1e−05
rs55877868
rs11715853
rs62273907
rs6828768
rs823424
rs7303158
rs7572229
rs10899911
rs2090816
rs6809952
rs78383039
rs7311004
rs2387977
rs35055552
rs8039785
All − MR Egger
All − IVW(MRE)
−1.5
−1.0
−0.5
0.0
0.5
1.0
MR effect size for
'Gut microbiota abundance (genus Parasutterella id.2892)' on 'Myosin−binding protein C, slow−type'

Batch 2−3955 : Gut microbiota abundance (genus Catenibacterium id.2153) on Myc proto−oncogene protein
with a threshold of 1e−05

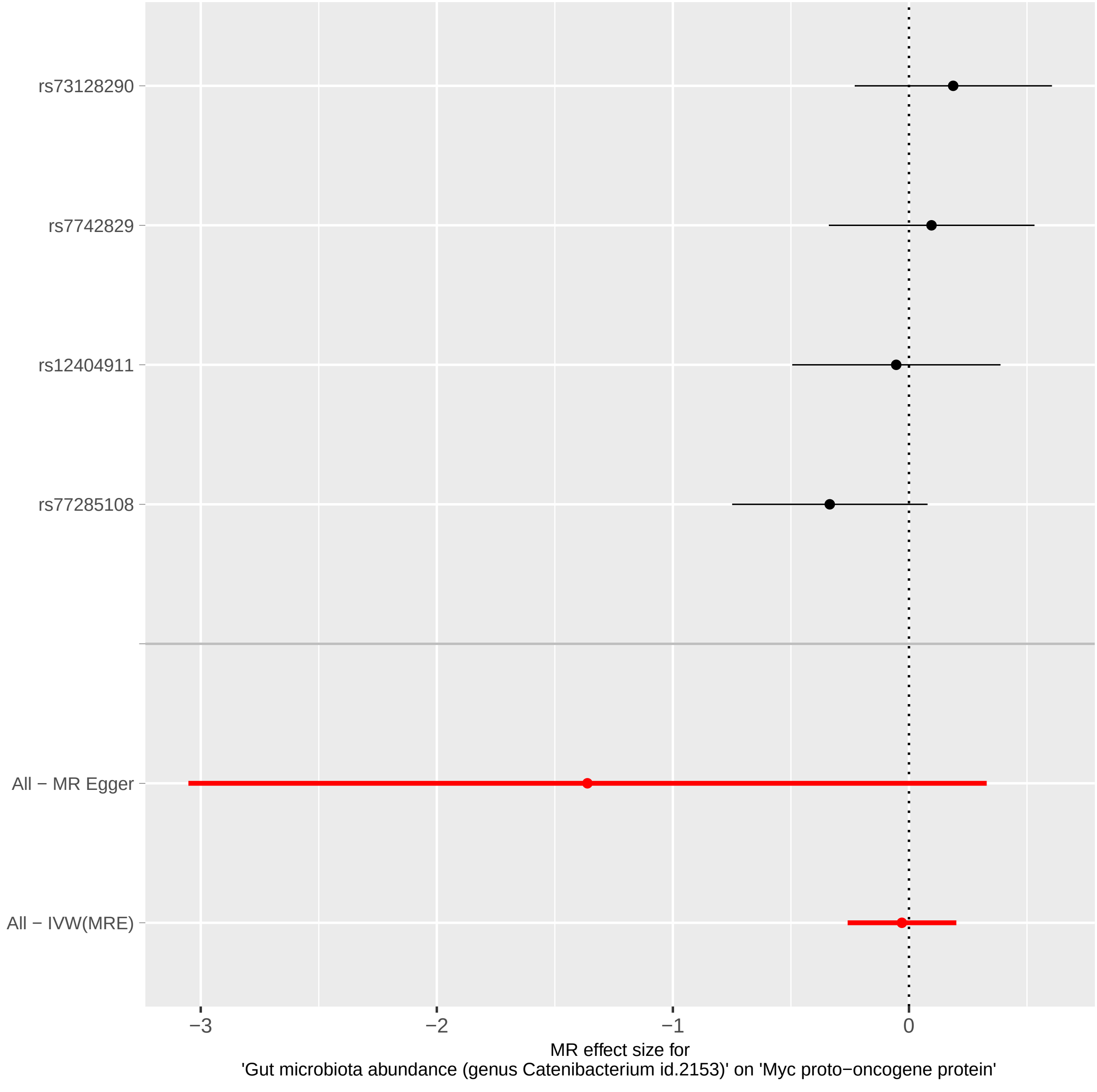

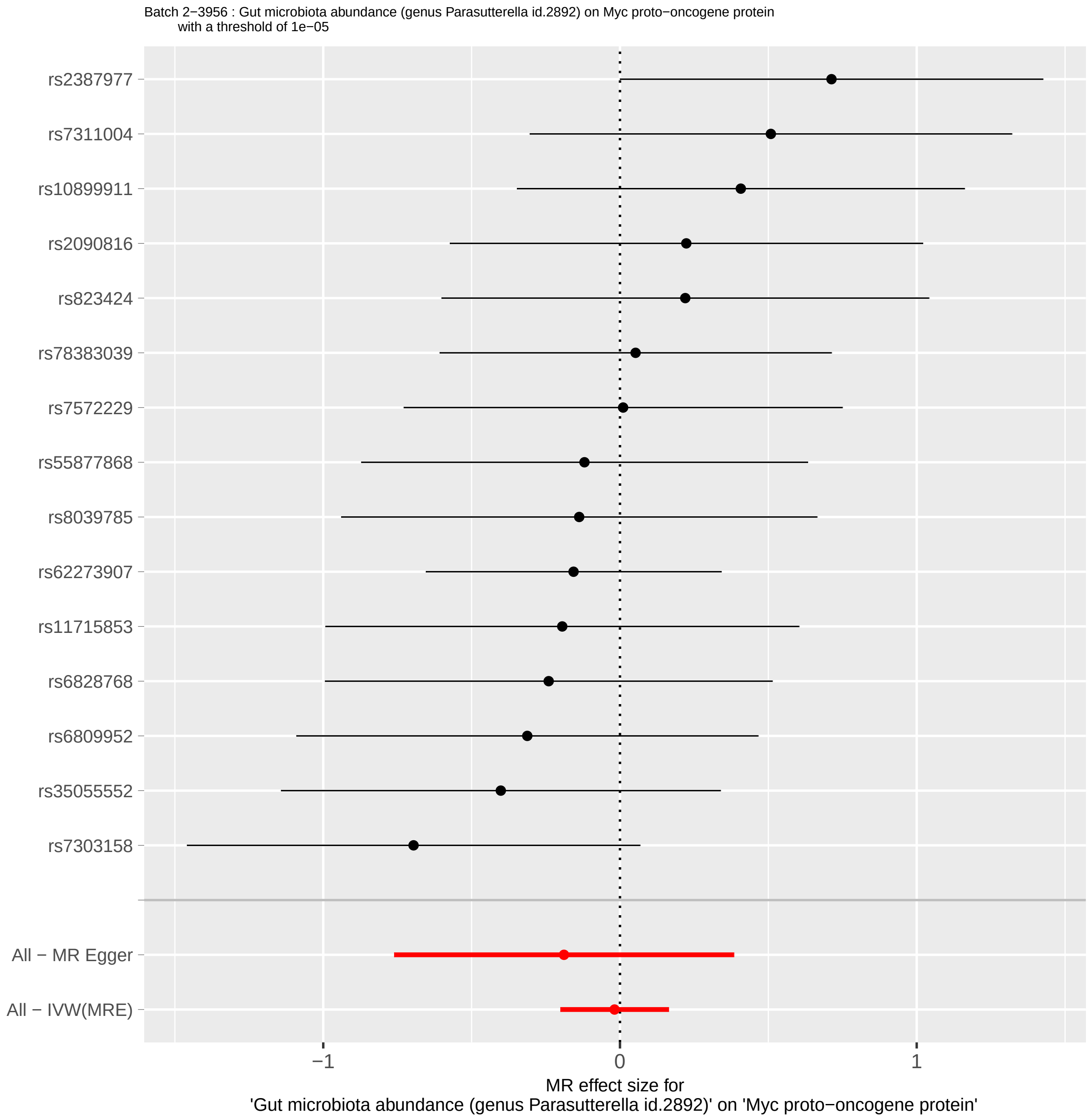

Batch 2−3956 : Gut microbiota abundance (genus Parasutterella id.2892) on Myc proto−oncogene protein
with a threshold of 1e−05
rs2387977
rs7311004
rs10899911
rs2090816
rs823424
rs78383039
rs7572229
rs55877868
rs8039785
rs62273907
rs11715853
rs6828768
rs6809952
rs35055552
rs7303158
All − MR Egger
All − IVW(MRE)
−1
0
1
MR effect size for
'Gut microbiota abundance (genus Parasutterella id.2892)' on 'Myc proto−oncogene protein'

Batch 2−3957 : Gut microbiota abundance (genus Catenibacterium id.2153) on Myc target protein 1
with a threshold of 1e−05

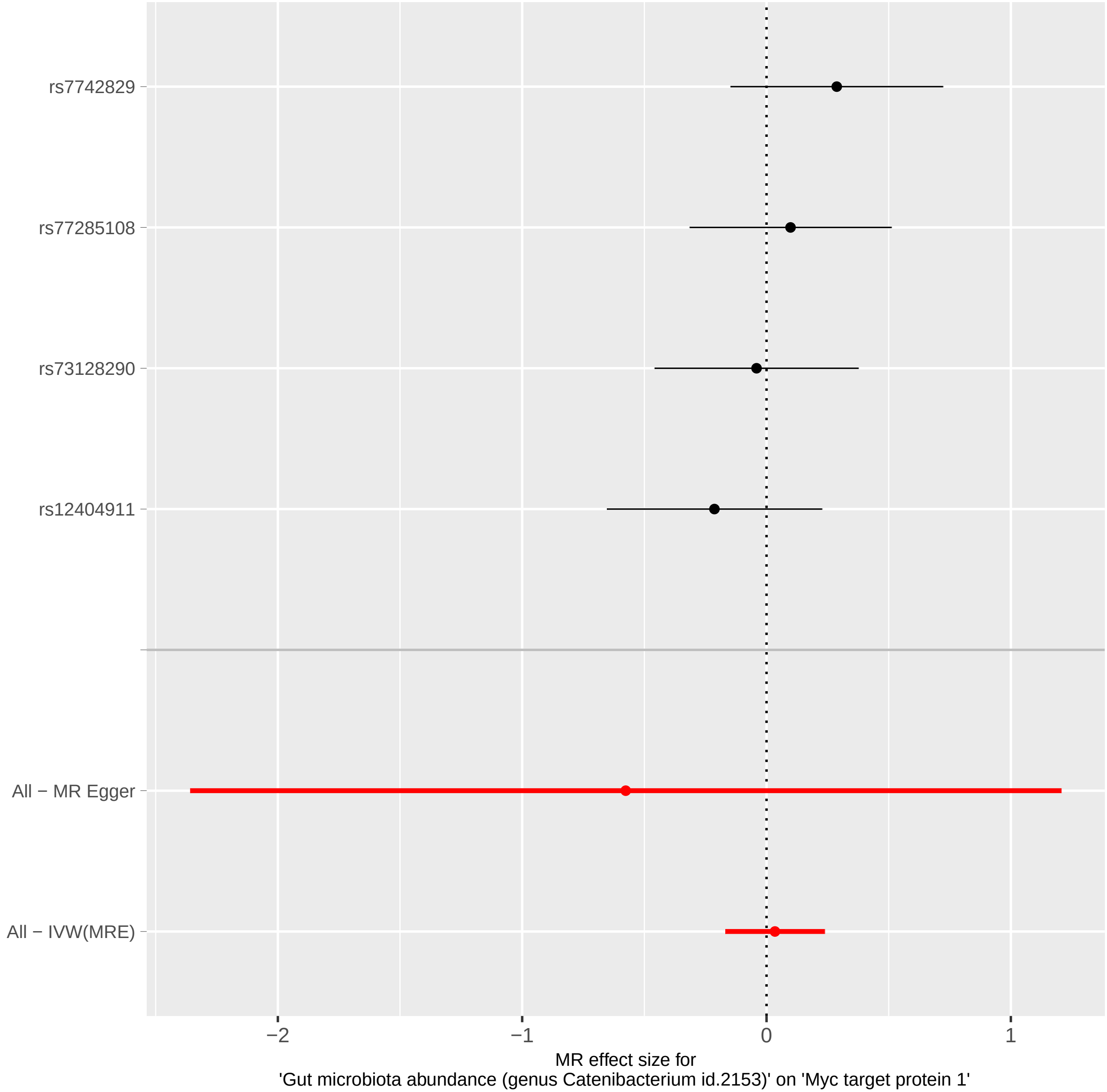

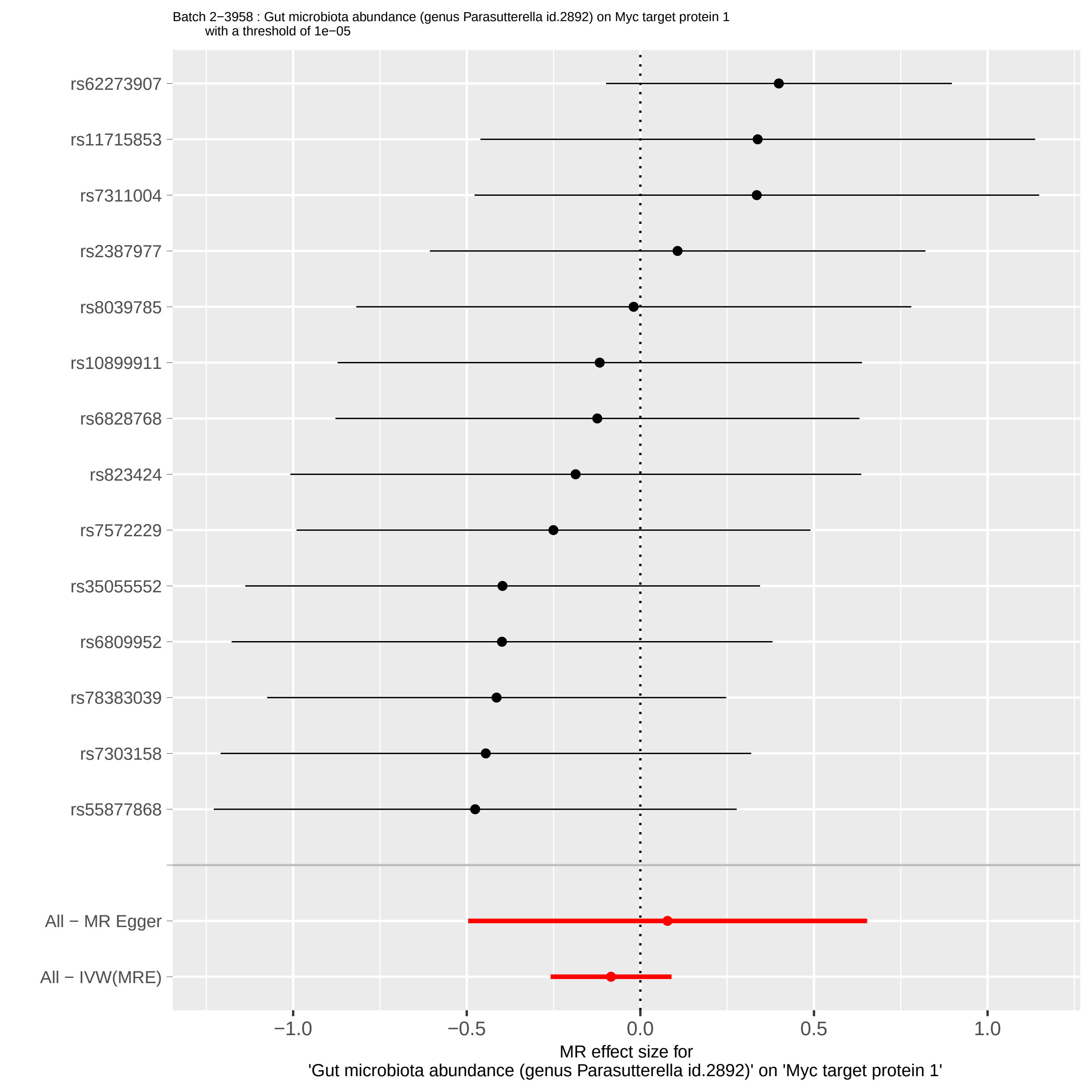

Batch 2−3958 : Gut microbiota abundance (genus Parasutterella id.2892) on Myc target protein 1
with a threshold of 1e−05
rs62273907
rs11715853
rs7311004
rs2387977
rs8039785
rs10899911
rs6828768
rs823424
rs7572229
rs35055552
rs6809952
rs78383039
rs7303158
rs55877868
All − MR Egger
All − IVW(MRE)
−1.0
−0.5
0.0
0.5
1.0
MR effect size for
'Gut microbiota abundance (genus Parasutterella id.2892)' on 'Myc target protein 1'

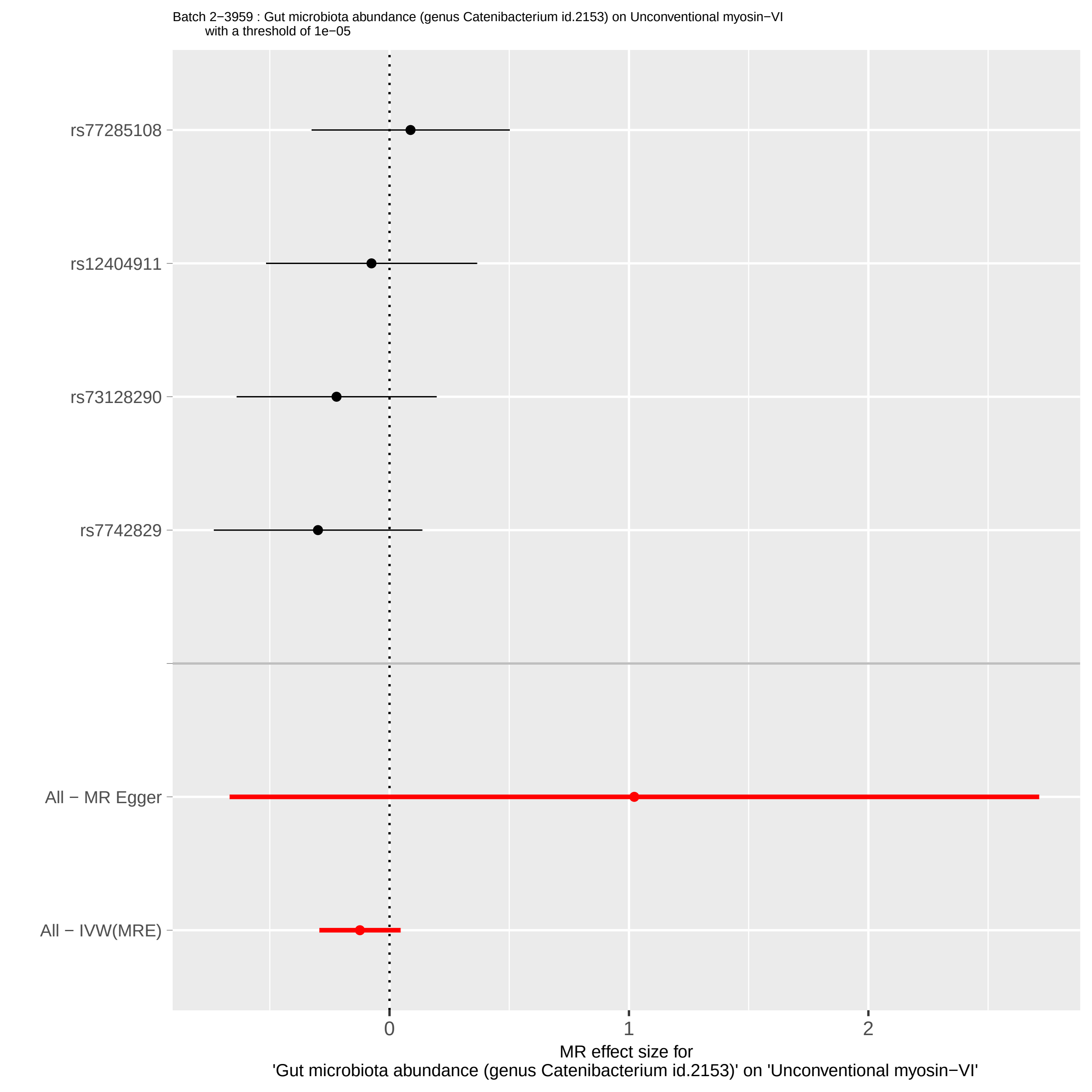
Batch 2−3959 : Gut microbiota abundance (genus Catenibacterium id.2153) on Unconventional myosin−VI
with a threshold of 1e−05
rs77285108
rs12404911
rs73128290
rs7742829
All − MR Egger
All − IVW(MRE)
0
1
2
MR effect size for
'Gut microbiota abundance (genus Catenibacterium id.2153)' on 'Unconventional myosin−VI'

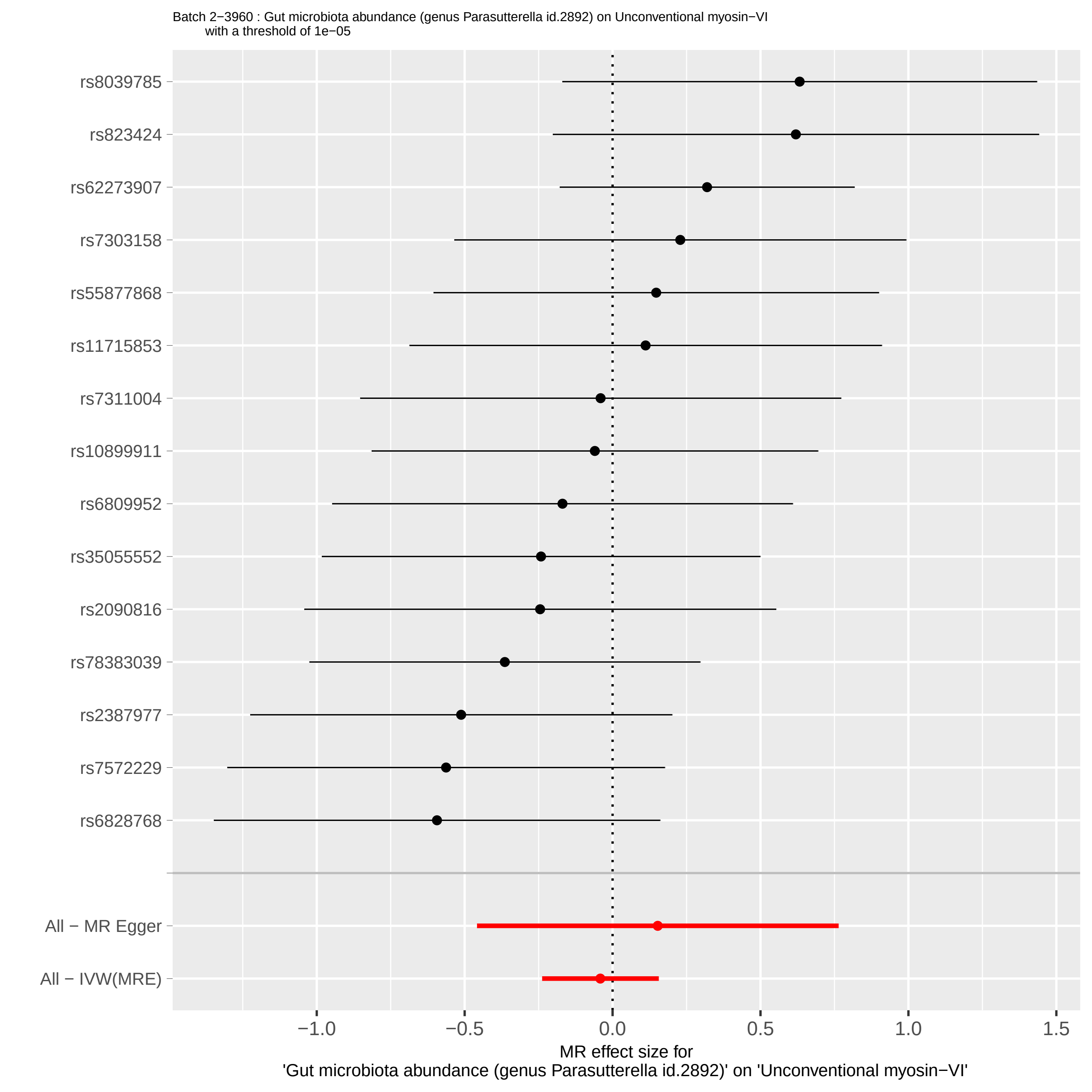
Batch 2−3960 : Gut microbiota abundance (genus Parasutterella id.2892) on Unconventional myosin−VI
with a threshold of 1e−05
rs8039785
rs823424
rs62273907
rs7303158
rs55877868
rs11715853
rs7311004
rs10899911
rs6809952
rs35055552
rs2090816
rs78383039
rs2387977
rs7572229
rs6828768
All − MR Egger
All − IVW(MRE)
−1.0
−0.5
0.0
0.5
1.0
1.5
MR effect size for
'Gut microbiota abundance (genus Parasutterella id.2892)' on 'Unconventional myosin−VI'

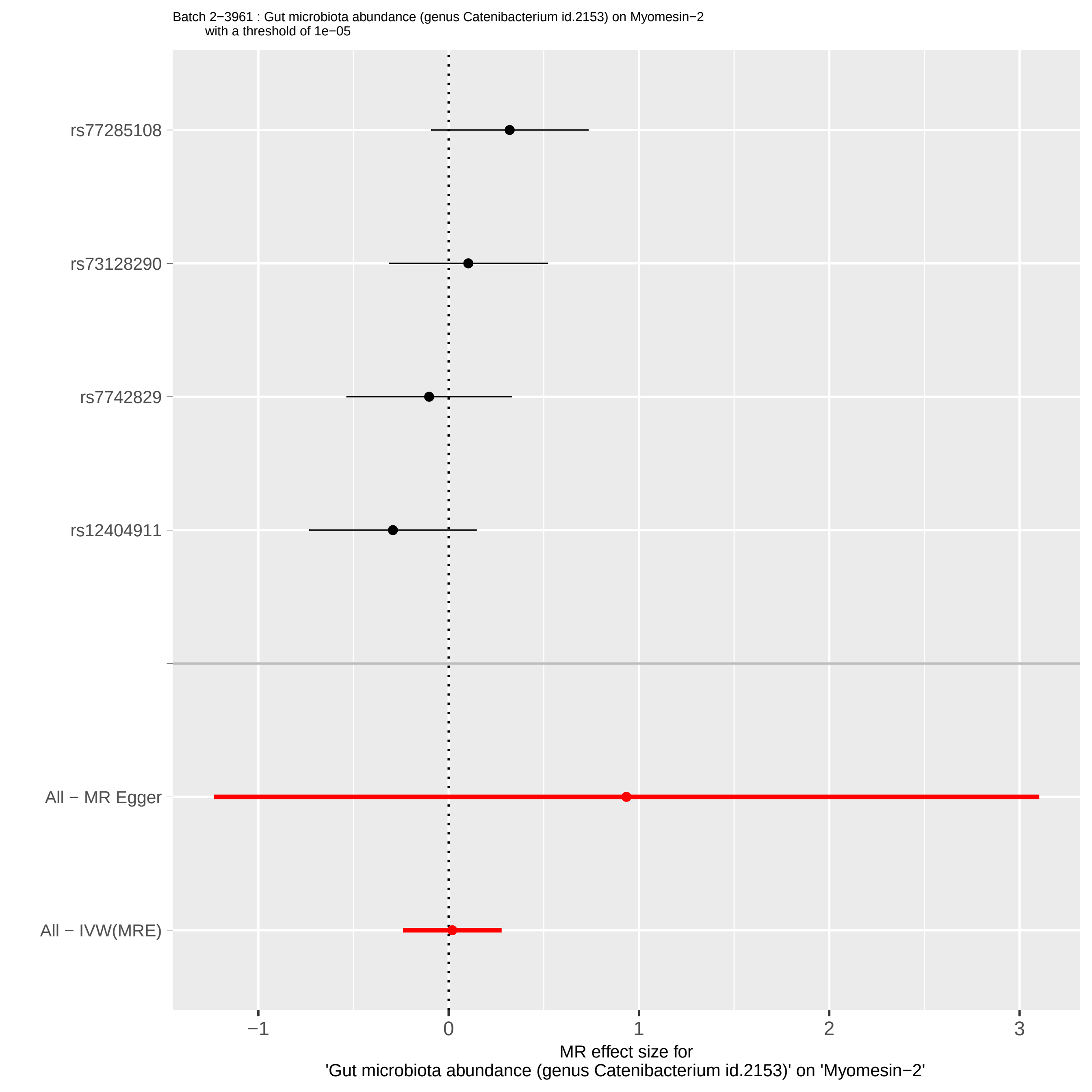

Batch 2−3961 : Gut microbiota abundance (genus Catenibacterium id.2153) on Myomesin−2
with a threshold of 1e−05
rs77285108
rs73128290
rs7742829
rs12404911
All − MR Egger
All − IVW(MRE)
−1
0
1
2
3
MR effect size for
'Gut microbiota abundance (genus Catenibacterium id.2153)' on 'Myomesin−2'

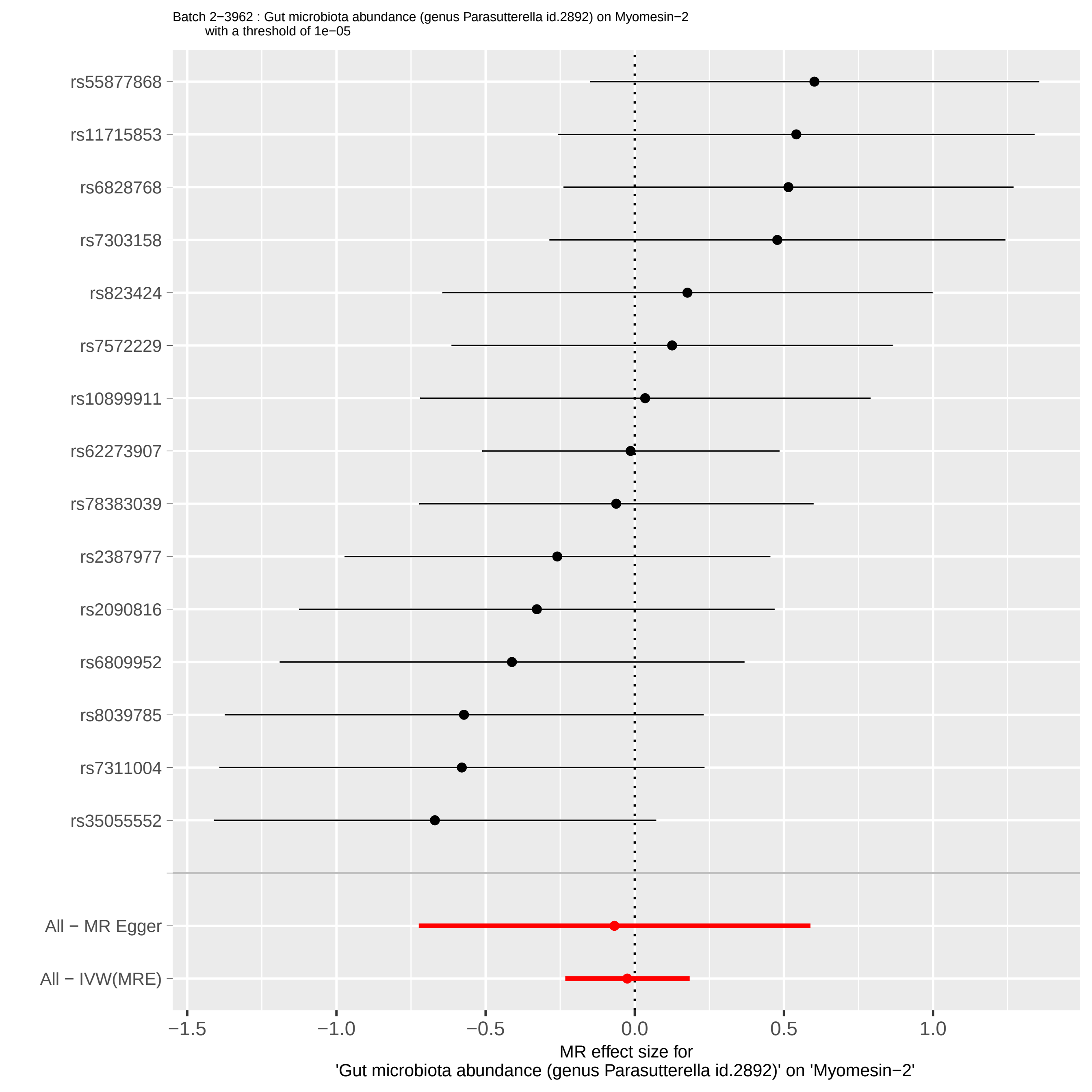

Batch 2−3962 : Gut microbiota abundance (genus Parasutterella id.2892) on Myomesin−2
with a threshold of 1e−05
rs55877868
rs11715853
rs6828768
rs7303158
rs823424
rs7572229
rs10899911
rs62273907
rs78383039
rs2387977
rs2090816
rs6809952
rs8039785
rs7311004
rs35055552
All − MR Egger
All − IVW(MRE)
−1.5
−1.0
−0.5
0.0
0.5
1.0
MR effect size for
'Gut microbiota abundance (genus Parasutterella id.2892)' on 'Myomesin−2'

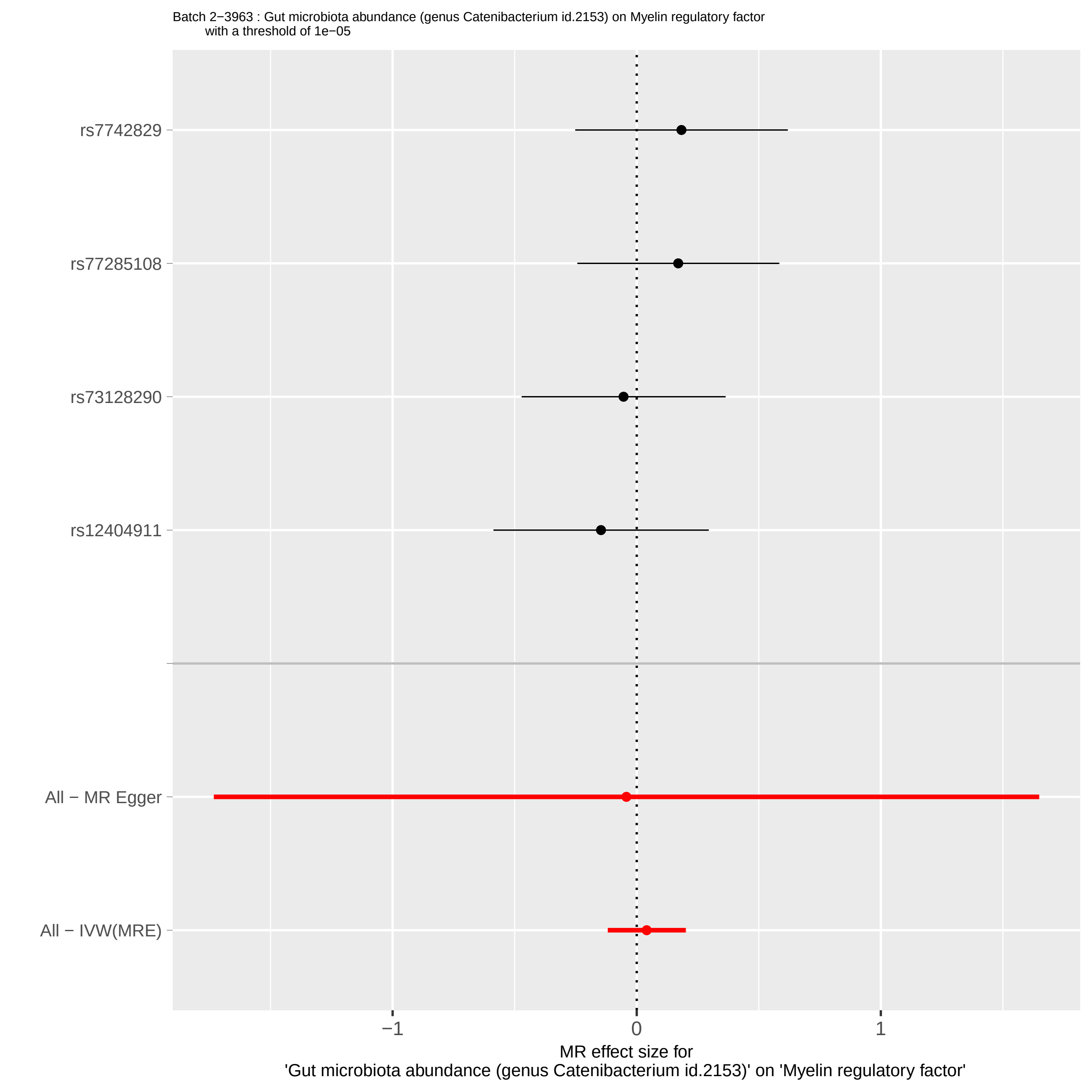
Batch 2−3963 : Gut microbiota abundance (genus Catenibacterium id.2153) on Myelin regulatory factor
with a threshold of 1e−05
rs7742829
rs77285108
rs73128290
rs12404911
All − MR Egger
All − IVW(MRE)
−1
0
1
MR effect size for
'Gut microbiota abundance (genus Catenibacterium id.2153)' on 'Myelin regulatory factor'

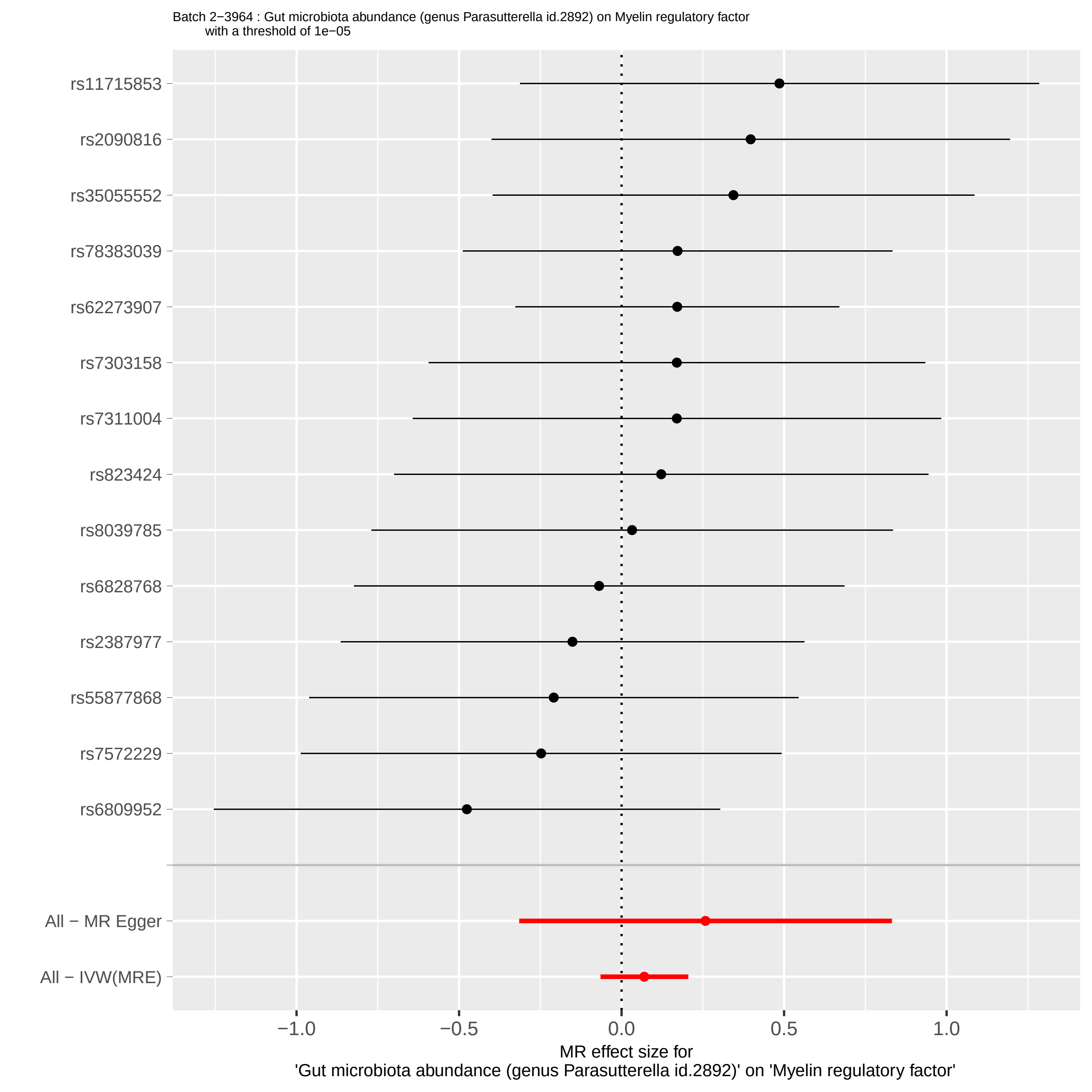

Batch 2−3964 : Gut microbiota abundance (genus Parasutterella id.2892) on Myelin regulatory factor
with a threshold of 1e−05
rs11715853
rs2090816
rs35055552
rs78383039
rs62273907
rs7303158
rs7311004
rs823424
rs8039785
rs6828768
rs2387977
rs55877868
rs7572229
rs6809952
All − MR Egger
All − IVW(MRE)
−1.0
−0.5
0.0
0.5
1.0
MR effect size for
'Gut microbiota abundance (genus Parasutterella id.2892)' on 'Myelin regulatory factor'

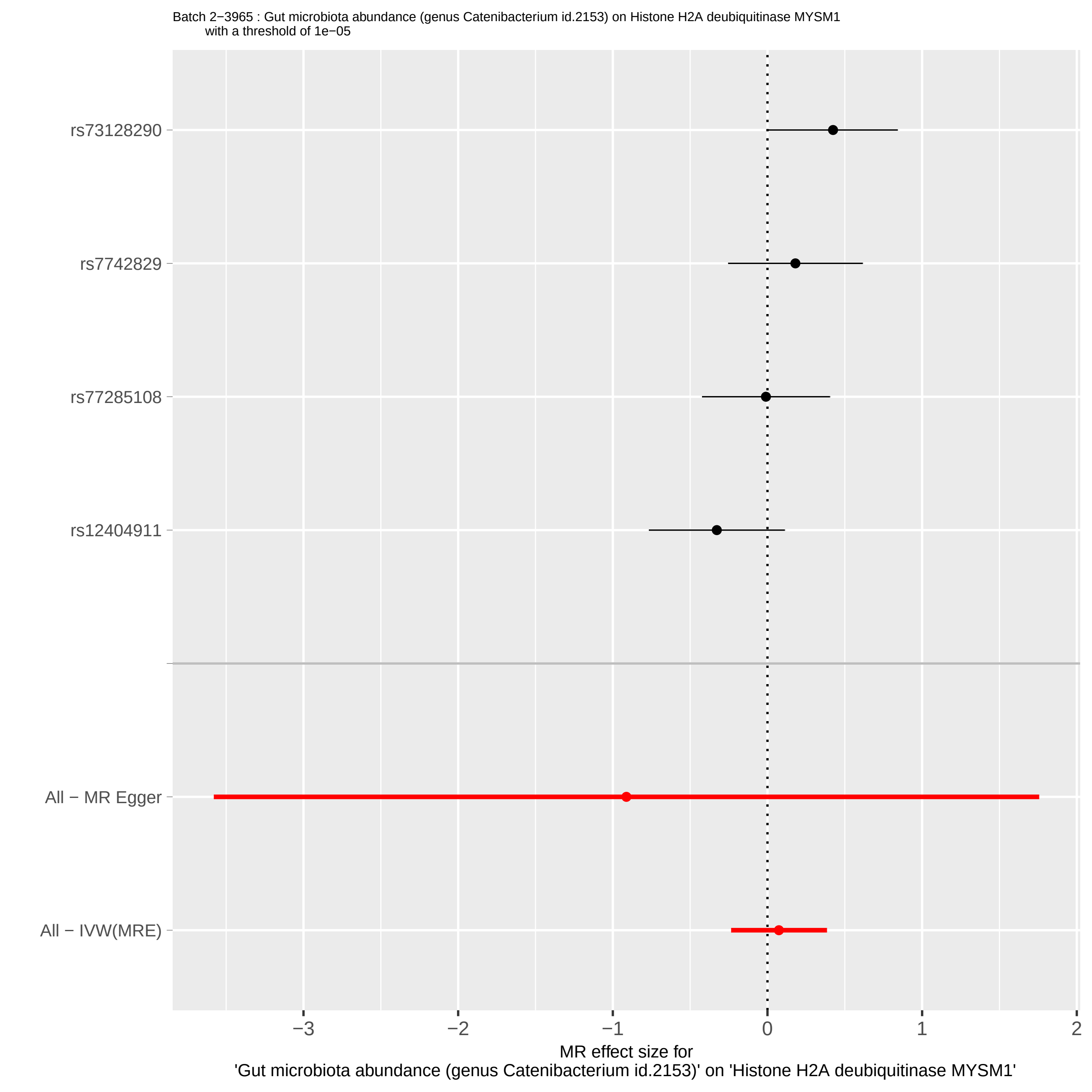
Batch 2−3965 : Gut microbiota abundance (genus Catenibacterium id.2153) on Histone H2A deubiquitinase MYSM1
with a threshold of 1e−05
rs73128290
rs7742829
rs77285108
rs12404911
All − MR Egger
All − IVW(MRE)
−3
−2
−1
0
1
2
MR effect size for
'Gut microbiota abundance (genus Catenibacterium id.2153)' on 'Histone H2A deubiquitinase MYSM1'

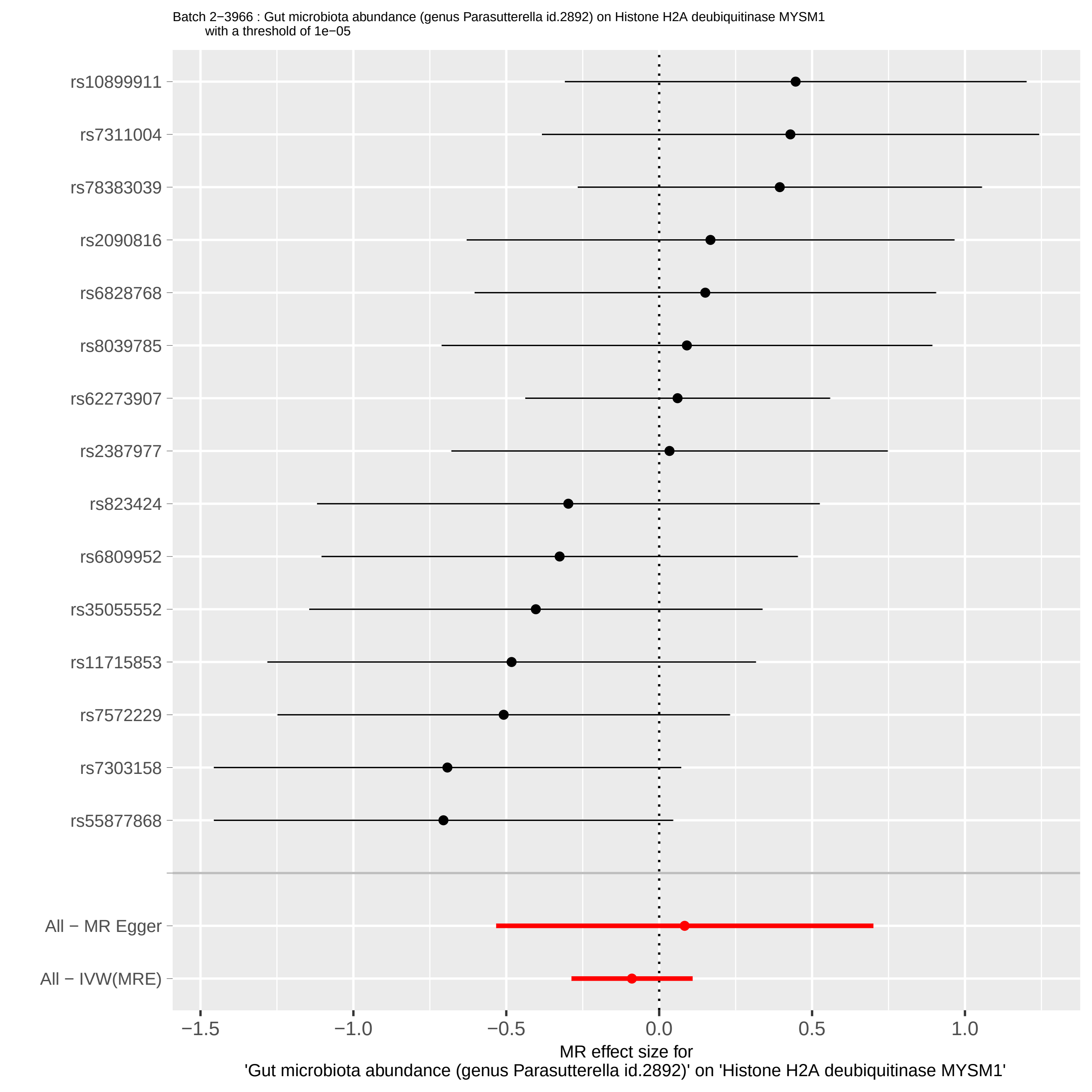

Batch 2−3966 : Gut microbiota abundance (genus Parasutterella id.2892) on Histone H2A deubiquitinase MYSM1
with a threshold of 1e−05
rs10899911
rs7311004
rs78383039
rs2090816
rs6828768
rs8039785
rs62273907
rs2387977
rs823424
rs6809952
rs35055552
rs11715853
rs7572229
rs7303158
rs55877868
All − MR Egger
All − IVW(MRE)
−1.5
−1.0
−0.5
0.0
0.5
1.0
MR effect size for
'Gut microbiota abundance (genus Parasutterella id.2892)' on 'Histone H2A deubiquitinase MYSM1'

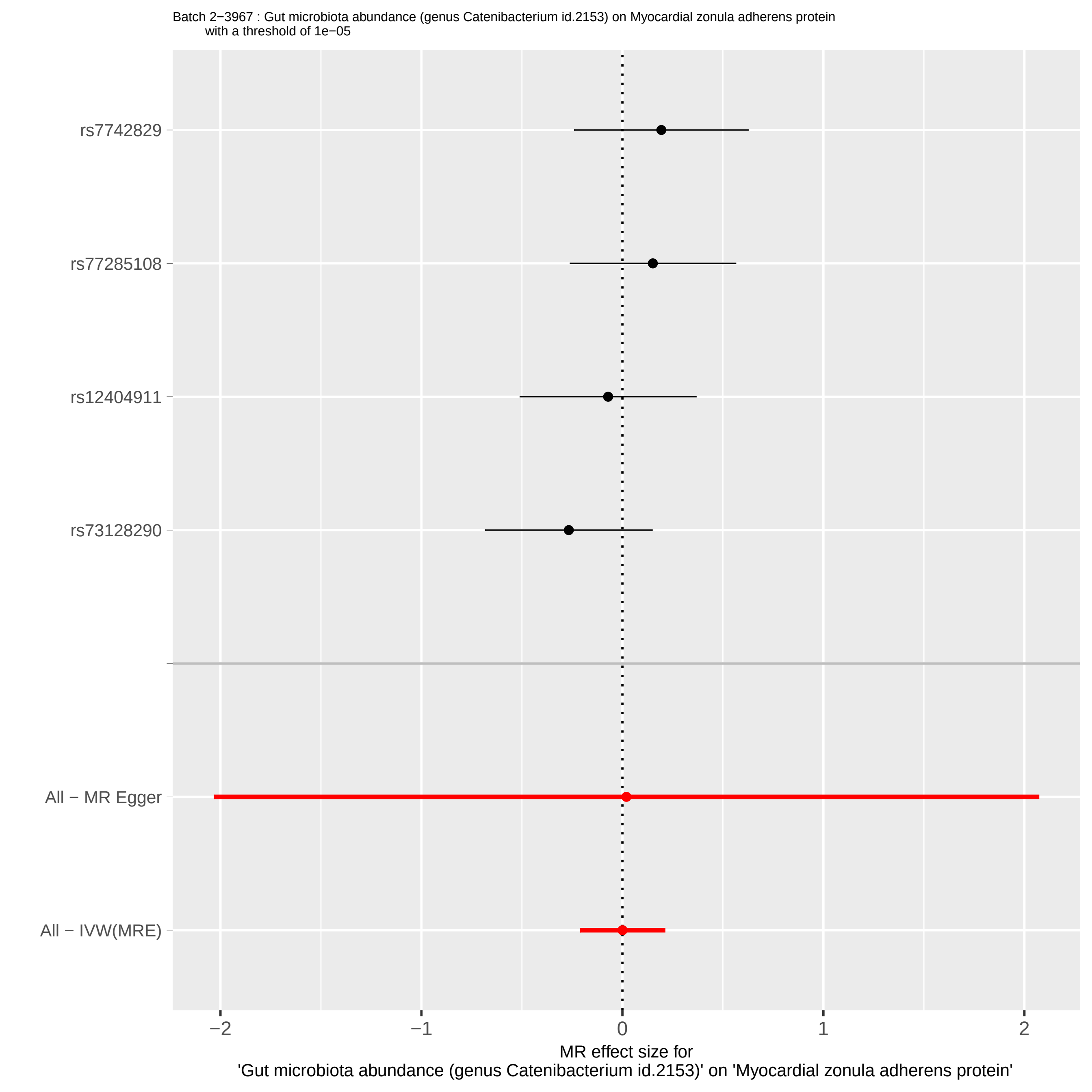
Batch 2−3967 : Gut microbiota abundance (genus Catenibacterium id.2153) on Myocardial zonula adherens protein
with a threshold of 1e−05
rs7742829
rs77285108
rs12404911
rs73128290
All − MR Egger
All − IVW(MRE)
−2
−1
0
1
2
MR effect size for
'Gut microbiota abundance (genus Catenibacterium id.2153)' on 'Myocardial zonula adherens protein'

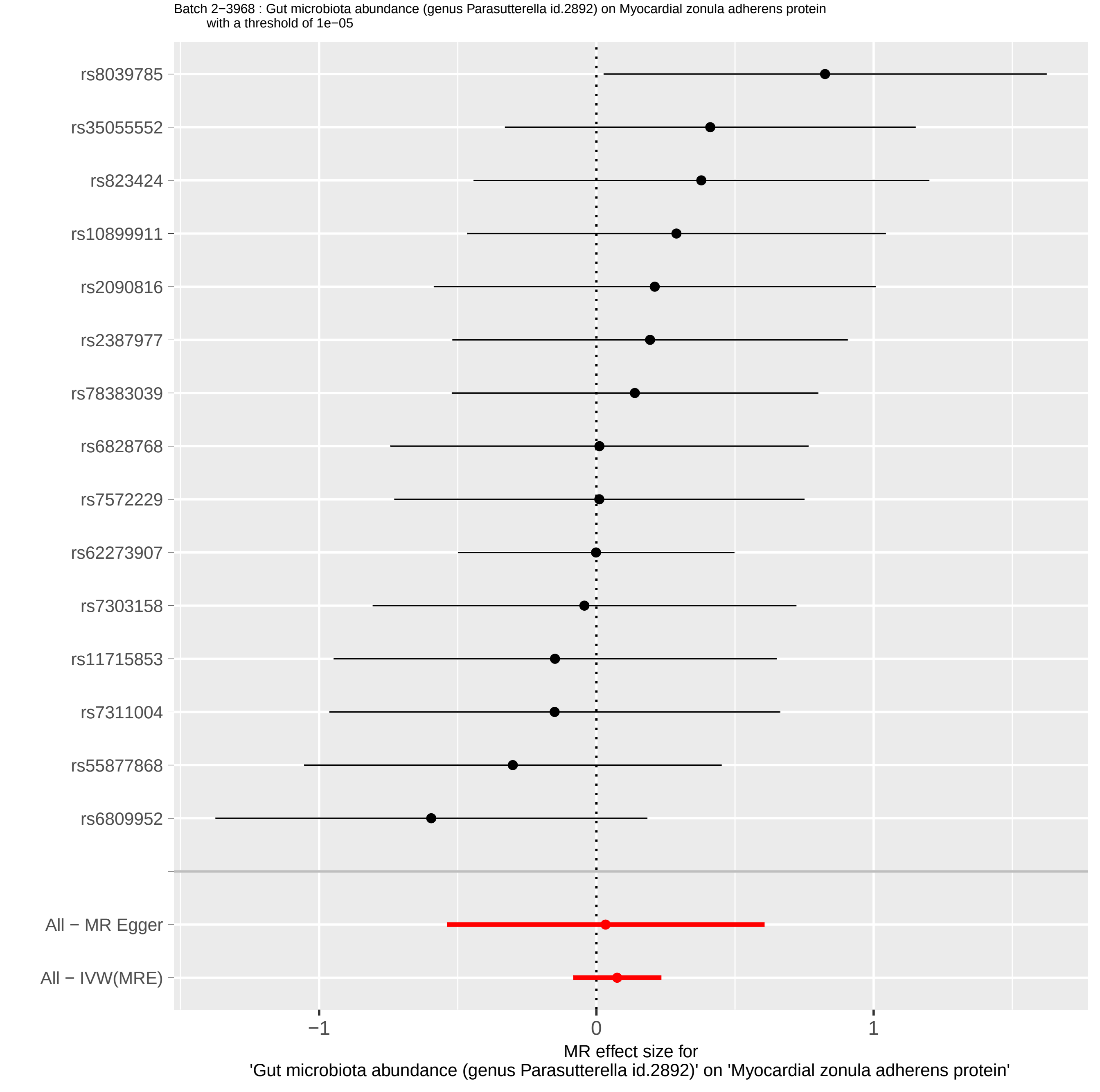

Batch 2−3968 : Gut microbiota abundance (genus Parasutterella id.2892) on Myocardial zonula adherens protein
with a threshold of 1e−05
rs8039785
rs35055552
rs823424
rs10899911
rs2090816
rs2387977
rs78383039
rs6828768
rs7572229
rs62273907
rs7303158
rs11715853
rs7311004
rs55877868
rs6809952
All − MR Egger
All − IVW(MRE)
−1
0
1
MR effect size for
'Gut microbiota abundance (genus Parasutterella id.2892)' on 'Myocardial zonula adherens protein'

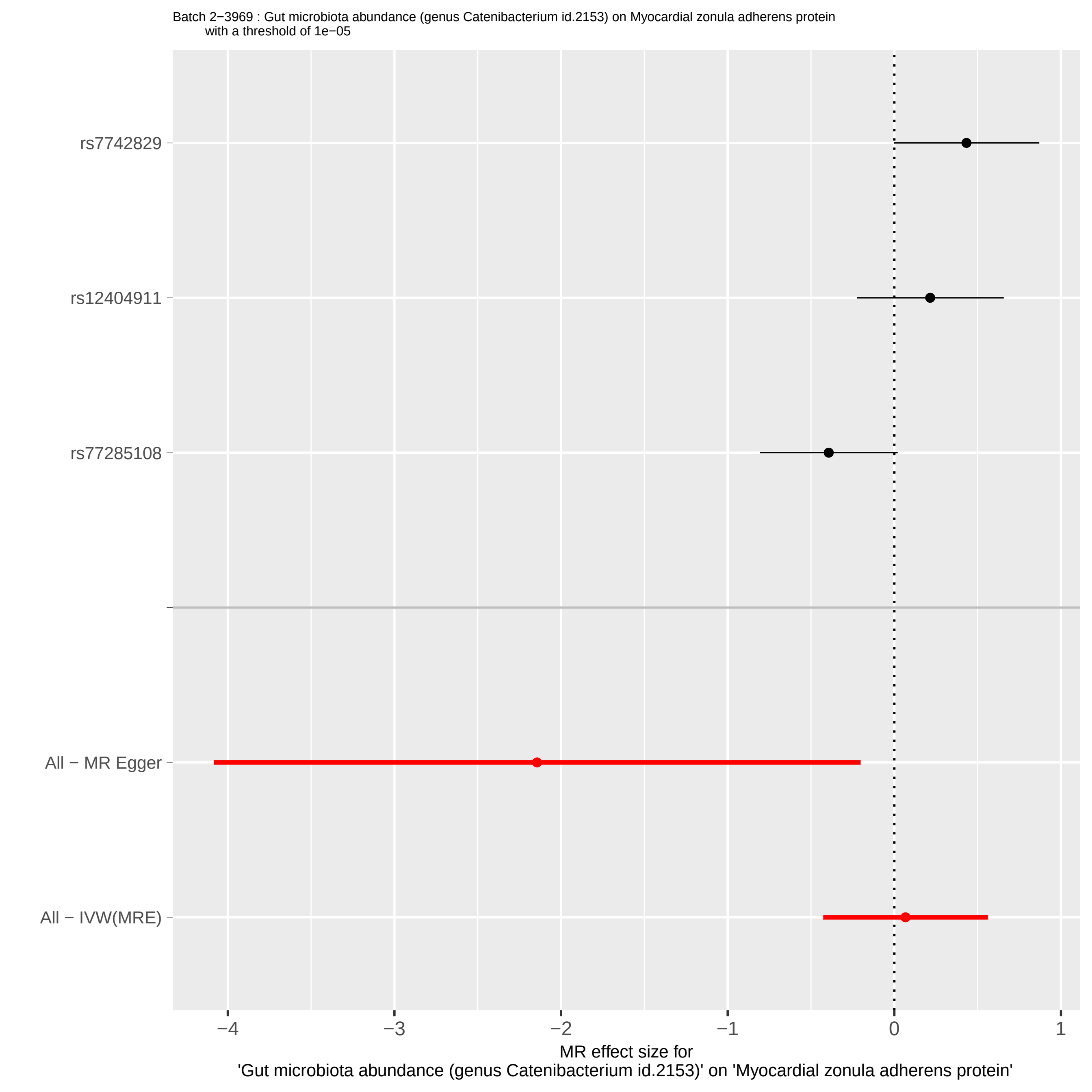
Batch 2−3969 : Gut microbiota abundance (genus Catenibacterium id.2153) on Myocardial zonula adherens protein
with a threshold of 1e−05
rs7742829
rs12404911
rs77285108
All − MR Egger
All − IVW(MRE)
−4
−3
−2
−1
0
1
MR effect size for
'Gut microbiota abundance (genus Catenibacterium id.2153)' on 'Myocardial zonula adherens protein'

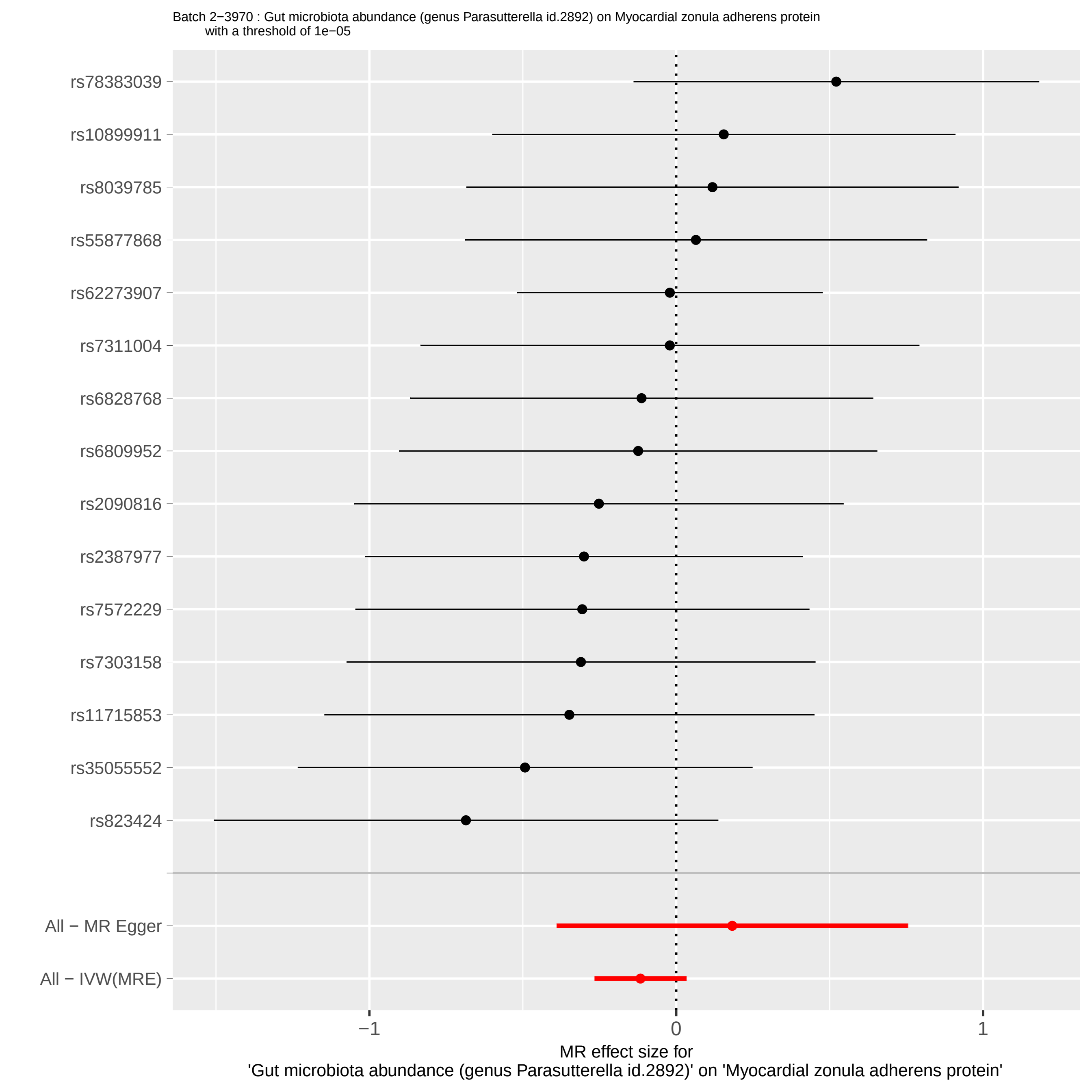

Batch 2−3970 : Gut microbiota abundance (genus Parasutterella id.2892) on Myocardial zonula adherens protein
with a threshold of 1e−05
rs78383039
rs10899911
rs8039785
rs55877868
rs62273907
rs7311004
rs6828768
rs6809952
rs2090816
rs2387977
rs7572229
rs7303158
rs11715853
rs35055552
rs823424
All − MR Egger
All − IVW(MRE)
−1
0
1
MR effect size for
'Gut microbiota abundance (genus Parasutterella id.2892)' on 'Myocardial zonula adherens protein'

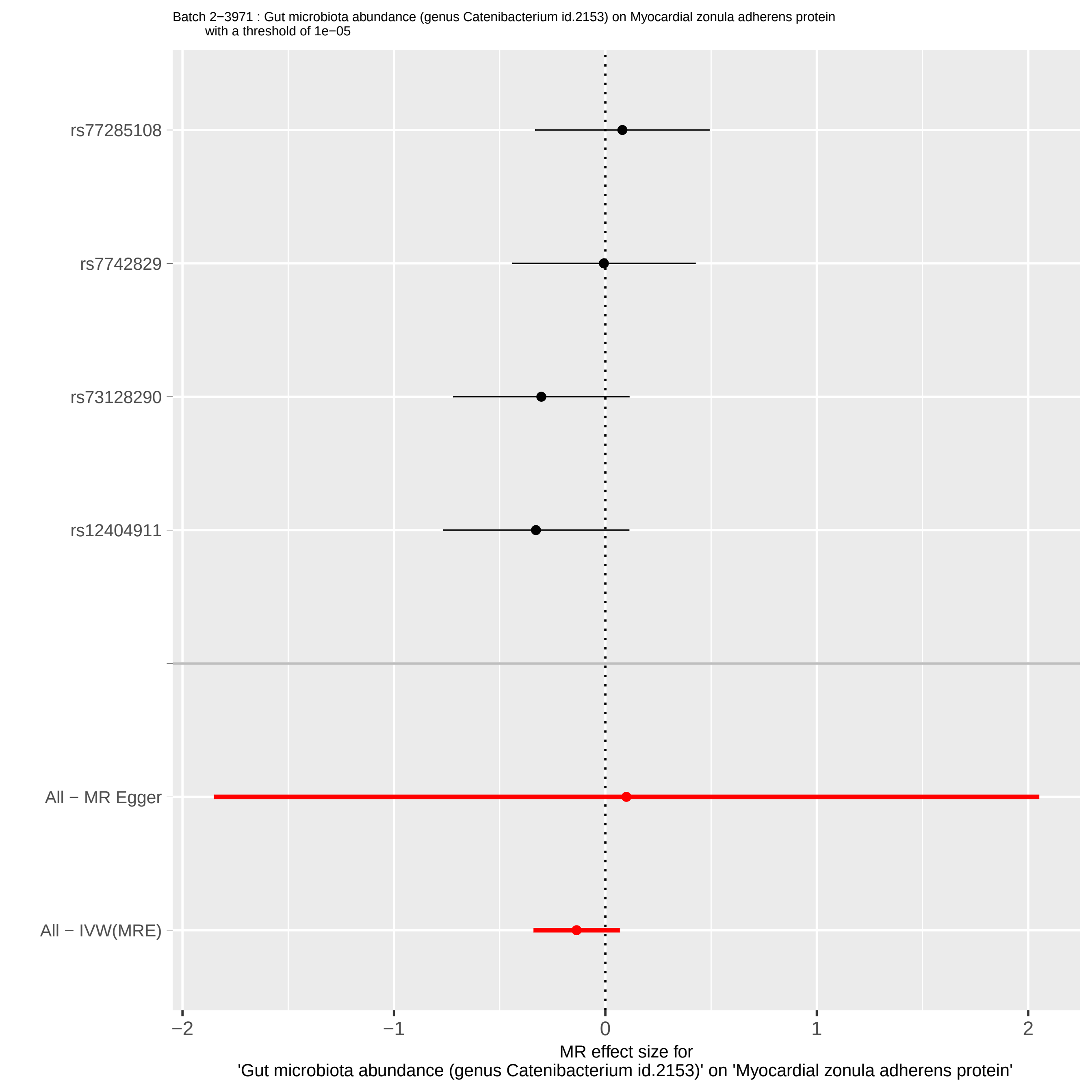
Batch 2−3971 : Gut microbiota abundance (genus Catenibacterium id.2153) on Myocardial zonula adherens protein
with a threshold of 1e−05
rs77285108
rs7742829
rs73128290
rs12404911
All − MR Egger
All − IVW(MRE)
−2
−1
0
1
2
MR effect size for
'Gut microbiota abundance (genus Catenibacterium id.2153)' on 'Myocardial zonula adherens protein'

Batch 2−3972 : Gut microbiota abundance (genus Parasutterella id.2892) on Myocardial zonula adherens protein
with a threshold of 1e−05

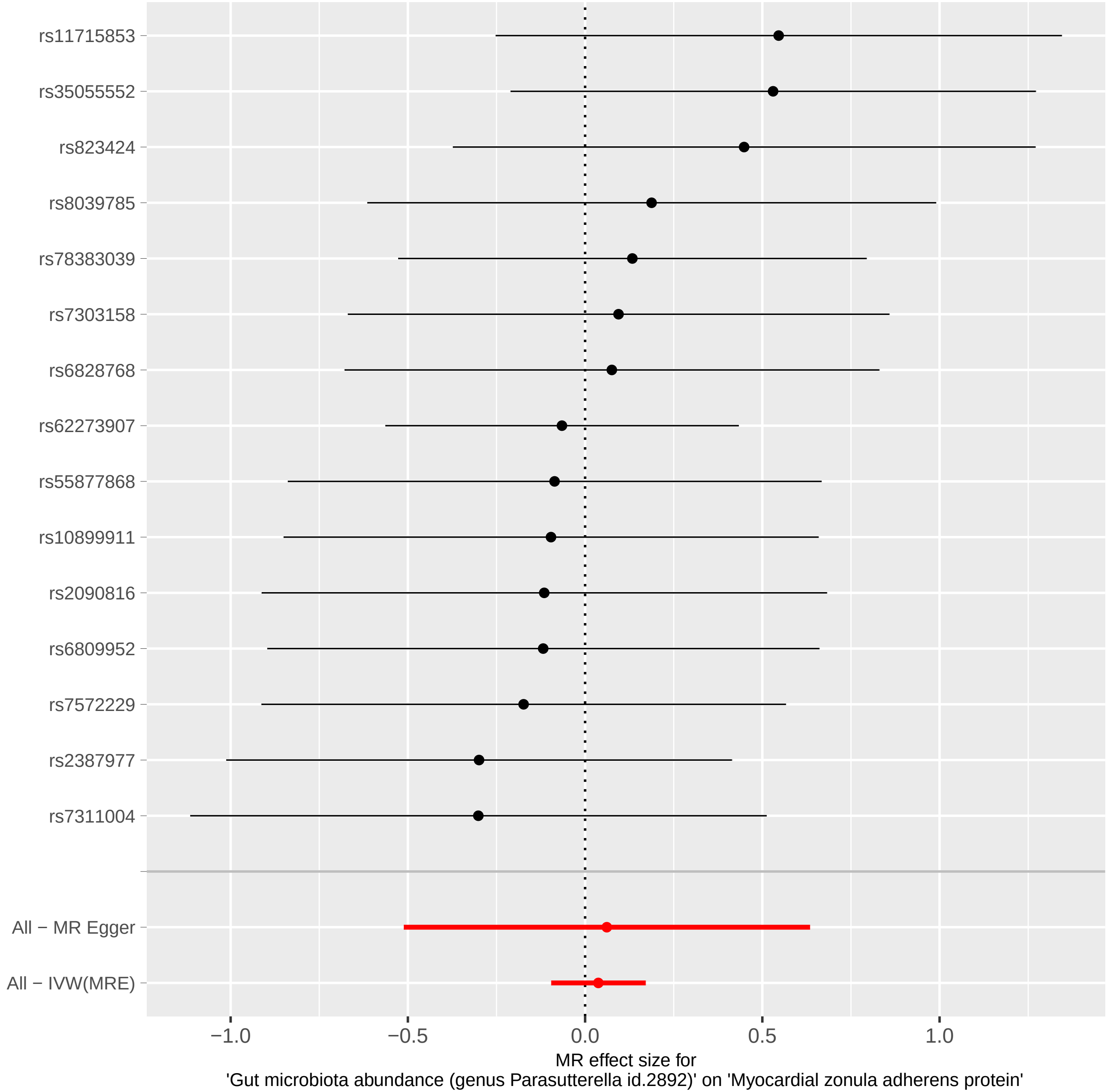

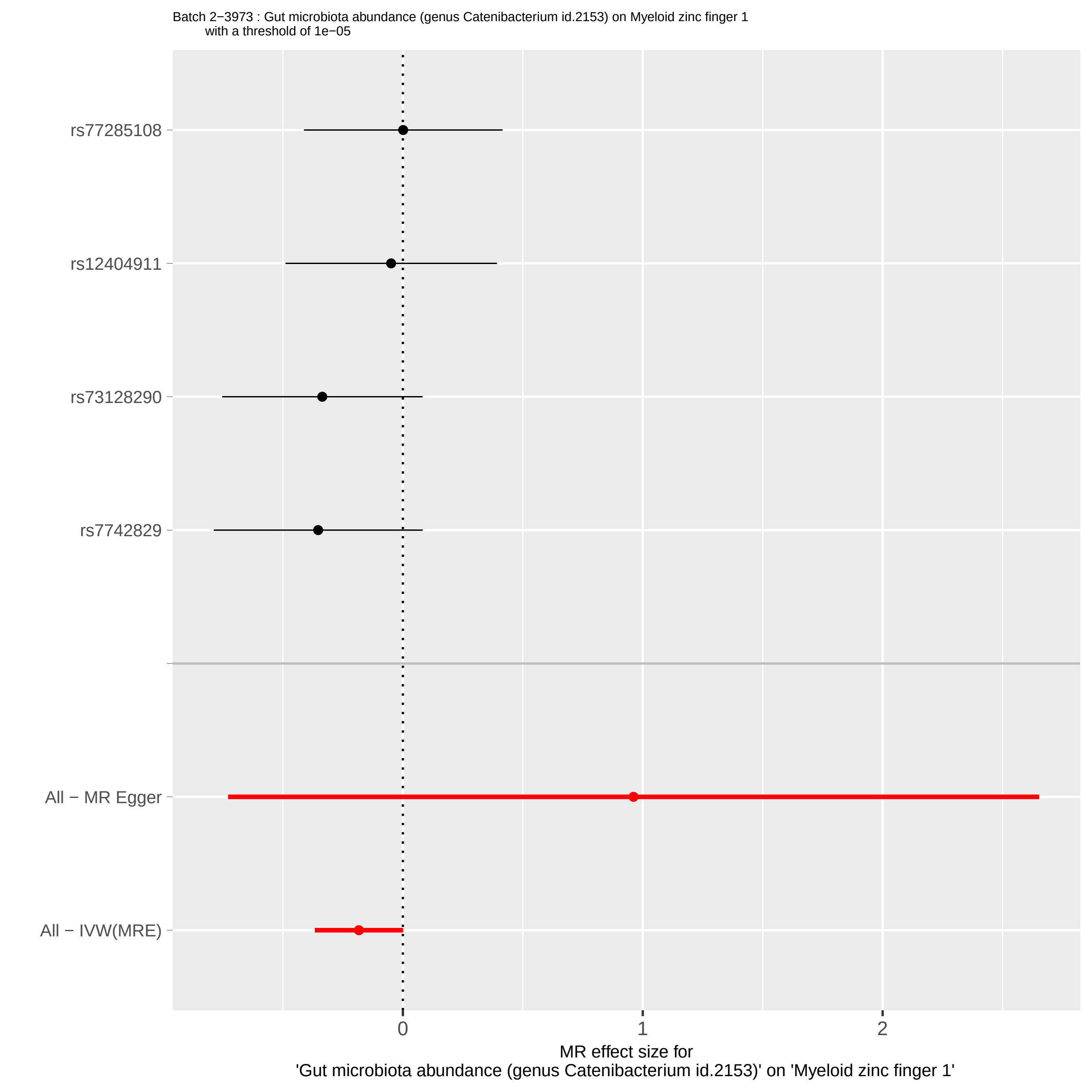

Batch 2−3973 : Gut microbiota abundance (genus Catenibacterium id.2153) on Myeloid zinc finger 1
with a threshold of 1e−05
rs77285108
rs12404911
rs73128290
rs7742829
All − MR Egger
All − IVW(MRE)
0
1
2
MR effect size for
'Gut microbiota abundance (genus Catenibacterium id.2153)' on 'Myeloid zinc finger 1'

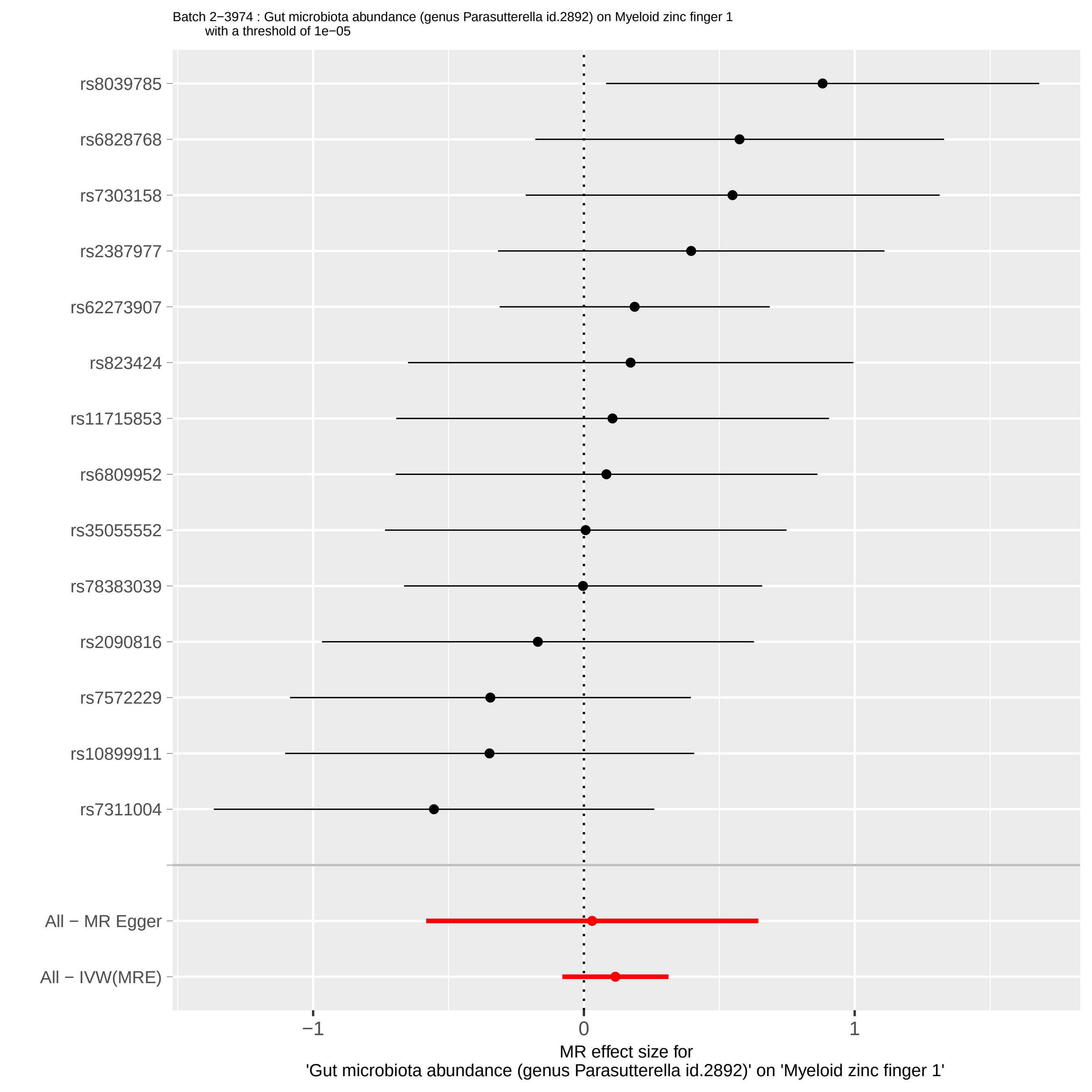

Batch 2−3974 : Gut microbiota abundance (genus Parasutterella id.2892) on Myeloid zinc finger 1
with a threshold of 1e−05
rs8039785
rs6828768
rs7303158
rs2387977
rs62273907
rs823424
rs11715853
rs6809952
rs35055552
rs78383039
rs2090816
rs7572229
rs10899911
rs7311004
All − MR Egger
All − IVW(MRE)
−1
0
1
MR effect size for
'Gut microbiota abundance (genus Parasutterella id.2892)' on 'Myeloid zinc finger 1'

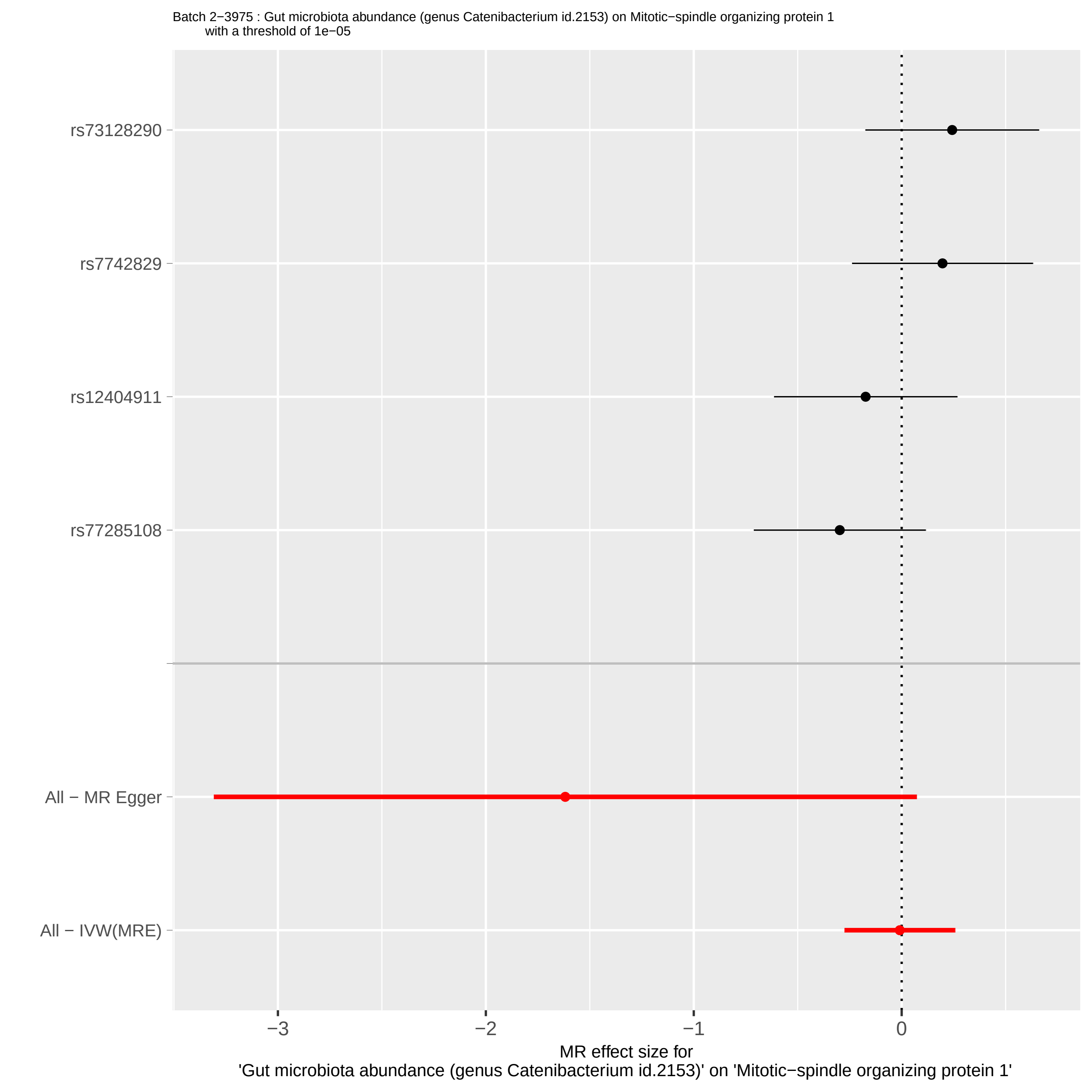
Batch 2−3975 : Gut microbiota abundance (genus Catenibacterium id.2153) on Mitotic−spindle organizing protein 1
with a threshold of 1e−05
rs73128290
rs7742829
rs12404911
rs77285108
All − MR Egger
All − IVW(MRE)
−3
−2
−1
0
MR effect size for
'Gut microbiota abundance (genus Catenibacterium id.2153)' on 'Mitotic−spindle organizing protein 1'

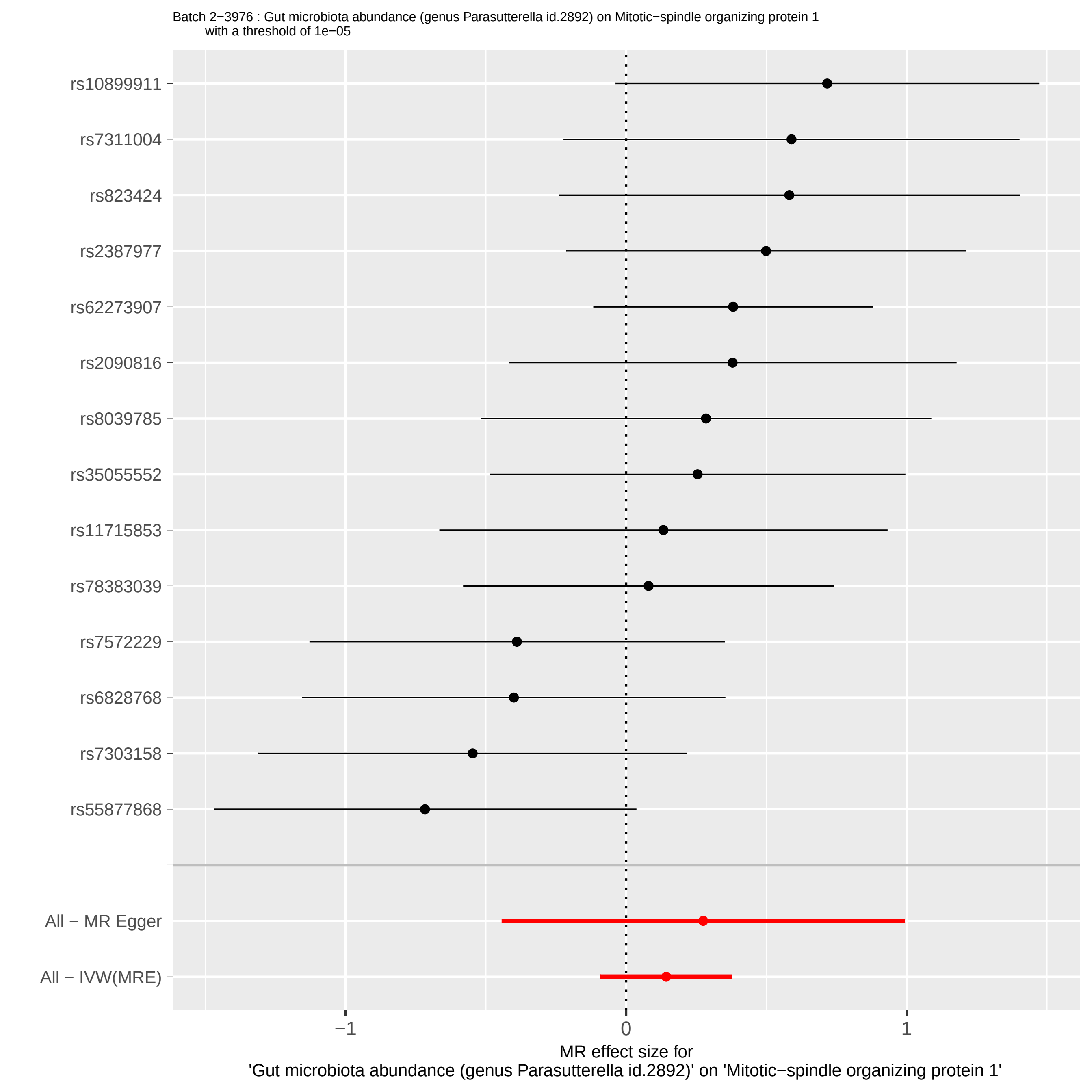

Batch 2−3976 : Gut microbiota abundance (genus Parasutterella id.2892) on Mitotic−spindle organizing protein 1
with a threshold of 1e−05
rs10899911
rs7311004
rs823424
rs2387977
rs62273907
rs2090816
rs8039785
rs35055552
rs11715853
rs78383039
rs7572229
rs6828768
rs7303158
rs55877868
All − MR Egger
All − IVW(MRE)
−1
0
1
MR effect size for
'Gut microbiota abundance (genus Parasutterella id.2892)' on 'Mitotic−spindle organizing protein 1'

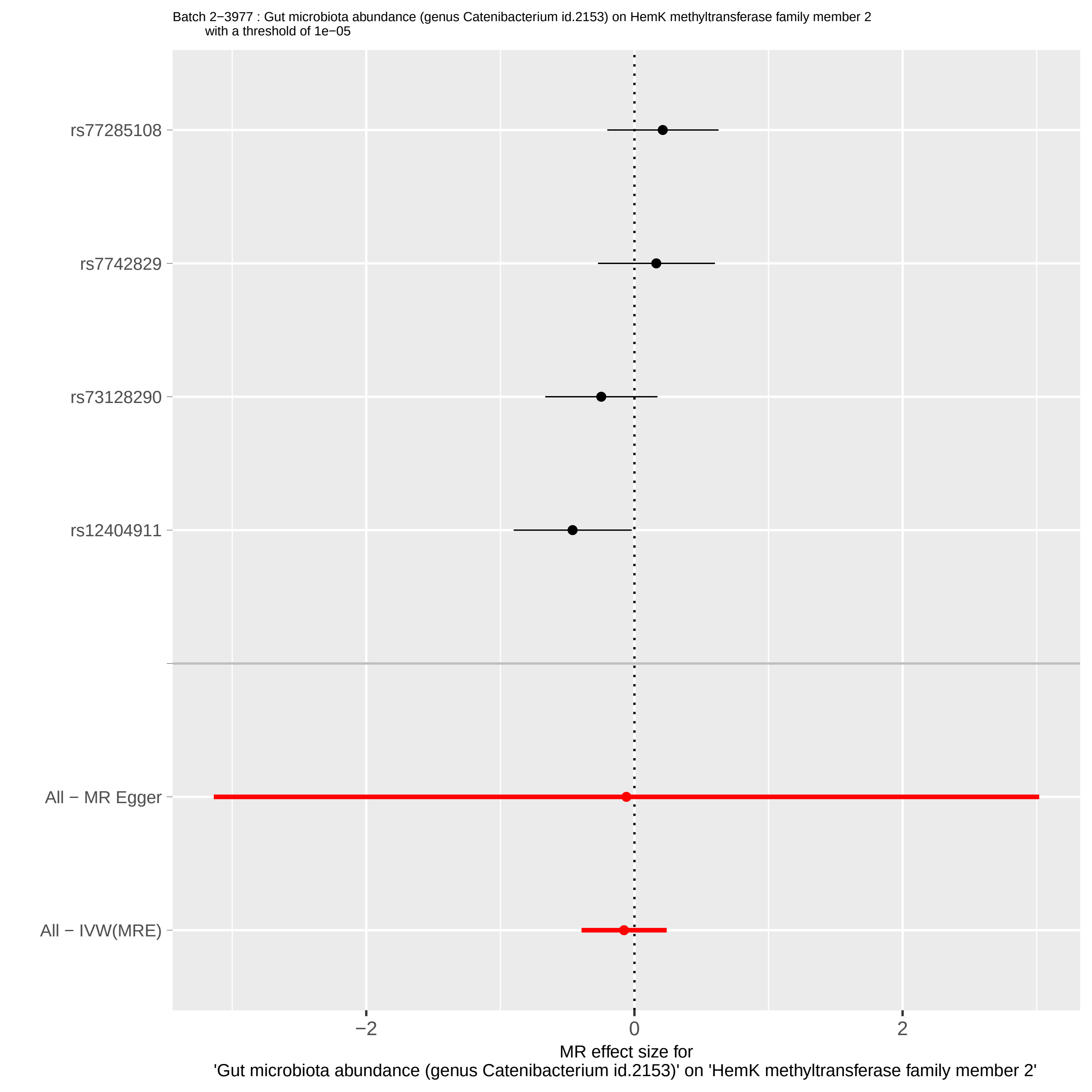
Batch 2−3977 : Gut microbiota abundance (genus Catenibacterium id.2153) on HemK methyltransferase family member 2
with a threshold of 1e−05
rs77285108
rs7742829
rs73128290
rs12404911
All − MR Egger
All − IVW(MRE)
−2
0
2
MR effect size for
'Gut microbiota abundance (genus Catenibacterium id.2153)' on 'HemK methyltransferase family member 2'

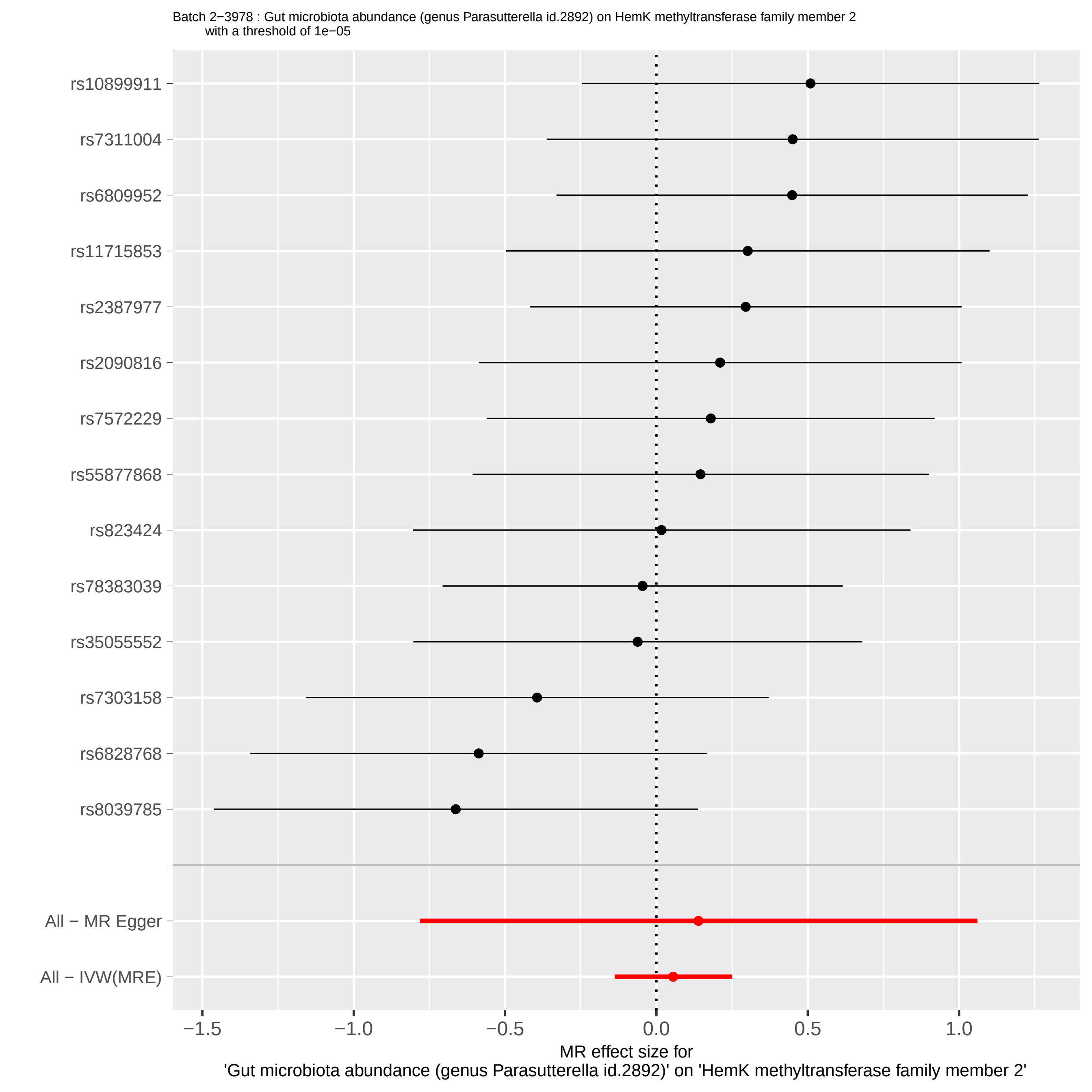
Batch 2−3978 : Gut microbiota abundance (genus Parasutterella id.2892) on HemK methyltransferase family member 2
with a threshold of 1e−05
rs10899911
rs7311004
rs6809952
rs11715853
rs2387977
rs2090816
rs7572229
rs55877868
rs823424
rs78383039
rs35055552
rs7303158
rs6828768
rs8039785
All − MR Egger
All − IVW(MRE)
−1.5
−1.0
−0.5
0.0
0.5
1.0
MR effect size for
'Gut microbiota abundance (genus Parasutterella id.2892)' on 'HemK methyltransferase family member 2'

Batch 2−3979 : Gut microbiota abundance (genus Catenibacterium id.2153) on N−acylethanolamine−hydrolyzing acid amidase
with a threshold of 1e−05

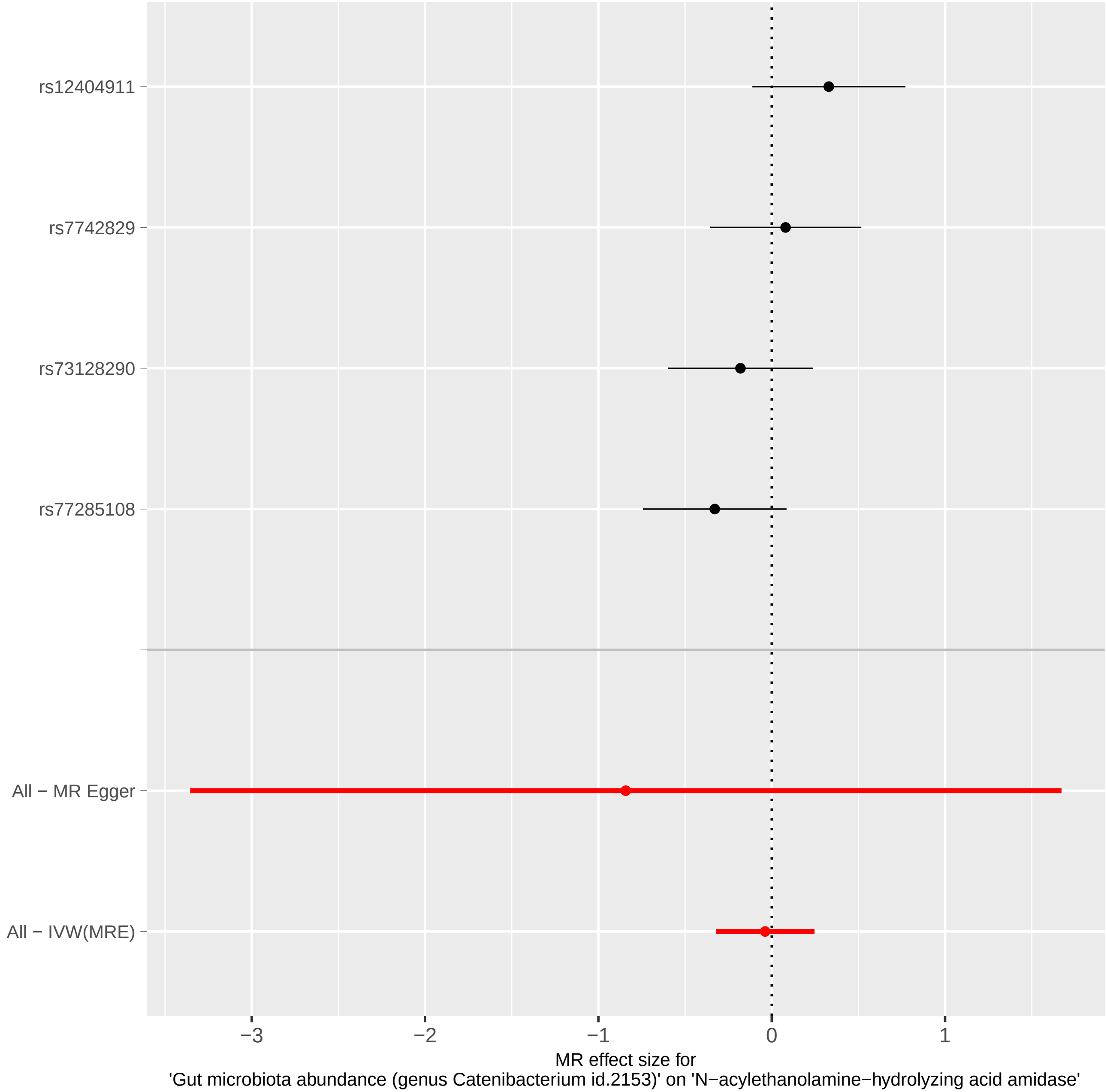

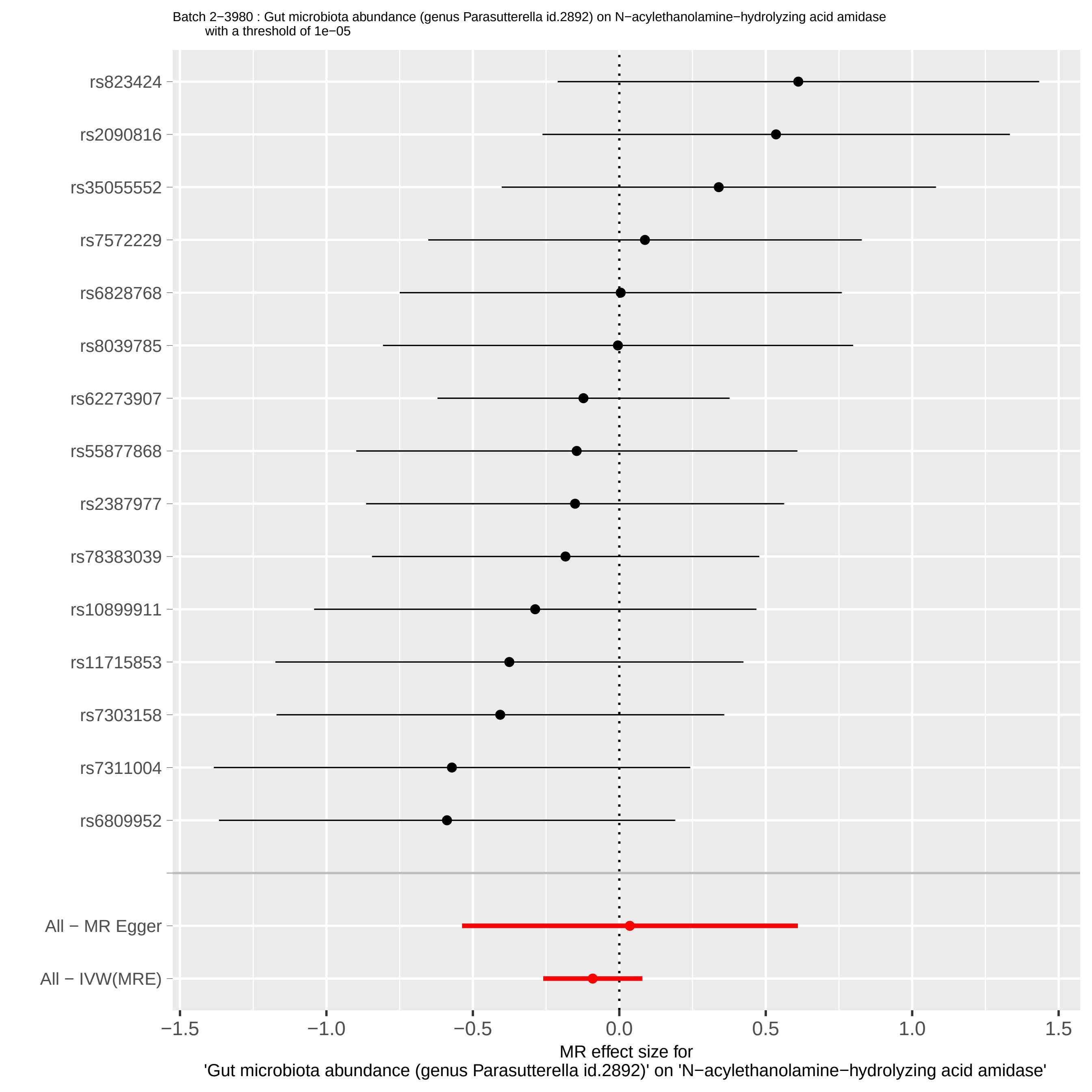
Batch 2−3980 : Gut microbiota abundance (genus Parasutterella id.2892) on N−acylethanolamine−hydrolyzing acid amidase with a threshold of 1e−05
rs823424
rs2090816
rs35055552
rs7572229
rs6828768
rs8039785
rs62273907
rs55877868
rs2387977
rs78383039
rs10899911
rs11715853
rs7303158
rs7311004
rs6809952
All − MR Egger
All − IVW(MRE)
−1.5
−1.0
−0.5
0.0
0.5
1.0
1.5
MR effect size for
'Gut microbiota abundance (genus Parasutterella id.2892)' on 'N−acylethanolamine−hydrolyzing acid amidase'

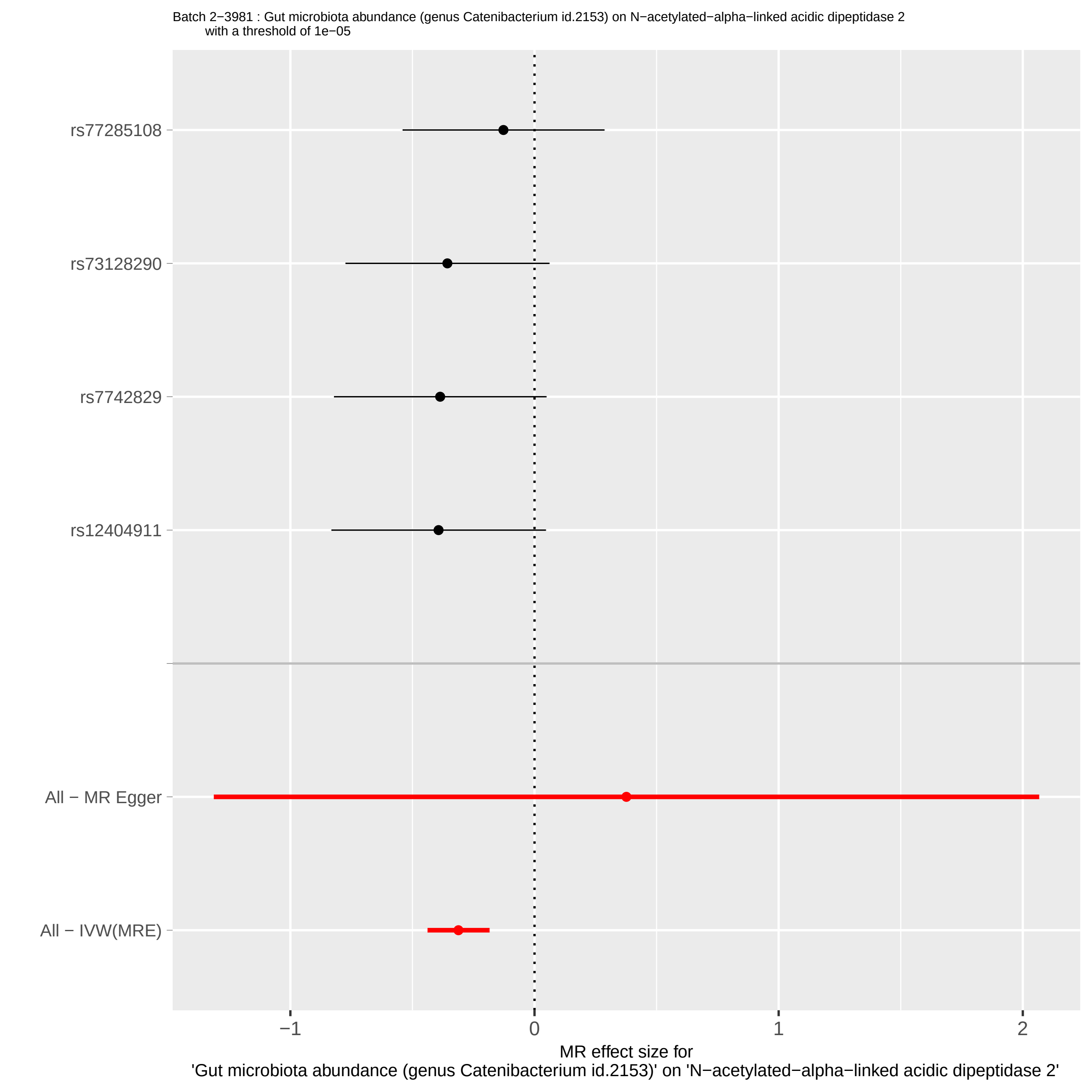

Batch 2−3981 : Gut microbiota abundance (genus Catenibacterium id.2153) on N−acetylated−alpha−linked acidic dipeptidase 2
with a threshold of 1e−05
rs77285108
rs73128290
rs7742829
rs12404911
All − MR Egger
All − IVW(MRE)
−1
0
1
2
MR effect size for
'Gut microbiota abundance (genus Catenibacterium id.2153)' on 'N−acetylated−alpha−linked acidic dipeptidase 2'

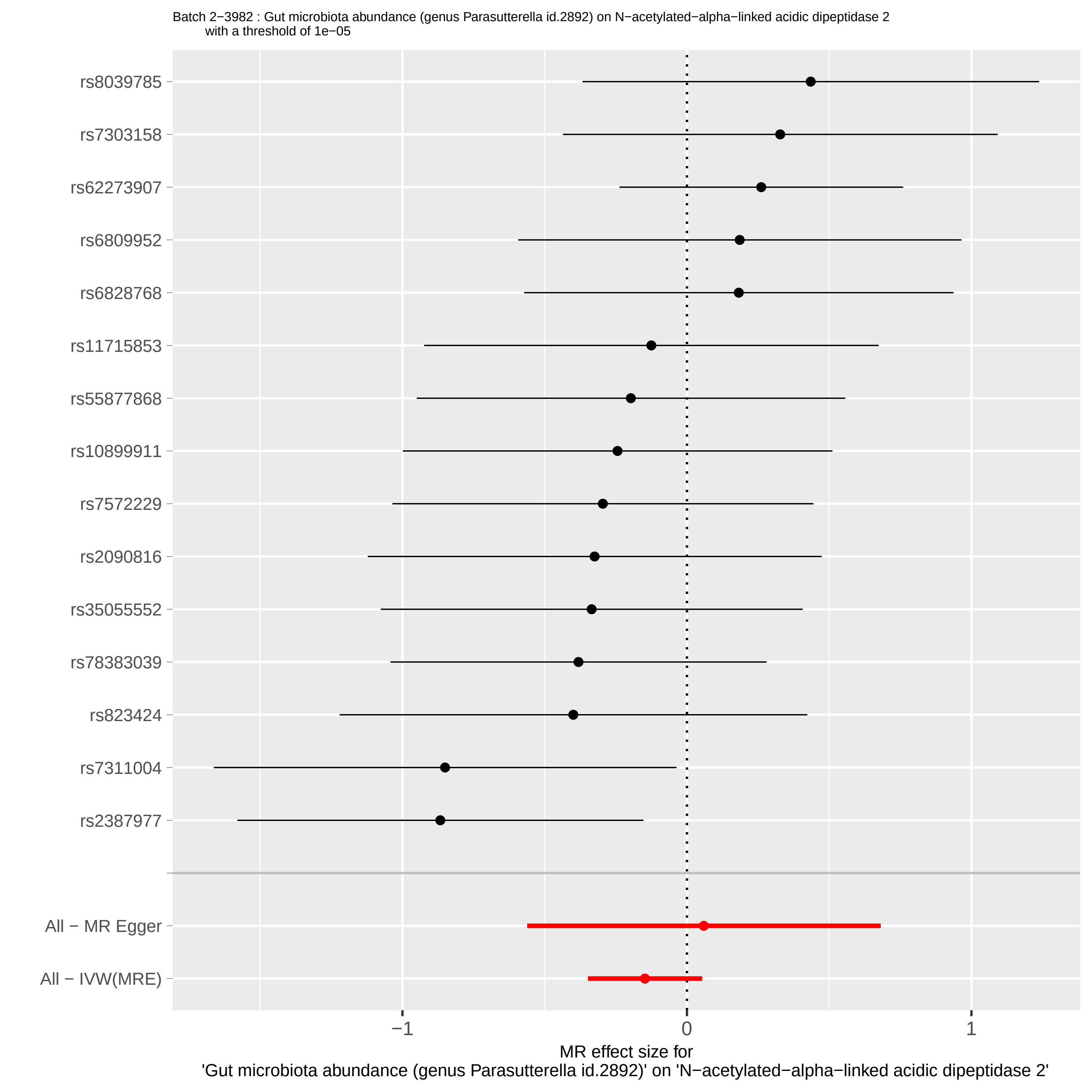

Batch 2−3982 : Gut microbiota abundance (genus Parasutterella id.2892) on N−acetylated−alpha−linked acidic dipeptidase 2
with a threshold of 1e−05
rs8039785
rs7303158
rs62273907
rs6809952
rs6828768
rs11715853
rs55877868
rs10899911
rs7572229
rs2090816
rs35055552
rs78383039
rs823424
rs7311004
rs2387977
All − MR Egger
All − IVW(MRE)
−1
0
1
MR effect size for
'Gut microbiota abundance (genus Parasutterella id.2892)' on 'N−acetylated−alpha−linked acidic dipeptidase 2'

Batch 2−3983 : Gut microbiota abundance (genus Catenibacterium id.2153) on NGFI−A−binding protein 1
with a threshold of 1e−05

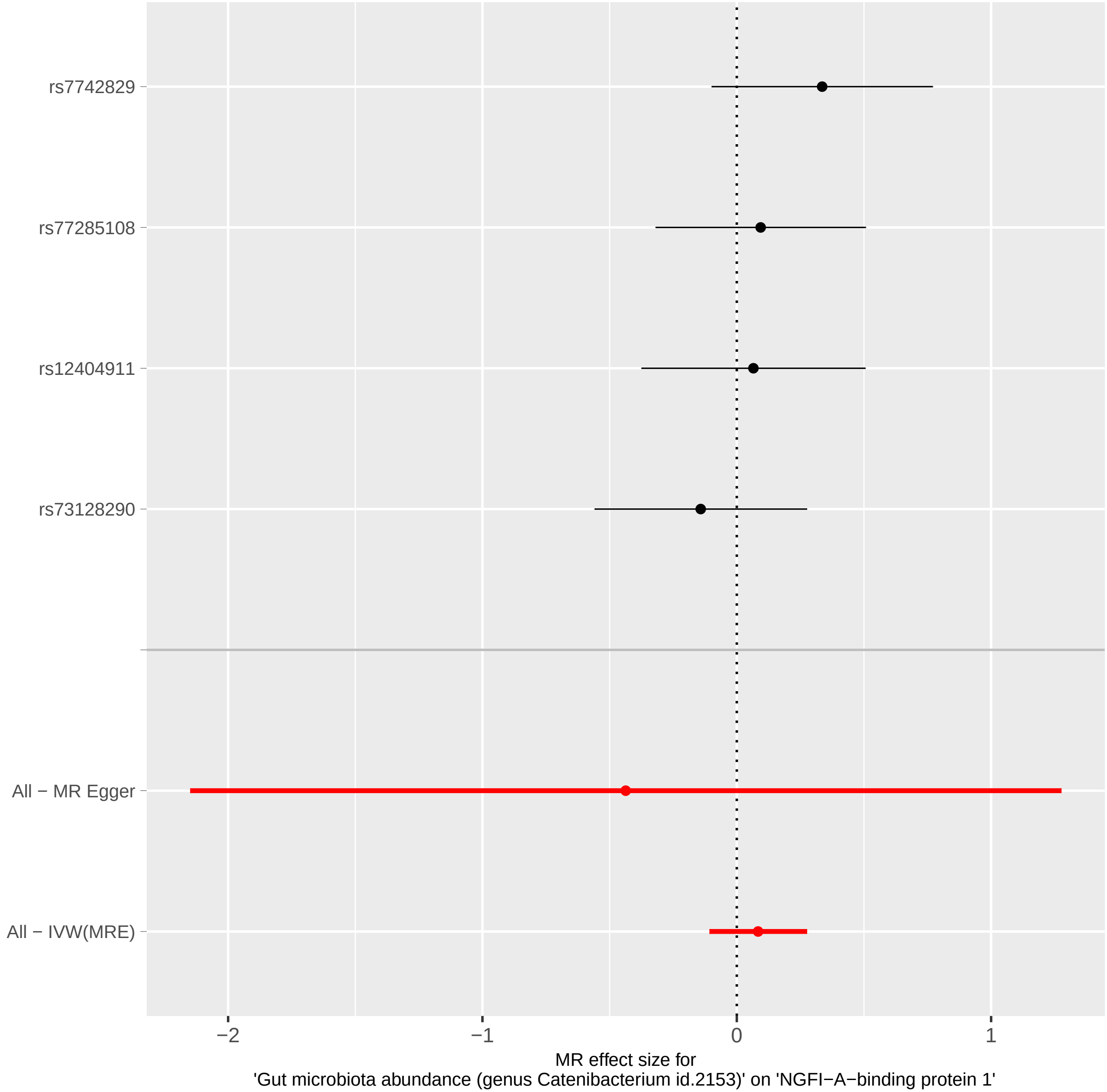

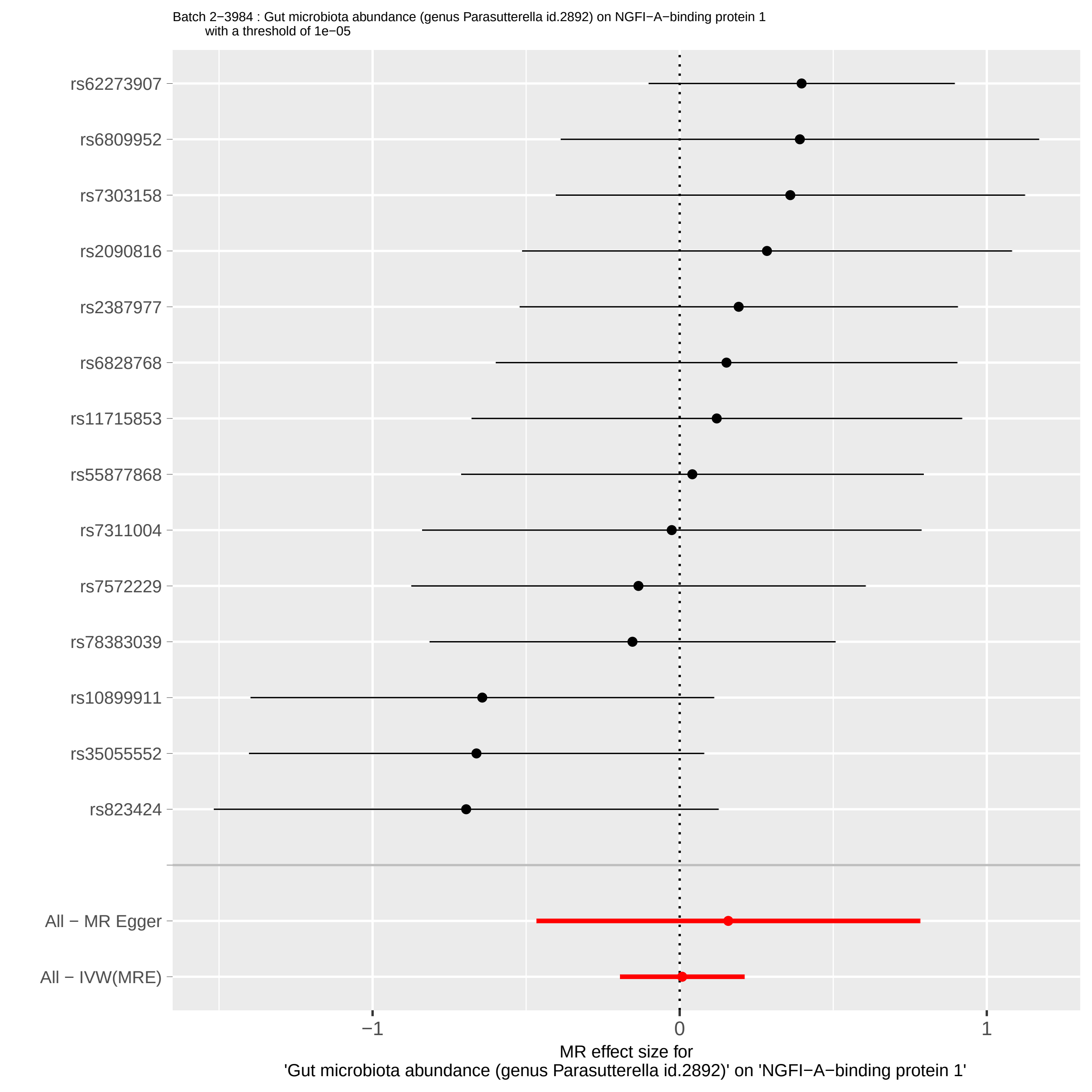

Batch 2−3984 : Gut microbiota abundance (genus Parasutterella id.2892) on NGFI−A−binding protein 1
with a threshold of 1e−05
rs62273907
rs6809952
rs7303158
rs2090816
rs2387977
rs6828768
rs11715853
rs55877868
rs7311004
rs7572229
rs78383039
rs10899911
rs35055552
rs823424
All − MR Egger
All − IVW(MRE)
−1
0
1
MR effect size for
'Gut microbiota abundance (genus Parasutterella id.2892)' on 'NGFI−A−binding protein 1'

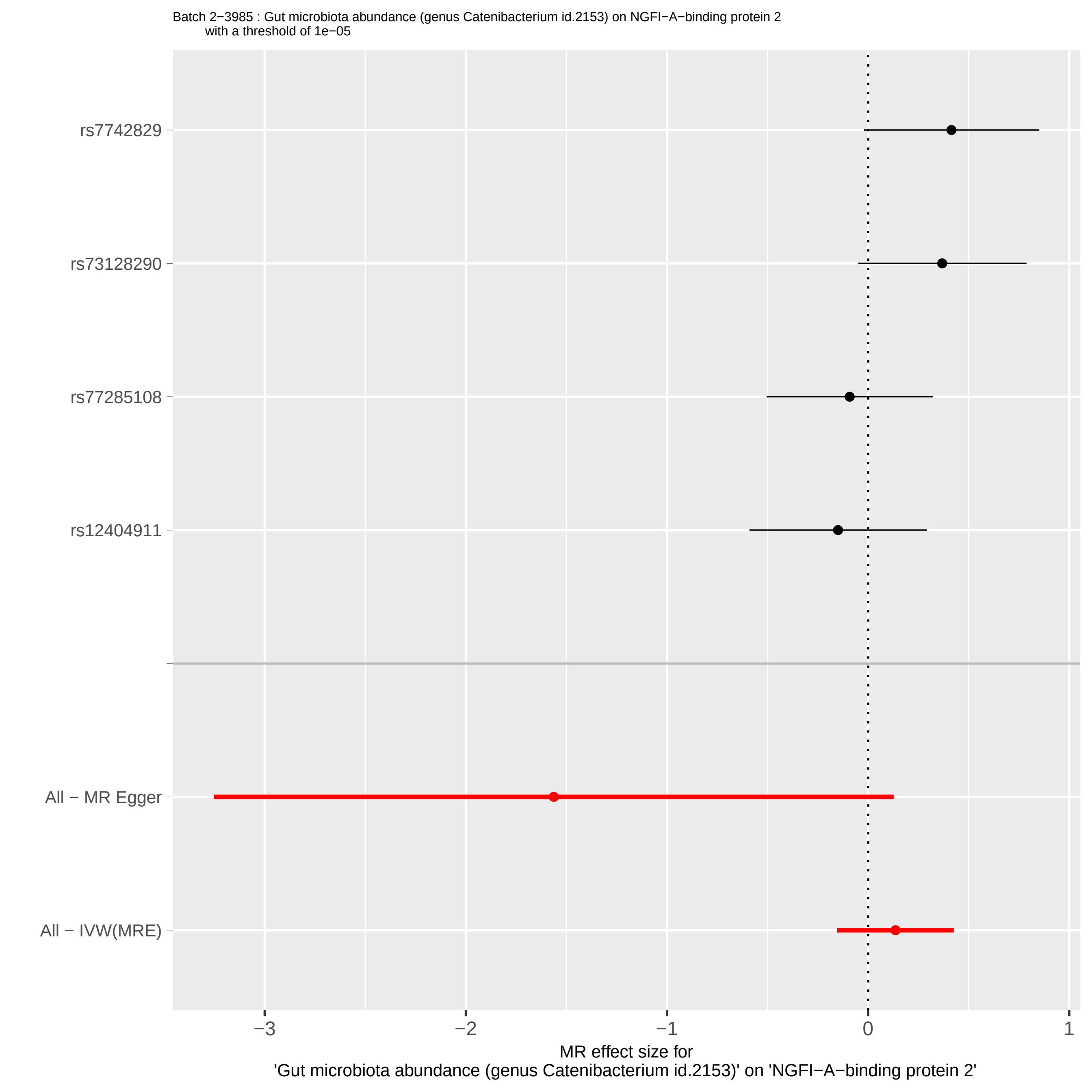

Batch 2−3985 : Gut microbiota abundance (genus Catenibacterium id.2153) on NGFI−A−binding protein 2
with a threshold of 1e−05
rs7742829
rs73128290
rs77285108
rs12404911
All − MR Egger
All − IVW(MRE)
−3
−2
−1
0
1
MR effect size for
'Gut microbiota abundance (genus Catenibacterium id.2153)' on 'NGFI−A−binding protein 2'

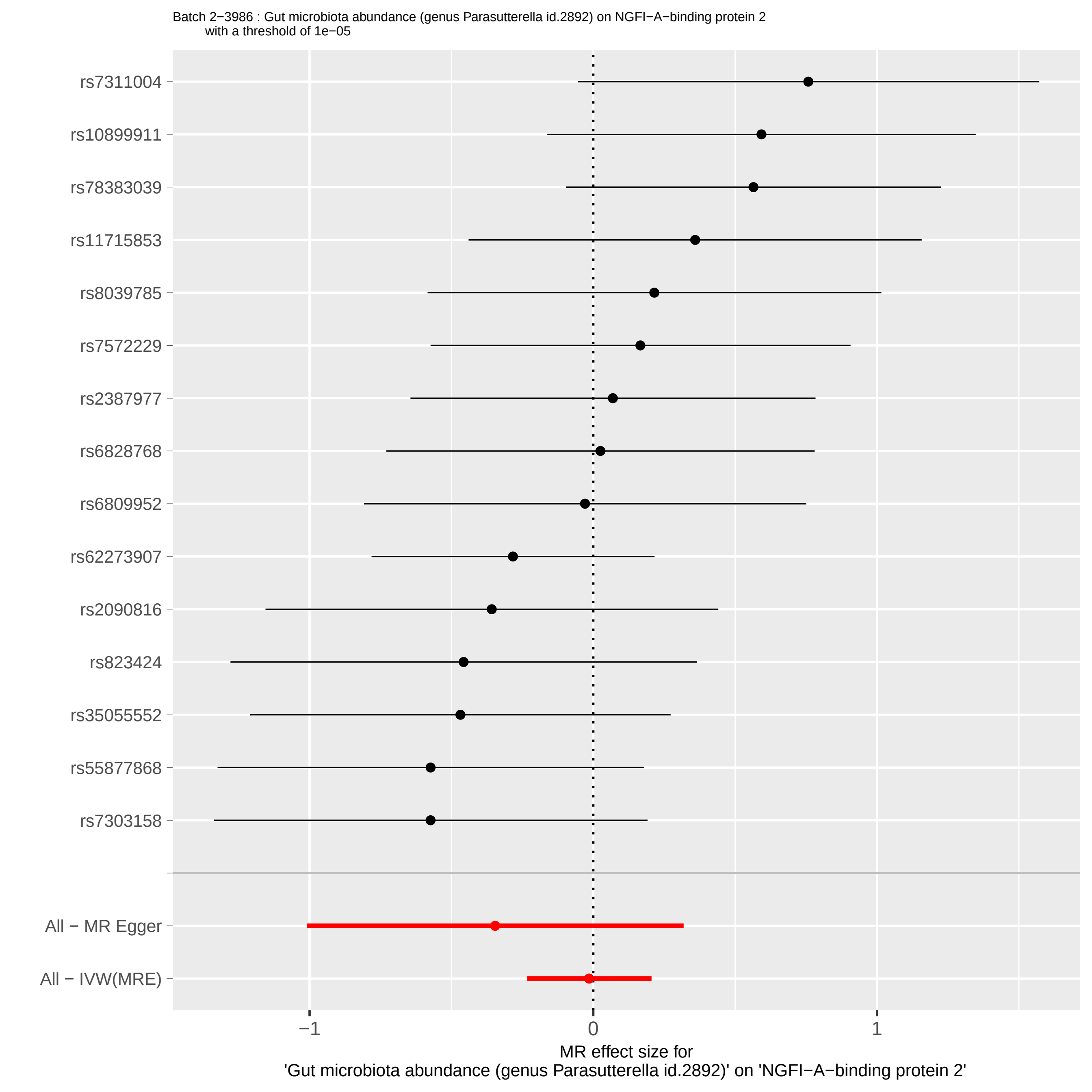

Batch 2−3986 : Gut microbiota abundance (genus Parasutterella id.2892) on NGFI−A−binding protein 2
with a threshold of 1e−05
rs7311004
rs10899911
rs78383039
rs11715853
rs8039785
rs7572229
rs2387977
rs6828768
rs6809952
rs62273907
rs2090816
rs823424
rs35055552
rs55877868
rs7303158
All − MR Egger
All − IVW(MRE)
−1
0
1
MR effect size for
'Gut microbiota abundance (genus Parasutterella id.2892)' on 'NGFI−A−binding protein 2'

Batch 2−3987 : Gut microbiota abundance (genus Catenibacterium id.2153) on NAD kinase
with a threshold of 1e−05

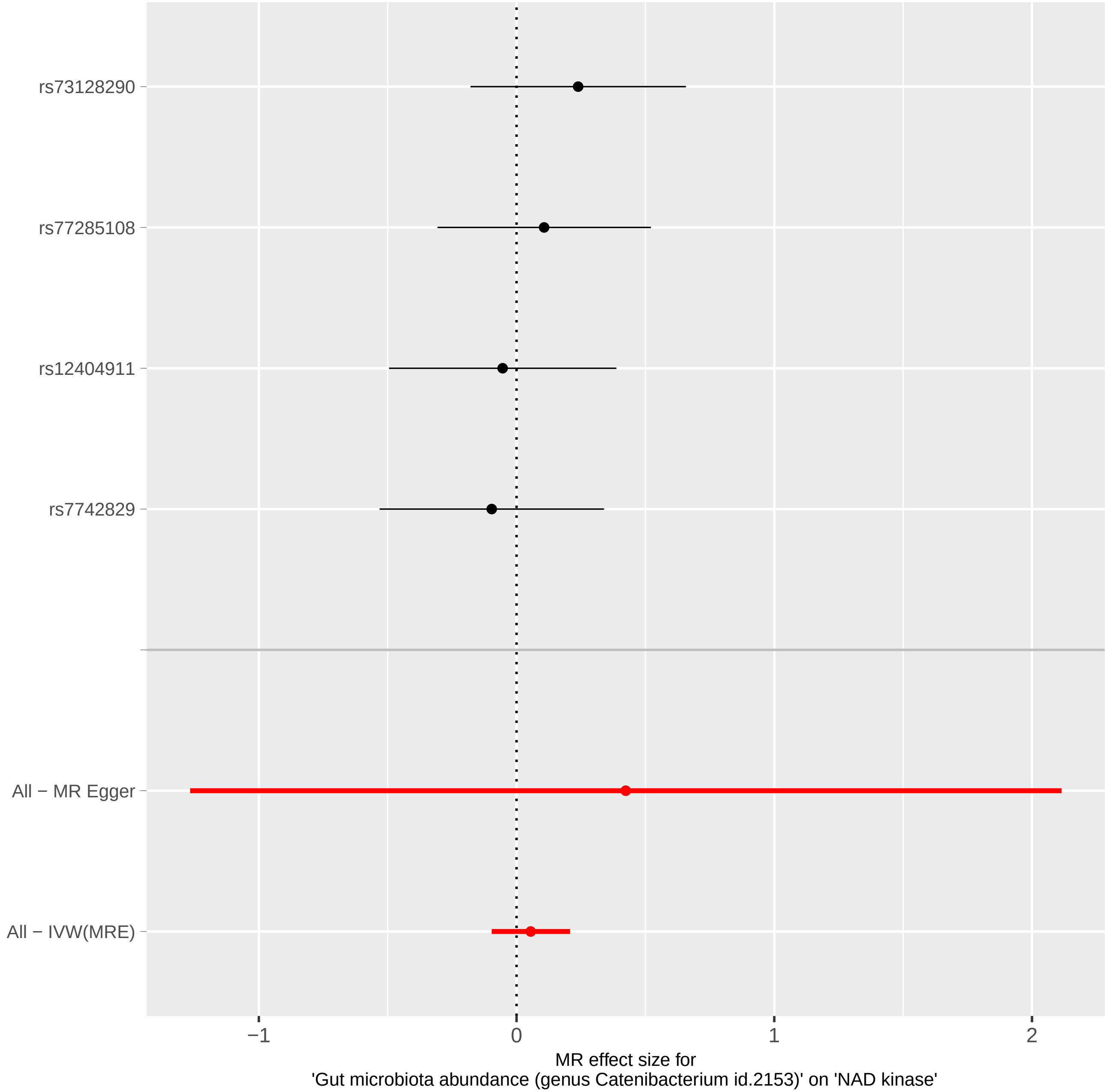

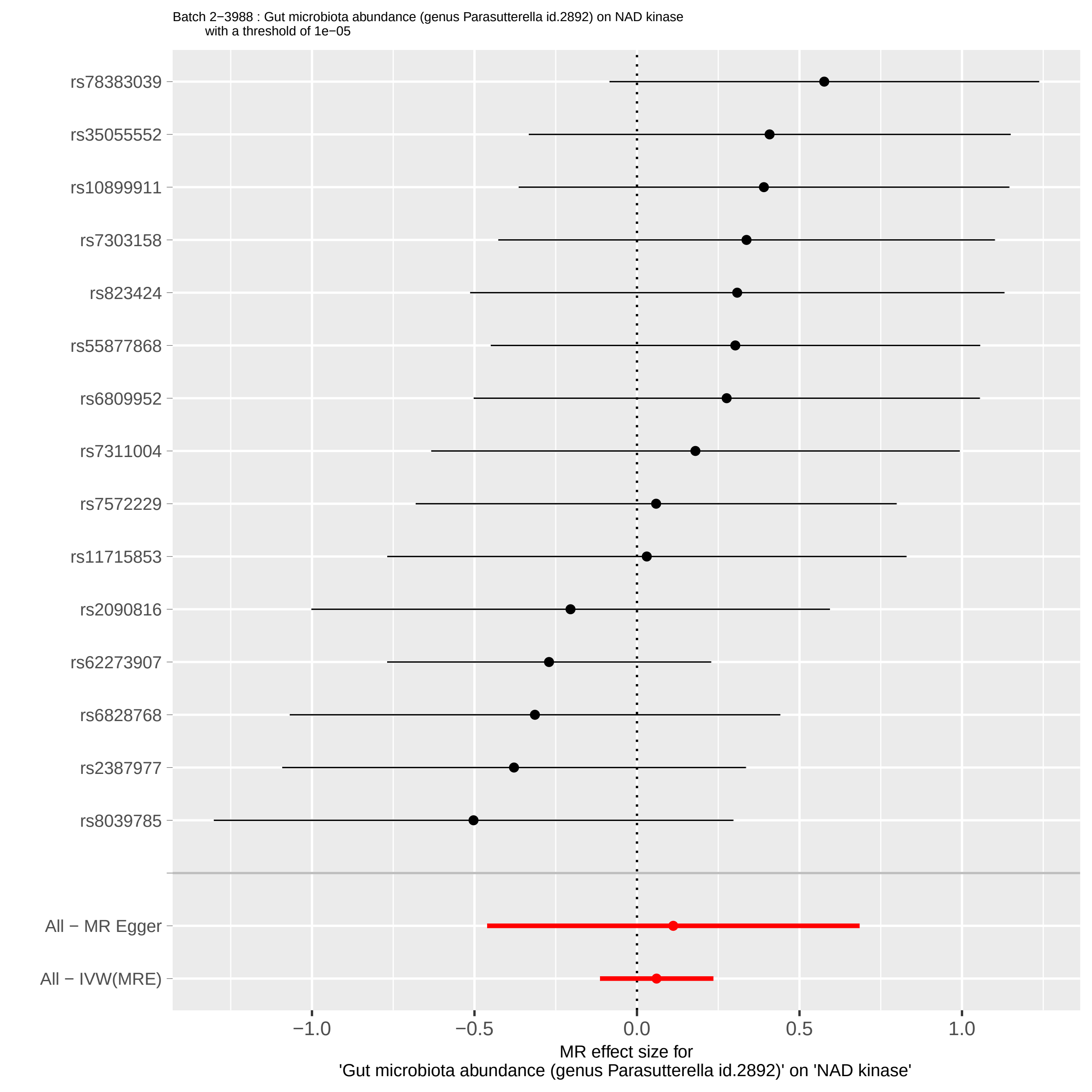

Batch 2−3988 : Gut microbiota abundance (genus Parasutterella id.2892) on NAD kinase
with a threshold of 1e−05
rs78383039
rs35055552
rs10899911
rs7303158
rs823424
rs55877868
rs6809952
rs7311004
rs7572229
rs11715853
rs2090816
rs62273907
rs6828768
rs2387977
rs8039785
All − MR Egger
All − IVW(MRE)
−1.0
−0.5
0.0
0.5
1.0
MR effect size for
'Gut microbiota abundance (genus Parasutterella id.2892)' on 'NAD kinase'

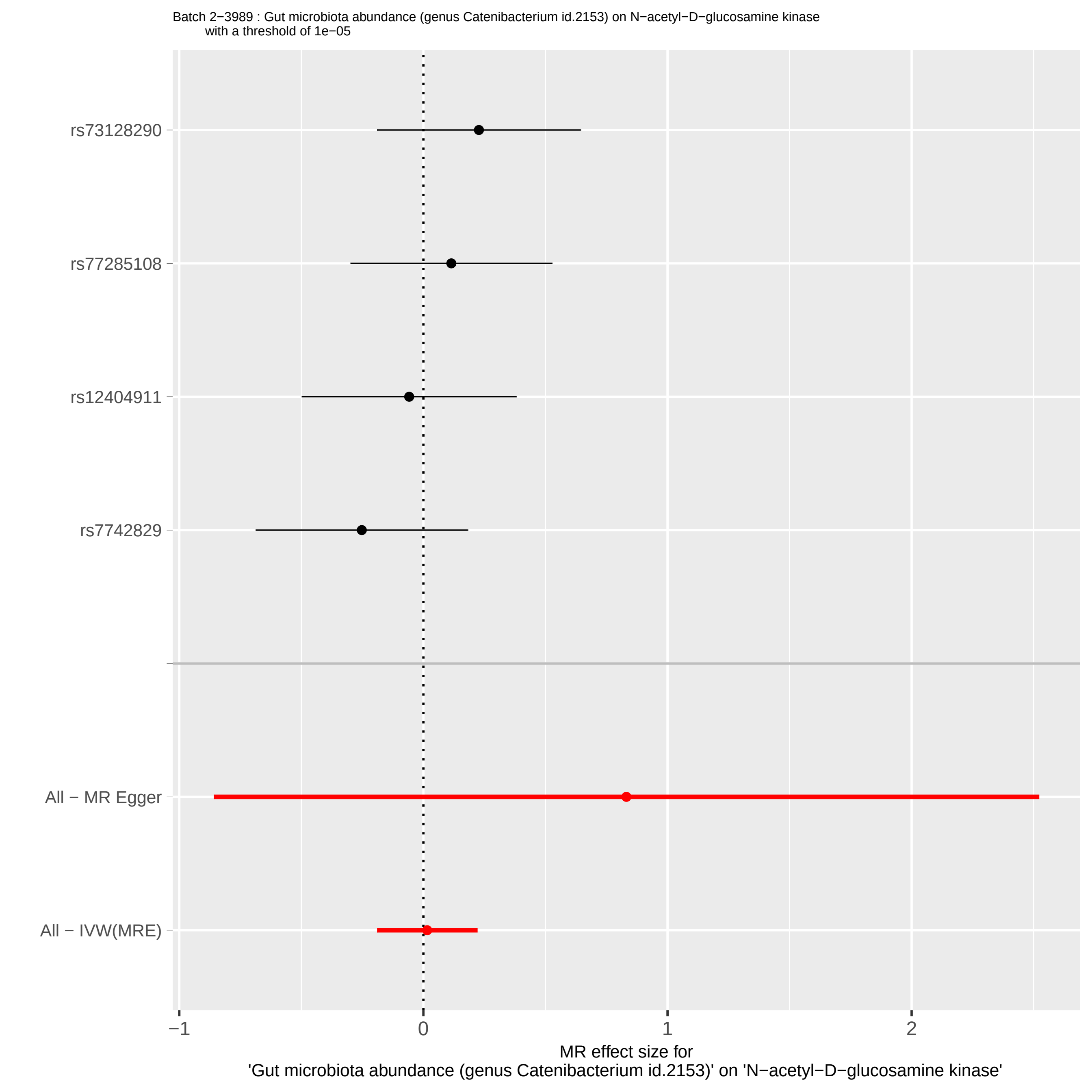
Batch 2−3989 : Gut microbiota abundance (genus Catenibacterium id.2153) on N−acetyl−D−glucosamine kinase
with a threshold of 1e−05
rs73128290
rs77285108
rs12404911
rs7742829
All − MR Egger
All − IVW(MRE)
−1
0
1
2
MR effect size for
'Gut microbiota abundance (genus Catenibacterium id.2153)' on 'N−acetyl−D−glucosamine kinase'

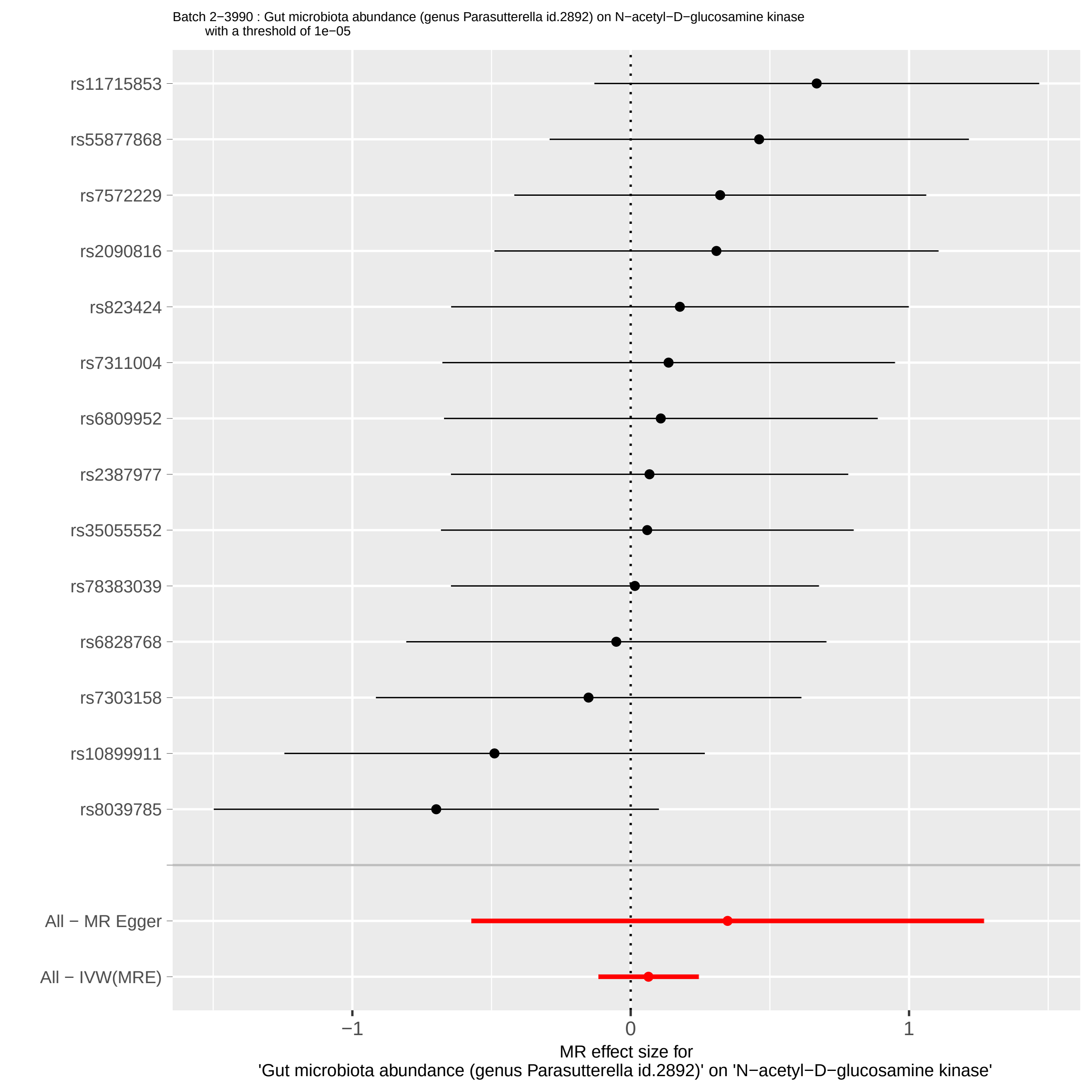

Batch 2−3990 : Gut microbiota abundance (genus Parasutterella id.2892) on N−acetyl−D−glucosamine kinase
with a threshold of 1e−05
rs11715853
rs55877868
rs7572229
rs2090816
rs823424
rs7311004
rs6809952
rs2387977
rs35055552
rs78383039
rs6828768
rs7303158
rs10899911
rs8039785
All − MR Egger
All − IVW(MRE)
−1
0
1
MR effect size for
'Gut microbiota abundance (genus Parasutterella id.2892)' on 'N−acetyl−D−glucosamine kinase'

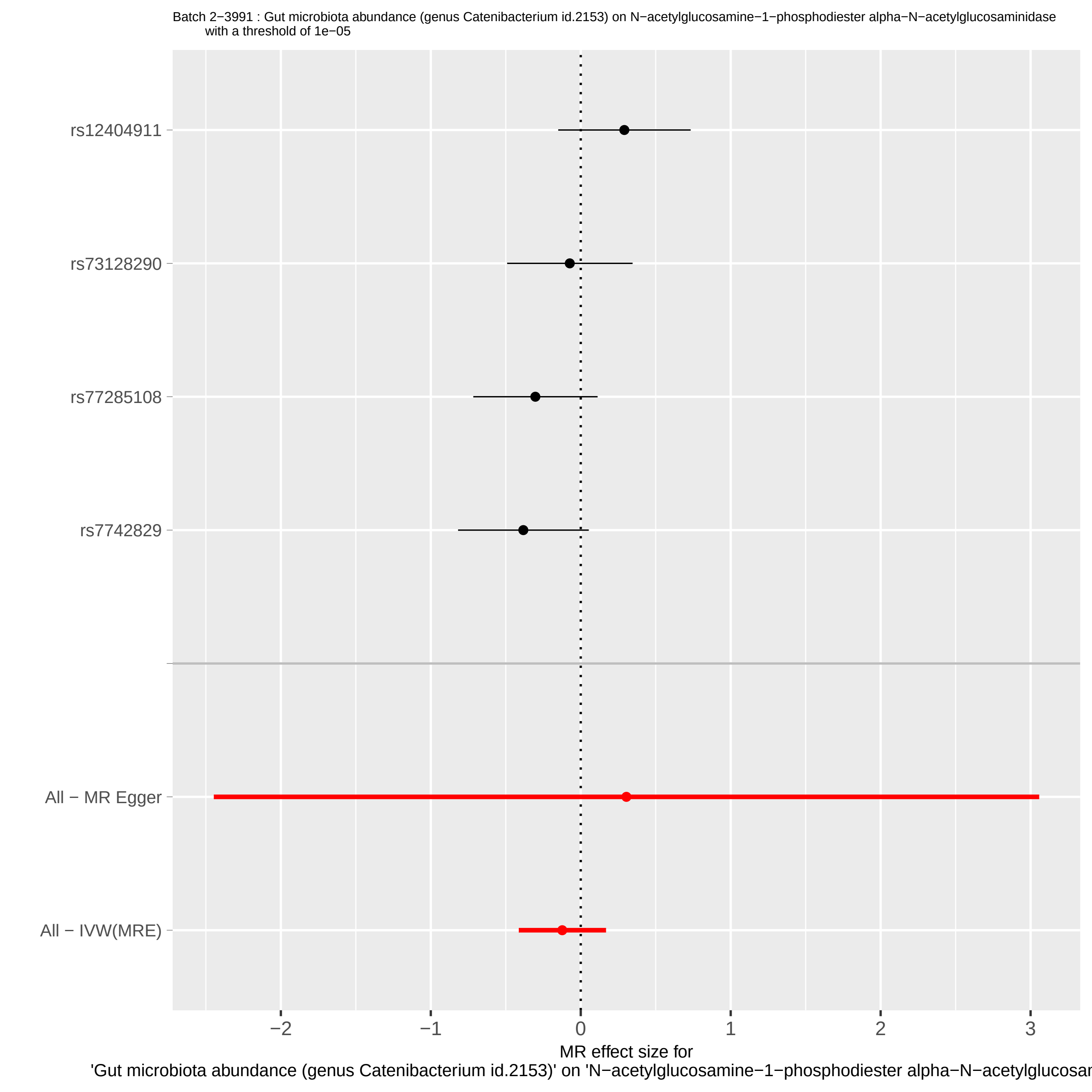
Batch 2−3991 : Gut microbiota abundance (genus Catenibacterium id.2153) on N−acetylglucosamine−1−phosphodiester alpha−N−acetylglucosaminidase
with a threshold of 1e−05
rs12404911
rs73128290
rs77285108
rs7742829
All − MR Egger
All − IVW(MRE)
−2
−1
0
1
2
3
MR effect size for
'Gut microbiota abundance (genus Catenibacterium id.2153)' on 'N−acetylglucosamine−1−phosphodiester alpha−N−acetylglucosar

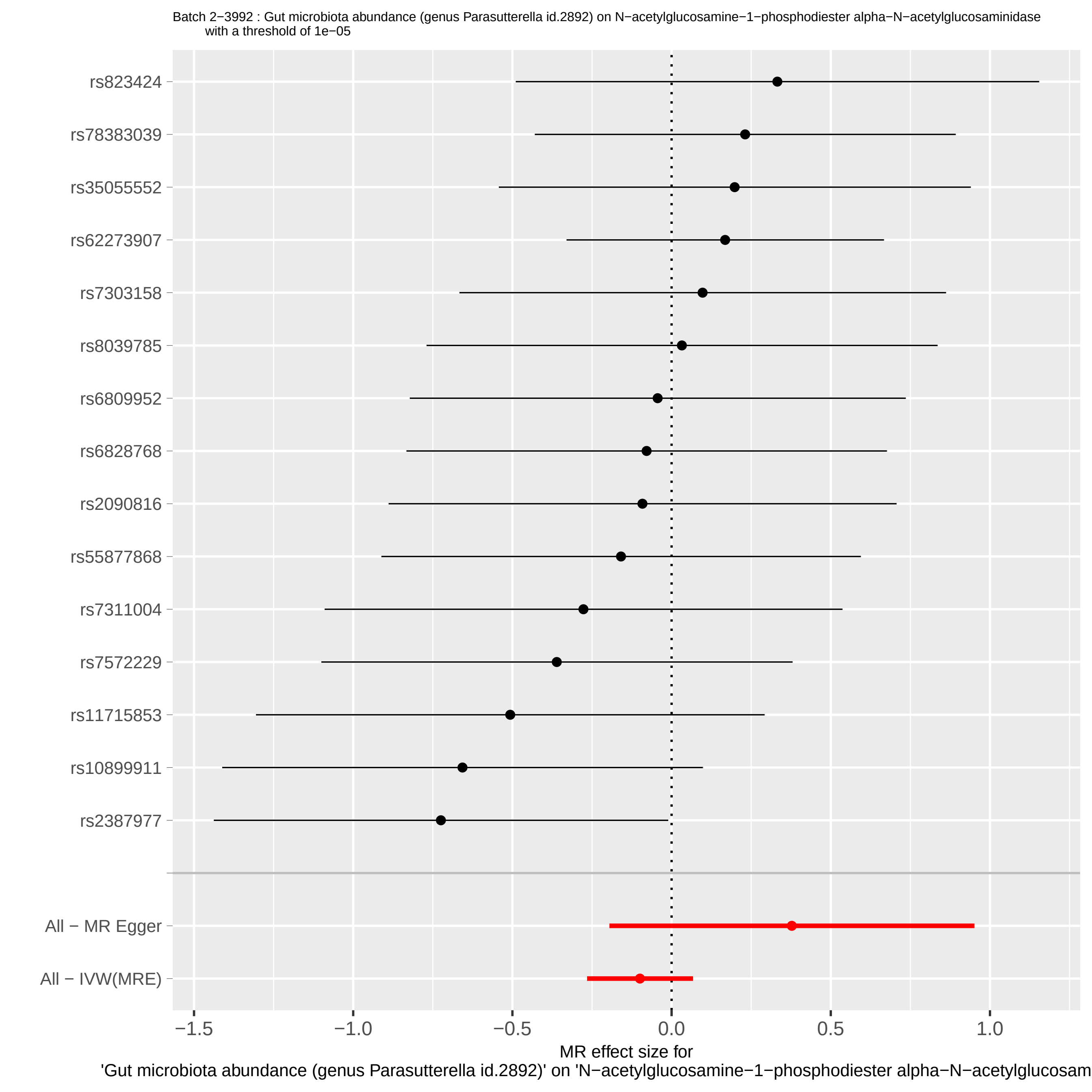

Batch 2−3992 : Gut microbiota abundance (genus Parasutterella id.2892) on N−acetylglucosamine−1−phosphodiester alpha−N−acetylglucosaminidase with a threshold of 1e−05
rs823424
rs78383039
rs35055552
rs62273907
rs7303158
rs8039785
rs6809952
rs6828768
rs2090816
rs55877868
rs7311004
rs7572229
rs11715853
rs10899911
rs2387977
All − MR Egger
All − IVW(MRE)
−1.5
−1.0
−0.5
0.0
0.5
1.0
MR effect size for
'Gut microbiota abundance (genus Parasutterella id.2892)' on 'N−acetylglucosamine−1−phosphodiester alpha−N−acetylglucosam

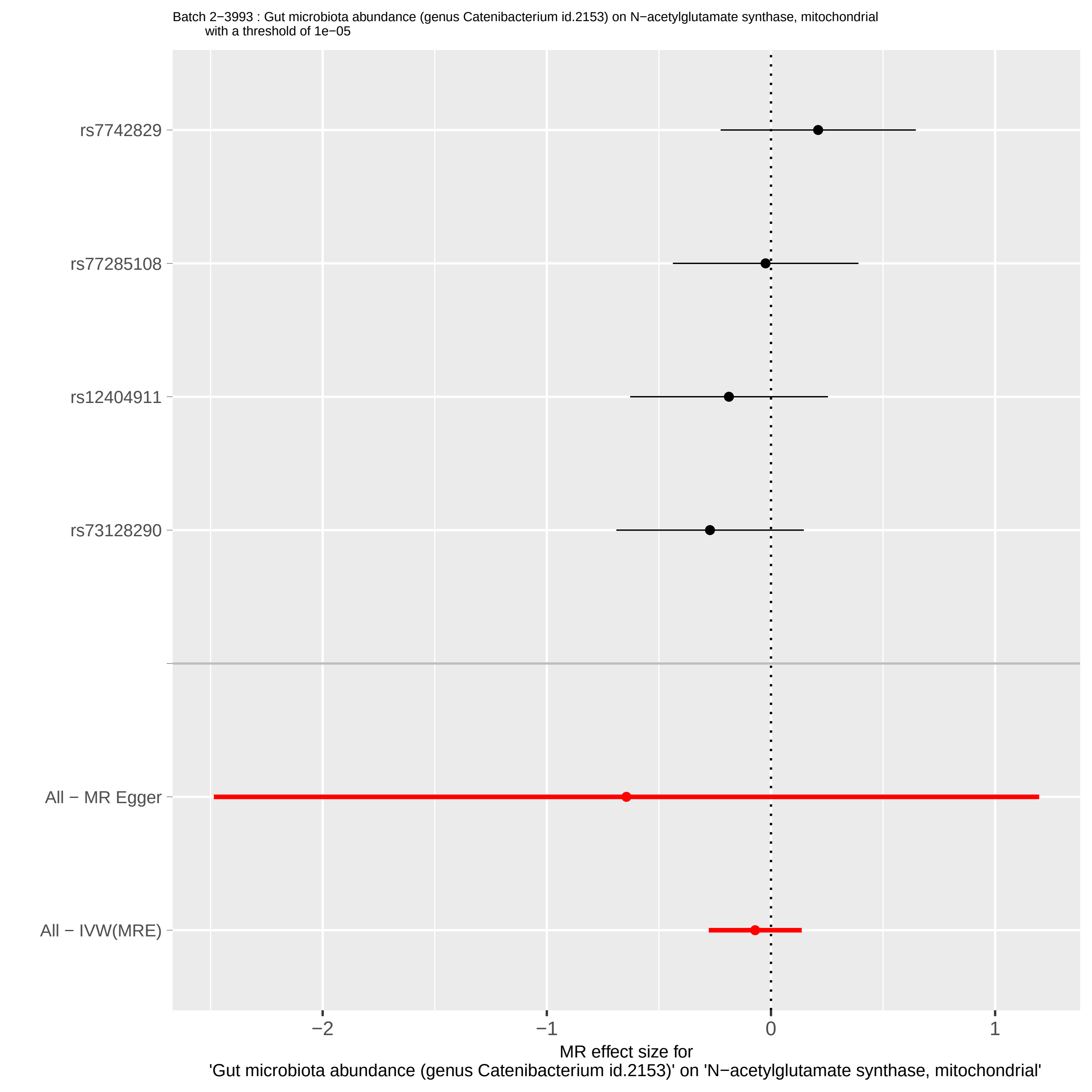
Batch 2−3993 : Gut microbiota abundance (genus Catenibacterium id.2153) on N−acetylglutamate synthase, mitochondrial
with a threshold of 1e−05
rs7742829
rs77285108
rs12404911
rs73128290
All − MR Egger
All − IVW(MRE)
−2
−1
0
1
MR effect size for
'Gut microbiota abundance (genus Catenibacterium id.2153)' on 'N−acetylglutamate synthase, mitochondrial'

Batch 2−3994 : Gut microbiota abundance (genus Parasutterella id.2892) on N−acetylglutamate synthase, mitochondrial
with a threshold of 1e−05

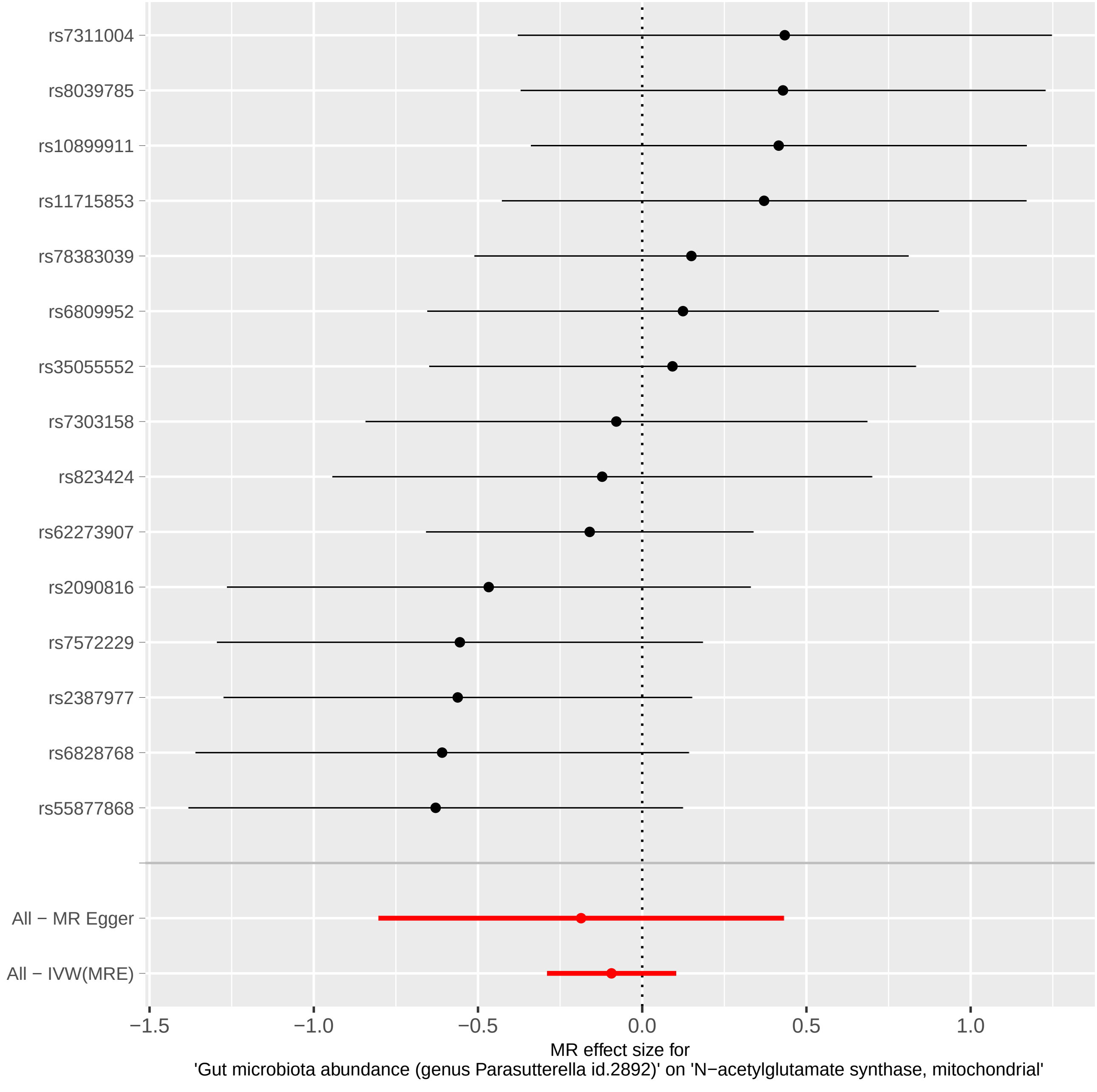

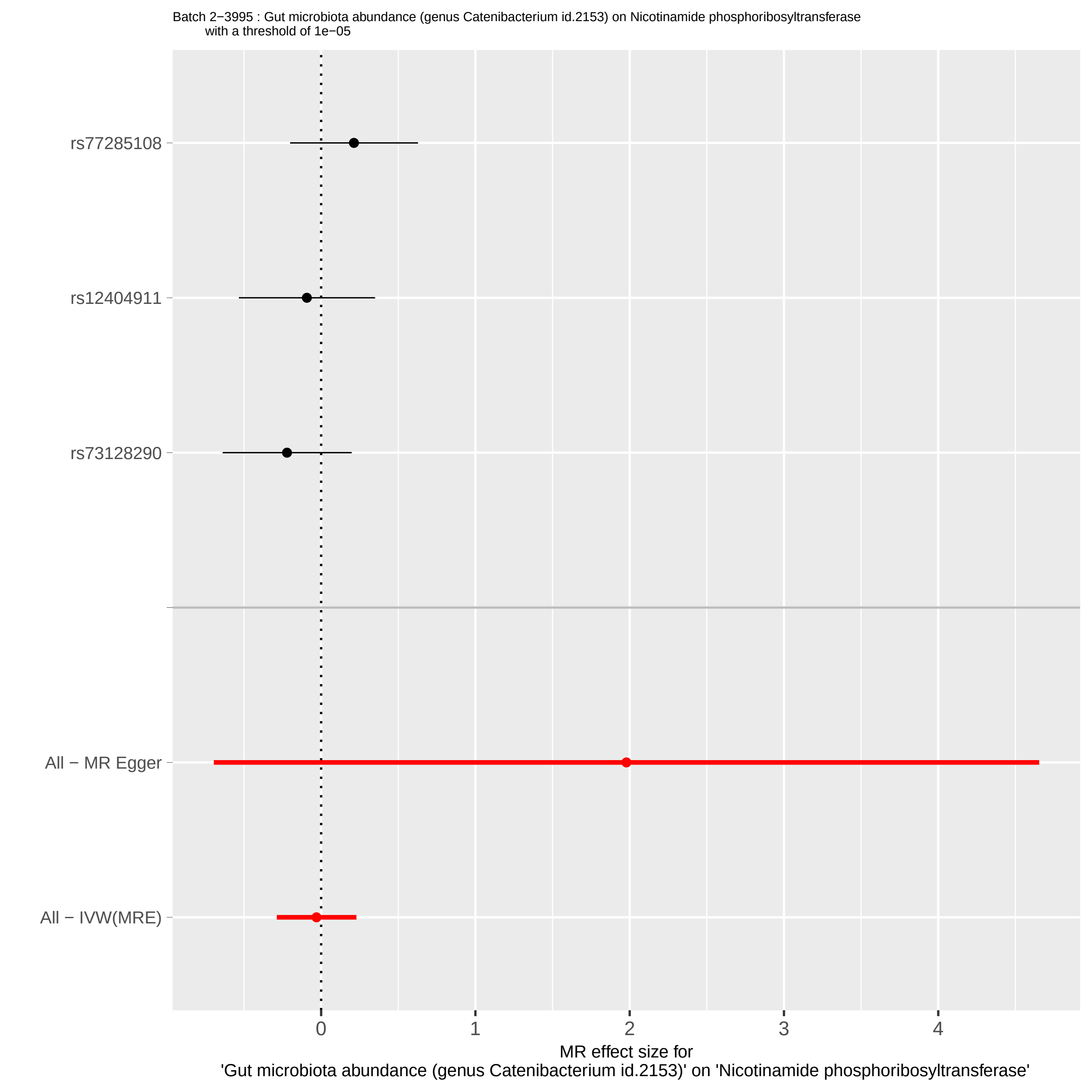

Batch 2−3995 : Gut microbiota abundance (genus Catenibacterium id.2153) on Nicotinamide phosphoribosyltransferase
with a threshold of 1e−05
rs77285108
rs12404911
rs73128290
All − MR Egger
All − IVW(MRE)
0
1
2
3
4
MR effect size for
'Gut microbiota abundance (genus Catenibacterium id.2153)' on 'Nicotinamide phosphoribosyltransferase'

Batch 2−3996 : Gut microbiota abundance (genus Parasutterella id.2892) on Nicotinamide phosphoribosyltransferase
with a threshold of 1e−05

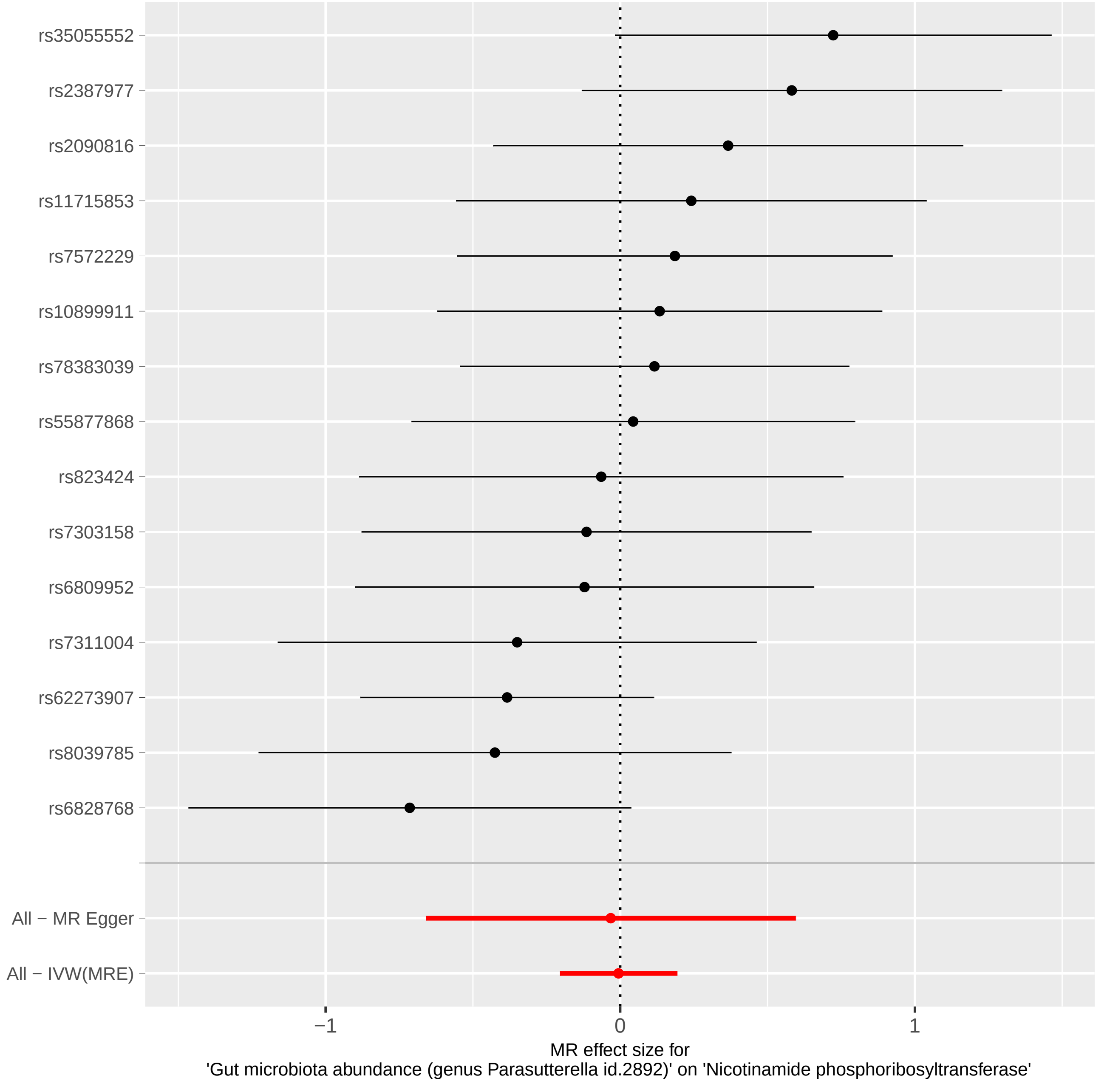

Batch 2−3997 : Gut microbiota abundance (genus Catenibacterium id.2153) on Nucleosome assembly protein 1−like 2
with a threshold of 1e−05

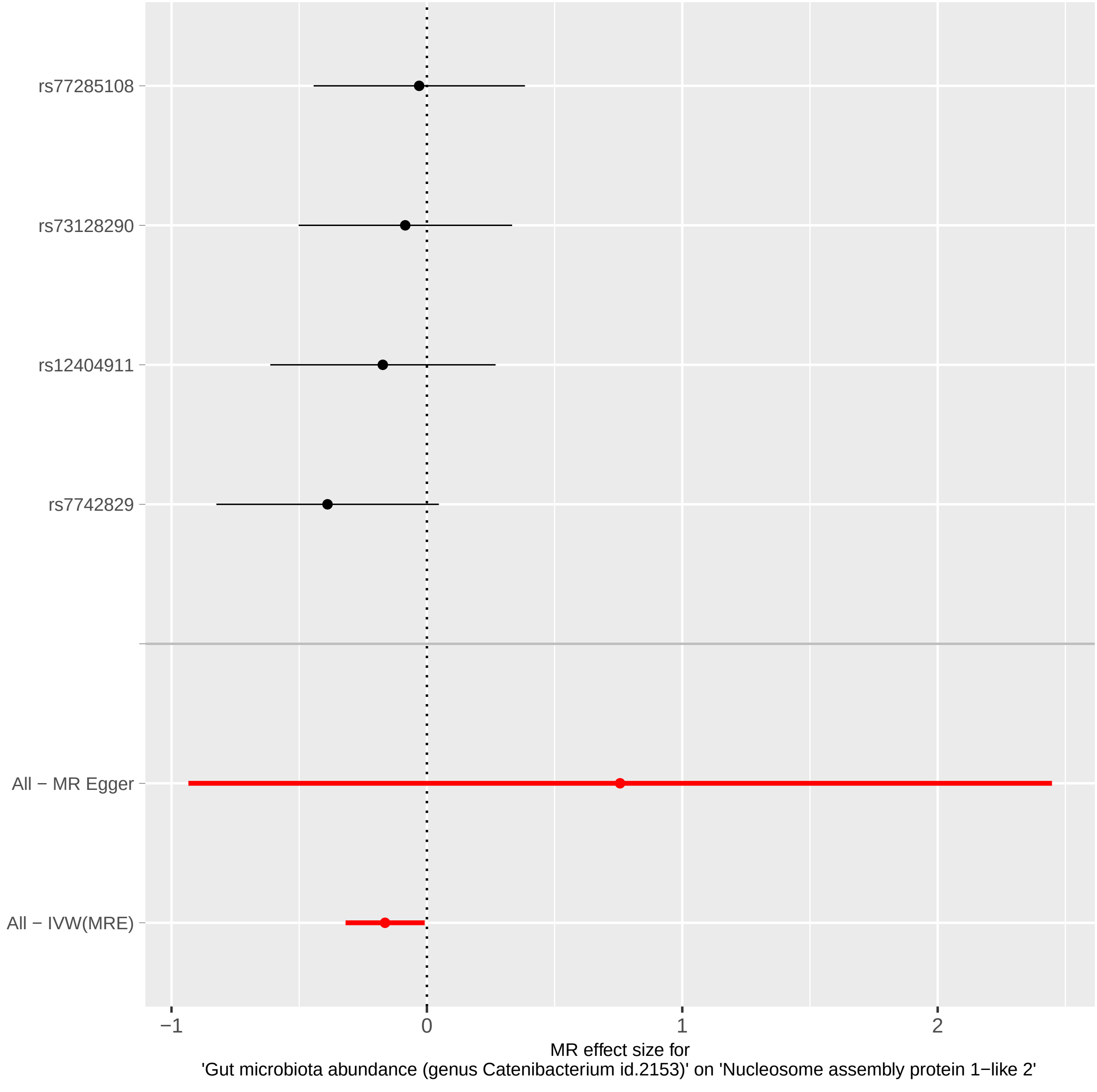

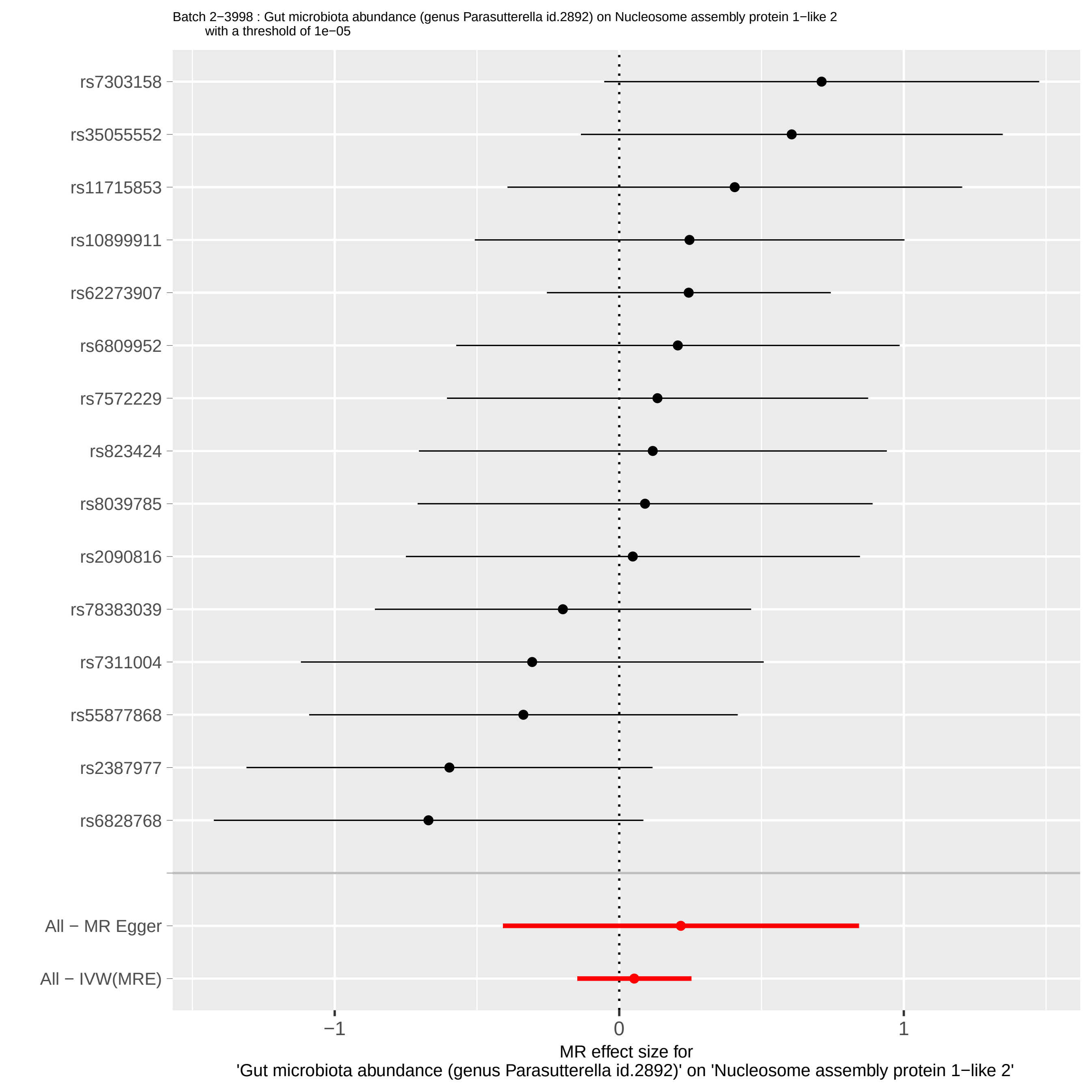

Batch 2−3998 : Gut microbiota abundance (genus Parasutterella id.2892) on Nucleosome assembly protein 1−like 2
with a threshold of 1e−05
rs7303158
rs35055552
rs11715853
rs10899911
rs62273907
rs6809952
rs7572229
rs823424
rs8039785
rs2090816
rs78383039
rs7311004
rs55877868
rs2387977
rs6828768
All − MR Egger
All − IVW(MRE)
−1
0
1
MR effect size for
'Gut microbiota abundance (genus Parasutterella id.2892)' on 'Nucleosome assembly protein 1−like 2'

Batch 2−3999 : Gut microbiota abundance (genus Catenibacterium id.2153) on Beta−soluble NSF attachment protein
with a threshold of 1e−05

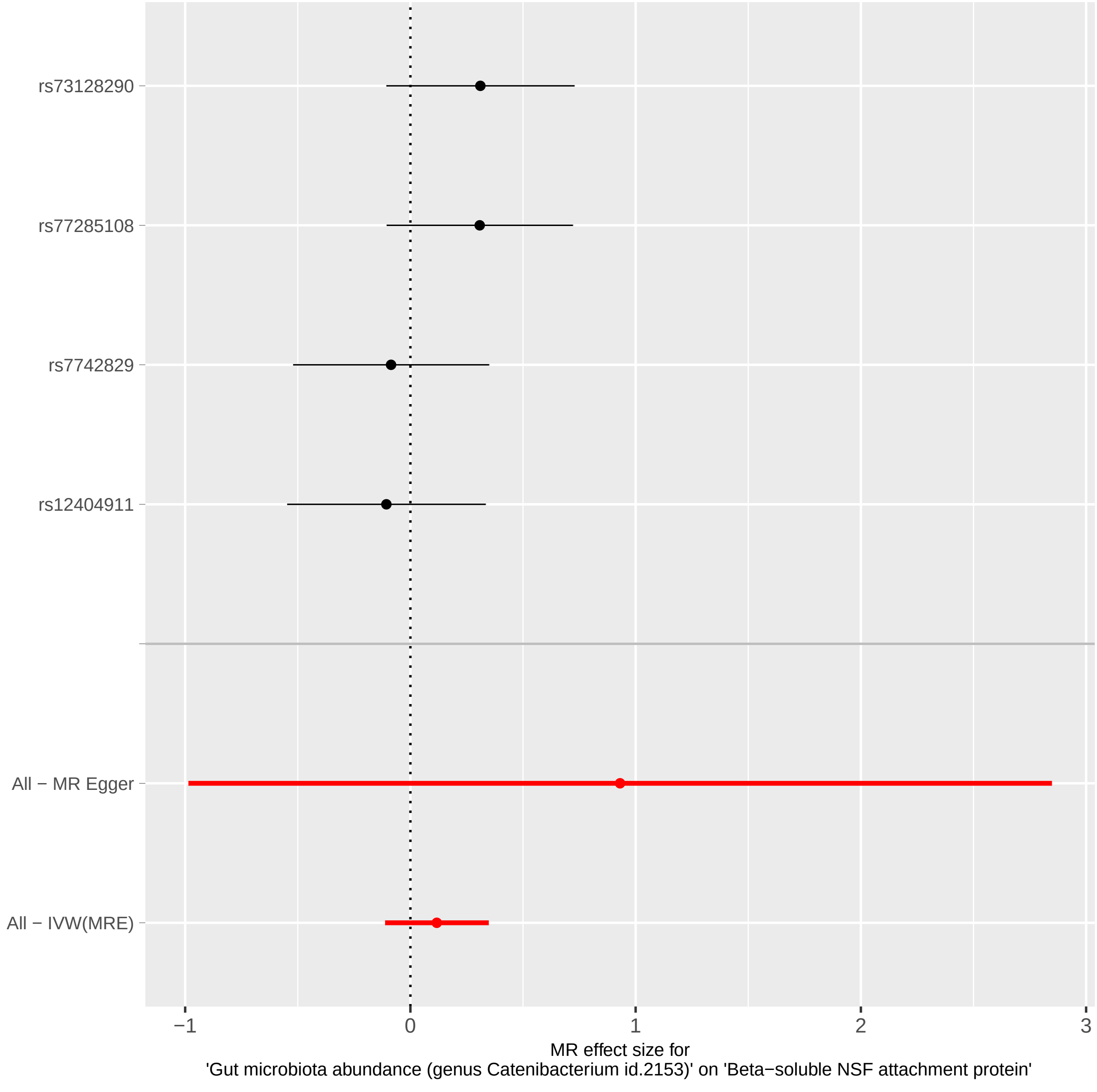

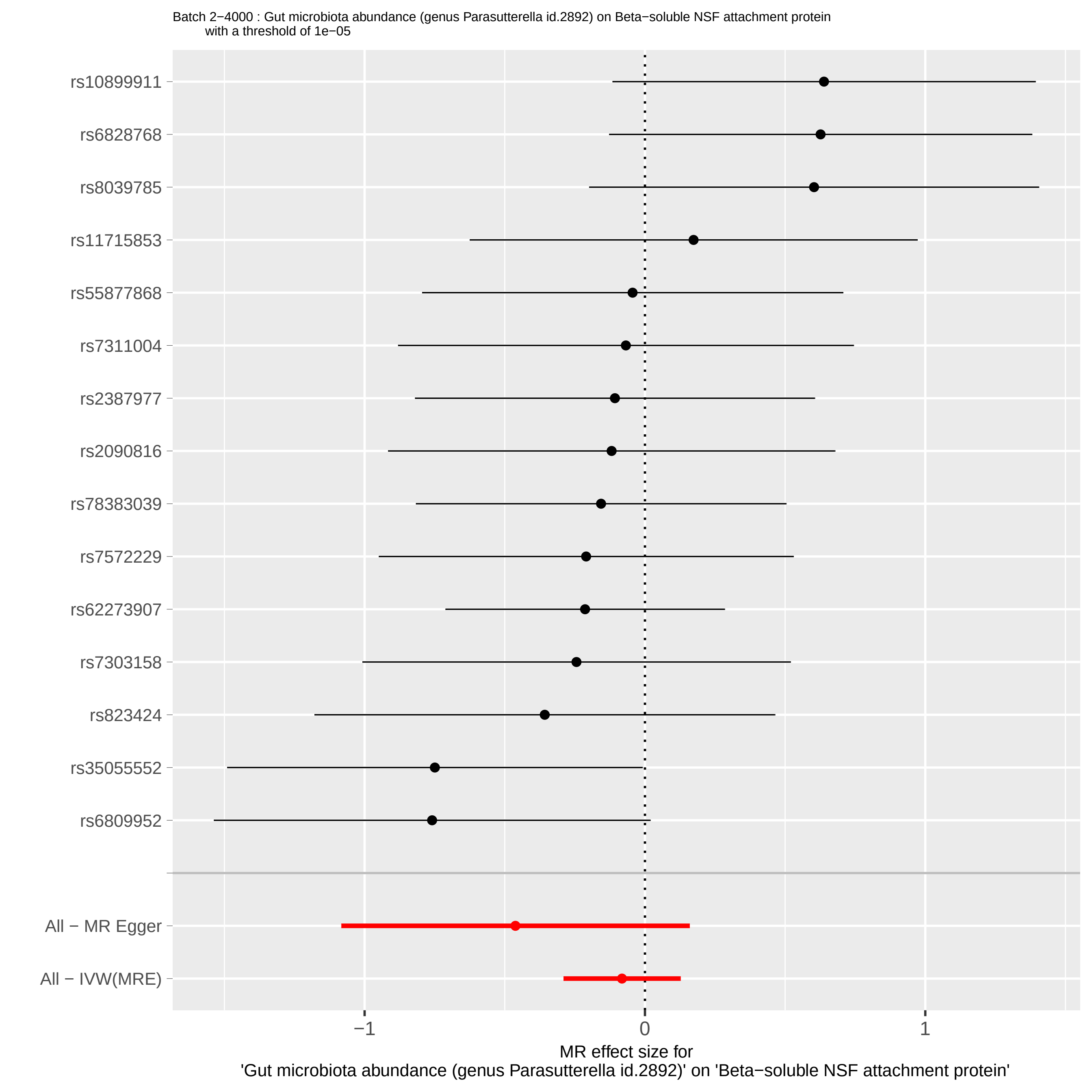

Batch 2−4000 : Gut microbiota abundance (genus Parasutterella id.2892) on Beta−soluble NSF attachment protein
with a threshold of 1e−05
rs10899911
rs6828768
rs8039785
rs11715853
rs55877868
rs7311004
rs2387977
rs2090816
rs78383039
rs7572229
rs62273907
rs7303158
rs823424
rs35055552
rs6809952
All − MR Egger
All − IVW(MRE)
−1
0
1
MR effect size for
'Gut microbiota abundance (genus Parasutterella id.2892)' on 'Beta−soluble NSF attachment protein'

Batch 2−4001 : Gut microbiota abundance (genus Catenibacterium id.2153) on Arylamine N−acetyltransferase 1
with a threshold of 1e−05

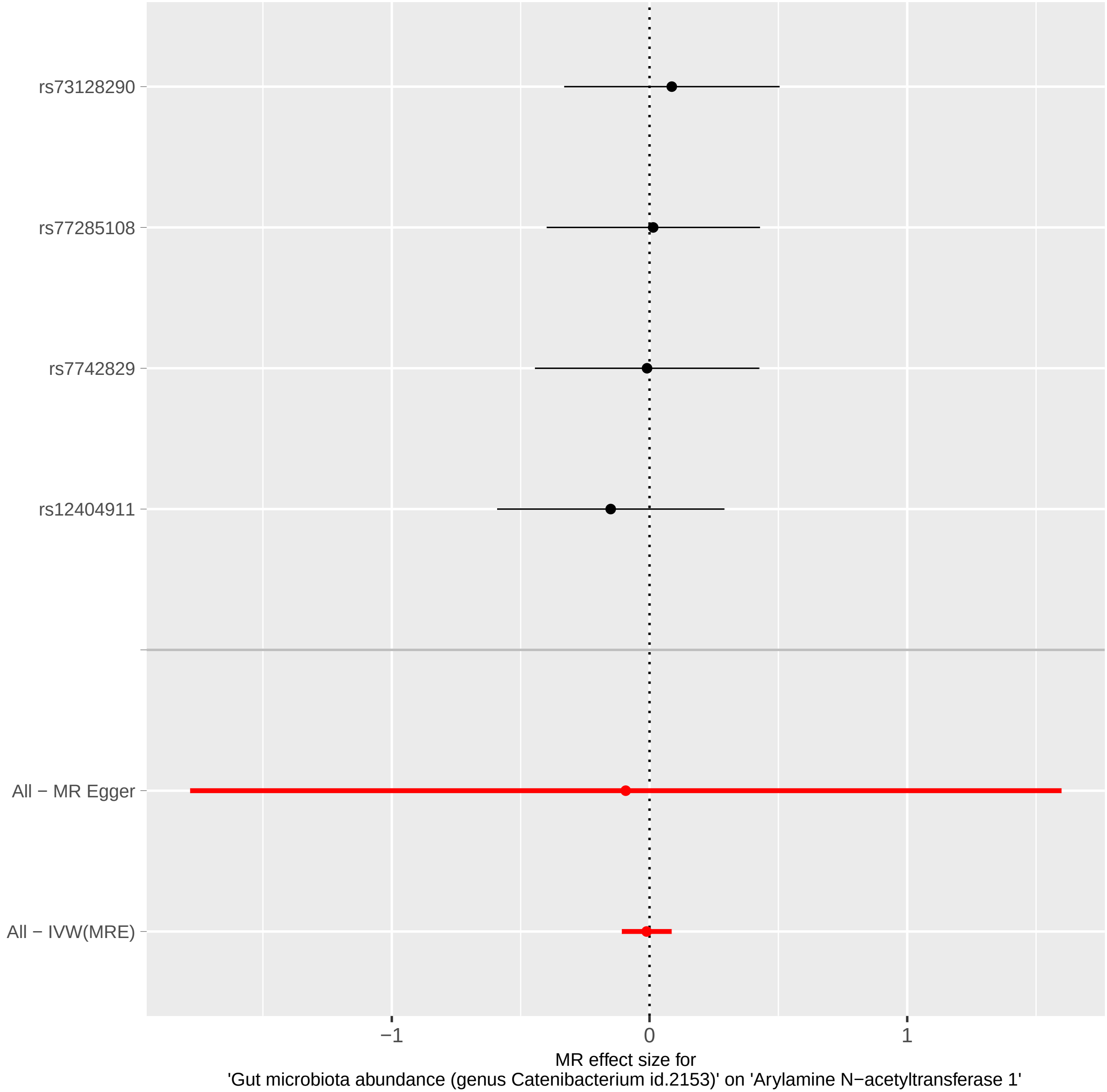

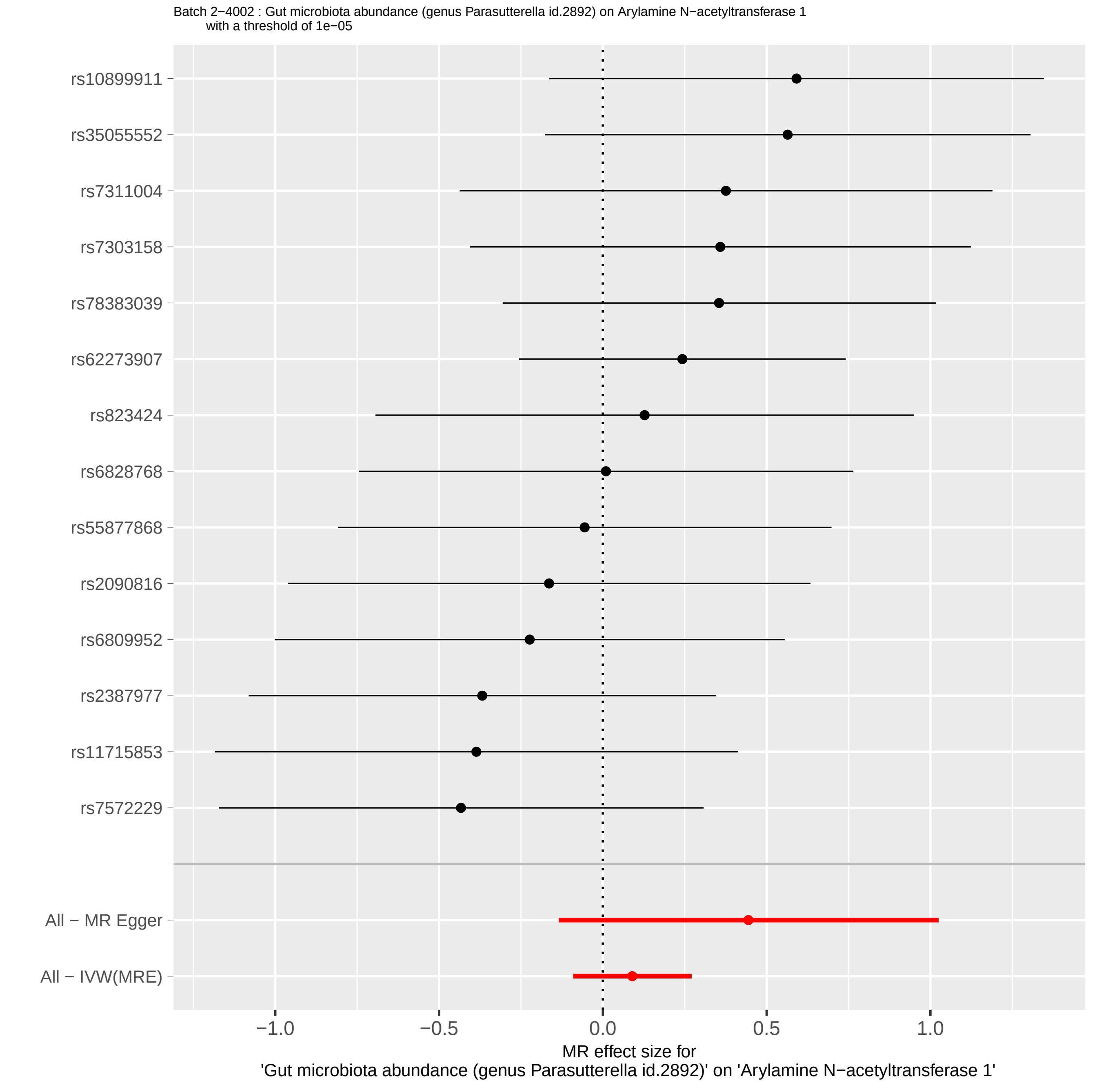
Batch 2−4002 : Gut microbiota abundance (genus Parasutterella id.2892) on Arylamine N−acetyltransferase 1
with a threshold of 1e−05
rs10899911
rs35055552
rs7311004
rs7303158
rs78383039
rs62273907
rs823424
rs6828768
rs55877868
rs2090816
rs6809952
rs2387977
rs11715853
rs7572229
All − MR Egger
All − IVW(MRE)
−1.0
−0.5
0.0
0.5
1.0
MR effect size for
'Gut microbiota abundance (genus Parasutterella id.2892)' on 'Arylamine N−acetyltransferase 1'

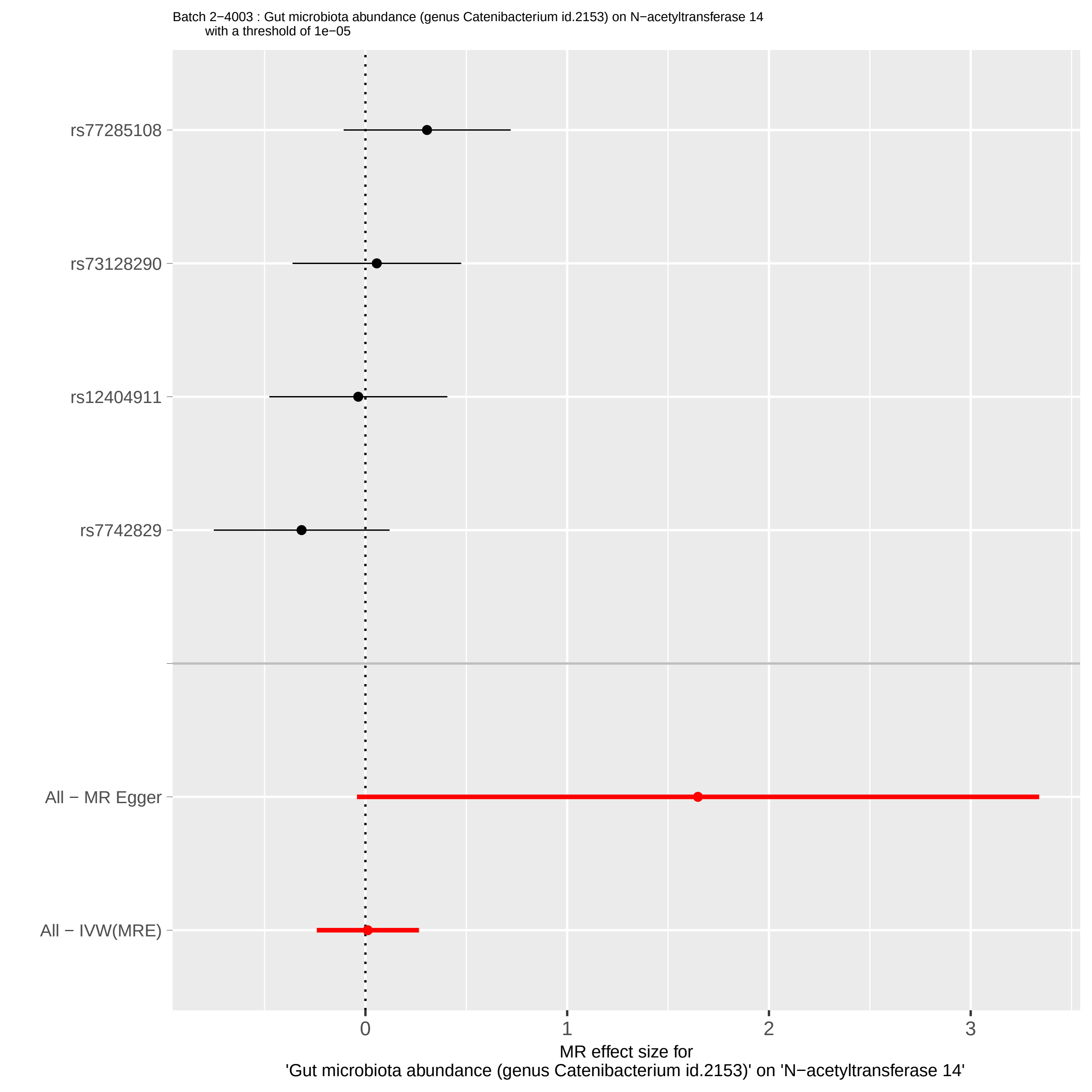
Batch 2−4003 : Gut microbiota abundance (genus Catenibacterium id.2153) on N−acetyltransferase 14
with a threshold of 1e−05
rs77285108
rs73128290
rs12404911
rs7742829
All − MR Egger
All − IVW(MRE)
0
1
2
3
MR effect size for
'Gut microbiota abundance (genus Catenibacterium id.2153)' on 'N−acetyltransferase 14'

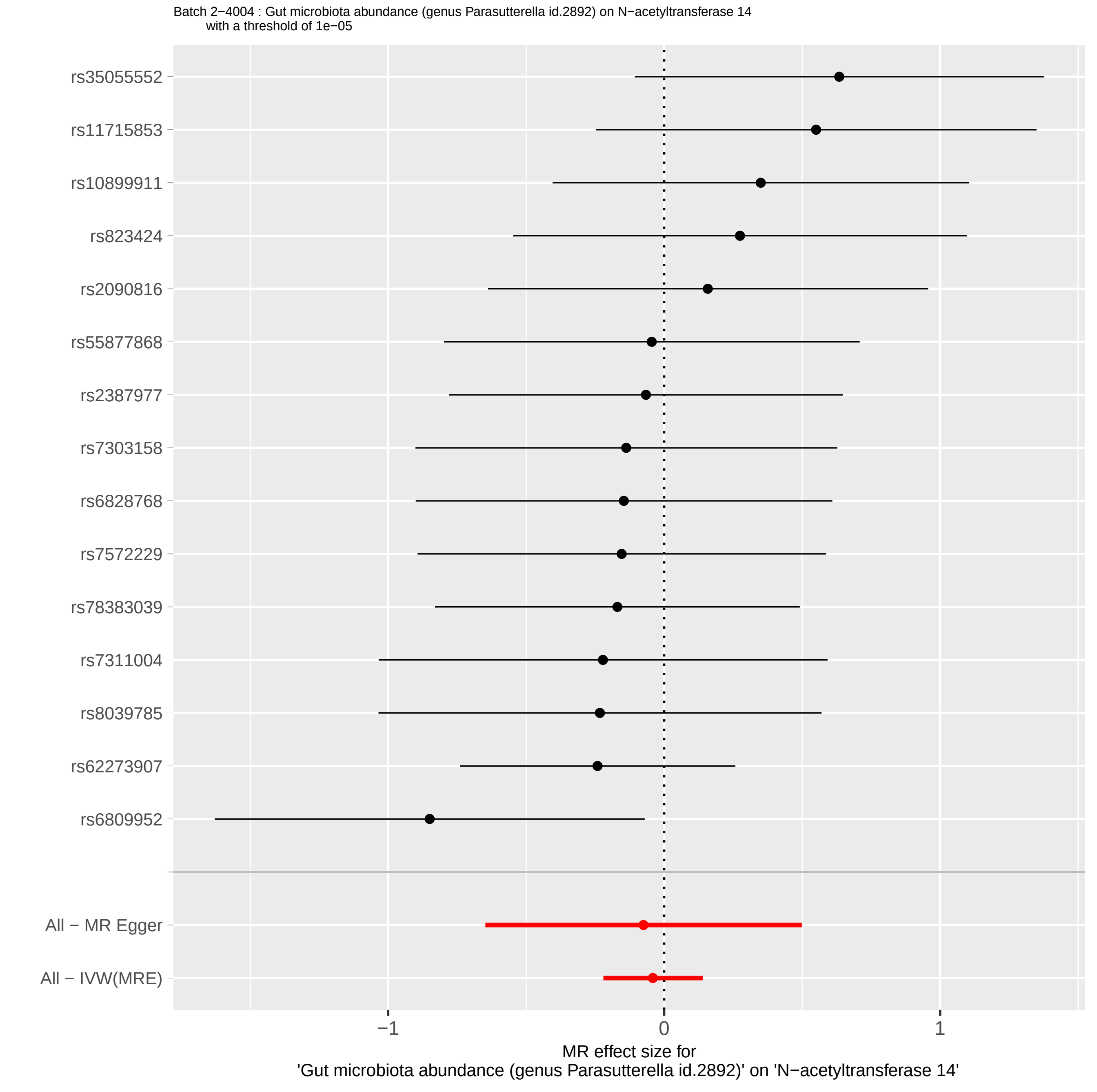
Batch 2−4004 : Gut microbiota abundance (genus Parasutterella id.2892) on N−acetyltransferase 14
with a threshold of 1e−05
rs35055552
rs11715853
rs10899911
rs823424
rs2090816
rs55877868
rs2387977
rs7303158
rs6828768
rs7572229
rs78383039
rs7311004
rs8039785
rs62273907
rs6809952
All − MR Egger
All − IVW(MRE)
−1
0
1
MR effect size for
'Gut microbiota abundance (genus Parasutterella id.2892)' on 'N−acetyltransferase 14'

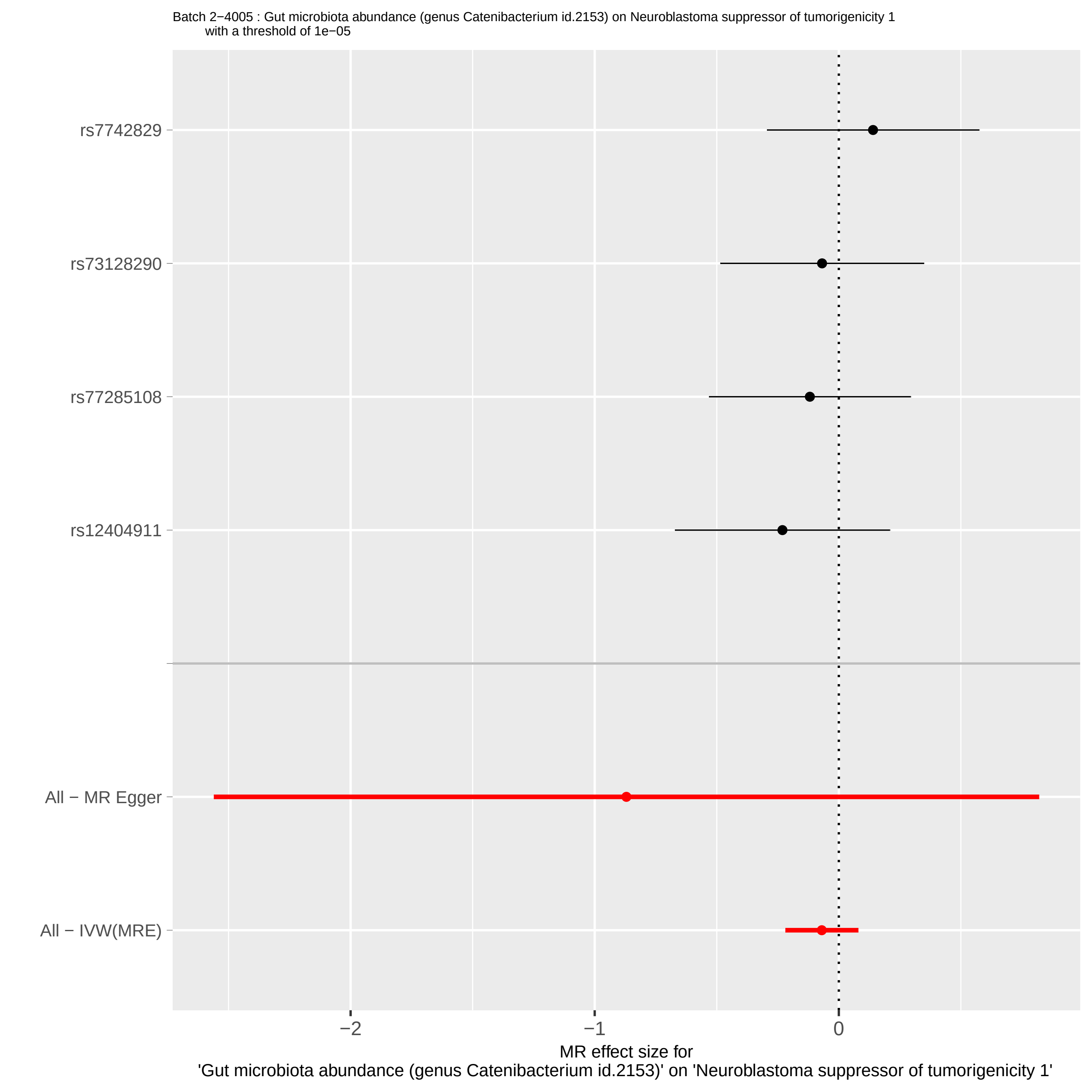

Batch 2−4005 : Gut microbiota abundance (genus Catenibacterium id.2153) on Neuroblastoma suppressor of tumorigenicity 1
with a threshold of 1e−05
rs7742829
rs73128290
rs77285108
rs12404911
All − MR Egger
All − IVW(MRE)
−2
−1
0
MR effect size for
'Gut microbiota abundance (genus Catenibacterium id.2153)' on 'Neuroblastoma suppressor of tumorigenicity 1'

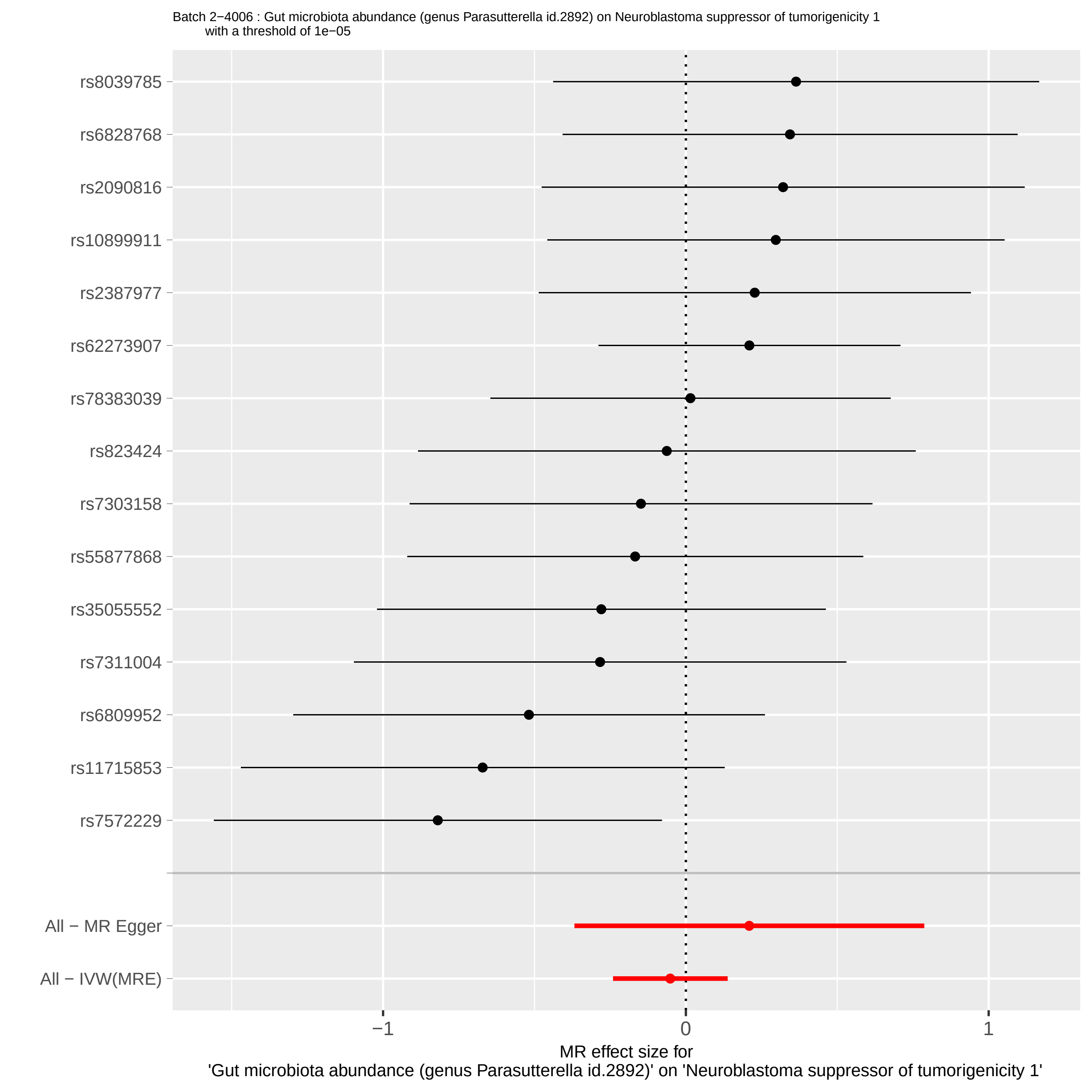

Batch 2−4006 : Gut microbiota abundance (genus Parasutterella id.2892) on Neuroblastoma suppressor of tumorigenicity 1
with a threshold of 1e−05
rs8039785
rs6828768
rs2090816
rs10899911
rs2387977
rs62273907
rs78383039
rs823424
rs7303158
rs55877868
rs35055552
rs7311004
rs6809952
rs11715853
rs7572229
All − MR Egger
All − IVW(MRE)
−1
0
1
MR effect size for
'Gut microbiota abundance (genus Parasutterella id.2892)' on 'Neuroblastoma suppressor of tumorigenicity 1'

Batch 2−4007 : Gut microbiota abundance (genus Catenibacterium id.2153) on Next to BRCA1 gene 1 protein
with a threshold of 1e−05

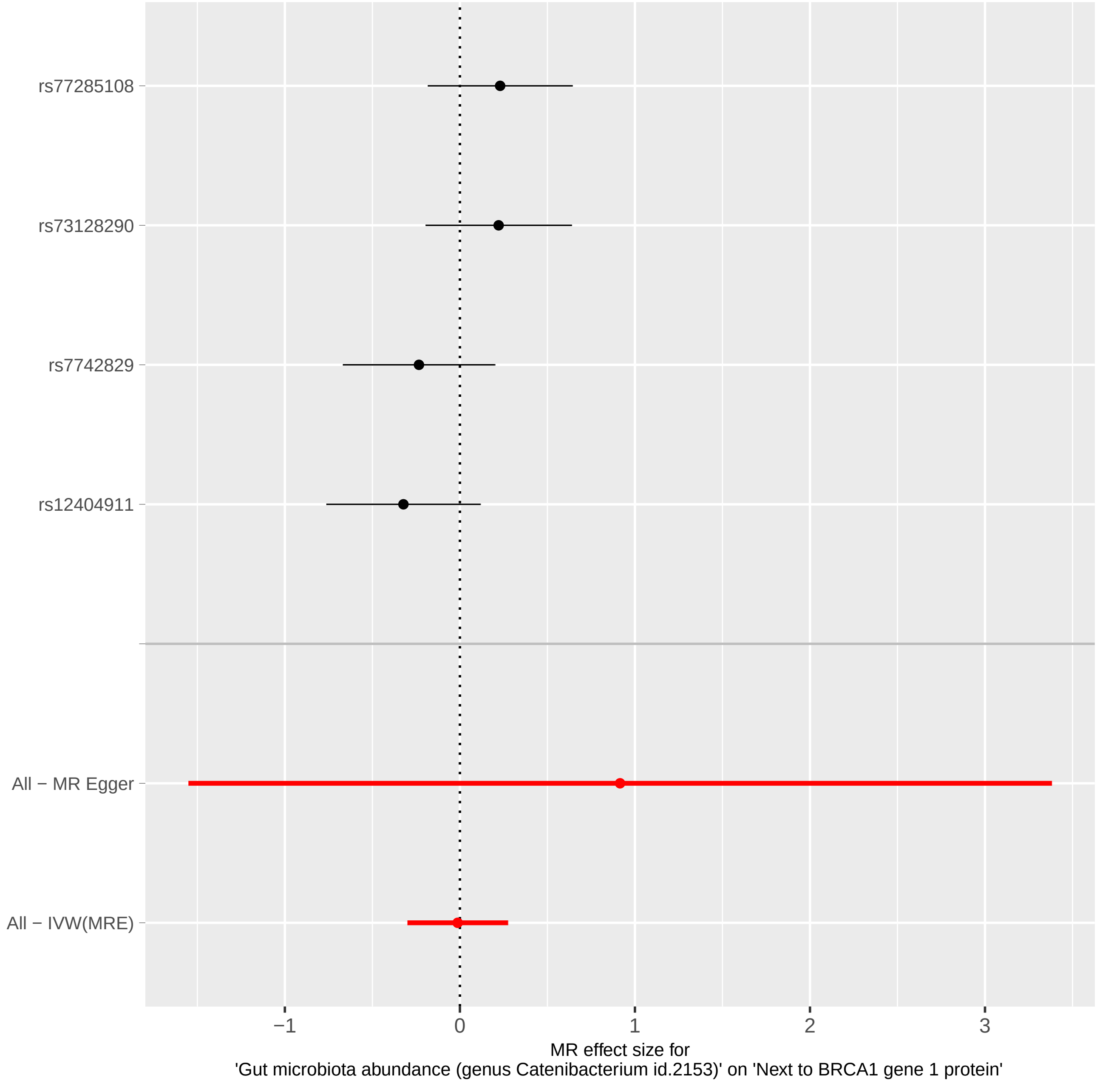

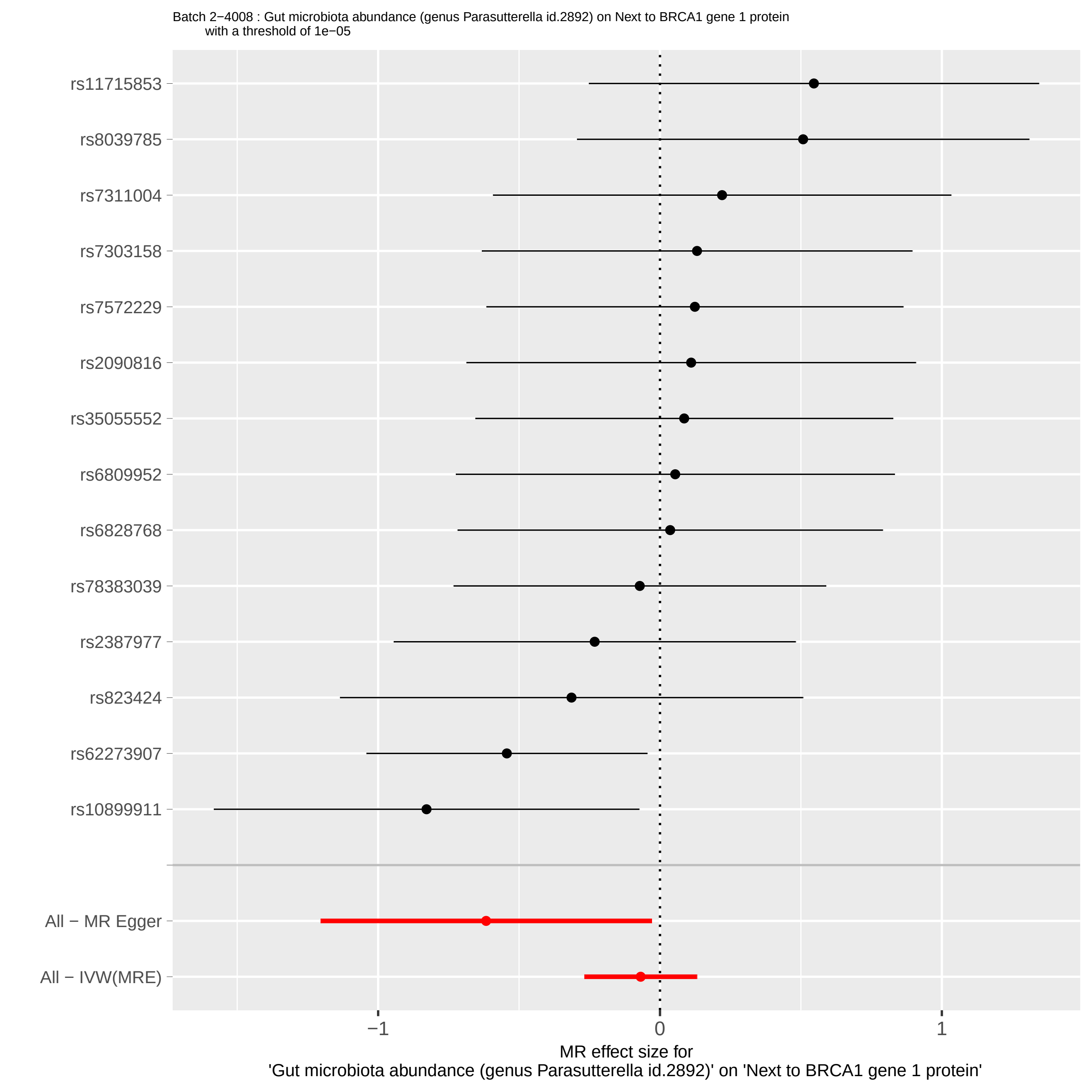

Batch 2−4008 : Gut microbiota abundance (genus Parasutterella id.2892) on Next to BRCA1 gene 1 protein
with a threshold of 1e−05
rs11715853
rs8039785
rs7311004
rs7303158
rs7572229
rs2090816
rs35055552
rs6809952
rs6828768
rs78383039
rs2387977
rs823424
rs62273907
rs10899911
All − MR Egger
All − IVW(MRE)
−1
0
1
MR effect size for
'Gut microbiota abundance (genus Parasutterella id.2892)' on 'Next to BRCA1 gene 1 protein'

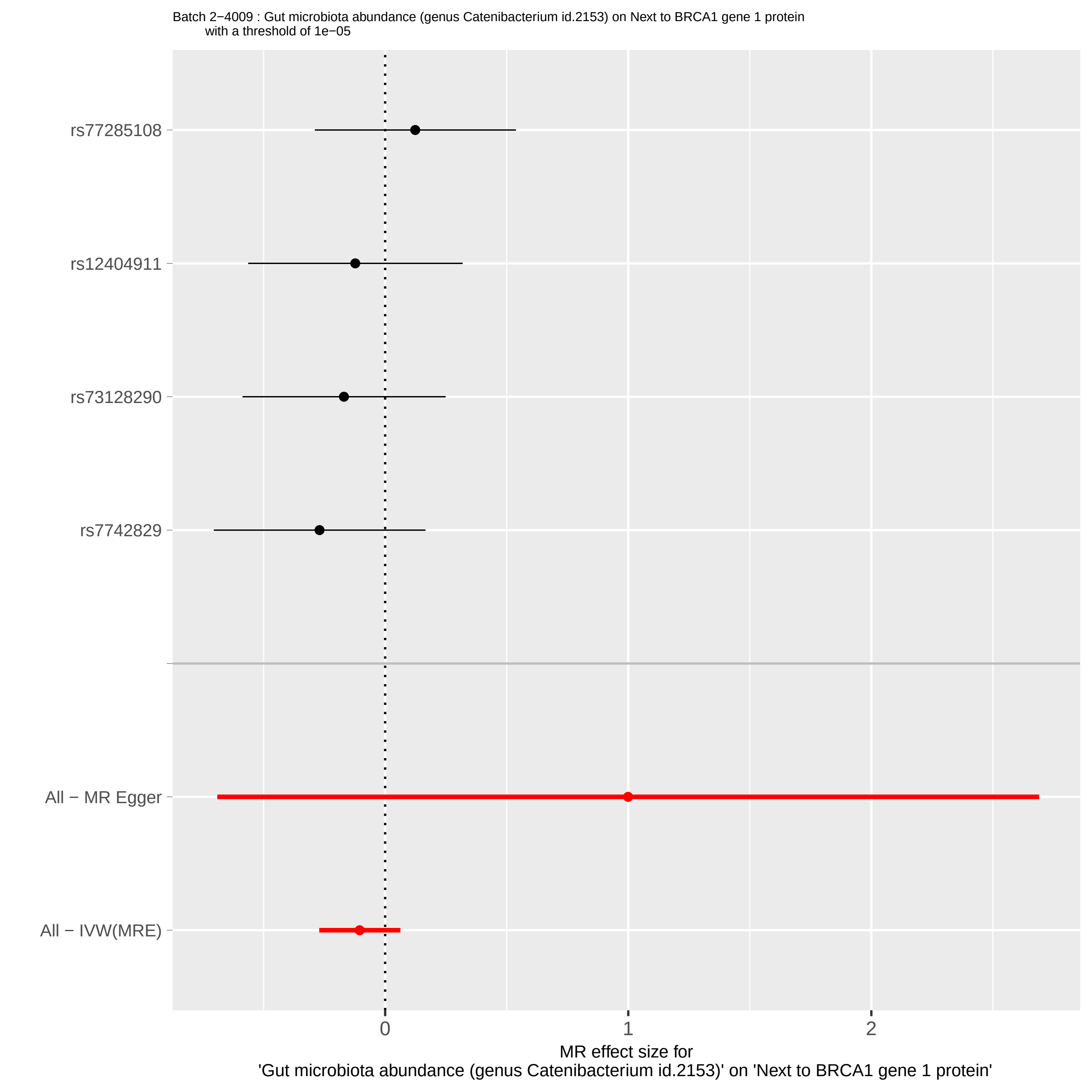

Batch 2−4009 : Gut microbiota abundance (genus Catenibacterium id.2153) on Next to BRCA1 gene 1 protein
with a threshold of 1e−05
rs77285108
rs12404911
rs73128290
rs7742829
All − MR Egger
All − IVW(MRE)
0
1
2
MR effect size for
'Gut microbiota abundance (genus Catenibacterium id.2153)' on 'Next to BRCA1 gene 1 protein'

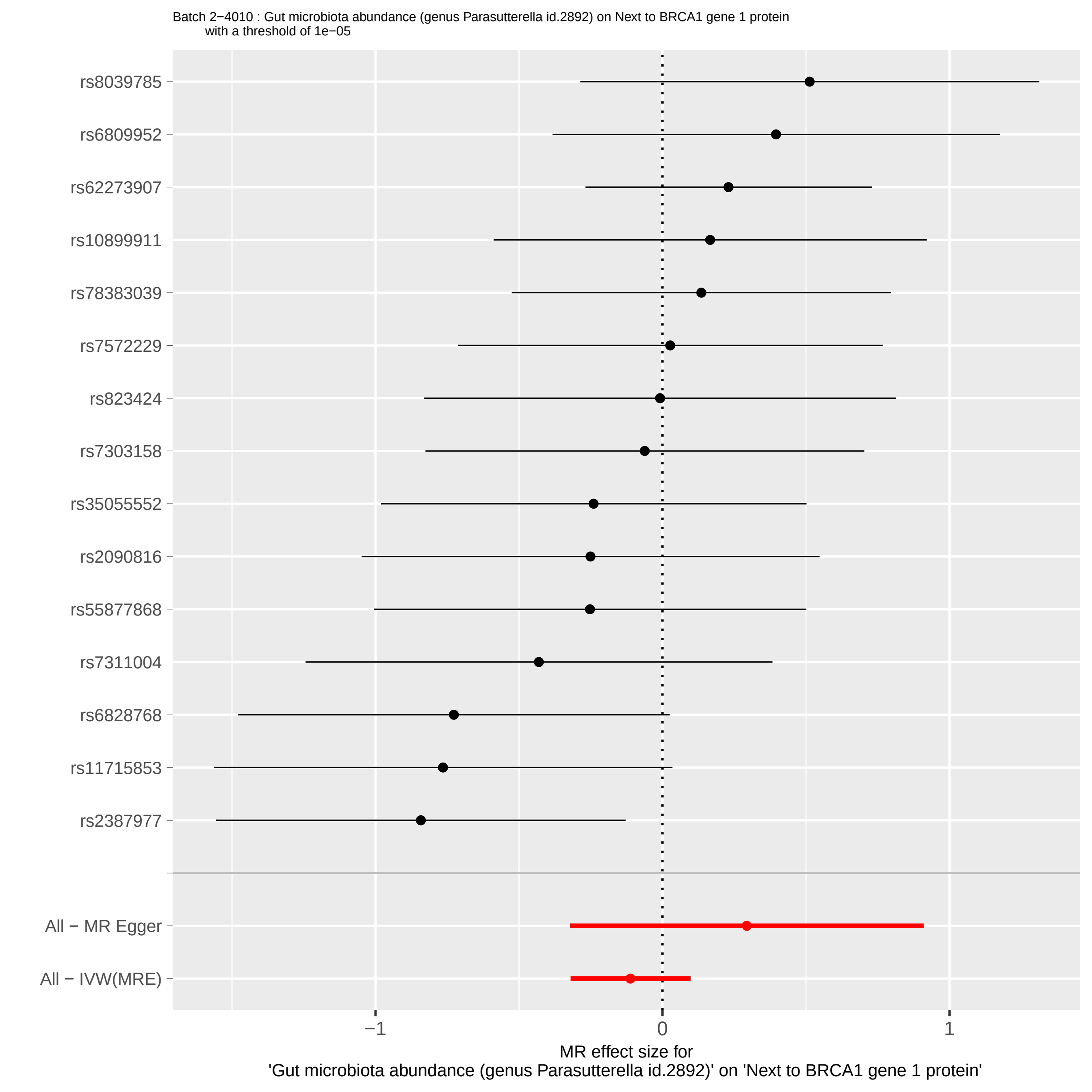
Batch 2−4010 : Gut microbiota abundance (genus Parasutterella id.2892) on Next to BRCA1 gene 1 protein
with a threshold of 1e−05
rs8039785
rs6809952
rs62273907
rs10899911
rs78383039
rs7572229
rs823424
rs7303158
rs35055552
rs2090816
rs55877868
rs7311004
rs6828768
rs11715853
rs2387977
All − MR Egger
All − IVW(MRE)
−1
0
1
MR effect size for
'Gut microbiota abundance (genus Parasutterella id.2892)' on 'Next to BRCA1 gene 1 protein'

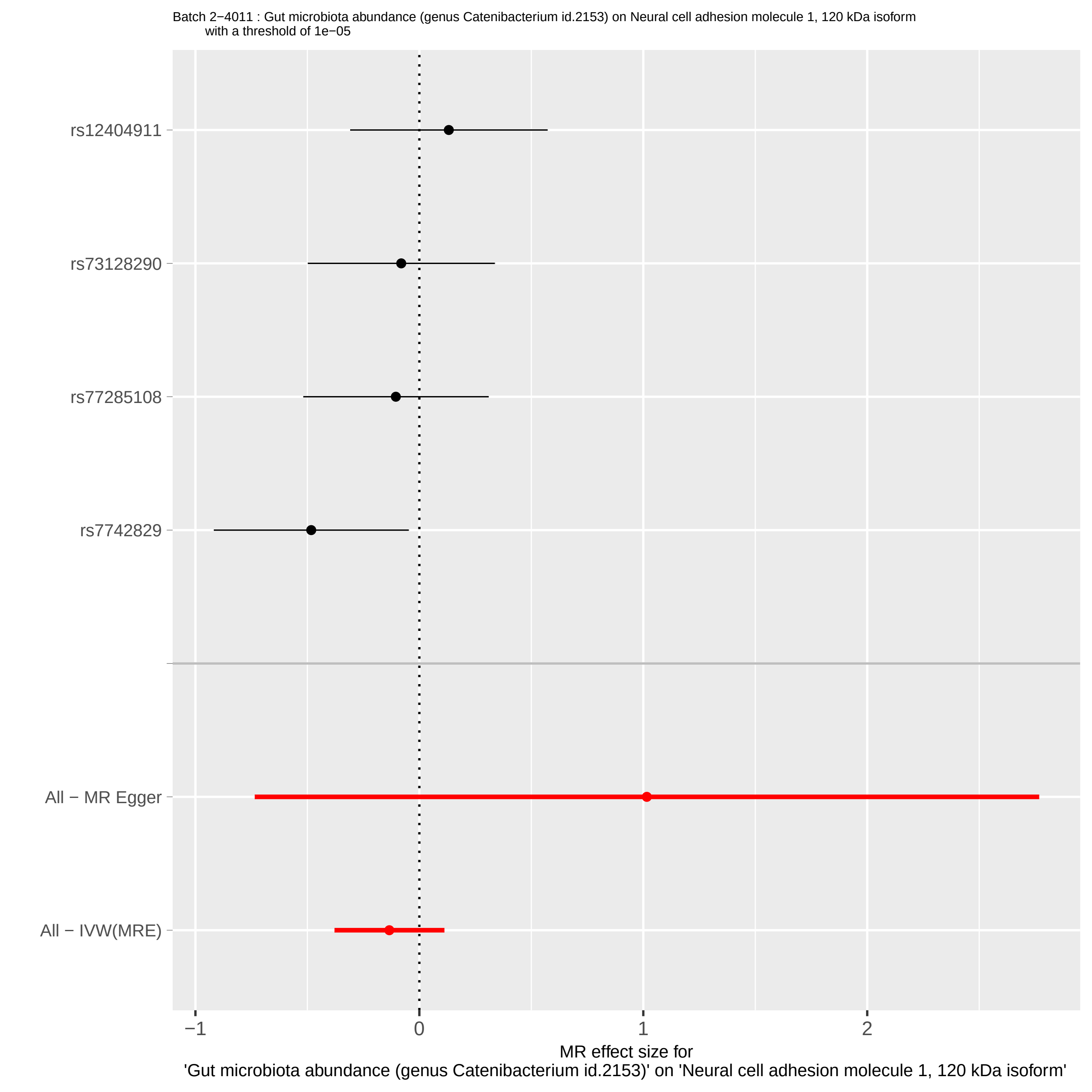

Batch 2−4011 : Gut microbiota abundance (genus Catenibacterium id.2153) on Neural cell adhesion molecule 1, 120 kDa isoform
with a threshold of 1e−05
rs12404911
rs73128290
rs77285108
rs7742829
All − MR Egger
All − IVW(MRE)
−1
0
1
2
MR effect size for
'Gut microbiota abundance (genus Catenibacterium id.2153)' on 'Neural cell adhesion molecule 1, 120 kDa isoform'

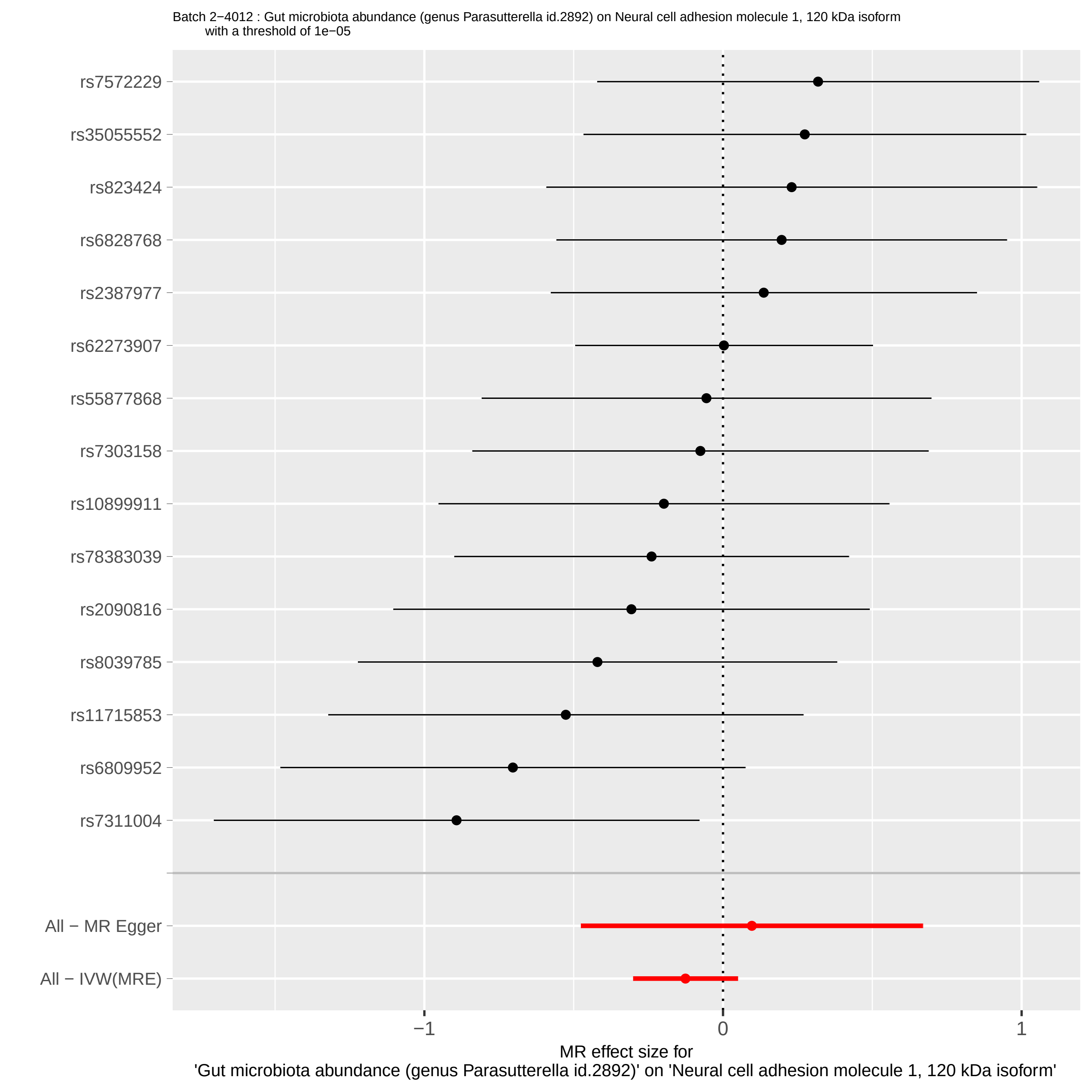

Batch 2−4012 : Gut microbiota abundance (genus Parasutterella id.2892) on Neural cell adhesion molecule 1, 120 kDa isoform
with a threshold of 1e−05
rs7572229
rs35055552
rs823424
rs6828768
rs2387977
rs62273907
rs55877868
rs7303158
rs10899911
rs78383039
rs2090816
rs8039785
rs11715853
rs6809952
rs7311004
All − MR Egger
All − IVW(MRE)
−1
0
1
MR effect size for
'Gut microbiota abundance (genus Parasutterella id.2892)' on 'Neural cell adhesion molecule 1, 120 kDa isoform'

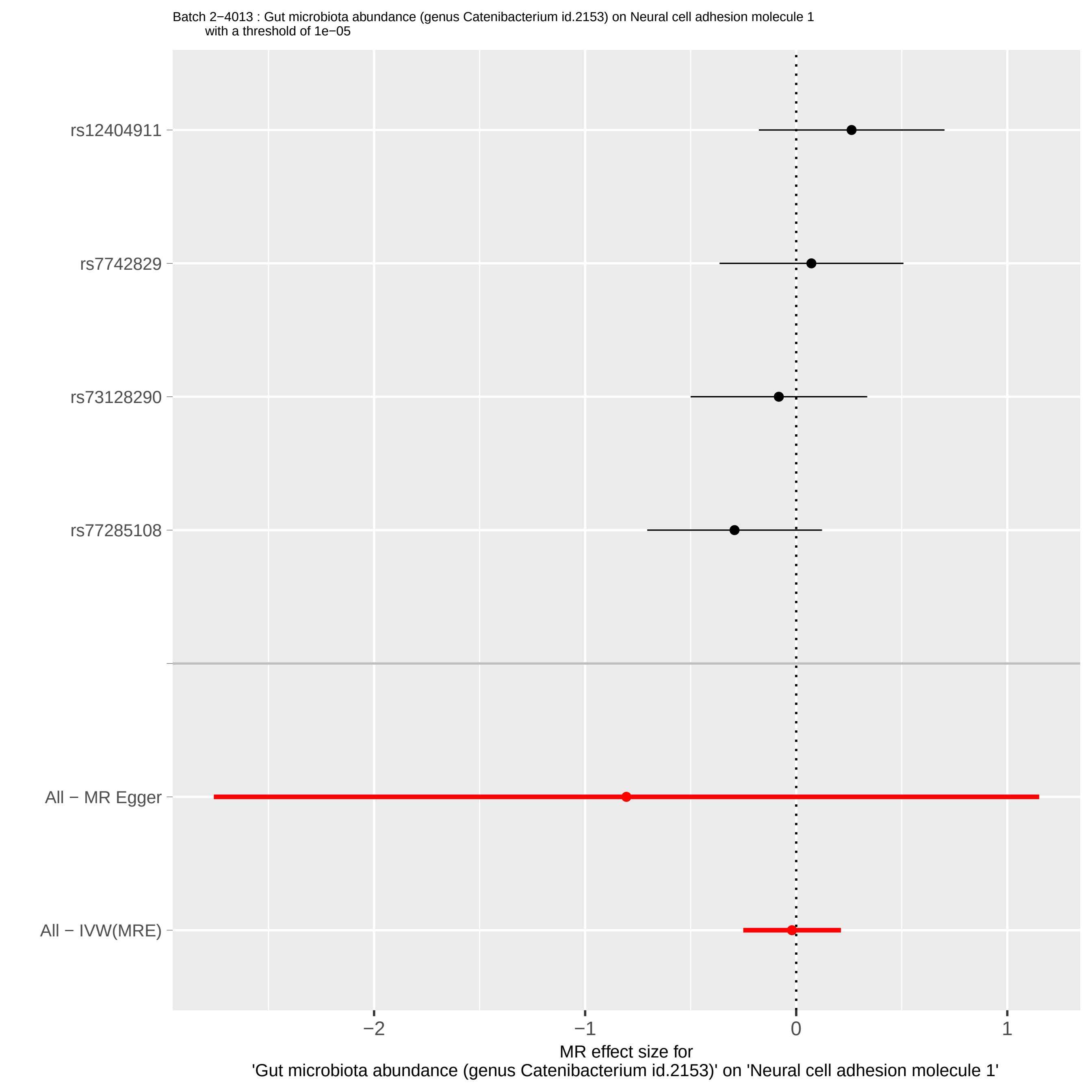
Batch 2−4013 : Gut microbiota abundance (genus Catenibacterium id.2153) on Neural cell adhesion molecule 1
with a threshold of 1e−05
rs12404911
rs7742829
rs73128290
rs77285108
All − MR Egger
All − IVW(MRE)
−2
−1
0
1
MR effect size for
'Gut microbiota abundance (genus Catenibacterium id.2153)' on 'Neural cell adhesion molecule 1'

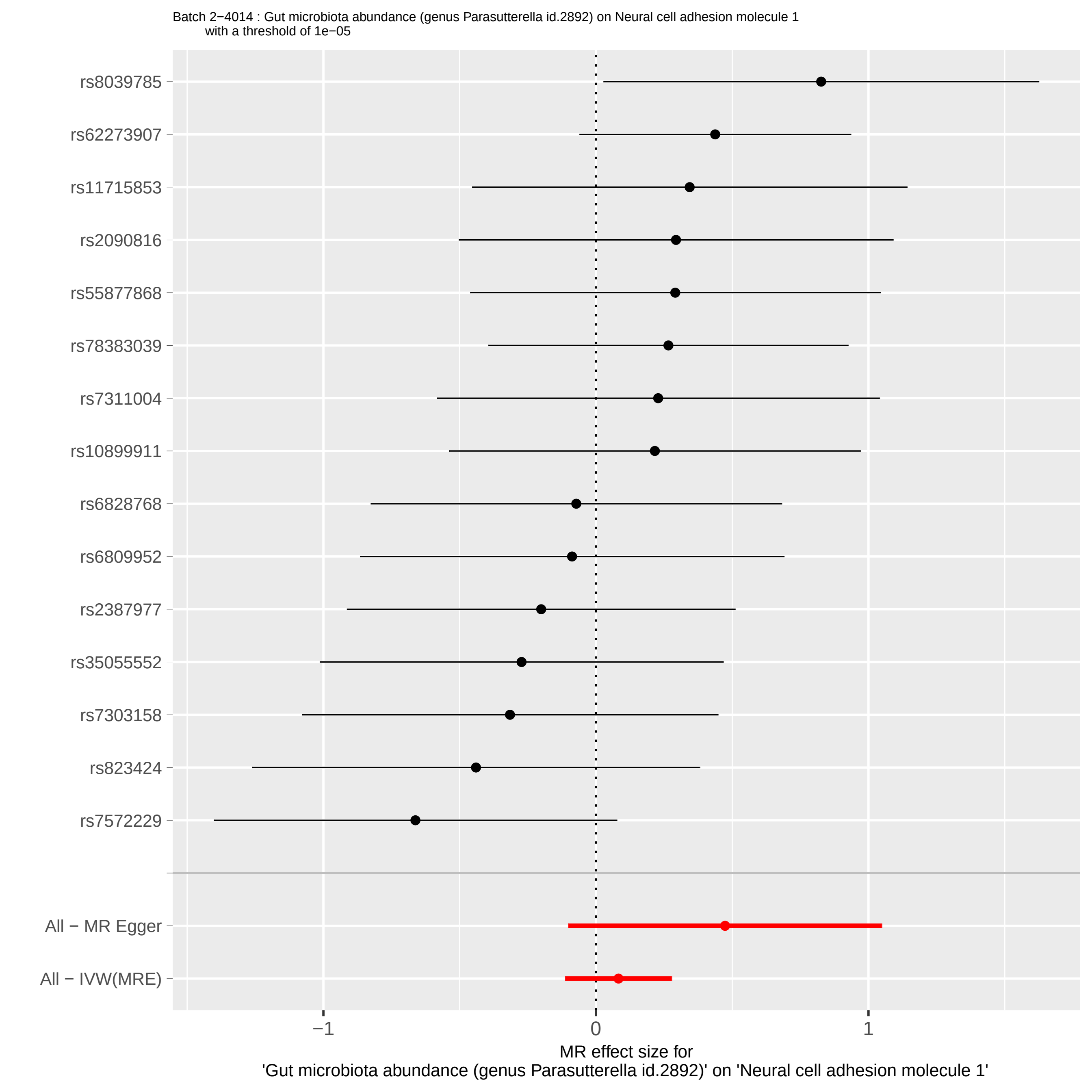

Batch 2−4014 : Gut microbiota abundance (genus Parasutterella id.2892) on Neural cell adhesion molecule 1
with a threshold of 1e−05
rs8039785
rs62273907
rs11715853
rs2090816
rs55877868
rs78383039
rs7311004
rs10899911
rs6828768
rs6809952
rs2387977
rs35055552
rs7303158
rs823424
rs7572229
All − MR Egger
All − IVW(MRE)
−1
0
1
MR effect size for
'Gut microbiota abundance (genus Parasutterella id.2892)' on 'Neural cell adhesion molecule 1'

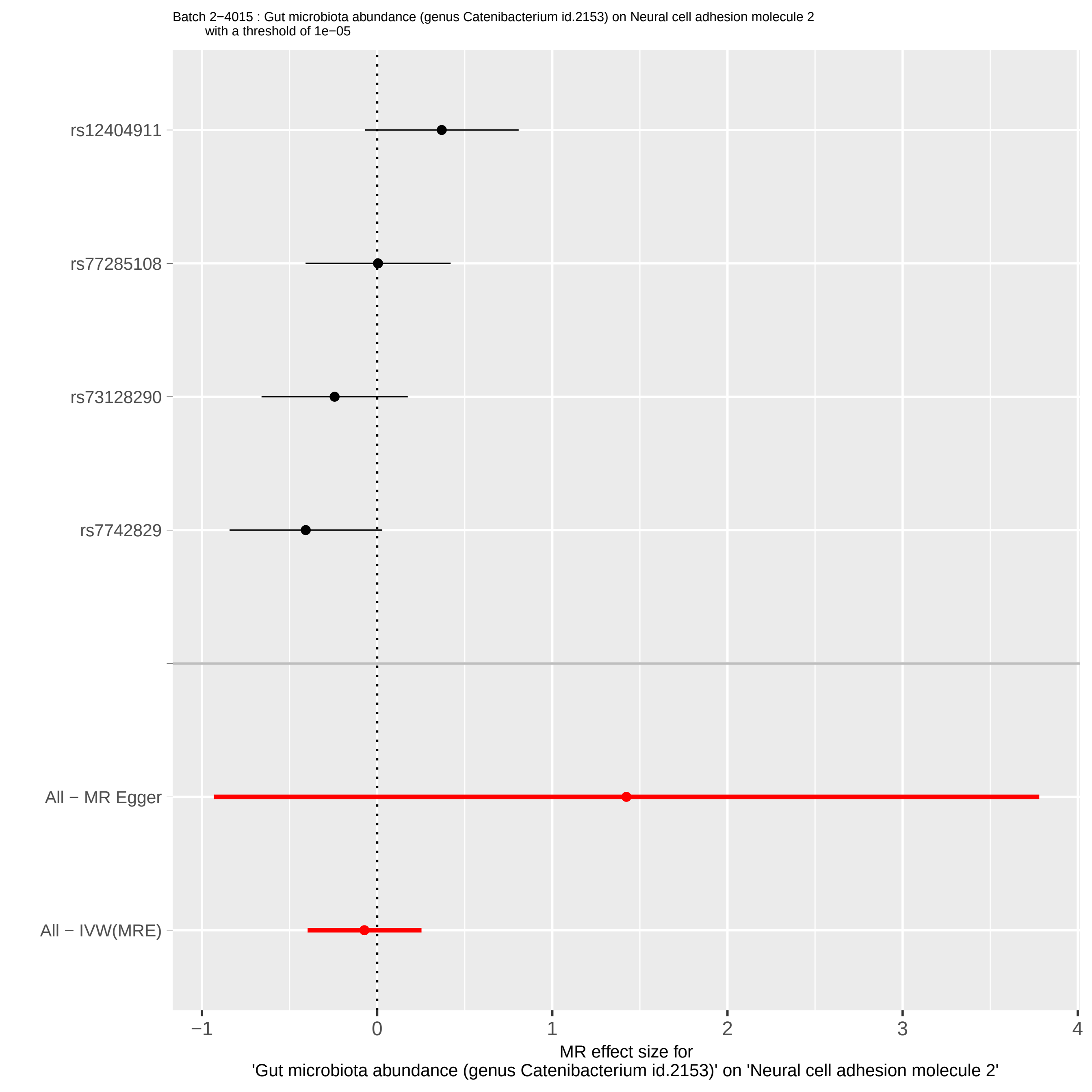

Batch 2−4015 : Gut microbiota abundance (genus Catenibacterium id.2153) on Neural cell adhesion molecule 2
with a threshold of 1e−05
rs12404911
rs77285108
rs73128290
rs7742829
All − MR Egger
All − IVW(MRE)
−1
0
1
2
3
4
MR effect size for
'Gut microbiota abundance (genus Catenibacterium id.2153)' on 'Neural cell adhesion molecule 2'

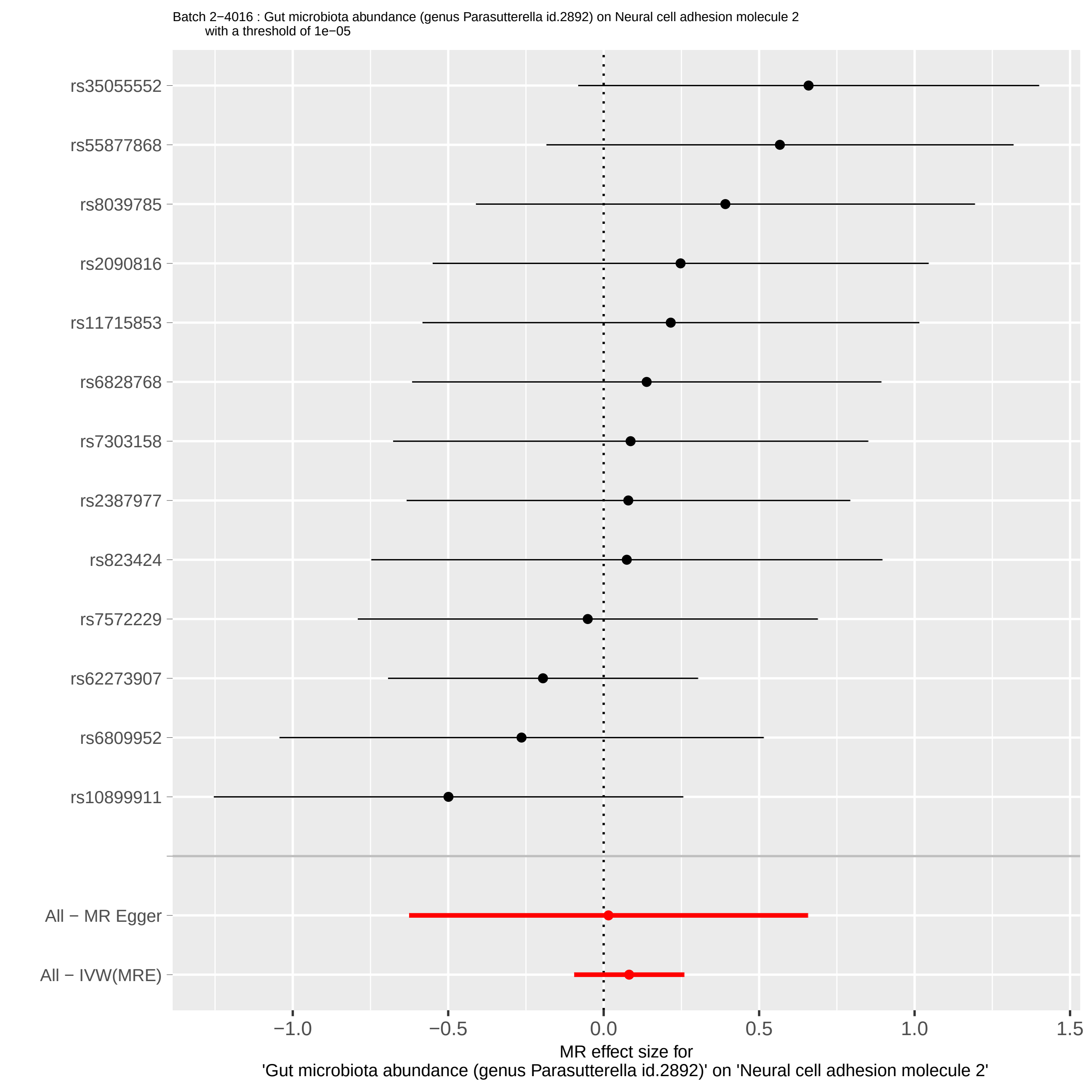

Batch 2−4016 : Gut microbiota abundance (genus Parasutterella id.2892) on Neural cell adhesion molecule 2
with a threshold of 1e−05
rs35055552
rs55877868
rs8039785
rs2090816
rs11715853
rs6828768
rs7303158
rs2387977
rs823424
rs7572229
rs62273907
rs6809952
rs10899911
All − MR Egger
All − IVW(MRE)
−1.0
−0.5
0.0
0.5
1.0
1.5
MR effect size for
'Gut microbiota abundance (genus Parasutterella id.2892)' on 'Neural cell adhesion molecule 2'

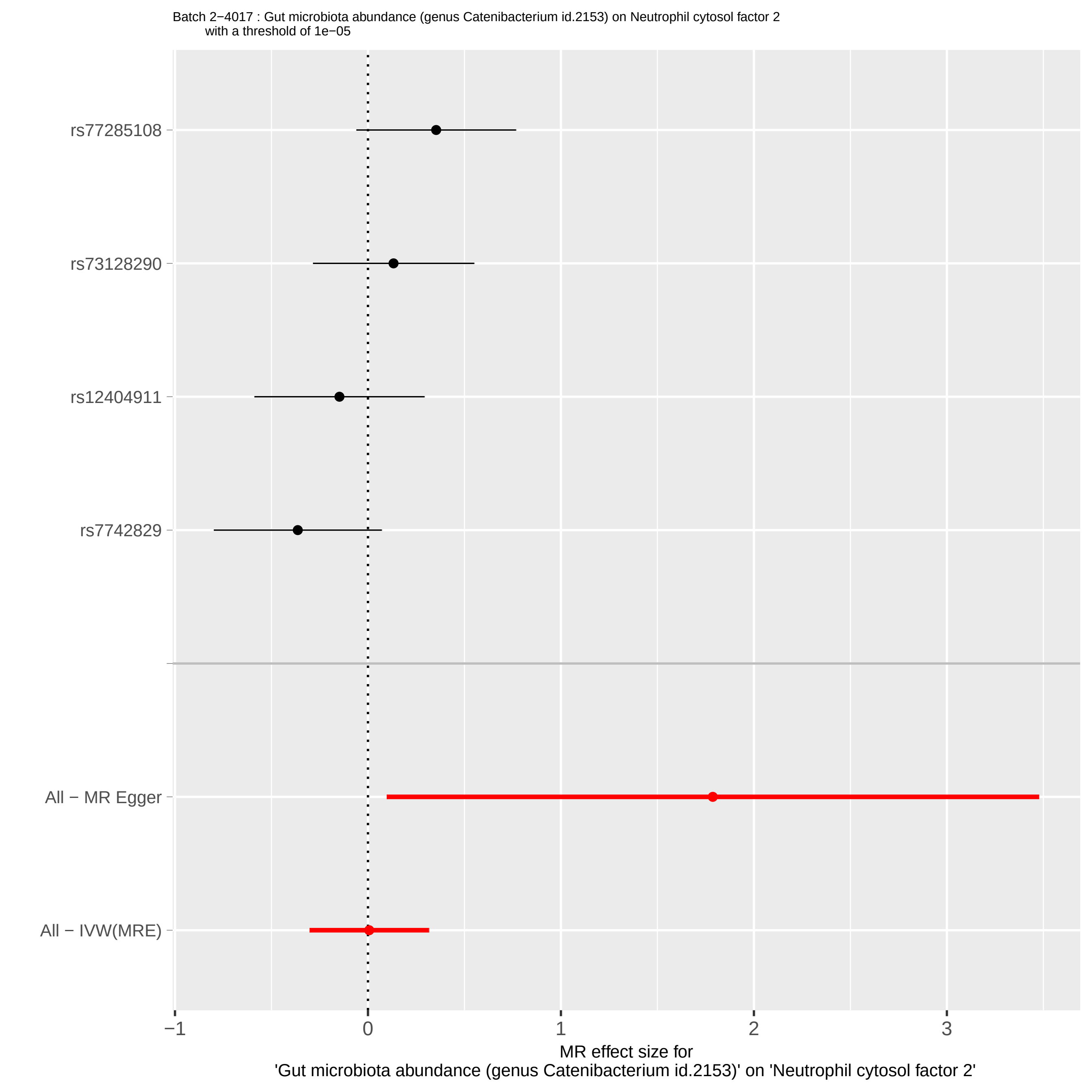

Batch 2−4017 : Gut microbiota abundance (genus Catenibacterium id.2153) on Neutrophil cytosol factor 2
with a threshold of 1e−05
rs77285108
rs73128290
rs12404911
rs7742829
All − MR Egger
All − IVW(MRE)
−1
0
1
2
3
MR effect size for
'Gut microbiota abundance (genus Catenibacterium id.2153)' on 'Neutrophil cytosol factor 2'

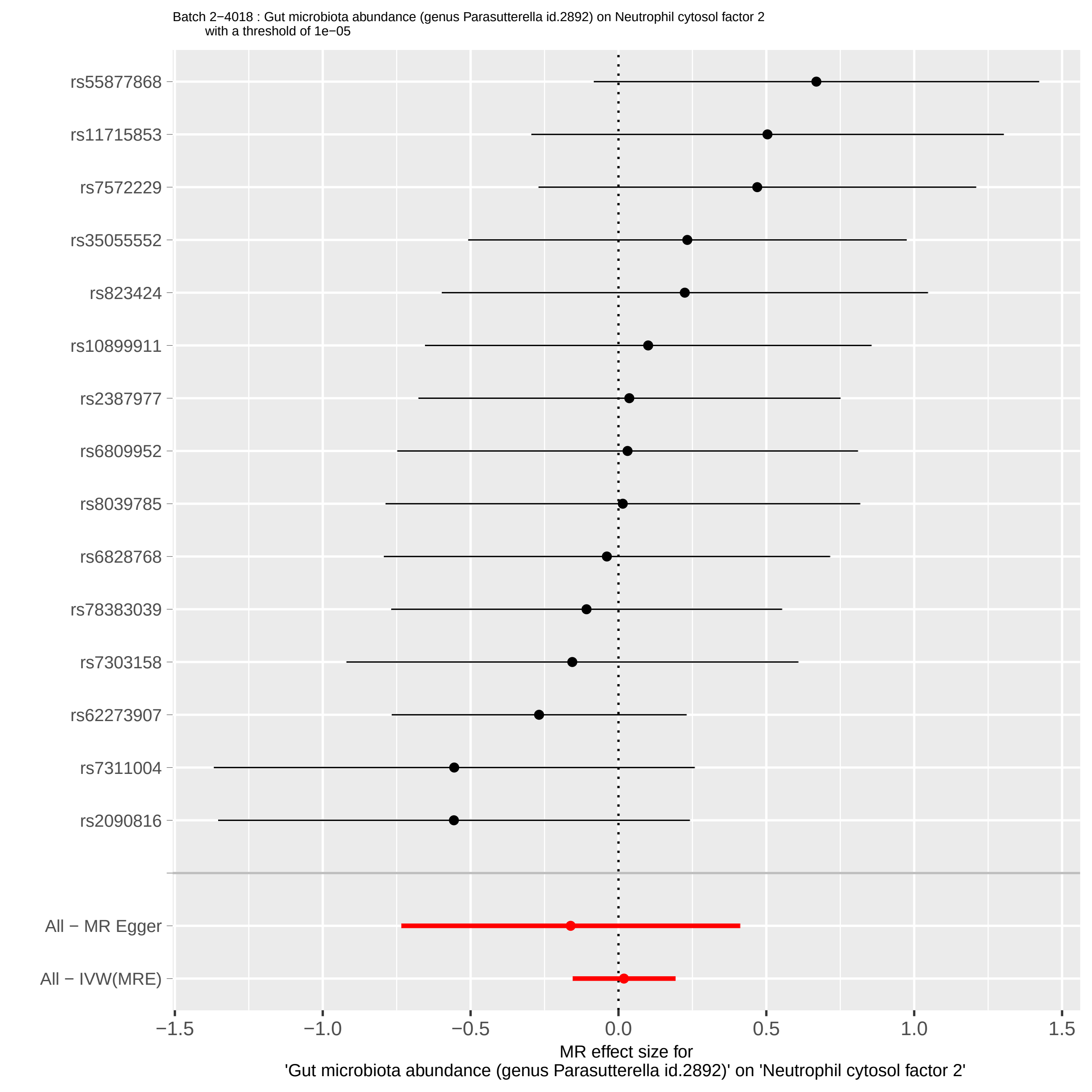
Batch 2−4018 : Gut microbiota abundance (genus Parasutterella id.2892) on Neutrophil cytosol factor 2
with a threshold of 1e−05
rs55877868
rs11715853
rs7572229
rs35055552
rs823424
rs10899911
rs2387977
rs6809952
rs8039785
rs6828768
rs78383039
rs7303158
rs62273907
rs7311004
rs2090816
All − MR Egger
All − IVW(MRE)
−1.5
−1.0
−0.5
0.0
0.5
1.0
1.5
MR effect size for
'Gut microbiota abundance (genus Parasutterella id.2892)' on 'Neutrophil cytosol factor 2'

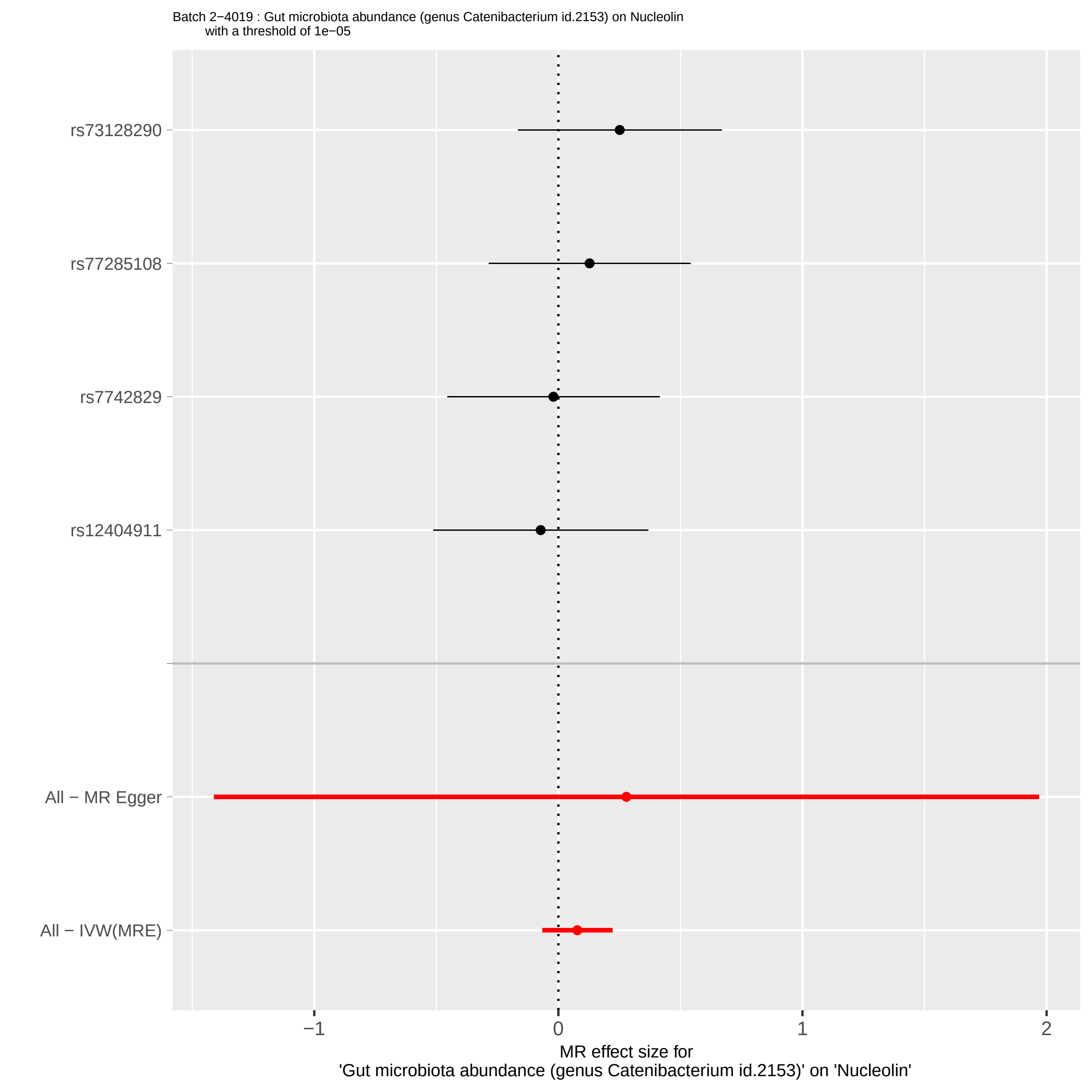
Batch 2−4019 : Gut microbiota abundance (genus Catenibacterium id.2153) on Nucleolin
with a threshold of 1e−05
rs73128290
rs77285108
rs7742829
rs12404911
All − MR Egger
All − IVW(MRE)
−1
0
1
2
MR effect size for
'Gut microbiota abundance (genus Catenibacterium id.2153)' on 'Nucleolin'

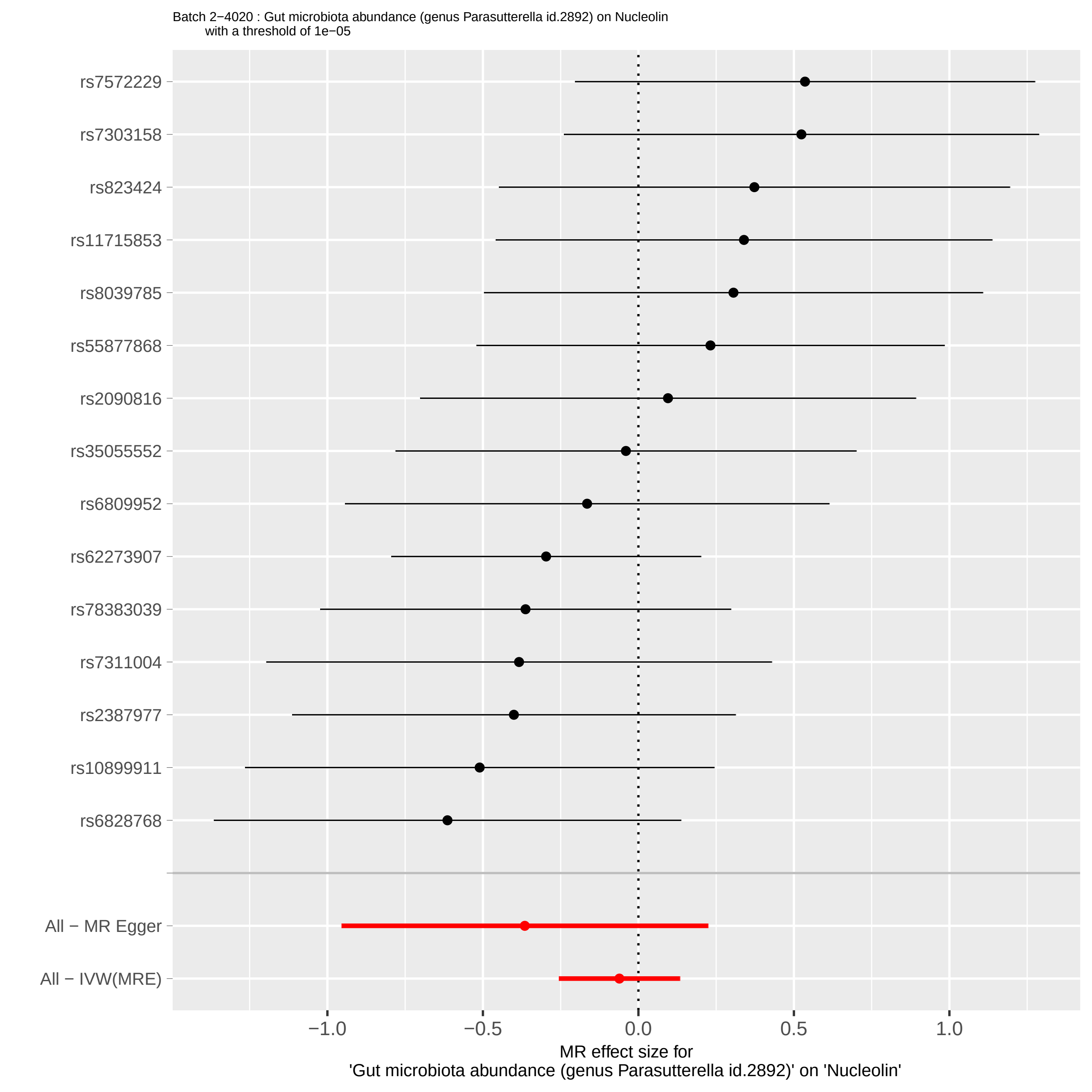
Batch 2−4020 : Gut microbiota abundance (genus Parasutterella id.2892) on Nucleolin
with a threshold of 1e−05
rs7572229
rs7303158
rs823424
rs11715853
rs8039785
rs55877868
rs2090816
rs35055552
rs6809952
rs62273907
rs78383039
rs7311004
rs2387977
rs10899911
rs6828768
All − MR Egger
All − IVW(MRE)
−1.0
−0.5
0.0
0.5
1.0
MR effect size for
'Gut microbiota abundance (genus Parasutterella id.2892)' on 'Nucleolin'

Batch 2−4021 : Gut microbiota abundance (genus Catenibacterium id.2153) on Noncompact myelin−associated protein
with a threshold of 1e−05

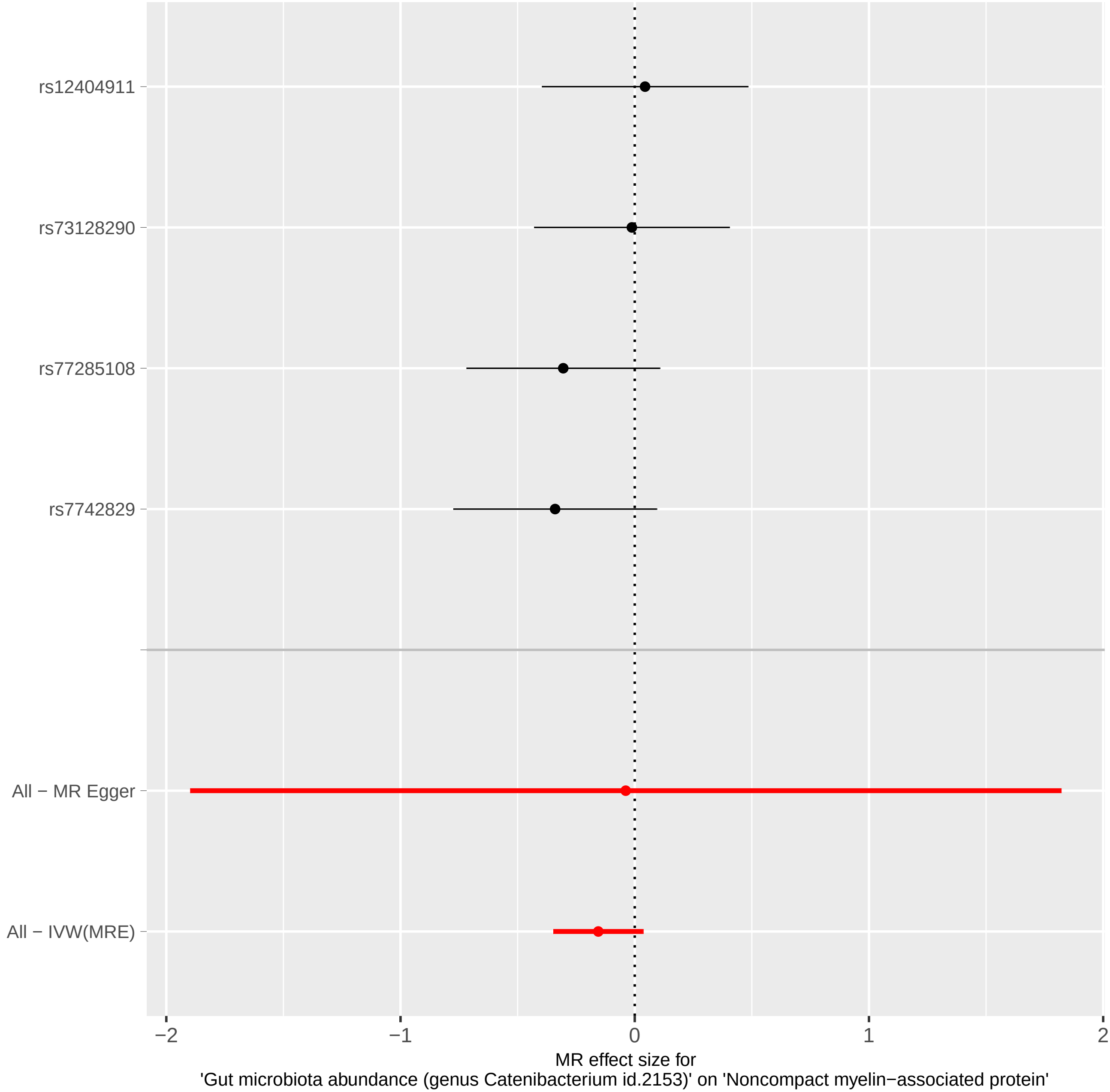

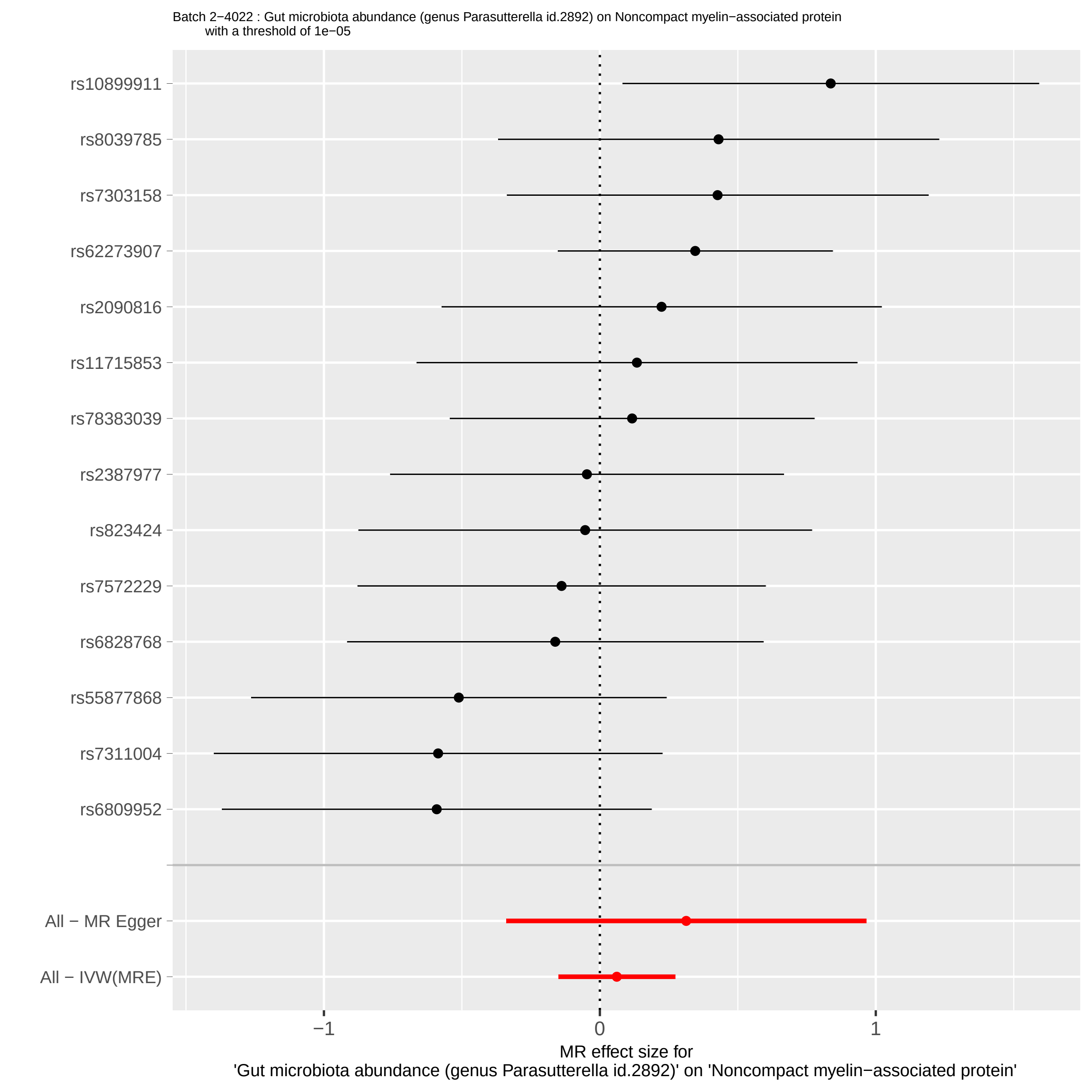

Batch 2−4022 : Gut microbiota abundance (genus Parasutterella id.2892) on Noncompact myelin−associated protein
with a threshold of 1e−05
rs10899911
rs8039785
rs7303158
rs62273907
rs2090816
rs11715853
rs78383039
rs2387977
rs823424
rs7572229
rs6828768
rs55877868
rs7311004
rs6809952
All − MR Egger
All − IVW(MRE)
−1
0
1
MR effect size for
'Gut microbiota abundance (genus Parasutterella id.2892)' on 'Noncompact myelin−associated protein'

Batch 2−4023 : Gut microbiota abundance (genus Catenibacterium id.2153) on Nuclear receptor coactivator 2
with a threshold of 1e−05

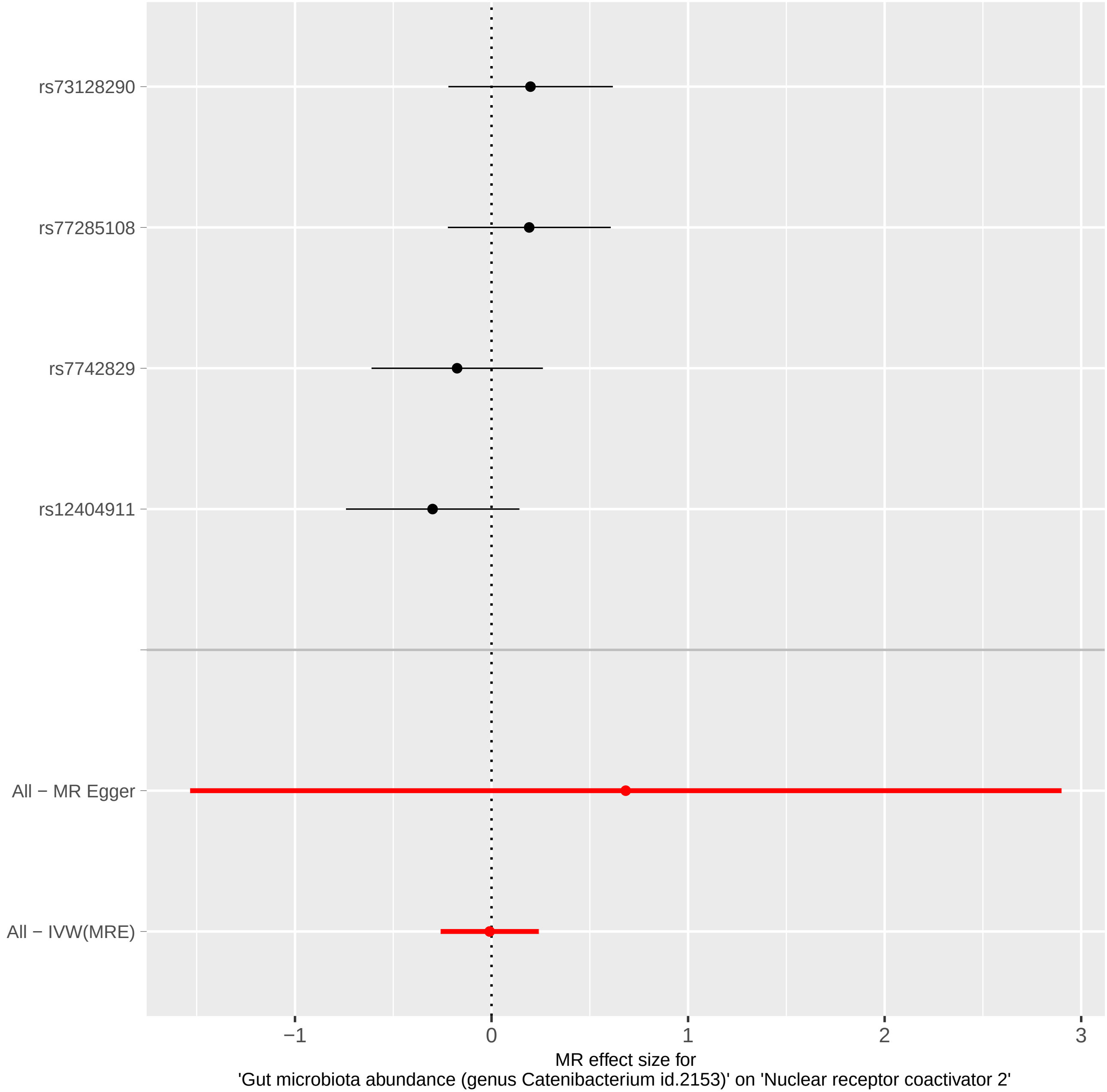

Batch 2−4024 : Gut microbiota abundance (genus Parasutterella id.2892) on Nuclear receptor coactivator 2
with a threshold of 1e−05

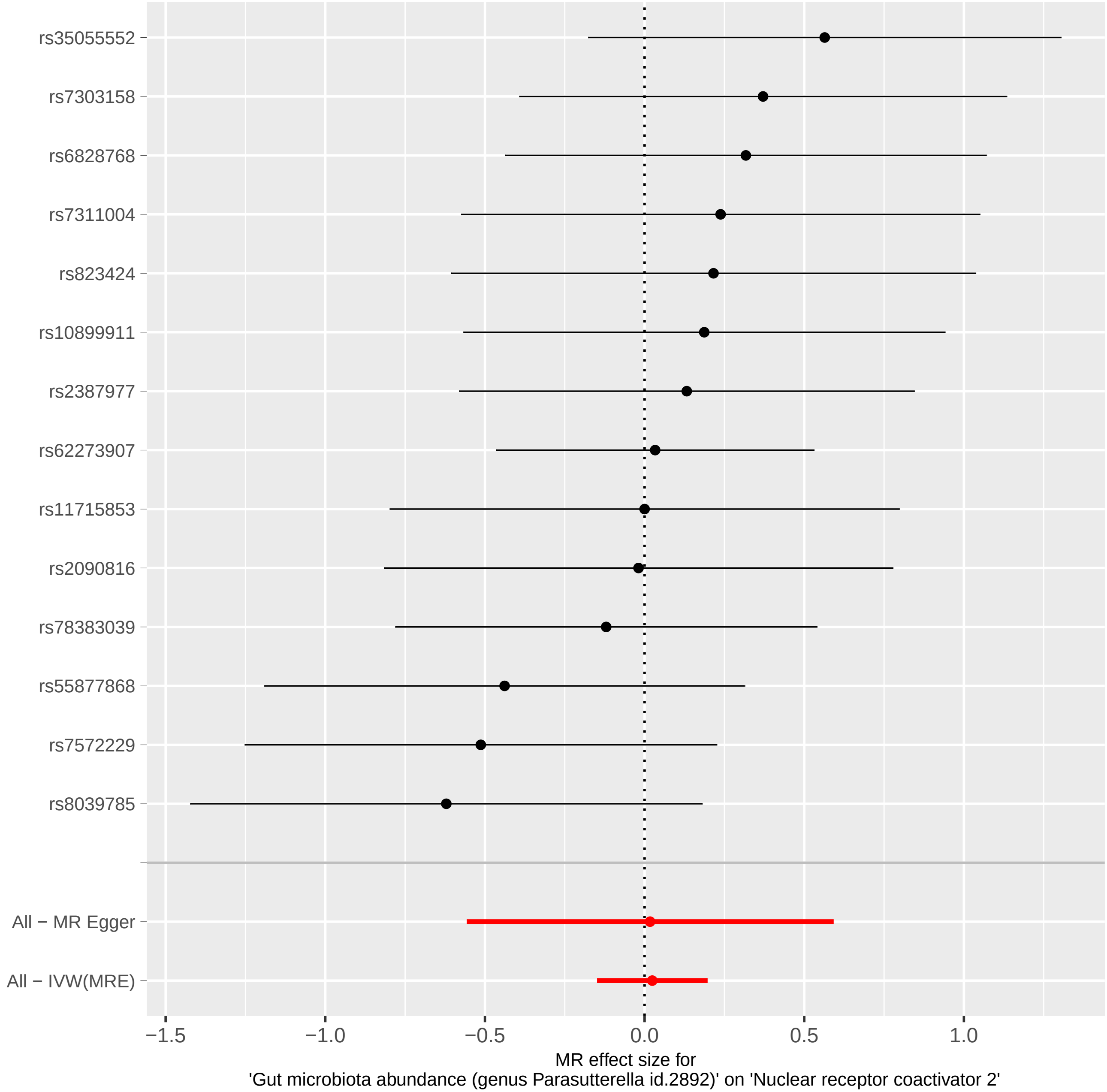

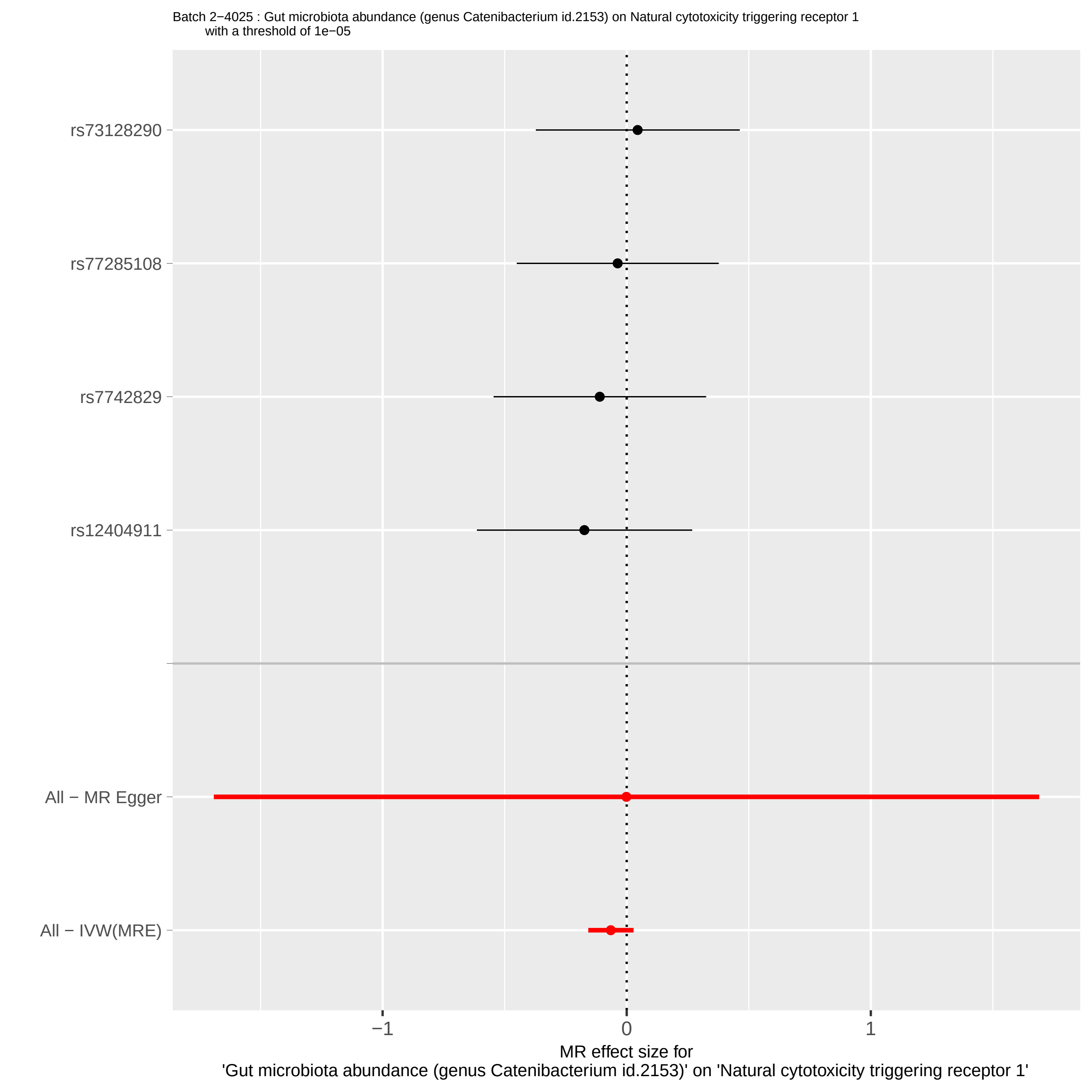

Batch 2−4025 : Gut microbiota abundance (genus Catenibacterium id.2153) on Natural cytotoxicity triggering receptor 1
with a threshold of 1e−05
rs73128290
rs77285108
rs7742829
rs12404911
All − MR Egger
All − IVW(MRE)
−1
0
1
MR effect size for
'Gut microbiota abundance (genus Catenibacterium id.2153)' on 'Natural cytotoxicity triggering receptor 1'

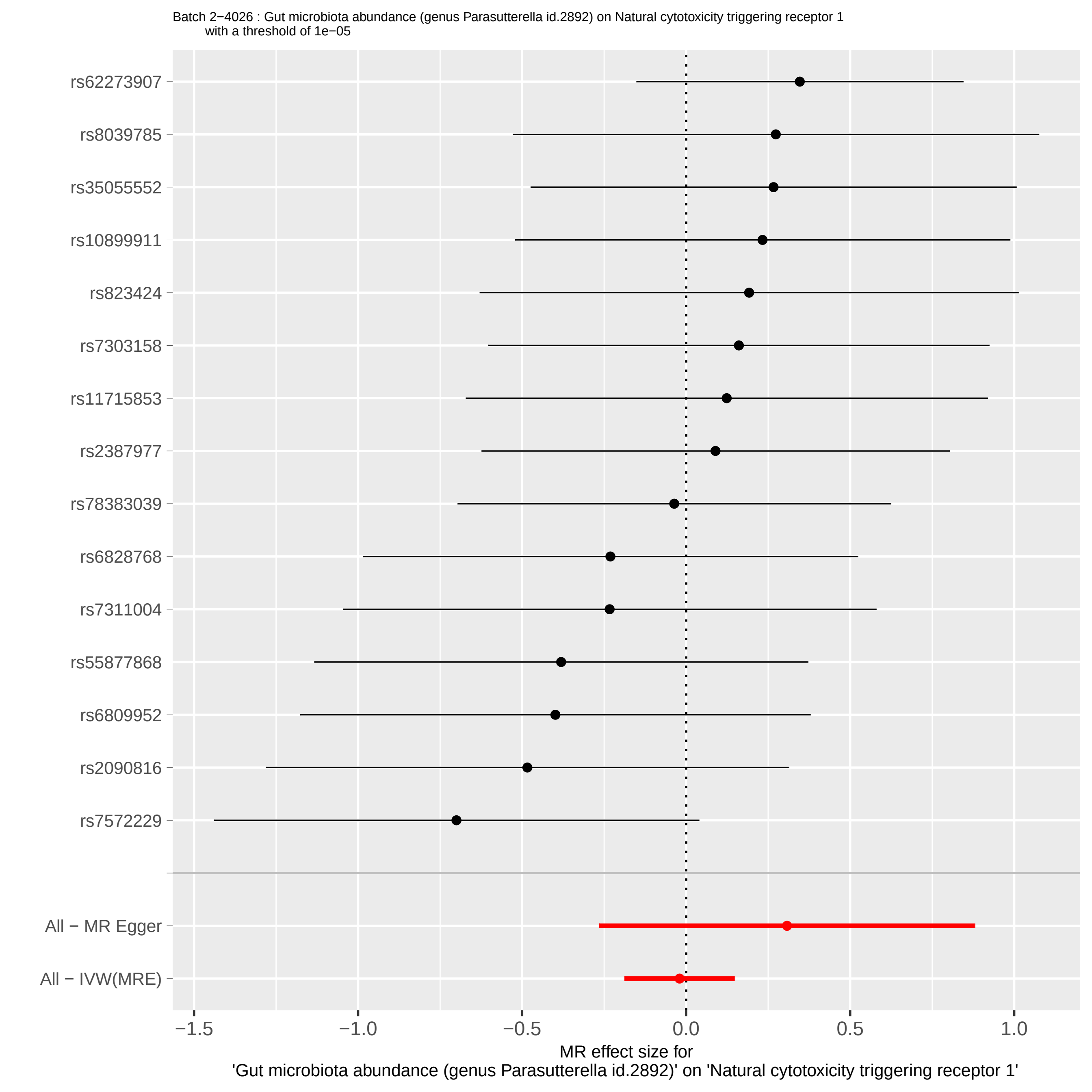

Batch 2−4026 : Gut microbiota abundance (genus Parasutterella id.2892) on Natural cytotoxicity triggering receptor 1
with a threshold of 1e−05
rs62273907
rs8039785
rs35055552
rs10899911
rs823424
rs7303158
rs11715853
rs2387977
rs78383039
rs6828768
rs7311004
rs55877868
rs6809952
rs2090816
rs7572229
All − MR Egger
All − IVW(MRE)
−1.5
−1.0
−0.5
0.0
0.5
1.0
MR effect size for
'Gut microbiota abundance (genus Parasutterella id.2892)' on 'Natural cytotoxicity triggering receptor 1'

Batch 2−4027 : Gut microbiota abundance (genus Catenibacterium id.2153) on Natural cytotoxicity triggering receptor 1
with a threshold of 1e−05

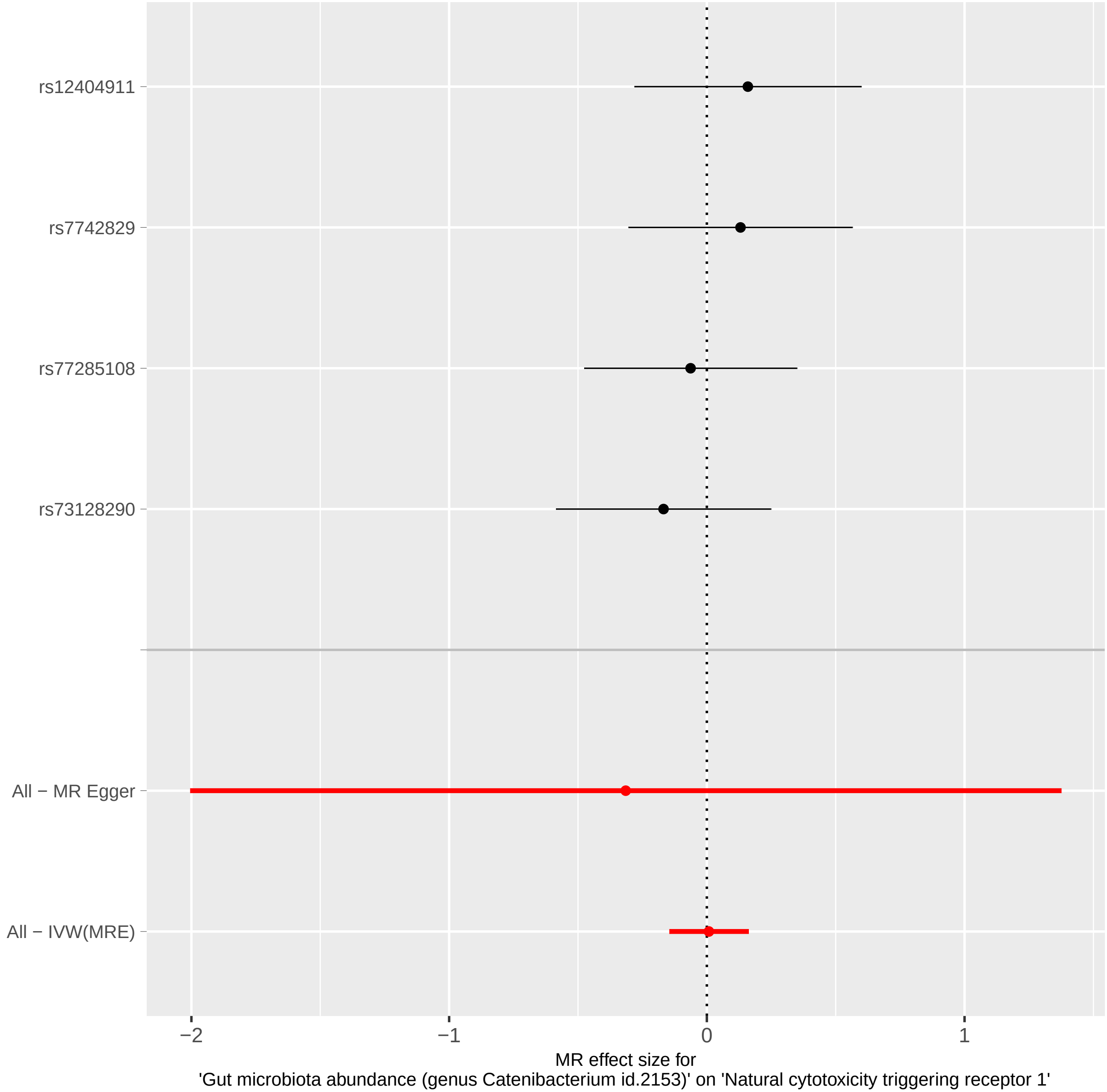

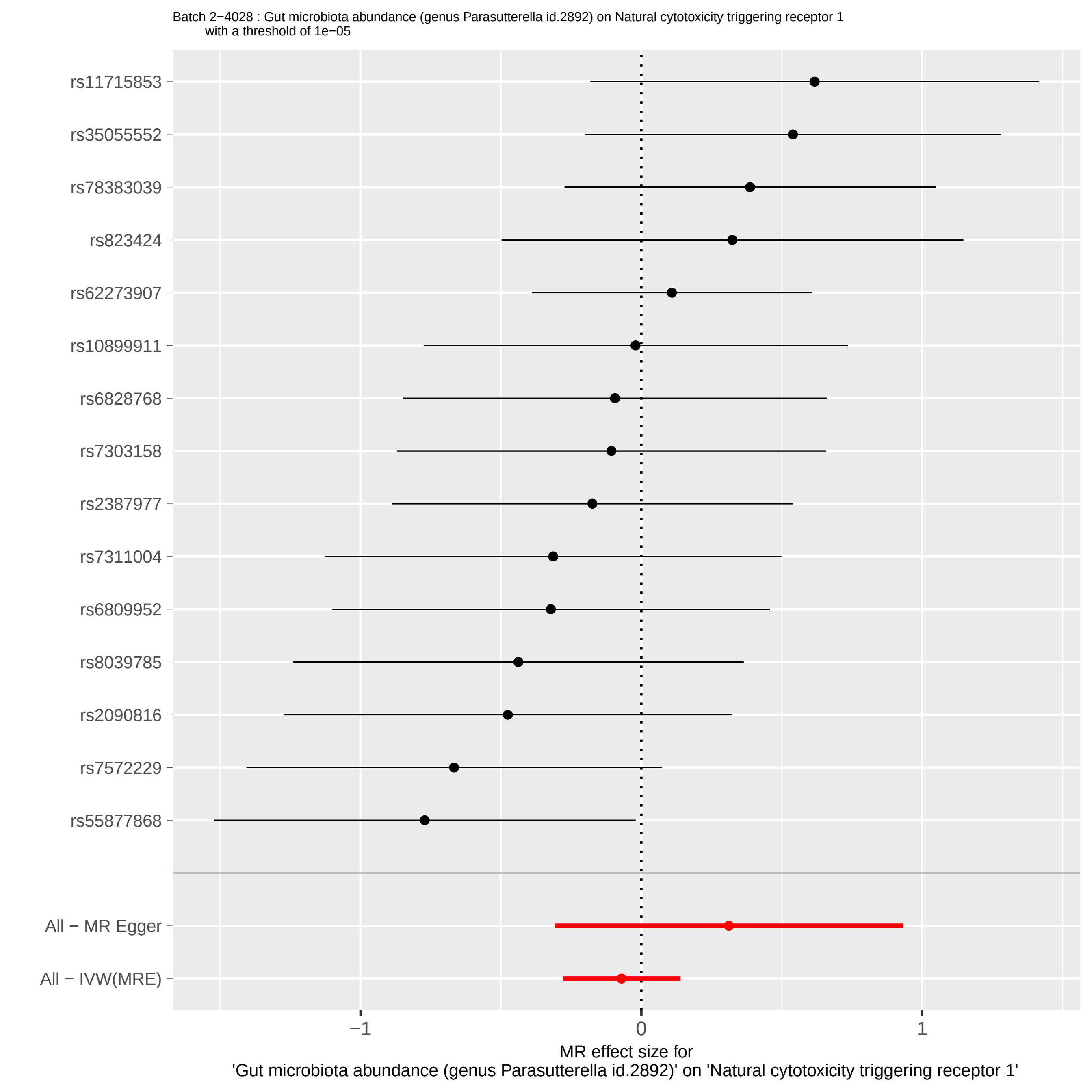
Batch 2−4028 : Gut microbiota abundance (genus Parasutterella id.2892) on Natural cytotoxicity triggering receptor 1
with a threshold of 1e−05
rs11715853
rs35055552
rs78383039
rs823424
rs62273907
rs10899911
rs6828768
rs7303158
rs2387977
rs7311004
rs6809952
rs8039785
rs2090816
rs7572229
rs55877868
All − MR Egger
All − IVW(MRE)
−1
0
1
MR effect size for
'Gut microbiota abundance (genus Parasutterella id.2892)' on 'Natural cytotoxicity triggering receptor 1'

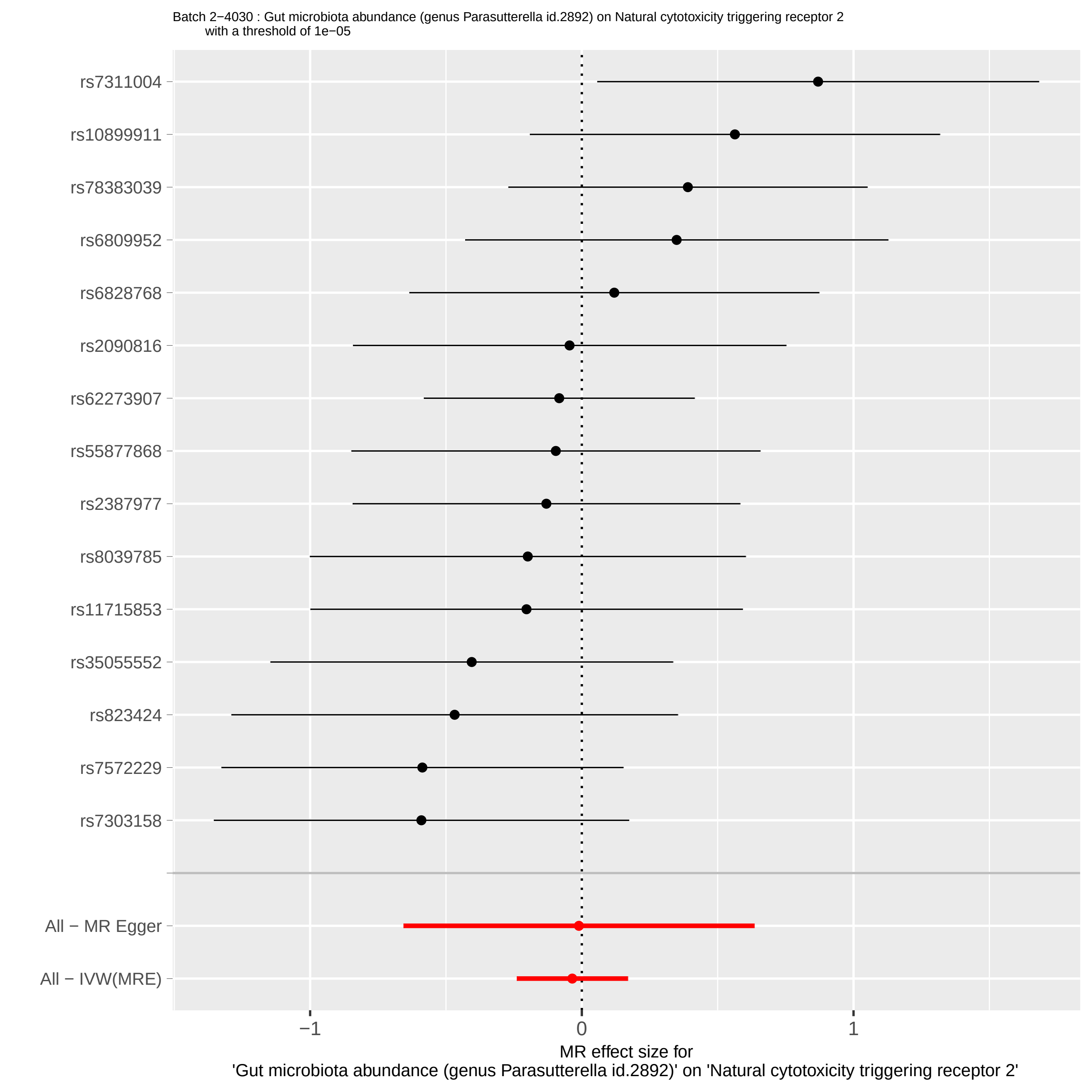

Batch 2−4030 : Gut microbiota abundance (genus Parasutterella id.2892) on Natural cytotoxicity triggering receptor 2
with a threshold of 1e−05
rs7311004
rs10899911
rs78383039
rs6809952
rs6828768
rs2090816
rs62273907
rs55877868
rs2387977
rs8039785
rs11715853
rs35055552
rs823424
rs7572229
rs7303158
All − MR Egger
All − IVW(MRE)
−1
0
1
MR effect size for
'Gut microbiota abundance (genus Parasutterella id.2892)' on 'Natural cytotoxicity triggering receptor 2'

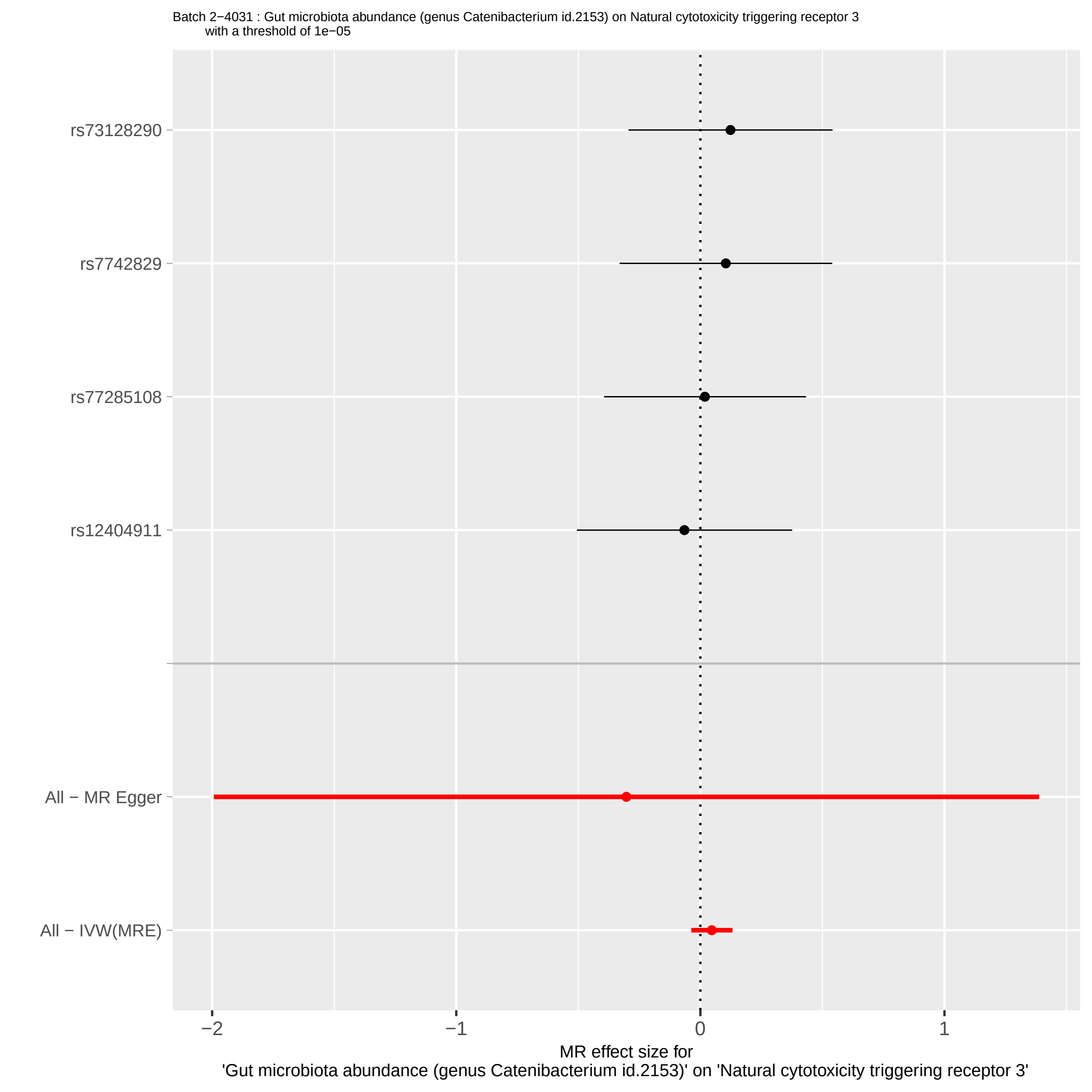

Batch 2−4031 : Gut microbiota abundance (genus Catenibacterium id.2153) on Natural cytotoxicity triggering receptor 3
with a threshold of 1e−05
rs73128290
rs7742829
rs77285108
rs12404911
All − MR Egger
All − IVW(MRE)
−2
−1
0
1
MR effect size for
'Gut microbiota abundance (genus Catenibacterium id.2153)' on 'Natural cytotoxicity triggering receptor 3'

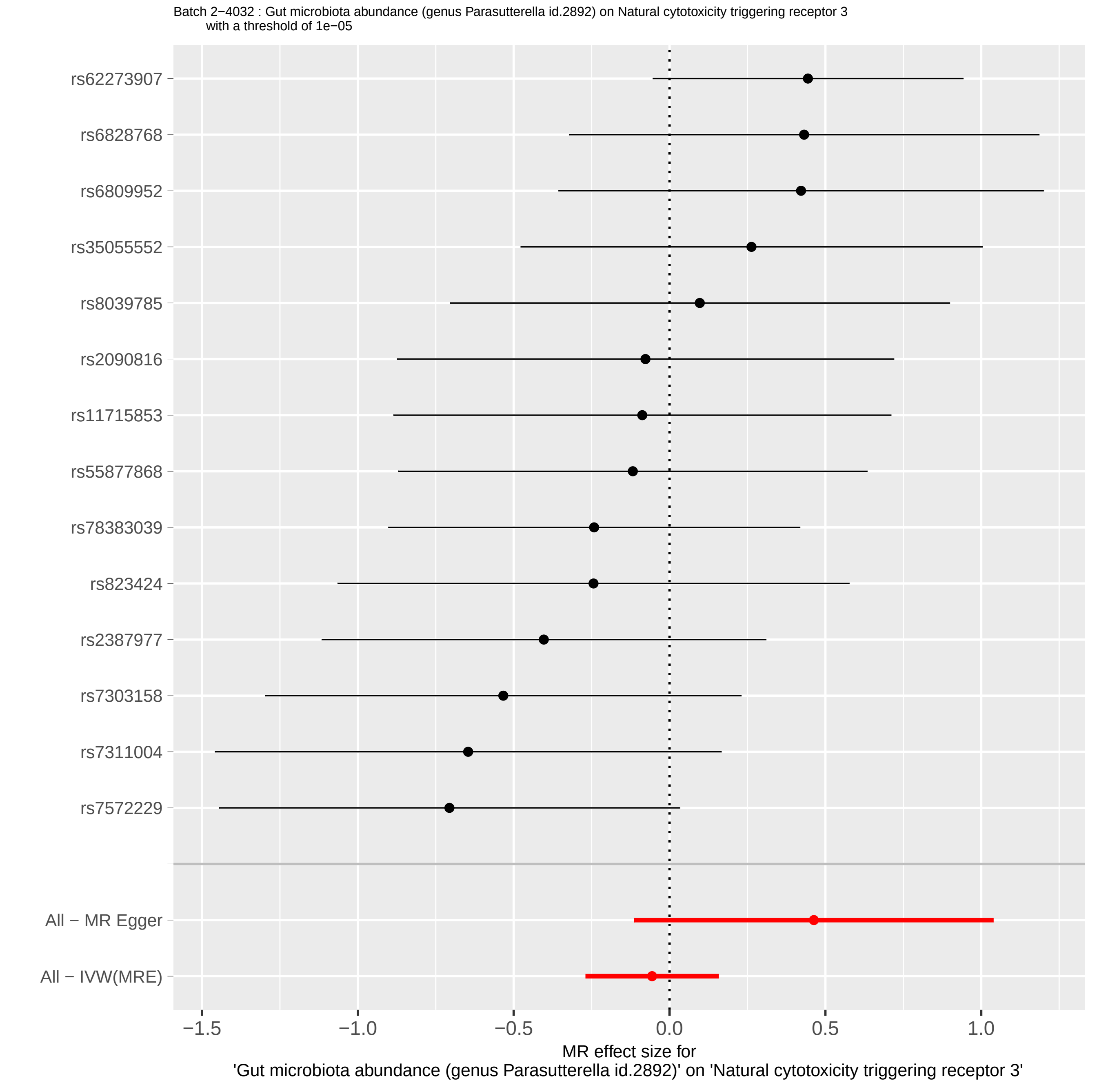
Batch 2−4032 : Gut microbiota abundance (genus Parasutterella id.2892) on Natural cytotoxicity triggering receptor 3
with a threshold of 1e−05
rs62273907
rs6828768
rs6809952
rs35055552
rs8039785
rs2090816
rs11715853
rs55877868
rs78383039
rs823424
rs2387977
rs7303158
rs7311004
rs7572229
All − MR Egger
All − IVW(MRE)
−1.5
−1.0
−0.5
0.0
0.5
1.0
MR effect size for
'Gut microbiota abundance (genus Parasutterella id.2892)' on 'Natural cytotoxicity triggering receptor 3'

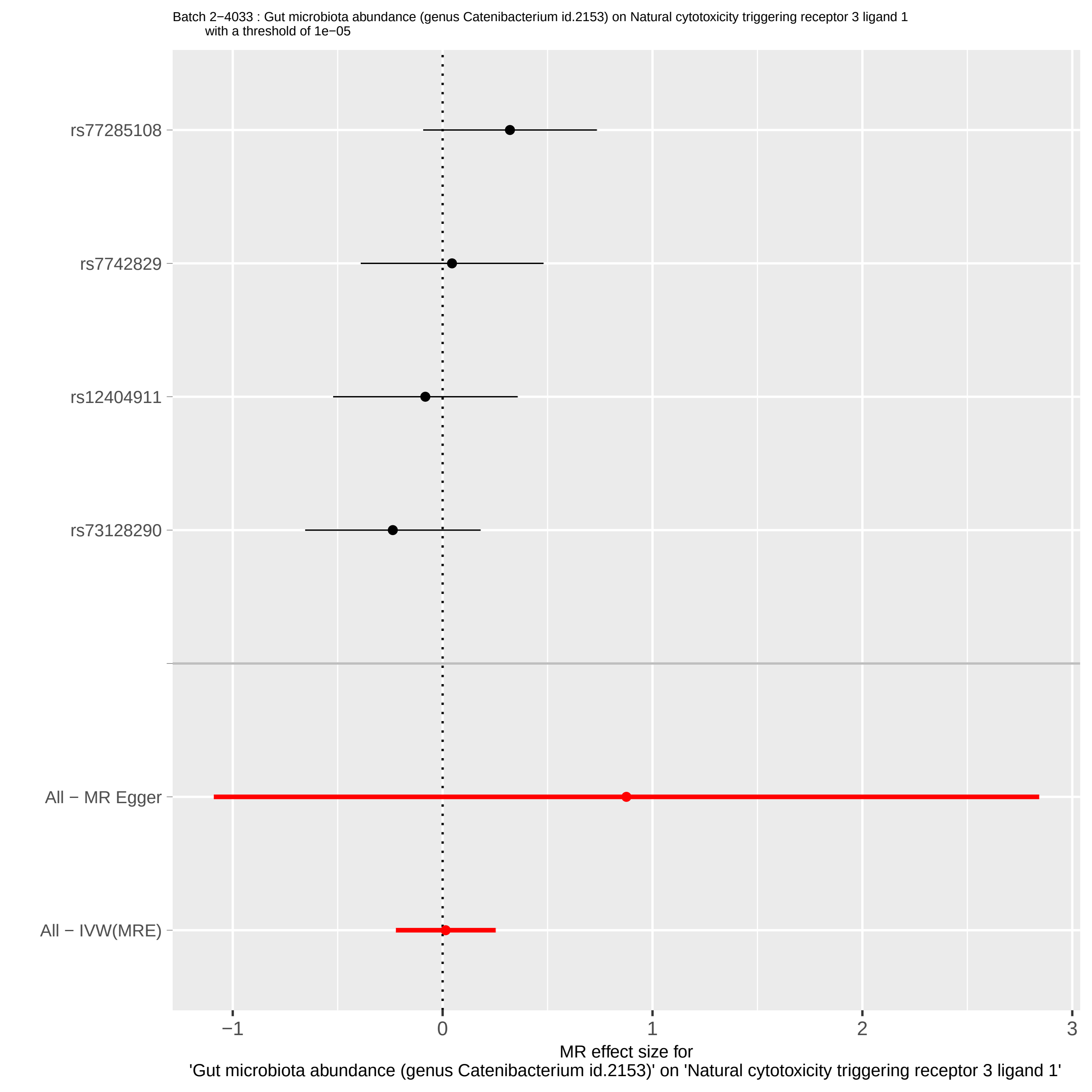

Batch 2−4033 : Gut microbiota abundance (genus Catenibacterium id.2153) on Natural cytotoxicity triggering receptor 3 ligand 1
with a threshold of 1e−05
rs77285108
rs7742829
rs12404911
rs73128290
All − MR Egger
All − IVW(MRE)
−1
0
1
2
3
MR effect size for
'Gut microbiota abundance (genus Catenibacterium id.2153)' on 'Natural cytotoxicity triggering receptor 3 ligand 1'

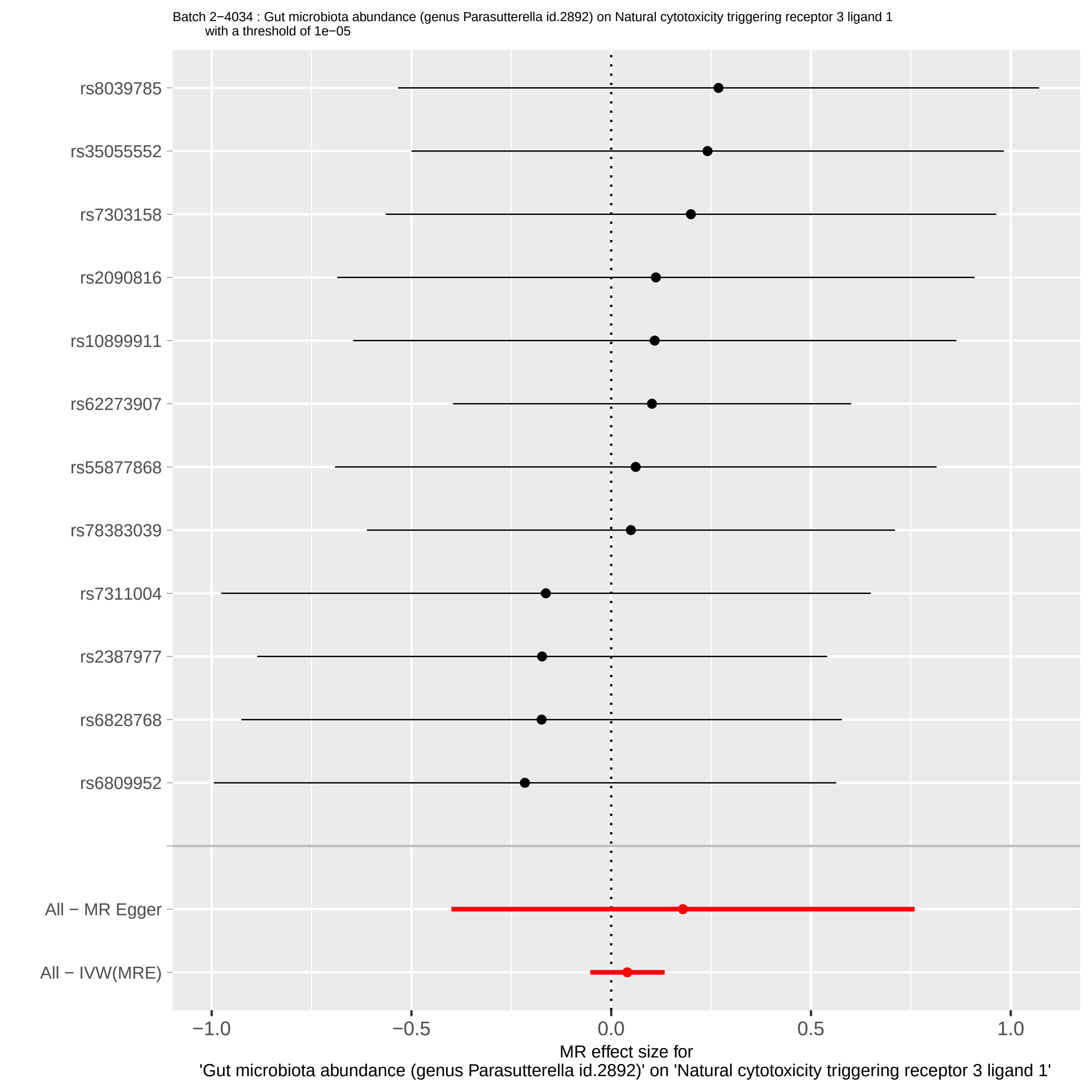

Batch 2−4034 : Gut microbiota abundance (genus Parasutterella id.2892) on Natural cytotoxicity triggering receptor 3 ligand 1
with a threshold of 1e−05
rs8039785
rs35055552
rs7303158
rs2090816
rs10899911
rs62273907
rs55877868
rs78383039
rs7311004
rs2387977
rs6828768
rs6809952
All − MR Egger
All − IVW(MRE)
−1.0
−0.5
0.0
0.5
1.0
MR effect size for
'Gut microbiota abundance (genus Parasutterella id.2892)' on 'Natural cytotoxicity triggering receptor 3 ligand 1'

Batch 2−4035 : Gut microbiota abundance (genus Catenibacterium id.2153) on Kinetochore protein NDC80 homolog
with a threshold of 1e−05

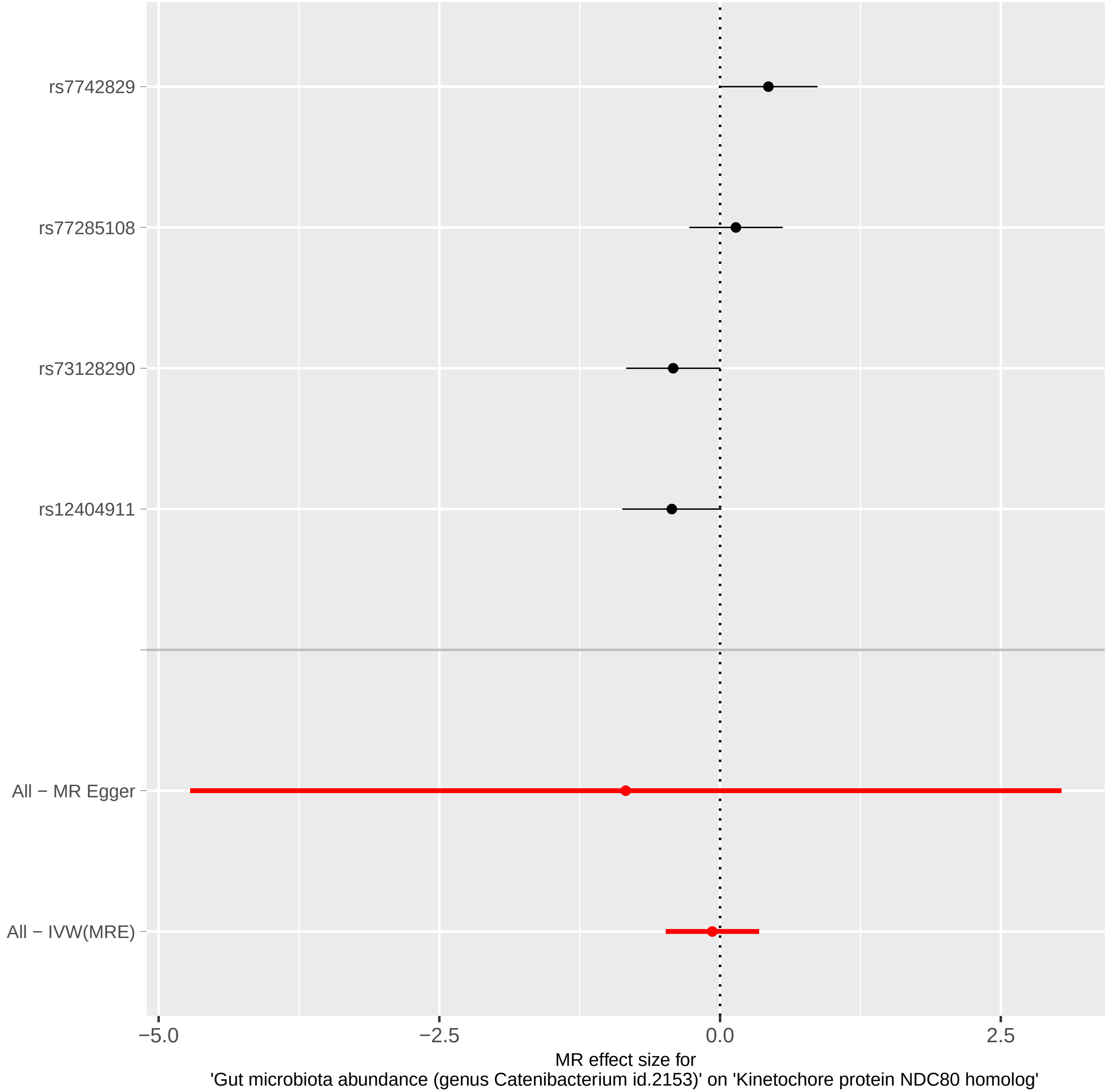

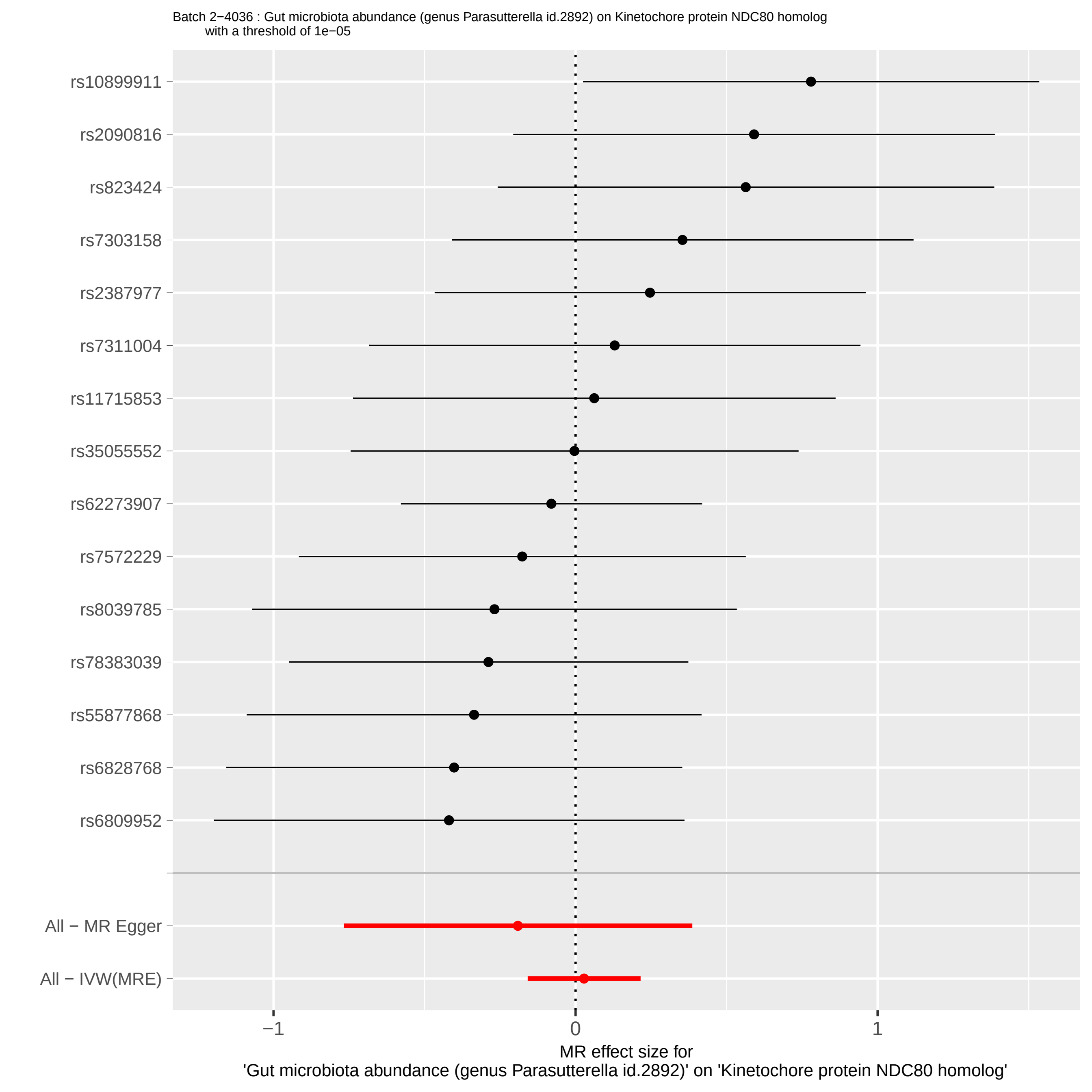
Batch 2−4036 : Gut microbiota abundance (genus Parasutterella id.2892) on Kinetochore protein NDC80 homolog
with a threshold of 1e−05
rs10899911
rs2090816
rs823424
rs7303158
rs2387977
rs7311004
rs11715853
rs35055552
rs62273907
rs7572229
rs8039785
rs78383039
rs55877868
rs6828768
rs6809952
All − MR Egger
All − IVW(MRE)
−1
0
1
MR effect size for
'Gut microbiota abundance (genus Parasutterella id.2892)' on 'Kinetochore protein NDC80 homolog'

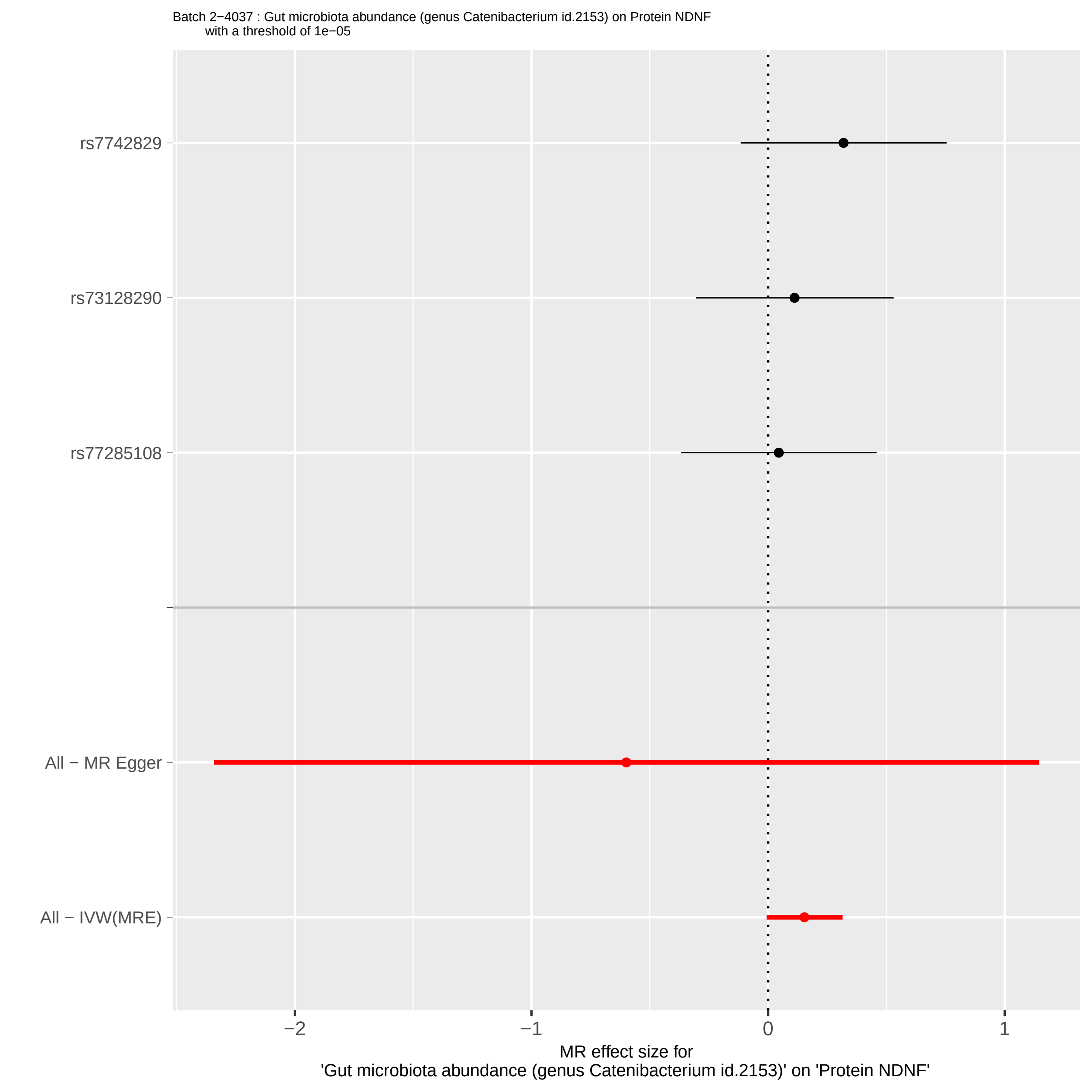

Batch 2−4037 : Gut microbiota abundance (genus Catenibacterium id.2153) on Protein NDNF
with a threshold of 1e−05
rs7742829
rs73128290
rs77285108
All − MR Egger
All − IVW(MRE)
−2
−1
0
1
MR effect size for
'Gut microbiota abundance (genus Catenibacterium id.2153)' on 'Protein NDNF'

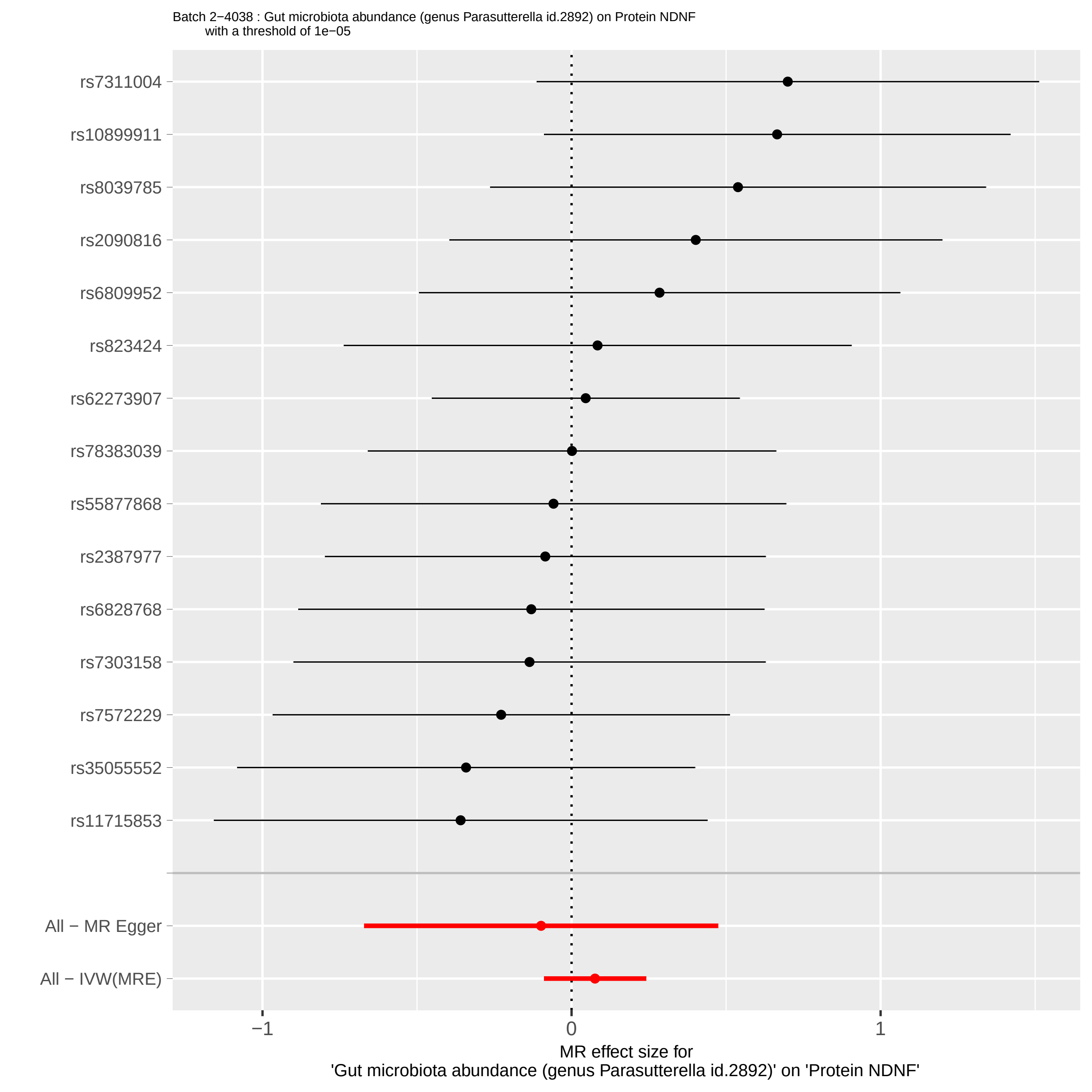

Batch 2−4038 : Gut microbiota abundance (genus Parasutterella id.2892) on Protein NDNF
with a threshold of 1e−05
rs7311004
rs10899911
rs8039785
rs2090816
rs6809952
rs823424
rs62273907
rs78383039
rs55877868
rs2387977
rs6828768
rs7303158
rs7572229
rs35055552
rs11715853
All − MR Egger
All − IVW(MRE)
−1
0
1
MR effect size for
'Gut microbiota abundance (genus Parasutterella id.2892)' on 'Protein NDNF'

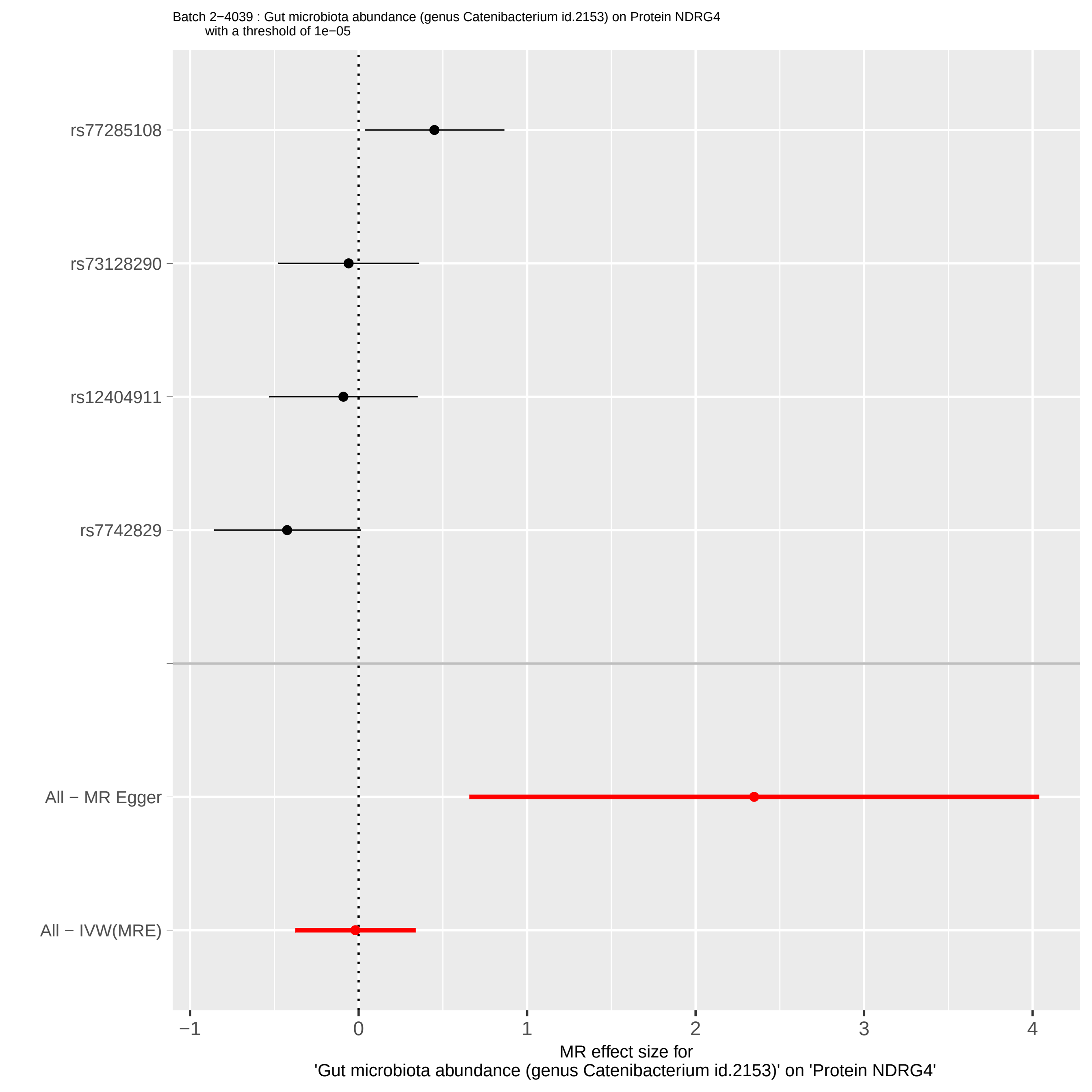

Batch 2−4039 : Gut microbiota abundance (genus Catenibacterium id.2153) on Protein NDRG4
with a threshold of 1e−05
rs77285108
rs73128290
rs12404911
rs7742829
All − MR Egger
All − IVW(MRE)
−1
0
1
2
3
4
MR effect size for
'Gut microbiota abundance (genus Catenibacterium id.2153)' on 'Protein NDRG4'

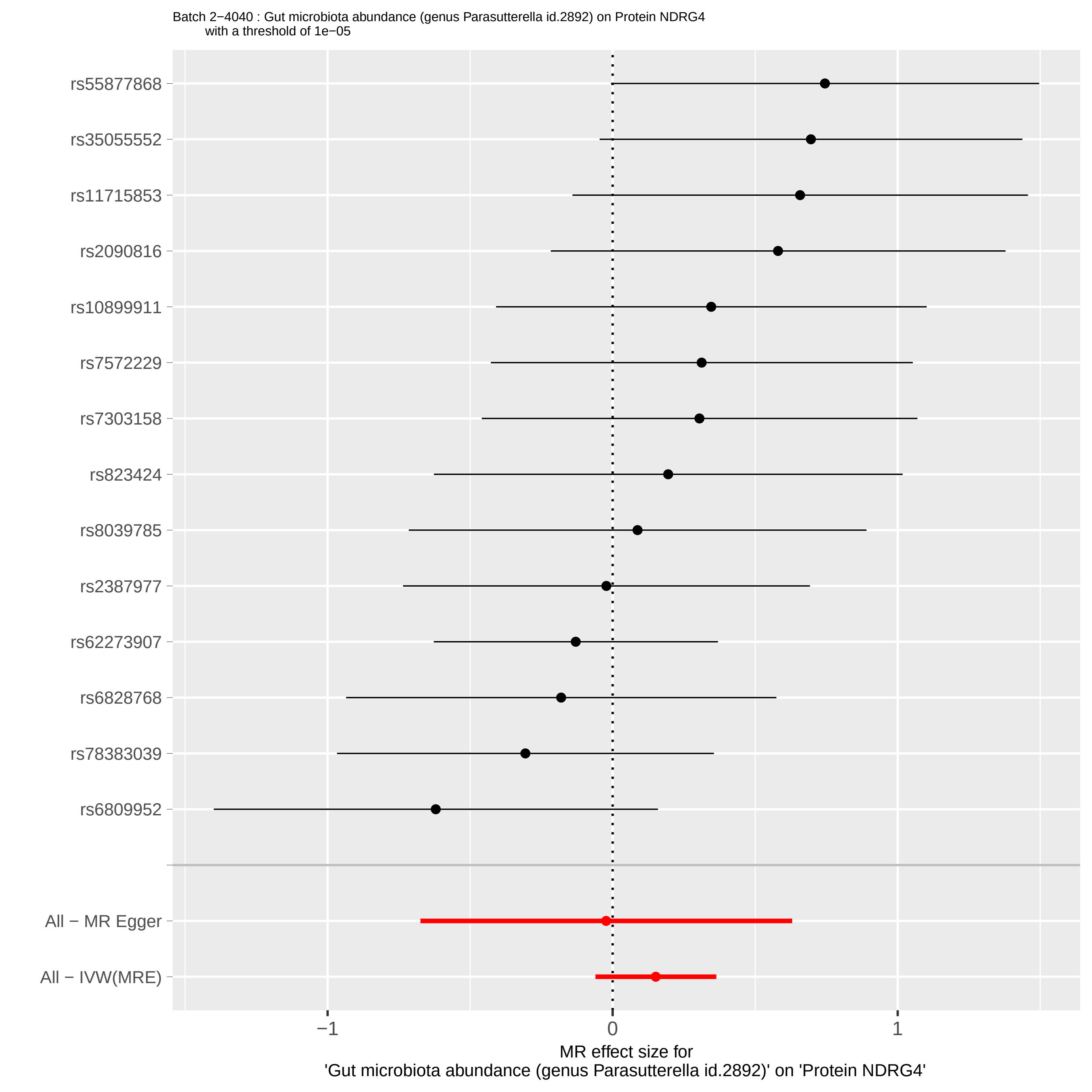
Batch 2−4040 : Gut microbiota abundance (genus Parasutterella id.2892) on Protein NDRG4
with a threshold of 1e−05
rs55877868
rs35055552
rs11715853
rs2090816
rs10899911
rs7572229
rs7303158
rs823424
rs8039785
rs2387977
rs62273907
rs6828768
rs78383039
rs6809952
All − MR Egger
All − IVW(MRE)
−1
0
1
MR effect size for
'Gut microbiota abundance (genus Parasutterella id.2892)' on 'Protein NDRG4'

Batch 2−4041 : Gut microbiota abundance (genus Catenibacterium id.2153) on Bifunctional heparan sulfate N−deacetylase/N−sulfotransferase 1
with a threshold of 1e−05

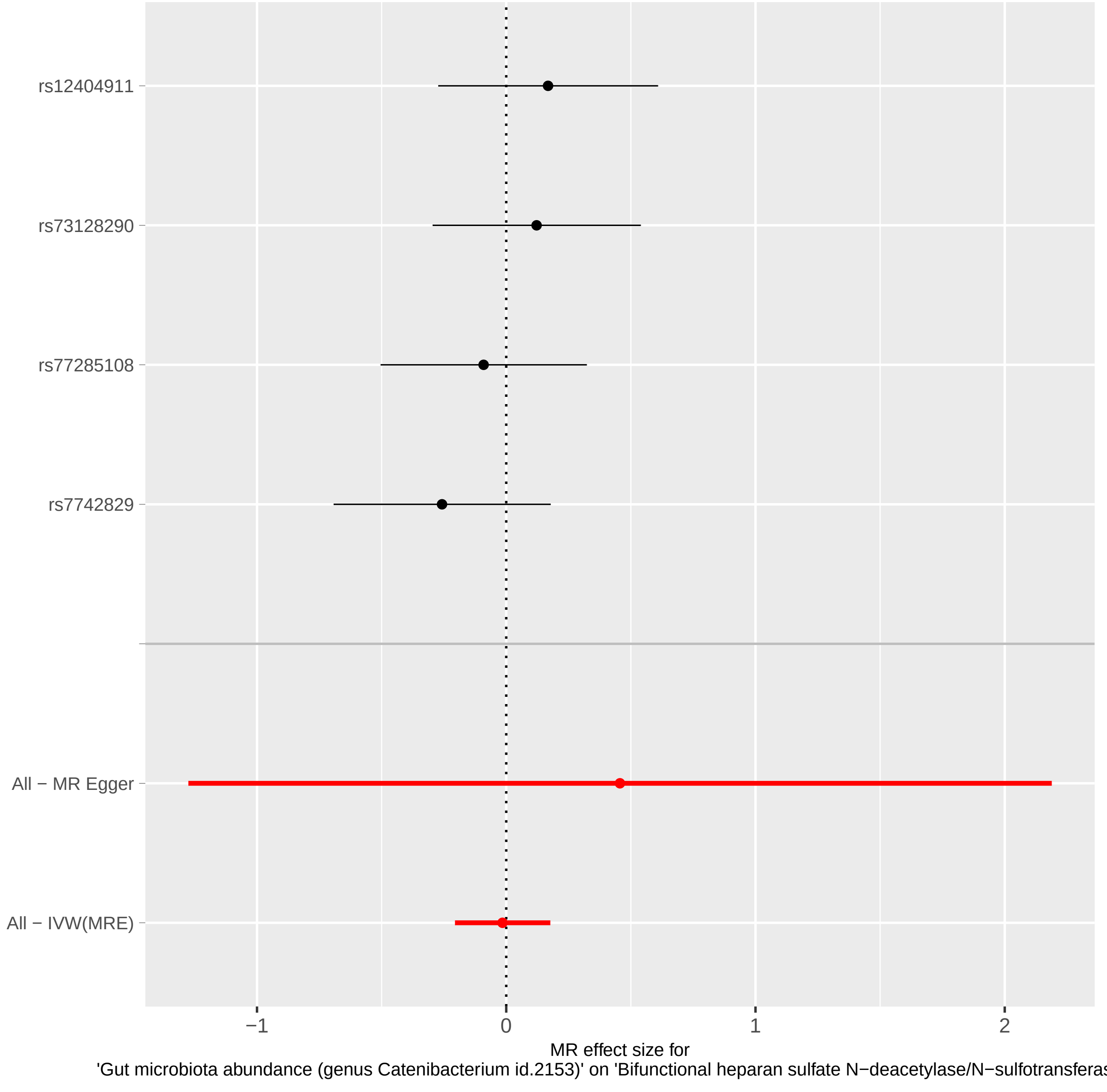

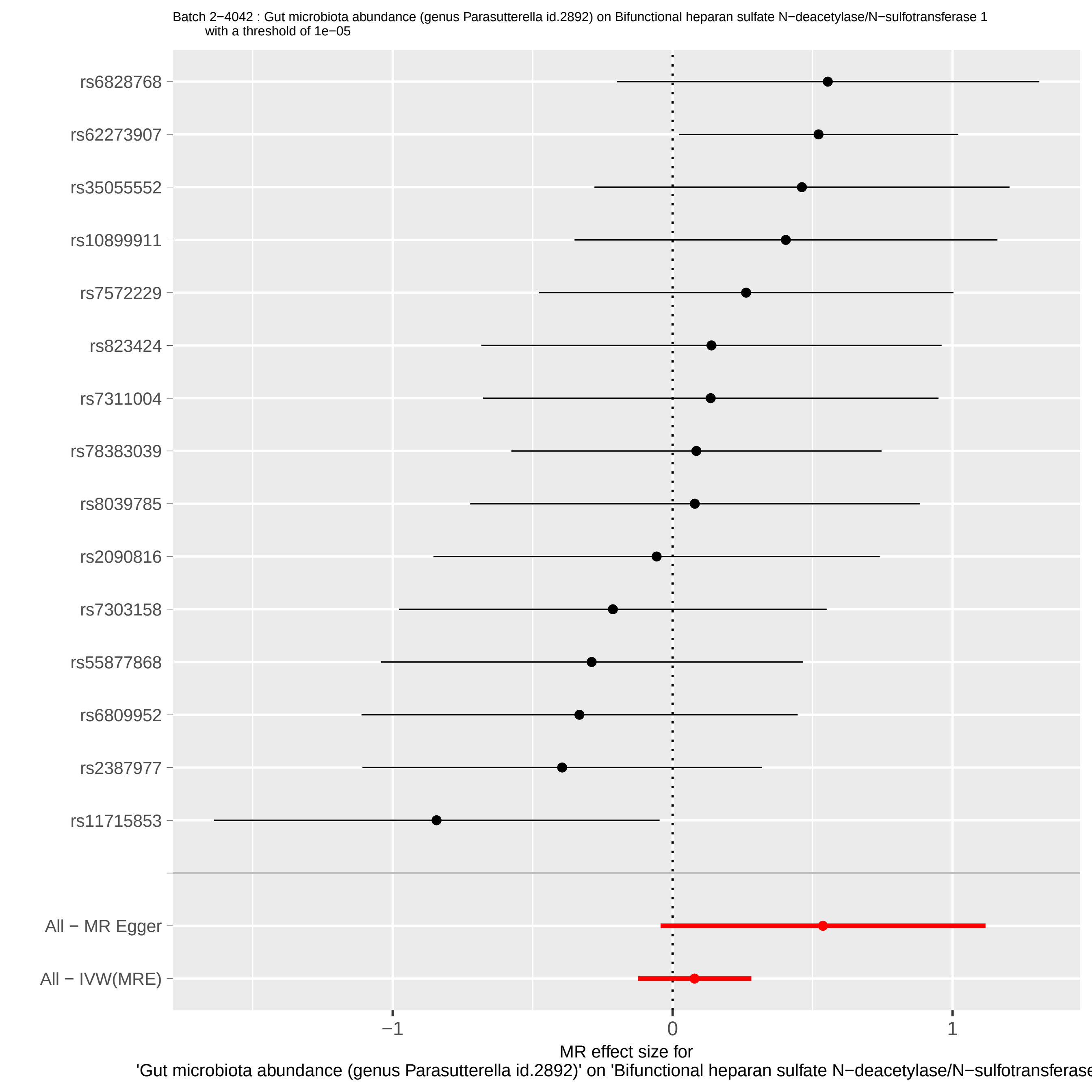

Batch 2−4042 : Gut microbiota abundance (genus Parasutterella id.2892) on Bifunctional heparan sulfate N−deacetylase/N−sulfotransferase 1
with a threshold of 1e−05
rs6828768
rs62273907
rs35055552
rs10899911
rs7572229
rs823424
rs7311004
rs78383039
rs8039785
rs2090816
rs7303158
rs55877868
rs6809952
rs2387977
rs11715853
All − MR Egger
All − IVW(MRE)
−1
0
1
MR effect size for
'Gut microbiota abundance (genus Parasutterella id.2892)' on 'Bifunctional heparan sulfate N−deacetylase/N−sulfotransferase

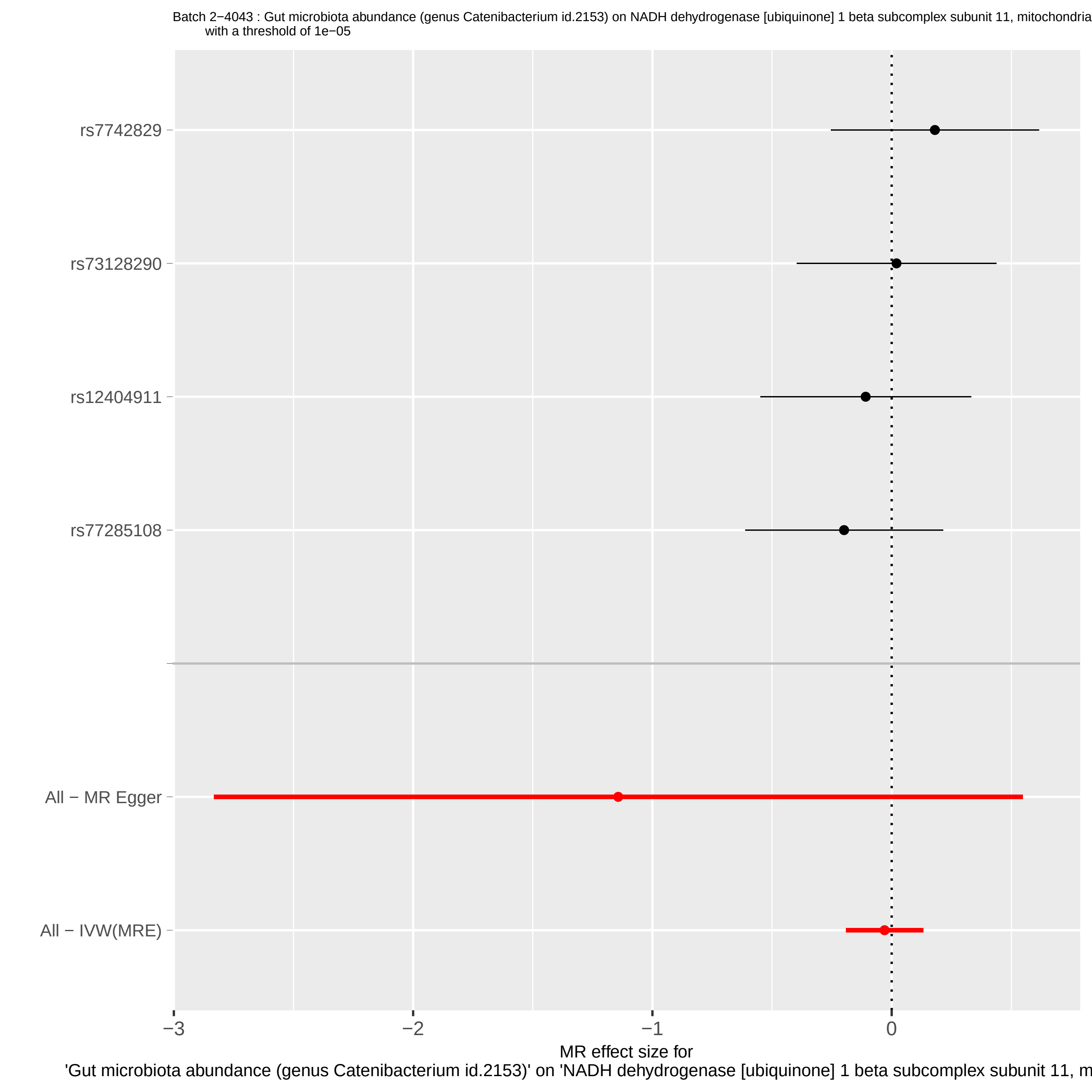

Batch 2−4043 : Gut microbiota abundance (genus Catenibacterium id.2153) on NADH dehydrogenase [ubiquinone] 1 beta subcomplex subunit 11, mitochondria
with a threshold of 1e−05
rs7742829
rs73128290
rs12404911
rs77285108
All − MR Egger
All − IVW(MRE)
−3
−2
−1
0
MR effect size for
'Gut microbiota abundance (genus Catenibacterium id.2153)' on 'NADH dehydrogenase [ubiquinone] 1 beta subcomplex subunit 11, m

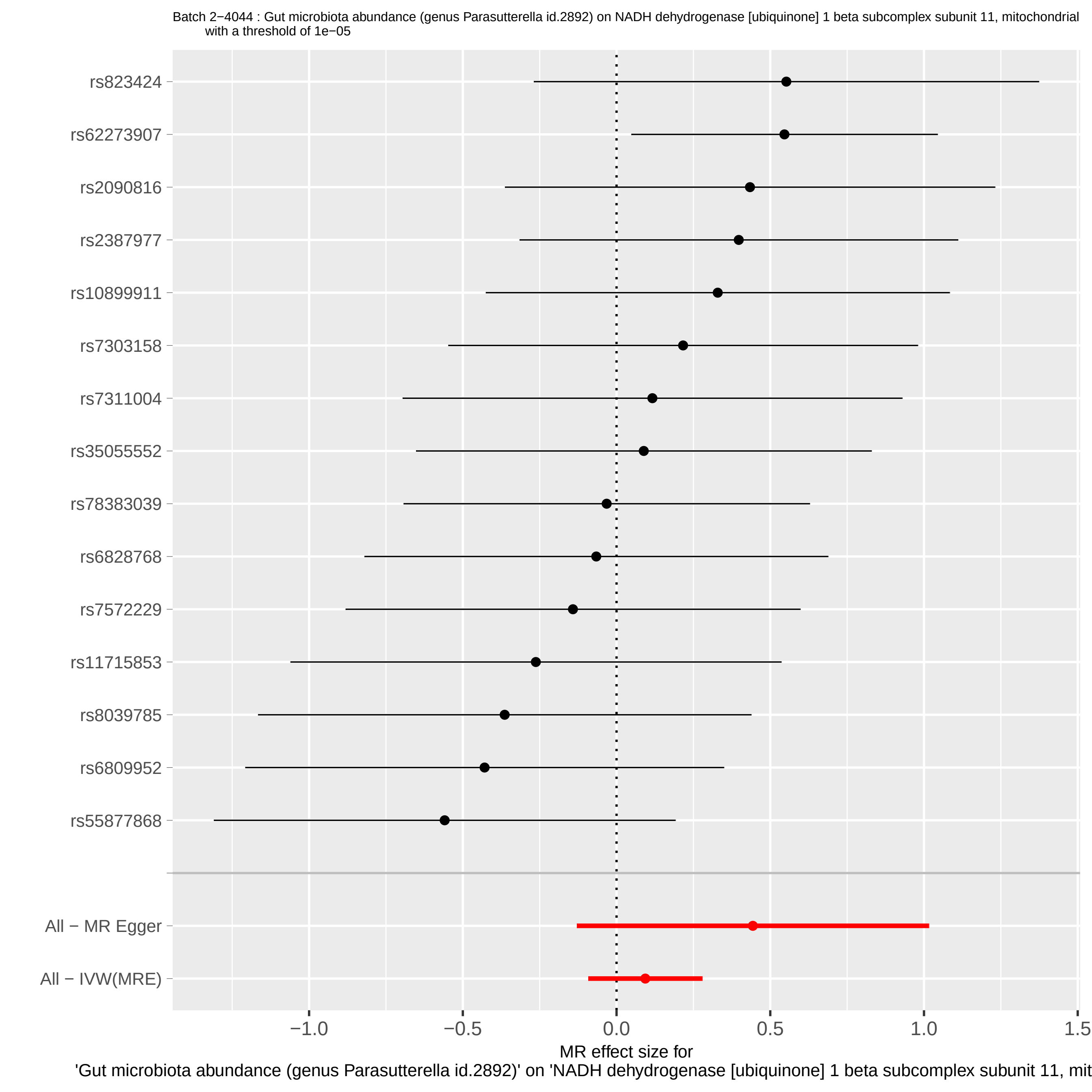

Batch 2−4044 : Gut microbiota abundance (genus Parasutterella id.2892) on NADH dehydrogenase [ubiquinone] 1 beta subcomplex subunit 11, mitochondrial
with a threshold of 1e−05
rs823424
rs62273907
rs2090816
rs2387977
rs10899911
rs7303158
rs7311004
rs35055552
rs78383039
rs6828768
rs7572229
rs11715853
rs8039785
rs6809952
rs55877868
All − MR Egger
All − IVW(MRE)
−1.0
−0.5
0.0
0.5
1.0
1.5
MR effect size for
'Gut microbiota abundance (genus Parasutterella id.2892)' on 'NADH dehydrogenase [ubiquinone] 1 beta subcomplex subunit 11, mit

Batch 2−4045 : Gut microbiota abundance (genus Catenibacterium id.2153) on NADH dehydrogenase [ubiquinone] 1 beta subcomplex subunit 4
with a threshold of 1e−05

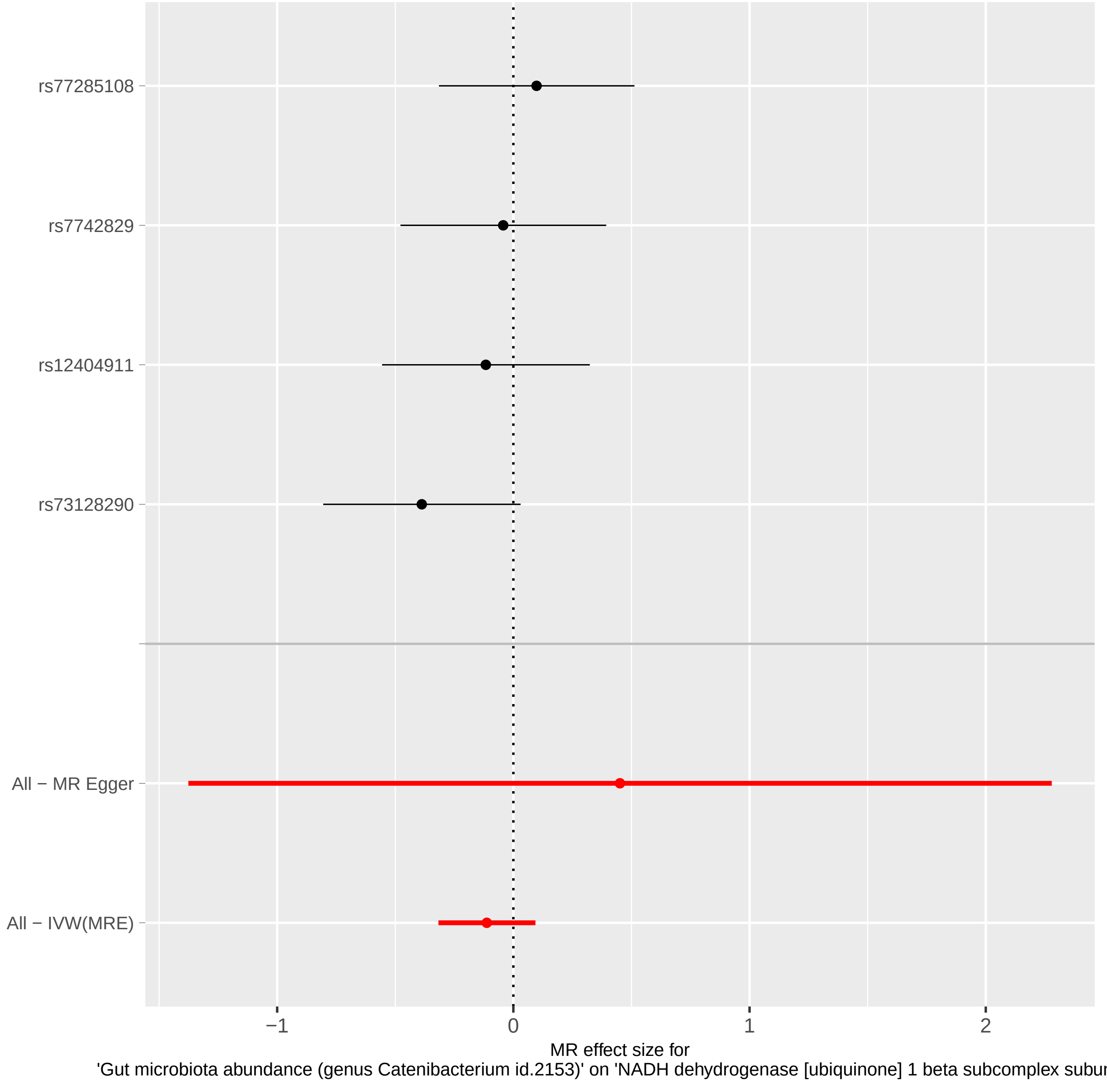

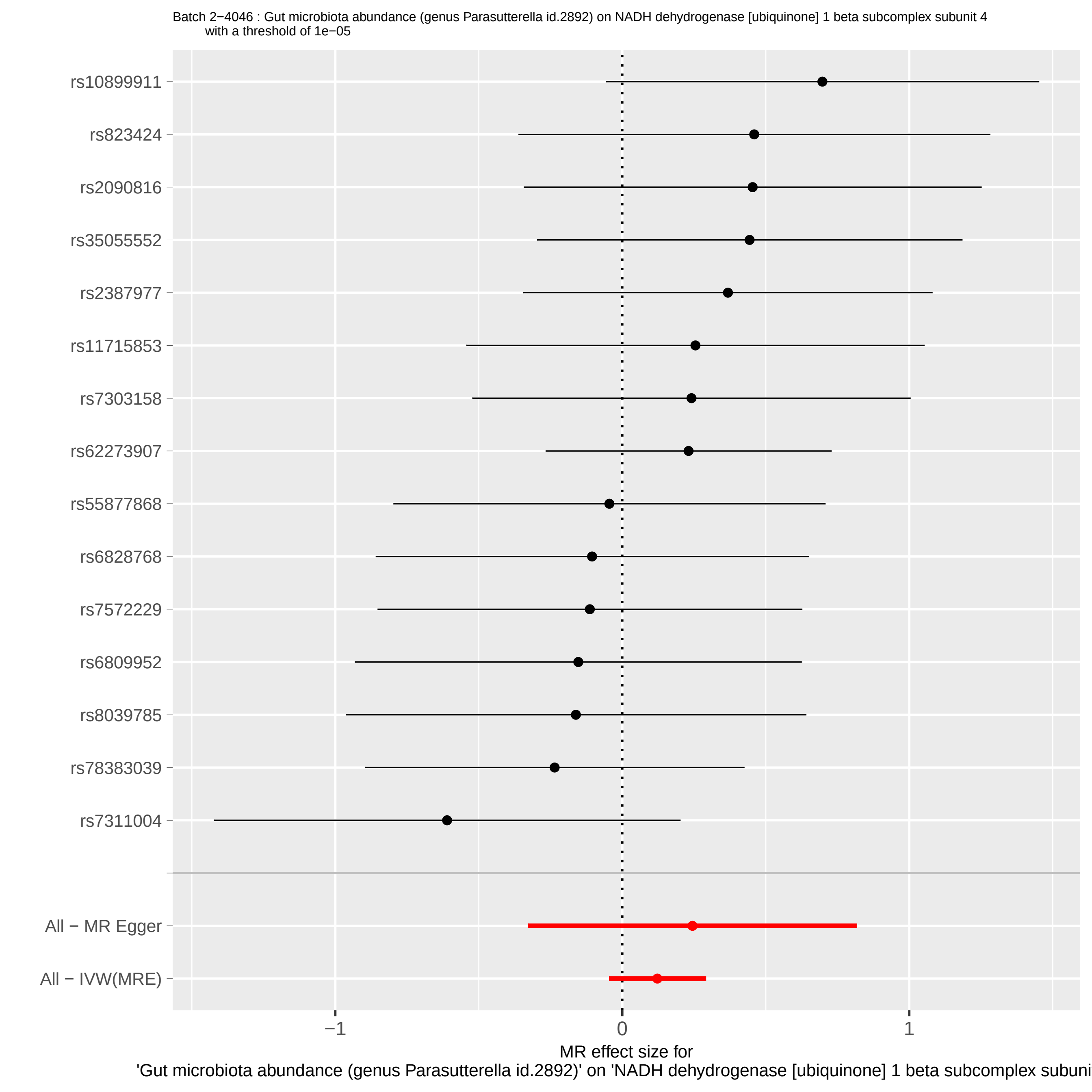
Batch 2−4046 : Gut microbiota abundance (genus Parasutterella id.2892) on NADH dehydrogenase [ubiquinone] 1 beta subcomplex subunit 4
with a threshold of 1e−05
rs10899911
rs823424
rs2090816
rs35055552
rs2387977
rs11715853
rs7303158
rs62273907
rs55877868
rs6828768
rs7572229
rs6809952
rs8039785
rs78383039
rs7311004
All − MR Egger
All − IVW(MRE)
−1
0
1
MR effect size for
'Gut microbiota abundance (genus Parasutterella id.2892)' on 'NADH dehydrogenase [ubiquinone] 1 beta subcomplex subuni

Batch 2−4047 : Gut microbiota abundance (genus Catenibacterium id.2153) on NADH dehydrogenase [ubiquinone] 1 beta subcomplex subunit 8, mitochondrial
with a threshold of 1e−05

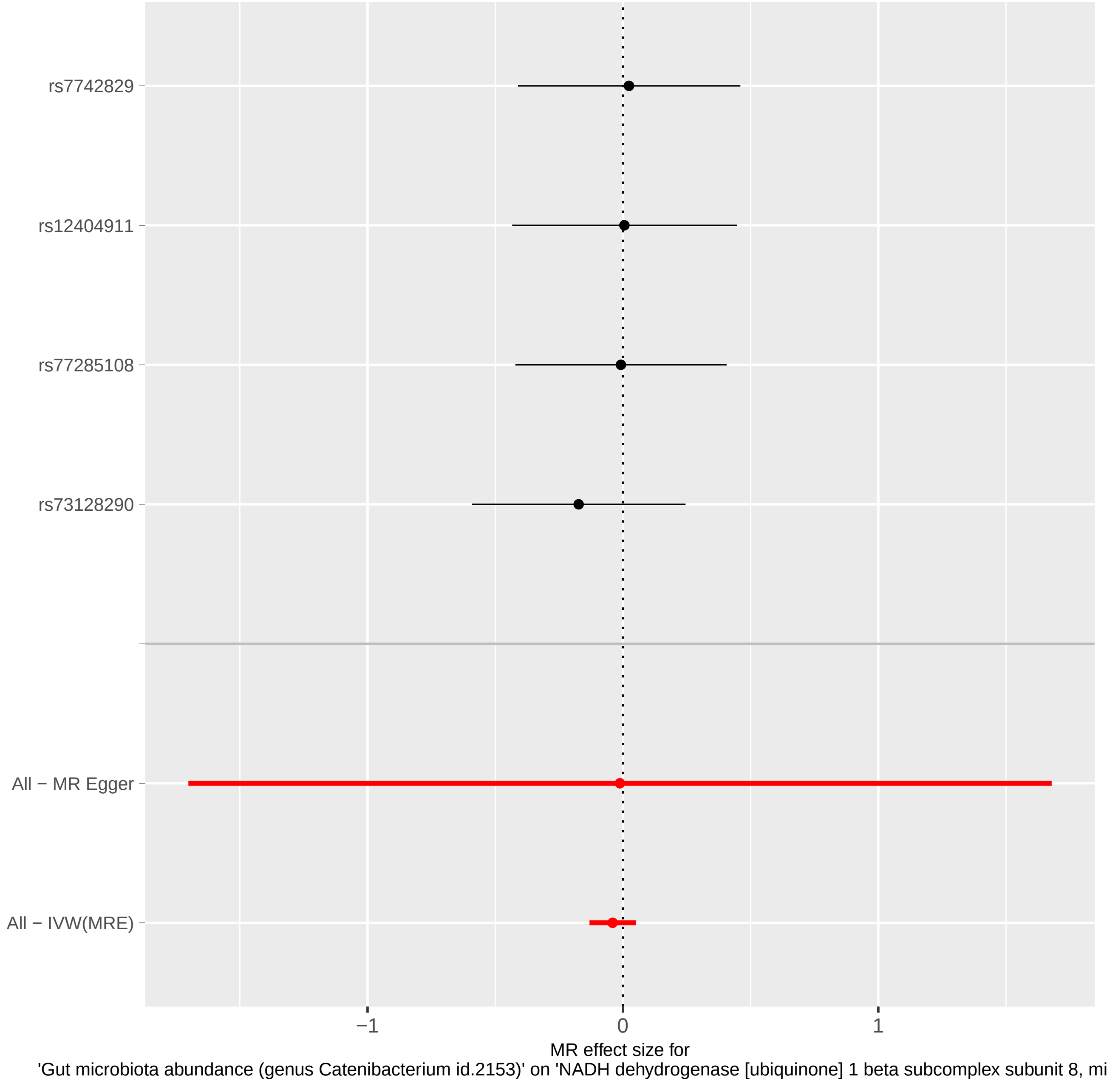

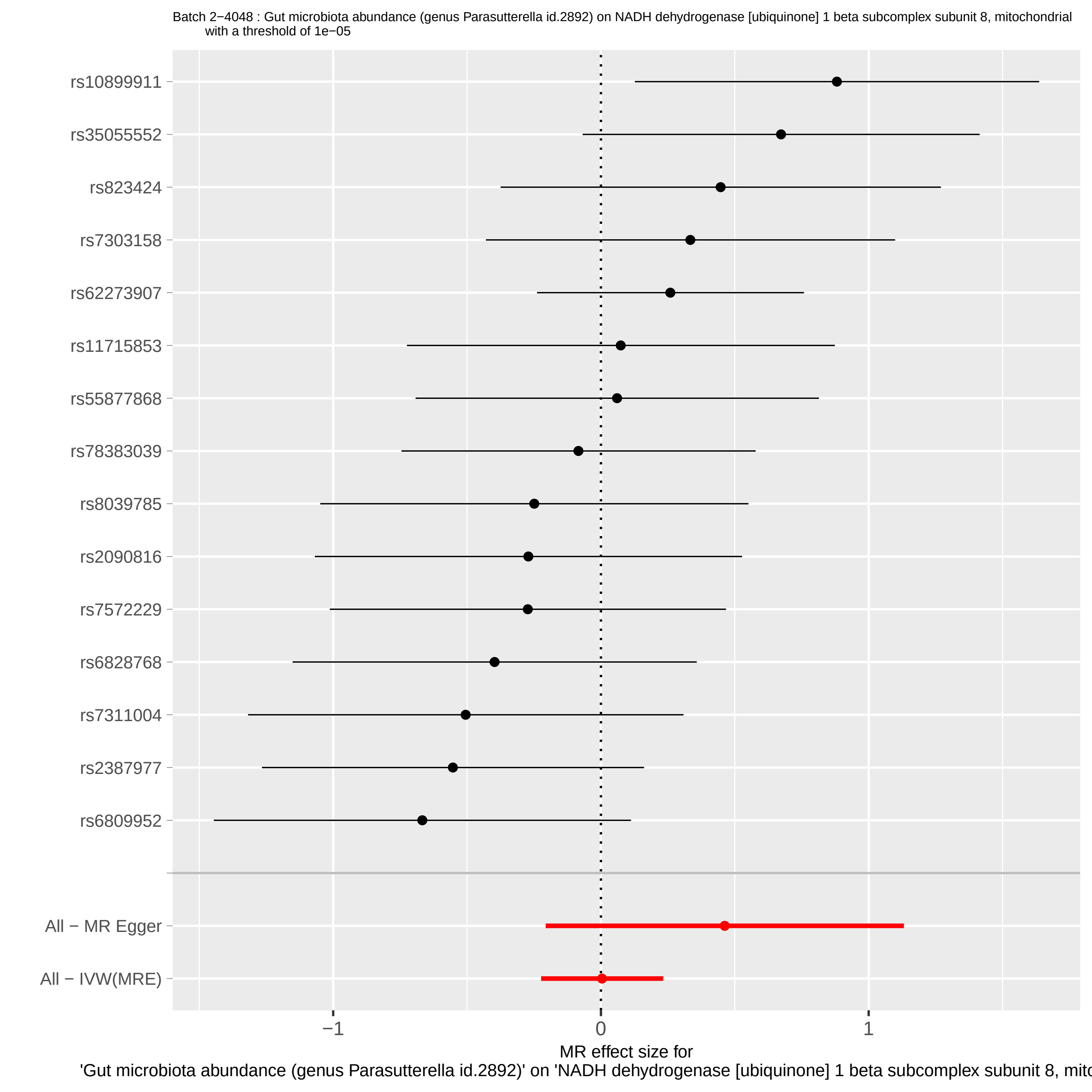

Batch 2−4048 : Gut microbiota abundance (genus Parasutterella id.2892) on NADH dehydrogenase [ubiquinone] 1 beta subcomplex subunit 8, mitochondrial with a threshold of 1e−05
rs10899911
rs35055552
rs823424
rs7303158
rs62273907
rs11715853
rs55877868
rs78383039
rs8039785
rs2090816
rs7572229
rs6828768
rs7311004
rs2387977
rs6809952
All − MR Egger
All − IVW(MRE)
−1
0
1
MR effect size for
'Gut microbiota abundance (genus Parasutterella id.2892)' on 'NADH dehydrogenase [ubiquinone] 1 beta subcomplex subunit 8, mito

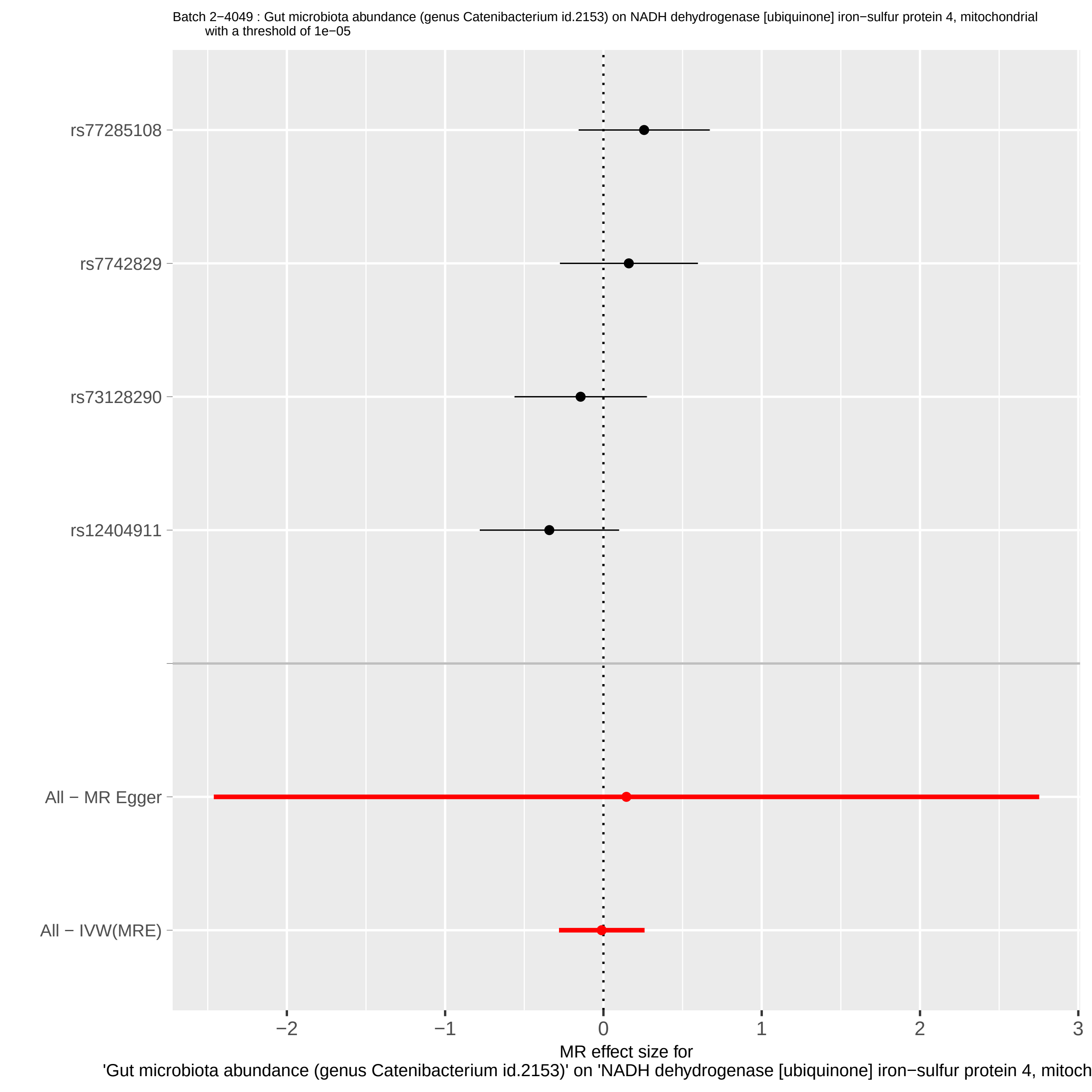

Batch 2−4049 : Gut microbiota abundance (genus Catenibacterium id.2153) on NADH dehydrogenase [ubiquinone] iron−sulfur protein 4, mitochondrial
with a threshold of 1e−05
rs77285108
rs7742829
rs73128290
rs12404911
All − MR Egger
All − IVW(MRE)
−2
−1
0
1
2
3
MR effect size for
'Gut microbiota abundance (genus Catenibacterium id.2153)' on 'NADH dehydrogenase [ubiquinone] iron−sulfur protein 4, mitoch

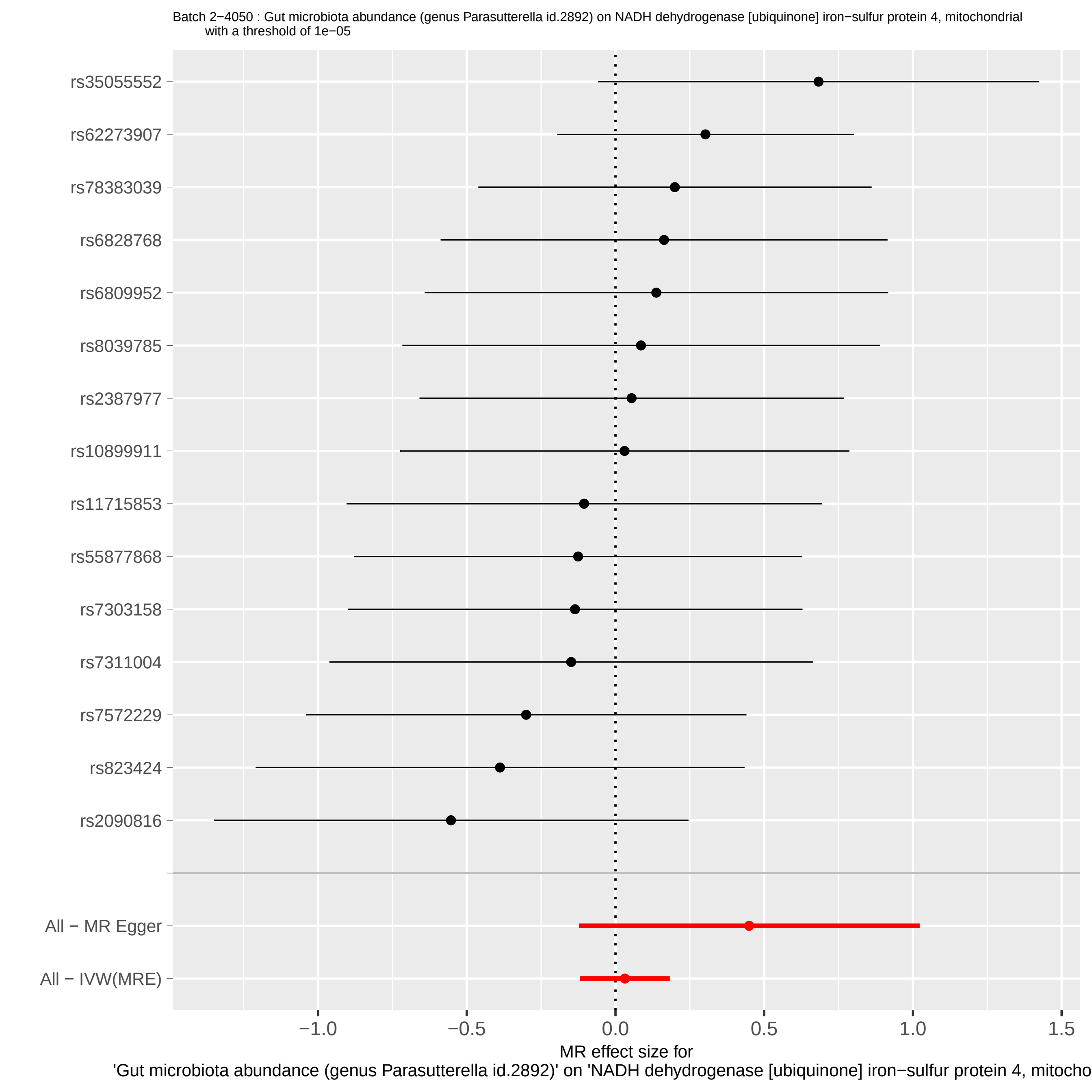

Batch 2−4050 : Gut microbiota abundance (genus Parasutterella id.2892) on NADH dehydrogenase [ubiquinone] iron−sulfur protein 4, mitochondrial with a threshold of 1e−05
rs35055552
rs62273907
rs78383039
rs6828768
rs6809952
rs8039785
rs2387977
rs10899911
rs11715853
rs55877868
rs7303158
rs7311004
rs7572229
rs823424
rs2090816
All − MR Egger
All − IVW(MRE)
−1.0
−0.5
0.0
0.5
1.0
1.5
MR effect size for
'Gut microbiota abundance (genus Parasutterella id.2892)' on 'NADH dehydrogenase [ubiquinone] iron−sulfur protein 4, mitocho

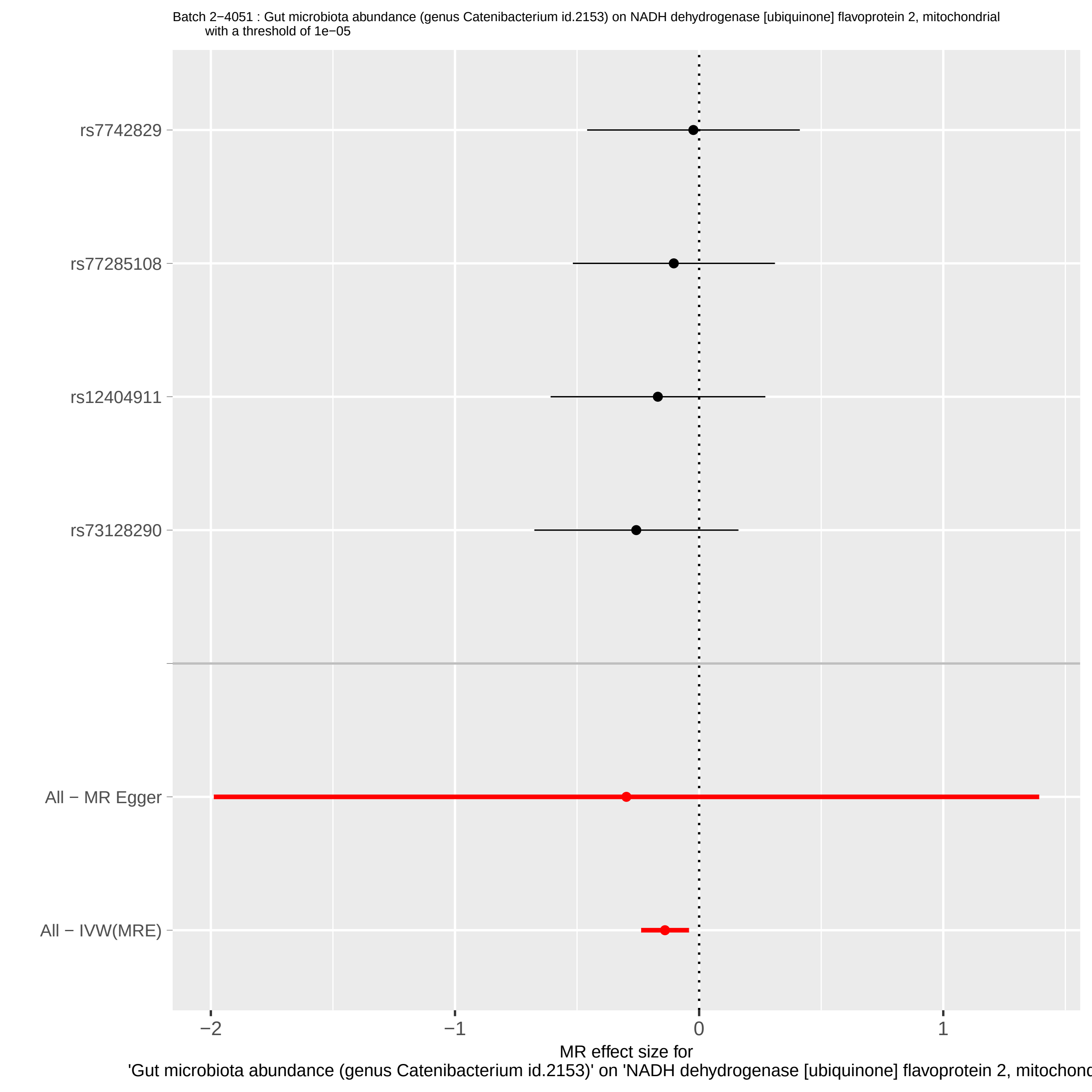

Batch 2−4051 : Gut microbiota abundance (genus Catenibacterium id.2153) on NADH dehydrogenase [ubiquinone] flavoprotein 2, mitochondrial
with a threshold of 1e−05
rs7742829
rs77285108
rs12404911
rs73128290
All − MR Egger
All − IVW(MRE)
−2
−1
0
1
MR effect size for
'Gut microbiota abundance (genus Catenibacterium id.2153)' on 'NADH dehydrogenase [ubiquinone] flavoprotein 2, mitochond

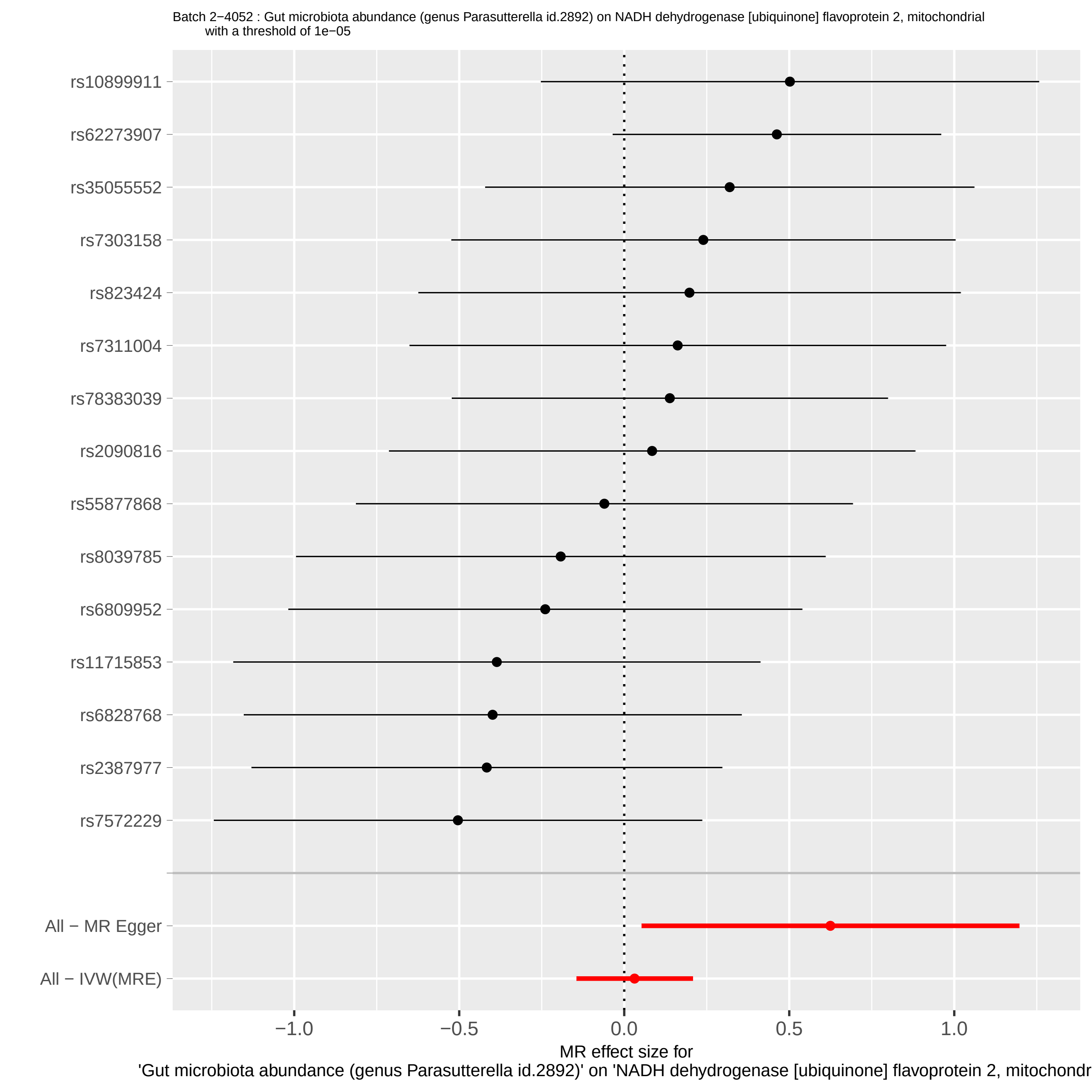

Batch 2−4052 : Gut microbiota abundance (genus Parasutterella id.2892) on NADH dehydrogenase [ubiquinone] flavoprotein 2, mitochondrial with a threshold of 1e−05
rs10899911
rs62273907
rs35055552
rs7303158
rs823424
rs7311004
rs78383039
rs2090816
rs55877868
rs8039785
rs6809952
rs11715853
rs6828768
rs2387977
rs7572229
All − MR Egger
All − IVW(MRE)
−1.0
−0.5
0.0
0.5
1.0
MR effect size for
'Gut microbiota abundance (genus Parasutterella id.2892)' on 'NADH dehydrogenase [ubiquinone] flavoprotein 2, mitochondr

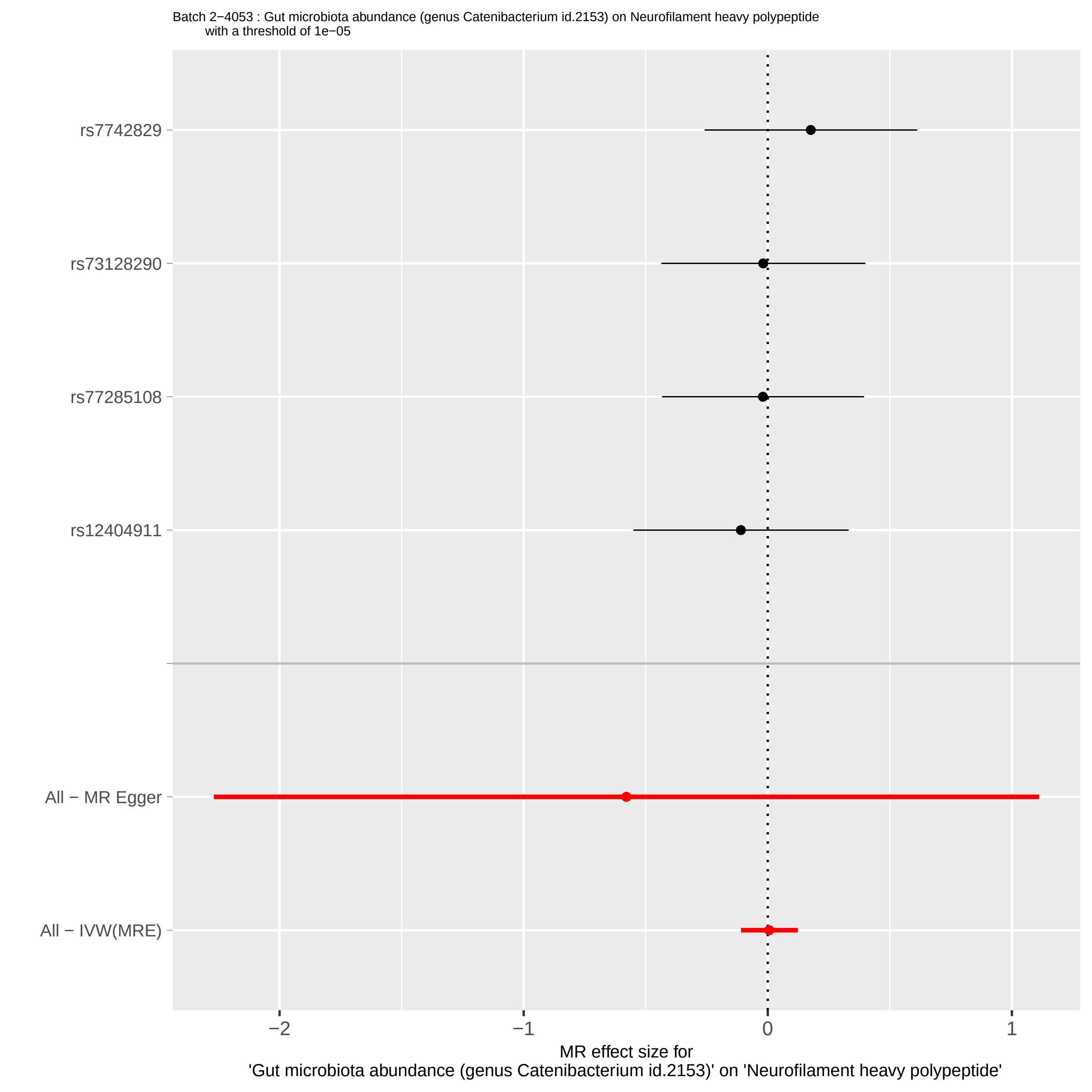

Batch 2−4053 : Gut microbiota abundance (genus Catenibacterium id.2153) on Neurofilament heavy polypeptide
with a threshold of 1e−05
rs7742829
rs73128290
rs77285108
rs12404911
All − MR Egger
All − IVW(MRE)
−2
−1
0
1
MR effect size for
'Gut microbiota abundance (genus Catenibacterium id.2153)' on 'Neurofilament heavy polypeptide'

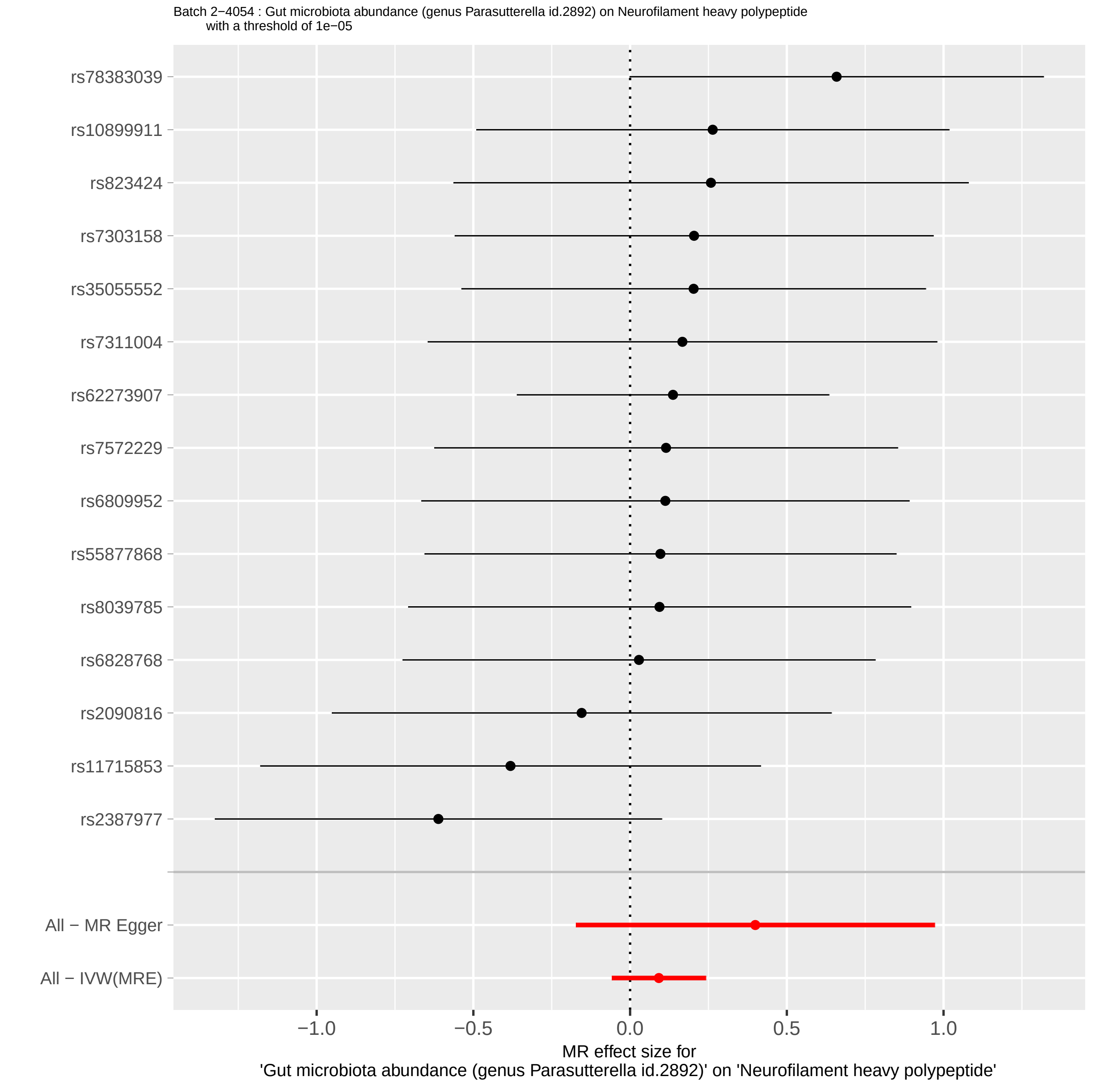

Batch 2−4054 : Gut microbiota abundance (genus Parasutterella id.2892) on Neurofilament heavy polypeptide
with a threshold of 1e−05
rs78383039
rs10899911
rs823424
rs7303158
rs35055552
rs7311004
rs62273907
rs7572229
rs6809952
rs55877868
rs8039785
rs6828768
rs2090816
rs11715853
rs2387977
All − MR Egger
All − IVW(MRE)
−1.0
−0.5
0.0
0.5
1.0
MR effect size for
'Gut microbiota abundance (genus Parasutterella id.2892)' on 'Neurofilament heavy polypeptide'

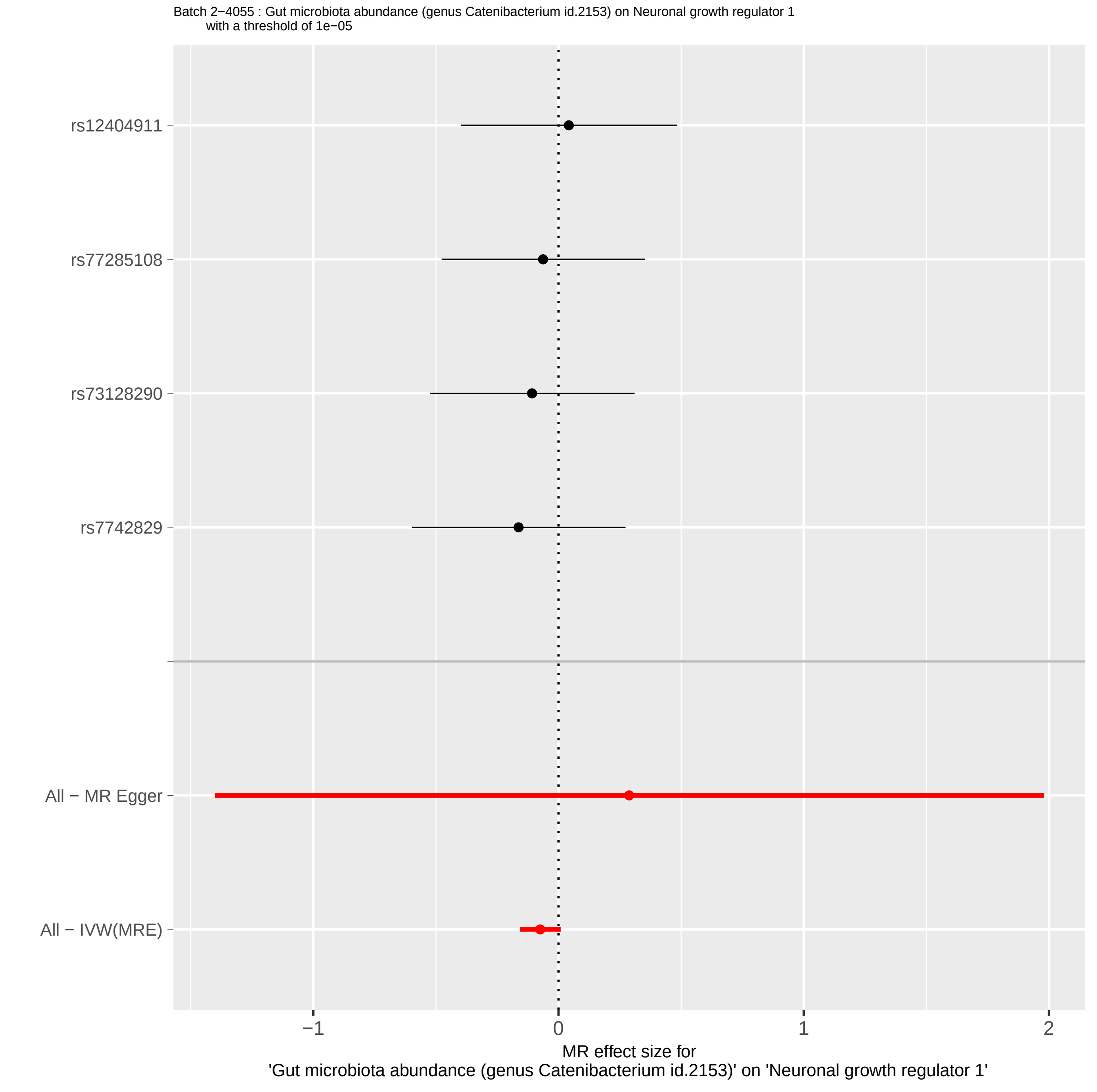
Batch 2−4055 : Gut microbiota abundance (genus Catenibacterium id.2153) on Neuronal growth regulator 1
with a threshold of 1e−05
rs12404911
rs77285108
rs73128290
rs7742829
All − MR Egger
All − IVW(MRE)
−1
0
1
2
MR effect size for
'Gut microbiota abundance (genus Catenibacterium id.2153)' on 'Neuronal growth regulator 1'

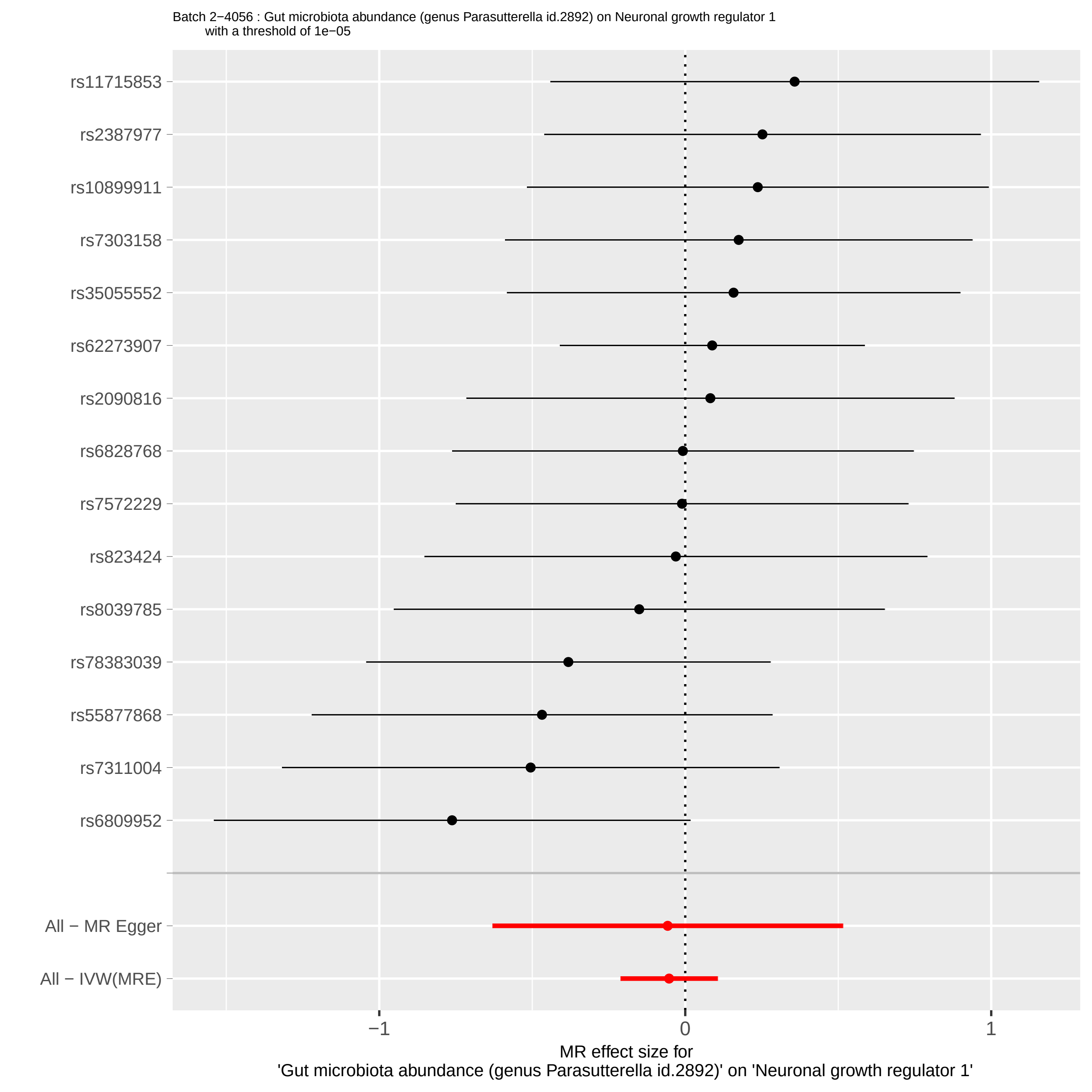
Batch 2−4056 : Gut microbiota abundance (genus Parasutterella id.2892) on Neuronal growth regulator 1
with a threshold of 1e−05
rs11715853
rs2387977
rs10899911
rs7303158
rs35055552
rs62273907
rs2090816
rs6828768
rs7572229
rs823424
rs8039785
rs78383039
rs55877868
rs7311004
rs6809952
All − MR Egger
All − IVW(MRE)
−1
0
1
MR effect size for
'Gut microbiota abundance (genus Parasutterella id.2892)' on 'Neuronal growth regulator 1'

Batch 2−4057 : Gut microbiota abundance (genus Catenibacterium id.2153) on Neuronal growth regulator 1
with a threshold of 1e−05

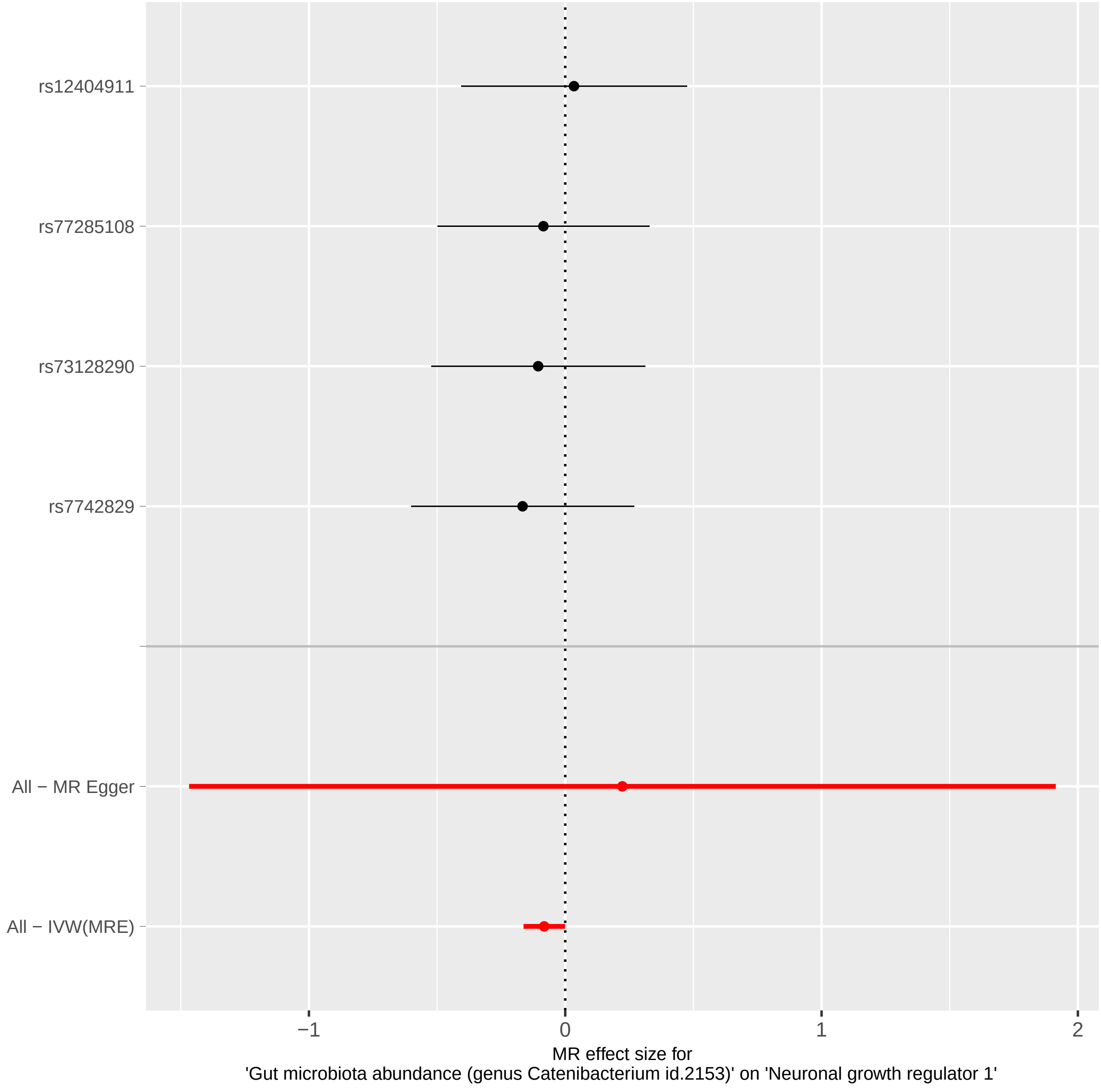

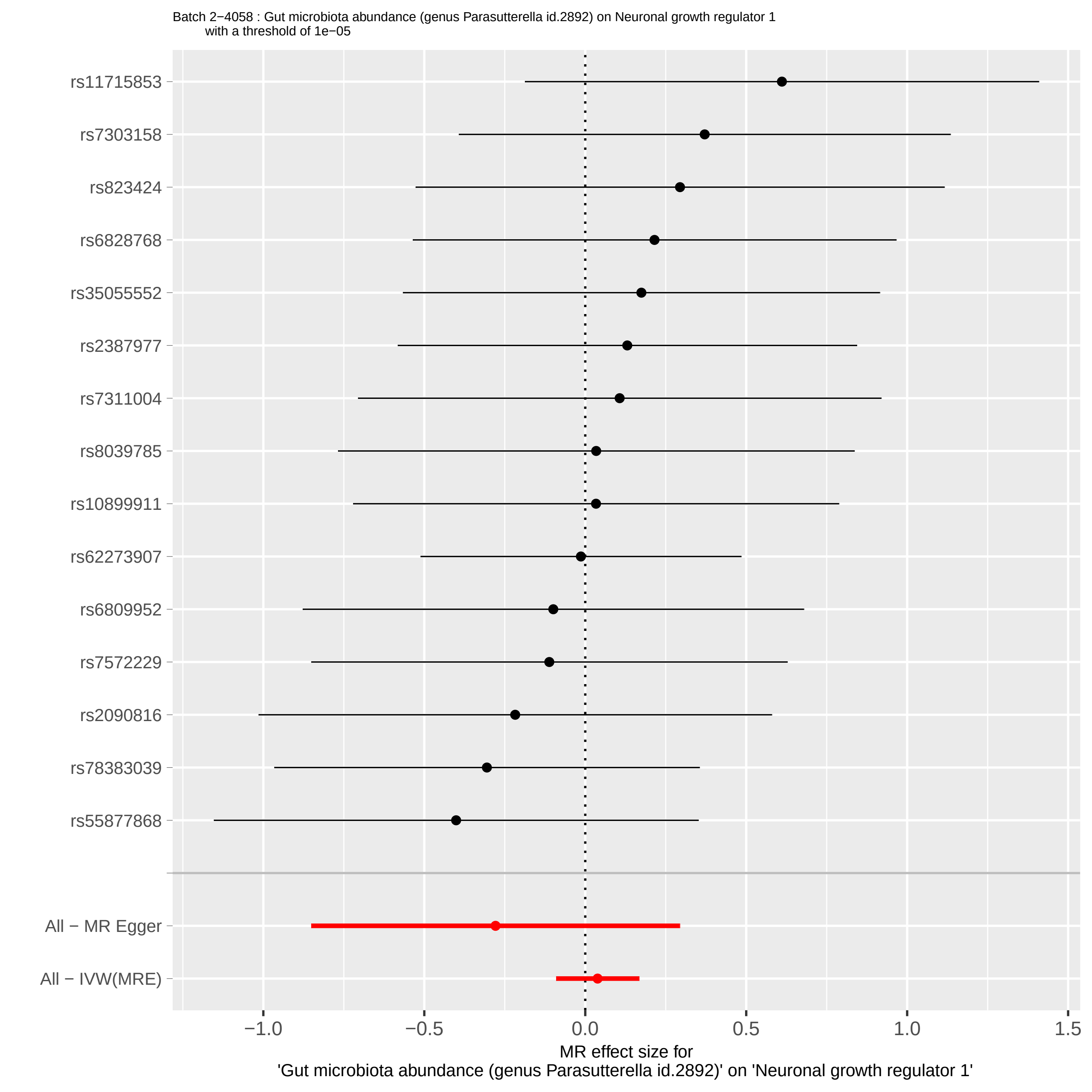

Batch 2−4058 : Gut microbiota abundance (genus Parasutterella id.2892) on Neuronal growth regulator 1
with a threshold of 1e−05
rs11715853
rs7303158
rs823424
rs6828768
rs35055552
rs2387977
rs7311004
rs8039785
rs10899911
rs62273907
rs6809952
rs7572229
rs2090816
rs78383039
rs55877868
All − MR Egger
All − IVW(MRE)
−1.0
−0.5
0.0
0.5
1.0
1.5
MR effect size for
'Gut microbiota abundance (genus Parasutterella id.2892)' on 'Neuronal growth regulator 1'

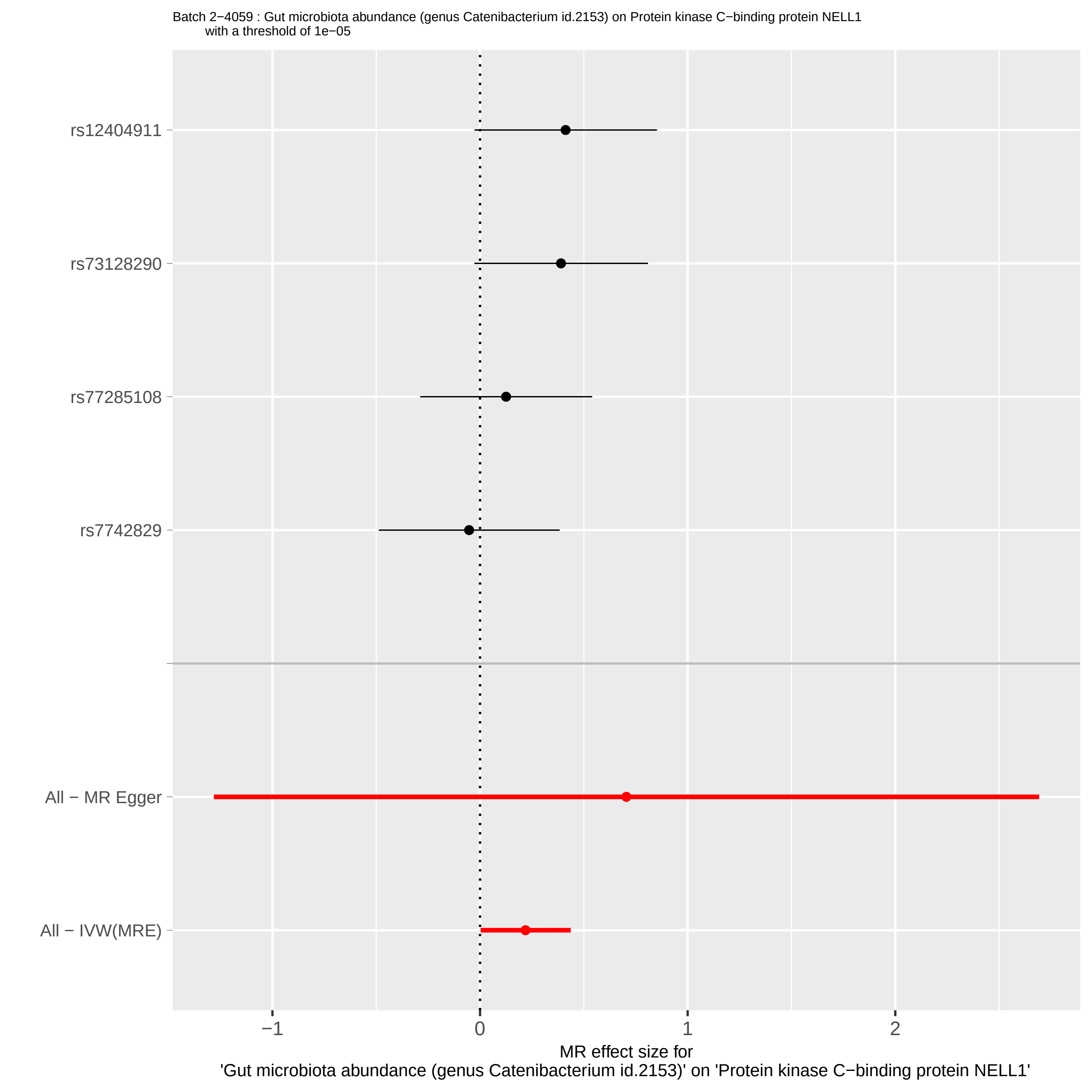

Batch 2−4059 : Gut microbiota abundance (genus Catenibacterium id.2153) on Protein kinase C−binding protein NELL1
with a threshold of 1e−05
rs12404911
rs73128290
rs77285108
rs7742829
All − MR Egger
All − IVW(MRE)
−1
0
1
2
MR effect size for
'Gut microbiota abundance (genus Catenibacterium id.2153)' on 'Protein kinase C−binding protein NELL1'

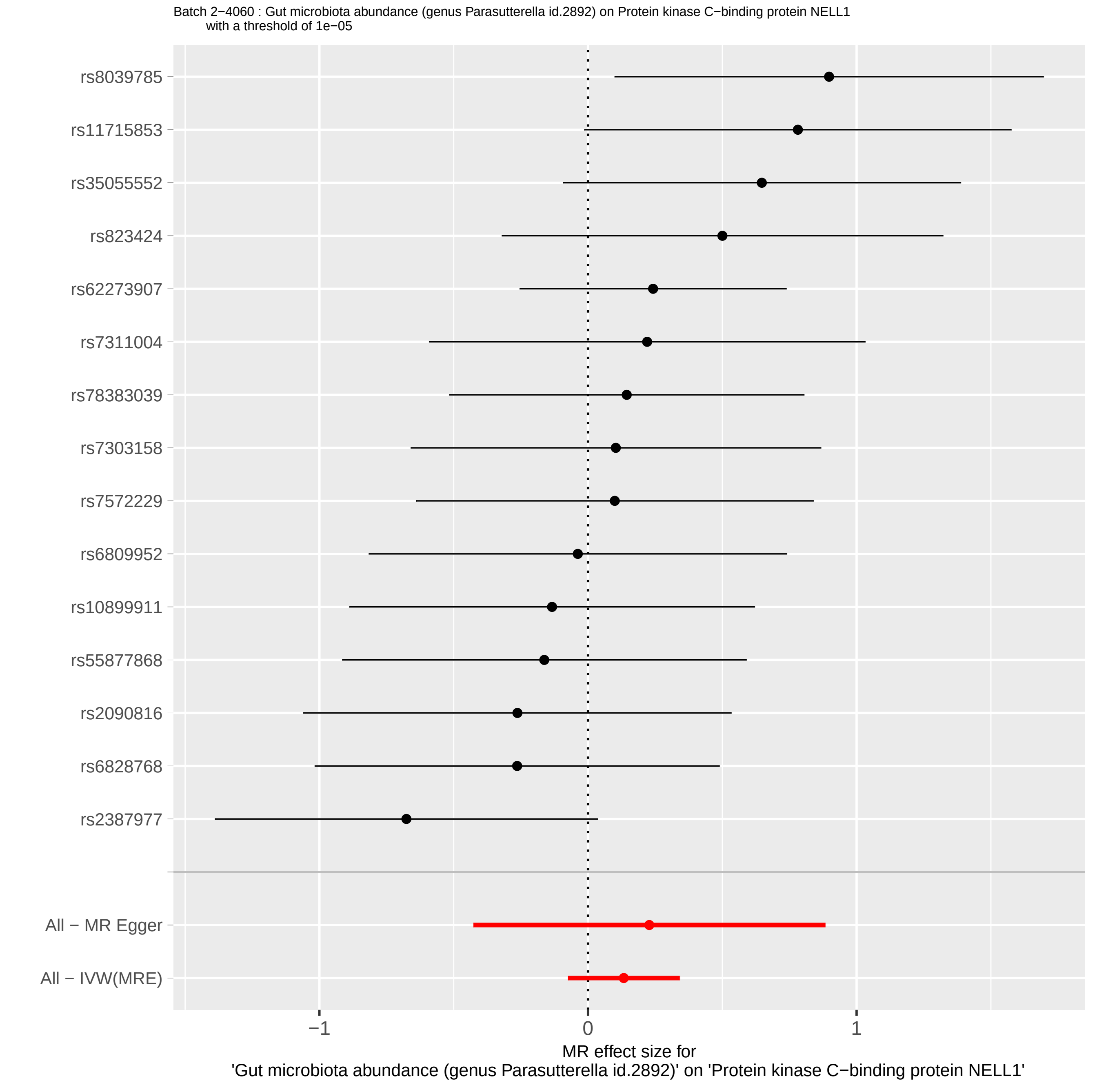

Batch 2−4060 : Gut microbiota abundance (genus Parasutterella id.2892) on Protein kinase C−binding protein NELL1
with a threshold of 1e−05
rs8039785
rs11715853
rs35055552
rs823424
rs62273907
rs7311004
rs78383039
rs7303158
rs7572229
rs6809952
rs10899911
rs55877868
rs2090816
rs6828768
rs2387977
All − MR Egger
All − IVW(MRE)
−1
0
1
MR effect size for
'Gut microbiota abundance (genus Parasutterella id.2892)' on 'Protein kinase C−binding protein NELL1'

Batch 2−4061 : Gut microbiota abundance (genus Catenibacterium id.2153) on Protein kinase C−binding protein NELL2
with a threshold of 1e−05

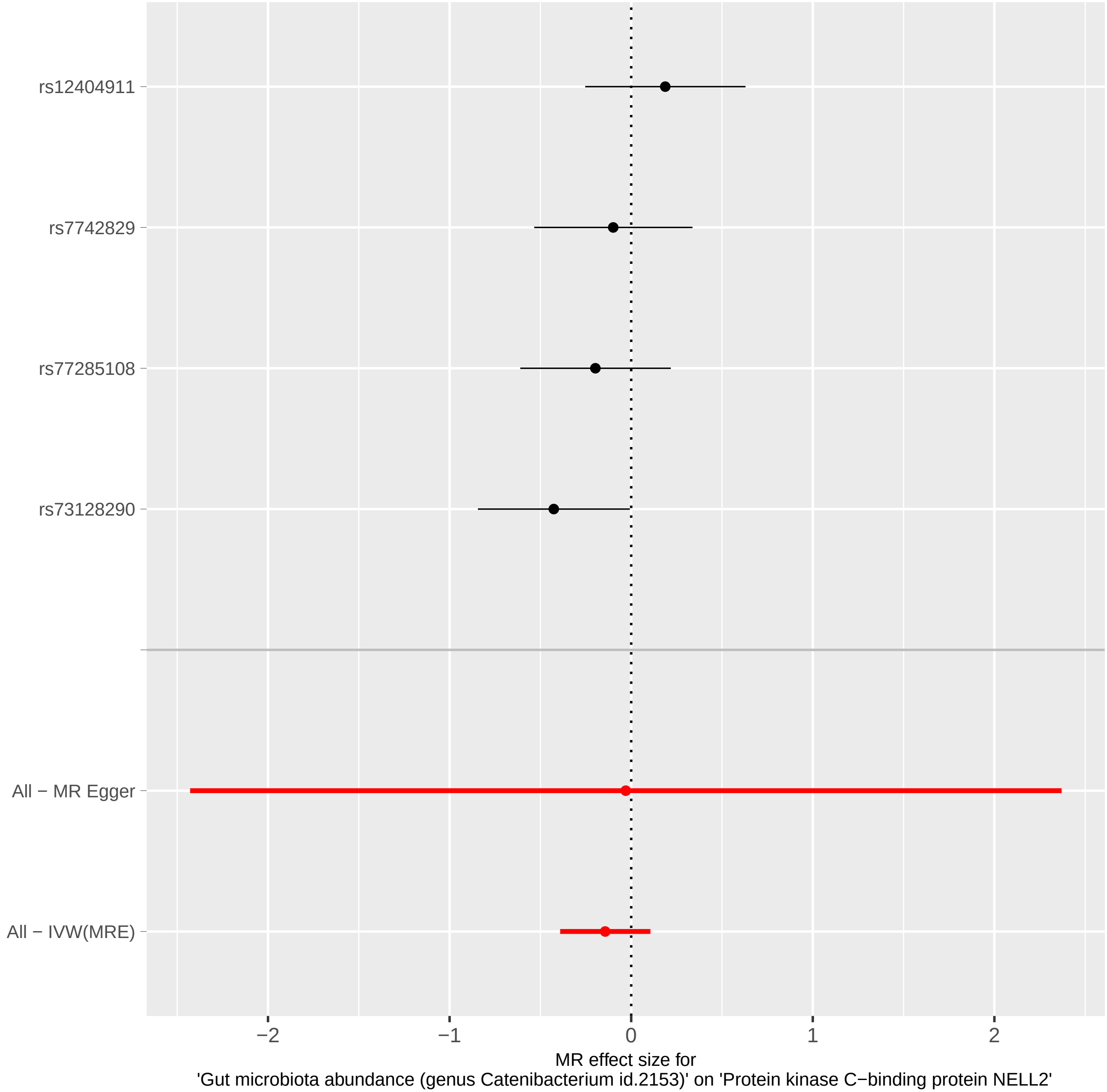

Batch 2−4062 : Gut microbiota abundance (genus Parasutterella id.2892) on Protein kinase C−binding protein NELL2
with a threshold of 1e−05

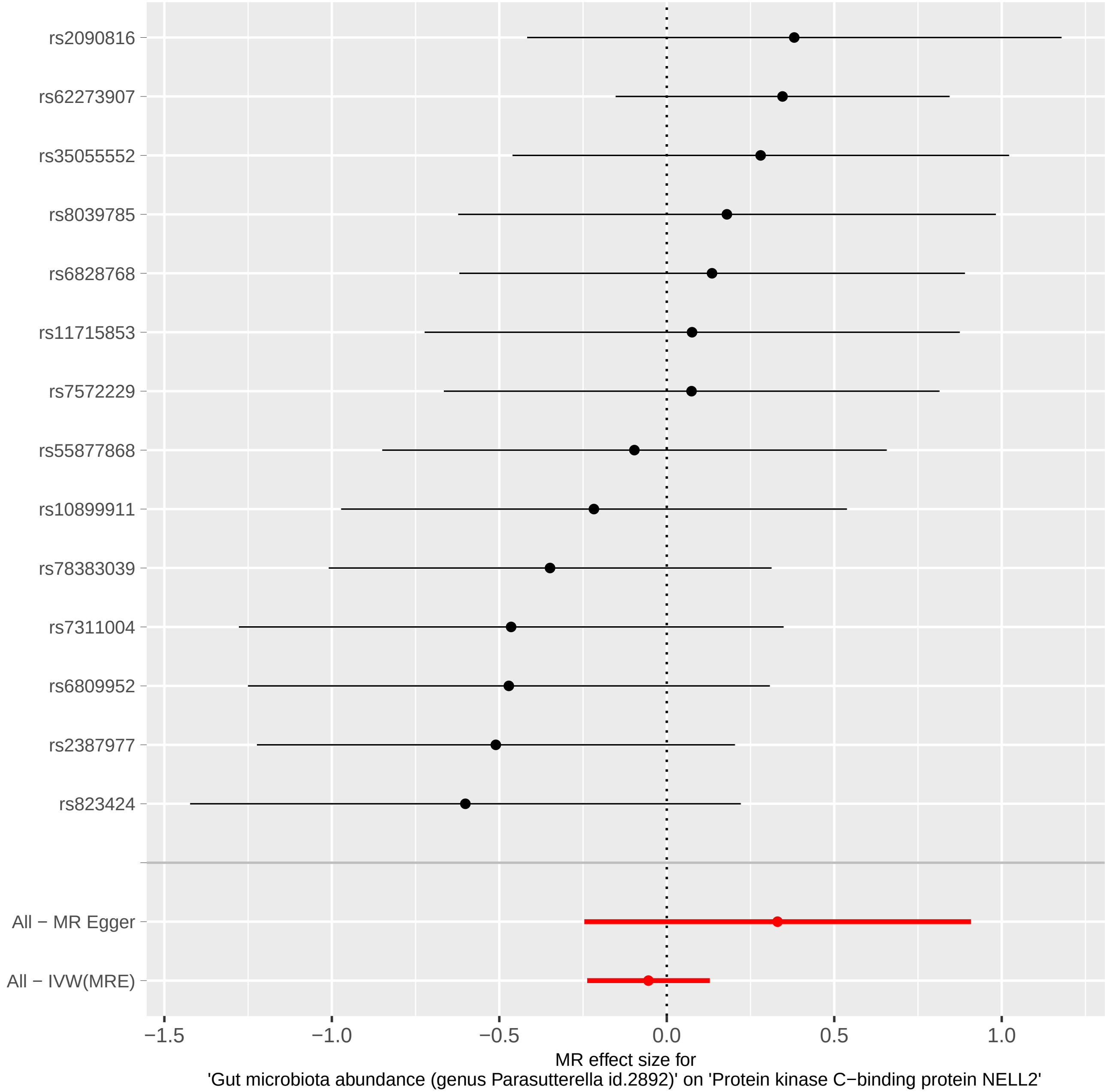

Batch 2−4063 : Gut microbiota abundance (genus Catenibacterium id.2153) on Neudesin
with a threshold of 1e−05

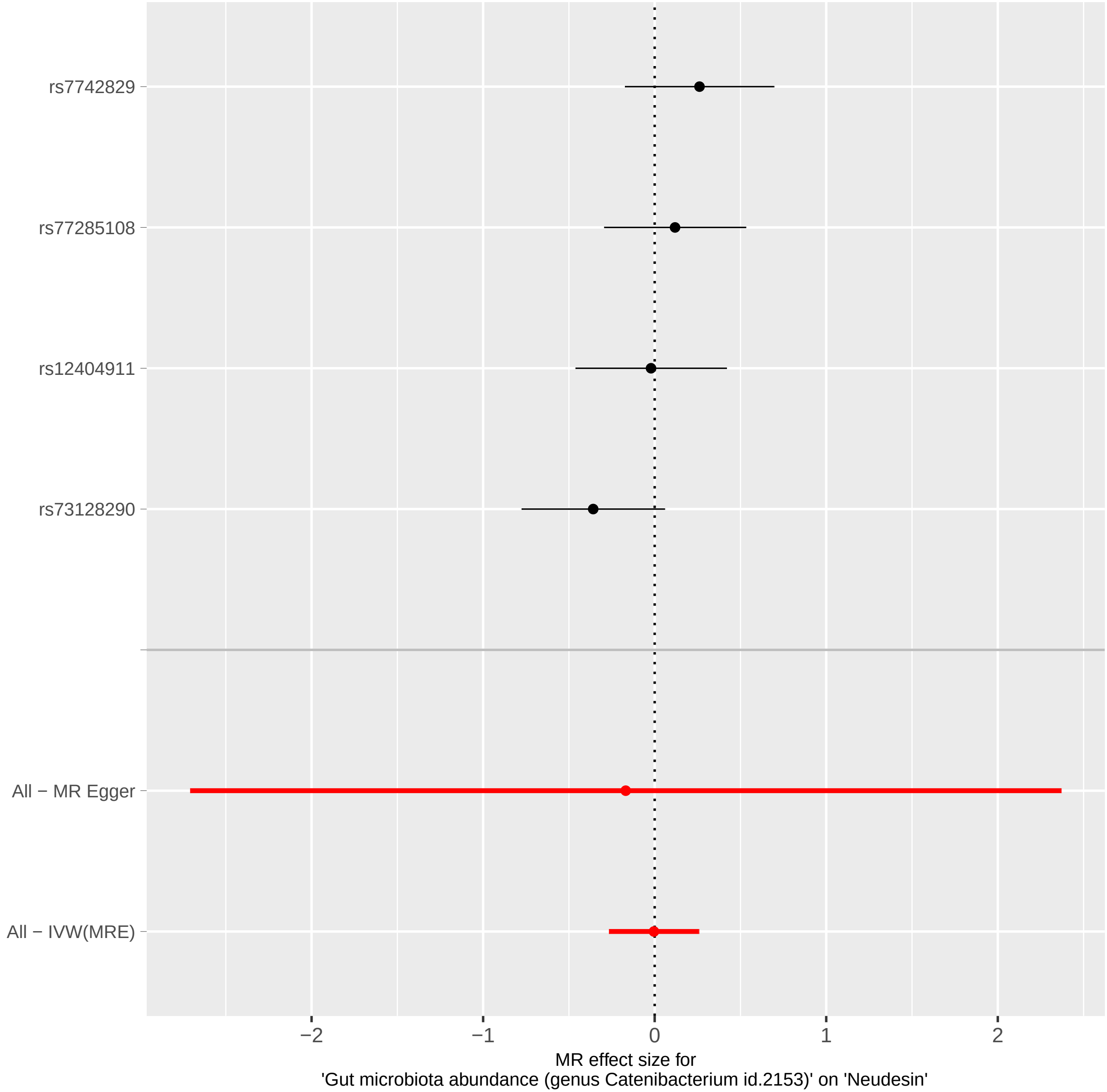

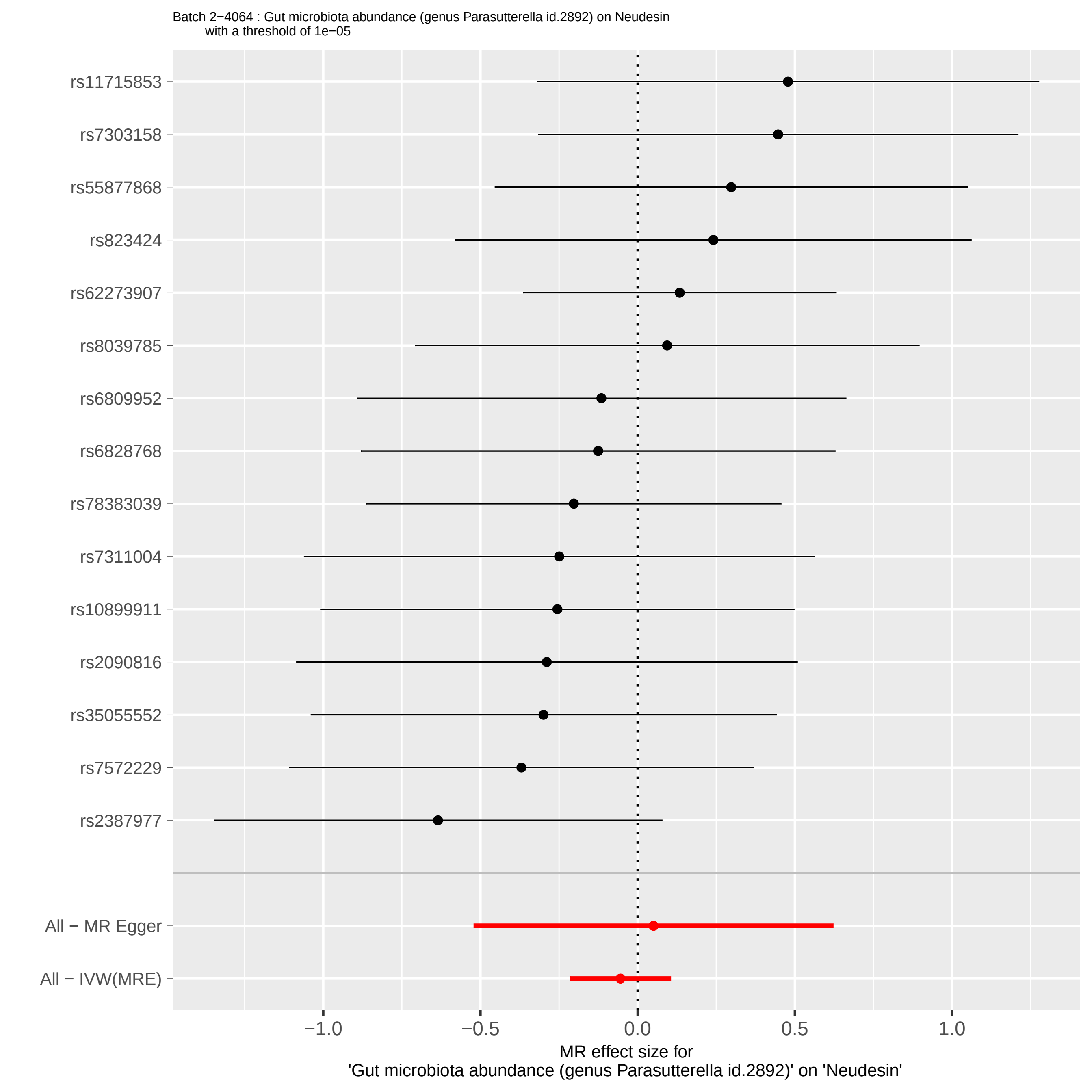

Batch 2−4064 : Gut microbiota abundance (genus Parasutterella id.2892) on Neudesin
with a threshold of 1e−05
rs11715853
rs7303158
rs55877868
rs823424
rs62273907
rs8039785
rs6809952
rs6828768
rs78383039
rs7311004
rs10899911
rs2090816
rs35055552
rs7572229
rs2387977
All − MR Egger
All − IVW(MRE)
−1.0
−0.5
0.0
0.5
1.0
MR effect size for
'Gut microbiota abundance (genus Parasutterella id.2892)' on 'Neudesin'

Batch 2−4065 : Gut microbiota abundance (genus Catenibacterium id.2153) on Neogenin
with a threshold of 1e−05

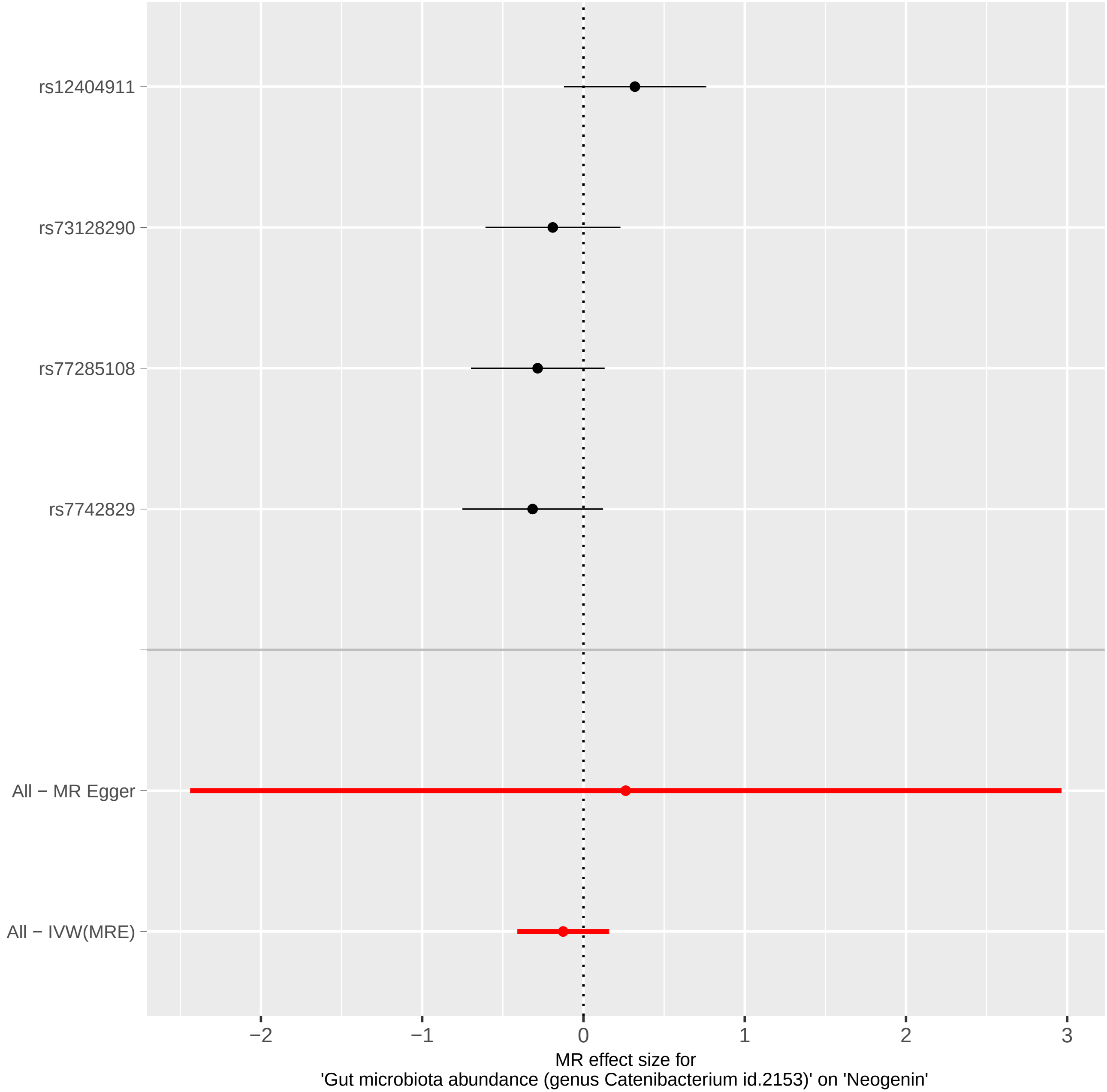

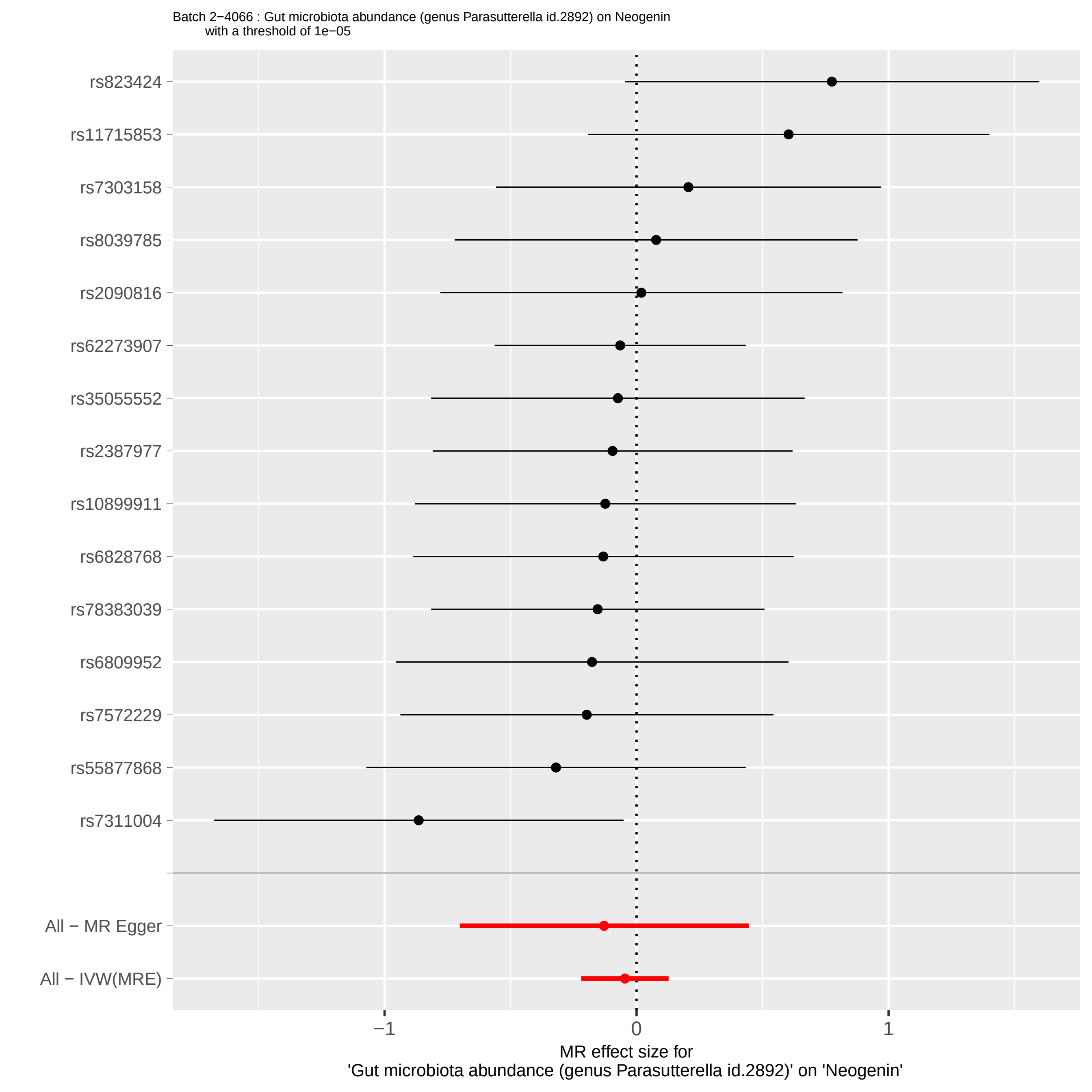

Batch 2−4066 : Gut microbiota abundance (genus Parasutterella id.2892) on Neogenin
with a threshold of 1e−05
rs823424
rs11715853
rs7303158
rs8039785
rs2090816
rs62273907
rs35055552
rs2387977
rs10899911
rs6828768
rs78383039
rs6809952
rs7572229
rs55877868
rs7311004
All − MR Egger
All − IVW(MRE)
−1
0
1
MR effect size for
'Gut microbiota abundance (genus Parasutterella id.2892)' on 'Neogenin'

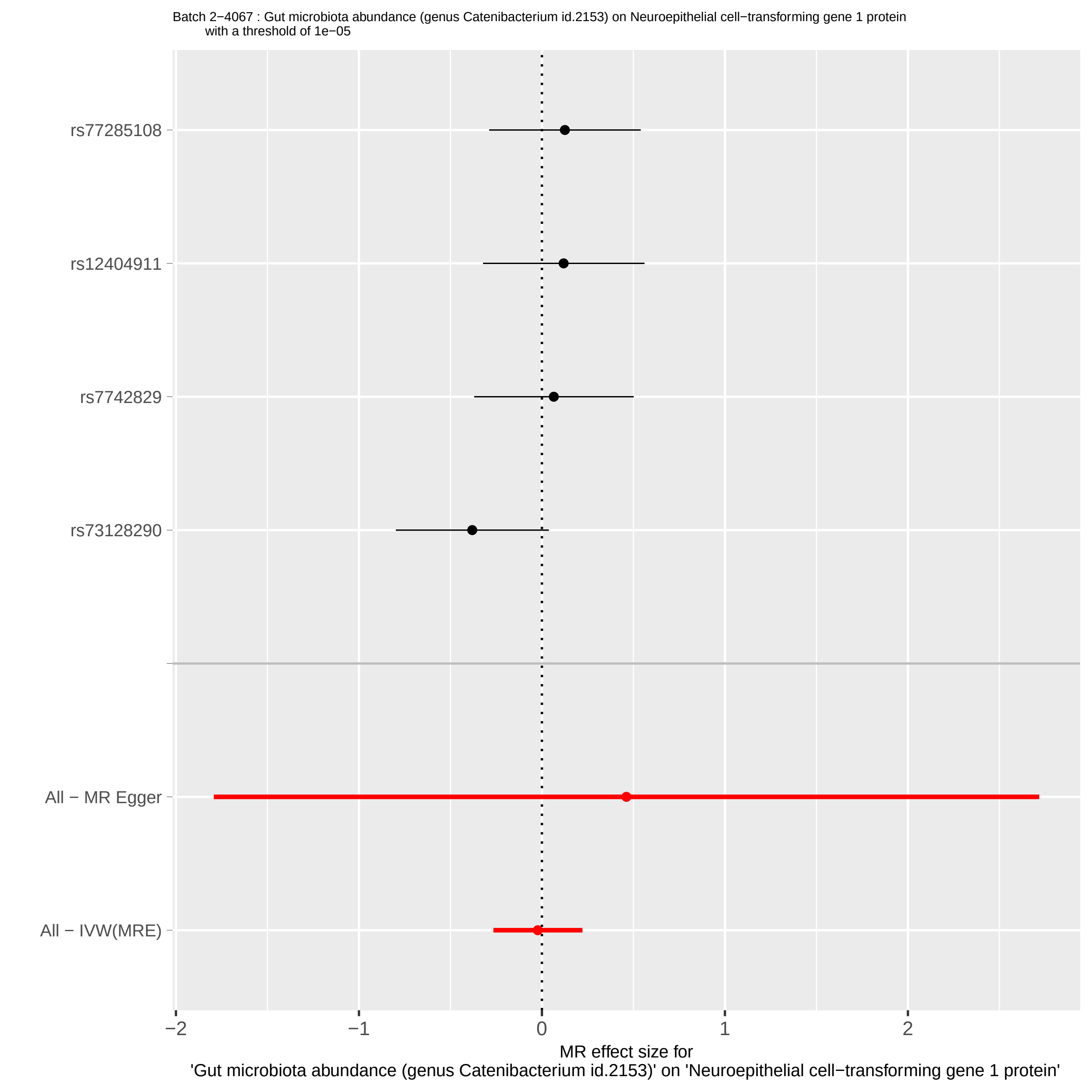

Batch 2−4067 : Gut microbiota abundance (genus Catenibacterium id.2153) on Neuroepithelial cell−transforming gene 1 protein
with a threshold of 1e−05
rs77285108
rs12404911
rs7742829
rs73128290
All − MR Egger
All − IVW(MRE)
−2
−1
0
1
2
MR effect size for
'Gut microbiota abundance (genus Catenibacterium id.2153)' on 'Neuroepithelial cell−transforming gene 1 protein'

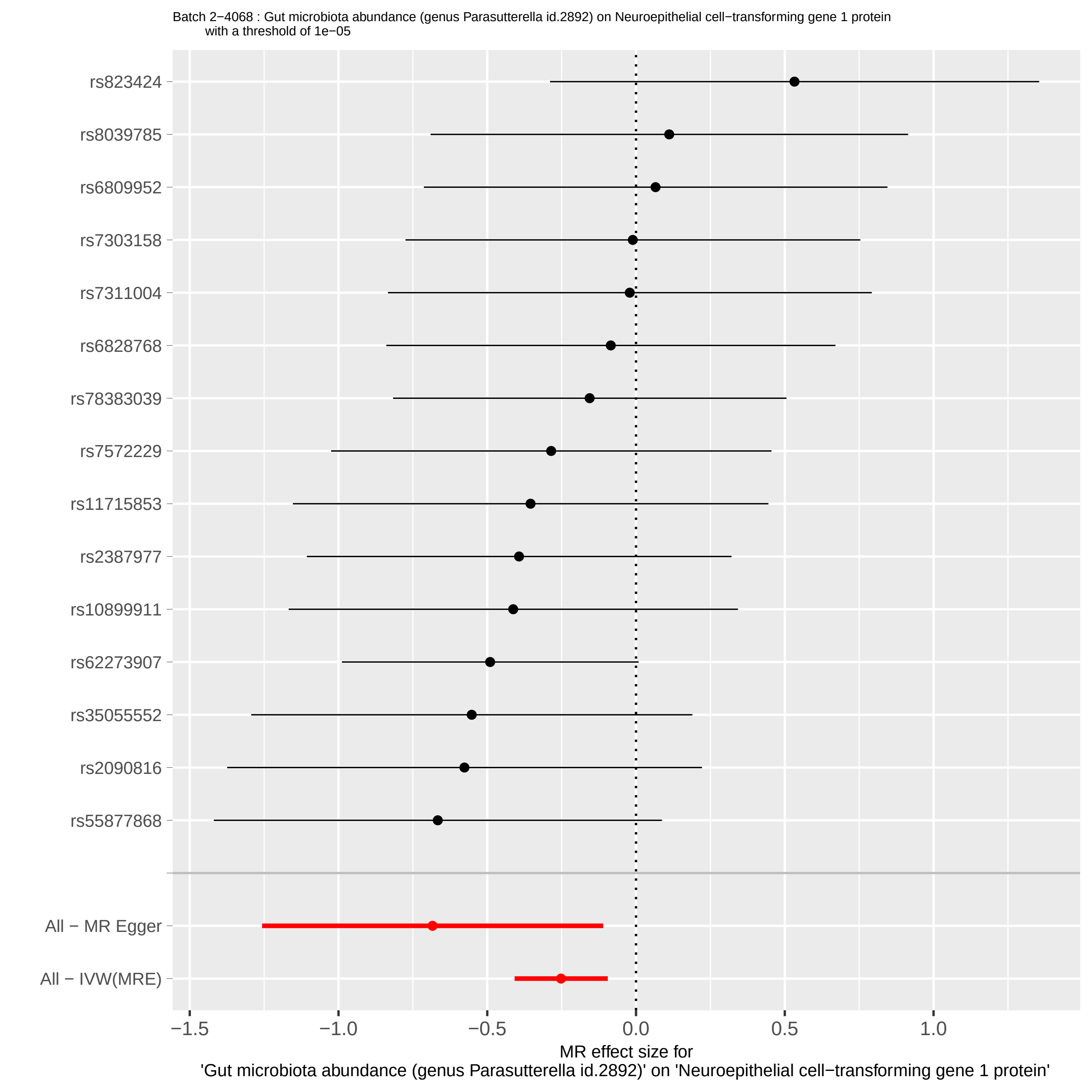
Batch 2−4068 : Gut microbiota abundance (genus Parasutterella id.2892) on Neuroepithelial cell−transforming gene 1 protein
with a threshold of 1e−05
rs823424
rs8039785
rs6809952
rs7303158
rs7311004
rs6828768
rs78383039
rs7572229
rs11715853
rs2387977
rs10899911
rs62273907
rs35055552
rs2090816
rs55877868
All − MR Egger
All − IVW(MRE)
−1.5
−1.0
−0.5
0.0
0.5
1.0
MR effect size for
'Gut microbiota abundance (genus Parasutterella id.2892)' on 'Neuroepithelial cell−transforming gene 1 protein'

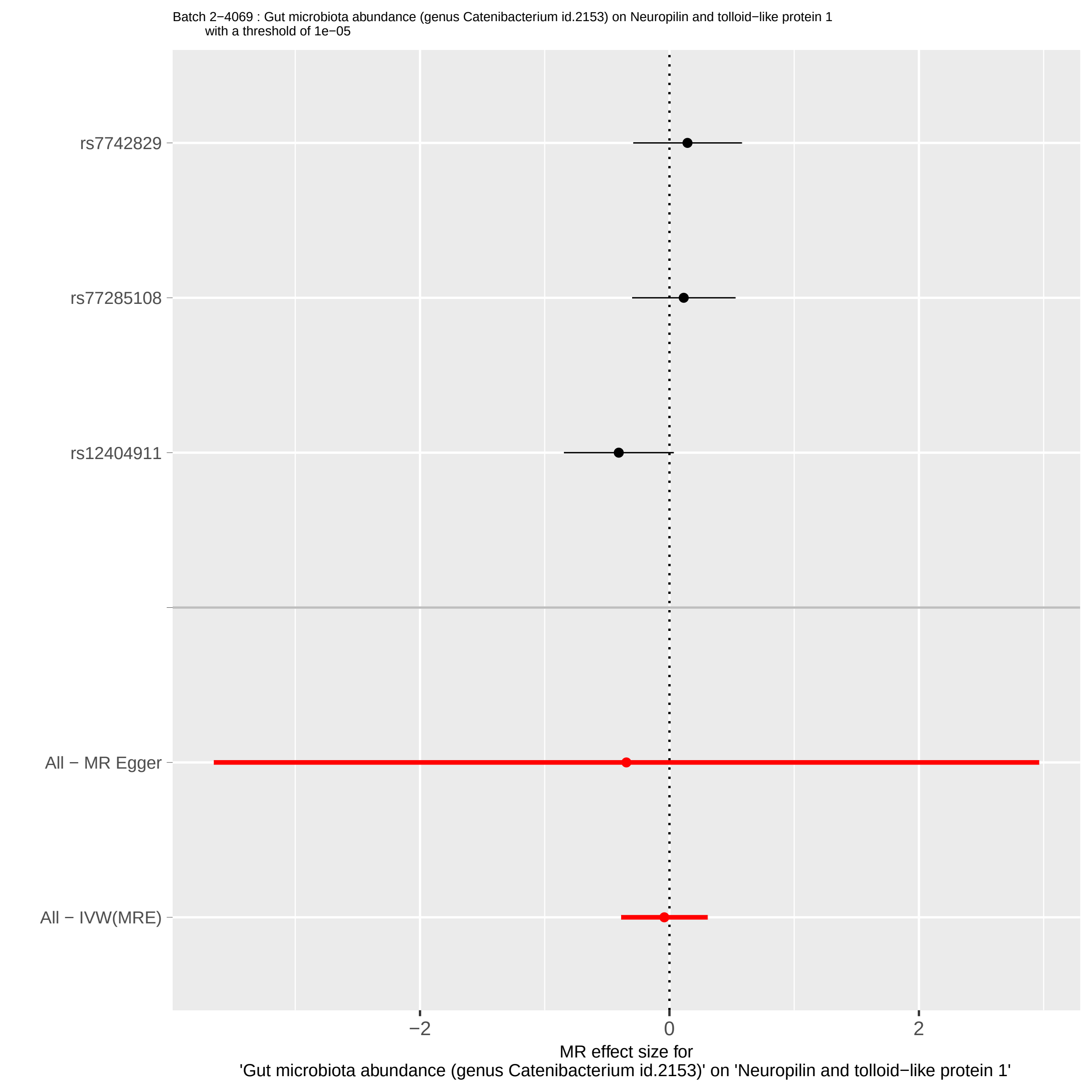

Batch 2−4069 : Gut microbiota abundance (genus Catenibacterium id.2153) on Neuropilin and tolloid−like protein 1
with a threshold of 1e−05
rs7742829
rs77285108
rs12404911
All − MR Egger
All − IVW(MRE)
−2
0
2
MR effect size for
'Gut microbiota abundance (genus Catenibacterium id.2153)' on 'Neuropilin and tolloid−like protein 1'

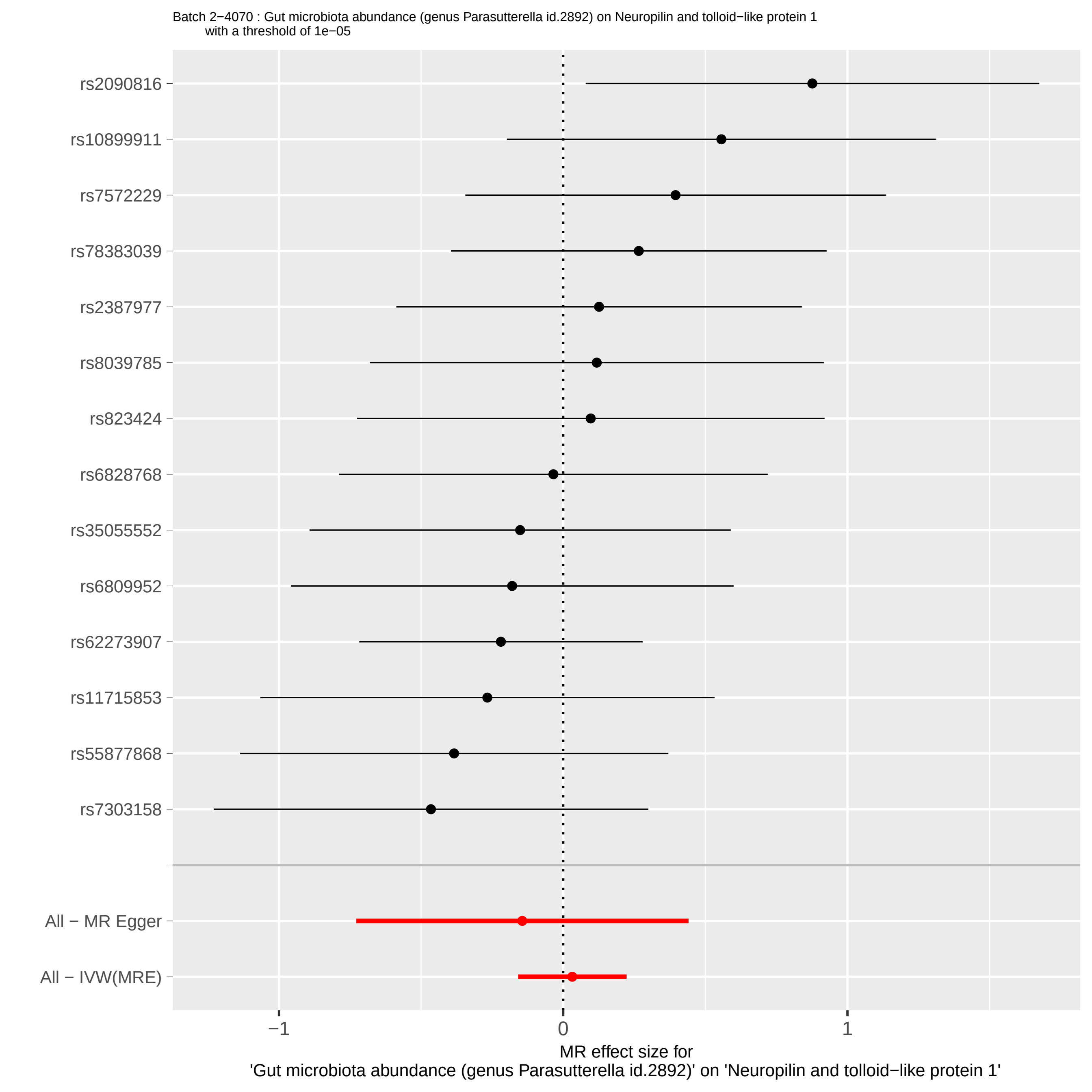

Batch 2−4070 : Gut microbiota abundance (genus Parasutterella id.2892) on Neuropilin and tolloid−like protein 1
with a threshold of 1e−05
rs2090816
rs10899911
rs7572229
rs78383039
rs2387977
rs8039785
rs823424
rs6828768
rs35055552
rs6809952
rs62273907
rs11715853
rs55877868
rs7303158
All − MR Egger
All − IVW(MRE)
−1
0
1
MR effect size for
'Gut microbiota abundance (genus Parasutterella id.2892)' on 'Neuropilin and tolloid−like protein 1'

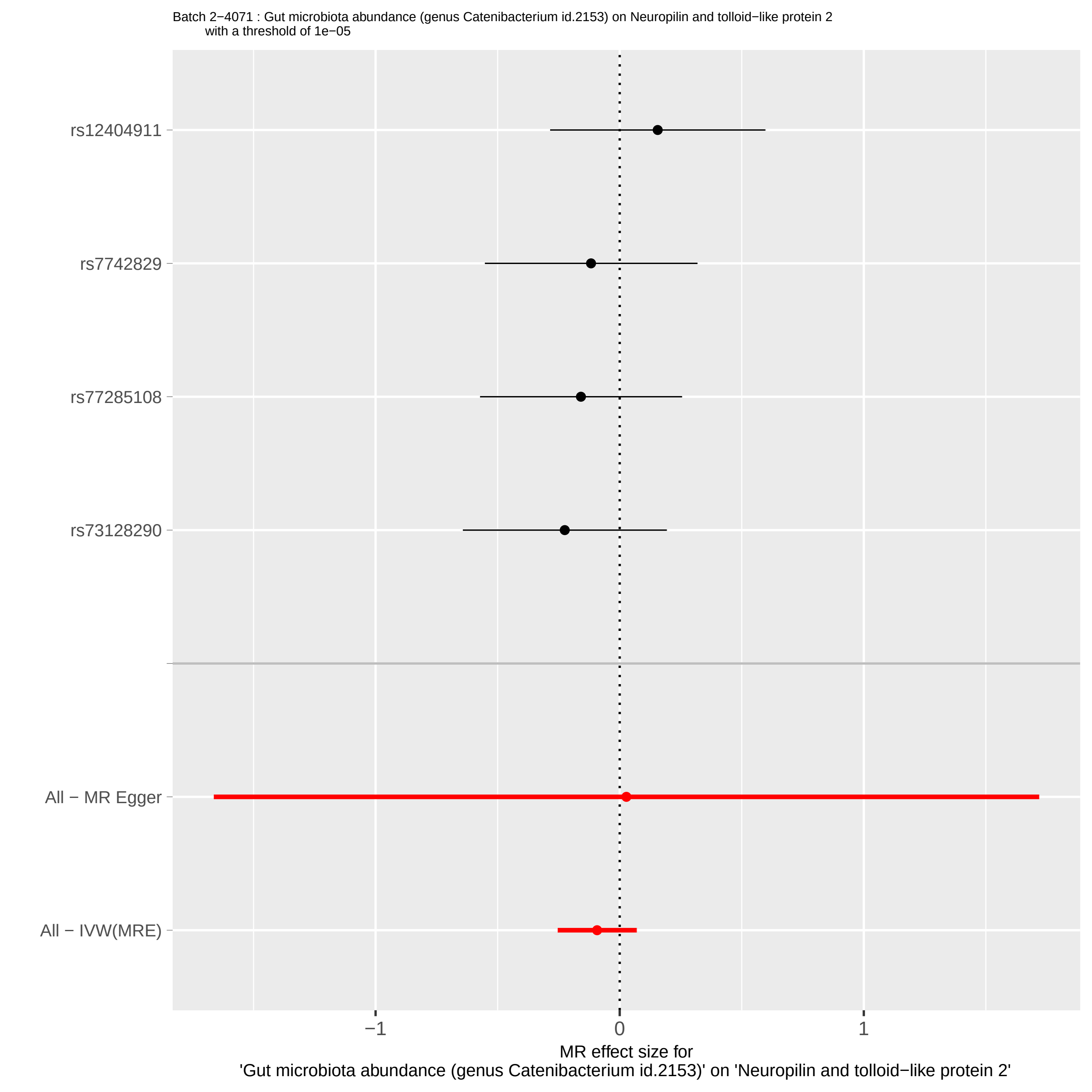
Batch 2−4071 : Gut microbiota abundance (genus Catenibacterium id.2153) on Neuropilin and tolloid−like protein 2
with a threshold of 1e−05
rs12404911
rs7742829
rs77285108
rs73128290
All − MR Egger
All − IVW(MRE)
−1
0
1
MR effect size for
'Gut microbiota abundance (genus Catenibacterium id.2153)' on 'Neuropilin and tolloid−like protein 2'

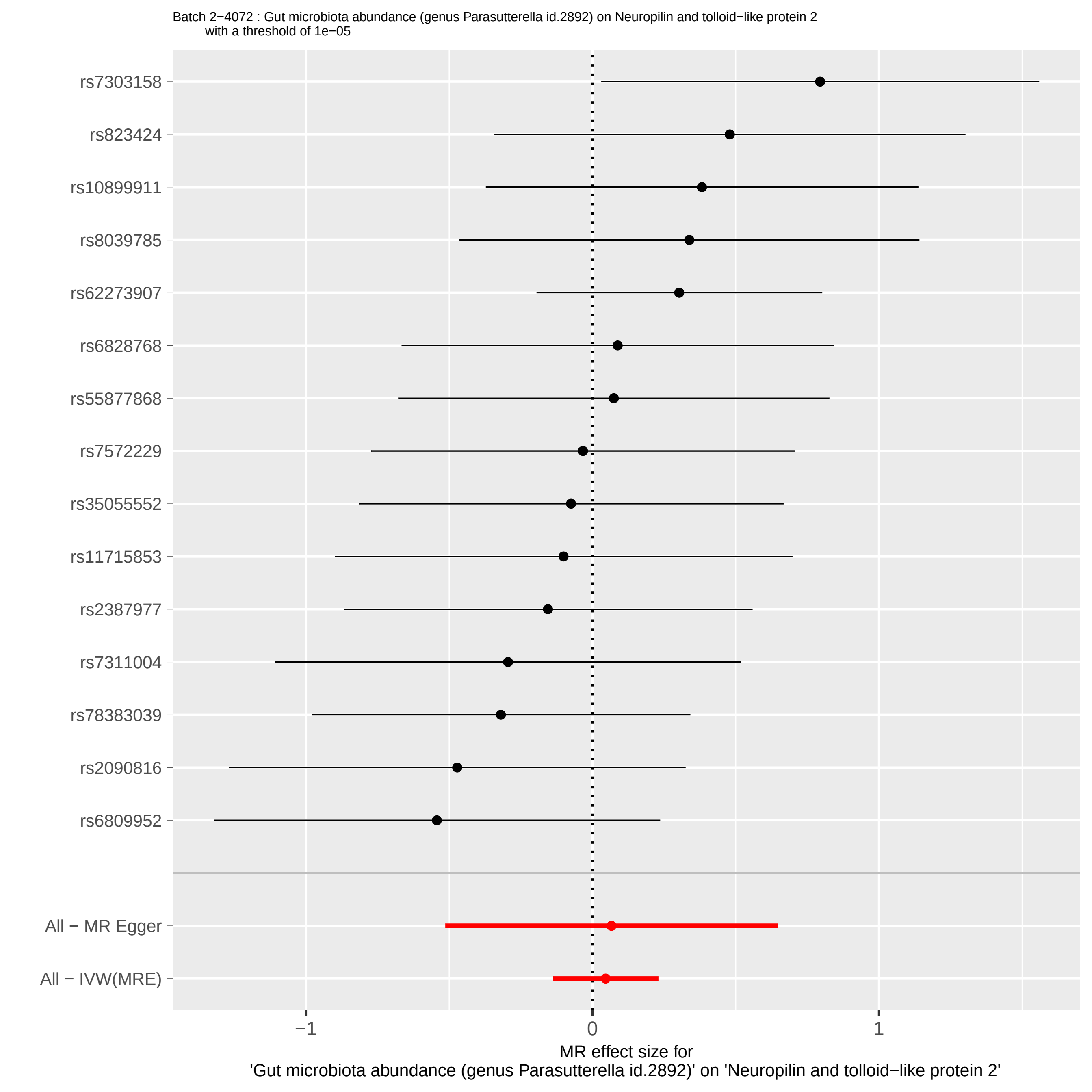
Batch 2−4072 : Gut microbiota abundance (genus Parasutterella id.2892) on Neuropilin and tolloid−like protein 2
with a threshold of 1e−05
rs7303158
rs823424
rs10899911
rs8039785
rs62273907
rs6828768
rs55877868
rs7572229
rs35055552
rs11715853
rs2387977
rs7311004
rs78383039
rs2090816
rs6809952
All − MR Egger
All − IVW(MRE)
−1
0
1
MR effect size for
'Gut microbiota abundance (genus Parasutterella id.2892)' on 'Neuropilin and tolloid−like protein 2'

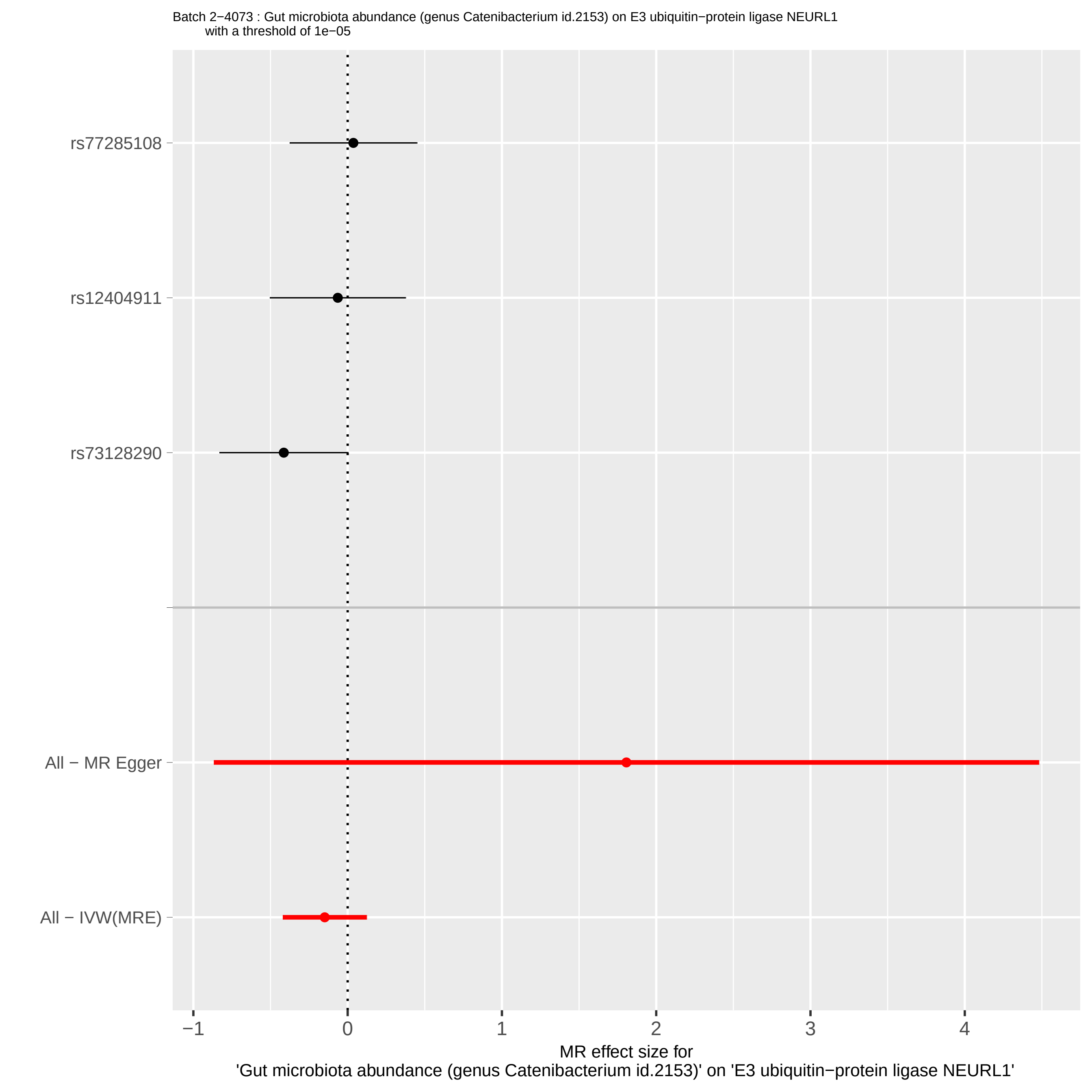

Batch 2−4073 : Gut microbiota abundance (genus Catenibacterium id.2153) on E3 ubiquitin−protein ligase NEURL1
with a threshold of 1e−05
rs77285108
rs12404911
rs73128290
All − MR Egger
All − IVW(MRE)
−1
0
1
2
3
4
MR effect size for
'Gut microbiota abundance (genus Catenibacterium id.2153)' on 'E3 ubiquitin−protein ligase NEURL1'

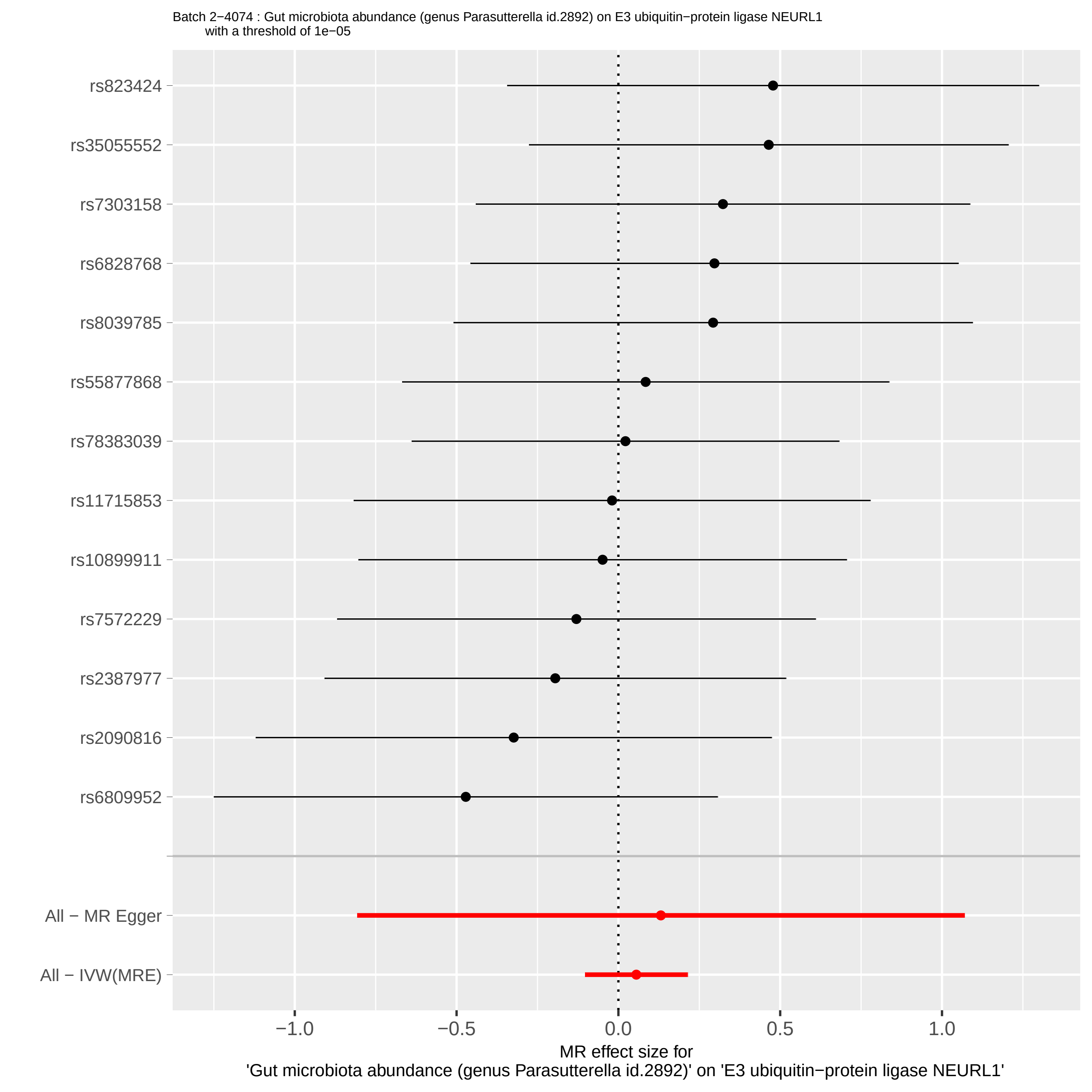

Batch 2−4074 : Gut microbiota abundance (genus Parasutterella id.2892) on E3 ubiquitin−protein ligase NEURL1
with a threshold of 1e−05
rs823424
rs35055552
rs7303158
rs6828768
rs8039785
rs55877868
rs78383039
rs11715853
rs10899911
rs7572229
rs2387977
rs2090816
rs6809952
All − MR Egger
All − IVW(MRE)
−1.0
−0.5
0.0
0.5
1.0
MR effect size for
'Gut microbiota abundance (genus Parasutterella id.2892)' on 'E3 ubiquitin−protein ligase NEURL1'

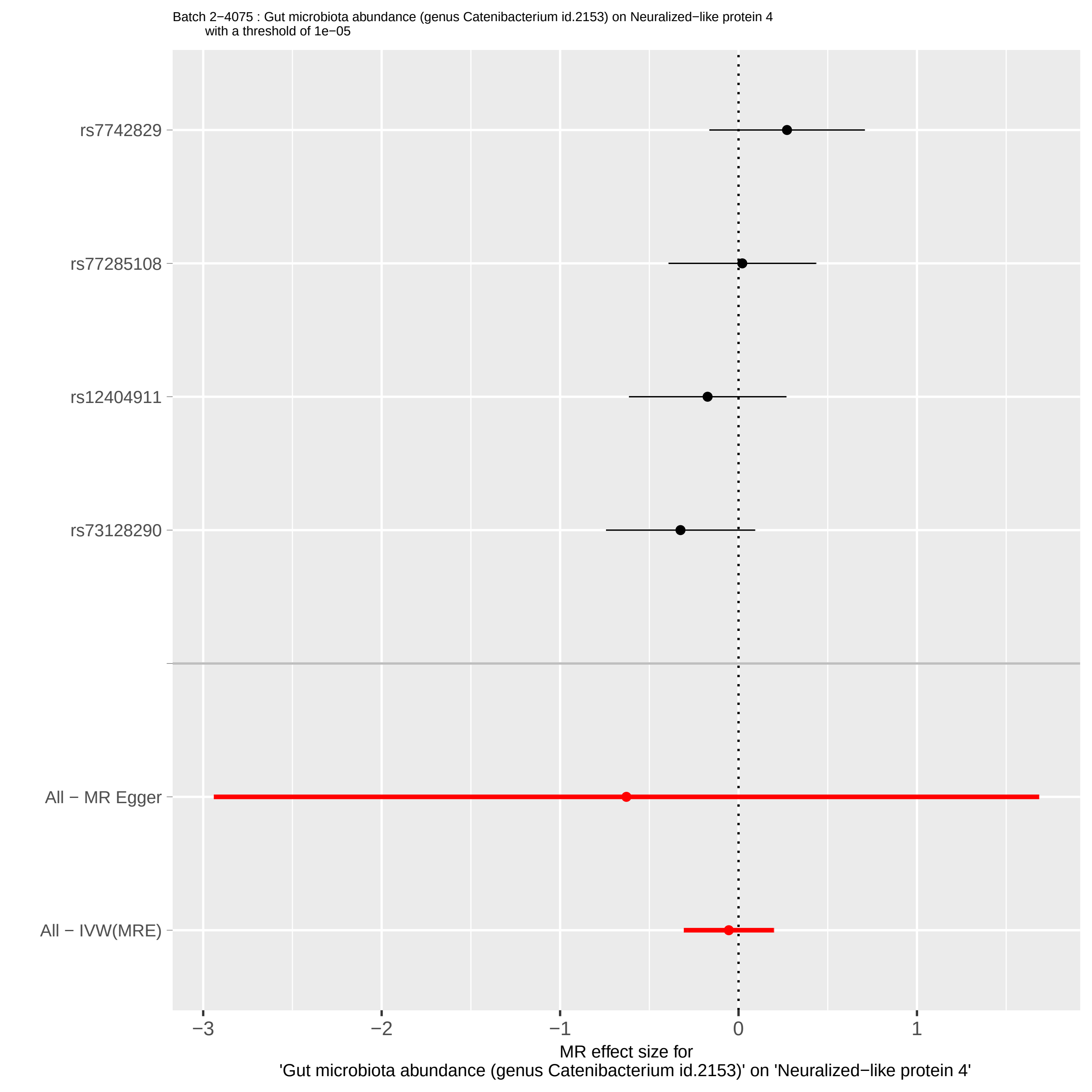
Batch 2−4075 : Gut microbiota abundance (genus Catenibacterium id.2153) on Neuralized−like protein 4
with a threshold of 1e−05
rs7742829
rs77285108
rs12404911
rs73128290
All − MR Egger
All − IVW(MRE)
−3
−2
−1
0
1
MR effect size for
'Gut microbiota abundance (genus Catenibacterium id.2153)' on 'Neuralized−like protein 4'

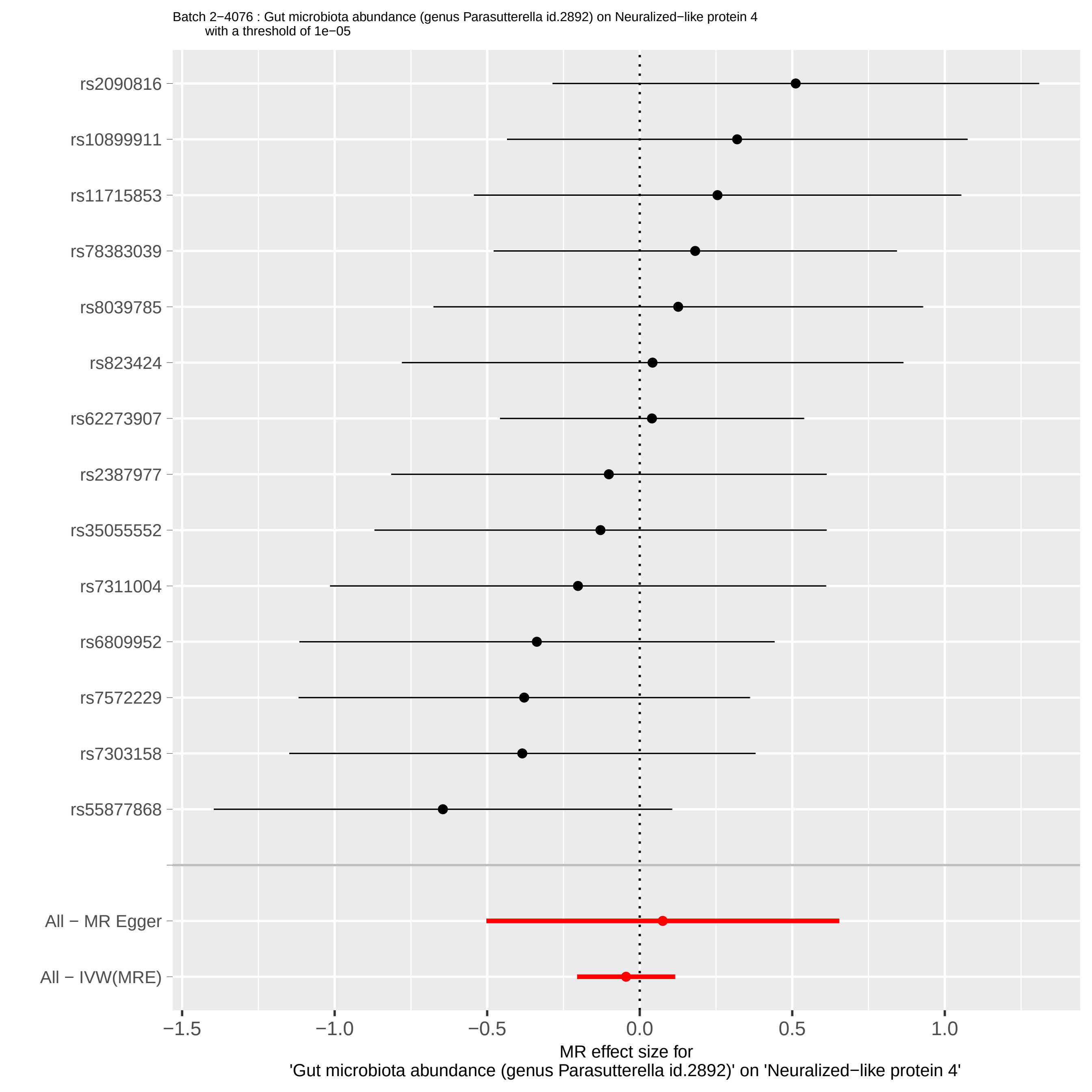

Batch 2−4076 : Gut microbiota abundance (genus Parasutterella id.2892) on Neuralized−like protein 4
with a threshold of 1e−05
rs2090816
rs10899911
rs11715853
rs78383039
rs8039785
rs823424
rs62273907
rs2387977
rs35055552
rs7311004
rs6809952
rs7572229
rs7303158
rs55877868
All − MR Egger
All − IVW(MRE)
−1.5
−1.0
−0.5
0.0
0.5
1.0
MR effect size for
'Gut microbiota abundance (genus Parasutterella id.2892)' on 'Neuralized−like protein 4'

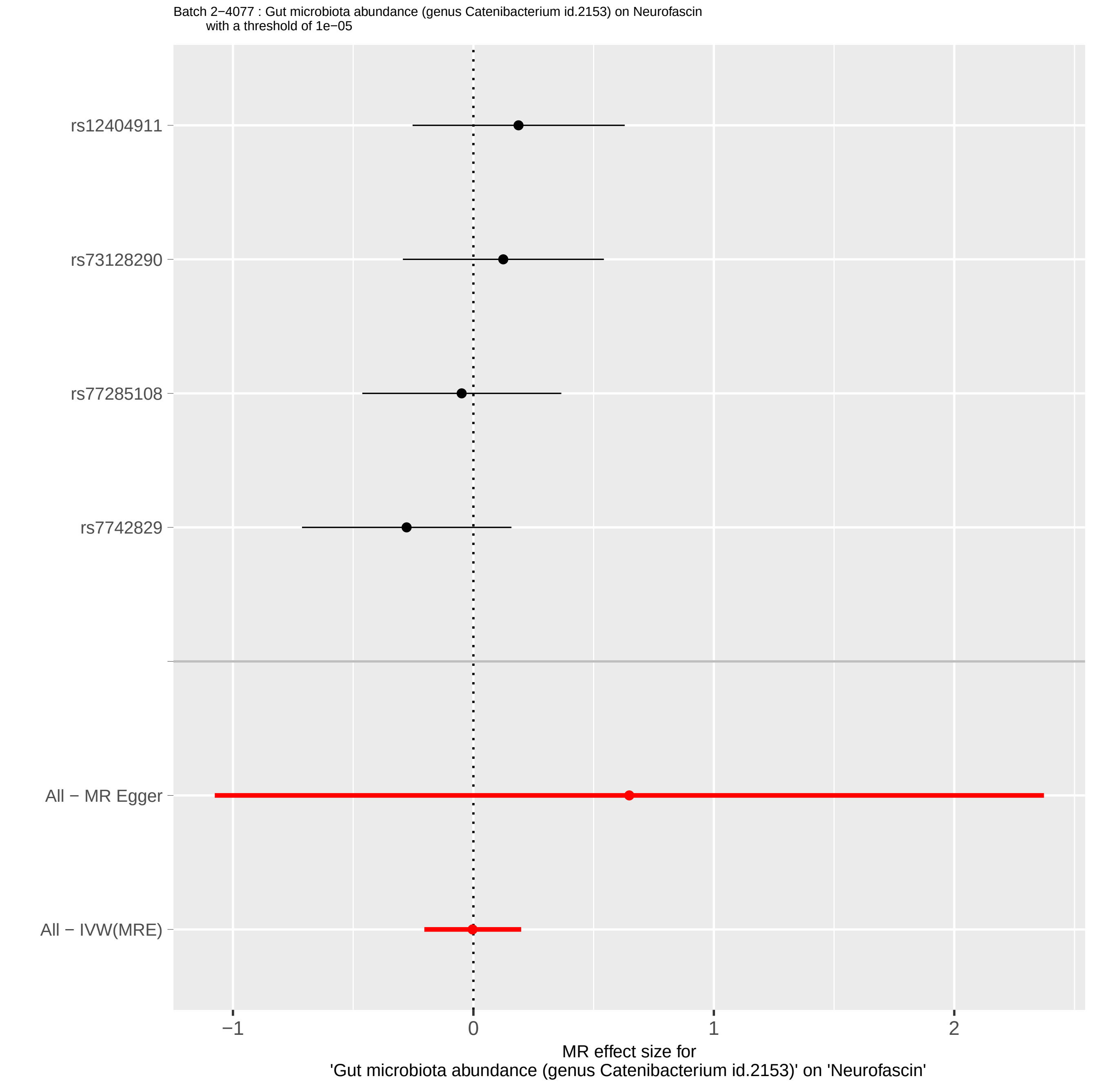
Batch 2−4077 : Gut microbiota abundance (genus Catenibacterium id.2153) on Neurofascin
with a threshold of 1e−05
rs12404911
rs73128290
rs77285108
rs7742829
All − MR Egger
All − IVW(MRE)
−1
0
1
2
MR effect size for
'Gut microbiota abundance (genus Catenibacterium id.2153)' on 'Neurofascin'

Batch 2−4078 : Gut microbiota abundance (genus Parasutterella id.2892) on Neurofascin
with a threshold of 1e−05

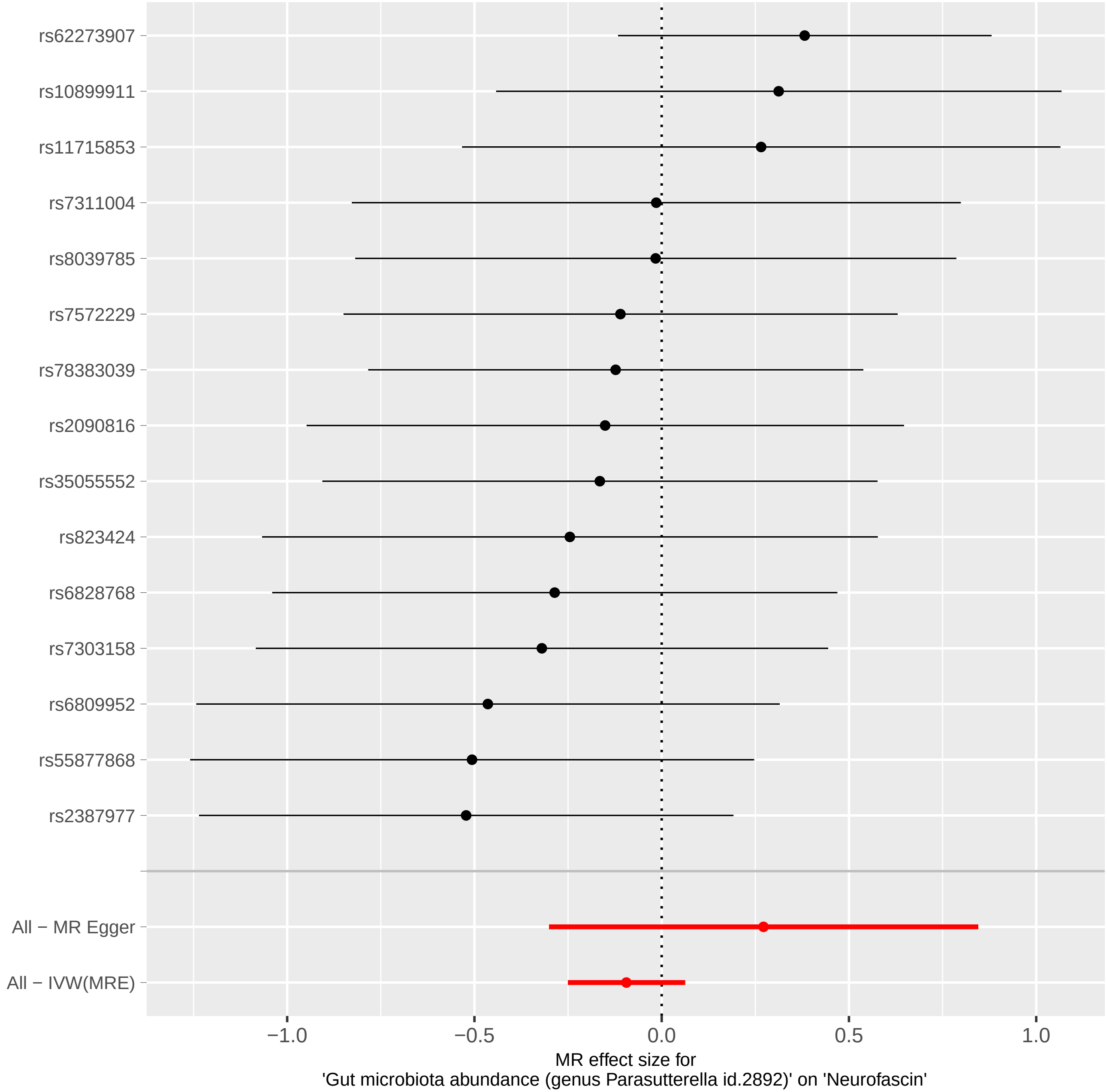

Batch 2−4079 : Gut microbiota abundance (genus Catenibacterium id.2153) on Nuclear factor erythroid 2−related factor 1
with a threshold of 1e−05

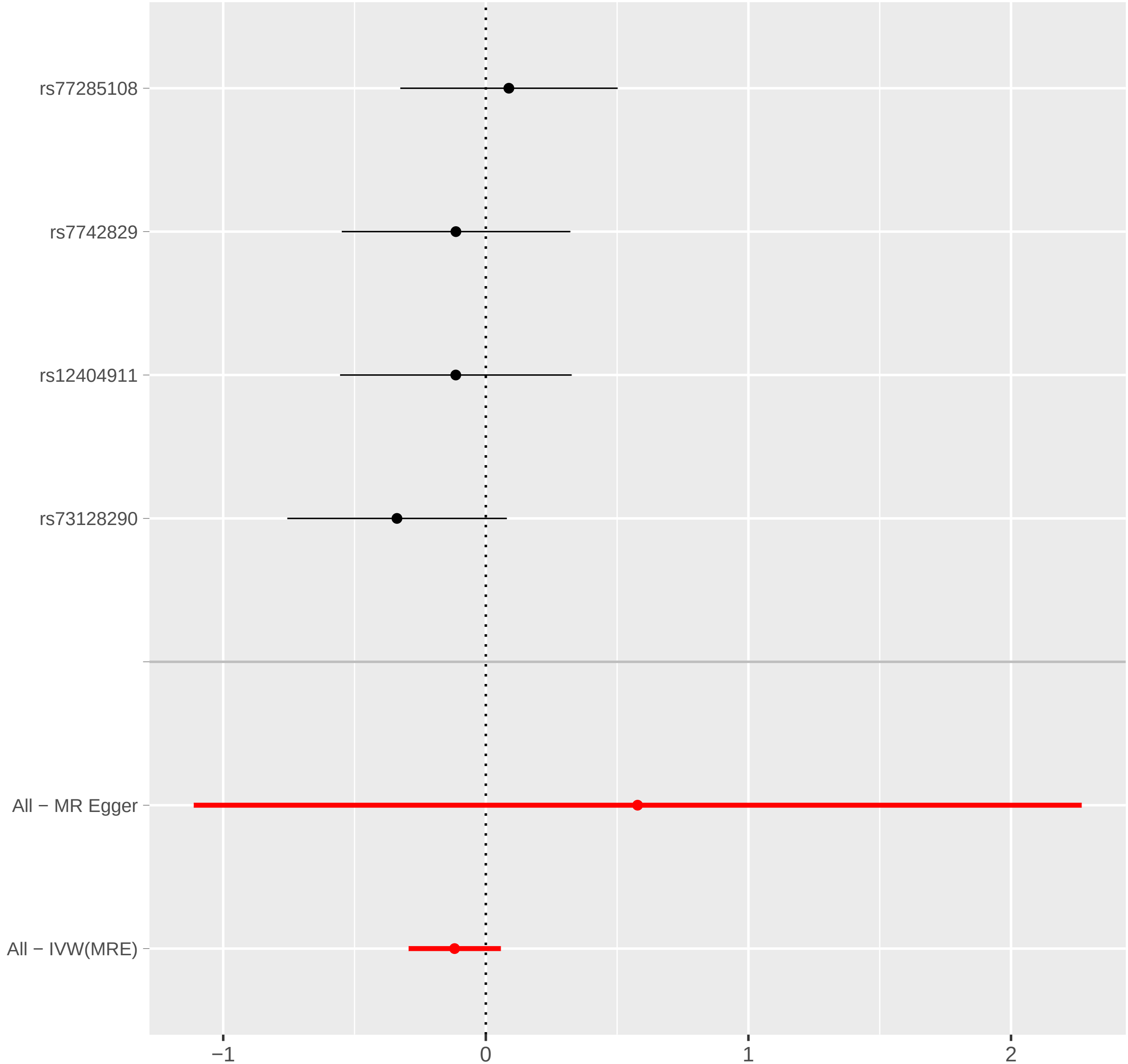

MR effect size for
'Gut microbiota abundance (genus Catenibacterium id.2153)' on 'Nuclear factor erythroid 2−related factor 1'

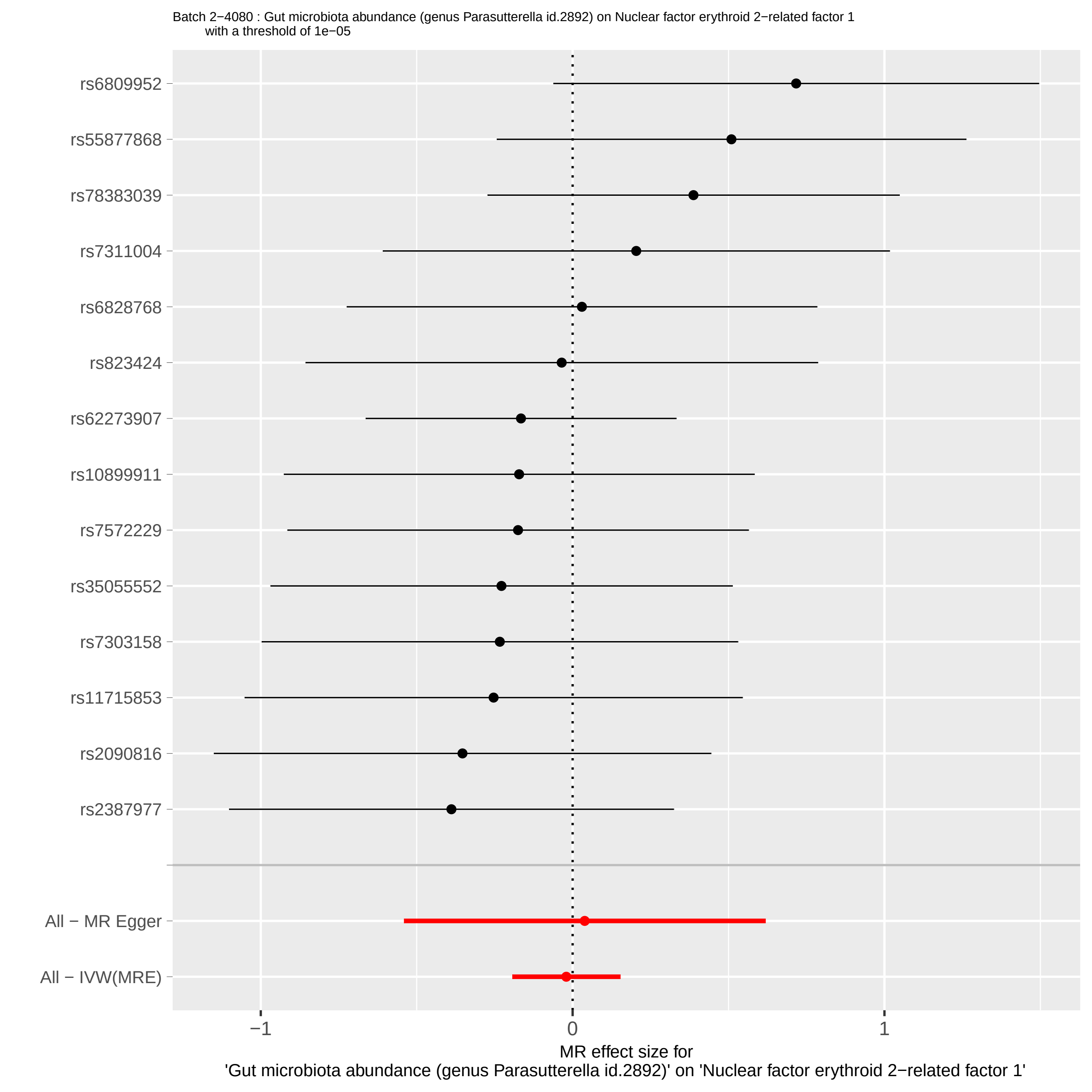

Batch 2−4080 : Gut microbiota abundance (genus Parasutterella id.2892) on Nuclear factor erythroid 2−related factor 1
with a threshold of 1e−05
rs6809952
rs55877868
rs78383039
rs7311004
rs6828768
rs823424
rs62273907
rs10899911
rs7572229
rs35055552
rs7303158
rs11715853
rs2090816
rs2387977
All − MR Egger
All − IVW(MRE)
−1
0
1
MR effect size for
'Gut microbiota abundance (genus Parasutterella id.2892)' on 'Nuclear factor erythroid 2−related factor 1'

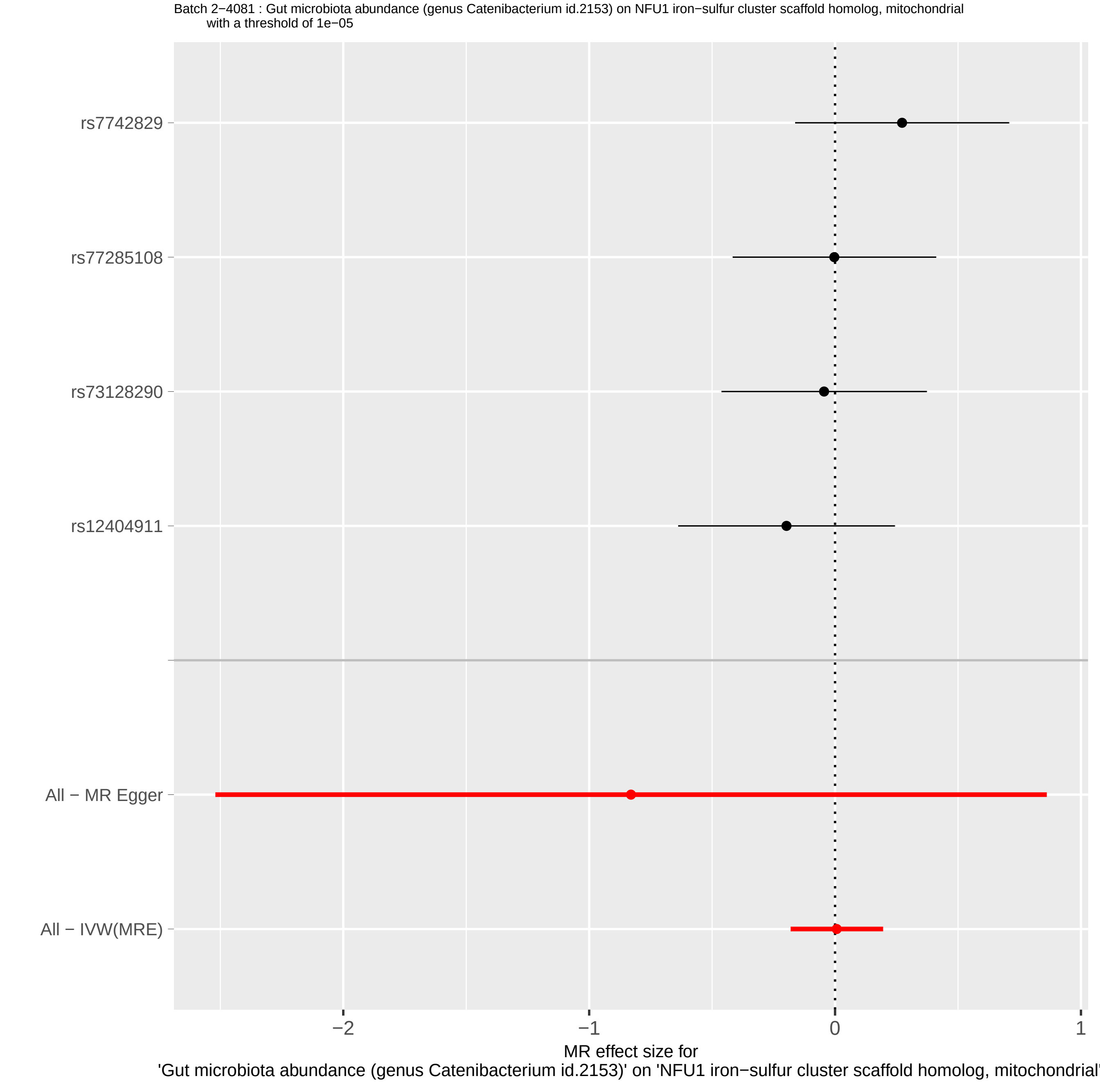

Batch 2−4081 : Gut microbiota abundance (genus Catenibacterium id.2153) on NFU1 iron−sulfur cluster scaffold homolog, mitochondrial
with a threshold of 1e−05
rs7742829
rs77285108
rs73128290
rs12404911
All − MR Egger
All − IVW(MRE)
−2
−1
0
1
MR effect size for
'Gut microbiota abundance (genus Catenibacterium id.2153)' on 'NFU1 iron−sulfur cluster scaffold homolog, mitochondrial'

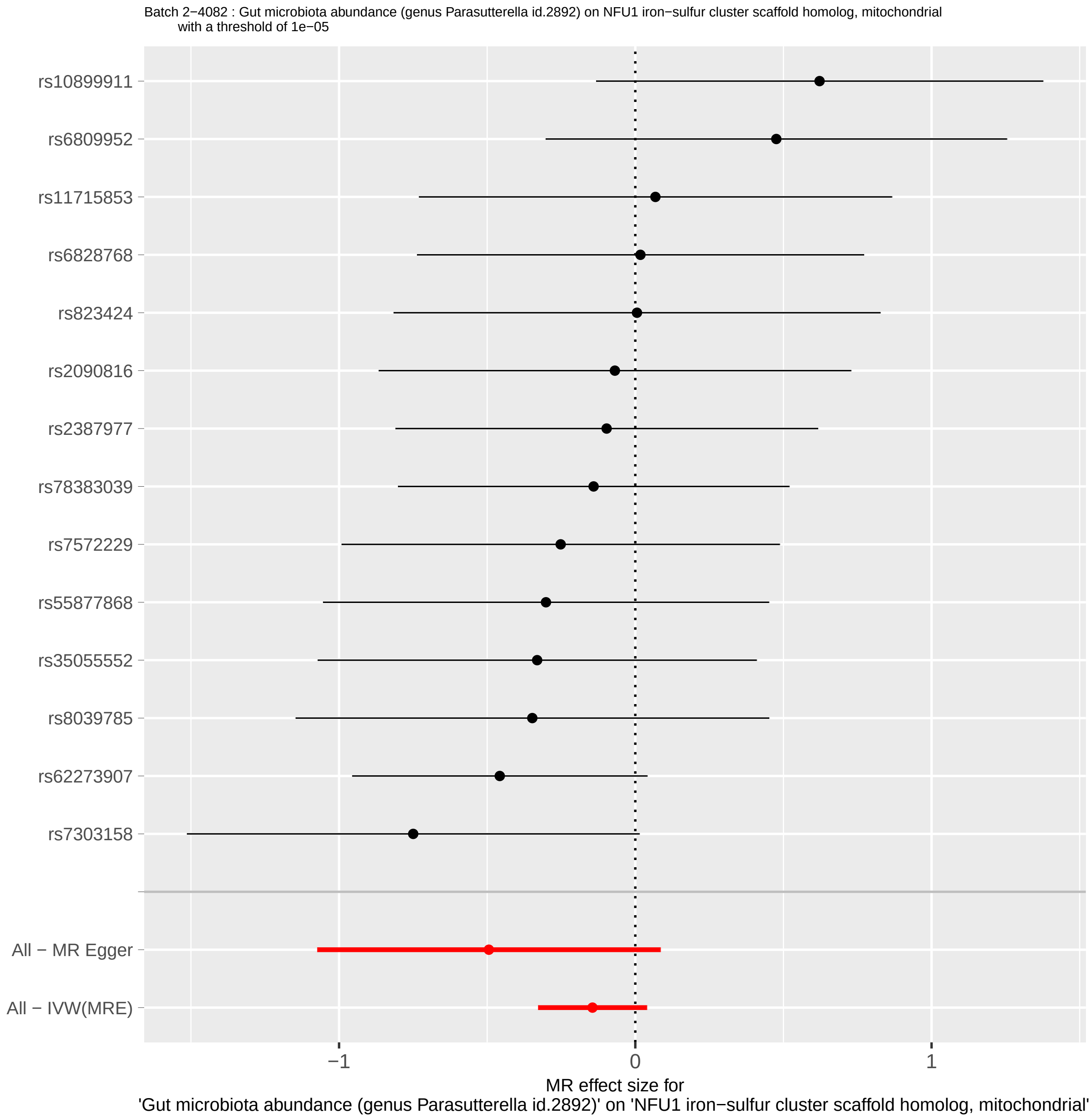

Batch 2−4082 : Gut microbiota abundance (genus Parasutterella id.2892) on NFU1 iron−sulfur cluster scaffold homolog, mitochondrial
with a threshold of 1e−05
rs10899911
rs6809952
rs11715853
rs6828768
rs823424
rs2090816
rs2387977
rs78383039
rs7572229
rs55877868
rs35055552
rs8039785
rs62273907
rs7303158
All − MR Egger
All − IVW(MRE)
−1
0
1
MR effect size for
'Gut microbiota abundance (genus Parasutterella id.2892)' on 'NFU1 iron−sulfur cluster scaffold homolog, mitochondrial'

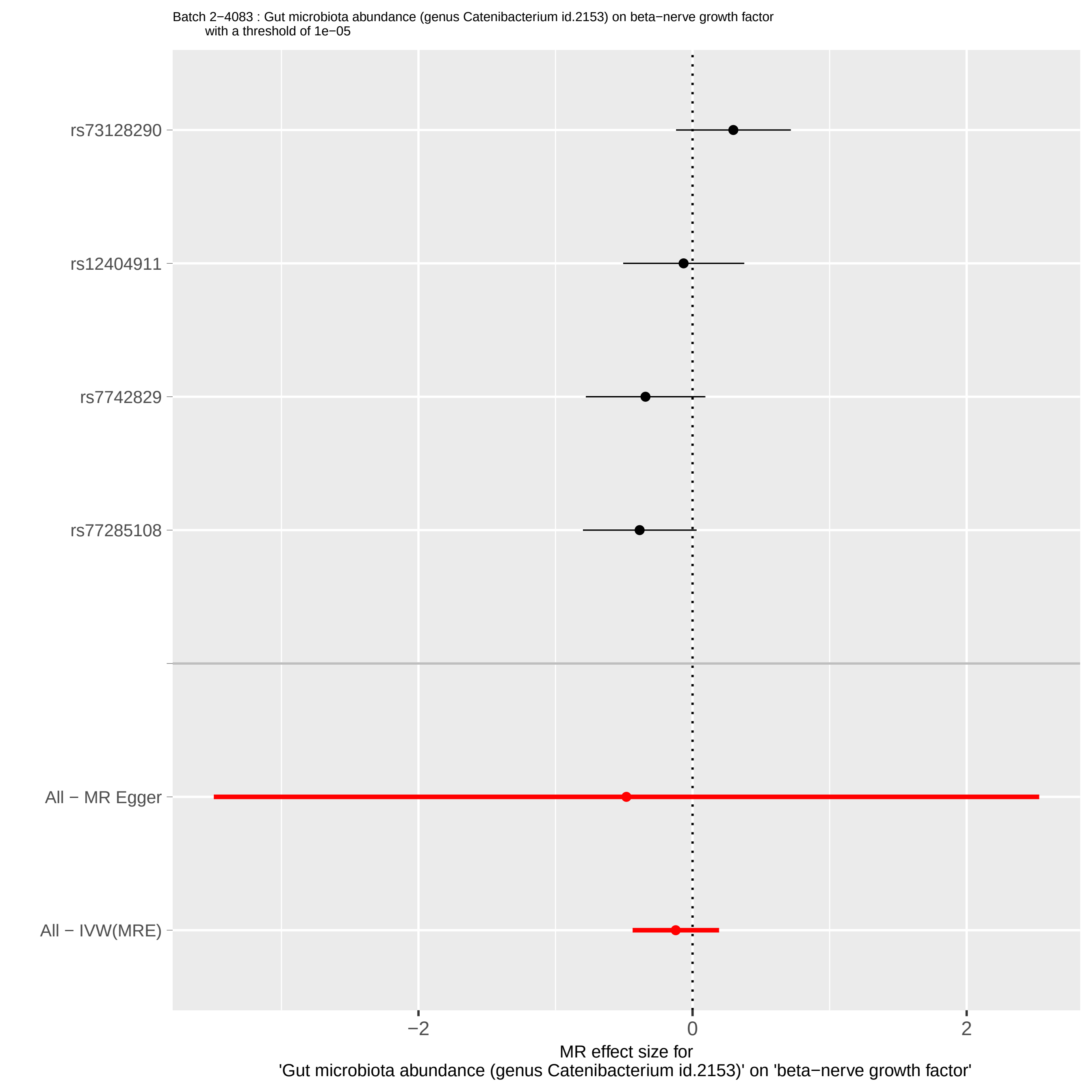
Batch 2−4083 : Gut microbiota abundance (genus Catenibacterium id.2153) on beta−nerve growth factor
with a threshold of 1e−05
rs73128290
rs12404911
rs7742829
rs77285108
All − MR Egger
All − IVW(MRE)
−2
0
2
MR effect size for
'Gut microbiota abundance (genus Catenibacterium id.2153)' on 'beta−nerve growth factor'

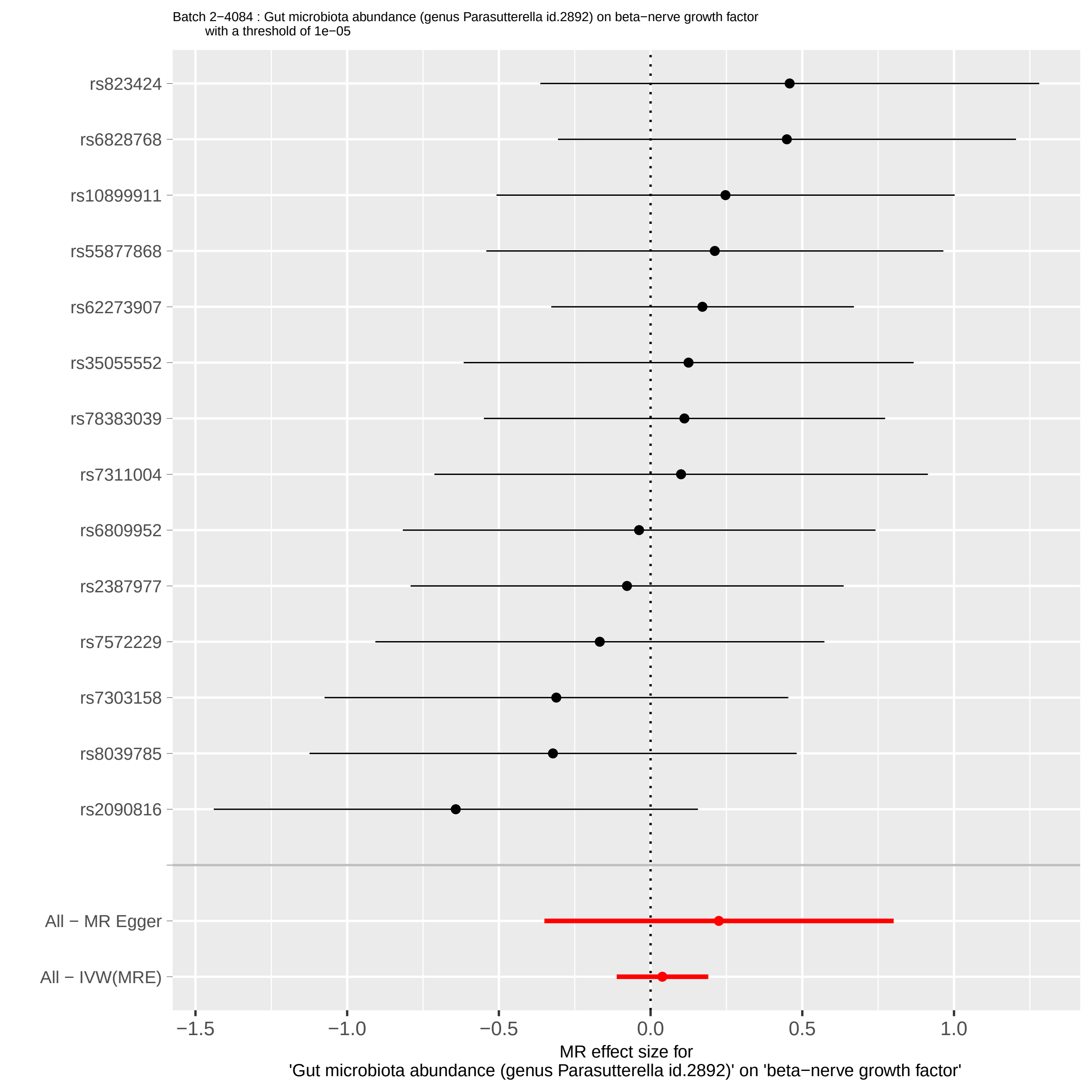

Batch 2−4084 : Gut microbiota abundance (genus Parasutterella id.2892) on beta−nerve growth factor
with a threshold of 1e−05
rs823424
rs6828768
rs10899911
rs55877868
rs62273907
rs35055552
rs78383039
rs7311004
rs6809952
rs2387977
rs7572229
rs7303158
rs8039785
rs2090816
All − MR Egger
All − IVW(MRE)
−1.5
−1.0
−0.5
0.0
0.5
1.0
MR effect size for
'Gut microbiota abundance (genus Parasutterella id.2892)' on 'beta−nerve growth factor'

Batch 2−4085 : Gut microbiota abundance (genus Catenibacterium id.2153) on Tumor necrosis factor receptor superfamily member 16
with a threshold of 1e−05

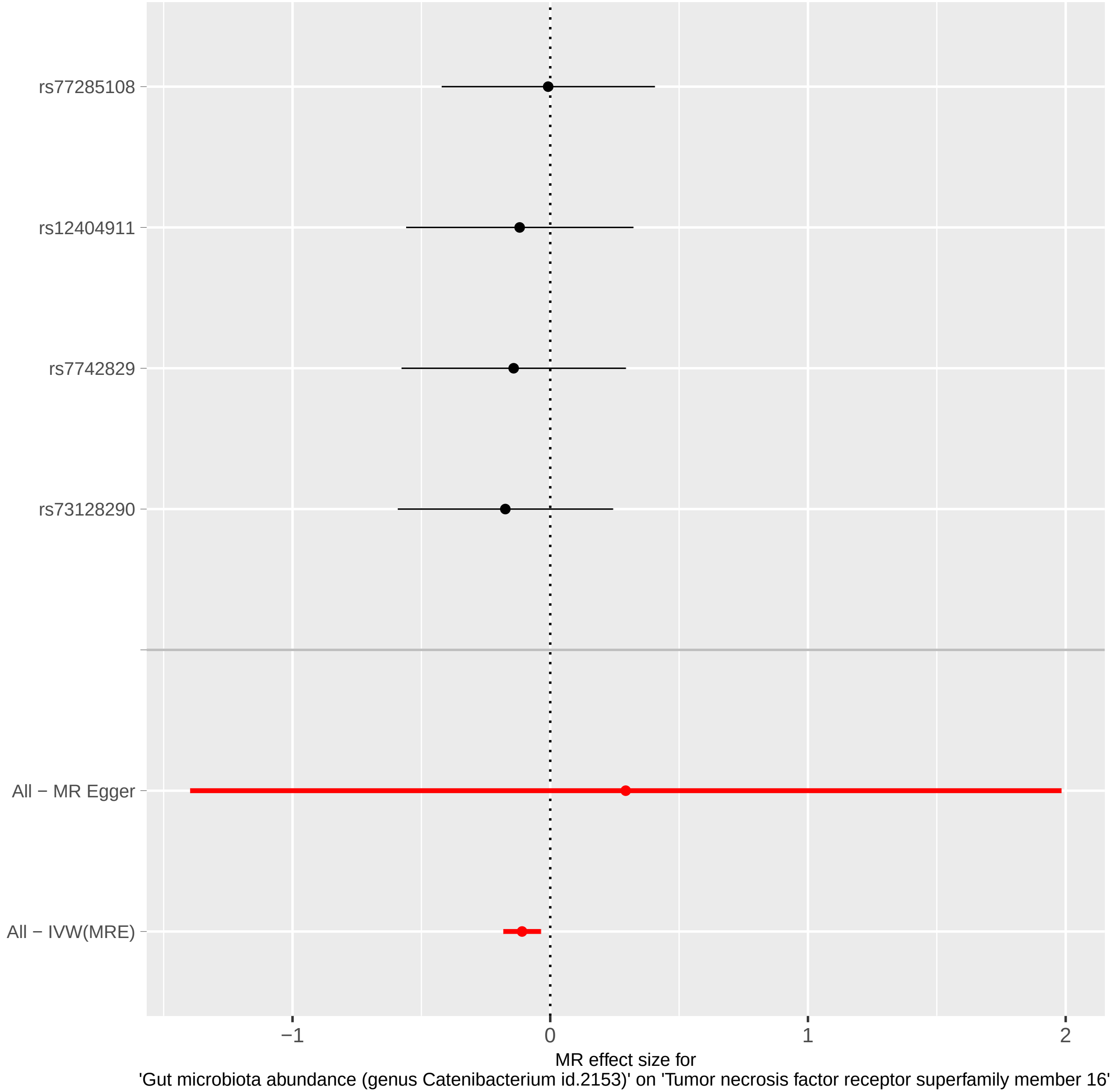

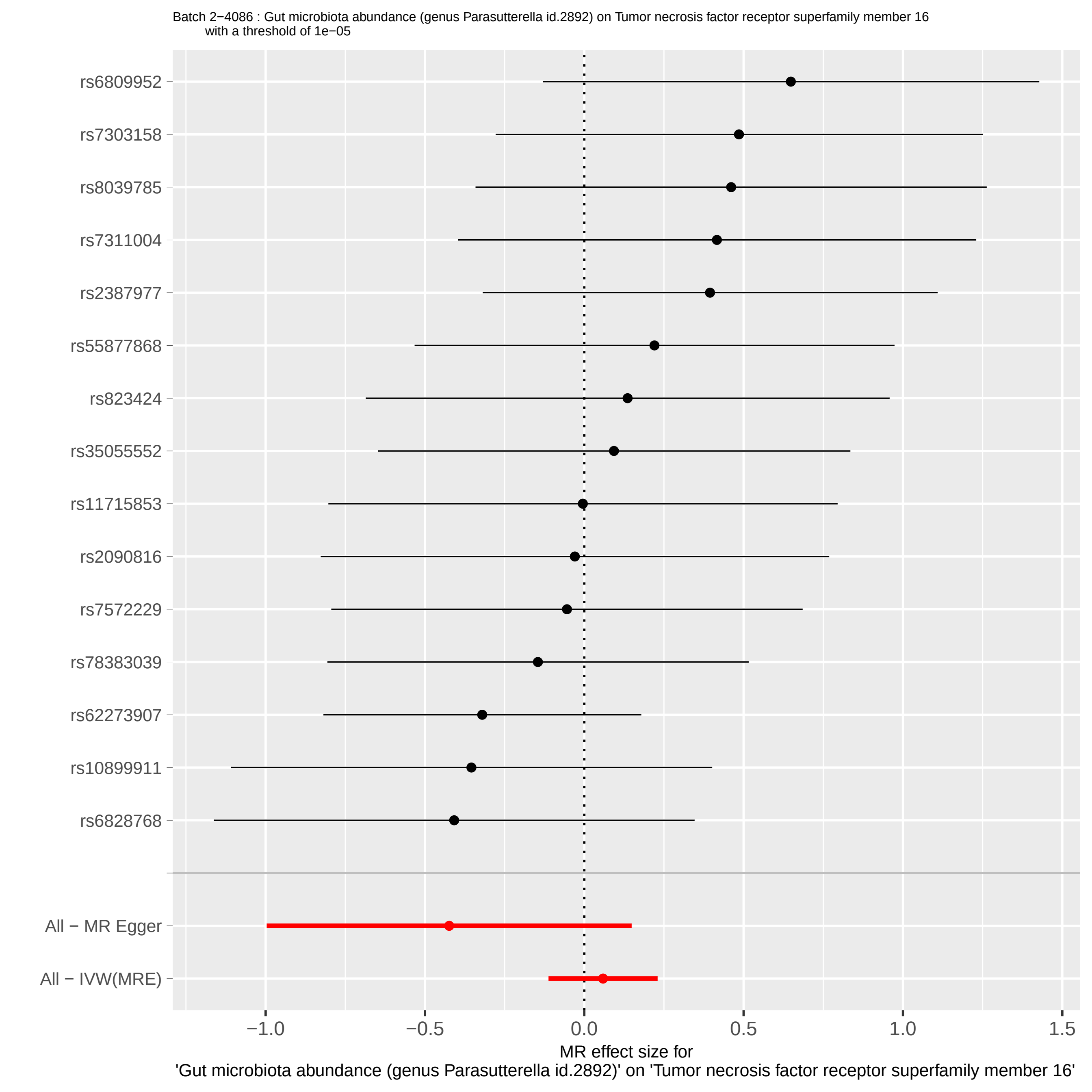

Batch 2−4086 : Gut microbiota abundance (genus Parasutterella id.2892) on Tumor necrosis factor receptor superfamily member 16
with a threshold of 1e−05
rs6809952
rs7303158
rs8039785
rs7311004
rs2387977
rs55877868
rs823424
rs35055552
rs11715853
rs2090816
rs7572229
rs78383039
rs62273907
rs10899911
rs6828768
All − MR Egger
All − IVW(MRE)
−1.0
−0.5
0.0
0.5
1.0
1.5
MR effect size for
'Gut microbiota abundance (genus Parasutterella id.2892)' on 'Tumor necrosis factor receptor superfamily member 16'

Batch 2−4087 : Gut microbiota abundance (genus Catenibacterium id.2153) on Tumor necrosis factor receptor superfamily member 16
with a threshold of 1e−05

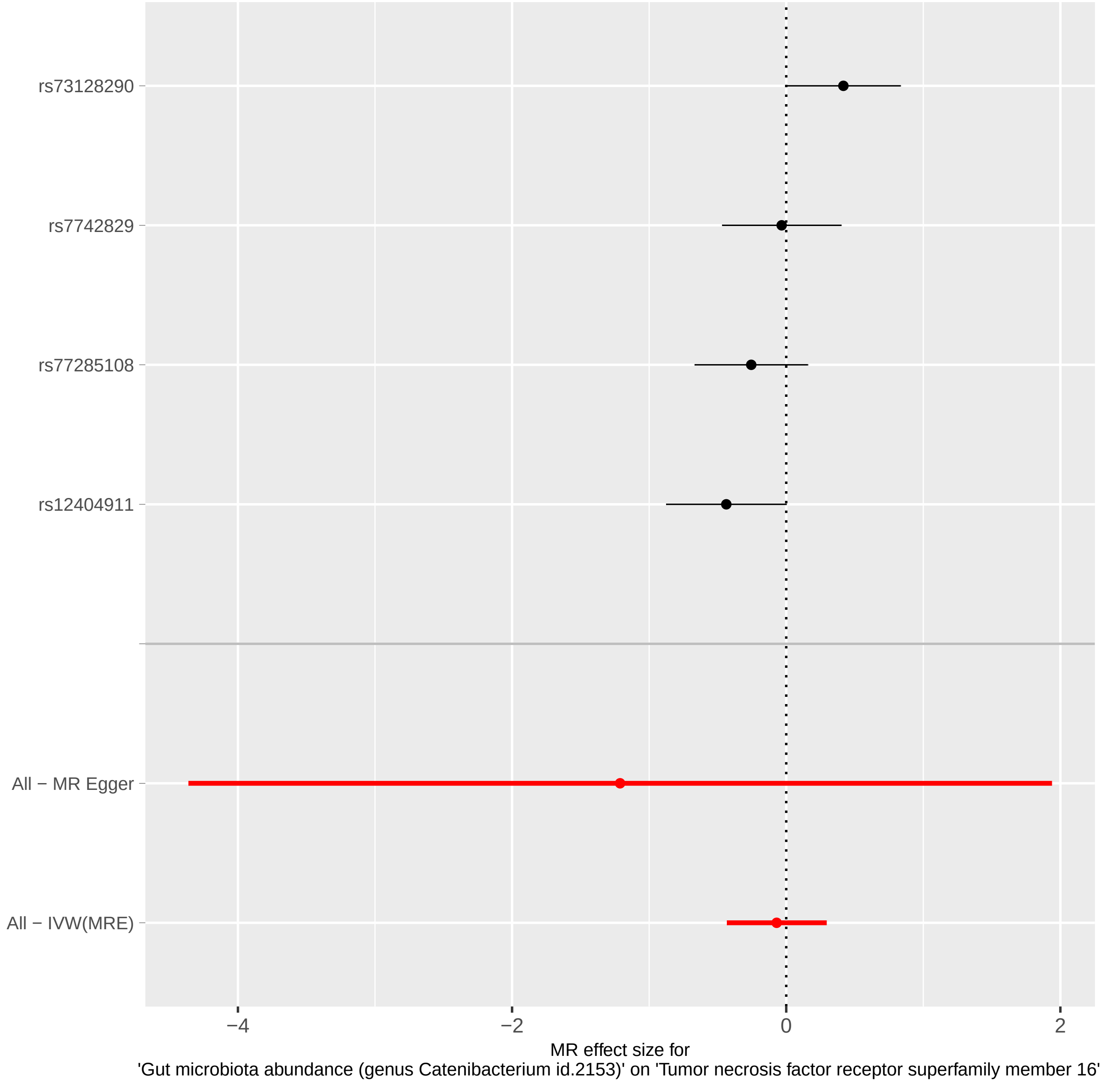

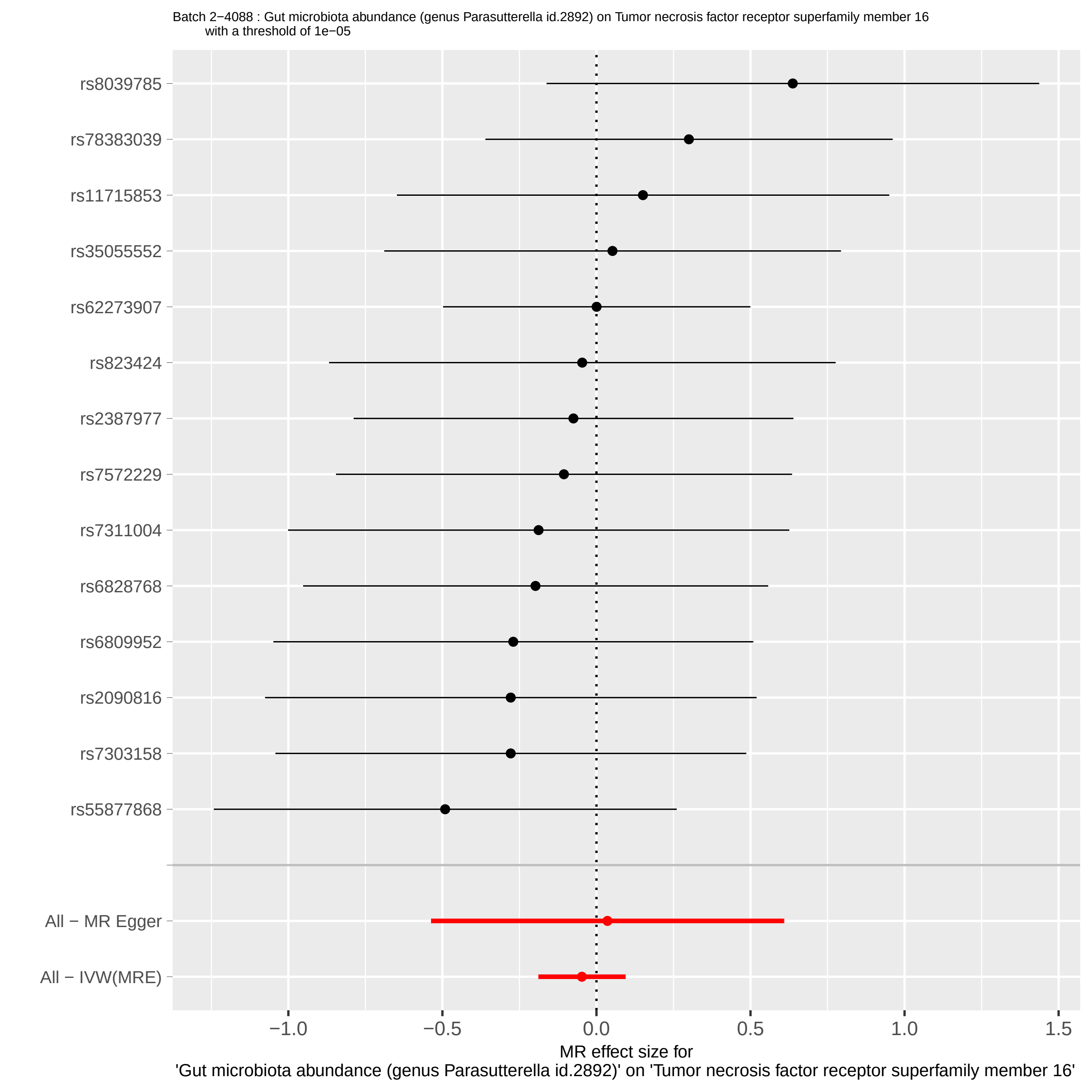

Batch 2−4088 : Gut microbiota abundance (genus Parasutterella id.2892) on Tumor necrosis factor receptor superfamily member 16
with a threshold of 1e−05
rs8039785
rs78383039
rs11715853
rs35055552
rs62273907
rs823424
rs2387977
rs7572229
rs7311004
rs6828768
rs6809952
rs2090816
rs7303158
rs55877868
All − MR Egger
All − IVW(MRE)
−1.0
−0.5
0.0
0.5
1.0
1.5
MR effect size for
'Gut microbiota abundance (genus Parasutterella id.2892)' on 'Tumor necrosis factor receptor superfamily member 16'

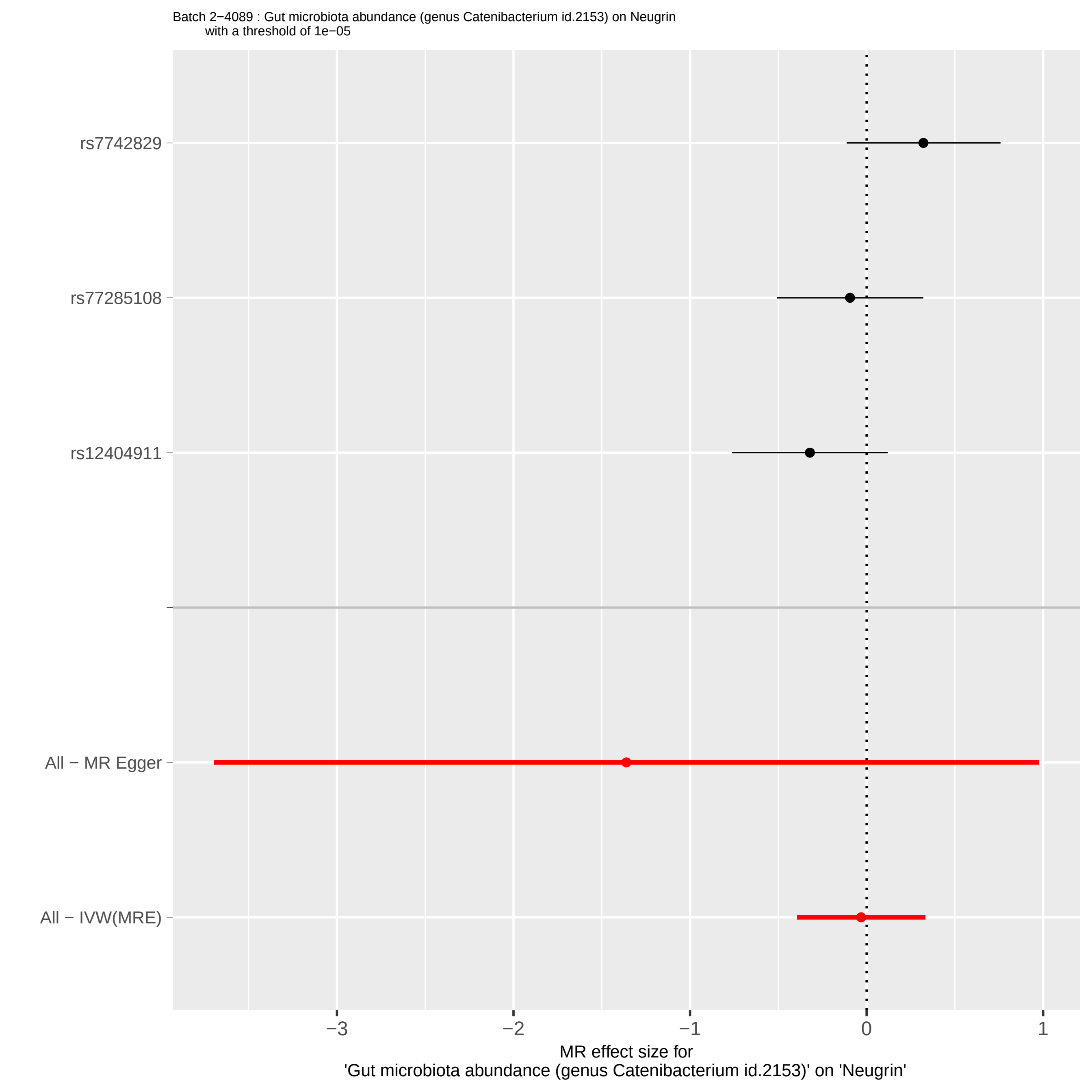

Batch 2−4089 : Gut microbiota abundance (genus Catenibacterium id.2153) on Neugrin
with a threshold of 1e−05
rs7742829
rs77285108
rs12404911
All − MR Egger
All − IVW(MRE)
−3
−2
−1
0
1
MR effect size for
'Gut microbiota abundance (genus Catenibacterium id.2153)' on 'Neugrin'

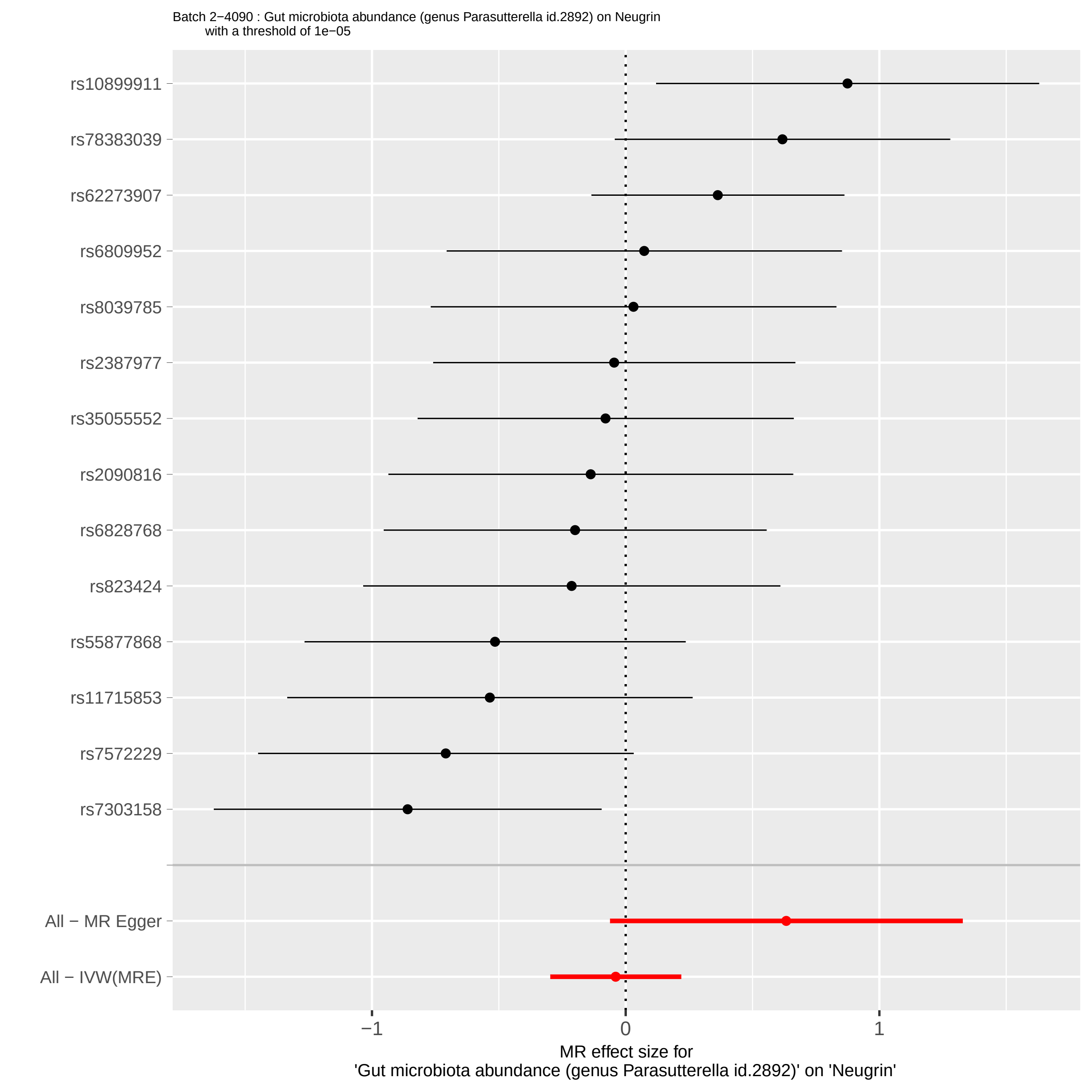

Batch 2−4090 : Gut microbiota abundance (genus Parasutterella id.2892) on Neugrin
with a threshold of 1e−05
rs10899911
rs78383039
rs62273907
rs6809952
rs8039785
rs2387977
rs35055552
rs2090816
rs6828768
rs823424
rs55877868
rs11715853
rs7572229
rs7303158
All − MR Egger
All − IVW(MRE)
−1
0
1
MR effect size for
'Gut microbiota abundance (genus Parasutterella id.2892)' on 'Neugrin'

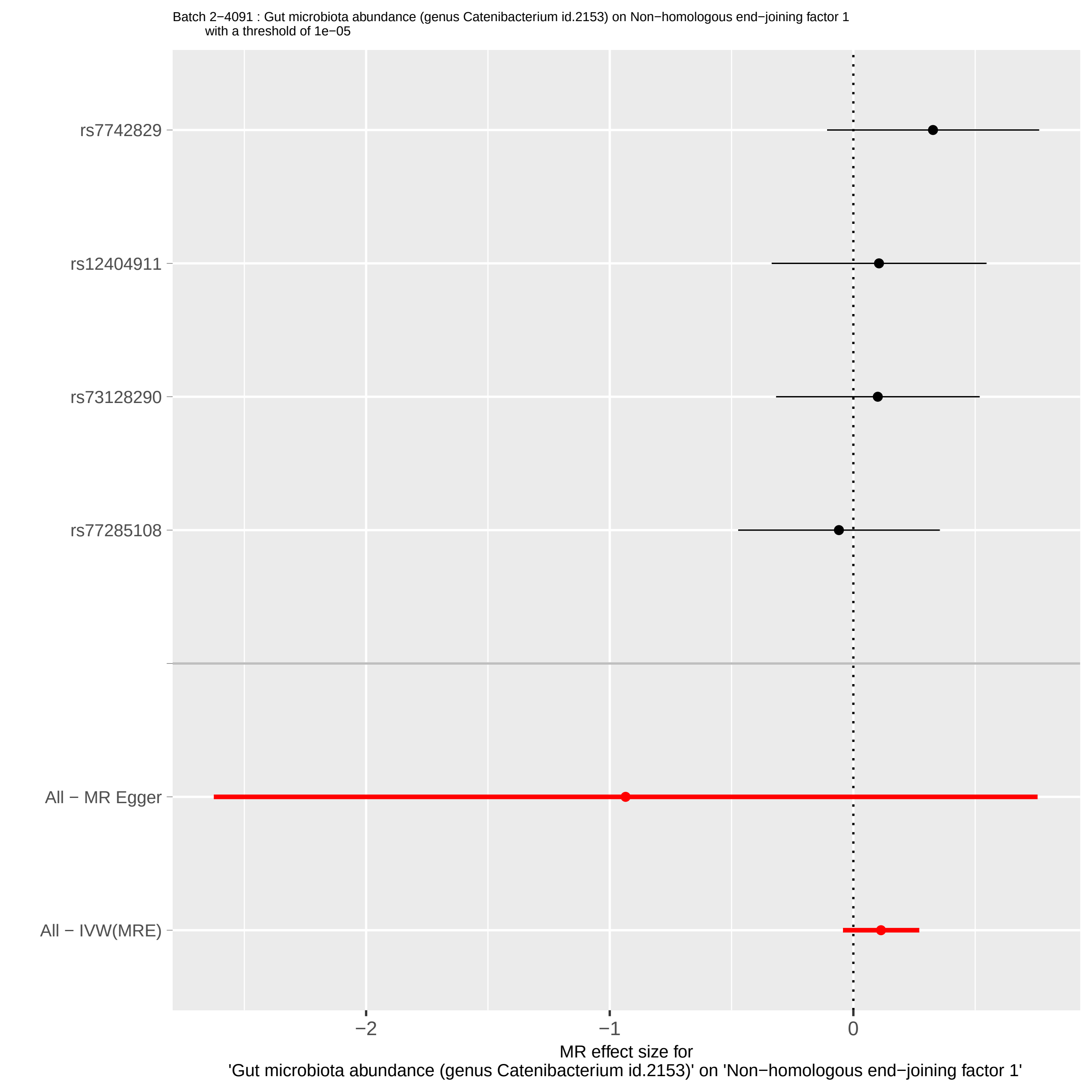

Batch 2−4091 : Gut microbiota abundance (genus Catenibacterium id.2153) on Non−homologous end−joining factor 1
with a threshold of 1e−05
rs7742829
rs12404911
rs73128290
rs77285108
All − MR Egger
All − IVW(MRE)
−2
−1
0
MR effect size for
'Gut microbiota abundance (genus Catenibacterium id.2153)' on 'Non−homologous end−joining factor 1'

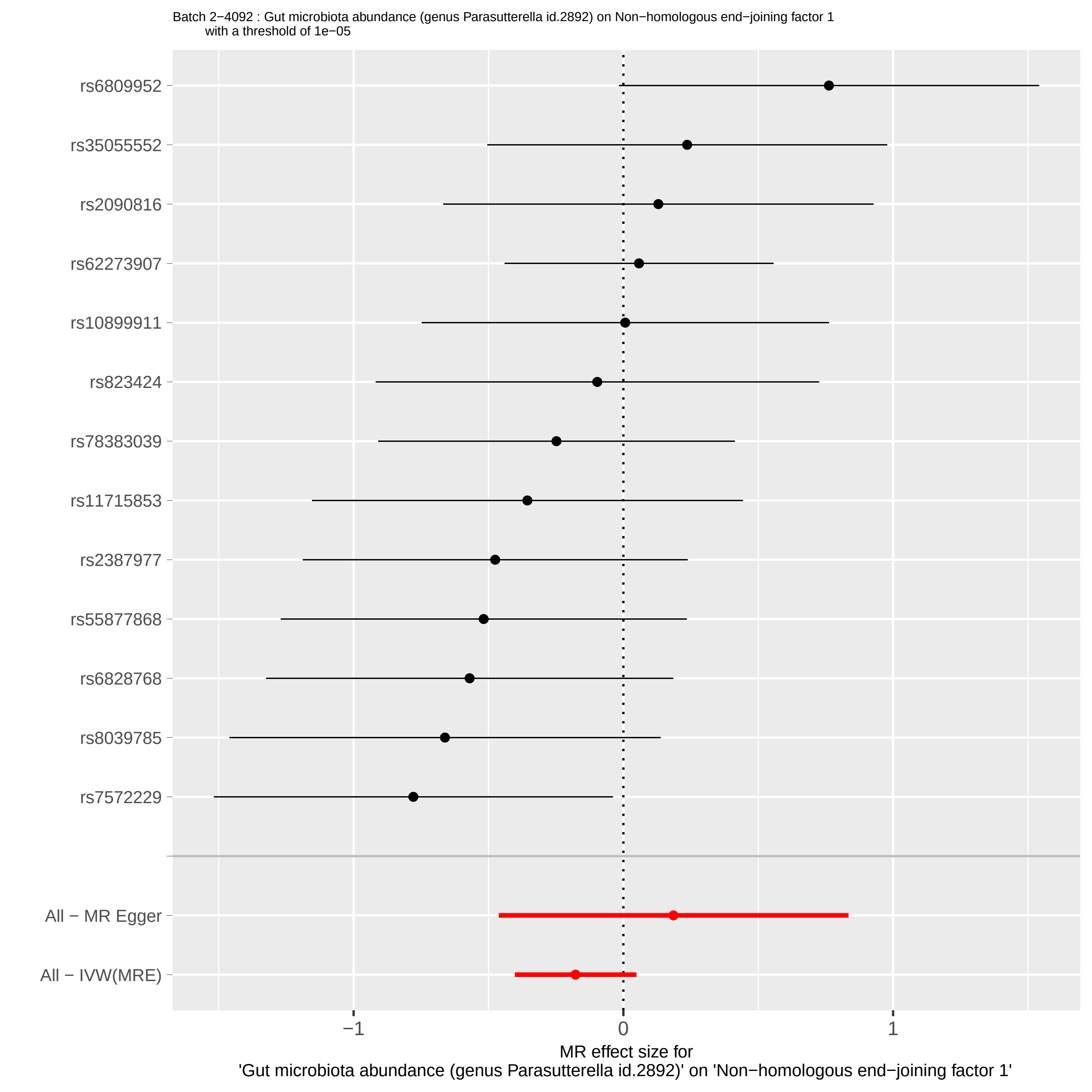

Batch 2−4092 : Gut microbiota abundance (genus Parasutterella id.2892) on Non−homologous end−joining factor 1
with a threshold of 1e−05
rs6809952
rs35055552
rs2090816
rs62273907
rs10899911
rs823424
rs78383039
rs11715853
rs2387977
rs55877868
rs6828768
rs8039785
rs7572229
All − MR Egger
All − IVW(MRE)
−1
0
1
MR effect size for
'Gut microbiota abundance (genus Parasutterella id.2892)' on 'Non−homologous end−joining factor 1'

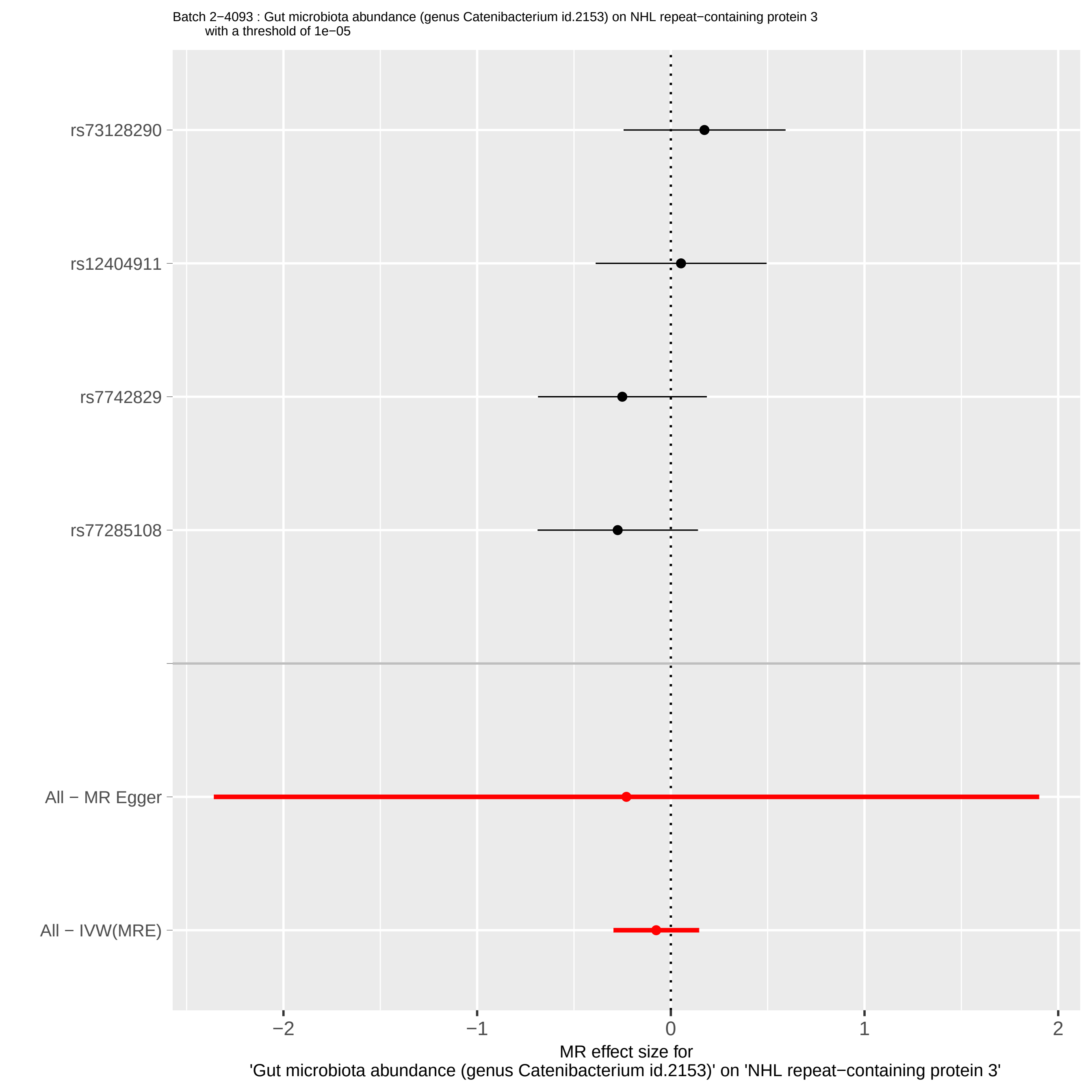
Batch 2−4093 : Gut microbiota abundance (genus Catenibacterium id.2153) on NHL repeat−containing protein 3
with a threshold of 1e−05
rs73128290
rs12404911
rs7742829
rs77285108
All − MR Egger
All − IVW(MRE)
−2
−1
0
1
2
MR effect size for
'Gut microbiota abundance (genus Catenibacterium id.2153)' on 'NHL repeat−containing protein 3'

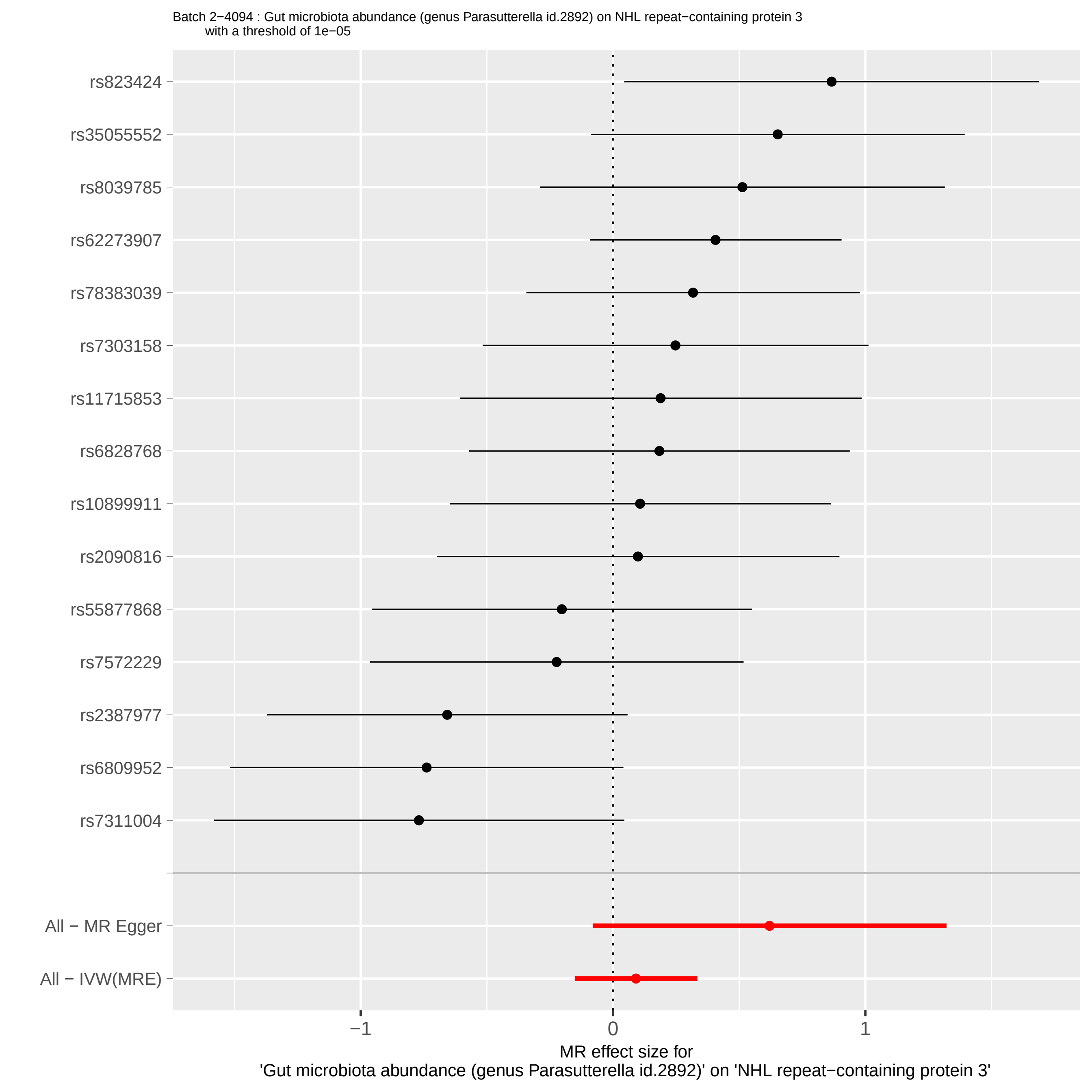
Batch 2−4094 : Gut microbiota abundance (genus Parasutterella id.2892) on NHL repeat−containing protein 3
with a threshold of 1e−05
rs823424
rs35055552
rs8039785
rs62273907
rs78383039
rs7303158
rs11715853
rs6828768
rs10899911
rs2090816
rs55877868
rs7572229
rs2387977
rs6809952
rs7311004
All − MR Egger
All − IVW(MRE)
−1
0
1
MR effect size for
'Gut microbiota abundance (genus Parasutterella id.2892)' on 'NHL repeat−containing protein 3'

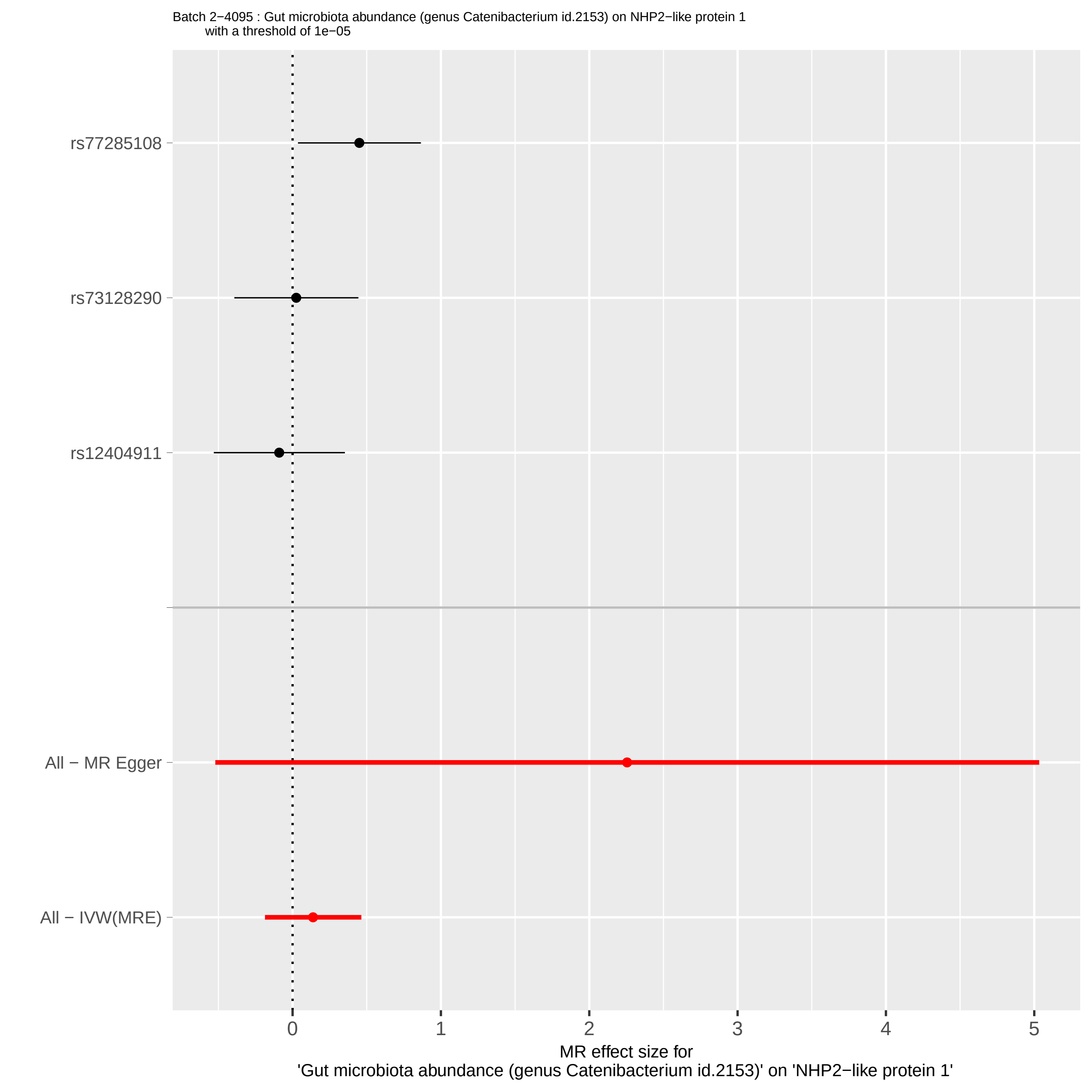

Batch 2−4095 : Gut microbiota abundance (genus Catenibacterium id.2153) on NHP2−like protein 1
with a threshold of 1e−05
rs77285108
rs73128290
rs12404911
All − MR Egger
All − IVW(MRE)
0
1
2
3
4
5
MR effect size for
'Gut microbiota abundance (genus Catenibacterium id.2153)' on 'NHP2−like protein 1'

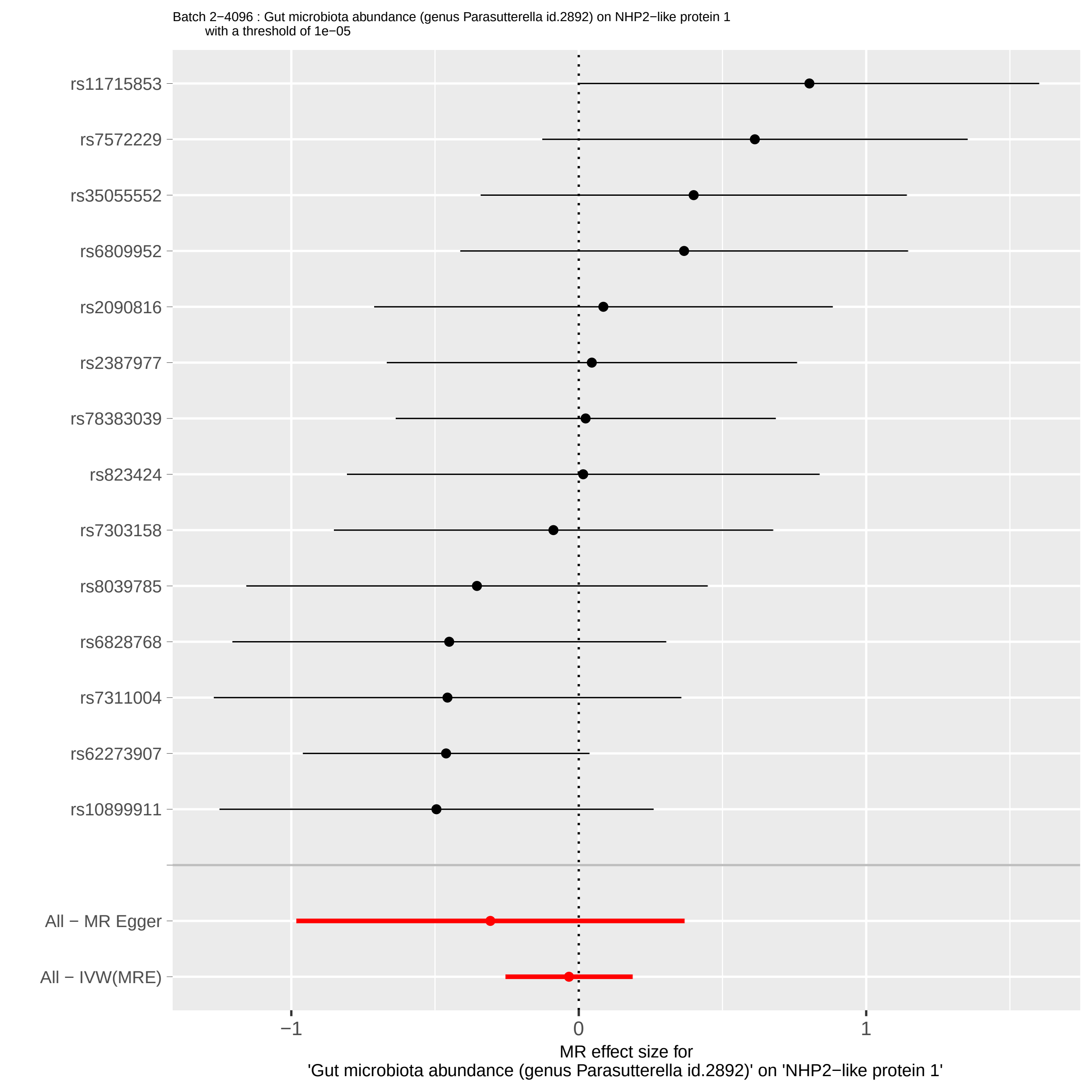

Batch 2−4096 : Gut microbiota abundance (genus Parasutterella id.2892) on NHP2−like protein 1
with a threshold of 1e−05
rs11715853
rs7572229
rs35055552
rs6809952
rs2090816
rs2387977
rs78383039
rs823424
rs7303158
rs8039785
rs6828768
rs7311004
rs62273907
rs10899911
All − MR Egger
All − IVW(MRE)
−1
0
1
MR effect size for
'Gut microbiota abundance (genus Parasutterella id.2892)' on 'NHP2−like protein 1'

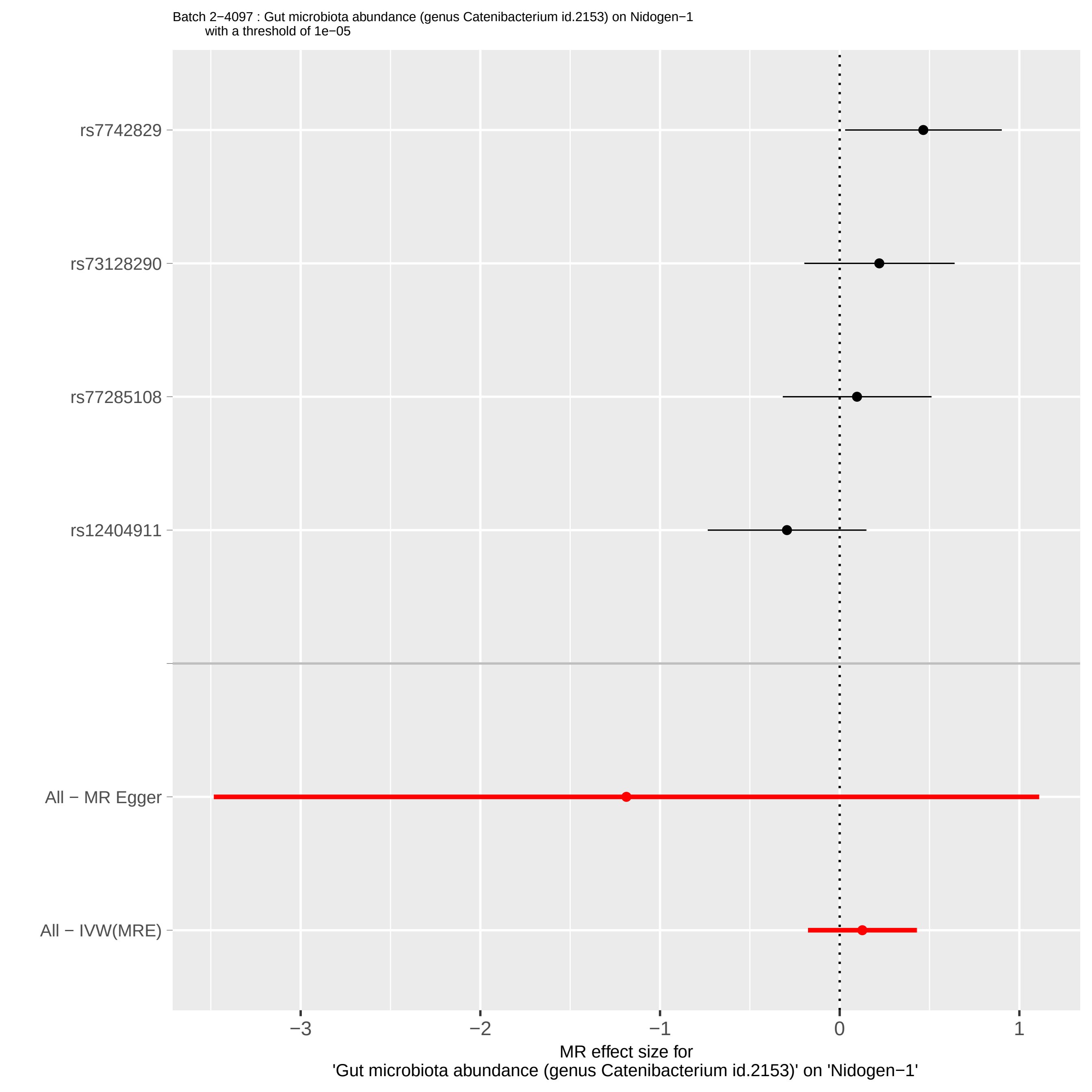

Batch 2−4097 : Gut microbiota abundance (genus Catenibacterium id.2153) on Nidogen−1
with a threshold of 1e−05
rs7742829
rs73128290
rs77285108
rs12404911
All − MR Egger
All − IVW(MRE)
−3
−2
−1
0
1
MR effect size for
'Gut microbiota abundance (genus Catenibacterium id.2153)' on 'Nidogen−1'

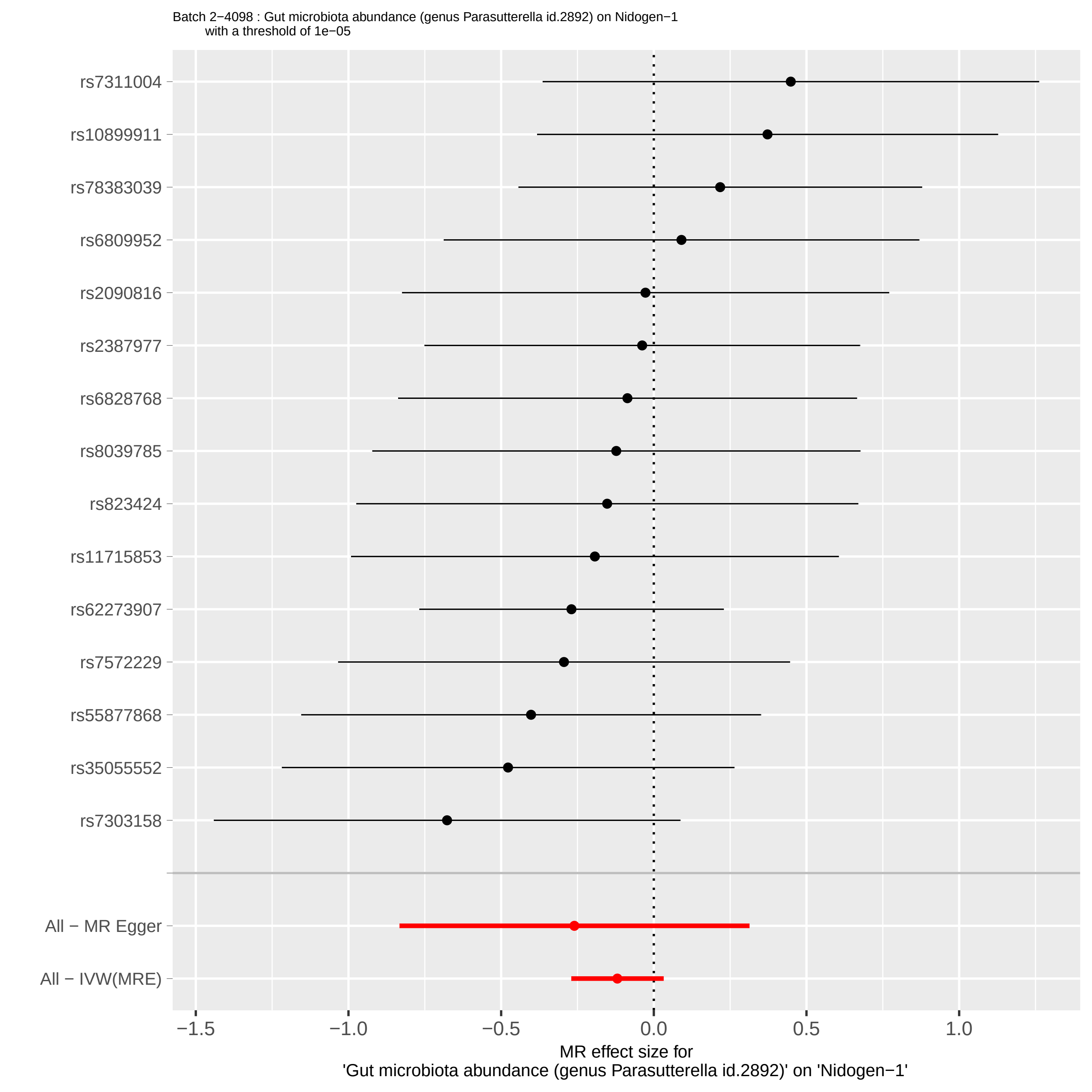
Batch 2−4098 : Gut microbiota abundance (genus Parasutterella id.2892) on Nidogen−1
with a threshold of 1e−05
rs7311004
rs10899911
rs78383039
rs6809952
rs2090816
rs2387977
rs6828768
rs8039785
rs823424
rs11715853
rs62273907
rs7572229
rs55877868
rs35055552
rs7303158
All − MR Egger
All − IVW(MRE)
−1.5
−1.0
−0.5
0.0
0.5
1.0
MR effect size for
'Gut microbiota abundance (genus Parasutterella id.2892)' on 'Nidogen−1'

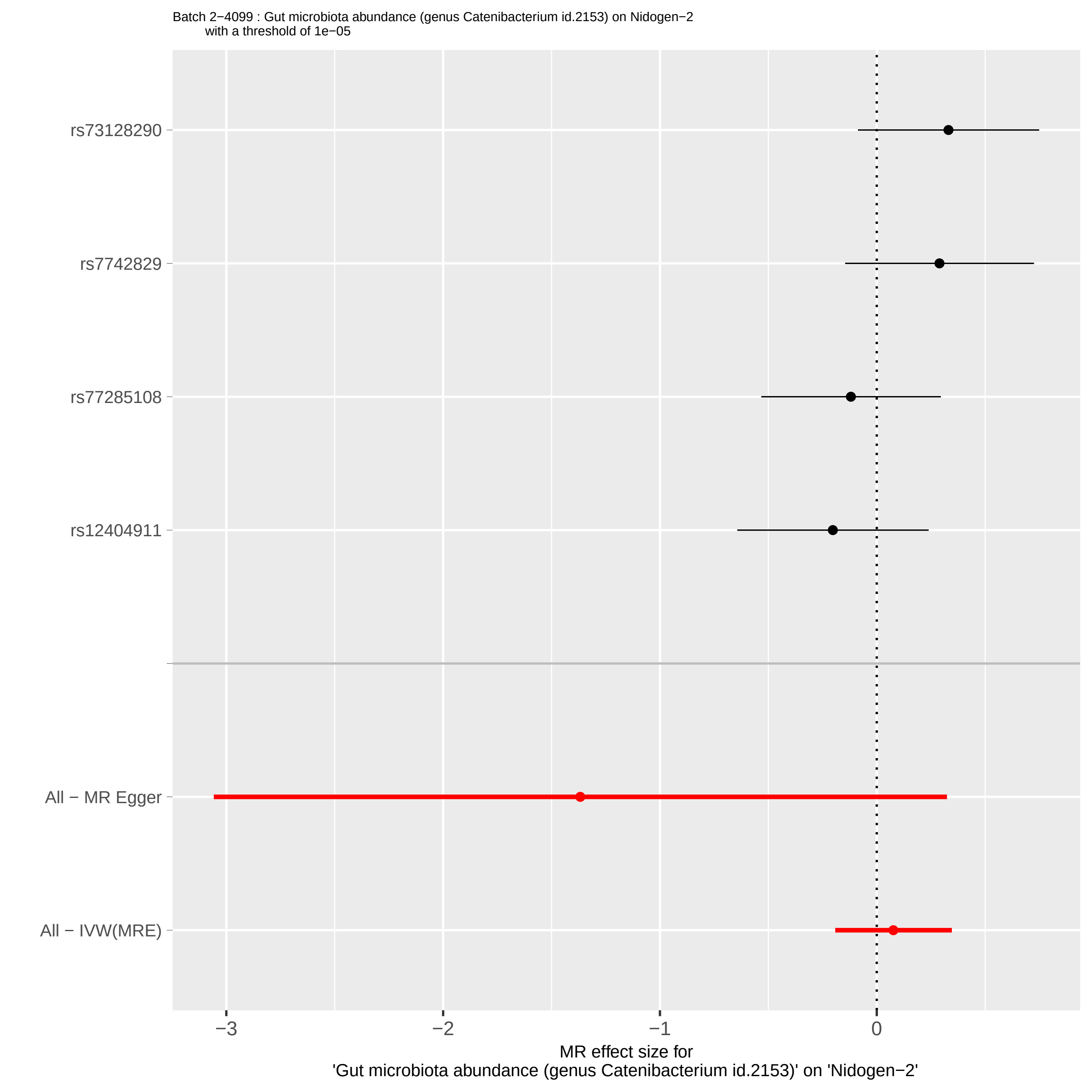

Batch 2−4099 : Gut microbiota abundance (genus Catenibacterium id.2153) on Nidogen−2
with a threshold of 1e−05
rs73128290
rs7742829
rs77285108
rs12404911
All − MR Egger
All − IVW(MRE)
−3
−2
−1
0
MR effect size for
'Gut microbiota abundance (genus Catenibacterium id.2153)' on 'Nidogen−2'

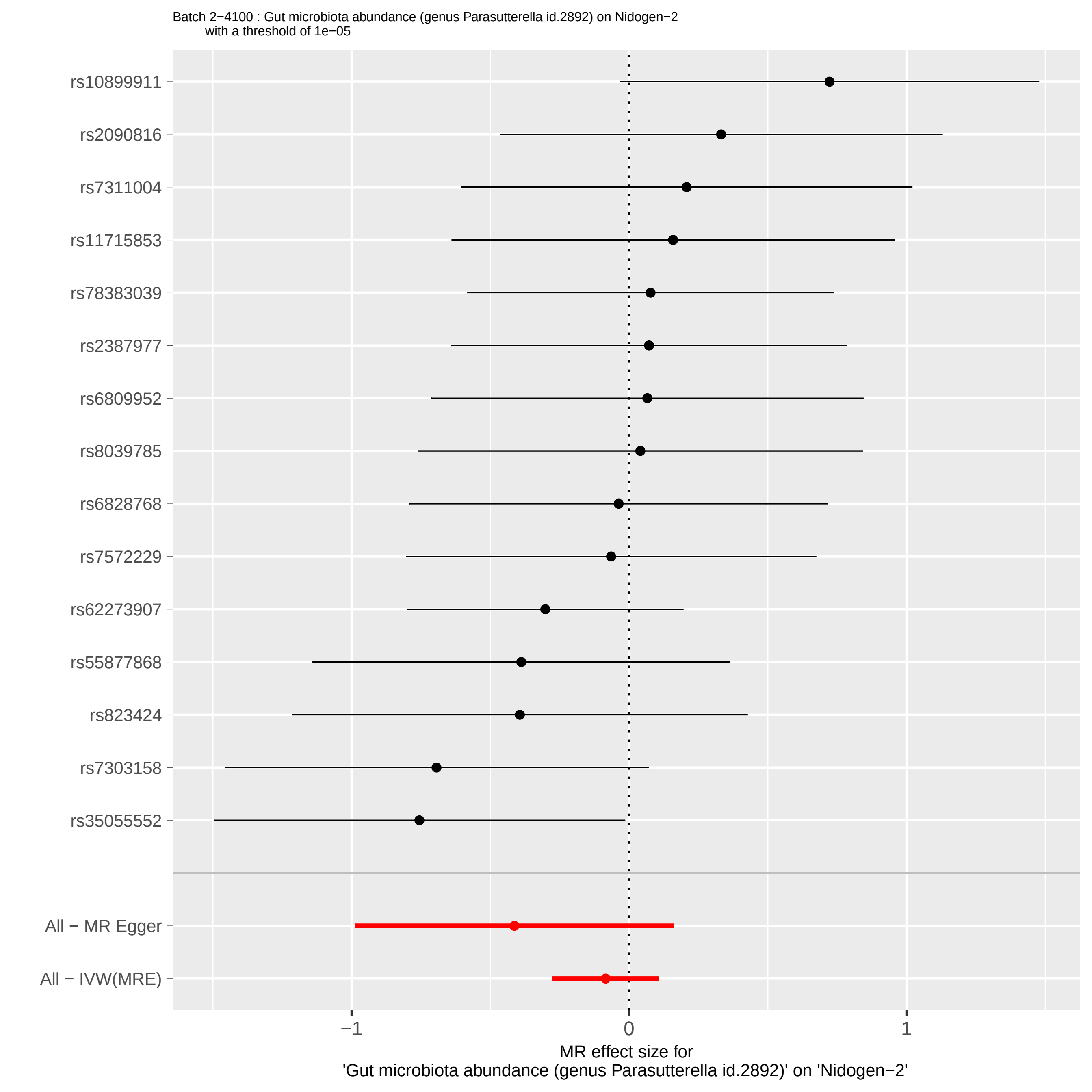

Batch 2−4100 : Gut microbiota abundance (genus Parasutterella id.2892) on Nidogen−2
with a threshold of 1e−05
rs10899911
rs2090816
rs7311004
rs11715853
rs78383039
rs2387977
rs6809952
rs8039785
rs6828768
rs7572229
rs62273907
rs55877868
rs823424
rs7303158
rs35055552
All − MR Egger
All − IVW(MRE)
−1
0
1
MR effect size for
'Gut microbiota abundance (genus Parasutterella id.2892)' on 'Nidogen−2'

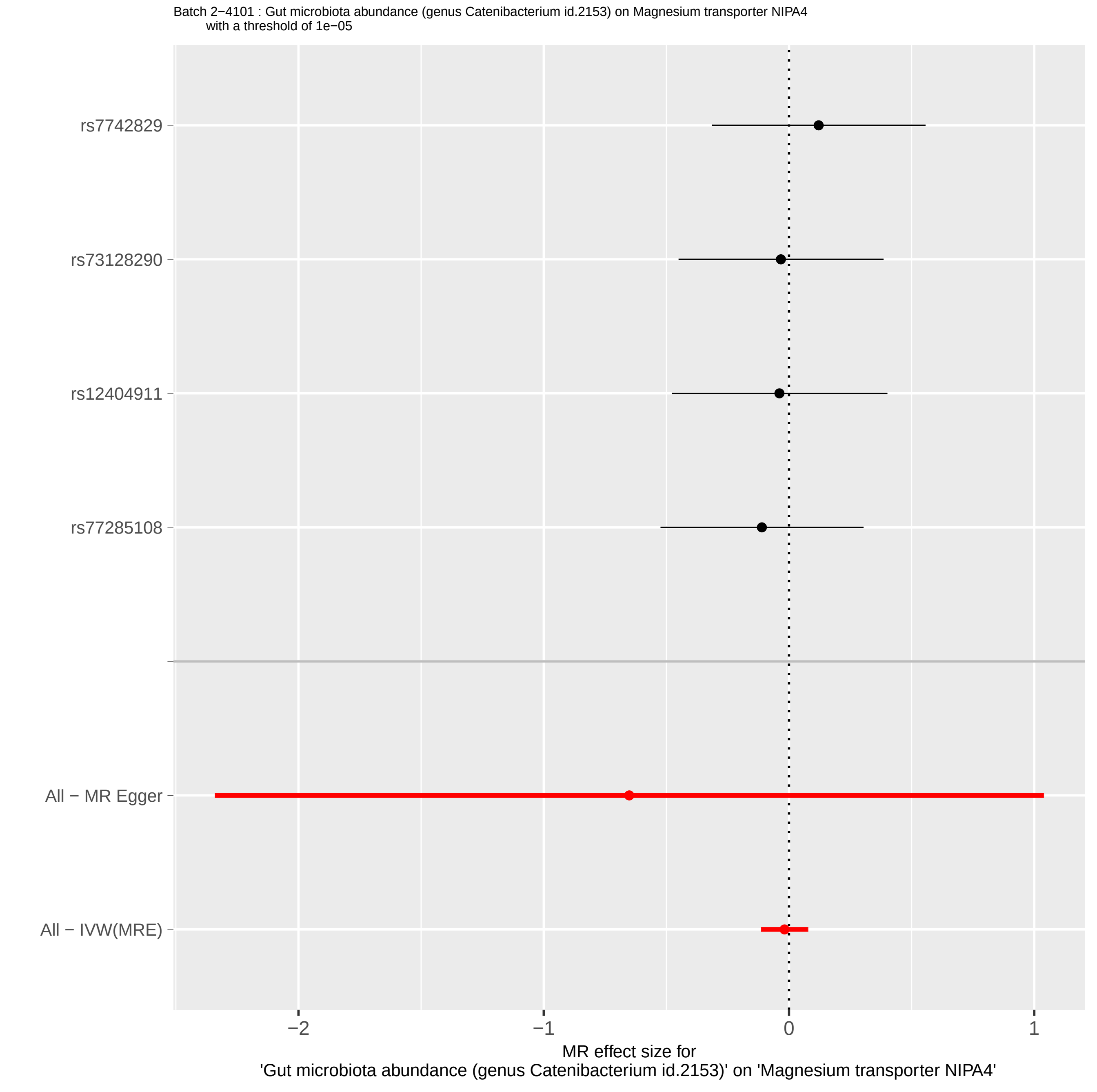
Batch 2−4101 : Gut microbiota abundance (genus Catenibacterium id.2153) on Magnesium transporter NIPA4
with a threshold of 1e−05
rs7742829
rs73128290
rs12404911
rs77285108
All − MR Egger
All − IVW(MRE)
−2
−1
0
1
MR effect size for
'Gut microbiota abundance (genus Catenibacterium id.2153)' on 'Magnesium transporter NIPA4'

Batch 2−4102 : Gut microbiota abundance (genus Parasutterella id.2892) on Magnesium transporter NIPA4
with a threshold of 1e−05

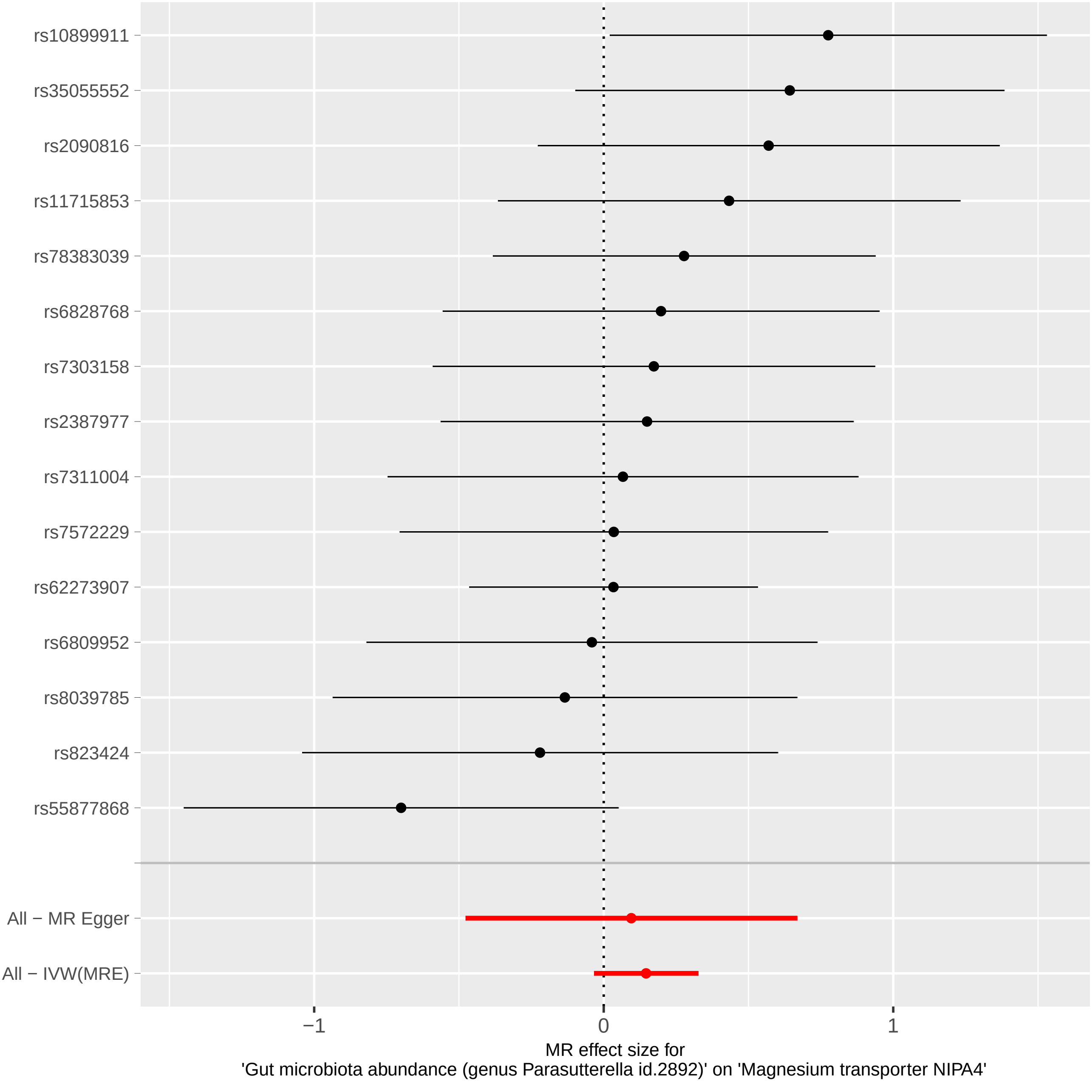

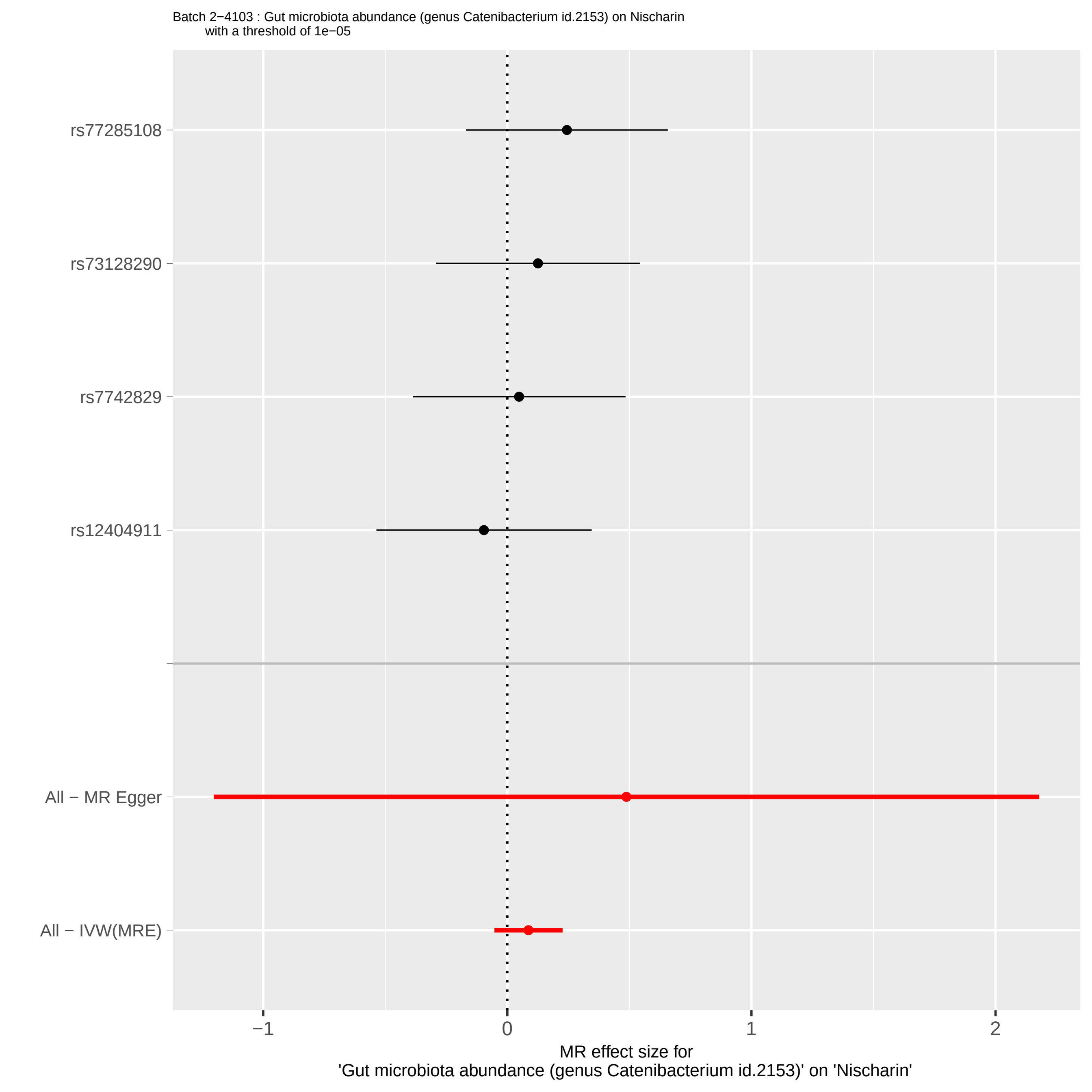

Batch 2−4103 : Gut microbiota abundance (genus Catenibacterium id.2153) on Nischarin
with a threshold of 1e−05
rs77285108
rs73128290
rs7742829
rs12404911
All − MR Egger
All − IVW(MRE)
−1
0
1
2
MR effect size for
'Gut microbiota abundance (genus Catenibacterium id.2153)' on 'Nischarin'

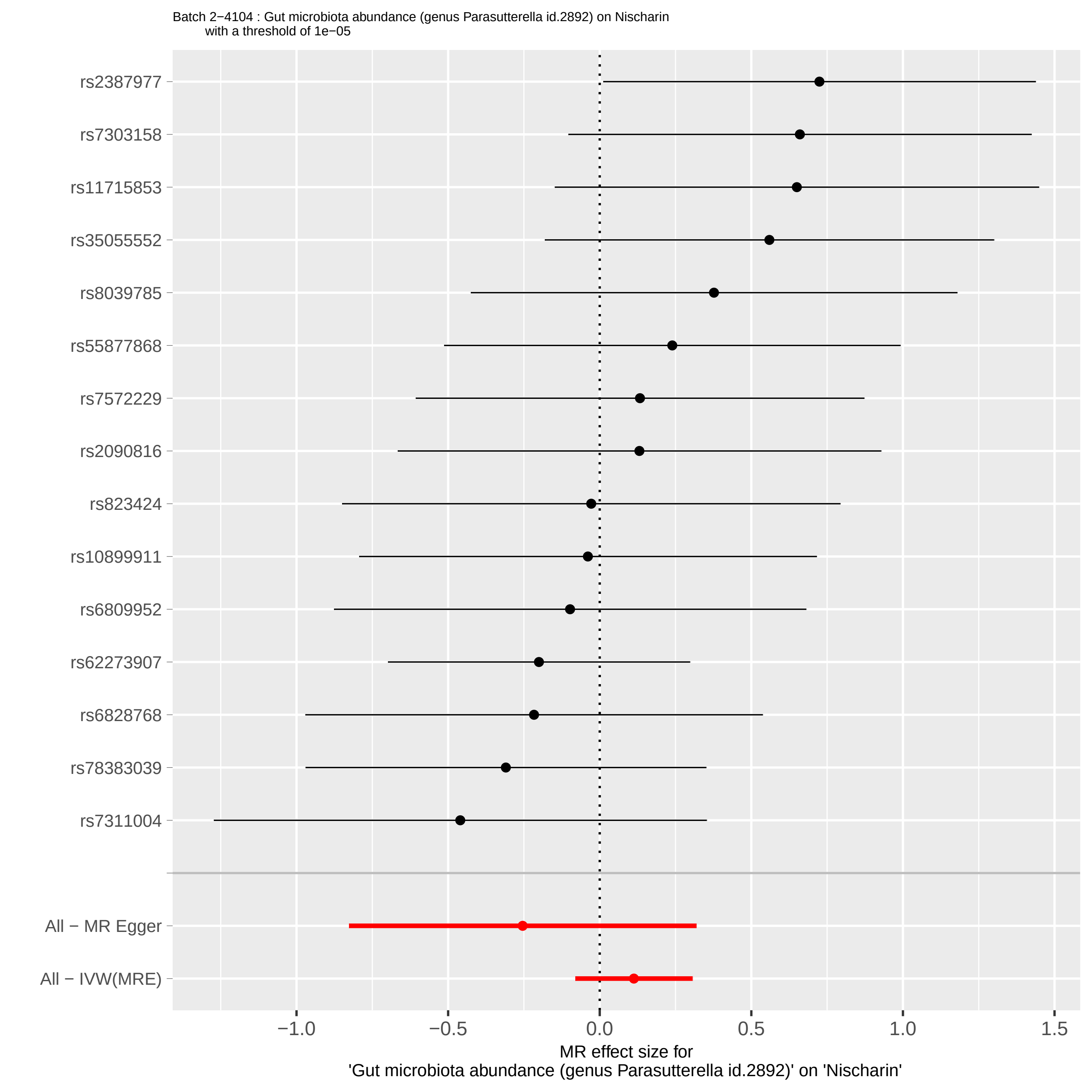
Batch 2−4104 : Gut microbiota abundance (genus Parasutterella id.2892) on Nischarin
with a threshold of 1e−05
rs2387977
rs7303158
rs11715853
rs35055552
rs8039785
rs55877868
rs7572229
rs2090816
rs823424
rs10899911
rs6809952
rs62273907
rs6828768
rs78383039
rs7311004
All − MR Egger
All − IVW(MRE)
−1.0
−0.5
0.0
0.5
1.0
1.5
MR effect size for
'Gut microbiota abundance (genus Parasutterella id.2892)' on 'Nischarin'

Batch 2−4105 : Gut microbiota abundance (genus Catenibacterium id.2153) on Neuroligin−1
with a threshold of 1e−05

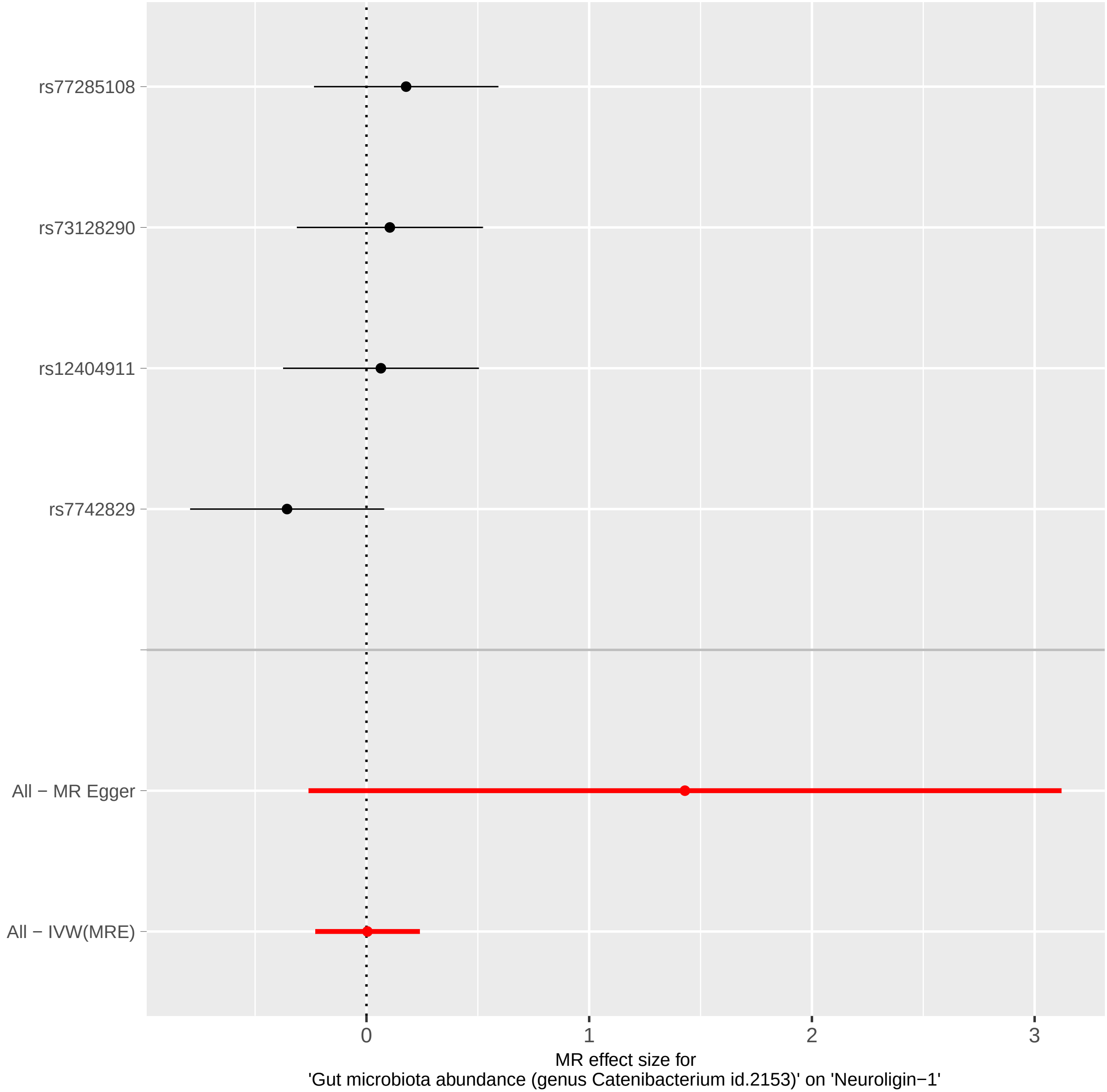

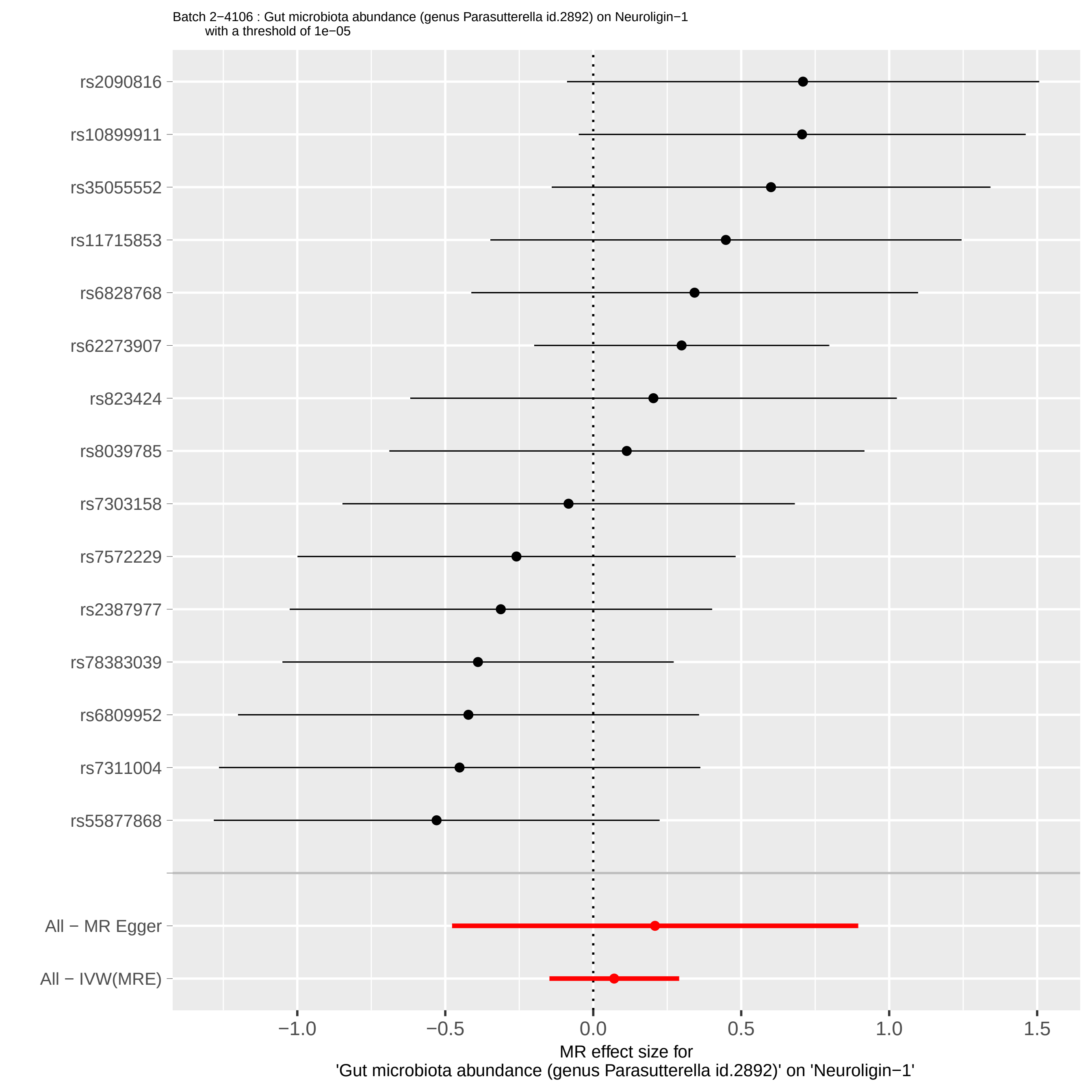

Batch 2−4106 : Gut microbiota abundance (genus Parasutterella id.2892) on Neuroligin−1
with a threshold of 1e−05
rs2090816
rs10899911
rs35055552
rs11715853
rs6828768
rs62273907
rs823424
rs8039785
rs7303158
rs7572229
rs2387977
rs78383039
rs6809952
rs7311004
rs55877868
All − MR Egger
All − IVW(MRE)
−1.0
−0.5
0.0
0.5
1.0
1.5
MR effect size for
'Gut microbiota abundance (genus Parasutterella id.2892)' on 'Neuroligin−1'

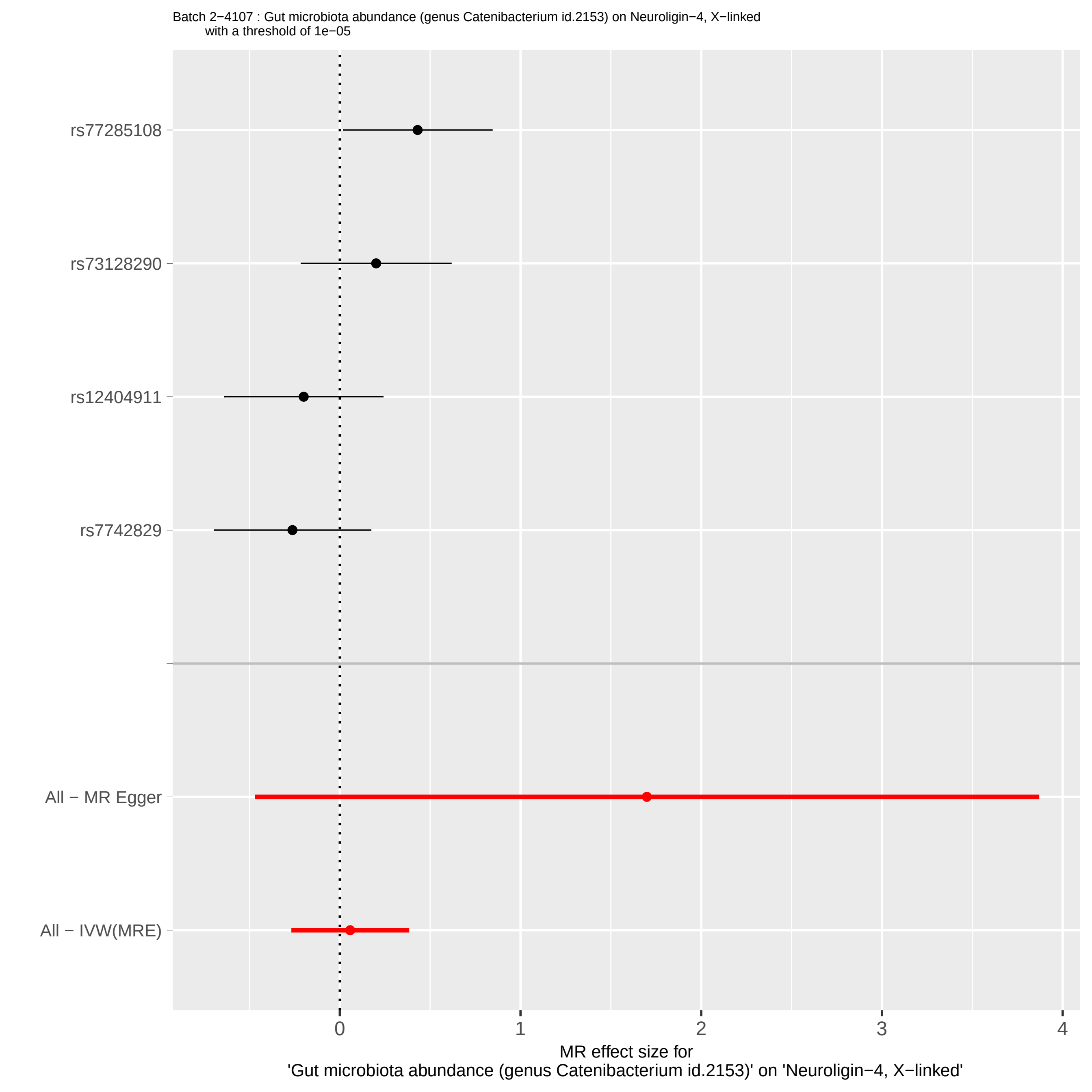

Batch 2−4107 : Gut microbiota abundance (genus Catenibacterium id.2153) on Neuroligin−4, X−linked
with a threshold of 1e−05
rs77285108
rs73128290
rs12404911
rs7742829
All − MR Egger
All − IVW(MRE)
0
1
2
3
4
MR effect size for
'Gut microbiota abundance (genus Catenibacterium id.2153)' on 'Neuroligin−4, X−linked'

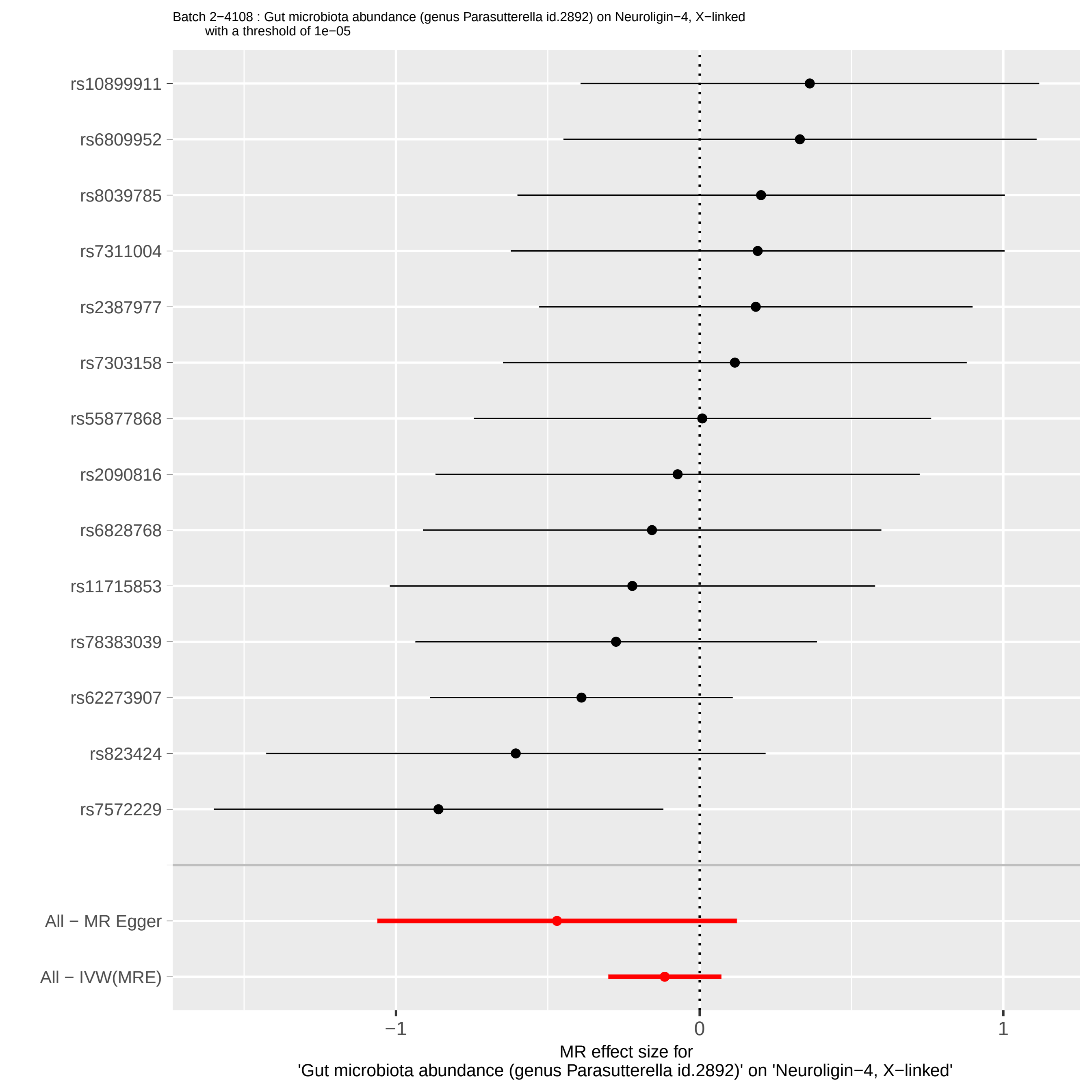
Batch 2−4108 : Gut microbiota abundance (genus Parasutterella id.2892) on Neuroligin−4, X−linked
with a threshold of 1e−05
rs10899911
rs6809952
rs8039785
rs7311004
rs2387977
rs7303158
rs55877868
rs2090816
rs6828768
rs11715853
rs78383039
rs62273907
rs823424
rs7572229
All − MR Egger
All − IVW(MRE)
−1
0
1
MR effect size for
'Gut microbiota abundance (genus Parasutterella id.2892)' on 'Neuroligin−4, X−linked'

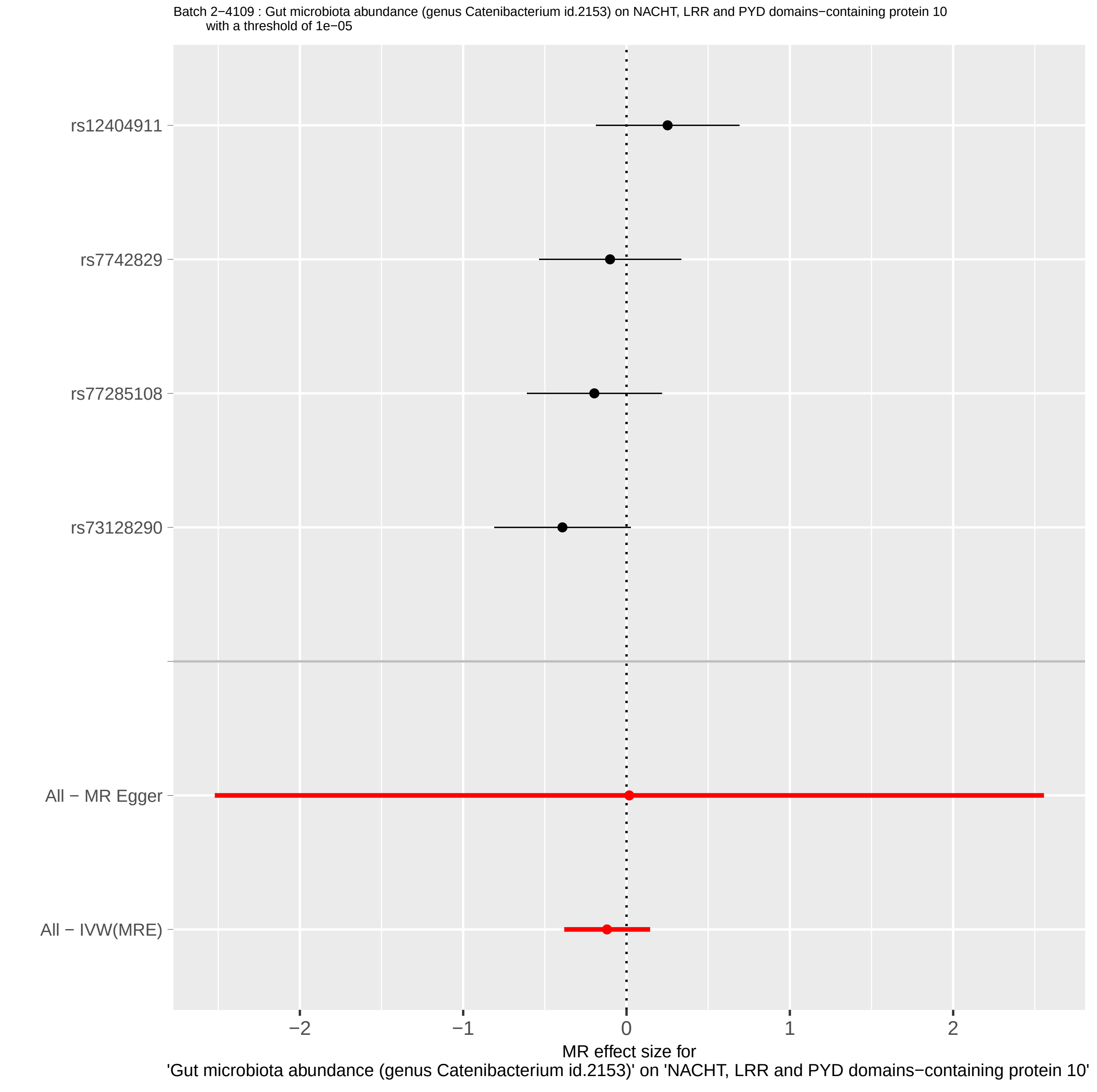

Batch 2−4109 : Gut microbiota abundance (genus Catenibacterium id.2153) on NACHT, LRR and PYD domains−containing protein 10
with a threshold of 1e−05
rs12404911
rs7742829
rs77285108
rs73128290
All − MR Egger
All − IVW(MRE)
−2
−1
0
1
2
MR effect size for
'Gut microbiota abundance (genus Catenibacterium id.2153)' on 'NACHT, LRR and PYD domains−containing protein 10'

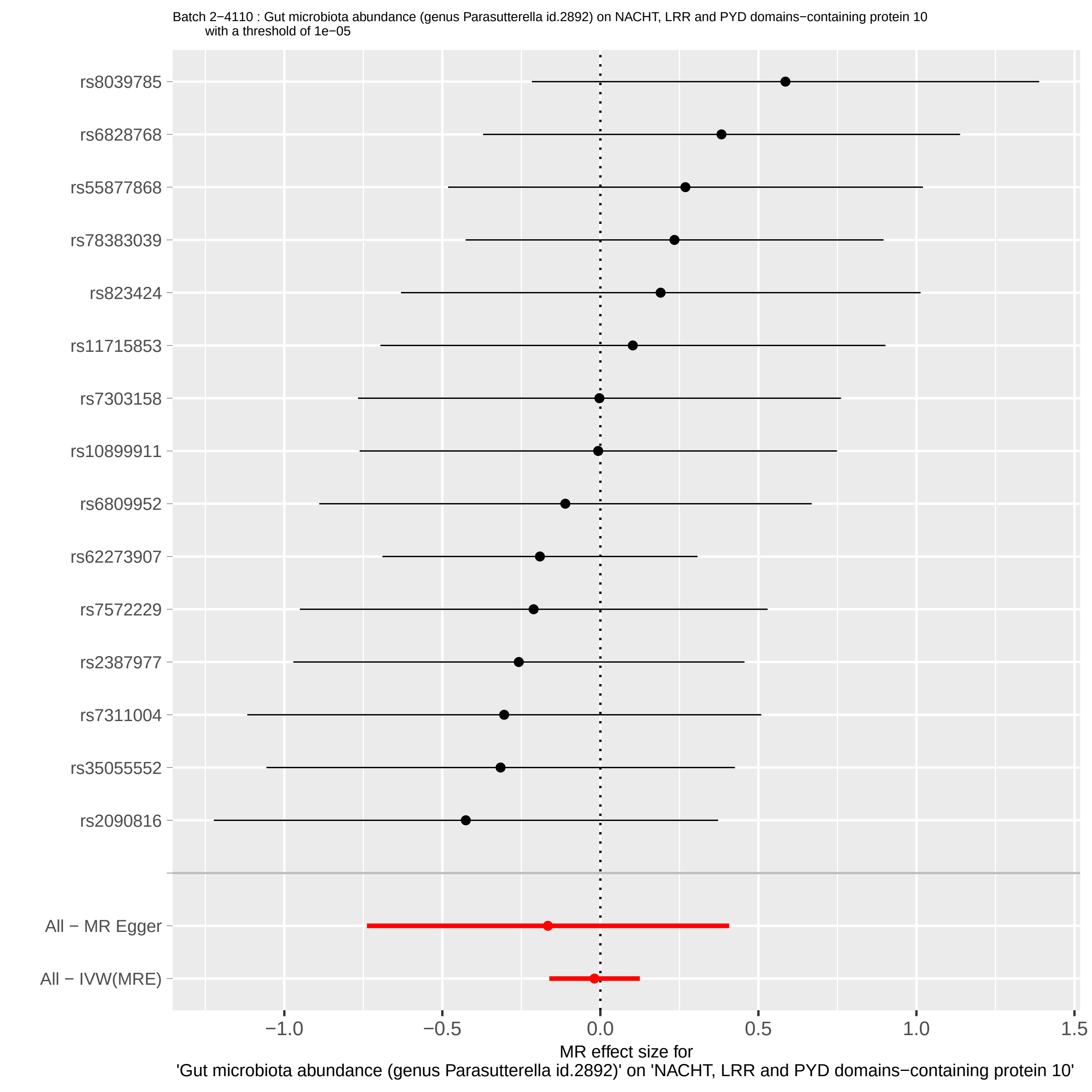

Batch 2−4110 : Gut microbiota abundance (genus Parasutterella id.2892) on NACHT, LRR and PYD domains−containing protein 10
with a threshold of 1e−05
rs8039785
rs6828768
rs55877868
rs78383039
rs823424
rs11715853
rs7303158
rs10899911
rs6809952
rs62273907
rs7572229
rs2387977
rs7311004
rs35055552
rs2090816
All − MR Egger
All − IVW(MRE)
−1.0
−0.5
0.0
0.5
1.0
1.5
MR effect size for
'Gut microbiota abundance (genus Parasutterella id.2892)' on 'NACHT, LRR and PYD domains−containing protein 10'

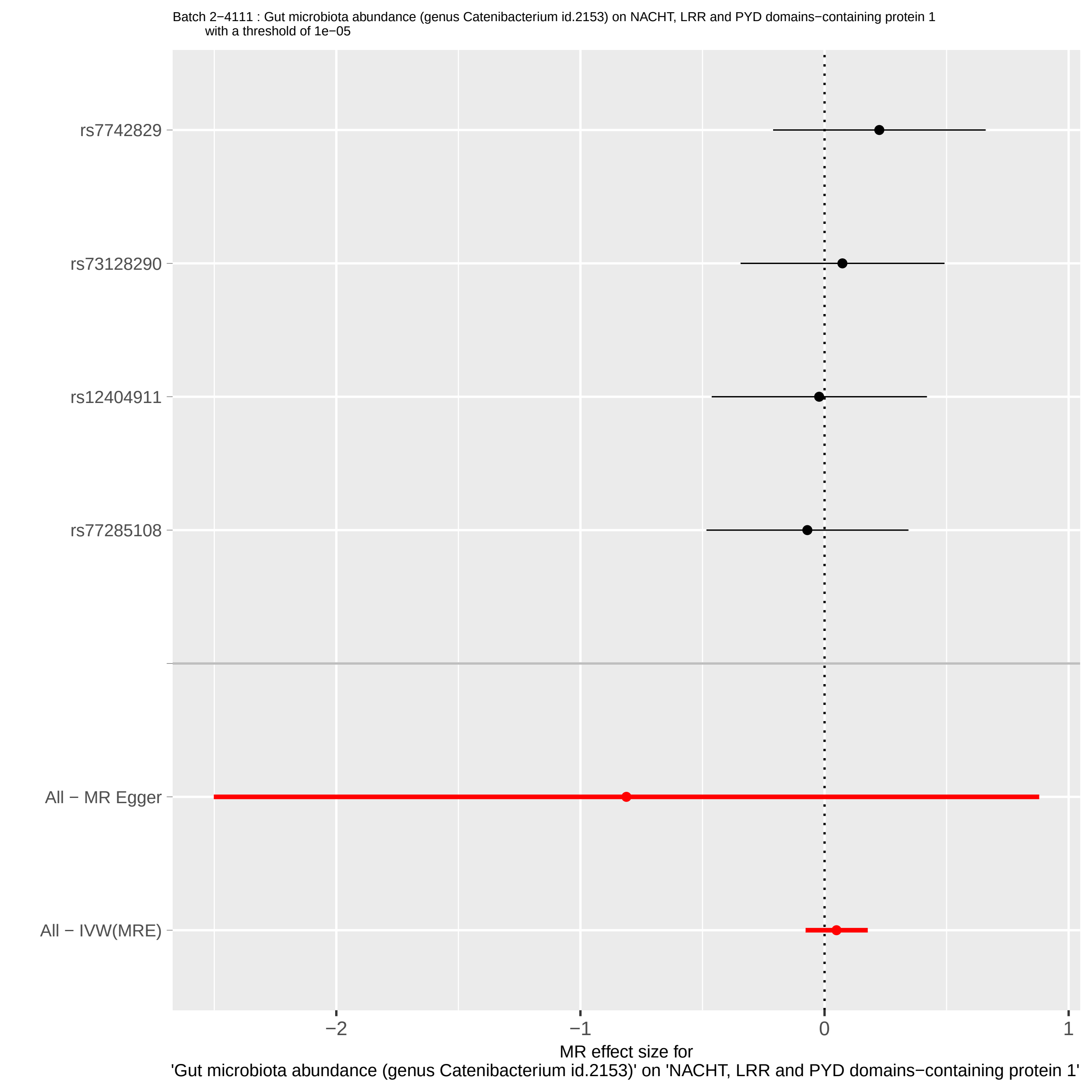
Batch 2−4111 : Gut microbiota abundance (genus Catenibacterium id.2153) on NACHT, LRR and PYD domains−containing protein 1
with a threshold of 1e−05
rs7742829
rs73128290
rs12404911
rs77285108
All − MR Egger
All − IVW(MRE)
−2
−1
0
1
MR effect size for
'Gut microbiota abundance (genus Catenibacterium id.2153)' on 'NACHT, LRR and PYD domains−containing protein 1'

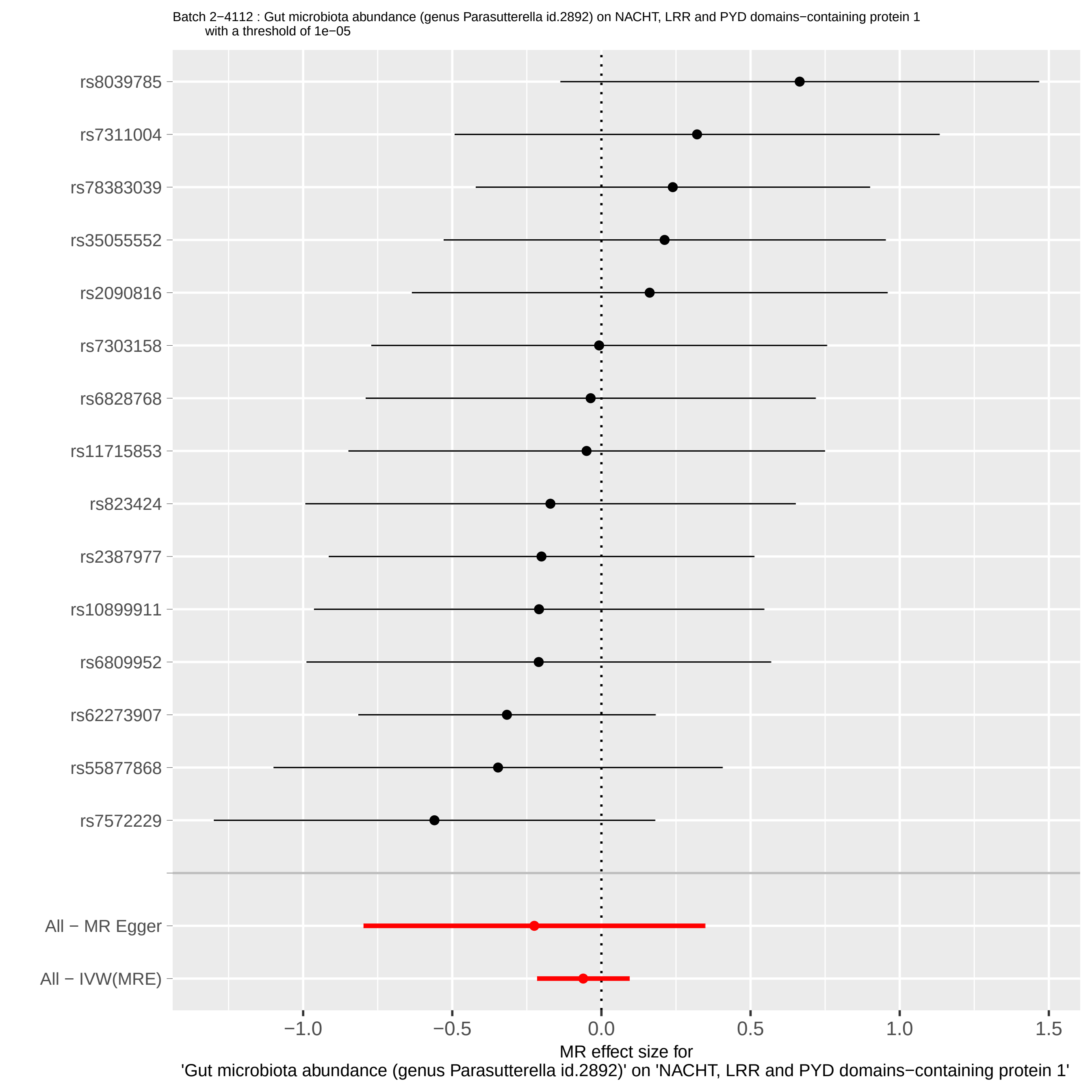

Batch 2−4112 : Gut microbiota abundance (genus Parasutterella id.2892) on NACHT, LRR and PYD domains−containing protein 1
with a threshold of 1e−05
rs8039785
rs7311004
rs78383039
rs35055552
rs2090816
rs7303158
rs6828768
rs11715853
rs823424
rs2387977
rs10899911
rs6809952
rs62273907
rs55877868
rs7572229
All − MR Egger
All − IVW(MRE)
−1.0
−0.5
0.0
0.5
1.0
1.5
MR effect size for
'Gut microbiota abundance (genus Parasutterella id.2892)' on 'NACHT, LRR and PYD domains−containing protein 1'

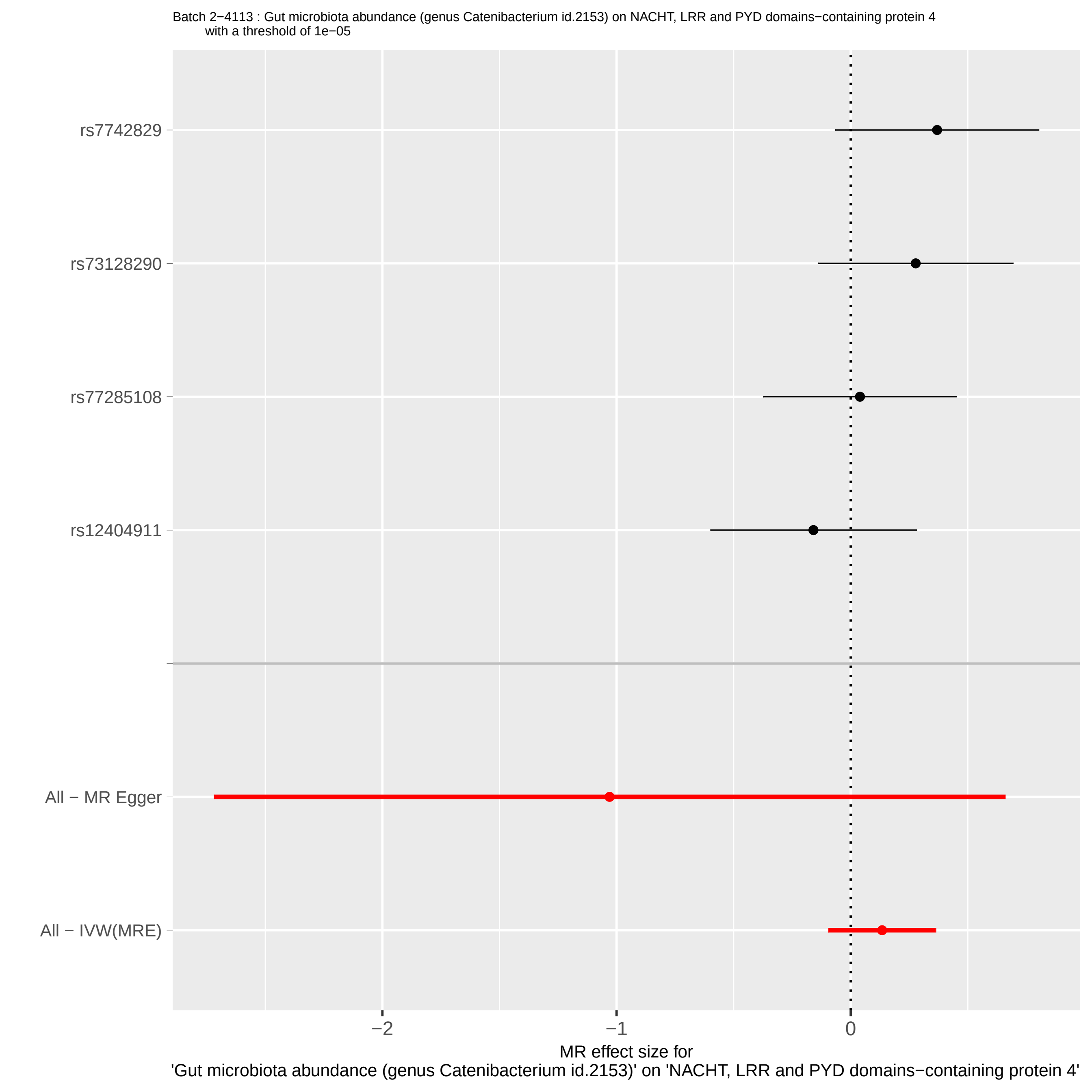

Batch 2−4113 : Gut microbiota abundance (genus Catenibacterium id.2153) on NACHT, LRR and PYD domains−containing protein 4
with a threshold of 1e−05
rs7742829
rs73128290
rs77285108
rs12404911
All − MR Egger
All − IVW(MRE)
−2
−1
0
MR effect size for
'Gut microbiota abundance (genus Catenibacterium id.2153)' on 'NACHT, LRR and PYD domains−containing protein 4'

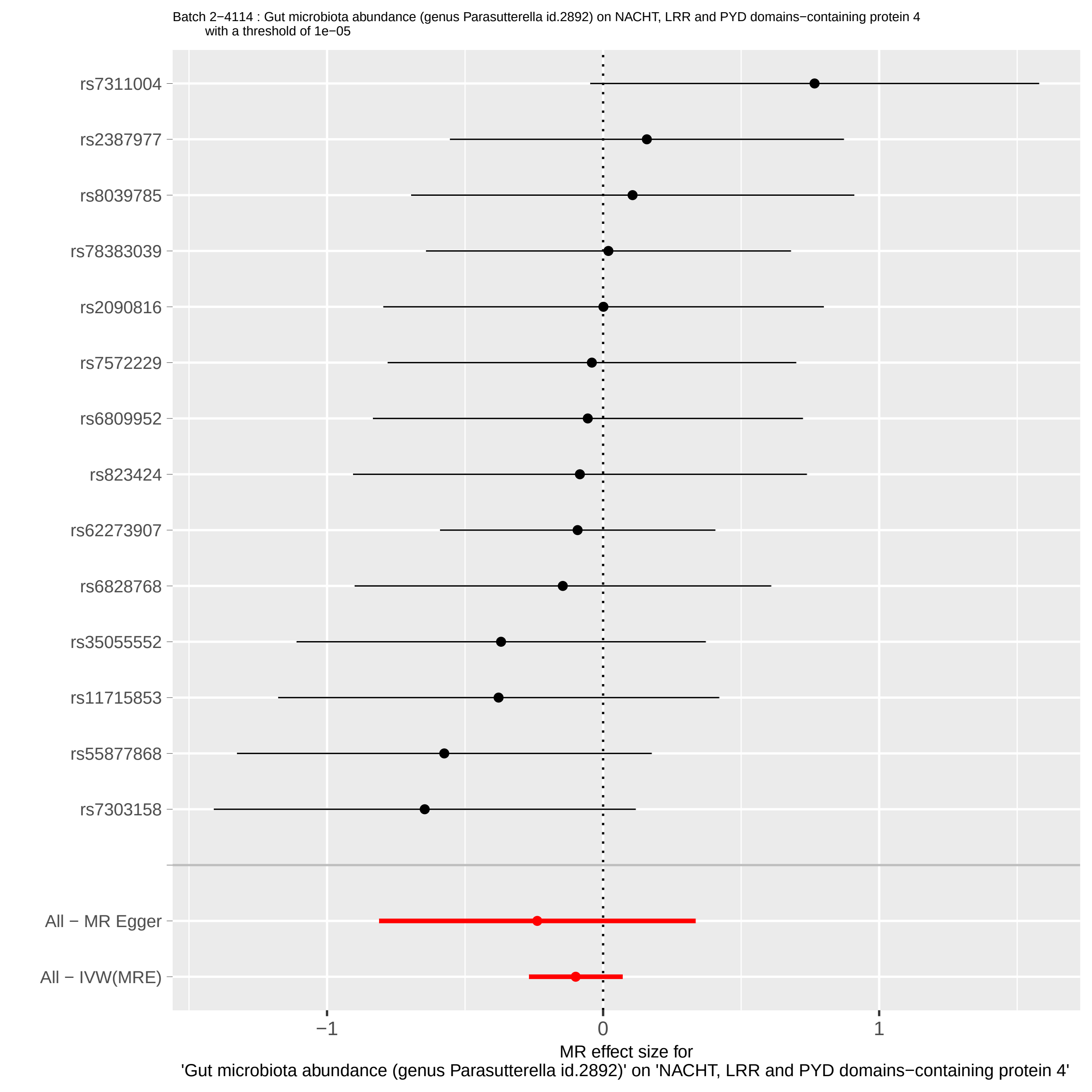
Batch 2−4114 : Gut microbiota abundance (genus Parasutterella id.2892) on NACHT, LRR and PYD domains−containing protein 4
with a threshold of 1e−05
rs7311004
rs2387977
rs8039785
rs78383039
rs2090816
rs7572229
rs6809952
rs823424
rs62273907
rs6828768
rs35055552
rs11715853
rs55877868
rs7303158
All − MR Egger
All − IVW(MRE)
−1
0
1
MR effect size for
'Gut microbiota abundance (genus Parasutterella id.2892)' on 'NACHT, LRR and PYD domains−containing protein 4'

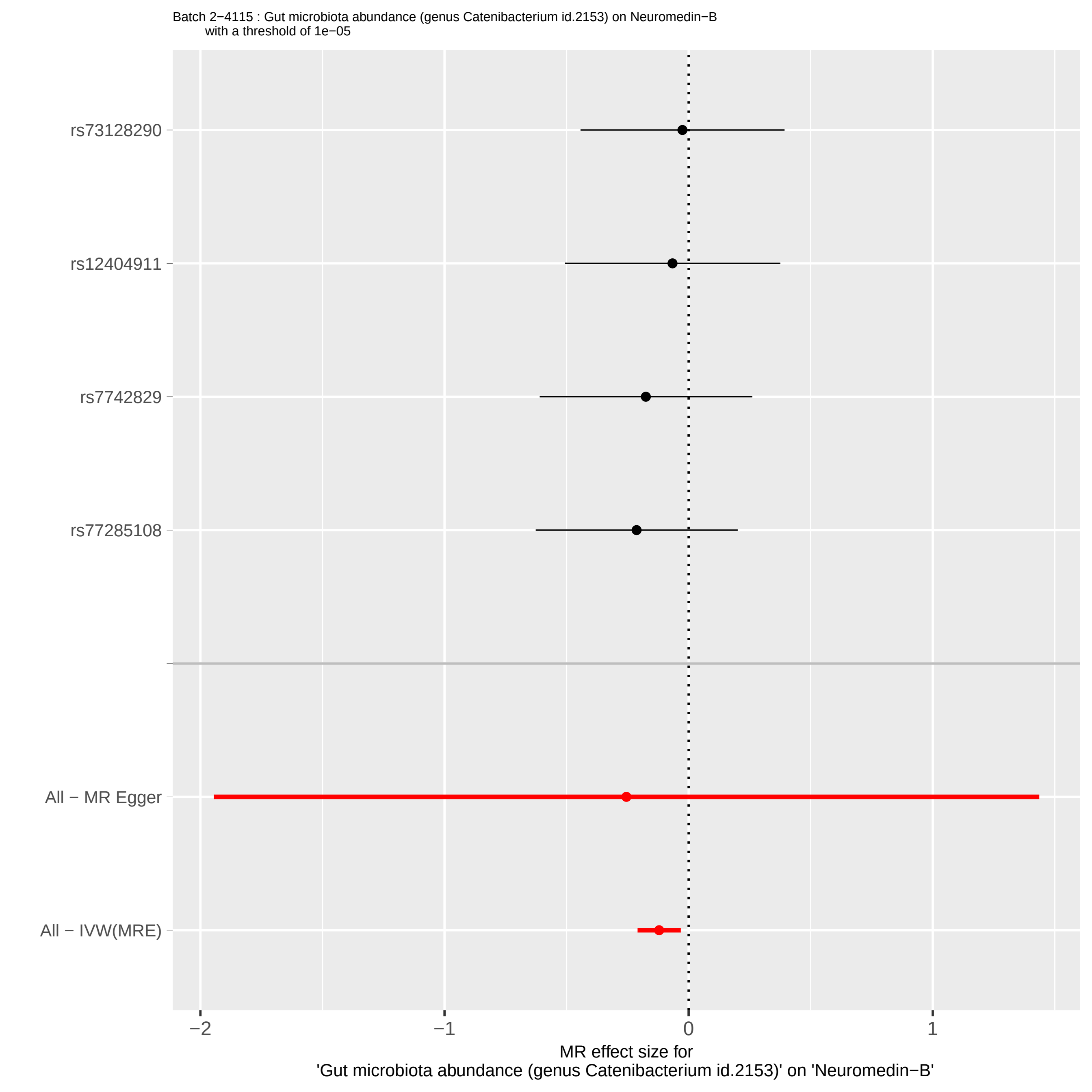

Batch 2−4115 : Gut microbiota abundance (genus Catenibacterium id.2153) on Neuromedin−B
with a threshold of 1e−05
rs73128290
rs12404911
rs7742829
rs77285108
All − MR Egger
All − IVW(MRE)
−2
−1
0
1
MR effect size for
'Gut microbiota abundance (genus Catenibacterium id.2153)' on 'Neuromedin−B'

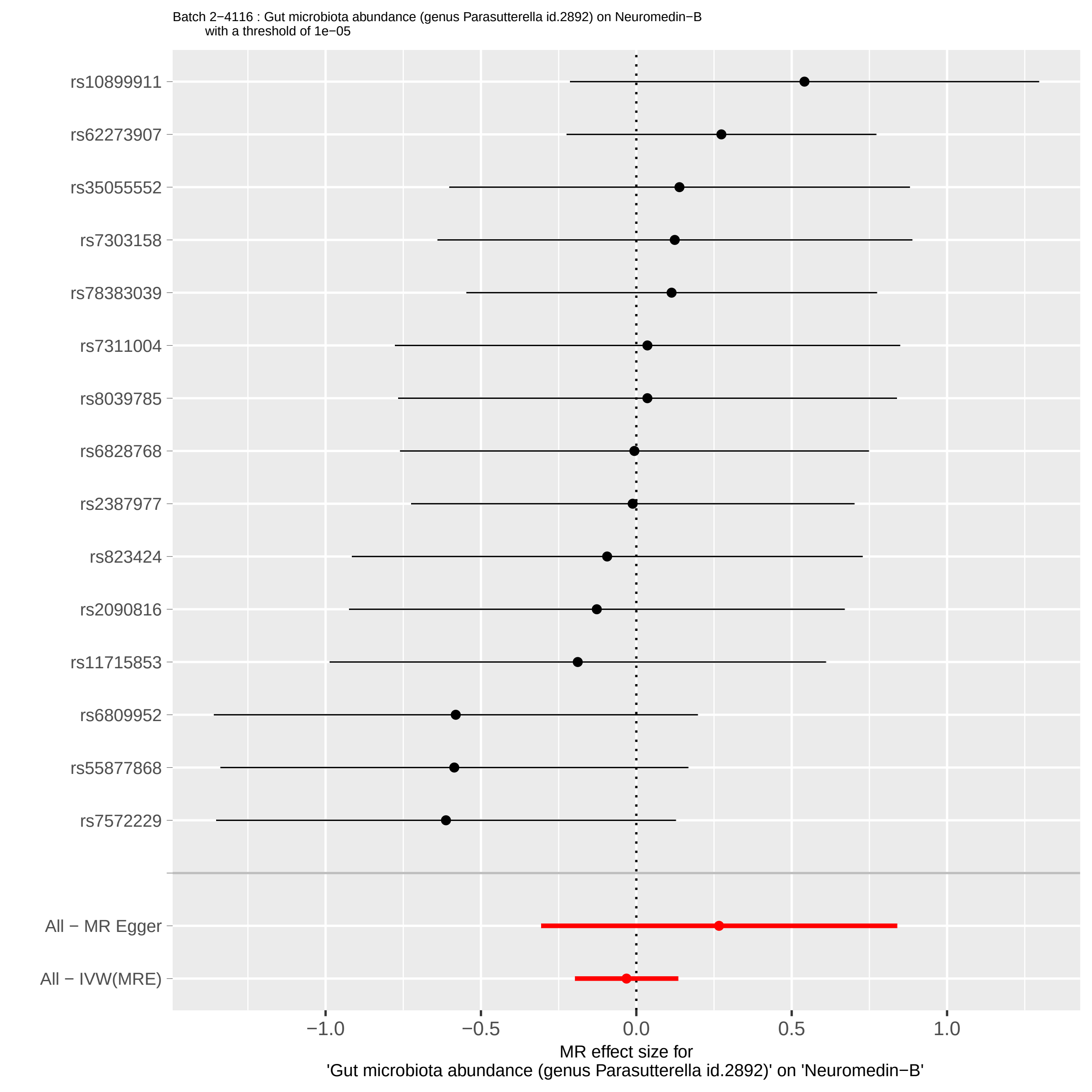

Batch 2−4116 : Gut microbiota abundance (genus Parasutterella id.2892) on Neuromedin−B
with a threshold of 1e−05
rs10899911
rs62273907
rs35055552
rs7303158
rs78383039
rs7311004
rs8039785
rs6828768
rs2387977
rs823424
rs2090816
rs11715853
rs6809952
rs55877868
rs7572229
All − MR Egger
All − IVW(MRE)
−1.0
−0.5
0.0
0.5
1.0
MR effect size for
'Gut microbiota abundance (genus Parasutterella id.2892)' on 'Neuromedin−B'

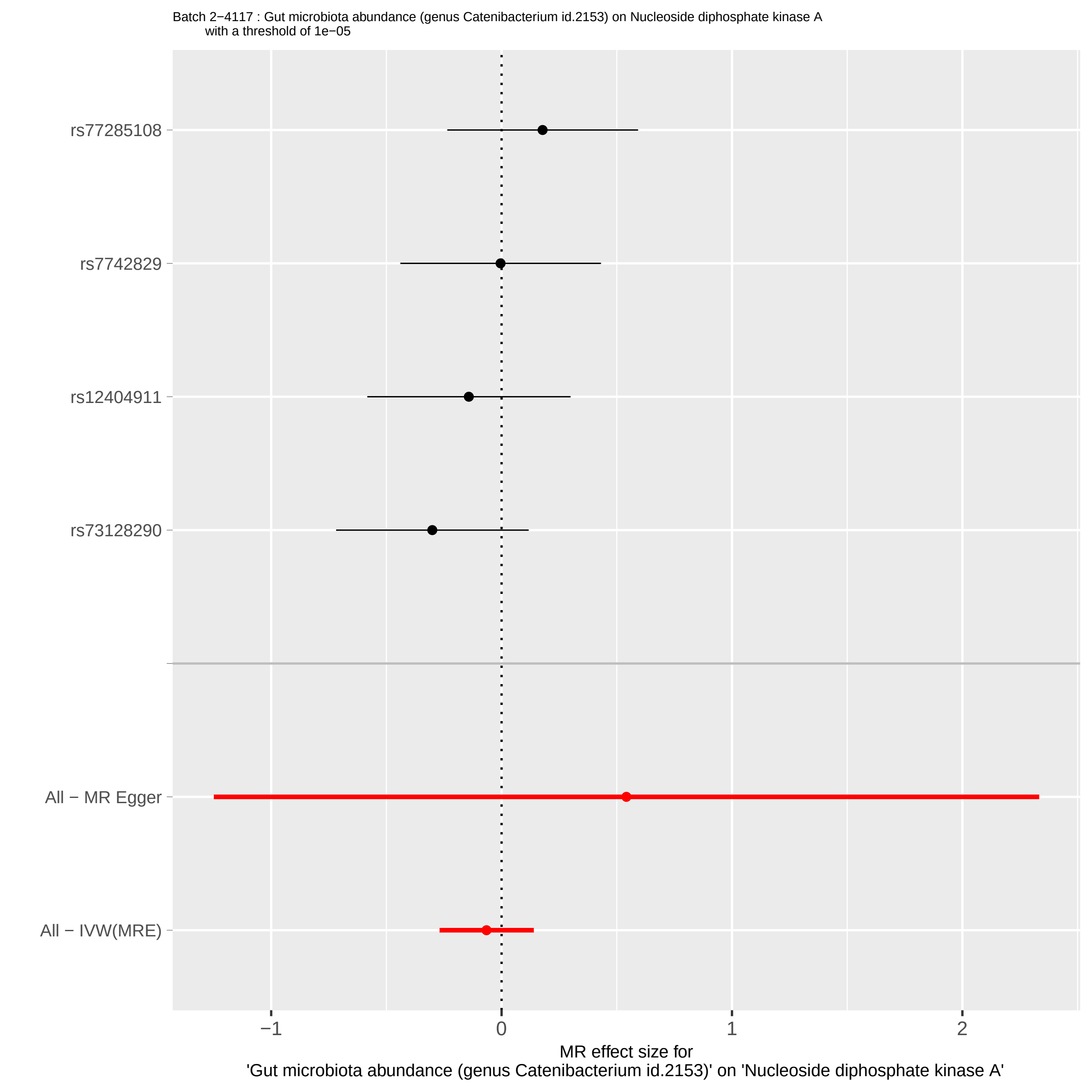

Batch 2−4117 : Gut microbiota abundance (genus Catenibacterium id.2153) on Nucleoside diphosphate kinase A
with a threshold of 1e−05
rs77285108
rs7742829
rs12404911
rs73128290
All − MR Egger
All − IVW(MRE)
−1
0
1
2
MR effect size for
'Gut microbiota abundance (genus Catenibacterium id.2153)' on 'Nucleoside diphosphate kinase A'

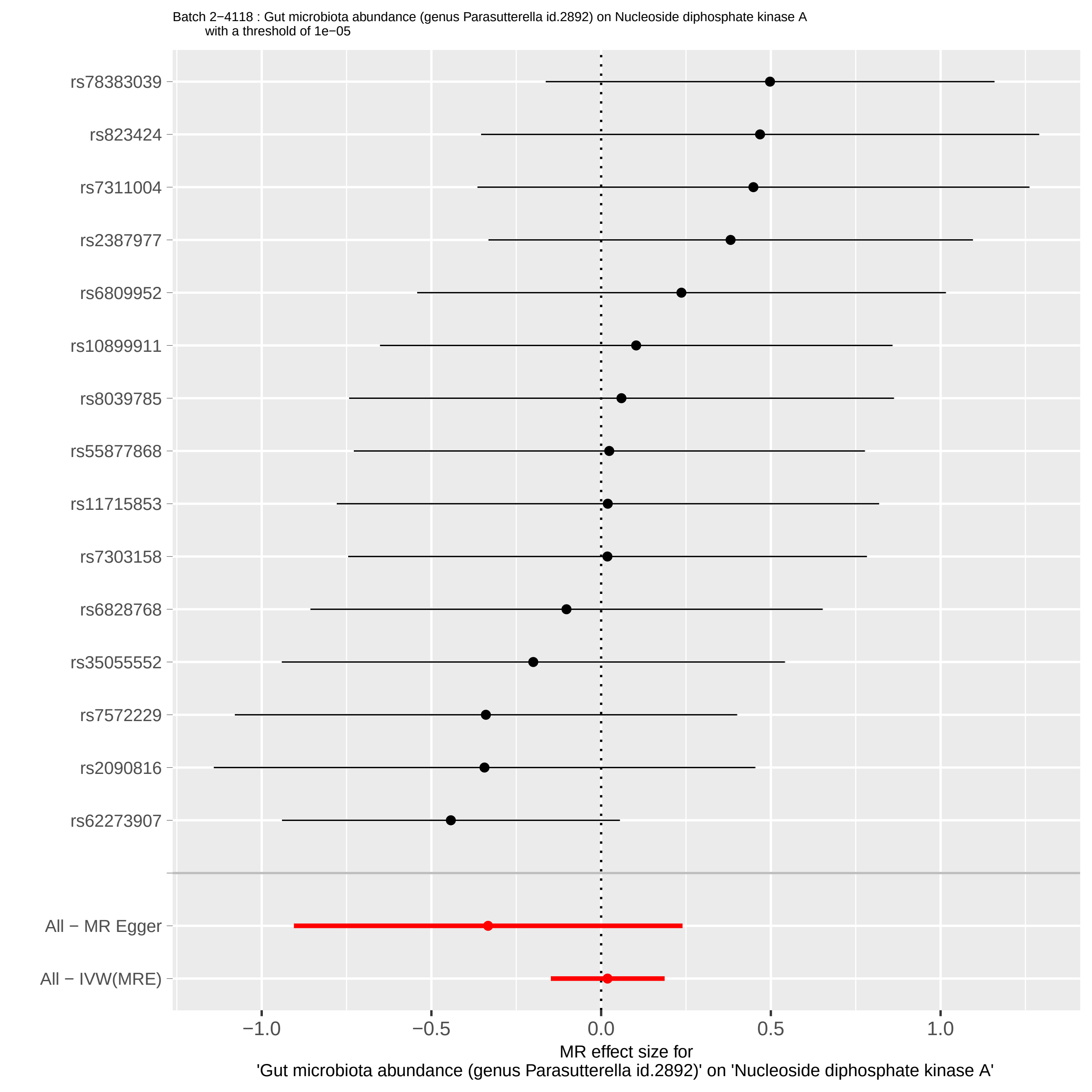

Batch 2−4118 : Gut microbiota abundance (genus Parasutterella id.2892) on Nucleoside diphosphate kinase A
with a threshold of 1e−05
rs78383039
rs823424
rs7311004
rs2387977
rs6809952
rs10899911
rs8039785
rs55877868
rs11715853
rs7303158
rs6828768
rs35055552
rs7572229
rs2090816
rs62273907
All − MR Egger
All − IVW(MRE)
−1.0
−0.5
0.0
0.5
1.0
MR effect size for
'Gut microbiota abundance (genus Parasutterella id.2892)' on 'Nucleoside diphosphate kinase A'

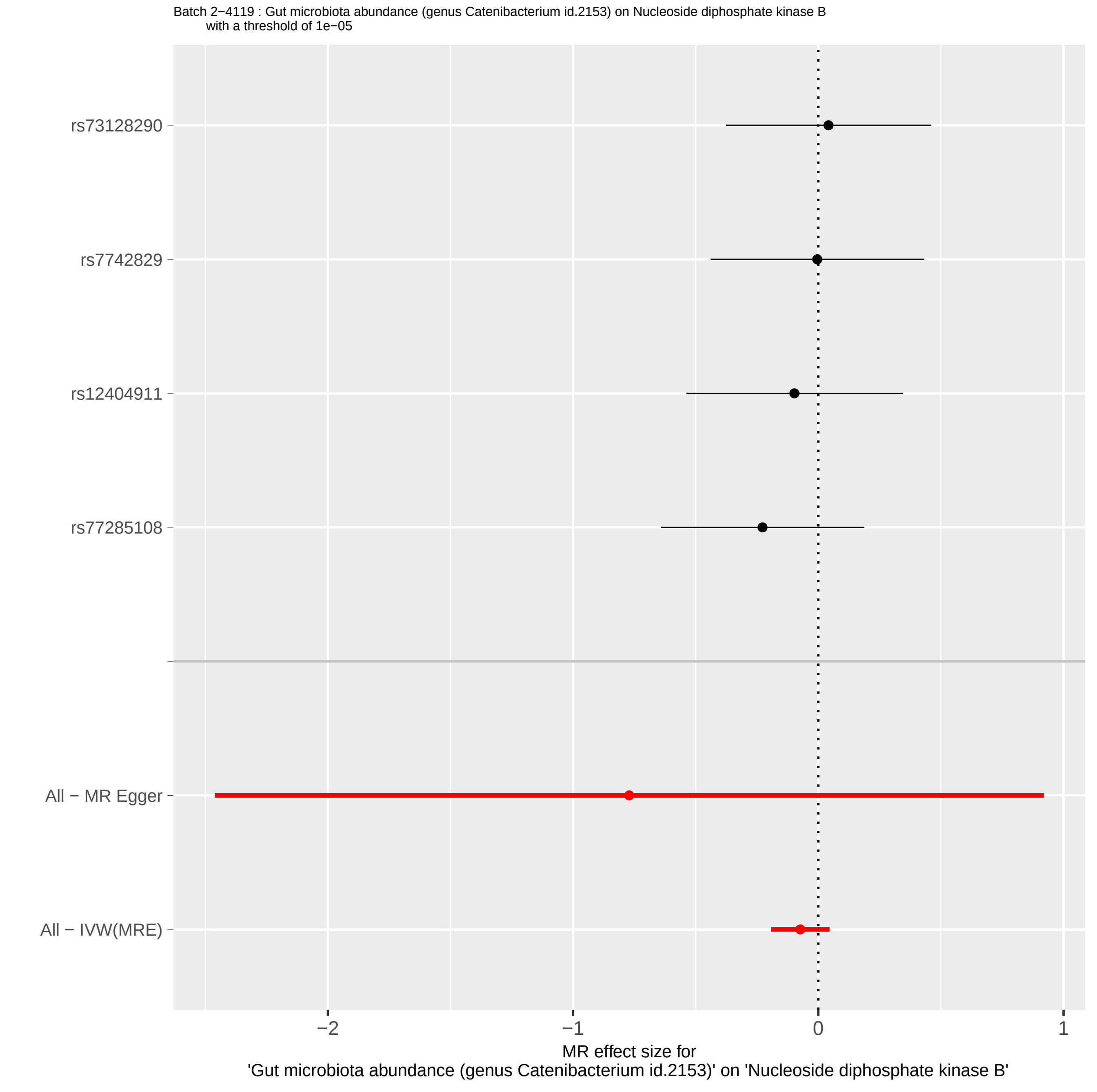

Batch 2−4119 : Gut microbiota abundance (genus Catenibacterium id.2153) on Nucleoside diphosphate kinase B
with a threshold of 1e−05
rs73128290
rs7742829
rs12404911
rs77285108
All − MR Egger
All − IVW(MRE)
−2
−1
0
1
MR effect size for
'Gut microbiota abundance (genus Catenibacterium id.2153)' on 'Nucleoside diphosphate kinase B'

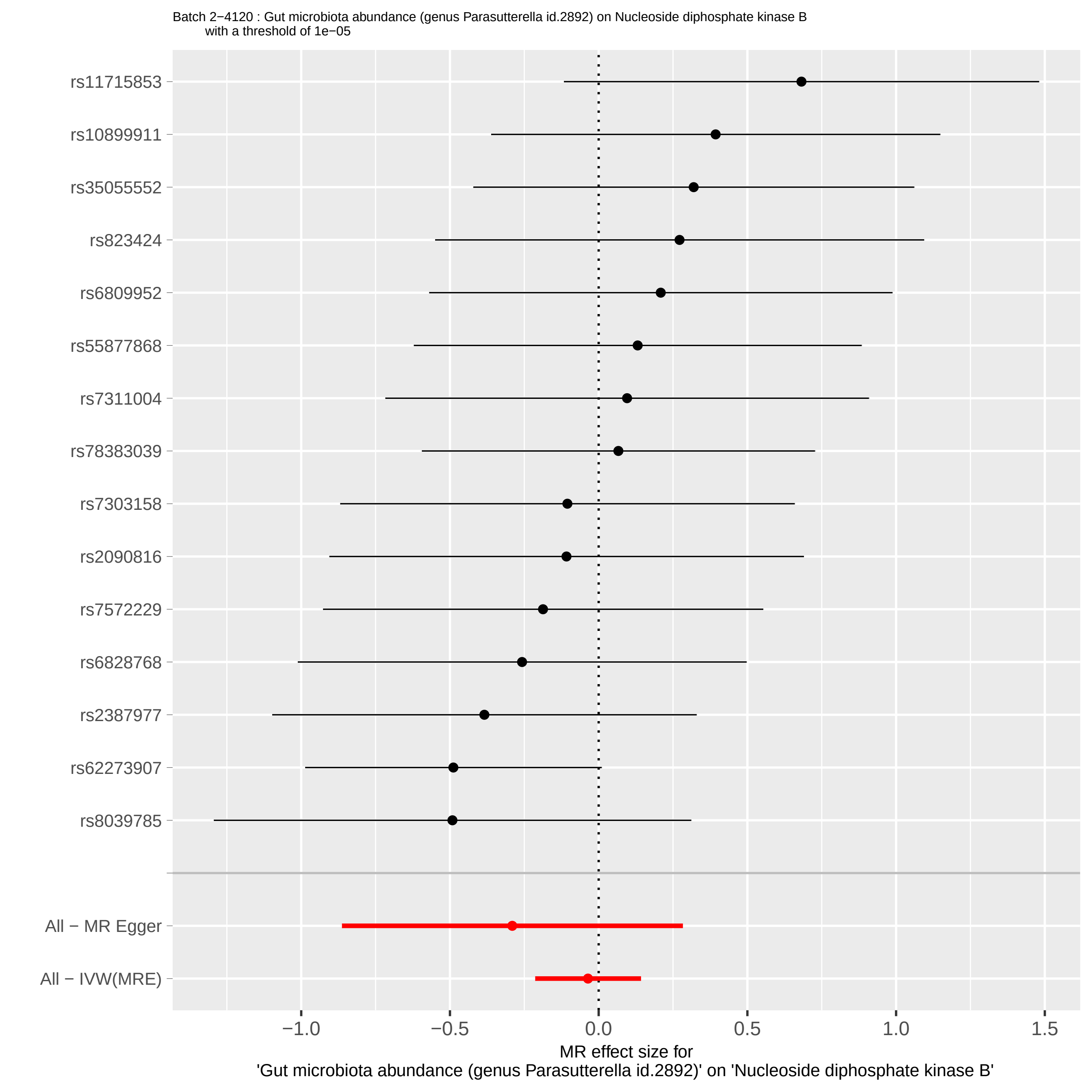

Batch 2−4120 : Gut microbiota abundance (genus Parasutterella id.2892) on Nucleoside diphosphate kinase B
with a threshold of 1e−05
rs11715853
rs10899911
rs35055552
rs823424
rs6809952
rs55877868
rs7311004
rs78383039
rs7303158
rs2090816
rs7572229
rs6828768
rs2387977
rs62273907
rs8039785
All − MR Egger
All − IVW(MRE)
−1.0
−0.5
0.0
0.5
1.0
1.5
MR effect size for
'Gut microbiota abundance (genus Parasutterella id.2892)' on 'Nucleoside diphosphate kinase B'

Batch 2−4121 : Gut microbiota abundance (genus Catenibacterium id.2153) on Normal mucosa of esophagus−specific gene 1 protein
with a threshold of 1e−05

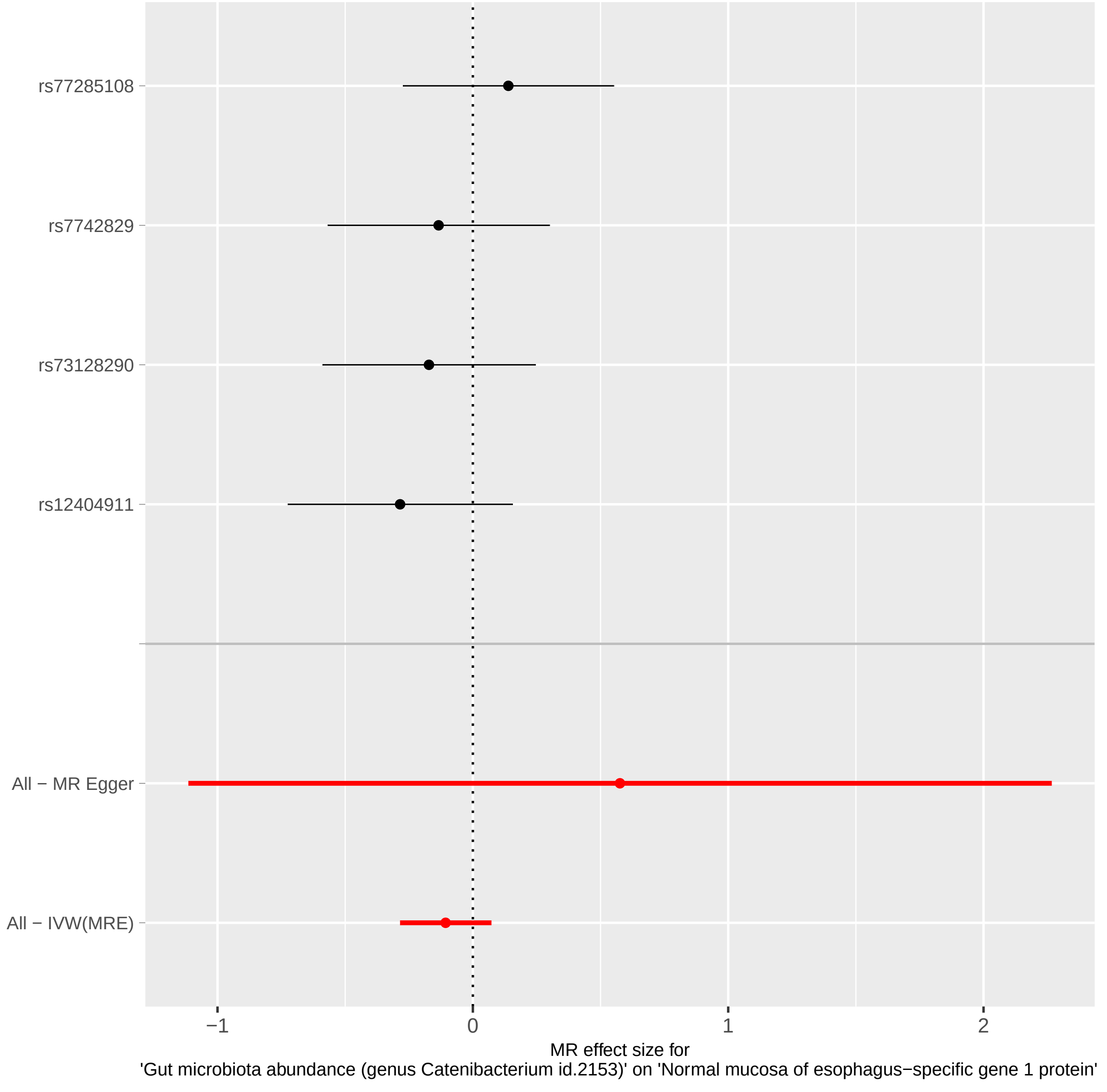

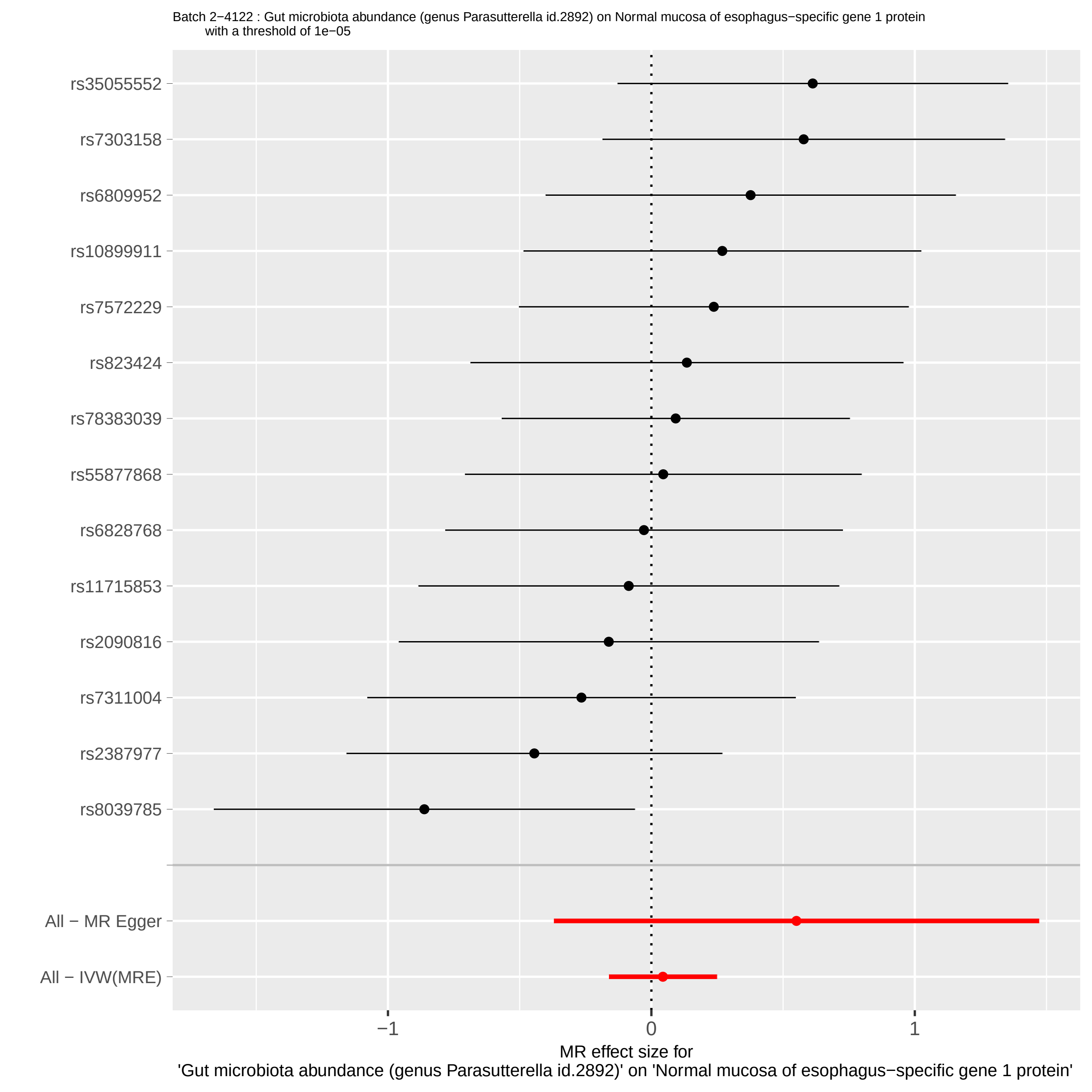

Batch 2−4122 : Gut microbiota abundance (genus Parasutterella id.2892) on Normal mucosa of esophagus−specific gene 1 protein
with a threshold of 1e−05
rs35055552
rs7303158
rs6809952
rs10899911
rs7572229
rs823424
rs78383039
rs55877868
rs6828768
rs11715853
rs2090816
rs7311004
rs2387977
rs8039785
All − MR Egger
All − IVW(MRE)
−1
0
1
MR effect size for
'Gut microbiota abundance (genus Parasutterella id.2892)' on 'Normal mucosa of esophagus−specific gene 1 protein'

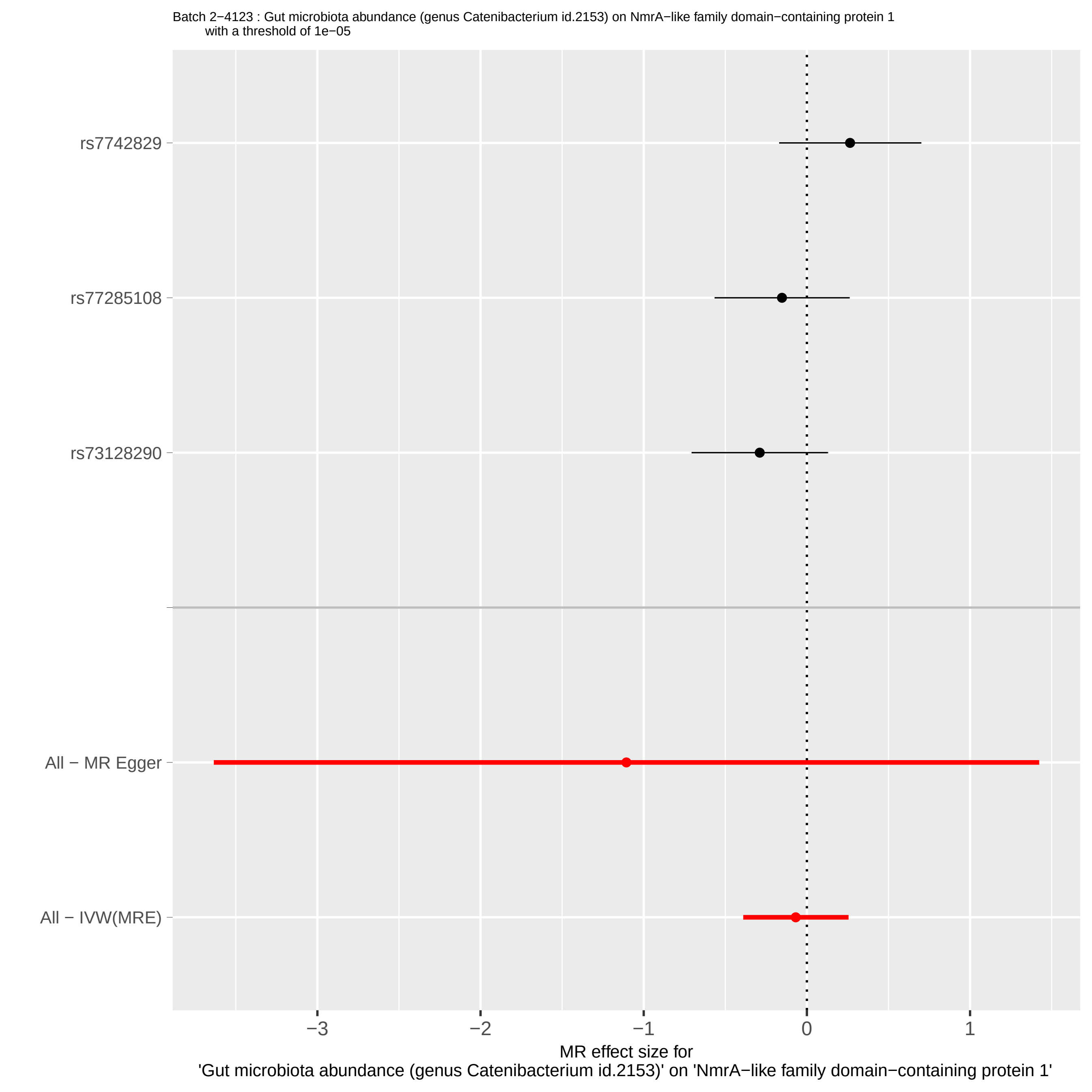

Batch 2−4123 : Gut microbiota abundance (genus Catenibacterium id.2153) on NmrA−like family domain−containing protein 1
with a threshold of 1e−05
rs7742829
rs77285108
rs73128290
All − MR Egger
All − IVW(MRE)
−3
−2
−1
0
1
MR effect size for
'Gut microbiota abundance (genus Catenibacterium id.2153)' on 'NmrA−like family domain−containing protein 1'

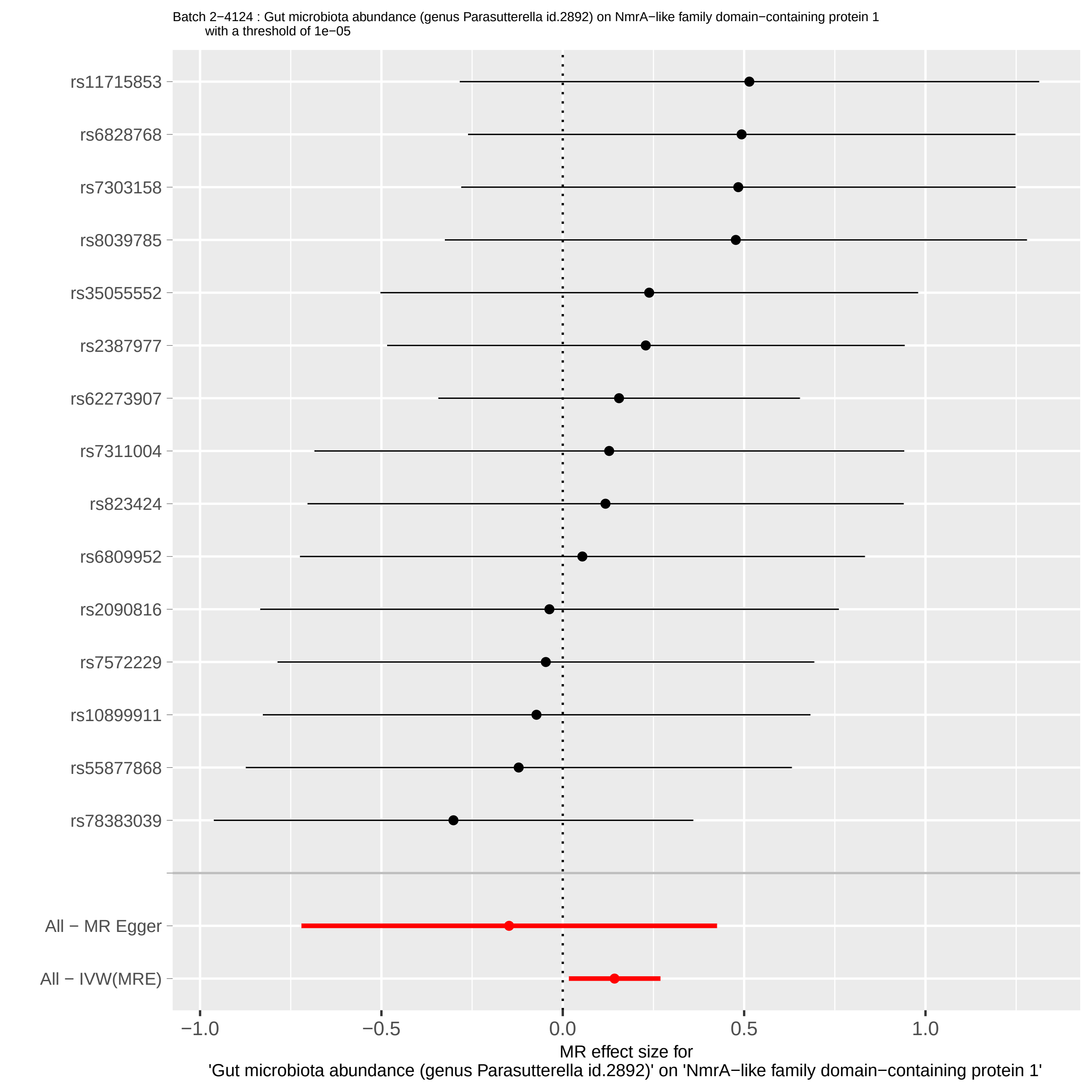
Batch 2−4124 : Gut microbiota abundance (genus Parasutterella id.2892) on NmrA−like family domain−containing protein 1
with a threshold of 1e−05
rs11715853
rs6828768
rs7303158
rs8039785
rs35055552
rs2387977
rs62273907
rs7311004
rs823424
rs6809952
rs2090816
rs7572229
rs10899911
rs55877868
rs78383039
All − MR Egger
All − IVW(MRE)
−1.0
−0.5
0.0
0.5
1.0
MR effect size for
'Gut microbiota abundance (genus Parasutterella id.2892)' on 'NmrA−like family domain−containing protein 1'

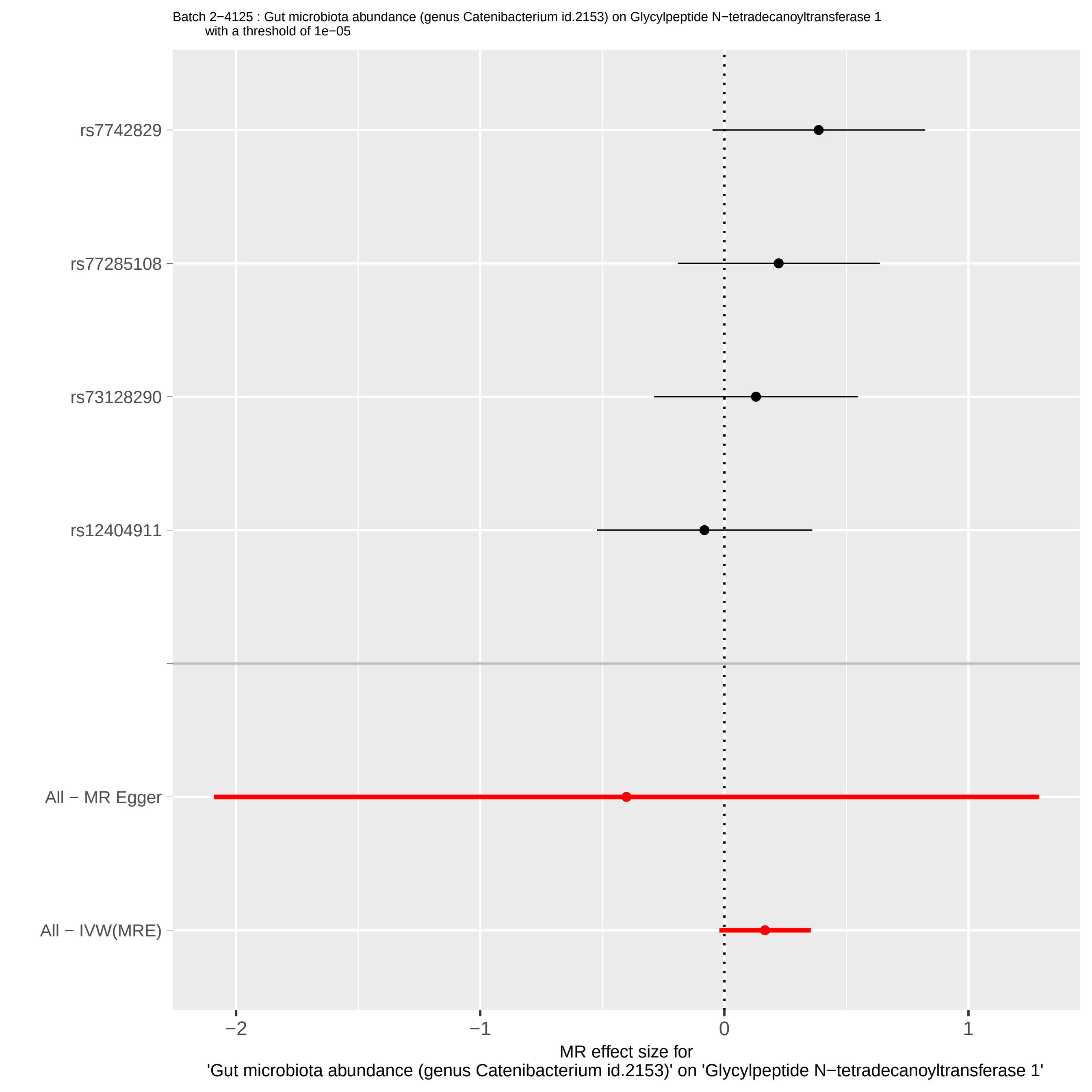

Batch 2−4125 : Gut microbiota abundance (genus Catenibacterium id.2153) on Glycylpeptide N−tetradecanoyltransferase 1
with a threshold of 1e−05
rs7742829
rs77285108
rs73128290
rs12404911
All − MR Egger
All − IVW(MRE)
−2
−1
0
1
MR effect size for
'Gut microbiota abundance (genus Catenibacterium id.2153)' on 'Glycylpeptide N−tetradecanoyltransferase 1'

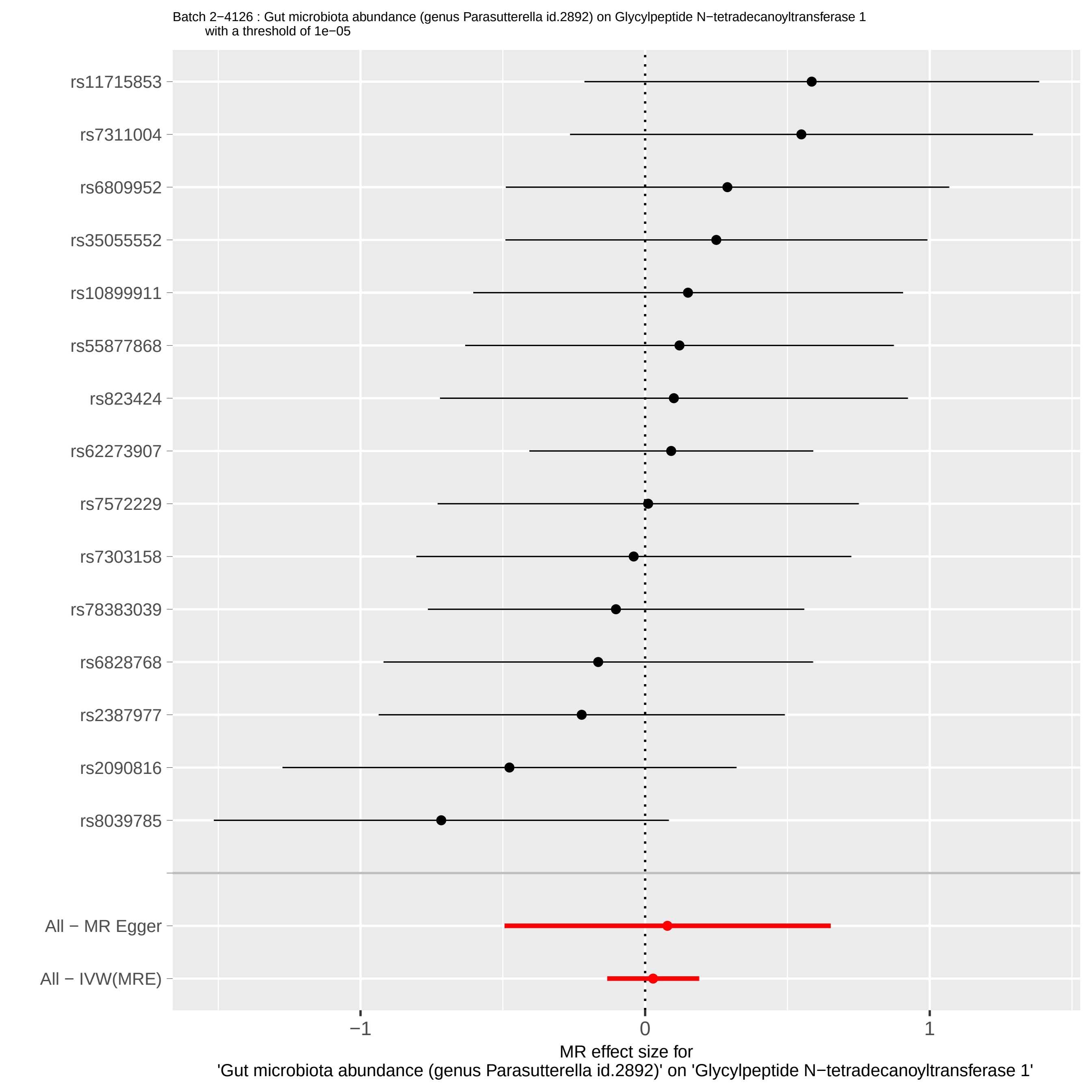

Batch 2−4126 : Gut microbiota abundance (genus Parasutterella id.2892) on Glycylpeptide N−tetradecanoyltransferase 1
with a threshold of 1e−05
rs11715853
rs7311004
rs6809952
rs35055552
rs10899911
rs55877868
rs823424
rs62273907
rs7572229
rs7303158
rs78383039
rs6828768
rs2387977
rs2090816
rs8039785
All − MR Egger
All − IVW(MRE)
−1
0
1
MR effect size for
'Gut microbiota abundance (genus Parasutterella id.2892)' on 'Glycylpeptide N−tetradecanoyltransferase 1'

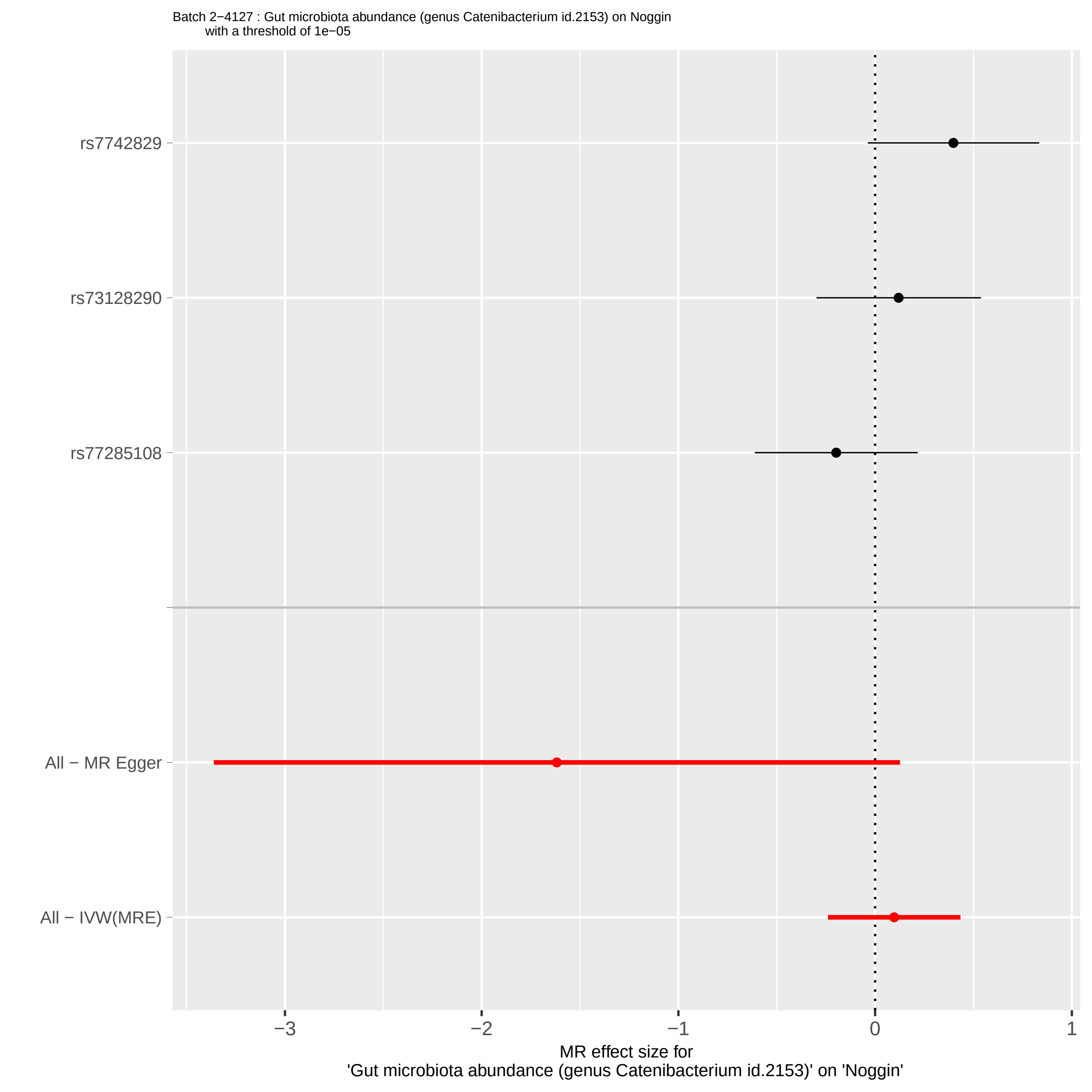
Batch 2−4127 : Gut microbiota abundance (genus Catenibacterium id.2153) on Noggin
with a threshold of 1e−05
rs7742829
rs73128290
rs77285108
All − MR Egger
All − IVW(MRE)
−3
−2
−1
0
1
MR effect size for
'Gut microbiota abundance (genus Catenibacterium id.2153)' on 'Noggin'

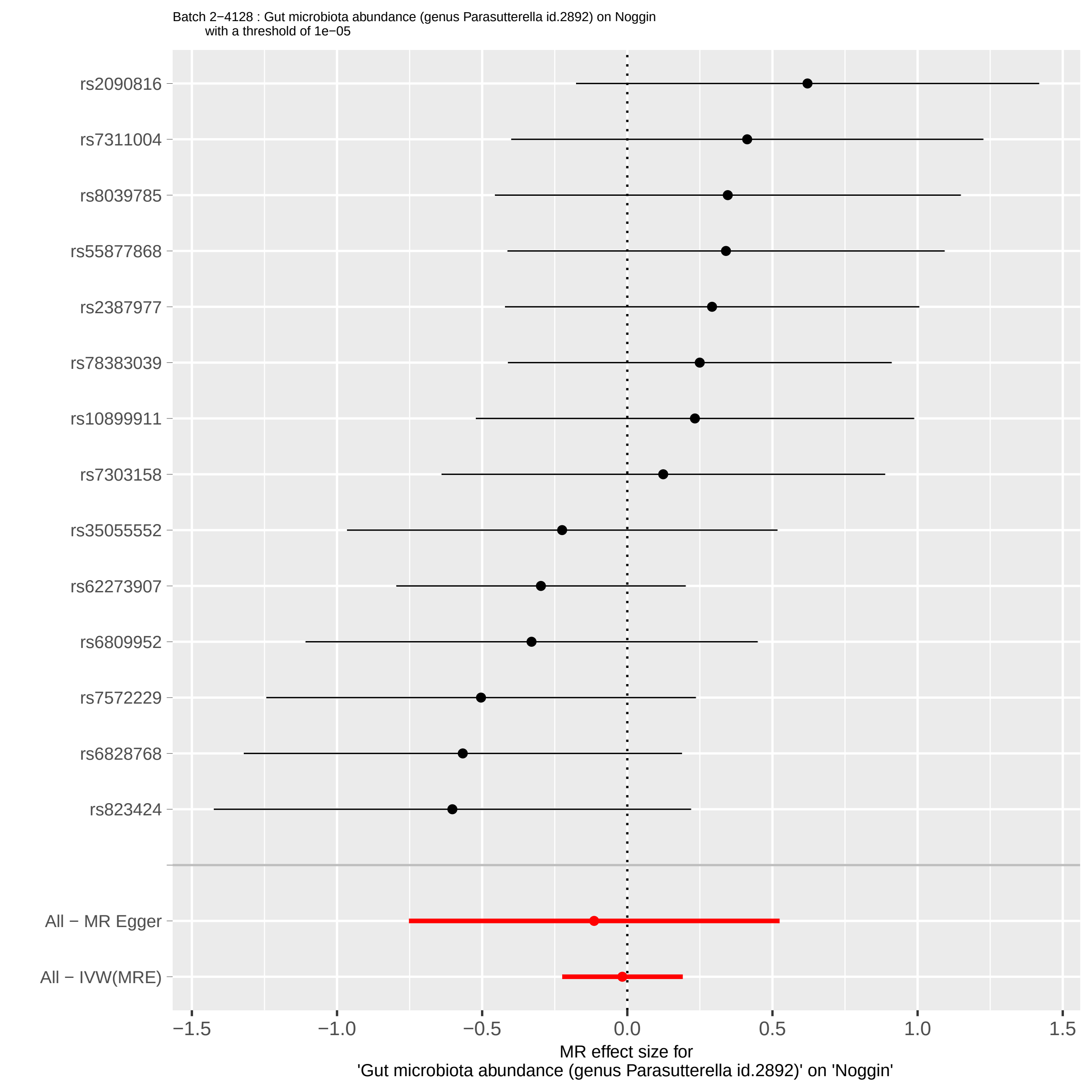
Batch 2−4128 : Gut microbiota abundance (genus Parasutterella id.2892) on Noggin
with a threshold of 1e−05
rs2090816
rs7311004
rs8039785
rs55877868
rs2387977
rs78383039
rs10899911
rs7303158
rs35055552
rs62273907
rs6809952
rs7572229
rs6828768
rs823424
All − MR Egger
All − IVW(MRE)
−1.5
−1.0
−0.5
0.0
0.5
1.0
1.5
MR effect size for
'Gut microbiota abundance (genus Parasutterella id.2892)' on 'Noggin'

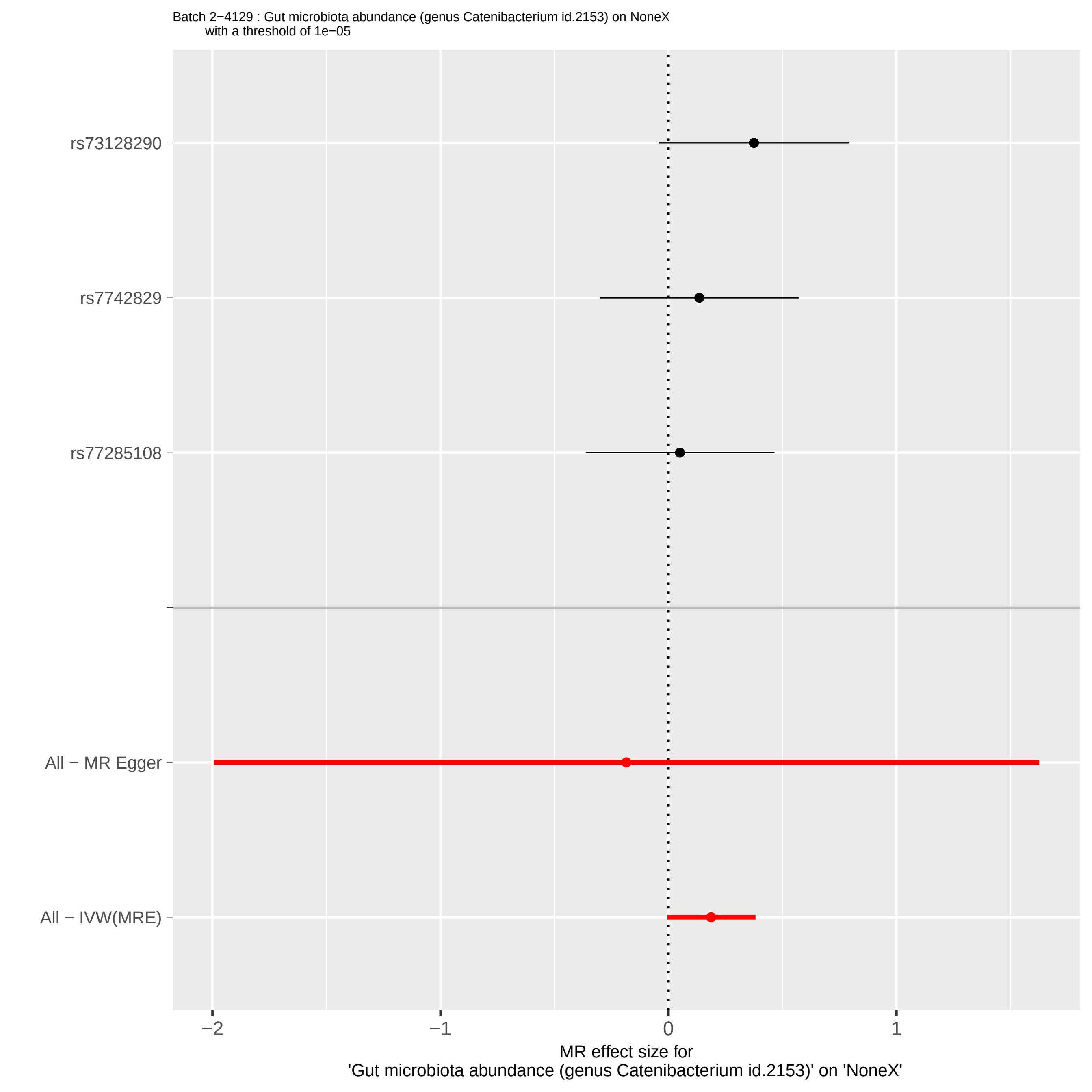
Batch 2−4129 : Gut microbiota abundance (genus Catenibacterium id.2153) on NoneX
with a threshold of 1e−05
rs73128290
rs7742829
rs77285108
All − MR Egger
All − IVW(MRE)
−2
−1
0
1
MR effect size for
'Gut microbiota abundance (genus Catenibacterium id.2153)' on 'NoneX'

Batch 2−4130 : Gut microbiota abundance (genus Parasutterella id.2892) on NoneX
with a threshold of 1e−05

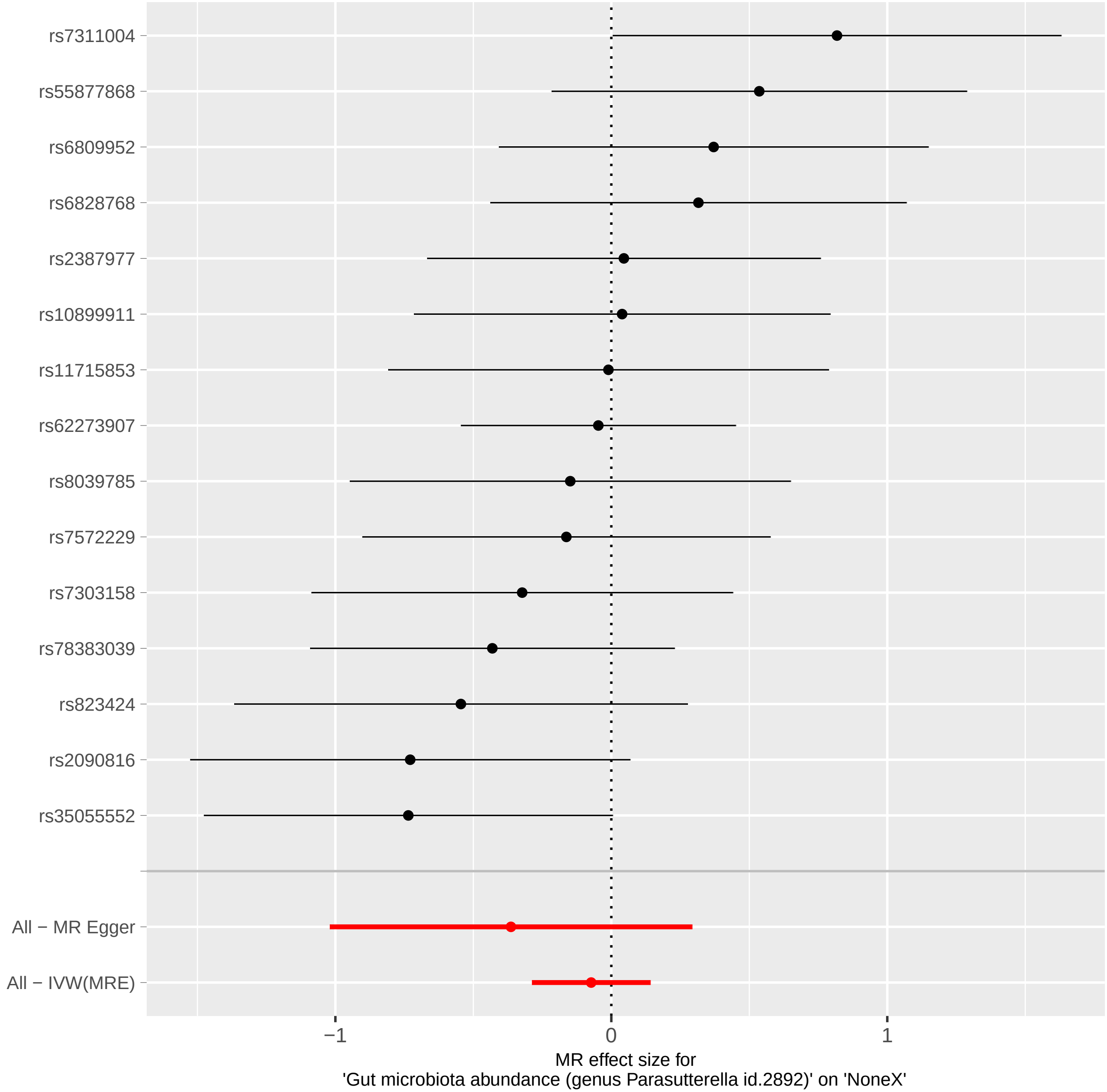

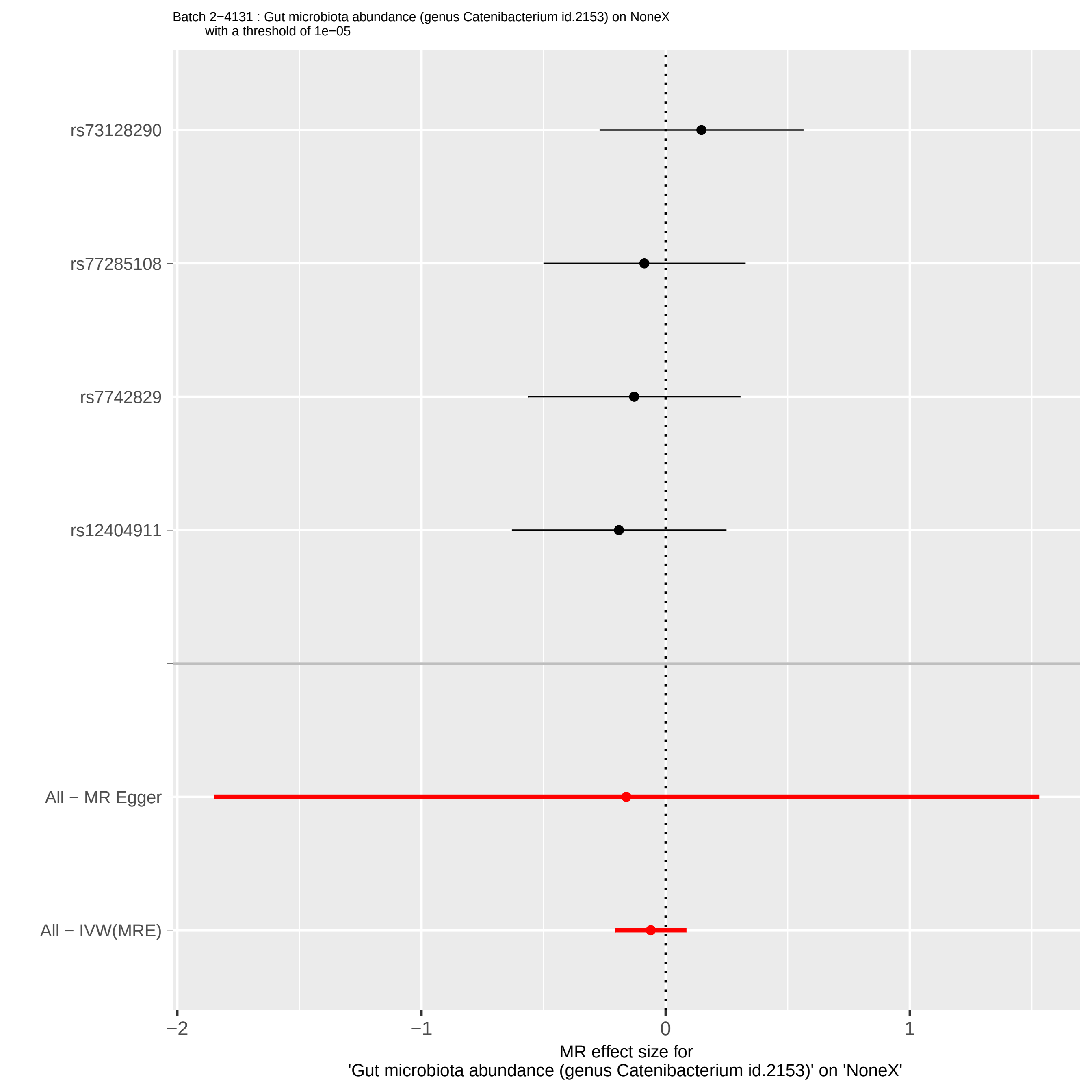

Batch 2−4131 : Gut microbiota abundance (genus Catenibacterium id.2153) on NoneX
with a threshold of 1e−05
rs73128290
rs77285108
rs7742829
rs12404911
All − MR Egger
All − IVW(MRE)
−2
−1
0
1
MR effect size for
'Gut microbiota abundance (genus Catenibacterium id.2153)' on 'NoneX'

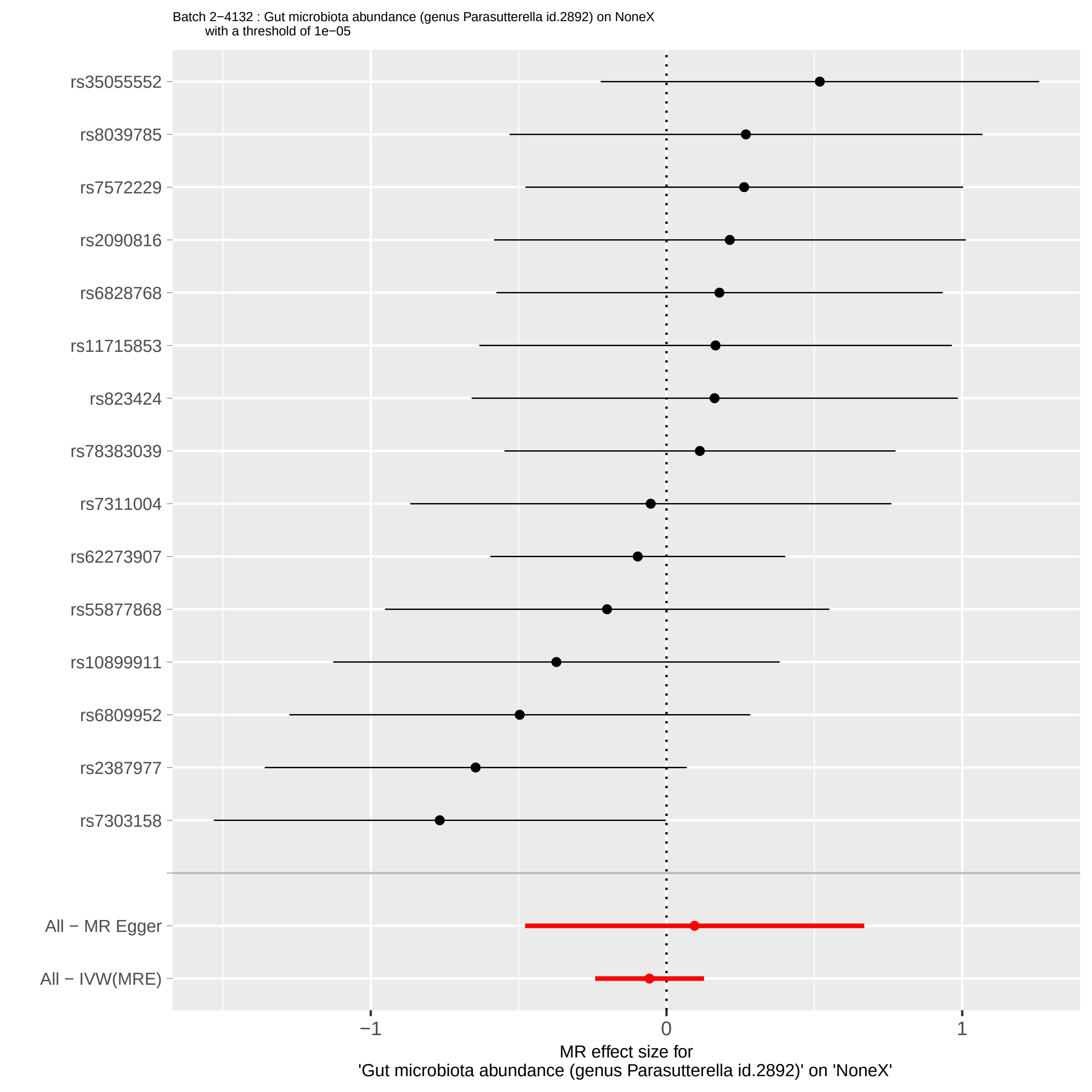

Batch 2−4132 : Gut microbiota abundance (genus Parasutterella id.2892) on NoneX
with a threshold of 1e−05
rs35055552
rs8039785
rs7572229
rs2090816
rs6828768
rs11715853
rs823424
rs78383039
rs7311004
rs62273907
rs55877868
rs10899911
rs6809952
rs2387977
rs7303158
All − MR Egger
All − IVW(MRE)
−1
0
1
MR effect size for
'Gut microbiota abundance (genus Parasutterella id.2892)' on 'NoneX'

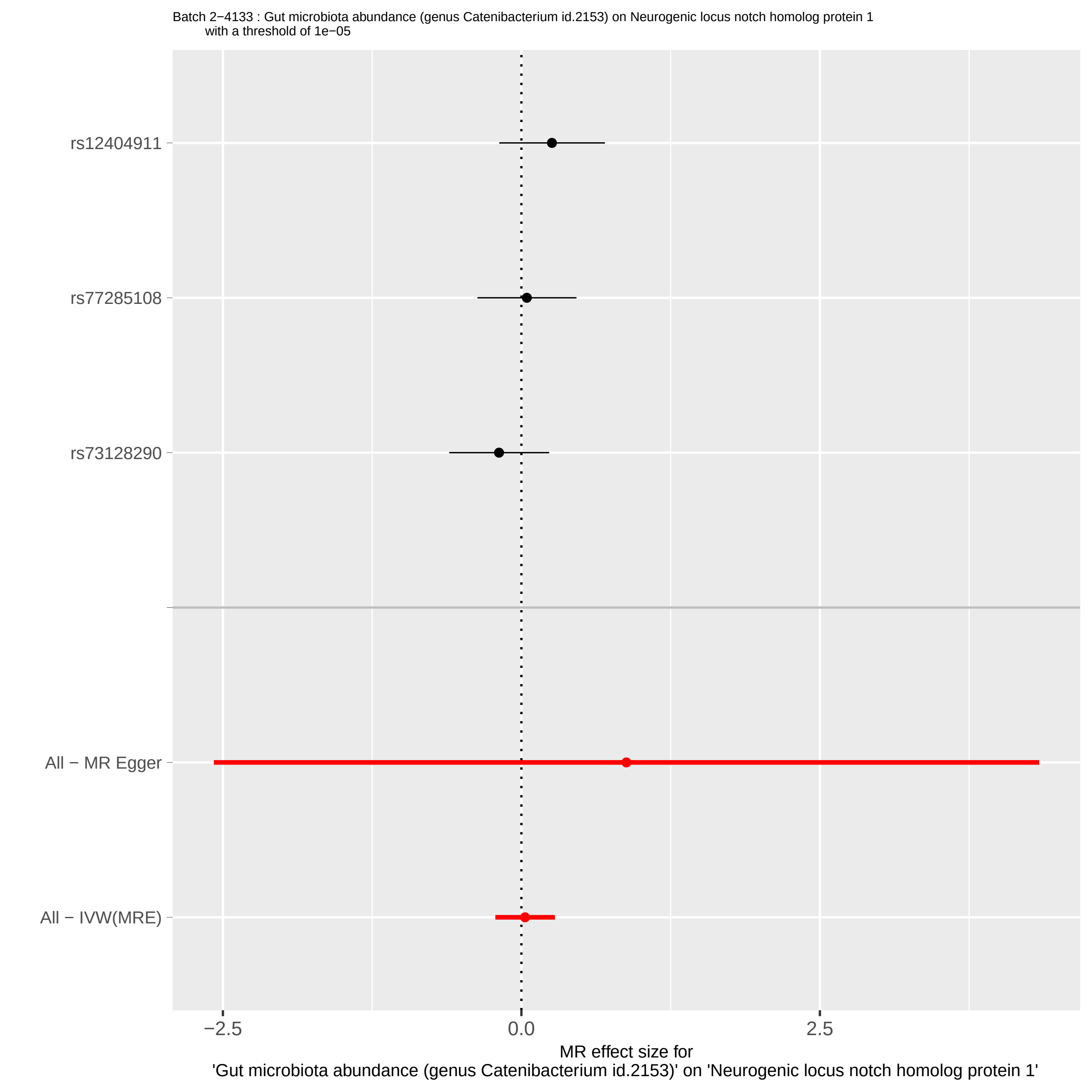

Batch 2−4133 : Gut microbiota abundance (genus Catenibacterium id.2153) on Neurogenic locus notch homolog protein 1
with a threshold of 1e−05
rs12404911
rs77285108
rs73128290
All − MR Egger
All − IVW(MRE)
−2.5
0.0
2.5
MR effect size for
'Gut microbiota abundance (genus Catenibacterium id.2153)' on 'Neurogenic locus notch homolog protein 1'

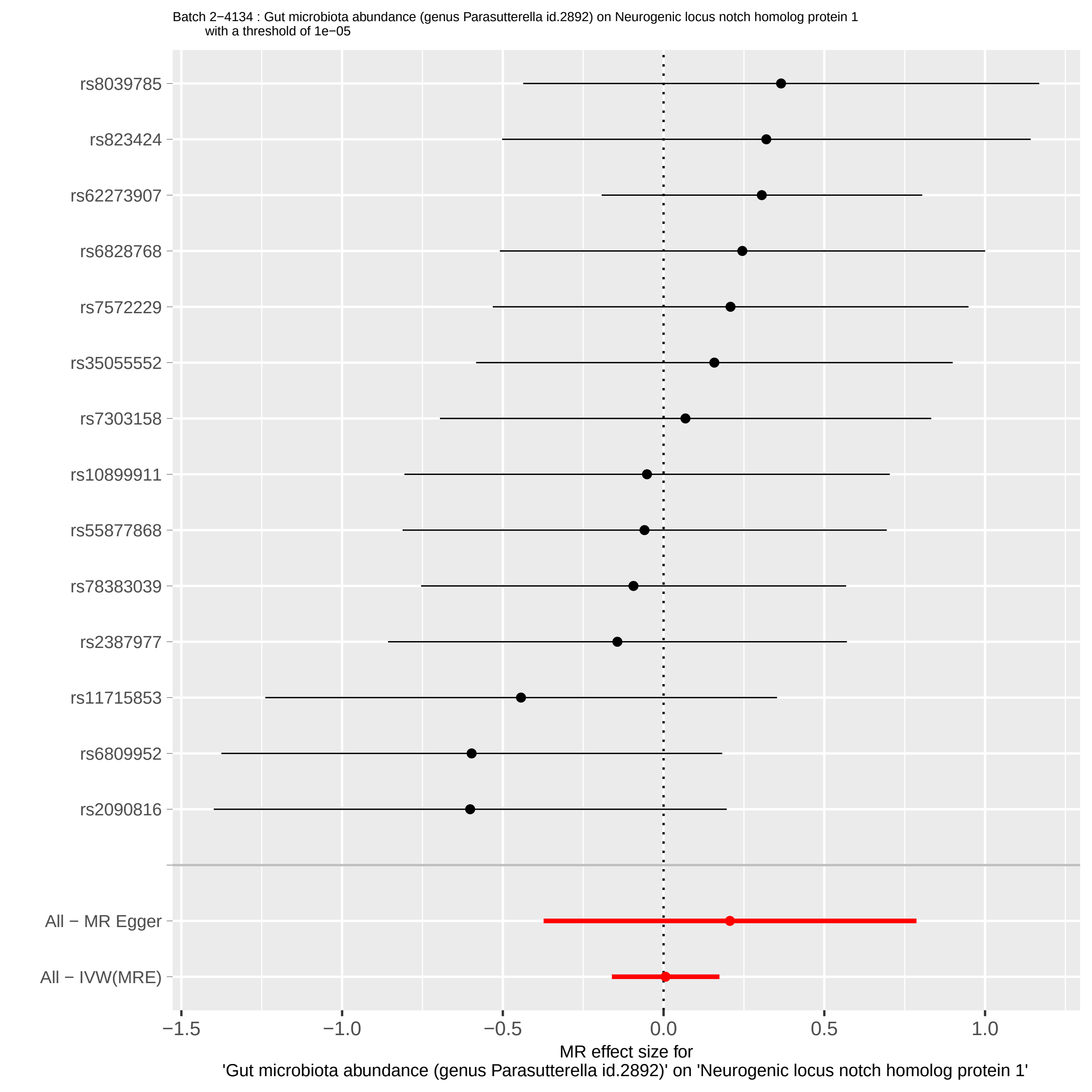
Batch 2−4134 : Gut microbiota abundance (genus Parasutterella id.2892) on Neurogenic locus notch homolog protein 1
with a threshold of 1e−05
rs8039785
rs823424
rs62273907
rs6828768
rs7572229
rs35055552
rs7303158
rs10899911
rs55877868
rs78383039
rs2387977
rs11715853
rs6809952
rs2090816
All − MR Egger
All − IVW(MRE)
−1.5
−1.0
−0.5
0.0
0.5
1.0
MR effect size for
'Gut microbiota abundance (genus Parasutterella id.2892)' on 'Neurogenic locus notch homolog protein 1'

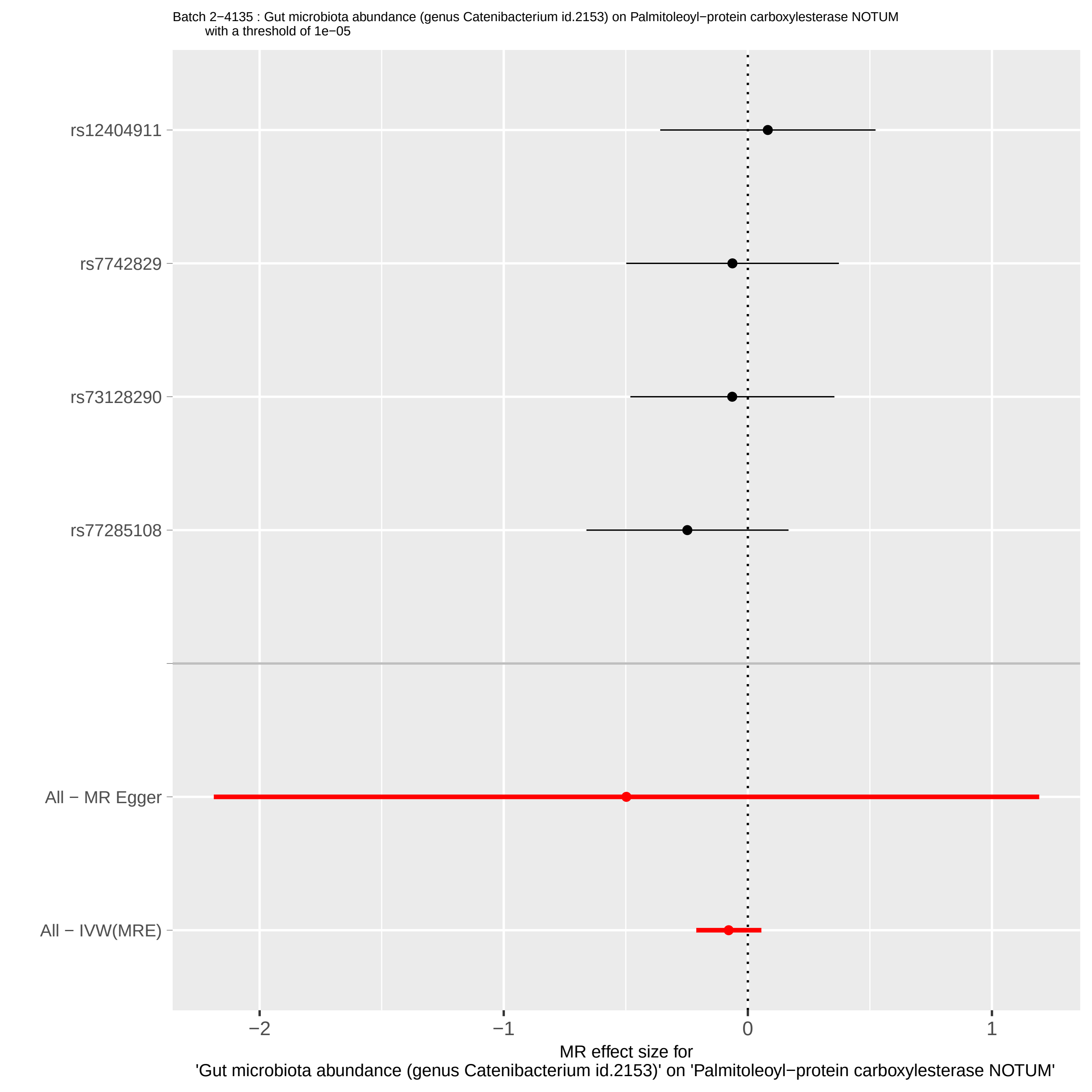
Batch 2−4135 : Gut microbiota abundance (genus Catenibacterium id.2153) on Palmitoleoyl−protein carboxylesterase NOTUM
with a threshold of 1e−05
rs12404911
rs7742829
rs73128290
rs77285108
All − MR Egger
All − IVW(MRE)
−2
−1
0
1
MR effect size for
'Gut microbiota abundance (genus Catenibacterium id.2153)' on 'Palmitoleoyl−protein carboxylesterase NOTUM'

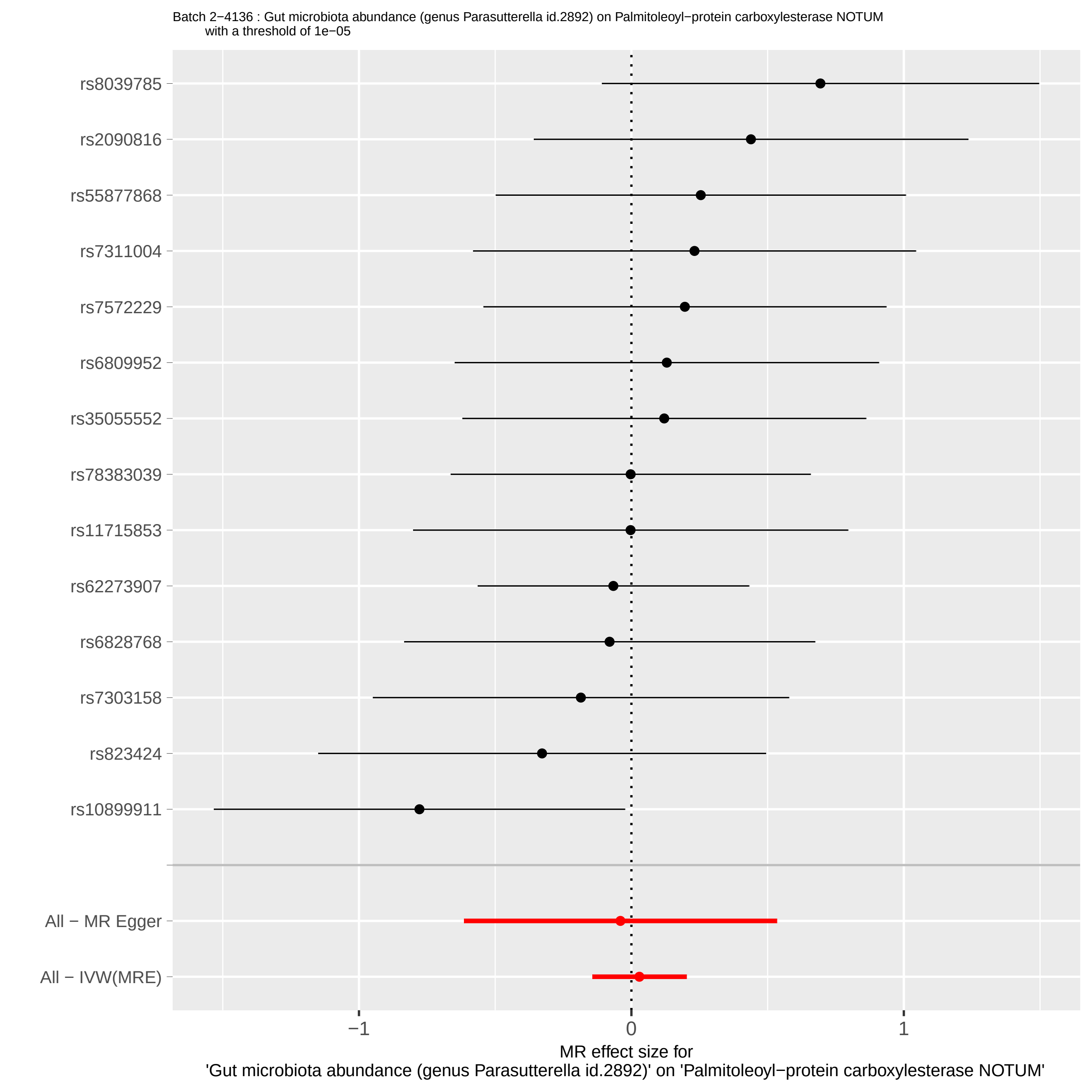
Batch 2−4136 : Gut microbiota abundance (genus Parasutterella id.2892) on Palmitoleoyl−protein carboxylesterase NOTUM
with a threshold of 1e−05
rs8039785
rs2090816
rs55877868
rs7311004
rs7572229
rs6809952
rs35055552
rs78383039
rs11715853
rs62273907
rs6828768
rs7303158
rs823424
rs10899911
All − MR Egger
All − IVW(MRE)
−1
0
1
MR effect size for
'Gut microbiota abundance (genus Parasutterella id.2892)' on 'Palmitoleoyl−protein carboxylesterase NOTUM'

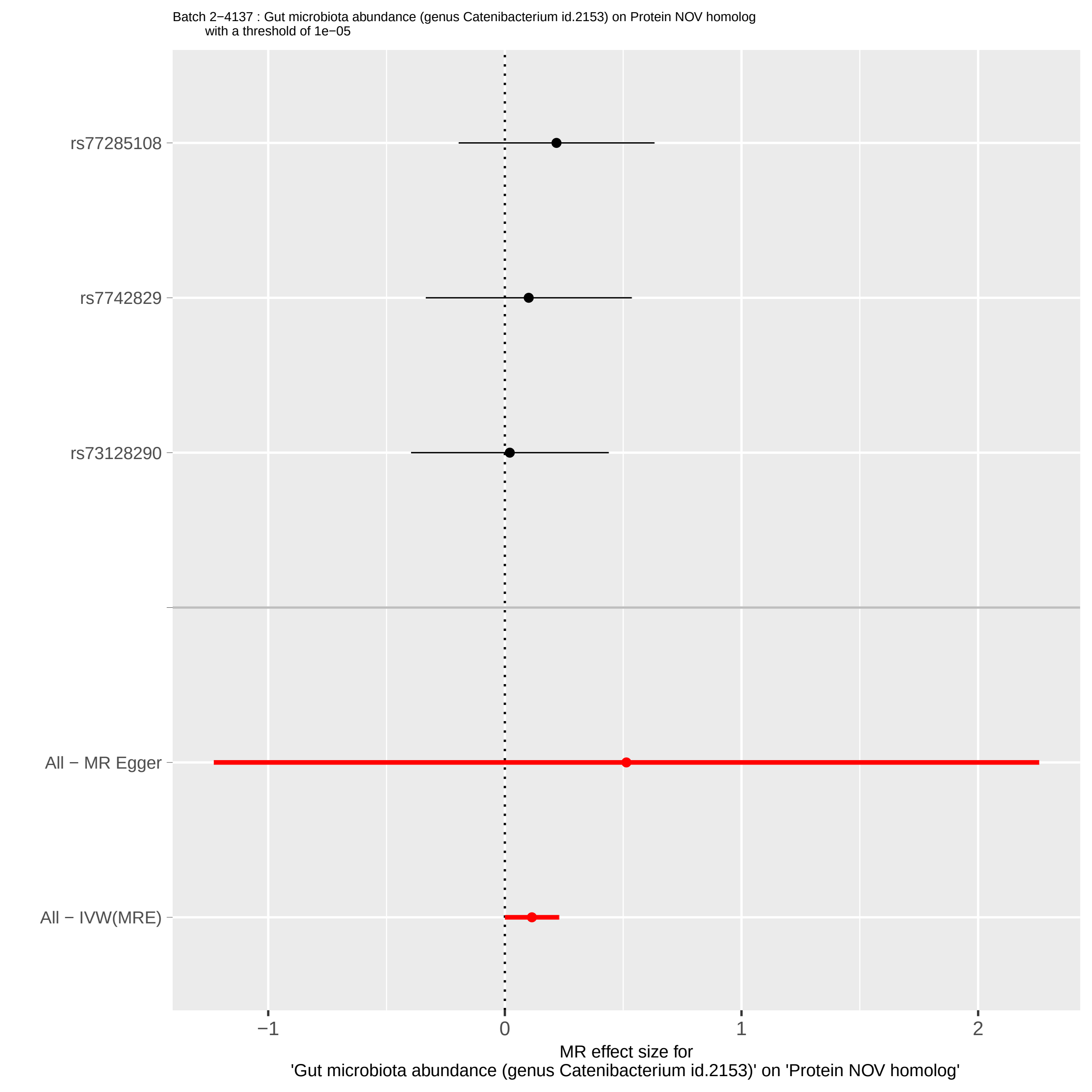
Batch 2−4137 : Gut microbiota abundance (genus Catenibacterium id.2153) on Protein NOV homolog
with a threshold of 1e−05
rs77285108
rs7742829
rs73128290
All − MR Egger
All − IVW(MRE)
−1
0
1
2
MR effect size for
'Gut microbiota abundance (genus Catenibacterium id.2153)' on 'Protein NOV homolog'

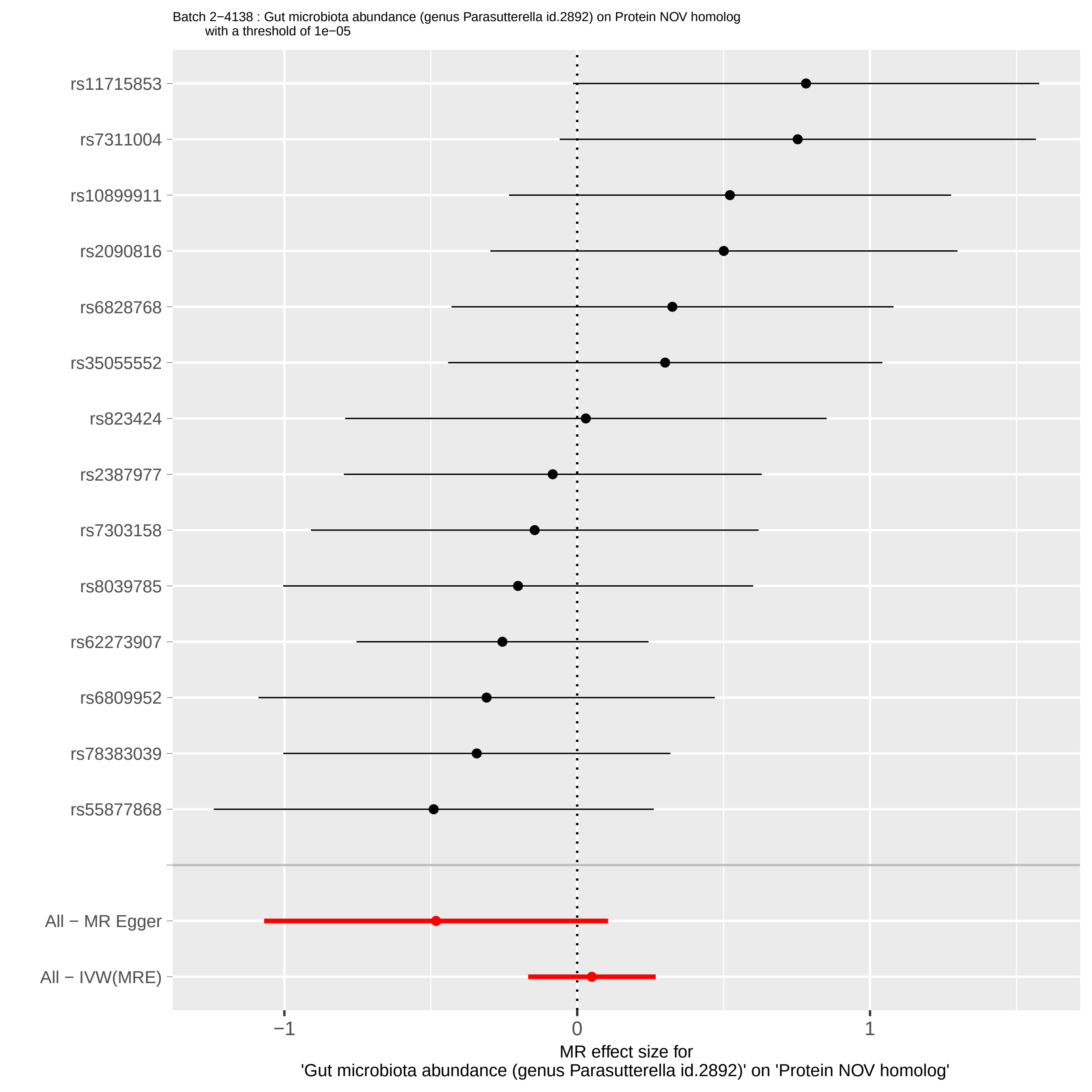
Batch 2−4138 : Gut microbiota abundance (genus Parasutterella id.2892) on Protein NOV homolog
with a threshold of 1e−05
rs11715853
rs7311004
rs10899911
rs2090816
rs6828768
rs35055552
rs823424
rs2387977
rs7303158
rs8039785
rs62273907
rs6809952
rs78383039
rs55877868
All − MR Egger
All − IVW(MRE)
−1
0
1
MR effect size for
'Gut microbiota abundance (genus Parasutterella id.2892)' on 'Protein NOV homolog'

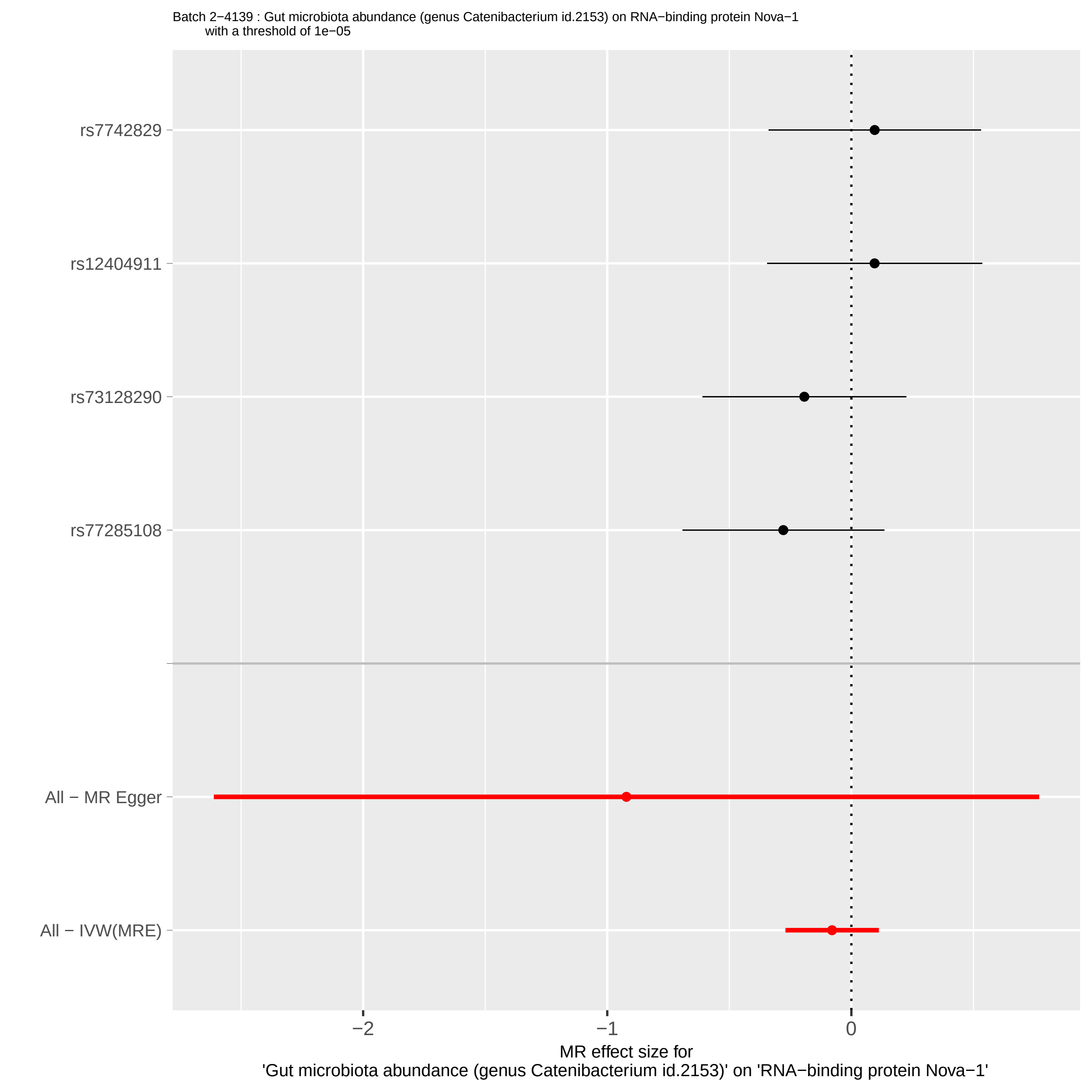
Batch 2−4139 : Gut microbiota abundance (genus Catenibacterium id.2153) on RNA−binding protein Nova−1
with a threshold of 1e−05
rs7742829
rs12404911
rs73128290
rs77285108
All − MR Egger
All − IVW(MRE)
−2
−1
0
MR effect size for
'Gut microbiota abundance (genus Catenibacterium id.2153)' on 'RNA−binding protein Nova−1'

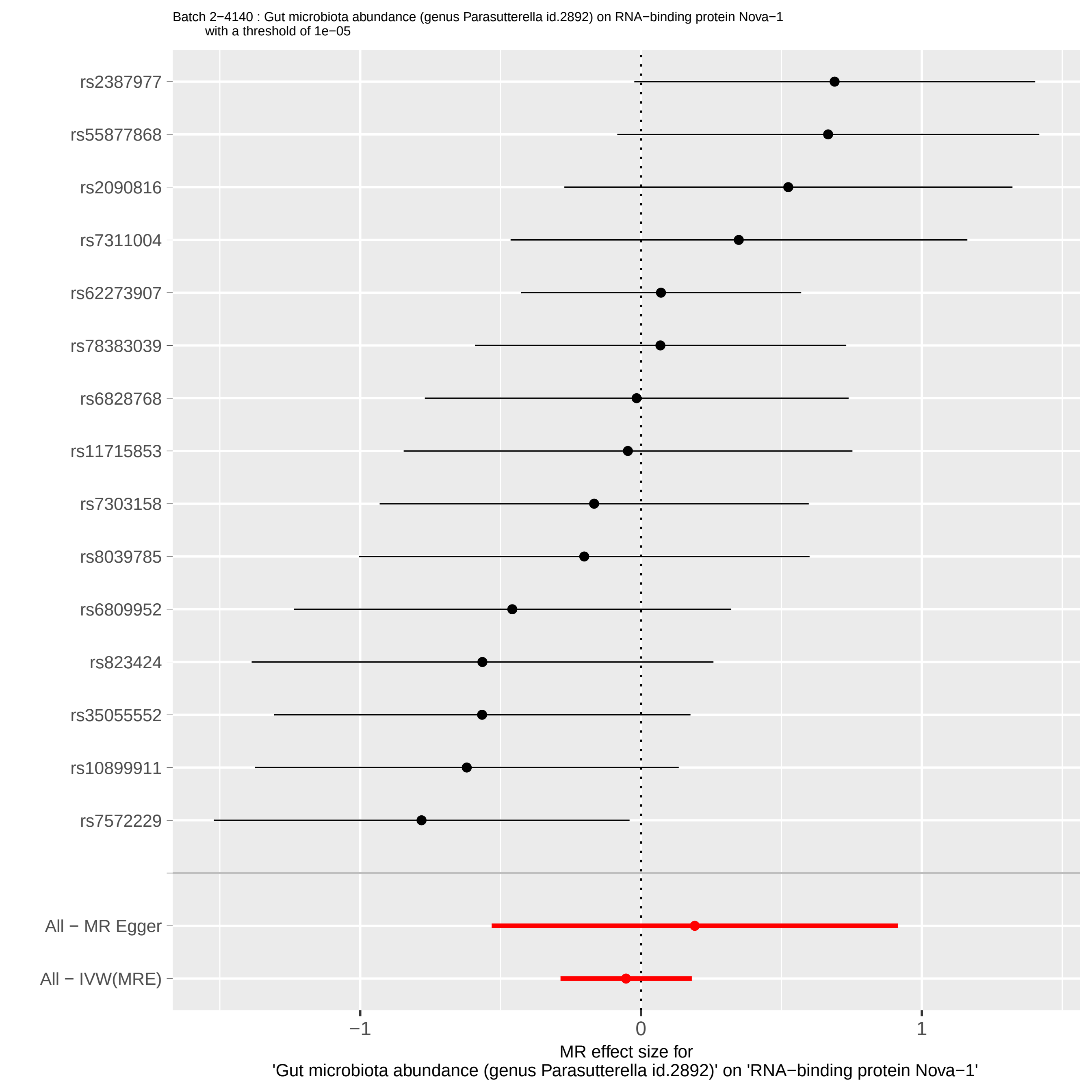

Batch 2−4140 : Gut microbiota abundance (genus Parasutterella id.2892) on RNA−binding protein Nova−1
with a threshold of 1e−05
rs2387977
rs55877868
rs2090816
rs7311004
rs62273907
rs78383039
rs6828768
rs11715853
rs7303158
rs8039785
rs6809952
rs823424
rs35055552
rs10899911
rs7572229
All − MR Egger
All − IVW(MRE)
−1
0
1
MR effect size for
'Gut microbiota abundance (genus Parasutterella id.2892)' on 'RNA−binding protein Nova−1'

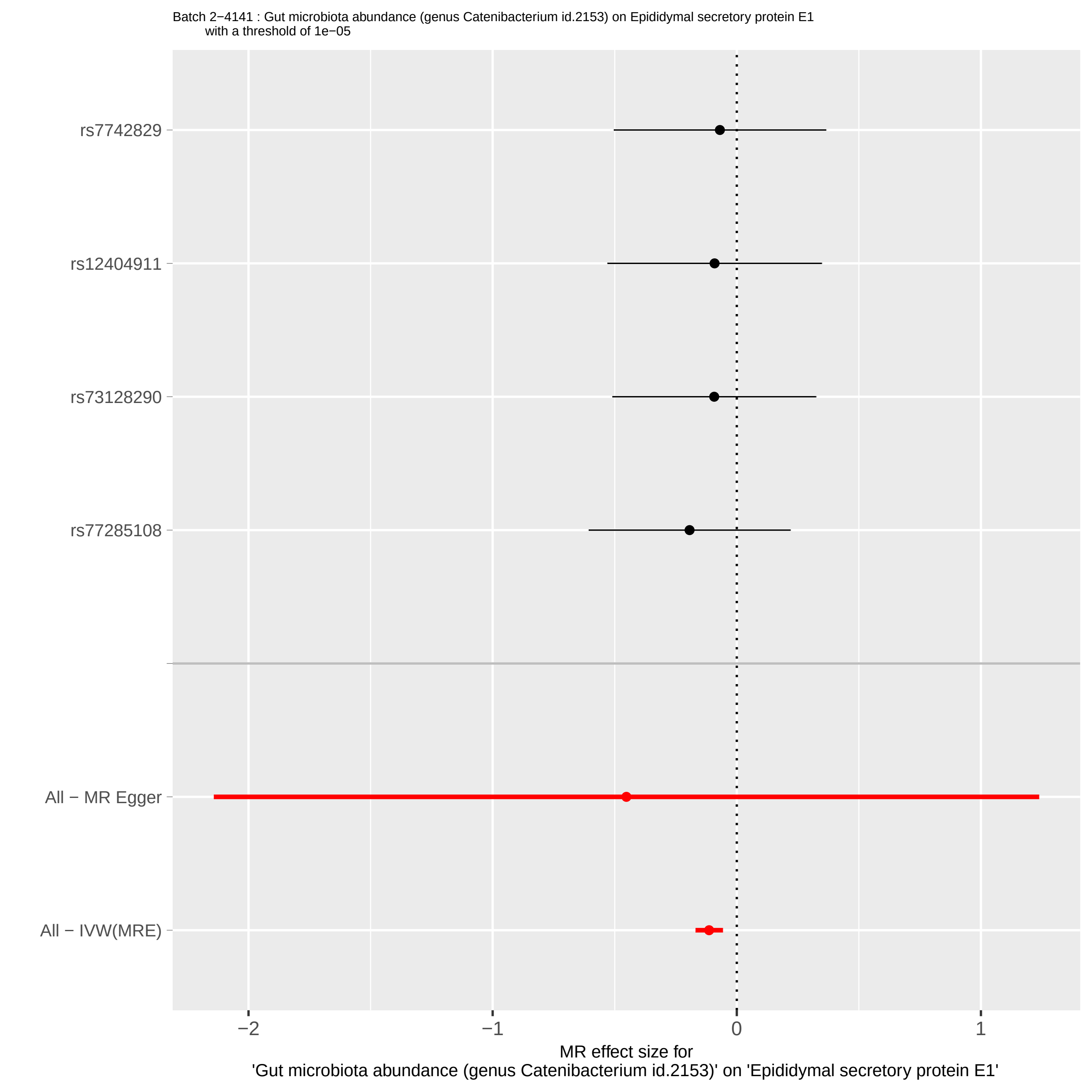

Batch 2−4141 : Gut microbiota abundance (genus Catenibacterium id.2153) on Epididymal secretory protein E1
with a threshold of 1e−05
rs7742829
rs12404911
rs73128290
rs77285108
All − MR Egger
All − IVW(MRE)
−2
−1
0
1
MR effect size for
'Gut microbiota abundance (genus Catenibacterium id.2153)' on 'Epididymal secretory protein E1'

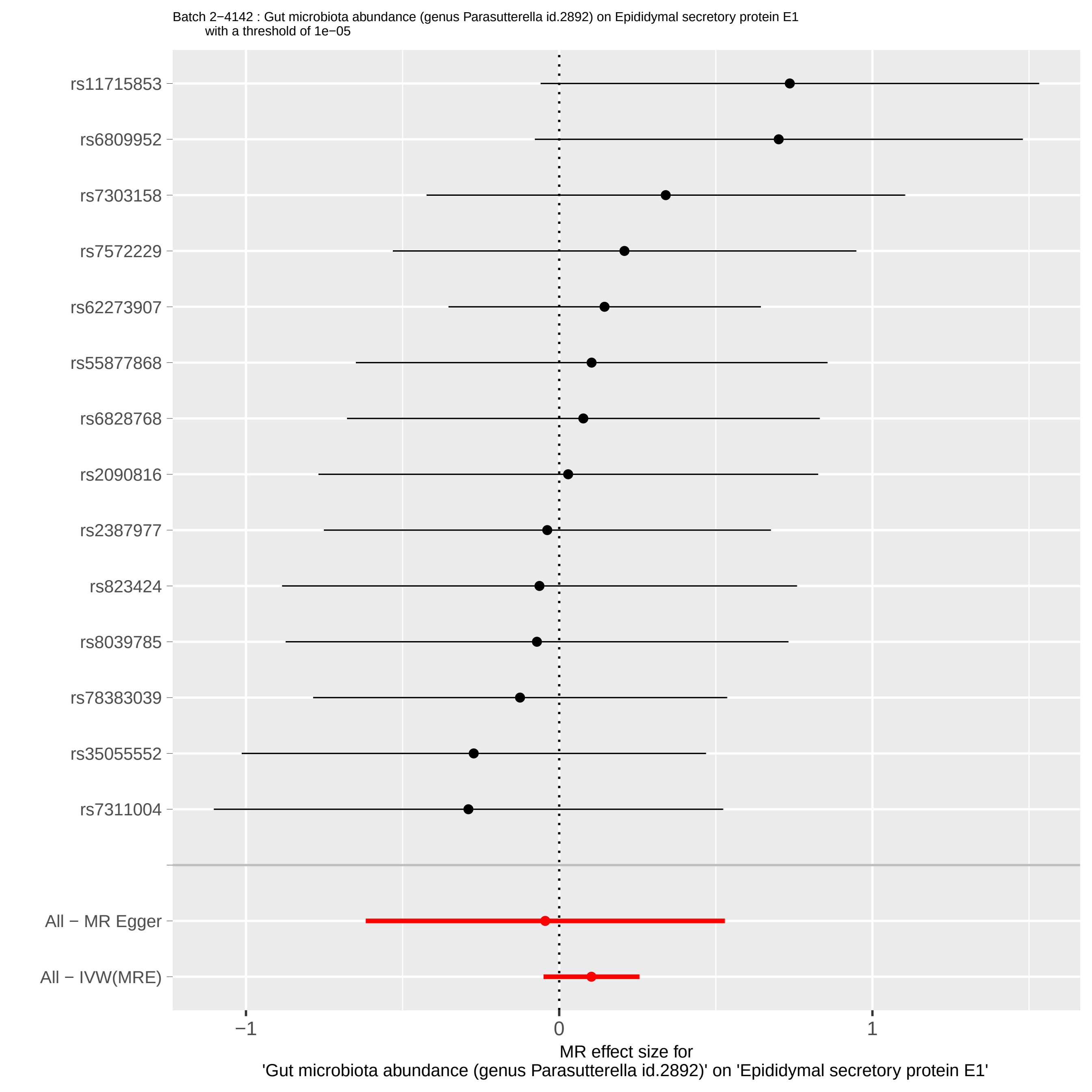

Batch 2−4142 : Gut microbiota abundance (genus Parasutterella id.2892) on Epididymal secretory protein E1
with a threshold of 1e−05
rs11715853
rs6809952
rs7303158
rs7572229
rs62273907
rs55877868
rs6828768
rs2090816
rs2387977
rs823424
rs8039785
rs78383039
rs35055552
rs7311004
All − MR Egger
All − IVW(MRE)
−1
0
1
MR effect size for
'Gut microbiota abundance (genus Parasutterella id.2892)' on 'Epididymal secretory protein E1'

Batch 2−4143 : Gut microbiota abundance (genus Catenibacterium id.2153) on Neural proliferation differentiation and control protein 1
with a threshold of 1e−05

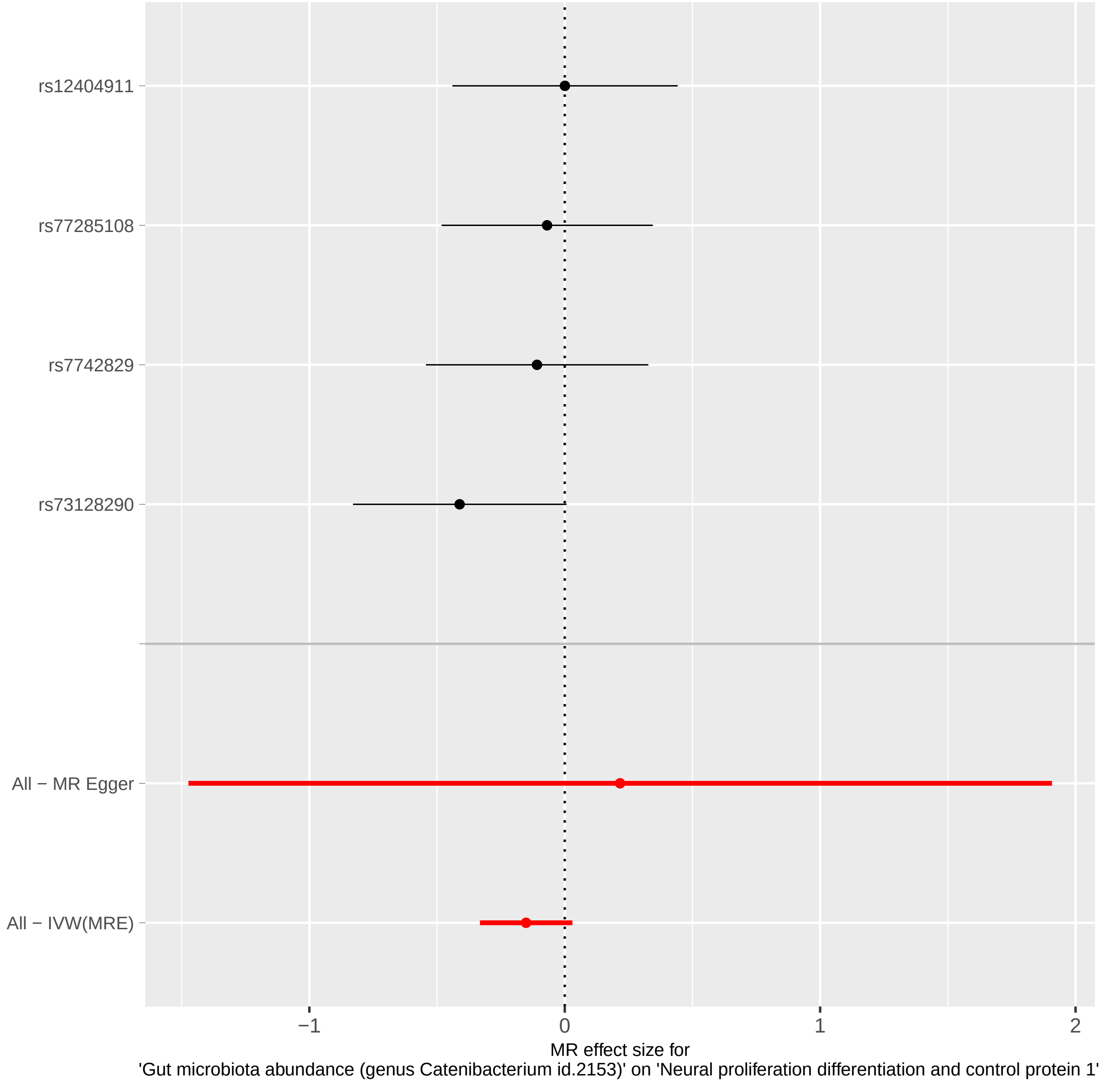

Batch 2−4144 : Gut microbiota abundance (genus Parasutterella id.2892) on Neural proliferation differentiation and control protein 1 with a threshold of 1e−05

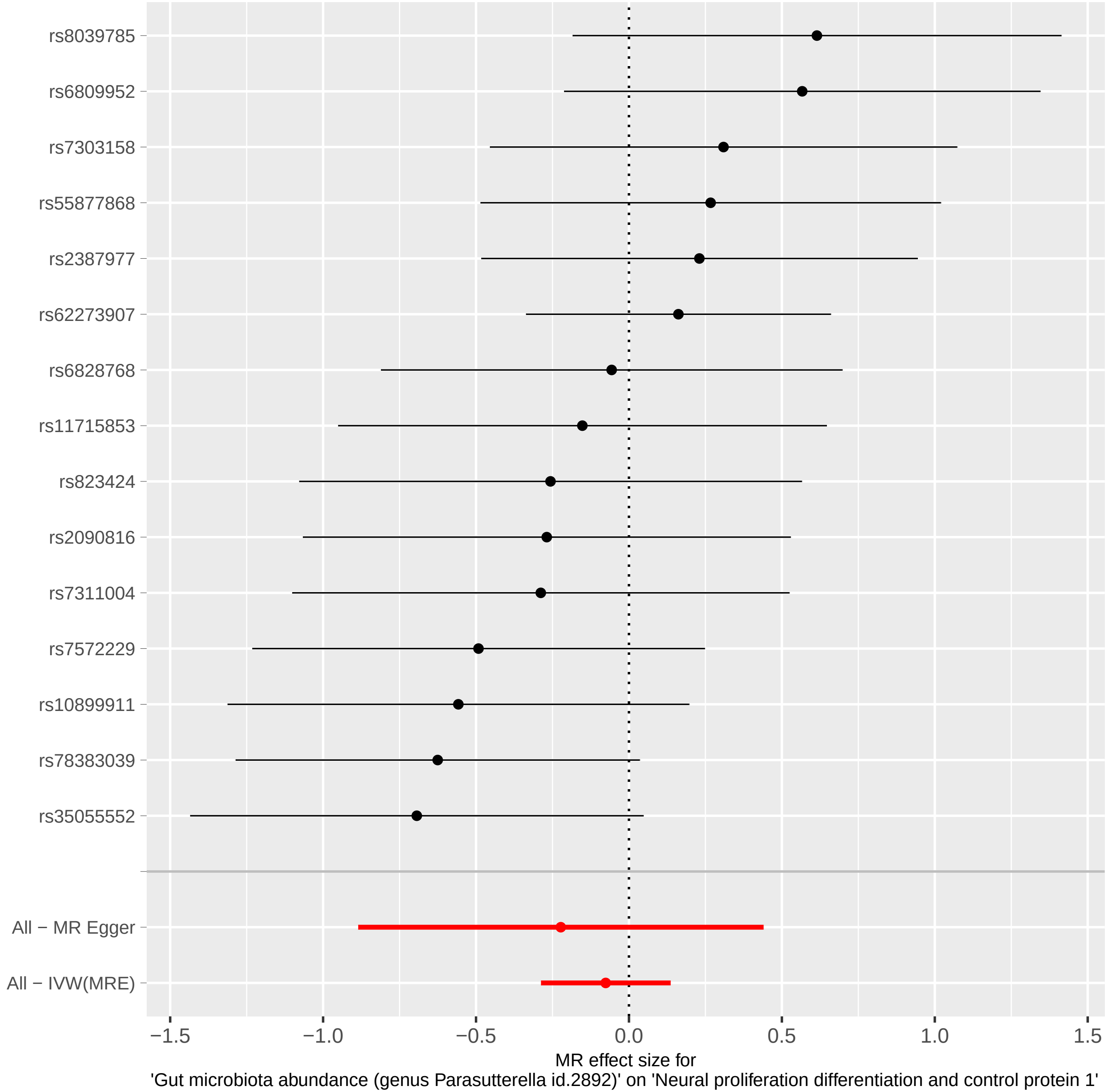

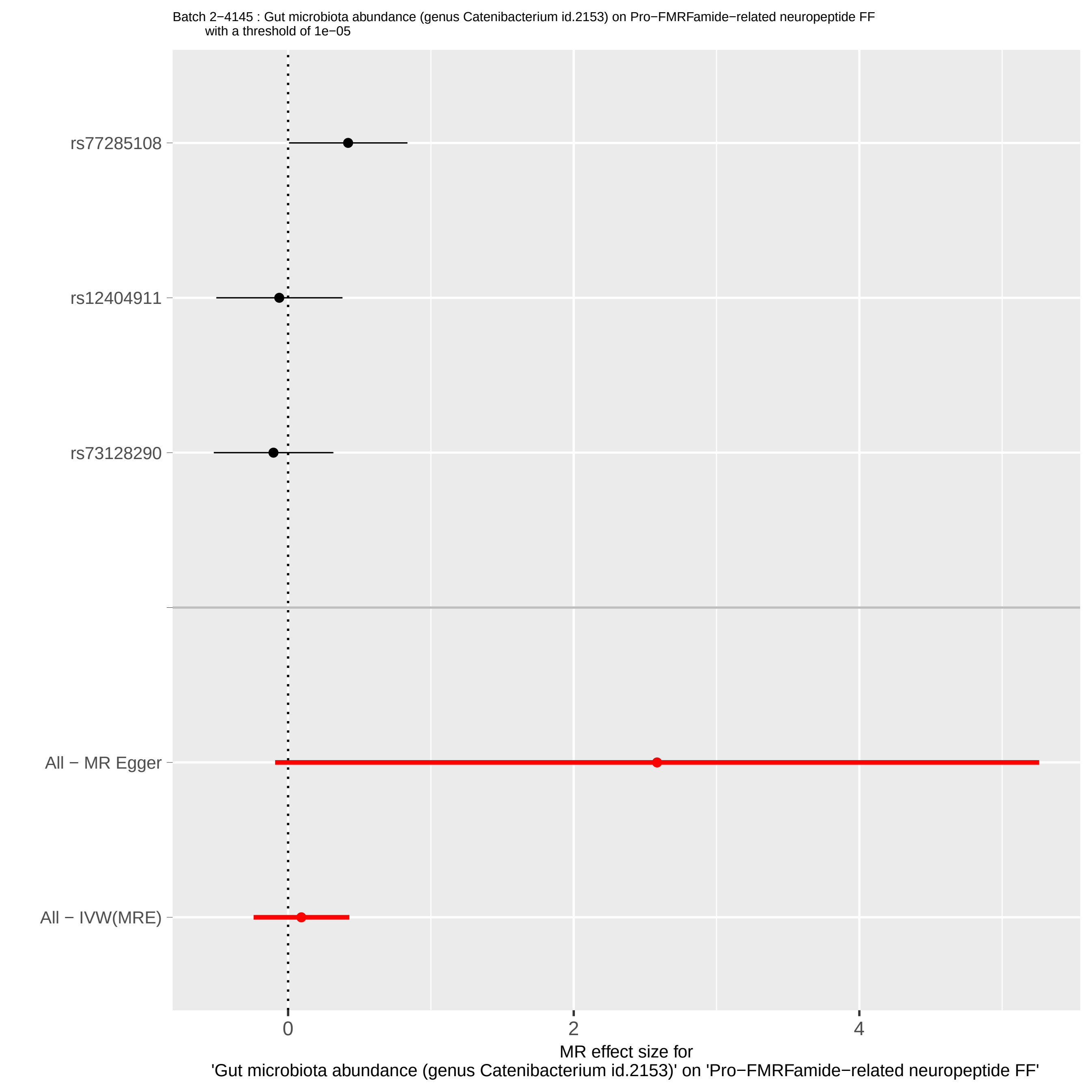
Batch 2−4145 : Gut microbiota abundance (genus Catenibacterium id.2153) on Pro−FMRFamide−related neuropeptide FF
with a threshold of 1e−05
rs77285108
rs12404911
rs73128290
All − MR Egger
All − IVW(MRE)
0
2
4
MR effect size for
'Gut microbiota abundance (genus Catenibacterium id.2153)' on 'Pro−FMRFamide−related neuropeptide FF'

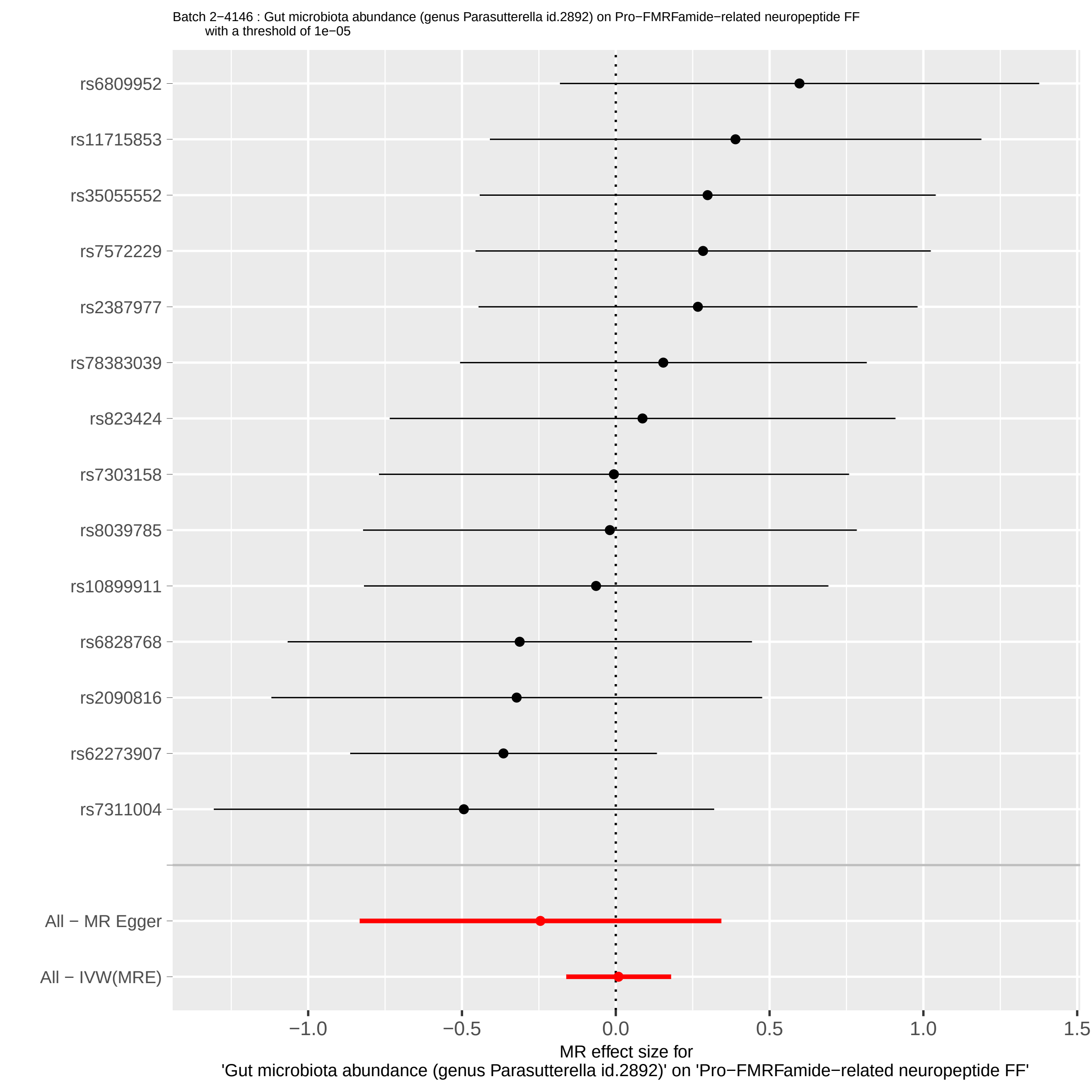
Batch 2−4146 : Gut microbiota abundance (genus Parasutterella id.2892) on Pro−FMRFamide−related neuropeptide FF
with a threshold of 1e−05
rs6809952
rs11715853
rs35055552
rs7572229
rs2387977
rs78383039
rs823424
rs7303158
rs8039785
rs10899911
rs6828768
rs2090816
rs62273907
rs7311004
All − MR Egger
All − IVW(MRE)
−1.0
−0.5
0.0
0.5
1.0
1.5
MR effect size for
'Gut microbiota abundance (genus Parasutterella id.2892)' on 'Pro−FMRFamide−related neuropeptide FF'

Batch 2−4147 : Gut microbiota abundance (genus Catenibacterium id.2153) on Nuclear pore complex−interacting protein family member B3
with a threshold of 1e−05

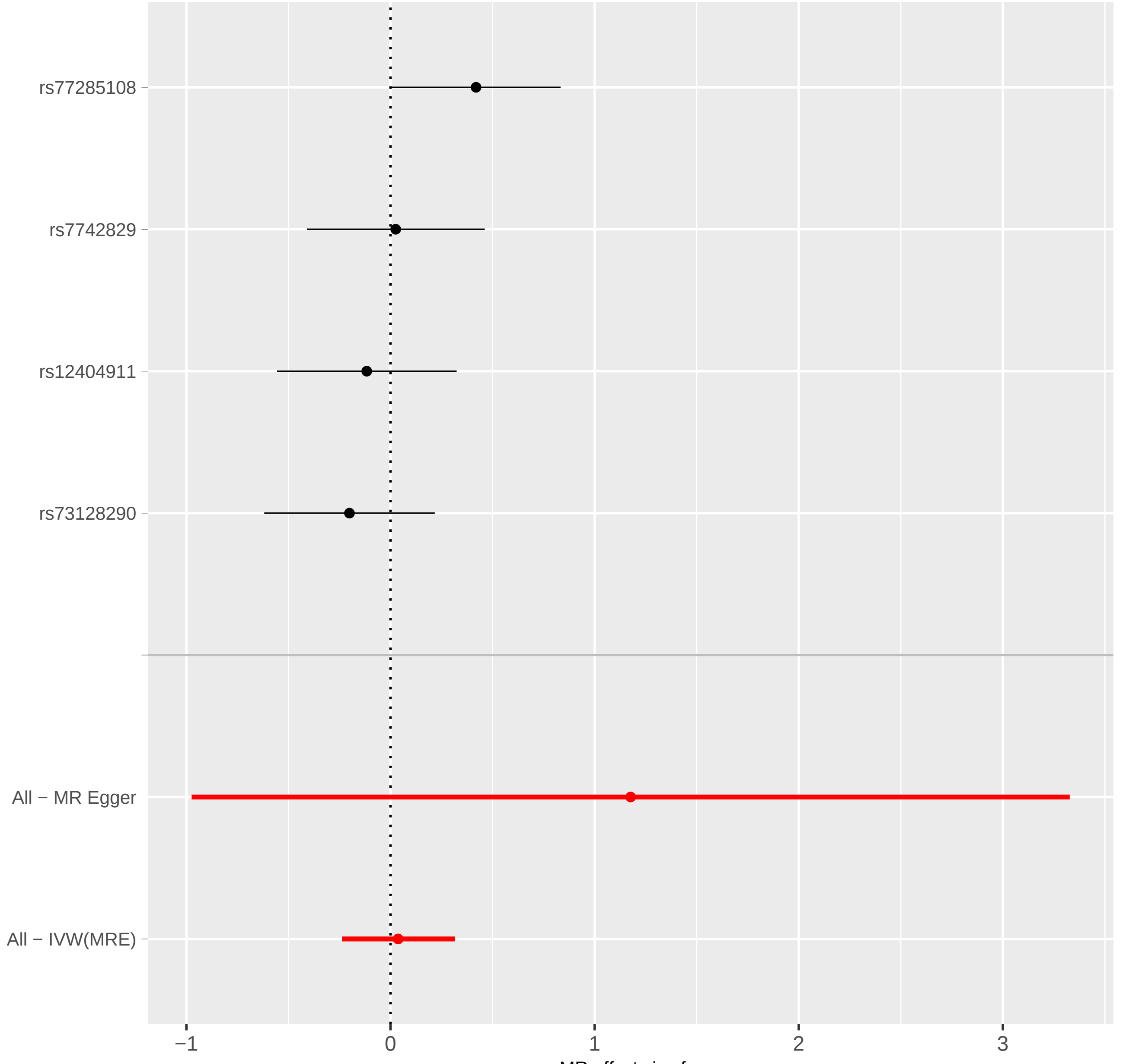

MR effect size for
'Gut microbiota abundance (genus Catenibacterium id.2153)' on 'Nuclear pore complex−interacting protein family member B

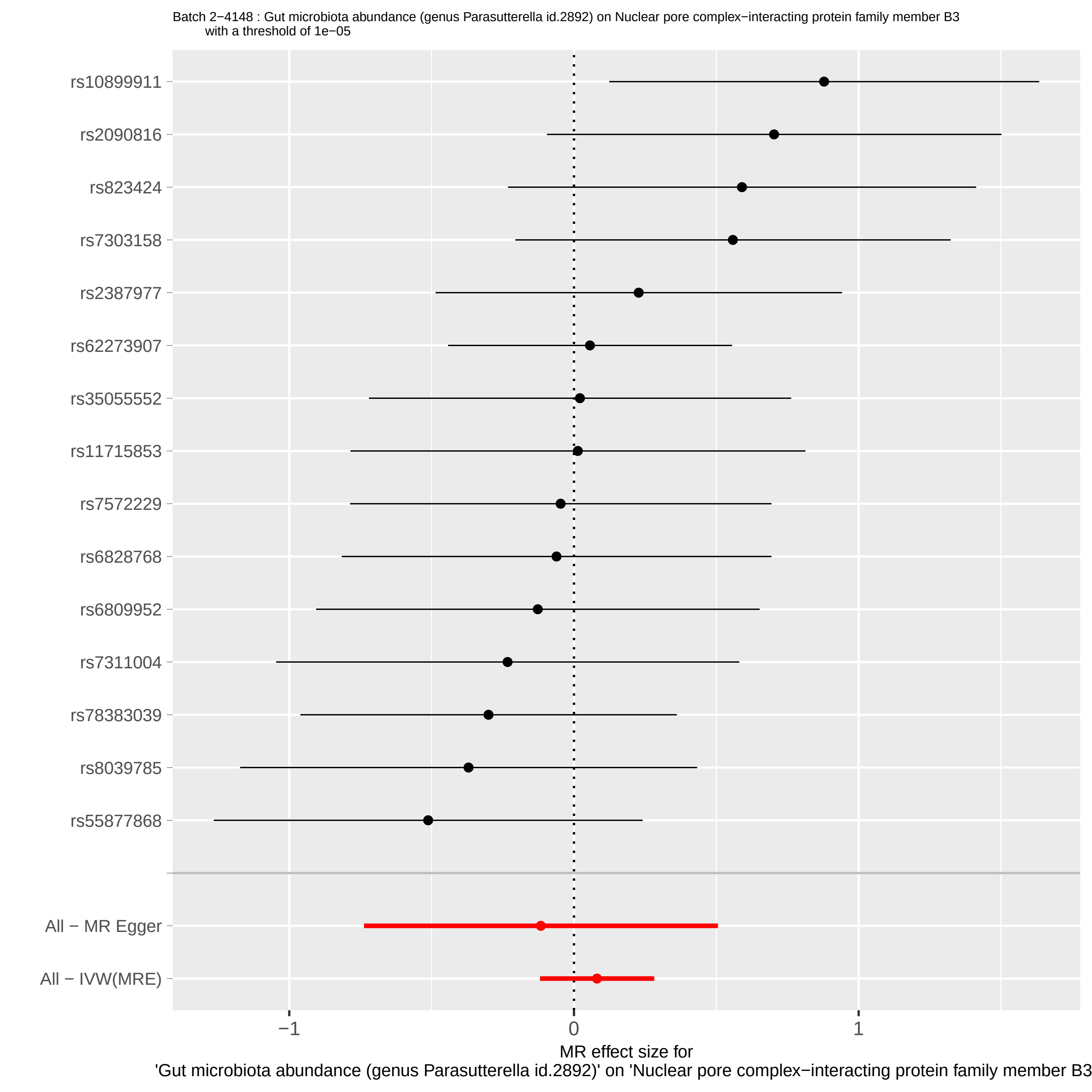
Batch 2−4148 : Gut microbiota abundance (genus Parasutterella id.2892) on Nuclear pore complex−interacting protein family member B3 with a threshold of 1e−05
rs10899911
rs2090816
rs823424
rs7303158
rs2387977
rs62273907
rs35055552
rs11715853
rs7572229
rs6828768
rs6809952
rs7311004
rs78383039
rs8039785
rs55877868
All − MR Egger
All − IVW(MRE)
−1
0
1
MR effect size for
'Gut microbiota abundance (genus Parasutterella id.2892)' on 'Nuclear pore complex−interacting protein family member B3

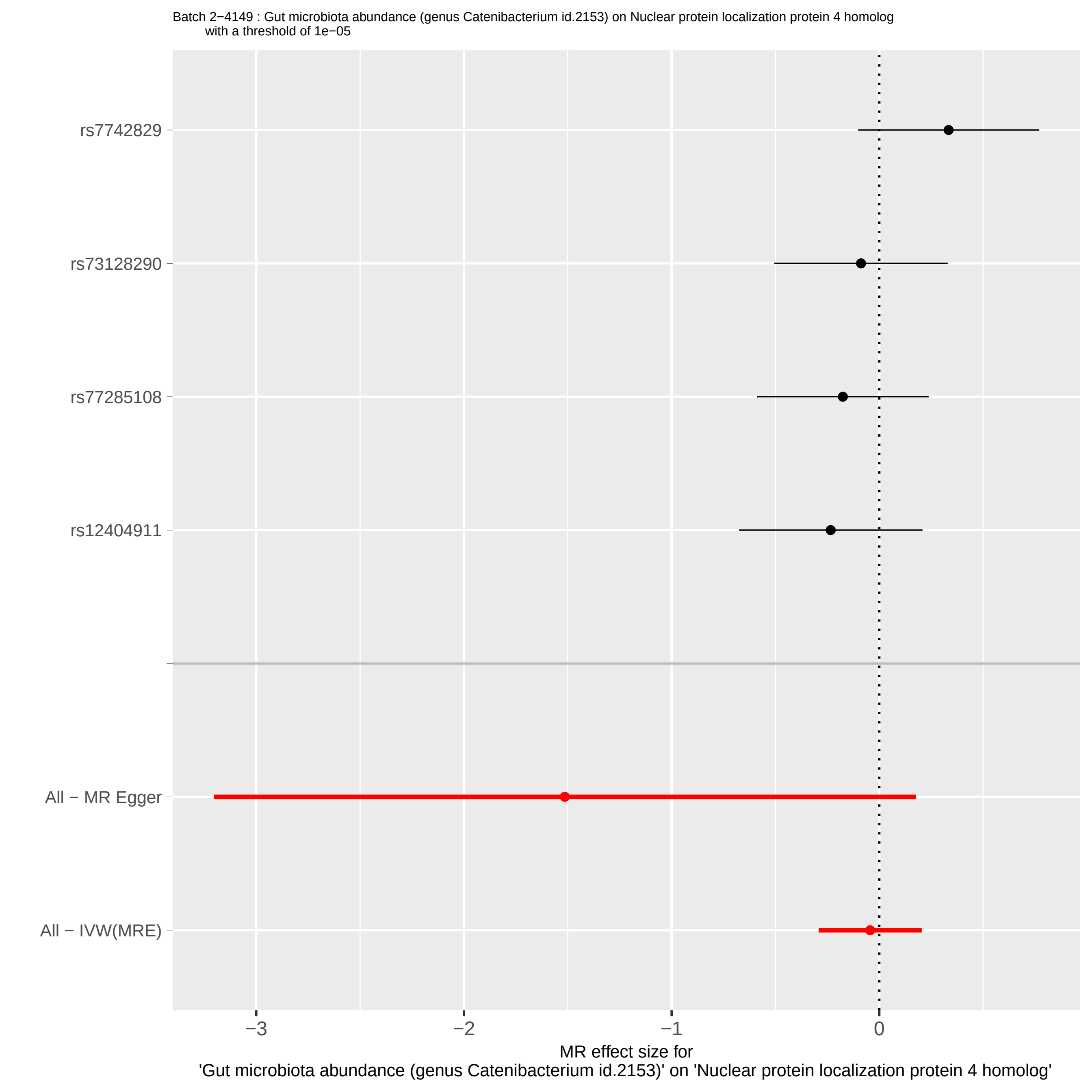
Batch 2−4149 : Gut microbiota abundance (genus Catenibacterium id.2153) on Nuclear protein localization protein 4 homolog
with a threshold of 1e−05
rs7742829
rs73128290
rs77285108
rs12404911
All − MR Egger
All − IVW(MRE)
−3
−2
−1
0
MR effect size for
'Gut microbiota abundance (genus Catenibacterium id.2153)' on 'Nuclear protein localization protein 4 homolog'

Batch 2−4150 : Gut microbiota abundance (genus Parasutterella id.2892) on Nuclear protein localization protein 4 homolog
with a threshold of 1e−05

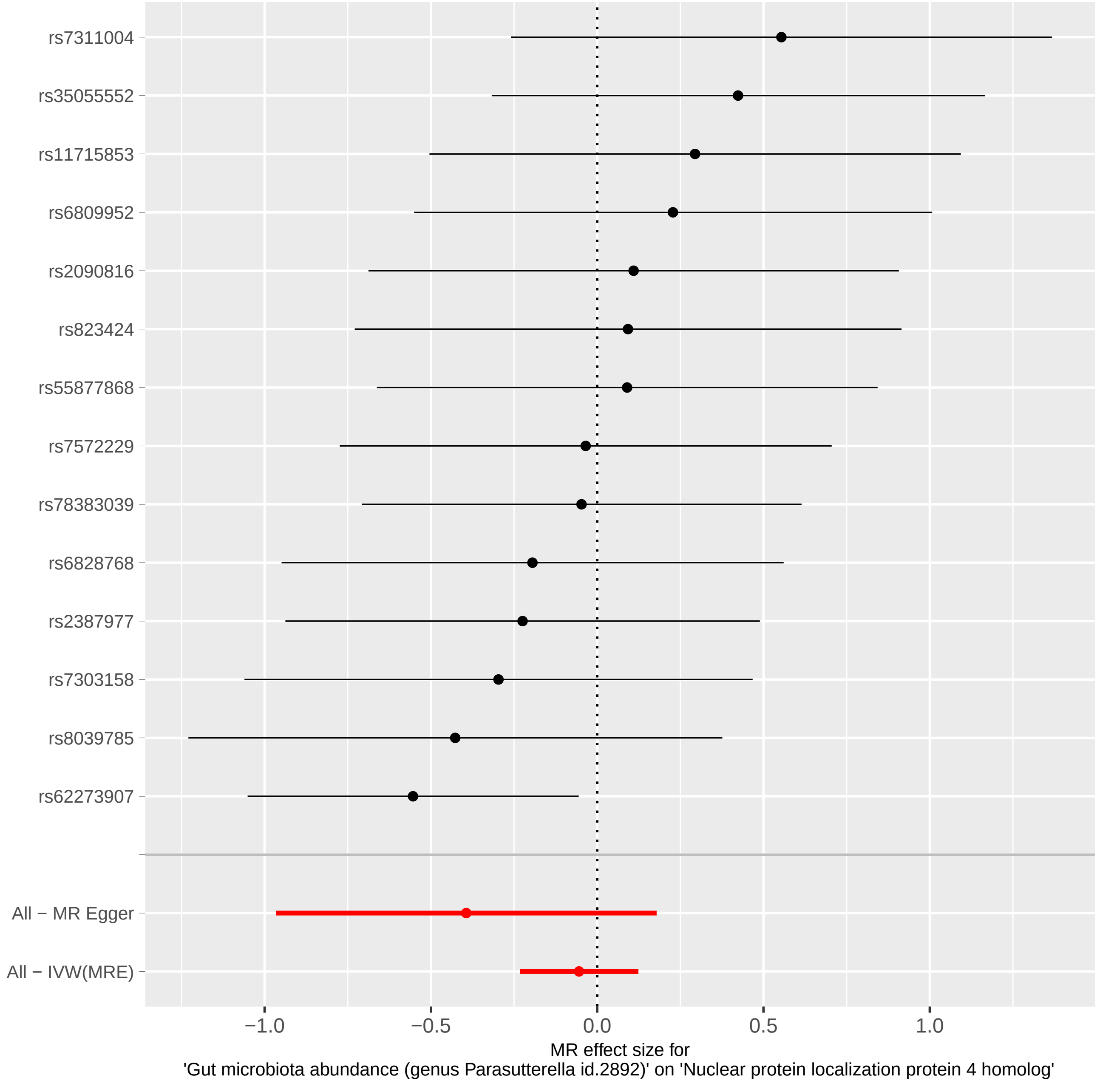

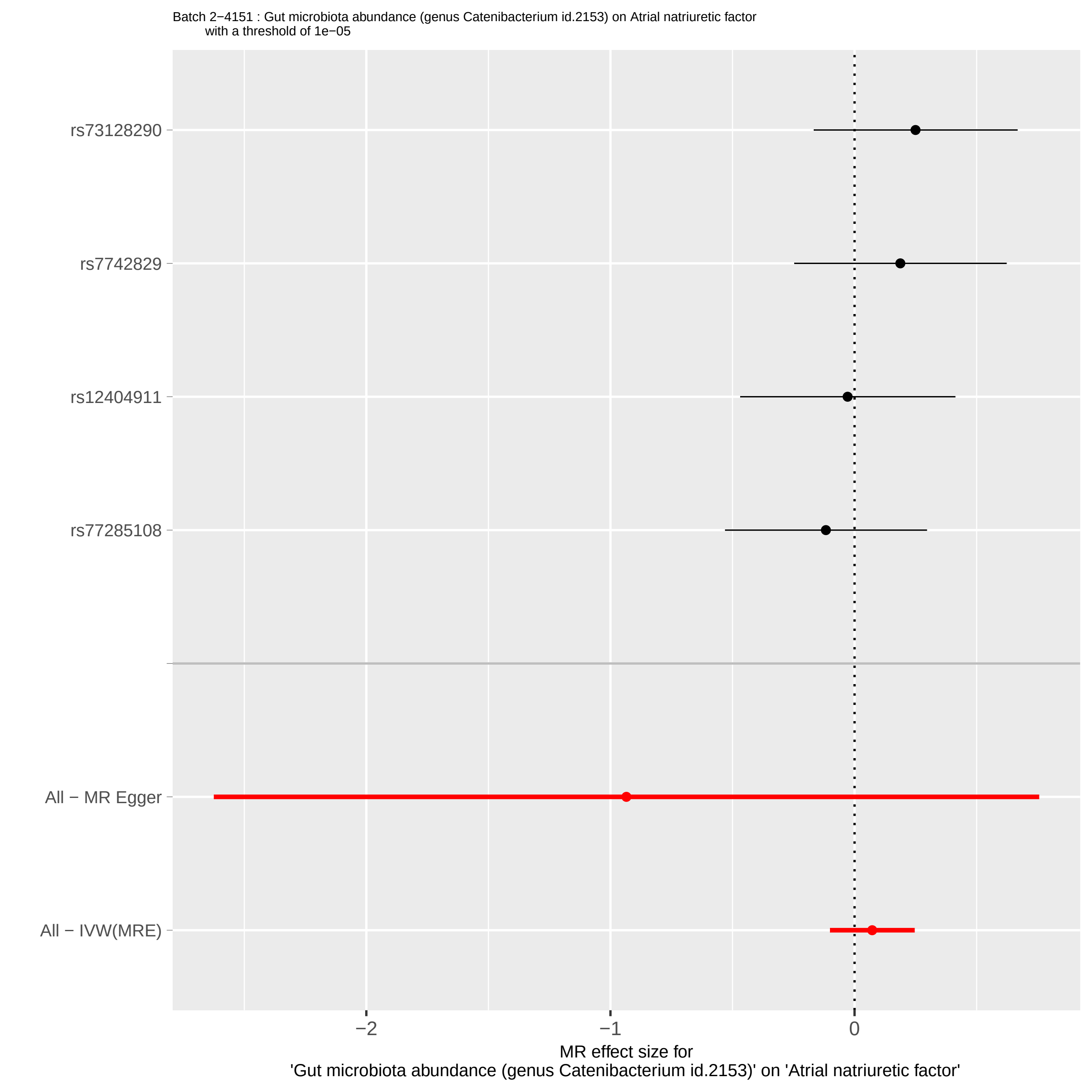

Batch 2−4151 : Gut microbiota abundance (genus Catenibacterium id.2153) on Atrial natriuretic factor
with a threshold of 1e−05
rs73128290
rs7742829
rs12404911
rs77285108
All − MR Egger
All − IVW(MRE)
−2
−1
0
MR effect size for
'Gut microbiota abundance (genus Catenibacterium id.2153)' on 'Atrial natriuretic factor'

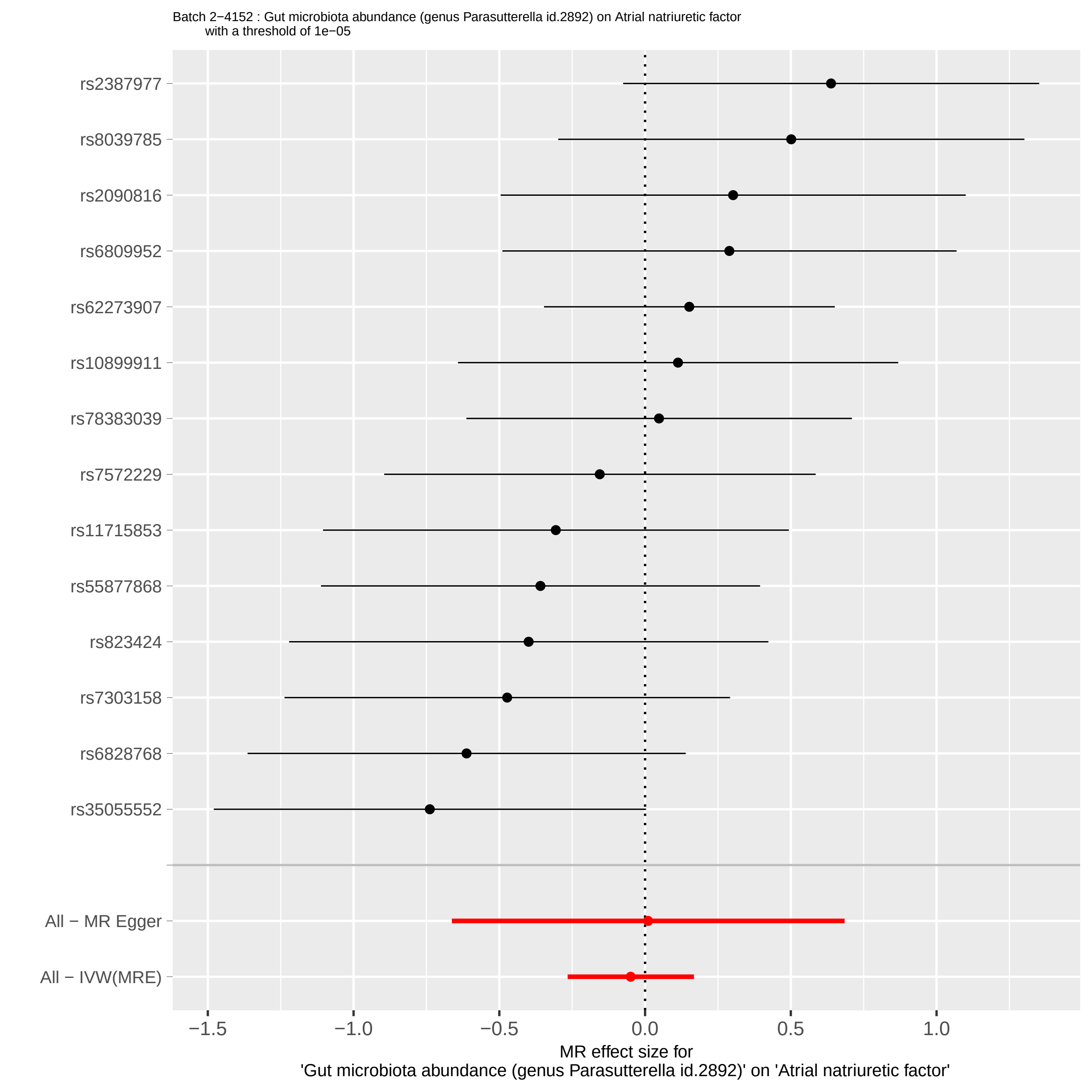

Batch 2−4152 : Gut microbiota abundance (genus Parasutterella id.2892) on Atrial natriuretic factor
with a threshold of 1e−05
rs2387977
rs8039785
rs2090816
rs6809952
rs62273907
rs10899911
rs78383039
rs7572229
rs11715853
rs55877868
rs823424
rs7303158
rs6828768
rs35055552
All − MR Egger
All − IVW(MRE)
−1.5
−1.0
−0.5
0.0
0.5
1.0
MR effect size for
'Gut microbiota abundance (genus Parasutterella id.2892)' on 'Atrial natriuretic factor'

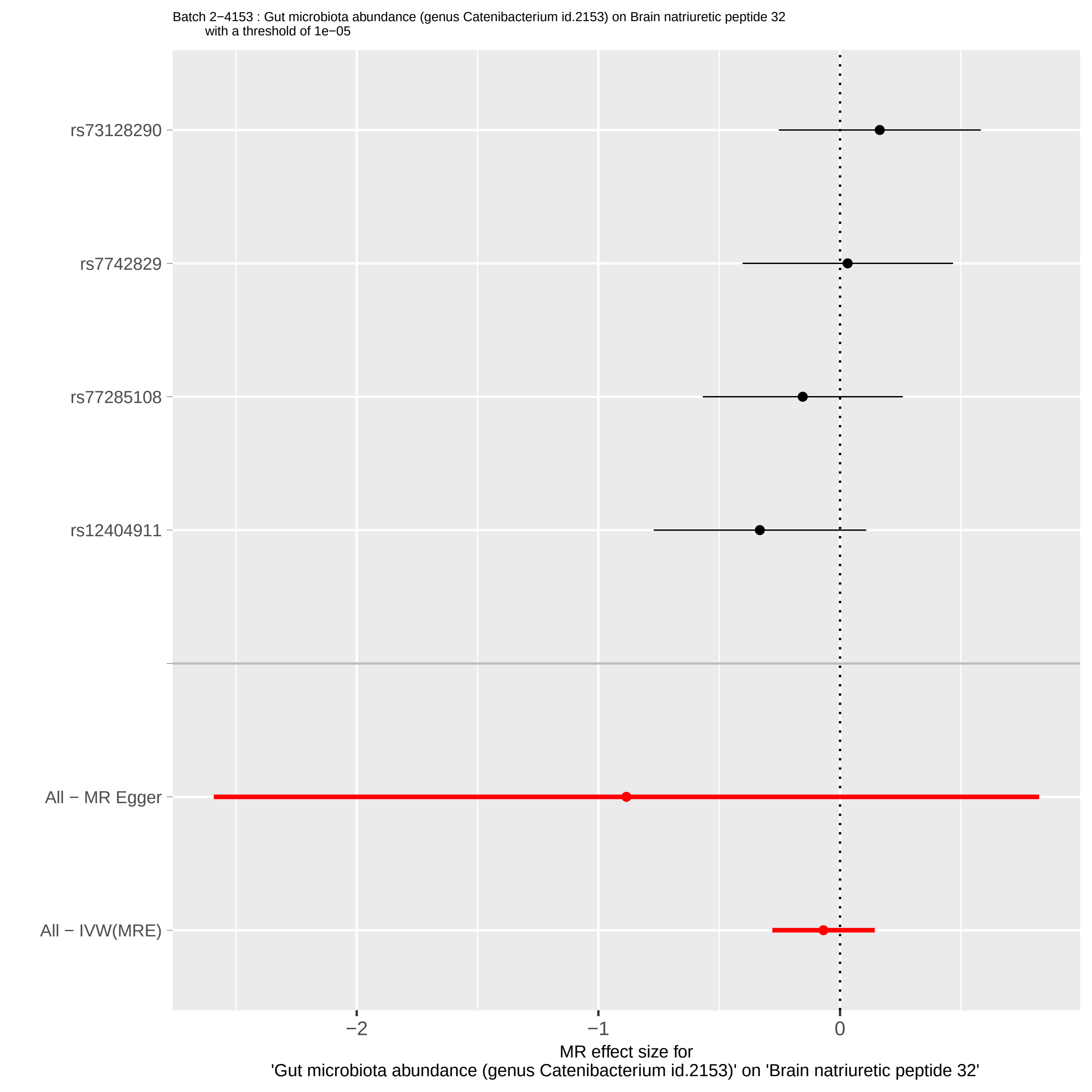
Batch 2−4153 : Gut microbiota abundance (genus Catenibacterium id.2153) on Brain natriuretic peptide 32
with a threshold of 1e−05
rs73128290
rs7742829
rs77285108
rs12404911
All − MR Egger
All − IVW(MRE)
−2
−1
0
MR effect size for
'Gut microbiota abundance (genus Catenibacterium id.2153)' on 'Brain natriuretic peptide 32'

Batch 2−4154 : Gut microbiota abundance (genus Parasutterella id.2892) on Brain natriuretic peptide 32
with a threshold of 1e−05

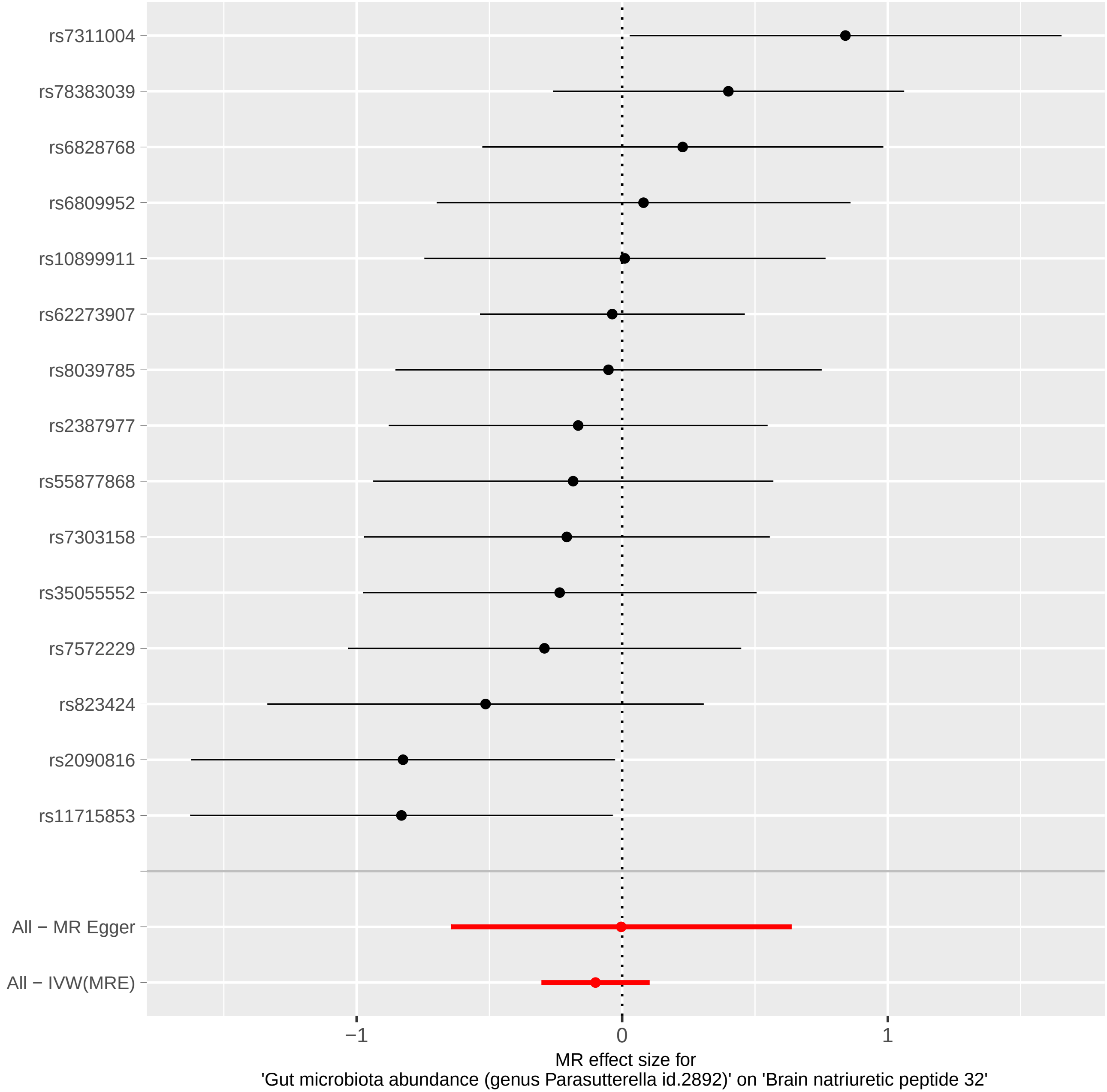

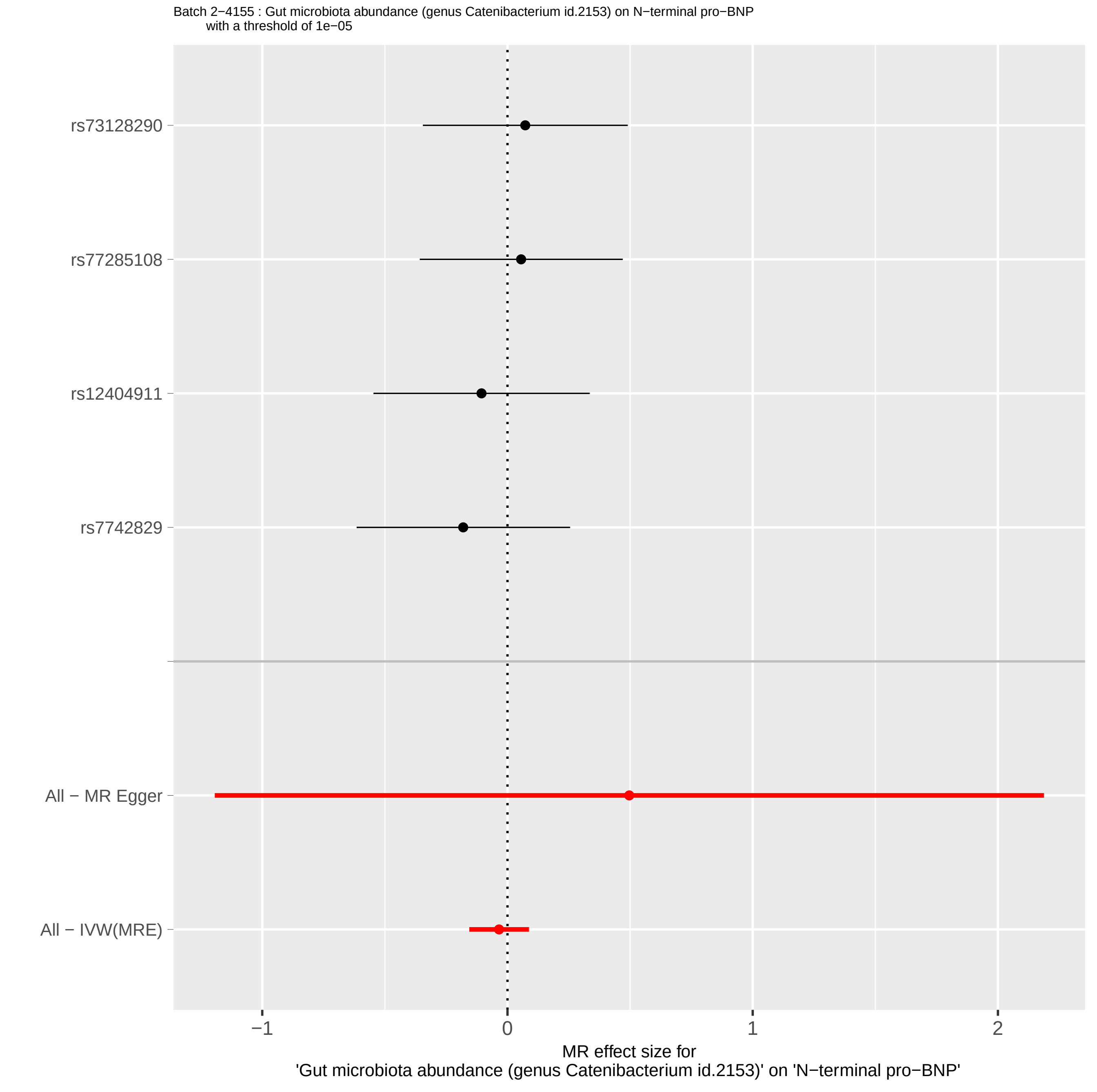

Batch 2−4155 : Gut microbiota abundance (genus Catenibacterium id.2153) on N−terminal pro−BNP
with a threshold of 1e−05
rs73128290
rs77285108
rs12404911
rs7742829
All − MR Egger
All − IVW(MRE)
−1
0
1
2
MR effect size for
'Gut microbiota abundance (genus Catenibacterium id.2153)' on 'N−terminal pro−BNP'

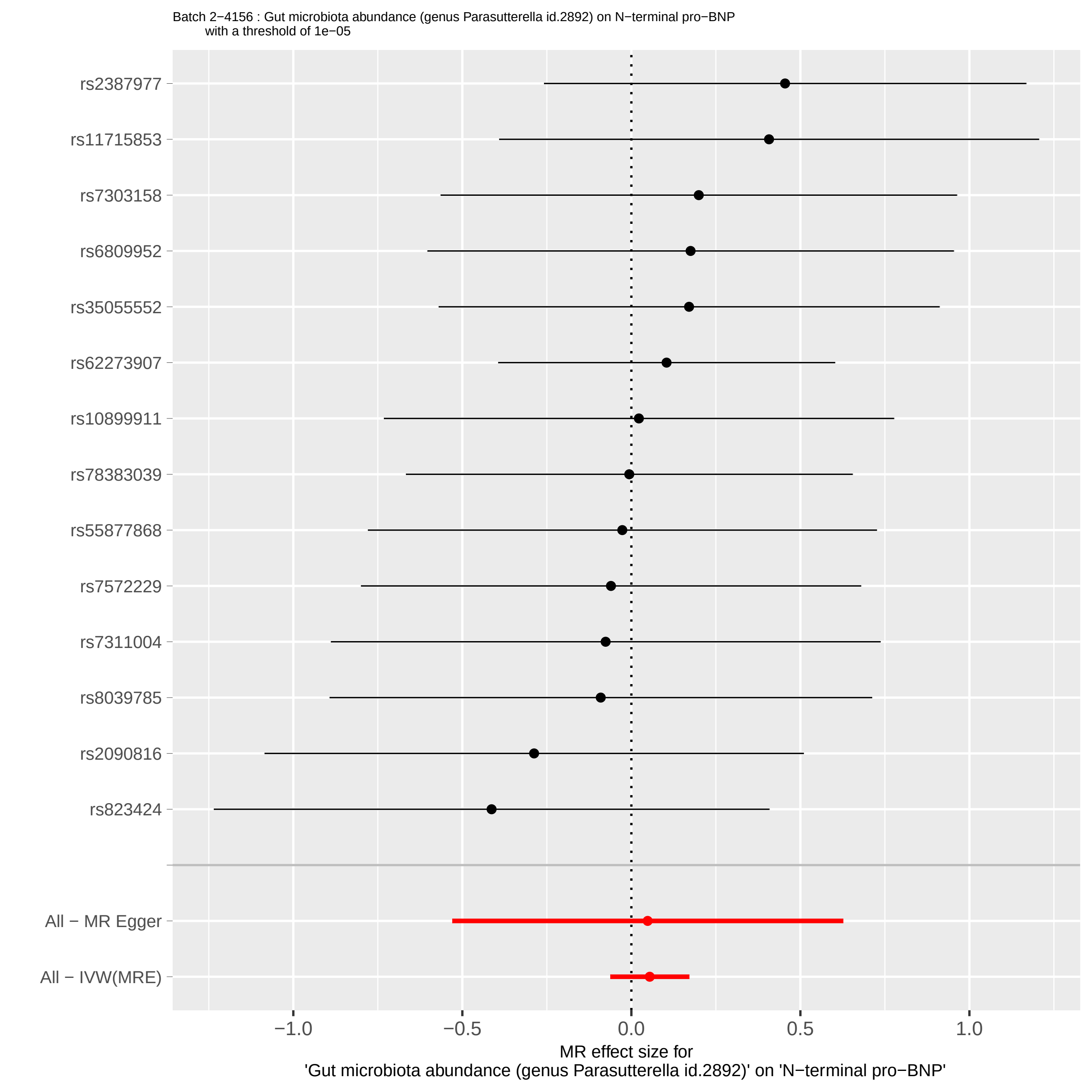

Batch 2−4156 : Gut microbiota abundance (genus Parasutterella id.2892) on N−terminal pro−BNP
with a threshold of 1e−05
rs2387977
rs11715853
rs7303158
rs6809952
rs35055552
rs62273907
rs10899911
rs78383039
rs55877868
rs7572229
rs7311004
rs8039785
rs2090816
rs823424
All − MR Egger
All − IVW(MRE)
−1.0
−0.5
0.0
0.5
1.0
MR effect size for
'Gut microbiota abundance (genus Parasutterella id.2892)' on 'N−terminal pro−BNP'

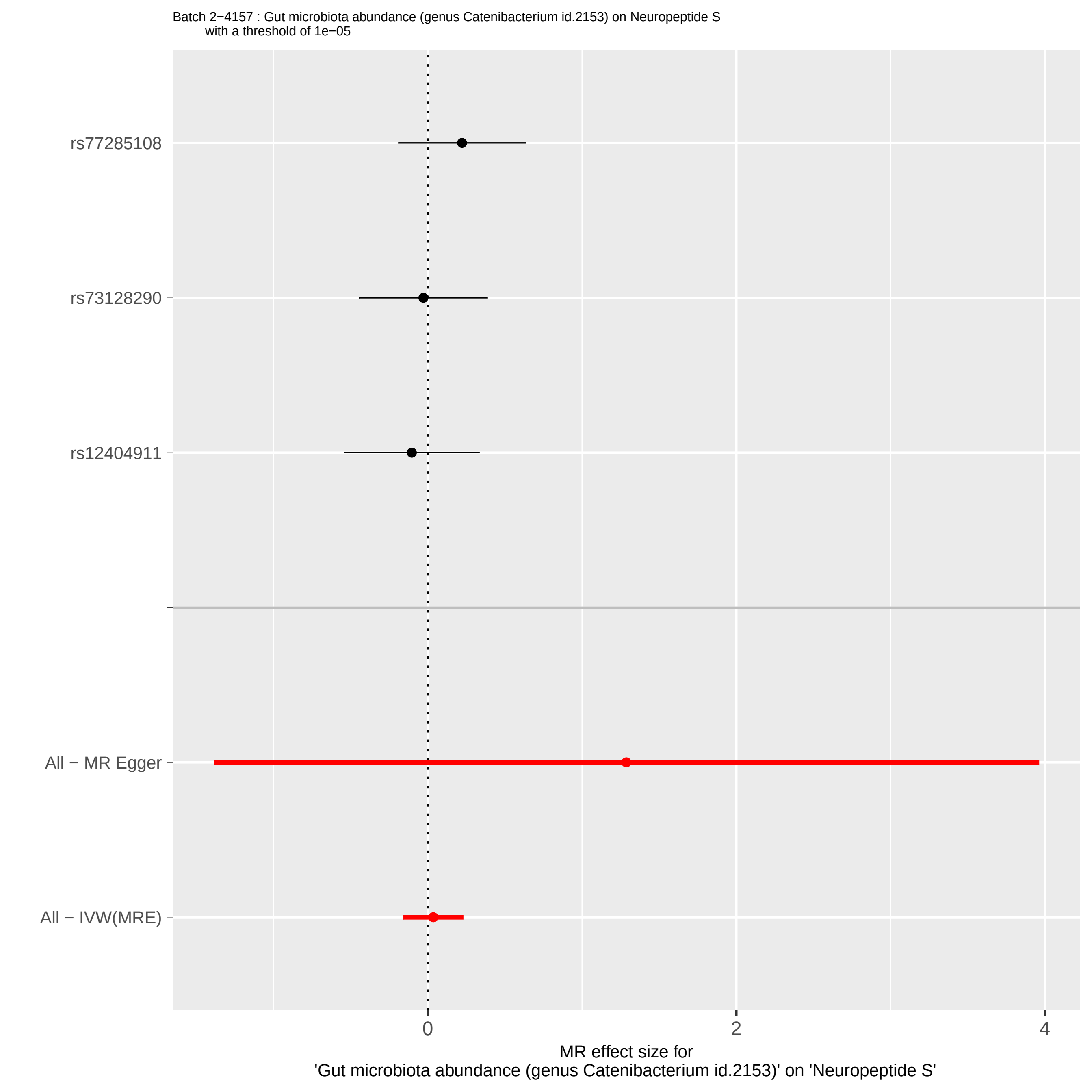

Batch 2−4157 : Gut microbiota abundance (genus Catenibacterium id.2153) on Neuropeptide S
with a threshold of 1e−05
rs77285108
rs73128290
rs12404911
All − MR Egger
All − IVW(MRE)
0
2
4
MR effect size for
'Gut microbiota abundance (genus Catenibacterium id.2153)' on 'Neuropeptide S'

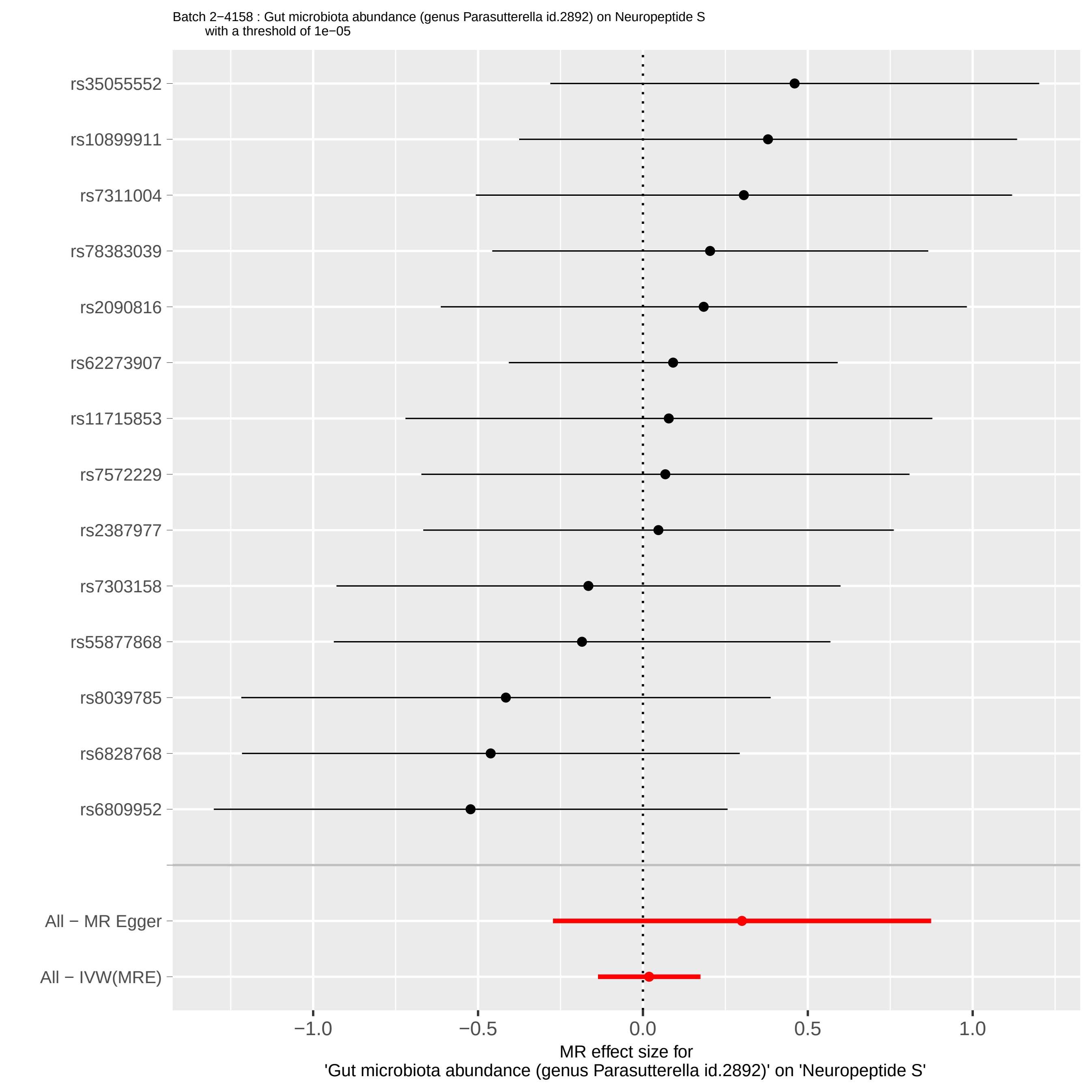

Batch 2−4158 : Gut microbiota abundance (genus Parasutterella id.2892) on Neuropeptide S
with a threshold of 1e−05
rs35055552
rs10899911
rs7311004
rs78383039
rs2090816
rs62273907
rs11715853
rs7572229
rs2387977
rs7303158
rs55877868
rs8039785
rs6828768
rs6809952
All − MR Egger
All − IVW(MRE)
−1.0
−0.5
0.0
0.5
1.0
MR effect size for
'Gut microbiota abundance (genus Parasutterella id.2892)' on 'Neuropeptide S'

Batch 2−4159 : Gut microbiota abundance (genus Catenibacterium id.2153) on Neuronal pentraxin−1
with a threshold of 1e−05

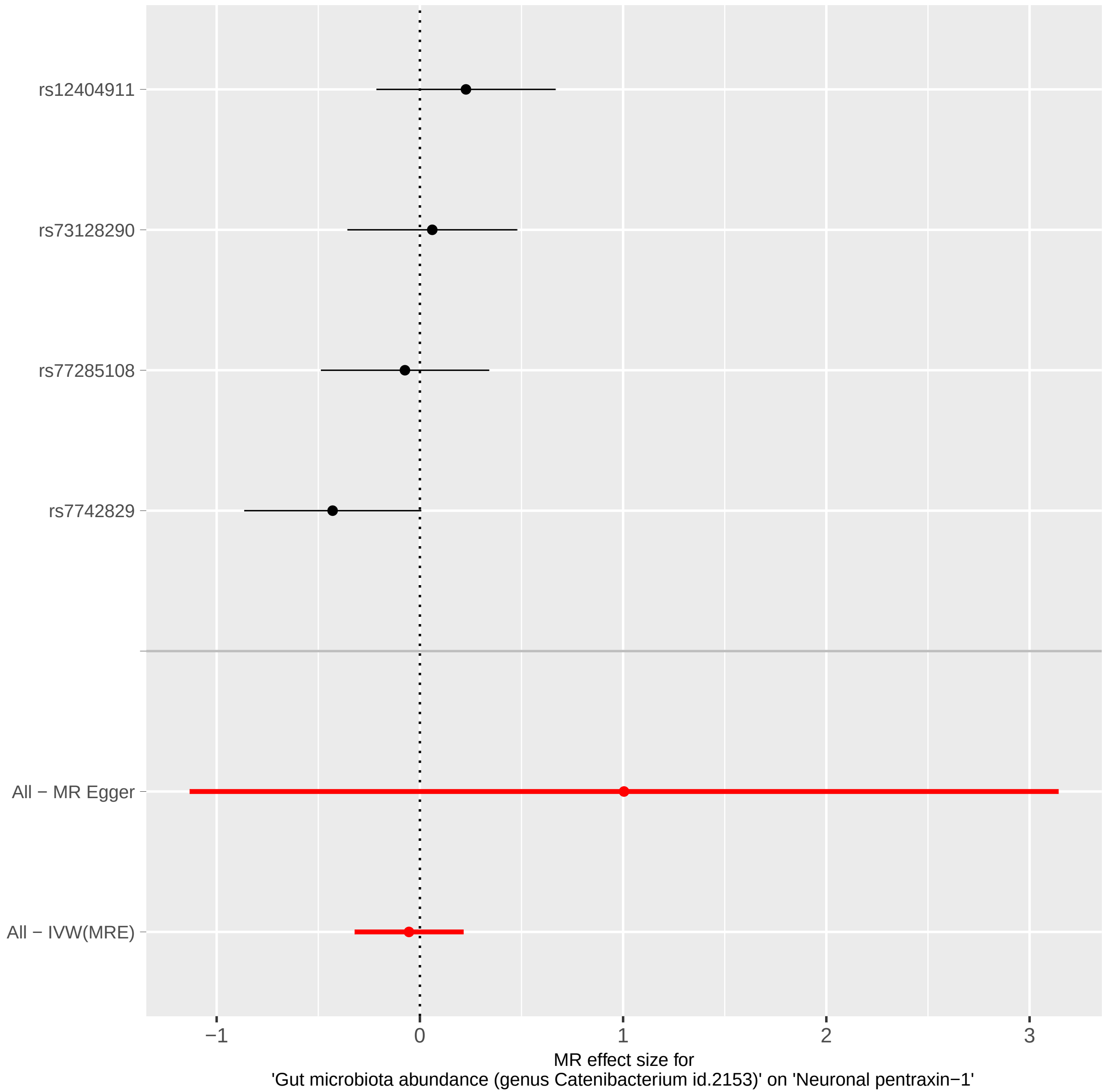

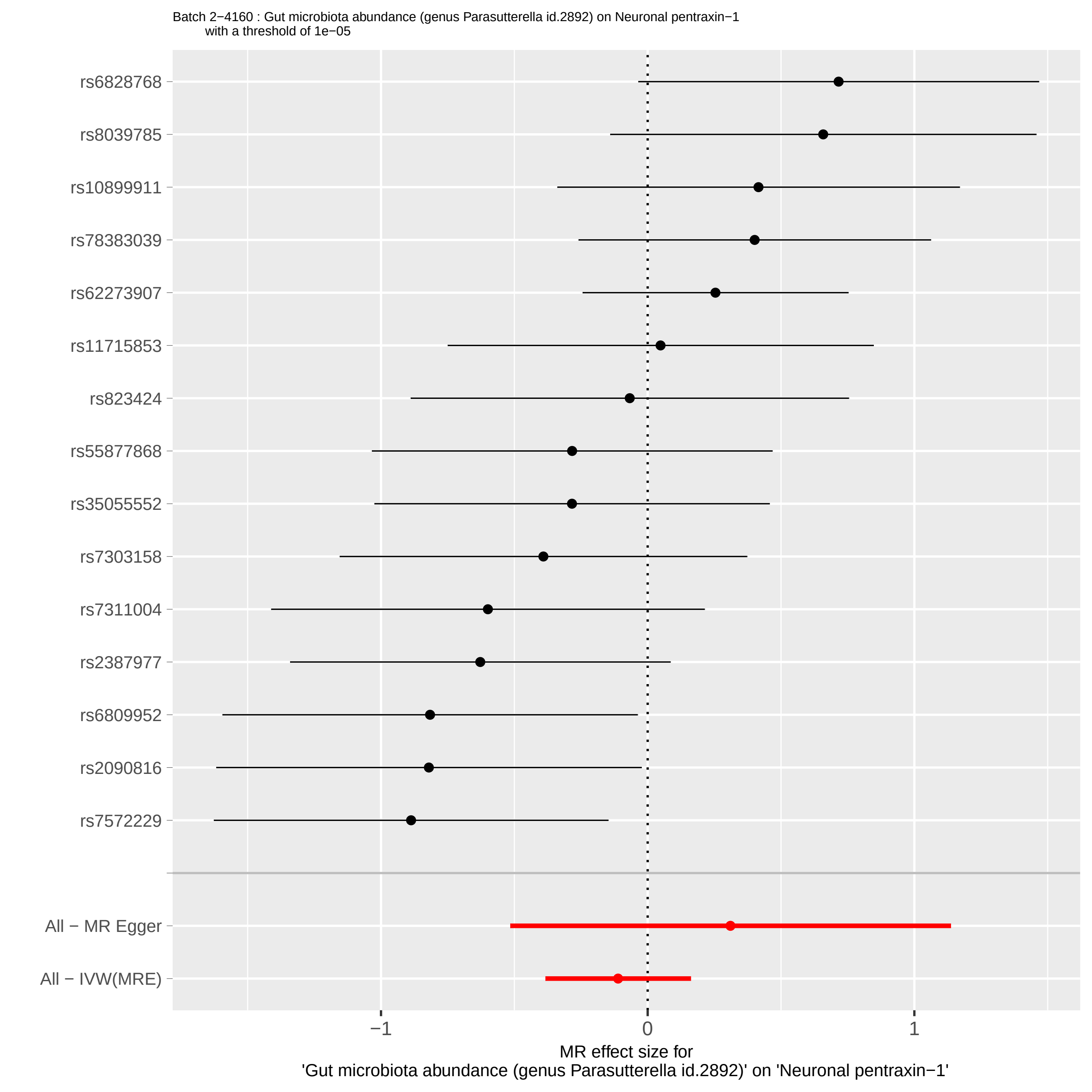
Batch 2−4160 : Gut microbiota abundance (genus Parasutterella id.2892) on Neuronal pentraxin−1
with a threshold of 1e−05
rs6828768
rs8039785
rs10899911
rs78383039
rs62273907
rs11715853
rs823424
rs55877868
rs35055552
rs7303158
rs7311004
rs2387977
rs6809952
rs2090816
rs7572229
All − MR Egger
All − IVW(MRE)
−1
0
1
MR effect size for
'Gut microbiota abundance (genus Parasutterella id.2892)' on 'Neuronal pentraxin−1'

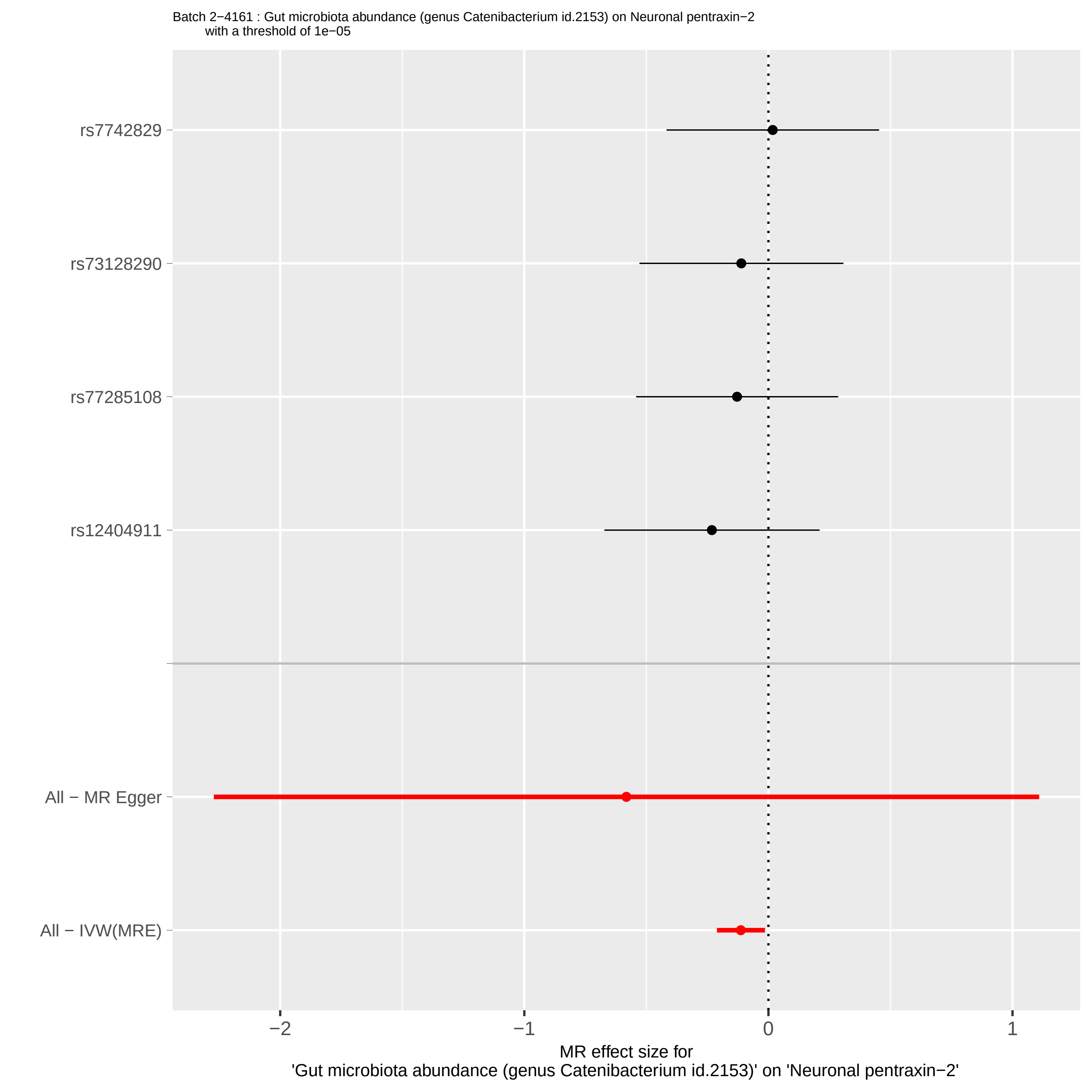

Batch 2−4161 : Gut microbiota abundance (genus Catenibacterium id.2153) on Neuronal pentraxin−2
with a threshold of 1e−05
rs7742829
rs73128290
rs77285108
rs12404911
All − MR Egger
All − IVW(MRE)
−2
−1
0
1
MR effect size for
'Gut microbiota abundance (genus Catenibacterium id.2153)' on 'Neuronal pentraxin−2'

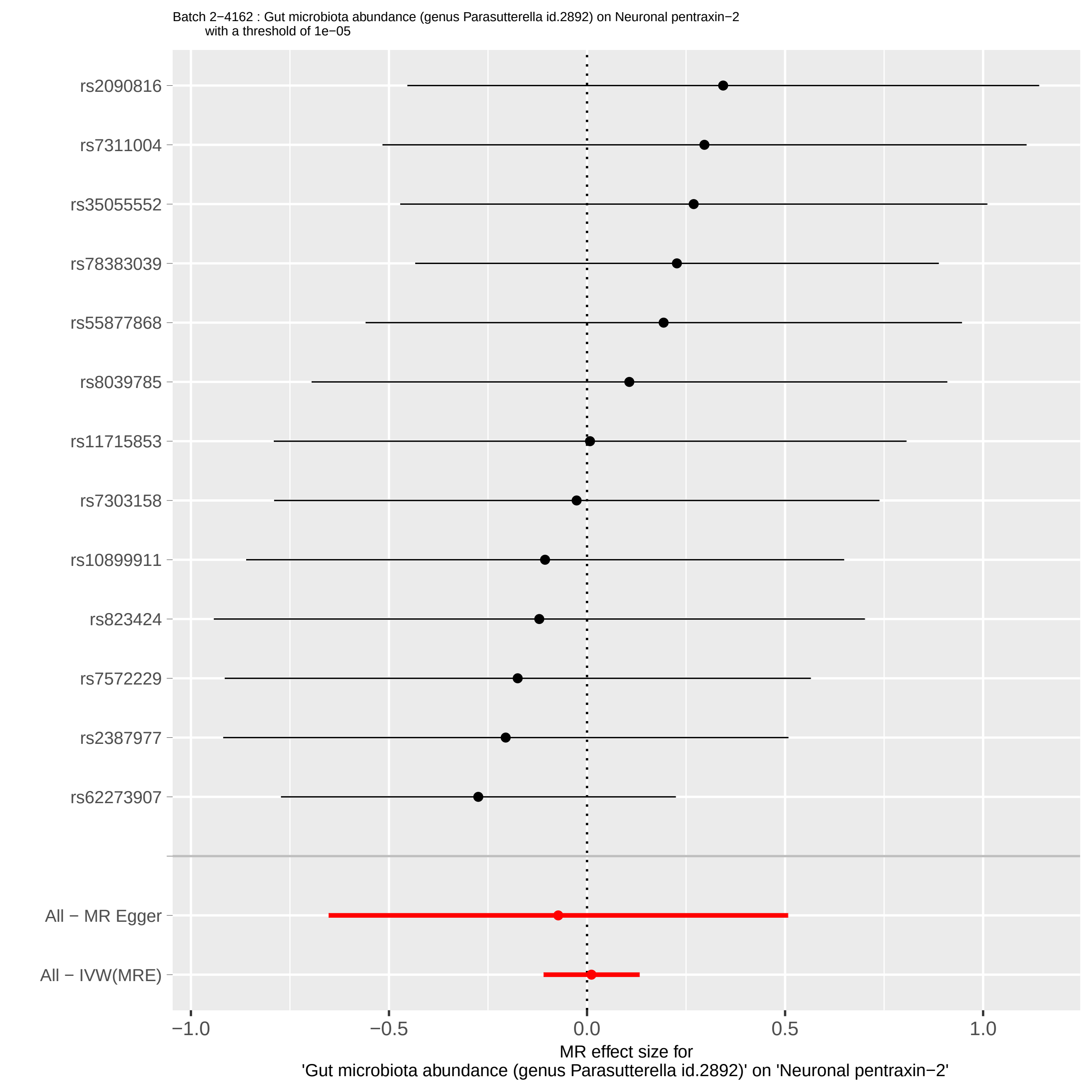
Batch 2−4162 : Gut microbiota abundance (genus Parasutterella id.2892) on Neuronal pentraxin−2
with a threshold of 1e−05
rs2090816
rs7311004
rs35055552
rs78383039
rs55877868
rs8039785
rs11715853
rs7303158
rs10899911
rs823424
rs7572229
rs2387977
rs62273907
All − MR Egger
All − IVW(MRE)
−1.0
−0.5
0.0
0.5
1.0
MR effect size for
'Gut microbiota abundance (genus Parasutterella id.2892)' on 'Neuronal pentraxin−2'

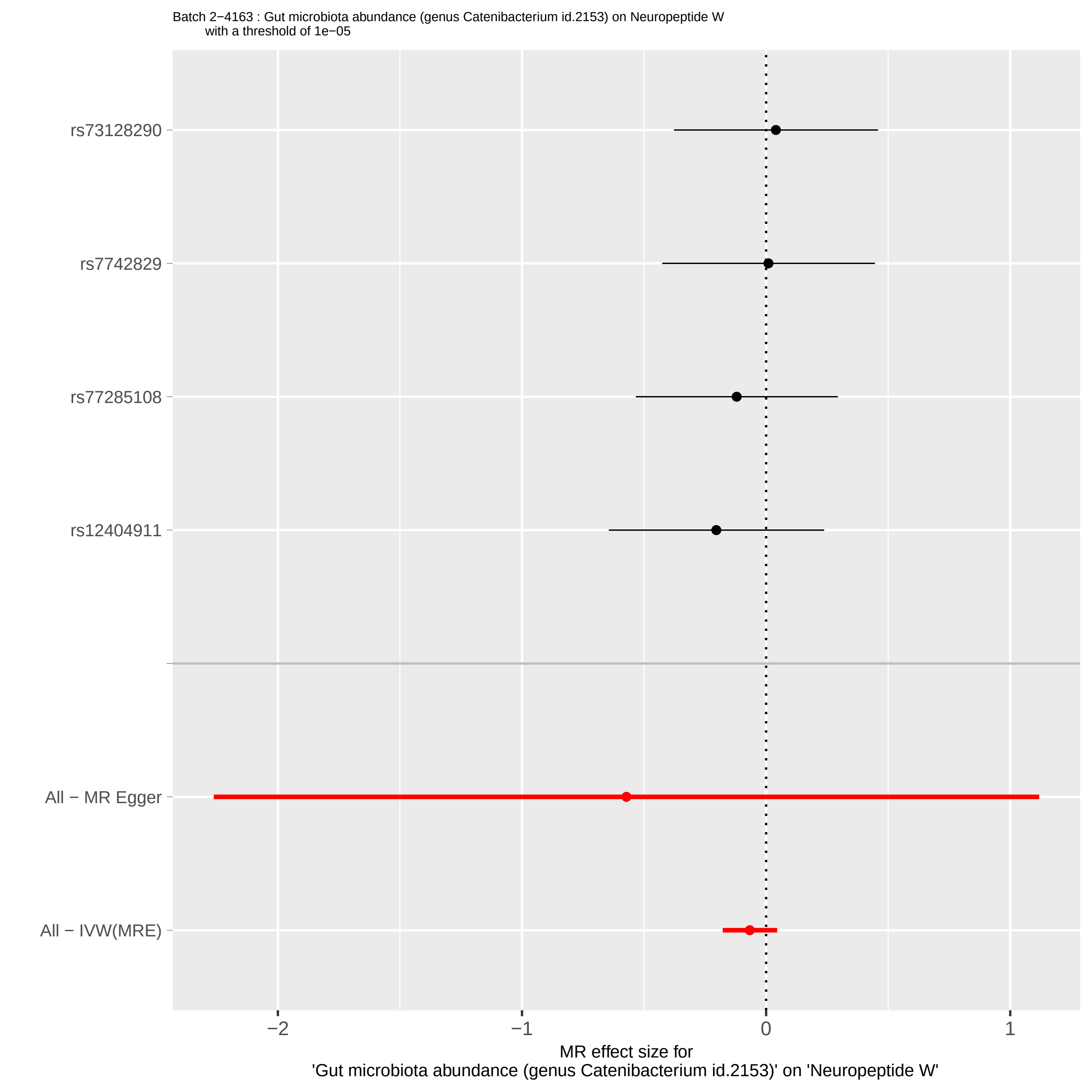

Batch 2−4163 : Gut microbiota abundance (genus Catenibacterium id.2153) on Neuropeptide W
with a threshold of 1e−05
rs73128290
rs7742829
rs77285108
rs12404911
All − MR Egger
All − IVW(MRE)
−2
−1
0
1
MR effect size for
'Gut microbiota abundance (genus Catenibacterium id.2153)' on 'Neuropeptide W'

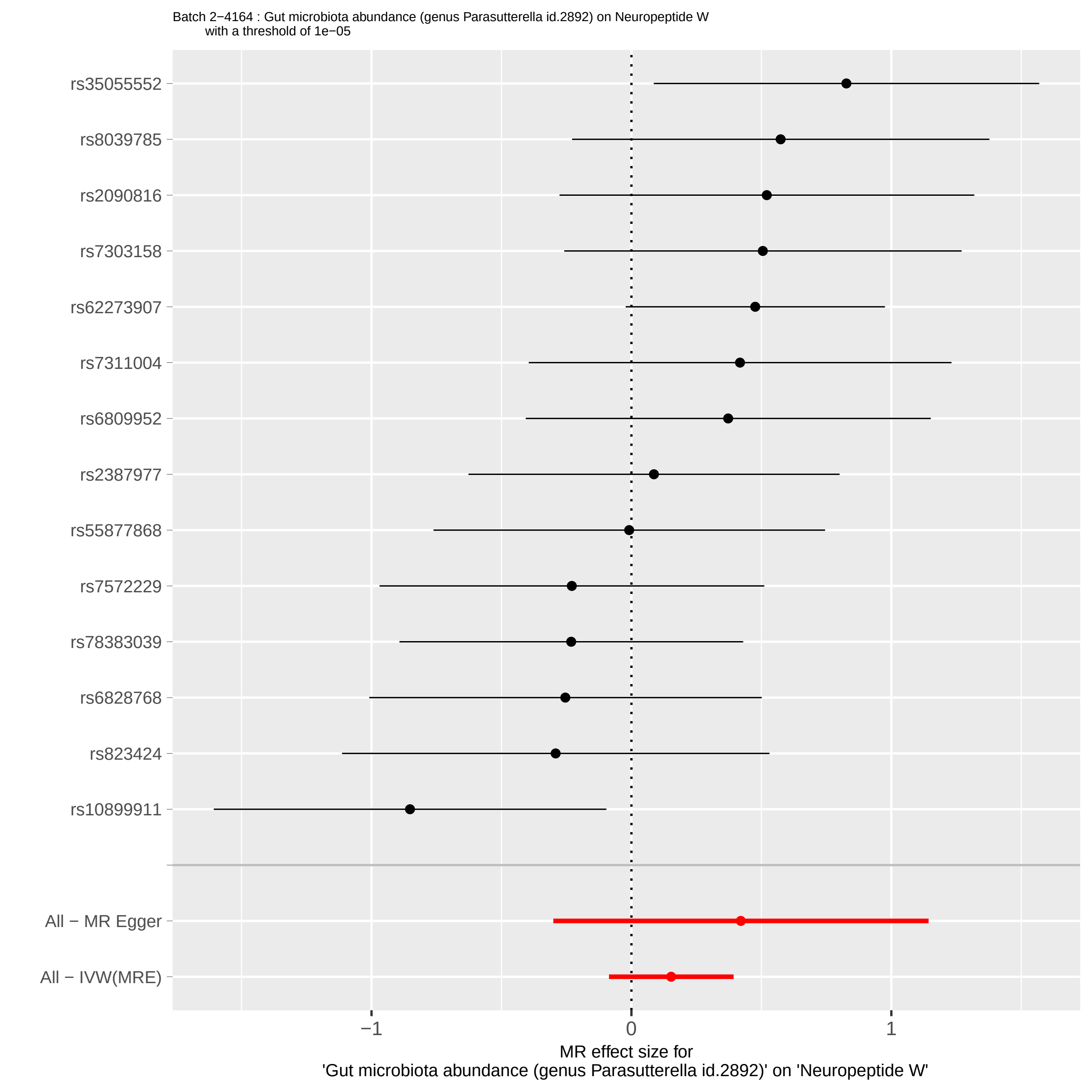

Batch 2−4164 : Gut microbiota abundance (genus Parasutterella id.2892) on Neuropeptide W
with a threshold of 1e−05
rs35055552
rs8039785
rs2090816
rs7303158
rs62273907
rs7311004
rs6809952
rs2387977
rs55877868
rs7572229
rs78383039
rs6828768
rs823424
rs10899911
All − MR Egger
All − IVW(MRE)
−1
0
1
MR effect size for
'Gut microbiota abundance (genus Parasutterella id.2892)' on 'Neuropeptide W'

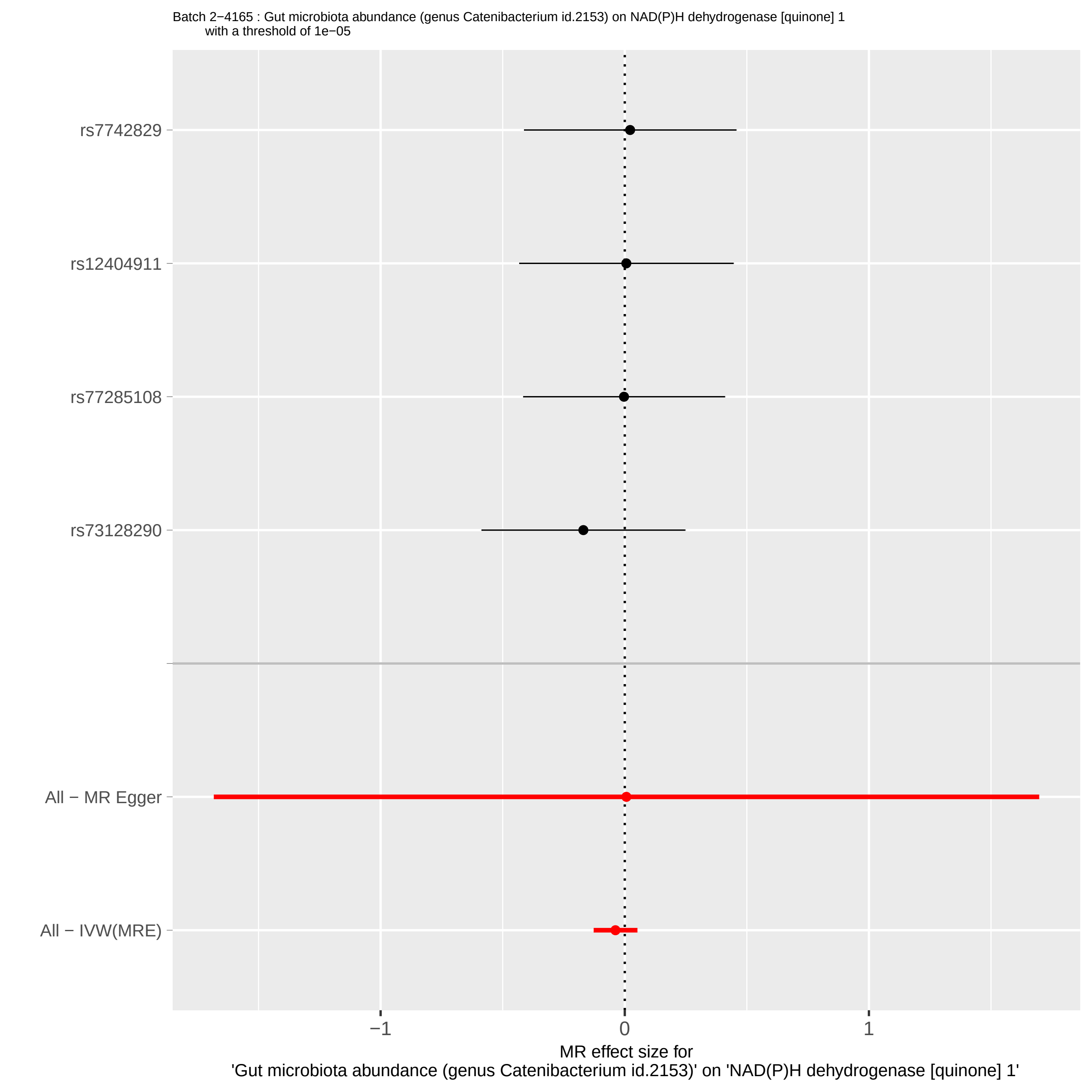

Batch 2−4165 : Gut microbiota abundance (genus Catenibacterium id.2153) on NAD(P)H dehydrogenase [quinone] 1
with a threshold of 1e−05
rs7742829
rs12404911
rs77285108
rs73128290
All − MR Egger
All − IVW(MRE)
−1
0
1
MR effect size for
'Gut microbiota abundance (genus Catenibacterium id.2153)' on 'NAD(P)H dehydrogenase [quinone] 1'

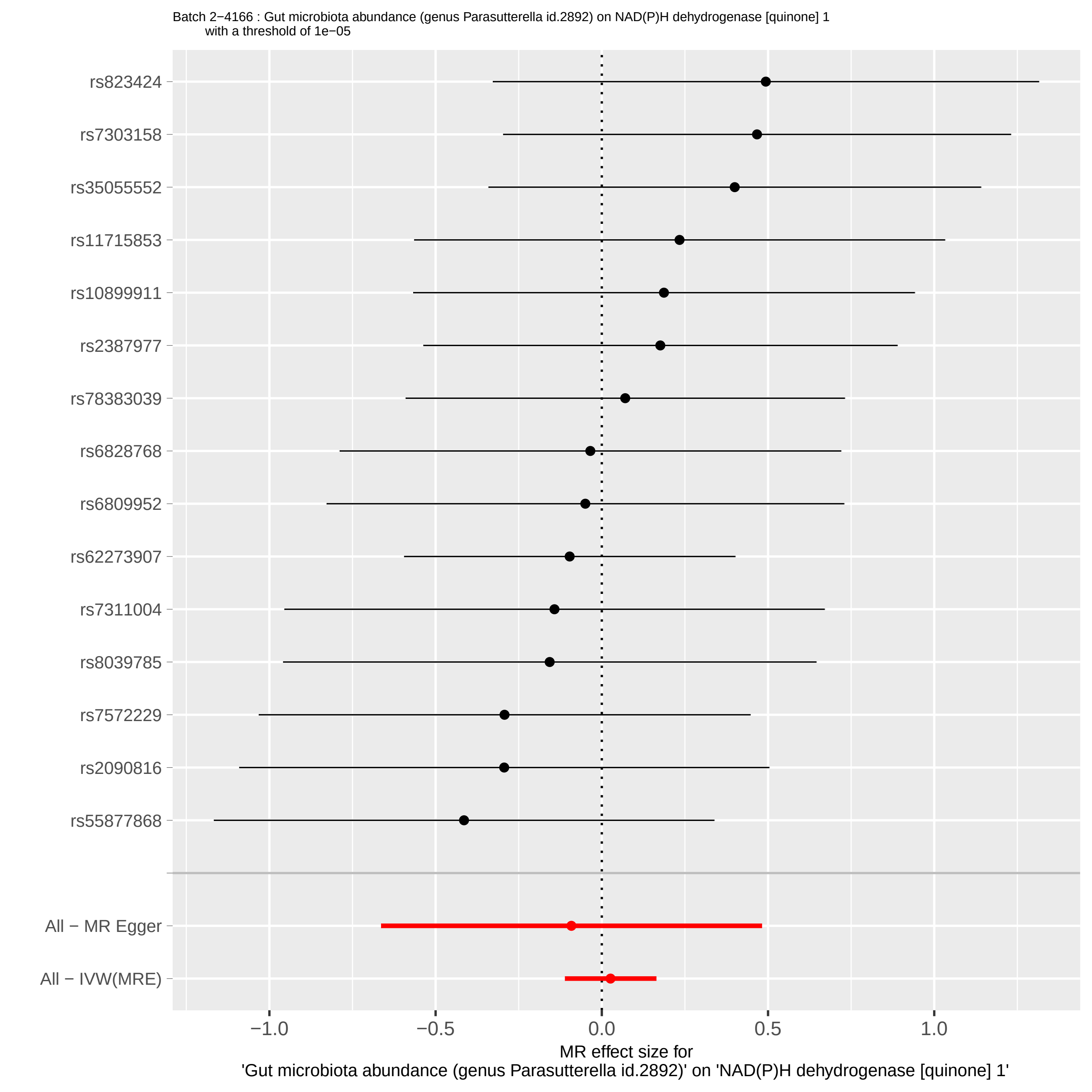
Batch 2−4166 : Gut microbiota abundance (genus Parasutterella id.2892) on NAD(P)H dehydrogenase [quinone] 1
with a threshold of 1e−05
rs823424
rs7303158
rs35055552
rs11715853
rs10899911
rs2387977
rs78383039
rs6828768
rs6809952
rs62273907
rs7311004
rs8039785
rs7572229
rs2090816
rs55877868
All − MR Egger
All − IVW(MRE)
−1.0
−0.5
0.0
0.5
1.0
MR effect size for
'Gut microbiota abundance (genus Parasutterella id.2892)' on 'NAD(P)H dehydrogenase [quinone] 1'

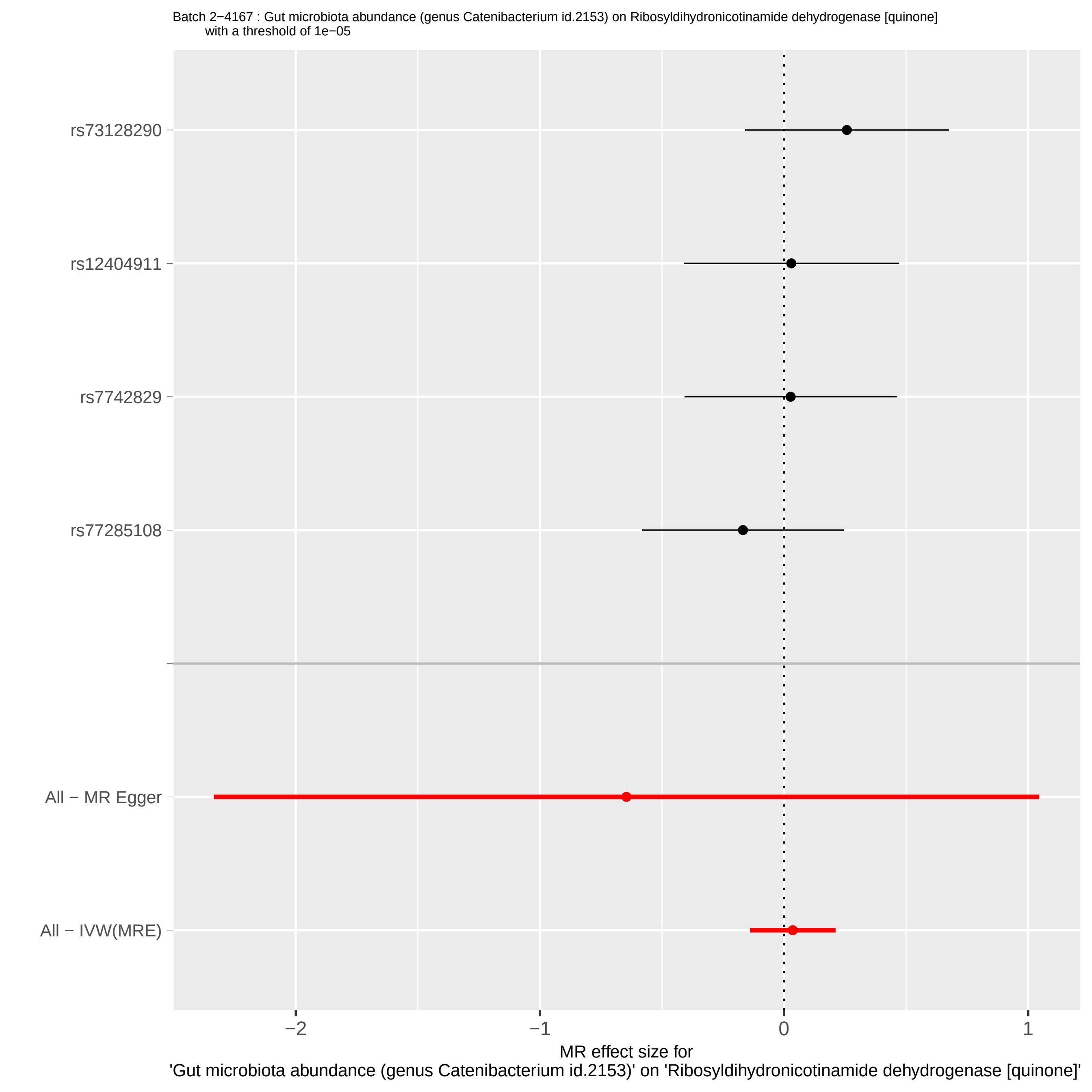
Batch 2−4167 : Gut microbiota abundance (genus Catenibacterium id.2153) on Ribosyldihydronicotinamide dehydrogenase [quinone]
with a threshold of 1e−05
rs73128290
rs12404911
rs7742829
rs77285108
All − MR Egger
All − IVW(MRE)
−2
−1
0
1
MR effect size for
'Gut microbiota abundance (genus Catenibacterium id.2153)' on 'Ribosyldihydronicotinamide dehydrogenase [quinone]'

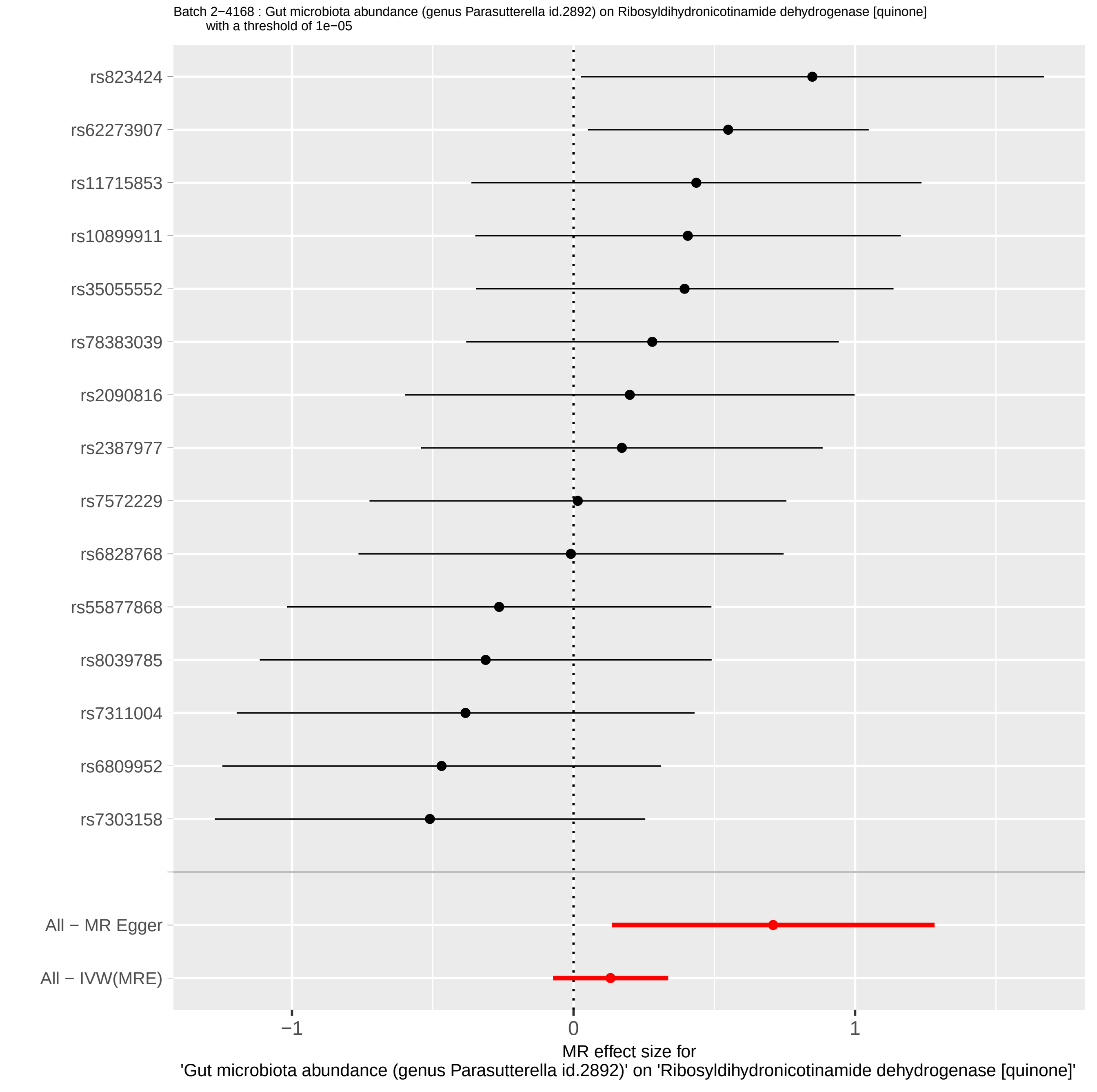
Batch 2−4168 : Gut microbiota abundance (genus Parasutterella id.2892) on Ribosyldihydronicotinamide dehydrogenase [quinone]
with a threshold of 1e−05
rs823424
rs62273907
rs11715853
rs10899911
rs35055552
rs78383039
rs2090816
rs2387977
rs7572229
rs6828768
rs55877868
rs8039785
rs7311004
rs6809952
rs7303158
All − MR Egger
All − IVW(MRE)
−1
0
1
MR effect size for
'Gut microbiota abundance (genus Parasutterella id.2892)' on 'Ribosyldihydronicotinamide dehydrogenase [quinone]'

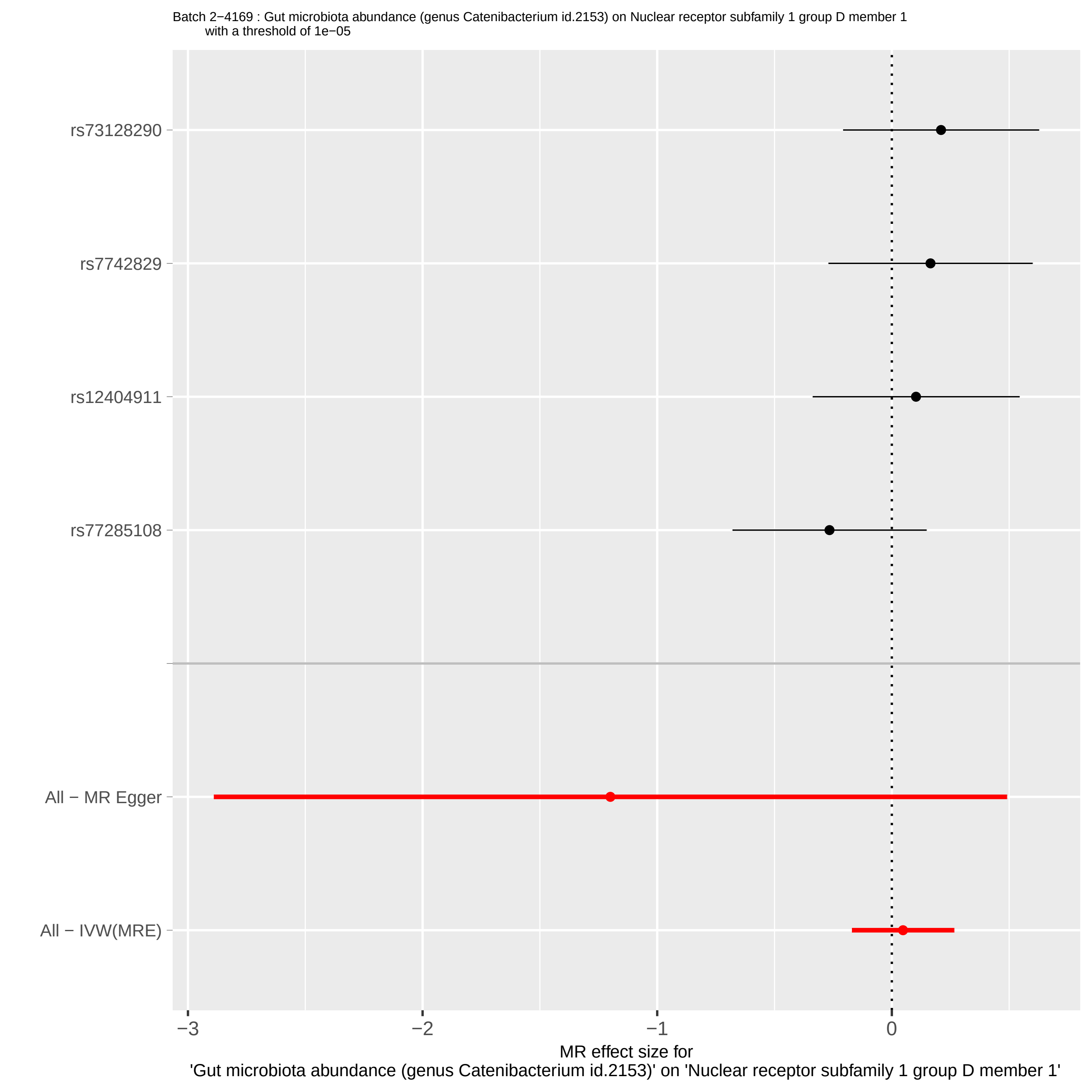

Batch 2−4169 : Gut microbiota abundance (genus Catenibacterium id.2153) on Nuclear receptor subfamily 1 group D member 1
with a threshold of 1e−05
rs73128290
rs7742829
rs12404911
rs77285108
All − MR Egger
All − IVW(MRE)
−3
−2
−1
0
MR effect size for
'Gut microbiota abundance (genus Catenibacterium id.2153)' on 'Nuclear receptor subfamily 1 group D member 1'

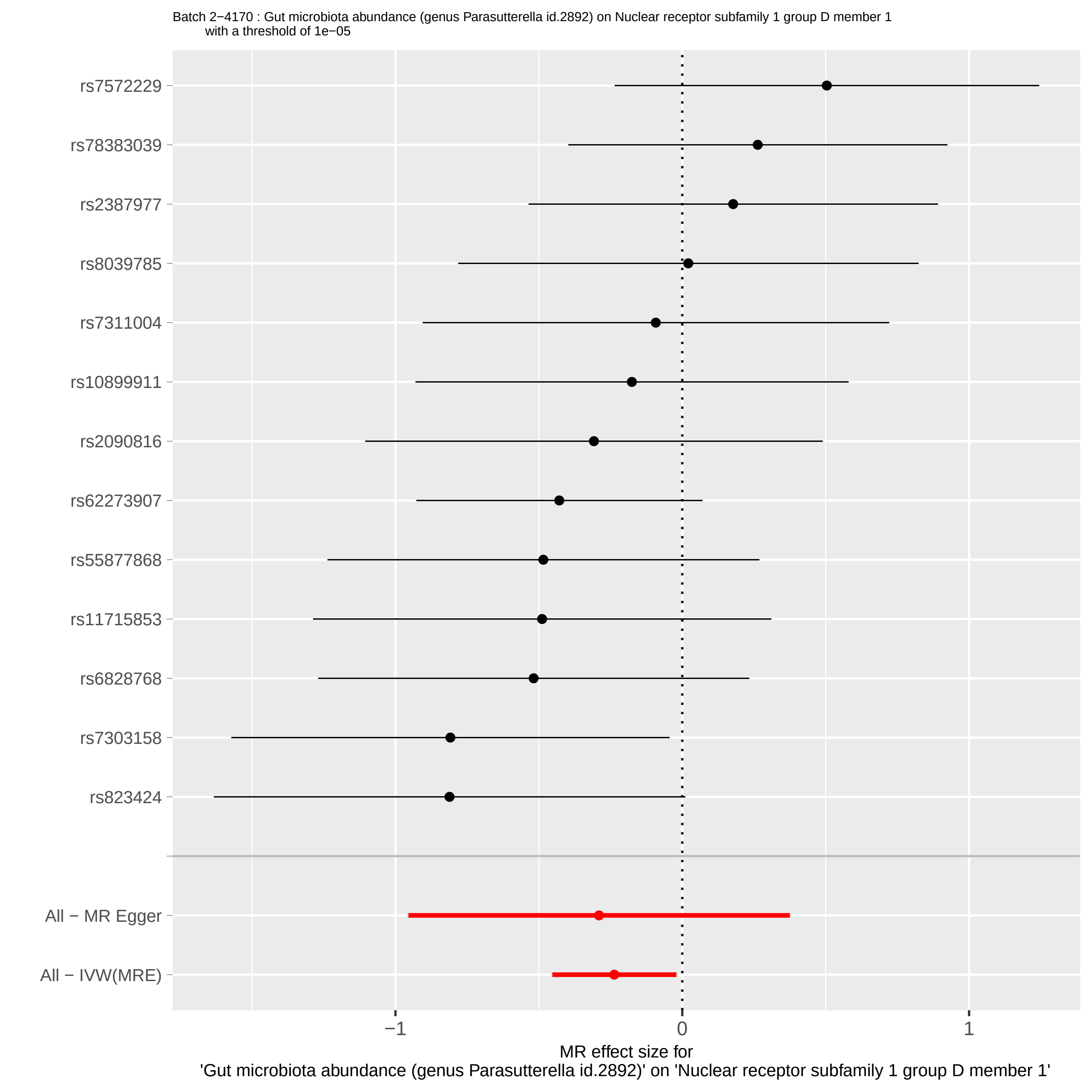
Batch 2−4170 : Gut microbiota abundance (genus Parasutterella id.2892) on Nuclear receptor subfamily 1 group D member 1
with a threshold of 1e−05
rs7572229
rs78383039
rs2387977
rs8039785
rs7311004
rs10899911
rs2090816
rs62273907
rs55877868
rs11715853
rs6828768
rs7303158
rs823424
All − MR Egger
All − IVW(MRE)
−1
0
1
MR effect size for
'Gut microbiota abundance (genus Parasutterella id.2892)' on 'Nuclear receptor subfamily 1 group D member 1'

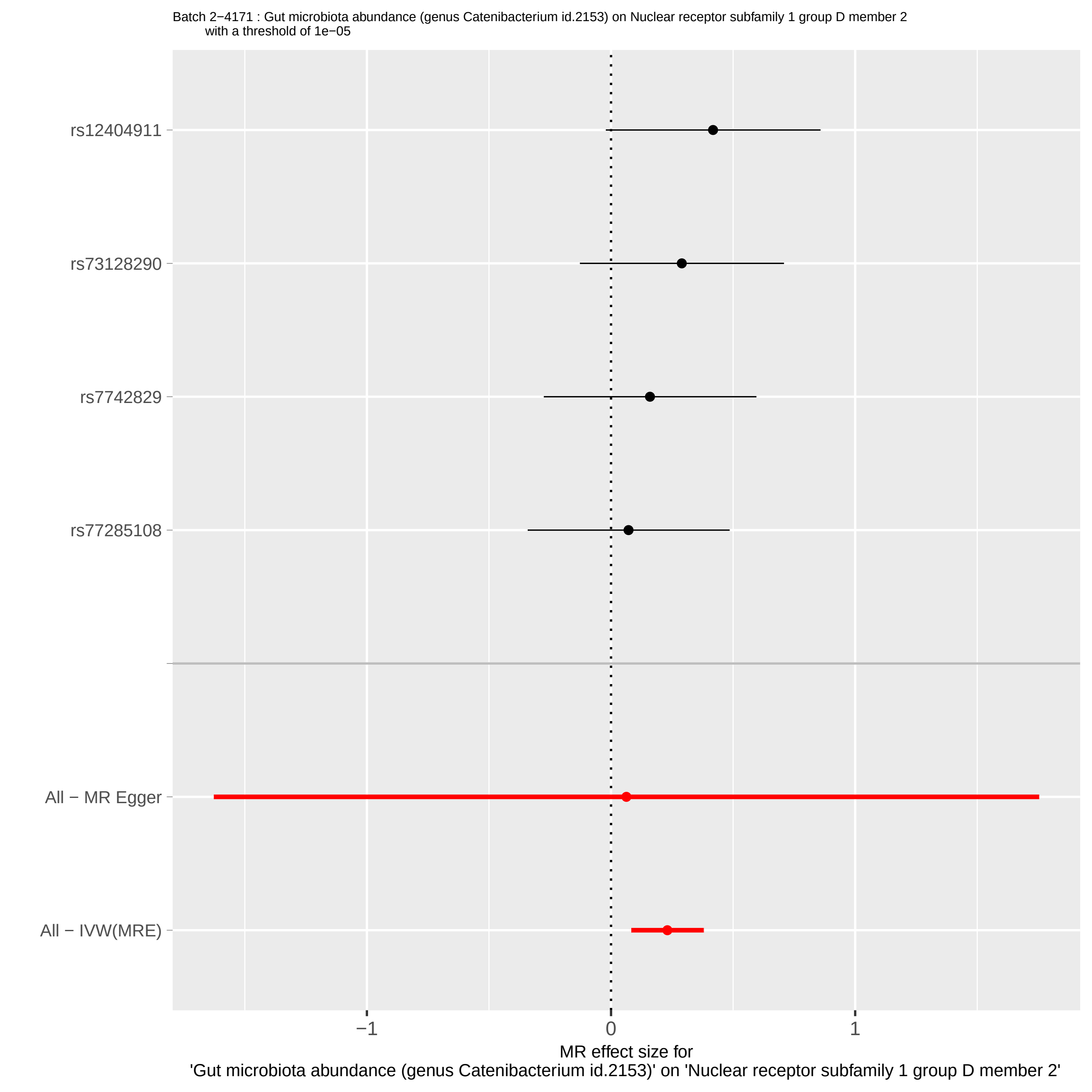
Batch 2−4171 : Gut microbiota abundance (genus Catenibacterium id.2153) on Nuclear receptor subfamily 1 group D member 2
with a threshold of 1e−05
rs12404911
rs73128290
rs7742829
rs77285108
All − MR Egger
All − IVW(MRE)
−1
0
1
MR effect size for
'Gut microbiota abundance (genus Catenibacterium id.2153)' on 'Nuclear receptor subfamily 1 group D member 2'

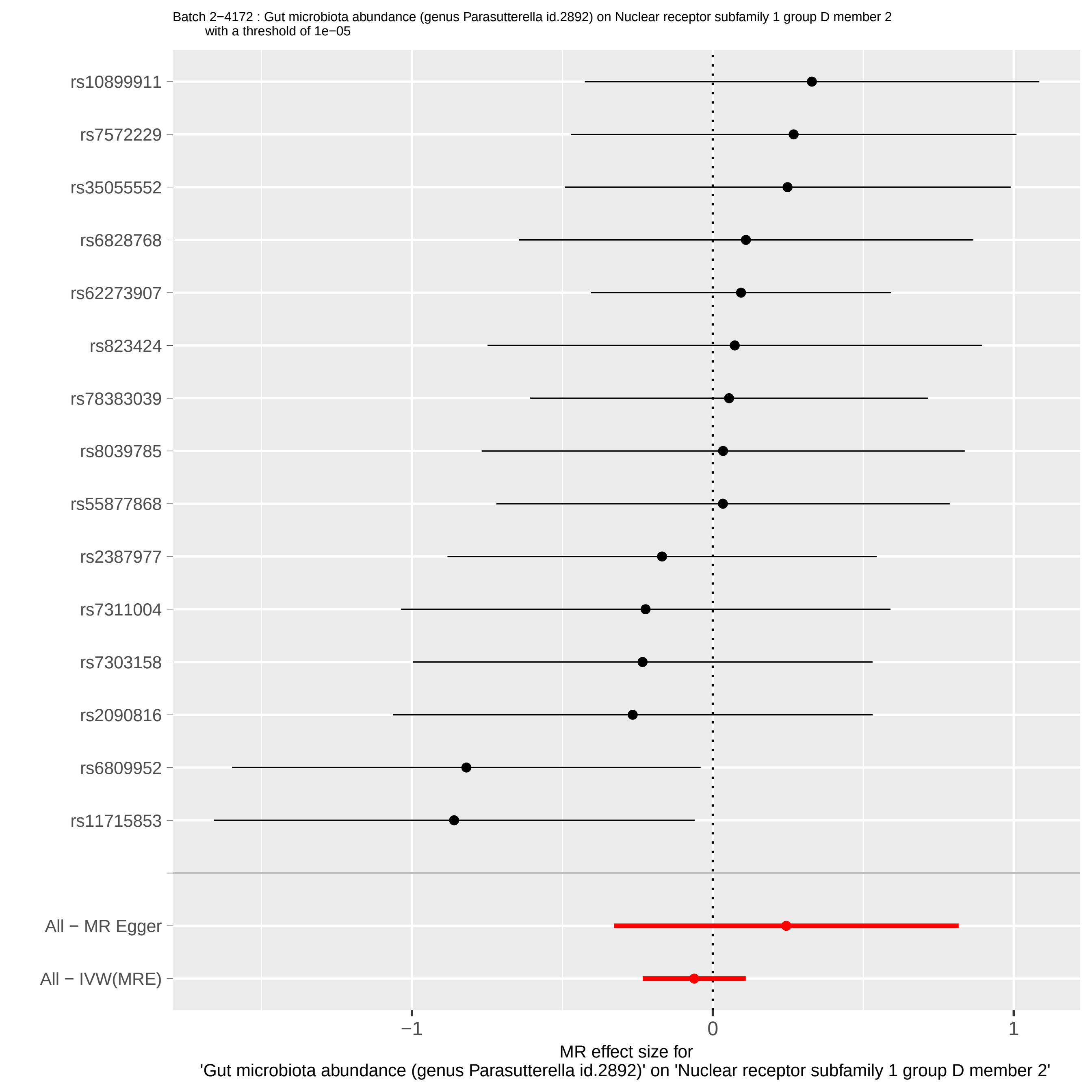

Batch 2−4172 : Gut microbiota abundance (genus Parasutterella id.2892) on Nuclear receptor subfamily 1 group D member 2
with a threshold of 1e−05
rs10899911
rs7572229
rs35055552
rs6828768
rs62273907
rs823424
rs78383039
rs8039785
rs55877868
rs2387977
rs7311004
rs7303158
rs2090816
rs6809952
rs11715853
All − MR Egger
All − IVW(MRE)
−1
0
1
MR effect size for
'Gut microbiota abundance (genus Parasutterella id.2892)' on 'Nuclear receptor subfamily 1 group D member 2'

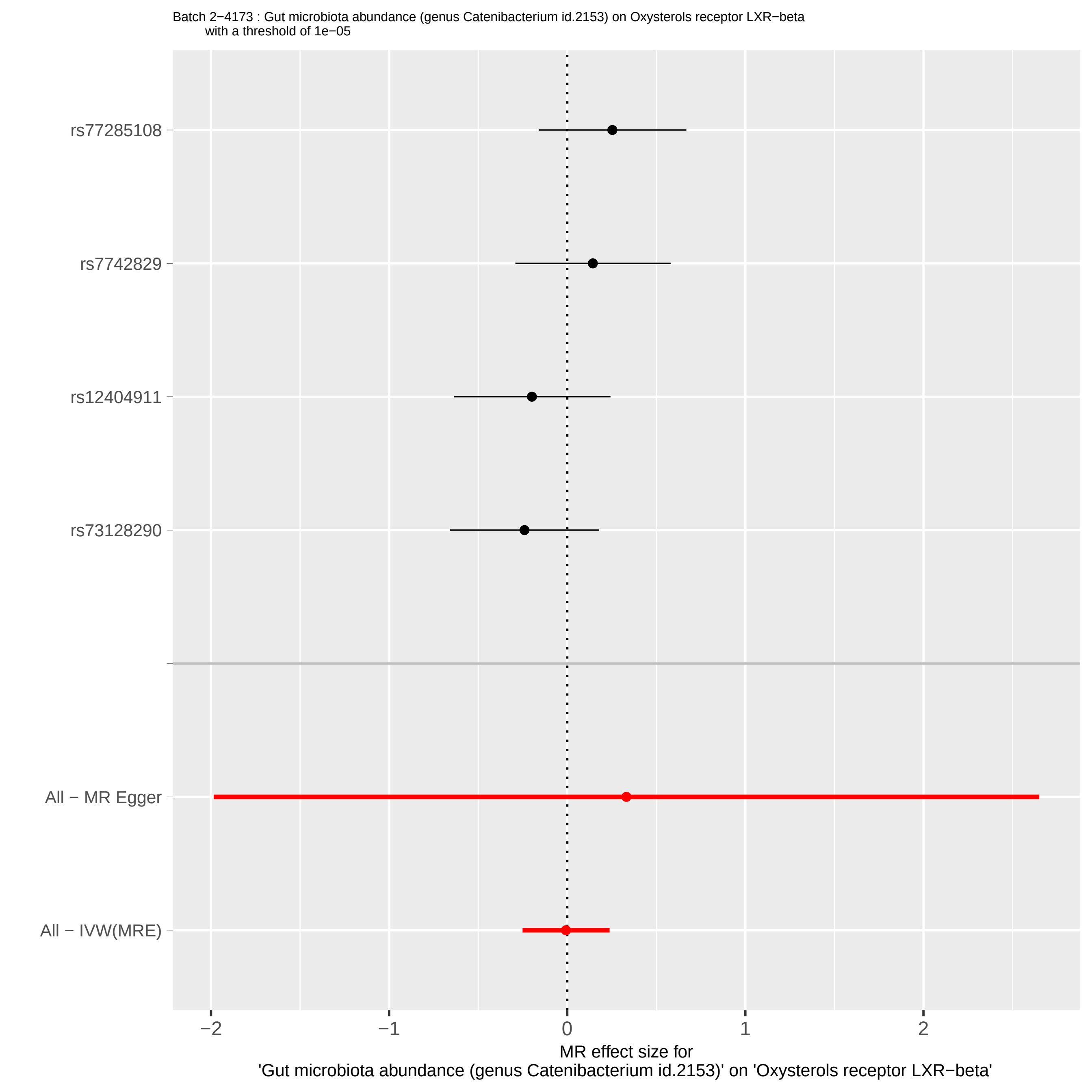

Batch 2−4173 : Gut microbiota abundance (genus Catenibacterium id.2153) on Oxysterols receptor LXR−beta
with a threshold of 1e−05
rs77285108
rs7742829
rs12404911
rs73128290
All − MR Egger
All − IVW(MRE)
−2
−1
0
1
2
MR effect size for
'Gut microbiota abundance (genus Catenibacterium id.2153)' on 'Oxysterols receptor LXR−beta'

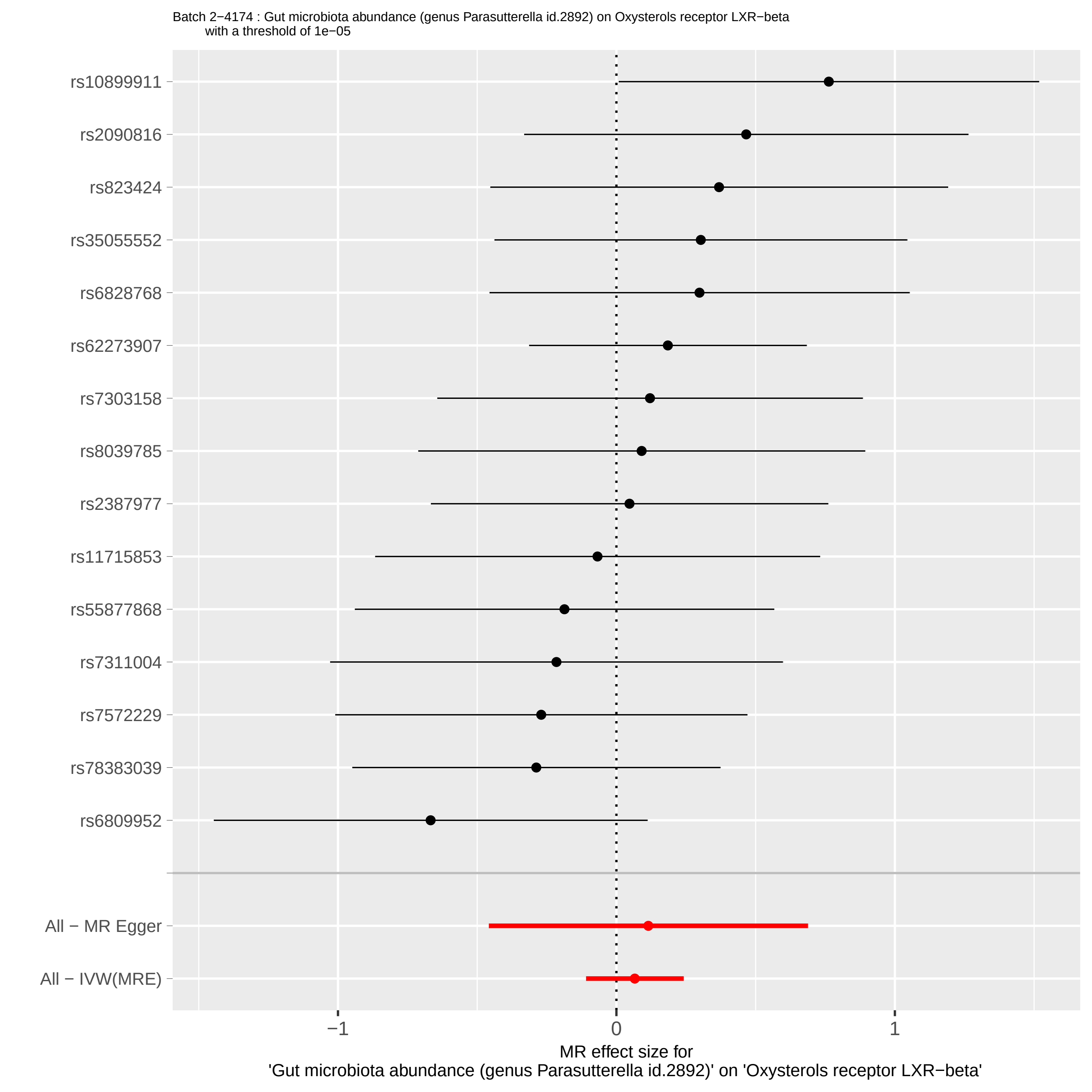
Batch 2−4174 : Gut microbiota abundance (genus Parasutterella id.2892) on Oxysterols receptor LXR−beta
with a threshold of 1e−05
rs10899911
rs2090816
rs823424
rs35055552
rs6828768
rs62273907
rs7303158
rs8039785
rs2387977
rs11715853
rs55877868
rs7311004
rs7572229
rs78383039
rs6809952
All − MR Egger
All − IVW(MRE)
−1
0
1
MR effect size for
'Gut microbiota abundance (genus Parasutterella id.2892)' on 'Oxysterols receptor LXR−beta'

Batch 2−4175 : Gut microbiota abundance (genus Catenibacterium id.2153) on Glucocorticoid receptor
with a threshold of 1e−05

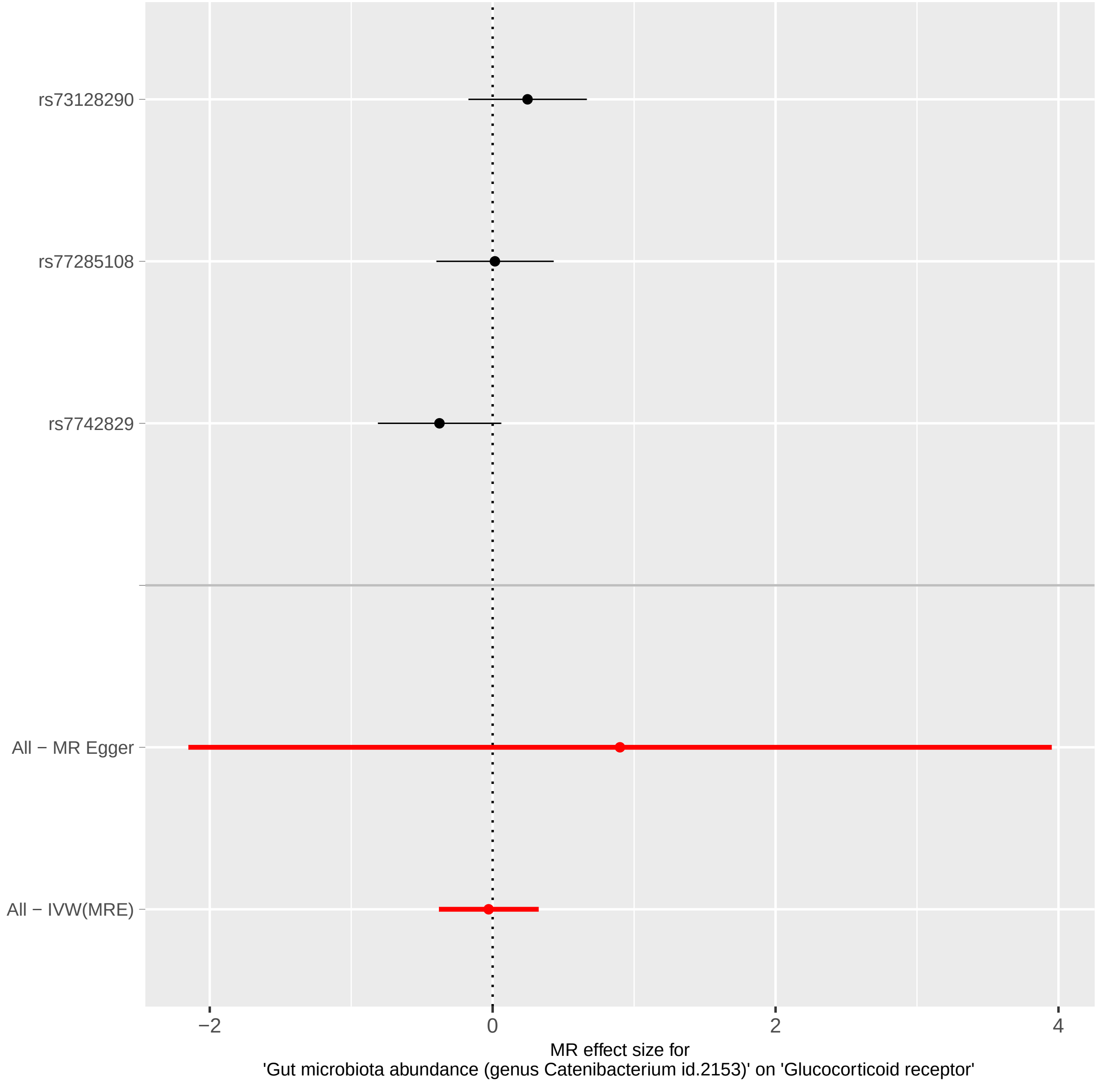

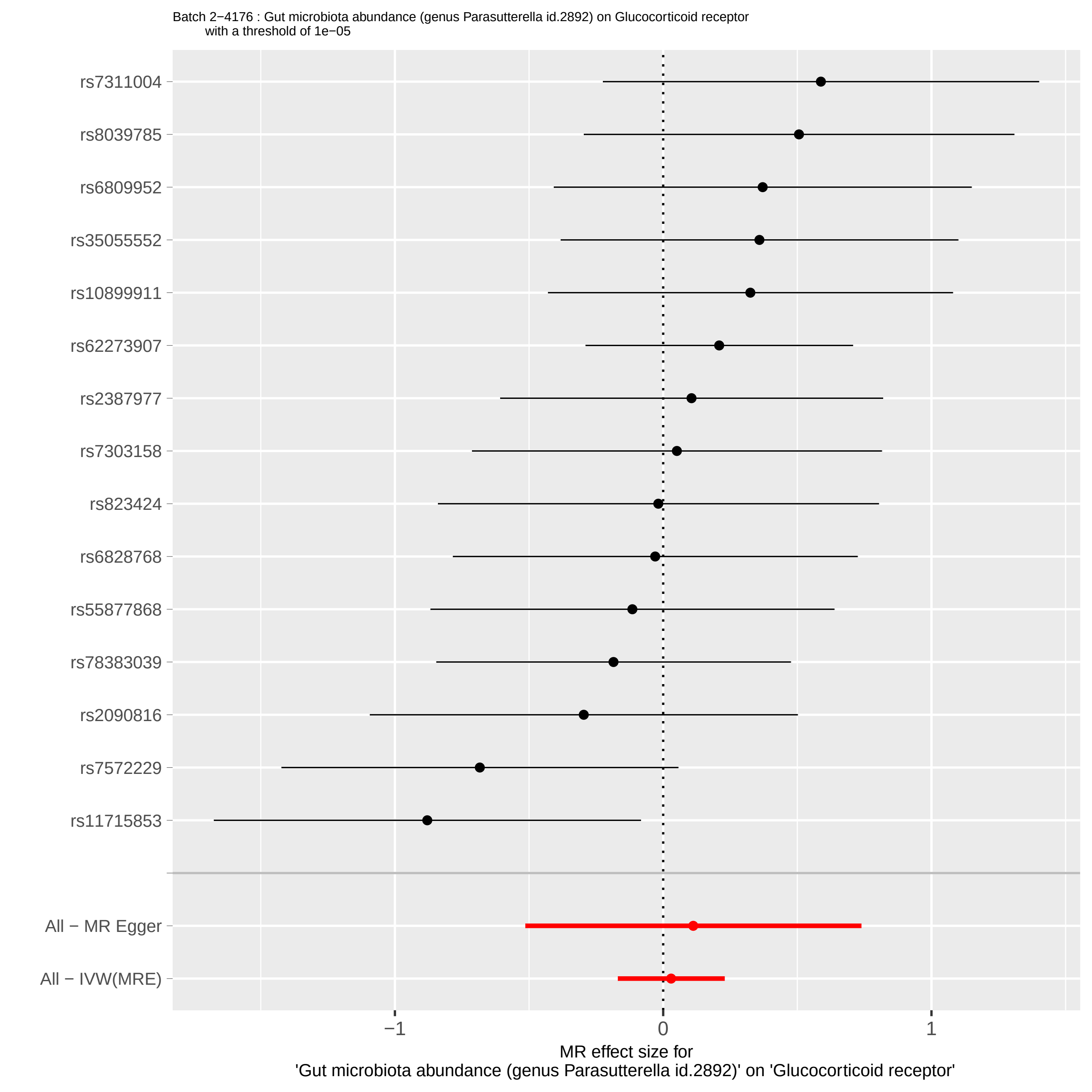

Batch 2−4176 : Gut microbiota abundance (genus Parasutterella id.2892) on Glucocorticoid receptor with a threshold of 1e−05
rs7311004
rs8039785
rs6809952
rs35055552
rs10899911
rs62273907
rs2387977
rs7303158
rs823424
rs6828768
rs55877868
rs78383039
rs2090816
rs7572229
rs11715853
All − MR Egger
All − IVW(MRE)
−1
0
1
MR effect size for
'Gut microbiota abundance (genus Parasutterella id.2892)' on 'Glucocorticoid receptor'

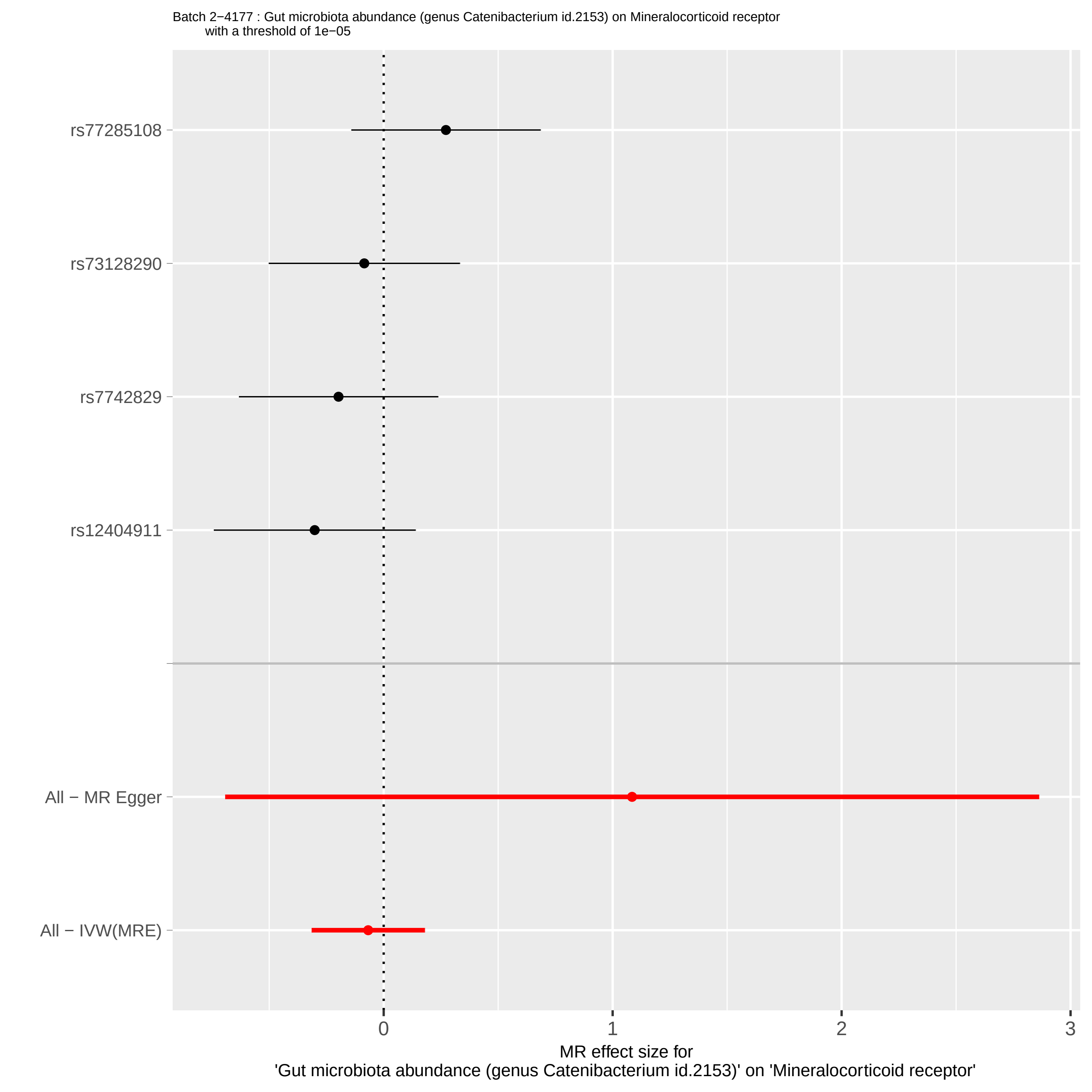
Batch 2−4177 : Gut microbiota abundance (genus Catenibacterium id.2153) on Mineralocorticoid receptor
with a threshold of 1e−05
rs77285108
rs73128290
rs7742829
rs12404911
All − MR Egger
All − IVW(MRE)
0
1
2
3
MR effect size for
'Gut microbiota abundance (genus Catenibacterium id.2153)' on 'Mineralocorticoid receptor'

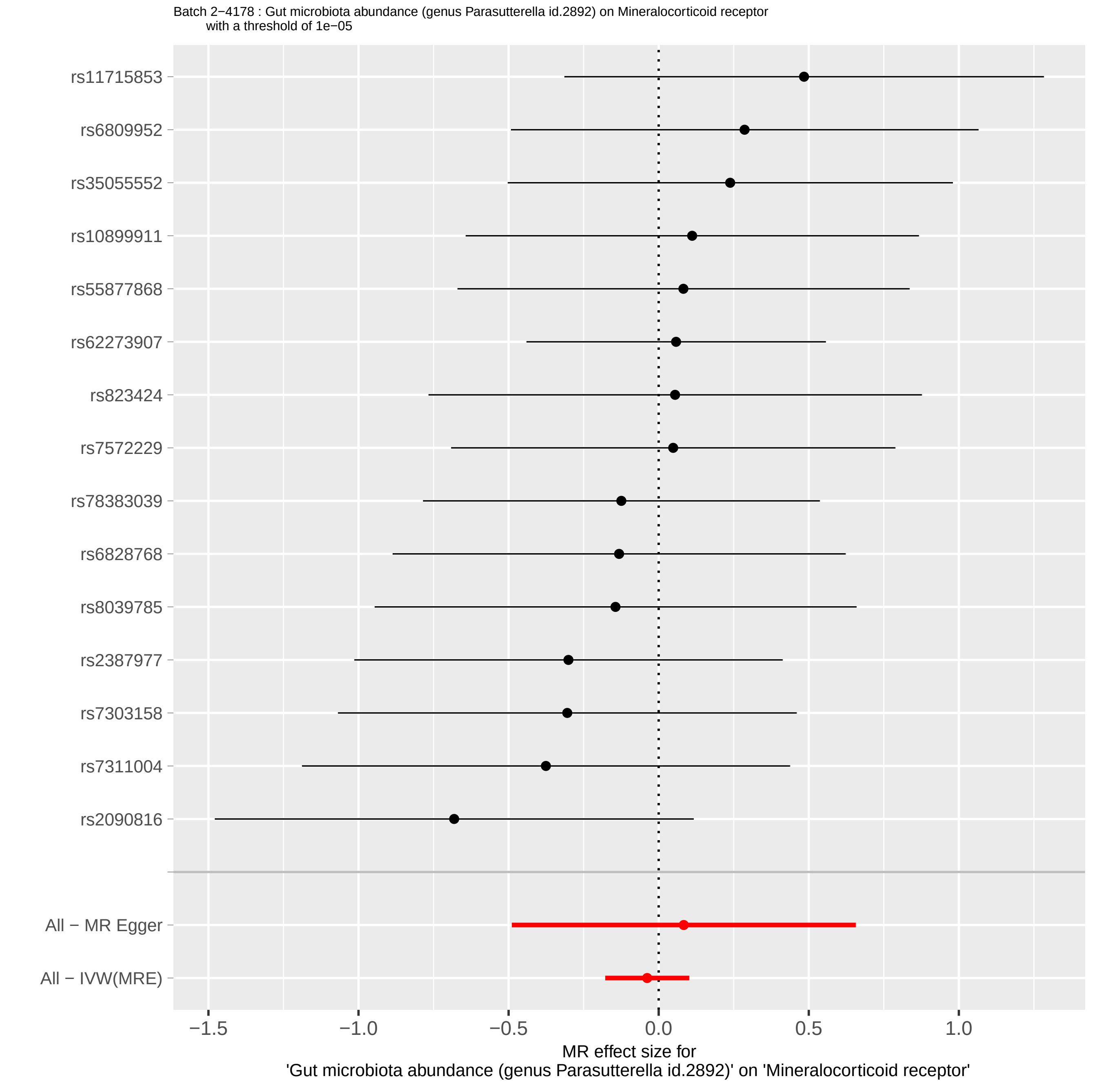

Batch 2−4178 : Gut microbiota abundance (genus Parasutterella id.2892) on Mineralocorticoid receptor
with a threshold of 1e−05
rs11715853
rs6809952
rs35055552
rs10899911
rs55877868
rs62273907
rs823424
rs7572229
rs78383039
rs6828768
rs8039785
rs2387977
rs7303158
rs7311004
rs2090816
All − MR Egger
All − IVW(MRE)
−1.5
−1.0
−0.5
0.0
0.5
1.0
MR effect size for
'Gut microbiota abundance (genus Parasutterella id.2892)' on 'Mineralocorticoid receptor'

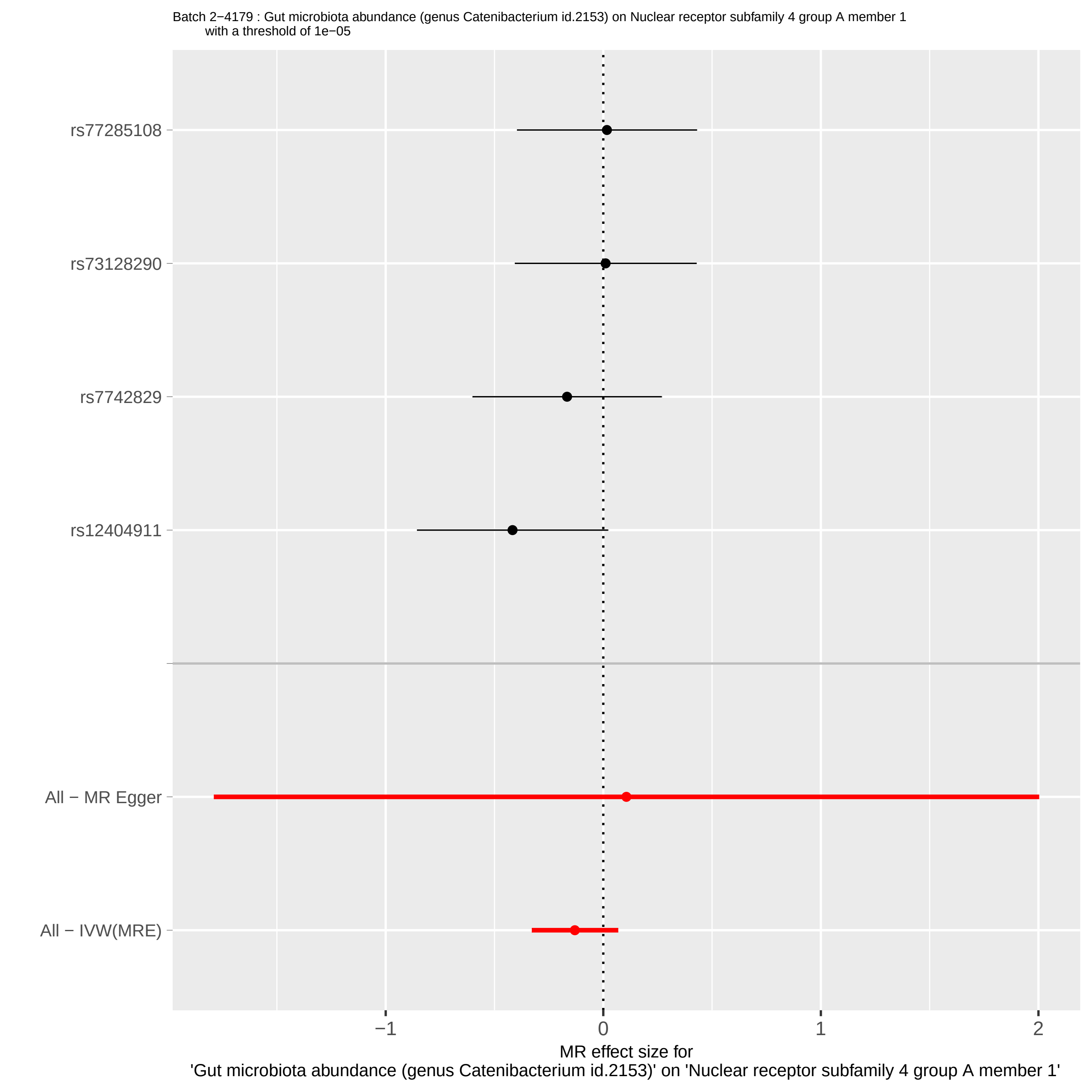

Batch 2−4179 : Gut microbiota abundance (genus Catenibacterium id.2153) on Nuclear receptor subfamily 4 group A member 1
with a threshold of 1e−05
rs77285108
rs73128290
rs7742829
rs12404911
All − MR Egger
All − IVW(MRE)
−1
0
1
2
MR effect size for
'Gut microbiota abundance (genus Catenibacterium id.2153)' on 'Nuclear receptor subfamily 4 group A member 1'

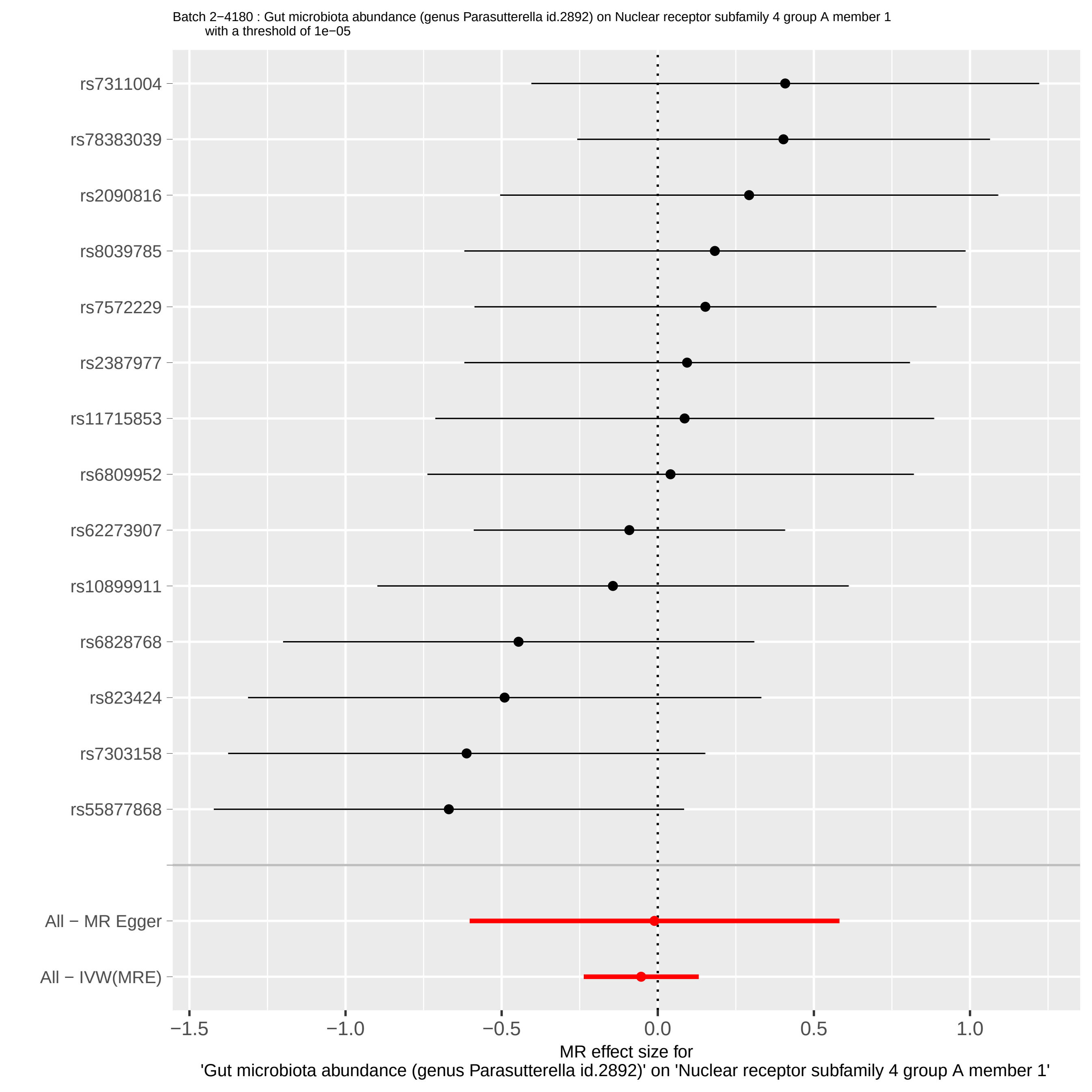
Batch 2−4180 : Gut microbiota abundance (genus Parasutterella id.2892) on Nuclear receptor subfamily 4 group A member 1
with a threshold of 1e−05
rs7311004
rs78383039
rs2090816
rs8039785
rs7572229
rs2387977
rs11715853
rs6809952
rs62273907
rs10899911
rs6828768
rs823424
rs7303158
rs55877868
All − MR Egger
All − IVW(MRE)
−1.5
−1.0
−0.5
0.0
0.5
1.0
MR effect size for
'Gut microbiota abundance (genus Parasutterella id.2892)' on 'Nuclear receptor subfamily 4 group A member 1'

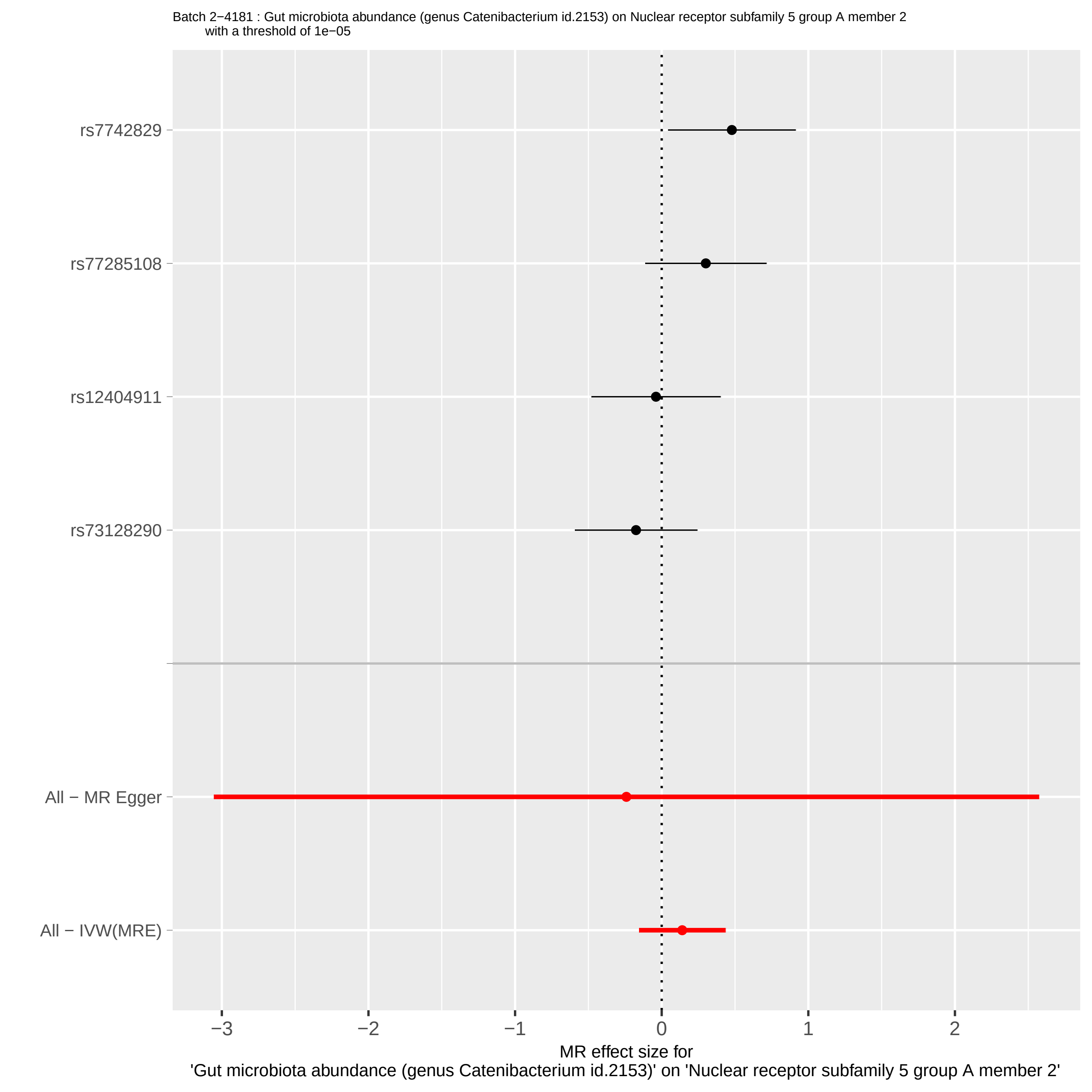

Batch 2−4181 : Gut microbiota abundance (genus Catenibacterium id.2153) on Nuclear receptor subfamily 5 group A member 2
with a threshold of 1e−05
rs7742829
rs77285108
rs12404911
rs73128290
All − MR Egger
All − IVW(MRE)
−3
−2
−1
0
1
2
MR effect size for
'Gut microbiota abundance (genus Catenibacterium id.2153)' on 'Nuclear receptor subfamily 5 group A member 2'

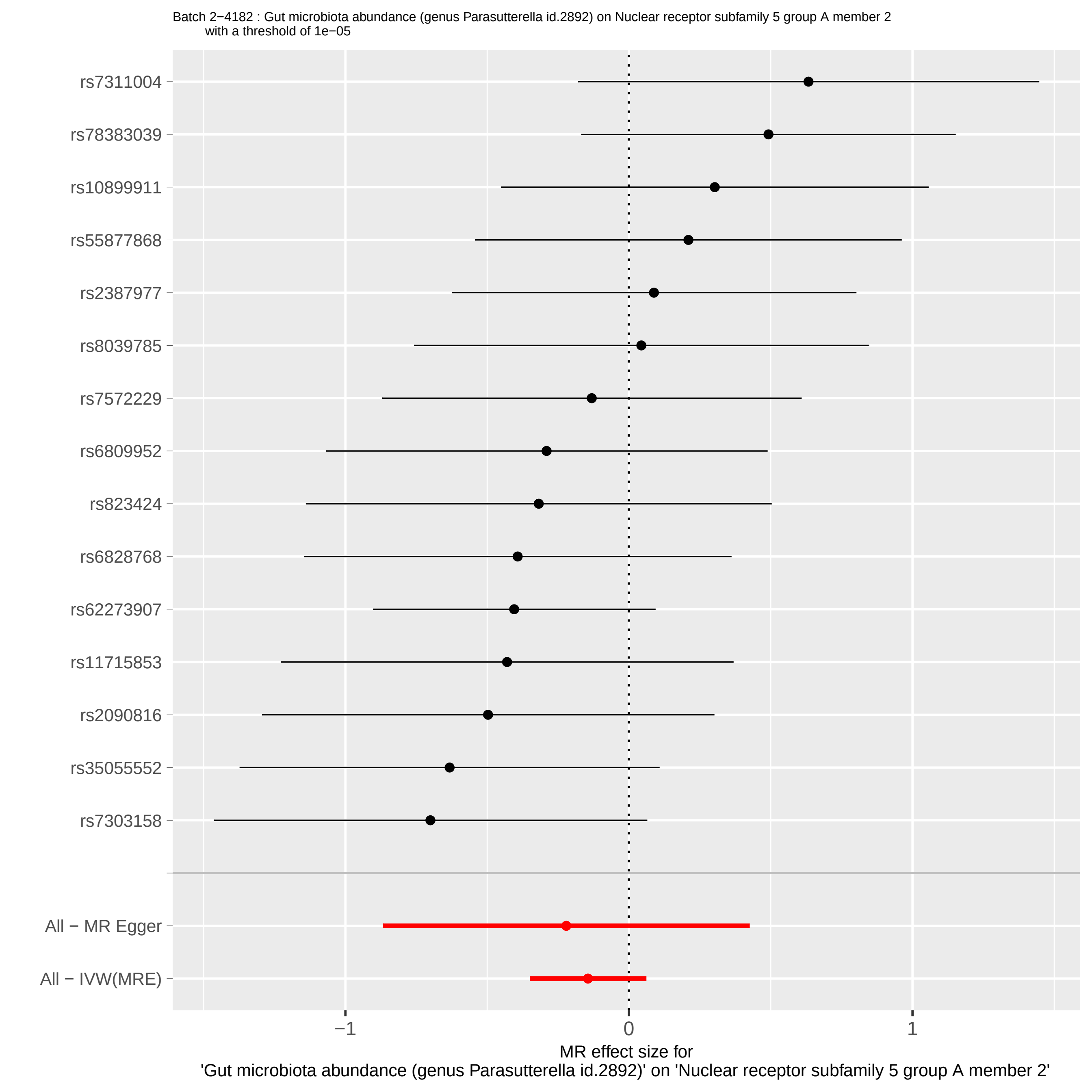

Batch 2−4182 : Gut microbiota abundance (genus Parasutterella id.2892) on Nuclear receptor subfamily 5 group A member 2
with a threshold of 1e−05
rs7311004
rs78383039
rs10899911
rs55877868
rs2387977
rs8039785
rs7572229
rs6809952
rs823424
rs6828768
rs62273907
rs11715853
rs2090816
rs35055552
rs7303158
All − MR Egger
All − IVW(MRE)
−1
0
1
MR effect size for
'Gut microbiota abundance (genus Parasutterella id.2892)' on 'Nuclear receptor subfamily 5 group A member 2'

Batch 2−4183 : Gut microbiota abundance (genus Catenibacterium id.2153) on Nutritionally−regulated adipose and cardiac enriched protein homolog with a threshold of 1e−05

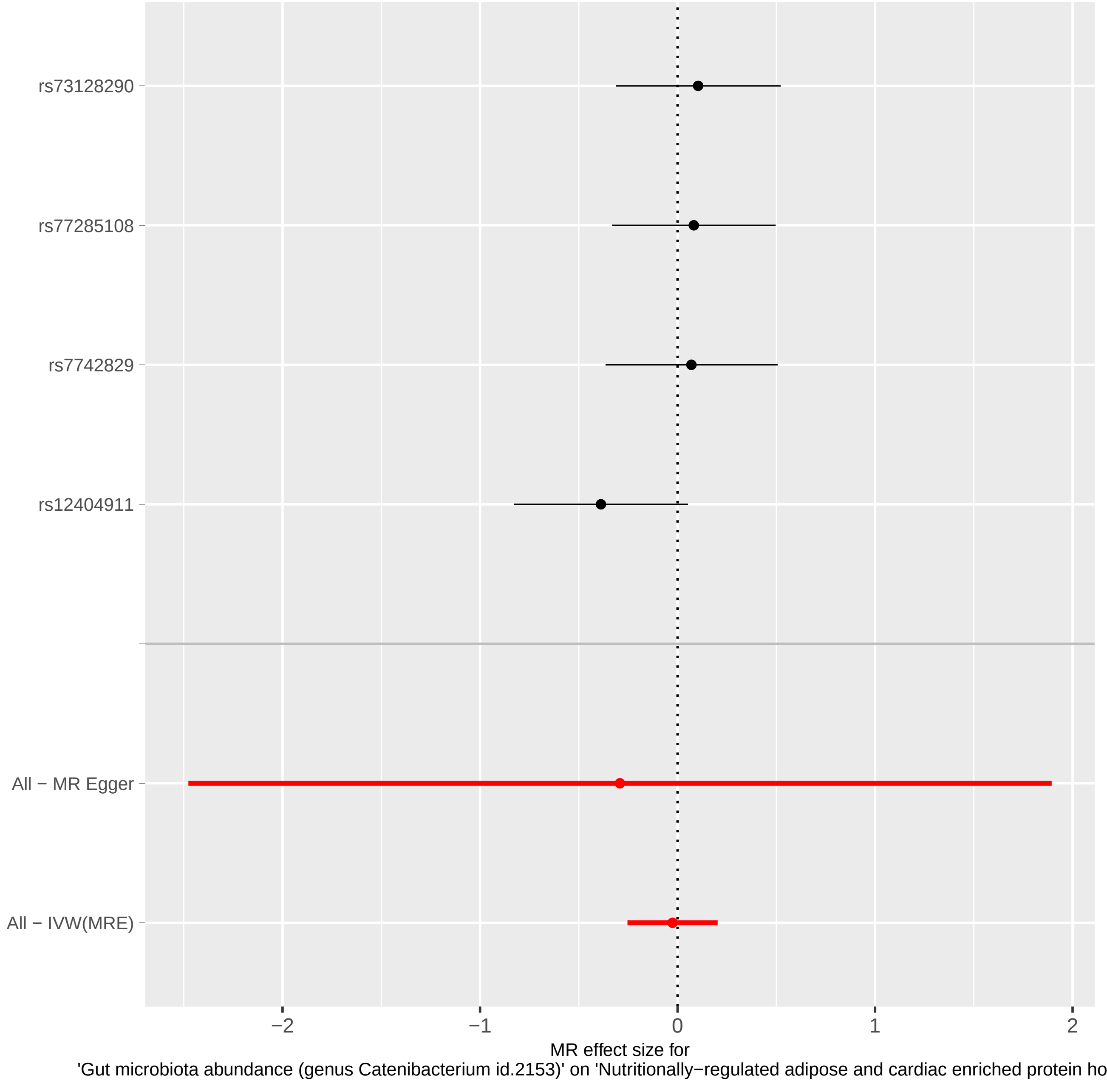

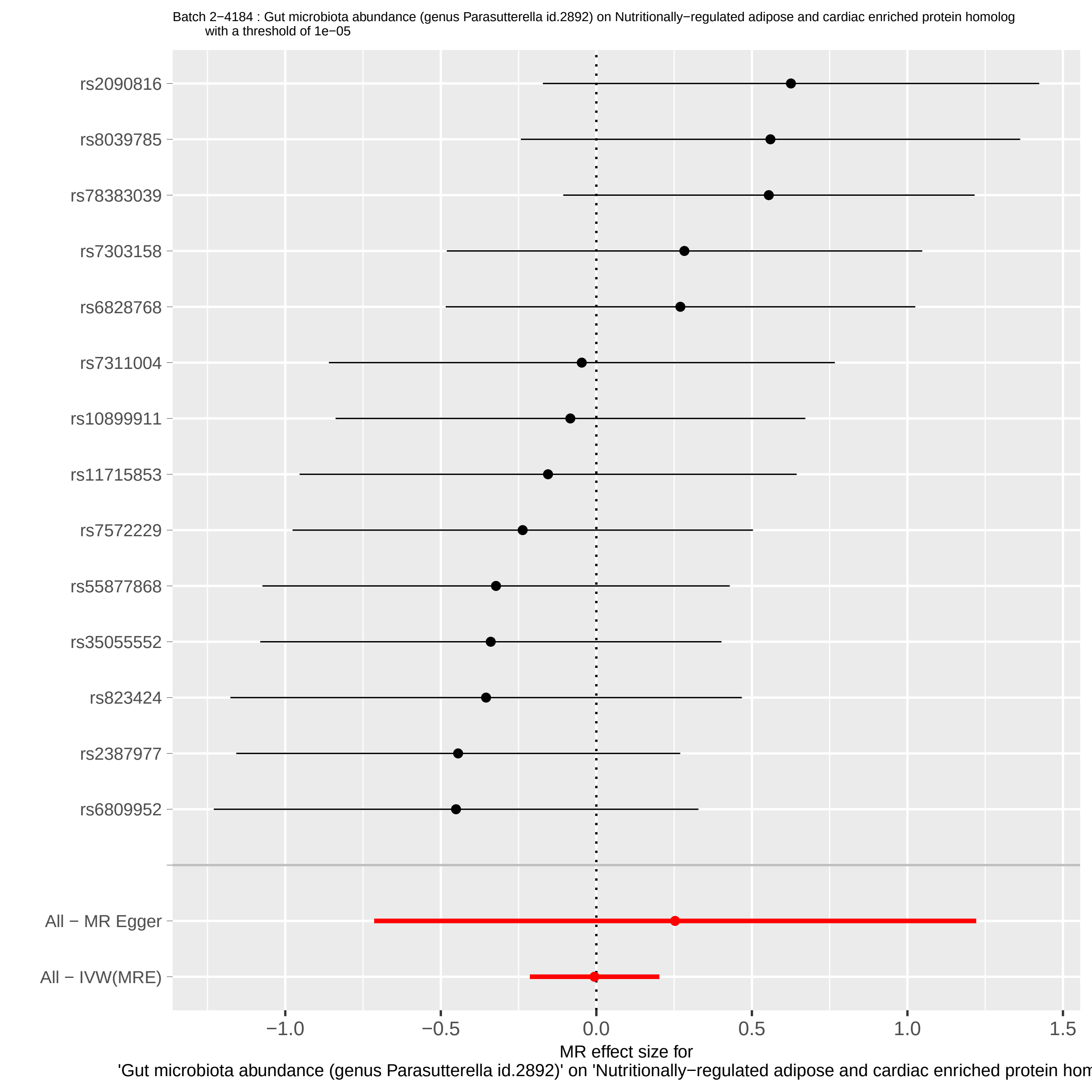
Batch 2−4184 : Gut microbiota abundance (genus Parasutterella id.2892) on Nutritionally−regulated adipose and cardiac enriched protein homolog
with a threshold of 1e−05
rs2090816
rs8039785
rs78383039
rs7303158
rs6828768
rs7311004
rs10899911
rs11715853
rs7572229
rs55877868
rs35055552
rs823424
rs2387977
rs6809952
All − MR Egger
All − IVW(MRE)
−1.0
−0.5
0.0
0.5
1.0
1.5
MR effect size for
'Gut microbiota abundance (genus Parasutterella id.2892)' on 'Nutritionally−regulated adipose and cardiac enriched protein hom

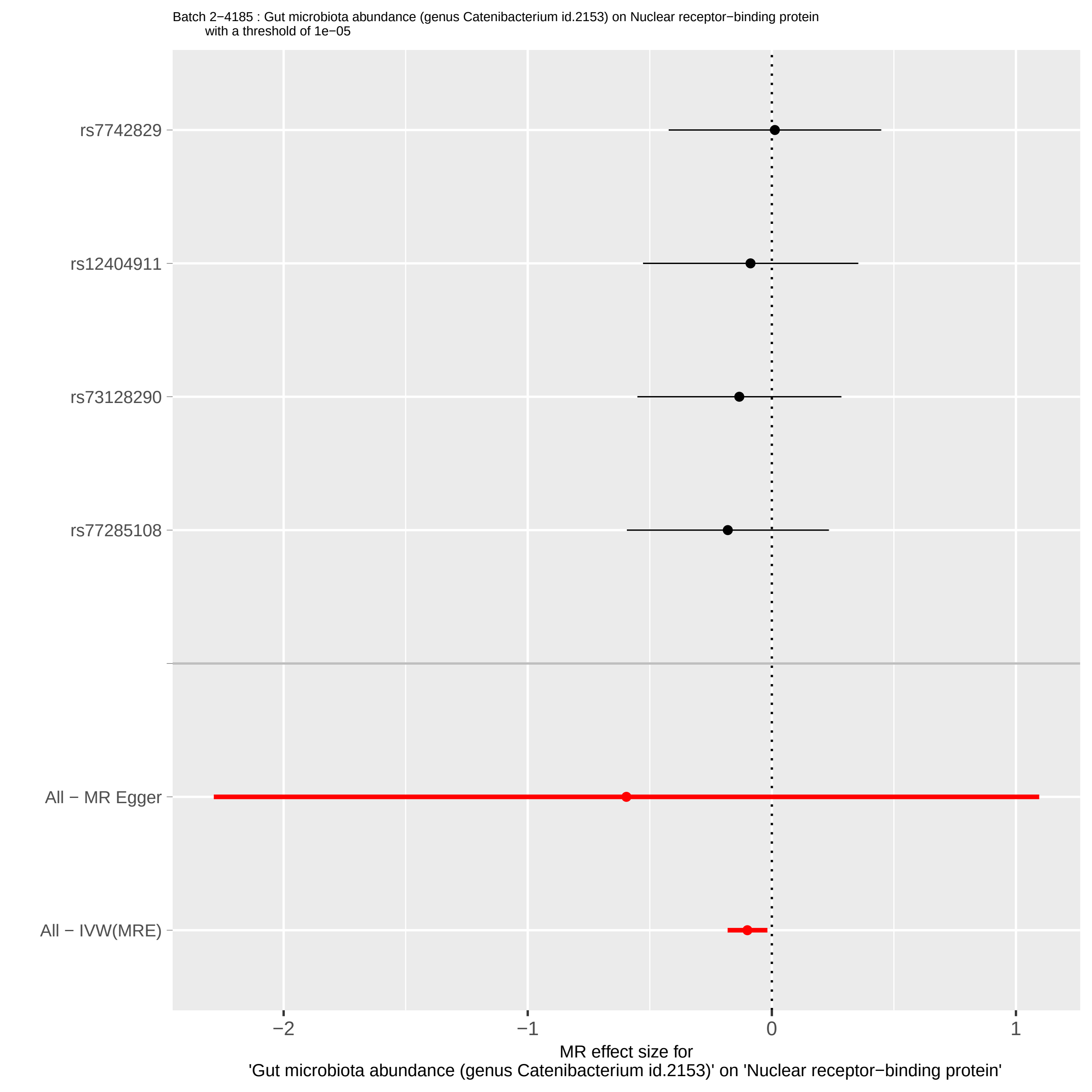

Batch 2−4185 : Gut microbiota abundance (genus Catenibacterium id.2153) on Nuclear receptor−binding protein
with a threshold of 1e−05
rs7742829
rs12404911
rs73128290
rs77285108
All − MR Egger
All − IVW(MRE)
−2
−1
0
1
MR effect size for
'Gut microbiota abundance (genus Catenibacterium id.2153)' on 'Nuclear receptor−binding protein'

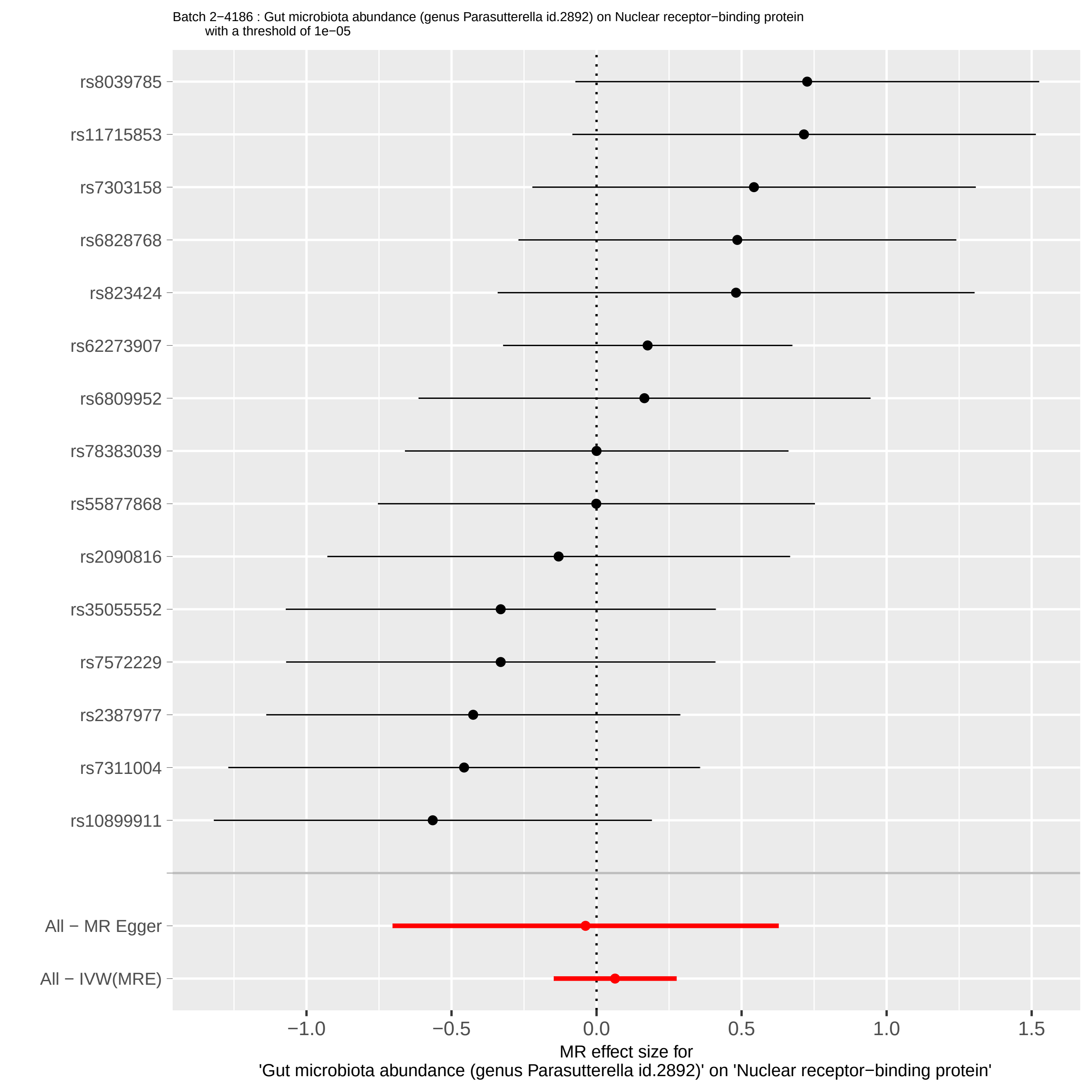
Batch 2−4186 : Gut microbiota abundance (genus Parasutterella id.2892) on Nuclear receptor−binding protein
with a threshold of 1e−05
rs8039785
rs11715853
rs7303158
rs6828768
rs823424
rs62273907
rs6809952
rs78383039
rs55877868
rs2090816
rs35055552
rs7572229
rs2387977
rs7311004
rs10899911
All − MR Egger
All − IVW(MRE)
−1.0
−0.5
0.0
0.5
1.0
1.5
MR effect size for
'Gut microbiota abundance (genus Parasutterella id.2892)' on 'Nuclear receptor−binding protein'

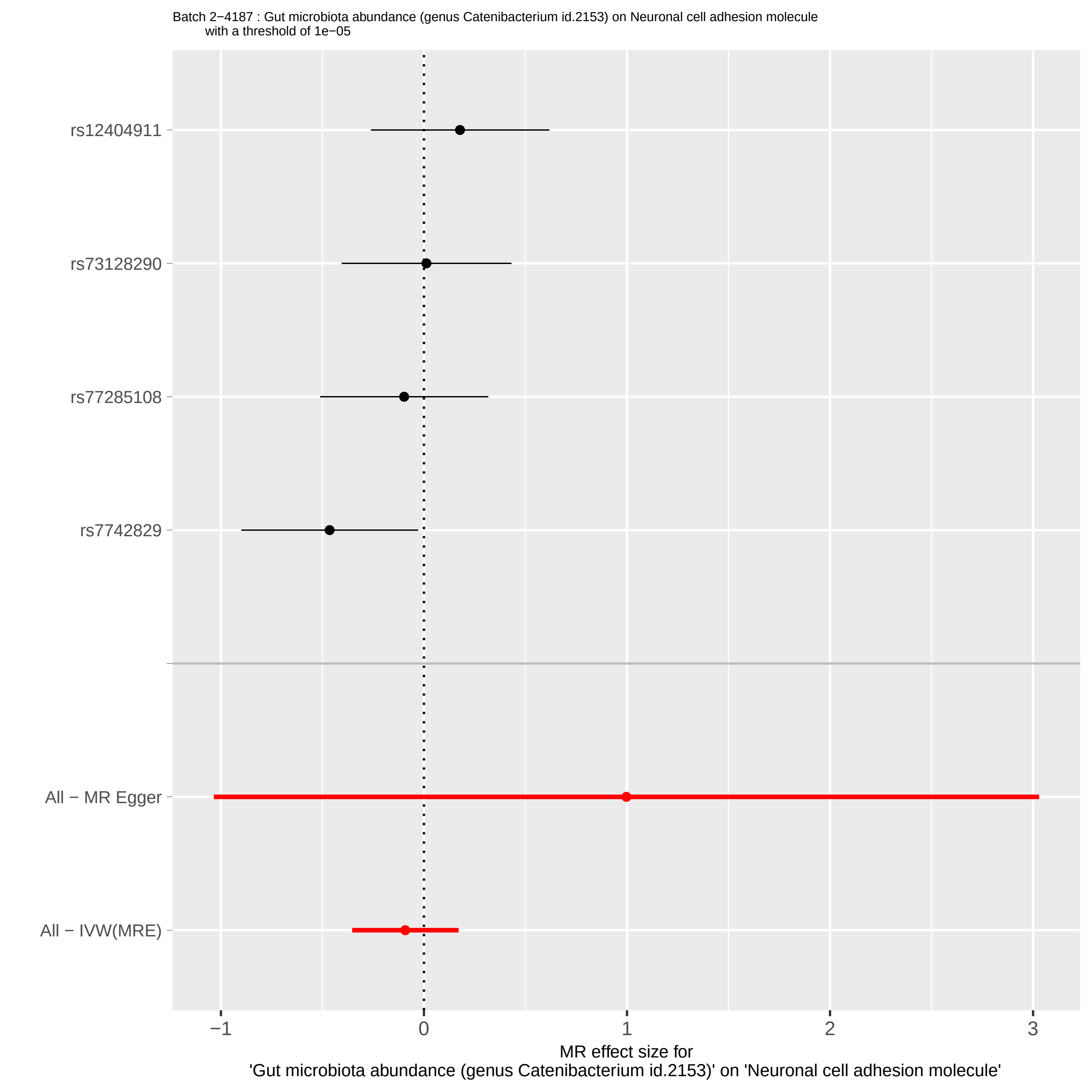

Batch 2−4187 : Gut microbiota abundance (genus Catenibacterium id.2153) on Neuronal cell adhesion molecule
with a threshold of 1e−05
rs12404911
rs73128290
rs77285108
rs7742829
All − MR Egger
All − IVW(MRE)
−1
0
1
2
3
MR effect size for
'Gut microbiota abundance (genus Catenibacterium id.2153)' on 'Neuronal cell adhesion molecule'

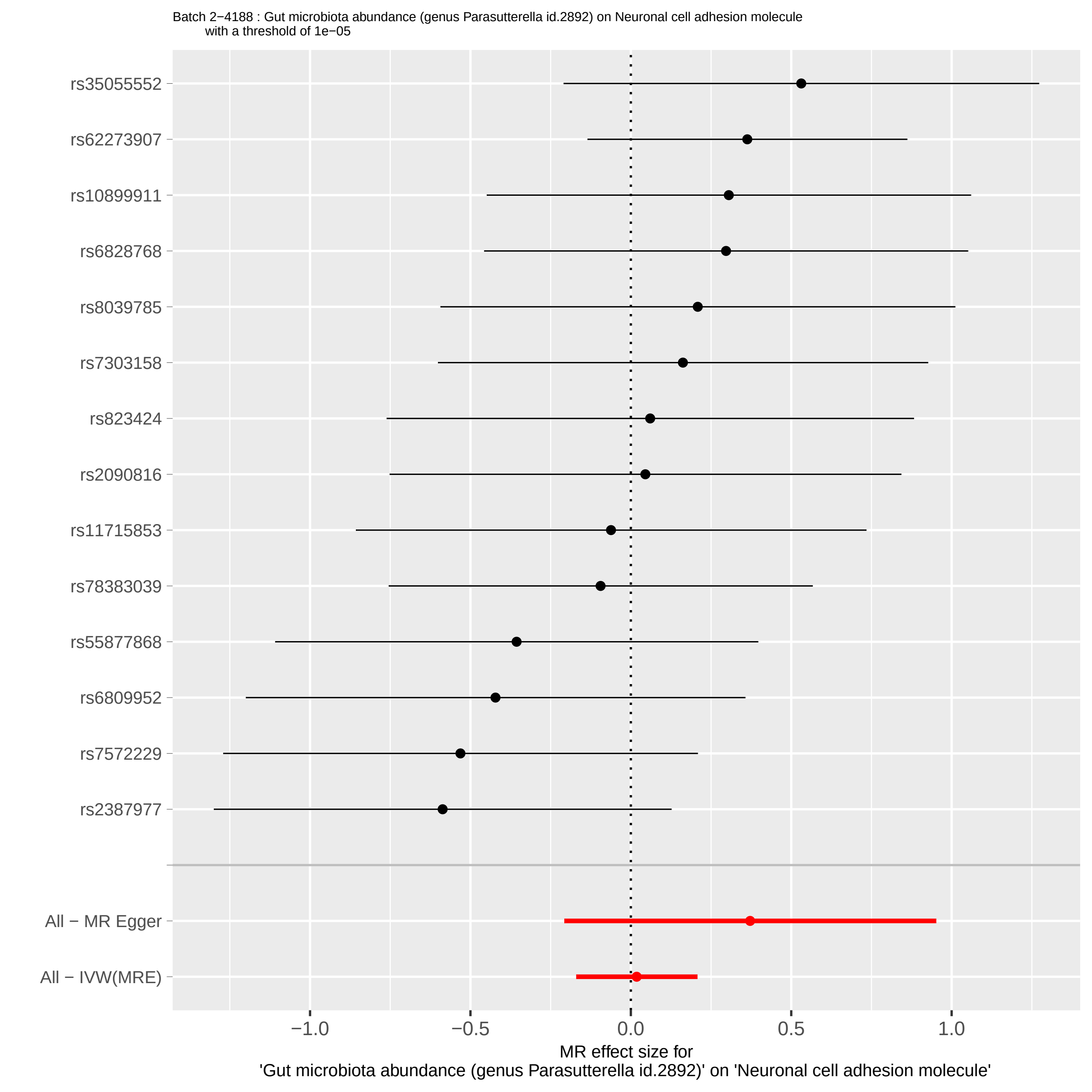

Batch 2−4188 : Gut microbiota abundance (genus Parasutterella id.2892) on Neuronal cell adhesion molecule
with a threshold of 1e−05
rs35055552
rs62273907
rs10899911
rs6828768
rs8039785
rs7303158
rs823424
rs2090816
rs11715853
rs78383039
rs55877868
rs6809952
rs7572229
rs2387977
All − MR Egger
All − IVW(MRE)
−1.0
−0.5
0.0
0.5
1.0
MR effect size for
'Gut microbiota abundance (genus Parasutterella id.2892)' on 'Neuronal cell adhesion molecule'

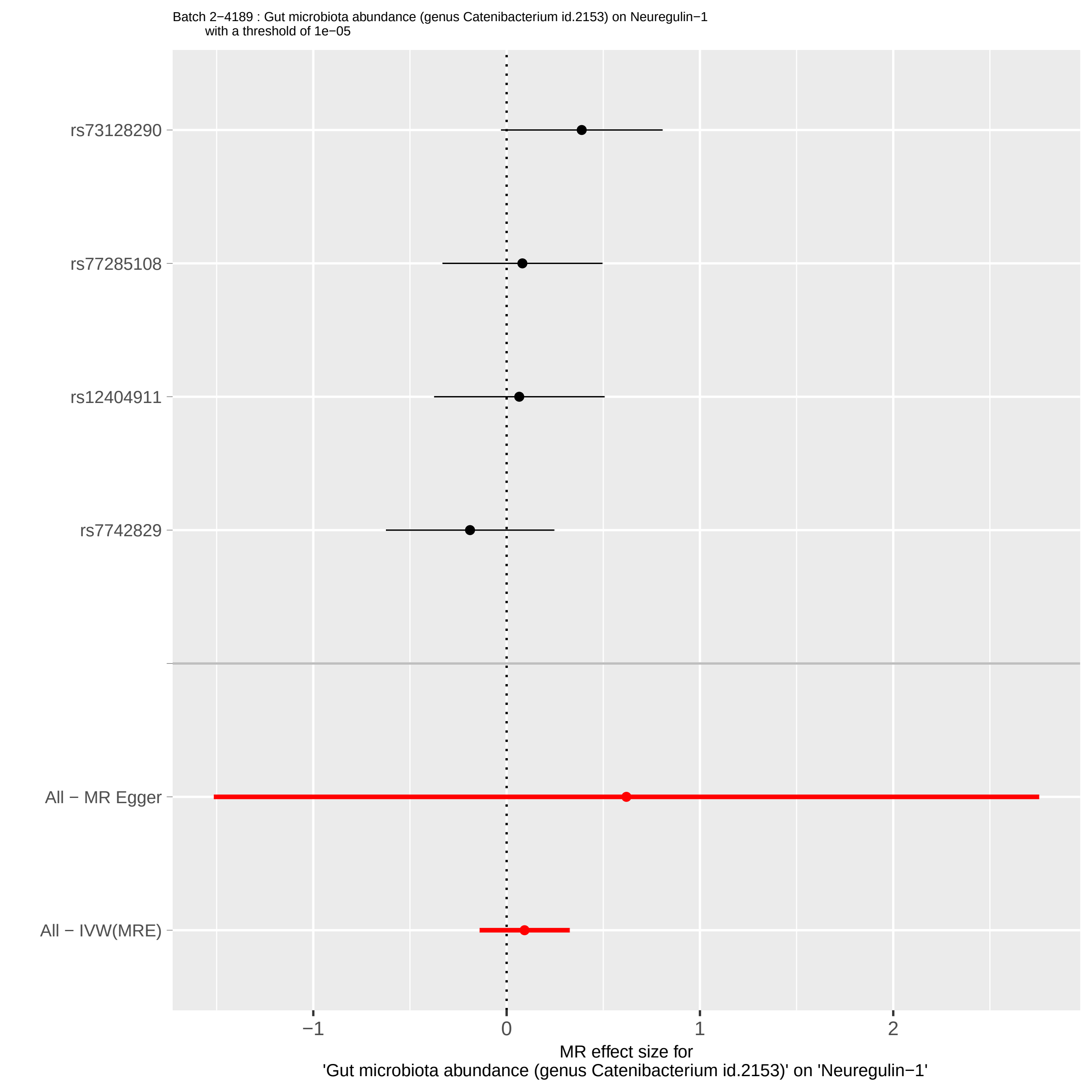

Batch 2−4189 : Gut microbiota abundance (genus Catenibacterium id.2153) on Neuregulin−1
with a threshold of 1e−05
rs73128290
rs77285108
rs12404911
rs7742829
All − MR Egger
All − IVW(MRE)
−1
0
1
2
MR effect size for
'Gut microbiota abundance (genus Catenibacterium id.2153)' on 'Neuregulin−1'

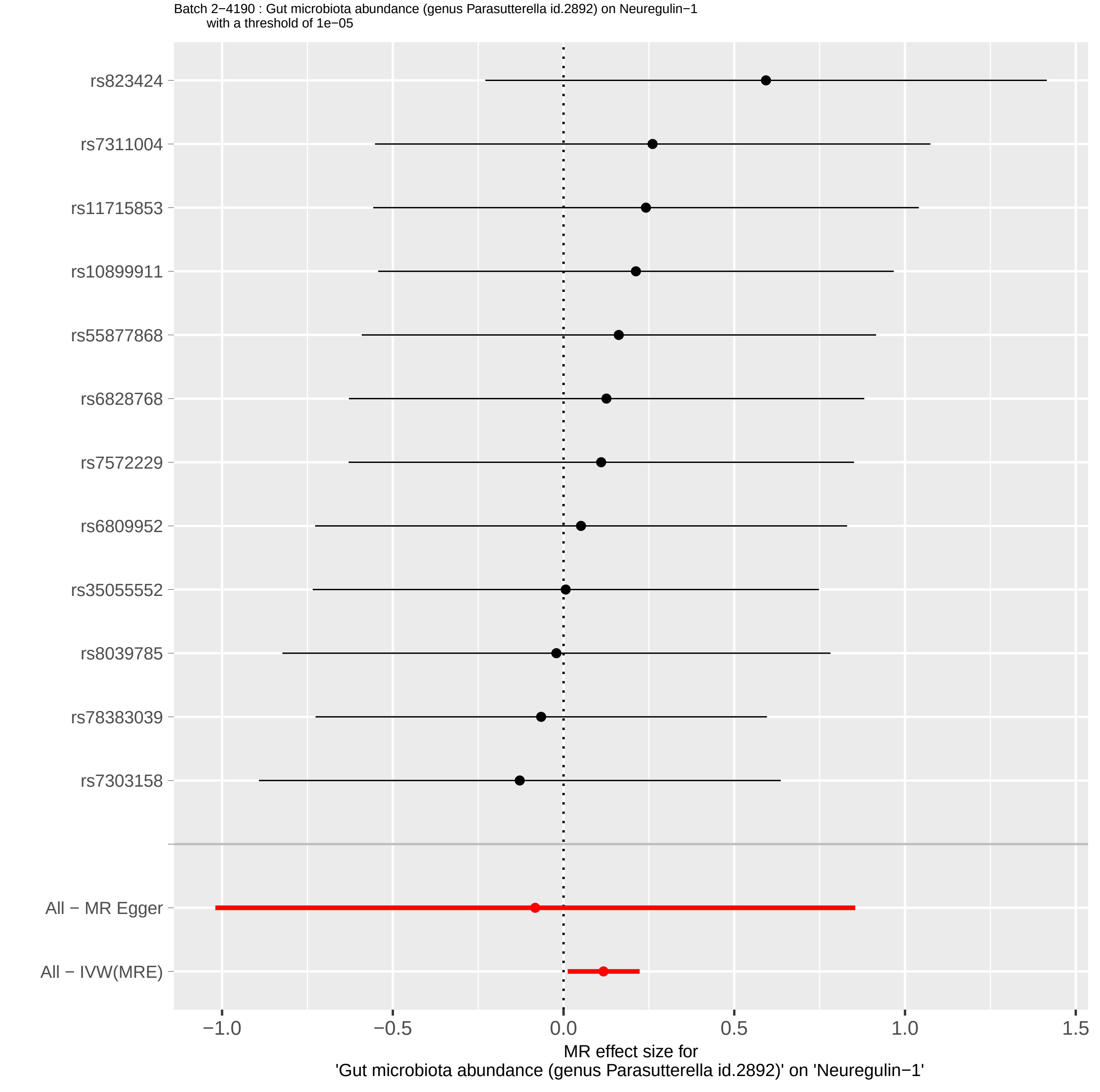

Batch 2−4190 : Gut microbiota abundance (genus Parasutterella id.2892) on Neuregulin−1
with a threshold of 1e−05
rs823424
rs7311004
rs11715853
rs10899911
rs55877868
rs6828768
rs7572229
rs6809952
rs35055552
rs8039785
rs78383039
rs7303158
All − MR Egger
All − IVW(MRE)
−1.0
−0.5
0.0
0.5
1.0
1.5
MR effect size for
'Gut microbiota abundance (genus Parasutterella id.2892)' on 'Neuregulin−1'

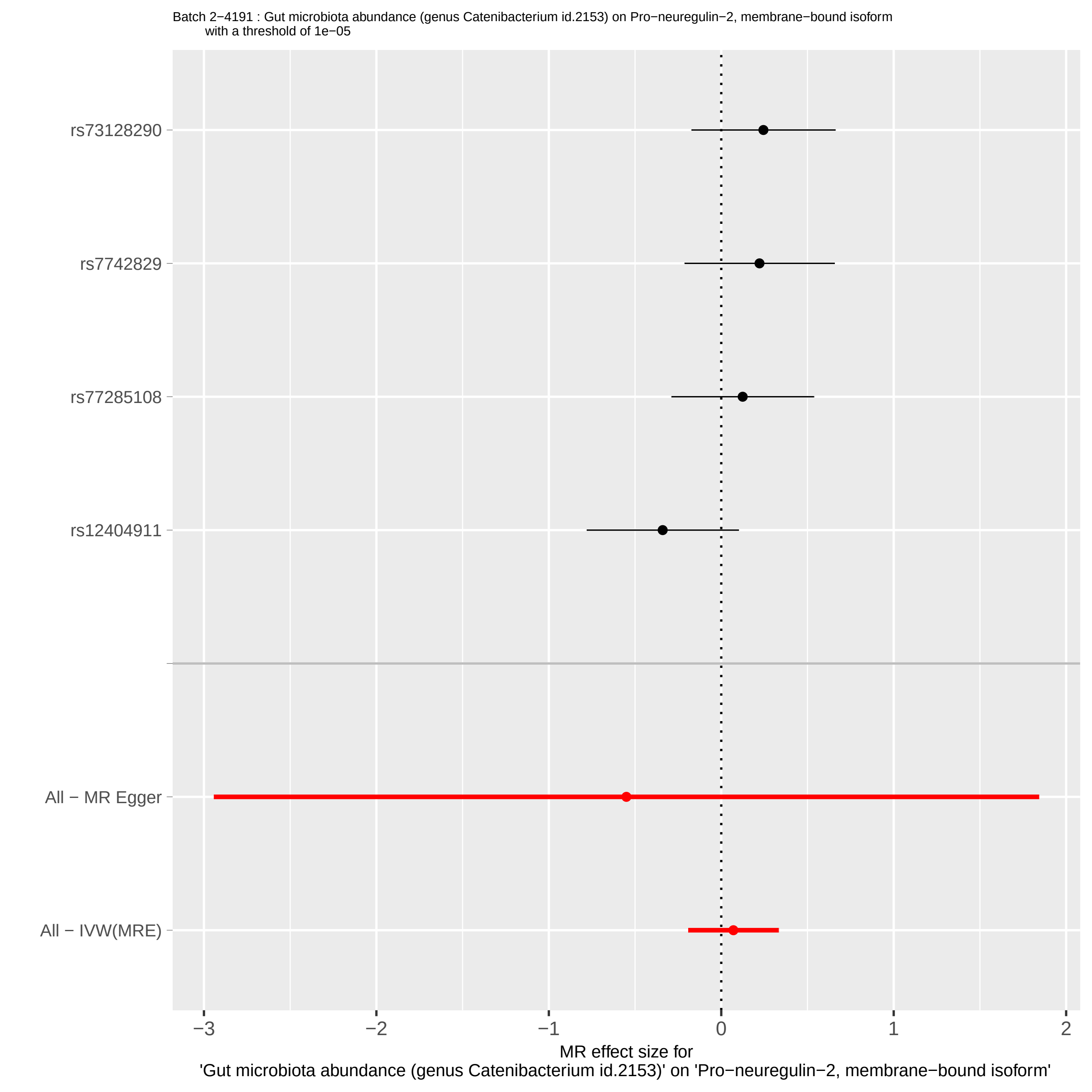
Batch 2−4191 : Gut microbiota abundance (genus Catenibacterium id.2153) on Pro−neuregulin−2, membrane−bound isoform
with a threshold of 1e−05
rs73128290
rs7742829
rs77285108
rs12404911
All − MR Egger
All − IVW(MRE)
−3
−2
−1
0
1
2
MR effect size for
'Gut microbiota abundance (genus Catenibacterium id.2153)' on 'Pro−neuregulin−2, membrane−bound isoform'

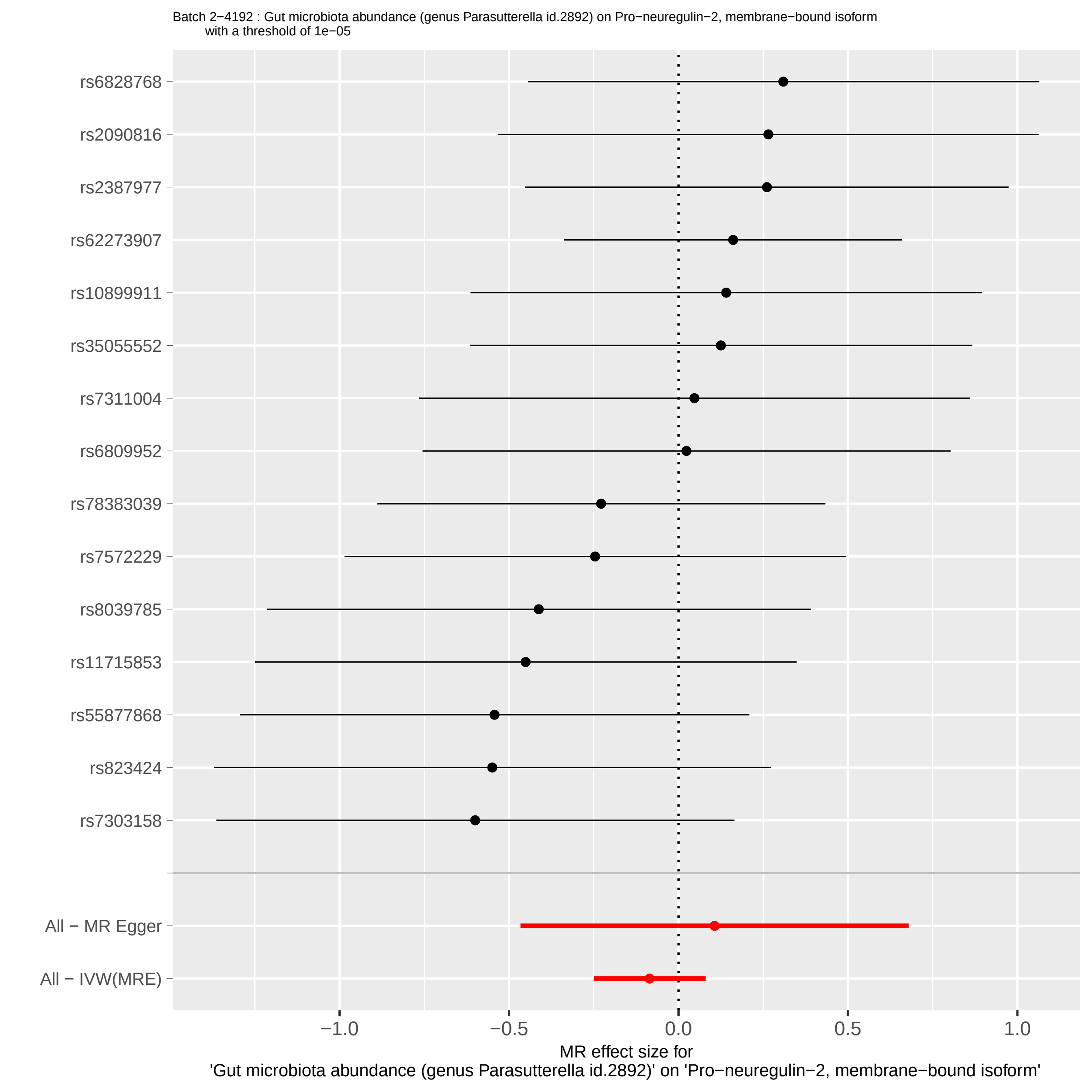
Batch 2−4192 : Gut microbiota abundance (genus Parasutterella id.2892) on Pro−neuregulin−2, membrane−bound isoform
with a threshold of 1e−05
rs6828768
rs2090816
rs2387977
rs62273907
rs10899911
rs35055552
rs7311004
rs6809952
rs78383039
rs7572229
rs8039785
rs11715853
rs55877868
rs823424
rs7303158
All − MR Egger
All − IVW(MRE)
−1.0
−0.5
0.0
0.5
1.0
MR effect size for
'Gut microbiota abundance (genus Parasutterella id.2892)' on 'Pro−neuregulin−2, membrane−bound isoform'

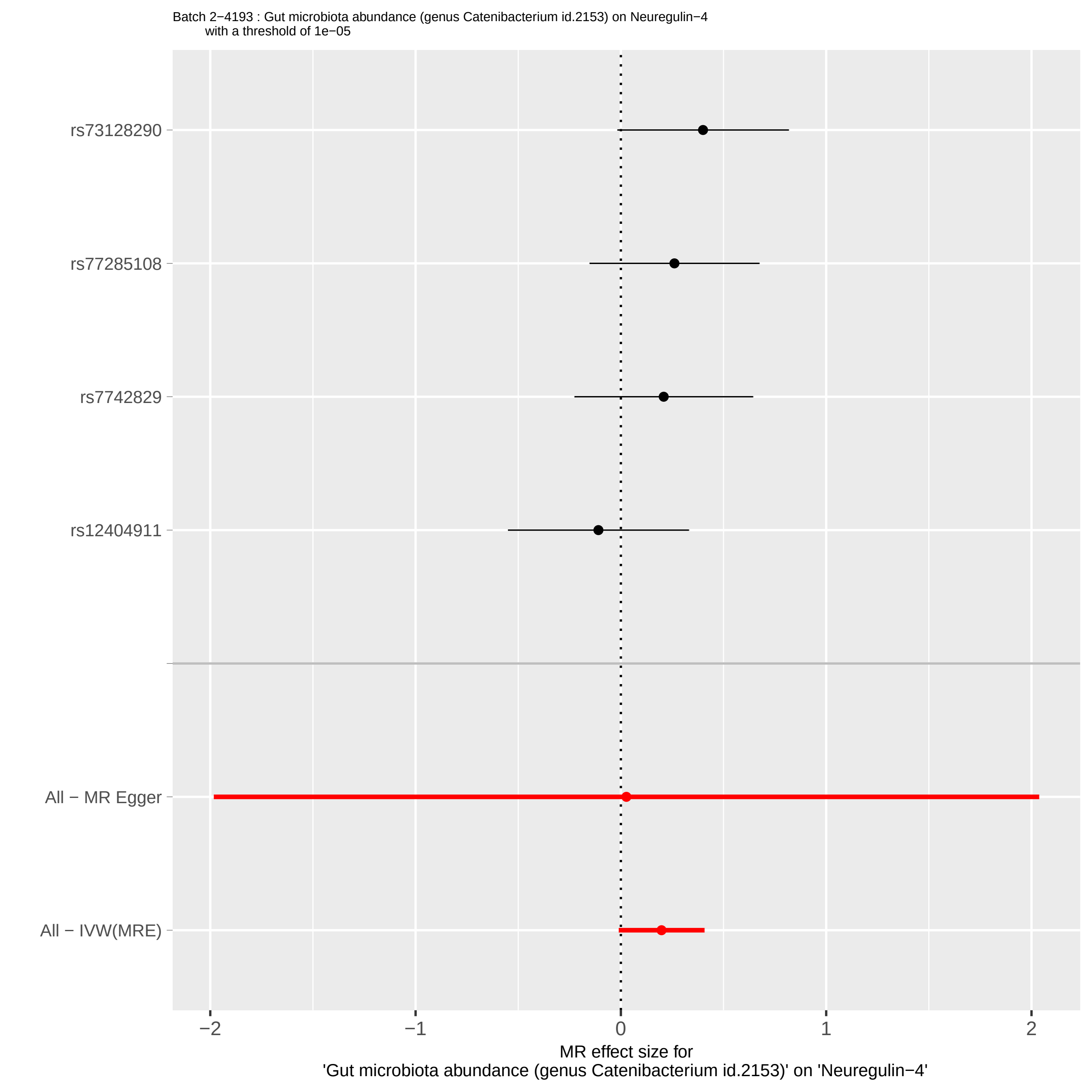
Batch 2−4193 : Gut microbiota abundance (genus Catenibacterium id.2153) on Neuregulin−4
with a threshold of 1e−05
rs73128290
rs77285108
rs7742829
rs12404911
All − MR Egger
All − IVW(MRE)
−2
−1
0
1
2
MR effect size for
'Gut microbiota abundance (genus Catenibacterium id.2153)' on 'Neuregulin−4'

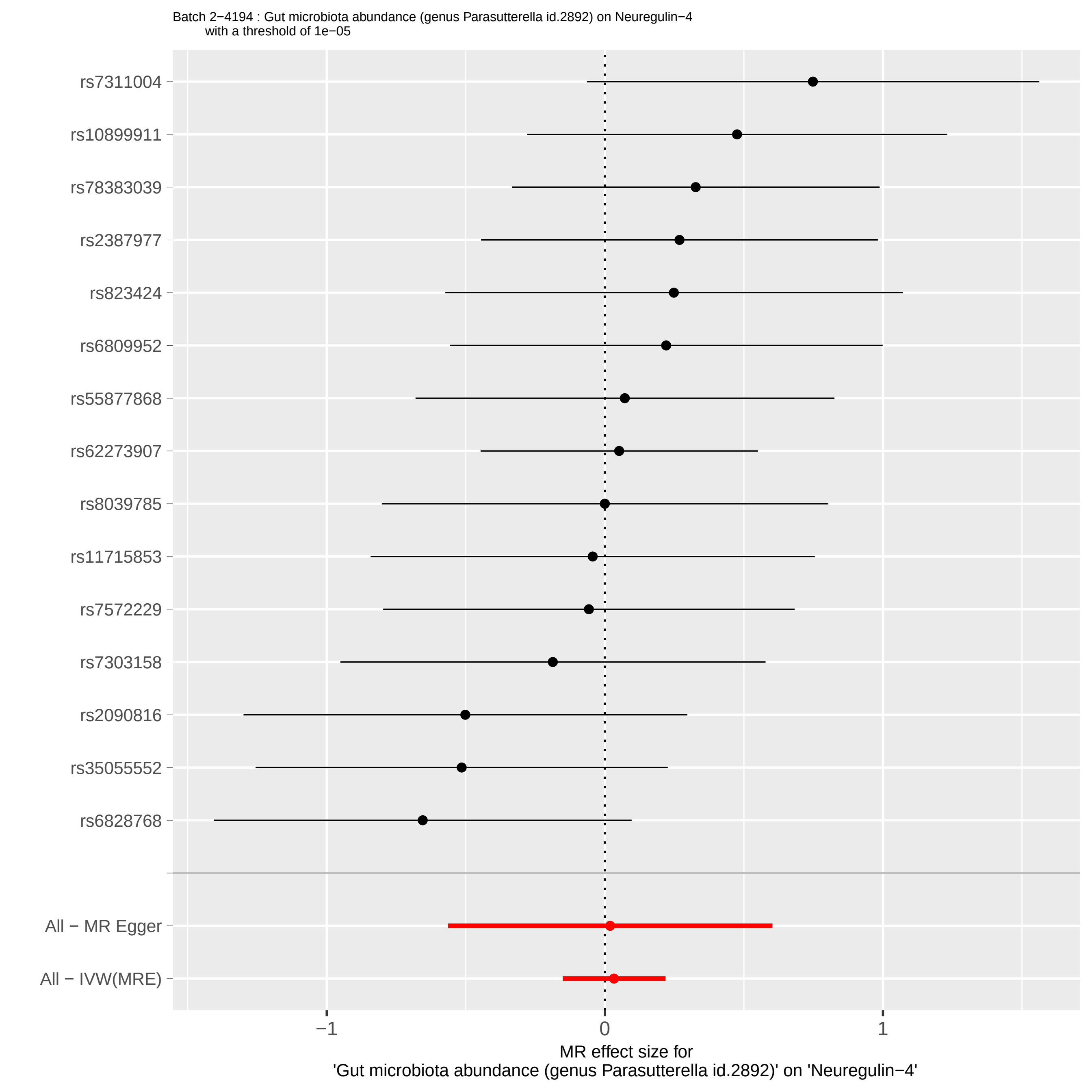

Batch 2−4194 : Gut microbiota abundance (genus Parasutterella id.2892) on Neuregulin−4
with a threshold of 1e−05
rs7311004
rs10899911
rs78383039
rs2387977
rs823424
rs6809952
rs55877868
rs62273907
rs8039785
rs11715853
rs7572229
rs7303158
rs2090816
rs35055552
rs6828768
All − MR Egger
All − IVW(MRE)
−1
0
1
MR effect size for
'Gut microbiota abundance (genus Parasutterella id.2892)' on 'Neuregulin−4'

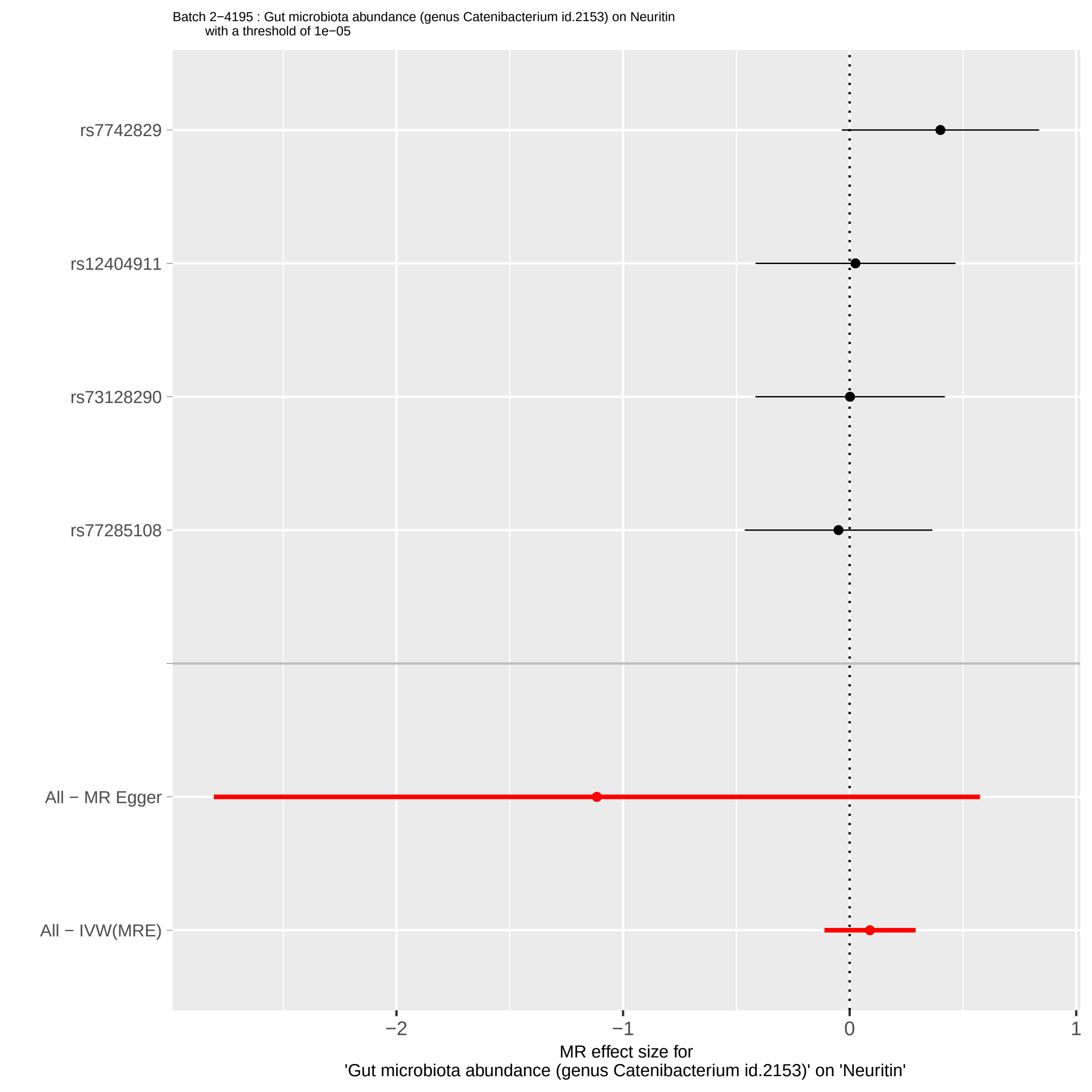

Batch 2−4195 : Gut microbiota abundance (genus Catenibacterium id.2153) on Neuritin
with a threshold of 1e−05
rs7742829
rs12404911
rs73128290
rs77285108
All − MR Egger
All − IVW(MRE)
−2
−1
0
1
MR effect size for
'Gut microbiota abundance (genus Catenibacterium id.2153)' on 'Neuritin'

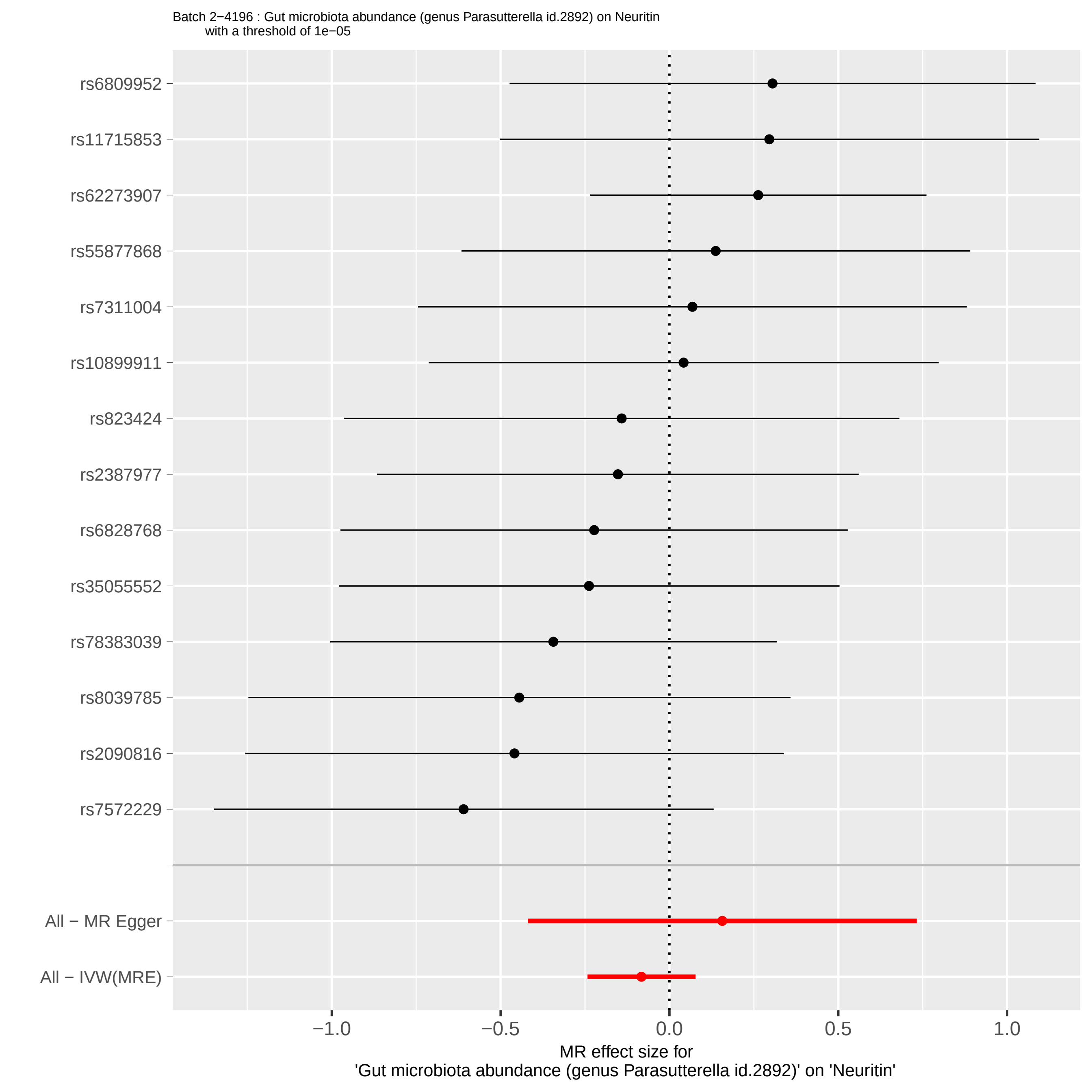
Batch 2−4196 : Gut microbiota abundance (genus Parasutterella id.2892) on Neuritin
with a threshold of 1e−05
rs6809952
rs11715853
rs62273907
rs55877868
rs7311004
rs10899911
rs823424
rs2387977
rs6828768
rs35055552
rs78383039
rs8039785
rs2090816
rs7572229
All − MR Egger
All − IVW(MRE)
−1.0
−0.5
0.0
0.5
1.0
MR effect size for
'Gut microbiota abundance (genus Parasutterella id.2892)' on 'Neuritin'

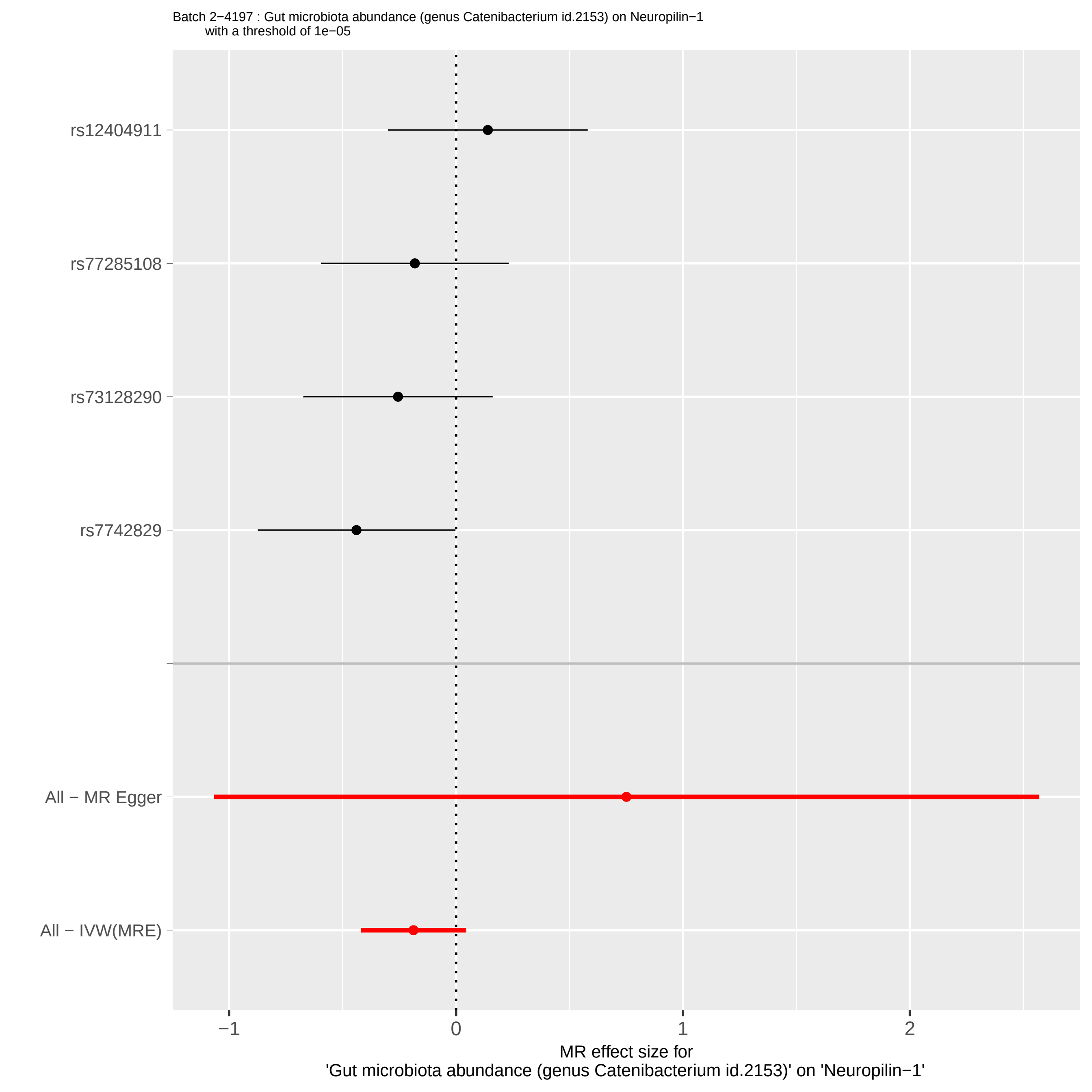

Batch 2−4197 : Gut microbiota abundance (genus Catenibacterium id.2153) on Neuropilin−1
with a threshold of 1e−05
rs12404911
rs77285108
rs73128290
rs7742829
All − MR Egger
All − IVW(MRE)
−1
0
1
2
MR effect size for
'Gut microbiota abundance (genus Catenibacterium id.2153)' on 'Neuropilin−1'

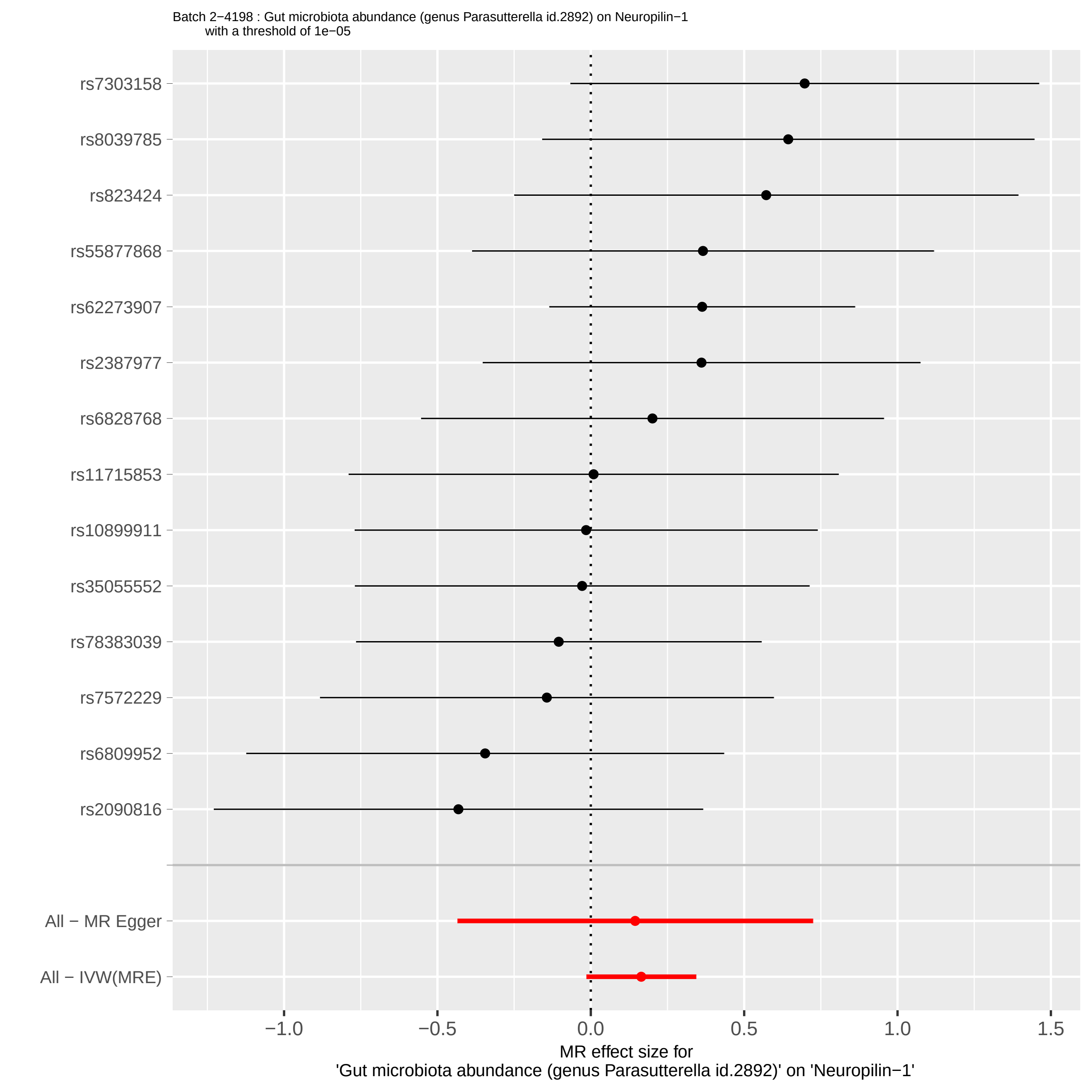

Batch 2−4198 : Gut microbiota abundance (genus Parasutterella id.2892) on Neuropilin−1
with a threshold of 1e−05
rs7303158
rs8039785
rs823424
rs55877868
rs62273907
rs2387977
rs6828768
rs11715853
rs10899911
rs35055552
rs78383039
rs7572229
rs6809952
rs2090816
All − MR Egger
All − IVW(MRE)
−1.0
−0.5
0.0
0.5
1.0
1.5
MR effect size for
'Gut microbiota abundance (genus Parasutterella id.2892)' on 'Neuropilin−1'

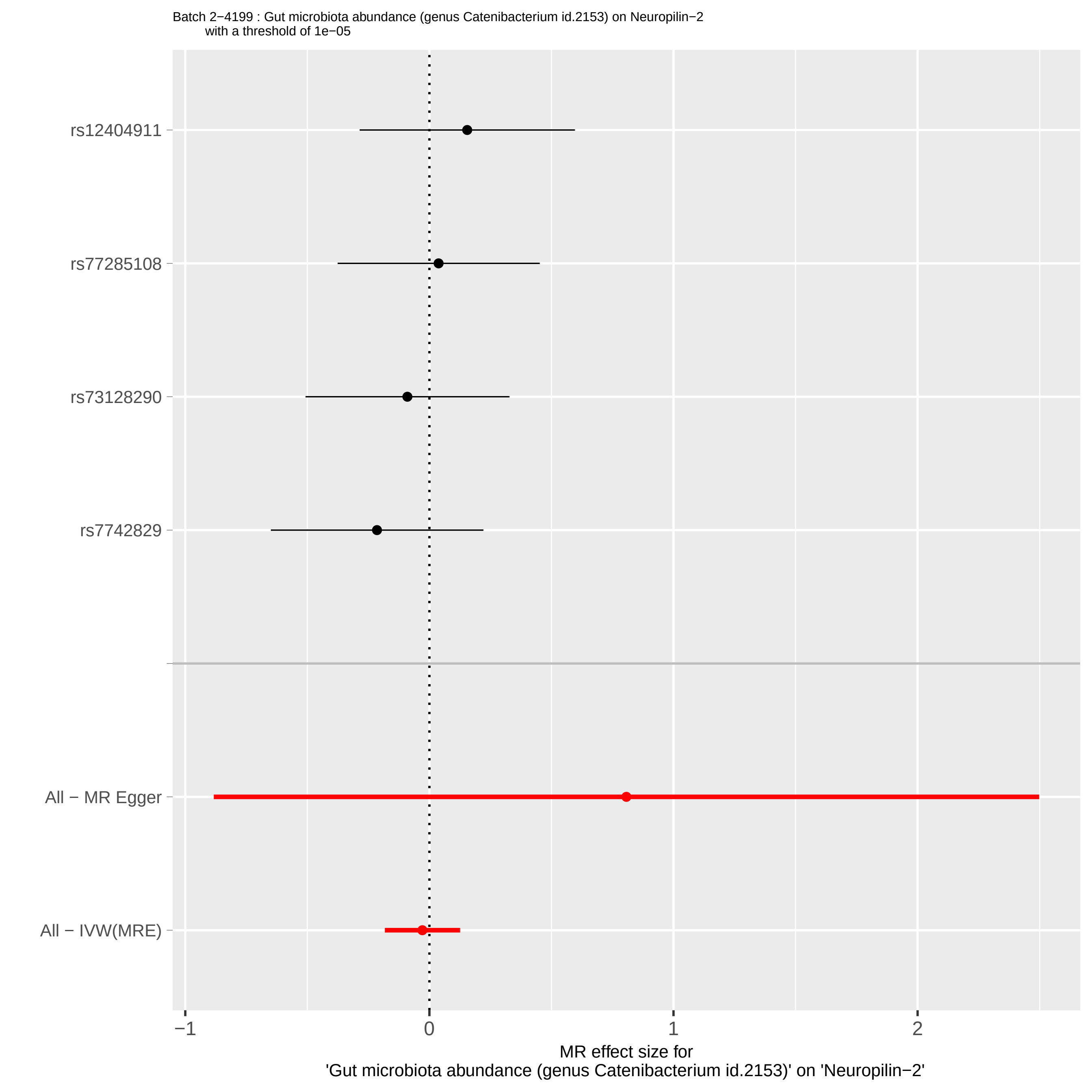
Batch 2−4199 : Gut microbiota abundance (genus Catenibacterium id.2153) on Neuropilin−2
with a threshold of 1e−05
rs12404911
rs77285108
rs73128290
rs7742829
All − MR Egger
All − IVW(MRE)
−1
0
1
2
MR effect size for
'Gut microbiota abundance (genus Catenibacterium id.2153)' on 'Neuropilin−2'

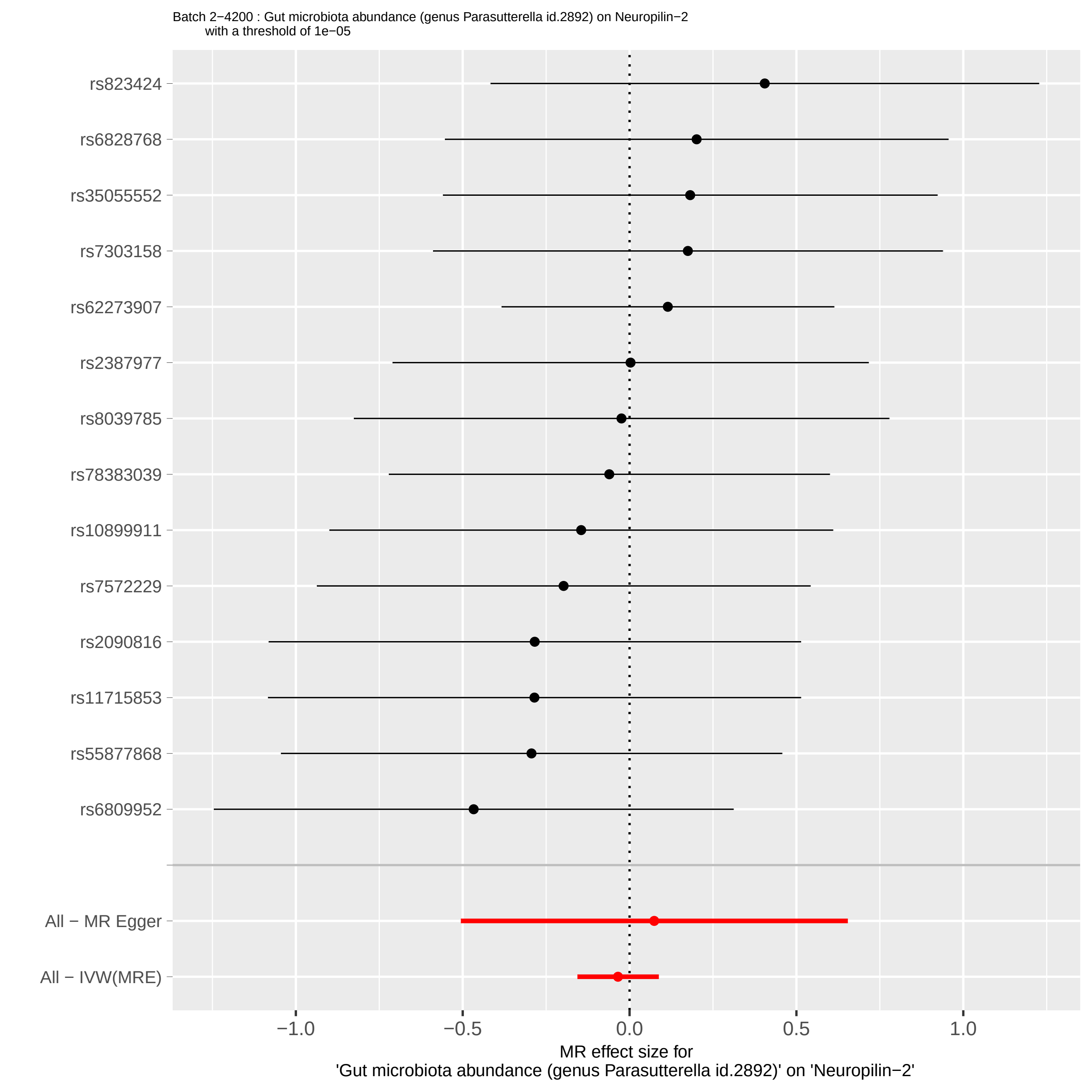

Batch 2−4200 : Gut microbiota abundance (genus Parasutterella id.2892) on Neuropilin−2
with a threshold of 1e−05
rs823424
rs6828768
rs35055552
rs7303158
rs62273907
rs2387977
rs8039785
rs78383039
rs10899911
rs7572229
rs2090816
rs11715853
rs55877868
rs6809952
All − MR Egger
All − IVW(MRE)
−1.0
−0.5
0.0
0.5
1.0
MR effect size for
'Gut microbiota abundance (genus Parasutterella id.2892)' on 'Neuropilin−2'

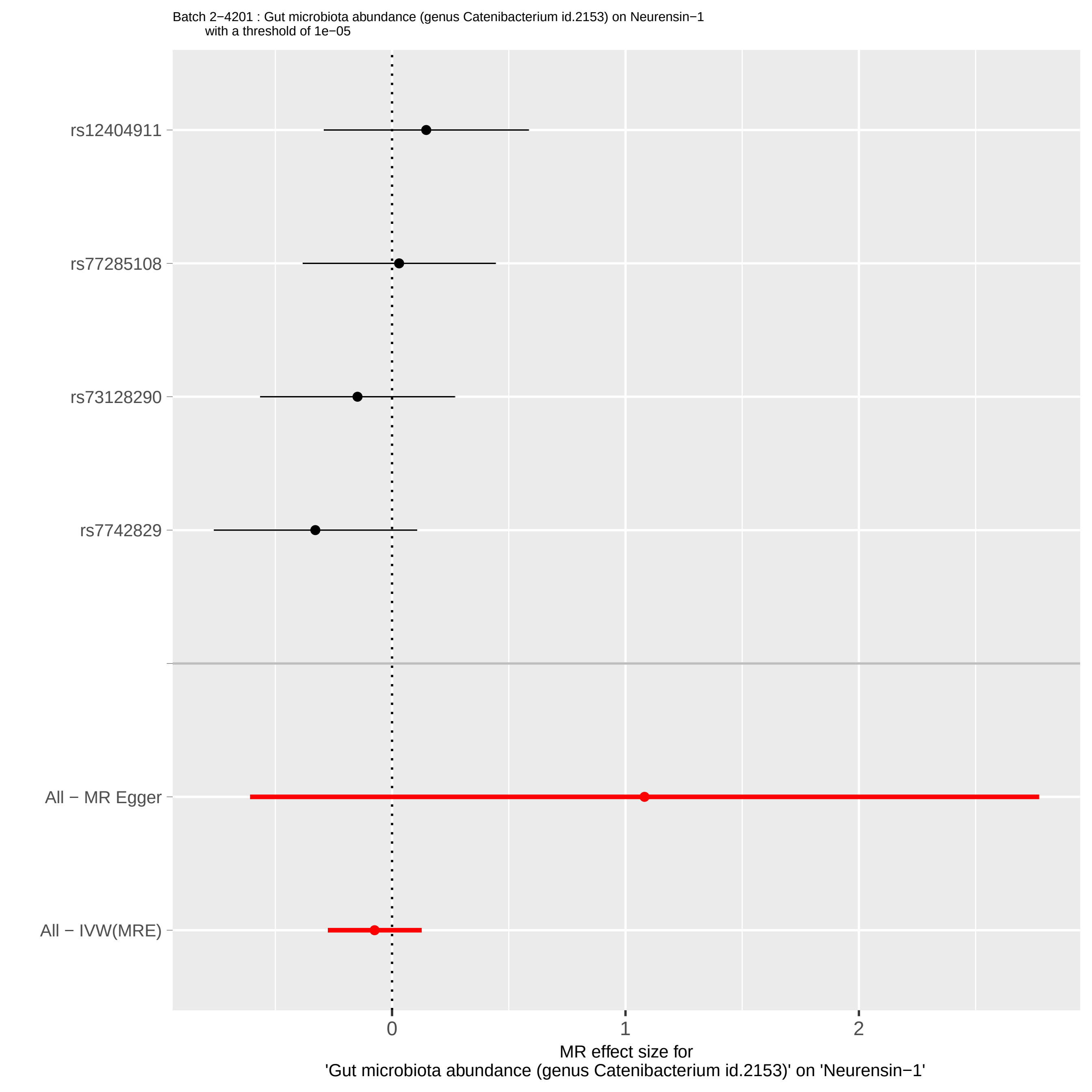
Batch 2−4201 : Gut microbiota abundance (genus Catenibacterium id.2153) on Neurensin−1
with a threshold of 1e−05
rs12404911
rs77285108
rs73128290
rs7742829
All − MR Egger
All − IVW(MRE)
0
1
2
MR effect size for
'Gut microbiota abundance (genus Catenibacterium id.2153)' on 'Neurensin−1'

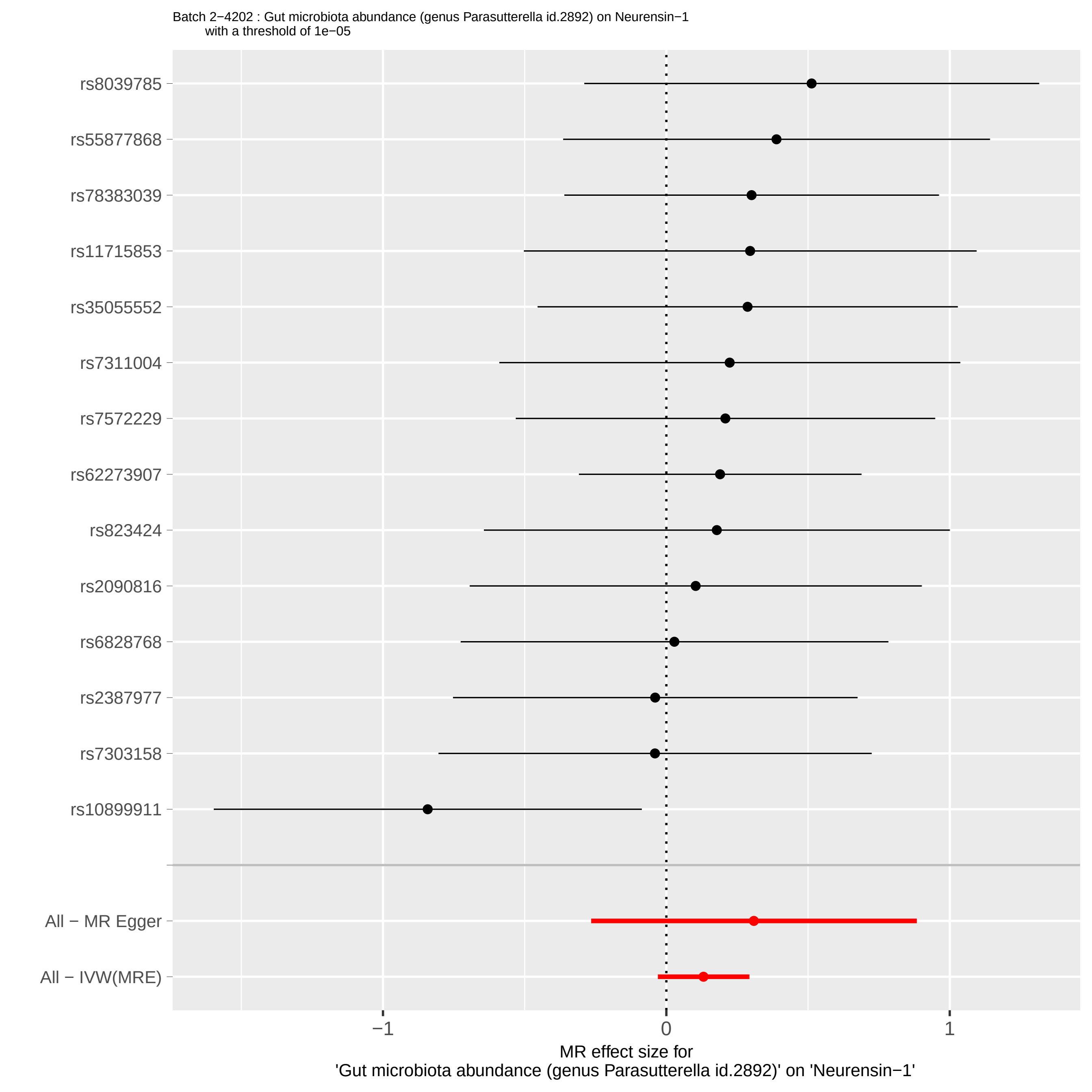

Batch 2−4202 : Gut microbiota abundance (genus Parasutterella id.2892) on Neurensin−1
with a threshold of 1e−05
rs8039785
rs55877868
rs78383039
rs11715853
rs35055552
rs7311004
rs7572229
rs62273907
rs823424
rs2090816
rs6828768
rs2387977
rs7303158
rs10899911
All − MR Egger
All − IVW(MRE)
−1
0
1
MR effect size for
'Gut microbiota abundance (genus Parasutterella id.2892)' on 'Neurensin−1'

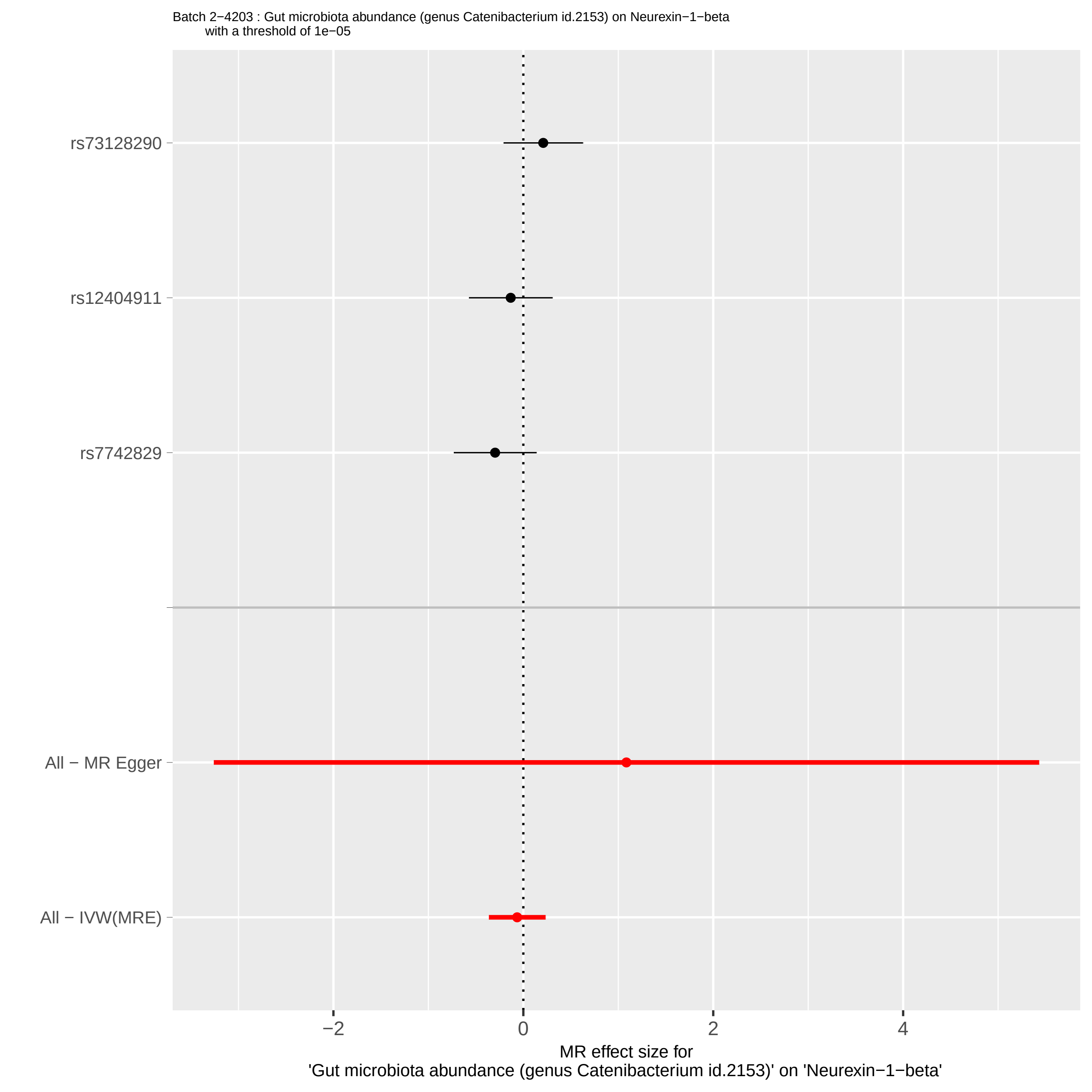

Batch 2−4203 : Gut microbiota abundance (genus Catenibacterium id.2153) on Neurexin−1−beta
with a threshold of 1e−05
rs73128290
rs12404911
rs7742829
All − MR Egger
All − IVW(MRE)
−2
0
2
4
MR effect size for
'Gut microbiota abundance (genus Catenibacterium id.2153)' on 'Neurexin−1−beta'

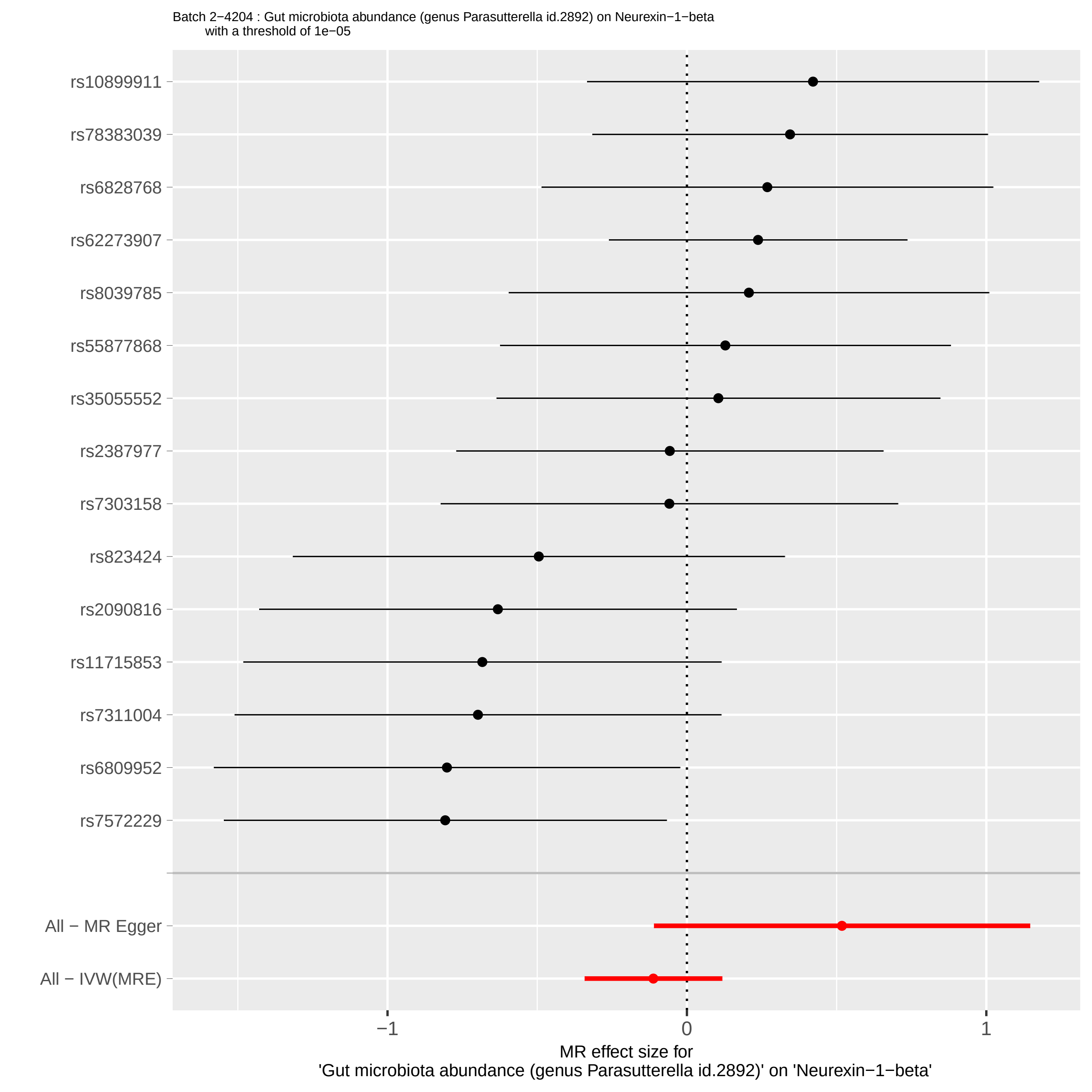

Batch 2−4204 : Gut microbiota abundance (genus Parasutterella id.2892) on Neurexin−1−beta
with a threshold of 1e−05
rs10899911
rs78383039
rs6828768
rs62273907
rs8039785
rs55877868
rs35055552
rs2387977
rs7303158
rs823424
rs2090816
rs11715853
rs7311004
rs6809952
rs7572229
All − MR Egger
All − IVW(MRE)
−1
0
1
MR effect size for
'Gut microbiota abundance (genus Parasutterella id.2892)' on 'Neurexin−1−beta'

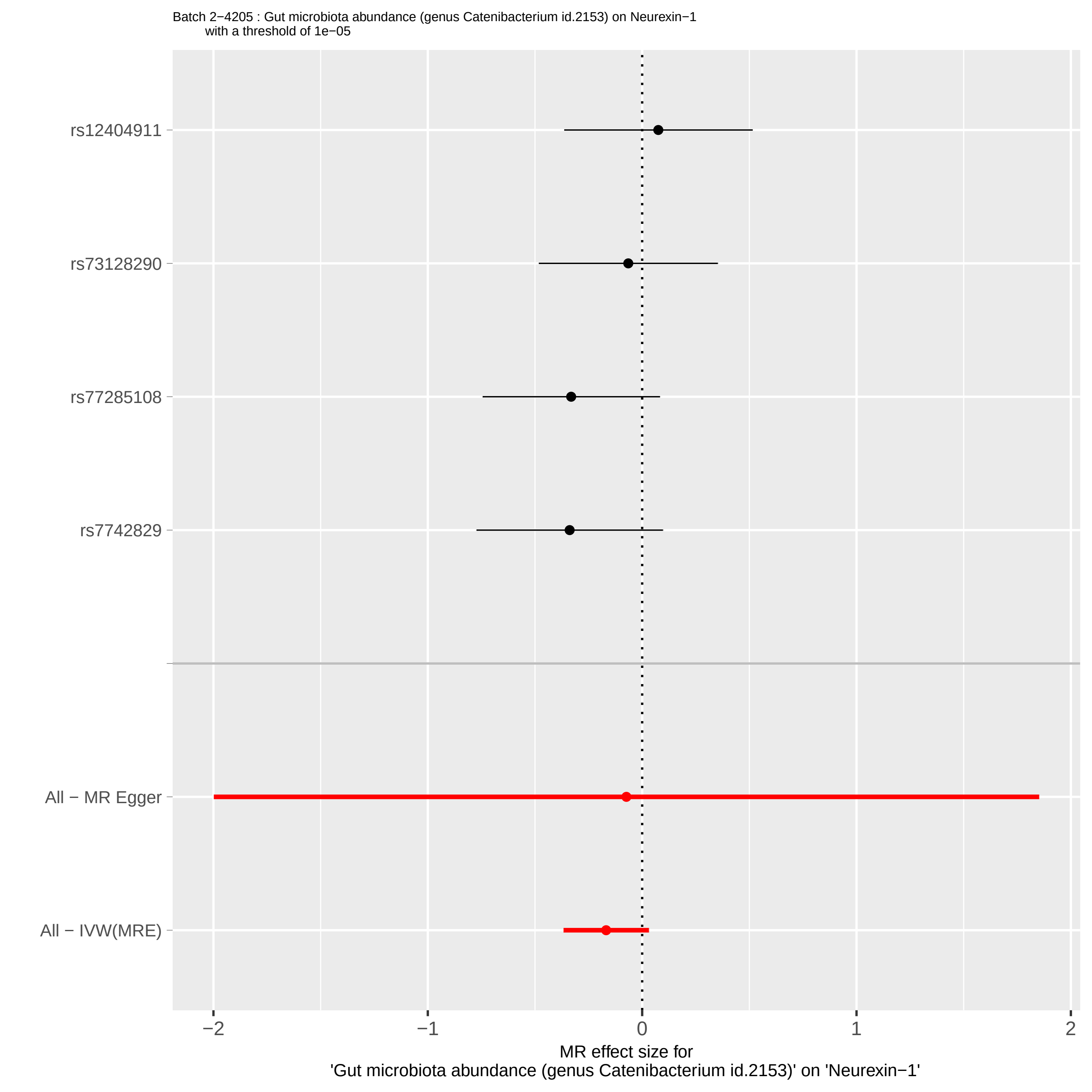
Batch 2−4205 : Gut microbiota abundance (genus Catenibacterium id.2153) on Neurexin−1
with a threshold of 1e−05
rs12404911
rs73128290
rs77285108
rs7742829
All − MR Egger
All − IVW(MRE)
−2
−1
0
1
2
MR effect size for
'Gut microbiota abundance (genus Catenibacterium id.2153)' on 'Neurexin−1'

Batch 2−4206 : Gut microbiota abundance (genus Parasutterella id.2892) on Neurexin−1
with a threshold of 1e−05

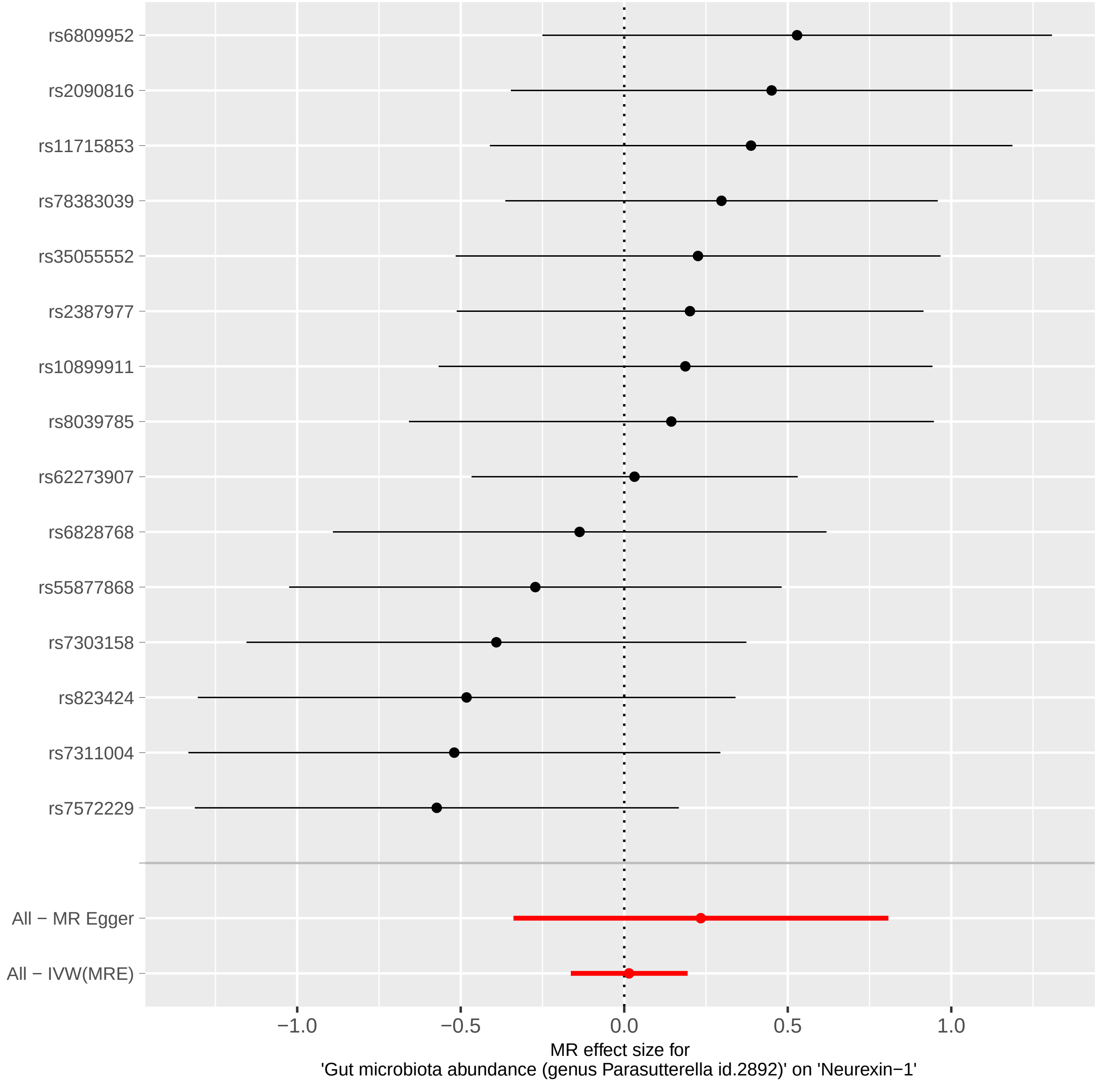

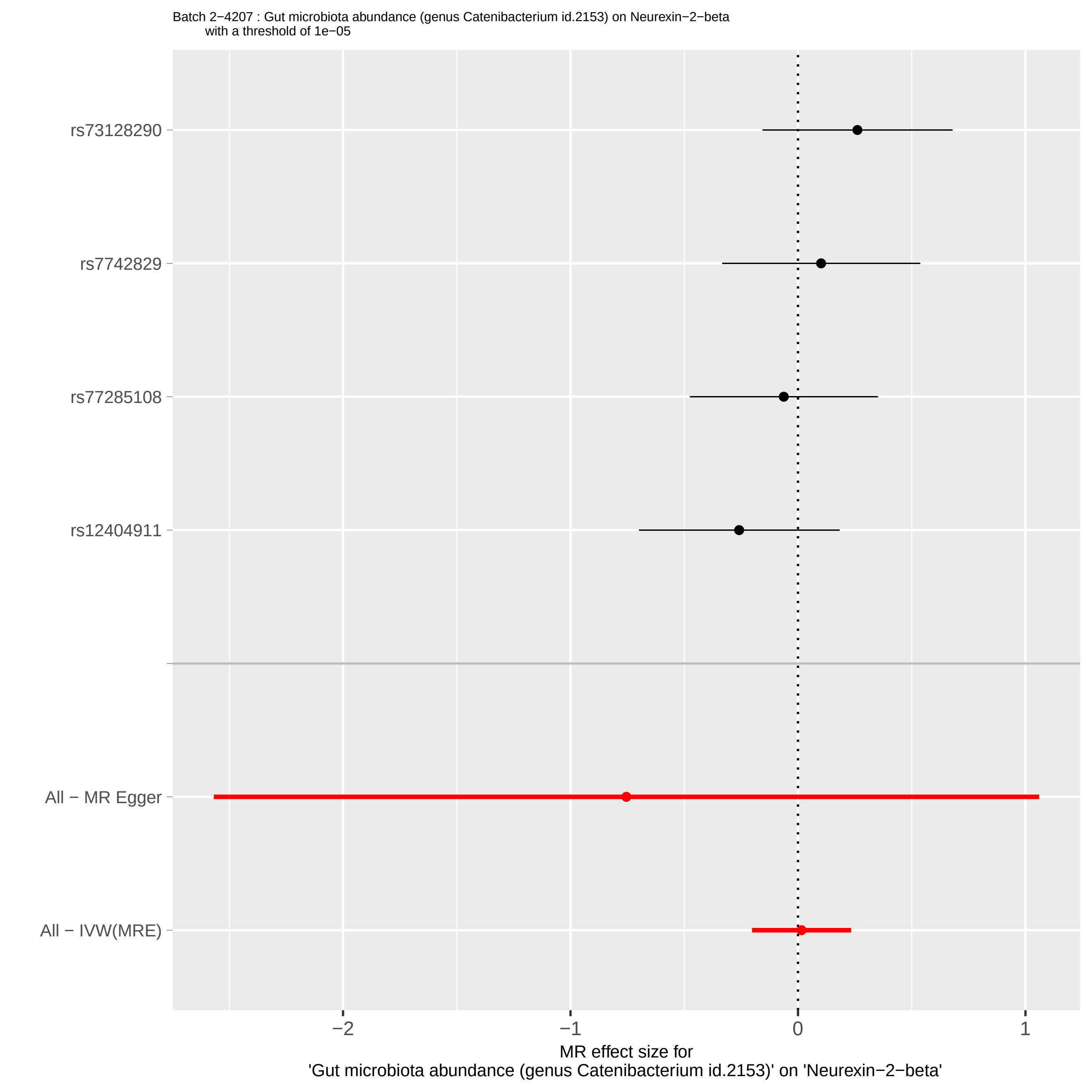

Batch 2−4207 : Gut microbiota abundance (genus Catenibacterium id.2153) on Neurexin−2−beta
with a threshold of 1e−05
rs73128290
rs7742829
rs77285108
rs12404911
All − MR Egger
All − IVW(MRE)
−2
−1
0
1
MR effect size for
'Gut microbiota abundance (genus Catenibacterium id.2153)' on 'Neurexin−2−beta'

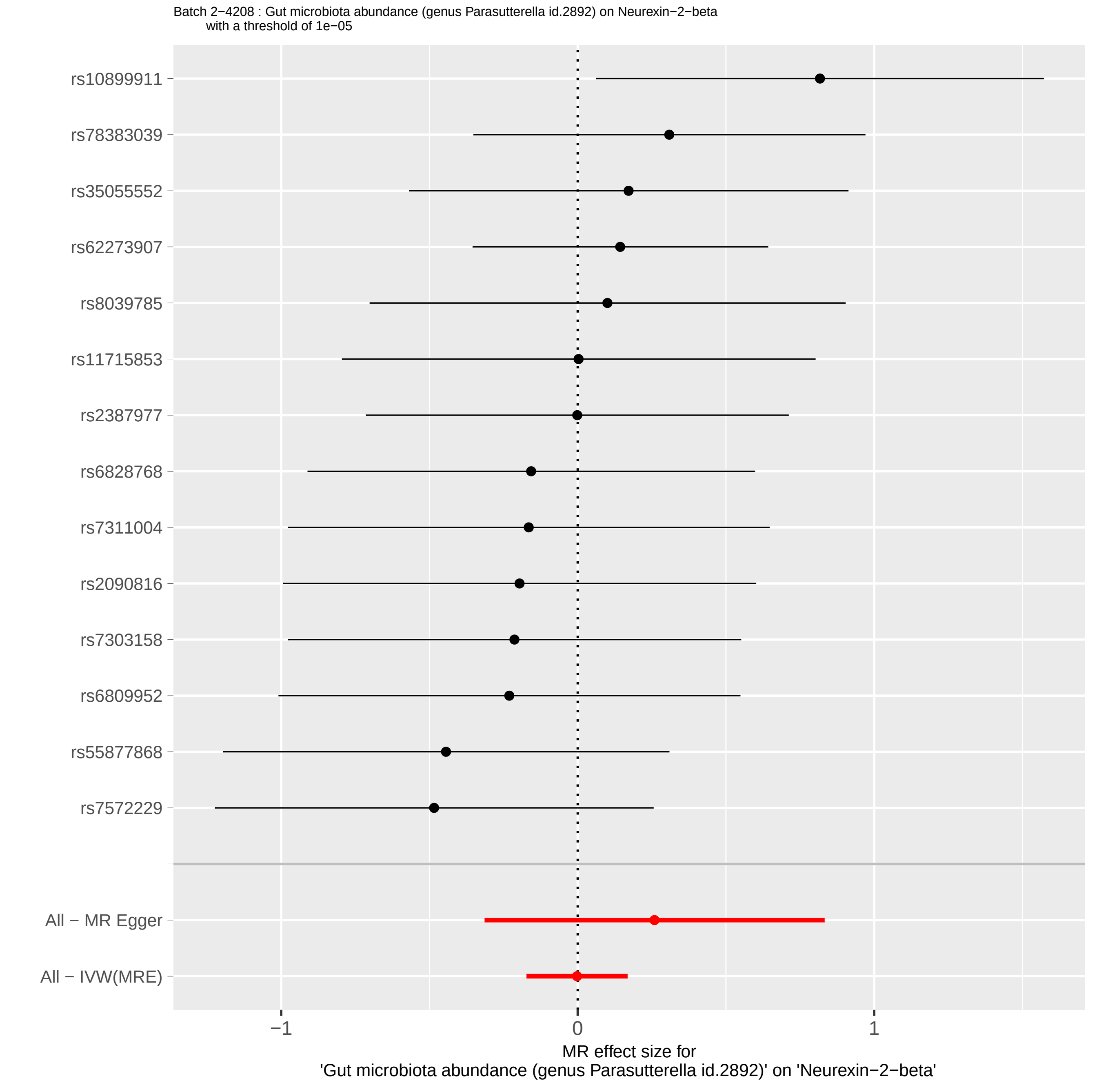

Batch 2−4208 : Gut microbiota abundance (genus Parasutterella id.2892) on Neurexin−2−beta
with a threshold of 1e−05
rs10899911
rs78383039
rs35055552
rs62273907
rs8039785
rs11715853
rs2387977
rs6828768
rs7311004
rs2090816
rs7303158
rs6809952
rs55877868
rs7572229
All − MR Egger
All − IVW(MRE)
−1
0
1
MR effect size for
'Gut microbiota abundance (genus Parasutterella id.2892)' on 'Neurexin−2−beta'

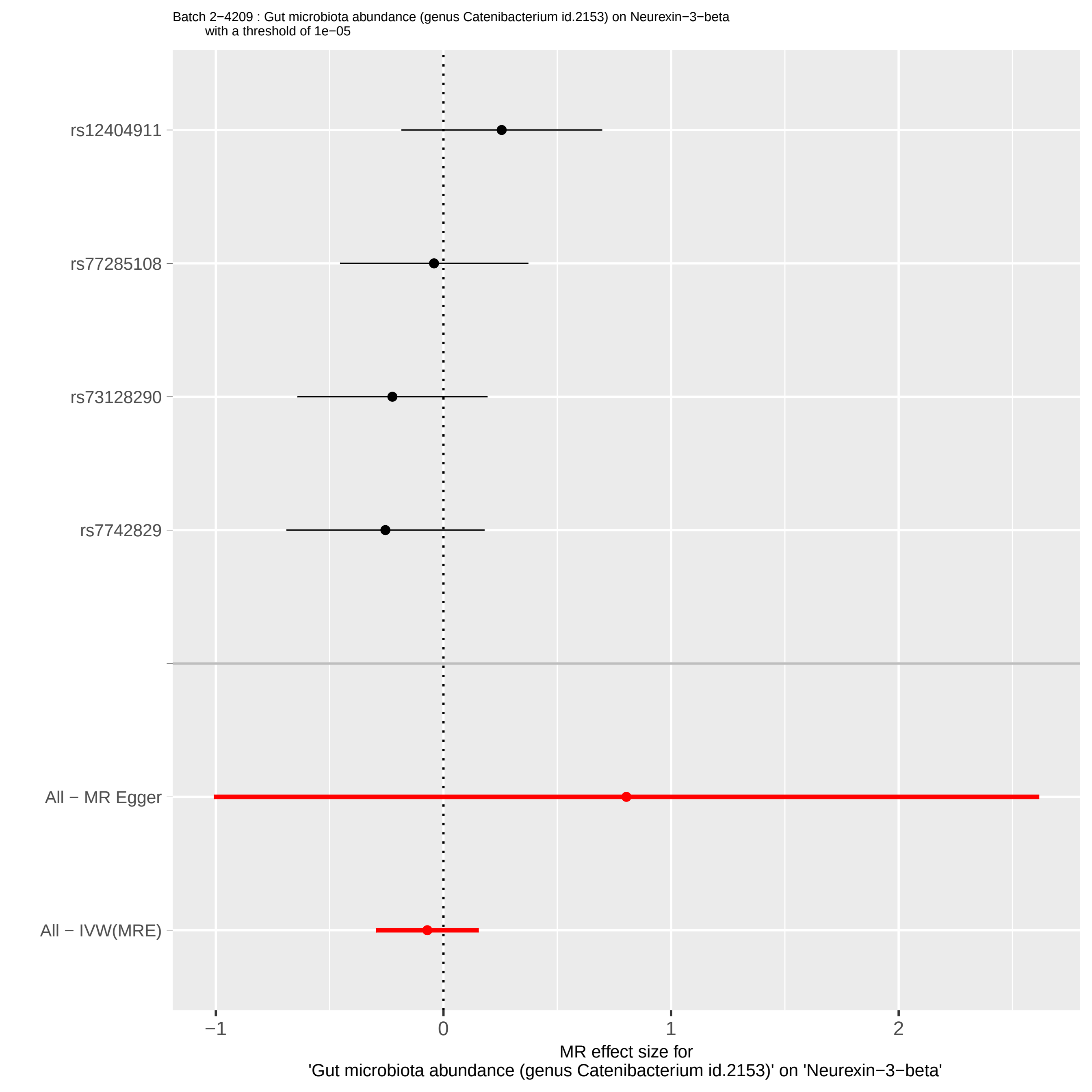

Batch 2−4209 : Gut microbiota abundance (genus Catenibacterium id.2153) on Neurexin−3−beta
with a threshold of 1e−05
rs12404911
rs77285108
rs73128290
rs7742829
All − MR Egger
All − IVW(MRE)
−1
0
1
2
MR effect size for
'Gut microbiota abundance (genus Catenibacterium id.2153)' on 'Neurexin−3−beta'

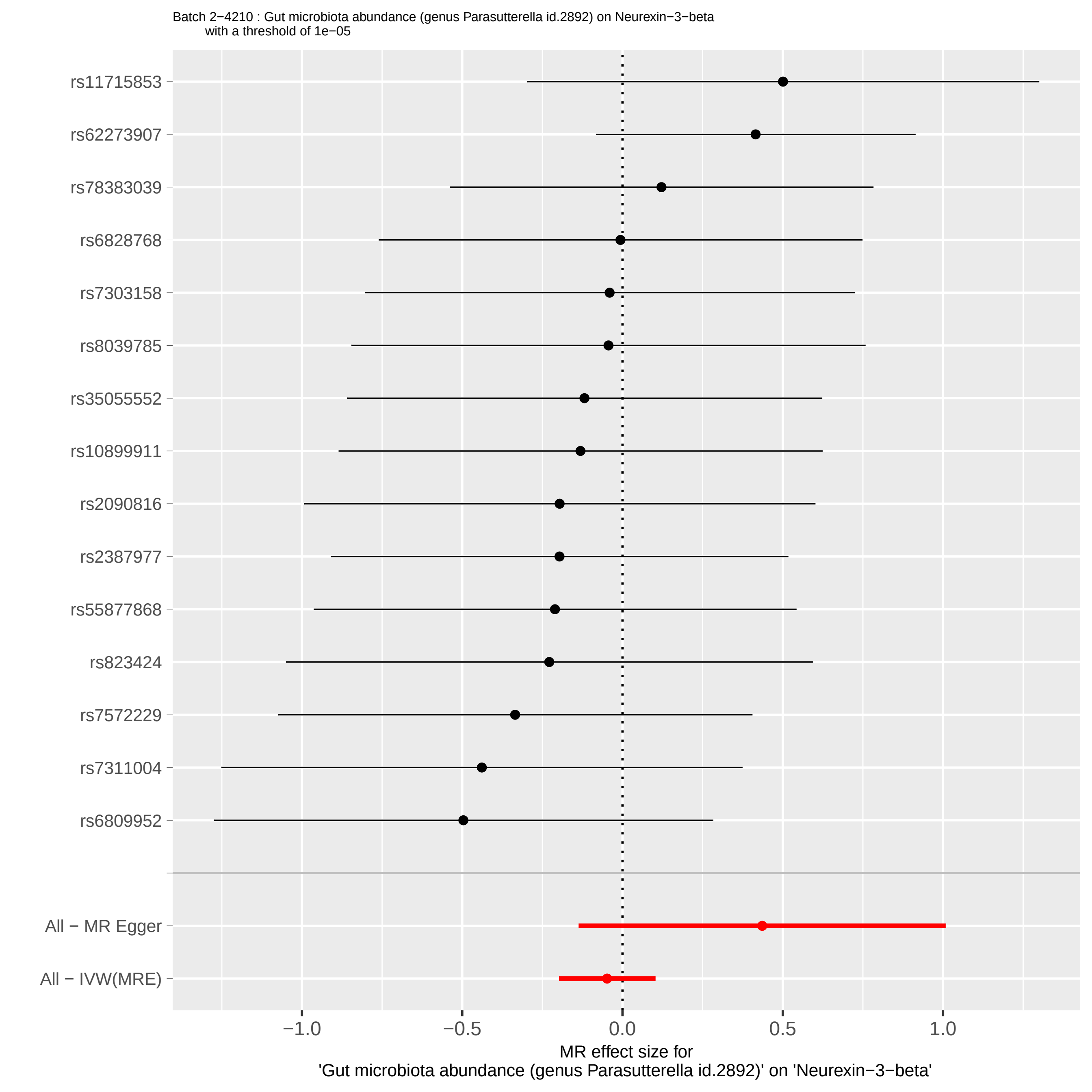

Batch 2−4210 : Gut microbiota abundance (genus Parasutterella id.2892) on Neurexin−3−beta
with a threshold of 1e−05
rs11715853
rs62273907
rs78383039
rs6828768
rs7303158
rs8039785
rs35055552
rs10899911
rs2090816
rs2387977
rs55877868
rs823424
rs7572229
rs7311004
rs6809952
All − MR Egger
All − IVW(MRE)
−1.0
−0.5
0.0
0.5
1.0
MR effect size for
'Gut microbiota abundance (genus Parasutterella id.2892)' on 'Neurexin−3−beta'

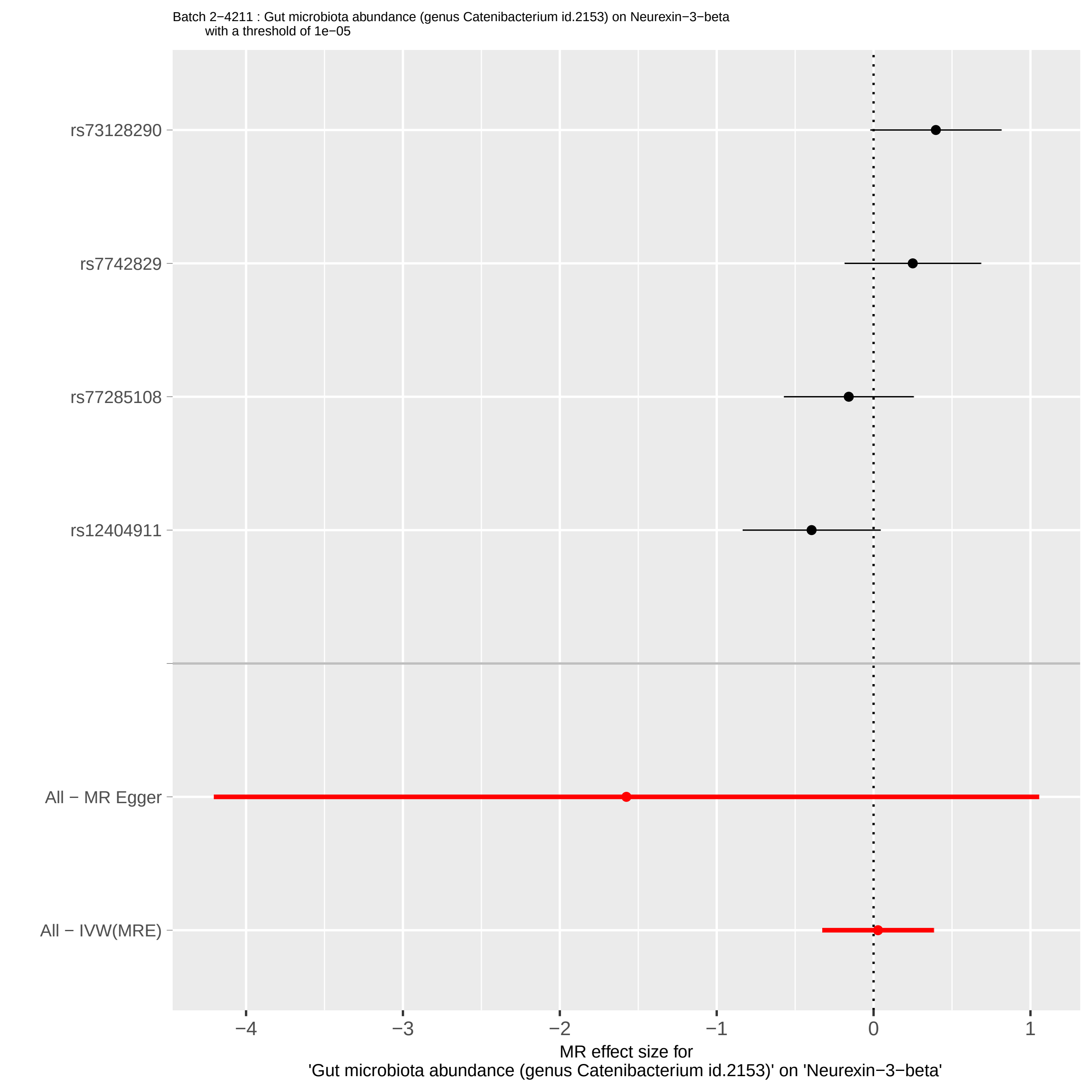
Batch 2−4211 : Gut microbiota abundance (genus Catenibacterium id.2153) on Neurexin−3−beta
with a threshold of 1e−05
rs73128290
rs7742829
rs77285108
rs12404911
All − MR Egger
All − IVW(MRE)
−4
−3
−2
−1
0
1
MR effect size for
'Gut microbiota abundance (genus Catenibacterium id.2153)' on 'Neurexin−3−beta'

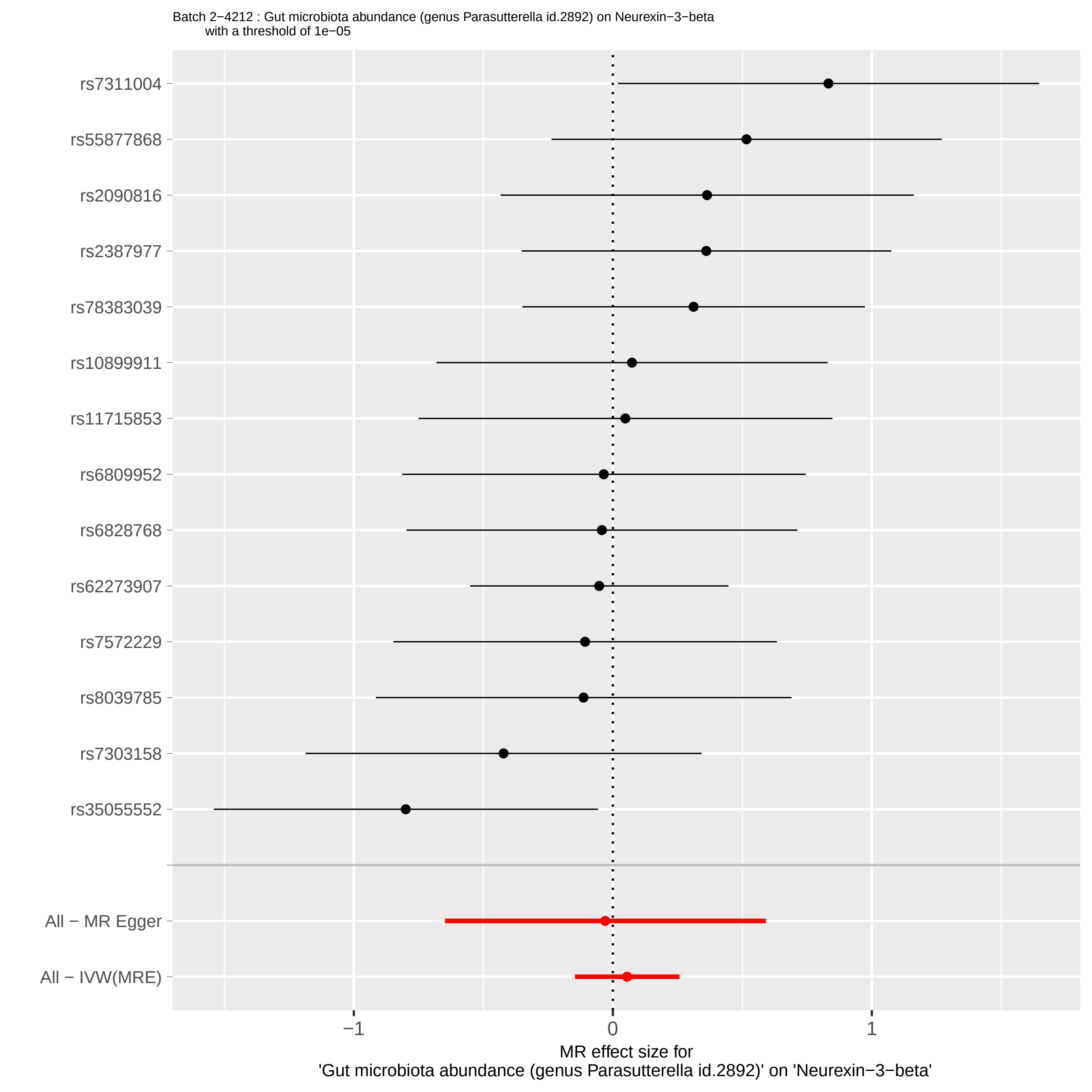

Batch 2−4212 : Gut microbiota abundance (genus Parasutterella id.2892) on Neurexin−3−beta
with a threshold of 1e−05
rs7311004
rs55877868
rs2090816
rs2387977
rs78383039
rs10899911
rs11715853
rs6809952
rs6828768
rs62273907
rs7572229
rs8039785
rs7303158
rs35055552
All − MR Egger
All − IVW(MRE)
−1
0
1
MR effect size for
'Gut microbiota abundance (genus Parasutterella id.2892)' on 'Neurexin−3−beta'

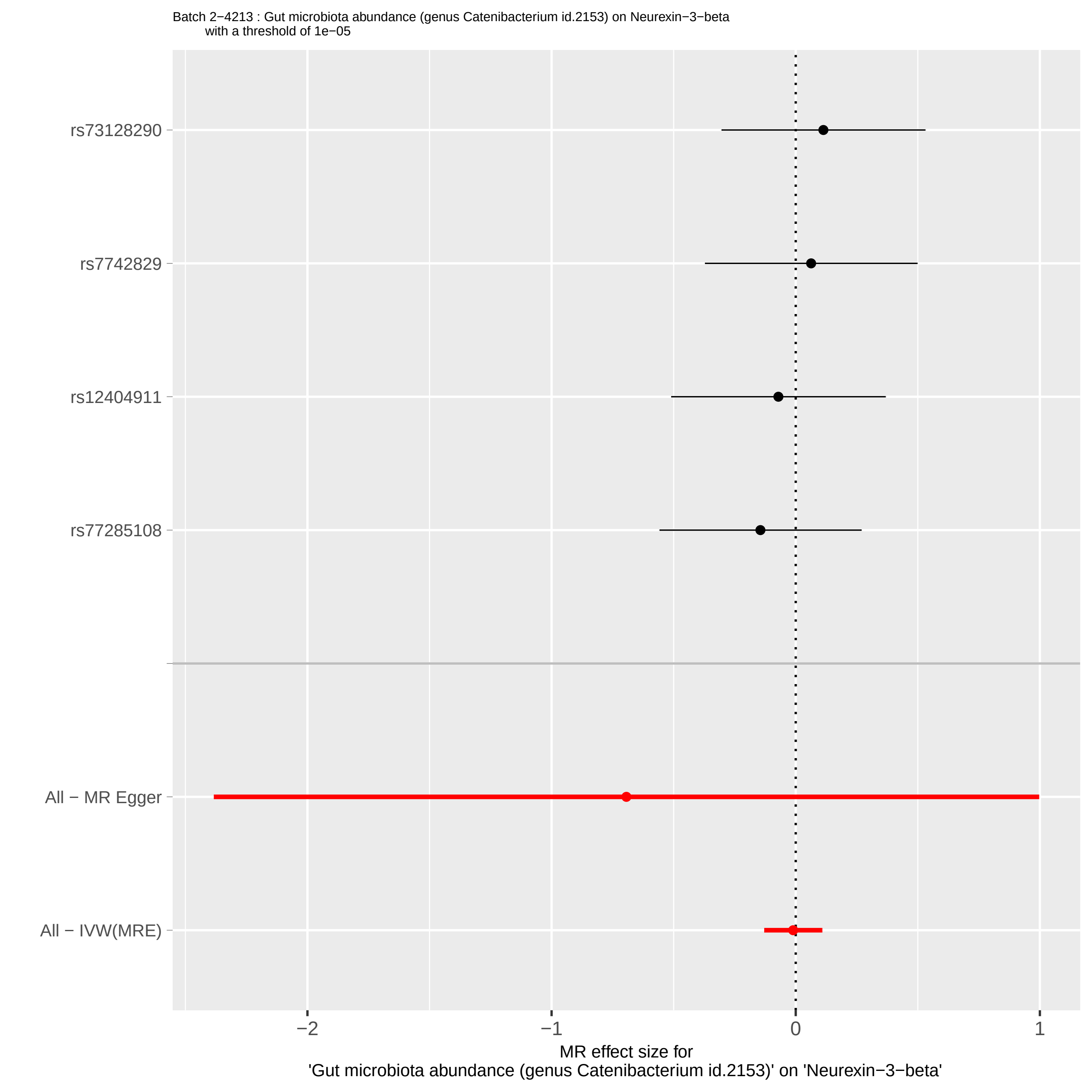
Batch 2−4213 : Gut microbiota abundance (genus Catenibacterium id.2153) on Neurexin−3−beta
with a threshold of 1e−05
rs73128290
rs7742829
rs12404911
rs77285108
All − MR Egger
All − IVW(MRE)
−2
−1
0
1
MR effect size for
'Gut microbiota abundance (genus Catenibacterium id.2153)' on 'Neurexin−3−beta'

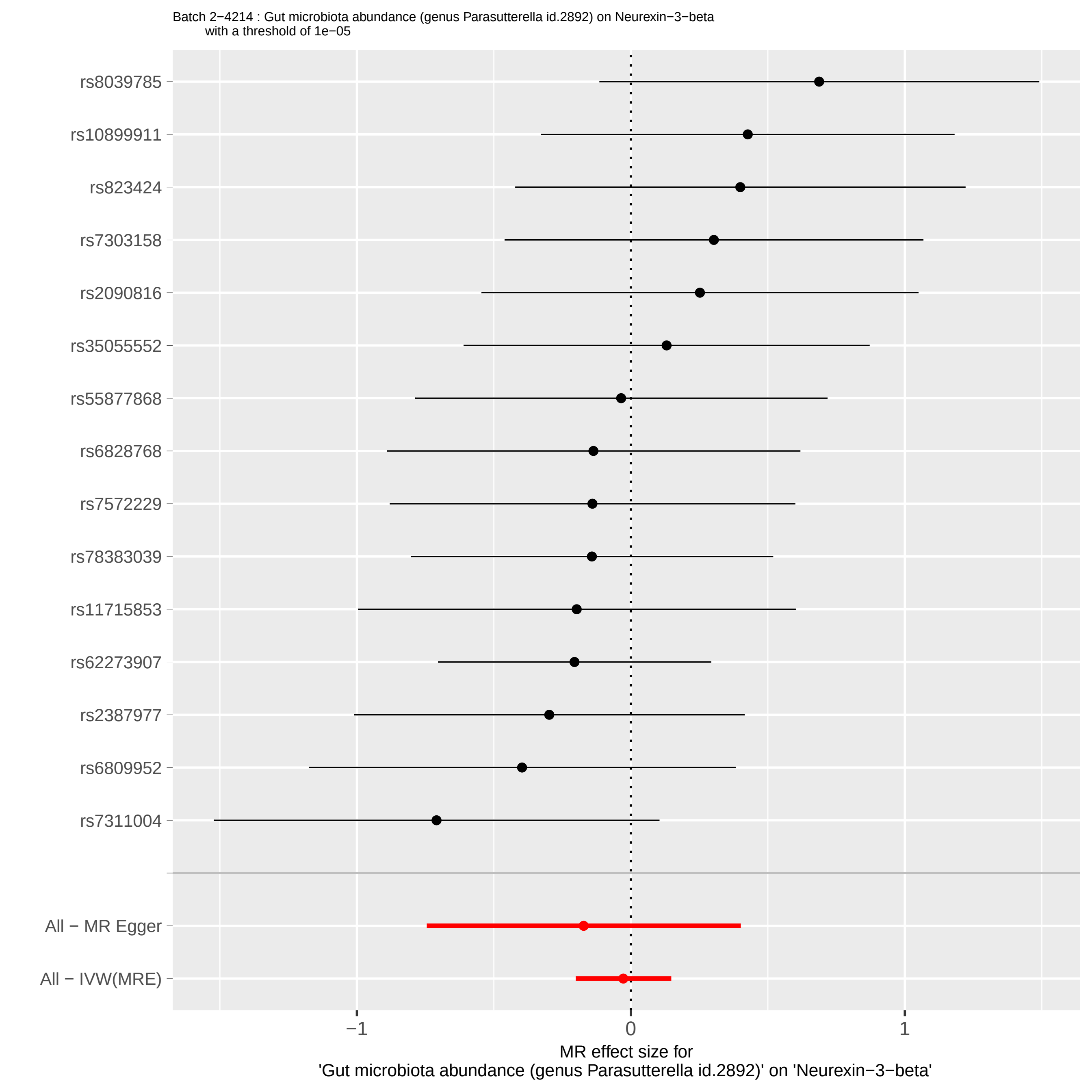

Batch 2−4214 : Gut microbiota abundance (genus Parasutterella id.2892) on Neurexin−3−beta
with a threshold of 1e−05
rs8039785
rs10899911
rs823424
rs7303158
rs2090816
rs35055552
rs55877868
rs6828768
rs7572229
rs78383039
rs11715853
rs62273907
rs2387977
rs6809952
rs7311004
All − MR Egger
All − IVW(MRE)
−1
0
1
MR effect size for
'Gut microbiota abundance (genus Parasutterella id.2892)' on 'Neurexin−3−beta'

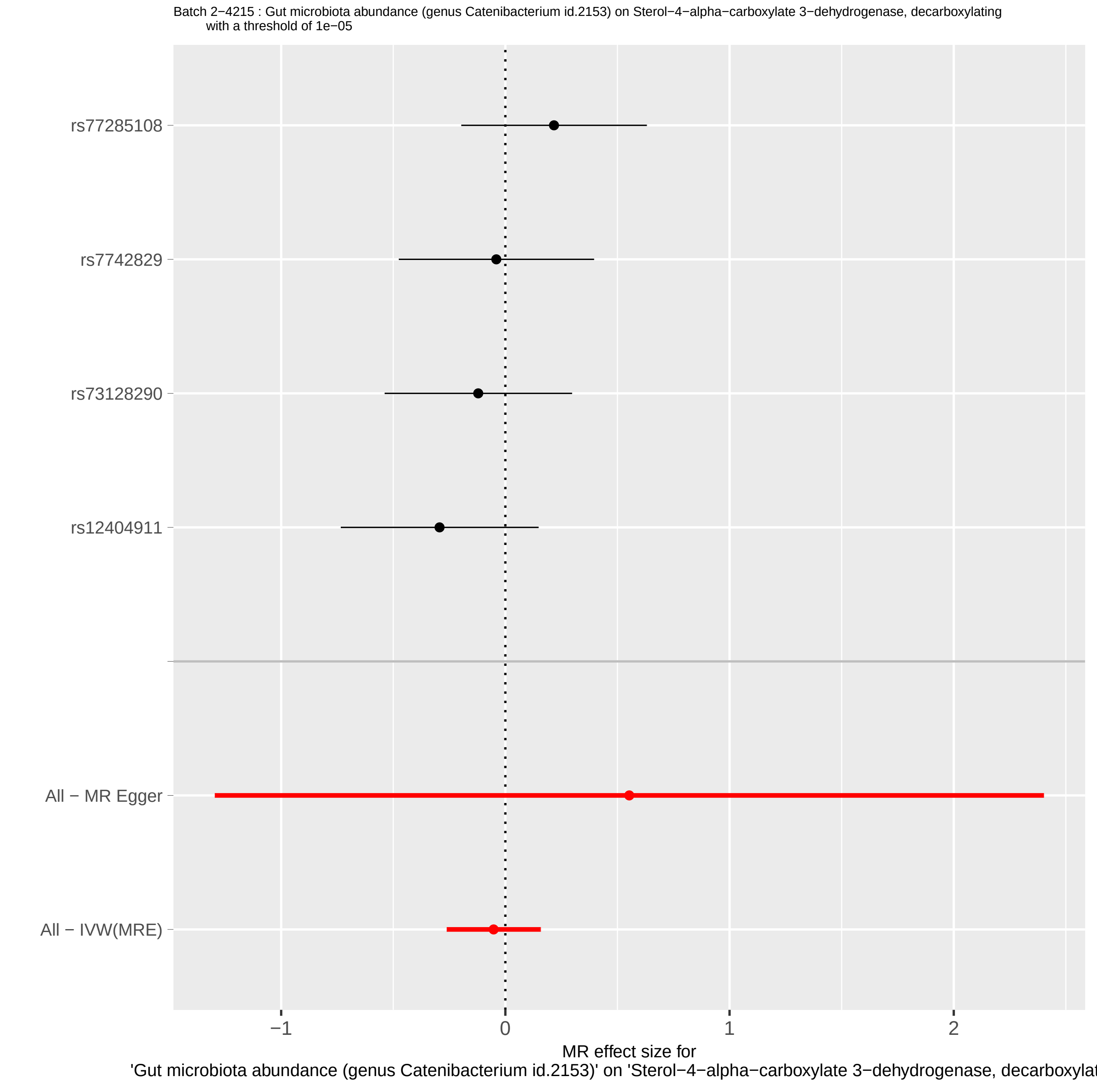

Batch 2−4215 : Gut microbiota abundance (genus Catenibacterium id.2153) on Sterol−4−alpha−carboxylate 3−dehydrogenase, decarboxylating
with a threshold of 1e−05
rs77285108
rs7742829
rs73128290
rs12404911
All − MR Egger
All − IVW(MRE)
−1
0
1
2
MR effect size for
'Gut microbiota abundance (genus Catenibacterium id.2153)' on 'Sterol−4−alpha−carboxylate 3−dehydrogenase, decarboxylat

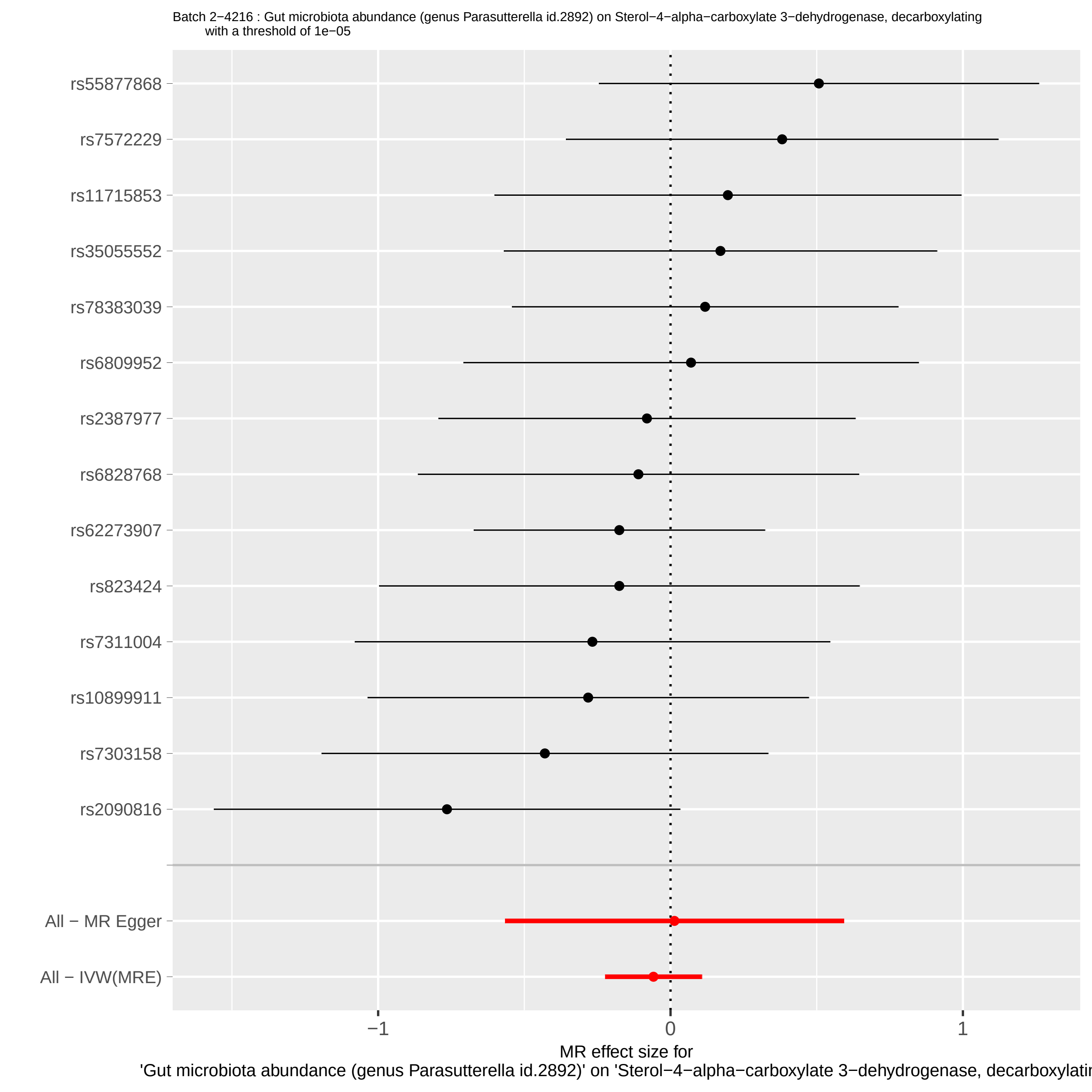

Batch 2−4216 : Gut microbiota abundance (genus Parasutterella id.2892) on Sterol−4−alpha−carboxylate 3−dehydrogenase, decarboxylating with a threshold of 1e−05
rs55877868
rs7572229
rs11715853
rs35055552
rs78383039
rs6809952
rs2387977
rs6828768
rs62273907
rs823424
rs7311004
rs10899911
rs7303158
rs2090816
All − MR Egger
All − IVW(MRE)
−1
0
1
MR effect size for
'Gut microbiota abundance (genus Parasutterella id.2892)' on 'Sterol−4−alpha−carboxylate 3−dehydrogenase, decarboxylatin

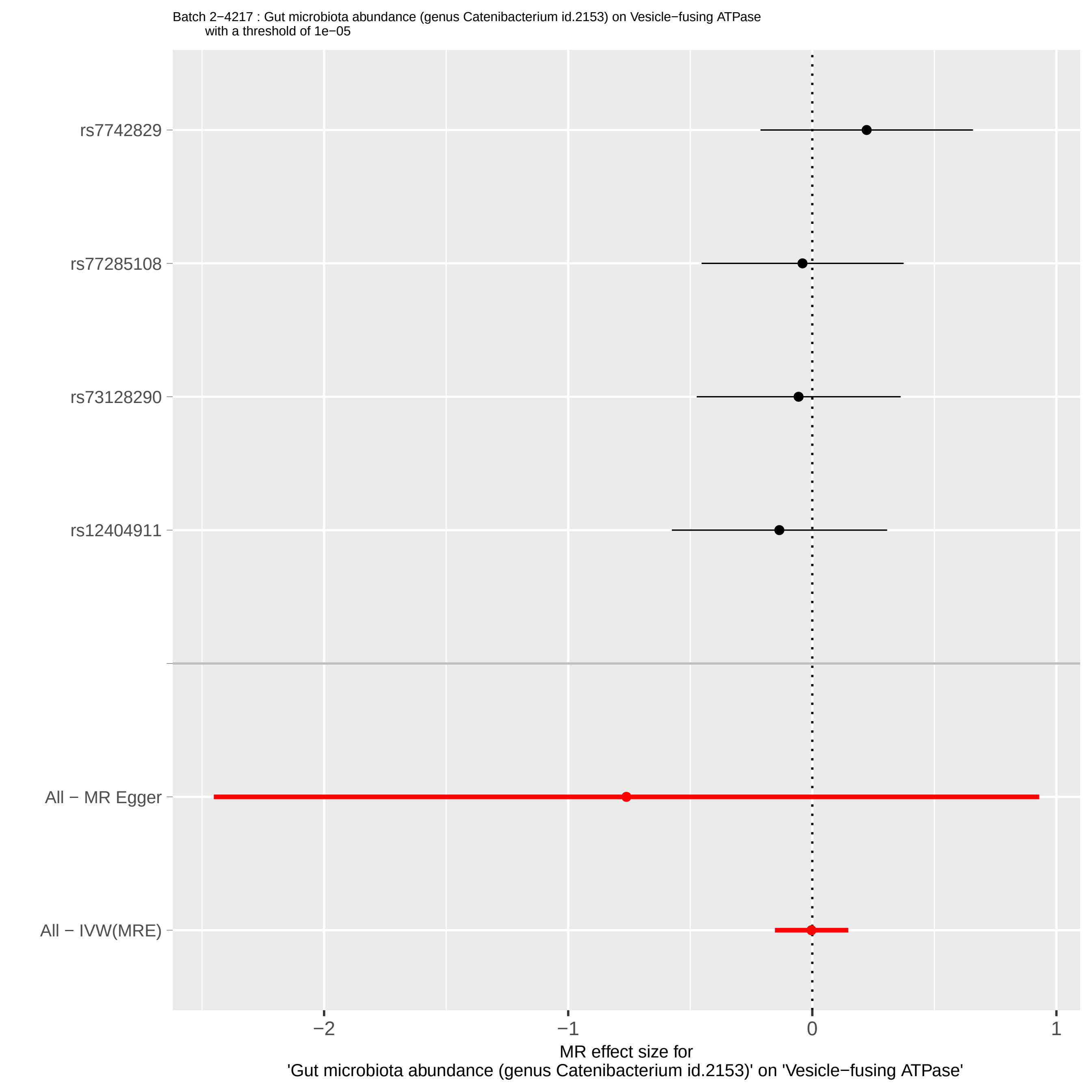

Batch 2−4217 : Gut microbiota abundance (genus Catenibacterium id.2153) on Vesicle−fusing ATPase
with a threshold of 1e−05
rs7742829
rs77285108
rs73128290
rs12404911
All − MR Egger
All − IVW(MRE)
−2
−1
0
1
MR effect size for
'Gut microbiota abundance (genus Catenibacterium id.2153)' on 'Vesicle−fusing ATPase'

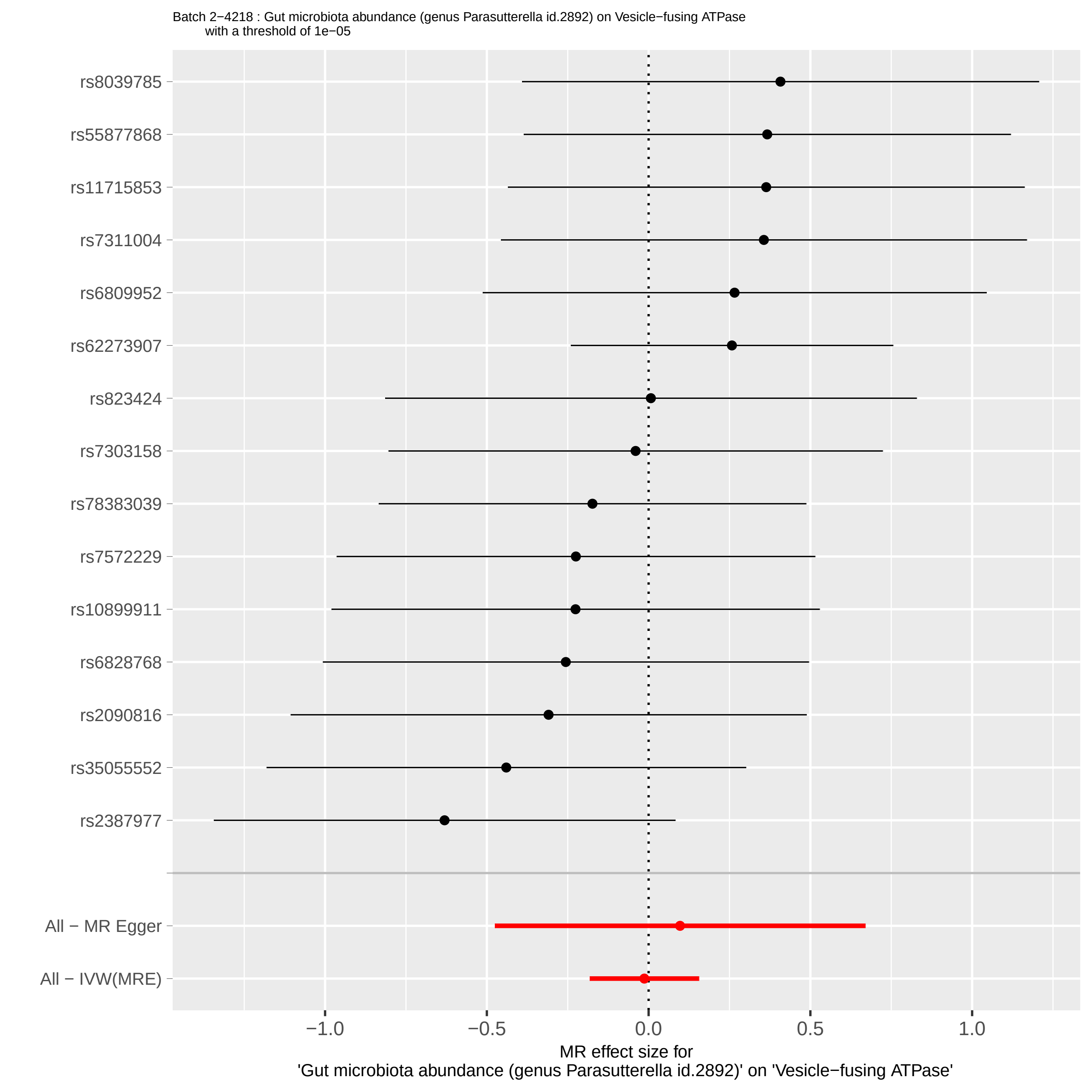

Batch 2−4218 : Gut microbiota abundance (genus Parasutterella id.2892) on Vesicle−fusing ATPase
with a threshold of 1e−05
rs8039785
rs55877868
rs11715853
rs7311004
rs6809952
rs62273907
rs823424
rs7303158
rs78383039
rs7572229
rs10899911
rs6828768
rs2090816
rs35055552
rs2387977
All − MR Egger
All − IVW(MRE)
−1.0
−0.5
0.0
0.5
1.0
MR effect size for
'Gut microbiota abundance (genus Parasutterella id.2892)' on 'Vesicle−fusing ATPase'

Batch 2−4219 : Gut microbiota abundance (genus Catenibacterium id.2153) on Neuron−specific protein family member 1
with a threshold of 1e−05

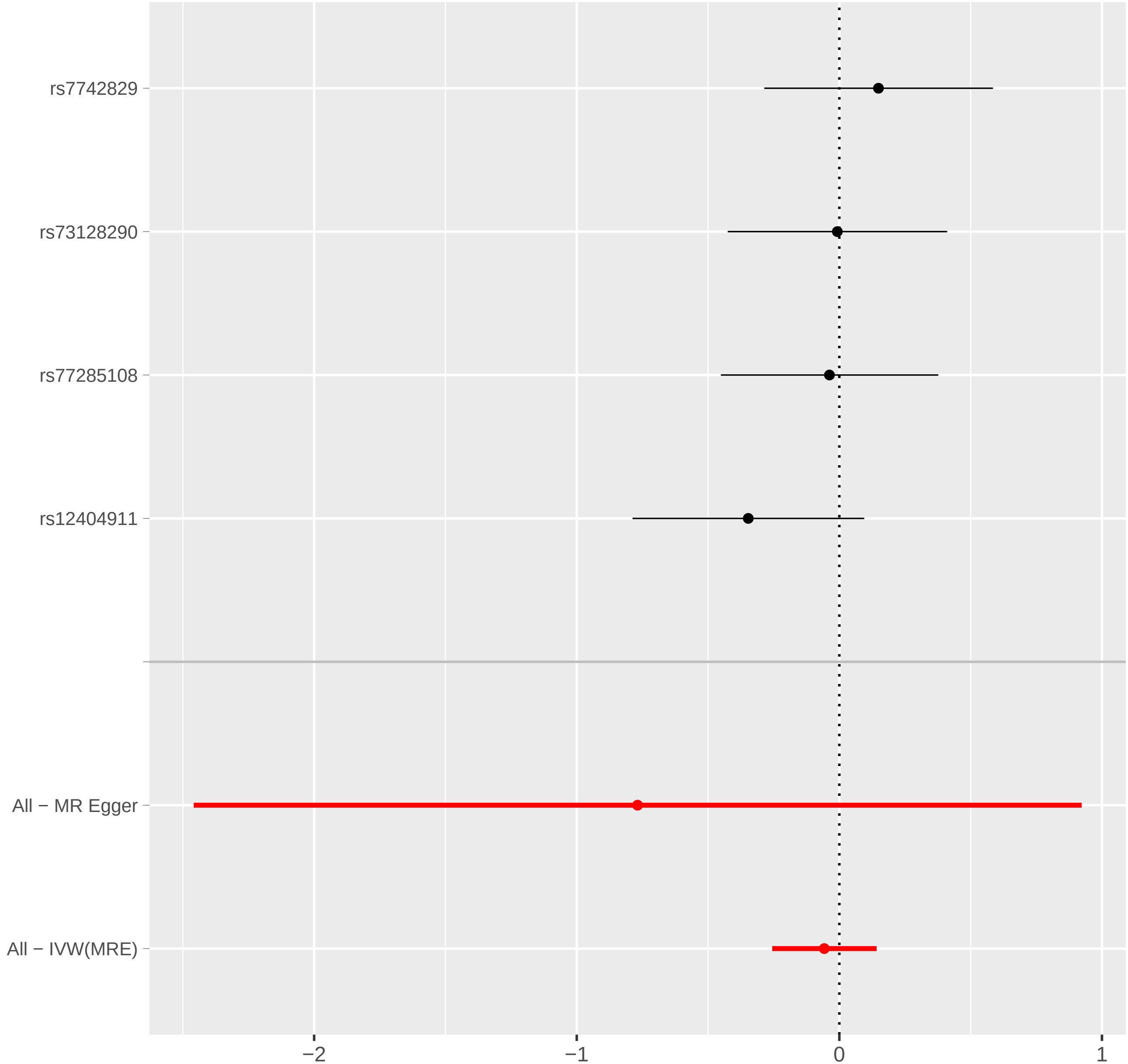

MR effect size for
'Gut microbiota abundance (genus Catenibacterium id.2153)' on 'Neuron−specific protein family member 1'

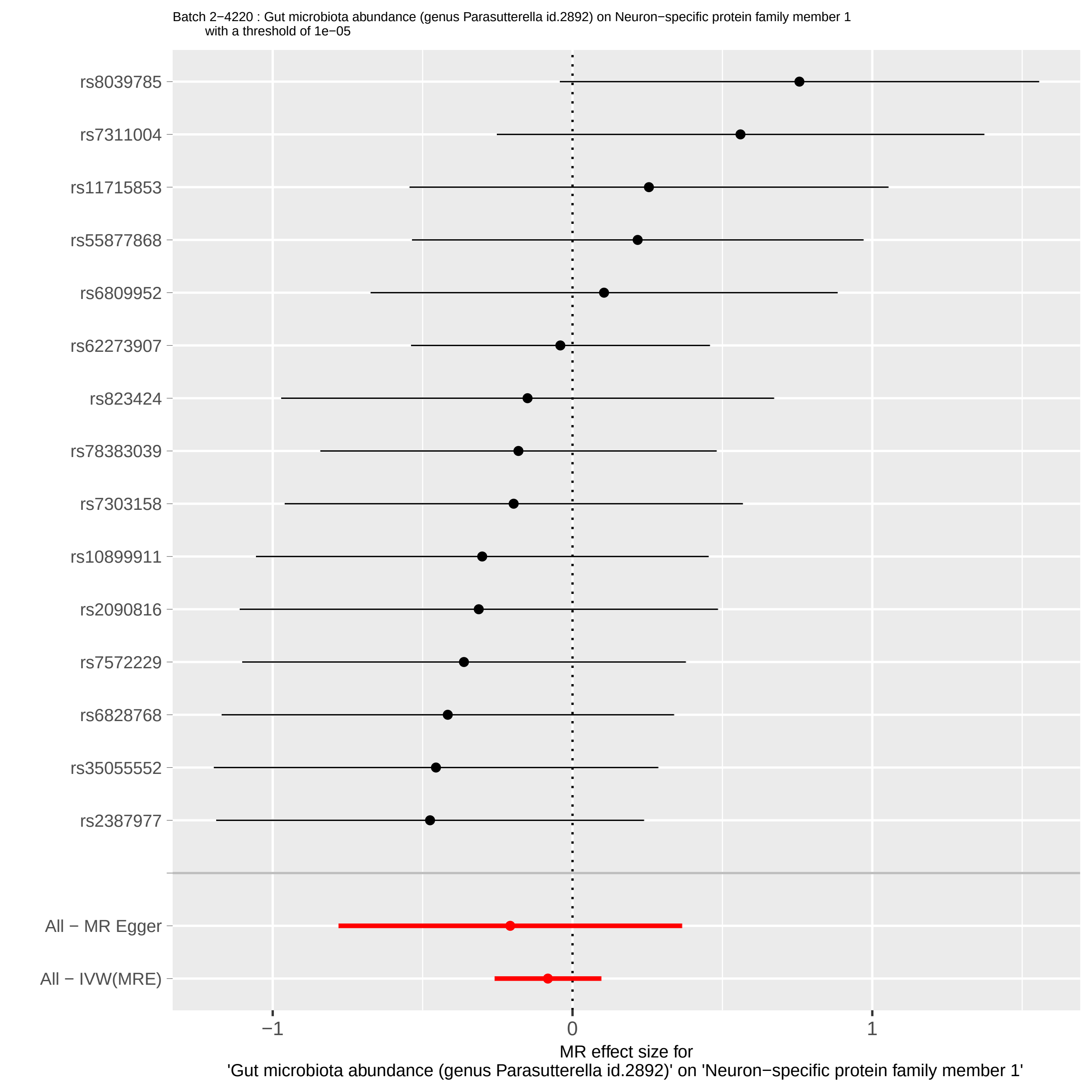

Batch 2−4220 : Gut microbiota abundance (genus Parasutterella id.2892) on Neuron−specific protein family member 1
with a threshold of 1e−05
rs8039785
rs7311004
rs11715853
rs55877868
rs6809952
rs62273907
rs823424
rs78383039
rs7303158
rs10899911
rs2090816
rs7572229
rs6828768
rs35055552
rs2387977
All − MR Egger
All − IVW(MRE)
−1
0
1
MR effect size for
'Gut microbiota abundance (genus Parasutterella id.2892)' on 'Neuron−specific protein family member 1'

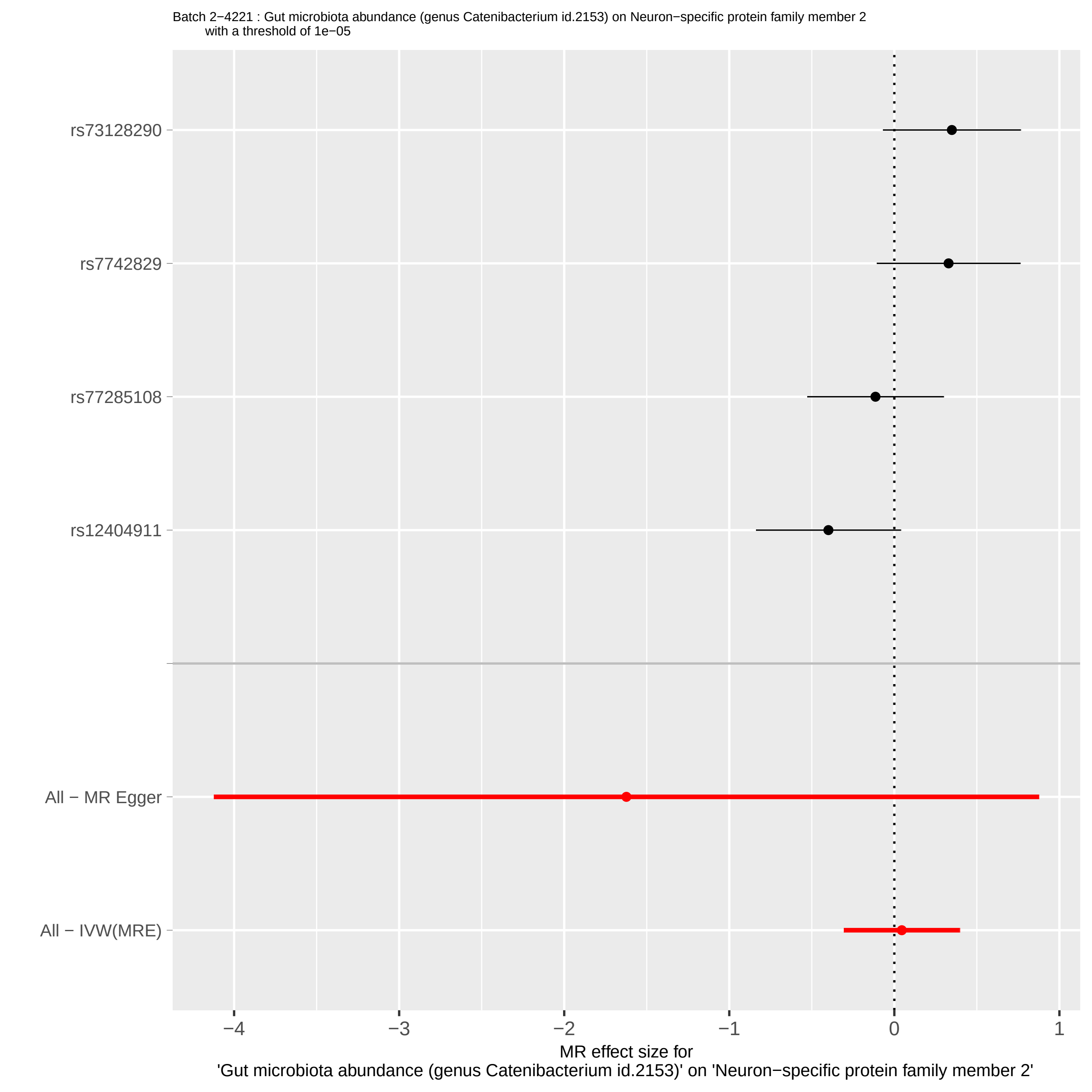

Batch 2−4221 : Gut microbiota abundance (genus Catenibacterium id.2153) on Neuron−specific protein family member 2
with a threshold of 1e−05
rs73128290
rs7742829
rs77285108
rs12404911
All − MR Egger
All − IVW(MRE)
−4
−3
−2
−1
0
1
MR effect size for
'Gut microbiota abundance (genus Catenibacterium id.2153)' on 'Neuron−specific protein family member 2'

Batch 2−4222 : Gut microbiota abundance (genus Parasutterella id.2892) on Neuron−specific protein family member 2
with a threshold of 1e−05

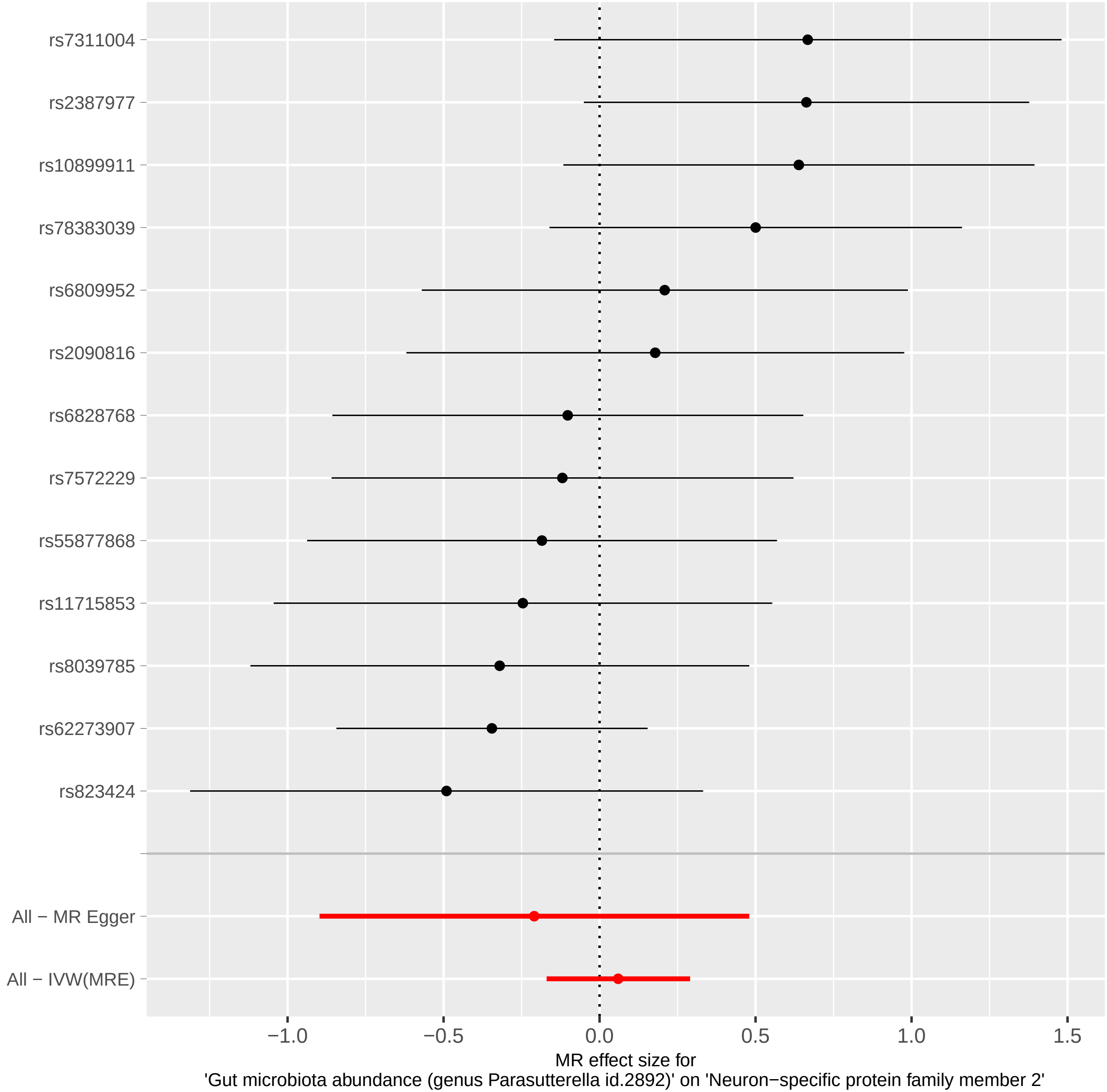

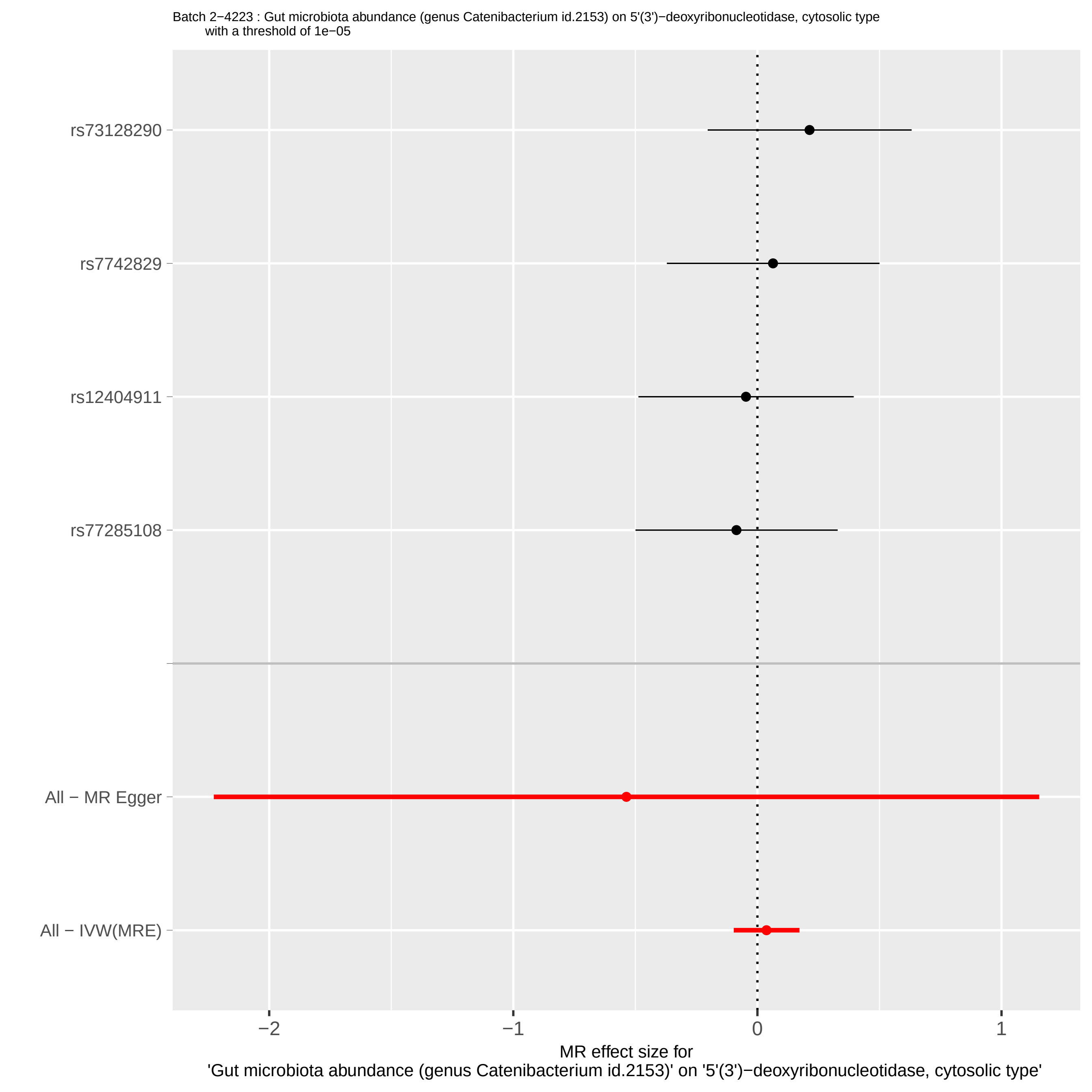
Batch 2−4223 : Gut microbiota abundance (genus Catenibacterium id.2153) on 5'(3')−deoxyribonucleotidase, cytosolic type
with a threshold of 1e−05
rs73128290
rs7742829
rs12404911
rs77285108
All − MR Egger
All − IVW(MRE)
−2
−1
0
1
MR effect size for
'Gut microbiota abundance (genus Catenibacterium id.2153)' on '5'(3')−deoxyribonucleotidase, cytosolic type'

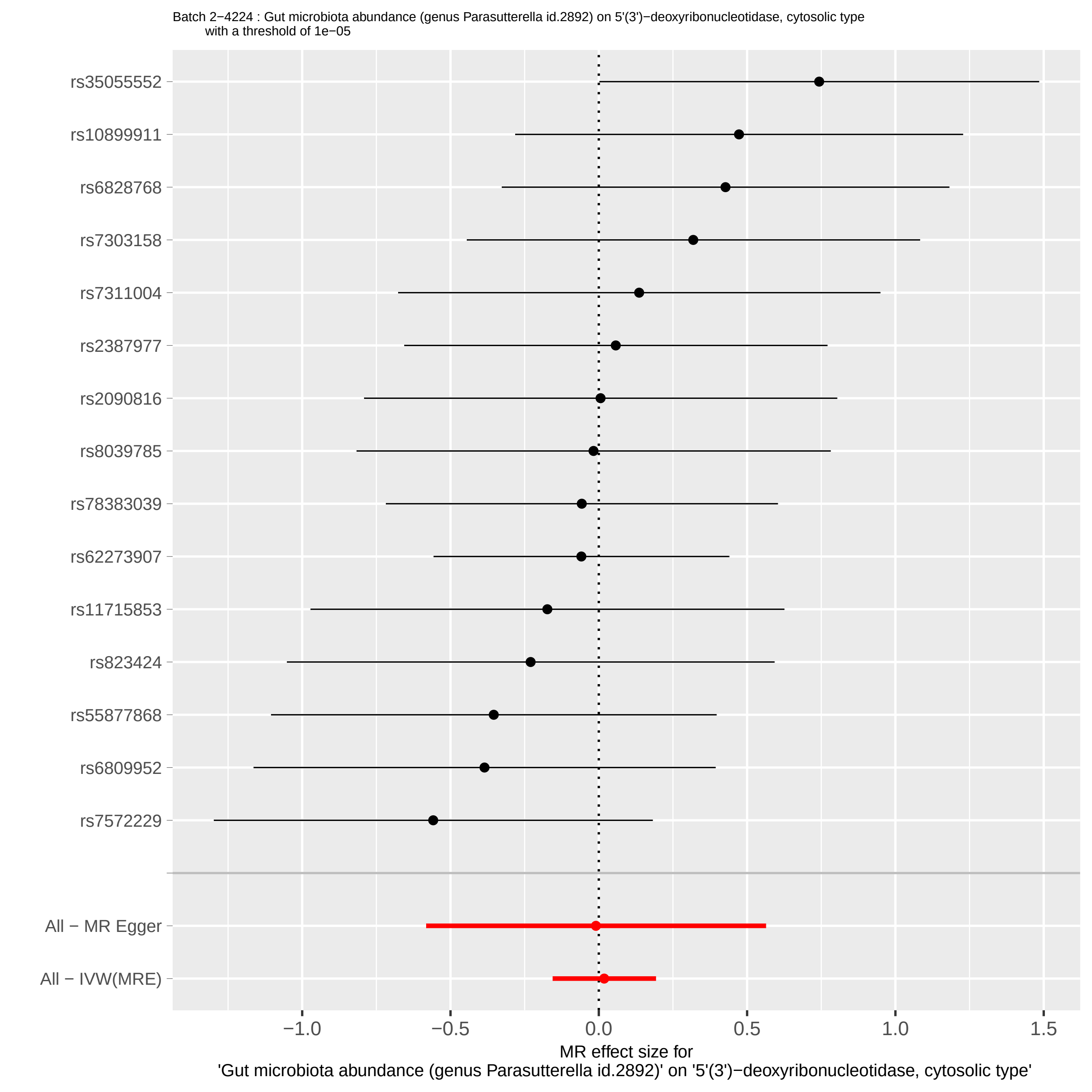
Batch 2−4224 : Gut microbiota abundance (genus Parasutterella id.2892) on 5'(3')−deoxyribonucleotidase, cytosolic type
with a threshold of 1e−05
rs35055552
rs10899911
rs6828768
rs7303158
rs7311004
rs2387977
rs2090816
rs8039785
rs78383039
rs62273907
rs11715853
rs823424
rs55877868
rs6809952
rs7572229
All − MR Egger
All − IVW(MRE)
−1.0
−0.5
0.0
0.5
1.0
1.5
MR effect size for
'Gut microbiota abundance (genus Parasutterella id.2892)' on '5'(3')−deoxyribonucleotidase, cytosolic type'

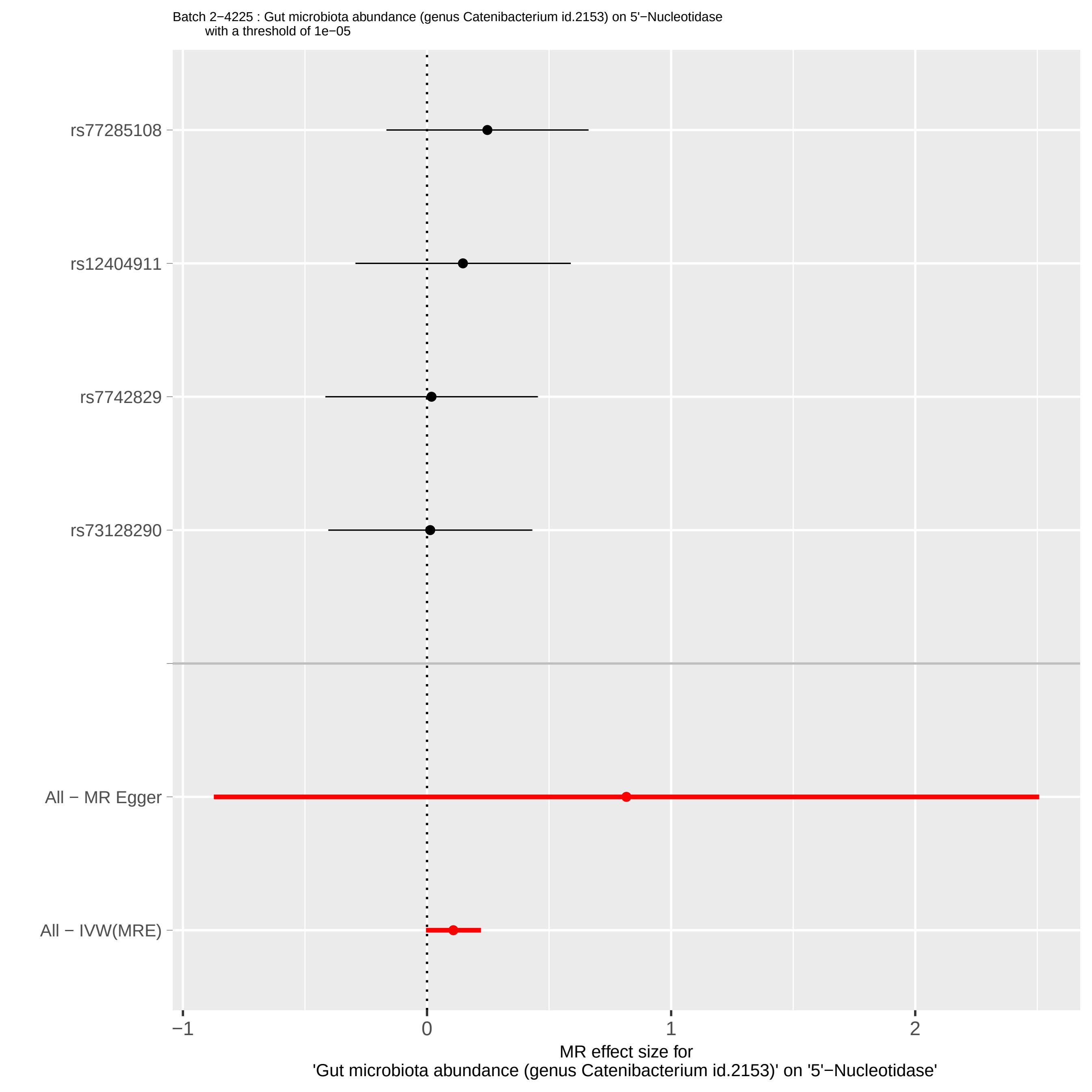
Batch 2−4225 : Gut microbiota abundance (genus Catenibacterium id.2153) on 5'−Nucleotidase
with a threshold of 1e−05
rs77285108
rs12404911
rs7742829
rs73128290
All − MR Egger
All − IVW(MRE)
−1
0
1
2
MR effect size for
'Gut microbiota abundance (genus Catenibacterium id.2153)' on '5'−Nucleotidase'

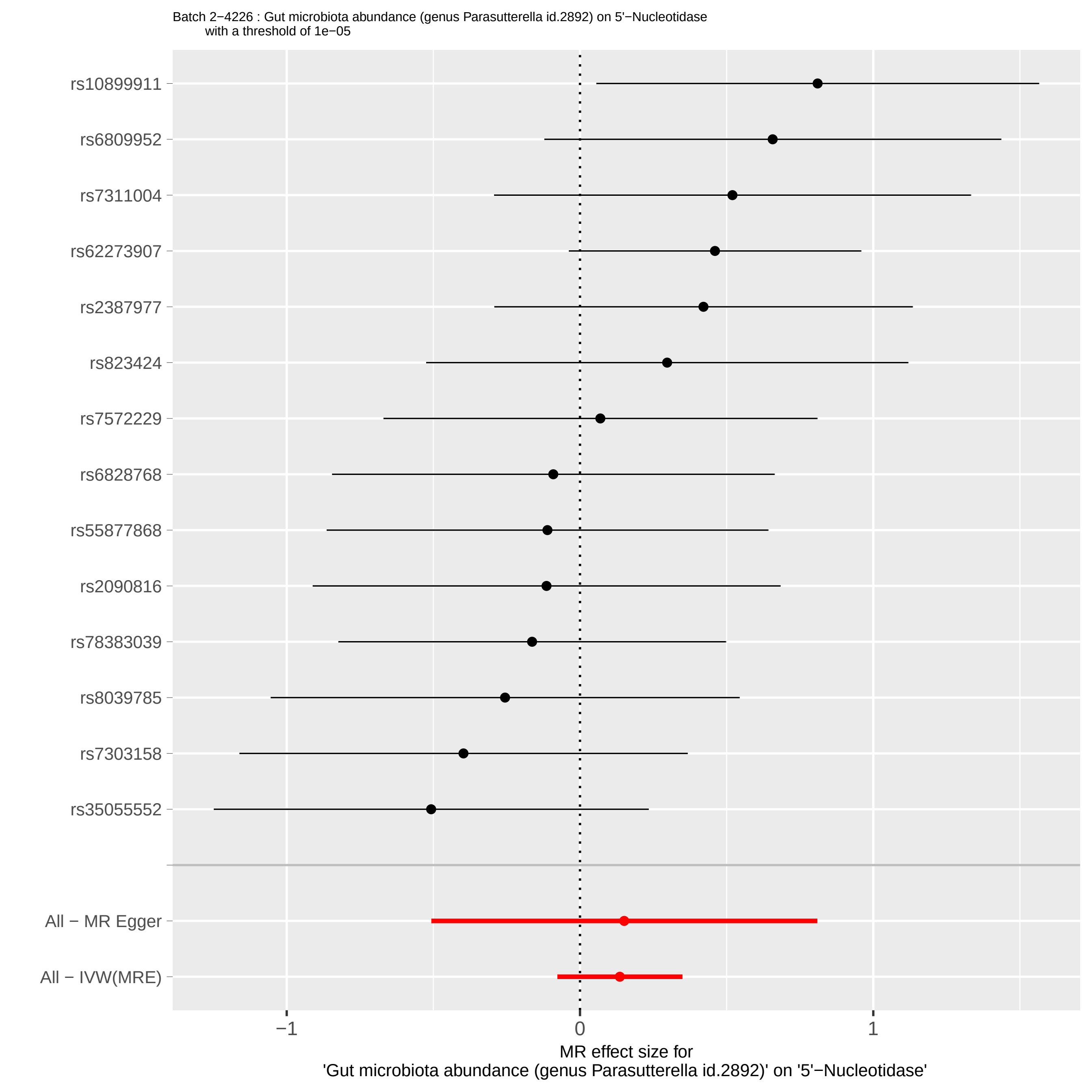
Batch 2−4226 : Gut microbiota abundance (genus Parasutterella id.2892) on 5'−Nucleotidase
with a threshold of 1e−05
rs10899911
rs6809952
rs7311004
rs62273907
rs2387977
rs823424
rs7572229
rs6828768
rs55877868
rs2090816
rs78383039
rs8039785
rs7303158
rs35055552
All − MR Egger
All − IVW(MRE)
−1
0
1
MR effect size for
'Gut microbiota abundance (genus Parasutterella id.2892)' on '5'−Nucleotidase'

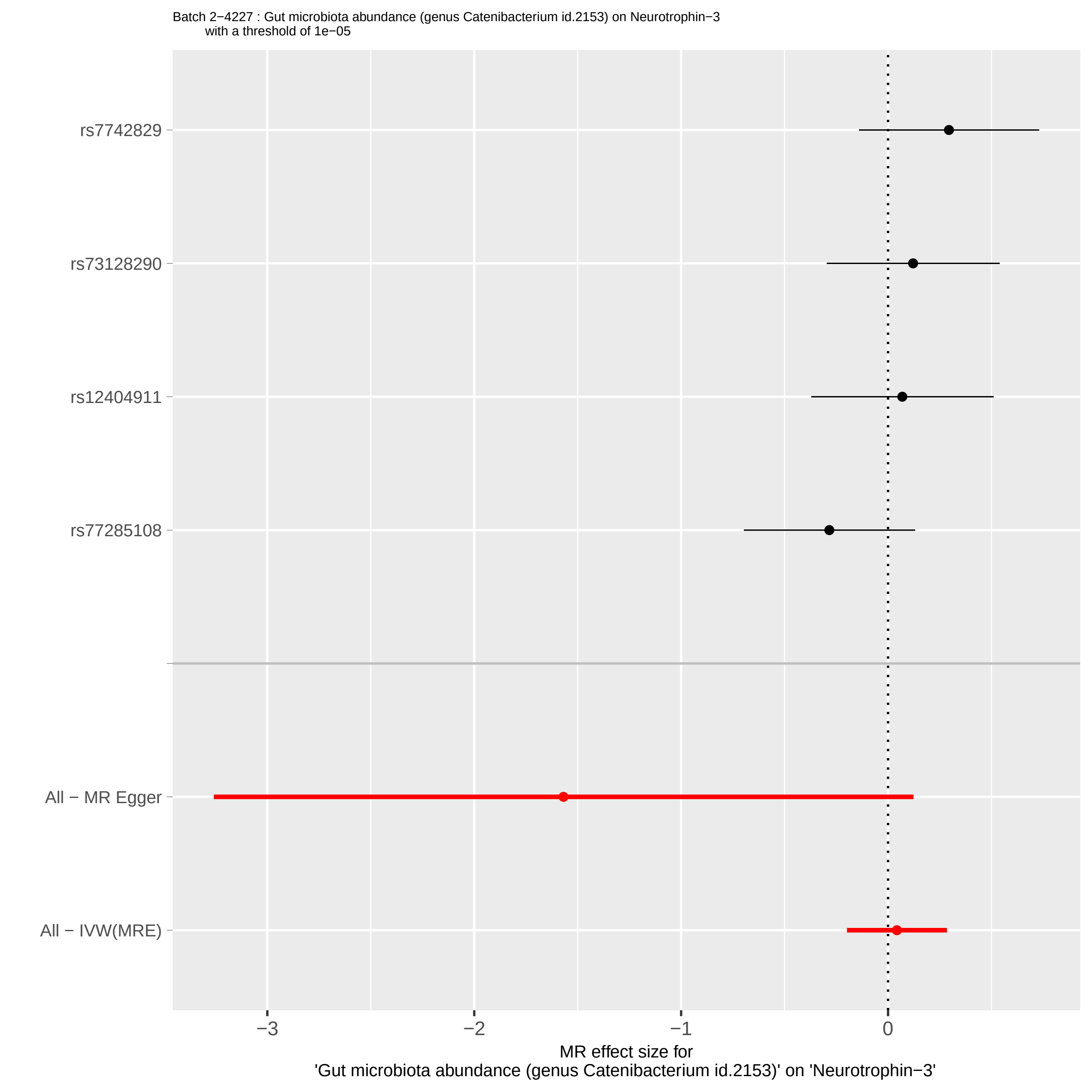
Batch 2−4227 : Gut microbiota abundance (genus Catenibacterium id.2153) on Neurotrophin−3
with a threshold of 1e−05
rs7742829
rs73128290
rs12404911
rs77285108
All − MR Egger
All − IVW(MRE)
−3
−2
−1
0
MR effect size for
'Gut microbiota abundance (genus Catenibacterium id.2153)' on 'Neurotrophin−3'

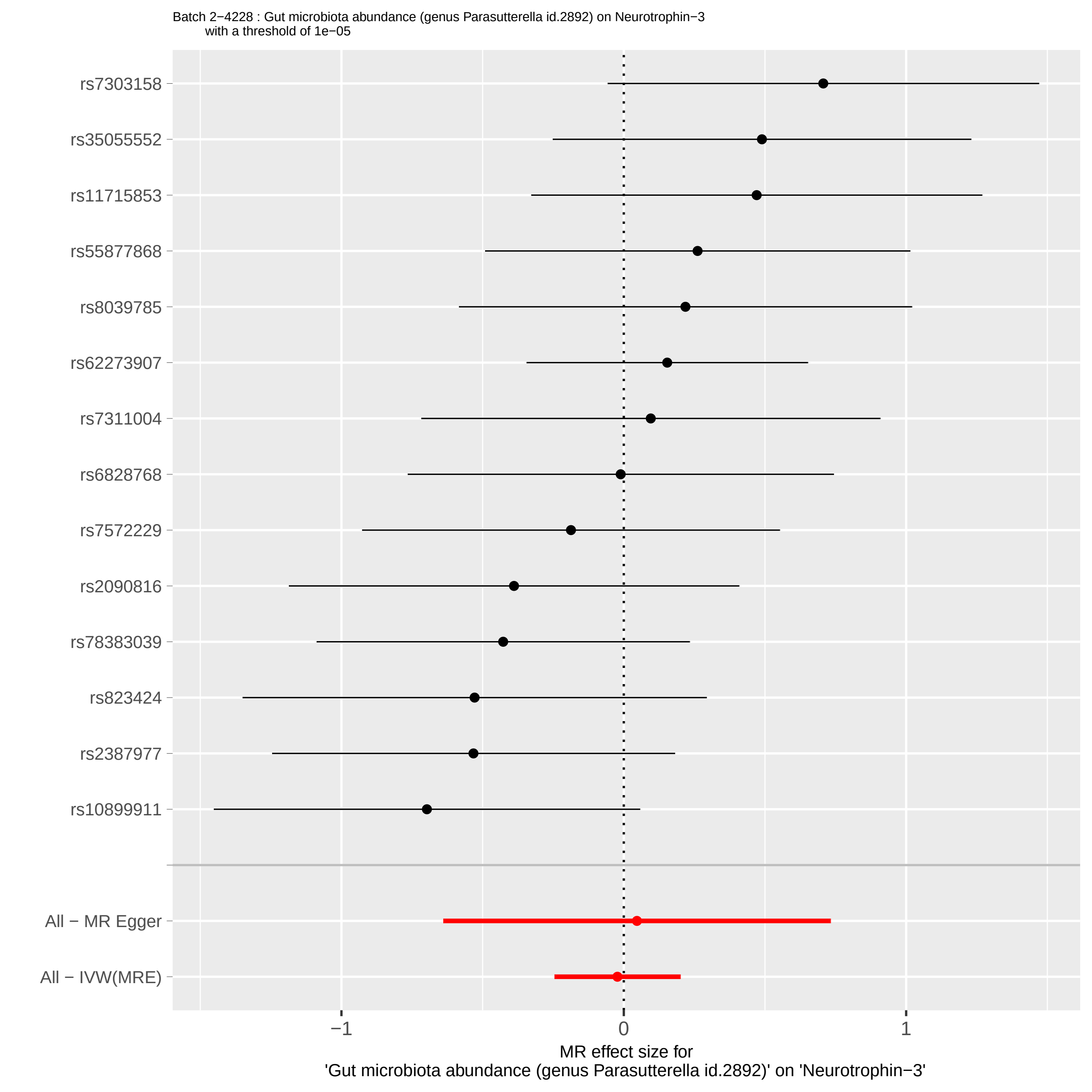

Batch 2−4228 : Gut microbiota abundance (genus Parasutterella id.2892) on Neurotrophin−3
with a threshold of 1e−05
rs7303158
rs35055552
rs11715853
rs55877868
rs8039785
rs62273907
rs7311004
rs6828768
rs7572229
rs2090816
rs78383039
rs823424
rs2387977
rs10899911
All − MR Egger
All − IVW(MRE)
−1
0
1
MR effect size for
'Gut microbiota abundance (genus Parasutterella id.2892)' on 'Neurotrophin−3'

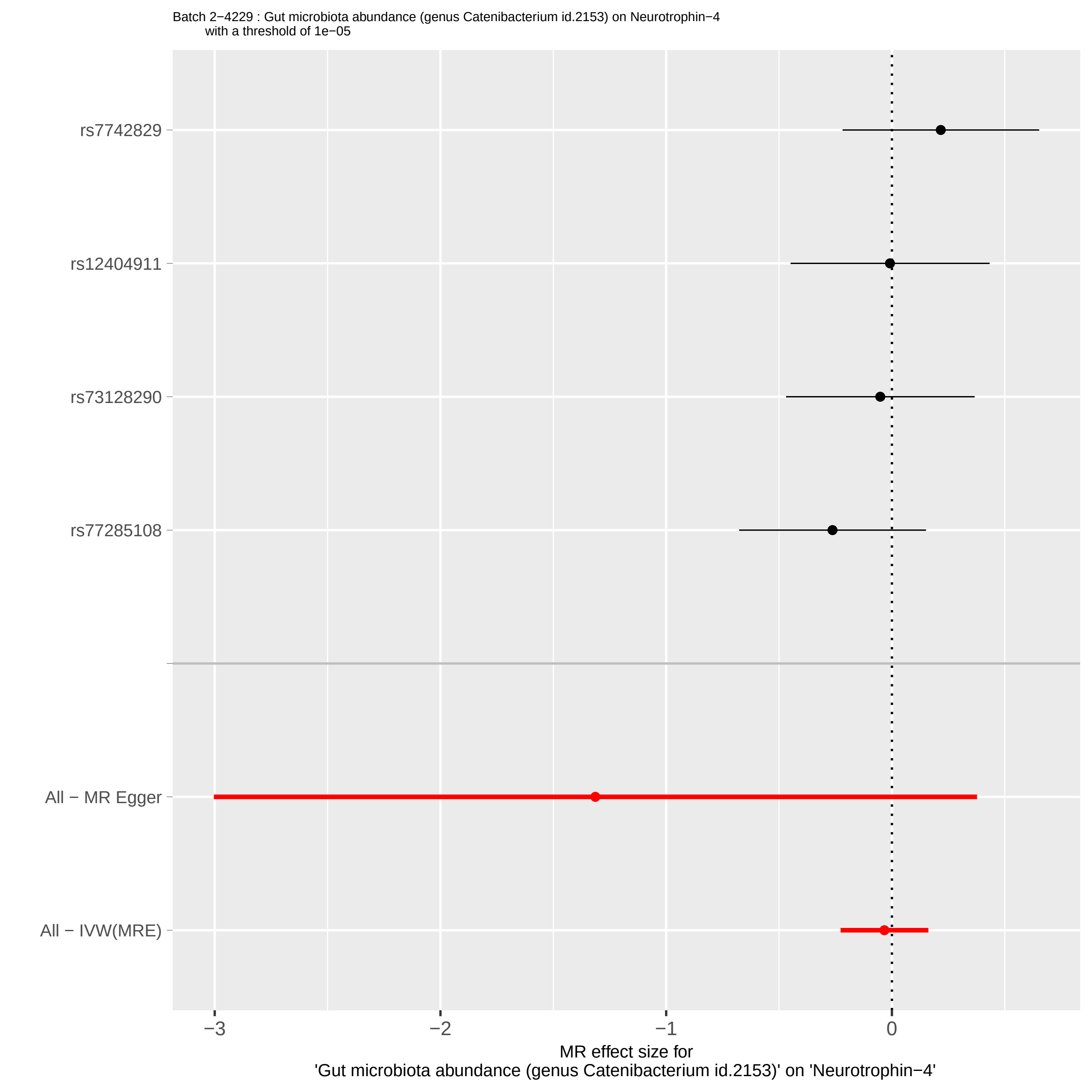

Batch 2−4229 : Gut microbiota abundance (genus Catenibacterium id.2153) on Neurotrophin−4
with a threshold of 1e−05
rs7742829
rs12404911
rs73128290
rs77285108
All − MR Egger
All − IVW(MRE)
−3
−2
−1
0
MR effect size for
'Gut microbiota abundance (genus Catenibacterium id.2153)' on 'Neurotrophin−4'

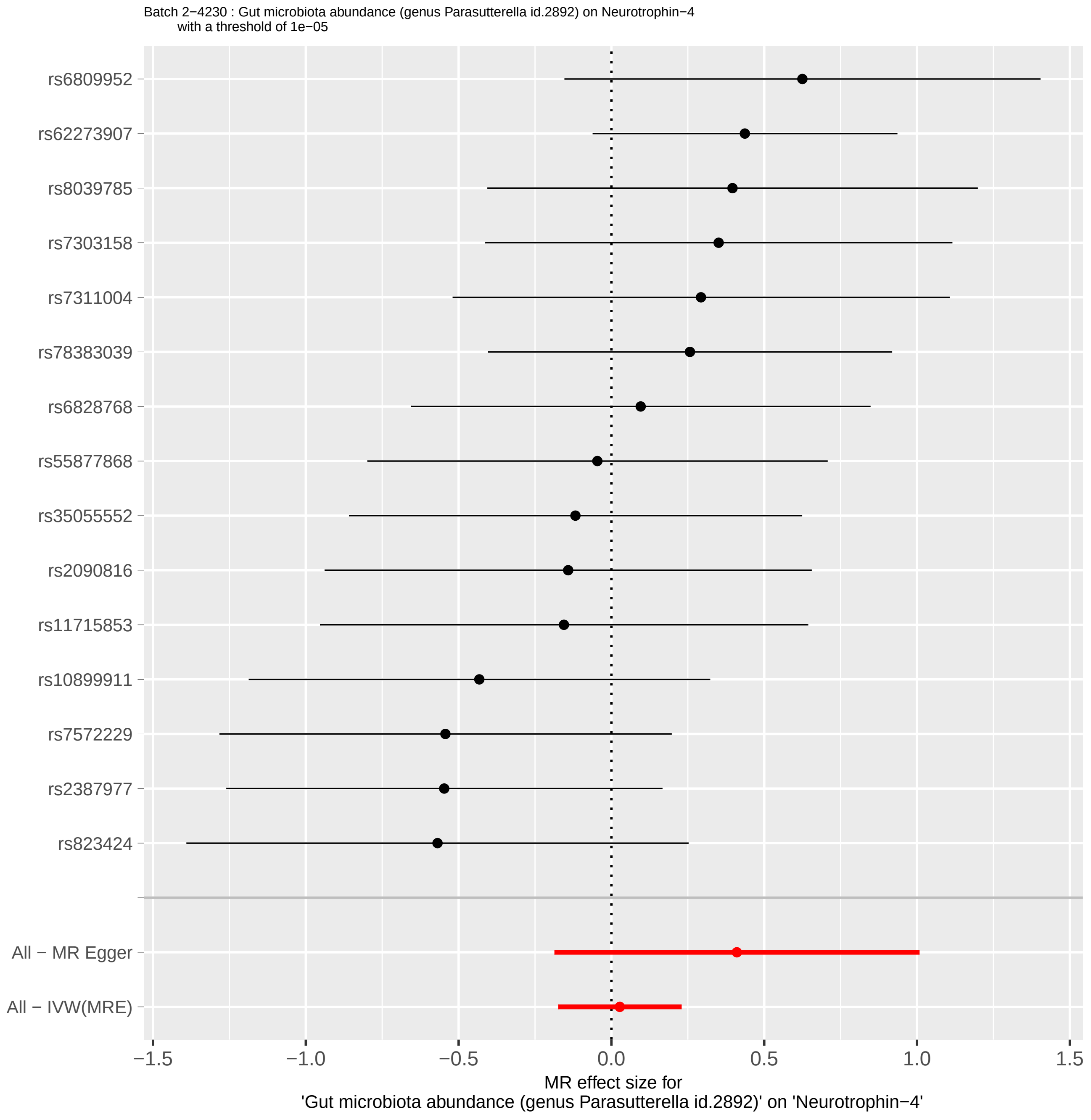

Batch 2−4230 : Gut microbiota abundance (genus Parasutterella id.2892) on Neurotrophin−4
with a threshold of 1e−05
rs6809952
rs62273907
rs8039785
rs7303158
rs7311004
rs78383039
rs6828768
rs55877868
rs35055552
rs2090816
rs11715853
rs10899911
rs7572229
rs2387977
rs823424
All − MR Egger
All − IVW(MRE)
−1.5
−1.0
−0.5
0.0
0.5
1.0
1.5
MR effect size for
'Gut microbiota abundance (genus Parasutterella id.2892)' on 'Neurotrophin−4'

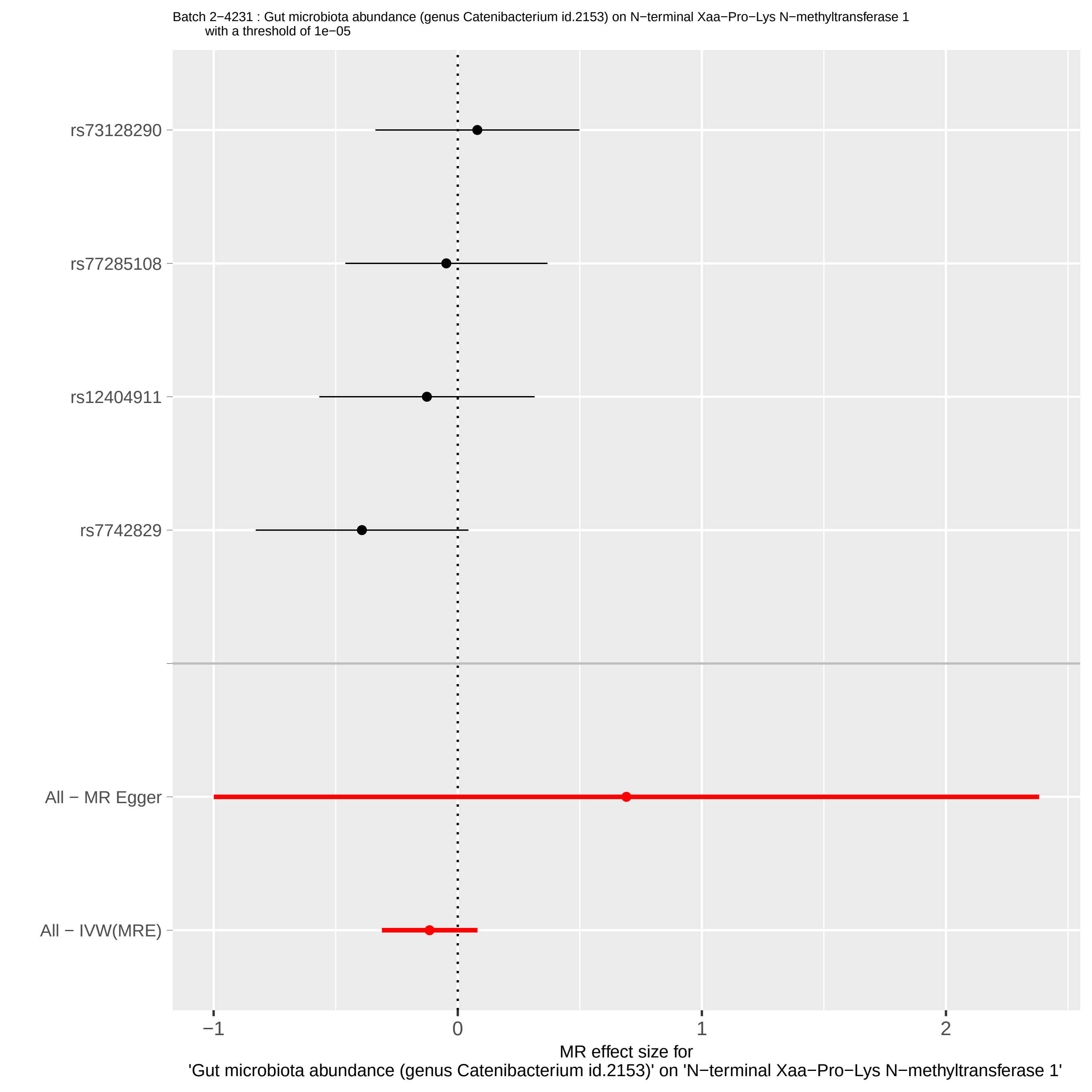
Batch 2−4231 : Gut microbiota abundance (genus Catenibacterium id.2153) on N−terminal Xaa−Pro−Lys N−methyltransferase 1
with a threshold of 1e−05
rs73128290
rs77285108
rs12404911
rs7742829
All − MR Egger
All − IVW(MRE)
−1
0
1
2
MR effect size for
'Gut microbiota abundance (genus Catenibacterium id.2153)' on 'N−terminal Xaa−Pro−Lys N−methyltransferase 1'

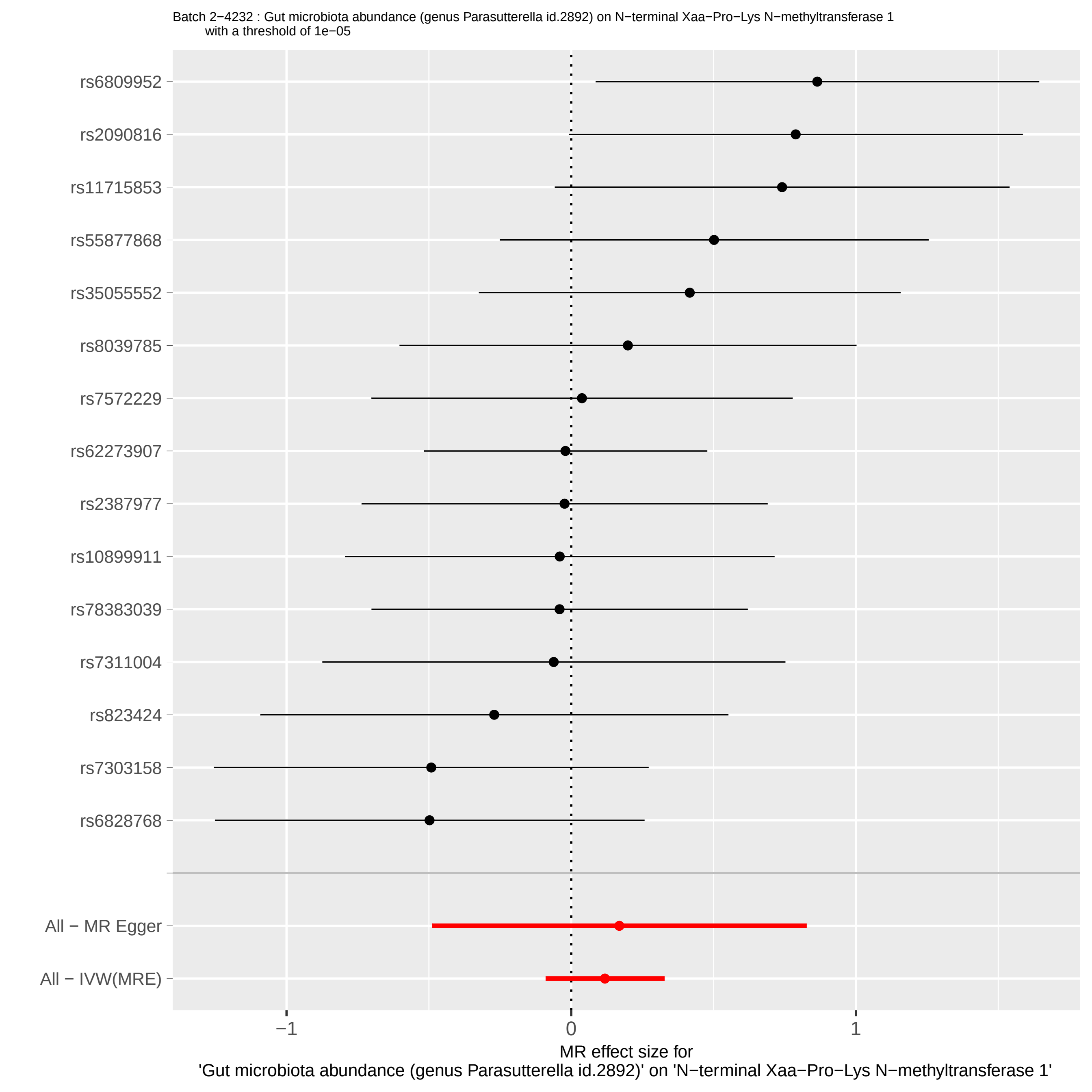
Batch 2−4232 : Gut microbiota abundance (genus Parasutterella id.2892) on N−terminal Xaa−Pro−Lys N−methyltransferase 1
with a threshold of 1e−05
rs6809952
rs2090816
rs11715853
rs55877868
rs35055552
rs8039785
rs7572229
rs62273907
rs2387977
rs10899911
rs78383039
rs7311004
rs823424
rs7303158
rs6828768
All − MR Egger
All − IVW(MRE)
−1
0
1
MR effect size for
'Gut microbiota abundance (genus Parasutterella id.2892)' on 'N−terminal Xaa−Pro−Lys N−methyltransferase 1'

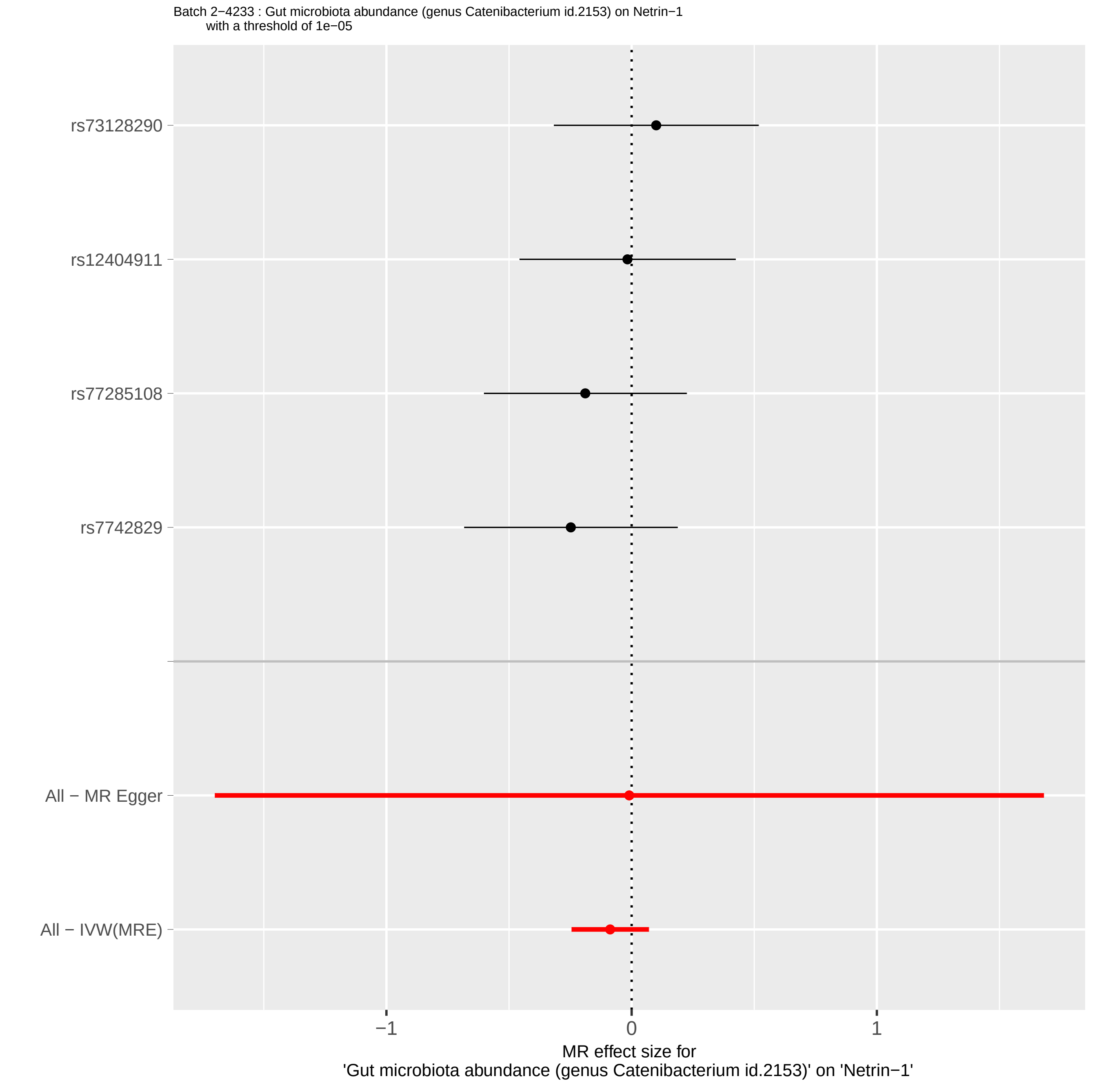

Batch 2−4233 : Gut microbiota abundance (genus Catenibacterium id.2153) on Netrin−1
with a threshold of 1e−05
rs73128290
rs12404911
rs77285108
rs7742829
All − MR Egger
All − IVW(MRE)
−1
0
1
MR effect size for
'Gut microbiota abundance (genus Catenibacterium id.2153)' on 'Netrin−1'

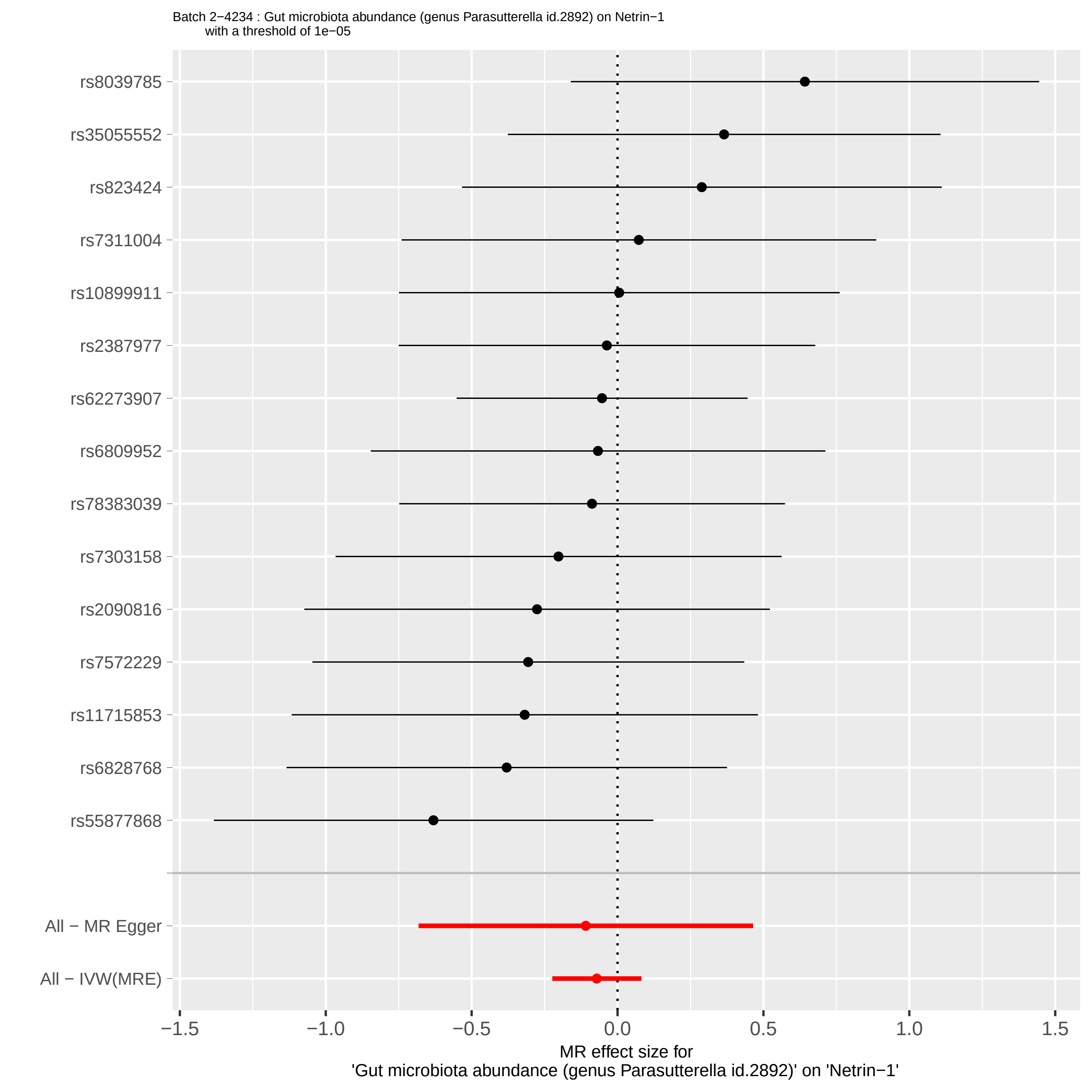

Batch 2−4234 : Gut microbiota abundance (genus Parasutterella id.2892) on Netrin−1
with a threshold of 1e−05
rs8039785
rs35055552
rs823424
rs7311004
rs10899911
rs2387977
rs62273907
rs6809952
rs78383039
rs7303158
rs2090816
rs7572229
rs11715853
rs6828768
rs55877868
All − MR Egger
All − IVW(MRE)
−1.5
−1.0
−0.5
0.0
0.5
1.0
1.5
MR effect size for
'Gut microbiota abundance (genus Parasutterella id.2892)' on 'Netrin−1'

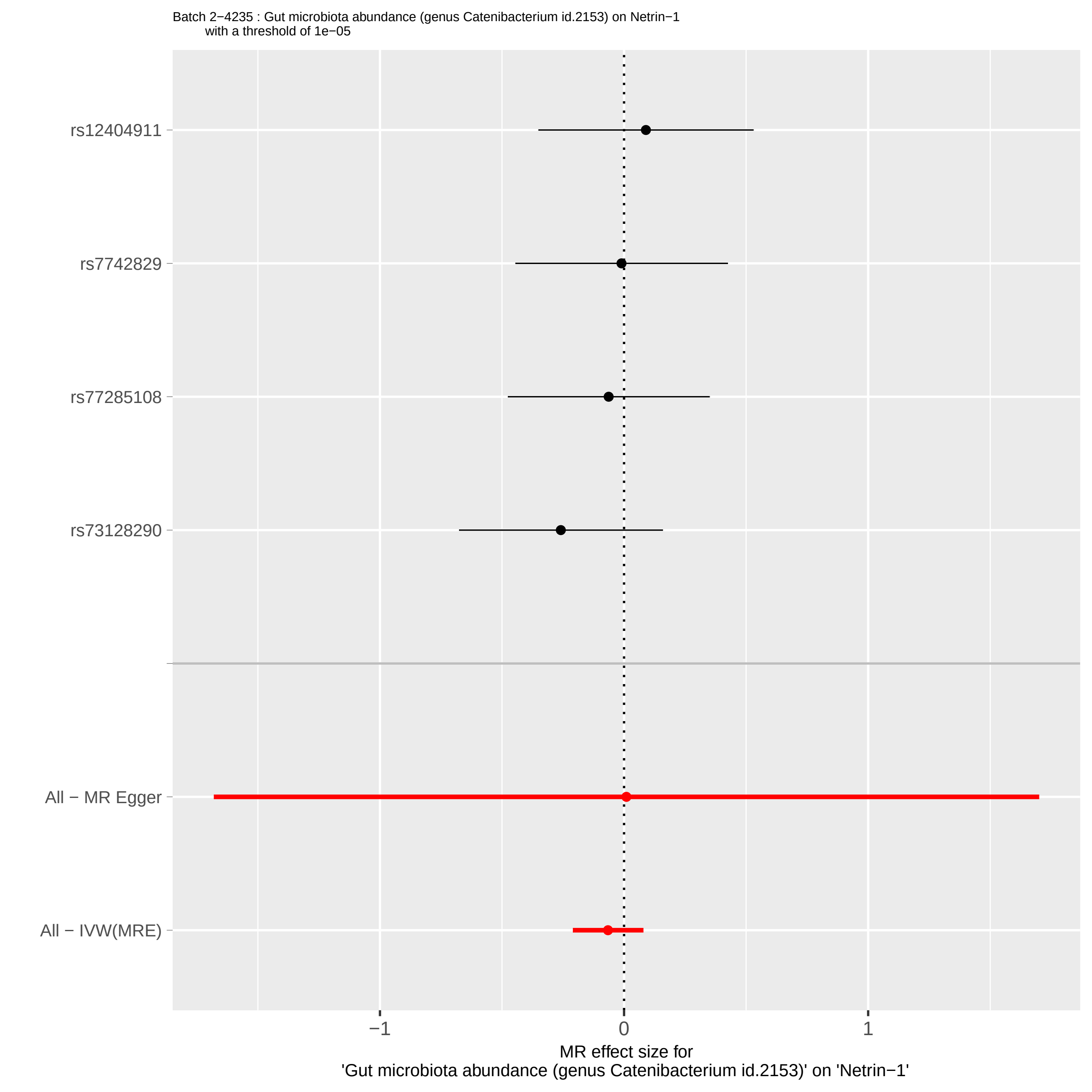
Batch 2−4235 : Gut microbiota abundance (genus Catenibacterium id.2153) on Netrin−1
with a threshold of 1e−05
rs12404911
rs7742829
rs77285108
rs73128290
All − MR Egger
All − IVW(MRE)
−1
0
1
MR effect size for
'Gut microbiota abundance (genus Catenibacterium id.2153)' on 'Netrin−1'

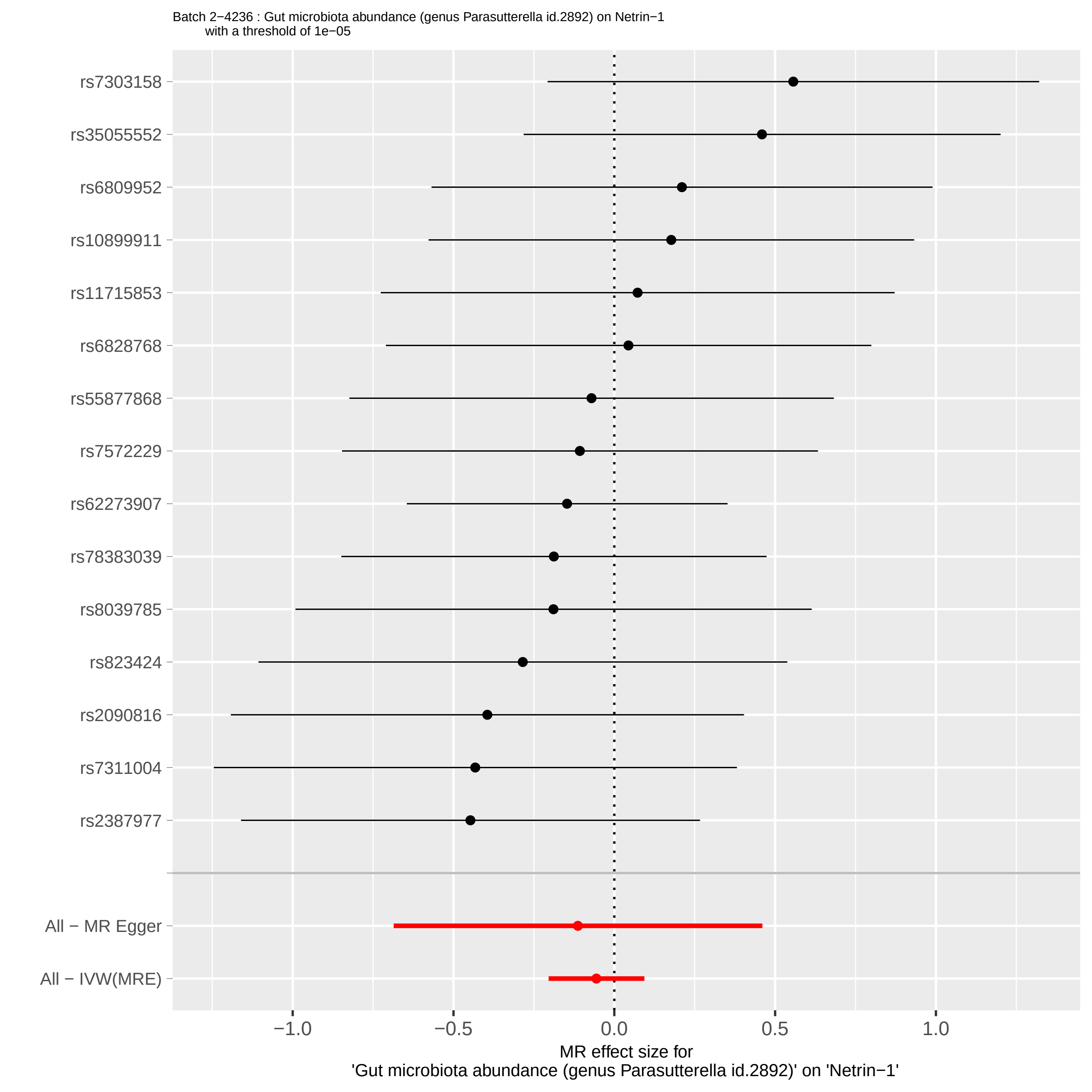
Batch 2−4236 : Gut microbiota abundance (genus Parasutterella id.2892) on Netrin−1
with a threshold of 1e−05
rs7303158
rs35055552
rs6809952
rs10899911
rs11715853
rs6828768
rs55877868
rs7572229
rs62273907
rs78383039
rs8039785
rs823424
rs2090816
rs7311004
rs2387977
All − MR Egger
All − IVW(MRE)
−1.0
−0.5
0.0
0.5
1.0
MR effect size for
'Gut microbiota abundance (genus Parasutterella id.2892)' on 'Netrin−1'

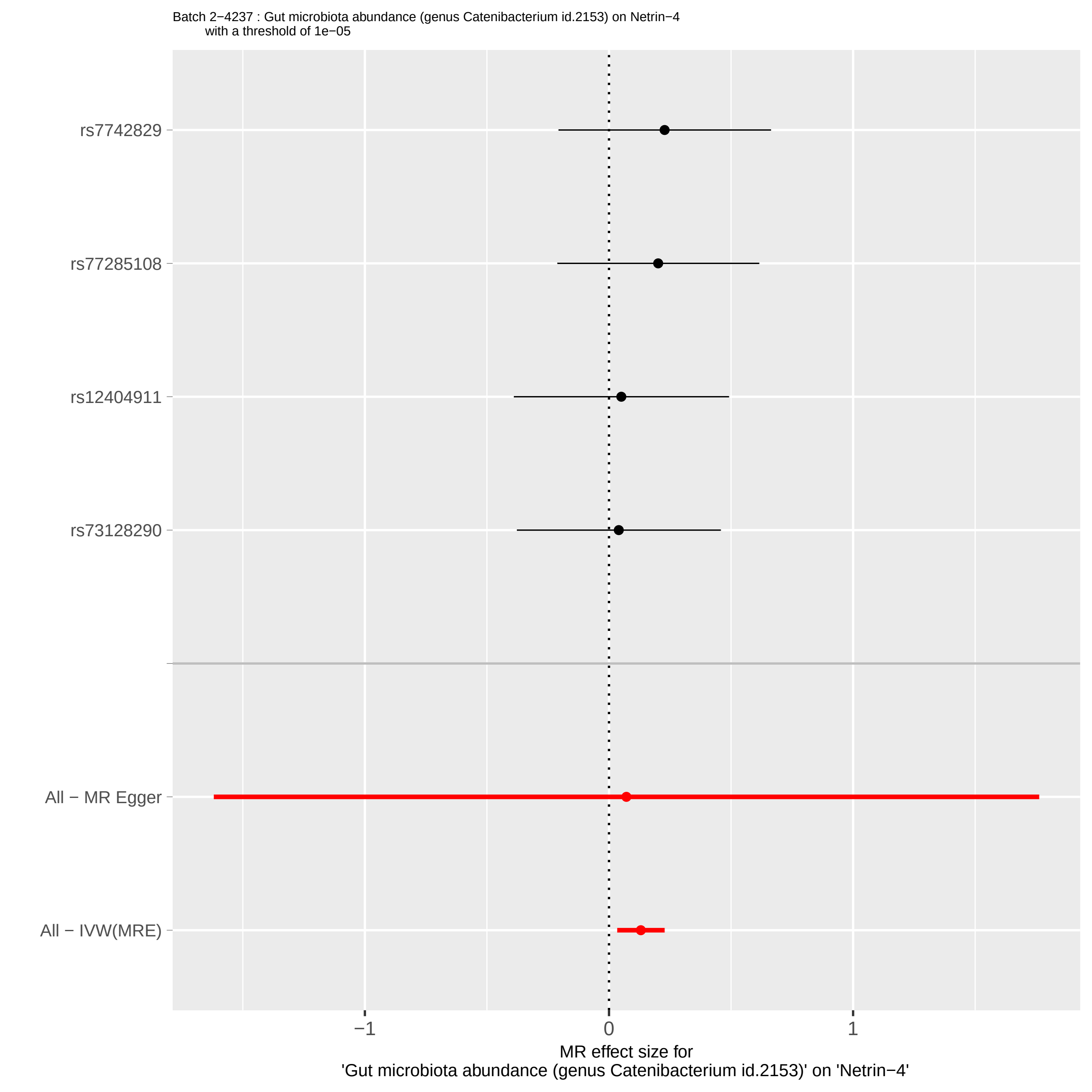

Batch 2−4237 : Gut microbiota abundance (genus Catenibacterium id.2153) on Netrin−4
with a threshold of 1e−05
rs7742829
rs77285108
rs12404911
rs73128290
All − MR Egger
All − IVW(MRE)
−1
0
1
MR effect size for
'Gut microbiota abundance (genus Catenibacterium id.2153)' on 'Netrin−4'

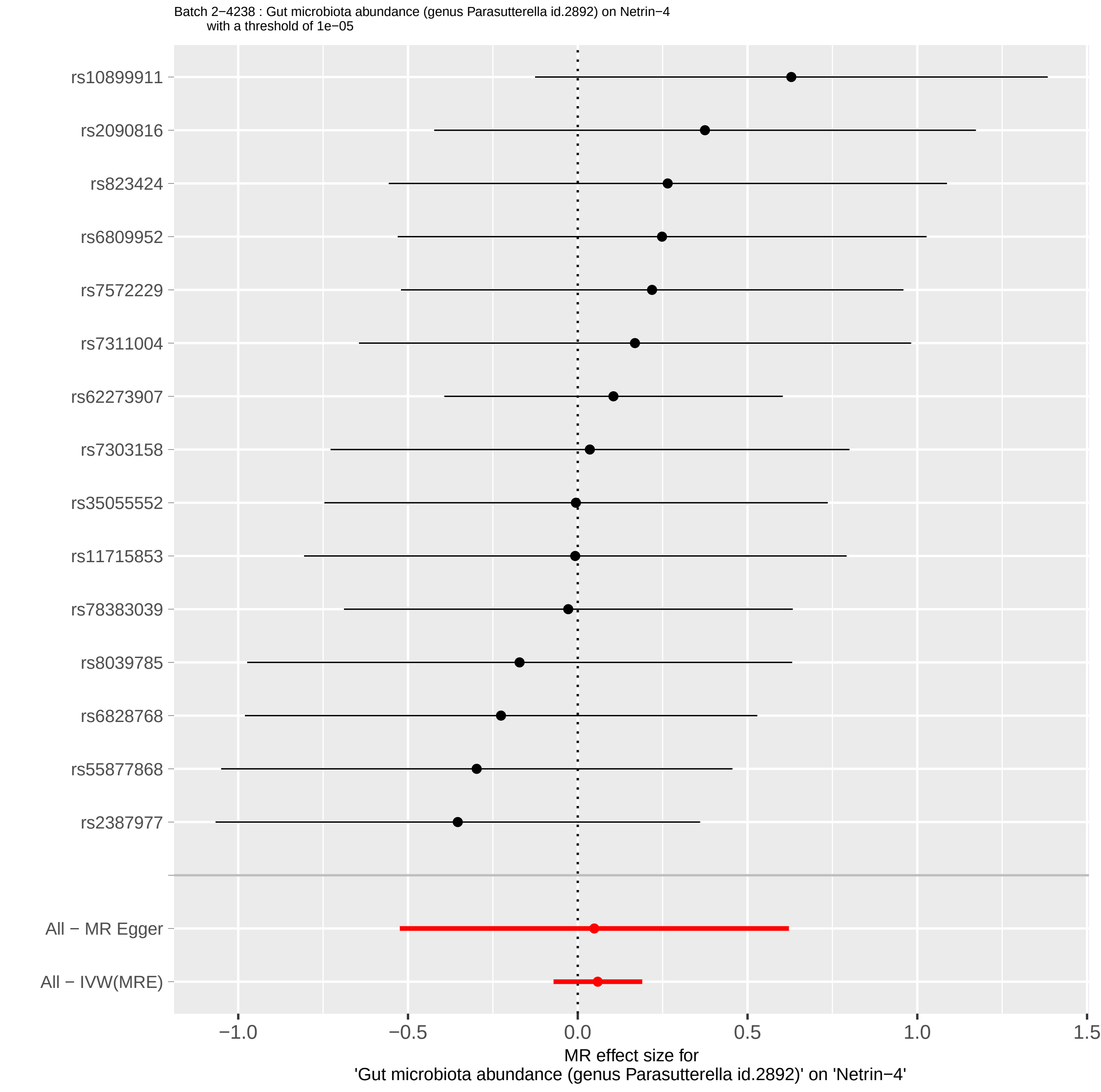
Batch 2−4238 : Gut microbiota abundance (genus Parasutterella id.2892) on Netrin−4
with a threshold of 1e−05
rs10899911
rs2090816
rs823424
rs6809952
rs7572229
rs7311004
rs62273907
rs7303158
rs35055552
rs11715853
rs78383039
rs8039785
rs6828768
rs55877868
rs2387977
All − MR Egger
All − IVW(MRE)
−1.0
−0.5
0.0
0.5
1.0
1.5
MR effect size for
'Gut microbiota abundance (genus Parasutterella id.2892)' on 'Netrin−4'

Batch 2−4239 : Gut microbiota abundance (genus Catenibacterium id.2153) on Netrin−G1
with a threshold of 1e−05

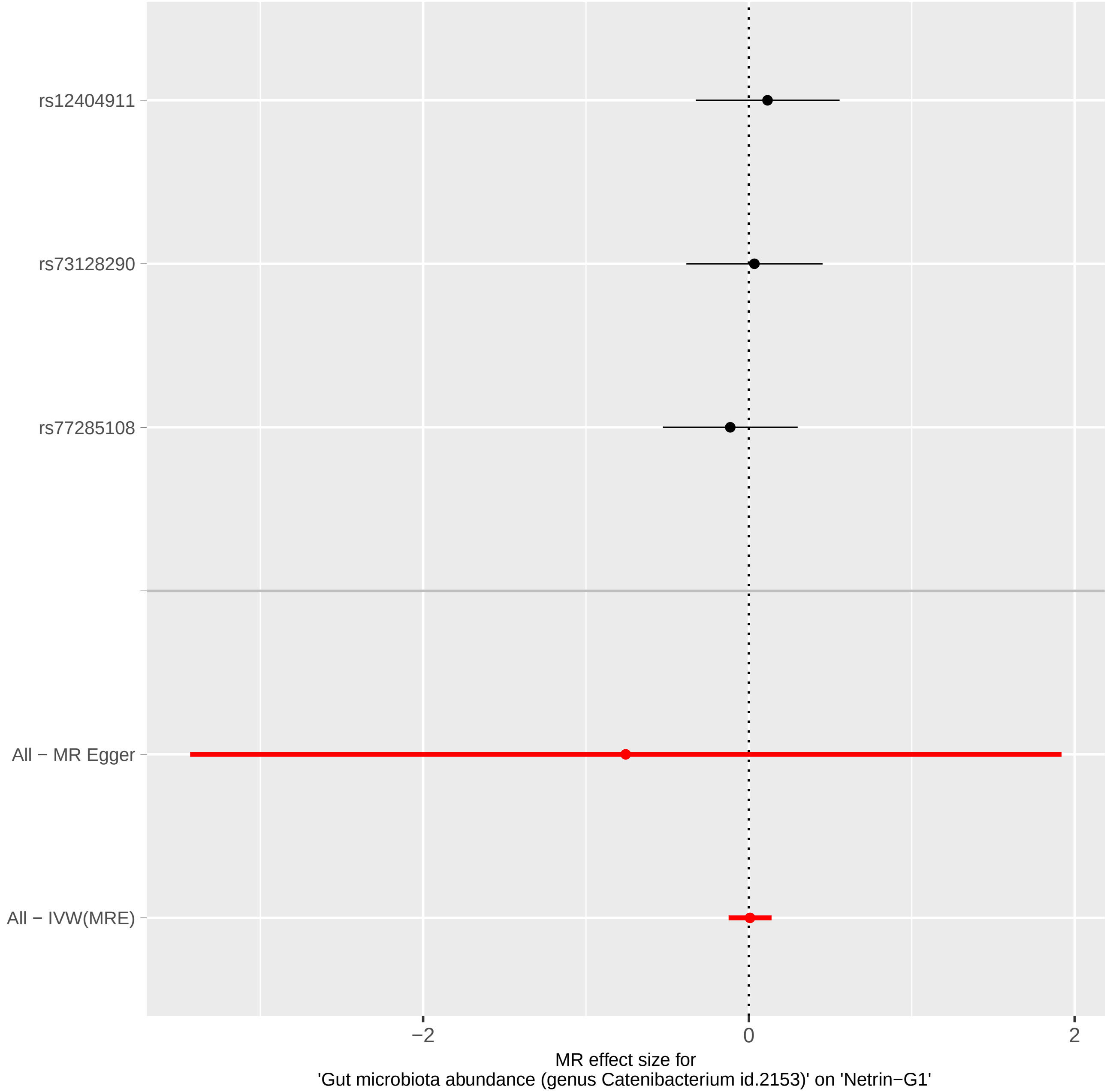

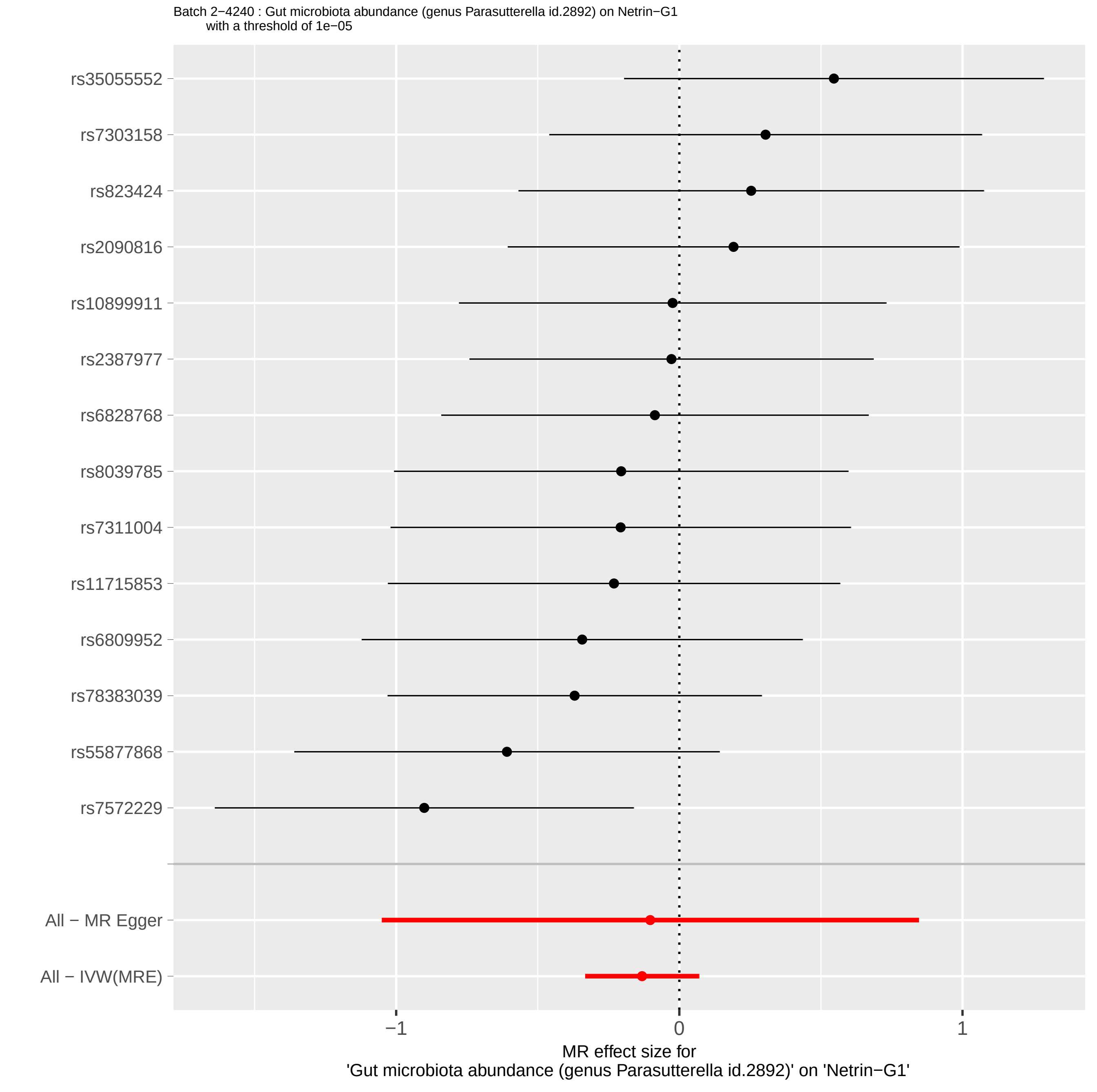

Batch 2−4240 : Gut microbiota abundance (genus Parasutterella id.2892) on Netrin−G1
with a threshold of 1e−05
rs35055552
rs7303158
rs823424
rs2090816
rs10899911
rs2387977
rs6828768
rs8039785
rs7311004
rs11715853
rs6809952
rs78383039
rs55877868
rs7572229
All − MR Egger
All − IVW(MRE)
−1
0
1
MR effect size for
'Gut microbiota abundance (genus Parasutterella id.2892)' on 'Netrin−G1'

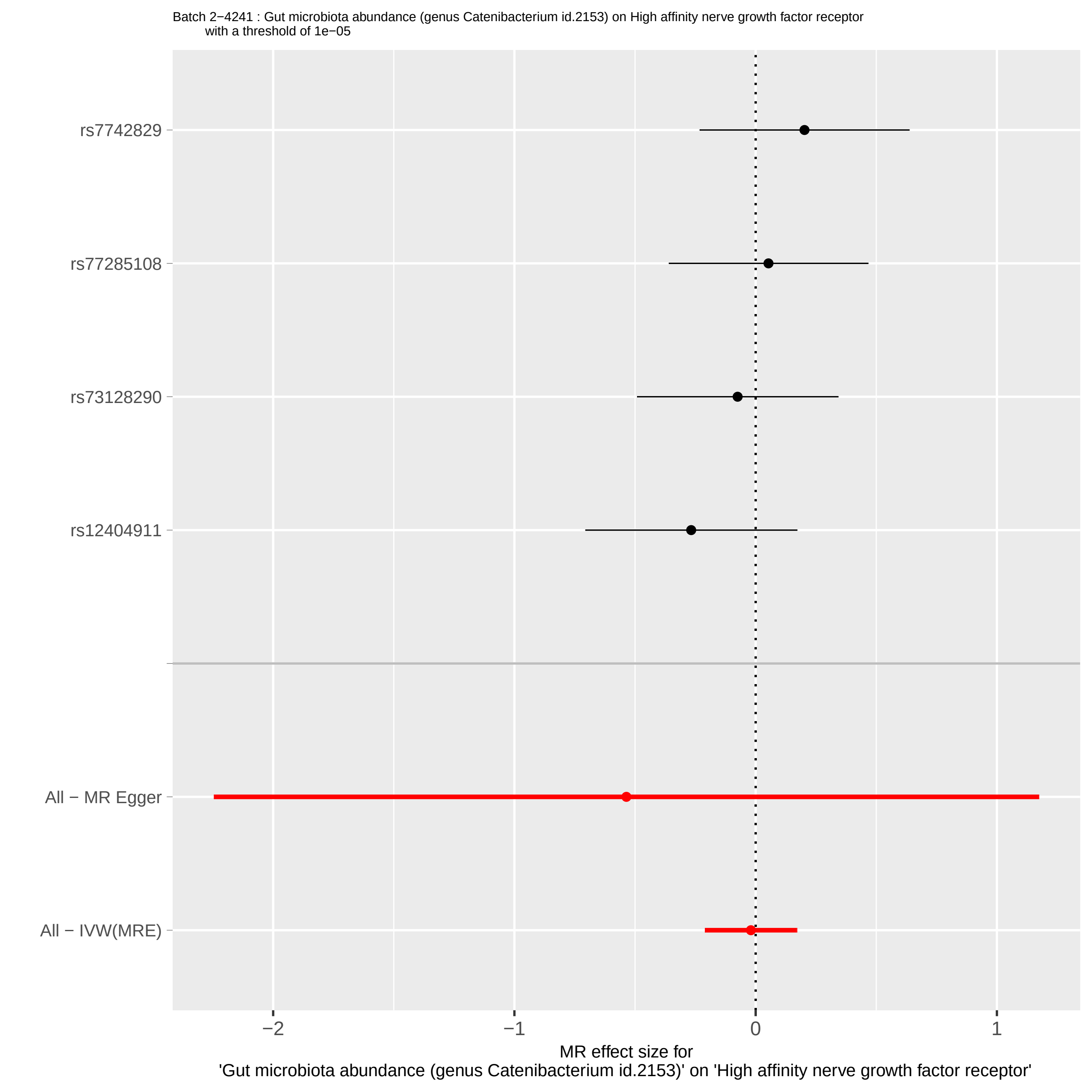
Batch 2−4241 : Gut microbiota abundance (genus Catenibacterium id.2153) on High affinity nerve growth factor receptor
with a threshold of 1e−05
rs7742829
rs77285108
rs73128290
rs12404911
All − MR Egger
All − IVW(MRE)
−2
−1
0
1
MR effect size for
'Gut microbiota abundance (genus Catenibacterium id.2153)' on 'High affinity nerve growth factor receptor'

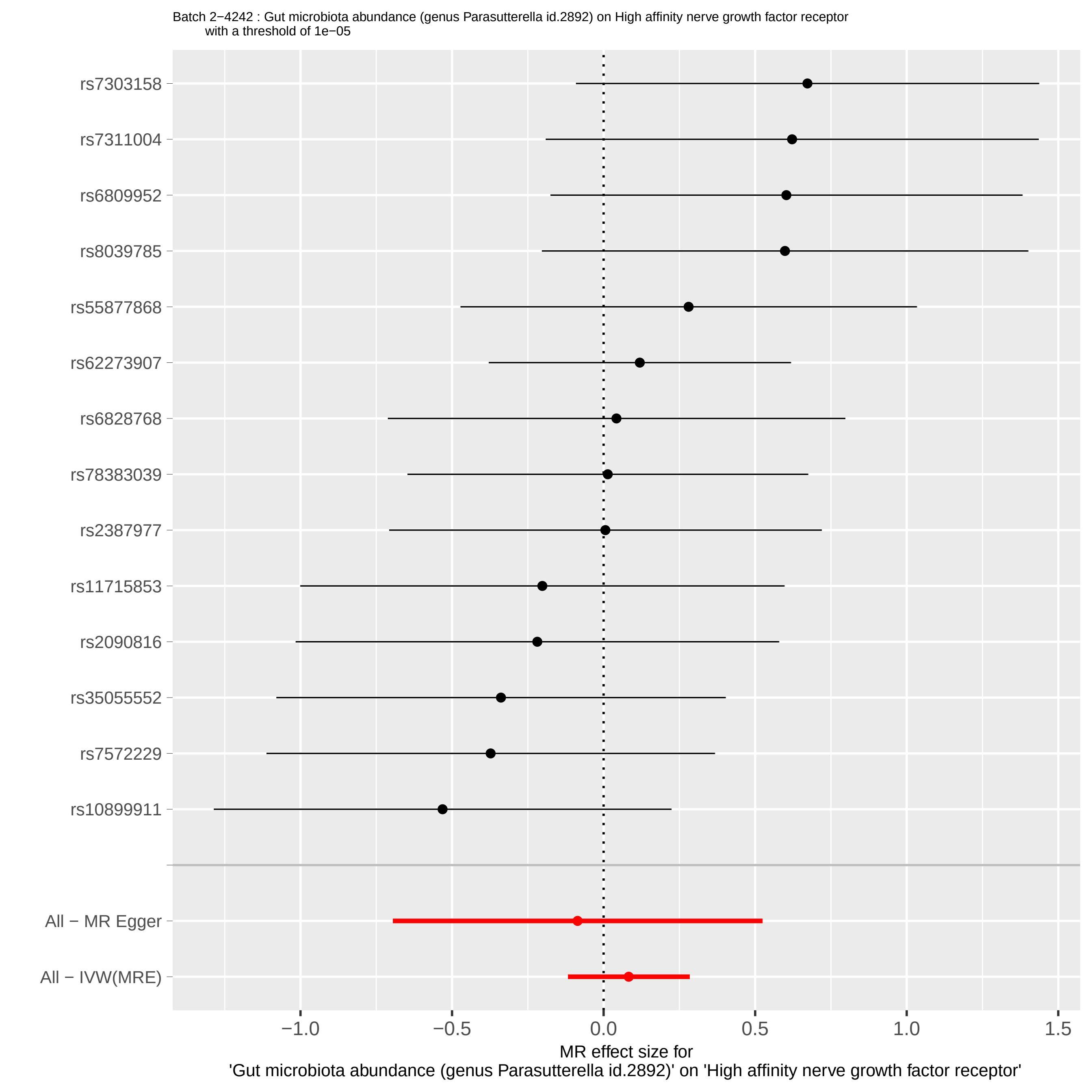

Batch 2−4242 : Gut microbiota abundance (genus Parasutterella id.2892) on High affinity nerve growth factor receptor
with a threshold of 1e−05
rs7303158
rs7311004
rs6809952
rs8039785
rs55877868
rs62273907
rs6828768
rs78383039
rs2387977
rs11715853
rs2090816
rs35055552
rs7572229
rs10899911
All − MR Egger
All − IVW(MRE)
−1.0
−0.5
0.0
0.5
1.0
1.5
MR effect size for
'Gut microbiota abundance (genus Parasutterella id.2892)' on 'High affinity nerve growth factor receptor'

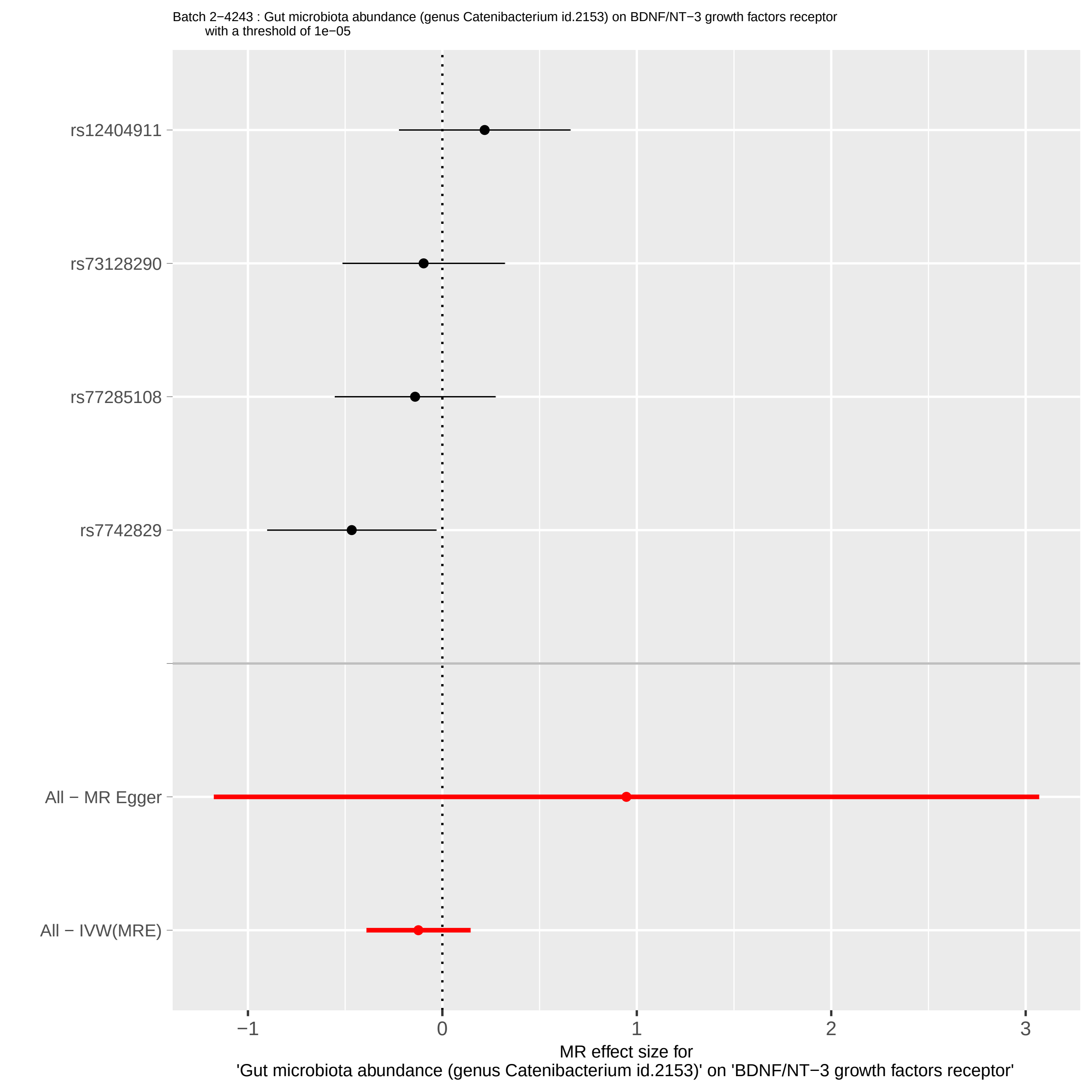
Batch 2−4243 : Gut microbiota abundance (genus Catenibacterium id.2153) on BDNF/NT−3 growth factors receptor
with a threshold of 1e−05
rs12404911
rs73128290
rs77285108
rs7742829
All − MR Egger
All − IVW(MRE)
−1
0
1
2
3
MR effect size for
'Gut microbiota abundance (genus Catenibacterium id.2153)' on 'BDNF/NT−3 growth factors receptor'

Batch 2−4244 : Gut microbiota abundance (genus Parasutterella id.2892) on BDNF/NT−3 growth factors receptor
with a threshold of 1e−05

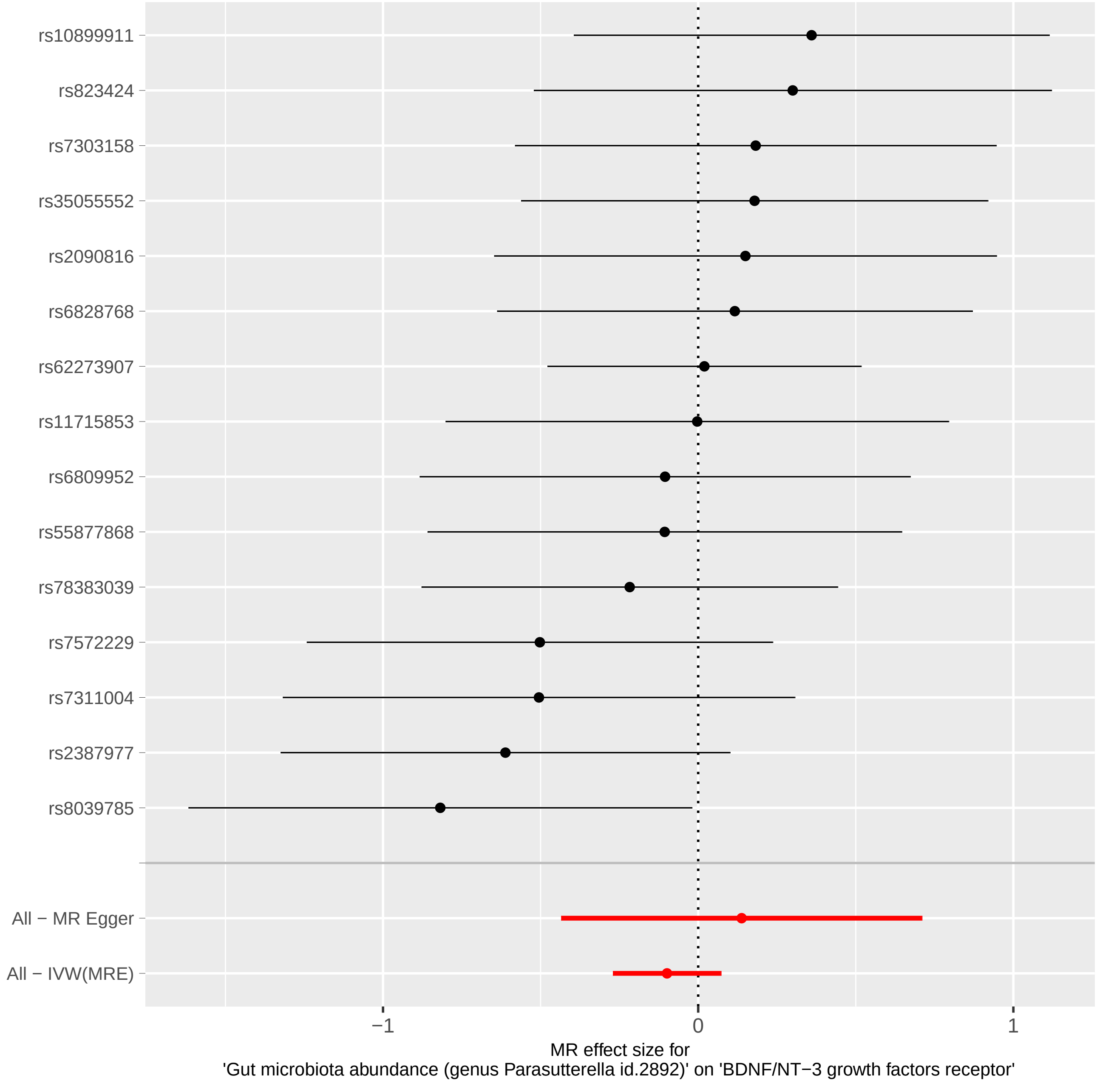

Batch 2−4245 : Gut microbiota abundance (genus Catenibacterium id.2153) on NT−3 growth factor receptor
with a threshold of 1e−05

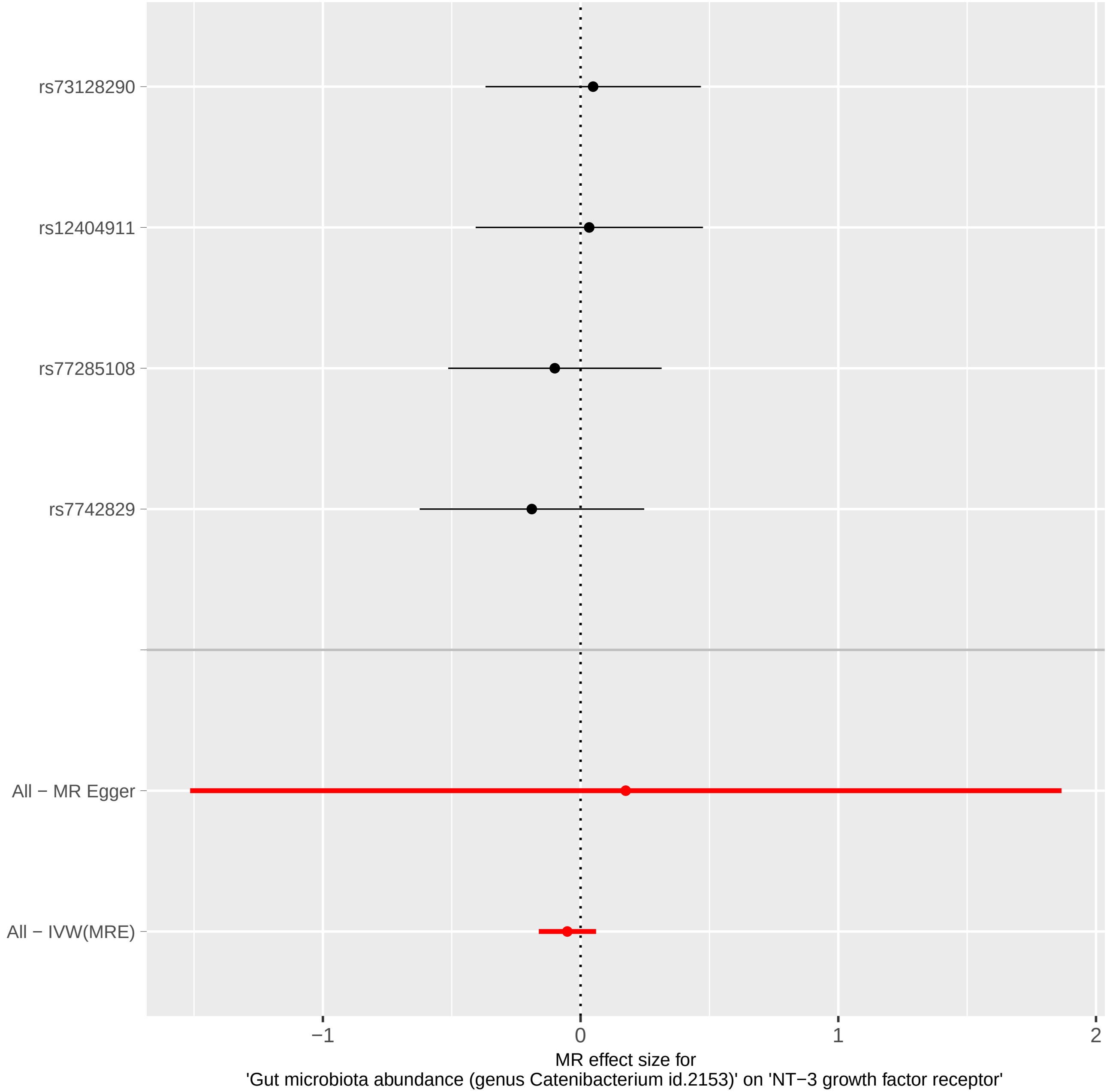

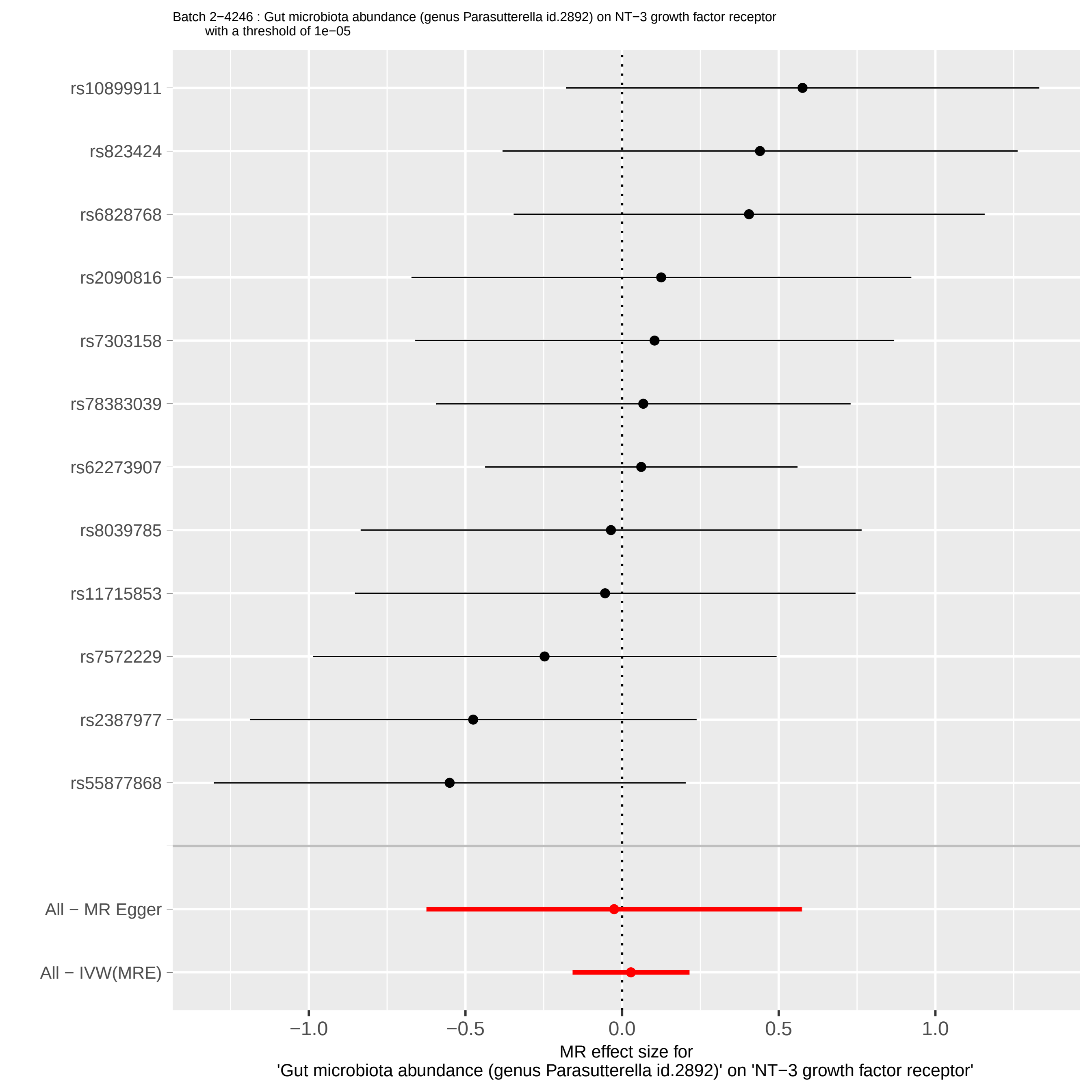

Batch 2−4246 : Gut microbiota abundance (genus Parasutterella id.2892) on NT−3 growth factor receptor
with a threshold of 1e−05
rs10899911
rs823424
rs6828768
rs2090816
rs7303158
rs78383039
rs62273907
rs8039785
rs11715853
rs7572229
rs2387977
rs55877868
All − MR Egger
All − IVW(MRE)
−1.0
−0.5
0.0
0.5
1.0
MR effect size for
'Gut microbiota abundance (genus Parasutterella id.2892)' on 'NT−3 growth factor receptor'

Batch 2−4247 : Gut microbiota abundance (genus Catenibacterium id.2153) on Neurotensin/neuromedin N
with a threshold of 1e−05

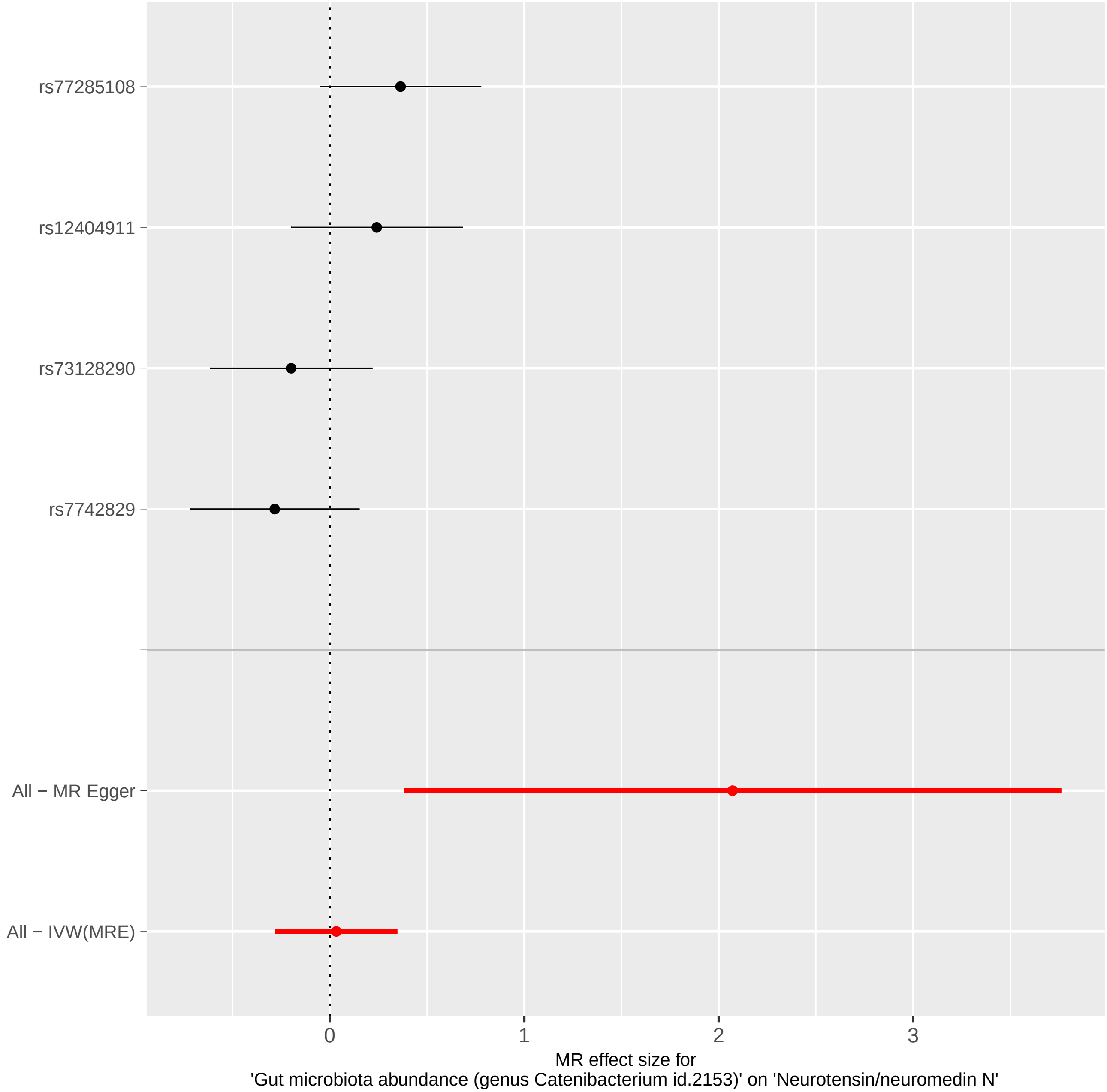

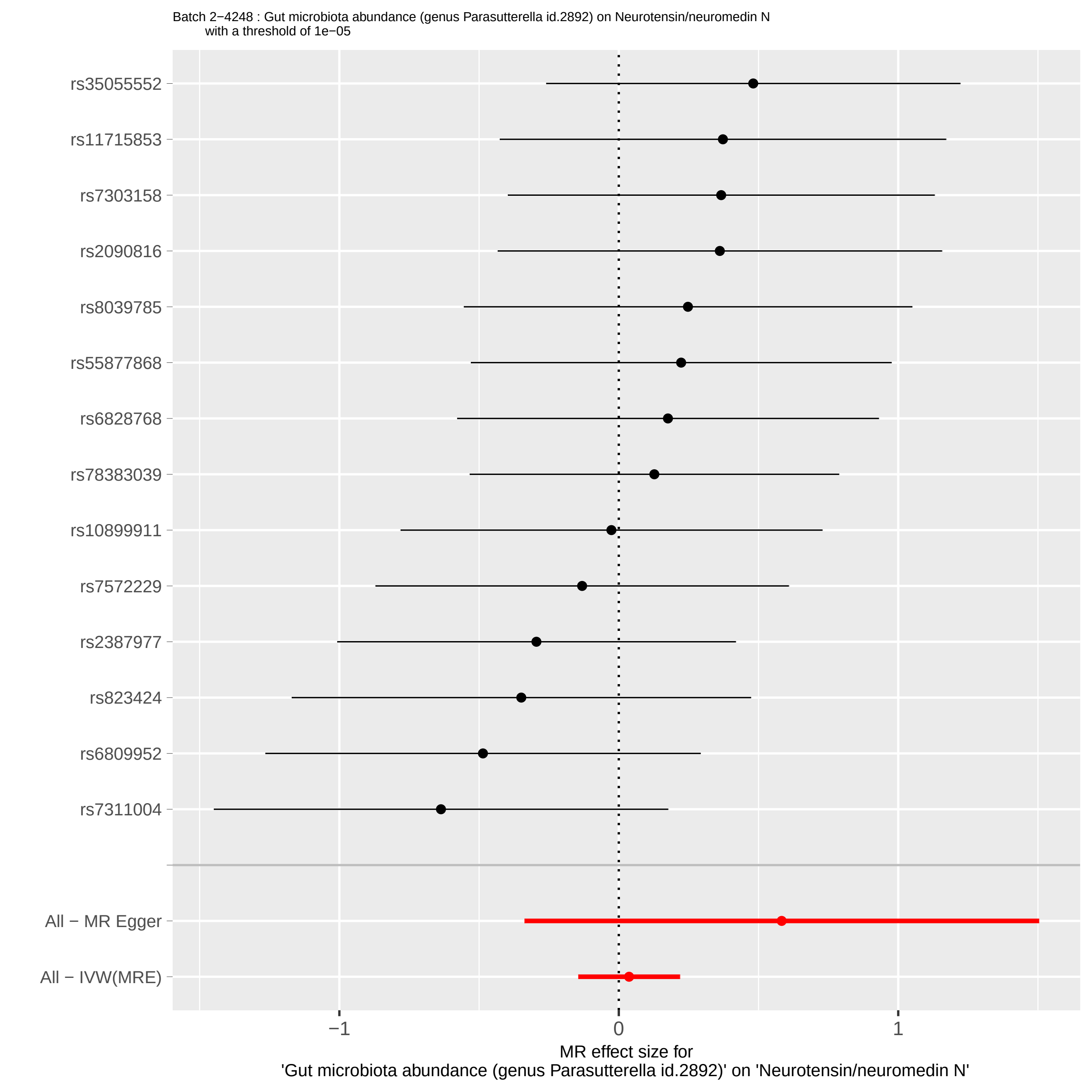

Batch 2−4248 : Gut microbiota abundance (genus Parasutterella id.2892) on Neurotensin/neuromedin N
with a threshold of 1e−05
rs35055552
rs11715853
rs7303158
rs2090816
rs8039785
rs55877868
rs6828768
rs78383039
rs10899911
rs7572229
rs2387977
rs823424
rs6809952
rs7311004
All − MR Egger
All − IVW(MRE)
−1
0
1
MR effect size for
'Gut microbiota abundance (genus Parasutterella id.2892)' on 'Neurotensin/neuromedin N'

Batch 2−4249 : Gut microbiota abundance (genus Catenibacterium id.2153) on Nuclear migration protein nudC
with a threshold of 1e−05

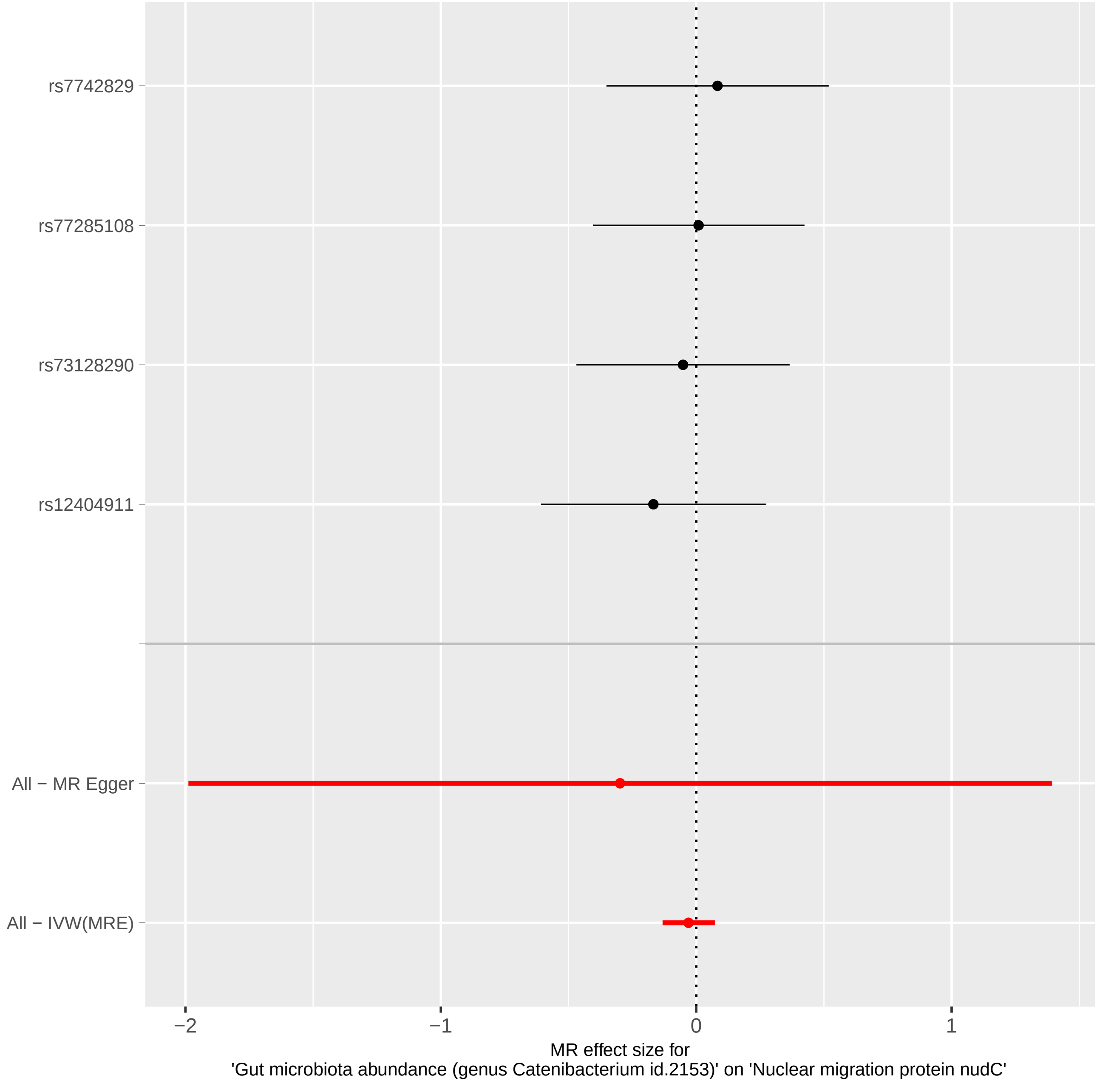

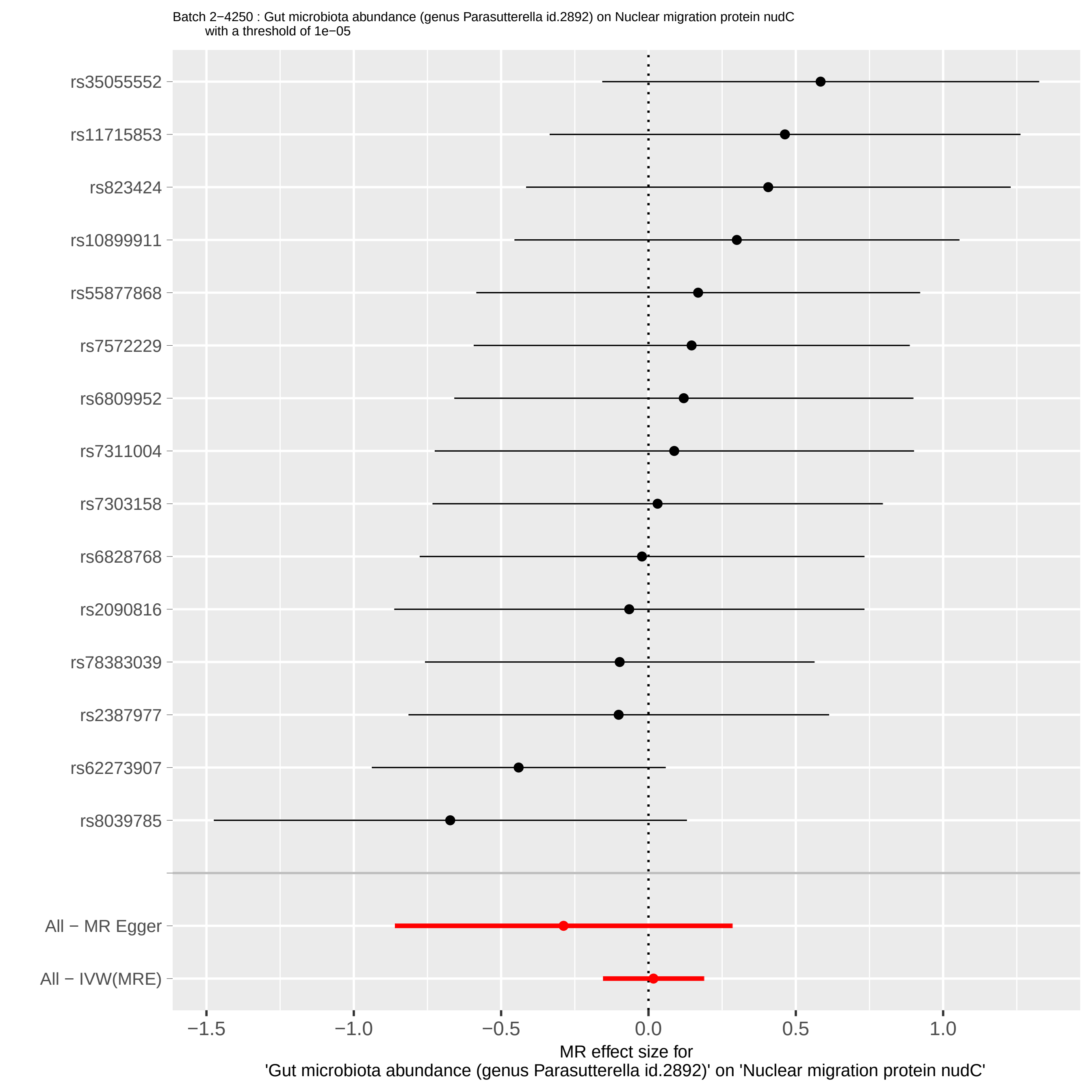

Batch 2−4250 : Gut microbiota abundance (genus Parasutterella id.2892) on Nuclear migration protein nudC
with a threshold of 1e−05
rs35055552
rs11715853
rs823424
rs10899911
rs55877868
rs7572229
rs6809952
rs7311004
rs7303158
rs6828768
rs2090816
rs78383039
rs2387977
rs62273907
rs8039785
All − MR Egger
All − IVW(MRE)
−1.5
−1.0
−0.5
0.0
0.5
1.0
MR effect size for
'Gut microbiota abundance (genus Parasutterella id.2892)' on 'Nuclear migration protein nudC'

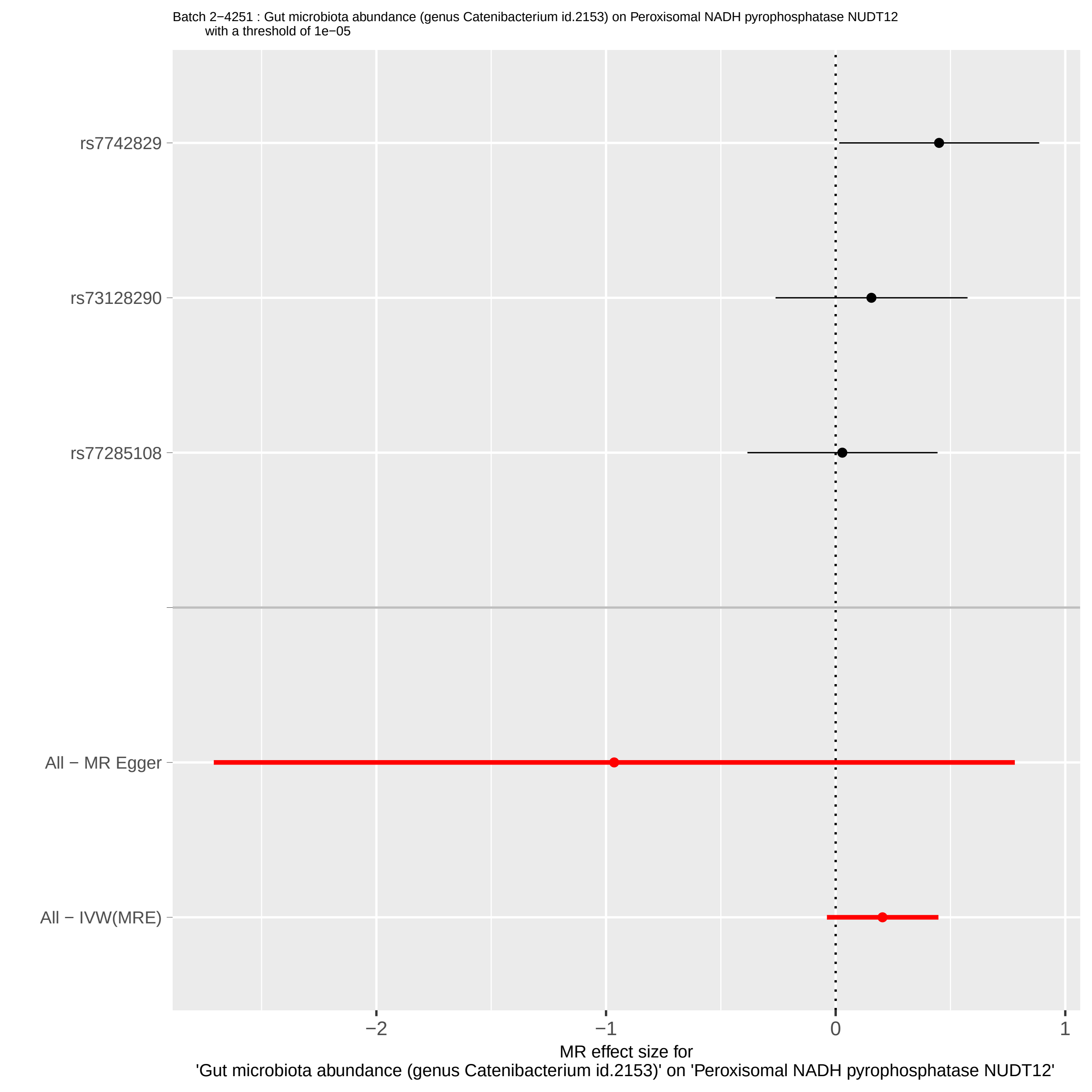

Batch 2−4251 : Gut microbiota abundance (genus Catenibacterium id.2153) on Peroxisomal NADH pyrophosphatase NUDT12
with a threshold of 1e−05
rs7742829
rs73128290
rs77285108
All − MR Egger
All − IVW(MRE)
−2
−1
0
1
MR effect size for
'Gut microbiota abundance (genus Catenibacterium id.2153)' on 'Peroxisomal NADH pyrophosphatase NUDT12'

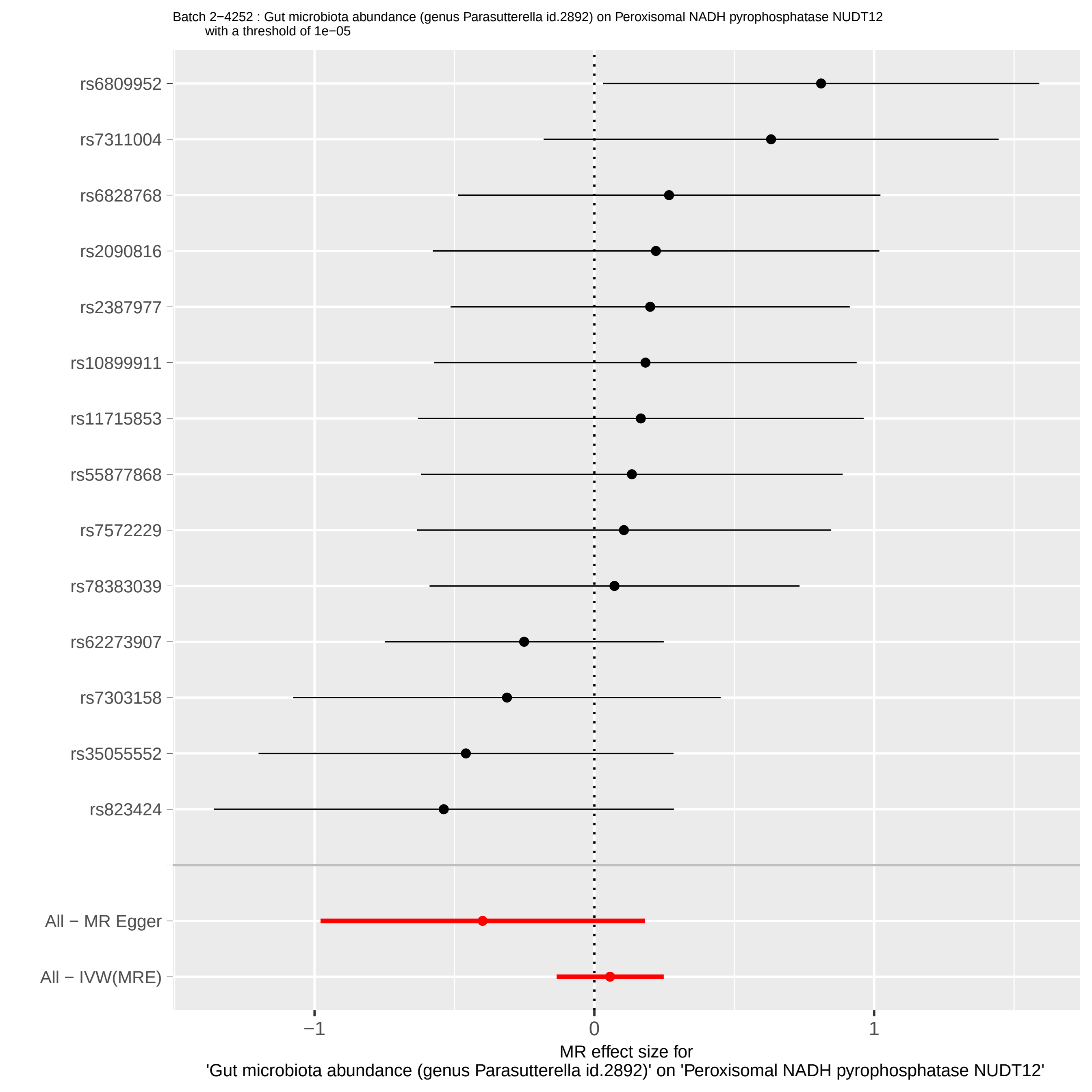

Batch 2−4252 : Gut microbiota abundance (genus Parasutterella id.2892) on Peroxisomal NADH pyrophosphatase NUDT12
with a threshold of 1e−05
rs6809952
rs7311004
rs6828768
rs2090816
rs2387977
rs10899911
rs11715853
rs55877868
rs7572229
rs78383039
rs62273907
rs7303158
rs35055552
rs823424
All − MR Egger
All − IVW(MRE)
−1
0
1
MR effect size for
'Gut microbiota abundance (genus Parasutterella id.2892)' on 'Peroxisomal NADH pyrophosphatase NUDT12'

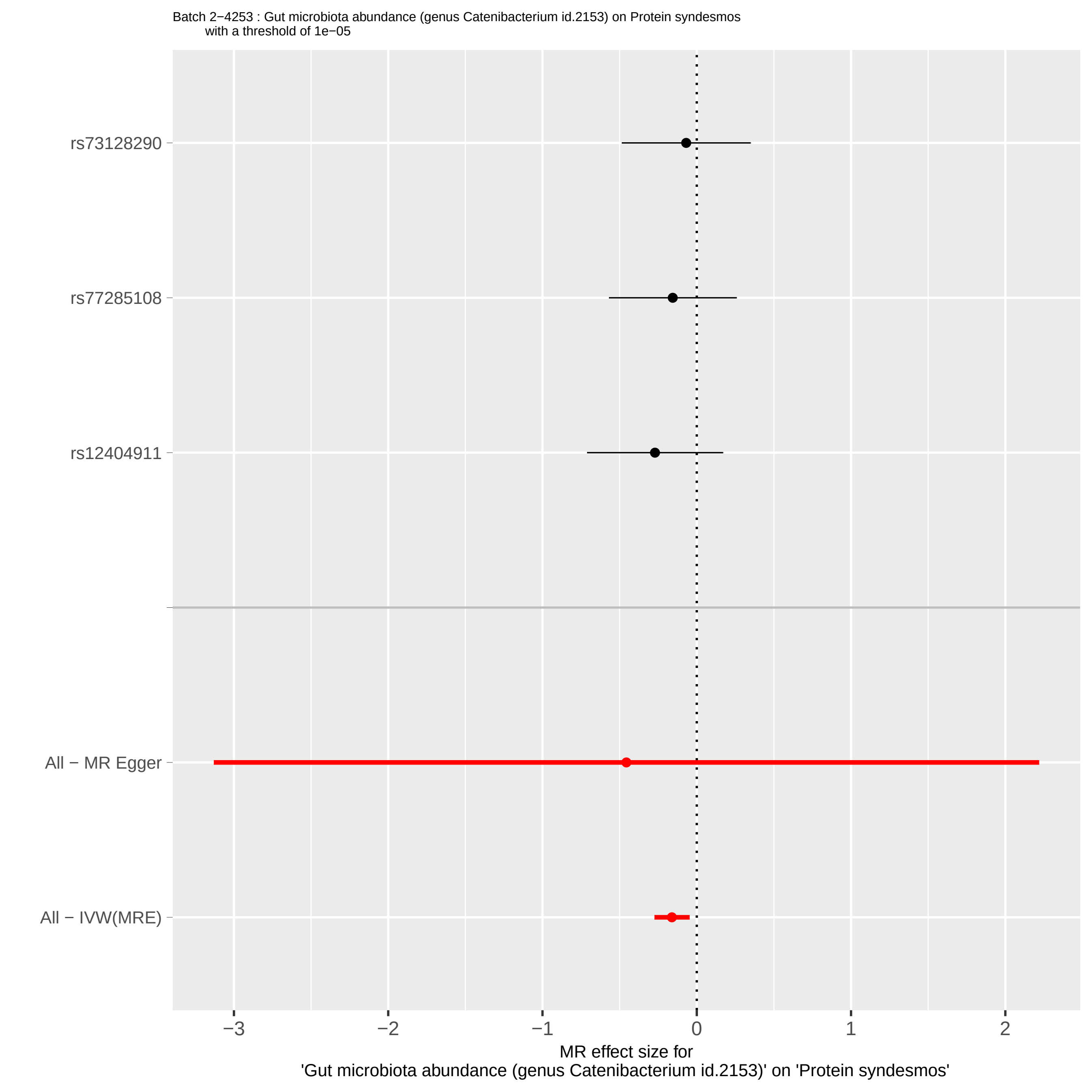
Batch 2−4253 : Gut microbiota abundance (genus Catenibacterium id.2153) on Protein syndesmos
with a threshold of 1e−05
rs73128290
rs77285108
rs12404911
All − MR Egger
All − IVW(MRE)
−3
−2
−1
0
1
2
MR effect size for
'Gut microbiota abundance (genus Catenibacterium id.2153)' on 'Protein syndesmos'

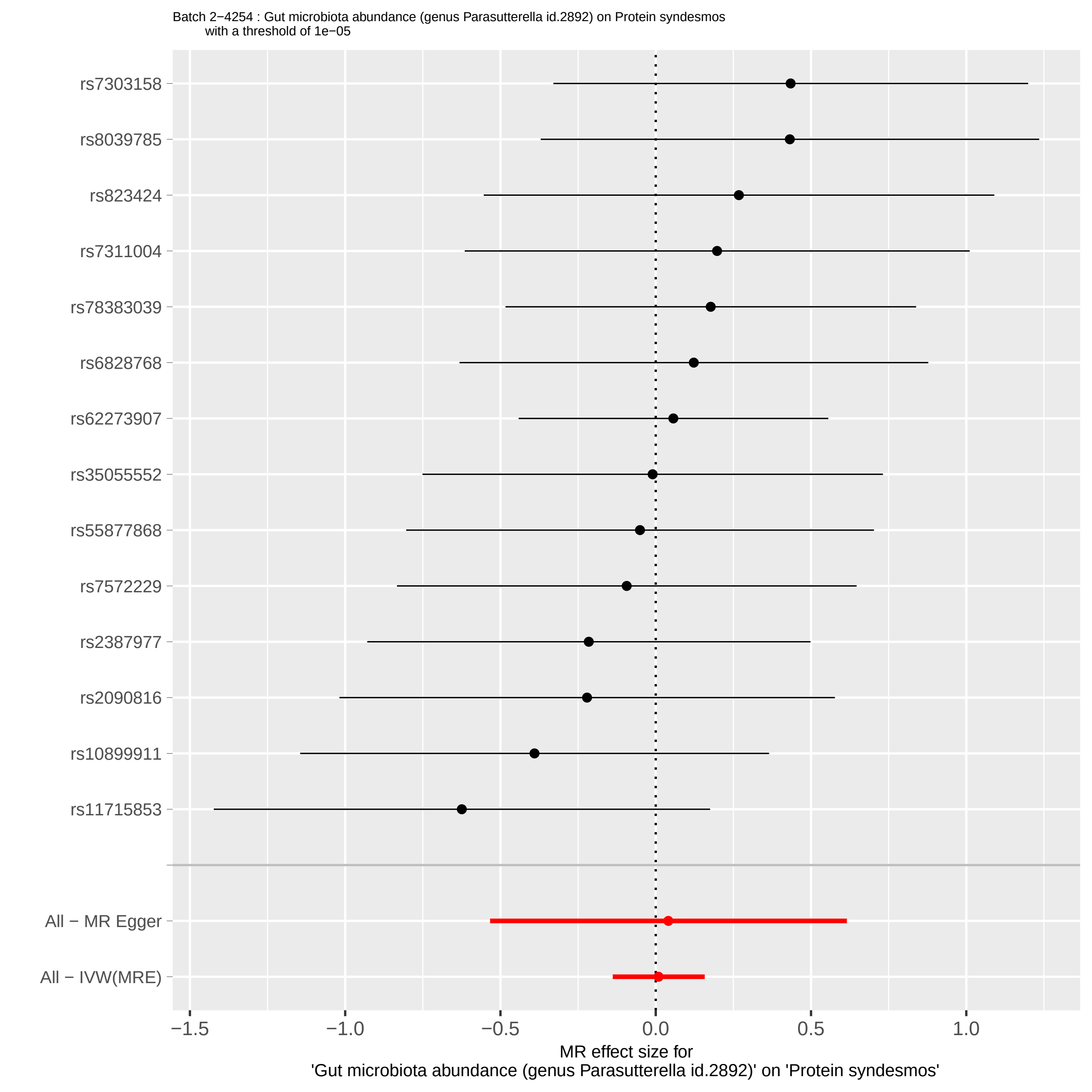

Batch 2−4254 : Gut microbiota abundance (genus Parasutterella id.2892) on Protein syndesmos
with a threshold of 1e−05
rs7303158
rs8039785
rs823424
rs7311004
rs78383039
rs6828768
rs62273907
rs35055552
rs55877868
rs7572229
rs2387977
rs2090816
rs10899911
rs11715853
All − MR Egger
All − IVW(MRE)
−1.5
−1.0
−0.5
0.0
0.5
1.0
MR effect size for
'Gut microbiota abundance (genus Parasutterella id.2892)' on 'Protein syndesmos'

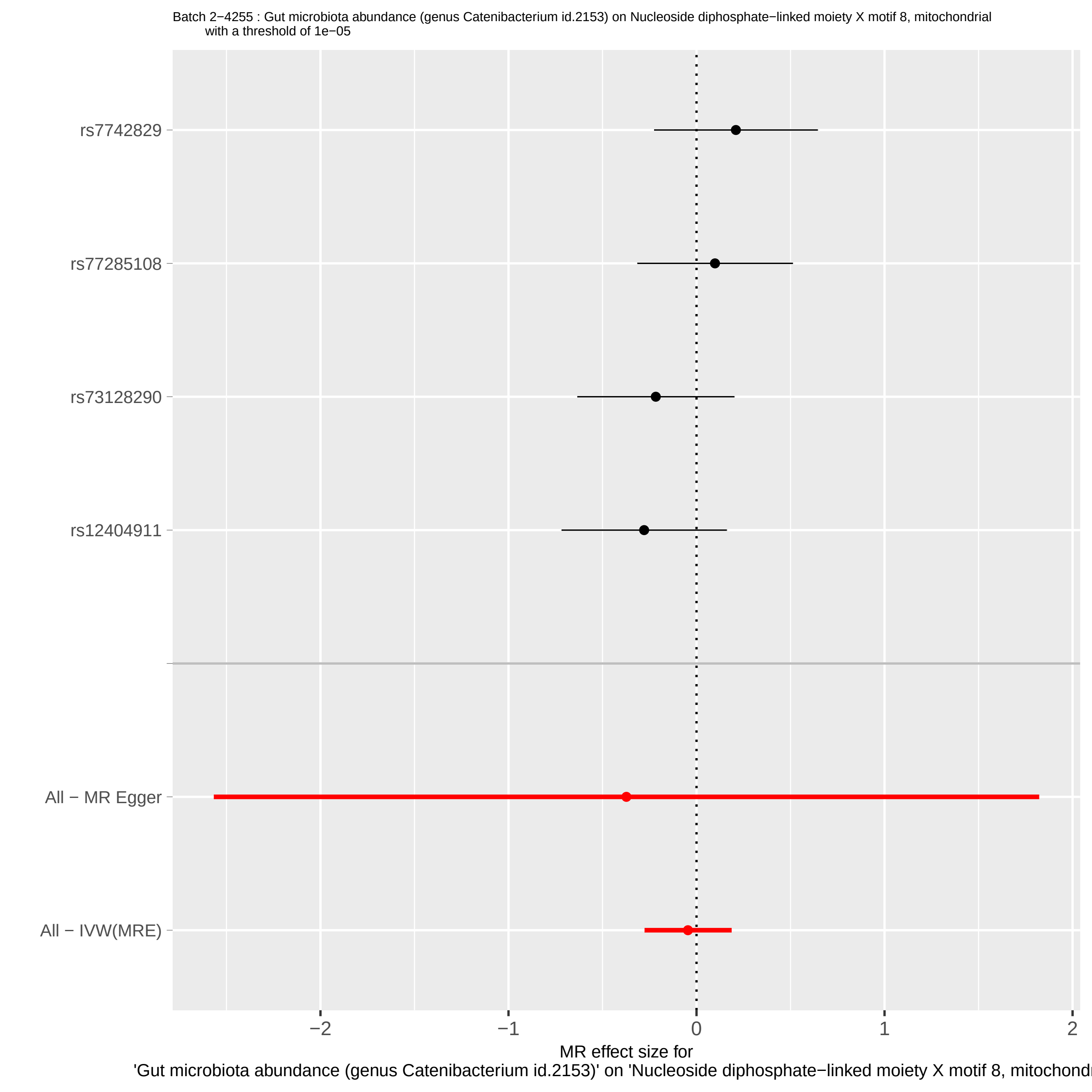

Batch 2−4255 : Gut microbiota abundance (genus Catenibacterium id.2153) on Nucleoside diphosphate−linked moiety X motif 8, mitochondrial
with a threshold of 1e−05
rs7742829
rs77285108
rs73128290
rs12404911
All − MR Egger
All − IVW(MRE)
−2
−1
0
1
2
MR effect size for
'Gut microbiota abundance (genus Catenibacterium id.2153)' on 'Nucleoside diphosphate−linked moiety X motif 8, mitochond

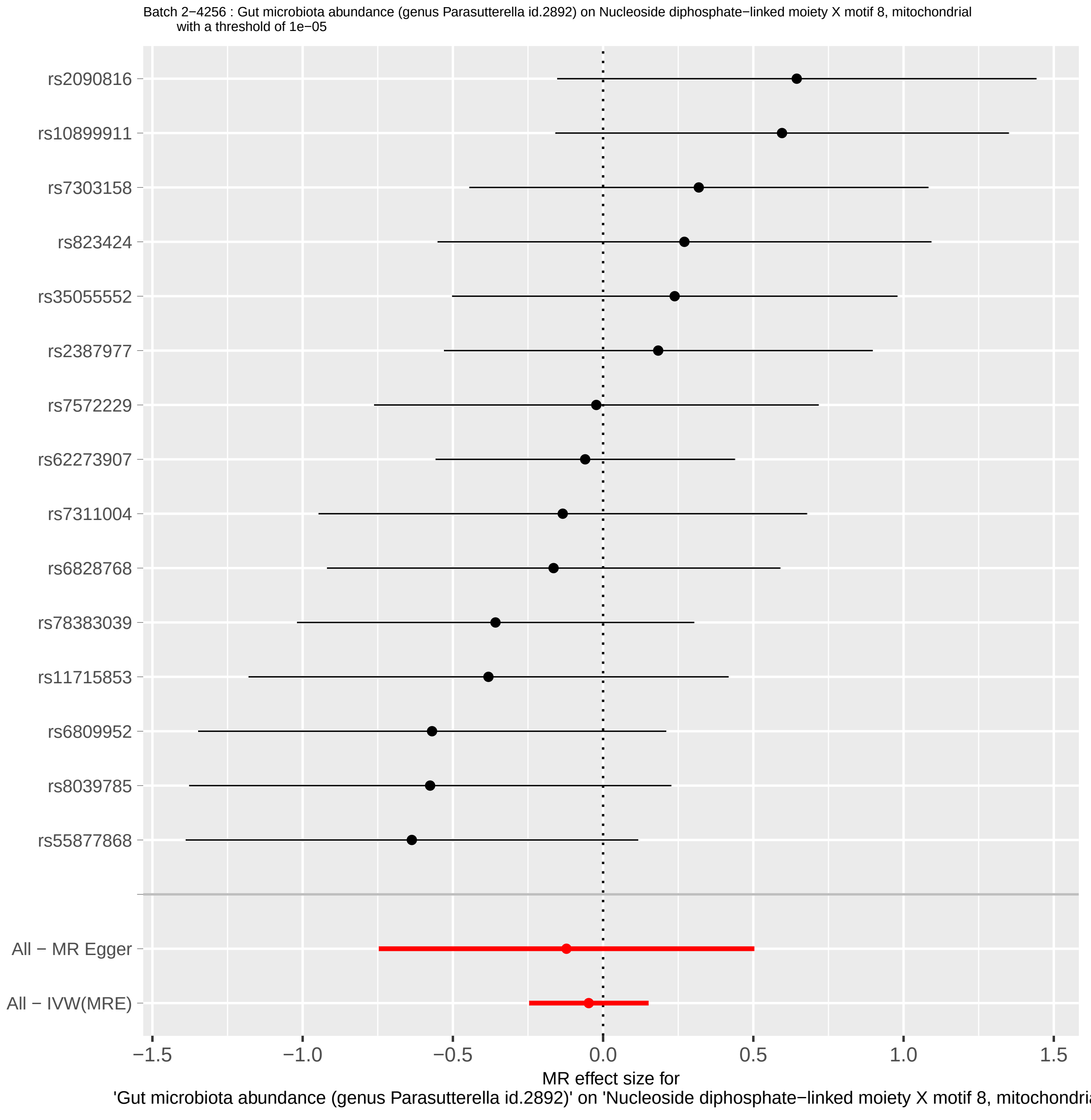
Batch 2−4256 : Gut microbiota abundance (genus Parasutterella id.2892) on Nucleoside diphosphate−linked moiety X motif 8, mitochondrial
with a threshold of 1e−05
rs2090816
rs10899911
rs7303158
rs823424
rs35055552
rs2387977
rs7572229
rs62273907
rs7311004
rs6828768
rs78383039
rs11715853
rs6809952
rs8039785
rs55877868
All − MR Egger
All − IVW(MRE)
−1.5
−1.0
−0.5
0.0
0.5
1.0
1.5
MR effect size for
'Gut microbiota abundance (genus Parasutterella id.2892)' on 'Nucleoside diphosphate−linked moiety X motif 8, mitochondria

Batch 2−4257 : Gut microbiota abundance (genus Catenibacterium id.2153) on ADP−ribose pyrophosphatase, mitochondrial
with a threshold of 1e−05

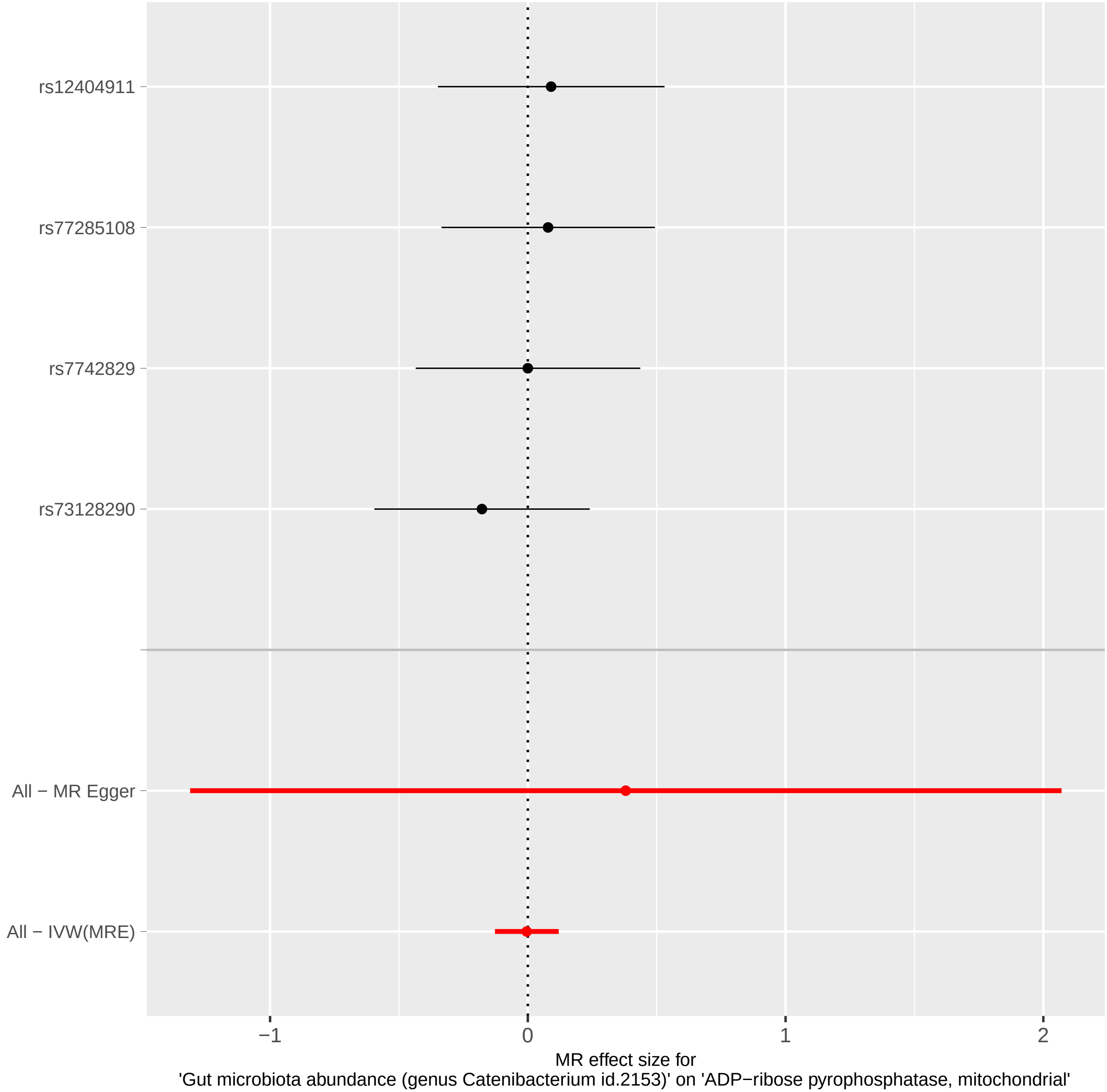

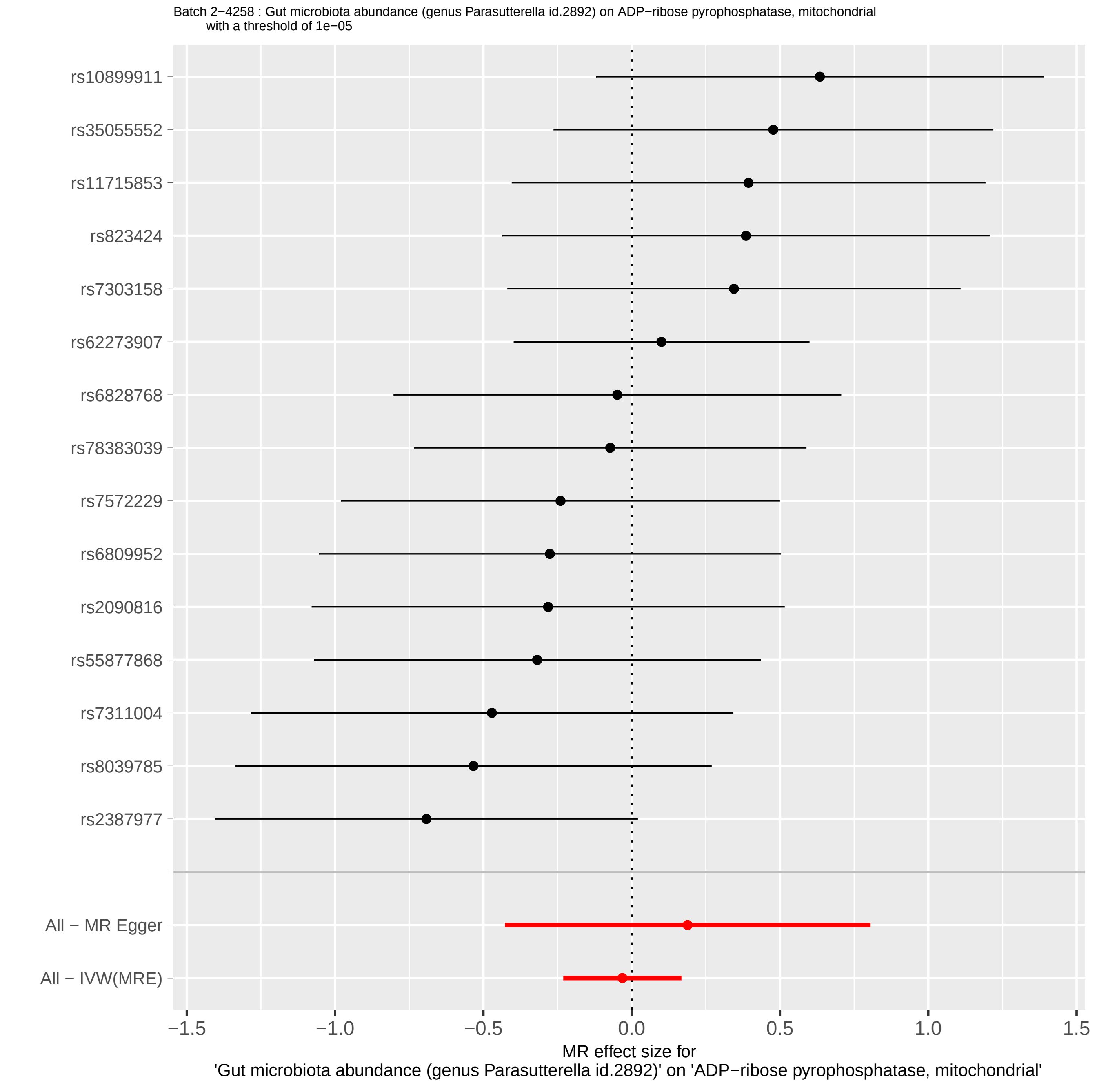

Batch 2−4258 : Gut microbiota abundance (genus Parasutterella id.2892) on ADP−ribose pyrophosphatase, mitochondrial
with a threshold of 1e−05
rs10899911
rs35055552
rs11715853
rs823424
rs7303158
rs62273907
rs6828768
rs78383039
rs7572229
rs6809952
rs2090816
rs55877868
rs7311004
rs8039785
rs2387977
All − MR Egger
All − IVW(MRE)
−1.5
−1.0
−0.5
0.0
0.5
1.0
1.5
MR effect size for
'Gut microbiota abundance (genus Parasutterella id.2892)' on 'ADP−ribose pyrophosphatase, mitochondrial'

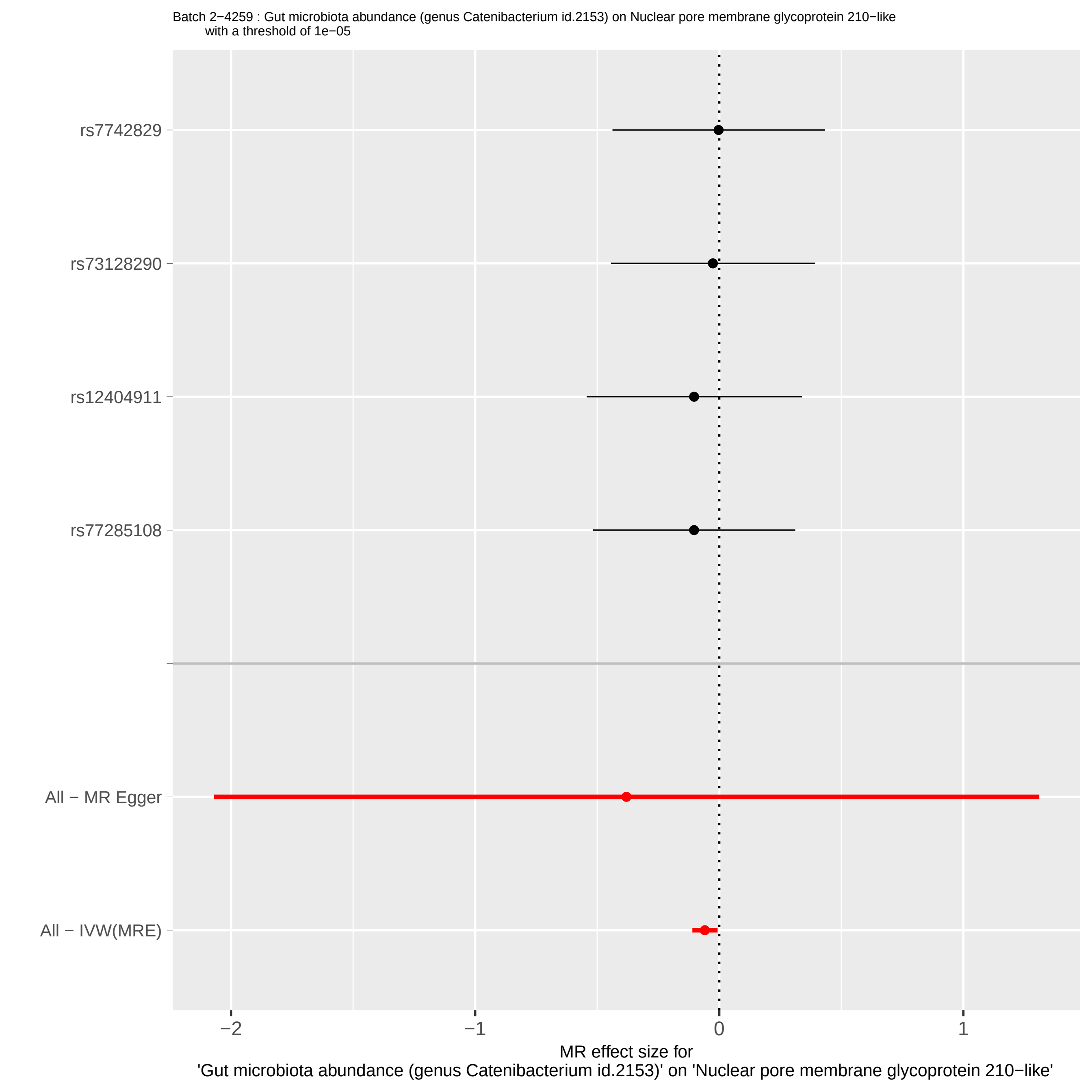

Batch 2−4259 : Gut microbiota abundance (genus Catenibacterium id.2153) on Nuclear pore membrane glycoprotein 210−like
with a threshold of 1e−05
rs7742829
rs73128290
rs12404911
rs77285108
All − MR Egger
All − IVW(MRE)
−2
−1
0
1
MR effect size for
'Gut microbiota abundance (genus Catenibacterium id.2153)' on 'Nuclear pore membrane glycoprotein 210−like'

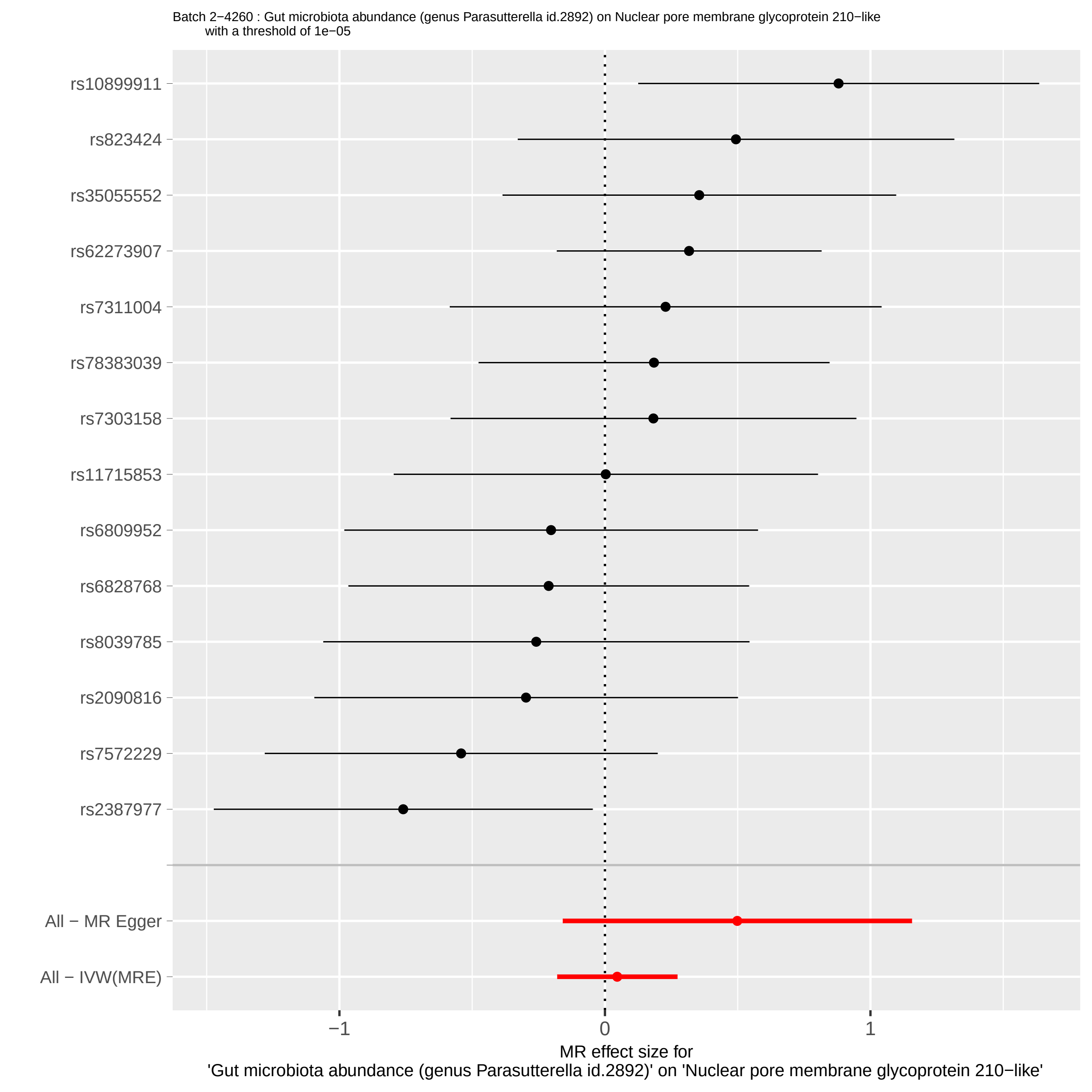
Batch 2−4260 : Gut microbiota abundance (genus Parasutterella id.2892) on Nuclear pore membrane glycoprotein 210−like
with a threshold of 1e−05
rs10899911
rs823424
rs35055552
rs62273907
rs7311004
rs78383039
rs7303158
rs11715853
rs6809952
rs6828768
rs8039785
rs2090816
rs7572229
rs2387977
All − MR Egger
All − IVW(MRE)
−1
0
1
MR effect size for
'Gut microbiota abundance (genus Parasutterella id.2892)' on 'Nuclear pore membrane glycoprotein 210−like'

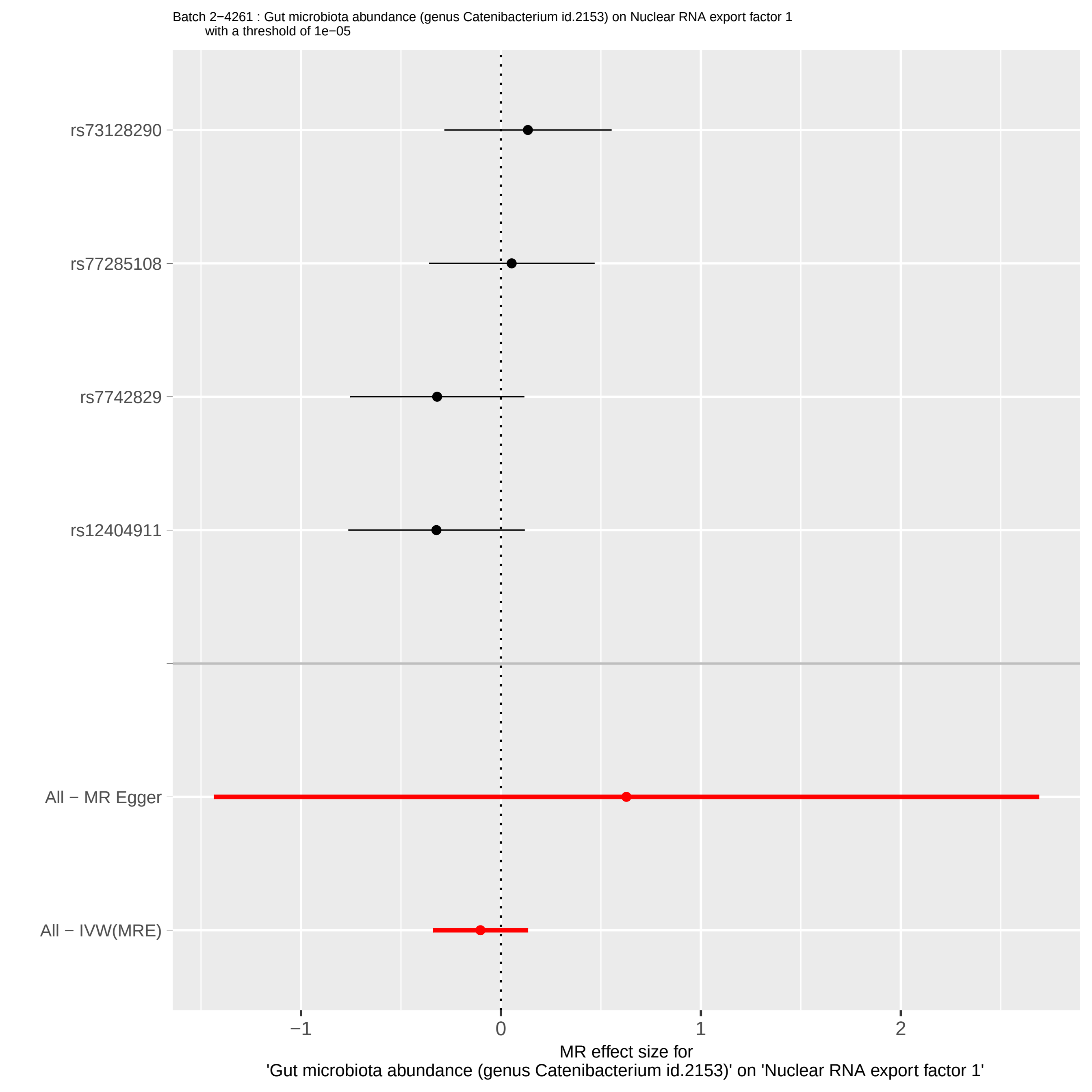

Batch 2−4261 : Gut microbiota abundance (genus Catenibacterium id.2153) on Nuclear RNA export factor 1
with a threshold of 1e−05
rs73128290
rs77285108
rs7742829
rs12404911
All − MR Egger
All − IVW(MRE)
−1
0
1
2
MR effect size for
'Gut microbiota abundance (genus Catenibacterium id.2153)' on 'Nuclear RNA export factor 1'

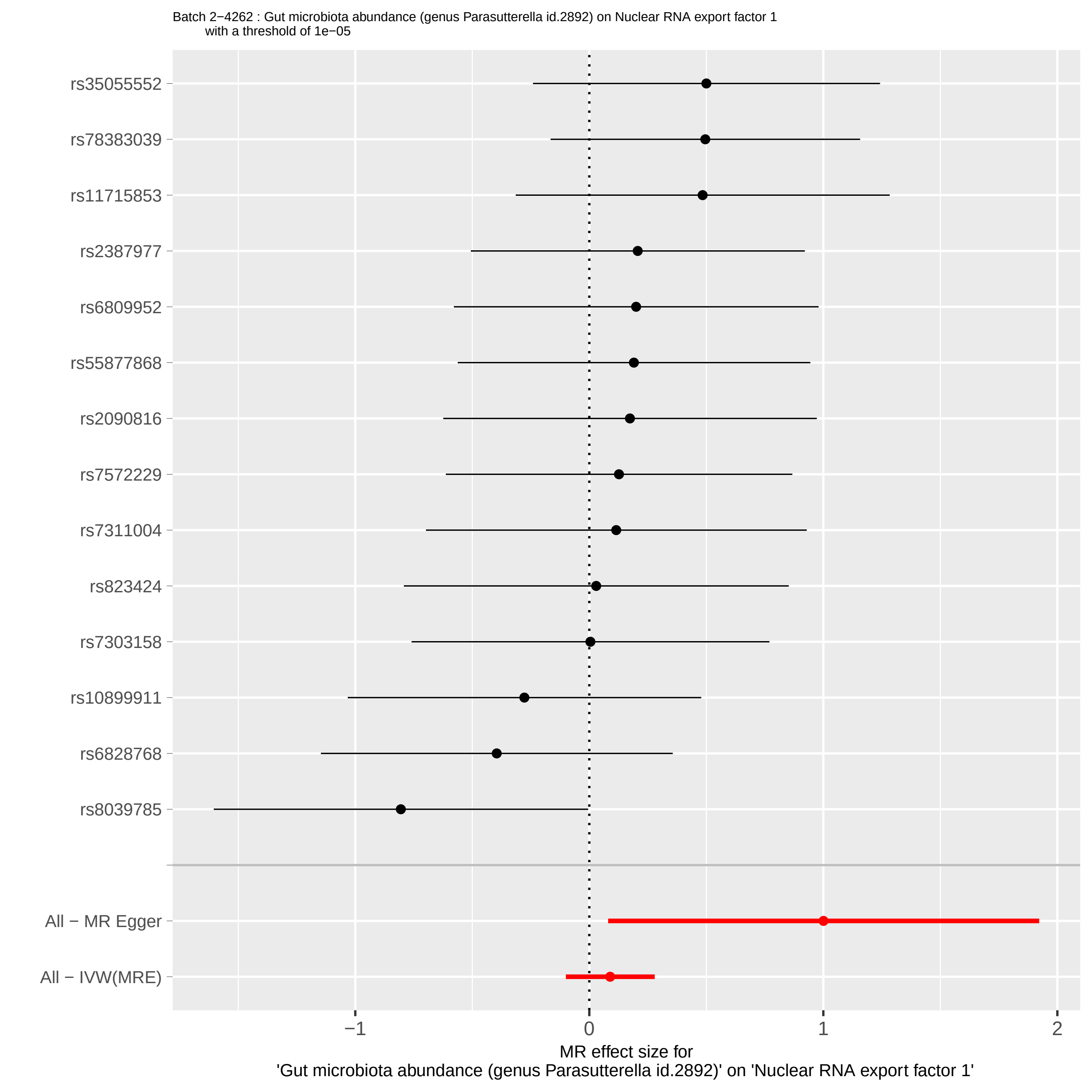

Batch 2−4262 : Gut microbiota abundance (genus Parasutterella id.2892) on Nuclear RNA export factor 1
with a threshold of 1e−05
rs35055552
rs78383039
rs11715853
rs2387977
rs6809952
rs55877868
rs2090816
rs7572229
rs7311004
rs823424
rs7303158
rs10899911
rs6828768
rs8039785
All − MR Egger
All − IVW(MRE)
−1
0
1
2
MR effect size for
'Gut microbiota abundance (genus Parasutterella id.2892)' on 'Nuclear RNA export factor 1'

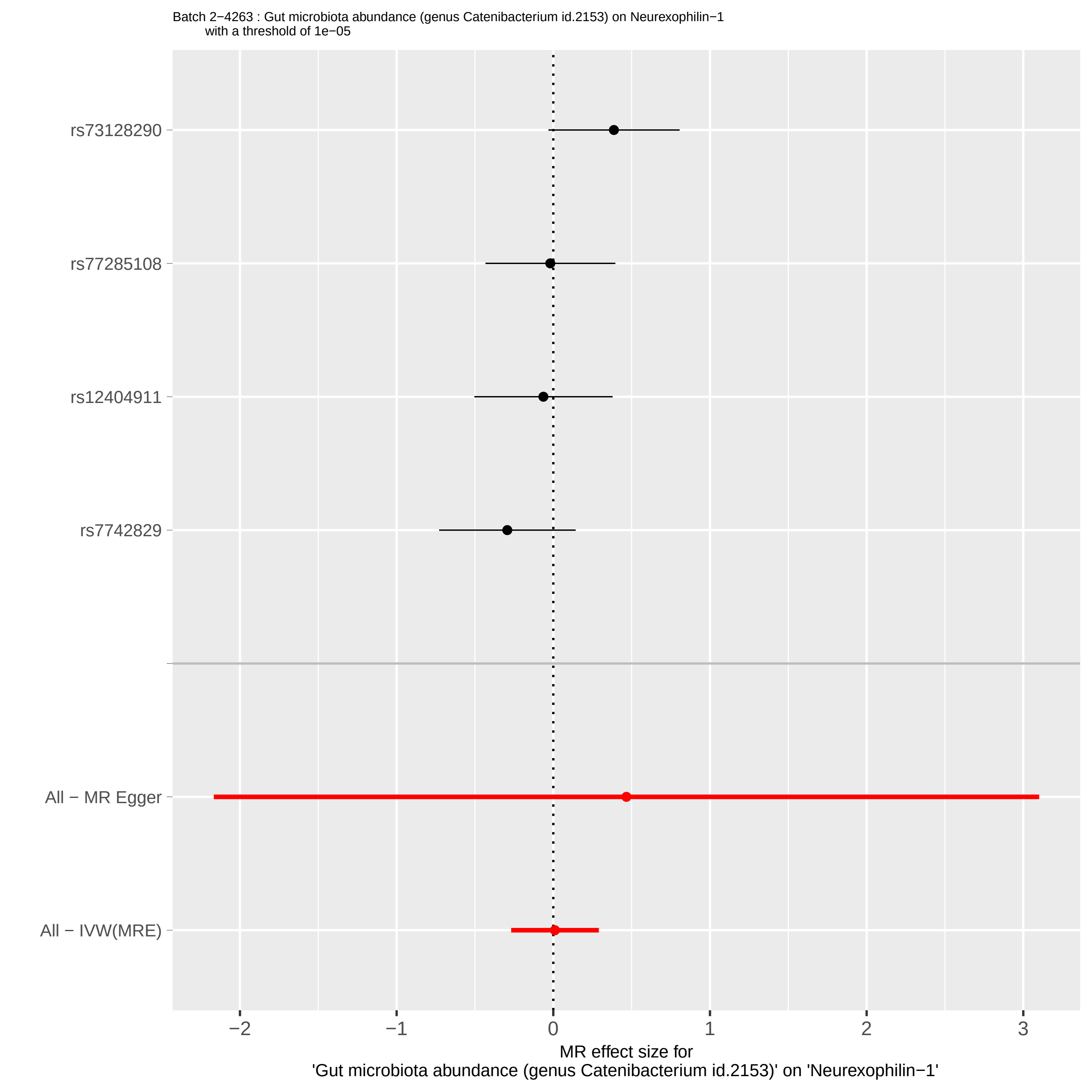
Batch 2−4263 : Gut microbiota abundance (genus Catenibacterium id.2153) on Neurexophilin−1
with a threshold of 1e−05
rs73128290
rs77285108
rs12404911
rs7742829
All − MR Egger
All − IVW(MRE)
−2
−1
0
1
2
3
MR effect size for
'Gut microbiota abundance (genus Catenibacterium id.2153)' on 'Neurexophilin−1'

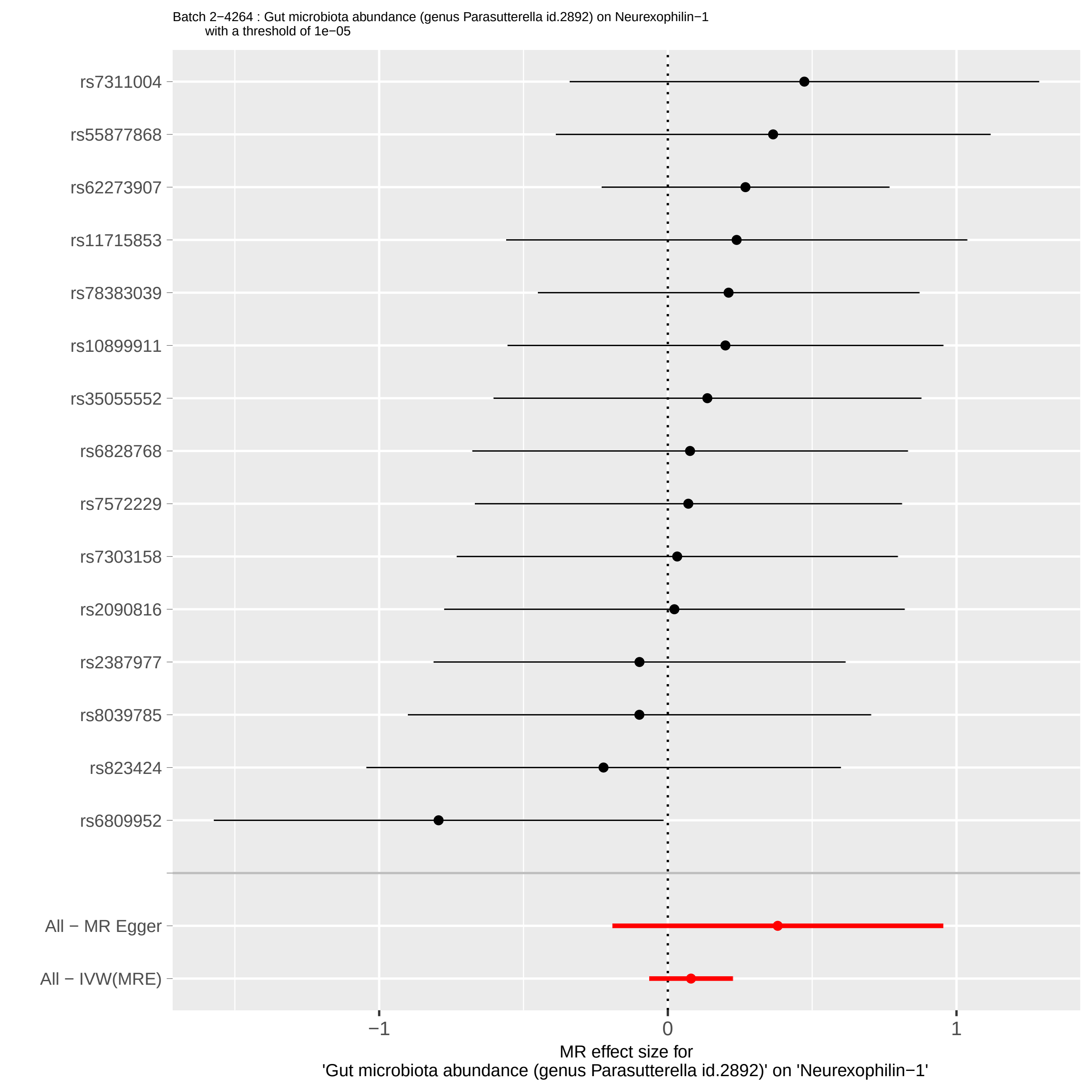
Batch 2−4264 : Gut microbiota abundance (genus Parasutterella id.2892) on Neurexophilin−1
with a threshold of 1e−05
rs7311004
rs55877868
rs62273907
rs11715853
rs78383039
rs10899911
rs35055552
rs6828768
rs7572229
rs7303158
rs2090816
rs2387977
rs8039785
rs823424
rs6809952
All − MR Egger
All − IVW(MRE)
−1
0
1
MR effect size for
'Gut microbiota abundance (genus Parasutterella id.2892)' on 'Neurexophilin−1'

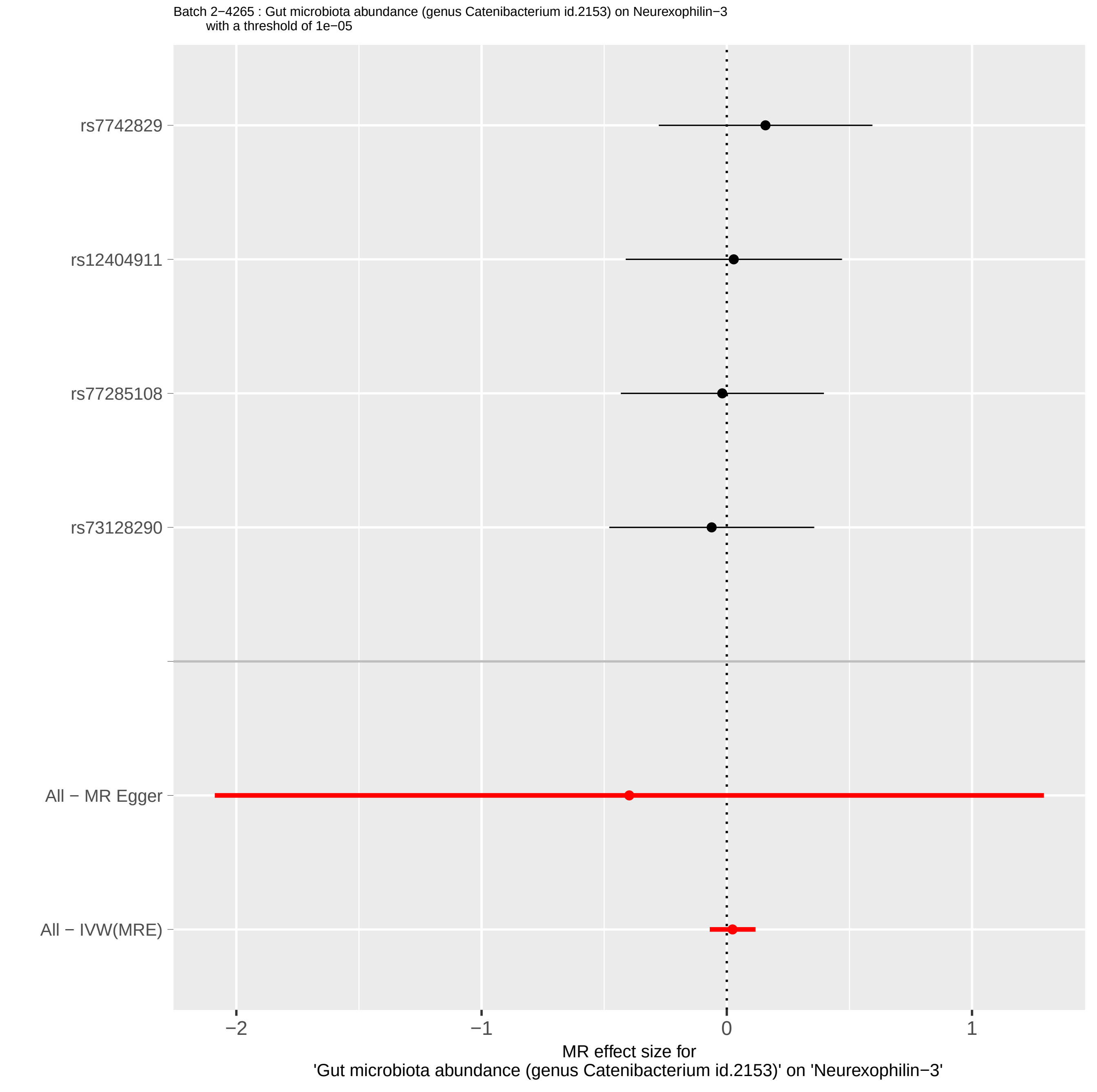
Batch 2−4265 : Gut microbiota abundance (genus Catenibacterium id.2153) on Neurexophilin−3
with a threshold of 1e−05
rs7742829
rs12404911
rs77285108
rs73128290
All − MR Egger
All − IVW(MRE)
−2
−1
0
1
MR effect size for
'Gut microbiota abundance (genus Catenibacterium id.2153)' on 'Neurexophilin−3'

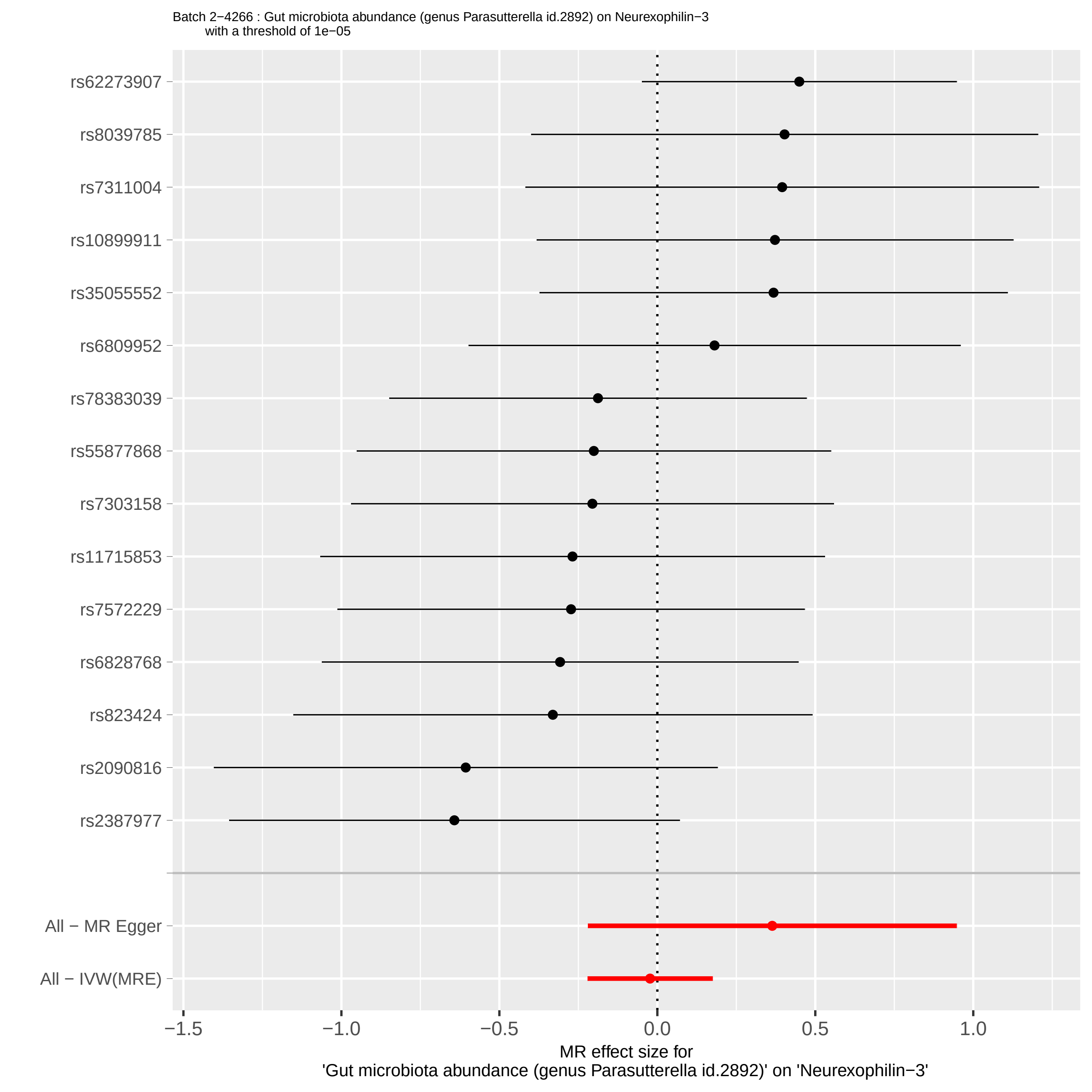

Batch 2−4266 : Gut microbiota abundance (genus Parasutterella id.2892) on Neurexophilin−3
with a threshold of 1e−05
rs62273907
rs8039785
rs7311004
rs10899911
rs35055552
rs6809952
rs78383039
rs55877868
rs7303158
rs11715853
rs7572229
rs6828768
rs823424
rs2090816
rs2387977
All − MR Egger
All − IVW(MRE)
−1.5
−1.0
−0.5
0.0
0.5
1.0
MR effect size for
'Gut microbiota abundance (genus Parasutterella id.2892)' on 'Neurexophilin−3'

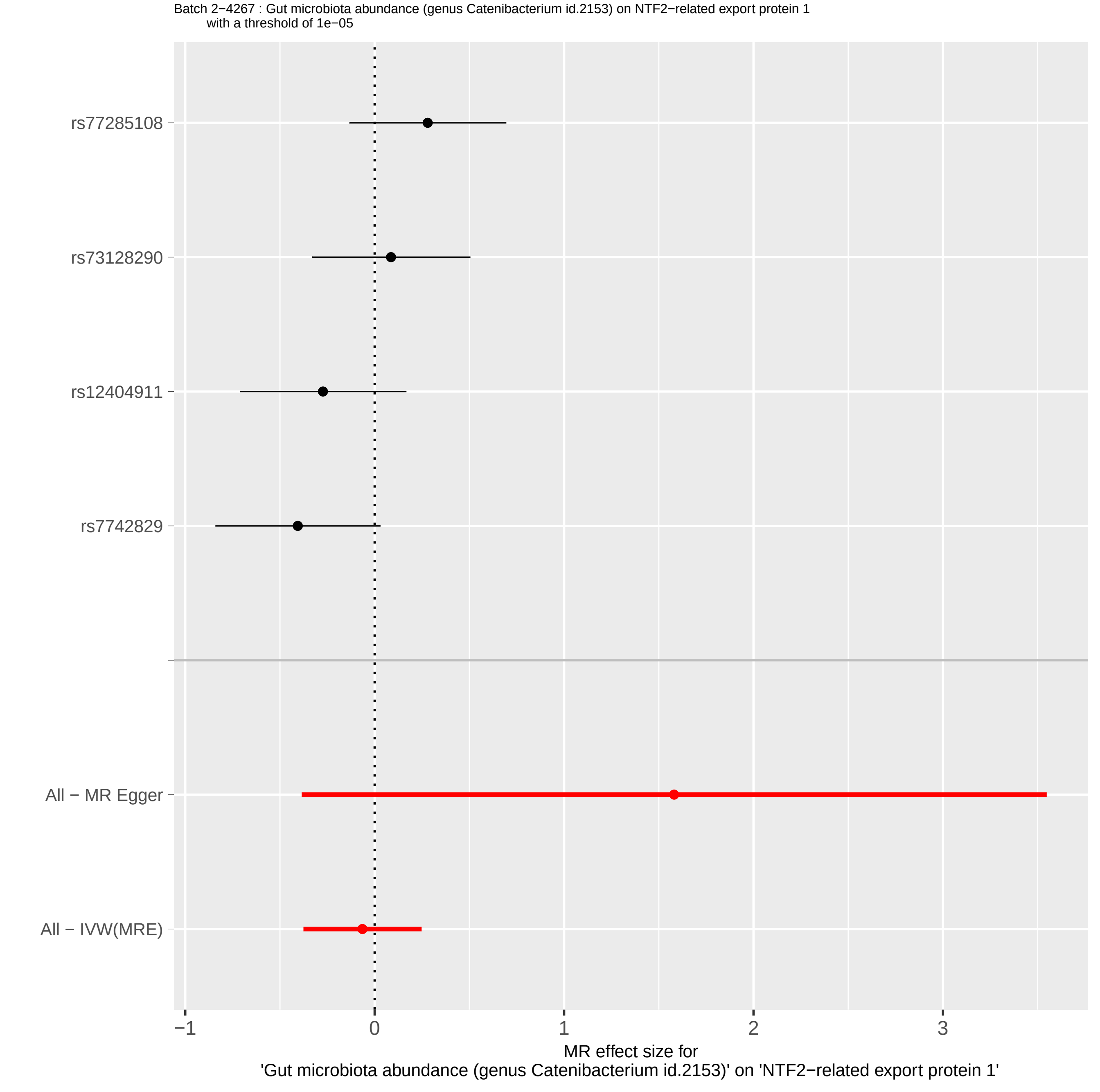

Batch 2−4267 : Gut microbiota abundance (genus Catenibacterium id.2153) on NTF2−related export protein 1
with a threshold of 1e−05
rs77285108
rs73128290
rs12404911
rs7742829
All − MR Egger
All − IVW(MRE)
−1
0
1
2
3
MR effect size for
'Gut microbiota abundance (genus Catenibacterium id.2153)' on 'NTF2−related export protein 1'

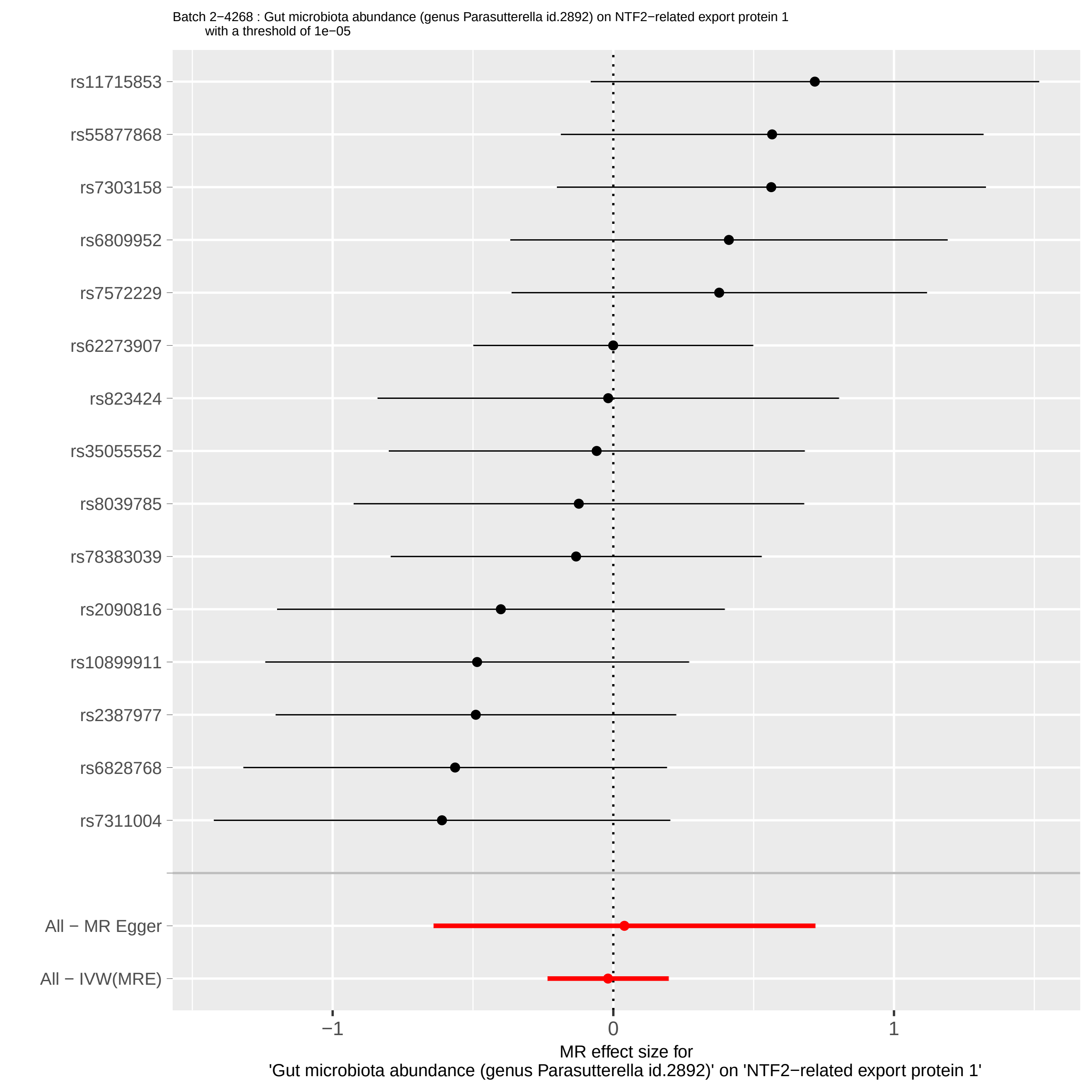
Batch 2−4268 : Gut microbiota abundance (genus Parasutterella id.2892) on NTF2−related export protein 1
with a threshold of 1e−05
rs11715853
rs55877868
rs7303158
rs6809952
rs7572229
rs62273907
rs823424
rs35055552
rs8039785
rs78383039
rs2090816
rs10899911
rs2387977
rs6828768
rs7311004
All − MR Egger
All − IVW(MRE)
−1
0
1
MR effect size for
'Gut microbiota abundance (genus Parasutterella id.2892)' on 'NTF2−related export protein 1'

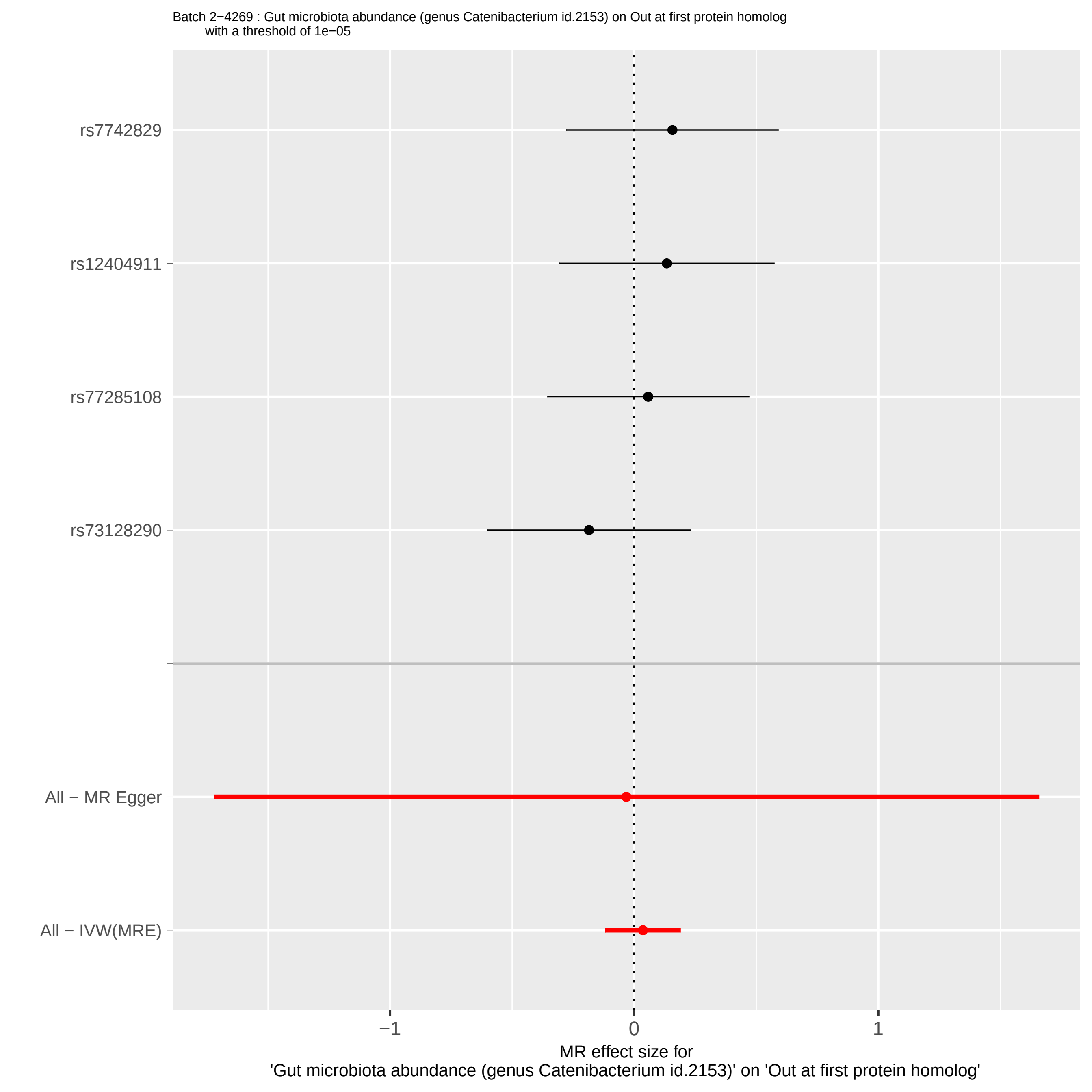
Batch 2−4269 : Gut microbiota abundance (genus Catenibacterium id.2153) on Out at first protein homolog
with a threshold of 1e−05
rs7742829
rs12404911
rs77285108
rs73128290
All − MR Egger
All − IVW(MRE)
−1
0
1
MR effect size for
'Gut microbiota abundance (genus Catenibacterium id.2153)' on 'Out at first protein homolog'

Batch 2−4270 : Gut microbiota abundance (genus Parasutterella id.2892) on Out at first protein homolog
with a threshold of 1e−05

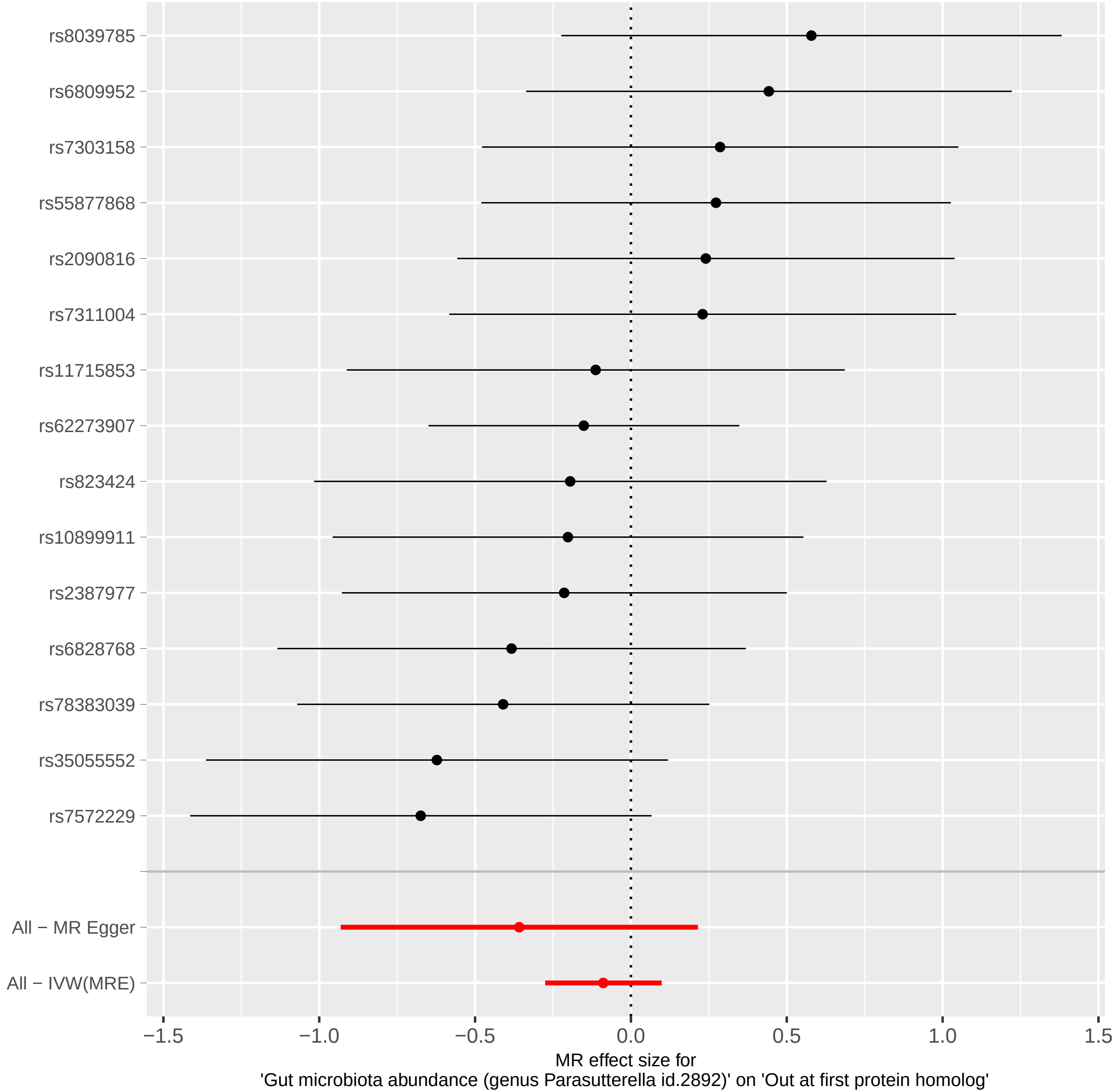

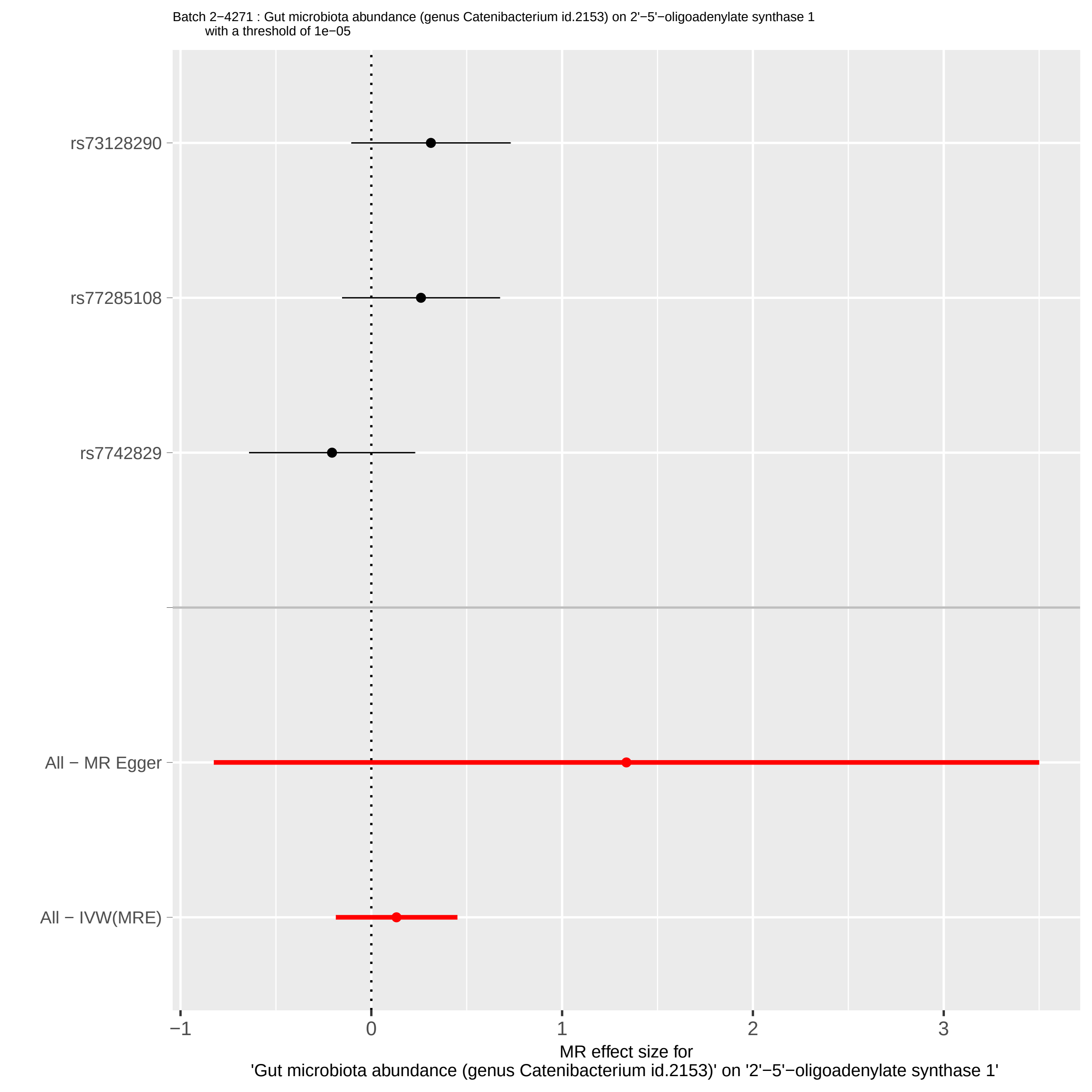

Batch 2−4271 : Gut microbiota abundance (genus Catenibacterium id.2153) on 2'−5'−oligoadenylate synthase 1
with a threshold of 1e−05
rs73128290
rs77285108
rs7742829
All − MR Egger
All − IVW(MRE)
−1
0
1
2
3
MR effect size for
'Gut microbiota abundance (genus Catenibacterium id.2153)' on '2'−5'−oligoadenylate synthase 1'

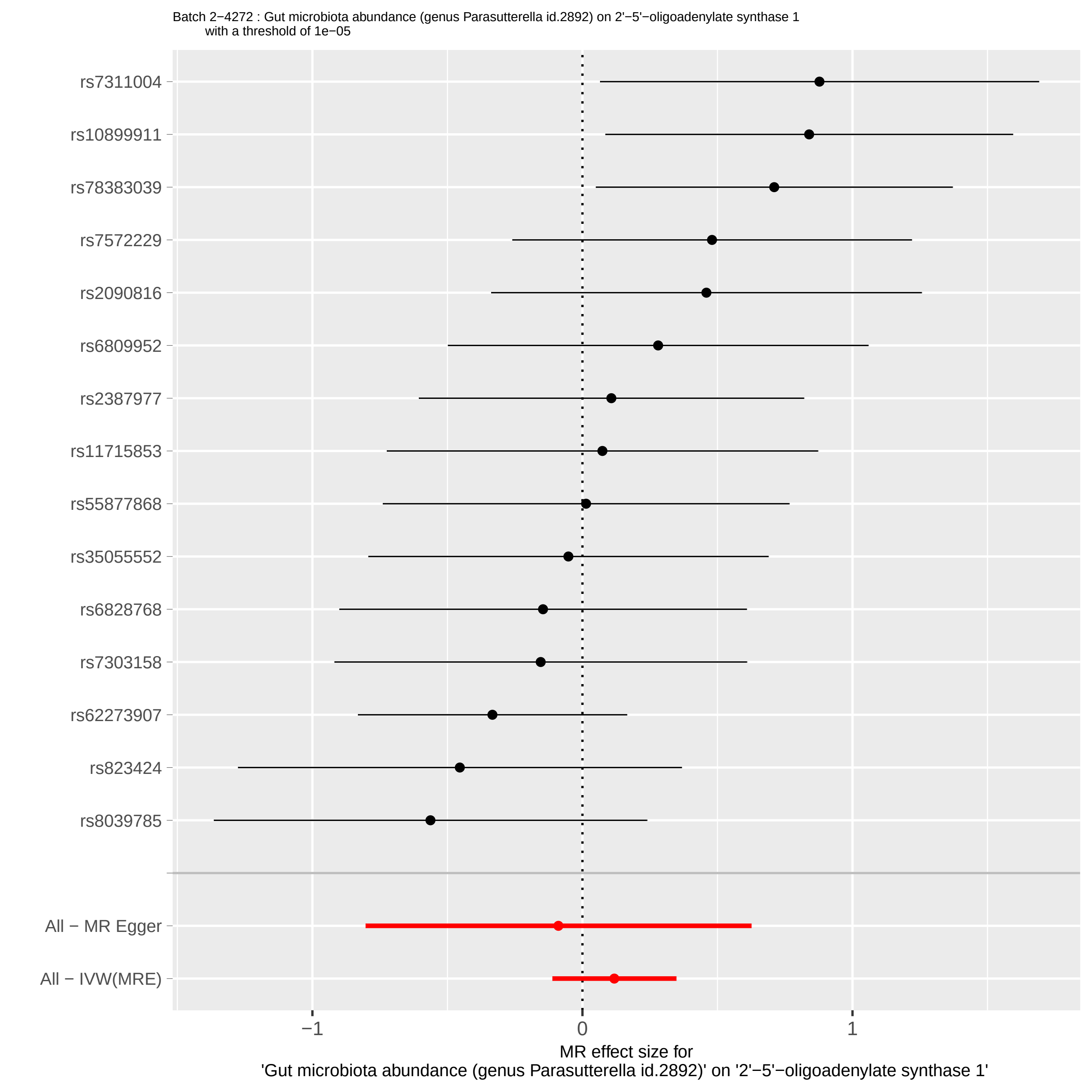

Batch 2−4272 : Gut microbiota abundance (genus Parasutterella id.2892) on 2'−5'−oligoadenylate synthase 1
with a threshold of 1e−05
rs7311004
rs10899911
rs78383039
rs7572229
rs2090816
rs6809952
rs2387977
rs11715853
rs55877868
rs35055552
rs6828768
rs7303158
rs62273907
rs823424
rs8039785
All − MR Egger
All − IVW(MRE)
−1
0
1
MR effect size for
'Gut microbiota abundance (genus Parasutterella id.2892)' on '2'−5'−oligoadenylate synthase 1'

Batch 2−4273 : Gut microbiota abundance (genus Catenibacterium id.2153) on Ornithine decarboxylase antizyme 1 with a threshold of 1e−05

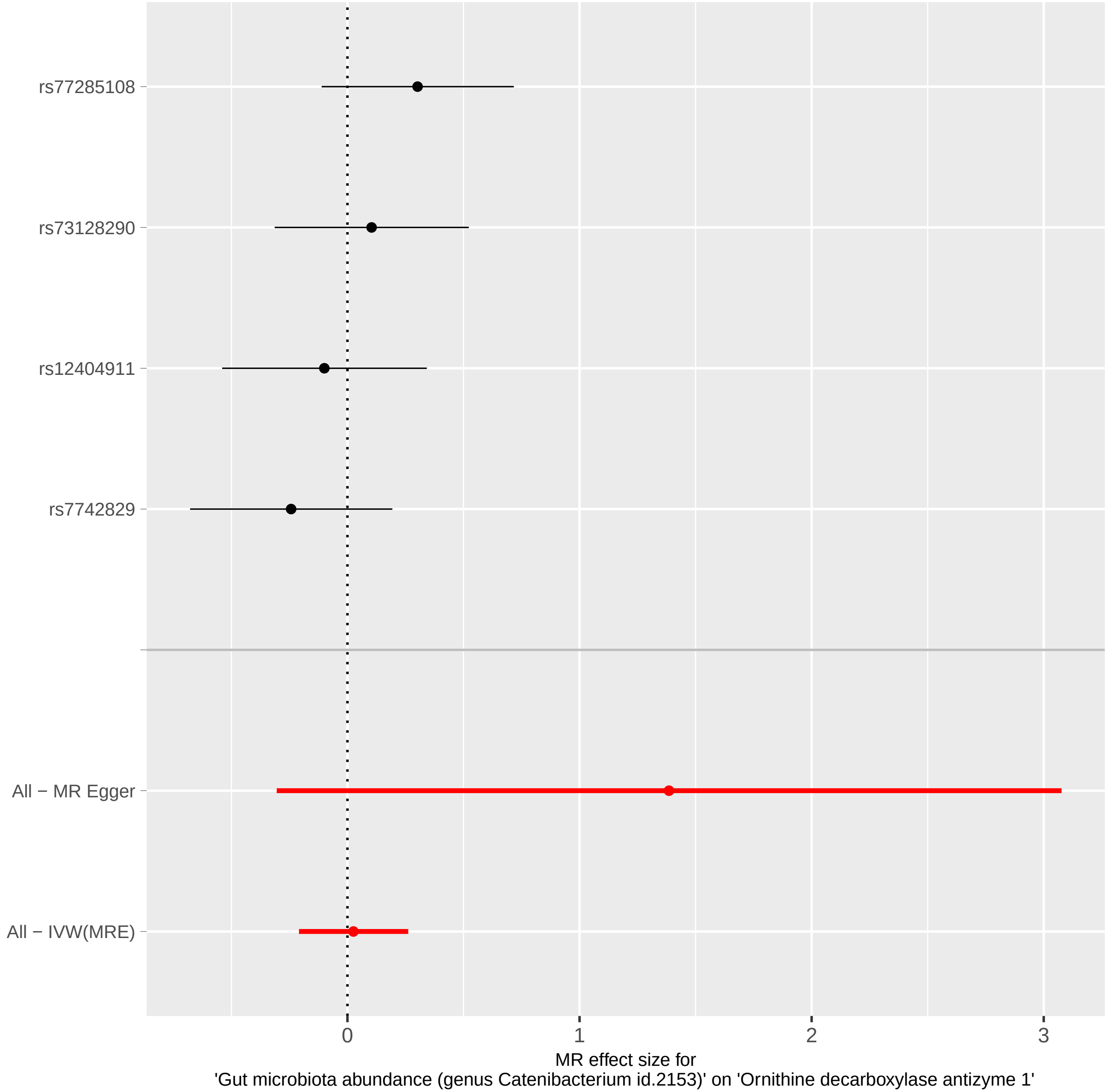

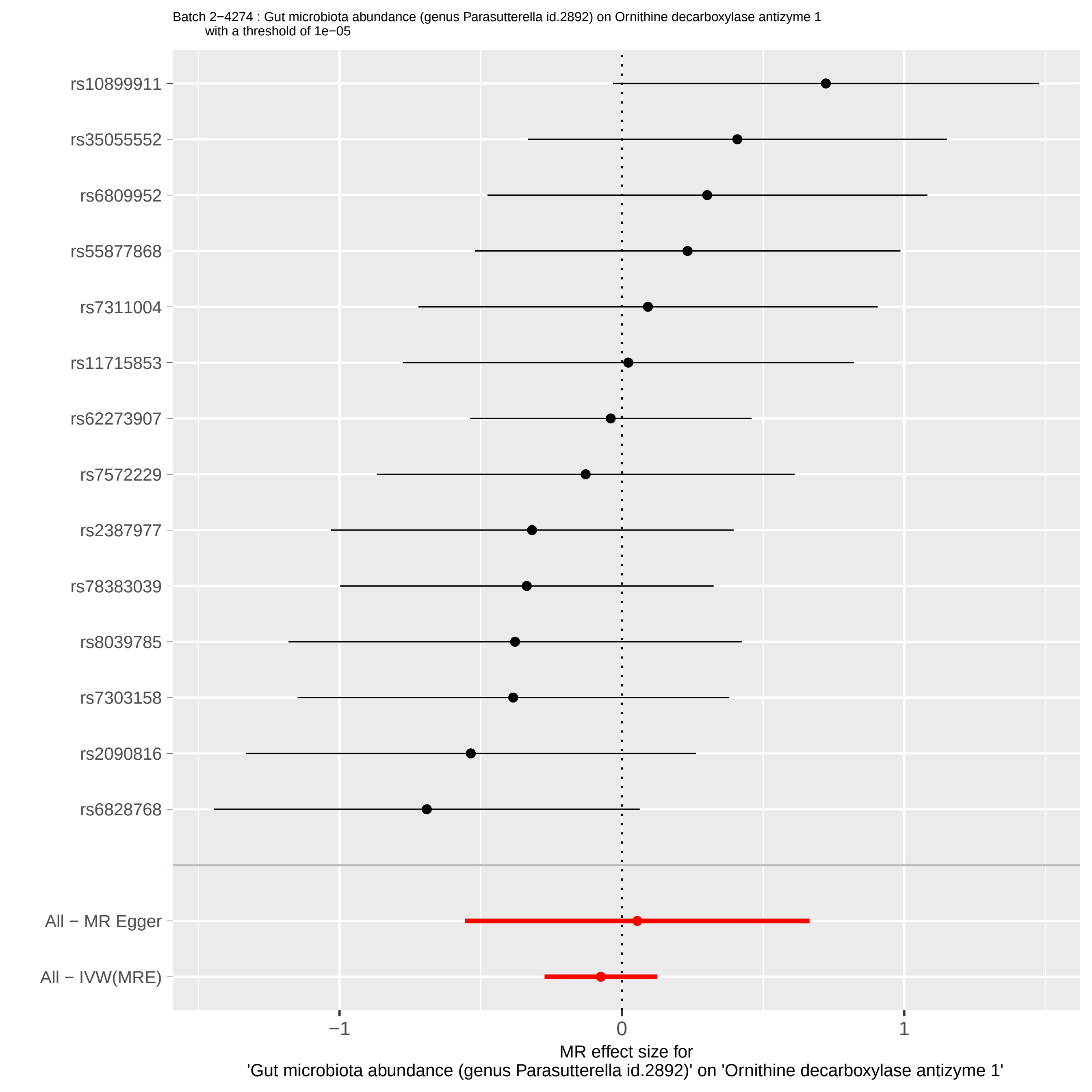

Batch 2−4274 : Gut microbiota abundance (genus Parasutterella id.2892) on Ornithine decarboxylase antizyme 1
with a threshold of 1e−05
rs10899911
rs35055552
rs6809952
rs55877868
rs7311004
rs11715853
rs62273907
rs7572229
rs2387977
rs78383039
rs8039785
rs7303158
rs2090816
rs6828768
All − MR Egger
All − IVW(MRE)
−1
0
1
MR effect size for
'Gut microbiota abundance (genus Parasutterella id.2892)' on 'Ornithine decarboxylase antizyme 1'

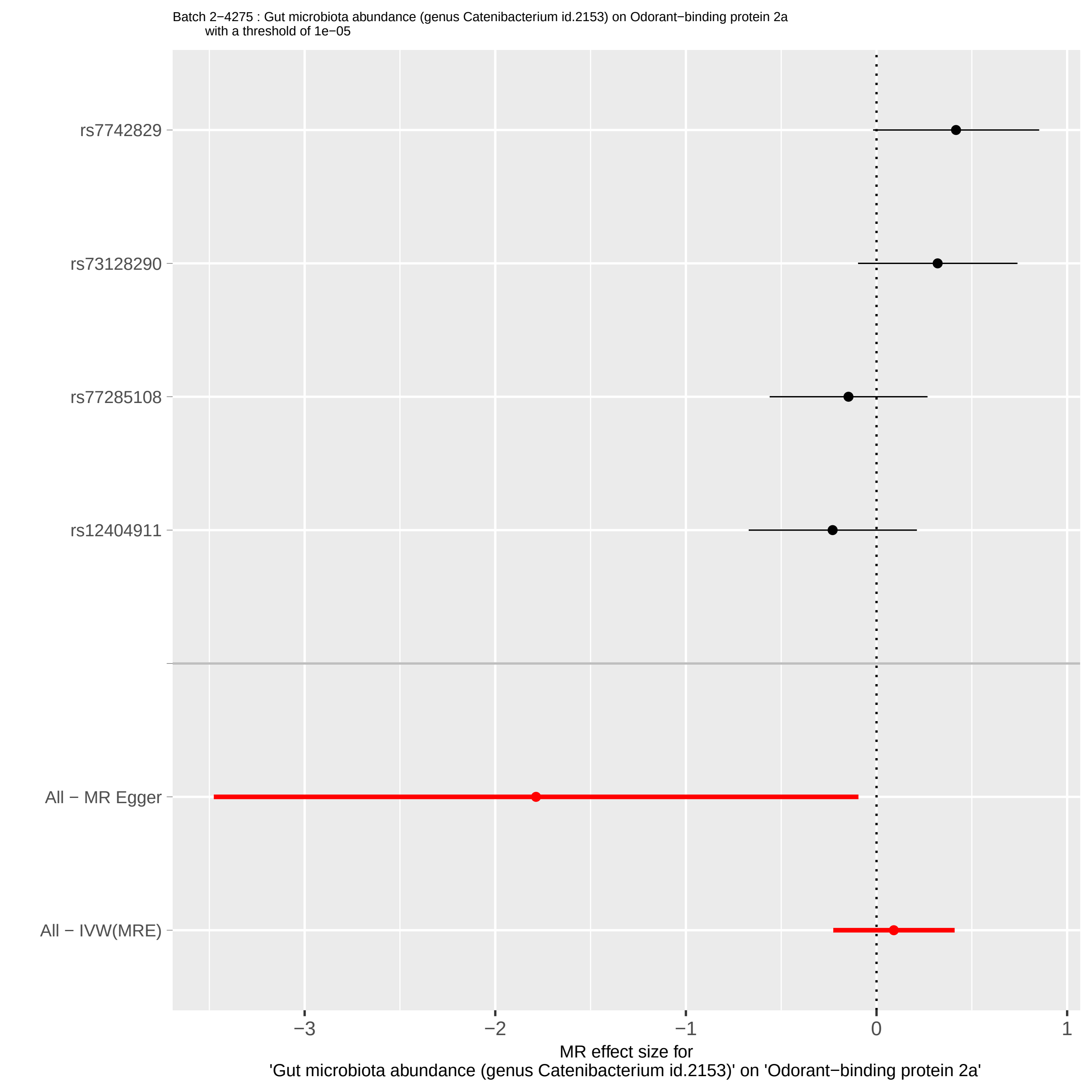

Batch 2−4275 : Gut microbiota abundance (genus Catenibacterium id.2153) on Odorant−binding protein 2a
with a threshold of 1e−05
rs7742829
rs73128290
rs77285108
rs12404911
All − MR Egger
All − IVW(MRE)
−3
−2
−1
0
1
MR effect size for
'Gut microbiota abundance (genus Catenibacterium id.2153)' on 'Odorant−binding protein 2a'

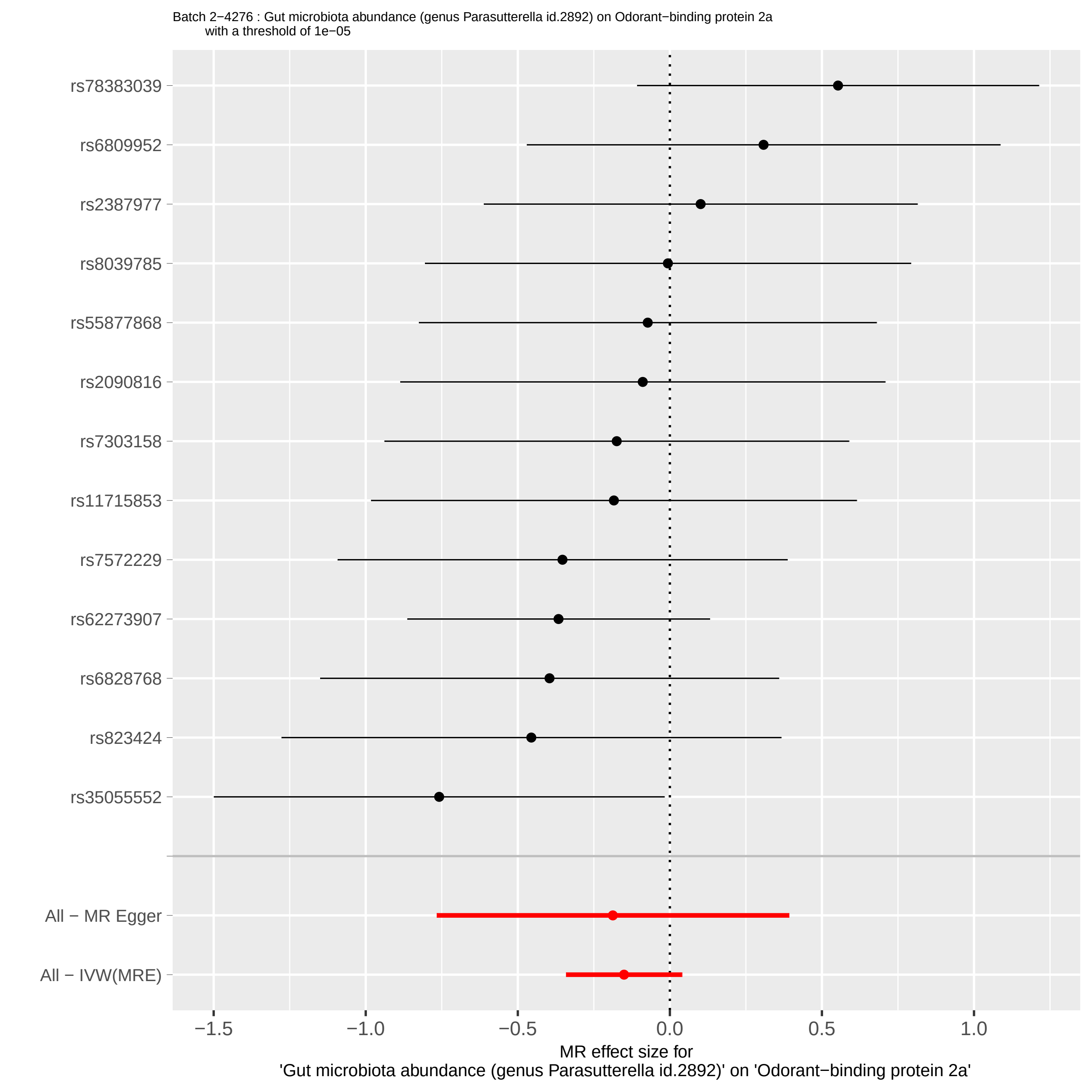

Batch 2−4276 : Gut microbiota abundance (genus Parasutterella id.2892) on Odorant−binding protein 2a
with a threshold of 1e−05
rs78383039
rs6809952
rs2387977
rs8039785
rs55877868
rs2090816
rs7303158
rs11715853
rs7572229
rs62273907
rs6828768
rs823424
rs35055552
All − MR Egger
All − IVW(MRE)
−1.5
−1.0
−0.5
0.0
0.5
1.0
MR effect size for
'Gut microbiota abundance (genus Parasutterella id.2892)' on 'Odorant−binding protein 2a'

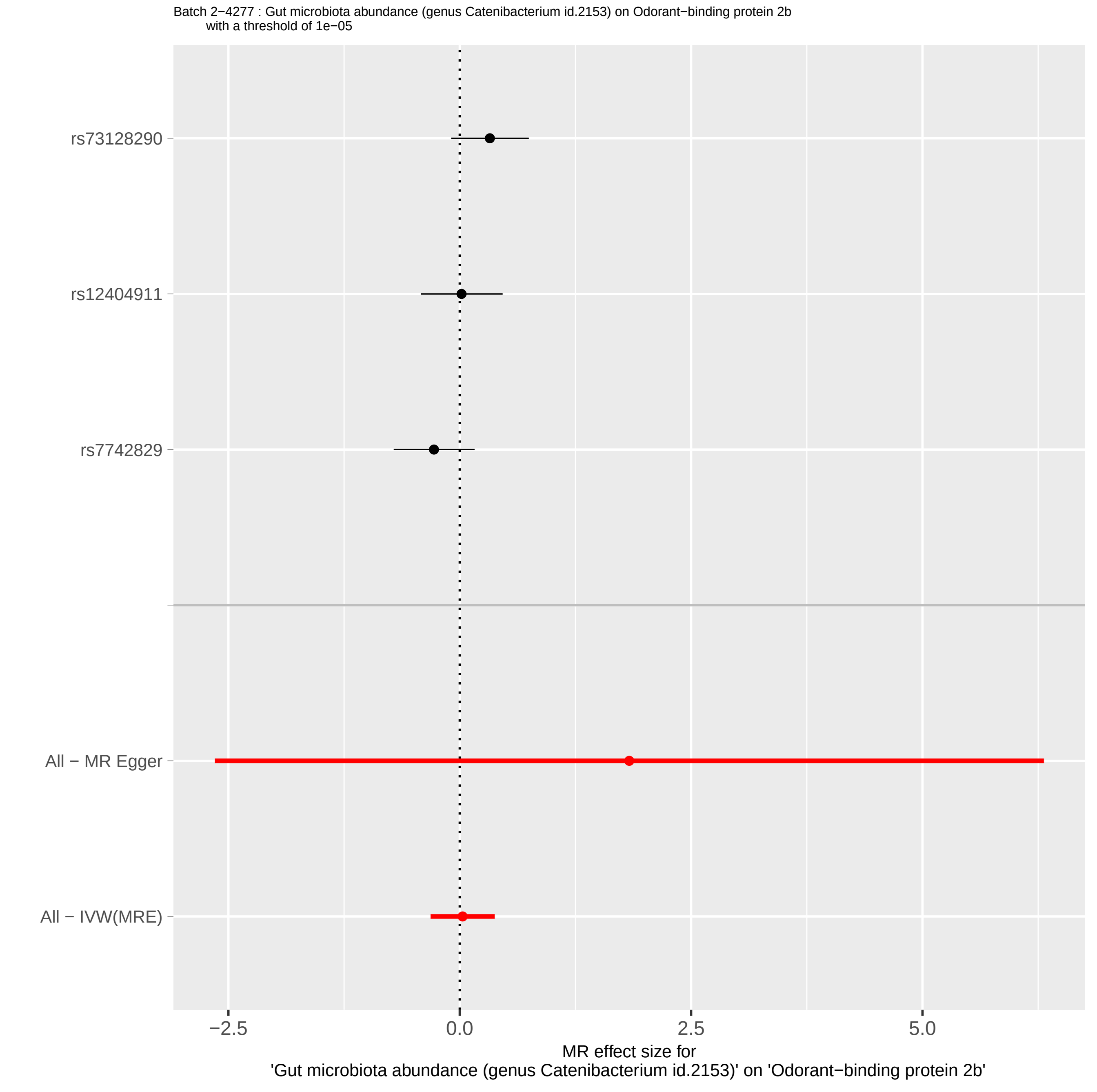

Batch 2−4277 : Gut microbiota abundance (genus Catenibacterium id.2153) on Odorant−binding protein 2b
with a threshold of 1e−05
rs73128290
rs12404911
rs7742829
All − MR Egger
All − IVW(MRE)
−2.5
0.0
2.5
5.0
MR effect size for
'Gut microbiota abundance (genus Catenibacterium id.2153)' on 'Odorant−binding protein 2b'

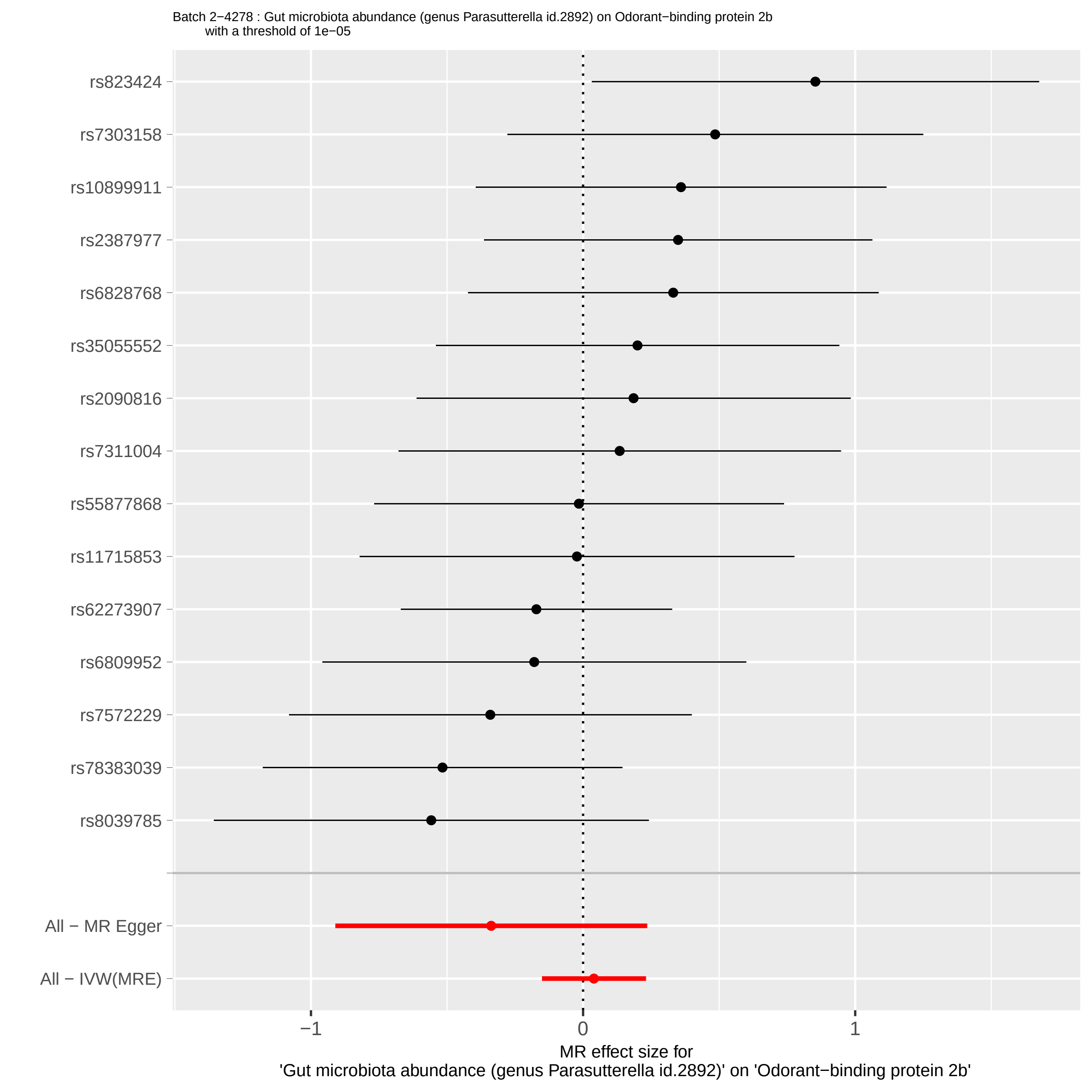

Batch 2−4278 : Gut microbiota abundance (genus Parasutterella id.2892) on Odorant−binding protein 2b
with a threshold of 1e−05
rs823424
rs7303158
rs10899911
rs2387977
rs6828768
rs35055552
rs2090816
rs7311004
rs55877868
rs11715853
rs62273907
rs6809952
rs7572229
rs78383039
rs8039785
All − MR Egger
All − IVW(MRE)
−1
0
1
MR effect size for
'Gut microbiota abundance (genus Parasutterella id.2892)' on 'Odorant−binding protein 2b'

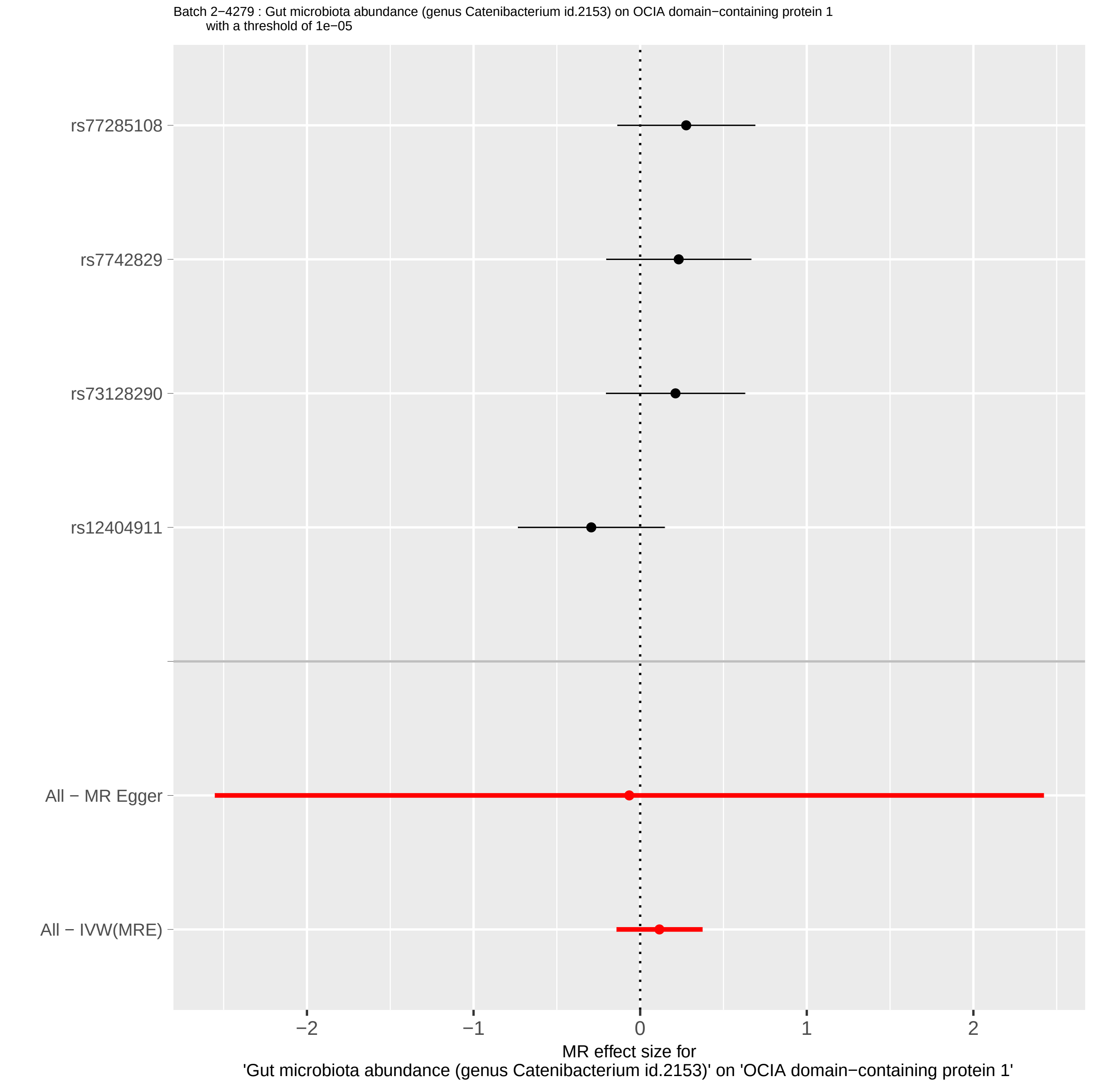
Batch 2−4279 : Gut microbiota abundance (genus Catenibacterium id.2153) on OCIA domain−containing protein 1
with a threshold of 1e−05
rs77285108
rs7742829
rs73128290
rs12404911
All − MR Egger
All − IVW(MRE)
−2
−1
0
1
2
MR effect size for
'Gut microbiota abundance (genus Catenibacterium id.2153)' on 'OCIA domain−containing protein 1'

Batch 2−4280 : Gut microbiota abundance (genus Parasutterella id.2892) on OCIA domain−containing protein 1
with a threshold of 1e−05

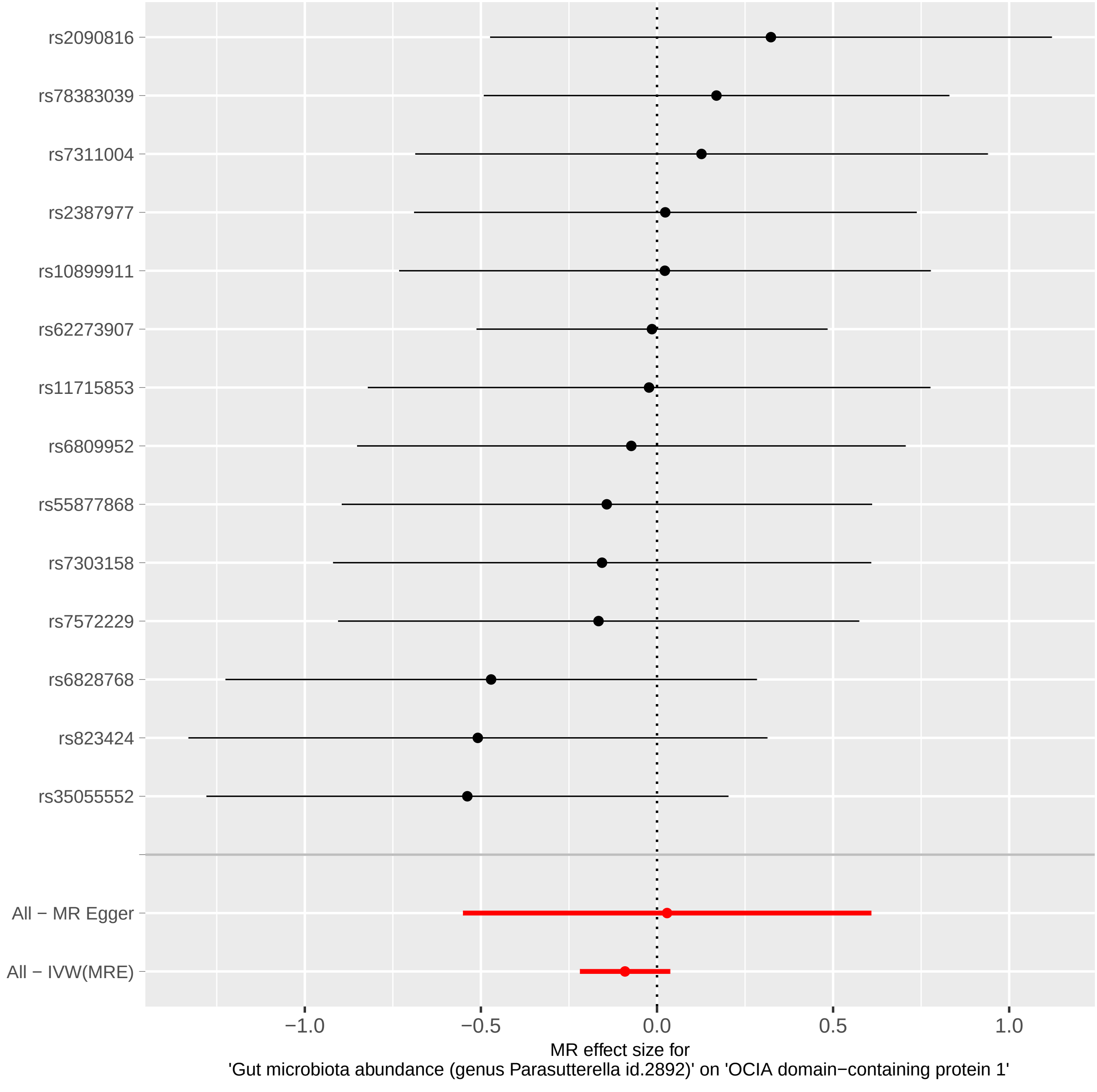

Batch 2−4281 : Gut microbiota abundance (genus Catenibacterium id.2153) on Inositol polyphosphate 5−phosphatase OCRL−1
with a threshold of 1e−05

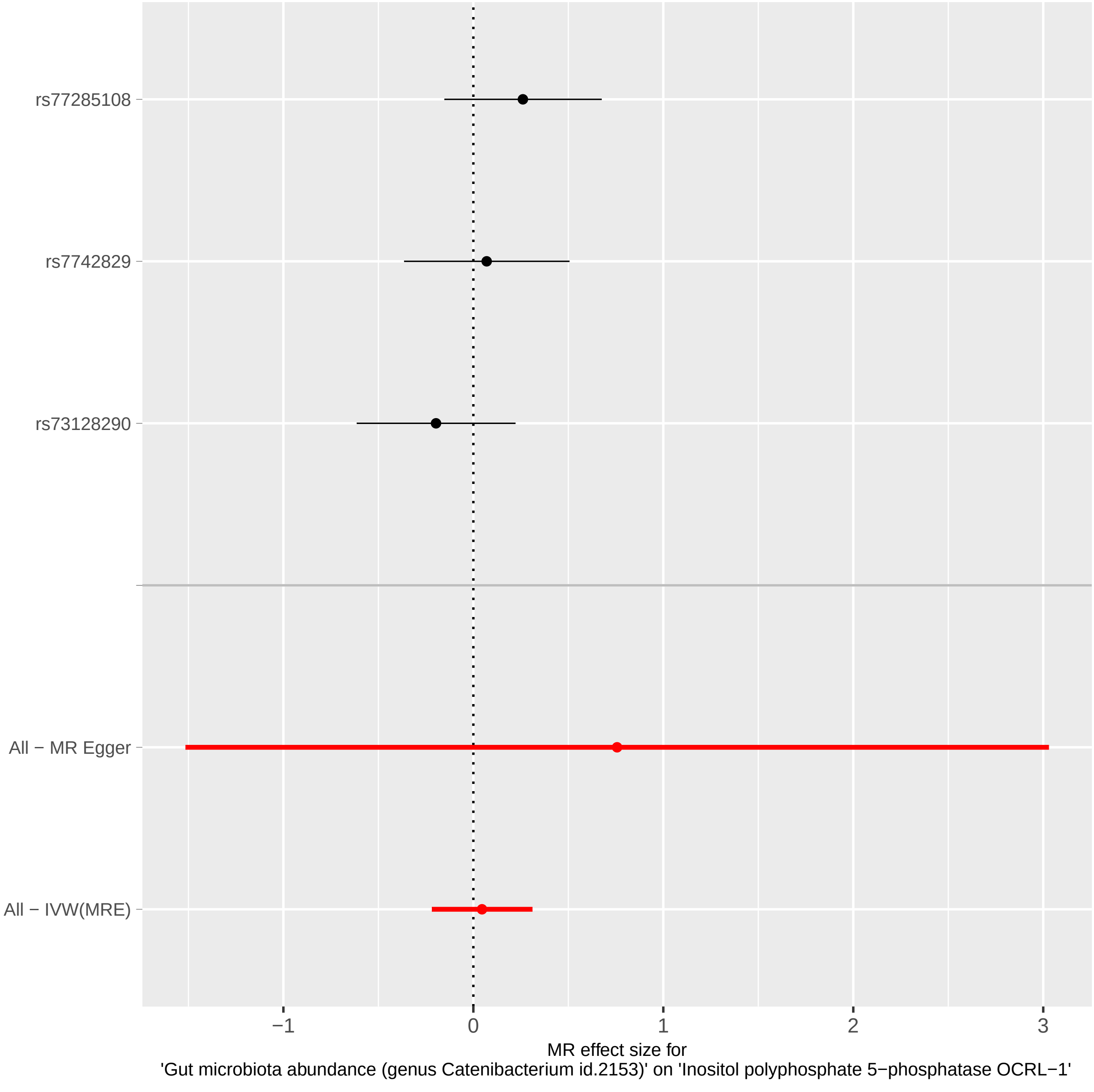

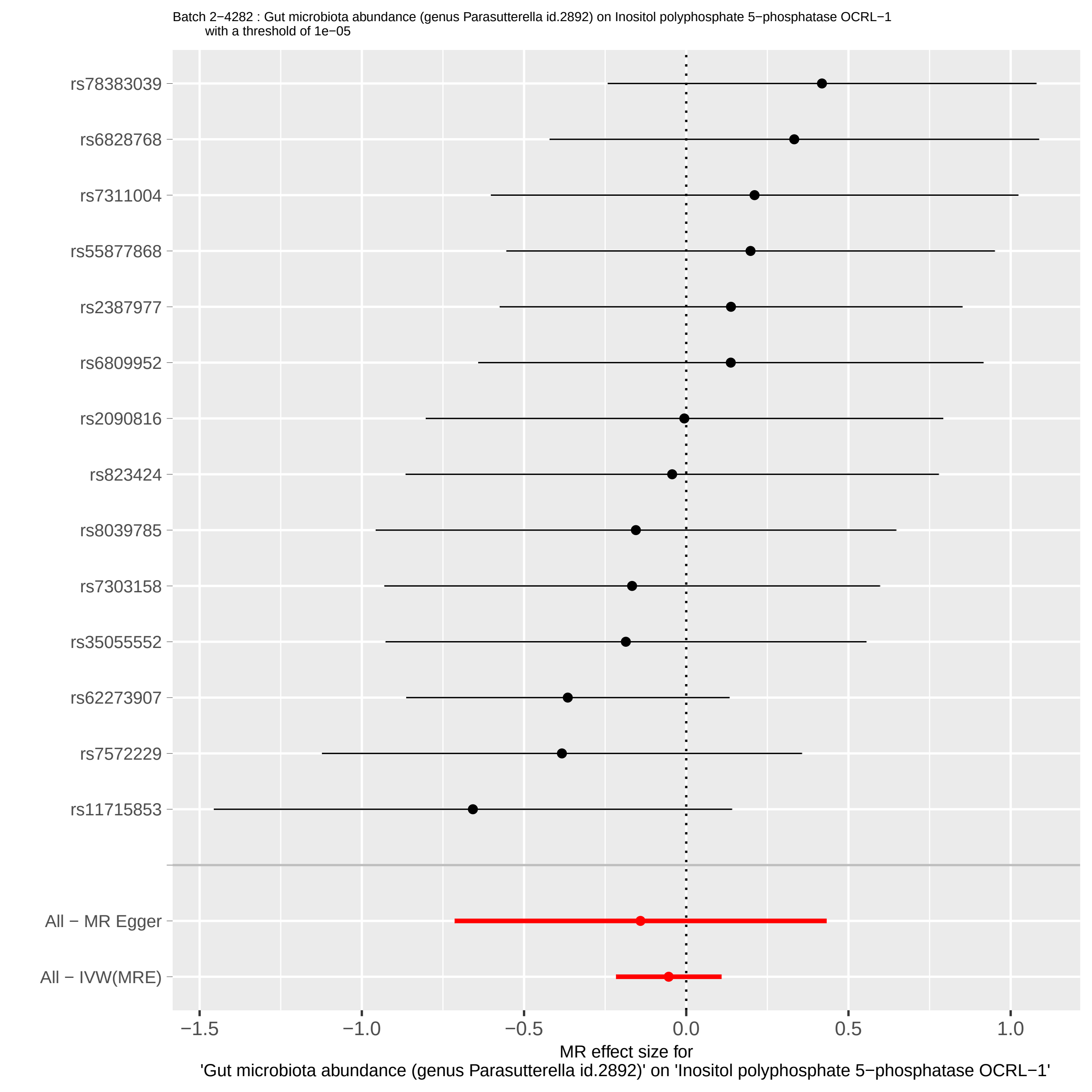

Batch 2−4282 : Gut microbiota abundance (genus Parasutterella id.2892) on Inositol polyphosphate 5−phosphatase OCRL−1
with a threshold of 1e−05
rs78383039
rs6828768
rs7311004
rs55877868
rs2387977
rs6809952
rs2090816
rs823424
rs8039785
rs7303158
rs35055552
rs62273907
rs7572229
rs11715853
All − MR Egger
All − IVW(MRE)
−1.5
−1.0
−0.5
0.0
0.5
1.0
MR effect size for
'Gut microbiota abundance (genus Parasutterella id.2892)' on 'Inositol polyphosphate 5−phosphatase OCRL−1'

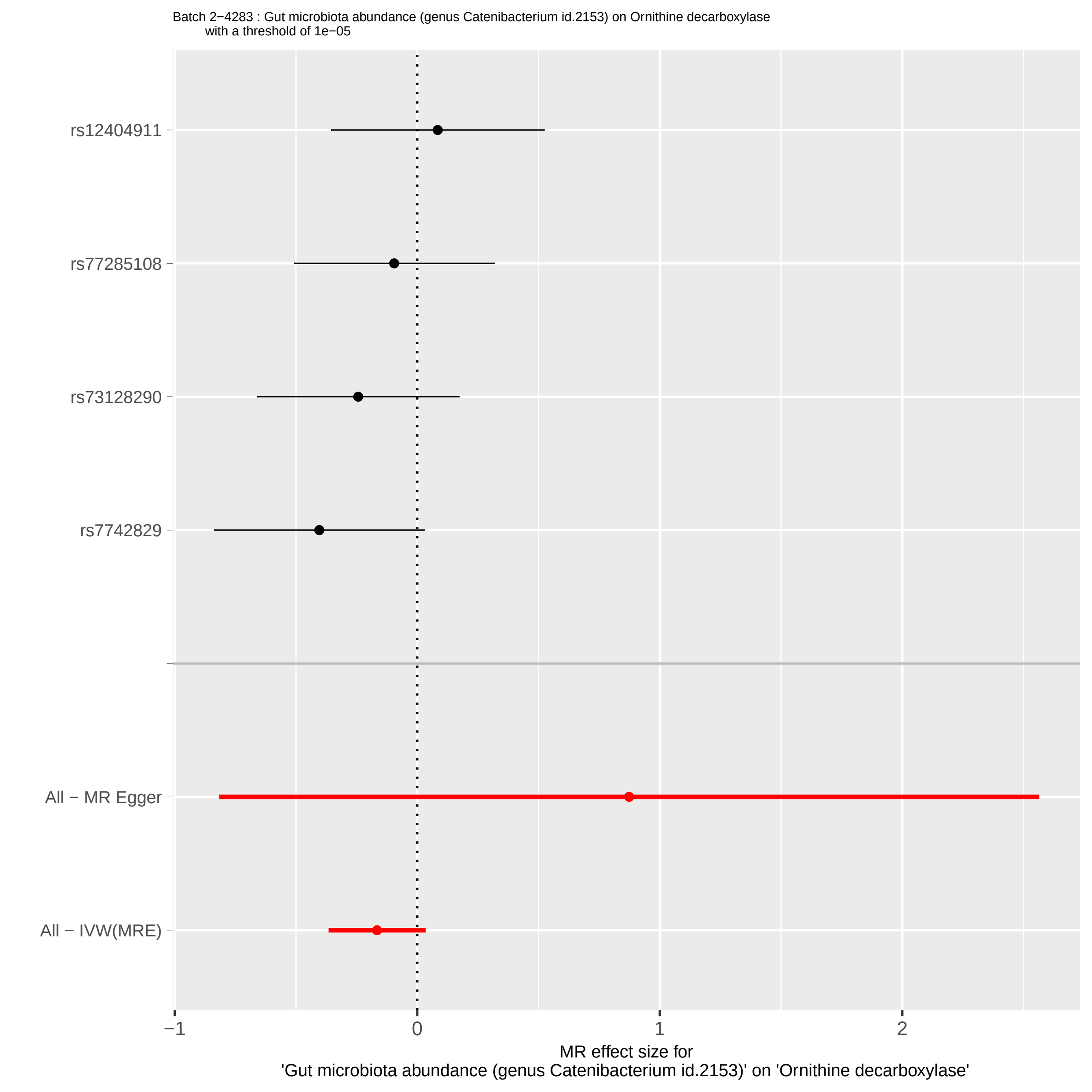

Batch 2−4283 : Gut microbiota abundance (genus Catenibacterium id.2153) on Ornithine decarboxylase
with a threshold of 1e−05
rs12404911
rs77285108
rs73128290
rs7742829
All − MR Egger
All − IVW(MRE)
−1
0
1
2
MR effect size for
'Gut microbiota abundance (genus Catenibacterium id.2153)' on 'Ornithine decarboxylase'

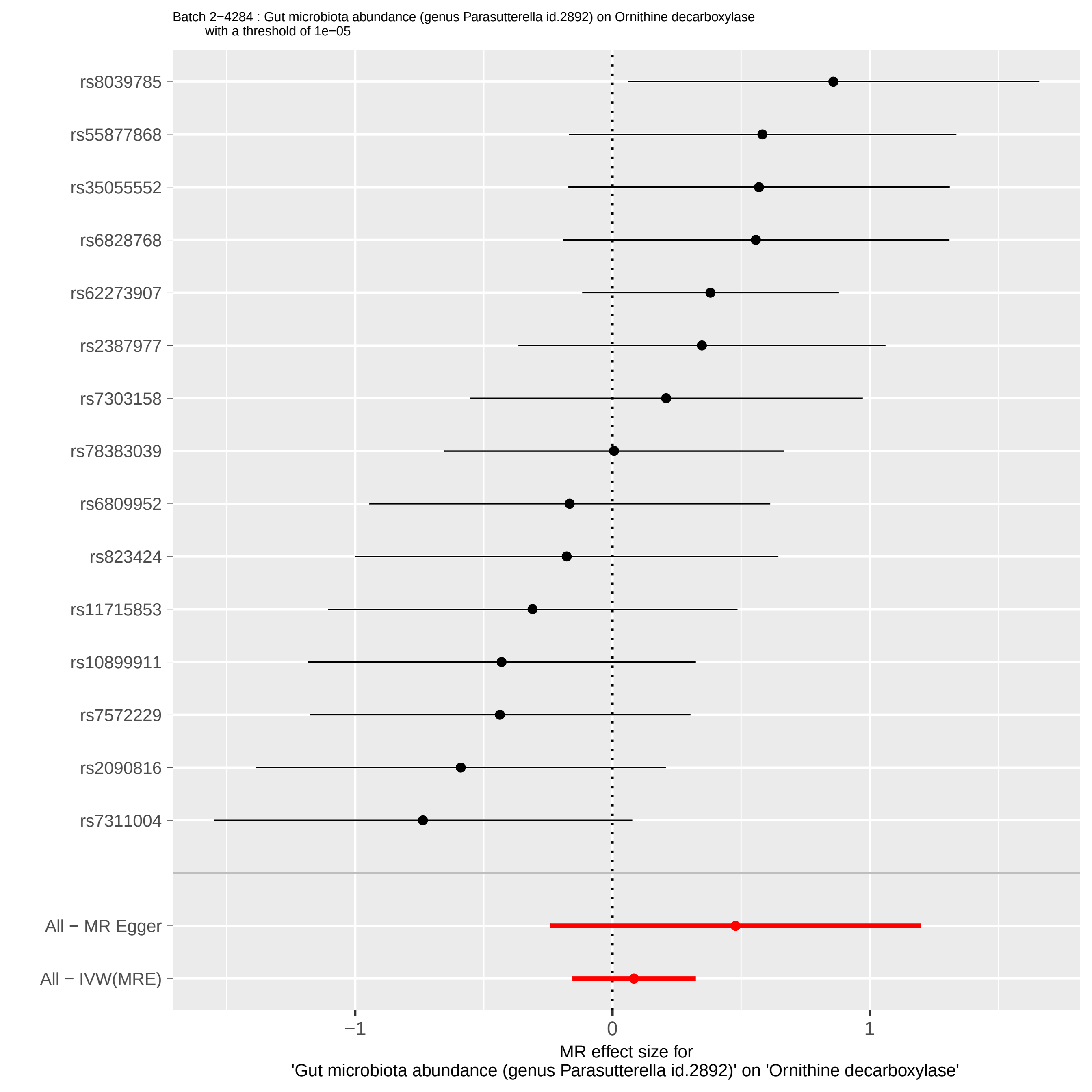
Batch 2−4284 : Gut microbiota abundance (genus Parasutterella id.2892) on Ornithine decarboxylase
with a threshold of 1e−05
rs8039785
rs55877868
rs35055552
rs6828768
rs62273907
rs2387977
rs7303158
rs78383039
rs6809952
rs823424
rs11715853
rs10899911
rs7572229
rs2090816
rs7311004
All − MR Egger
All − IVW(MRE)
−1
0
1
MR effect size for
'Gut microbiota abundance (genus Parasutterella id.2892)' on 'Ornithine decarboxylase'

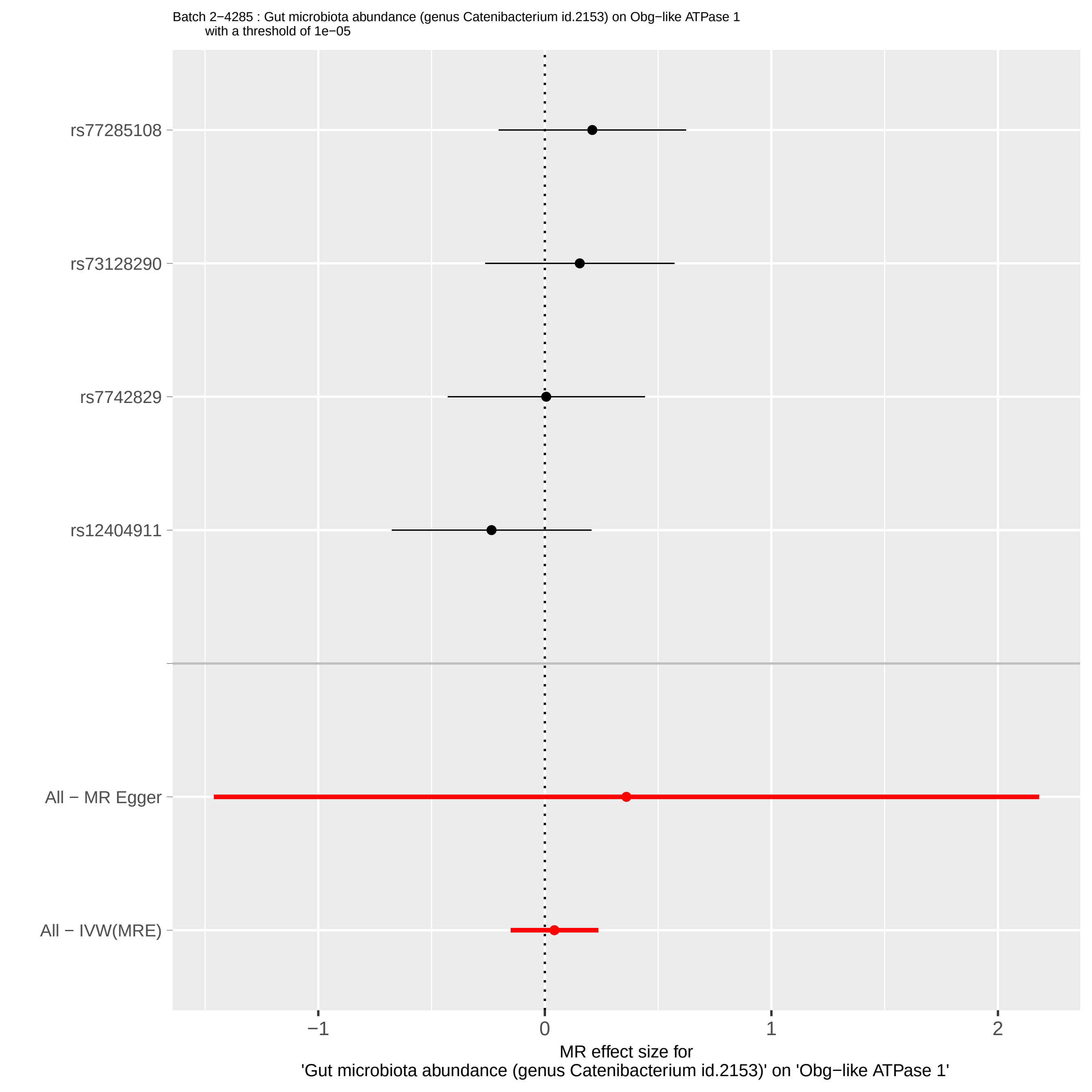
Batch 2−4285 : Gut microbiota abundance (genus Catenibacterium id.2153) on Obg−like ATPase 1
with a threshold of 1e−05
rs77285108
rs73128290
rs7742829
rs12404911
All − MR Egger
All − IVW(MRE)
−1
0
1
2
MR effect size for
'Gut microbiota abundance (genus Catenibacterium id.2153)' on 'Obg−like ATPase 1'

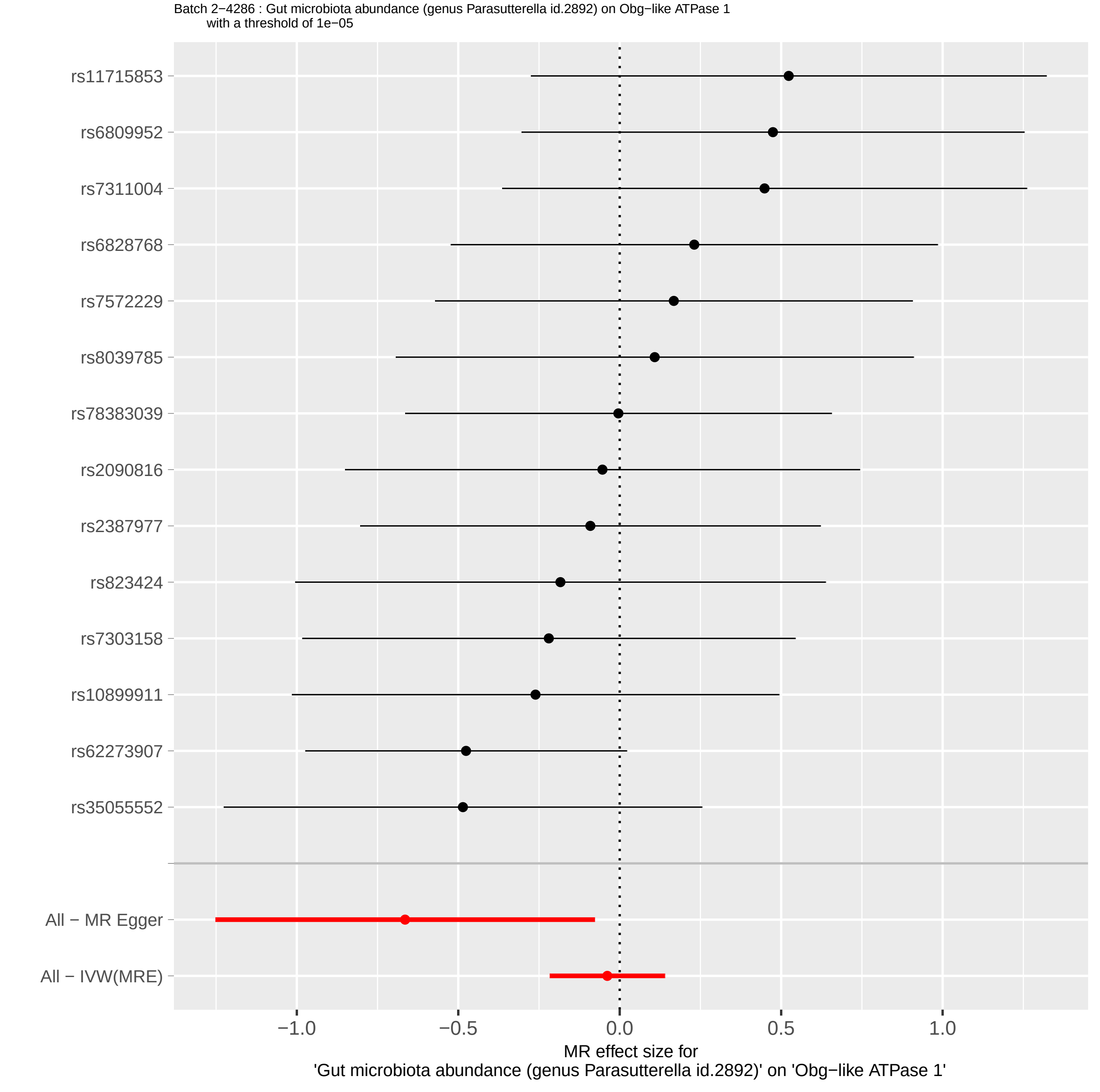

Batch 2−4286 : Gut microbiota abundance (genus Parasutterella id.2892) on Obg−like ATPase 1
with a threshold of 1e−05
rs11715853
rs6809952
rs7311004
rs6828768
rs7572229
rs8039785
rs78383039
rs2090816
rs2387977
rs823424
rs7303158
rs10899911
rs62273907
rs35055552
All − MR Egger
All − IVW(MRE)
−1.0
−0.5
0.0
0.5
1.0
MR effect size for
'Gut microbiota abundance (genus Parasutterella id.2892)' on 'Obg−like ATPase 1'

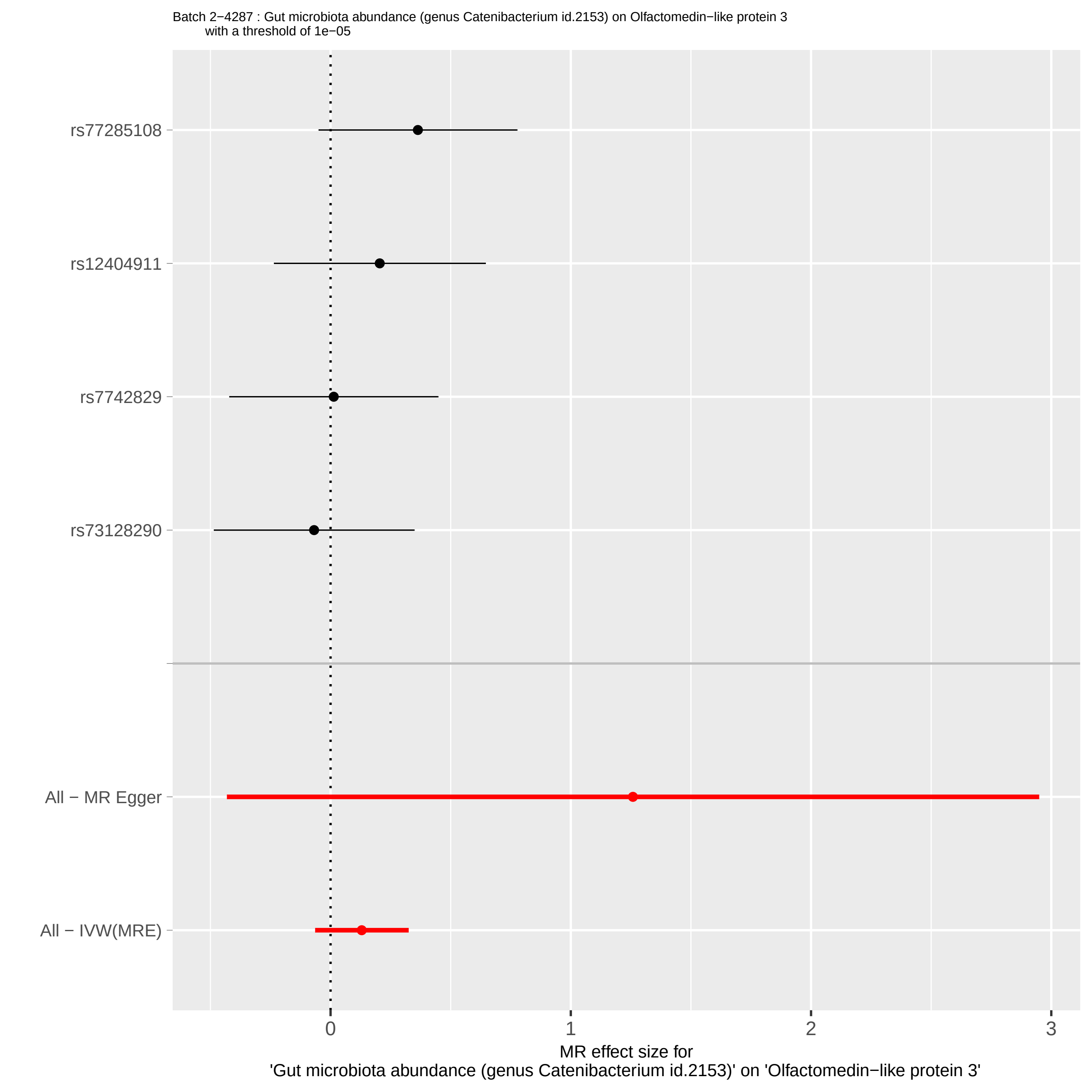

Batch 2−4287 : Gut microbiota abundance (genus Catenibacterium id.2153) on Olfactomedin−like protein 3
with a threshold of 1e−05
rs77285108
rs12404911
rs7742829
rs73128290
All − MR Egger
All − IVW(MRE)
0
1
2
3
MR effect size for
'Gut microbiota abundance (genus Catenibacterium id.2153)' on 'Olfactomedin−like protein 3'

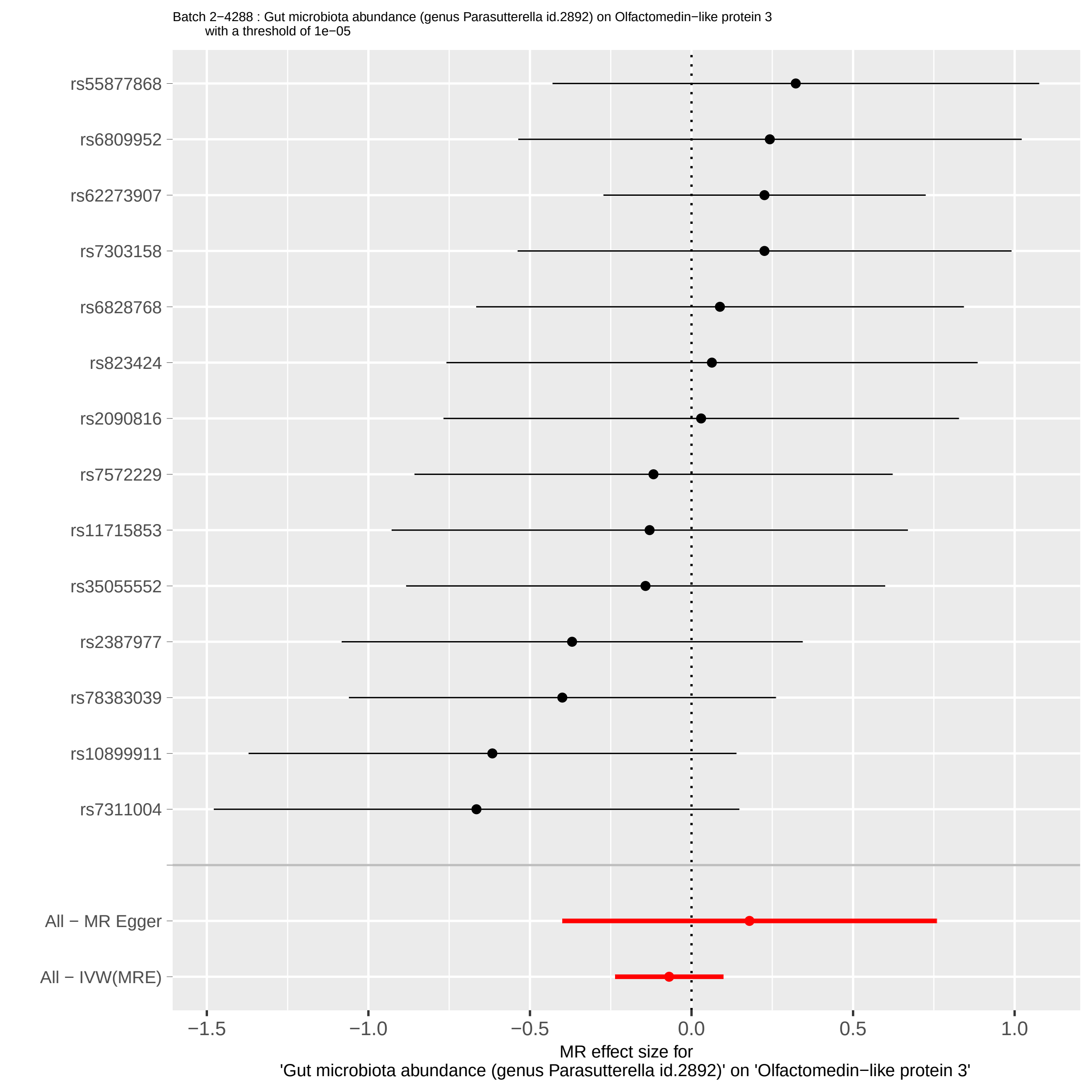

Batch 2−4288 : Gut microbiota abundance (genus Parasutterella id.2892) on Olfactomedin−like protein 3
with a threshold of 1e−05
rs55877868
rs6809952
rs62273907
rs7303158
rs6828768
rs823424
rs2090816
rs7572229
rs11715853
rs35055552
rs2387977
rs78383039
rs10899911
rs7311004
All − MR Egger
All − IVW(MRE)
−1.5
−1.0
−0.5
0.0
0.5
1.0
MR effect size for
'Gut microbiota abundance (genus Parasutterella id.2892)' on 'Olfactomedin−like protein 3'

Batch 2−4289 : Gut microbiota abundance (genus Catenibacterium id.2153) on Olfactomedin−like protein 3
with a threshold of 1e−05

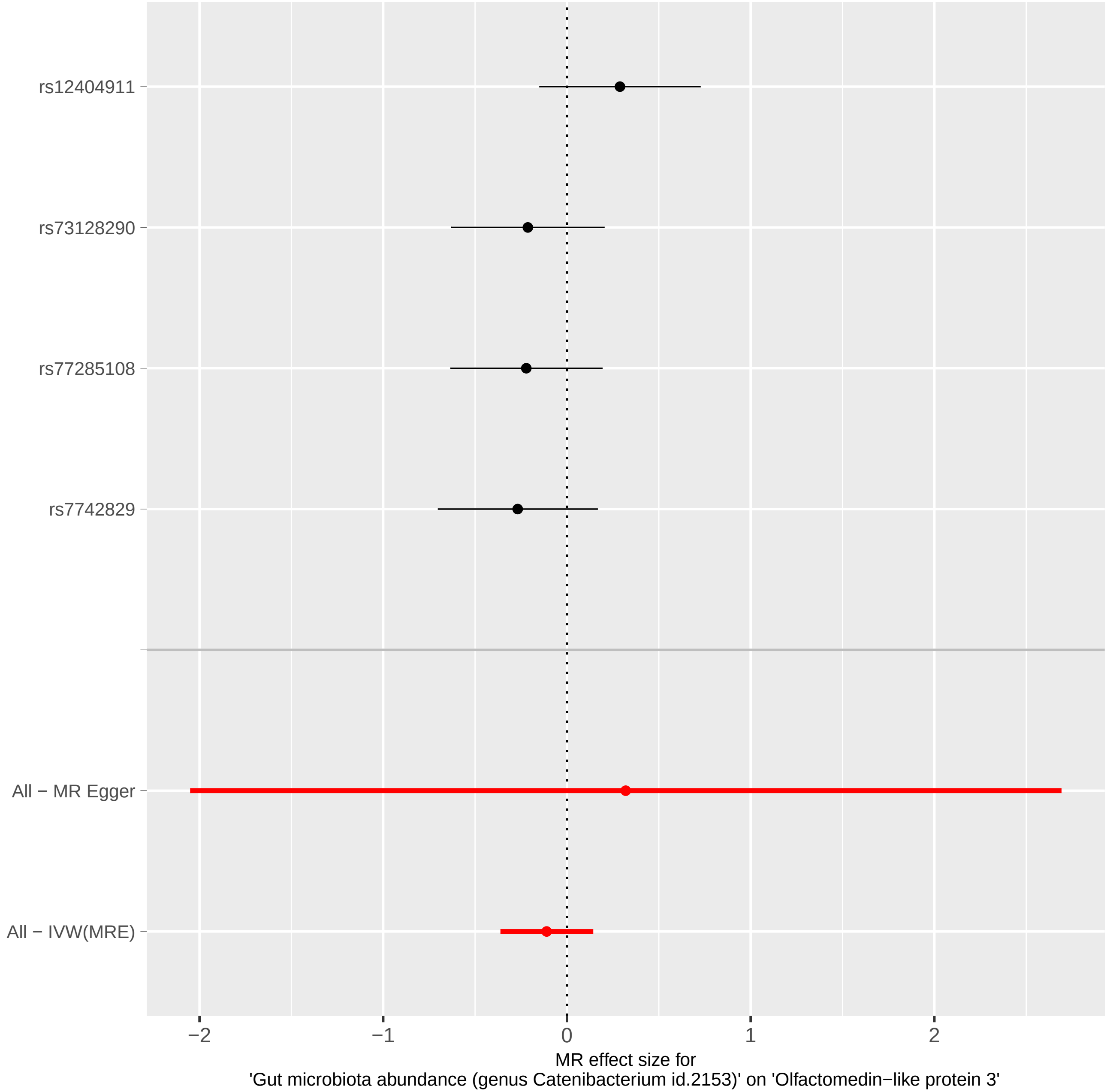

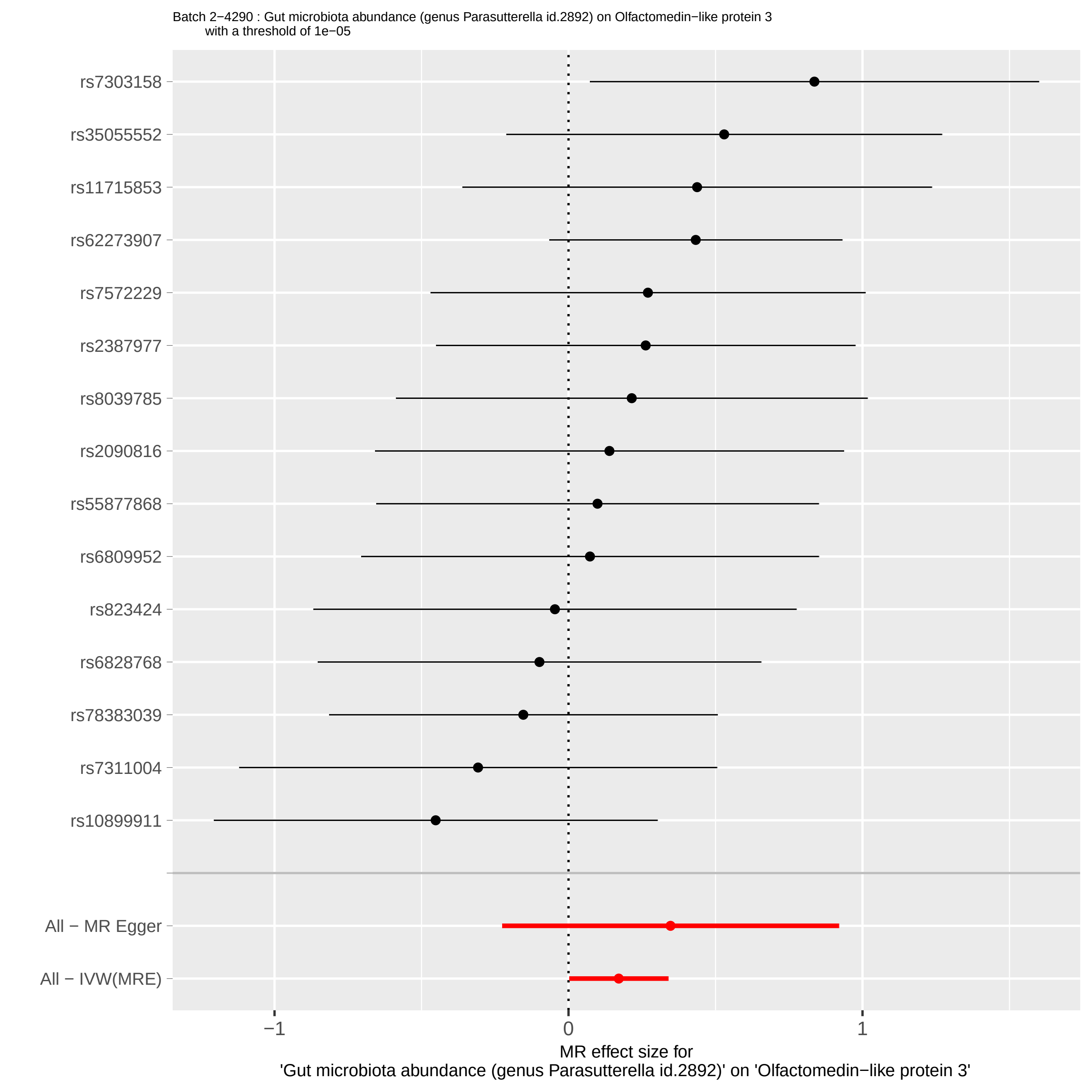

Batch 2−4290 : Gut microbiota abundance (genus Parasutterella id.2892) on Olfactomedin−like protein 3
with a threshold of 1e−05
rs7303158
rs35055552
rs11715853
rs62273907
rs7572229
rs2387977
rs8039785
rs2090816
rs55877868
rs6809952
rs823424
rs6828768
rs78383039
rs7311004
rs10899911
All − MR Egger
All − IVW(MRE)
−1
0
1
MR effect size for
'Gut microbiota abundance (genus Parasutterella id.2892)' on 'Olfactomedin−like protein 3'

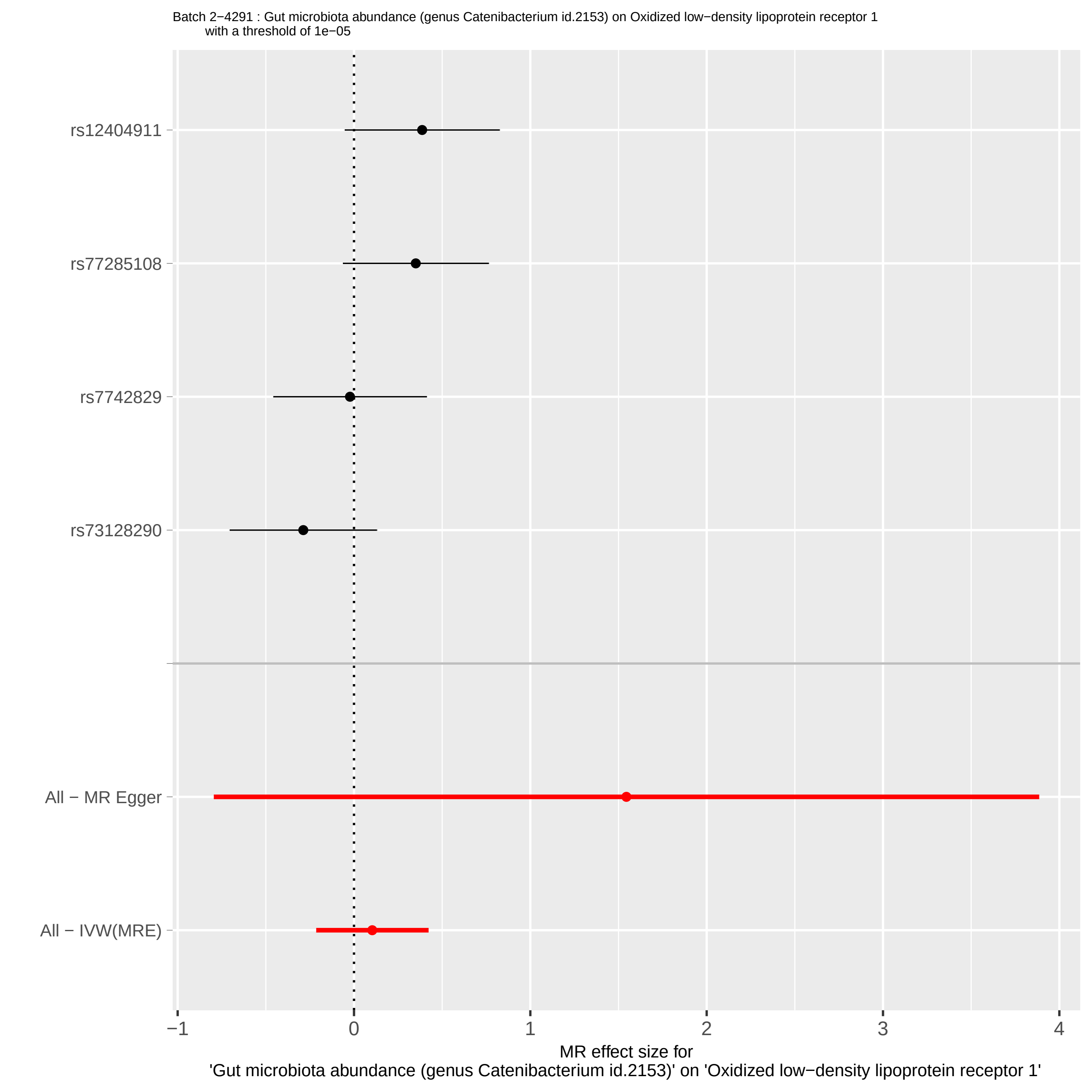
Batch 2−4291 : Gut microbiota abundance (genus Catenibacterium id.2153) on Oxidized low−density lipoprotein receptor 1
with a threshold of 1e−05
rs12404911
rs77285108
rs7742829
rs73128290
All − MR Egger
All − IVW(MRE)
−1
0
1
2
3
4
MR effect size for
'Gut microbiota abundance (genus Catenibacterium id.2153)' on 'Oxidized low−density lipoprotein receptor 1'

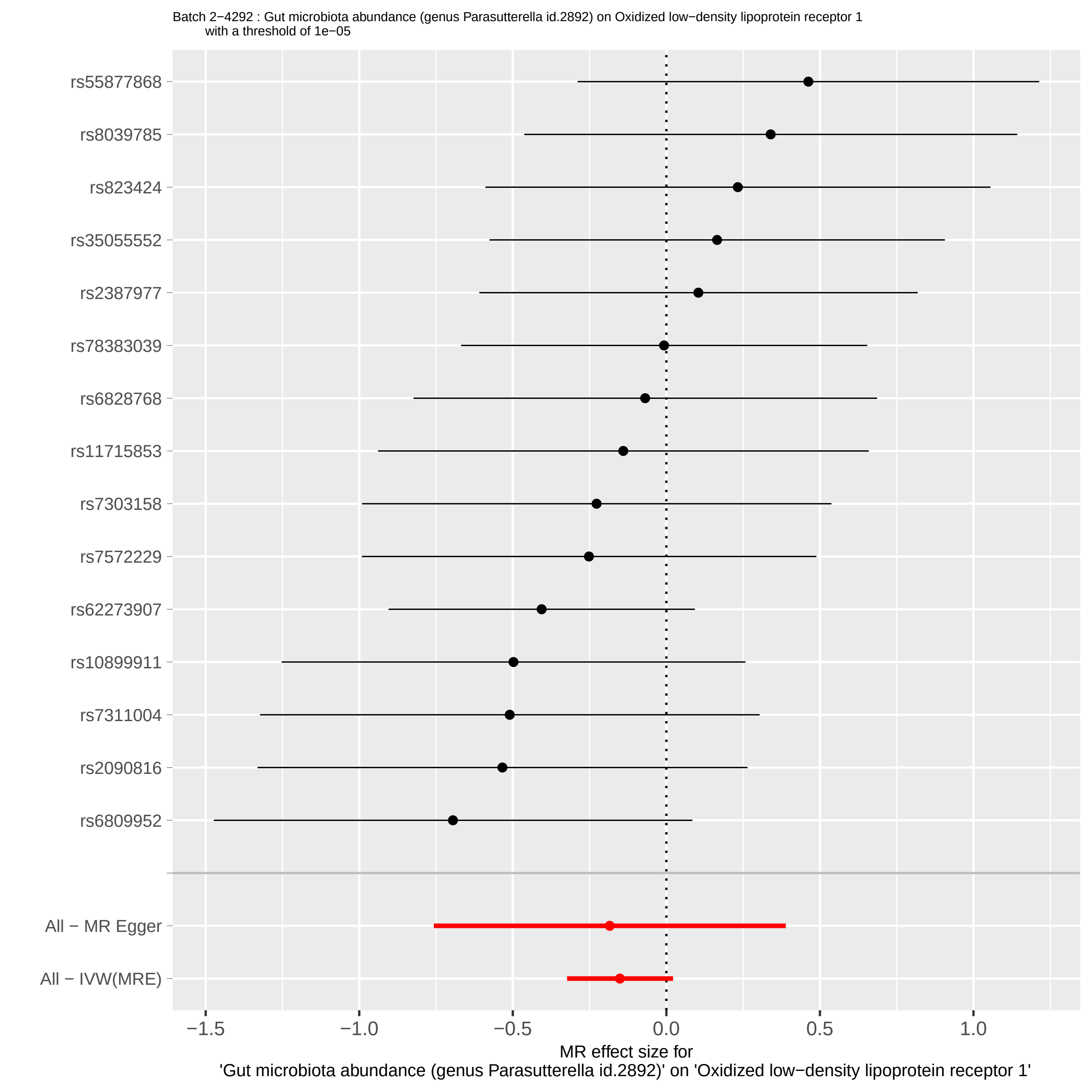
Batch 2−4292 : Gut microbiota abundance (genus Parasutterella id.2892) on Oxidized low−density lipoprotein receptor 1
with a threshold of 1e−05
rs55877868
rs8039785
rs823424
rs35055552
rs2387977
rs78383039
rs6828768
rs11715853
rs7303158
rs7572229
rs62273907
rs10899911
rs7311004
rs2090816
rs6809952
All − MR Egger
All − IVW(MRE)
−1.5
−1.0
−0.5
0.0
0.5
1.0
MR effect size for
'Gut microbiota abundance (genus Parasutterella id.2892)' on 'Oxidized low−density lipoprotein receptor 1'

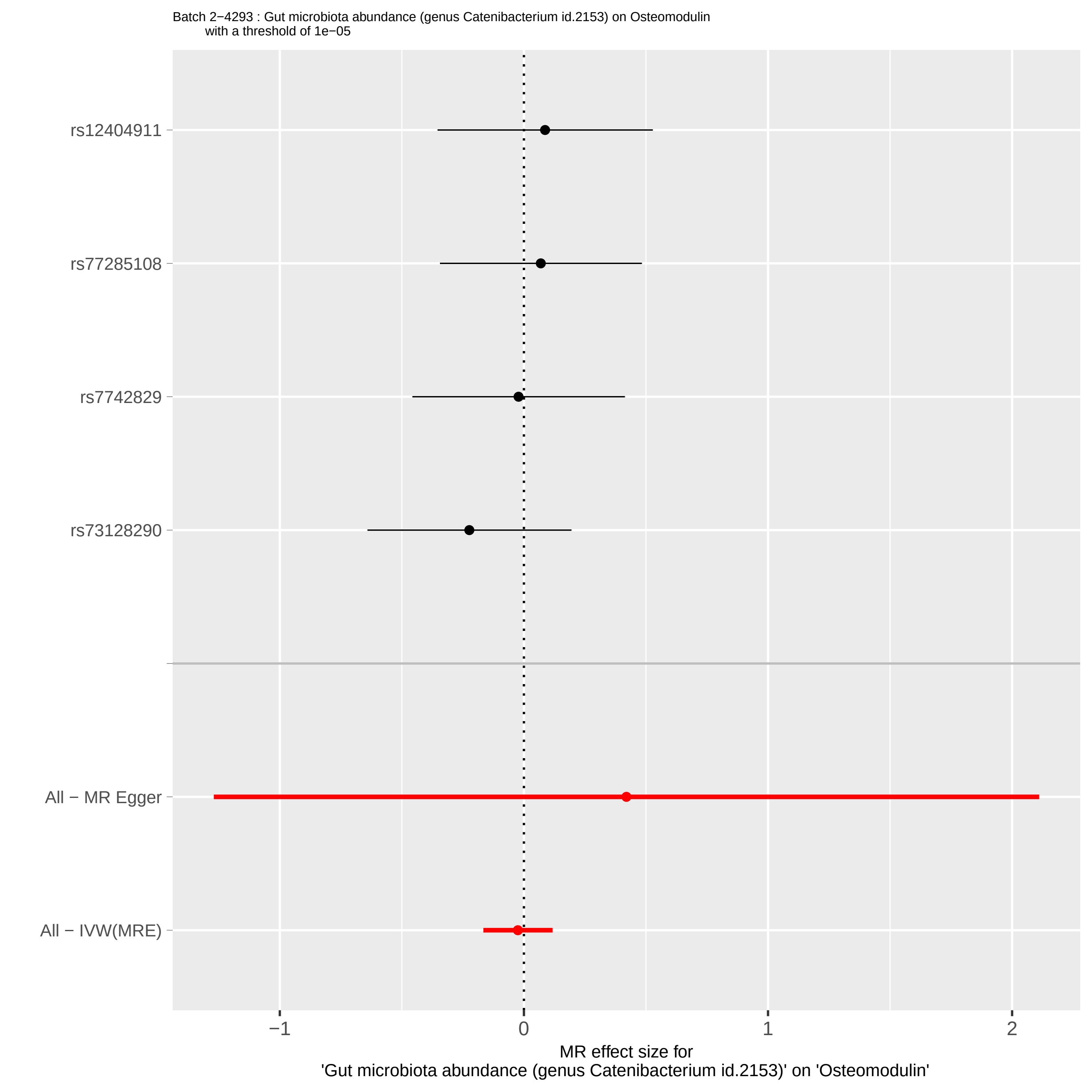
Batch 2−4293 : Gut microbiota abundance (genus Catenibacterium id.2153) on Osteomodulin
with a threshold of 1e−05
rs12404911
rs77285108
rs7742829
rs73128290
All − MR Egger
All − IVW(MRE)
−1
0
1
2
MR effect size for
'Gut microbiota abundance (genus Catenibacterium id.2153)' on 'Osteomodulin'

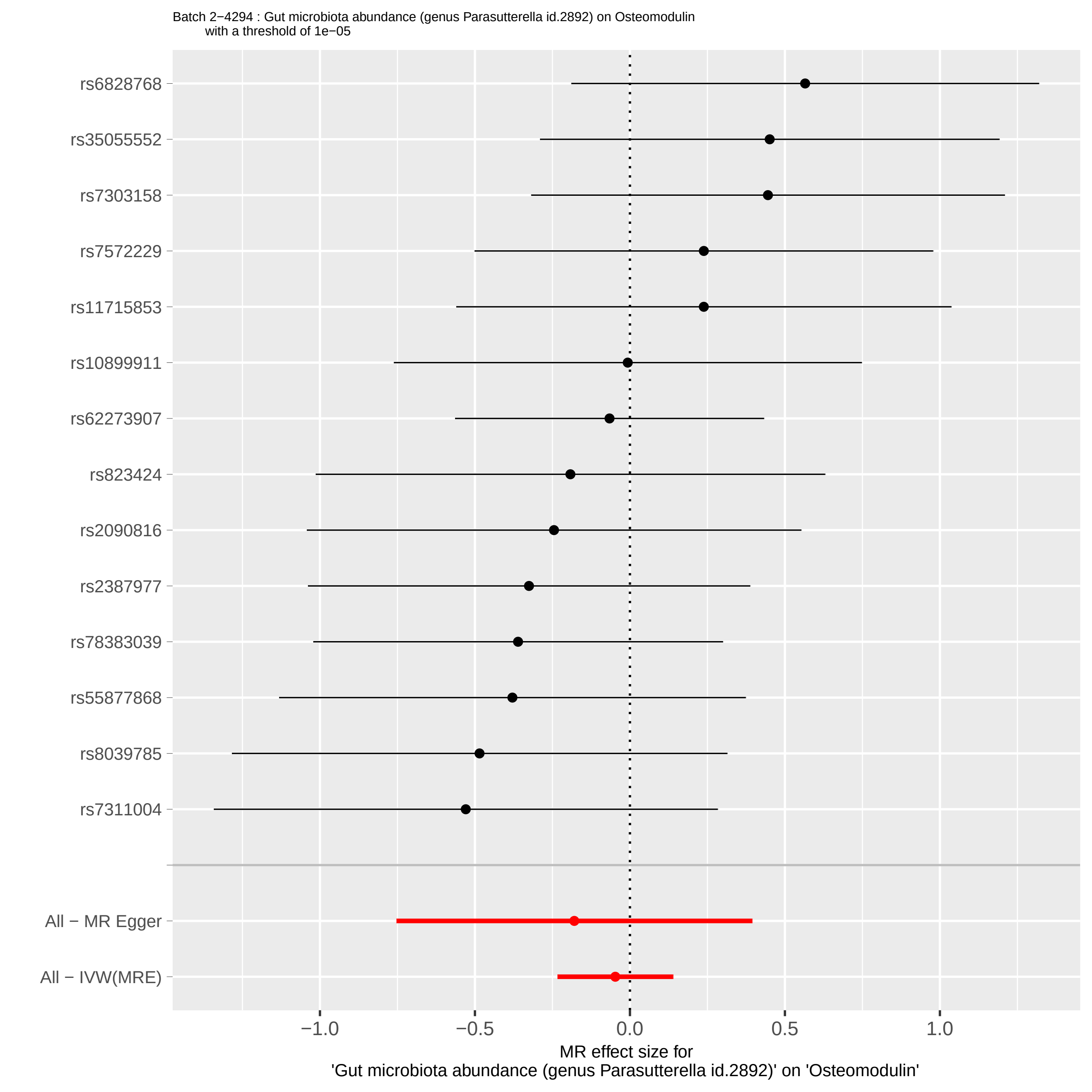

Batch 2−4294 : Gut microbiota abundance (genus Parasutterella id.2892) on Osteomodulin
with a threshold of 1e−05
rs6828768
rs35055552
rs7303158
rs7572229
rs11715853
rs10899911
rs62273907
rs823424
rs2090816
rs2387977
rs78383039
rs55877868
rs8039785
rs7311004
All − MR Egger
All − IVW(MRE)
−1.0
−0.5
0.0
0.5
1.0
MR effect size for
'Gut microbiota abundance (genus Parasutterella id.2892)' on 'Osteomodulin'

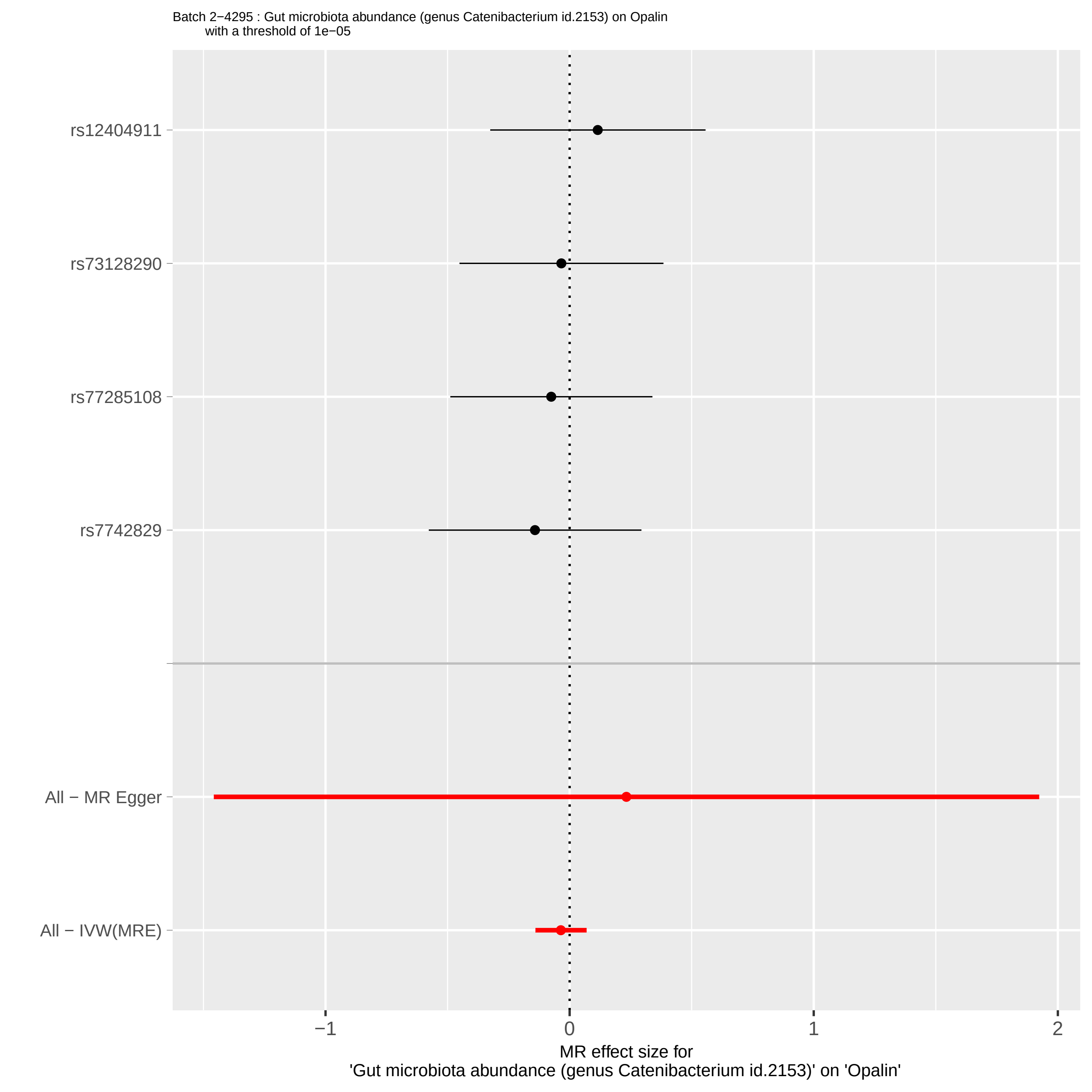

Batch 2−4295 : Gut microbiota abundance (genus Catenibacterium id.2153) on Opalin
with a threshold of 1e−05
rs12404911
rs73128290
rs77285108
rs7742829
All − MR Egger
All − IVW(MRE)
−1
0
1
2
MR effect size for
'Gut microbiota abundance (genus Catenibacterium id.2153)' on 'Opalin'

Batch 2−4296 : Gut microbiota abundance (genus Parasutterella id.2892) on Opalin
with a threshold of 1e−05

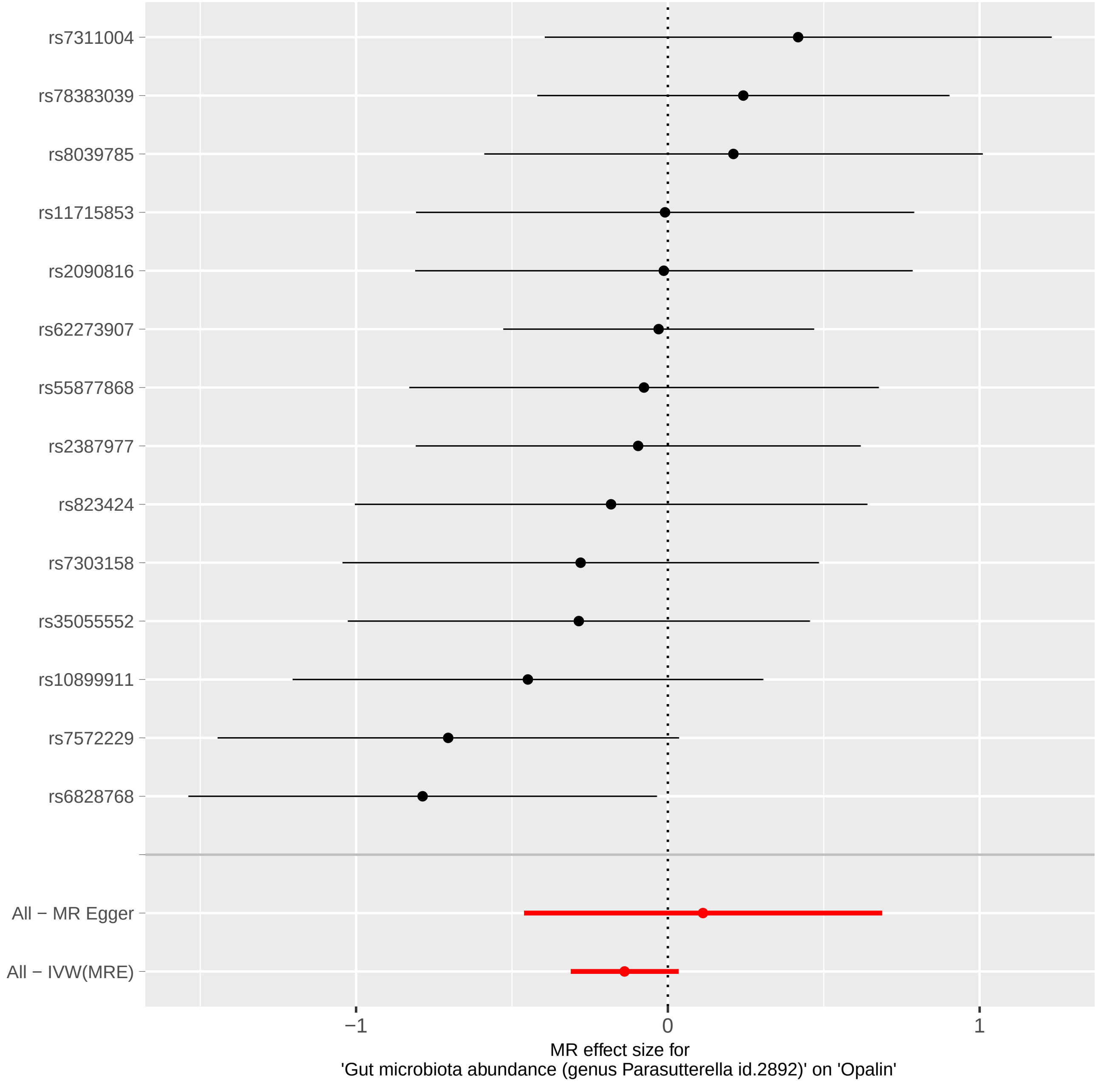

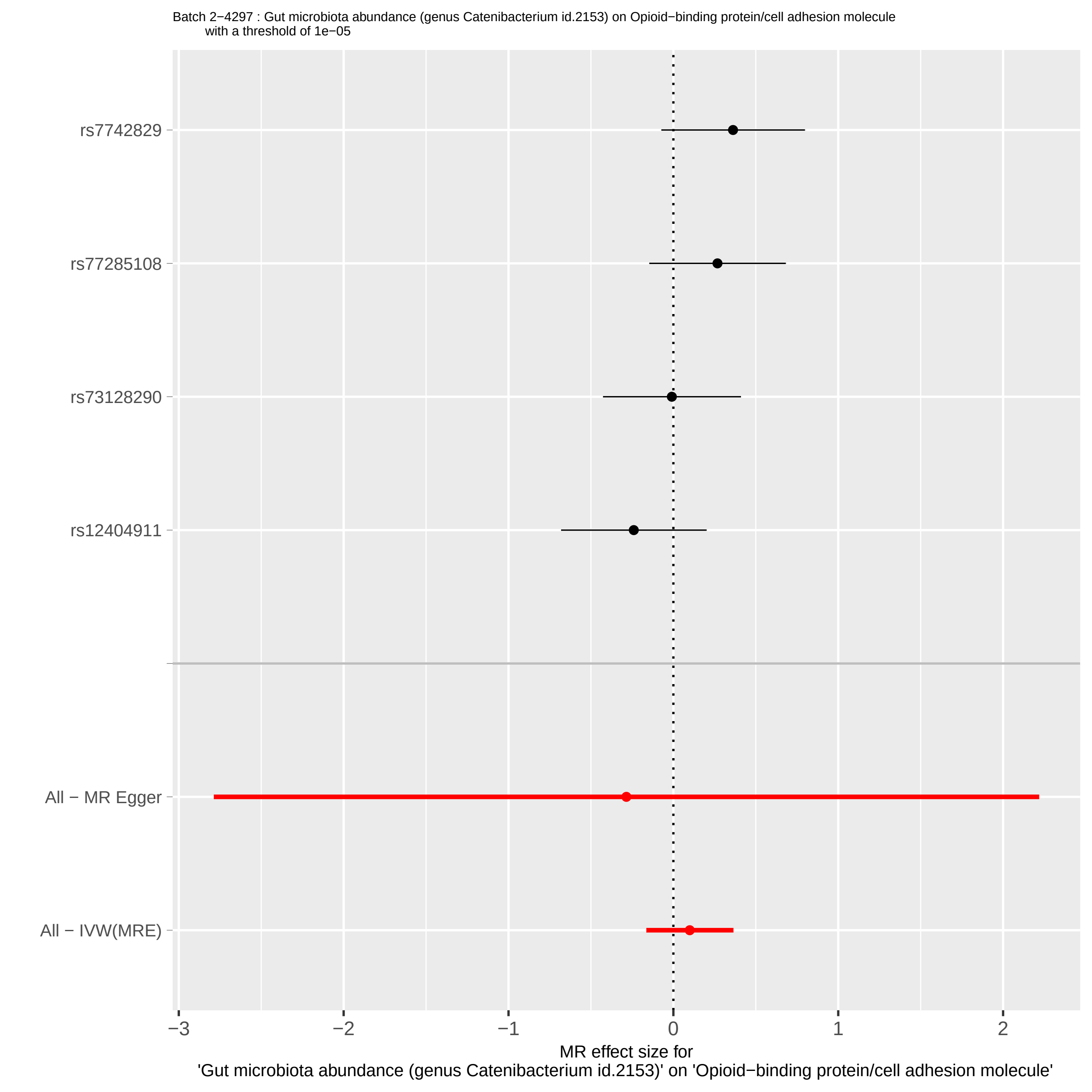

Batch 2−4297 : Gut microbiota abundance (genus Catenibacterium id.2153) on Opioid−binding protein/cell adhesion molecule
with a threshold of 1e−05
rs7742829
rs77285108
rs73128290
rs12404911
All − MR Egger
All − IVW(MRE)
−3
−2
−1
0
1
2
MR effect size for
'Gut microbiota abundance (genus Catenibacterium id.2153)' on 'Opioid−binding protein/cell adhesion molecule'

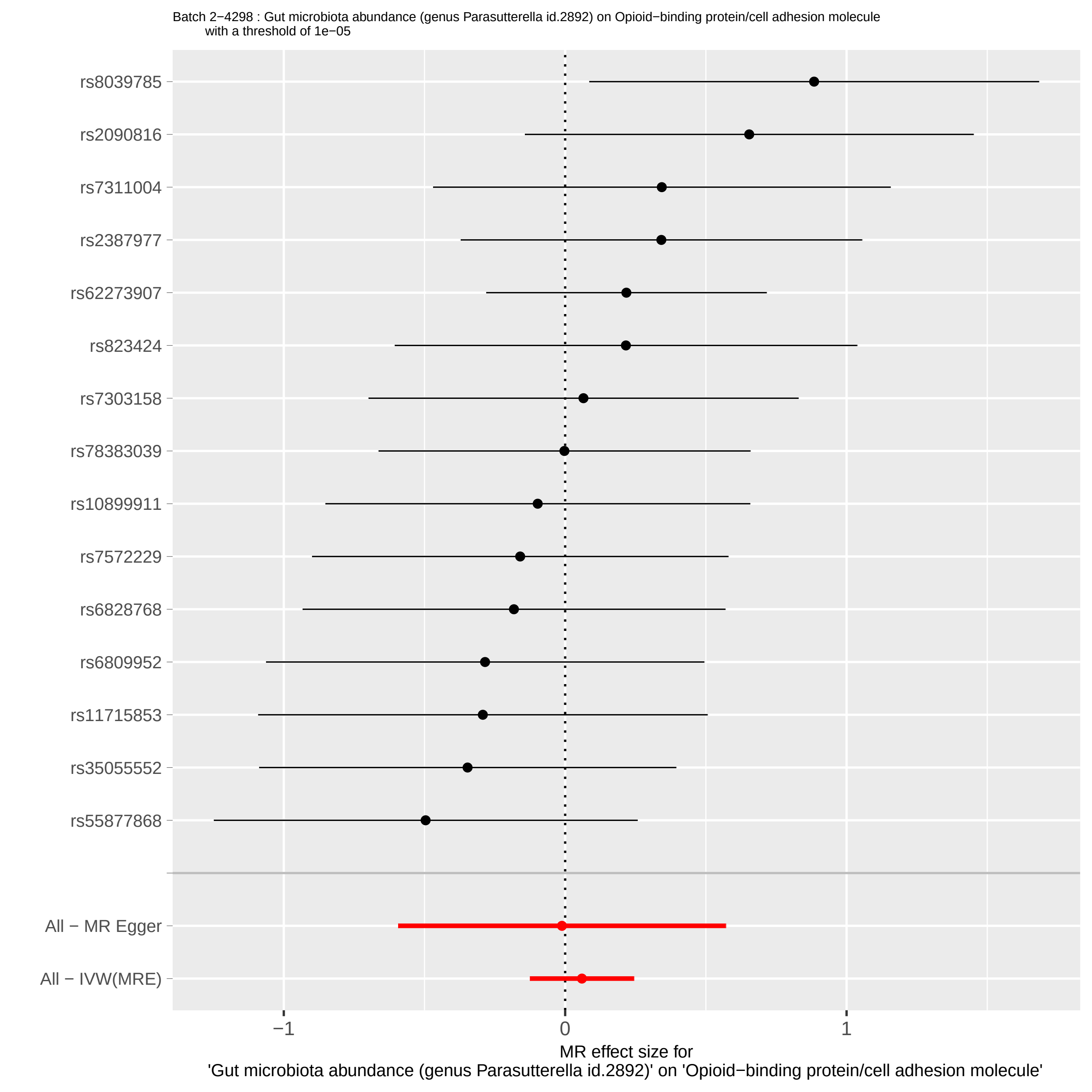

Batch 2−4298 : Gut microbiota abundance (genus Parasutterella id.2892) on Opioid−binding protein/cell adhesion molecule with a threshold of 1e−05
rs8039785
rs2090816
rs7311004
rs2387977
rs62273907
rs823424
rs7303158
rs78383039
rs10899911
rs7572229
rs6828768
rs6809952
rs11715853
rs35055552
rs55877868
All − MR Egger
All − IVW(MRE)
−1
0
1
MR effect size for
'Gut microbiota abundance (genus Parasutterella id.2892)' on 'Opioid−binding protein/cell adhesion molecule'

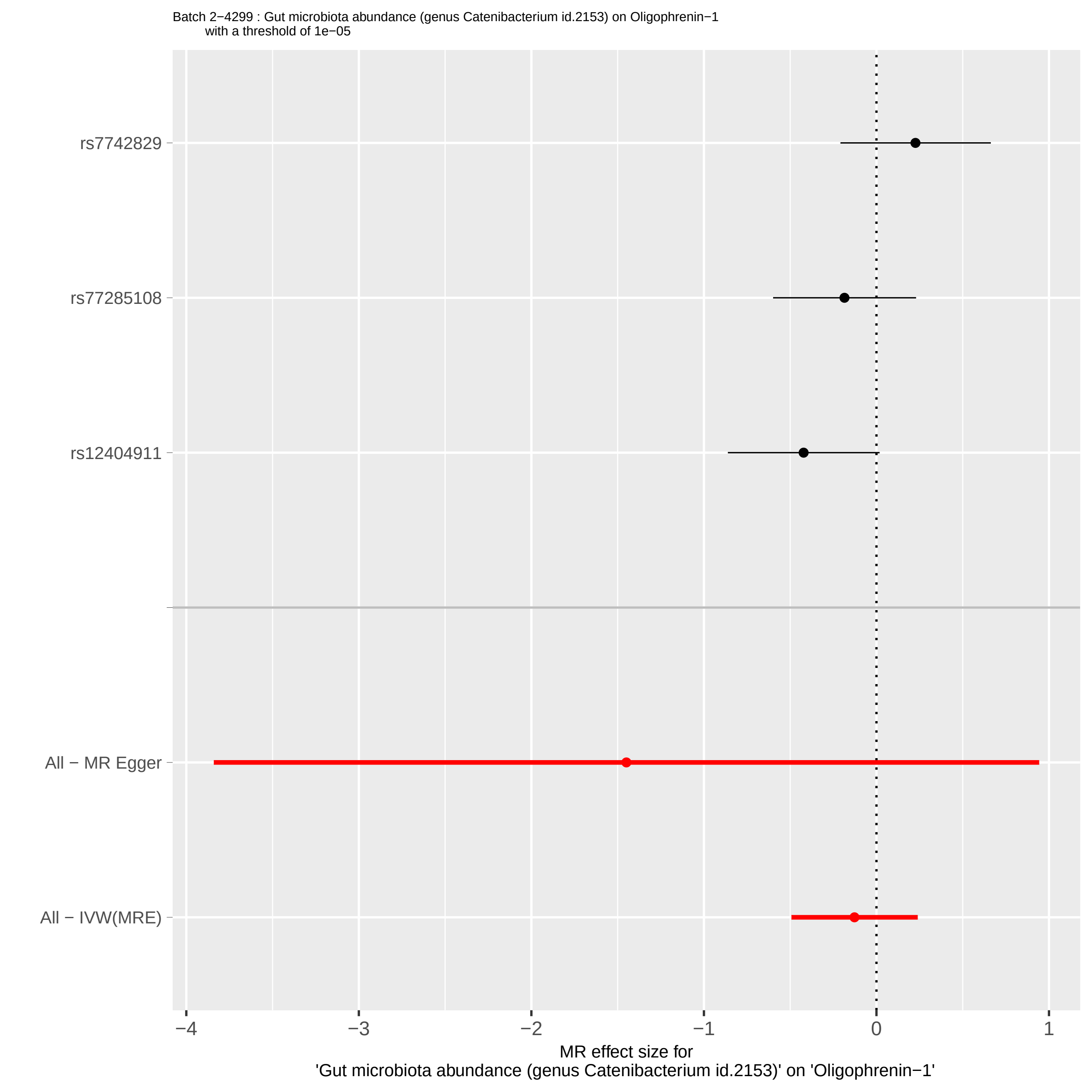

Batch 2−4299 : Gut microbiota abundance (genus Catenibacterium id.2153) on Oligophrenin−1
with a threshold of 1e−05
rs7742829
rs77285108
rs12404911
All − MR Egger
All − IVW(MRE)
−4
−3
−2
−1
0
1
MR effect size for
'Gut microbiota abundance (genus Catenibacterium id.2153)' on 'Oligophrenin−1'

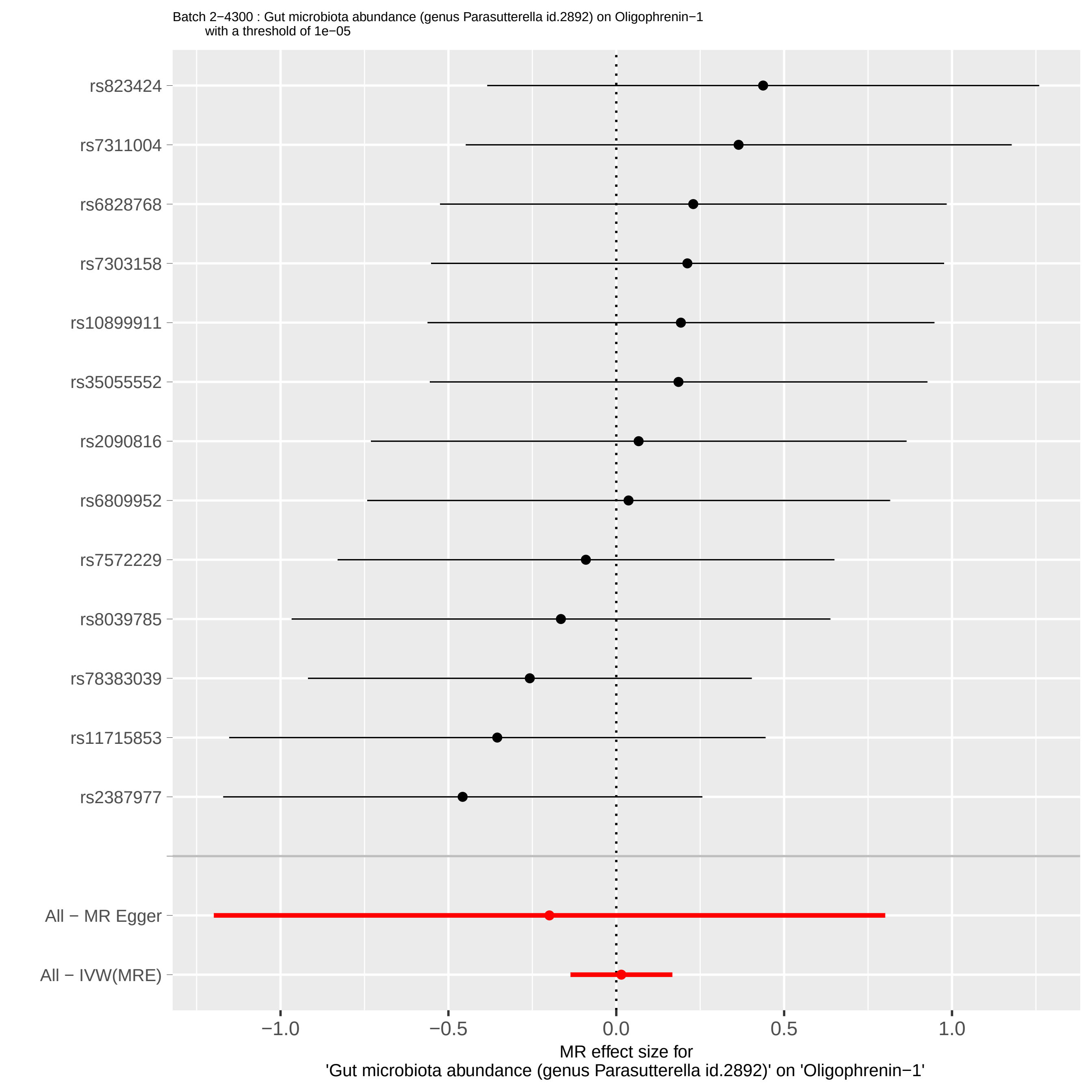
Batch 2−4300 : Gut microbiota abundance (genus Parasutterella id.2892) on Oligophrenin−1
with a threshold of 1e−05
rs823424
rs7311004
rs6828768
rs7303158
rs10899911
rs35055552
rs2090816
rs6809952
rs7572229
rs8039785
rs78383039
rs11715853
rs2387977
All − MR Egger
All − IVW(MRE)
−1.0
−0.5
0.0
0.5
1.0
MR effect size for
'Gut microbiota abundance (genus Parasutterella id.2892)' on 'Oligophrenin−1'

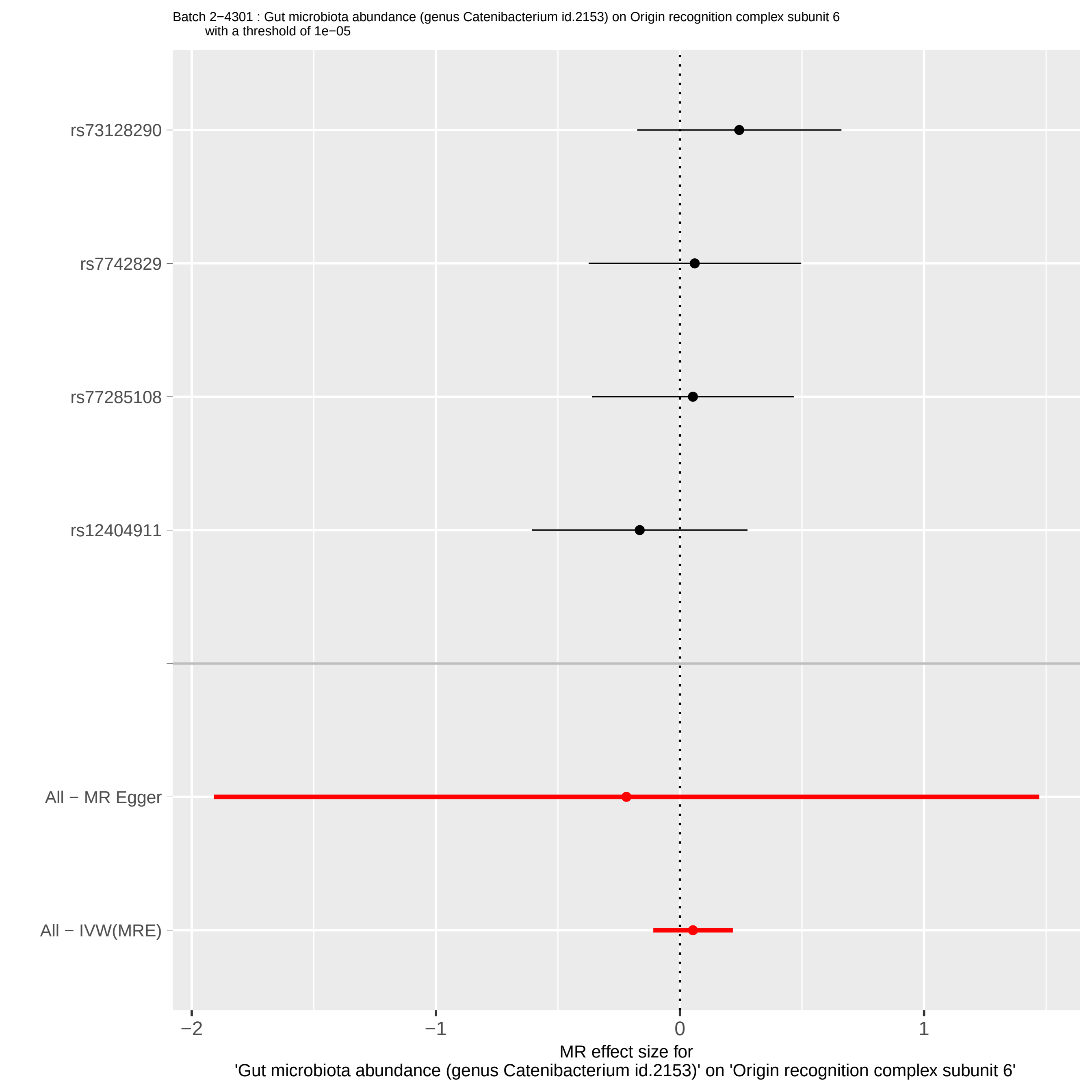

Batch 2−4301 : Gut microbiota abundance (genus Catenibacterium id.2153) on Origin recognition complex subunit 6
with a threshold of 1e−05
rs73128290
rs7742829
rs77285108
rs12404911
All − MR Egger
All − IVW(MRE)
−2
−1
0
1
MR effect size for
'Gut microbiota abundance (genus Catenibacterium id.2153)' on 'Origin recognition complex subunit 6'

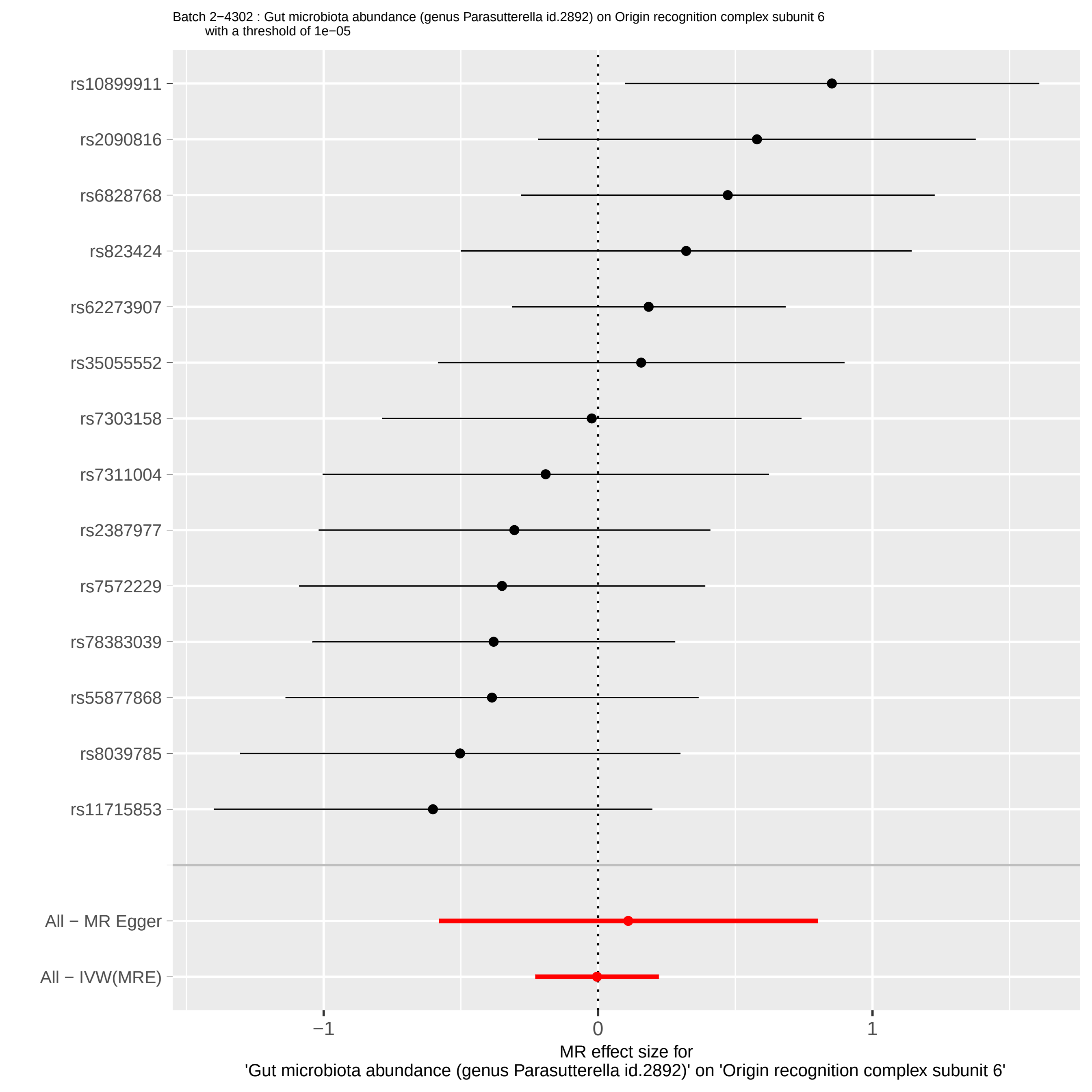

Batch 2−4302 : Gut microbiota abundance (genus Parasutterella id.2892) on Origin recognition complex subunit 6
with a threshold of 1e−05
rs10899911
rs2090816
rs6828768
rs823424
rs62273907
rs35055552
rs7303158
rs7311004
rs2387977
rs7572229
rs78383039
rs55877868
rs8039785
rs11715853
All − MR Egger
All − IVW(MRE)
−1
0
1
MR effect size for
'Gut microbiota abundance (genus Parasutterella id.2892)' on 'Origin recognition complex subunit 6'

Batch 2−4303 : Gut microbiota abundance (genus Catenibacterium id.2153) on Oxysterol−binding protein−related protein 11
with a threshold of 1e−05

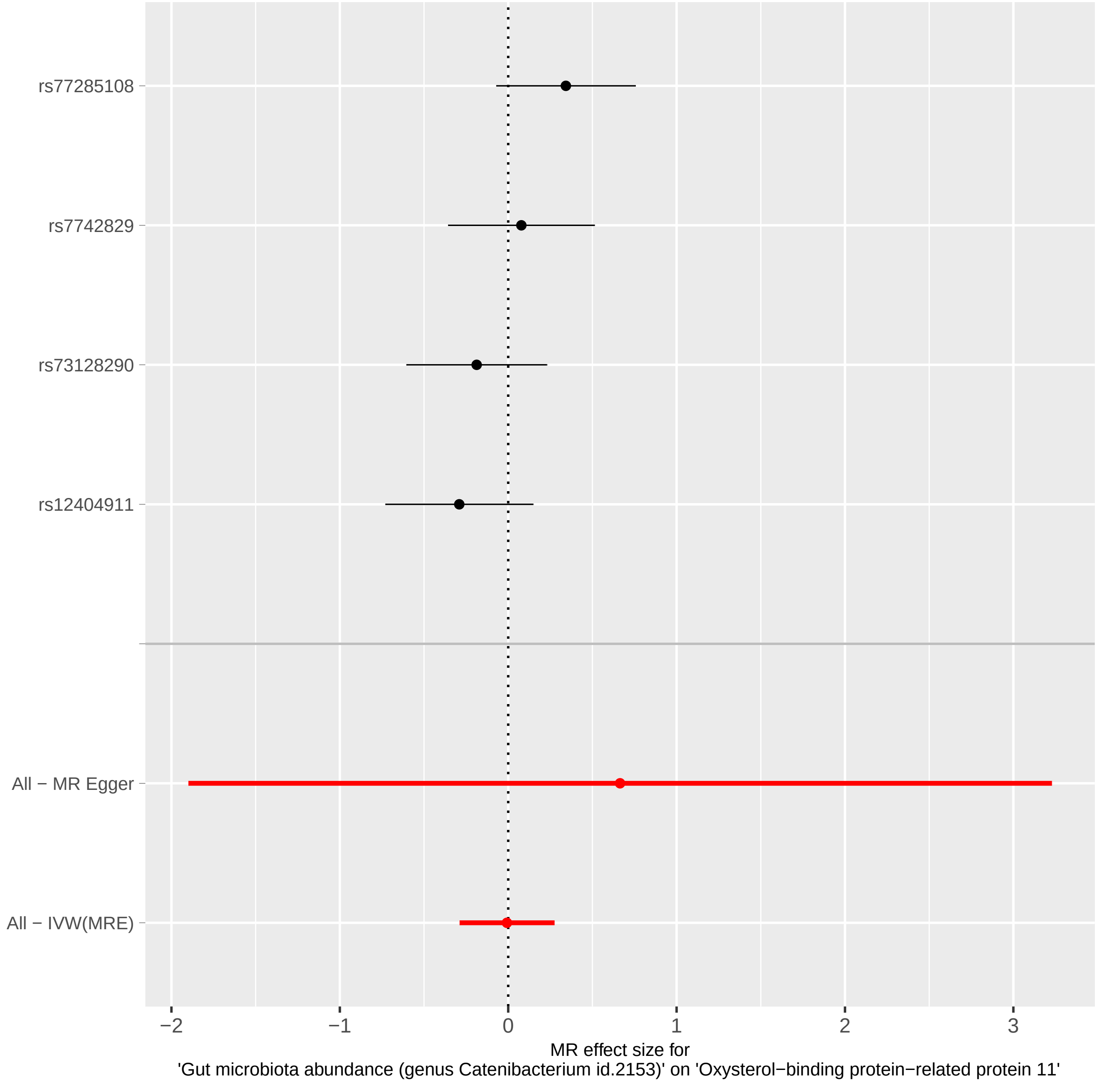

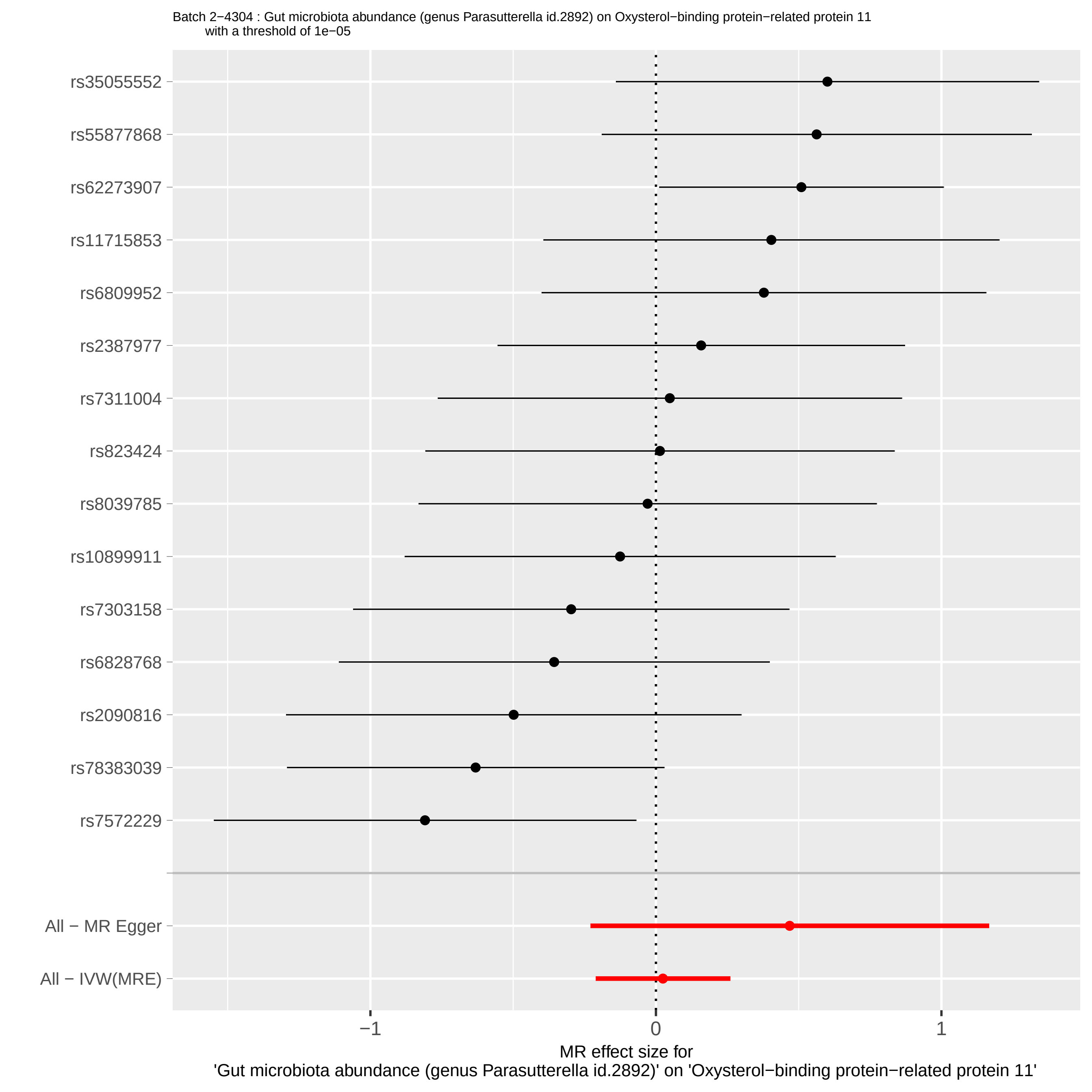
Batch 2−4304 : Gut microbiota abundance (genus Parasutterella id.2892) on Oxysterol−binding protein−related protein 11
with a threshold of 1e−05
rs35055552
rs55877868
rs62273907
rs11715853
rs6809952
rs2387977
rs7311004
rs823424
rs8039785
rs10899911
rs7303158
rs6828768
rs2090816
rs78383039
rs7572229
All − MR Egger
All − IVW(MRE)
−1
0
1
MR effect size for
'Gut microbiota abundance (genus Parasutterella id.2892)' on 'Oxysterol−binding protein−related protein 11'

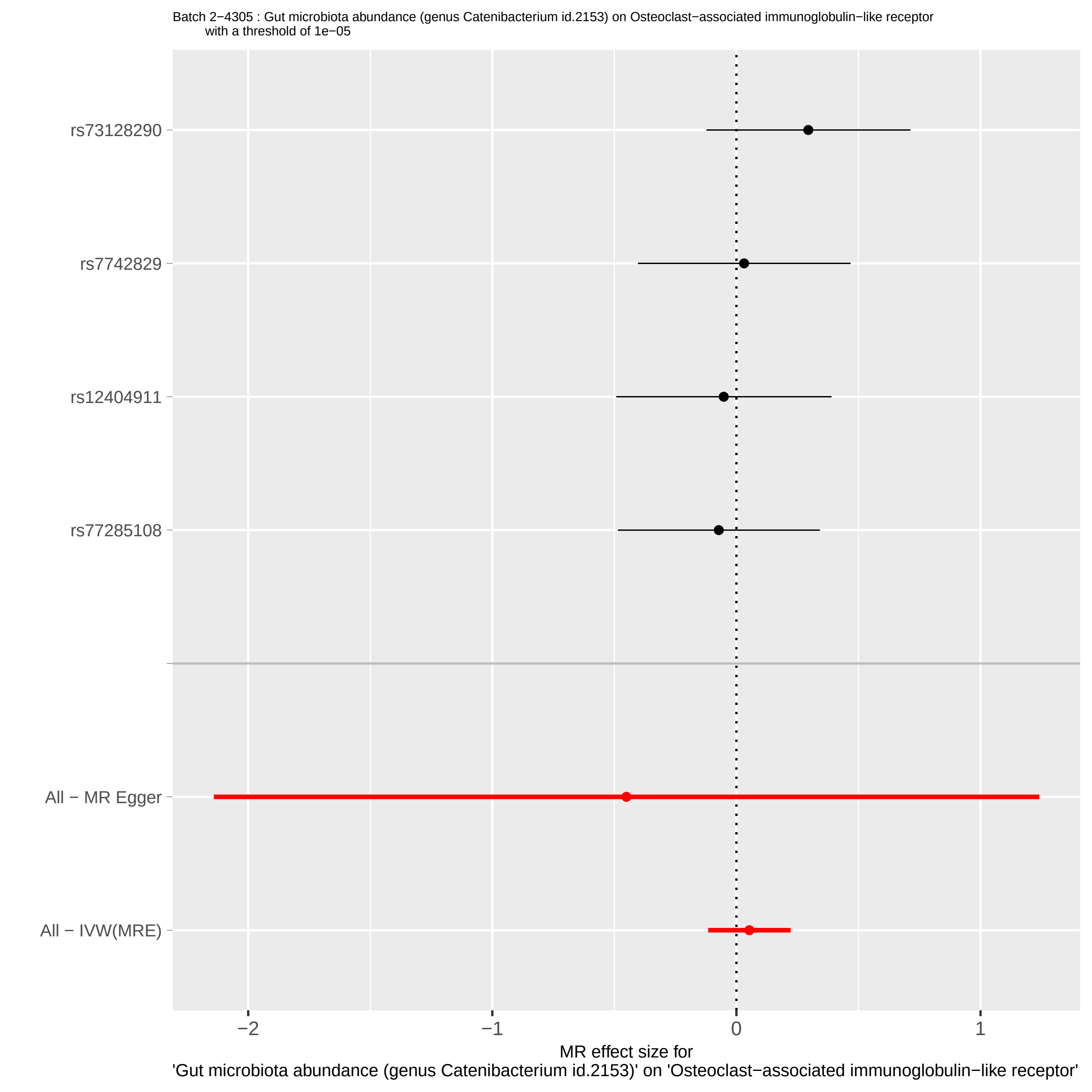
Batch 2−4305 : Gut microbiota abundance (genus Catenibacterium id.2153) on Osteoclast−associated immunoglobulin−like receptor
with a threshold of 1e−05
rs73128290
rs7742829
rs12404911
rs77285108
All − MR Egger
All − IVW(MRE)
−2
−1
0
1
MR effect size for
'Gut microbiota abundance (genus Catenibacterium id.2153)' on 'Osteoclast−associated immunoglobulin−like receptor'

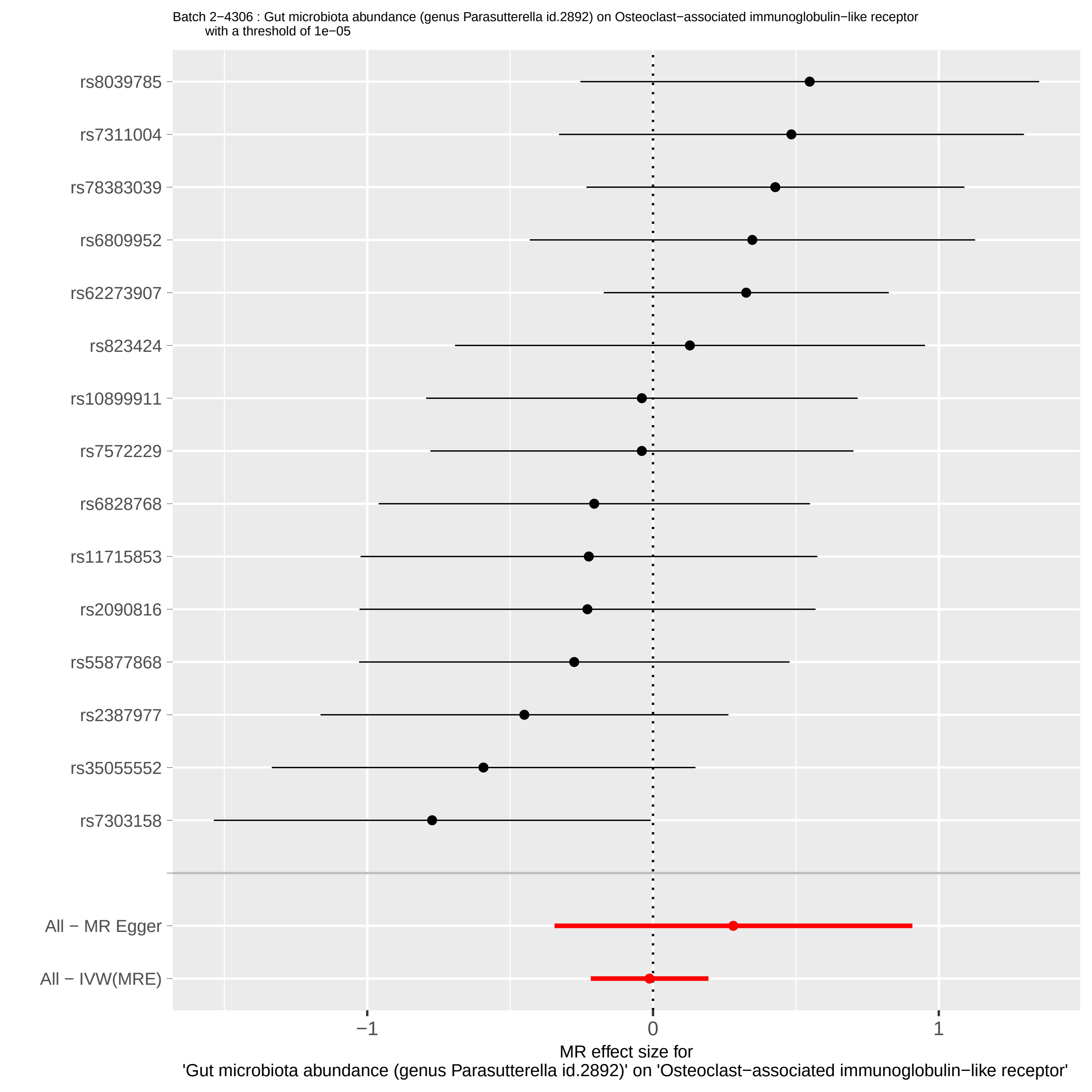
Batch 2−4306 : Gut microbiota abundance (genus Parasutterella id.2892) on Osteoclast−associated immunoglobulin−like receptor
with a threshold of 1e−05
rs8039785
rs7311004
rs78383039
rs6809952
rs62273907
rs823424
rs10899911
rs7572229
rs6828768
rs11715853
rs2090816
rs55877868
rs2387977
rs35055552
rs7303158
All − MR Egger
All − IVW(MRE)
−1
0
1
MR effect size for
'Gut microbiota abundance (genus Parasutterella id.2892)' on 'Osteoclast−associated immunoglobulin−like receptor'

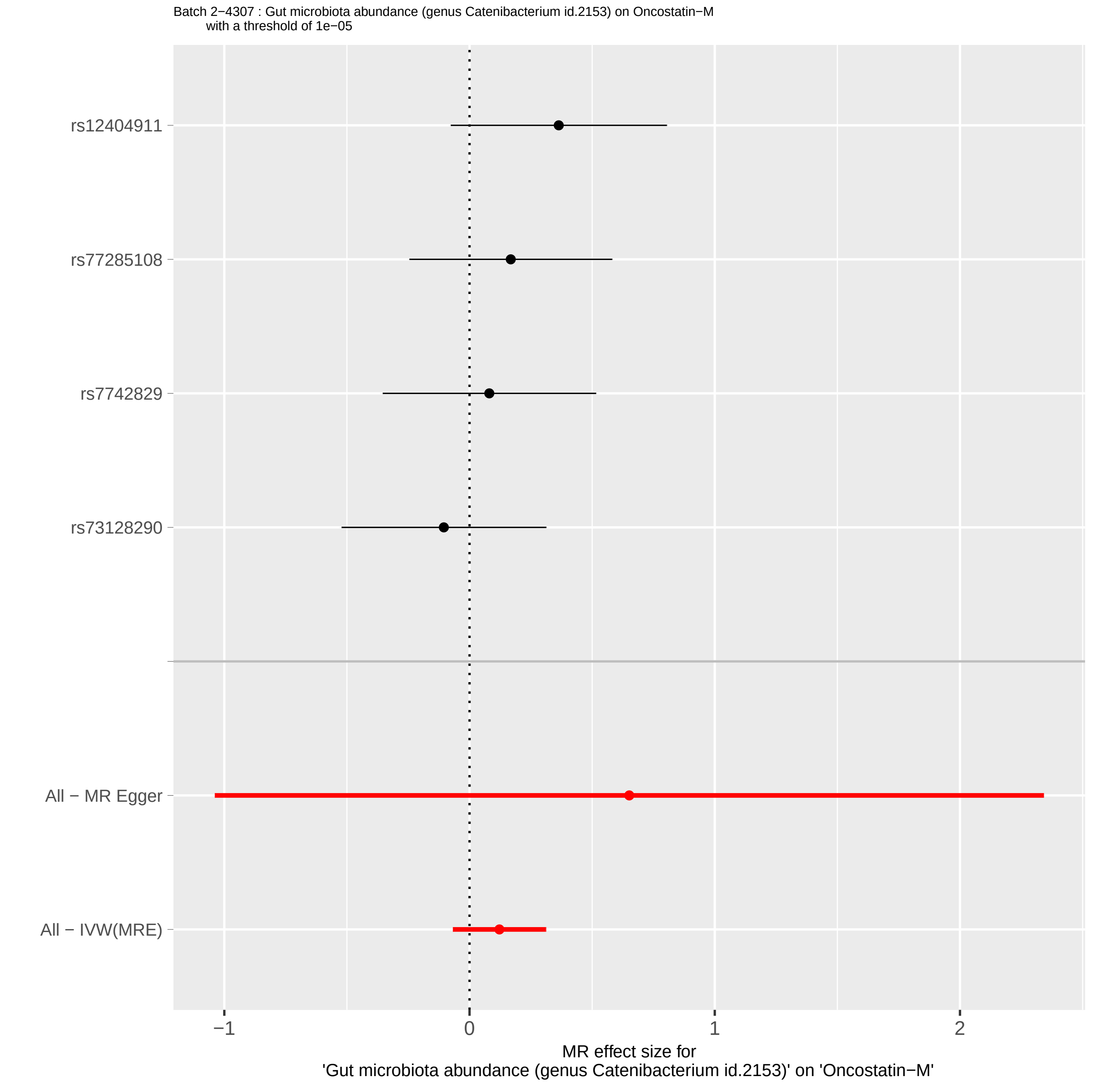

Batch 2−4307 : Gut microbiota abundance (genus Catenibacterium id.2153) on Oncostatin−M
with a threshold of 1e−05
rs12404911
rs77285108
rs7742829
rs73128290
All − MR Egger
All − IVW(MRE)
−1
0
1
2
MR effect size for
'Gut microbiota abundance (genus Catenibacterium id.2153)' on 'Oncostatin−M'

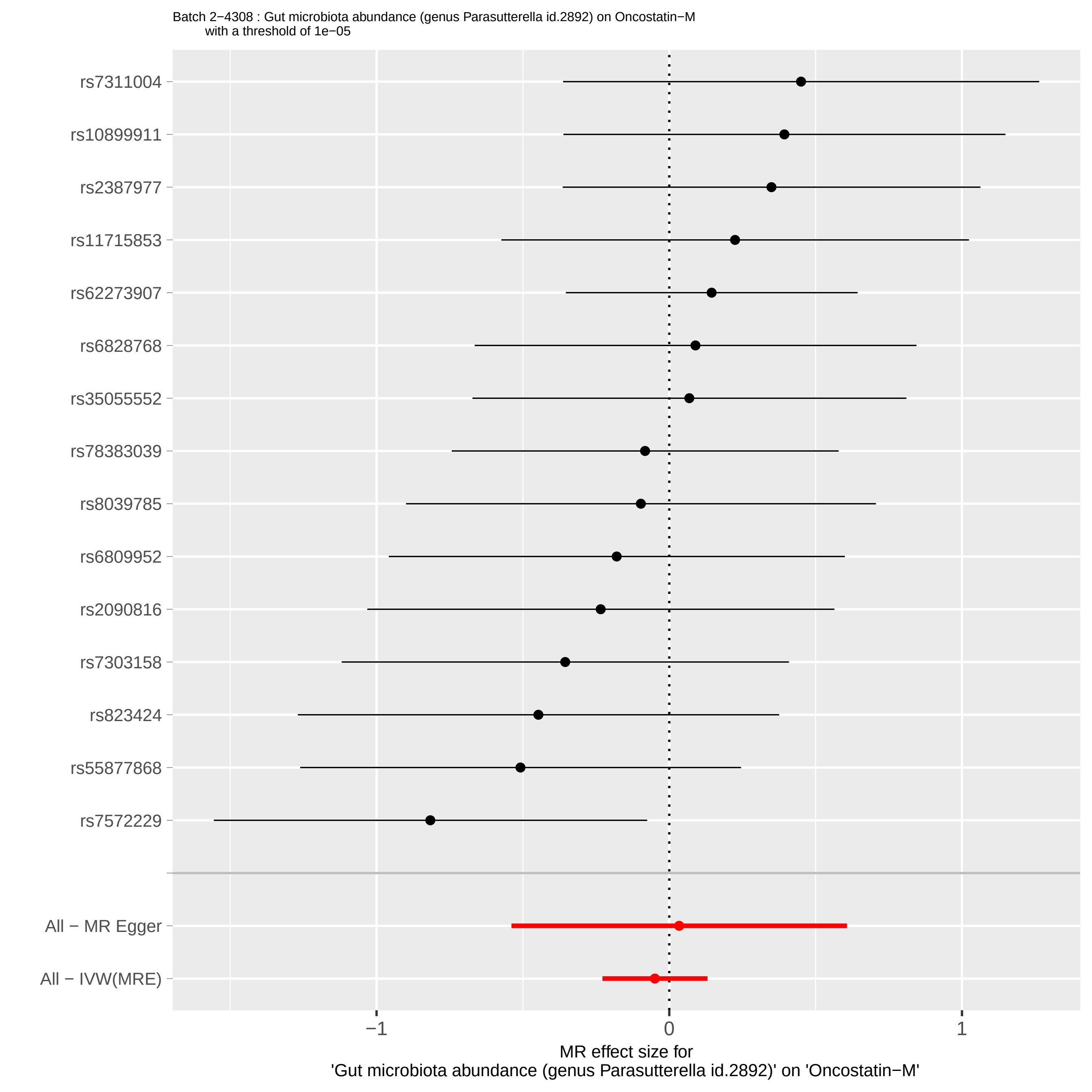
Batch 2−4308 : Gut microbiota abundance (genus Parasutterella id.2892) on Oncostatin−M
with a threshold of 1e−05
rs7311004
rs10899911
rs2387977
rs11715853
rs62273907
rs6828768
rs35055552
rs78383039
rs8039785
rs6809952
rs2090816
rs7303158
rs823424
rs55877868
rs7572229
All − MR Egger
All − IVW(MRE)
−1
0
1
MR effect size for
'Gut microbiota abundance (genus Parasutterella id.2892)' on 'Oncostatin−M'

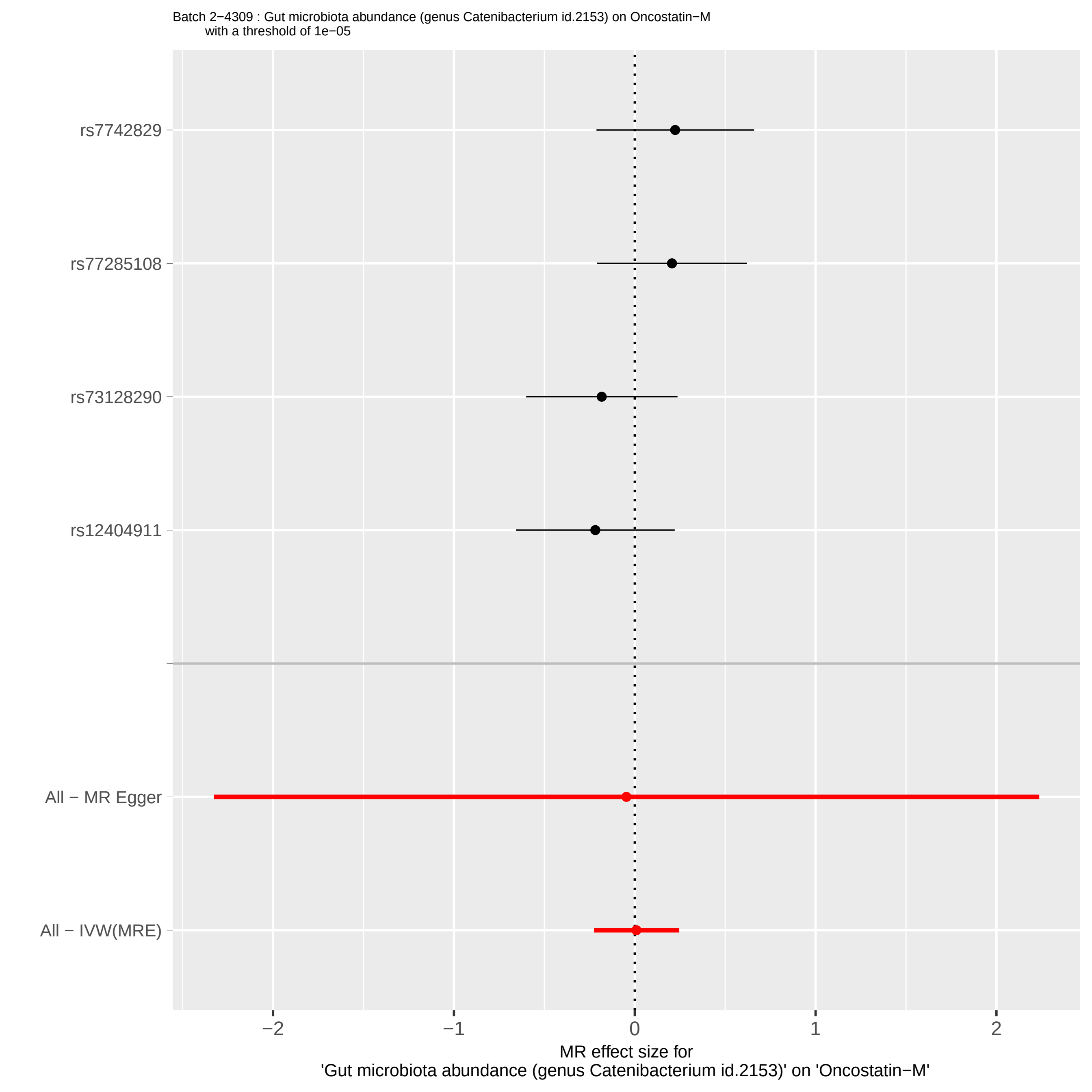
Batch 2−4309 : Gut microbiota abundance (genus Catenibacterium id.2153) on Oncostatin−M
with a threshold of 1e−05
rs7742829
rs77285108
rs73128290
rs12404911
All − MR Egger
All − IVW(MRE)
−2
−1
0
1
2
MR effect size for
'Gut microbiota abundance (genus Catenibacterium id.2153)' on 'Oncostatin−M'

Batch 2−4310 : Gut microbiota abundance (genus Parasutterella id.2892) on Oncostatin−M
with a threshold of 1e−05

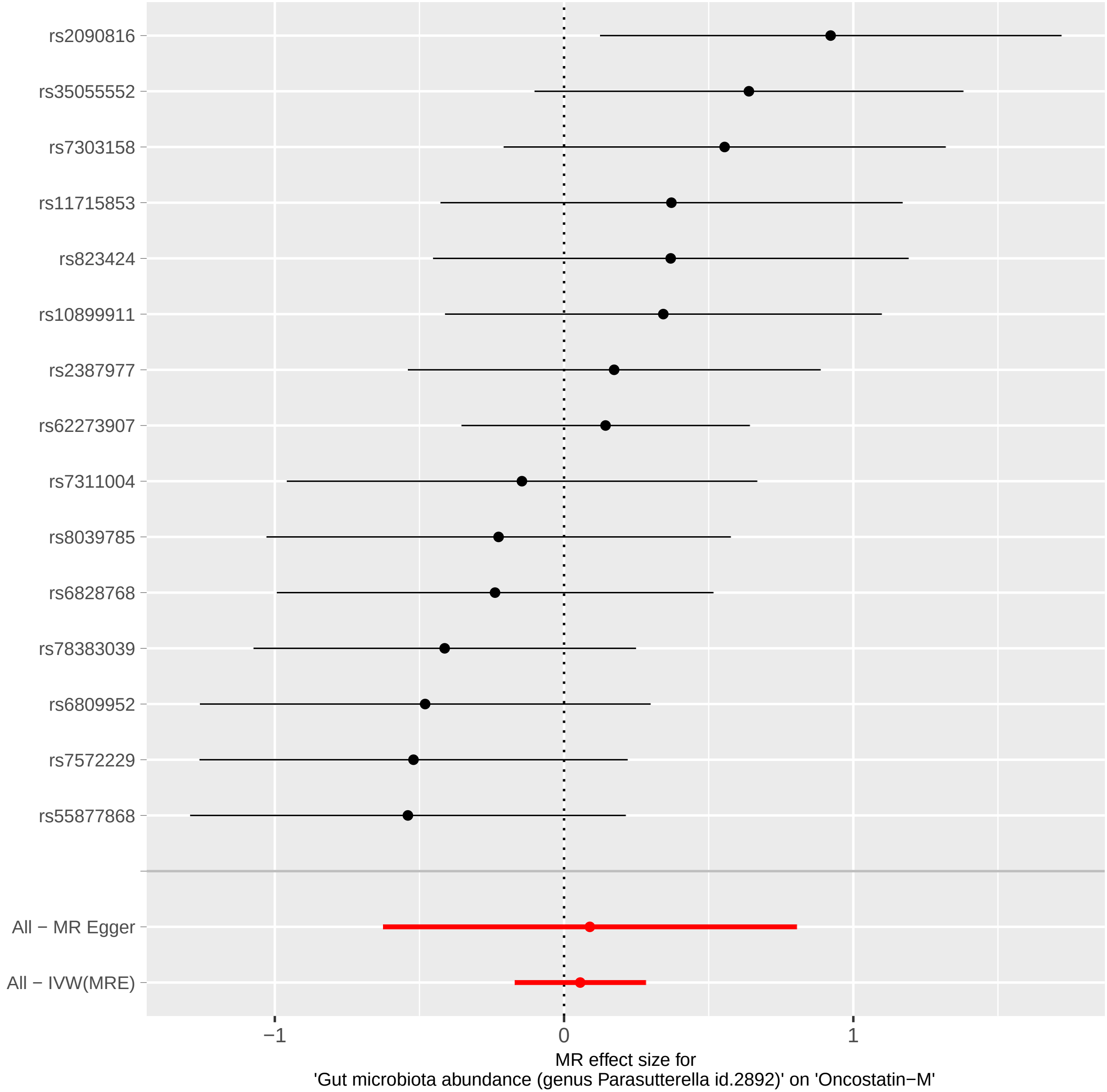

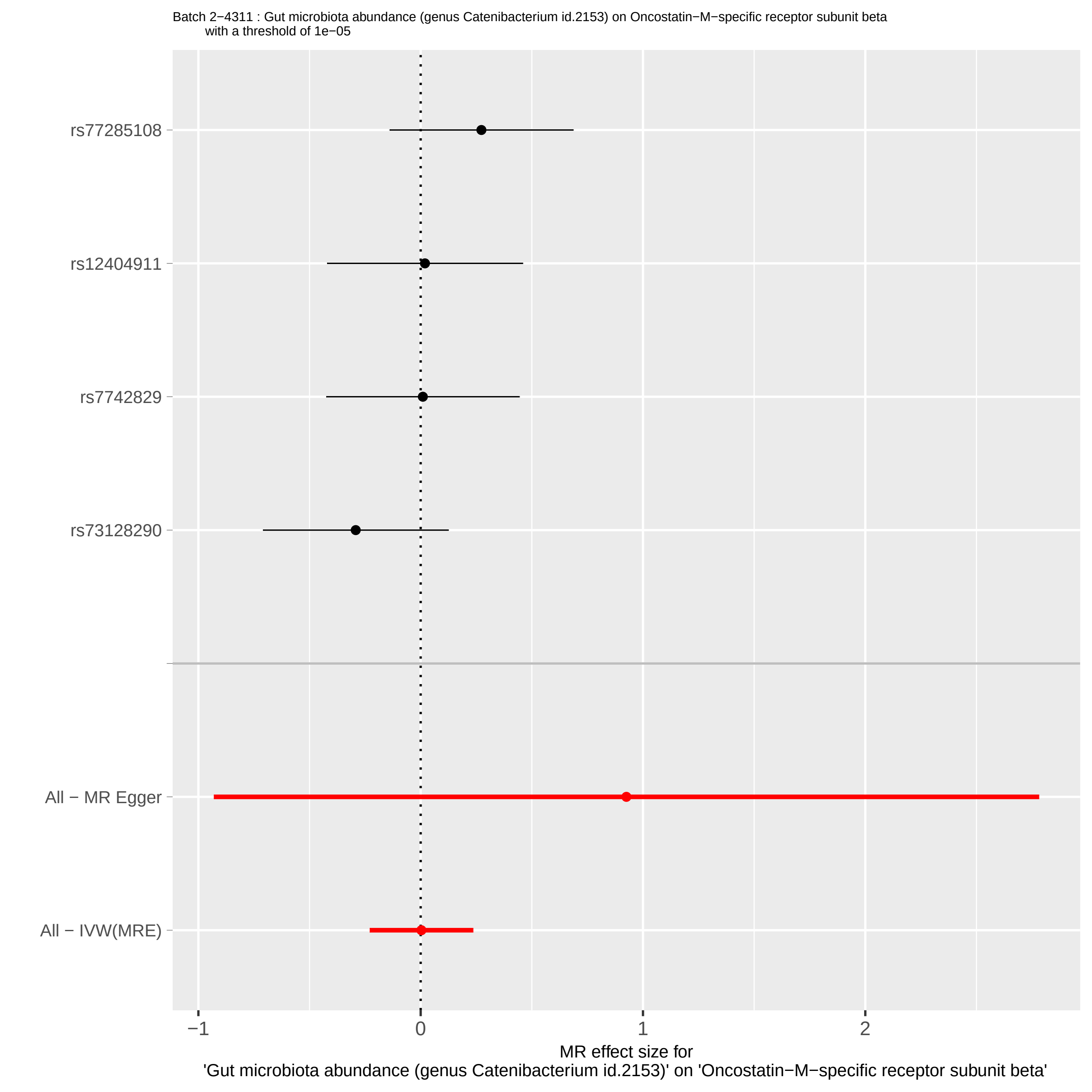

Batch 2−4311 : Gut microbiota abundance (genus Catenibacterium id.2153) on Oncostatin−M−specific receptor subunit beta
with a threshold of 1e−05
rs77285108
rs12404911
rs7742829
rs73128290
All − MR Egger
All − IVW(MRE)
−1
0
1
2
MR effect size for
'Gut microbiota abundance (genus Catenibacterium id.2153)' on 'Oncostatin−M−specific receptor subunit beta'

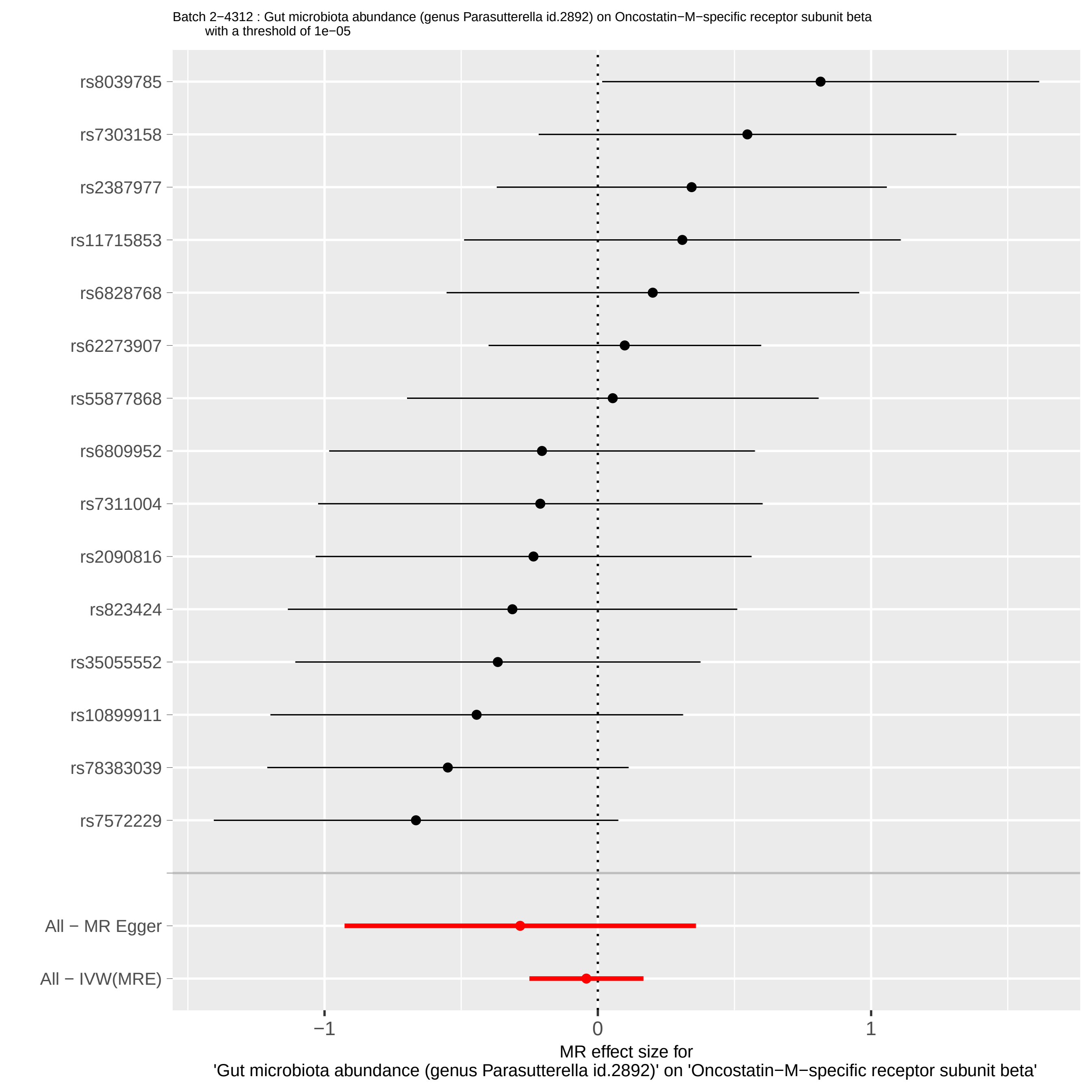
Batch 2−4312 : Gut microbiota abundance (genus Parasutterella id.2892) on Oncostatin−M−specific receptor subunit beta
with a threshold of 1e−05
rs8039785
rs7303158
rs2387977
rs11715853
rs6828768
rs62273907
rs55877868
rs6809952
rs7311004
rs2090816
rs823424
rs35055552
rs10899911
rs78383039
rs7572229
All − MR Egger
All − IVW(MRE)
−1
0
1
MR effect size for
'Gut microbiota abundance (genus Parasutterella id.2892)' on 'Oncostatin−M−specific receptor subunit beta'

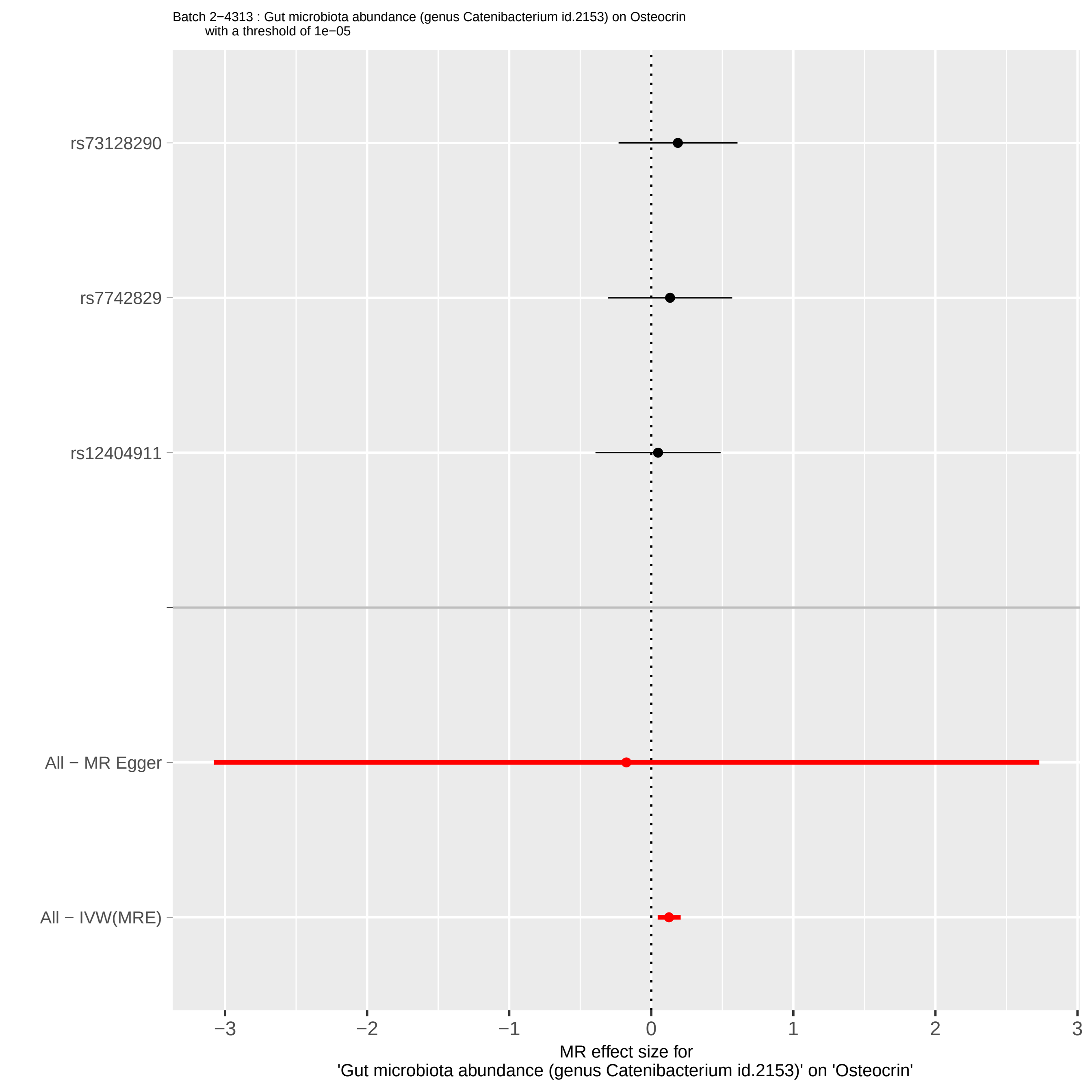

Batch 2−4313 : Gut microbiota abundance (genus Catenibacterium id.2153) on Osteocrin
with a threshold of 1e−05
rs73128290
rs7742829
rs12404911
All − MR Egger
All − IVW(MRE)
−3
−2
−1
0
1
2
3
MR effect size for
'Gut microbiota abundance (genus Catenibacterium id.2153)' on 'Osteocrin'

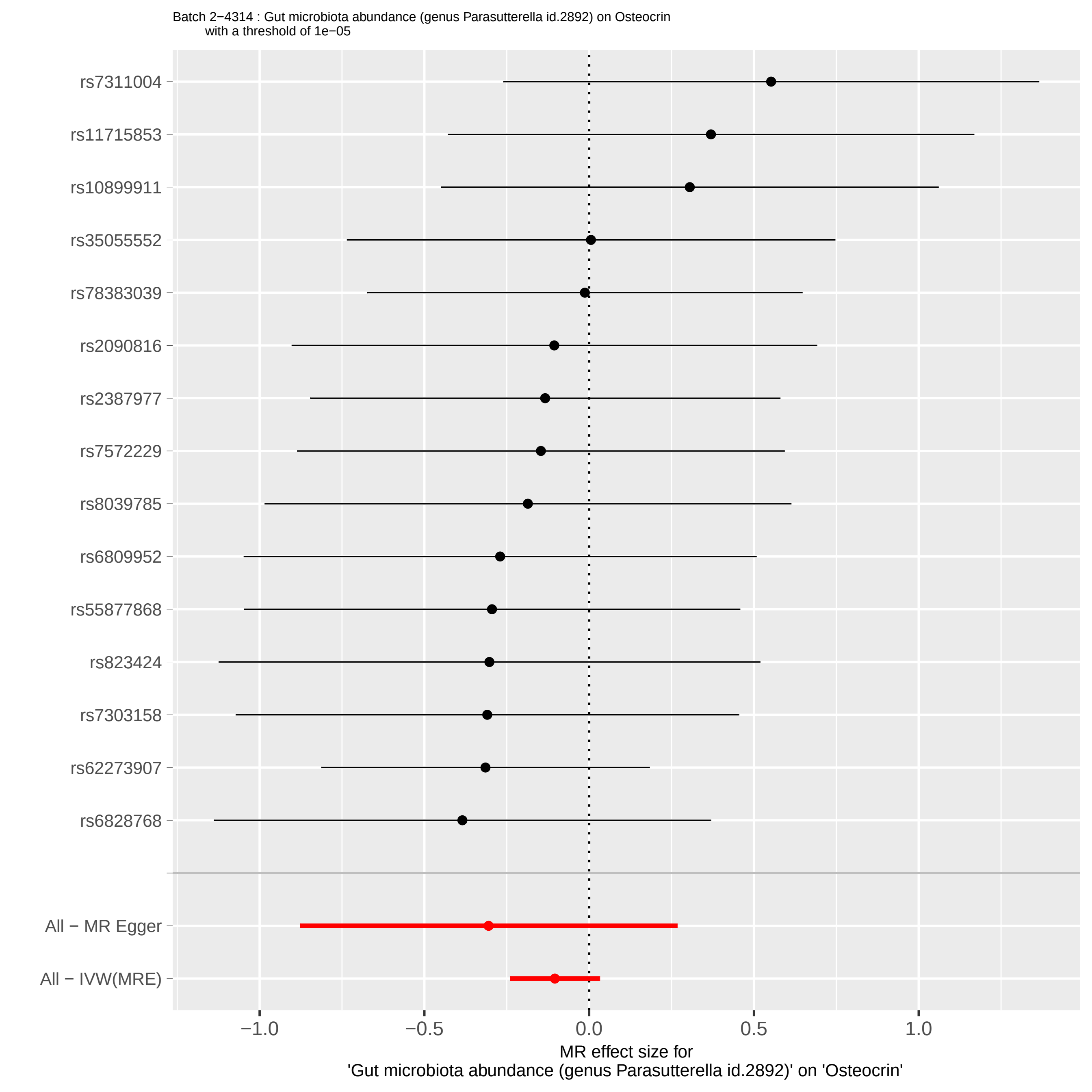
Batch 2−4314 : Gut microbiota abundance (genus Parasutterella id.2892) on Osteocrin
with a threshold of 1e−05
rs7311004
rs11715853
rs10899911
rs35055552
rs78383039
rs2090816
rs2387977
rs7572229
rs8039785
rs6809952
rs55877868
rs823424
rs7303158
rs62273907
rs6828768
All − MR Egger
All − IVW(MRE)
−1.0
−0.5
0.0
0.5
1.0
MR effect size for
'Gut microbiota abundance (genus Parasutterella id.2892)' on 'Osteocrin'

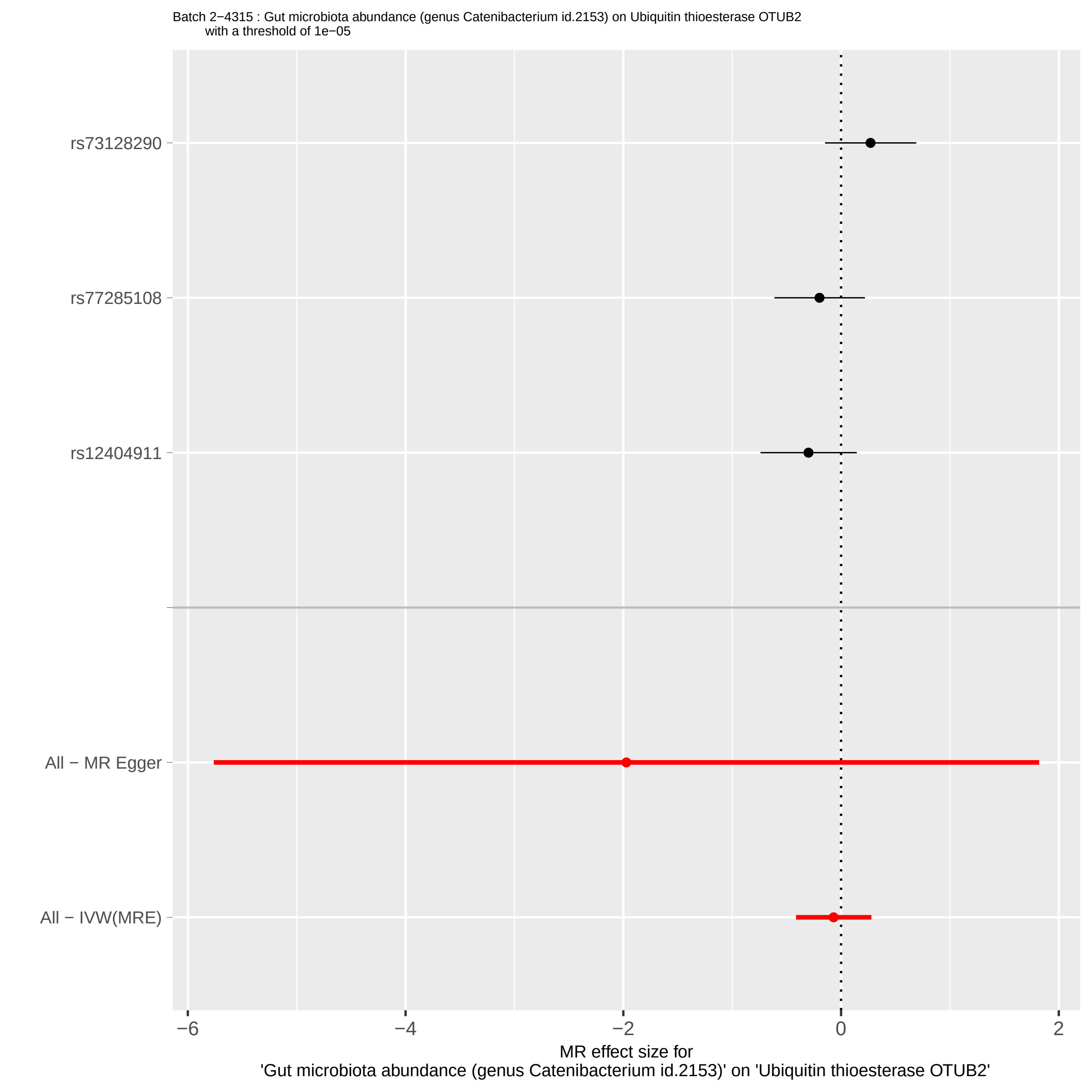

Batch 2−4315 : Gut microbiota abundance (genus Catenibacterium id.2153) on Ubiquitin thioesterase OTUB2
with a threshold of 1e−05
rs73128290
rs77285108
rs12404911
All − MR Egger
All − IVW(MRE)
−6
−4
−2
0
2
MR effect size for
'Gut microbiota abundance (genus Catenibacterium id.2153)' on 'Ubiquitin thioesterase OTUB2'

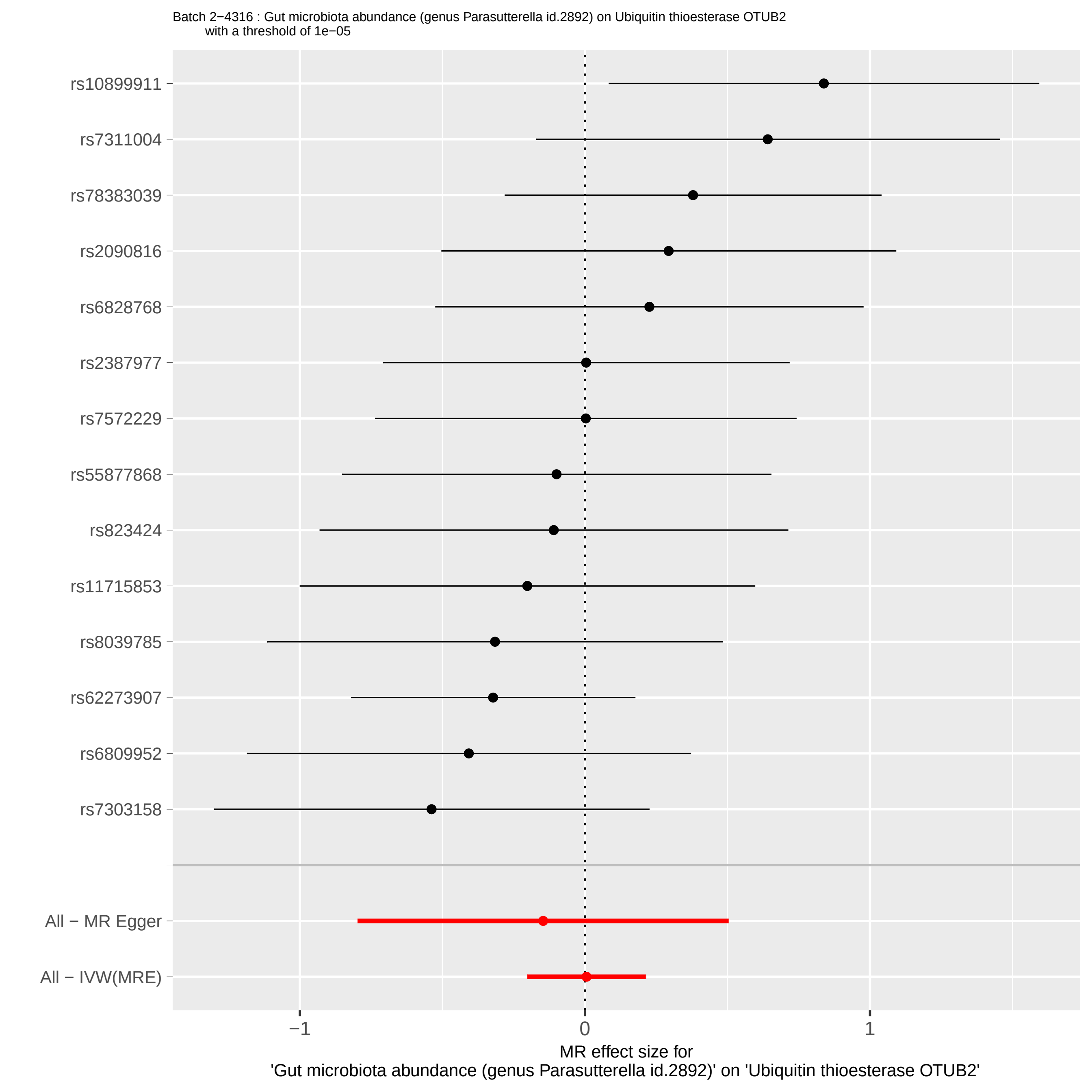

Batch 2−4316 : Gut microbiota abundance (genus Parasutterella id.2892) on Ubiquitin thioesterase OTUB2
with a threshold of 1e−05
rs10899911
rs7311004
rs78383039
rs2090816
rs6828768
rs2387977
rs7572229
rs55877868
rs823424
rs11715853
rs8039785
rs62273907
rs6809952
rs7303158
All − MR Egger
All − IVW(MRE)
−1
0
1
MR effect size for
'Gut microbiota abundance (genus Parasutterella id.2892)' on 'Ubiquitin thioesterase OTUB2'

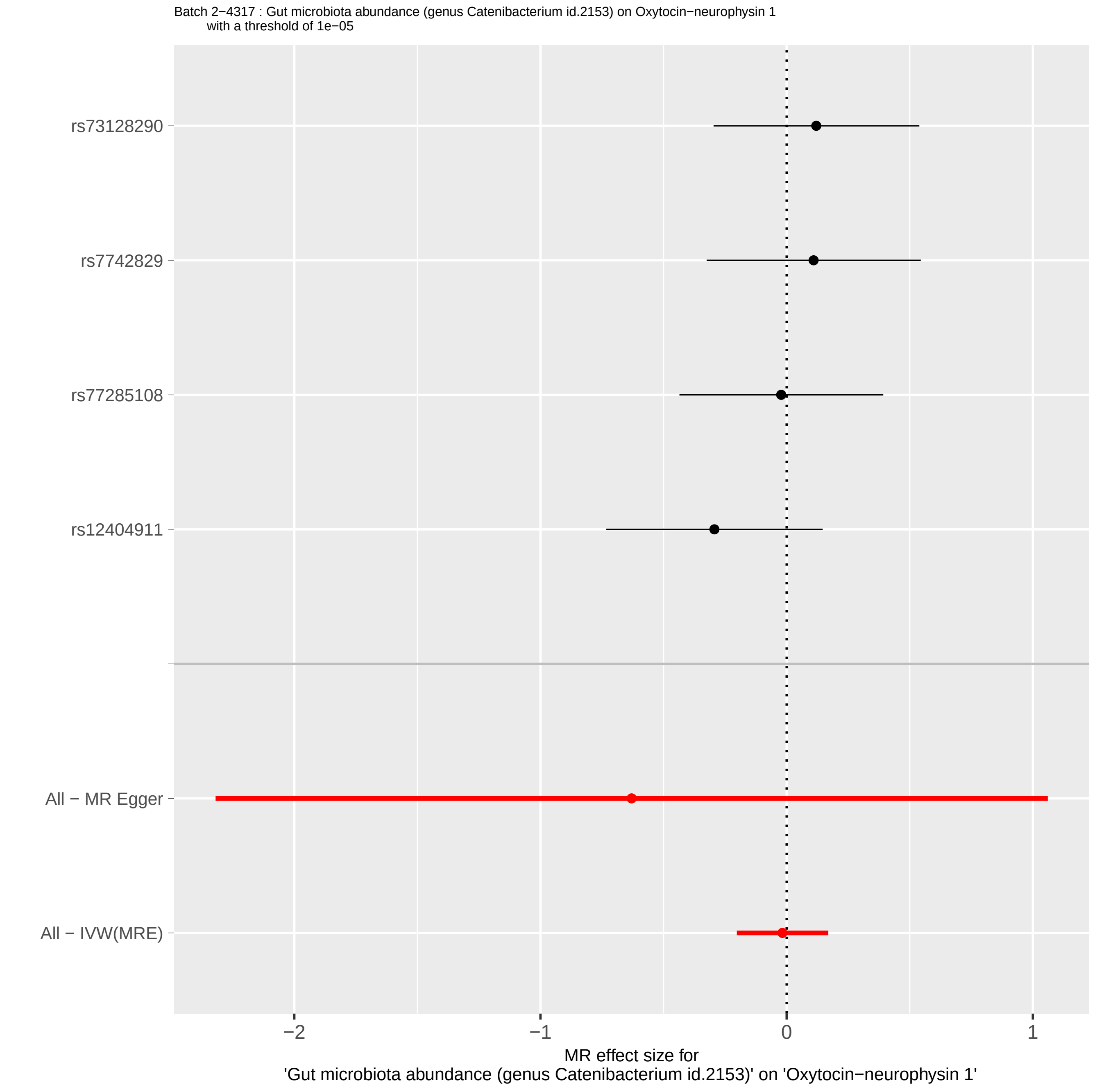

Batch 2−4317 : Gut microbiota abundance (genus Catenibacterium id.2153) on Oxytocin−neurophysin 1
with a threshold of 1e−05
rs73128290
rs7742829
rs77285108
rs12404911
All − MR Egger
All − IVW(MRE)
−2
−1
0
1
MR effect size for
'Gut microbiota abundance (genus Catenibacterium id.2153)' on 'Oxytocin−neurophysin 1'

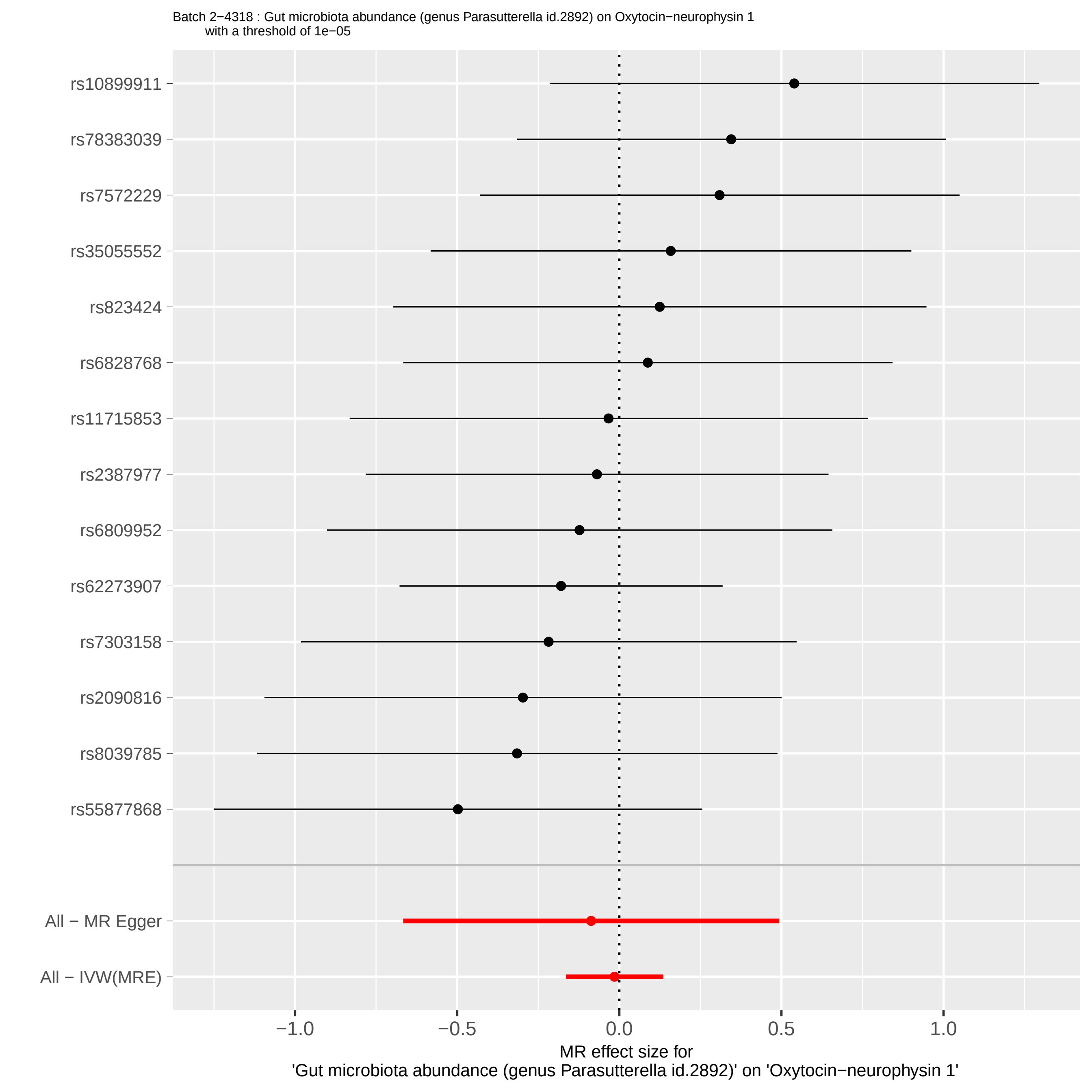

Batch 2−4318 : Gut microbiota abundance (genus Parasutterella id.2892) on Oxytocin−neurophysin 1
with a threshold of 1e−05
rs10899911
rs78383039
rs7572229
rs35055552
rs823424
rs6828768
rs11715853
rs2387977
rs6809952
rs62273907
rs7303158
rs2090816
rs8039785
rs55877868
All − MR Egger
All − IVW(MRE)
−1.0
−0.5
0.0
0.5
1.0
MR effect size for
'Gut microbiota abundance (genus Parasutterella id.2892)' on 'Oxytocin−neurophysin 1'

Batch 2−4319 : Gut microbiota abundance (genus Catenibacterium id.2153) on P2X purinoceptor 6
with a threshold of 1e−05

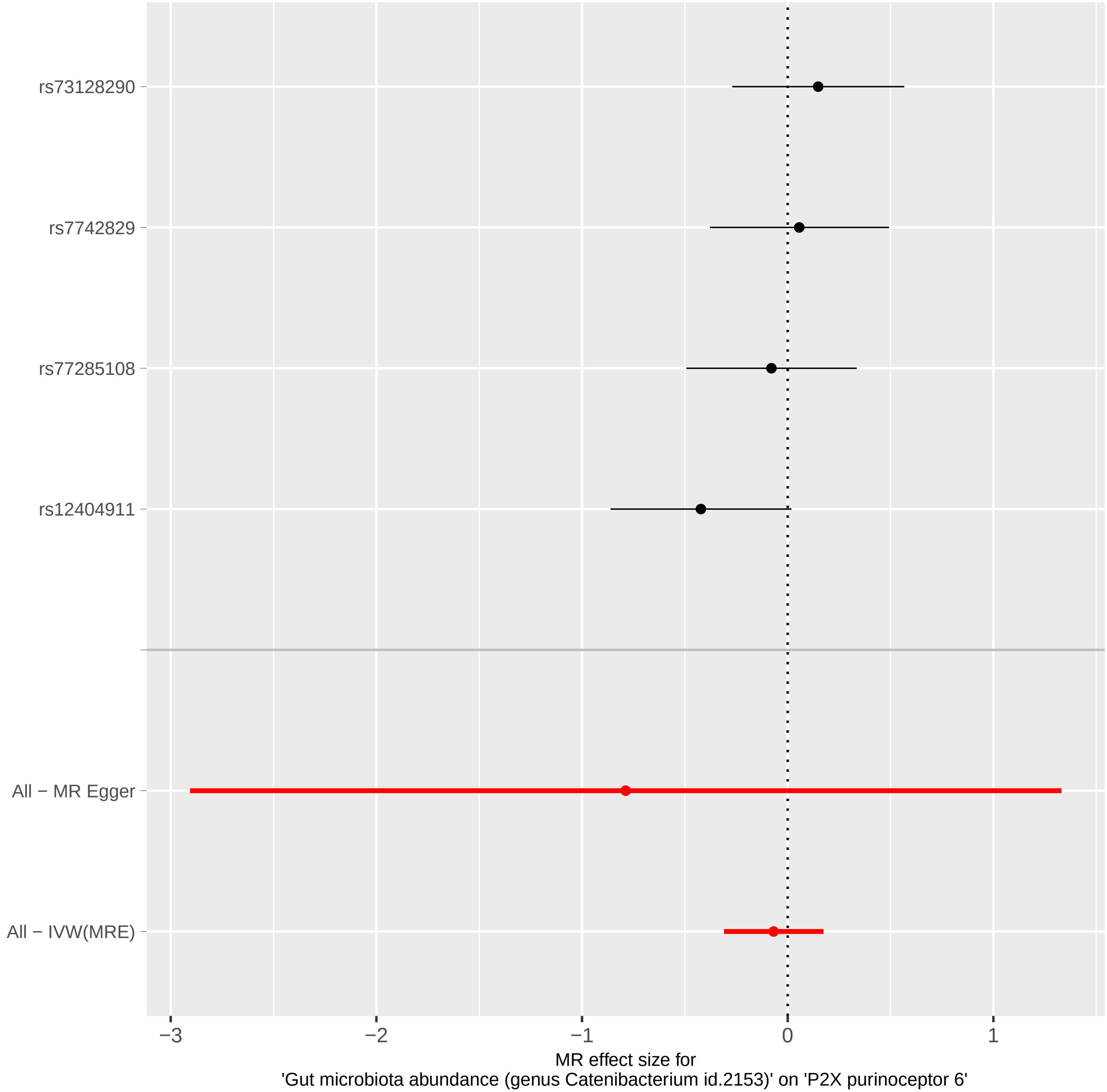

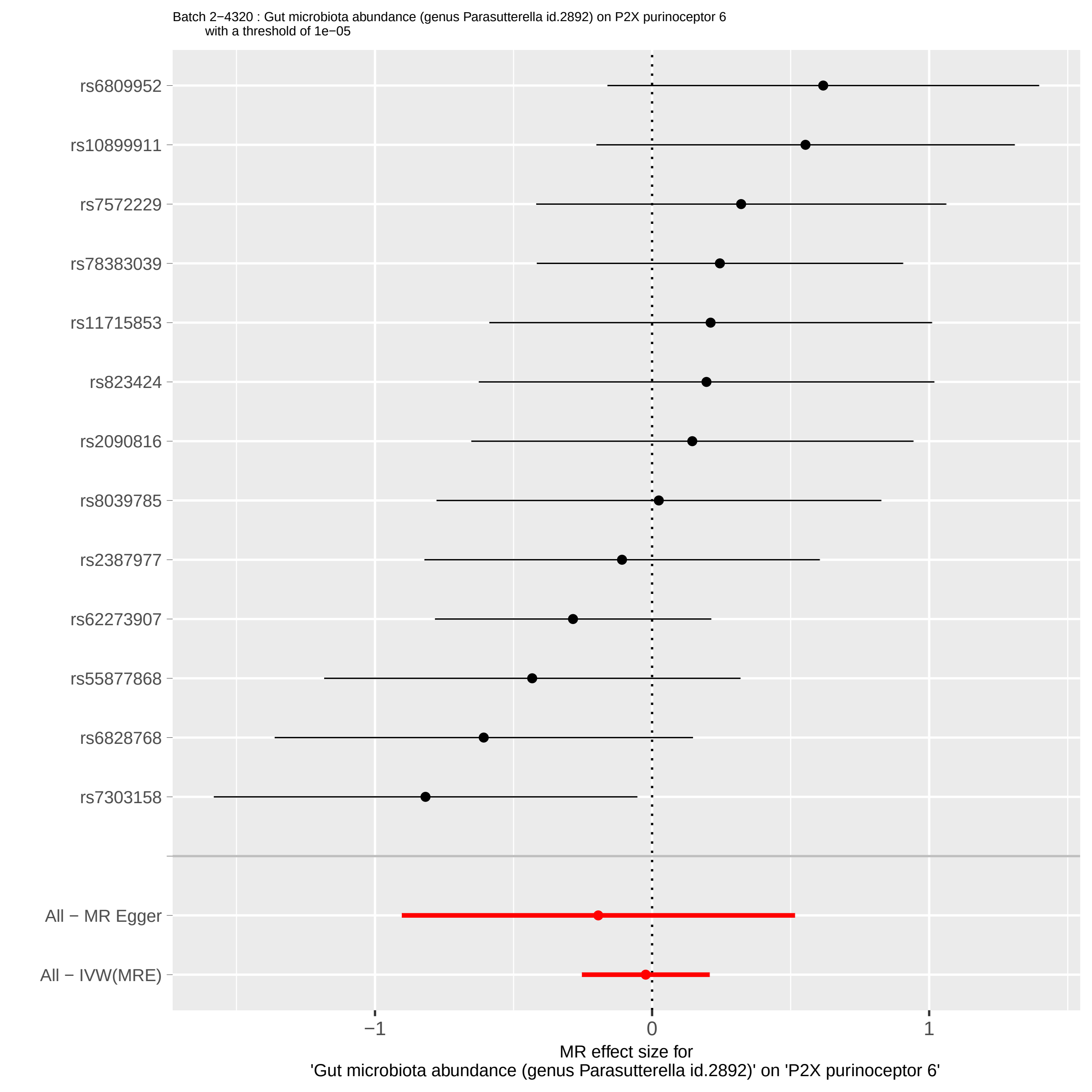
Batch 2−4320 : Gut microbiota abundance (genus Parasutterella id.2892) on P2X purinoceptor 6
with a threshold of 1e−05
rs6809952
rs10899911
rs7572229
rs78383039
rs11715853
rs823424
rs2090816
rs8039785
rs2387977
rs62273907
rs55877868
rs6828768
rs7303158
All − MR Egger
All − IVW(MRE)
−1
0
1
MR effect size for
'Gut microbiota abundance (genus Parasutterella id.2892)' on 'P2X purinoceptor 6'

Batch 2−4321 : Gut microbiota abundance (genus Catenibacterium id.2153) on Prolyl 4−hydroxylase subunit alpha−1
with a threshold of 1e−05

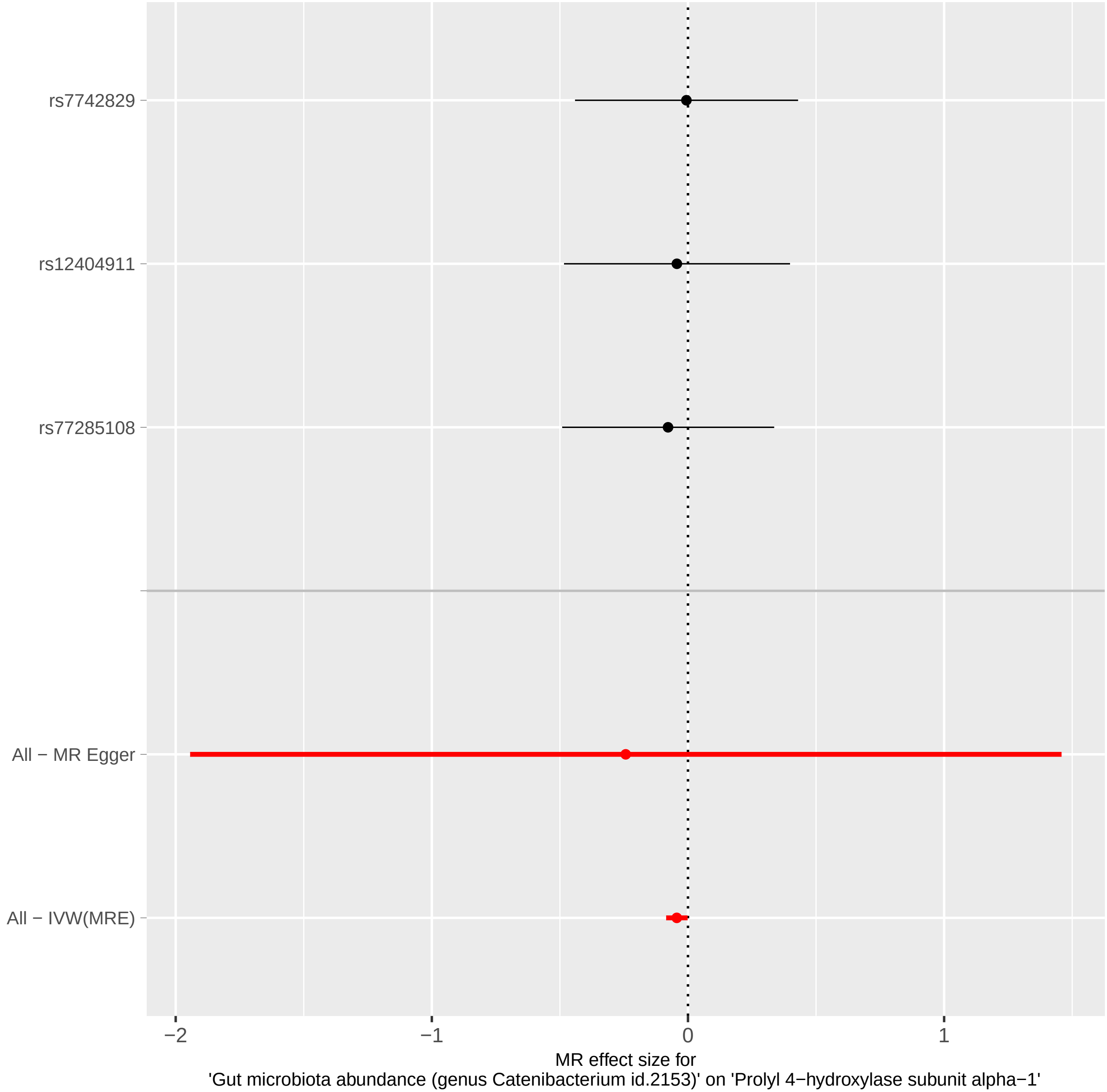

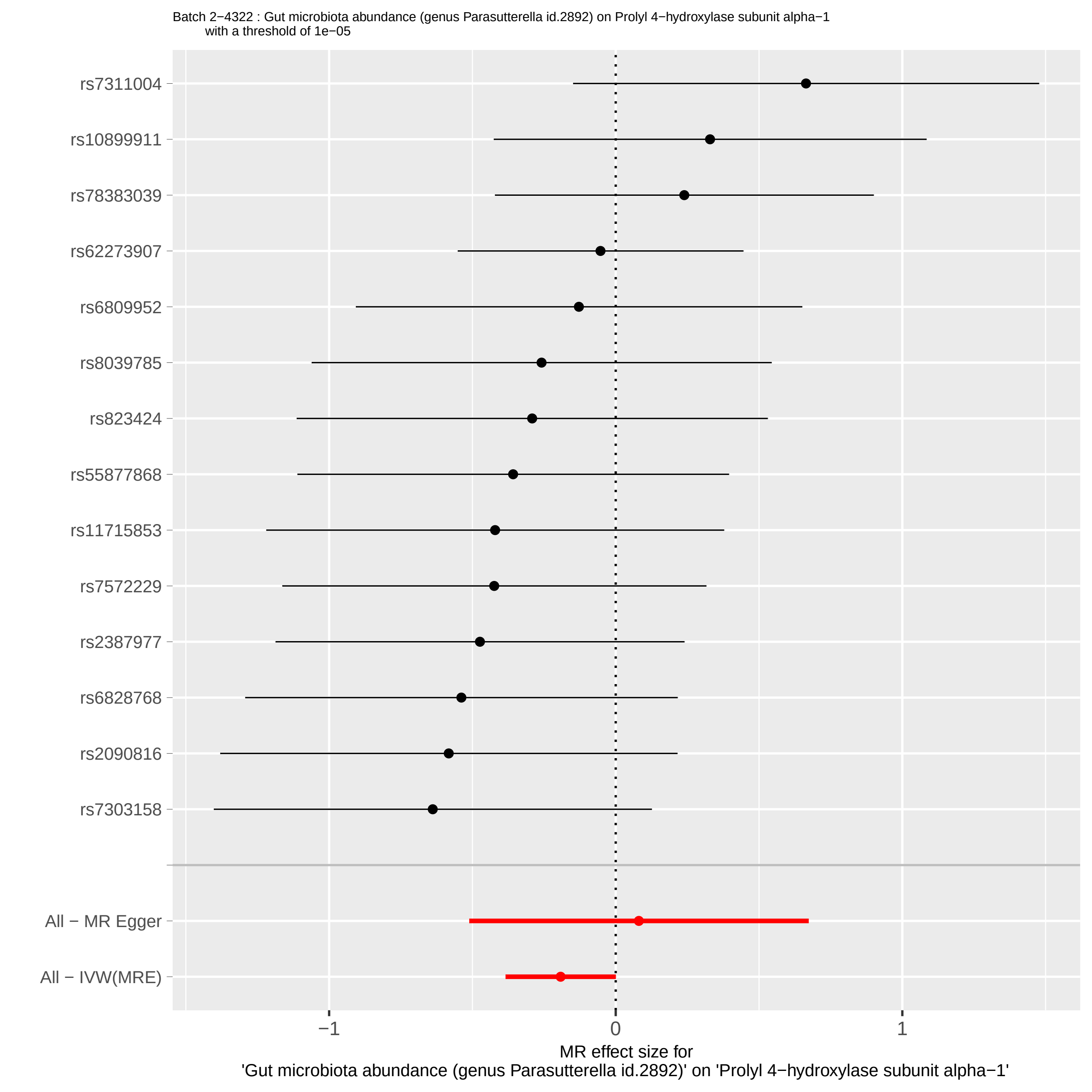

Batch 2−4322 : Gut microbiota abundance (genus Parasutterella id.2892) on Prolyl 4−hydroxylase subunit alpha−1
with a threshold of 1e−05
rs7311004
rs10899911
rs78383039
rs62273907
rs6809952
rs8039785
rs823424
rs55877868
rs11715853
rs7572229
rs2387977
rs6828768
rs2090816
rs7303158
All − MR Egger
All − IVW(MRE)
−1
0
1
MR effect size for
'Gut microbiota abundance (genus Parasutterella id.2892)' on 'Prolyl 4−hydroxylase subunit alpha−1'

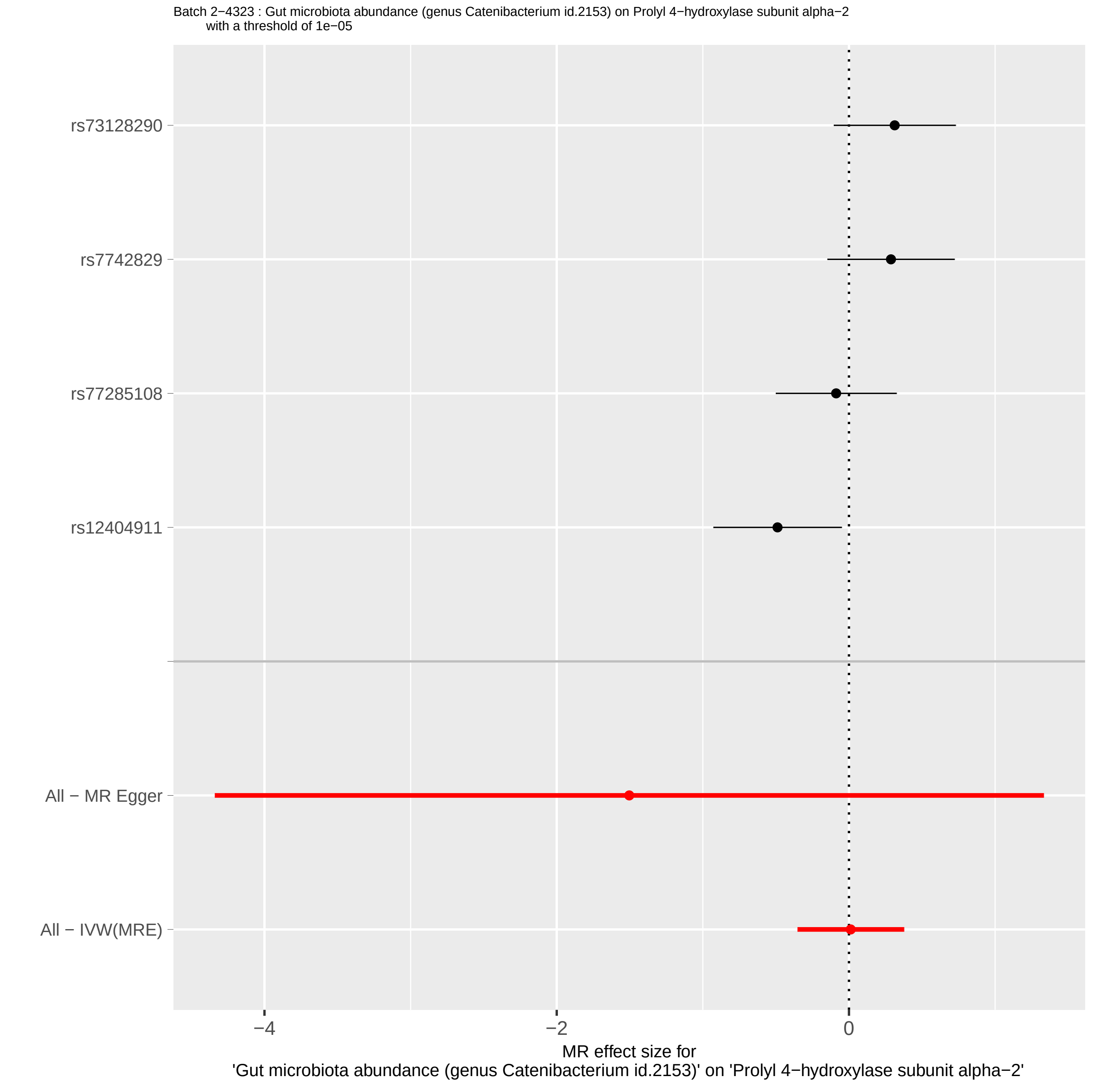

Batch 2−4323 : Gut microbiota abundance (genus Catenibacterium id.2153) on Prolyl 4−hydroxylase subunit alpha−2
with a threshold of 1e−05
rs73128290
rs7742829
rs77285108
rs12404911
All − MR Egger
All − IVW(MRE)
−4
−2
0
MR effect size for
'Gut microbiota abundance (genus Catenibacterium id.2153)' on 'Prolyl 4−hydroxylase subunit alpha−2'

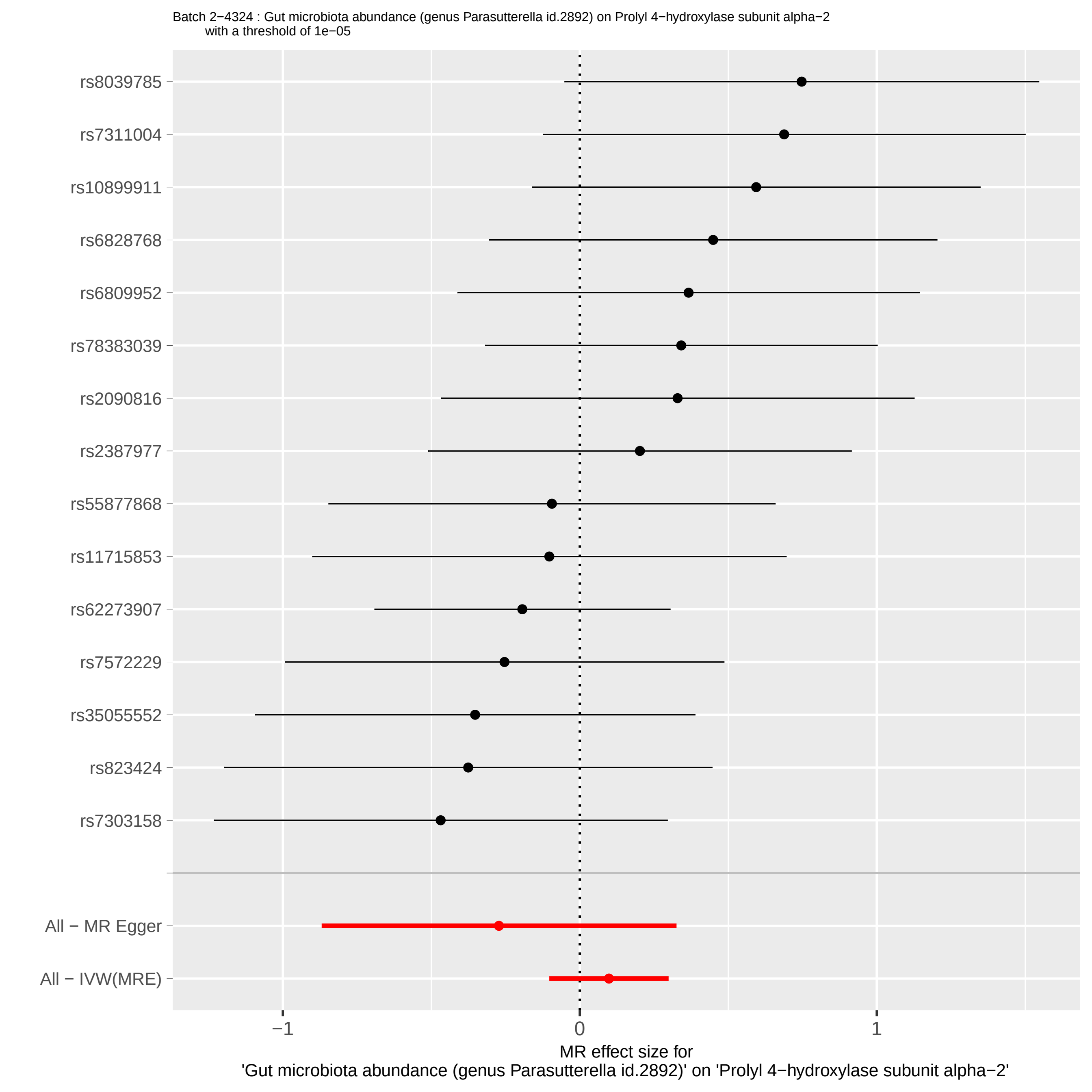
Batch 2−4324 : Gut microbiota abundance (genus Parasutterella id.2892) on Prolyl 4−hydroxylase subunit alpha−2
with a threshold of 1e−05
rs8039785
rs7311004
rs10899911
rs6828768
rs6809952
rs78383039
rs2090816
rs2387977
rs55877868
rs11715853
rs62273907
rs7572229
rs35055552
rs823424
rs7303158
All − MR Egger
All − IVW(MRE)
−1
0
1
MR effect size for
'Gut microbiota abundance (genus Parasutterella id.2892)' on 'Prolyl 4−hydroxylase subunit alpha−2'

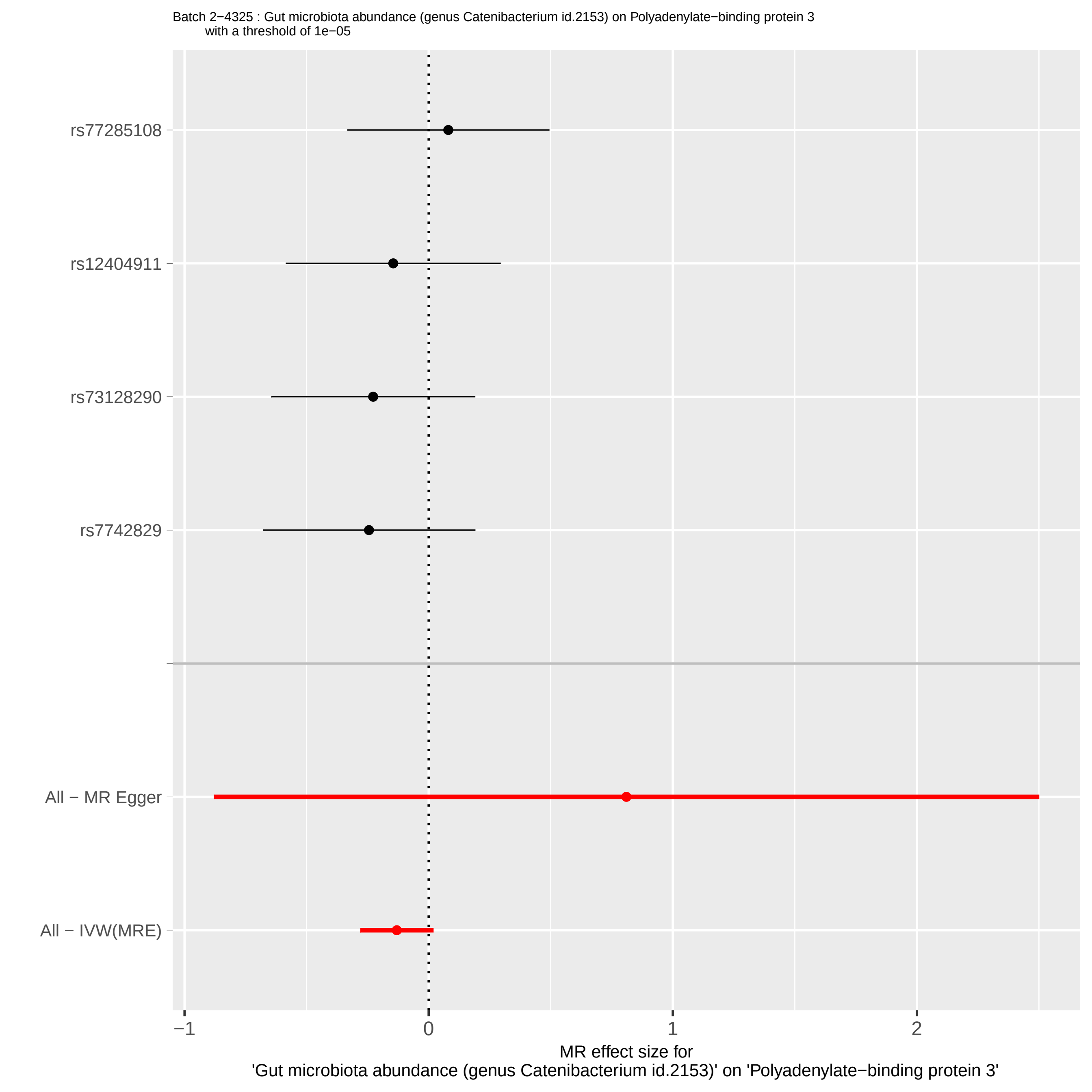

Batch 2−4325 : Gut microbiota abundance (genus Catenibacterium id.2153) on Polyadenylate−binding protein 3
with a threshold of 1e−05
rs77285108
rs12404911
rs73128290
rs7742829
All − MR Egger
All − IVW(MRE)
−1
0
1
2
MR effect size for
'Gut microbiota abundance (genus Catenibacterium id.2153)' on 'Polyadenylate−binding protein 3'

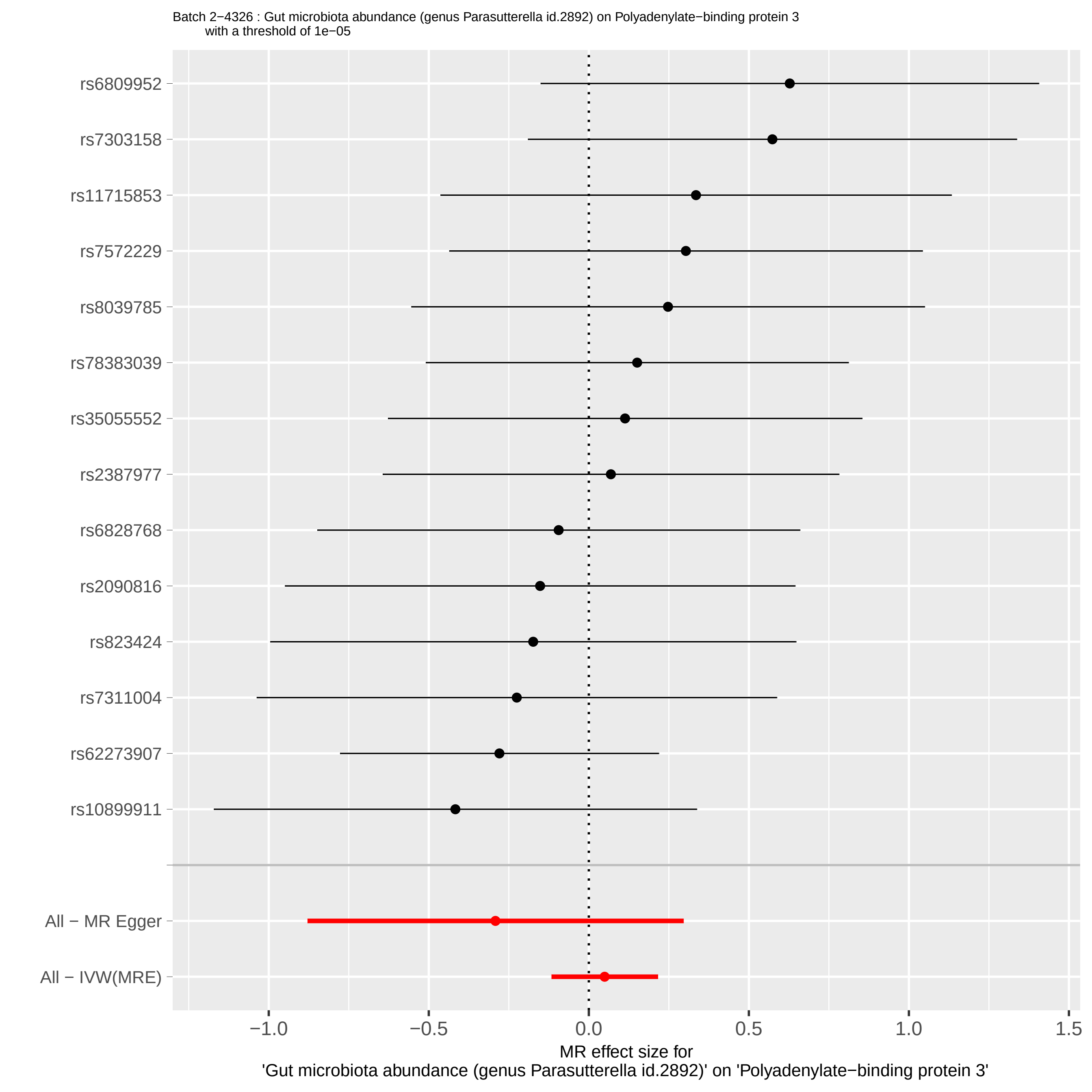

Batch 2−4326 : Gut microbiota abundance (genus Parasutterella id.2892) on Polyadenylate−binding protein 3
with a threshold of 1e−05
rs6809952
rs7303158
rs11715853
rs7572229
rs8039785
rs78383039
rs35055552
rs2387977
rs6828768
rs2090816
rs823424
rs7311004
rs62273907
rs10899911
All − MR Egger
All − IVW(MRE)
−1.0
−0.5
0.0
0.5
1.0
1.5
MR effect size for
'Gut microbiota abundance (genus Parasutterella id.2892)' on 'Polyadenylate−binding protein 3'

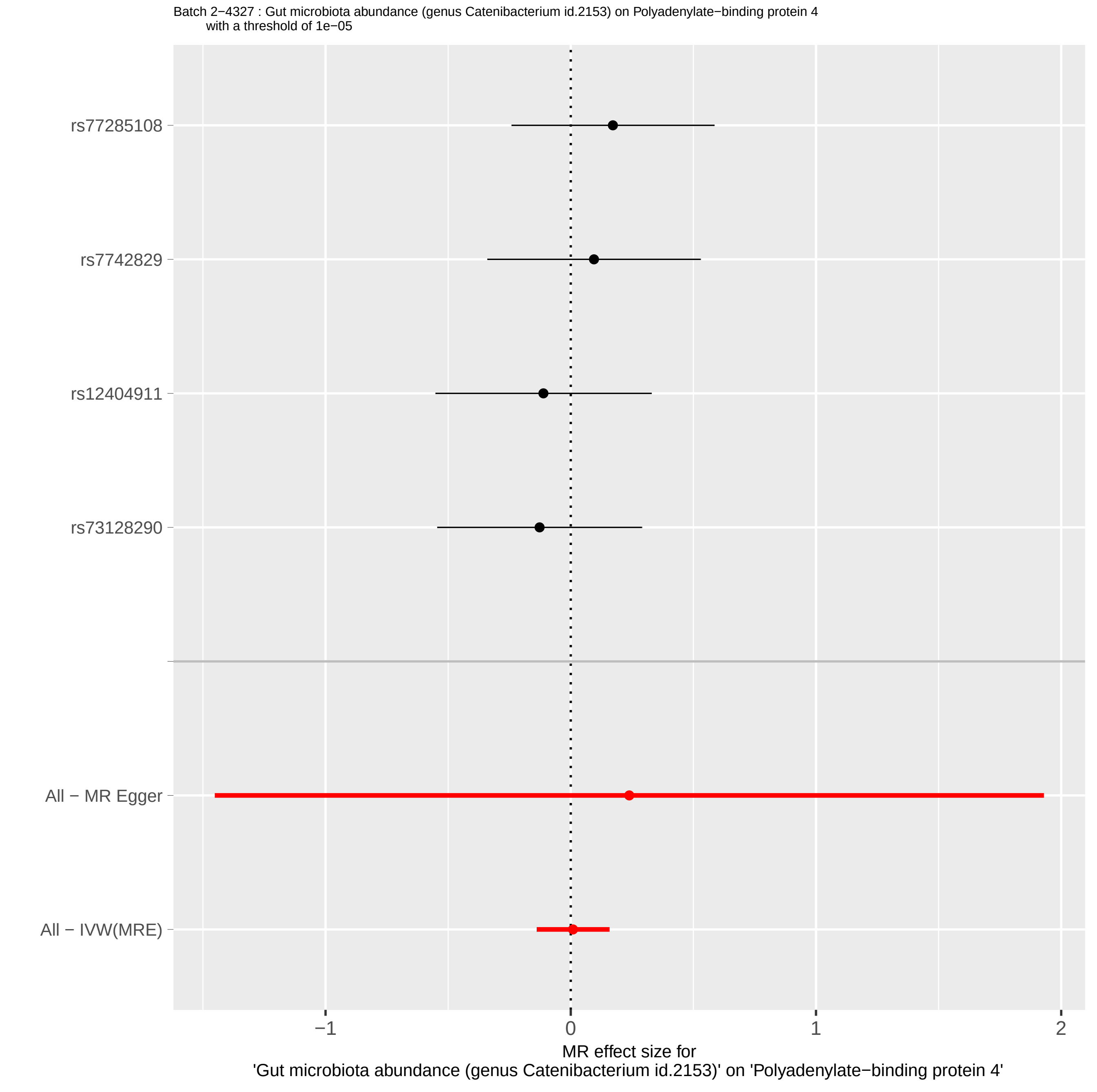

Batch 2−4327 : Gut microbiota abundance (genus Catenibacterium id.2153) on Polyadenylate−binding protein 4
with a threshold of 1e−05
rs77285108
rs7742829
rs12404911
rs73128290
All − MR Egger
All − IVW(MRE)
−1
0
1
2
MR effect size for
'Gut microbiota abundance (genus Catenibacterium id.2153)' on 'Polyadenylate−binding protein 4'

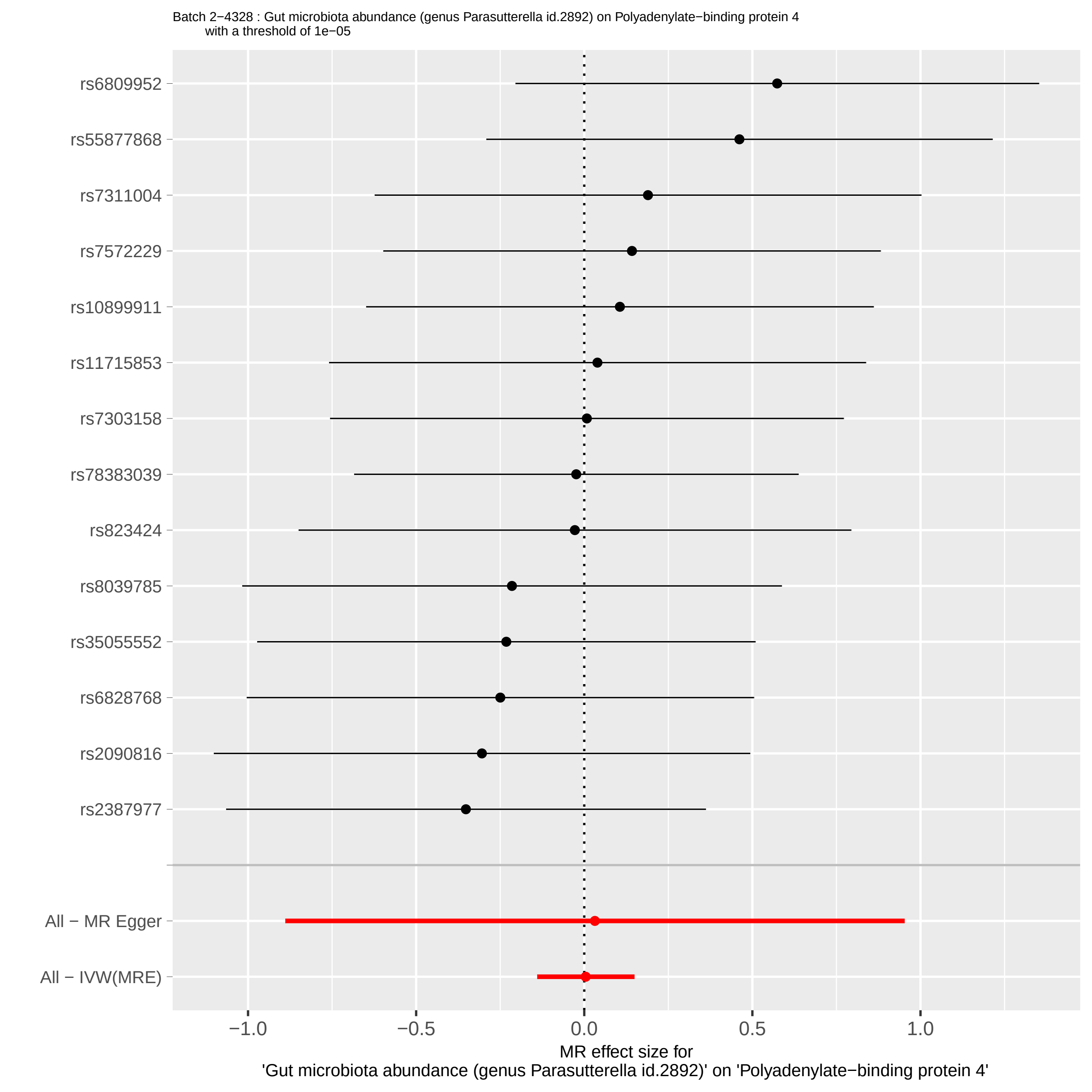

Batch 2−4328 : Gut microbiota abundance (genus Parasutterella id.2892) on Polyadenylate−binding protein 4
with a threshold of 1e−05
rs6809952
rs55877868
rs7311004
rs7572229
rs10899911
rs11715853
rs7303158
rs78383039
rs823424
rs8039785
rs35055552
rs6828768
rs2090816
rs2387977
All − MR Egger
All − IVW(MRE)
−1.0
−0.5
0.0
0.5
1.0
MR effect size for
'Gut microbiota abundance (genus Parasutterella id.2892)' on 'Polyadenylate−binding protein 4'

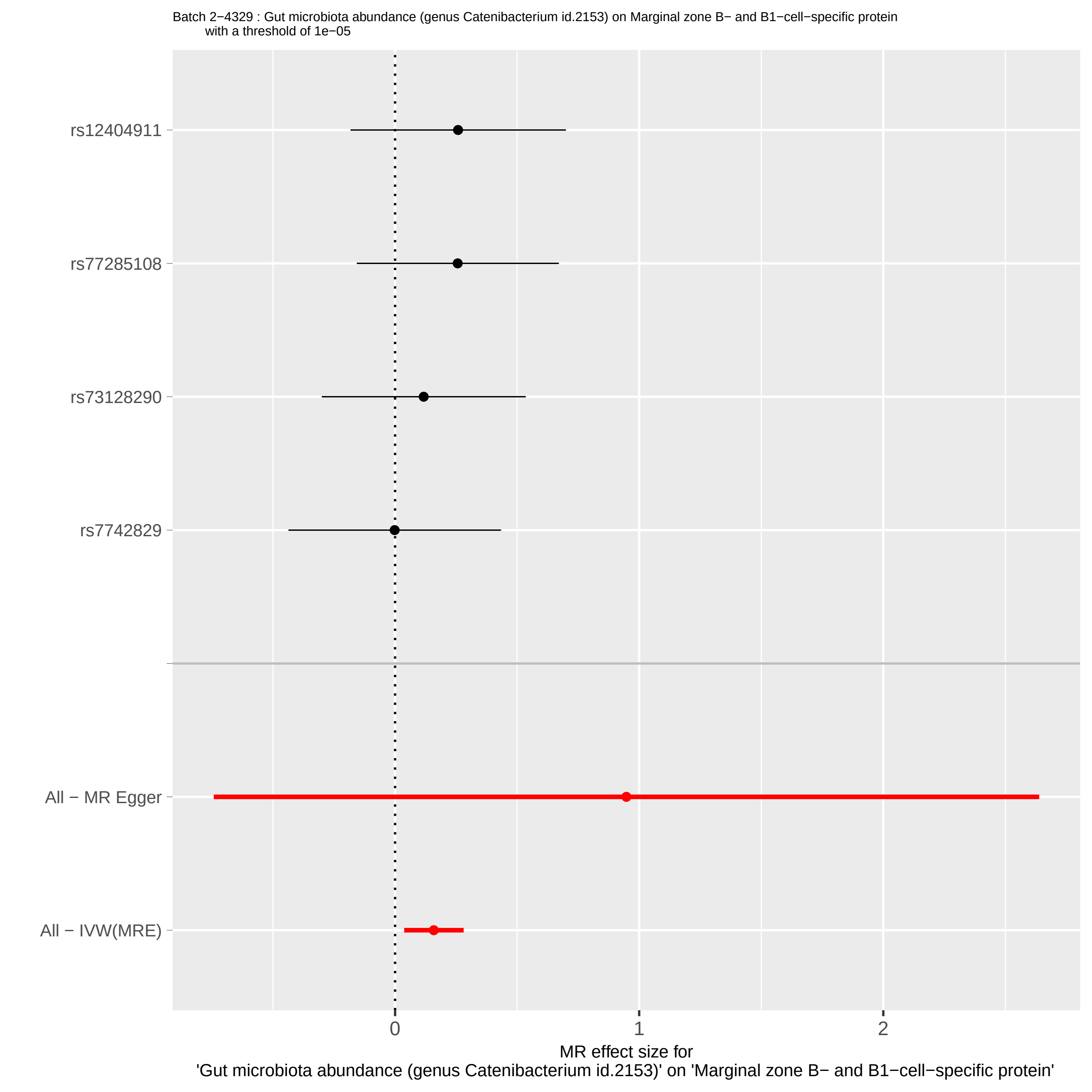
Batch 2−4329 : Gut microbiota abundance (genus Catenibacterium id.2153) on Marginal zone B− and B1−cell−specific protein with a threshold of 1e−05
rs12404911
rs77285108
rs73128290
rs7742829
All − MR Egger
All − IVW(MRE)
0
1
2
MR effect size for
'Gut microbiota abundance (genus Catenibacterium id.2153)' on 'Marginal zone B− and B1−cell−specific protein'

Batch 2−4330 : Gut microbiota abundance (genus Parasutterella id.2892) on Marginal zone B− and B1−cell−specific protein
with a threshold of 1e−05

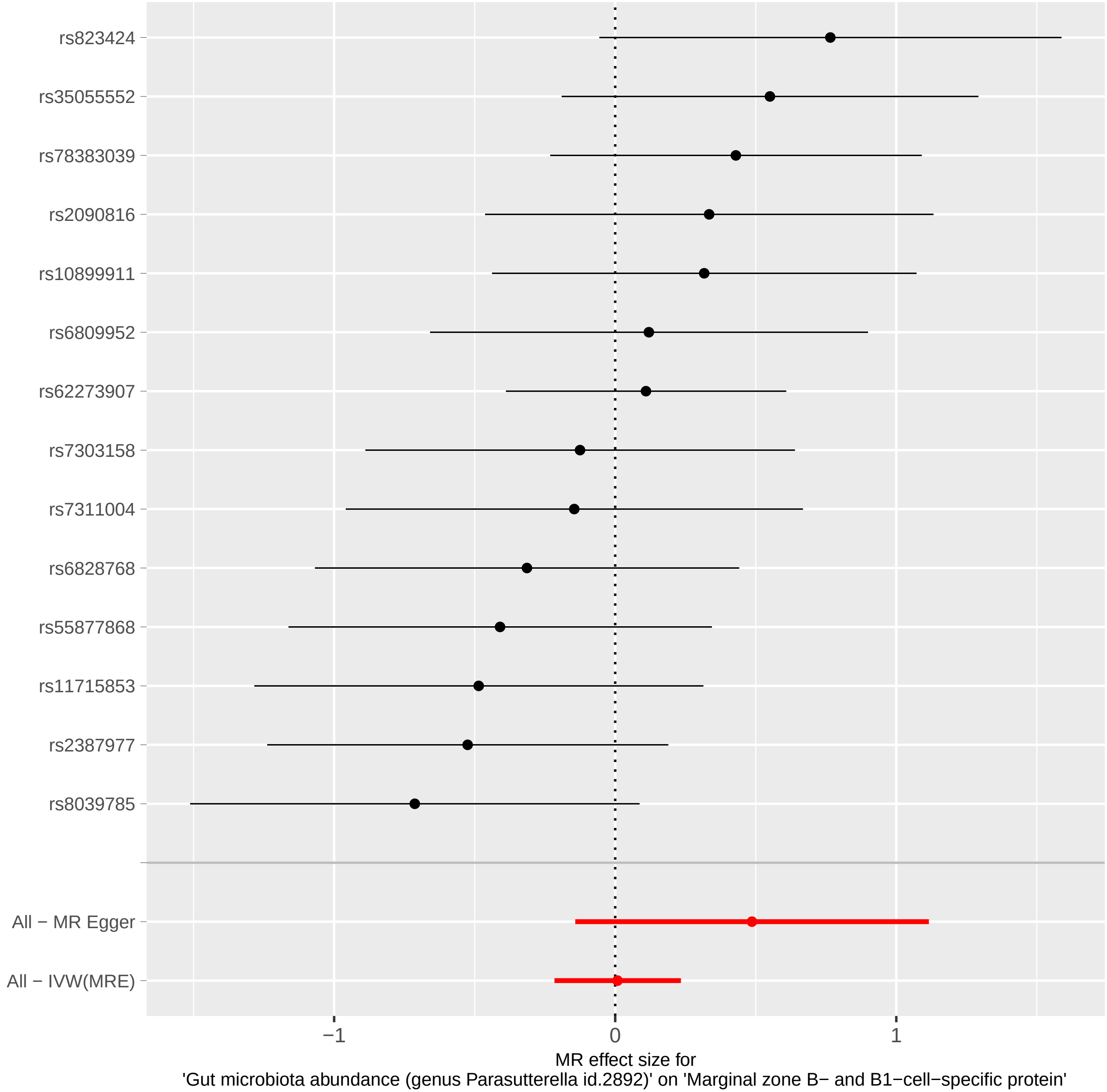

Batch 2−4331 : Gut microbiota abundance (genus Catenibacterium id.2153) on Platelet−activating factor acetylhydrolase IB subunit beta
with a threshold of 1e−05

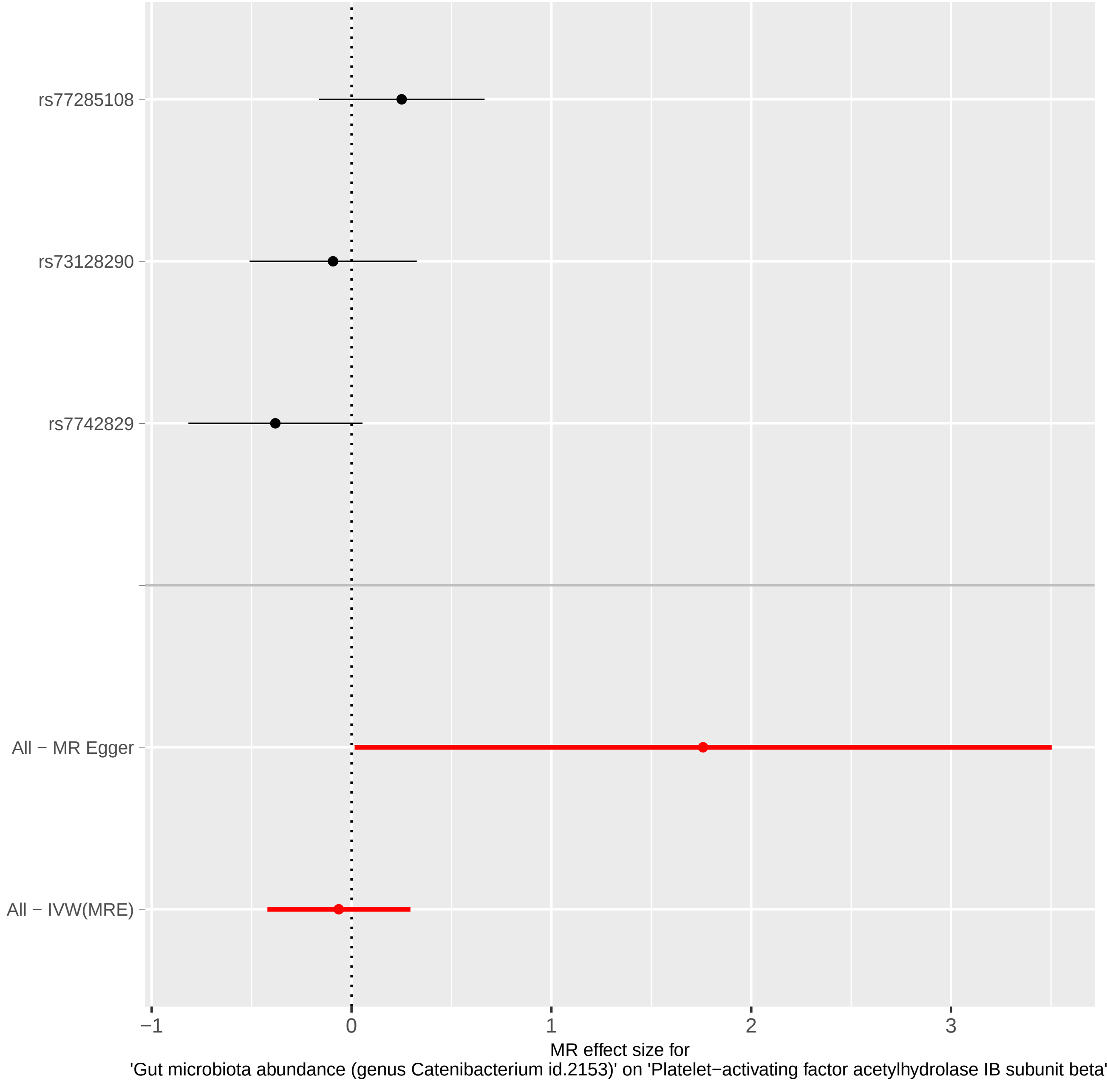

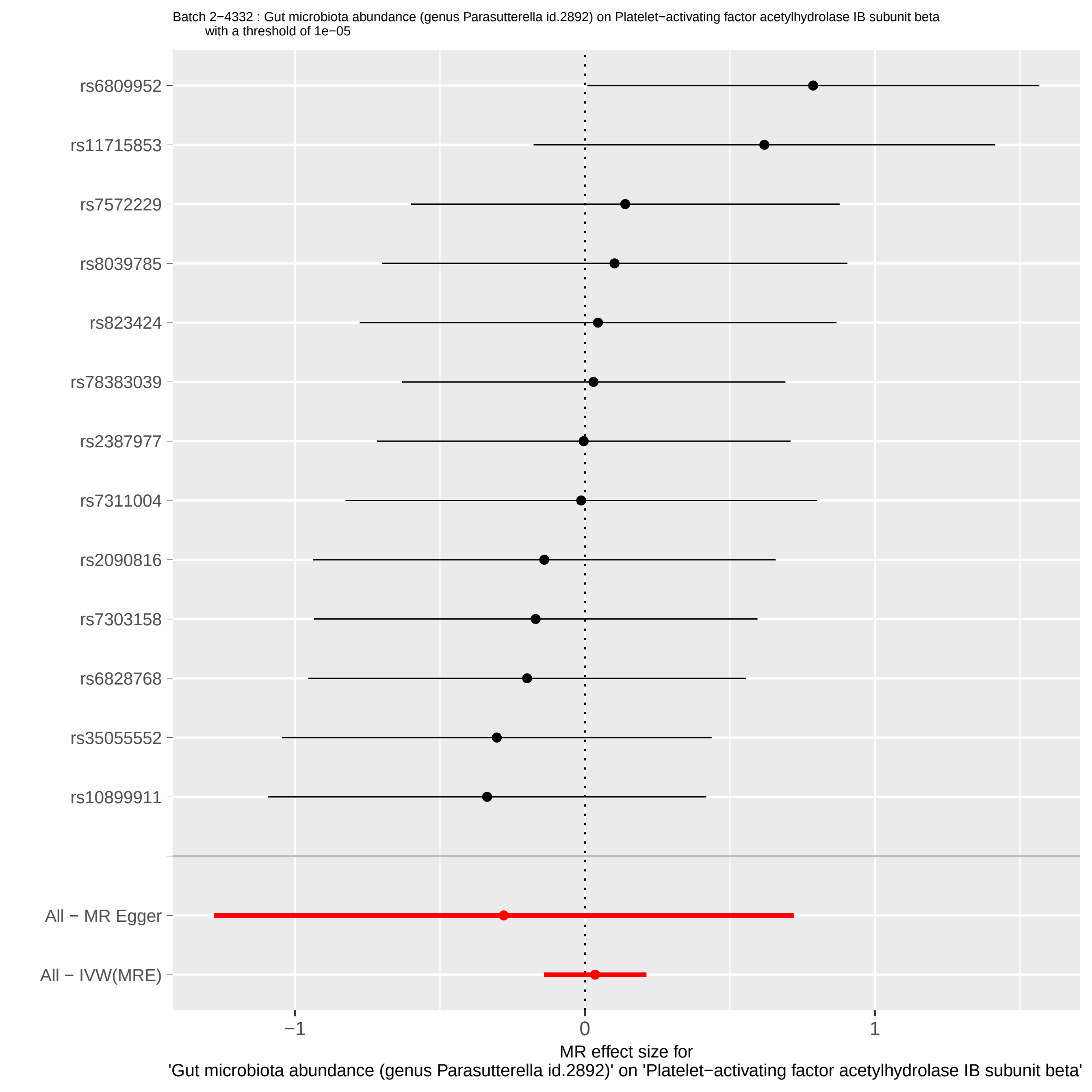
Batch 2−4332 : Gut microbiota abundance (genus Parasutterella id.2892) on Platelet−activating factor acetylhydrolase IB subunit beta
with a threshold of 1e−05
rs6809952
rs11715853
rs7572229
rs8039785
rs823424
rs78383039
rs2387977
rs7311004
rs2090816
rs7303158
rs6828768
rs35055552
rs10899911
All − MR Egger
All − IVW(MRE)
−1
0
1
MR effect size for
'Gut microbiota abundance (genus Parasutterella id.2892)' on 'Platelet−activating factor acetylhydrolase IB subunit beta'

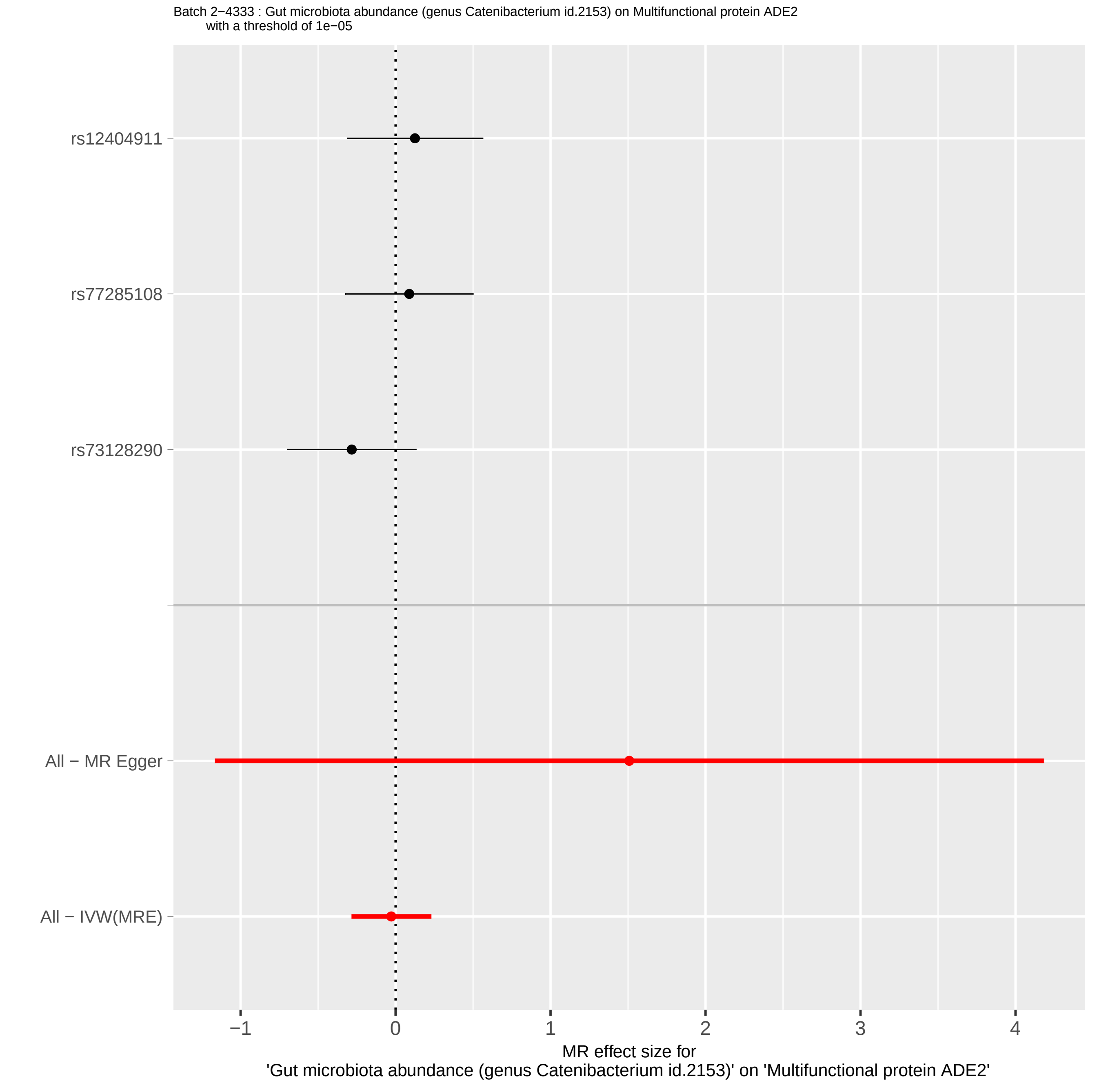
Batch 2−4333 : Gut microbiota abundance (genus Catenibacterium id.2153) on Multifunctional protein ADE2
with a threshold of 1e−05
rs12404911
rs77285108
rs73128290
All − MR Egger
All − IVW(MRE)
−1
0
1
2
3
4
MR effect size for
'Gut microbiota abundance (genus Catenibacterium id.2153)' on 'Multifunctional protein ADE2'

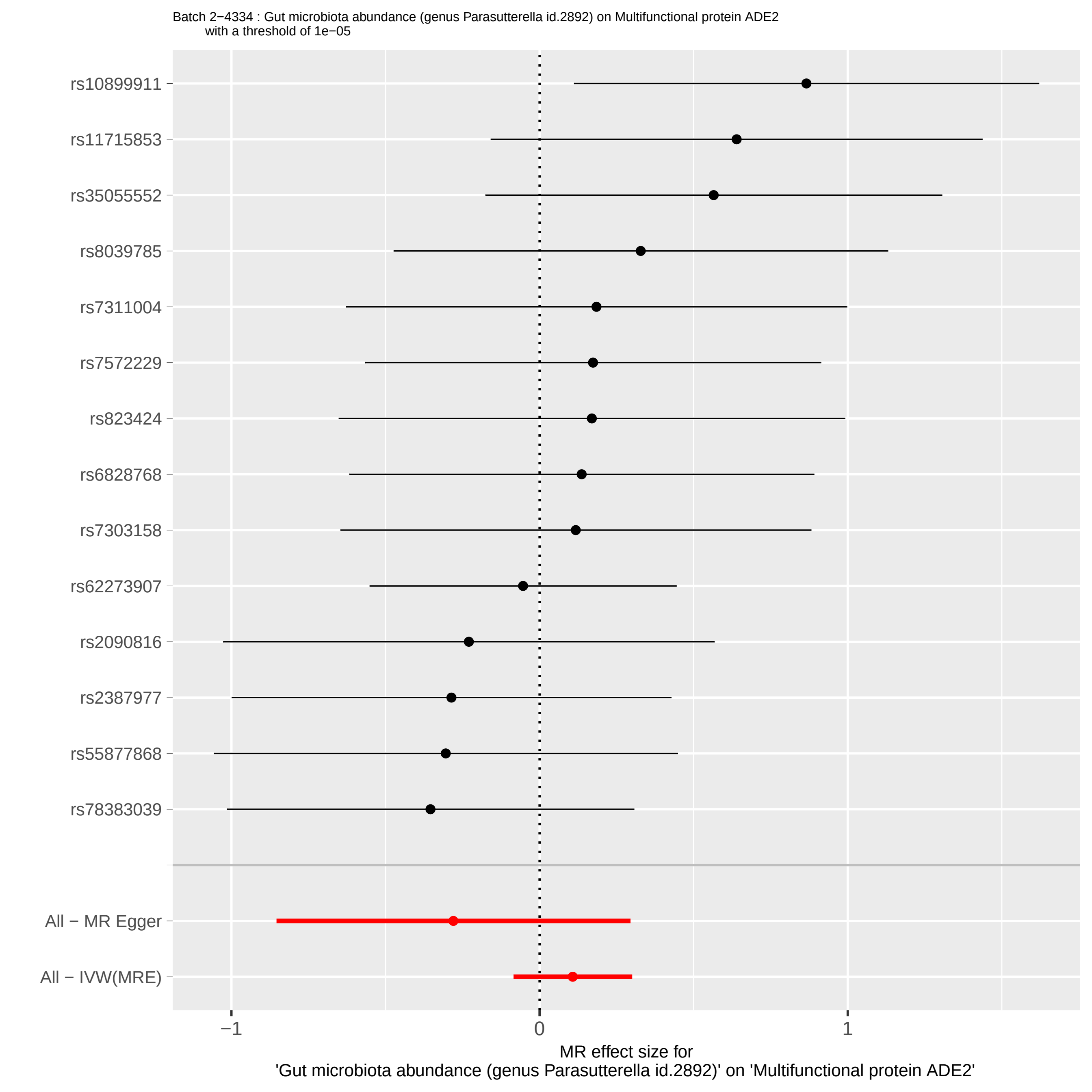
Batch 2−4334 : Gut microbiota abundance (genus Parasutterella id.2892) on Multifunctional protein ADE2
with a threshold of 1e−05
rs10899911
rs11715853
rs35055552
rs8039785
rs7311004
rs7572229
rs823424
rs6828768
rs7303158
rs62273907
rs2090816
rs2387977
rs55877868
rs78383039
All − MR Egger
All − IVW(MRE)
−1
0
1
MR effect size for
'Gut microbiota abundance (genus Parasutterella id.2892)' on 'Multifunctional protein ADE2'

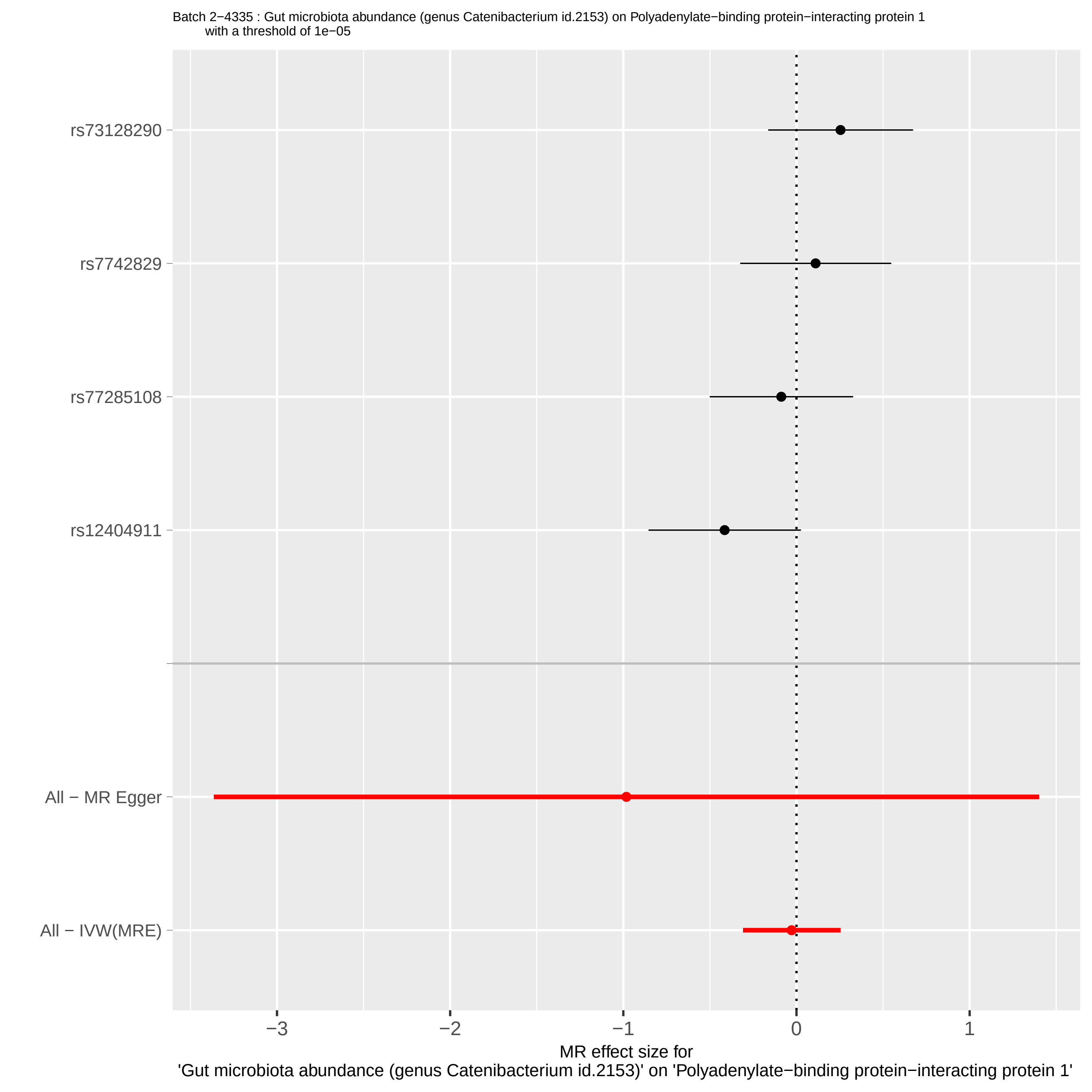
Batch 2−4335 : Gut microbiota abundance (genus Catenibacterium id.2153) on Polyadenylate−binding protein−interacting protein 1
with a threshold of 1e−05
rs73128290
rs7742829
rs77285108
rs12404911
All − MR Egger
All − IVW(MRE)
−3
−2
−1
0
1
MR effect size for
'Gut microbiota abundance (genus Catenibacterium id.2153)' on 'Polyadenylate−binding protein−interacting protein 1'

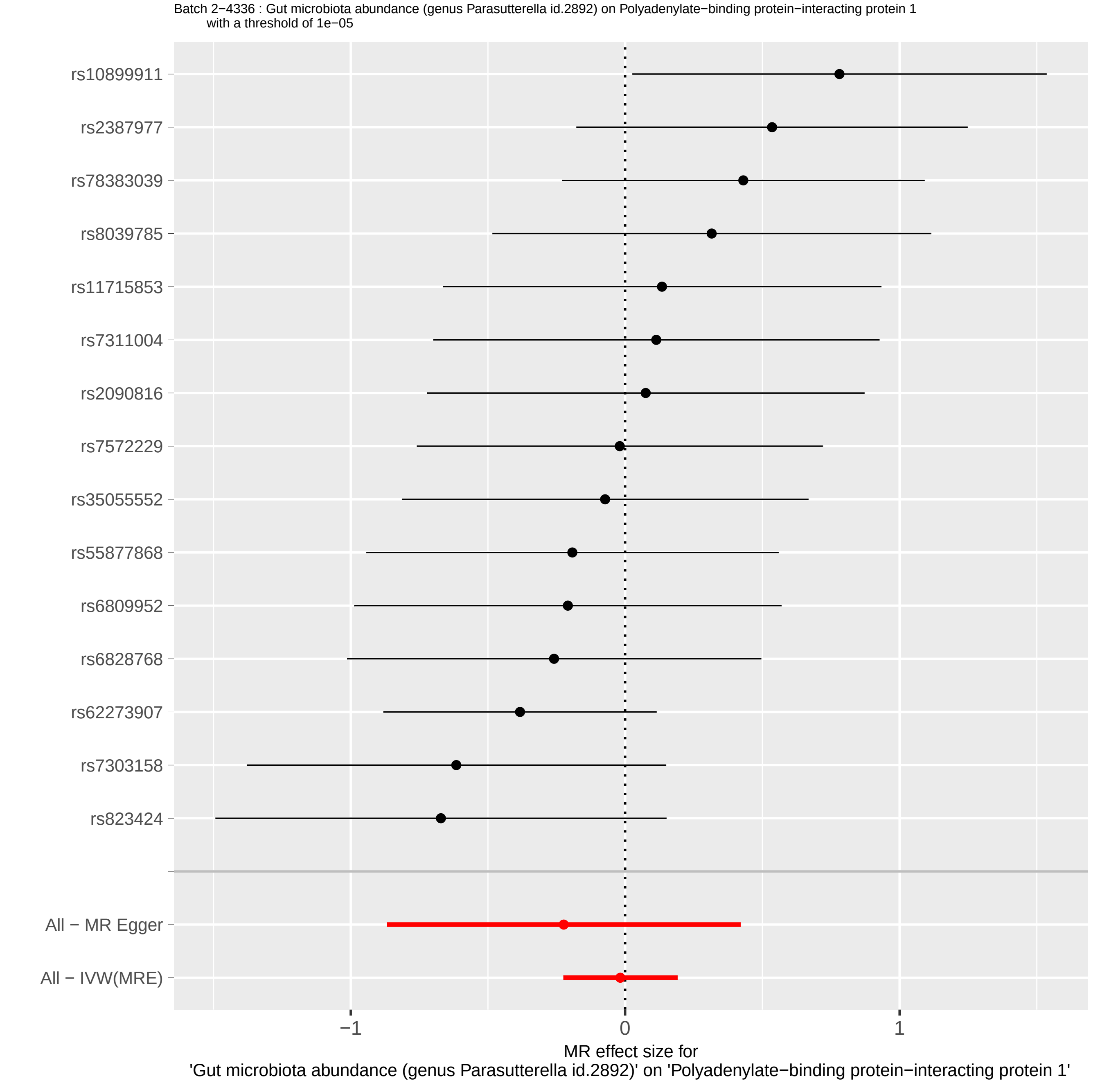

Batch 2−4336 : Gut microbiota abundance (genus Parasutterella id.2892) on Polyadenylate−binding protein−interacting protein 1
with a threshold of 1e−05
rs10899911
rs2387977
rs78383039
rs8039785
rs11715853
rs7311004
rs2090816
rs7572229
rs35055552
rs55877868
rs6809952
rs6828768
rs62273907
rs7303158
rs823424
All − MR Egger
All − IVW(MRE)
−1
0
1
MR effect size for
'Gut microbiota abundance (genus Parasutterella id.2892)' on 'Polyadenylate−binding protein−interacting protein 1'

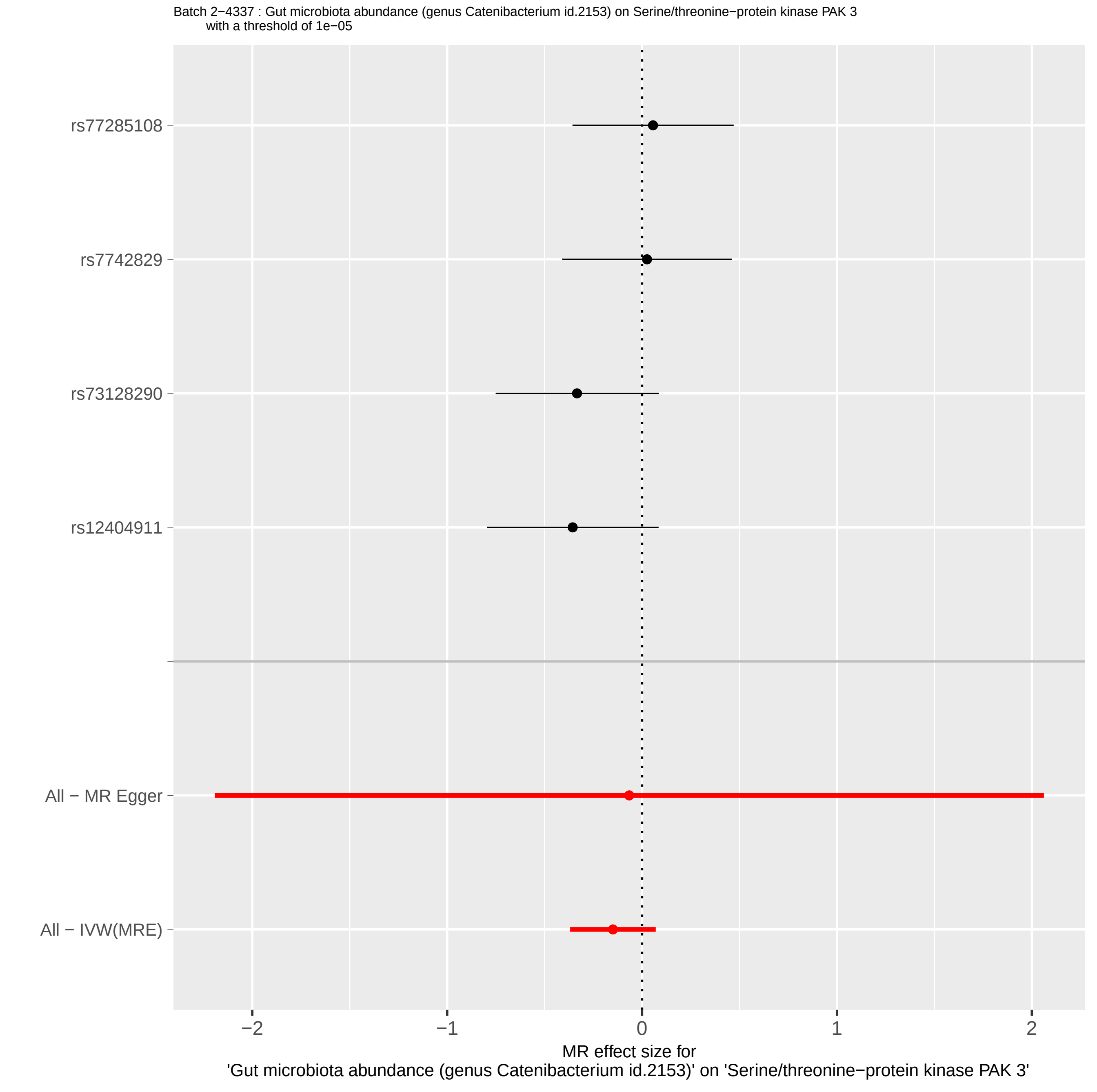

Batch 2−4337 : Gut microbiota abundance (genus Catenibacterium id.2153) on Serine/threonine−protein kinase PAK 3
with a threshold of 1e−05
rs77285108
rs7742829
rs73128290
rs12404911
All − MR Egger
All − IVW(MRE)
−2
−1
0
1
2
MR effect size for
'Gut microbiota abundance (genus Catenibacterium id.2153)' on 'Serine/threonine−protein kinase PAK 3'

Batch 2−4338 : Gut microbiota abundance (genus Parasutterella id.2892) on Serine/threonine−protein kinase PAK 3
with a threshold of 1e−05

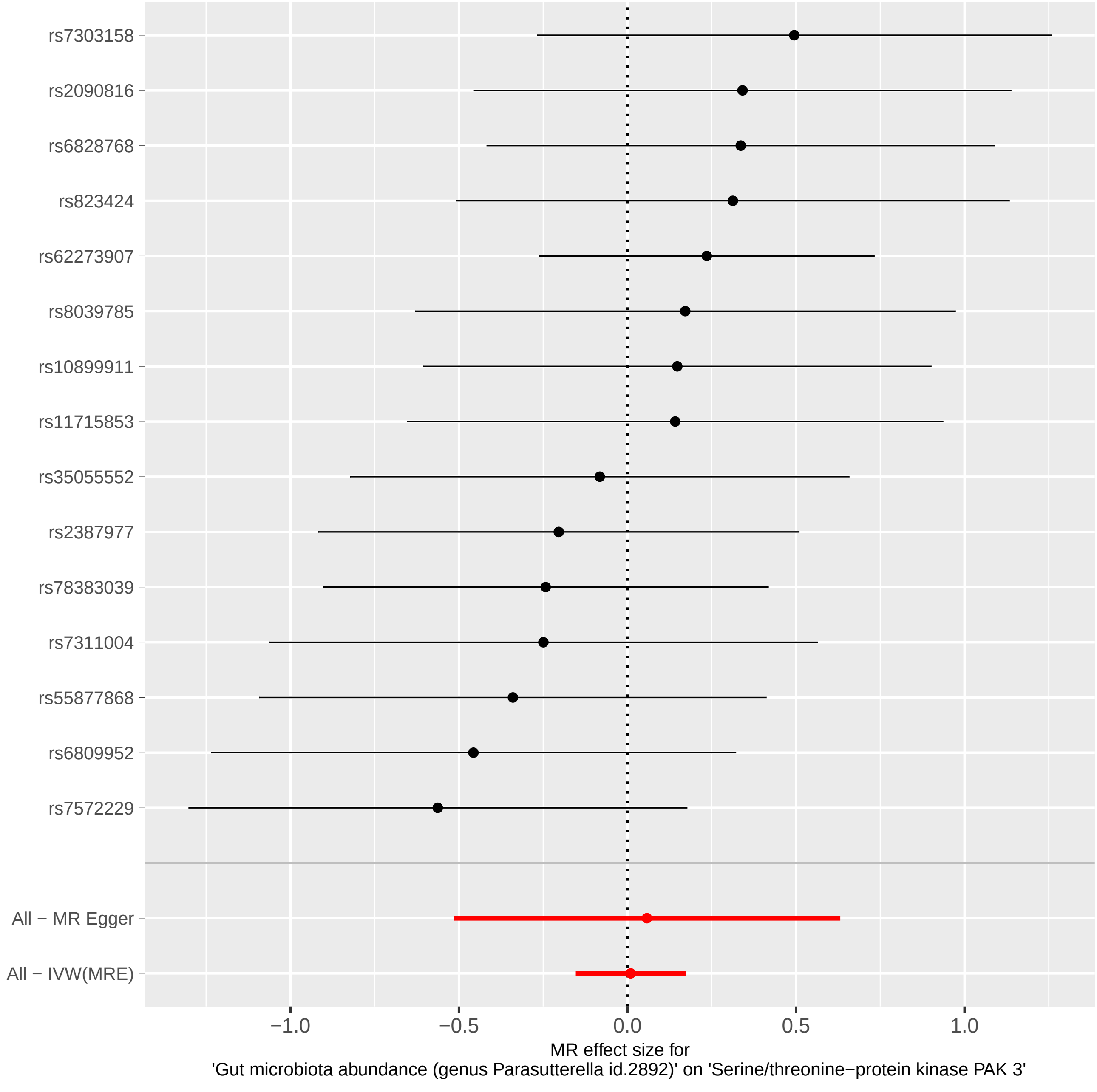

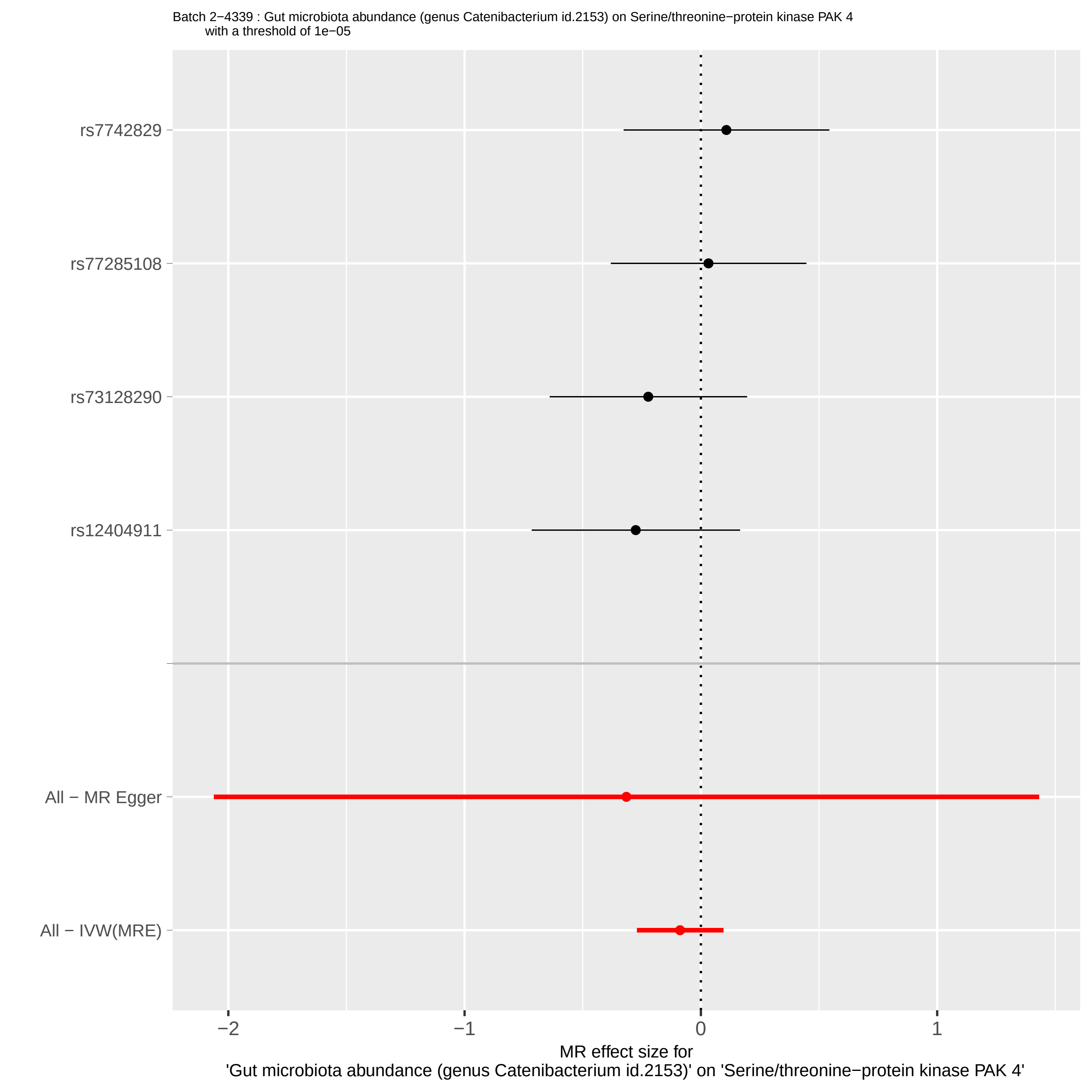

Batch 2−4339 : Gut microbiota abundance (genus Catenibacterium id.2153) on Serine/threonine−protein kinase PAK 4
with a threshold of 1e−05
rs7742829
rs77285108
rs73128290
rs12404911
All − MR Egger
All − IVW(MRE)
−2
−1
0
1
MR effect size for
'Gut microbiota abundance (genus Catenibacterium id.2153)' on 'Serine/threonine−protein kinase PAK 4'

Batch 2−4340 : Gut microbiota abundance (genus Parasutterella id.2892) on Serine/threonine−protein kinase PAK 4
with a threshold of 1e−05

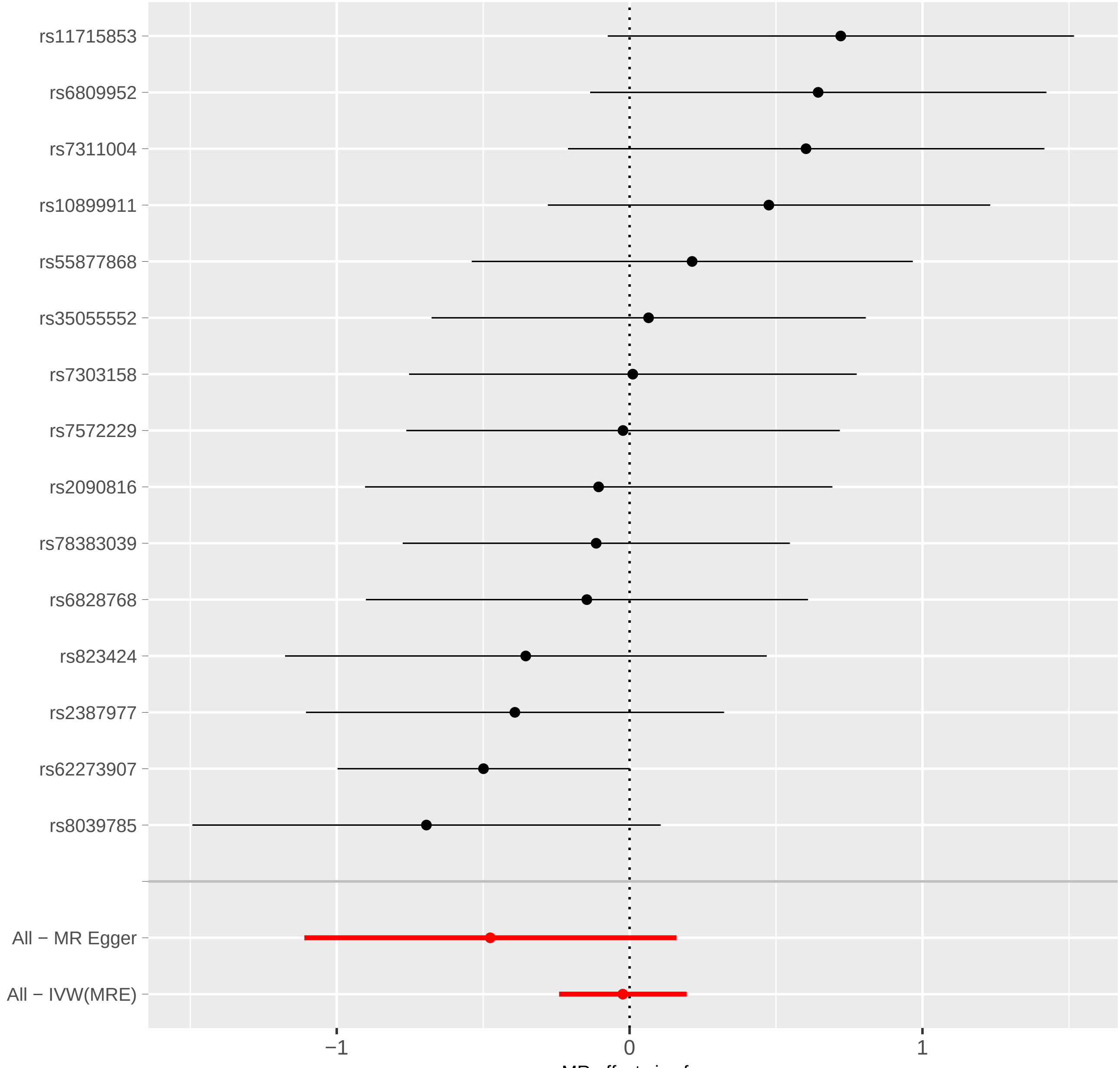

MR effect size for
'Gut microbiota abundance (genus Parasutterella id.2892)' on 'Serine/threonine−protein kinase PAK 4'

Batch 2−4341 : Gut microbiota abundance (genus Catenibacterium id.2153) on Serine/threonine−protein kinase PAK 7
with a threshold of 1e−05

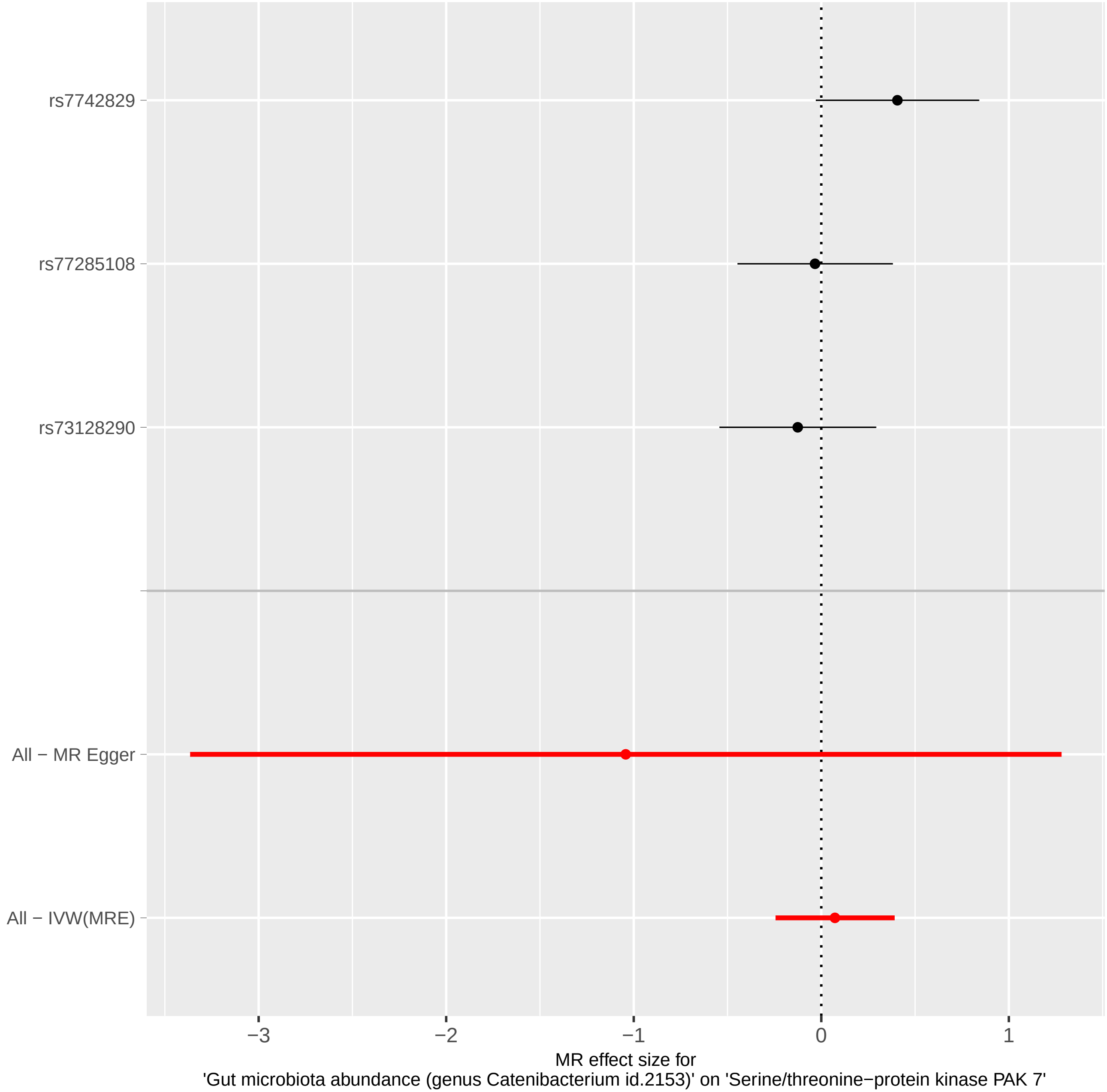

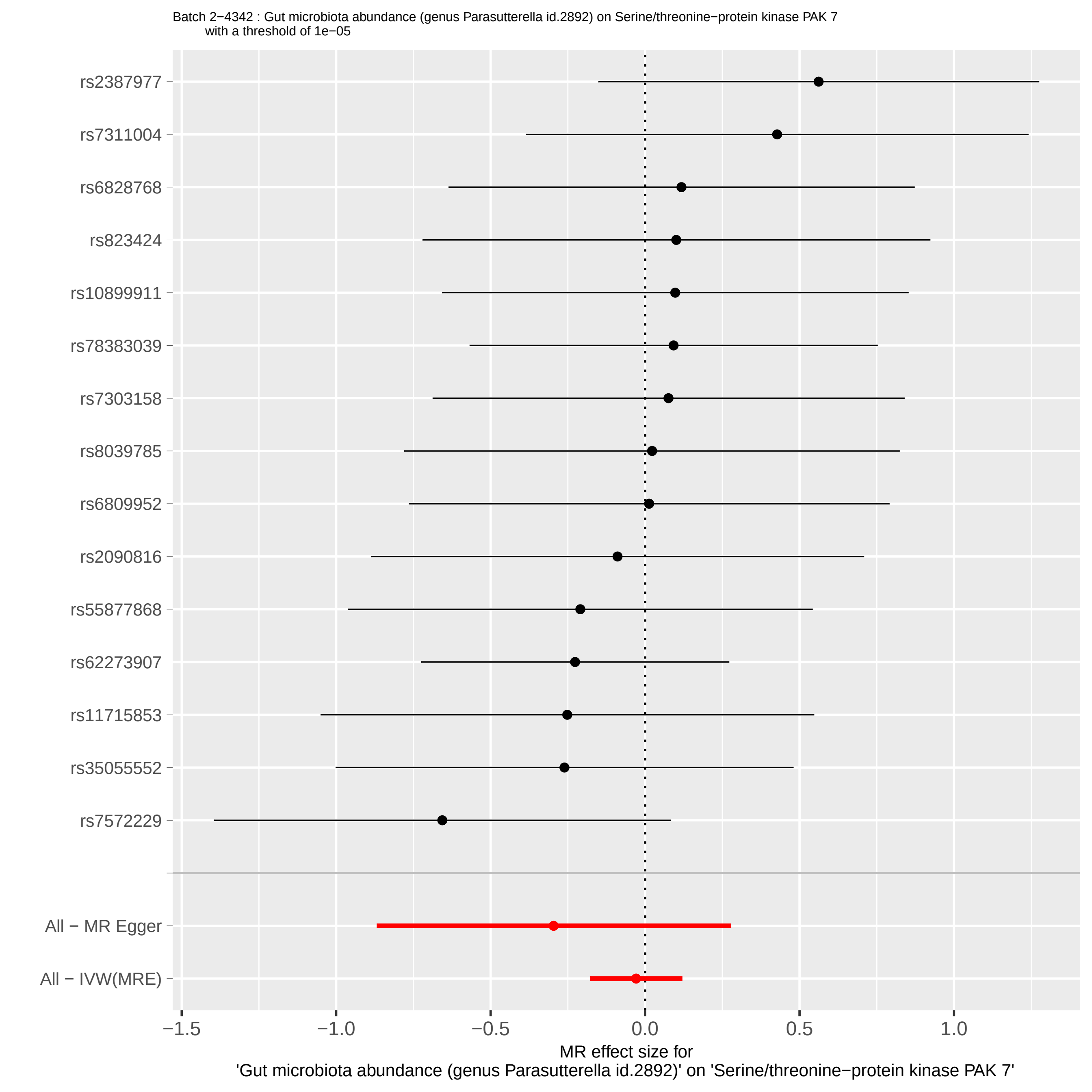

Batch 2−4342 : Gut microbiota abundance (genus Parasutterella id.2892) on Serine/threonine−protein kinase PAK 7
with a threshold of 1e−05
rs2387977
rs7311004
rs6828768
rs823424
rs10899911
rs78383039
rs7303158
rs8039785
rs6809952
rs2090816
rs55877868
rs62273907
rs11715853
rs35055552
rs7572229
All − MR Egger
All − IVW(MRE)
−1.5
−1.0
−0.5
0.0
0.5
1.0
MR effect size for
'Gut microbiota abundance (genus Parasutterella id.2892)' on 'Serine/threonine−protein kinase PAK 7'

Batch 2−4343 : Gut microbiota abundance (genus Catenibacterium id.2153) on Peptidyl−glycine alpha−amidating monooxygenase
with a threshold of 1e−05

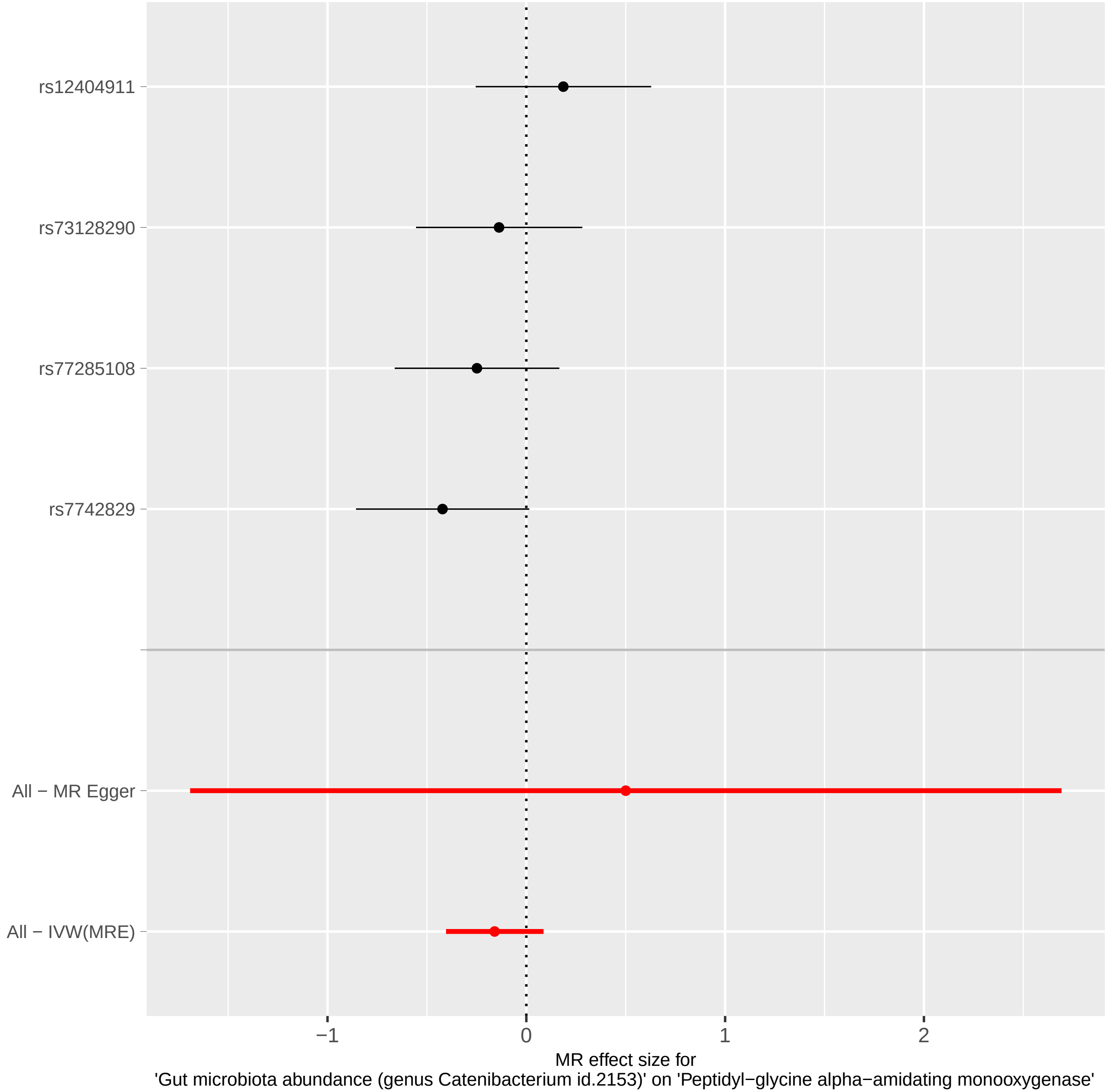

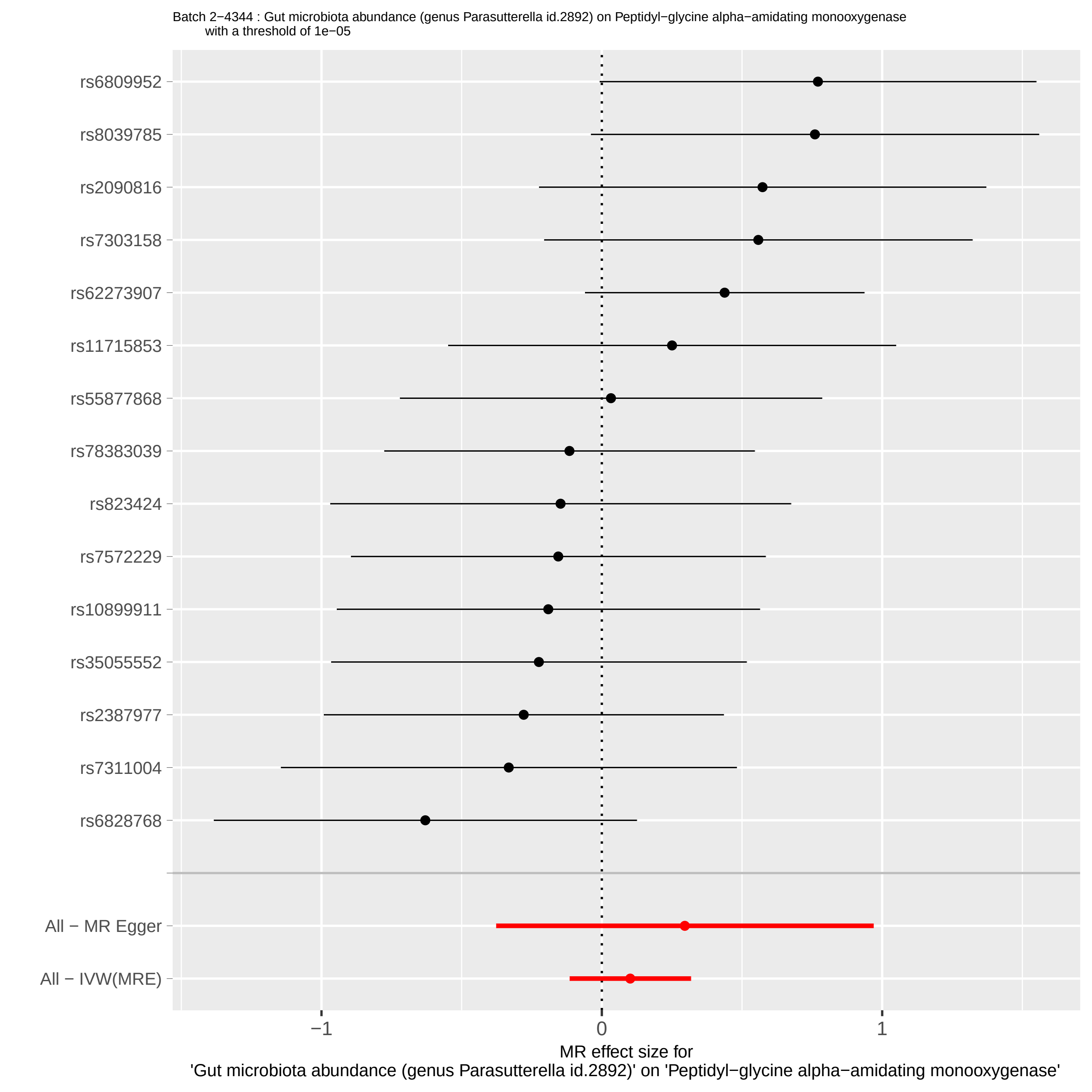

Batch 2−4344 : Gut microbiota abundance (genus Parasutterella id.2892) on Peptidyl−glycine alpha−amidating monooxygenase
with a threshold of 1e−05
rs6809952
rs8039785
rs2090816
rs7303158
rs62273907
rs11715853
rs55877868
rs78383039
rs823424
rs7572229
rs10899911
rs35055552
rs2387977
rs7311004
rs6828768
All − MR Egger
All − IVW(MRE)
−1
0
1
MR effect size for
'Gut microbiota abundance (genus Parasutterella id.2892)' on 'Peptidyl−glycine alpha−amidating monooxygenase'

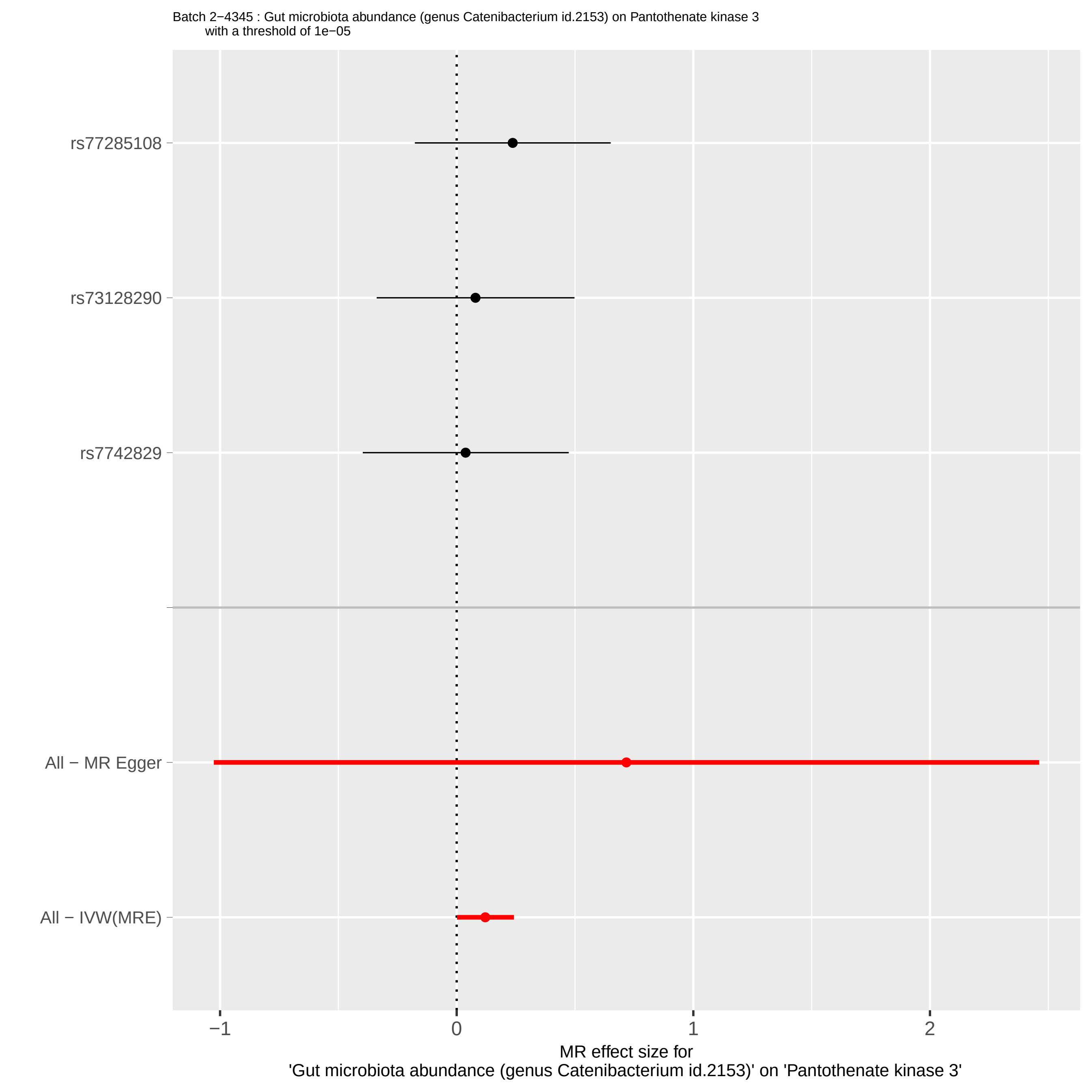

Batch 2−4345 : Gut microbiota abundance (genus Catenibacterium id.2153) on Pantothenate kinase 3
with a threshold of 1e−05
rs77285108
rs73128290
rs7742829
All − MR Egger
All − IVW(MRE)
−1
0
1
2
MR effect size for
'Gut microbiota abundance (genus Catenibacterium id.2153)' on 'Pantothenate kinase 3'

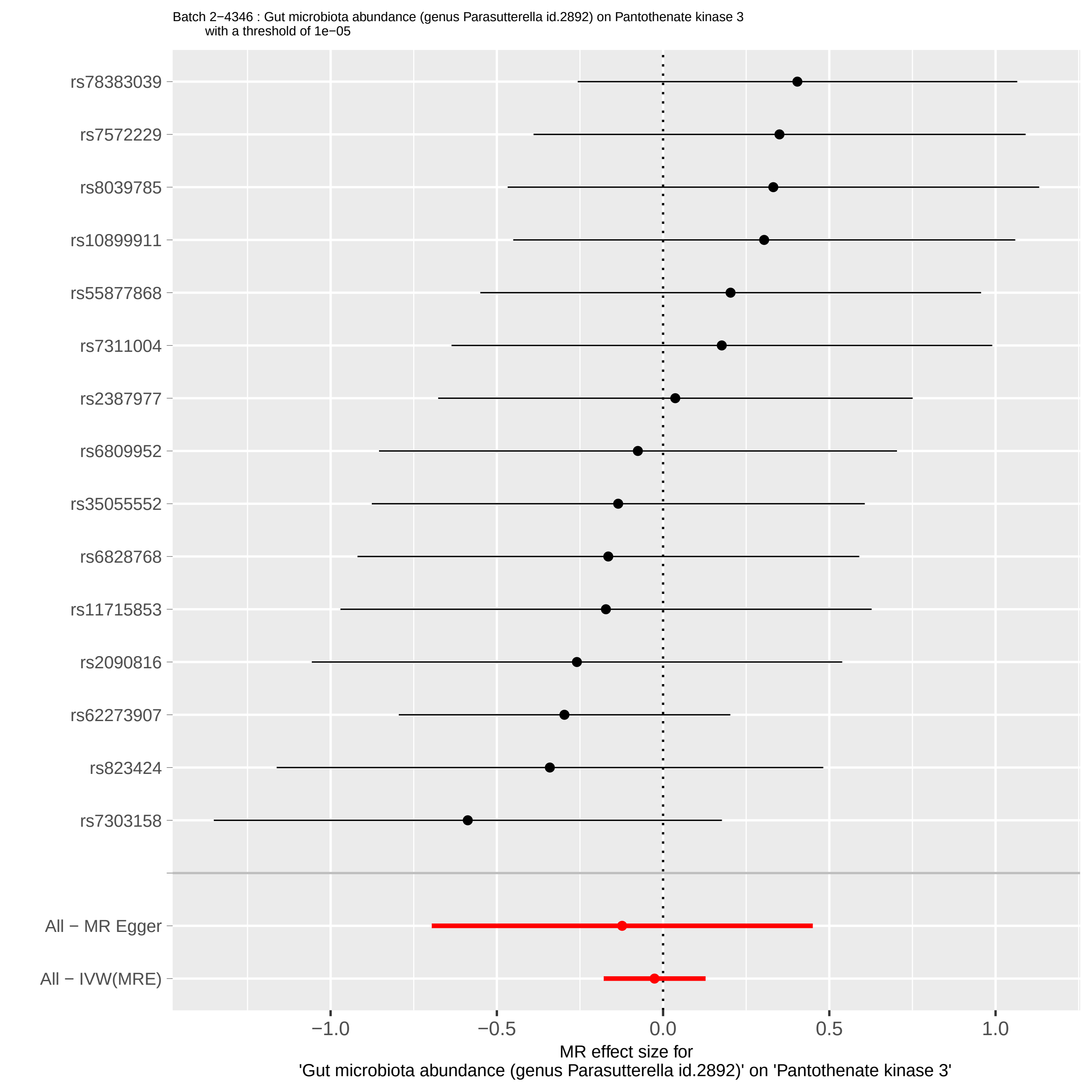

Batch 2−4346 : Gut microbiota abundance (genus Parasutterella id.2892) on Pantothenate kinase 3
with a threshold of 1e−05
rs78383039
rs7572229
rs8039785
rs10899911
rs55877868
rs7311004
rs2387977
rs6809952
rs35055552
rs6828768
rs11715853
rs2090816
rs62273907
rs823424
rs7303158
All − MR Egger
All − IVW(MRE)
−1.0
−0.5
0.0
0.5
1.0
MR effect size for
'Gut microbiota abundance (genus Parasutterella id.2892)' on 'Pantothenate kinase 3'

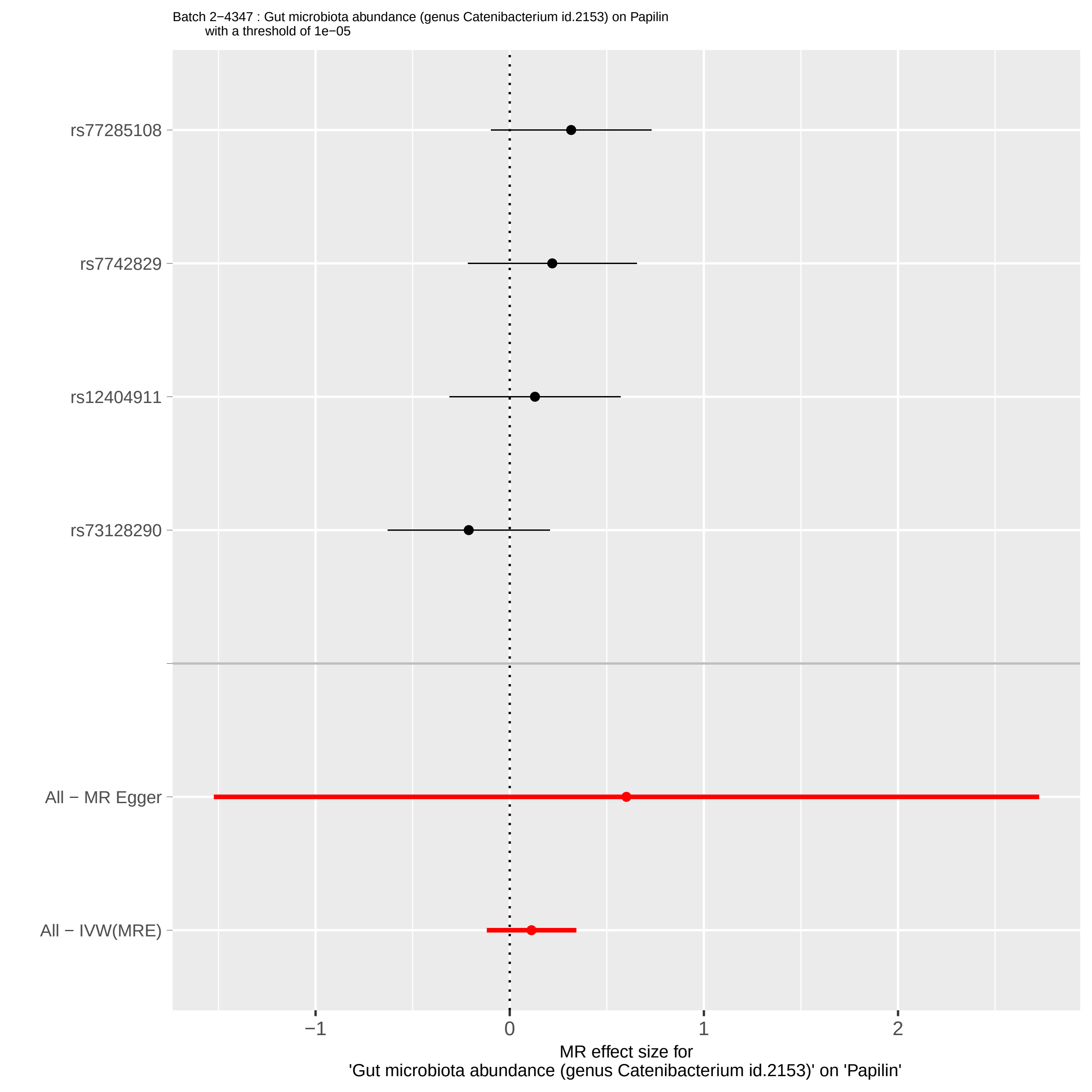

Batch 2−4347 : Gut microbiota abundance (genus Catenibacterium id.2153) on Papilin
with a threshold of 1e−05
rs77285108
rs7742829
rs12404911
rs73128290
All − MR Egger
All − IVW(MRE)
−1
0
1
2
MR effect size for
'Gut microbiota abundance (genus Catenibacterium id.2153)' on 'Papilin'

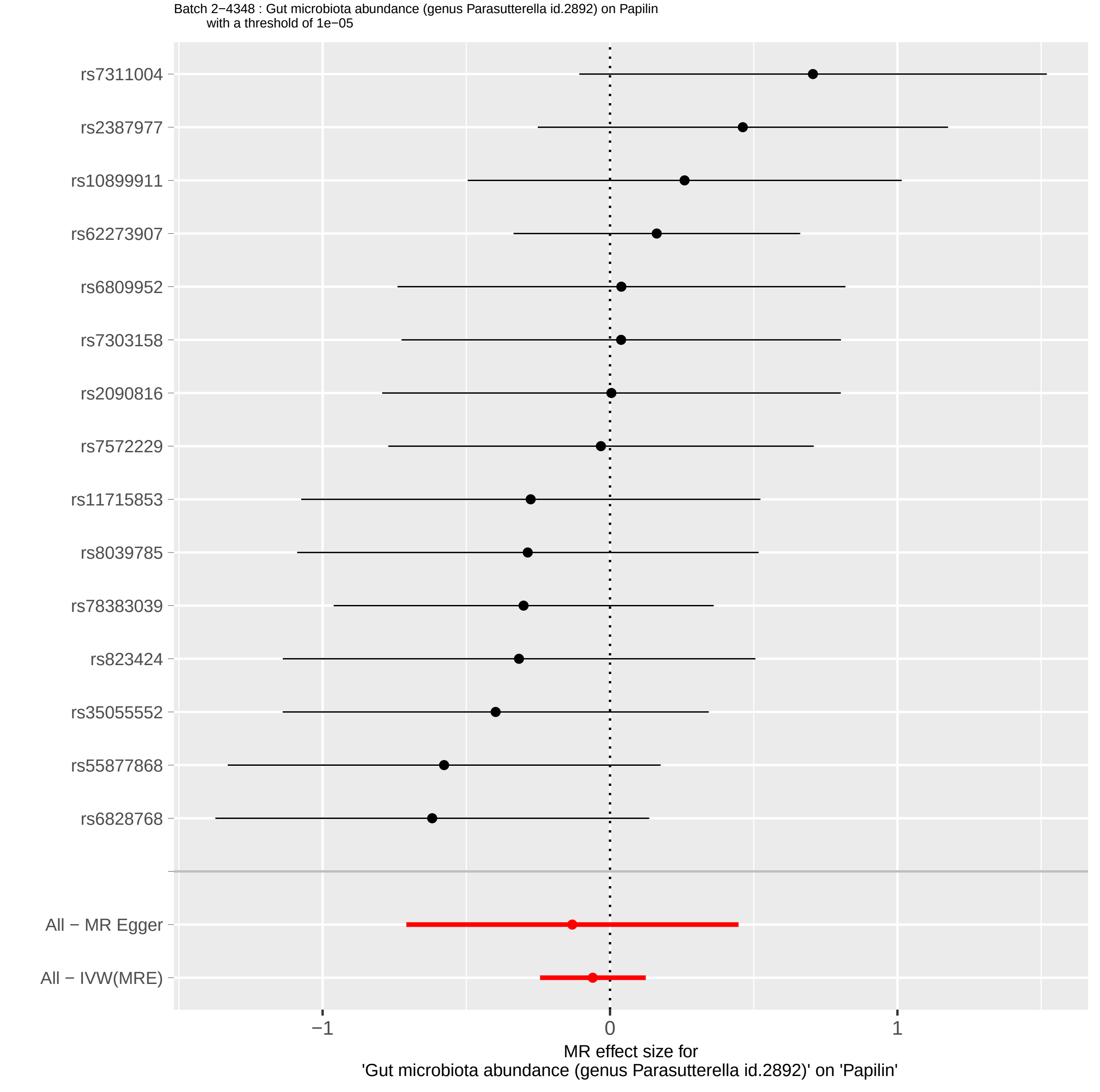

Batch 2−4348 : Gut microbiota abundance (genus Parasutterella id.2892) on Papilin
with a threshold of 1e−05
rs7311004
rs2387977
rs10899911
rs62273907
rs6809952
rs7303158
rs2090816
rs7572229
rs11715853
rs8039785
rs78383039
rs823424
rs35055552
rs55877868
rs6828768
All − MR Egger
All − IVW(MRE)
−1
0
1
MR effect size for
'Gut microbiota abundance (genus Parasutterella id.2892)' on 'Papilin'

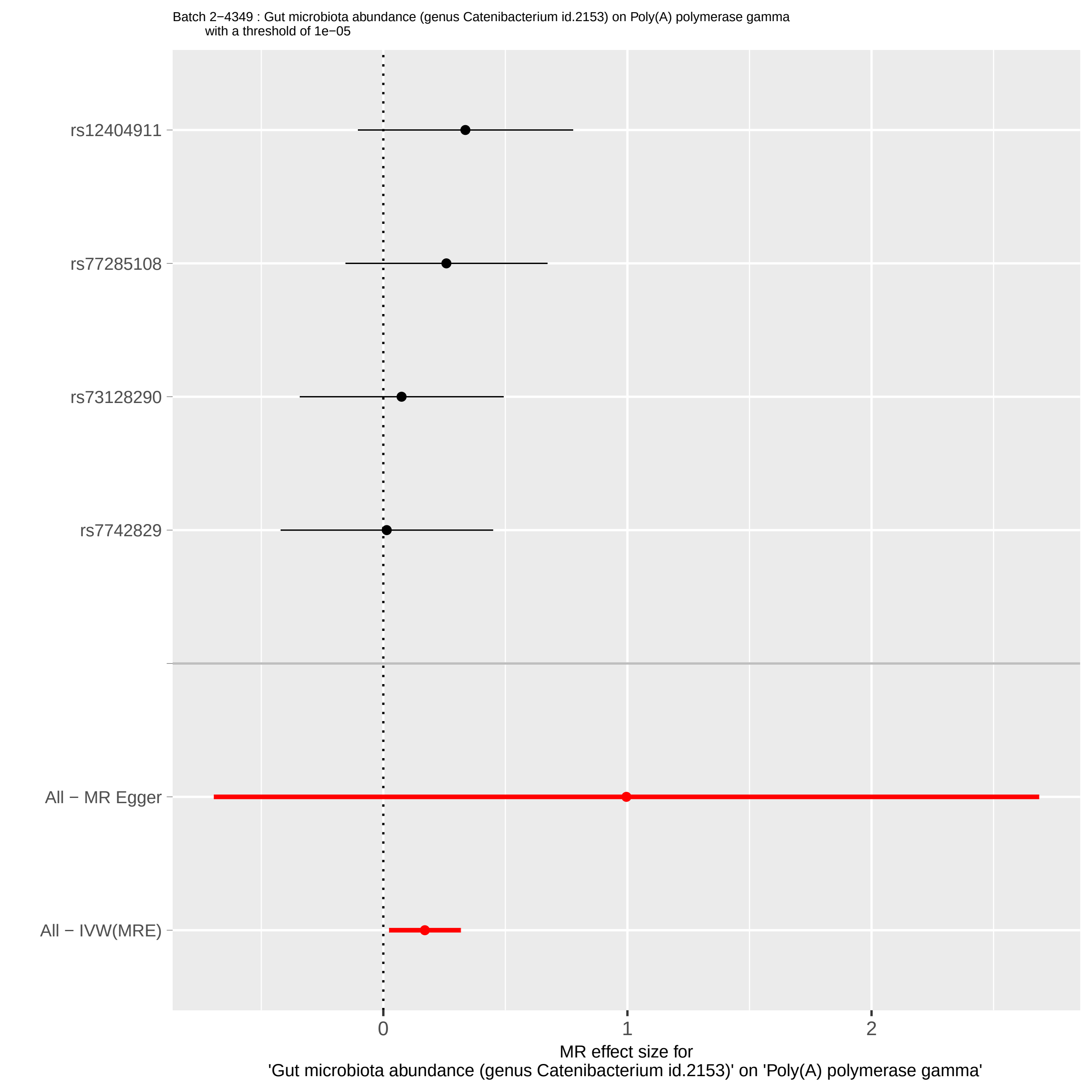

Batch 2−4349 : Gut microbiota abundance (genus Catenibacterium id.2153) on Poly(A) polymerase gamma
with a threshold of 1e−05
rs12404911
rs77285108
rs73128290
rs7742829
All − MR Egger
All − IVW(MRE)
0
1
2
MR effect size for
'Gut microbiota abundance (genus Catenibacterium id.2153)' on 'Poly(A) polymerase gamma'

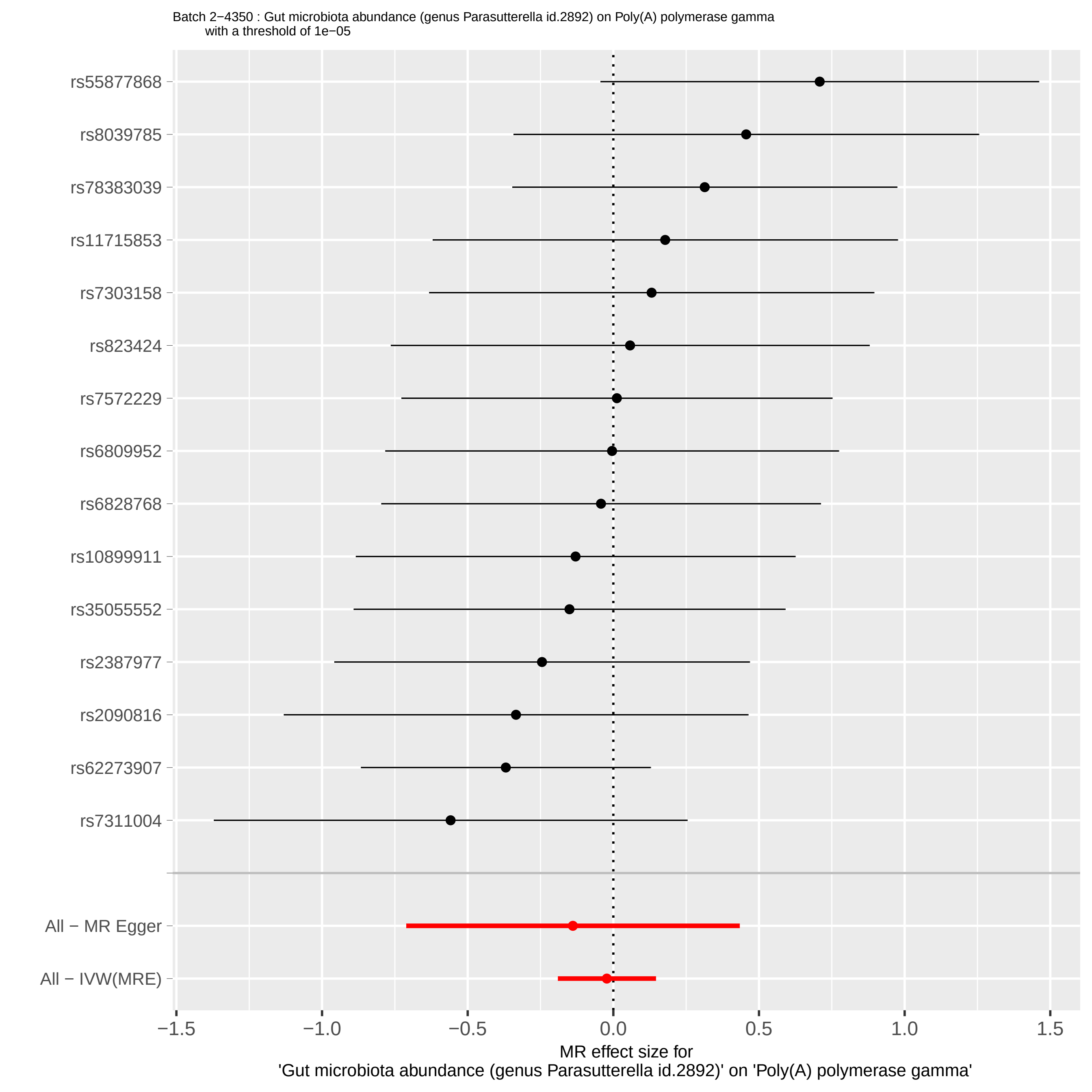

Batch 2−4350 : Gut microbiota abundance (genus Parasutterella id.2892) on Poly(A) polymerase gamma
with a threshold of 1e−05
rs55877868
rs8039785
rs78383039
rs11715853
rs7303158
rs823424
rs7572229
rs6809952
rs6828768
rs10899911
rs35055552
rs2387977
rs2090816
rs62273907
rs7311004
All − MR Egger
All − IVW(MRE)
−1.5
−1.0
−0.5
0.0
0.5
1.0
1.5
MR effect size for
'Gut microbiota abundance (genus Parasutterella id.2892)' on 'Poly(A) polymerase gamma'

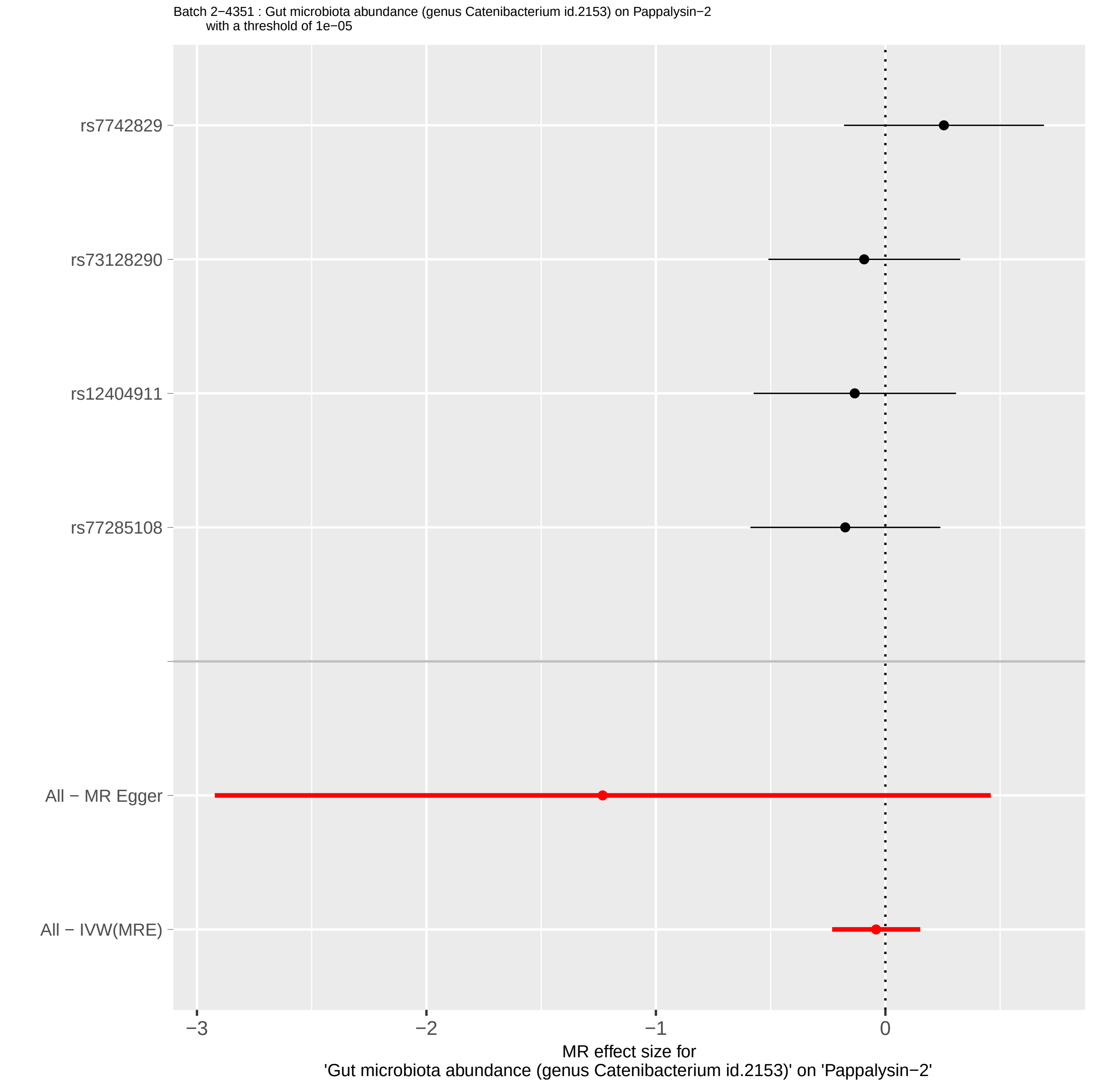
Batch 2−4351 : Gut microbiota abundance (genus Catenibacterium id.2153) on Pappalysin−2
with a threshold of 1e−05
rs7742829
rs73128290
rs12404911
rs77285108
All − MR Egger
All − IVW(MRE)
−3
−2
−1
0
MR effect size for
'Gut microbiota abundance (genus Catenibacterium id.2153)' on 'Pappalysin−2'

Batch 2−4352 : Gut microbiota abundance (genus Parasutterella id.2892) on Pappalysin−2
with a threshold of 1e−05

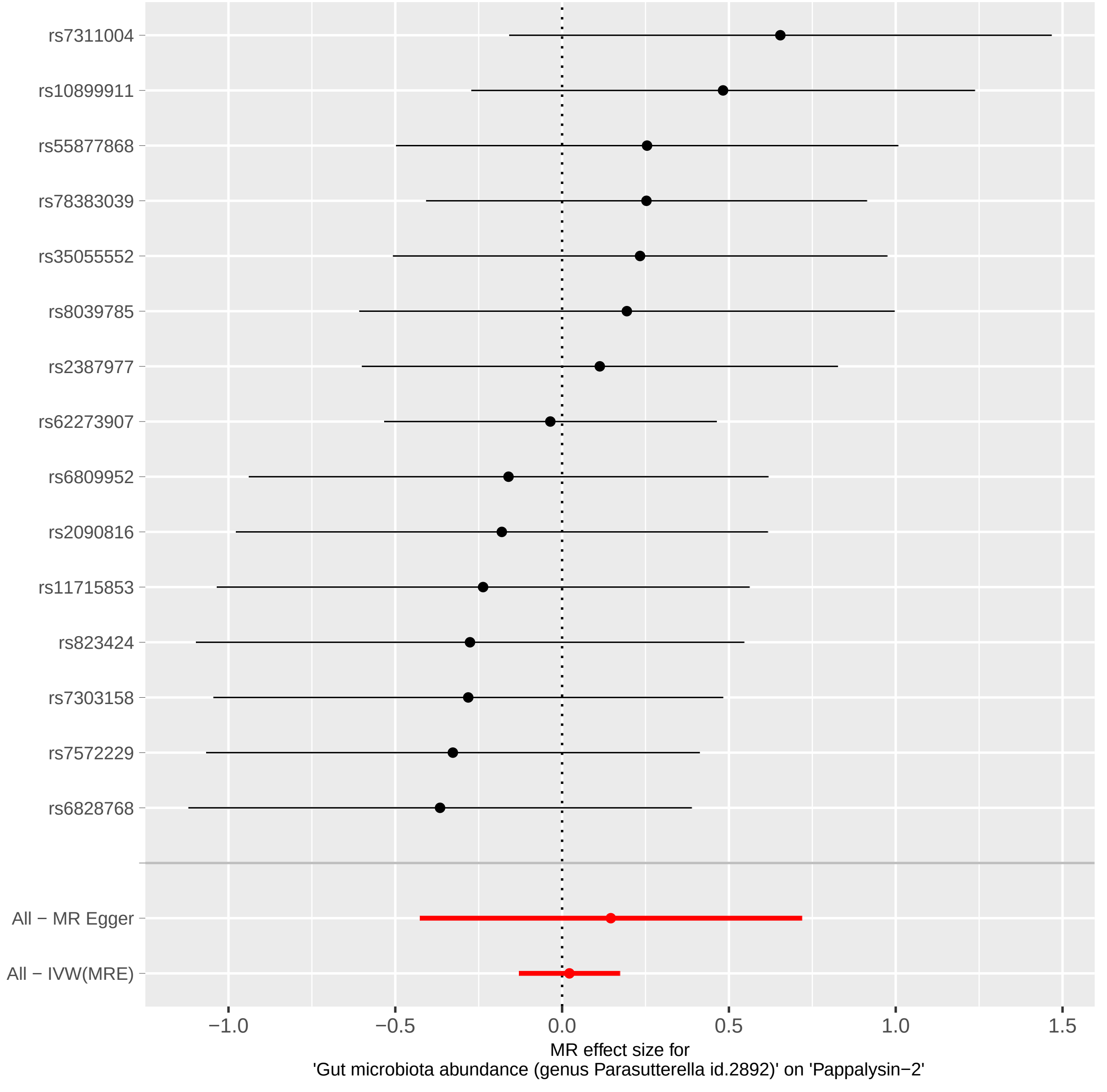

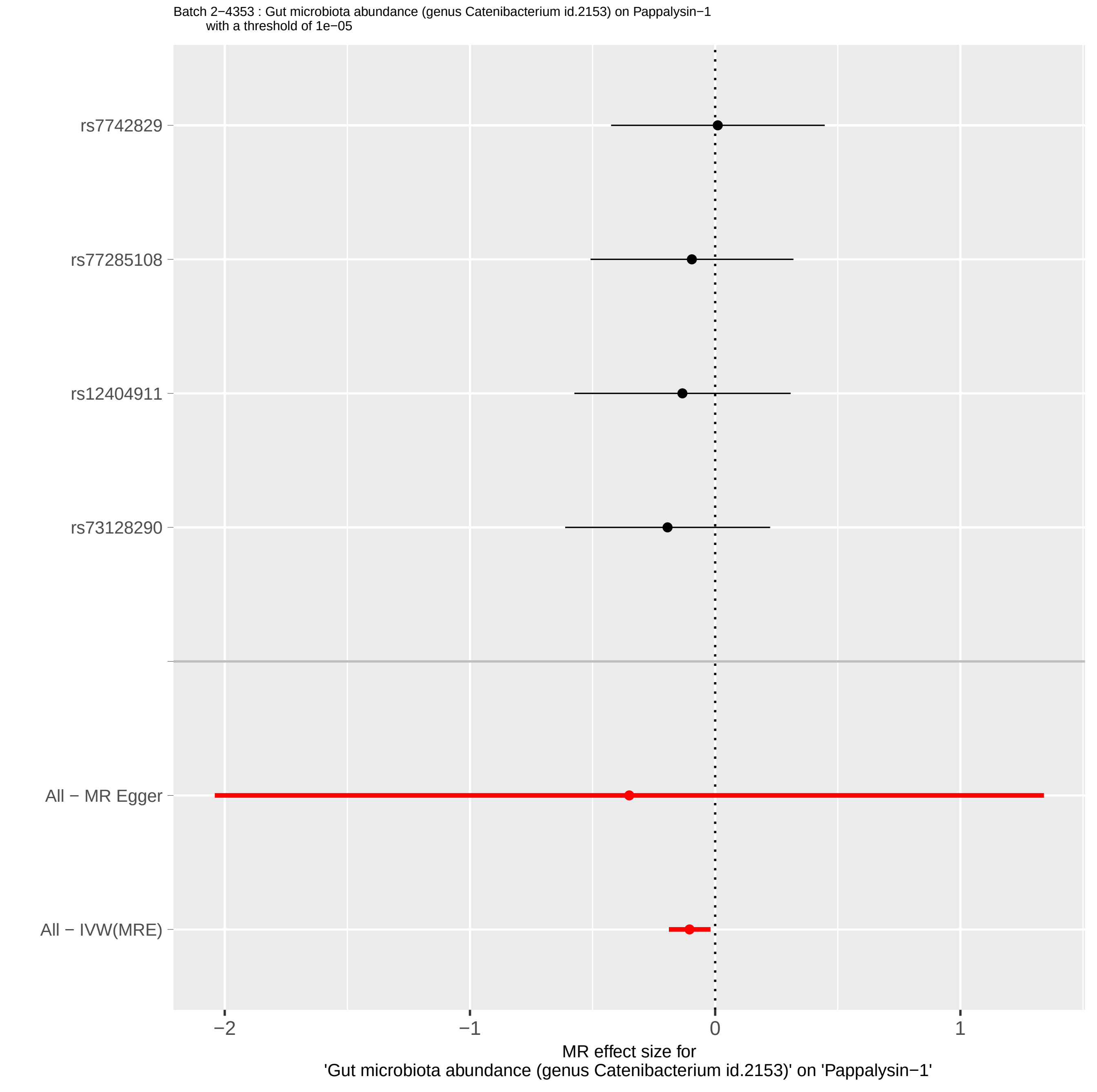

Batch 2−4353 : Gut microbiota abundance (genus Catenibacterium id.2153) on Pappalysin−1
with a threshold of 1e−05
rs7742829
rs77285108
rs12404911
rs73128290
All − MR Egger
All − IVW(MRE)
−2
−1
0
1
MR effect size for
'Gut microbiota abundance (genus Catenibacterium id.2153)' on 'Pappalysin−1'

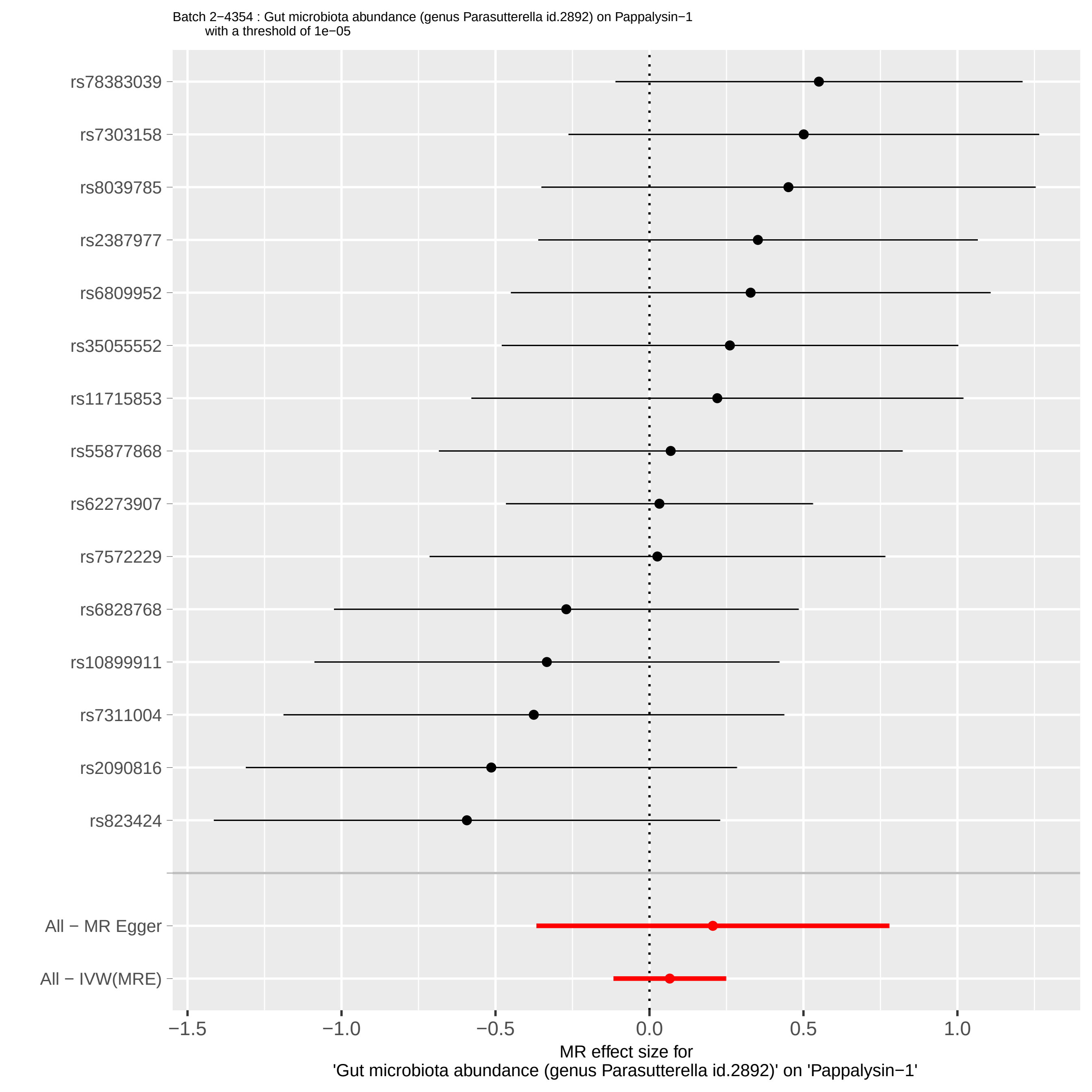

Batch 2−4354 : Gut microbiota abundance (genus Parasutterella id.2892) on Pappalysin−1
with a threshold of 1e−05
rs78383039
rs7303158
rs8039785
rs2387977
rs6809952
rs35055552
rs11715853
rs55877868
rs62273907
rs7572229
rs6828768
rs10899911
rs7311004
rs2090816
rs823424
All − MR Egger
All − IVW(MRE)
−1.5
−1.0
−0.5
0.0
0.5
1.0
MR effect size for
'Gut microbiota abundance (genus Parasutterella id.2892)' on 'Pappalysin−1'

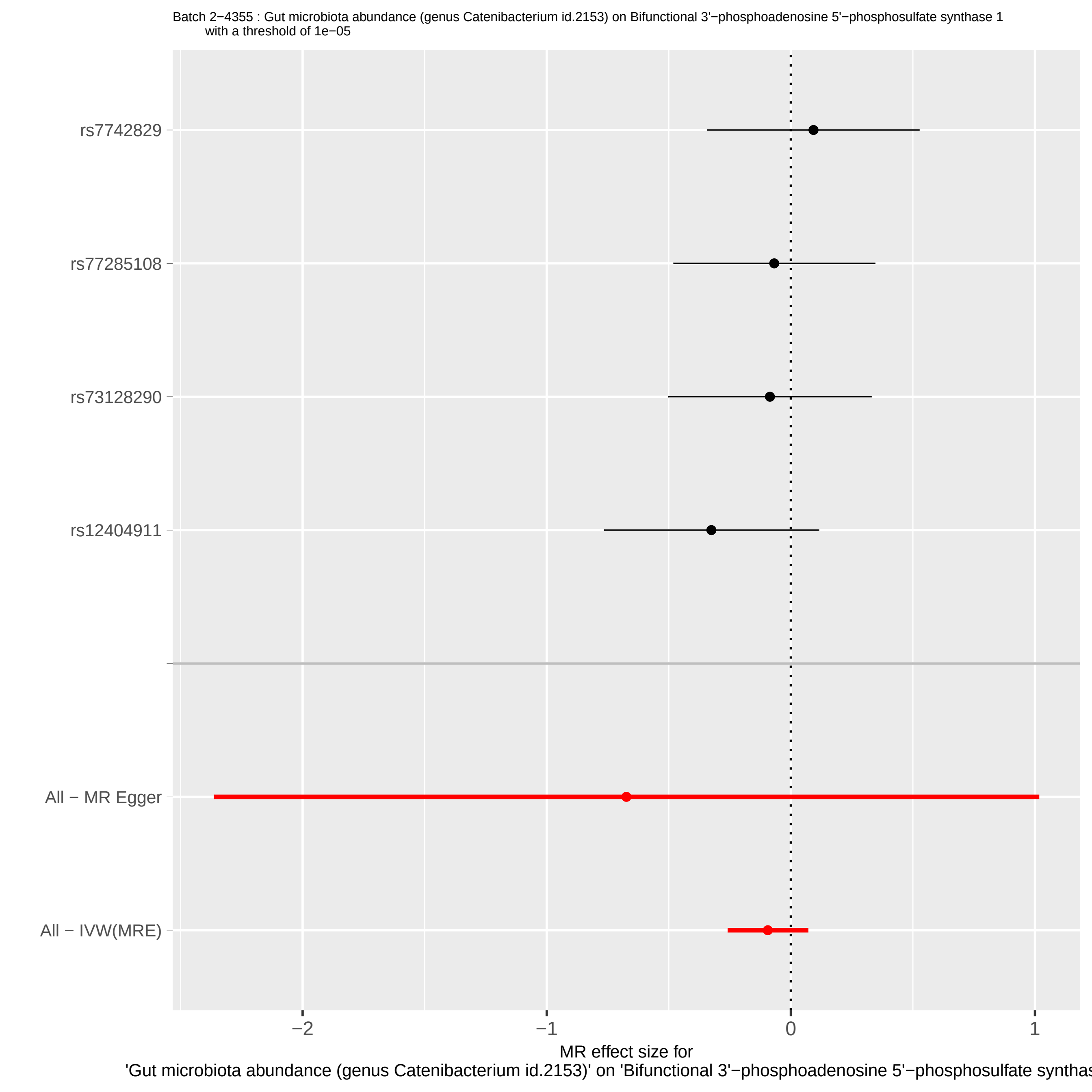
Batch 2−4355 : Gut microbiota abundance (genus Catenibacterium id.2153) on Bifunctional 3'−phosphoadenosine 5'−phosphosulfate synthase 1
with a threshold of 1e−05
rs7742829
rs77285108
rs73128290
rs12404911
All − MR Egger
All − IVW(MRE)
−2
−1
0
1
MR effect size for
'Gut microbiota abundance (genus Catenibacterium id.2153)' on 'Bifunctional 3'−phosphoadenosine 5'−phosphosulfate synthas

Batch 2−4356 : Gut microbiota abundance (genus Parasutterella id.2892) on Bifunctional 3'−phosphoadenosine 5'−phosphosulfate synthase 1 with a threshold of 1e−05

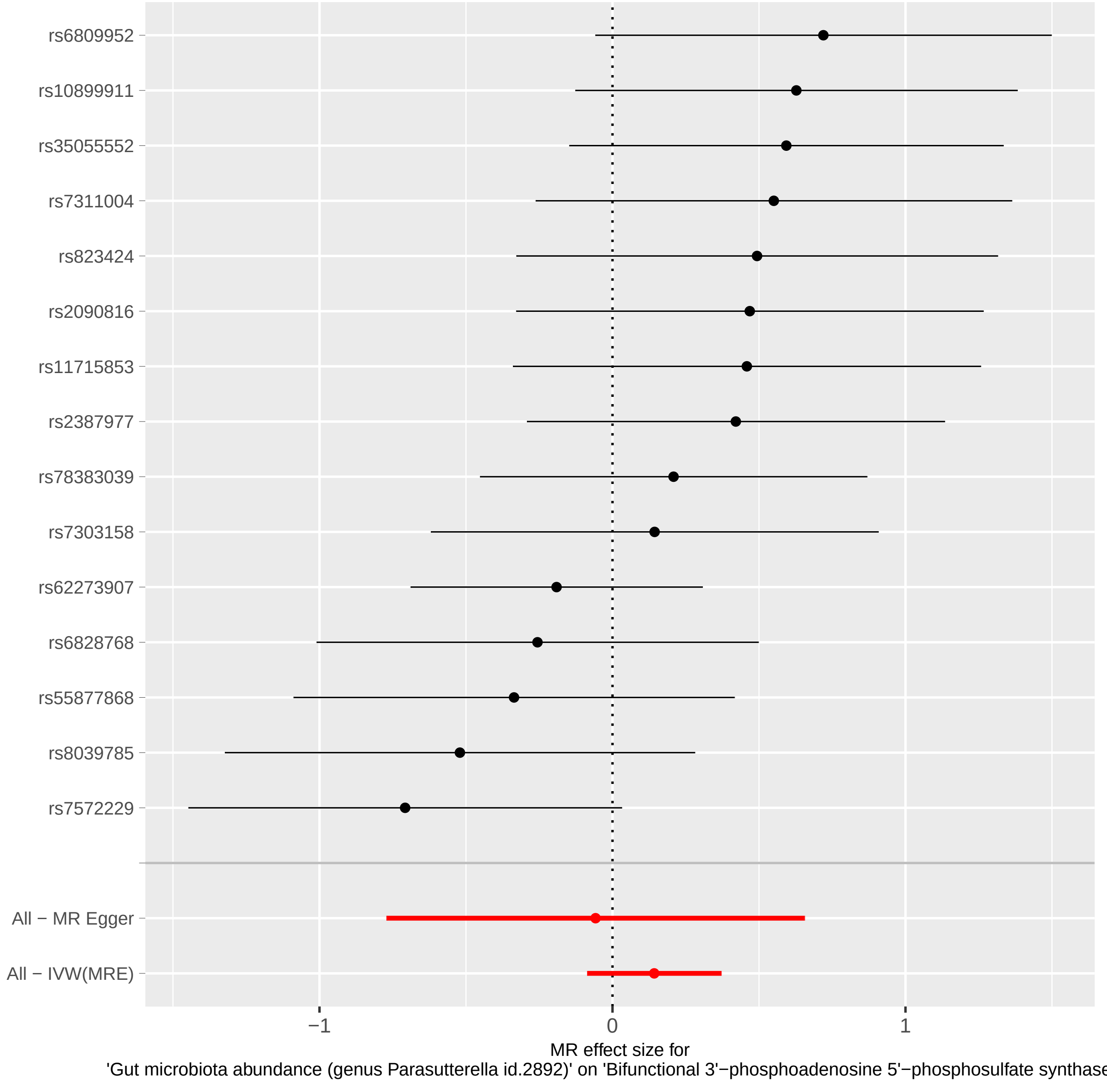

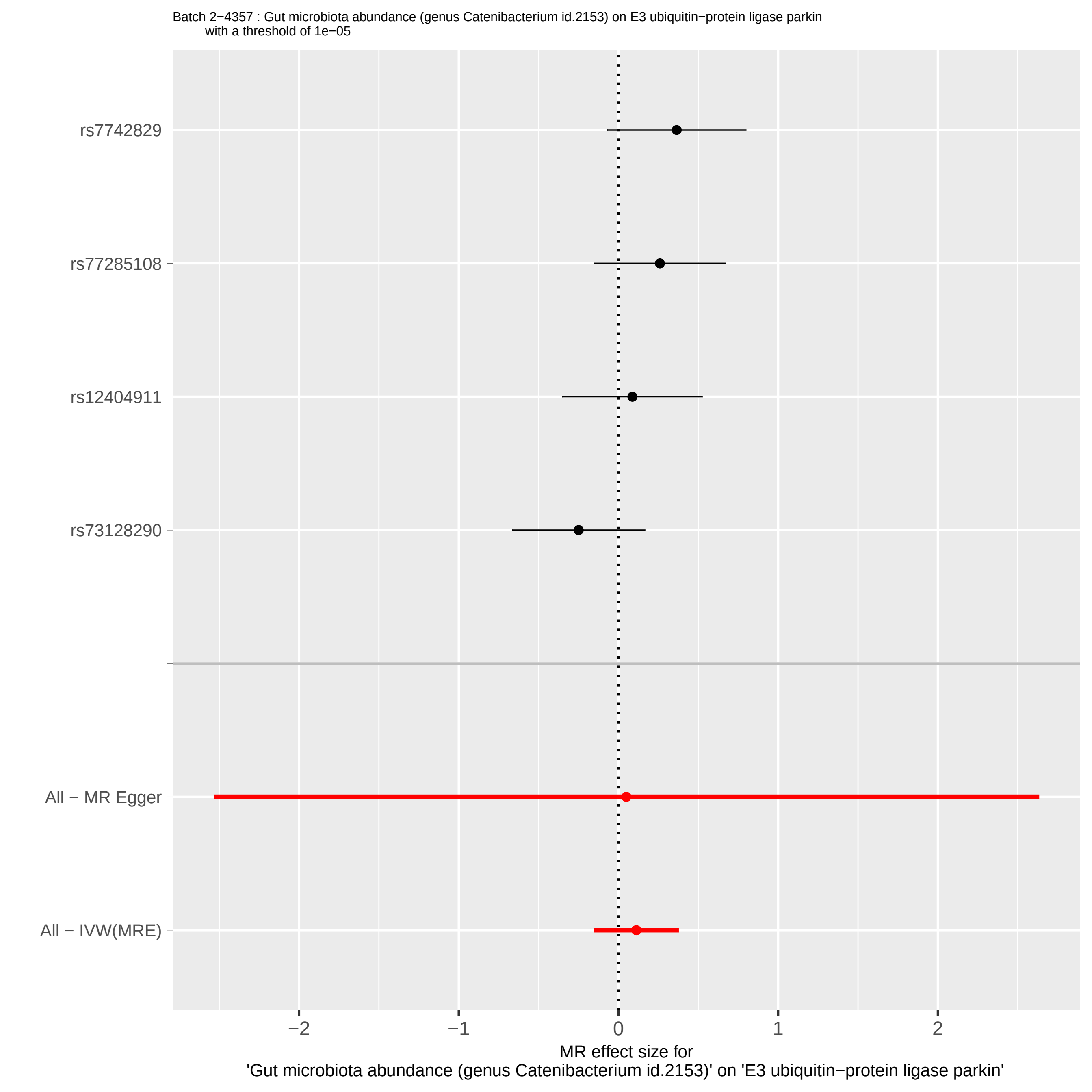

Batch 2−4357 : Gut microbiota abundance (genus Catenibacterium id.2153) on E3 ubiquitin−protein ligase parkin
with a threshold of 1e−05
rs7742829
rs77285108
rs12404911
rs73128290
All − MR Egger
All − IVW(MRE)
−2
−1
0
1
2
MR effect size for
'Gut microbiota abundance (genus Catenibacterium id.2153)' on 'E3 ubiquitin−protein ligase parkin'

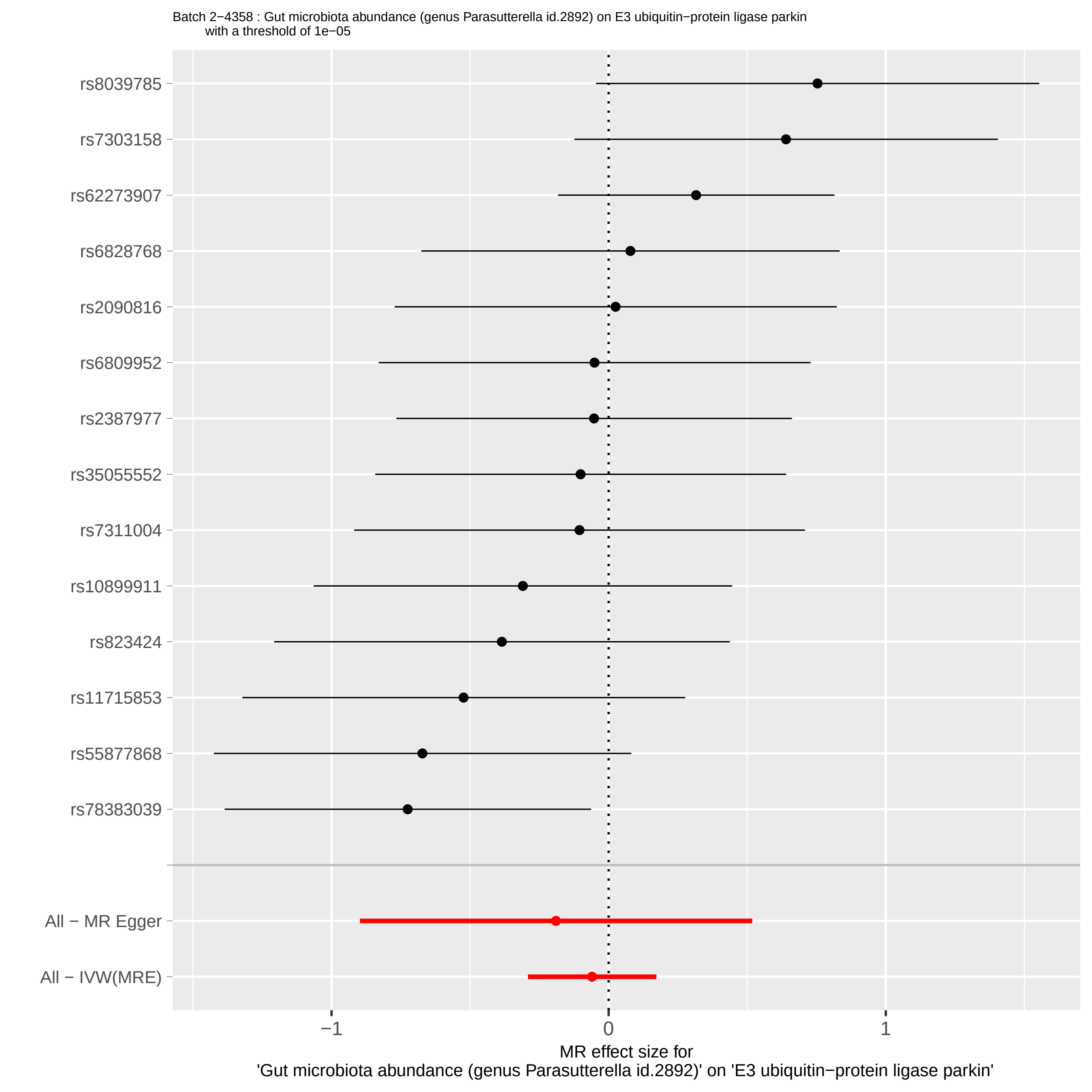
Batch 2−4358 : Gut microbiota abundance (genus Parasutterella id.2892) on E3 ubiquitin−protein ligase parkin
with a threshold of 1e−05
rs8039785
rs7303158
rs62273907
rs6828768
rs2090816
rs6809952
rs2387977
rs35055552
rs7311004
rs10899911
rs823424
rs11715853
rs55877868
rs78383039
All − MR Egger
All − IVW(MRE)
−1
0
1
MR effect size for
'Gut microbiota abundance (genus Parasutterella id.2892)' on 'E3 ubiquitin−protein ligase parkin'

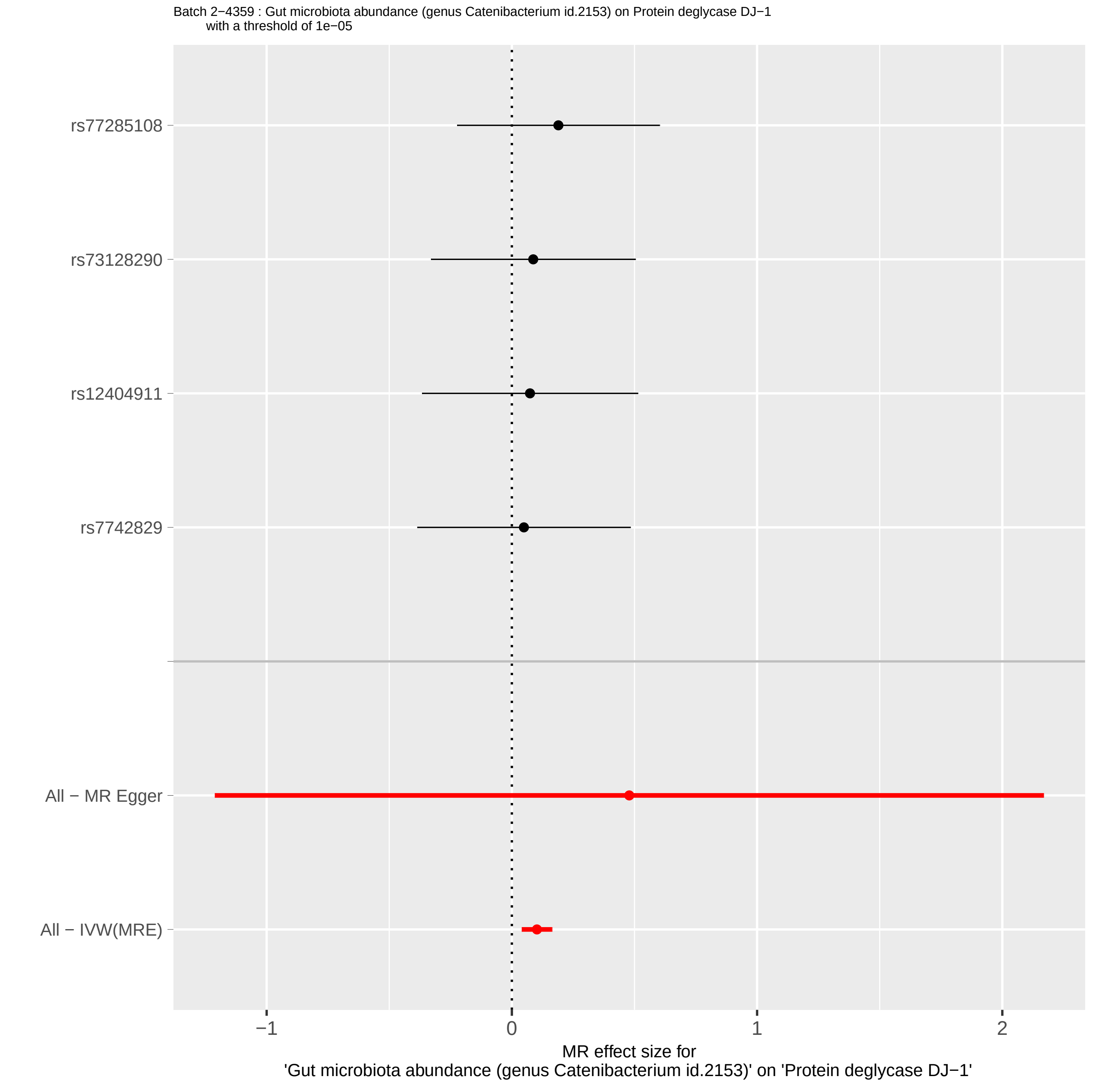
Batch 2−4359 : Gut microbiota abundance (genus Catenibacterium id.2153) on Protein deglycase DJ−1
with a threshold of 1e−05
rs77285108
rs73128290
rs12404911
rs7742829
All − MR Egger
All − IVW(MRE)
−1
0
1
2
MR effect size for
'Gut microbiota abundance (genus Catenibacterium id.2153)' on 'Protein deglycase DJ−1'

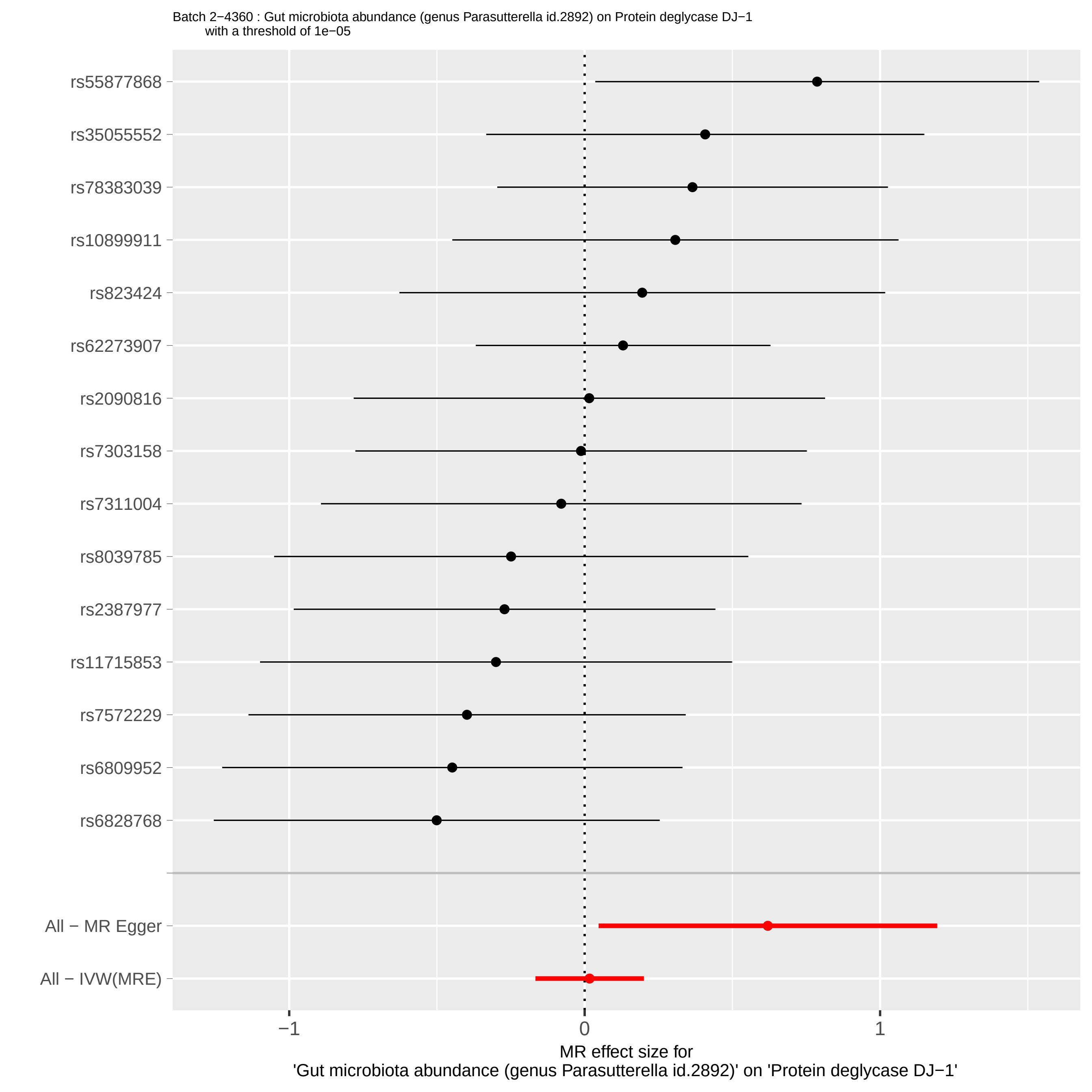

Batch 2−4360 : Gut microbiota abundance (genus Parasutterella id.2892) on Protein deglycase DJ−1
with a threshold of 1e−05
rs55877868
rs35055552
rs78383039
rs10899911
rs823424
rs62273907
rs2090816
rs7303158
rs7311004
rs8039785
rs2387977
rs11715853
rs7572229
rs6809952
rs6828768
All − MR Egger
All − IVW(MRE)
−1
0
1
MR effect size for
'Gut microbiota abundance (genus Parasutterella id.2892)' on 'Protein deglycase DJ−1'

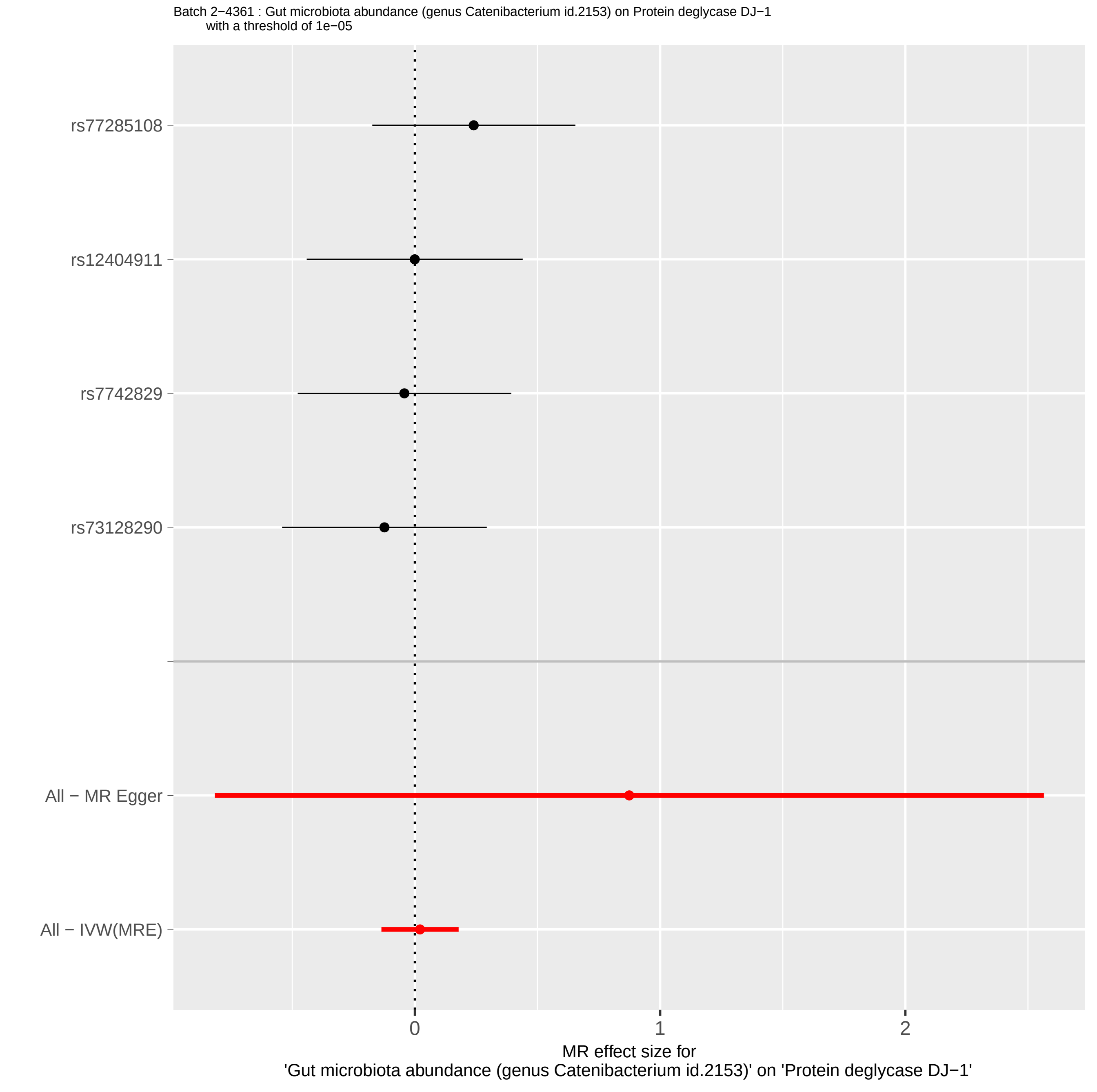

Batch 2−4361 : Gut microbiota abundance (genus Catenibacterium id.2153) on Protein deglycase DJ−1
with a threshold of 1e−05
rs77285108
rs12404911
rs7742829
rs73128290
All − MR Egger
All − IVW(MRE)
0
1
2
MR effect size for
'Gut microbiota abundance (genus Catenibacterium id.2153)' on 'Protein deglycase DJ−1'

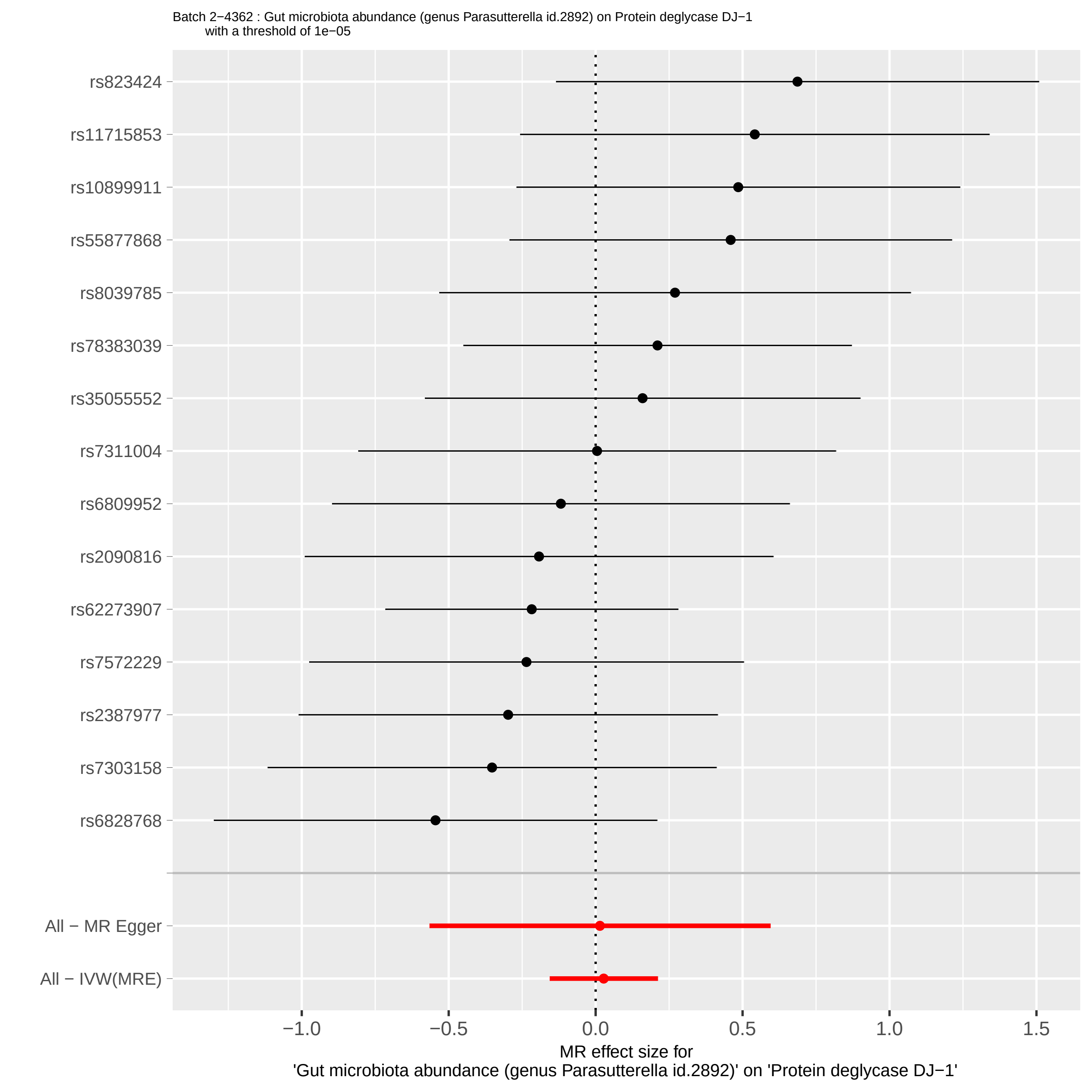
Batch 2−4362 : Gut microbiota abundance (genus Parasutterella id.2892) on Protein deglycase DJ−1
with a threshold of 1e−05
rs823424
rs11715853
rs10899911
rs55877868
rs8039785
rs78383039
rs35055552
rs7311004
rs6809952
rs2090816
rs62273907
rs7572229
rs2387977
rs7303158
rs6828768
All − MR Egger
All − IVW(MRE)
−1.0
−0.5
0.0
0.5
1.0
1.5
MR effect size for
'Gut microbiota abundance (genus Parasutterella id.2892)' on 'Protein deglycase DJ−1'

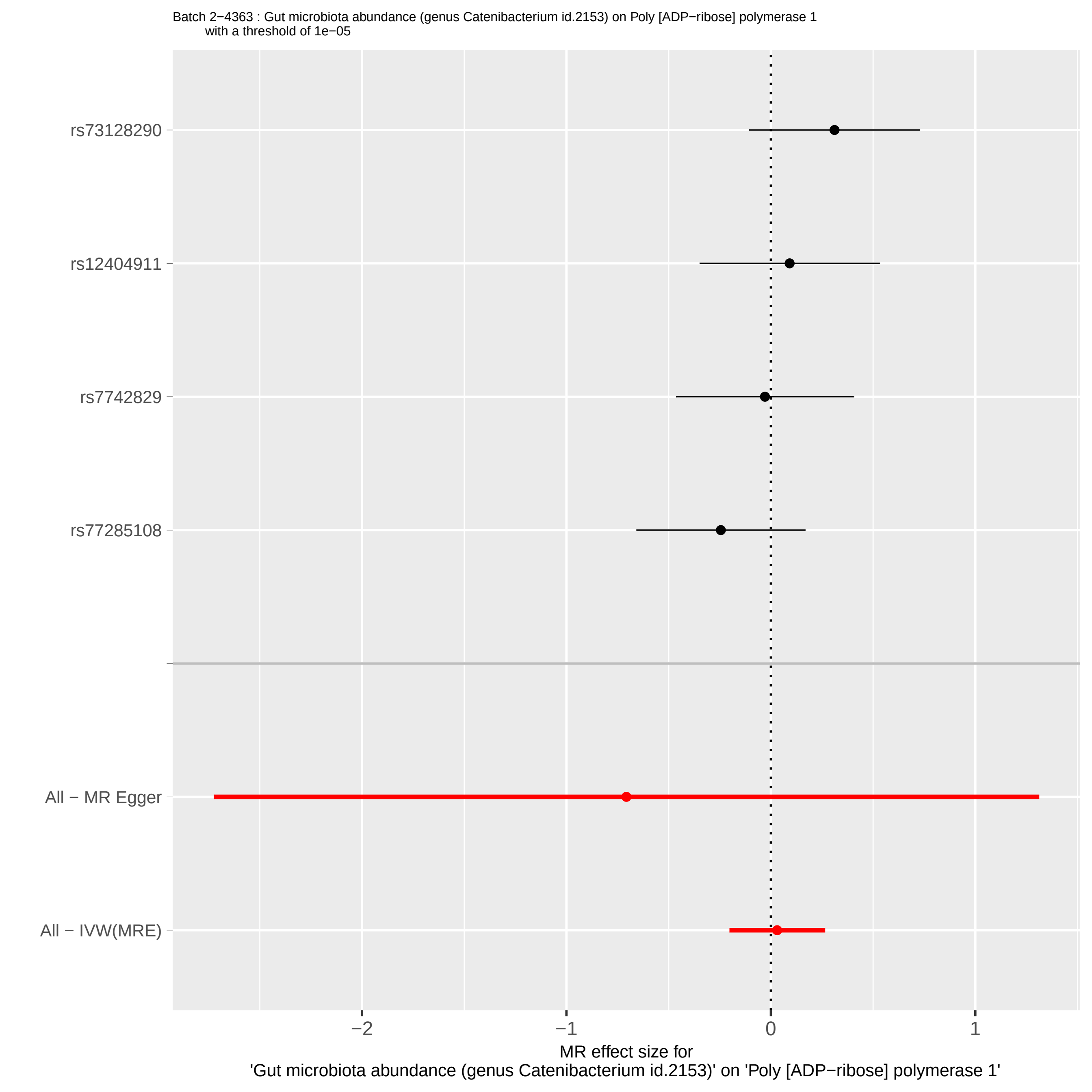

Batch 2−4363 : Gut microbiota abundance (genus Catenibacterium id.2153) on Poly [ADP−ribose] polymerase 1
with a threshold of 1e−05
rs73128290
rs12404911
rs7742829
rs77285108
All − MR Egger
All − IVW(MRE)
−2
−1
0
1
MR effect size for
'Gut microbiota abundance (genus Catenibacterium id.2153)' on 'Poly [ADP−ribose] polymerase 1'

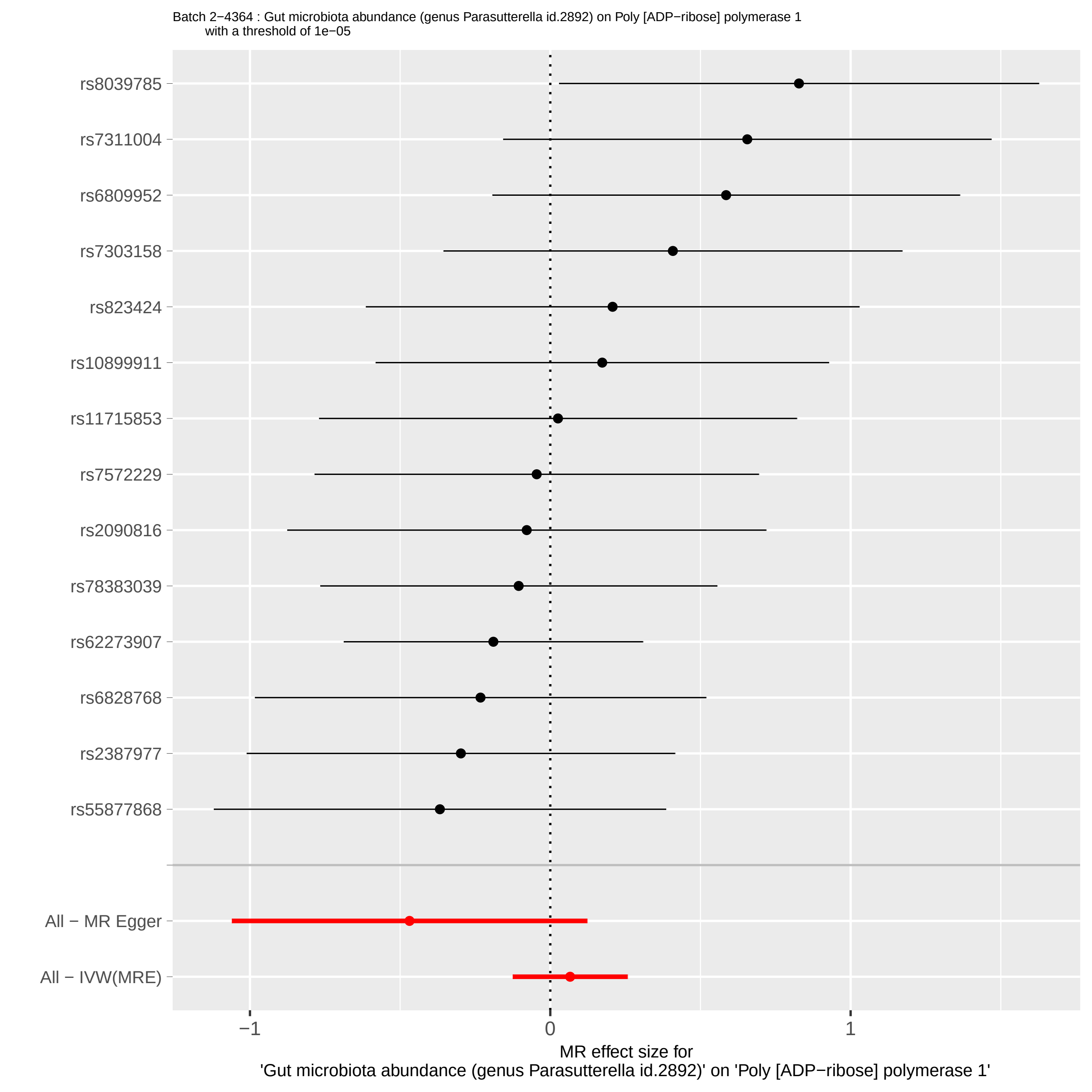

Batch 2−4364 : Gut microbiota abundance (genus Parasutterella id.2892) on Poly [ADP−ribose] polymerase 1
with a threshold of 1e−05
rs8039785
rs7311004
rs6809952
rs7303158
rs823424
rs10899911
rs11715853
rs7572229
rs2090816
rs78383039
rs62273907
rs6828768
rs2387977
rs55877868
All − MR Egger
All − IVW(MRE)
−1
0
1
MR effect size for
'Gut microbiota abundance (genus Parasutterella id.2892)' on 'Poly [ADP−ribose] polymerase 1'

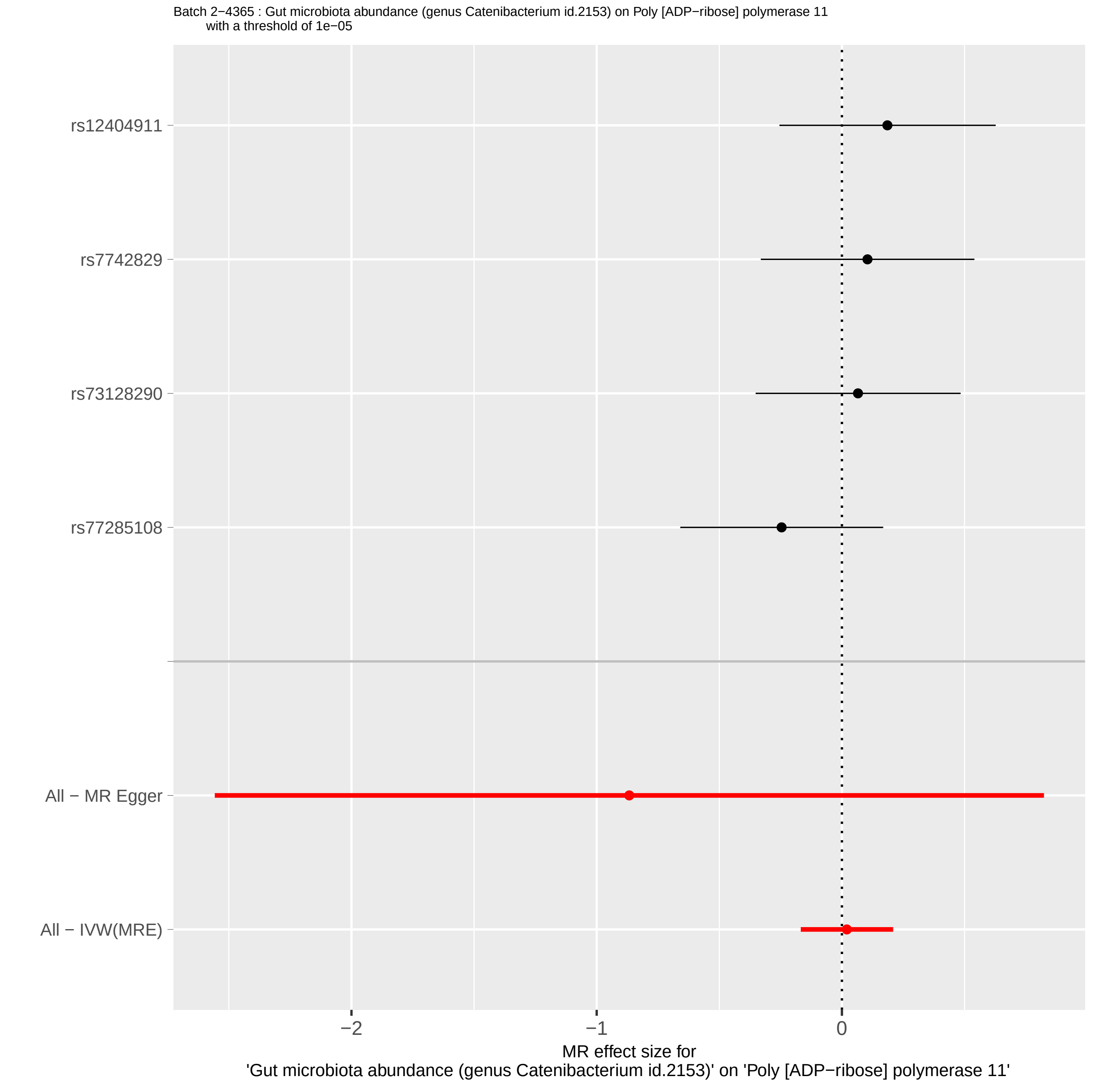
Batch 2−4365 : Gut microbiota abundance (genus Catenibacterium id.2153) on Poly [ADP−ribose] polymerase 11
with a threshold of 1e−05
rs12404911
rs7742829
rs73128290
rs77285108
All − MR Egger
All − IVW(MRE)
−2
−1
0
MR effect size for
'Gut microbiota abundance (genus Catenibacterium id.2153)' on 'Poly [ADP−ribose] polymerase 11

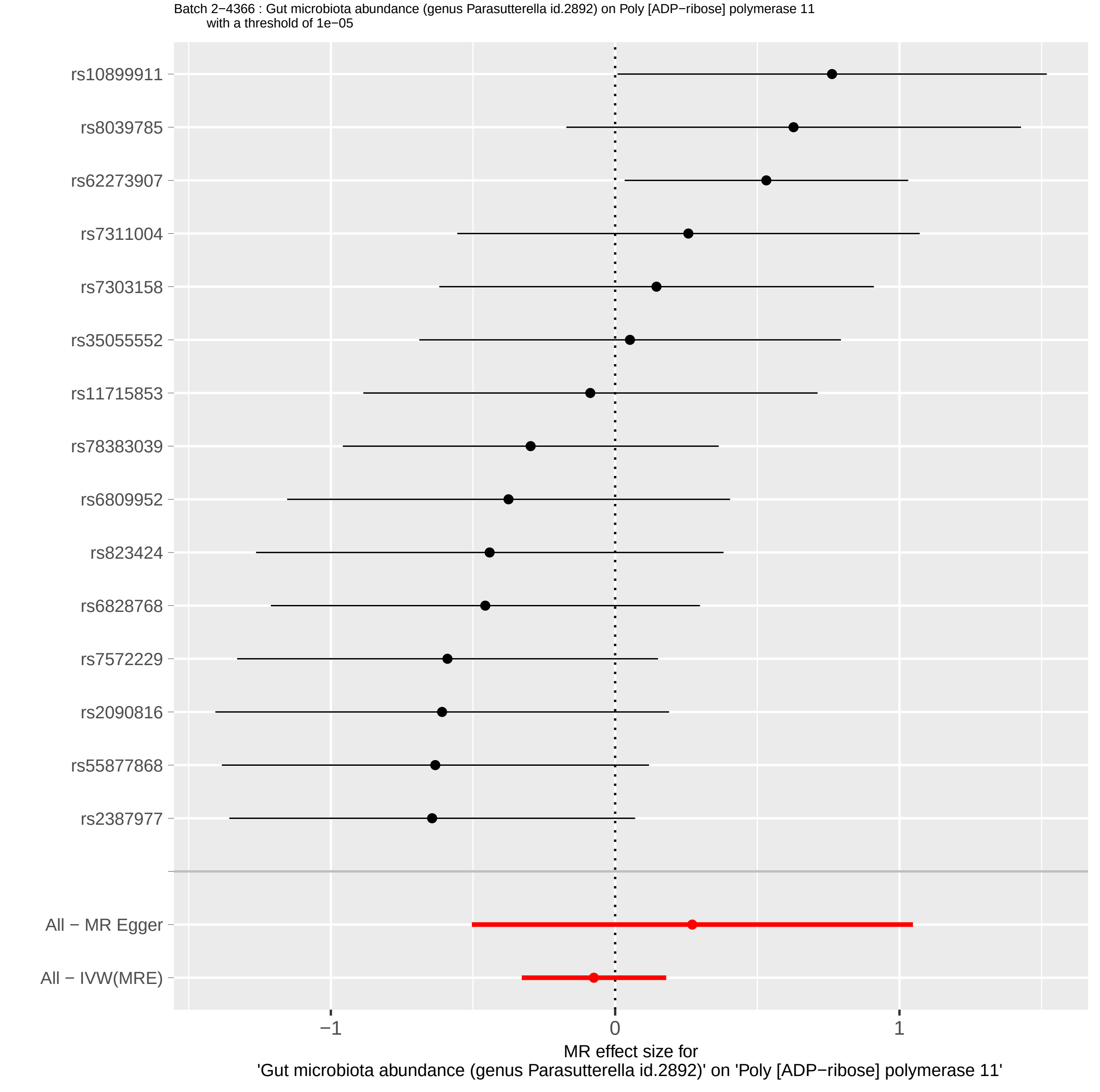
Batch 2−4366 : Gut microbiota abundance (genus Parasutterella id.2892) on Poly [ADP−ribose] polymerase 11
with a threshold of 1e−05
rs10899911
rs8039785
rs62273907
rs7311004
rs7303158
rs35055552
rs11715853
rs78383039
rs6809952
rs823424
rs6828768
rs7572229
rs2090816
rs55877868
rs2387977
All − MR Egger
All − IVW(MRE)
−1
0
1
MR effect size for
'Gut microbiota abundance (genus Parasutterella id.2892)' on 'Poly [ADP−ribose] polymerase 11'

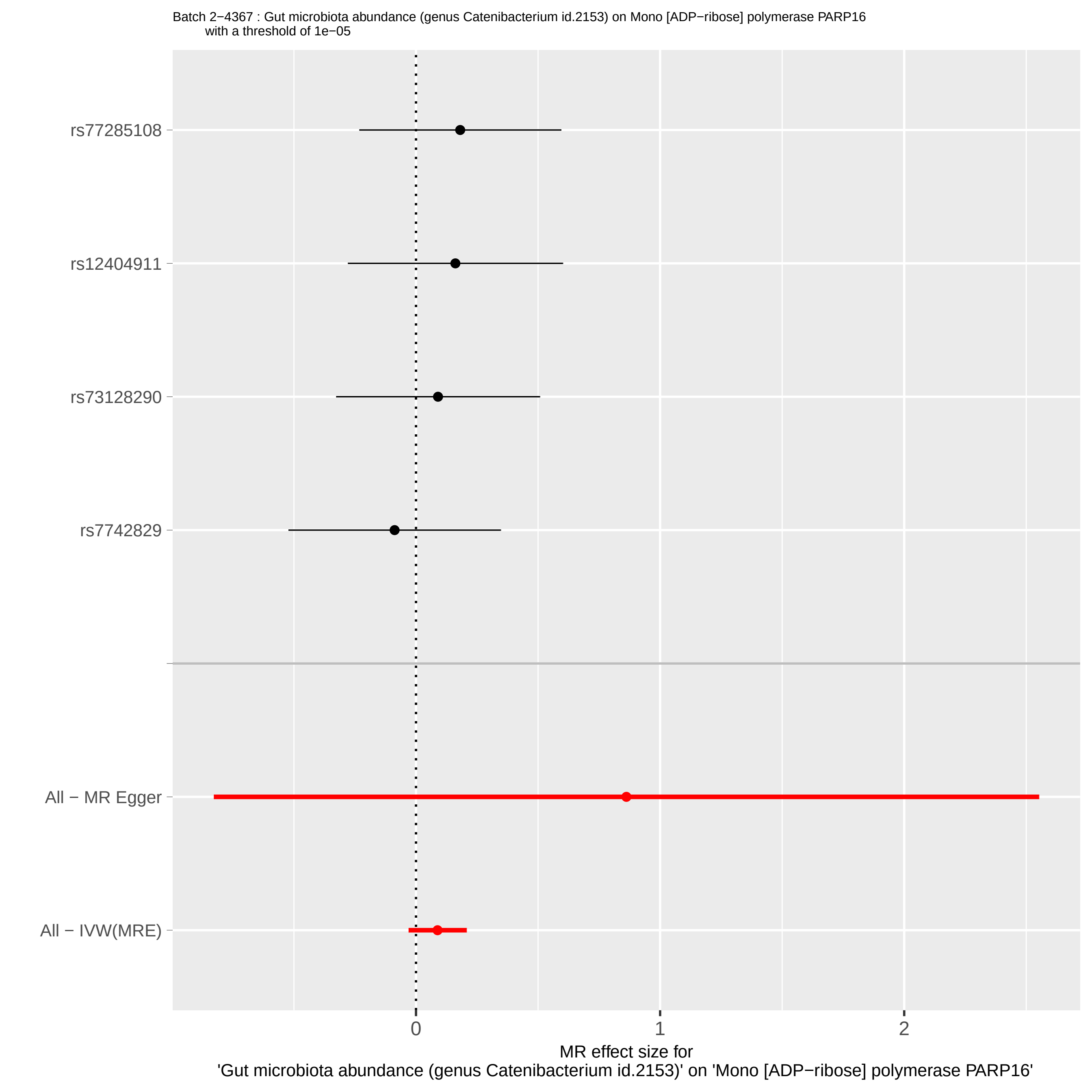

Batch 2−4367 : Gut microbiota abundance (genus Catenibacterium id.2153) on Mono [ADP−ribose] polymerase PARP16
with a threshold of 1e−05
rs77285108
rs12404911
rs73128290
rs7742829
All − MR Egger
All − IVW(MRE)
0
1
2
MR effect size for
'Gut microbiota abundance (genus Catenibacterium id.2153)' on 'Mono [ADP−ribose] polymerase PARP16'

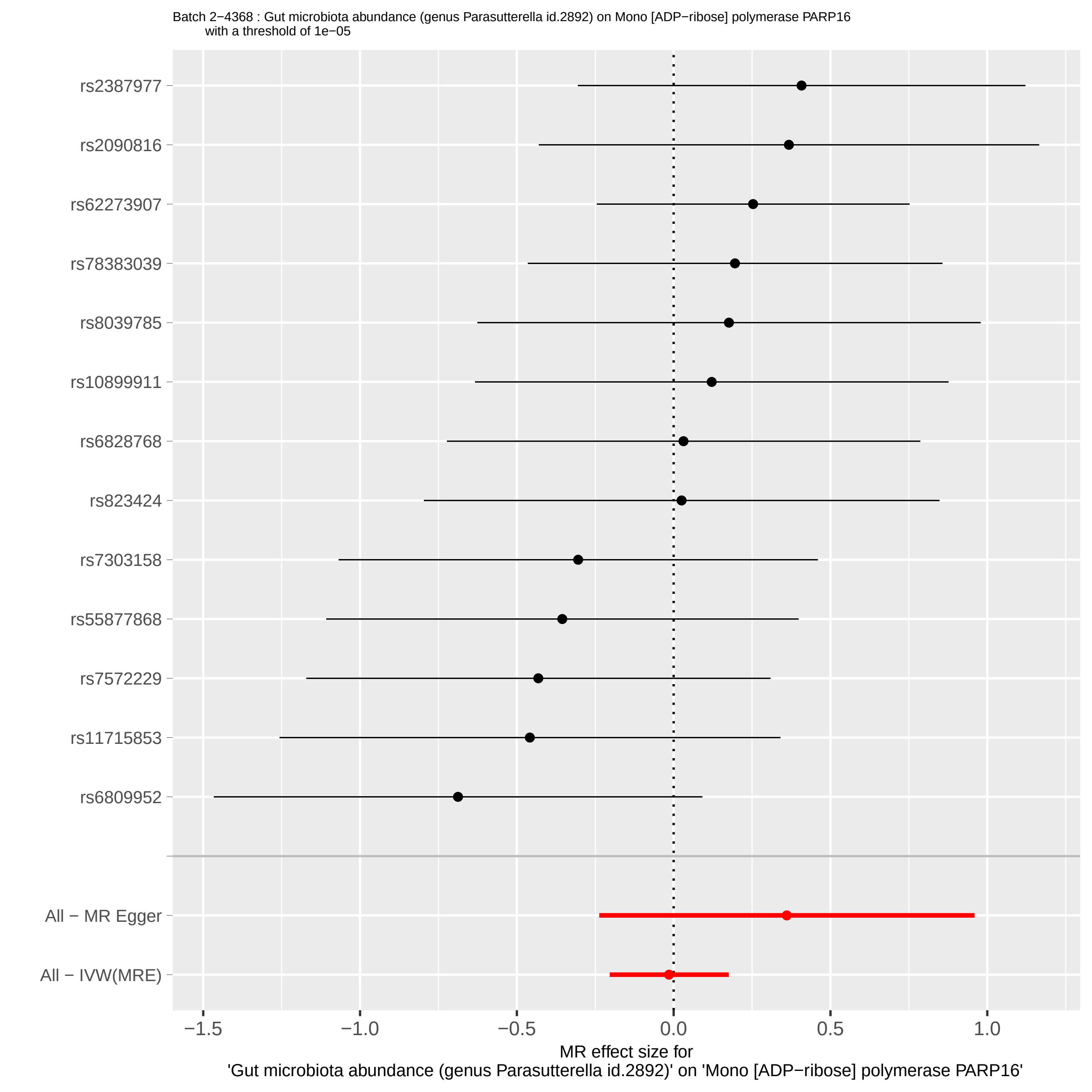

Batch 2−4368 : Gut microbiota abundance (genus Parasutterella id.2892) on Mono [ADP−ribose] polymerase PARP16
with a threshold of 1e−05
rs2387977
rs2090816
rs62273907
rs78383039
rs8039785
rs10899911
rs6828768
rs823424
rs7303158
rs55877868
rs7572229
rs11715853
rs6809952
All − MR Egger
All − IVW(MRE)
−1.5
−1.0
−0.5
0.0
0.5
1.0
MR effect size for
'Gut microbiota abundance (genus Parasutterella id.2892)' on 'Mono [ADP−ribose] polymerase PARP16'

Batch 2−4369 : Gut microbiota abundance (genus Catenibacterium id.2153) on Alpha−parvin
with a threshold of 1e−05

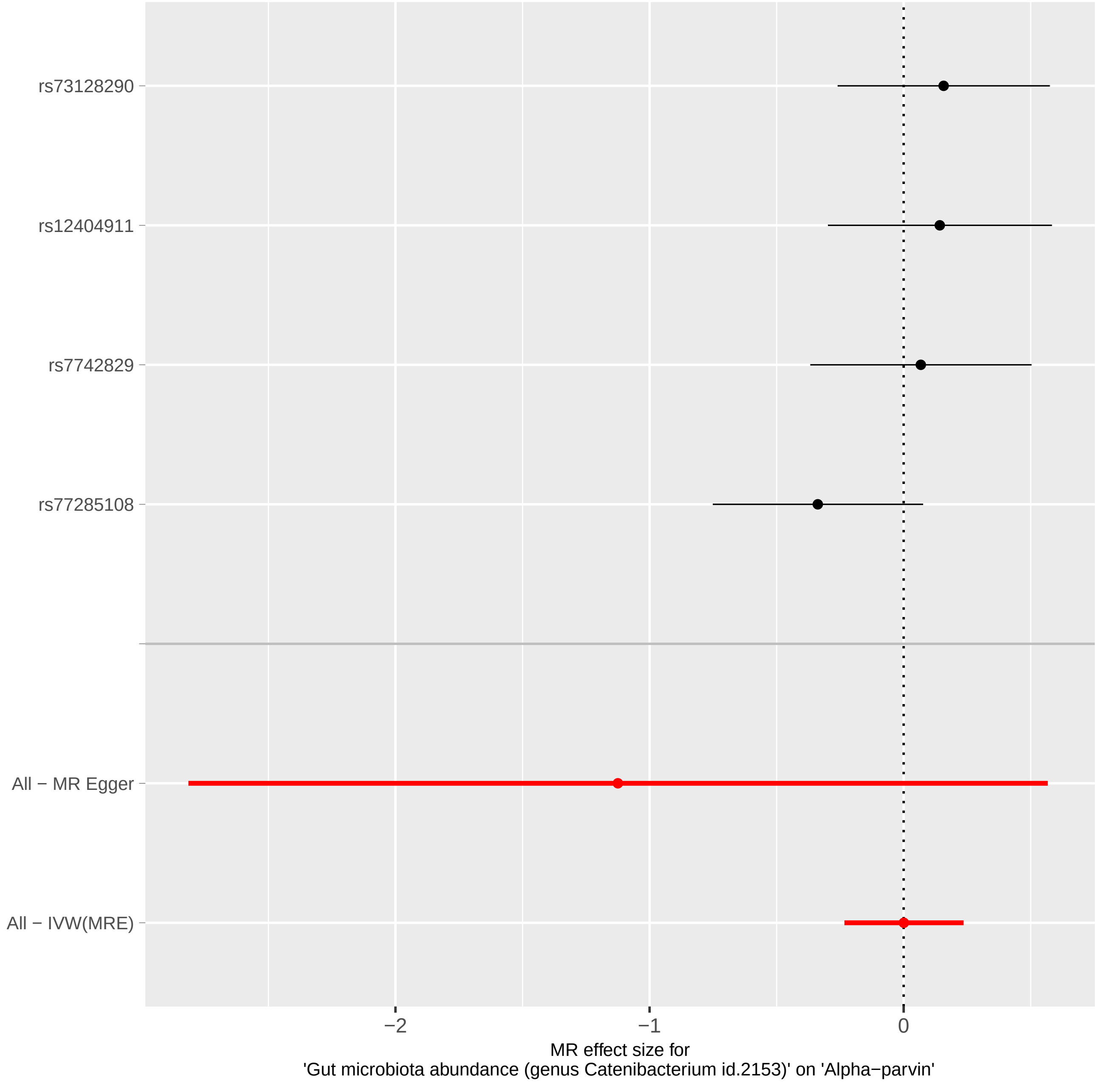

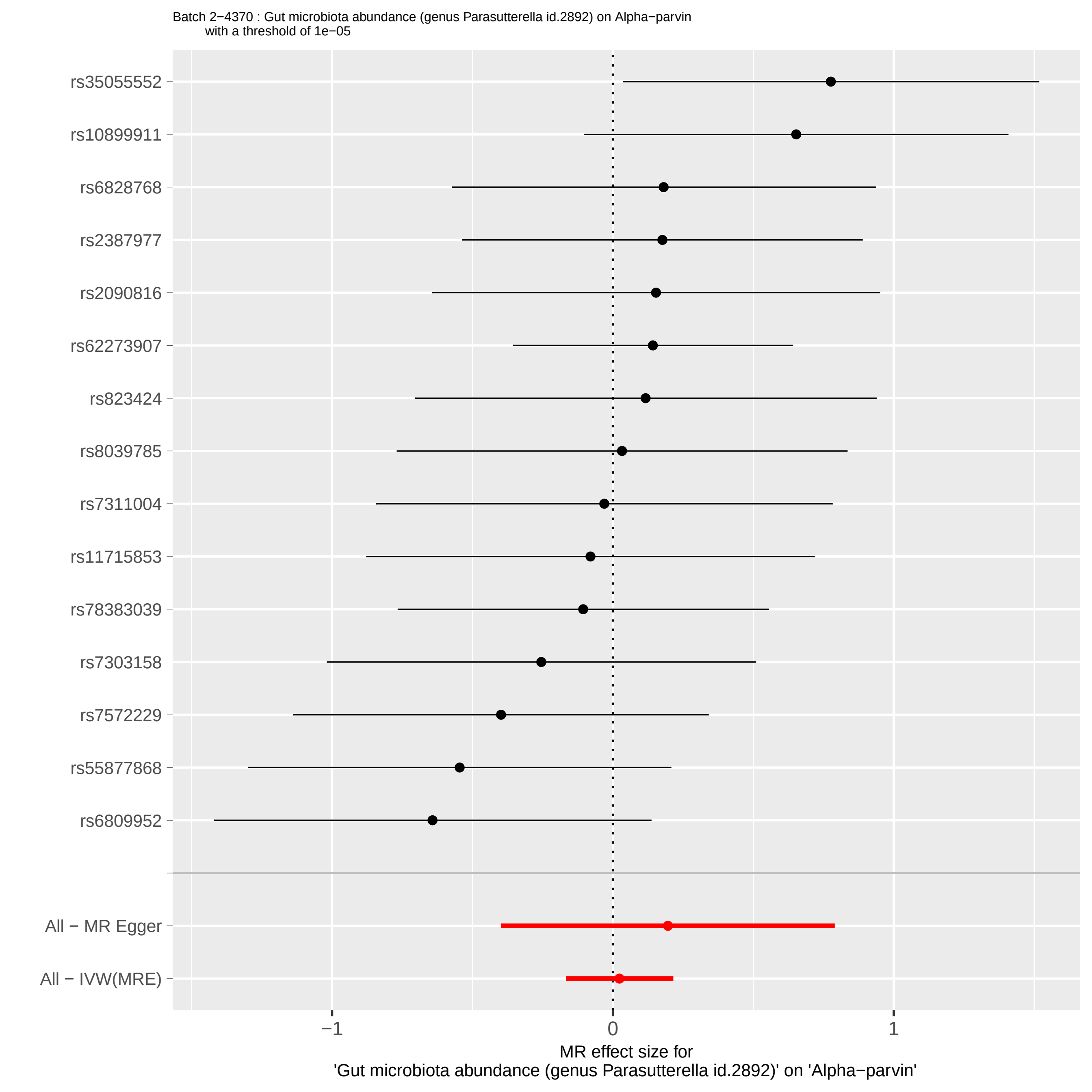

Batch 2−4370 : Gut microbiota abundance (genus Parasutterella id.2892) on Alpha−parvin
with a threshold of 1e−05
rs35055552
rs10899911
rs6828768
rs2387977
rs2090816
rs62273907
rs823424
rs8039785
rs7311004
rs11715853
rs78383039
rs7303158
rs7572229
rs55877868
rs6809952
All − MR Egger
All − IVW(MRE)
−1
0
1
MR effect size for
'Gut microbiota abundance (genus Parasutterella id.2892)' on 'Alpha−parvin'

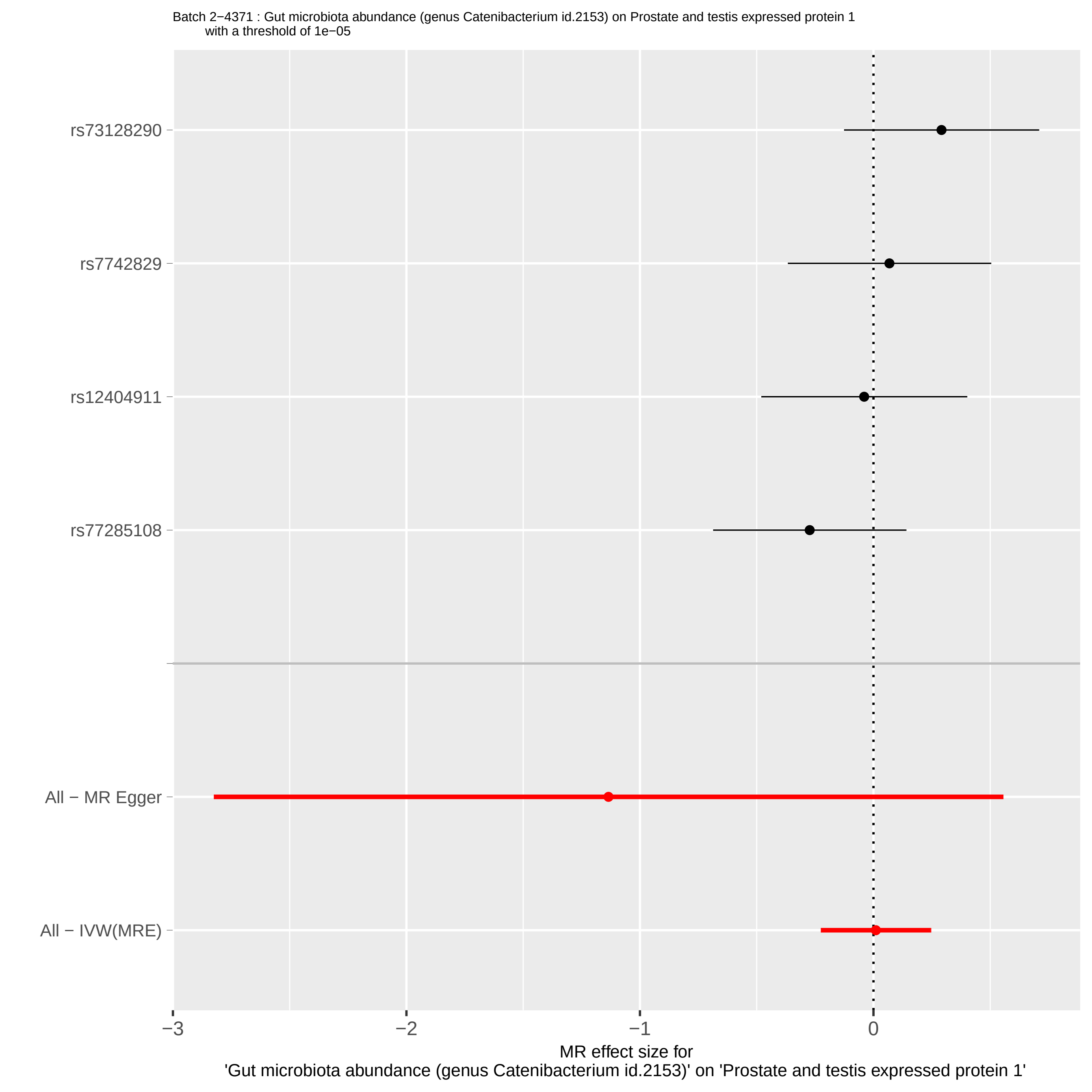

Batch 2−4371 : Gut microbiota abundance (genus Catenibacterium id.2153) on Prostate and testis expressed protein 1
with a threshold of 1e−05
rs73128290
rs7742829
rs12404911
rs77285108
All − MR Egger
All − IVW(MRE)
−3
−2
−1
0
MR effect size for
'Gut microbiota abundance (genus Catenibacterium id.2153)' on 'Prostate and testis expressed protein 1'

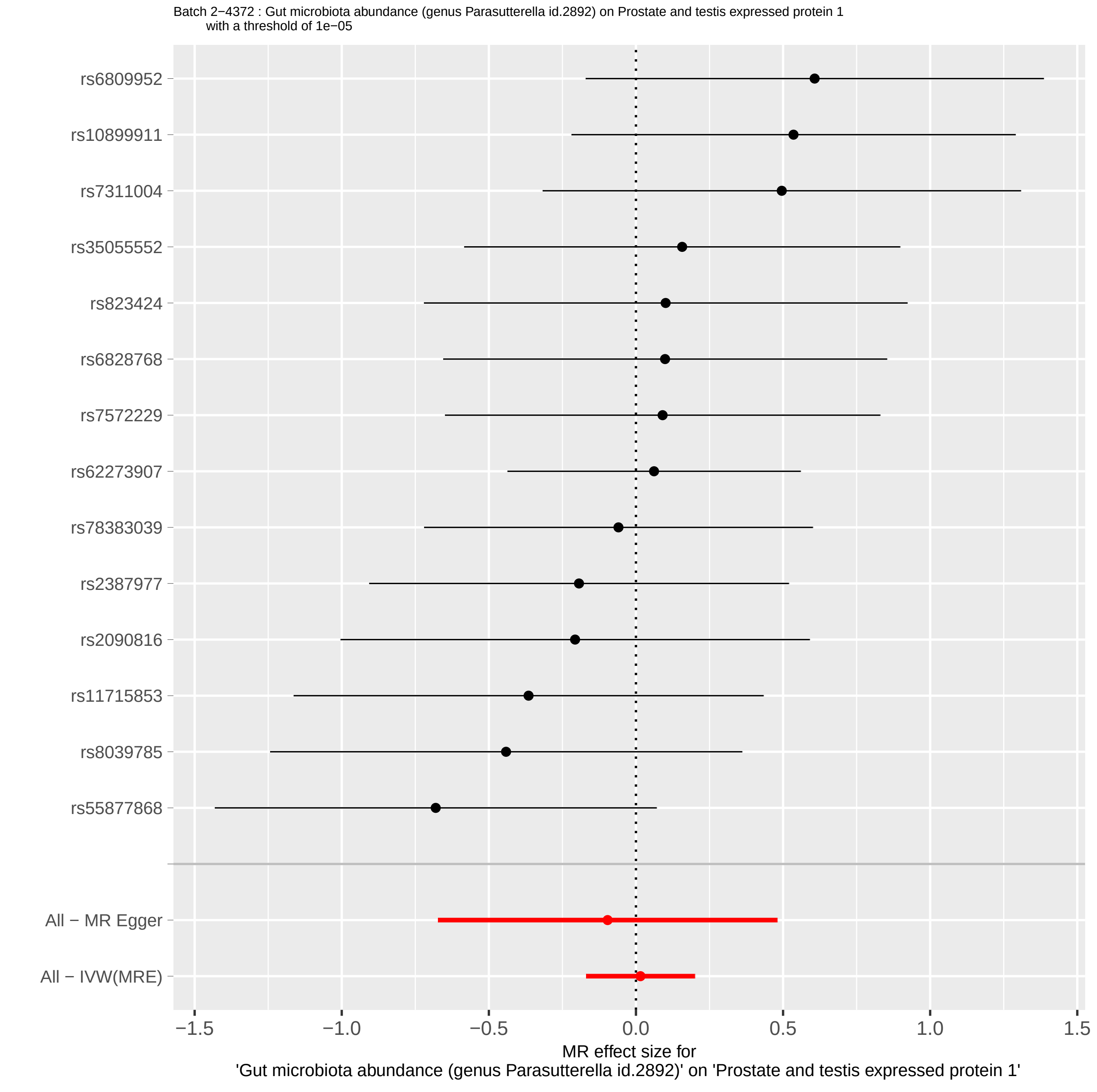

Batch 2−4372 : Gut microbiota abundance (genus Parasutterella id.2892) on Prostate and testis expressed protein 1
with a threshold of 1e−05
rs6809952
rs10899911
rs7311004
rs35055552
rs823424
rs6828768
rs7572229
rs62273907
rs78383039
rs2387977
rs2090816
rs11715853
rs8039785
rs55877868
All − MR Egger
All − IVW(MRE)
−1.5
−1.0
−0.5
0.0
0.5
1.0
1.5
MR effect size for
'Gut microbiota abundance (genus Parasutterella id.2892)' on 'Prostate and testis expressed protein 1'

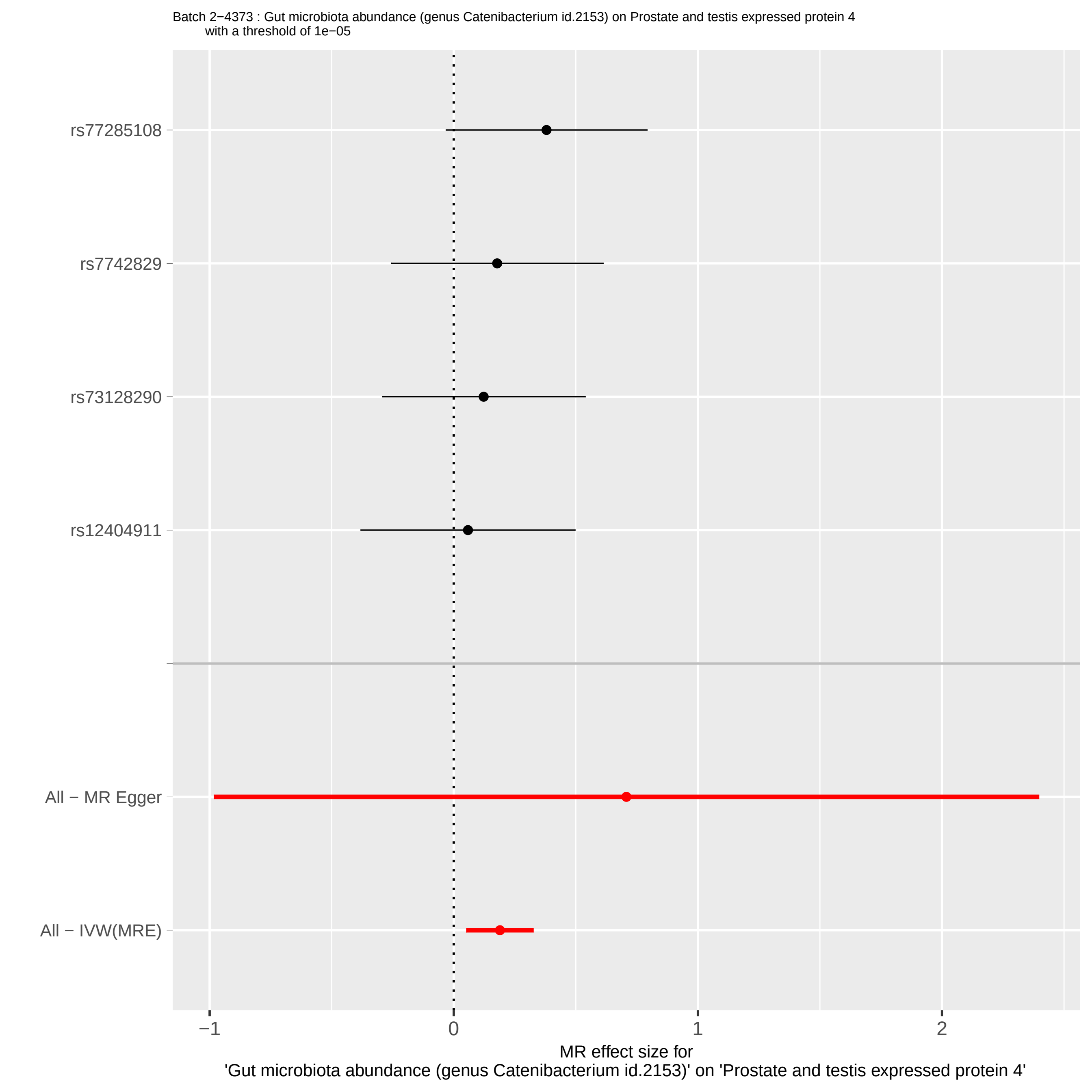
Batch 2−4373 : Gut microbiota abundance (genus Catenibacterium id.2153) on Prostate and testis expressed protein 4
with a threshold of 1e−05
rs77285108
rs7742829
rs73128290
rs12404911
All − MR Egger
All − IVW(MRE)
−1
0
1
2
MR effect size for
'Gut microbiota abundance (genus Catenibacterium id.2153)' on 'Prostate and testis expressed protein 4'

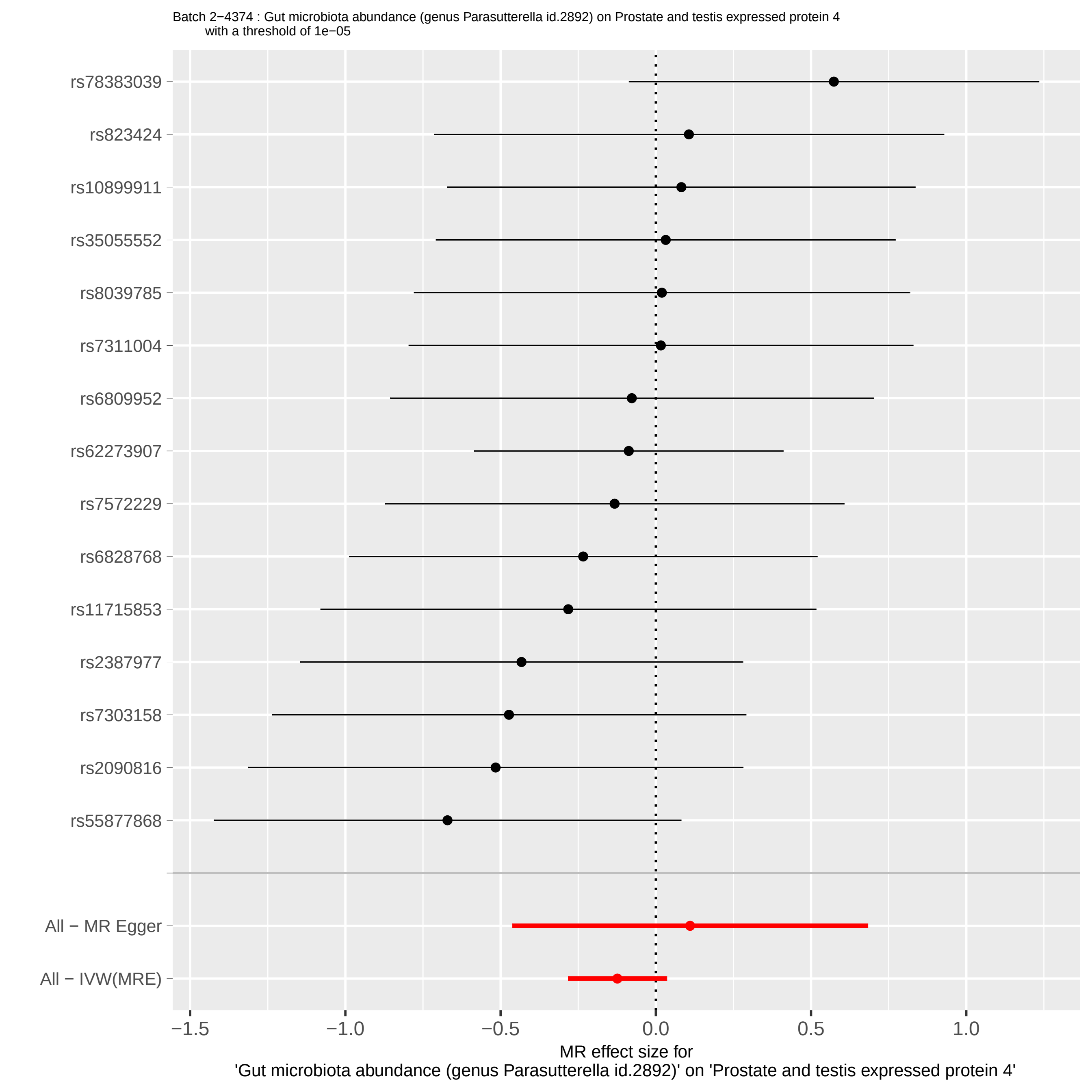
Batch 2−4374 : Gut microbiota abundance (genus Parasutterella id.2892) on Prostate and testis expressed protein 4
with a threshold of 1e−05
rs78383039
rs823424
rs10899911
rs35055552
rs8039785
rs7311004
rs6809952
rs62273907
rs7572229
rs6828768
rs11715853
rs2387977
rs7303158
rs2090816
rs55877868
All − MR Egger
All − IVW(MRE)
−1.5
−1.0
−0.5
0.0
0.5
1.0
MR effect size for
'Gut microbiota abundance (genus Parasutterella id.2892)' on 'Prostate and testis expressed protein 4'

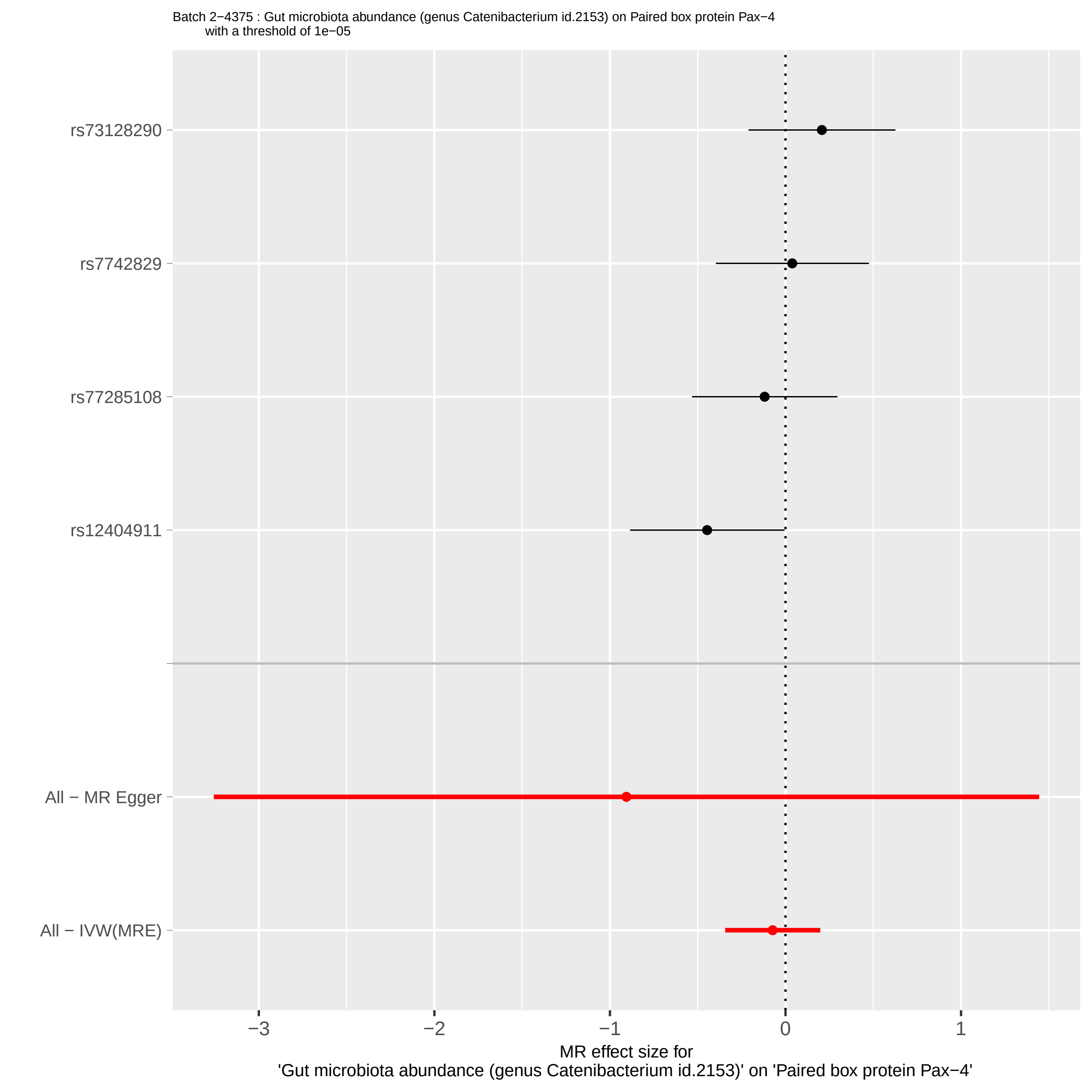

Batch 2−4375 : Gut microbiota abundance (genus Catenibacterium id.2153) on Paired box protein Pax−4
with a threshold of 1e−05
rs73128290
rs7742829
rs77285108
rs12404911
All − MR Egger
All − IVW(MRE)
−3
−2
−1
0
1
MR effect size for
'Gut microbiota abundance (genus Catenibacterium id.2153)' on 'Paired box protein Pax−4'

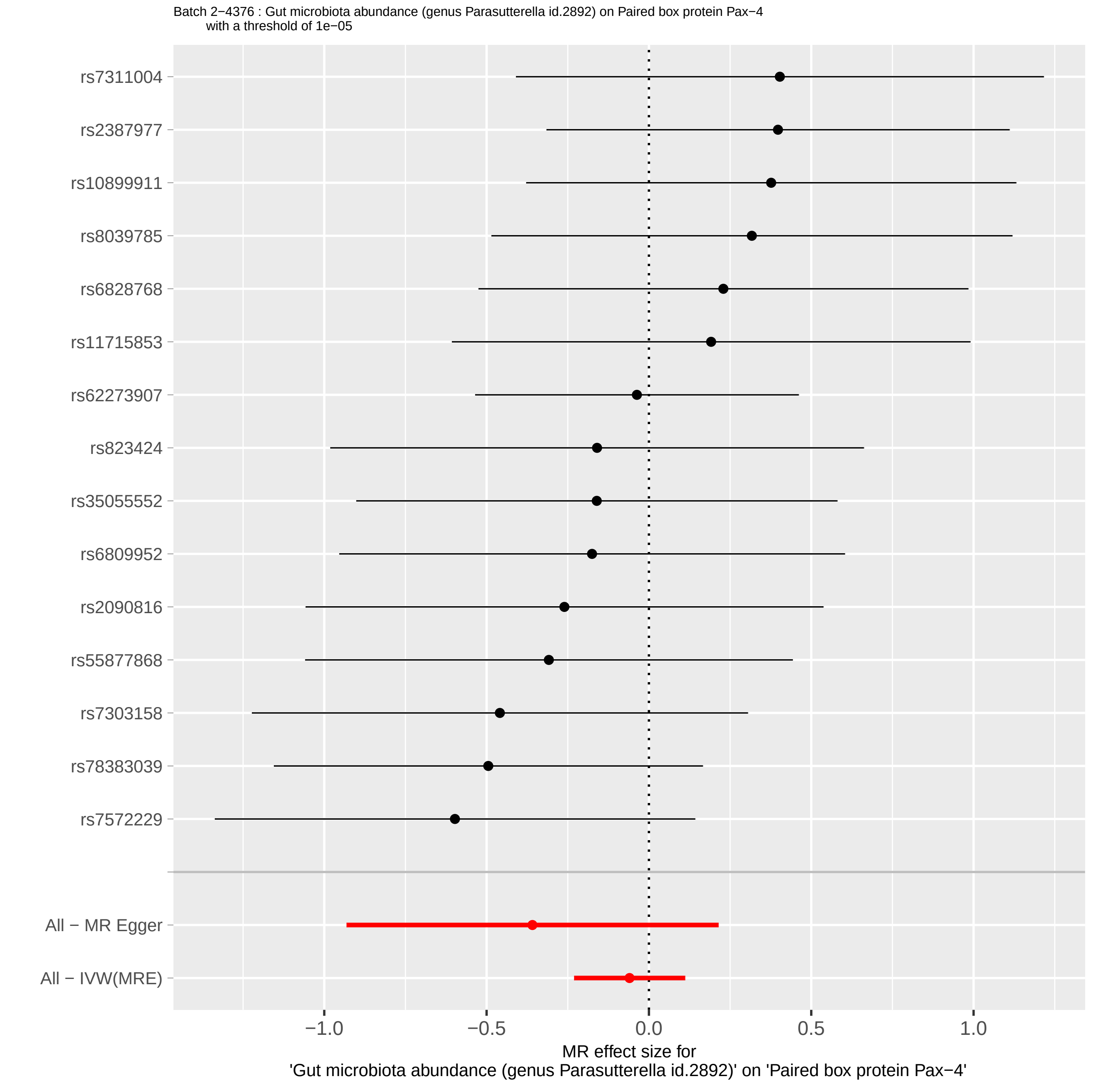
Batch 2−4376 : Gut microbiota abundance (genus Parasutterella id.2892) on Paired box protein Pax−4
with a threshold of 1e−05
rs7311004
rs2387977
rs10899911
rs8039785
rs6828768
rs11715853
rs62273907
rs823424
rs35055552
rs6809952
rs2090816
rs55877868
rs7303158
rs78383039
rs7572229
All − MR Egger
All − IVW(MRE)
−1.0
−0.5
0.0
0.5
1.0
MR effect size for
'Gut microbiota abundance (genus Parasutterella id.2892)' on 'Paired box protein Pax−4'

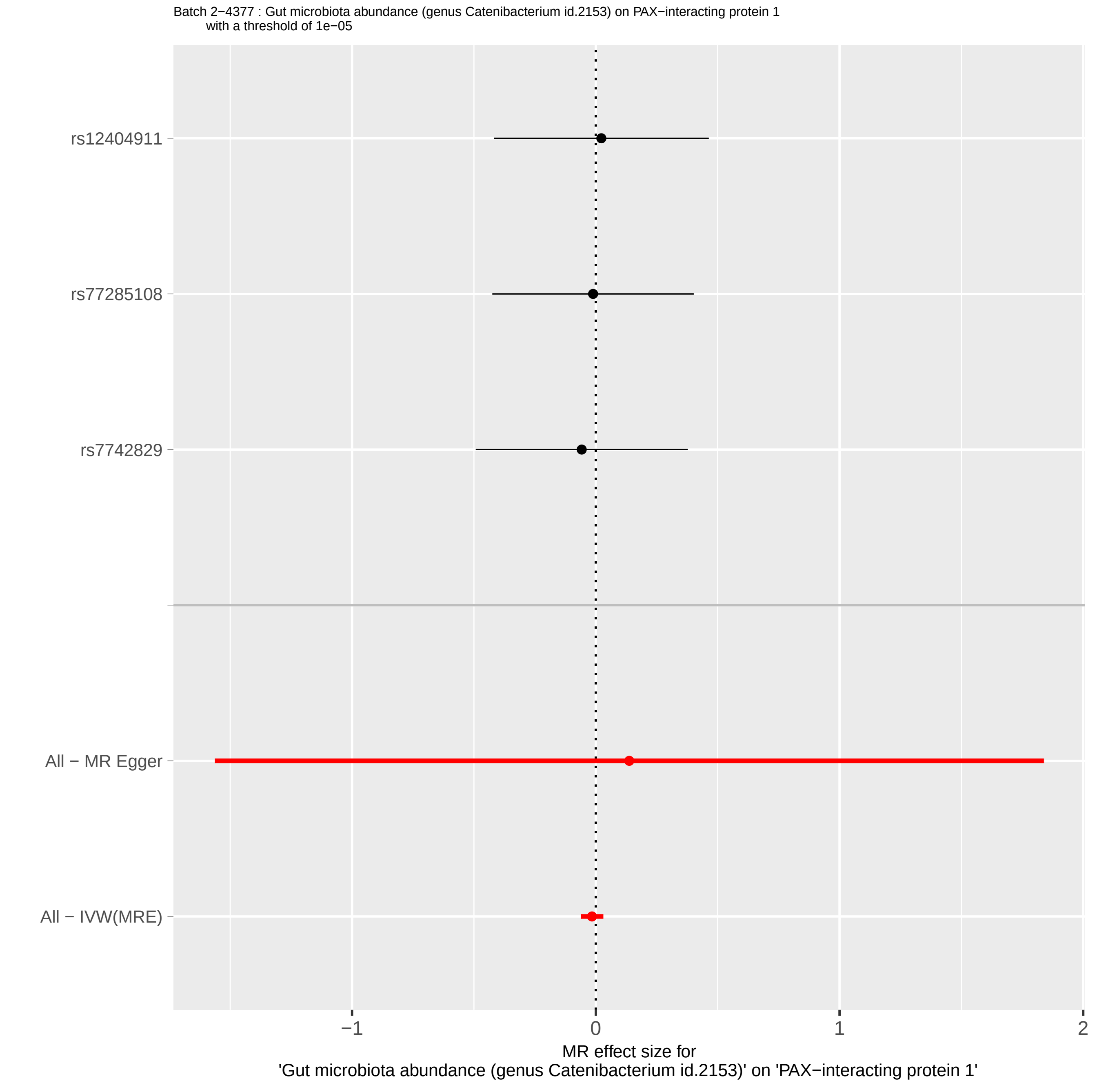

Batch 2−4377 : Gut microbiota abundance (genus Catenibacterium id.2153) on PAX−interacting protein 1
with a threshold of 1e−05
rs12404911
rs77285108
rs7742829
All − MR Egger
All − IVW(MRE)
−1
0
1
2
MR effect size for
'Gut microbiota abundance (genus Catenibacterium id.2153)' on 'PAX−interacting protein 1'

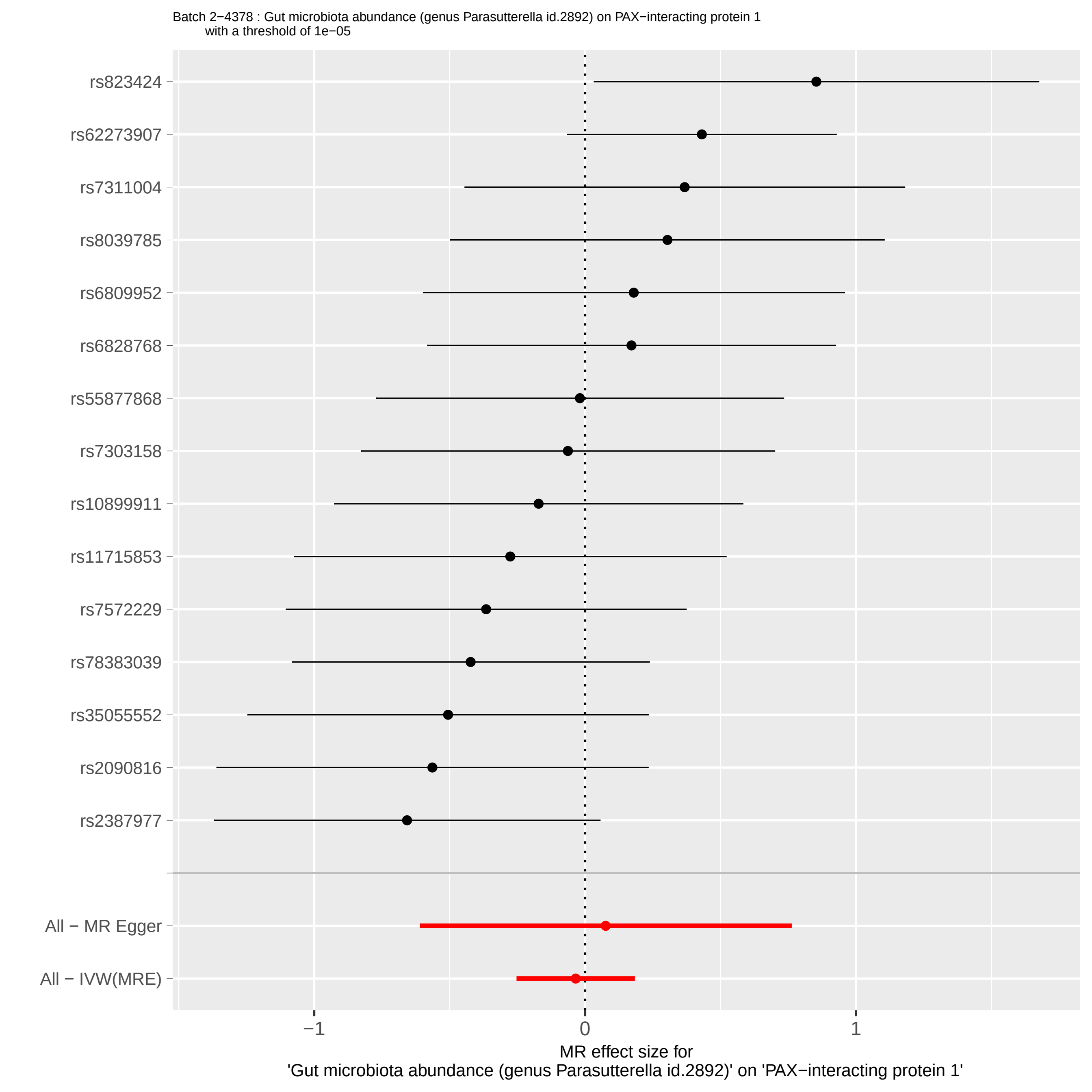
Batch 2−4378 : Gut microbiota abundance (genus Parasutterella id.2892) on PAX−interacting protein 1
with a threshold of 1e−05
rs823424
rs62273907
rs7311004
rs8039785
rs6809952
rs6828768
rs55877868
rs7303158
rs10899911
rs11715853
rs7572229
rs78383039
rs35055552
rs2090816
rs2387977
All − MR Egger
All − IVW(MRE)
−1
0
1
MR effect size for
'Gut microbiota abundance (genus Parasutterella id.2892)' on 'PAX−interacting protein 1'

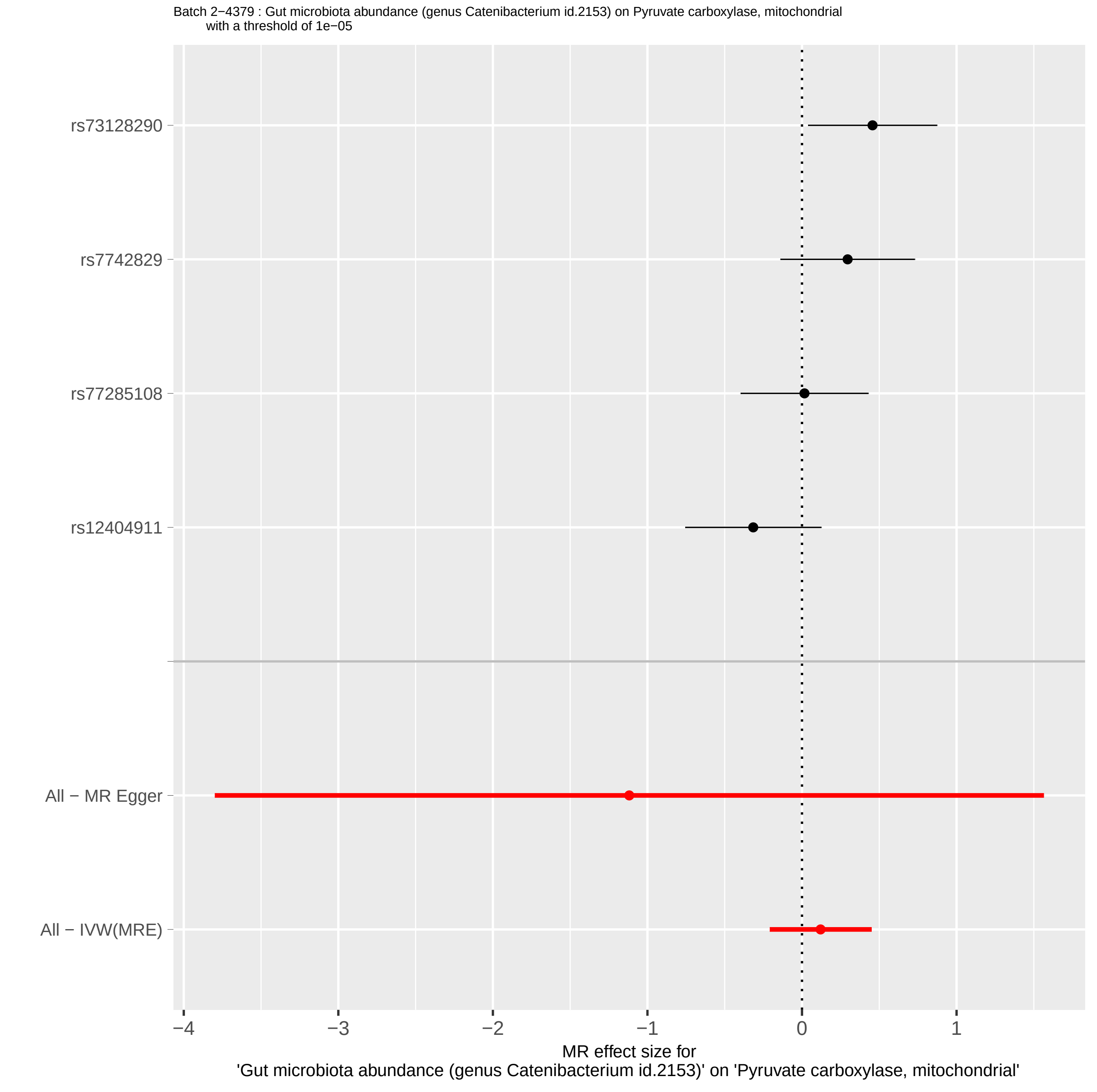

Batch 2−4379 : Gut microbiota abundance (genus Catenibacterium id.2153) on Pyruvate carboxylase, mitochondrial
with a threshold of 1e−05
rs73128290
rs7742829
rs77285108
rs12404911
All − MR Egger
All − IVW(MRE)
−4
−3
−2
−1
0
1
MR effect size for
'Gut microbiota abundance (genus Catenibacterium id.2153)' on 'Pyruvate carboxylase, mitochondrial'

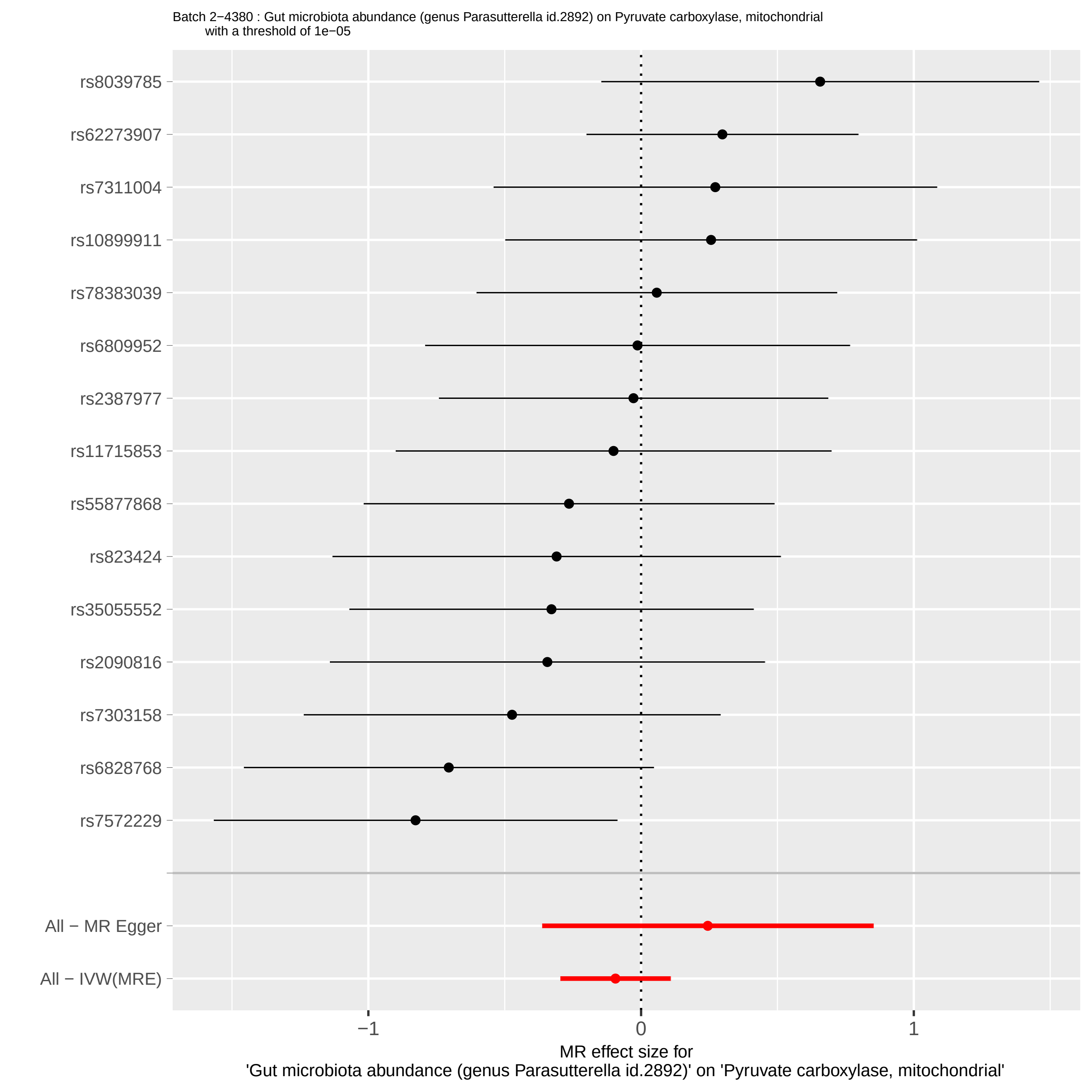

Batch 2−4380 : Gut microbiota abundance (genus Parasutterella id.2892) on Pyruvate carboxylase, mitochondrial
with a threshold of 1e−05
rs8039785
rs62273907
rs7311004
rs10899911
rs78383039
rs6809952
rs2387977
rs11715853
rs55877868
rs823424
rs35055552
rs2090816
rs7303158
rs6828768
rs7572229
All − MR Egger
All − IVW(MRE)
−1
0
1
MR effect size for
'Gut microbiota abundance (genus Parasutterella id.2892)' on 'Pyruvate carboxylase, mitochondrial'

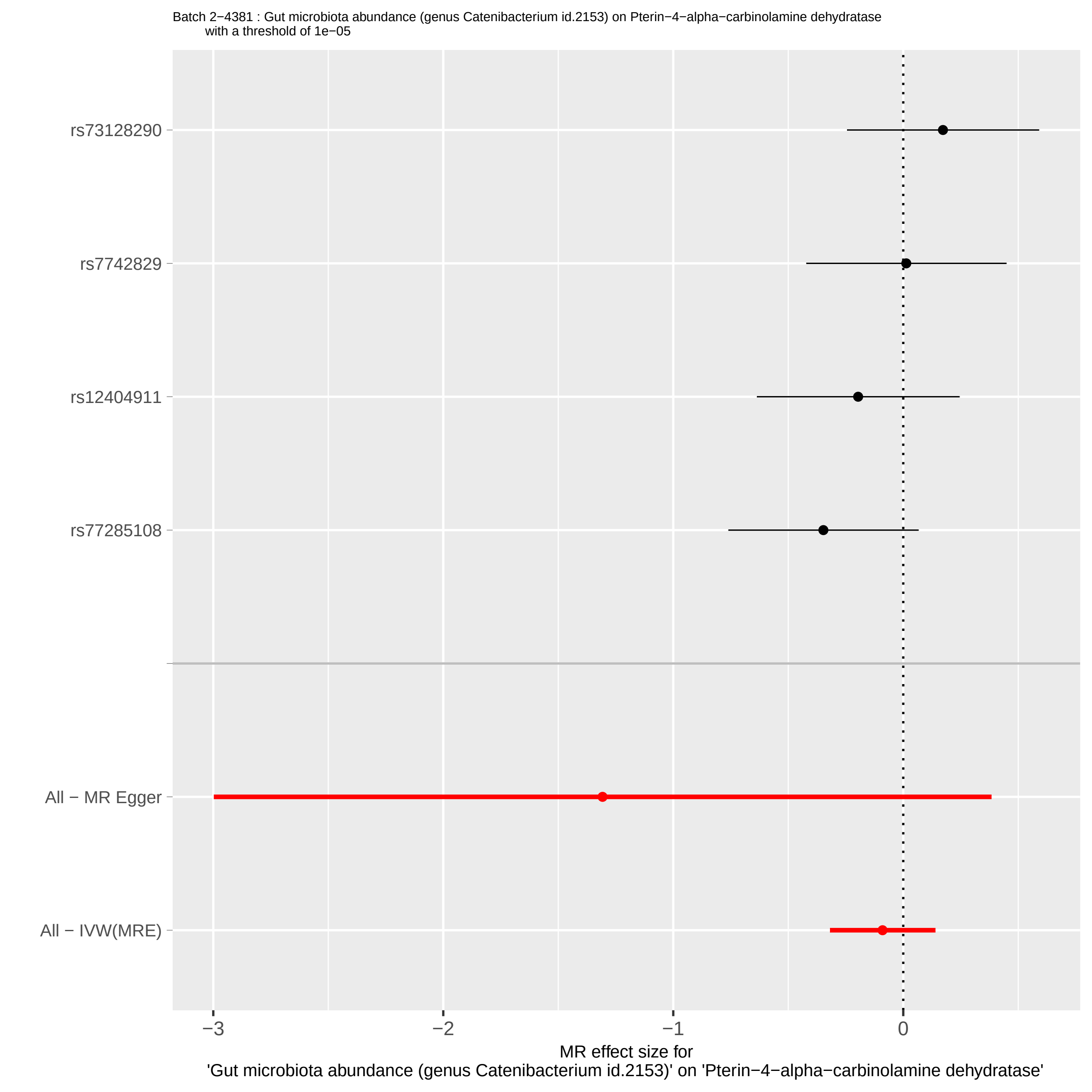
Batch 2−4381 : Gut microbiota abundance (genus Catenibacterium id.2153) on Pterin−4−alpha−carbinolamine dehydratase
with a threshold of 1e−05
rs73128290
rs7742829
rs12404911
rs77285108
All − MR Egger
All − IVW(MRE)
−3
−2
−1
0
MR effect size for
'Gut microbiota abundance (genus Catenibacterium id.2153)' on 'Pterin−4−alpha−carbinolamine dehydratase'

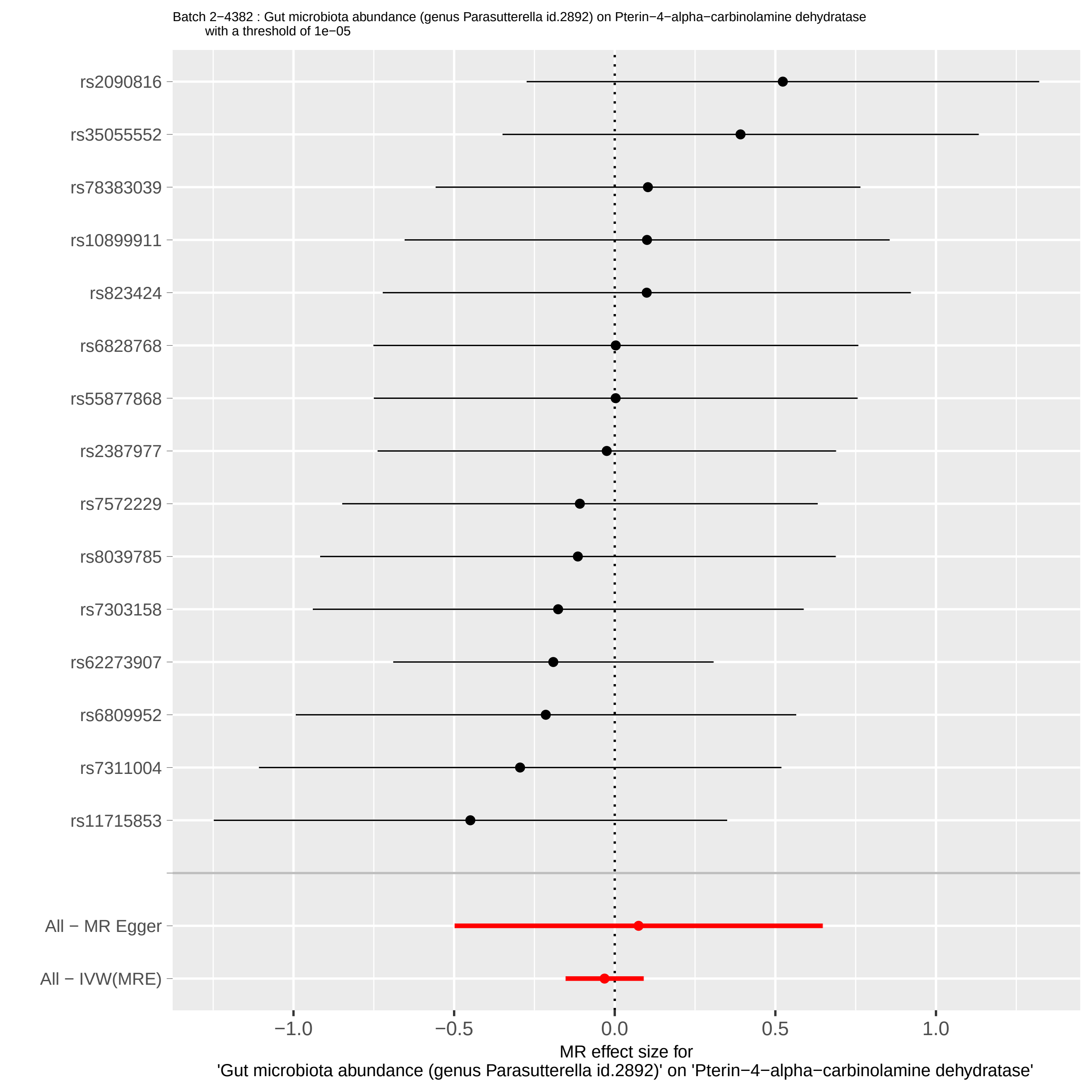
Batch 2−4382 : Gut microbiota abundance (genus Parasutterella id.2892) on Pterin−4−alpha−carbinolamine dehydratase
with a threshold of 1e−05
rs2090816
rs35055552
rs78383039
rs10899911
rs823424
rs6828768
rs55877868
rs2387977
rs7572229
rs8039785
rs7303158
rs62273907
rs6809952
rs7311004
rs11715853
All − MR Egger
All − IVW(MRE)
−1.0
−0.5
0.0
0.5
1.0
MR effect size for
'Gut microbiota abundance (genus Parasutterella id.2892)' on 'Pterin−4−alpha−carbinolamine dehydratase'

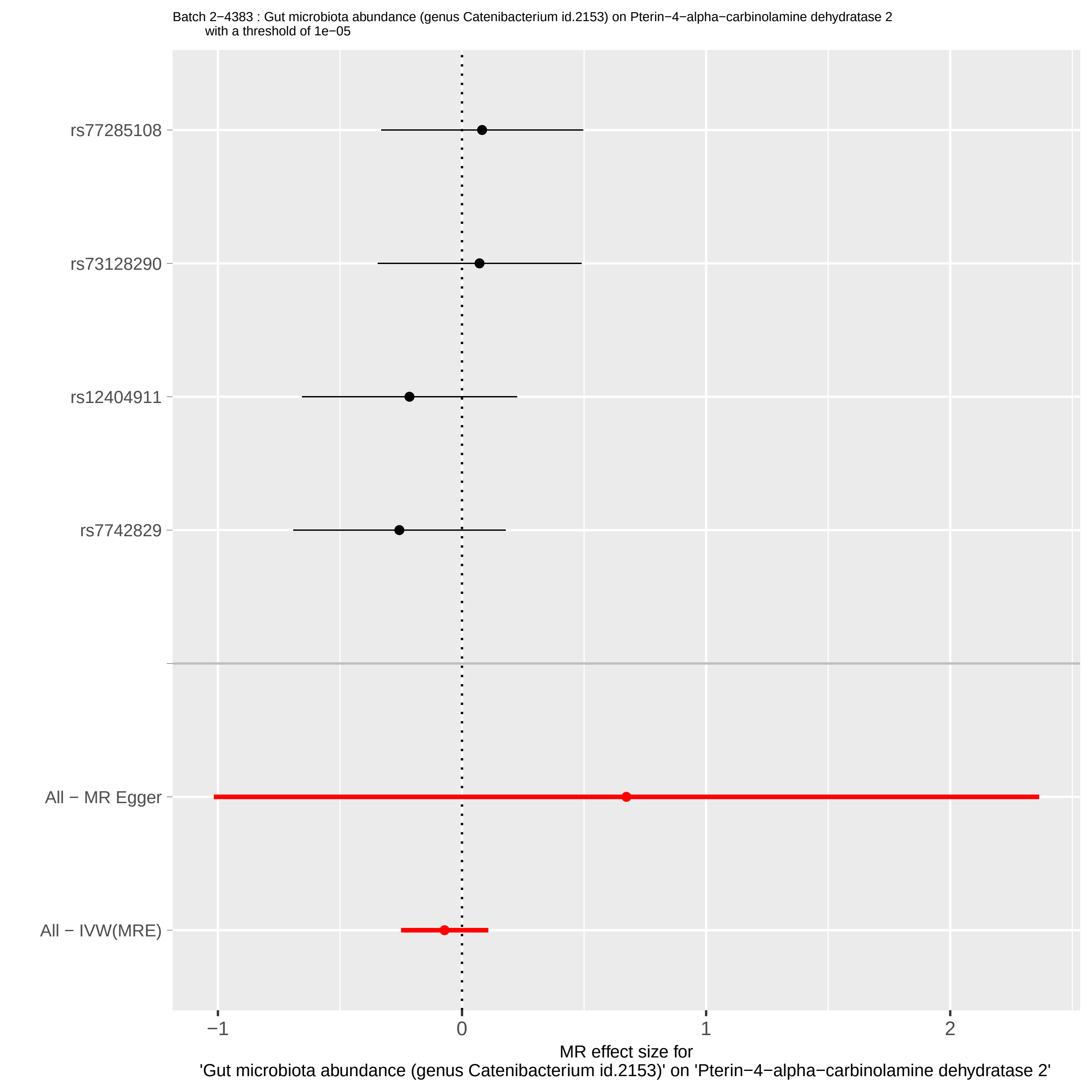

Batch 2−4383 : Gut microbiota abundance (genus Catenibacterium id.2153) on Pterin−4−alpha−carbinolamine dehydratase 2
with a threshold of 1e−05
rs77285108
rs73128290
rs12404911
rs7742829
All − MR Egger
All − IVW(MRE)
−1
0
1
2
MR effect size for
'Gut microbiota abundance (genus Catenibacterium id.2153)' on 'Pterin−4−alpha−carbinolamine dehydratase 2'

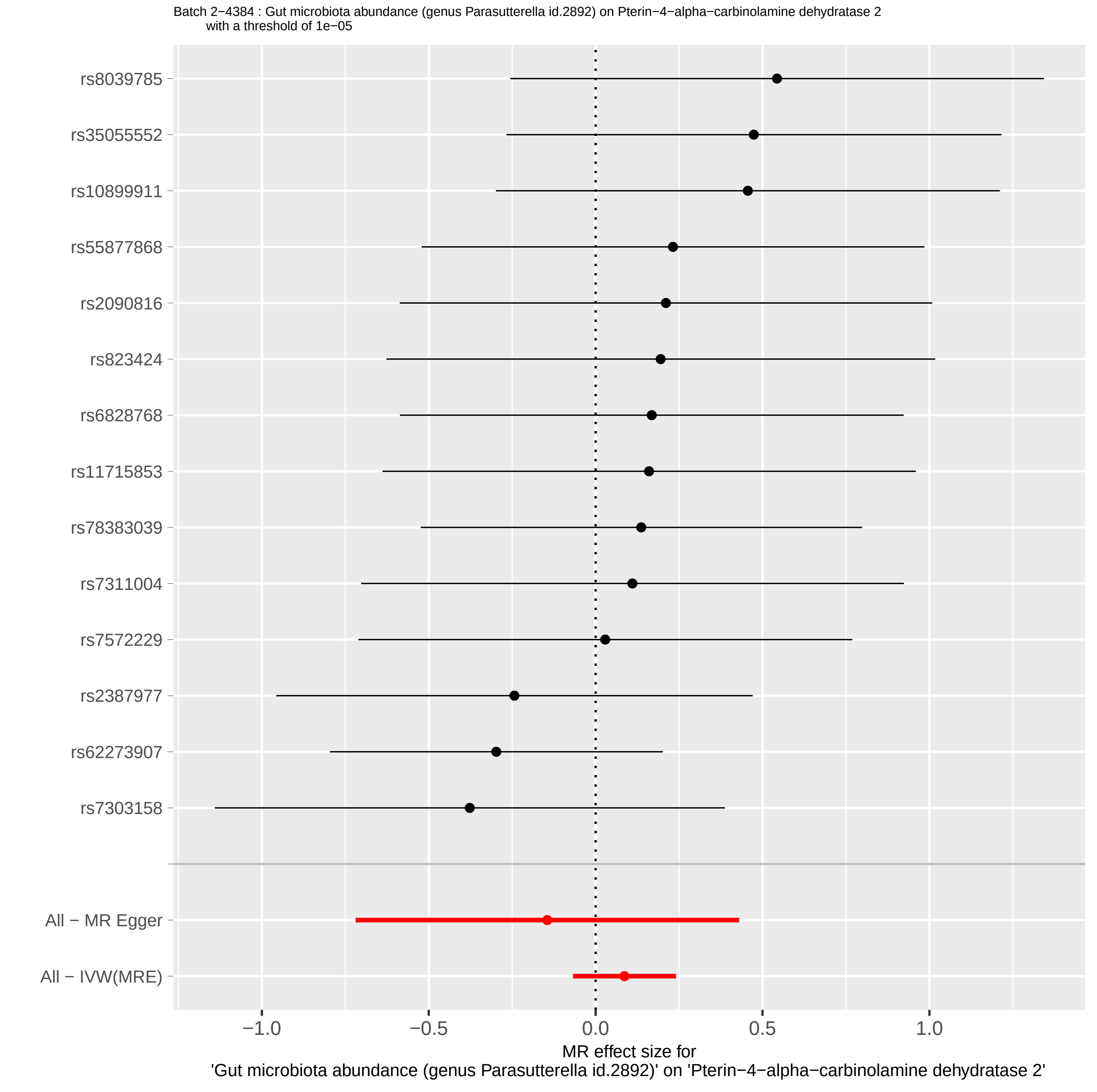
Batch 2−4384 : Gut microbiota abundance (genus Parasutterella id.2892) on Pterin−4−alpha−carbinolamine dehydratase 2
with a threshold of 1e−05
rs8039785
rs35055552
rs10899911
rs55877868
rs2090816
rs823424
rs6828768
rs11715853
rs78383039
rs7311004
rs7572229
rs2387977
rs62273907
rs7303158
All − MR Egger
All − IVW(MRE)
−1.0
−0.5
0.0
0.5
1.0
MR effect size for
'Gut microbiota abundance (genus Parasutterella id.2892)' on 'Pterin−4−alpha−carbinolamine dehydratase 2'

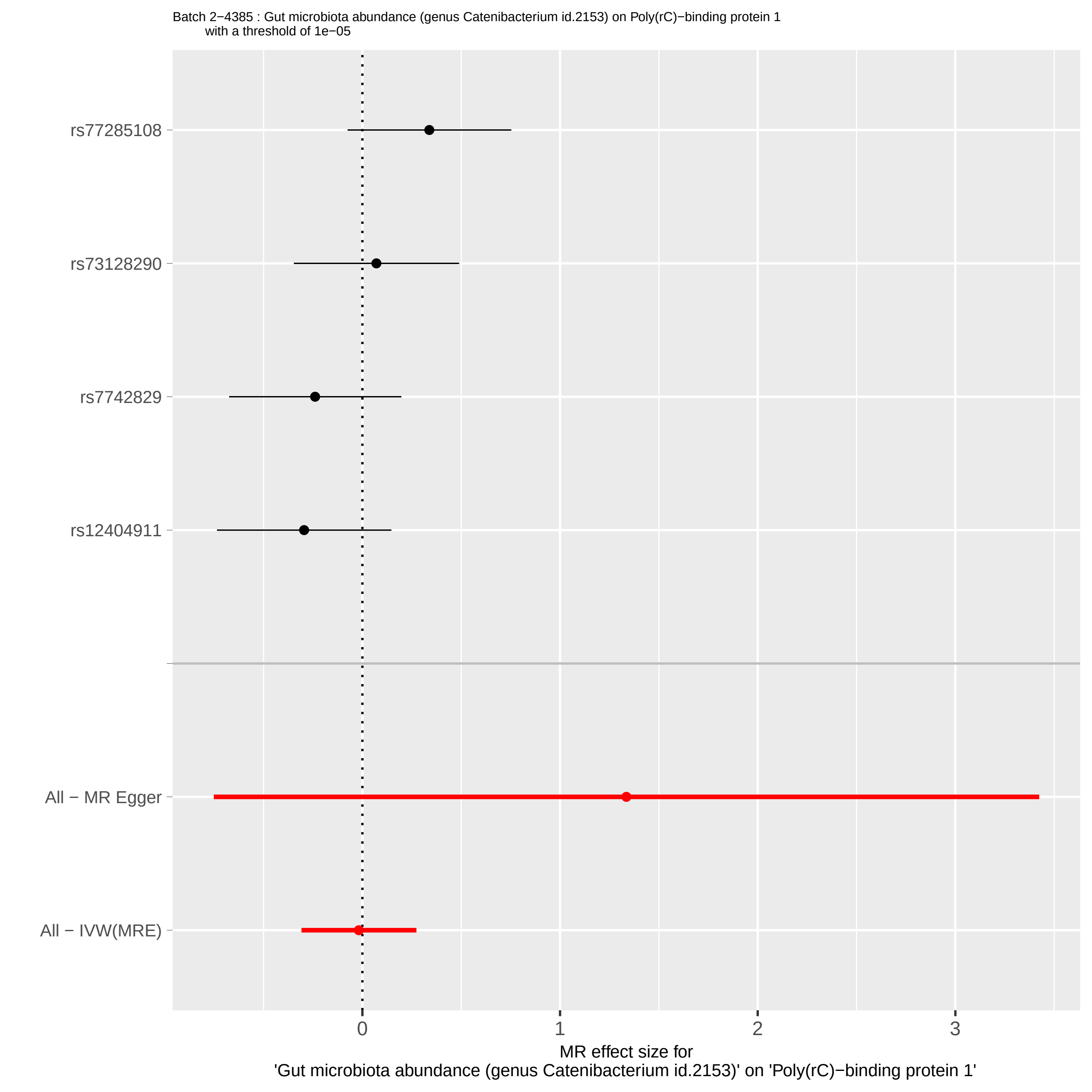

Batch 2−4385 : Gut microbiota abundance (genus Catenibacterium id.2153) on Poly(rC)−binding protein 1
with a threshold of 1e−05
rs77285108
rs73128290
rs7742829
rs12404911
All − MR Egger
All − IVW(MRE)
0
1
2
3
MR effect size for
'Gut microbiota abundance (genus Catenibacterium id.2153)' on 'Poly(rC)−binding protein 1'

Batch 2−4386 : Gut microbiota abundance (genus Parasutterella id.2892) on Poly(rC)−binding protein 1
with a threshold of 1e−05

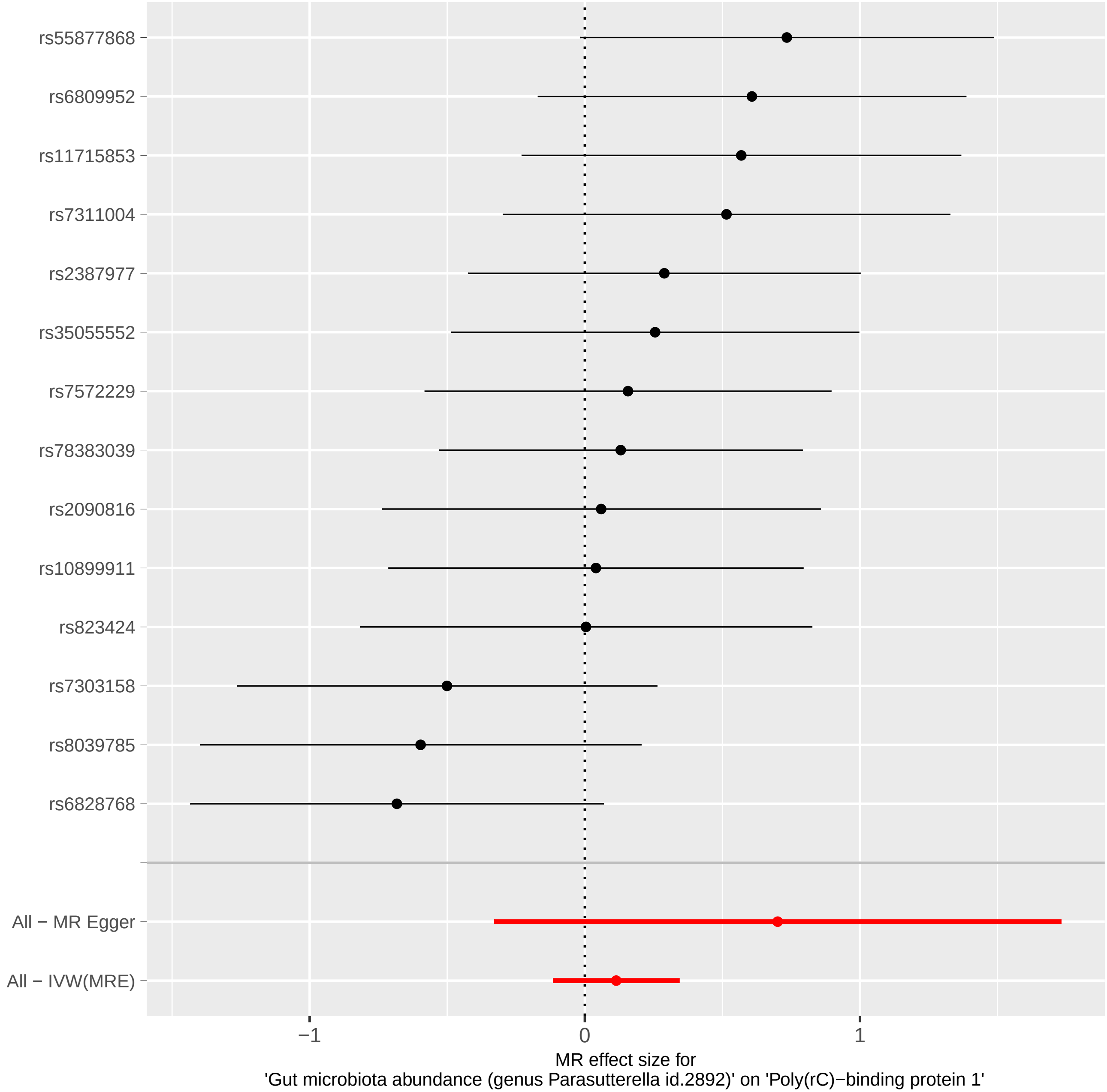

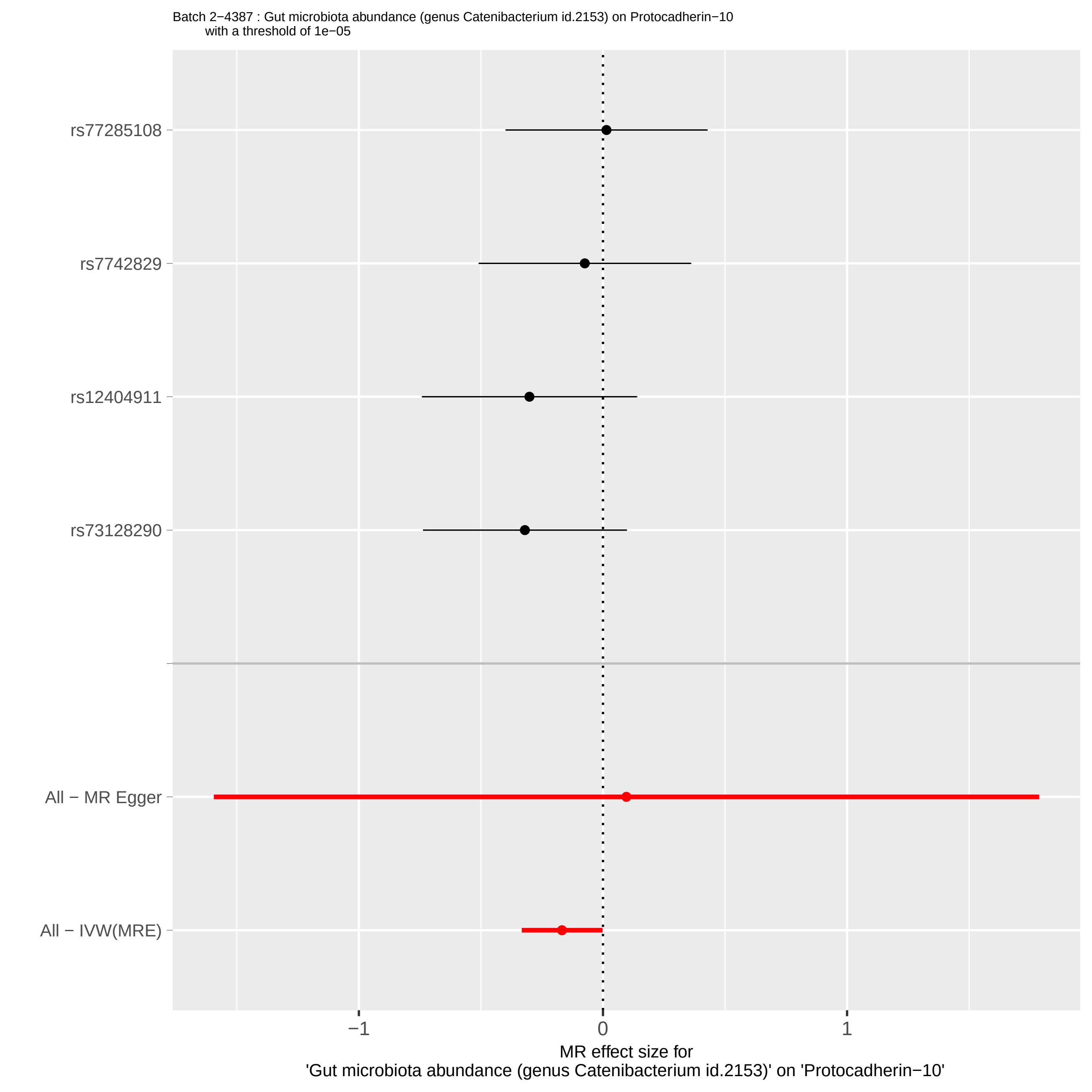
Batch 2−4387 : Gut microbiota abundance (genus Catenibacterium id.2153) on Protocadherin−10
with a threshold of 1e−05
rs77285108
rs7742829
rs12404911
rs73128290
All − MR Egger
All − IVW(MRE)
−1
0
1
MR effect size for
'Gut microbiota abundance (genus Catenibacterium id.2153)' on 'Protocadherin−10'

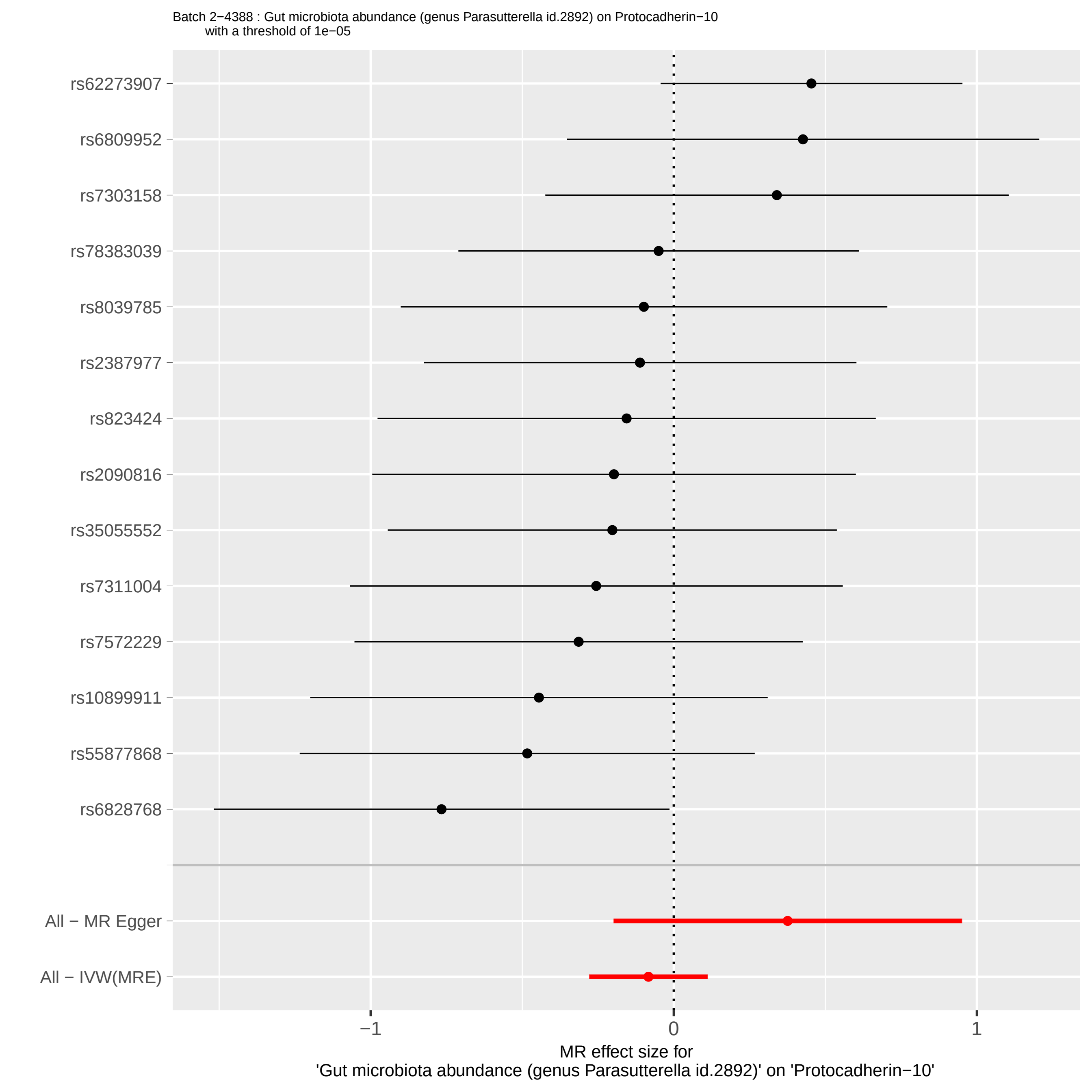
Batch 2−4388 : Gut microbiota abundance (genus Parasutterella id.2892) on Protocadherin−10
with a threshold of 1e−05
rs62273907
rs6809952
rs7303158
rs78383039
rs8039785
rs2387977
rs823424
rs2090816
rs35055552
rs7311004
rs7572229
rs10899911
rs55877868
rs6828768
All − MR Egger
All − IVW(MRE)
−1
0
1
MR effect size for
'Gut microbiota abundance (genus Parasutterella id.2892)' on 'Protocadherin−10'

Batch 2−4389 : Gut microbiota abundance (genus Catenibacterium id.2153) on Protocadherin−10
with a threshold of 1e−05

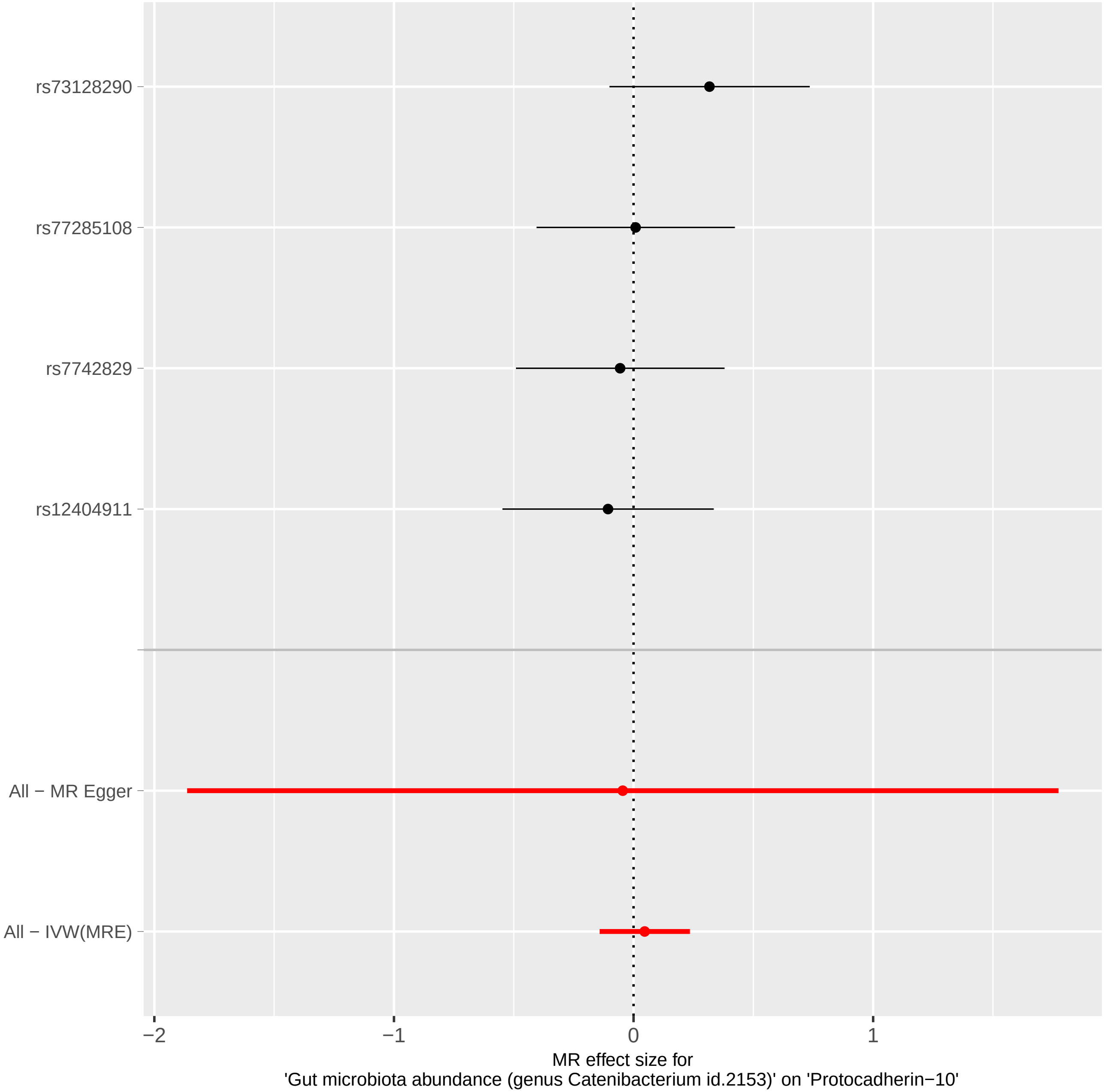

Batch 2−4390 : Gut microbiota abundance (genus Parasutterella id.2892) on Protocadherin−10
with a threshold of 1e−05

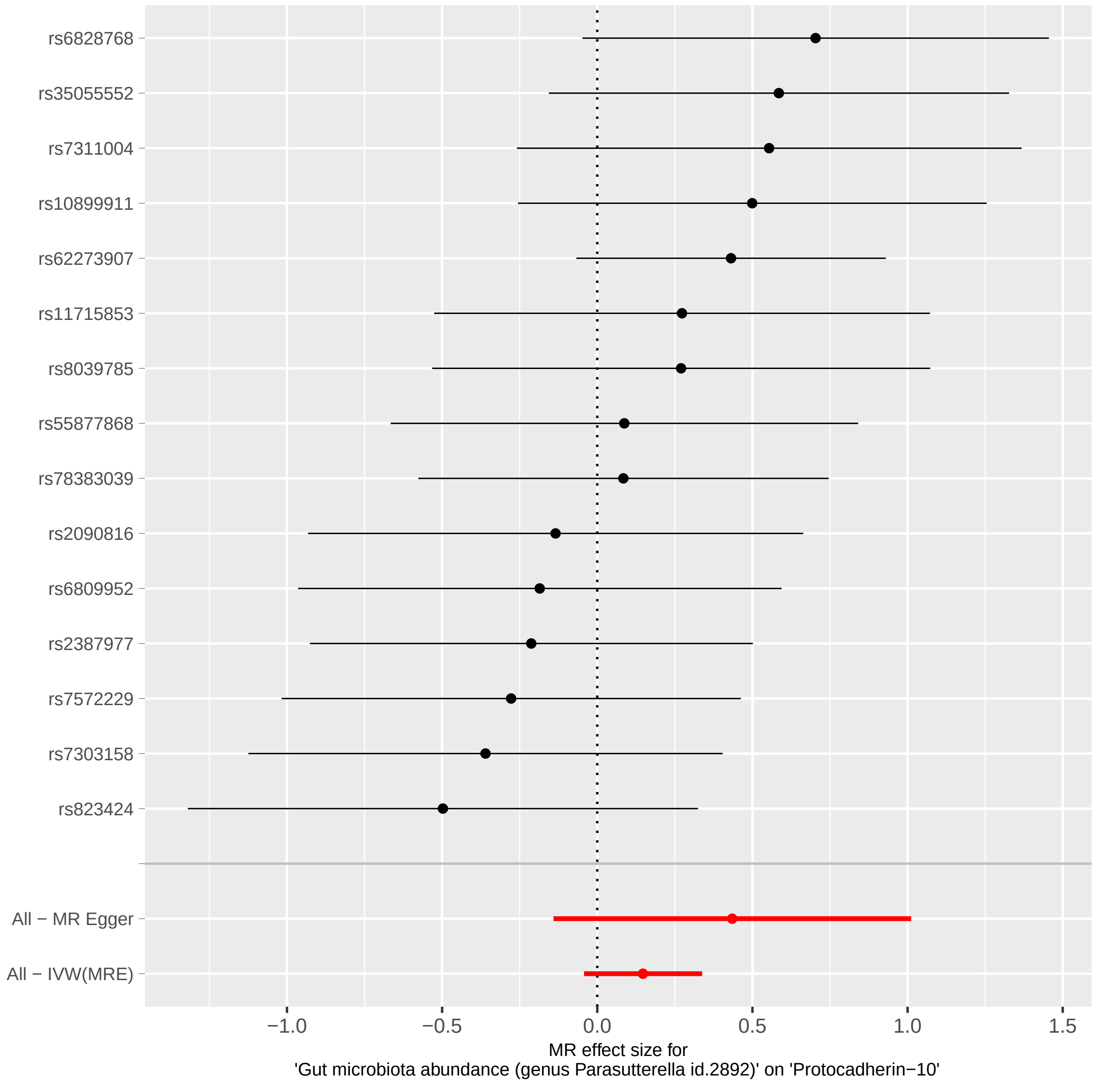

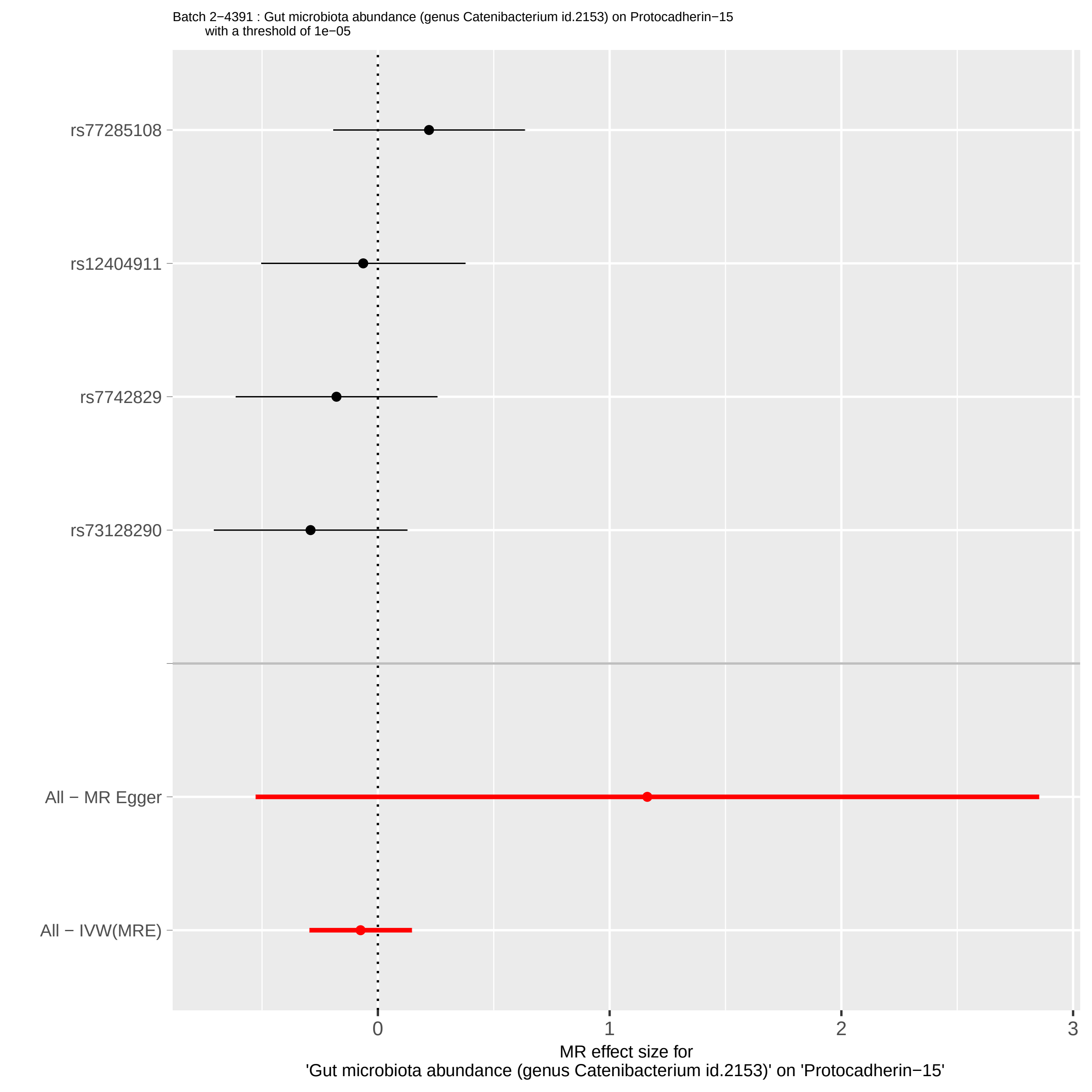
Batch 2−4391 : Gut microbiota abundance (genus Catenibacterium id.2153) on Protocadherin−15
with a threshold of 1e−05
rs77285108
rs12404911
rs7742829
rs73128290
All − MR Egger
All − IVW(MRE)
0
1
2
3
MR effect size for
'Gut microbiota abundance (genus Catenibacterium id.2153)' on 'Protocadherin−15'

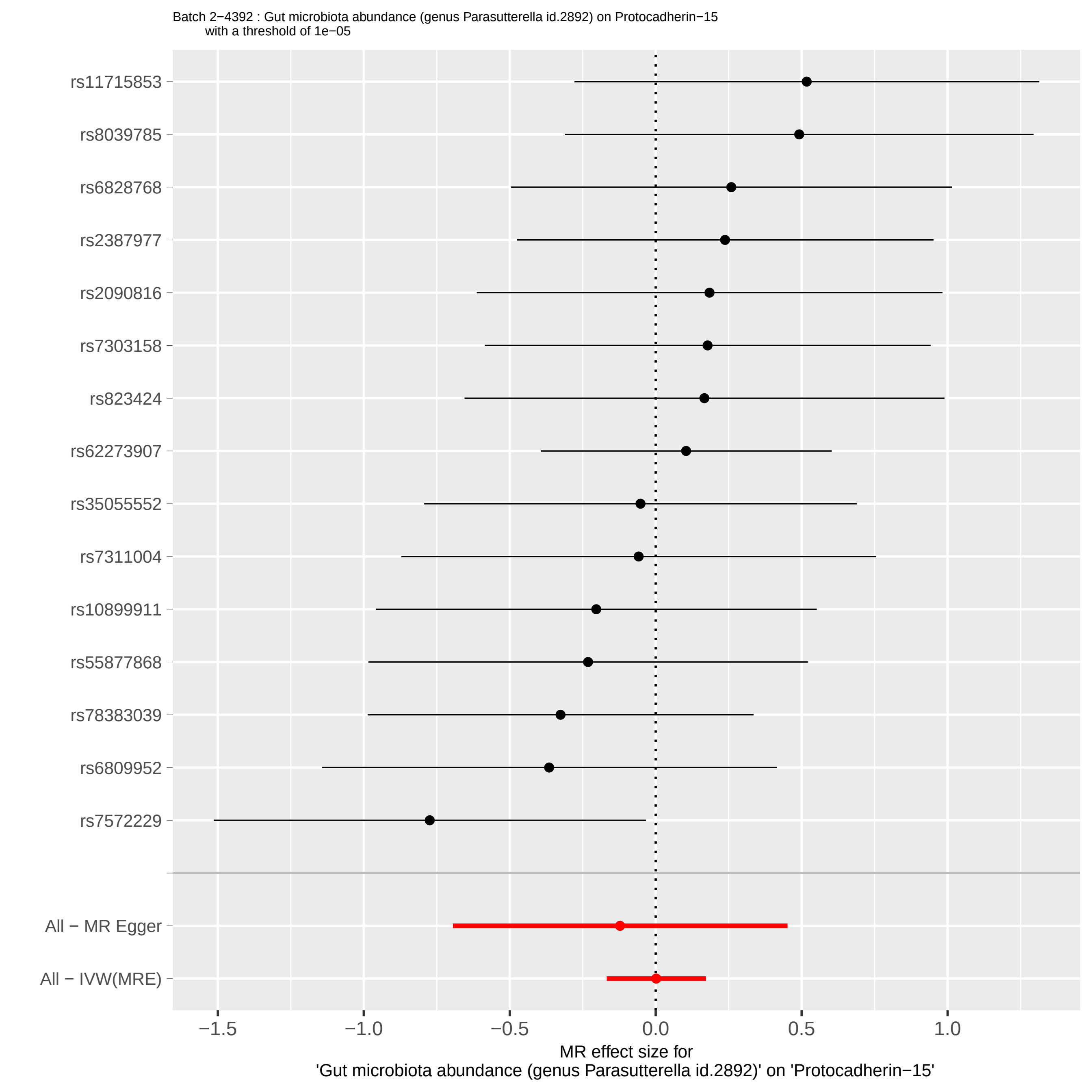

Batch 2−4392 : Gut microbiota abundance (genus Parasutterella id.2892) on Protocadherin−15
with a threshold of 1e−05
rs11715853
rs8039785
rs6828768
rs2387977
rs2090816
rs7303158
rs823424
rs62273907
rs35055552
rs7311004
rs10899911
rs55877868
rs78383039
rs6809952
rs7572229
All − MR Egger
All − IVW(MRE)
−1.5
−1.0
−0.5
0.0
0.5
1.0
MR effect size for
'Gut microbiota abundance (genus Parasutterella id.2892)' on 'Protocadherin−15'

Batch 2−4393 : Gut microbiota abundance (genus Catenibacterium id.2153) on Protocadherin−9
with a threshold of 1e−05

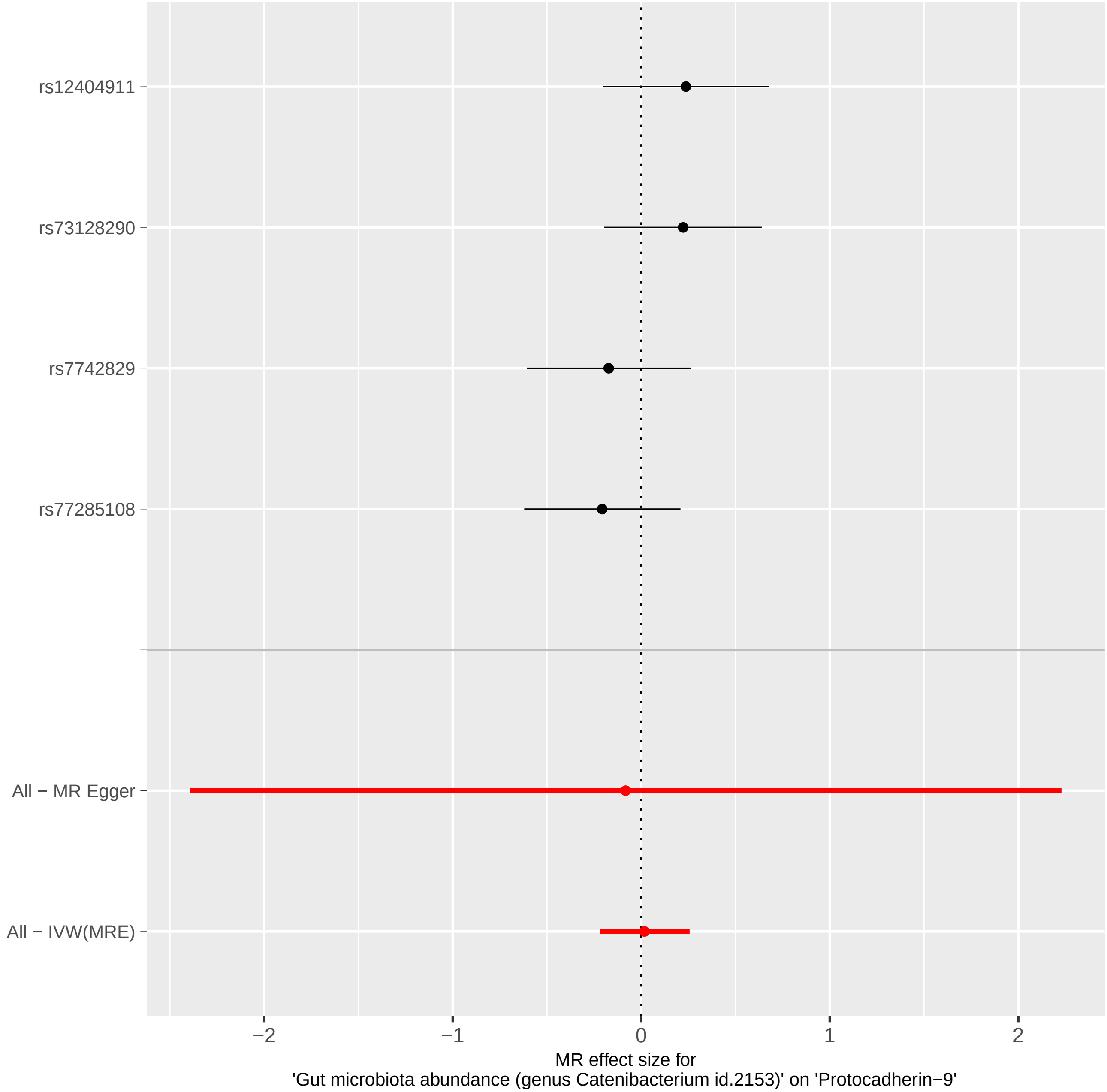

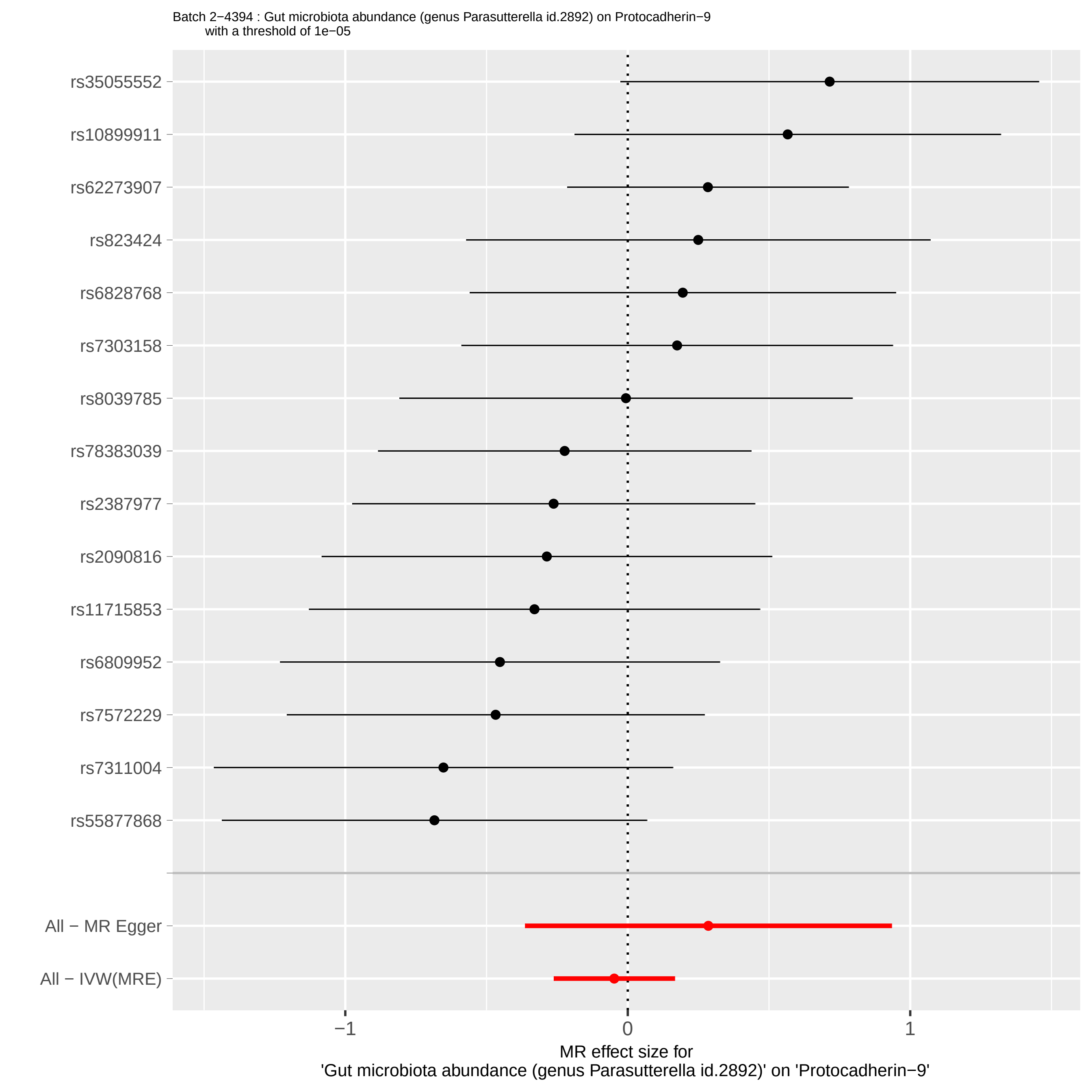
Batch 2−4394 : Gut microbiota abundance (genus Parasutterella id.2892) on Protocadherin−9
with a threshold of 1e−05
rs35055552
rs10899911
rs62273907
rs823424
rs6828768
rs7303158
rs8039785
rs78383039
rs2387977
rs2090816
rs11715853
rs6809952
rs7572229
rs7311004
rs55877868
All − MR Egger
All − IVW(MRE)
−1
0
1
MR effect size for
'Gut microbiota abundance (genus Parasutterella id.2892)' on 'Protocadherin−9'

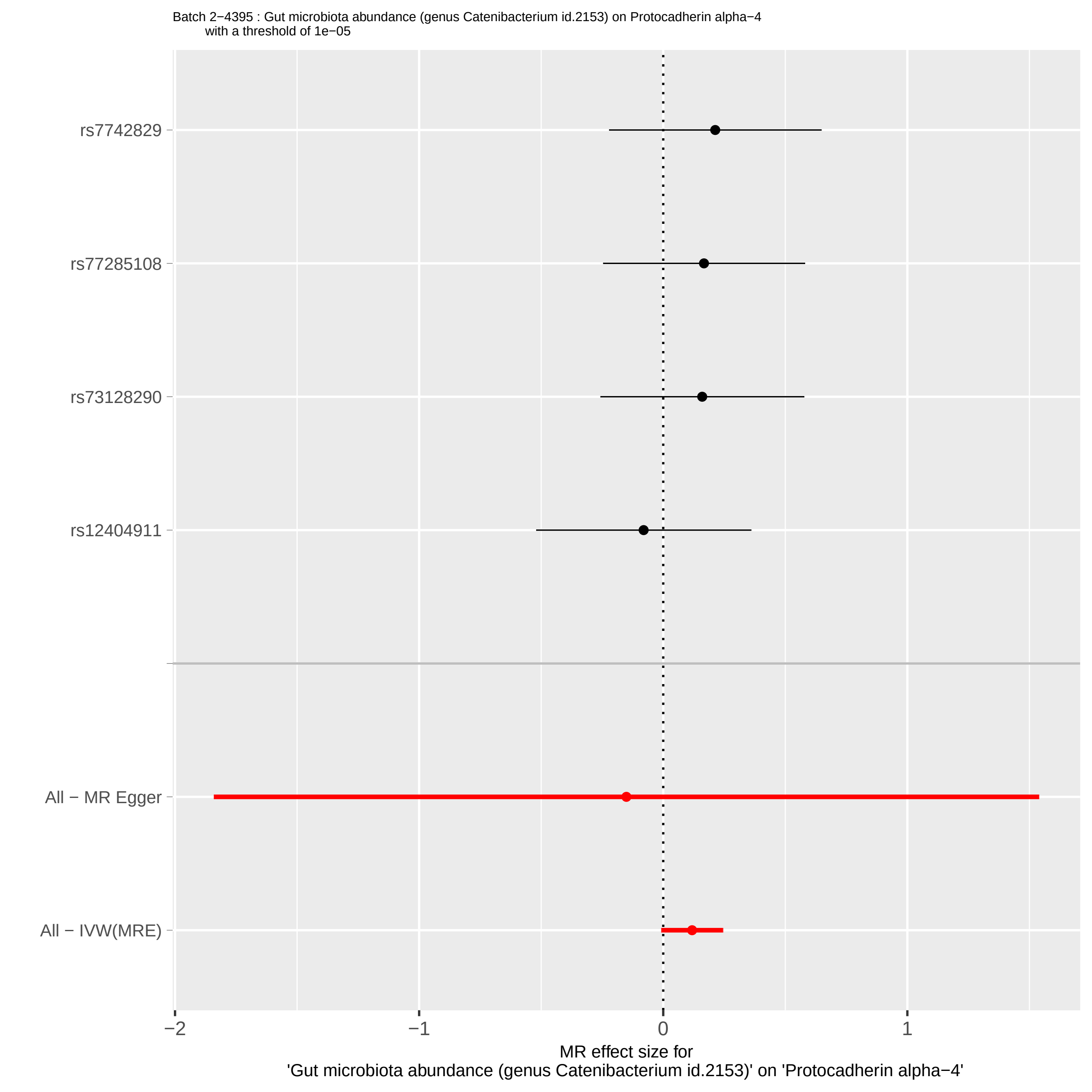

Batch 2−4395 : Gut microbiota abundance (genus Catenibacterium id.2153) on Protocadherin alpha−4
with a threshold of 1e−05
rs7742829
rs77285108
rs73128290
rs12404911
All − MR Egger
All − IVW(MRE)
−2
−1
0
1
MR effect size for
'Gut microbiota abundance (genus Catenibacterium id.2153)' on 'Protocadherin alpha−4'

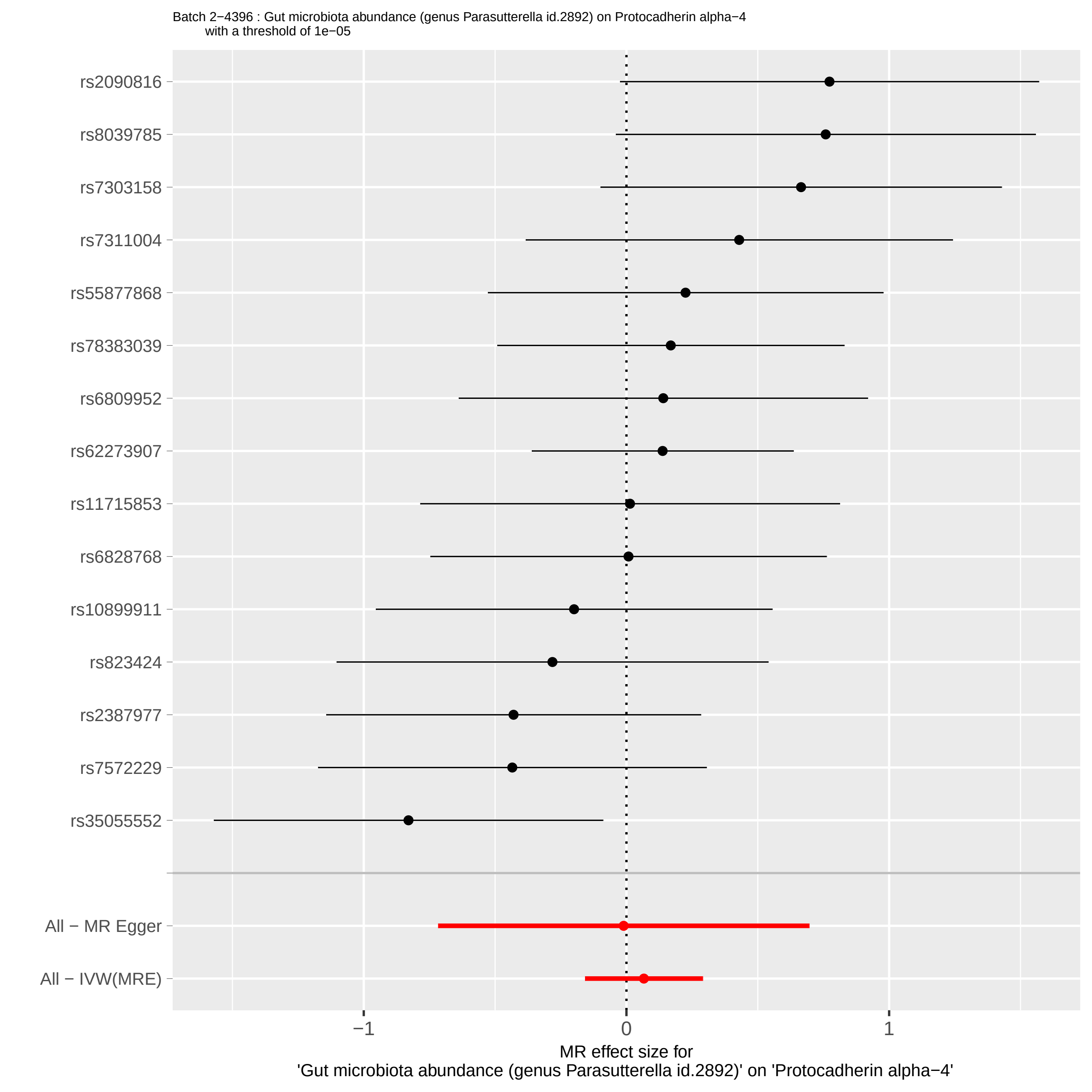

Batch 2−4396 : Gut microbiota abundance (genus Parasutterella id.2892) on Protocadherin alpha−4
with a threshold of 1e−05
rs2090816
rs8039785
rs7303158
rs7311004
rs55877868
rs78383039
rs6809952
rs62273907
rs11715853
rs6828768
rs10899911
rs823424
rs2387977
rs7572229
rs35055552
All − MR Egger
All − IVW(MRE)
−1
0
1
MR effect size for
'Gut microbiota abundance (genus Parasutterella id.2892)' on 'Protocadherin alpha−4'

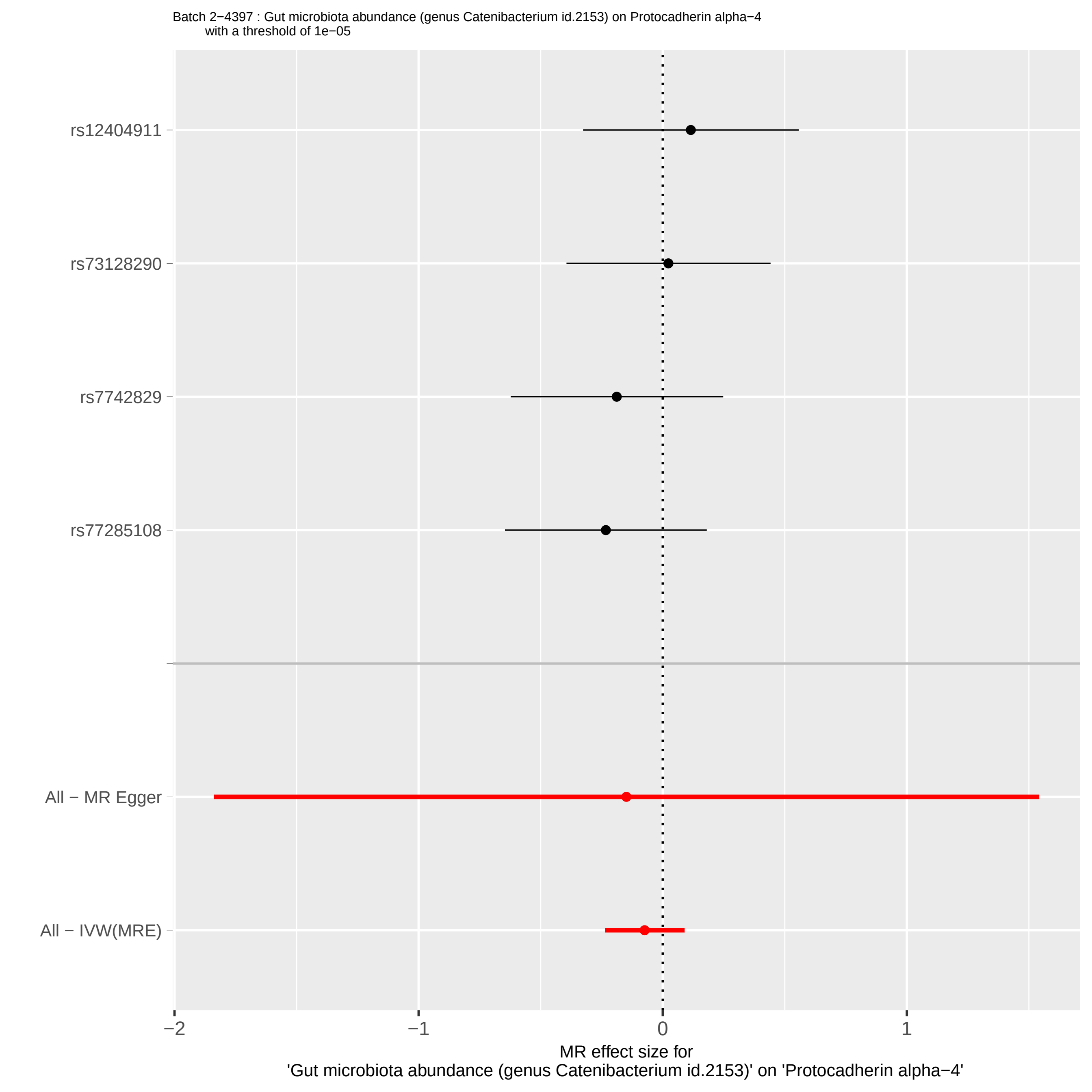

Batch 2−4397 : Gut microbiota abundance (genus Catenibacterium id.2153) on Protocadherin alpha−4
with a threshold of 1e−05
rs12404911
rs73128290
rs7742829
rs77285108
All − MR Egger
All − IVW(MRE)
−2
−1
0
1
MR effect size for
'Gut microbiota abundance (genus Catenibacterium id.2153)' on 'Protocadherin alpha−4'

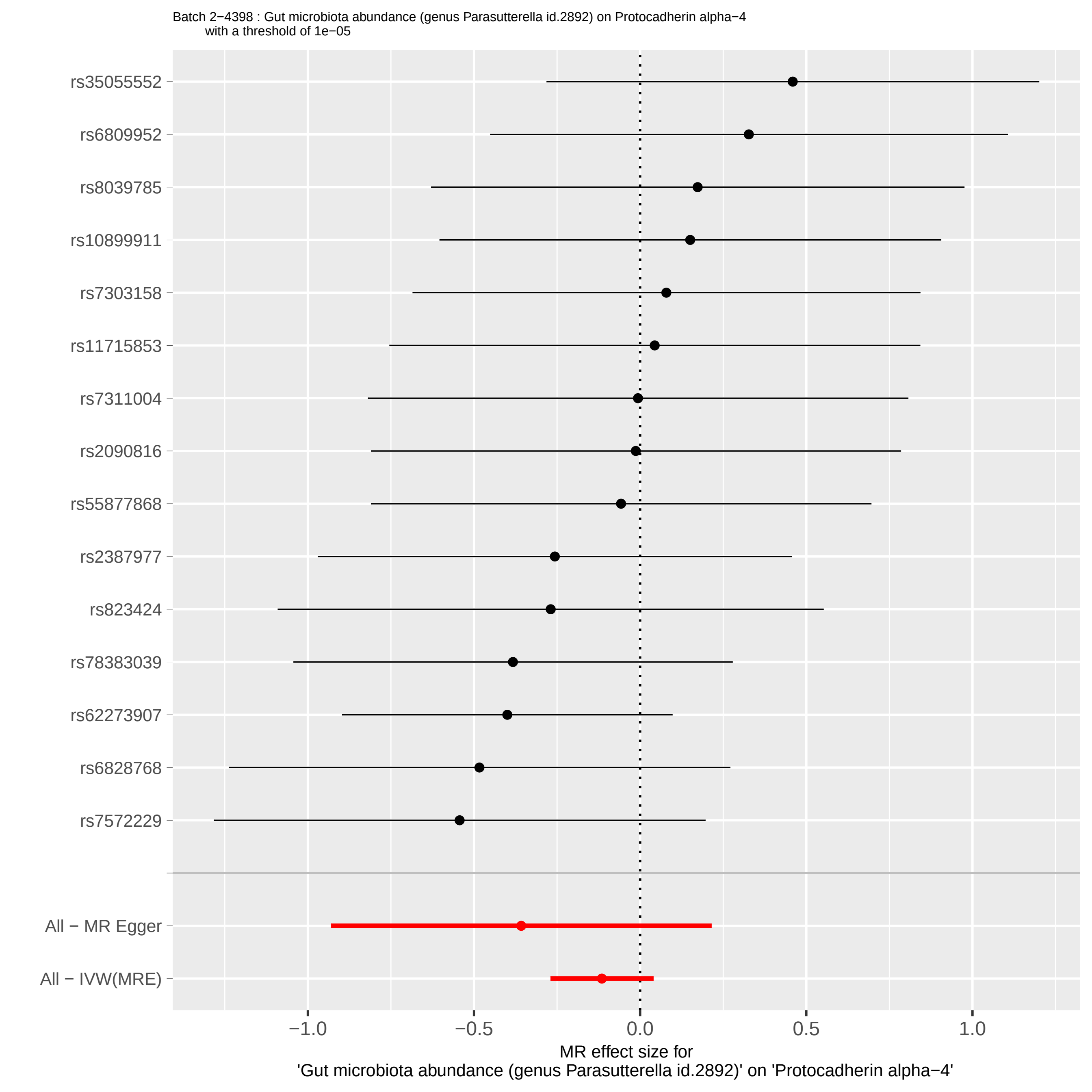

Batch 2−4398 : Gut microbiota abundance (genus Parasutterella id.2892) on Protocadherin alpha−4
with a threshold of 1e−05
rs35055552
rs6809952
rs8039785
rs10899911
rs7303158
rs11715853
rs7311004
rs2090816
rs55877868
rs2387977
rs823424
rs78383039
rs62273907
rs6828768
rs7572229
All − MR Egger
All − IVW(MRE)
−1.0
−0.5
0.0
0.5
1.0
MR effect size for
'Gut microbiota abundance (genus Parasutterella id.2892)' on 'Protocadherin alpha−4'

Batch 2−4399 : Gut microbiota abundance (genus Catenibacterium id.2153) on Protocadherin alpha−7
with a threshold of 1e−05

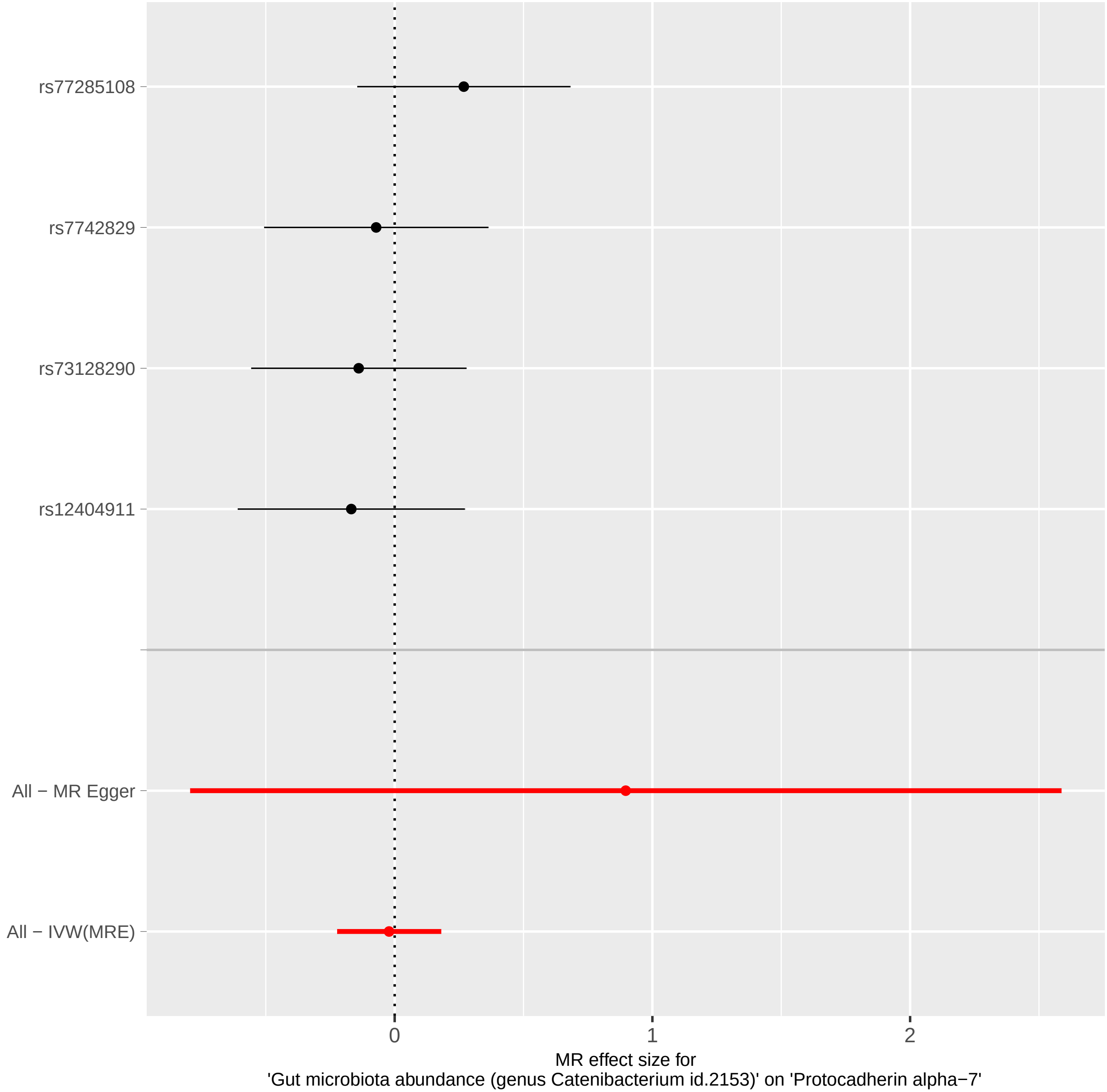

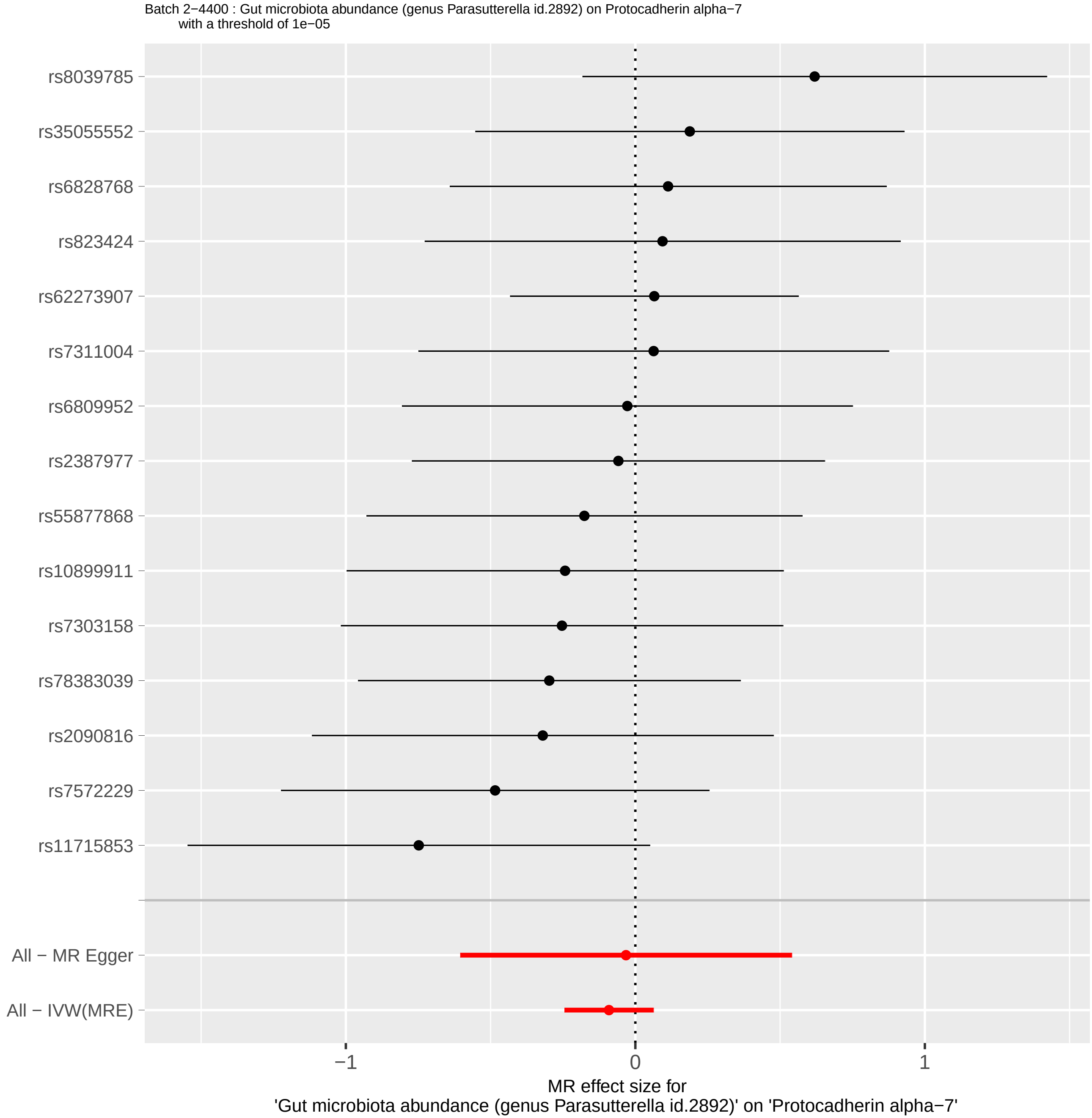

Batch 2−4400 : Gut microbiota abundance (genus Parasutterella id.2892) on Protocadherin alpha−7
with a threshold of 1e−05
rs8039785
rs35055552
rs6828768
rs823424
rs62273907
rs7311004
rs6809952
rs2387977
rs55877868
rs10899911
rs7303158
rs78383039
rs2090816
rs7572229
rs11715853
All − MR Egger
All − IVW(MRE)
−1
0
1
MR effect size for
'Gut microbiota abundance (genus Parasutterella id.2892)' on 'Protocadherin alpha−7'

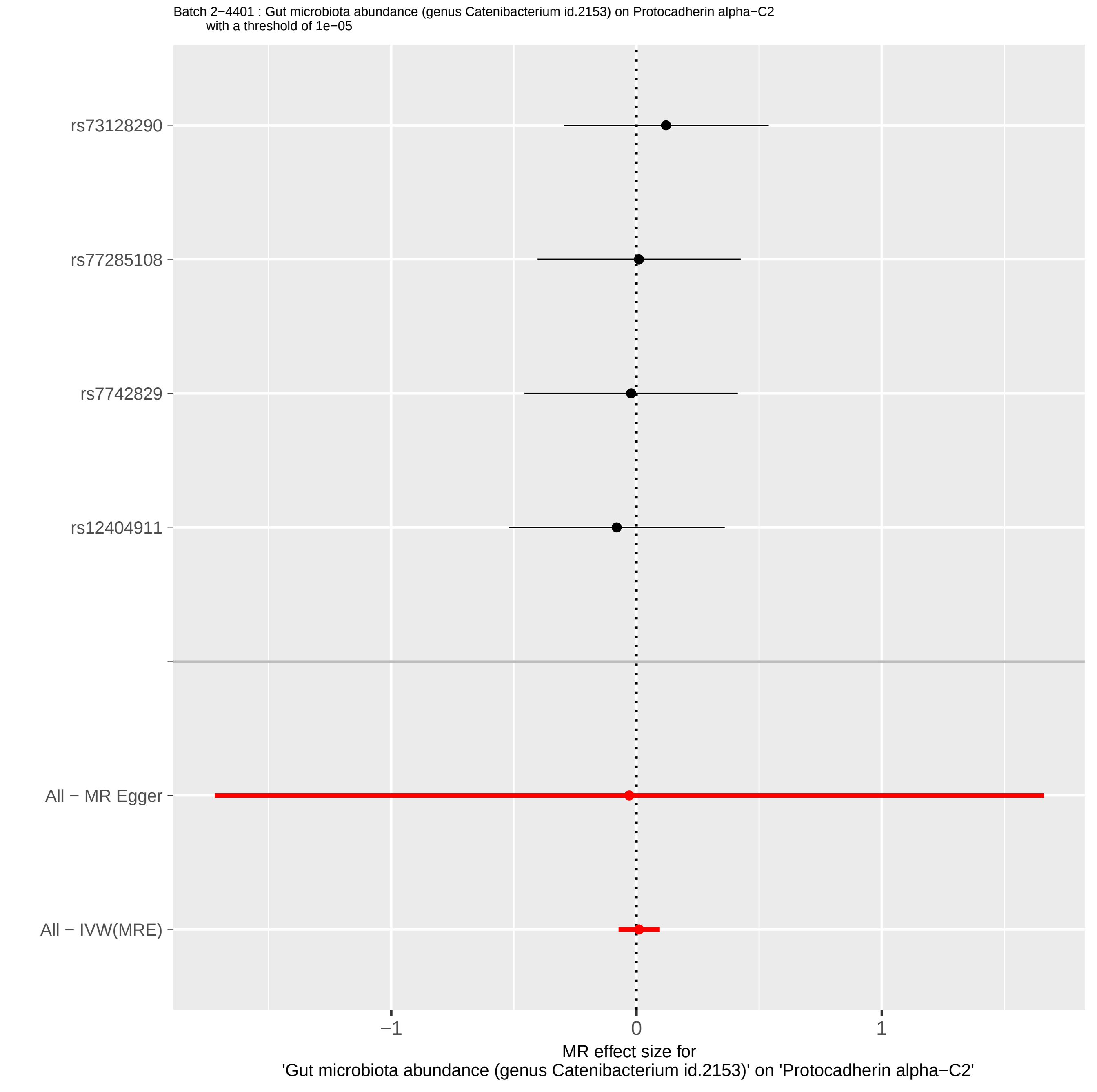
Batch 2−4401 : Gut microbiota abundance (genus Catenibacterium id.2153) on Protocadherin alpha−C2
with a threshold of 1e−05
rs73128290
rs77285108
rs7742829
rs12404911
All − MR Egger
All − IVW(MRE)
−1
0
1
MR effect size for
'Gut microbiota abundance (genus Catenibacterium id.2153)' on 'Protocadherin alpha−C2'

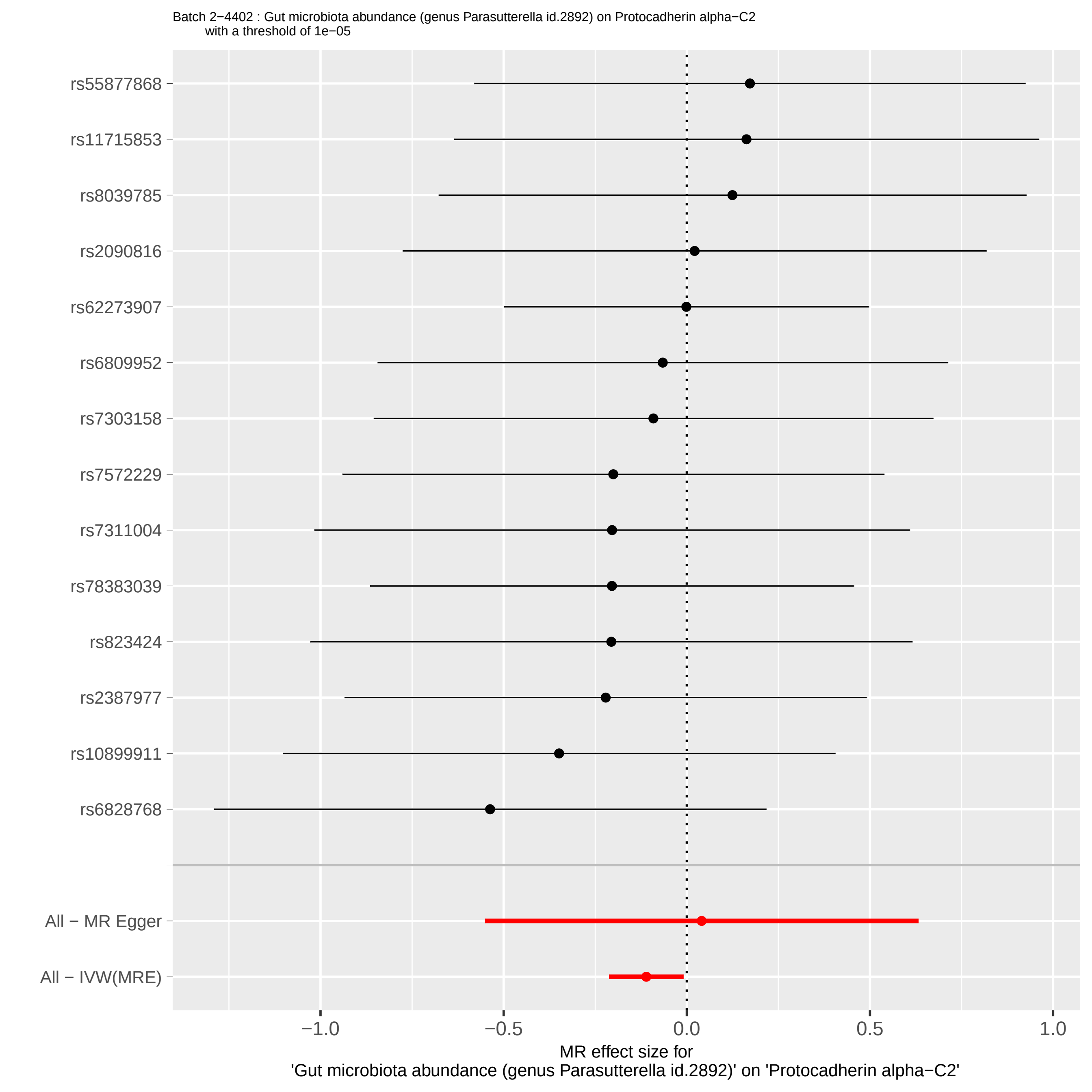

Batch 2−4402 : Gut microbiota abundance (genus Parasutterella id.2892) on Protocadherin alpha−C2
with a threshold of 1e−05
rs55877868
rs11715853
rs8039785
rs2090816
rs62273907
rs6809952
rs7303158
rs7572229
rs7311004
rs78383039
rs823424
rs2387977
rs10899911
rs6828768
All − MR Egger
All − IVW(MRE)
−1.0
−0.5
0.0
0.5
1.0
MR effect size for
'Gut microbiota abundance (genus Parasutterella id.2892)' on 'Protocadherin alpha−C2'

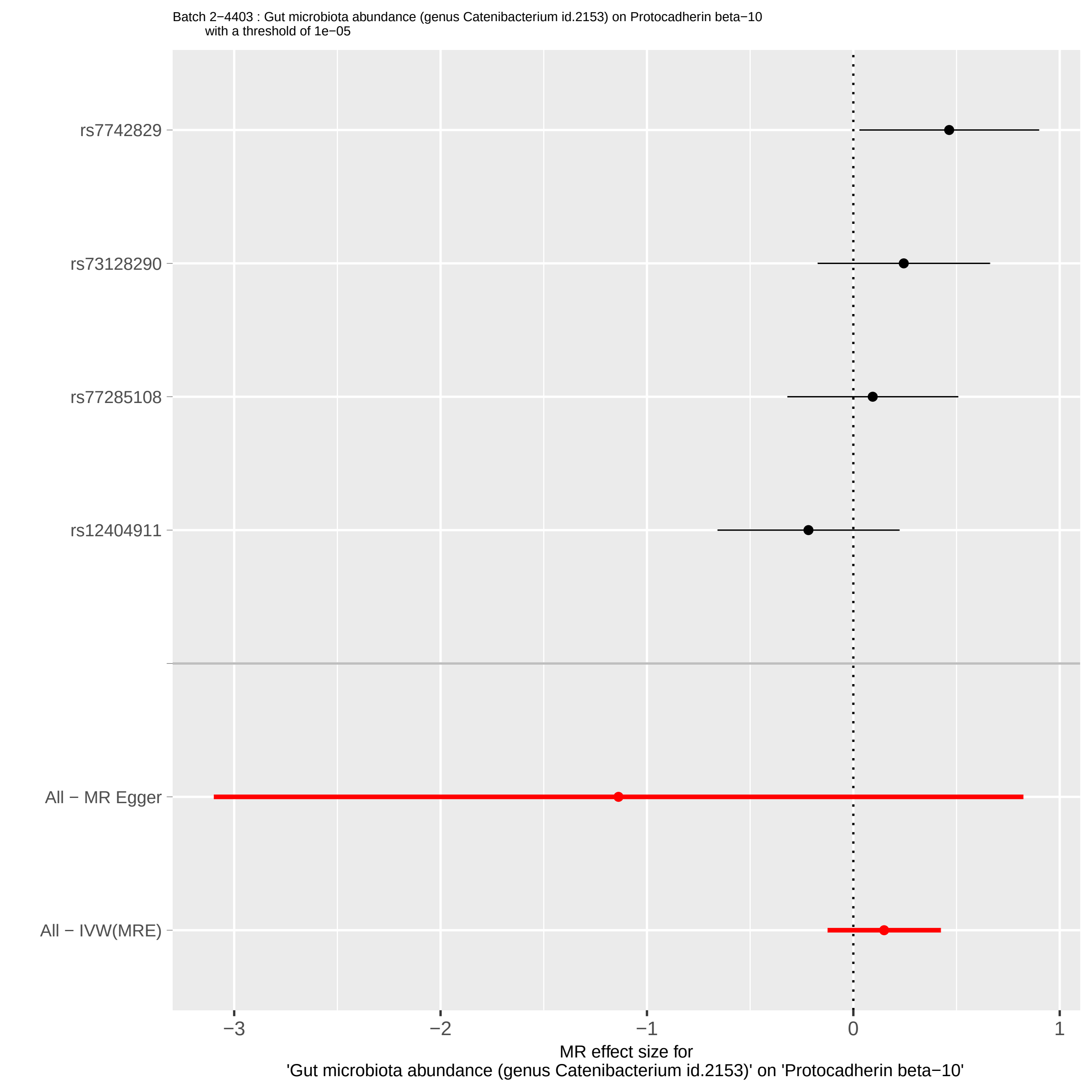

Batch 2−4403 : Gut microbiota abundance (genus Catenibacterium id.2153) on Protocadherin beta−10
with a threshold of 1e−05
rs7742829
rs73128290
rs77285108
rs12404911
All − MR Egger
All − IVW(MRE)
−3
−2
−1
0
1
MR effect size for
'Gut microbiota abundance (genus Catenibacterium id.2153)' on 'Protocadherin beta−10'

Batch 2−4404 : Gut microbiota abundance (genus Parasutterella id.2892) on Protocadherin beta−10
with a threshold of 1e−05

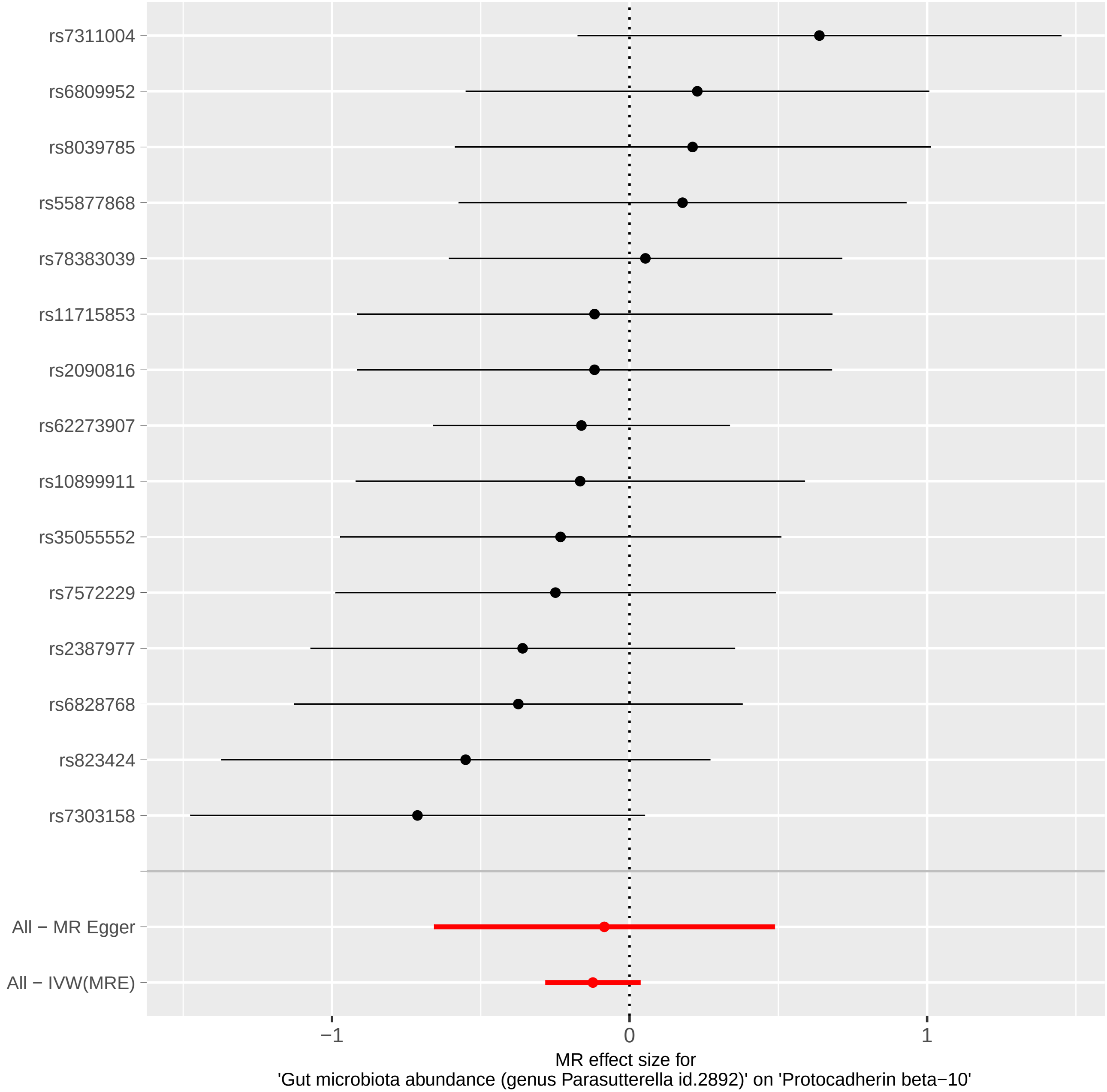

Batch 2−4405 : Gut microbiota abundance (genus Catenibacterium id.2153) on Protocadherin beta−1
with a threshold of 1e−05

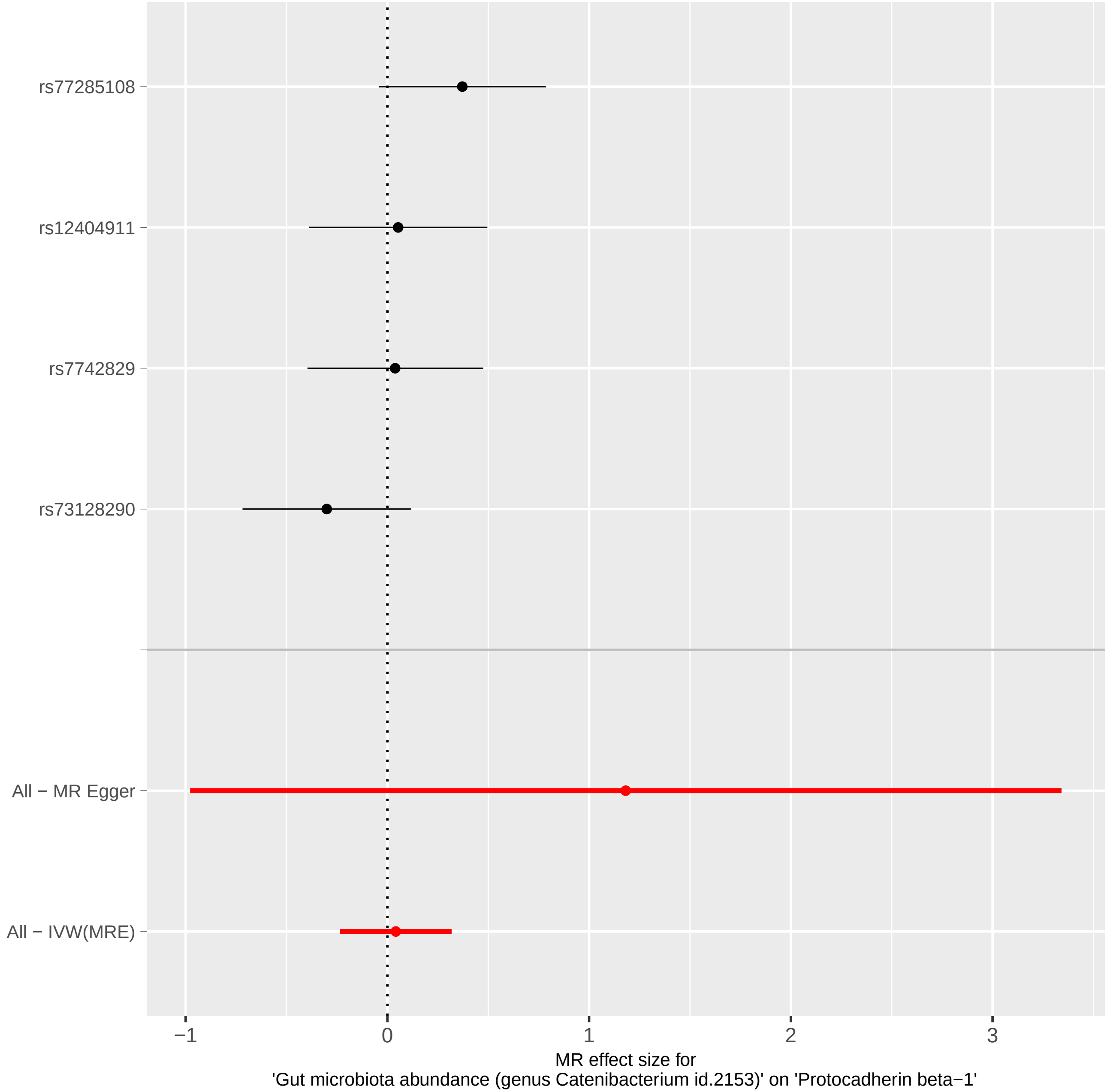

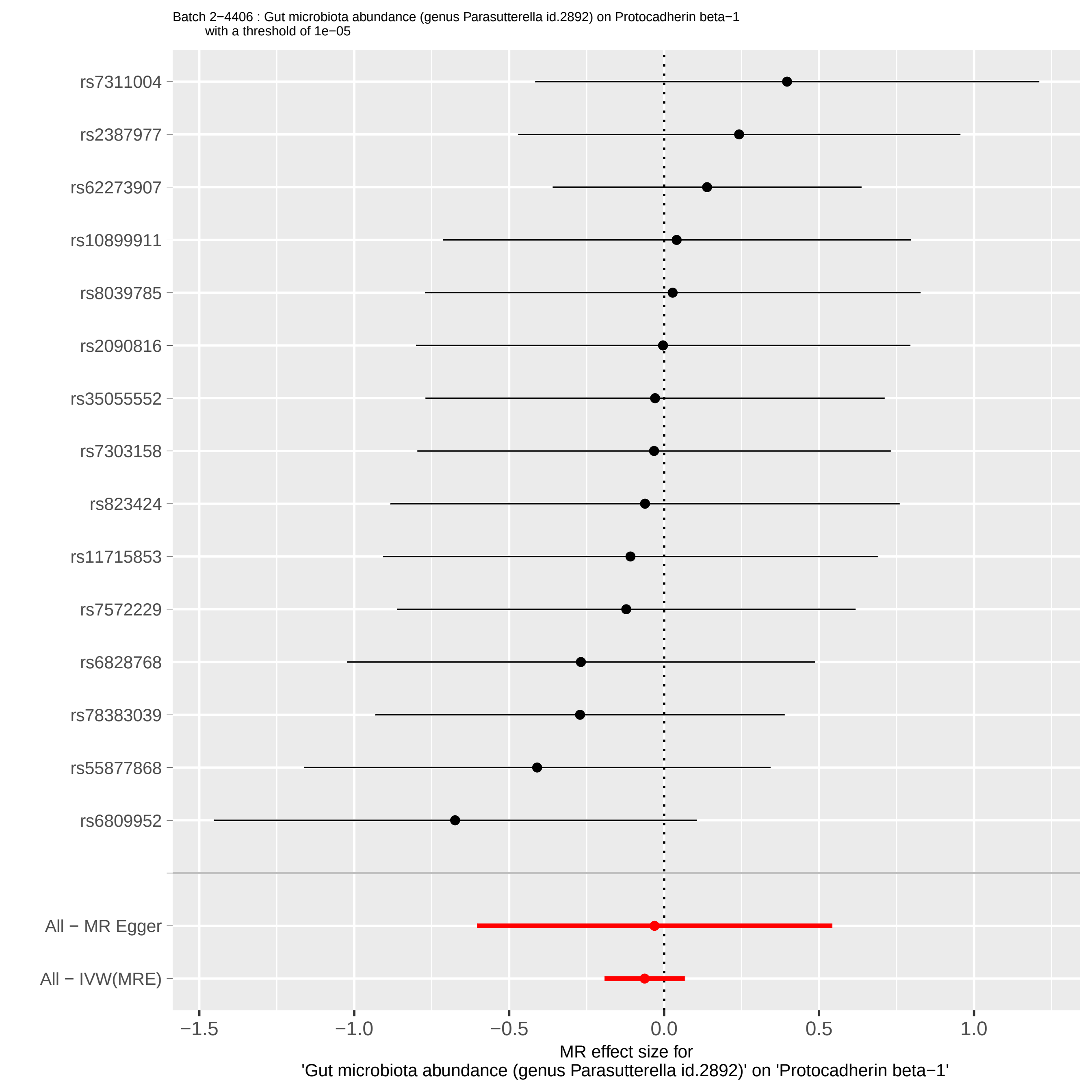

Batch 2−4406 : Gut microbiota abundance (genus Parasutterella id.2892) on Protocadherin beta−1
with a threshold of 1e−05
rs7311004
rs2387977
rs62273907
rs10899911
rs8039785
rs2090816
rs35055552
rs7303158
rs823424
rs11715853
rs7572229
rs6828768
rs78383039
rs55877868
rs6809952
All − MR Egger
All − IVW(MRE)
−1.5
−1.0
−0.5
0.0
0.5
1.0
MR effect size for
'Gut microbiota abundance (genus Parasutterella id.2892)' on 'Protocadherin beta−1'

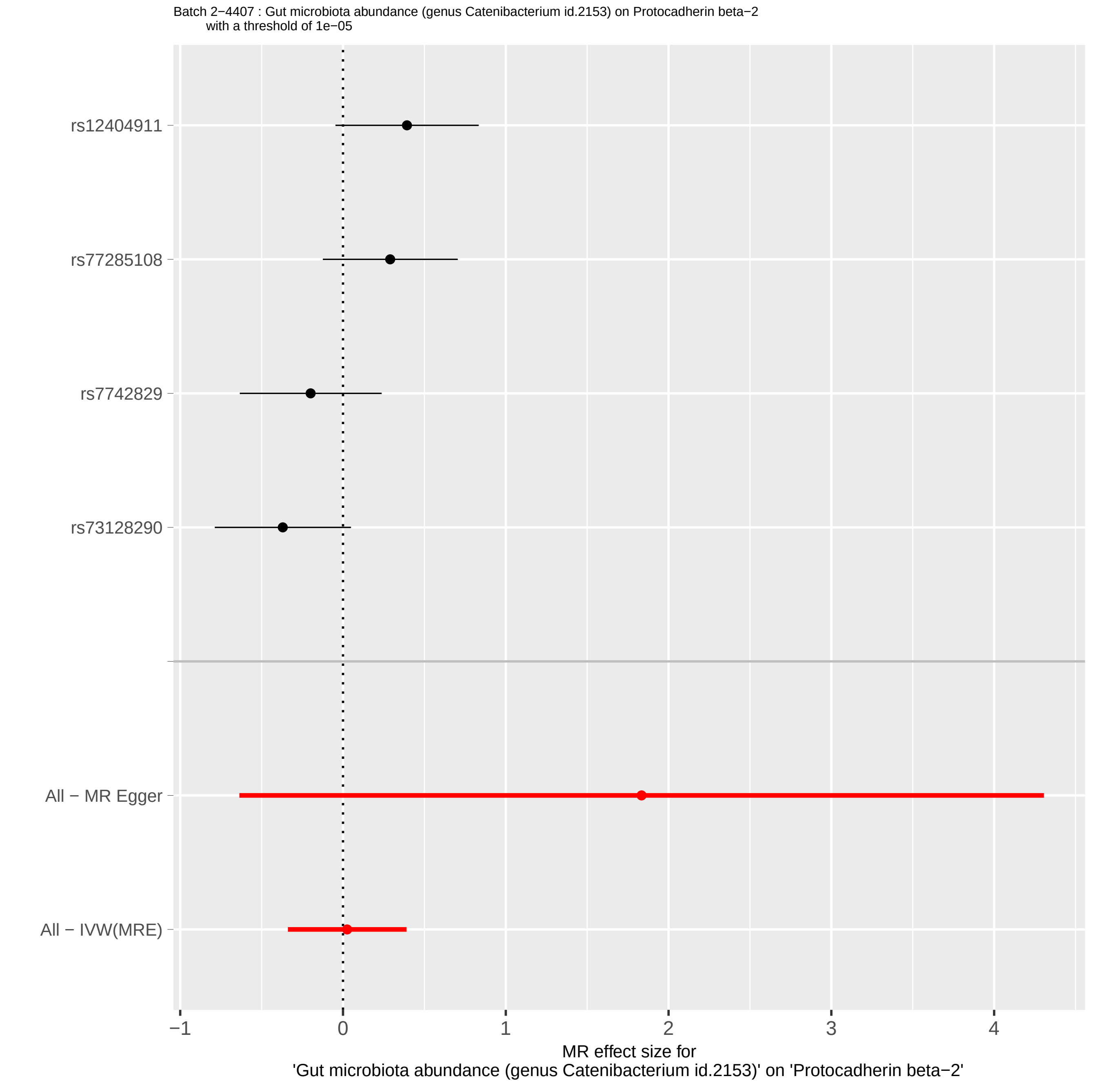

Batch 2−4407 : Gut microbiota abundance (genus Catenibacterium id.2153) on Protocadherin beta−2
with a threshold of 1e−05
rs12404911
rs77285108
rs7742829
rs73128290
All − MR Egger
All − IVW(MRE)
−1
0
1
2
3
4
MR effect size for
'Gut microbiota abundance (genus Catenibacterium id.2153)' on 'Protocadherin beta−2'

Batch 2−4408 : Gut microbiota abundance (genus Parasutterella id.2892) on Protocadherin beta−2
with a threshold of 1e−05

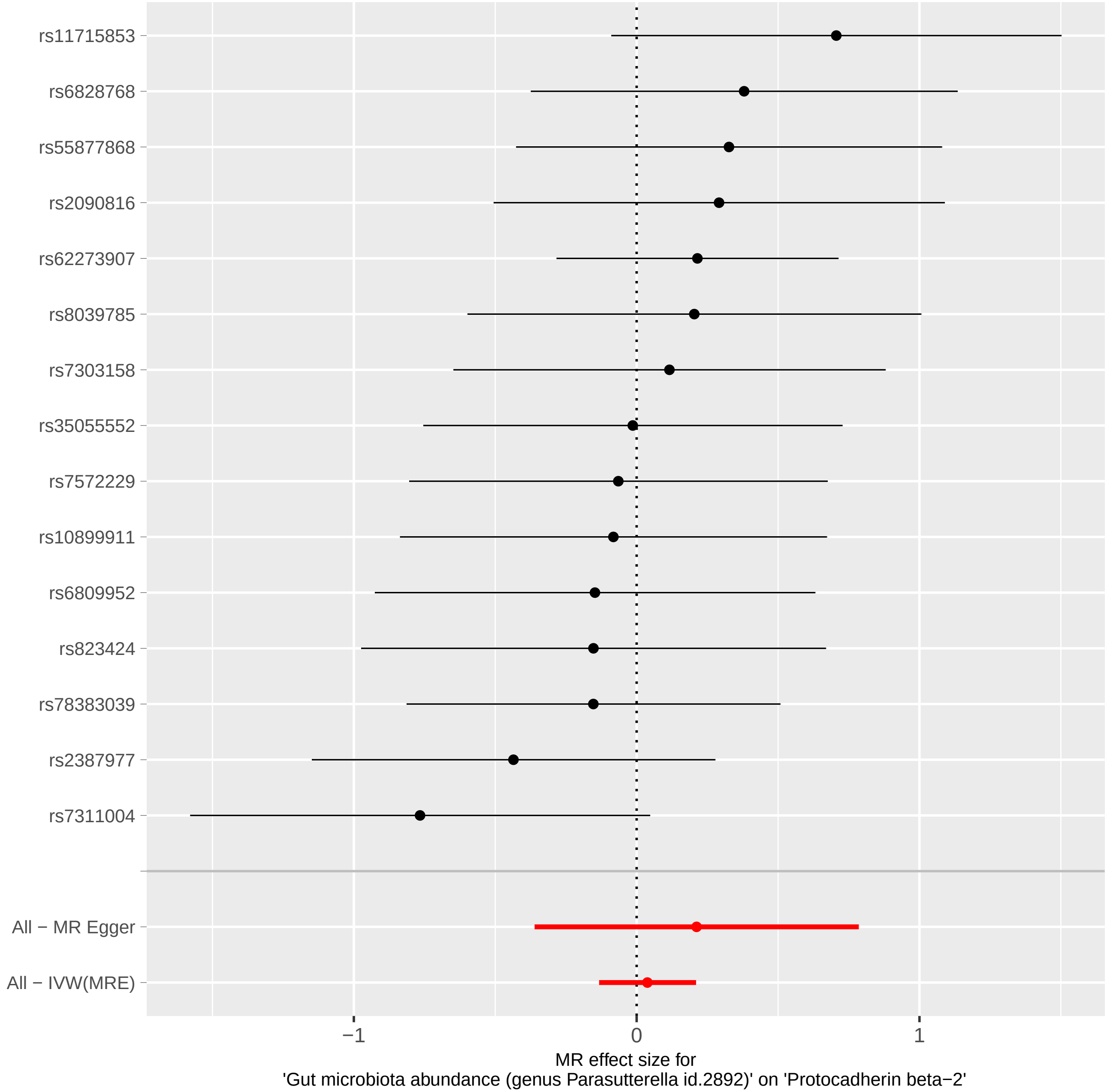

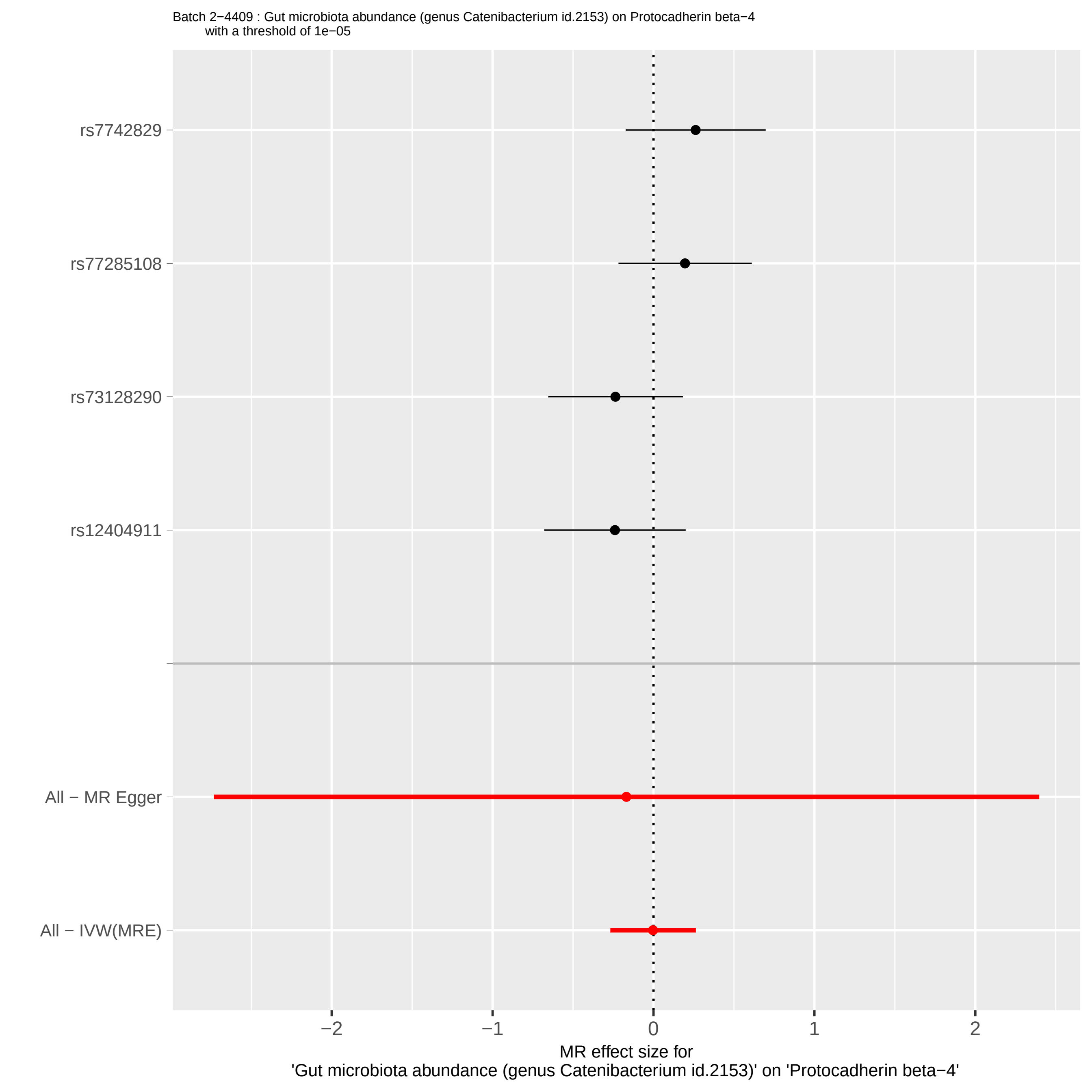

Batch 2−4409 : Gut microbiota abundance (genus Catenibacterium id.2153) on Protocadherin beta−4
with a threshold of 1e−05
rs7742829
rs77285108
rs73128290
rs12404911
All − MR Egger
All − IVW(MRE)
−2
−1
0
1
2
MR effect size for
'Gut microbiota abundance (genus Catenibacterium id.2153)' on 'Protocadherin beta−4'

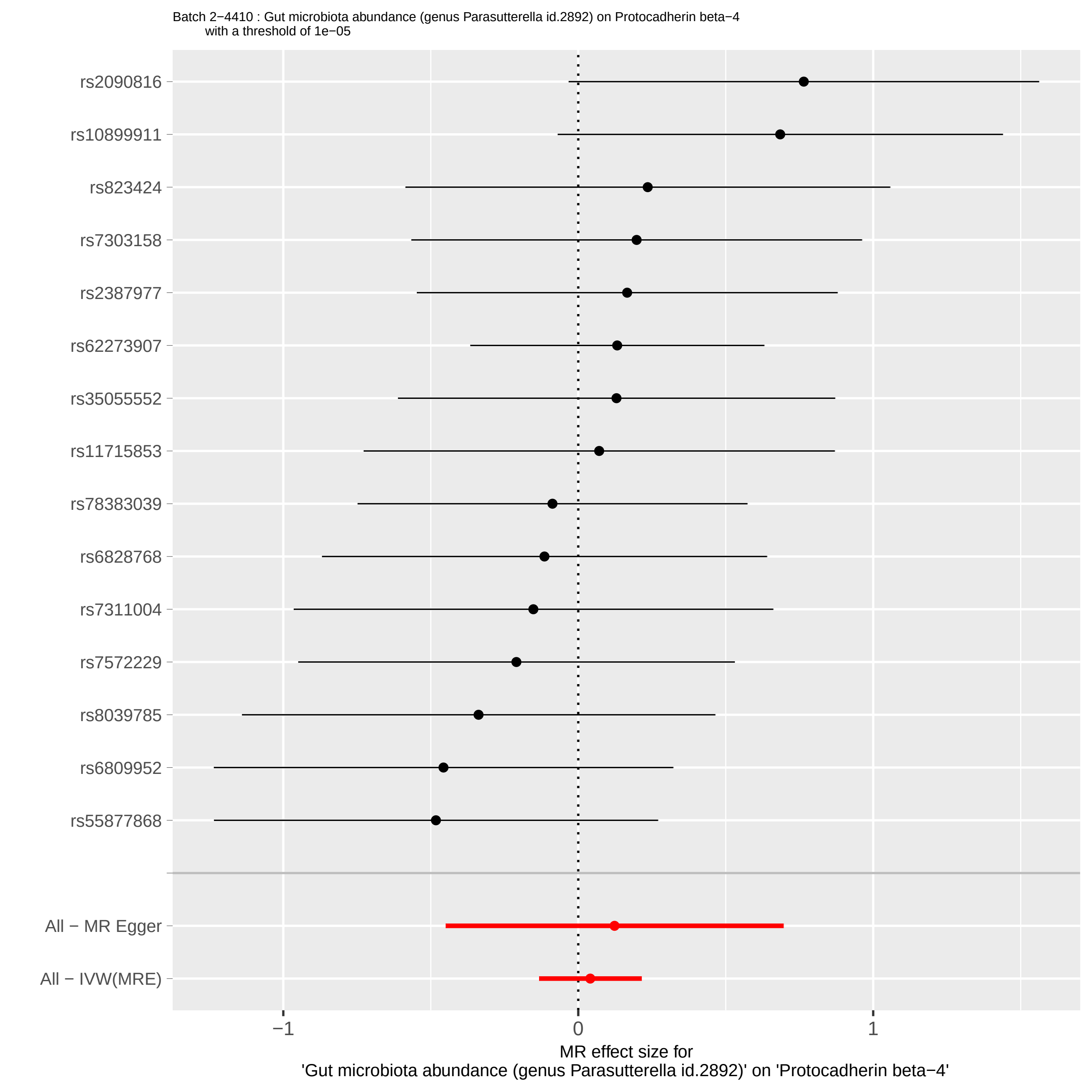

Batch 2−4410 : Gut microbiota abundance (genus Parasutterella id.2892) on Protocadherin beta−4
with a threshold of 1e−05
rs2090816
rs10899911
rs823424
rs7303158
rs2387977
rs62273907
rs35055552
rs11715853
rs78383039
rs6828768
rs7311004
rs7572229
rs8039785
rs6809952
rs55877868
All − MR Egger
All − IVW(MRE)
−1
0
1
MR effect size for
'Gut microbiota abundance (genus Parasutterella id.2892)' on 'Protocadherin beta−4'

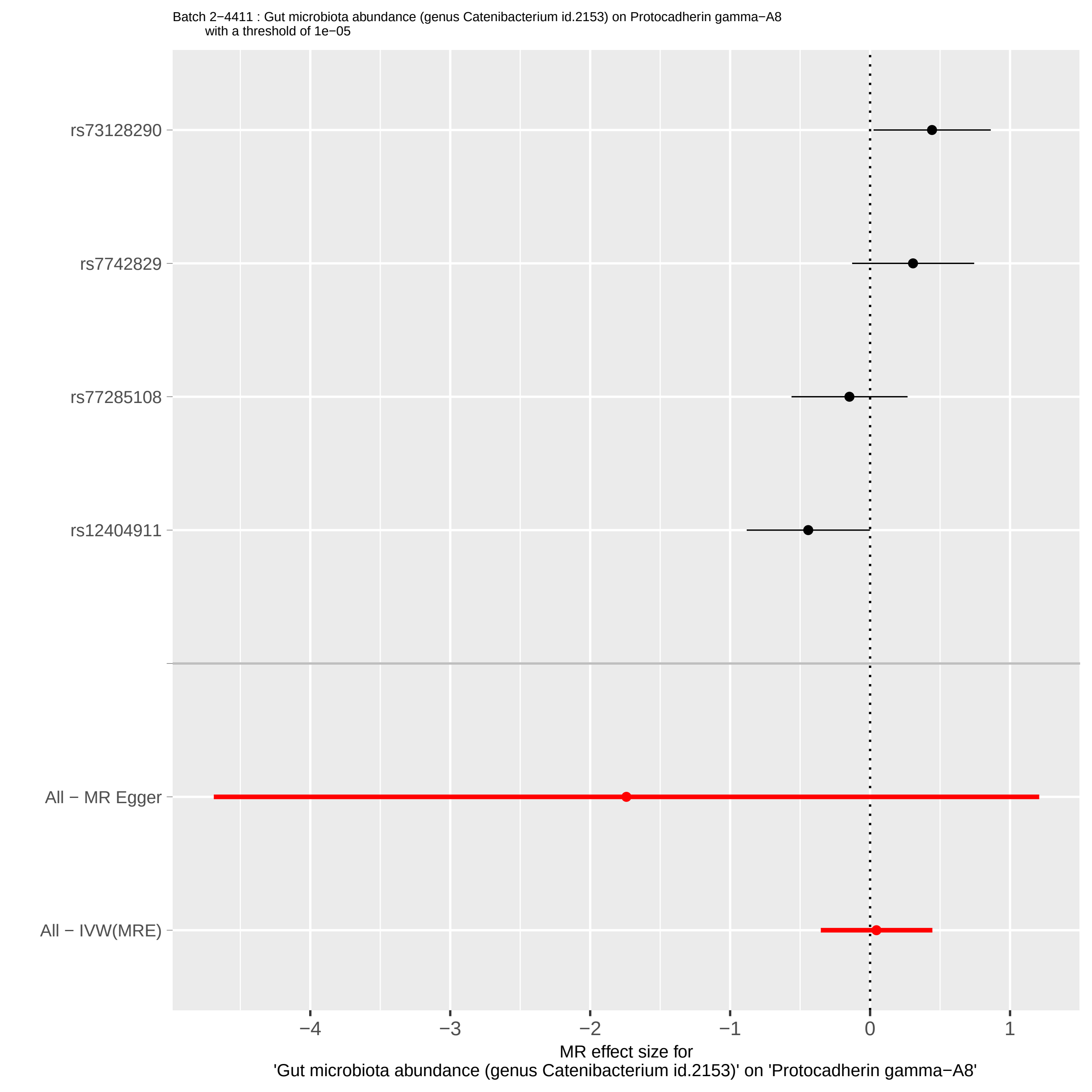
Batch 2−4411 : Gut microbiota abundance (genus Catenibacterium id.2153) on Protocadherin gamma−A8
with a threshold of 1e−05
rs73128290
rs7742829
rs77285108
rs12404911
All − MR Egger
All − IVW(MRE)
−4
−3
−2
−1
0
1
MR effect size for
'Gut microbiota abundance (genus Catenibacterium id.2153)' on 'Protocadherin gamma−A8'

Batch 2−4412 : Gut microbiota abundance (genus Parasutterella id.2892) on Protocadherin gamma−A8
with a threshold of 1e−05

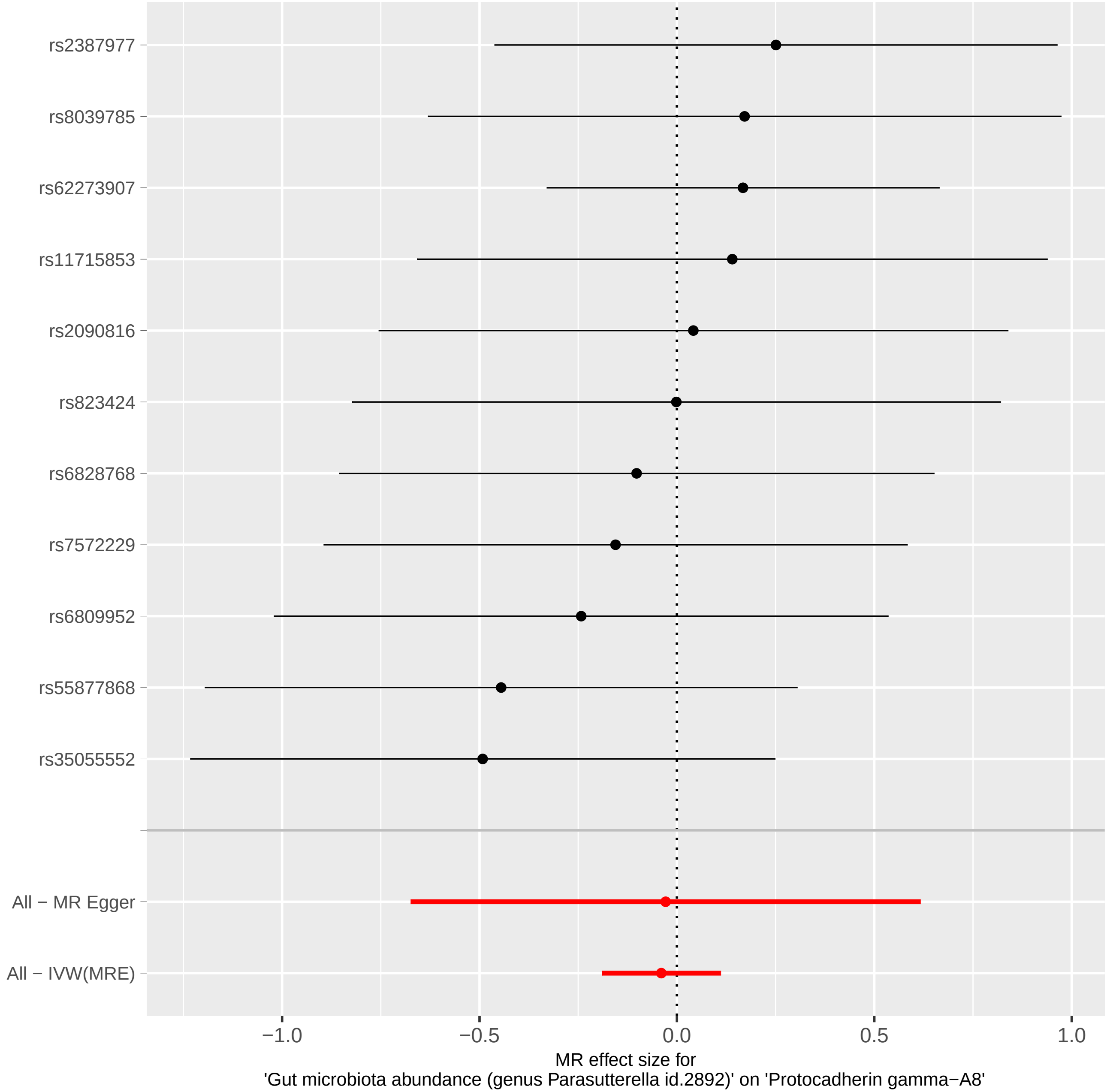

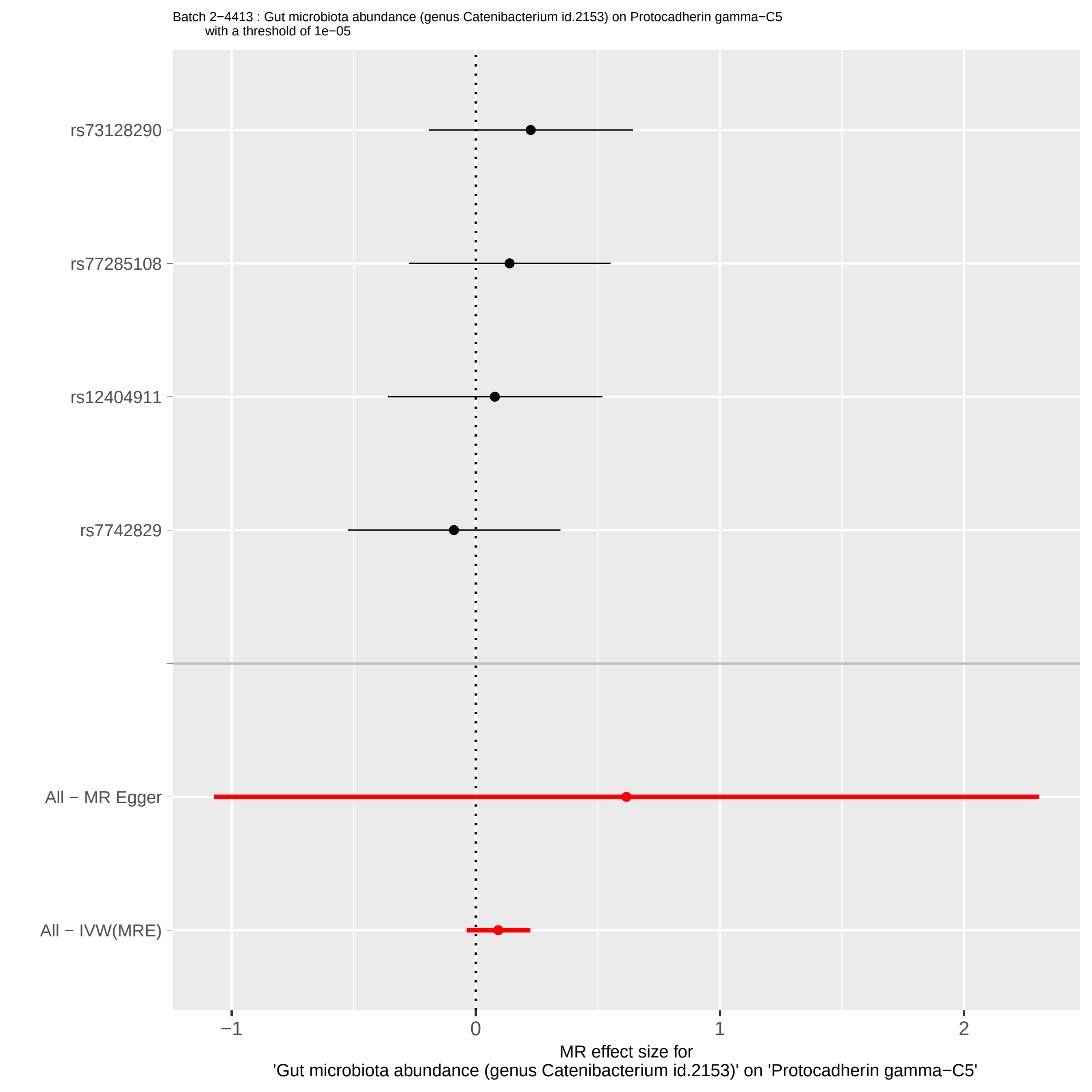
Batch 2−4413 : Gut microbiota abundance (genus Catenibacterium id.2153) on Protocadherin gamma−C5
with a threshold of 1e−05
rs73128290
rs77285108
rs12404911
rs7742829
All − MR Egger
All − IVW(MRE)
−1
0
1
2
MR effect size for
'Gut microbiota abundance (genus Catenibacterium id.2153)' on 'Protocadherin gamma−C5'

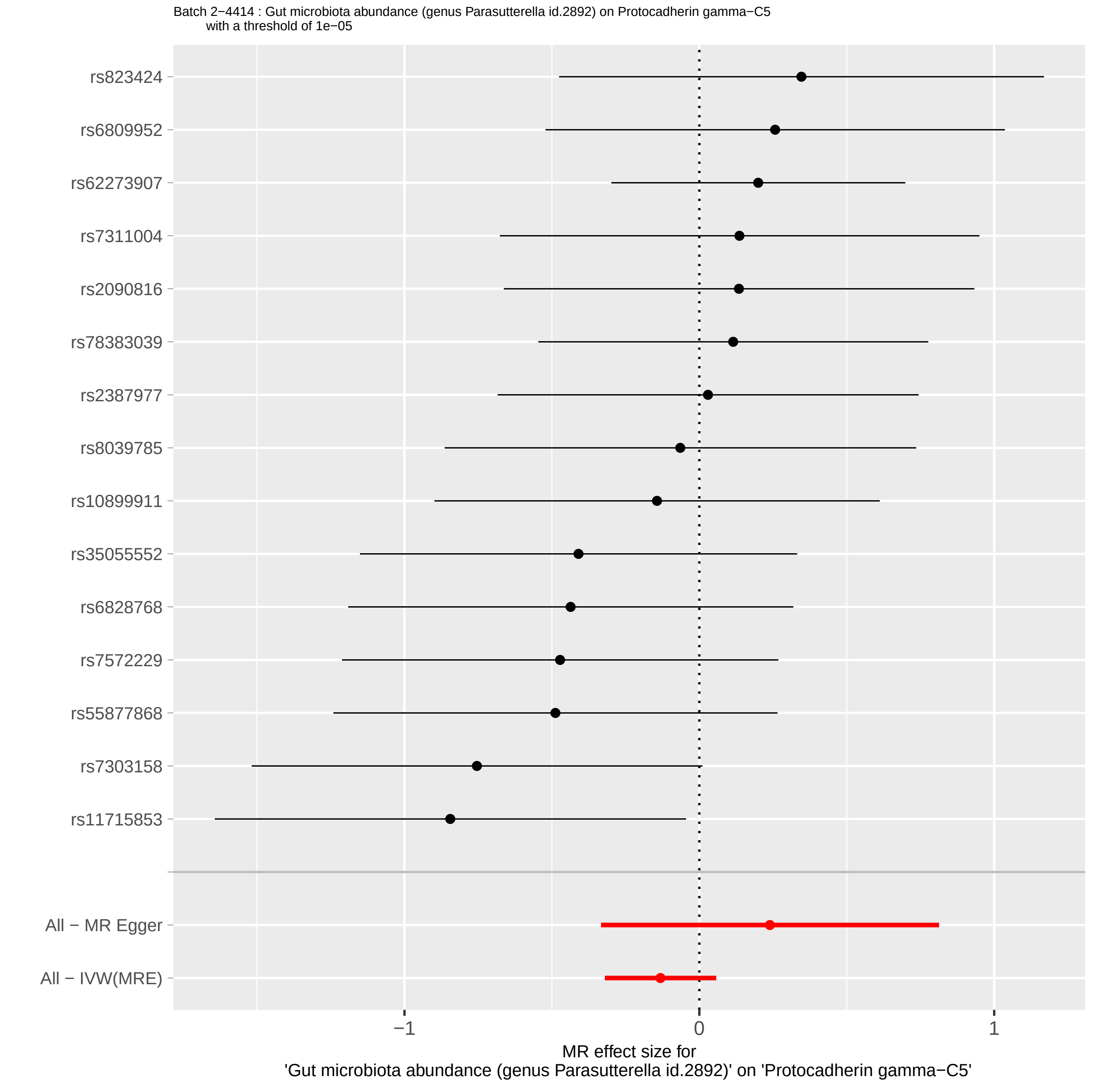
Batch 2−4414 : Gut microbiota abundance (genus Parasutterella id.2892) on Protocadherin gamma−C5
with a threshold of 1e−05
rs823424
rs6809952
rs62273907
rs7311004
rs2090816
rs78383039
rs2387977
rs8039785
rs10899911
rs35055552
rs6828768
rs7572229
rs55877868
rs7303158
rs11715853
All − MR Egger
All − IVW(MRE)
−1
0
1
MR effect size for
'Gut microbiota abundance (genus Parasutterella id.2892)' on 'Protocadherin gamma−C5'

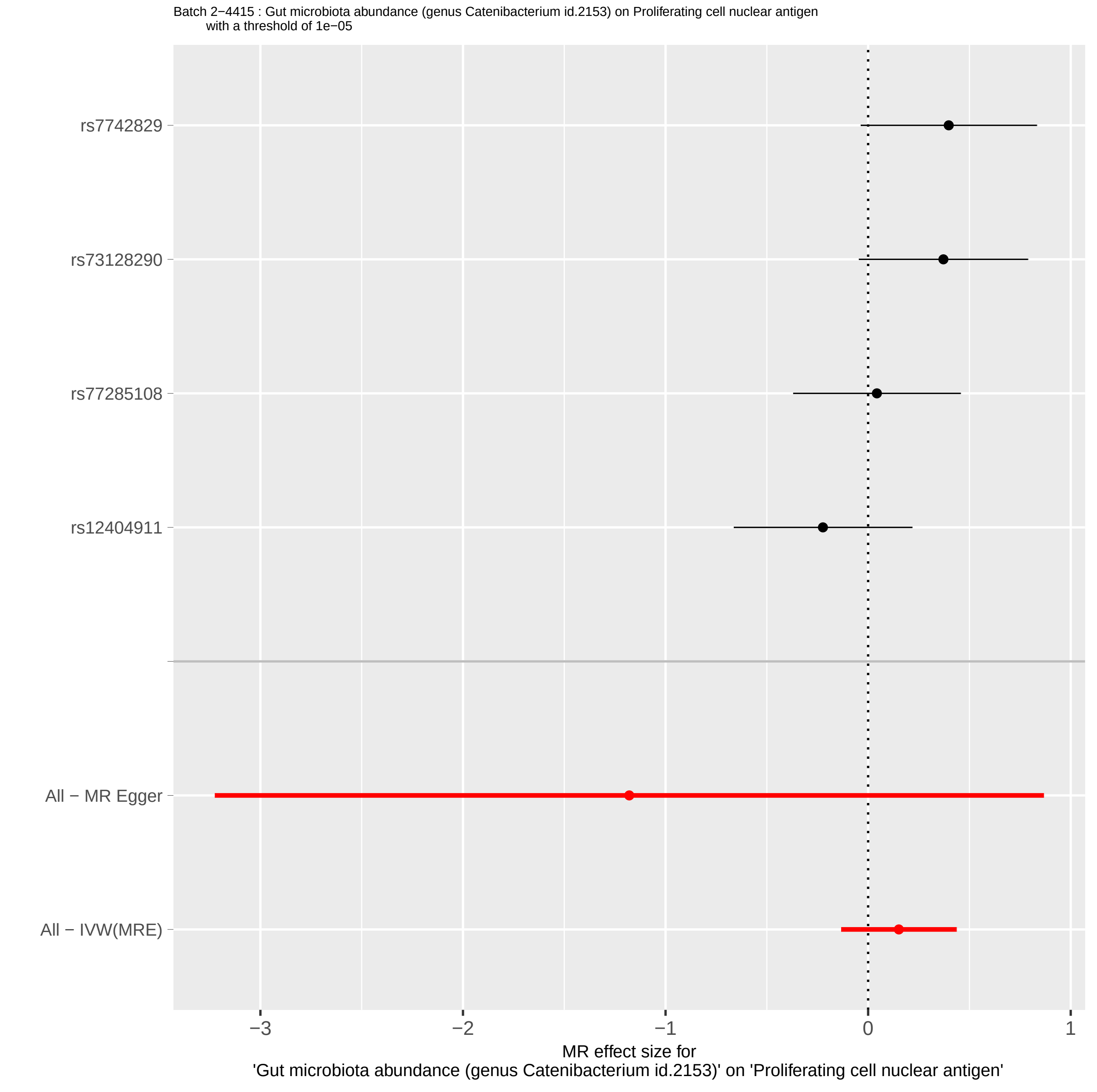

Batch 2−4415 : Gut microbiota abundance (genus Catenibacterium id.2153) on Proliferating cell nuclear antigen
with a threshold of 1e−05
rs7742829
rs73128290
rs77285108
rs12404911
All − MR Egger
All − IVW(MRE)
−3
−2
−1
0
1
MR effect size for
'Gut microbiota abundance (genus Catenibacterium id.2153)' on 'Proliferating cell nuclear antigen'

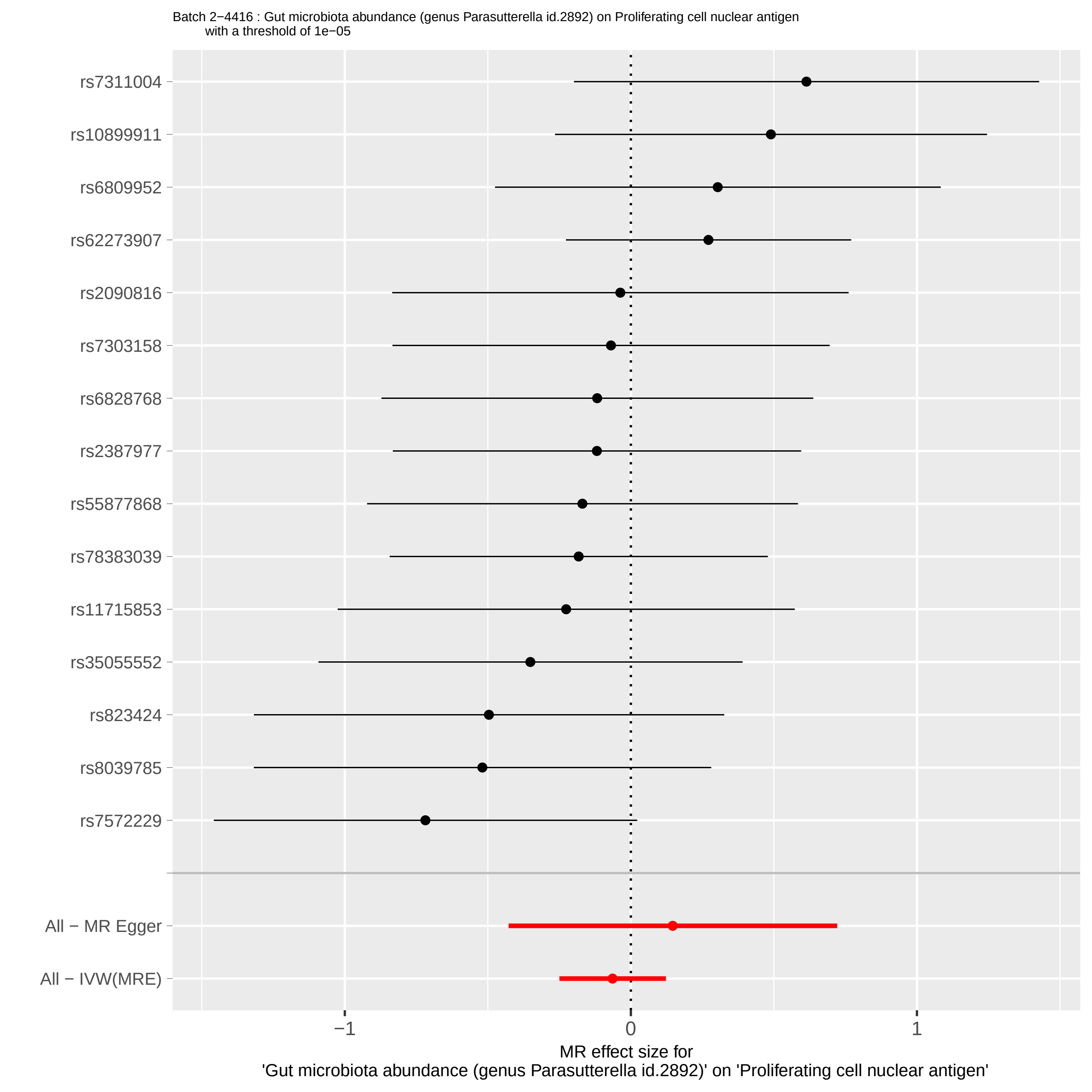

Batch 2−4416 : Gut microbiota abundance (genus Parasutterella id.2892) on Proliferating cell nuclear antigen
with a threshold of 1e−05
rs7311004
rs10899911
rs6809952
rs62273907
rs2090816
rs7303158
rs6828768
rs2387977
rs55877868
rs78383039
rs11715853
rs35055552
rs823424
rs8039785
rs7572229
All − MR Egger
All − IVW(MRE)
−1
0
1
MR effect size for
'Gut microbiota abundance (genus Parasutterella id.2892)' on 'Proliferating cell nuclear antigen'

Batch 2−4417 : Gut microbiota abundance (genus Catenibacterium id.2153) on Procollagen C−endopeptidase enhancer 1
with a threshold of 1e−05

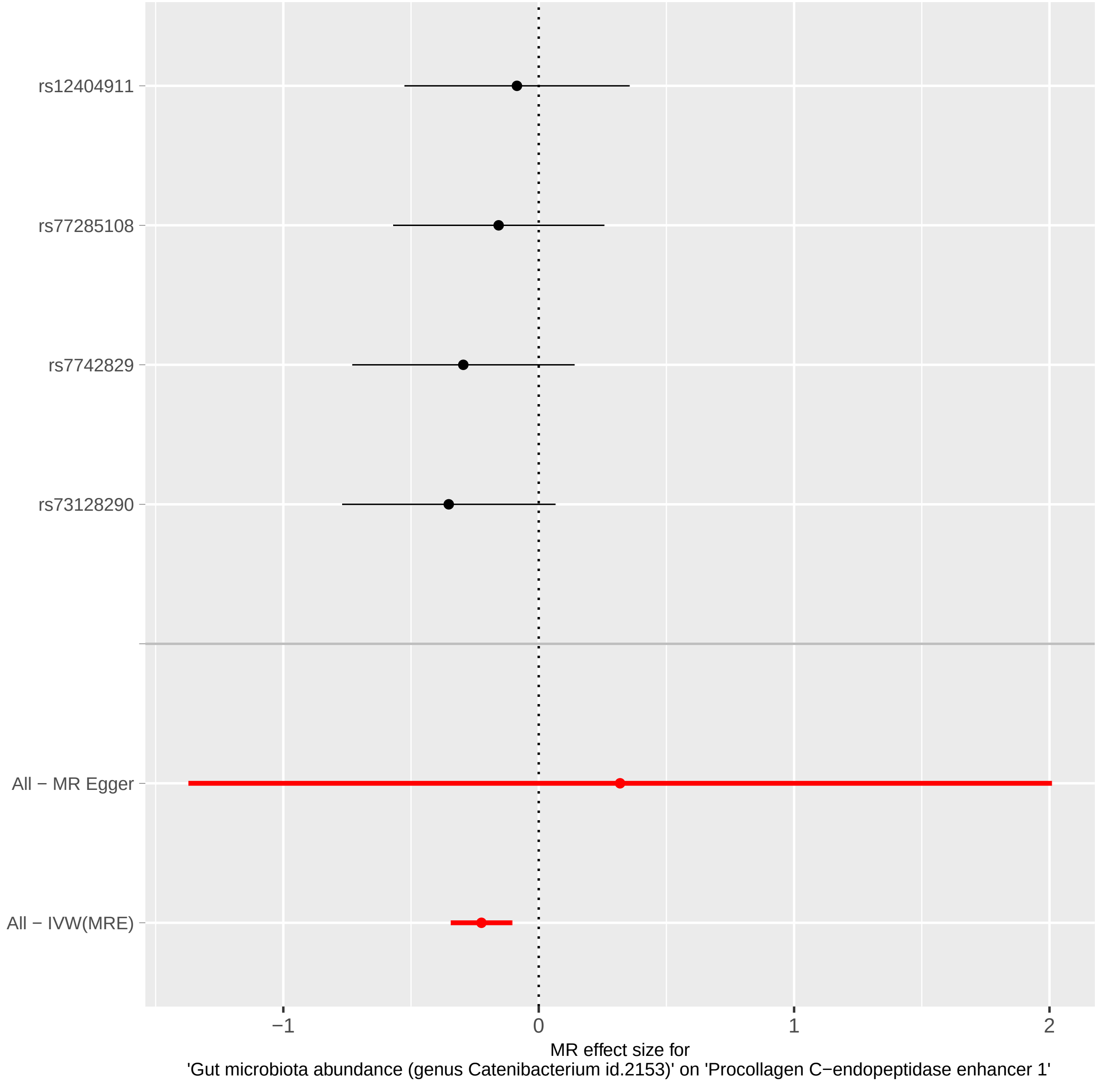

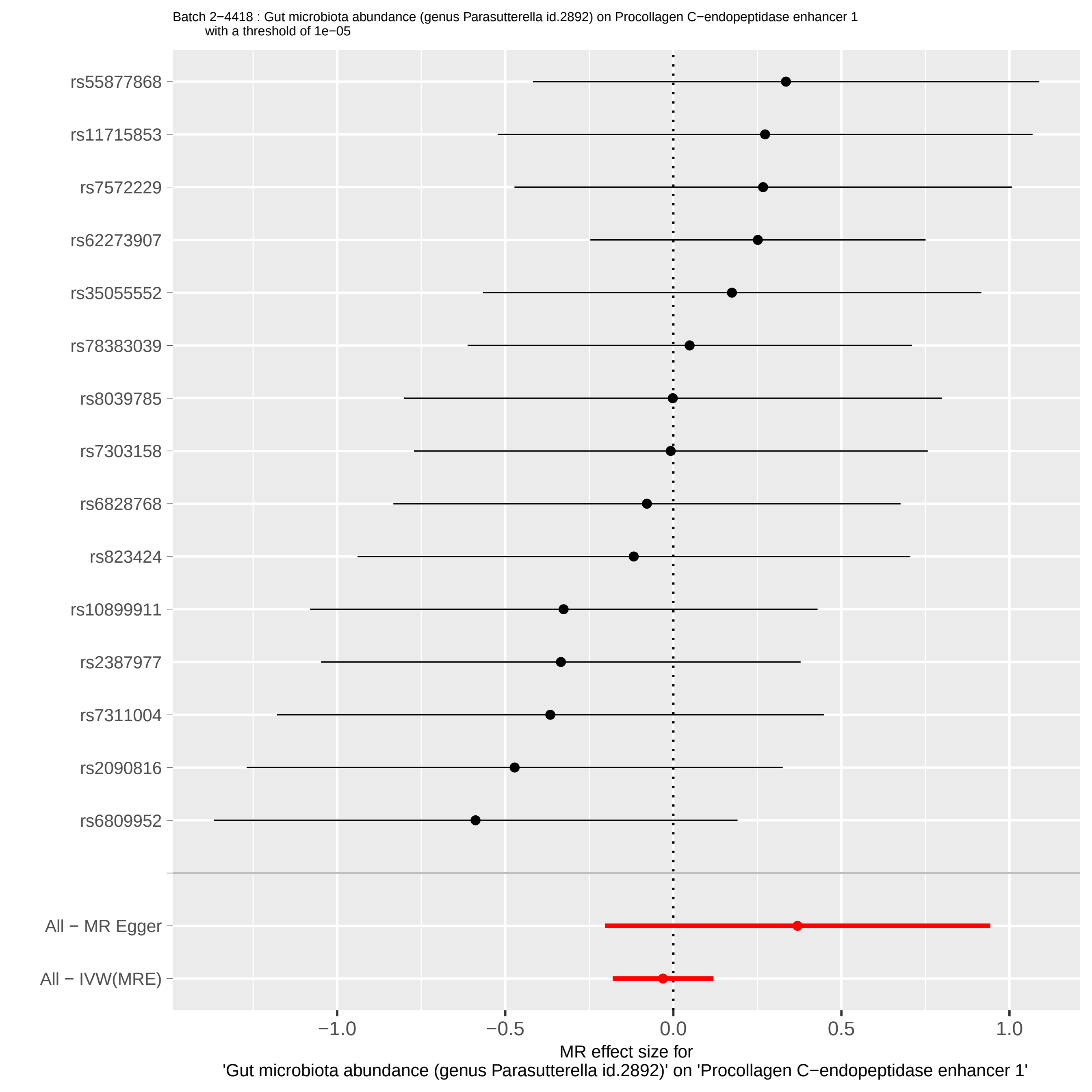
Batch 2−4418 : Gut microbiota abundance (genus Parasutterella id.2892) on Procollagen C−endopeptidase enhancer 1
with a threshold of 1e−05
rs55877868
rs11715853
rs7572229
rs62273907
rs35055552
rs78383039
rs8039785
rs7303158
rs6828768
rs823424
rs10899911
rs2387977
rs7311004
rs2090816
rs6809952
All − MR Egger
All − IVW(MRE)
−1.0
−0.5
0.0
0.5
1.0
MR effect size for
'Gut microbiota abundance (genus Parasutterella id.2892)' on 'Procollagen C−endopeptidase enhancer 1'

Batch 2−4419 : Gut microbiota abundance (genus Catenibacterium id.2153) on Procollagen C−endopeptidase enhancer 2
with a threshold of 1e−05

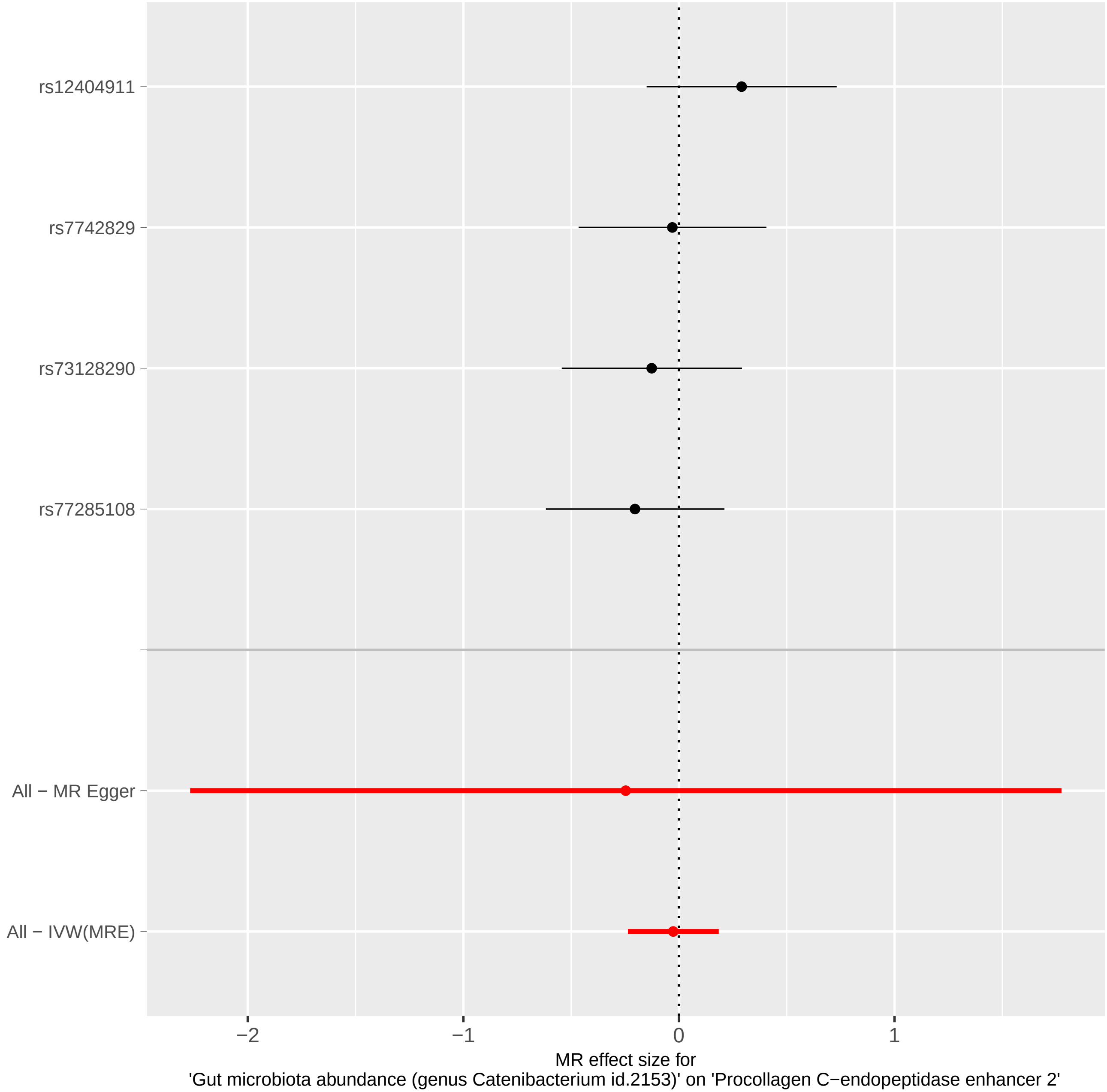

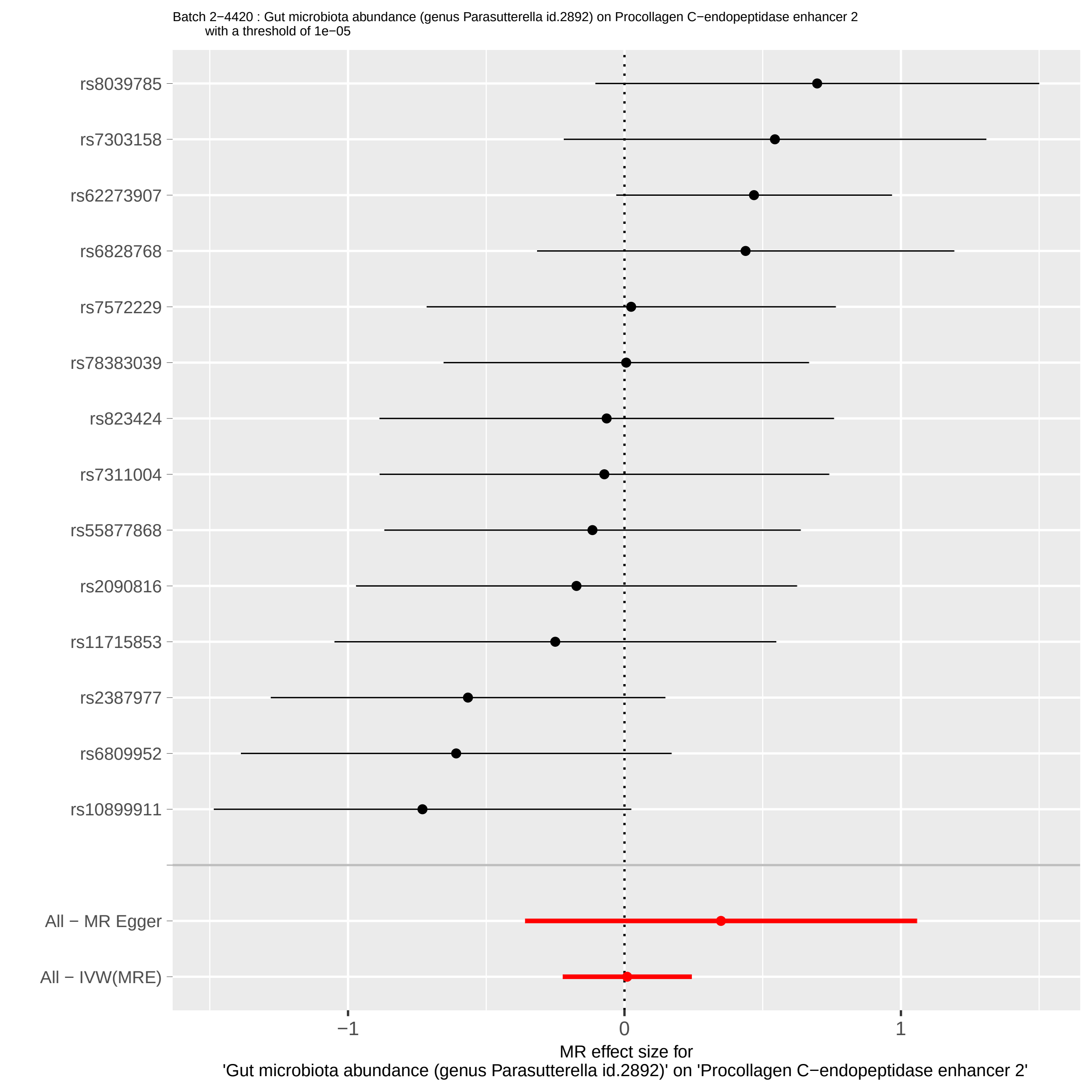

Batch 2−4420 : Gut microbiota abundance (genus Parasutterella id.2892) on Procollagen C−endopeptidase enhancer 2
with a threshold of 1e−05
rs8039785
rs7303158
rs62273907
rs6828768
rs7572229
rs78383039
rs823424
rs7311004
rs55877868
rs2090816
rs11715853
rs2387977
rs6809952
rs10899911
All − MR Egger
All − IVW(MRE)
−1
0
1
MR effect size for
'Gut microbiota abundance (genus Parasutterella id.2892)' on 'Procollagen C−endopeptidase enhancer 2'

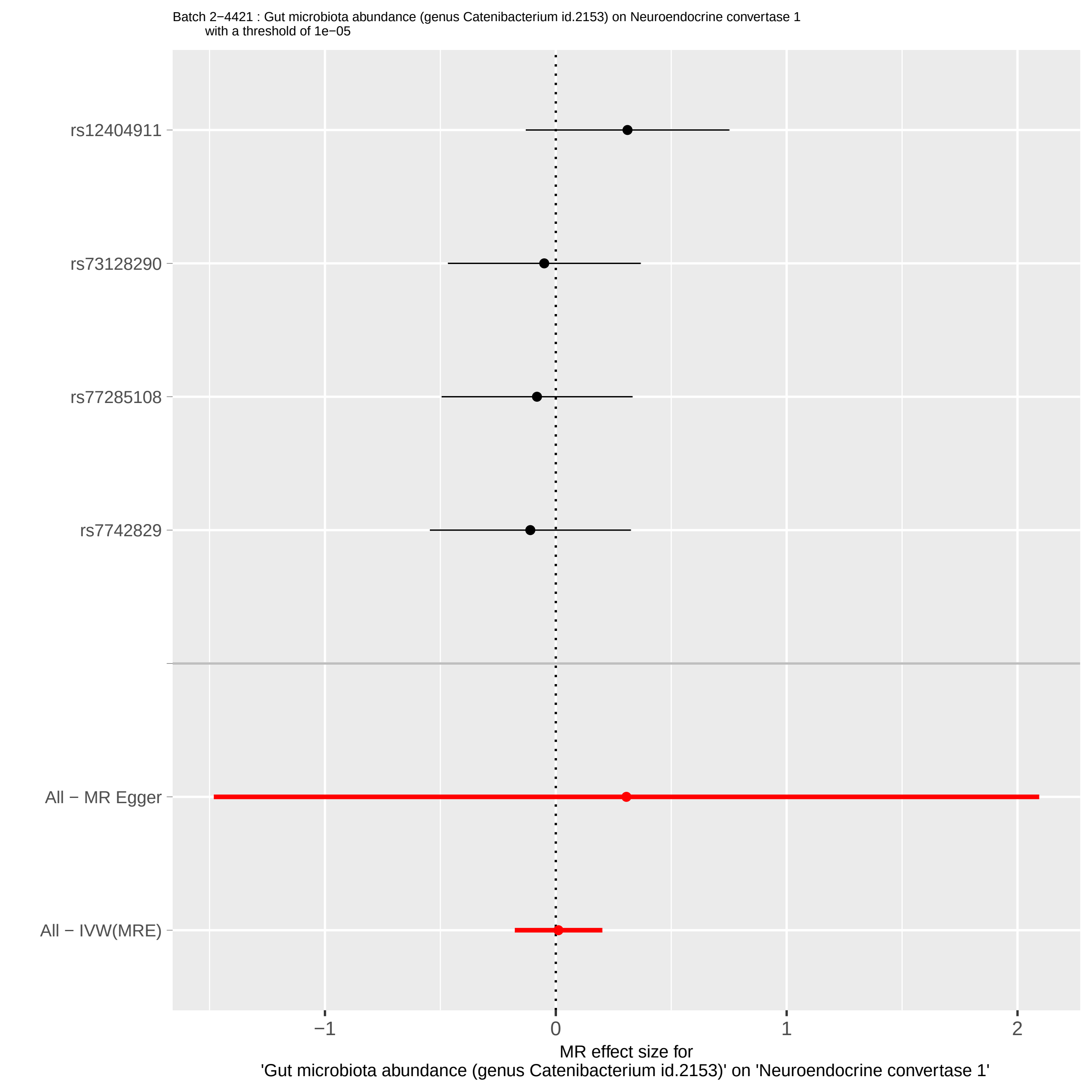

Batch 2−4421 : Gut microbiota abundance (genus Catenibacterium id.2153) on Neuroendocrine convertase 1
with a threshold of 1e−05
rs12404911
rs73128290
rs77285108
rs7742829
All − MR Egger
All − IVW(MRE)
−1
0
1
2
MR effect size for
'Gut microbiota abundance (genus Catenibacterium id.2153)' on 'Neuroendocrine convertase 1'

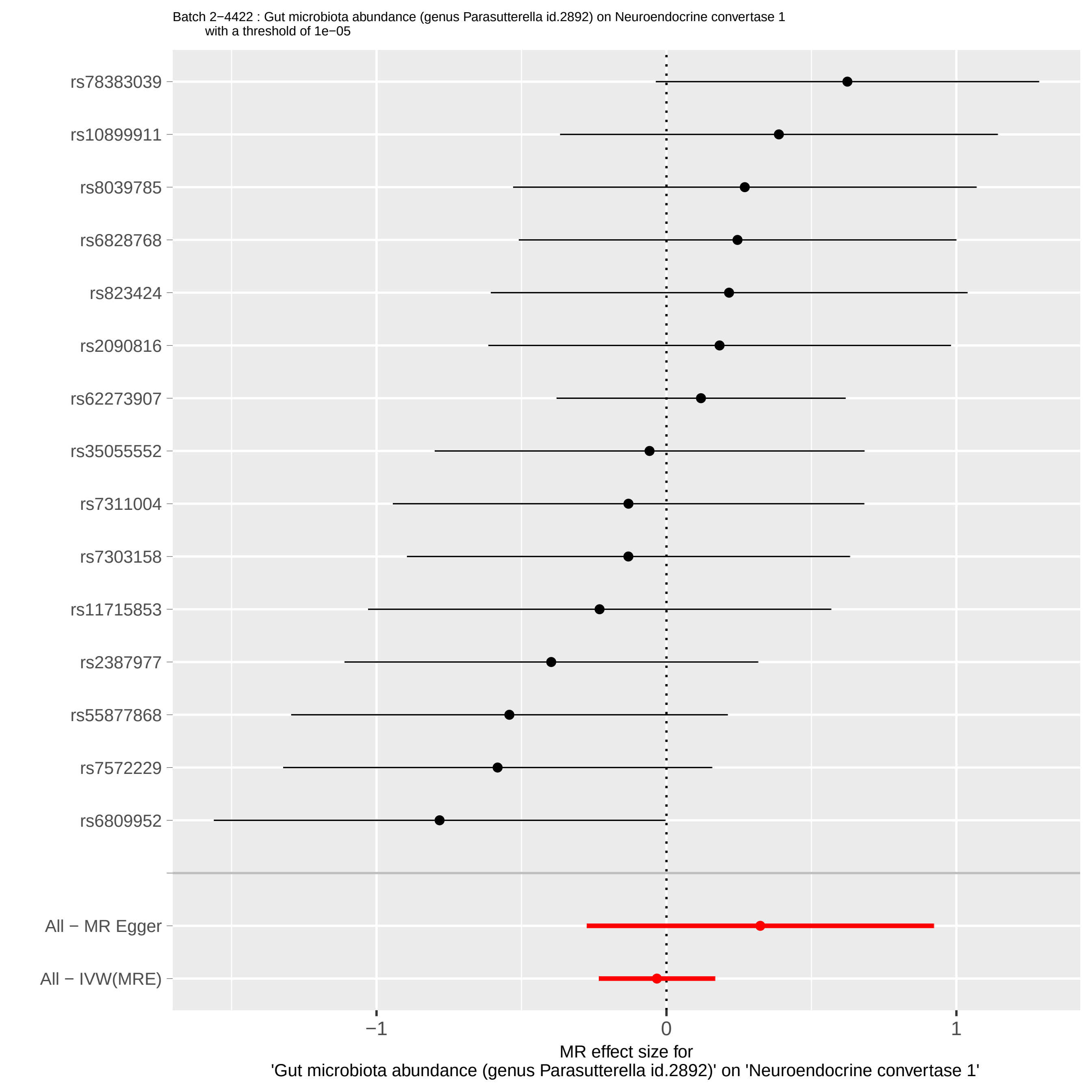

Batch 2−4422 : Gut microbiota abundance (genus Parasutterella id.2892) on Neuroendocrine convertase 1
with a threshold of 1e−05
rs78383039
rs10899911
rs8039785
rs6828768
rs823424
rs2090816
rs62273907
rs35055552
rs7311004
rs7303158
rs11715853
rs2387977
rs55877868
rs7572229
rs6809952
All − MR Egger
All − IVW(MRE)
−1
0
1
MR effect size for
'Gut microbiota abundance (genus Parasutterella id.2892)' on 'Neuroendocrine convertase 1'

Batch 2−4423 : Gut microbiota abundance (genus Catenibacterium id.2153) on Proprotein convertase subtilisin/kexin type 7
with a threshold of 1e−05

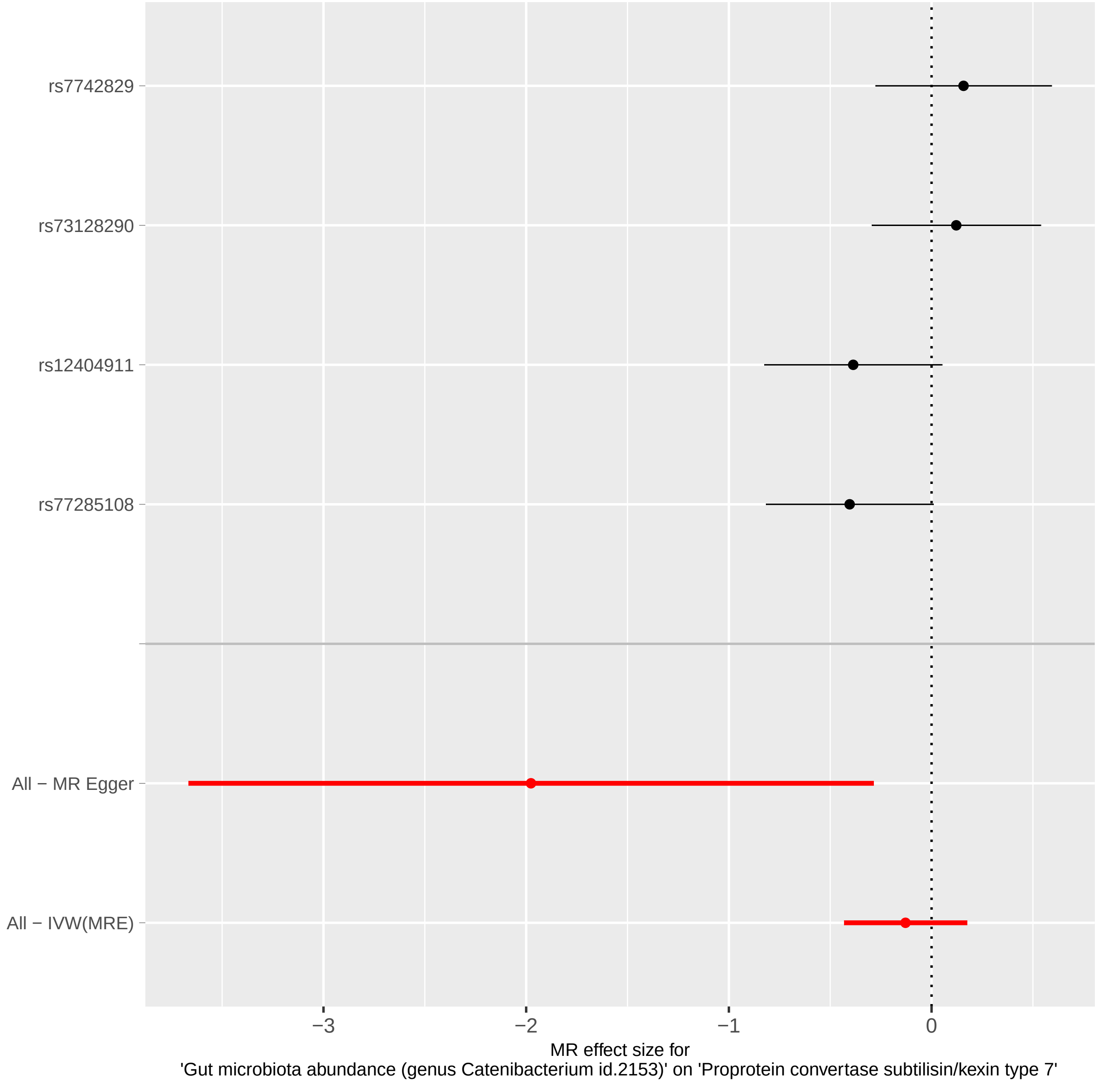

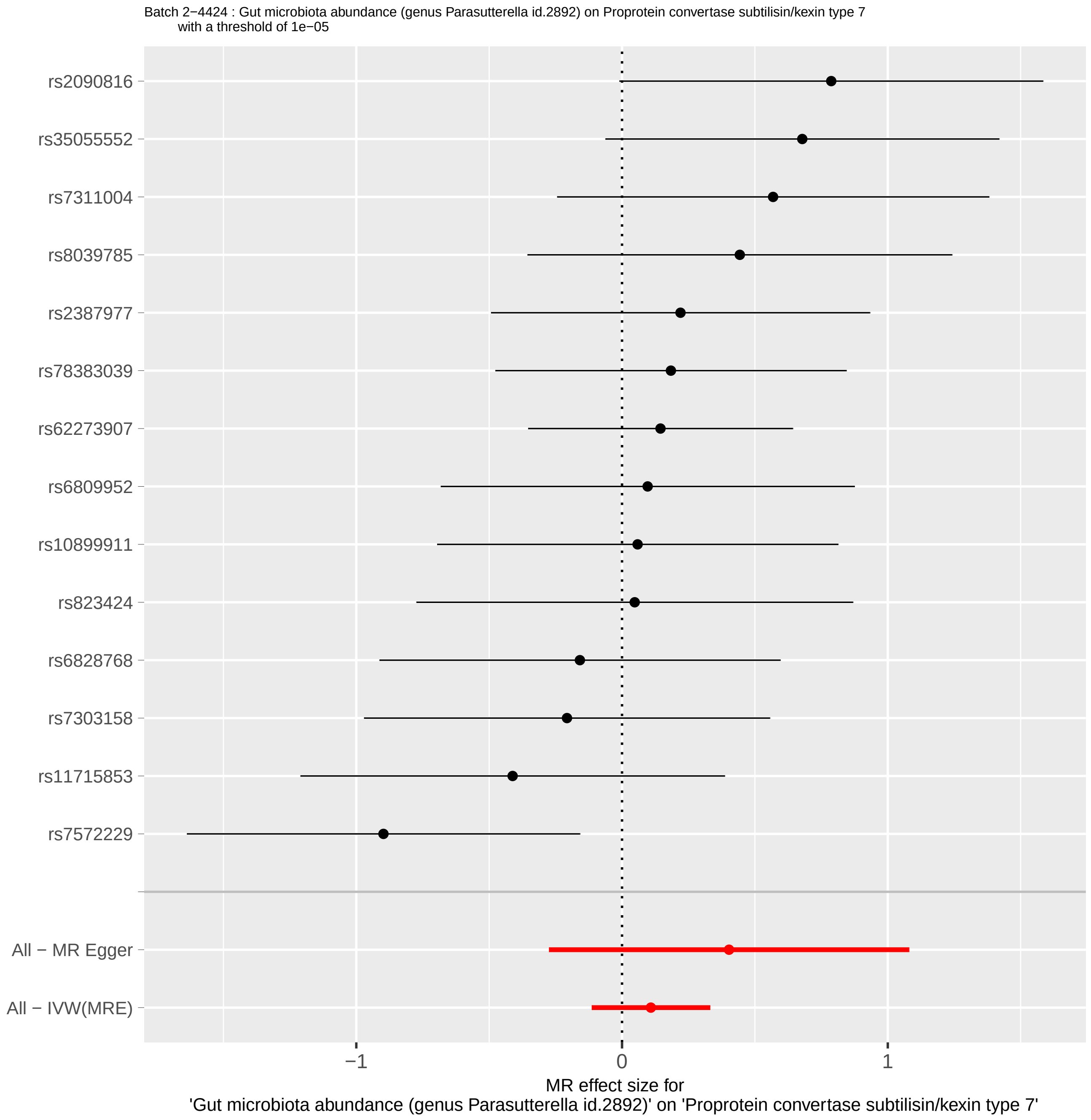

Batch 2−4424 : Gut microbiota abundance (genus Parasutterella id.2892) on Proprotein convertase subtilisin/kexin type 7
with a threshold of 1e−05
rs2090816
rs35055552
rs7311004
rs8039785
rs2387977
rs78383039
rs62273907
rs6809952
rs10899911
rs823424
rs6828768
rs7303158
rs11715853
rs7572229
All − MR Egger
All − IVW(MRE)
−1
0
1
MR effect size for
'Gut microbiota abundance (genus Parasutterella id.2892)' on 'Proprotein convertase subtilisin/kexin type 7'

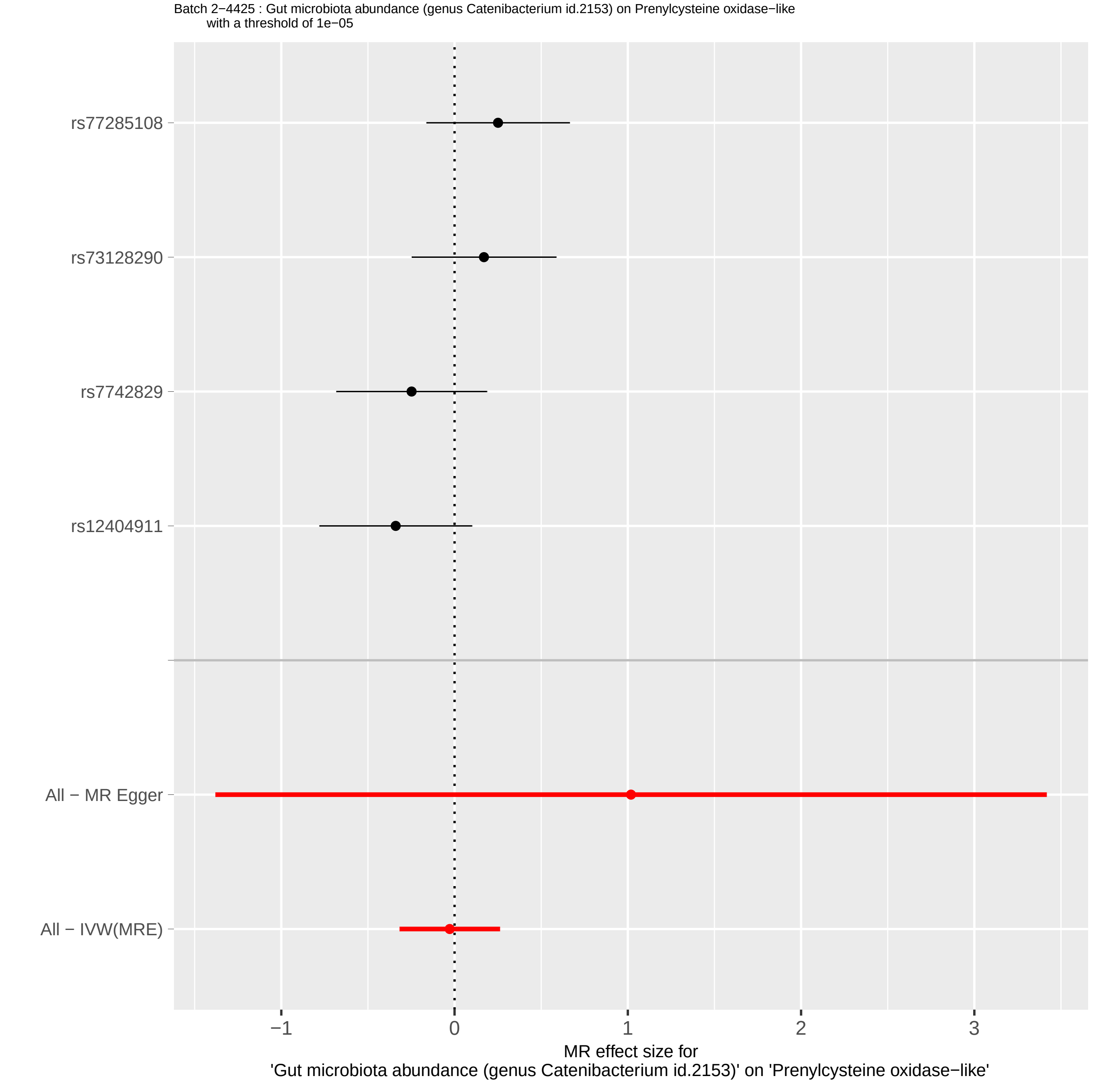
Batch 2−4425 : Gut microbiota abundance (genus Catenibacterium id.2153) on Prenylcysteine oxidase−like
with a threshold of 1e−05
rs77285108
rs73128290
rs7742829
rs12404911
All − MR Egger
All − IVW(MRE)
−1
0
1
2
3
MR effect size for
'Gut microbiota abundance (genus Catenibacterium id.2153)' on 'Prenylcysteine oxidase−like'

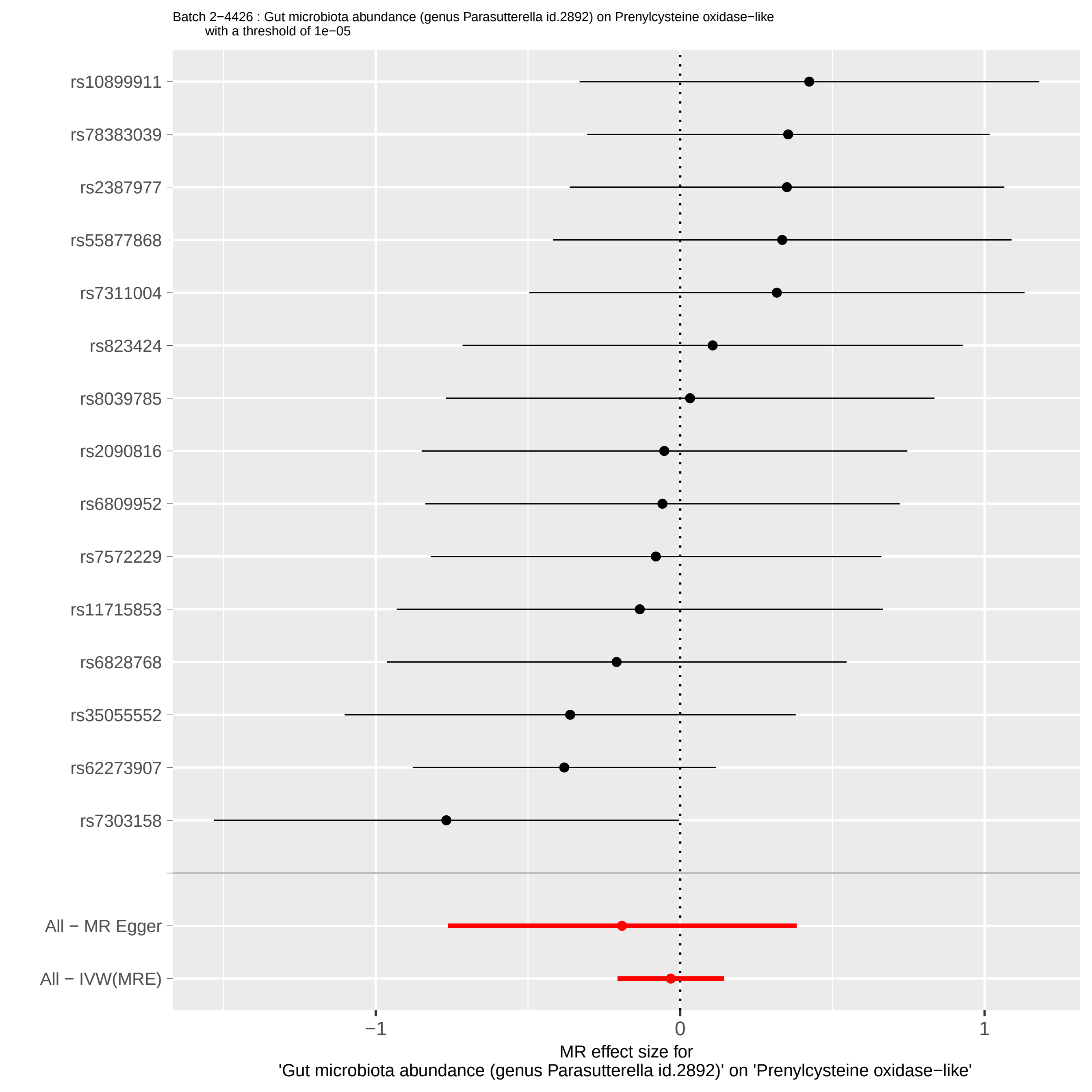
Batch 2−4426 : Gut microbiota abundance (genus Parasutterella id.2892) on Prenylcysteine oxidase−like
with a threshold of 1e−05
rs10899911
rs78383039
rs2387977
rs55877868
rs7311004
rs823424
rs8039785
rs2090816
rs6809952
rs7572229
rs11715853
rs6828768
rs35055552
rs62273907
rs7303158
All − MR Egger
All − IVW(MRE)
−1
0
1
MR effect size for
'Gut microbiota abundance (genus Parasutterella id.2892)' on 'Prenylcysteine oxidase−like'

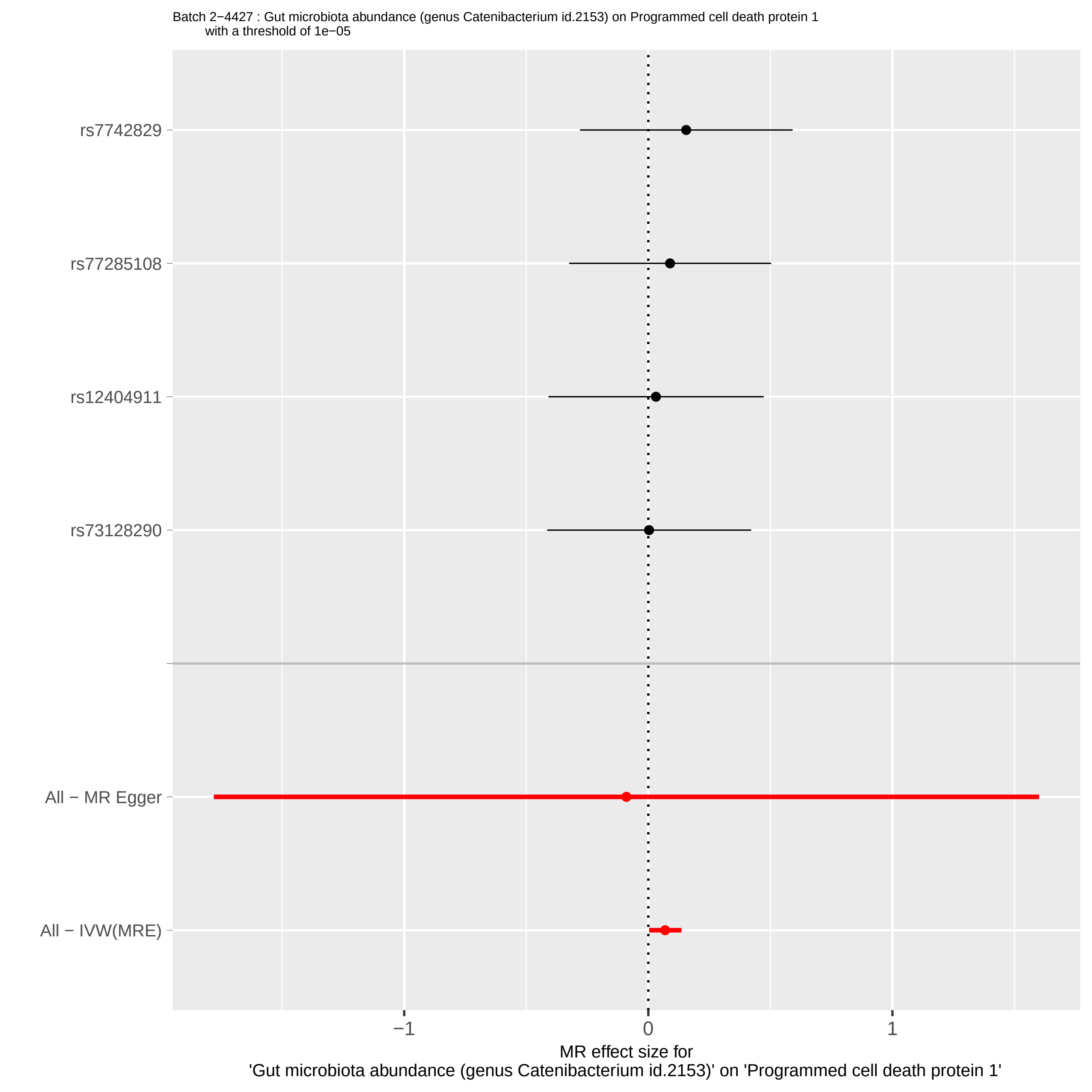
Batch 2−4427 : Gut microbiota abundance (genus Catenibacterium id.2153) on Programmed cell death protein 1
with a threshold of 1e−05
rs7742829
rs77285108
rs12404911
rs73128290
All − MR Egger
All − IVW(MRE)
−1
0
1
MR effect size for
'Gut microbiota abundance (genus Catenibacterium id.2153)' on 'Programmed cell death protein 1'

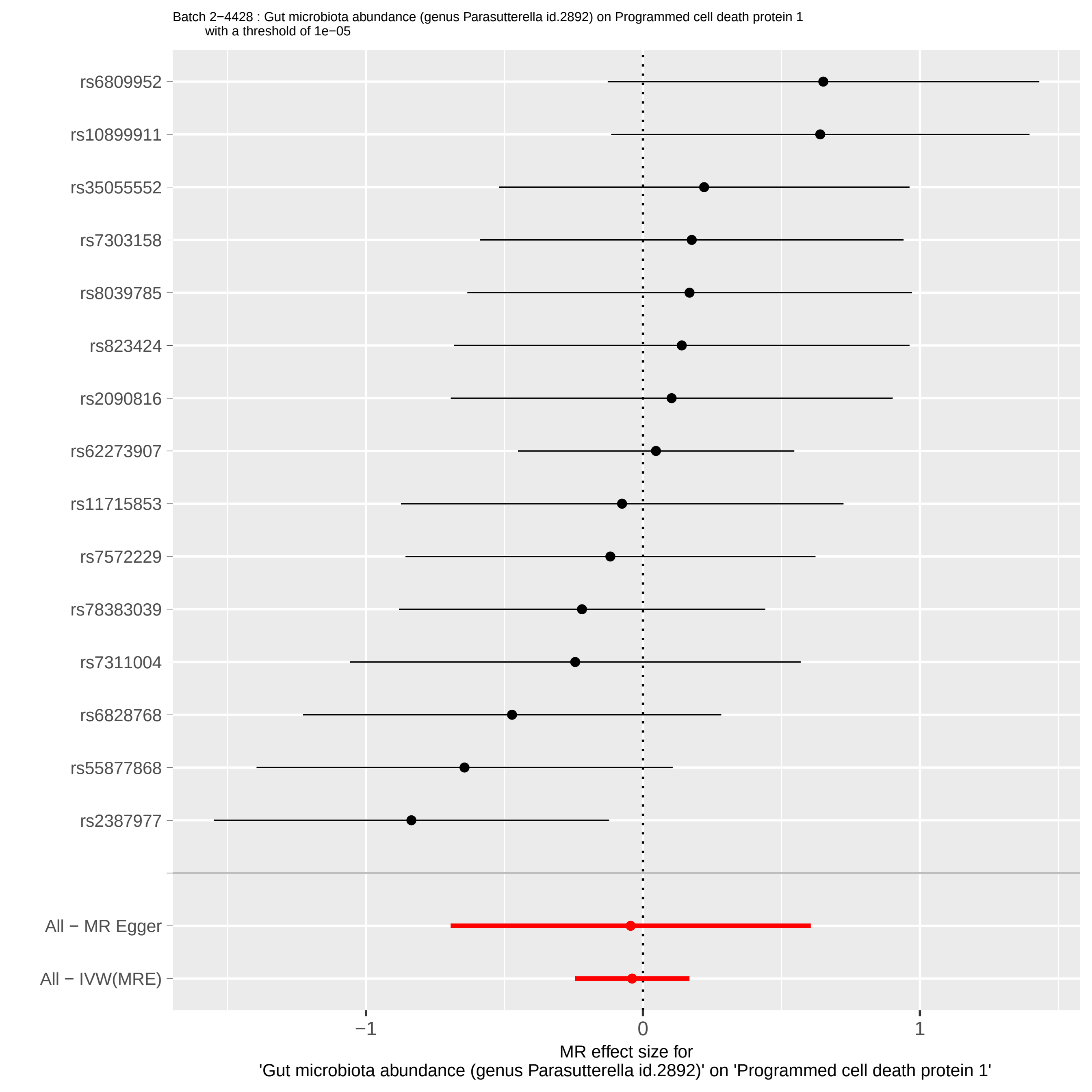
Batch 2−4428 : Gut microbiota abundance (genus Parasutterella id.2892) on Programmed cell death protein 1
with a threshold of 1e−05
rs6809952
rs10899911
rs35055552
rs7303158
rs8039785
rs823424
rs2090816
rs62273907
rs11715853
rs7572229
rs78383039
rs7311004
rs6828768
rs55877868
rs2387977
All − MR Egger
All − IVW(MRE)
−1
0
1
MR effect size for
'Gut microbiota abundance (genus Parasutterella id.2892)' on 'Programmed cell death protein 1'

Batch 2−4429 : Gut microbiota abundance (genus Catenibacterium id.2153) on Programmed cell death 1 ligand 2
with a threshold of 1e−05

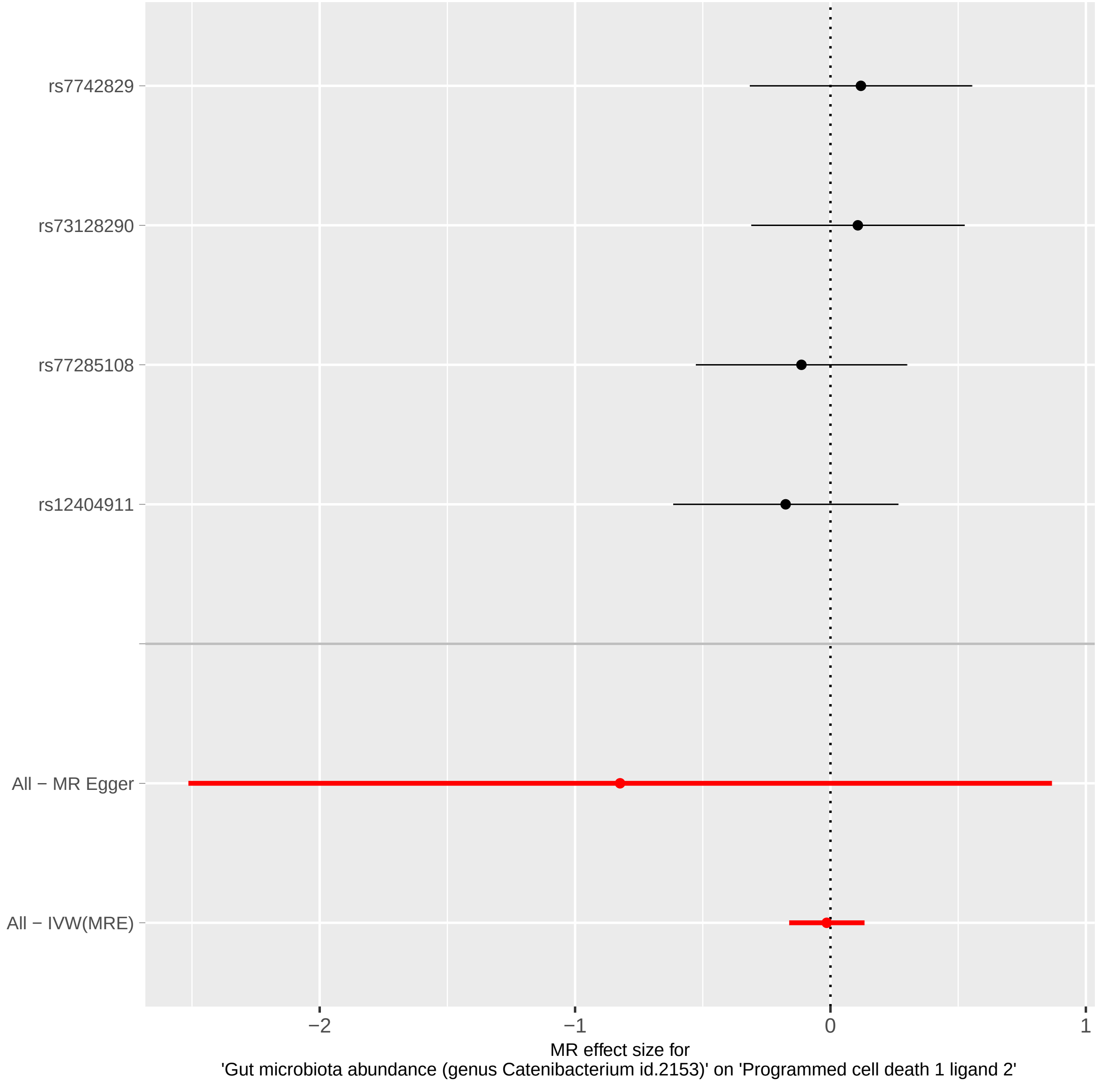

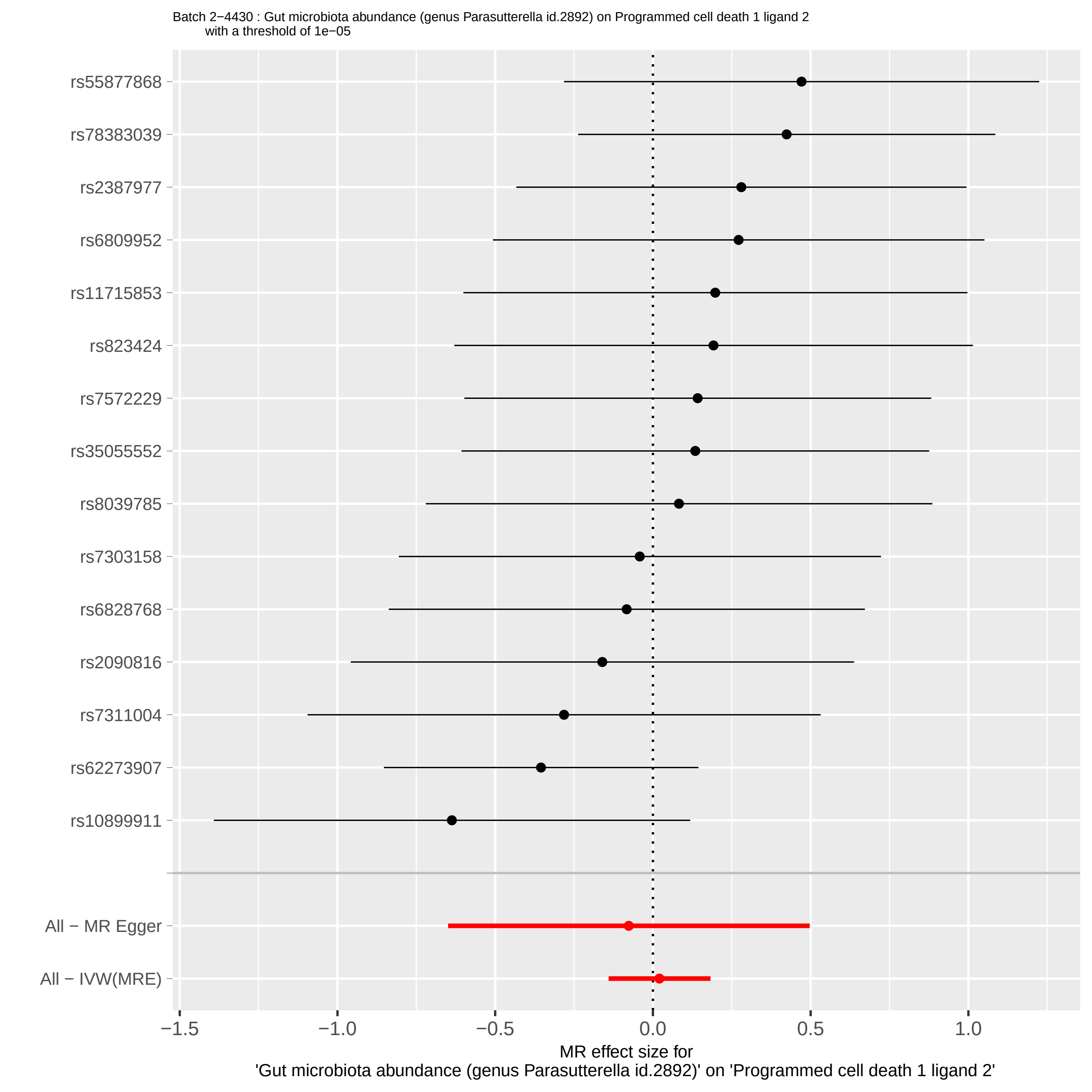

Batch 2−4430 : Gut microbiota abundance (genus Parasutterella id.2892) on Programmed cell death 1 ligand 2
with a threshold of 1e−05
rs55877868
rs78383039
rs2387977
rs6809952
rs11715853
rs823424
rs7572229
rs35055552
rs8039785
rs7303158
rs6828768
rs2090816
rs7311004
rs62273907
rs10899911
All − MR Egger
All − IVW(MRE)
−1.5
−1.0
−0.5
0.0
0.5
1.0
MR effect size for
'Gut microbiota abundance (genus Parasutterella id.2892)' on 'Programmed cell death 1 ligand 2'

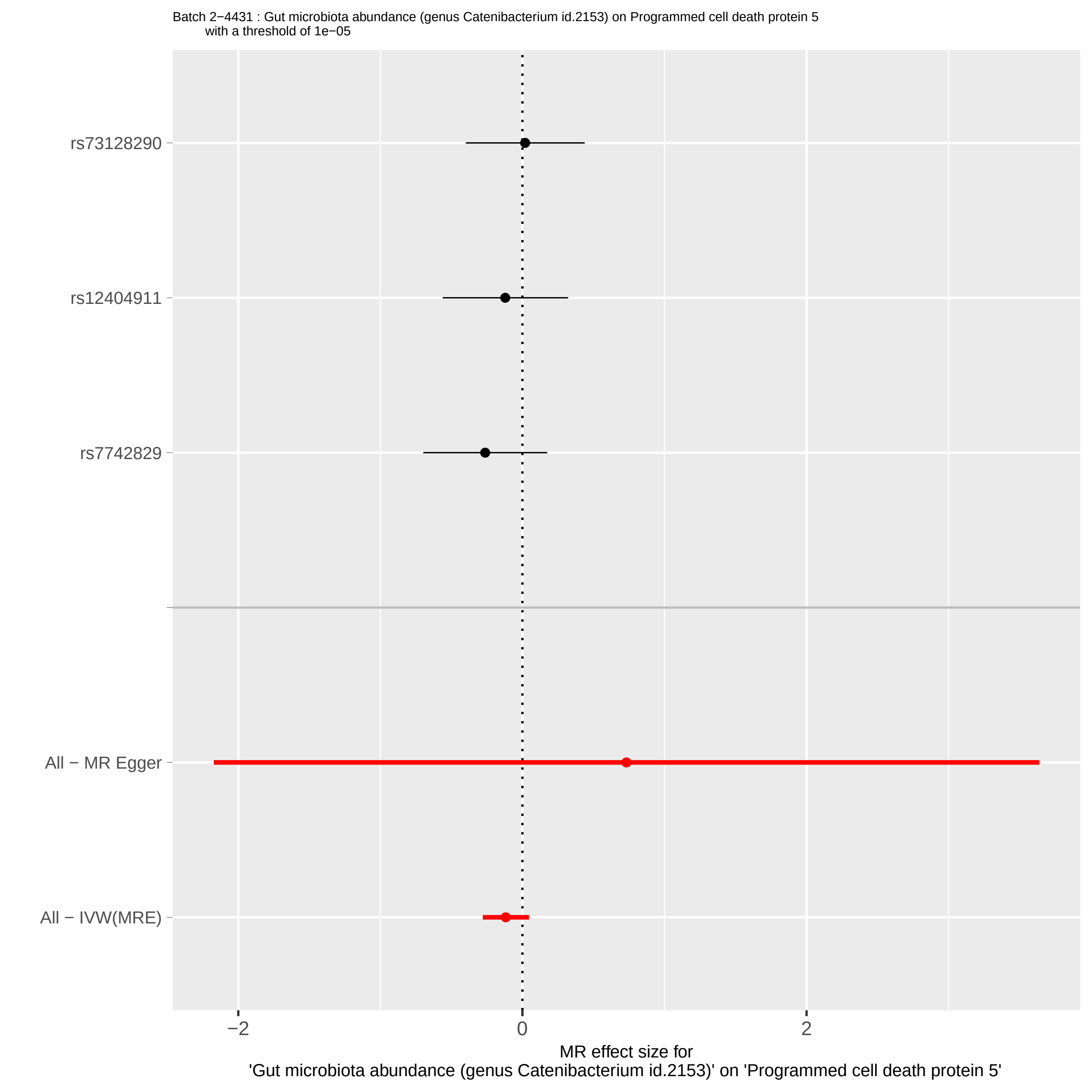
Batch 2−4431 : Gut microbiota abundance (genus Catenibacterium id.2153) on Programmed cell death protein 5
with a threshold of 1e−05
rs73128290
rs12404911
rs7742829
All − MR Egger
All − IVW(MRE)
−2
0
2
MR effect size for
'Gut microbiota abundance (genus Catenibacterium id.2153)' on 'Programmed cell death protein 5'

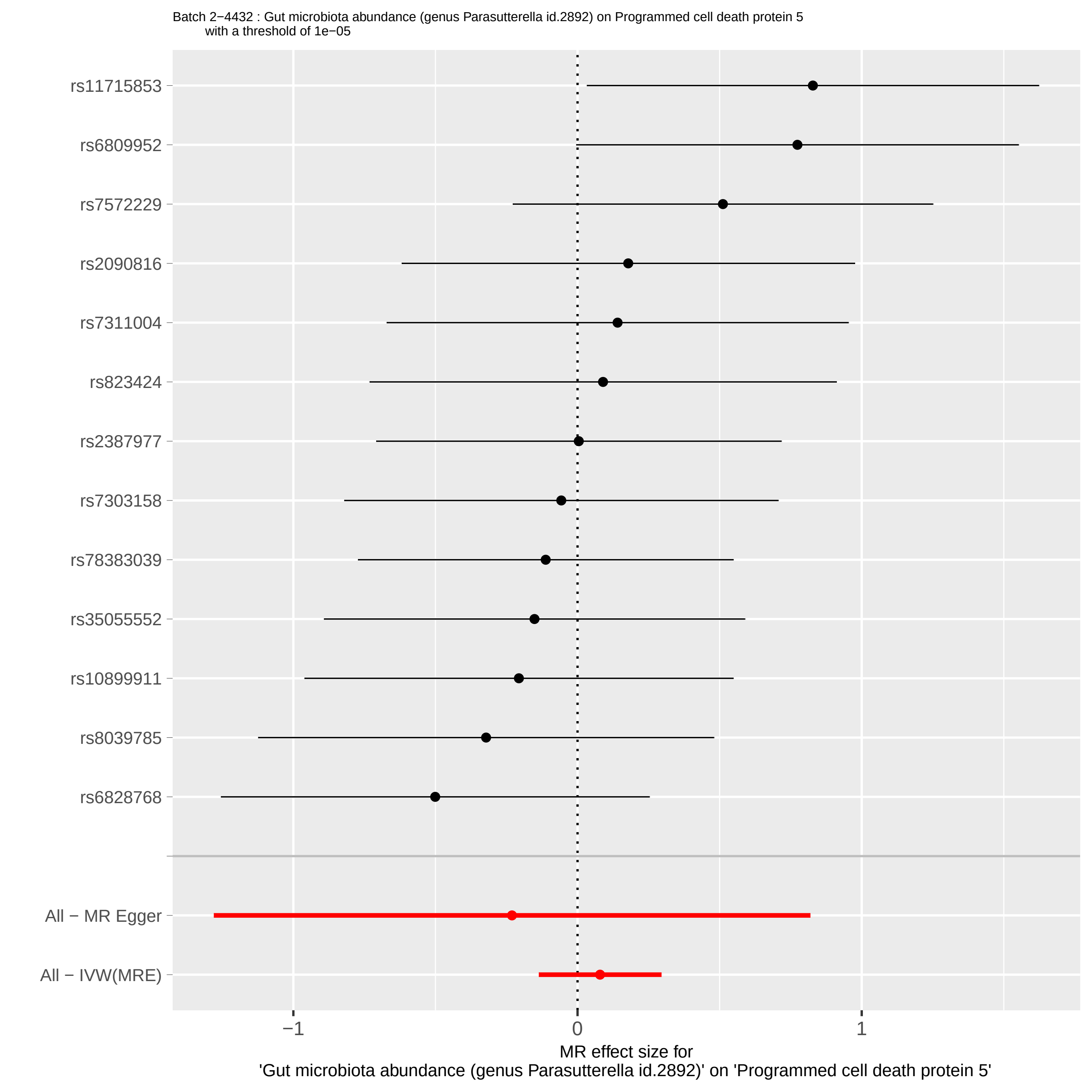

Batch 2−4432 : Gut microbiota abundance (genus Parasutterella id.2892) on Programmed cell death protein 5
with a threshold of 1e−05
rs11715853
rs6809952
rs7572229
rs2090816
rs7311004
rs823424
rs2387977
rs7303158
rs78383039
rs35055552
rs10899911
rs8039785
rs6828768
All − MR Egger
All − IVW(MRE)
−1
0
1
MR effect size for
'Gut microbiota abundance (genus Parasutterella id.2892)' on 'Programmed cell death protein 5'

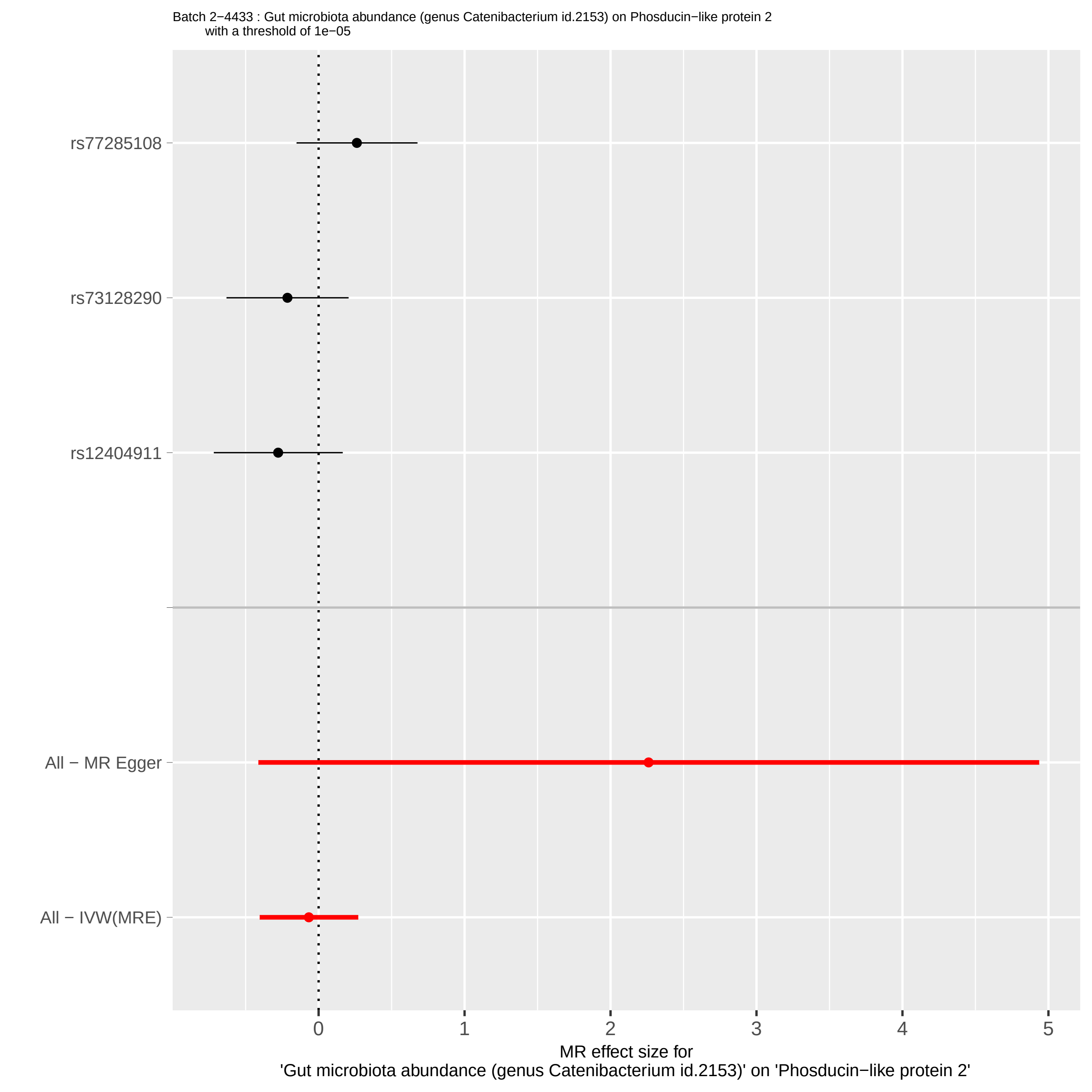
Batch 2−4433 : Gut microbiota abundance (genus Catenibacterium id.2153) on Phosducin−like protein 2
with a threshold of 1e−05
rs77285108
rs73128290
rs12404911
All − MR Egger
All − IVW(MRE)
0
1
2
3
4
5
MR effect size for
'Gut microbiota abundance (genus Catenibacterium id.2153)' on 'Phosducin−like protein 2'

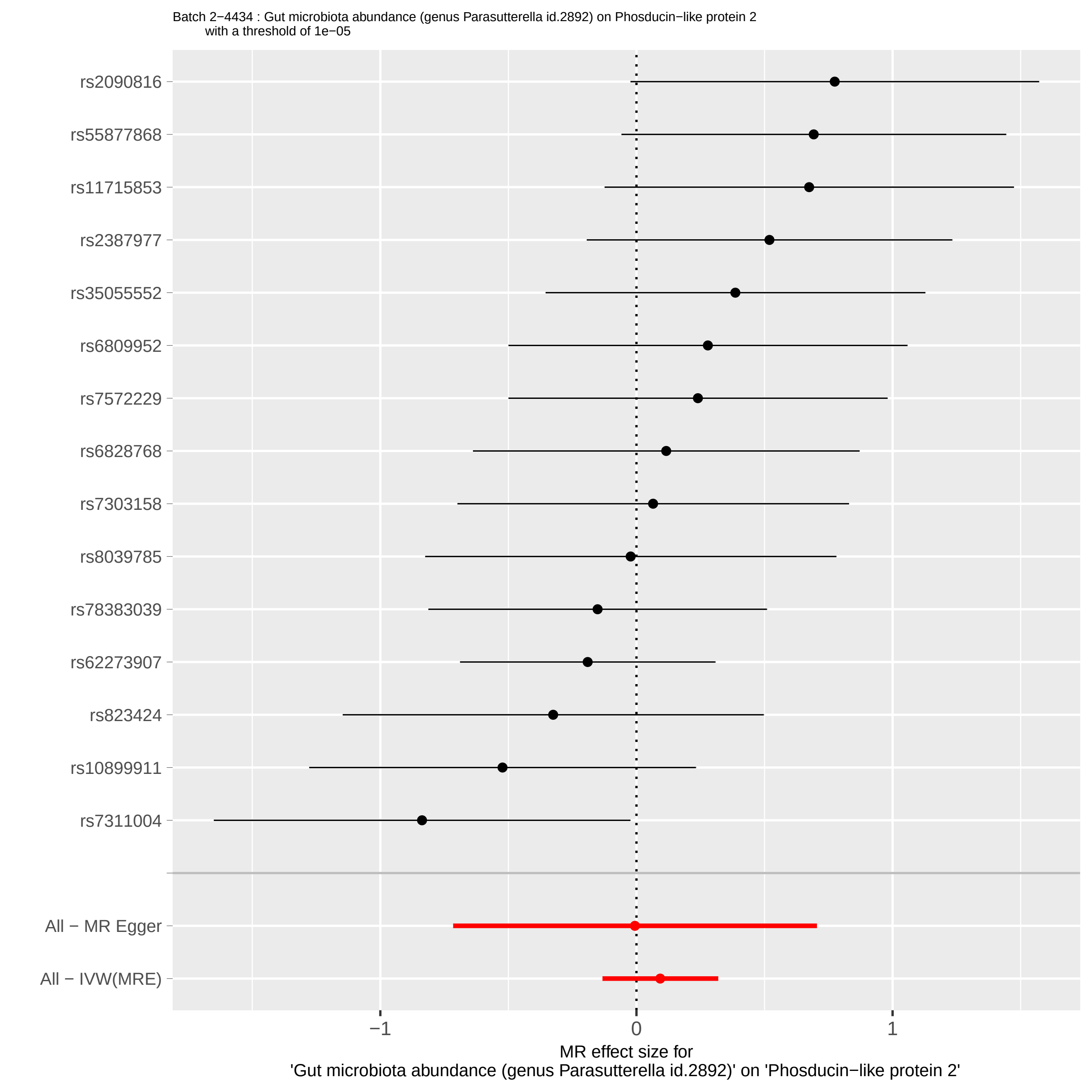

Batch 2−4434 : Gut microbiota abundance (genus Parasutterella id.2892) on Phosducin−like protein 2
with a threshold of 1e−05
rs2090816
rs55877868
rs11715853
rs2387977
rs35055552
rs6809952
rs7572229
rs6828768
rs7303158
rs8039785
rs78383039
rs62273907
rs823424
rs10899911
rs7311004
All − MR Egger
All − IVW(MRE)
−1
0
1
MR effect size for
'Gut microbiota abundance (genus Parasutterella id.2892)' on 'Phosducin−like protein 2'

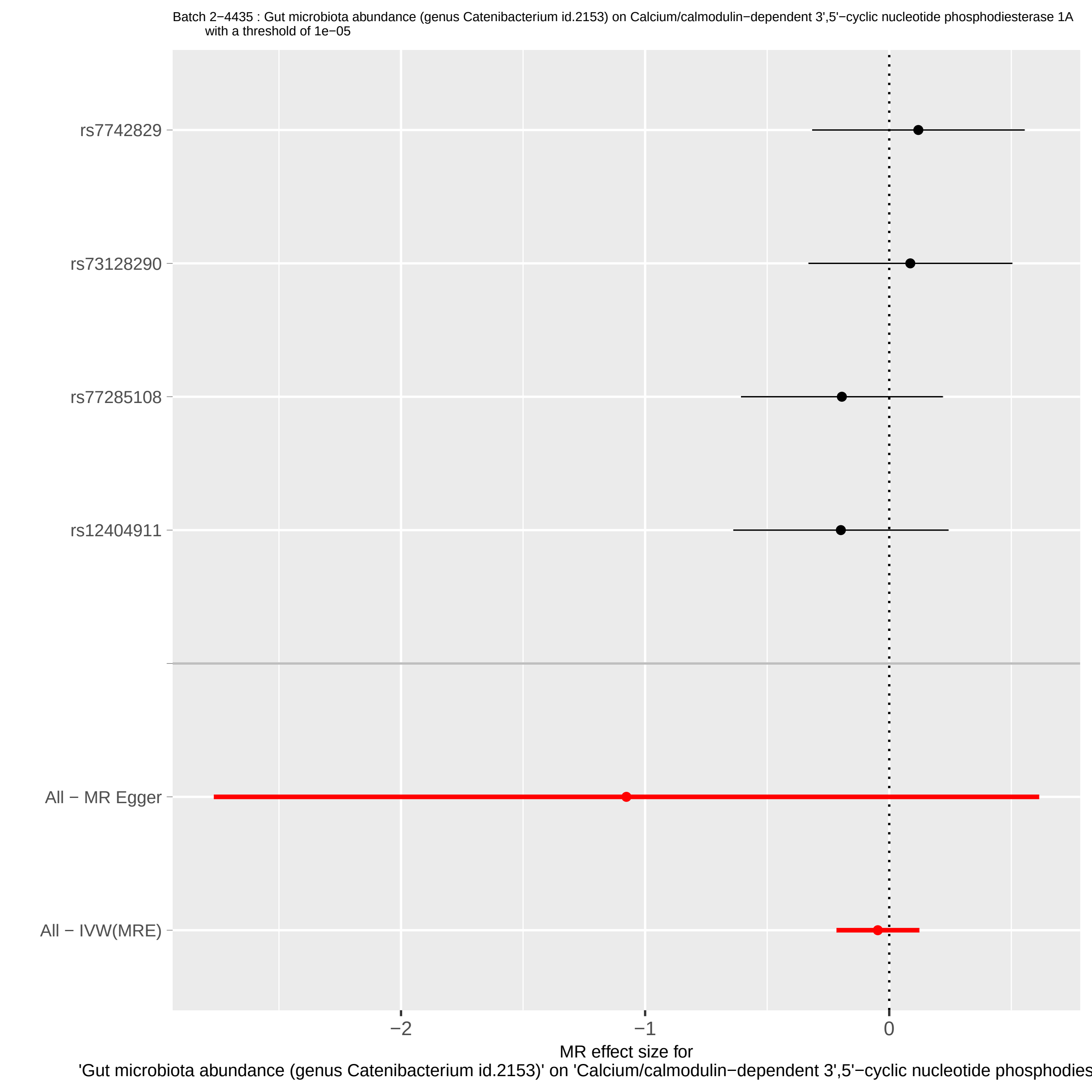
Batch 2−4435 : Gut microbiota abundance (genus Catenibacterium id.2153) on Calcium/calmodulin−dependent 3',5'−cyclic nucleotide phosphodiesterase 1A with a threshold of 1e−05
rs7742829
rs73128290
rs77285108
rs12404911
All − MR Egger
All − IVW(MRE)
−2
−1
0
MR effect size for
'Gut microbiota abundance (genus Catenibacterium id.2153)' on 'Calcium/calmodulin−dependent 3',5'−cyclic nucleotide phosphodies

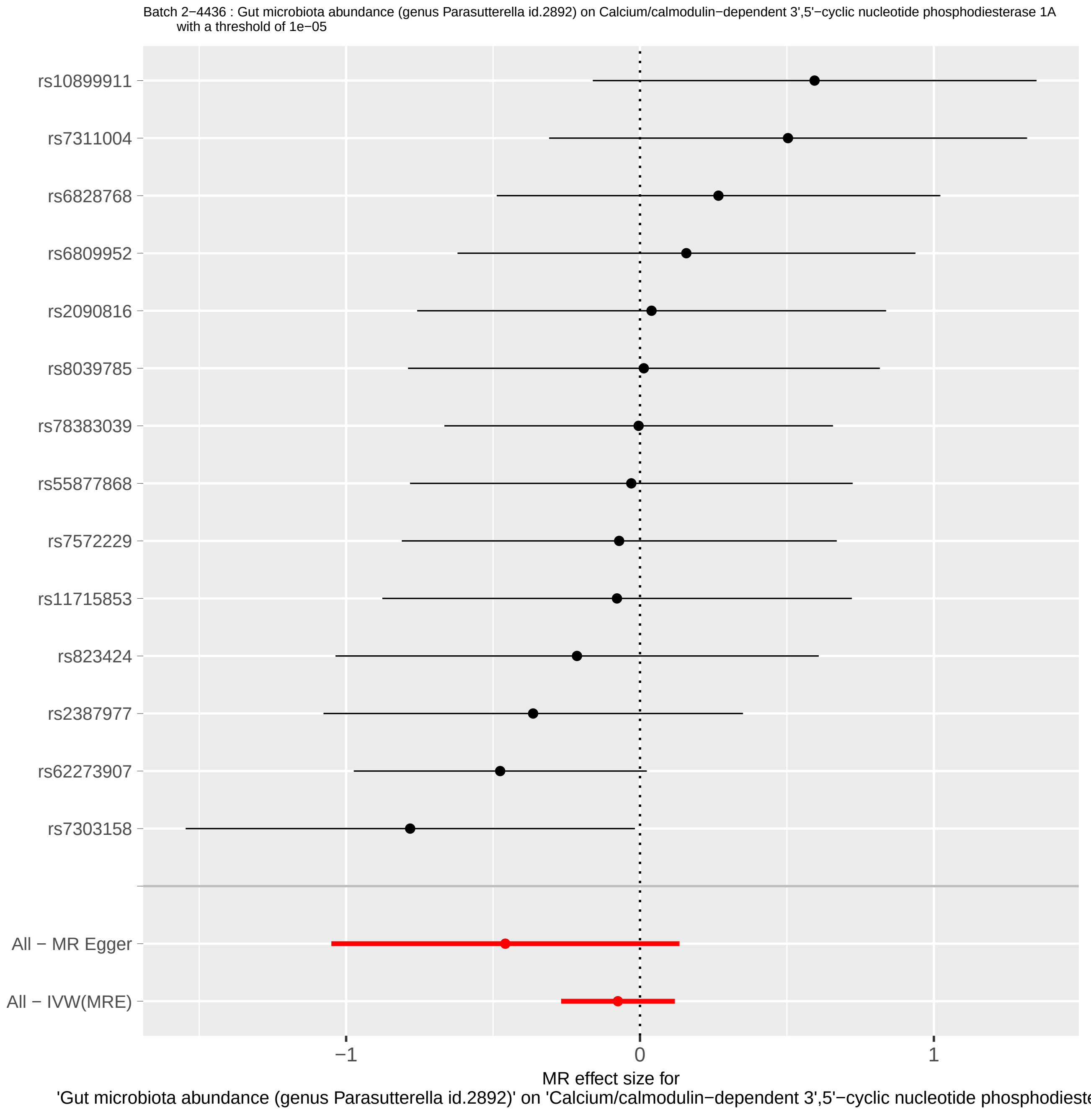
Batch 2−4436 : Gut microbiota abundance (genus Parasutterella id.2892) on Calcium/calmodulin−dependent 3',5'−cyclic nucleotide phosphodiesterase 1A with a threshold of 1e−05
rs10899911
rs7311004
rs6828768
rs6809952
rs2090816
rs8039785
rs78383039
rs55877868
rs7572229
rs11715853
rs823424
rs2387977
rs62273907
rs7303158
All − MR Egger
All − IVW(MRE)
−1
0
1
MR effect size for
'Gut microbiota abundance (genus Parasutterella id.2892)' on 'Calcium/calmodulin−dependent 3',5'−cyclic nucleotide phosphodieste

Batch 2−4437 : Gut microbiota abundance (genus Catenibacterium id.2153) on cGMP−dependent 3',5'−cyclic phosphodiesterase
with a threshold of 1e−05

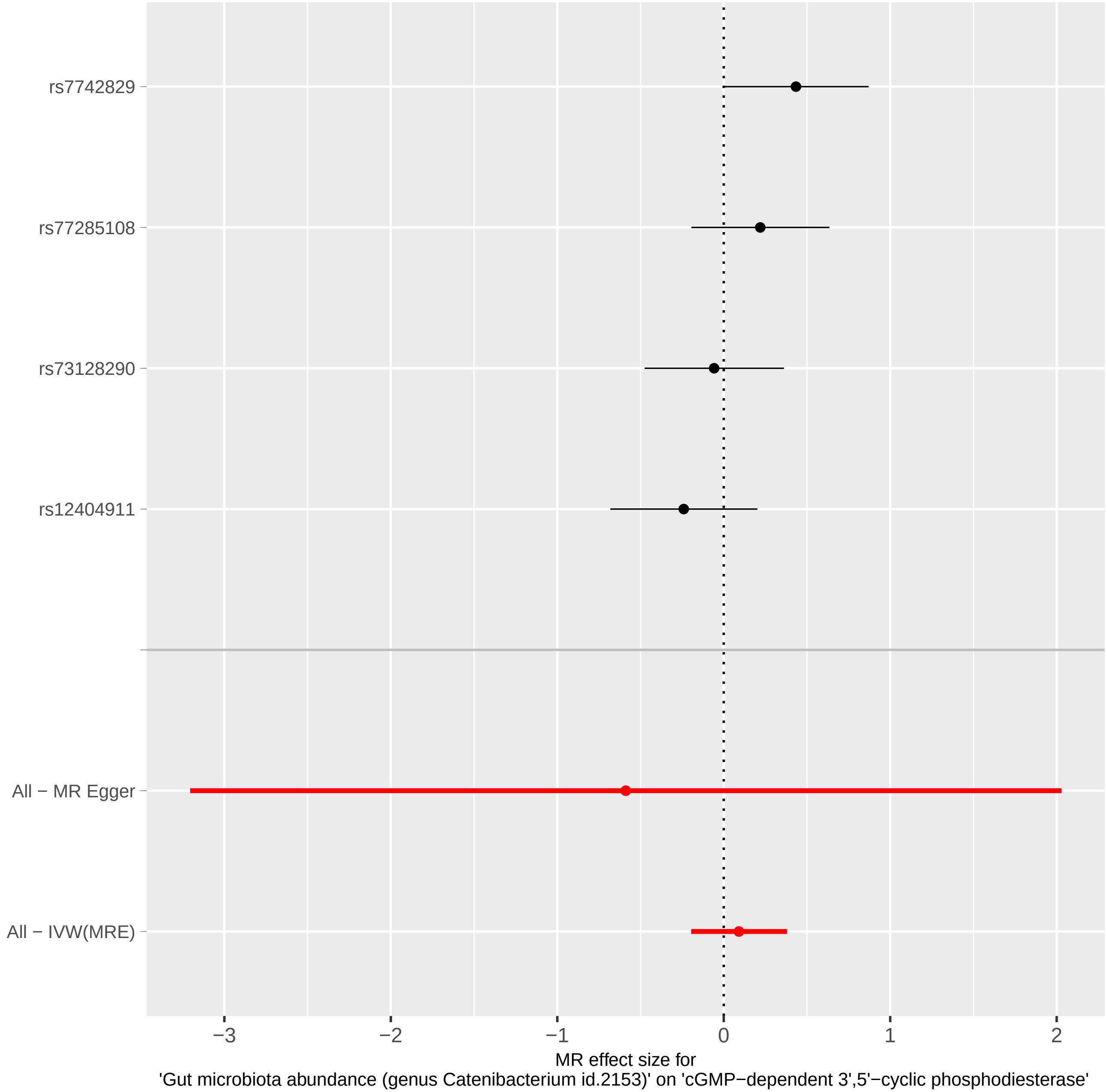

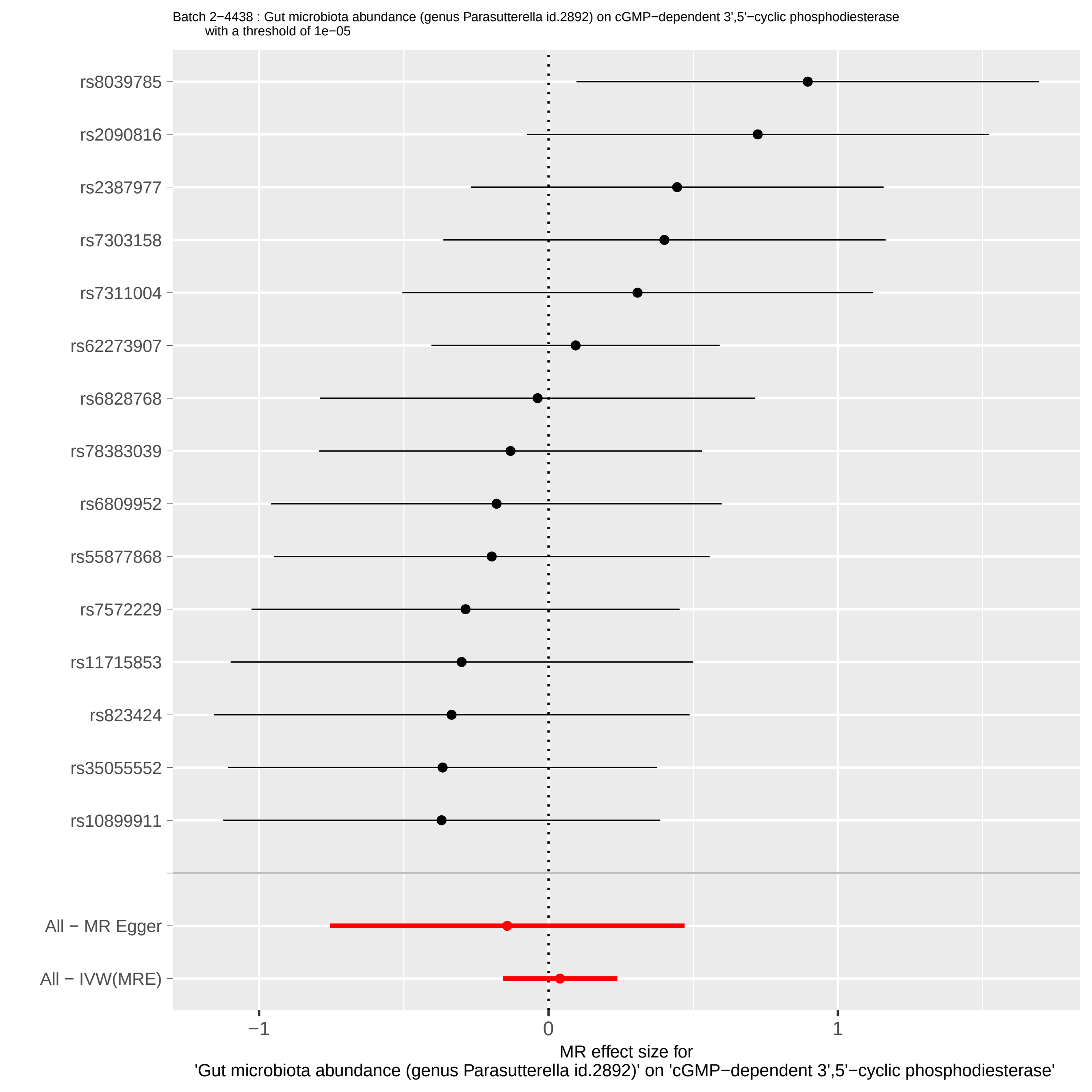
Batch 2−4438 : Gut microbiota abundance (genus Parasutterella id.2892) on cGMP−dependent 3',5'−cyclic phosphodiesterase
with a threshold of 1e−05
rs8039785
rs2090816
rs2387977
rs7303158
rs7311004
rs62273907
rs6828768
rs78383039
rs6809952
rs55877868
rs7572229
rs11715853
rs823424
rs35055552
rs10899911
All − MR Egger
All − IVW(MRE)
−1
0
1
MR effect size for
'Gut microbiota abundance (genus Parasutterella id.2892)' on 'cGMP−dependent 3',5'−cyclic phosphodiesterase'

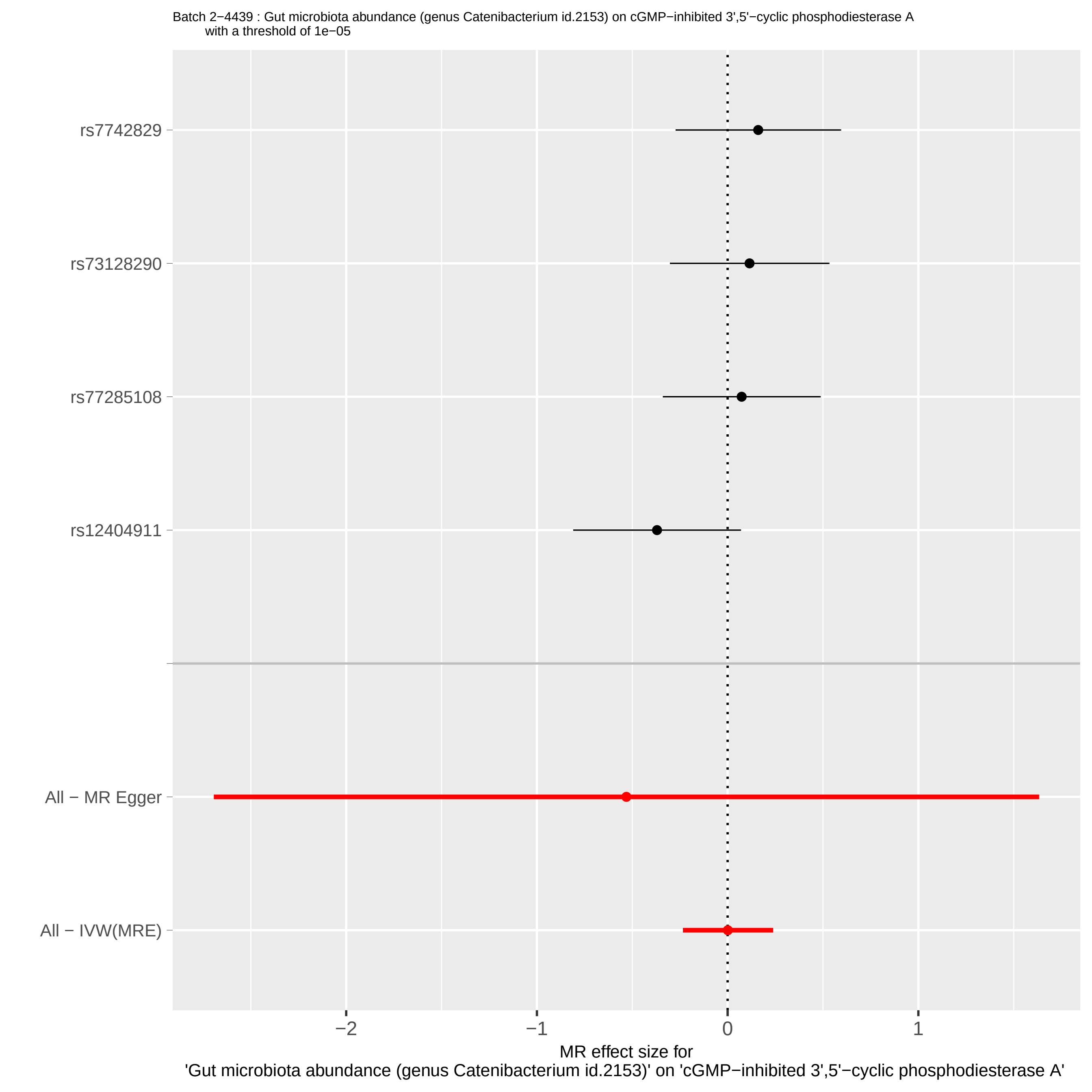
Batch 2−4439 : Gut microbiota abundance (genus Catenibacterium id.2153) on cGMP−inhibited 3',5'−cyclic phosphodiesterase A
with a threshold of 1e−05
rs7742829
rs73128290
rs77285108
rs12404911
All − MR Egger
All − IVW(MRE)
−2
−1
0
1
MR effect size for
'Gut microbiota abundance (genus Catenibacterium id.2153)' on 'cGMP−inhibited 3',5'−cyclic phosphodiesterase A'

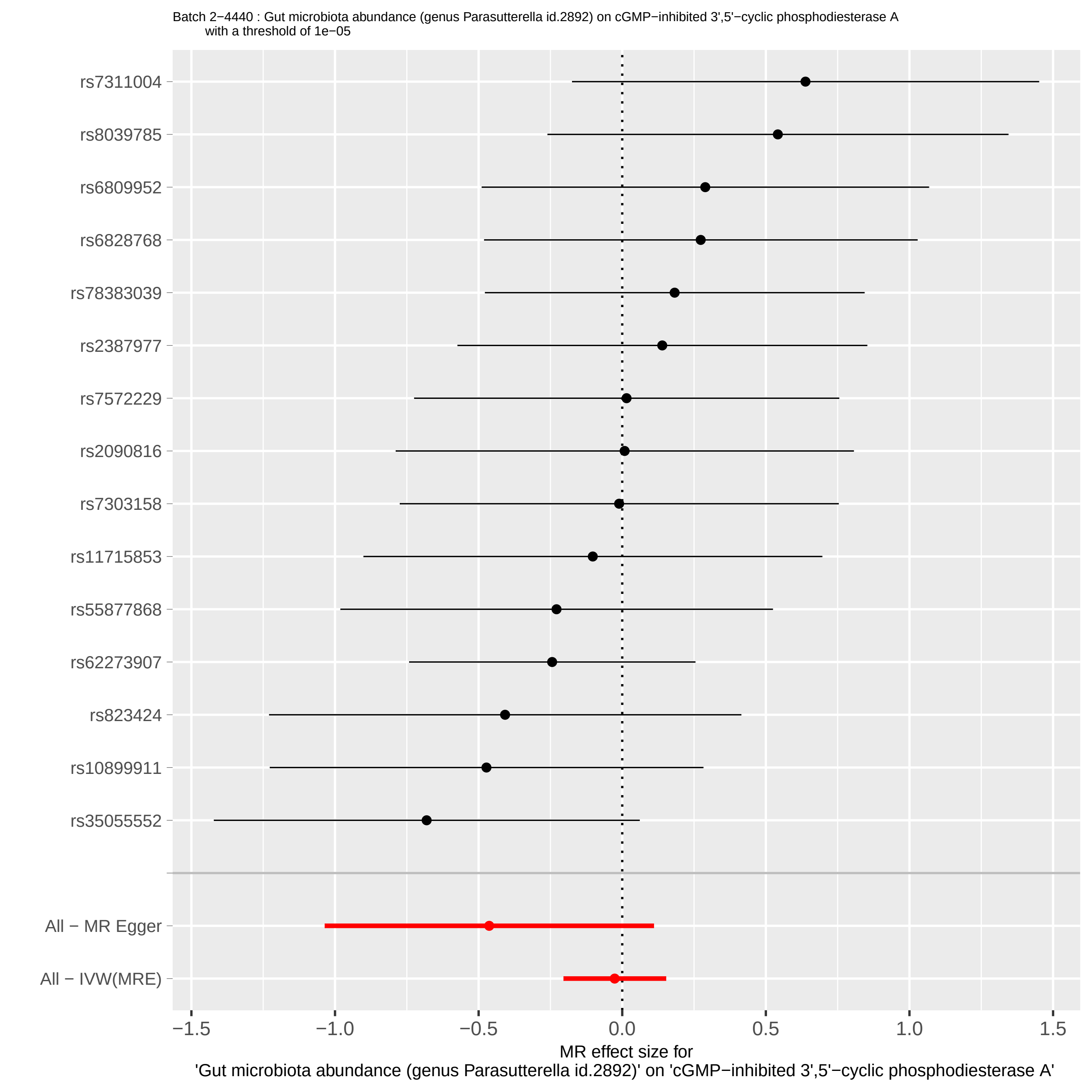

Batch 2−4440 : Gut microbiota abundance (genus Parasutterella id.2892) on cGMP−inhibited 3',5'−cyclic phosphodiesterase A
with a threshold of 1e−05
rs7311004
rs8039785
rs6809952
rs6828768
rs78383039
rs2387977
rs7572229
rs2090816
rs7303158
rs11715853
rs55877868
rs62273907
rs823424
rs10899911
rs35055552
All − MR Egger
All − IVW(MRE)
−1.5
−1.0
−0.5
0.0
0.5
1.0
1.5
MR effect size for
'Gut microbiota abundance (genus Parasutterella id.2892)' on 'cGMP−inhibited 3',5'−cyclic phosphodiesterase A'

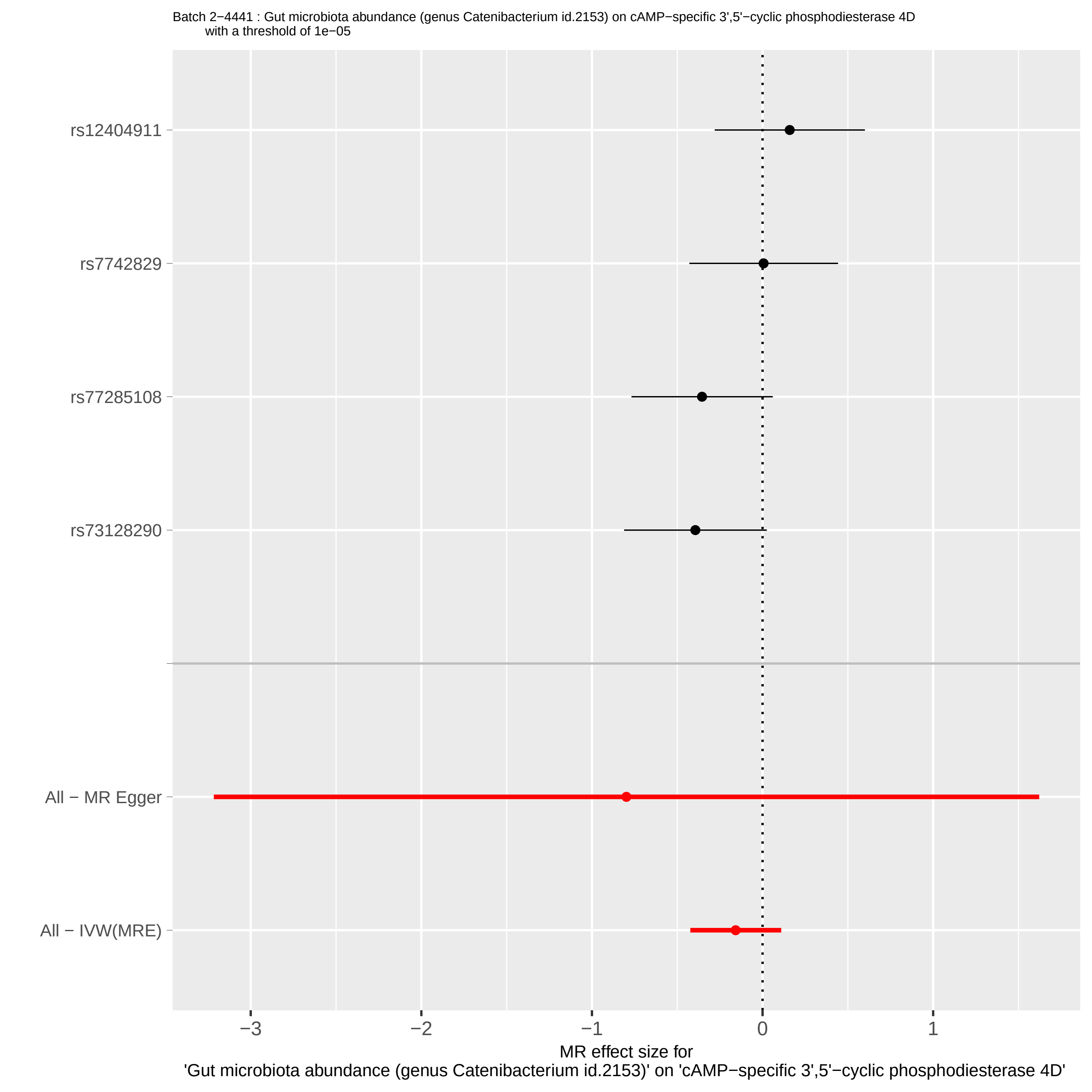

Batch 2−4441 : Gut microbiota abundance (genus Catenibacterium id.2153) on cAMP−specific 3',5'−cyclic phosphodiesterase 4D with a threshold of 1e−05
rs12404911
rs7742829
rs77285108
rs73128290
All − MR Egger
All − IVW(MRE)
−3
−2
−1
0
1
MR effect size for
'Gut microbiota abundance (genus Catenibacterium id.2153)' on 'cAMP−specific 3',5'−cyclic phosphodiesterase 4D'

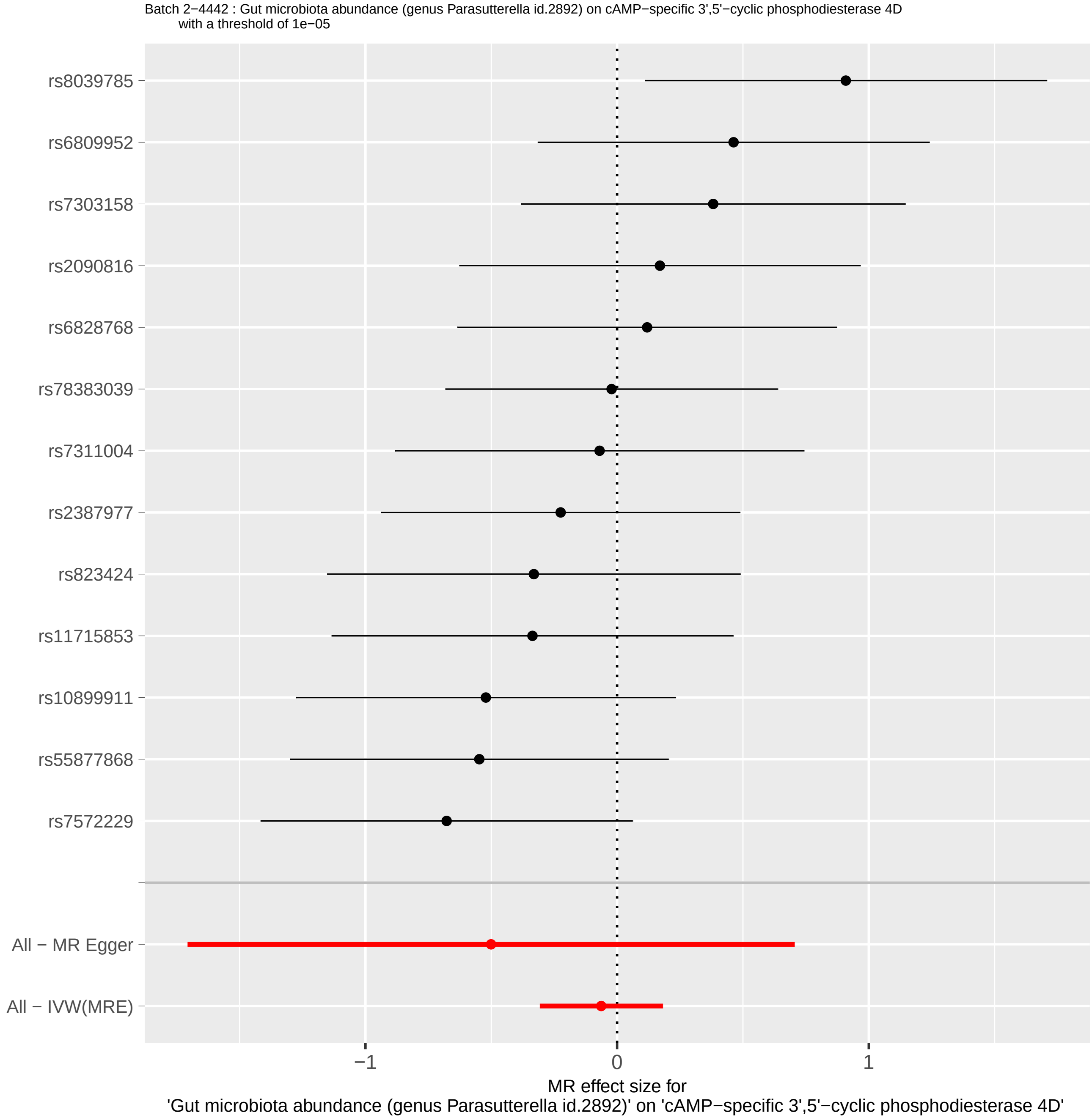
Batch 2−4442 : Gut microbiota abundance (genus Parasutterella id.2892) on cAMP−specific 3',5'−cyclic phosphodiesterase 4D
with a threshold of 1e−05
rs8039785
rs6809952
rs7303158
rs2090816
rs6828768
rs78383039
rs7311004
rs2387977
rs823424
rs11715853
rs10899911
rs55877868
rs7572229
All − MR Egger
All − IVW(MRE)
−1
0
1
MR effect size for
'Gut microbiota abundance (genus Parasutterella id.2892)' on 'cAMP−specific 3',5'−cyclic phosphodiesterase 4D'

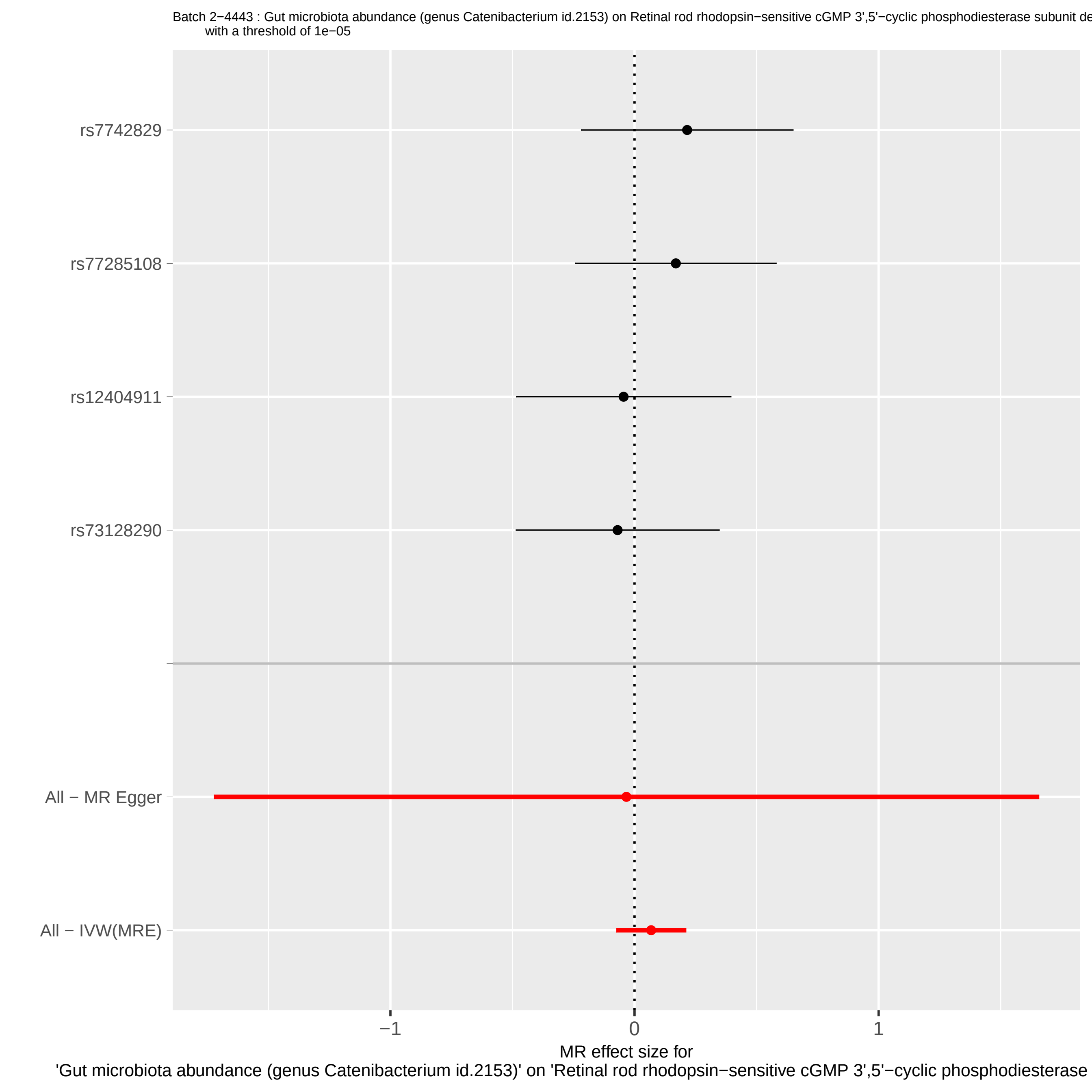
Batch 2−4443 : Gut microbiota abundance (genus Catenibacterium id.2153) on Retinal rod rhodopsin−sensitive cGMP 3',5'−cyclic phosphodiesterase subunit de
with a threshold of 1e−05
rs7742829
rs77285108
rs12404911
rs73128290
All − MR Egger
All − IVW(MRE)
−1
0
1
MR effect size for
'Gut microbiota abundance (genus Catenibacterium id.2153)' on 'Retinal rod rhodopsin−sensitive cGMP 3',5'−cyclic phosphodiesterase

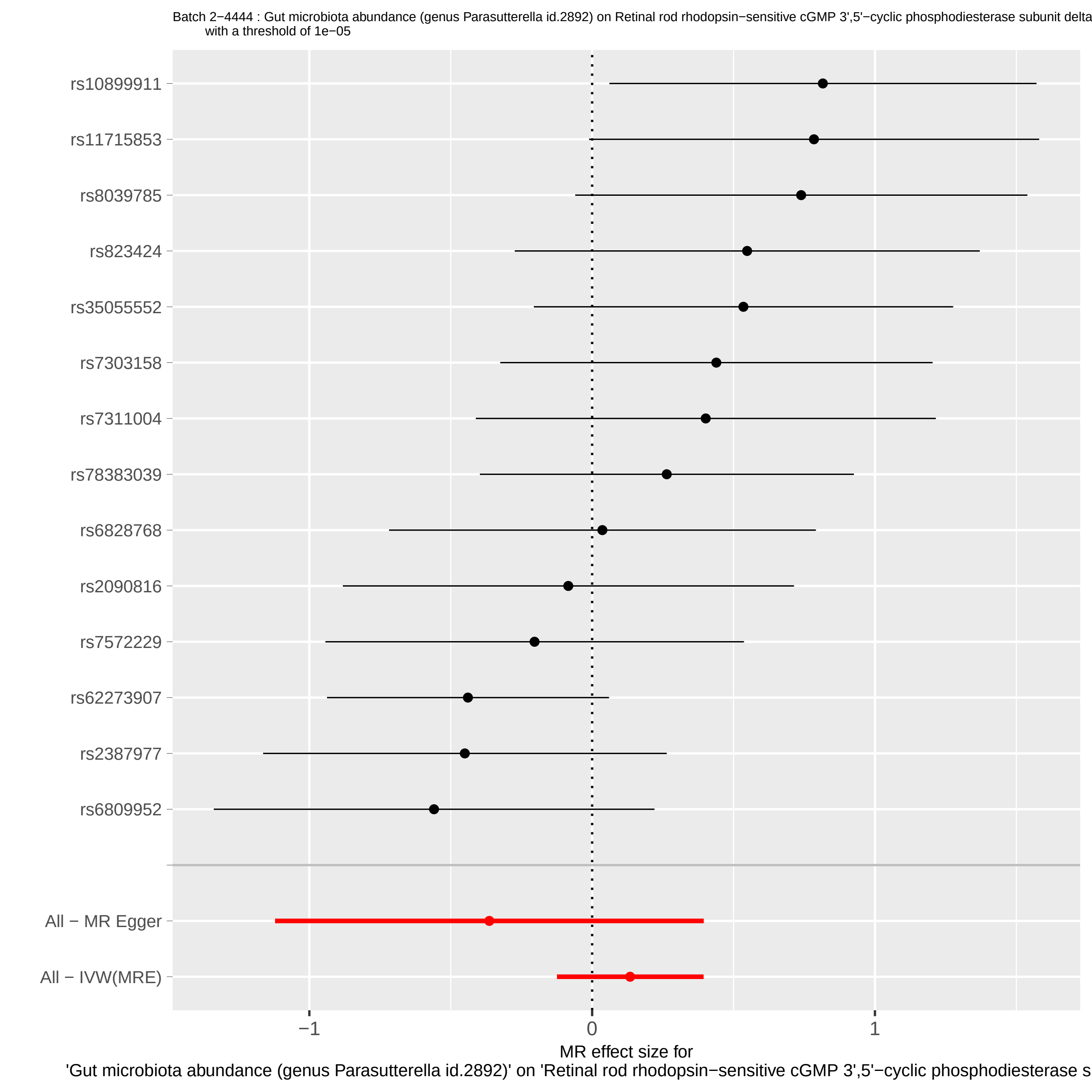

Batch 2−4444 : Gut microbiota abundance (genus Parasutterella id.2892) on Retinal rod rhodopsin−sensitive cGMP 3',5'−cyclic phosphodiesterase subunit delta
with a threshold of 1e−05
rs10899911
rs11715853
rs8039785
rs823424
rs35055552
rs7303158
rs7311004
rs78383039
rs6828768
rs2090816
rs7572229
rs62273907
rs2387977
rs6809952
All − MR Egger
All − IVW(MRE)
−1
0
1
MR effect size for
'Gut microbiota abundance (genus Parasutterella id.2892)' on 'Retinal rod rhodopsin−sensitive cGMP 3',5'−cyclic phosphodiesterase s

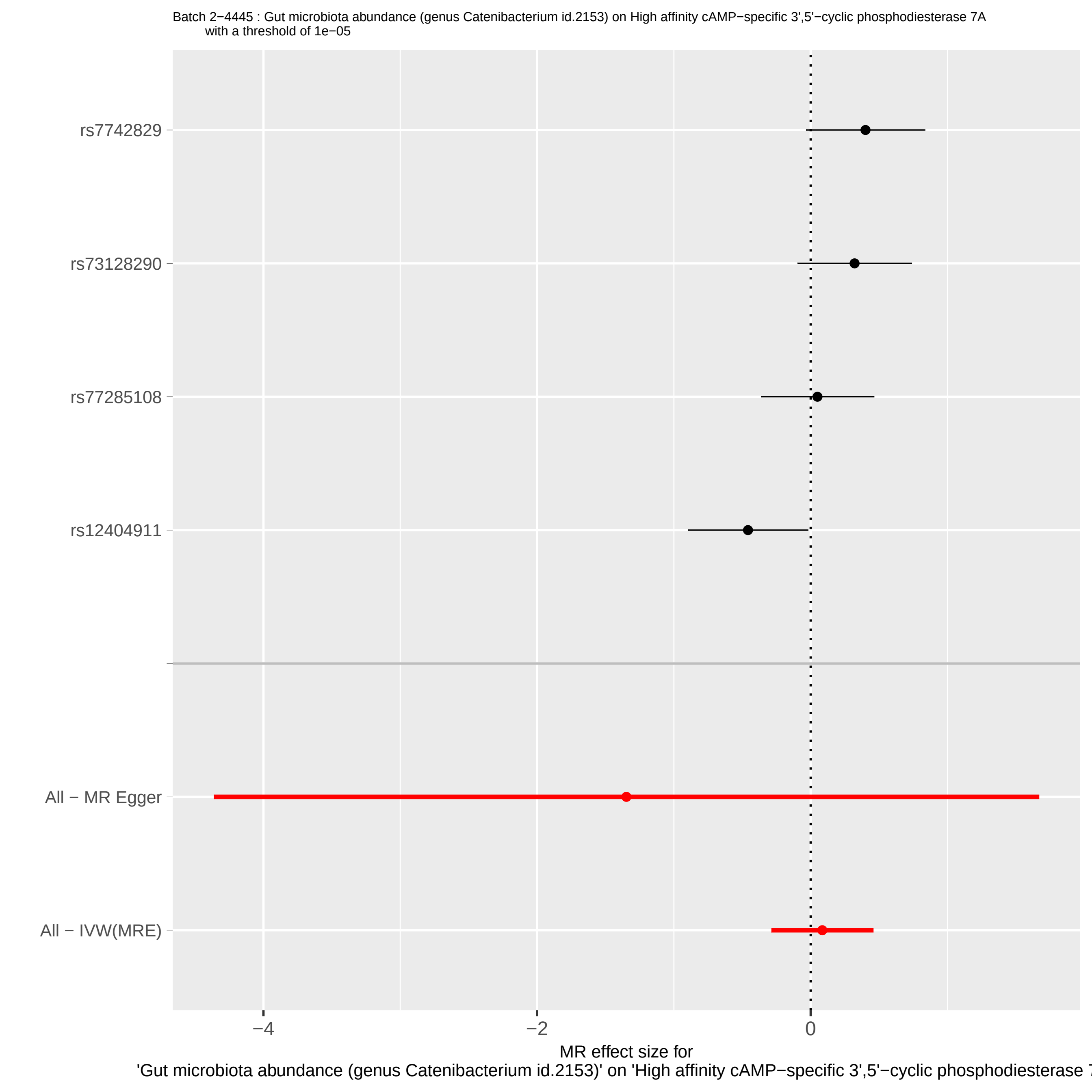
Batch 2−4445 : Gut microbiota abundance (genus Catenibacterium id.2153) on High affinity cAMP−specific 3',5'−cyclic phosphodiesterase 7A
with a threshold of 1e−05
rs7742829
rs73128290
rs77285108
rs12404911
All − MR Egger
All − IVW(MRE)
−4
−2
0
MR effect size for
'Gut microbiota abundance (genus Catenibacterium id.2153)' on 'High affinity cAMP−specific 3',5'−cyclic phosphodiesterase 7

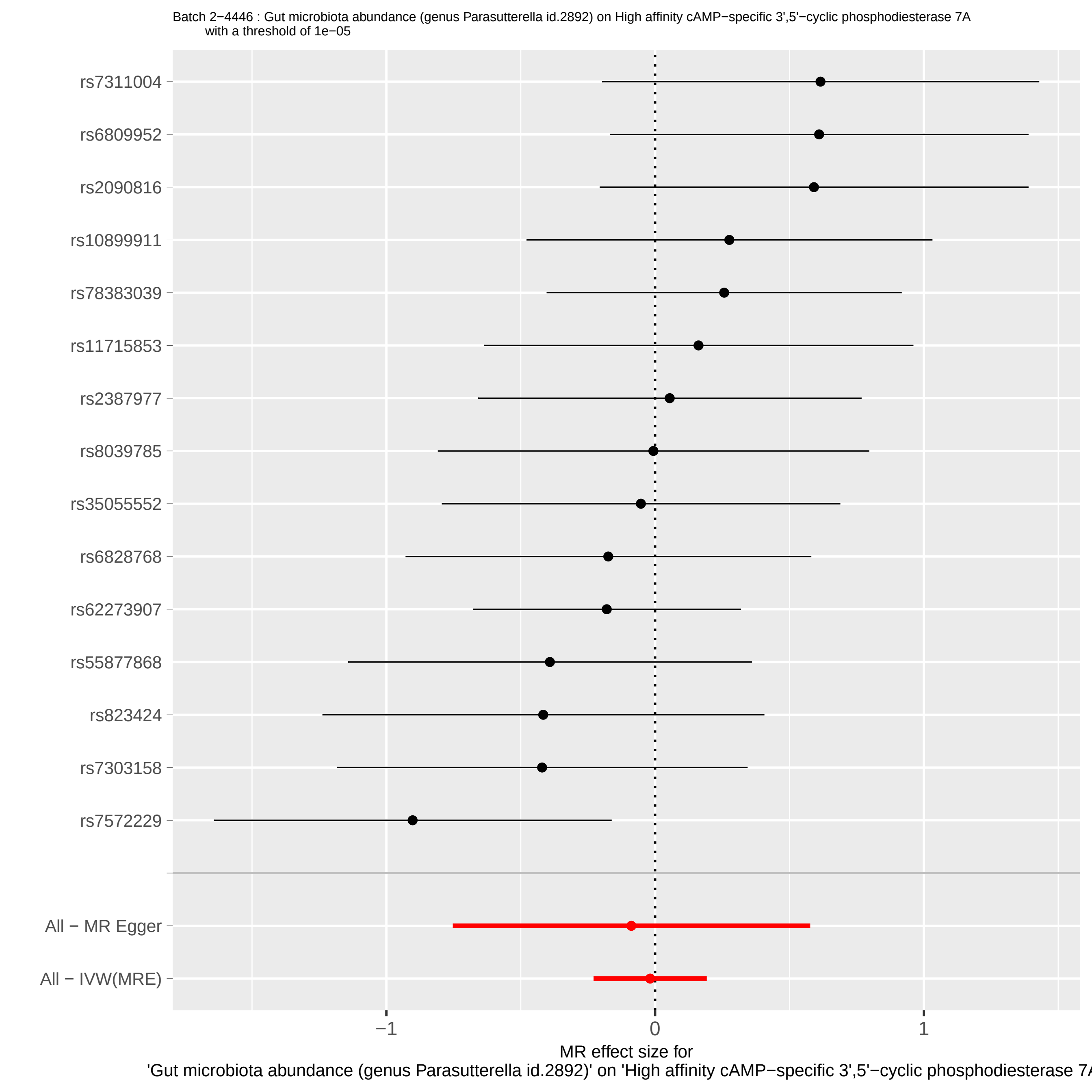

Batch 2−4446 : Gut microbiota abundance (genus Parasutterella id.2892) on High affinity cAMP−specific 3',5'−cyclic phosphodiesterase 7A
with a threshold of 1e−05
rs7311004
rs6809952
rs2090816
rs10899911
rs78383039
rs11715853
rs2387977
rs8039785
rs35055552
rs6828768
rs62273907
rs55877868
rs823424
rs7303158
rs7572229
All − MR Egger
All − IVW(MRE)
−1
0
1
MR effect size for
'Gut microbiota abundance (genus Parasutterella id.2892)' on 'High affinity cAMP−specific 3',5'−cyclic phosphodiesterase 7A

Batch 2−4447 : Gut microbiota abundance (genus Catenibacterium id.2153) on High affinity cGMP−specific 3',5'−cyclic phosphodiesterase 9A
with a threshold of 1e−05

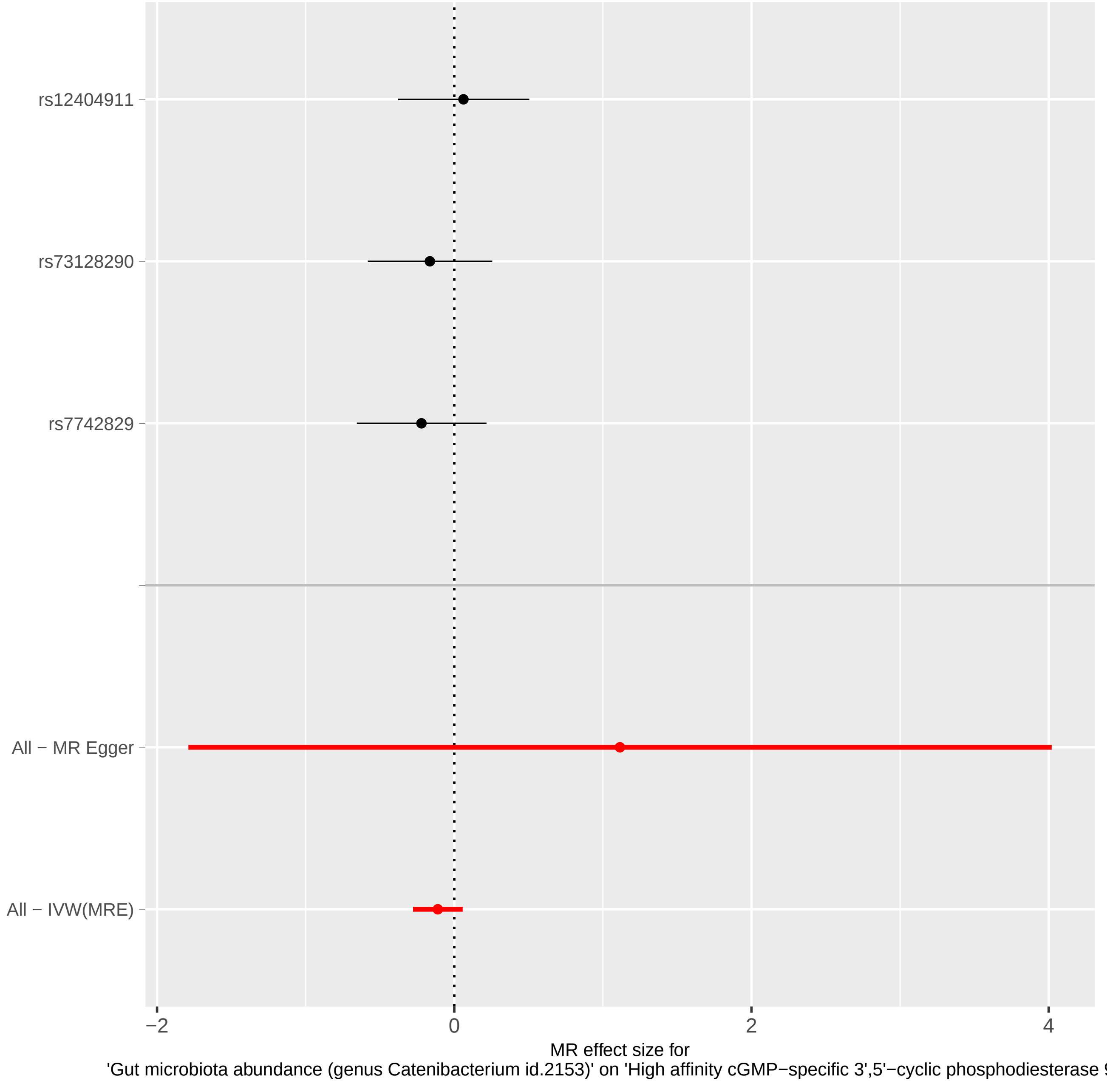

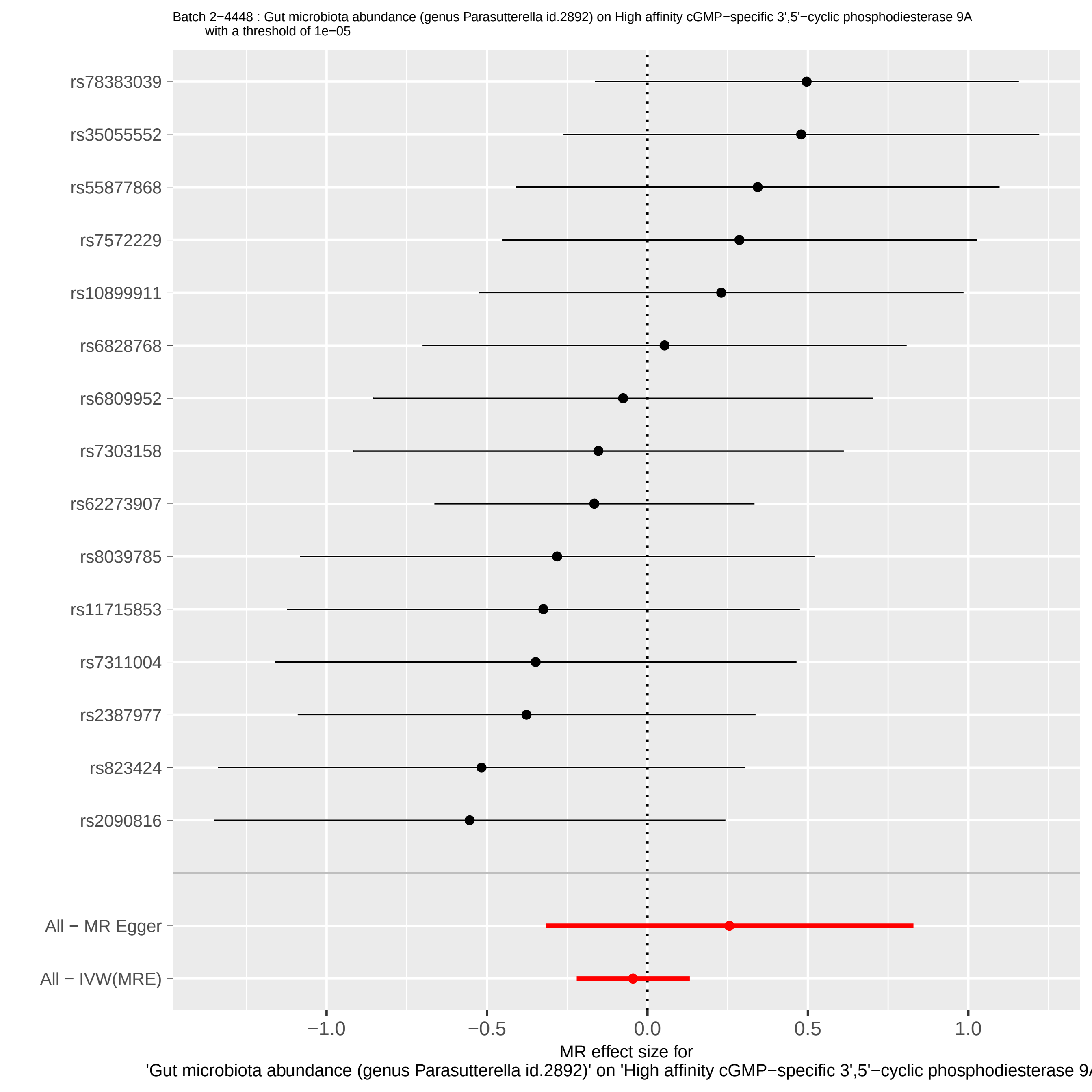

Batch 2−4448 : Gut microbiota abundance (genus Parasutterella id.2892) on High affinity cGMP−specific 3',5'−cyclic phosphodiesterase 9A
with a threshold of 1e−05
rs78383039
rs35055552
rs55877868
rs7572229
rs10899911
rs6828768
rs6809952
rs7303158
rs62273907
rs8039785
rs11715853
rs7311004
rs2387977
rs823424
rs2090816
All − MR Egger
All − IVW(MRE)
−1.0
−0.5
0.0
0.5
1.0
MR effect size for
'Gut microbiota abundance (genus Parasutterella id.2892)' on 'High affinity cGMP−specific 3',5'−cyclic phosphodiesterase 9A

Batch 2−4449 : Gut microbiota abundance (genus Catenibacterium id.2153) on Platelet−derived growth factor subunit A
with a threshold of 1e−05

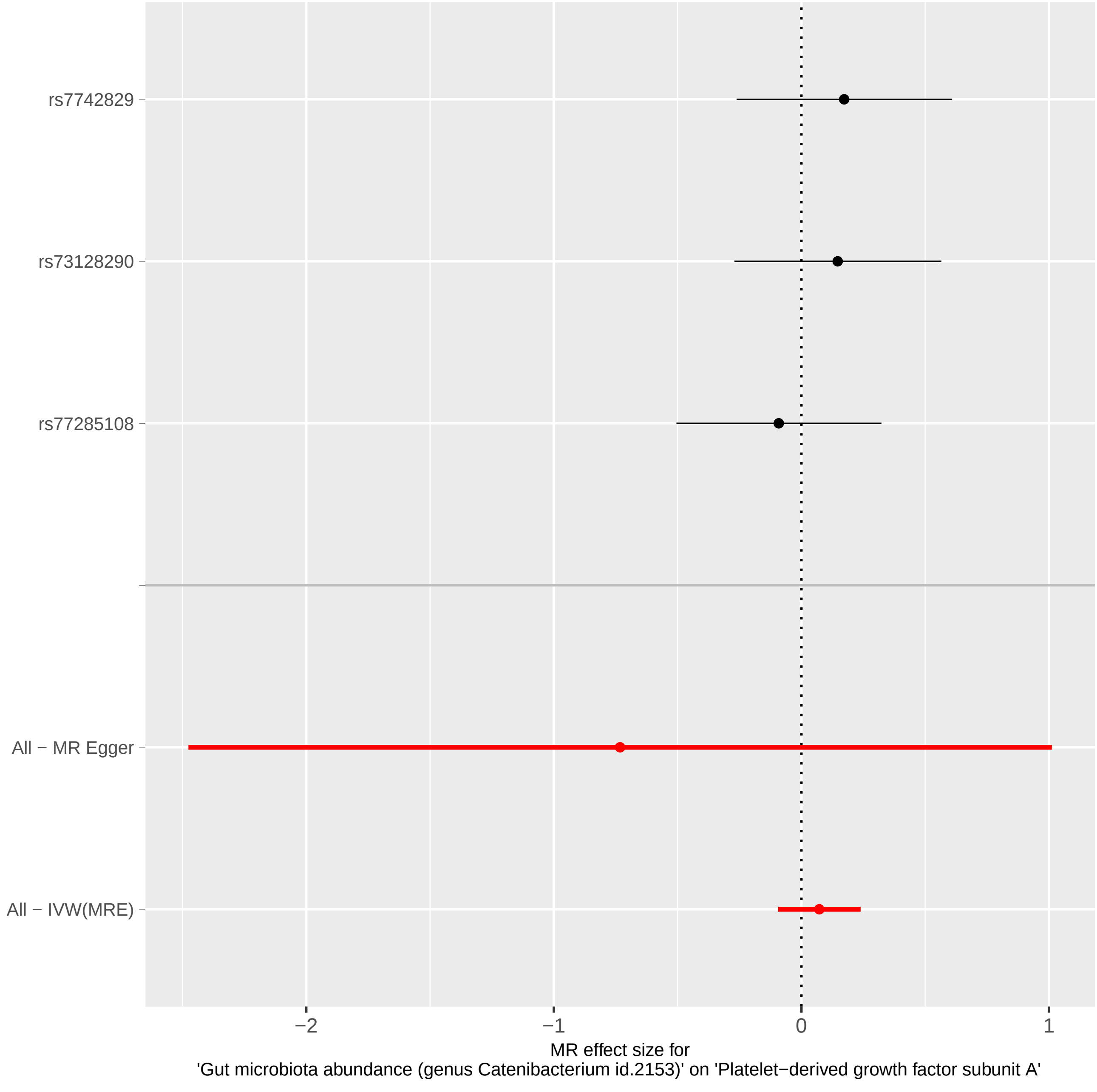

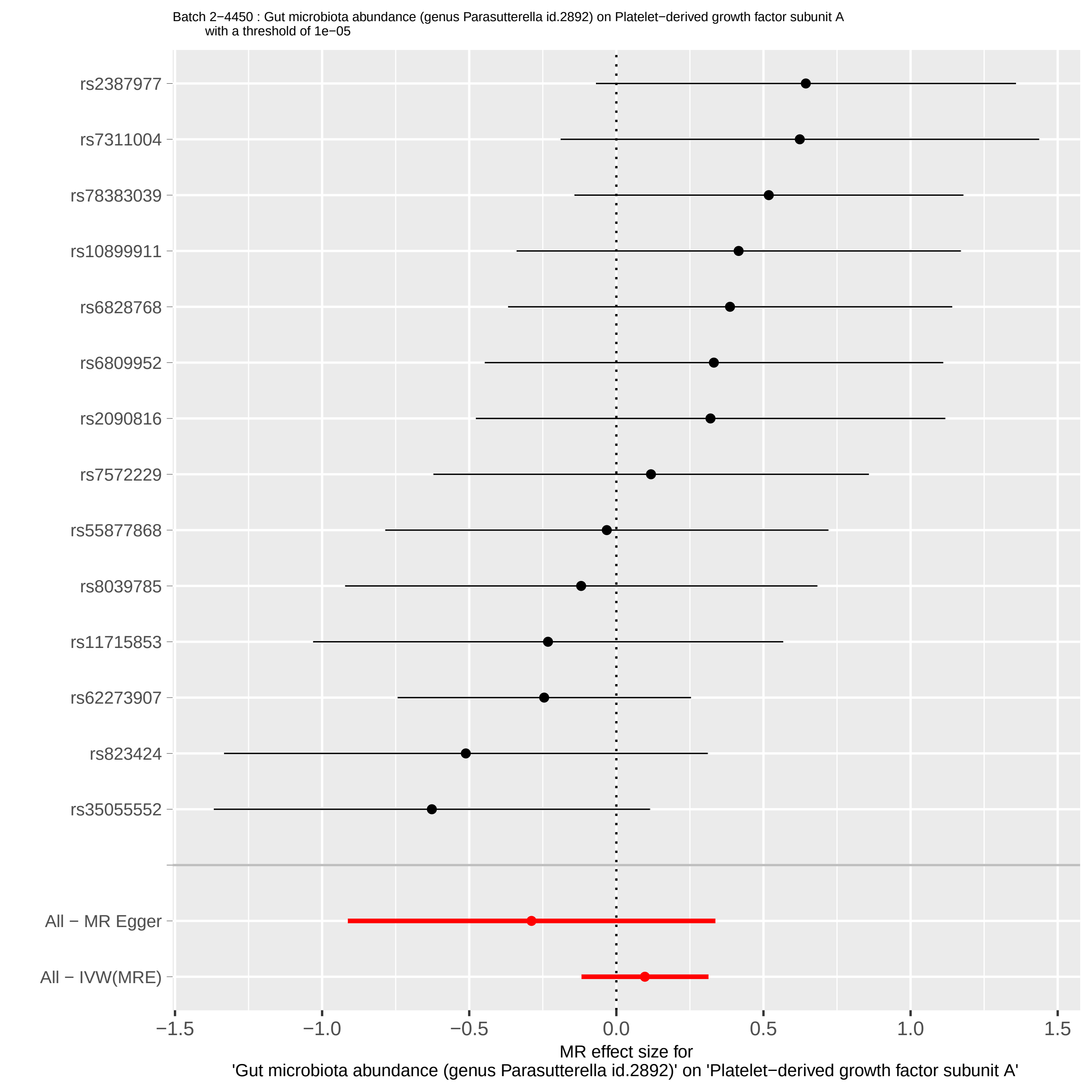
Batch 2−4450 : Gut microbiota abundance (genus Parasutterella id.2892) on Platelet−derived growth factor subunit A
with a threshold of 1e−05
rs2387977
rs7311004
rs78383039
rs10899911
rs6828768
rs6809952
rs2090816
rs7572229
rs55877868
rs8039785
rs11715853
rs62273907
rs823424
rs35055552
All − MR Egger
All − IVW(MRE)
−1.5
−1.0
−0.5
0.0
0.5
1.0
1.5
MR effect size for
'Gut microbiota abundance (genus Parasutterella id.2892)' on 'Platelet−derived growth factor subunit A'

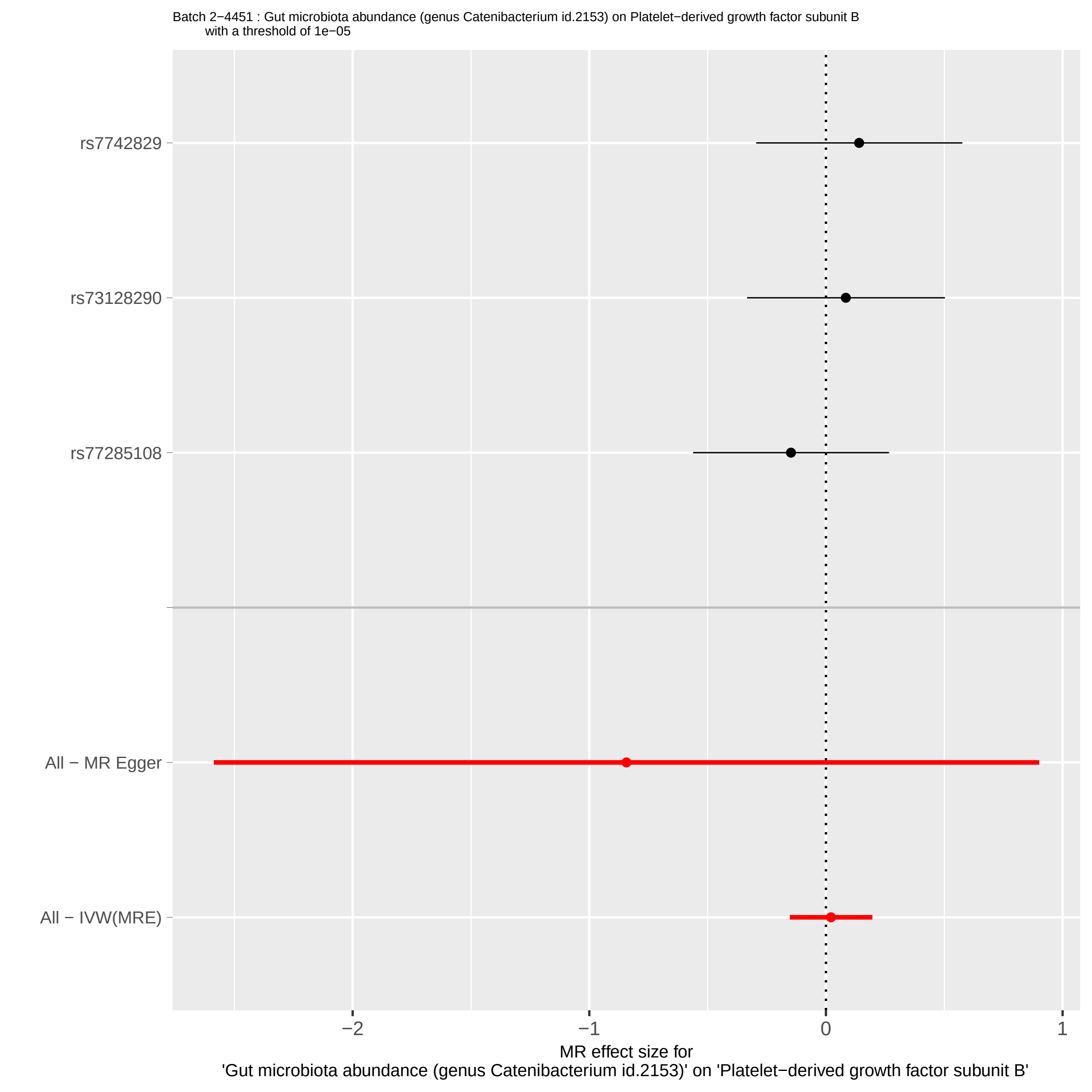

Batch 2−4451 : Gut microbiota abundance (genus Catenibacterium id.2153) on Platelet−derived growth factor subunit B
with a threshold of 1e−05
rs7742829
rs73128290
rs77285108
All − MR Egger
All − IVW(MRE)
−2
−1
0
1
MR effect size for
'Gut microbiota abundance (genus Catenibacterium id.2153)' on 'Platelet−derived growth factor subunit B'

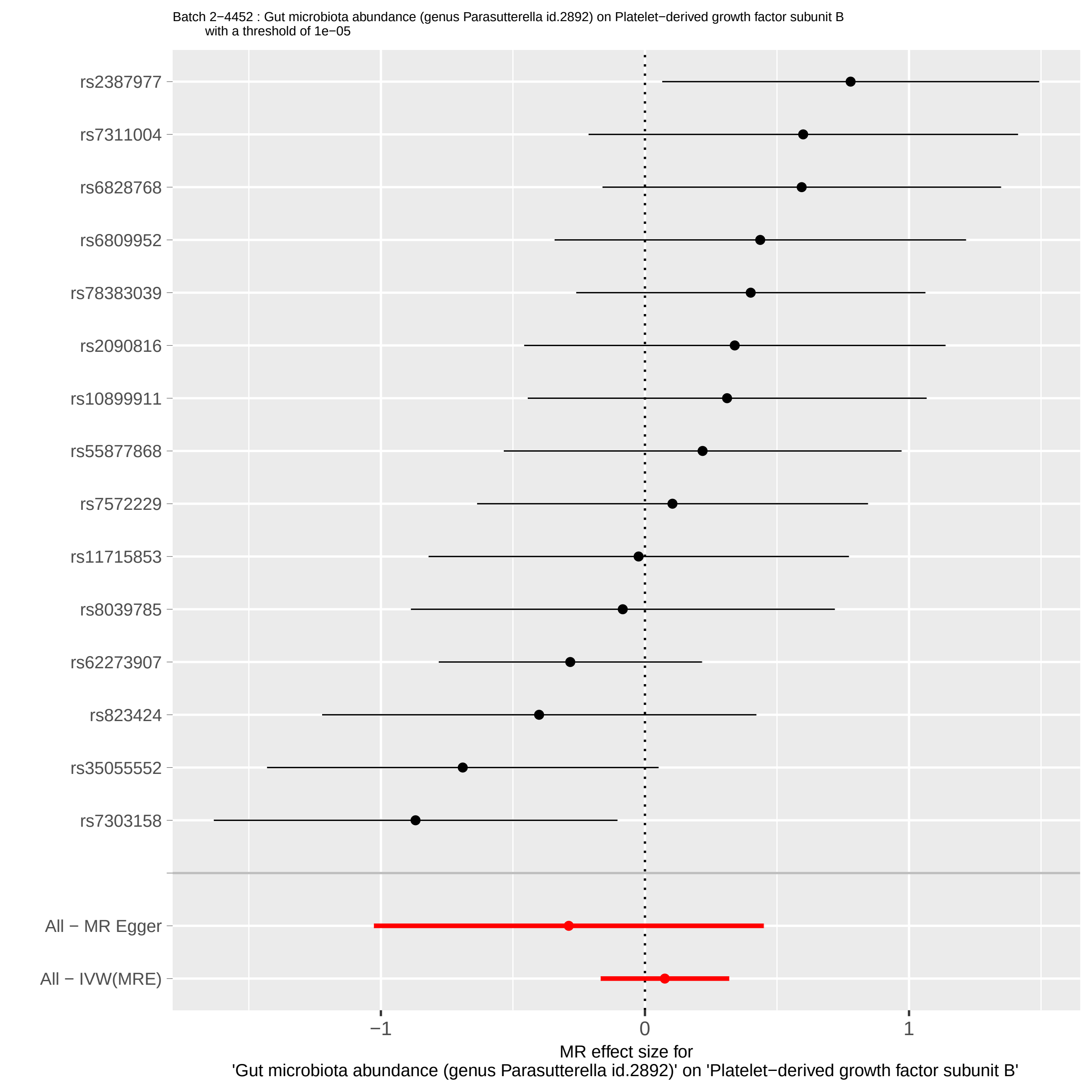
Batch 2−4452 : Gut microbiota abundance (genus Parasutterella id.2892) on Platelet−derived growth factor subunit B
with a threshold of 1e−05
rs2387977
rs7311004
rs6828768
rs6809952
rs78383039
rs2090816
rs10899911
rs55877868
rs7572229
rs11715853
rs8039785
rs62273907
rs823424
rs35055552
rs7303158
All − MR Egger
All − IVW(MRE)
−1
0
1
MR effect size for
'Gut microbiota abundance (genus Parasutterella id.2892)' on 'Platelet−derived growth factor subunit B'

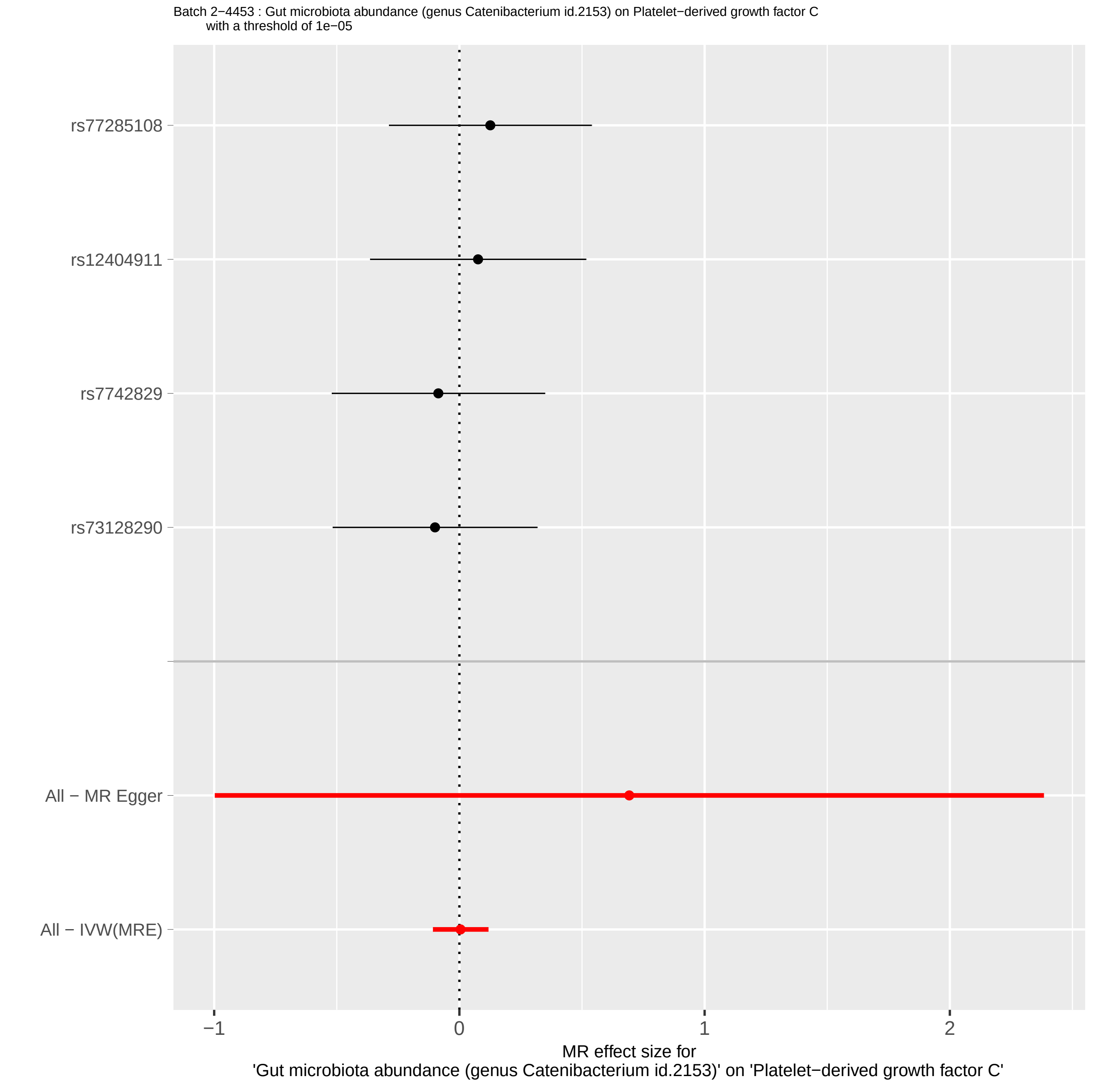
Batch 2−4453 : Gut microbiota abundance (genus Catenibacterium id.2153) on Platelet−derived growth factor C
with a threshold of 1e−05
rs77285108
rs12404911
rs7742829
rs73128290
All − MR Egger
All − IVW(MRE)
−1
0
1
2
MR effect size for
'Gut microbiota abundance (genus Catenibacterium id.2153)' on 'Platelet−derived growth factor C'

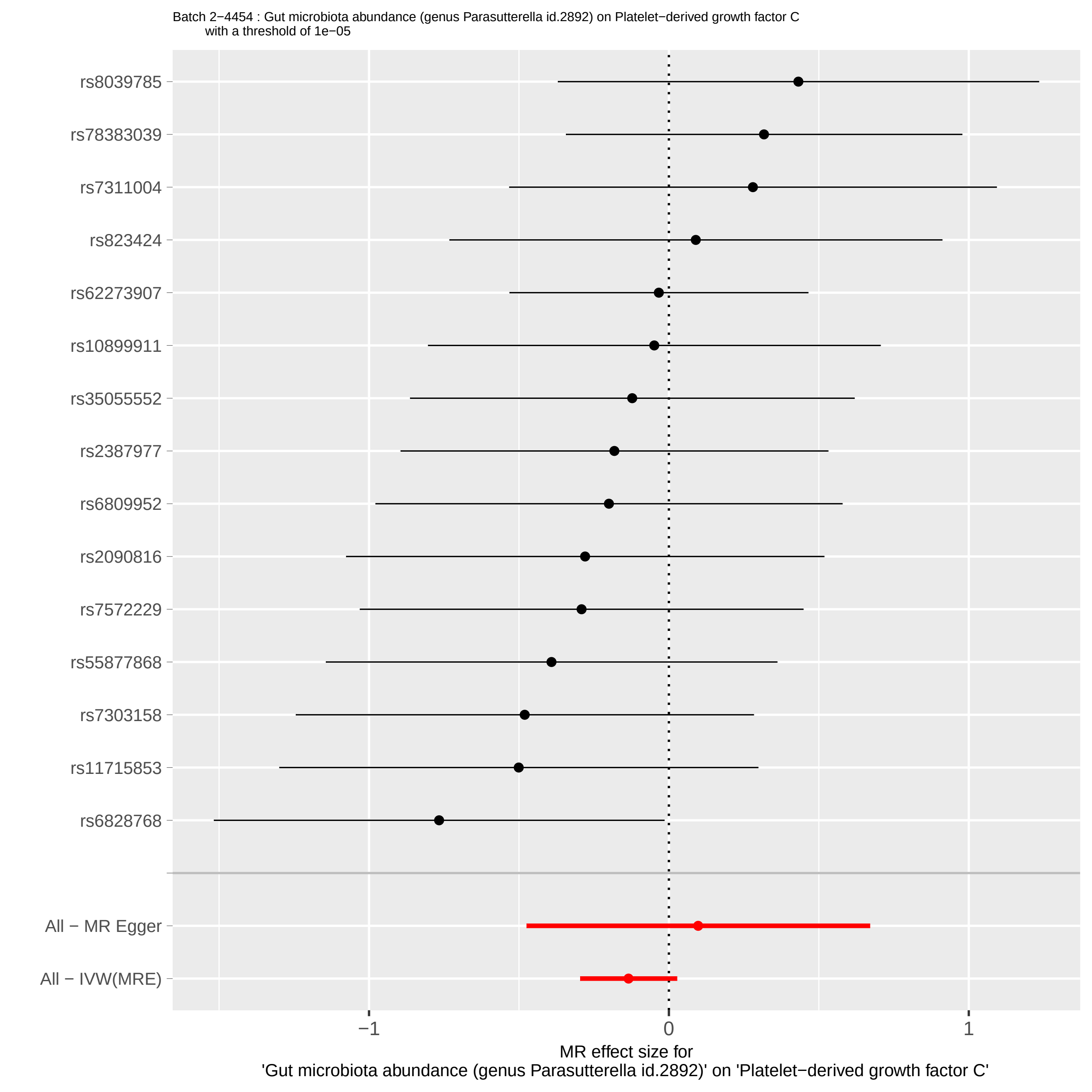

Batch 2−4454 : Gut microbiota abundance (genus Parasutterella id.2892) on Platelet−derived growth factor C
with a threshold of 1e−05
rs8039785
rs78383039
rs7311004
rs823424
rs62273907
rs10899911
rs35055552
rs2387977
rs6809952
rs2090816
rs7572229
rs55877868
rs7303158
rs11715853
rs6828768
All − MR Egger
All − IVW(MRE)
−1
0
1
MR effect size for
'Gut microbiota abundance (genus Parasutterella id.2892)' on 'Platelet−derived growth factor C'

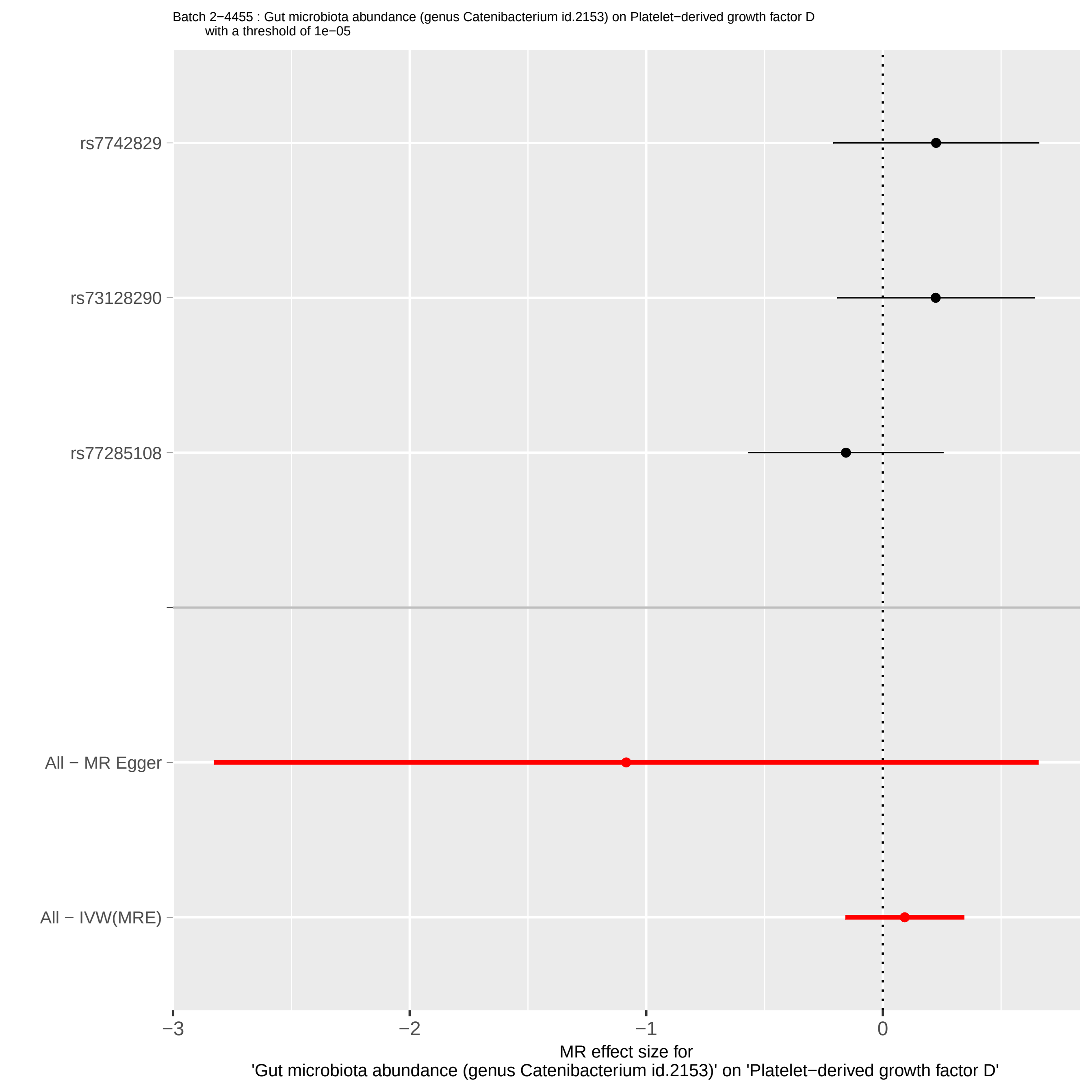

Batch 2−4455 : Gut microbiota abundance (genus Catenibacterium id.2153) on Platelet−derived growth factor D
with a threshold of 1e−05
rs7742829
rs73128290
rs77285108
All − MR Egger
All − IVW(MRE)
−3
−2
−1
0
MR effect size for
'Gut microbiota abundance (genus Catenibacterium id.2153)' on 'Platelet−derived growth factor D'

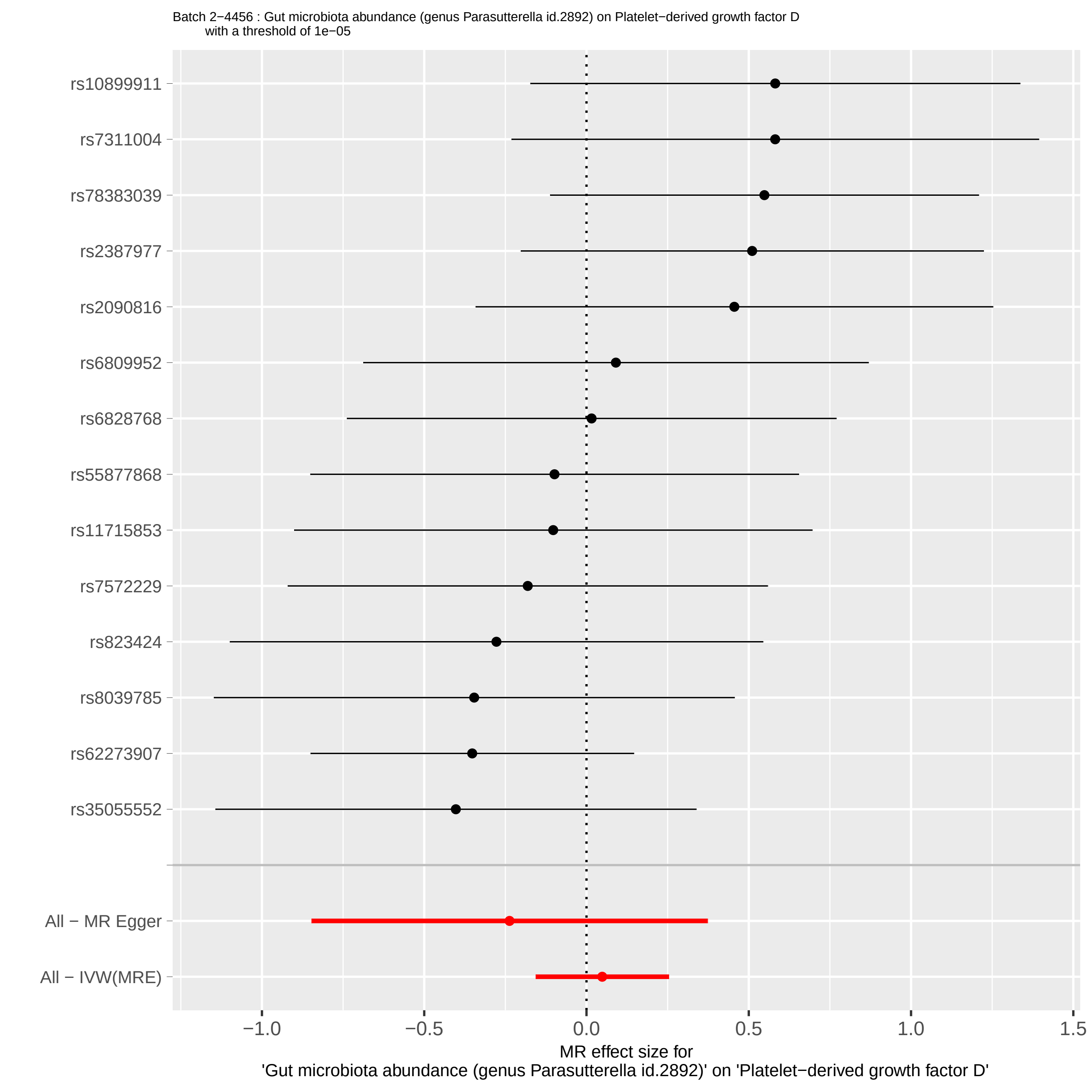

Batch 2−4456 : Gut microbiota abundance (genus Parasutterella id.2892) on Platelet−derived growth factor D
with a threshold of 1e−05
rs10899911
rs7311004
rs78383039
rs2387977
rs2090816
rs6809952
rs6828768
rs55877868
rs11715853
rs7572229
rs823424
rs8039785
rs62273907
rs35055552
All − MR Egger
All − IVW(MRE)
−1.0
−0.5
0.0
0.5
1.0
1.5
MR effect size for
'Gut microbiota abundance (genus Parasutterella id.2892)' on 'Platelet−derived growth factor D'

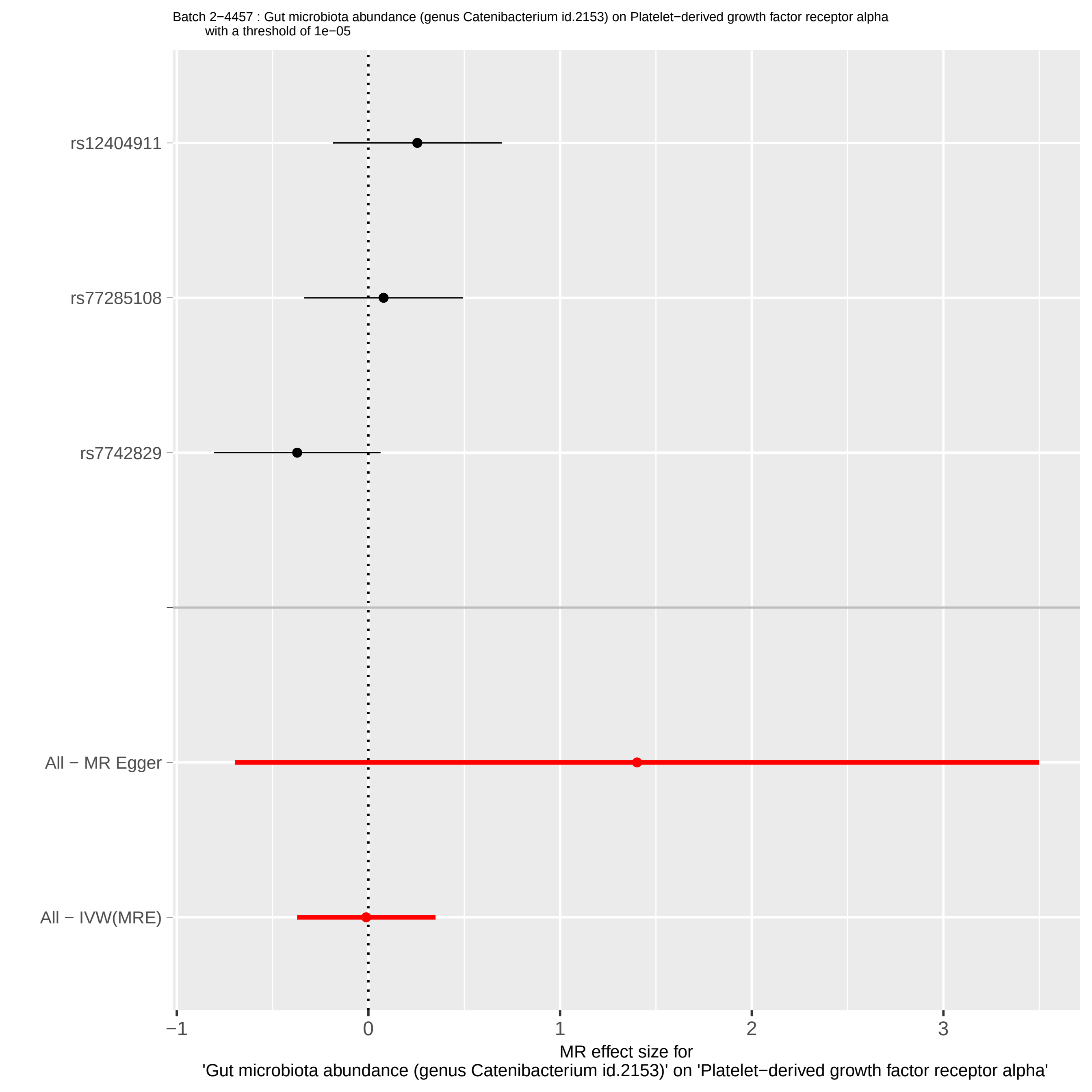
Batch 2−4457 : Gut microbiota abundance (genus Catenibacterium id.2153) on Platelet−derived growth factor receptor alpha
with a threshold of 1e−05
rs12404911
rs77285108
rs7742829
All − MR Egger
All − IVW(MRE)
−1
0
1
2
3
MR effect size for
'Gut microbiota abundance (genus Catenibacterium id.2153)' on 'Platelet−derived growth factor receptor alpha'

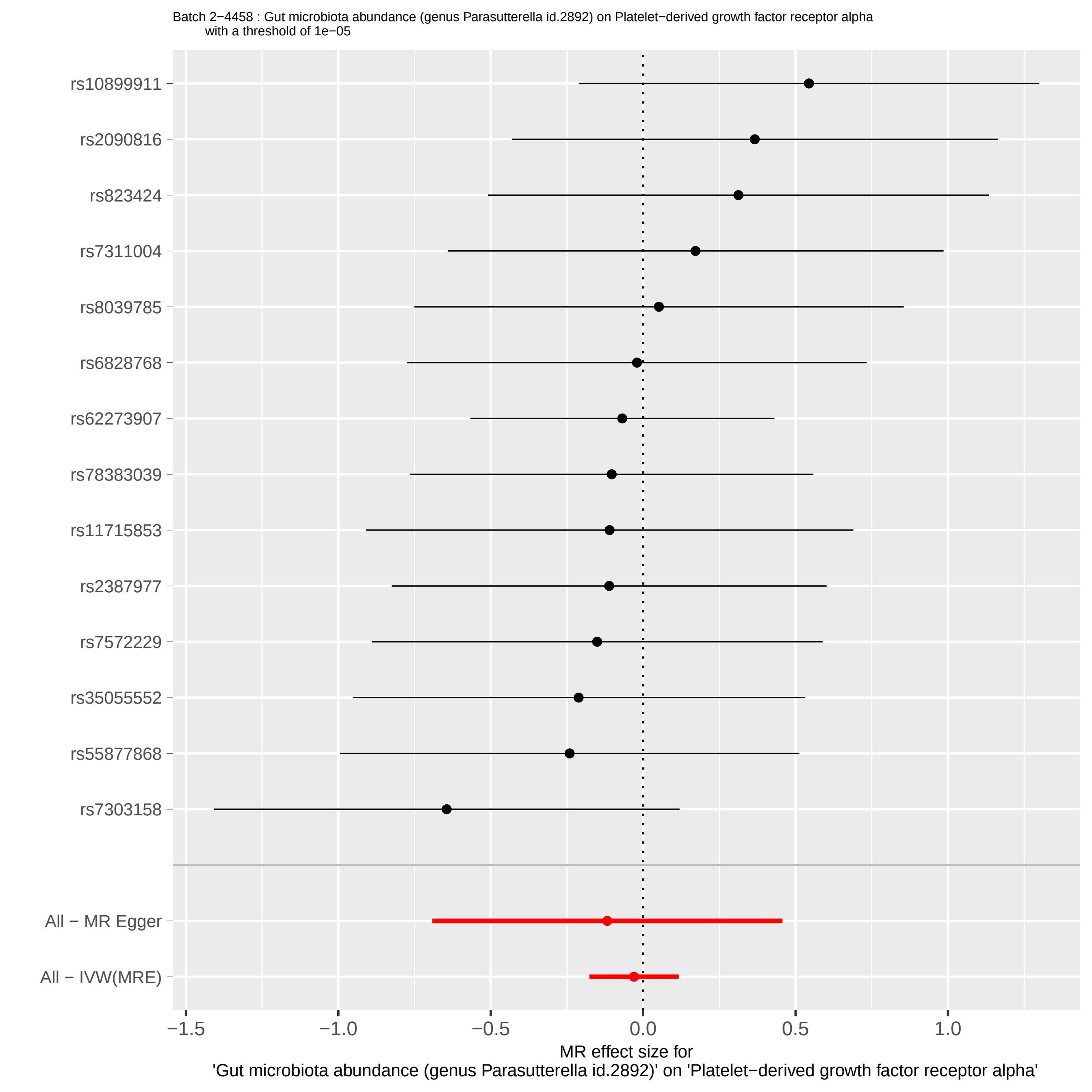
Batch 2−4458 : Gut microbiota abundance (genus Parasutterella id.2892) on Platelet−derived growth factor receptor alpha
with a threshold of 1e−05
rs10899911
rs2090816
rs823424
rs7311004
rs8039785
rs6828768
rs62273907
rs78383039
rs11715853
rs2387977
rs7572229
rs35055552
rs55877868
rs7303158
All − MR Egger
All − IVW(MRE)
−1.5
−1.0
−0.5
0.0
0.5
1.0
MR effect size for
'Gut microbiota abundance (genus Parasutterella id.2892)' on 'Platelet−derived growth factor receptor alpha'

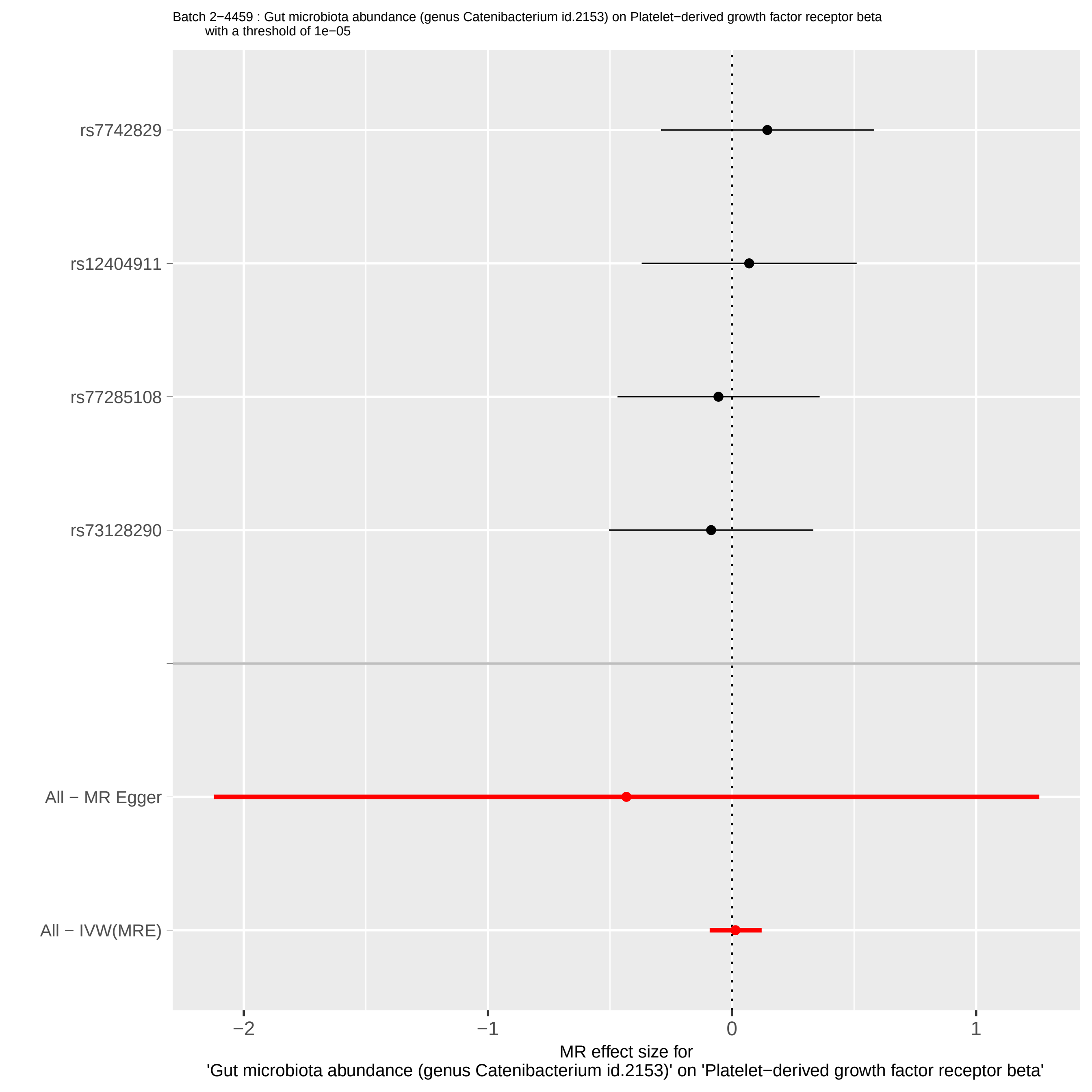

Batch 2−4459 : Gut microbiota abundance (genus Catenibacterium id.2153) on Platelet−derived growth factor receptor beta
with a threshold of 1e−05
rs7742829
rs12404911
rs77285108
rs73128290
All − MR Egger
All − IVW(MRE)
−2
−1
0
1
MR effect size for
'Gut microbiota abundance (genus Catenibacterium id.2153)' on 'Platelet−derived growth factor receptor beta'

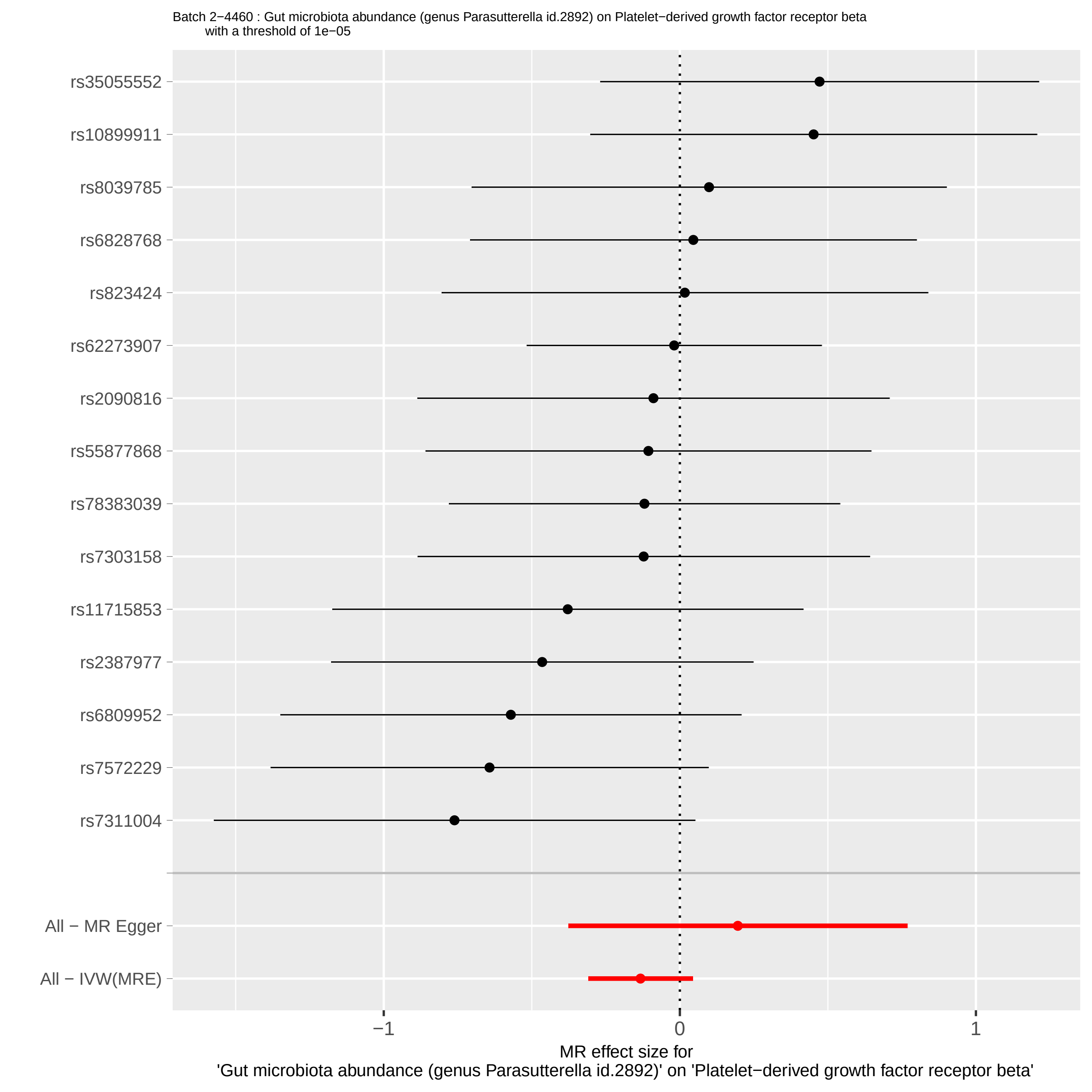

Batch 2−4460 : Gut microbiota abundance (genus Parasutterella id.2892) on Platelet−derived growth factor receptor beta
with a threshold of 1e−05
rs35055552
rs10899911
rs8039785
rs6828768
rs823424
rs62273907
rs2090816
rs55877868
rs78383039
rs7303158
rs11715853
rs2387977
rs6809952
rs7572229
rs7311004
All − MR Egger
All − IVW(MRE)
−1
0
1
MR effect size for
'Gut microbiota abundance (genus Parasutterella id.2892)' on 'Platelet−derived growth factor receptor beta'

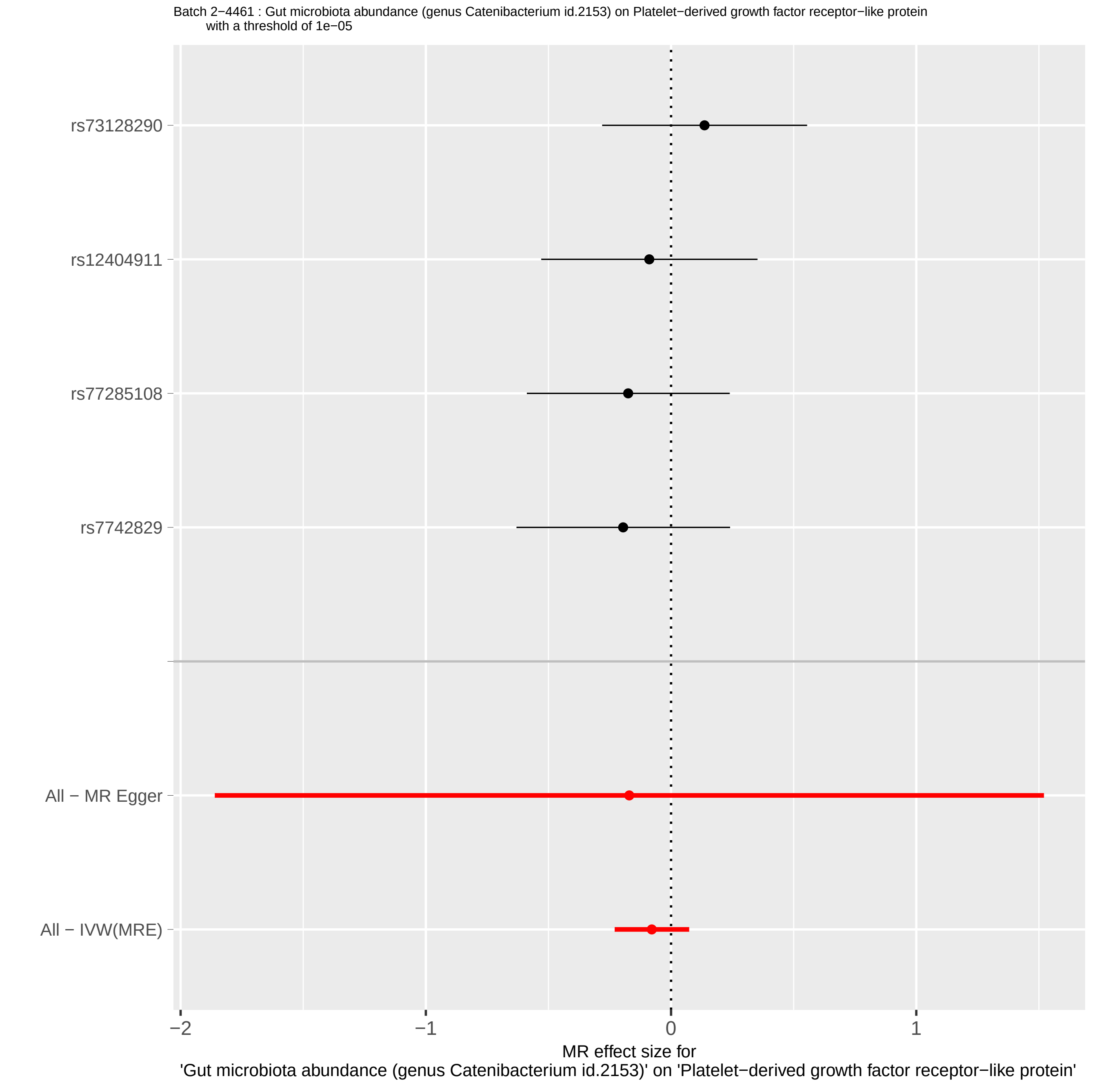
Batch 2−4461 : Gut microbiota abundance (genus Catenibacterium id.2153) on Platelet−derived growth factor receptor−like protein
with a threshold of 1e−05
rs73128290
rs12404911
rs77285108
rs7742829
All − MR Egger
All − IVW(MRE)
−2
−1
0
1
MR effect size for
'Gut microbiota abundance (genus Catenibacterium id.2153)' on 'Platelet−derived growth factor receptor−like protein'

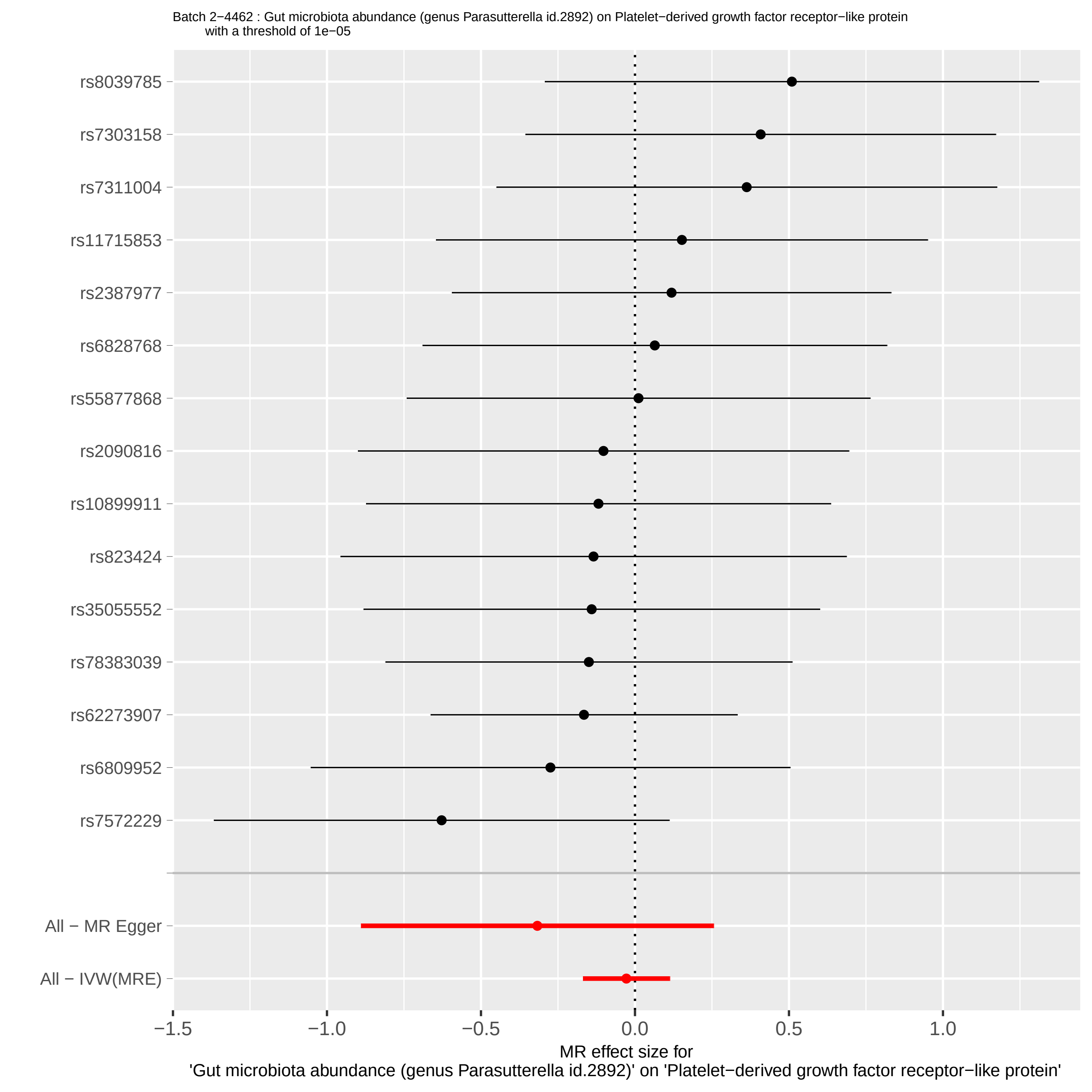

Batch 2−4462 : Gut microbiota abundance (genus Parasutterella id.2892) on Platelet−derived growth factor receptor−like protein
with a threshold of 1e−05
rs8039785
rs7303158
rs7311004
rs11715853
rs2387977
rs6828768
rs55877868
rs2090816
rs10899911
rs823424
rs35055552
rs78383039
rs62273907
rs6809952
rs7572229
All − MR Egger
All − IVW(MRE)
−1.5
−1.0
−0.5
0.0
0.5
1.0
MR effect size for
'Gut microbiota abundance (genus Parasutterella id.2892)' on 'Platelet−derived growth factor receptor−like protein'

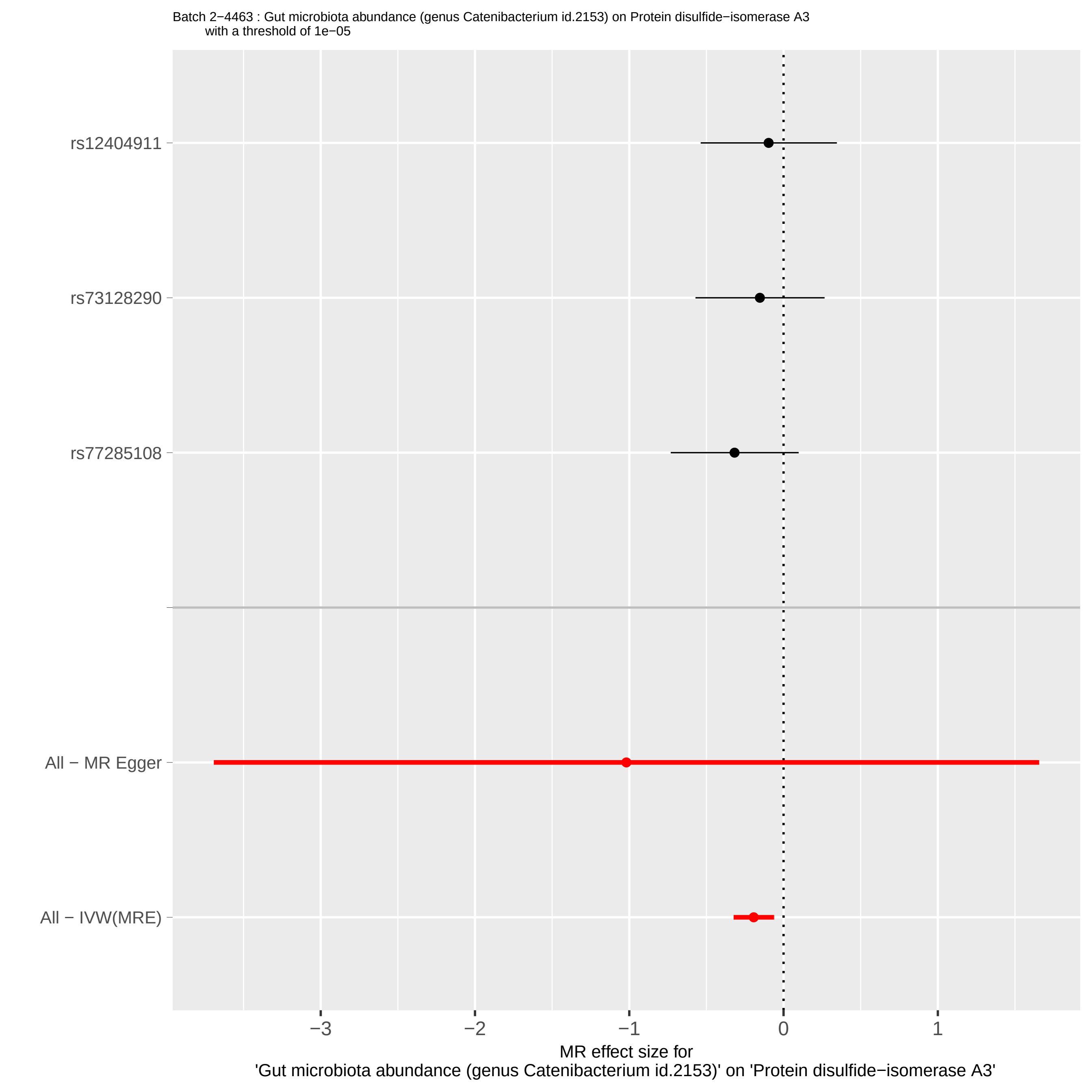

Batch 2−4463 : Gut microbiota abundance (genus Catenibacterium id.2153) on Protein disulfide−isomerase A3
with a threshold of 1e−05
rs12404911
rs73128290
rs77285108
All − MR Egger
All − IVW(MRE)
−3
−2
−1
0
1
MR effect size for
'Gut microbiota abundance (genus Catenibacterium id.2153)' on 'Protein disulfide−isomerase A3'

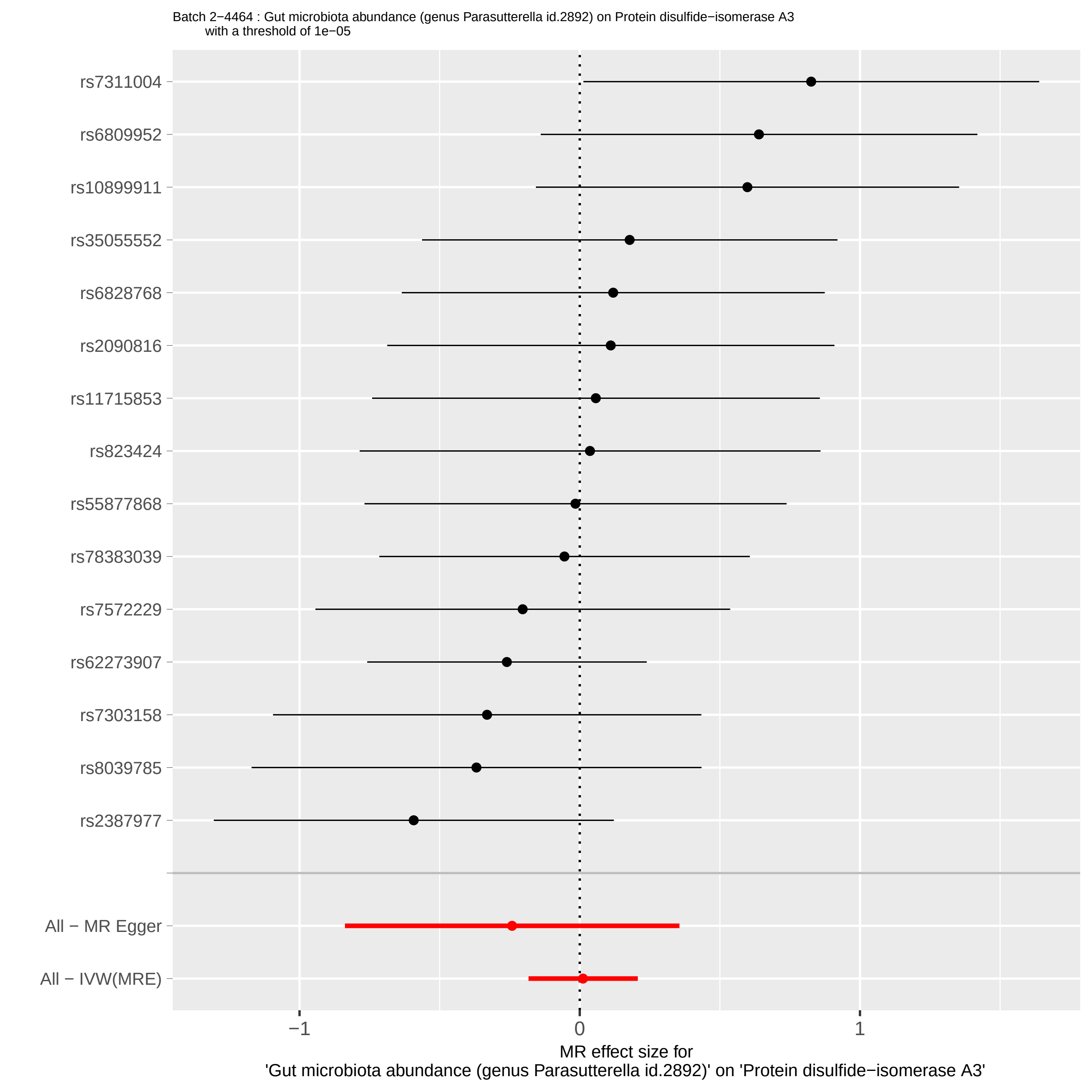
Batch 2−4464 : Gut microbiota abundance (genus Parasutterella id.2892) on Protein disulfide−isomerase A3
with a threshold of 1e−05
rs7311004
rs6809952
rs10899911
rs35055552
rs6828768
rs2090816
rs11715853
rs823424
rs55877868
rs78383039
rs7572229
rs62273907
rs7303158
rs8039785
rs2387977
All − MR Egger
All − IVW(MRE)
−1
0
1
MR effect size for
'Gut microbiota abundance (genus Parasutterella id.2892)' on 'Protein disulfide−isomerase A3'

Batch 2−4465 : Gut microbiota abundance (genus Catenibacterium id.2153) on Protein disulfide−isomerase A5
with a threshold of 1e−05

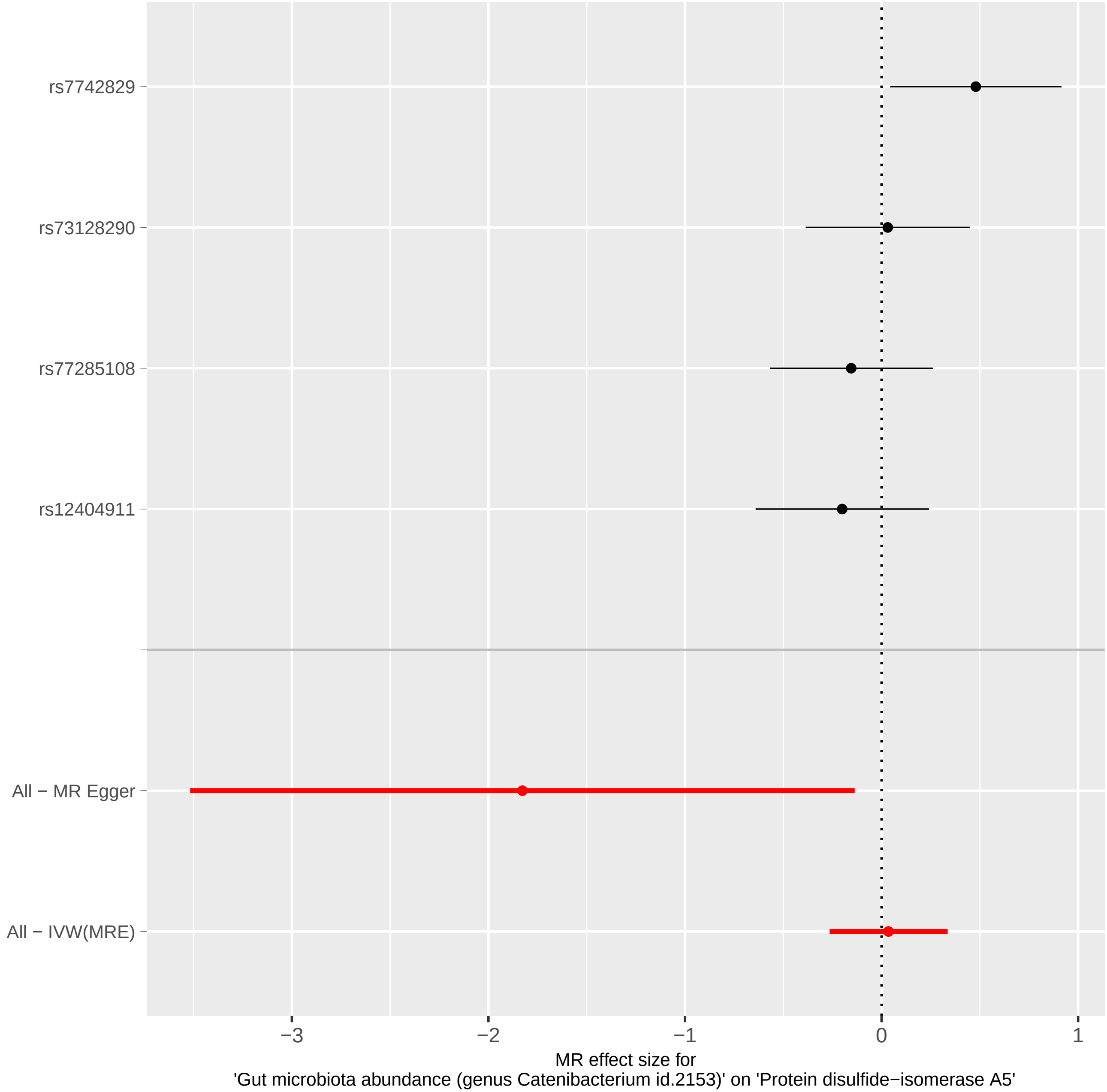

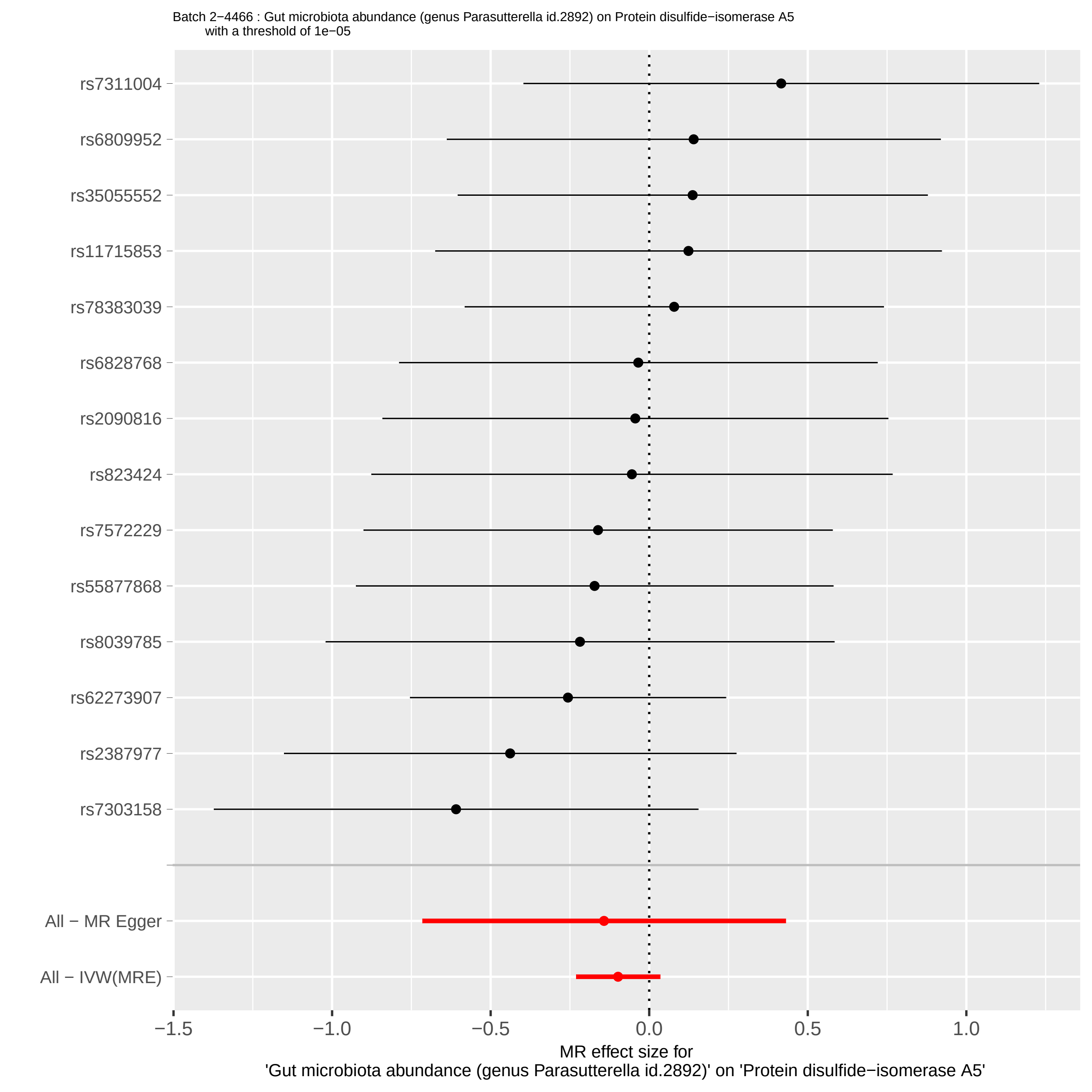

Batch 2−4466 : Gut microbiota abundance (genus Parasutterella id.2892) on Protein disulfide−isomerase A5
with a threshold of 1e−05
rs7311004
rs6809952
rs35055552
rs11715853
rs78383039
rs6828768
rs2090816
rs823424
rs7572229
rs55877868
rs8039785
rs62273907
rs2387977
rs7303158
All − MR Egger
All − IVW(MRE)
−1.5
−1.0
−0.5
0.0
0.5
1.0
MR effect size for
'Gut microbiota abundance (genus Parasutterella id.2892)' on 'Protein disulfide−isomerase A5'

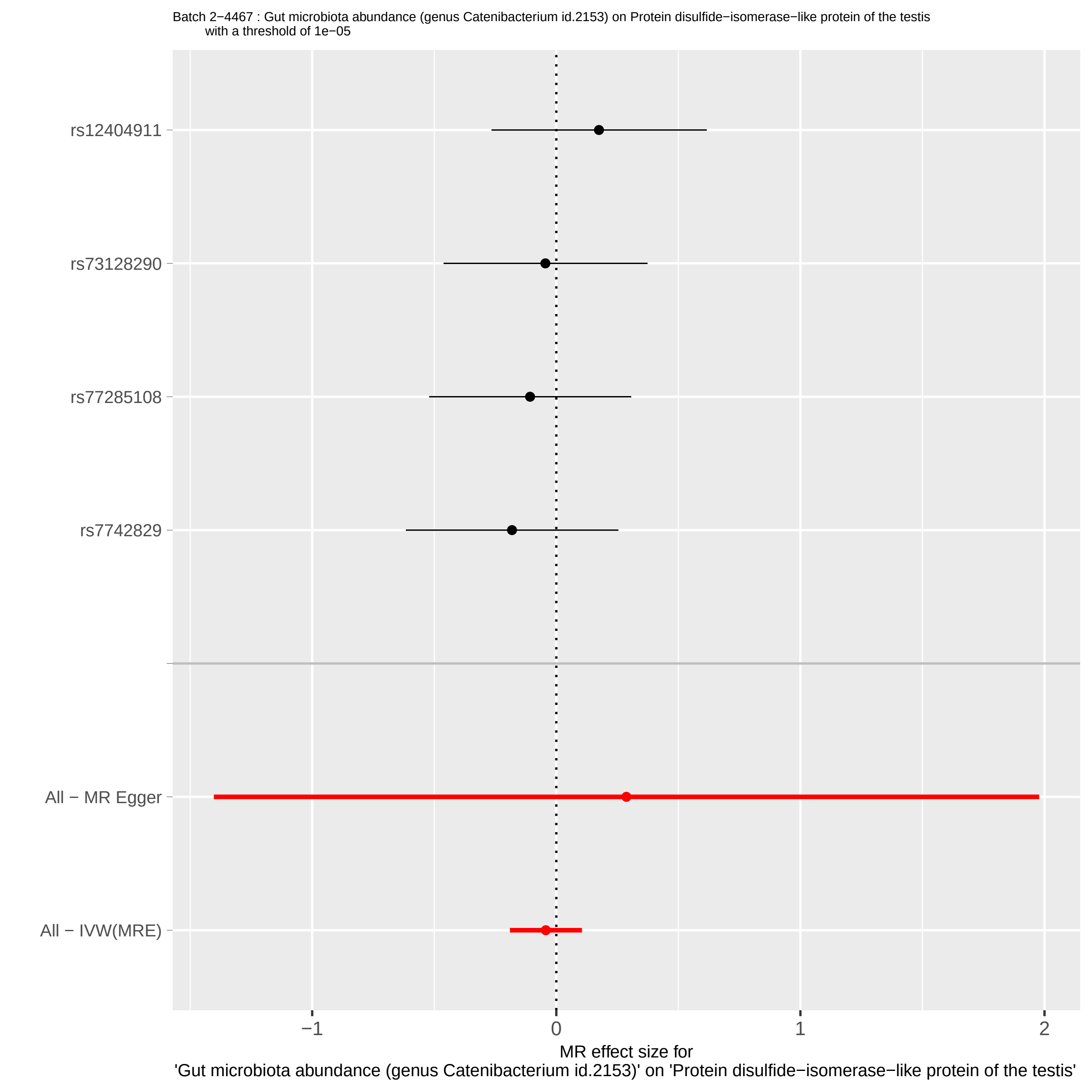

Batch 2−4467 : Gut microbiota abundance (genus Catenibacterium id.2153) on Protein disulfide−isomerase−like protein of the testis
with a threshold of 1e−05
rs12404911
rs73128290
rs77285108
rs7742829
All − MR Egger
All − IVW(MRE)
−1
0
1
2
MR effect size for
'Gut microbiota abundance (genus Catenibacterium id.2153)' on 'Protein disulfide−isomerase−like protein of the testis'

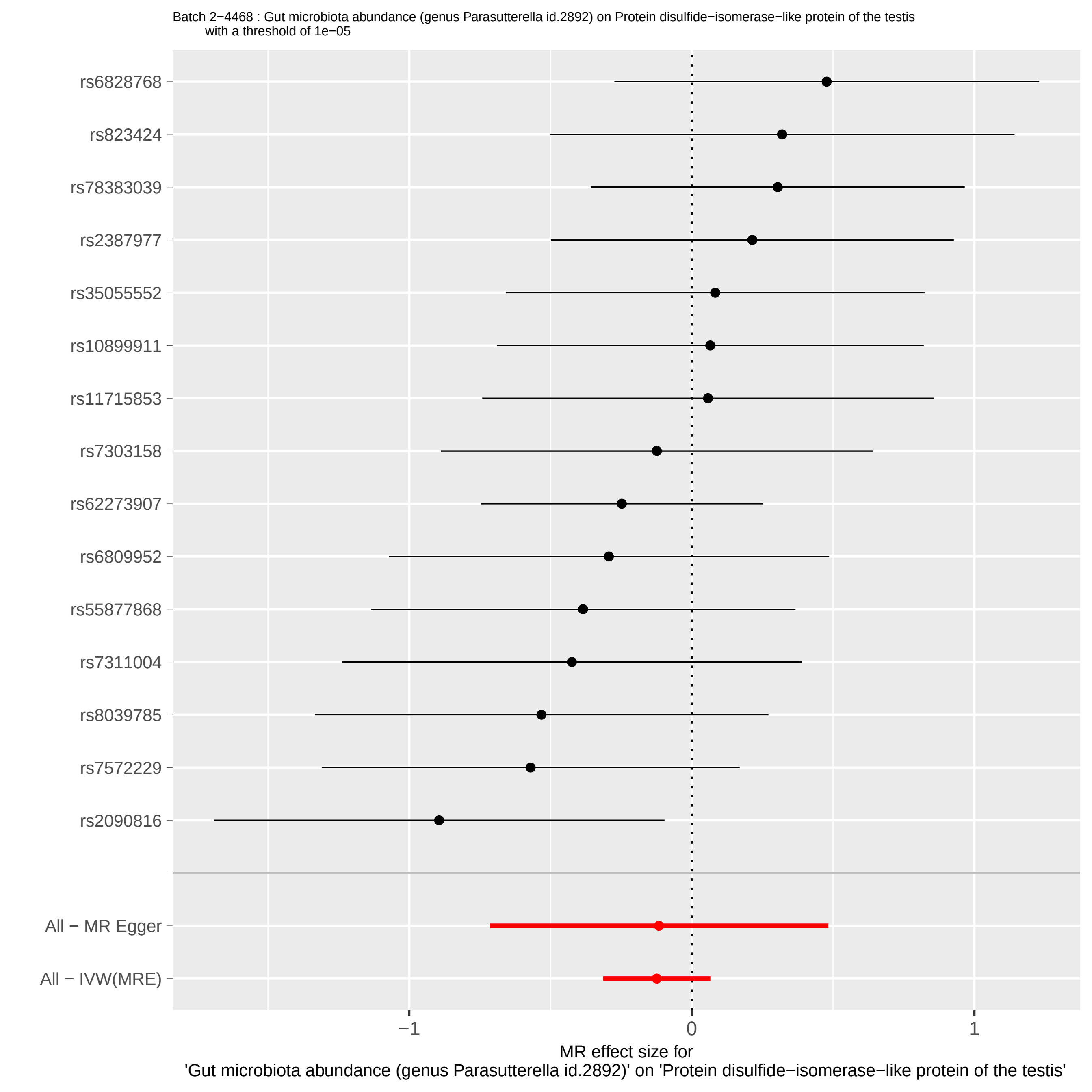
Batch 2−4468 : Gut microbiota abundance (genus Parasutterella id.2892) on Protein disulfide−isomerase−like protein of the testis
with a threshold of 1e−05
rs6828768
rs823424
rs78383039
rs2387977
rs35055552
rs10899911
rs11715853
rs7303158
rs62273907
rs6809952
rs55877868
rs7311004
rs8039785
rs7572229
rs2090816
All − MR Egger
All − IVW(MRE)
−1
0
1
MR effect size for
'Gut microbiota abundance (genus Parasutterella id.2892)' on 'Protein disulfide−isomerase−like protein of the testis'

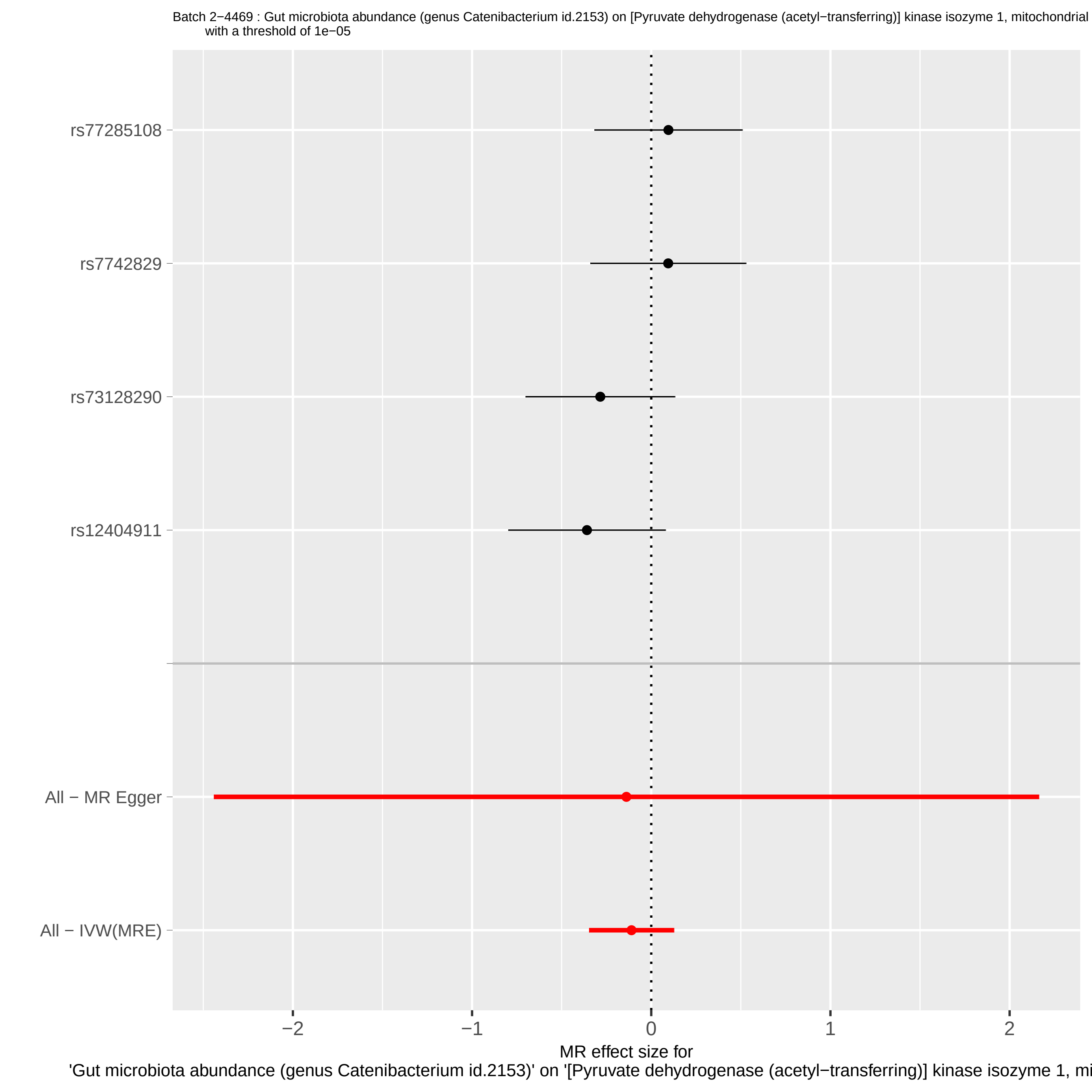

Batch 2−4469 : Gut microbiota abundance (genus Catenibacterium id.2153) on [Pyruvate dehydrogenase (acetyl−transferring)] kinase isozyme 1, mitochondrial
with a threshold of 1e−05
rs77285108
rs7742829
rs73128290
rs12404911
All − MR Egger
All − IVW(MRE)
−2
−1
0
1
2
MR effect size for
'Gut microbiota abundance (genus Catenibacterium id.2153)' on '[Pyruvate dehydrogenase (acetyl−transferring)] kinase isozyme 1, mi

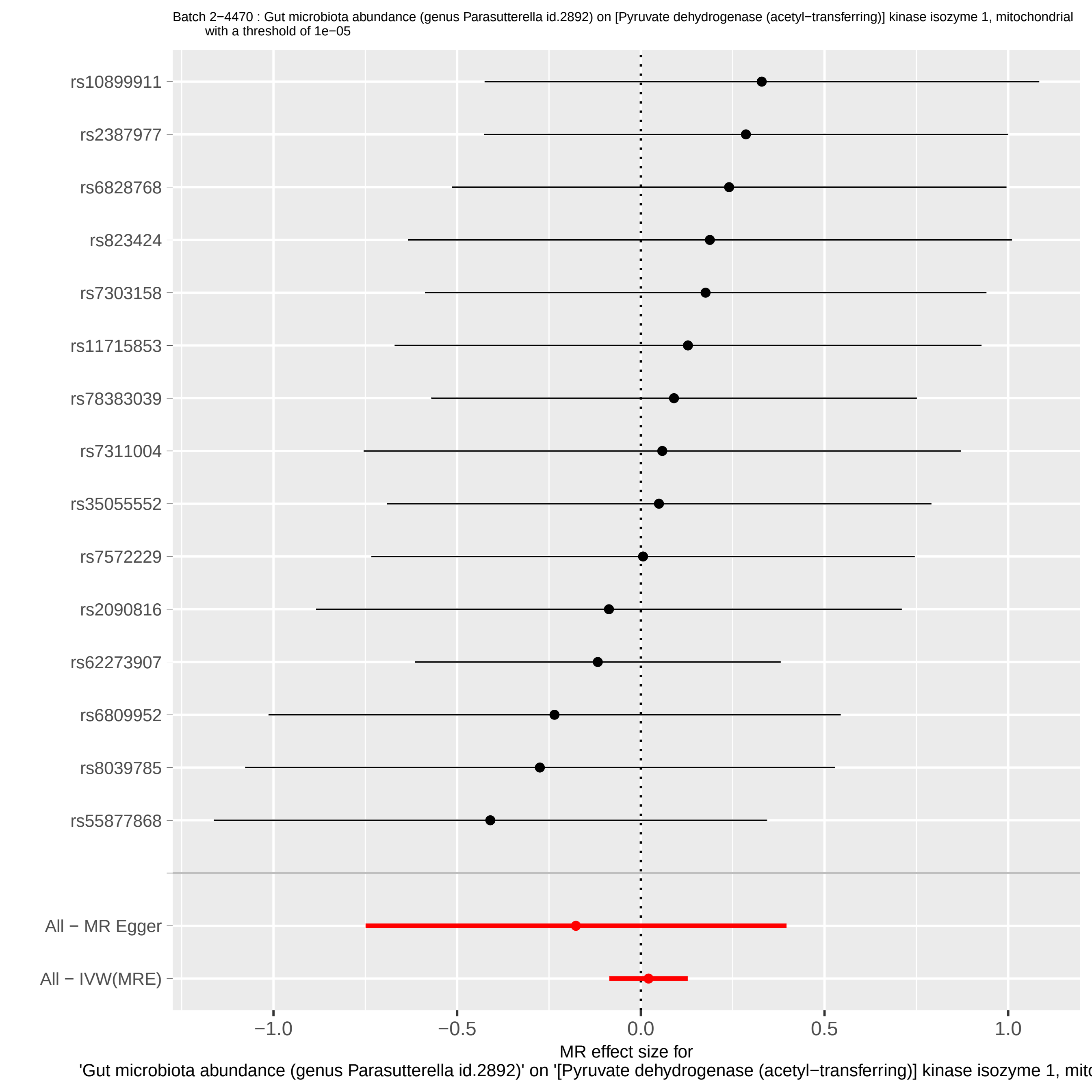
Batch 2−4470 : Gut microbiota abundance (genus Parasutterella id.2892) on [Pyruvate dehydrogenase (acetyl−transferring)] kinase isozyme 1, mitochondrial with a threshold of 1e−05
rs10899911
rs2387977
rs6828768
rs823424
rs7303158
rs11715853
rs78383039
rs7311004
rs35055552
rs7572229
rs2090816
rs62273907
rs6809952
rs8039785
rs55877868
All − MR Egger
All − IVW(MRE)
−1.0
−0.5
0.0
0.5
1.0
MR effect size for
'Gut microbiota abundance (genus Parasutterella id.2892)' on '[Pyruvate dehydrogenase (acetyl−transferring)] kinase isozyme 1, mito

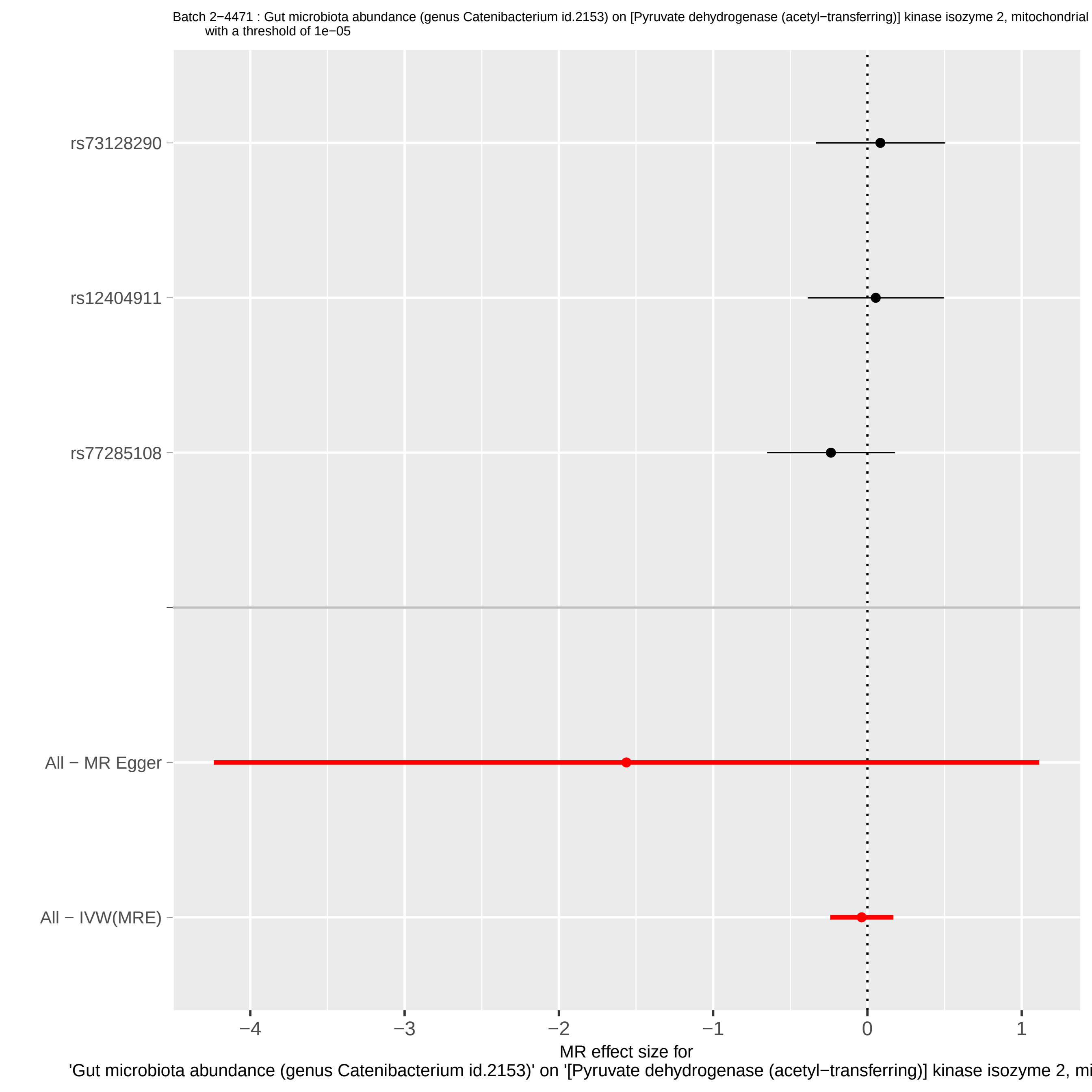
Batch 2−4471 : Gut microbiota abundance (genus Catenibacterium id.2153) on [Pyruvate dehydrogenase (acetyl−transferring)] kinase isozyme 2, mitochondrial
with a threshold of 1e−05
rs73128290
rs12404911
rs77285108
All − MR Egger
All − IVW(MRE)
−4
−3
−2
−1
0
1
MR effect size for
'Gut microbiota abundance (genus Catenibacterium id.2153)' on '[Pyruvate dehydrogenase (acetyl−transferring)] kinase isozyme 2, mi

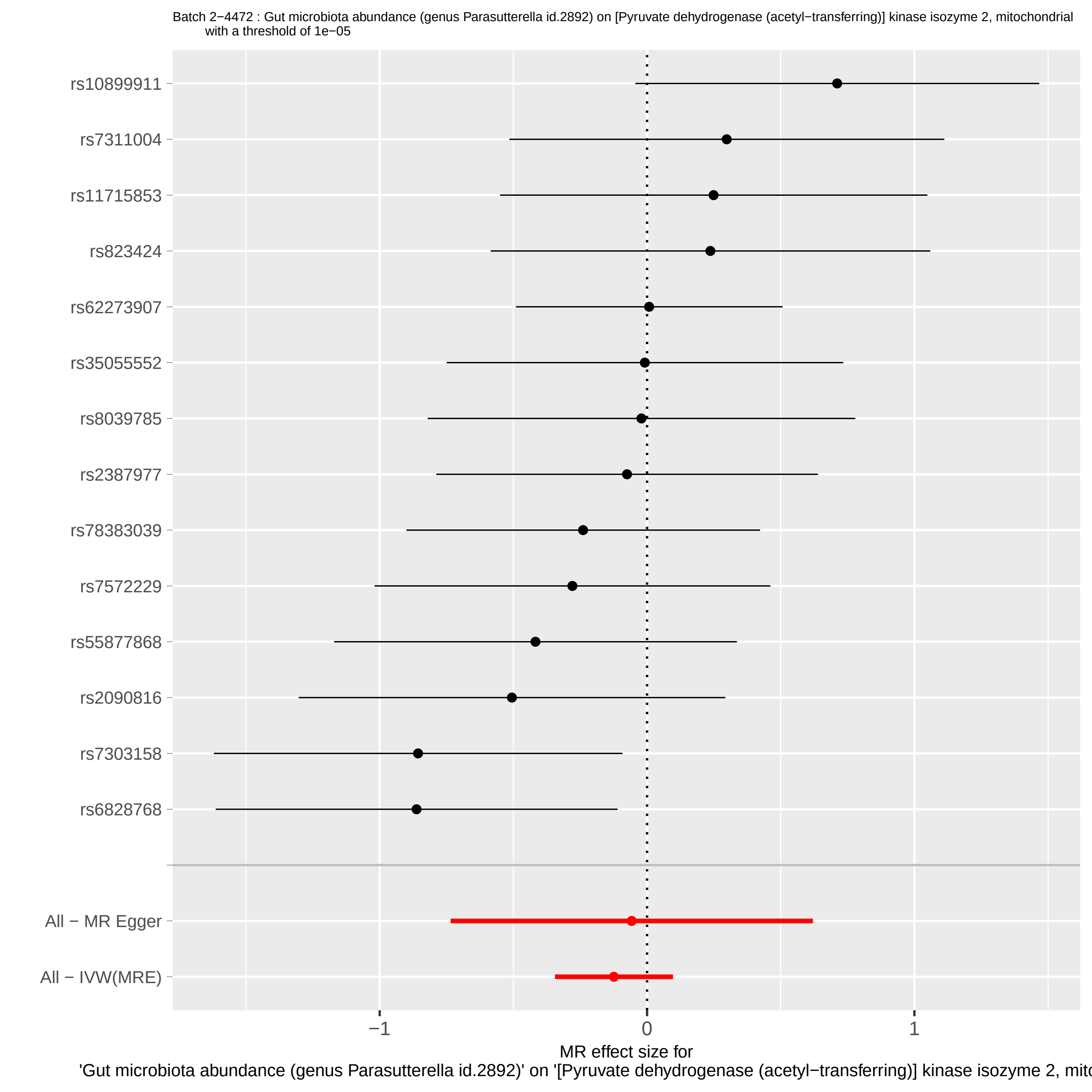

Batch 2−4472 : Gut microbiota abundance (genus Parasutterella id.2892) on [Pyruvate dehydrogenase (acetyl−transferring)] kinase isozyme 2, mitochondrial with a threshold of 1e−05
rs10899911
rs7311004
rs11715853
rs823424
rs62273907
rs35055552
rs8039785
rs2387977
rs78383039
rs7572229
rs55877868
rs2090816
rs7303158
rs6828768
All − MR Egger
All − IVW(MRE)
−1
0
1
MR effect size for
'Gut microbiota abundance (genus Parasutterella id.2892)' on '[Pyruvate dehydrogenase (acetyl−transferring)] kinase isozyme 2, mito

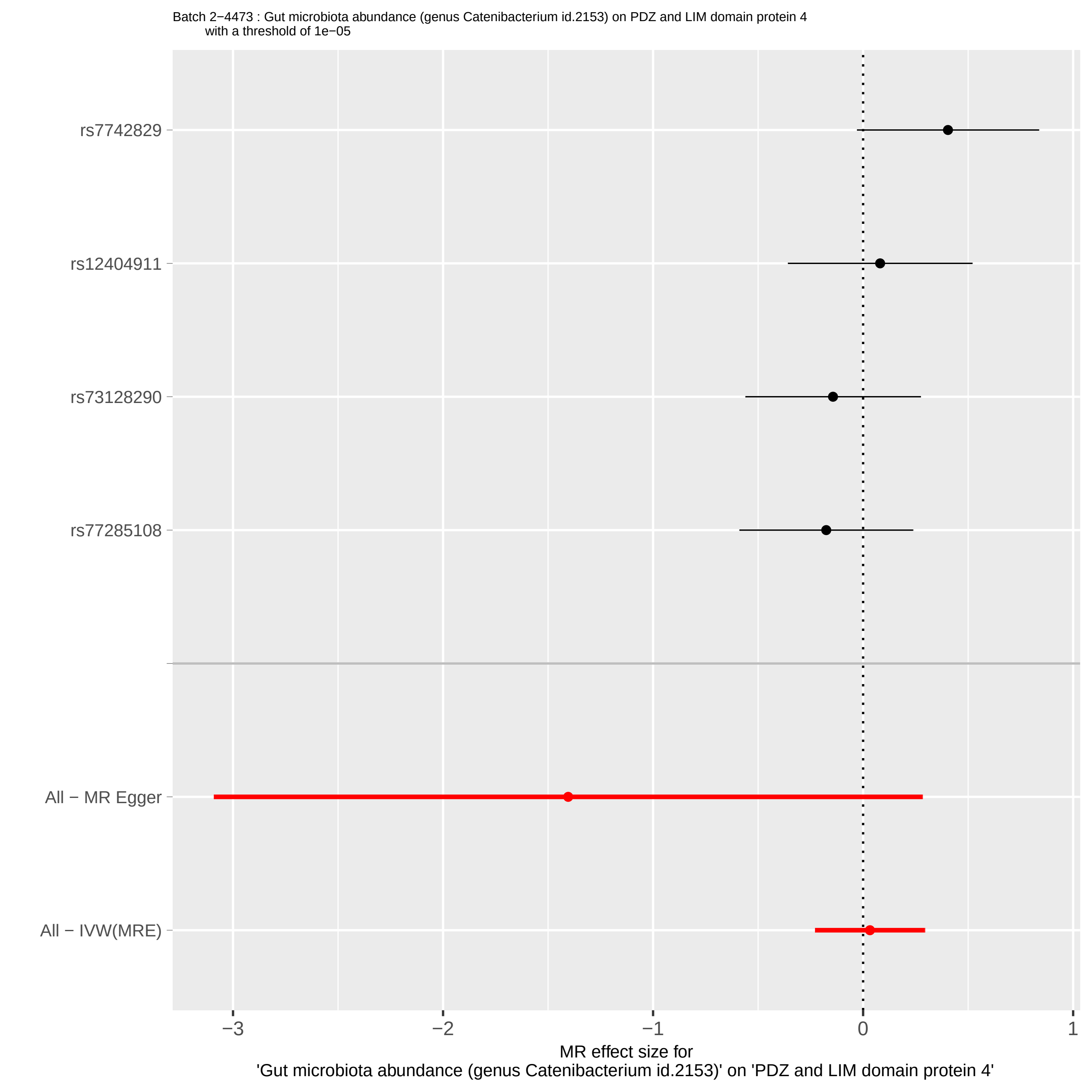

Batch 2−4473 : Gut microbiota abundance (genus Catenibacterium id.2153) on PDZ and LIM domain protein 4
with a threshold of 1e−05
rs7742829
rs12404911
rs73128290
rs77285108
All − MR Egger
All − IVW(MRE)
−3
−2
−1
0
1
MR effect size for
'Gut microbiota abundance (genus Catenibacterium id.2153)' on 'PDZ and LIM domain protein 4'

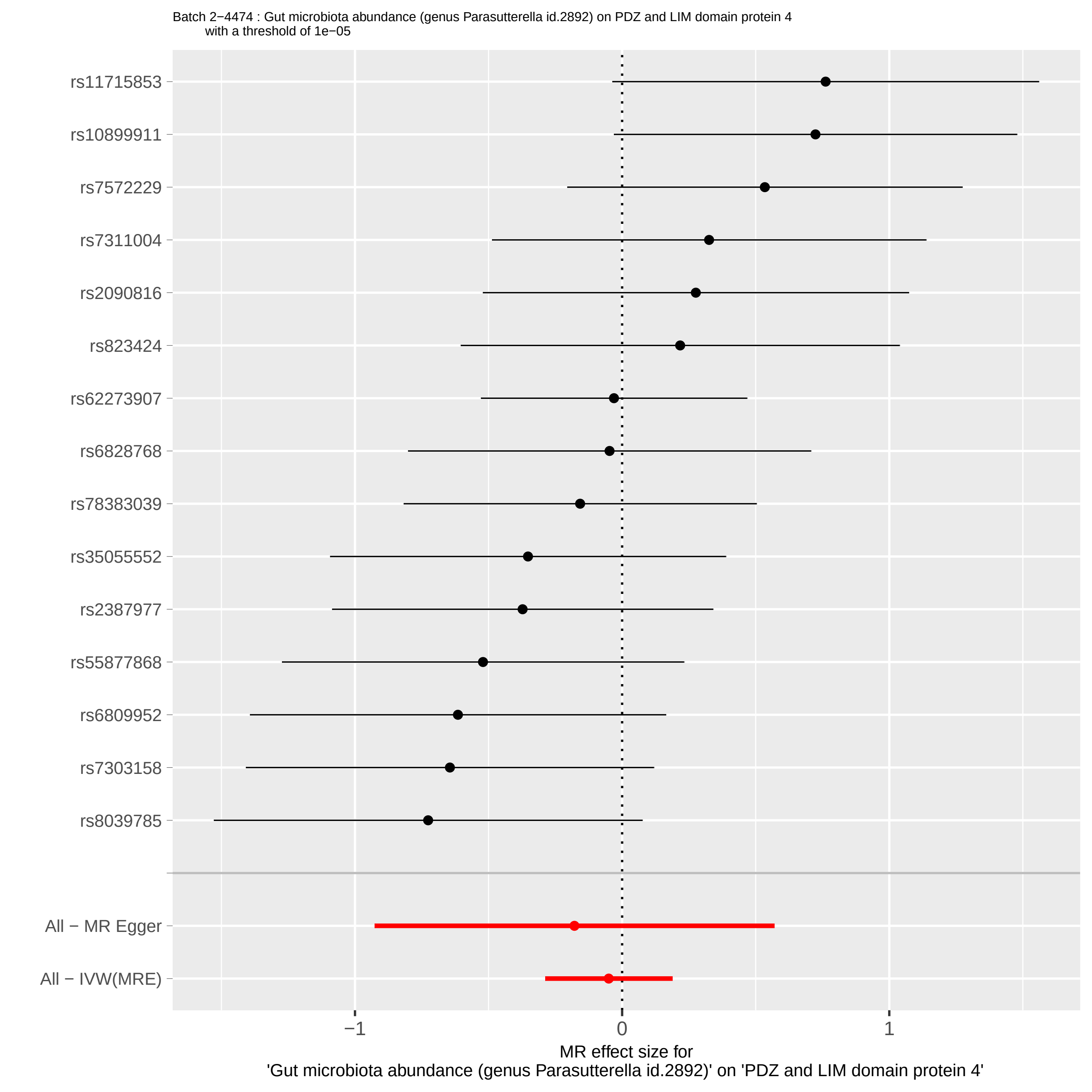

Batch 2−4474 : Gut microbiota abundance (genus Parasutterella id.2892) on PDZ and LIM domain protein 4
with a threshold of 1e−05
rs11715853
rs10899911
rs7572229
rs7311004
rs2090816
rs823424
rs62273907
rs6828768
rs78383039
rs35055552
rs2387977
rs55877868
rs6809952
rs7303158
rs8039785
All − MR Egger
All − IVW(MRE)
−1
0
1
MR effect size for
'Gut microbiota abundance (genus Parasutterella id.2892)' on 'PDZ and LIM domain protein 4'

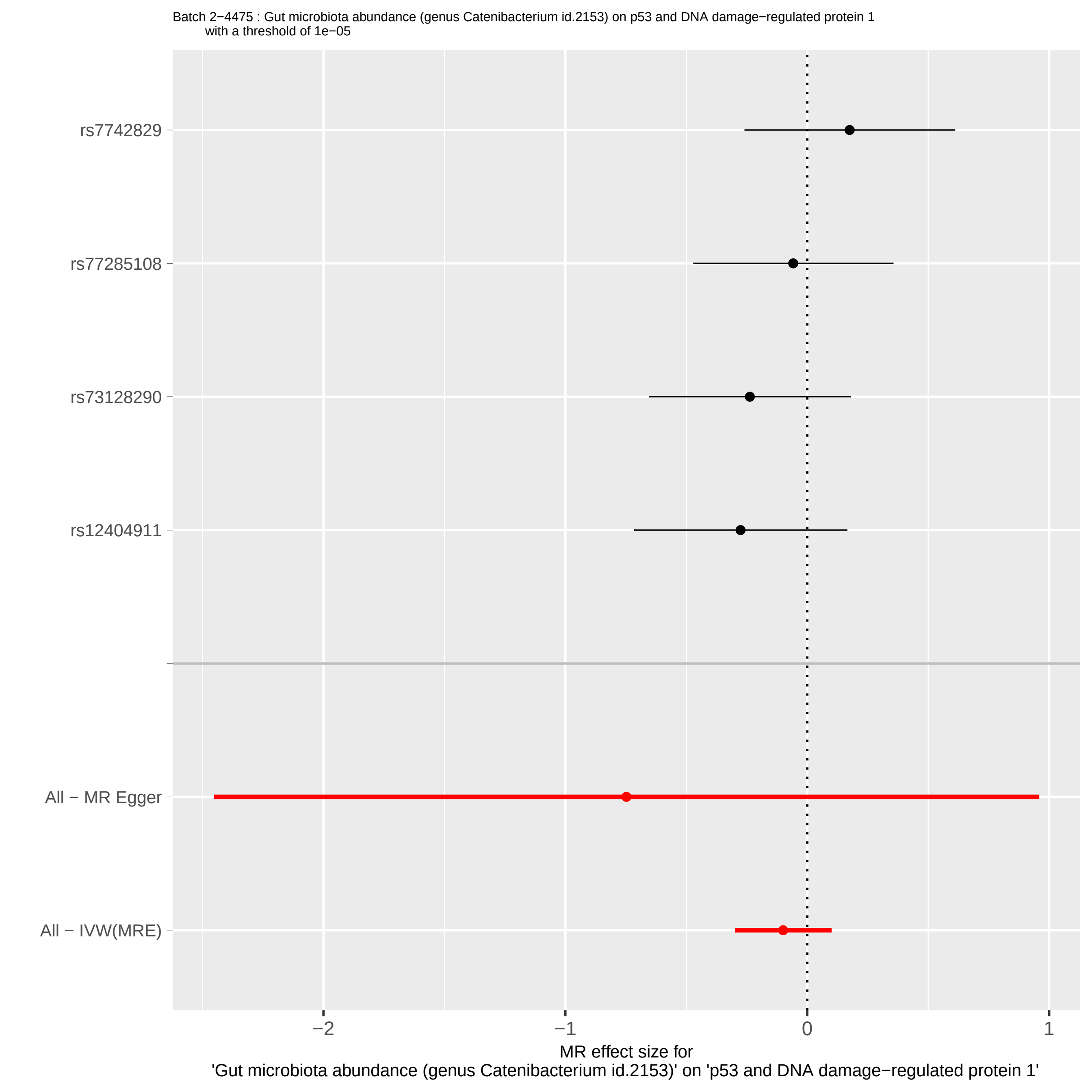
Batch 2−4475 : Gut microbiota abundance (genus Catenibacterium id.2153) on p53 and DNA damage−regulated protein 1
with a threshold of 1e−05
rs7742829
rs77285108
rs73128290
rs12404911
All − MR Egger
All − IVW(MRE)
−2
−1
0
1
MR effect size for
'Gut microbiota abundance (genus Catenibacterium id.2153)' on 'p53 and DNA damage−regulated protein 1'

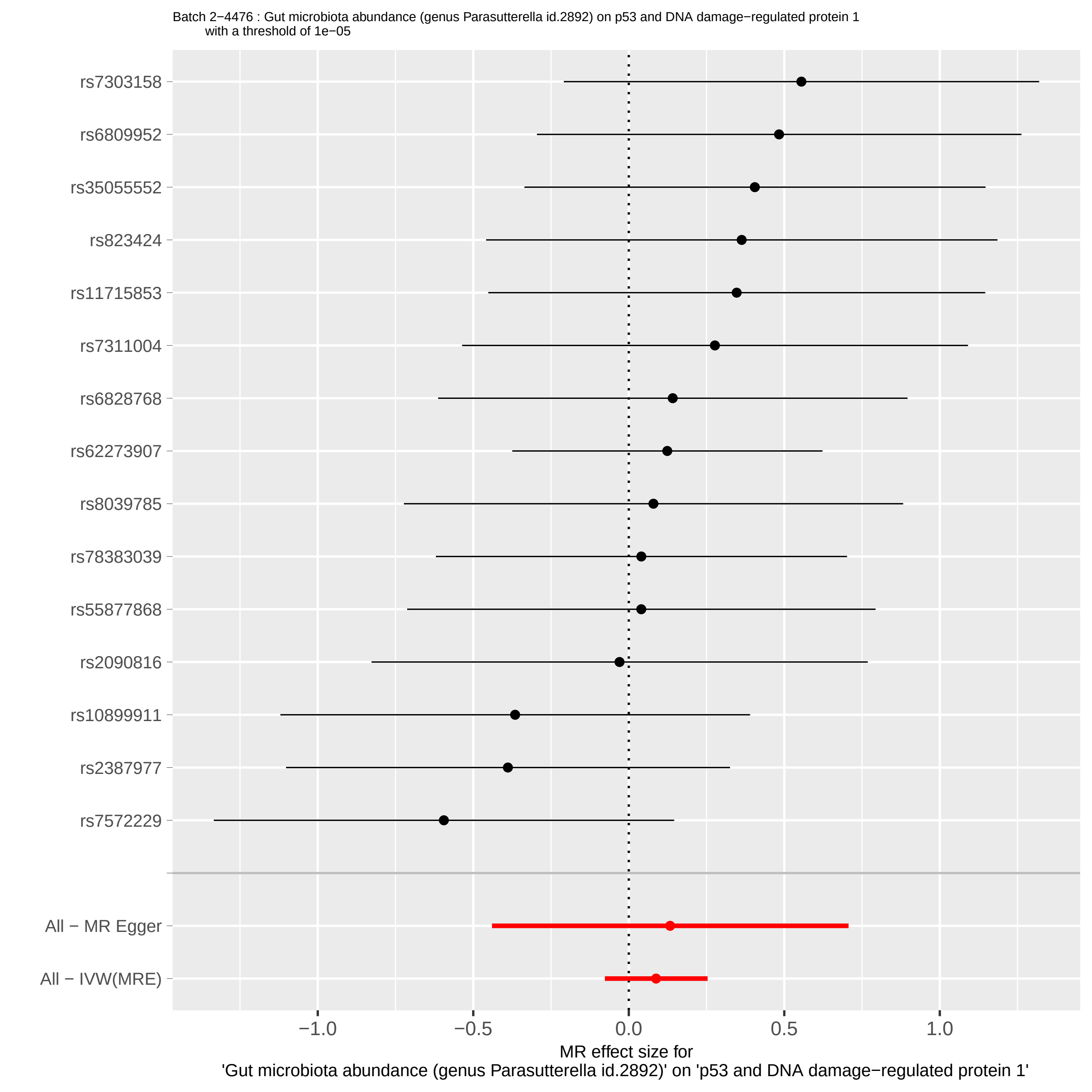

Batch 2−4476 : Gut microbiota abundance (genus Parasutterella id.2892) on p53 and DNA damage−regulated protein 1
with a threshold of 1e−05
rs7303158
rs6809952
rs35055552
rs823424
rs11715853
rs7311004
rs6828768
rs62273907
rs8039785
rs78383039
rs55877868
rs2090816
rs10899911
rs2387977
rs7572229
All − MR Egger
All − IVW(MRE)
−1.0
−0.5
0.0
0.5
1.0
MR effect size for
'Gut microbiota abundance (genus Parasutterella id.2892)' on 'p53 and DNA damage−regulated protein 1'

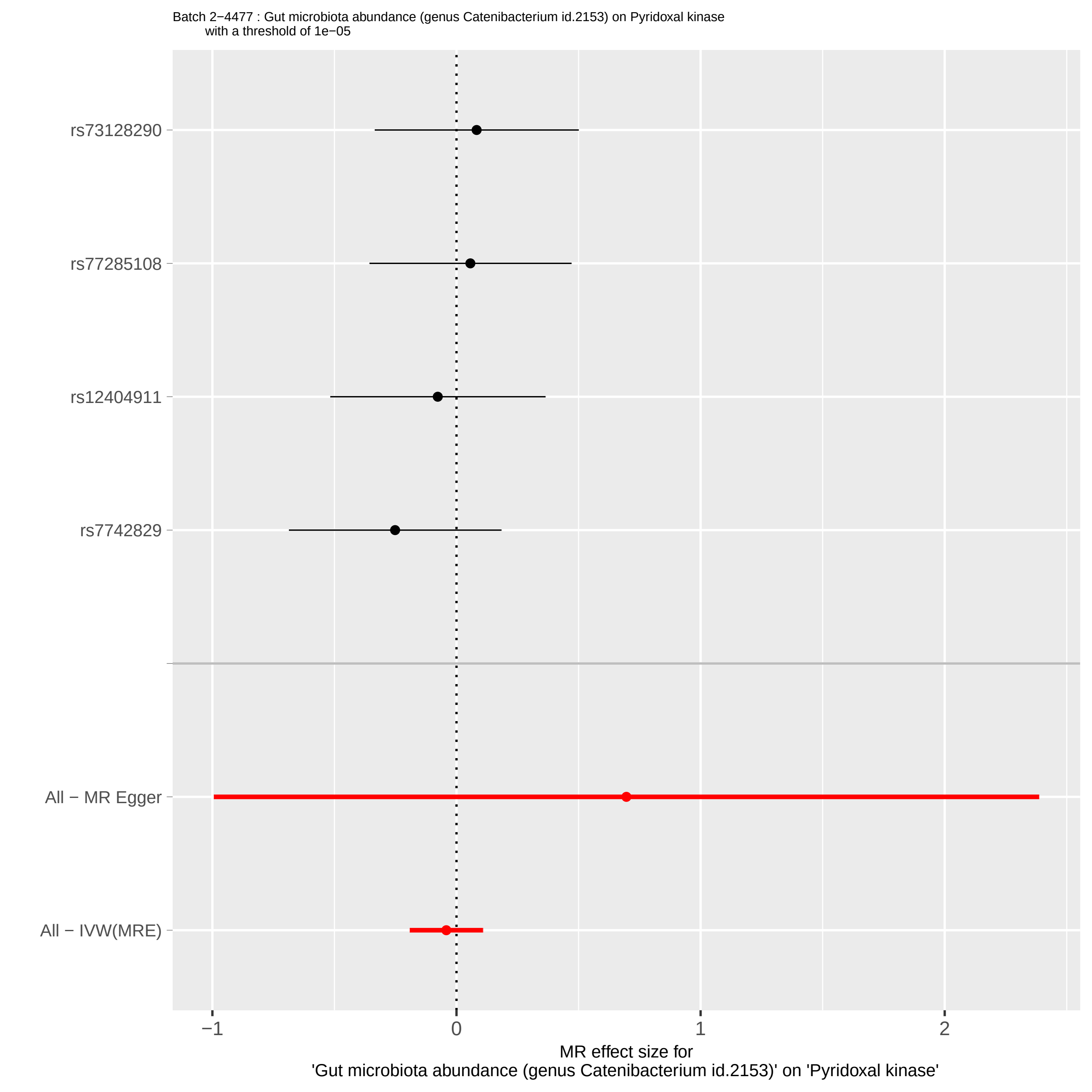

Batch 2−4477 : Gut microbiota abundance (genus Catenibacterium id.2153) on Pyridoxal kinase
with a threshold of 1e−05
rs73128290
rs77285108
rs12404911
rs7742829
All − MR Egger
All − IVW(MRE)
−1
0
1
2
MR effect size for
'Gut microbiota abundance (genus Catenibacterium id.2153)' on 'Pyridoxal kinase'

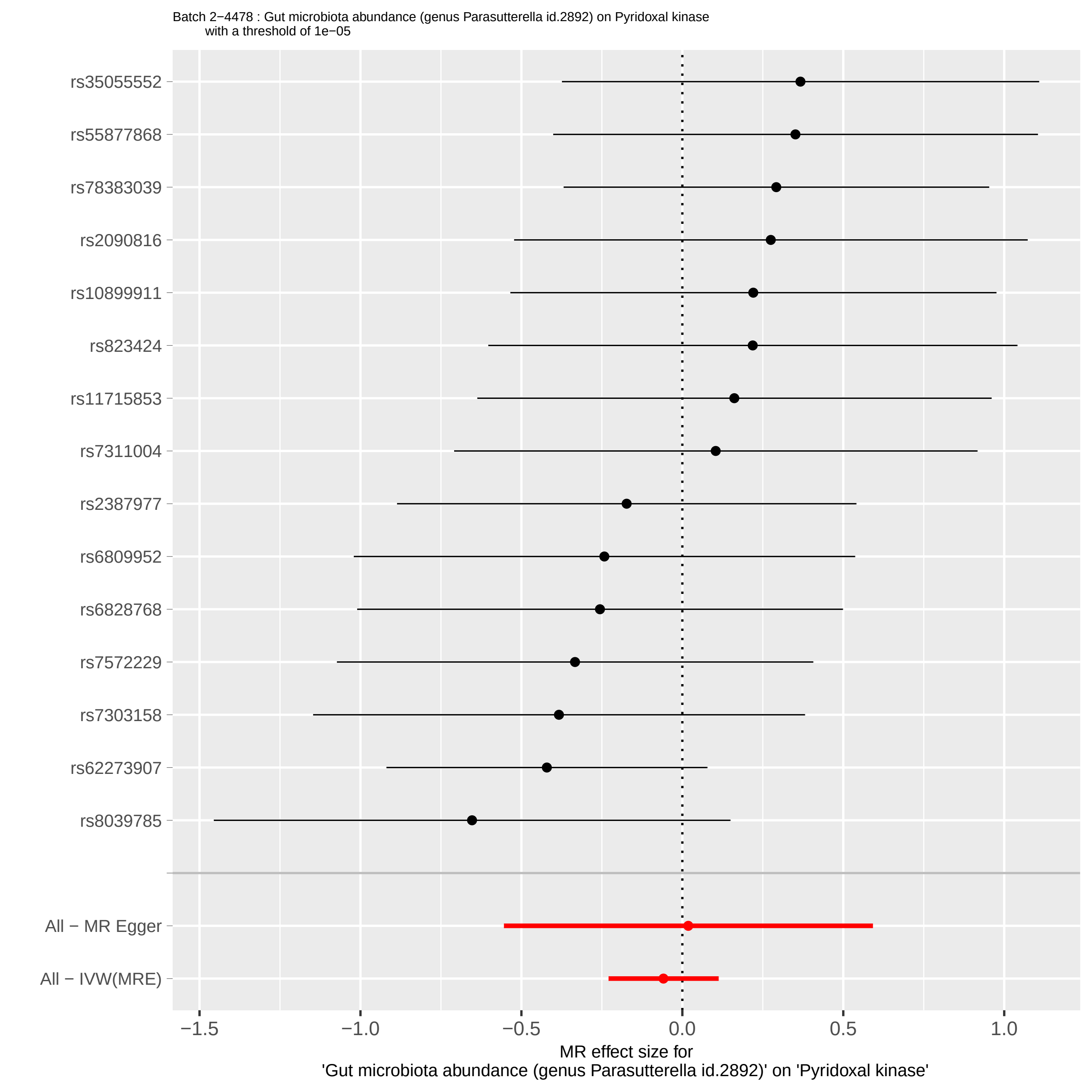
Batch 2−4478 : Gut microbiota abundance (genus Parasutterella id.2892) on Pyridoxal kinase
with a threshold of 1e−05
rs35055552
rs55877868
rs78383039
rs2090816
rs10899911
rs823424
rs11715853
rs7311004
rs2387977
rs6809952
rs6828768
rs7572229
rs7303158
rs62273907
rs8039785
All − MR Egger
All − IVW(MRE)
−1.5
−1.0
−0.5
0.0
0.5
1.0
MR effect size for
'Gut microbiota abundance (genus Parasutterella id.2892)' on 'Pyridoxal kinase'

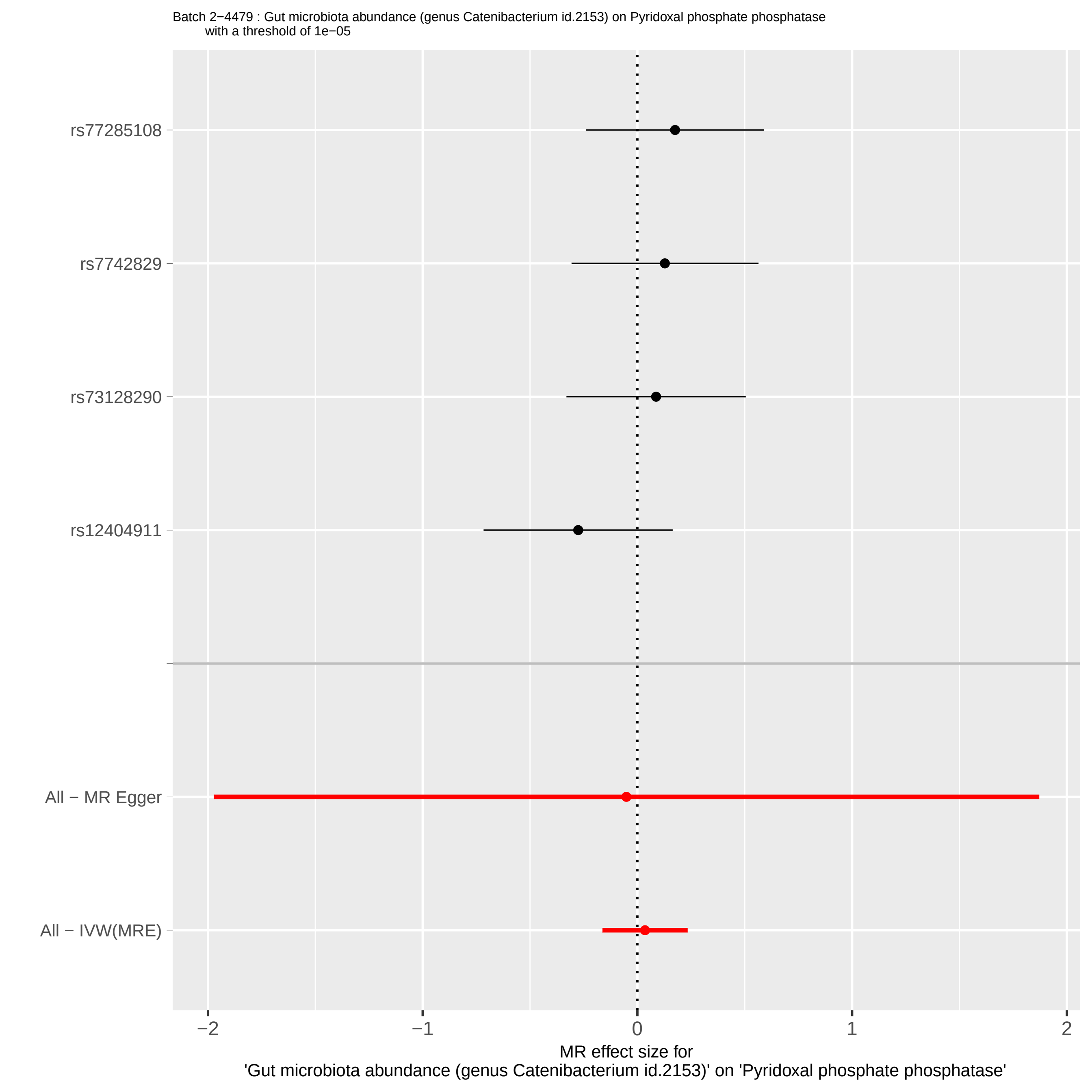

Batch 2−4479 : Gut microbiota abundance (genus Catenibacterium id.2153) on Pyridoxal phosphate phosphatase
with a threshold of 1e−05
rs77285108
rs7742829
rs73128290
rs12404911
All − MR Egger
All − IVW(MRE)
−2
−1
0
1
2
MR effect size for
'Gut microbiota abundance (genus Catenibacterium id.2153)' on 'Pyridoxal phosphate phosphatase'

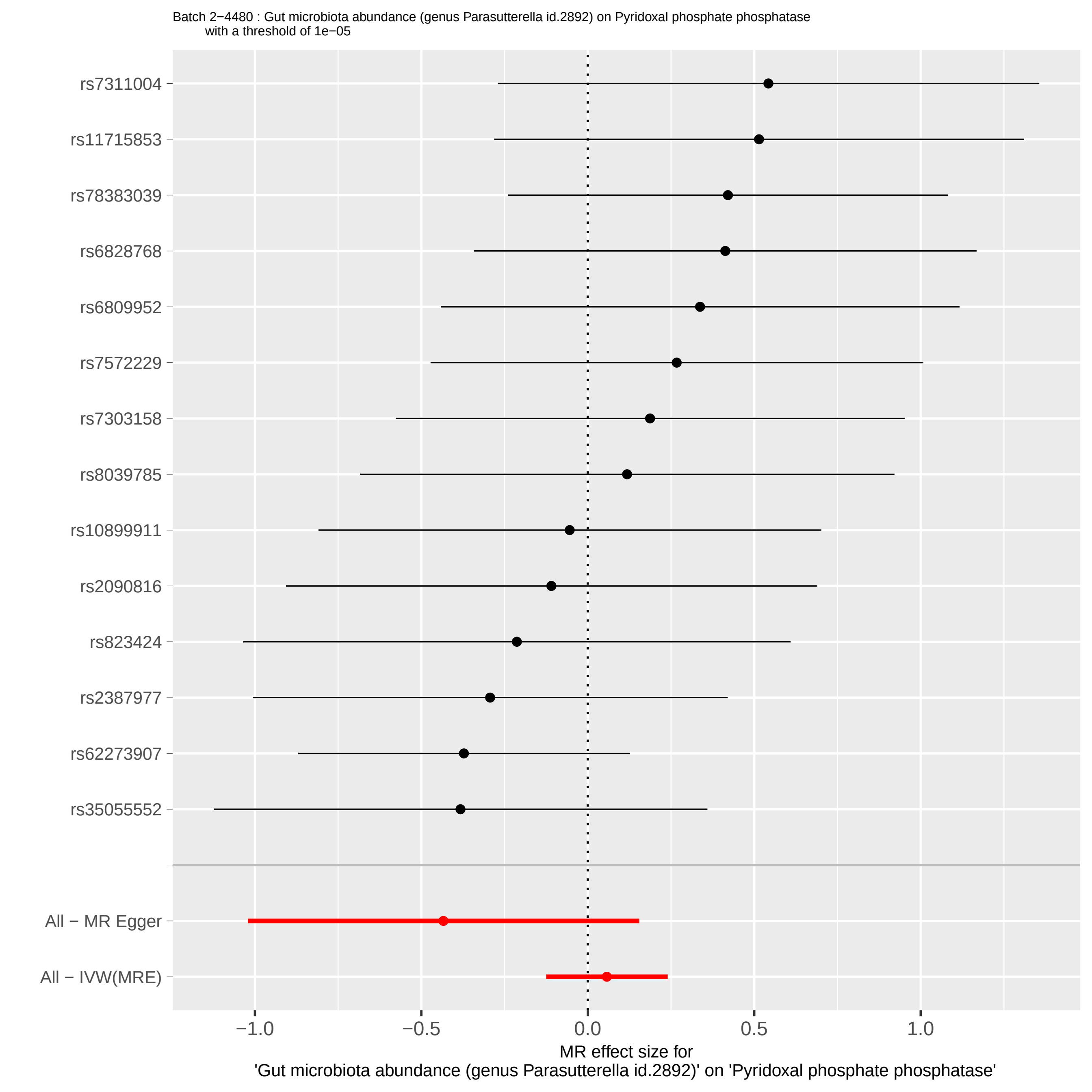

Batch 2−4480 : Gut microbiota abundance (genus Parasutterella id.2892) on Pyridoxal phosphate phosphatase
with a threshold of 1e−05
rs7311004
rs11715853
rs78383039
rs6828768
rs6809952
rs7572229
rs7303158
rs8039785
rs10899911
rs2090816
rs823424
rs2387977
rs62273907
rs35055552
All − MR Egger
All − IVW(MRE)
−1.0
−0.5
0.0
0.5
1.0
MR effect size for
'Gut microbiota abundance (genus Parasutterella id.2892)' on 'Pyridoxal phosphate phosphatase'

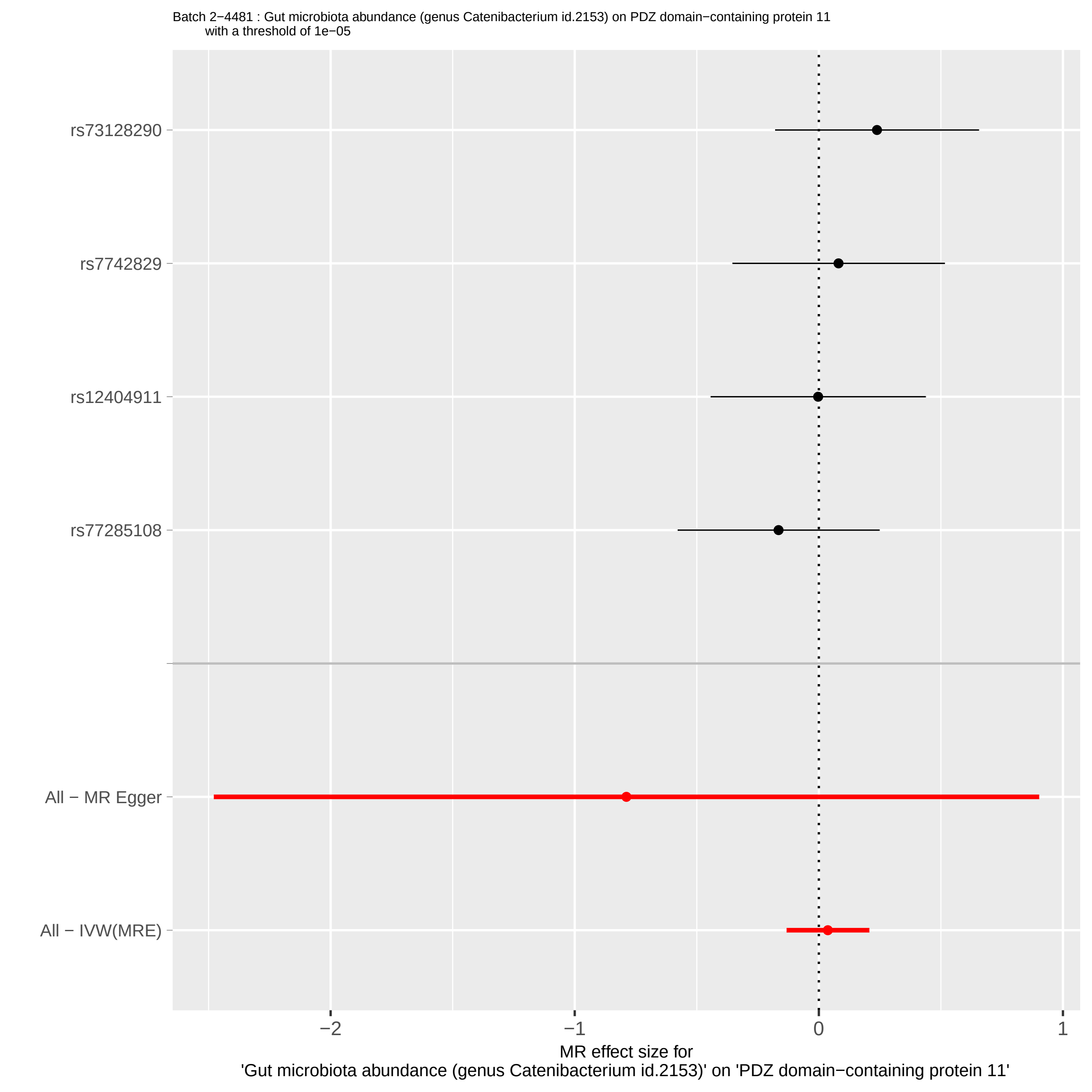

Batch 2−4481 : Gut microbiota abundance (genus Catenibacterium id.2153) on PDZ domain−containing protein 11
with a threshold of 1e−05
rs73128290
rs7742829
rs12404911
rs77285108
All − MR Egger
All − IVW(MRE)
−2
−1
0
1
MR effect size for
'Gut microbiota abundance (genus Catenibacterium id.2153)' on 'PDZ domain−containing protein 11'

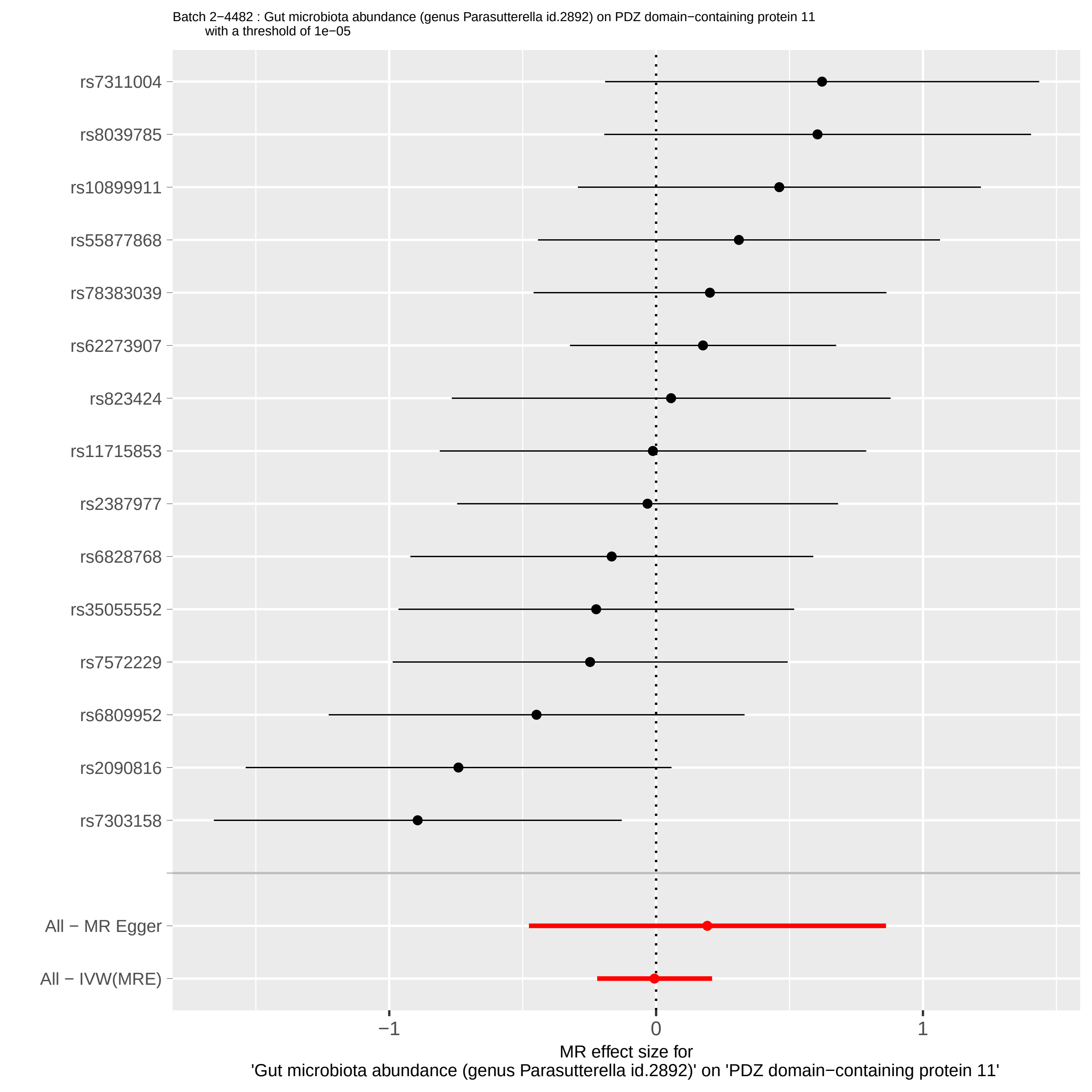

Batch 2−4482 : Gut microbiota abundance (genus Parasutterella id.2892) on PDZ domain−containing protein 11
with a threshold of 1e−05
rs7311004
rs8039785
rs10899911
rs55877868
rs78383039
rs62273907
rs823424
rs11715853
rs2387977
rs6828768
rs35055552
rs7572229
rs6809952
rs2090816
rs7303158
All − MR Egger
All − IVW(MRE)
−1
0
1
MR effect size for
'Gut microbiota abundance (genus Parasutterella id.2892)' on 'PDZ domain−containing protein 11'

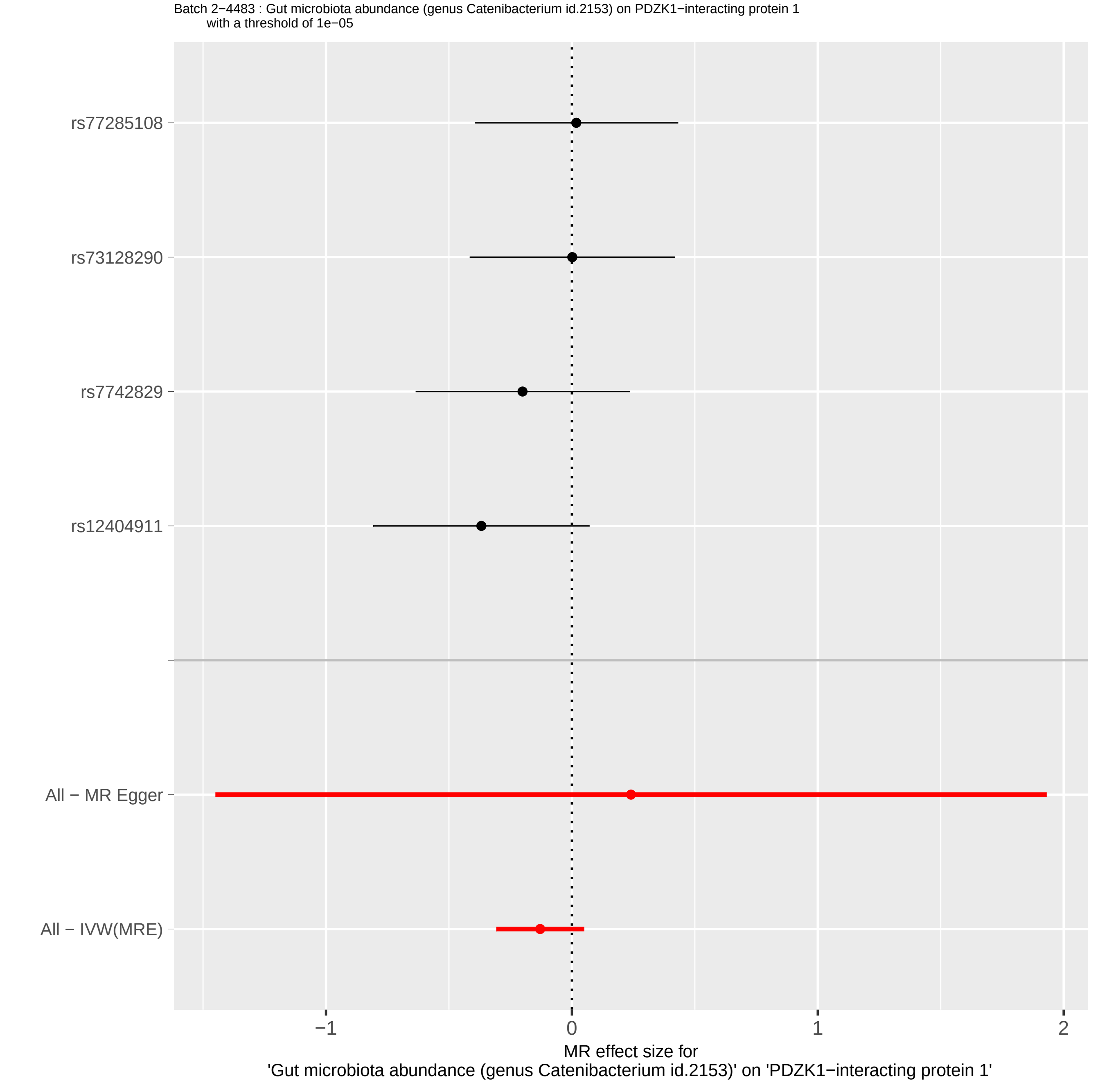

Batch 2−4483 : Gut microbiota abundance (genus Catenibacterium id.2153) on PDZK1−interacting protein 1
with a threshold of 1e−05
rs77285108
rs73128290
rs7742829
rs12404911
All − MR Egger
All − IVW(MRE)
−1
0
1
2
MR effect size for
'Gut microbiota abundance (genus Catenibacterium id.2153)' on 'PDZK1−interacting protein 1'

Batch 2−4484 : Gut microbiota abundance (genus Parasutterella id.2892) on PDZK1−interacting protein 1
with a threshold of 1e−05

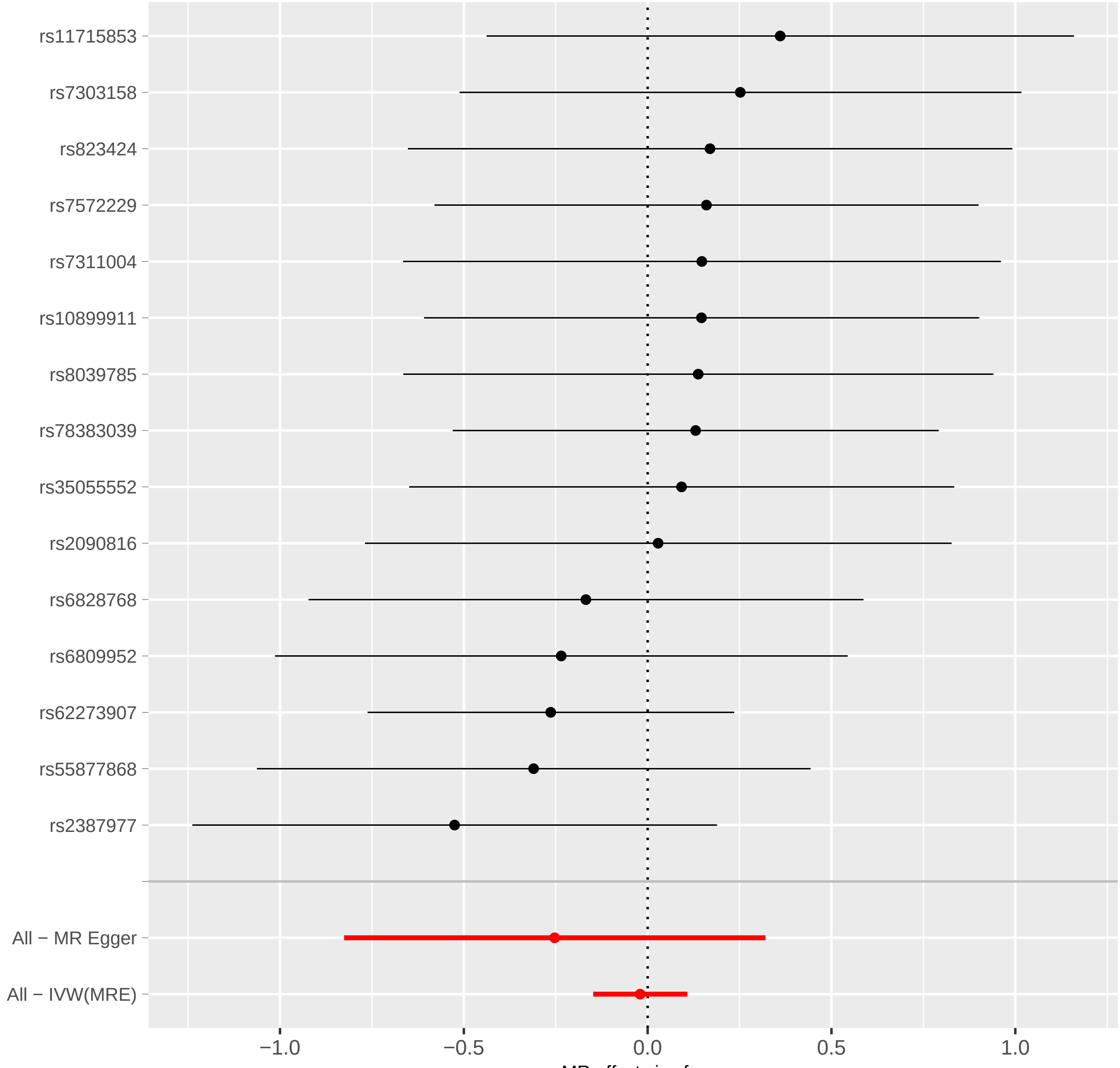

MR effect size for
'Gut microbiota abundance (genus Parasutterella id.2892)' on 'PDZK1−interacting protein 1'

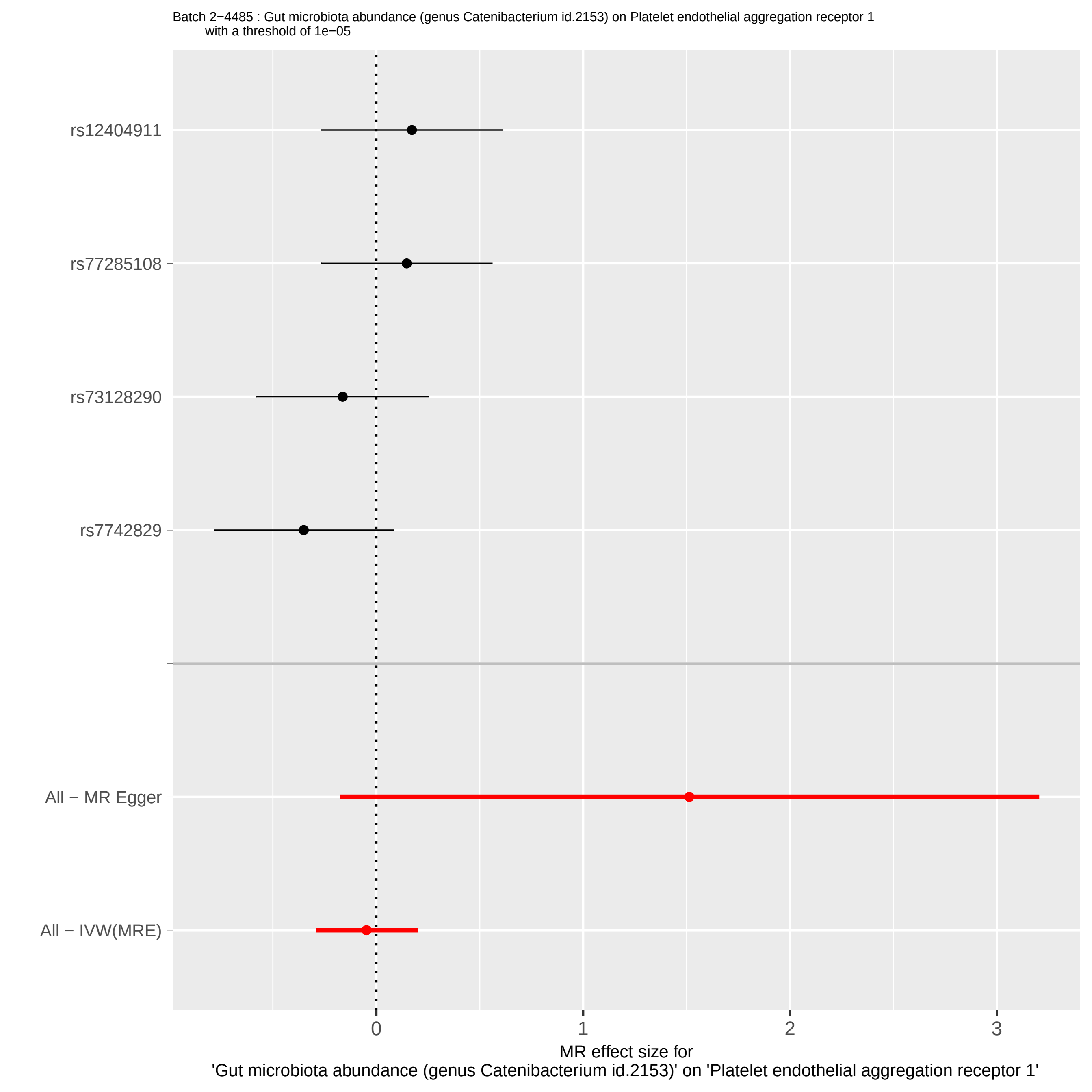
Batch 2−4485 : Gut microbiota abundance (genus Catenibacterium id.2153) on Platelet endothelial aggregation receptor 1
with a threshold of 1e−05
rs12404911
rs77285108
rs73128290
rs7742829
All − MR Egger
All − IVW(MRE)
0
1
2
3
MR effect size for
'Gut microbiota abundance (genus Catenibacterium id.2153)' on 'Platelet endothelial aggregation receptor 1'

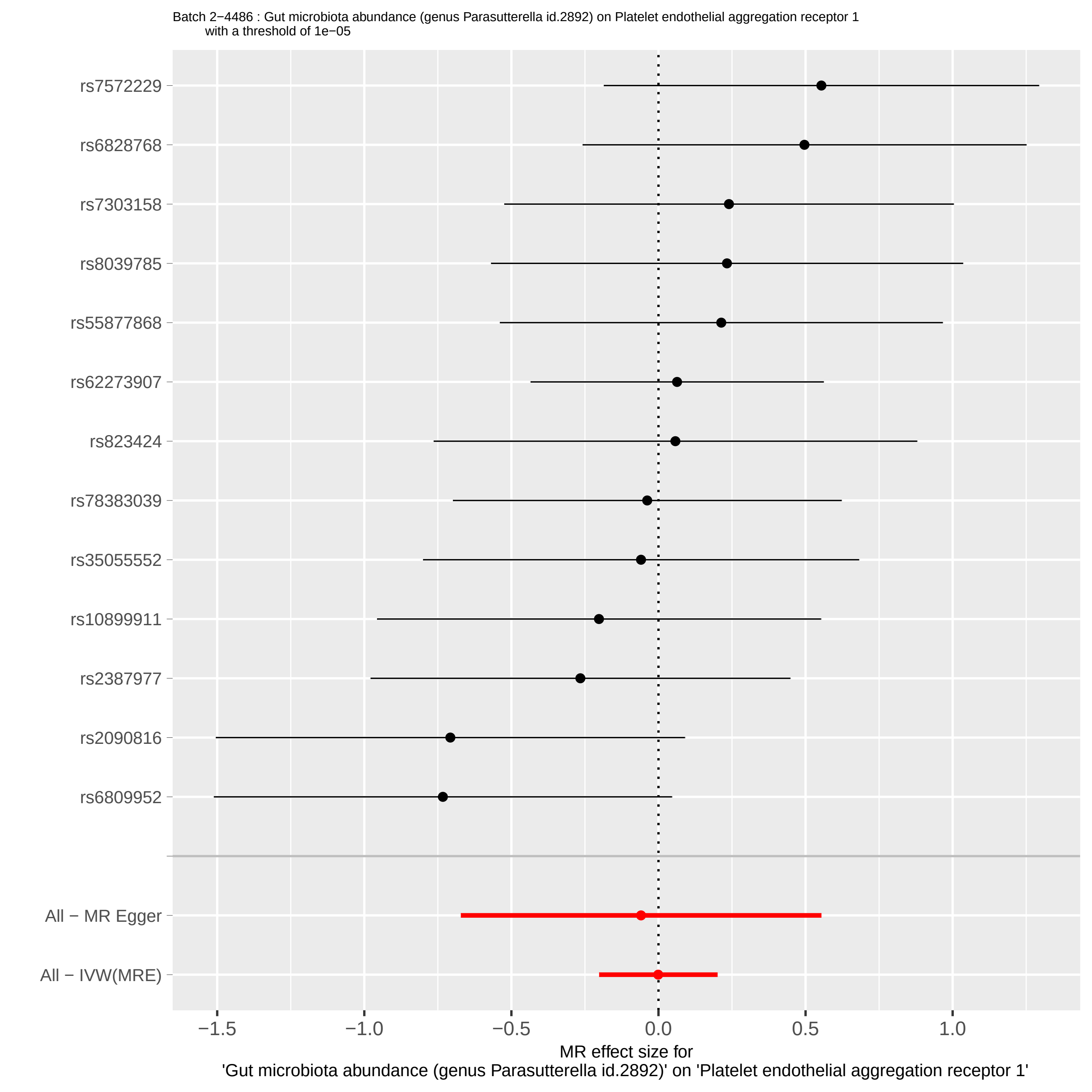

Batch 2−4486 : Gut microbiota abundance (genus Parasutterella id.2892) on Platelet endothelial aggregation receptor 1
with a threshold of 1e−05
rs7572229
rs6828768
rs7303158
rs8039785
rs55877868
rs62273907
rs823424
rs78383039
rs35055552
rs10899911
rs2387977
rs2090816
rs6809952
All − MR Egger
All − IVW(MRE)
−1.5
−1.0
−0.5
0.0
0.5
1.0
MR effect size for
'Gut microbiota abundance (genus Parasutterella id.2892)' on 'Platelet endothelial aggregation receptor 1'

Batch 2−4487 : Gut microbiota abundance (genus Catenibacterium id.2153) on Platelet endothelial aggregation receptor 1
with a threshold of 1e−05

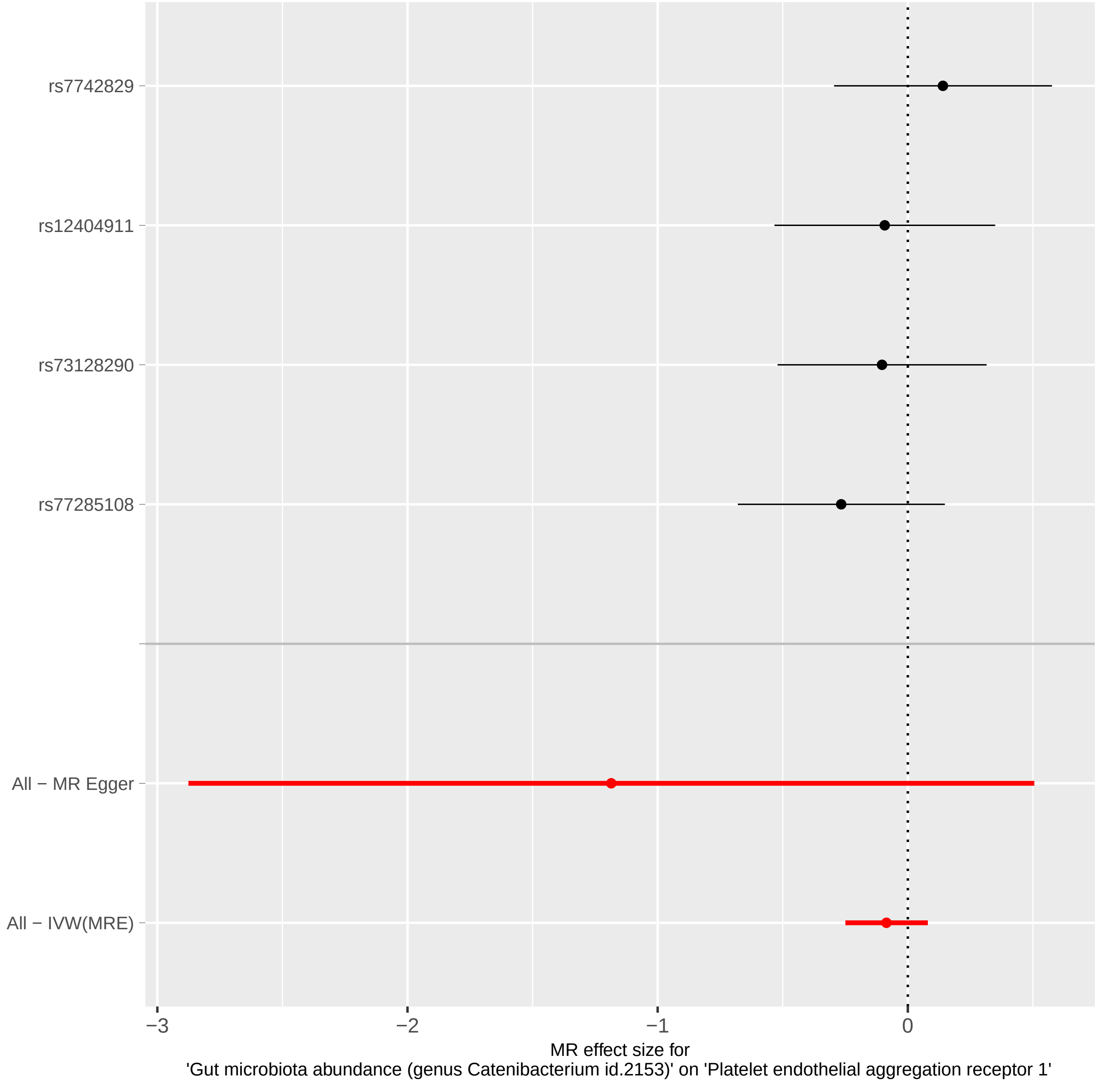

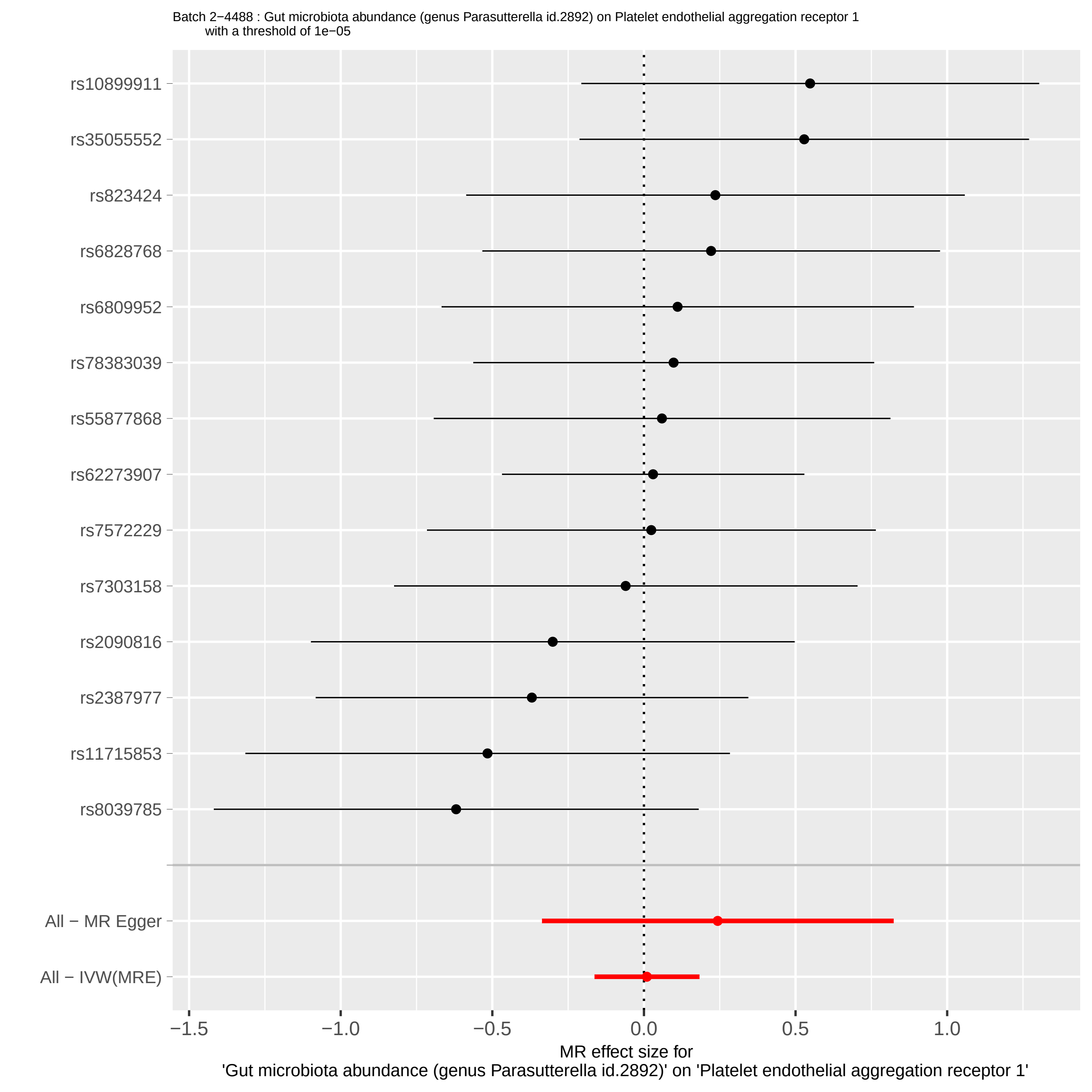

Batch 2−4488 : Gut microbiota abundance (genus Parasutterella id.2892) on Platelet endothelial aggregation receptor 1
with a threshold of 1e−05
rs10899911
rs35055552
rs823424
rs6828768
rs6809952
rs78383039
rs55877868
rs62273907
rs7572229
rs7303158
rs2090816
rs2387977
rs11715853
rs8039785
All − MR Egger
All − IVW(MRE)
−1.5
−1.0
−0.5
0.0
0.5
1.0
MR effect size for
'Gut microbiota abundance (genus Parasutterella id.2892)' on 'Platelet endothelial aggregation receptor 1'

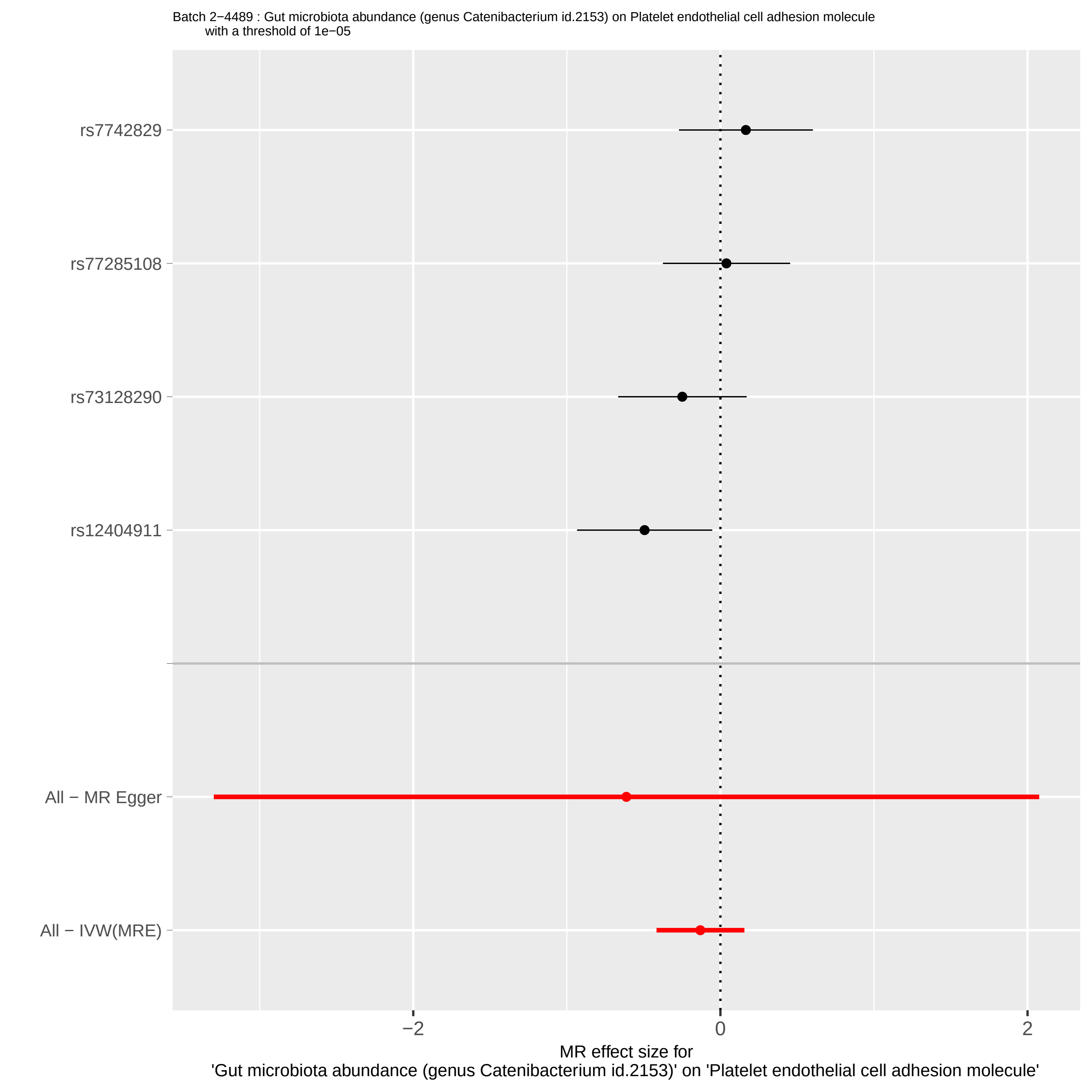

Batch 2−4489 : Gut microbiota abundance (genus Catenibacterium id.2153) on Platelet endothelial cell adhesion molecule
with a threshold of 1e−05
rs7742829
rs77285108
rs73128290
rs12404911
All − MR Egger
All − IVW(MRE)
−2
0
2
MR effect size for
'Gut microbiota abundance (genus Catenibacterium id.2153)' on 'Platelet endothelial cell adhesion molecule'

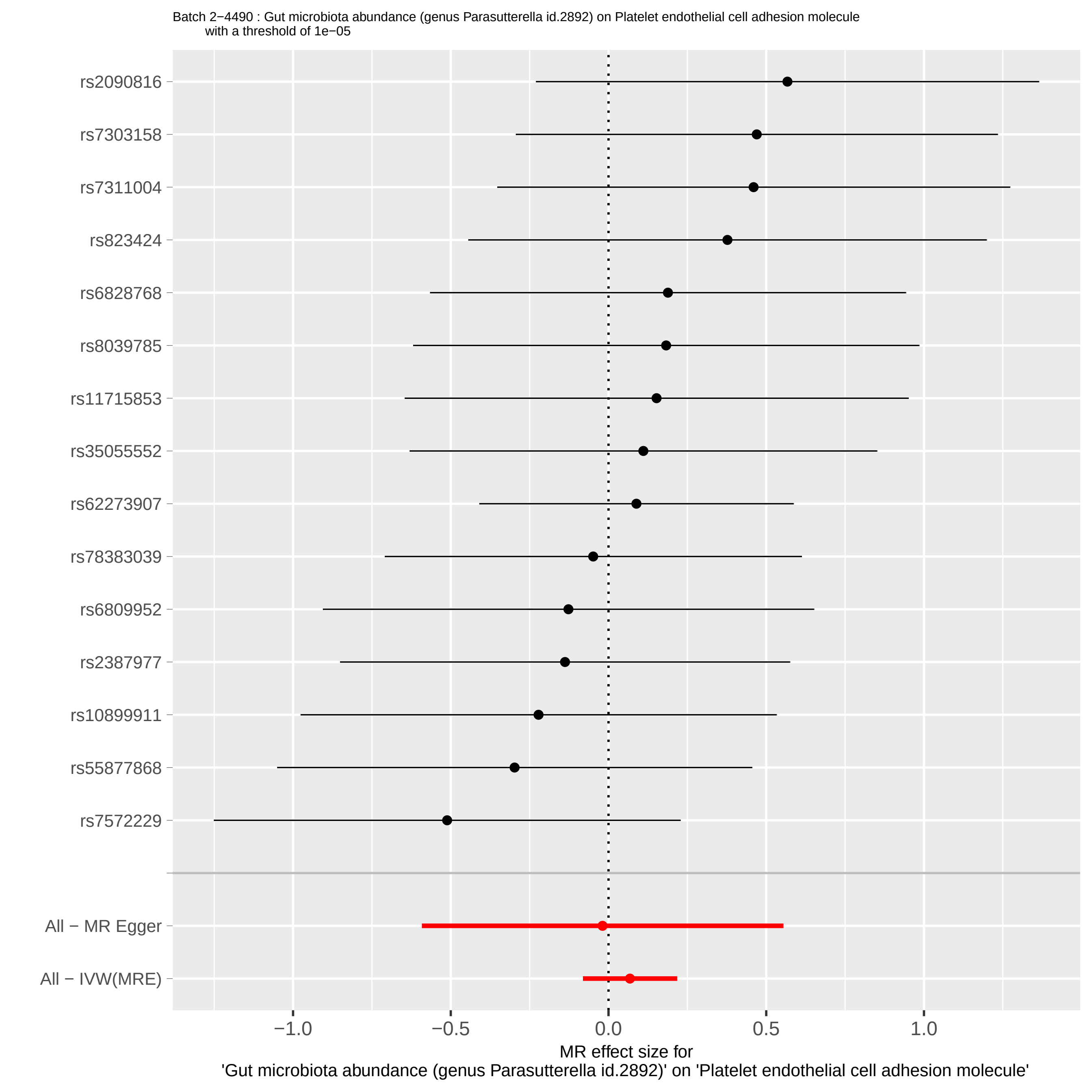

Batch 2−4490 : Gut microbiota abundance (genus Parasutterella id.2892) on Platelet endothelial cell adhesion molecule
with a threshold of 1e−05
rs2090816
rs7303158
rs7311004
rs823424
rs6828768
rs8039785
rs11715853
rs35055552
rs62273907
rs78383039
rs6809952
rs2387977
rs10899911
rs55877868
rs7572229
All − MR Egger
All − IVW(MRE)
−1.0
−0.5
0.0
0.5
1.0
MR effect size for
'Gut microbiota abundance (genus Parasutterella id.2892)' on 'Platelet endothelial cell adhesion molecule'

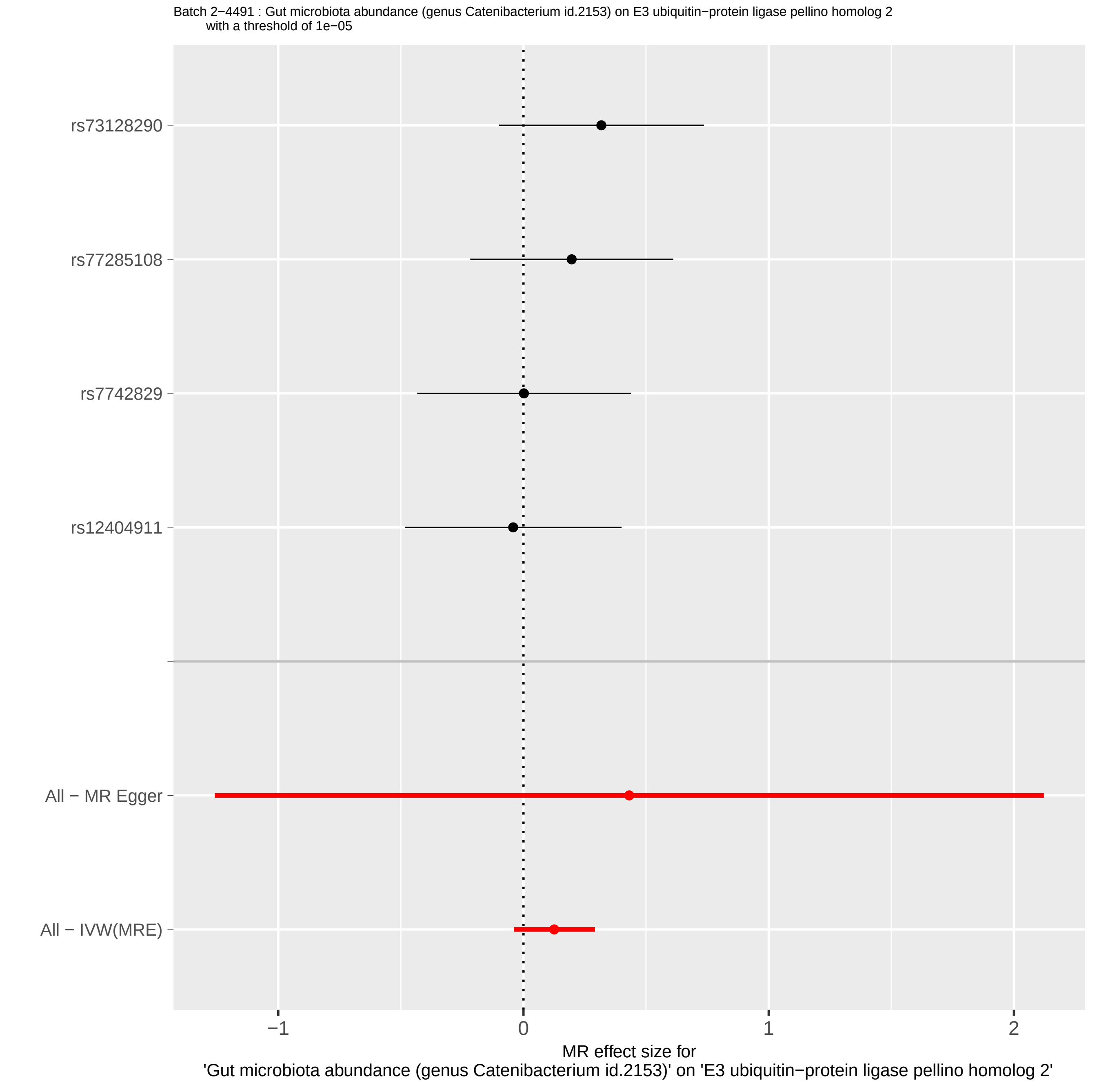
Batch 2−4491 : Gut microbiota abundance (genus Catenibacterium id.2153) on E3 ubiquitin−protein ligase pellino homolog 2
with a threshold of 1e−05
rs73128290
rs77285108
rs7742829
rs12404911
All − MR Egger
All − IVW(MRE)
−1
0
1
2
MR effect size for
'Gut microbiota abundance (genus Catenibacterium id.2153)' on 'E3 ubiquitin−protein ligase pellino homolog 2'

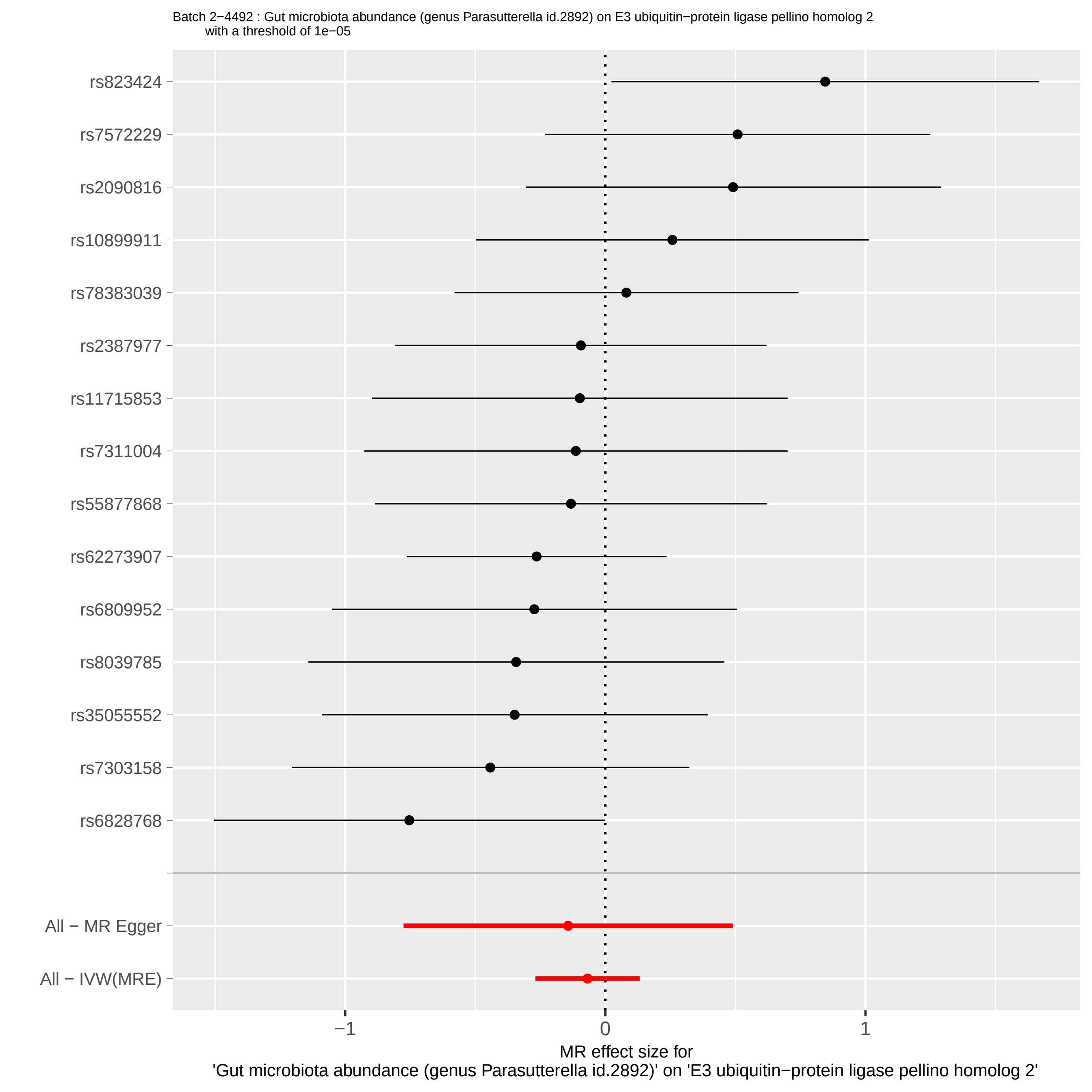

Batch 2−4492 : Gut microbiota abundance (genus Parasutterella id.2892) on E3 ubiquitin−protein ligase pellino homolog 2
with a threshold of 1e−05
rs823424
rs7572229
rs2090816
rs10899911
rs78383039
rs2387977
rs11715853
rs7311004
rs55877868
rs62273907
rs6809952
rs8039785
rs35055552
rs7303158
rs6828768
All − MR Egger
All − IVW(MRE)
−1
0
1
MR effect size for
'Gut microbiota abundance (genus Parasutterella id.2892)' on 'E3 ubiquitin−protein ligase pellino homolog 2'

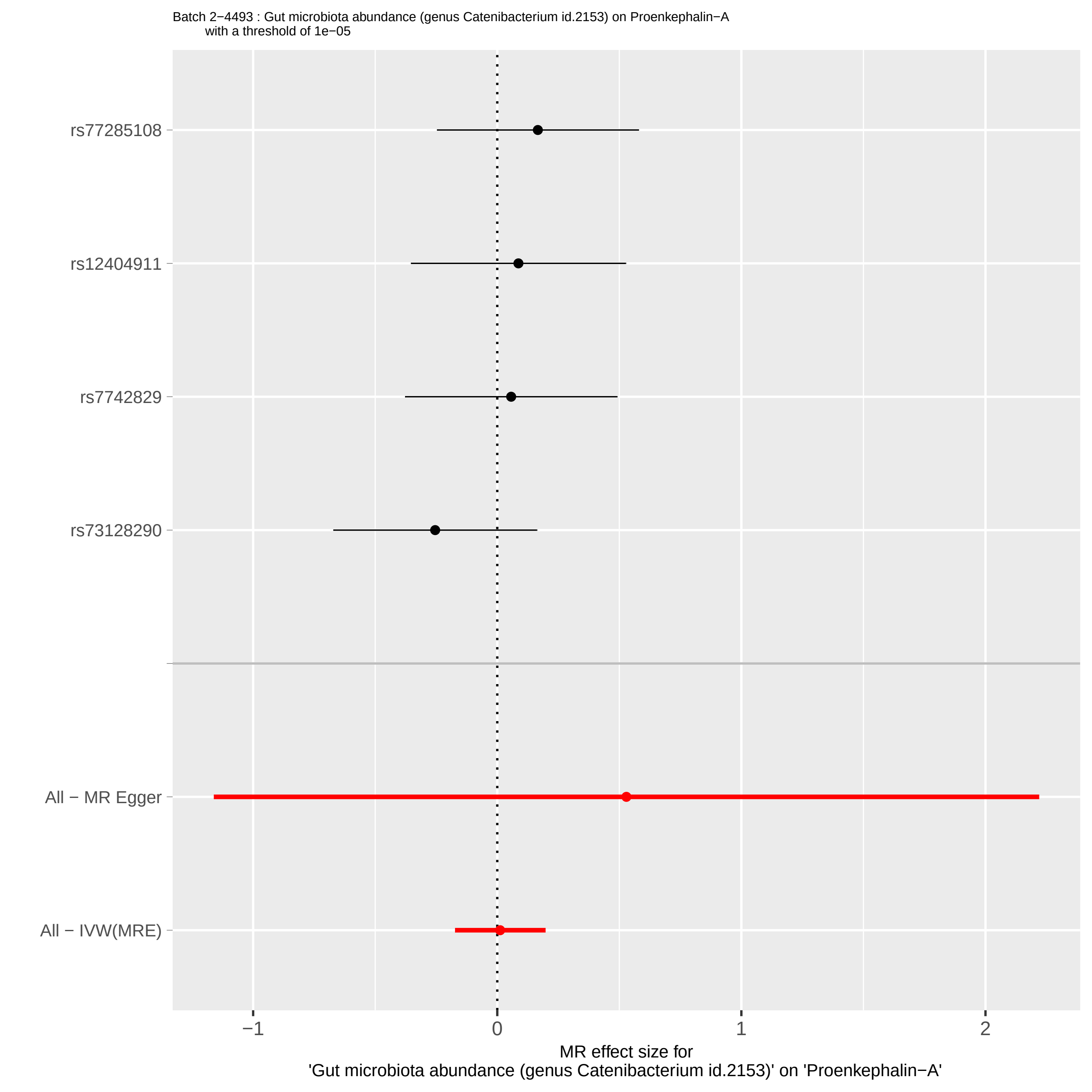

Batch 2−4493 : Gut microbiota abundance (genus Catenibacterium id.2153) on Proenkephalin−A
with a threshold of 1e−05
rs77285108
rs12404911
rs7742829
rs73128290
All − MR Egger
All − IVW(MRE)
−1
0
1
2
MR effect size for
'Gut microbiota abundance (genus Catenibacterium id.2153)' on 'Proenkephalin−A'

Batch 2−4494 : Gut microbiota abundance (genus Parasutterella id.2892) on Proenkephalin−A
with a threshold of 1e−05

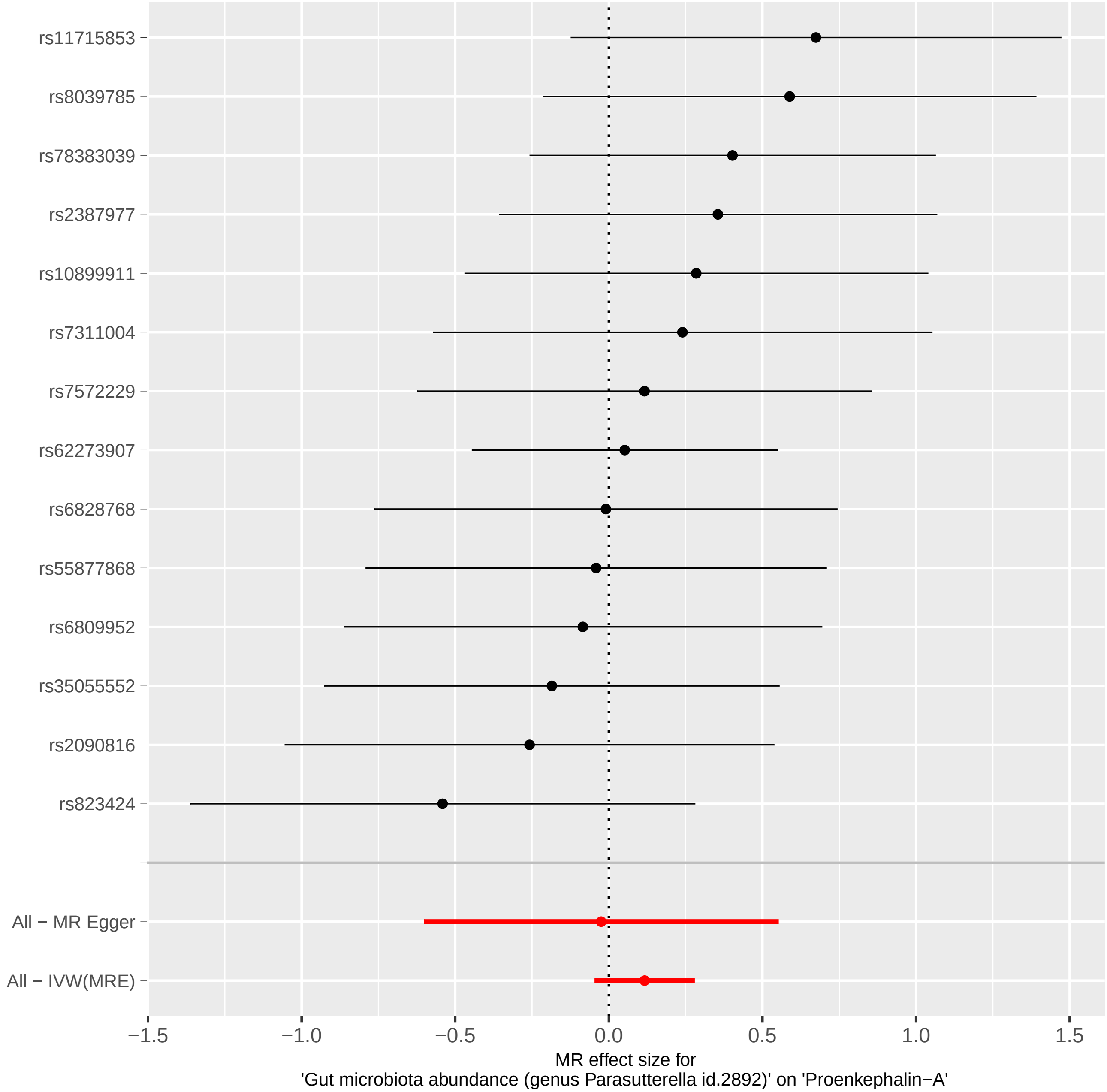

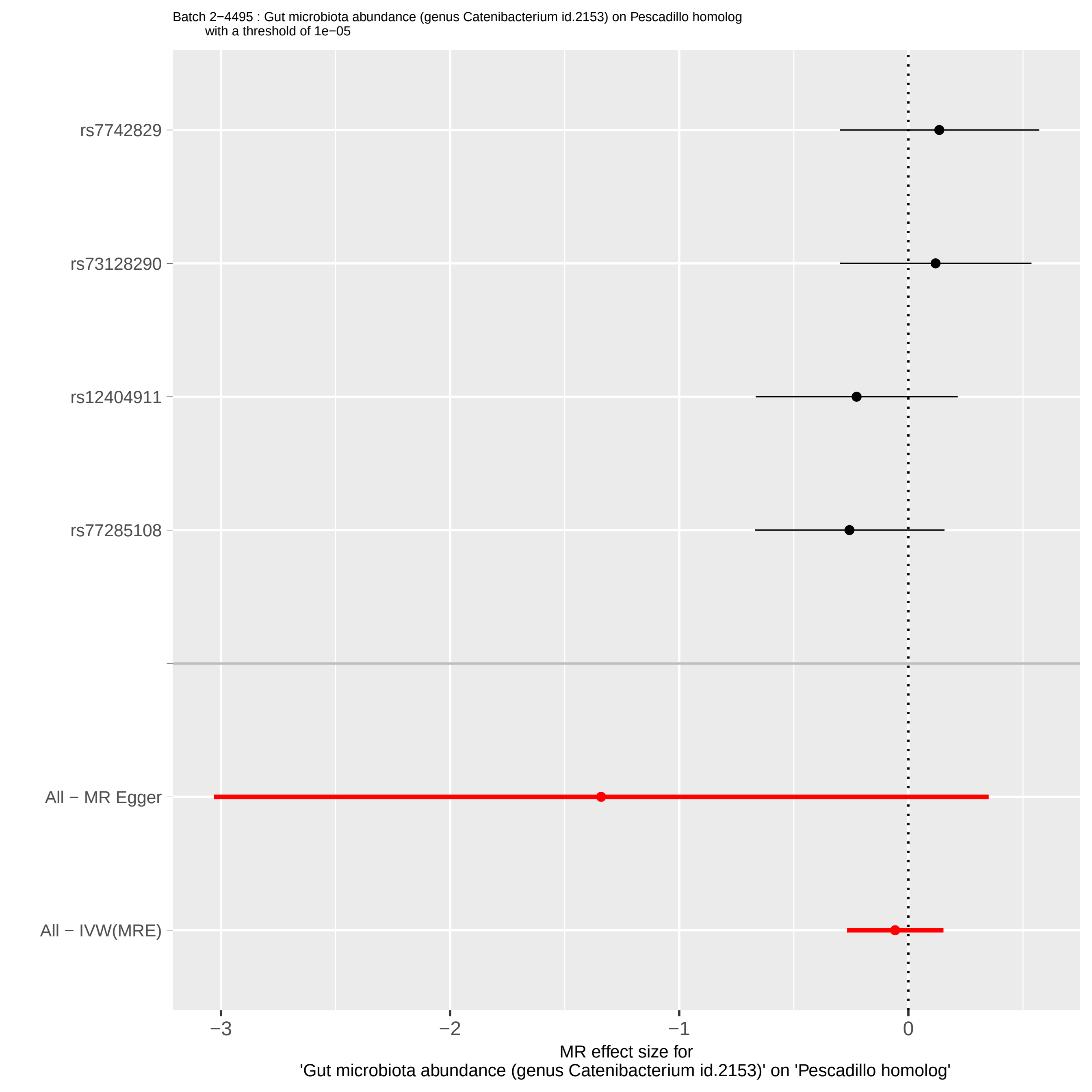

Batch 2−4495 : Gut microbiota abundance (genus Catenibacterium id.2153) on Pescadillo homolog
with a threshold of 1e−05
rs7742829
rs73128290
rs12404911
rs77285108
All − MR Egger
All − IVW(MRE)
−3
−2
−1
0
MR effect size for
'Gut microbiota abundance (genus Catenibacterium id.2153)' on 'Pescadillo homolog'

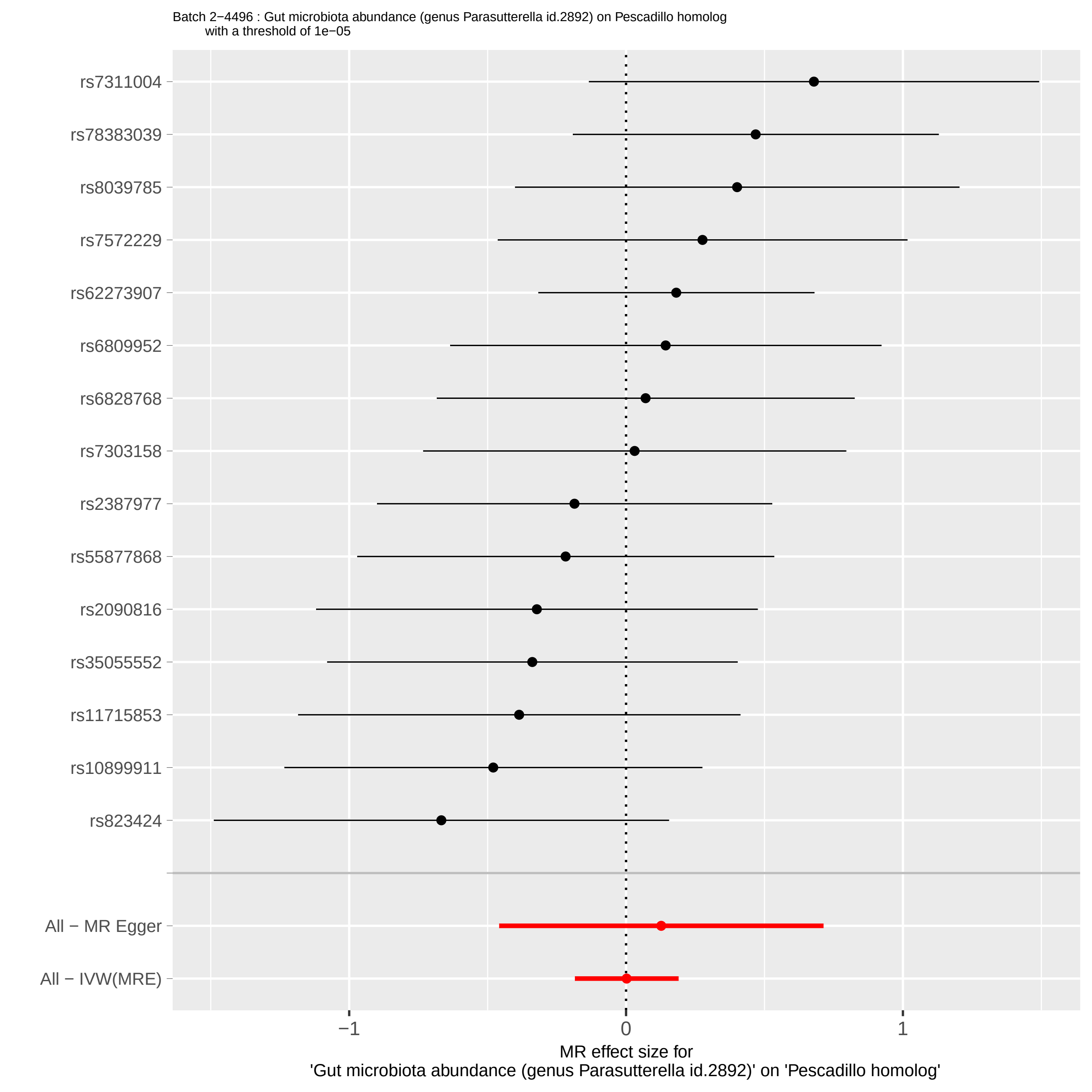

Batch 2−4496 : Gut microbiota abundance (genus Parasutterella id.2892) on Pescadillo homolog
with a threshold of 1e−05
rs7311004
rs78383039
rs8039785
rs7572229
rs62273907
rs6809952
rs6828768
rs7303158
rs2387977
rs55877868
rs2090816
rs35055552
rs11715853
rs10899911
rs823424
All − MR Egger
All − IVW(MRE)
−1
0
1
MR effect size for
'Gut microbiota abundance (genus Parasutterella id.2892)' on 'Pescadillo homolog'

Batch 2−4497 : Gut microbiota abundance (genus Catenibacterium id.2153) on Peroxisomal membrane protein PEX14
with a threshold of 1e−05

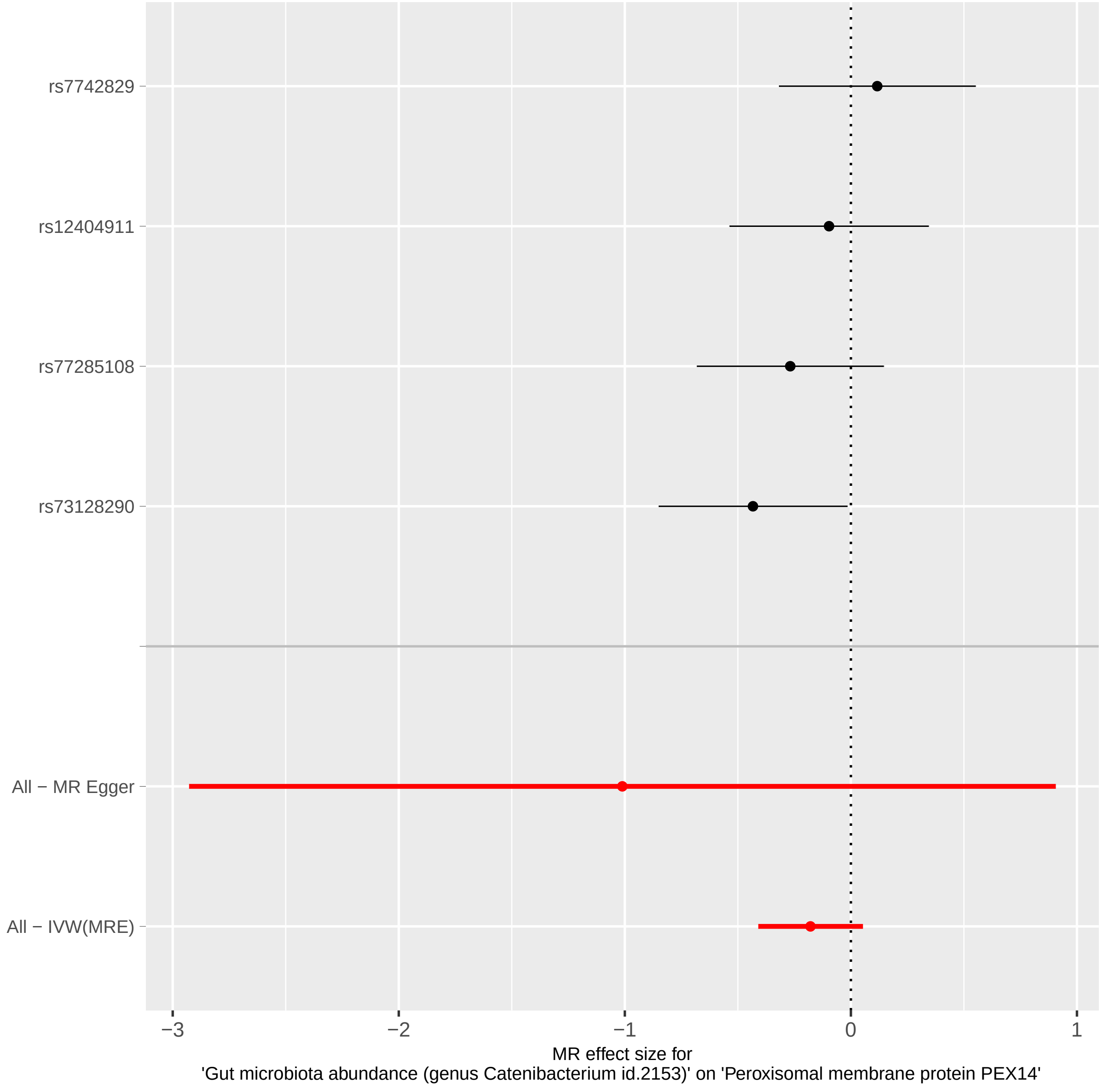

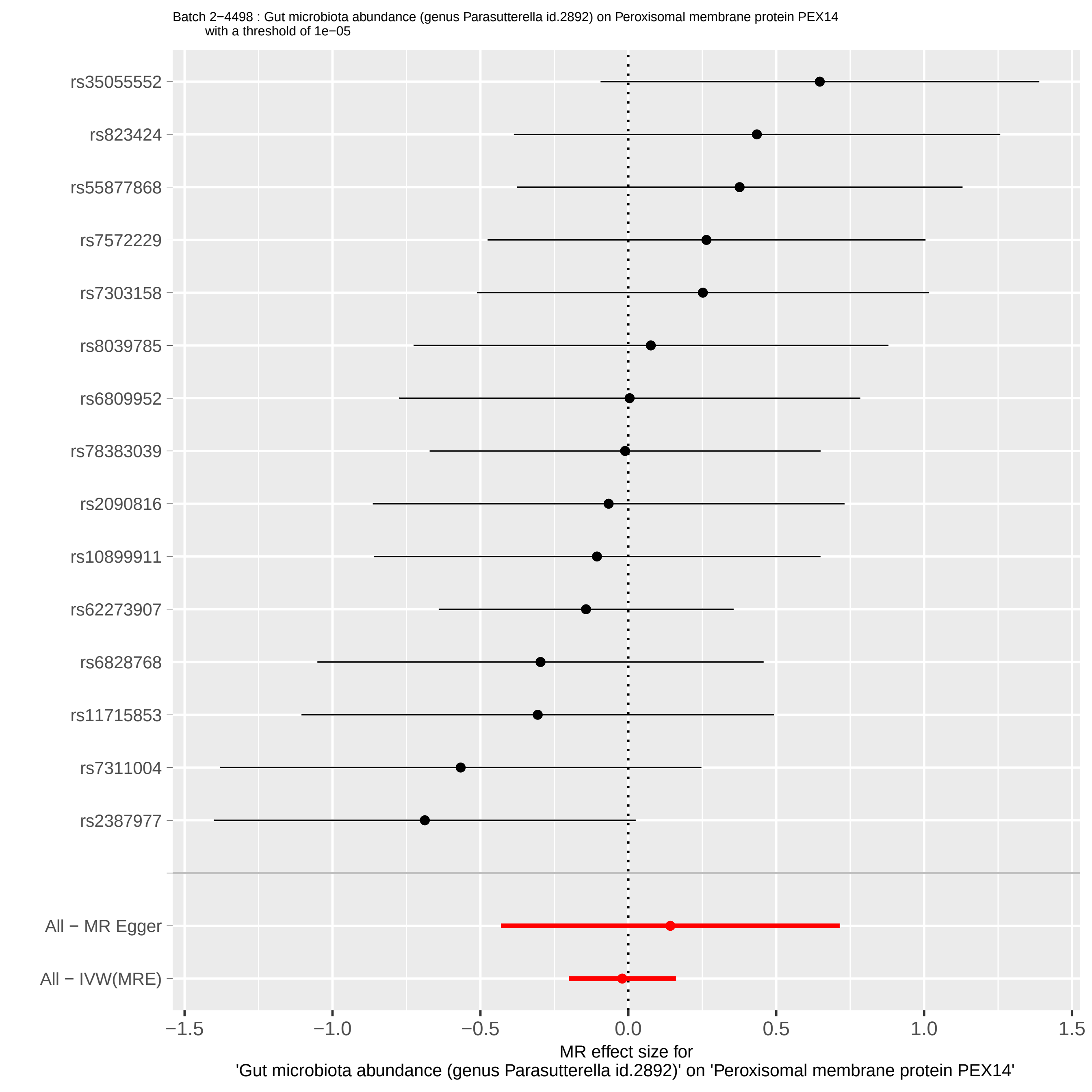

Batch 2−4498 : Gut microbiota abundance (genus Parasutterella id.2892) on Peroxisomal membrane protein PEX14
with a threshold of 1e−05
rs35055552
rs823424
rs55877868
rs7572229
rs7303158
rs8039785
rs6809952
rs78383039
rs2090816
rs10899911
rs62273907
rs6828768
rs11715853
rs7311004
rs2387977
All − MR Egger
All − IVW(MRE)
−1.5
−1.0
−0.5
0.0
0.5
1.0
1.5
MR effect size for
'Gut microbiota abundance (genus Parasutterella id.2892)' on 'Peroxisomal membrane protein PEX14'

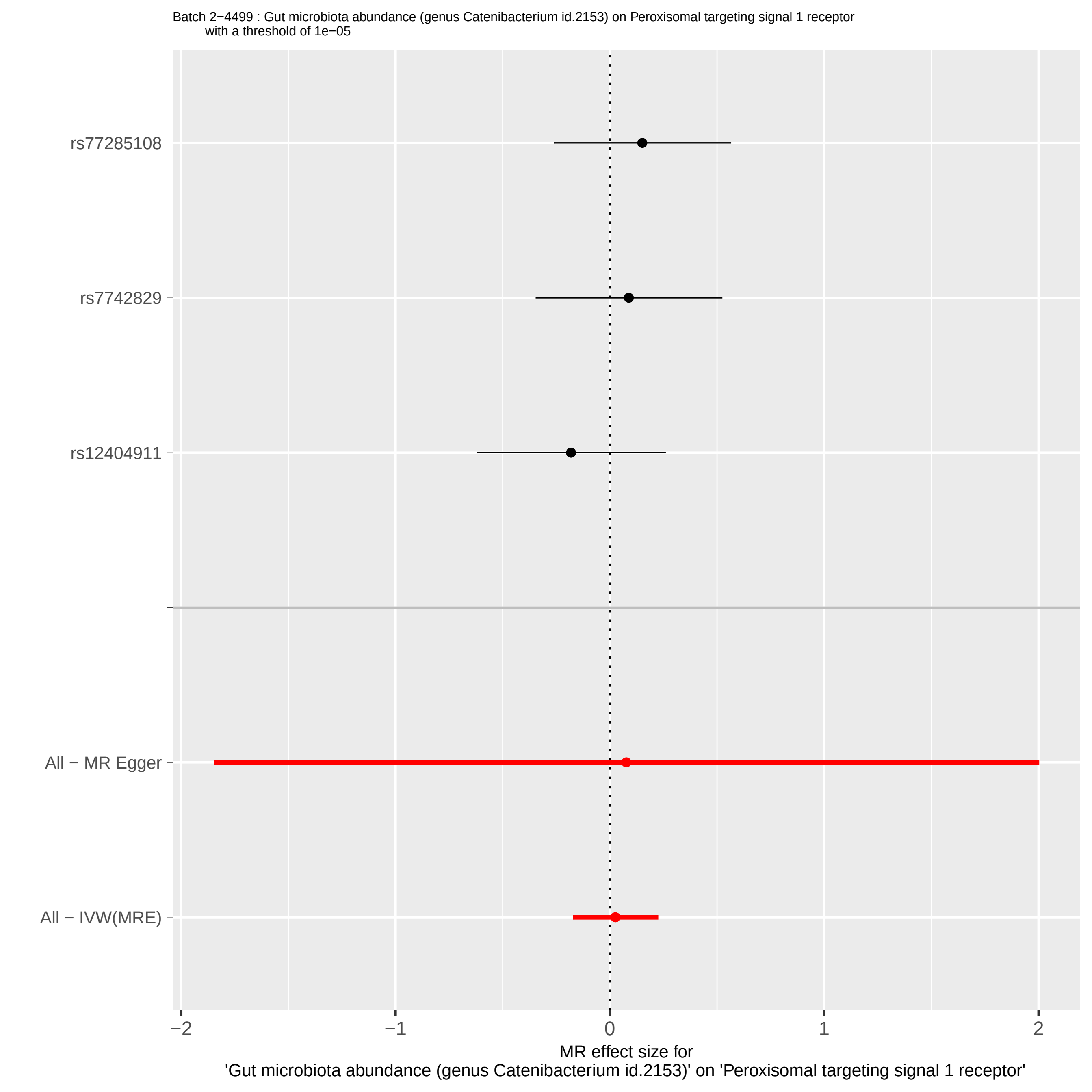
Batch 2−4499 : Gut microbiota abundance (genus Catenibacterium id.2153) on Peroxisomal targeting signal 1 receptor
with a threshold of 1e−05
rs77285108
rs7742829
rs12404911
All − MR Egger
All − IVW(MRE)
−2
−1
0
1
2
MR effect size for
'Gut microbiota abundance (genus Catenibacterium id.2153)' on 'Peroxisomal targeting signal 1 receptor'

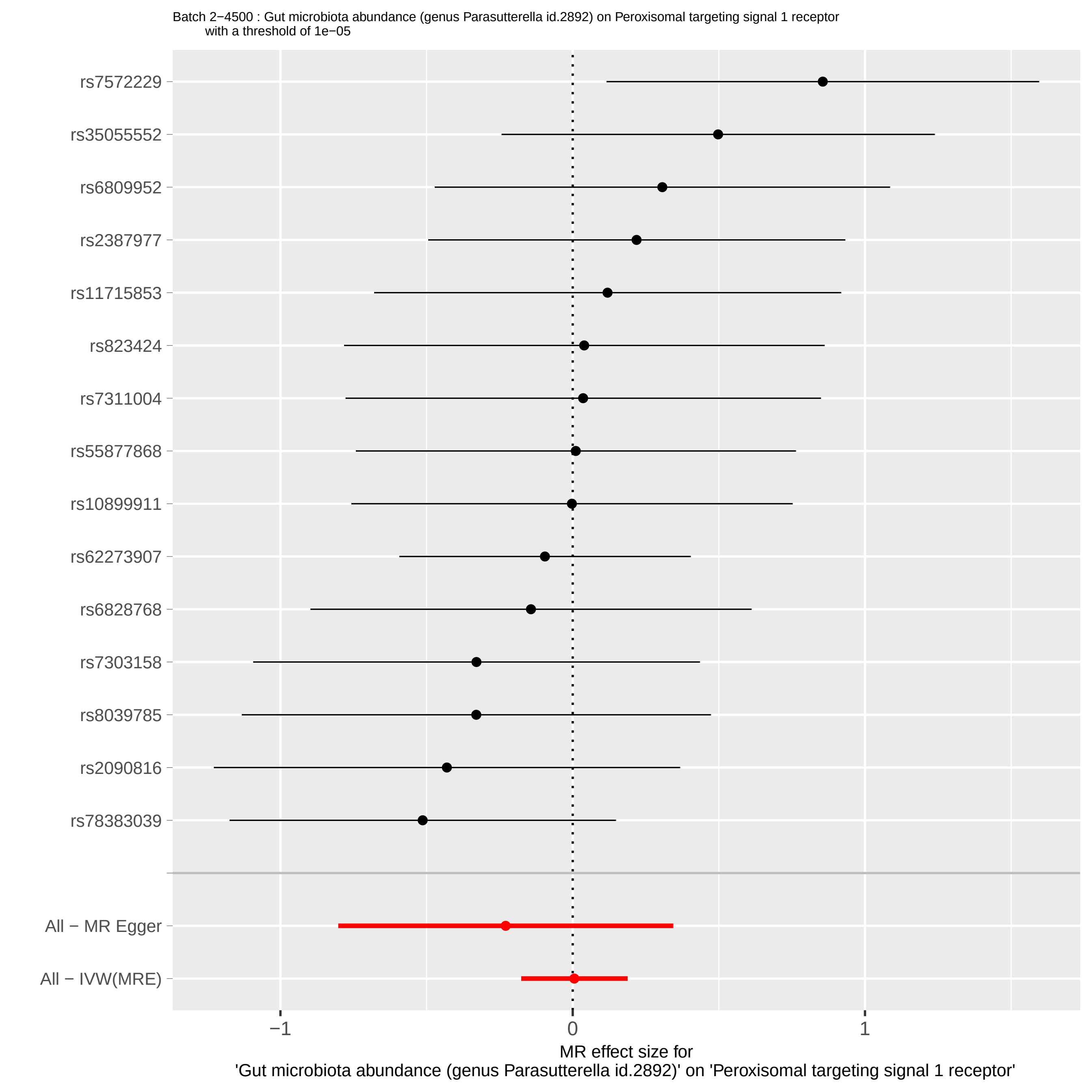

Batch 2−4500 : Gut microbiota abundance (genus Parasutterella id.2892) on Peroxisomal targeting signal 1 receptor
with a threshold of 1e−05
rs7572229
rs35055552
rs6809952
rs2387977
rs11715853
rs823424
rs7311004
rs55877868
rs10899911
rs62273907
rs6828768
rs7303158
rs8039785
rs2090816
rs78383039
All − MR Egger
All − IVW(MRE)
−1
0
1
MR effect size for
'Gut microbiota abundance (genus Parasutterella id.2892)' on 'Peroxisomal targeting signal 1 receptor'

Batch 2−4501 : Gut microbiota abundance (genus Catenibacterium id.2153) on Platelet factor 4 variant with a threshold of 1e−05

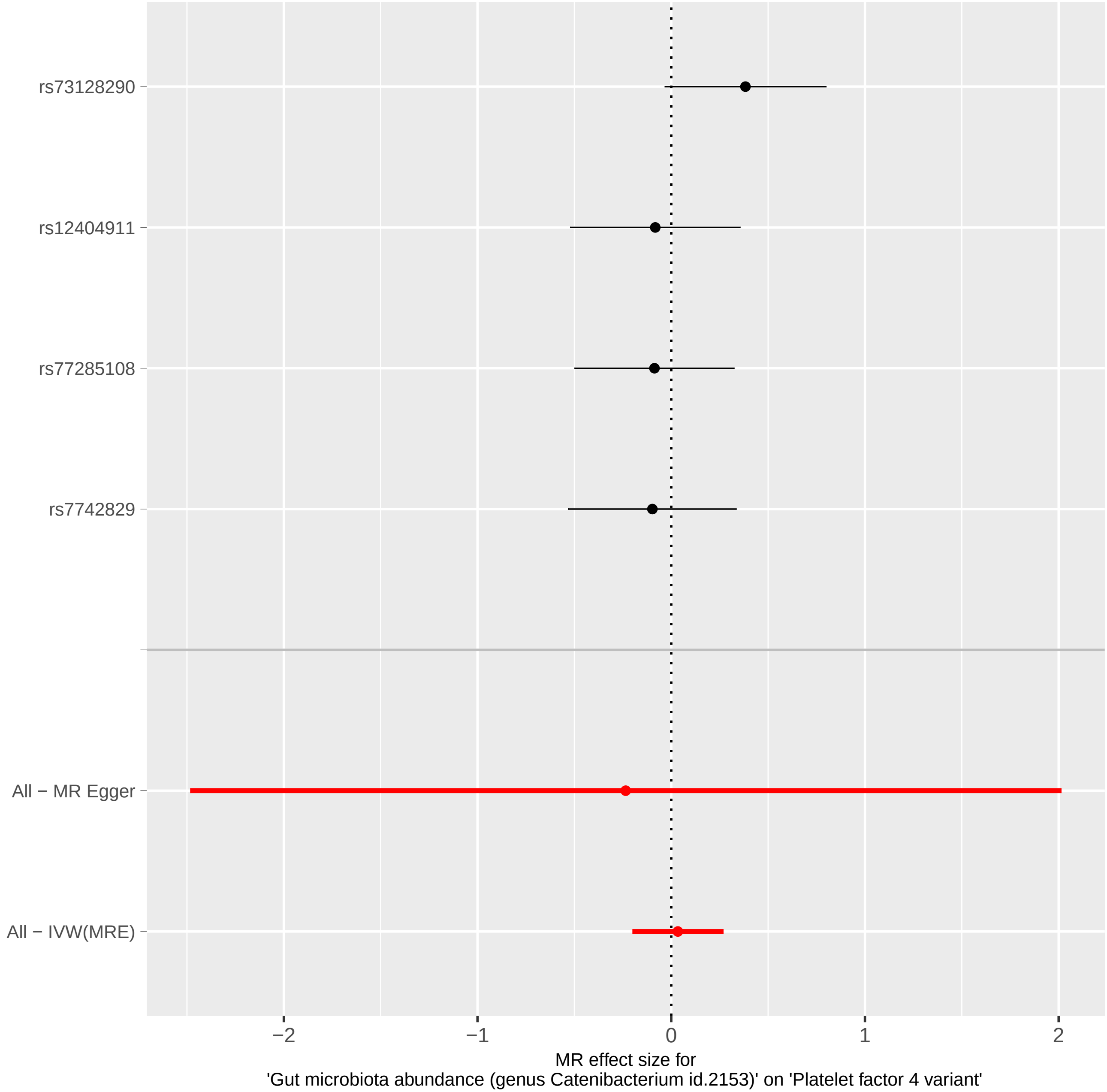

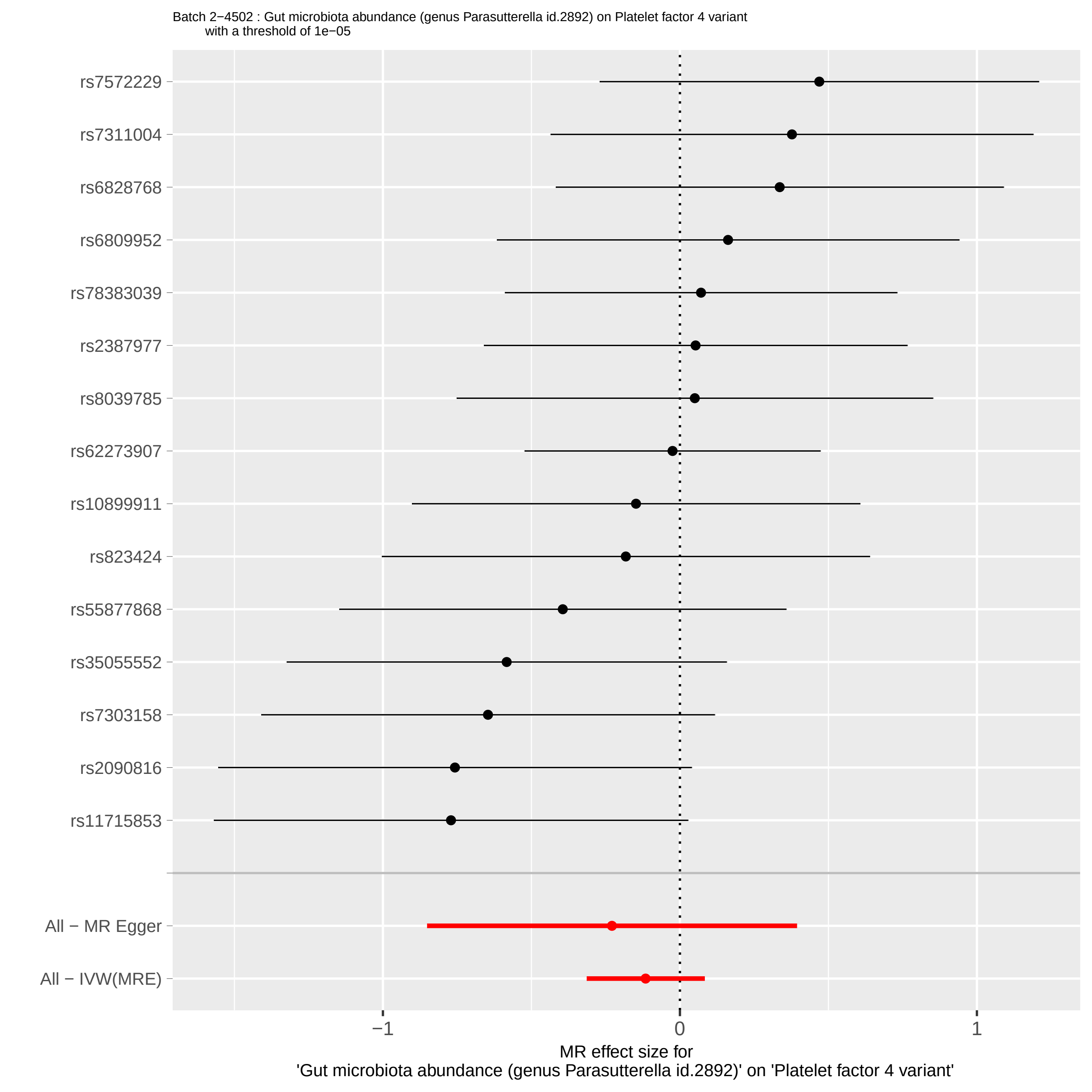
Batch 2−4502 : Gut microbiota abundance (genus Parasutterella id.2892) on Platelet factor 4 variant
with a threshold of 1e−05
rs7572229
rs7311004
rs6828768
rs6809952
rs78383039
rs2387977
rs8039785
rs62273907
rs10899911
rs823424
rs55877868
rs35055552
rs7303158
rs2090816
rs11715853
All − MR Egger
All − IVW(MRE)
−1
0
1
MR effect size for
'Gut microbiota abundance (genus Parasutterella id.2892)' on 'Platelet factor 4 variant'

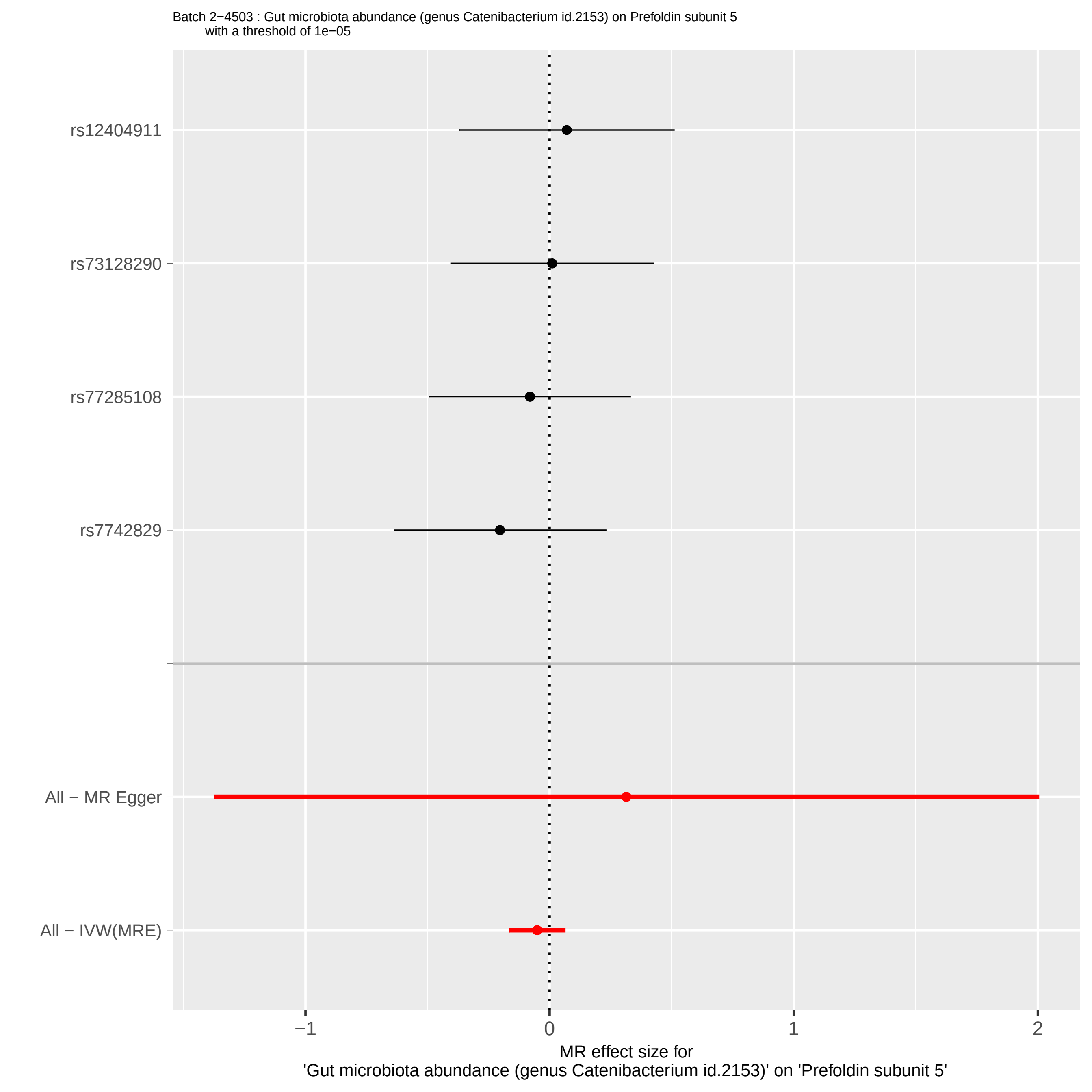

Batch 2−4503 : Gut microbiota abundance (genus Catenibacterium id.2153) on Prefoldin subunit 5
with a threshold of 1e−05
rs12404911
rs73128290
rs77285108
rs7742829
All − MR Egger
All − IVW(MRE)
−1
0
1
2
MR effect size for
'Gut microbiota abundance (genus Catenibacterium id.2153)' on 'Prefoldin subunit 5'

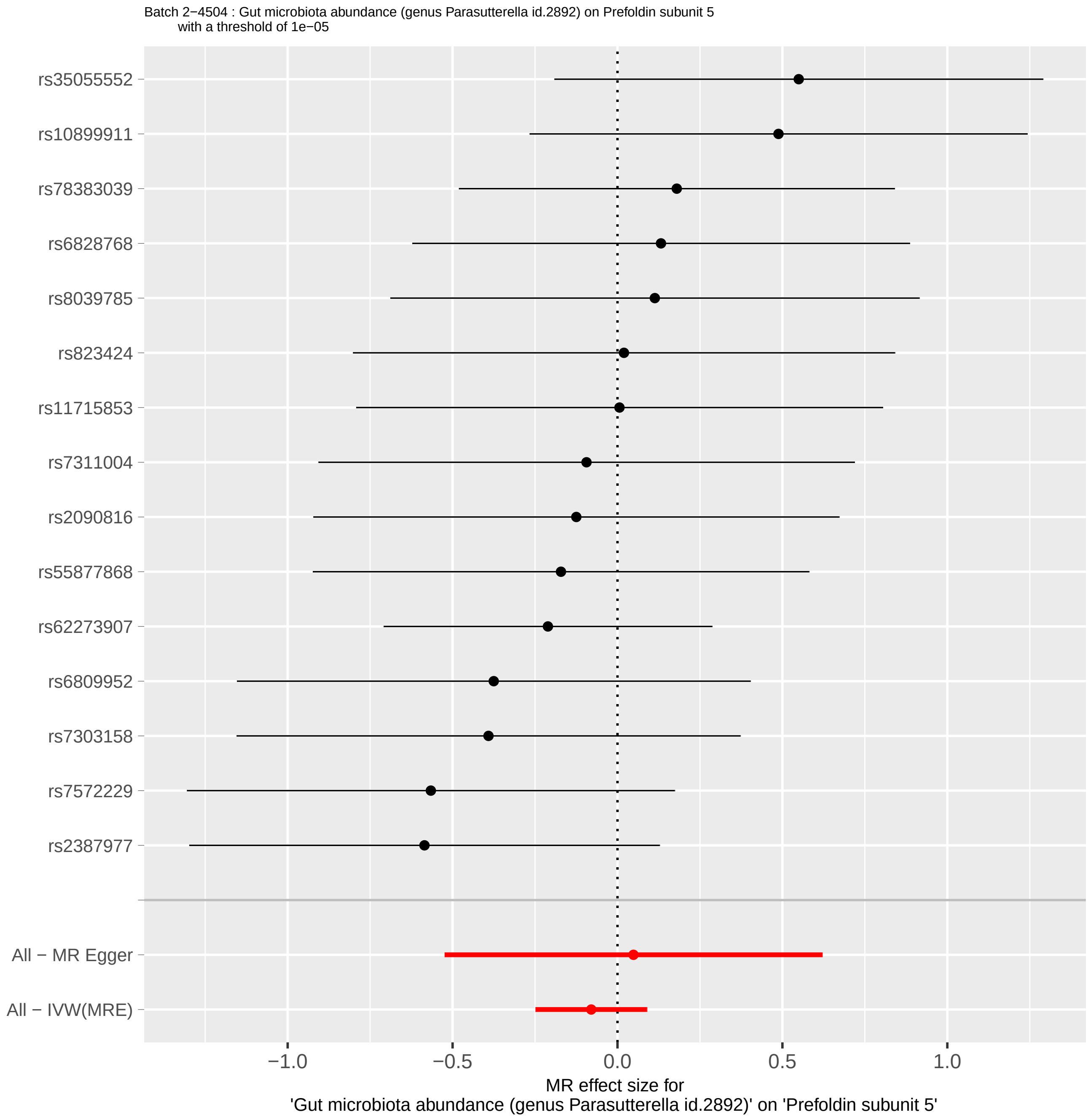

Batch 2−4504 : Gut microbiota abundance (genus Parasutterella id.2892) on Prefoldin subunit 5
with a threshold of 1e−05
rs35055552
rs10899911
rs78383039
rs6828768
rs8039785
rs823424
rs11715853
rs7311004
rs2090816
rs55877868
rs62273907
rs6809952
rs7303158
rs7572229
rs2387977
All − MR Egger
All − IVW(MRE)
−1.0
−0.5
0.0
0.5
1.0
MR effect size for
'Gut microbiota abundance (genus Parasutterella id.2892)' on 'Prefoldin subunit 5'

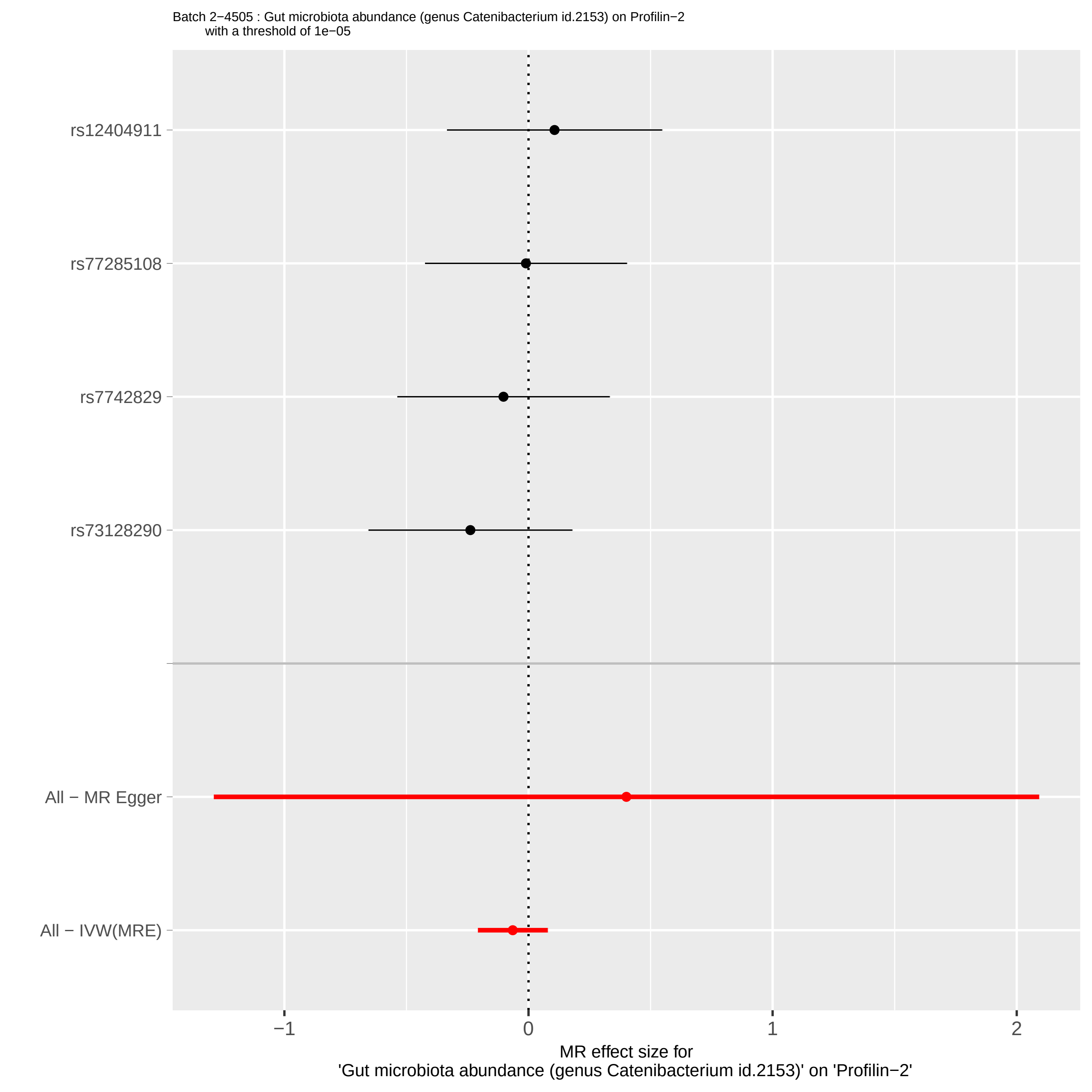
Batch 2−4505 : Gut microbiota abundance (genus Catenibacterium id.2153) on Profilin−2
with a threshold of 1e−05
rs12404911
rs77285108
rs7742829
rs73128290
All − MR Egger
All − IVW(MRE)
−1
0
1
2
MR effect size for
'Gut microbiota abundance (genus Catenibacterium id.2153)' on 'Profilin−2'

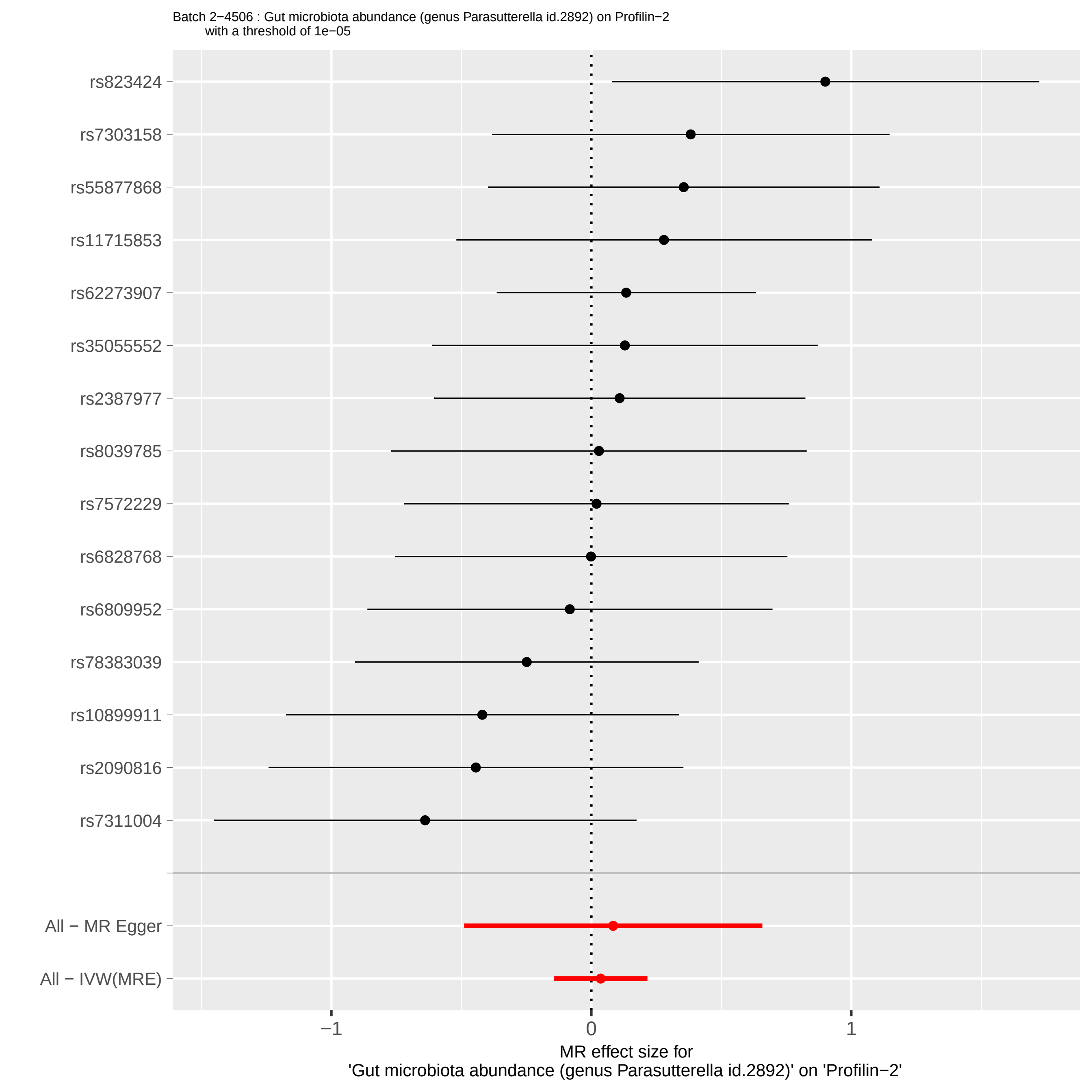
Batch 2−4506 : Gut microbiota abundance (genus Parasutterella id.2892) on Profilin−2
with a threshold of 1e−05
rs823424
rs7303158
rs55877868
rs11715853
rs62273907
rs35055552
rs2387977
rs8039785
rs7572229
rs6828768
rs6809952
rs78383039
rs10899911
rs2090816
rs7311004
All − MR Egger
All − IVW(MRE)
−1
0
1
MR effect size for
'Gut microbiota abundance (genus Parasutterella id.2892)' on 'Profilin−2'

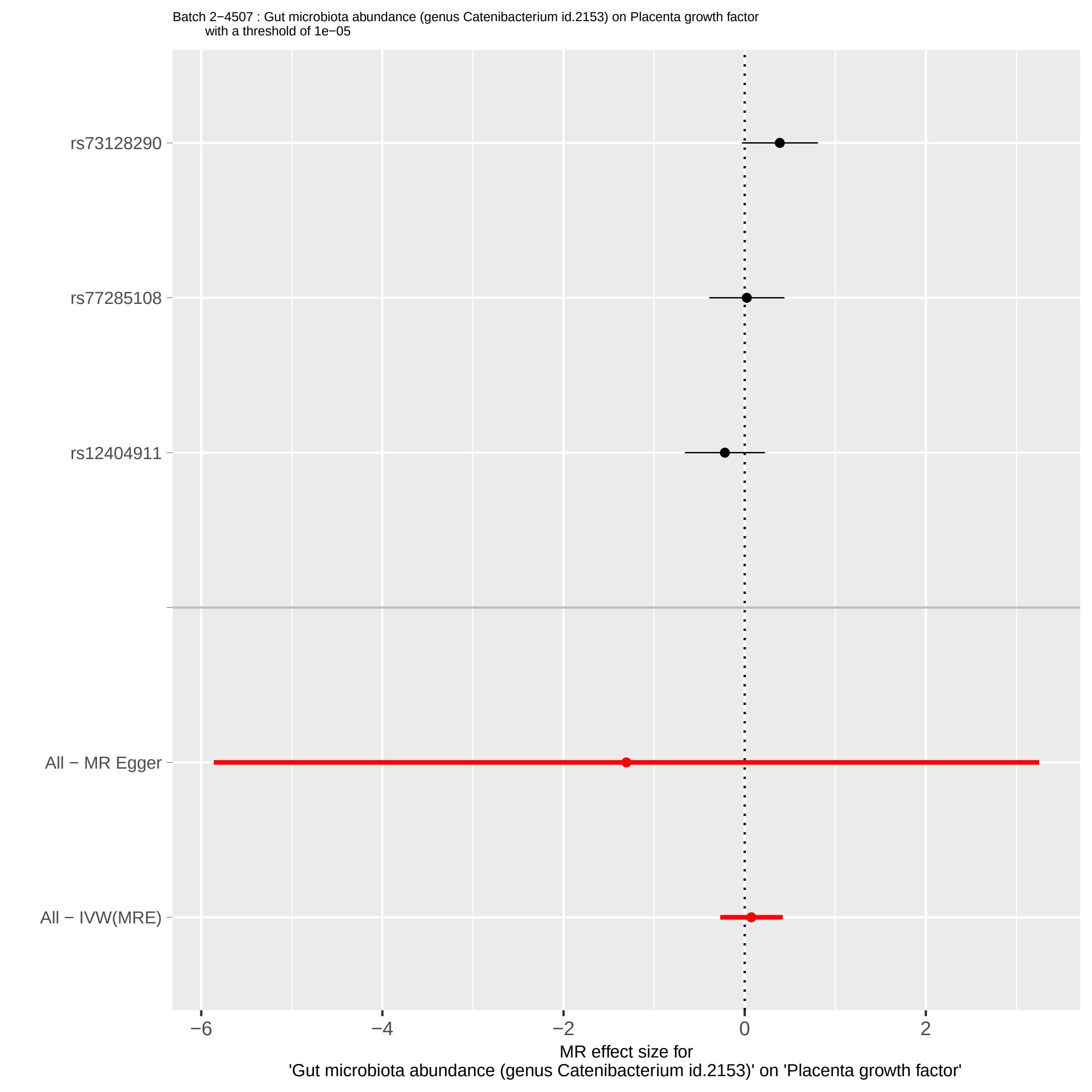
Batch 2−4507 : Gut microbiota abundance (genus Catenibacterium id.2153) on Placenta growth factor
with a threshold of 1e−05
rs73128290
rs77285108
rs12404911
All − MR Egger
All − IVW(MRE)
−6
−4
−2
0
2
MR effect size for
'Gut microbiota abundance (genus Catenibacterium id.2153)' on 'Placenta growth factor'

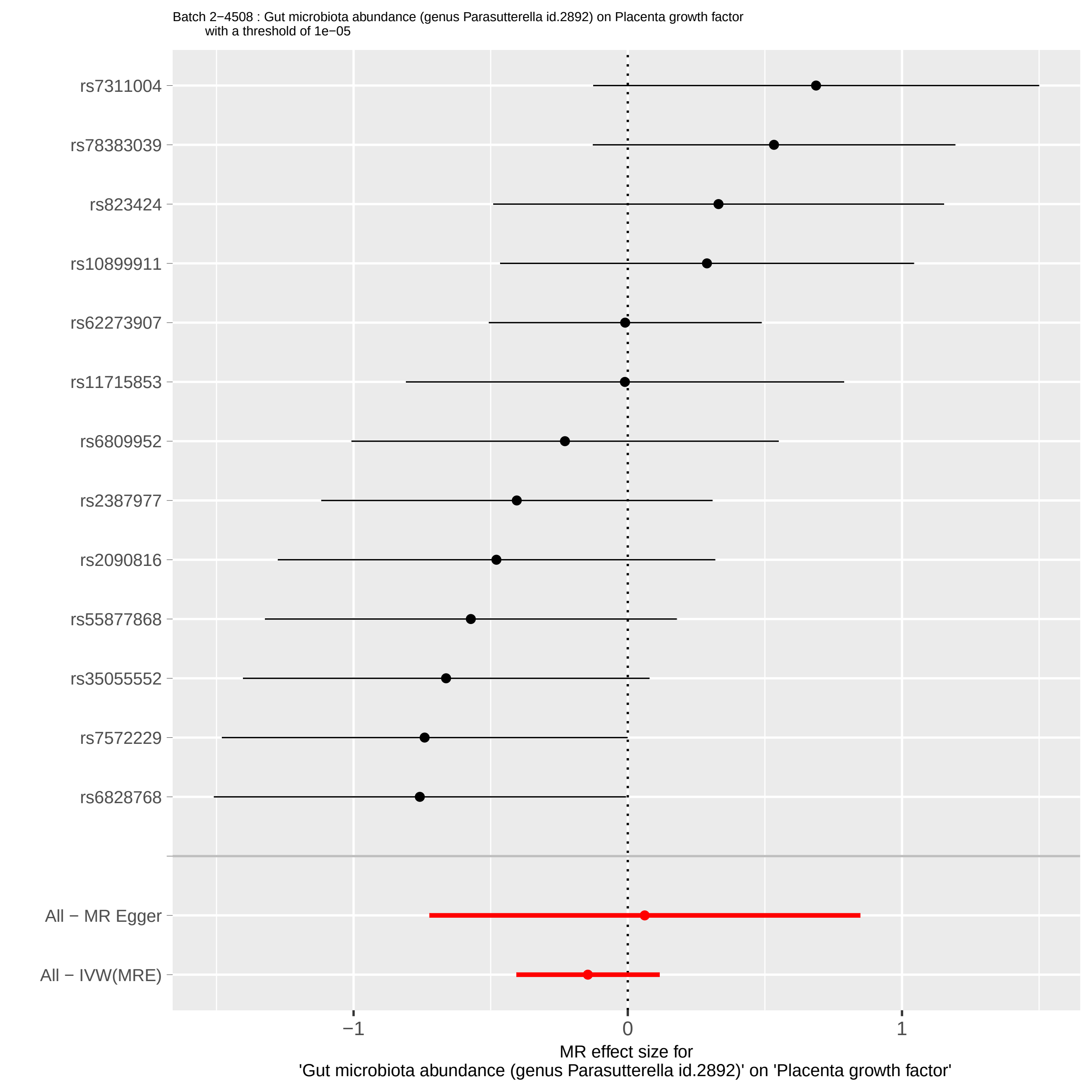

Batch 2−4508 : Gut microbiota abundance (genus Parasutterella id.2892) on Placenta growth factor
with a threshold of 1e−05
rs7311004
rs78383039
rs823424
rs10899911
rs62273907
rs11715853
rs6809952
rs2387977
rs2090816
rs55877868
rs35055552
rs7572229
rs6828768
All − MR Egger
All − IVW(MRE)
−1
0
1
MR effect size for
'Gut microbiota abundance (genus Parasutterella id.2892)' on 'Placenta growth factor'

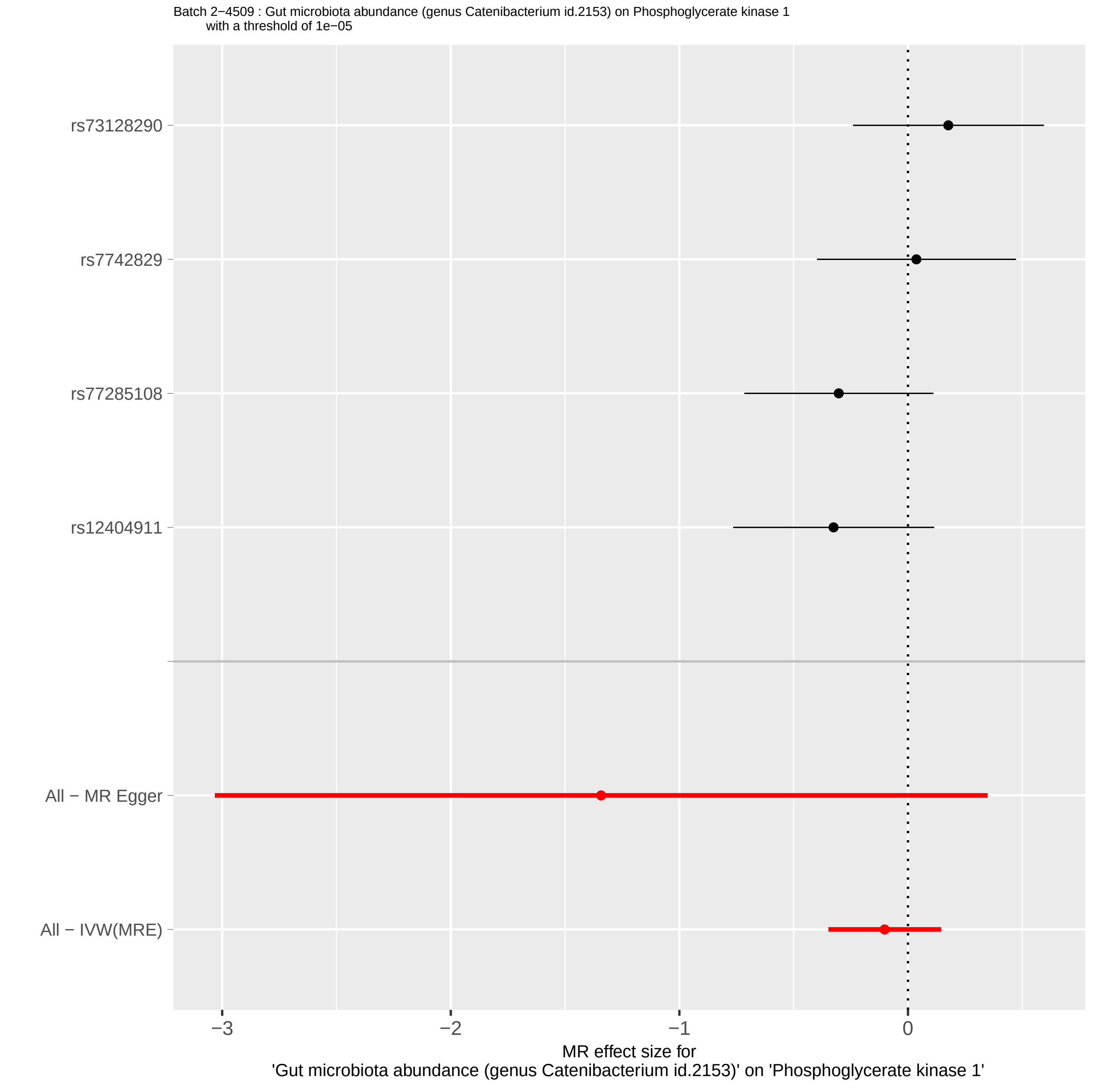
Batch 2−4509 : Gut microbiota abundance (genus Catenibacterium id.2153) on Phosphoglycerate kinase 1
with a threshold of 1e−05
rs73128290
rs7742829
rs77285108
rs12404911
All − MR Egger
All − IVW(MRE)
−3
−2
−1
0
MR effect size for
'Gut microbiota abundance (genus Catenibacterium id.2153)' on 'Phosphoglycerate kinase 1'

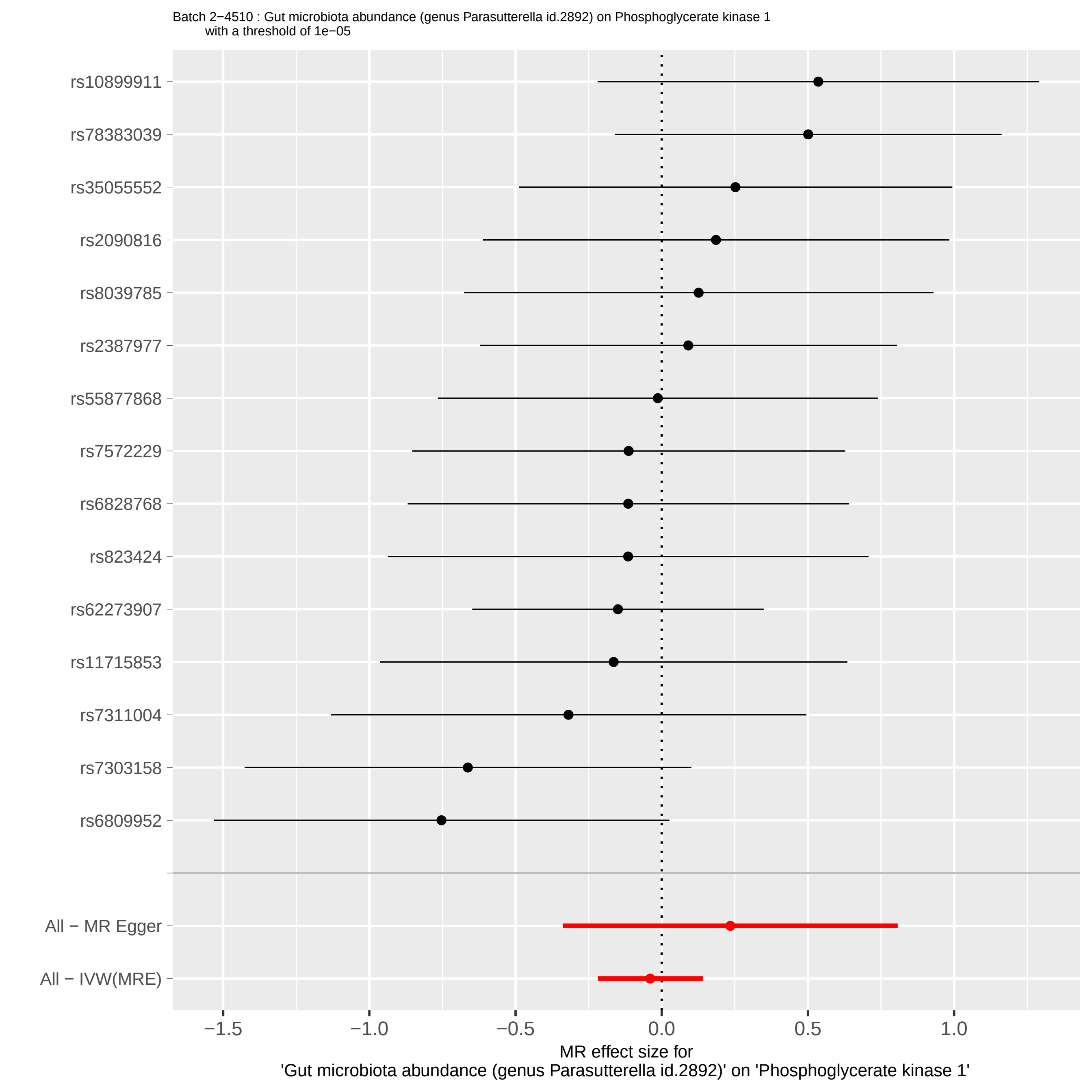

Batch 2−4510 : Gut microbiota abundance (genus Parasutterella id.2892) on Phosphoglycerate kinase 1
with a threshold of 1e−05
rs10899911
rs78383039
rs35055552
rs2090816
rs8039785
rs2387977
rs55877868
rs7572229
rs6828768
rs823424
rs62273907
rs11715853
rs7311004
rs7303158
rs6809952
All − MR Egger
All − IVW(MRE)
−1.5
−1.0
−0.5
0.0
0.5
1.0
MR effect size for
'Gut microbiota abundance (genus Parasutterella id.2892)' on 'Phosphoglycerate kinase 1'

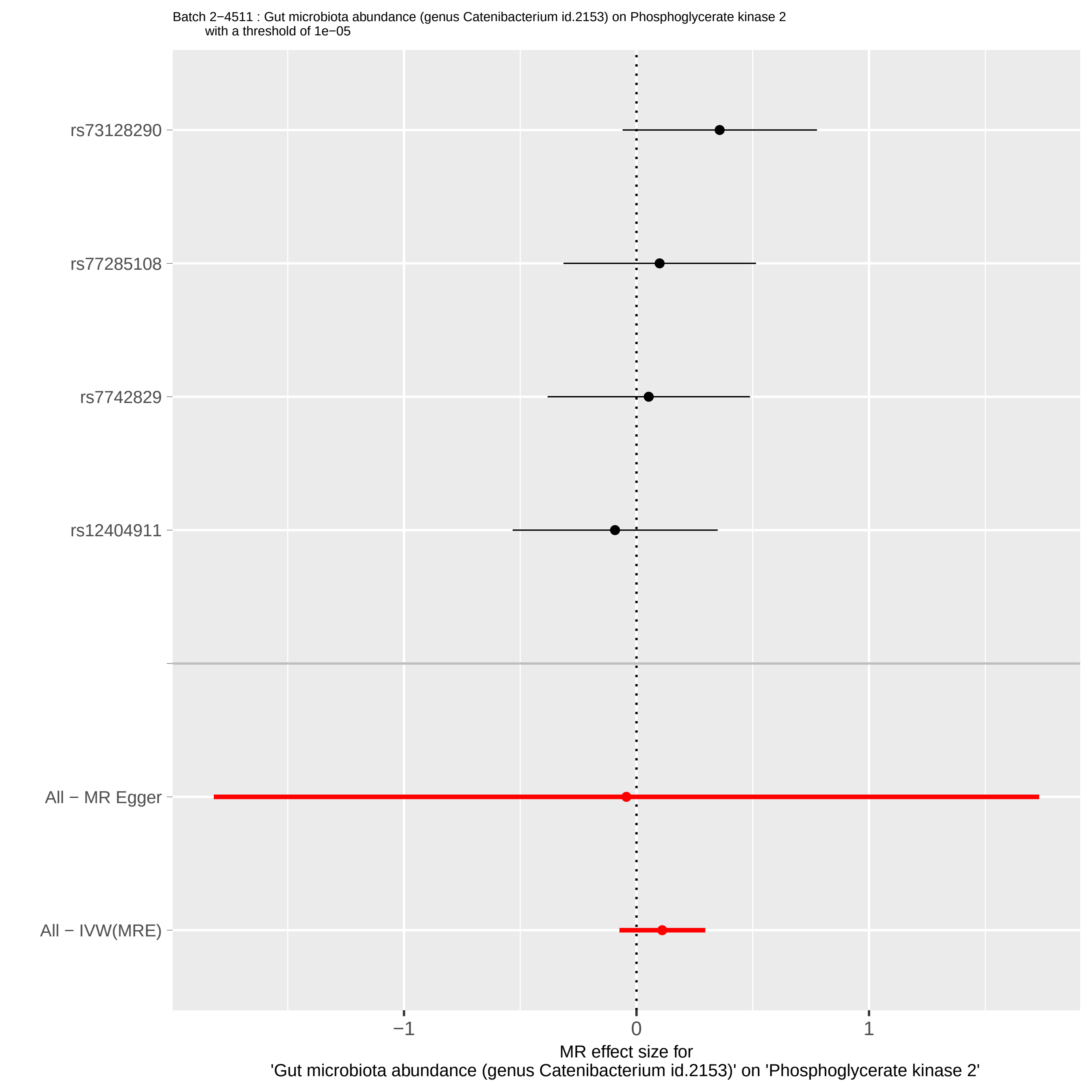

Batch 2−4511 : Gut microbiota abundance (genus Catenibacterium id.2153) on Phosphoglycerate kinase 2
with a threshold of 1e−05
rs73128290
rs77285108
rs7742829
rs12404911
All − MR Egger
All − IVW(MRE)
−1
0
1
MR effect size for
'Gut microbiota abundance (genus Catenibacterium id.2153)' on 'Phosphoglycerate kinase 2'

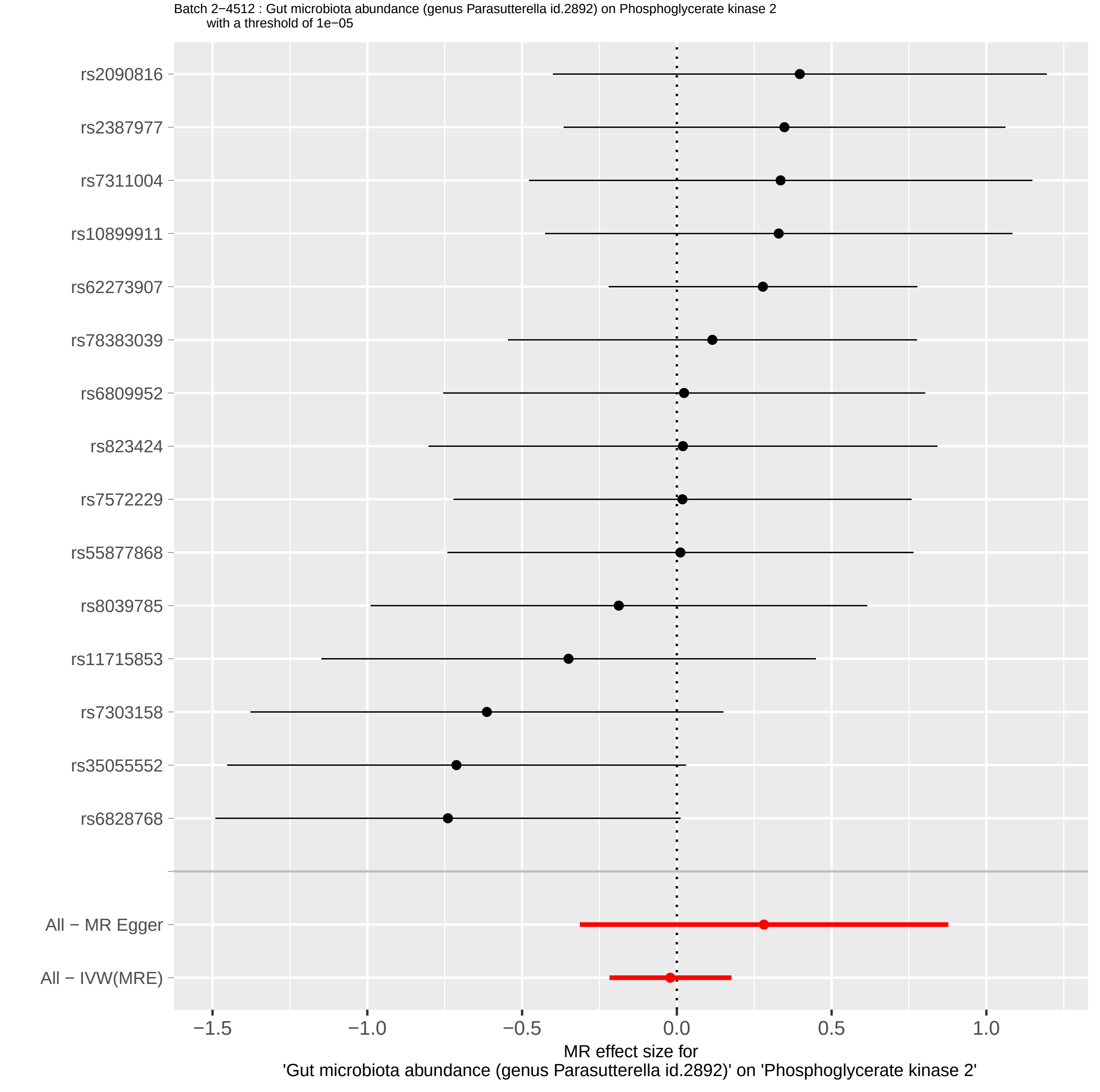

Batch 2−4512 : Gut microbiota abundance (genus Parasutterella id.2892) on Phosphoglycerate kinase 2
with a threshold of 1e−05
rs2090816
rs2387977
rs7311004
rs10899911
rs62273907
rs78383039
rs6809952
rs823424
rs7572229
rs55877868
rs8039785
rs11715853
rs7303158
rs35055552
rs6828768
All − MR Egger
All − IVW(MRE)
−1.5
−1.0
−0.5
0.0
0.5
1.0
MR effect size for
'Gut microbiota abundance (genus Parasutterella id.2892)' on 'Phosphoglycerate kinase 2'

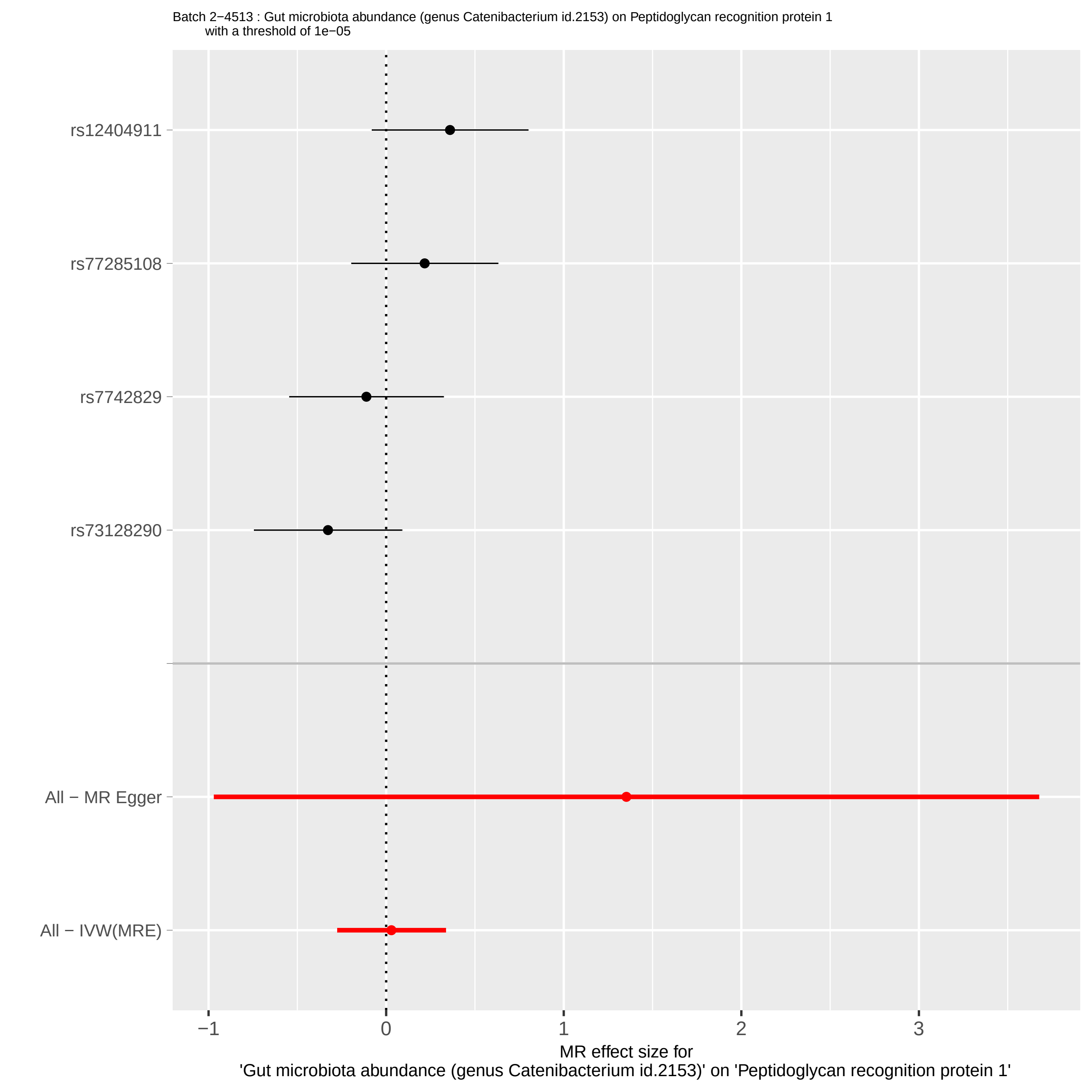
Batch 2−4513 : Gut microbiota abundance (genus Catenibacterium id.2153) on Peptidoglycan recognition protein 1
with a threshold of 1e−05
rs12404911
rs77285108
rs7742829
rs73128290
All − MR Egger
All − IVW(MRE)
−1
0
1
2
3
MR effect size for
'Gut microbiota abundance (genus Catenibacterium id.2153)' on 'Peptidoglycan recognition protein 1'

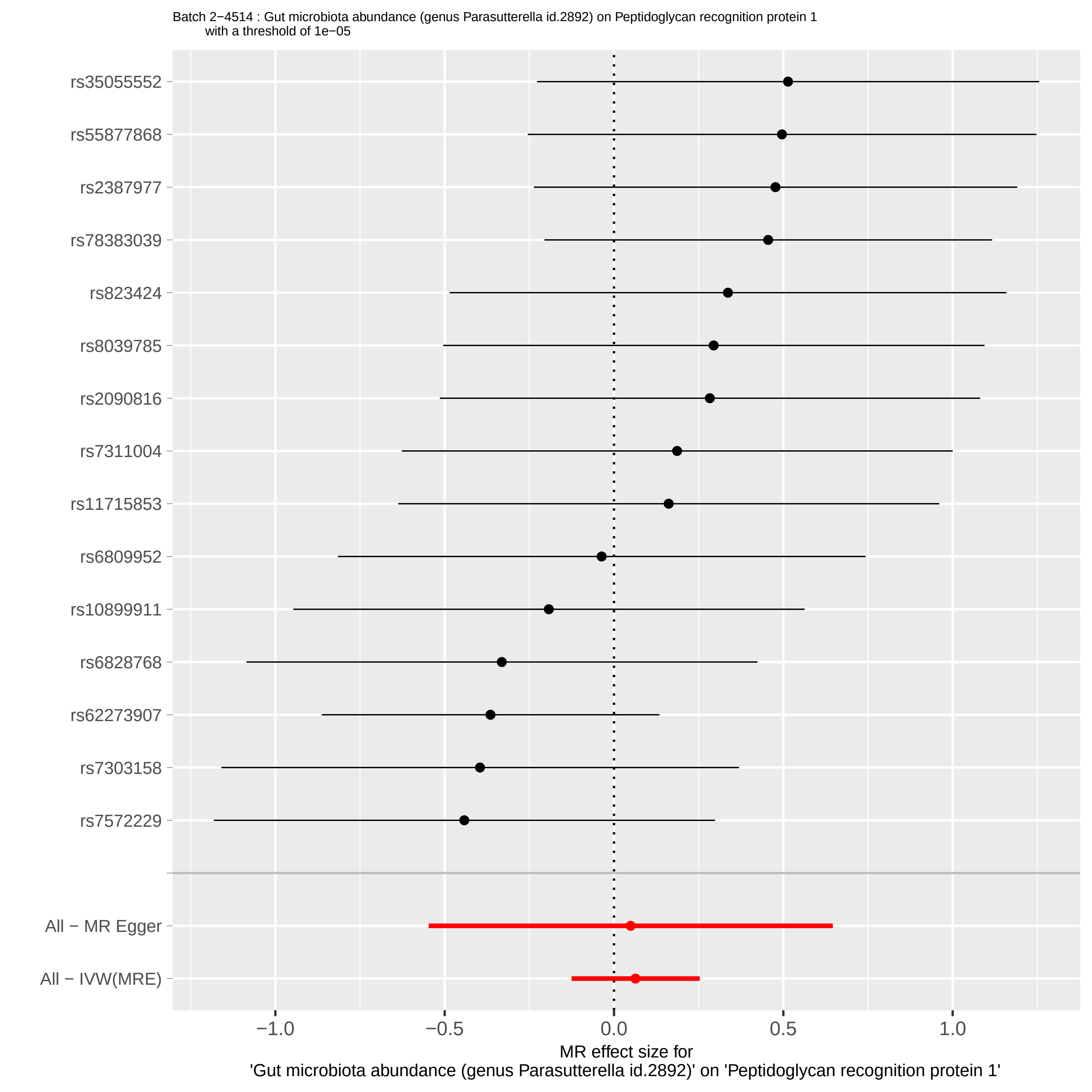
Batch 2−4514 : Gut microbiota abundance (genus Parasutterella id.2892) on Peptidoglycan recognition protein 1
with a threshold of 1e−05
rs35055552
rs55877868
rs2387977
rs78383039
rs823424
rs8039785
rs2090816
rs7311004
rs11715853
rs6809952
rs10899911
rs6828768
rs62273907
rs7303158
rs7572229
All − MR Egger
All − IVW(MRE)
−1.0
−0.5
0.0
0.5
1.0
MR effect size for
'Gut microbiota abundance (genus Parasutterella id.2892)' on 'Peptidoglycan recognition protein 1'

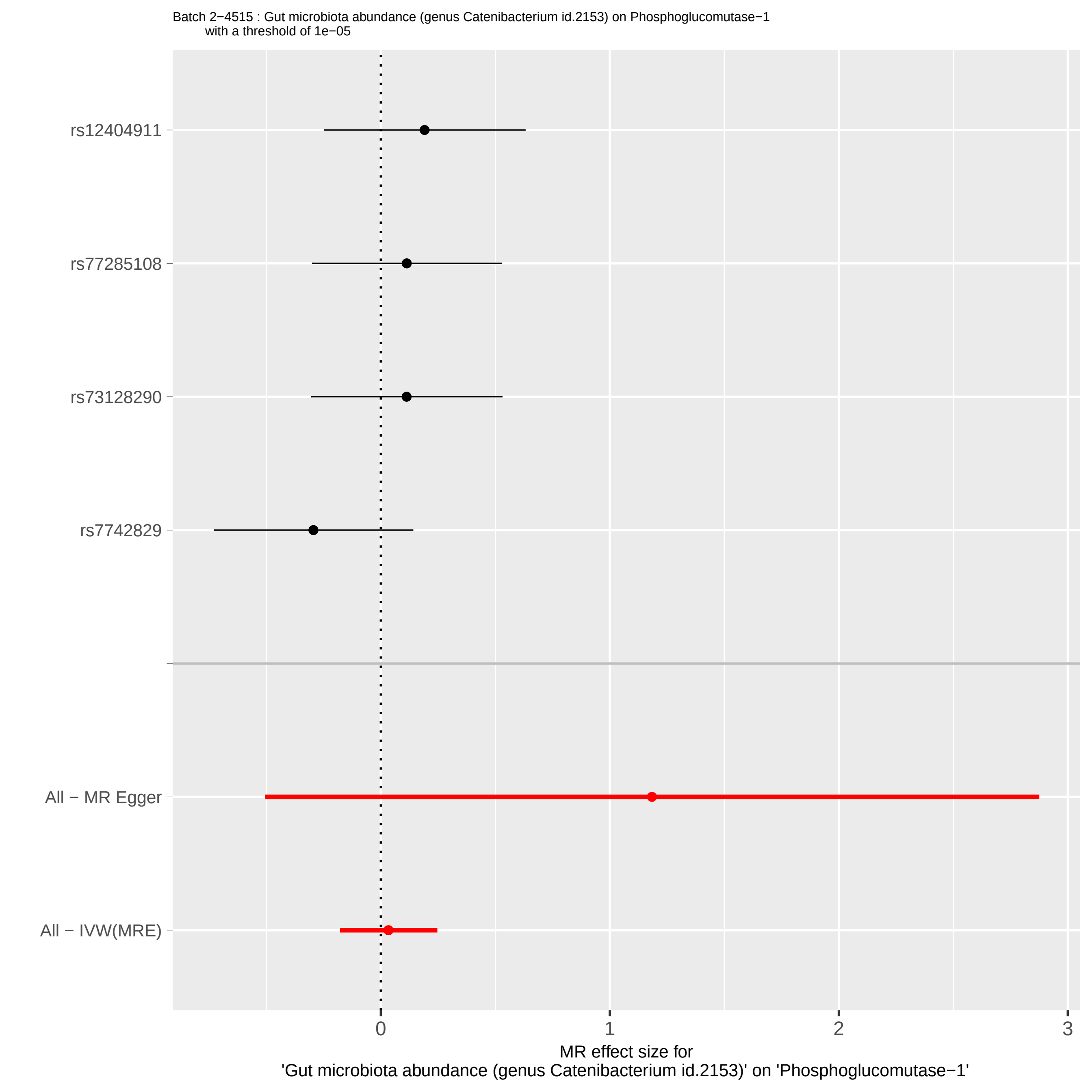
Batch 2−4515 : Gut microbiota abundance (genus Catenibacterium id.2153) on Phosphoglucomutase−1
with a threshold of 1e−05
rs12404911
rs77285108
rs73128290
rs7742829
All − MR Egger
All − IVW(MRE)
0
1
2
3
MR effect size for
'Gut microbiota abundance (genus Catenibacterium id.2153)' on 'Phosphoglucomutase−1'

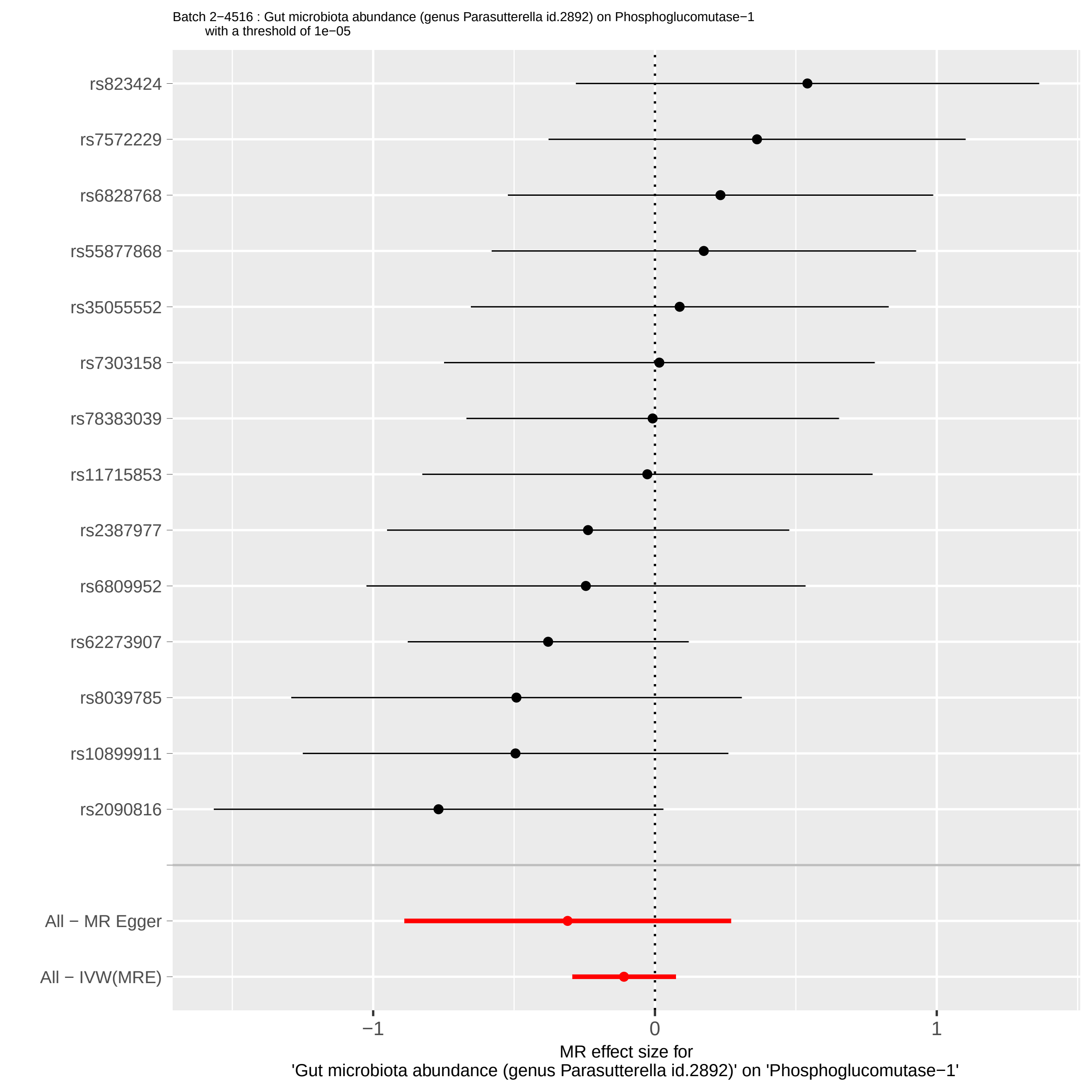

Batch 2−4516 : Gut microbiota abundance (genus Parasutterella id.2892) on Phosphoglucomutase−1
with a threshold of 1e−05
rs823424
rs7572229
rs6828768
rs55877868
rs35055552
rs7303158
rs78383039
rs11715853
rs2387977
rs6809952
rs62273907
rs8039785
rs10899911
rs2090816
All − MR Egger
All − IVW(MRE)
−1
0
1
MR effect size for
'Gut microbiota abundance (genus Parasutterella id.2892)' on 'Phosphoglucomutase−1'

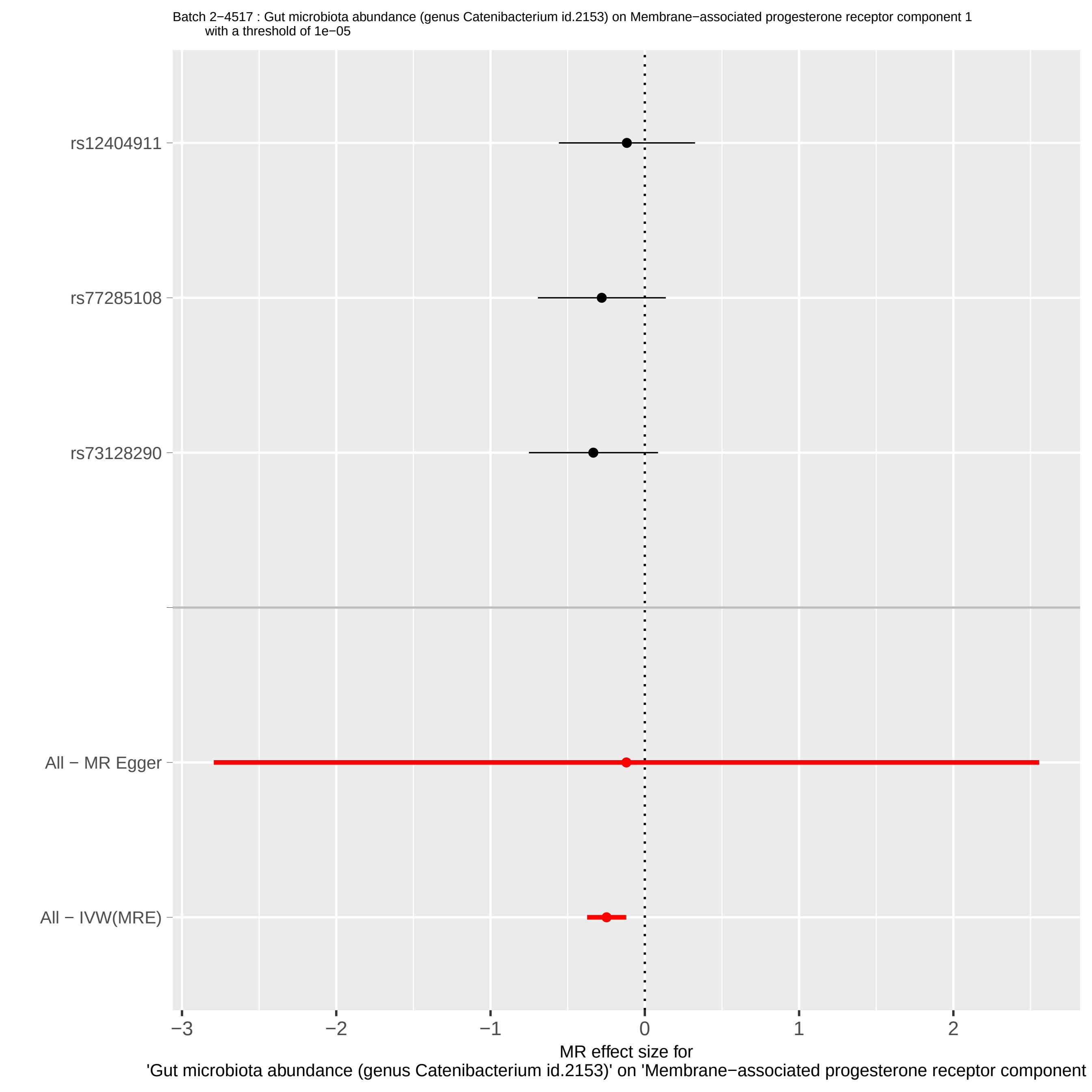

Batch 2−4517 : Gut microbiota abundance (genus Catenibacterium id.2153) on Membrane−associated progesterone receptor component 1
with a threshold of 1e−05
rs12404911
rs77285108
rs73128290
All − MR Egger
All − IVW(MRE)
−3
−2
−1
0
1
2
MR effect size for
'Gut microbiota abundance (genus Catenibacterium id.2153)' on 'Membrane−associated progesterone receptor component

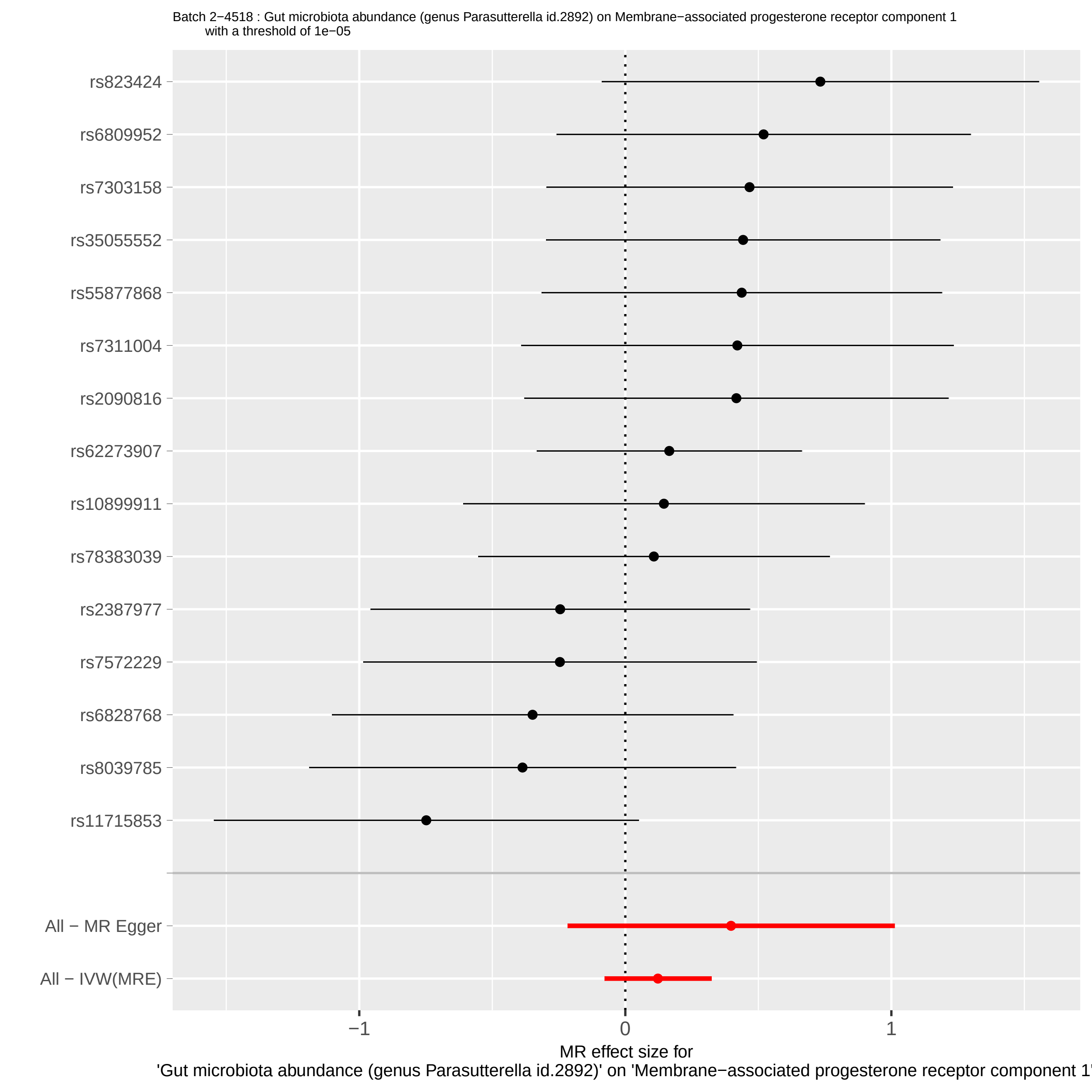

Batch 2−4518 : Gut microbiota abundance (genus Parasutterella id.2892) on Membrane−associated progesterone receptor component 1 with a threshold of 1e−05
rs823424
rs6809952
rs7303158
rs35055552
rs55877868
rs7311004
rs2090816
rs62273907
rs10899911
rs78383039
rs2387977
rs7572229
rs6828768
rs8039785
rs11715853
All − MR Egger
All − IVW(MRE)
−1
0
1
MR effect size for
'Gut microbiota abundance (genus Parasutterella id.2892)' on 'Membrane−associated progesterone receptor component 1'

Batch 2−4520 : Gut microbiota abundance (genus Parasutterella id.2892) on Membrane−associated progesterone receptor component 2
with a threshold of 1e−05

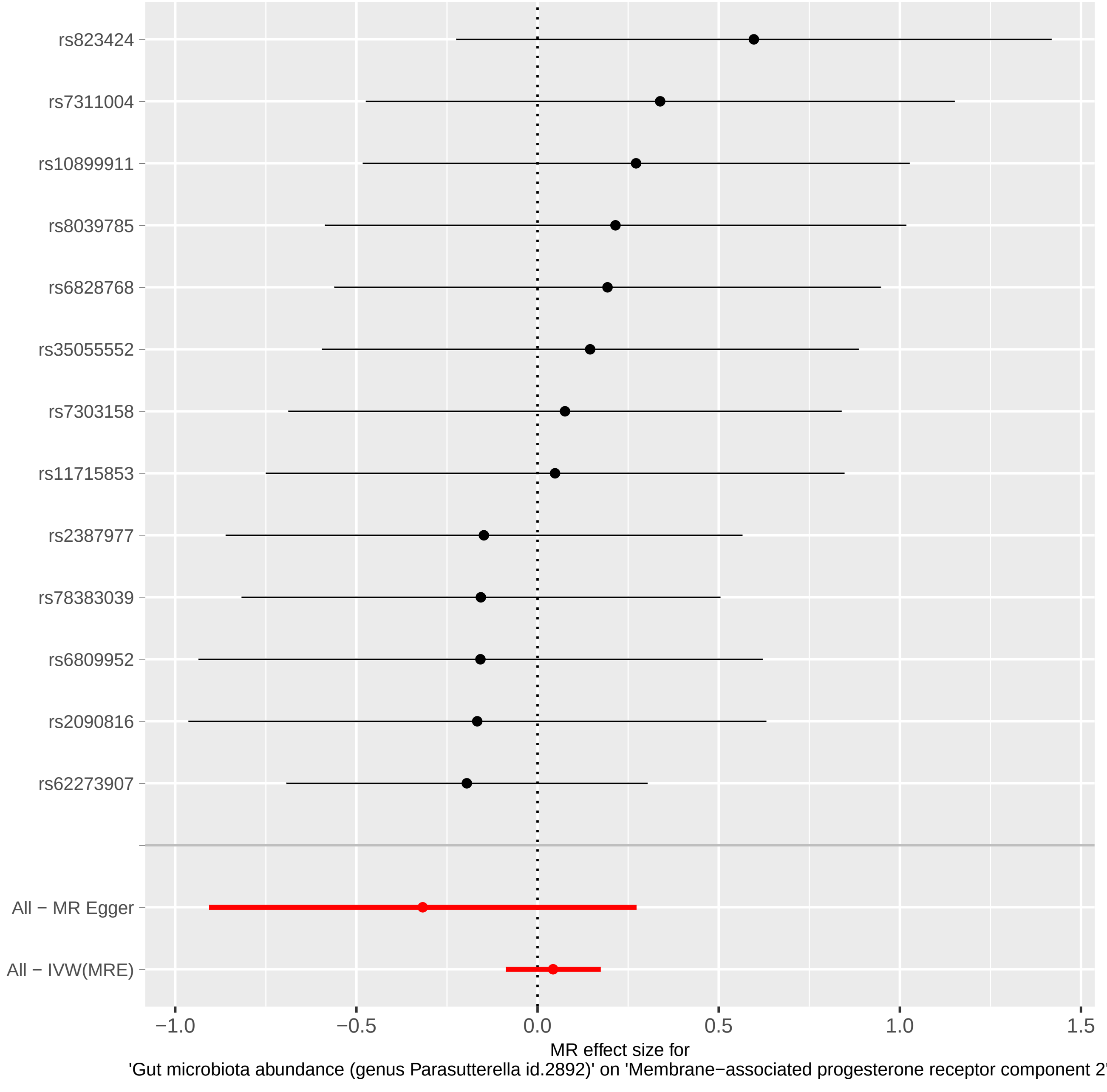

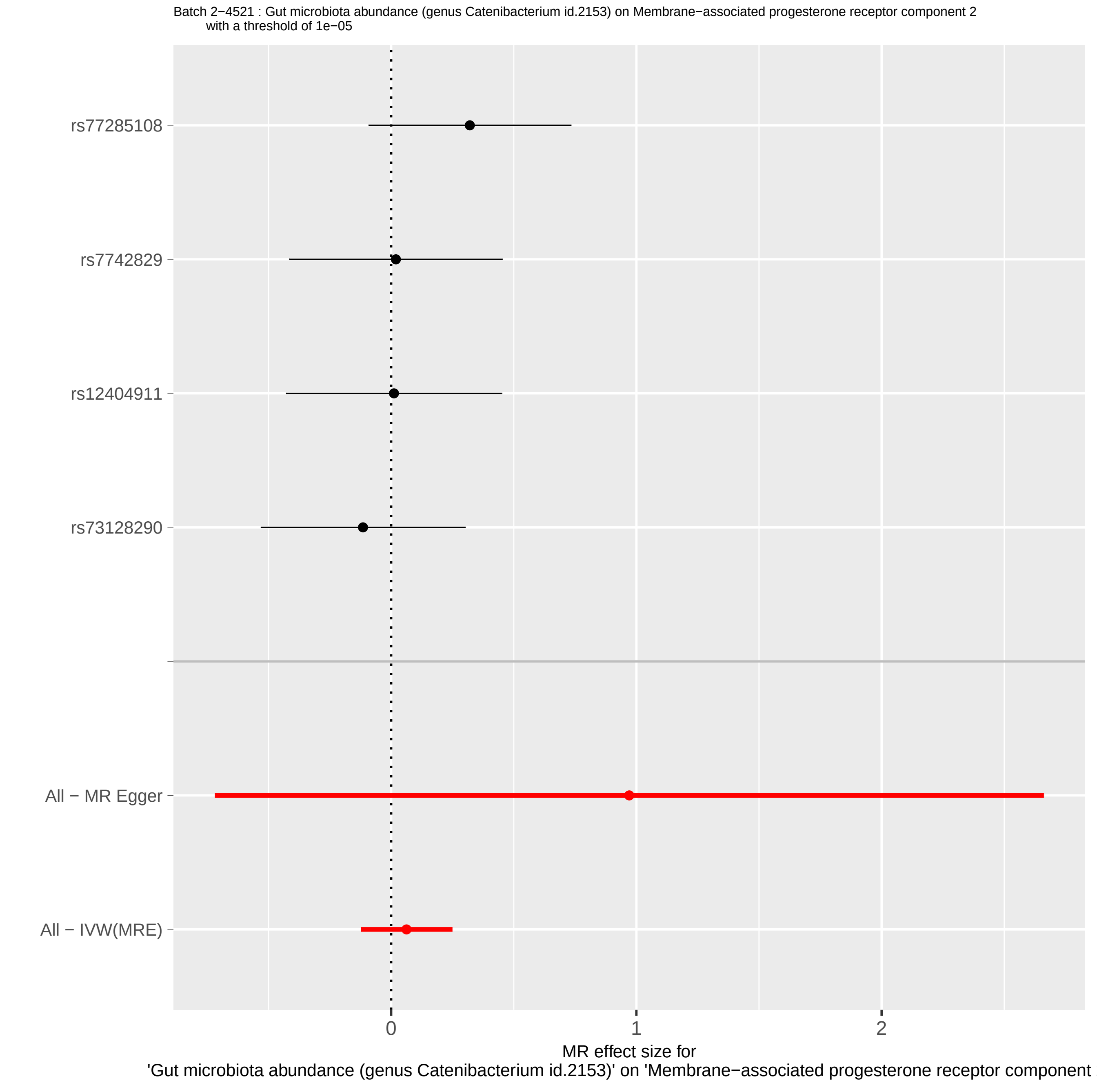
Batch 2−4521 : Gut microbiota abundance (genus Catenibacterium id.2153) on Membrane−associated progesterone receptor component 2
with a threshold of 1e−05
rs77285108
rs7742829
rs12404911
rs73128290
All − MR Egger
All − IVW(MRE)
0
1
2
MR effect size for
'Gut microbiota abundance (genus Catenibacterium id.2153)' on 'Membrane−associated progesterone receptor component

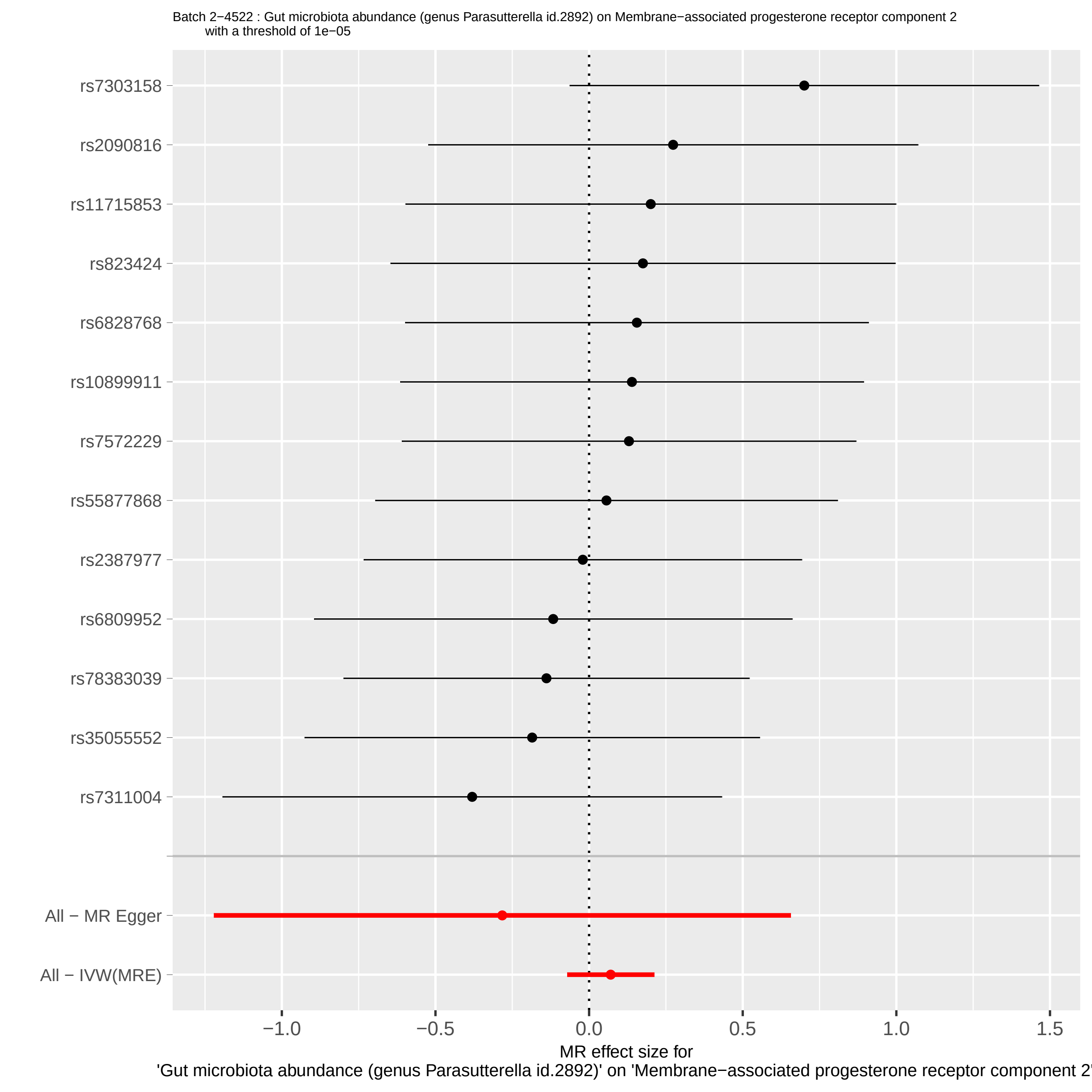
Batch 2−4522 : Gut microbiota abundance (genus Parasutterella id.2892) on Membrane−associated progesterone receptor component 2
with a threshold of 1e−05
rs7303158
rs2090816
rs11715853
rs823424
rs6828768
rs10899911
rs7572229
rs55877868
rs2387977
rs6809952
rs78383039
rs35055552
rs7311004
All − MR Egger
All − IVW(MRE)
−1.0
−0.5
0.0
0.5
1.0
1.5
MR effect size for
'Gut microbiota abundance (genus Parasutterella id.2892)' on 'Membrane−associated progesterone receptor component 2'

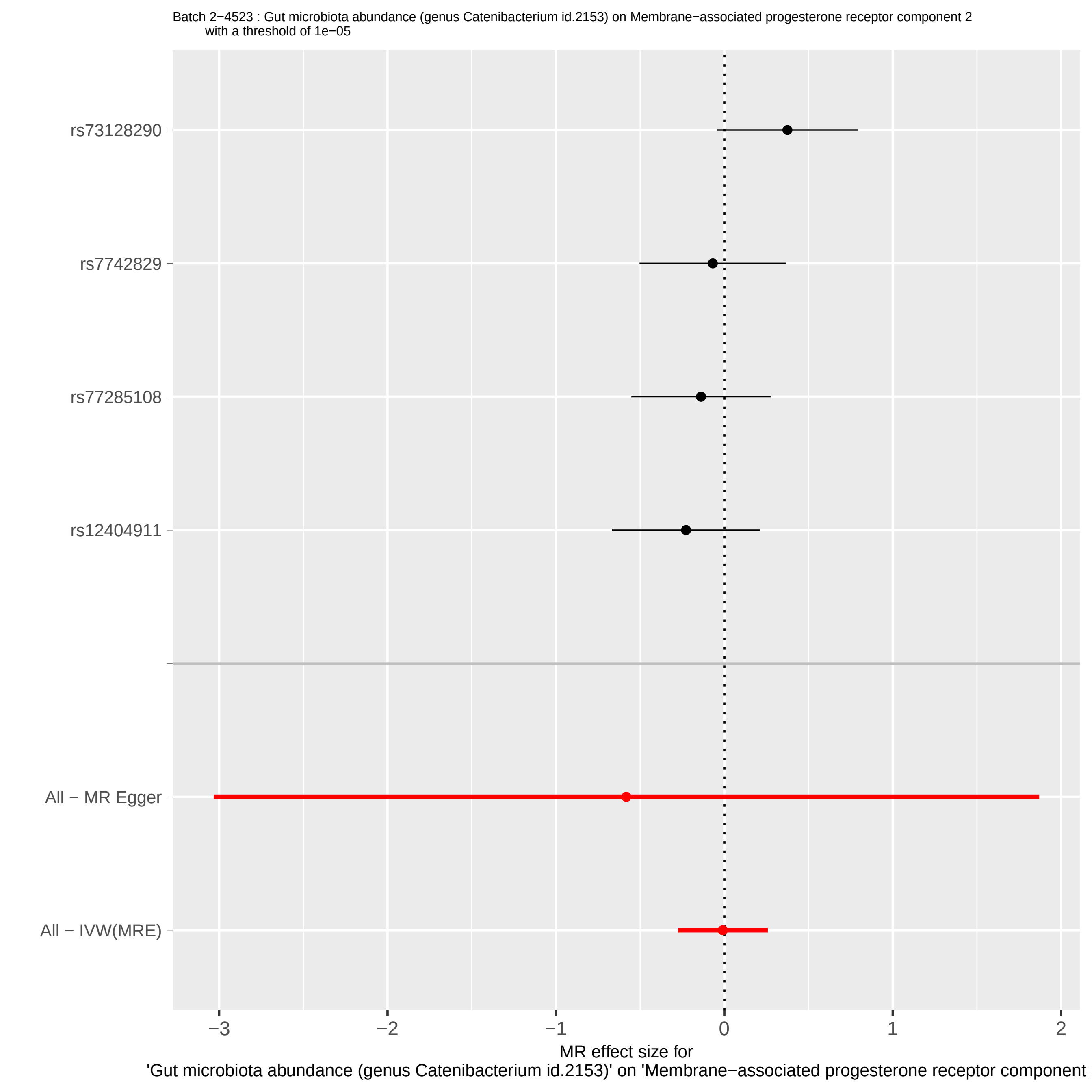

Batch 2−4523 : Gut microbiota abundance (genus Catenibacterium id.2153) on Membrane−associated progesterone receptor component 2
with a threshold of 1e−05
rs73128290
rs7742829
rs77285108
rs12404911
All − MR Egger
All − IVW(MRE)
−3
−2
−1
0
1
2
MR effect size for
'Gut microbiota abundance (genus Catenibacterium id.2153)' on 'Membrane−associated progesterone receptor component

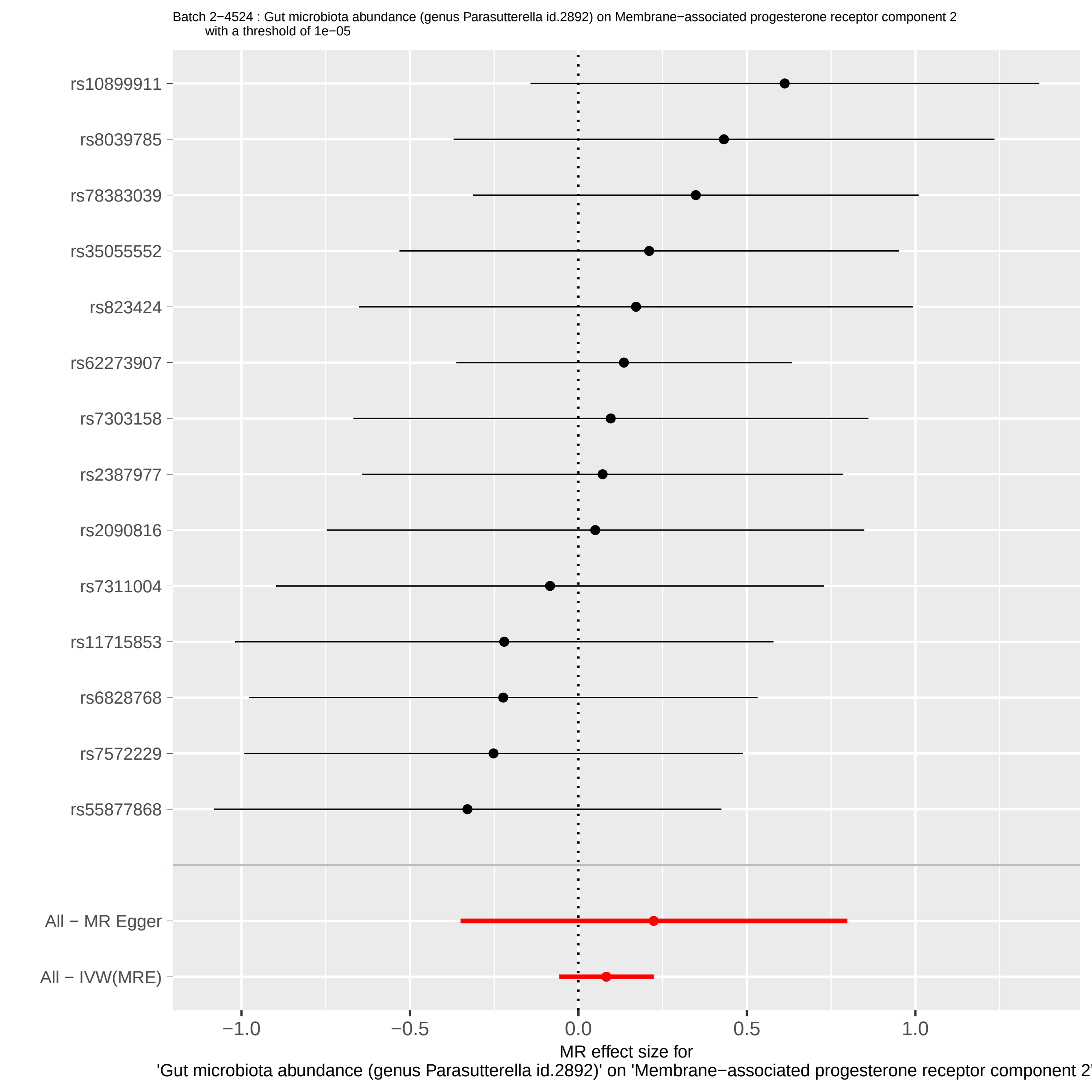

Batch 2−4524 : Gut microbiota abundance (genus Parasutterella id.2892) on Membrane−associated progesterone receptor component 2 with a threshold of 1e−05
rs10899911
rs8039785
rs78383039
rs35055552
rs823424
rs62273907
rs7303158
rs2387977
rs2090816
rs7311004
rs11715853
rs6828768
rs7572229
rs55877868
All − MR Egger
All − IVW(MRE)
−1.0
−0.5
0.0
0.5
1.0
MR effect size for
'Gut microbiota abundance (genus Parasutterella id.2892)' on 'Membrane−associated progesterone receptor component 2'

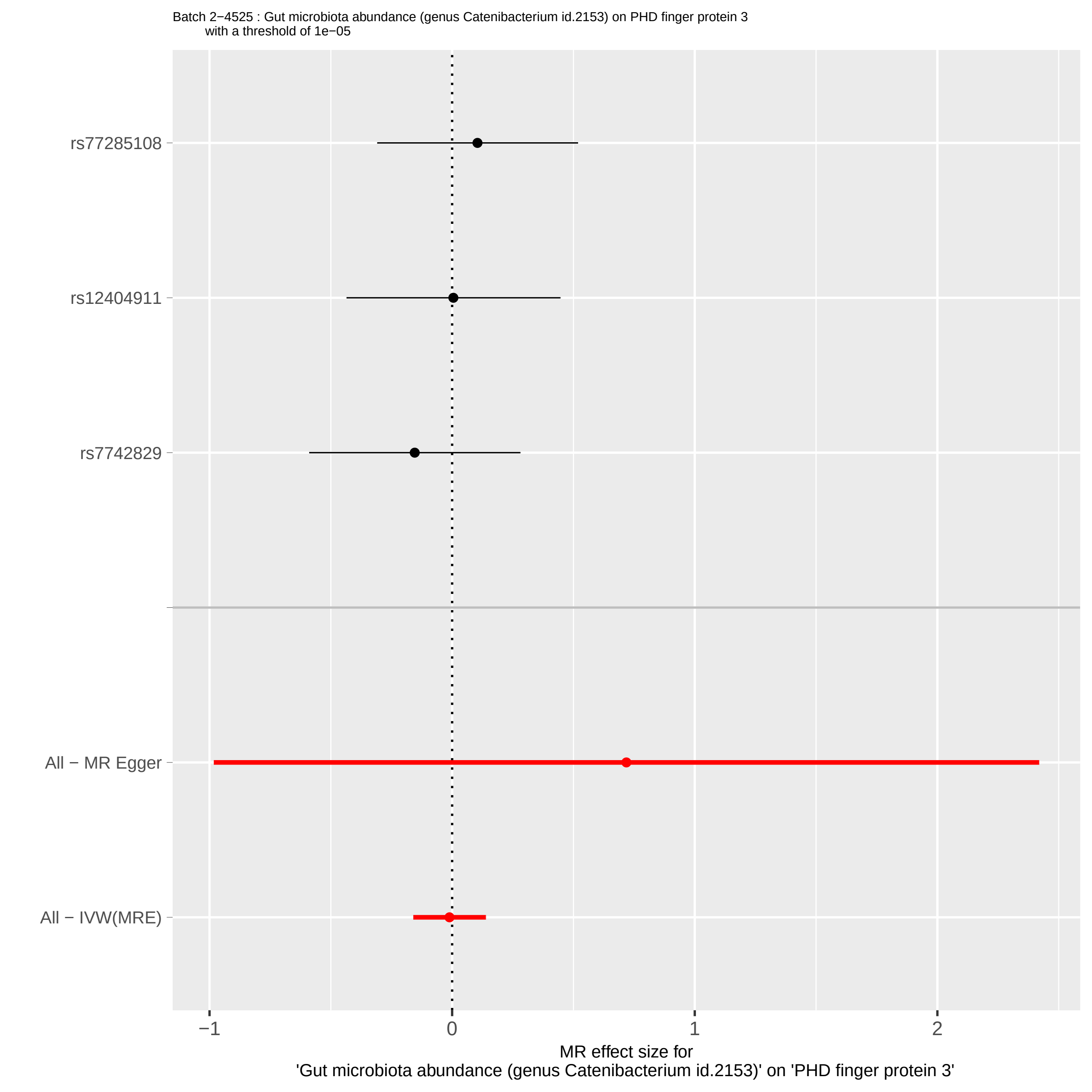

Batch 2−4525 : Gut microbiota abundance (genus Catenibacterium id.2153) on PHD finger protein 3
with a threshold of 1e−05
rs77285108
rs12404911
rs7742829
All − MR Egger
All − IVW(MRE)
−1
0
1
2
MR effect size for
'Gut microbiota abundance (genus Catenibacterium id.2153)' on 'PHD finger protein 3'

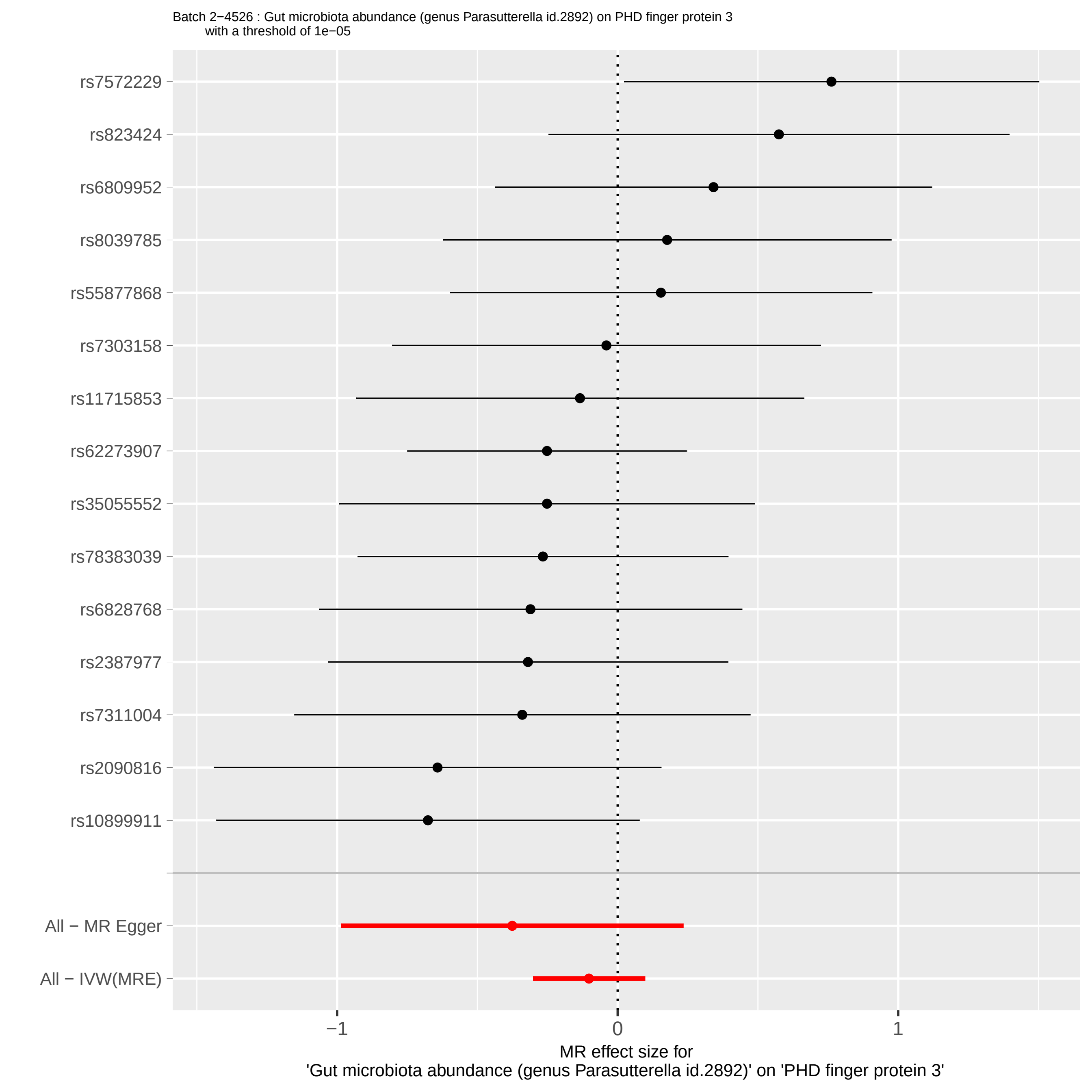
Batch 2−4526 : Gut microbiota abundance (genus Parasutterella id.2892) on PHD finger protein 3
with a threshold of 1e−05
rs7572229
rs823424
rs6809952
rs8039785
rs55877868
rs7303158
rs11715853
rs62273907
rs35055552
rs78383039
rs6828768
rs2387977
rs7311004
rs2090816
rs10899911
All − MR Egger
All − IVW(MRE)
−1
0
1
MR effect size for
'Gut microbiota abundance (genus Parasutterella id.2892)' on 'PHD finger protein 3'

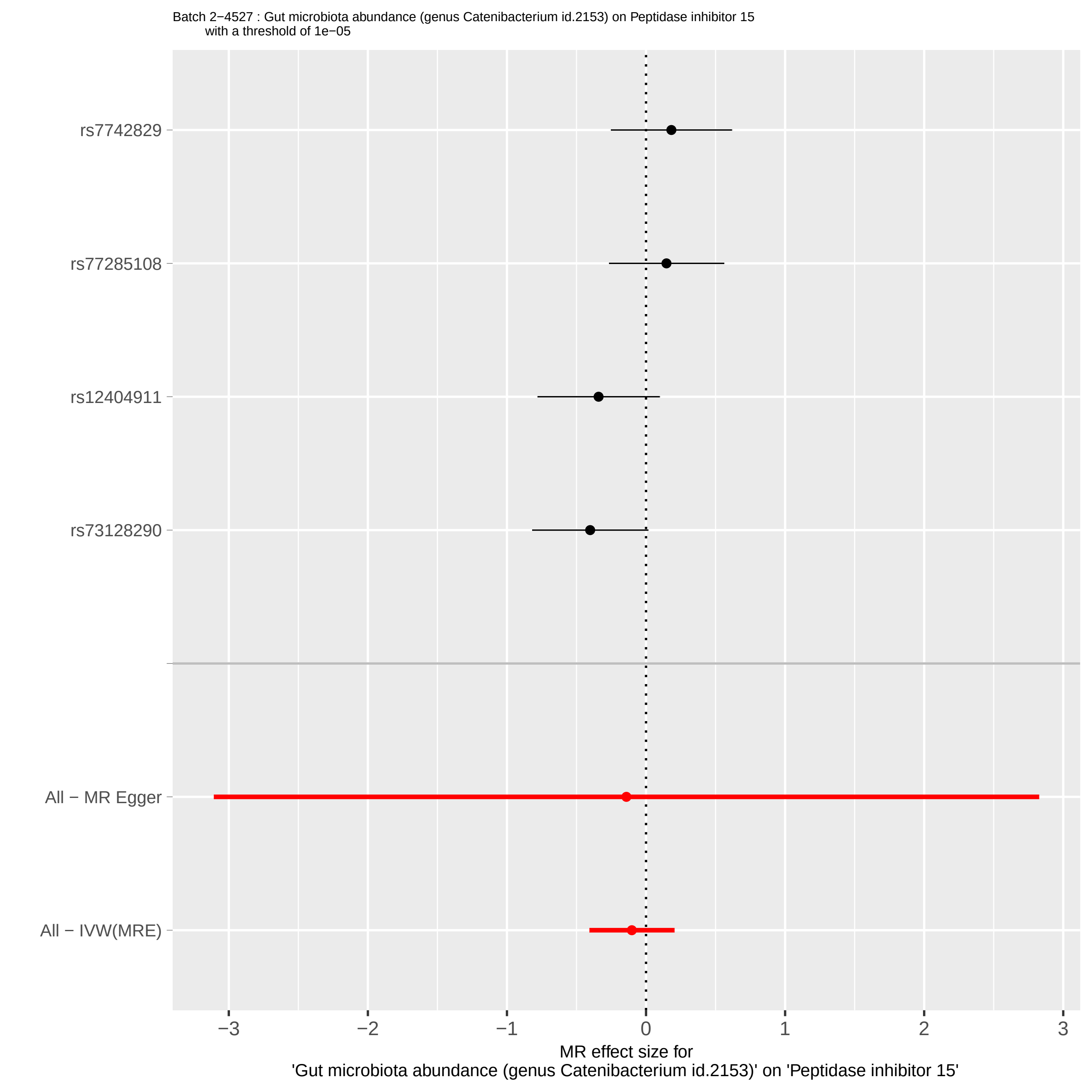

Batch 2−4527 : Gut microbiota abundance (genus Catenibacterium id.2153) on Peptidase inhibitor 15
with a threshold of 1e−05
rs7742829
rs77285108
rs12404911
rs73128290
All − MR Egger
All − IVW(MRE)
−3
−2
−1
0
1
2
3
MR effect size for
'Gut microbiota abundance (genus Catenibacterium id.2153)' on 'Peptidase inhibitor 15'

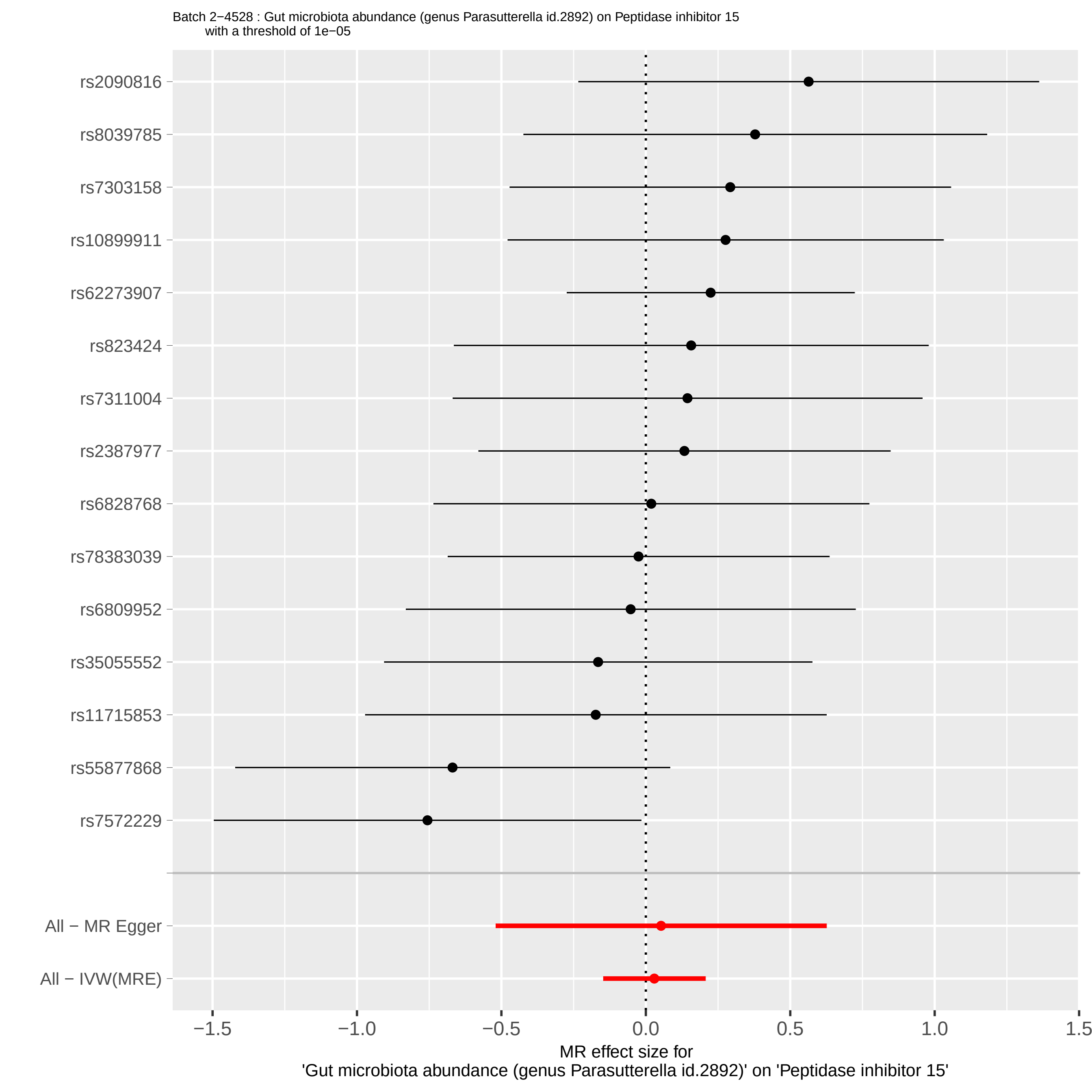

Batch 2−4528 : Gut microbiota abundance (genus Parasutterella id.2892) on Peptidase inhibitor 15
with a threshold of 1e−05
rs2090816
rs8039785
rs7303158
rs10899911
rs62273907
rs823424
rs7311004
rs2387977
rs6828768
rs78383039
rs6809952
rs35055552
rs11715853
rs55877868
rs7572229
All − MR Egger
All − IVW(MRE)
−1.5
−1.0
−0.5
0.0
0.5
1.0
1.5
MR effect size for
'Gut microbiota abundance (genus Parasutterella id.2892)' on 'Peptidase inhibitor 15'

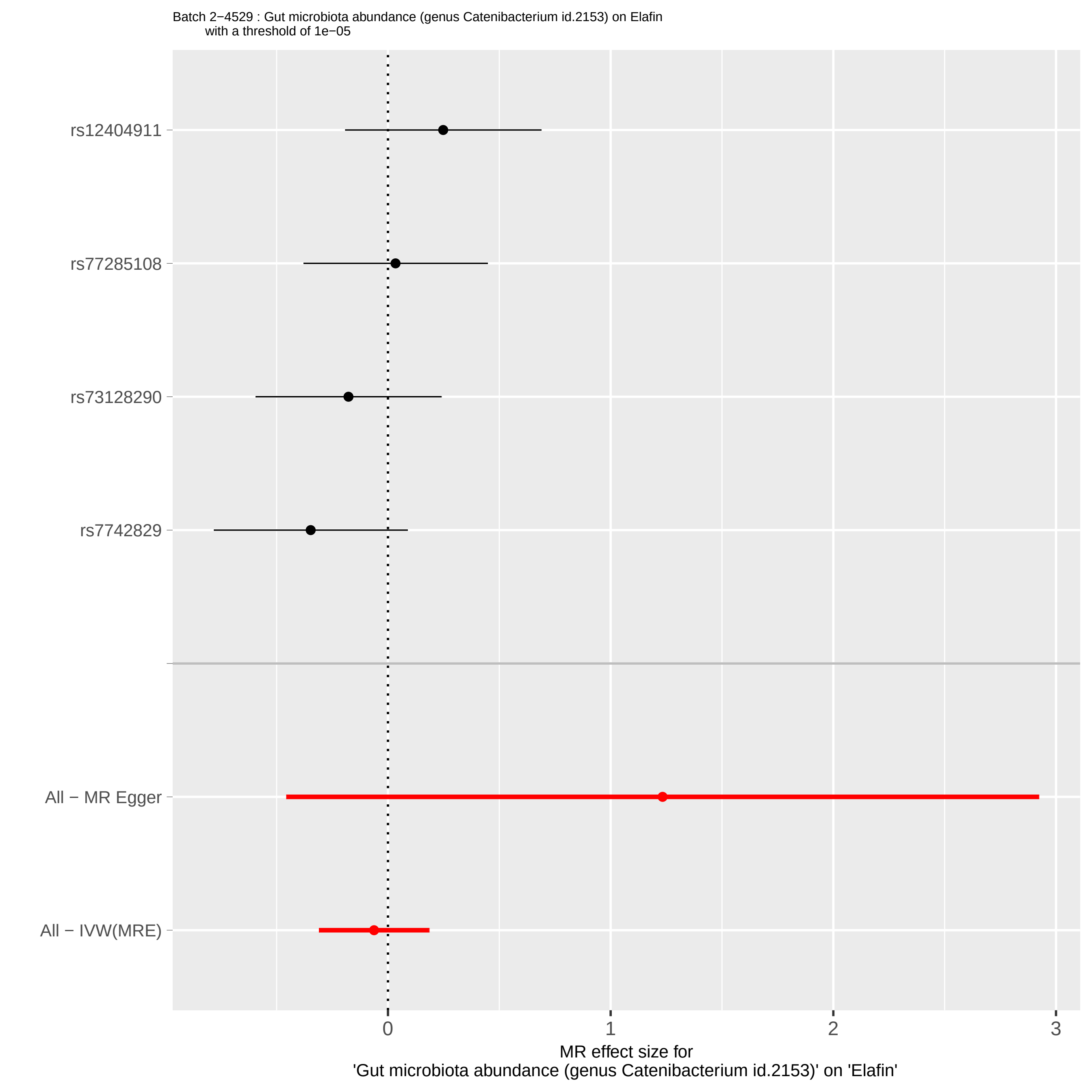
Batch 2−4529 : Gut microbiota abundance (genus Catenibacterium id.2153) on Elafin
with a threshold of 1e−05
rs12404911
rs77285108
rs73128290
rs7742829
All − MR Egger
All − IVW(MRE)
0
1
2
3
MR effect size for
'Gut microbiota abundance (genus Catenibacterium id.2153)' on 'Elafin'

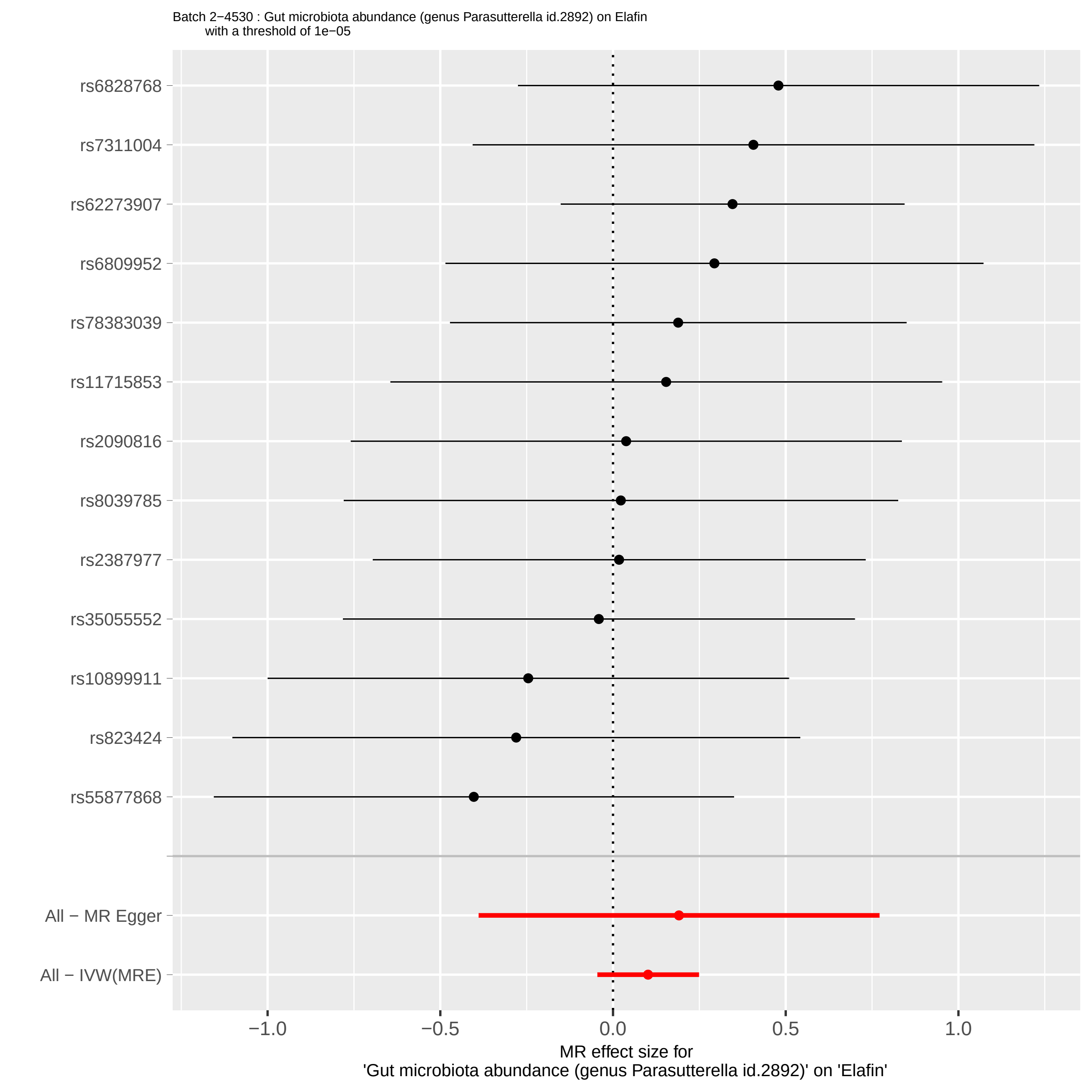
Batch 2−4530 : Gut microbiota abundance (genus Parasutterella id.2892) on Elafin
with a threshold of 1e−05
rs6828768
rs7311004
rs62273907
rs6809952
rs78383039
rs11715853
rs2090816
rs8039785
rs2387977
rs35055552
rs10899911
rs823424
rs55877868
All − MR Egger
All − IVW(MRE)
−1.0
−0.5
0.0
0.5
1.0
MR effect size for
'Gut microbiota abundance (genus Parasutterella id.2892)' on 'Elafin'

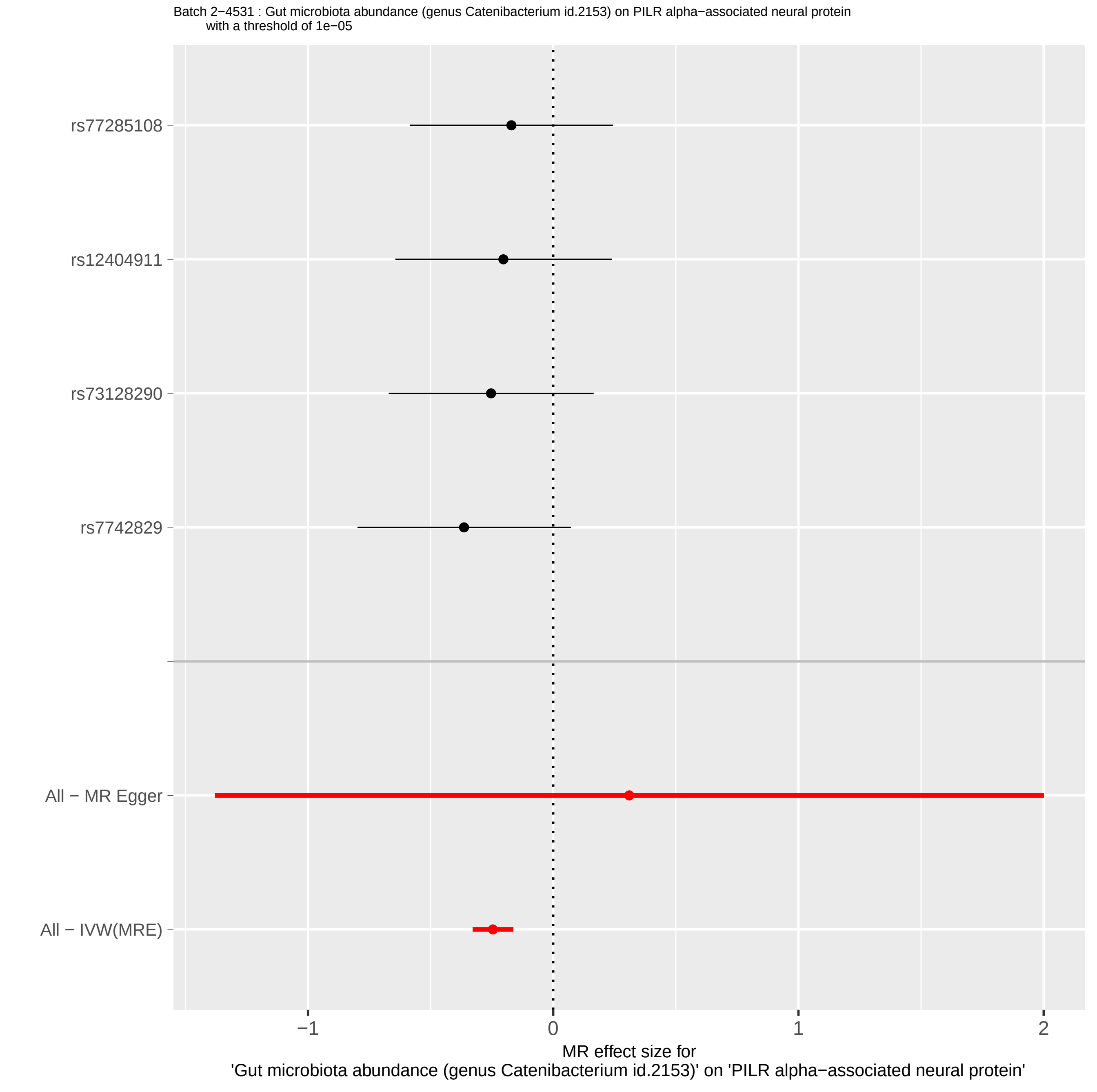
Batch 2−4531 : Gut microbiota abundance (genus Catenibacterium id.2153) on PILR alpha−associated neural protein
with a threshold of 1e−05
rs77285108
rs12404911
rs73128290
rs7742829
All − MR Egger
All − IVW(MRE)
−1
0
1
2
MR effect size for
'Gut microbiota abundance (genus Catenibacterium id.2153)' on 'PILR alpha−associated neural protein'

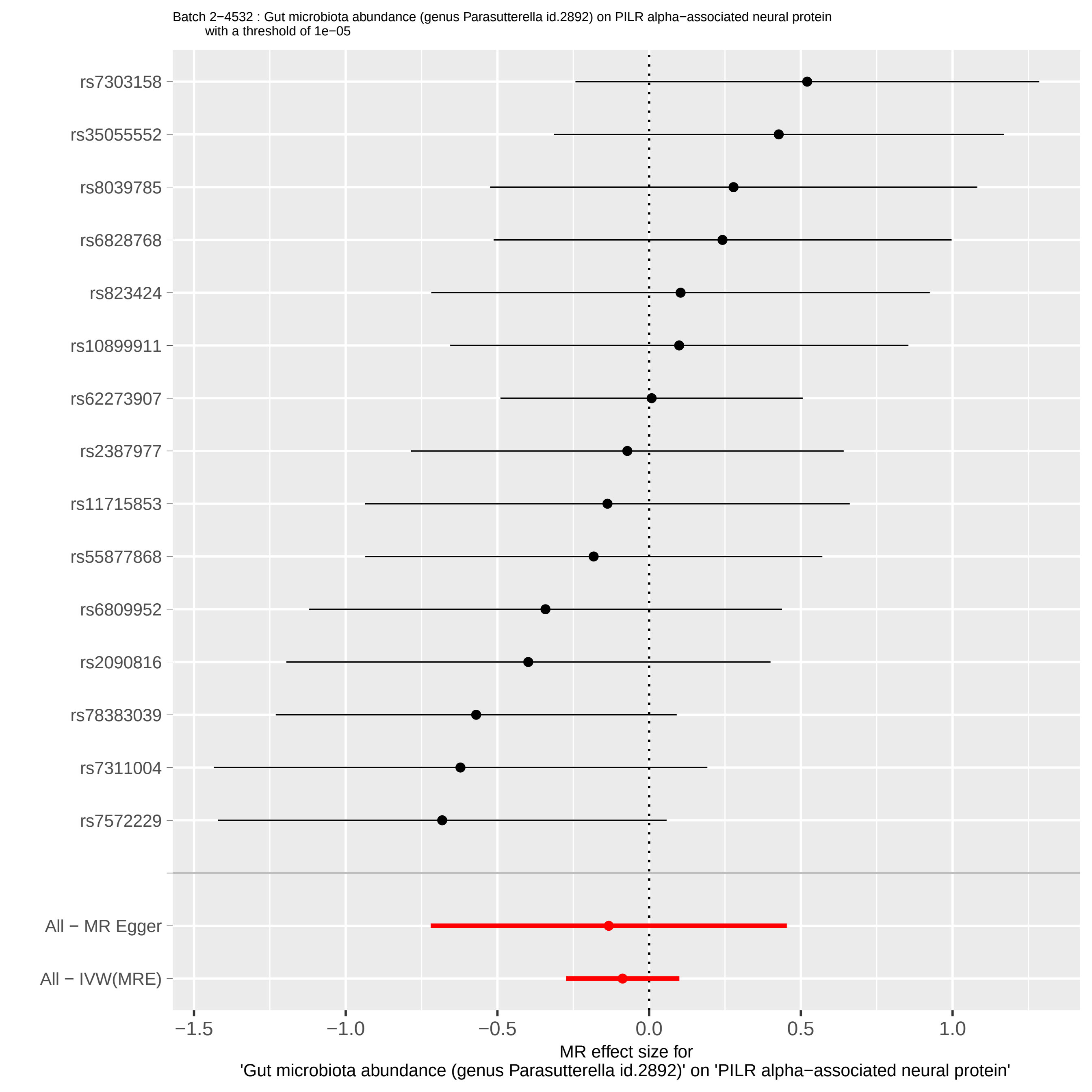

Batch 2−4532 : Gut microbiota abundance (genus Parasutterella id.2892) on PILR alpha−associated neural protein
with a threshold of 1e−05
rs7303158
rs35055552
rs8039785
rs6828768
rs823424
rs10899911
rs62273907
rs2387977
rs11715853
rs55877868
rs6809952
rs2090816
rs78383039
rs7311004
rs7572229
All − MR Egger
All − IVW(MRE)
−1.5
−1.0
−0.5
0.0
0.5
1.0
MR effect size for
'Gut microbiota abundance (genus Parasutterella id.2892)' on 'PILR alpha−associated neural protein'

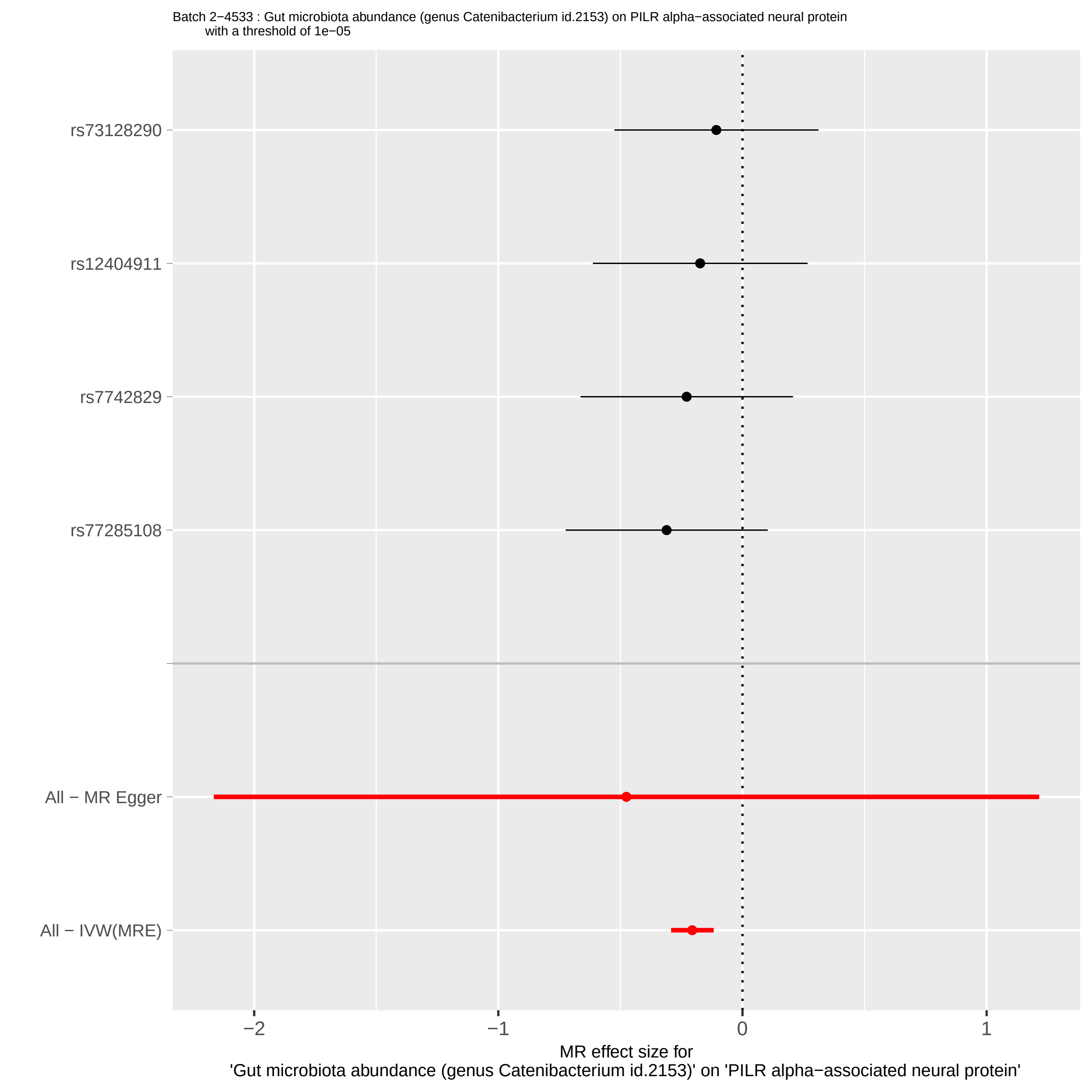

Batch 2−4533 : Gut microbiota abundance (genus Catenibacterium id.2153) on PILR alpha−associated neural protein
with a threshold of 1e−05
rs73128290
rs12404911
rs7742829
rs77285108
All − MR Egger
All − IVW(MRE)
−2
−1
0
1
MR effect size for
'Gut microbiota abundance (genus Catenibacterium id.2153)' on 'PILR alpha−associated neural protein'

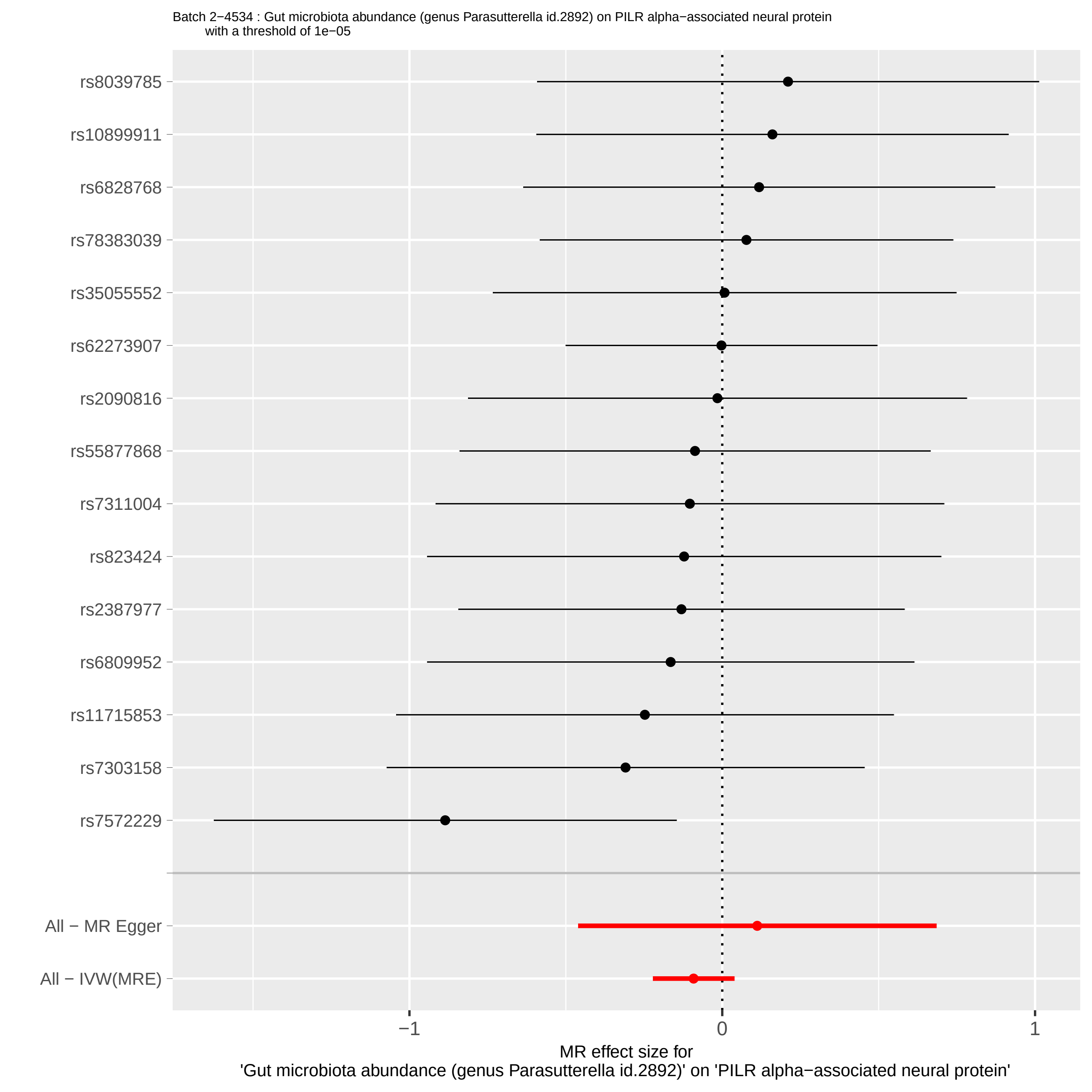

Batch 2−4534 : Gut microbiota abundance (genus Parasutterella id.2892) on PILR alpha−associated neural protein
with a threshold of 1e−05
rs8039785
rs10899911
rs6828768
rs78383039
rs35055552
rs62273907
rs2090816
rs55877868
rs7311004
rs823424
rs2387977
rs6809952
rs11715853
rs7303158
rs7572229
All − MR Egger
All − IVW(MRE)
−1
0
1
MR effect size for
'Gut microbiota abundance (genus Parasutterella id.2892)' on 'PILR alpha−associated neural protein'

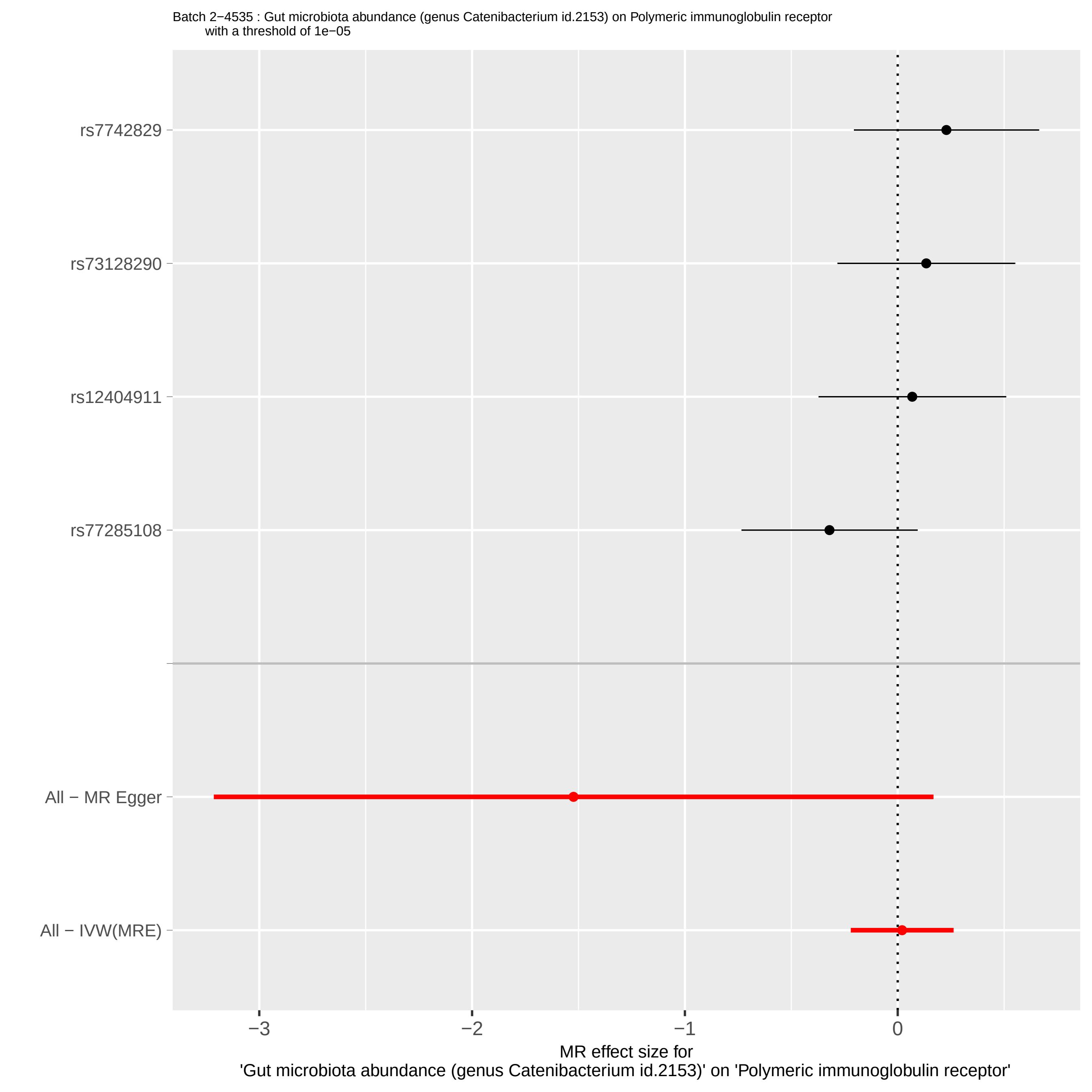
Batch 2−4535 : Gut microbiota abundance (genus Catenibacterium id.2153) on Polymeric immunoglobulin receptor
with a threshold of 1e−05
rs7742829
rs73128290
rs12404911
rs77285108
All − MR Egger
All − IVW(MRE)
−3
−2
−1
0
MR effect size for
'Gut microbiota abundance (genus Catenibacterium id.2153)' on 'Polymeric immunoglobulin receptor'

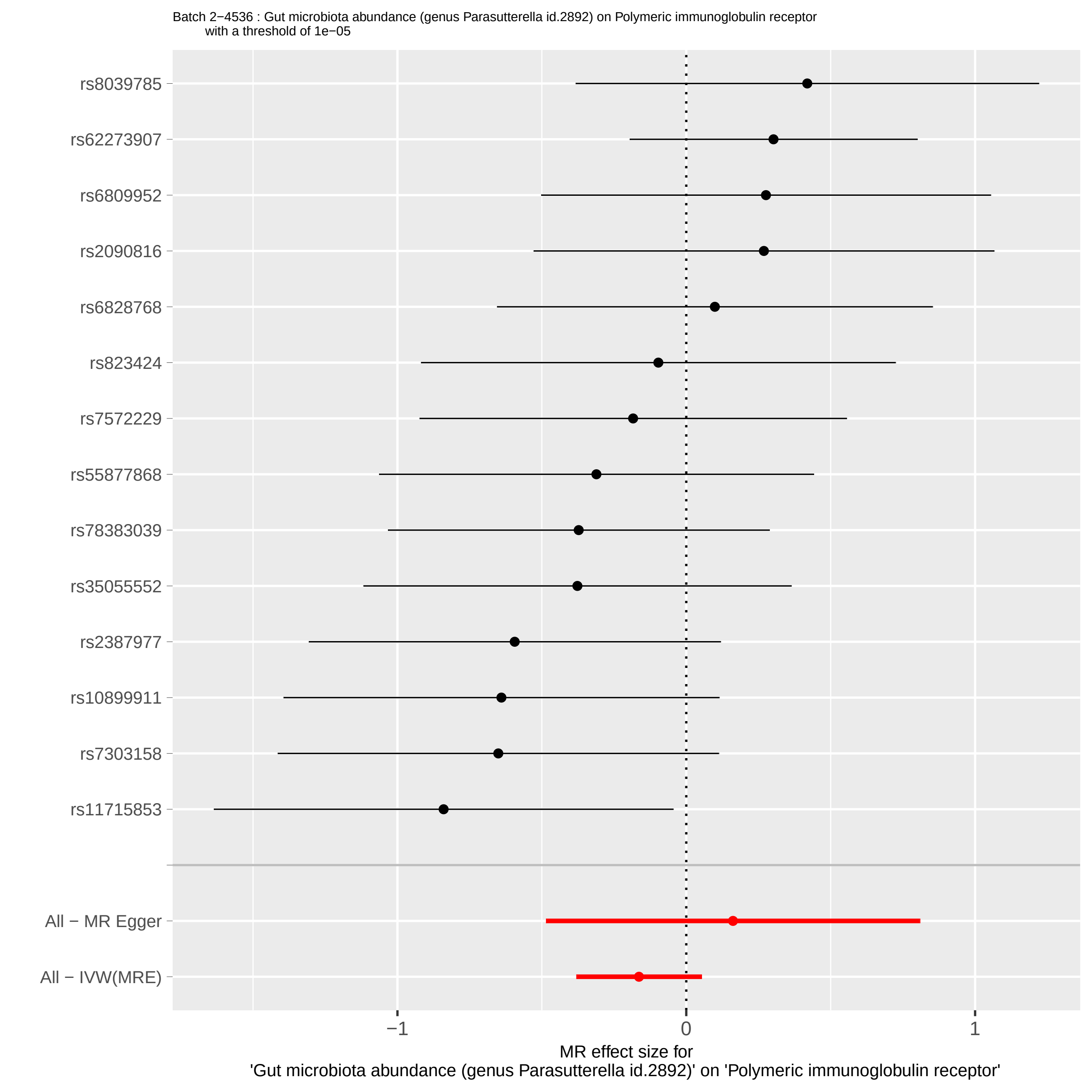

Batch 2−4536 : Gut microbiota abundance (genus Parasutterella id.2892) on Polymeric immunoglobulin receptor
with a threshold of 1e−05
rs8039785
rs62273907
rs6809952
rs2090816
rs6828768
rs823424
rs7572229
rs55877868
rs78383039
rs35055552
rs2387977
rs10899911
rs7303158
rs11715853
All − MR Egger
All − IVW(MRE)
−1
0
1
MR effect size for
'Gut microbiota abundance (genus Parasutterella id.2892)' on 'Polymeric immunoglobulin receptor

Batch 2−4537 : Gut microbiota abundance (genus Catenibacterium id.2153) on Phosphatidylinositol 4−phosphate 3−kinase C2 domain−containing subunit alpha
with a threshold of 1e−05

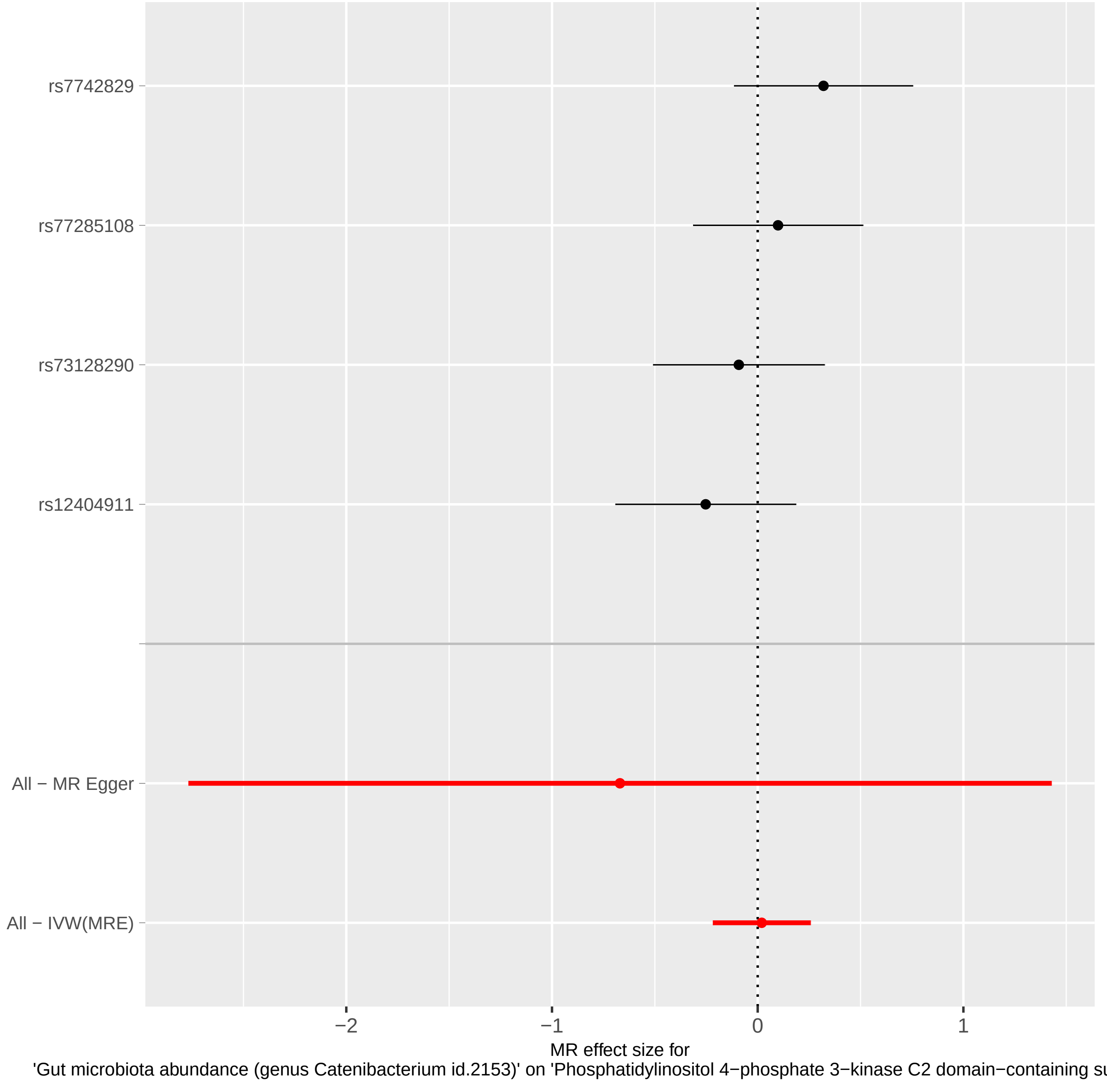

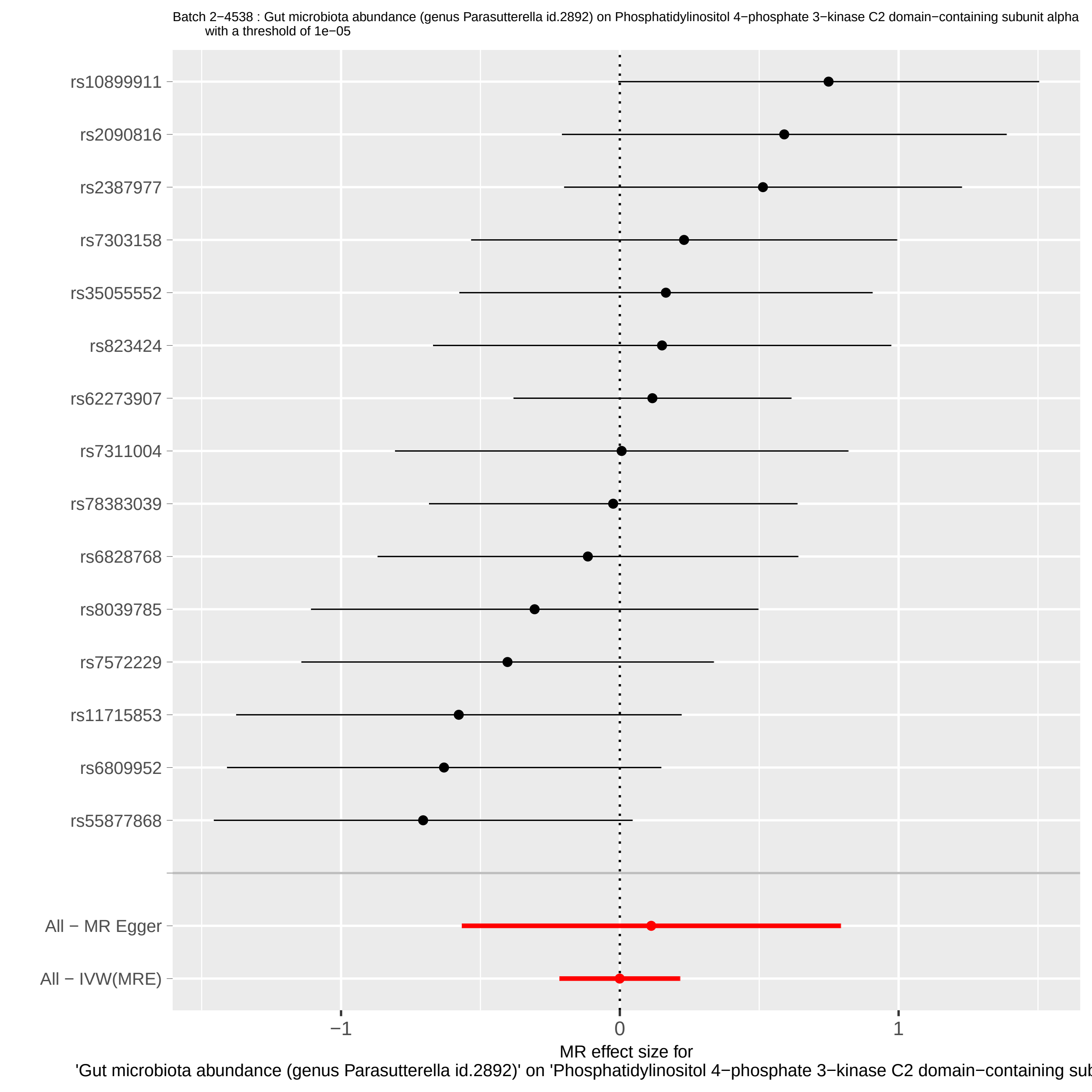

Batch 2−4538 : Gut microbiota abundance (genus Parasutterella id.2892) on Phosphatidylinositol 4−phosphate 3−kinase C2 domain−containing subunit alpha
with a threshold of 1e−05
rs10899911
rs2090816
rs2387977
rs7303158
rs35055552
rs823424
rs62273907
rs7311004
rs78383039
rs6828768
rs8039785
rs7572229
rs11715853
rs6809952
rs55877868
All − MR Egger
All − IVW(MRE)
−1
0
1
MR effect size for
'Gut microbiota abundance (genus Parasutterella id.2892)' on 'Phosphatidylinositol 4−phosphate 3−kinase C2 domain−containing sub

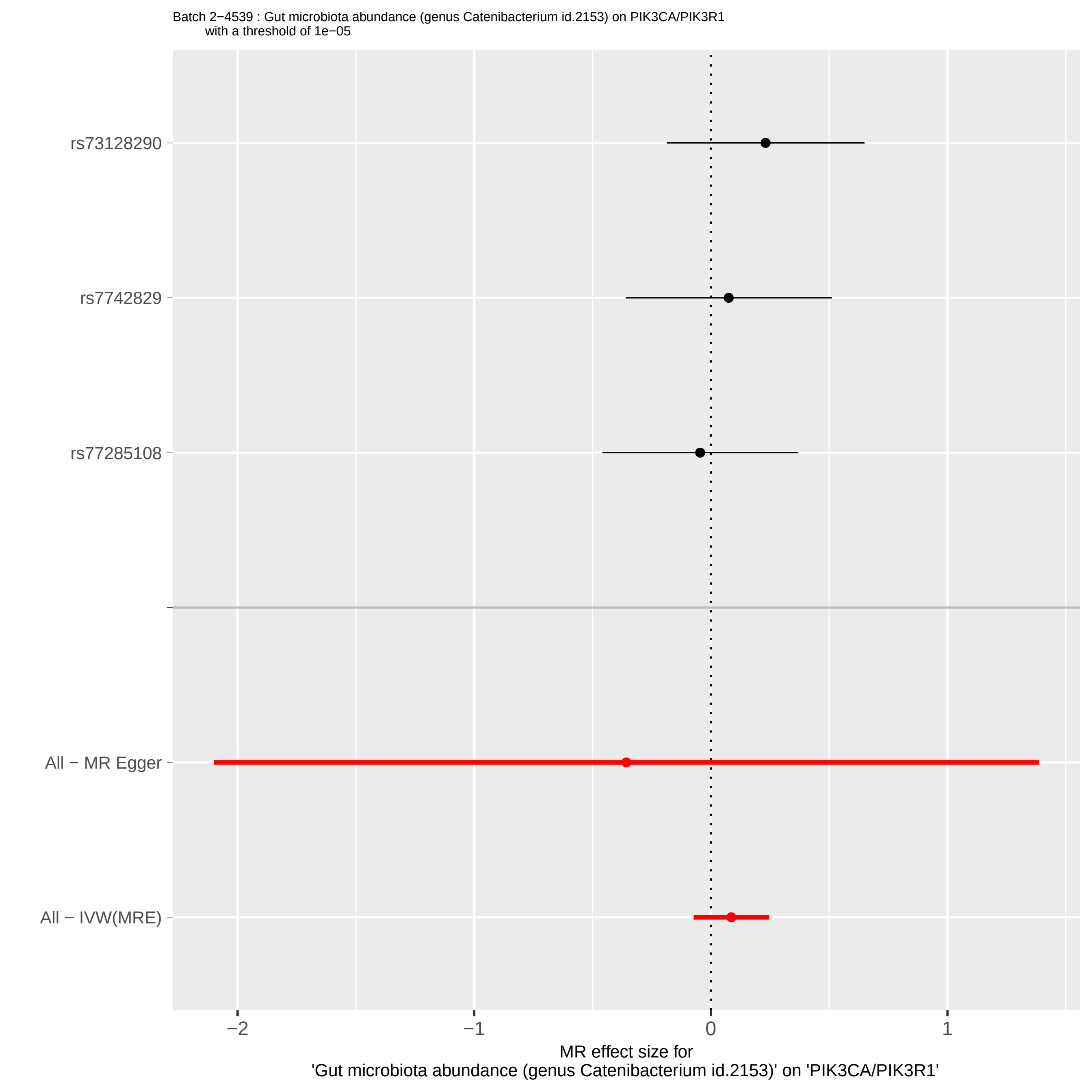

Batch 2−4539 : Gut microbiota abundance (genus Catenibacterium id.2153) on PIK3CA/PIK3R1
with a threshold of 1e−05
rs73128290
rs7742829
rs77285108
All − MR Egger
All − IVW(MRE)
−2
−1
0
1
MR effect size for
'Gut microbiota abundance (genus Catenibacterium id.2153)' on 'PIK3CA/PIK3R1'

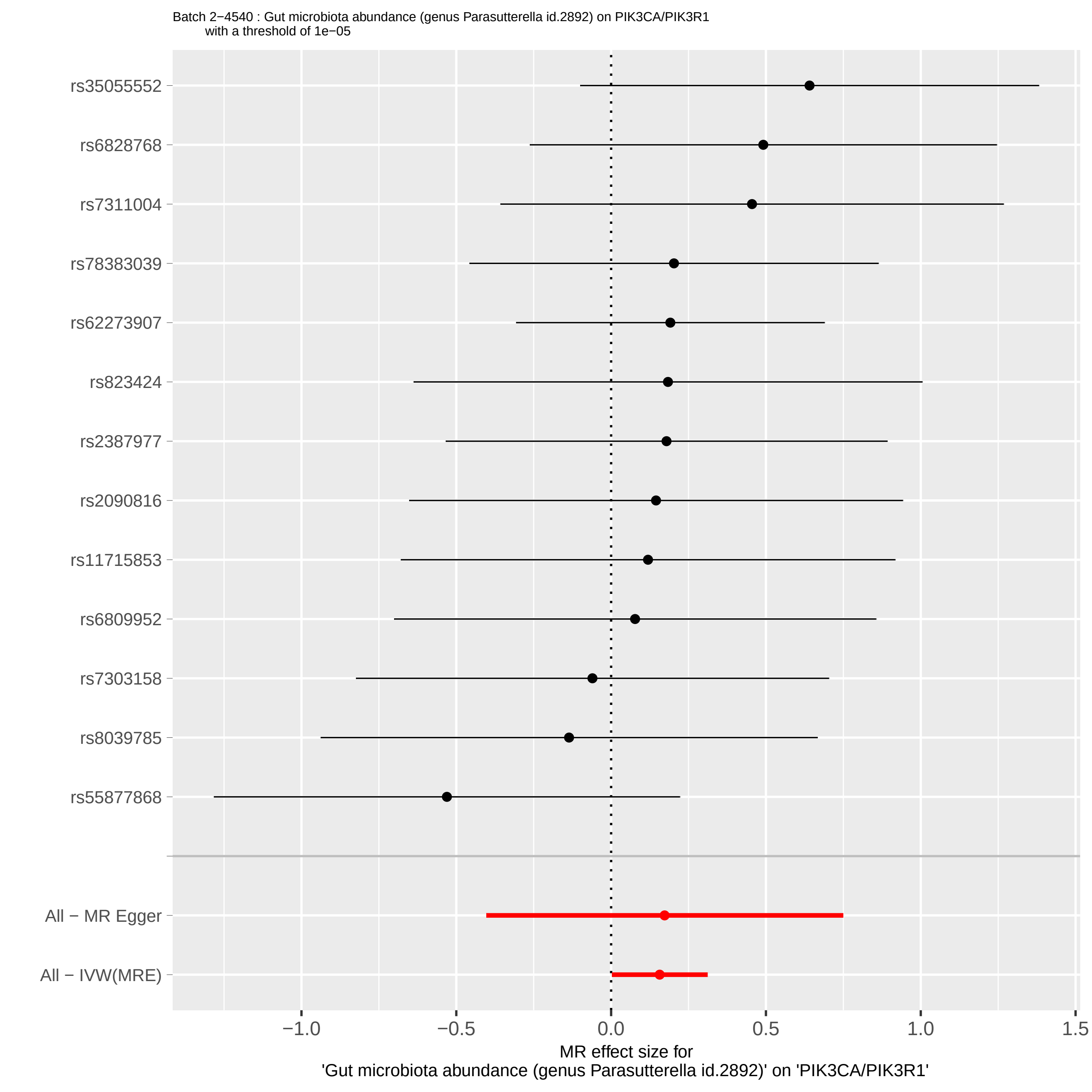

Batch 2−4540 : Gut microbiota abundance (genus Parasutterella id.2892) on PIK3CA/PIK3R1
with a threshold of 1e−05
rs35055552
rs6828768
rs7311004
rs78383039
rs62273907
rs823424
rs2387977
rs2090816
rs11715853
rs6809952
rs7303158
rs8039785
rs55877868
All − MR Egger
All − IVW(MRE)
−1.0
−0.5
0.0
0.5
1.0
1.5
MR effect size for
'Gut microbiota abundance (genus Parasutterella id.2892)' on 'PIK3CA/PIK3R1'

Batch 2−4541 : Gut microbiota abundance (genus Catenibacterium id.2153) on Phosphatidylinositol 4,5−bisphosphate 3−kinase catalytic subunit gamma isoform
with a threshold of 1e−05

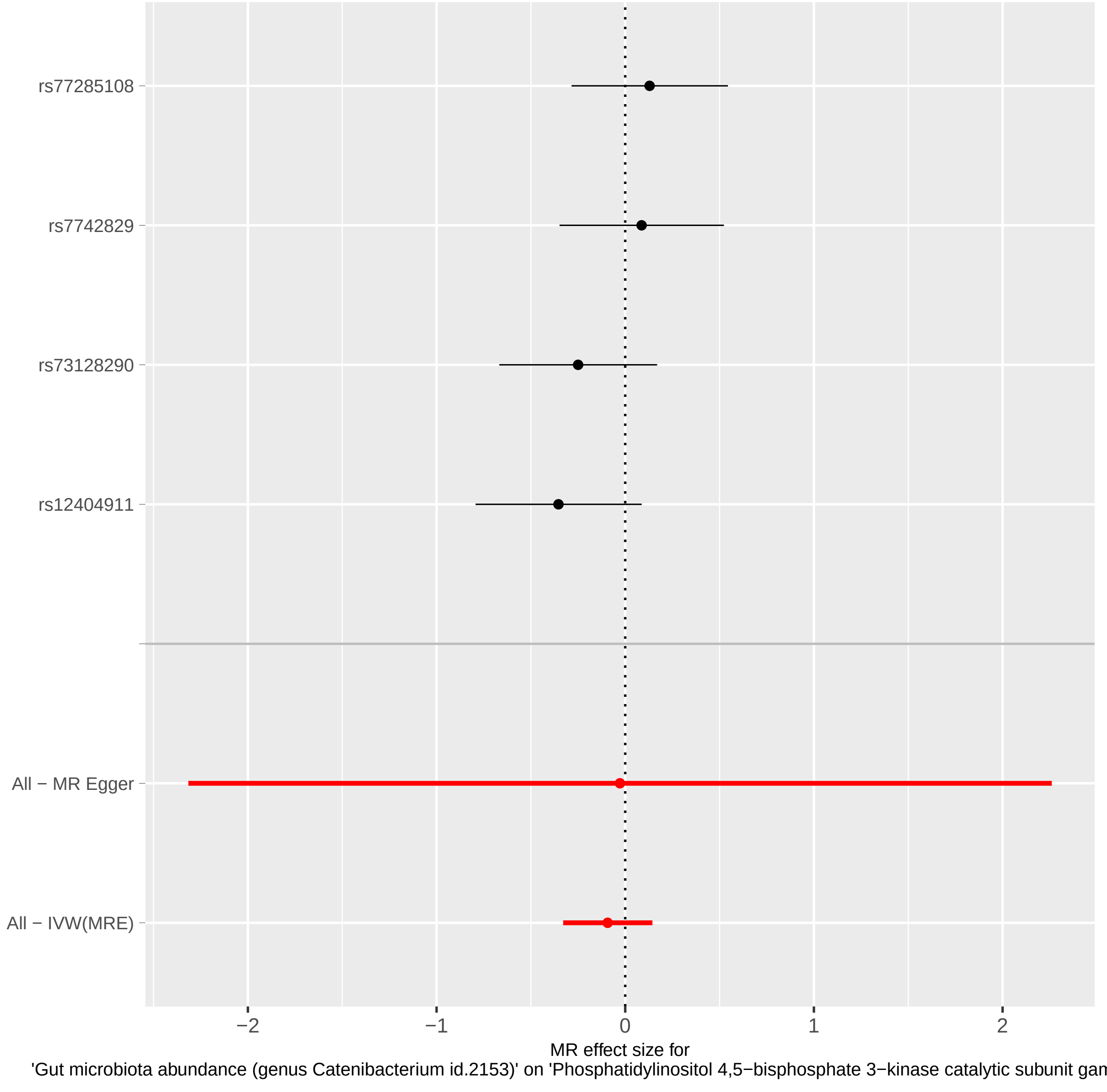

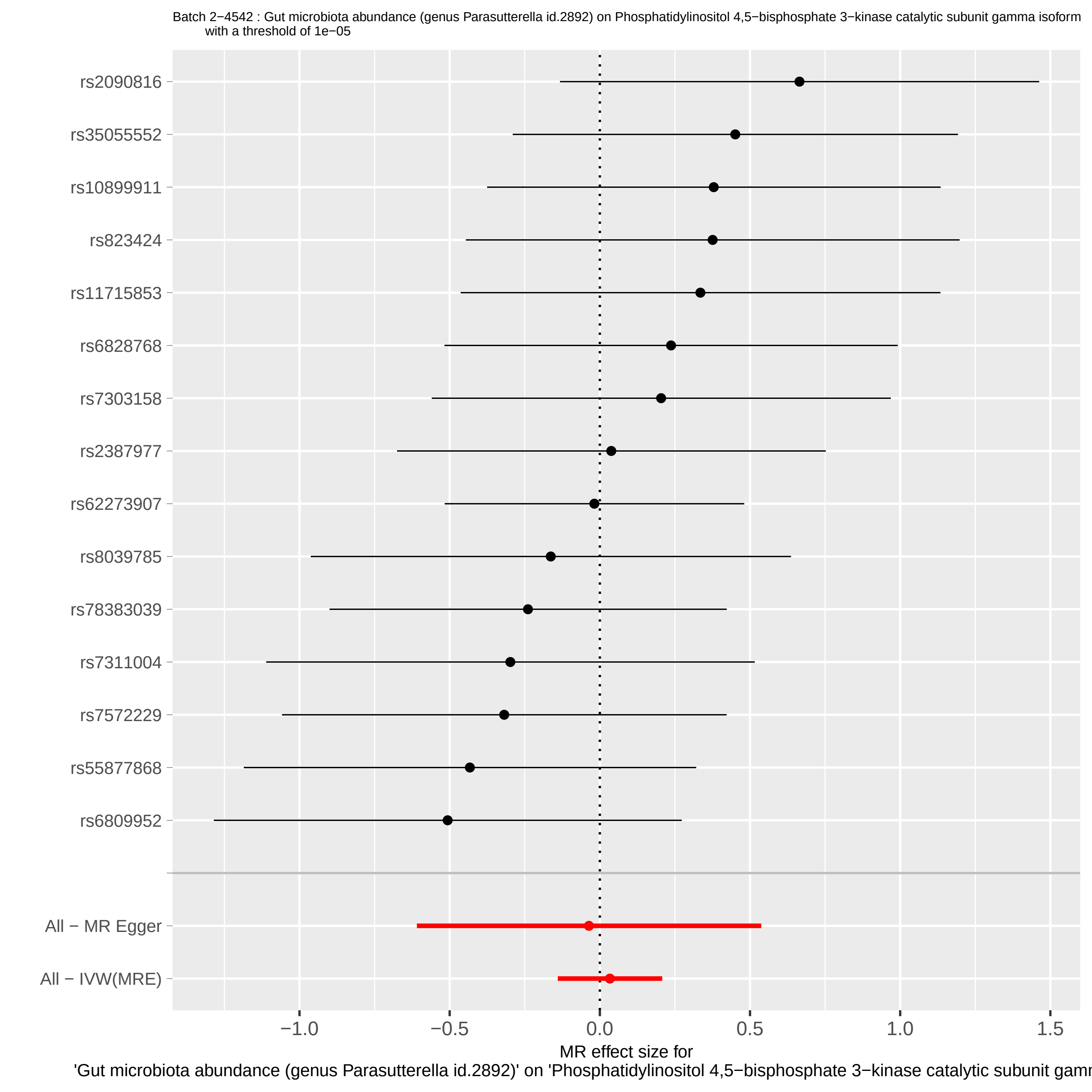

Batch 2−4542 : Gut microbiota abundance (genus Parasutterella id.2892) on Phosphatidylinositol 4,5−bisphosphate 3−kinase catalytic subunit gamma isoform
with a threshold of 1e−05
rs2090816
rs35055552
rs10899911
rs823424
rs11715853
rs6828768
rs7303158
rs2387977
rs62273907
rs8039785
rs78383039
rs7311004
rs7572229
rs55877868
rs6809952
All − MR Egger
All − IVW(MRE)
−1.0
−0.5
0.0
0.5
1.0
1.5
MR effect size for
'Gut microbiota abundance (genus Parasutterella id.2892)' on 'Phosphatidylinositol 4,5−bisphosphate 3−kinase catalytic subunit gamm

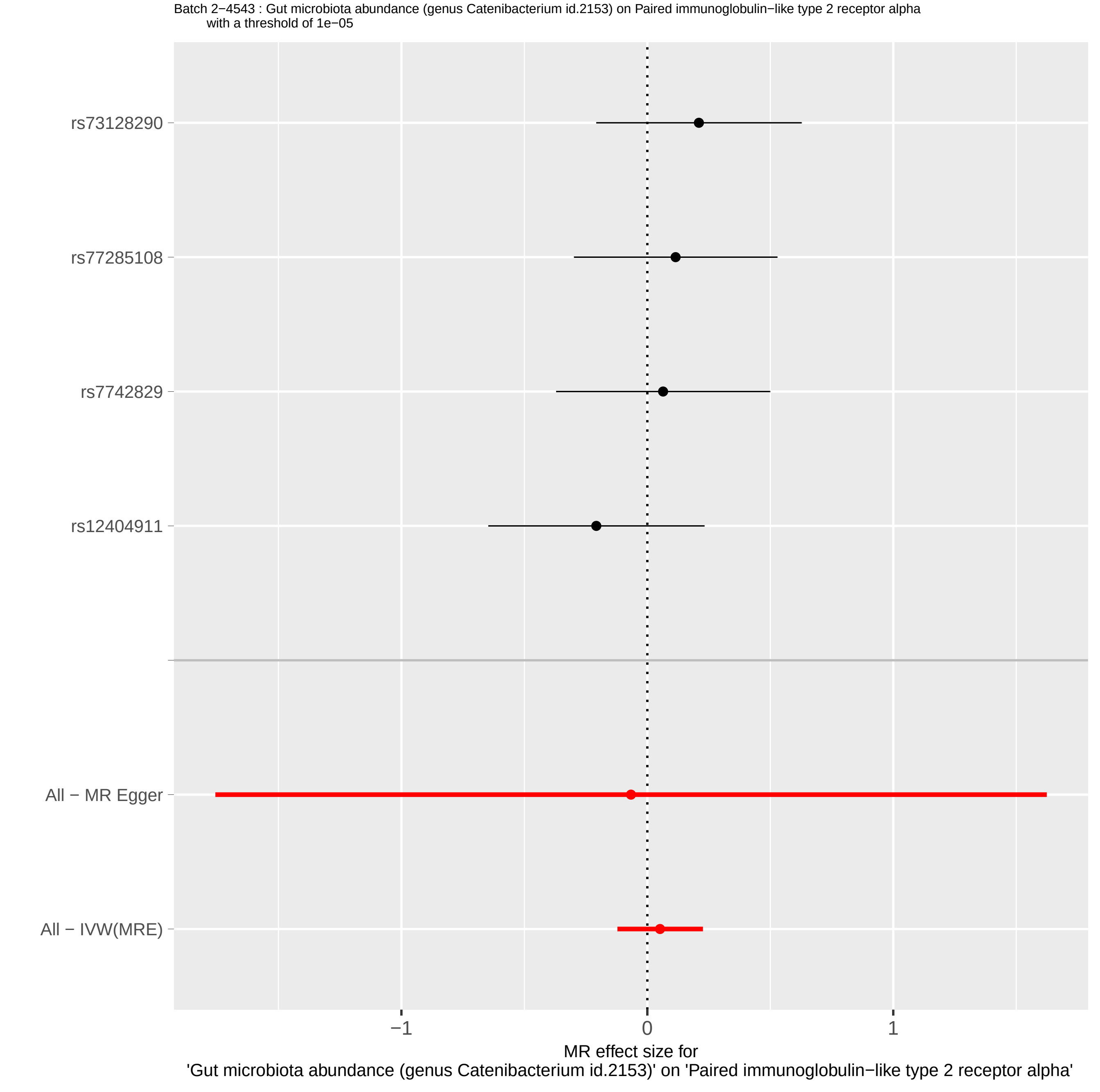

Batch 2−4543 : Gut microbiota abundance (genus Catenibacterium id.2153) on Paired immunoglobulin−like type 2 receptor alpha
with a threshold of 1e−05
rs73128290
rs77285108
rs7742829
rs12404911
All − MR Egger
All − IVW(MRE)
−1
0
1
MR effect size for
'Gut microbiota abundance (genus Catenibacterium id.2153)' on 'Paired immunoglobulin−like type 2 receptor alpha'

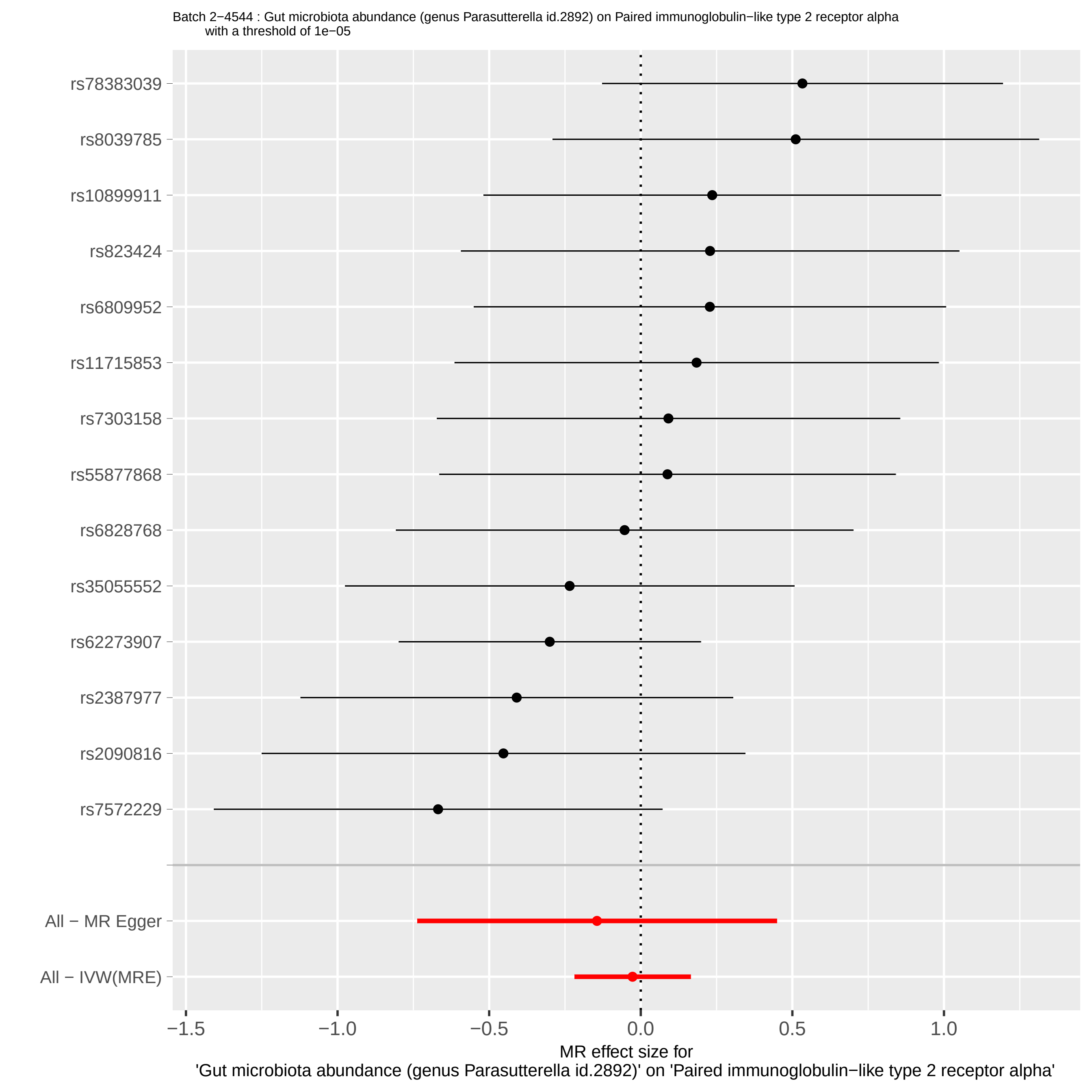

Batch 2−4544 : Gut microbiota abundance (genus Parasutterella id.2892) on Paired immunoglobulin−like type 2 receptor alpha
with a threshold of 1e−05
rs78383039
rs8039785
rs10899911
rs823424
rs6809952
rs11715853
rs7303158
rs55877868
rs6828768
rs35055552
rs62273907
rs2387977
rs2090816
rs7572229
All − MR Egger
All − IVW(MRE)
−1.5
−1.0
−0.5
0.0
0.5
1.0
MR effect size for
'Gut microbiota abundance (genus Parasutterella id.2892)' on 'Paired immunoglobulin−like type 2 receptor alpha'

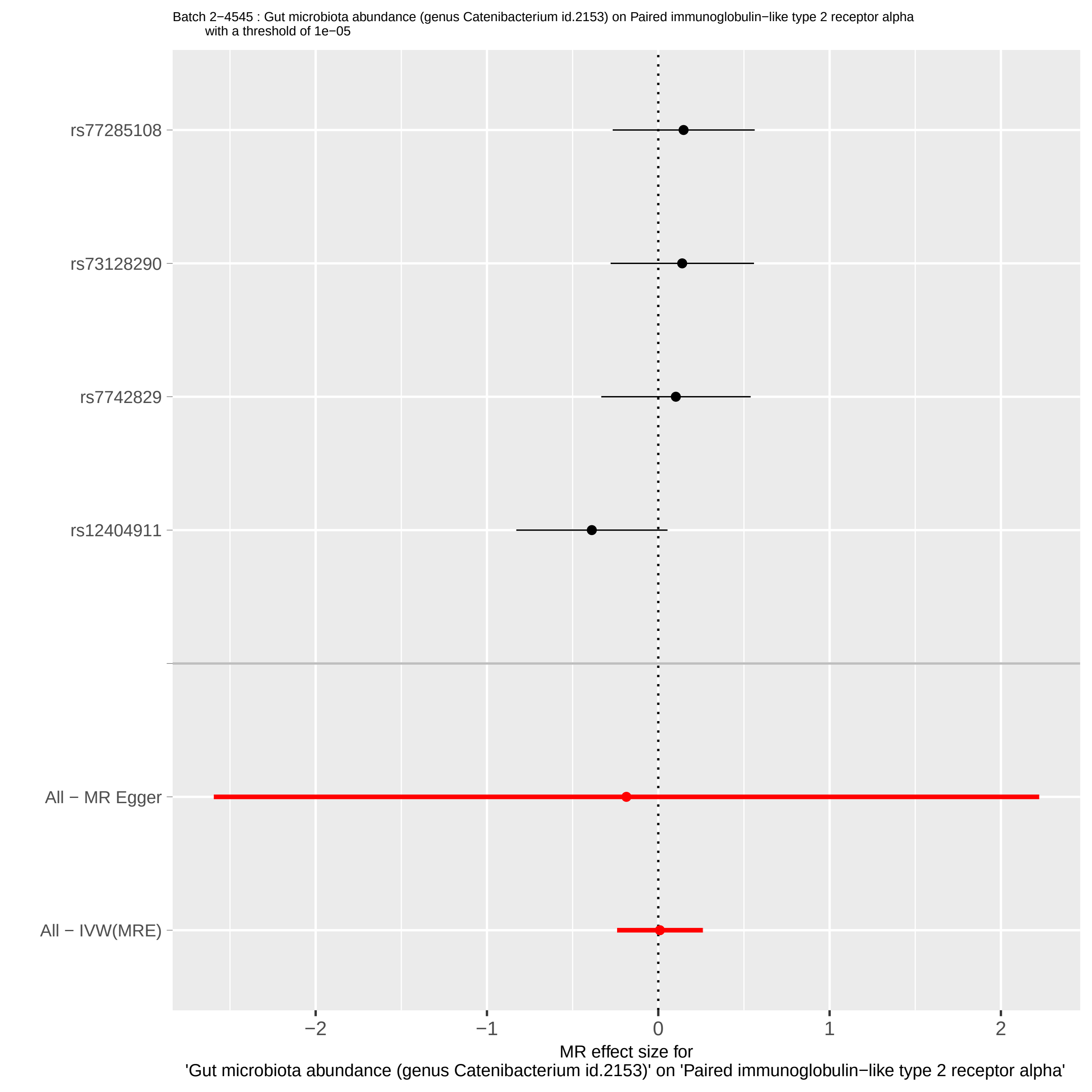

Batch 2−4545 : Gut microbiota abundance (genus Catenibacterium id.2153) on Paired immunoglobulin−like type 2 receptor alpha
with a threshold of 1e−05
rs77285108
rs73128290
rs7742829
rs12404911
All − MR Egger
All − IVW(MRE)
−2
−1
0
1
2
MR effect size for
'Gut microbiota abundance (genus Catenibacterium id.2153)' on 'Paired immunoglobulin−like type 2 receptor alpha'

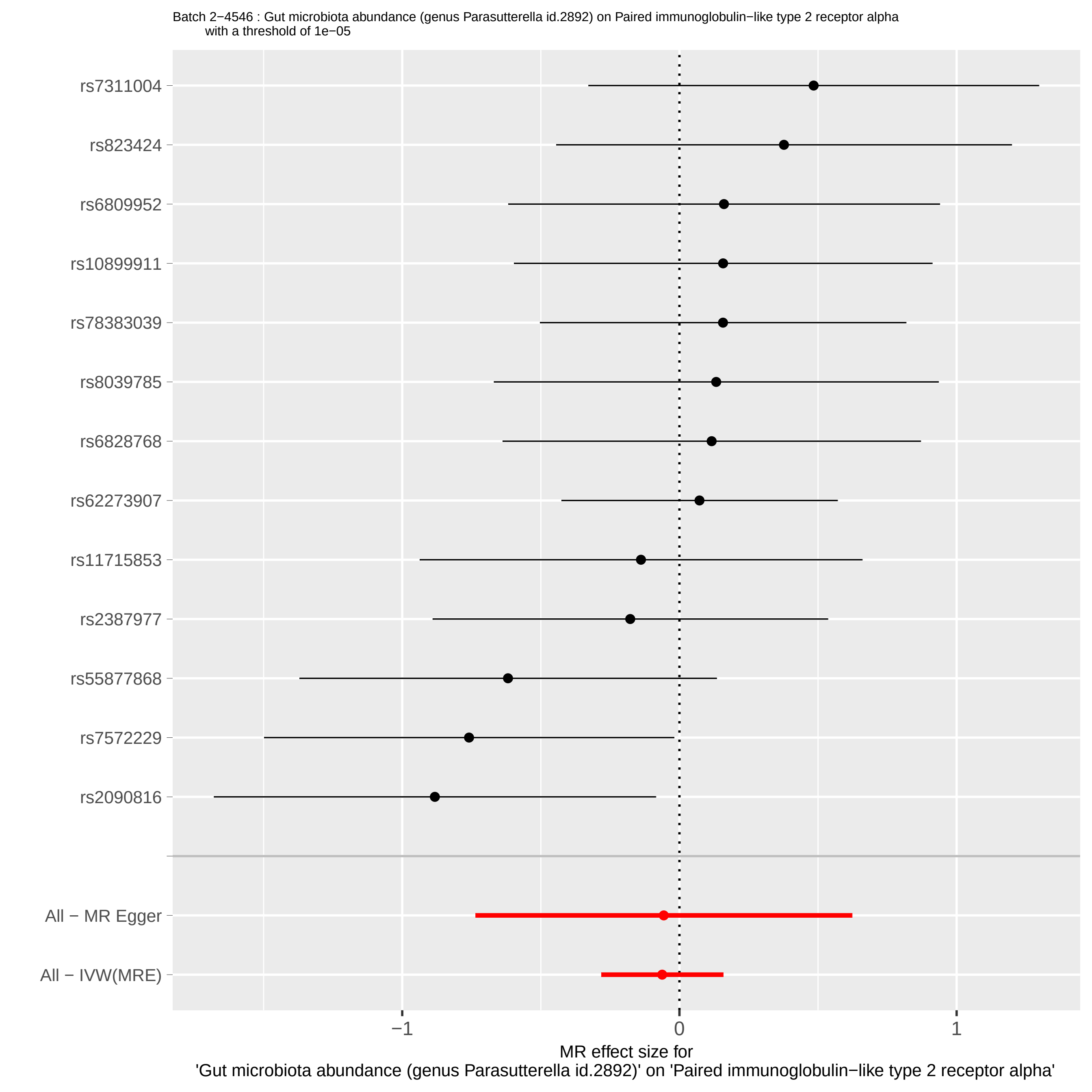

Batch 2−4546 : Gut microbiota abundance (genus Parasutterella id.2892) on Paired immunoglobulin−like type 2 receptor alpha
with a threshold of 1e−05
rs7311004
rs823424
rs6809952
rs10899911
rs78383039
rs8039785
rs6828768
rs62273907
rs11715853
rs2387977
rs55877868
rs7572229
rs2090816
All − MR Egger
All − IVW(MRE)
−1
0
1
MR effect size for
'Gut microbiota abundance (genus Parasutterella id.2892)' on 'Paired immunoglobulin−like type 2 receptor alpha'

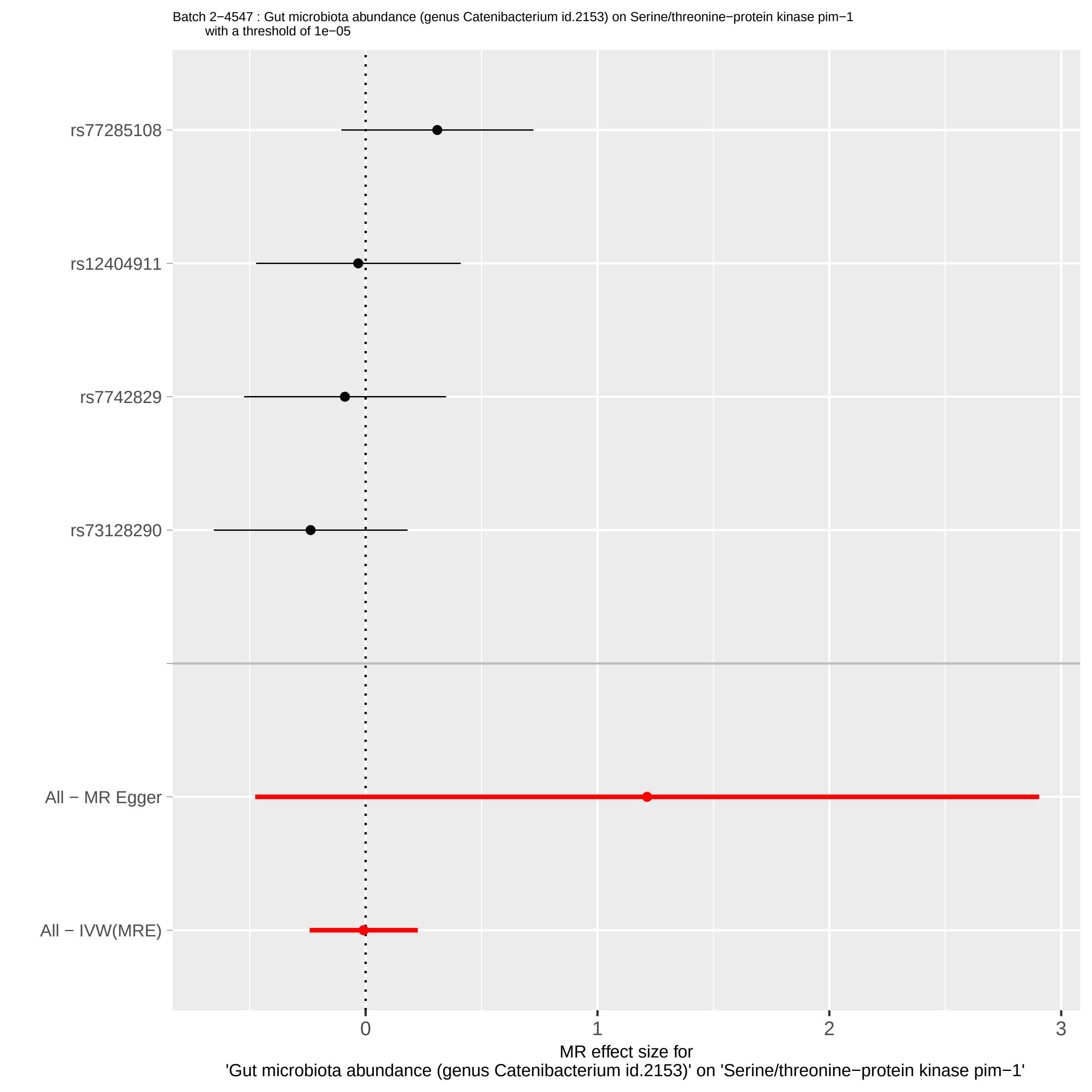
Batch 2−4547 : Gut microbiota abundance (genus Catenibacterium id.2153) on Serine/threonine−protein kinase pim−1
with a threshold of 1e−05
rs77285108
rs12404911
rs7742829
rs73128290
All − MR Egger
All − IVW(MRE)
0
1
2
3
MR effect size for
'Gut microbiota abundance (genus Catenibacterium id.2153)' on 'Serine/threonine−protein kinase pim−1'

Batch 2−4548 : Gut microbiota abundance (genus Parasutterella id.2892) on Serine/threonine−protein kinase pim−1
with a threshold of 1e−05

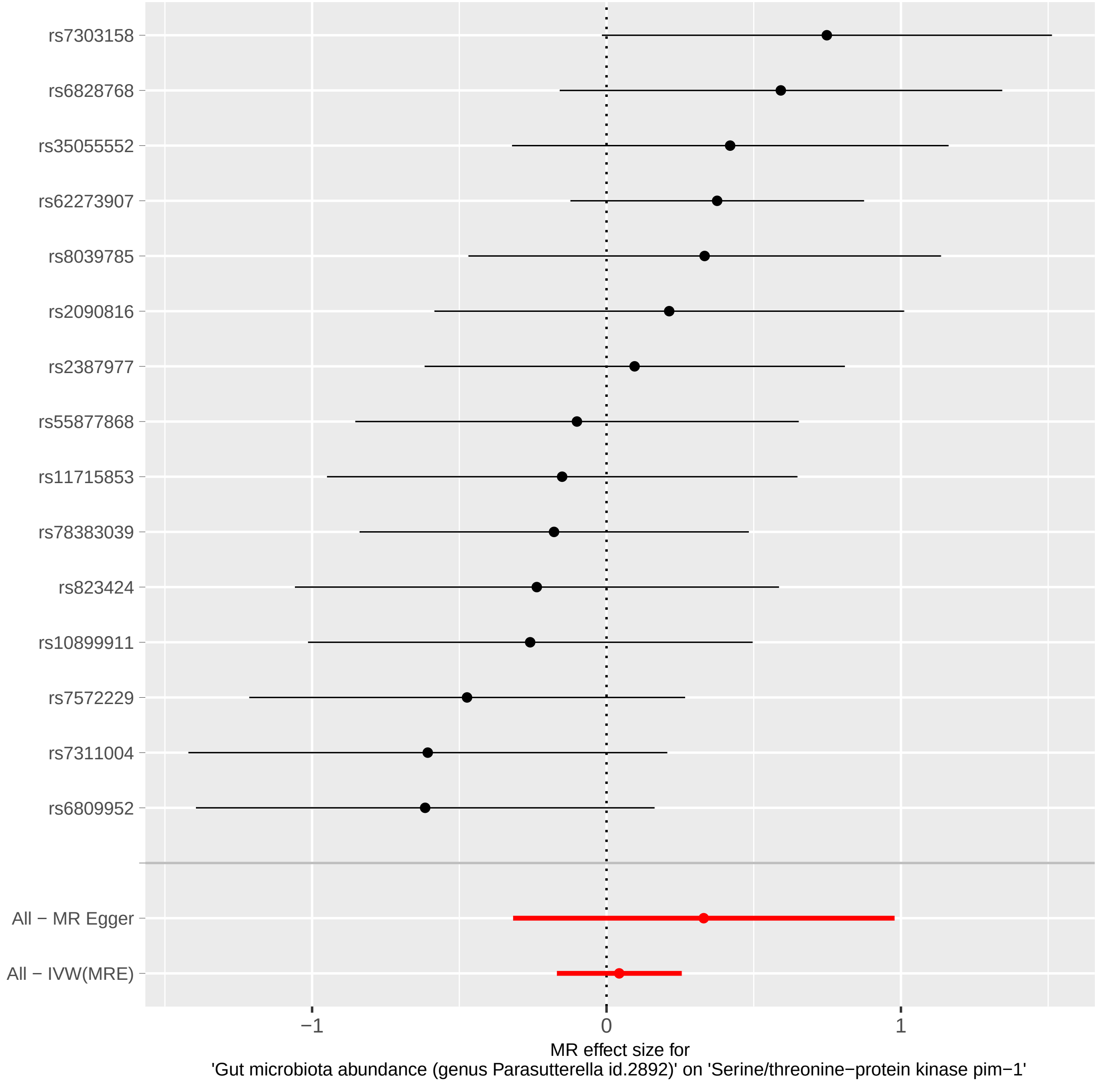

Batch 2−4549 : Gut microbiota abundance (genus Catenibacterium id.2153) on Peptidyl−prolyl cis−trans isomerase NIMA−interacting 4 with a threshold of 1e−05

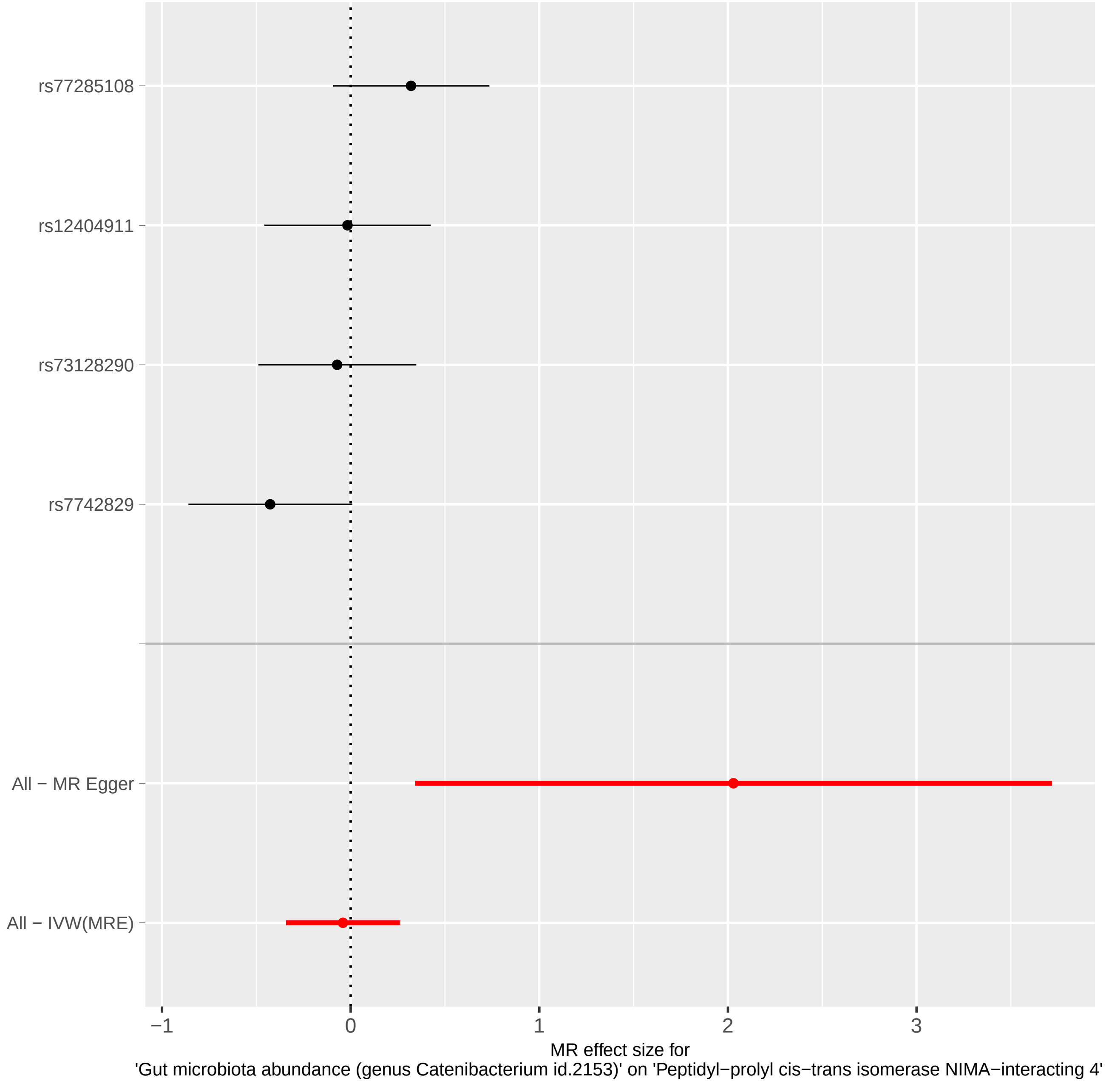

Batch 2−4550 : Gut microbiota abundance (genus Parasutterella id.2892) on Peptidyl−prolyl cis−trans isomerase NIMA−interacting 4
with a threshold of 1e−05

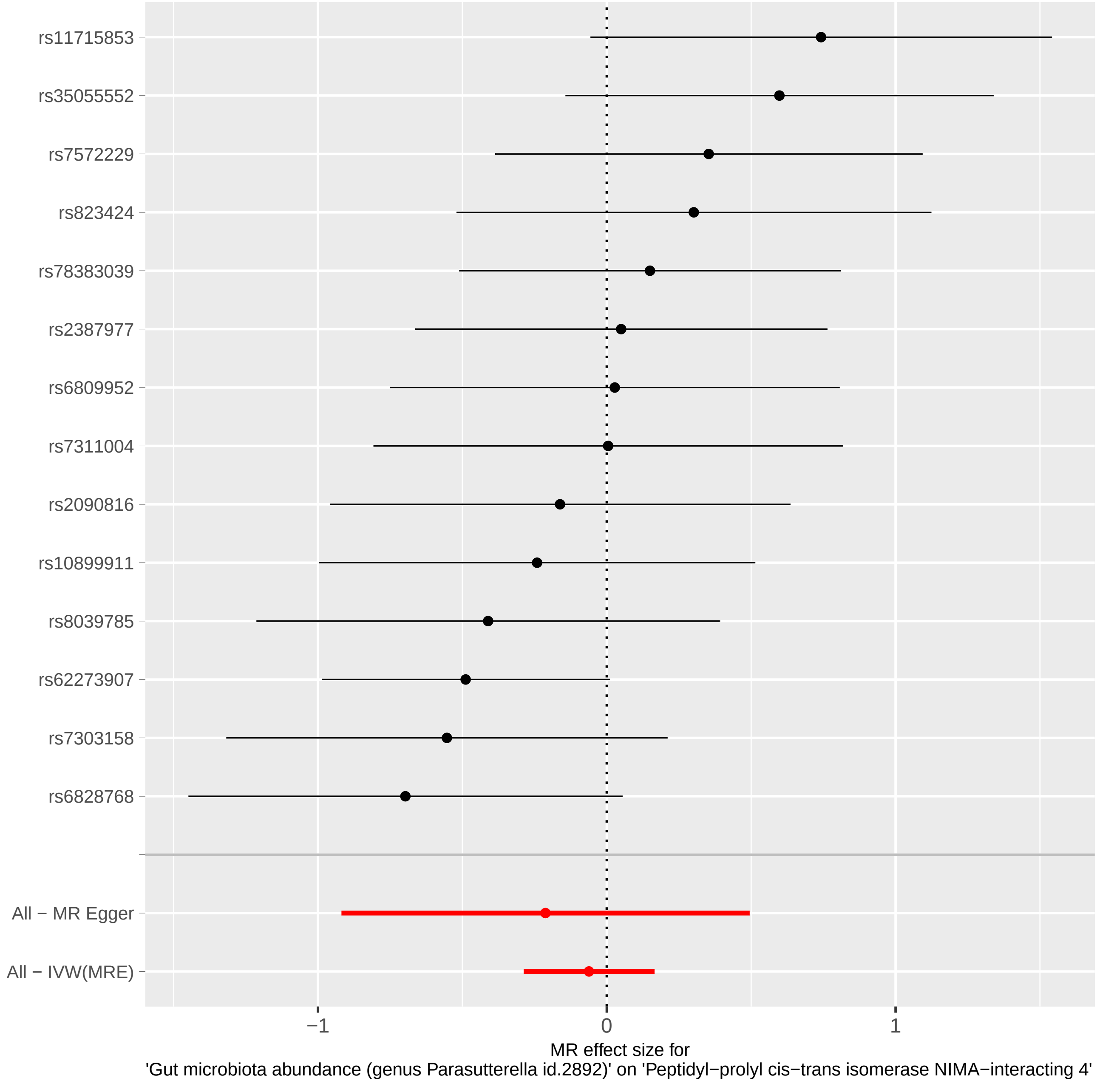

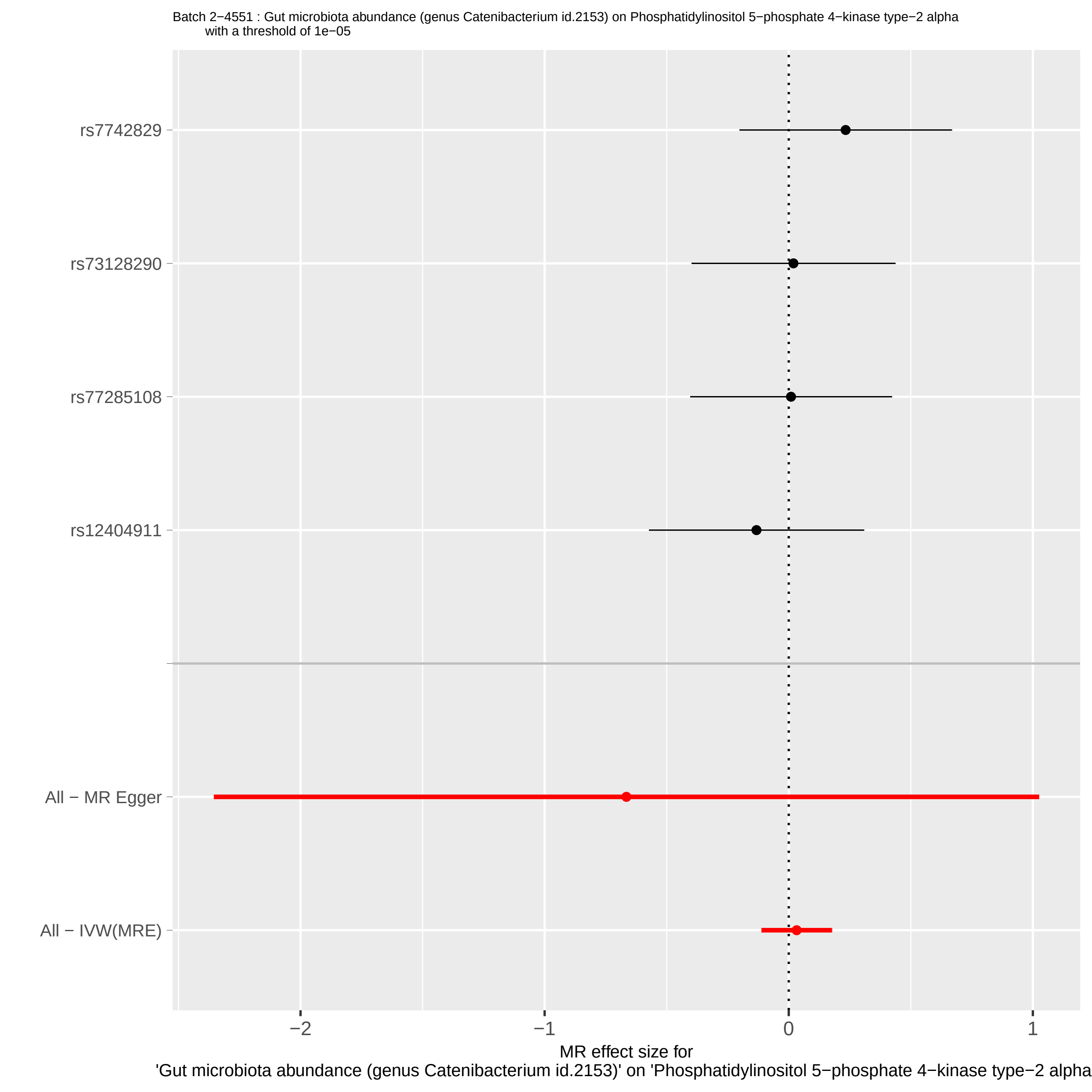

Batch 2−4551 : Gut microbiota abundance (genus Catenibacterium id.2153) on Phosphatidylinositol 5−phosphate 4−kinase type−2 alpha
with a threshold of 1e−05
rs7742829
rs73128290
rs77285108
rs12404911
All − MR Egger
All − IVW(MRE)
−2
−1
0
1
MR effect size for
'Gut microbiota abundance (genus Catenibacterium id.2153)' on 'Phosphatidylinositol 5−phosphate 4−kinase type−2 alpha

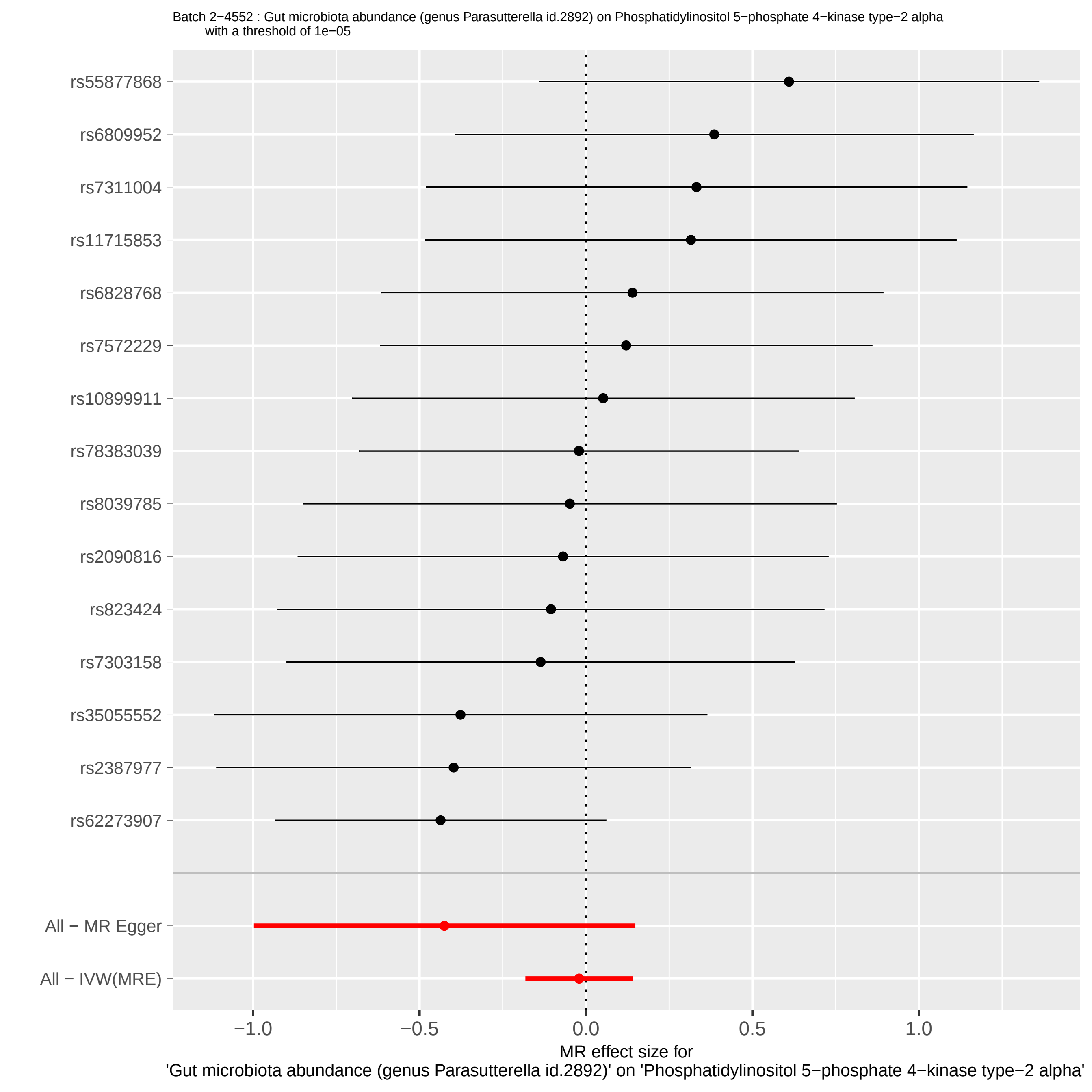

Batch 2−4552 : Gut microbiota abundance (genus Parasutterella id.2892) on Phosphatidylinositol 5−phosphate 4−kinase type−2 alpha
with a threshold of 1e−05
rs55877868
rs6809952
rs7311004
rs11715853
rs6828768
rs7572229
rs10899911
rs78383039
rs8039785
rs2090816
rs823424
rs7303158
rs35055552
rs2387977
rs62273907
All − MR Egger
All − IVW(MRE)
−1.0
−0.5
0.0
0.5
1.0
MR effect size for
'Gut microbiota abundance (genus Parasutterella id.2892)' on 'Phosphatidylinositol 5−phosphate 4−kinase type−2 alpha'

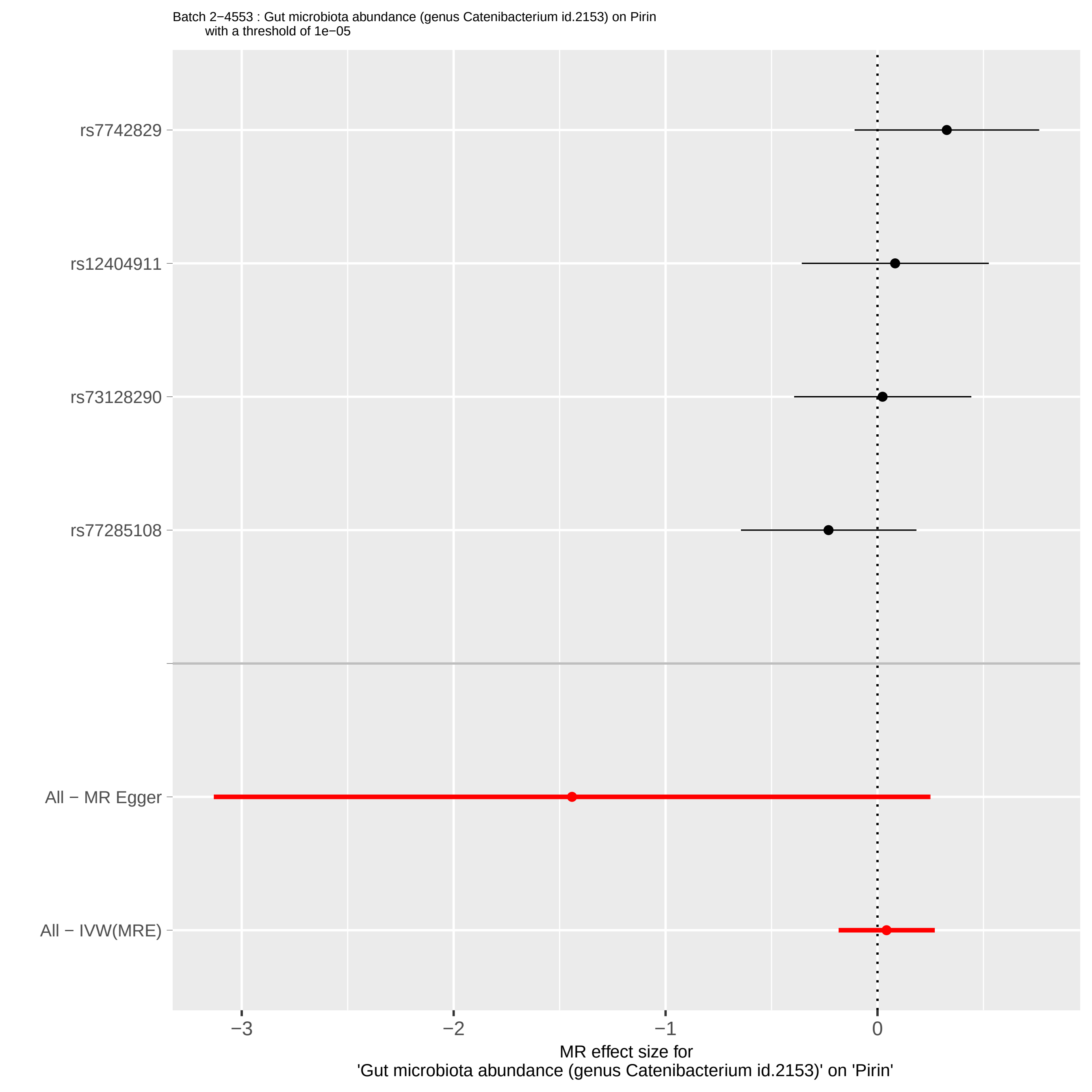

Batch 2−4553 : Gut microbiota abundance (genus Catenibacterium id.2153) on Pirin
with a threshold of 1e−05
rs7742829
rs12404911
rs73128290
rs77285108
All − MR Egger
All − IVW(MRE)
−3
−2
−1
0
MR effect size for
'Gut microbiota abundance (genus Catenibacterium id.2153)' on 'Pirin'

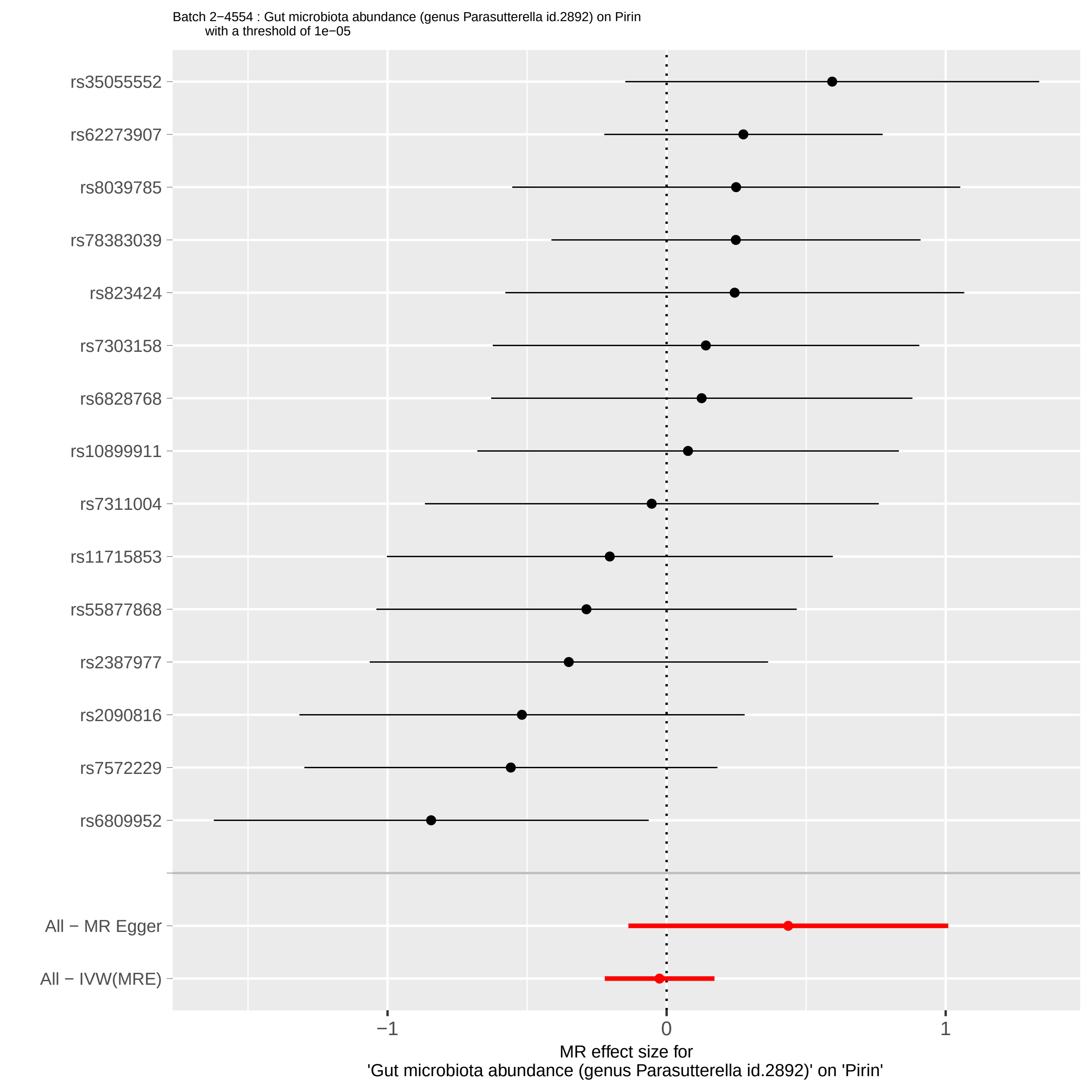

Batch 2−4554 : Gut microbiota abundance (genus Parasutterella id.2892) on Pirin
with a threshold of 1e−05
rs35055552
rs62273907
rs8039785
rs78383039
rs823424
rs7303158
rs6828768
rs10899911
rs7311004
rs11715853
rs55877868
rs2387977
rs2090816
rs7572229
rs6809952
All − MR Egger
All − IVW(MRE)
−1
0
1
MR effect size for
'Gut microbiota abundance (genus Parasutterella id.2892)' on 'Pirin'

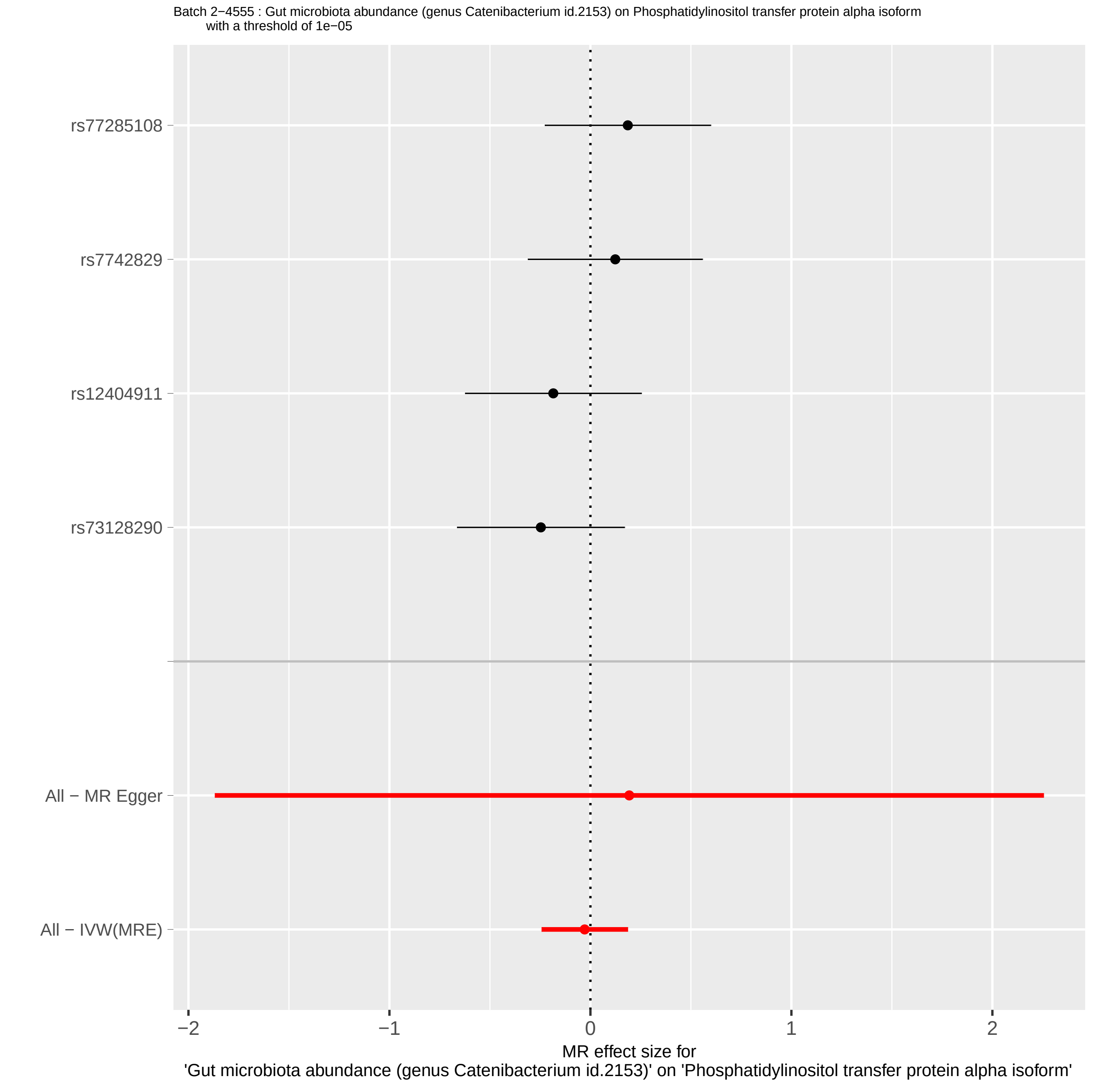

Batch 2−4555 : Gut microbiota abundance (genus Catenibacterium id.2153) on Phosphatidylinositol transfer protein alpha isoform
with a threshold of 1e−05
rs77285108
rs7742829
rs12404911
rs73128290
All − MR Egger
All − IVW(MRE)
−2
−1
0
1
2
MR effect size for
'Gut microbiota abundance (genus Catenibacterium id.2153)' on 'Phosphatidylinositol transfer protein alpha isoform'

Batch 2−4556 : Gut microbiota abundance (genus Parasutterella id.2892) on Phosphatidylinositol transfer protein alpha isoform with a threshold of 1e−05

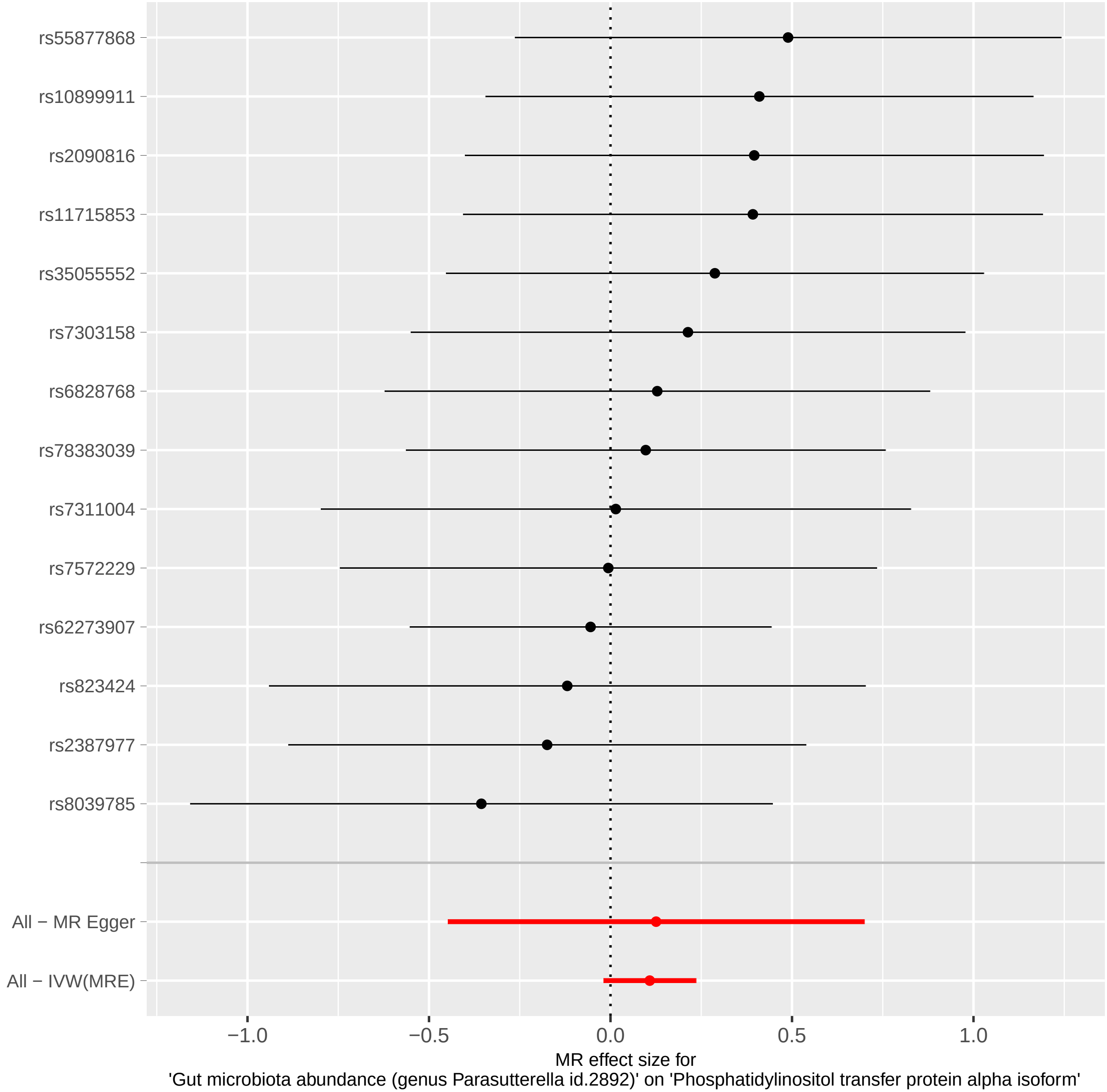

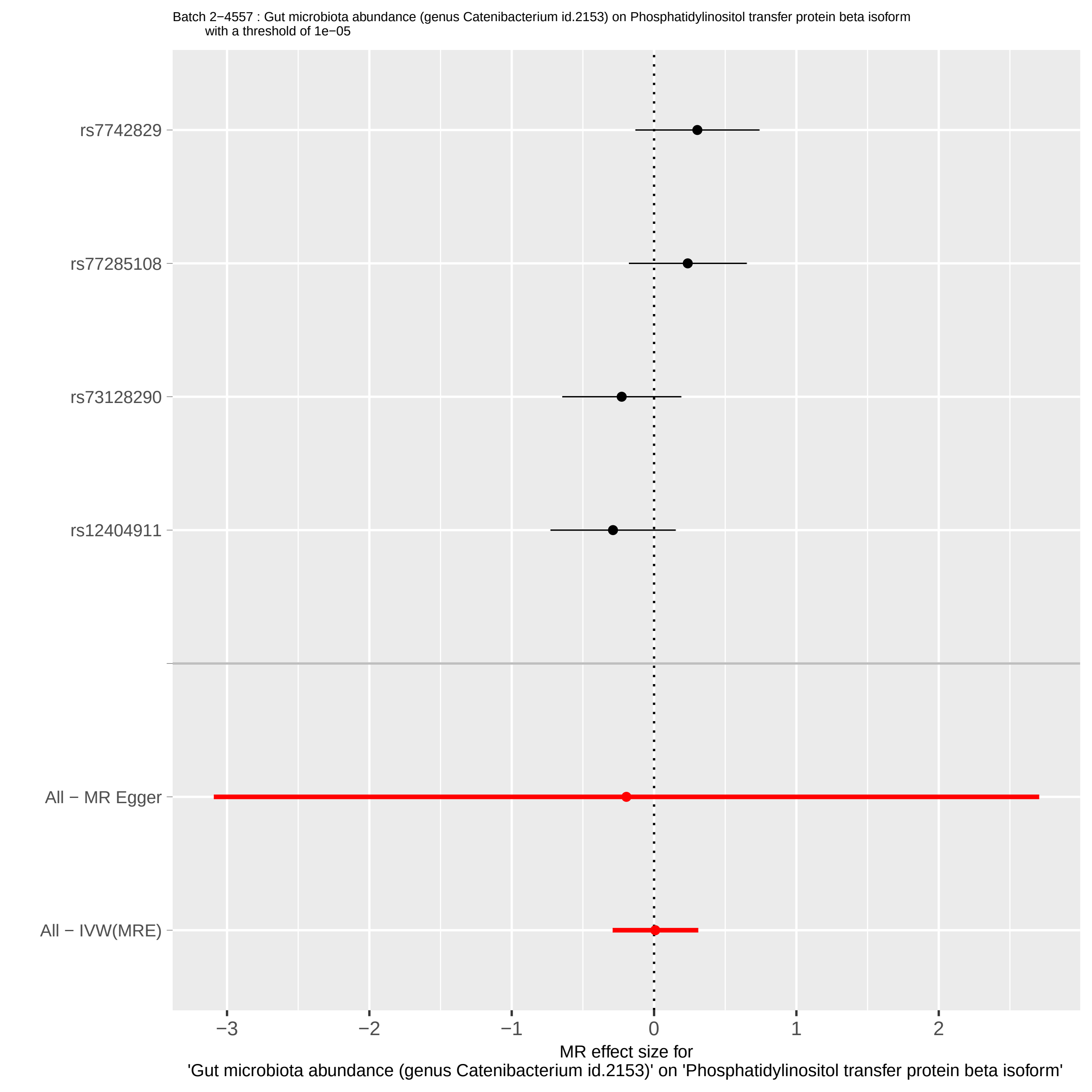

Batch 2−4557 : Gut microbiota abundance (genus Catenibacterium id.2153) on Phosphatidylinositol transfer protein beta isoform with a threshold of 1e−05
rs7742829
rs77285108
rs73128290
rs12404911
All − MR Egger
All − IVW(MRE)
−3
−2
−1
0
1
2
MR effect size for 'Gut microbiota abundance (genus Catenibacterium id.2153)' on 'Phosphatidylinositol transfer protein beta isoform'

Batch 2−4558 : Gut microbiota abundance (genus Parasutterella id.2892) on Phosphatidylinositol transfer protein beta isoform with a threshold of 1e−05

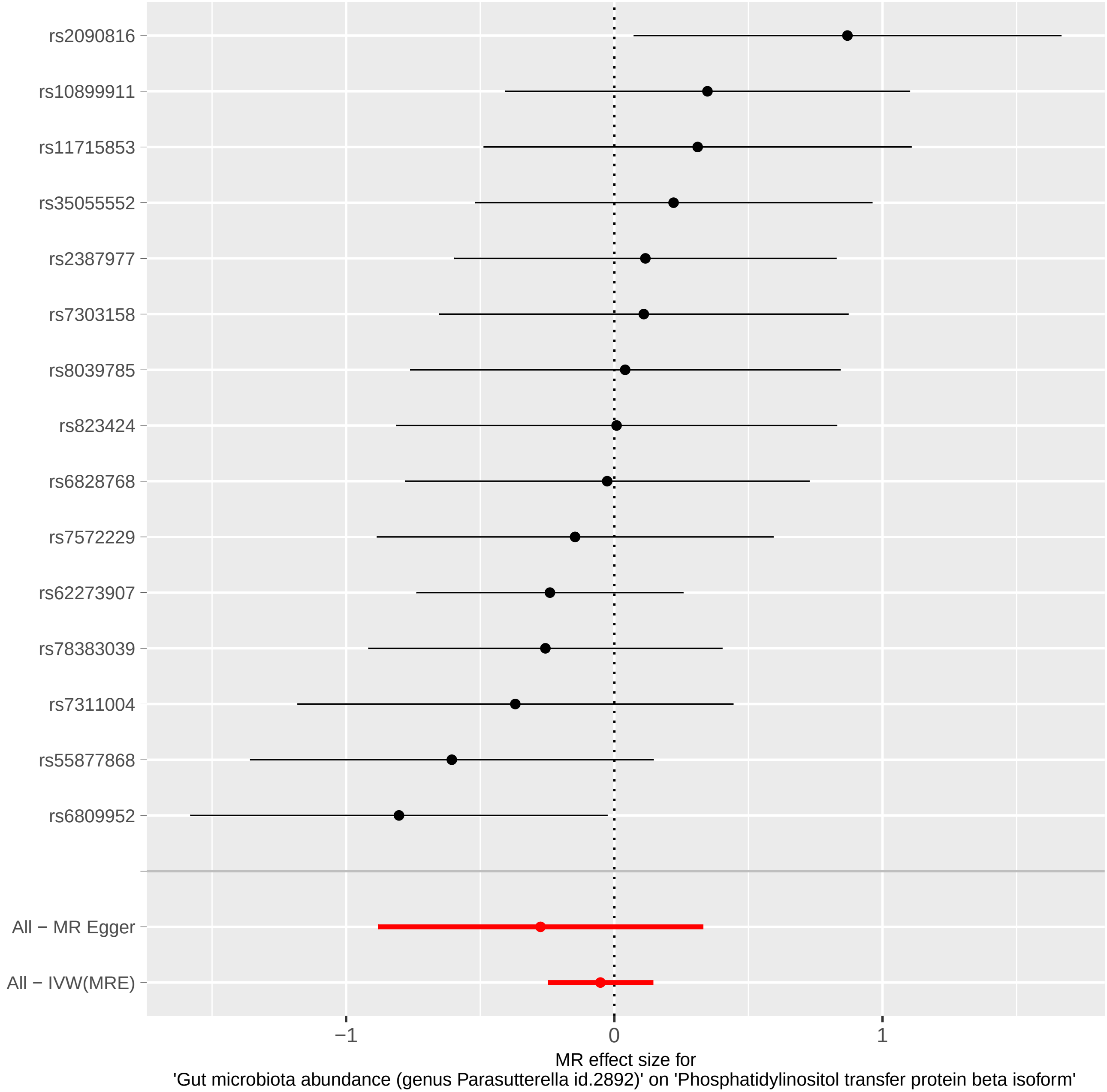

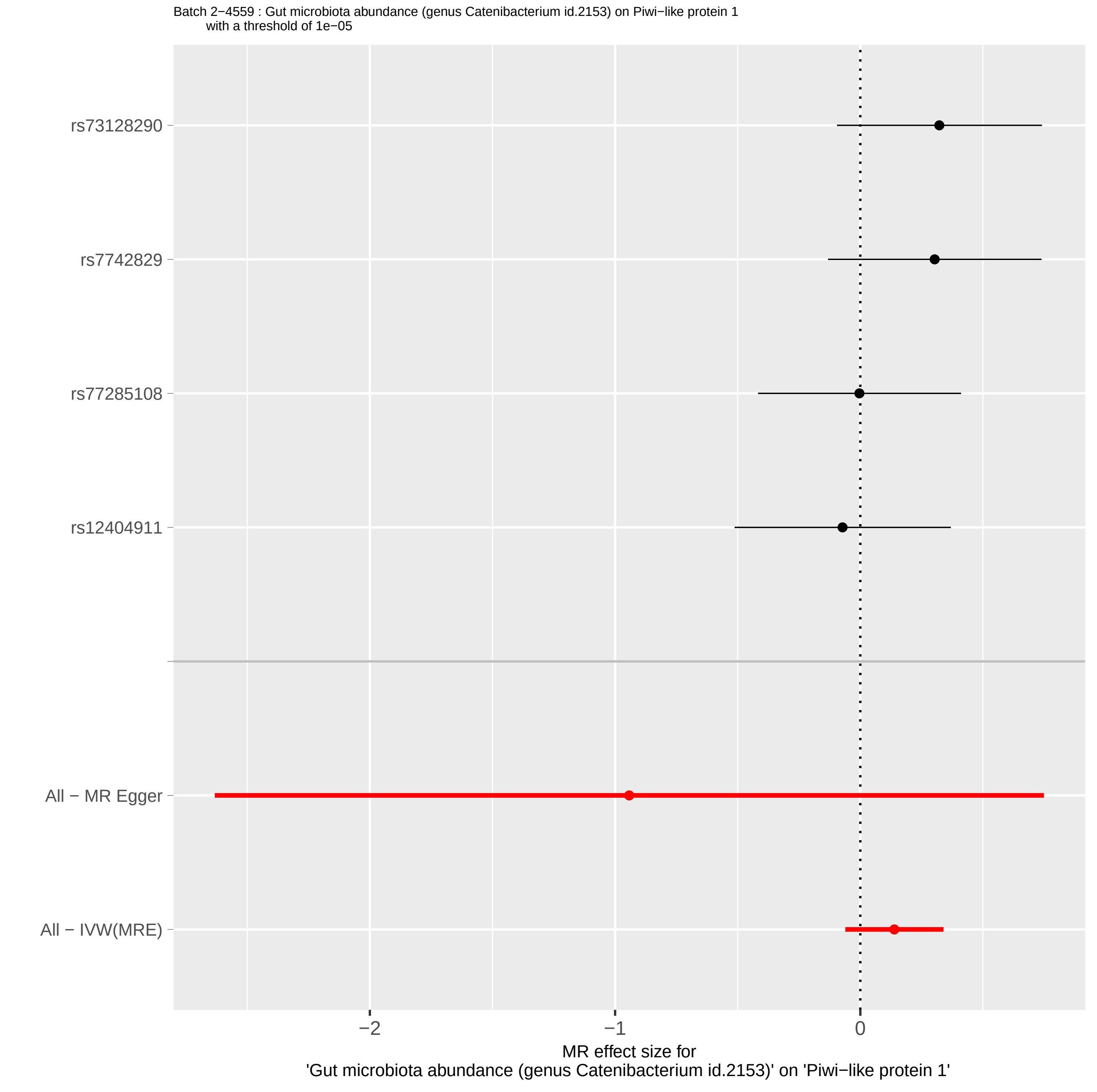

Batch 2−4559 : Gut microbiota abundance (genus Catenibacterium id.2153) on Piwi−like protein 1
with a threshold of 1e−05
rs73128290
rs7742829
rs77285108
rs12404911
All − MR Egger
All − IVW(MRE)
−2
−1
0
MR effect size for
'Gut microbiota abundance (genus Catenibacterium id.2153)' on 'Piwi−like protein 1'

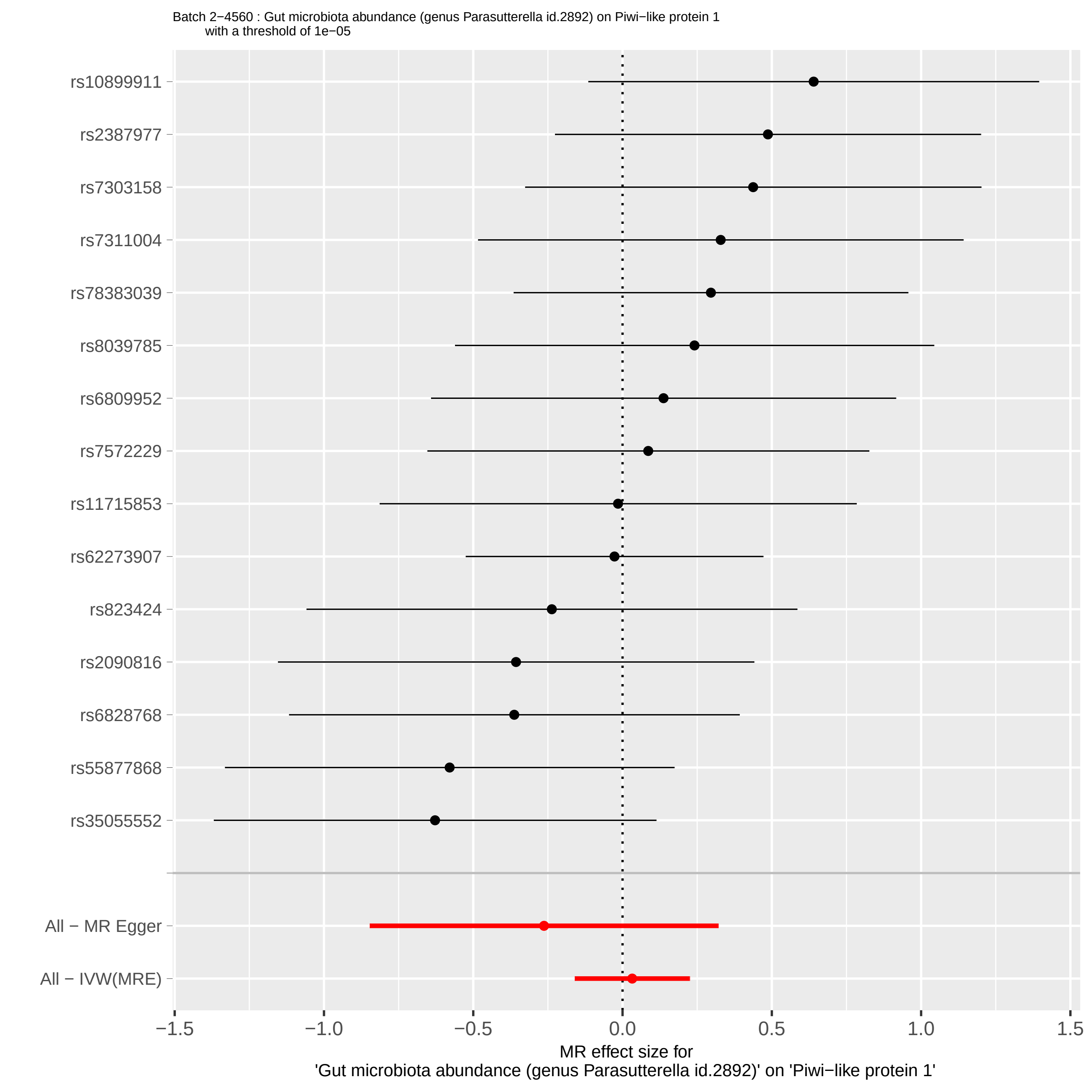

Batch 2−4560 : Gut microbiota abundance (genus Parasutterella id.2892) on Piwi−like protein 1
with a threshold of 1e−05
rs10899911
rs2387977
rs7303158
rs7311004
rs78383039
rs8039785
rs6809952
rs7572229
rs11715853
rs62273907
rs823424
rs2090816
rs6828768
rs55877868
rs35055552
All − MR Egger
All − IVW(MRE)
−1.5
−1.0
−0.5
0.0
0.5
1.0
1.5
MR effect size for
'Gut microbiota abundance (genus Parasutterella id.2892)' on 'Piwi−like protein 1'

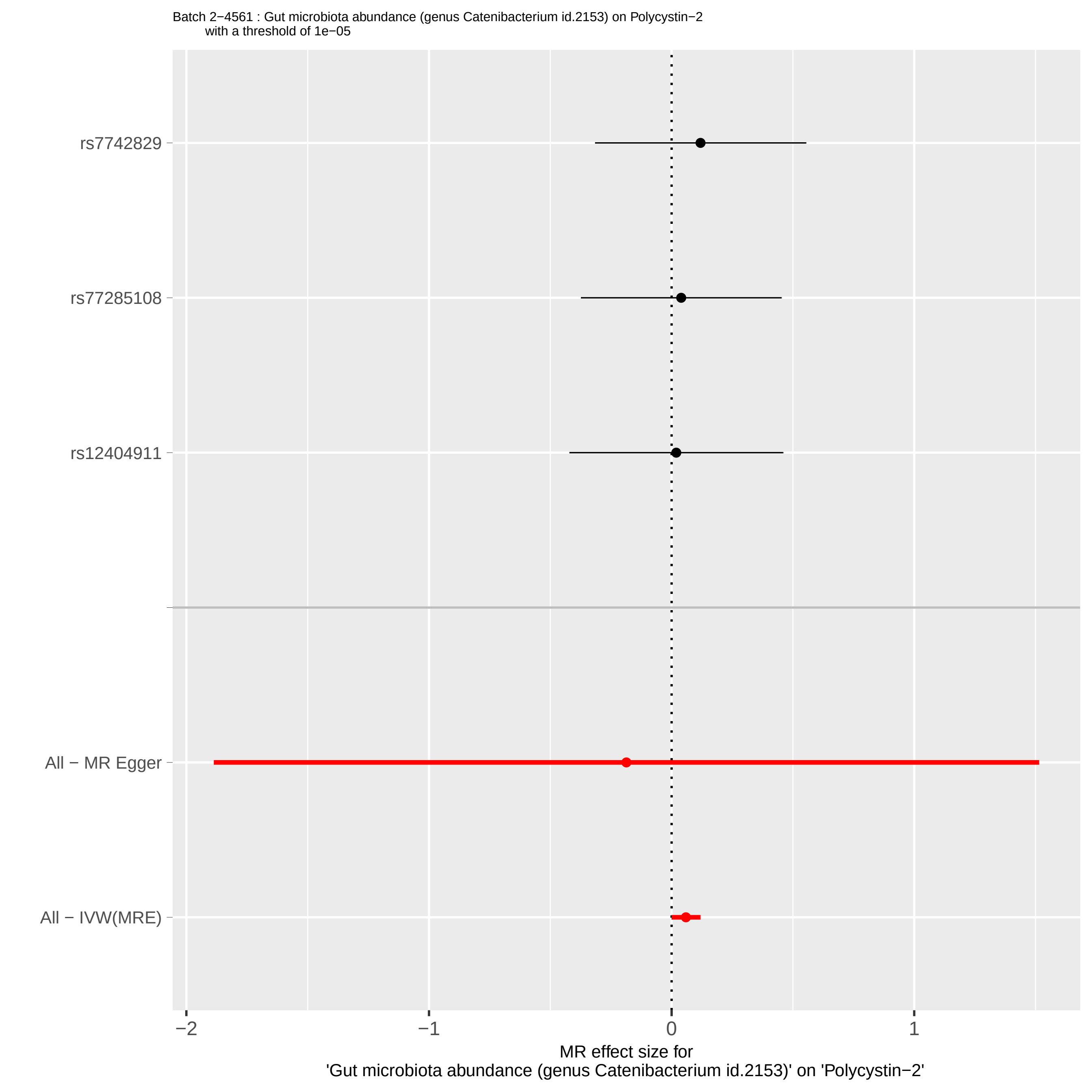
Batch 2−4561 : Gut microbiota abundance (genus Catenibacterium id.2153) on Polycystin−2
with a threshold of 1e−05
rs7742829
rs77285108
rs12404911
All − MR Egger
All − IVW(MRE)
−2
−1
0
1
MR effect size for
'Gut microbiota abundance (genus Catenibacterium id.2153)' on 'Polycystin−2'

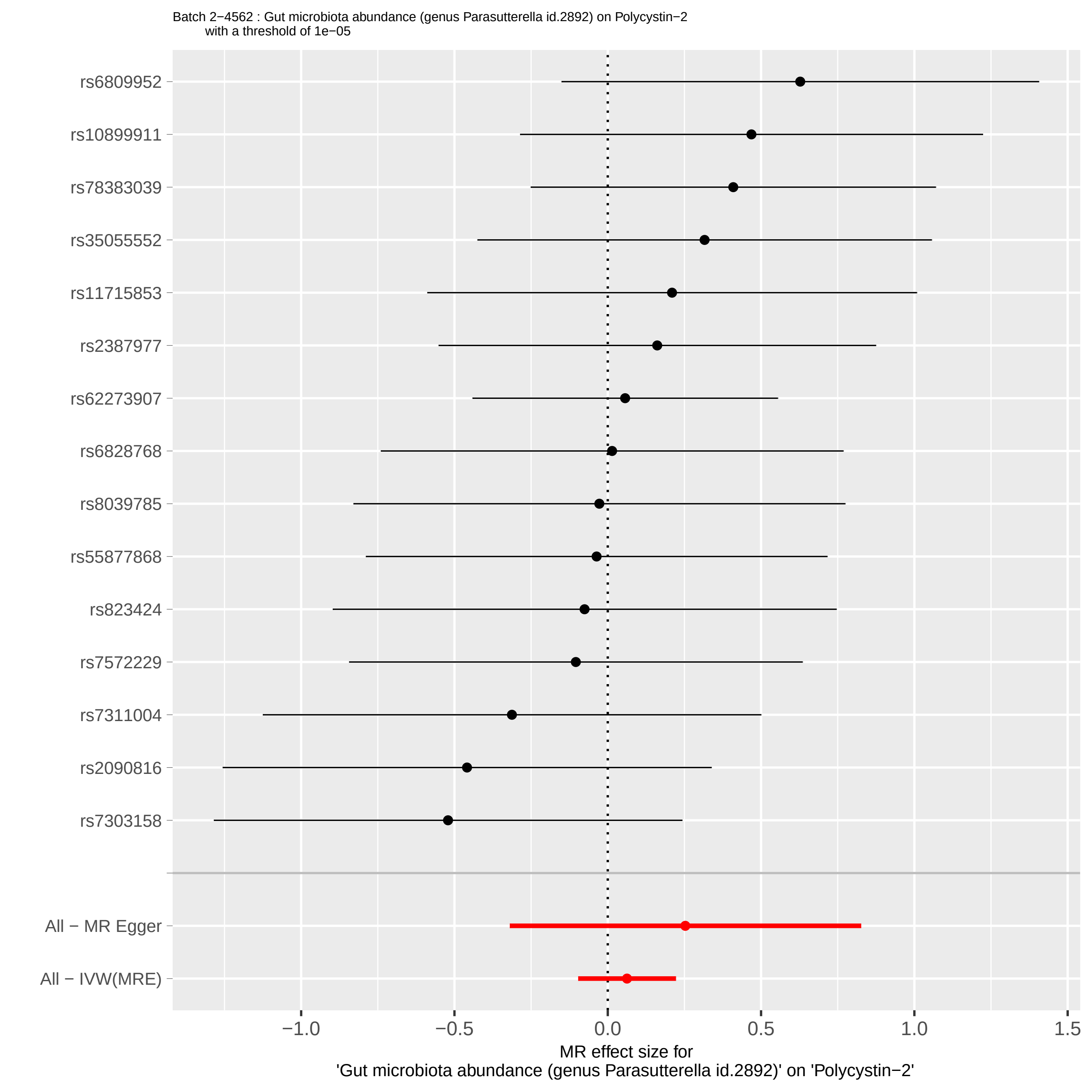

Batch 2−4562 : Gut microbiota abundance (genus Parasutterella id.2892) on Polycystin−2
with a threshold of 1e−05
rs6809952
rs10899911
rs78383039
rs35055552
rs11715853
rs2387977
rs62273907
rs6828768
rs8039785
rs55877868
rs823424
rs7572229
rs7311004
rs2090816
rs7303158
All − MR Egger
All − IVW(MRE)
−1.0
−0.5
0.0
0.5
1.0
1.5
MR effect size for
'Gut microbiota abundance (genus Parasutterella id.2892)' on 'Polycystin−2'

Batch 2−4563 : Gut microbiota abundance (genus Catenibacterium id.2153) on Pyruvate kinase PKLR
with a threshold of 1e−05

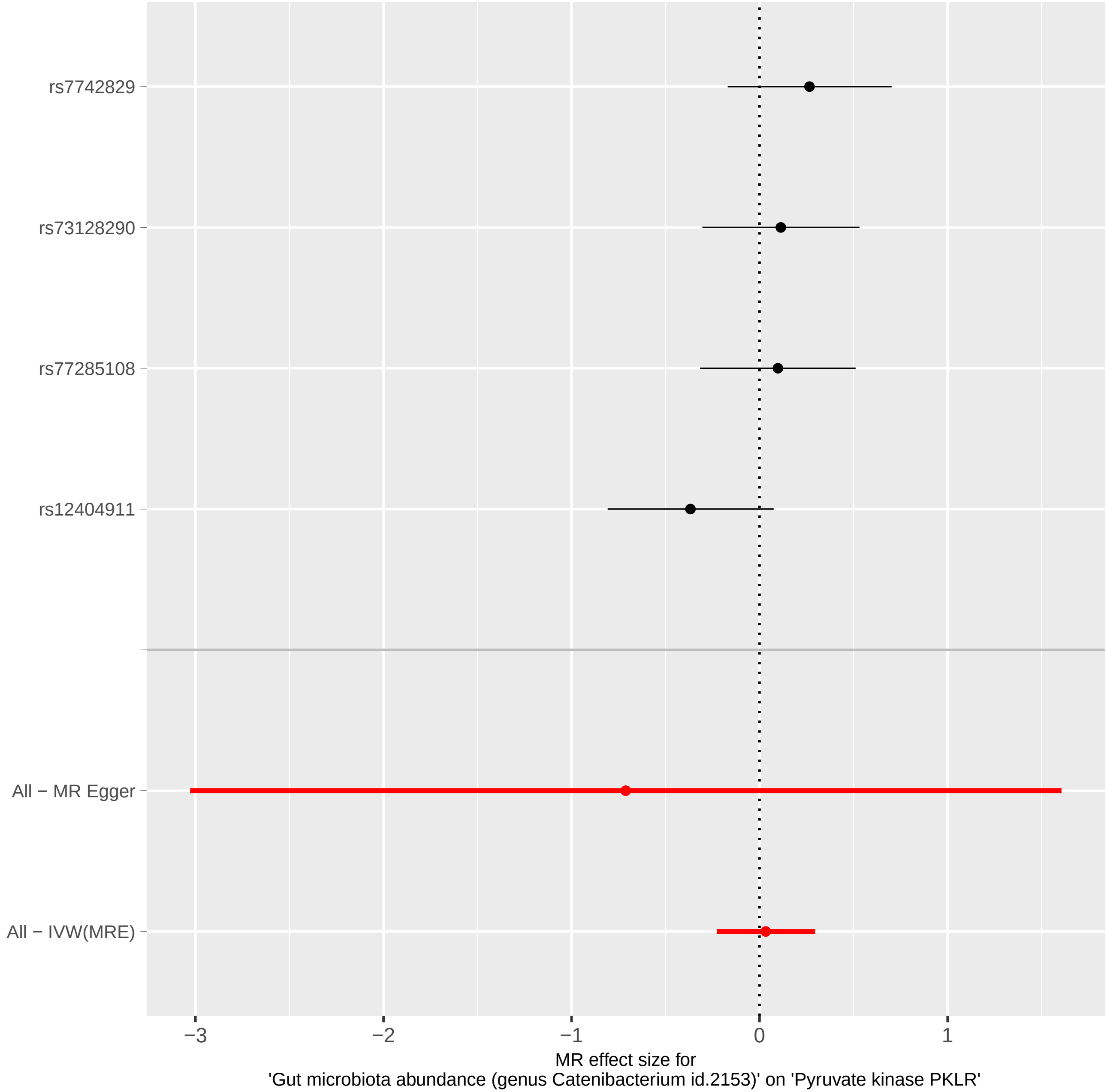

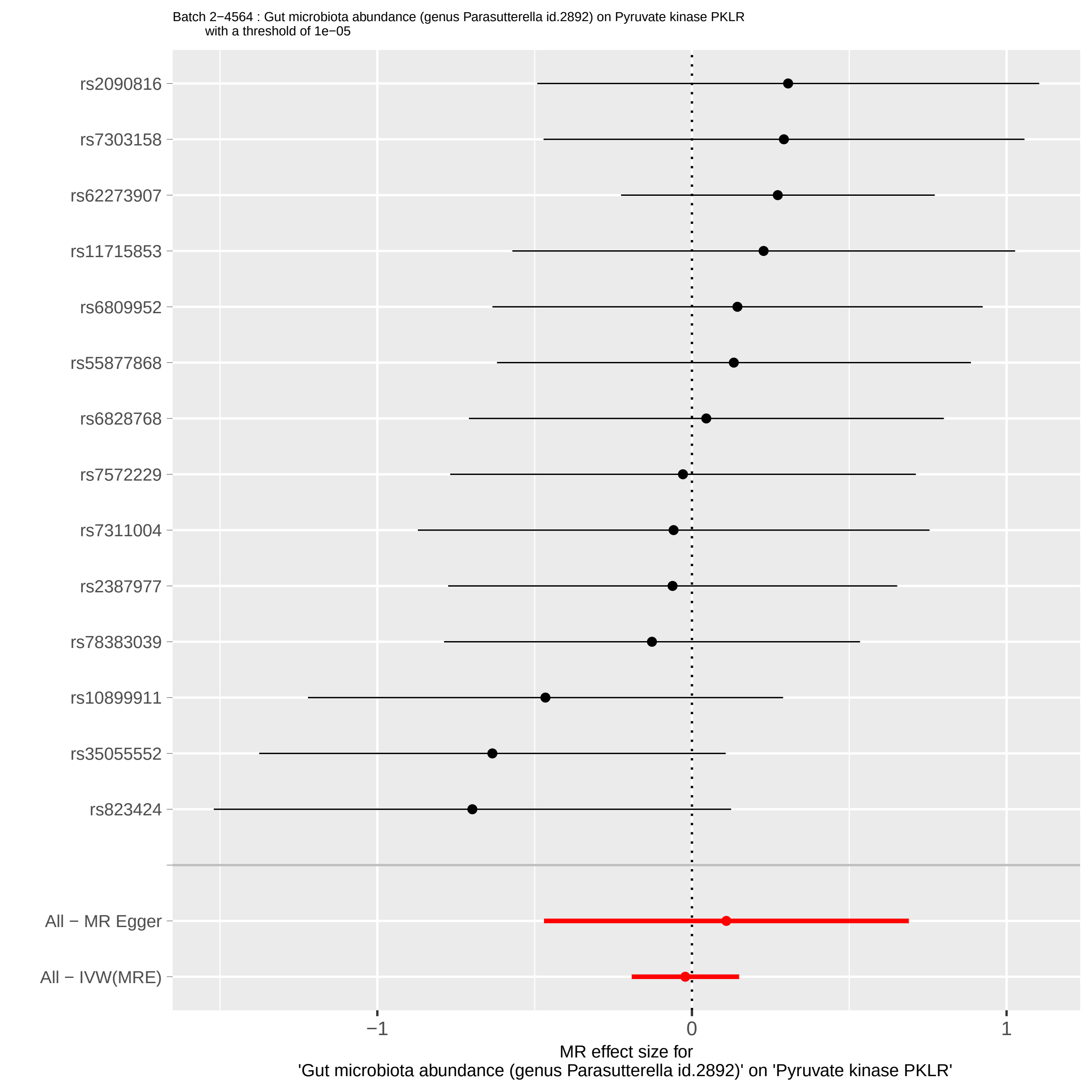

Batch 2−4564 : Gut microbiota abundance (genus Parasutterella id.2892) on Pyruvate kinase PKLR
with a threshold of 1e−05
rs2090816
rs7303158
rs62273907
rs11715853
rs6809952
rs55877868
rs6828768
rs7572229
rs7311004
rs2387977
rs78383039
rs10899911
rs35055552
rs823424
All − MR Egger
All − IVW(MRE)
−1
0
1
MR effect size for
'Gut microbiota abundance (genus Parasutterella id.2892)' on 'Pyruvate kinase PKLR'

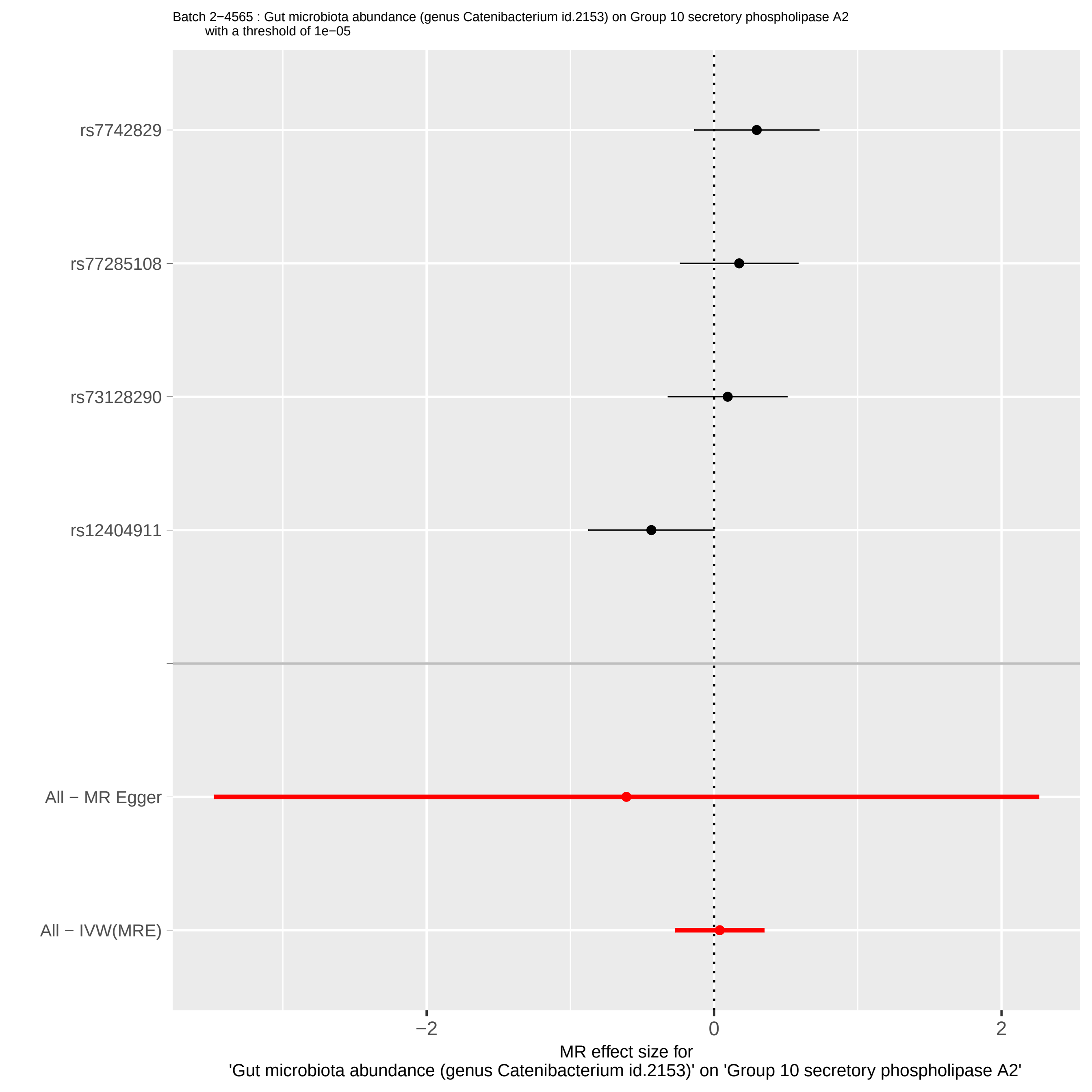
Batch 2−4565 : Gut microbiota abundance (genus Catenibacterium id.2153) on Group 10 secretory phospholipase A2
with a threshold of 1e−05
rs7742829
rs77285108
rs73128290
rs12404911
All − MR Egger
All − IVW(MRE)
−2
0
2
MR effect size for
'Gut microbiota abundance (genus Catenibacterium id.2153)' on 'Group 10 secretory phospholipase A2'

Batch 2−4566 : Gut microbiota abundance (genus Parasutterella id.2892) on Group 10 secretory phospholipase A2
with a threshold of 1e−05

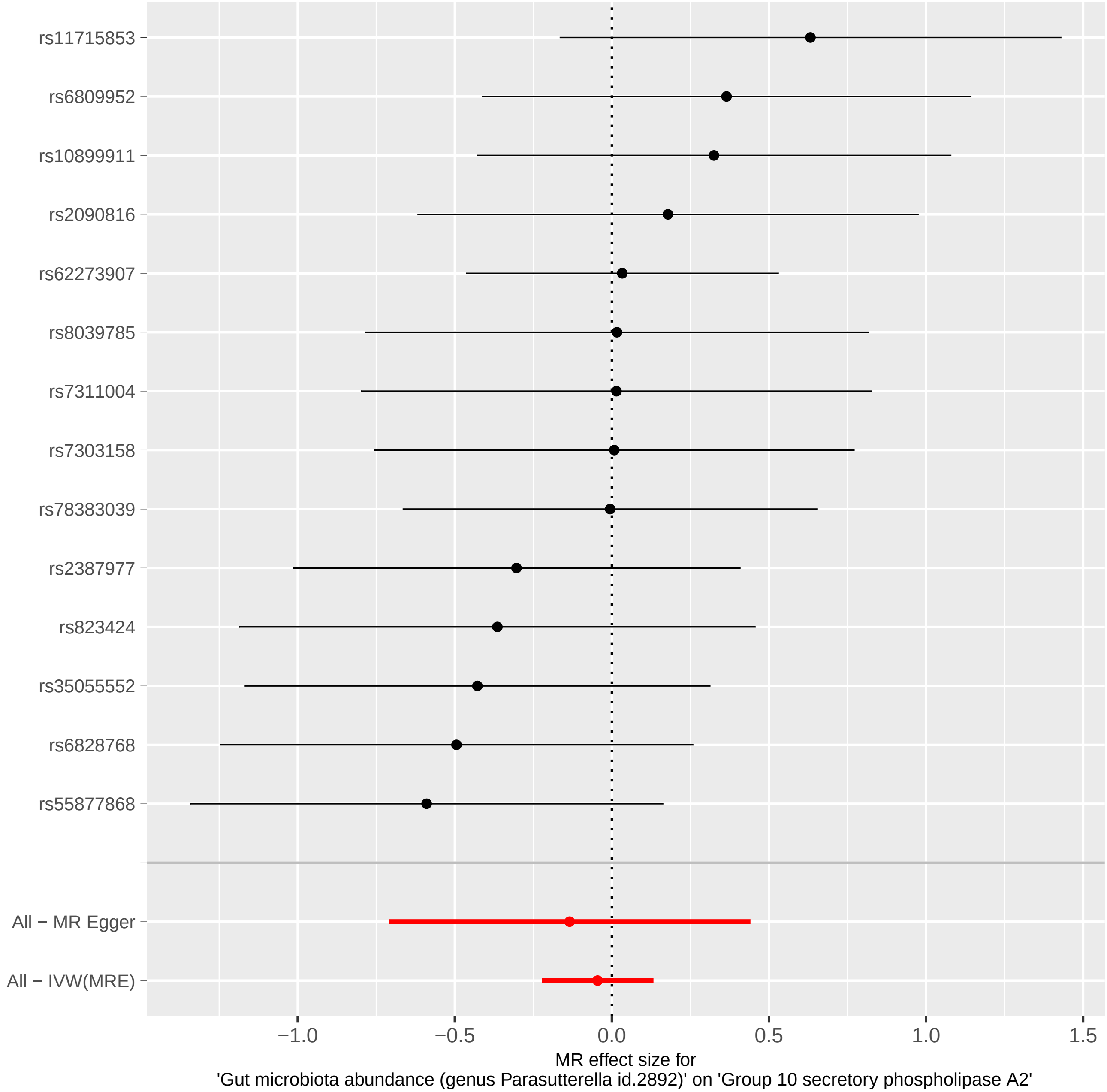

Batch 2−4567 : Gut microbiota abundance (genus Catenibacterium id.2153) on Group XIIB secretory phospholipase A2−like protein
with a threshold of 1e−05

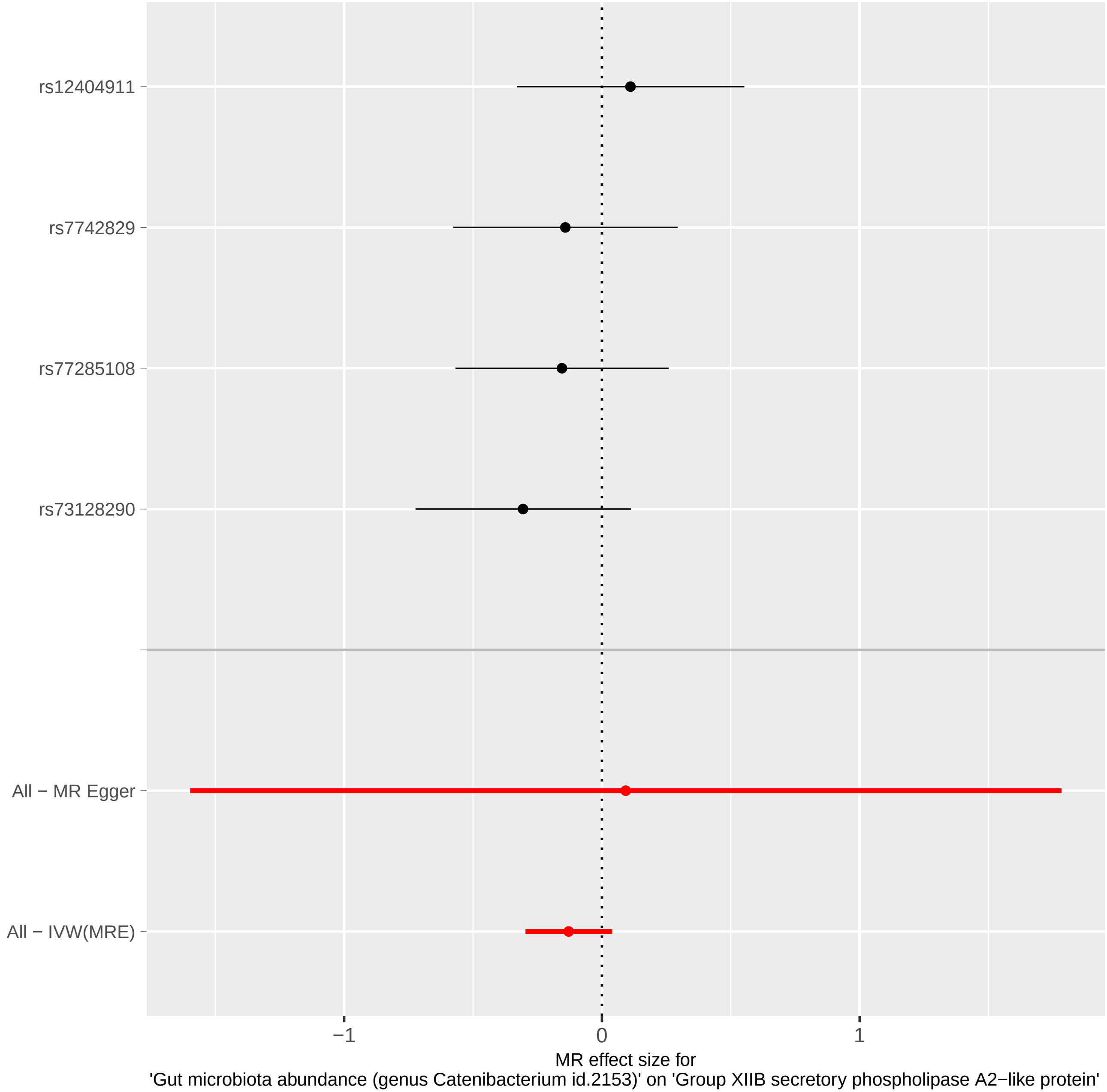

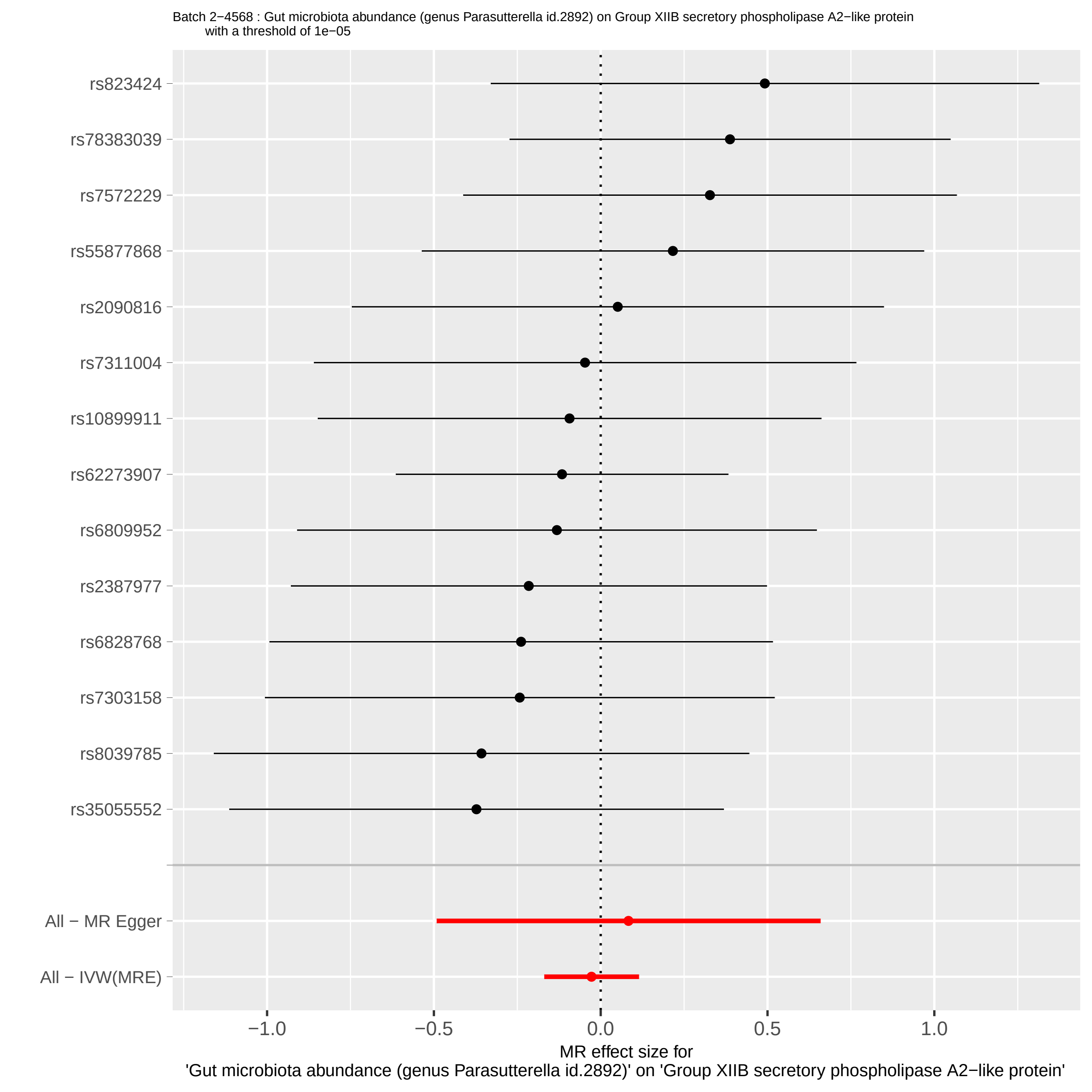

Batch 2−4568 : Gut microbiota abundance (genus Parasutterella id.2892) on Group XIIB secretory phospholipase A2−like protein
with a threshold of 1e−05
rs823424
rs78383039
rs7572229
rs55877868
rs2090816
rs7311004
rs10899911
rs62273907
rs6809952
rs2387977
rs6828768
rs7303158
rs8039785
rs35055552
All − MR Egger
All − IVW(MRE)
−1.0
−0.5
0.0
0.5
1.0
MR effect size for
'Gut microbiota abundance (genus Parasutterella id.2892)' on 'Group XIIB secretory phospholipase A2−like protein'

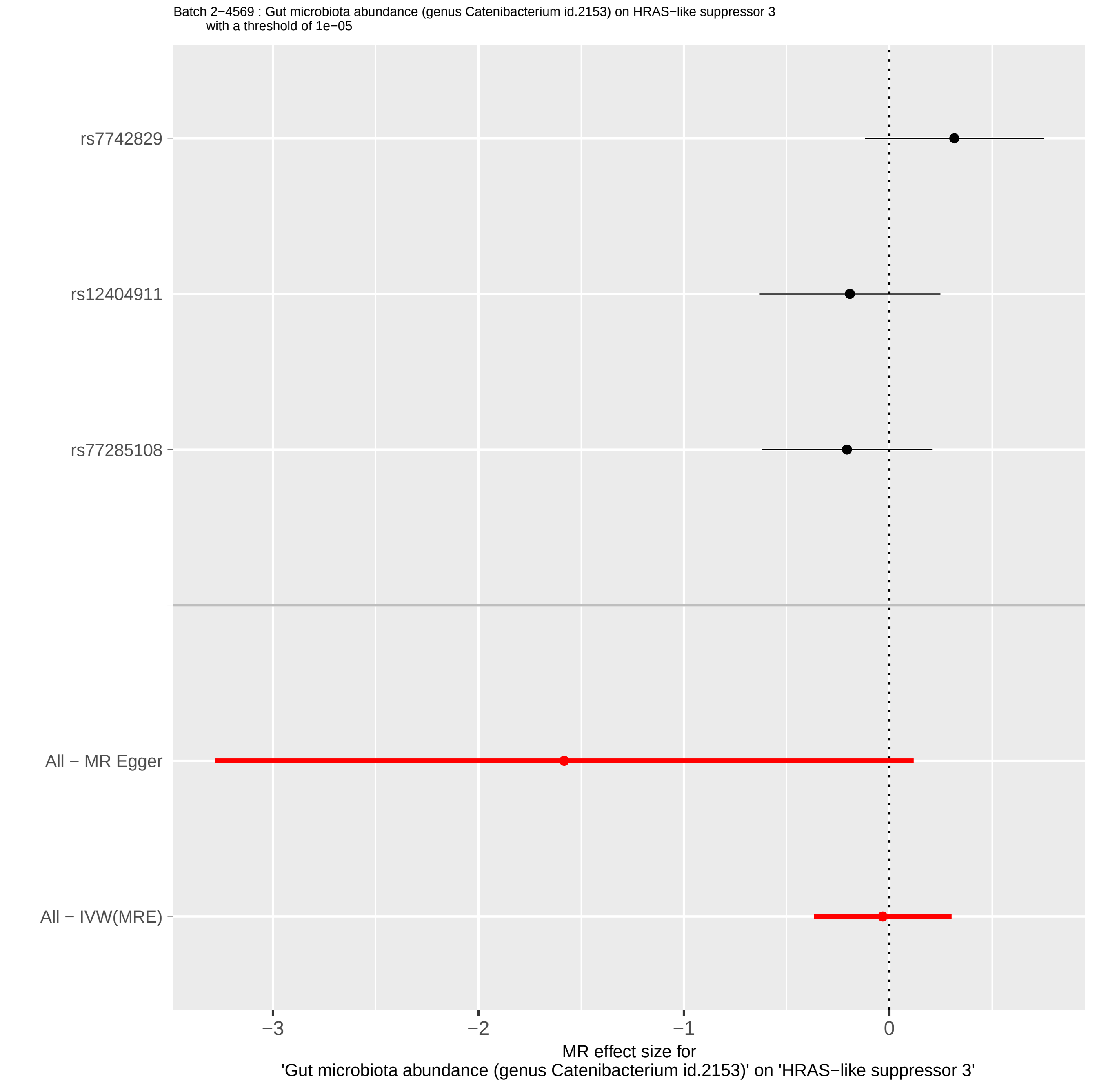

Batch 2−4569 : Gut microbiota abundance (genus Catenibacterium id.2153) on HRAS−like suppressor 3
with a threshold of 1e−05
rs7742829
rs12404911
rs77285108
All − MR Egger
All − IVW(MRE)
−3
−2
−1
0
MR effect size for
'Gut microbiota abundance (genus Catenibacterium id.2153)' on 'HRAS−like suppressor 3'

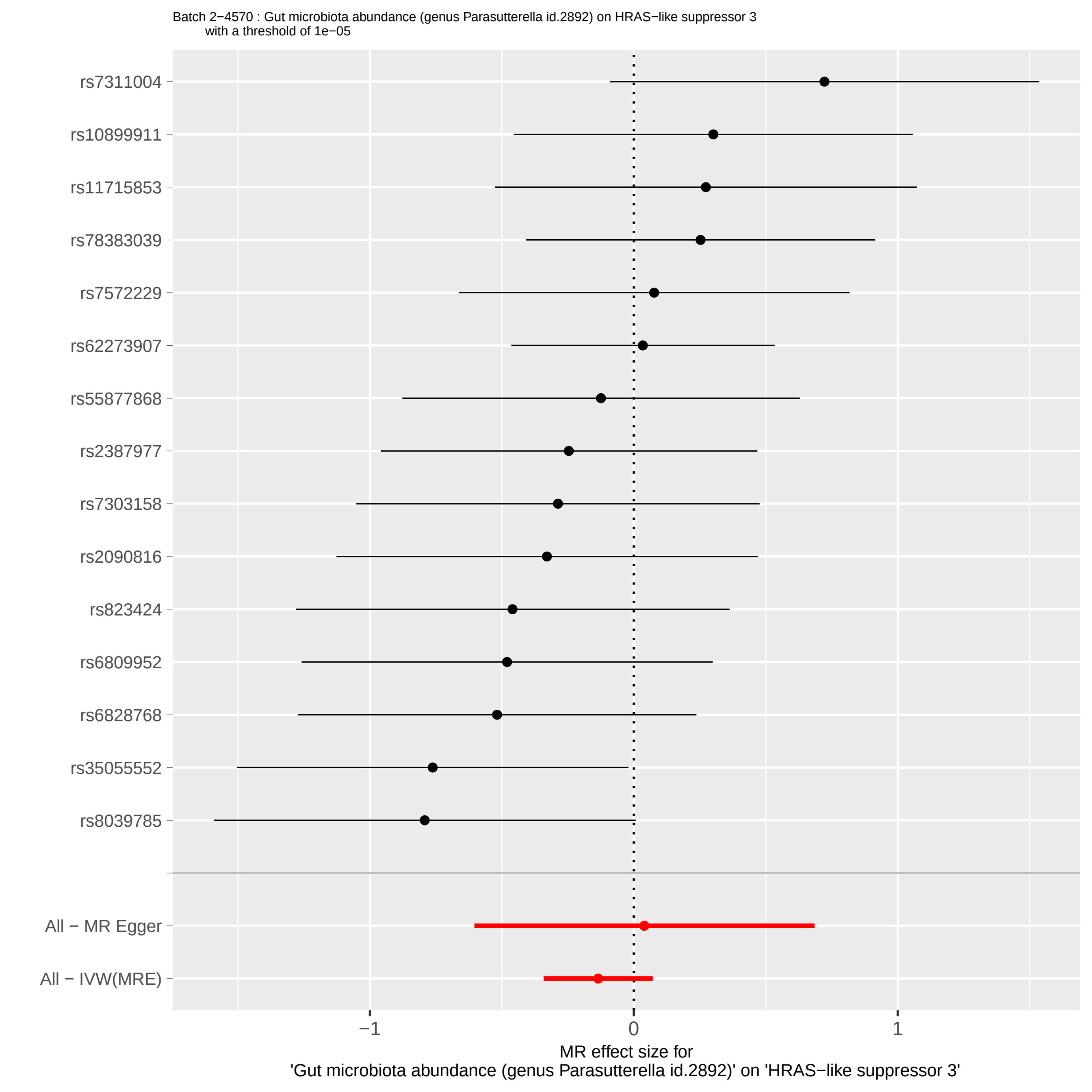

Batch 2−4570 : Gut microbiota abundance (genus Parasutterella id.2892) on HRAS−like suppressor 3
with a threshold of 1e−05
rs7311004
rs10899911
rs11715853
rs78383039
rs7572229
rs62273907
rs55877868
rs2387977
rs7303158
rs2090816
rs823424
rs6809952
rs6828768
rs35055552
rs8039785
All − MR Egger
All − IVW(MRE)
−1
0
1
MR effect size for
'Gut microbiota abundance (genus Parasutterella id.2892)' on 'HRAS−like suppressor 3'

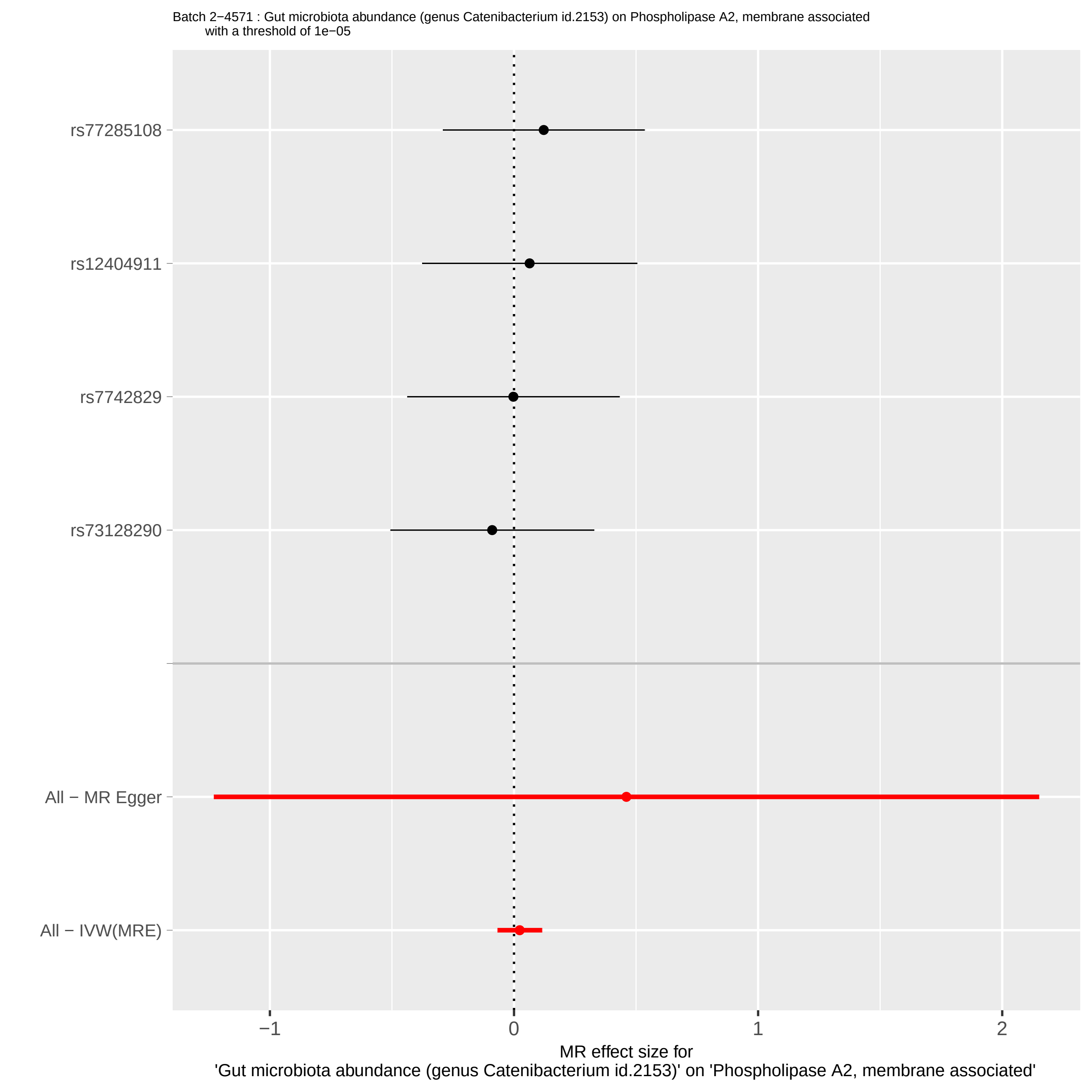
Batch 2−4571 : Gut microbiota abundance (genus Catenibacterium id.2153) on Phospholipase A2, membrane associated
with a threshold of 1e−05
rs77285108
rs12404911
rs7742829
rs73128290
All − MR Egger
All − IVW(MRE)
−1
0
1
2
MR effect size for
'Gut microbiota abundance (genus Catenibacterium id.2153)' on 'Phospholipase A2, membrane associated'

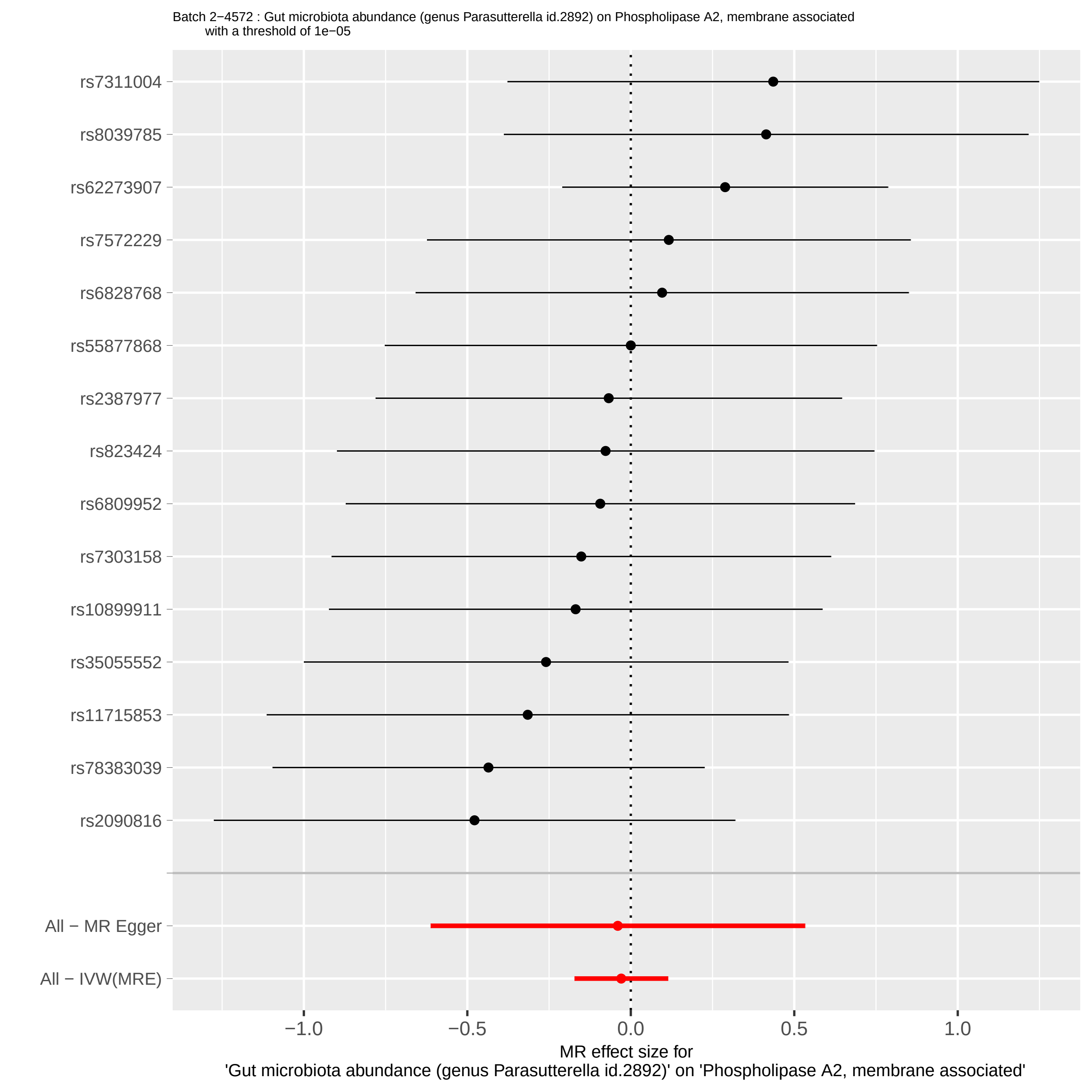
Batch 2−4572 : Gut microbiota abundance (genus Parasutterella id.2892) on Phospholipase A2, membrane associated
with a threshold of 1e−05
rs7311004
rs8039785
rs62273907
rs7572229
rs6828768
rs55877868
rs2387977
rs823424
rs6809952
rs7303158
rs10899911
rs35055552
rs11715853
rs78383039
rs2090816
All − MR Egger
All − IVW(MRE)
−1.0
−0.5
0.0
0.5
1.0
MR effect size for
'Gut microbiota abundance (genus Parasutterella id.2892)' on 'Phospholipase A2, membrane associated'

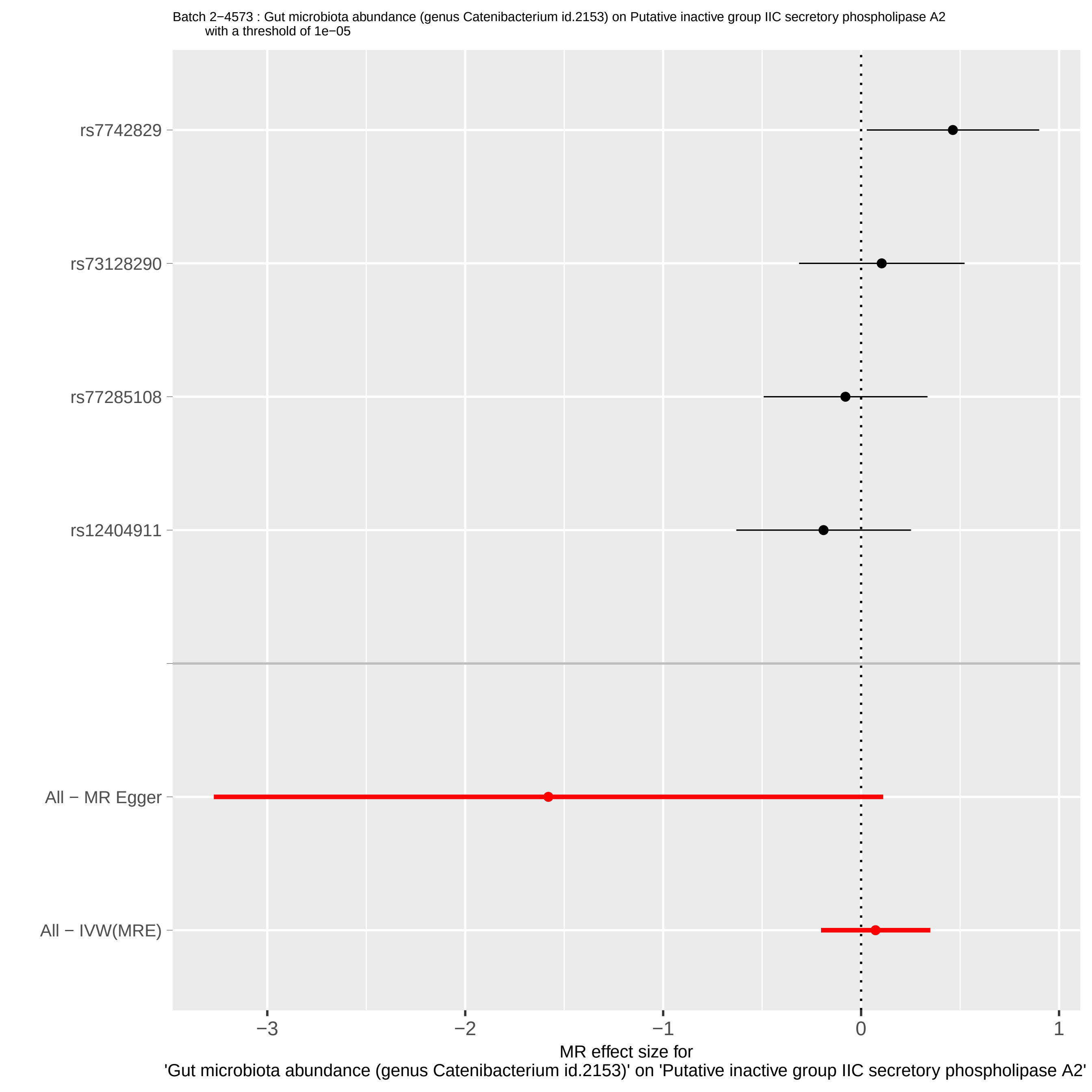

Batch 2−4573 : Gut microbiota abundance (genus Catenibacterium id.2153) on Putative inactive group IIC secretory phospholipase A2
with a threshold of 1e−05
rs7742829
rs73128290
rs77285108
rs12404911
All − MR Egger
All − IVW(MRE)
−3
−2
−1
0
1
MR effect size for
'Gut microbiota abundance (genus Catenibacterium id.2153)' on 'Putative inactive group IIC secretory phospholipase A2'

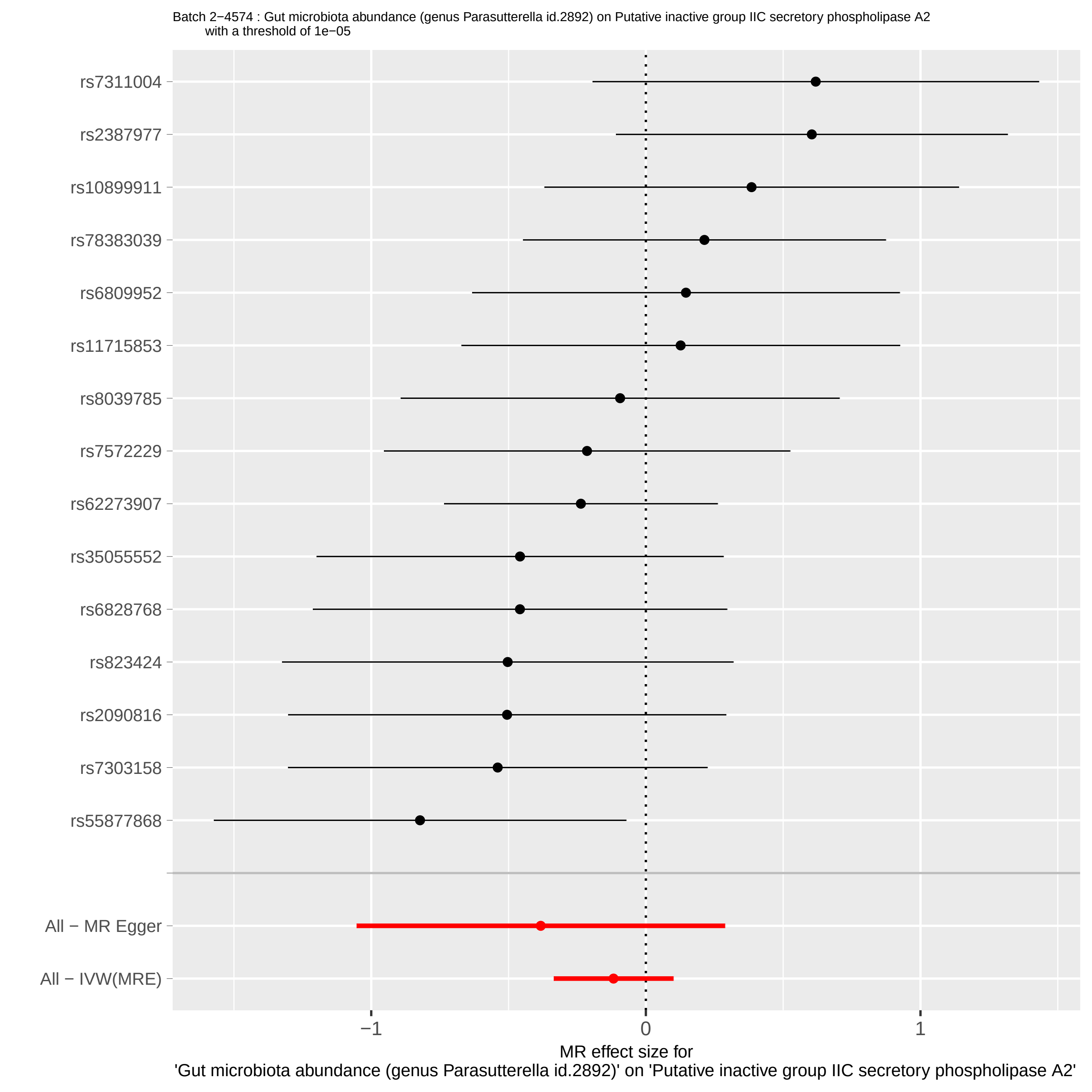
Batch 2−4574 : Gut microbiota abundance (genus Parasutterella id.2892) on Putative inactive group IIC secretory phospholipase A2
with a threshold of 1e−05
rs7311004
rs2387977
rs10899911
rs78383039
rs6809952
rs11715853
rs8039785
rs7572229
rs62273907
rs35055552
rs6828768
rs823424
rs2090816
rs7303158
rs55877868
All − MR Egger
All − IVW(MRE)
−1
0
1
MR effect size for
'Gut microbiota abundance (genus Parasutterella id.2892)' on 'Putative inactive group IIC secretory phospholipase A2'

Batch 2−4575 : Gut microbiota abundance (genus Catenibacterium id.2153) on Group IIE secretory phospholipase A2
with a threshold of 1e−05

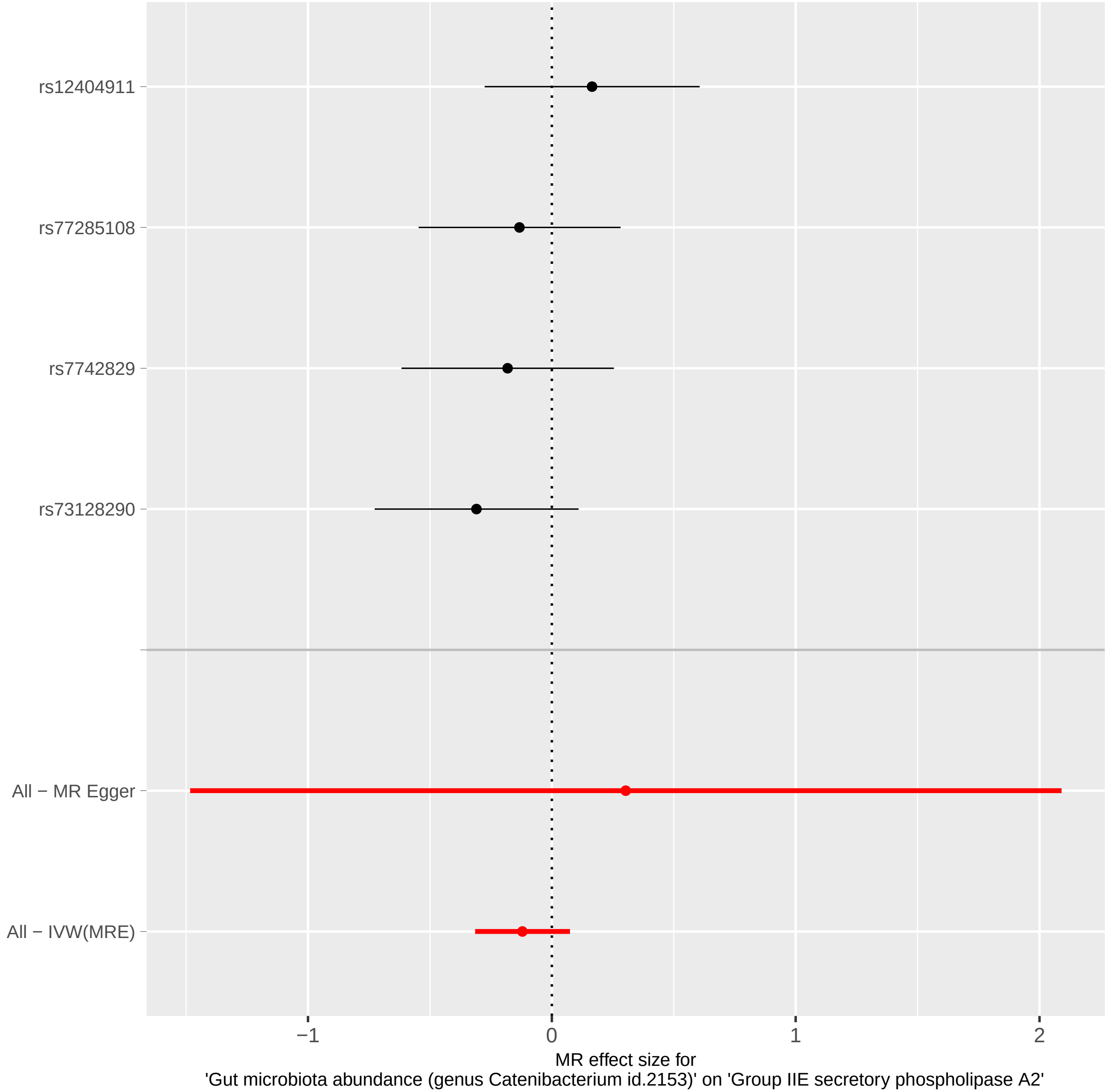

Batch 2−4576 : Gut microbiota abundance (genus Parasutterella id.2892) on Group IIE secretory phospholipase A2
with a threshold of 1e−05

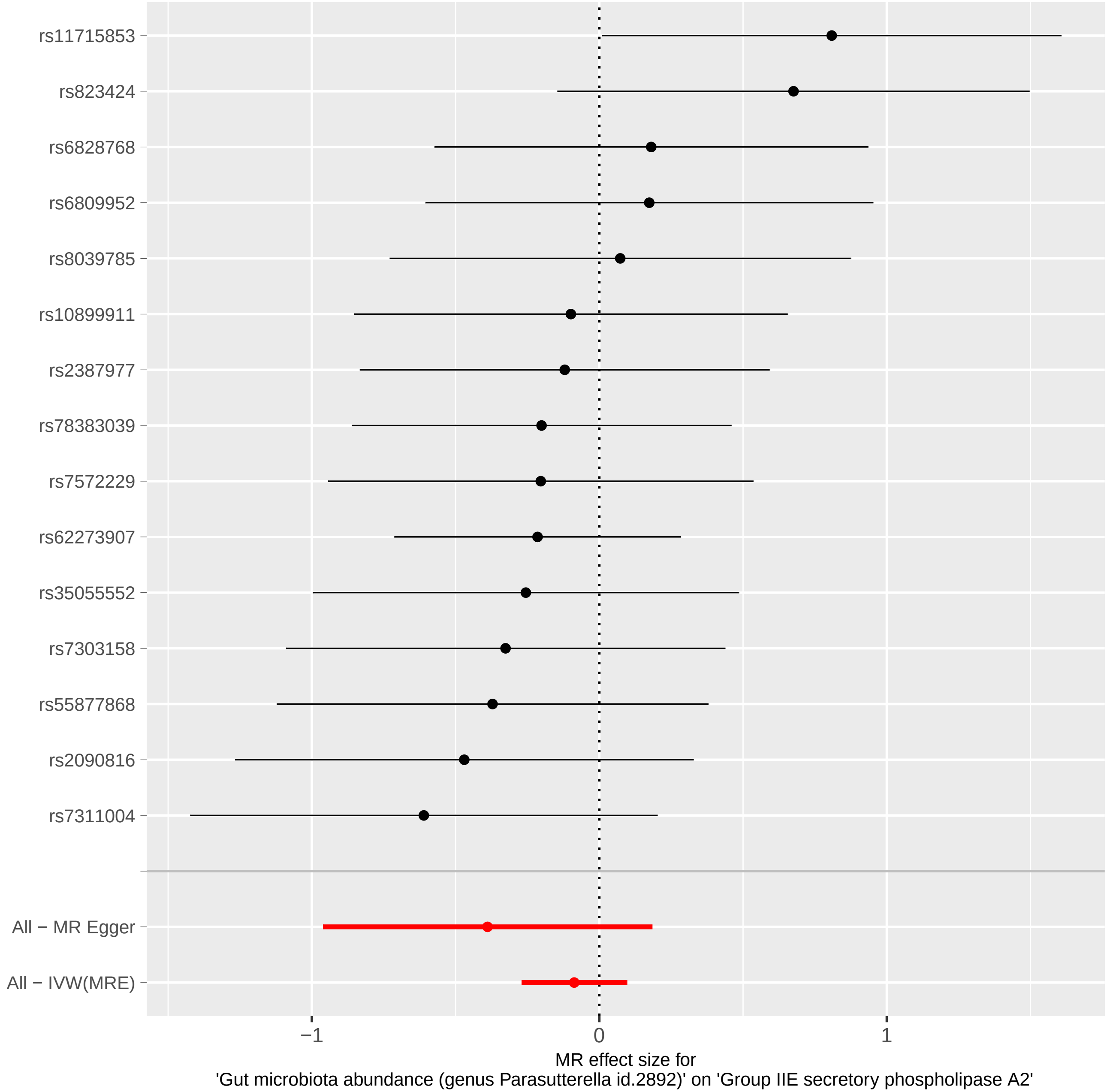

Batch 2−4577 : Gut microbiota abundance (genus Catenibacterium id.2153) on Group IIE secretory phospholipase A2 with a threshold of 1e−05

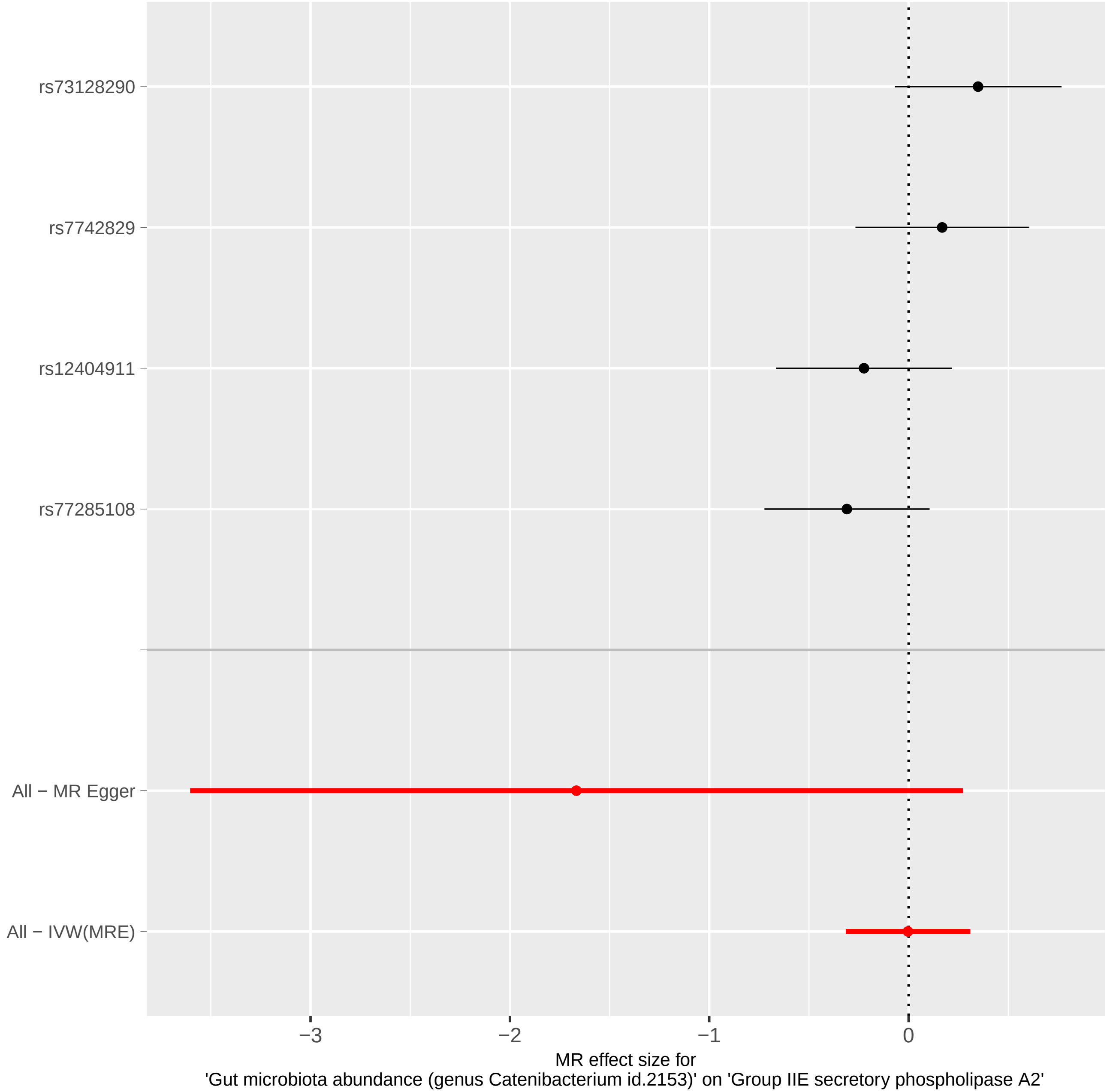

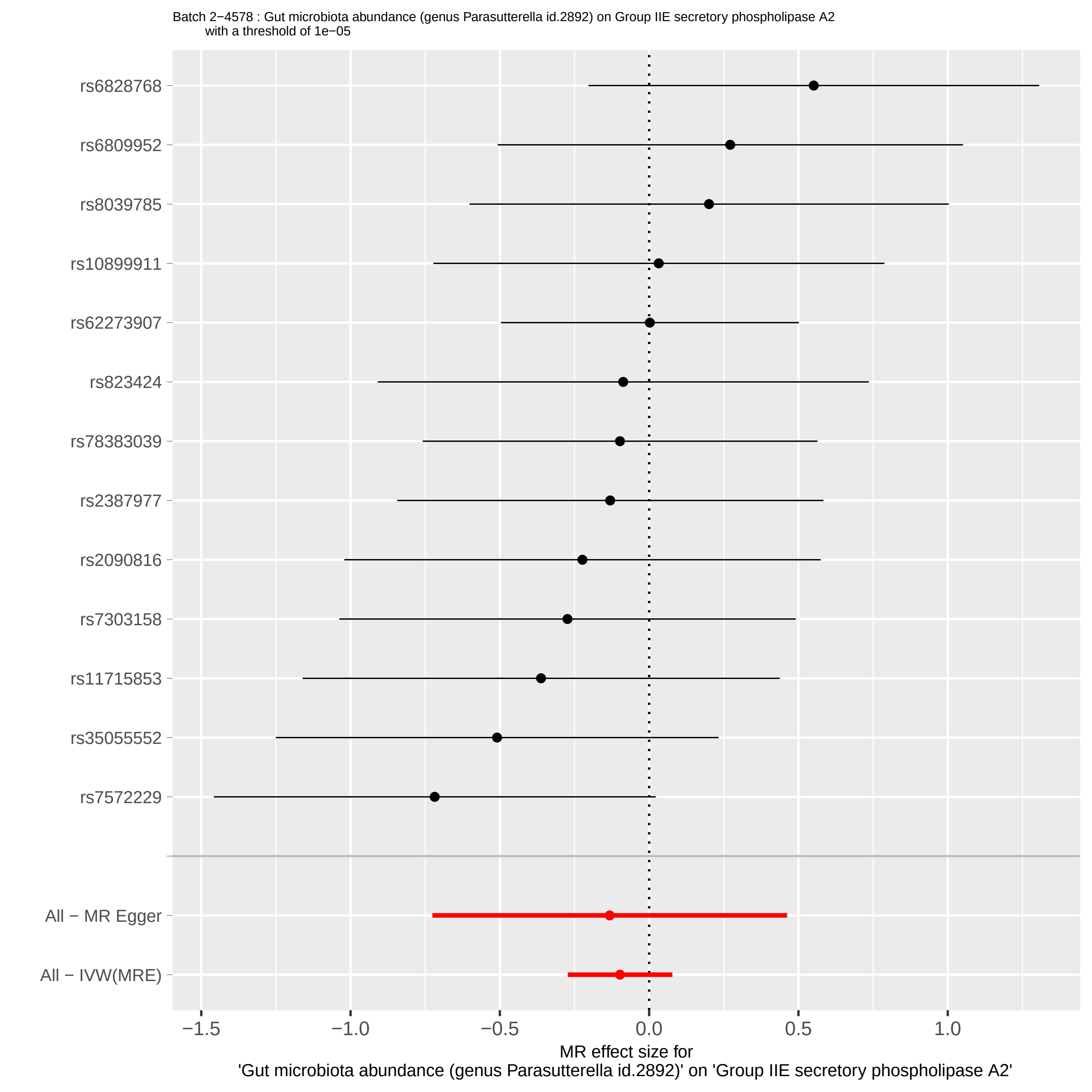

Batch 2−4578 : Gut microbiota abundance (genus Parasutterella id.2892) on Group IIE secretory phospholipase A2
with a threshold of 1e−05
rs6828768
rs6809952
rs8039785
rs10899911
rs62273907
rs823424
rs78383039
rs2387977
rs2090816
rs7303158
rs11715853
rs35055552
rs7572229
All − MR Egger
All − IVW(MRE)
−1.5
−1.0
−0.5
0.0
0.5
1.0
MR effect size for
'Gut microbiota abundance (genus Parasutterella id.2892)' on 'Group IIE secretory phospholipase A2'

Batch 2−4579 : Gut microbiota abundance (genus Catenibacterium id.2153) on Calcium−dependent phospholipase A2
with a threshold of 1e−05

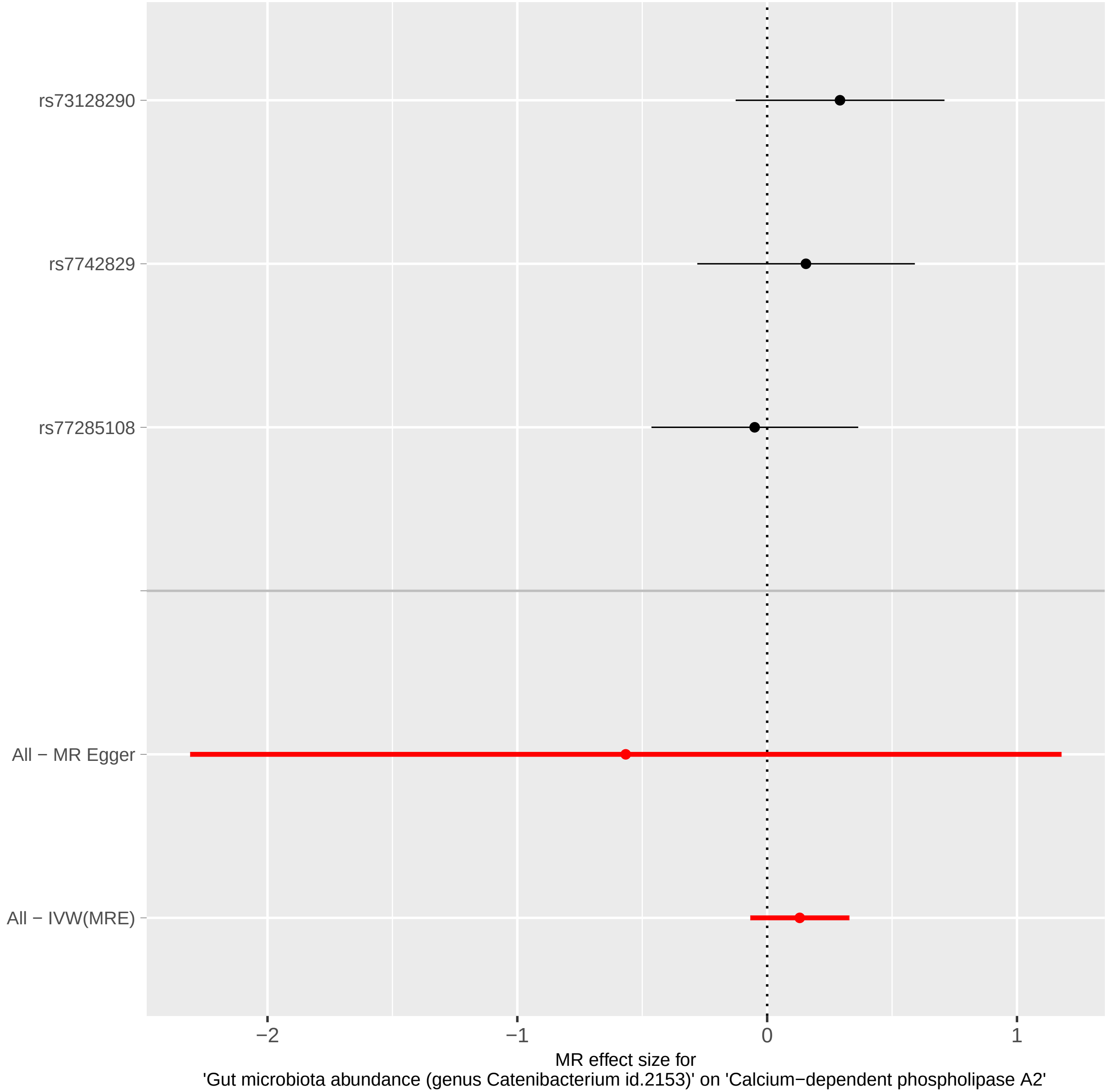

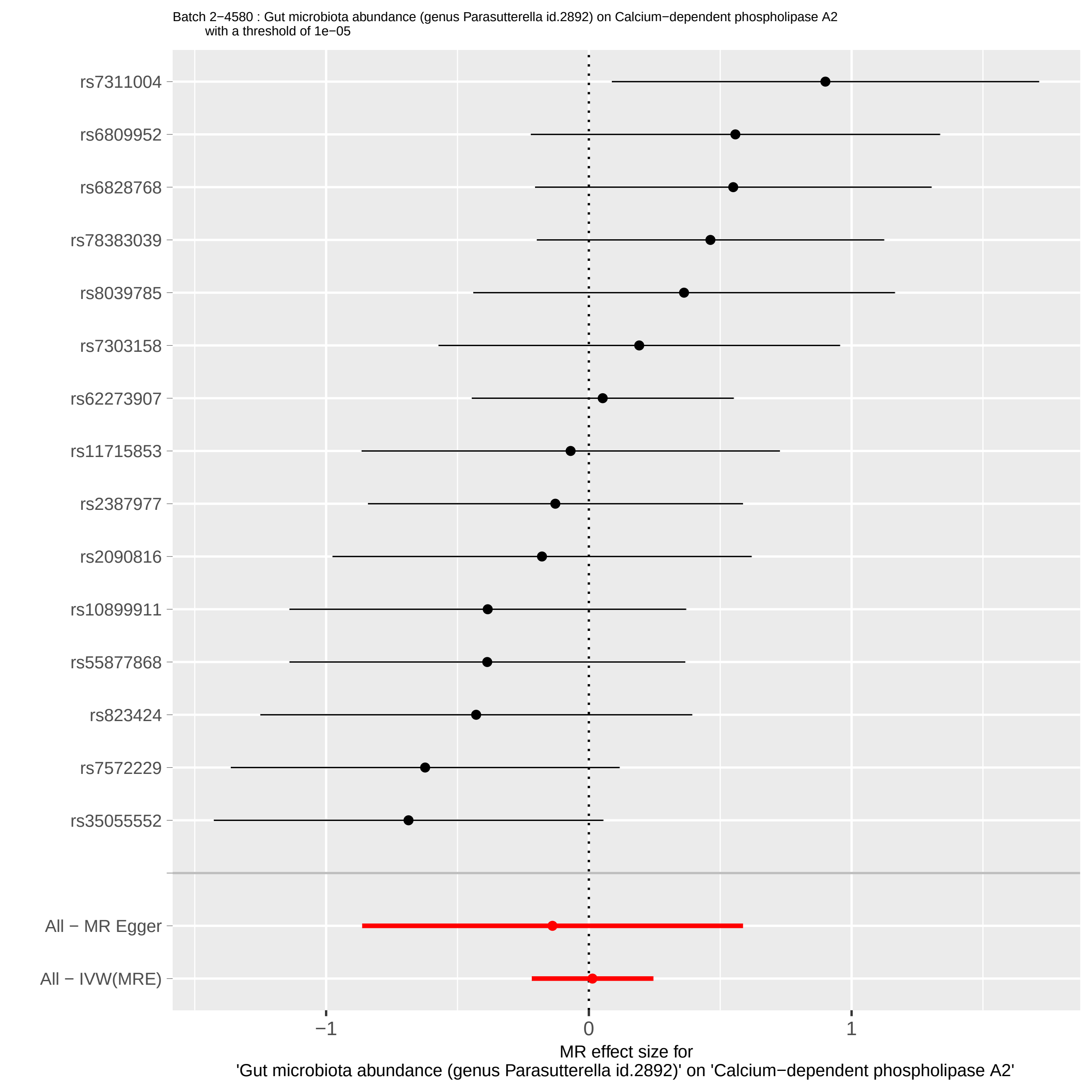

Batch 2−4580 : Gut microbiota abundance (genus Parasutterella id.2892) on Calcium−dependent phospholipase A2
with a threshold of 1e−05
rs7311004
rs6809952
rs6828768
rs78383039
rs8039785
rs7303158
rs62273907
rs11715853
rs2387977
rs2090816
rs10899911
rs55877868
rs823424
rs7572229
rs35055552
All − MR Egger
All − IVW(MRE)
−1
0
1
MR effect size for
'Gut microbiota abundance (genus Parasutterella id.2892)' on 'Calcium−dependent phospholipase A2'

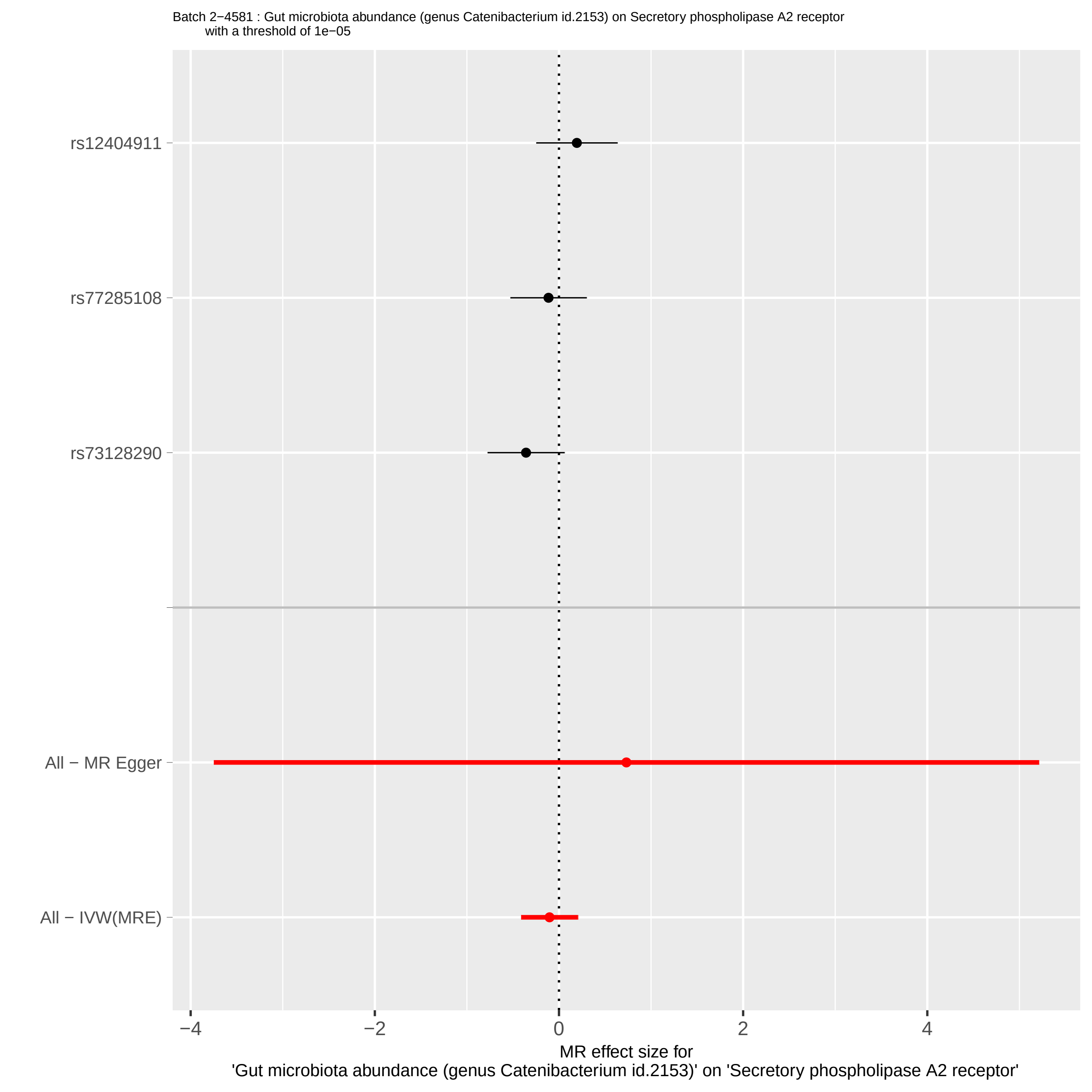

Batch 2−4581 : Gut microbiota abundance (genus Catenibacterium id.2153) on Secretory phospholipase A2 receptor
with a threshold of 1e−05
rs12404911
rs77285108
rs73128290
All − MR Egger
All − IVW(MRE)
−4
−2
0
2
4
MR effect size for
'Gut microbiota abundance (genus Catenibacterium id.2153)' on 'Secretory phospholipase A2 receptor'

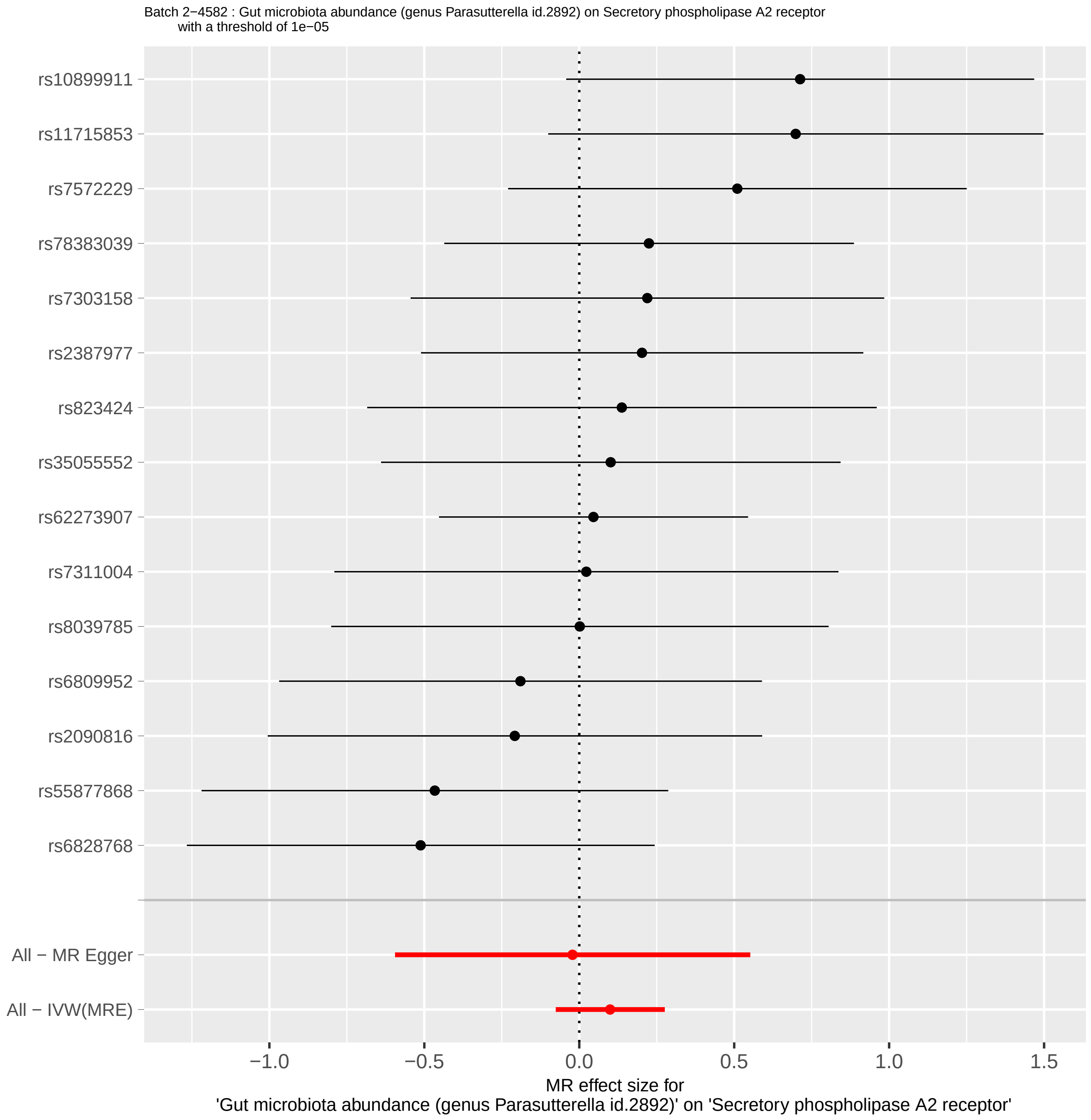

Batch 2−4582 : Gut microbiota abundance (genus Parasutterella id.2892) on Secretory phospholipase A2 receptor
with a threshold of 1e−05
rs10899911
rs11715853
rs7572229
rs78383039
rs7303158
rs2387977
rs823424
rs35055552
rs62273907
rs7311004
rs8039785
rs6809952
rs2090816
rs55877868
rs6828768
All − MR Egger
All − IVW(MRE)
−1.0
−0.5
0.0
0.5
1.0
1.5
MR effect size for
'Gut microbiota abundance (genus Parasutterella id.2892)' on 'Secretory phospholipase A2 receptor'

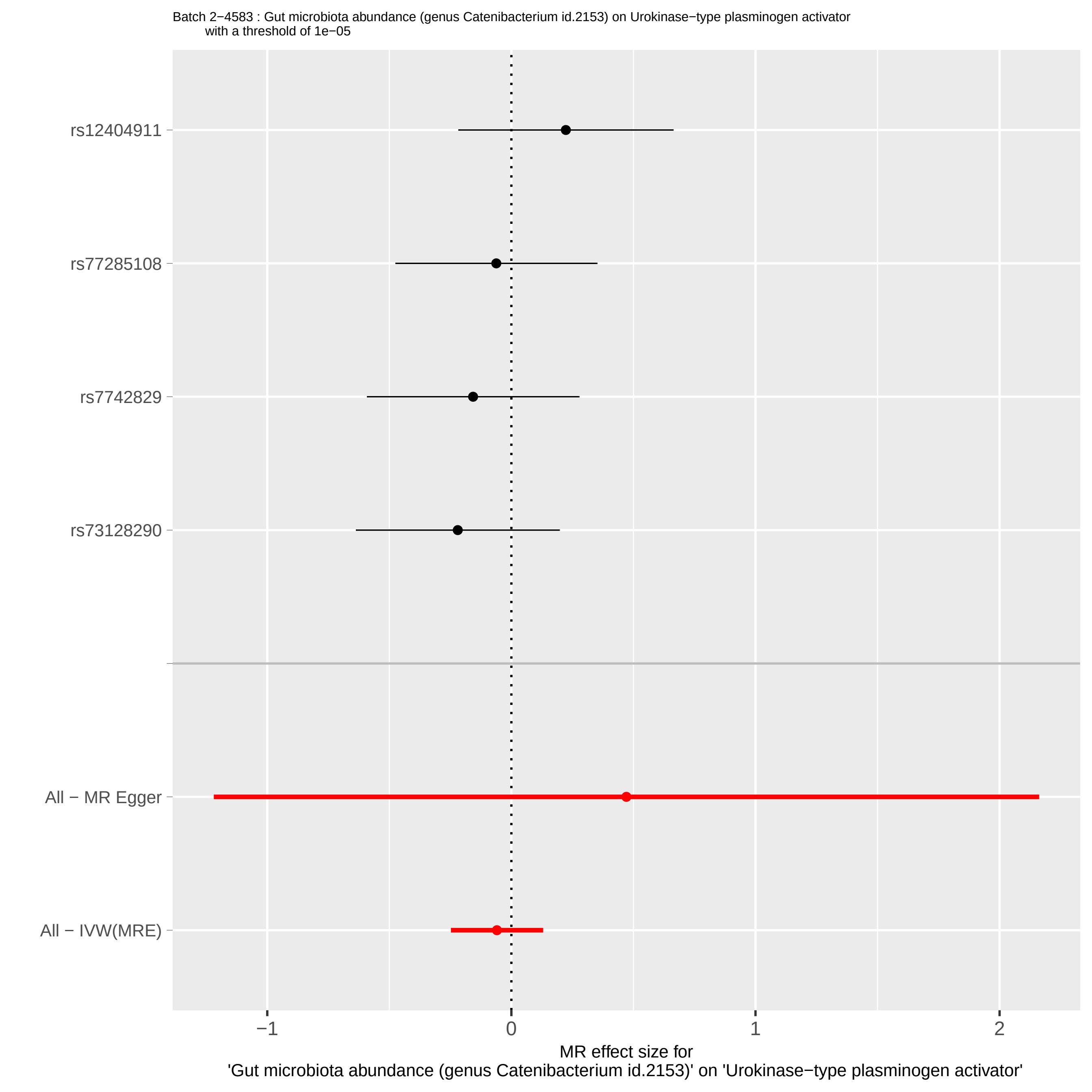

Batch 2−4583 : Gut microbiota abundance (genus Catenibacterium id.2153) on Urokinase−type plasminogen activator
with a threshold of 1e−05
rs12404911
rs77285108
rs7742829
rs73128290
All − MR Egger
All − IVW(MRE)
−1
0
1
2
MR effect size for
'Gut microbiota abundance (genus Catenibacterium id.2153)' on 'Urokinase−type plasminogen activator'

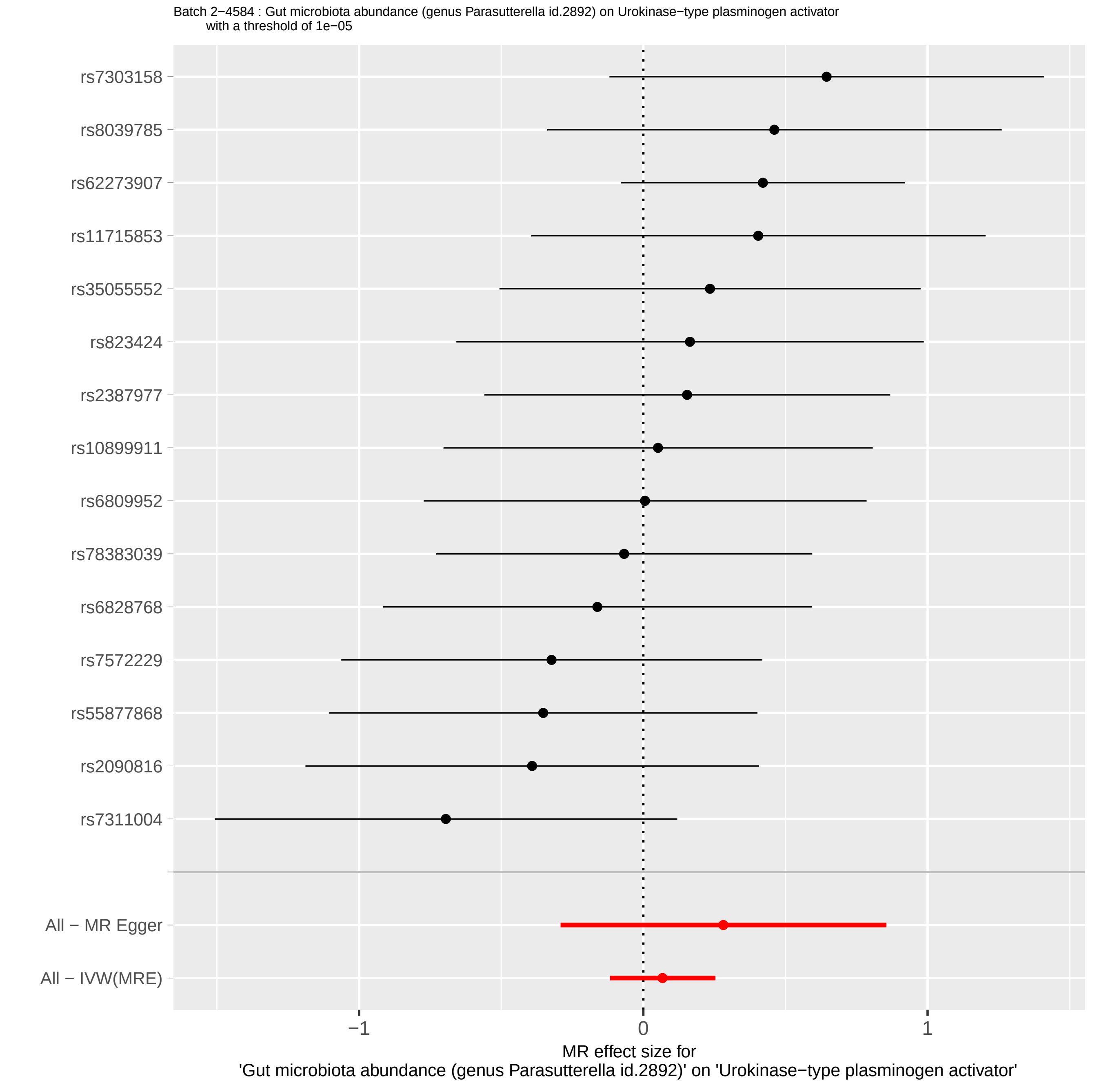

Batch 2−4584 : Gut microbiota abundance (genus Parasutterella id.2892) on Urokinase−type plasminogen activator
with a threshold of 1e−05
rs7303158
rs8039785
rs62273907
rs11715853
rs35055552
rs823424
rs2387977
rs10899911
rs6809952
rs78383039
rs6828768
rs7572229
rs55877868
rs2090816
rs7311004
All − MR Egger
All − IVW(MRE)
−1
0
1
MR effect size for
'Gut microbiota abundance (genus Parasutterella id.2892)' on 'Urokinase−type plasminogen activator'

Batch 2−4585 : Gut microbiota abundance (genus Catenibacterium id.2153) on Urokinase plasminogen activator surface receptor
with a threshold of 1e−05

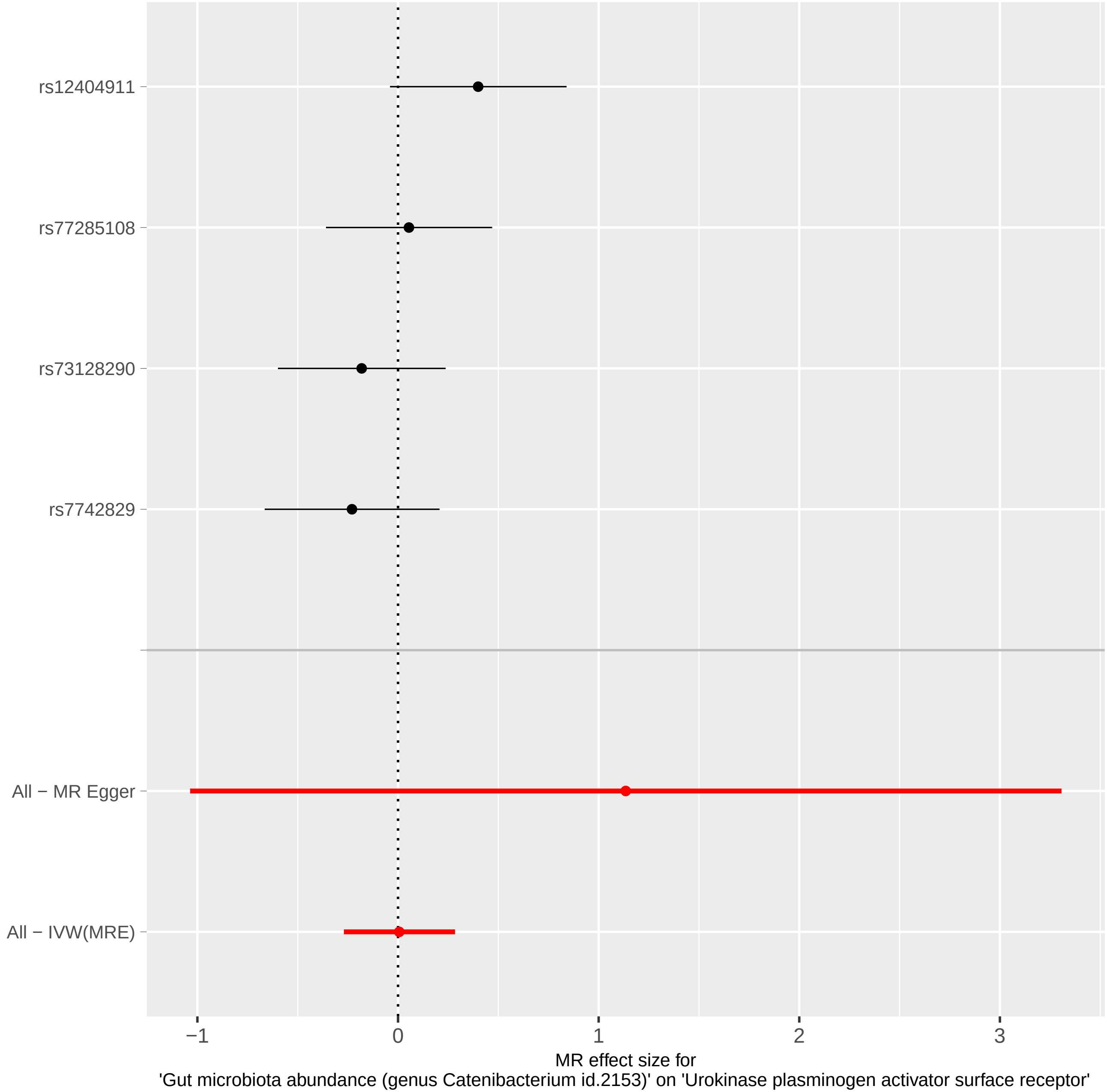

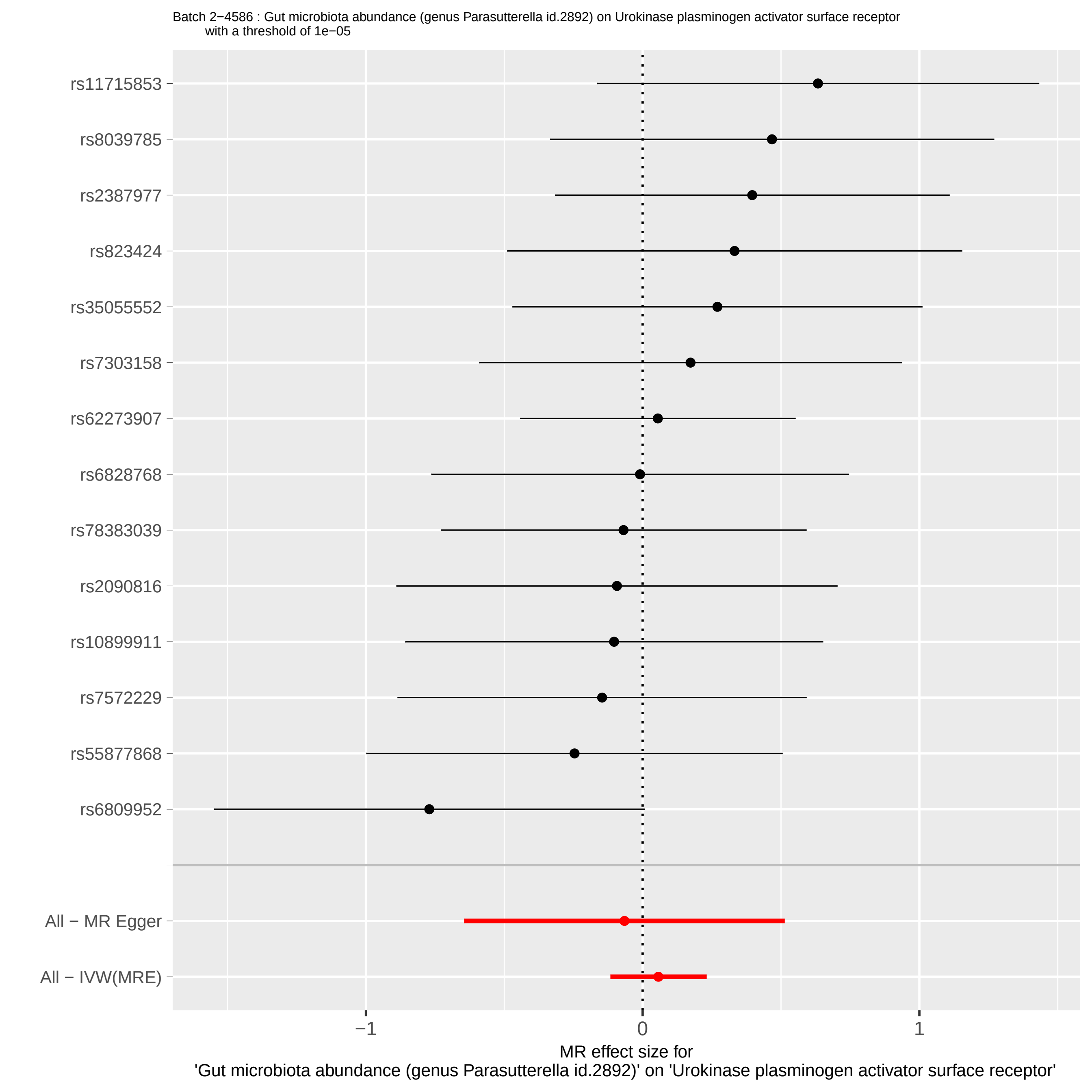

Batch 2−4586 : Gut microbiota abundance (genus Parasutterella id.2892) on Urokinase plasminogen activator surface receptor
with a threshold of 1e−05
rs11715853
rs8039785
rs2387977
rs823424
rs35055552
rs7303158
rs62273907
rs6828768
rs78383039
rs2090816
rs10899911
rs7572229
rs55877868
rs6809952
All − MR Egger
All − IVW(MRE)
−1
0
1
MR effect size for
'Gut microbiota abundance (genus Parasutterella id.2892)' on 'Urokinase plasminogen activator surface receptor'

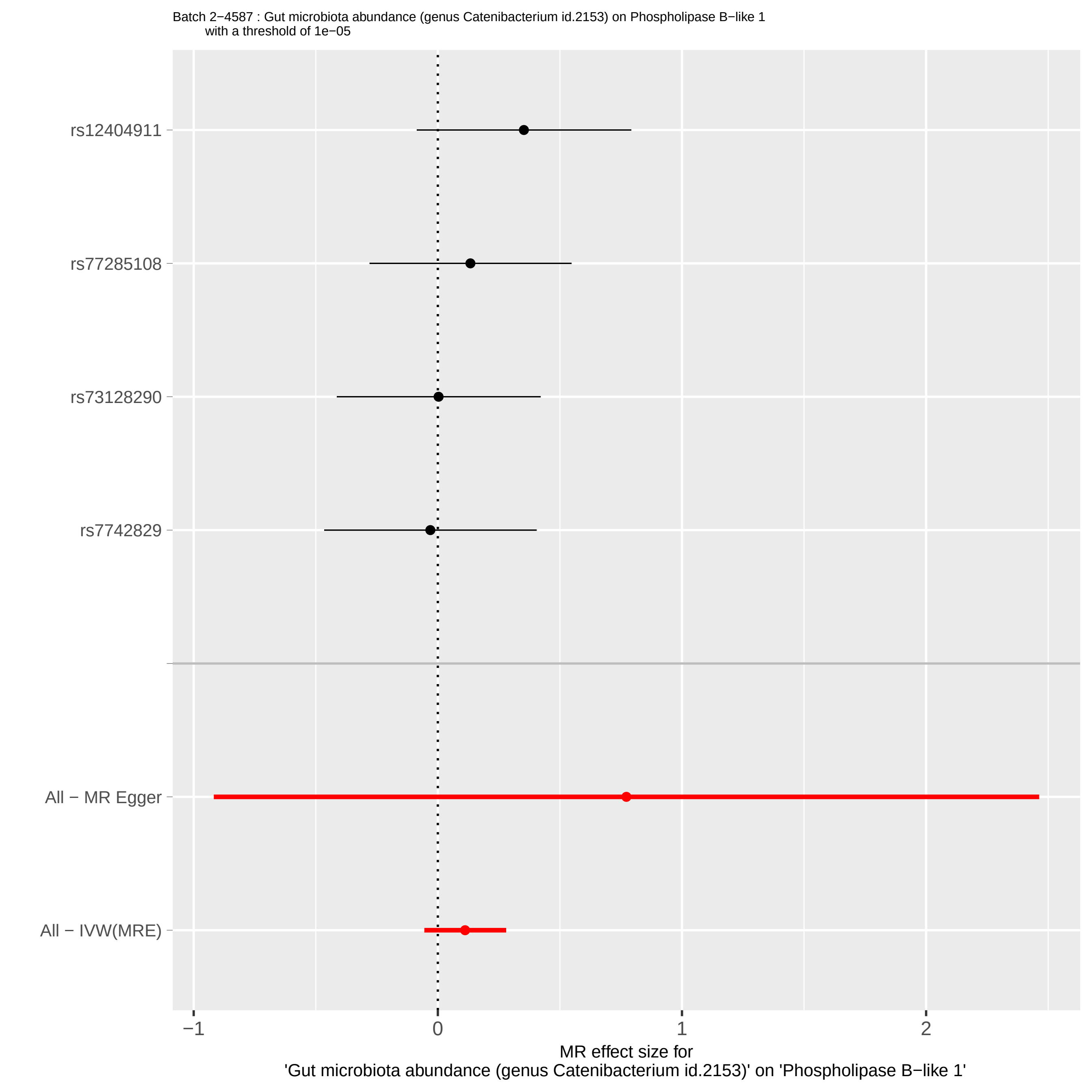

Batch 2−4587 : Gut microbiota abundance (genus Catenibacterium id.2153) on Phospholipase B−like 1
with a threshold of 1e−05
rs12404911
rs77285108
rs73128290
rs7742829
All − MR Egger
All − IVW(MRE)
−1
0
1
2
MR effect size for
'Gut microbiota abundance (genus Catenibacterium id.2153)' on 'Phospholipase B−like 1'

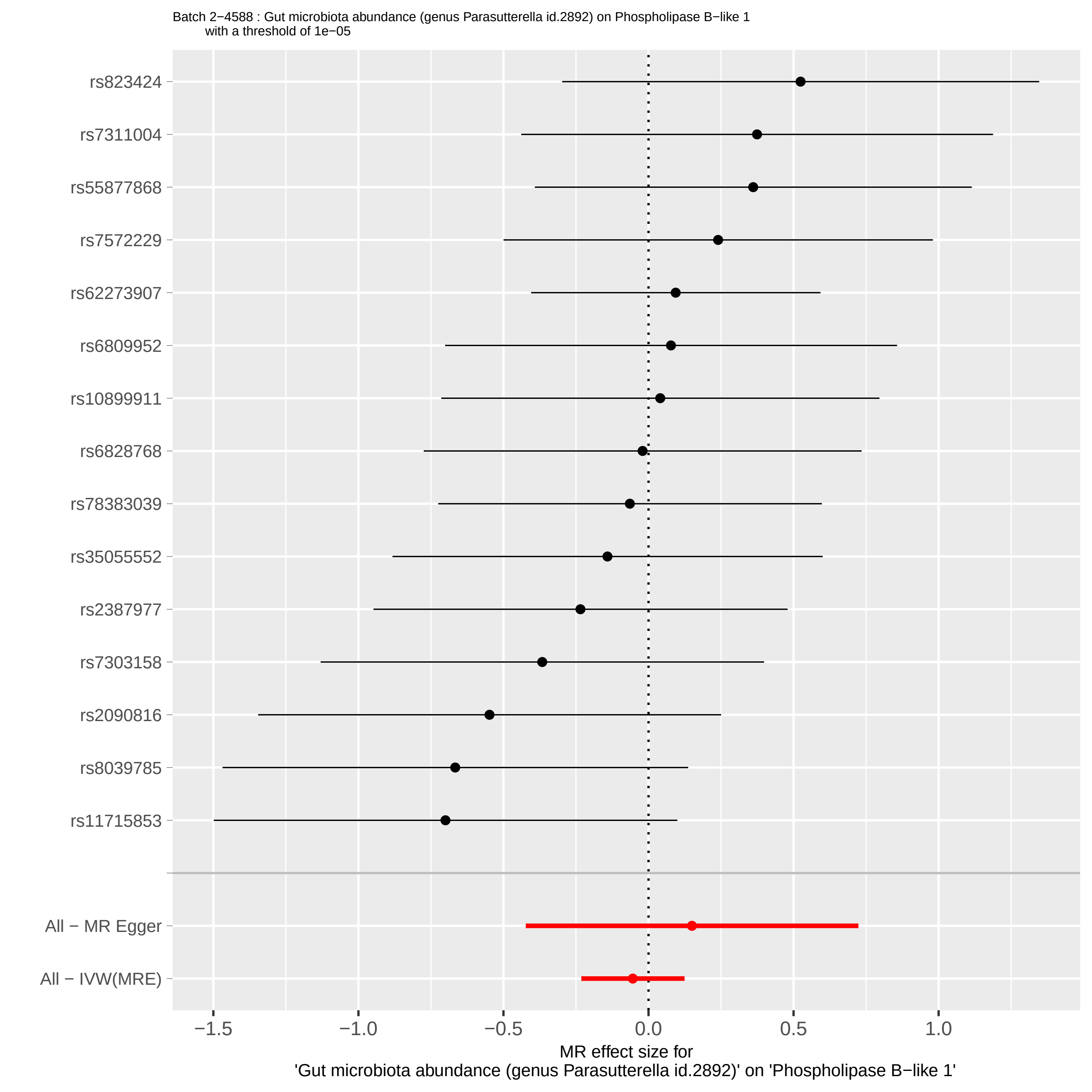

Batch 2−4588 : Gut microbiota abundance (genus Parasutterella id.2892) on Phospholipase B−like 1
with a threshold of 1e−05
rs823424
rs7311004
rs55877868
rs7572229
rs62273907
rs6809952
rs10899911
rs6828768
rs78383039
rs35055552
rs2387977
rs7303158
rs2090816
rs8039785
rs11715853
All − MR Egger
All − IVW(MRE)
−1.5
−1.0
−0.5
0.0
0.5
1.0
MR effect size for
'Gut microbiota abundance (genus Parasutterella id.2892)' on 'Phospholipase B−like 1'

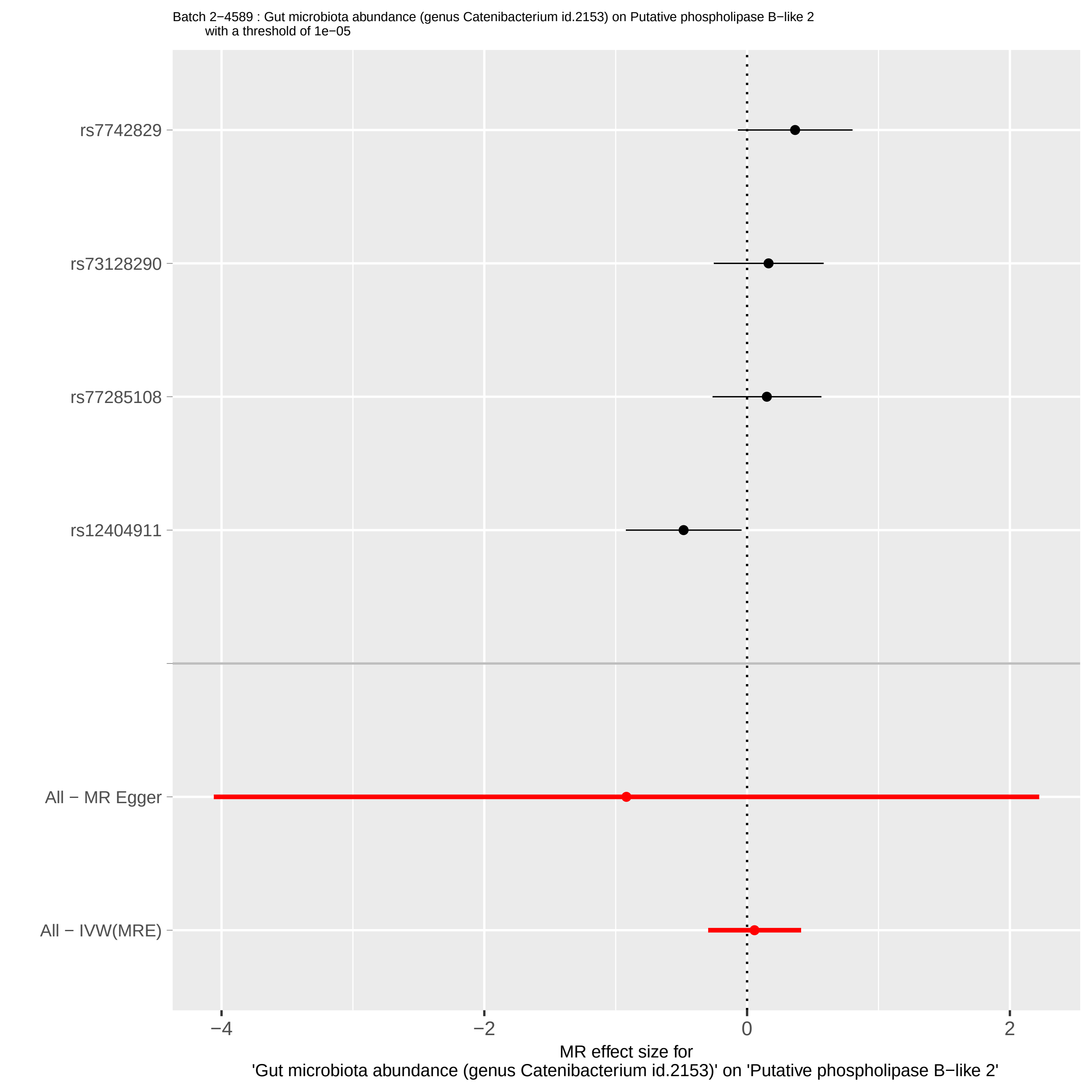

Batch 2−4589 : Gut microbiota abundance (genus Catenibacterium id.2153) on Putative phospholipase B−like 2
with a threshold of 1e−05
rs7742829
rs73128290
rs77285108
rs12404911
All − MR Egger
All − IVW(MRE)
−4
−2
0
2
MR effect size for
'Gut microbiota abundance (genus Catenibacterium id.2153)' on 'Putative phospholipase B−like 2'

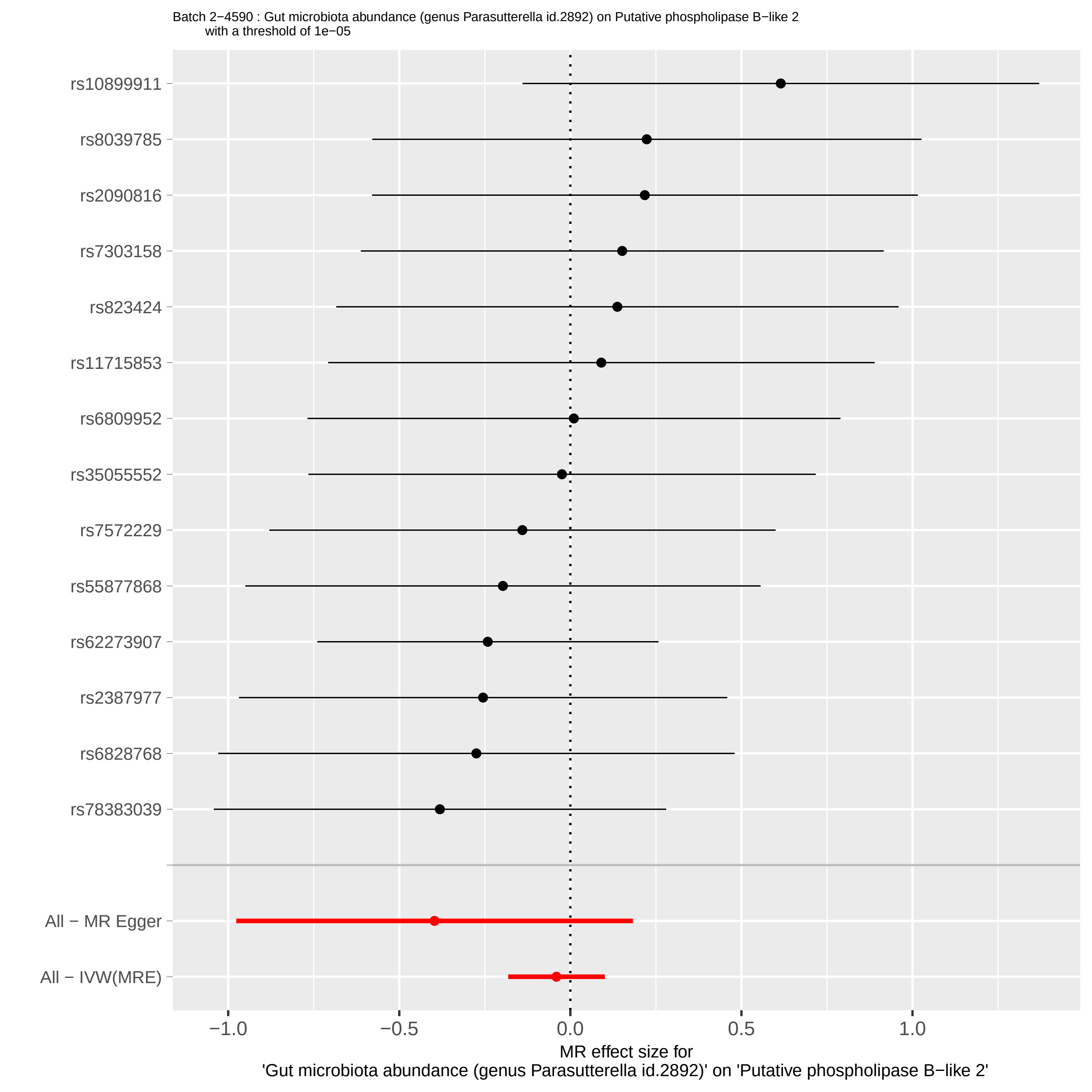
Batch 2−4590 : Gut microbiota abundance (genus Parasutterella id.2892) on Putative phospholipase B−like 2
with a threshold of 1e−05
rs10899911
rs8039785
rs2090816
rs7303158
rs823424
rs11715853
rs6809952
rs35055552
rs7572229
rs55877868
rs62273907
rs2387977
rs6828768
rs78383039
All − MR Egger
All − IVW(MRE)
−1.0
−0.5
0.0
0.5
1.0
MR effect size for
'Gut microbiota abundance (genus Parasutterella id.2892)' on 'Putative phospholipase B−like 2'

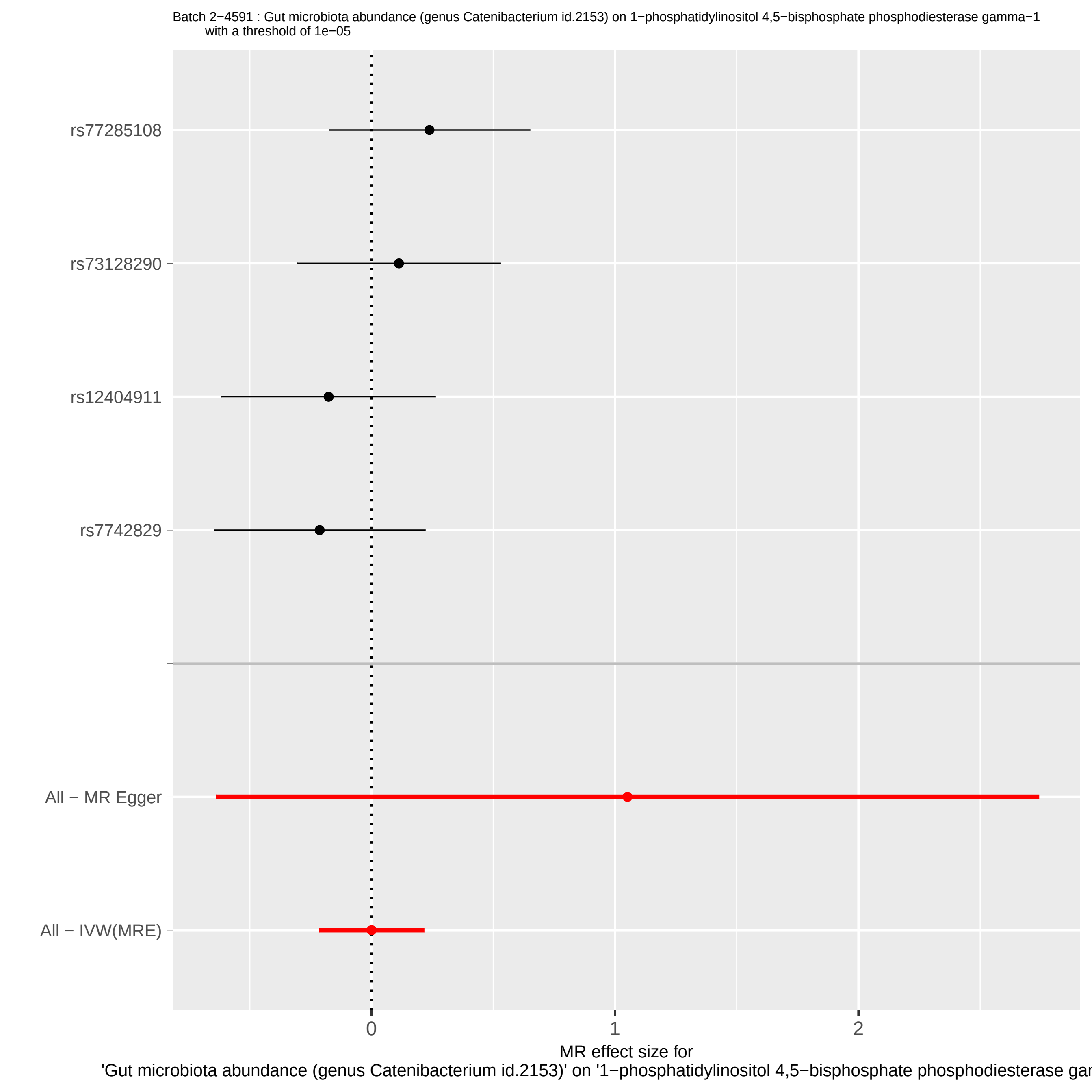

Batch 2−4591 : Gut microbiota abundance (genus Catenibacterium id.2153) on 1−phosphatidylinositol 4,5−bisphosphate phosphodiesterase gamma−1 with a threshold of 1e−05
rs77285108
rs73128290
rs12404911
rs7742829
All − MR Egger
All − IVW(MRE)
0
1
2
MR effect size for
'Gut microbiota abundance (genus Catenibacterium id.2153)' on '1−phosphatidylinositol 4,5−bisphosphate phosphodiesterase gar

Batch 2−4592 : Gut microbiota abundance (genus Parasutterella id.2892) on 1−phosphatidylinositol 4,5−bisphosphate phosphodiesterase gamma−1 with a threshold of 1e−05

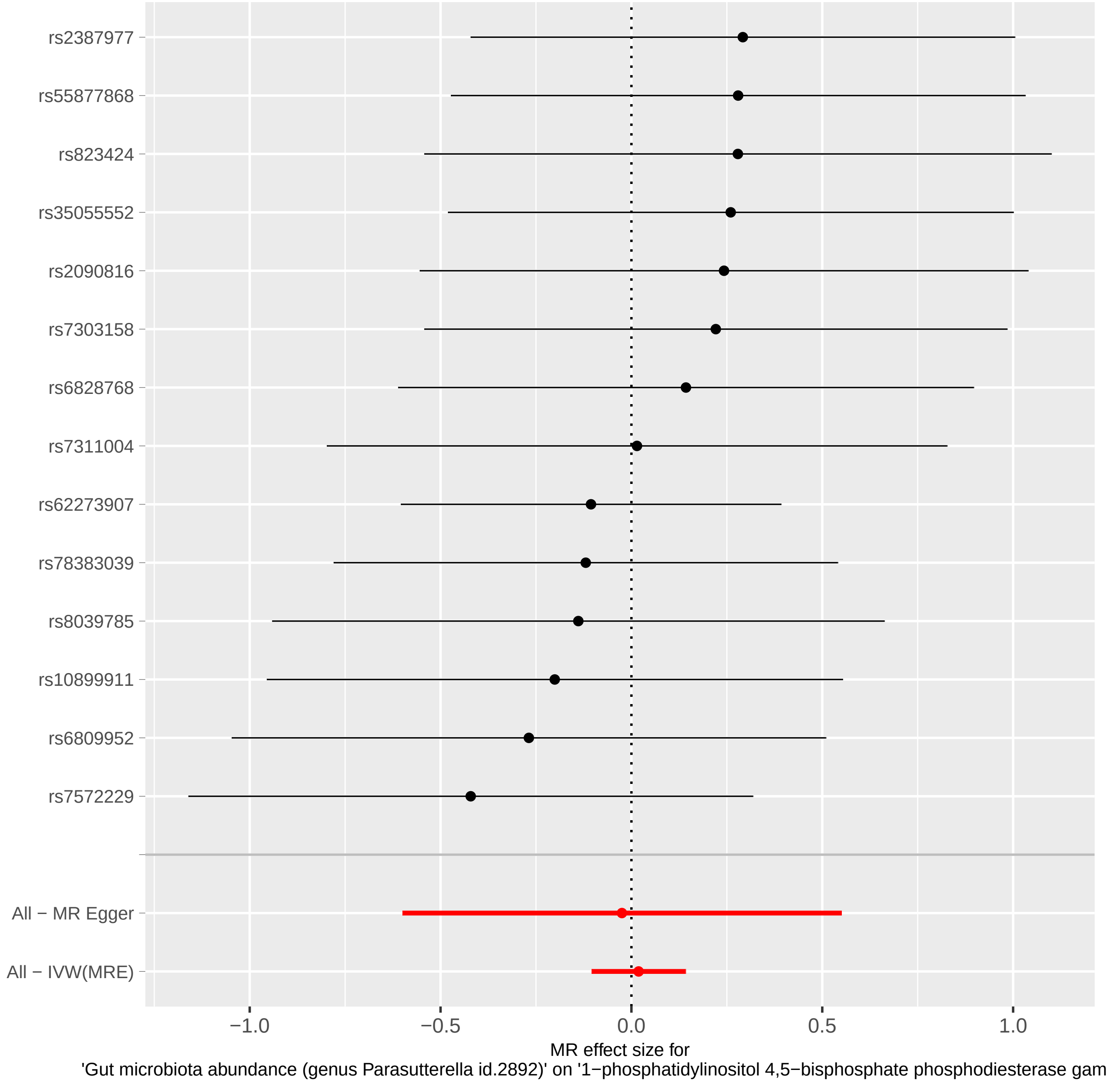

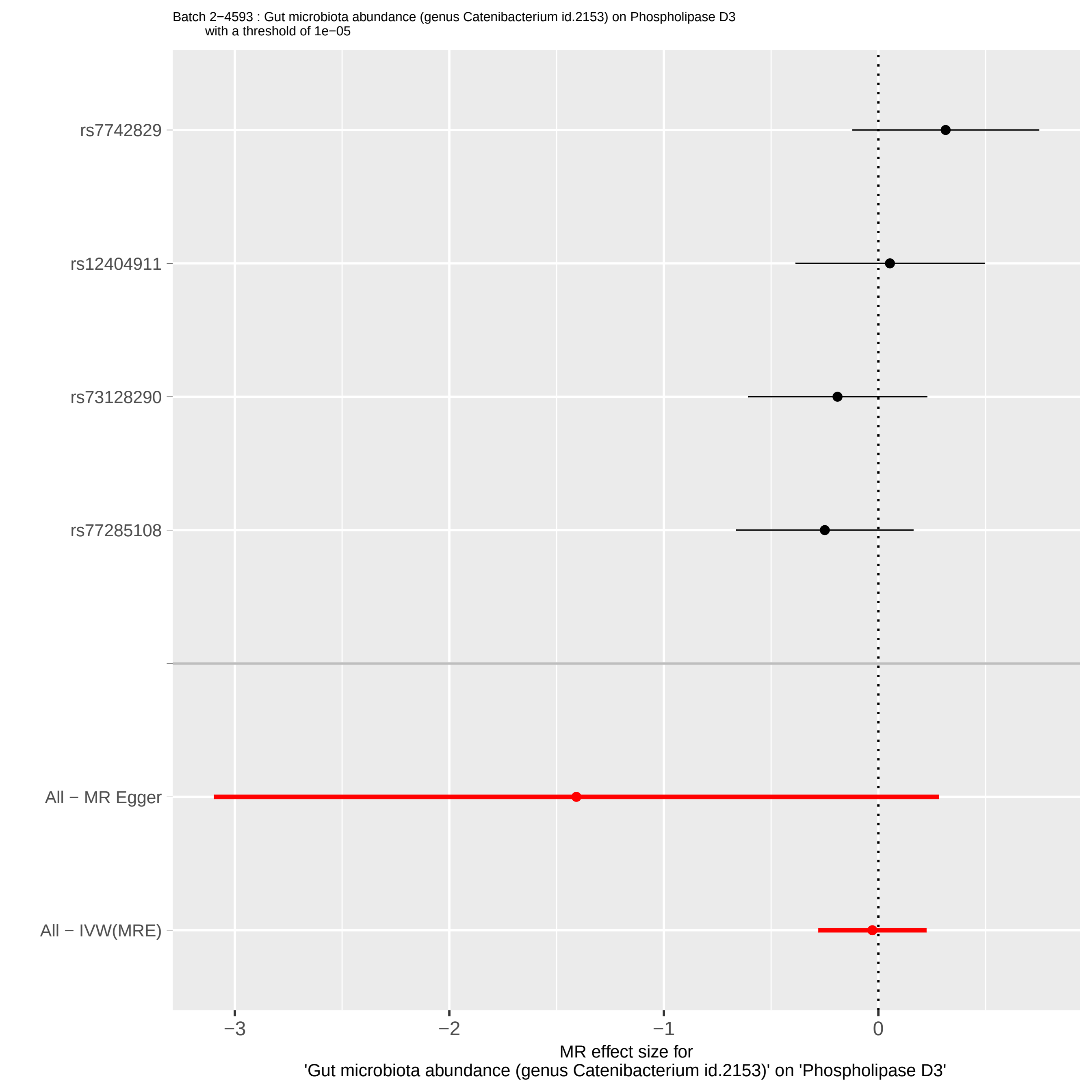
Batch 2−4593 : Gut microbiota abundance (genus Catenibacterium id.2153) on Phospholipase D3
with a threshold of 1e−05
rs7742829
rs12404911
rs73128290
rs77285108
All − MR Egger
All − IVW(MRE)
−3
−2
−1
0
MR effect size for
'Gut microbiota abundance (genus Catenibacterium id.2153)' on 'Phospholipase D3'

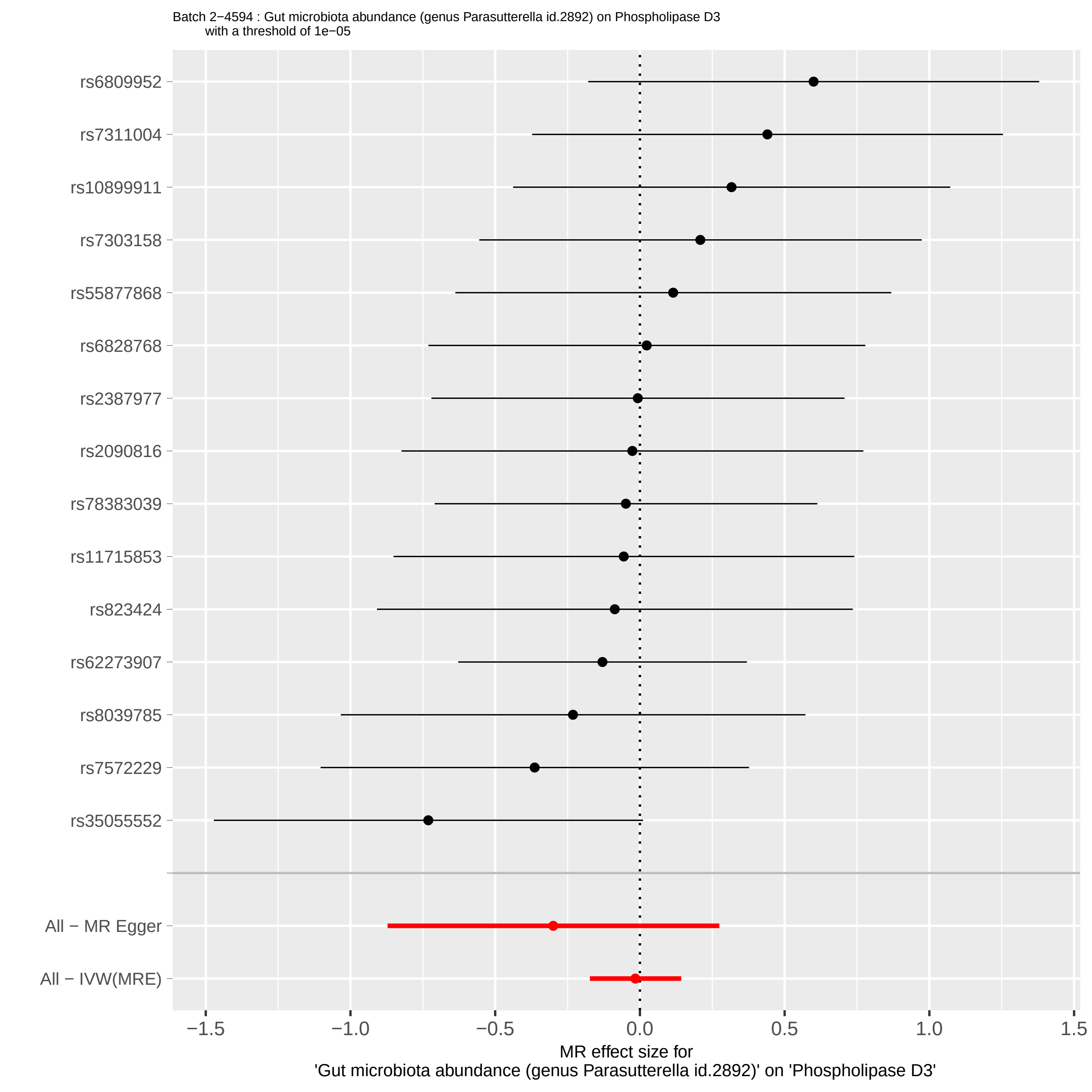

Batch 2−4594 : Gut microbiota abundance (genus Parasutterella id.2892) on Phospholipase D3
with a threshold of 1e−05
rs6809952
rs7311004
rs10899911
rs7303158
rs55877868
rs6828768
rs2387977
rs2090816
rs78383039
rs11715853
rs823424
rs62273907
rs8039785
rs7572229
rs35055552
All − MR Egger
All − IVW(MRE)
−1.5
−1.0
−0.5
0.0
0.5
1.0
1.5
MR effect size for
'Gut microbiota abundance (genus Parasutterella id.2892)' on 'Phospholipase D3'

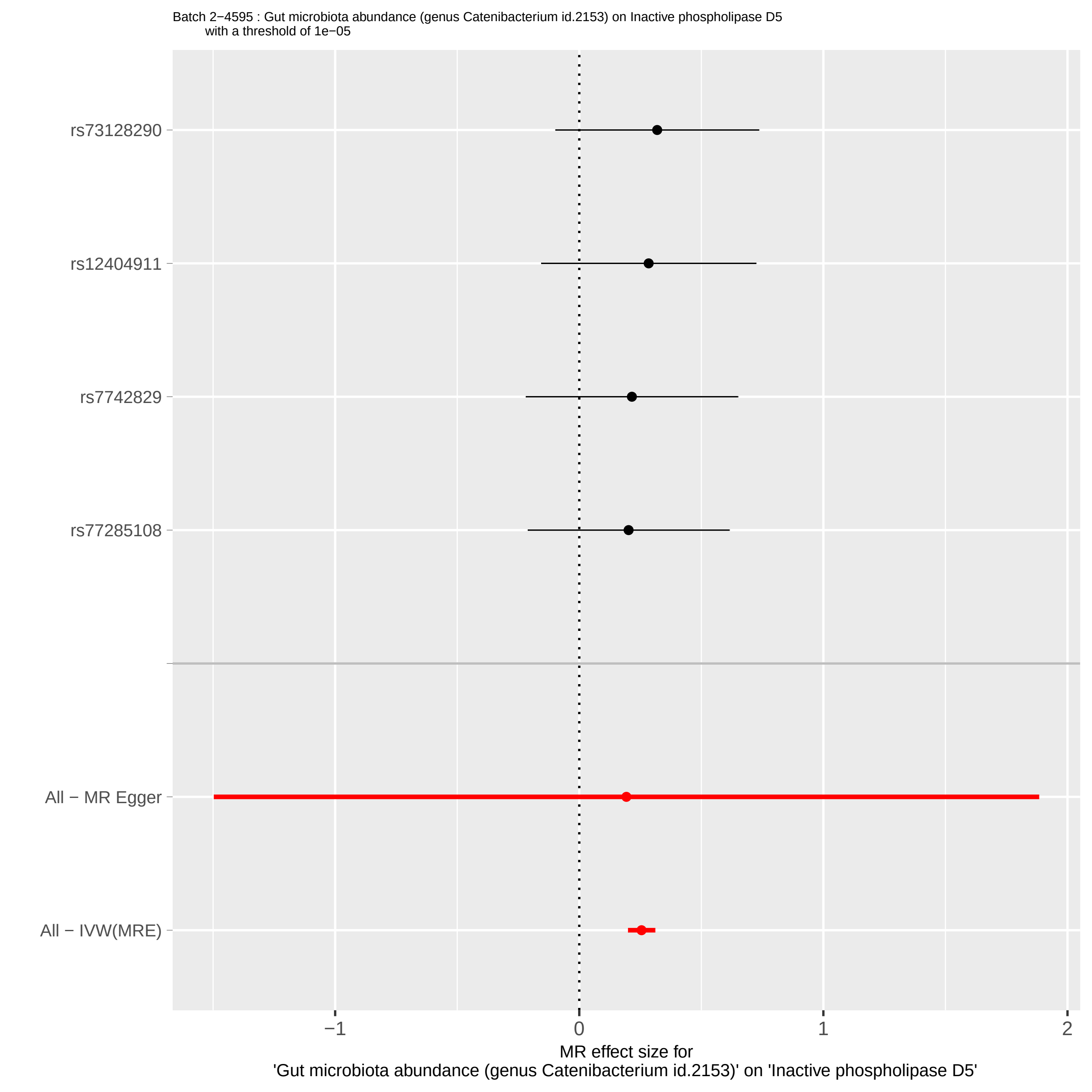
Batch 2−4595 : Gut microbiota abundance (genus Catenibacterium id.2153) on Inactive phospholipase D5
with a threshold of 1e−05
rs73128290
rs12404911
rs7742829
rs77285108
All − MR Egger
All − IVW(MRE)
−1
0
1
2
MR effect size for
'Gut microbiota abundance (genus Catenibacterium id.2153)' on 'Inactive phospholipase D5'

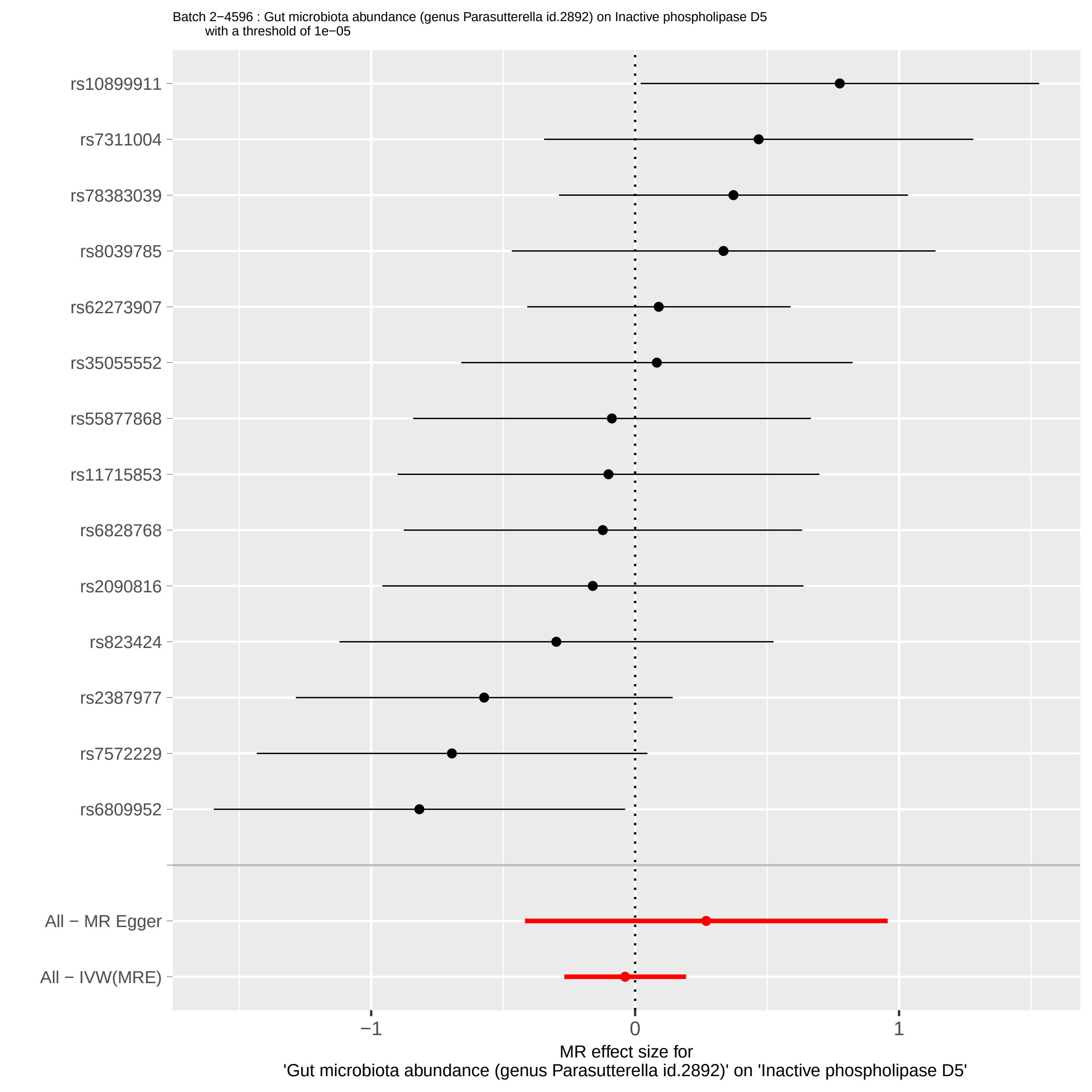

Batch 2−4596 : Gut microbiota abundance (genus Parasutterella id.2892) on Inactive phospholipase D5
with a threshold of 1e−05
rs10899911
rs7311004
rs78383039
rs8039785
rs62273907
rs35055552
rs55877868
rs11715853
rs6828768
rs2090816
rs823424
rs2387977
rs7572229
rs6809952
All − MR Egger
All − IVW(MRE)
−1
0
1
MR effect size for
'Gut microbiota abundance (genus Parasutterella id.2892)' on 'Inactive phospholipase D5'

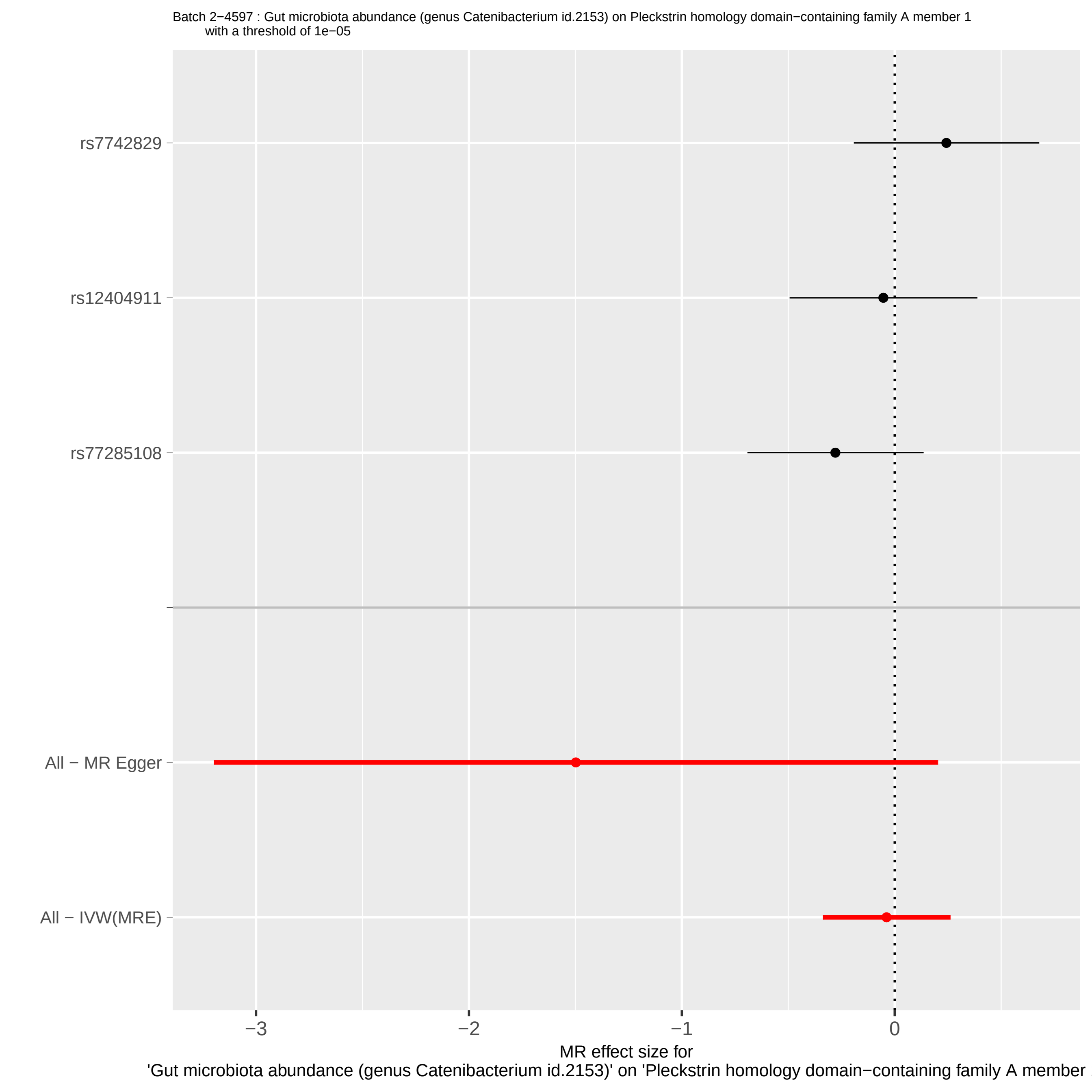
Batch 2−4597 : Gut microbiota abundance (genus Catenibacterium id.2153) on Pleckstrin homology domain−containing family A member 1
with a threshold of 1e−05
rs7742829
rs12404911
rs77285108
All − MR Egger
All − IVW(MRE)
−3
−2
−1
0
MR effect size for
'Gut microbiota abundance (genus Catenibacterium id.2153)' on 'Pleckstrin homology domain−containing family A member

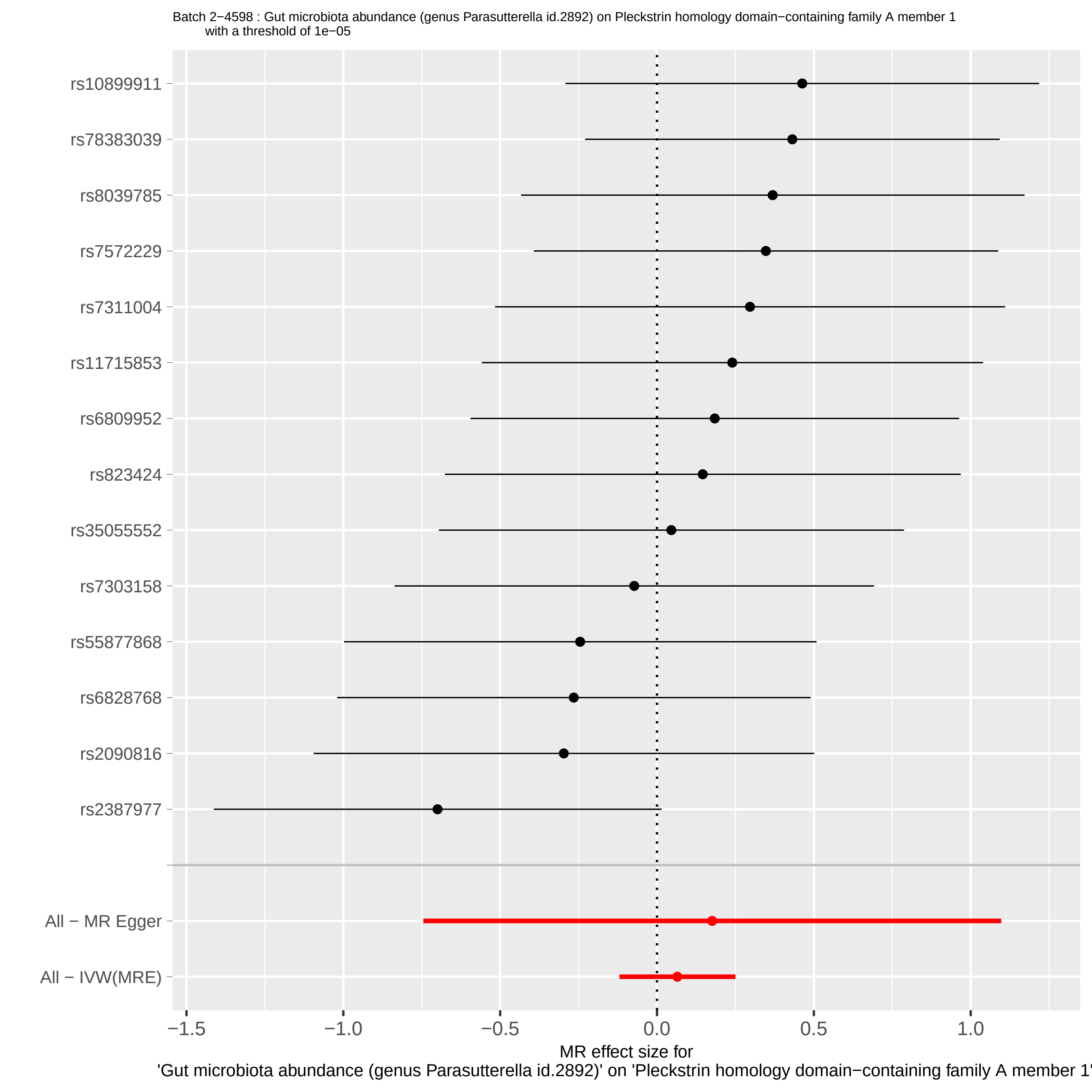

Batch 2−4598 : Gut microbiota abundance (genus Parasutterella id.2892) on Pleckstrin homology domain−containing family A member 1
with a threshold of 1e−05
rs10899911
rs78383039
rs8039785
rs7572229
rs7311004
rs11715853
rs6809952
rs823424
rs35055552
rs7303158
rs55877868
rs6828768
rs2090816
rs2387977
All − MR Egger
All − IVW(MRE)
−1.5
−1.0
−0.5
0.0
0.5
1.0
MR effect size for
'Gut microbiota abundance (genus Parasutterella id.2892)' on 'Pleckstrin homology domain−containing family A member 1'

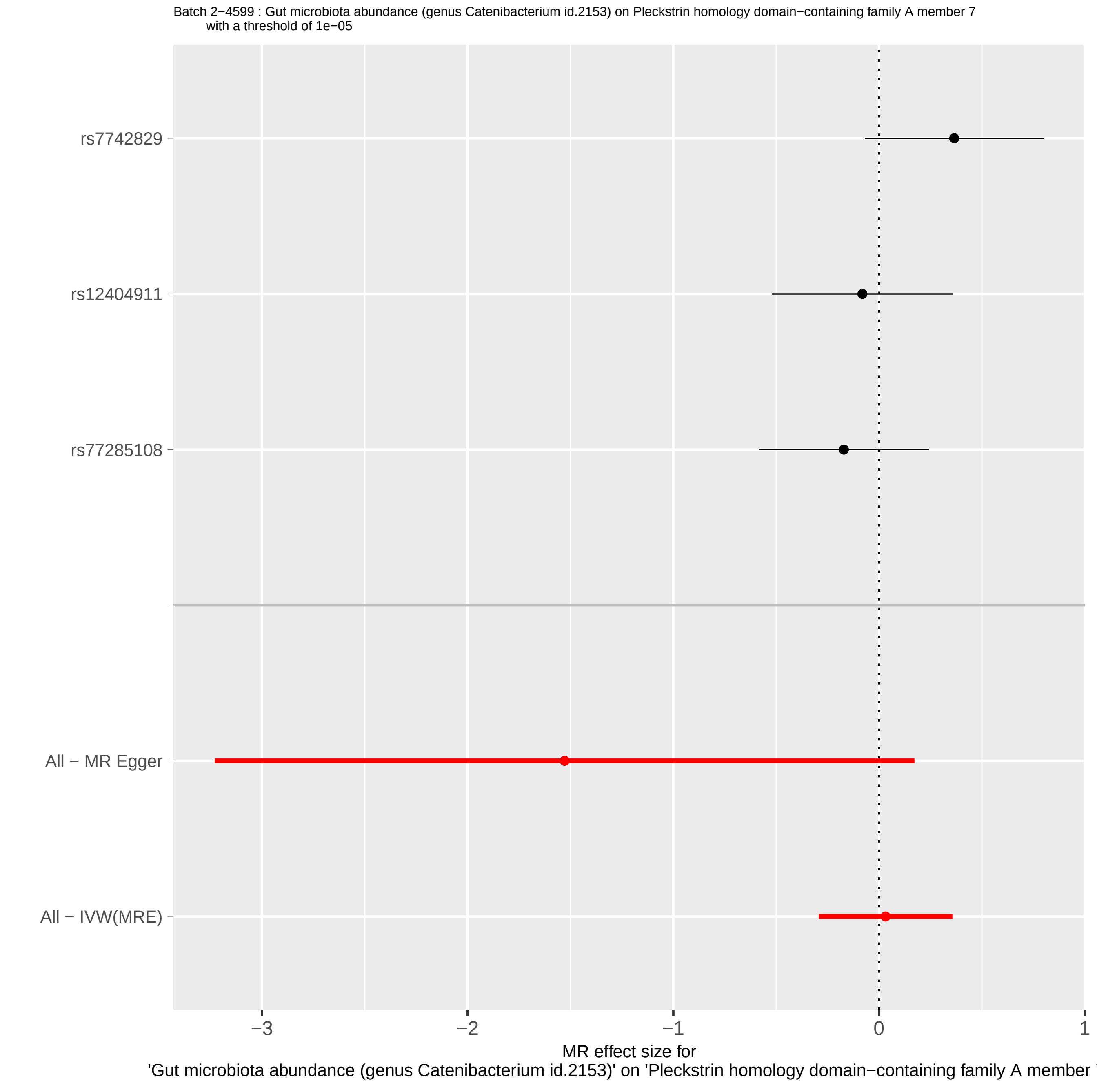
Batch 2−4599 : Gut microbiota abundance (genus Catenibacterium id.2153) on Pleckstrin homology domain−containing family A member 7
with a threshold of 1e−05
rs7742829
rs12404911
rs77285108
All − MR Egger
All − IVW(MRE)
−3
−2
−1
0
1
MR effect size for
'Gut microbiota abundance (genus Catenibacterium id.2153)' on 'Pleckstrin homology domain−containing family A member 7'

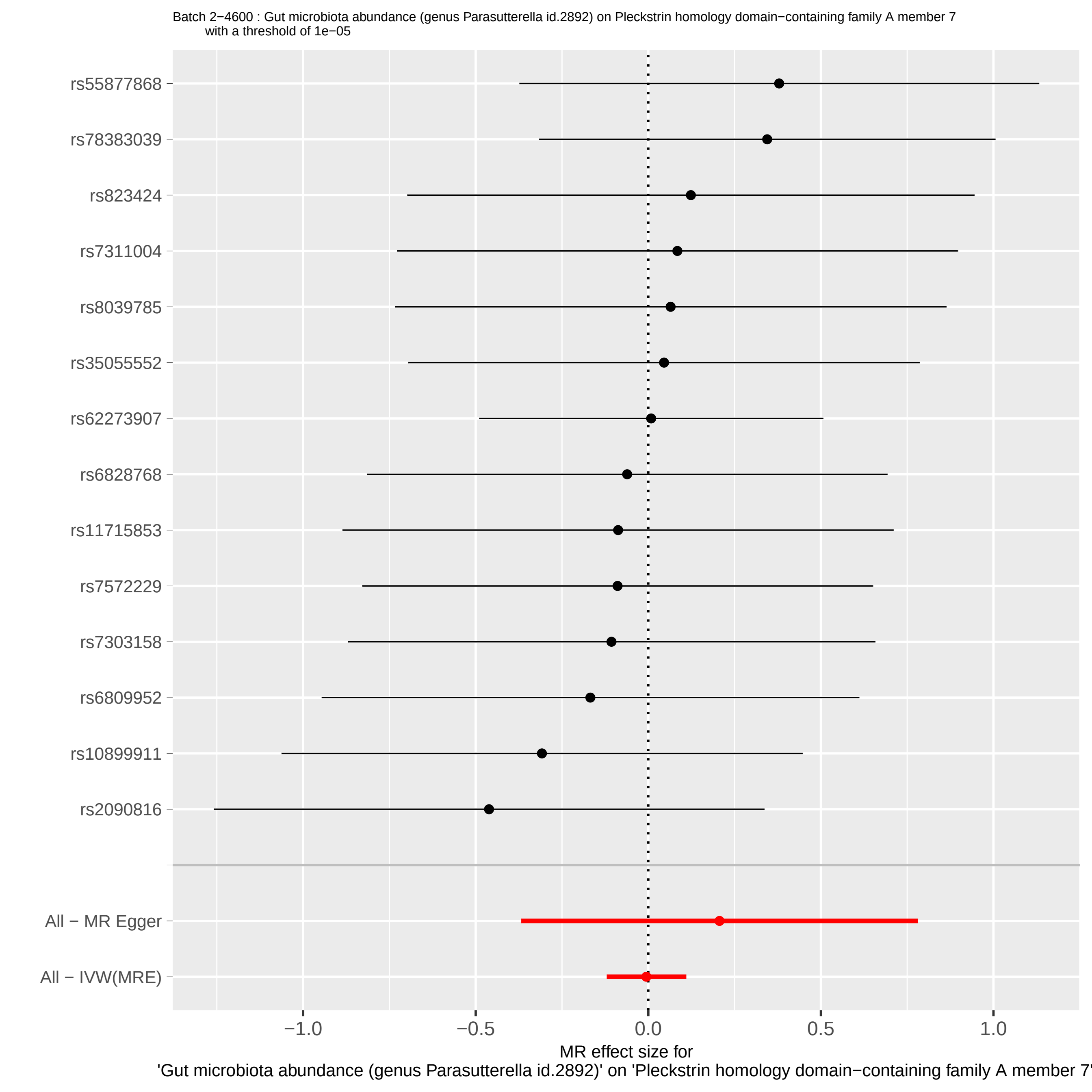
Batch 2−4600 : Gut microbiota abundance (genus Parasutterella id.2892) on Pleckstrin homology domain−containing family A member 7
with a threshold of 1e−05
rs55877868
rs78383039
rs823424
rs7311004
rs8039785
rs35055552
rs62273907
rs6828768
rs11715853
rs7572229
rs7303158
rs6809952
rs10899911
rs2090816
All − MR Egger
All − IVW(MRE)
−1.0
−0.5
0.0
0.5
1.0
MR effect size for
'Gut microbiota abundance (genus Parasutterella id.2892)' on 'Pleckstrin homology domain−containing family A member 7'

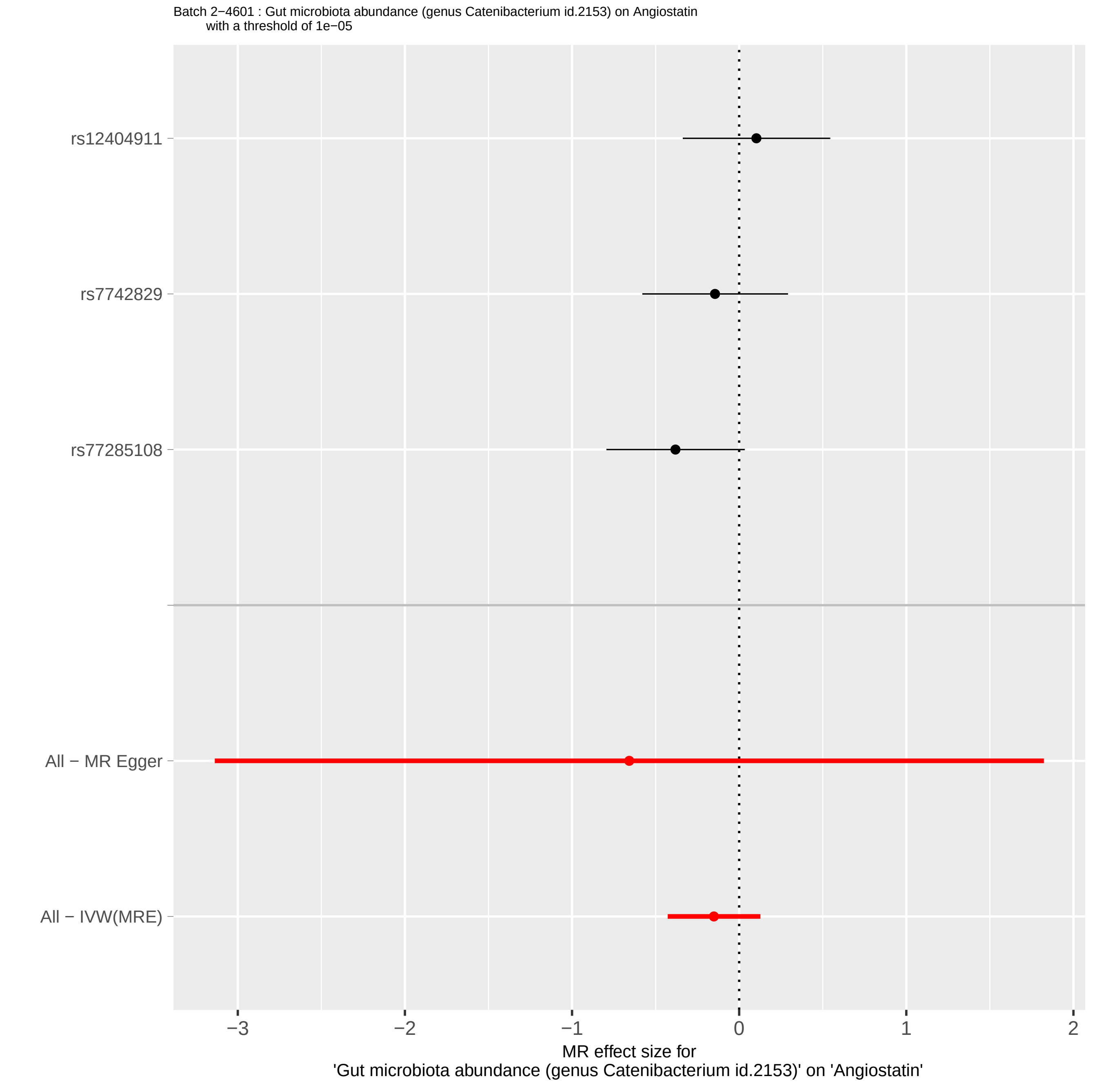

Batch 2−4601 : Gut microbiota abundance (genus Catenibacterium id.2153) on Angiostatin
with a threshold of 1e−05
rs12404911
rs7742829
rs77285108
All − MR Egger
All − IVW(MRE)
−3
−2
−1
0
1
2
MR effect size for
'Gut microbiota abundance (genus Catenibacterium id.2153)' on 'Angiostatin'

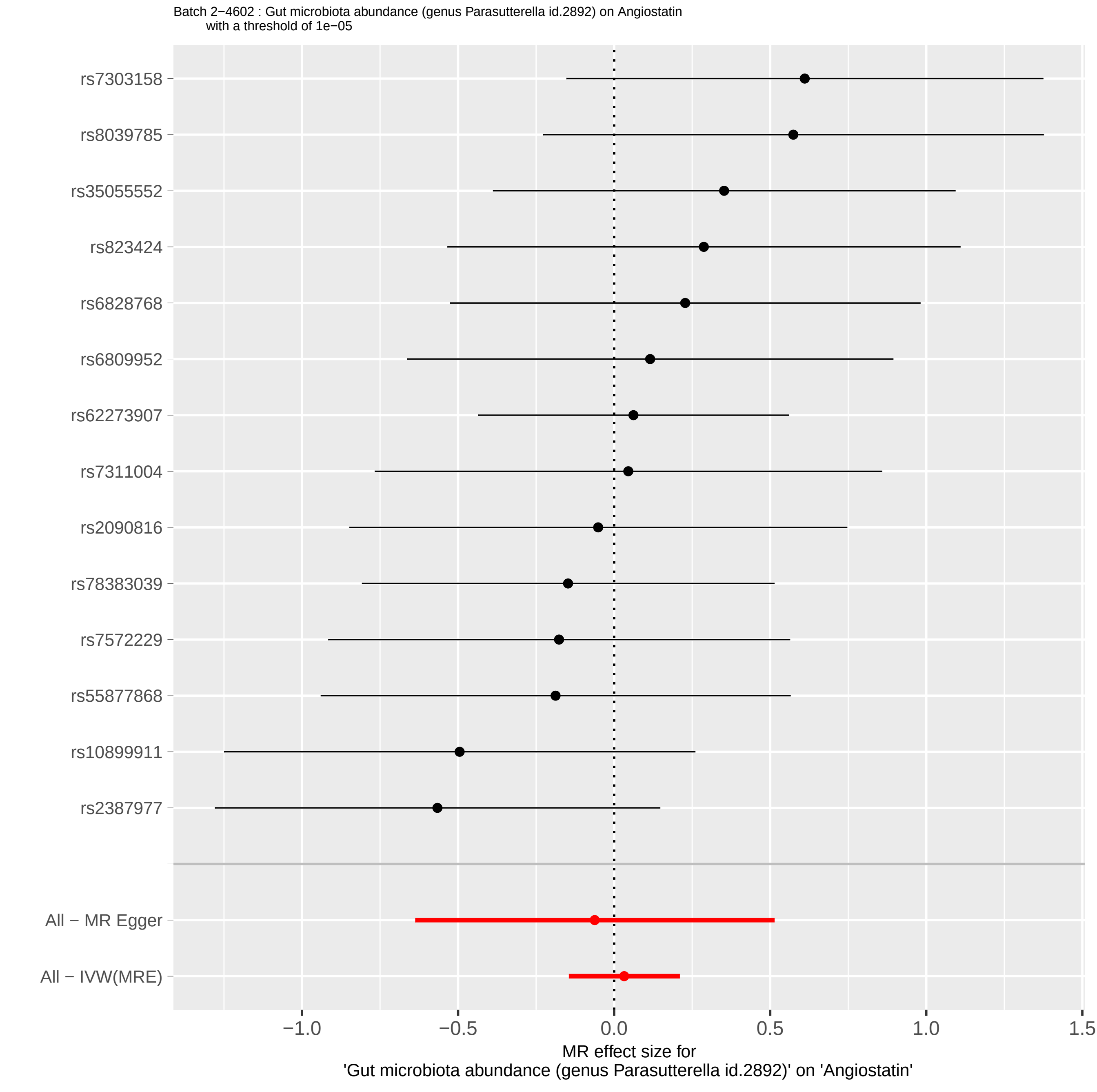
Batch 2−4602 : Gut microbiota abundance (genus Parasutterella id.2892) on Angiostatin
with a threshold of 1e−05
rs7303158
rs8039785
rs35055552
rs823424
rs6828768
rs6809952
rs62273907
rs7311004
rs2090816
rs78383039
rs7572229
rs55877868
rs10899911
rs2387977
All − MR Egger
All − IVW(MRE)
−1.0
−0.5
0.0
0.5
1.0
1.5
MR effect size for
'Gut microbiota abundance (genus Parasutterella id.2892)' on 'Angiostatin'

Batch 2−4603 : Gut microbiota abundance (genus Catenibacterium id.2153) on Plasmin
with a threshold of 1e−05

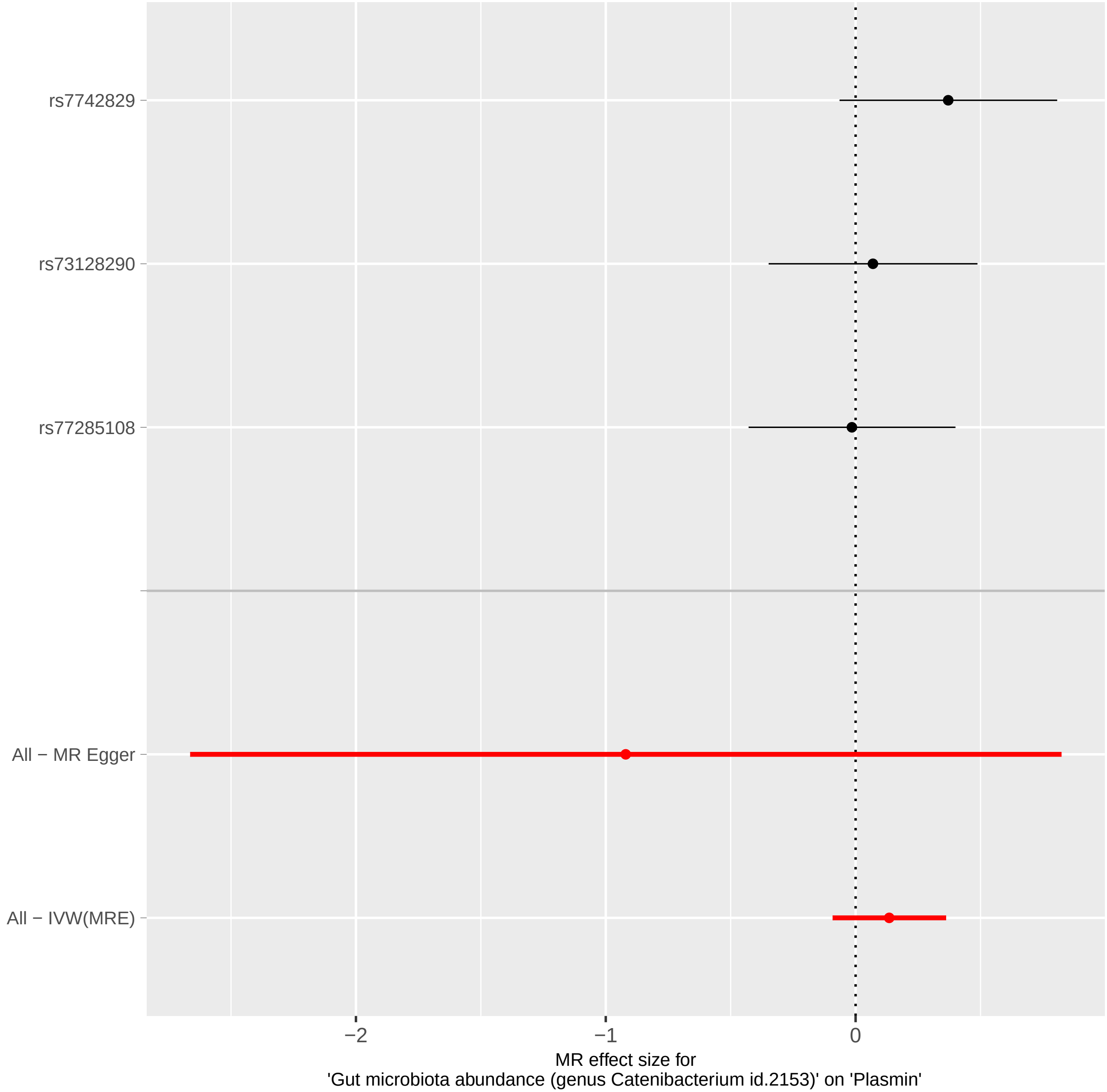

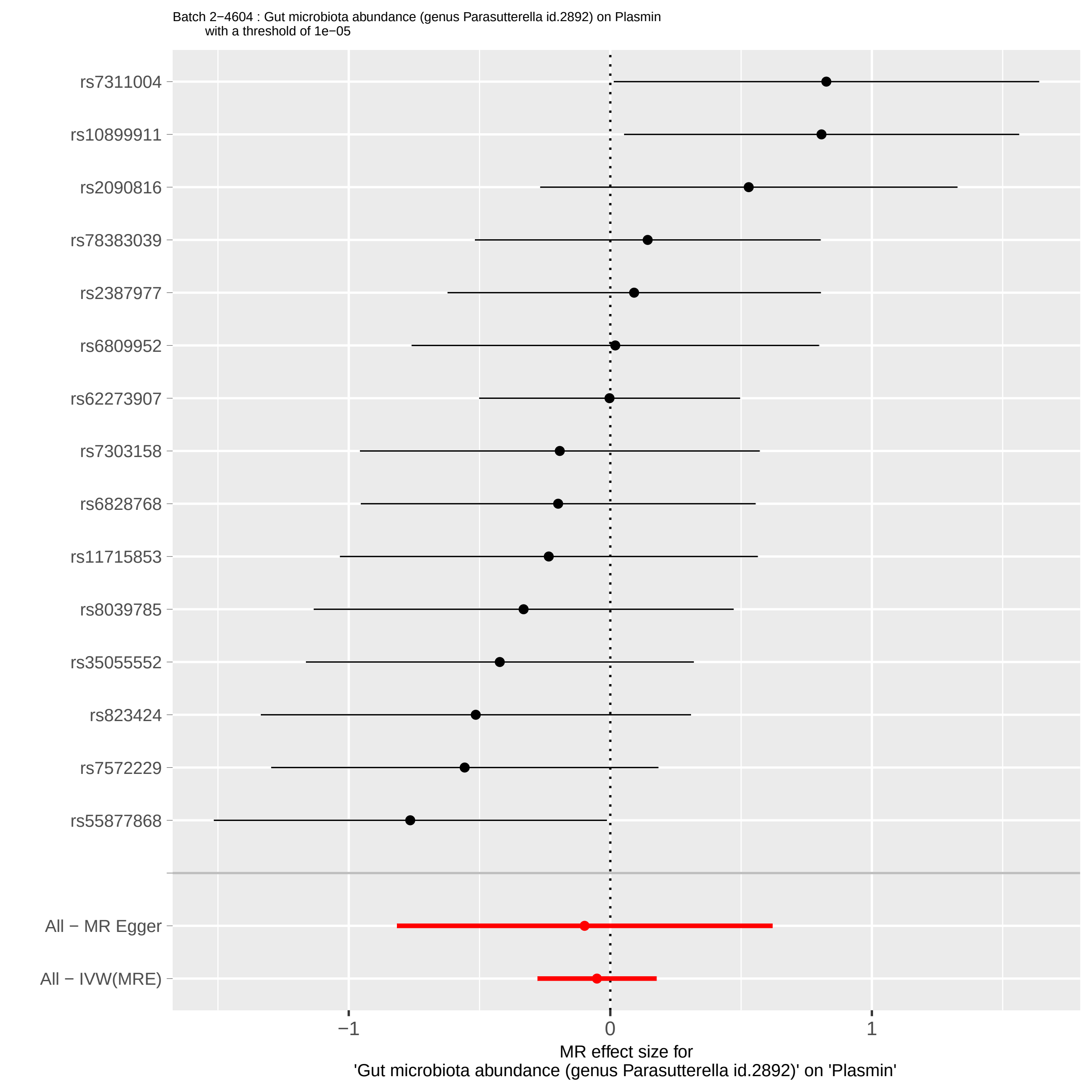

Batch 2−4604 : Gut microbiota abundance (genus Parasutterella id.2892) on Plasmin
with a threshold of 1e−05
rs7311004
rs10899911
rs2090816
rs78383039
rs2387977
rs6809952
rs62273907
rs7303158
rs6828768
rs11715853
rs8039785
rs35055552
rs823424
rs7572229
rs55877868
All − MR Egger
All − IVW(MRE)
−1
0
1
MR effect size for
'Gut microbiota abundance (genus Parasutterella id.2892)' on 'Plasmin'

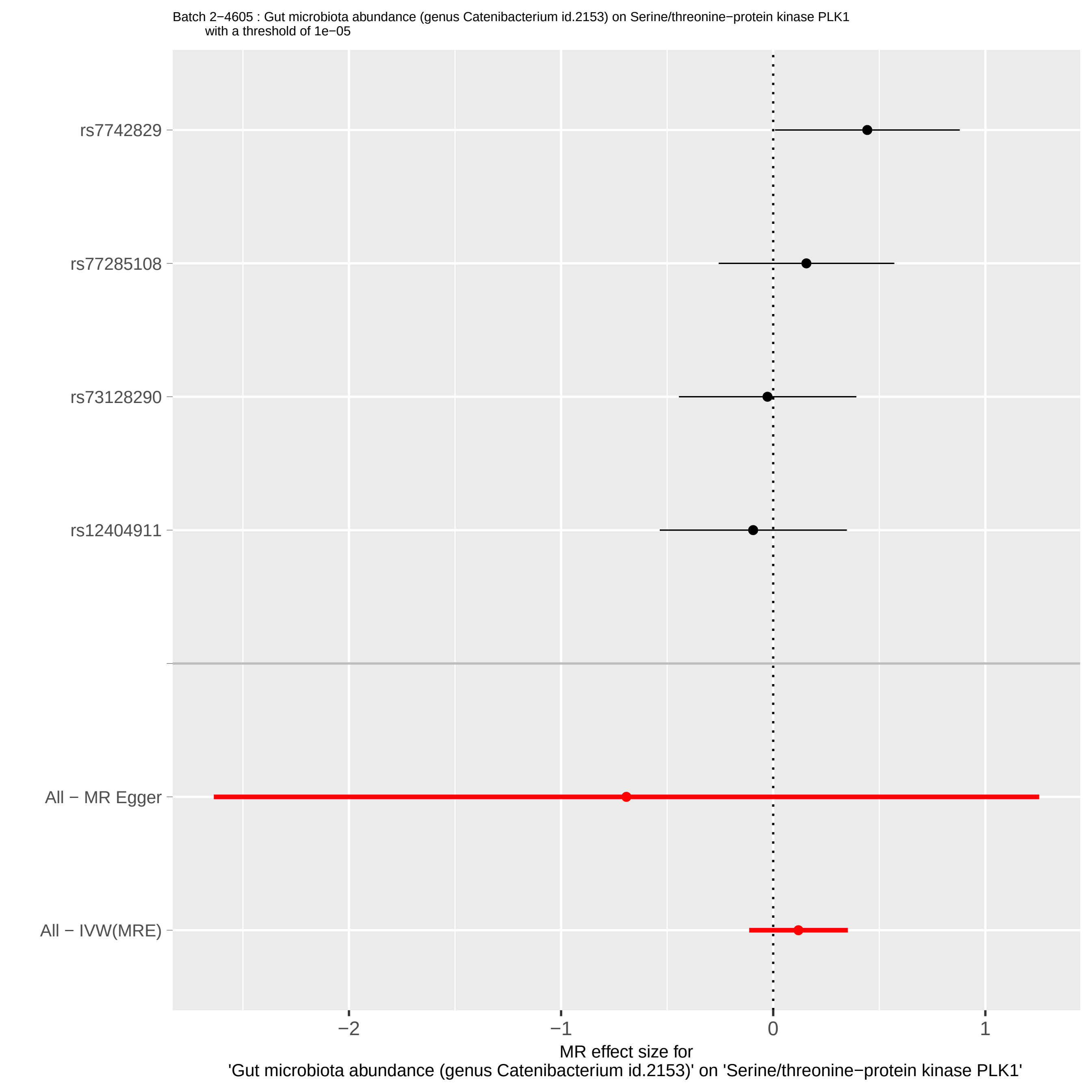
Batch 2−4605 : Gut microbiota abundance (genus Catenibacterium id.2153) on Serine/threonine−protein kinase PLK1
with a threshold of 1e−05
rs7742829
rs77285108
rs73128290
rs12404911
All − MR Egger
All − IVW(MRE)
−2
−1
0
1
MR effect size for
'Gut microbiota abundance (genus Catenibacterium id.2153)' on 'Serine/threonine−protein kinase PLK1'

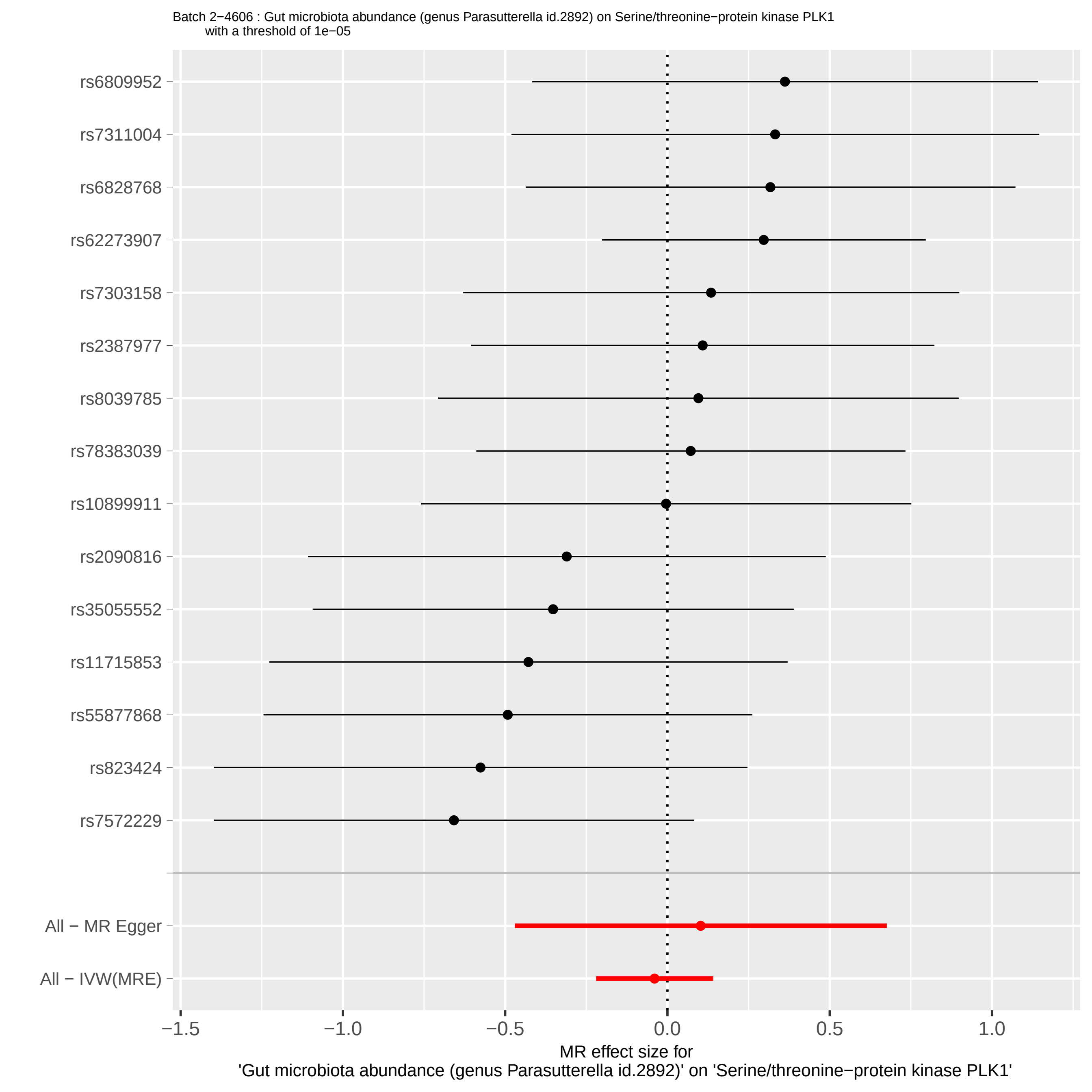
Batch 2−4606 : Gut microbiota abundance (genus Parasutterella id.2892) on Serine/threonine−protein kinase PLK1
with a threshold of 1e−05
rs6809952
rs7311004
rs6828768
rs62273907
rs7303158
rs2387977
rs8039785
rs78383039
rs10899911
rs2090816
rs35055552
rs11715853
rs55877868
rs823424
rs7572229
All − MR Egger
All − IVW(MRE)
−1.5
−1.0
−0.5
0.0
0.5
1.0
MR effect size for
'Gut microbiota abundance (genus Parasutterella id.2892)' on 'Serine/threonine−protein kinase PLK1'

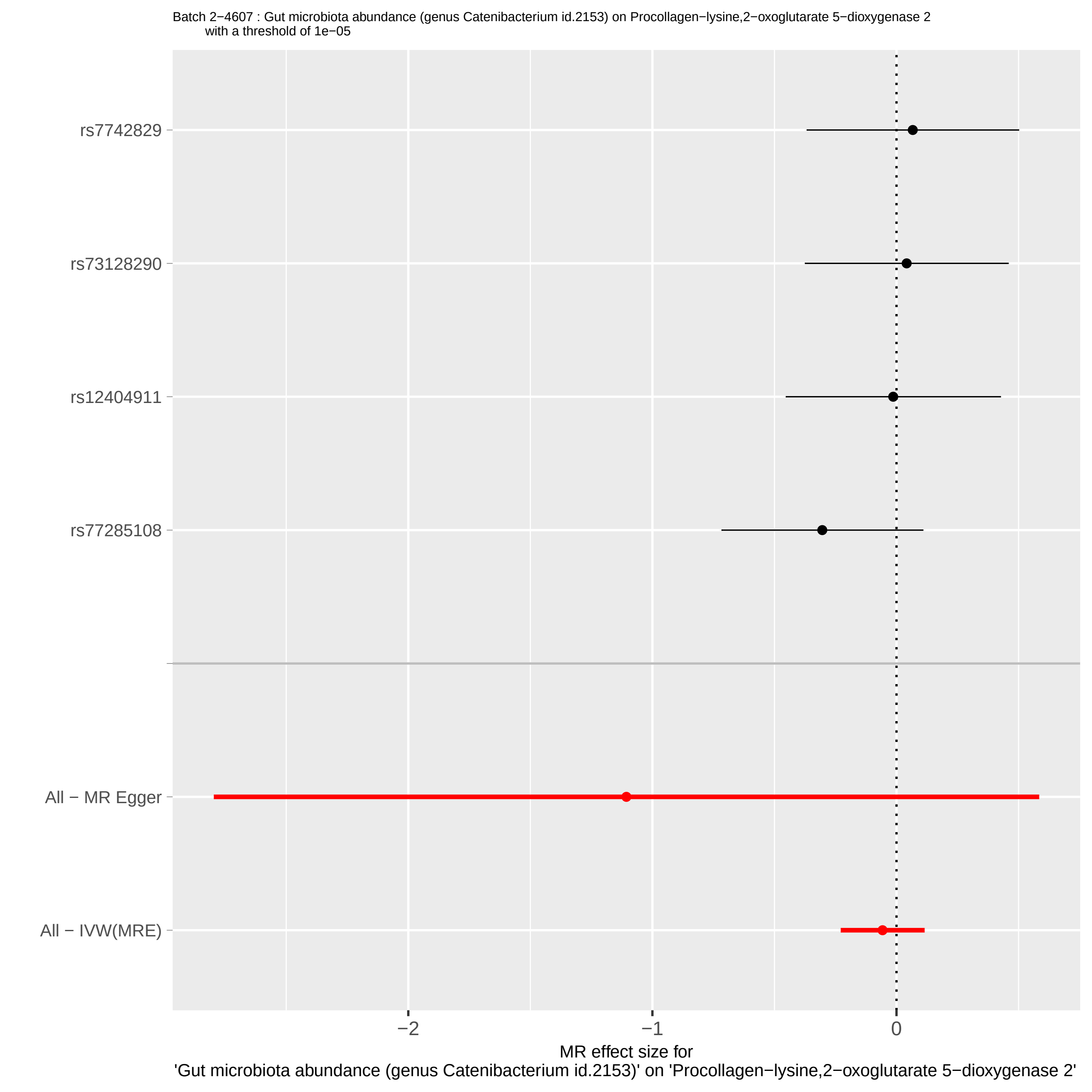
Batch 2−4607 : Gut microbiota abundance (genus Catenibacterium id.2153) on Procollagen−lysine,2−oxoglutarate 5−dioxygenase 2
with a threshold of 1e−05
rs7742829
rs73128290
rs12404911
rs77285108
All − MR Egger
All − IVW(MRE)
−2
−1
0
MR effect size for
'Gut microbiota abundance (genus Catenibacterium id.2153)' on 'Procollagen−lysine,2−oxoglutarate 5−dioxygenase 2'

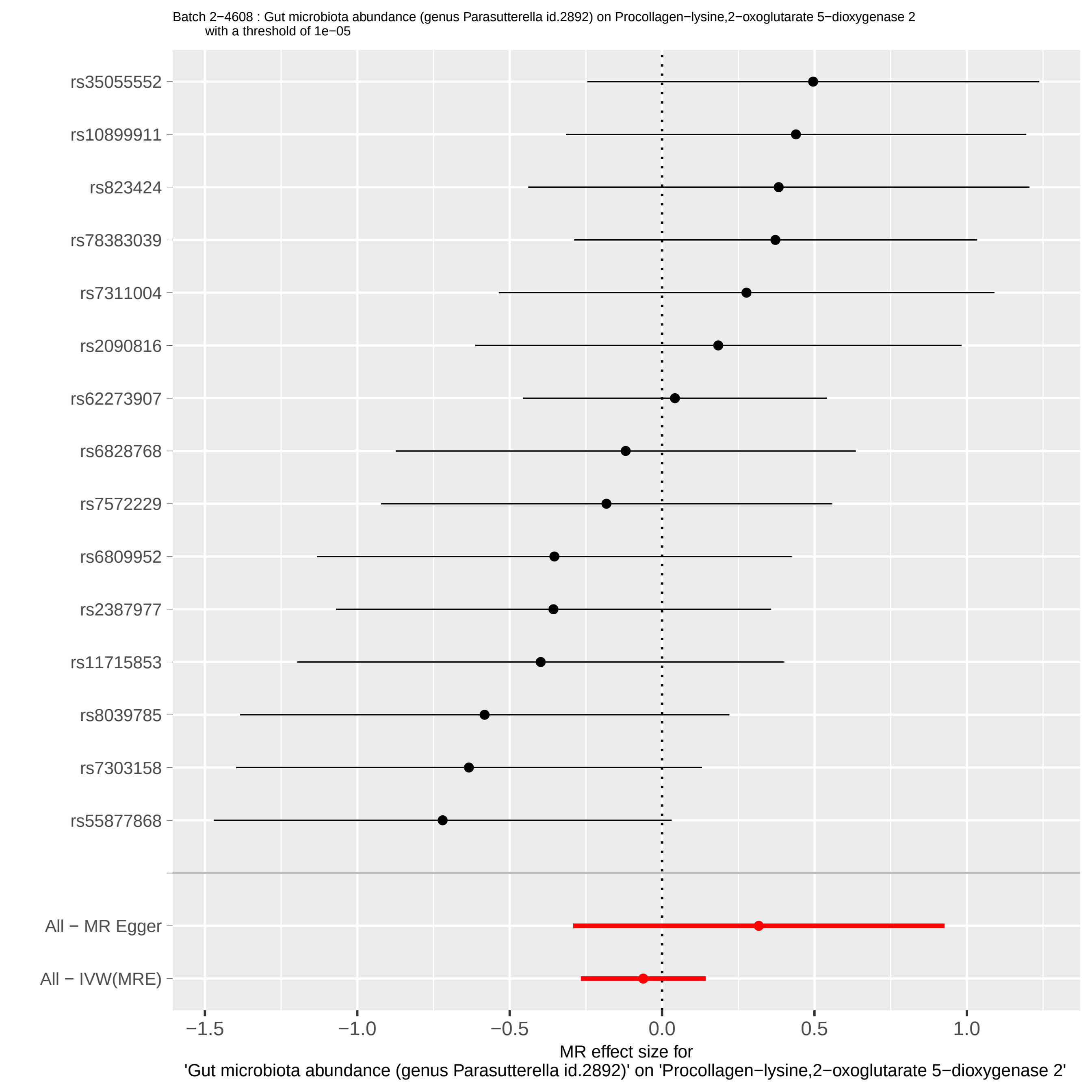
Batch 2−4608 : Gut microbiota abundance (genus Parasutterella id.2892) on Procollagen−lysine,2−oxoglutarate 5−dioxygenase 2
with a threshold of 1e−05
rs35055552
rs10899911
rs823424
rs78383039
rs7311004
rs2090816
rs62273907
rs6828768
rs7572229
rs6809952
rs2387977
rs11715853
rs8039785
rs7303158
rs55877868
All − MR Egger
All − IVW(MRE)
−1.5
−1.0
−0.5
0.0
0.5
1.0
MR effect size for
'Gut microbiota abundance (genus Parasutterella id.2892)' on 'Procollagen−lysine,2−oxoglutarate 5−dioxygenase 2'

Batch 2−4609 : Gut microbiota abundance (genus Catenibacterium id.2153) on Procollagen−lysine,2−oxoglutarate 5−dioxygenase 3
with a threshold of 1e−05

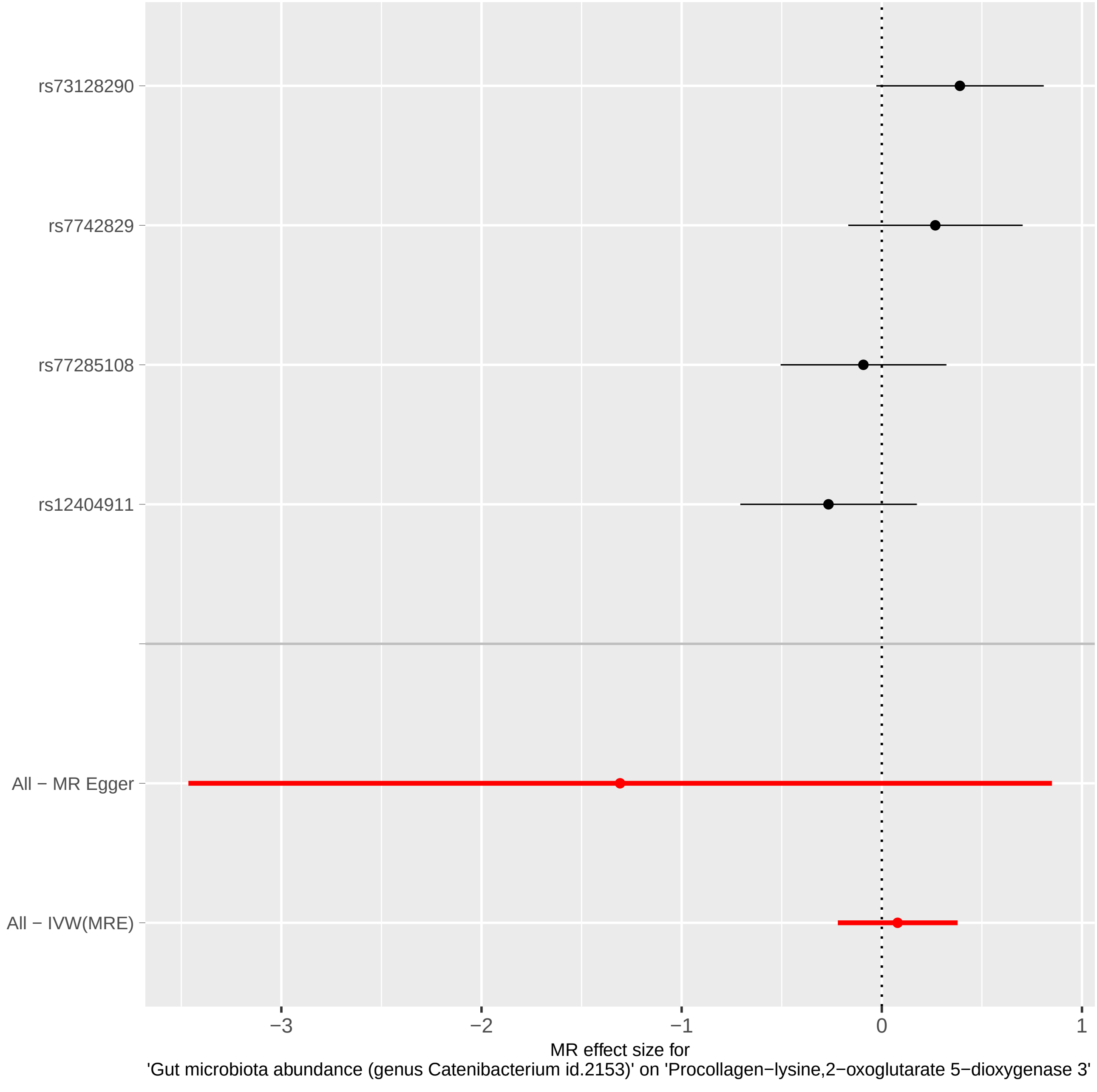

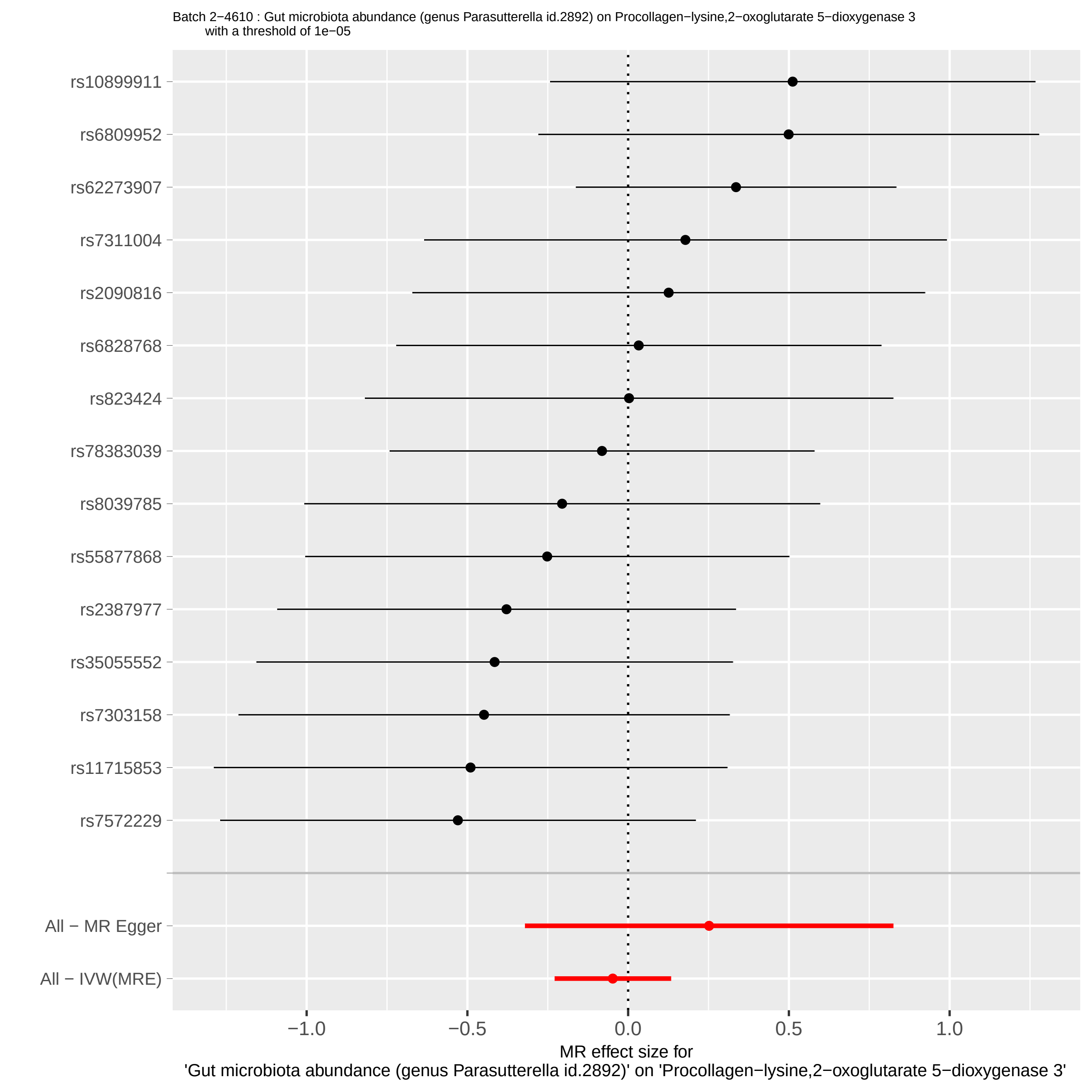

Batch 2−4610 : Gut microbiota abundance (genus Parasutterella id.2892) on Procollagen−lysine,2−oxoglutarate 5−dioxygenase 3
with a threshold of 1e−05
rs10899911
rs6809952
rs62273907
rs7311004
rs2090816
rs6828768
rs823424
rs78383039
rs8039785
rs55877868
rs2387977
rs35055552
rs7303158
rs11715853
rs7572229
All − MR Egger
All − IVW(MRE)
−1.0
−0.5
0.0
0.5
1.0
MR effect size for
'Gut microbiota abundance (genus Parasutterella id.2892)' on 'Procollagen−lysine,2−oxoglutarate 5−dioxygenase 3'

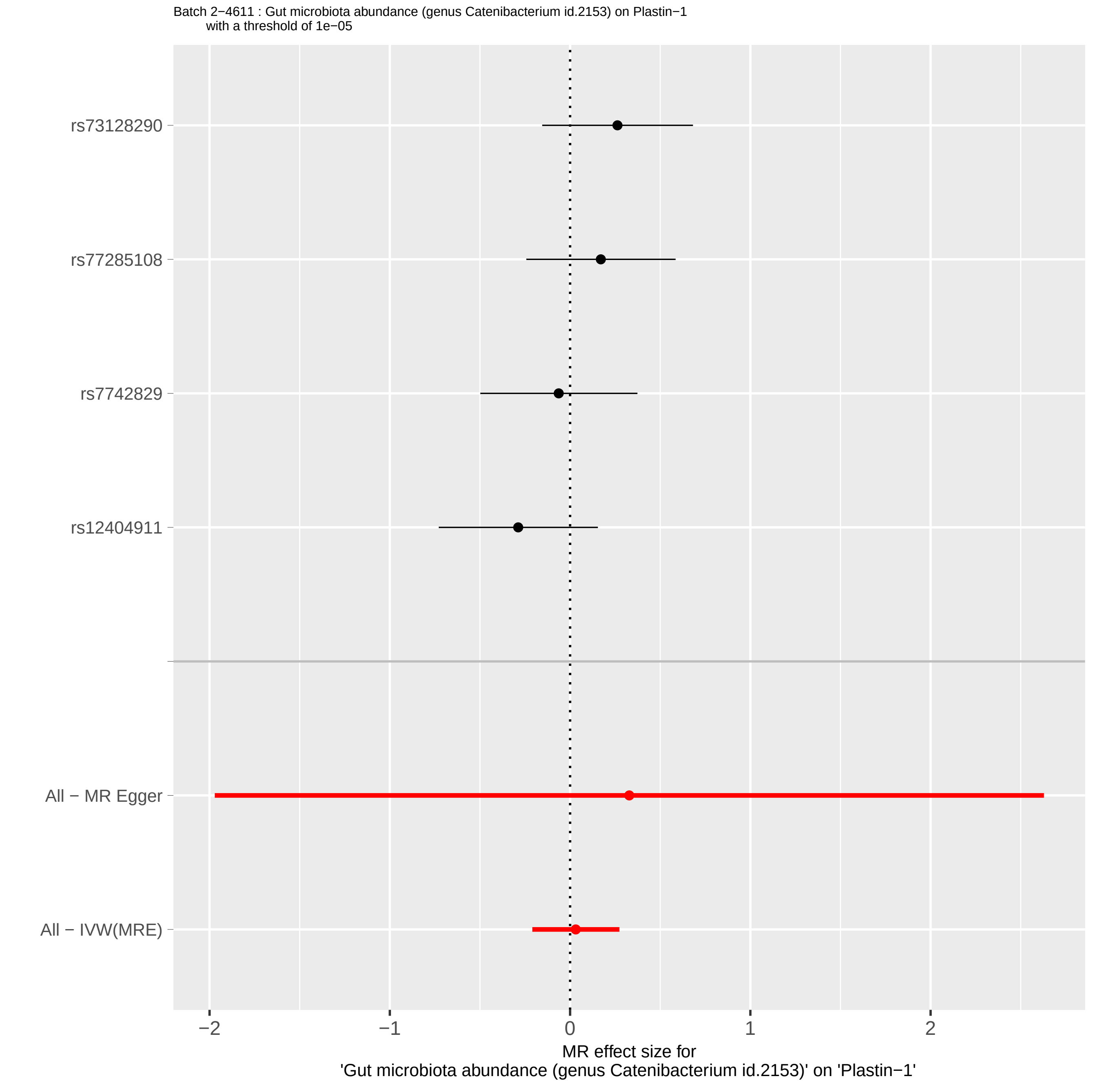

Batch 2−4611 : Gut microbiota abundance (genus Catenibacterium id.2153) on Plastin−1
with a threshold of 1e−05
rs73128290
rs77285108
rs7742829
rs12404911
All − MR Egger
All − IVW(MRE)
−2
−1
0
1
2
MR effect size for
'Gut microbiota abundance (genus Catenibacterium id.2153)' on 'Plastin−1'

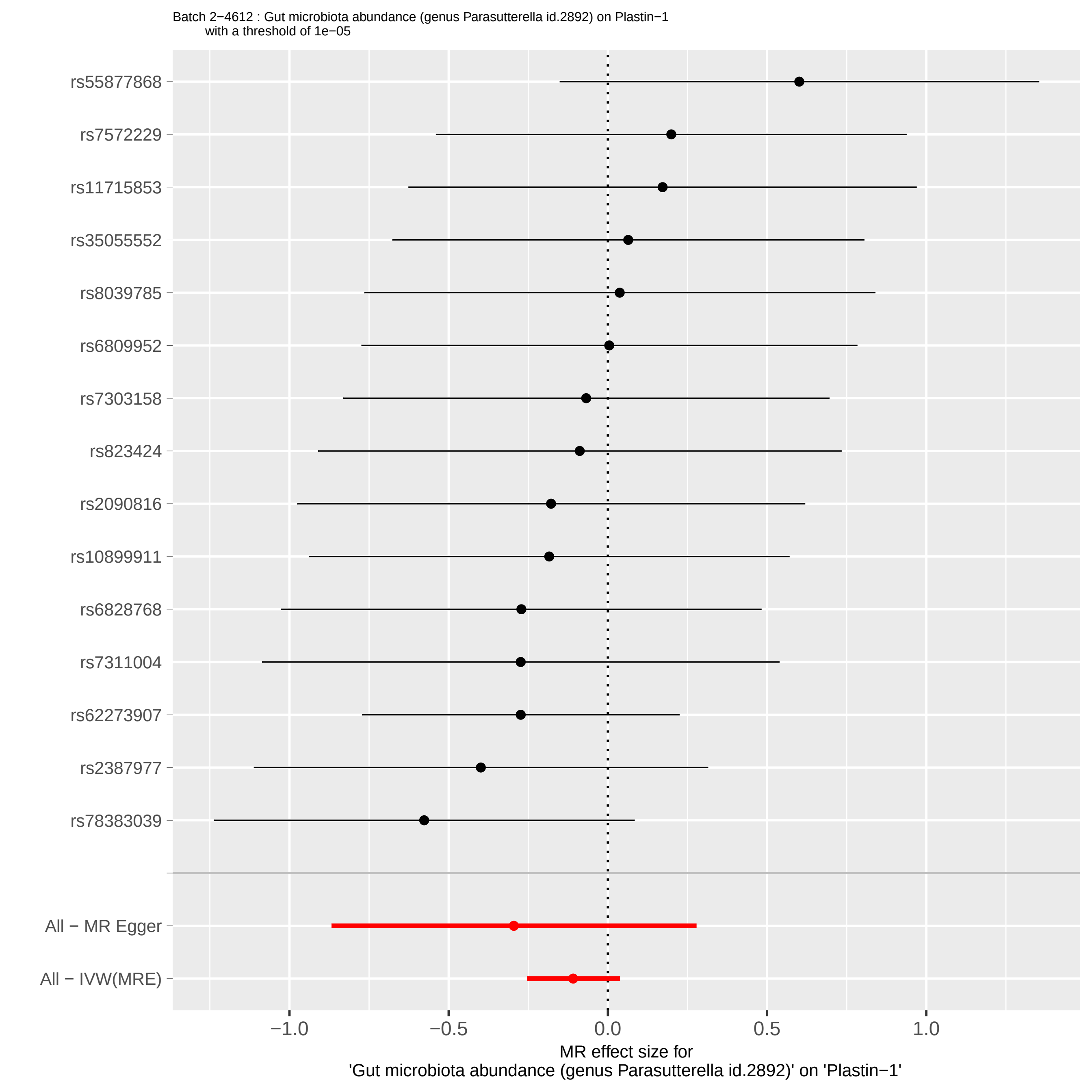

Batch 2−4612 : Gut microbiota abundance (genus Parasutterella id.2892) on Plastin−1
with a threshold of 1e−05
rs55877868
rs7572229
rs11715853
rs35055552
rs8039785
rs6809952
rs7303158
rs823424
rs2090816
rs10899911
rs6828768
rs7311004
rs62273907
rs2387977
rs78383039
All − MR Egger
All − IVW(MRE)
−1.0
−0.5
0.0
0.5
1.0
MR effect size for
'Gut microbiota abundance (genus Parasutterella id.2892)' on 'Plastin−1'

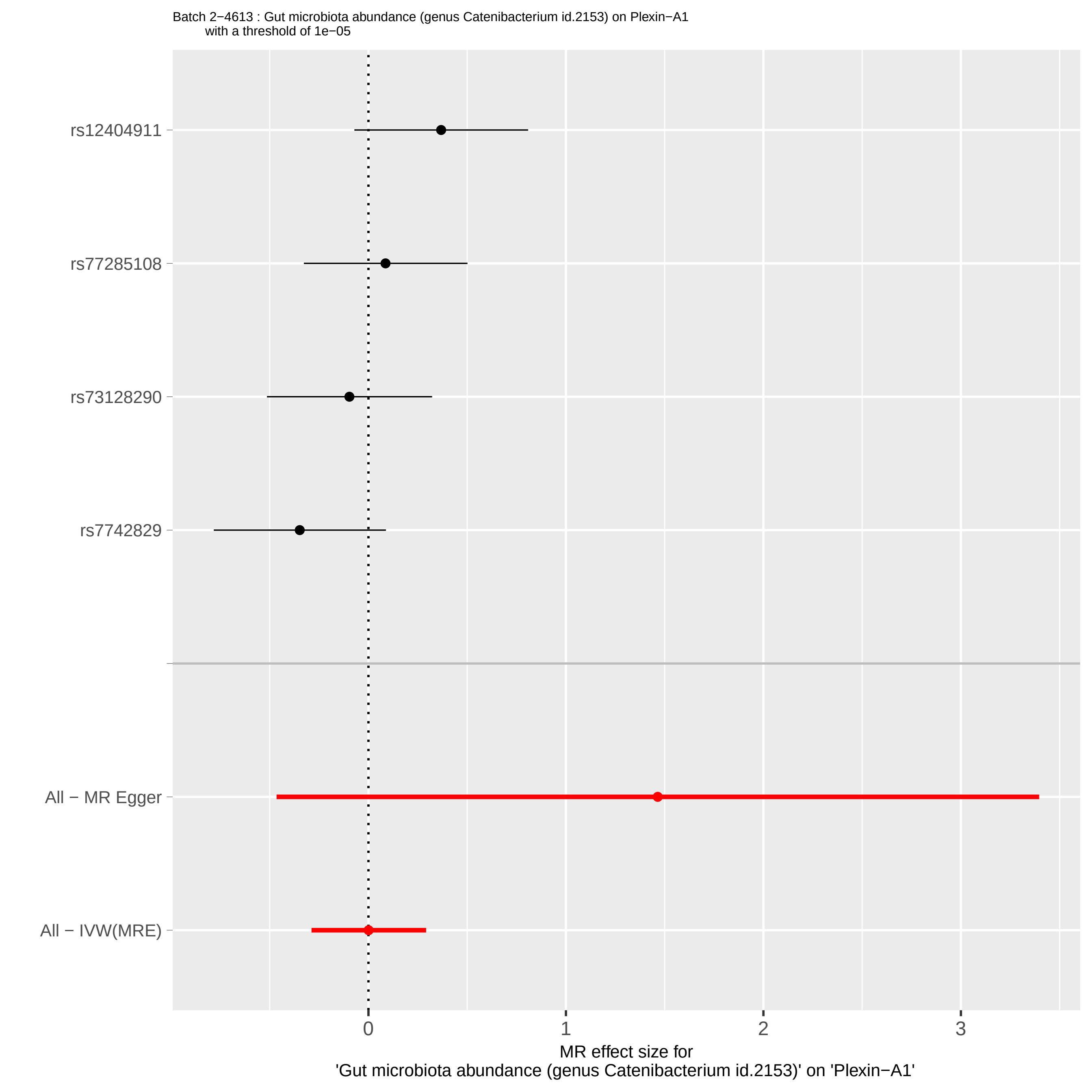
Batch 2−4613 : Gut microbiota abundance (genus Catenibacterium id.2153) on Plexin−A1
with a threshold of 1e−05
rs12404911
rs77285108
rs73128290
rs7742829
All − MR Egger
All − IVW(MRE)
0
1
2
3
MR effect size for
'Gut microbiota abundance (genus Catenibacterium id.2153)' on 'Plexin−A1'

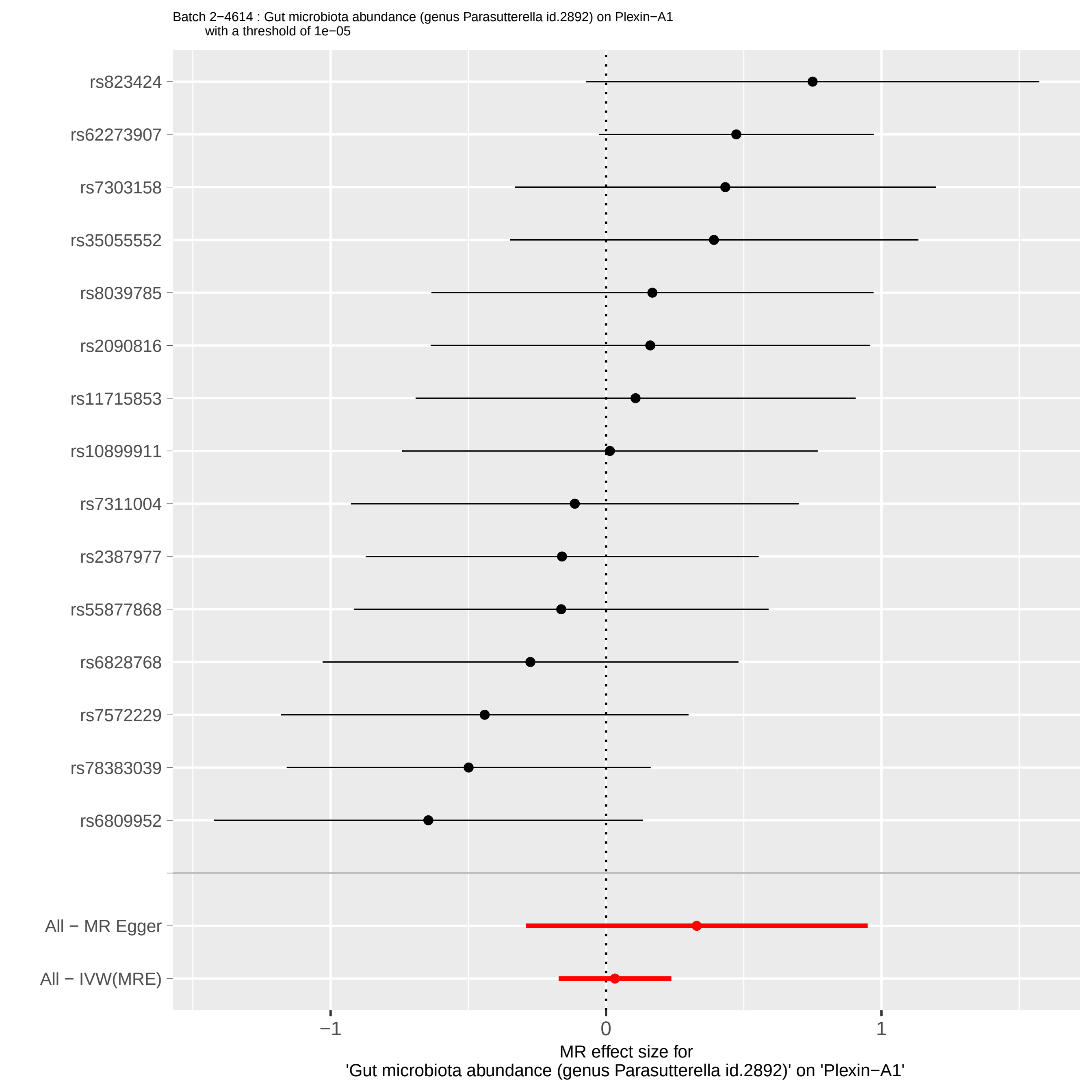
Batch 2−4614 : Gut microbiota abundance (genus Parasutterella id.2892) on Plexin−A1
with a threshold of 1e−05
rs823424
rs62273907
rs7303158
rs35055552
rs8039785
rs2090816
rs11715853
rs10899911
rs7311004
rs2387977
rs55877868
rs6828768
rs7572229
rs78383039
rs6809952
All − MR Egger
All − IVW(MRE)
−1
0
1
MR effect size for
'Gut microbiota abundance (genus Parasutterella id.2892)' on 'Plexin−A1'

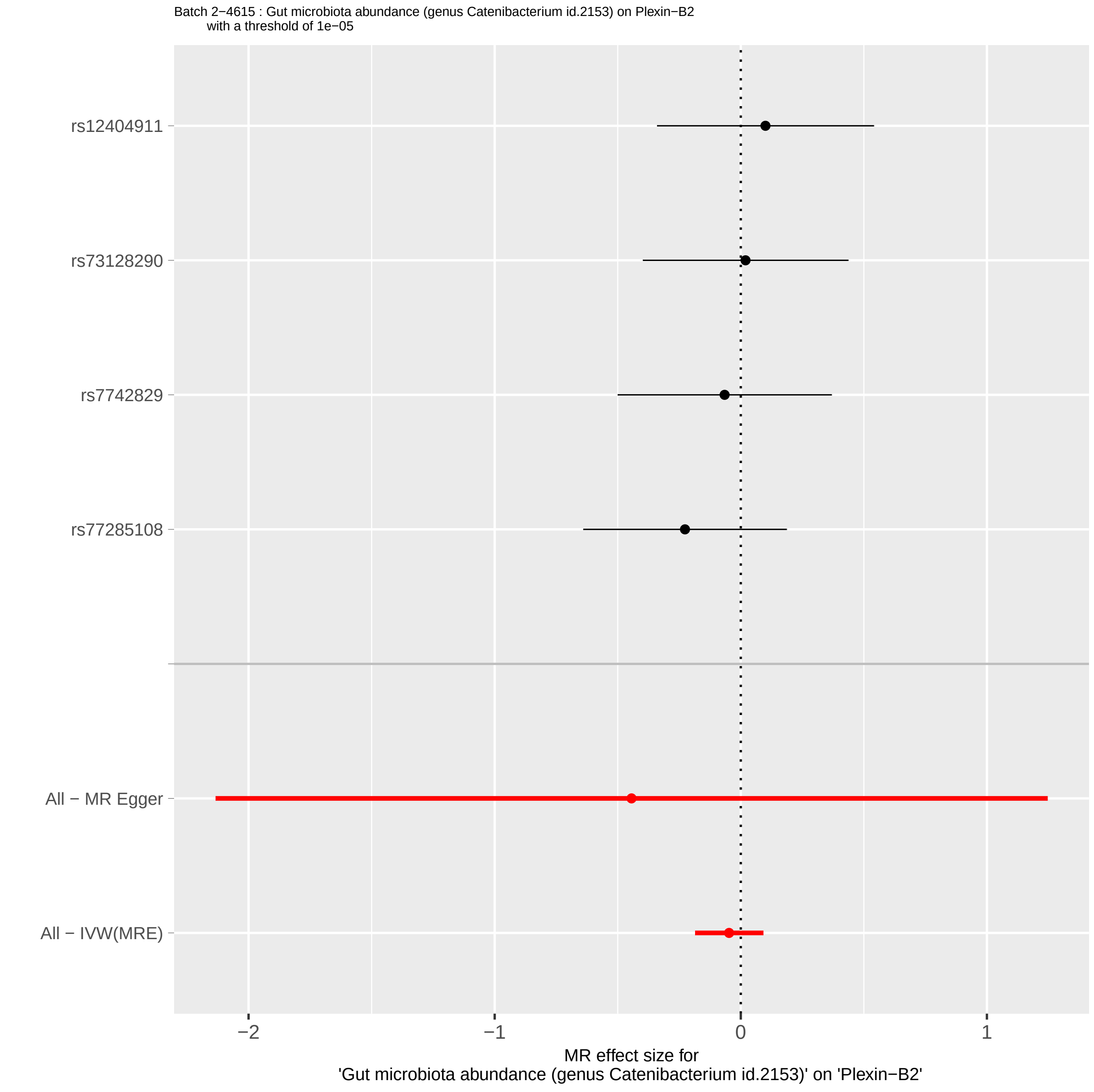
Batch 2−4615 : Gut microbiota abundance (genus Catenibacterium id.2153) on Plexin−B2
with a threshold of 1e−05
rs12404911
rs73128290
rs7742829
rs77285108
All − MR Egger
All − IVW(MRE)
−2
−1
0
1
MR effect size for
'Gut microbiota abundance (genus Catenibacterium id.2153)' on 'Plexin−B2'

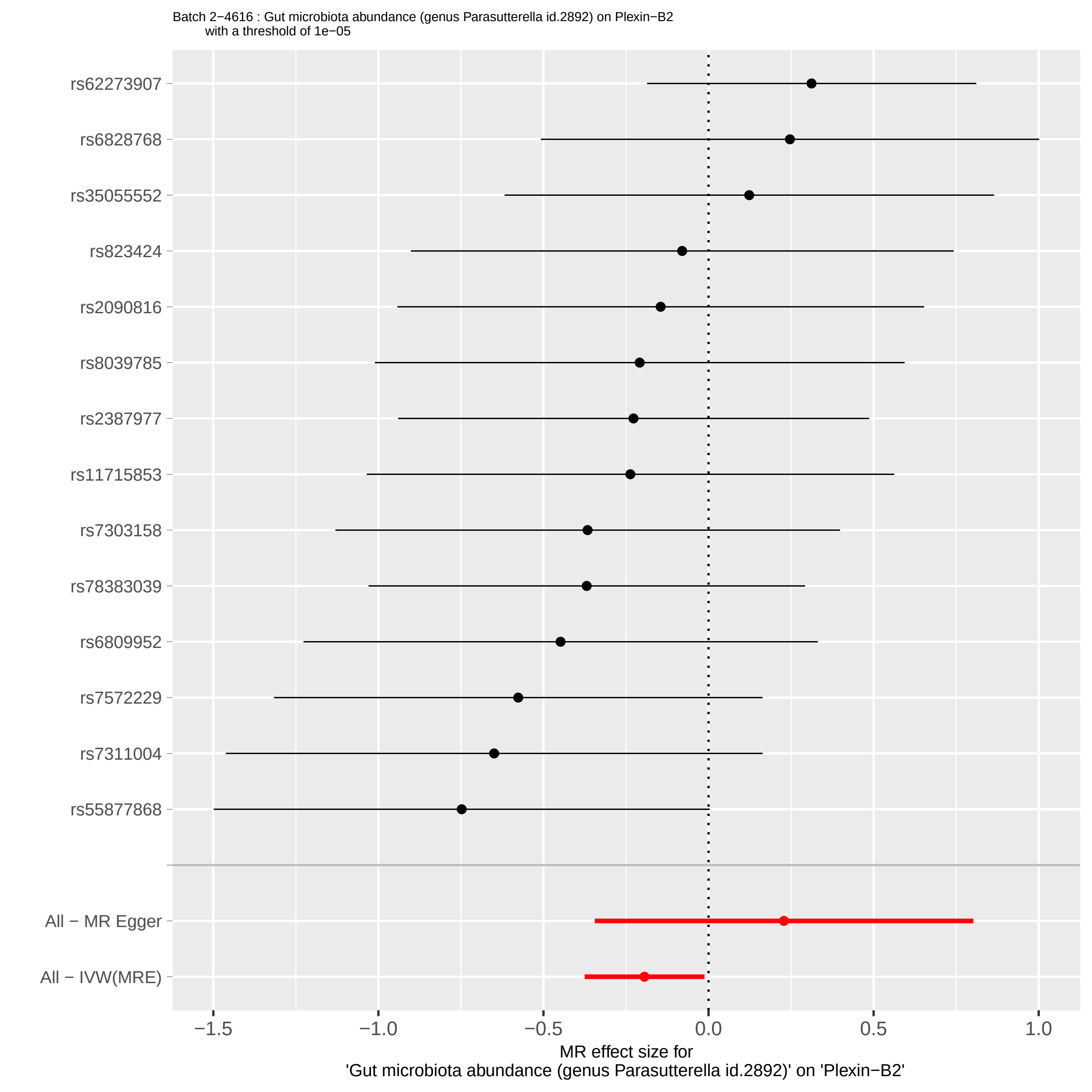
Batch 2−4616 : Gut microbiota abundance (genus Parasutterella id.2892) on Plexin−B2
with a threshold of 1e−05
rs62273907
rs6828768
rs35055552
rs823424
rs2090816
rs8039785
rs2387977
rs11715853
rs7303158
rs78383039
rs6809952
rs7572229
rs7311004
rs55877868
All − MR Egger
All − IVW(MRE)
−1.5
−1.0
−0.5
0.0
0.5
1.0
MR effect size for
'Gut microbiota abundance (genus Parasutterella id.2892)' on 'Plexin−B2'

Batch 2−4617 : Gut microbiota abundance (genus Catenibacterium id.2153) on Plexin−C1
with a threshold of 1e−05

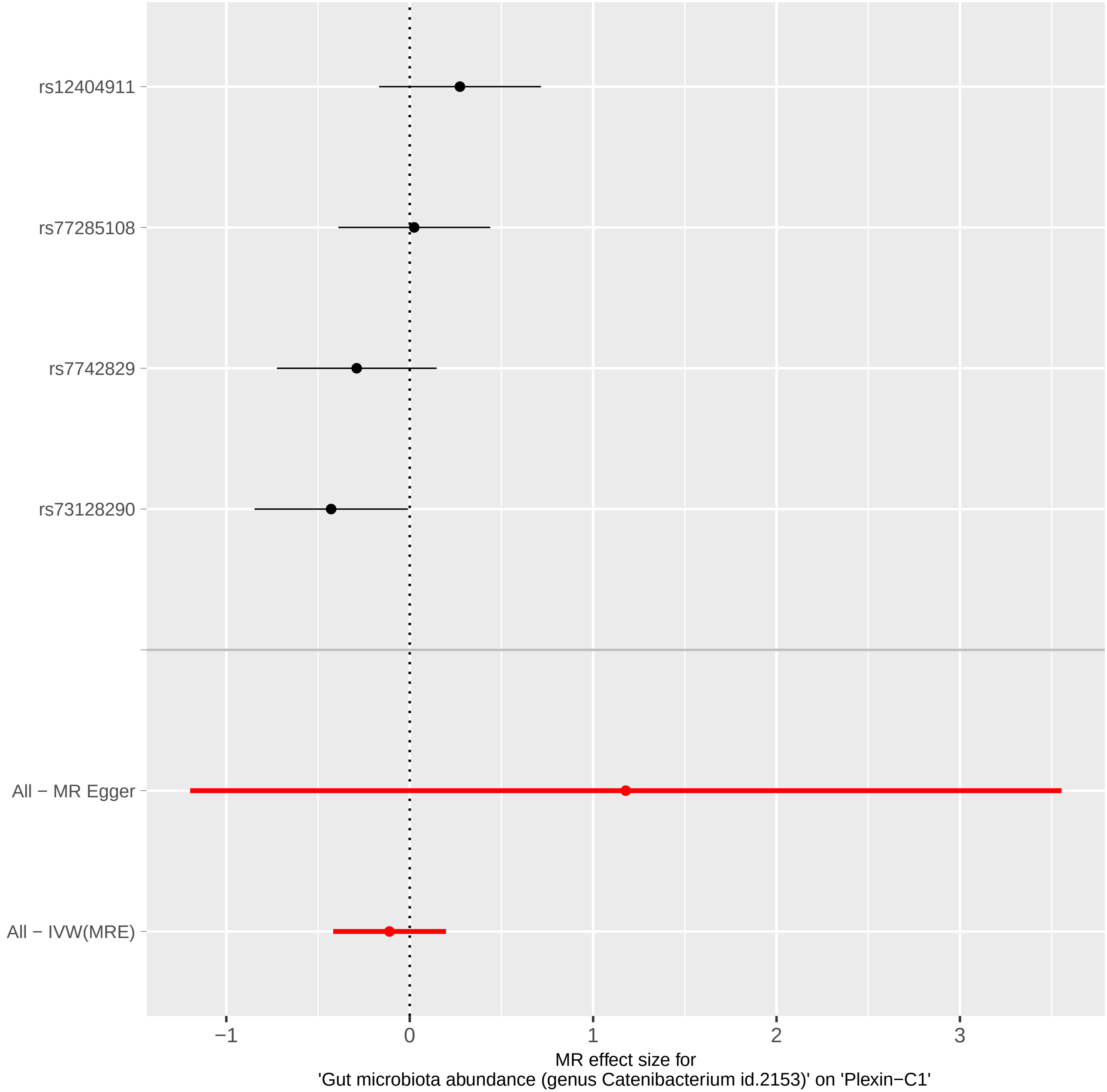

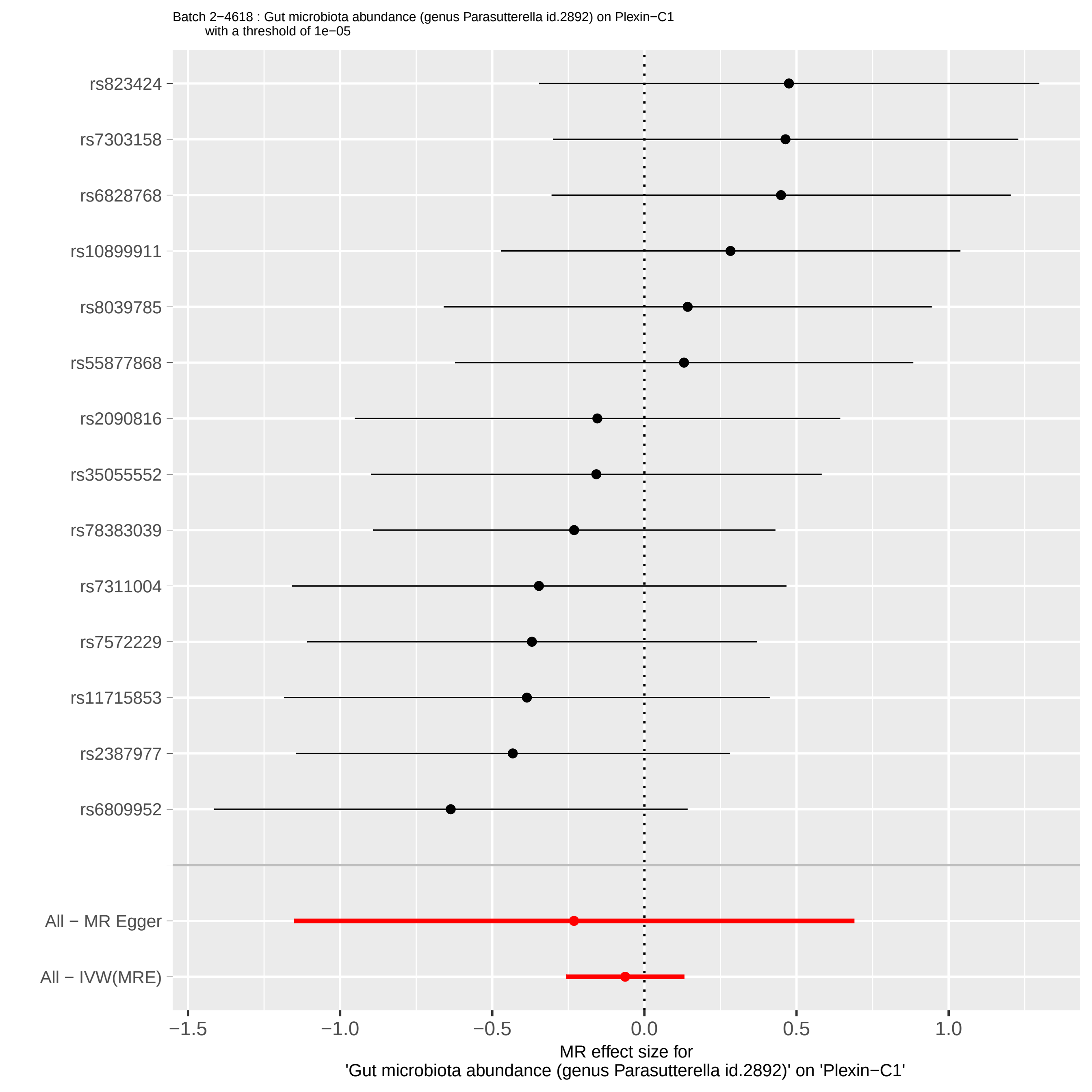

Batch 2−4618 : Gut microbiota abundance (genus Parasutterella id.2892) on Plexin−C1
with a threshold of 1e−05
rs823424
rs7303158
rs6828768
rs10899911
rs8039785
rs55877868
rs2090816
rs35055552
rs78383039
rs7311004
rs7572229
rs11715853
rs2387977
rs6809952
All − MR Egger
All − IVW(MRE)
−1.5
−1.0
−0.5
0.0
0.5
1.0
MR effect size for
'Gut microbiota abundance (genus Parasutterella id.2892)' on 'Plexin−C1'

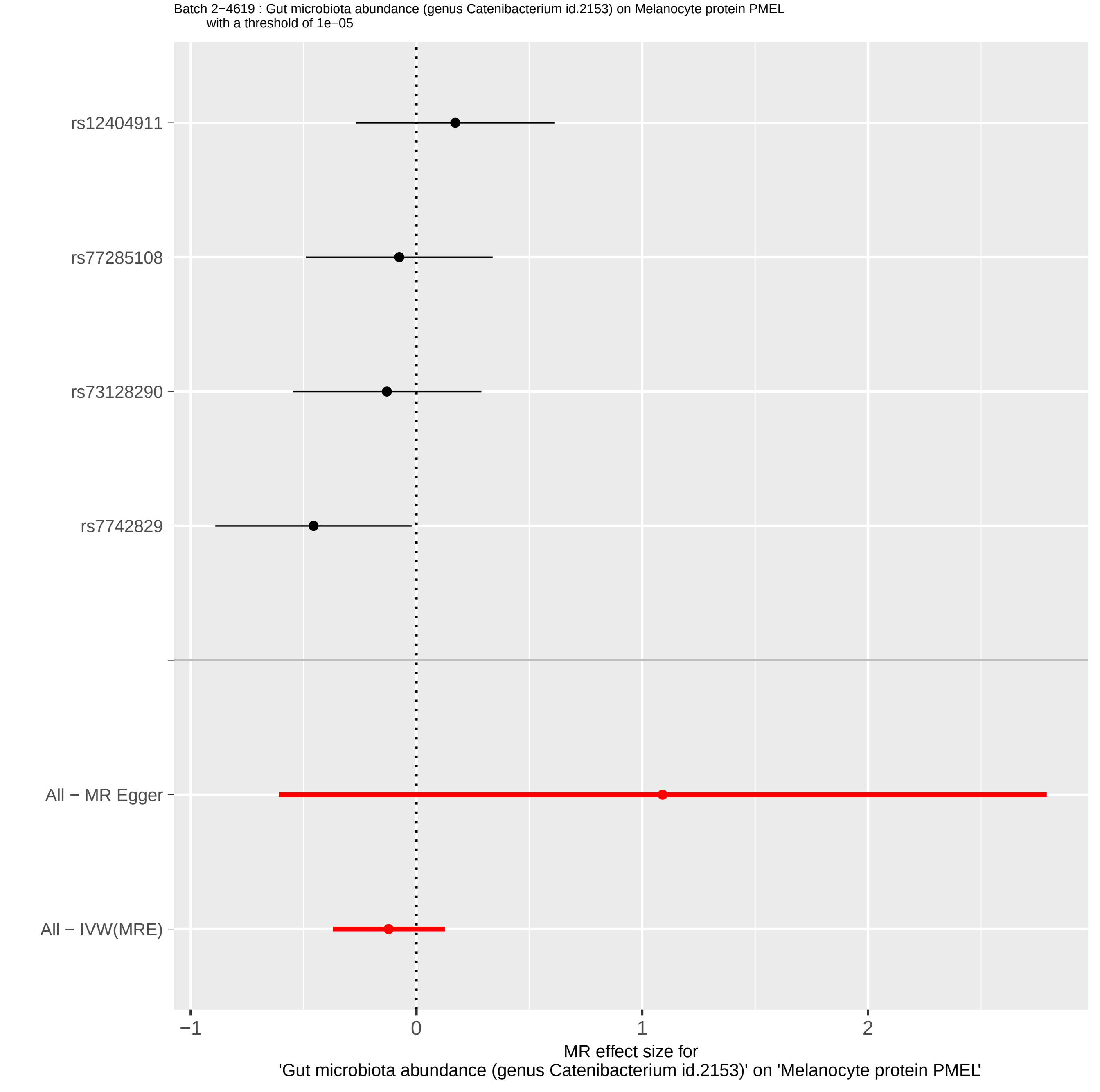

Batch 2−4619 : Gut microbiota abundance (genus Catenibacterium id.2153) on Melanocyte protein PMEL
with a threshold of 1e−05
rs12404911
rs77285108
rs73128290
rs7742829
All − MR Egger
All − IVW(MRE)
−1
0
1
2
MR effect size for
'Gut microbiota abundance (genus Catenibacterium id.2153)' on 'Melanocyte protein PMEL'

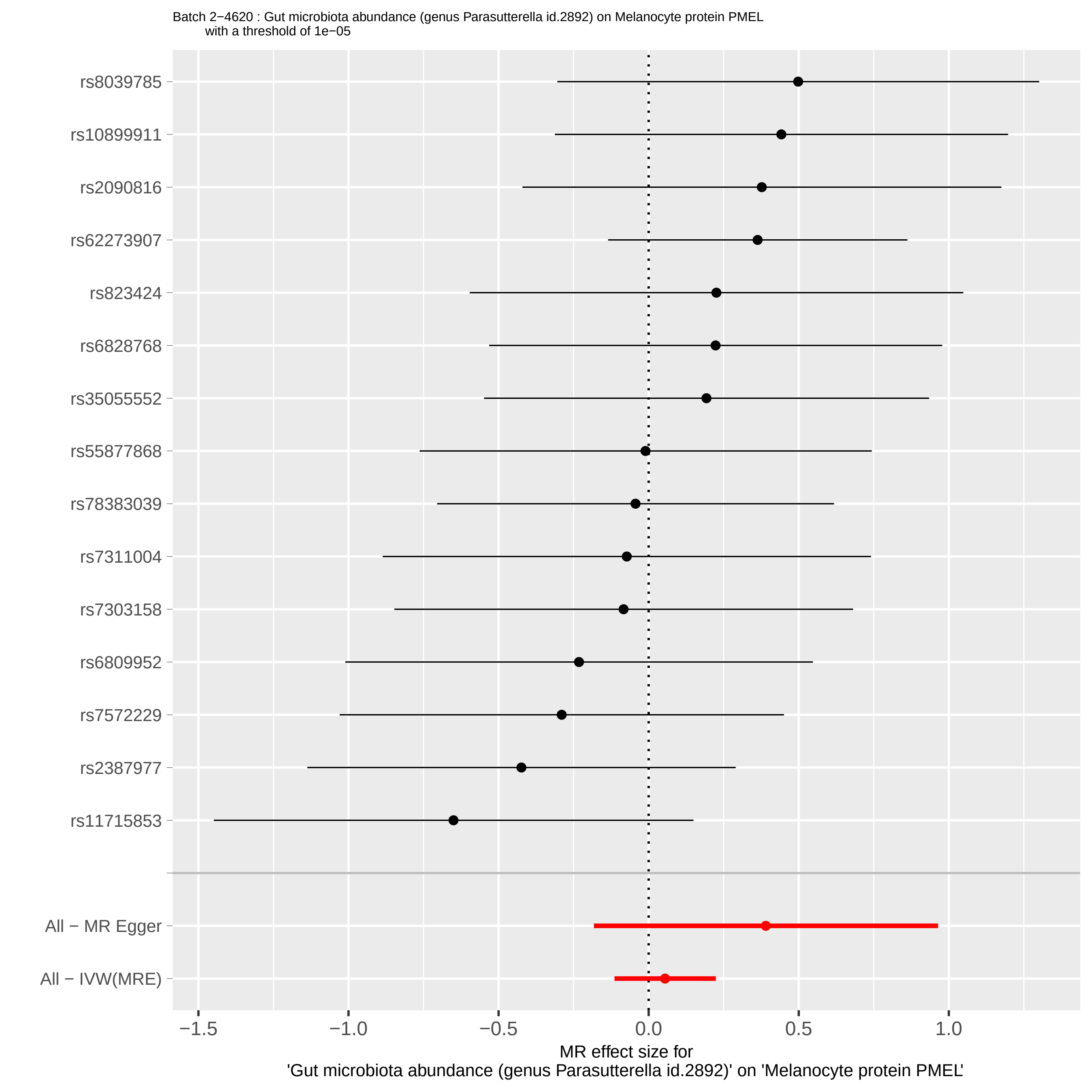

Batch 2−4620 : Gut microbiota abundance (genus Parasutterella id.2892) on Melanocyte protein PMEL
with a threshold of 1e−05
rs8039785
rs10899911
rs2090816
rs62273907
rs823424
rs6828768
rs35055552
rs55877868
rs78383039
rs7311004
rs7303158
rs6809952
rs7572229
rs2387977
rs11715853
All − MR Egger
All − IVW(MRE)
−1.5
−1.0
−0.5
0.0
0.5
1.0
MR effect size for
'Gut microbiota abundance (genus Parasutterella id.2892)' on 'Melanocyte protein PMEL'

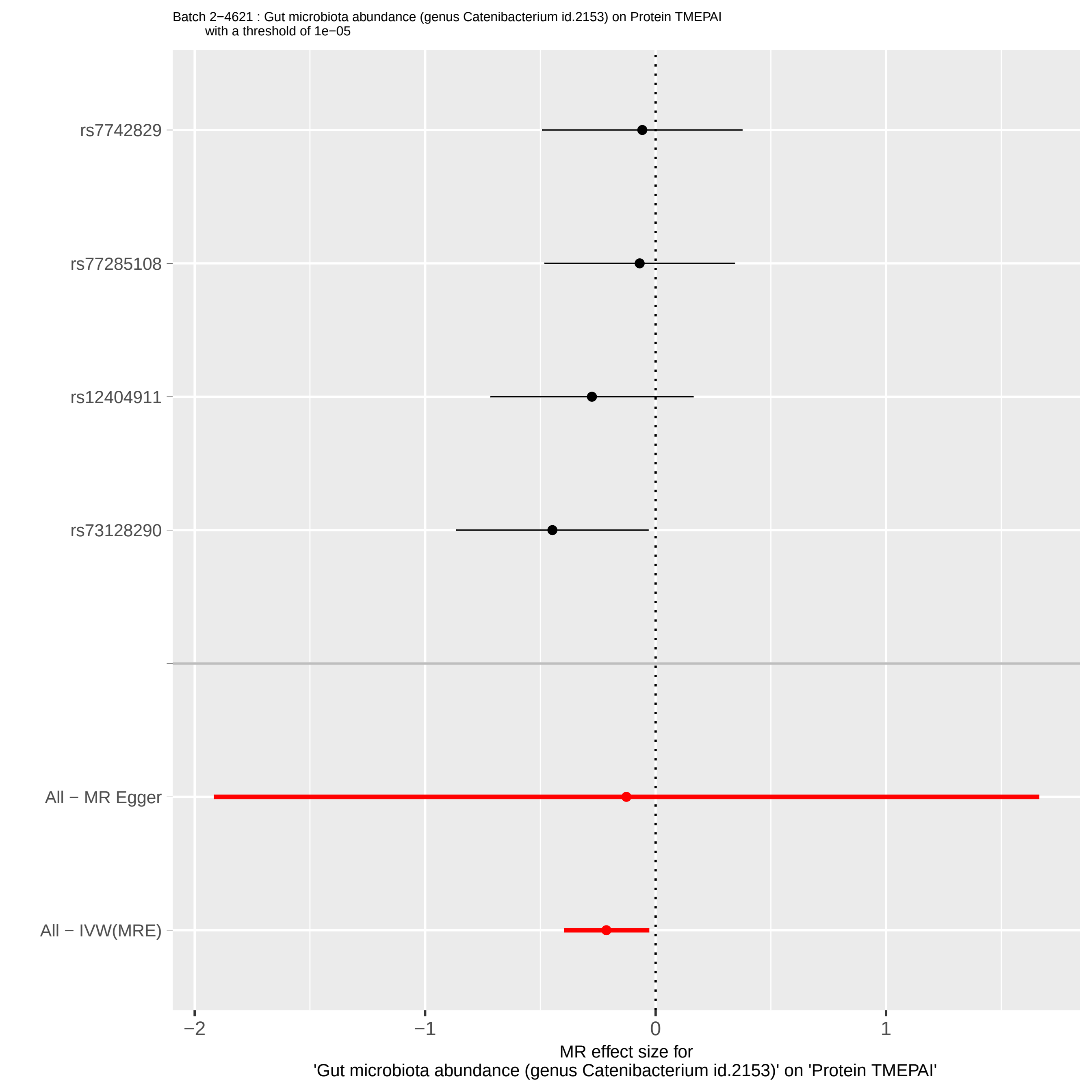
Batch 2−4621 : Gut microbiota abundance (genus Catenibacterium id.2153) on Protein TMEPAI
with a threshold of 1e−05
rs7742829
rs77285108
rs12404911
rs73128290
All − MR Egger
All − IVW(MRE)
−2
−1
0
1
MR effect size for
'Gut microbiota abundance (genus Catenibacterium id.2153)' on 'Protein TMEPAI'

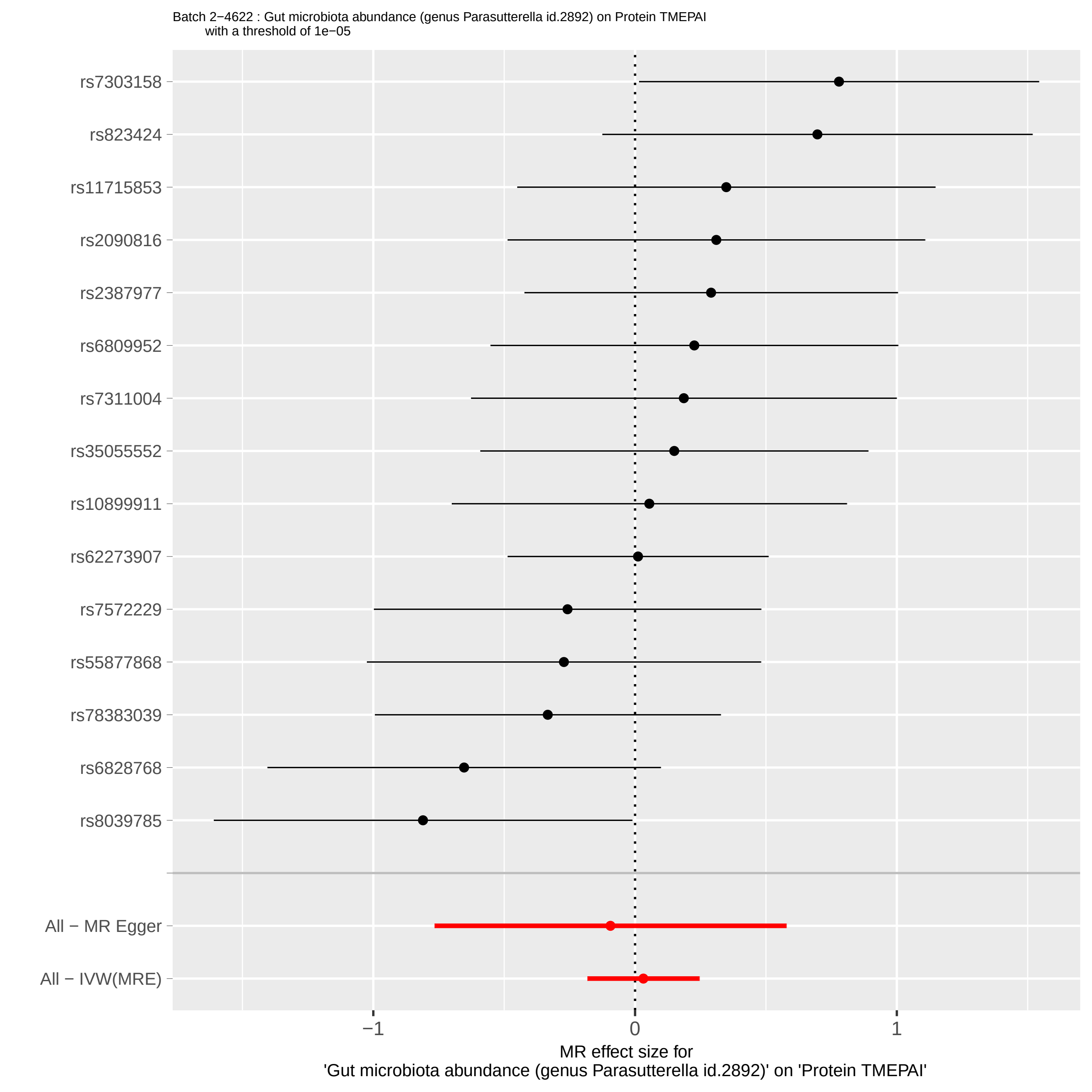
Batch 2−4622 : Gut microbiota abundance (genus Parasutterella id.2892) on Protein TMEPAI
with a threshold of 1e−05
rs7303158
rs823424
rs11715853
rs2090816
rs2387977
rs6809952
rs7311004
rs35055552
rs10899911
rs62273907
rs7572229
rs55877868
rs78383039
rs6828768
rs8039785
All − MR Egger
All − IVW(MRE)
−1
0
1
MR effect size for
'Gut microbiota abundance (genus Parasutterella id.2892)' on 'Protein TMEPAI'

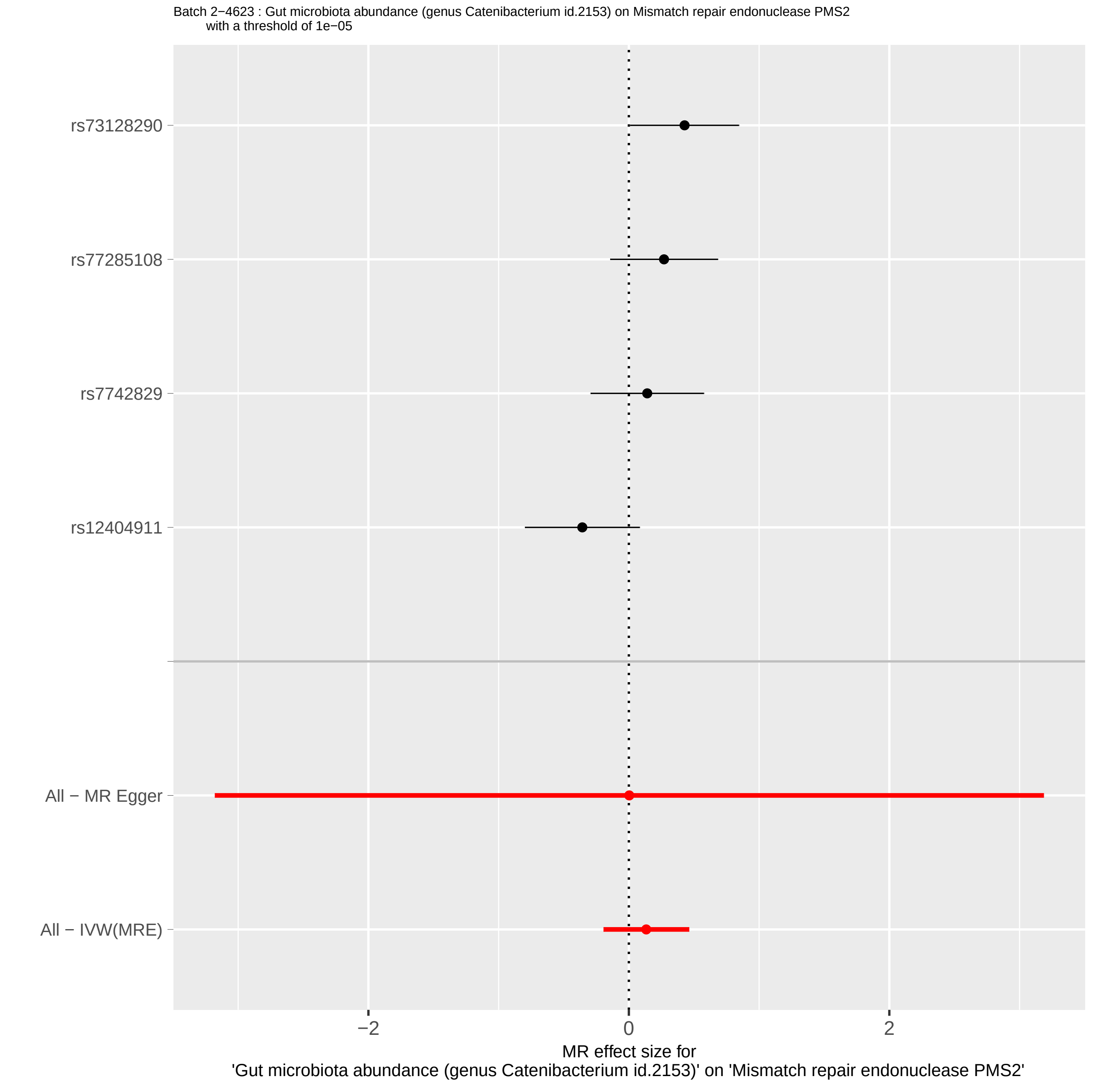

Batch 2−4623 : Gut microbiota abundance (genus Catenibacterium id.2153) on Mismatch repair endonuclease PMS2
with a threshold of 1e−05
rs73128290
rs77285108
rs7742829
rs12404911
All − MR Egger
All − IVW(MRE)
−2
0
2
MR effect size for
'Gut microbiota abundance (genus Catenibacterium id.2153)' on 'Mismatch repair endonuclease PMS2'

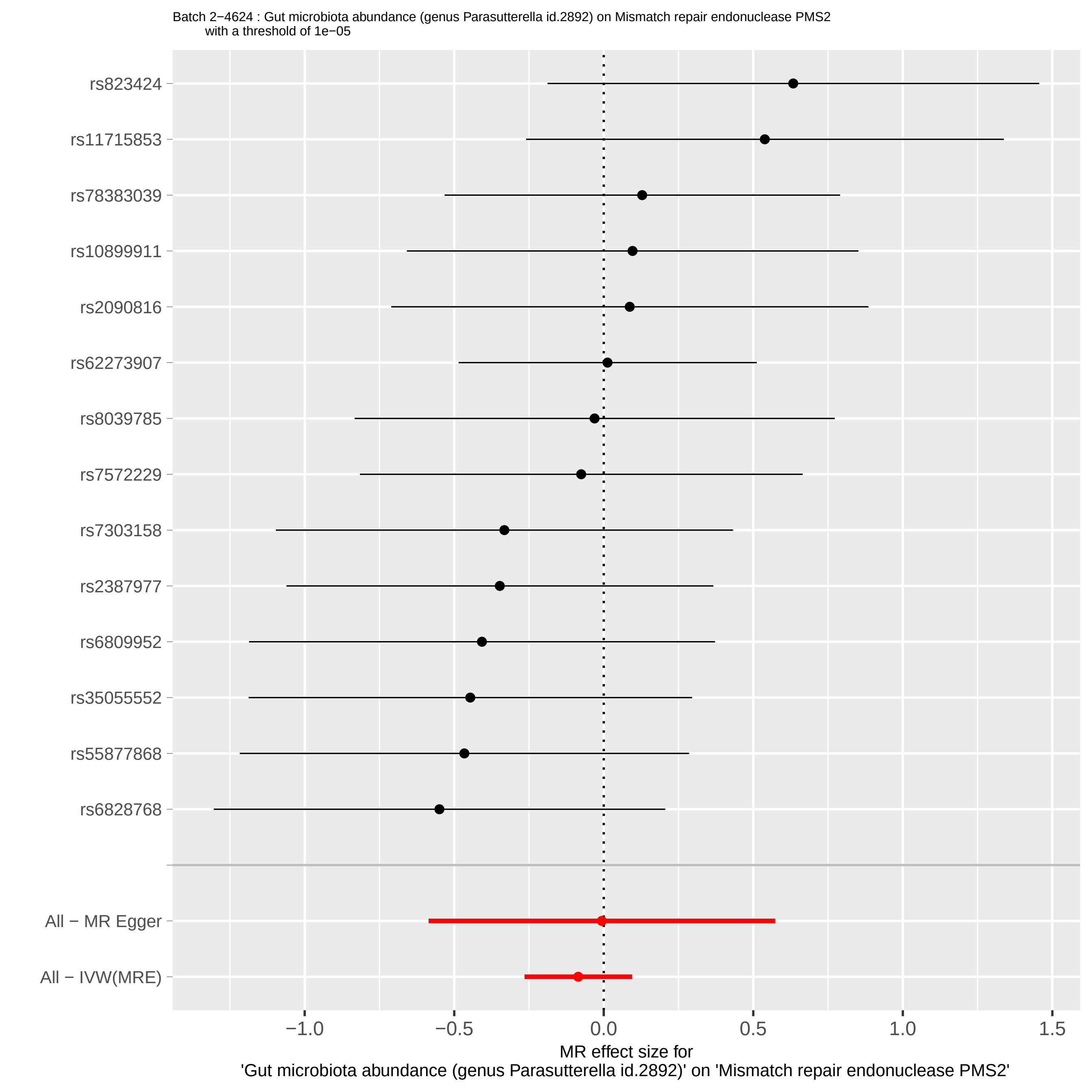

Batch 2−4624 : Gut microbiota abundance (genus Parasutterella id.2892) on Mismatch repair endonuclease PMS2
with a threshold of 1e−05
rs823424
rs11715853
rs78383039
rs10899911
rs2090816
rs62273907
rs8039785
rs7572229
rs7303158
rs2387977
rs6809952
rs35055552
rs55877868
rs6828768
All − MR Egger
All − IVW(MRE)
−1.0
−0.5
0.0
0.5
1.0
1.5
MR effect size for
'Gut microbiota abundance (genus Parasutterella id.2892)' on 'Mismatch repair endonuclease PMS2'

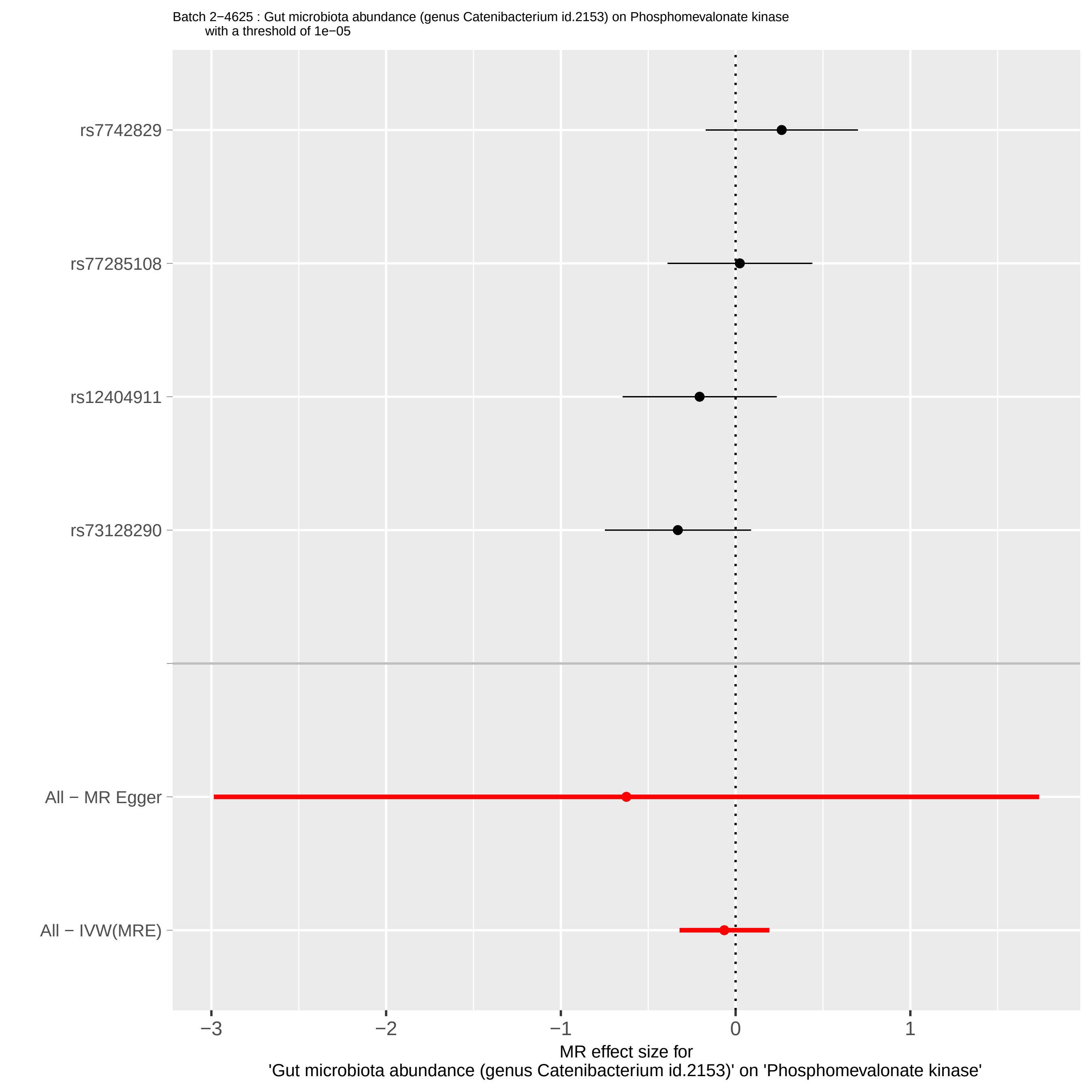

Batch 2−4625 : Gut microbiota abundance (genus Catenibacterium id.2153) on Phosphomevalonate kinase
with a threshold of 1e−05
rs7742829
rs77285108
rs12404911
rs73128290
All − MR Egger
All − IVW(MRE)
−3
−2
−1
0
1
MR effect size for
'Gut microbiota abundance (genus Catenibacterium id.2153)' on 'Phosphomevalonate kinase'

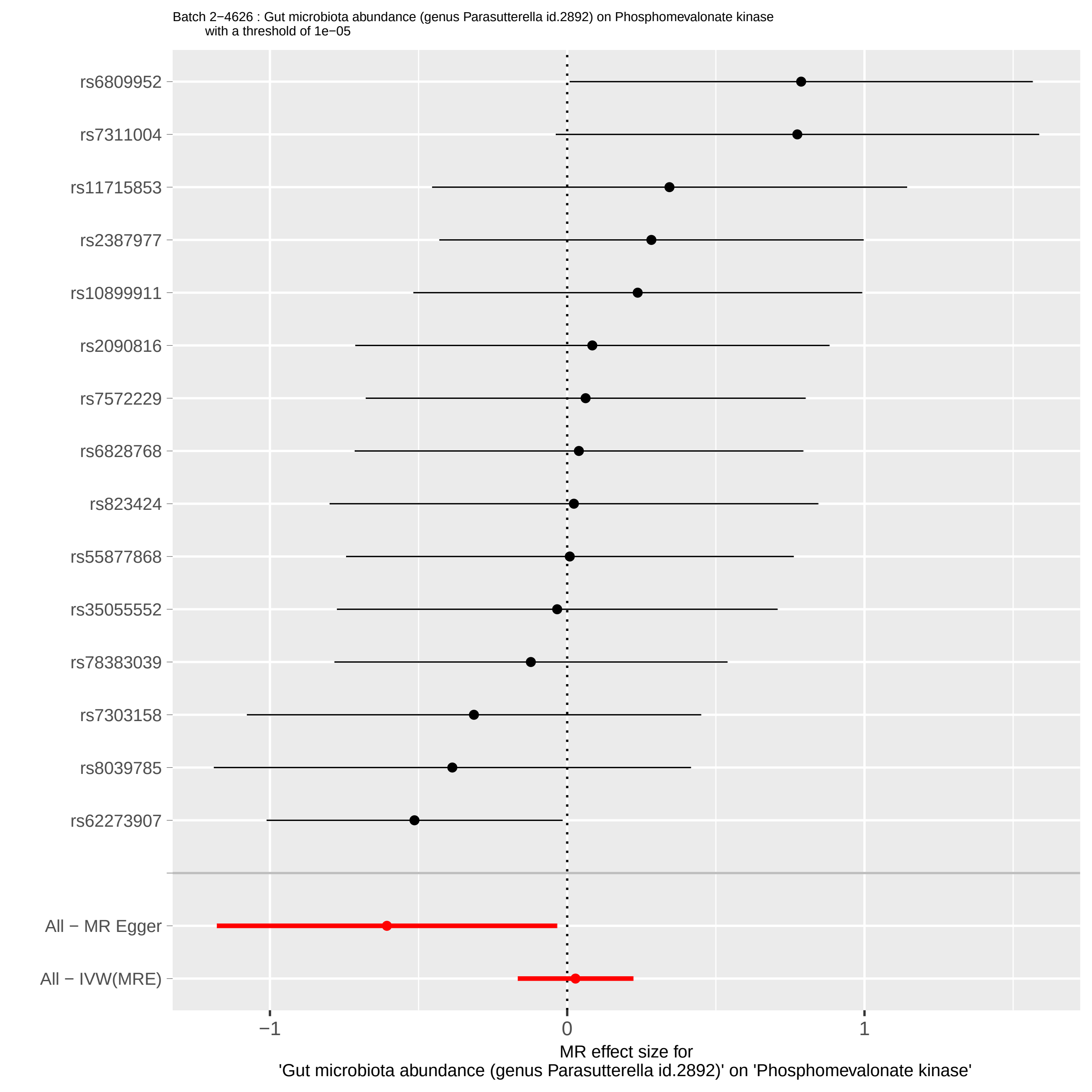

Batch 2−4626 : Gut microbiota abundance (genus Parasutterella id.2892) on Phosphomevalonate kinase
with a threshold of 1e−05
rs6809952
rs7311004
rs11715853
rs2387977
rs10899911
rs2090816
rs7572229
rs6828768
rs823424
rs55877868
rs35055552
rs78383039
rs7303158
rs8039785
rs62273907
All − MR Egger
All − IVW(MRE)
−1
0
1
MR effect size for
'Gut microbiota abundance (genus Parasutterella id.2892)' on 'Phosphomevalonate kinase'

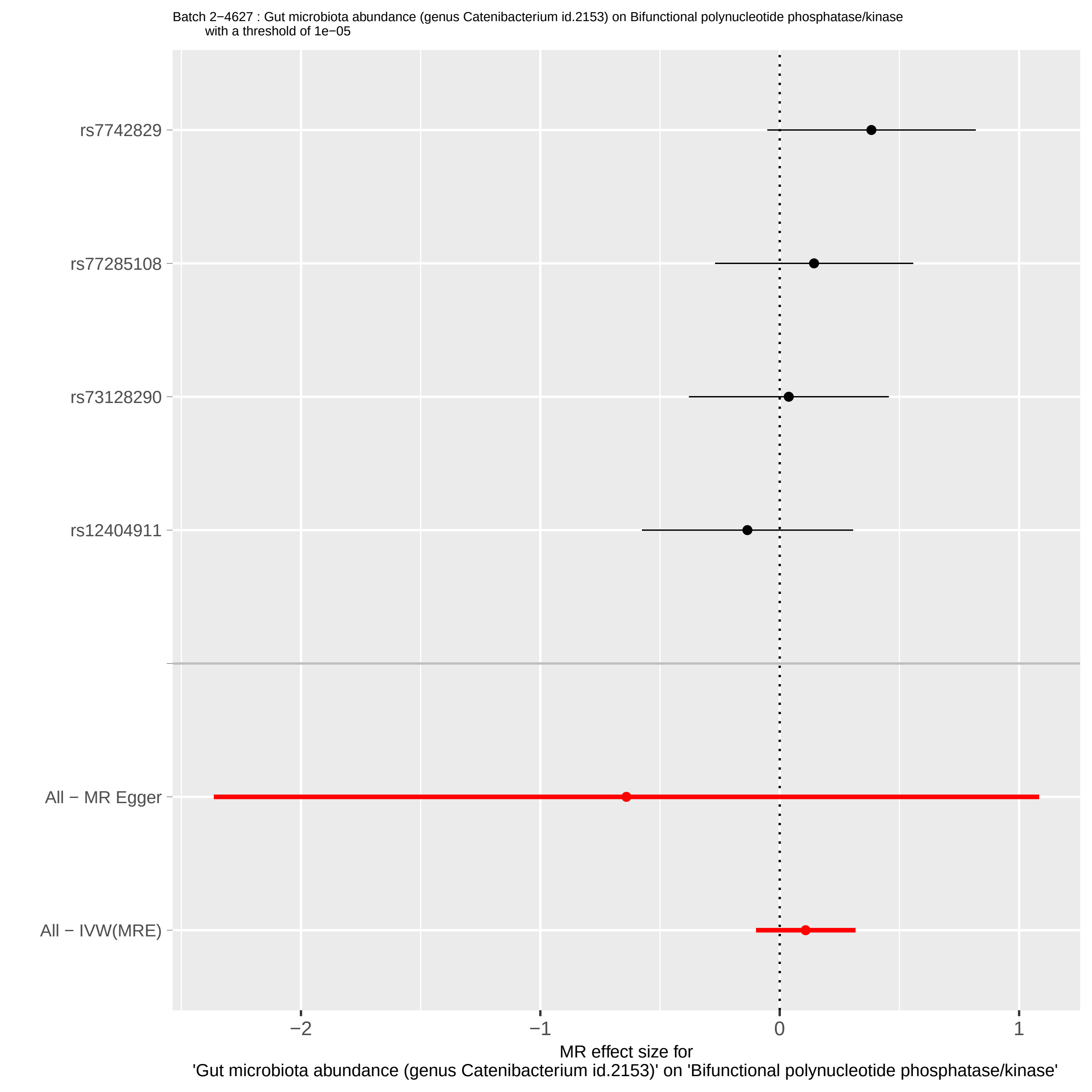
Batch 2−4627 : Gut microbiota abundance (genus Catenibacterium id.2153) on Bifunctional polynucleotide phosphatase/kinase
with a threshold of 1e−05
rs7742829
rs77285108
rs73128290
rs12404911
All − MR Egger
All − IVW(MRE)
−2
−1
0
1
MR effect size for
'Gut microbiota abundance (genus Catenibacterium id.2153)' on 'Bifunctional polynucleotide phosphatase/kinase'

Batch 2−4628 : Gut microbiota abundance (genus Parasutterella id.2892) on Bifunctional polynucleotide phosphatase/kinase
with a threshold of 1e−05

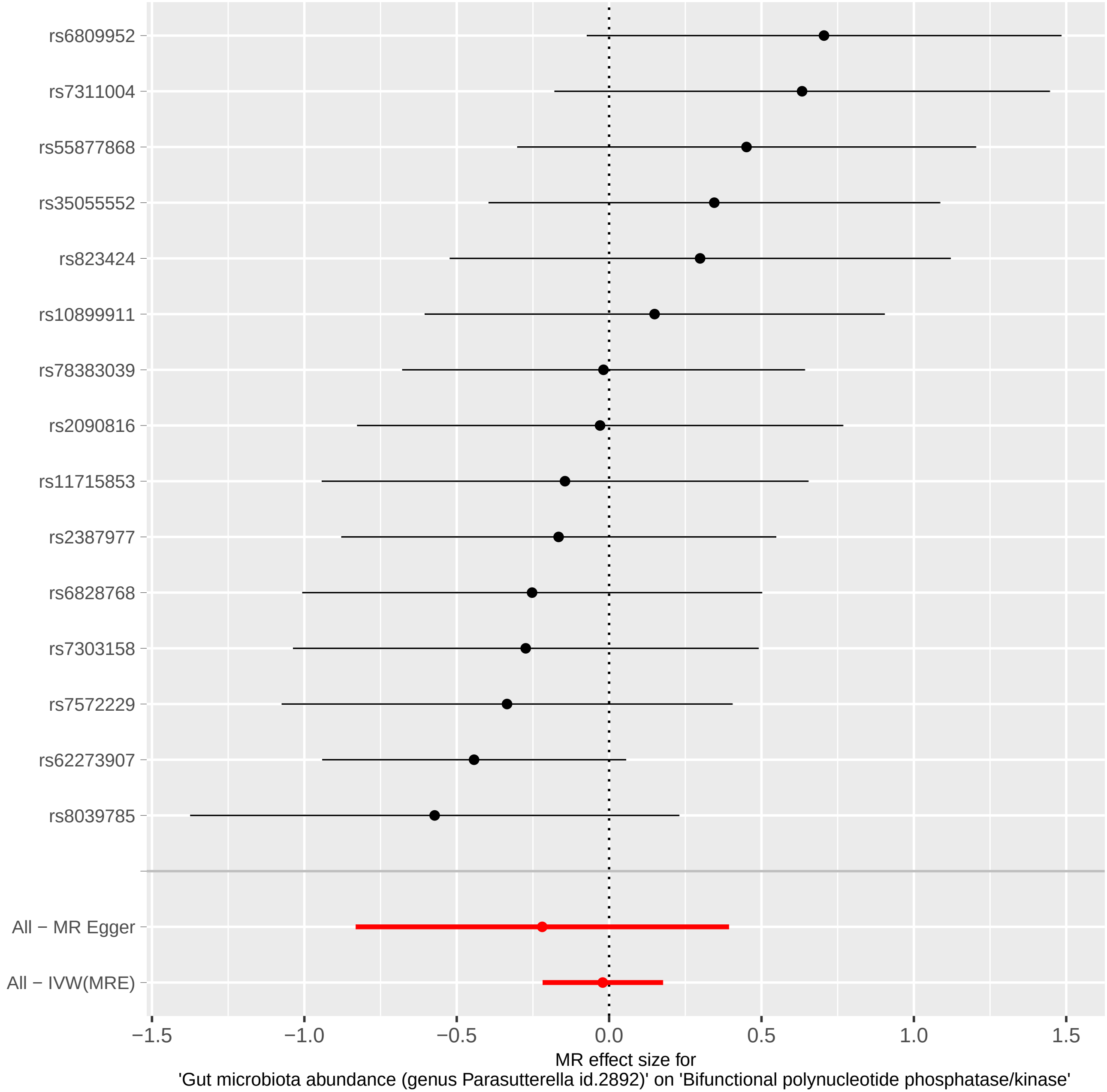

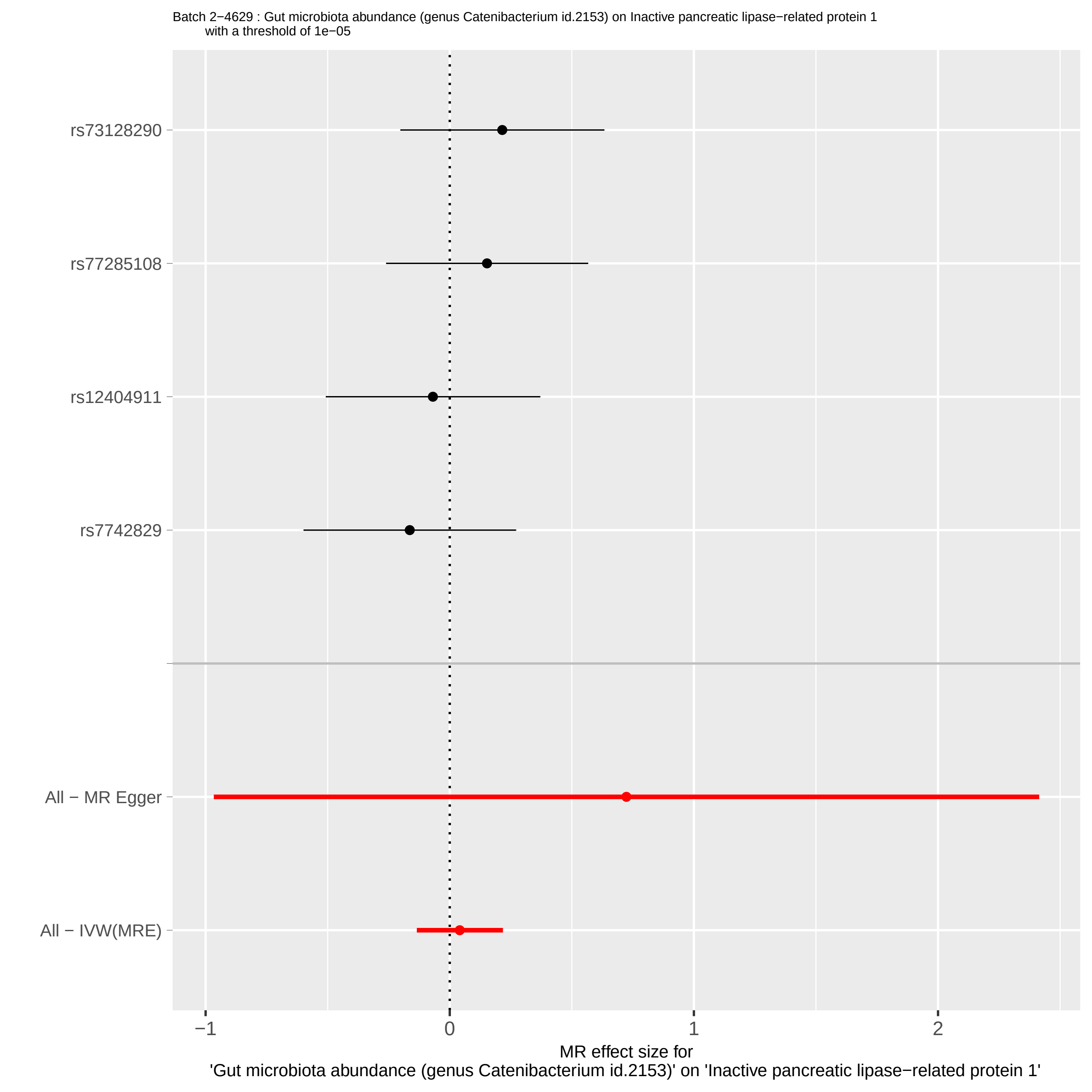
Batch 2−4629 : Gut microbiota abundance (genus Catenibacterium id.2153) on Inactive pancreatic lipase−related protein 1
with a threshold of 1e−05
rs73128290
rs77285108
rs12404911
rs7742829
All − MR Egger
All − IVW(MRE)
−1
0
1
2
MR effect size for
'Gut microbiota abundance (genus Catenibacterium id.2153)' on 'Inactive pancreatic lipase−related protein 1'

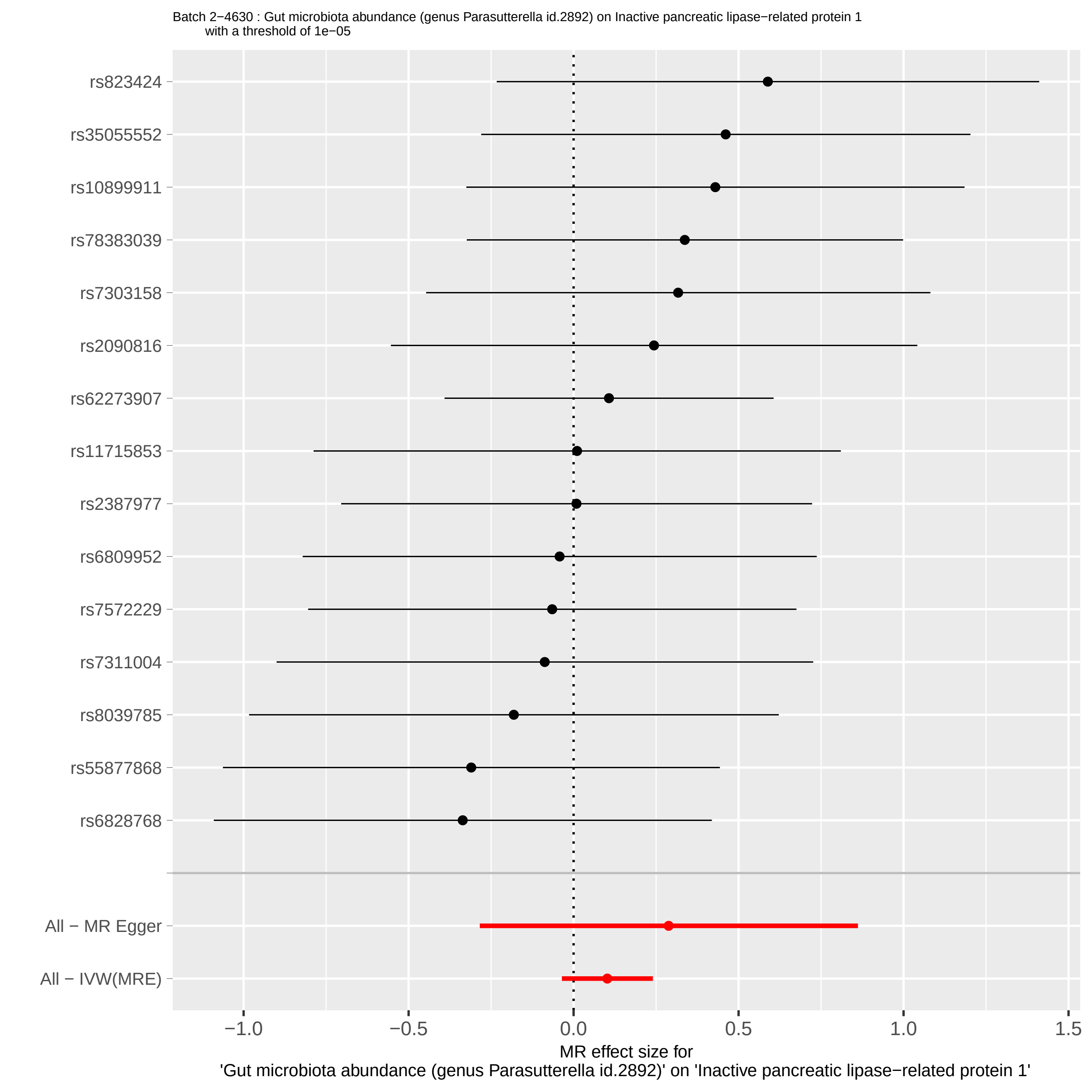

Batch 2−4630 : Gut microbiota abundance (genus Parasutterella id.2892) on Inactive pancreatic lipase−related protein 1
with a threshold of 1e−05
rs823424
rs35055552
rs10899911
rs78383039
rs7303158
rs2090816
rs62273907
rs11715853
rs2387977
rs6809952
rs7572229
rs7311004
rs8039785
rs55877868
rs6828768
All − MR Egger
All − IVW(MRE)
−1.0
−0.5
0.0
0.5
1.0
1.5
MR effect size for
'Gut microbiota abundance (genus Parasutterella id.2892)' on 'Inactive pancreatic lipase−related protein 1'

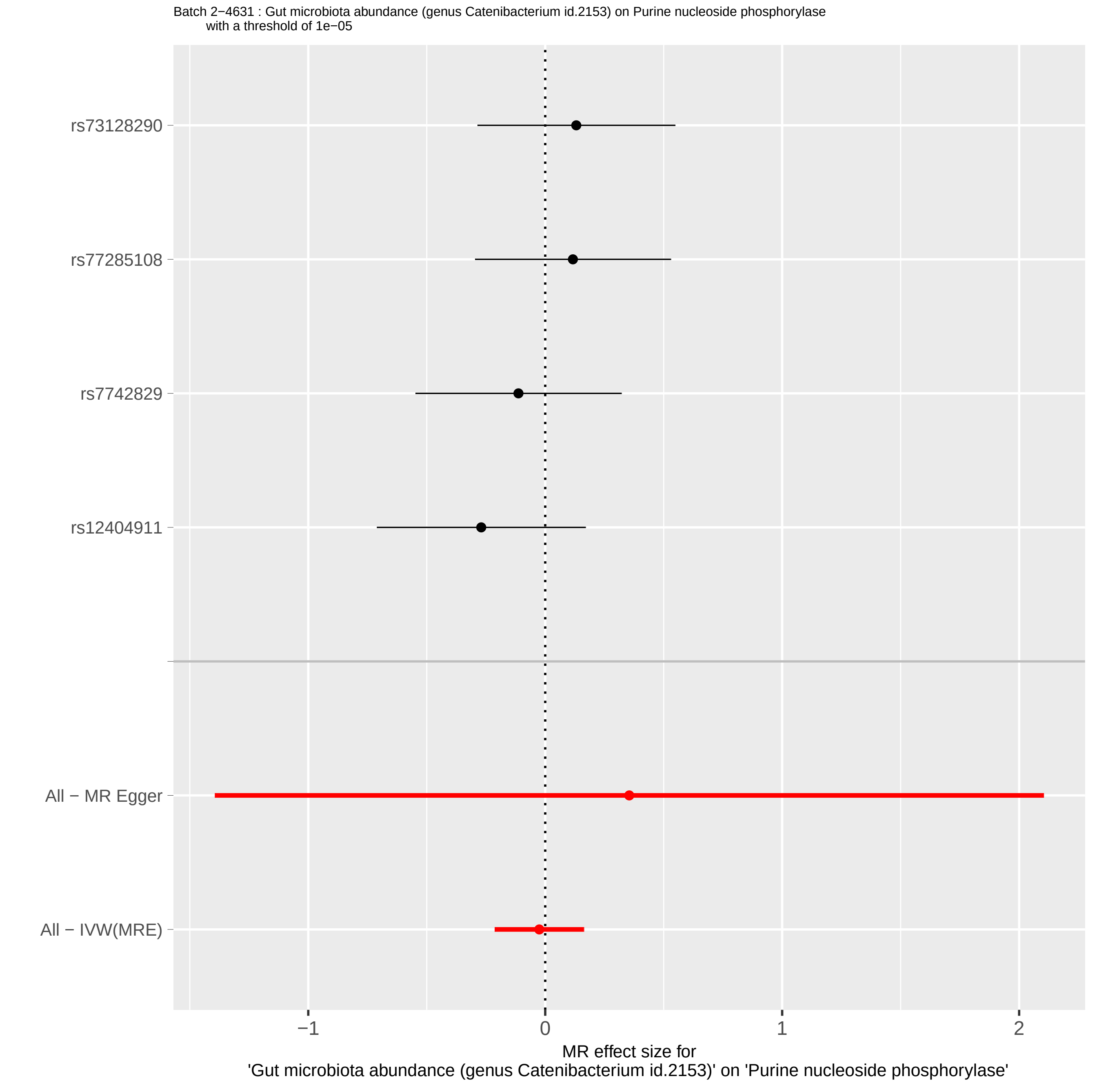

Batch 2−4631 : Gut microbiota abundance (genus Catenibacterium id.2153) on Purine nucleoside phosphorylase
with a threshold of 1e−05
rs73128290
rs77285108
rs7742829
rs12404911
All − MR Egger
All − IVW(MRE)
−1
0
1
2
MR effect size for
'Gut microbiota abundance (genus Catenibacterium id.2153)' on 'Purine nucleoside phosphorylase'

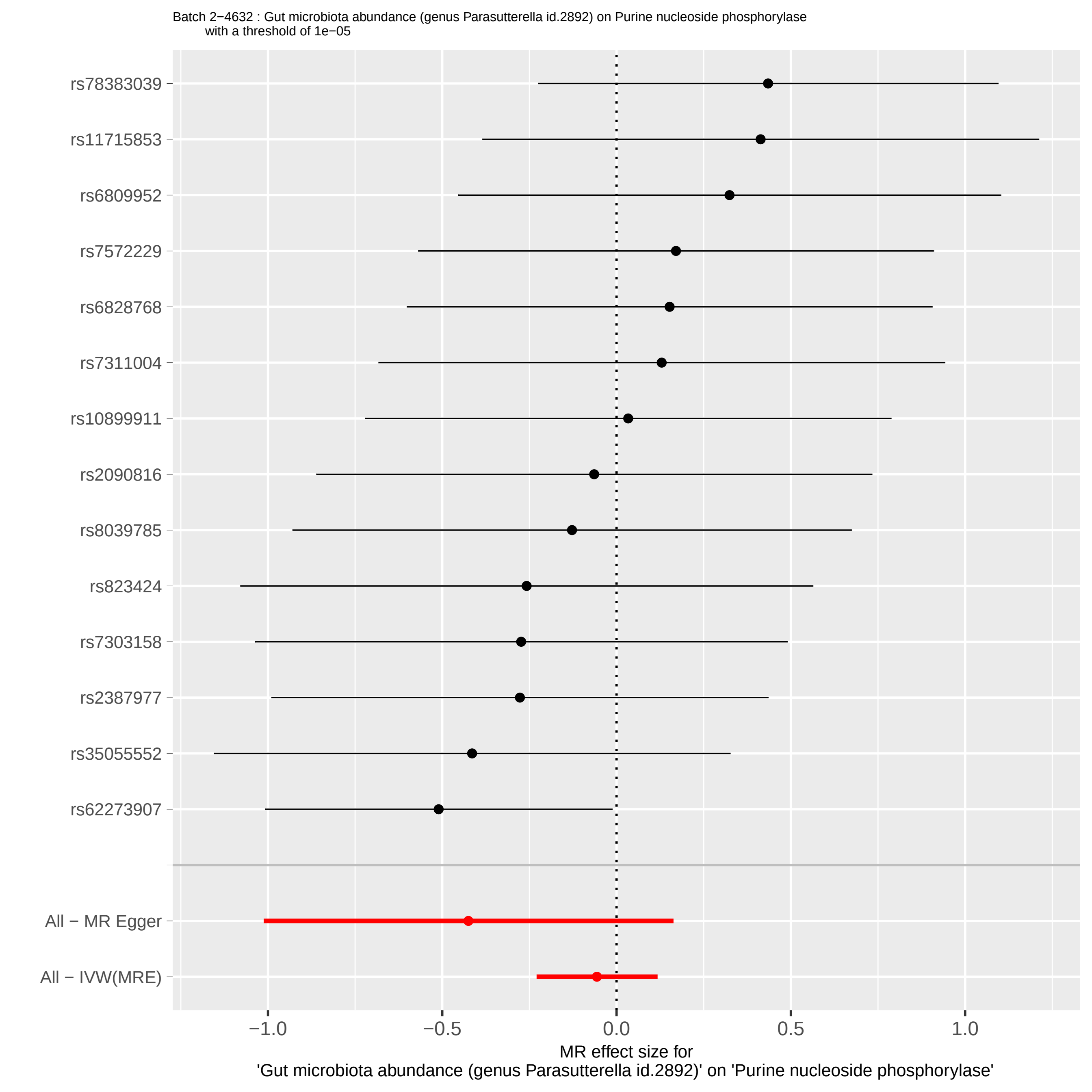
Batch 2−4632 : Gut microbiota abundance (genus Parasutterella id.2892) on Purine nucleoside phosphorylase
with a threshold of 1e−05
rs78383039
rs11715853
rs6809952
rs7572229
rs6828768
rs7311004
rs10899911
rs2090816
rs8039785
rs823424
rs7303158
rs2387977
rs35055552
rs62273907
All − MR Egger
All − IVW(MRE)
−1.0
−0.5
0.0
0.5
1.0
MR effect size for
'Gut microbiota abundance (genus Parasutterella id.2892)' on 'Purine nucleoside phosphorylase'

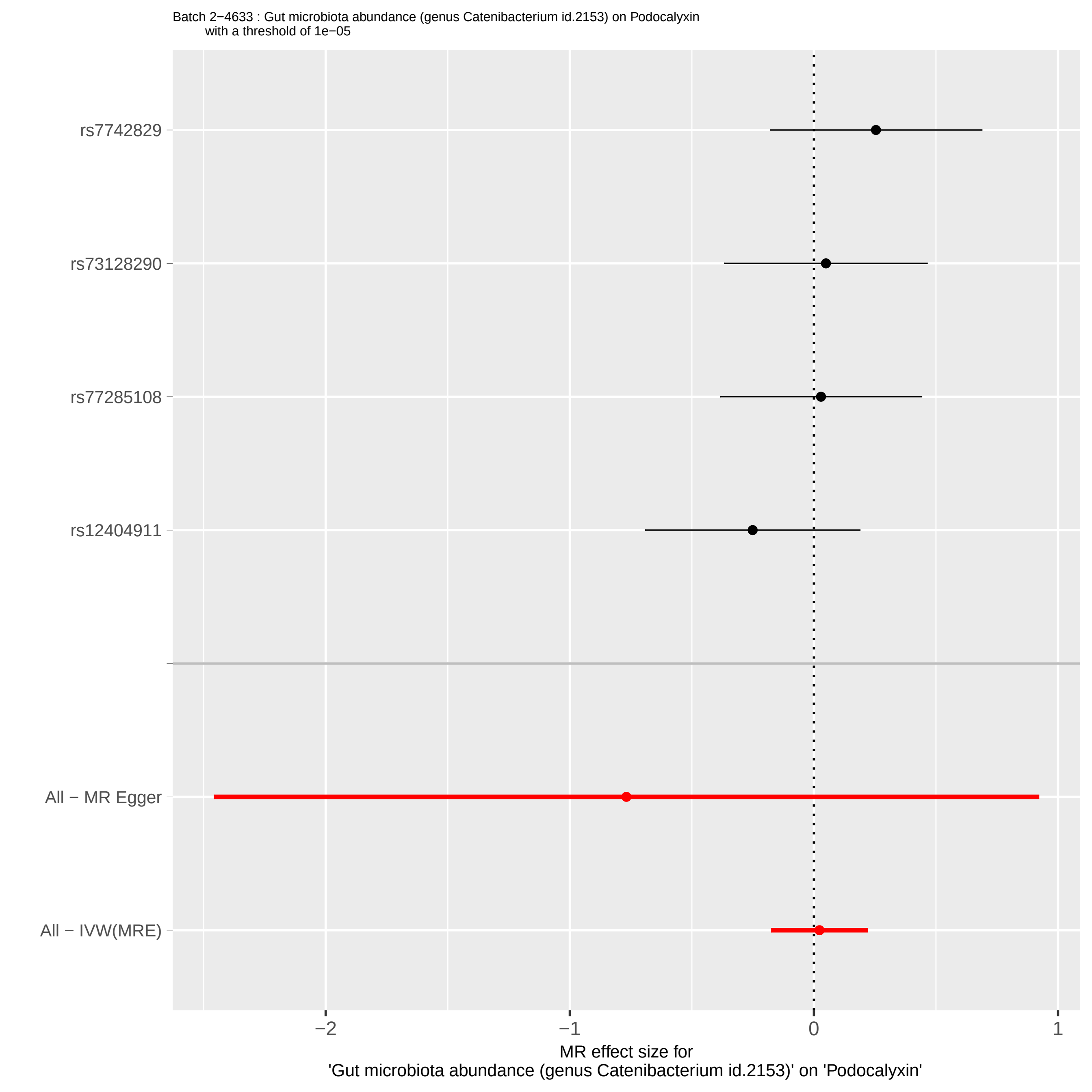

Batch 2−4633 : Gut microbiota abundance (genus Catenibacterium id.2153) on Podocalyxin
with a threshold of 1e−05
rs7742829
rs73128290
rs77285108
rs12404911
All − MR Egger
All − IVW(MRE)
−2
−1
0
1
MR effect size for
'Gut microbiota abundance (genus Catenibacterium id.2153)' on 'Podocalyxin'

Batch 2−4634 : Gut microbiota abundance (genus Parasutterella id.2892) on Podocalyxin
with a threshold of 1e−05

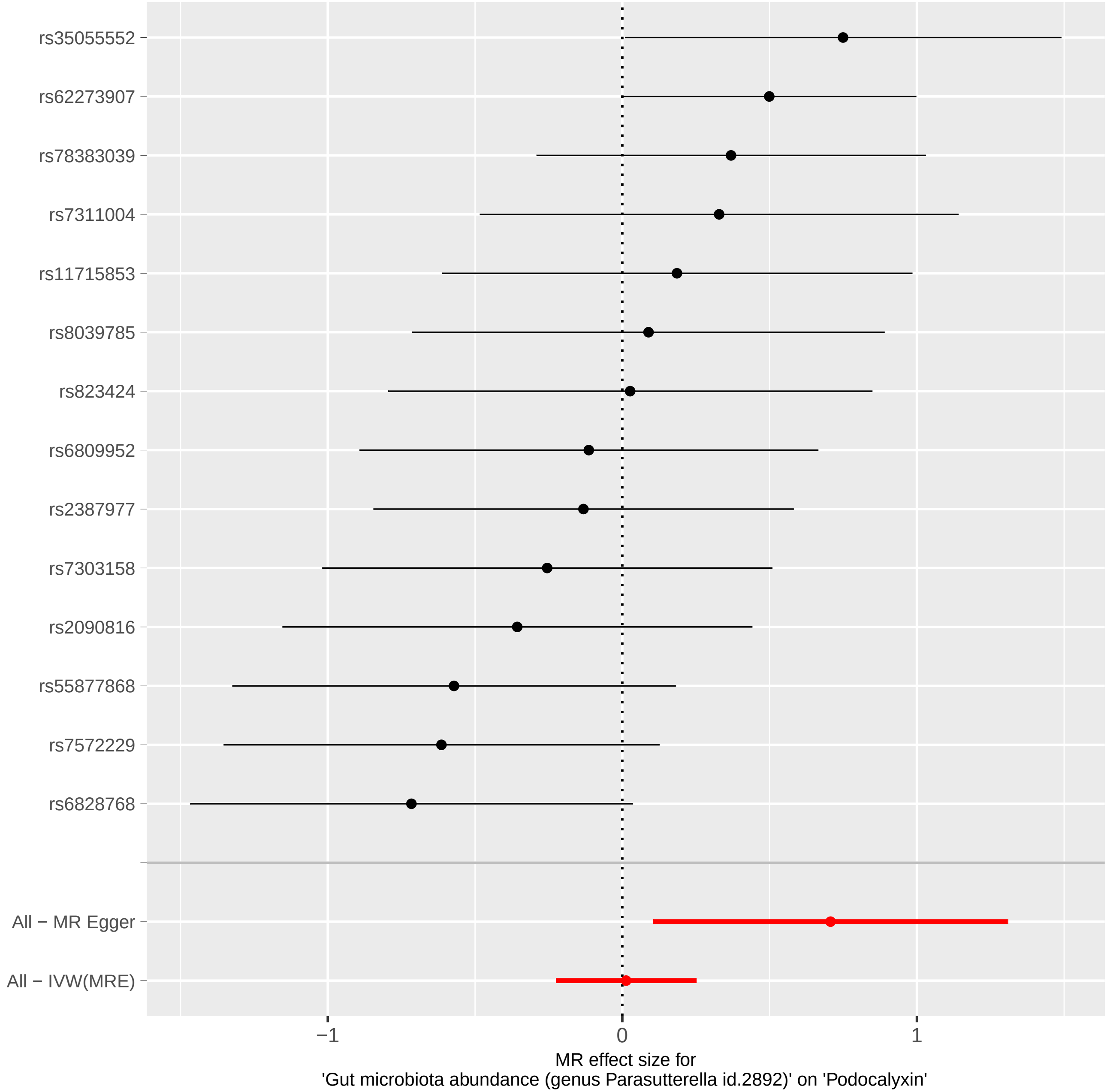

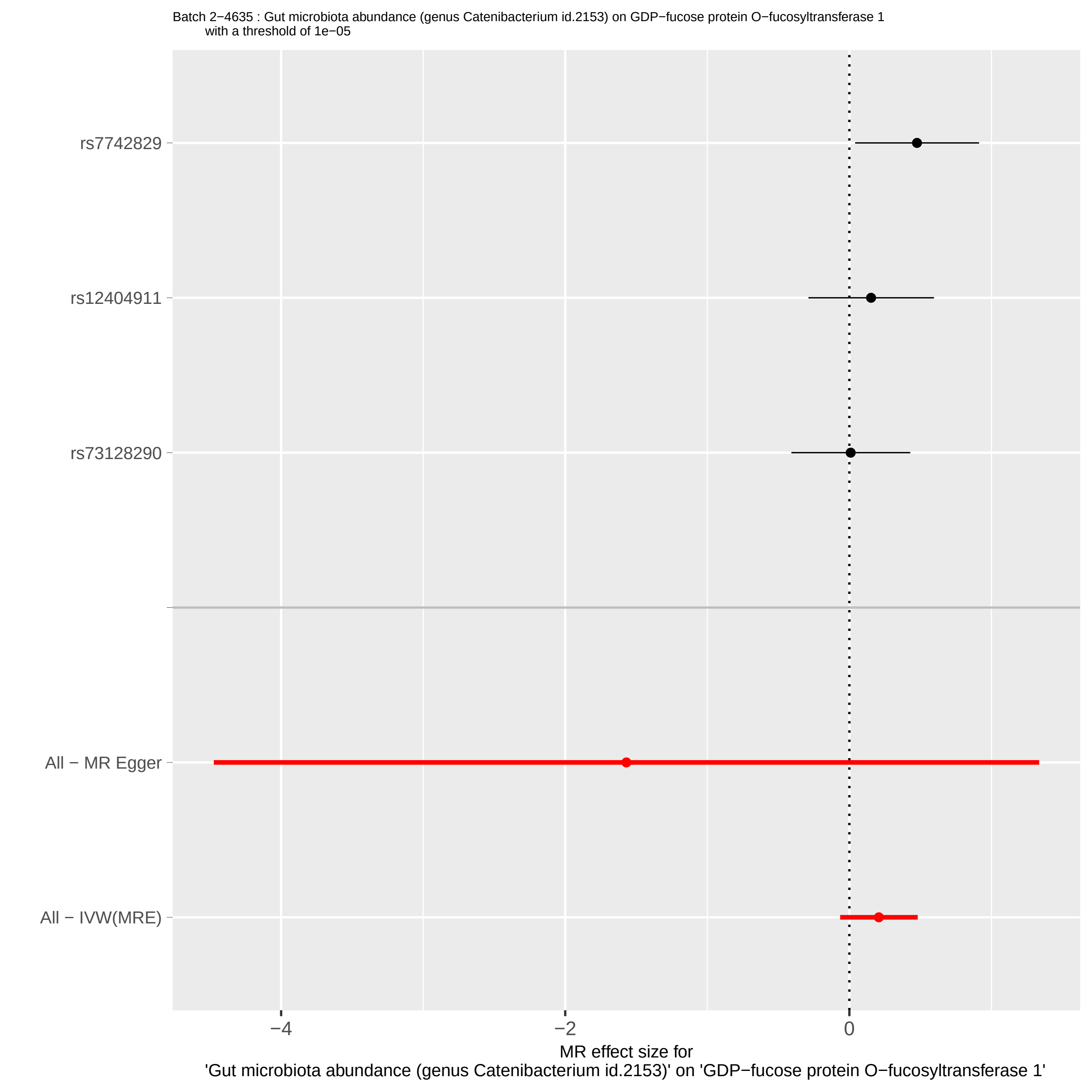

Batch 2−4635 : Gut microbiota abundance (genus Catenibacterium id.2153) on GDP−fucose protein O−fucosyltransferase 1
with a threshold of 1e−05
rs7742829
rs12404911
rs73128290
All − MR Egger
All − IVW(MRE)
−4
−2
0
MR effect size for
'Gut microbiota abundance (genus Catenibacterium id.2153)' on 'GDP−fucose protein O−fucosyltransferase 1'

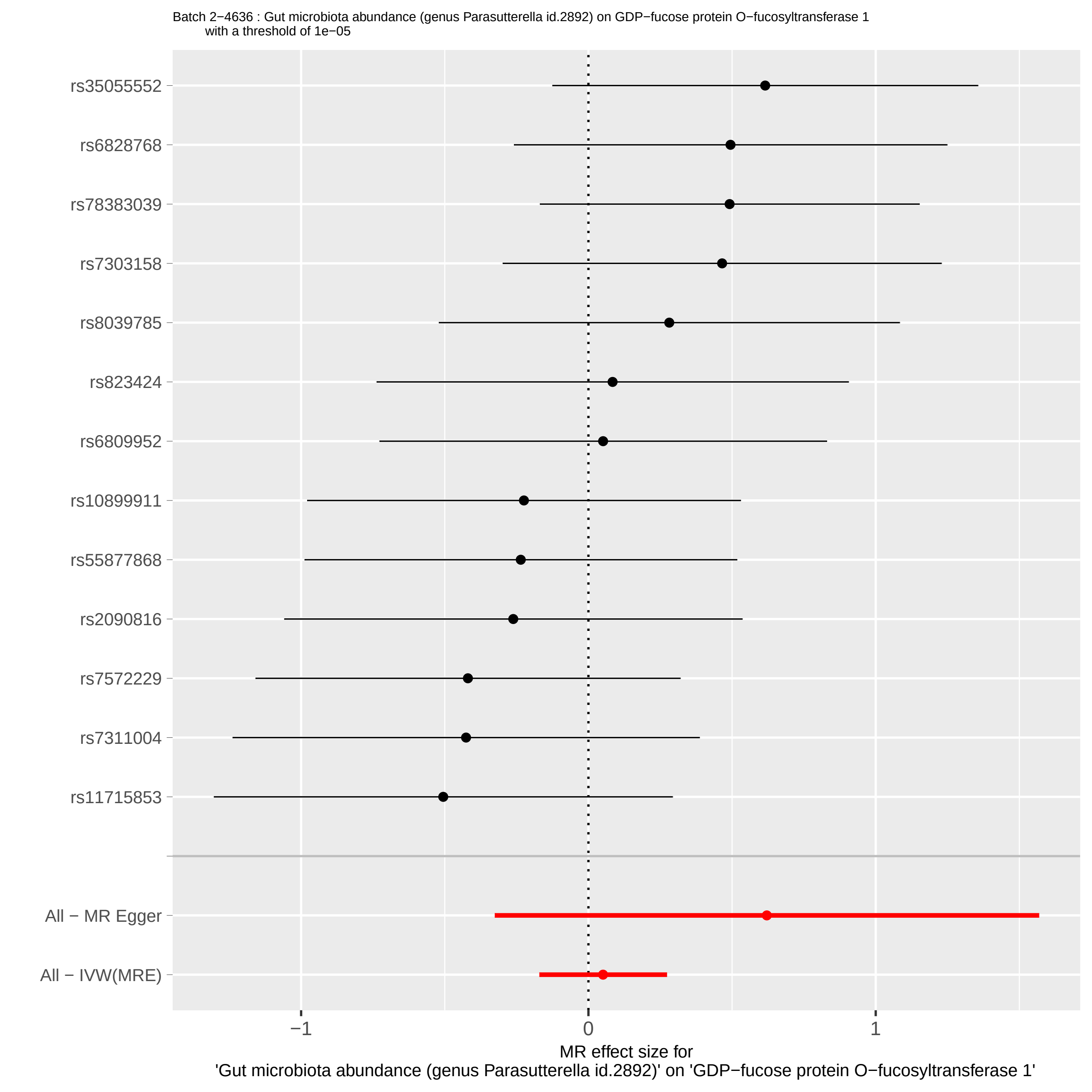
Batch 2−4636 : Gut microbiota abundance (genus Parasutterella id.2892) on GDP−fucose protein O−fucosyltransferase 1
with a threshold of 1e−05
rs35055552
rs6828768
rs78383039
rs7303158
rs8039785
rs823424
rs6809952
rs10899911
rs55877868
rs2090816
rs7572229
rs7311004
rs11715853
All − MR Egger
All − IVW(MRE)
−1
0
1
MR effect size for
'Gut microbiota abundance (genus Parasutterella id.2892)' on 'GDP−fucose protein O−fucosyltransferase 1'

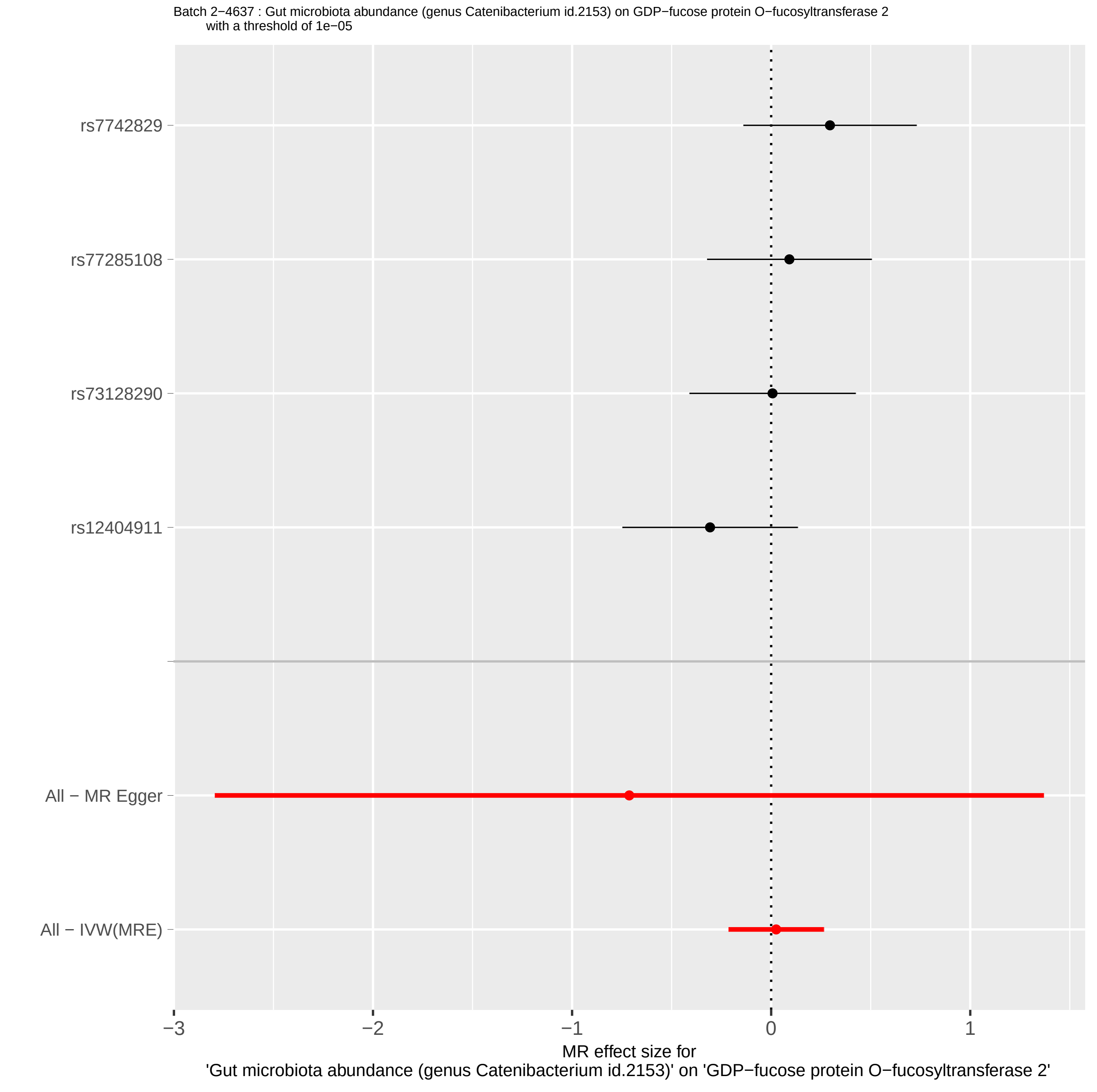

Batch 2−4637 : Gut microbiota abundance (genus Catenibacterium id.2153) on GDP−fucose protein O−fucosyltransferase 2
with a threshold of 1e−05
rs7742829
rs77285108
rs73128290
rs12404911
All − MR Egger
All − IVW(MRE)
−3
−2
−1
0
1
MR effect size for
'Gut microbiota abundance (genus Catenibacterium id.2153)' on 'GDP−fucose protein O−fucosyltransferase 2'

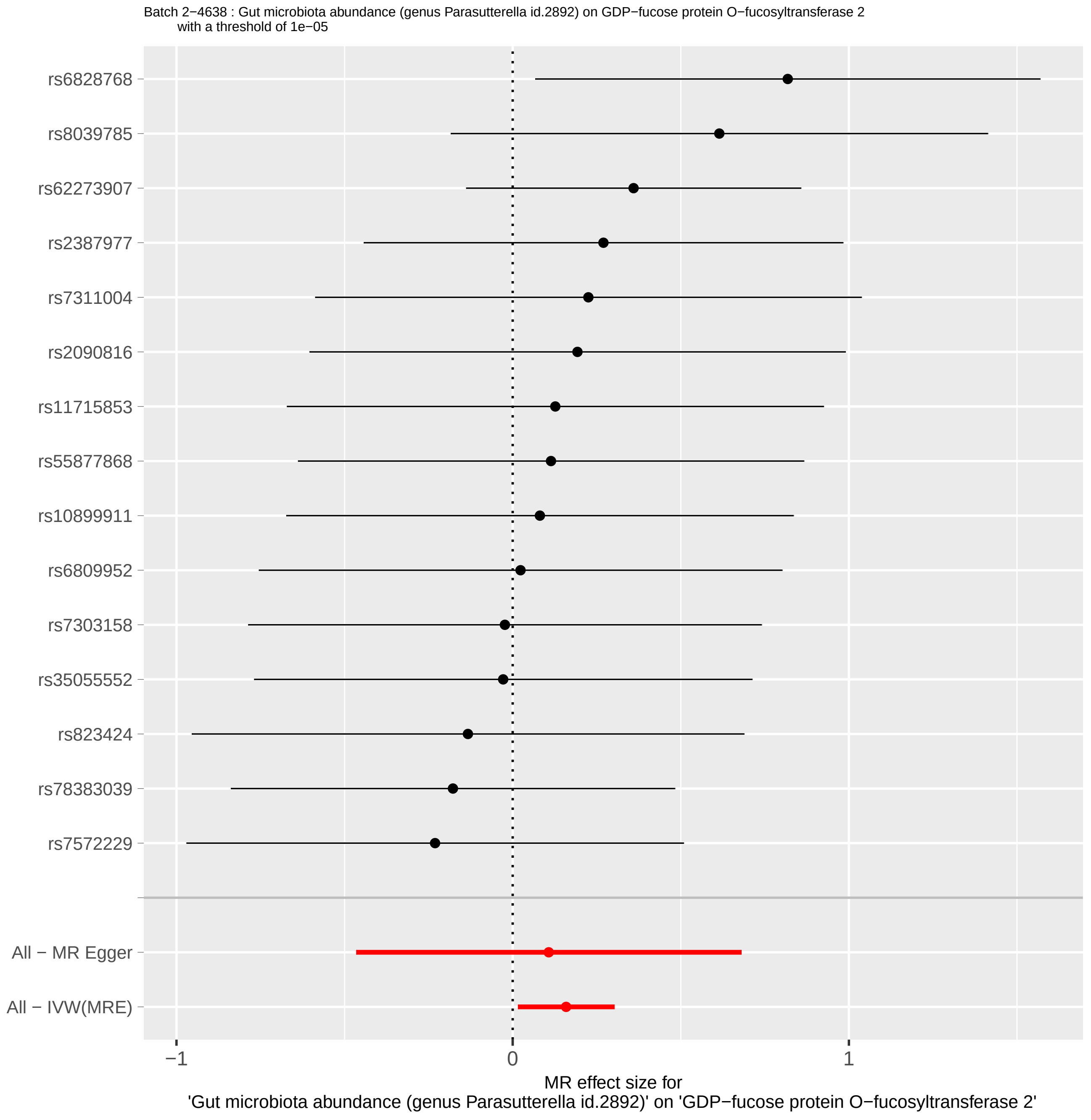

Batch 2−4638 : Gut microbiota abundance (genus Parasutterella id.2892) on GDP−fucose protein O−fucosyltransferase 2
with a threshold of 1e−05
rs6828768
rs8039785
rs62273907
rs2387977
rs7311004
rs2090816
rs11715853
rs55877868
rs10899911
rs6809952
rs7303158
rs35055552
rs823424
rs78383039
rs7572229
All − MR Egger
All − IVW(MRE)
−1
0
1
MR effect size for
'Gut microbiota abundance (genus Parasutterella id.2892)' on 'GDP−fucose protein O−fucosyltransferase 2'

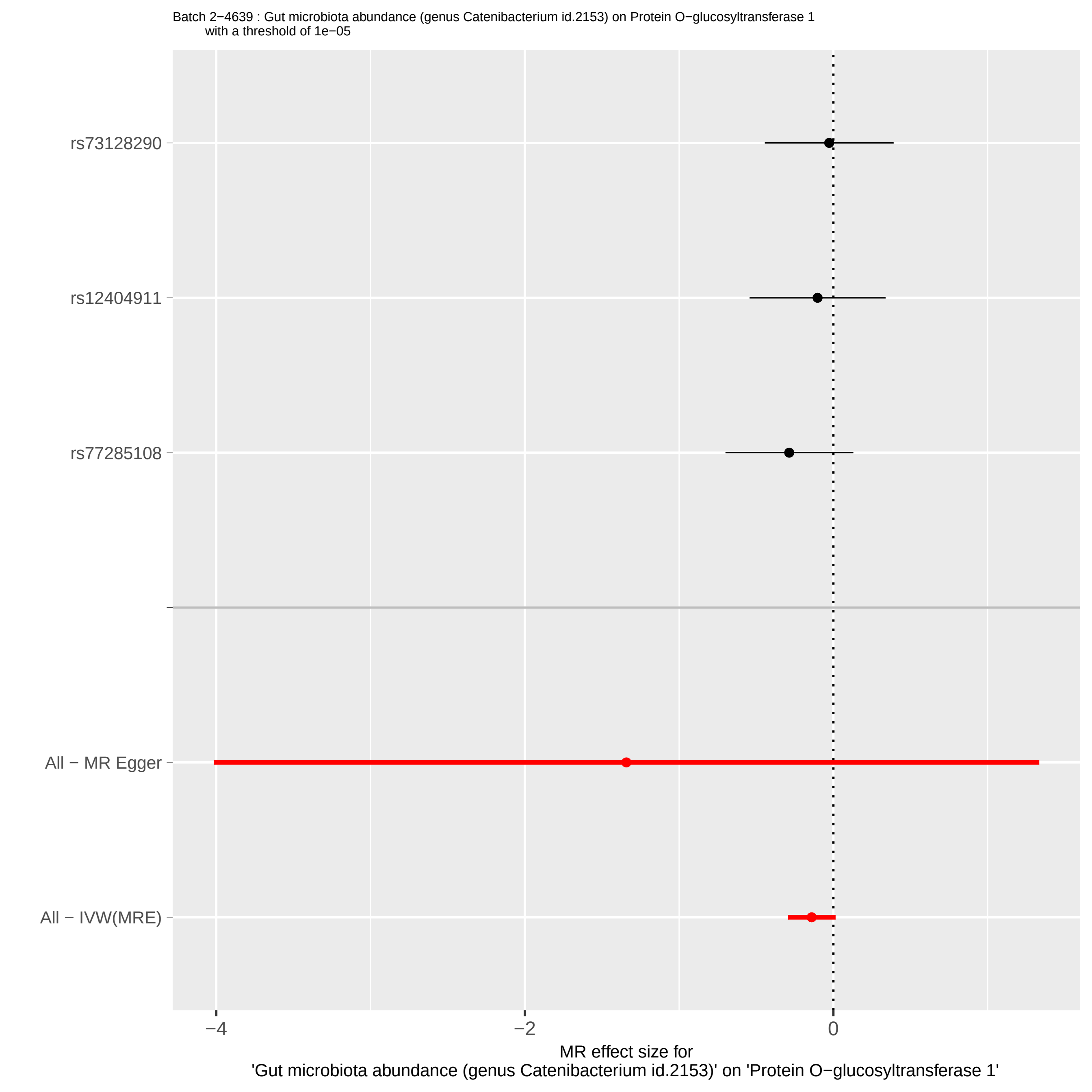

Batch 2−4639 : Gut microbiota abundance (genus Catenibacterium id.2153) on Protein O−glucosyltransferase 1
with a threshold of 1e−05
rs73128290
rs12404911
rs77285108
All − MR Egger
All − IVW(MRE)
−4
−2
0
MR effect size for
'Gut microbiota abundance (genus Catenibacterium id.2153)' on 'Protein O−glucosyltransferase 1'

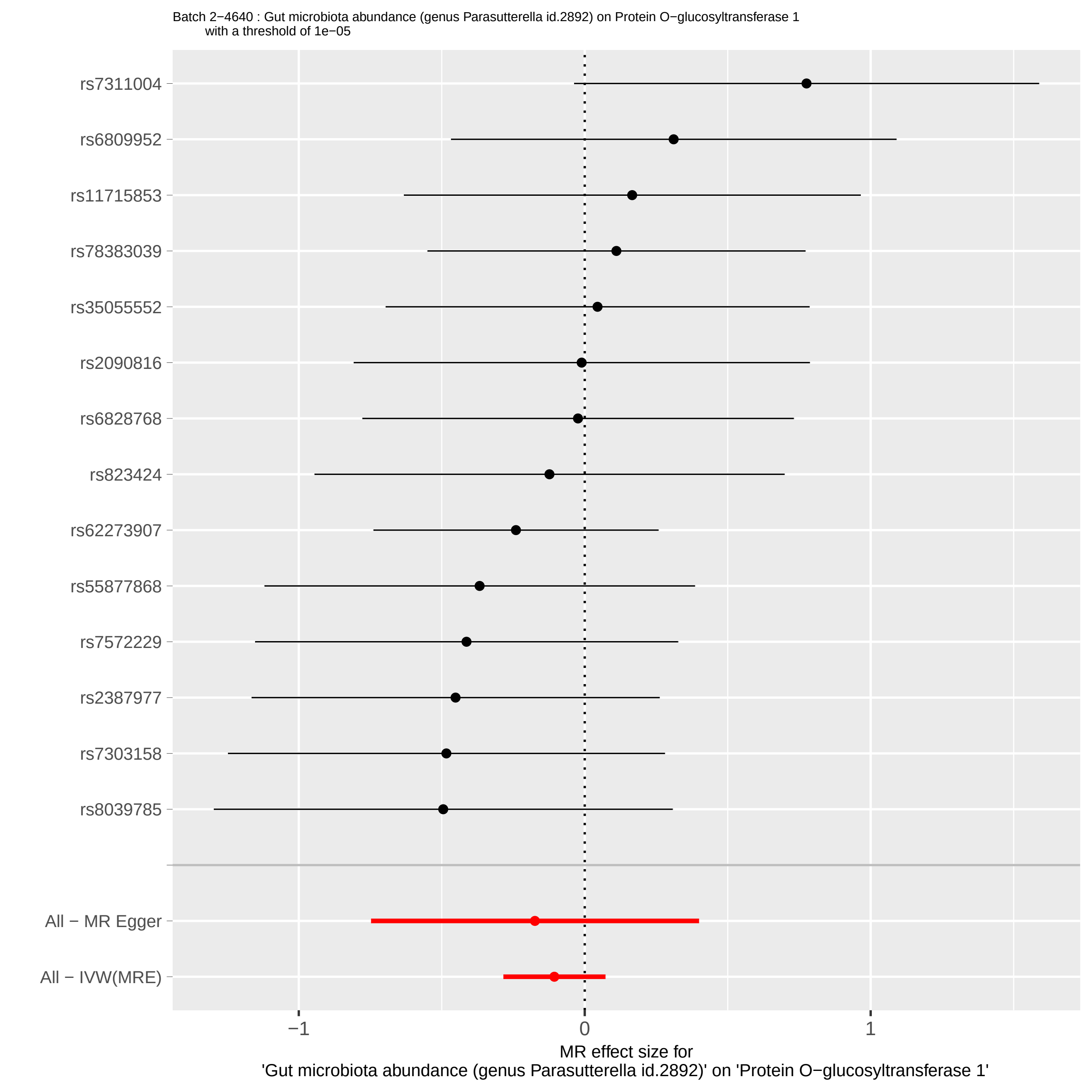

Batch 2−4640 : Gut microbiota abundance (genus Parasutterella id.2892) on Protein O−glucosyltransferase 1
with a threshold of 1e−05
rs7311004
rs6809952
rs11715853
rs78383039
rs35055552
rs2090816
rs6828768
rs823424
rs62273907
rs55877868
rs7572229
rs2387977
rs7303158
rs8039785
All − MR Egger
All − IVW(MRE)
−1
0
1
MR effect size for
'Gut microbiota abundance (genus Parasutterella id.2892)' on 'Protein O−glucosyltransferase 1'

Batch 2−4641 : Gut microbiota abundance (genus Catenibacterium id.2153) on DNA polymerase eta
with a threshold of 1e−05

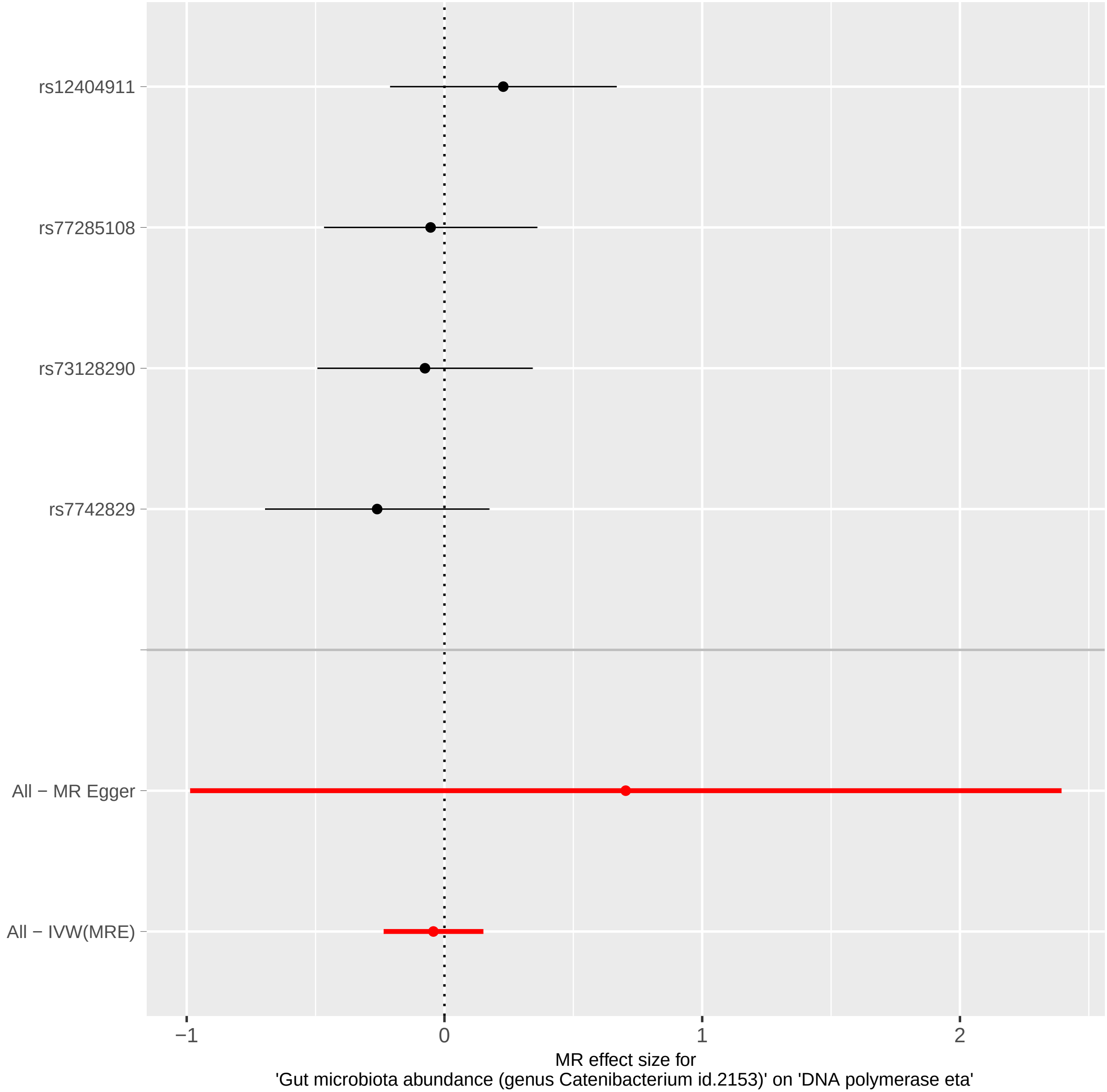

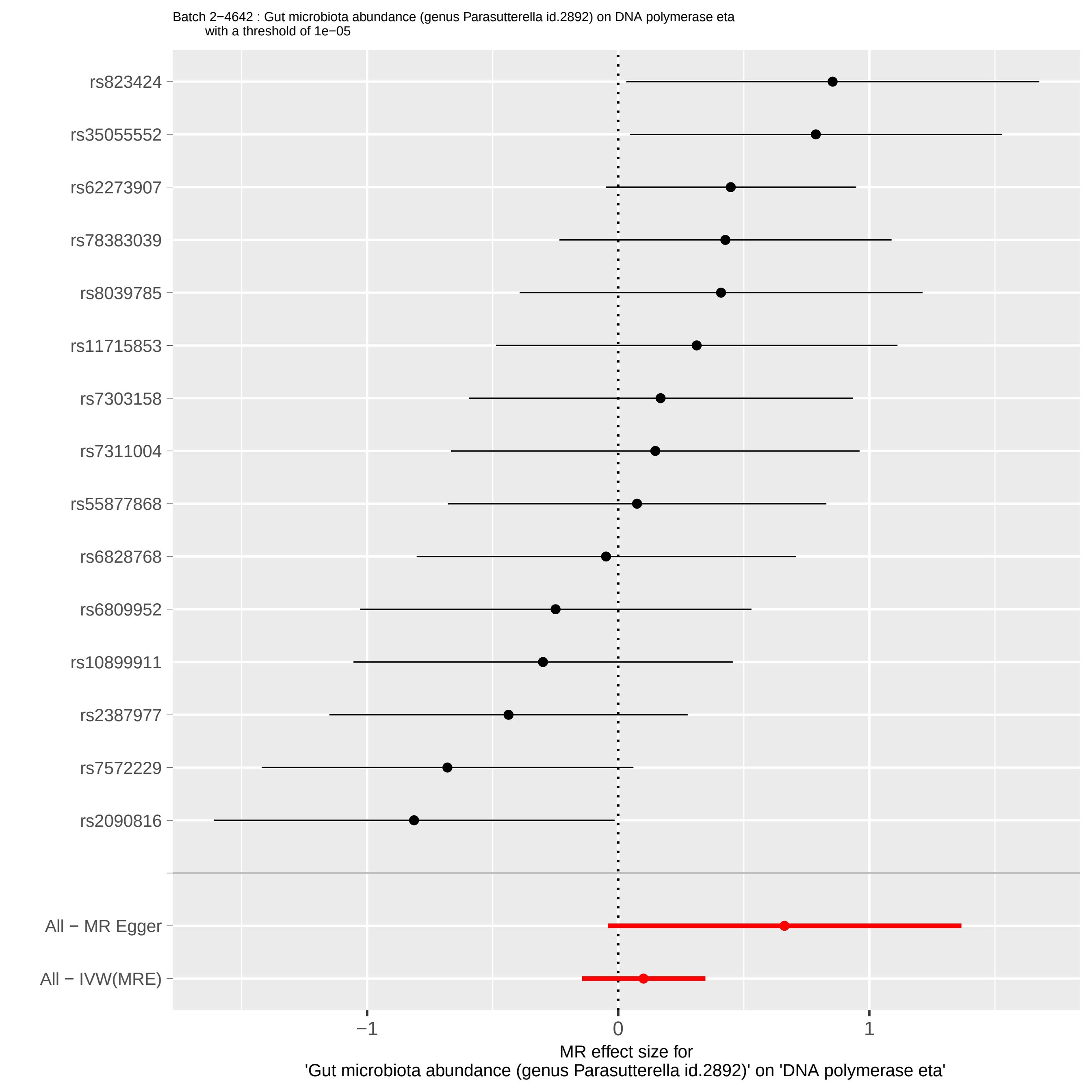

Batch 2−4642 : Gut microbiota abundance (genus Parasutterella id.2892) on DNA polymerase eta
with a threshold of 1e−05
rs823424
rs35055552
rs62273907
rs78383039
rs8039785
rs11715853
rs7303158
rs7311004
rs55877868
rs6828768
rs6809952
rs10899911
rs2387977
rs7572229
rs2090816
All − MR Egger
All − IVW(MRE)
−1
0
1
MR effect size for
'Gut microbiota abundance (genus Parasutterella id.2892)' on 'DNA polymerase eta'

Batch 2−4643 : Gut microbiota abundance (genus Catenibacterium id.2153) on DNA polymerase iota
with a threshold of 1e−05

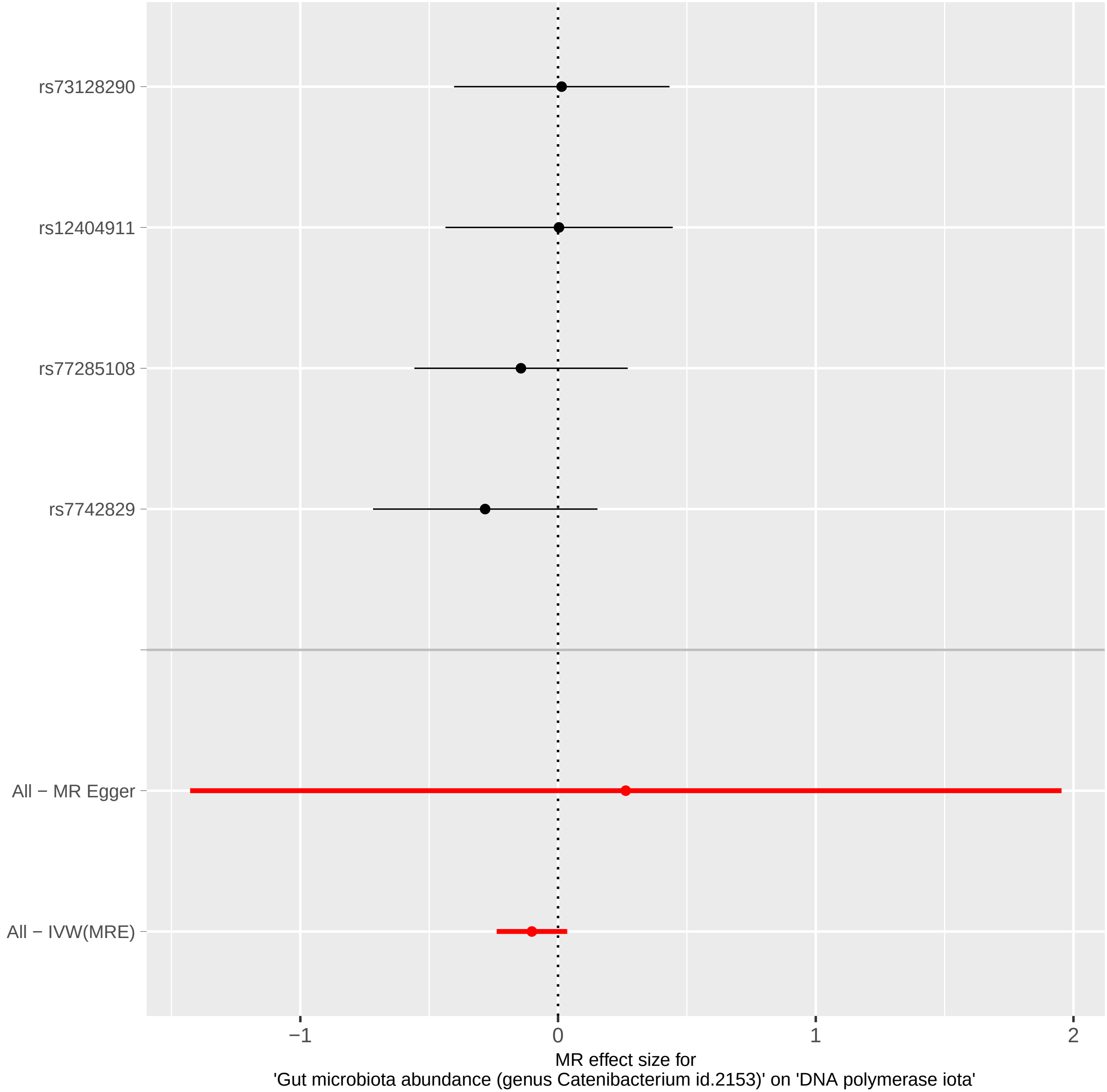

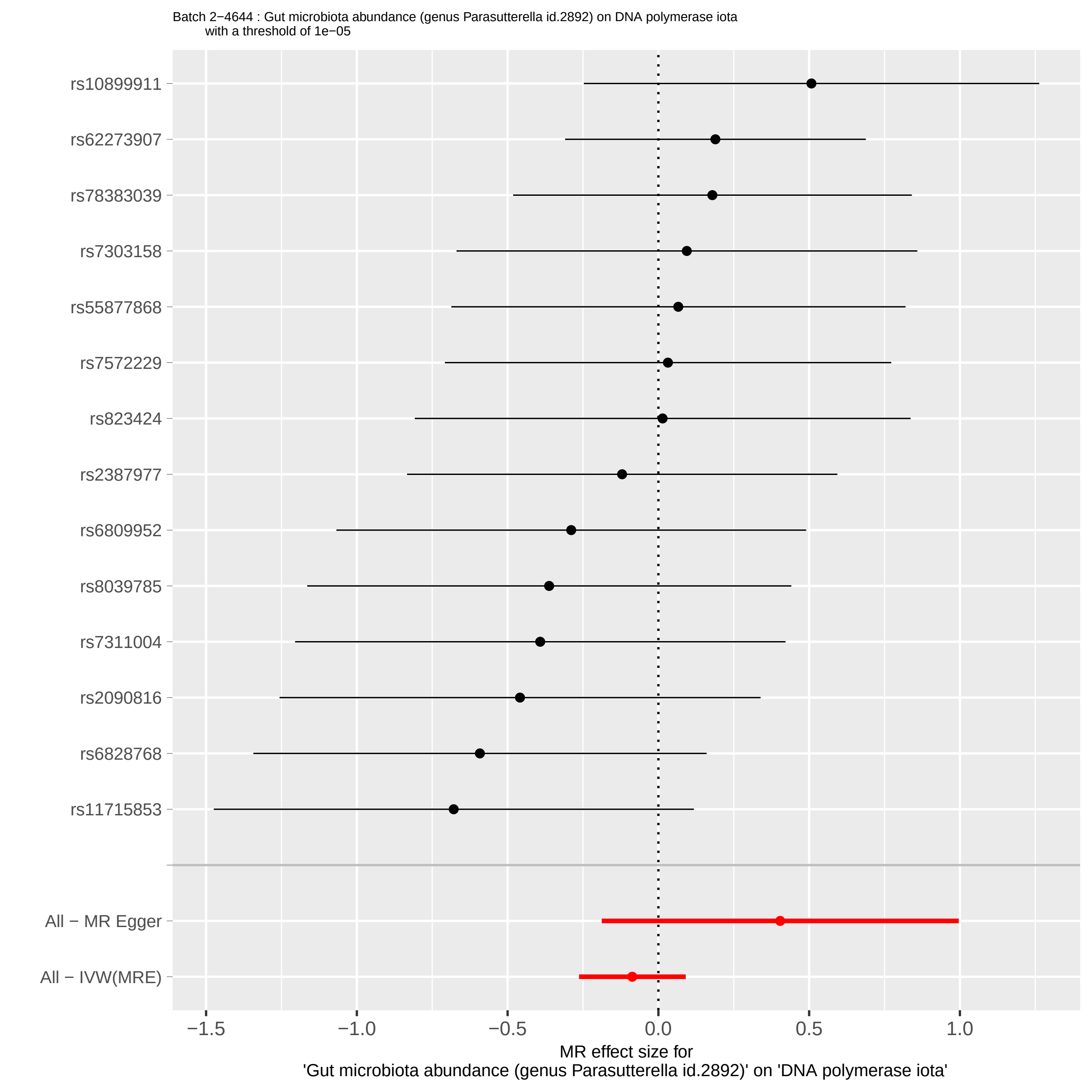
Batch 2−4644 : Gut microbiota abundance (genus Parasutterella id.2892) on DNA polymerase iota
with a threshold of 1e−05
rs10899911
rs62273907
rs78383039
rs7303158
rs55877868
rs7572229
rs823424
rs2387977
rs6809952
rs8039785
rs7311004
rs2090816
rs6828768
rs11715853
All − MR Egger
All − IVW(MRE)
−1.5
−1.0
−0.5
0.0
0.5
1.0
MR effect size for
'Gut microbiota abundance (genus Parasutterella id.2892)' on 'DNA polymerase iota'

Batch 2−4645 : Gut microbiota abundance (genus Catenibacterium id.2153) on DNA−directed DNA/RNA polymerase mu with a threshold of 1e−05

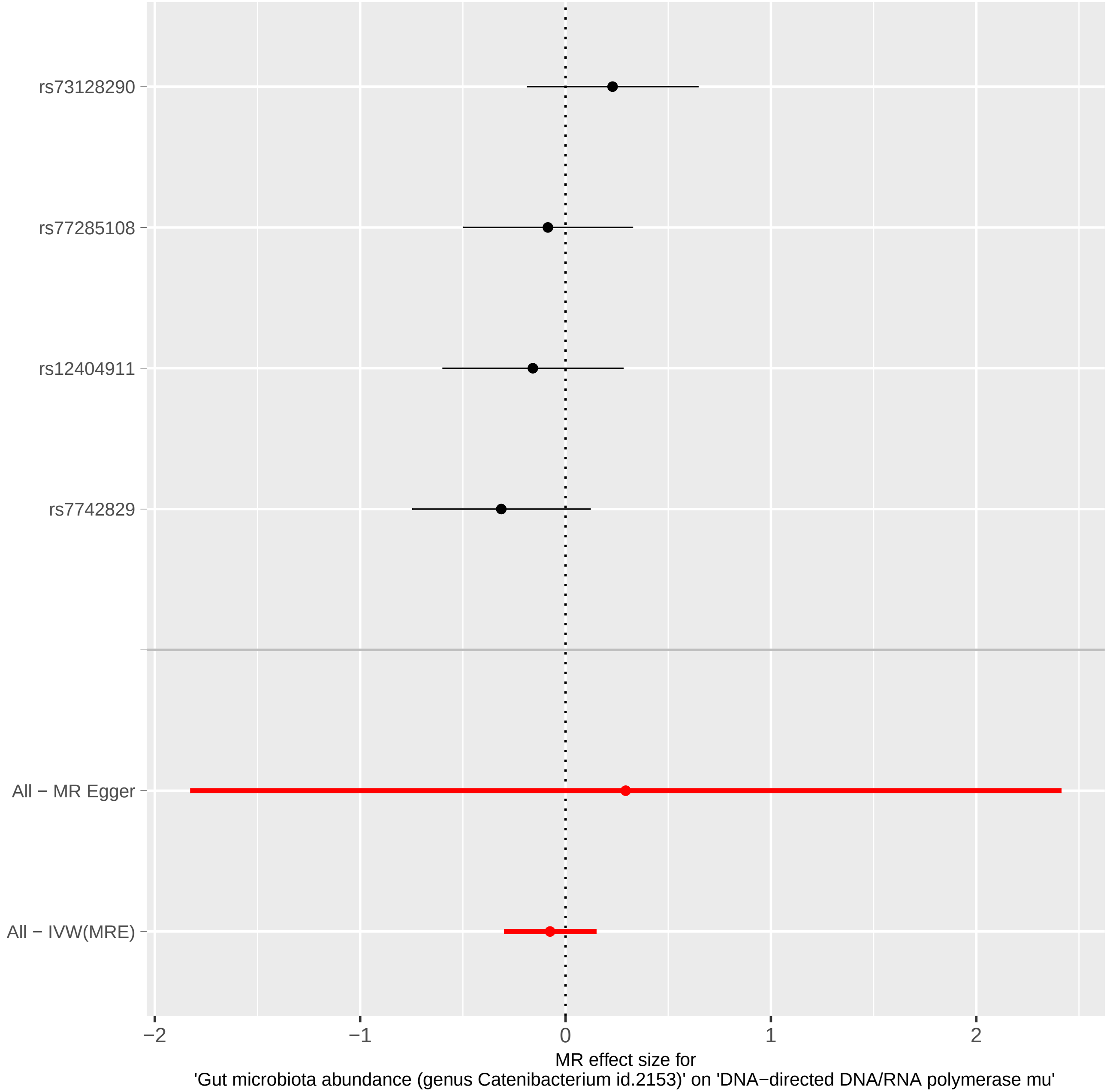

Batch 2−4646 : Gut microbiota abundance (genus Parasutterella id.2892) on DNA−directed DNA/RNA polymerase mu
with a threshold of 1e−05

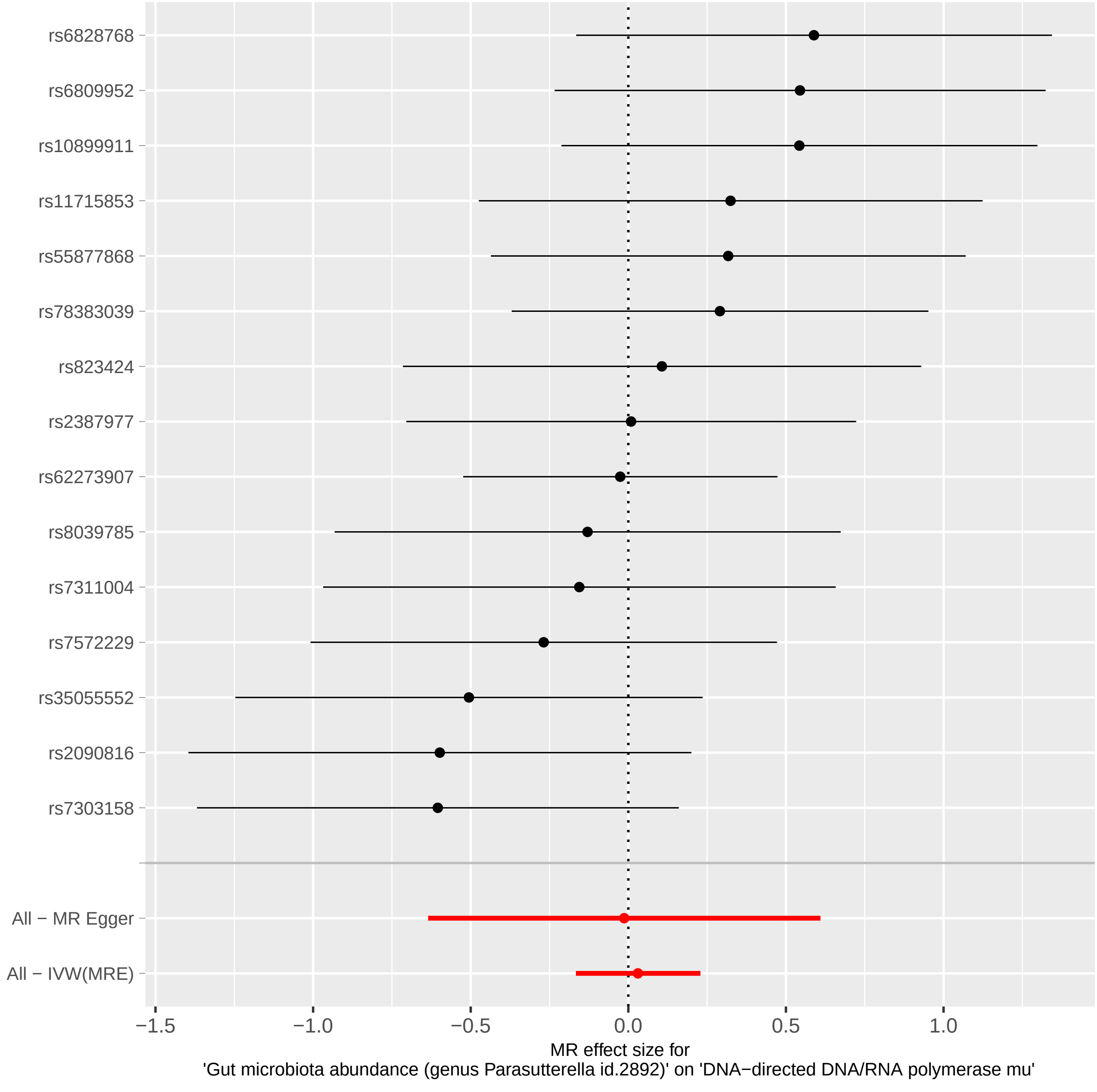

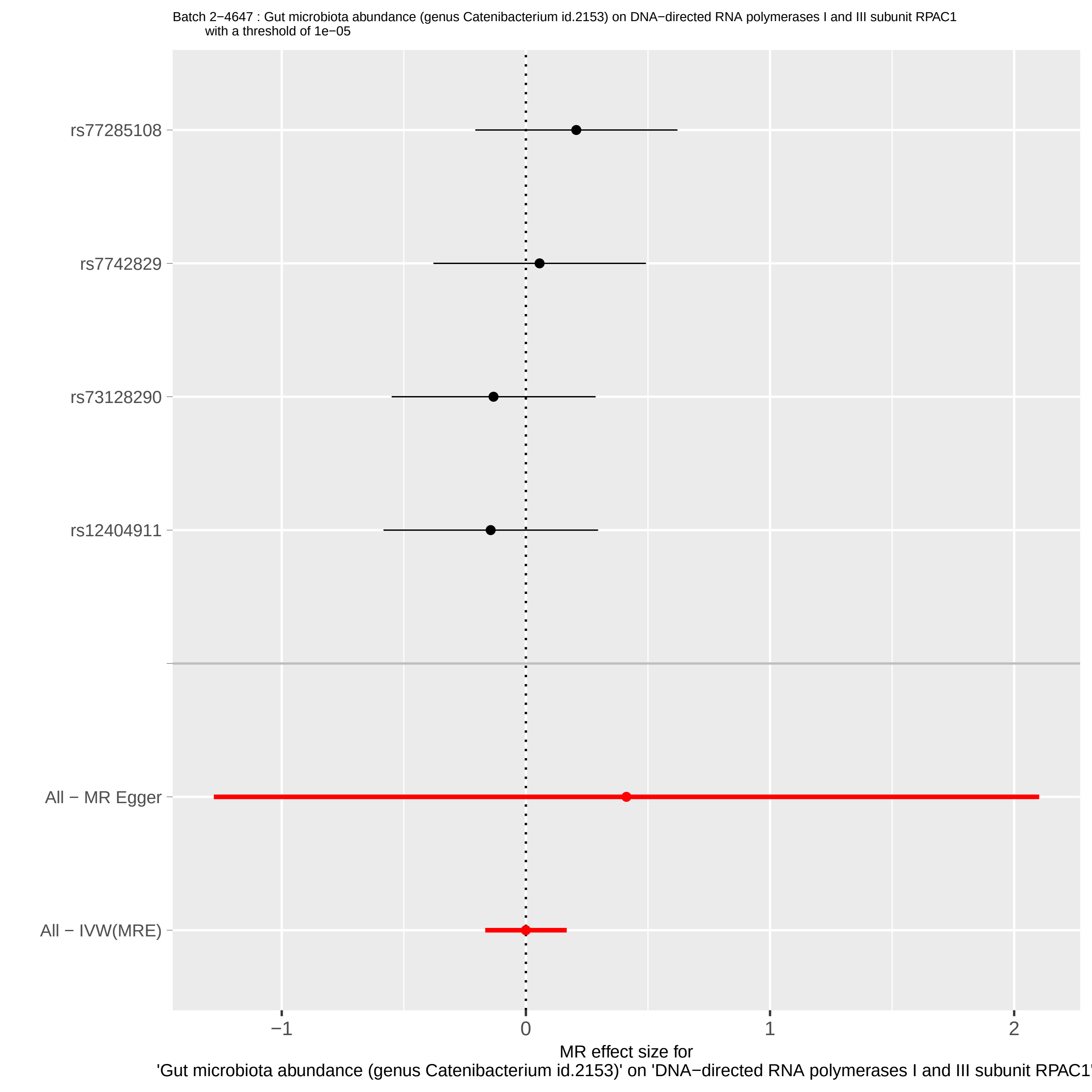
Batch 2−4647 : Gut microbiota abundance (genus Catenibacterium id.2153) on DNA−directed RNA polymerases I and III subunit RPAC1
with a threshold of 1e−05
rs77285108
rs7742829
rs73128290
rs12404911
All − MR Egger
All − IVW(MRE)
−1
0
1
2
MR effect size for
'Gut microbiota abundance (genus Catenibacterium id.2153)' on 'DNA−directed RNA polymerases I and III subunit RPAC1'

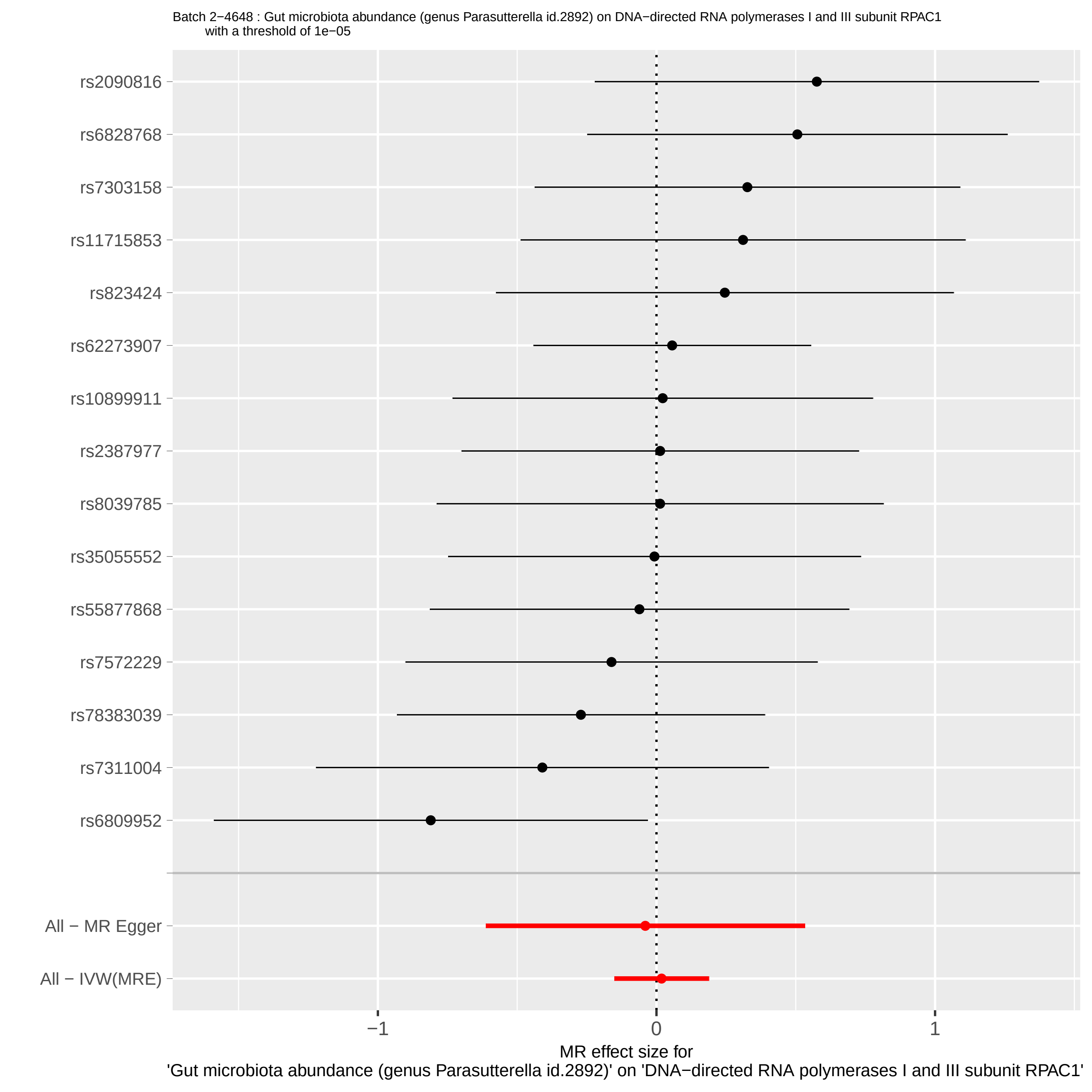

Batch 2−4648 : Gut microbiota abundance (genus Parasutterella id.2892) on DNA−directed RNA polymerases I and III subunit RPAC1
with a threshold of 1e−05
rs2090816
rs6828768
rs7303158
rs11715853
rs823424
rs62273907
rs10899911
rs2387977
rs8039785
rs35055552
rs55877868
rs7572229
rs78383039
rs7311004
rs6809952
All − MR Egger
All − IVW(MRE)
−1
0
1
MR effect size for
'Gut microbiota abundance (genus Parasutterella id.2892)' on 'DNA−directed RNA polymerases I and III subunit RPAC1'

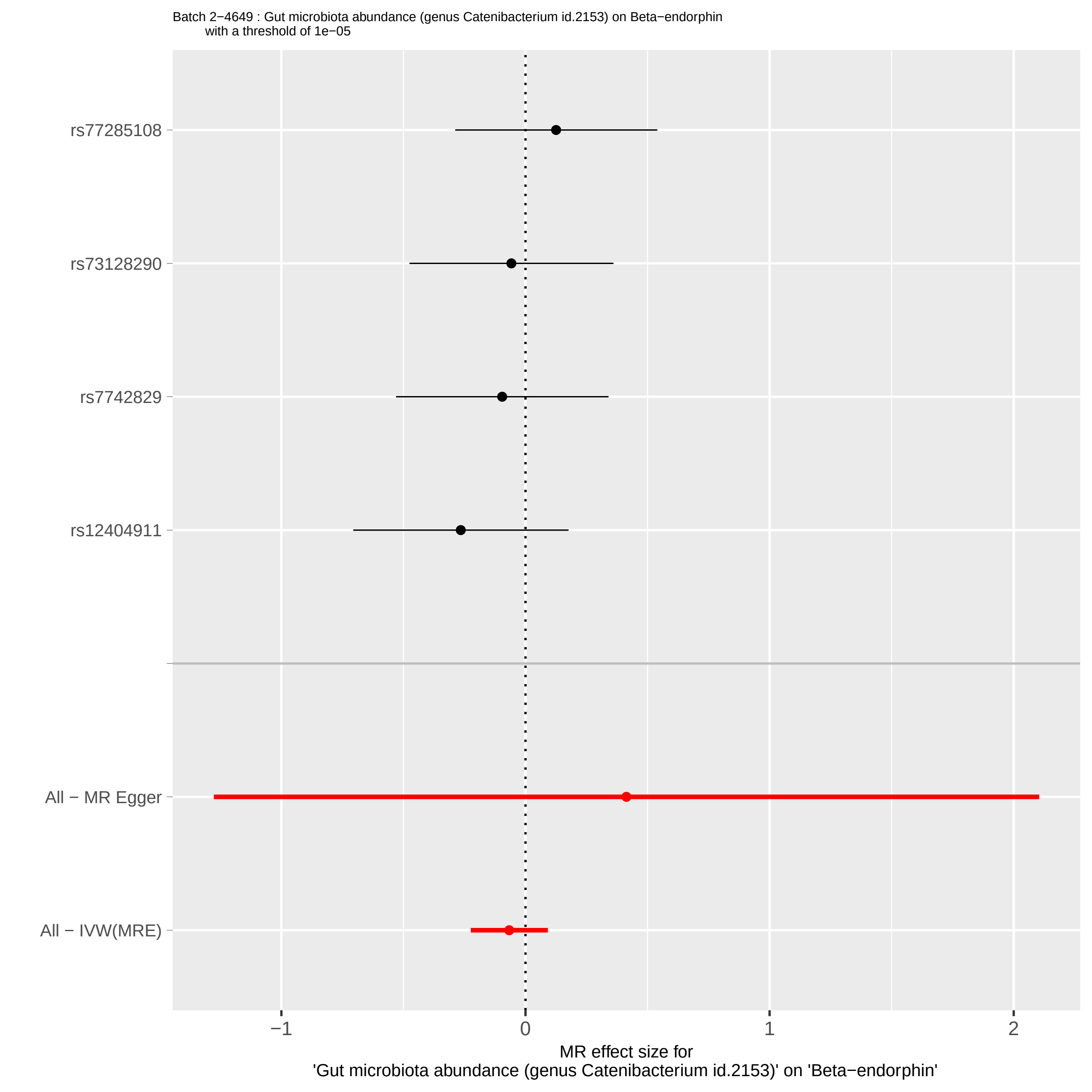
Batch 2−4649 : Gut microbiota abundance (genus Catenibacterium id.2153) on Beta−endorphin
with a threshold of 1e−05
rs77285108
rs73128290
rs7742829
rs12404911
All − MR Egger
All − IVW(MRE)
−1
0
1
2
MR effect size for
'Gut microbiota abundance (genus Catenibacterium id.2153)' on 'Beta−endorphin'

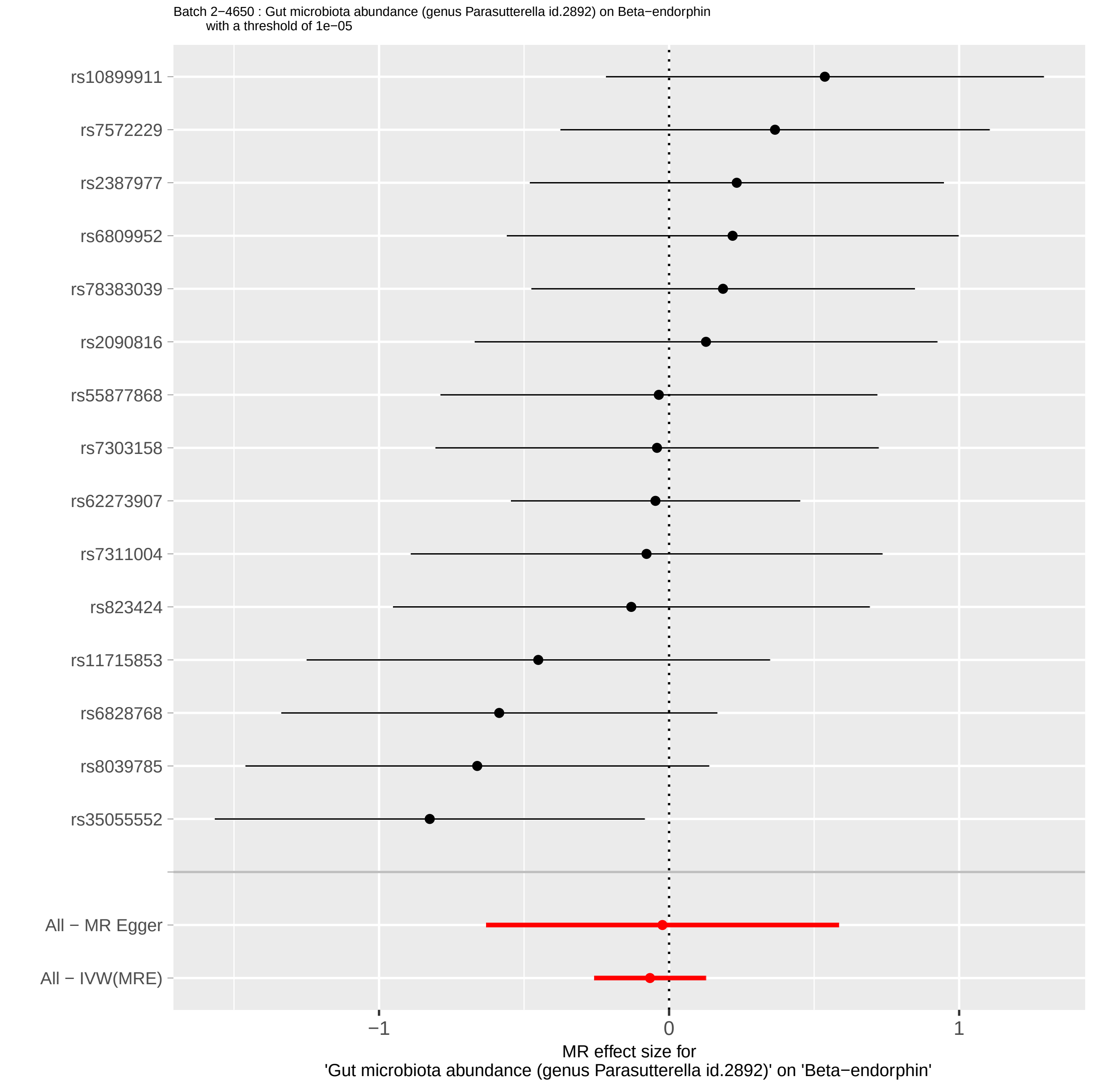
Batch 2−4650 : Gut microbiota abundance (genus Parasutterella id.2892) on Beta−endorphin
with a threshold of 1e−05
rs10899911
rs7572229
rs2387977
rs6809952
rs78383039
rs2090816
rs55877868
rs7303158
rs62273907
rs7311004
rs823424
rs11715853
rs6828768
rs8039785
rs35055552
All − MR Egger
All − IVW(MRE)
−1
0
1
MR effect size for
'Gut microbiota abundance (genus Parasutterella id.2892)' on 'Beta−endorphin'

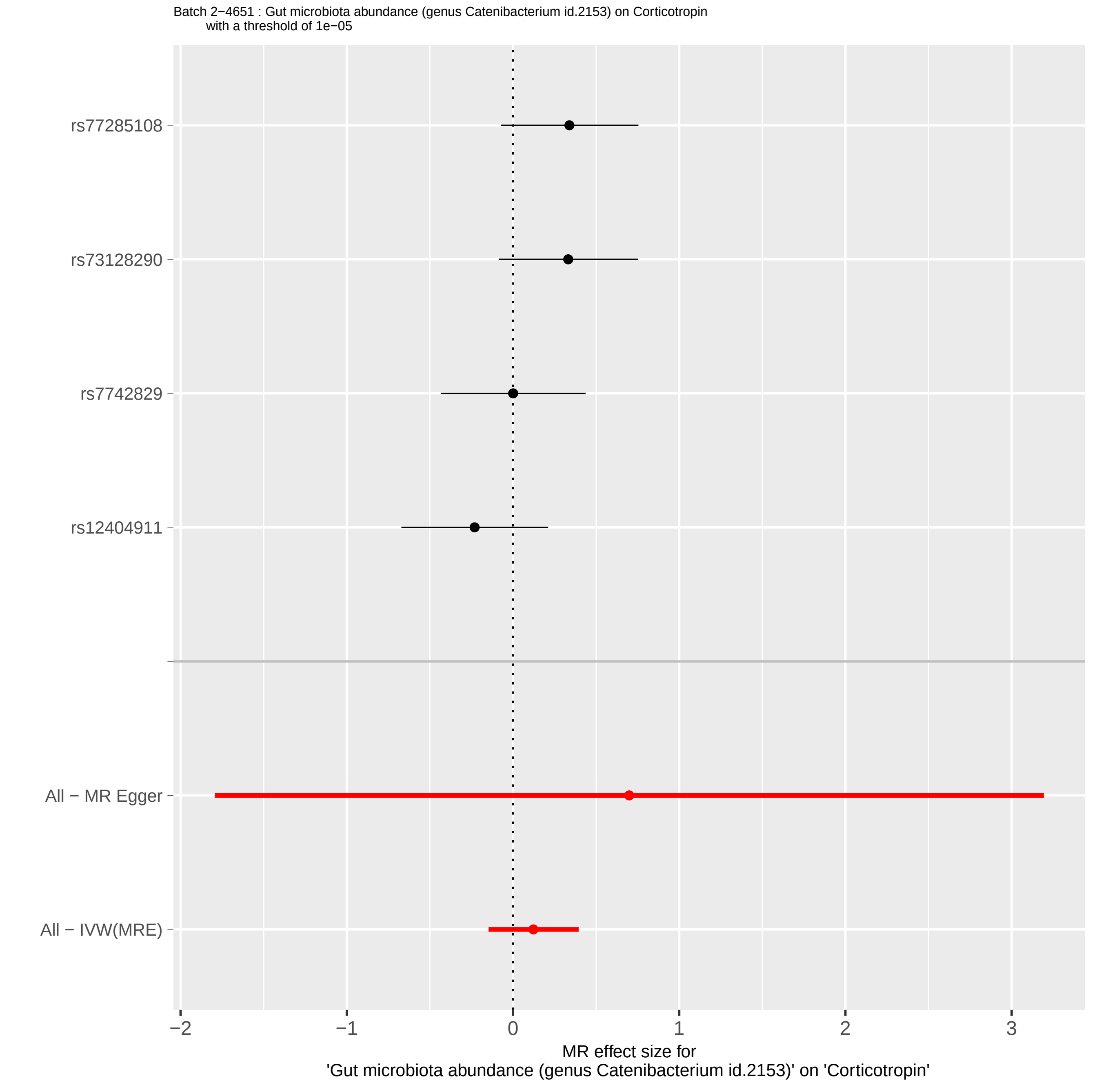

Batch 2−4651 : Gut microbiota abundance (genus Catenibacterium id.2153) on Corticotropin
with a threshold of 1e−05
rs77285108
rs73128290
rs7742829
rs12404911
All − MR Egger
All − IVW(MRE)
−2
−1
0
1
2
3
MR effect size for
'Gut microbiota abundance (genus Catenibacterium id.2153)' on 'Corticotropin'

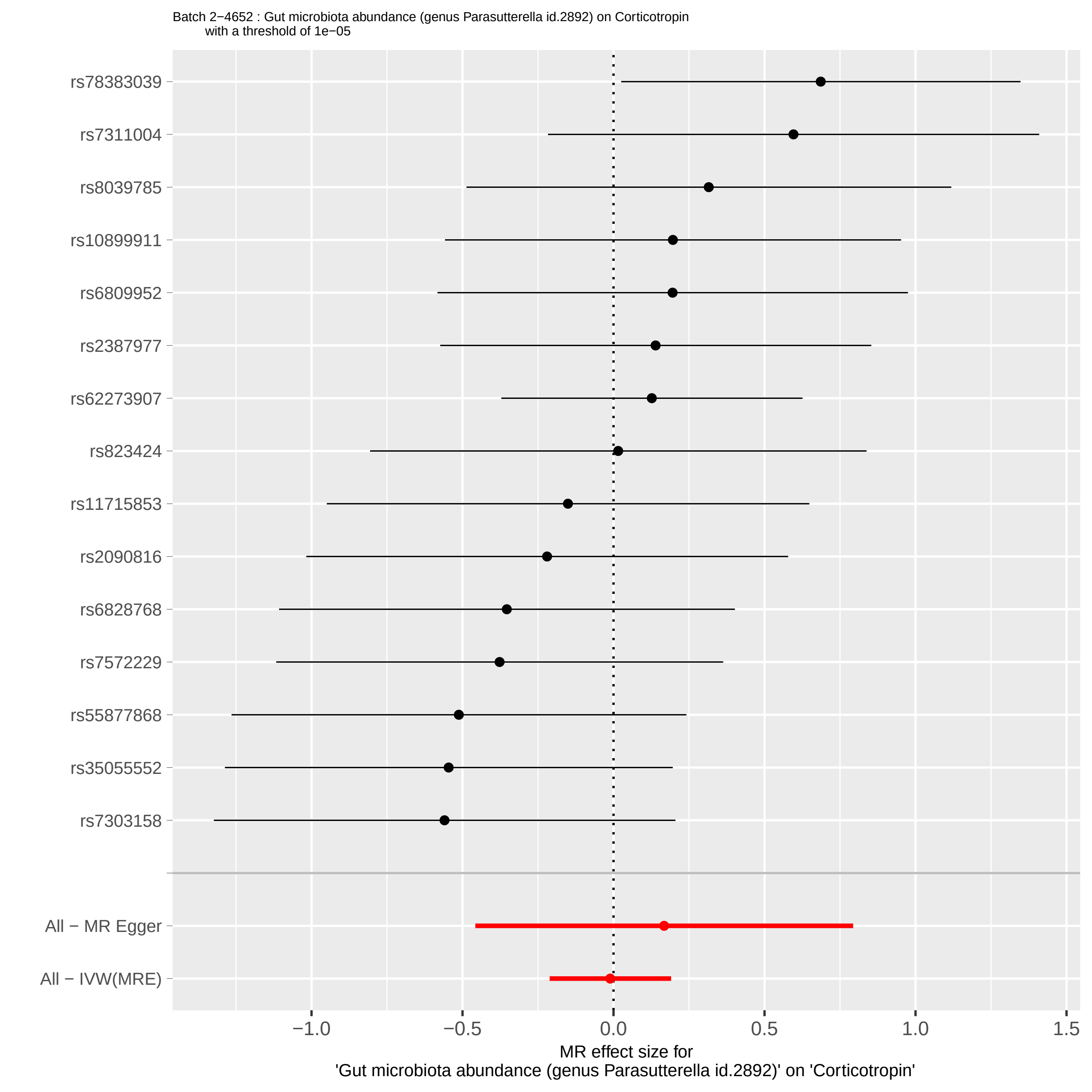

Batch 2−4652 : Gut microbiota abundance (genus Parasutterella id.2892) on Corticotropin
with a threshold of 1e−05
rs78383039
rs7311004
rs8039785
rs10899911
rs6809952
rs2387977
rs62273907
rs823424
rs11715853
rs2090816
rs6828768
rs7572229
rs55877868
rs35055552
rs7303158
All − MR Egger
All − IVW(MRE)
−1.0
−0.5
0.0
0.5
1.0
1.5
MR effect size for
'Gut microbiota abundance (genus Parasutterella id.2892)' on 'Corticotropin'

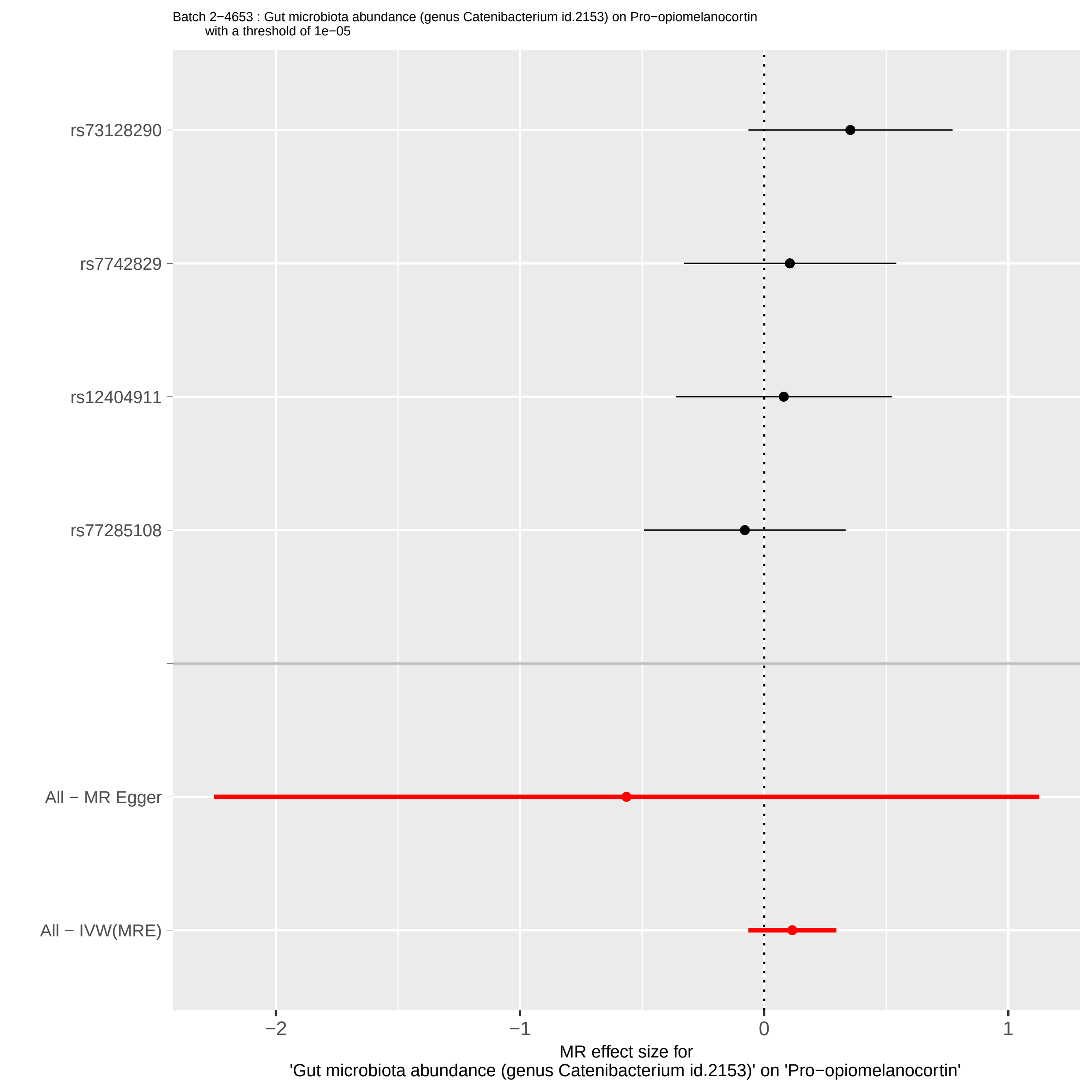
Batch 2−4653 : Gut microbiota abundance (genus Catenibacterium id.2153) on Pro−opiomelanocortin
with a threshold of 1e−05
rs73128290
rs7742829
rs12404911
rs77285108
All − MR Egger
All − IVW(MRE)
−2
−1
0
1
MR effect size for
'Gut microbiota abundance (genus Catenibacterium id.2153)' on 'Pro−opiomelanocortin'

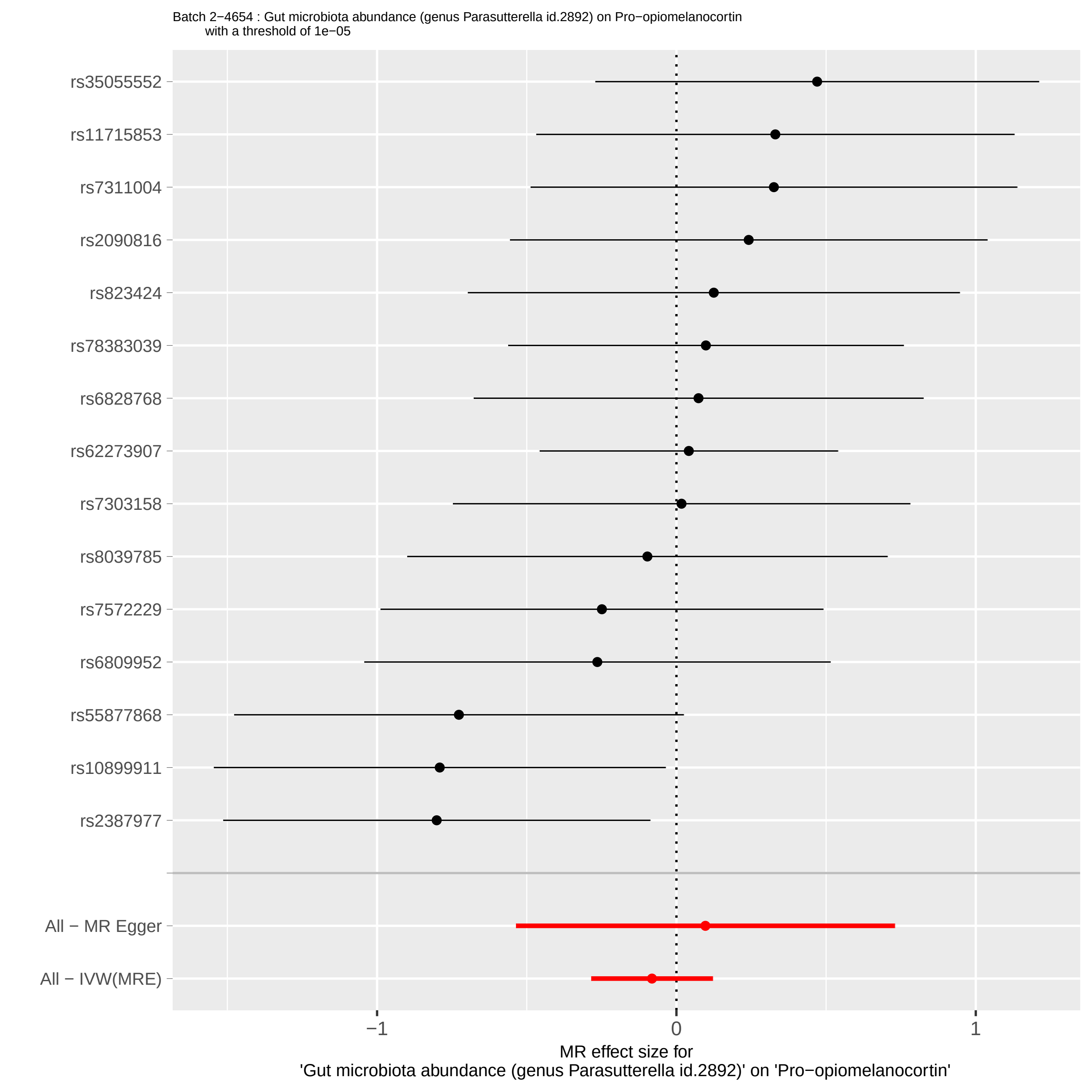

Batch 2−4654 : Gut microbiota abundance (genus Parasutterella id.2892) on Pro−opiomelanocortin
with a threshold of 1e−05
rs35055552
rs11715853
rs7311004
rs2090816
rs823424
rs78383039
rs6828768
rs62273907
rs7303158
rs8039785
rs7572229
rs6809952
rs55877868
rs10899911
rs2387977
All − MR Egger
All − IVW(MRE)
−1
0
1
MR effect size for
'Gut microbiota abundance (genus Parasutterella id.2892)' on 'Pro−opiomelanocortin'

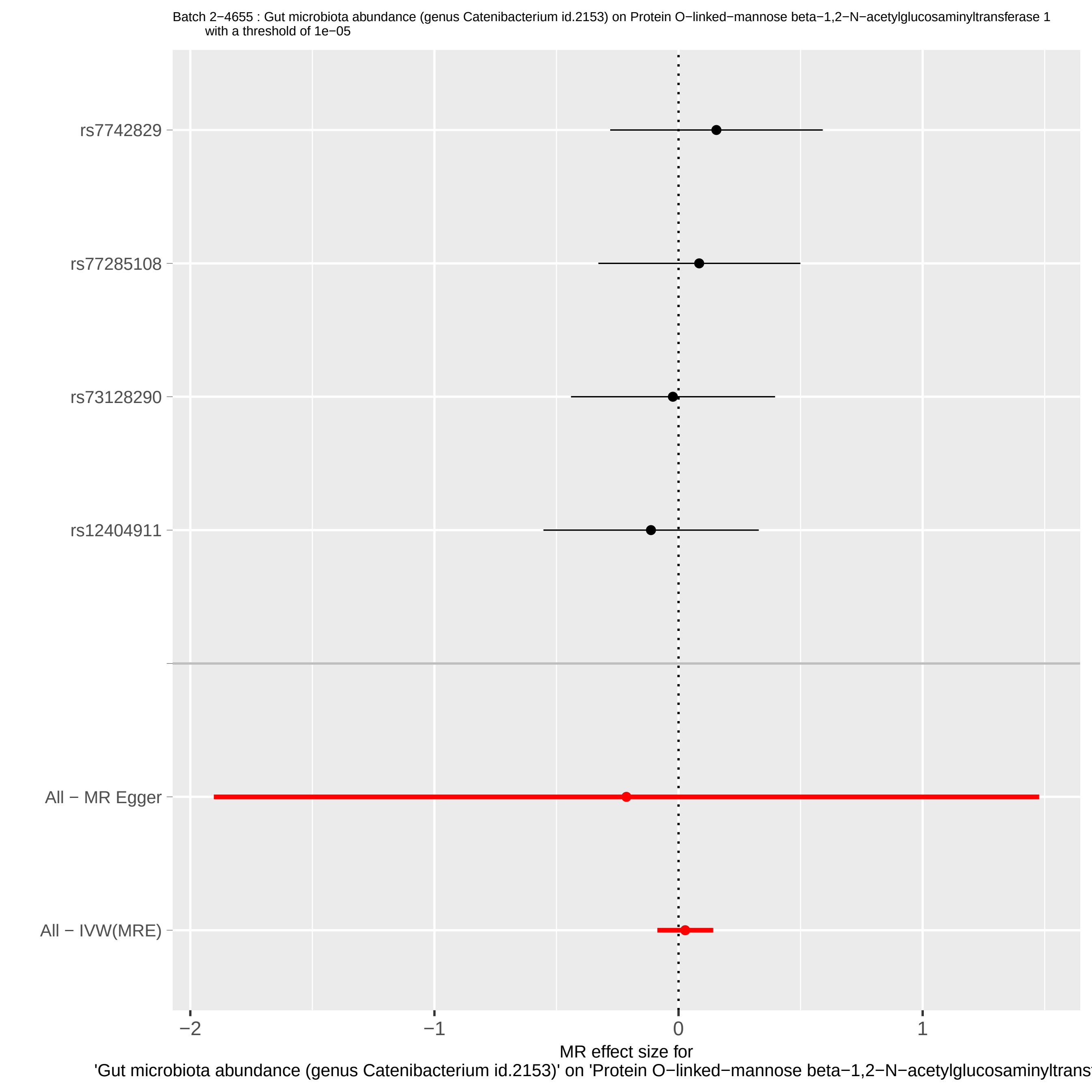

Batch 2−4655 : Gut microbiota abundance (genus Catenibacterium id.2153) on Protein O−linked−mannose beta−1,2−N−acetylglucosaminyltransferase 1
with a threshold of 1e−05
rs7742829
rs77285108
rs73128290
rs12404911
All − MR Egger
All − IVW(MRE)
−2
−1
0
1
MR effect size for
'Gut microbiota abundance (genus Catenibacterium id.2153)' on 'Protein O−linked−mannose beta−1,2−N−acetylglucosaminyltrans

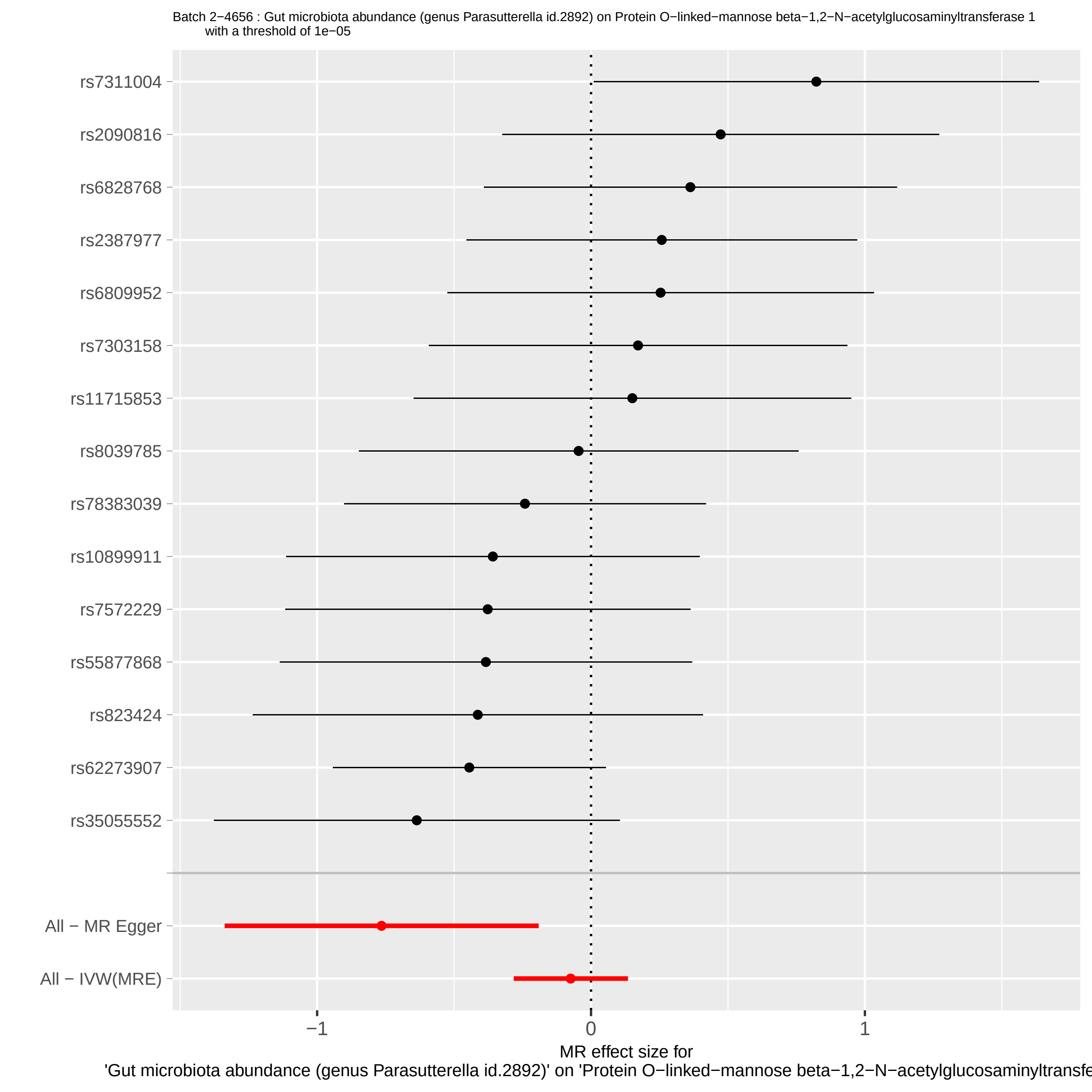

Batch 2−4656 : Gut microbiota abundance (genus Parasutterella id.2892) on Protein O−linked−mannose beta−1,2−N−acetylglucosaminyltransferase 1
with a threshold of 1e−05
rs7311004
rs2090816
rs6828768
rs2387977
rs6809952
rs7303158
rs11715853
rs8039785
rs78383039
rs10899911
rs7572229
rs55877868
rs823424
rs62273907
rs35055552
All − MR Egger
All − IVW(MRE)
−1
0
1
MR effect size for
'Gut microbiota abundance (genus Parasutterella id.2892)' on 'Protein O−linked−mannose beta−1,2−N−acetylglucosaminyltransfe

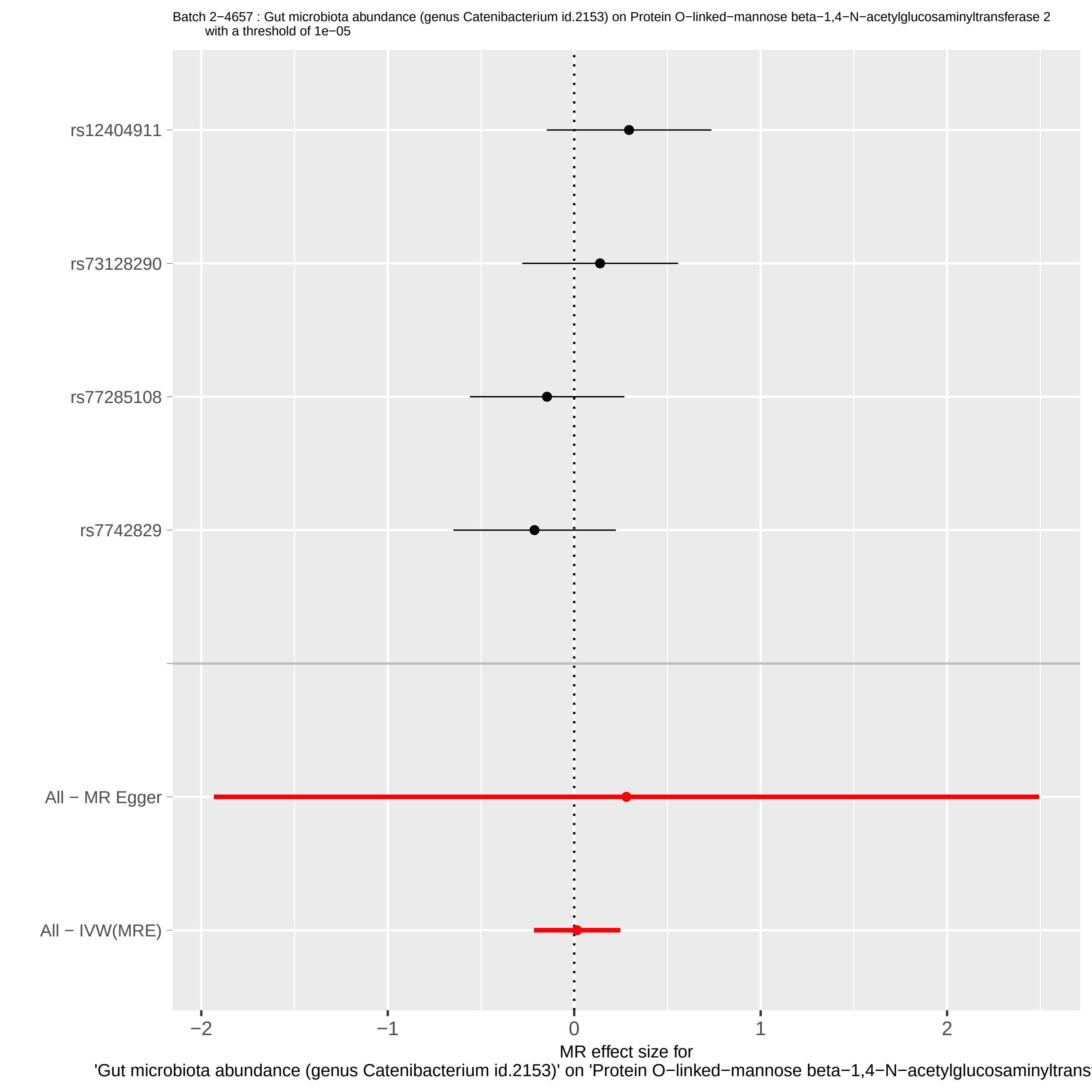

Batch 2−4657 : Gut microbiota abundance (genus Catenibacterium id.2153) on Protein O−linked−mannose beta−1,4−N−acetylglucosaminyltransferase 2
with a threshold of 1e−05
rs12404911
rs73128290
rs77285108
rs7742829
All − MR Egger
All − IVW(MRE)
−2
−1
0
1
2
MR effect size for
'Gut microbiota abundance (genus Catenibacterium id.2153)' on 'Protein O−linked−mannose beta−1,4−N−acetylglucosaminyltransf

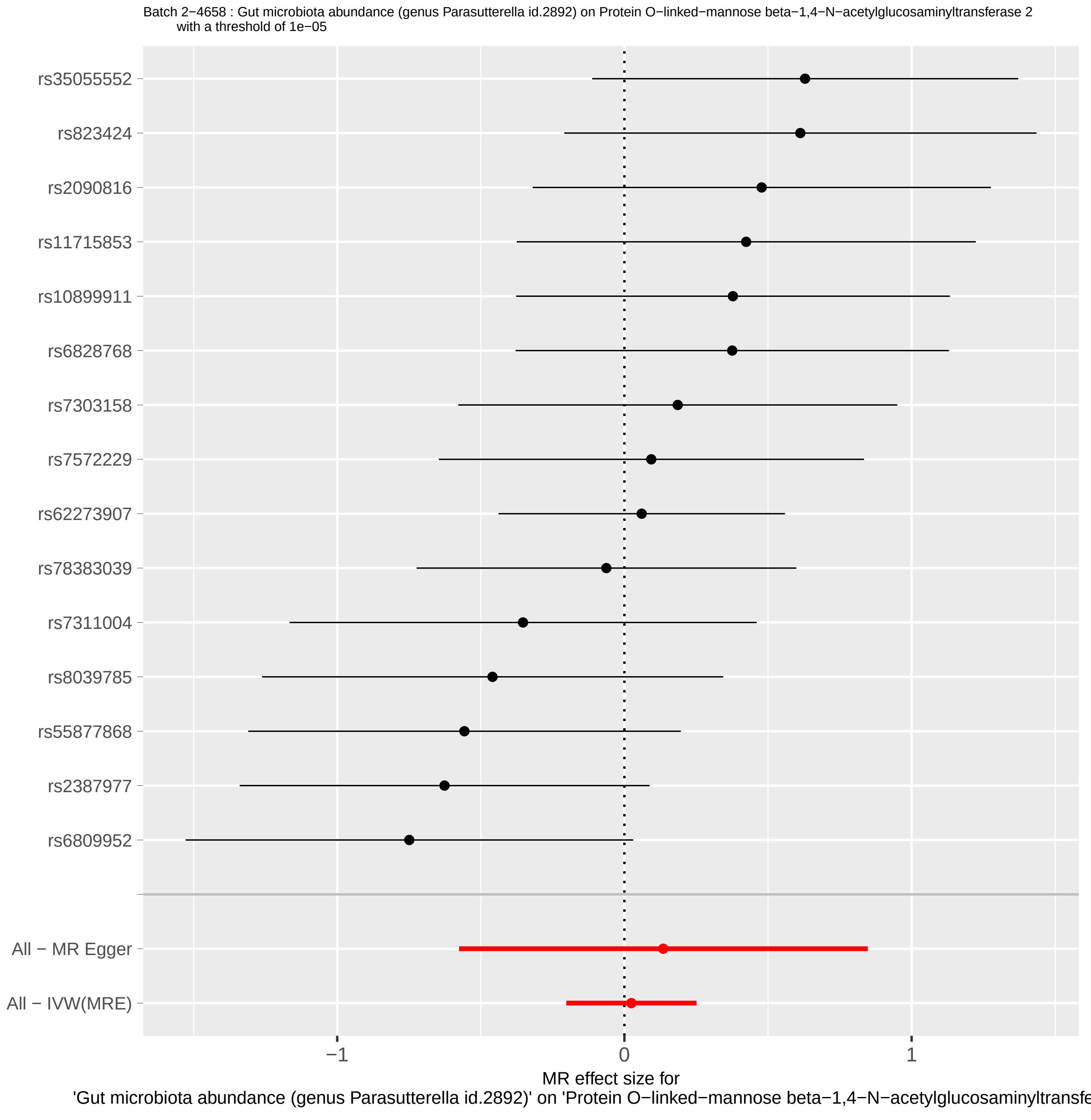

Batch 2−4658 : Gut microbiota abundance (genus Parasutterella id.2892) on Protein O−linked−mannose beta−1,4−N−acetylglucosaminyltransferase 2
with a threshold of 1e−05
rs35055552
rs823424
rs2090816
rs11715853
rs10899911
rs6828768
rs7303158
rs7572229
rs62273907
rs78383039
rs7311004
rs8039785
rs55877868
rs2387977
rs6809952
All − MR Egger
All − IVW(MRE)
−1
0
1
MR effect size for
'Gut microbiota abundance (genus Parasutterella id.2892)' on 'Protein O−linked−mannose beta−1,4−N−acetylglucosaminyltransfe

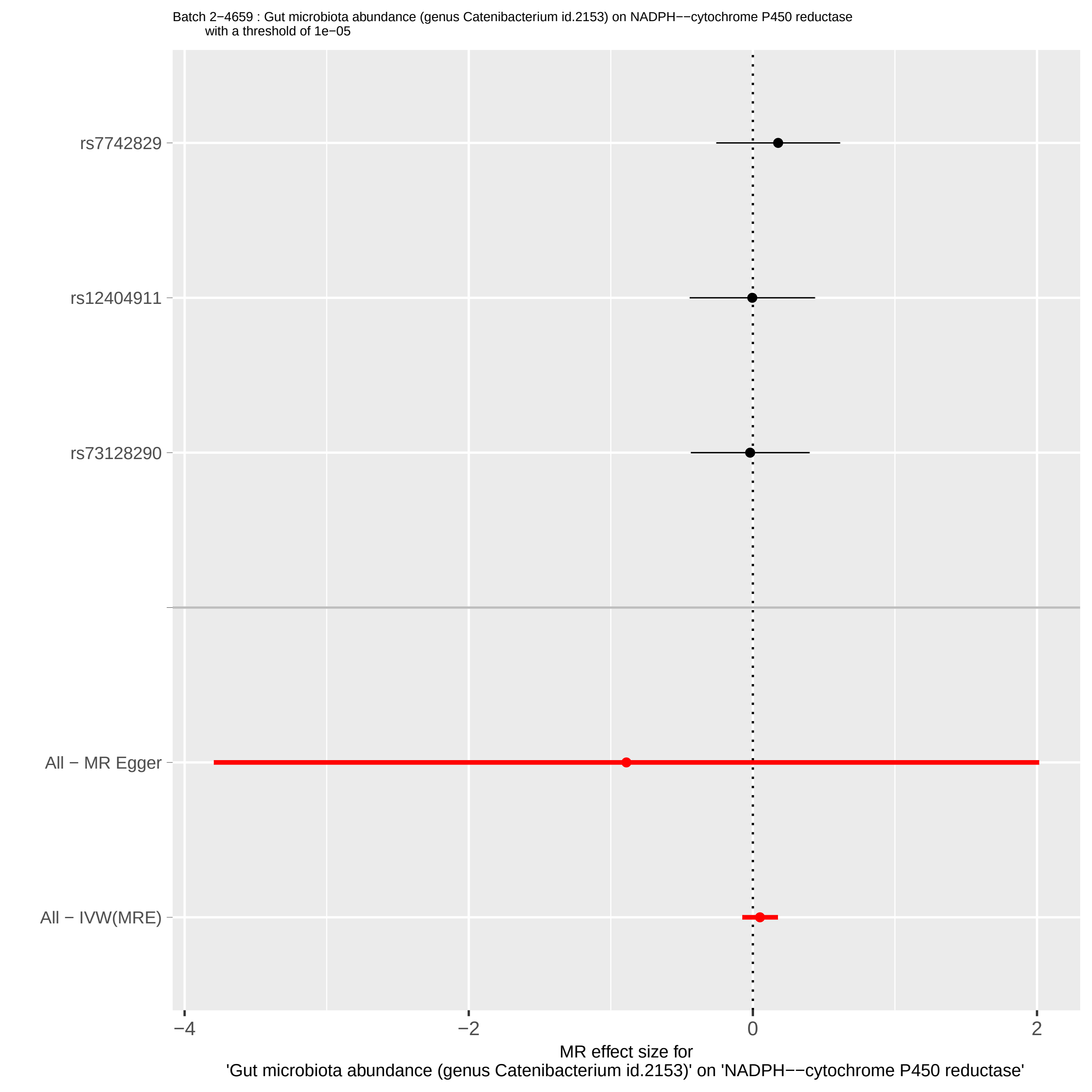

Batch 2−4659 : Gut microbiota abundance (genus Catenibacterium id.2153) on NADPH−−cytochrome P450 reductase
with a threshold of 1e−05
rs7742829
rs12404911
rs73128290
All − MR Egger
All − IVW(MRE)
−4
−2
0
2
MR effect size for
'Gut microbiota abundance (genus Catenibacterium id.2153)' on 'NADPH−−cytochrome P450 reductase'

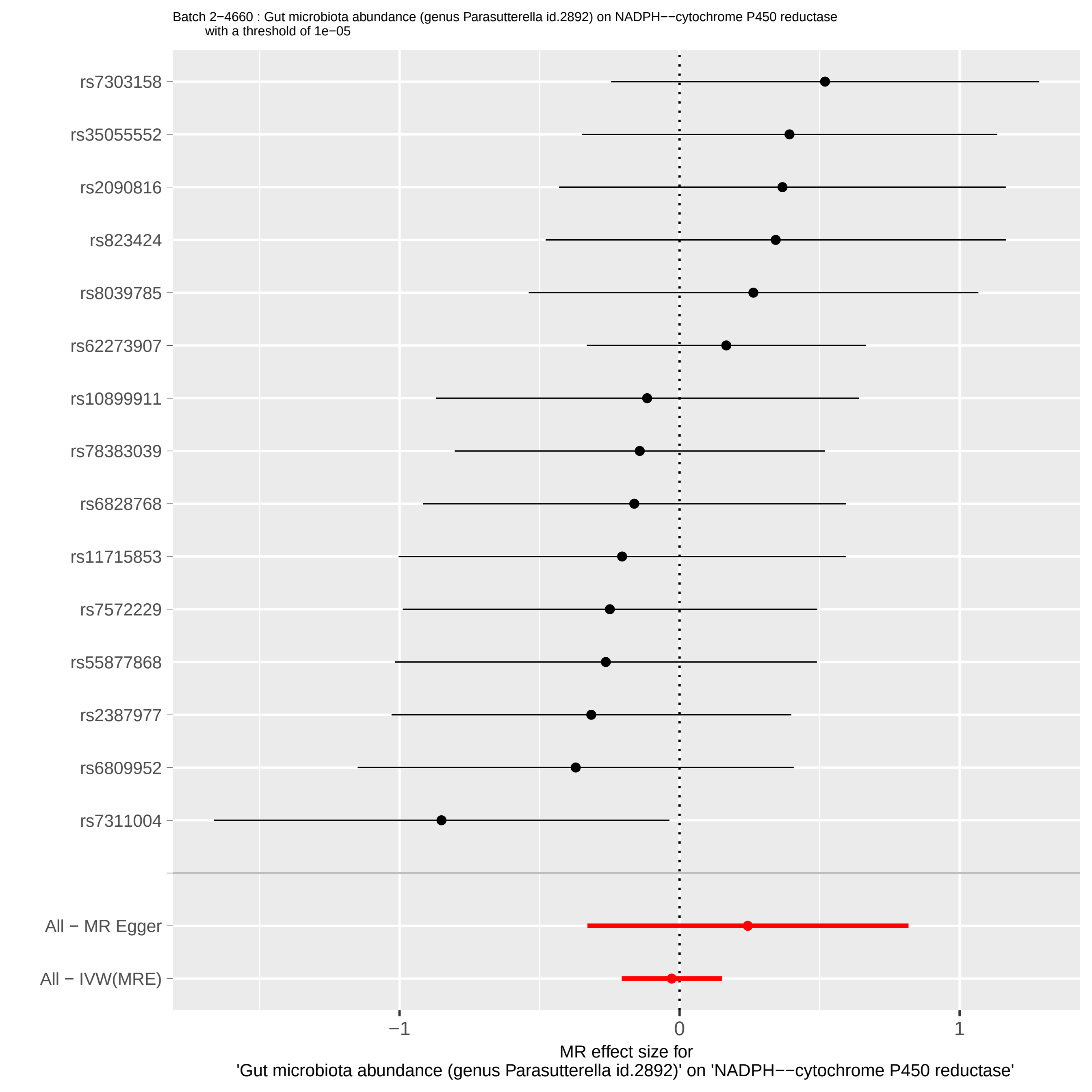

Batch 2−4660 : Gut microbiota abundance (genus Parasutterella id.2892) on NADPH−−cytochrome P450 reductase
with a threshold of 1e−05
rs7303158
rs35055552
rs2090816
rs823424
rs8039785
rs62273907
rs10899911
rs78383039
rs6828768
rs11715853
rs7572229
rs55877868
rs2387977
rs6809952
rs7311004
All − MR Egger
All − IVW(MRE)
−1
0
1
MR effect size for
'Gut microbiota abundance (genus Parasutterella id.2892)' on 'NADPH−−cytochrome P450 reductase'

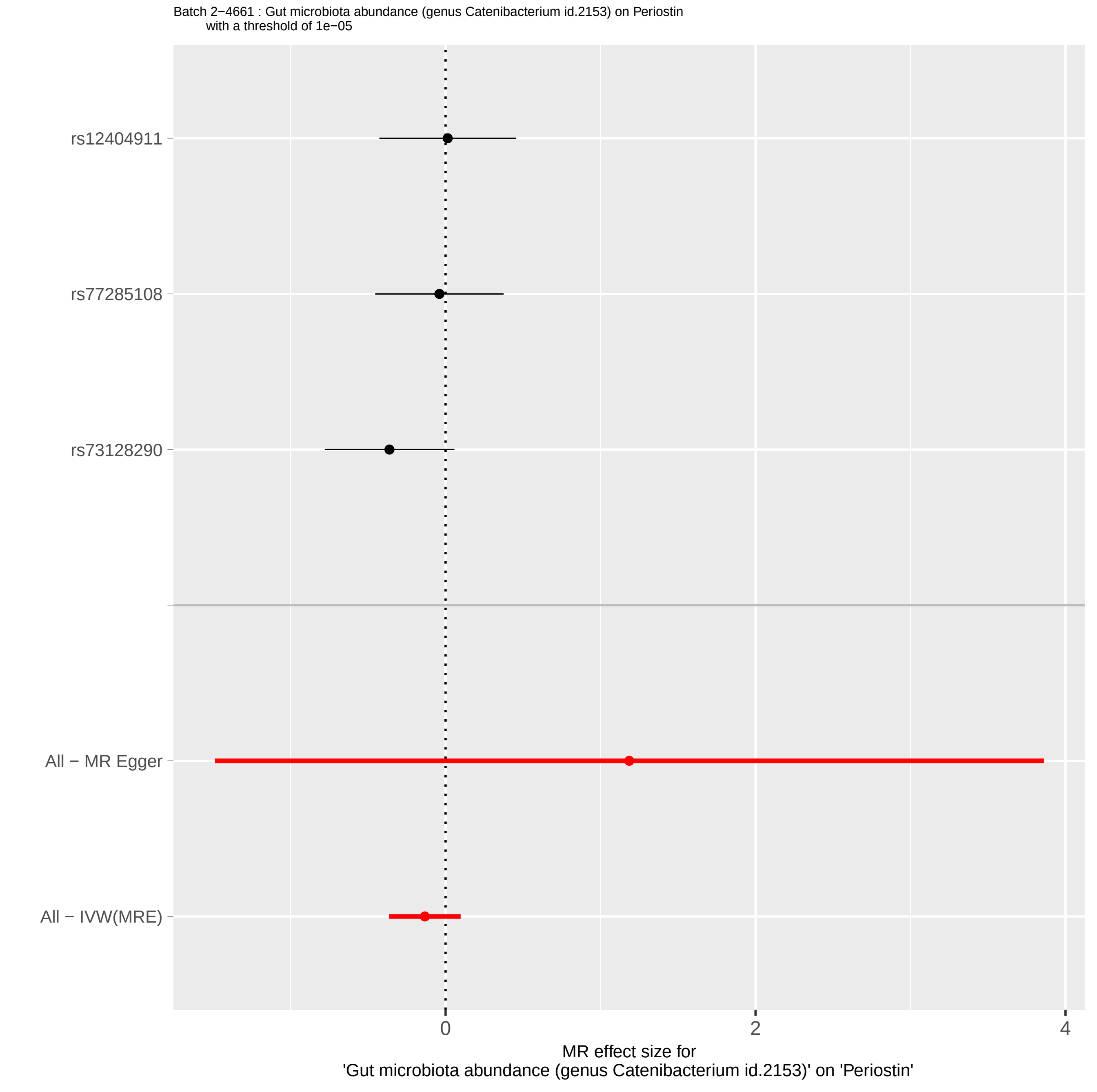
Batch 2−4661 : Gut microbiota abundance (genus Catenibacterium id.2153) on Periostin
with a threshold of 1e−05
rs12404911
rs77285108
rs73128290
All − MR Egger
All − IVW(MRE)
0
2
4
MR effect size for
'Gut microbiota abundance (genus Catenibacterium id.2153)' on 'Periostin'

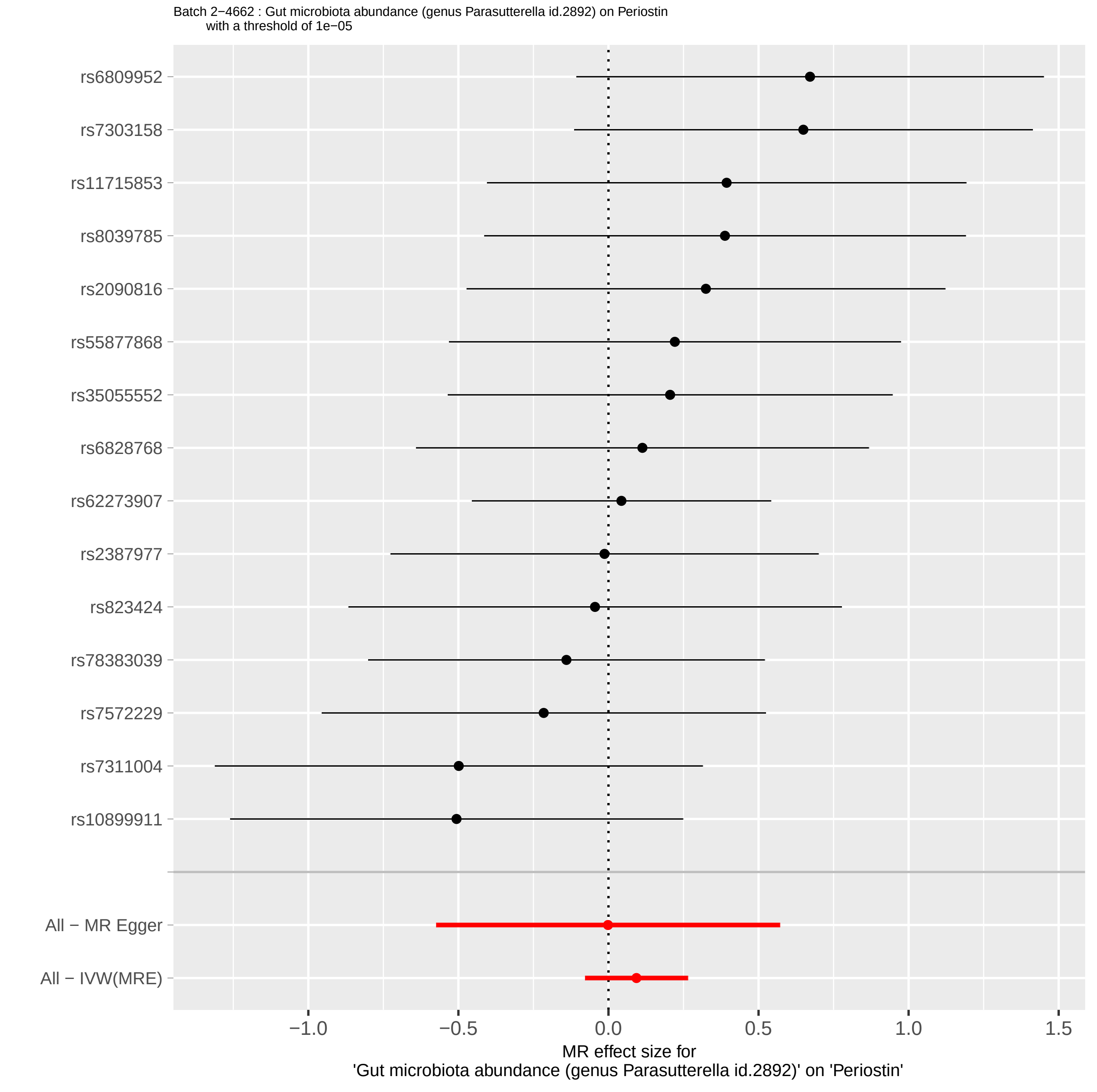

Batch 2−4662 : Gut microbiota abundance (genus Parasutterella id.2892) on Periostin
with a threshold of 1e−05
rs6809952
rs7303158
rs11715853
rs8039785
rs2090816
rs55877868
rs35055552
rs6828768
rs62273907
rs2387977
rs823424
rs78383039
rs7572229
rs7311004
rs10899911
All − MR Egger
All − IVW(MRE)
−1.0
−0.5
0.0
0.5
1.0
1.5
MR effect size for
'Gut microbiota abundance (genus Parasutterella id.2892)' on 'Periostin'

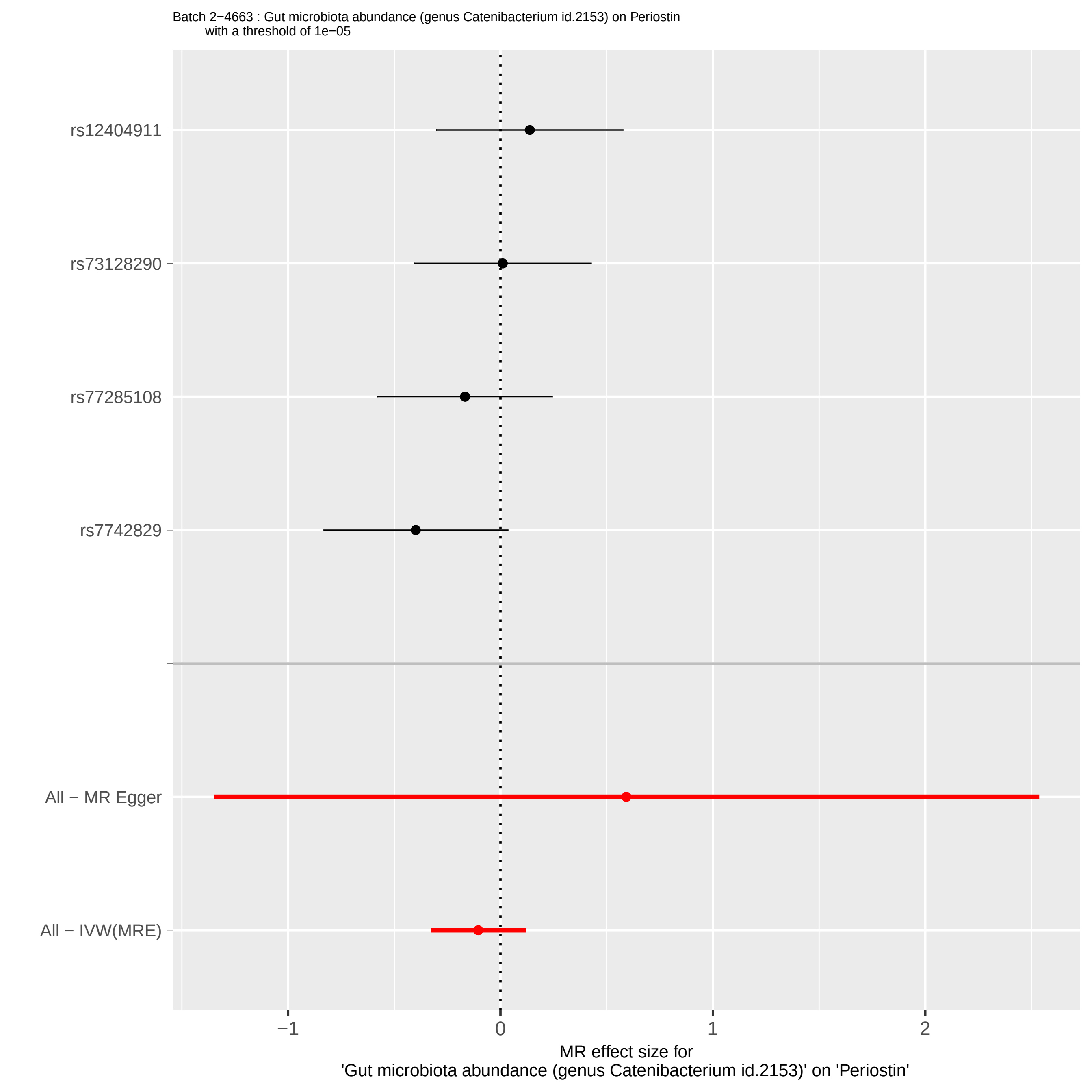

Batch 2−4663 : Gut microbiota abundance (genus Catenibacterium id.2153) on Periostin
with a threshold of 1e−05
rs12404911
rs73128290
rs77285108
rs7742829
All − MR Egger
All − IVW(MRE)
−1
0
1
2
MR effect size for
'Gut microbiota abundance (genus Catenibacterium id.2153)' on 'Periostin'

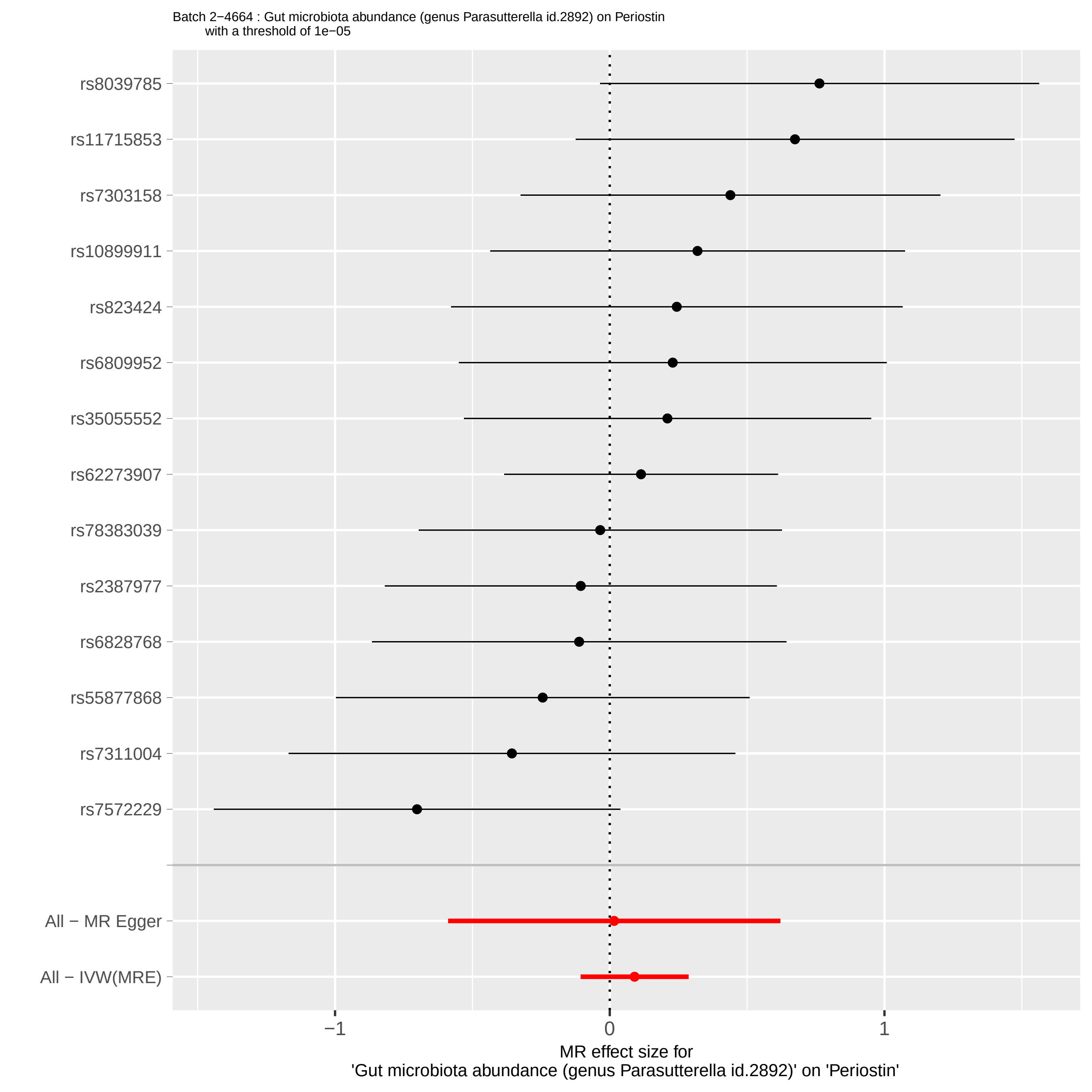
Batch 2−4664 : Gut microbiota abundance (genus Parasutterella id.2892) on Periostin
with a threshold of 1e−05
rs8039785
rs11715853
rs7303158
rs10899911
rs823424
rs6809952
rs35055552
rs62273907
rs78383039
rs2387977
rs6828768
rs55877868
rs7311004
rs7572229
All − MR Egger
All − IVW(MRE)
−1
0
1
MR effect size for
'Gut microbiota abundance (genus Parasutterella id.2892)' on 'Periostin'

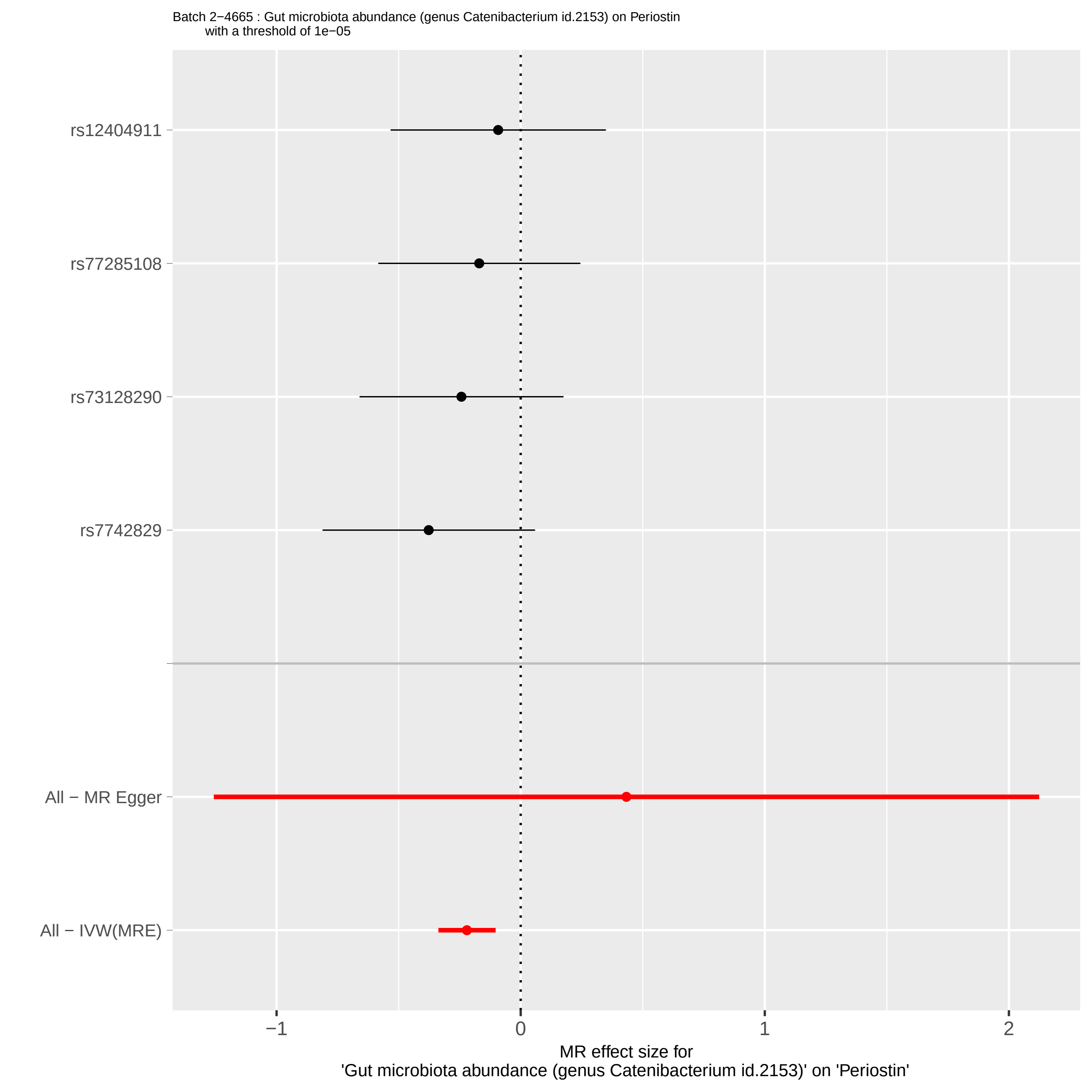

Batch 2−4665 : Gut microbiota abundance (genus Catenibacterium id.2153) on Periostin
with a threshold of 1e−05
rs12404911
rs77285108
rs73128290
rs7742829
All − MR Egger
All − IVW(MRE)
−1
0
1
2
MR effect size for
'Gut microbiota abundance (genus Catenibacterium id.2153)' on 'Periostin'

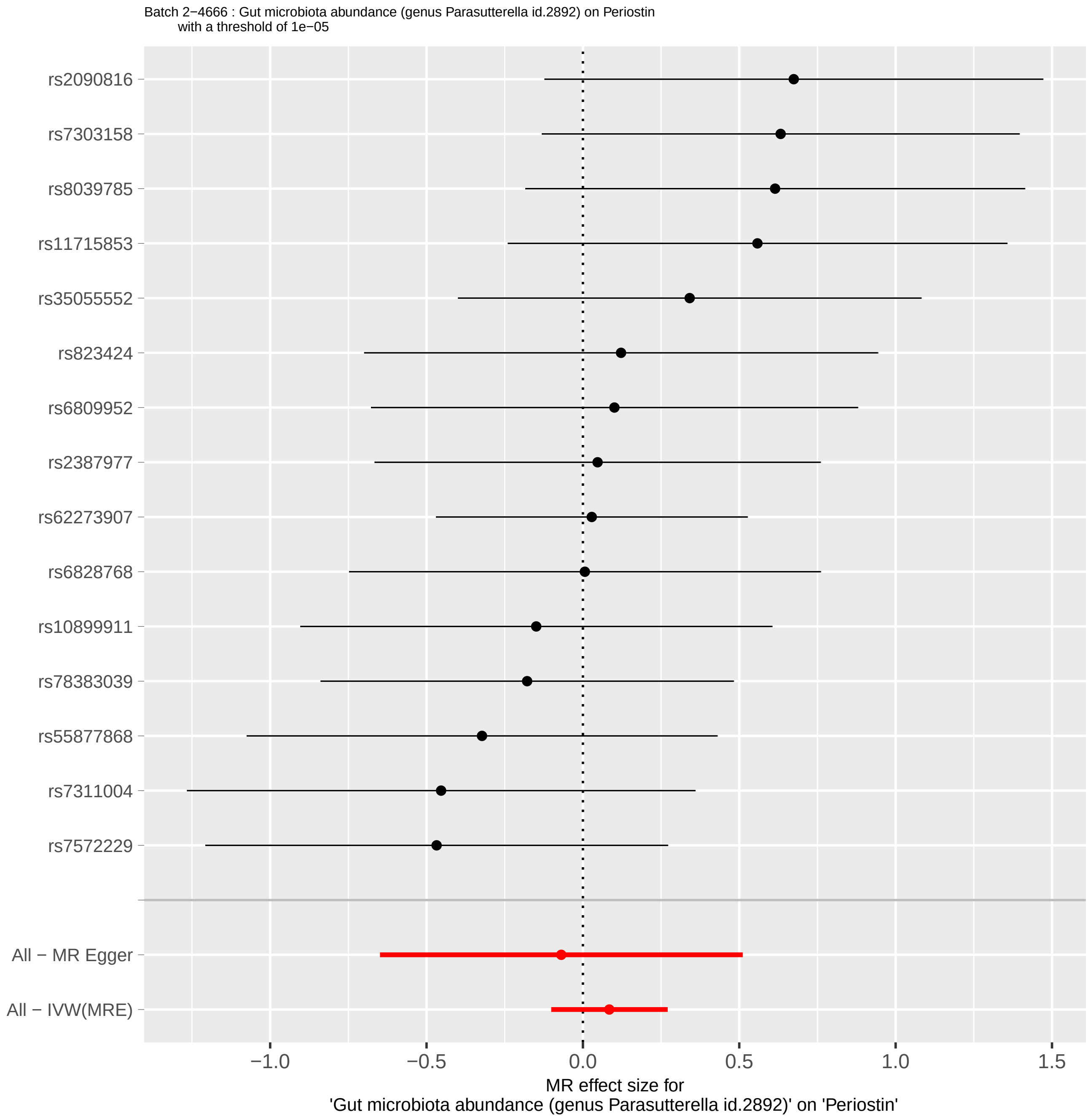

Batch 2−4666 : Gut microbiota abundance (genus Parasutterella id.2892) on Periostin
with a threshold of 1e−05
rs2090816
rs7303158
rs8039785
rs11715853
rs35055552
rs823424
rs6809952
rs2387977
rs62273907
rs6828768
rs10899911
rs78383039
rs55877868
rs7311004
rs7572229
All − MR Egger
All − IVW(MRE)
−1.0
−0.5
0.0
0.5
1.0
1.5
MR effect size for
'Gut microbiota abundance (genus Parasutterella id.2892)' on 'Periostin'

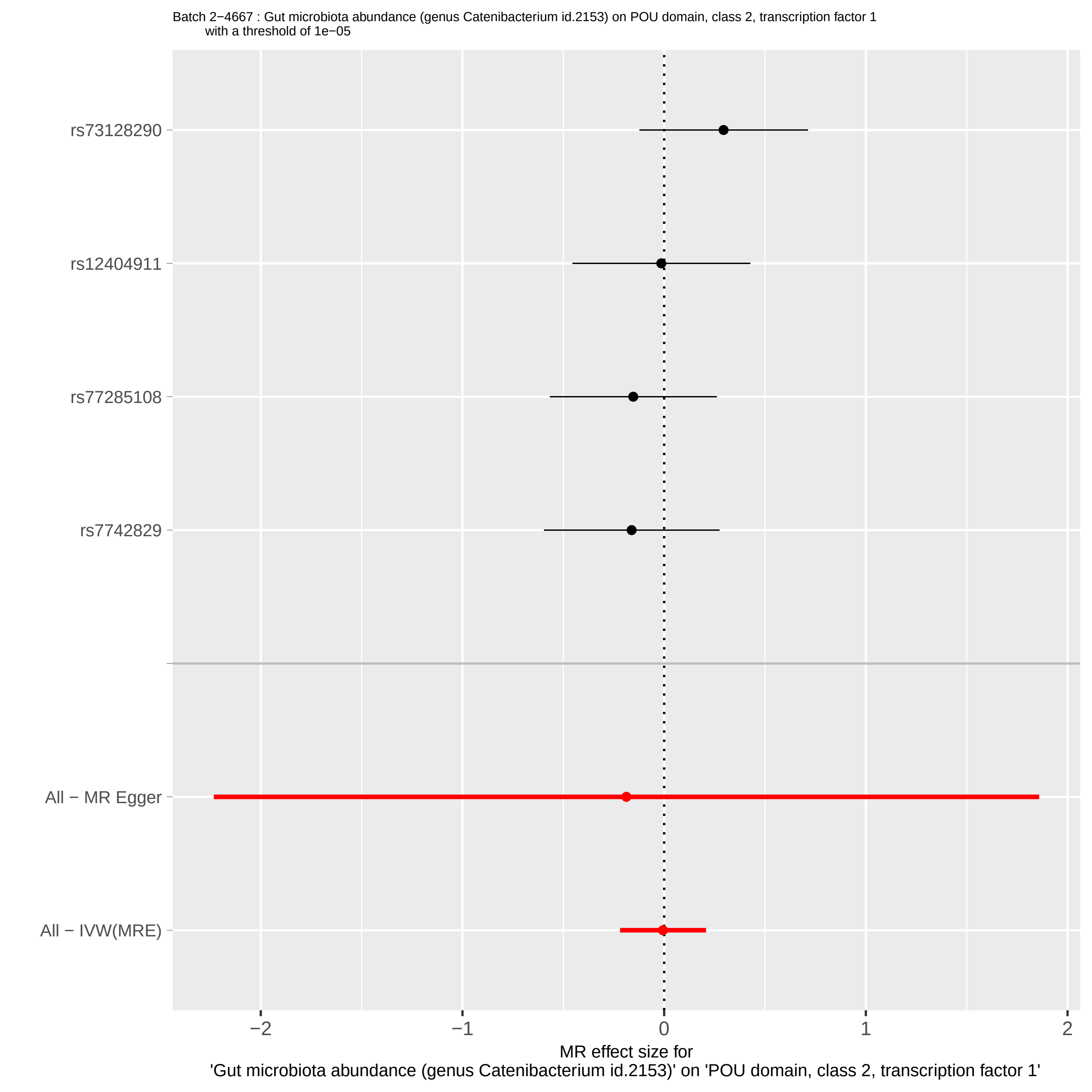
Batch 2−4667 : Gut microbiota abundance (genus Catenibacterium id.2153) on POU domain, class 2, transcription factor 1
with a threshold of 1e−05
rs73128290
rs12404911
rs77285108
rs7742829
All − MR Egger
All − IVW(MRE)
−2
−1
0
1
2
MR effect size for
'Gut microbiota abundance (genus Catenibacterium id.2153)' on 'POU domain, class 2, transcription factor 1'

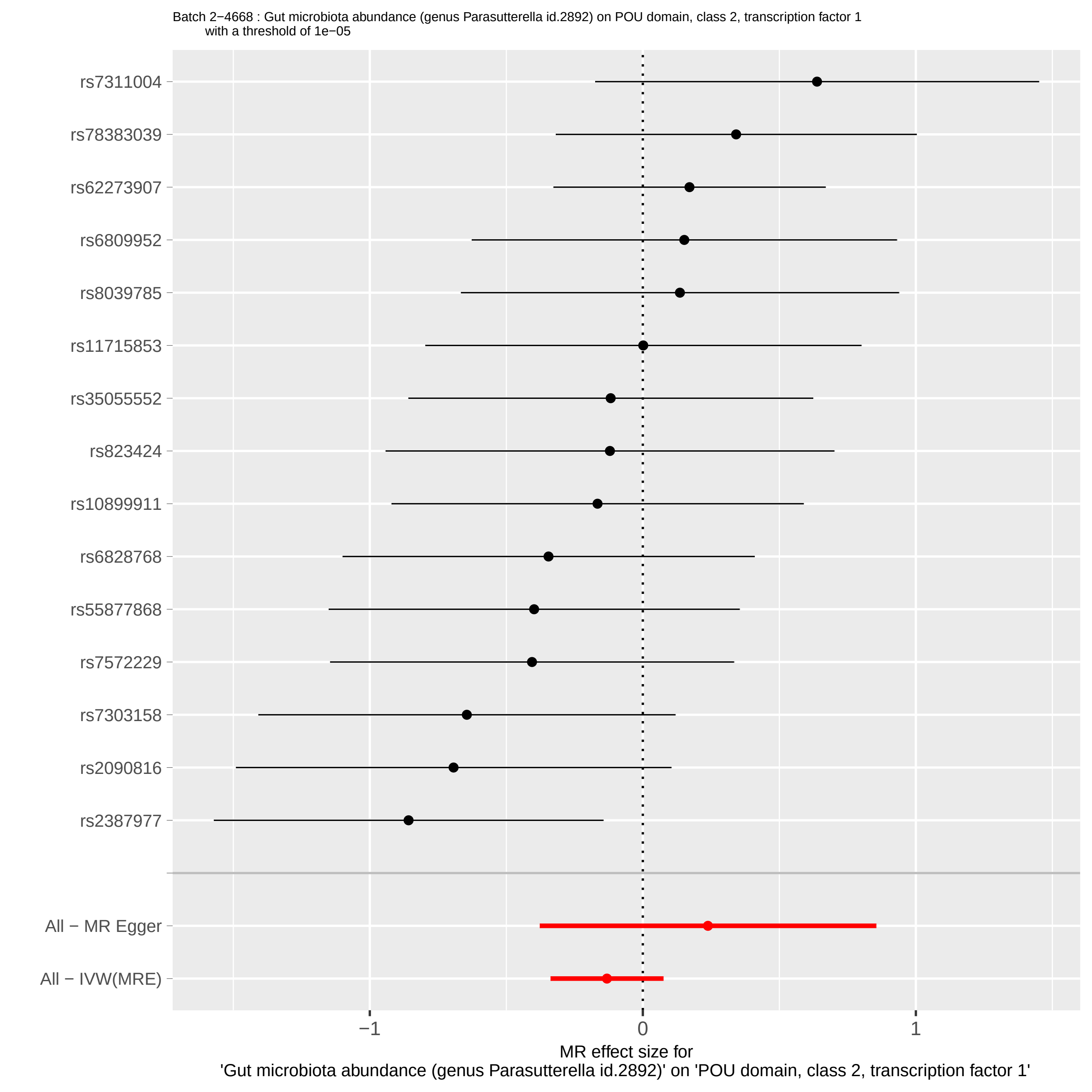

Batch 2−4668 : Gut microbiota abundance (genus Parasutterella id.2892) on POU domain, class 2, transcription factor 1
with a threshold of 1e−05
rs7311004
rs78383039
rs62273907
rs6809952
rs8039785
rs11715853
rs35055552
rs823424
rs10899911
rs6828768
rs55877868
rs7572229
rs7303158
rs2090816
rs2387977
All − MR Egger
All − IVW(MRE)
−1
0
1
MR effect size for
'Gut microbiota abundance (genus Parasutterella id.2892)' on 'POU domain, class 2, transcription factor 1'

Batch 2−4669 : Gut microbiota abundance (genus Catenibacterium id.2153) on Phosphatidate phosphatase PPAPDC1A
with a threshold of 1e−05

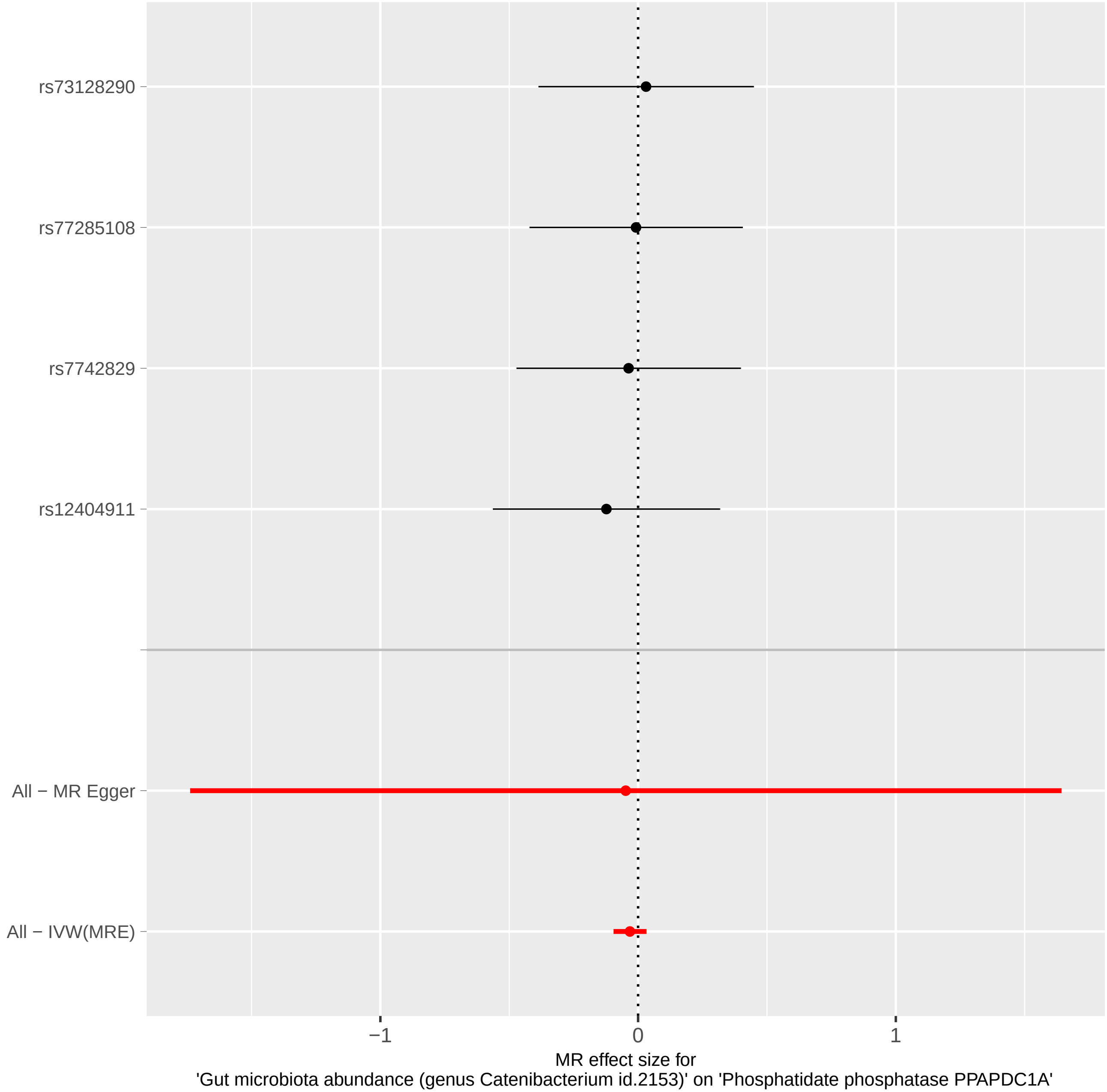

Batch 2−4670 : Gut microbiota abundance (genus Parasutterella id.2892) on Phosphatidate phosphatase PPAPDC1A
with a threshold of 1e−05

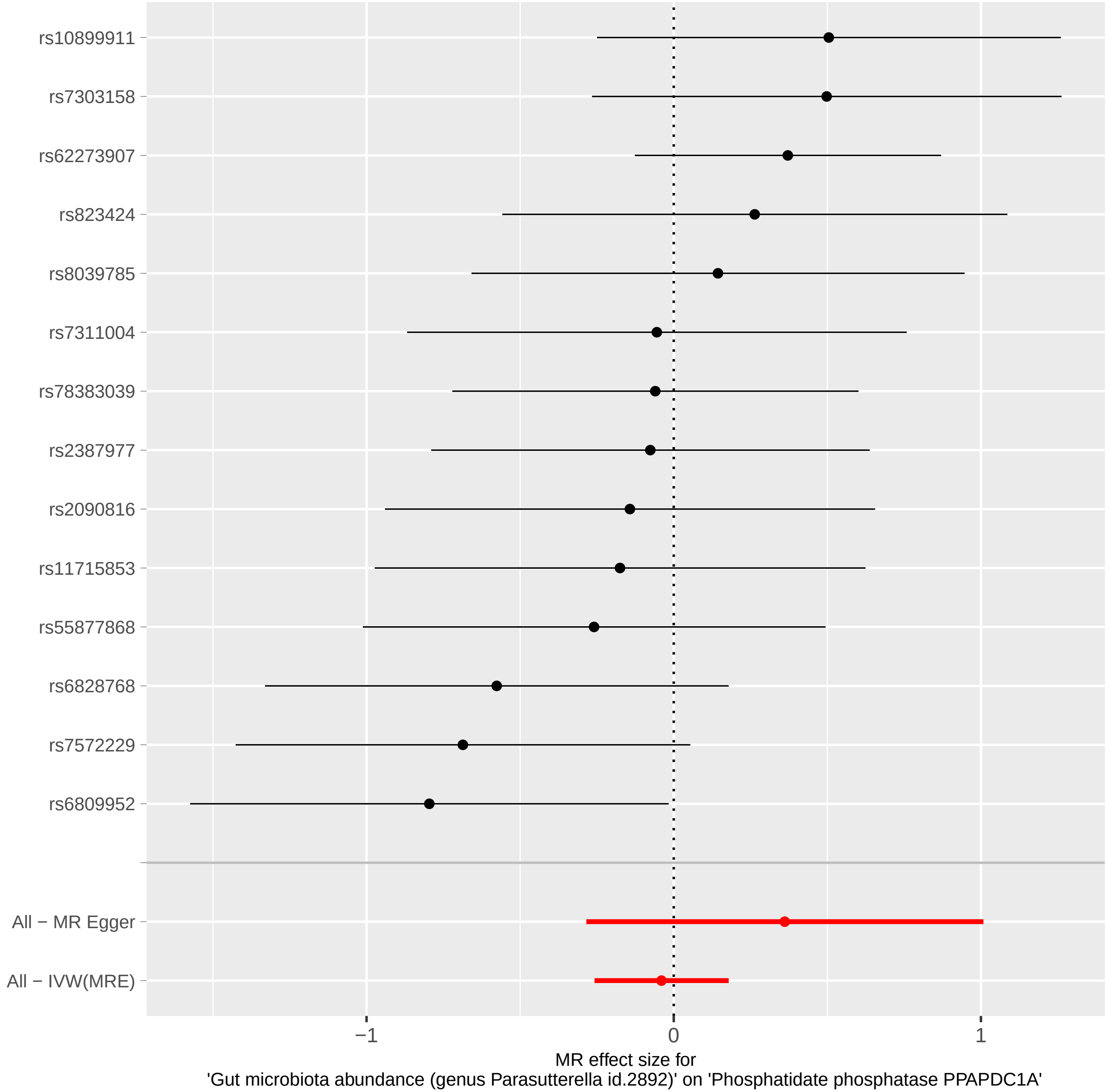

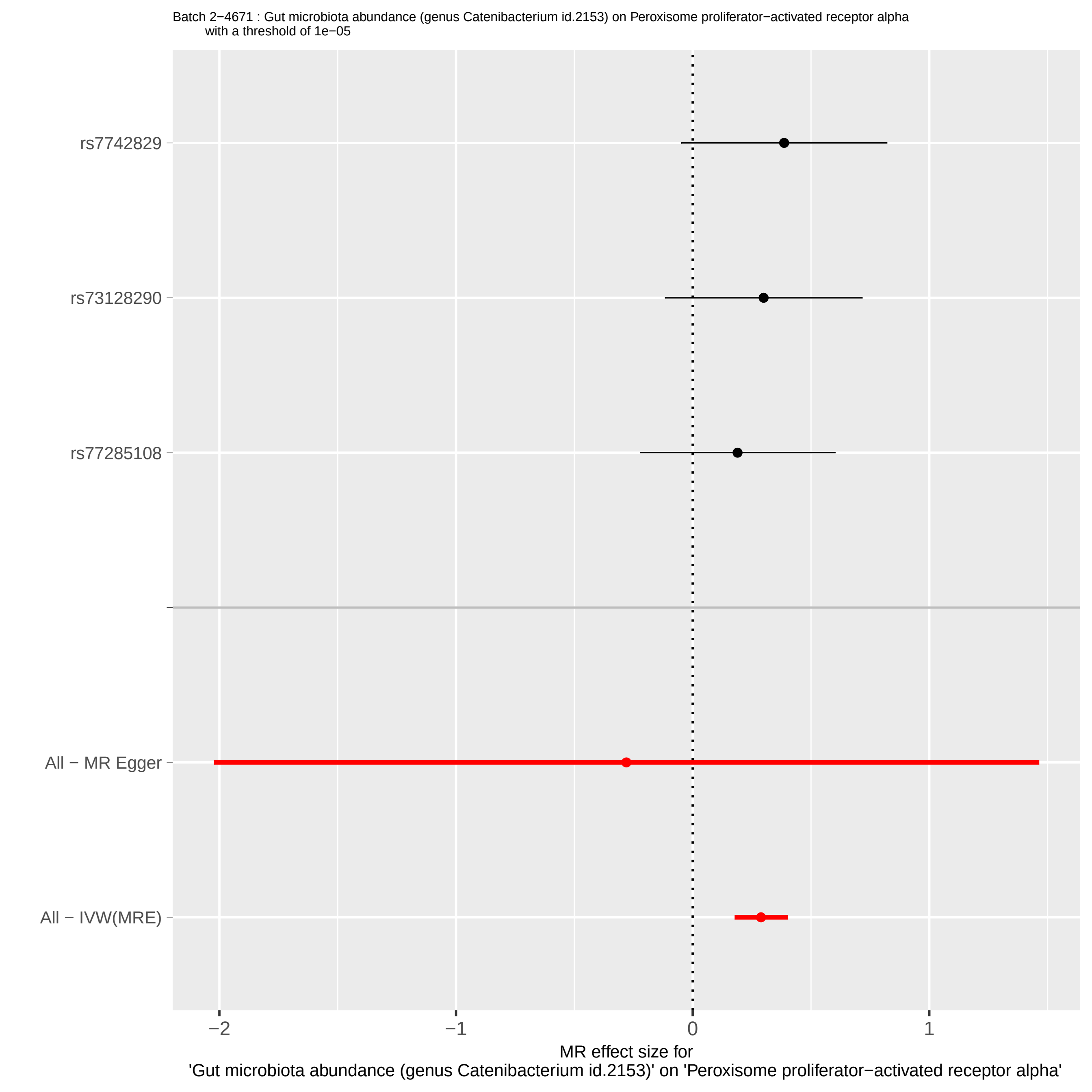

Batch 2−4671 : Gut microbiota abundance (genus Catenibacterium id.2153) on Peroxisome proliferator−activated receptor alpha
with a threshold of 1e−05
rs7742829
rs73128290
rs77285108
All − MR Egger
All − IVW(MRE)
−2
−1
0
1
MR effect size for
'Gut microbiota abundance (genus Catenibacterium id.2153)' on 'Peroxisome proliferator−activated receptor alpha'

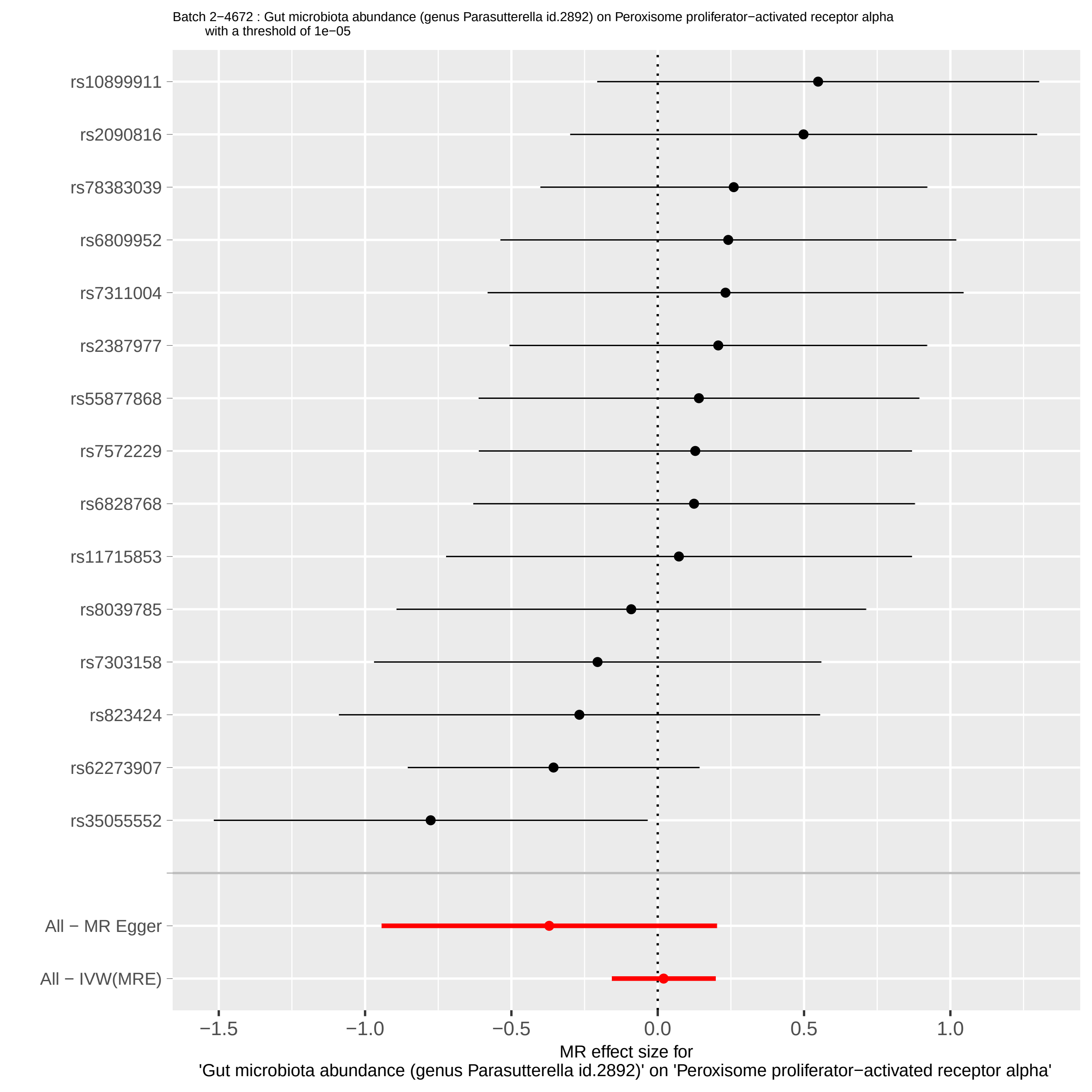

Batch 2−4672 : Gut microbiota abundance (genus Parasutterella id.2892) on Peroxisome proliferator−activated receptor alpha
with a threshold of 1e−05
rs10899911
rs2090816
rs78383039
rs6809952
rs7311004
rs2387977
rs55877868
rs7572229
rs6828768
rs11715853
rs8039785
rs7303158
rs823424
rs62273907
rs35055552
All − MR Egger
All − IVW(MRE)
−1.5
−1.0
−0.5
0.0
0.5
1.0
MR effect size for
'Gut microbiota abundance (genus Parasutterella id.2892)' on 'Peroxisome proliferator−activated receptor alpha'

Batch 2−4673 : Gut microbiota abundance (genus Catenibacterium id.2153) on Neutrophil−activating peptide 2
with a threshold of 1e−05

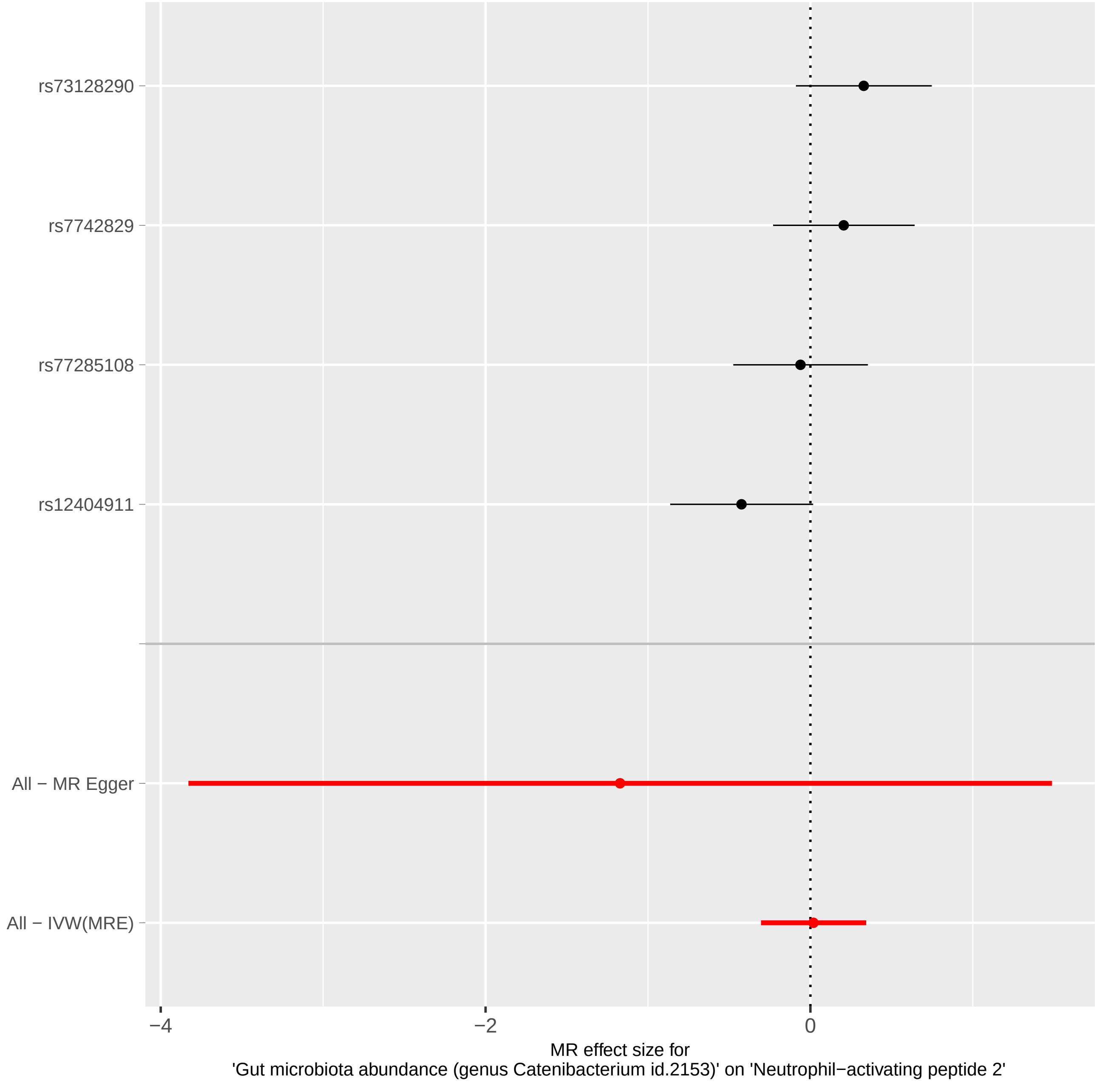

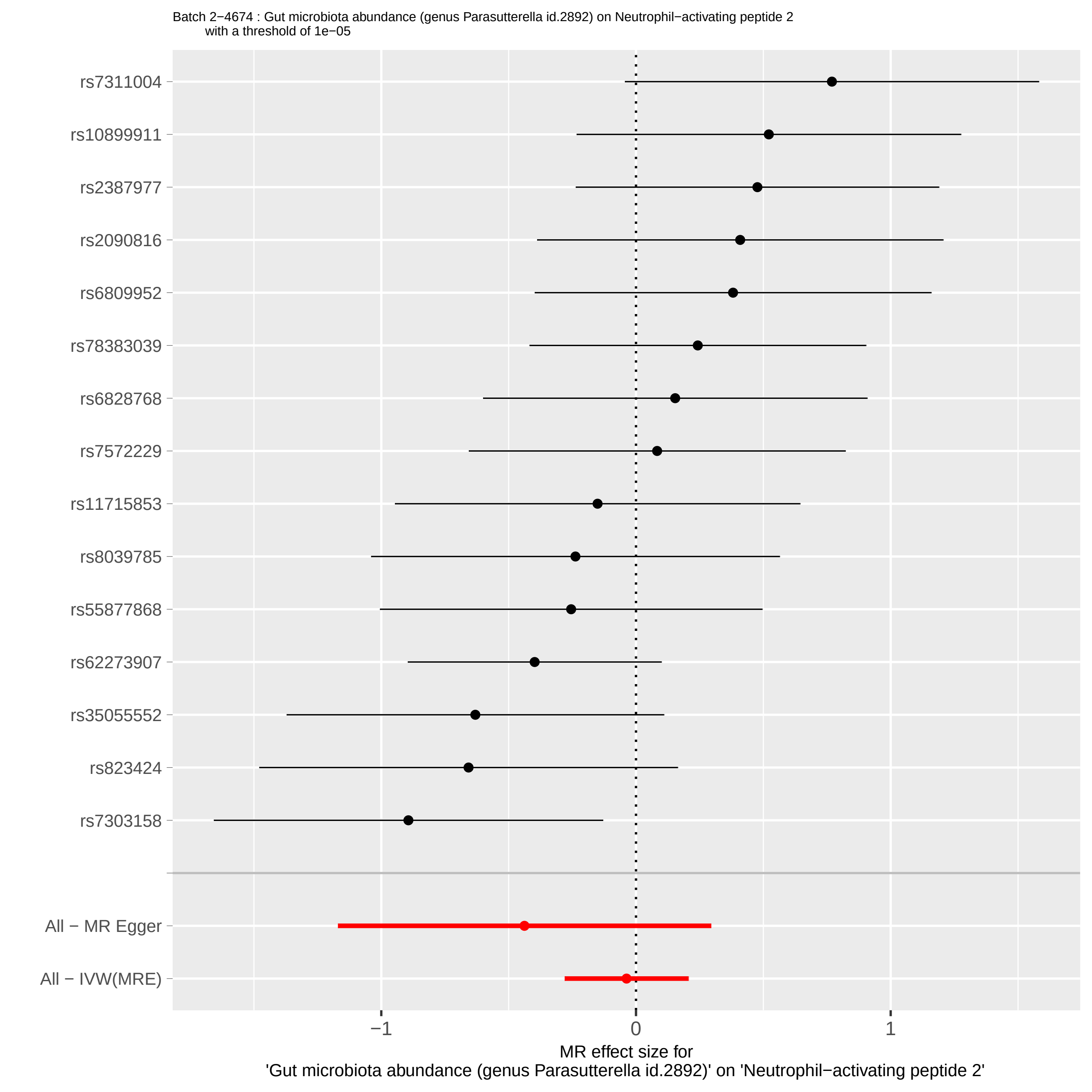
Batch 2−4674 : Gut microbiota abundance (genus Parasutterella id.2892) on Neutrophil−activating peptide 2
with a threshold of 1e−05
rs7311004
rs10899911
rs2387977
rs2090816
rs6809952
rs78383039
rs6828768
rs7572229
rs11715853
rs8039785
rs55877868
rs62273907
rs35055552
rs823424
rs7303158
All − MR Egger
All − IVW(MRE)
−1
0
1
MR effect size for
'Gut microbiota abundance (genus Parasutterella id.2892)' on 'Neutrophil−activating peptide 2'

Batch 2−4675 : Gut microbiota abundance (genus Catenibacterium id.2153) on Phosphopantothenoylcysteine decarboxylase
with a threshold of 1e−05

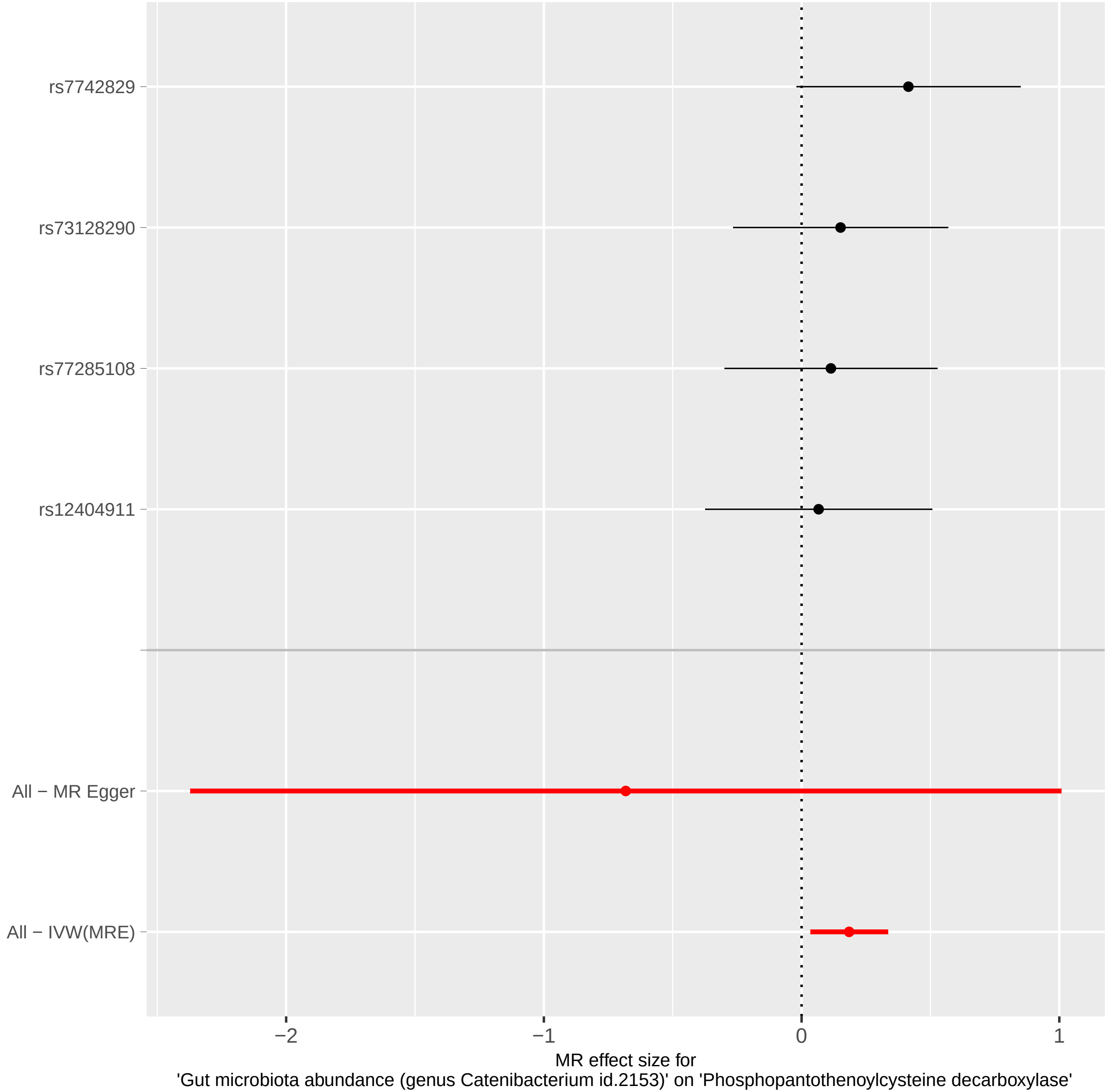

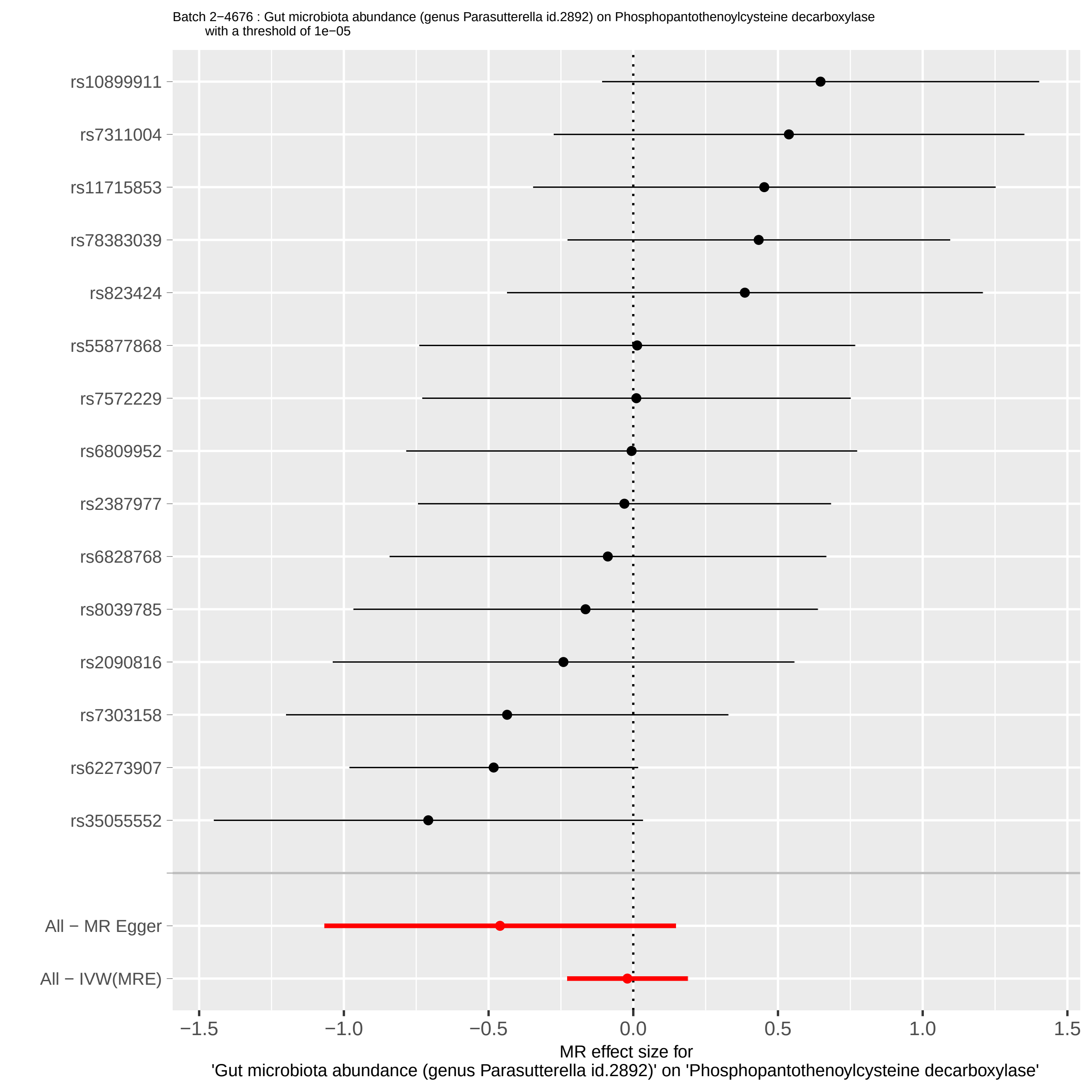
Batch 2−4676 : Gut microbiota abundance (genus Parasutterella id.2892) on Phosphopantothenoylcysteine decarboxylase
with a threshold of 1e−05
rs10899911
rs7311004
rs11715853
rs78383039
rs823424
rs55877868
rs7572229
rs6809952
rs2387977
rs6828768
rs8039785
rs2090816
rs7303158
rs62273907
rs35055552
All − MR Egger
All − IVW(MRE)
−1.5
−1.0
−0.5
0.0
0.5
1.0
1.5
MR effect size for
'Gut microbiota abundance (genus Parasutterella id.2892)' on 'Phosphopantothenoylcysteine decarboxylase'

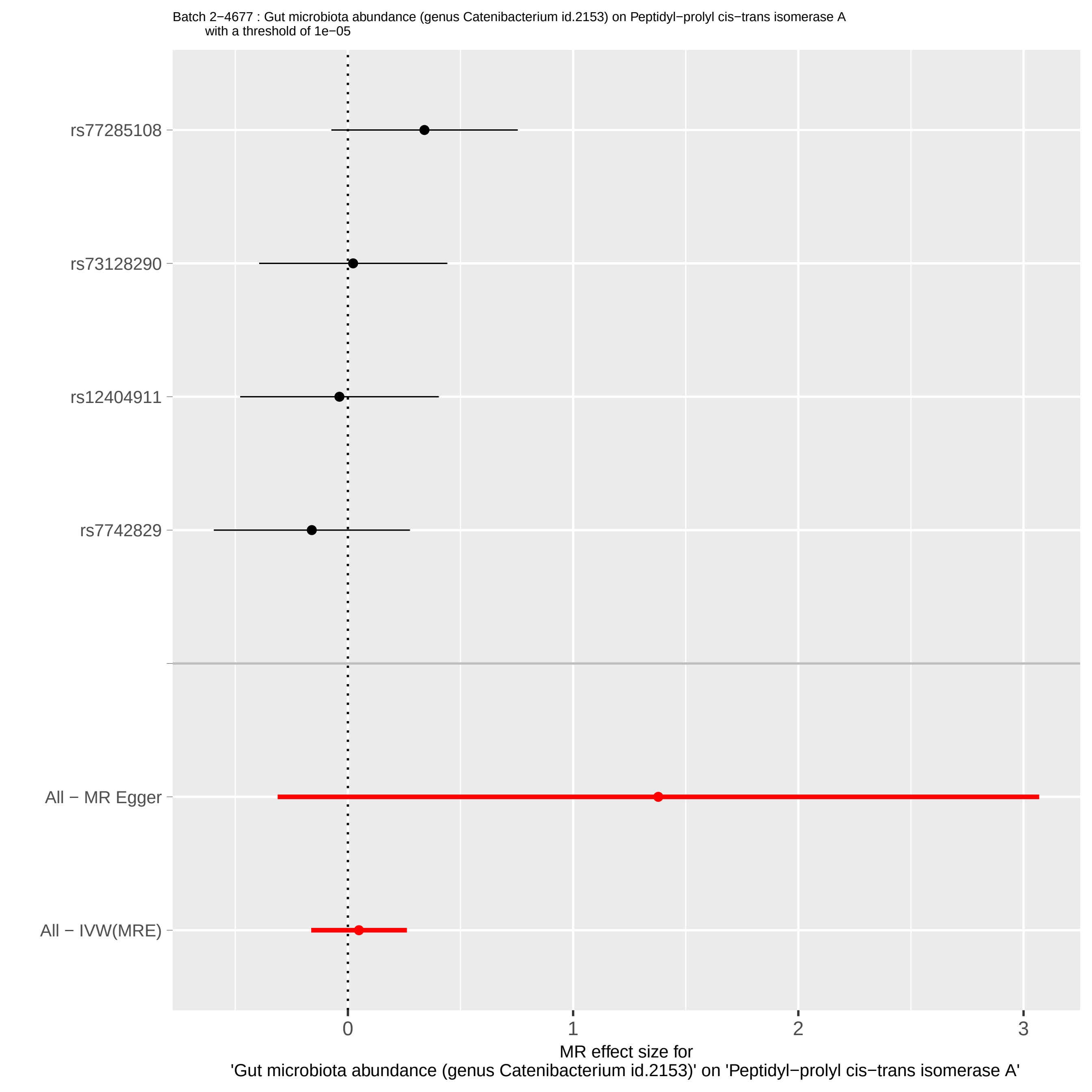

Batch 2−4677 : Gut microbiota abundance (genus Catenibacterium id.2153) on Peptidyl−prolyl cis−trans isomerase A
with a threshold of 1e−05
rs77285108
rs73128290
rs12404911
rs7742829
All − MR Egger
All − IVW(MRE)
0
1
2
3
MR effect size for
'Gut microbiota abundance (genus Catenibacterium id.2153)' on 'Peptidyl−prolyl cis−trans isomerase A'

Batch 2−4678 : Gut microbiota abundance (genus Parasutterella id.2892) on Peptidyl−prolyl cis−trans isomerase A
with a threshold of 1e−05

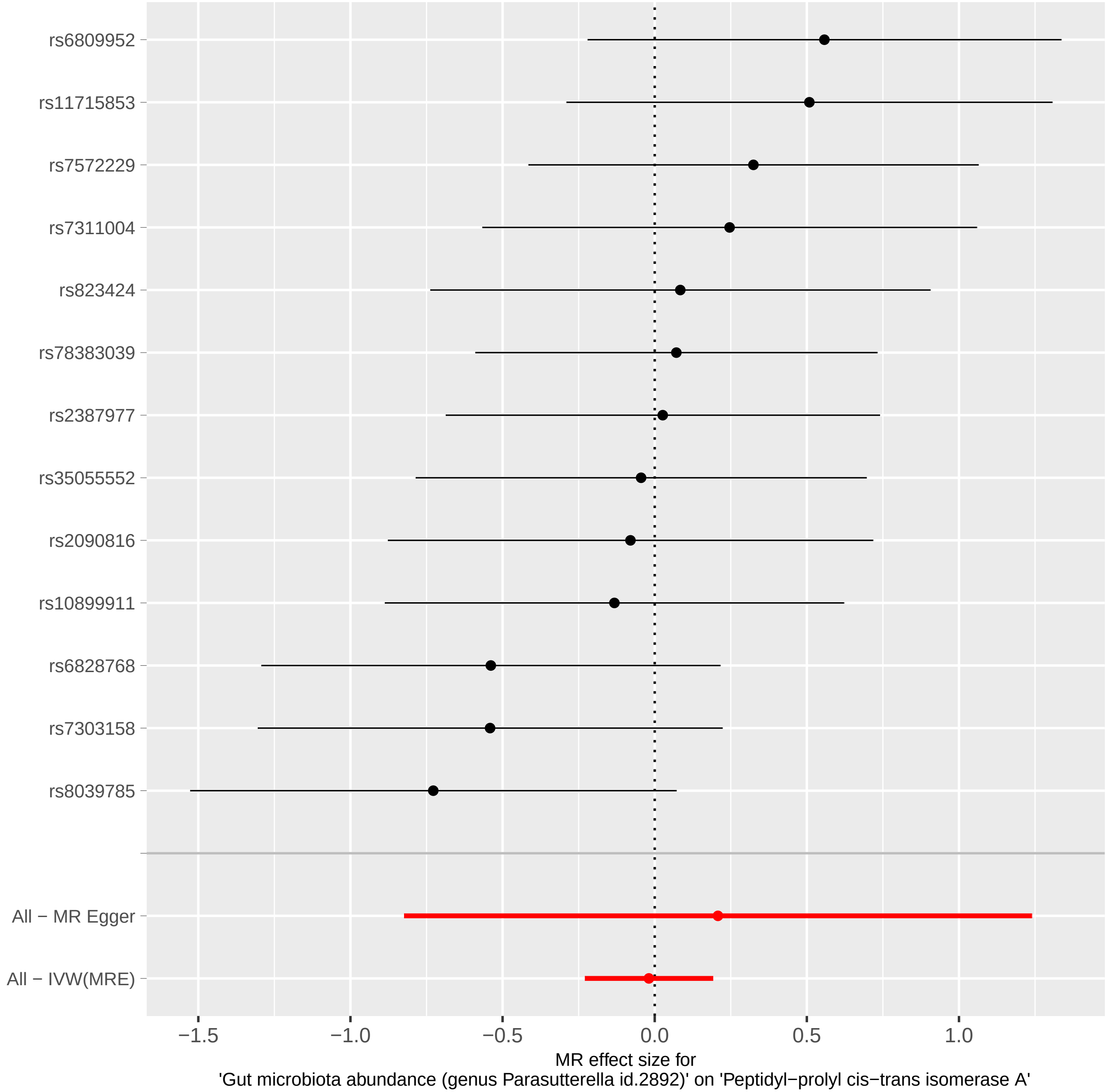

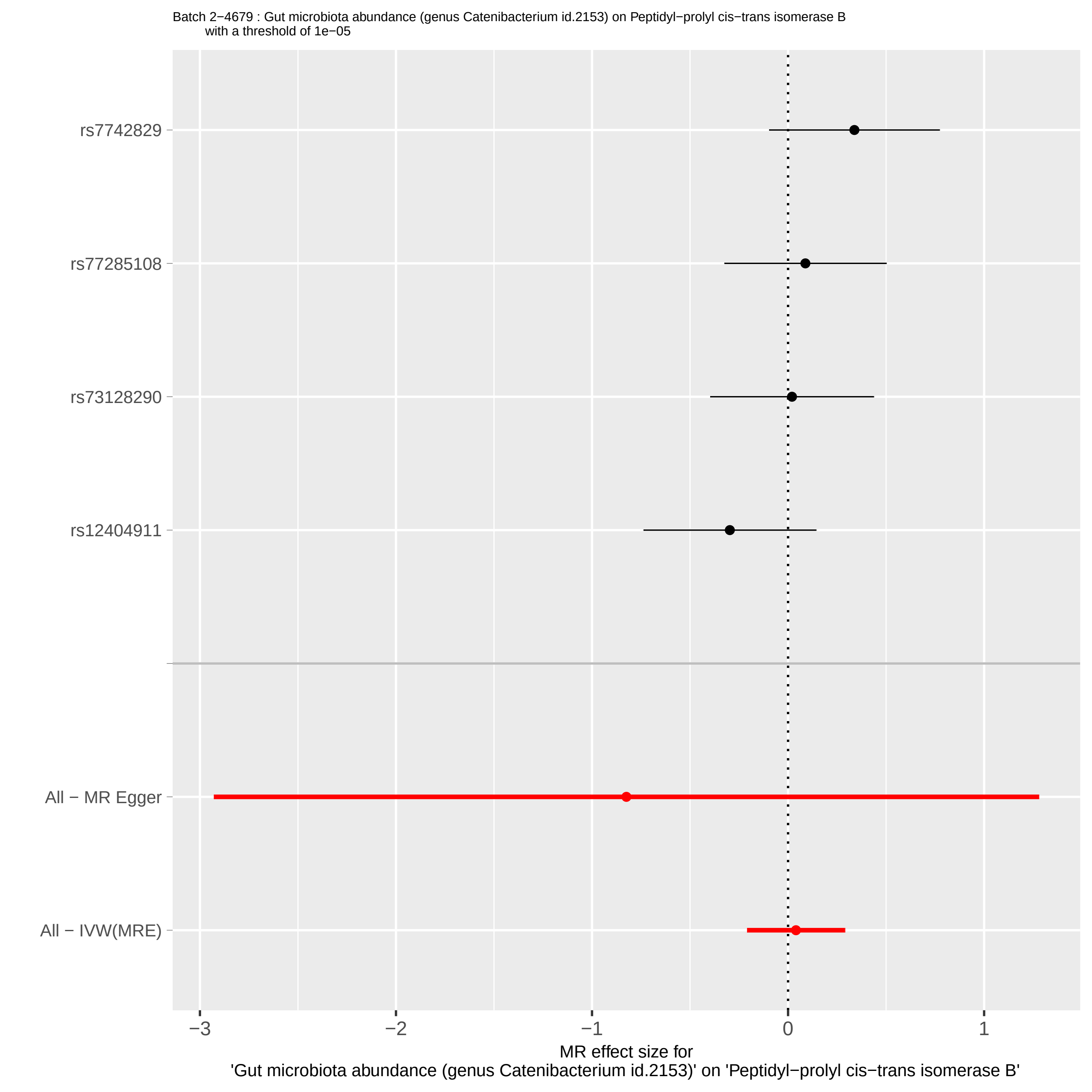
Batch 2−4679 : Gut microbiota abundance (genus Catenibacterium id.2153) on Peptidyl−prolyl cis−trans isomerase B
with a threshold of 1e−05
rs7742829
rs77285108
rs73128290
rs12404911
All − MR Egger
All − IVW(MRE)
−3
−2
−1
0
1
MR effect size for
'Gut microbiota abundance (genus Catenibacterium id.2153)' on 'Peptidyl−prolyl cis−trans isomerase B'

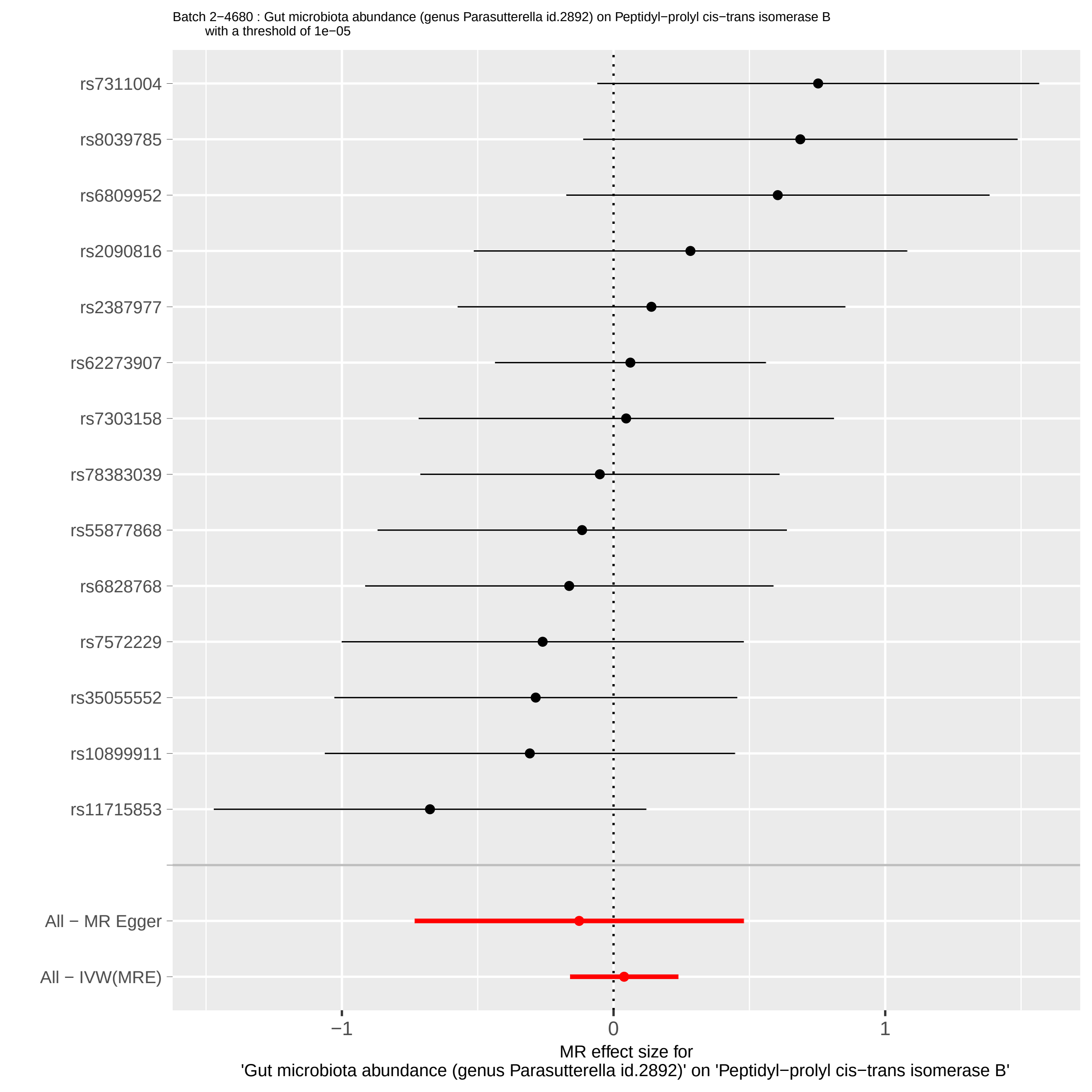
Batch 2−4680 : Gut microbiota abundance (genus Parasutterella id.2892) on Peptidyl−prolyl cis−trans isomerase B
with a threshold of 1e−05
rs7311004
rs8039785
rs6809952
rs2090816
rs2387977
rs62273907
rs7303158
rs78383039
rs55877868
rs6828768
rs7572229
rs35055552
rs10899911
rs11715853
All − MR Egger
All − IVW(MRE)
−1
0
1
MR effect size for
'Gut microbiota abundance (genus Parasutterella id.2892)' on 'Peptidyl−prolyl cis−trans isomerase B'

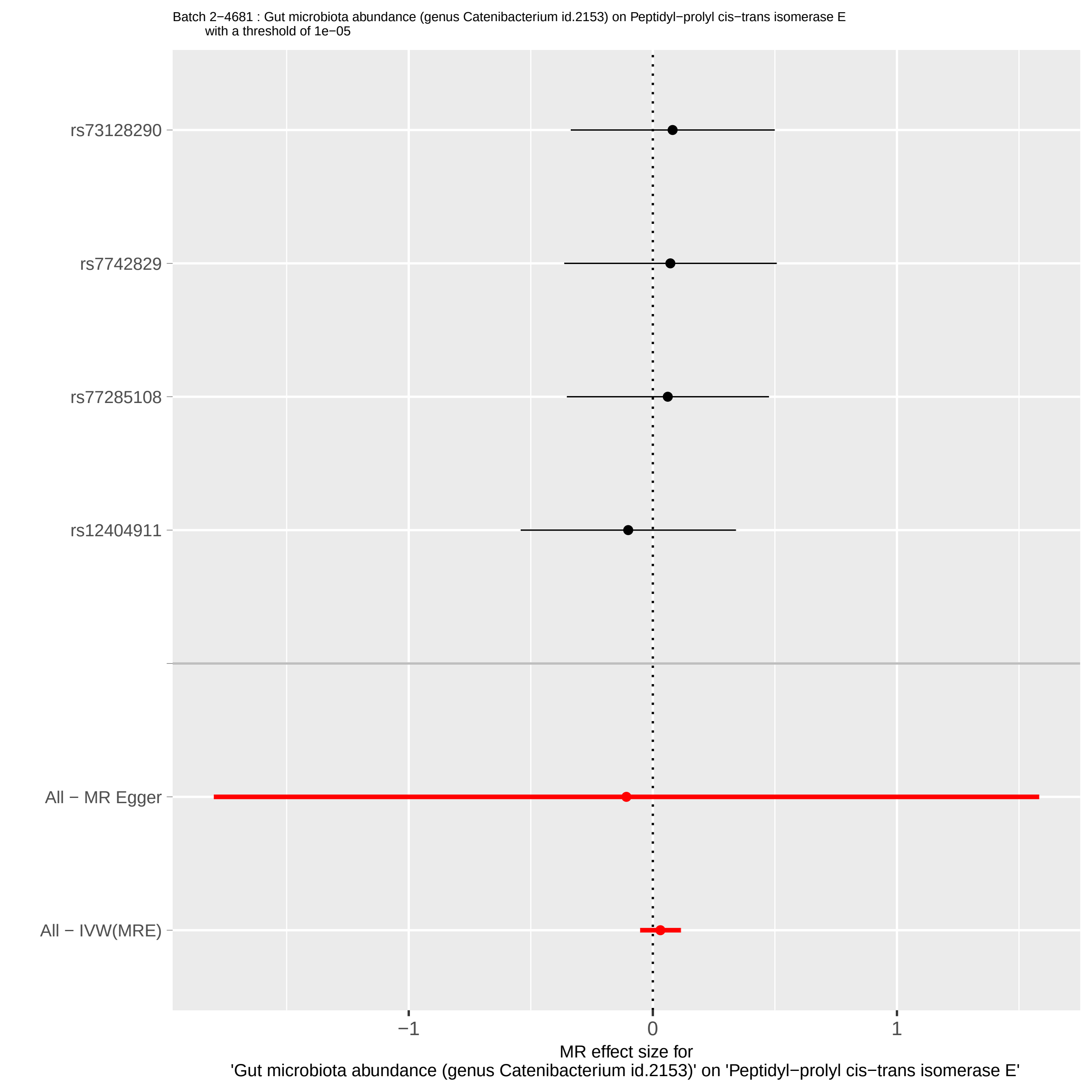

Batch 2−4681 : Gut microbiota abundance (genus Catenibacterium id.2153) on Peptidyl−prolyl cis−trans isomerase E
with a threshold of 1e−05
rs73128290
rs7742829
rs77285108
rs12404911
All − MR Egger
All − IVW(MRE)
−1
0
1
MR effect size for
'Gut microbiota abundance (genus Catenibacterium id.2153)' on 'Peptidyl−prolyl cis−trans isomerase E'

Batch 2−4682 : Gut microbiota abundance (genus Parasutterella id.2892) on Peptidyl−prolyl cis−trans isomerase E
with a threshold of 1e−05

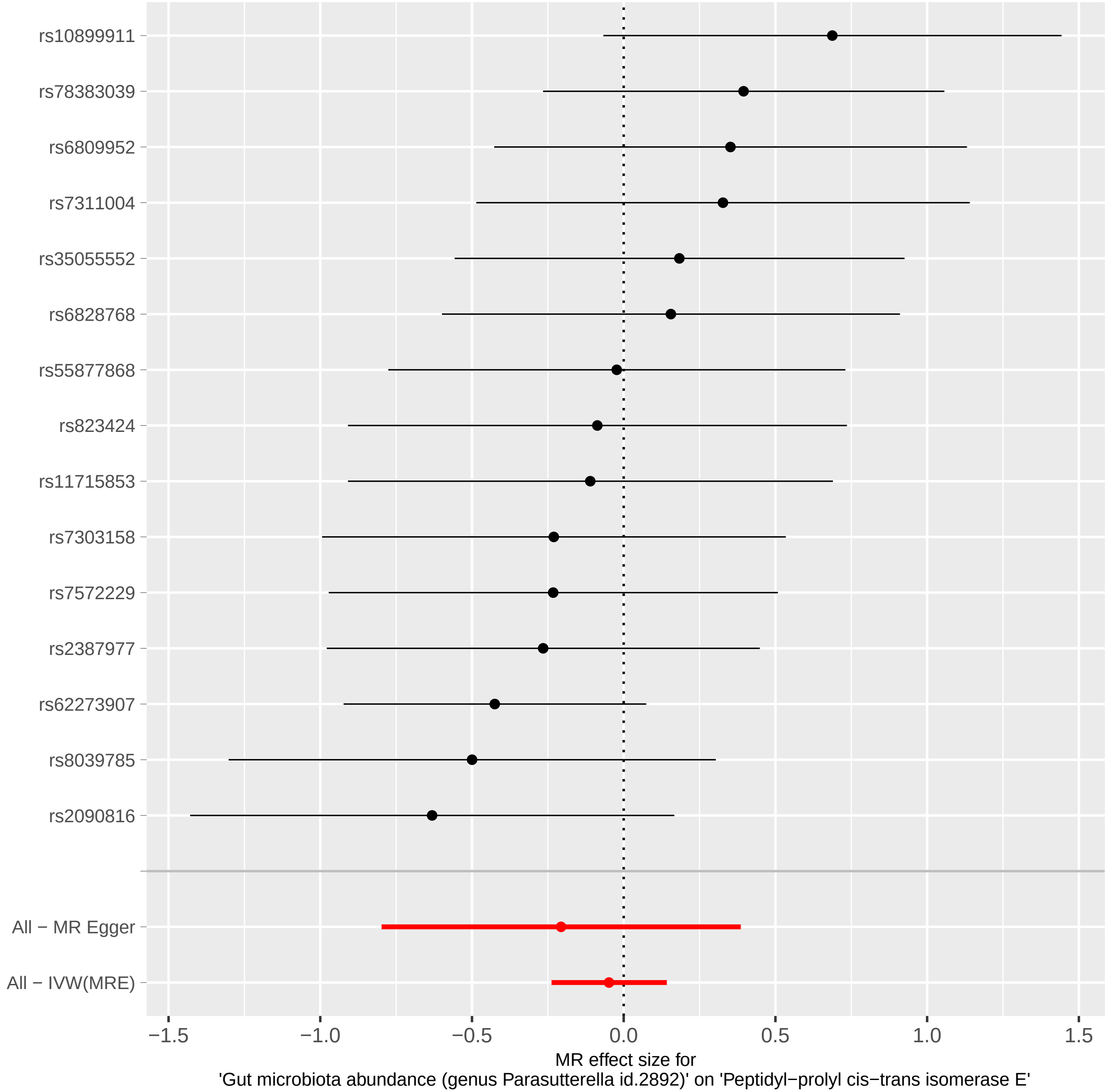

Batch 2−4683 : Gut microbiota abundance (genus Catenibacterium id.2153) on Peptidyl−prolyl cis−trans isomerase H with a threshold of 1e−05

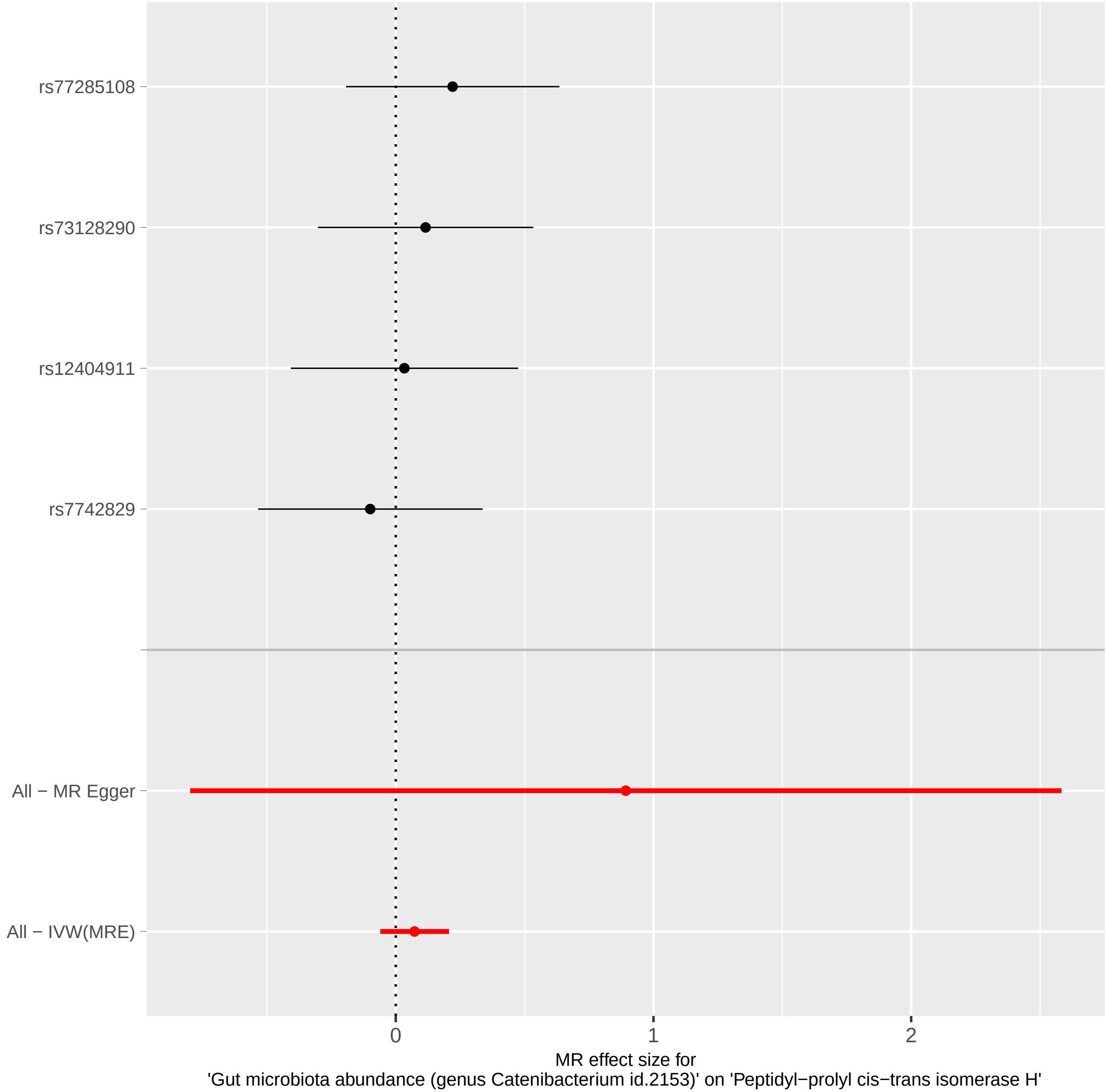

Batch 2−4684 : Gut microbiota abundance (genus Parasutterella id.2892) on Peptidyl−prolyl cis−trans isomerase H with a threshold of 1e−05

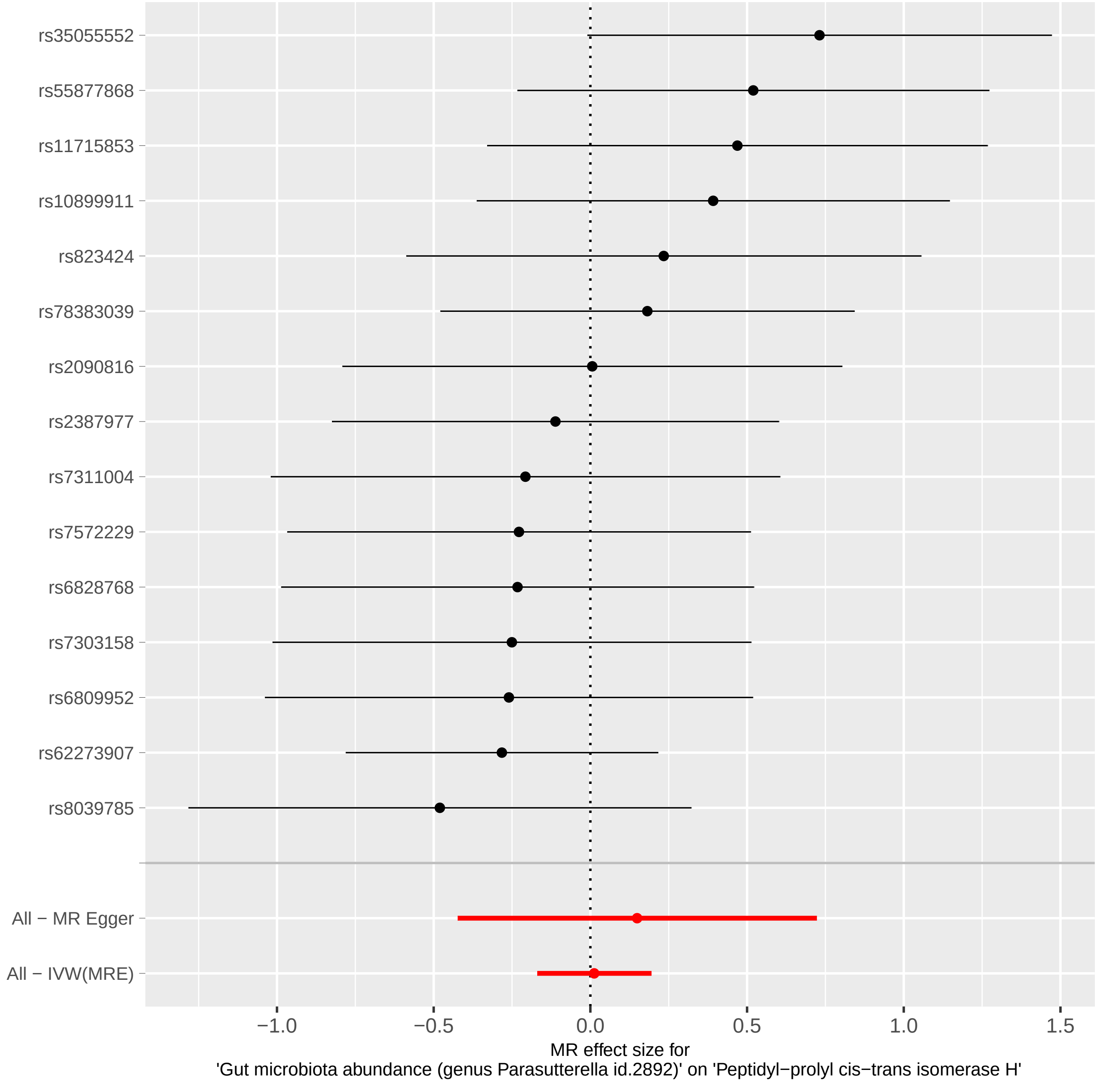

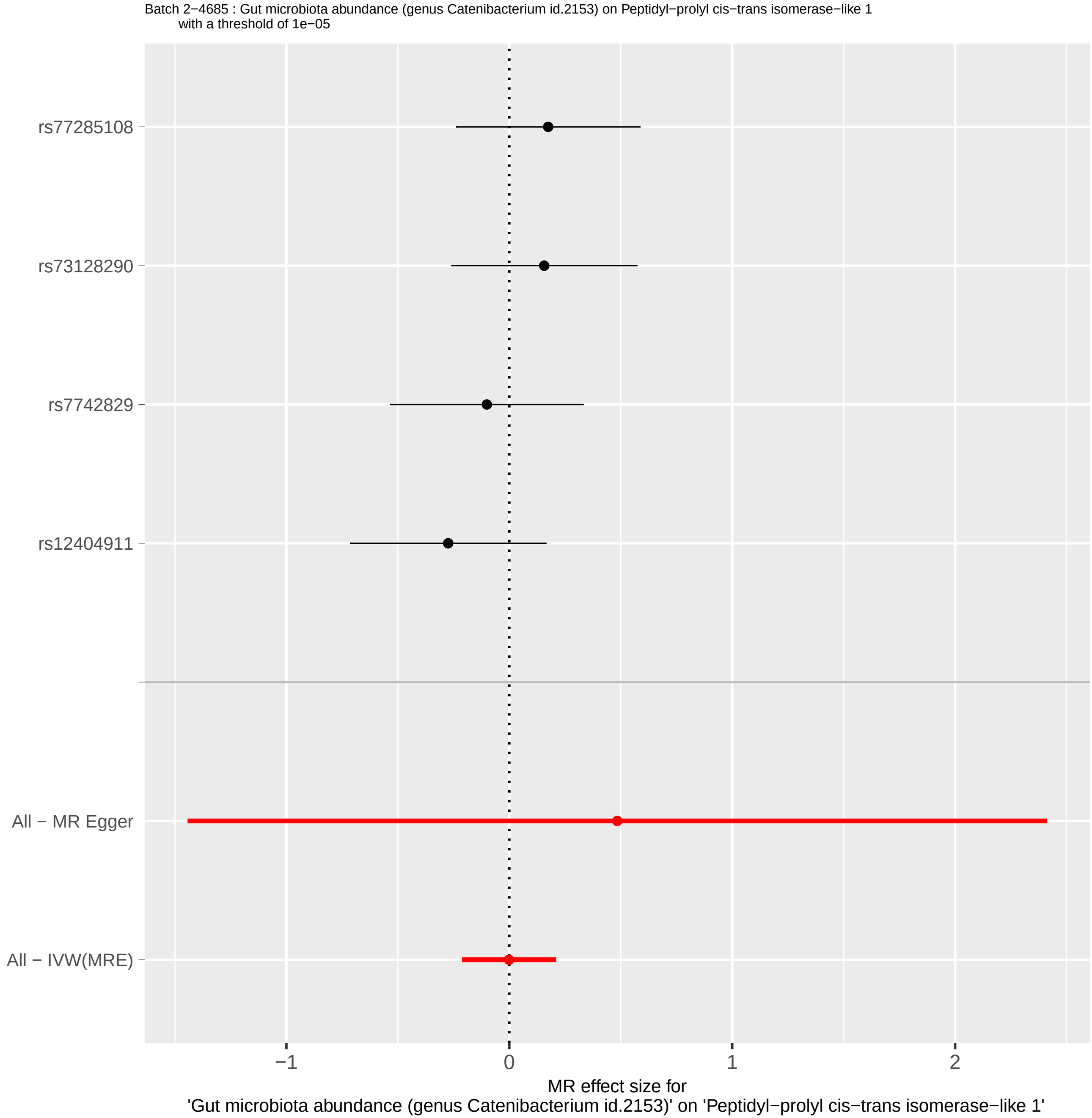

Batch 2−4685 : Gut microbiota abundance (genus Catenibacterium id.2153) on Peptidyl−prolyl cis−trans isomerase−like 1
with a threshold of 1e−05
rs77285108
rs73128290
rs7742829
rs12404911
All − MR Egger
All − IVW(MRE)
−1
0
1
2
MR effect size for
'Gut microbiota abundance (genus Catenibacterium id.2153)' on 'Peptidyl−prolyl cis−trans isomerase−like 1'

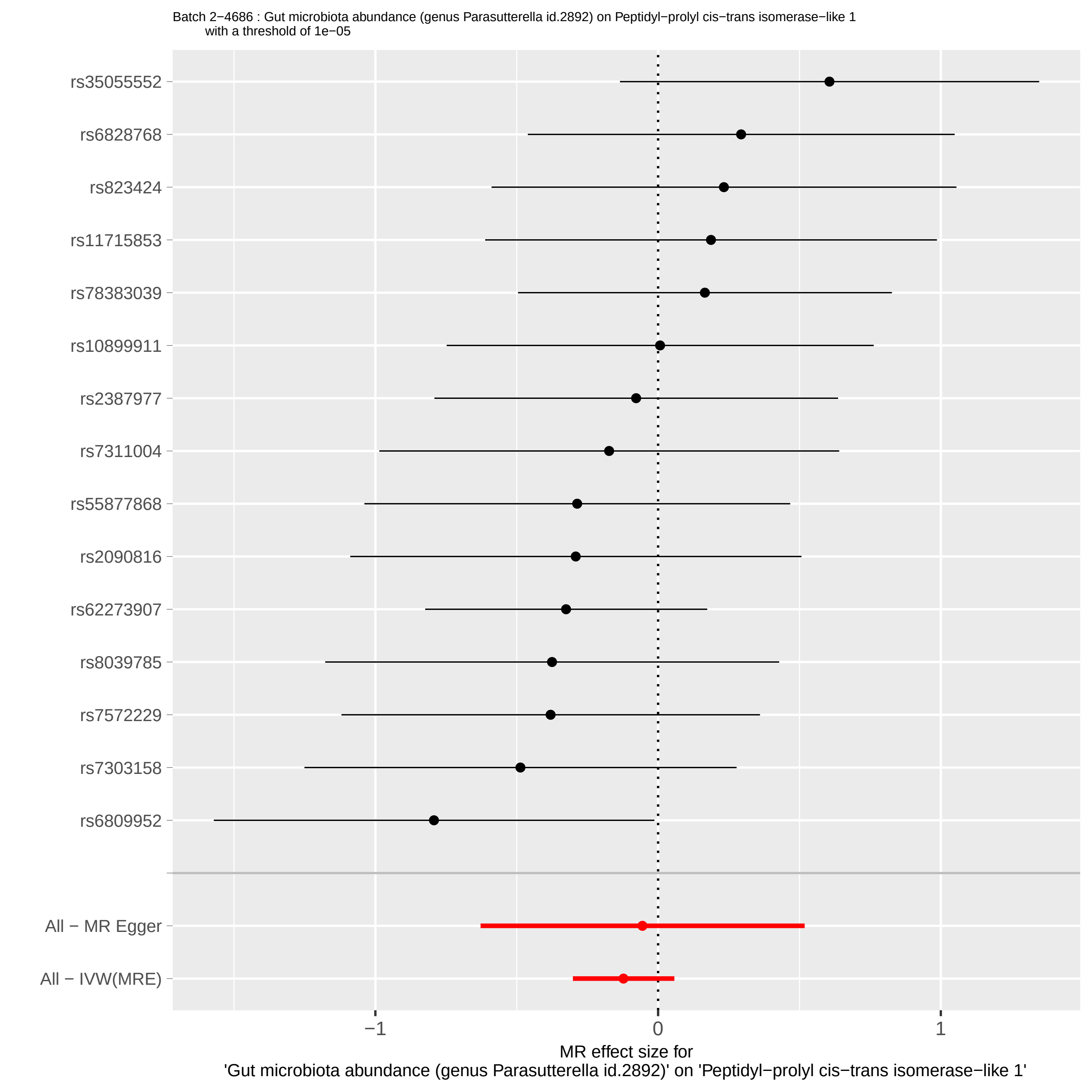

Batch 2−4686 : Gut microbiota abundance (genus Parasutterella id.2892) on Peptidyl−prolyl cis−trans isomerase−like 1
with a threshold of 1e−05
rs35055552
rs6828768
rs823424
rs11715853
rs78383039
rs10899911
rs2387977
rs7311004
rs55877868
rs2090816
rs62273907
rs8039785
rs7572229
rs7303158
rs6809952
All − MR Egger
All − IVW(MRE)
−1
0
1
MR effect size for
'Gut microbiota abundance (genus Parasutterella id.2892)' on 'Peptidyl−prolyl cis−trans isomerase−like 1'

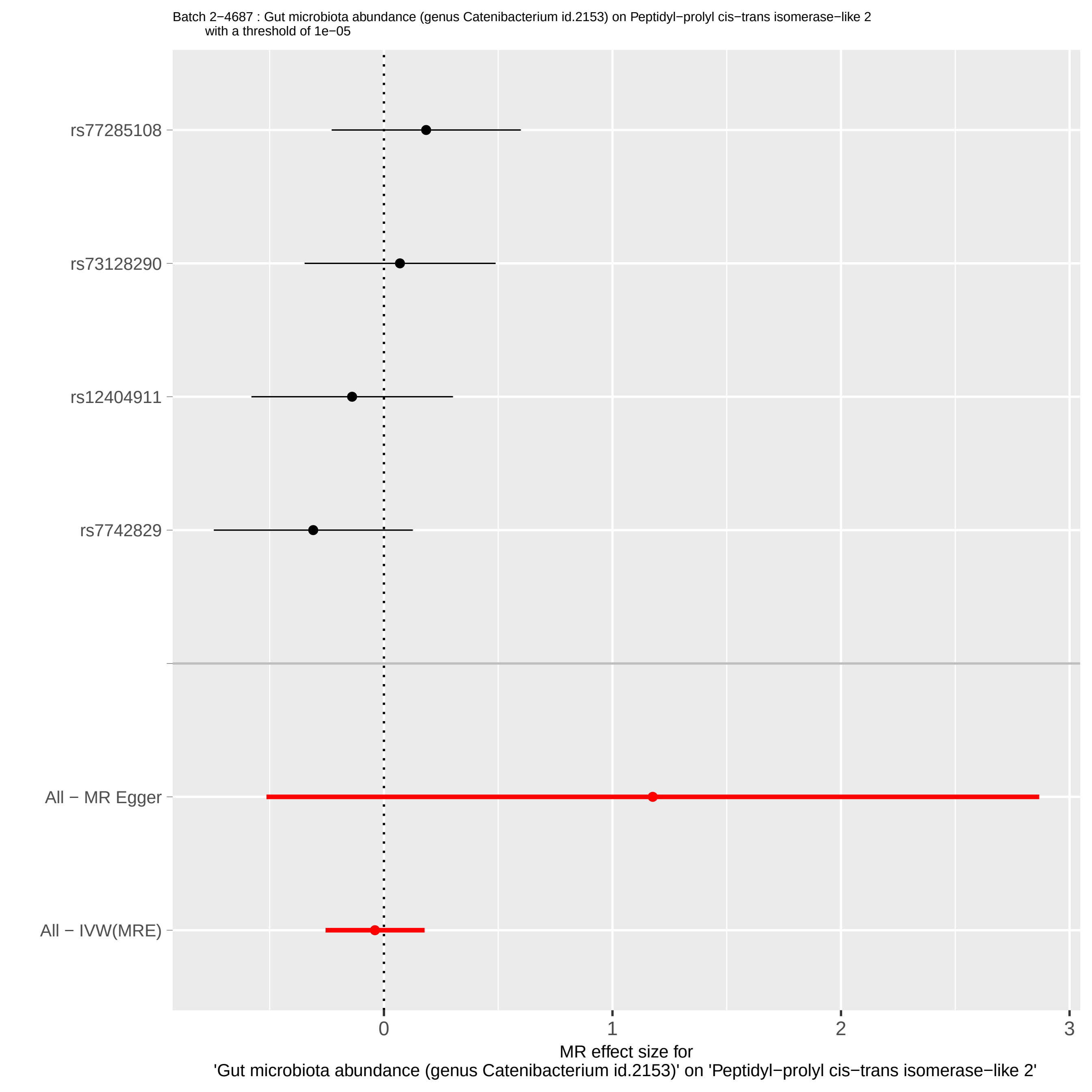
Batch 2−4687 : Gut microbiota abundance (genus Catenibacterium id.2153) on Peptidyl−prolyl cis−trans isomerase−like 2
with a threshold of 1e−05
rs77285108
rs73128290
rs12404911
rs7742829
All − MR Egger
All − IVW(MRE)
0
1
2
3
MR effect size for
'Gut microbiota abundance (genus Catenibacterium id.2153)' on 'Peptidyl−prolyl cis−trans isomerase−like 2'

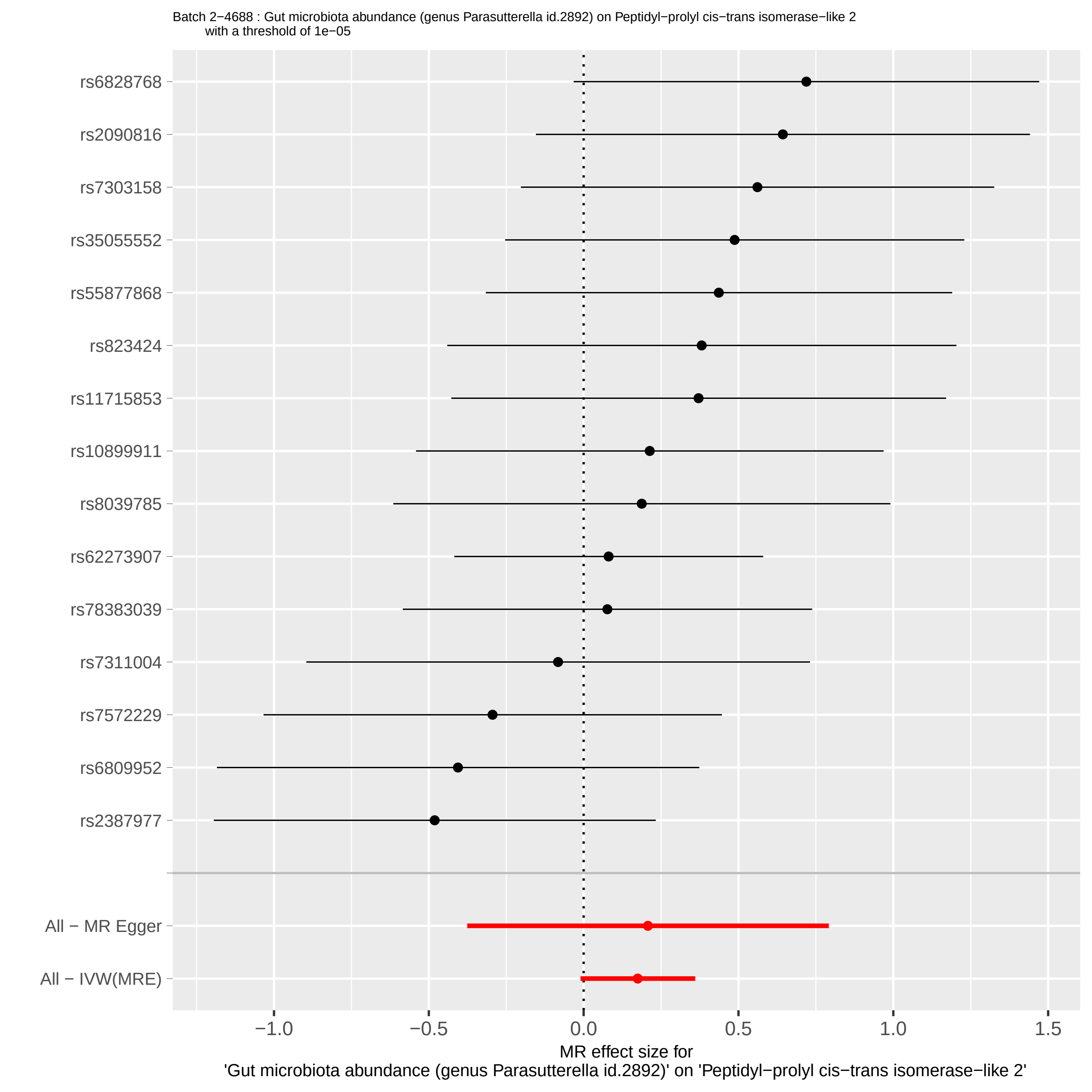

Batch 2−4688 : Gut microbiota abundance (genus Parasutterella id.2892) on Peptidyl−prolyl cis−trans isomerase−like 2
with a threshold of 1e−05
rs6828768
rs2090816
rs7303158
rs35055552
rs55877868
rs823424
rs11715853
rs10899911
rs8039785
rs62273907
rs78383039
rs7311004
rs7572229
rs6809952
rs2387977
All − MR Egger
All − IVW(MRE)
−1.0
−0.5
0.0
0.5
1.0
1.5
MR effect size for
'Gut microbiota abundance (genus Parasutterella id.2892)' on 'Peptidyl−prolyl cis−trans isomerase−like 2'

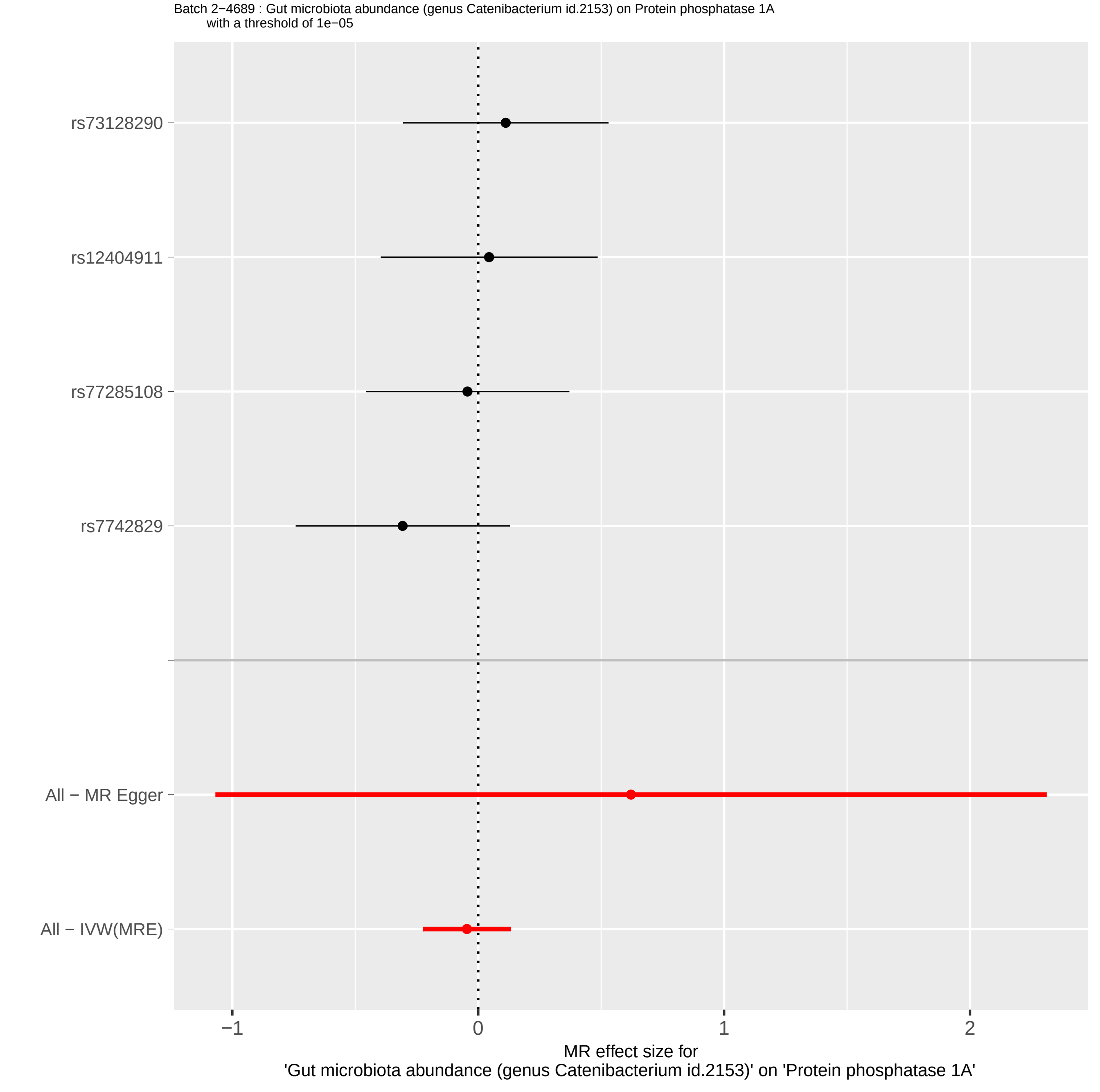
Batch 2−4689 : Gut microbiota abundance (genus Catenibacterium id.2153) on Protein phosphatase 1A
with a threshold of 1e−05
rs73128290
rs12404911
rs77285108
rs7742829
All − MR Egger
All − IVW(MRE)
−1
0
1
2
MR effect size for
'Gut microbiota abundance (genus Catenibacterium id.2153)' on 'Protein phosphatase 1A'

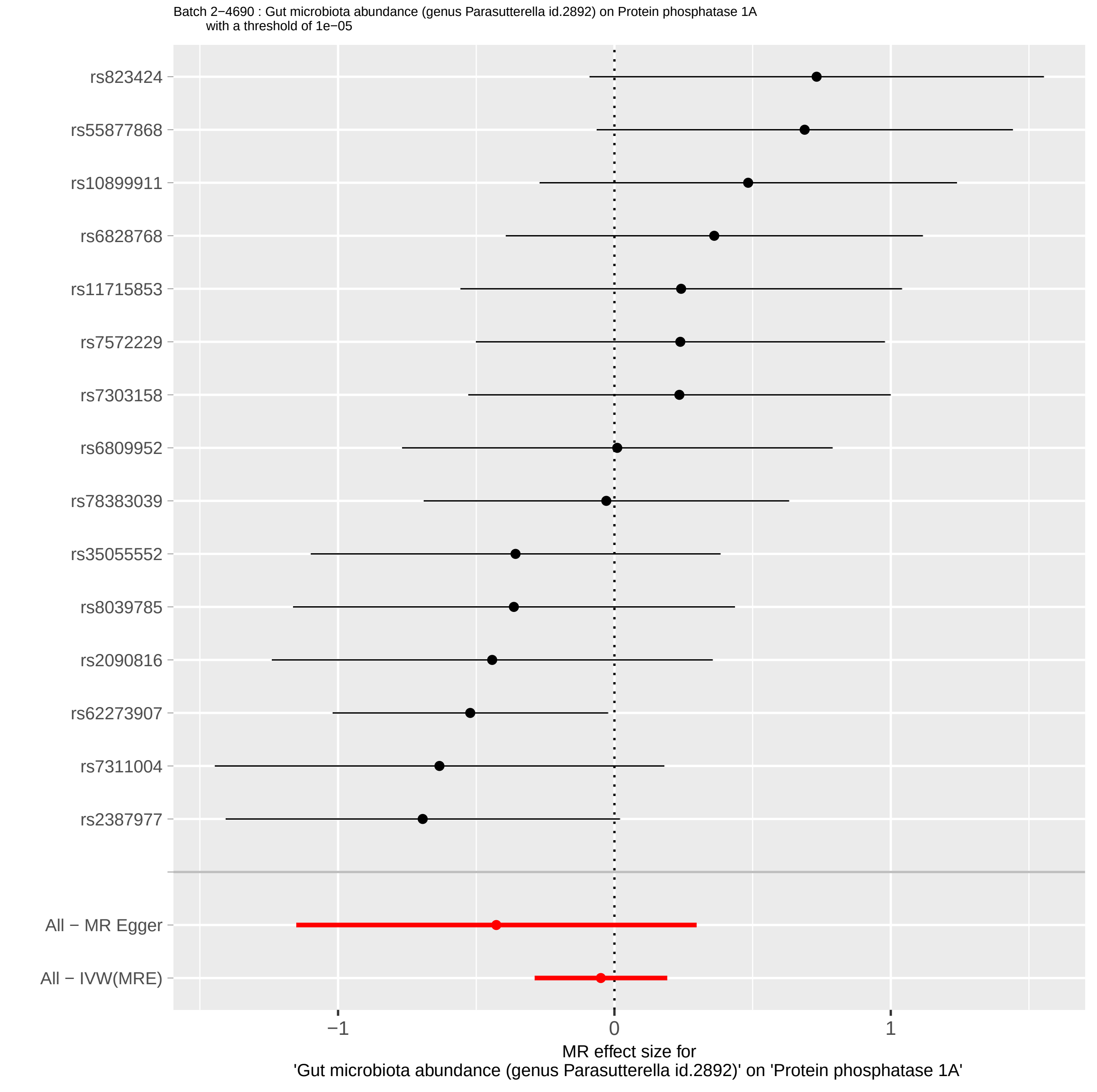

Batch 2−4690 : Gut microbiota abundance (genus Parasutterella id.2892) on Protein phosphatase 1A
with a threshold of 1e−05
rs823424
rs55877868
rs10899911
rs6828768
rs11715853
rs7572229
rs7303158
rs6809952
rs78383039
rs35055552
rs8039785
rs2090816
rs62273907
rs7311004
rs2387977
All − MR Egger
All − IVW(MRE)
−1
0
1
MR effect size for
'Gut microbiota abundance (genus Parasutterella id.2892)' on 'Protein phosphatase 1A'

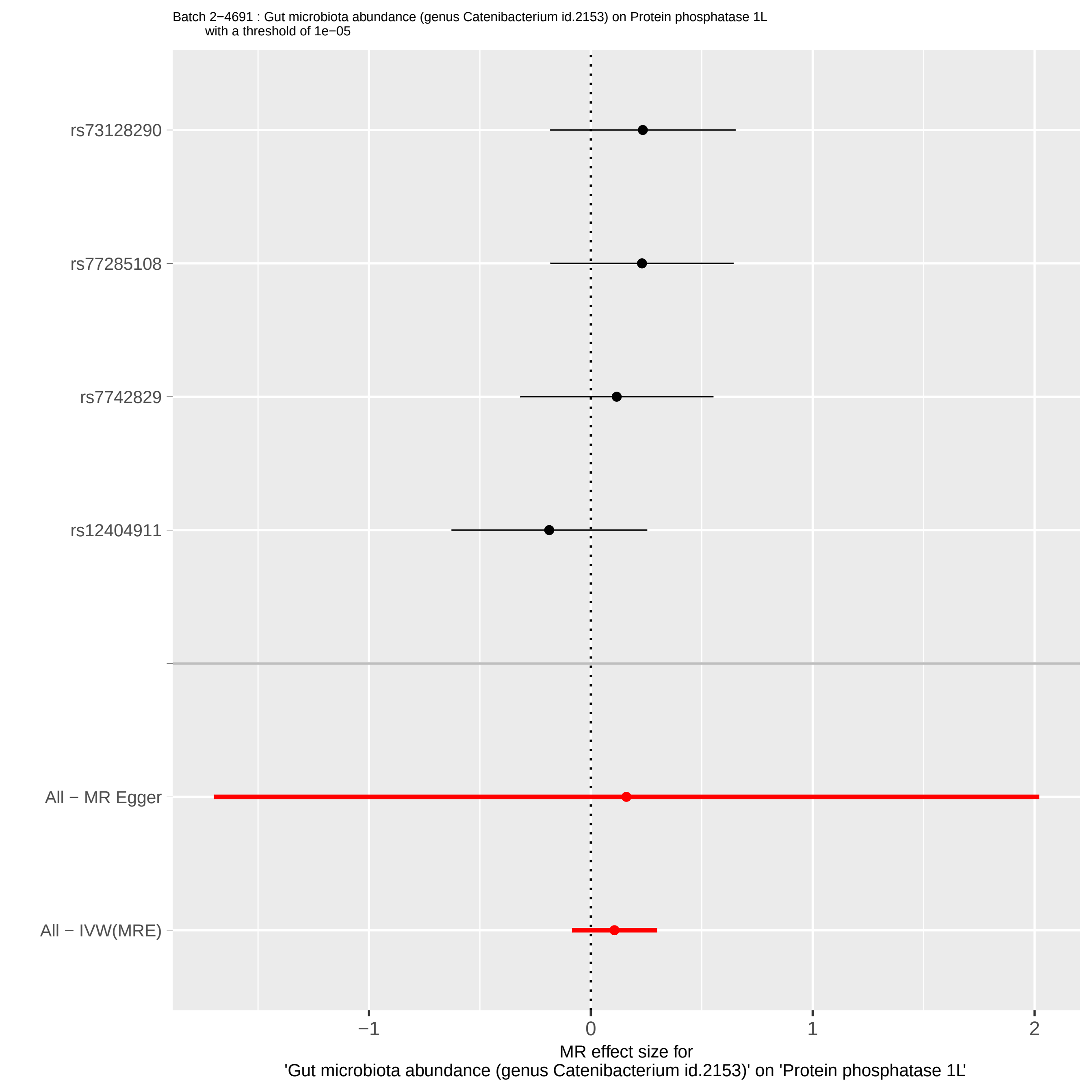

Batch 2−4691 : Gut microbiota abundance (genus Catenibacterium id.2153) on Protein phosphatase 1L
with a threshold of 1e−05
rs73128290
rs77285108
rs7742829
rs12404911
All − MR Egger
All − IVW(MRE)
−1
0
1
2
MR effect size for
'Gut microbiota abundance (genus Catenibacterium id.2153)' on 'Protein phosphatase 1L'

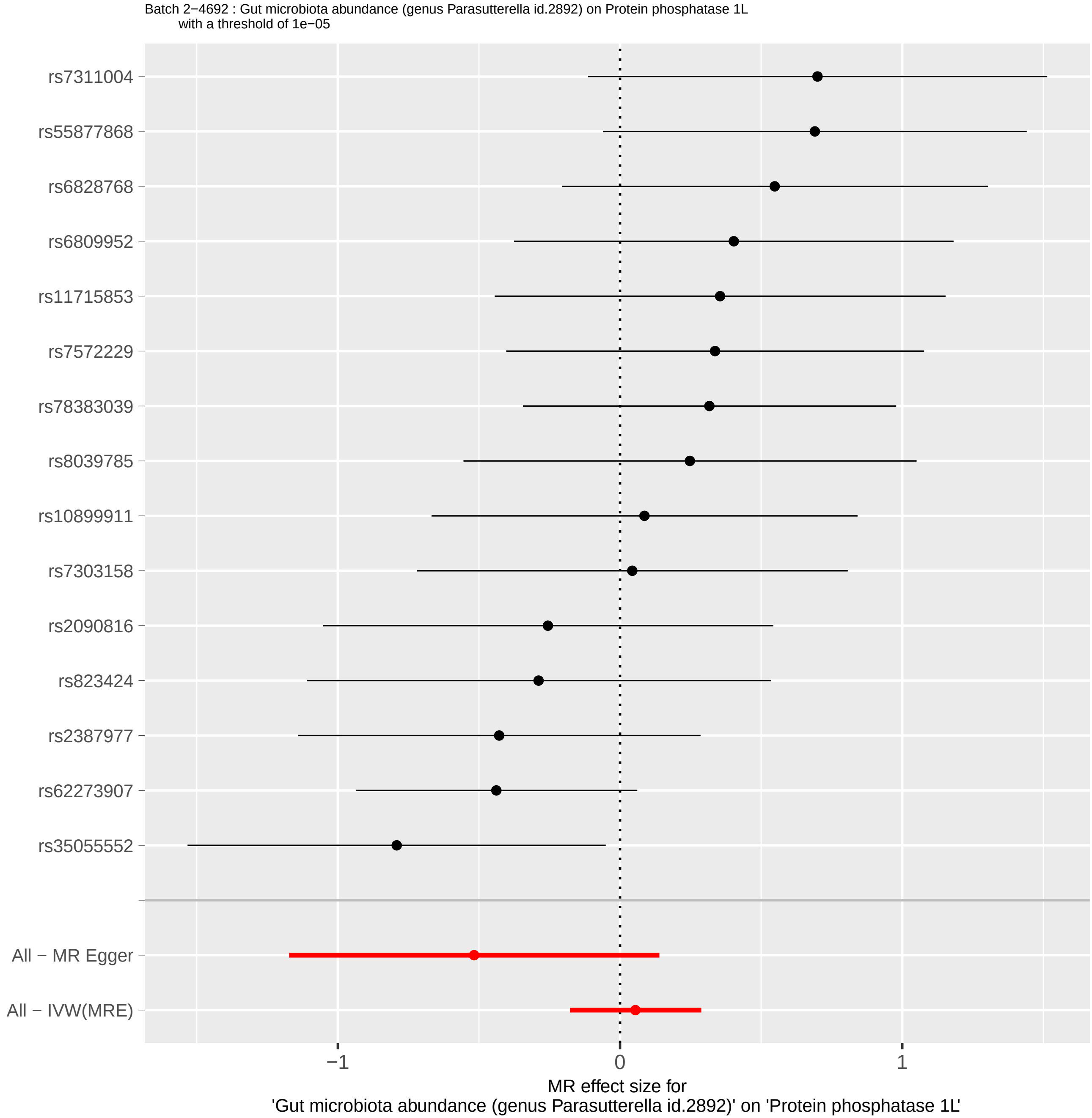

Batch 2−4692 : Gut microbiota abundance (genus Parasutterella id.2892) on Protein phosphatase 1L
with a threshold of 1e−05
rs7311004
rs55877868
rs6828768
rs6809952
rs11715853
rs7572229
rs78383039
rs8039785
rs10899911
rs7303158
rs2090816
rs823424
rs2387977
rs62273907
rs35055552
All − MR Egger
All − IVW(MRE)
−1
0
1
MR effect size for
'Gut microbiota abundance (genus Parasutterella id.2892)' on 'Protein phosphatase 1L'

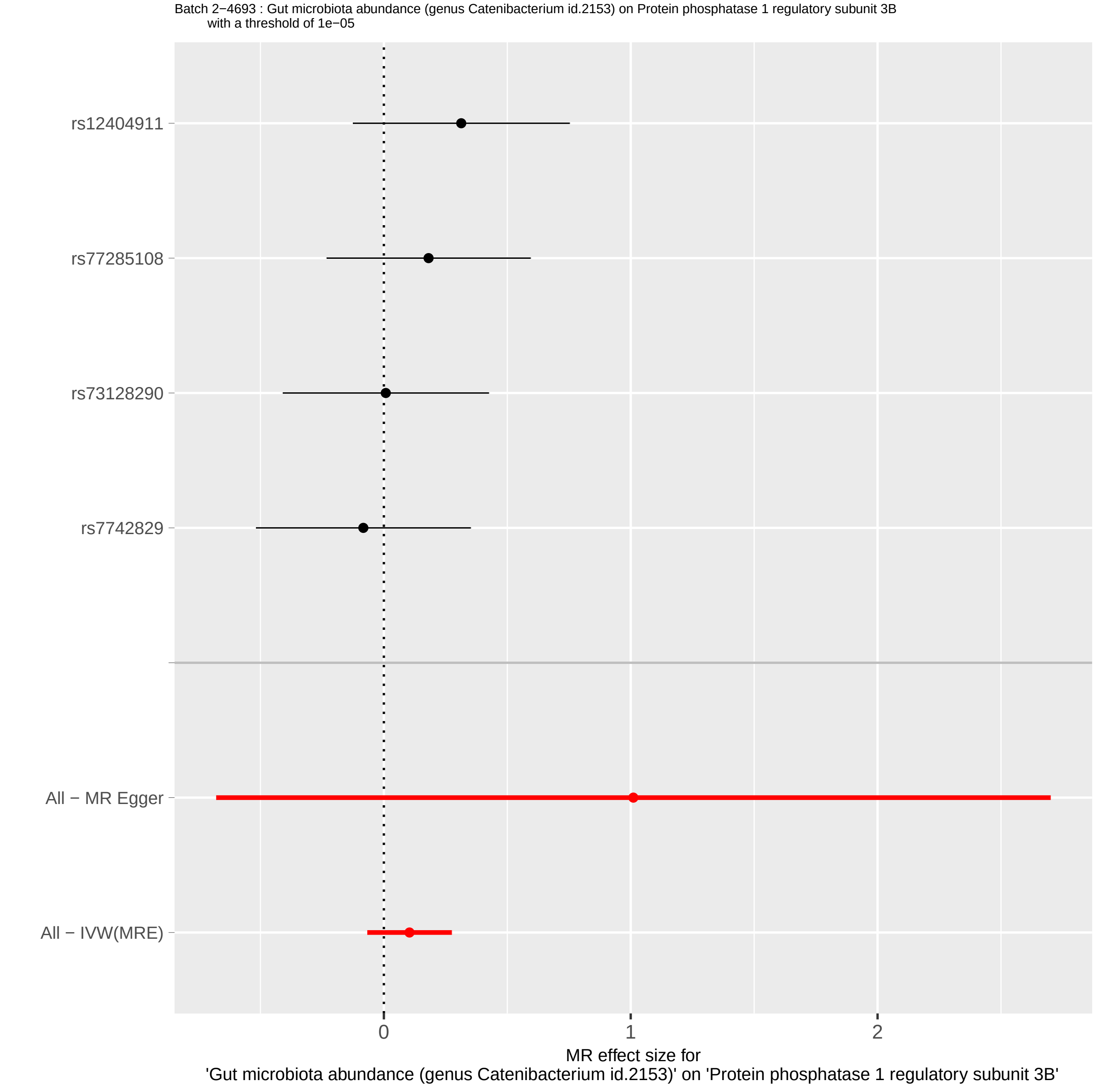

Batch 2−4693 : Gut microbiota abundance (genus Catenibacterium id.2153) on Protein phosphatase 1 regulatory subunit 3B
with a threshold of 1e−05
rs12404911
rs77285108
rs73128290
rs7742829
All − MR Egger
All − IVW(MRE)
0
1
2
MR effect size for
'Gut microbiota abundance (genus Catenibacterium id.2153)' on 'Protein phosphatase 1 regulatory subunit 3B'

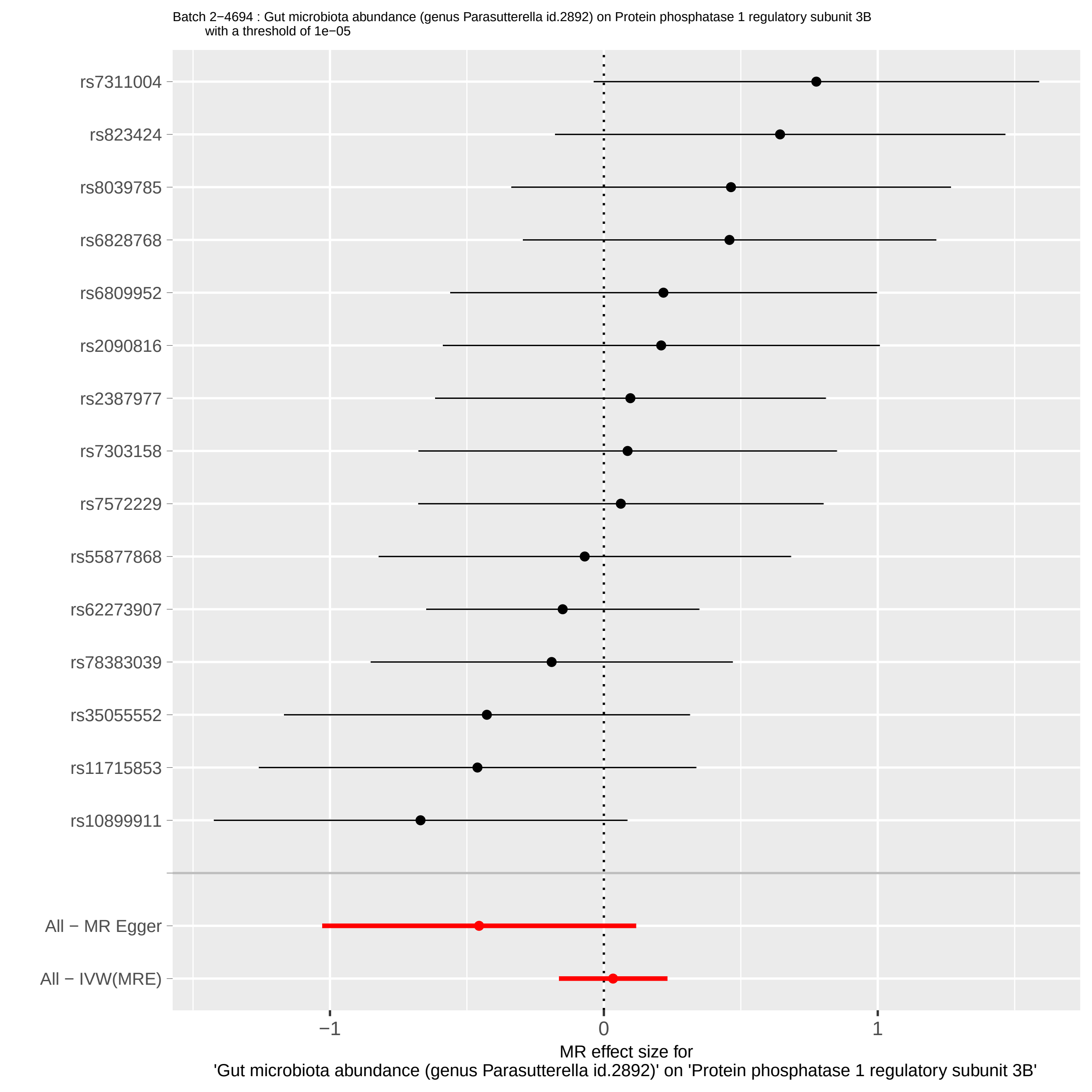

Batch 2−4694 : Gut microbiota abundance (genus Parasutterella id.2892) on Protein phosphatase 1 regulatory subunit 3B
with a threshold of 1e−05
rs7311004
rs823424
rs8039785
rs6828768
rs6809952
rs2090816
rs2387977
rs7303158
rs7572229
rs55877868
rs62273907
rs78383039
rs35055552
rs11715853
rs10899911
All − MR Egger
All − IVW(MRE)
−1
0
1
MR effect size for
'Gut microbiota abundance (genus Parasutterella id.2892)' on 'Protein phosphatase 1 regulatory subunit 3B'

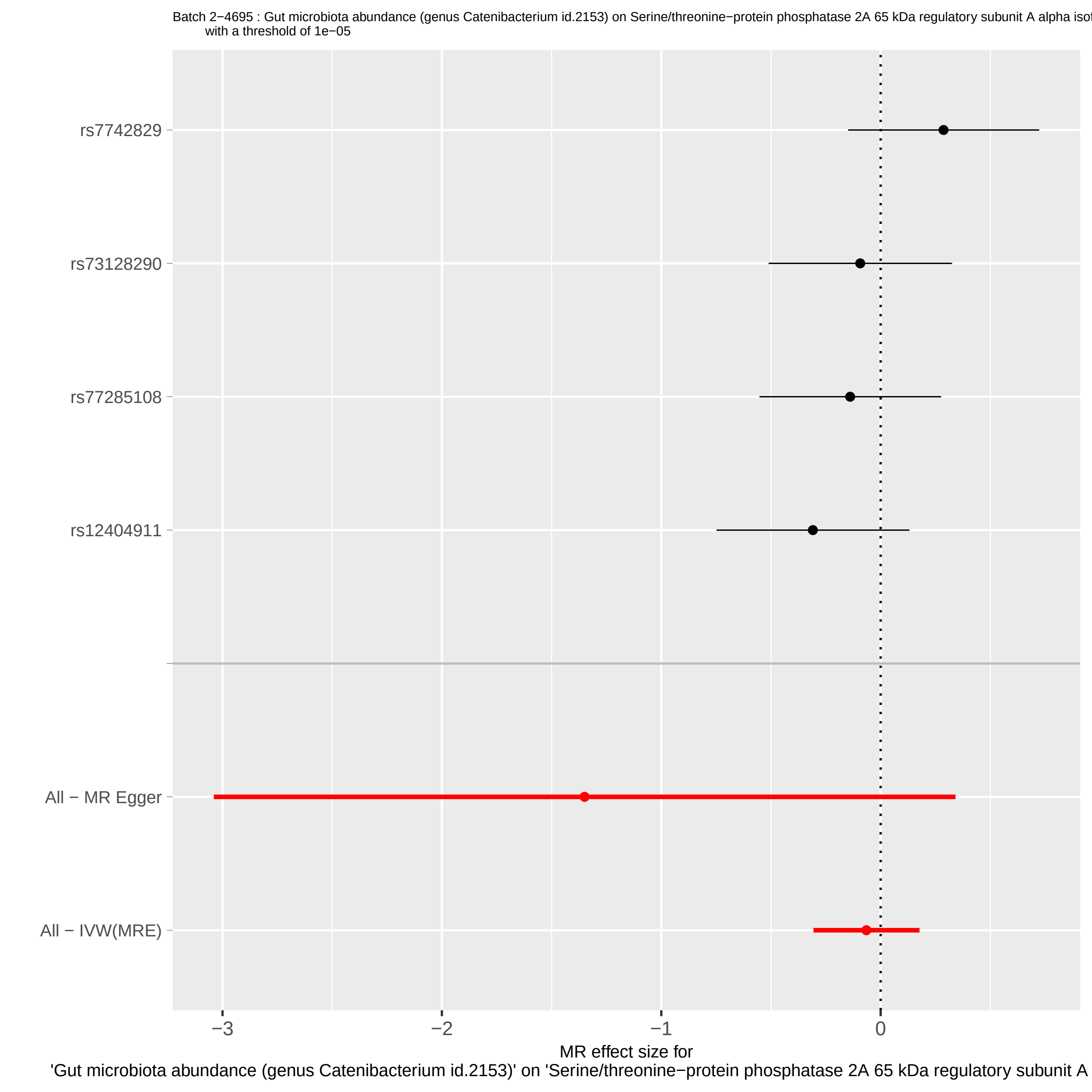
Batch 2−4695 : Gut microbiota abundance (genus Catenibacterium id.2153) on Serine/threonine−protein phosphatase 2A 65 kDa regulatory subunit A alpha isof
with a threshold of 1e−05
rs7742829
rs73128290
rs77285108
rs12404911
All − MR Egger
All − IVW(MRE)
−3
−2
−1
0
MR effect size for
'Gut microbiota abundance (genus Catenibacterium id.2153)' on 'Serine/threonine−protein phosphatase 2A 65 kDa regulatory subunit A

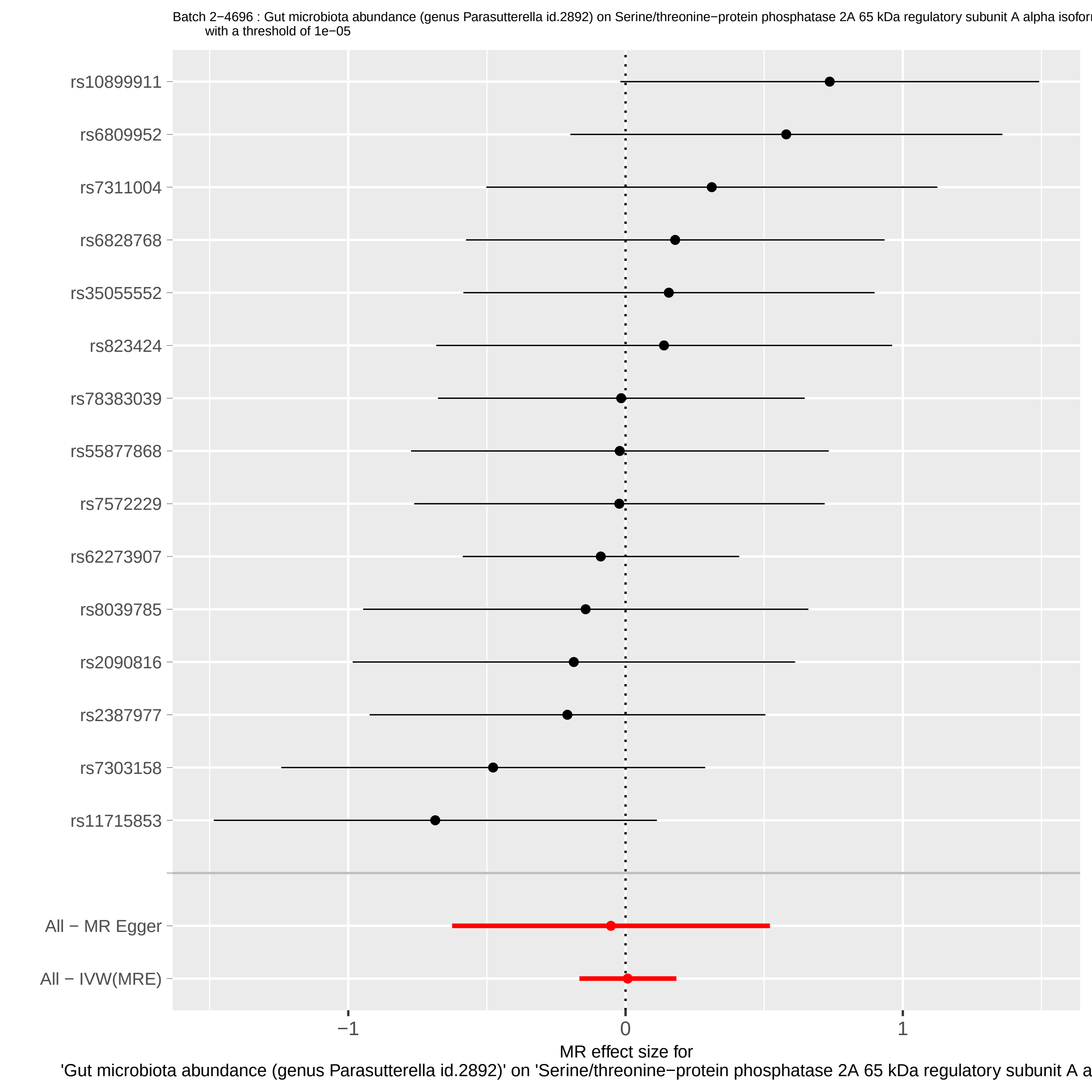

Batch 2−4696 : Gut microbiota abundance (genus Parasutterella id.2892) on Serine/threonine−protein phosphatase 2A 65 kDa regulatory subunit A alpha isofor
with a threshold of 1e−05
rs10899911
rs6809952
rs7311004
rs6828768
rs35055552
rs823424
rs78383039
rs55877868
rs7572229
rs62273907
rs8039785
rs2090816
rs2387977
rs7303158
rs11715853
All − MR Egger
All − IVW(MRE)
−1
0
1
MR effect size for
'Gut microbiota abundance (genus Parasutterella id.2892)' on 'Serine/threonine−protein phosphatase 2A 65 kDa regulatory subunit A a

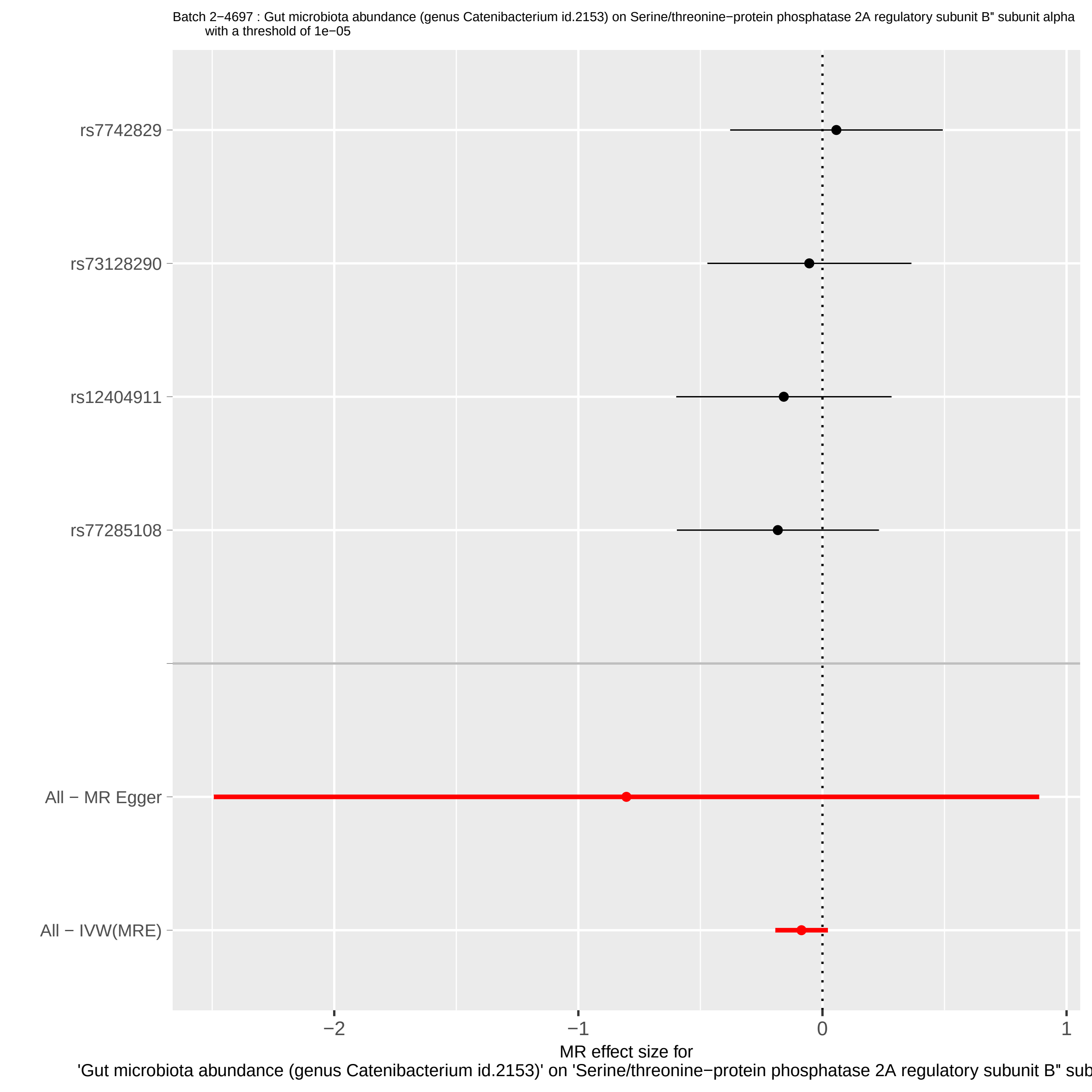

Batch 2−4697 : Gut microbiota abundance (genus Catenibacterium id.2153) on Serine/threonine−protein phosphatase 2A regulatory subunit B" subunit alpha
with a threshold of 1e−05
rs7742829
rs73128290
rs12404911
rs77285108
All − MR Egger
All − IVW(MRE)
−2
−1
0
1
MR effect size for
'Gut microbiota abundance (genus Catenibacterium id.2153)' on 'Serine/threonine−protein phosphatase 2A regulatory subunit B" sub

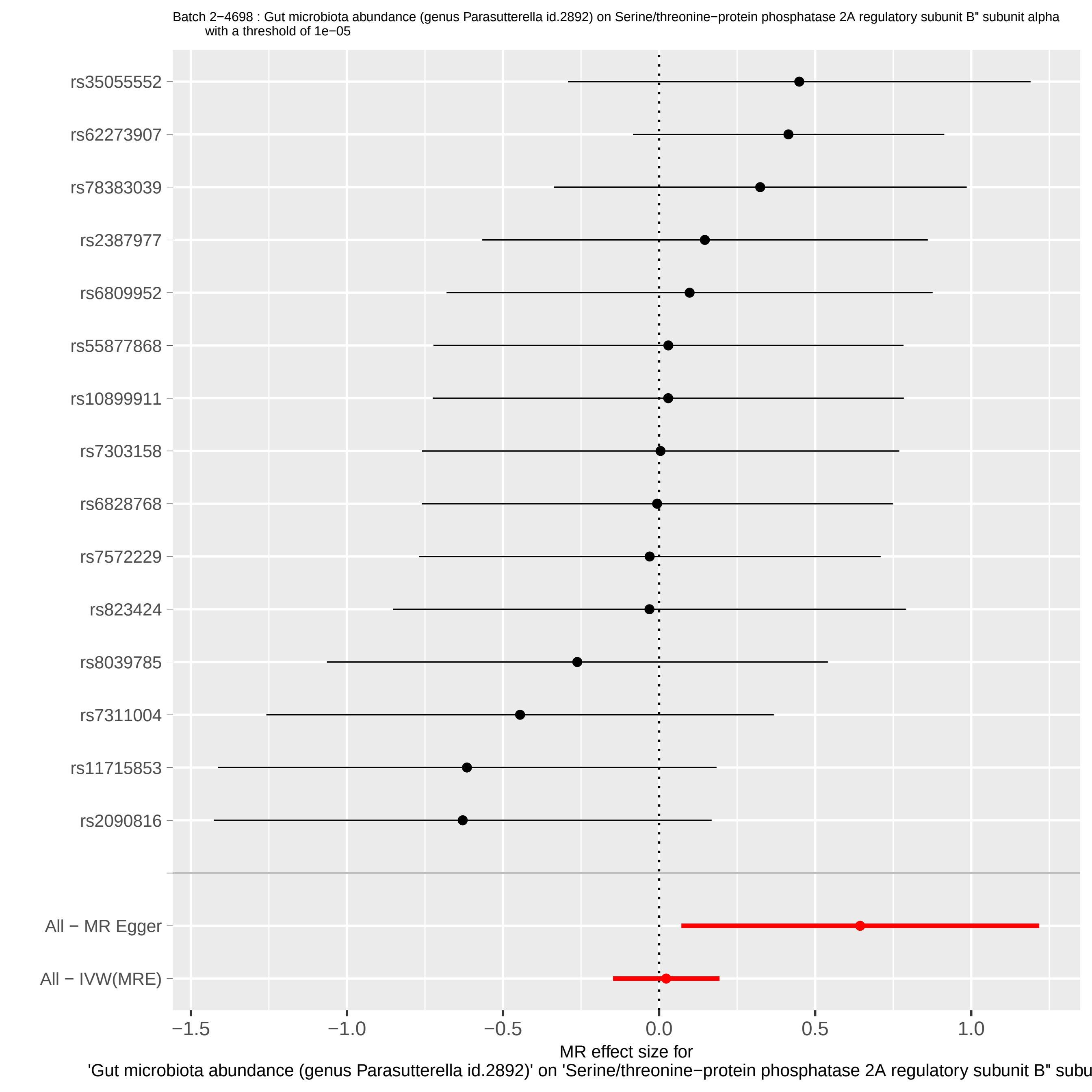

Batch 2−4698 : Gut microbiota abundance (genus Parasutterella id.2892) on Serine/threonine−protein phosphatase 2A regulatory subunit B" subunit alpha
with a threshold of 1e−05
rs35055552
rs62273907
rs78383039
rs2387977
rs6809952
rs55877868
rs10899911
rs7303158
rs6828768
rs7572229
rs823424
rs8039785
rs7311004
rs11715853
rs2090816
All − MR Egger
All − IVW(MRE)
−1.5
−1.0
−0.5
0.0
0.5
1.0
MR effect size for
'Gut microbiota abundance (genus Parasutterella id.2892)' on 'Serine/threonine−protein phosphatase 2A regulatory subunit B" subu

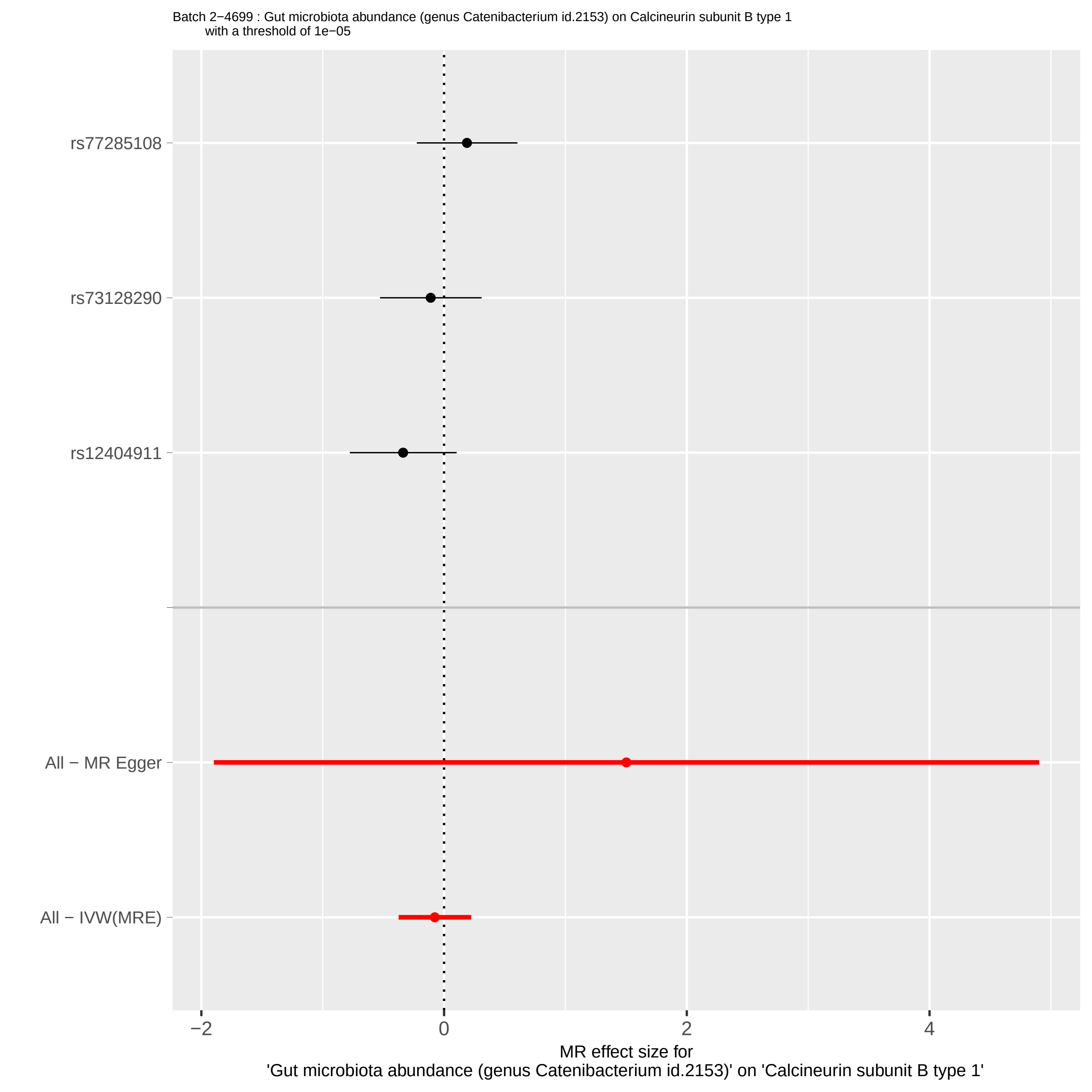

Batch 2−4699 : Gut microbiota abundance (genus Catenibacterium id.2153) on Calcineurin subunit B type 1
with a threshold of 1e−05
rs77285108
rs73128290
rs12404911
All − MR Egger
All − IVW(MRE)
−2
0
2
4
MR effect size for
'Gut microbiota abundance (genus Catenibacterium id.2153)' on 'Calcineurin subunit B type 1'

Batch 2−4700 : Gut microbiota abundance (genus Parasutterella id.2892) on Calcineurin subunit B type 1
with a threshold of 1e−05

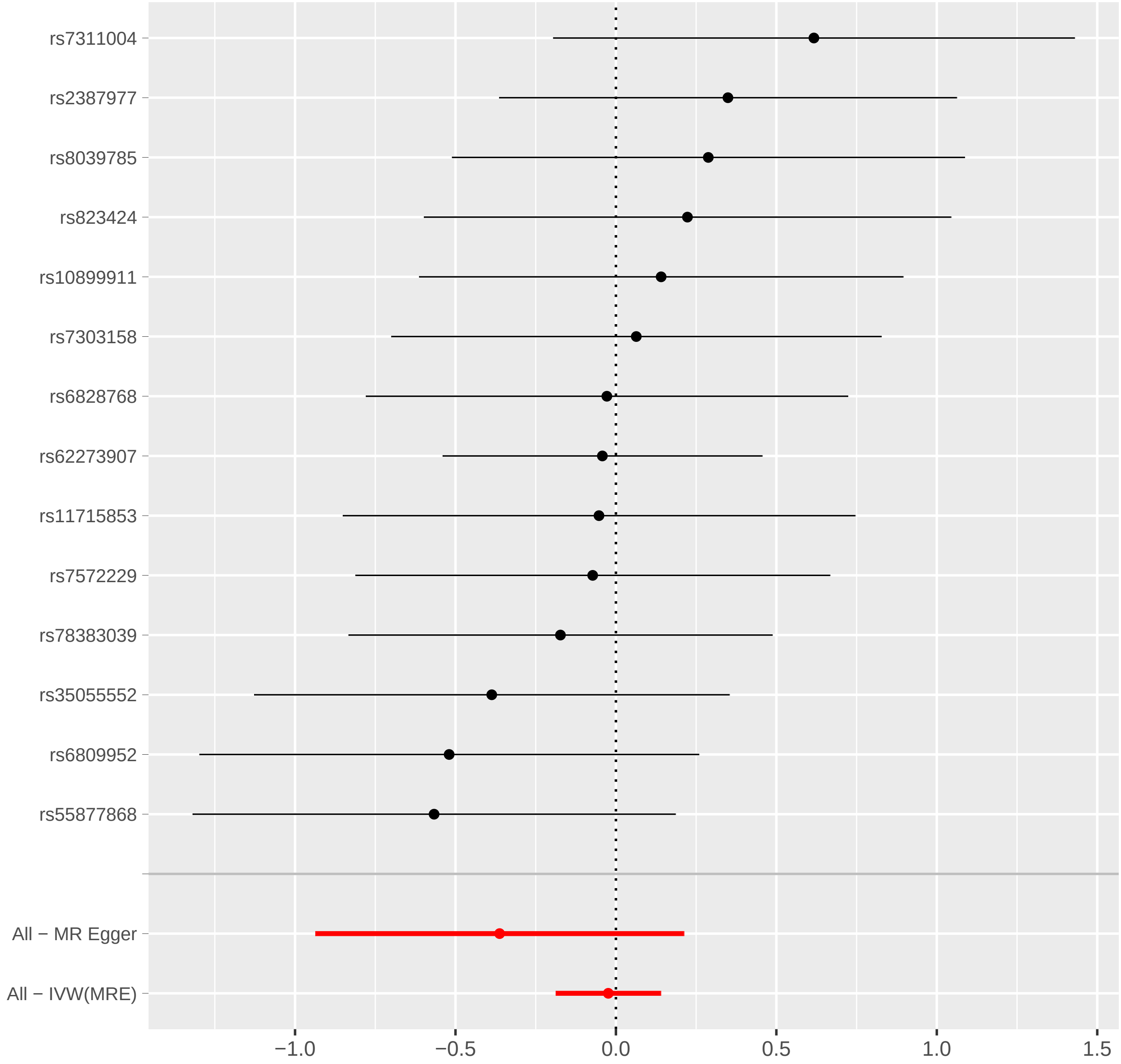

MR effect size for
'Gut microbiota abundance (genus Parasutterella id.2892)' on 'Calcineurin subunit B type 1'

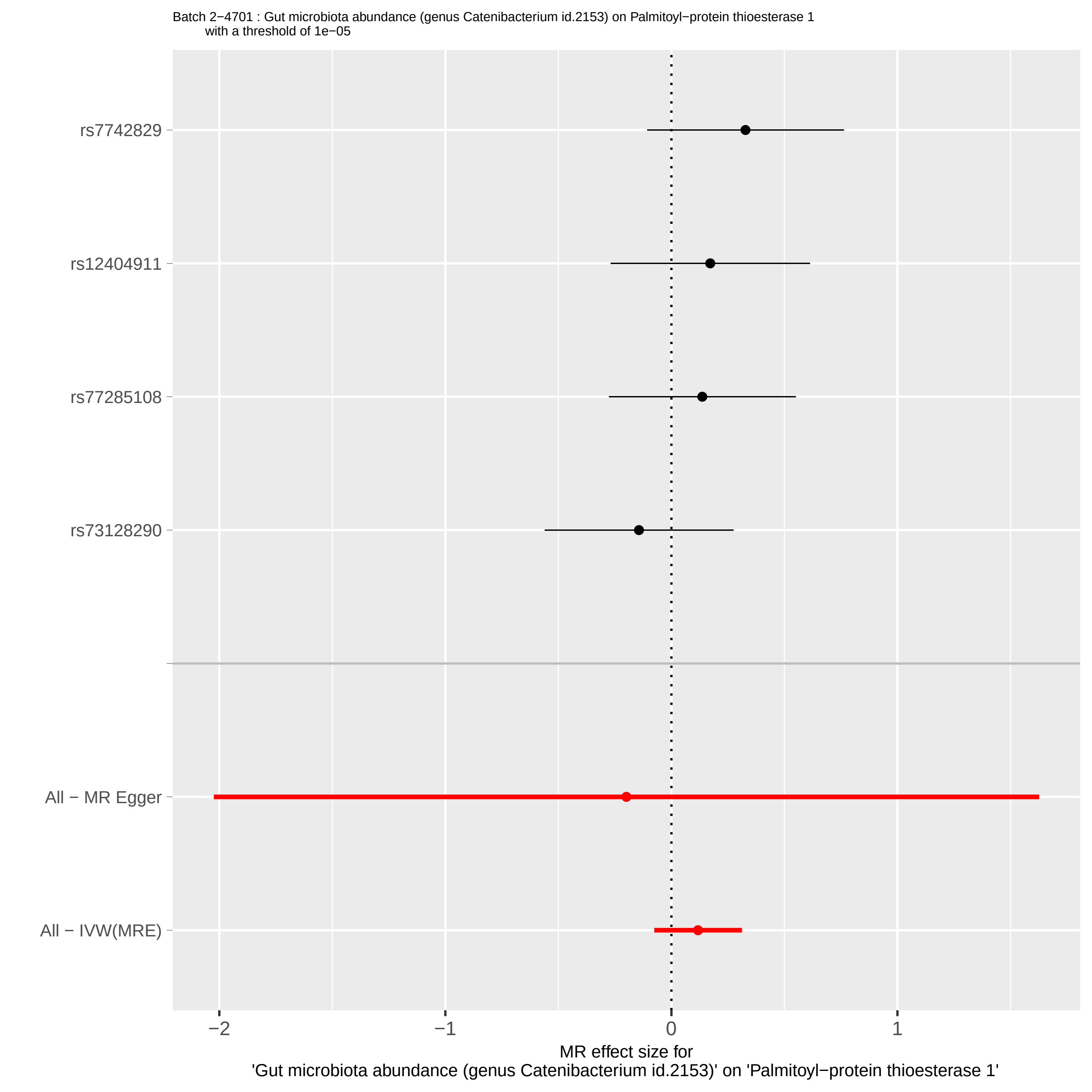
Batch 2−4701 : Gut microbiota abundance (genus Catenibacterium id.2153) on Palmitoyl−protein thioesterase 1
with a threshold of 1e−05
rs7742829
rs12404911
rs77285108
rs73128290
All − MR Egger
All − IVW(MRE)
−2
−1
0
1
MR effect size for
'Gut microbiota abundance (genus Catenibacterium id.2153)' on 'Palmitoyl−protein thioesterase 1'

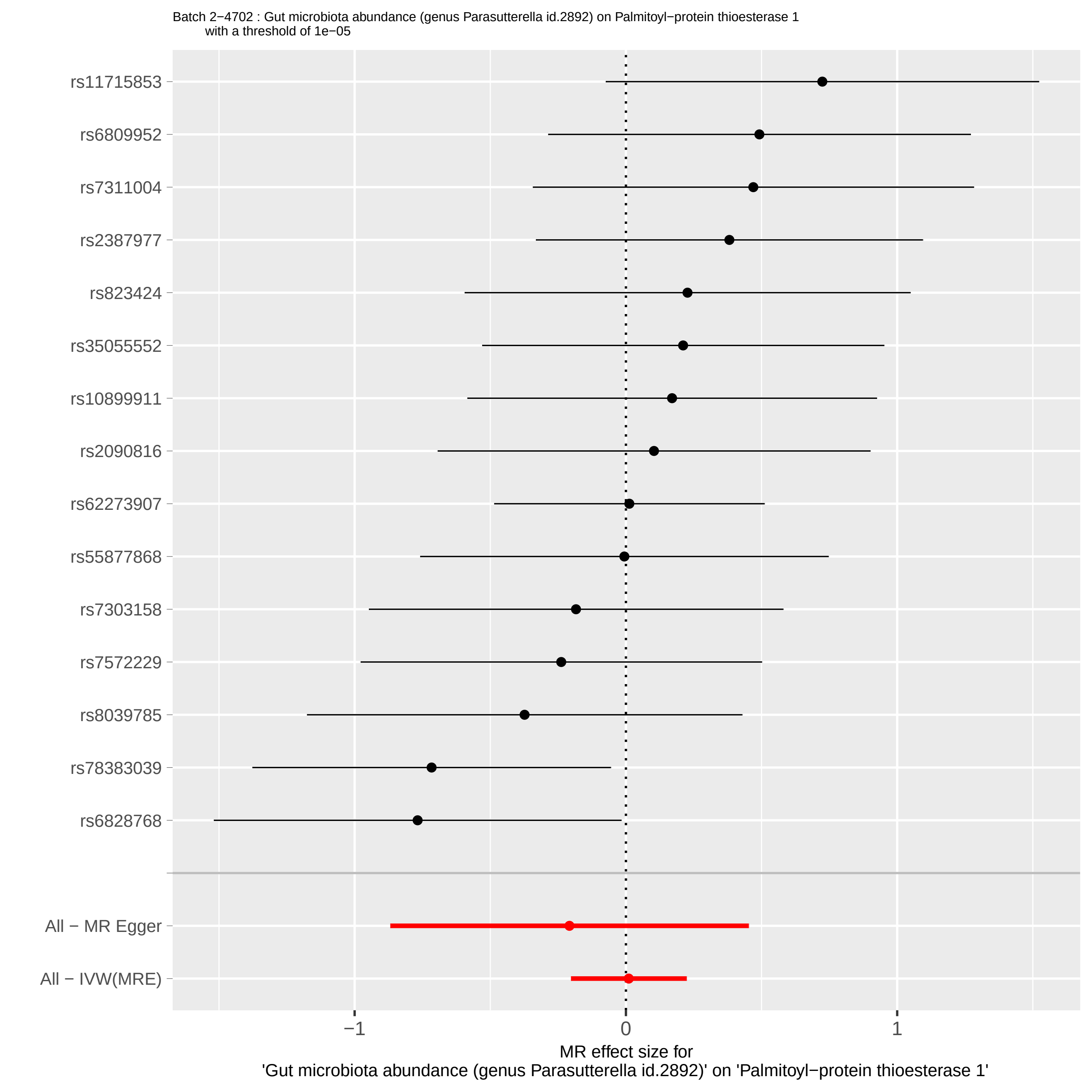

Batch 2−4702 : Gut microbiota abundance (genus Parasutterella id.2892) on Palmitoyl−protein thioesterase 1
with a threshold of 1e−05
rs11715853
rs6809952
rs7311004
rs2387977
rs823424
rs35055552
rs10899911
rs2090816
rs62273907
rs55877868
rs7303158
rs7572229
rs8039785
rs78383039
rs6828768
All − MR Egger
All − IVW(MRE)
−1
0
1
MR effect size for
'Gut microbiota abundance (genus Parasutterella id.2892)' on 'Palmitoyl−protein thioesterase 1'

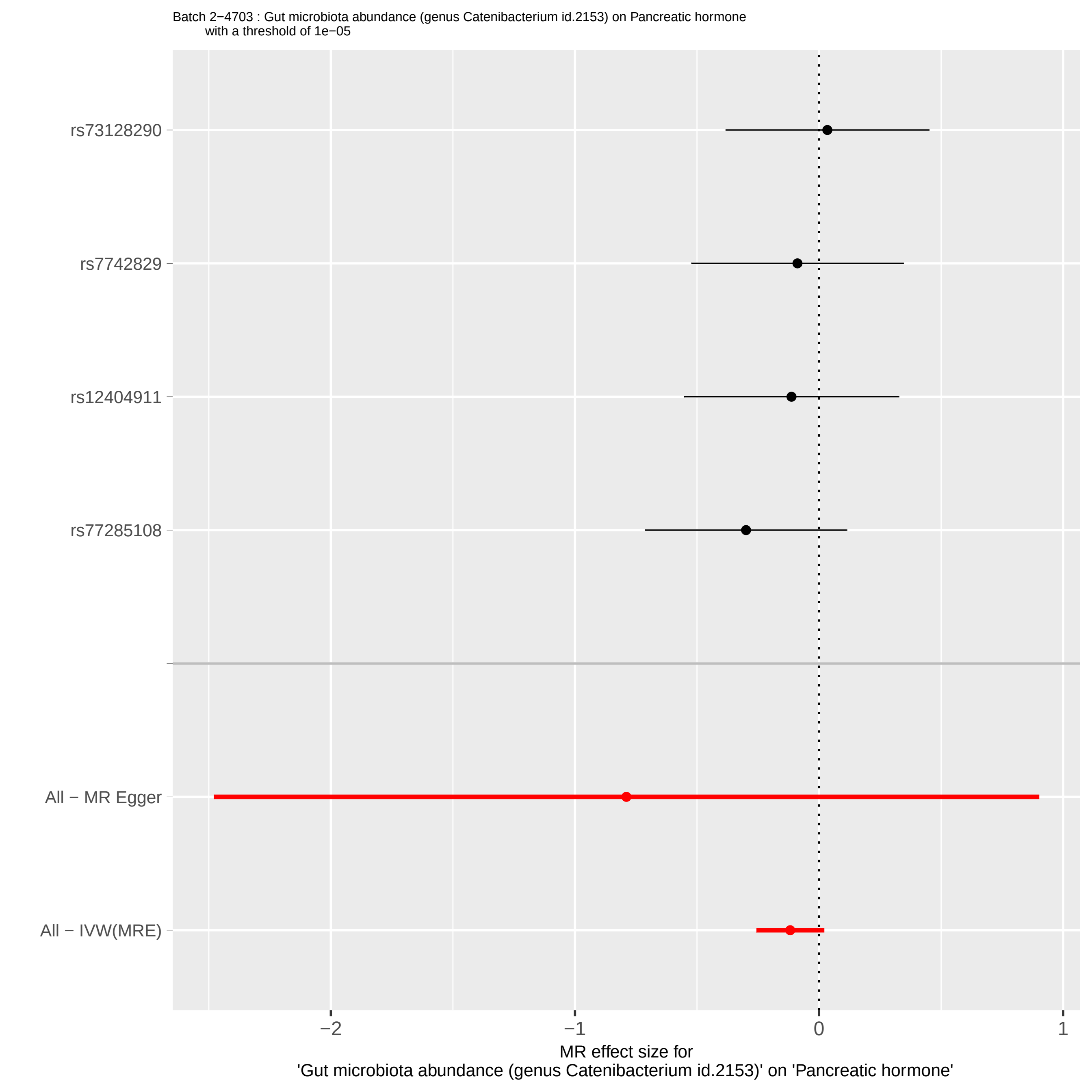

Batch 2−4703 : Gut microbiota abundance (genus Catenibacterium id.2153) on Pancreatic hormone
with a threshold of 1e−05
rs73128290
rs7742829
rs12404911
rs77285108
All − MR Egger
All − IVW(MRE)
−2
−1
0
1
MR effect size for
'Gut microbiota abundance (genus Catenibacterium id.2153)' on 'Pancreatic hormone'

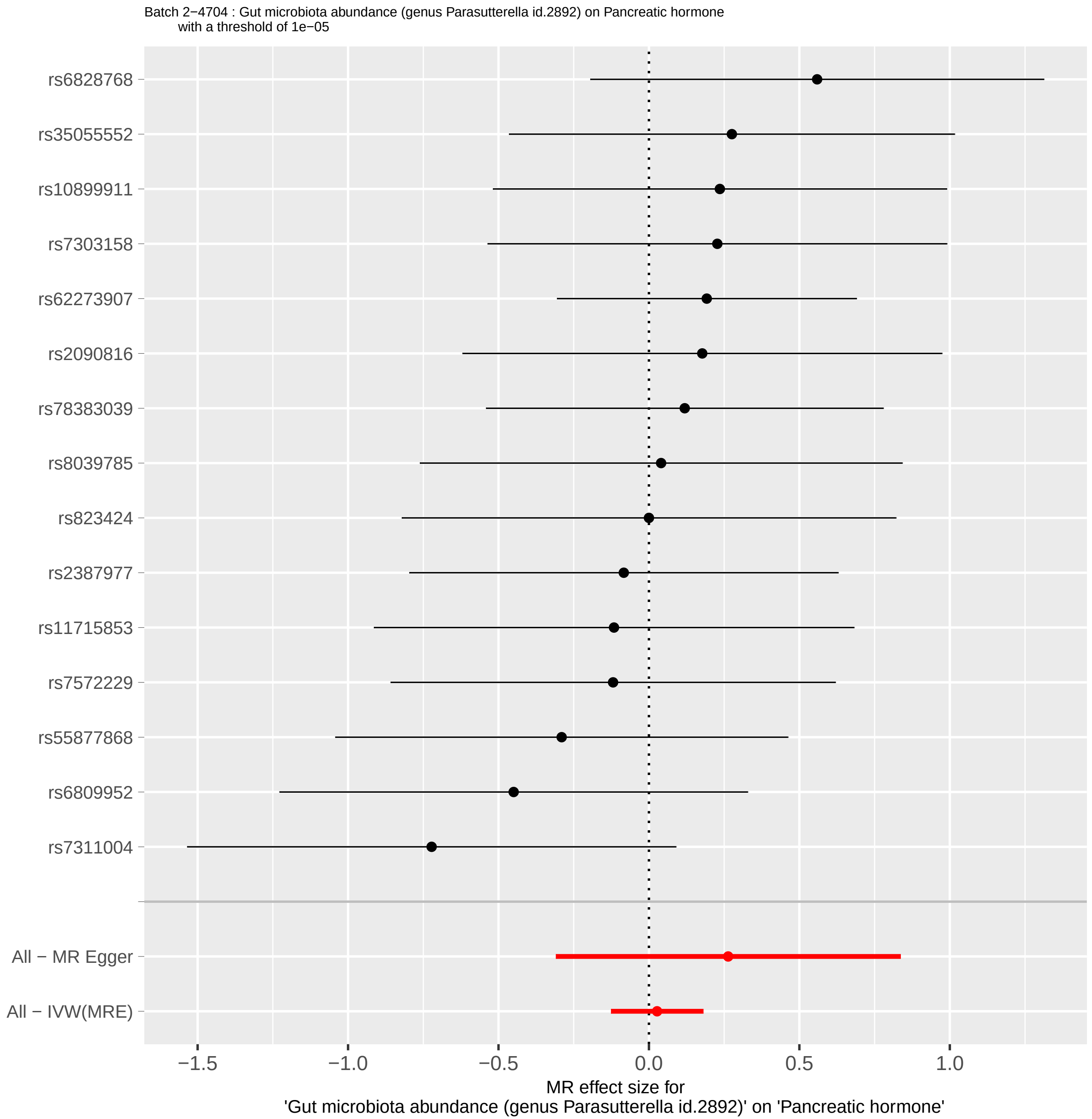

Batch 2−4704 : Gut microbiota abundance (genus Parasutterella id.2892) on Pancreatic hormone
with a threshold of 1e−05
rs6828768
rs35055552
rs10899911
rs7303158
rs62273907
rs2090816
rs78383039
rs8039785
rs823424
rs2387977
rs11715853
rs7572229
rs55877868
rs6809952
rs7311004
All − MR Egger
All − IVW(MRE)
−1.5
−1.0
−0.5
0.0
0.5
1.0
MR effect size for
'Gut microbiota abundance (genus Parasutterella id.2892)' on 'Pancreatic hormone'

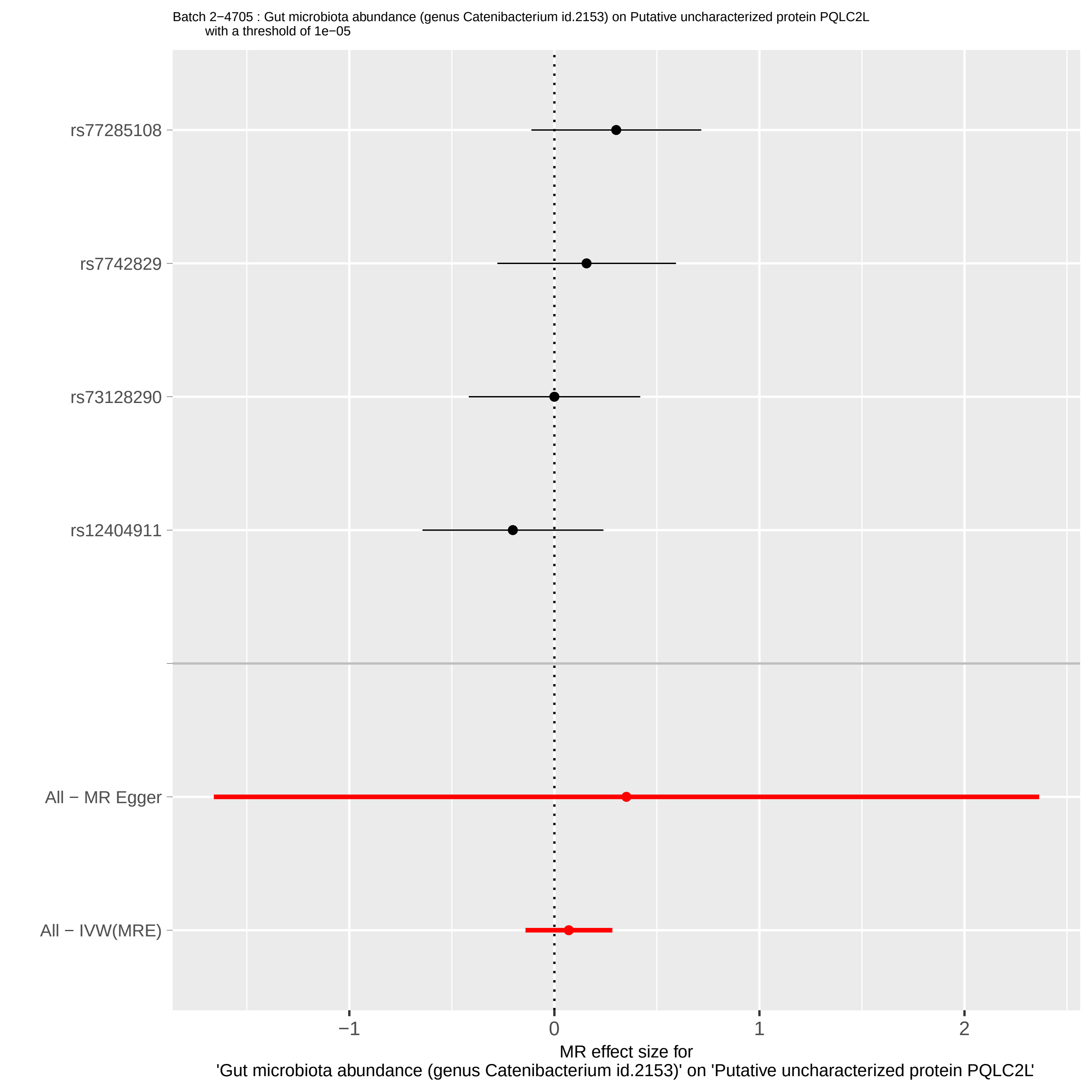
Batch 2−4705 : Gut microbiota abundance (genus Catenibacterium id.2153) on Putative uncharacterized protein PQLC2L
with a threshold of 1e−05
rs77285108
rs7742829
rs73128290
rs12404911
All − MR Egger
All − IVW(MRE)
−1
0
1
2
MR effect size for
'Gut microbiota abundance (genus Catenibacterium id.2153)' on 'Putative uncharacterized protein PQLC2L'

Batch 2−4706 : Gut microbiota abundance (genus Parasutterella id.2892) on Putative uncharacterized protein PQLC2L
with a threshold of 1e−05

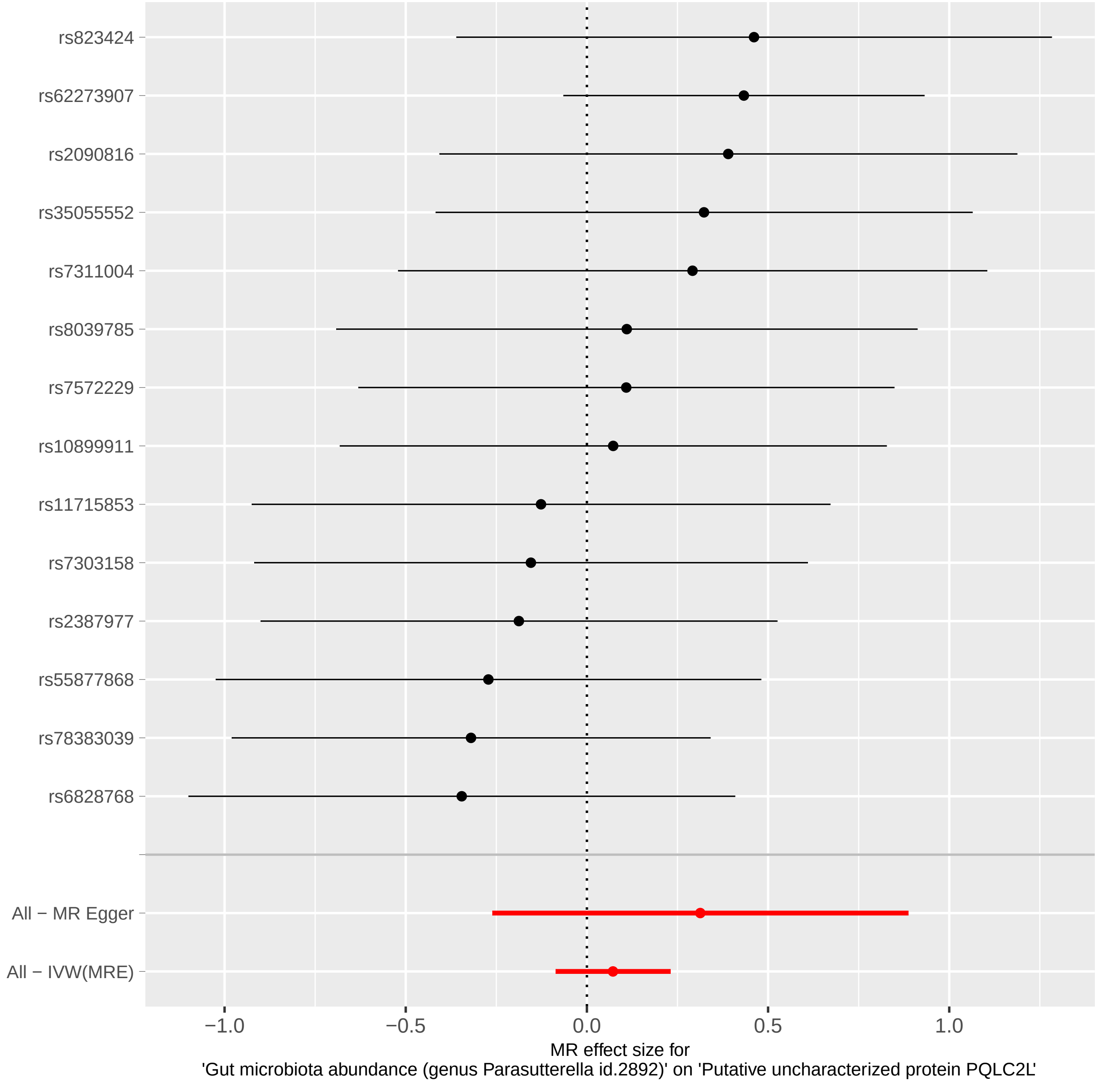

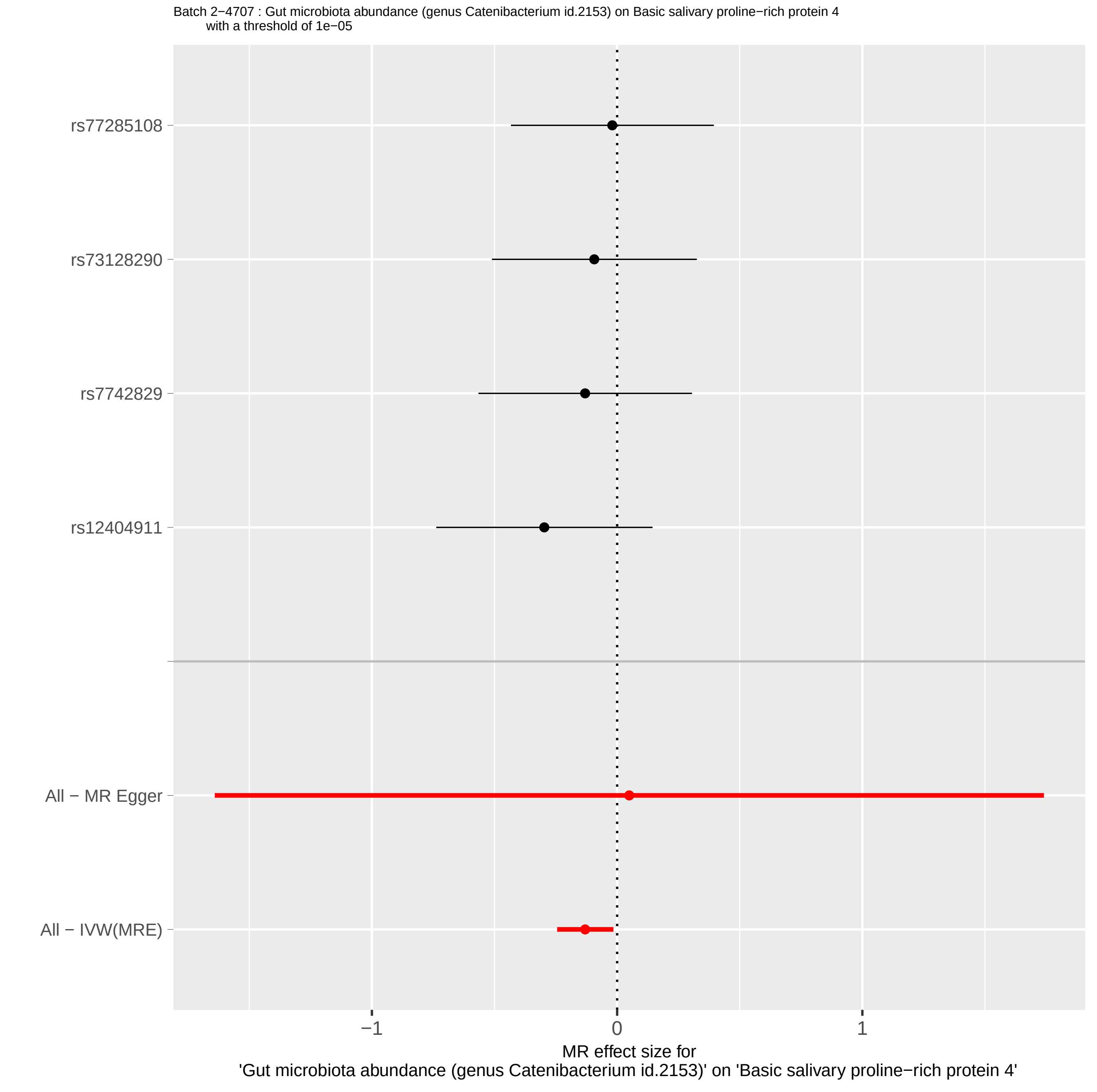

Batch 2−4707 : Gut microbiota abundance (genus Catenibacterium id.2153) on Basic salivary proline−rich protein 4
with a threshold of 1e−05
rs77285108
rs73128290
rs7742829
rs12404911
All − MR Egger
All − IVW(MRE)
−1
0
1
MR effect size for
'Gut microbiota abundance (genus Catenibacterium id.2153)' on 'Basic salivary proline−rich protein 4'

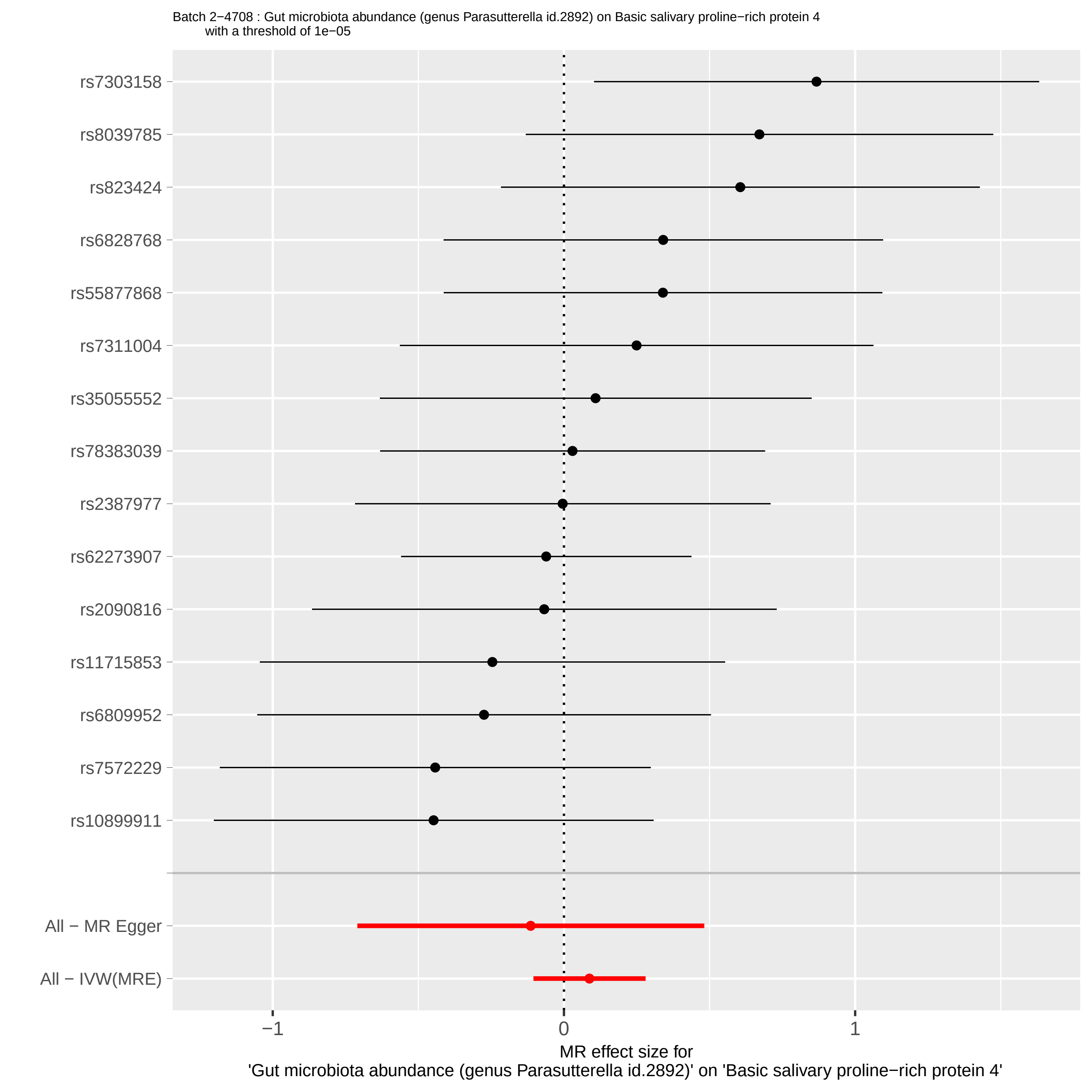
Batch 2−4708 : Gut microbiota abundance (genus Parasutterella id.2892) on Basic salivary proline−rich protein 4
with a threshold of 1e−05
rs7303158
rs8039785
rs823424
rs6828768
rs55877868
rs7311004
rs35055552
rs78383039
rs2387977
rs62273907
rs2090816
rs11715853
rs6809952
rs7572229
rs10899911
All − MR Egger
All − IVW(MRE)
−1
0
1
MR effect size for
'Gut microbiota abundance (genus Parasutterella id.2892)' on 'Basic salivary proline−rich protein 4'

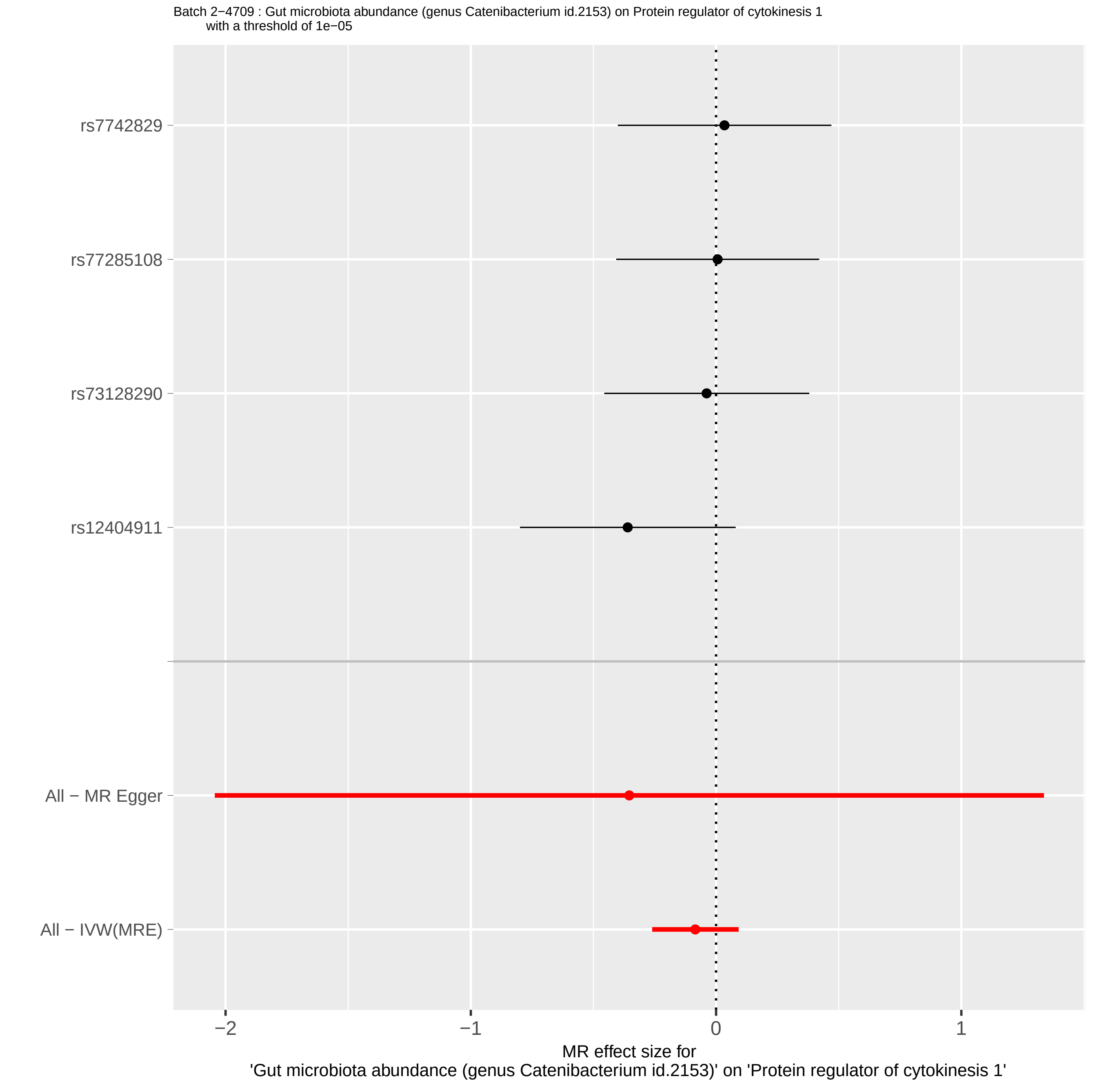
Batch 2−4709 : Gut microbiota abundance (genus Catenibacterium id.2153) on Protein regulator of cytokinesis 1
with a threshold of 1e−05
rs7742829
rs77285108
rs73128290
rs12404911
All − MR Egger
All − IVW(MRE)
−2
−1
0
1
MR effect size for
'Gut microbiota abundance (genus Catenibacterium id.2153)' on 'Protein regulator of cytokinesis 1'

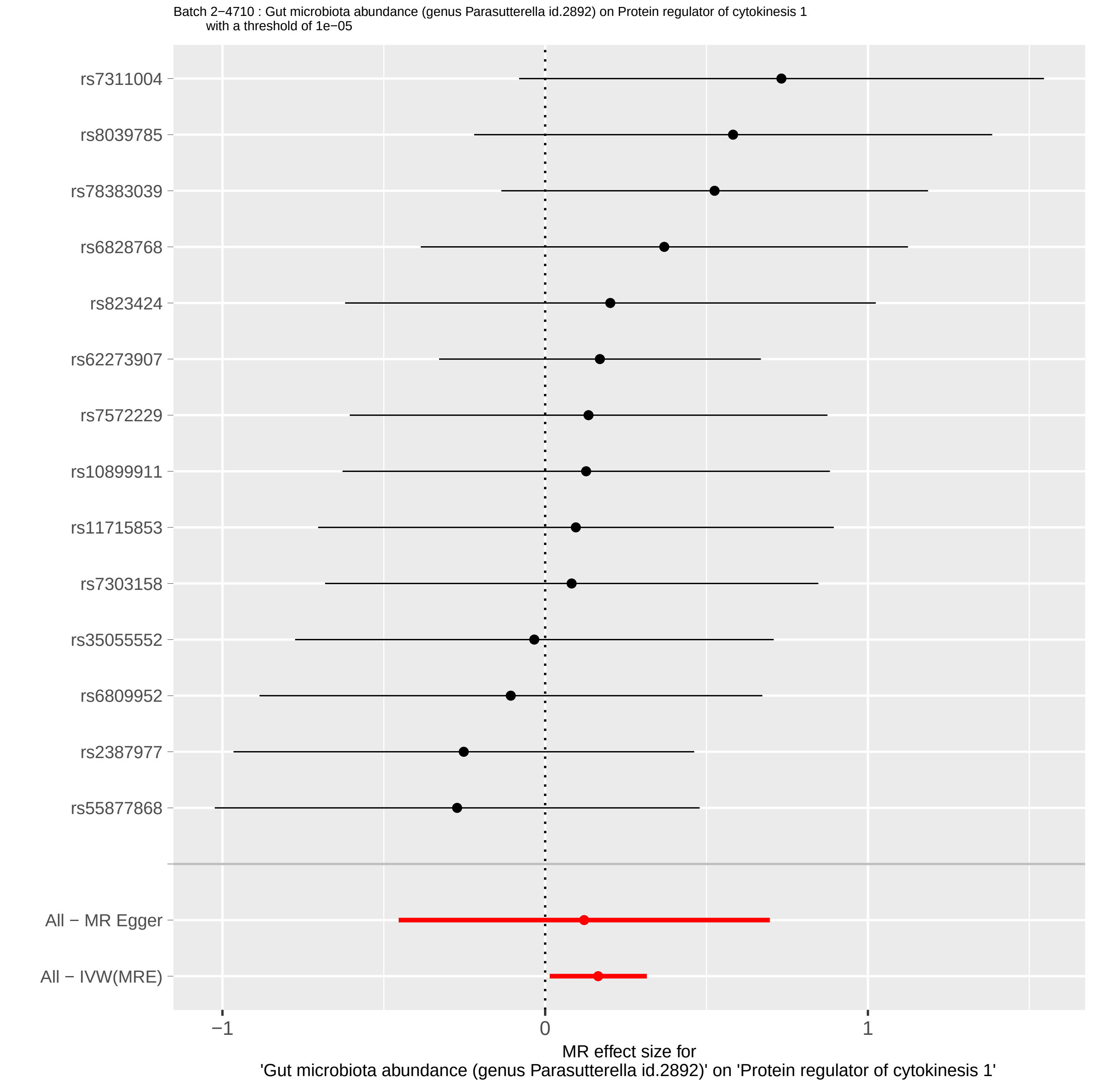

Batch 2−4710 : Gut microbiota abundance (genus Parasutterella id.2892) on Protein regulator of cytokinesis 1
with a threshold of 1e−05
rs7311004
rs8039785
rs78383039
rs6828768
rs823424
rs62273907
rs7572229
rs10899911
rs11715853
rs7303158
rs35055552
rs6809952
rs2387977
rs55877868
All − MR Egger
All − IVW(MRE)
−1
0
1
MR effect size for
'Gut microbiota abundance (genus Parasutterella id.2892)' on 'Protein regulator of cytokinesis 1'

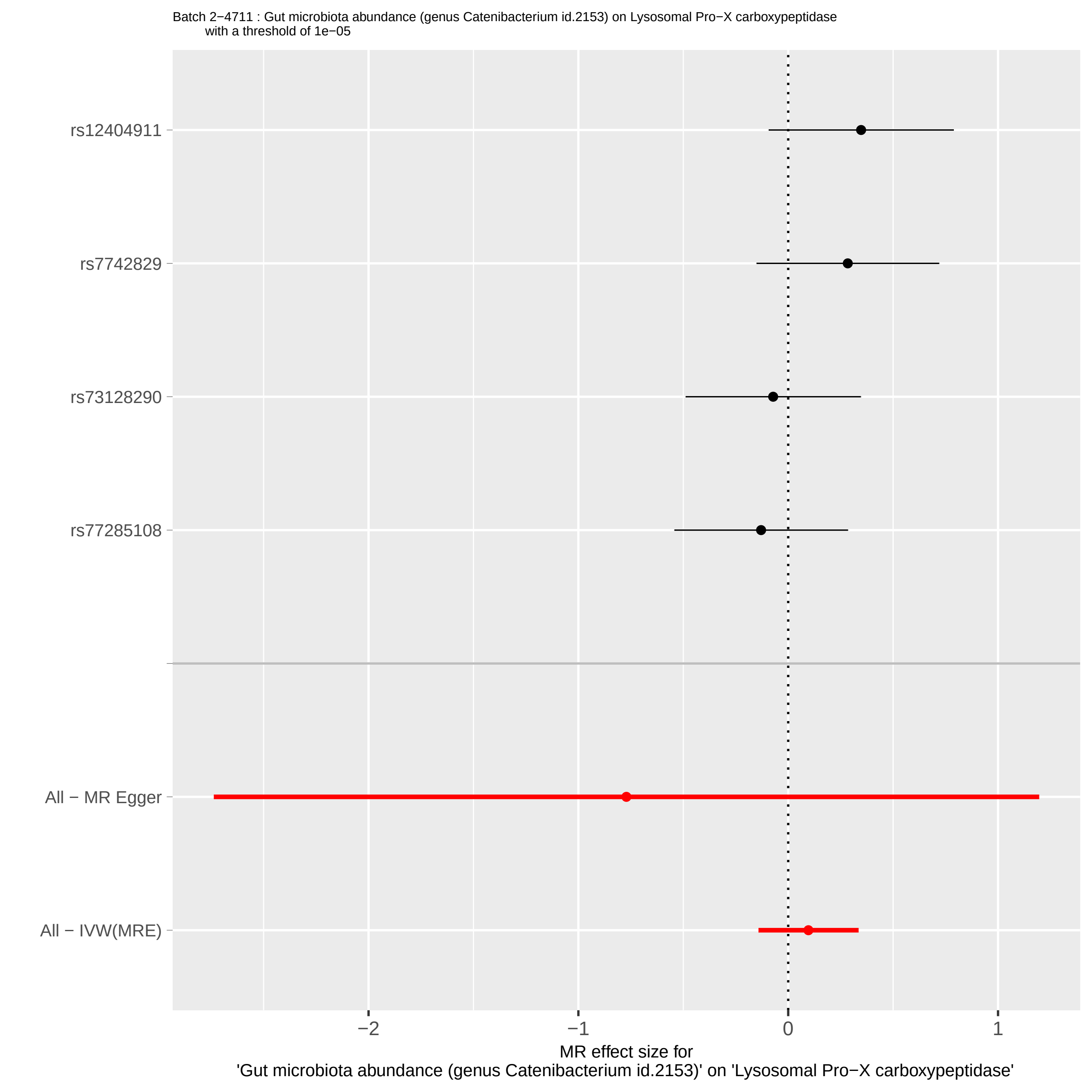
Batch 2−4711 : Gut microbiota abundance (genus Catenibacterium id.2153) on Lysosomal Pro−X carboxypeptidase
with a threshold of 1e−05
rs12404911
rs7742829
rs73128290
rs77285108
All − MR Egger
All − IVW(MRE)
−2
−1
0
1
MR effect size for
'Gut microbiota abundance (genus Catenibacterium id.2153)' on 'Lysosomal Pro−X carboxypeptidase'

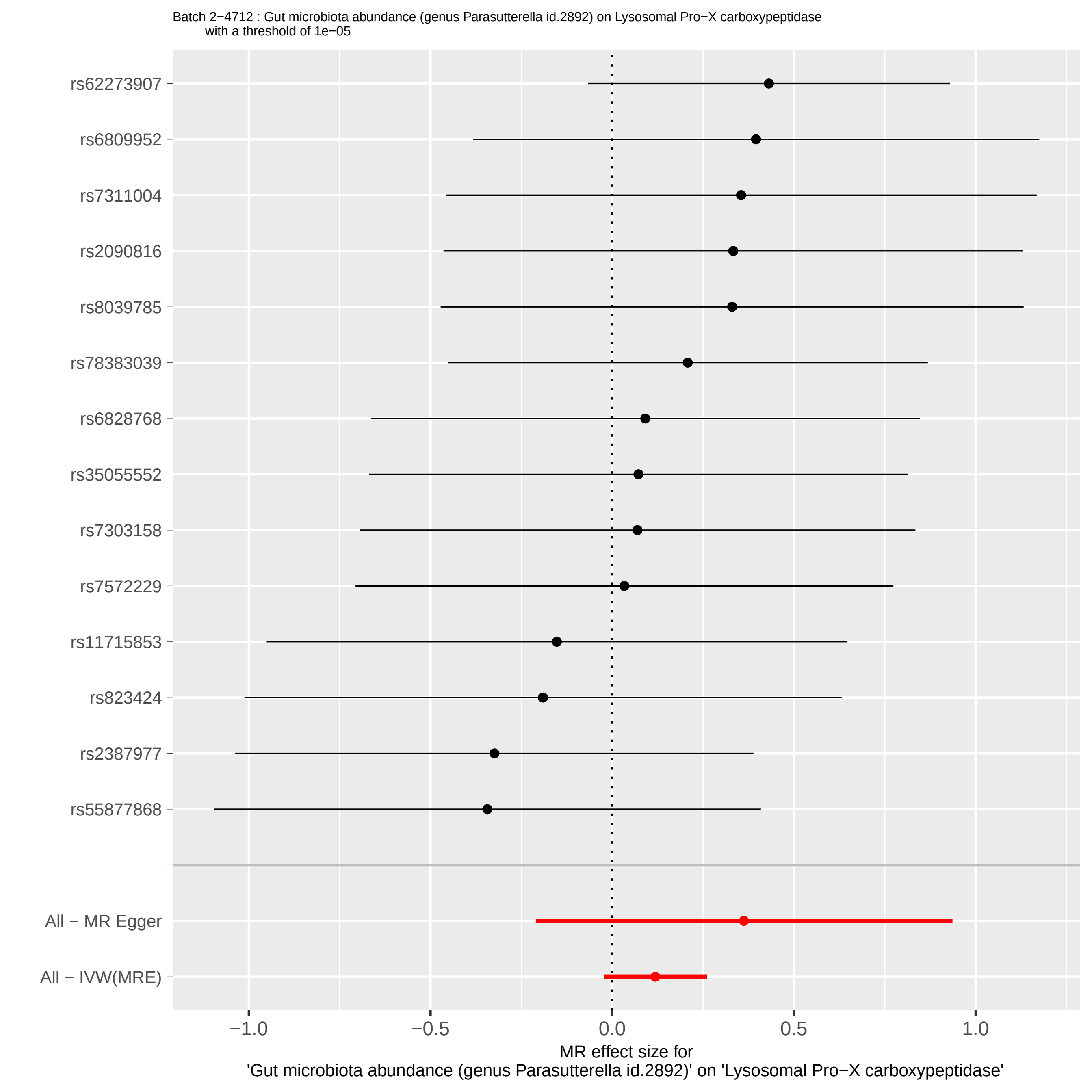

Batch 2−4712 : Gut microbiota abundance (genus Parasutterella id.2892) on Lysosomal Pro−X carboxypeptidase
with a threshold of 1e−05
rs62273907
rs6809952
rs7311004
rs2090816
rs8039785
rs78383039
rs6828768
rs35055552
rs7303158
rs7572229
rs11715853
rs823424
rs2387977
rs55877868
All − MR Egger
All − IVW(MRE)
−1.0
−0.5
0.0
0.5
1.0
MR effect size for
'Gut microbiota abundance (genus Parasutterella id.2892)' on 'Lysosomal Pro−X carboxypeptidase'

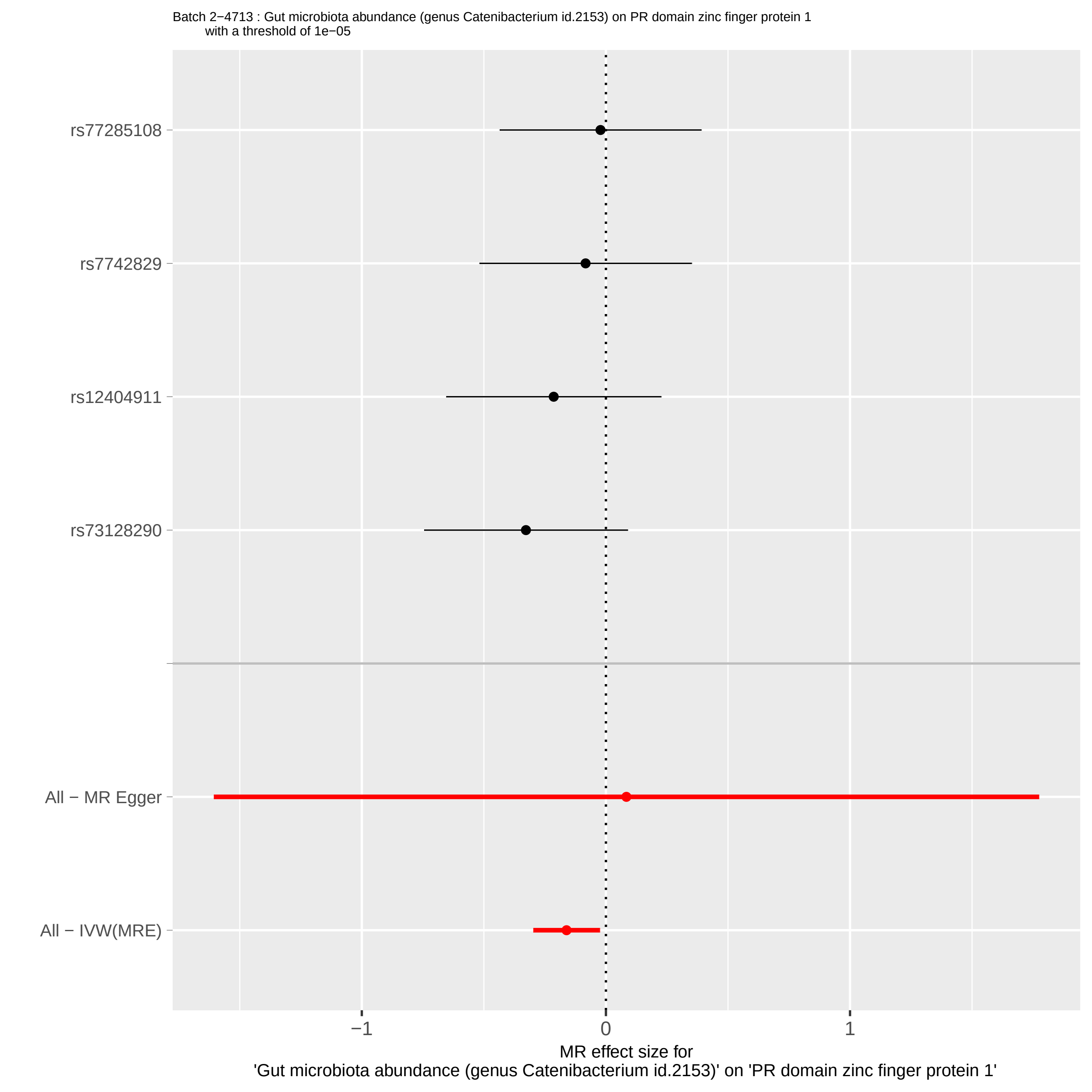
Batch 2−4713 : Gut microbiota abundance (genus Catenibacterium id.2153) on PR domain zinc finger protein 1
with a threshold of 1e−05
rs77285108
rs7742829
rs12404911
rs73128290
All − MR Egger
All − IVW(MRE)
−1
0
1
MR effect size for
'Gut microbiota abundance (genus Catenibacterium id.2153)' on 'PR domain zinc finger protein 1'

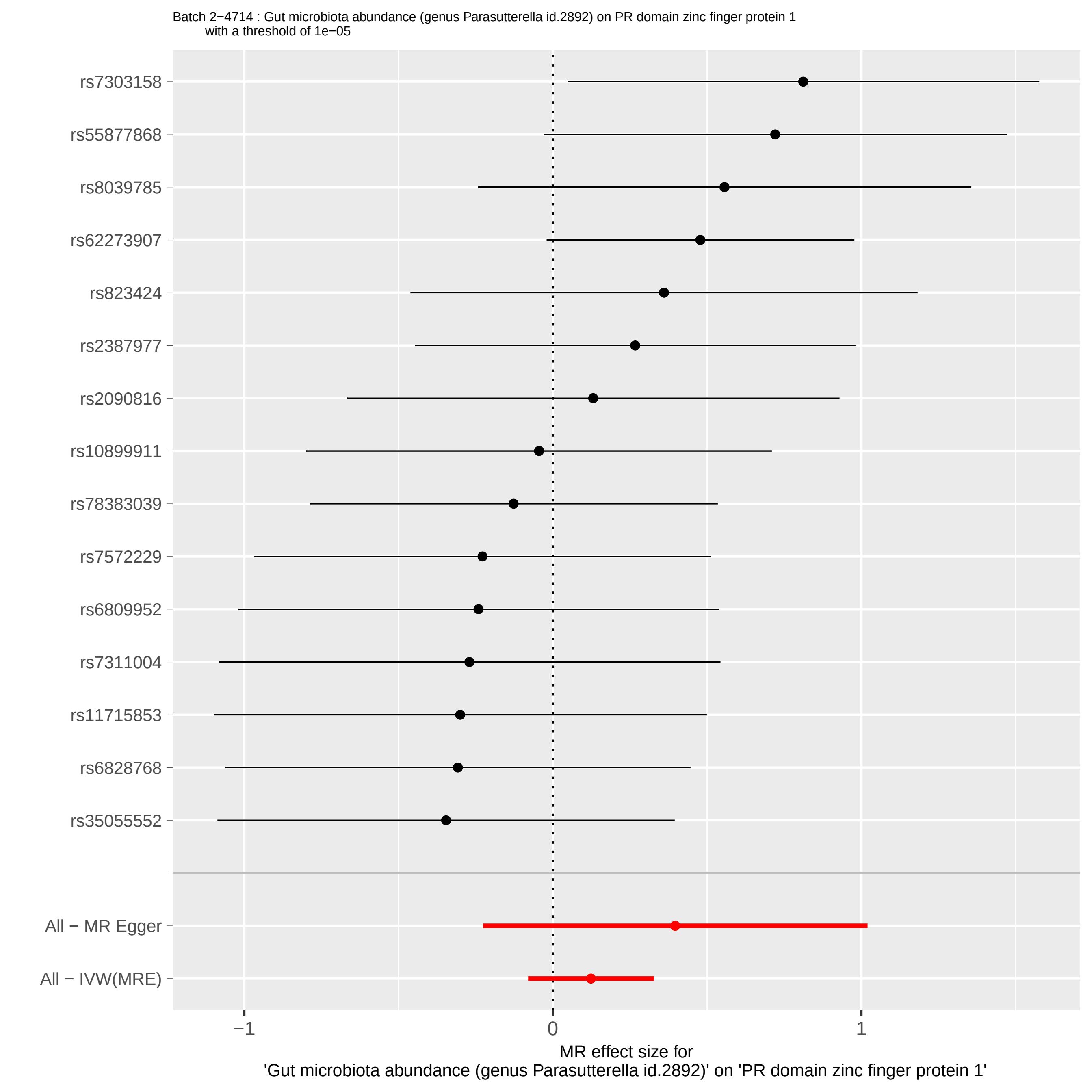

Batch 2−4714 : Gut microbiota abundance (genus Parasutterella id.2892) on PR domain zinc finger protein 1
with a threshold of 1e−05
rs7303158
rs55877868
rs8039785
rs62273907
rs823424
rs2387977
rs2090816
rs10899911
rs78383039
rs7572229
rs6809952
rs7311004
rs11715853
rs6828768
rs35055552
All − MR Egger
All − IVW(MRE)
−1
0
1
MR effect size for
'Gut microbiota abundance (genus Parasutterella id.2892)' on 'PR domain zinc finger protein 1'

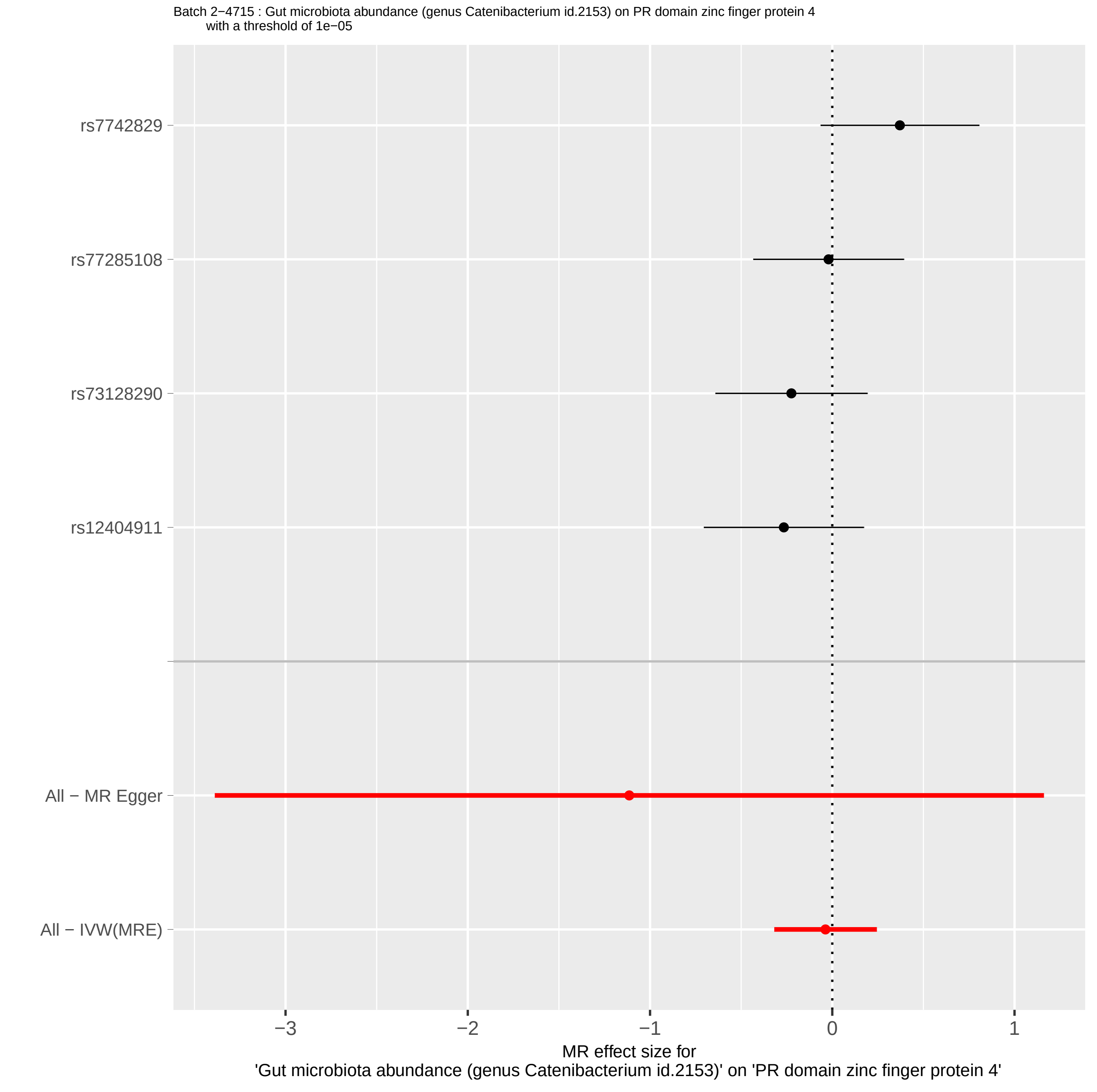
Batch 2−4715 : Gut microbiota abundance (genus Catenibacterium id.2153) on PR domain zinc finger protein 4
with a threshold of 1e−05
rs7742829
rs77285108
rs73128290
rs12404911
All − MR Egger
All − IVW(MRE)
−3
−2
−1
0
1
MR effect size for
'Gut microbiota abundance (genus Catenibacterium id.2153)' on 'PR domain zinc finger protein 4'

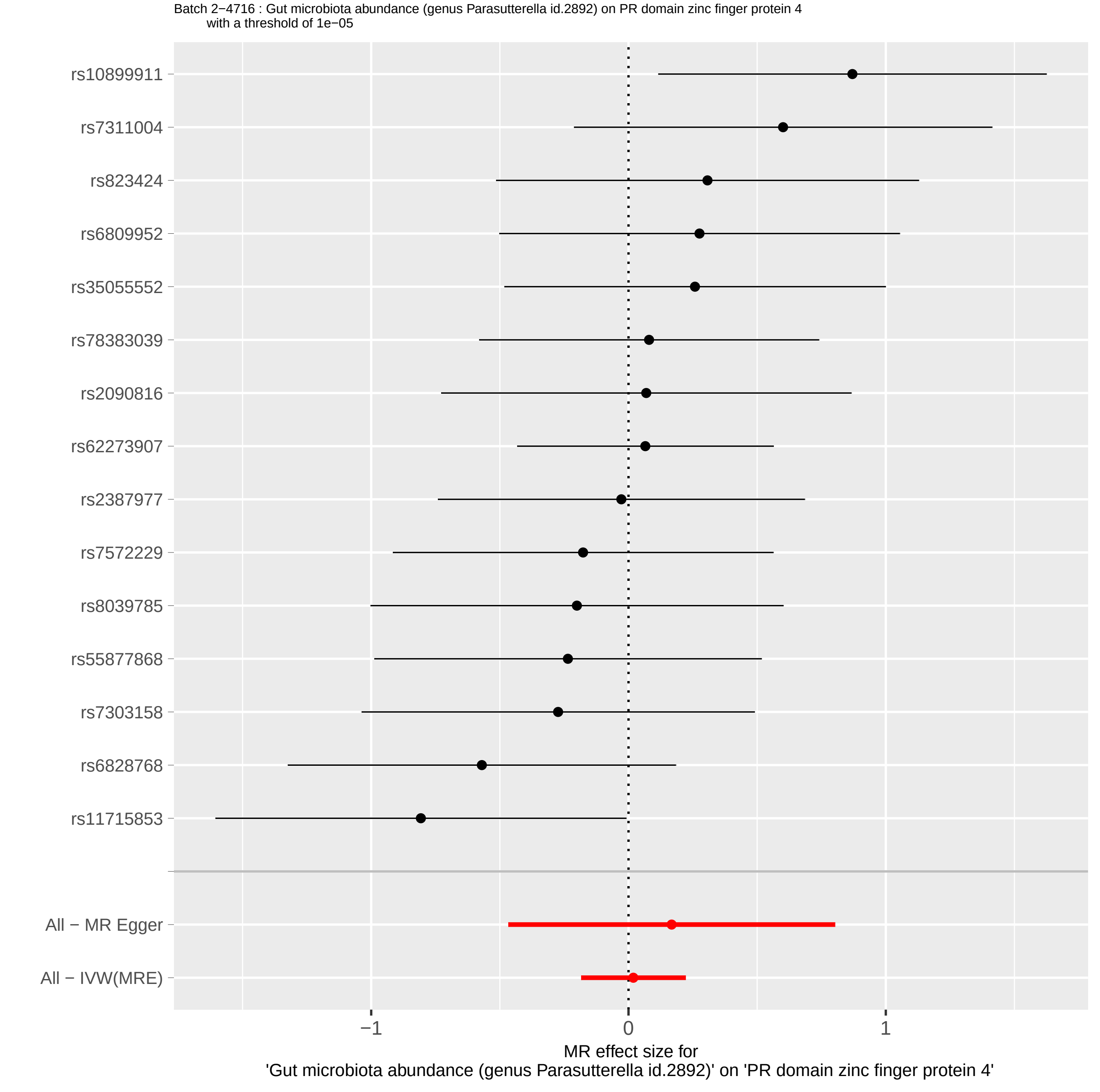

Batch 2−4716 : Gut microbiota abundance (genus Parasutterella id.2892) on PR domain zinc finger protein 4
with a threshold of 1e−05
rs10899911
rs7311004
rs823424
rs6809952
rs35055552
rs78383039
rs2090816
rs62273907
rs2387977
rs7572229
rs8039785
rs55877868
rs7303158
rs6828768
rs11715853
All − MR Egger
All − IVW(MRE)
−1
0
1
MR effect size for
'Gut microbiota abundance (genus Parasutterella id.2892)' on 'PR domain zinc finger protein 4'

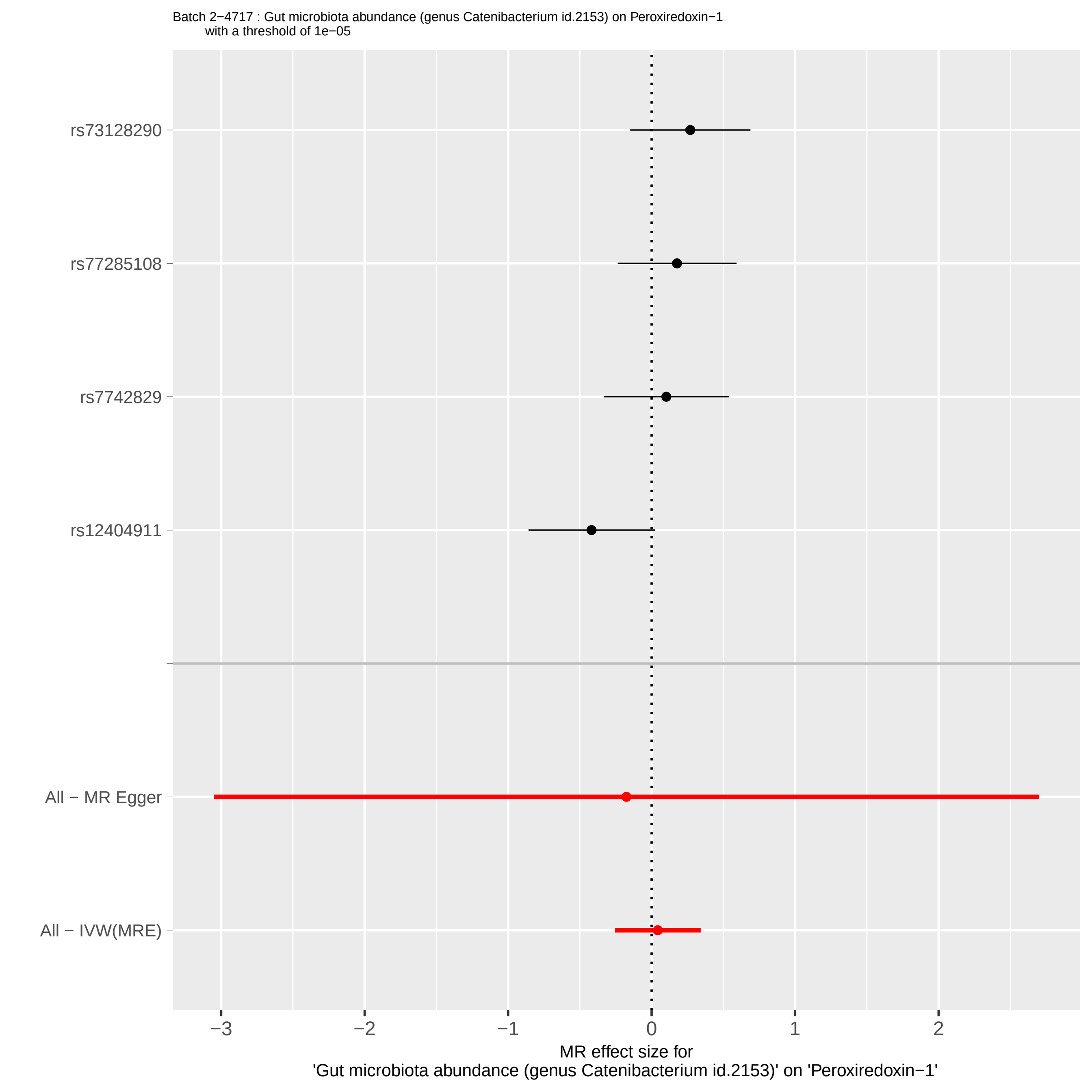
Batch 2−4717 : Gut microbiota abundance (genus Catenibacterium id.2153) on Peroxiredoxin−1
with a threshold of 1e−05
rs73128290
rs77285108
rs7742829
rs12404911
All − MR Egger
All − IVW(MRE)
−3
−2
−1
0
1
2
MR effect size for
'Gut microbiota abundance (genus Catenibacterium id.2153)' on 'Peroxiredoxin−1'

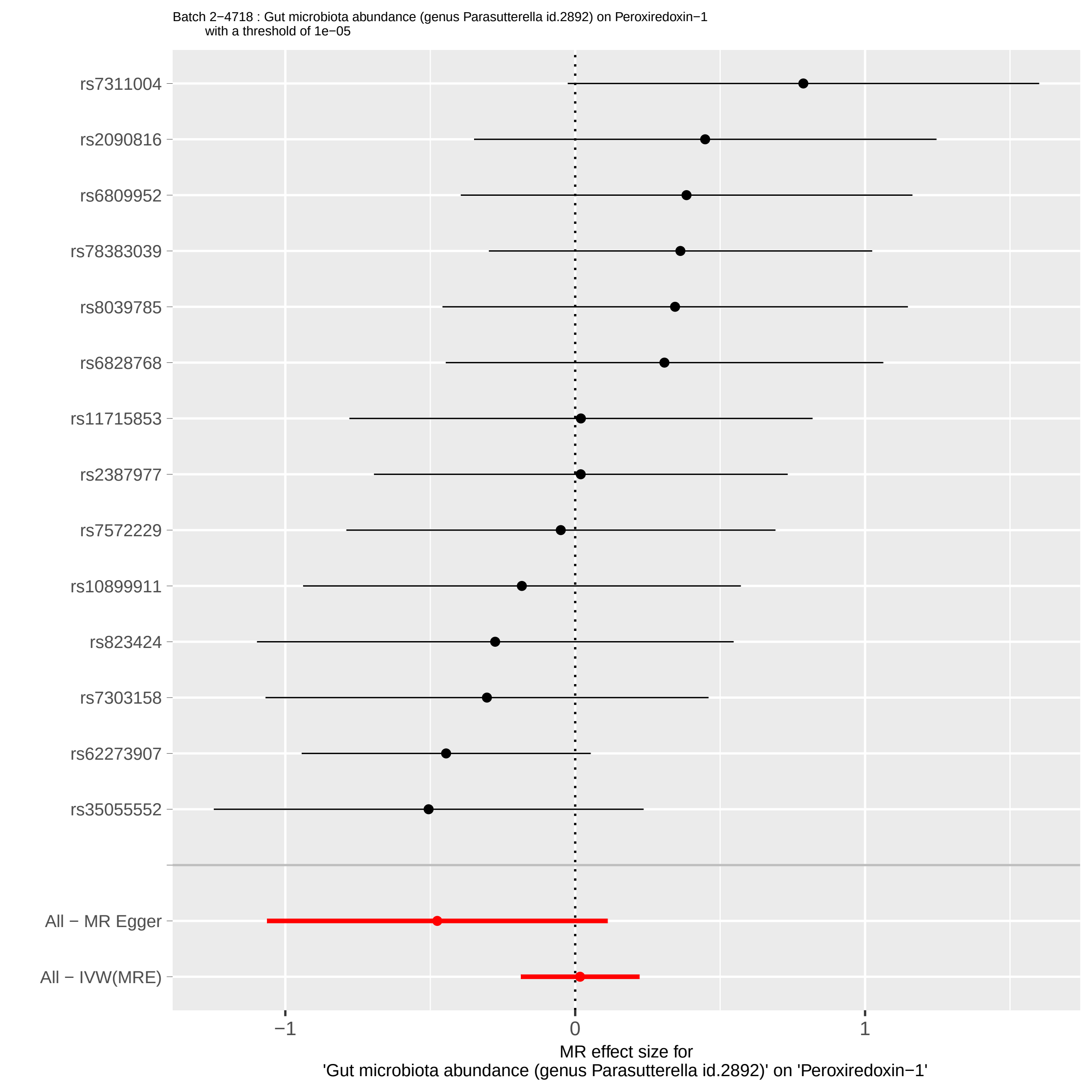
Batch 2−4718 : Gut microbiota abundance (genus Parasutterella id.2892) on Peroxiredoxin−1
with a threshold of 1e−05
rs7311004
rs2090816
rs6809952
rs78383039
rs8039785
rs6828768
rs11715853
rs2387977
rs7572229
rs10899911
rs823424
rs7303158
rs62273907
rs35055552
All − MR Egger
All − IVW(MRE)
−1
0
1
MR effect size for
'Gut microbiota abundance (genus Parasutterella id.2892)' on 'Peroxiredoxin−1'

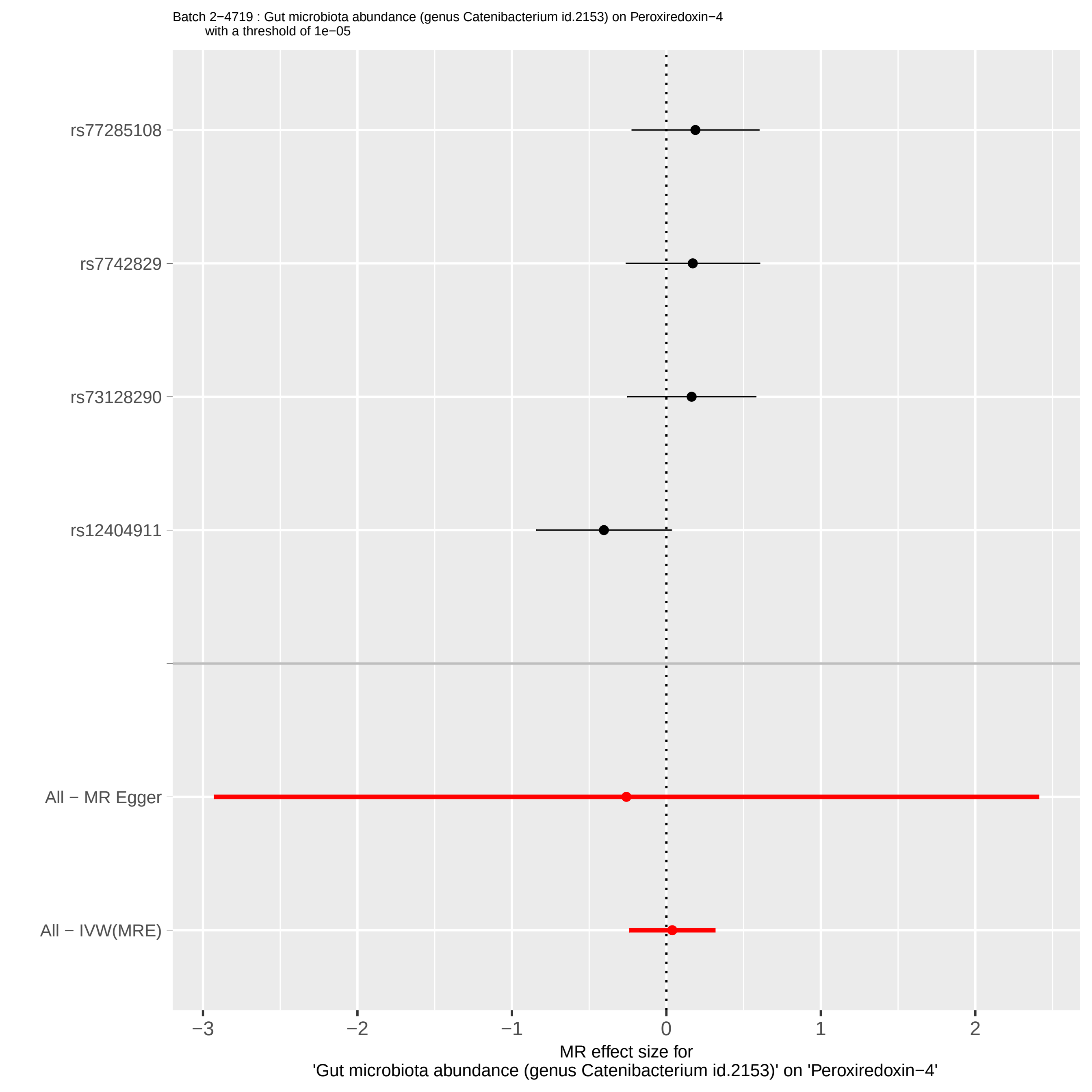

Batch 2−4719 : Gut microbiota abundance (genus Catenibacterium id.2153) on Peroxiredoxin−4
with a threshold of 1e−05
rs77285108
rs7742829
rs73128290
rs12404911
All − MR Egger
All − IVW(MRE)
−3
−2
−1
0
1
2
MR effect size for
'Gut microbiota abundance (genus Catenibacterium id.2153)' on 'Peroxiredoxin−4'

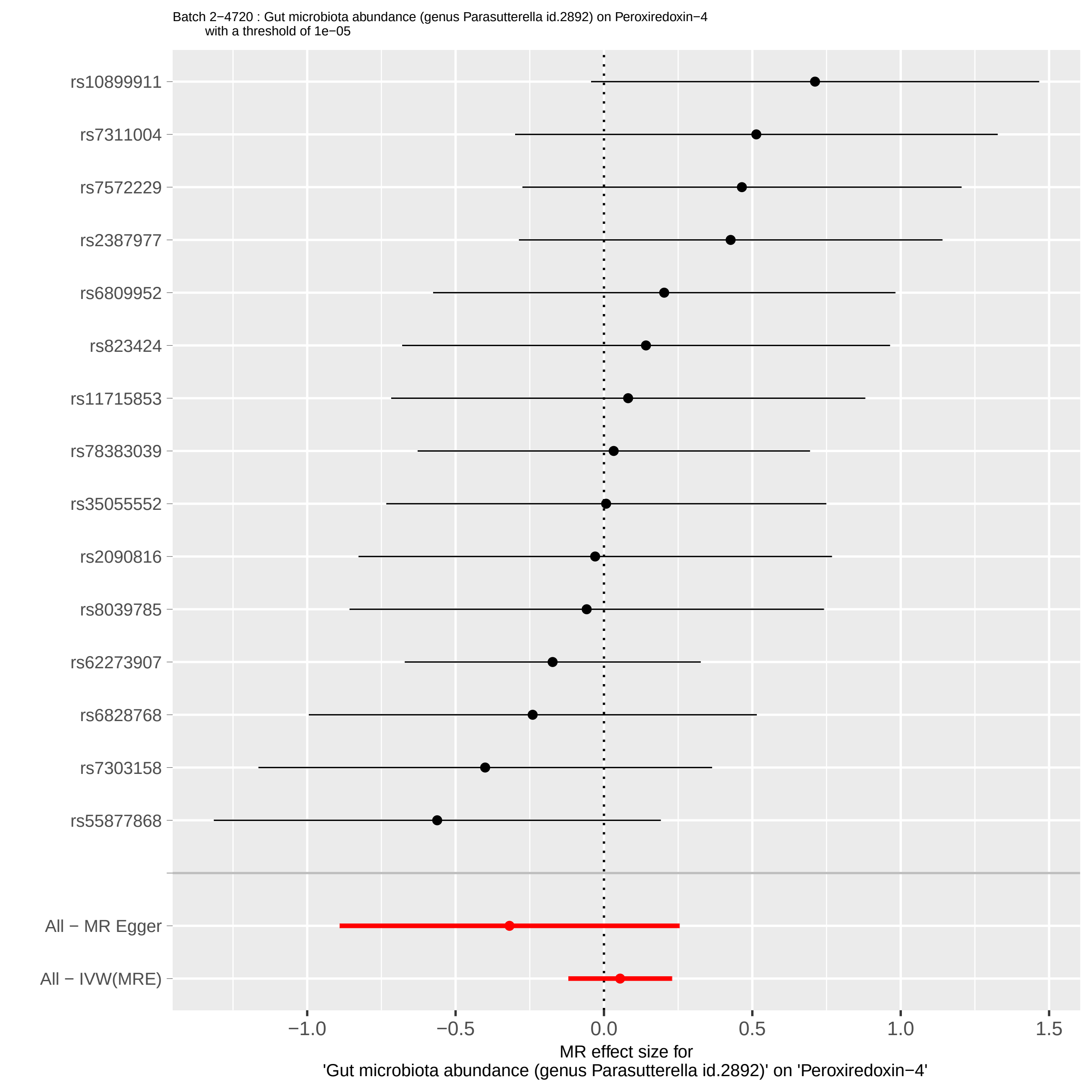
Batch 2−4720 : Gut microbiota abundance (genus Parasutterella id.2892) on Peroxiredoxin−4
with a threshold of 1e−05
rs10899911
rs7311004
rs7572229
rs2387977
rs6809952
rs823424
rs11715853
rs78383039
rs35055552
rs2090816
rs8039785
rs62273907
rs6828768
rs7303158
rs55877868
All − MR Egger
All − IVW(MRE)
−1.0
−0.5
0.0
0.5
1.0
1.5
MR effect size for
'Gut microbiota abundance (genus Parasutterella id.2892)' on 'Peroxiredoxin−4'

Batch 2−4721 : Gut microbiota abundance (genus Catenibacterium id.2153) on Prolargin
with a threshold of 1e−05

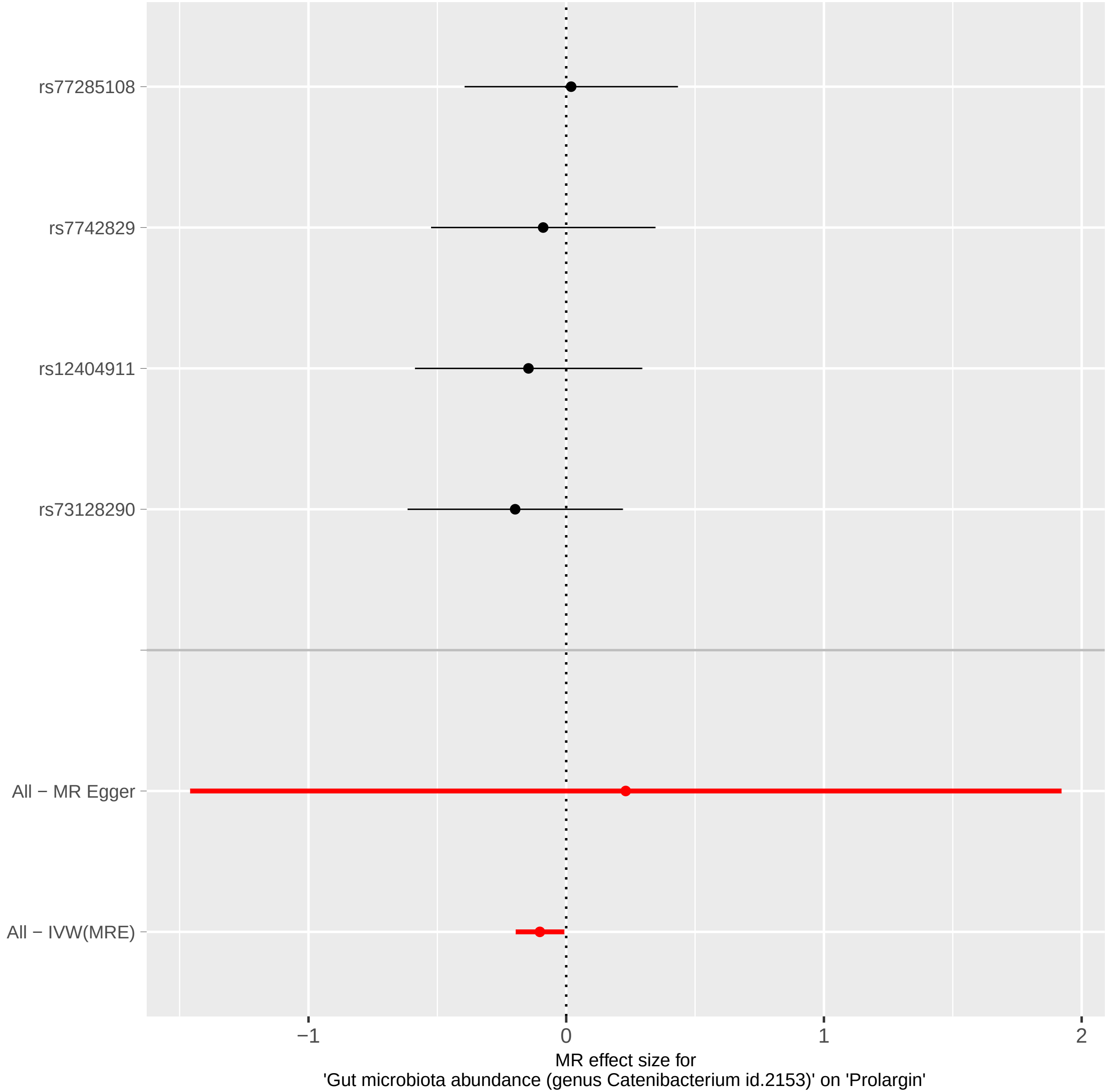

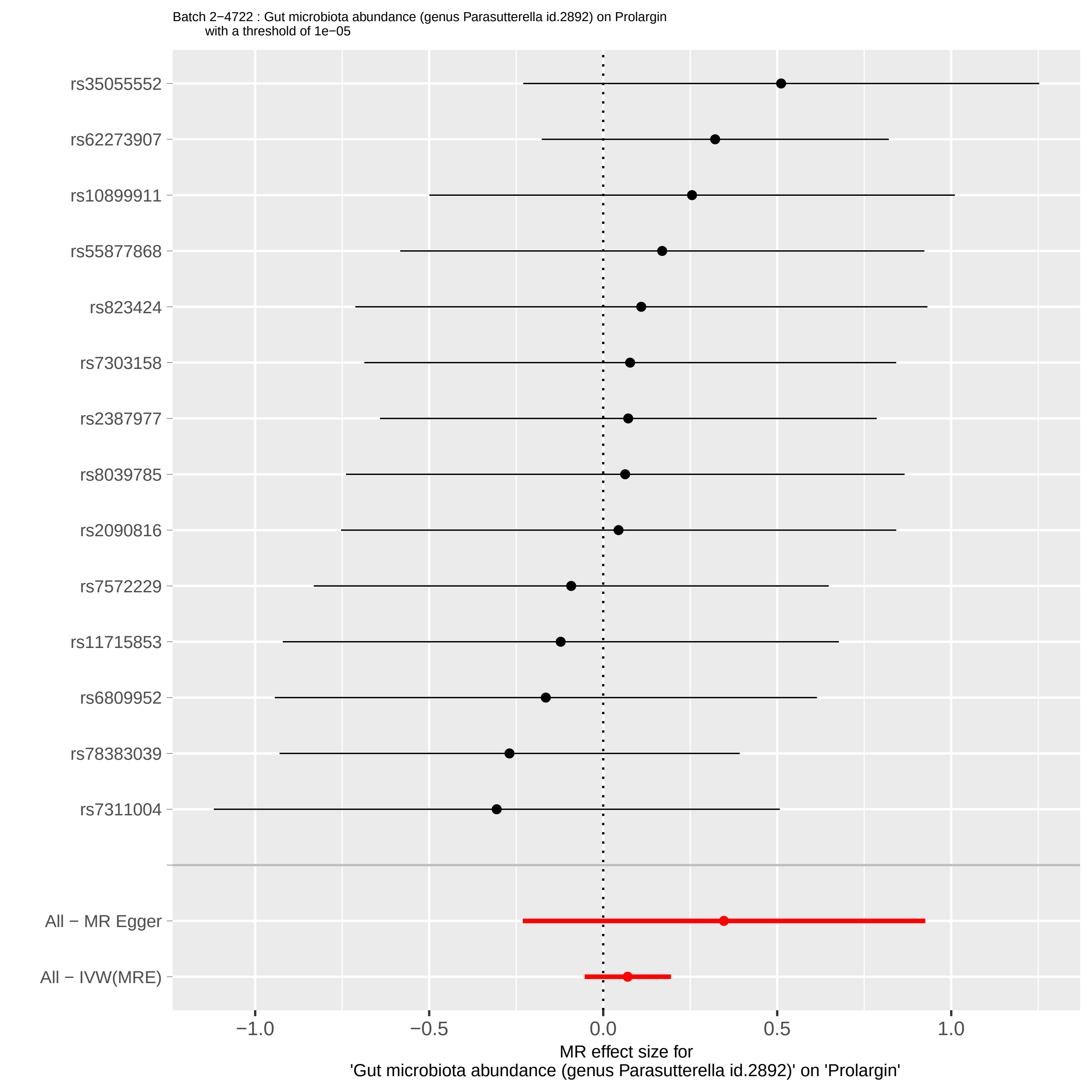

Batch 2−4722 : Gut microbiota abundance (genus Parasutterella id.2892) on Prolargin
with a threshold of 1e−05
rs35055552
rs62273907
rs10899911
rs55877868
rs823424
rs7303158
rs2387977
rs8039785
rs2090816
rs7572229
rs11715853
rs6809952
rs78383039
rs7311004
All − MR Egger
All − IVW(MRE)
−1.0
−0.5
0.0
0.5
1.0
MR effect size for
'Gut microbiota abundance (genus Parasutterella id.2892)' on 'Prolargin'

Batch 2−4723 : Gut microbiota abundance (genus Catenibacterium id.2153) on Proteoglycan 3
with a threshold of 1e−05

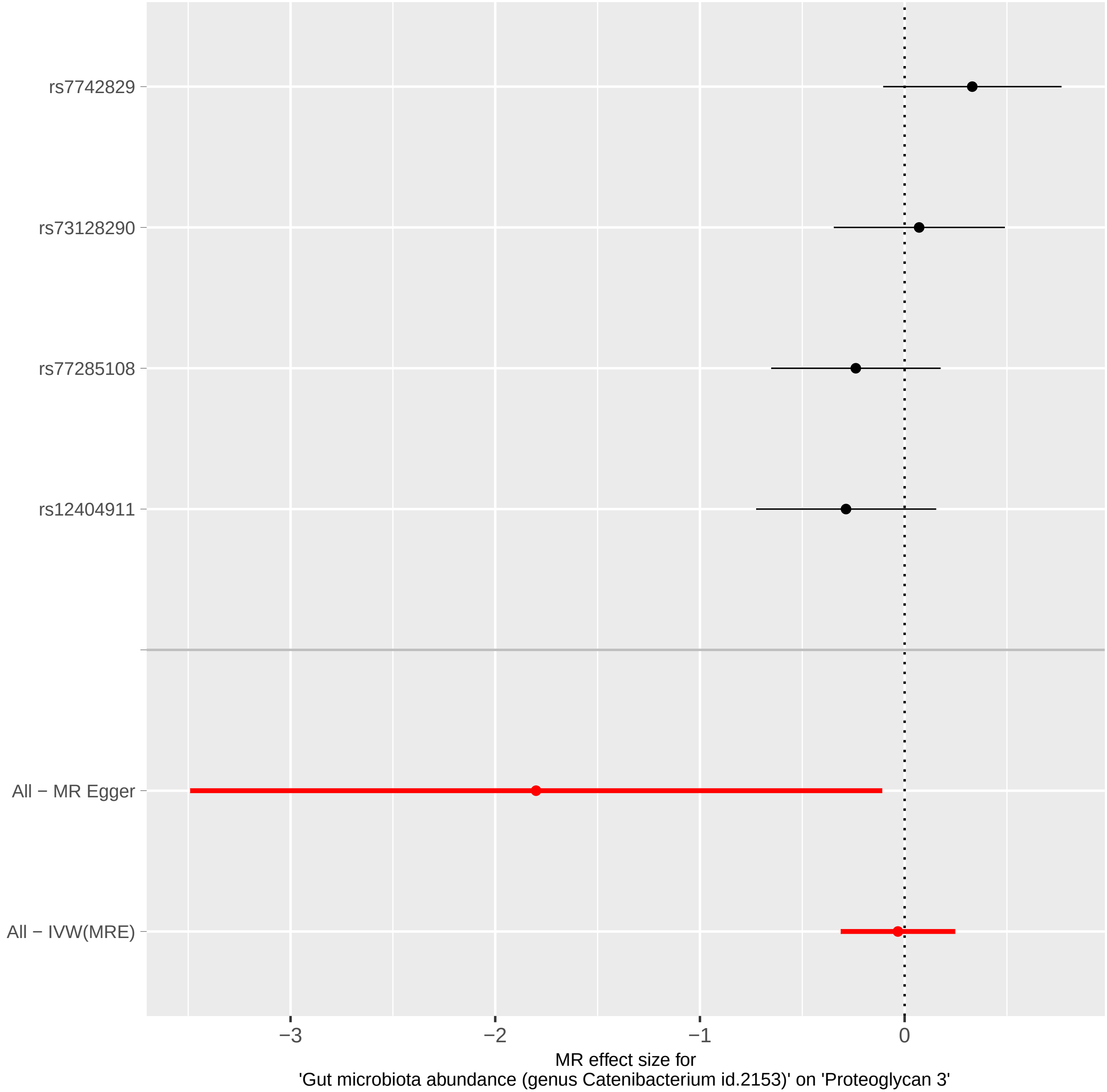

Batch 2−4724 : Gut microbiota abundance (genus Parasutterella id.2892) on Proteoglycan 3
with a threshold of 1e−05

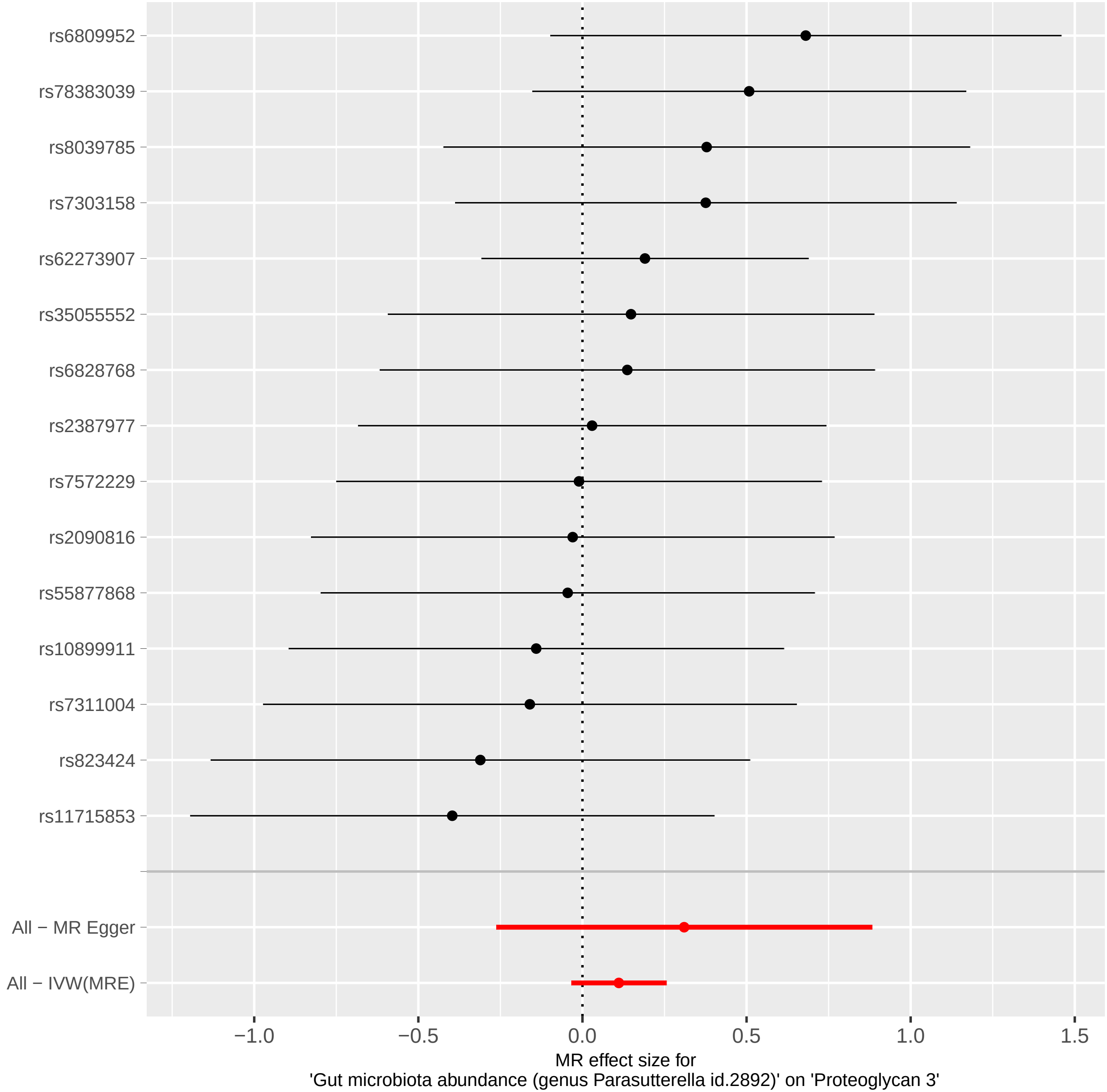

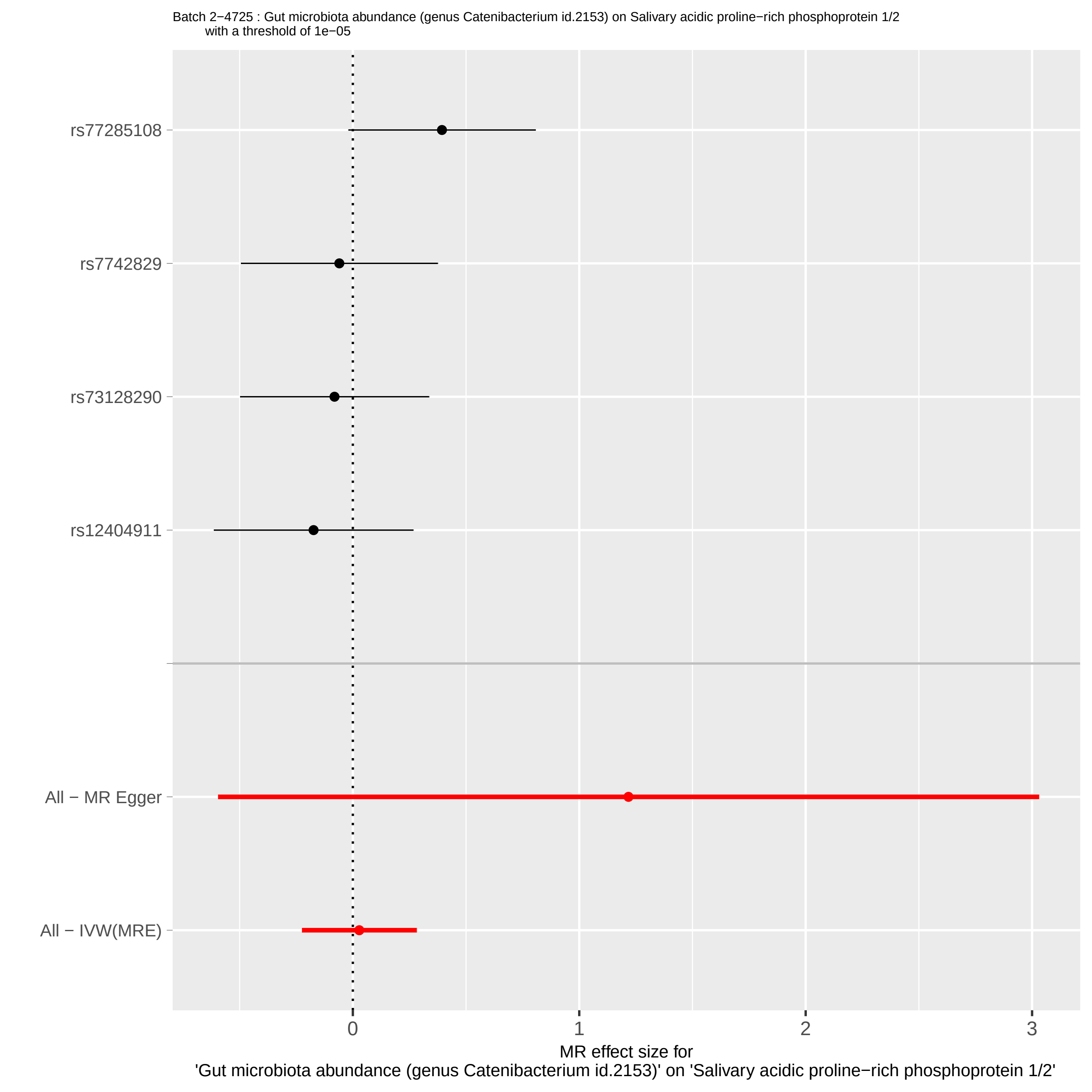

Batch 2−4725 : Gut microbiota abundance (genus Catenibacterium id.2153) on Salivary acidic proline−rich phosphoprotein 1/2
with a threshold of 1e−05
rs77285108
rs7742829
rs73128290
rs12404911
All − MR Egger
All − IVW(MRE)
0
1
2
3
MR effect size for
'Gut microbiota abundance (genus Catenibacterium id.2153)' on 'Salivary acidic proline−rich phosphoprotein 1/2'

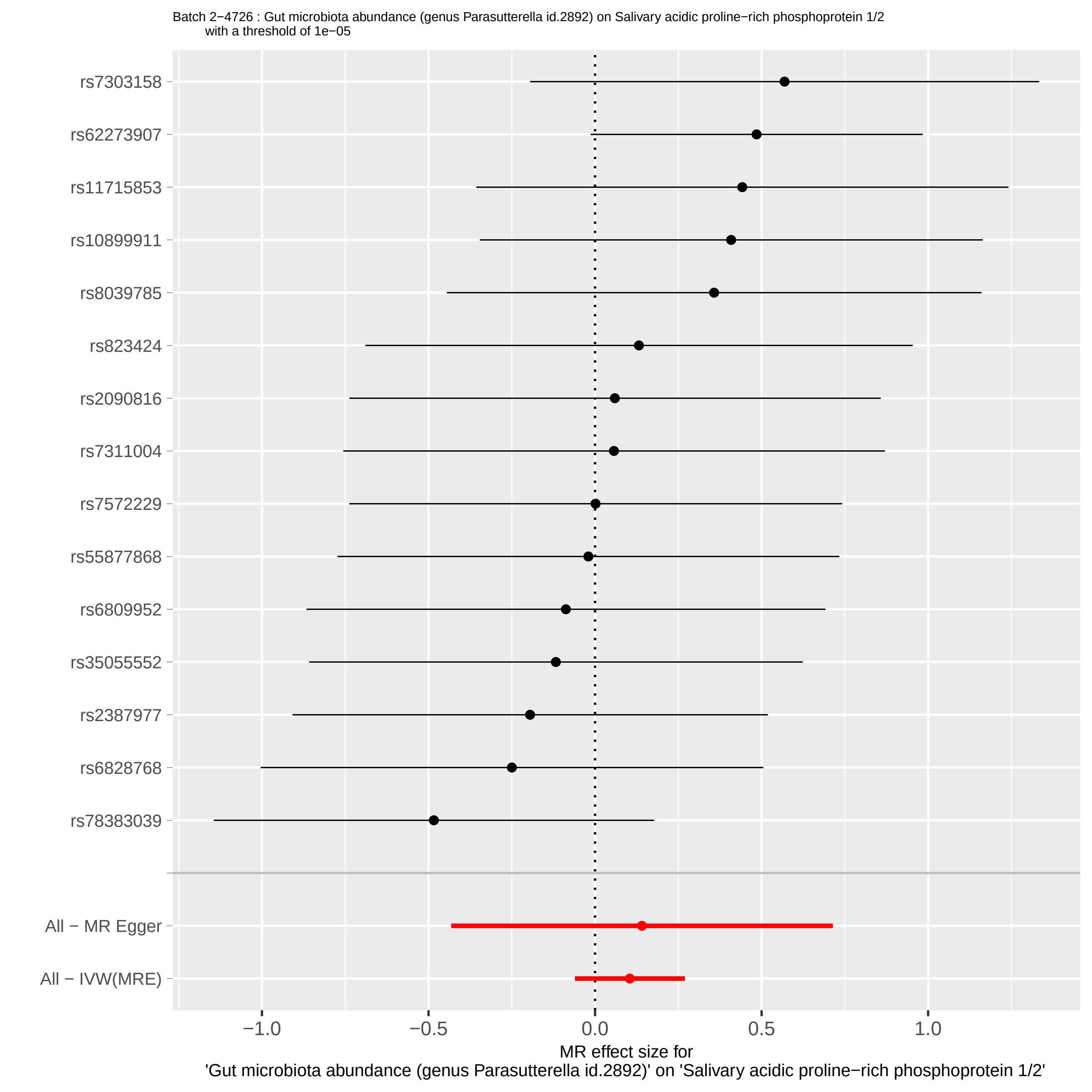
Batch 2−4726 : Gut microbiota abundance (genus Parasutterella id.2892) on Salivary acidic proline−rich phosphoprotein 1/2
with a threshold of 1e−05
rs7303158
rs62273907
rs11715853
rs10899911
rs8039785
rs823424
rs2090816
rs7311004
rs7572229
rs55877868
rs6809952
rs35055552
rs2387977
rs6828768
rs78383039
All − MR Egger
All − IVW(MRE)
−1.0
−0.5
0.0
0.5
1.0
MR effect size for
'Gut microbiota abundance (genus Parasutterella id.2892)' on 'Salivary acidic proline−rich phosphoprotein 1/2'

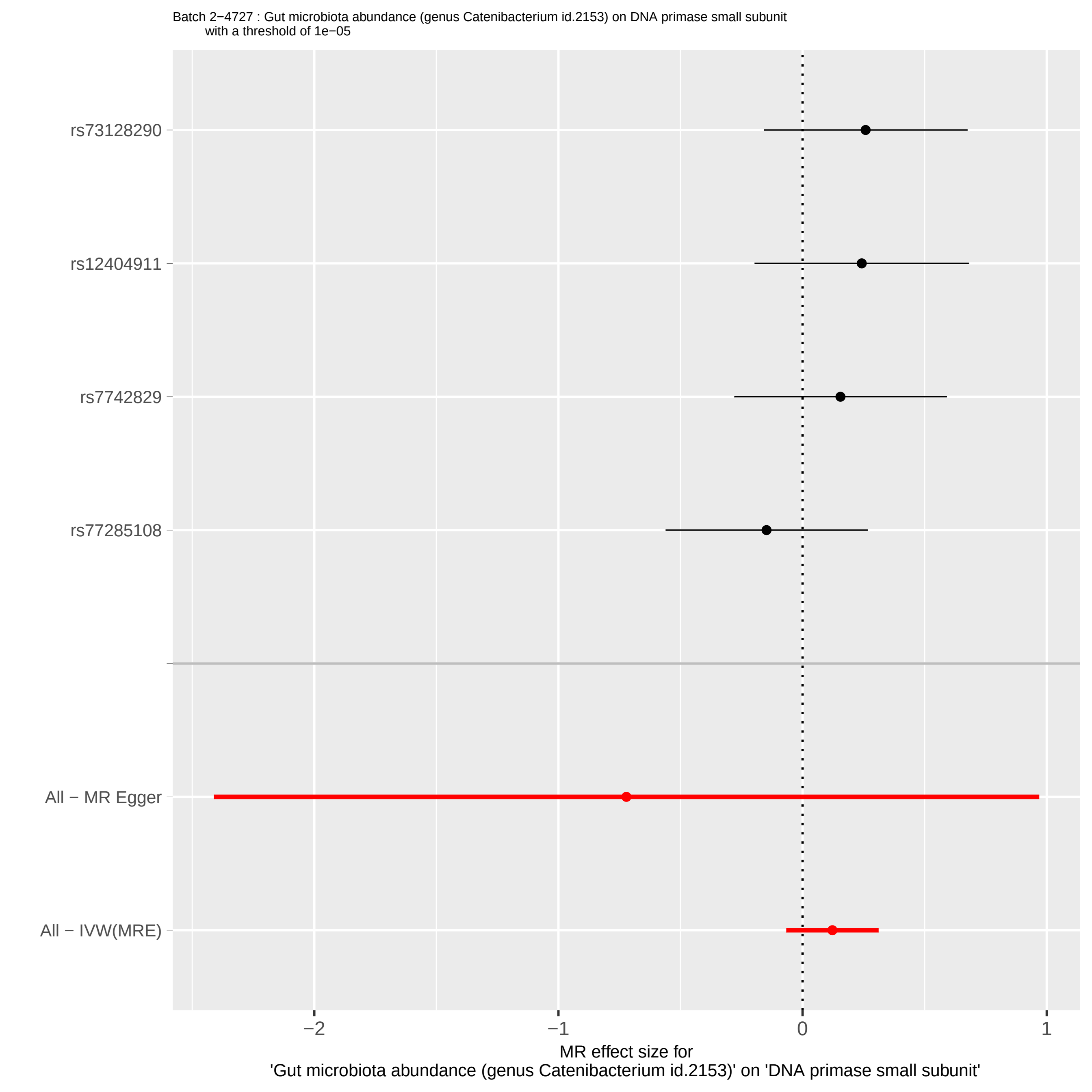
Batch 2−4727 : Gut microbiota abundance (genus Catenibacterium id.2153) on DNA primase small subunit
with a threshold of 1e−05
rs73128290
rs12404911
rs7742829
rs77285108
All − MR Egger
All − IVW(MRE)
−2
−1
0
1
MR effect size for
'Gut microbiota abundance (genus Catenibacterium id.2153)' on 'DNA primase small subunit'

Batch 2−4728 : Gut microbiota abundance (genus Parasutterella id.2892) on DNA primase small subunit
with a threshold of 1e−05

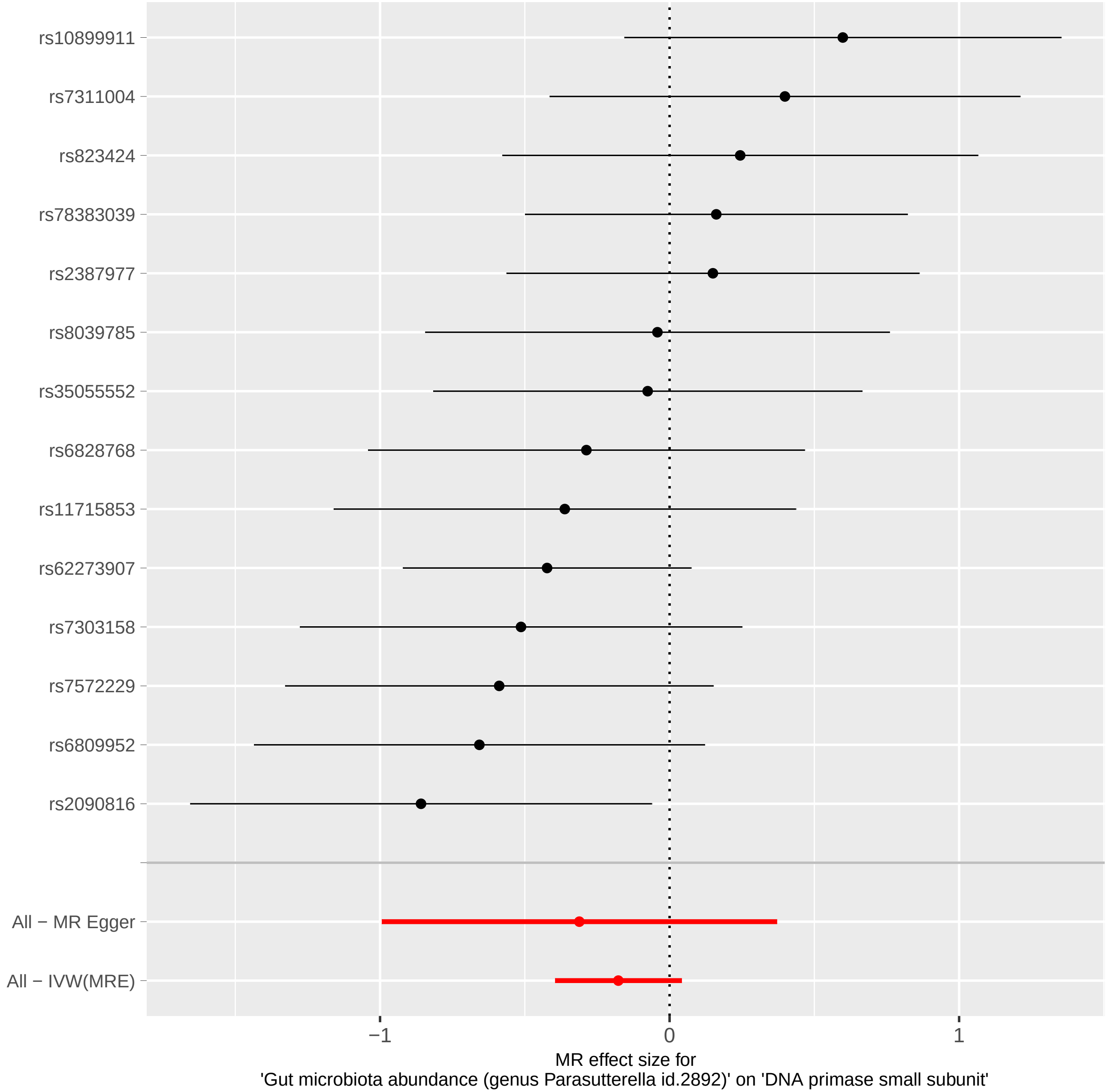

Batch 2−4729 : Gut microbiota abundance (genus Catenibacterium id.2153) on AMP Kinase (alpha1beta1gamma1) with a threshold of 1e−05

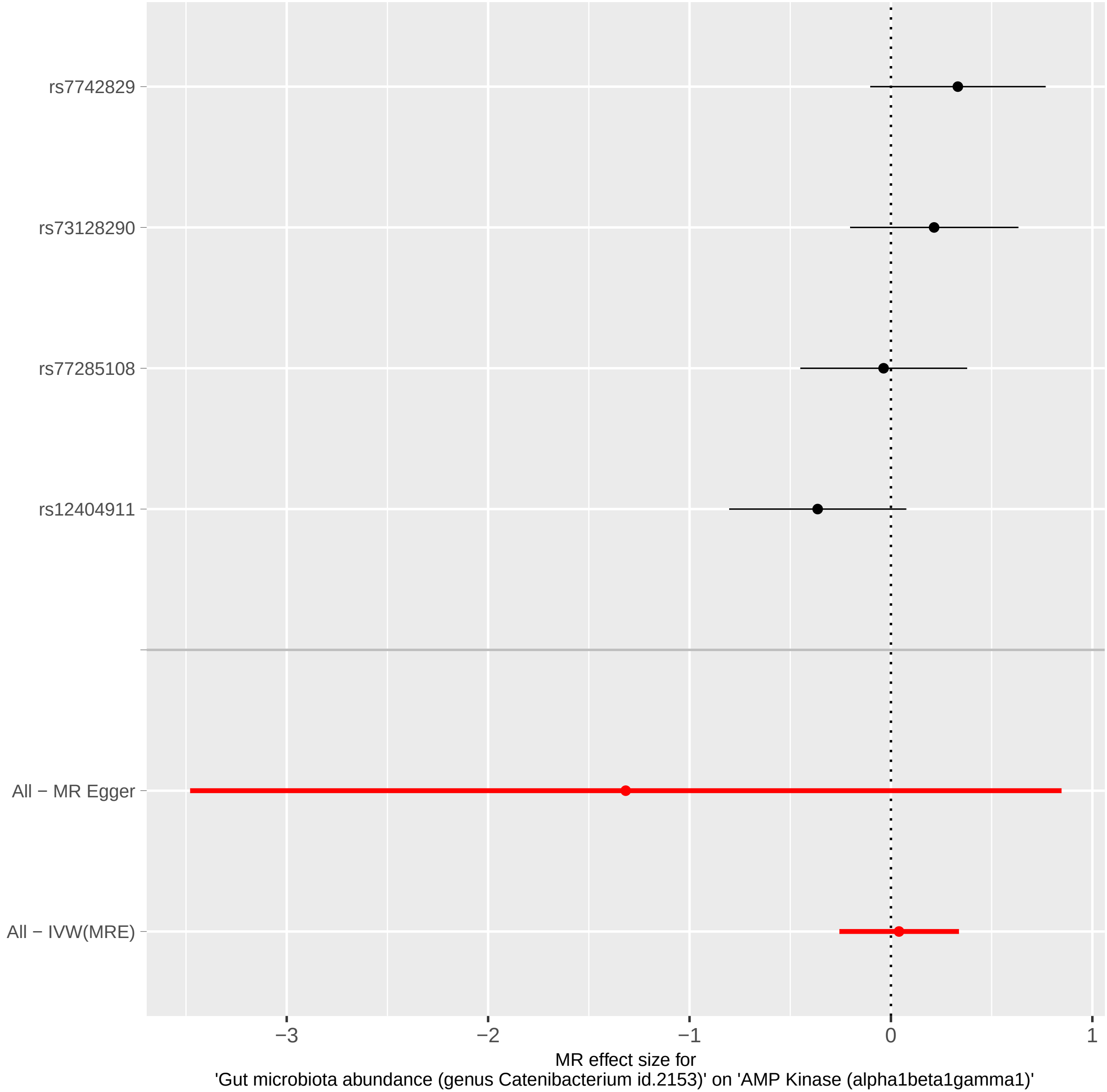

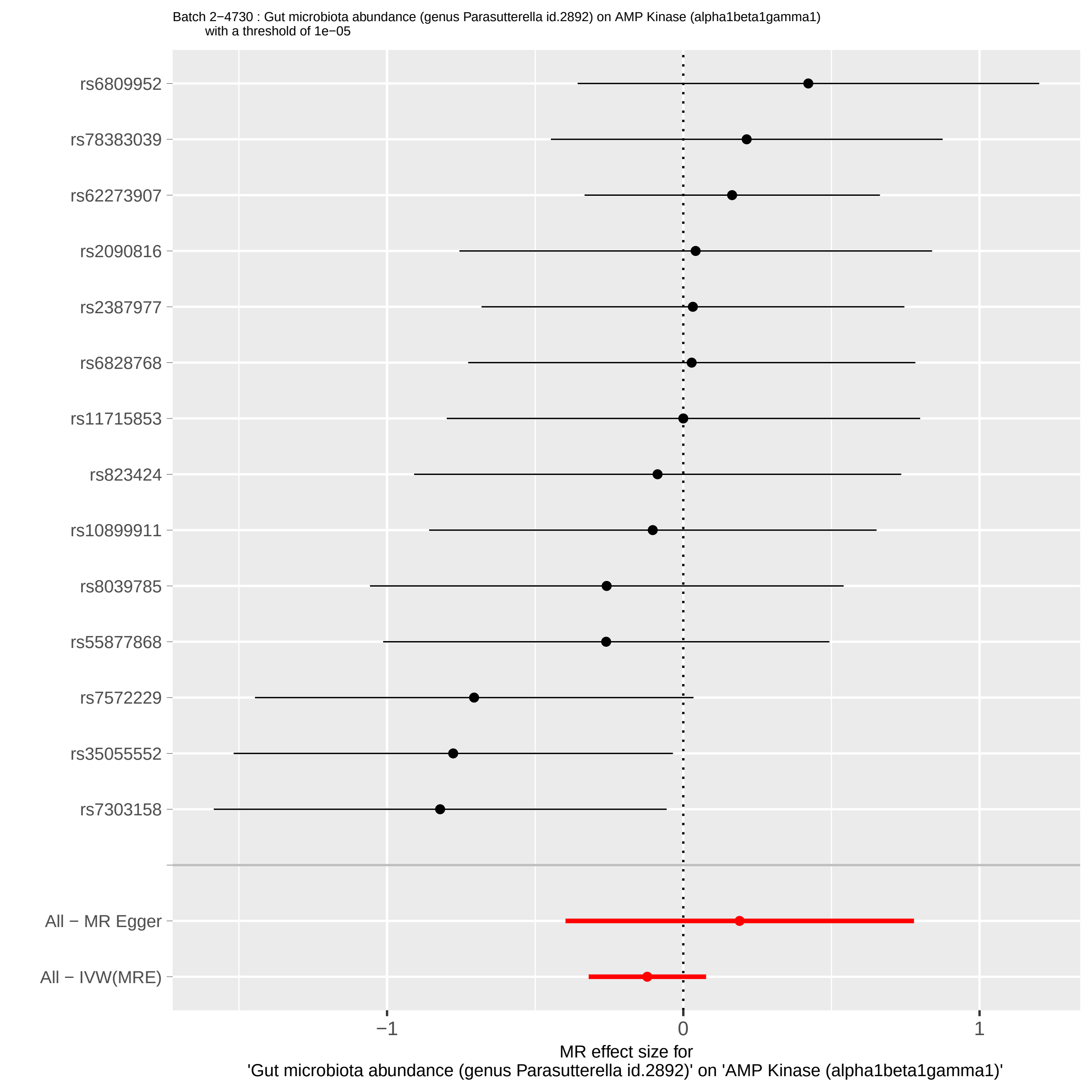
Batch 2−4730 : Gut microbiota abundance (genus Parasutterella id.2892) on AMP Kinase (alpha1beta1gamma1)
with a threshold of 1e−05
rs6809952
rs78383039
rs62273907
rs2090816
rs2387977
rs6828768
rs11715853
rs823424
rs10899911
rs8039785
rs55877868
rs7572229
rs35055552
rs7303158
All − MR Egger
All − IVW(MRE)
−1
0
1
MR effect size for
'Gut microbiota abundance (genus Parasutterella id.2892)' on 'AMP Kinase (alpha1beta1gamma1)'

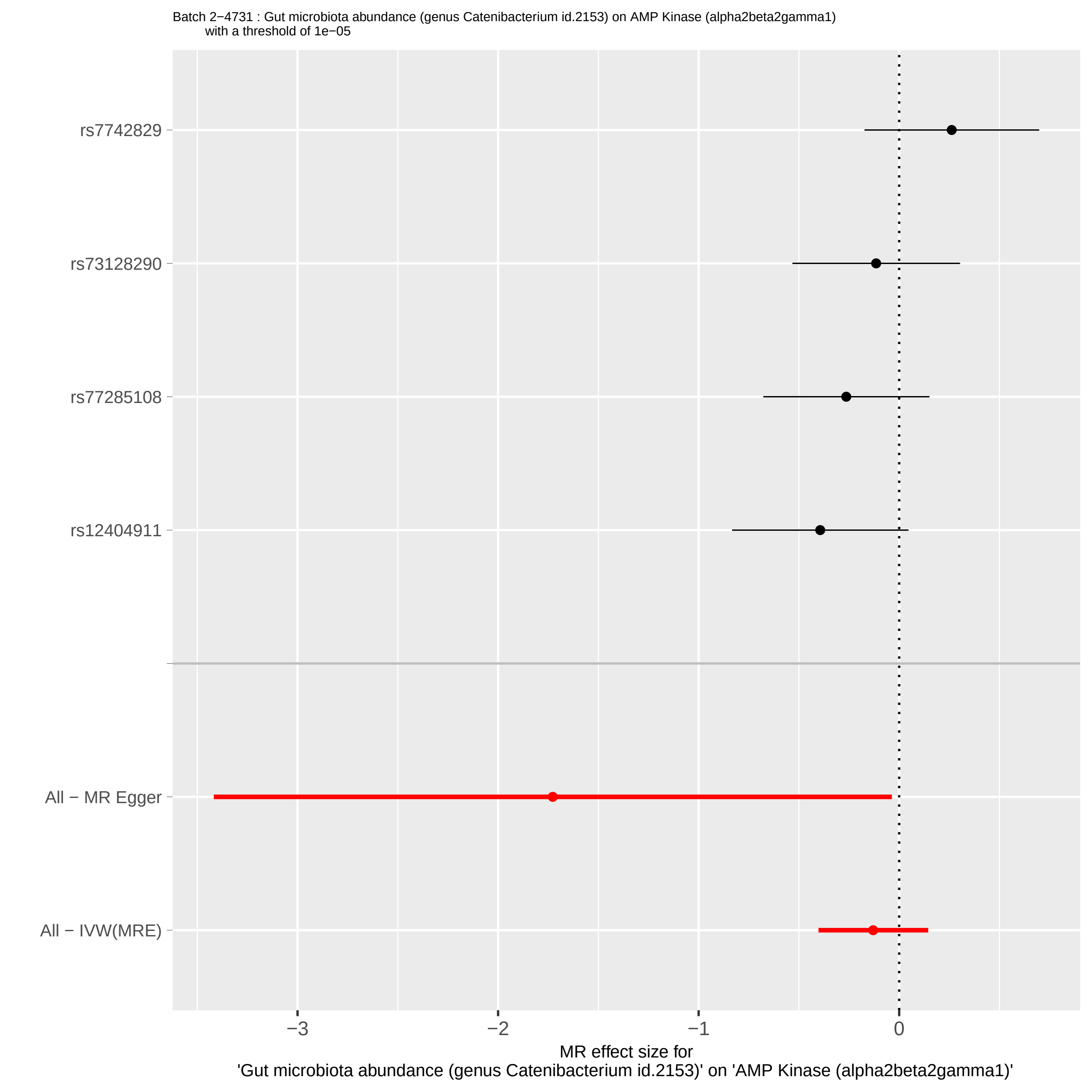

Batch 2−4731 : Gut microbiota abundance (genus Catenibacterium id.2153) on AMP Kinase (alpha2beta2gamma1)
with a threshold of 1e−05
rs7742829
rs73128290
rs77285108
rs12404911
All − MR Egger
All − IVW(MRE)
−3
−2
−1
0
MR effect size for
'Gut microbiota abundance (genus Catenibacterium id.2153)' on 'AMP Kinase (alpha2beta2gamma1)'

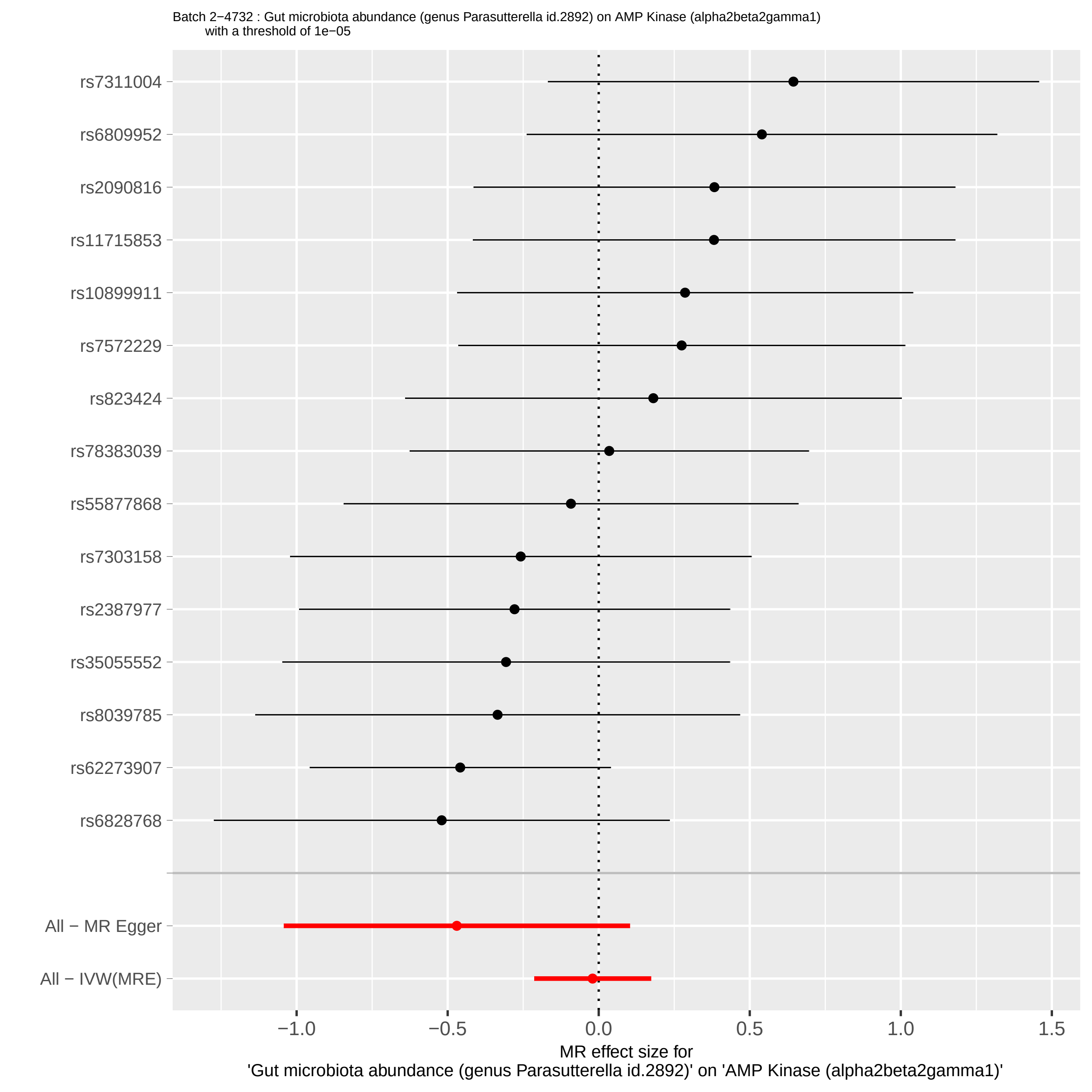

Batch 2−4732 : Gut microbiota abundance (genus Parasutterella id.2892) on AMP Kinase (alpha2beta2gamma1)
with a threshold of 1e−05
rs7311004
rs6809952
rs2090816
rs11715853
rs10899911
rs7572229
rs823424
rs78383039
rs55877868
rs7303158
rs2387977
rs35055552
rs8039785
rs62273907
rs6828768
All − MR Egger
All − IVW(MRE)
−1.0
−0.5
0.0
0.5
1.0
1.5
MR effect size for
'Gut microbiota abundance (genus Parasutterella id.2892)' on 'AMP Kinase (alpha2beta2gamma1)'

Batch 2−4733 : Gut microbiota abundance (genus Catenibacterium id.2153) on cAMP−dependent protein kinase catalytic subunit alpha with a threshold of 1e−05

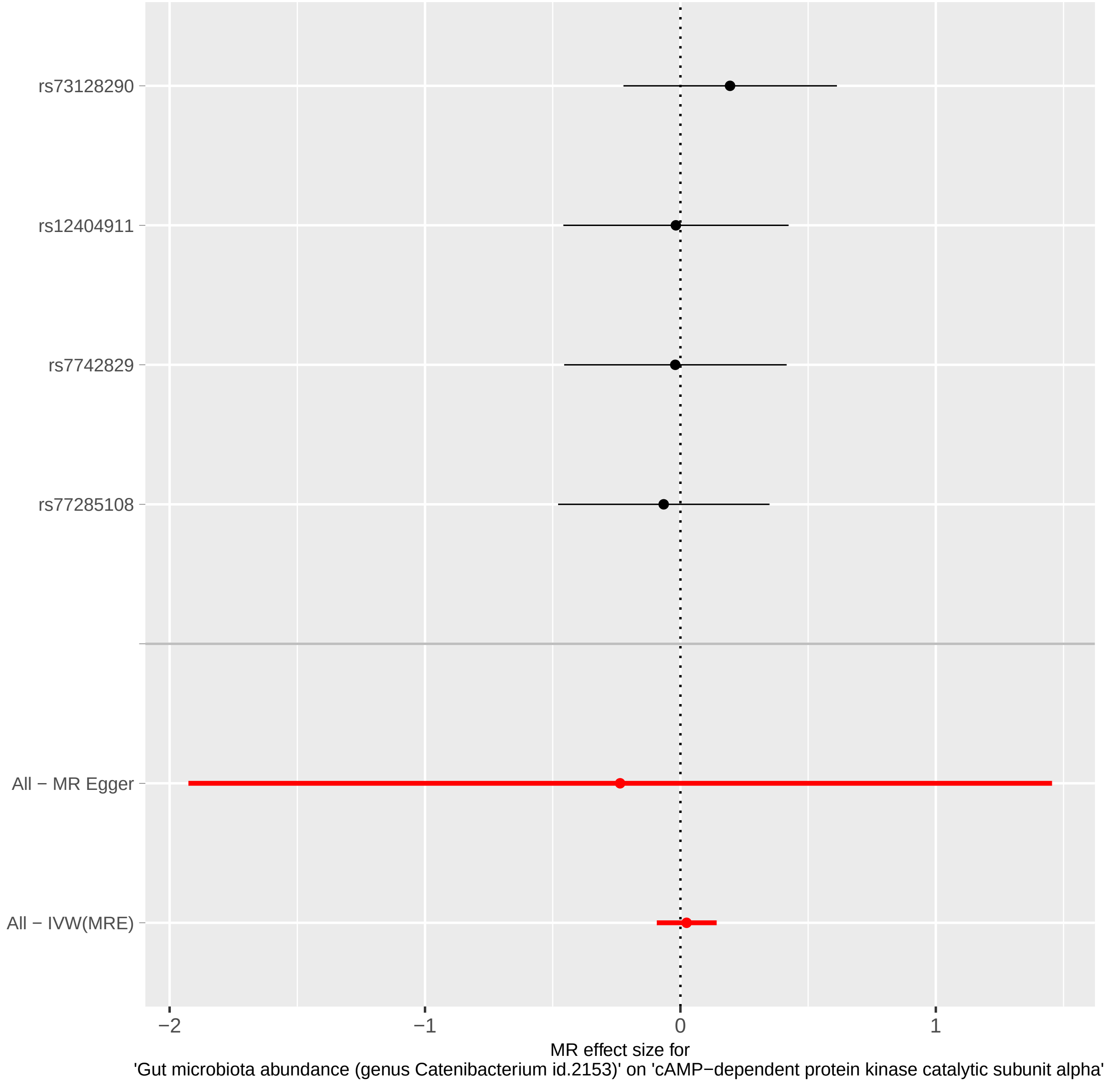

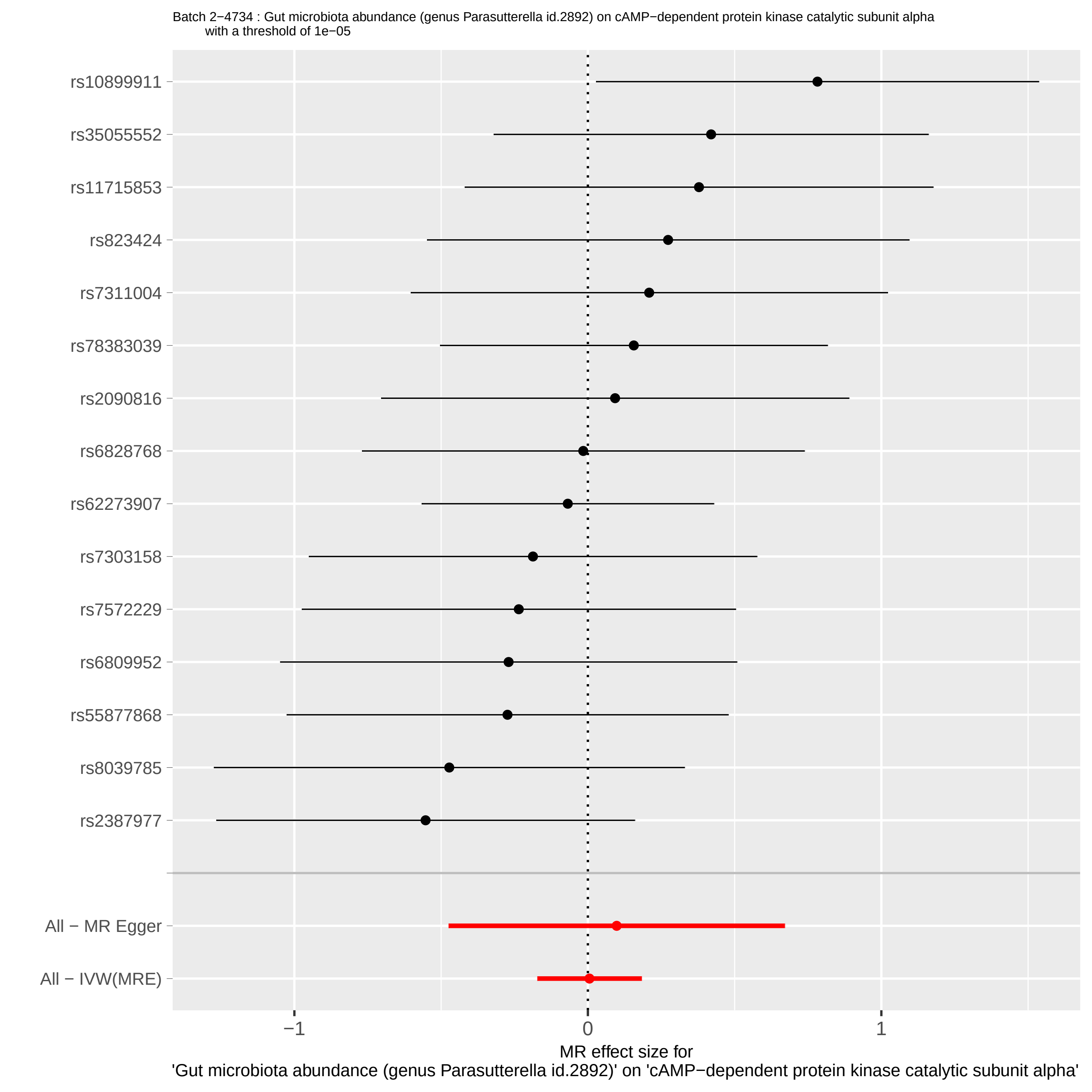

Batch 2−4734 : Gut microbiota abundance (genus Parasutterella id.2892) on cAMP−dependent protein kinase catalytic subunit alpha with a threshold of 1e−05
rs10899911
rs35055552
rs11715853
rs823424
rs7311004
rs78383039
rs2090816
rs6828768
rs62273907
rs7303158
rs7572229
rs6809952
rs55877868
rs8039785
rs2387977
All − MR Egger
All − IVW(MRE)
−1
0
1
MR effect size for
'Gut microbiota abundance (genus Parasutterella id.2892)' on 'cAMP−dependent protein kinase catalytic subunit alpha'

Batch 2−4735 : Gut microbiota abundance (genus Catenibacterium id.2153) on cAMP−dependent protein kinase type I−beta regulatory subunit
with a threshold of 1e−05

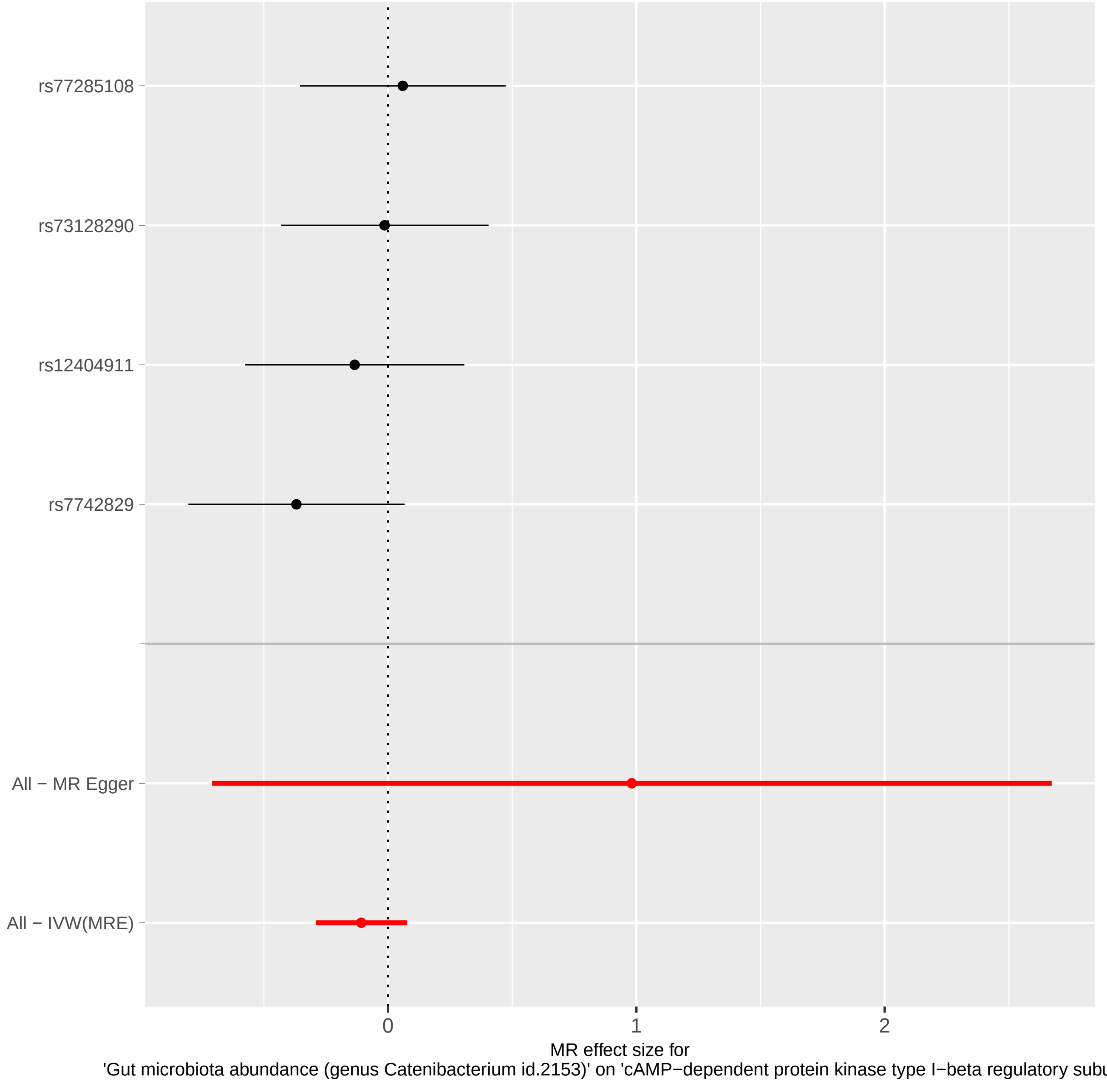

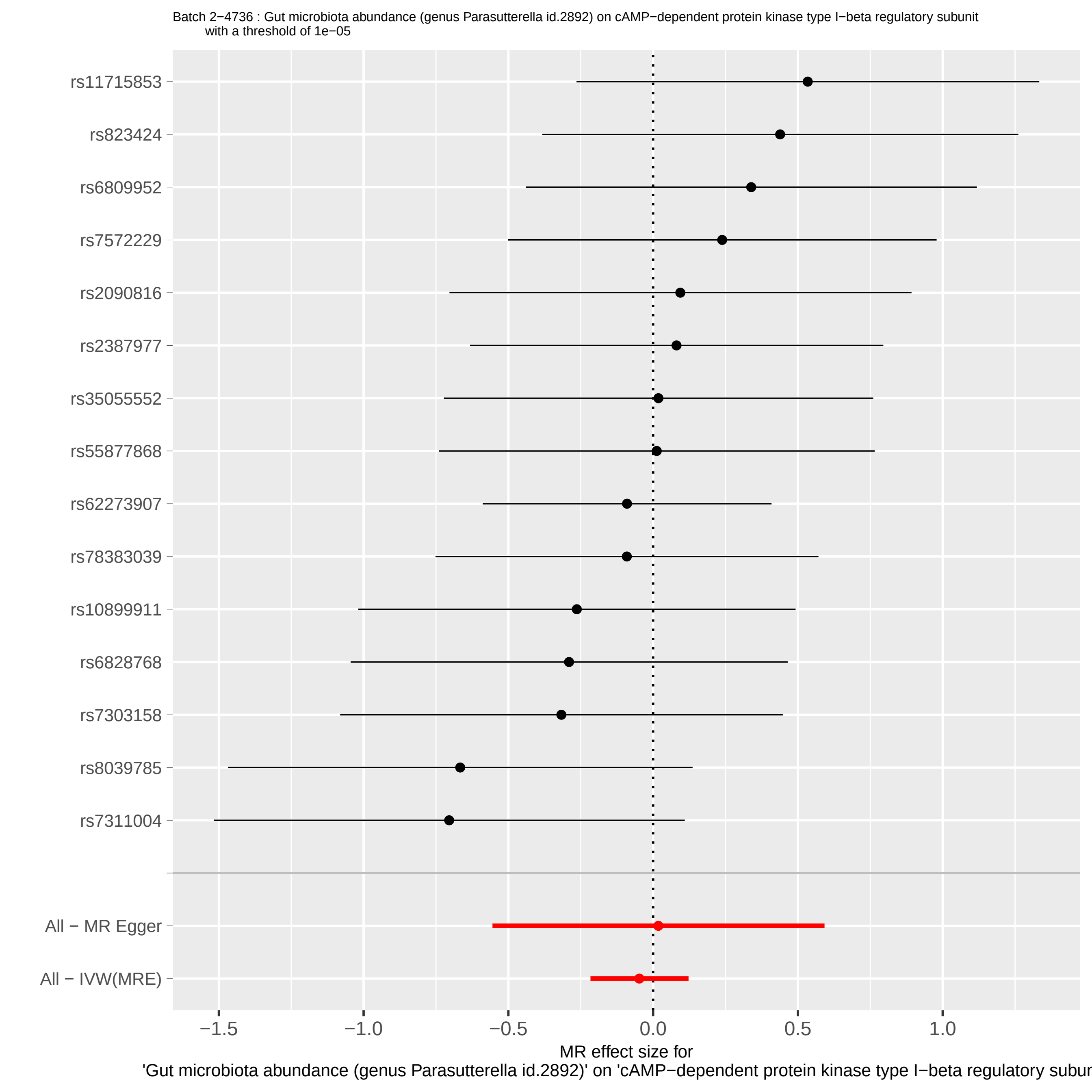

Batch 2−4736 : Gut microbiota abundance (genus Parasutterella id.2892) on cAMP−dependent protein kinase type I−beta regulatory subunit
with a threshold of 1e−05
rs11715853
rs823424
rs6809952
rs7572229
rs2090816
rs2387977
rs35055552
rs55877868
rs62273907
rs78383039
rs10899911
rs6828768
rs7303158
rs8039785
rs7311004
All − MR Egger
All − IVW(MRE)
−1.5
−1.0
−0.5
0.0
0.5
1.0
MR effect size for
'Gut microbiota abundance (genus Parasutterella id.2892)' on 'cAMP−dependent protein kinase type I−beta regulatory subur

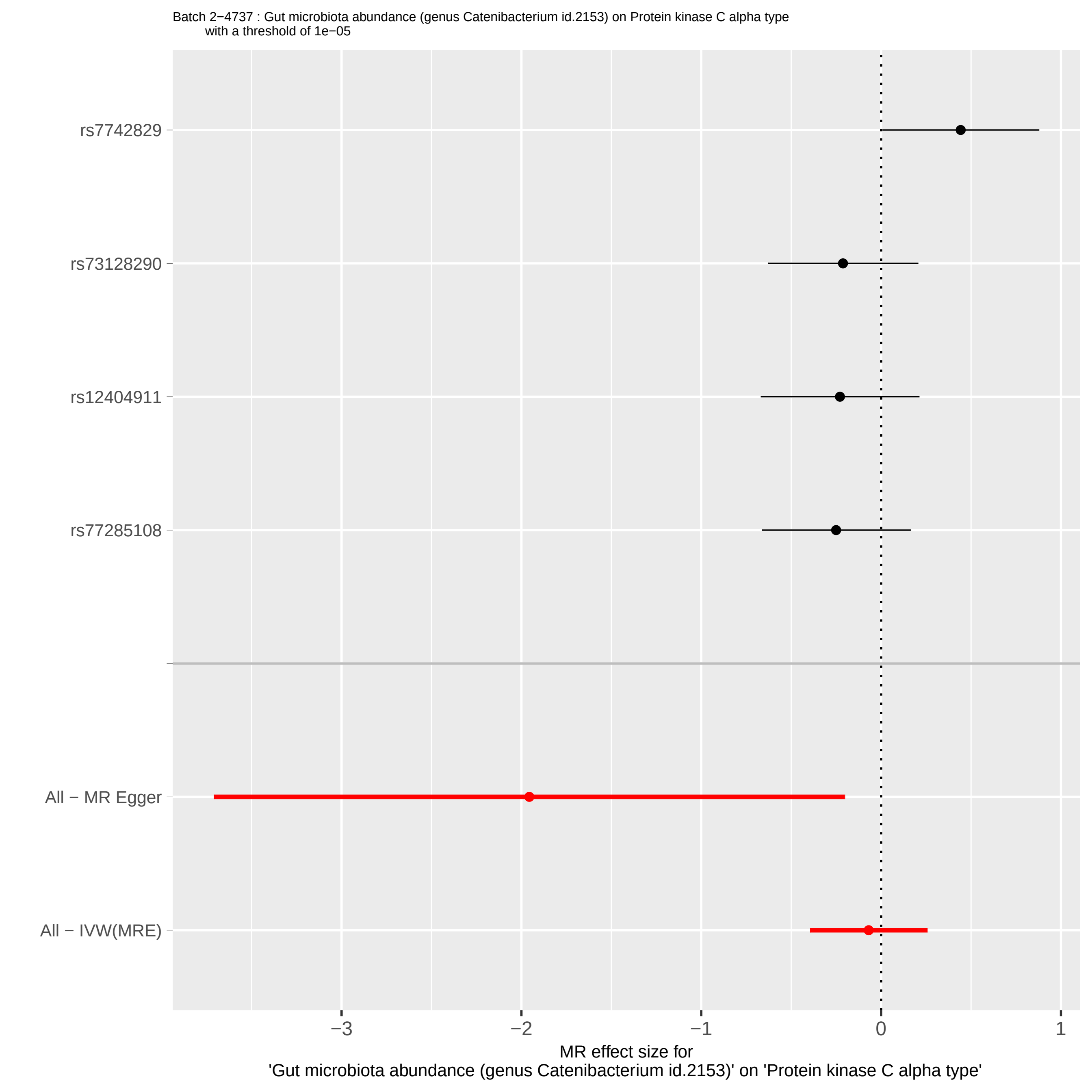
Batch 2−4737 : Gut microbiota abundance (genus Catenibacterium id.2153) on Protein kinase C alpha type
with a threshold of 1e−05
rs7742829
rs73128290
rs12404911
rs77285108
All − MR Egger
All − IVW(MRE)
−3
−2
−1
0
1
MR effect size for
'Gut microbiota abundance (genus Catenibacterium id.2153)' on 'Protein kinase C alpha type'

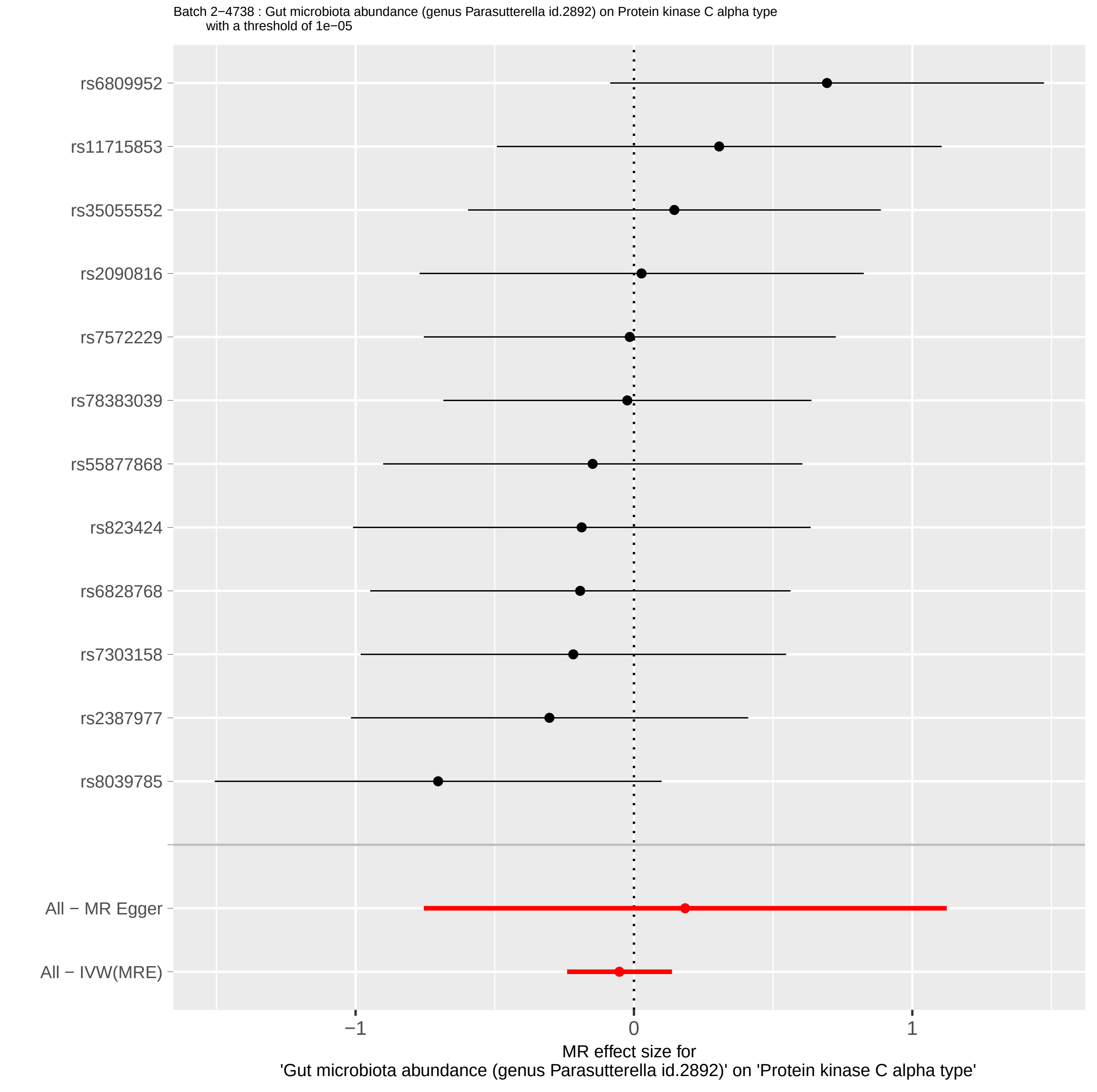

Batch 2−4738 : Gut microbiota abundance (genus Parasutterella id.2892) on Protein kinase C alpha type
with a threshold of 1e−05
rs6809952
rs11715853
rs35055552
rs2090816
rs7572229
rs78383039
rs55877868
rs823424
rs6828768
rs7303158
rs2387977
rs8039785
All − MR Egger
All − IVW(MRE)
−1
0
1
MR effect size for
'Gut microbiota abundance (genus Parasutterella id.2892)' on 'Protein kinase C alpha type'

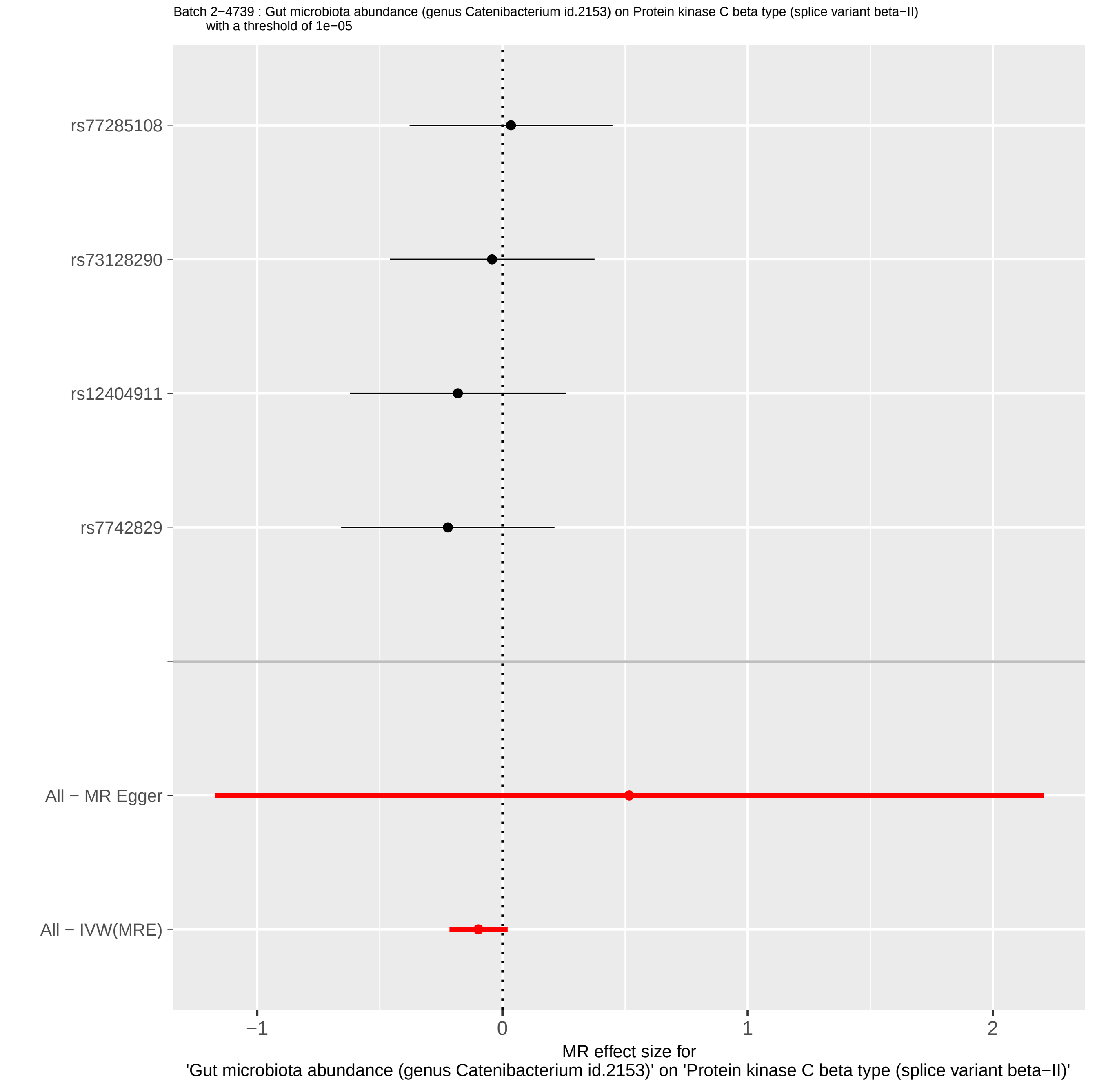

Batch 2−4739 : Gut microbiota abundance (genus Catenibacterium id.2153) on Protein kinase C beta type (splice variant beta−II) with a threshold of 1e−05
rs77285108
rs73128290
rs12404911
rs7742829
All − MR Egger
All − IVW(MRE)
−1
0
1
2
MR effect size for
'Gut microbiota abundance (genus Catenibacterium id.2153)' on 'Protein kinase C beta type (splice variant beta−II)'

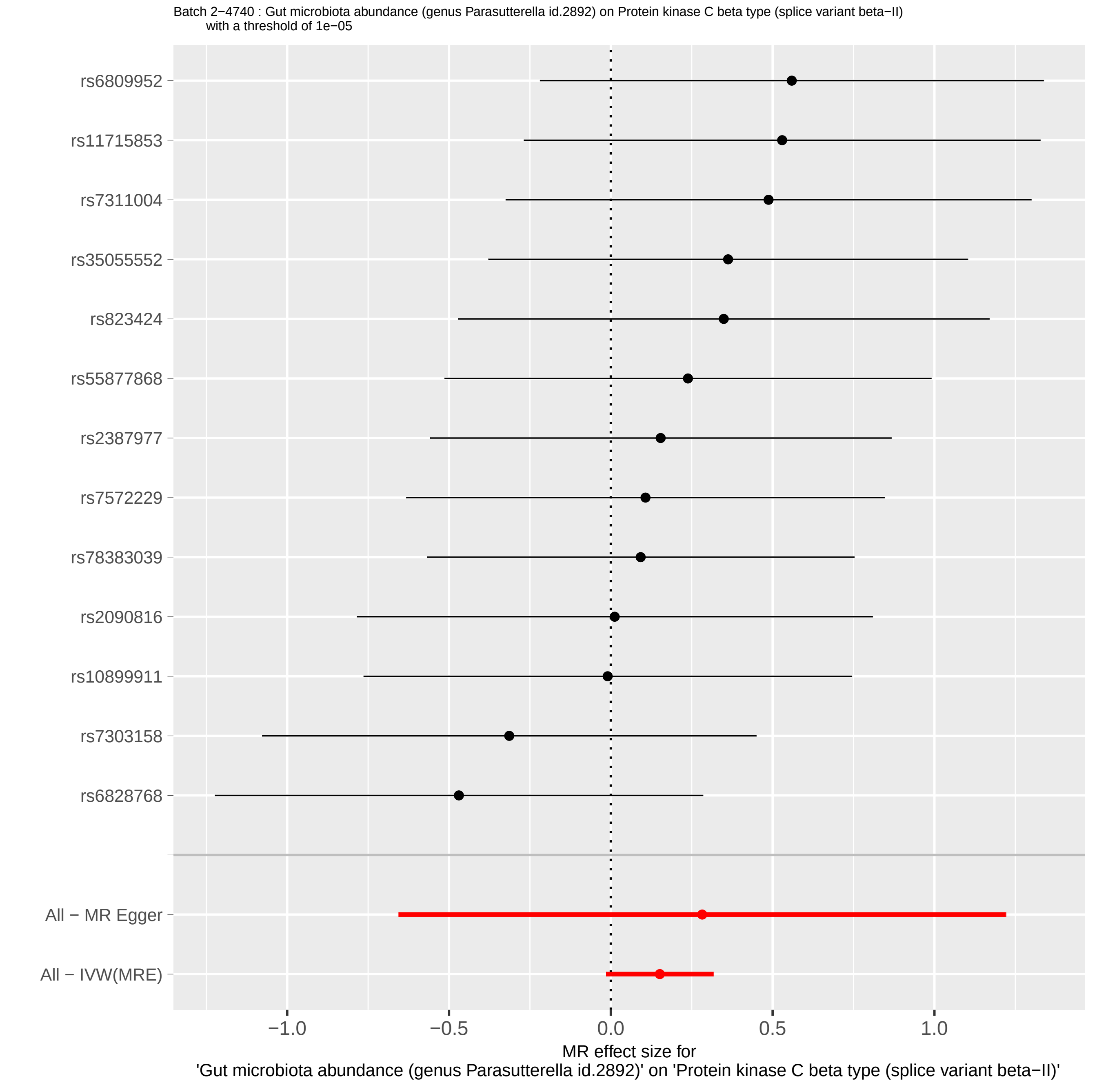

Batch 2−4740 : Gut microbiota abundance (genus Parasutterella id.2892) on Protein kinase C beta type (splice variant beta−II) with a threshold of 1e−05
rs6809952
rs11715853
rs7311004
rs35055552
rs823424
rs55877868
rs2387977
rs7572229
rs78383039
rs2090816
rs10899911
rs7303158
rs6828768
All − MR Egger
All − IVW(MRE)
−1.0
−0.5
0.0
0.5
1.0
MR effect size for
'Gut microbiota abundance (genus Parasutterella id.2892)' on 'Protein kinase C beta type (splice variant beta−II)'

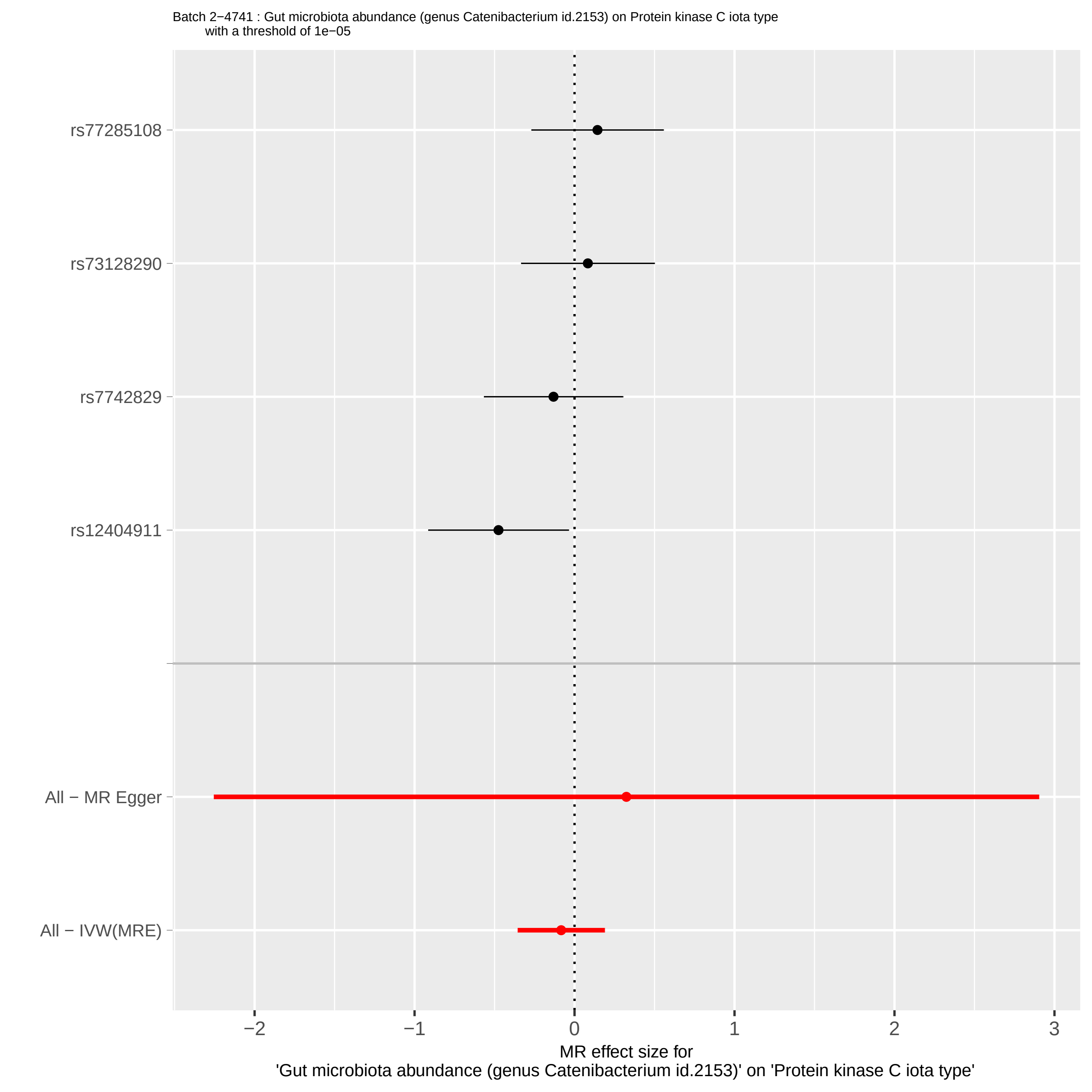

Batch 2−4741 : Gut microbiota abundance (genus Catenibacterium id.2153) on Protein kinase C iota type
with a threshold of 1e−05
rs77285108
rs73128290
rs7742829
rs12404911
All − MR Egger
All − IVW(MRE)
−2
−1
0
1
2
3
MR effect size for
'Gut microbiota abundance (genus Catenibacterium id.2153)' on 'Protein kinase C iota type'

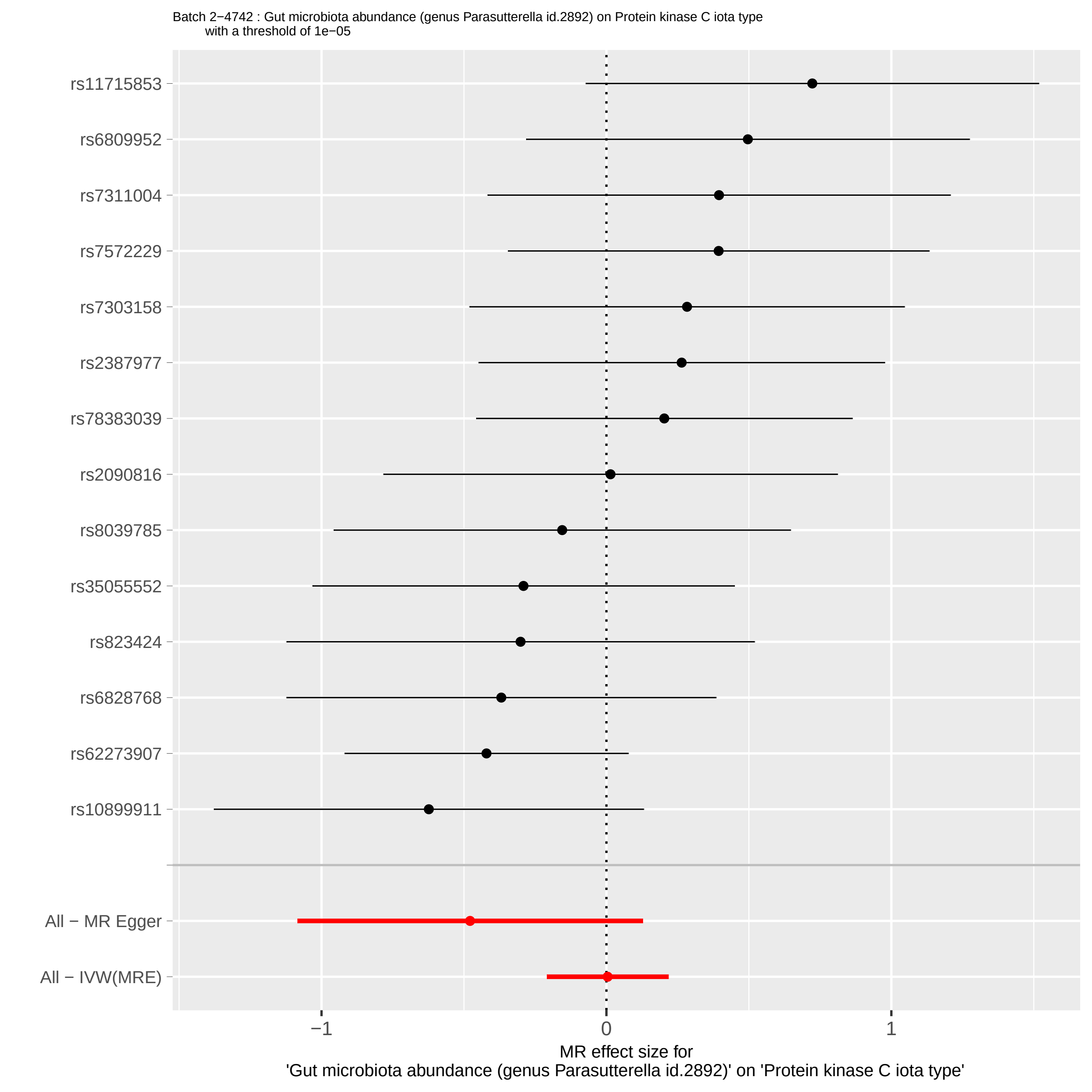
Batch 2−4742 : Gut microbiota abundance (genus Parasutterella id.2892) on Protein kinase C iota type
with a threshold of 1e−05
rs11715853
rs6809952
rs7311004
rs7572229
rs7303158
rs2387977
rs78383039
rs2090816
rs8039785
rs35055552
rs823424
rs6828768
rs62273907
rs10899911
All − MR Egger
All − IVW(MRE)
−1
0
1
MR effect size for
'Gut microbiota abundance (genus Parasutterella id.2892)' on 'Protein kinase C iota type'

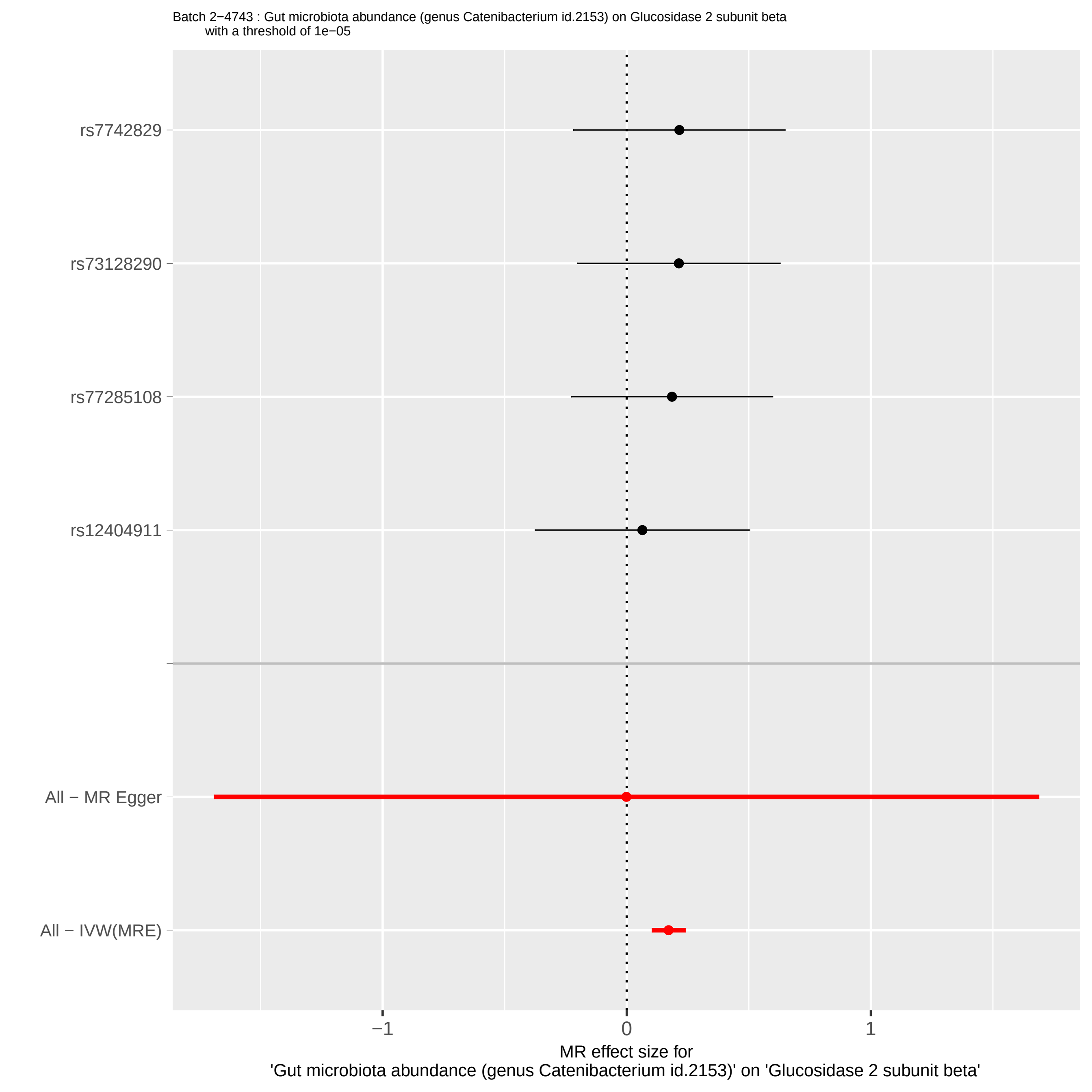
Batch 2−4743 : Gut microbiota abundance (genus Catenibacterium id.2153) on Glucosidase 2 subunit beta
with a threshold of 1e−05
rs7742829
rs73128290
rs77285108
rs12404911
All − MR Egger
All − IVW(MRE)
−1
0
1
MR effect size for
'Gut microbiota abundance (genus Catenibacterium id.2153)' on 'Glucosidase 2 subunit beta'

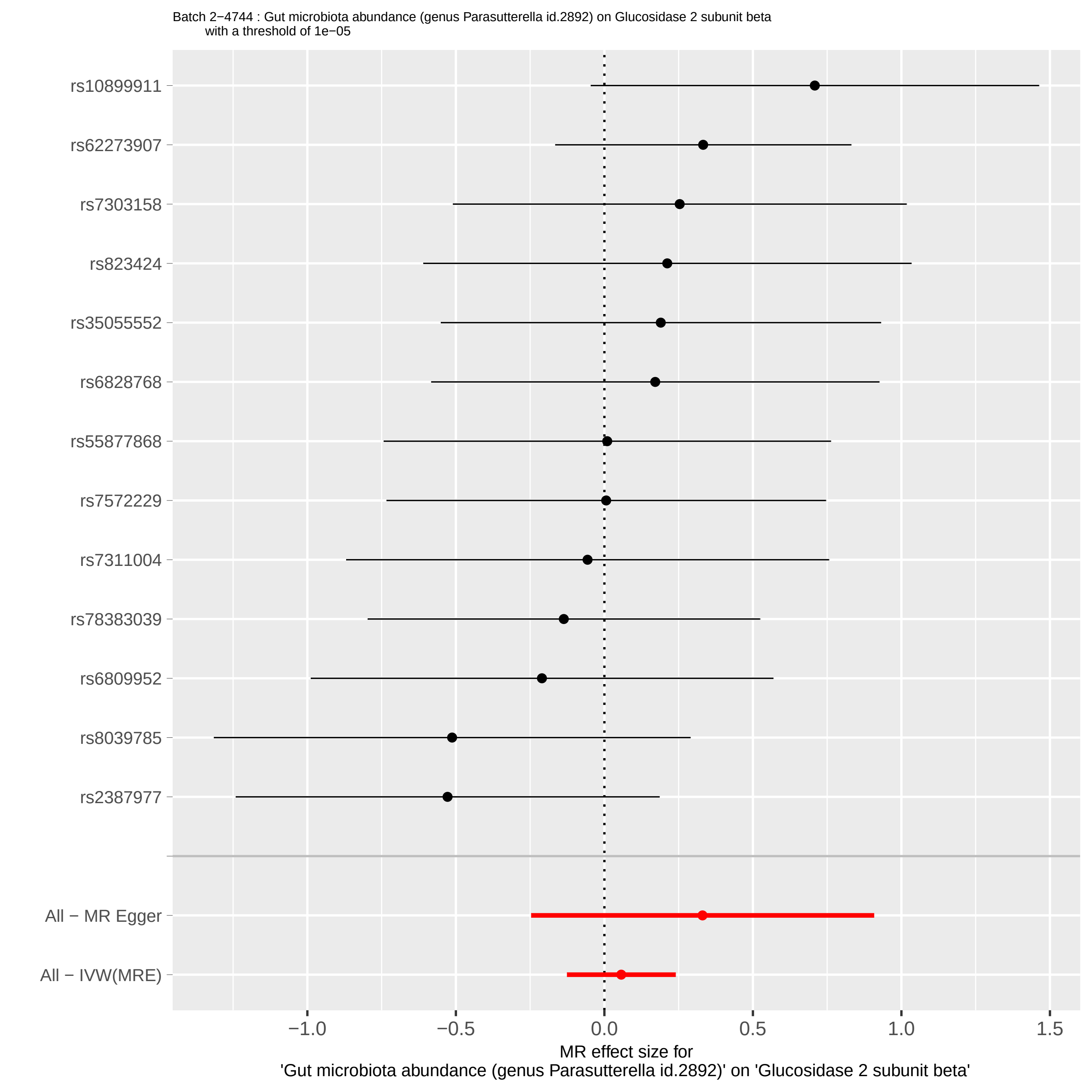

Batch 2−4744 : Gut microbiota abundance (genus Parasutterella id.2892) on Glucosidase 2 subunit beta
with a threshold of 1e−05
rs10899911
rs62273907
rs7303158
rs823424
rs35055552
rs6828768
rs55877868
rs7572229
rs7311004
rs78383039
rs6809952
rs8039785
rs2387977
All − MR Egger
All − IVW(MRE)
−1.0
−0.5
0.0
0.5
1.0
1.5
MR effect size for
'Gut microbiota abundance (genus Parasutterella id.2892)' on 'Glucosidase 2 subunit beta'

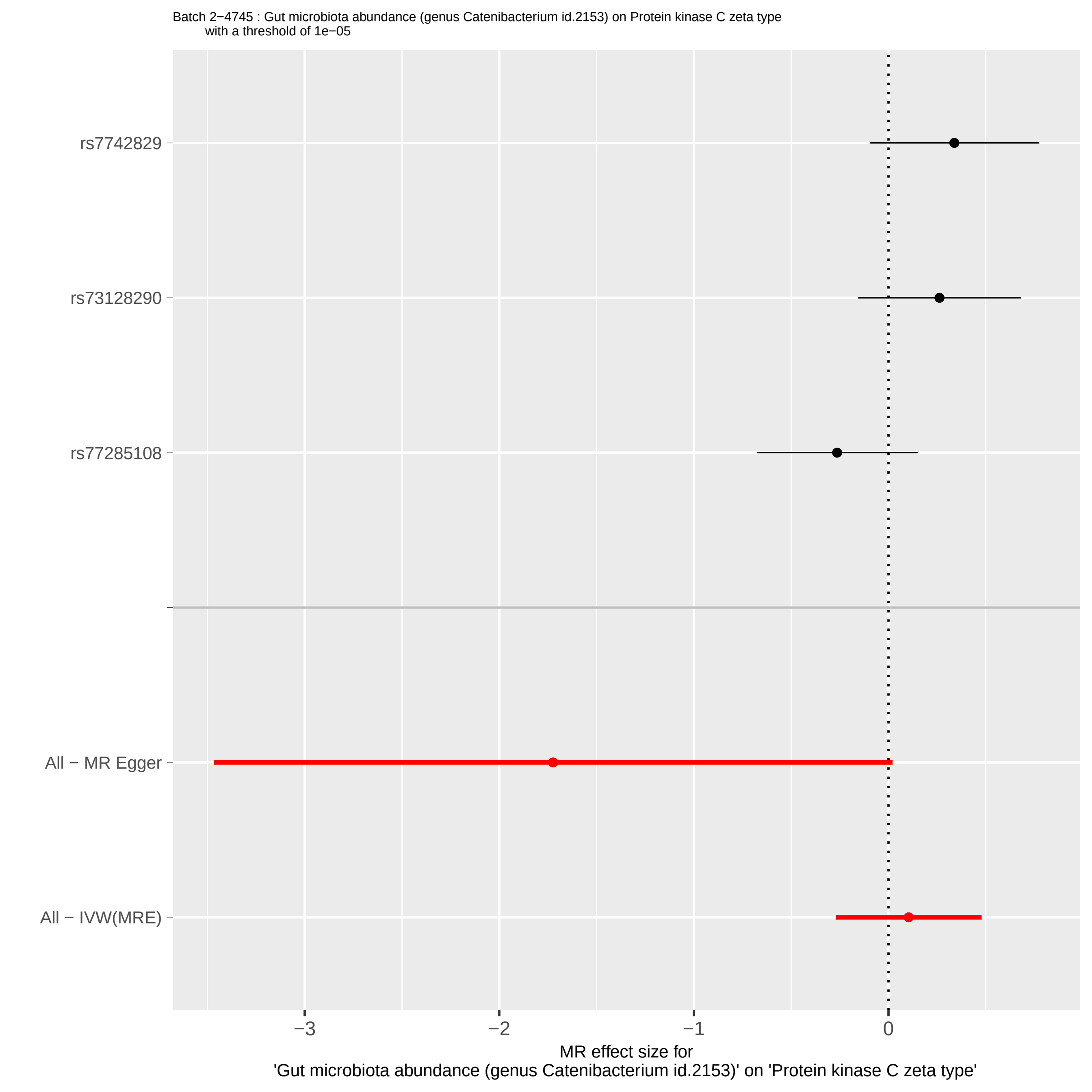

Batch 2−4745 : Gut microbiota abundance (genus Catenibacterium id.2153) on Protein kinase C zeta type
with a threshold of 1e−05
rs7742829
rs73128290
rs77285108
All − MR Egger
All − IVW(MRE)
−3
−2
−1
0
MR effect size for
'Gut microbiota abundance (genus Catenibacterium id.2153)' on 'Protein kinase C zeta type'

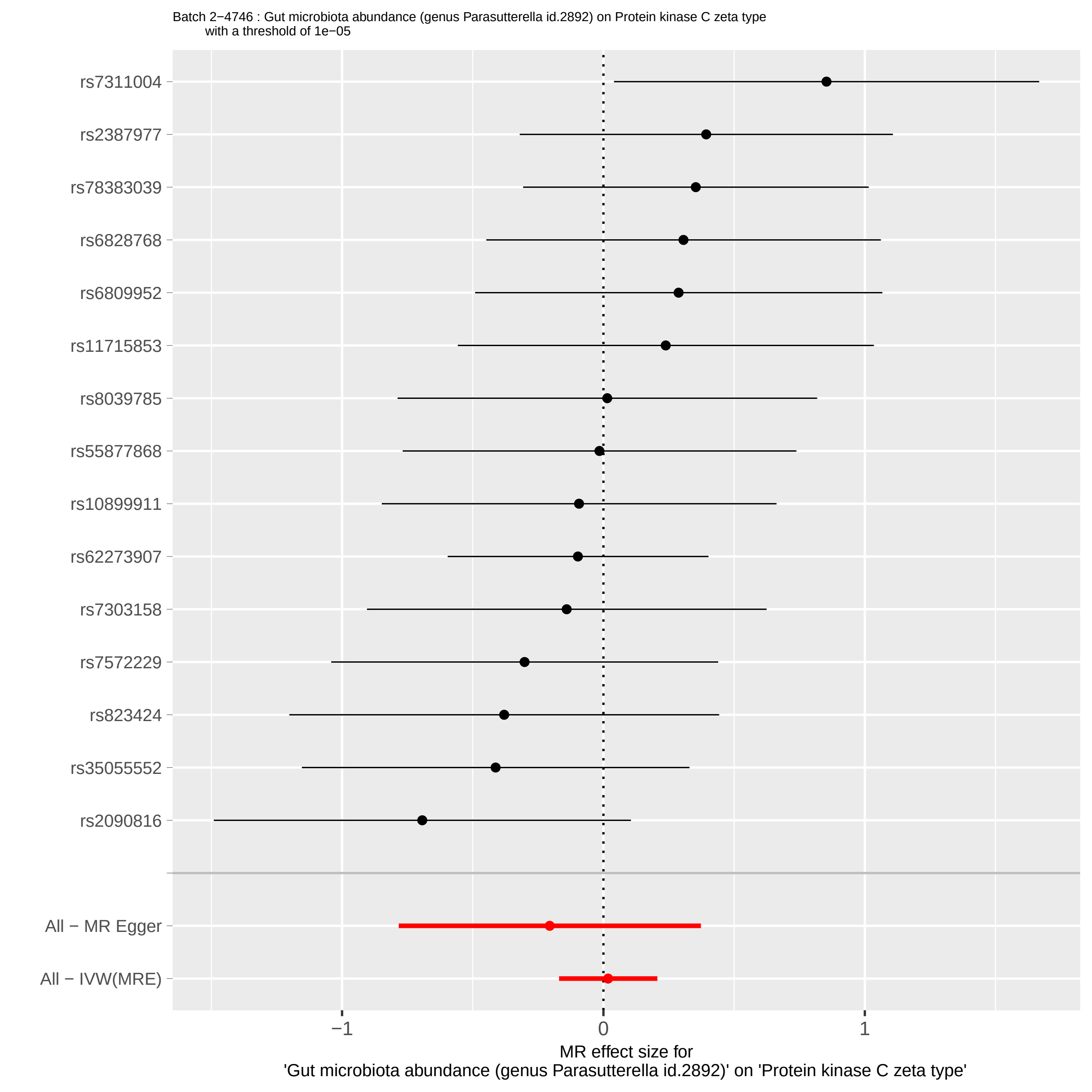
Batch 2−4746 : Gut microbiota abundance (genus Parasutterella id.2892) on Protein kinase C zeta type
with a threshold of 1e−05
rs7311004
rs2387977
rs78383039
rs6828768
rs6809952
rs11715853
rs8039785
rs55877868
rs10899911
rs62273907
rs7303158
rs7572229
rs823424
rs35055552
rs2090816
All − MR Egger
All − IVW(MRE)
−1
0
1
MR effect size for
'Gut microbiota abundance (genus Parasutterella id.2892)' on 'Protein kinase C zeta type'

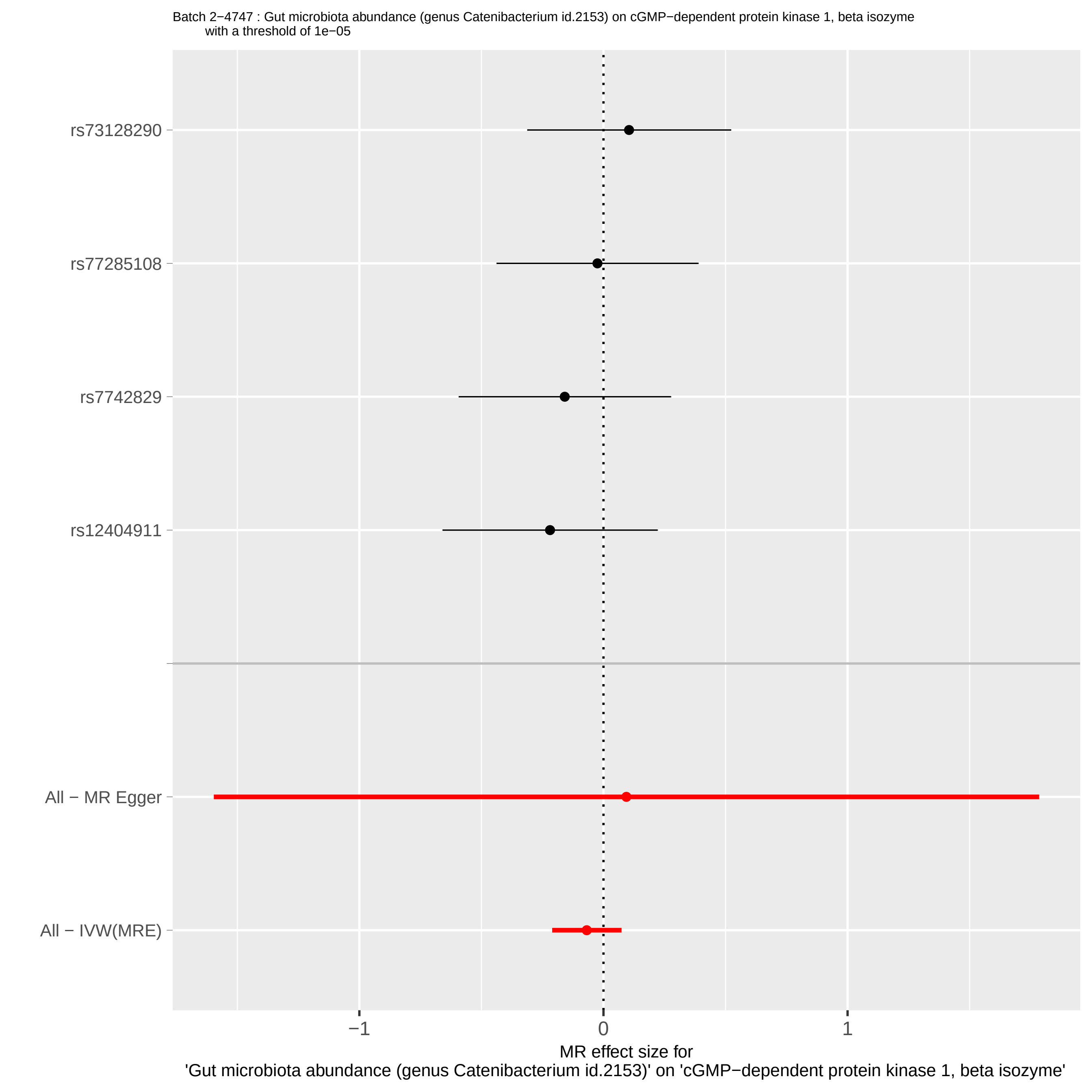
Batch 2−4747 : Gut microbiota abundance (genus Catenibacterium id.2153) on cGMP−dependent protein kinase 1, beta isozyme
with a threshold of 1e−05
rs73128290
rs77285108
rs7742829
rs12404911
All − MR Egger
All − IVW(MRE)
−1
0
1
MR effect size for
'Gut microbiota abundance (genus Catenibacterium id.2153)' on 'cGMP−dependent protein kinase 1, beta isozyme'

Batch 2−4748 : Gut microbiota abundance (genus Parasutterella id.2892) on cGMP−dependent protein kinase 1, beta isozyme
with a threshold of 1e−05

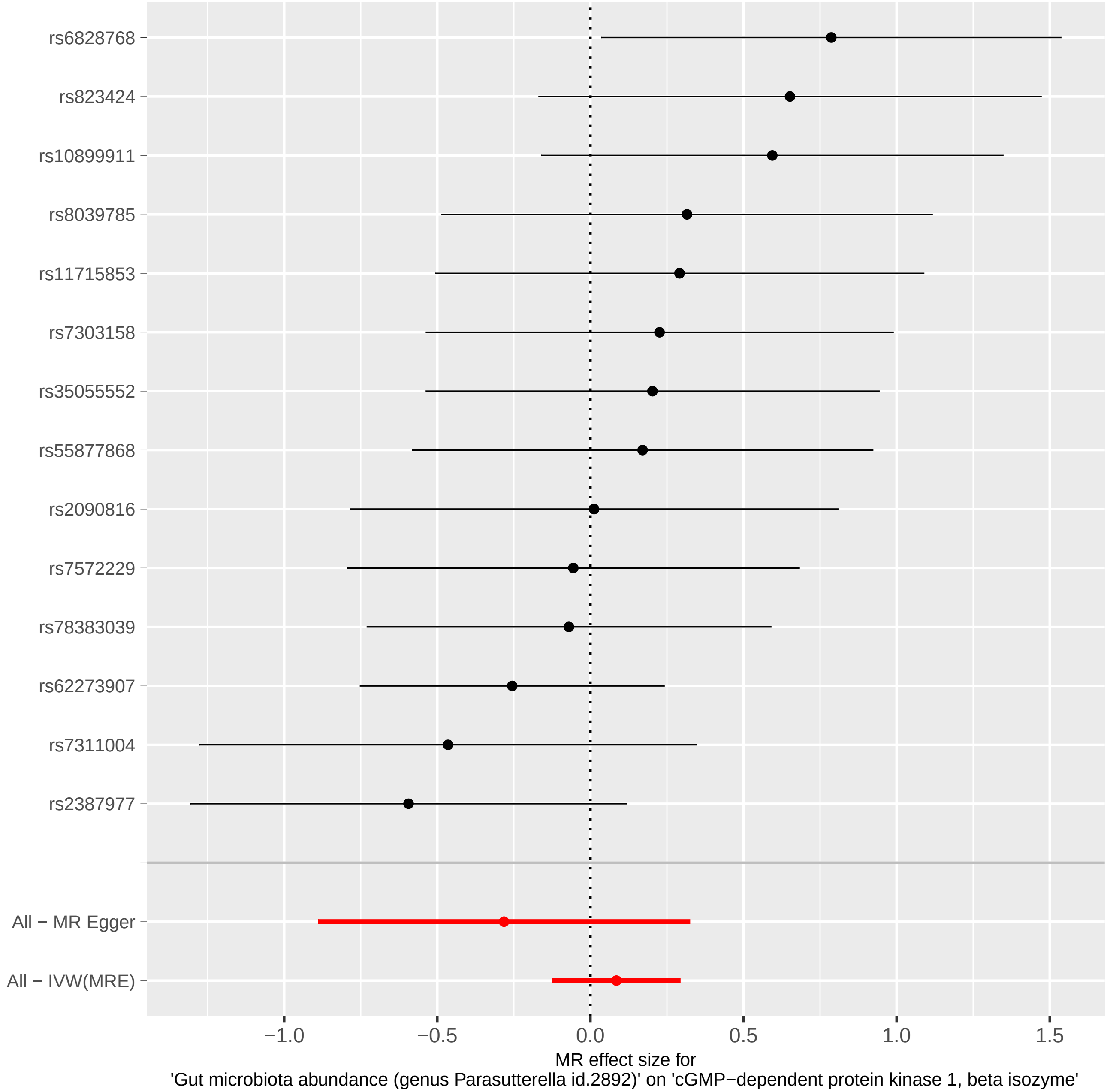

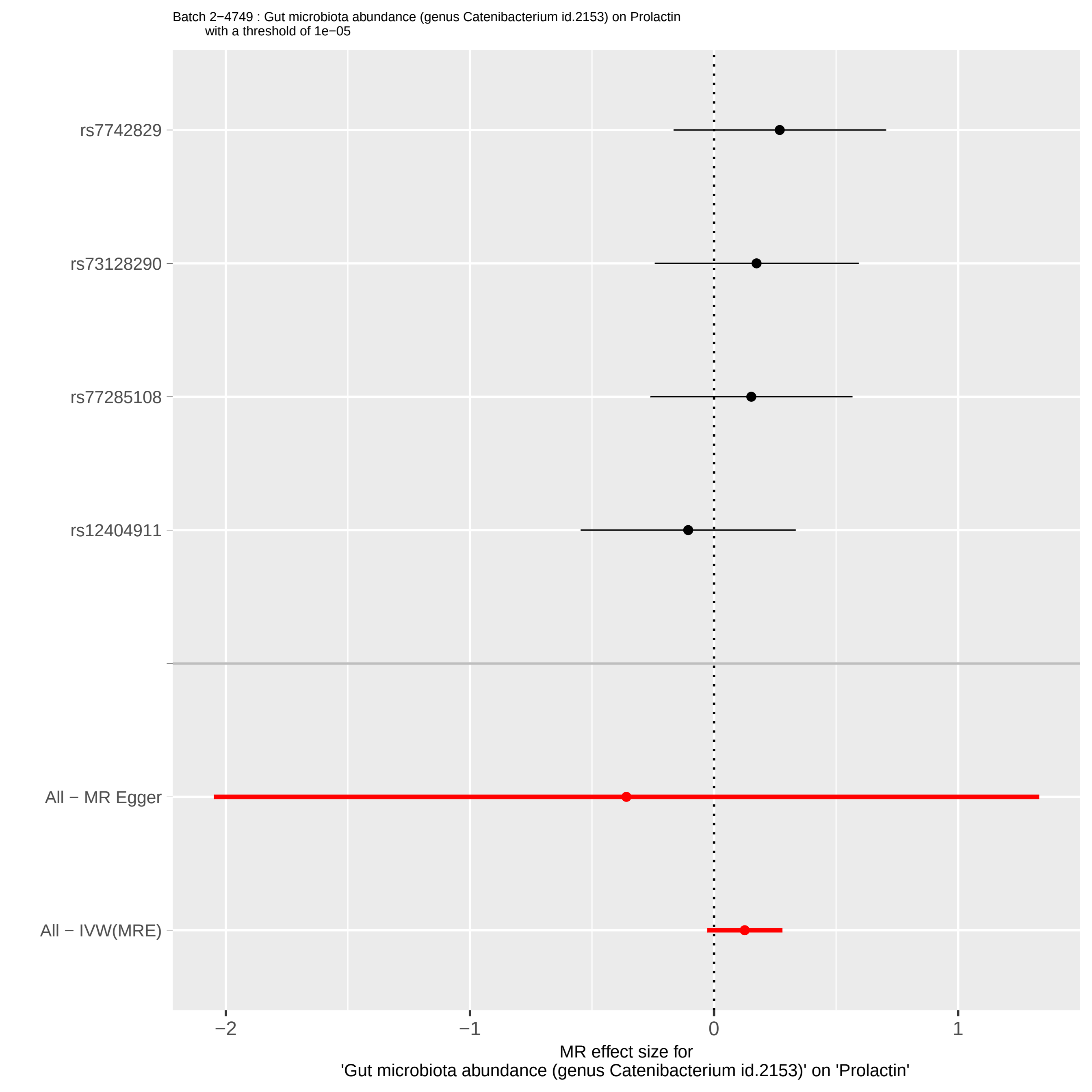
Batch 2−4749 : Gut microbiota abundance (genus Catenibacterium id.2153) on Prolactin
with a threshold of 1e−05
rs7742829
rs73128290
rs77285108
rs12404911
All − MR Egger
All − IVW(MRE)
−2
−1
0
1
MR effect size for
'Gut microbiota abundance (genus Catenibacterium id.2153)' on 'Prolactin'

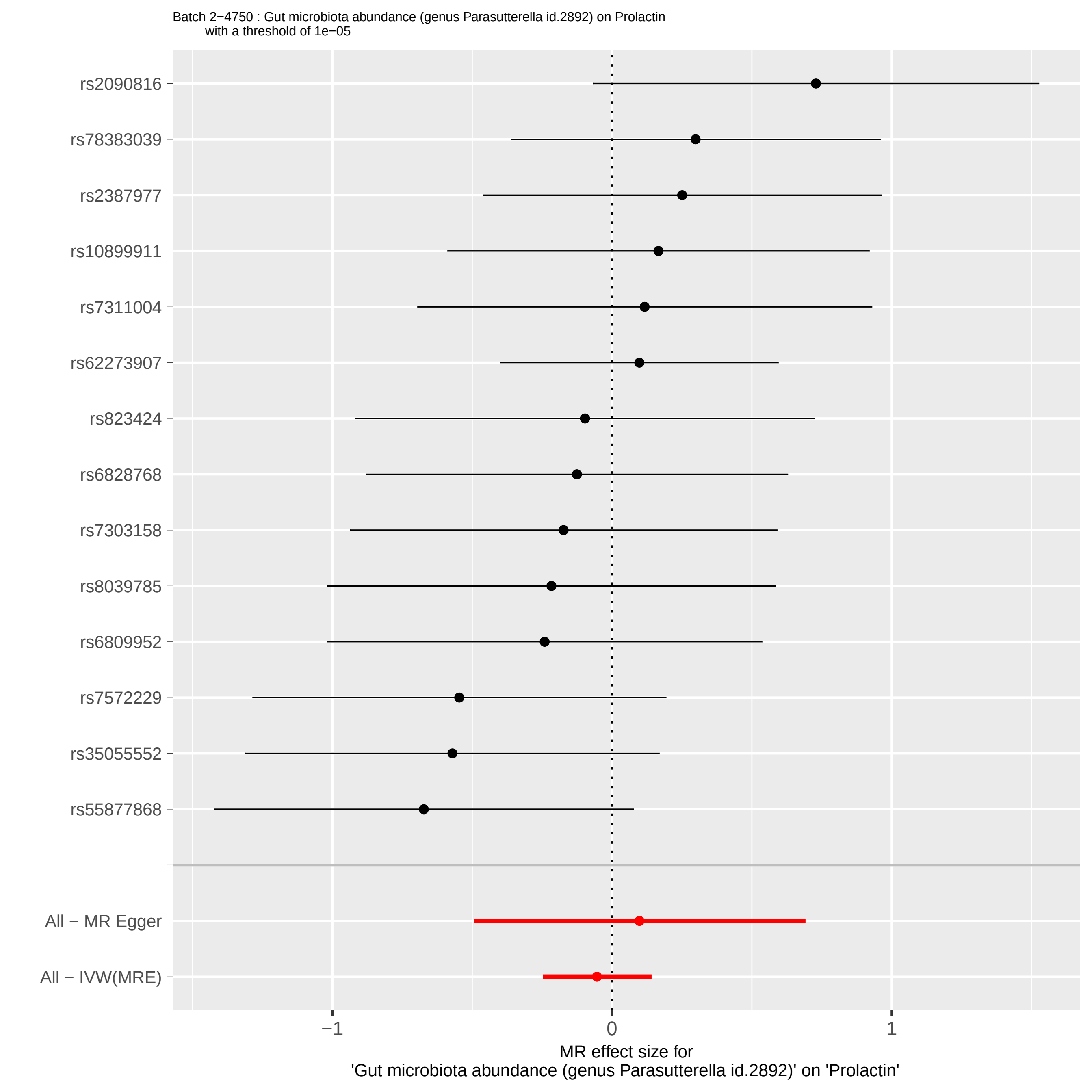

Batch 2−4750 : Gut microbiota abundance (genus Parasutterella id.2892) on Prolactin
with a threshold of 1e−05
rs2090816
rs78383039
rs2387977
rs10899911
rs7311004
rs62273907
rs823424
rs6828768
rs7303158
rs8039785
rs6809952
rs7572229
rs35055552
rs55877868
All − MR Egger
All − IVW(MRE)
−1
0
1
MR effect size for
'Gut microbiota abundance (genus Parasutterella id.2892)' on 'Prolactin'

Batch 2−4751 : Gut microbiota abundance (genus Catenibacterium id.2153) on Prolactin−releasing peptide
with a threshold of 1e−05

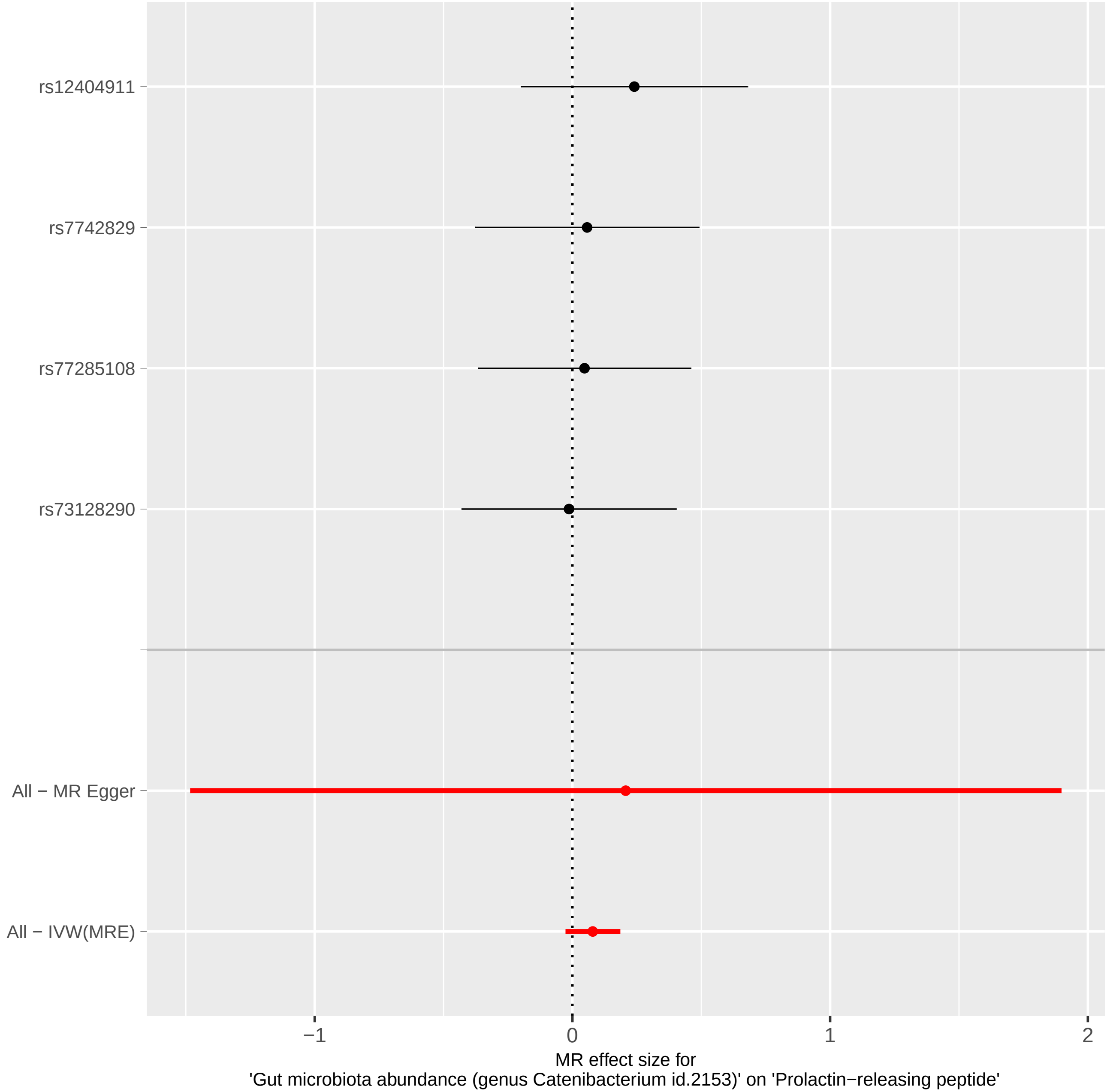

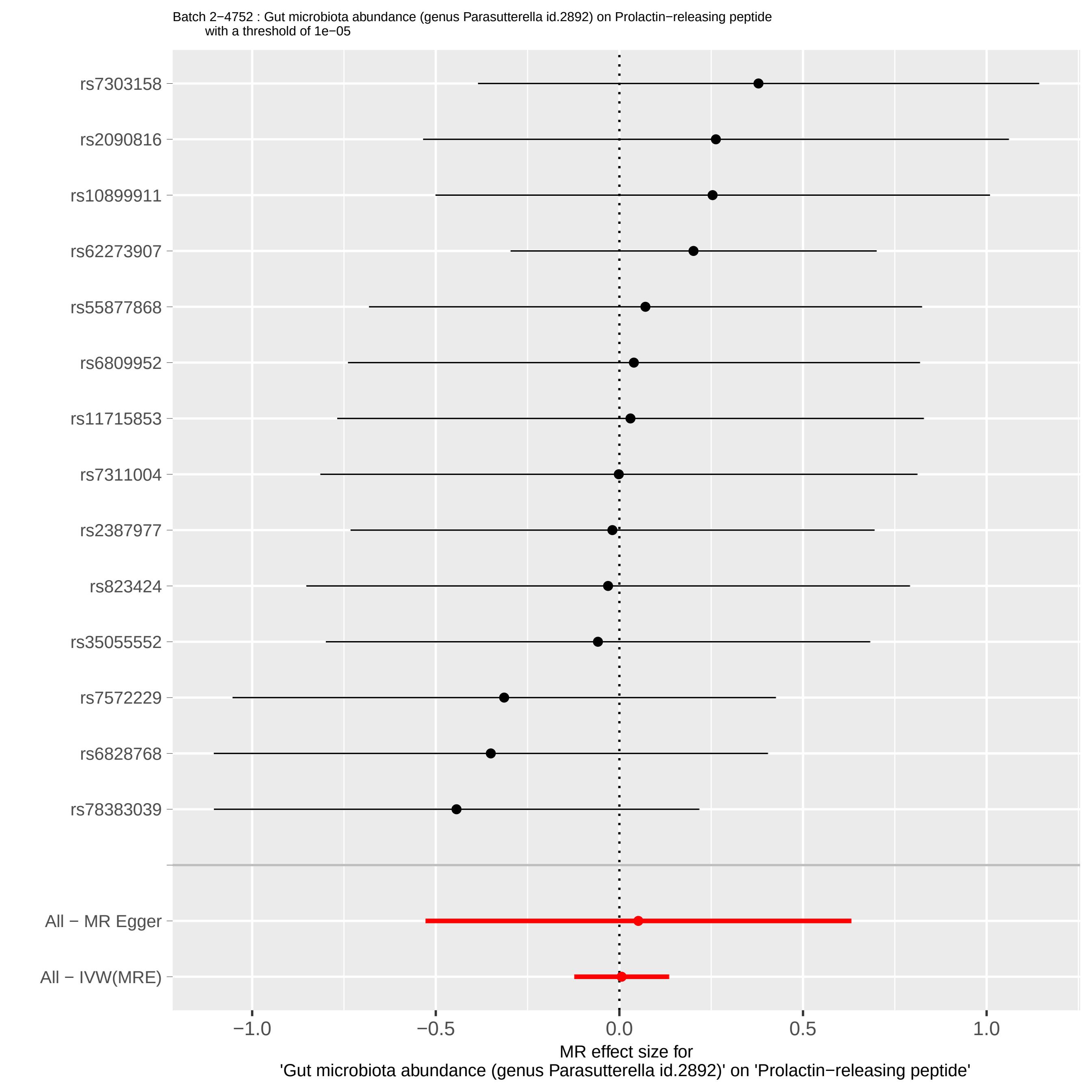
Batch 2−4752 : Gut microbiota abundance (genus Parasutterella id.2892) on Prolactin−releasing peptide
with a threshold of 1e−05
rs7303158
rs2090816
rs10899911
rs62273907
rs55877868
rs6809952
rs11715853
rs7311004
rs2387977
rs823424
rs35055552
rs7572229
rs6828768
rs78383039
All − MR Egger
All − IVW(MRE)
−1.0
−0.5
0.0
0.5
1.0
MR effect size for
'Gut microbiota abundance (genus Parasutterella id.2892)' on 'Prolactin−releasing peptide'

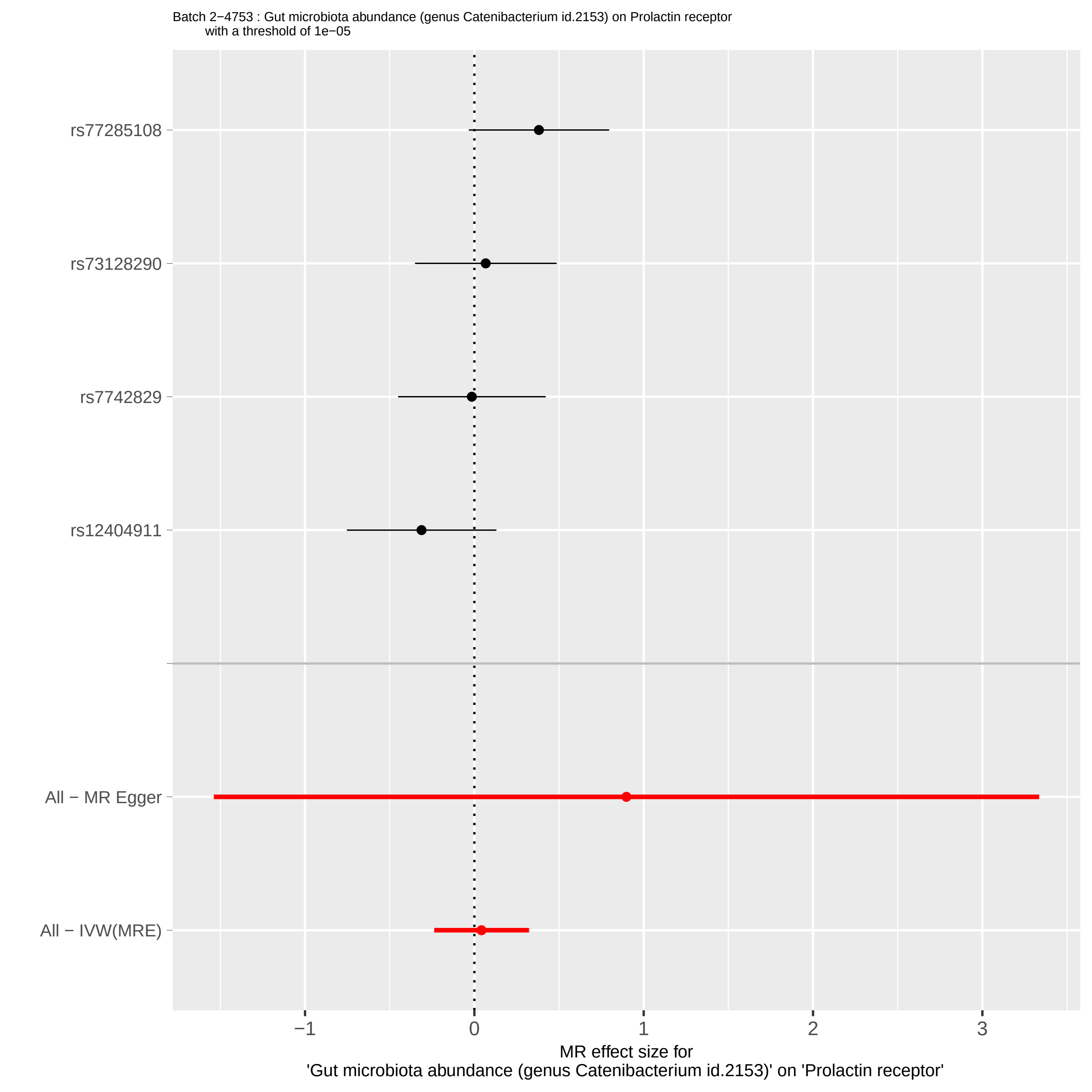
Batch 2−4753 : Gut microbiota abundance (genus Catenibacterium id.2153) on Prolactin receptor
with a threshold of 1e−05
rs77285108
rs73128290
rs7742829
rs12404911
All − MR Egger
All − IVW(MRE)
−1
0
1
2
3
MR effect size for
'Gut microbiota abundance (genus Catenibacterium id.2153)' on 'Prolactin receptor'

Batch 2−4754 : Gut microbiota abundance (genus Parasutterella id.2892) on Prolactin receptor
with a threshold of 1e−05

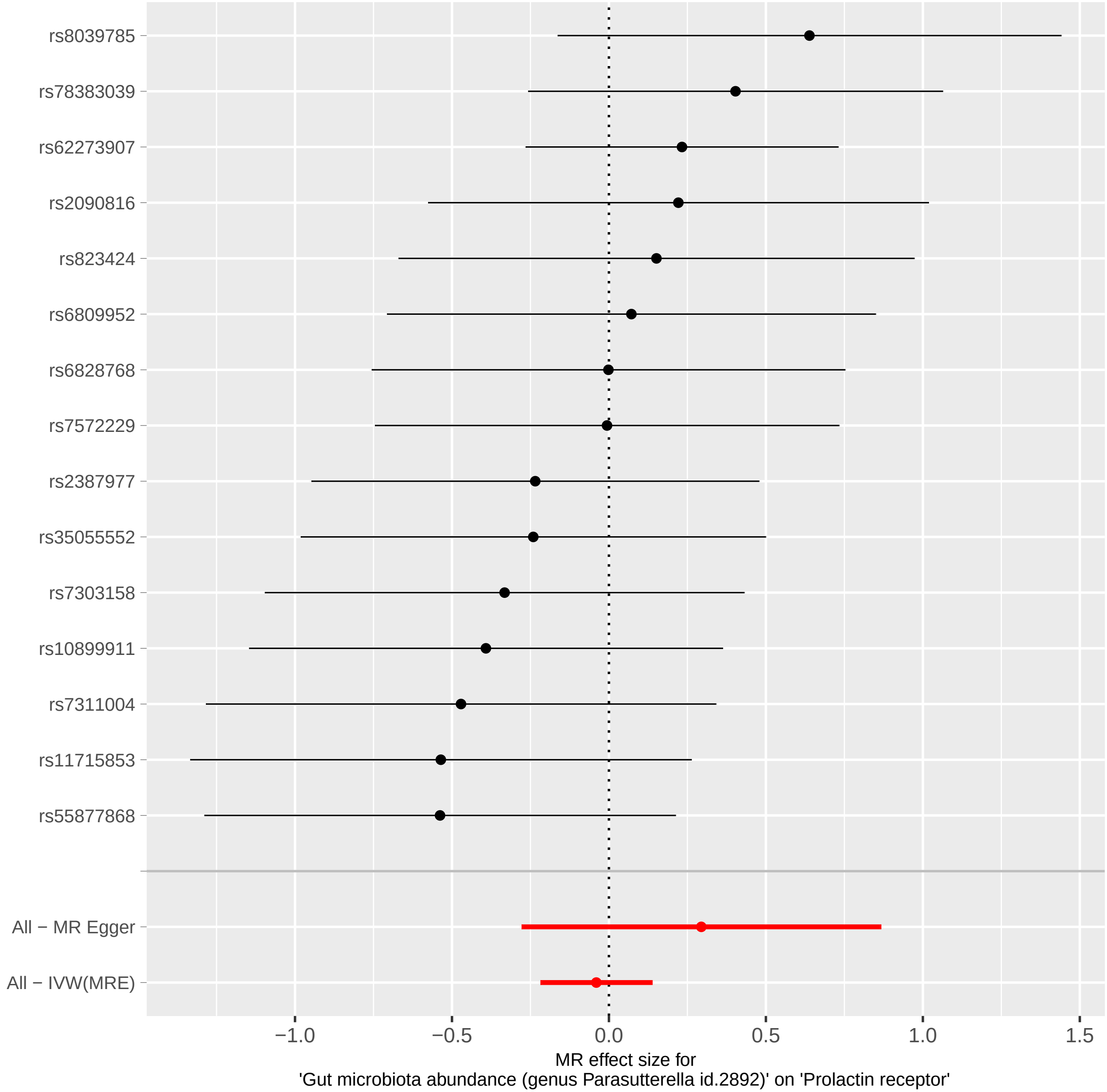

Batch 2−4755 : Gut microbiota abundance (genus Catenibacterium id.2153) on Protein arginine N−methyltransferase 3
with a threshold of 1e−05

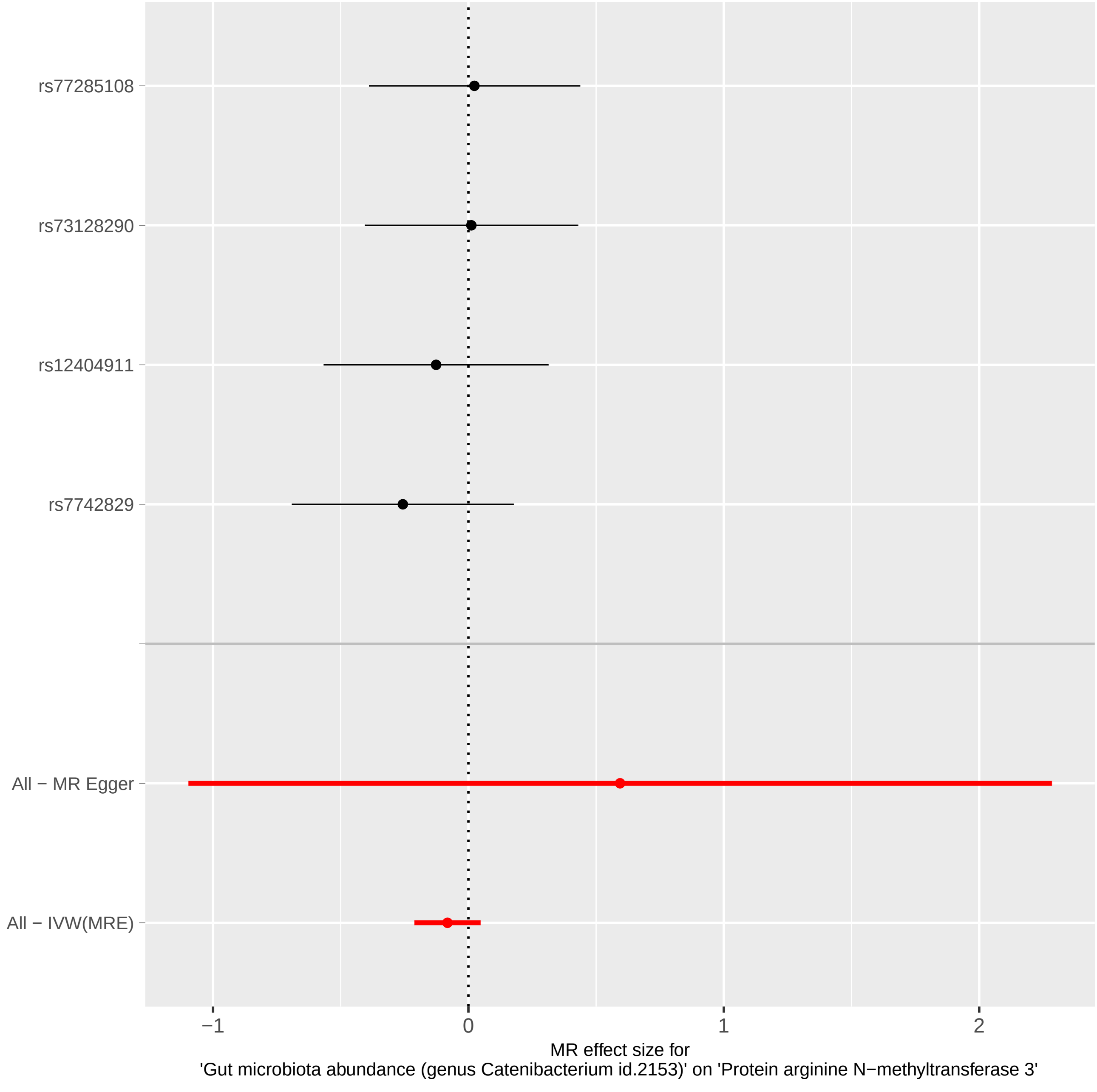

Batch 2−4756 : Gut microbiota abundance (genus Parasutterella id.2892) on Protein arginine N−methyltransferase 3
with a threshold of 1e−05

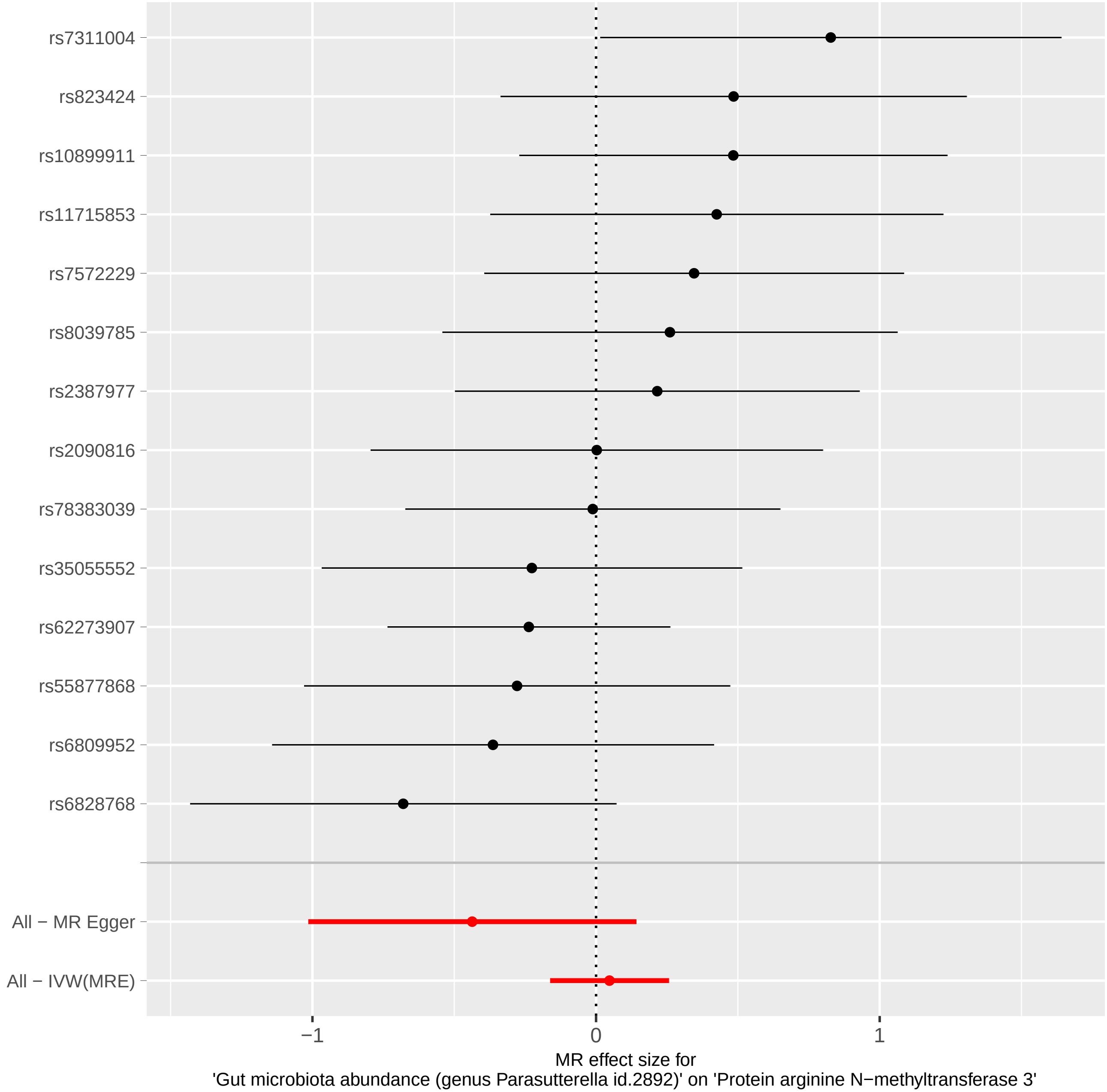

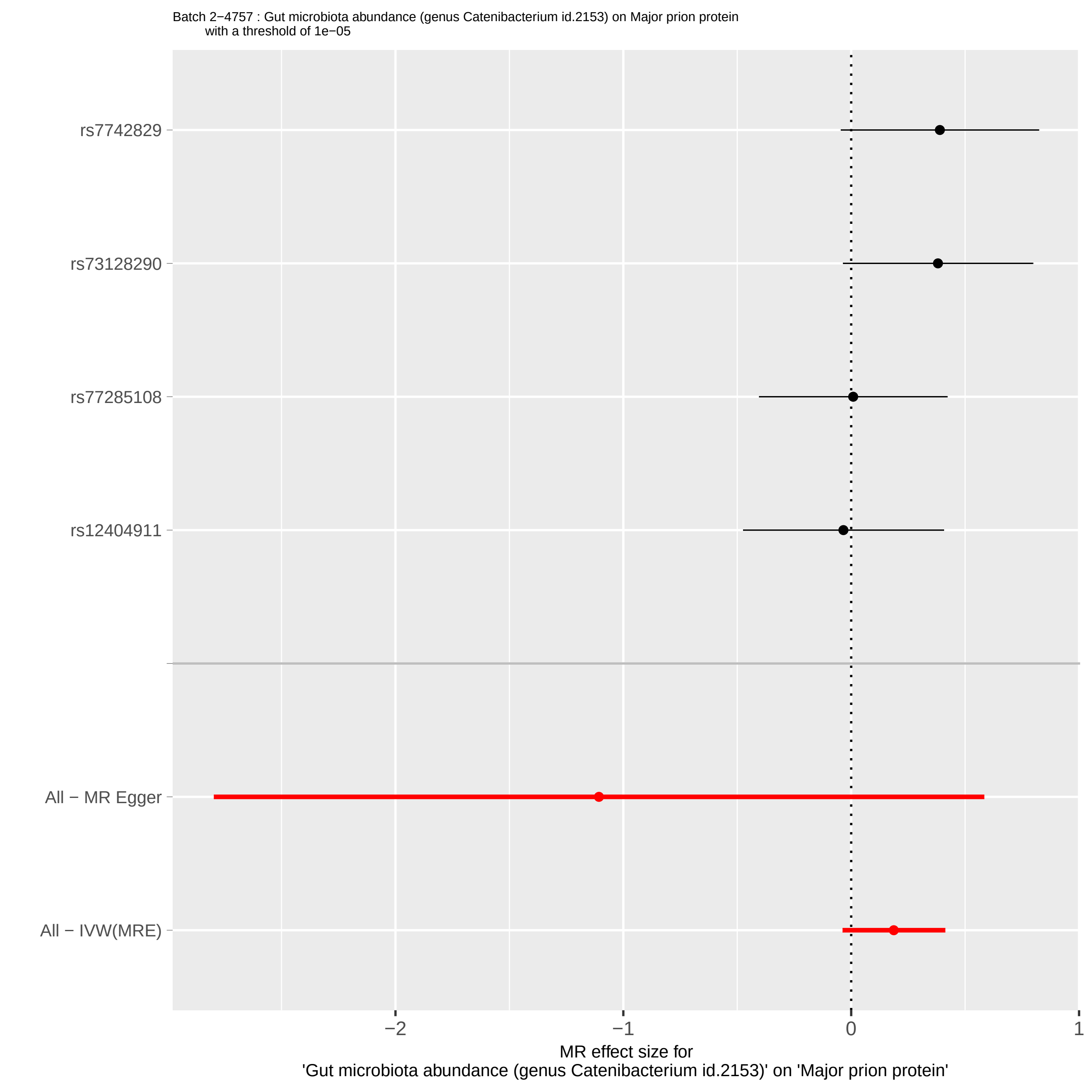

Batch 2−4757 : Gut microbiota abundance (genus Catenibacterium id.2153) on Major prion protein
with a threshold of 1e−05
rs7742829
rs73128290
rs77285108
rs12404911
All − MR Egger
All − IVW(MRE)
−2
−1
0
1
MR effect size for
'Gut microbiota abundance (genus Catenibacterium id.2153)' on 'Major prion protein'

Batch 2−4758 : Gut microbiota abundance (genus Parasutterella id.2892) on Major prion protein
with a threshold of 1e−05

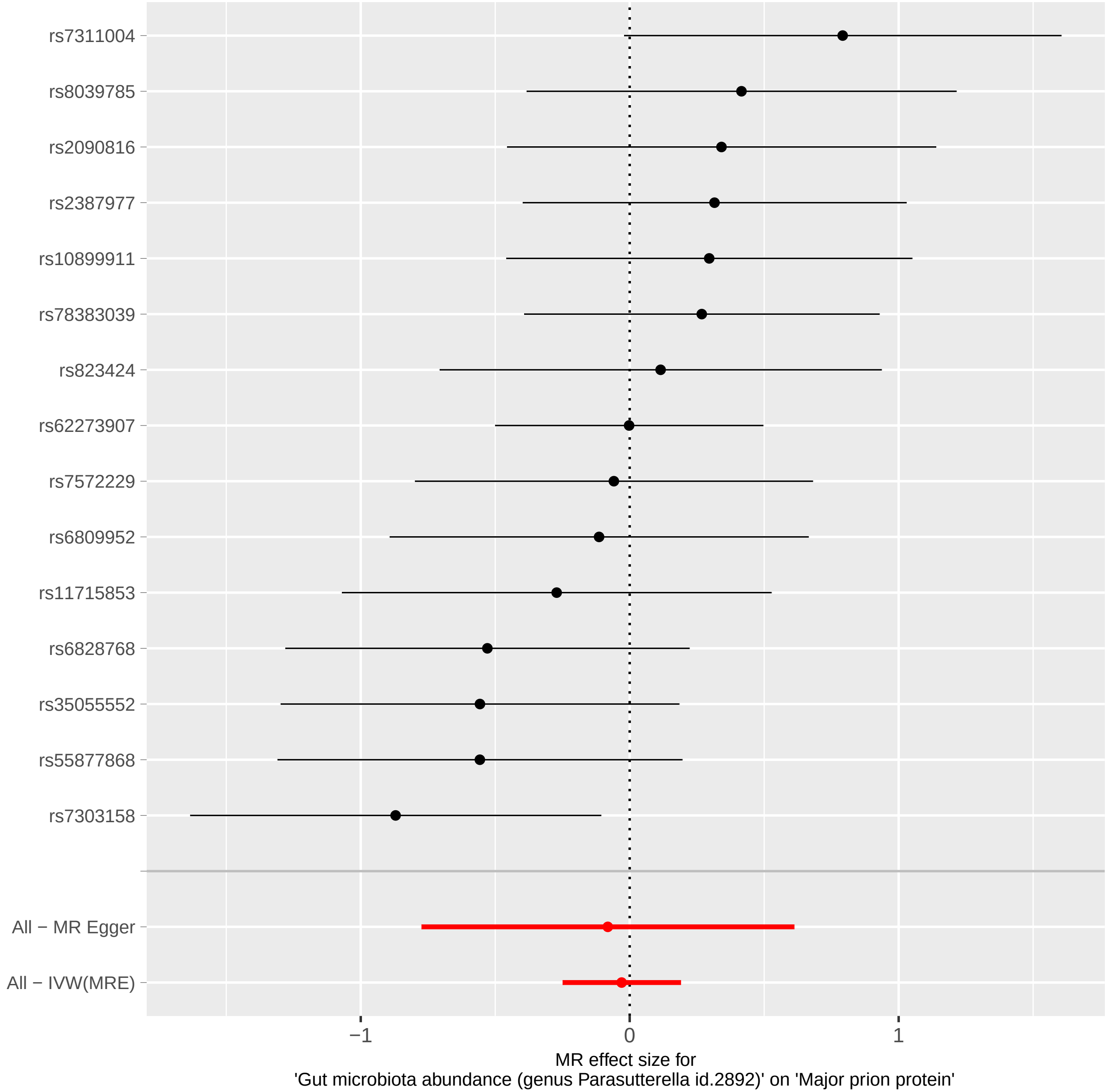

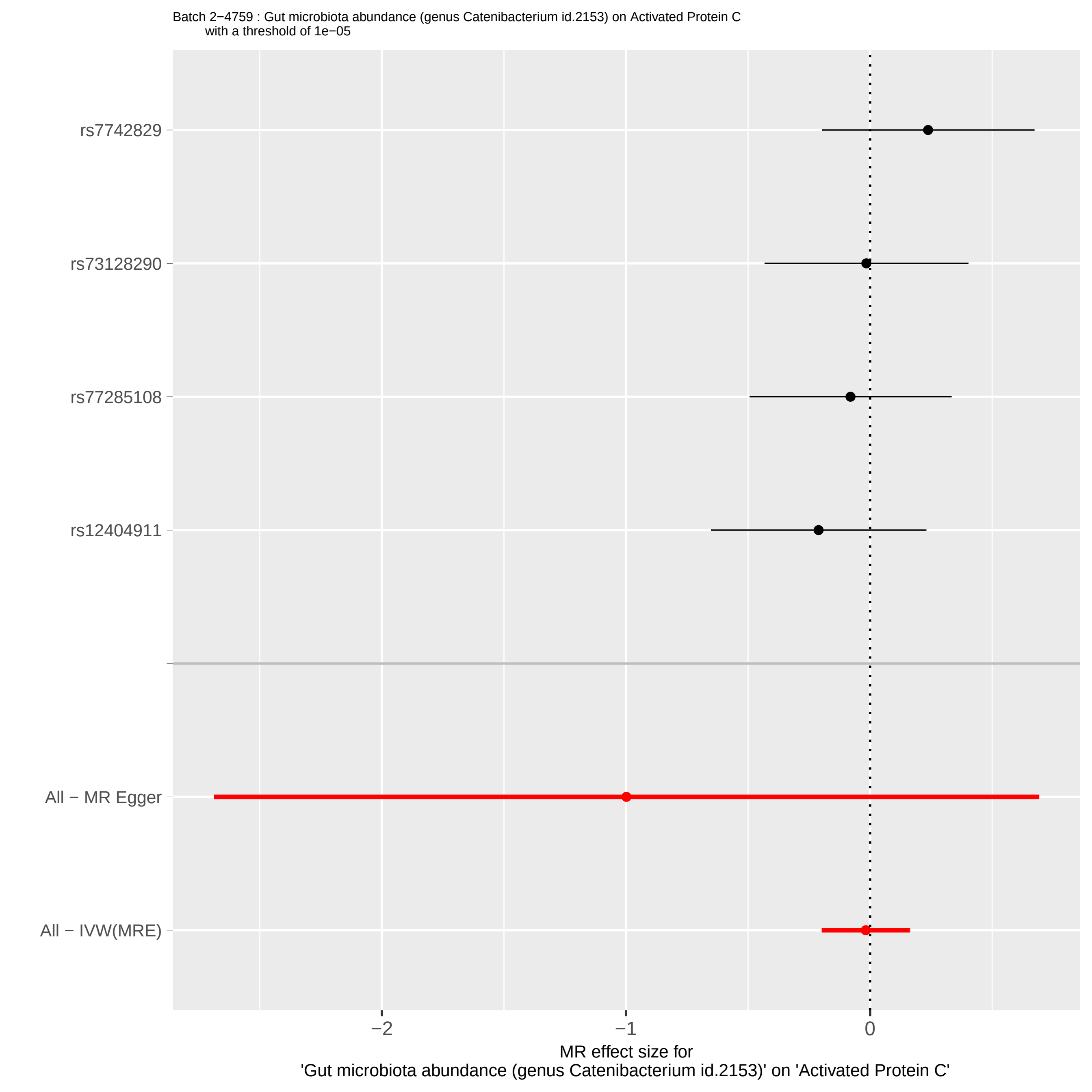
Batch 2−4759 : Gut microbiota abundance (genus Catenibacterium id.2153) on Activated Protein C
with a threshold of 1e−05
rs7742829
rs73128290
rs77285108
rs12404911
All − MR Egger
All − IVW(MRE)
−2
−1
0
MR effect size for
'Gut microbiota abundance (genus Catenibacterium id.2153)' on 'Activated Protein C'

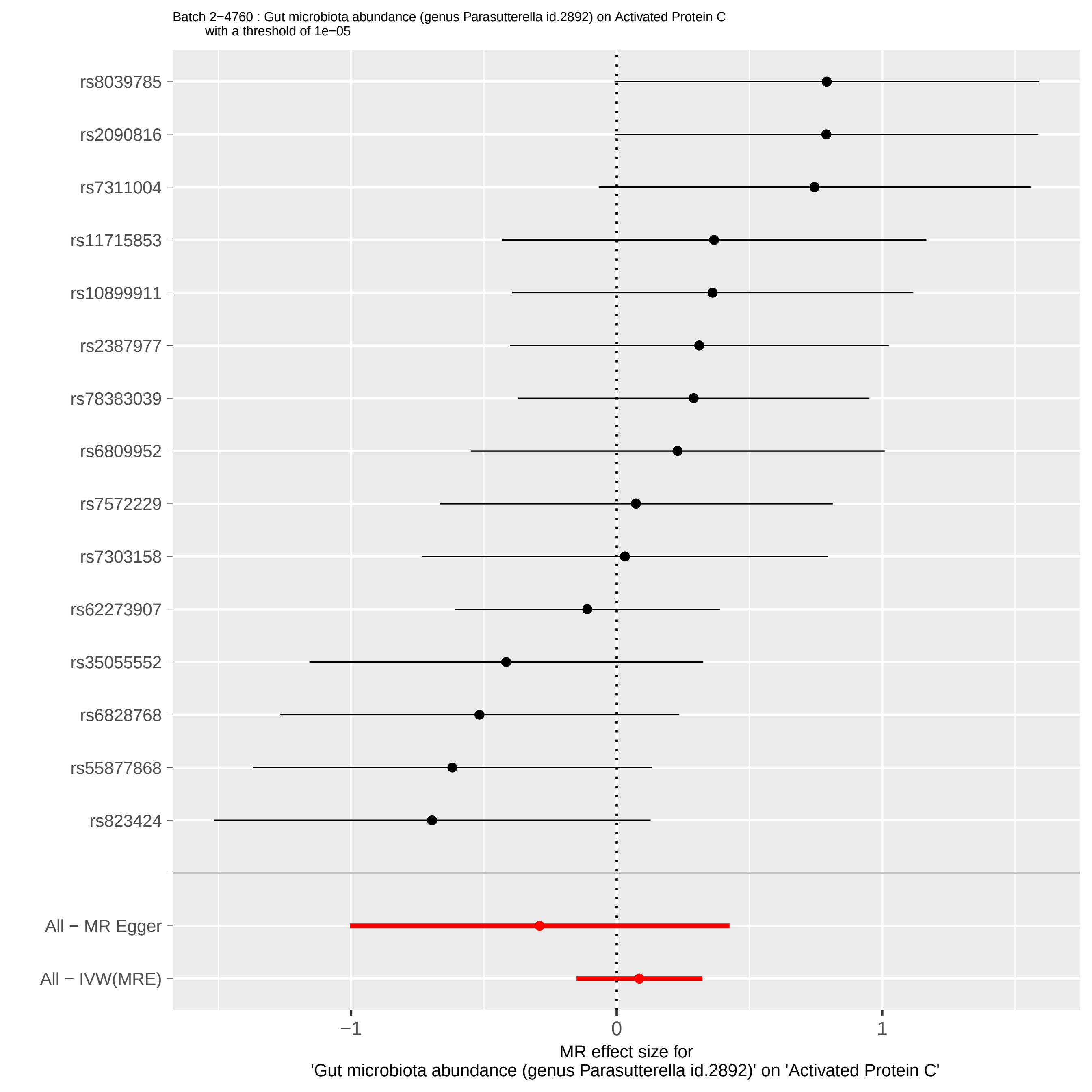

Batch 2−4760 : Gut microbiota abundance (genus Parasutterella id.2892) on Activated Protein C
with a threshold of 1e−05
rs8039785
rs2090816
rs7311004
rs11715853
rs10899911
rs2387977
rs78383039
rs6809952
rs7572229
rs7303158
rs62273907
rs35055552
rs6828768
rs55877868
rs823424
All − MR Egger
All − IVW(MRE)
−1
0
1
MR effect size for
'Gut microbiota abundance (genus Parasutterella id.2892)' on 'Activated Protein C'

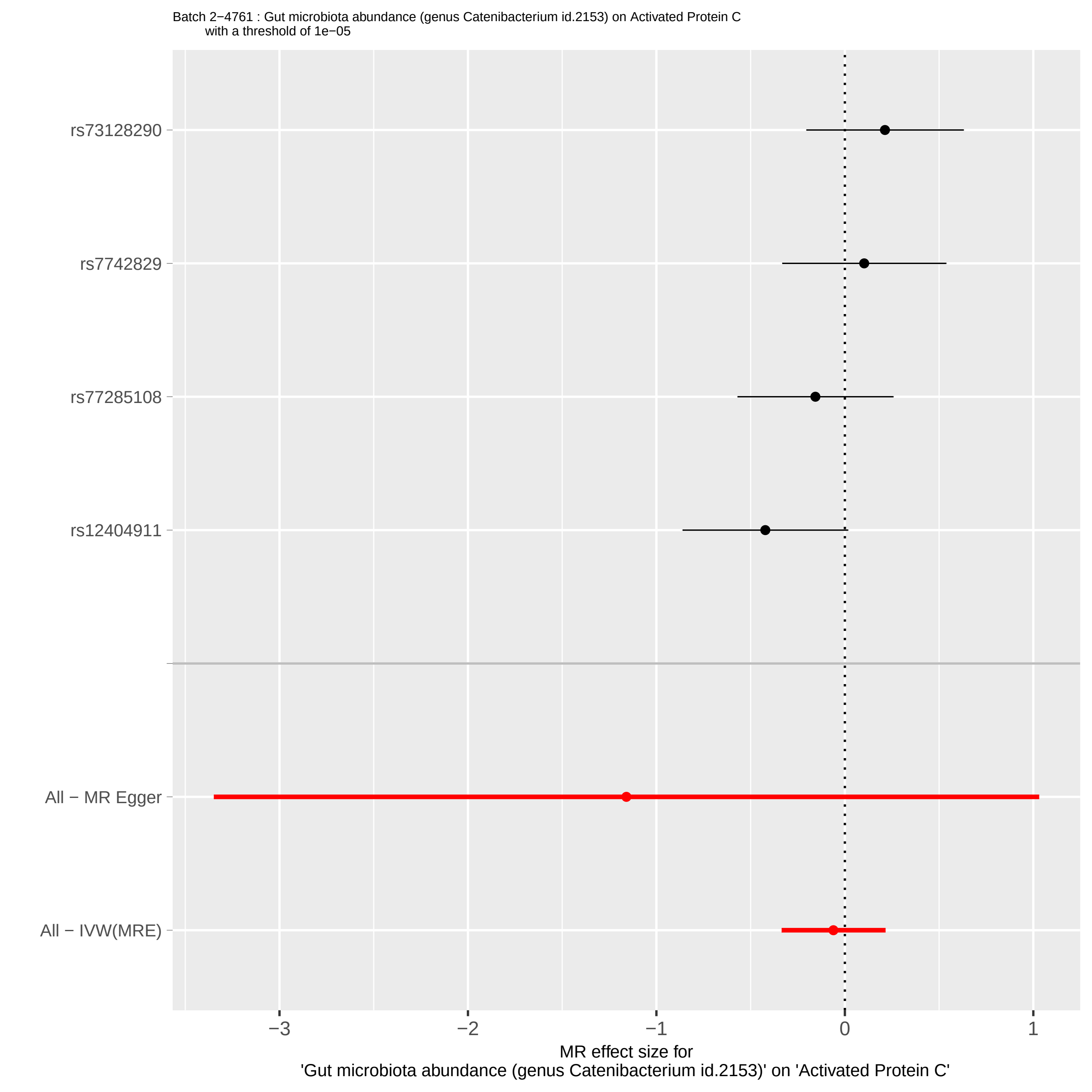

Batch 2−4761 : Gut microbiota abundance (genus Catenibacterium id.2153) on Activated Protein C
with a threshold of 1e−05
rs73128290
rs7742829
rs77285108
rs12404911
All − MR Egger
All − IVW(MRE)
−3
−2
−1
0
1
MR effect size for
'Gut microbiota abundance (genus Catenibacterium id.2153)' on 'Activated Protein C'

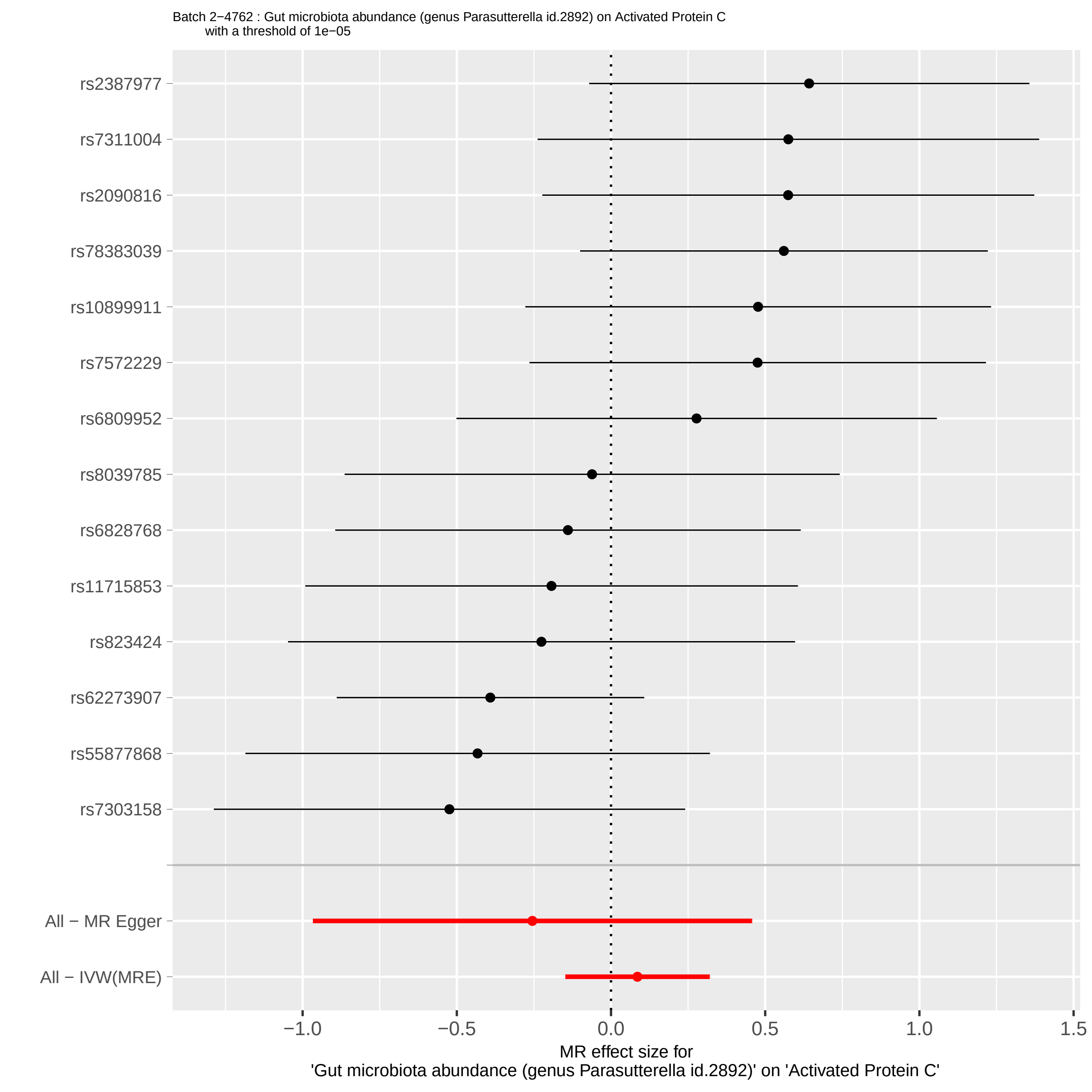

Batch 2−4762 : Gut microbiota abundance (genus Parasutterella id.2892) on Activated Protein C
with a threshold of 1e−05
rs2387977
rs7311004
rs2090816
rs78383039
rs10899911
rs7572229
rs6809952
rs8039785
rs6828768
rs11715853
rs823424
rs62273907
rs55877868
rs7303158
All − MR Egger
All − IVW(MRE)
−1.0
−0.5
0.0
0.5
1.0
1.5
MR effect size for
'Gut microbiota abundance (genus Parasutterella id.2892)' on 'Activated Protein C'

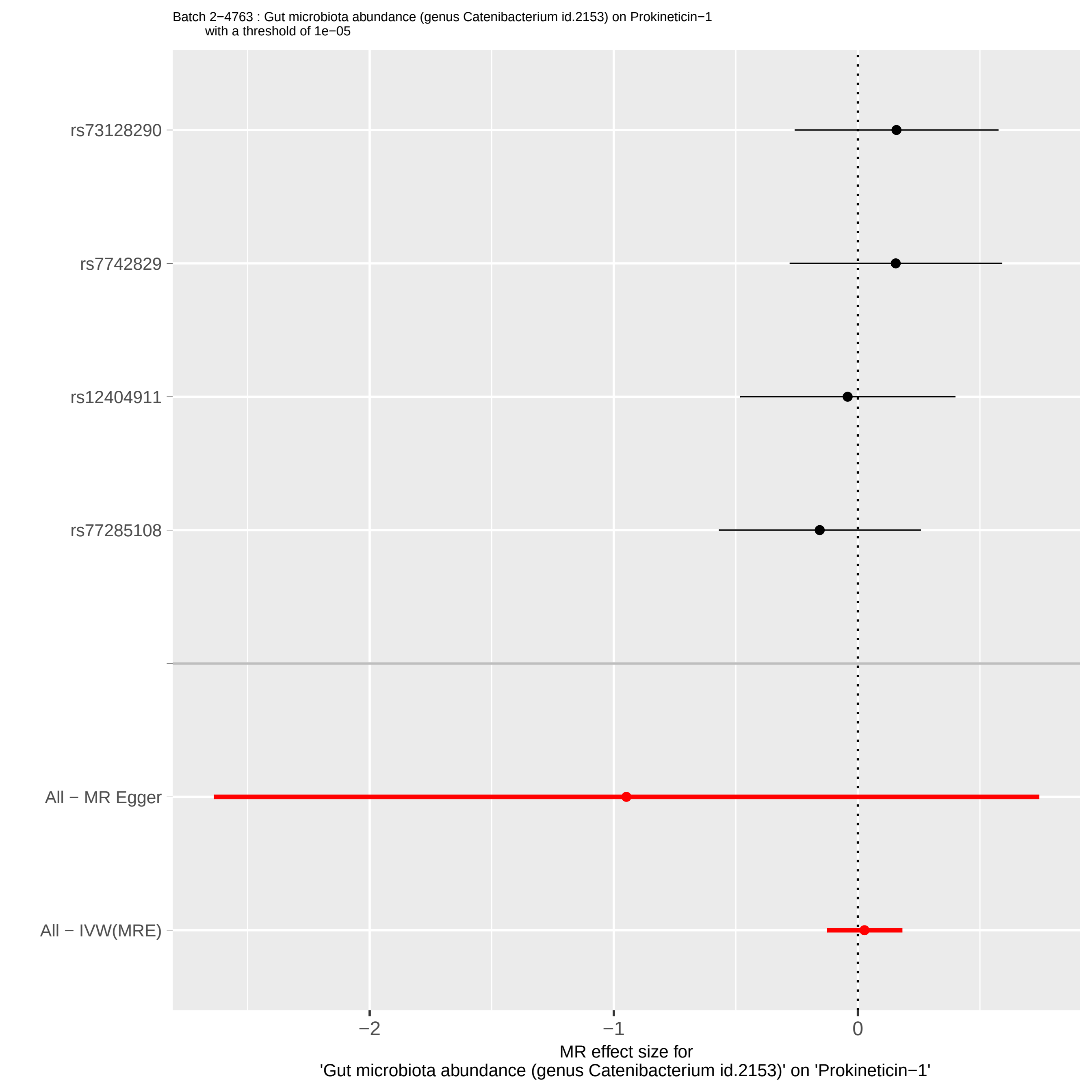
Batch 2−4763 : Gut microbiota abundance (genus Catenibacterium id.2153) on Prokineticin−1
with a threshold of 1e−05
rs73128290
rs7742829
rs12404911
rs77285108
All − MR Egger
All − IVW(MRE)
−2
−1
0
MR effect size for
'Gut microbiota abundance (genus Catenibacterium id.2153)' on 'Prokineticin−1'

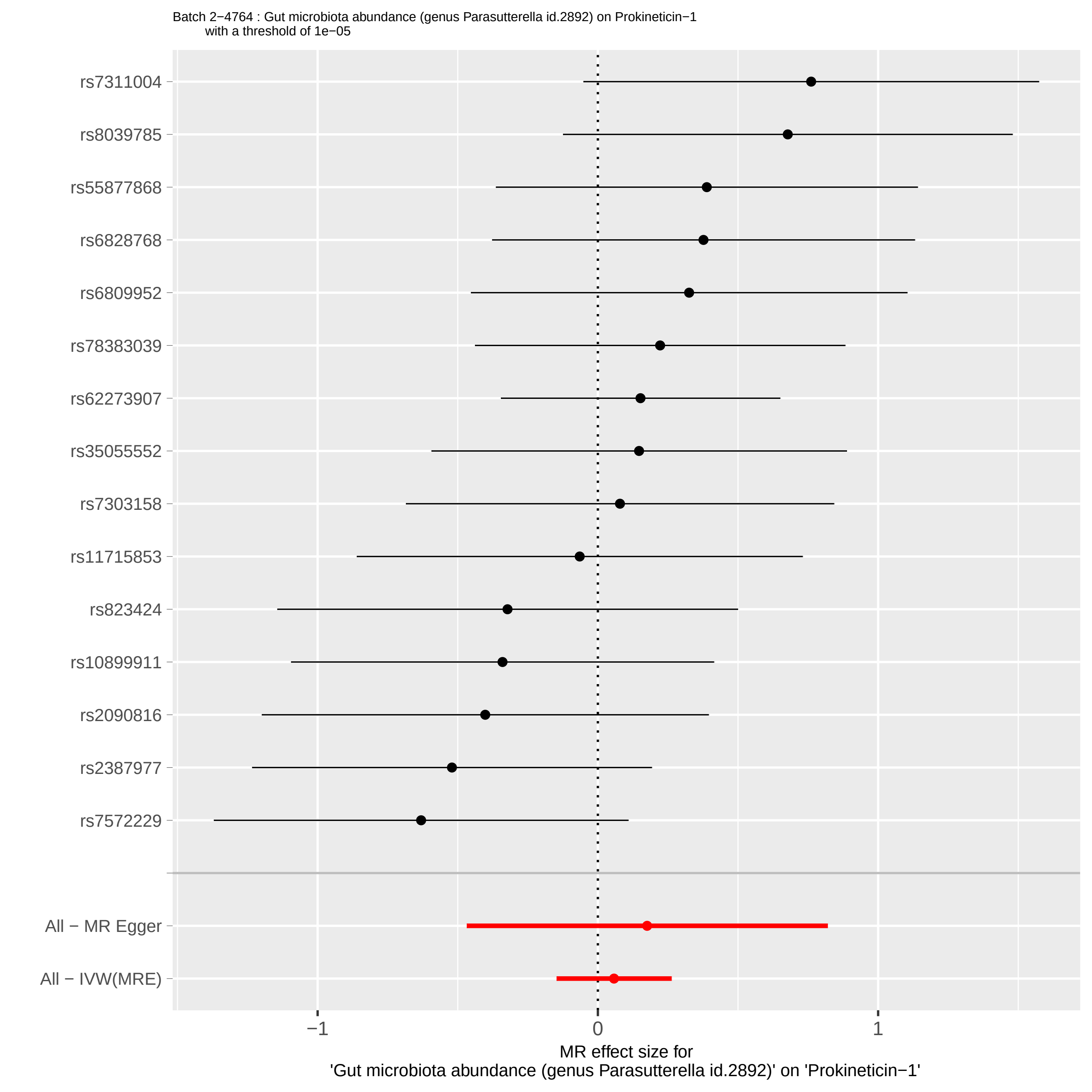

Batch 2−4764 : Gut microbiota abundance (genus Parasutterella id.2892) on Prokineticin−1
with a threshold of 1e−05
rs7311004
rs8039785
rs55877868
rs6828768
rs6809952
rs78383039
rs62273907
rs35055552
rs7303158
rs11715853
rs823424
rs10899911
rs2090816
rs2387977
rs7572229
All − MR Egger
All − IVW(MRE)
−1
0
1
MR effect size for
'Gut microbiota abundance (genus Parasutterella id.2892)' on 'Prokineticin−1'

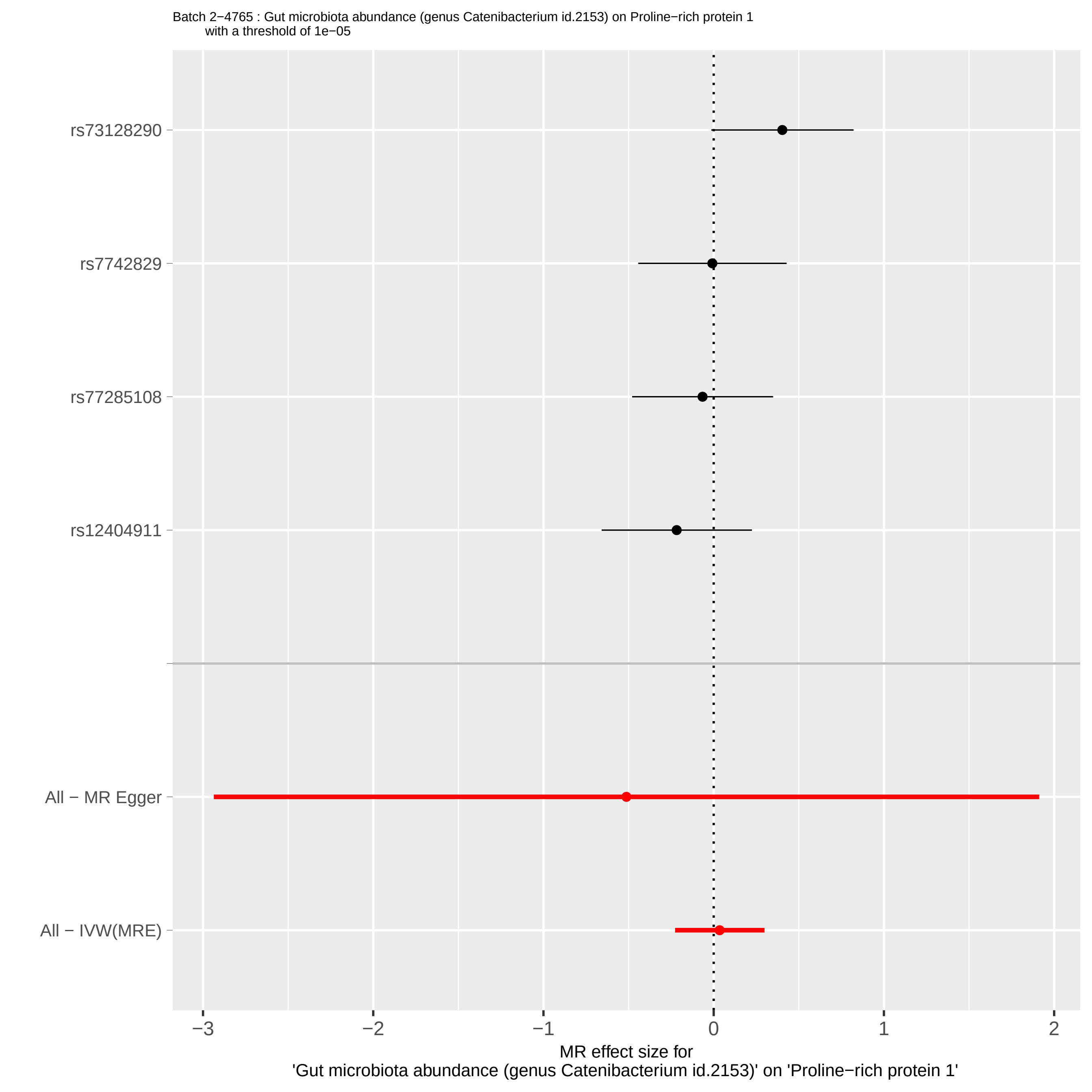

Batch 2−4765 : Gut microbiota abundance (genus Catenibacterium id.2153) on Proline−rich protein 1
with a threshold of 1e−05
rs73128290
rs7742829
rs77285108
rs12404911
All − MR Egger
All − IVW(MRE)
−3
−2
−1
0
1
2
MR effect size for
'Gut microbiota abundance (genus Catenibacterium id.2153)' on 'Proline−rich protein 1'

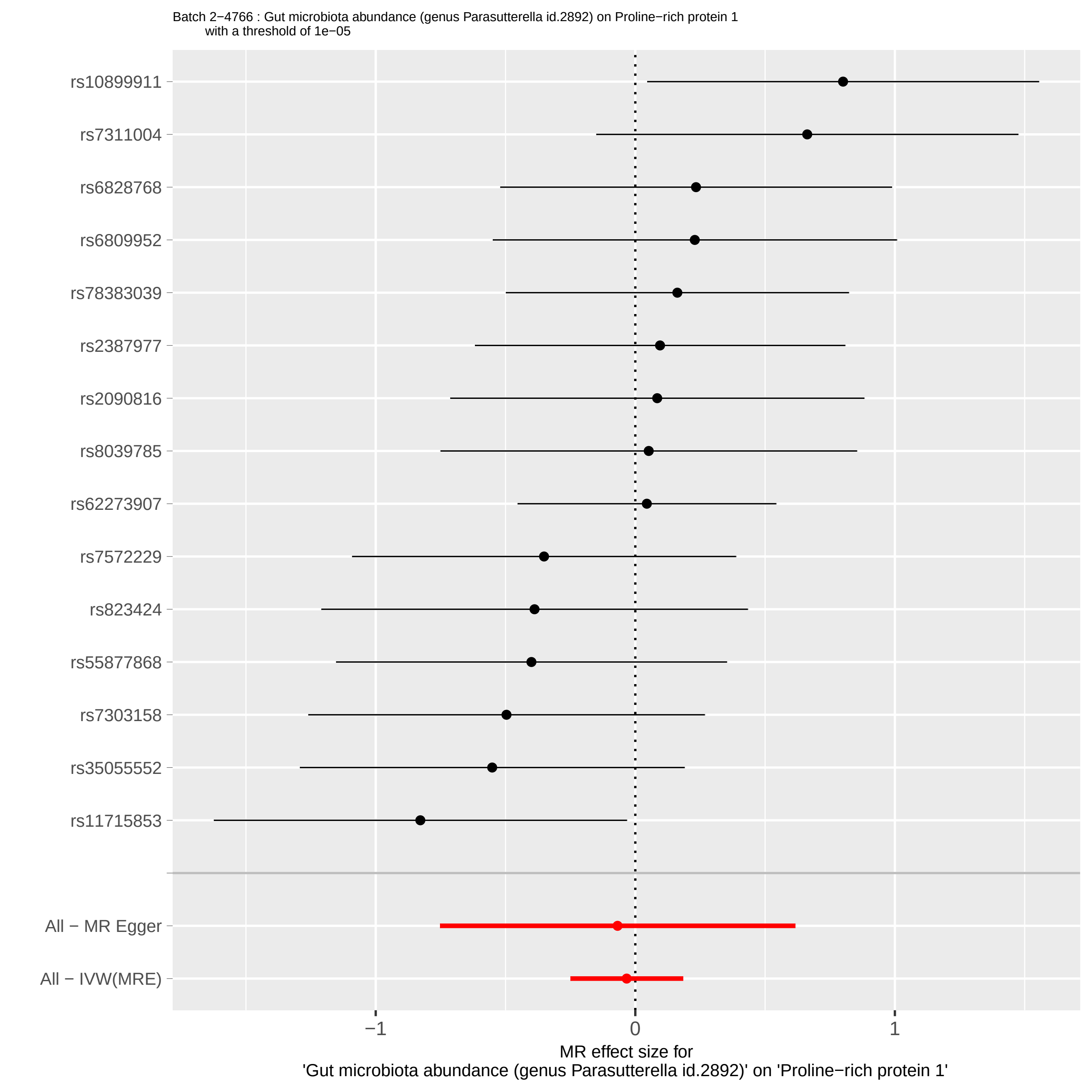

Batch 2−4766 : Gut microbiota abundance (genus Parasutterella id.2892) on Proline−rich protein 1
with a threshold of 1e−05
rs10899911
rs7311004
rs6828768
rs6809952
rs78383039
rs2387977
rs2090816
rs8039785
rs62273907
rs7572229
rs823424
rs55877868
rs7303158
rs35055552
rs11715853
All − MR Egger
All − IVW(MRE)
−1
0
1
MR effect size for
'Gut microbiota abundance (genus Parasutterella id.2892)' on 'Proline−rich protein 1'

Batch 2−4767 : Gut microbiota abundance (genus Catenibacterium id.2153) on Pre−mRNA−processing factor 6
with a threshold of 1e−05

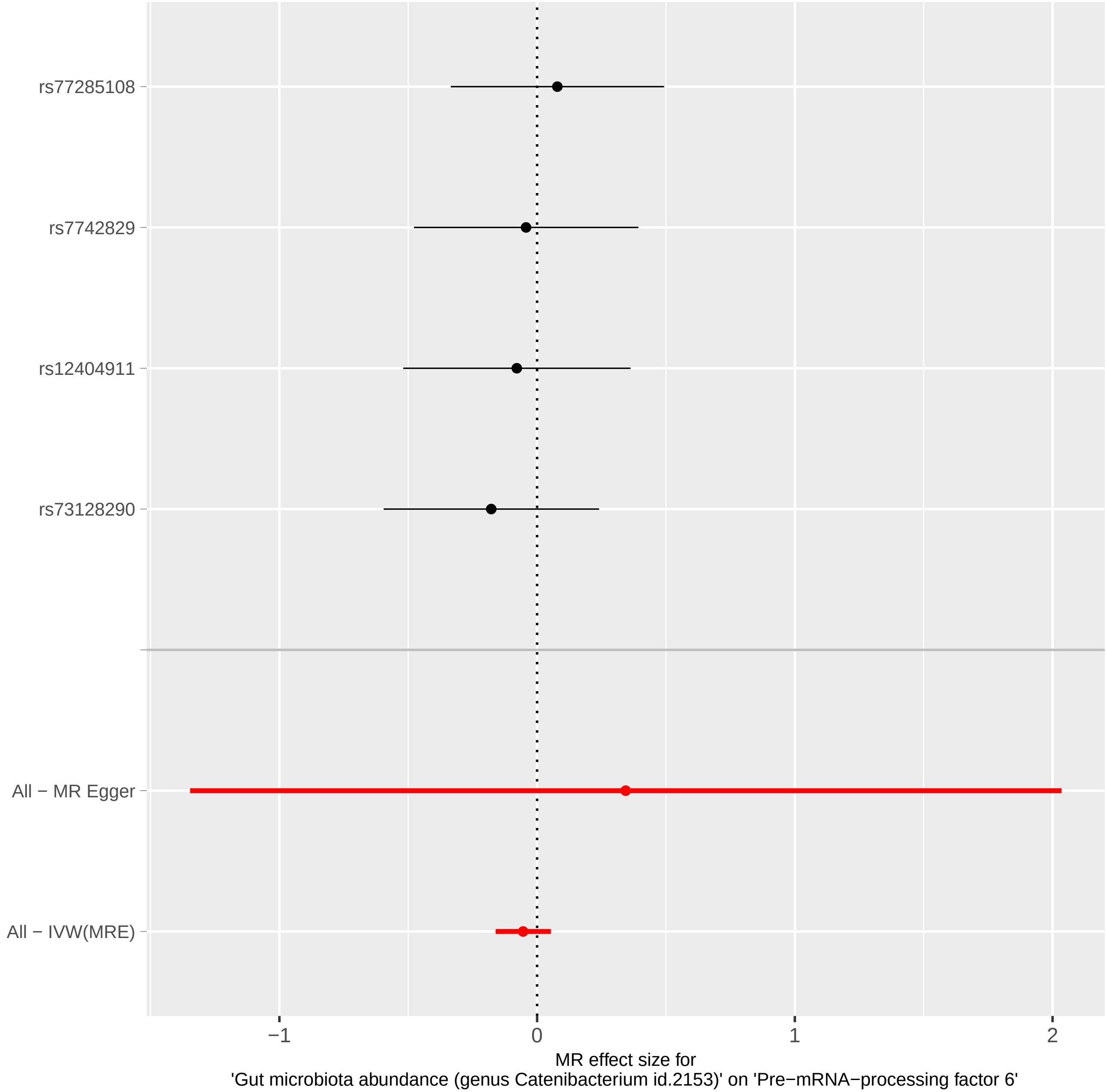

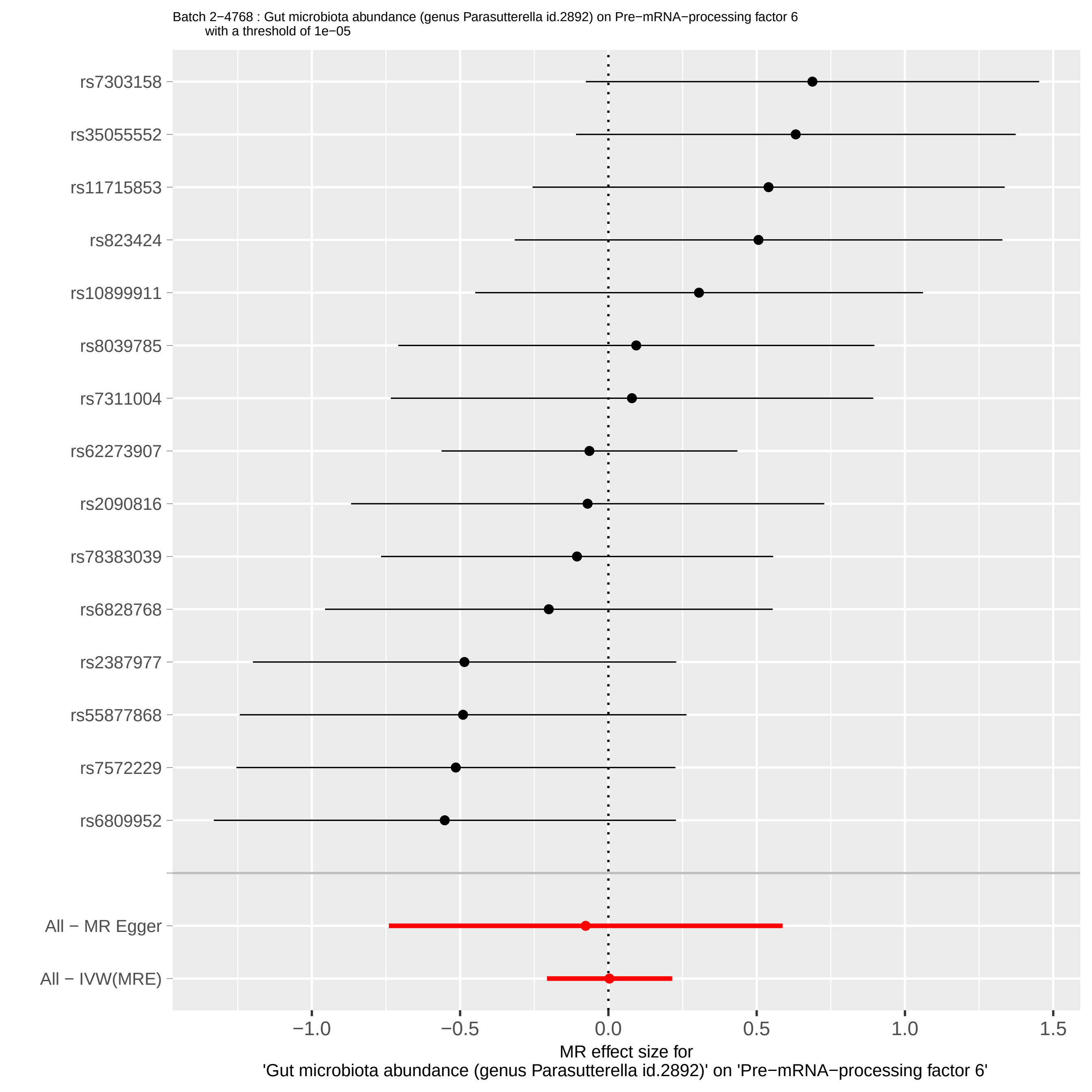

Batch 2−4768 : Gut microbiota abundance (genus Parasutterella id.2892) on Pre−mRNA−processing factor 6
with a threshold of 1e−05
rs7303158
rs35055552
rs11715853
rs823424
rs10899911
rs8039785
rs7311004
rs62273907
rs2090816
rs78383039
rs6828768
rs2387977
rs55877868
rs7572229
rs6809952
All − MR Egger
All − IVW(MRE)
−1.0
−0.5
0.0
0.5
1.0
1.5
MR effect size for
'Gut microbiota abundance (genus Parasutterella id.2892)' on 'Pre−mRNA−processing factor 6'

Batch 2−4769 : Gut microbiota abundance (genus Catenibacterium id.2153) on Phosphoribosyl pyrophosphate synthase−associated protein 1
with a threshold of 1e−05

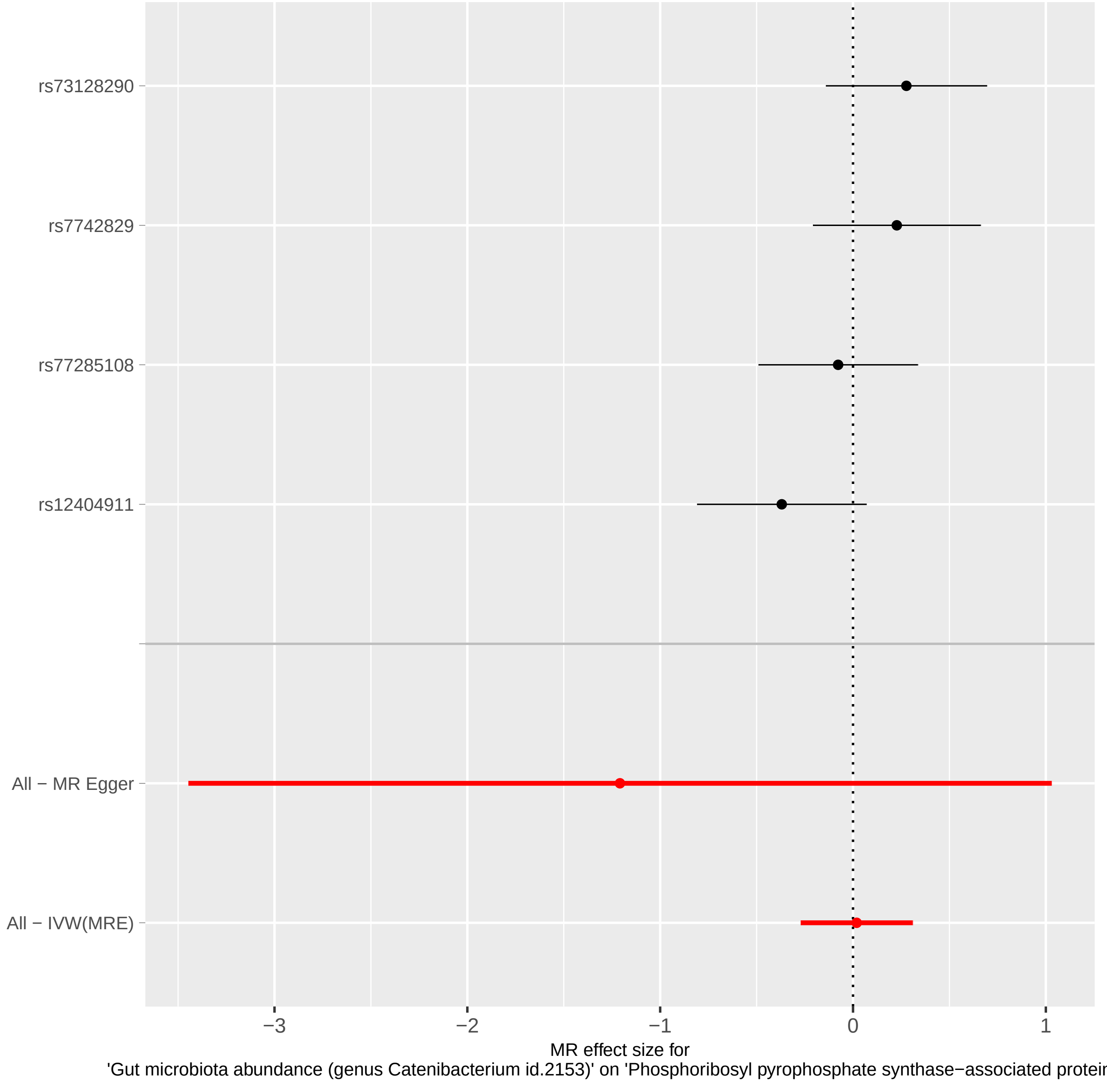

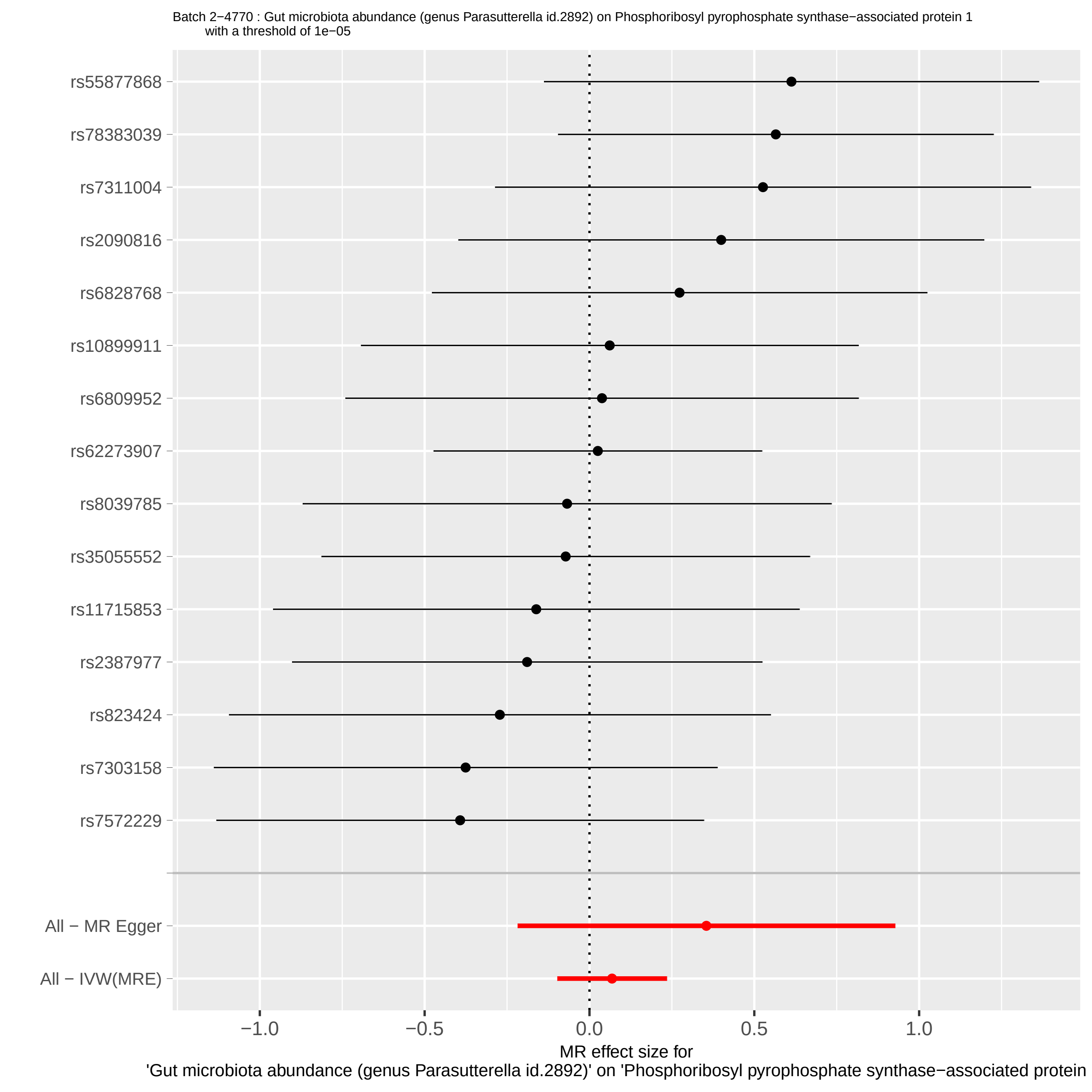
Batch 2−4770 : Gut microbiota abundance (genus Parasutterella id.2892) on Phosphoribosyl pyrophosphate synthase−associated protein 1
with a threshold of 1e−05
rs55877868
rs78383039
rs7311004
rs2090816
rs6828768
rs10899911
rs6809952
rs62273907
rs8039785
rs35055552
rs11715853
rs2387977
rs823424
rs7303158
rs7572229
All − MR Egger
All − IVW(MRE)
−1.0
−0.5
0.0
0.5
1.0
MR effect size for
'Gut microbiota abundance (genus Parasutterella id.2892)' on 'Phosphoribosyl pyrophosphate synthase−associated protein

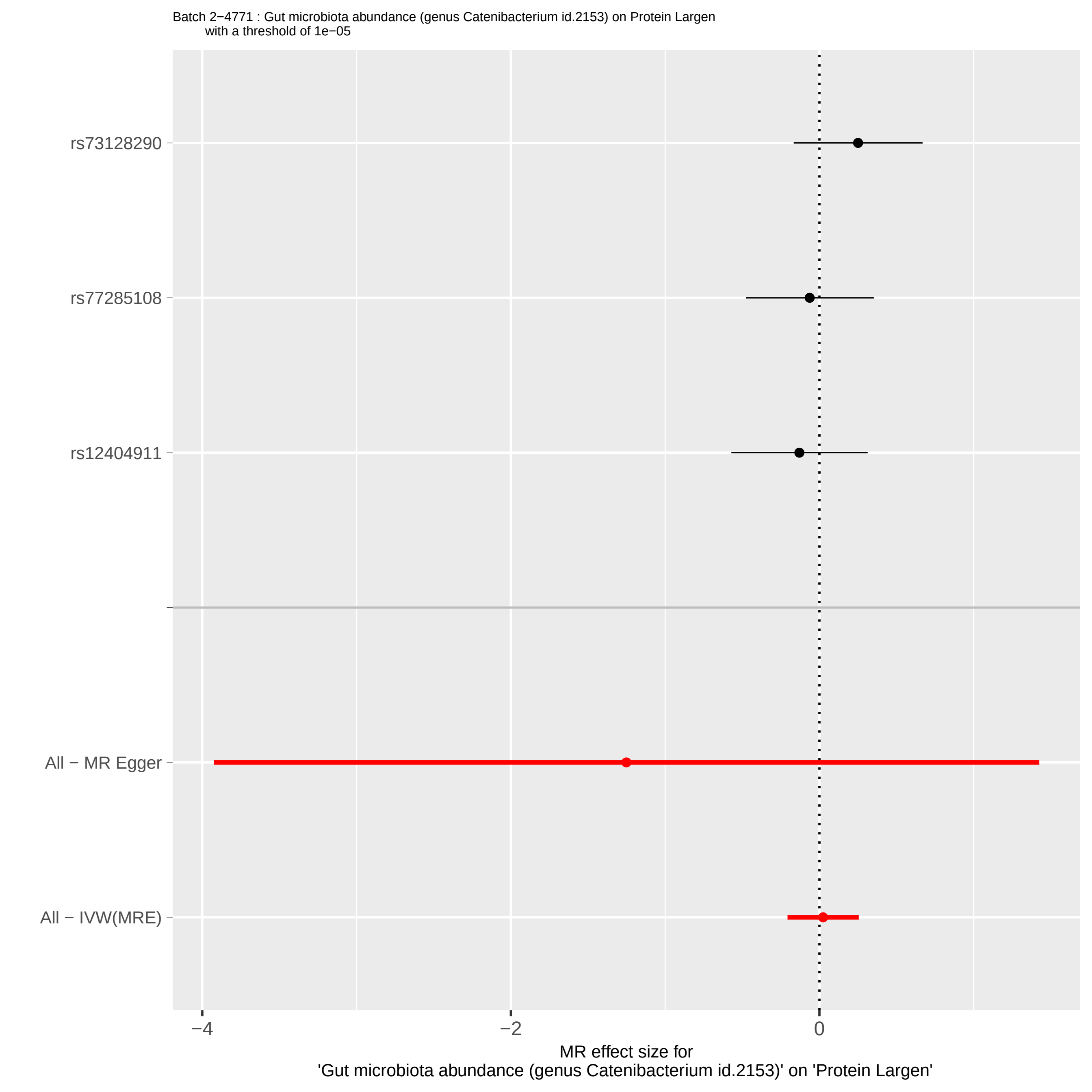
Batch 2−4771 : Gut microbiota abundance (genus Catenibacterium id.2153) on Protein Largen
with a threshold of 1e−05
rs73128290
rs77285108
rs12404911
All − MR Egger
All − IVW(MRE)
−4
−2
0
MR effect size for
'Gut microbiota abundance (genus Catenibacterium id.2153)' on 'Protein Largen'

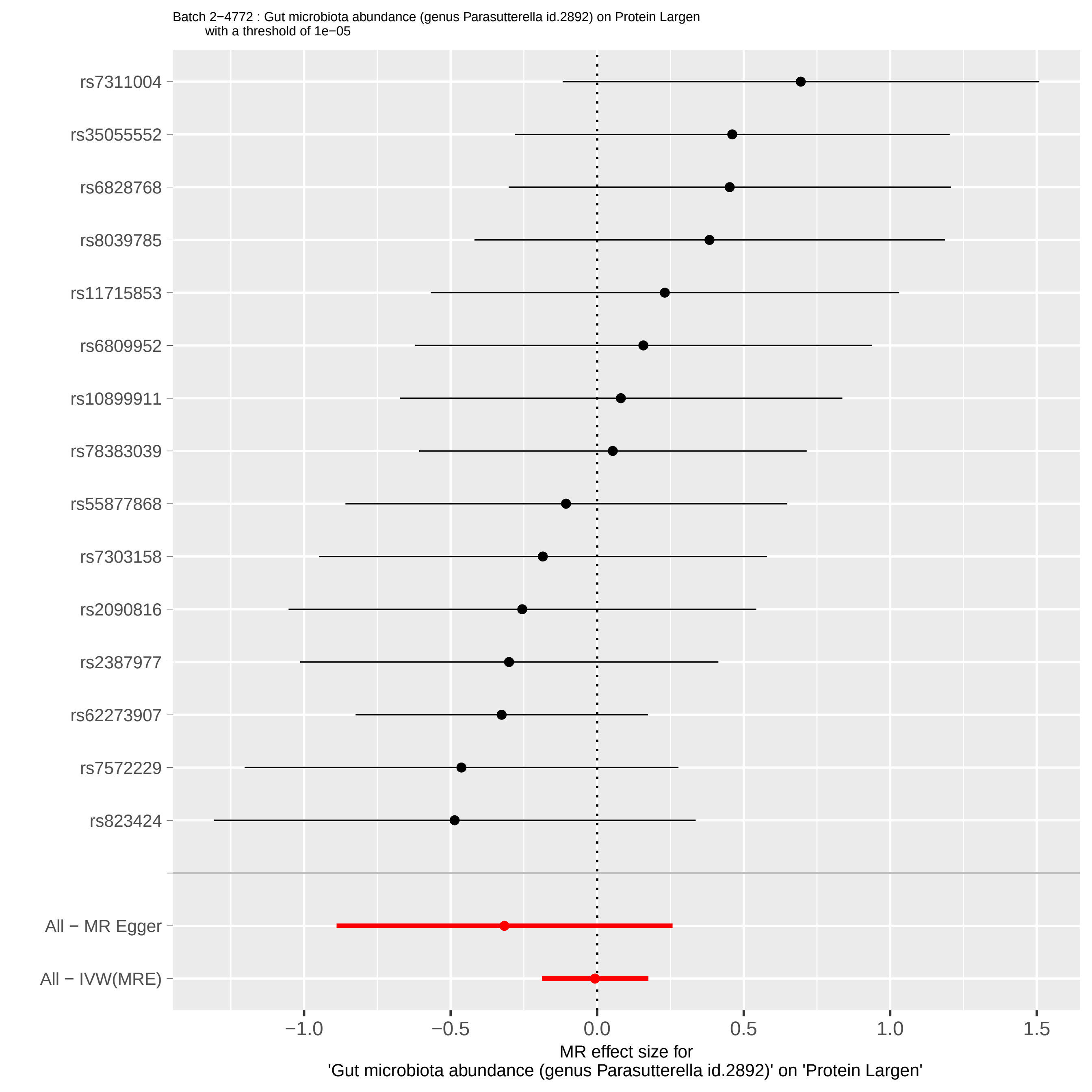

Batch 2−4772 : Gut microbiota abundance (genus Parasutterella id.2892) on Protein Largen
with a threshold of 1e−05
rs7311004
rs35055552
rs6828768
rs8039785
rs11715853
rs6809952
rs10899911
rs78383039
rs55877868
rs7303158
rs2090816
rs2387977
rs62273907
rs7572229
rs823424
All − MR Egger
All − IVW(MRE)
−1.0
−0.5
0.0
0.5
1.0
1.5
MR effect size for
'Gut microbiota abundance (genus Parasutterella id.2892)' on 'Protein Largen'

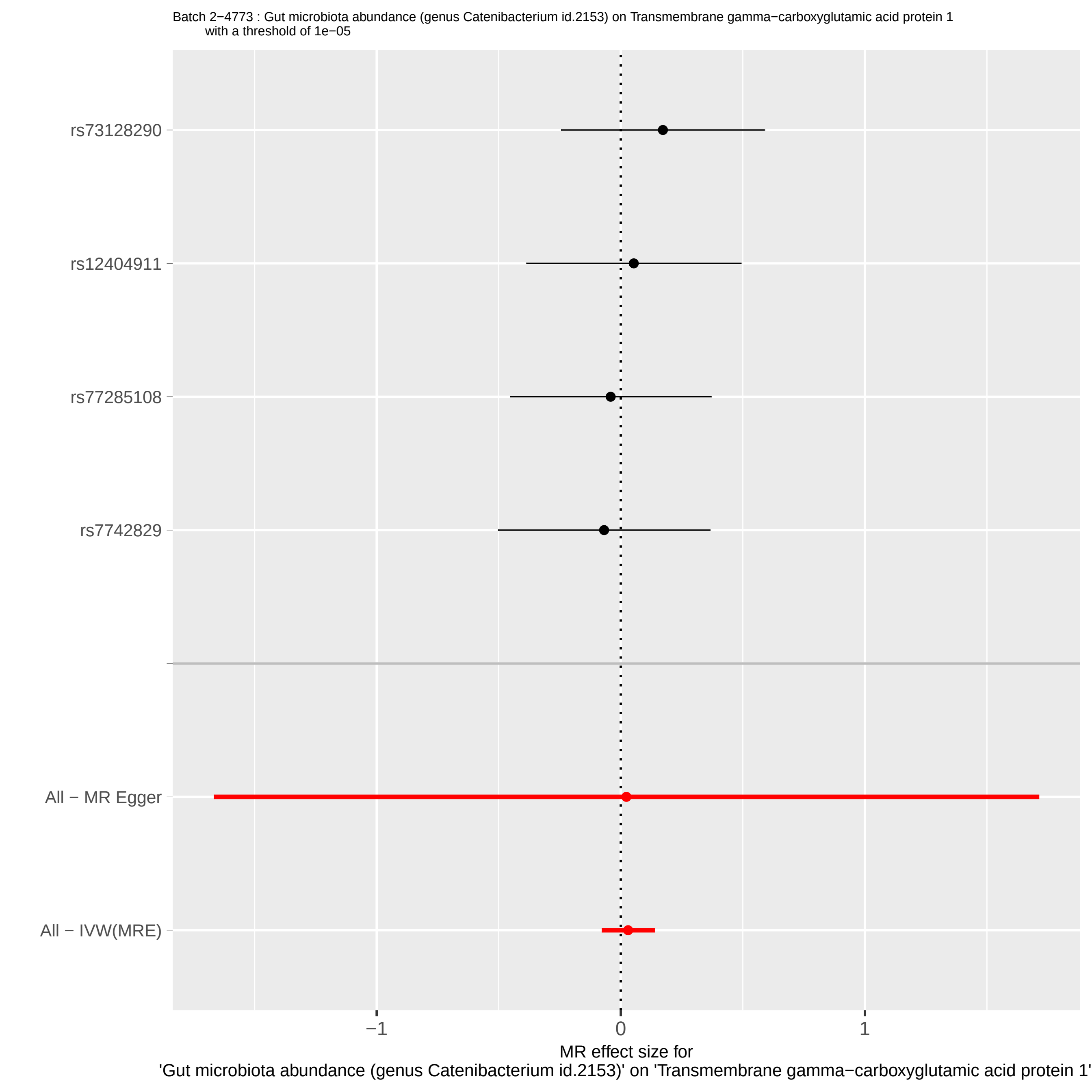

Batch 2−4773 : Gut microbiota abundance (genus Catenibacterium id.2153) on Transmembrane gamma−carboxyglutamic acid protein 1
with a threshold of 1e−05
rs73128290
rs12404911
rs77285108
rs7742829
All − MR Egger
All − IVW(MRE)
−1
0
1
MR effect size for
'Gut microbiota abundance (genus Catenibacterium id.2153)' on 'Transmembrane gamma−carboxyglutamic acid protein 1'

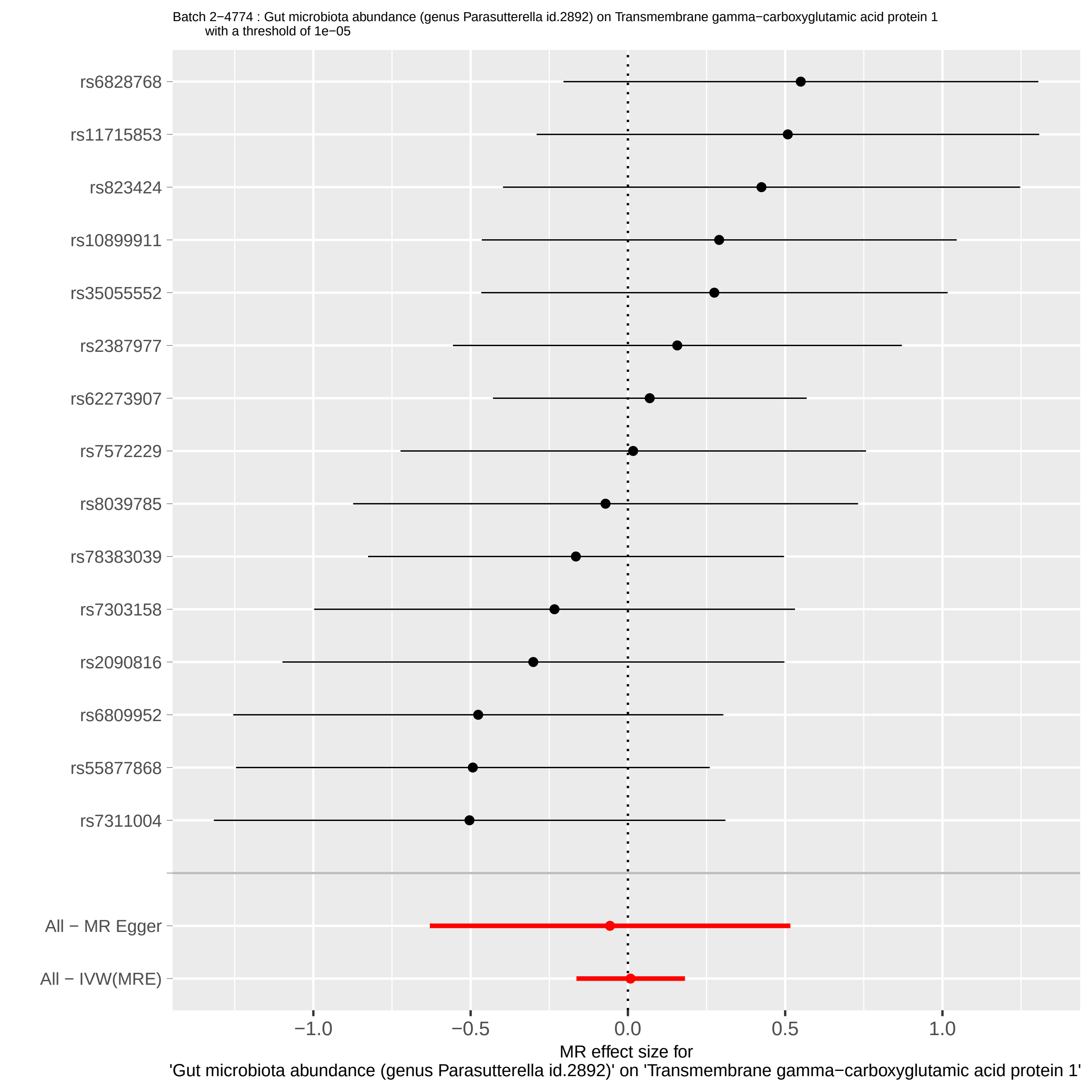

Batch 2−4774 : Gut microbiota abundance (genus Parasutterella id.2892) on Transmembrane gamma−carboxyglutamic acid protein 1
with a threshold of 1e−05
rs6828768
rs11715853
rs823424
rs10899911
rs35055552
rs2387977
rs62273907
rs7572229
rs8039785
rs78383039
rs7303158
rs2090816
rs6809952
rs55877868
rs7311004
All − MR Egger
All − IVW(MRE)
−1.0
−0.5
0.0
0.5
1.0
MR effect size for
'Gut microbiota abundance (genus Parasutterella id.2892)' on 'Transmembrane gamma−carboxyglutamic acid protein 1'

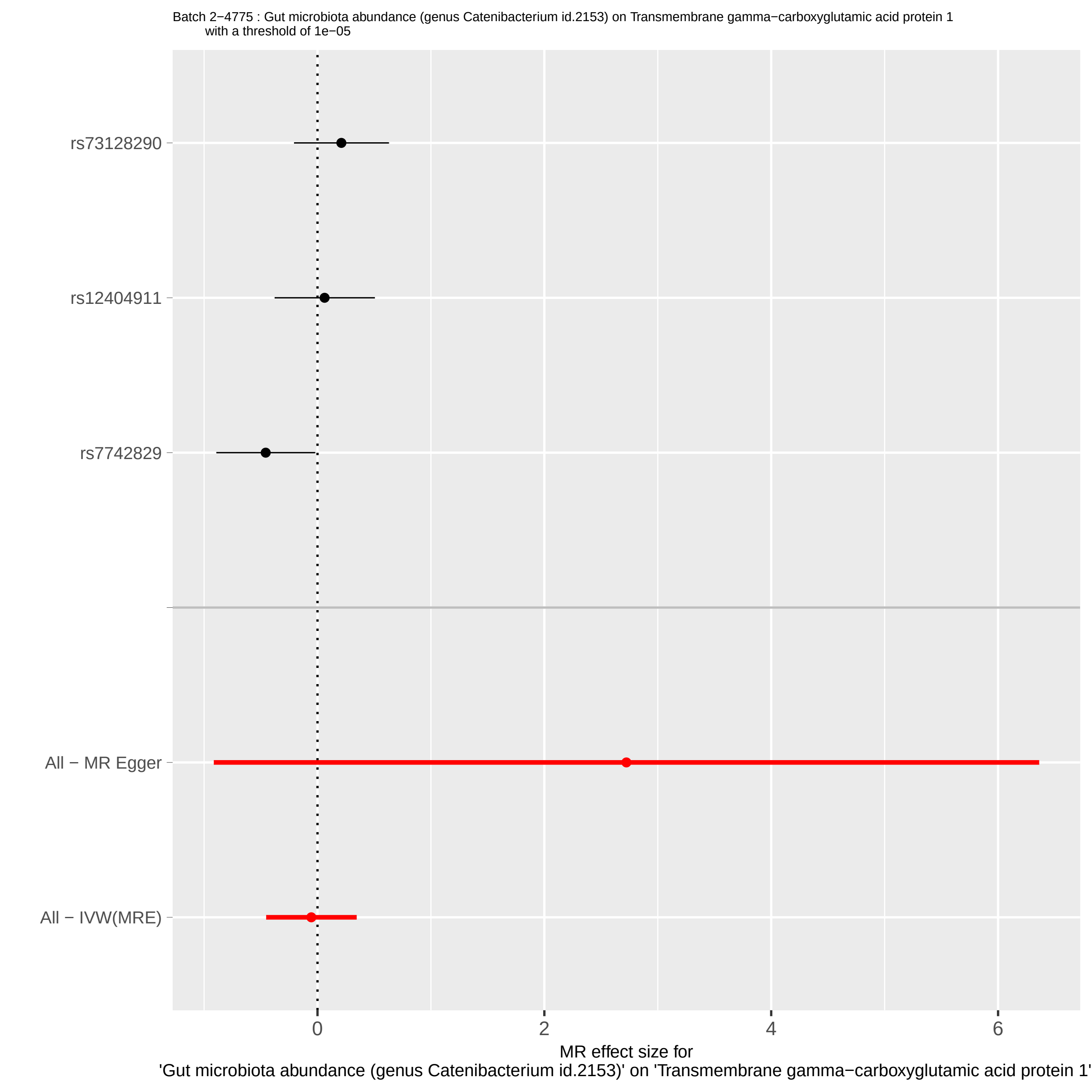
Batch 2−4775 : Gut microbiota abundance (genus Catenibacterium id.2153) on Transmembrane gamma−carboxyglutamic acid protein 1
with a threshold of 1e−05
rs73128290
rs12404911
rs7742829
All − MR Egger
All − IVW(MRE)
0
2
4
6
MR effect size for
'Gut microbiota abundance (genus Catenibacterium id.2153)' on 'Transmembrane gamma−carboxyglutamic acid protein 1'

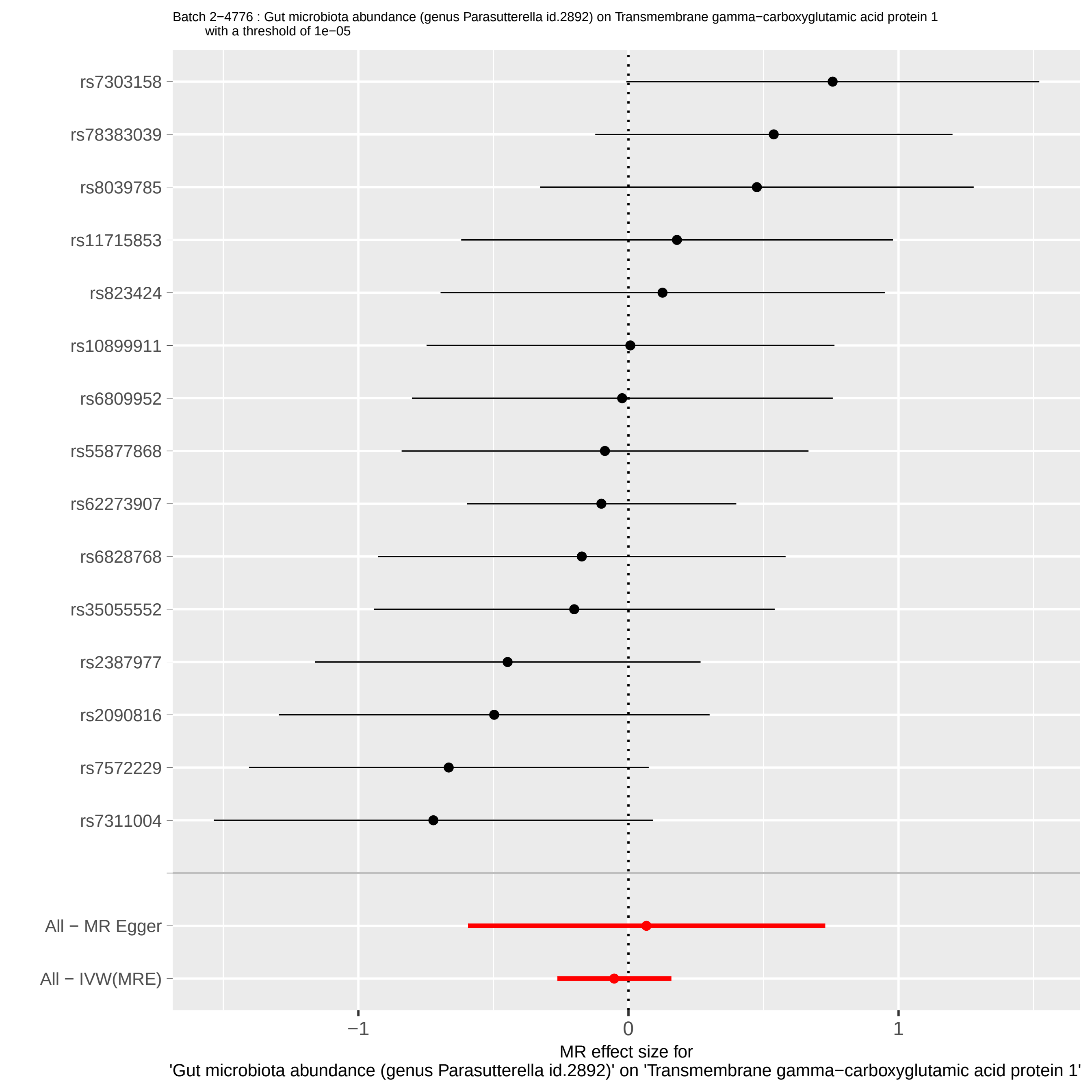

Batch 2−4776 : Gut microbiota abundance (genus Parasutterella id.2892) on Transmembrane gamma−carboxyglutamic acid protein 1
with a threshold of 1e−05
rs7303158
rs78383039
rs8039785
rs11715853
rs823424
rs10899911
rs6809952
rs55877868
rs62273907
rs6828768
rs35055552
rs2387977
rs2090816
rs7572229
rs7311004
All − MR Egger
All − IVW(MRE)
−1
0
1
MR effect size for
'Gut microbiota abundance (genus Parasutterella id.2892)' on 'Transmembrane gamma−carboxyglutamic acid protein 1'

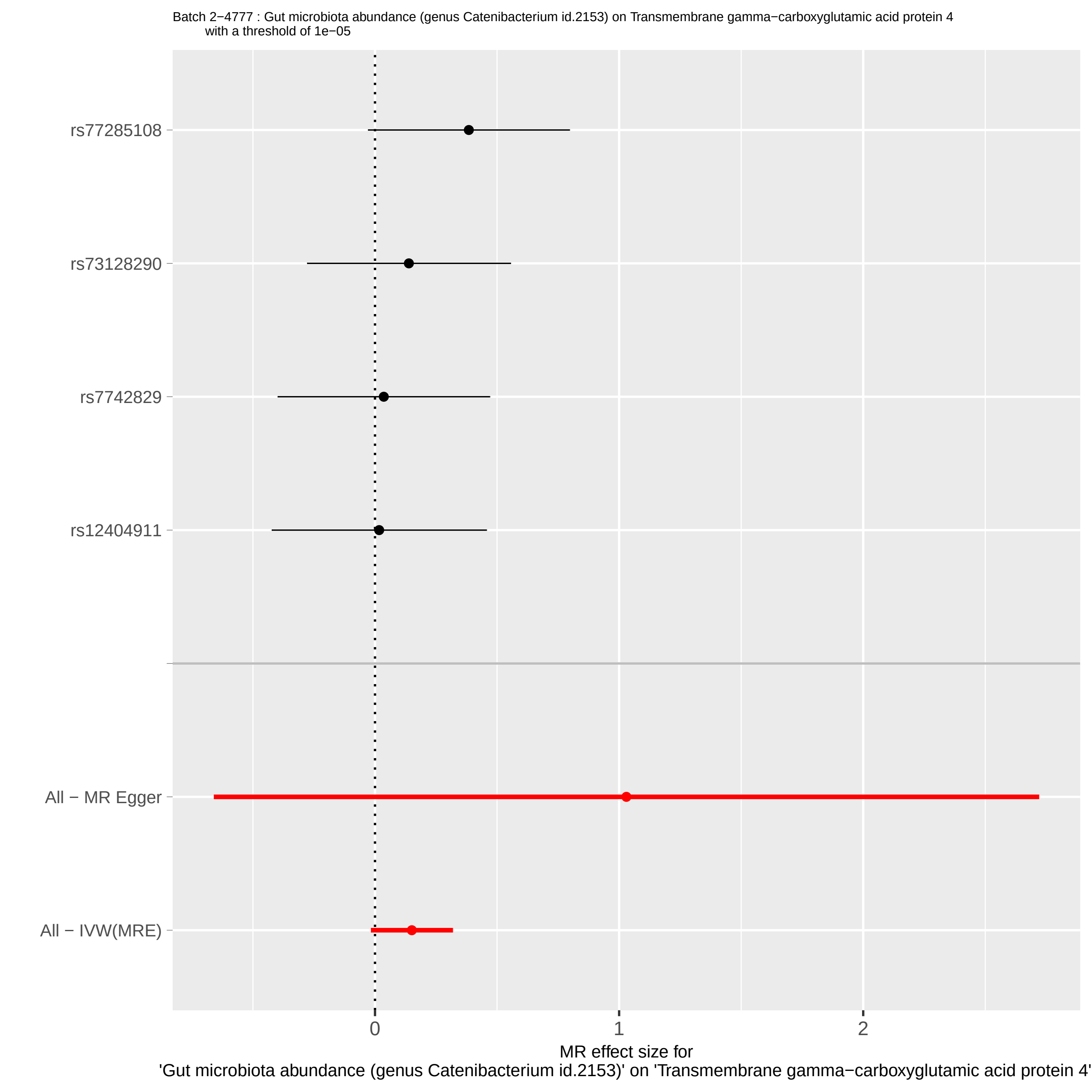
Batch 2−4777 : Gut microbiota abundance (genus Catenibacterium id.2153) on Transmembrane gamma−carboxyglutamic acid protein 4
with a threshold of 1e−05
rs77285108
rs73128290
rs7742829
rs12404911
All − MR Egger
All − IVW(MRE)
0
1
2
MR effect size for
'Gut microbiota abundance (genus Catenibacterium id.2153)' on 'Transmembrane gamma−carboxyglutamic acid protein 4'

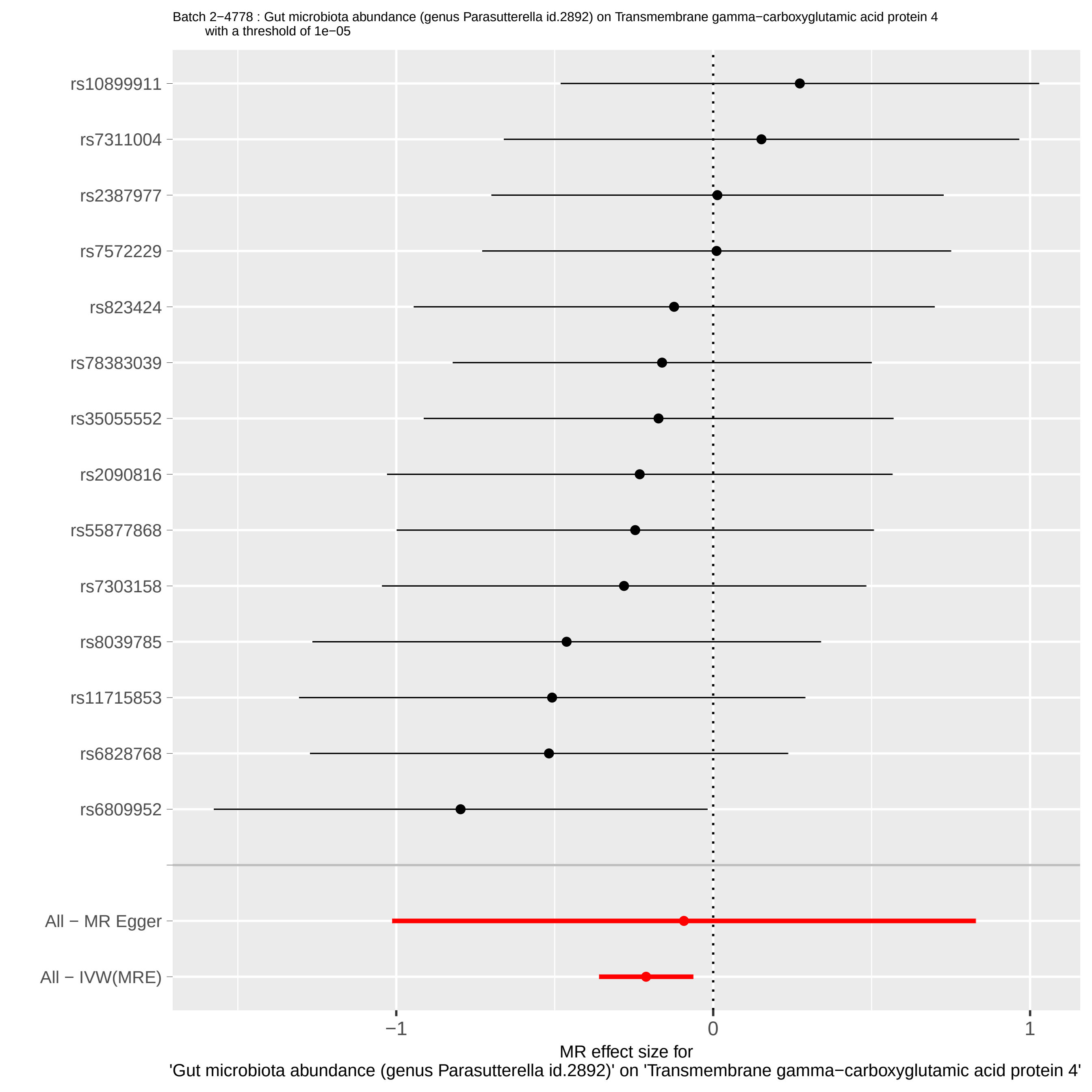

Batch 2−4778 : Gut microbiota abundance (genus Parasutterella id.2892) on Transmembrane gamma−carboxyglutamic acid protein 4
with a threshold of 1e−05
rs10899911
rs7311004
rs2387977
rs7572229
rs823424
rs78383039
rs35055552
rs2090816
rs55877868
rs7303158
rs8039785
rs11715853
rs6828768
rs6809952
All − MR Egger
All − IVW(MRE)
−1
0
1
MR effect size for
'Gut microbiota abundance (genus Parasutterella id.2892)' on 'Transmembrane gamma−carboxyglutamic acid protein 4'

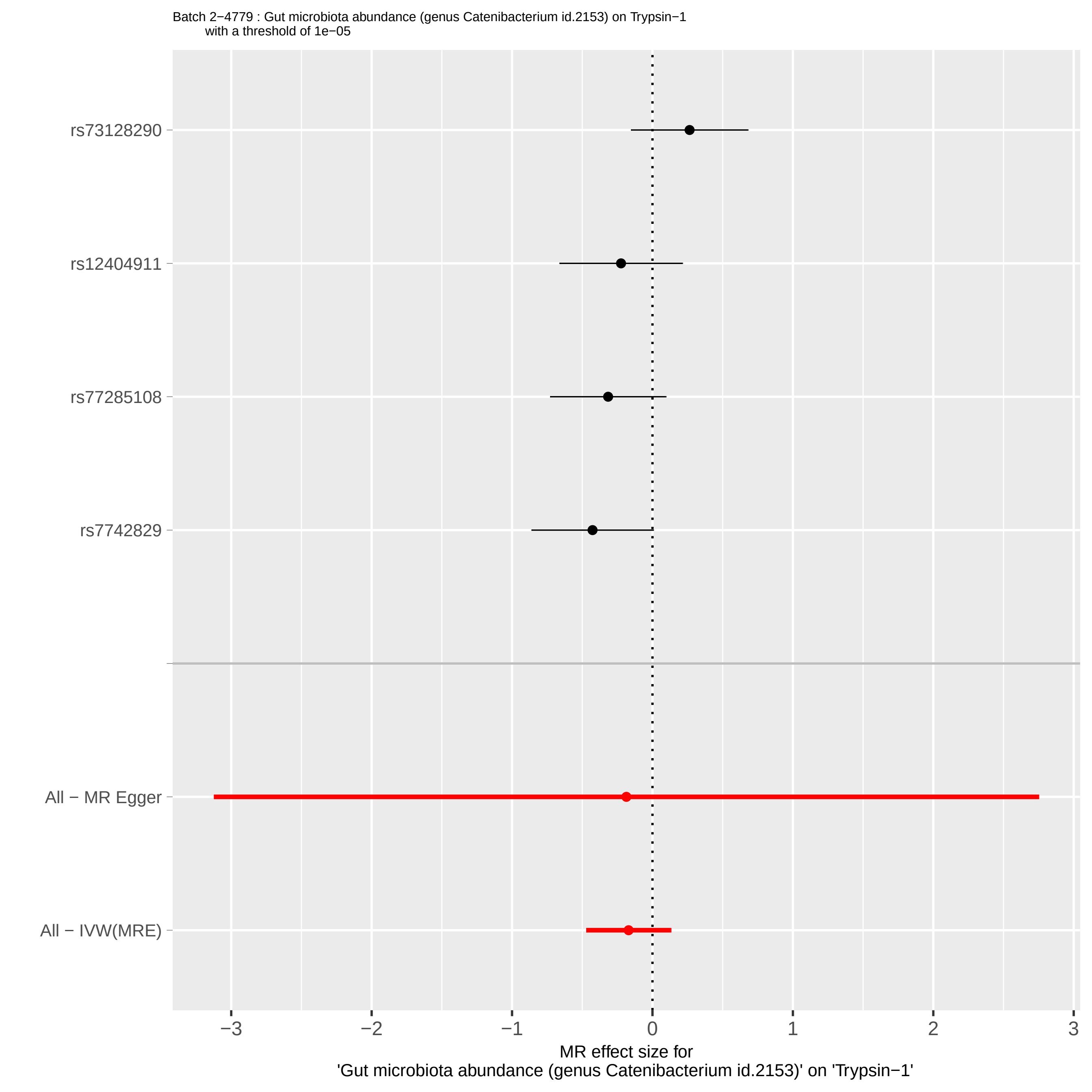
Batch 2−4779 : Gut microbiota abundance (genus Catenibacterium id.2153) on Trypsin−1
with a threshold of 1e−05
rs73128290
rs12404911
rs77285108
rs7742829
All − MR Egger
All − IVW(MRE)
−3
−2
−1
0
1
2
3
MR effect size for
'Gut microbiota abundance (genus Catenibacterium id.2153)' on 'Trypsin−1'

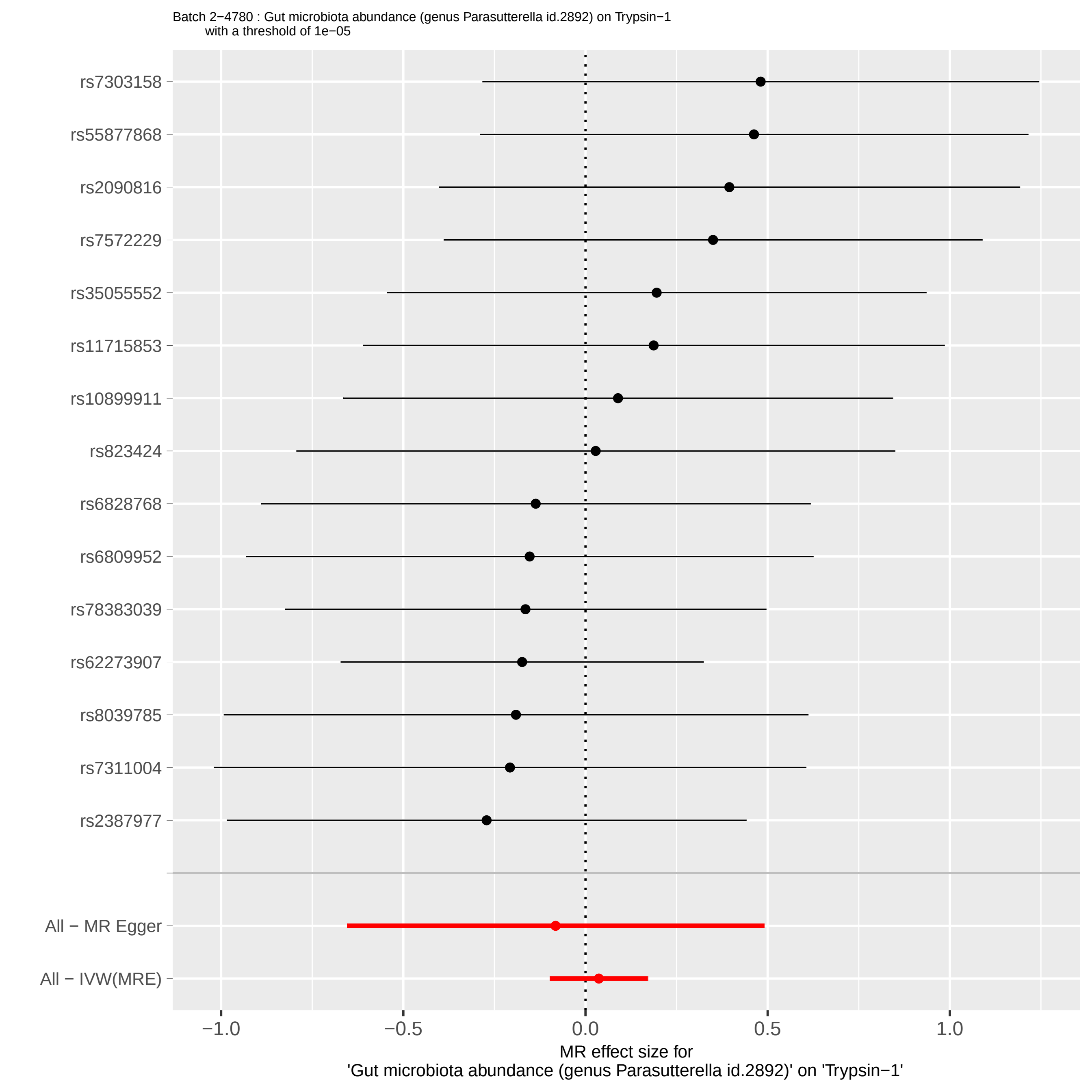

Batch 2−4780 : Gut microbiota abundance (genus Parasutterella id.2892) on Trypsin−1
with a threshold of 1e−05
rs7303158
rs55877868
rs2090816
rs7572229
rs35055552
rs11715853
rs10899911
rs823424
rs6828768
rs6809952
rs78383039
rs62273907
rs8039785
rs7311004
rs2387977
All − MR Egger
All − IVW(MRE)
−1.0
−0.5
0.0
0.5
1.0
MR effect size for
'Gut microbiota abundance (genus Parasutterella id.2892)' on 'Trypsin−1'

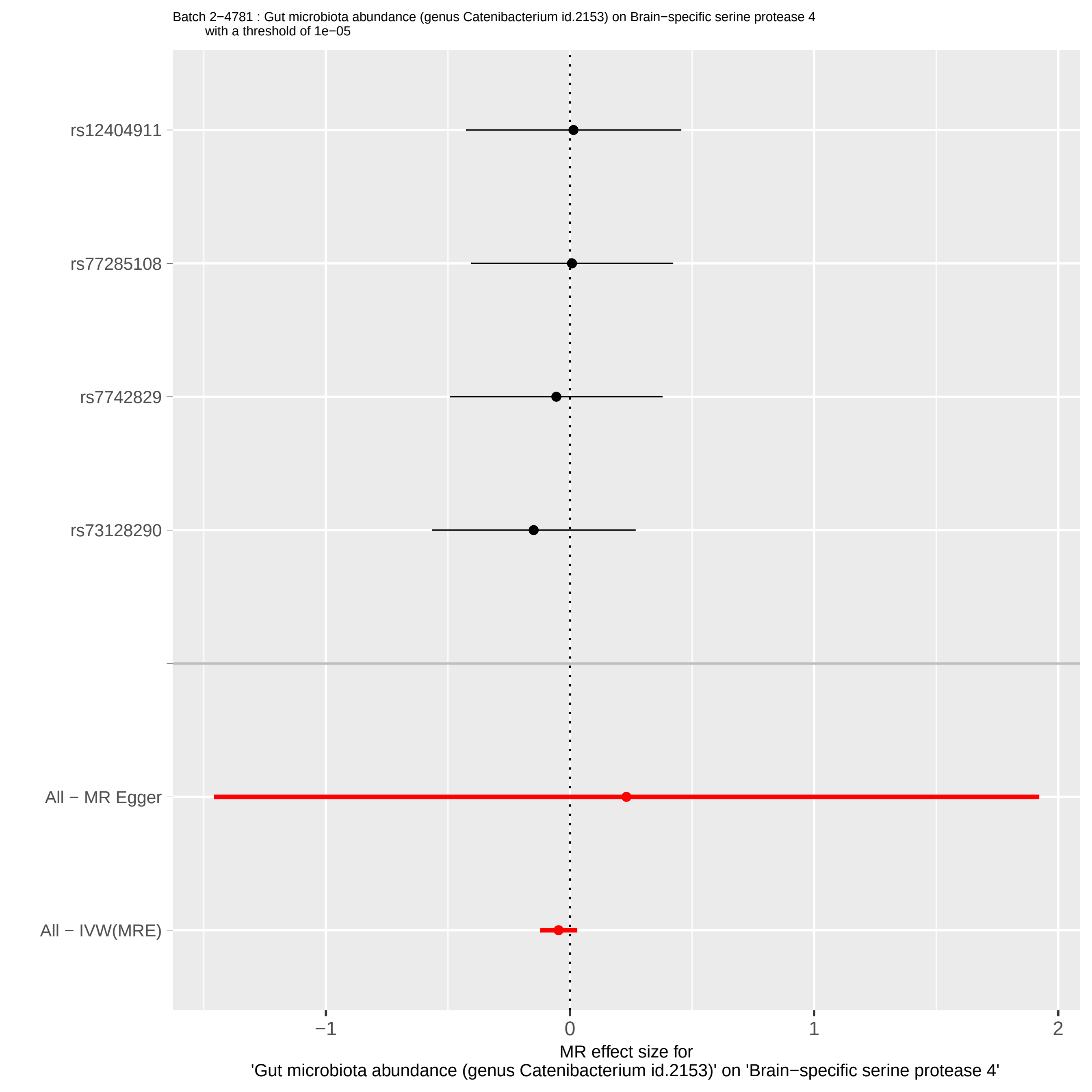

Batch 2−4781 : Gut microbiota abundance (genus Catenibacterium id.2153) on Brain−specific serine protease 4
with a threshold of 1e−05
rs12404911
rs77285108
rs7742829
rs73128290
All − MR Egger
All − IVW(MRE)
−1
0
1
2
MR effect size for
'Gut microbiota abundance (genus Catenibacterium id.2153)' on 'Brain−specific serine protease 4'

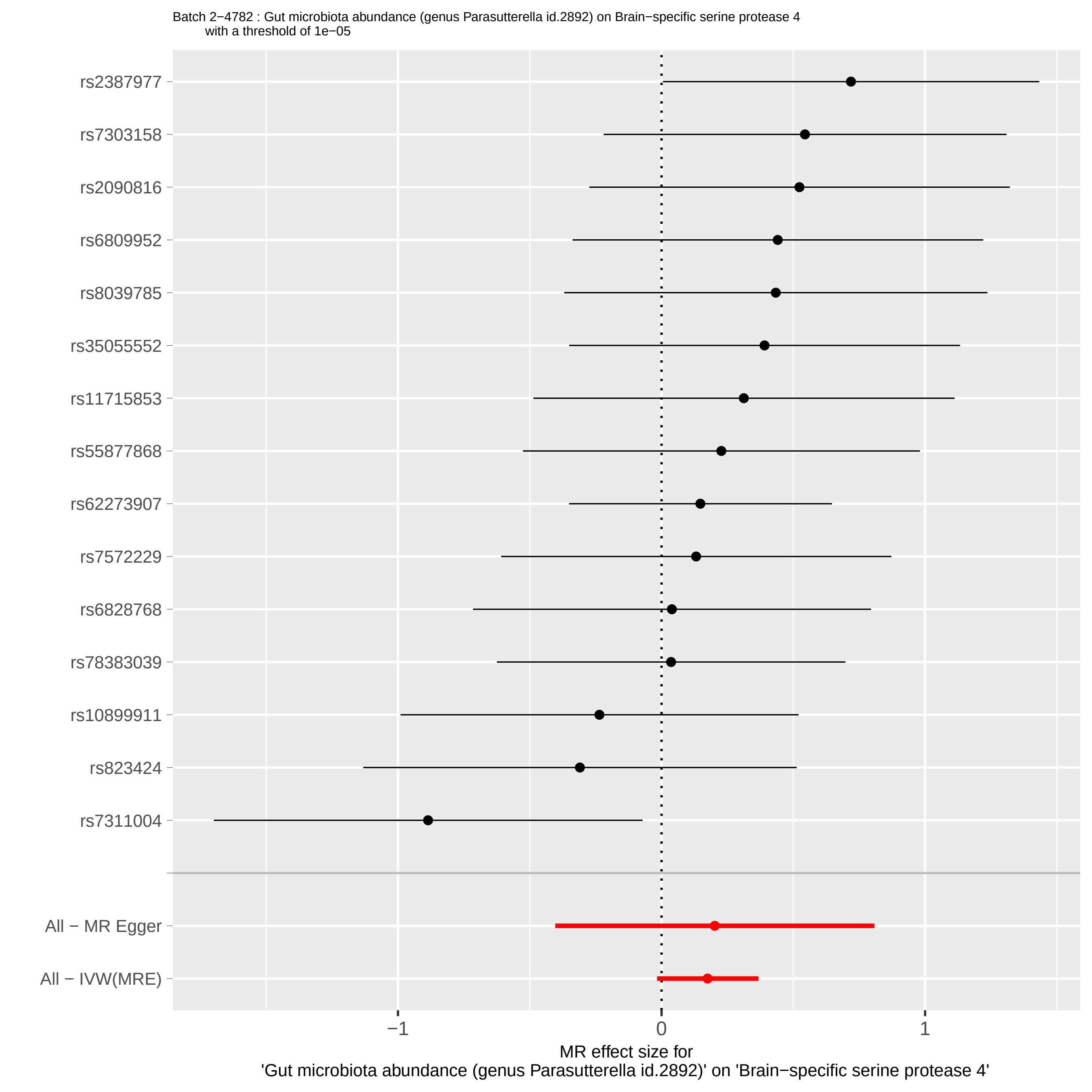
Batch 2−4782 : Gut microbiota abundance (genus Parasutterella id.2892) on Brain−specific serine protease 4
with a threshold of 1e−05
rs2387977
rs7303158
rs2090816
rs6809952
rs8039785
rs35055552
rs11715853
rs55877868
rs62273907
rs7572229
rs6828768
rs78383039
rs10899911
rs823424
rs7311004
All − MR Egger
All − IVW(MRE)
−1
0
1
MR effect size for
'Gut microbiota abundance (genus Parasutterella id.2892)' on 'Brain−specific serine protease 4'

Batch 2−4783 : Gut microbiota abundance (genus Catenibacterium id.2153) on Trypsin−2
with a threshold of 1e−05

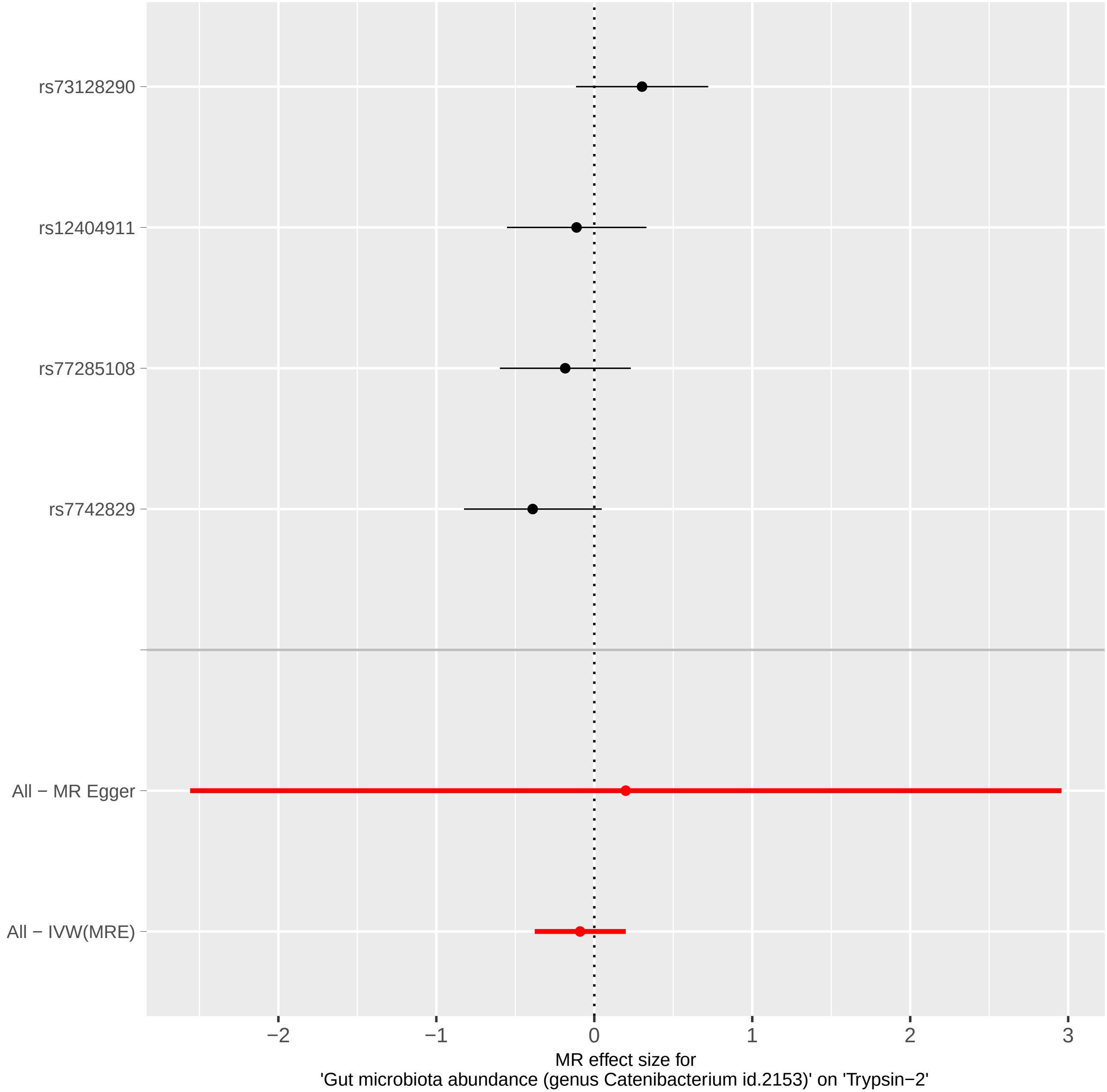

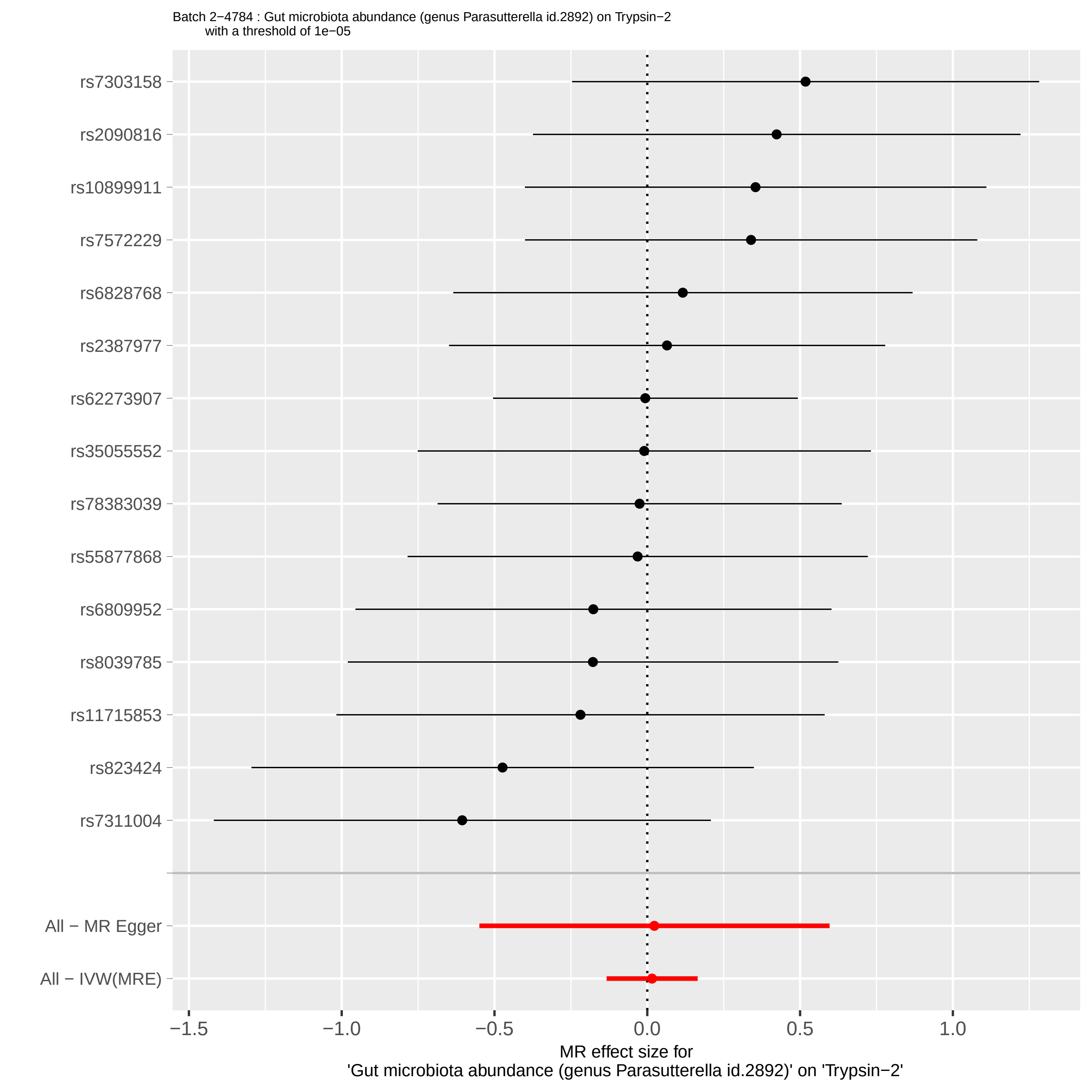
Batch 2−4784 : Gut microbiota abundance (genus Parasutterella id.2892) on Trypsin−2
with a threshold of 1e−05
rs7303158
rs2090816
rs10899911
rs7572229
rs6828768
rs2387977
rs62273907
rs35055552
rs78383039
rs55877868
rs6809952
rs8039785
rs11715853
rs823424
rs7311004
All − MR Egger
All − IVW(MRE)
−1.5
−1.0
−0.5
0.0
0.5
1.0
MR effect size for
'Gut microbiota abundance (genus Parasutterella id.2892)' on 'Trypsin−2'

Batch 2−4785 : Gut microbiota abundance (genus Catenibacterium id.2153) on Trypsin−3
with a threshold of 1e−05

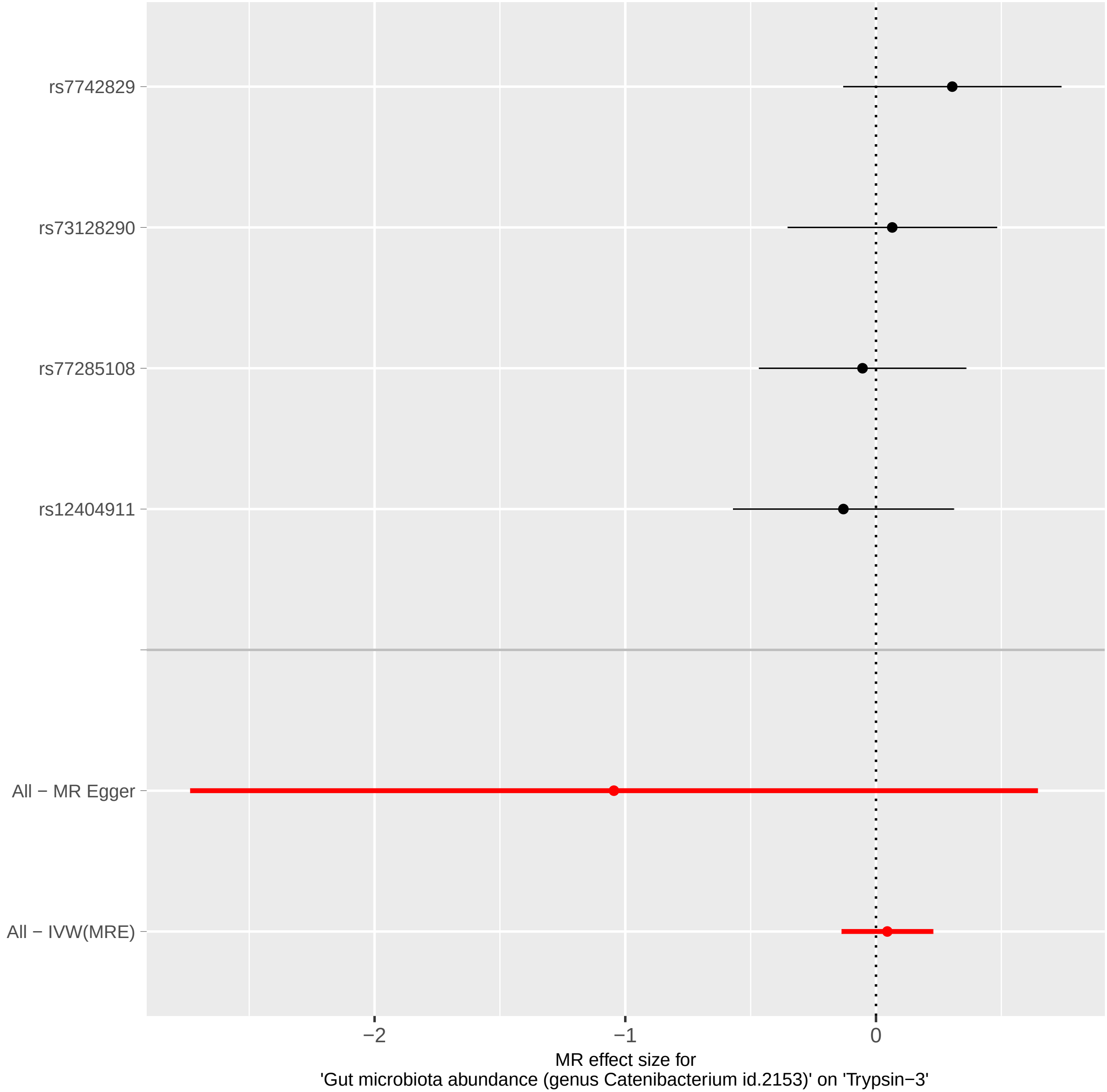

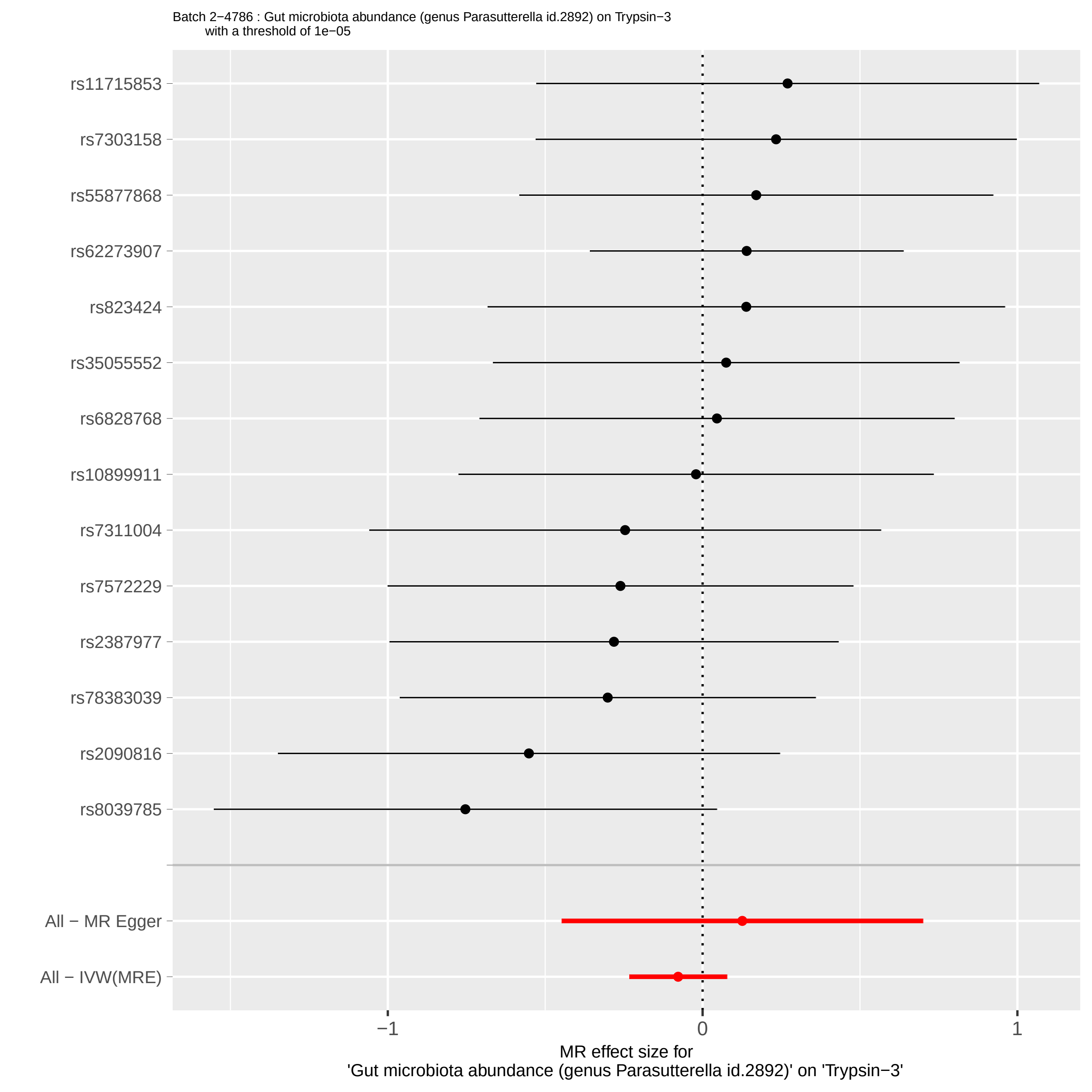

Batch 2−4786 : Gut microbiota abundance (genus Parasutterella id.2892) on Trypsin−3
with a threshold of 1e−05
rs11715853
rs7303158
rs55877868
rs62273907
rs823424
rs35055552
rs6828768
rs10899911
rs7311004
rs7572229
rs2387977
rs78383039
rs2090816
rs8039785
All − MR Egger
All − IVW(MRE)
−1
0
1
MR effect size for
'Gut microbiota abundance (genus Parasutterella id.2892)' on 'Trypsin−3'

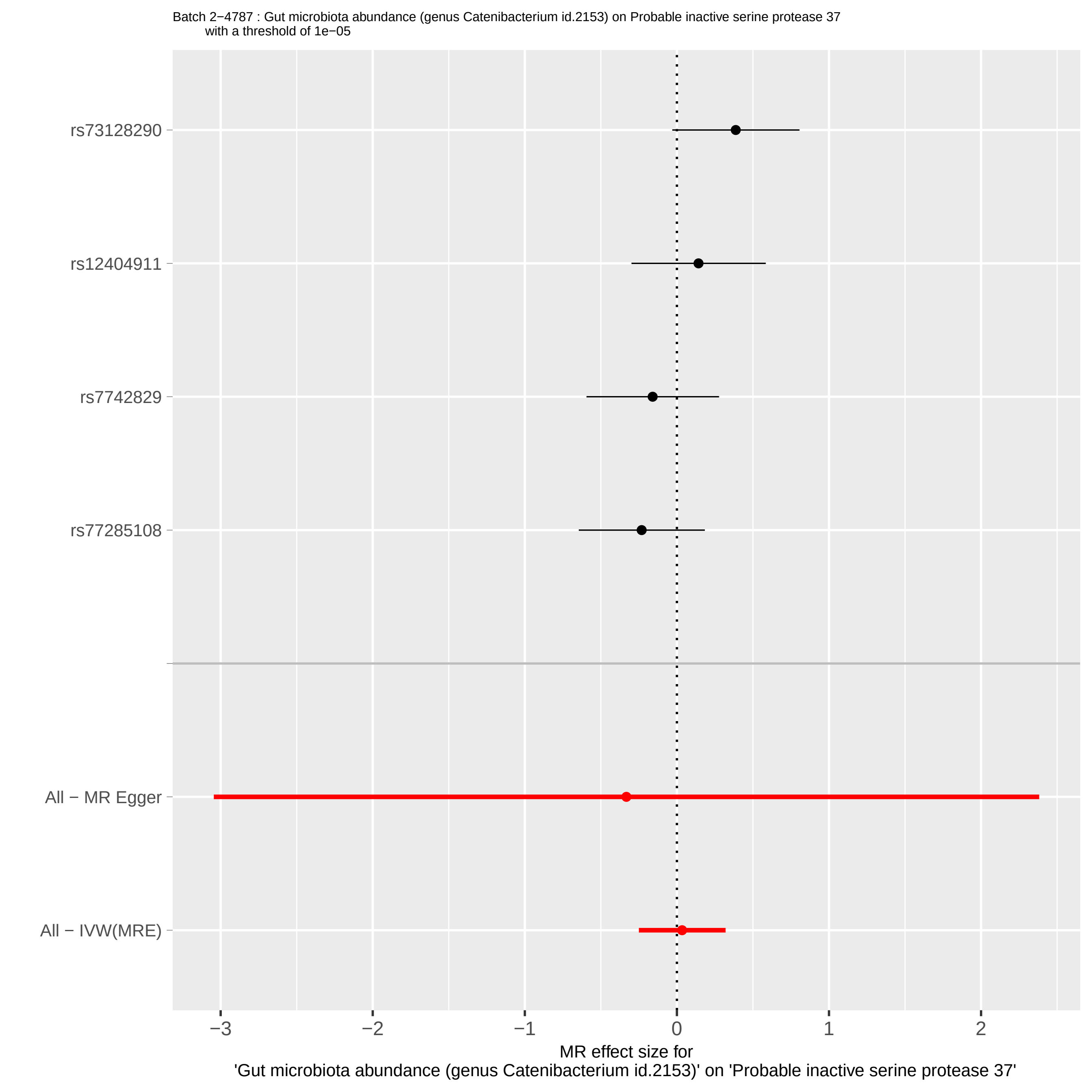

Batch 2−4787 : Gut microbiota abundance (genus Catenibacterium id.2153) on Probable inactive serine protease 37
with a threshold of 1e−05
rs73128290
rs12404911
rs7742829
rs77285108
All − MR Egger
All − IVW(MRE)
−3
−2
−1
0
1
2
MR effect size for
'Gut microbiota abundance (genus Catenibacterium id.2153)' on 'Probable inactive serine protease 37'

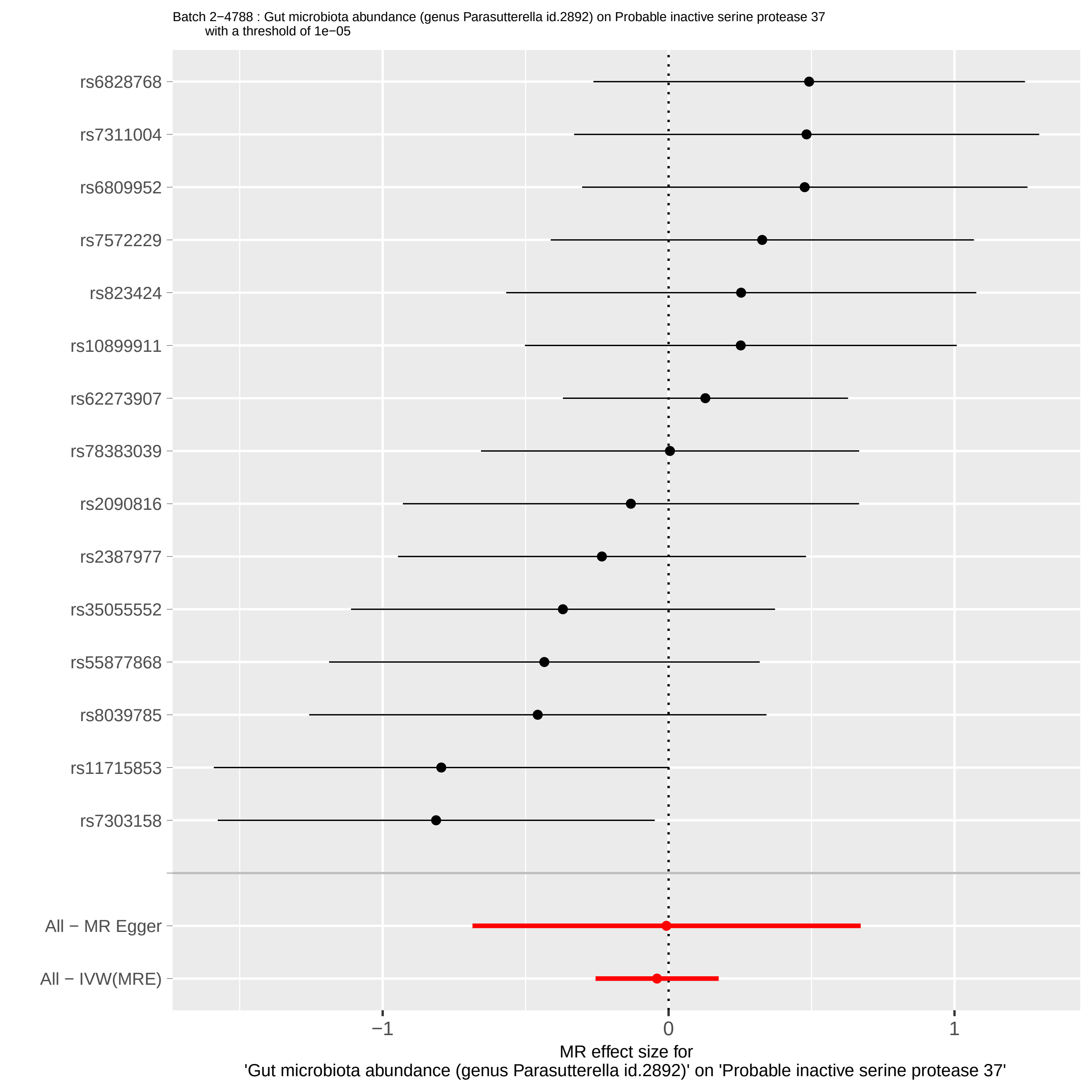

Batch 2−4788 : Gut microbiota abundance (genus Parasutterella id.2892) on Probable inactive serine protease 37
with a threshold of 1e−05
rs6828768
rs7311004
rs6809952
rs7572229
rs823424
rs10899911
rs62273907
rs78383039
rs2090816
rs2387977
rs35055552
rs55877868
rs8039785
rs11715853
rs7303158
All − MR Egger
All − IVW(MRE)
−1
0
1
MR effect size for
'Gut microbiota abundance (genus Parasutterella id.2892)' on 'Probable inactive serine protease 37'

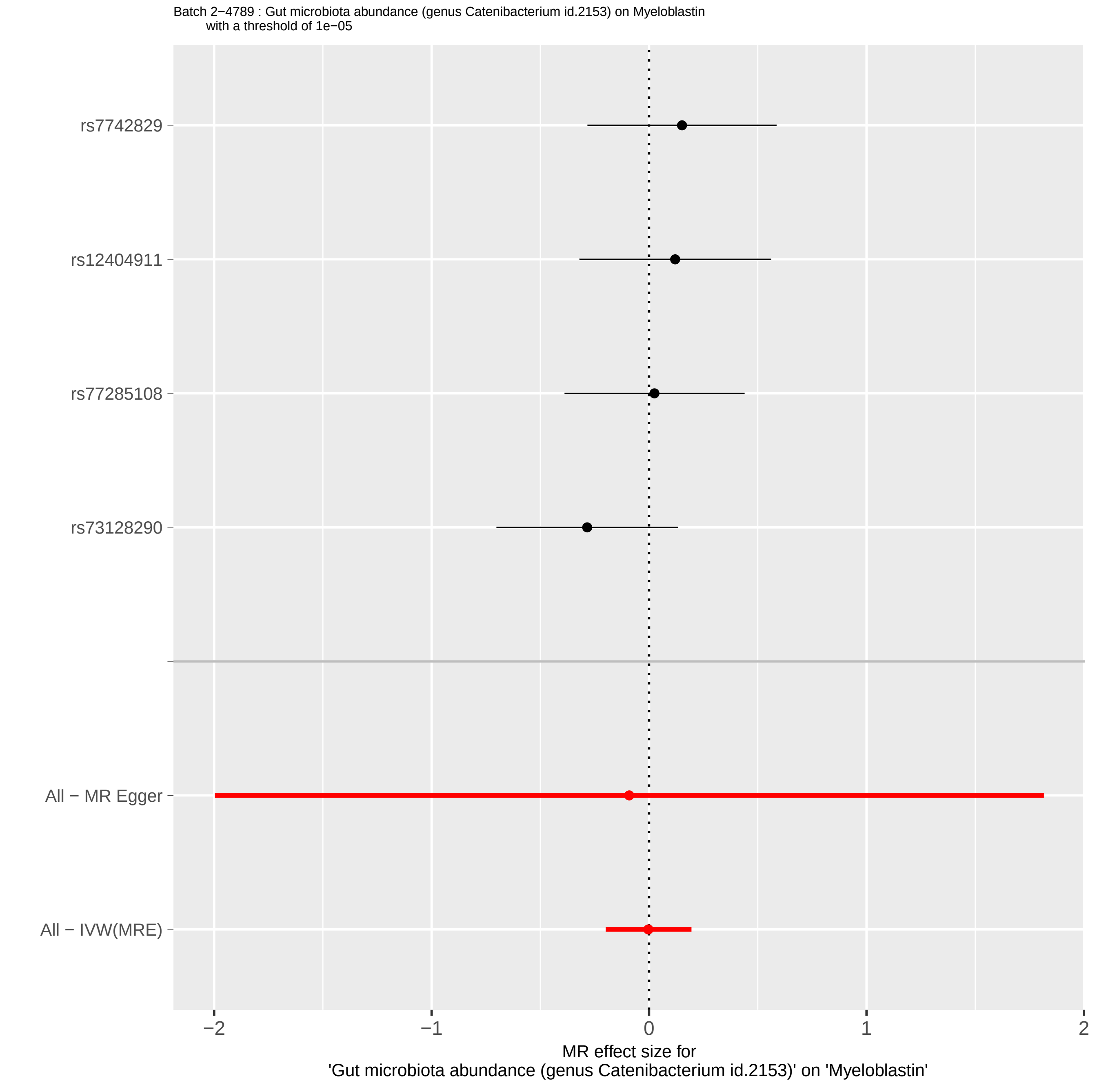

Batch 2−4789 : Gut microbiota abundance (genus Catenibacterium id.2153) on Myeloblastin
with a threshold of 1e−05
rs7742829
rs12404911
rs77285108
rs73128290
All − MR Egger
All − IVW(MRE)
−2
−1
0
1
2
MR effect size for
'Gut microbiota abundance (genus Catenibacterium id.2153)' on 'Myeloblastin'

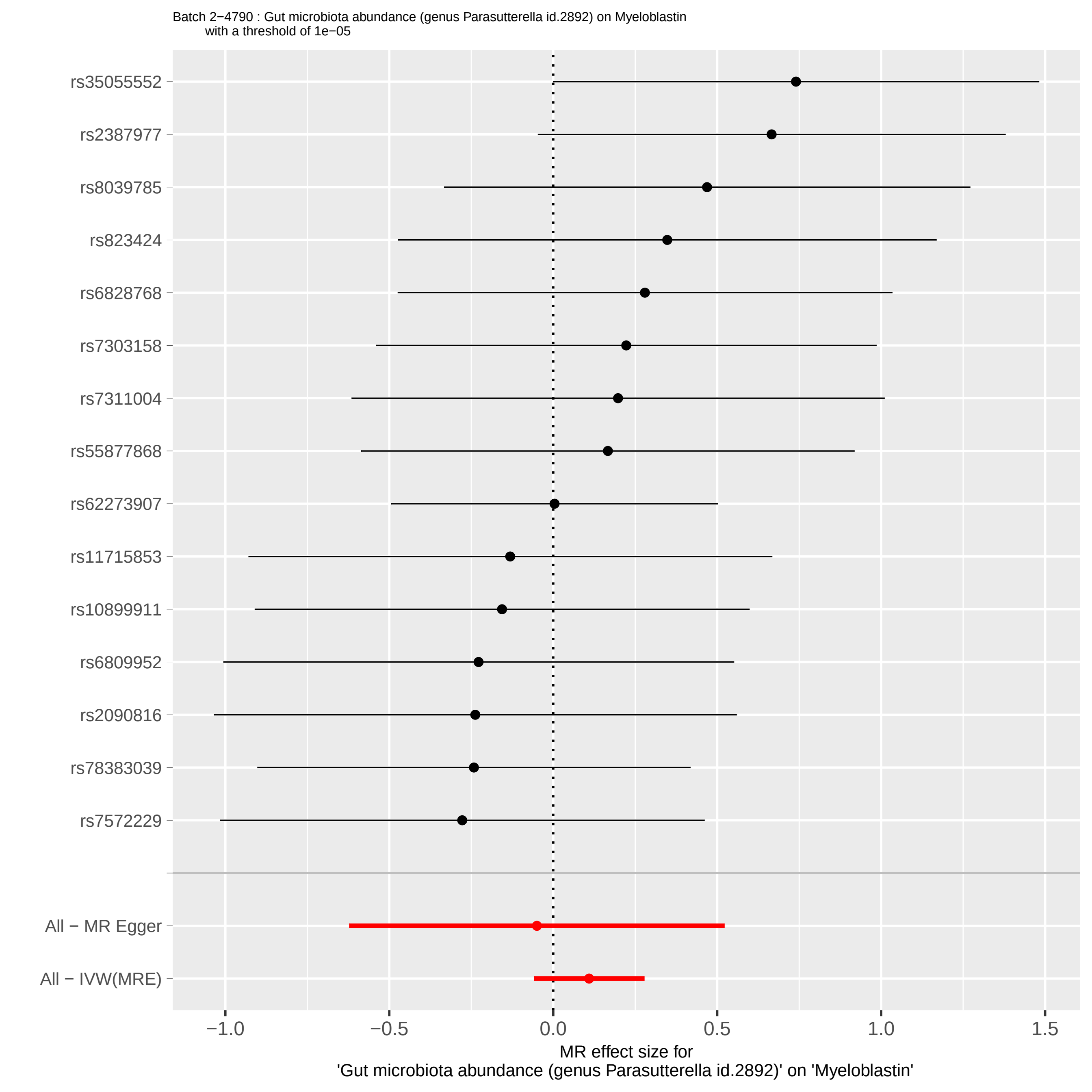
Batch 2−4790 : Gut microbiota abundance (genus Parasutterella id.2892) on Myeloblastin
with a threshold of 1e−05
rs35055552
rs2387977
rs8039785
rs823424
rs6828768
rs7303158
rs7311004
rs55877868
rs62273907
rs11715853
rs10899911
rs6809952
rs2090816
rs78383039
rs7572229
All − MR Egger
All − IVW(MRE)
−1.0
−0.5
0.0
0.5
1.0
1.5
MR effect size for
'Gut microbiota abundance (genus Parasutterella id.2892)' on 'Myeloblastin'

Batch 2−4791 : Gut microbiota abundance (genus Catenibacterium id.2153) on Myeloblastin
with a threshold of 1e−05

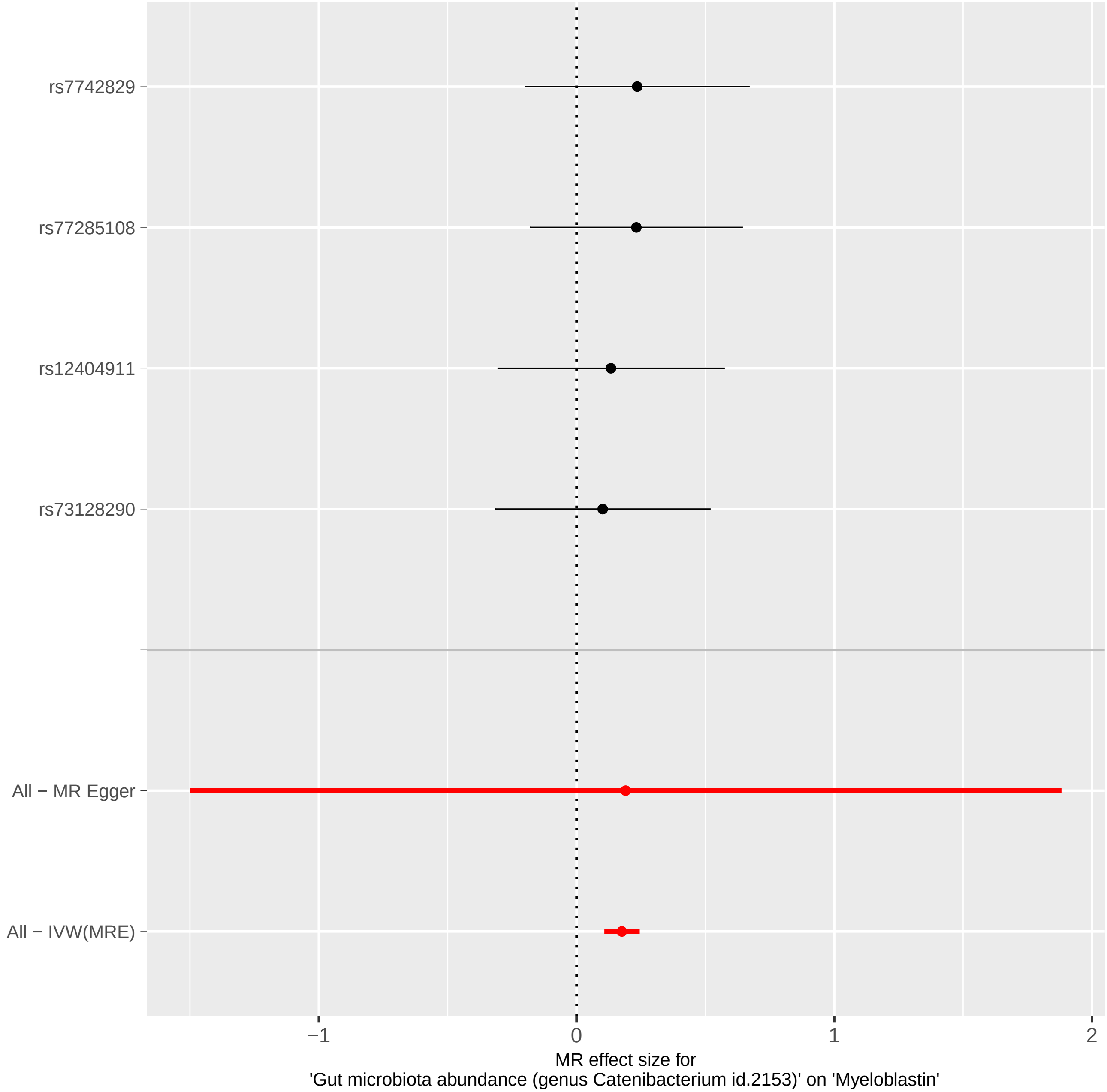

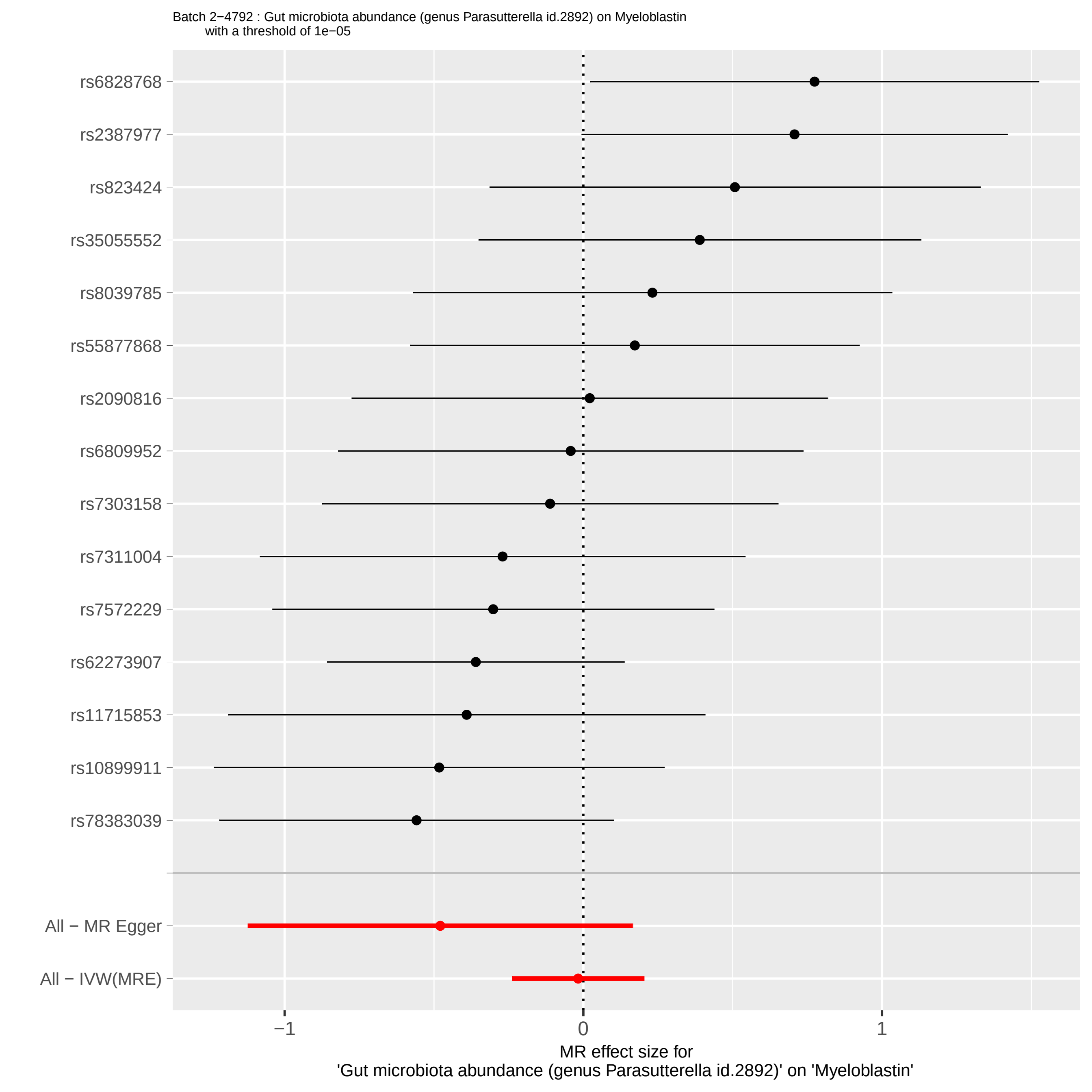
Batch 2−4792 : Gut microbiota abundance (genus Parasutterella id.2892) on Myeloblastin
with a threshold of 1e−05
rs6828768
rs2387977
rs823424
rs35055552
rs8039785
rs55877868
rs2090816
rs6809952
rs7303158
rs7311004
rs7572229
rs62273907
rs11715853
rs10899911
rs78383039
All − MR Egger
All − IVW(MRE)
−1
0
1
MR effect size for
'Gut microbiota abundance (genus Parasutterella id.2892)' on 'Myeloblastin'

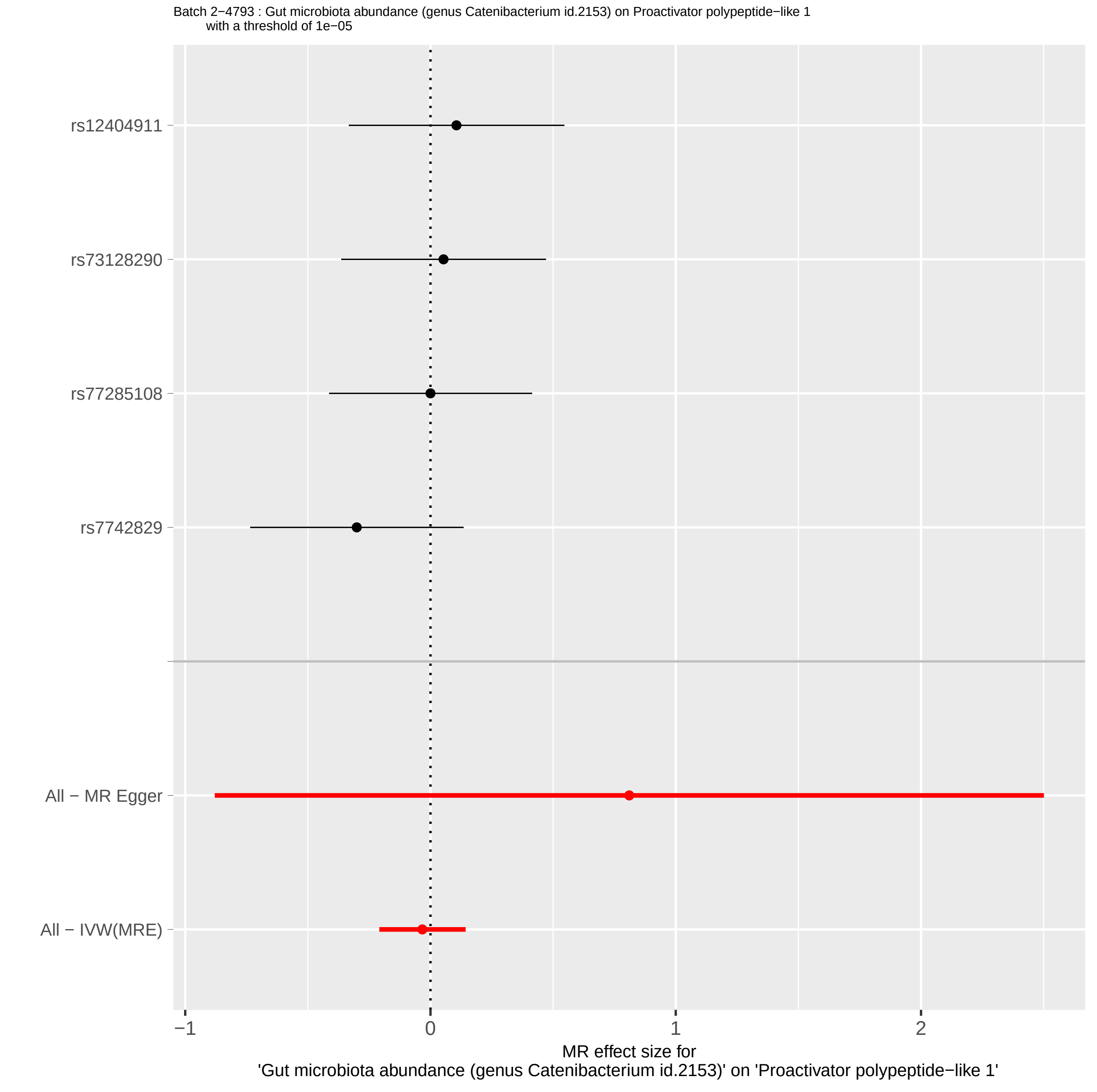
Batch 2−4793 : Gut microbiota abundance (genus Catenibacterium id.2153) on Proactivator polypeptide−like 1
with a threshold of 1e−05
rs12404911
rs73128290
rs77285108
rs7742829
All − MR Egger
All − IVW(MRE)
−1
0
1
2
MR effect size for
'Gut microbiota abundance (genus Catenibacterium id.2153)' on 'Proactivator polypeptide−like 1'

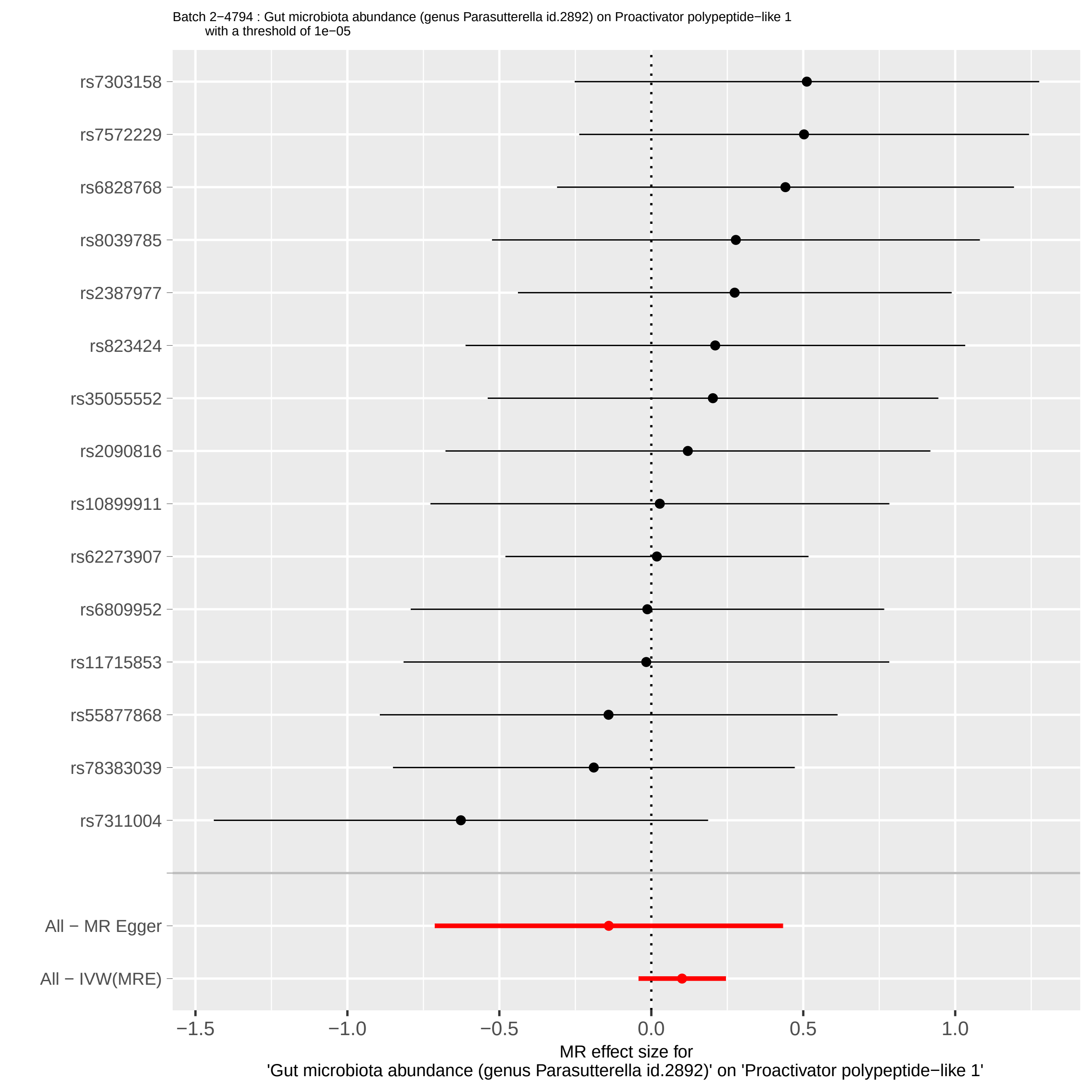
Batch 2−4794 : Gut microbiota abundance (genus Parasutterella id.2892) on Proactivator polypeptide−like 1
with a threshold of 1e−05
rs7303158
rs7572229
rs6828768
rs8039785
rs2387977
rs823424
rs35055552
rs2090816
rs10899911
rs62273907
rs6809952
rs11715853
rs55877868
rs78383039
rs7311004
All − MR Egger
All − IVW(MRE)
−1.5
−1.0
−0.5
0.0
0.5
1.0
MR effect size for
'Gut microbiota abundance (genus Parasutterella id.2892)' on 'Proactivator polypeptide−like 1'

Batch 2−4795 : Gut microbiota abundance (genus Catenibacterium id.2153) on PH and SEC7 domain−containing protein 1
with a threshold of 1e−05

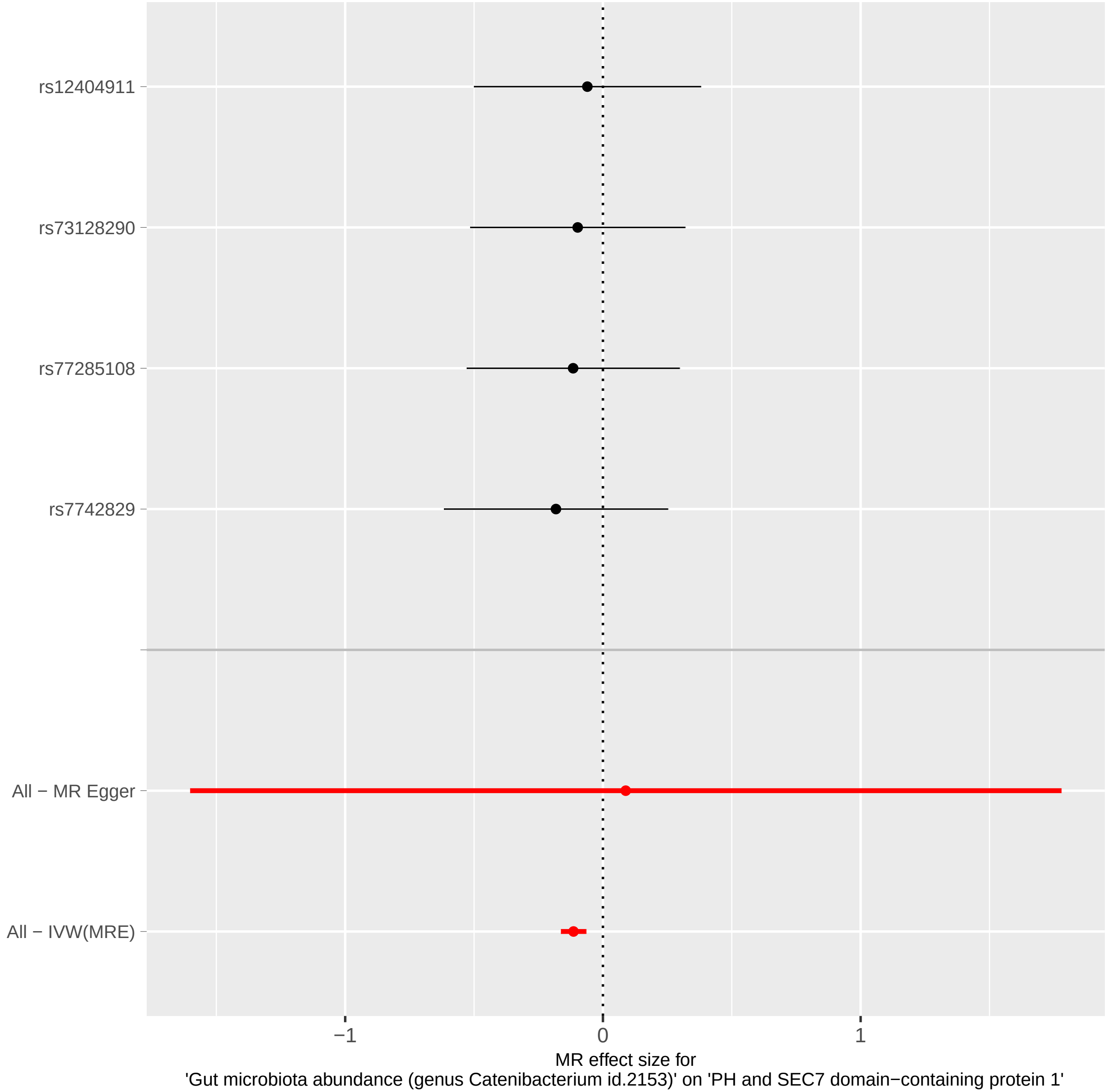

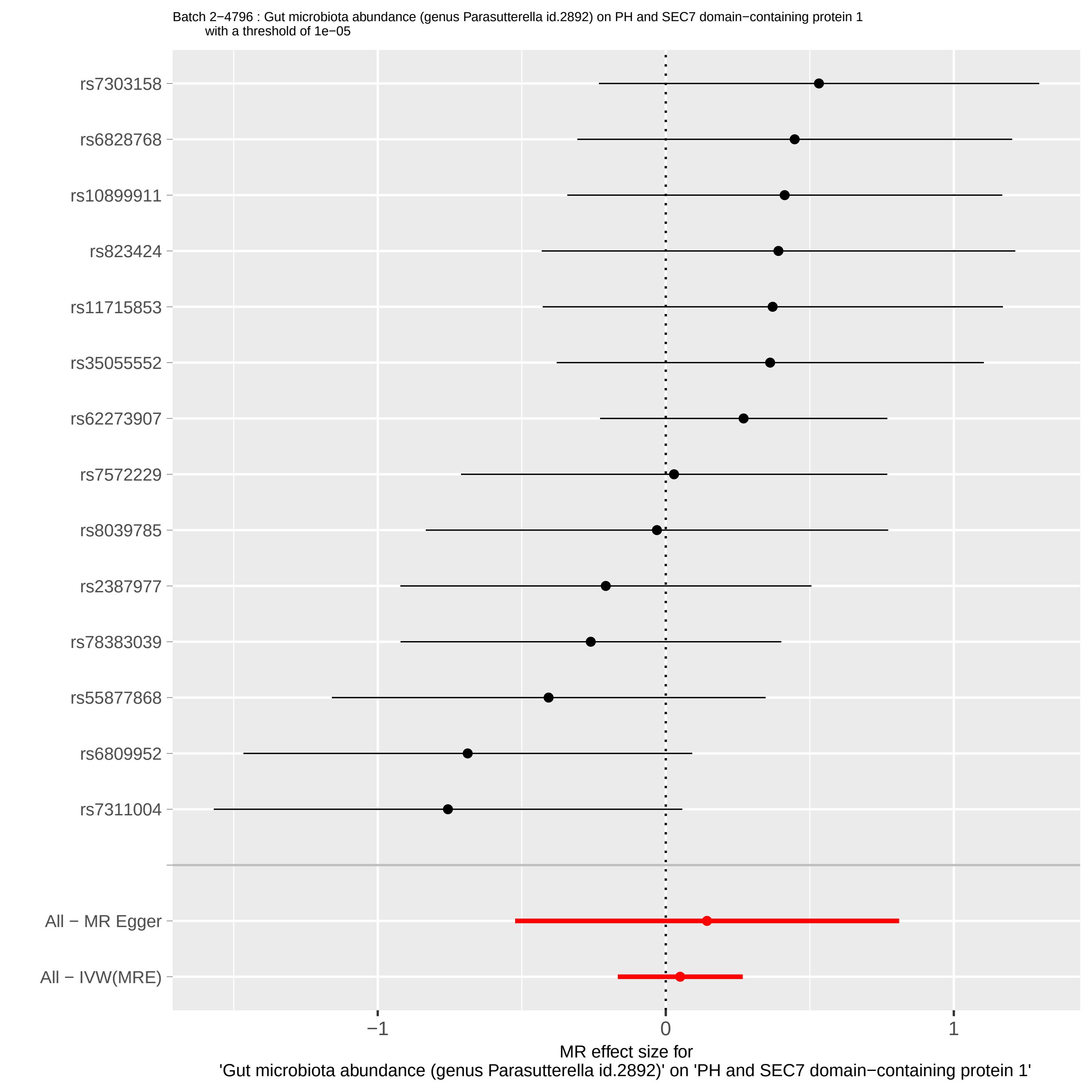

Batch 2−4796 : Gut microbiota abundance (genus Parasutterella id.2892) on PH and SEC7 domain−containing protein 1
with a threshold of 1e−05
rs7303158
rs6828768
rs10899911
rs823424
rs11715853
rs35055552
rs62273907
rs7572229
rs8039785
rs2387977
rs78383039
rs55877868
rs6809952
rs7311004
All − MR Egger
All − IVW(MRE)
−1
0
1
MR effect size for
'Gut microbiota abundance (genus Parasutterella id.2892)' on 'PH and SEC7 domain−containing protein 1'

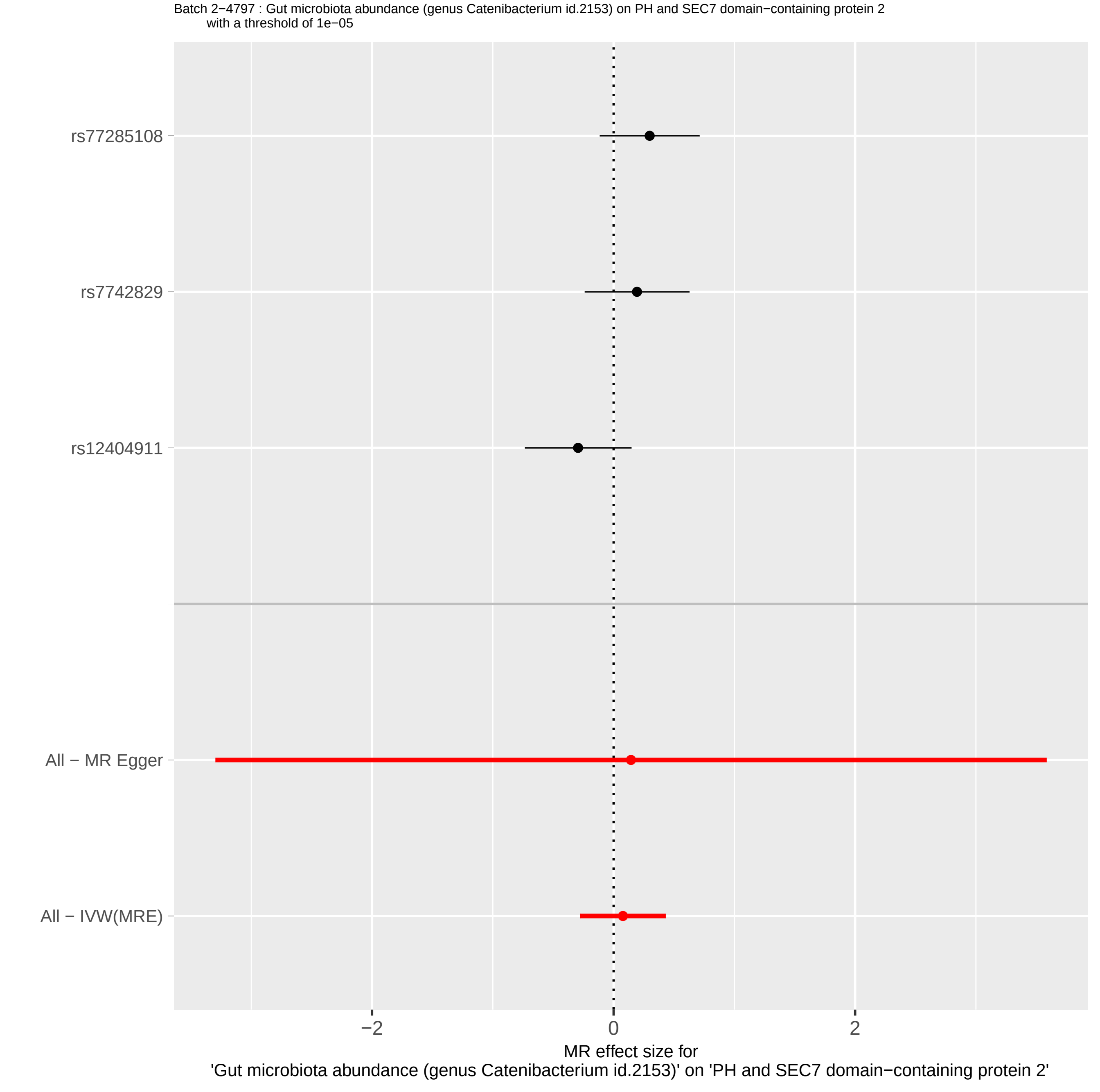

Batch 2−4797 : Gut microbiota abundance (genus Catenibacterium id.2153) on PH and SEC7 domain−containing protein 2
with a threshold of 1e−05
rs77285108
rs7742829
rs12404911
All − MR Egger
All − IVW(MRE)
−2
0
2
MR effect size for
'Gut microbiota abundance (genus Catenibacterium id.2153)' on 'PH and SEC7 domain−containing protein 2'

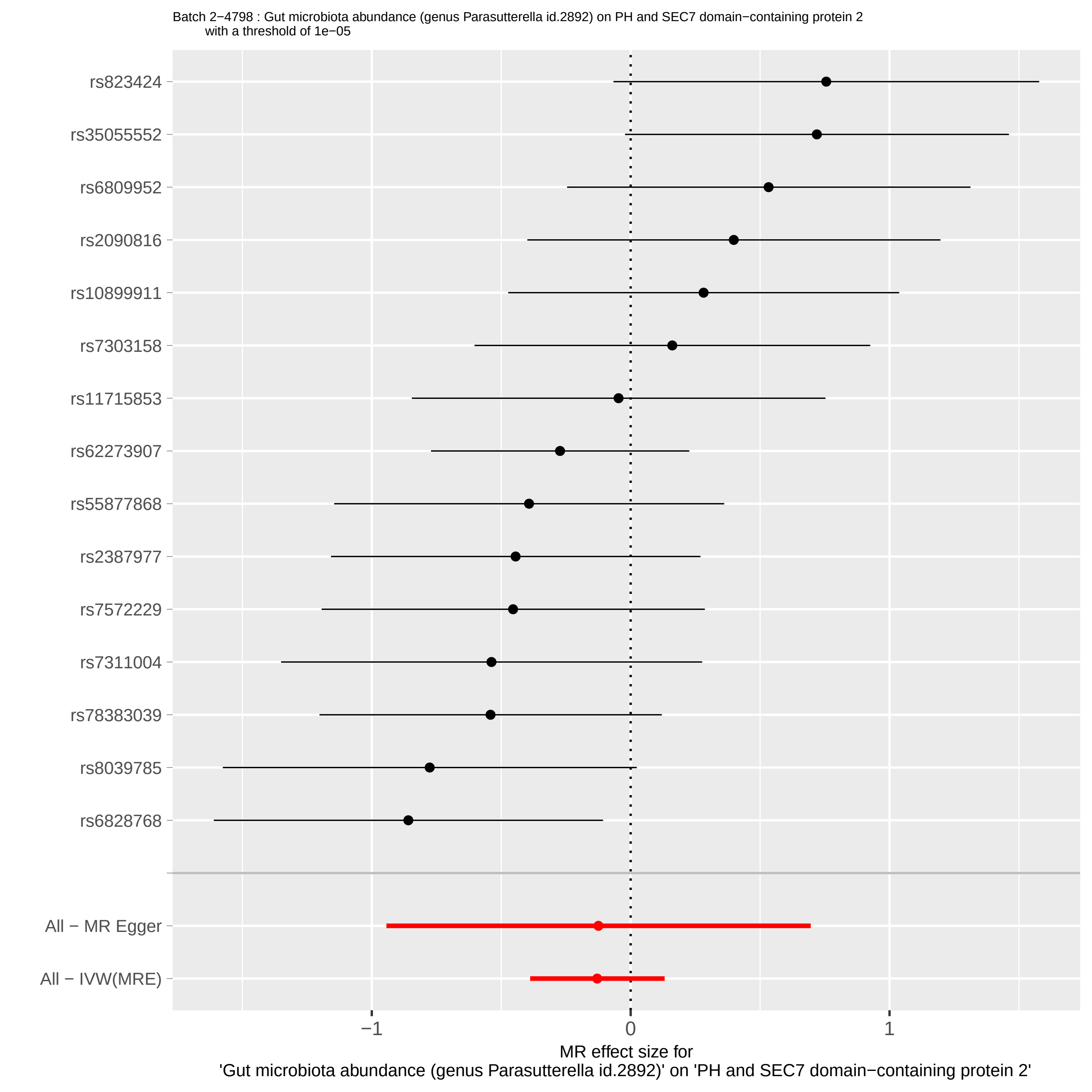

Batch 2−4798 : Gut microbiota abundance (genus Parasutterella id.2892) on PH and SEC7 domain−containing protein 2
with a threshold of 1e−05
rs823424
rs35055552
rs6809952
rs2090816
rs10899911
rs7303158
rs11715853
rs62273907
rs55877868
rs2387977
rs7572229
rs7311004
rs78383039
rs8039785
rs6828768
All − MR Egger
All − IVW(MRE)
−1
0
1
MR effect size for
'Gut microbiota abundance (genus Parasutterella id.2892)' on 'PH and SEC7 domain−containing protein 2'

Batch 2−4799 : Gut microbiota abundance (genus Catenibacterium id.2153) on Pregnancy−specific beta−1−glycoprotein 11
with a threshold of 1e−05

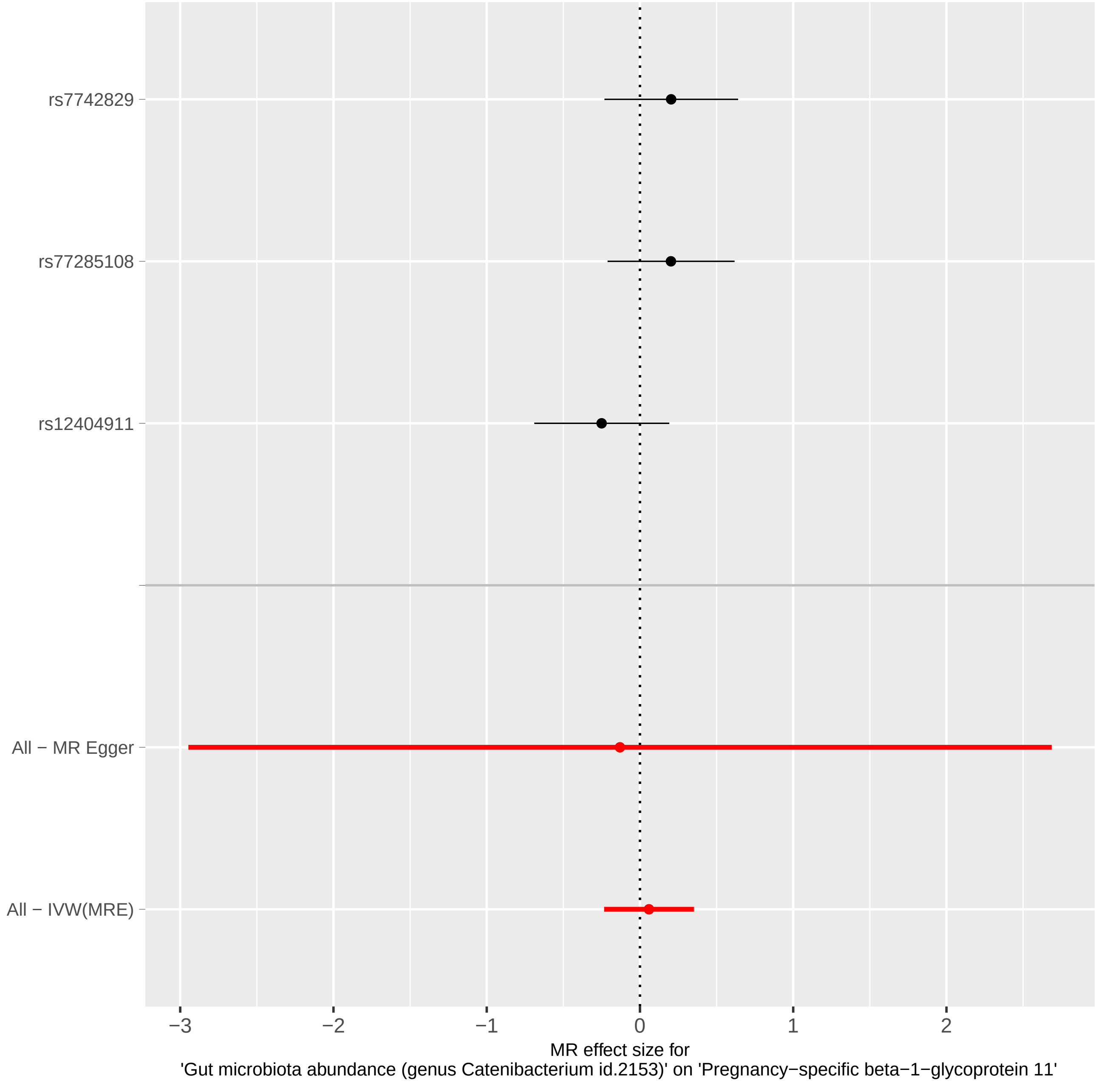

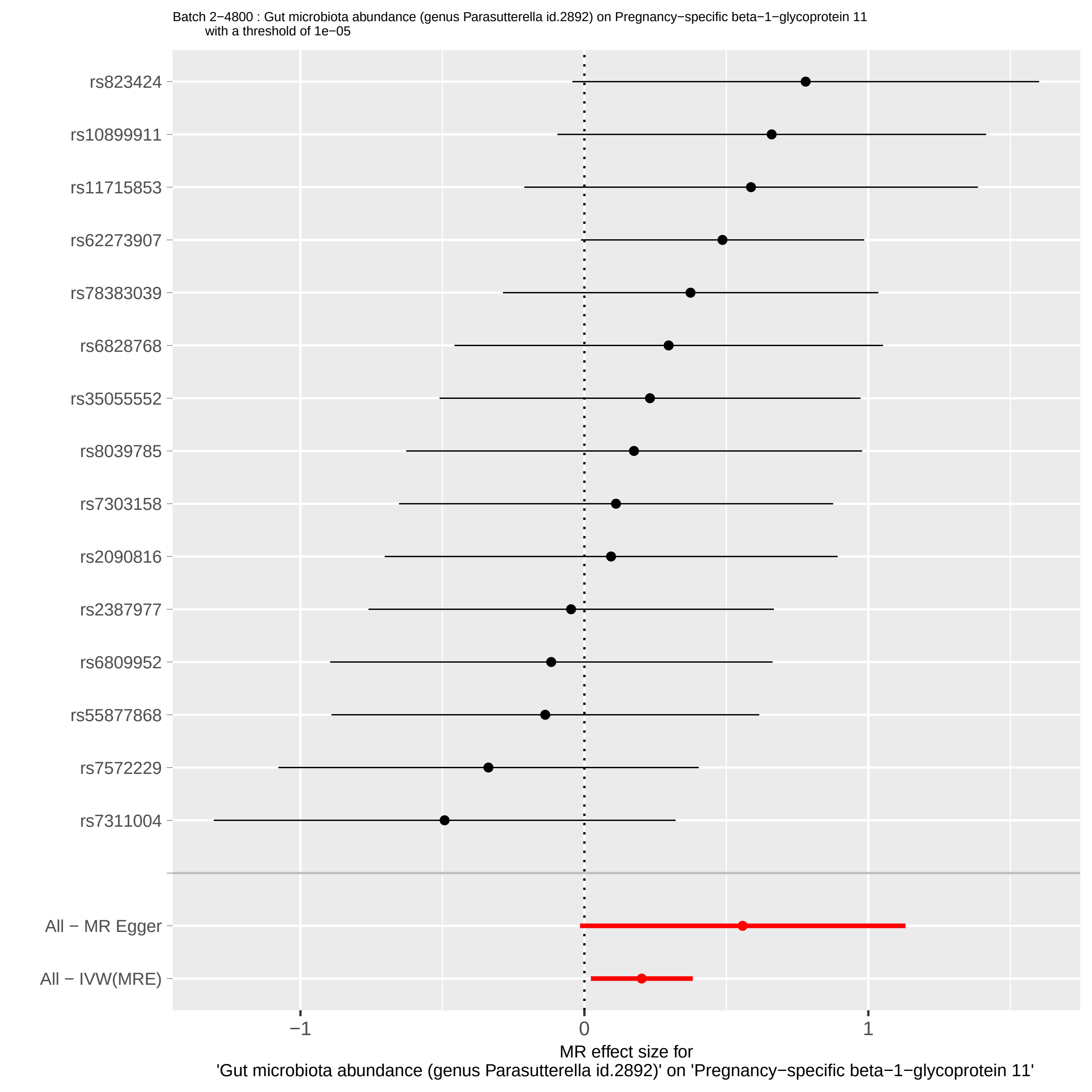

Batch 2−4800 : Gut microbiota abundance (genus Parasutterella id.2892) on Pregnancy−specific beta−1−glycoprotein 11
with a threshold of 1e−05
rs823424
rs10899911
rs11715853
rs62273907
rs78383039
rs6828768
rs35055552
rs8039785
rs7303158
rs2090816
rs2387977
rs6809952
rs55877868
rs7572229
rs7311004
All − MR Egger
All − IVW(MRE)
−1
0
1
MR effect size for
'Gut microbiota abundance (genus Parasutterella id.2892)' on 'Pregnancy−specific beta−1−glycoprotein 11'

Batch 2−4801 : Gut microbiota abundance (genus Catenibacterium id.2153) on Pregnancy−specific beta−1−glycoprotein 2
with a threshold of 1e−05

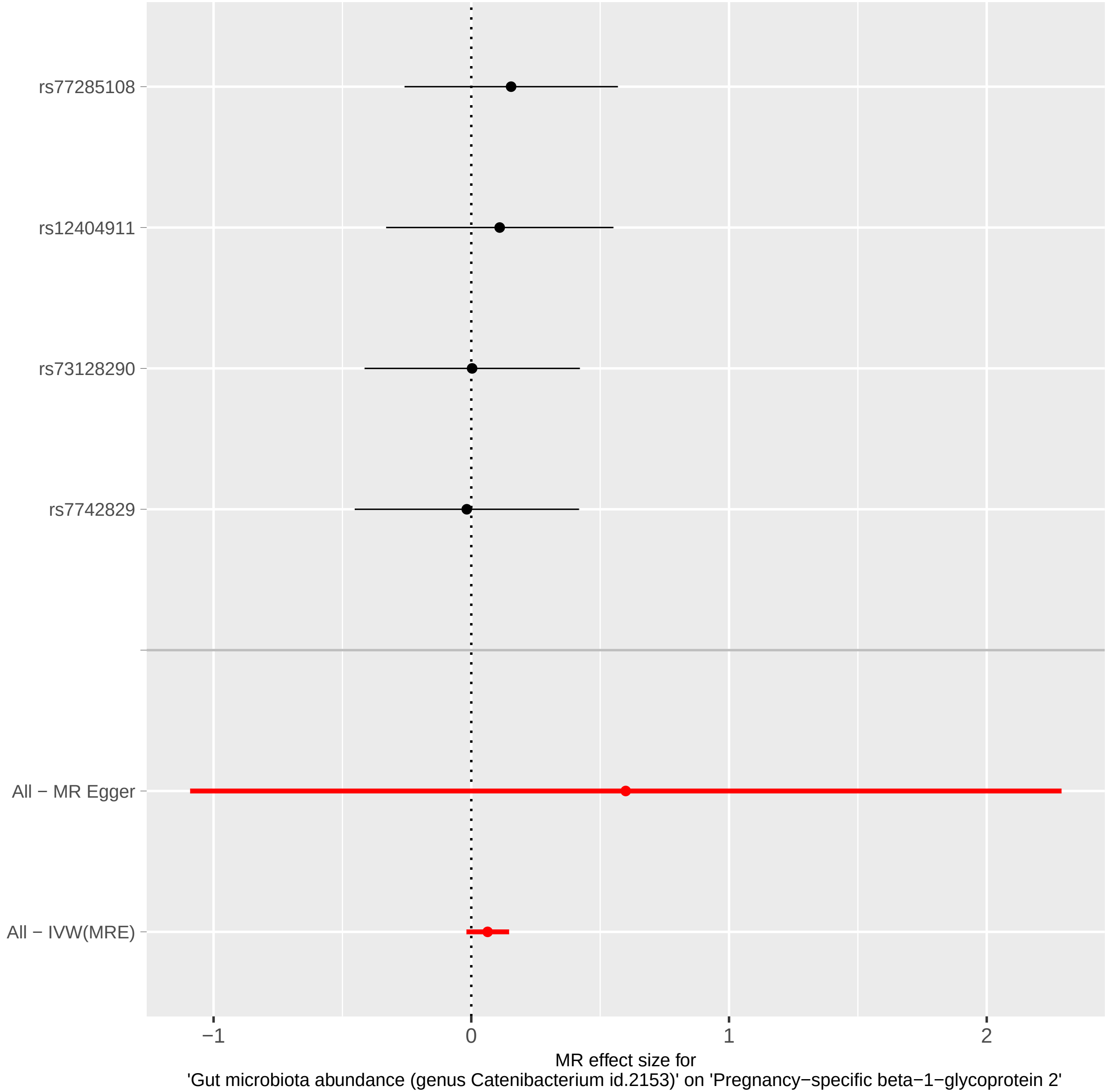

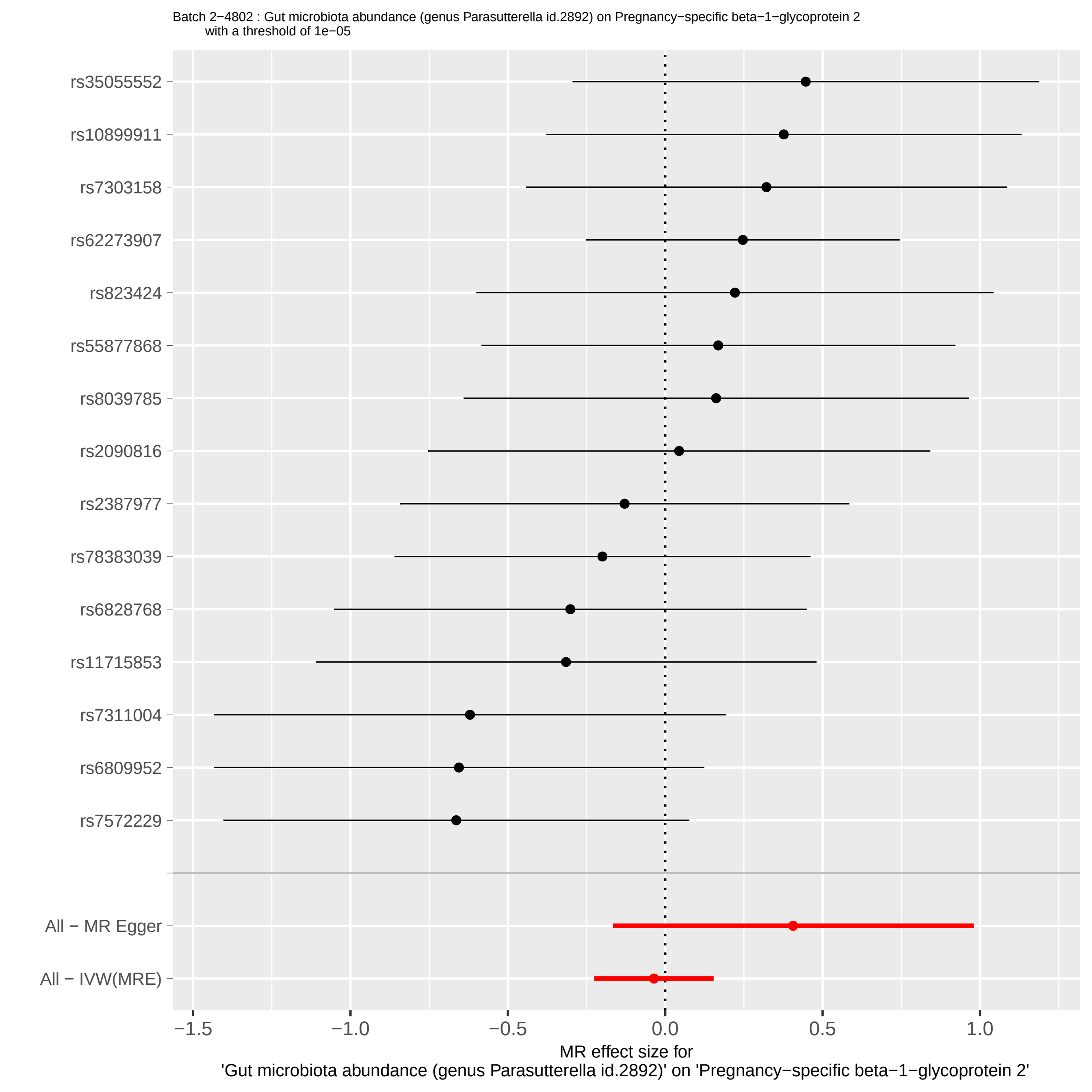
Batch 2−4802 : Gut microbiota abundance (genus Parasutterella id.2892) on Pregnancy−specific beta−1−glycoprotein 2
with a threshold of 1e−05
rs35055552
rs10899911
rs7303158
rs62273907
rs823424
rs55877868
rs8039785
rs2090816
rs2387977
rs78383039
rs6828768
rs11715853
rs7311004
rs6809952
rs7572229
All − MR Egger
All − IVW(MRE)
−1.5
−1.0
−0.5
0.0
0.5
1.0
MR effect size for
'Gut microbiota abundance (genus Parasutterella id.2892)' on 'Pregnancy−specific beta−1−glycoprotein 2'

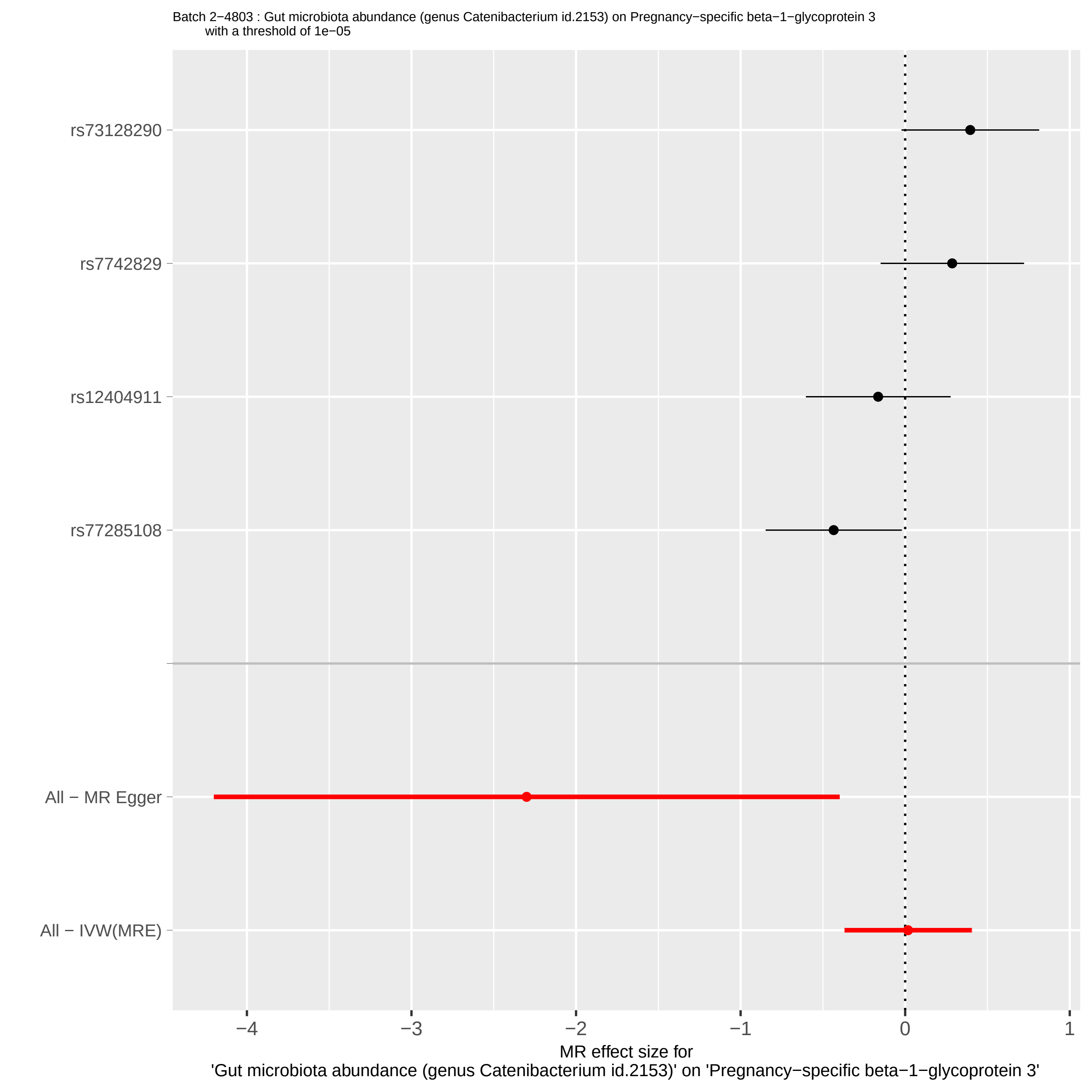
Batch 2−4803 : Gut microbiota abundance (genus Catenibacterium id.2153) on Pregnancy−specific beta−1−glycoprotein 3
with a threshold of 1e−05
rs73128290
rs7742829
rs12404911
rs77285108
All − MR Egger
All − IVW(MRE)
−4
−3
−2
−1
0
1
MR effect size for
'Gut microbiota abundance (genus Catenibacterium id.2153)' on 'Pregnancy−specific beta−1−glycoprotein 3'

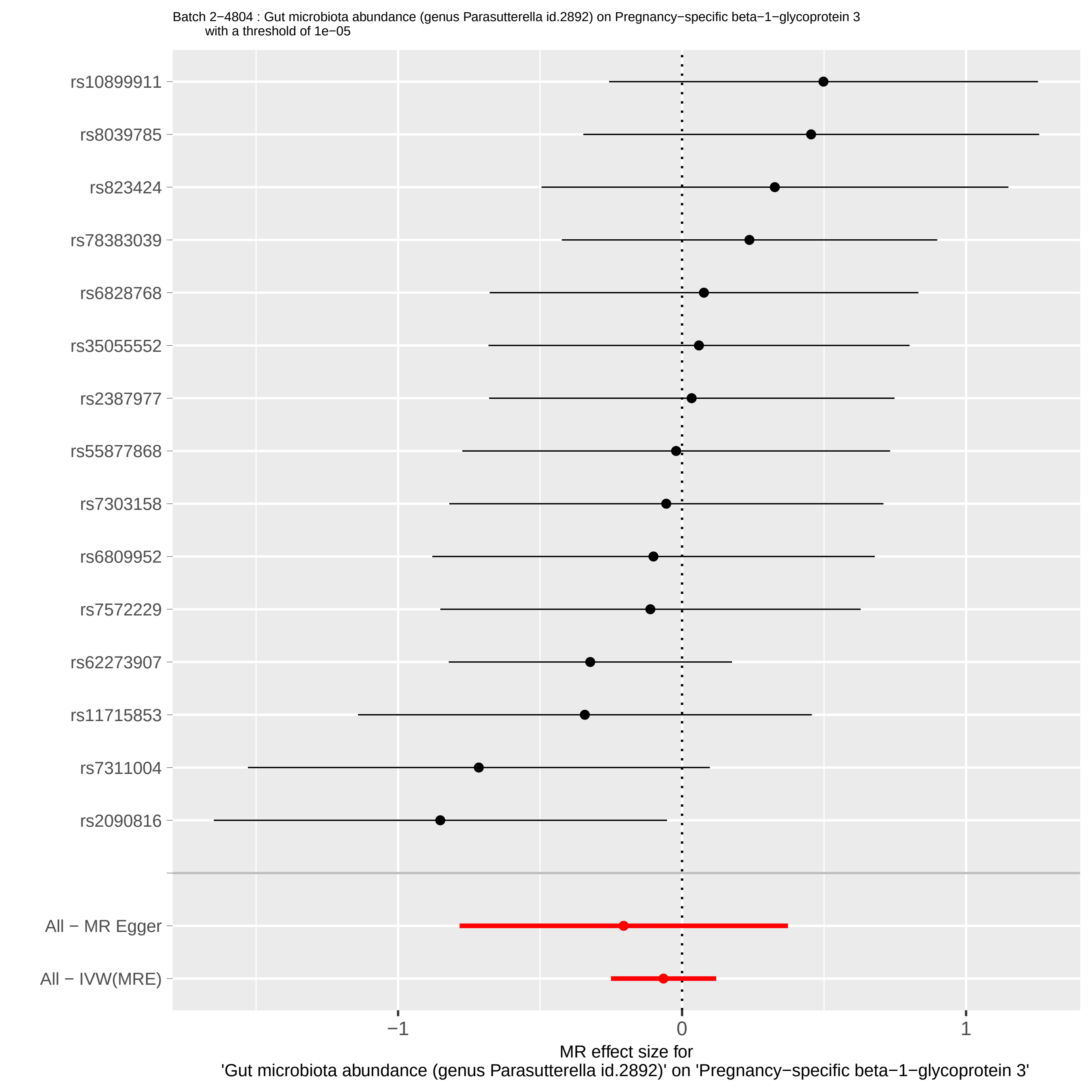

Batch 2−4804 : Gut microbiota abundance (genus Parasutterella id.2892) on Pregnancy−specific beta−1−glycoprotein 3
with a threshold of 1e−05
rs10899911
rs8039785
rs823424
rs78383039
rs6828768
rs35055552
rs2387977
rs55877868
rs7303158
rs6809952
rs7572229
rs62273907
rs11715853
rs7311004
rs2090816
All − MR Egger
All − IVW(MRE)
−1
0
1
MR effect size for
'Gut microbiota abundance (genus Parasutterella id.2892)' on 'Pregnancy−specific beta−1−glycoprotein 3'

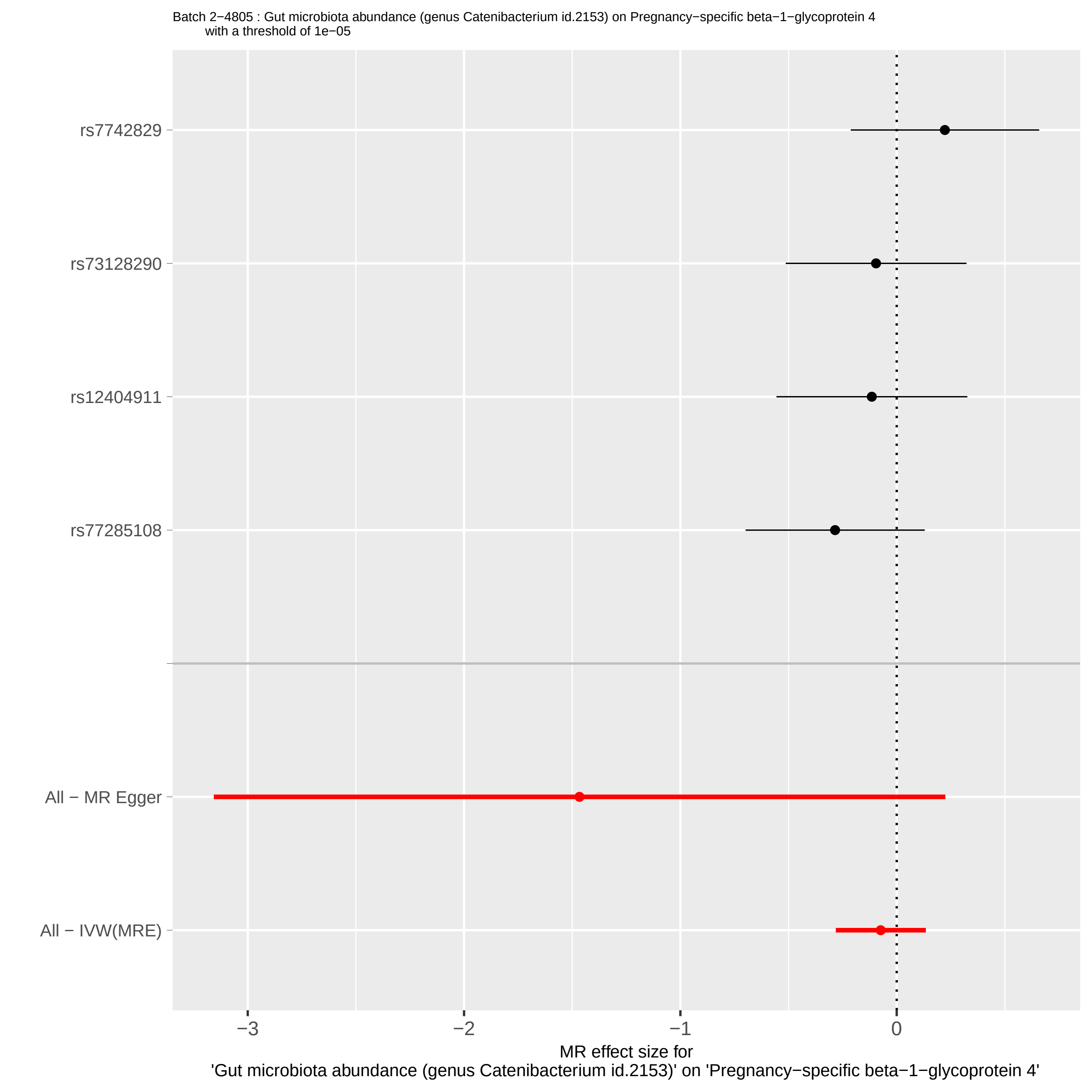

Batch 2−4805 : Gut microbiota abundance (genus Catenibacterium id.2153) on Pregnancy−specific beta−1−glycoprotein 4
with a threshold of 1e−05
rs7742829
rs73128290
rs12404911
rs77285108
All − MR Egger
All − IVW(MRE)
−3
−2
−1
0
MR effect size for
'Gut microbiota abundance (genus Catenibacterium id.2153)' on 'Pregnancy−specific beta−1−glycoprotein 4'

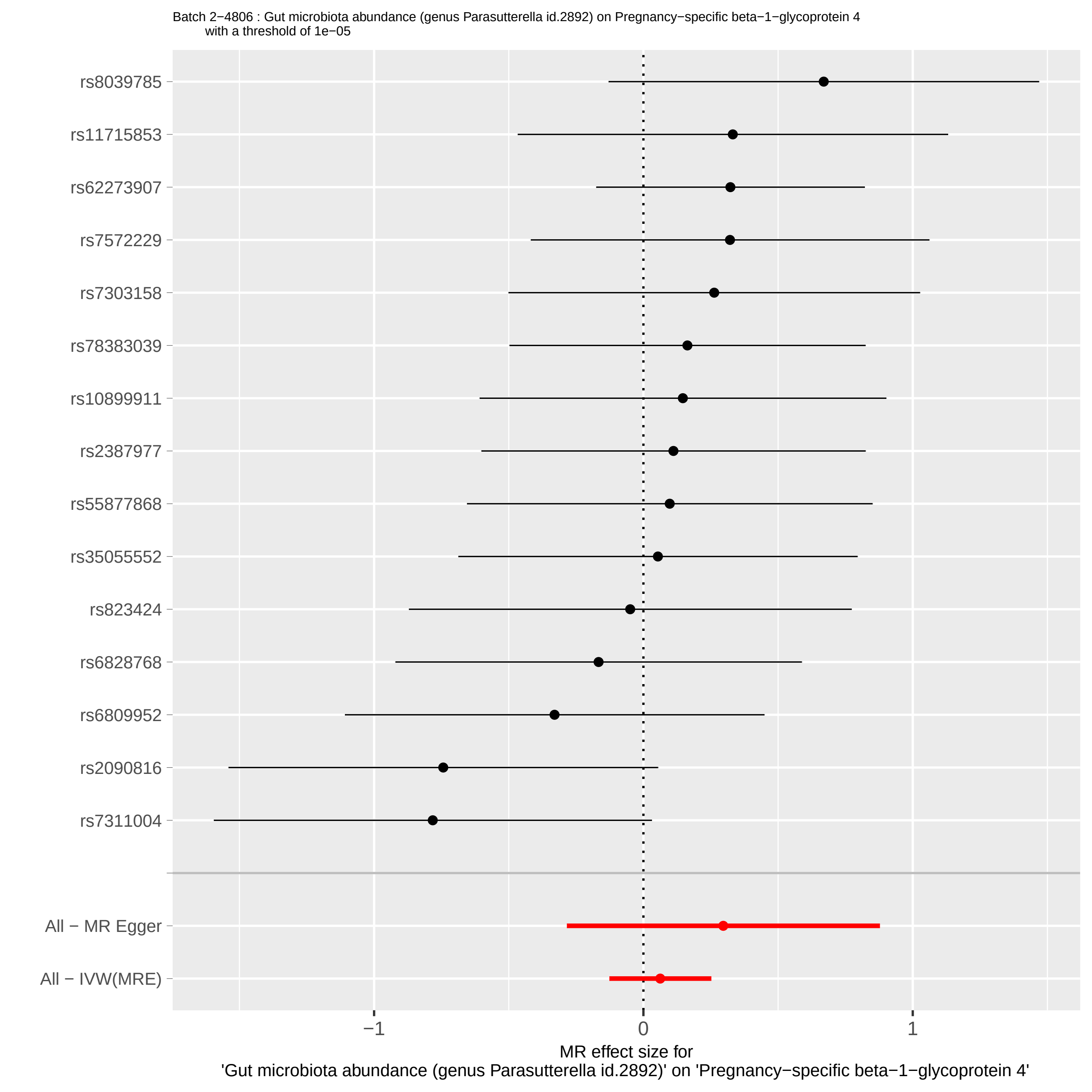

Batch 2−4806 : Gut microbiota abundance (genus Parasutterella id.2892) on Pregnancy−specific beta−1−glycoprotein 4
with a threshold of 1e−05
rs8039785
rs11715853
rs62273907
rs7572229
rs7303158
rs78383039
rs10899911
rs2387977
rs55877868
rs35055552
rs823424
rs6828768
rs6809952
rs2090816
rs7311004
All − MR Egger
All − IVW(MRE)
−1
0
1
MR effect size for
'Gut microbiota abundance (genus Parasutterella id.2892)' on 'Pregnancy−specific beta−1−glycoprotein 4'

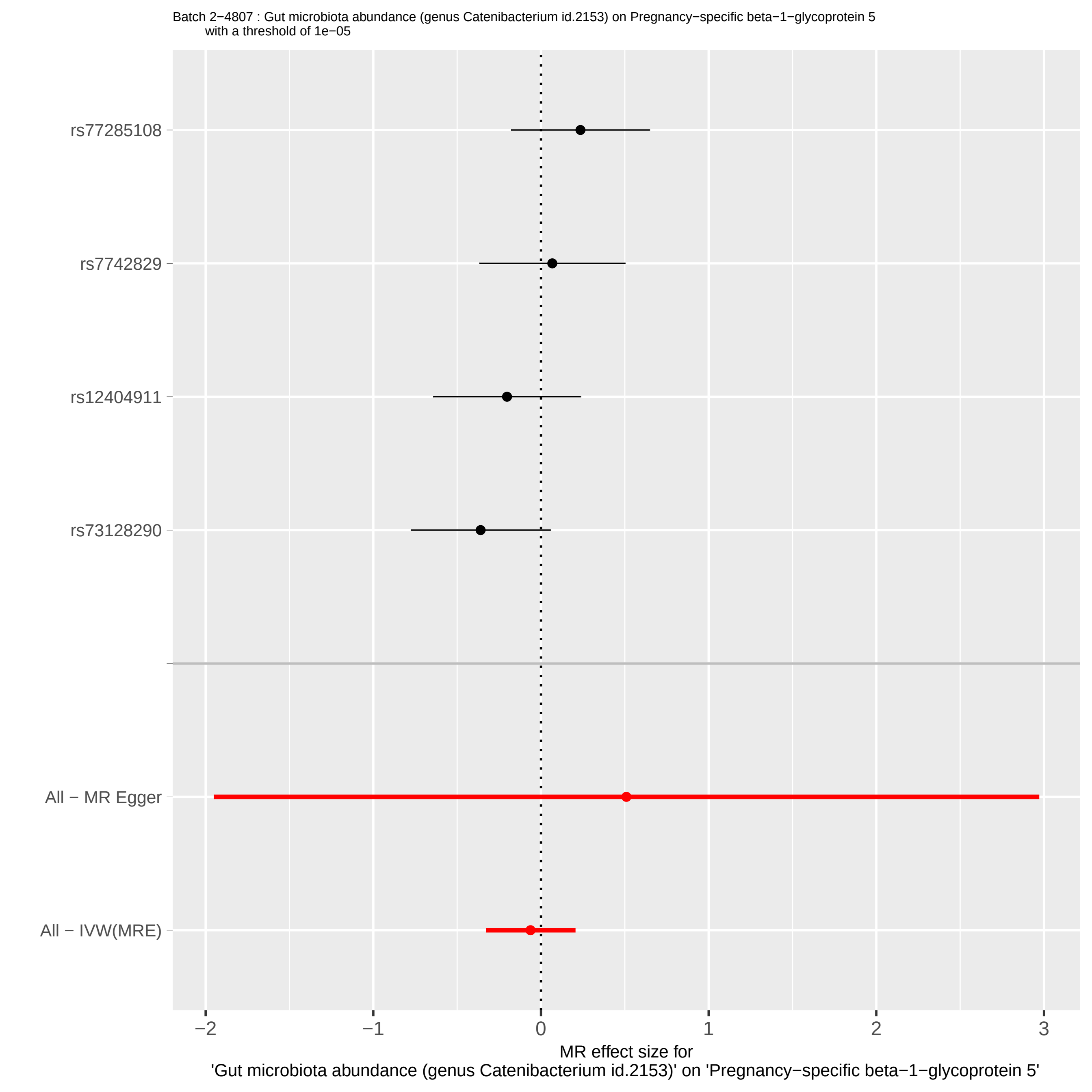

Batch 2−4807 : Gut microbiota abundance (genus Catenibacterium id.2153) on Pregnancy−specific beta−1−glycoprotein 5
with a threshold of 1e−05
rs77285108
rs7742829
rs12404911
rs73128290
All − MR Egger
All − IVW(MRE)
−2
−1
0
1
2
3
MR effect size for
'Gut microbiota abundance (genus Catenibacterium id.2153)' on 'Pregnancy−specific beta−1−glycoprotein 5'

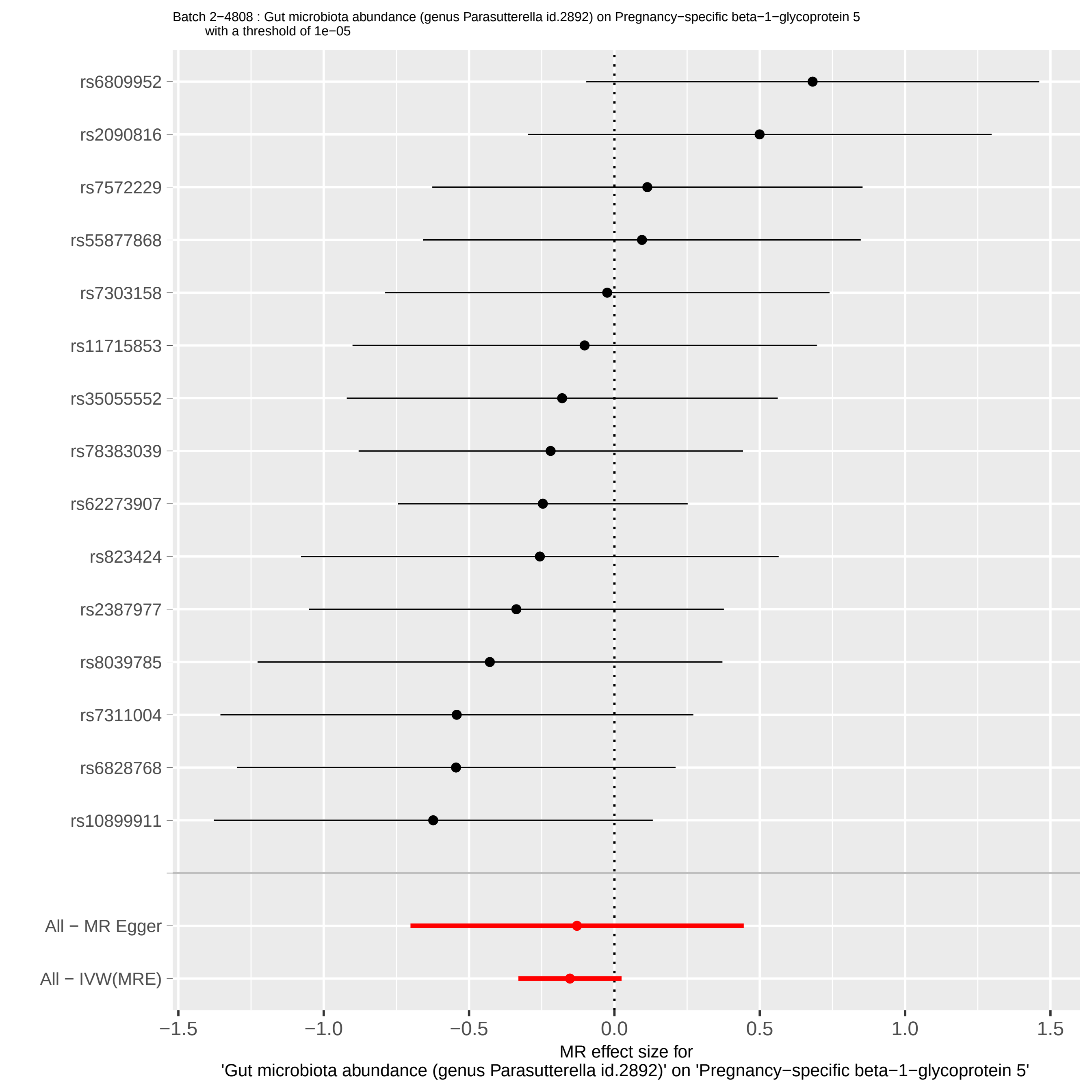
Batch 2−4808 : Gut microbiota abundance (genus Parasutterella id.2892) on Pregnancy−specific beta−1−glycoprotein 5
with a threshold of 1e−05
rs6809952
rs2090816
rs7572229
rs55877868
rs7303158
rs11715853
rs35055552
rs78383039
rs62273907
rs823424
rs2387977
rs8039785
rs7311004
rs6828768
rs10899911
All − MR Egger
All − IVW(MRE)
−1.5
−1.0
−0.5
0.0
0.5
1.0
1.5
MR effect size for
'Gut microbiota abundance (genus Parasutterella id.2892)' on 'Pregnancy−specific beta−1−glycoprotein 5'

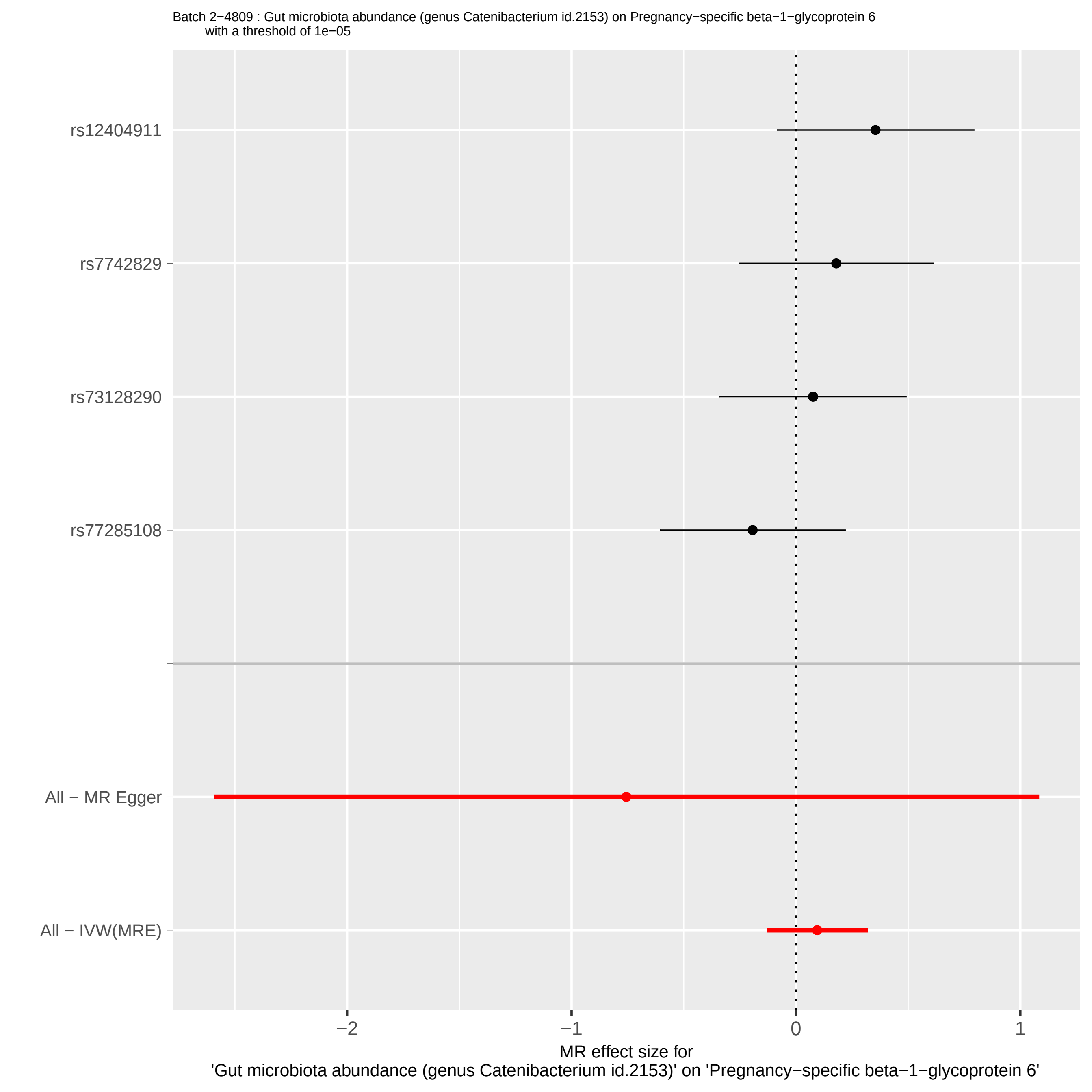
Batch 2−4809 : Gut microbiota abundance (genus Catenibacterium id.2153) on Pregnancy−specific beta−1−glycoprotein 6
with a threshold of 1e−05
rs12404911
rs7742829
rs73128290
rs77285108
All − MR Egger
All − IVW(MRE)
−2
−1
0
1
MR effect size for
'Gut microbiota abundance (genus Catenibacterium id.2153)' on 'Pregnancy−specific beta−1−glycoprotein 6'

Batch 2−4810 : Gut microbiota abundance (genus Parasutterella id.2892) on Pregnancy−specific beta−1−glycoprotein 6
with a threshold of 1e−05

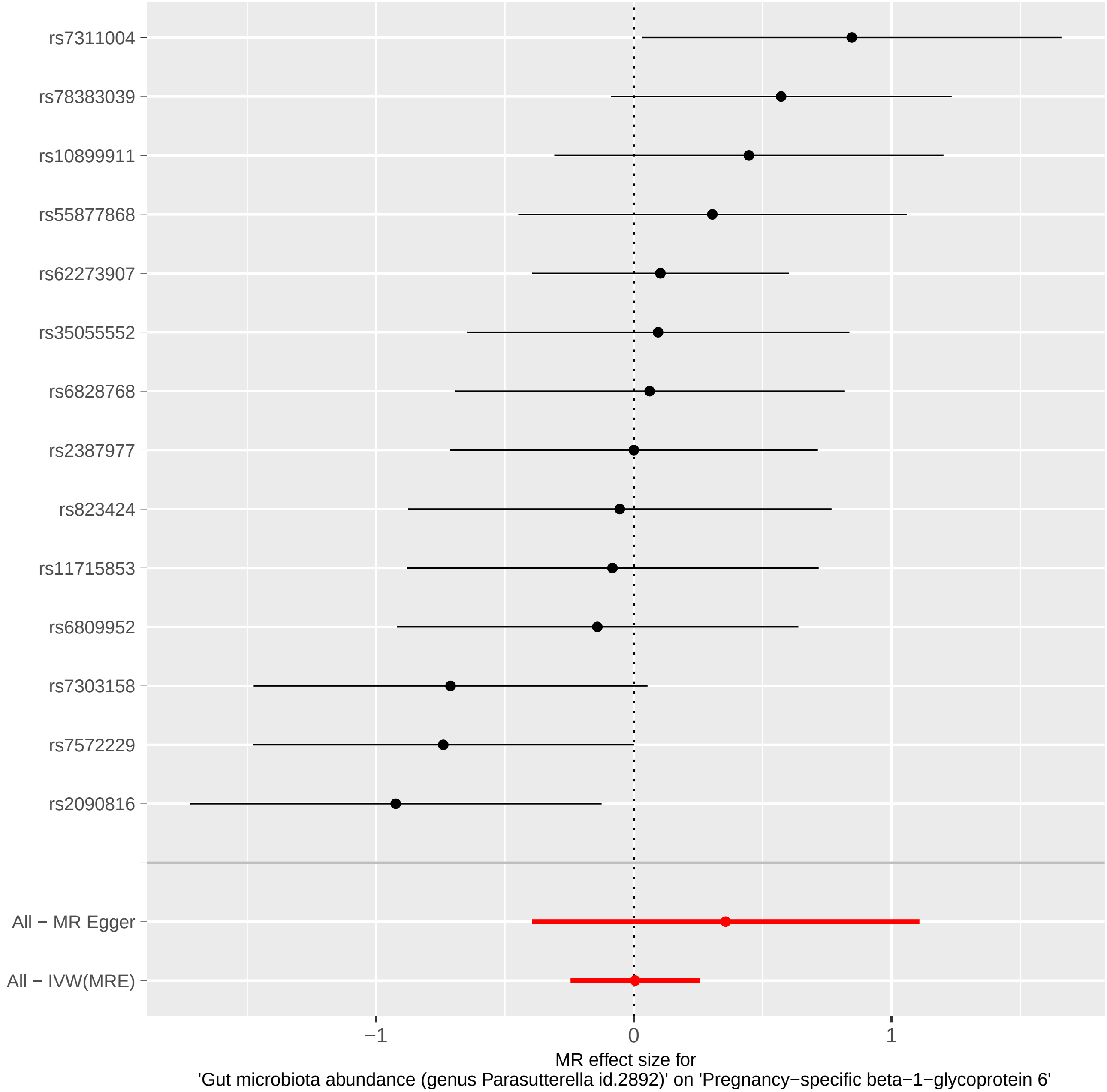

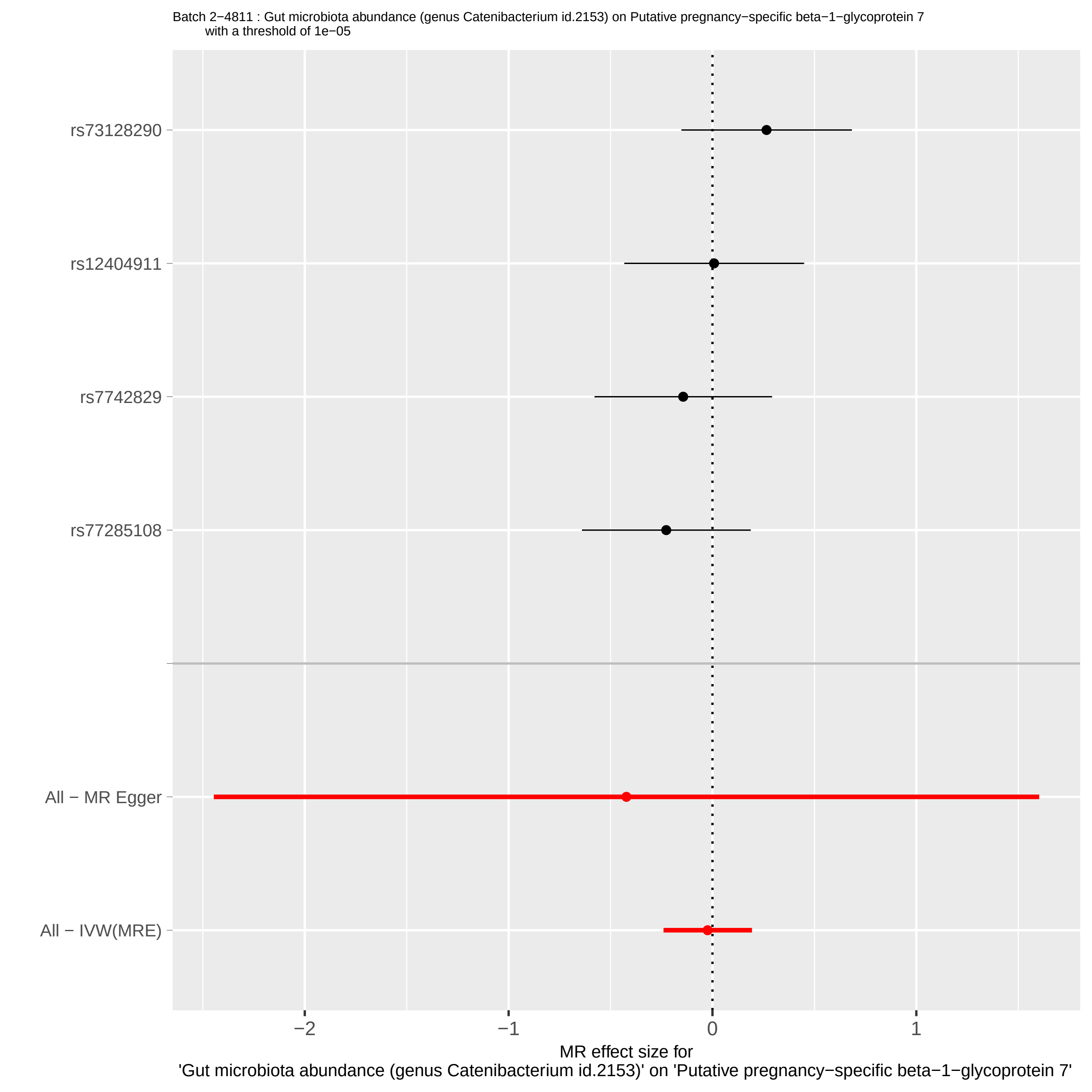
Batch 2−4811 : Gut microbiota abundance (genus Catenibacterium id.2153) on Putative pregnancy−specific beta−1−glycoprotein 7
with a threshold of 1e−05
rs73128290
rs12404911
rs7742829
rs77285108
All − MR Egger
All − IVW(MRE)
−2
−1
0
1
MR effect size for
'Gut microbiota abundance (genus Catenibacterium id.2153)' on 'Putative pregnancy−specific beta−1−glycoprotein 7'

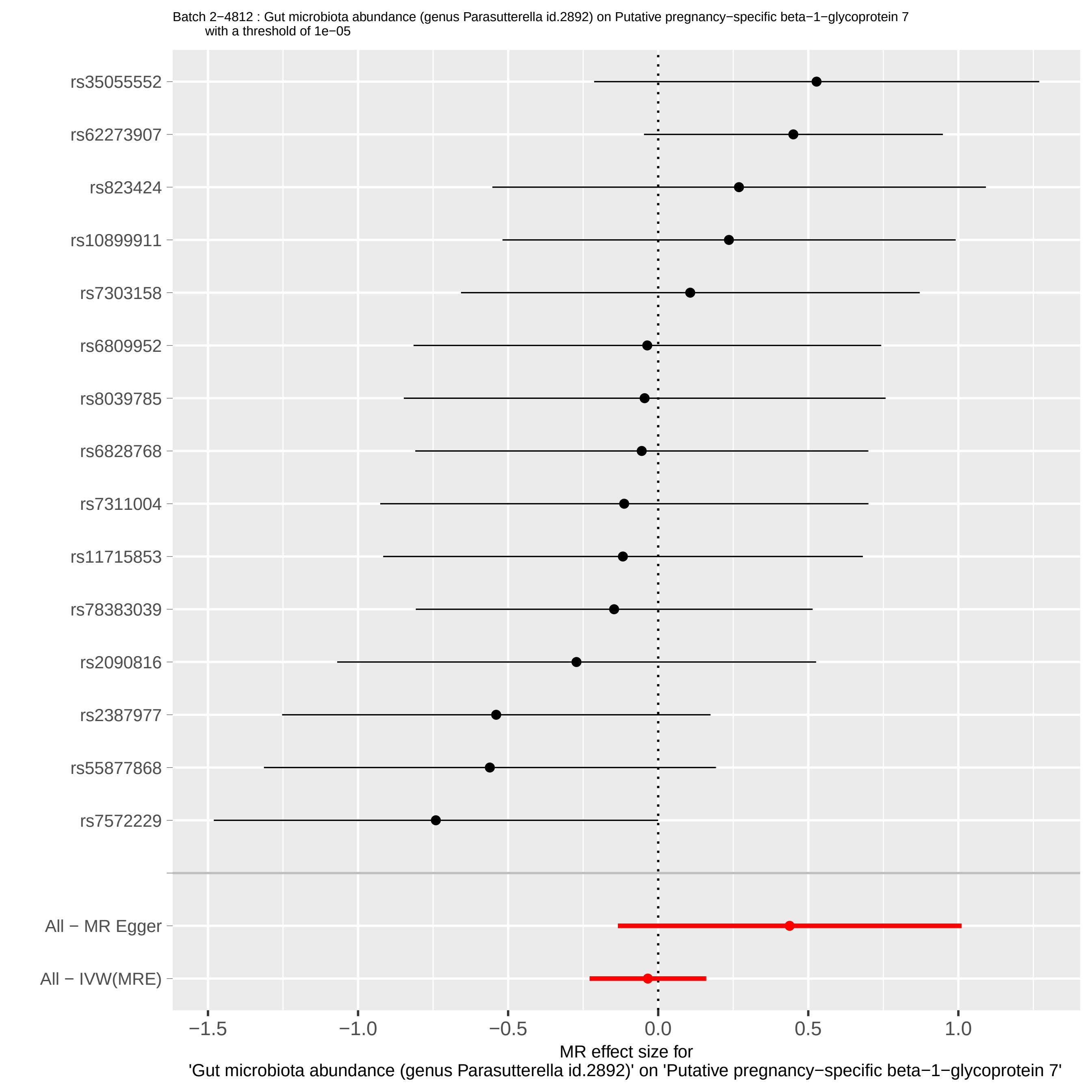

Batch 2−4812 : Gut microbiota abundance (genus Parasutterella id.2892) on Putative pregnancy−specific beta−1−glycoprotein 7
with a threshold of 1e−05
rs35055552
rs62273907
rs823424
rs10899911
rs7303158
rs6809952
rs8039785
rs6828768
rs7311004
rs11715853
rs78383039
rs2090816
rs2387977
rs55877868
rs7572229
All − MR Egger
All − IVW(MRE)
−1.5
−1.0
−0.5
0.0
0.5
1.0
MR effect size for
'Gut microbiota abundance (genus Parasutterella id.2892)' on 'Putative pregnancy−specific beta−1−glycoprotein 7'

Batch 2−4813 : Gut microbiota abundance (genus Catenibacterium id.2153) on Pregnancy−specific beta−1−glycoprotein 8
with a threshold of 1e−05

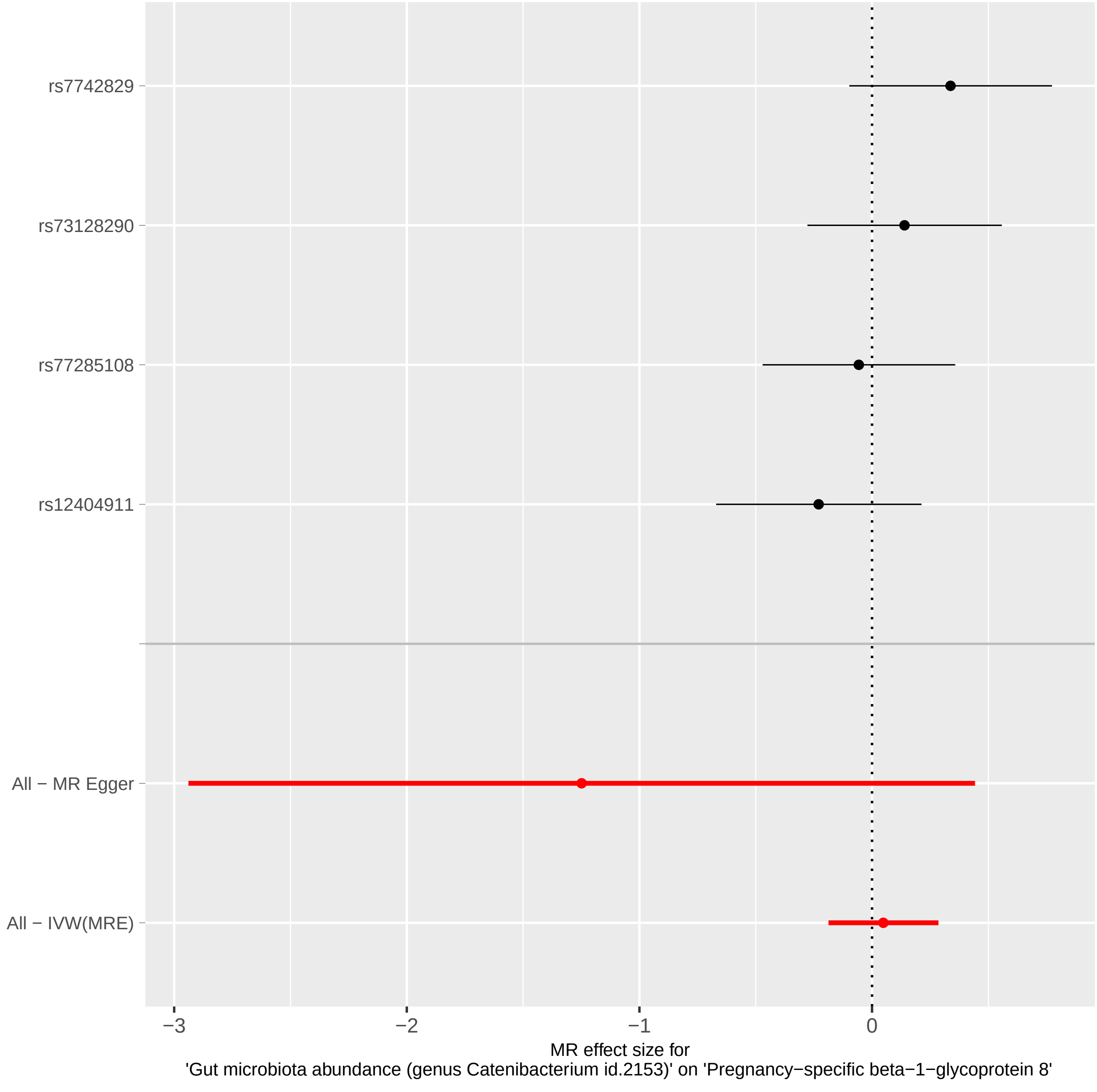

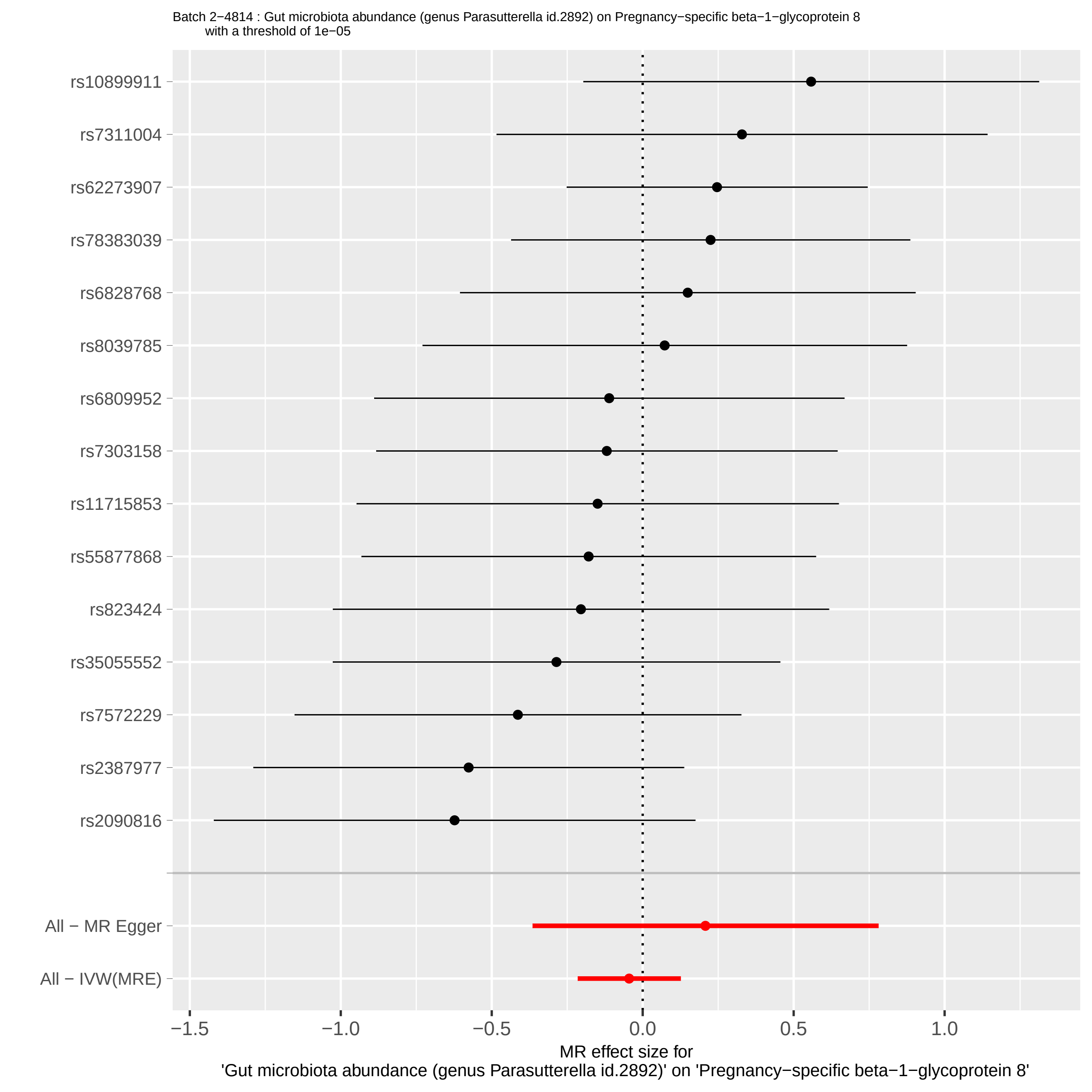

Batch 2−4814 : Gut microbiota abundance (genus Parasutterella id.2892) on Pregnancy−specific beta−1−glycoprotein 8
with a threshold of 1e−05
rs10899911
rs7311004
rs62273907
rs78383039
rs6828768
rs8039785
rs6809952
rs7303158
rs11715853
rs55877868
rs823424
rs35055552
rs7572229
rs2387977
rs2090816
All − MR Egger
All − IVW(MRE)
−1.5
−1.0
−0.5
0.0
0.5
1.0
MR effect size for
'Gut microbiota abundance (genus Parasutterella id.2892)' on 'Pregnancy−specific beta−1−glycoprotein 8'

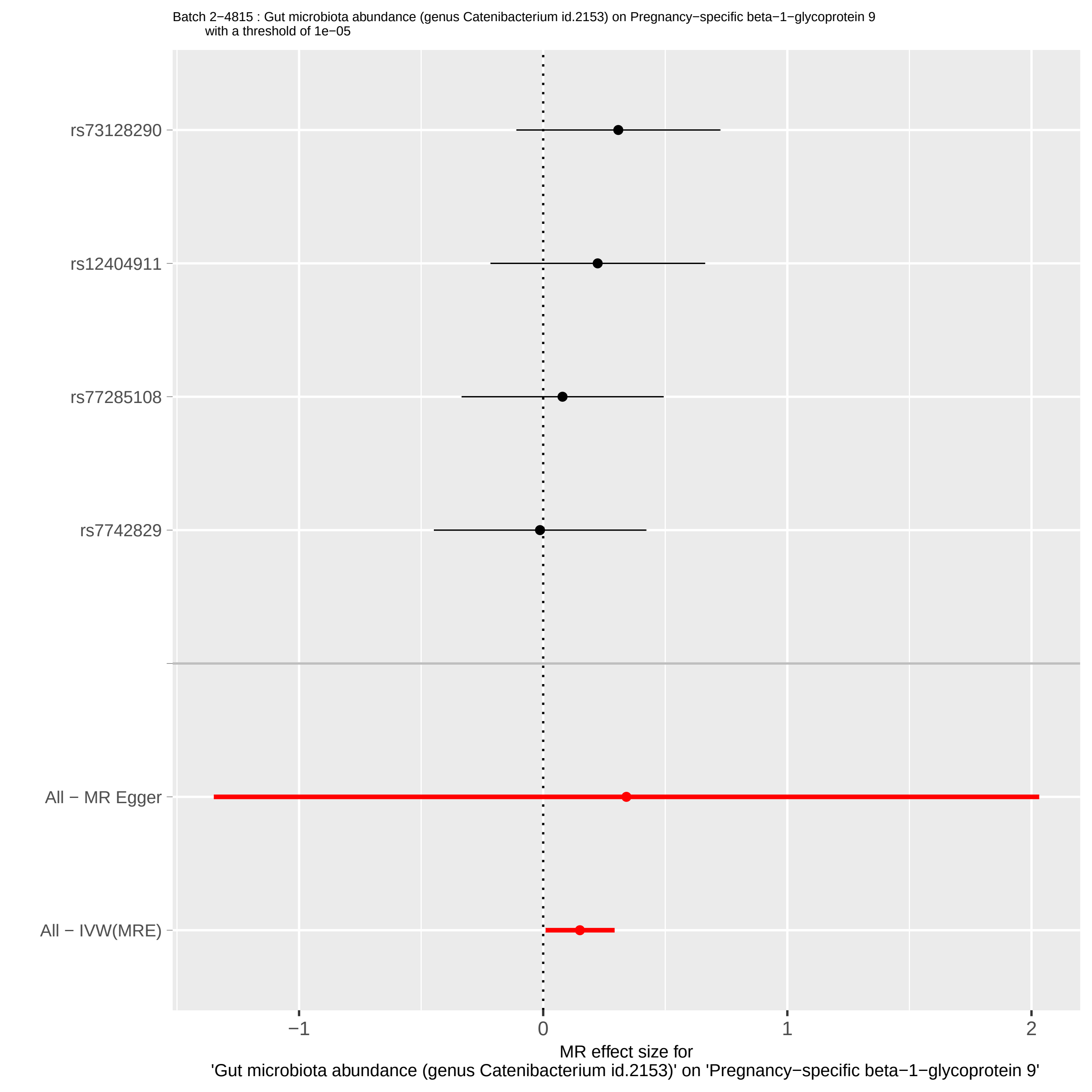
Batch 2−4815 : Gut microbiota abundance (genus Catenibacterium id.2153) on Pregnancy−specific beta−1−glycoprotein 9
with a threshold of 1e−05
rs73128290
rs12404911
rs77285108
rs7742829
All − MR Egger
All − IVW(MRE)
−1
0
1
2
MR effect size for
'Gut microbiota abundance (genus Catenibacterium id.2153)' on 'Pregnancy−specific beta−1−glycoprotein 9'

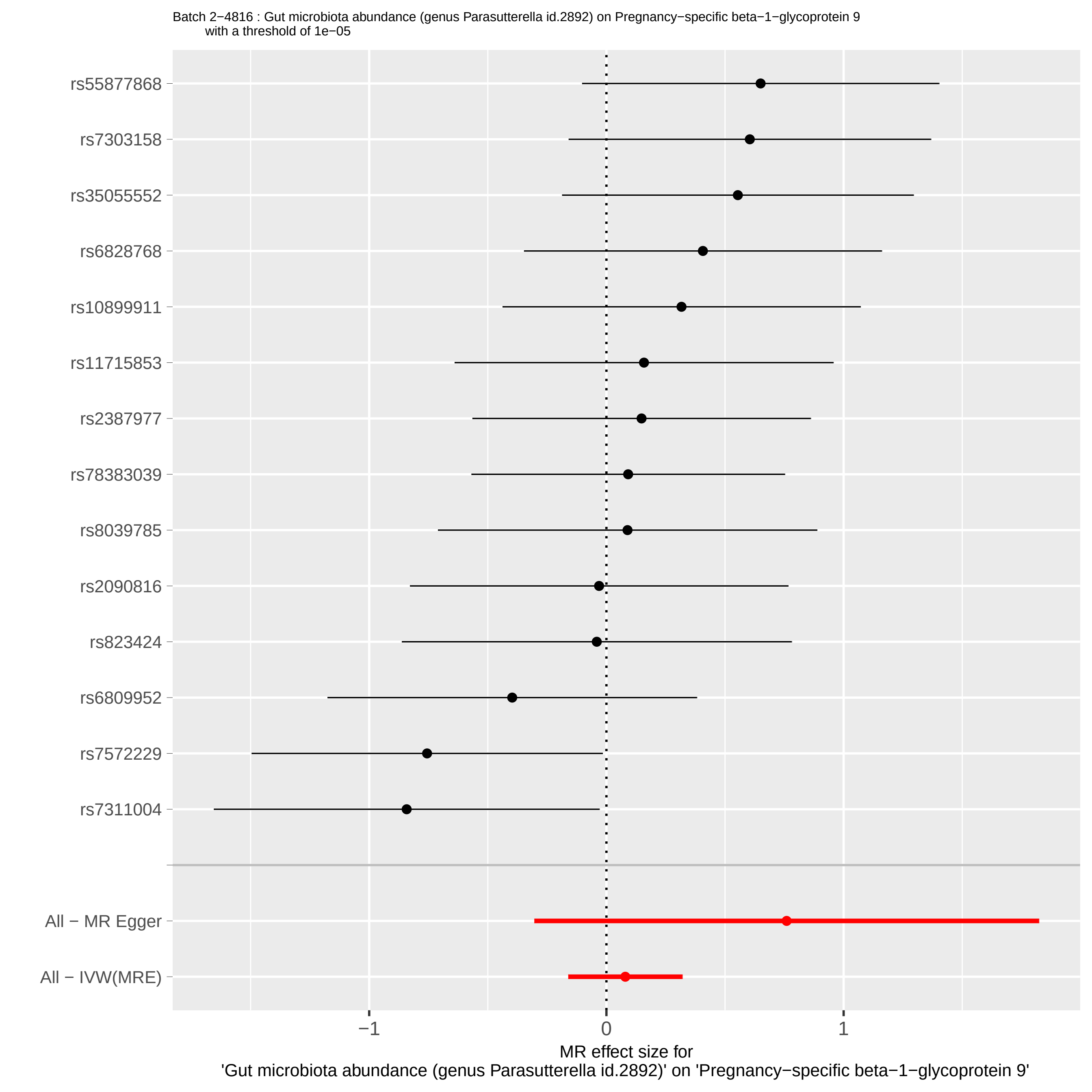

Batch 2−4816 : Gut microbiota abundance (genus Parasutterella id.2892) on Pregnancy−specific beta−1−glycoprotein 9
with a threshold of 1e−05
rs55877868
rs7303158
rs35055552
rs6828768
rs10899911
rs11715853
rs2387977
rs78383039
rs8039785
rs2090816
rs823424
rs6809952
rs7572229
rs7311004
All − MR Egger
All − IVW(MRE)
−1
0
1
MR effect size for
'Gut microbiota abundance (genus Parasutterella id.2892)' on 'Pregnancy−specific beta−1−glycoprotein 9'

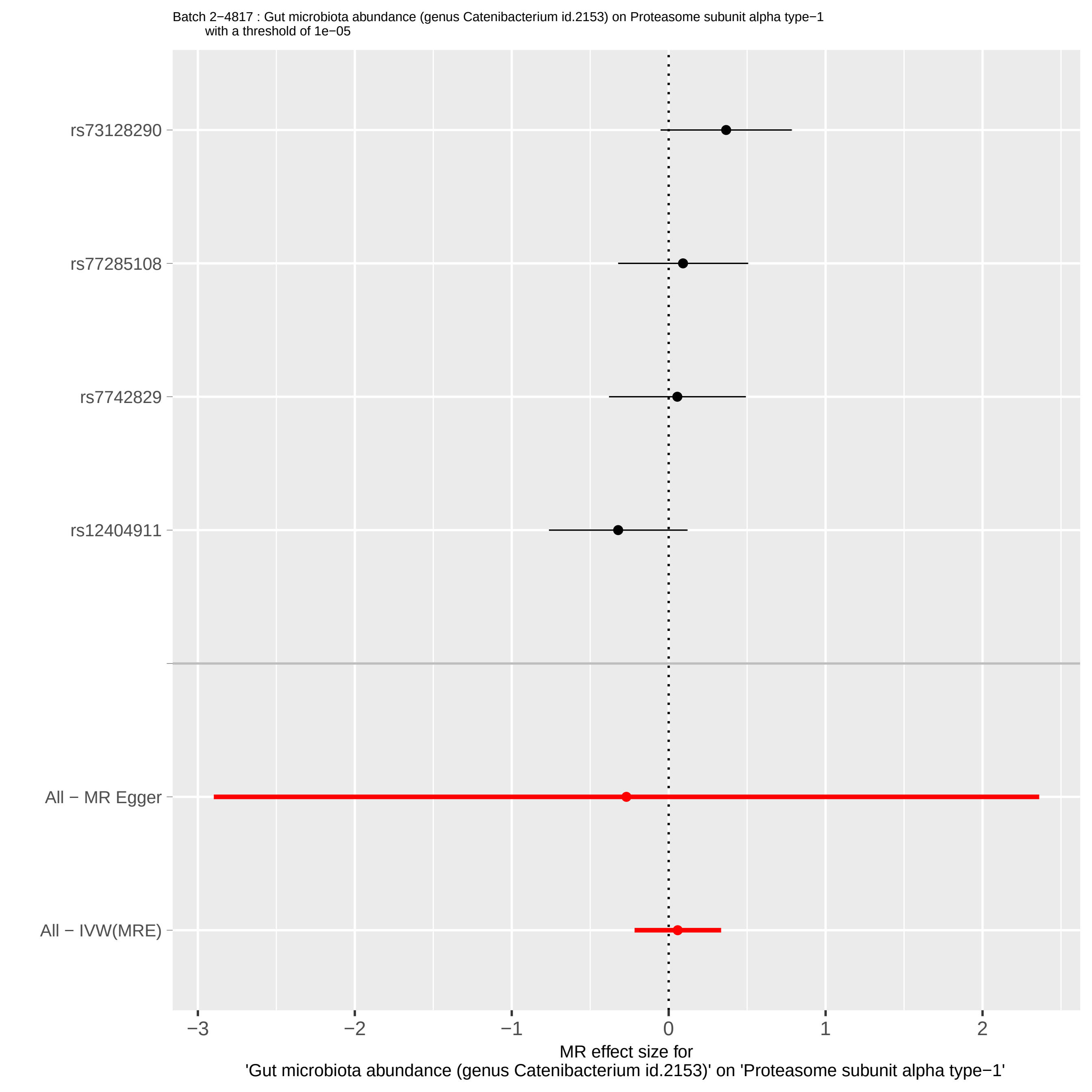

Batch 2−4817 : Gut microbiota abundance (genus Catenibacterium id.2153) on Proteasome subunit alpha type−1
with a threshold of 1e−05
rs73128290
rs77285108
rs7742829
rs12404911
All − MR Egger
All − IVW(MRE)
−3
−2
−1
0
1
2
MR effect size for
'Gut microbiota abundance (genus Catenibacterium id.2153)' on 'Proteasome subunit alpha type−1'

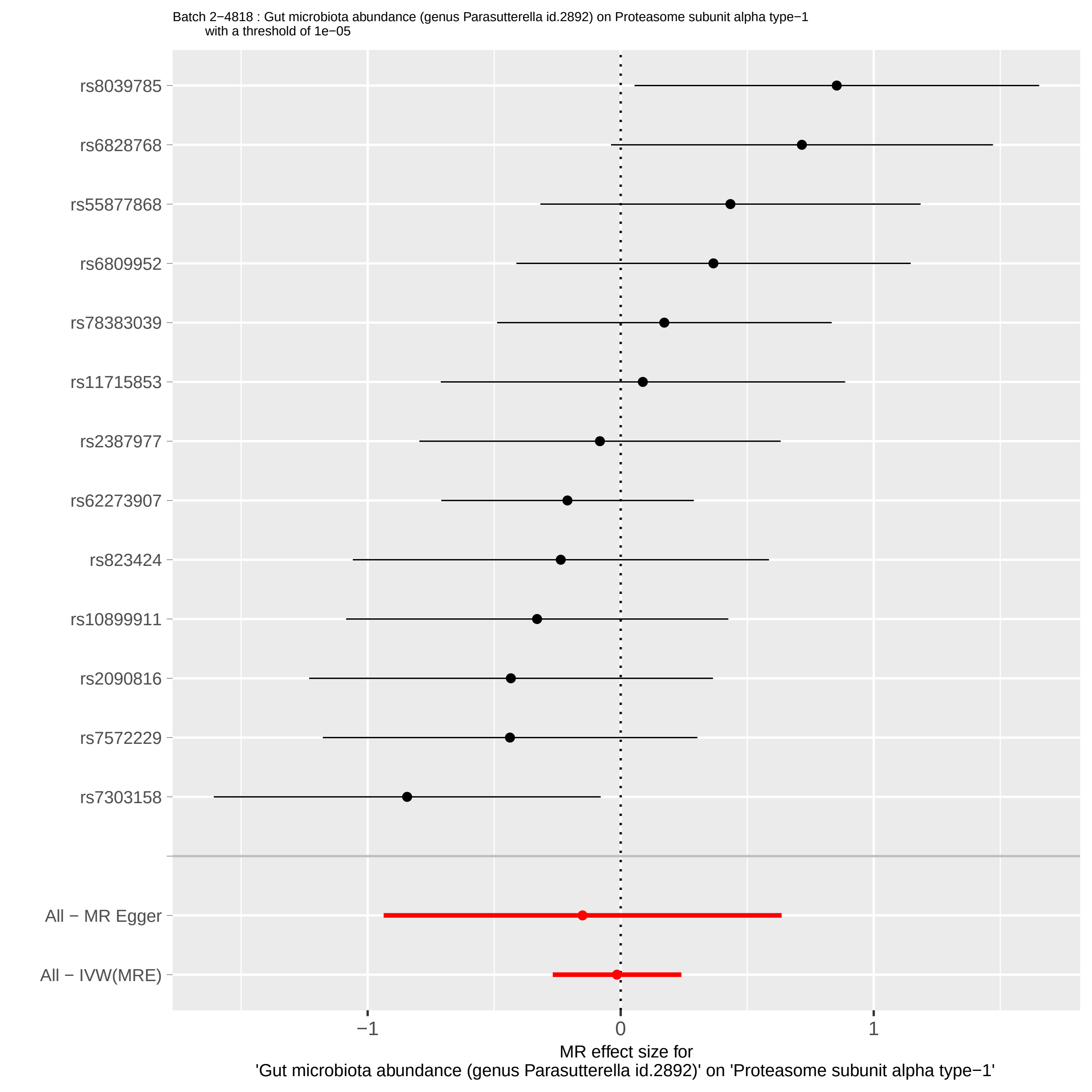

Batch 2−4818 : Gut microbiota abundance (genus Parasutterella id.2892) on Proteasome subunit alpha type−1
with a threshold of 1e−05
rs8039785
rs6828768
rs55877868
rs6809952
rs78383039
rs11715853
rs2387977
rs62273907
rs823424
rs10899911
rs2090816
rs7572229
rs7303158
All − MR Egger
All − IVW(MRE)
−1
0
1
MR effect size for
'Gut microbiota abundance (genus Parasutterella id.2892)' on 'Proteasome subunit alpha type−1'

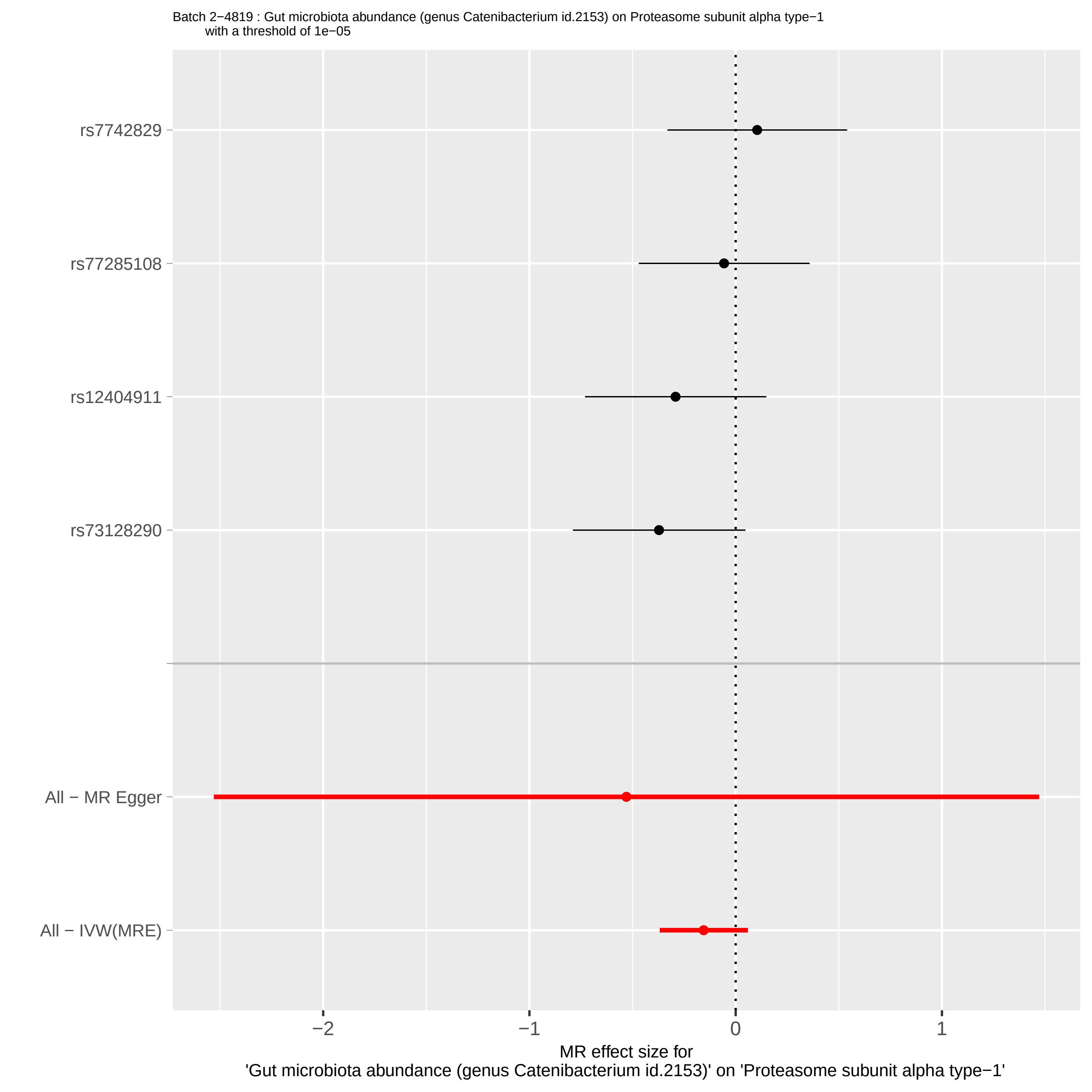
Batch 2−4819 : Gut microbiota abundance (genus Catenibacterium id.2153) on Proteasome subunit alpha type−1
with a threshold of 1e−05
rs7742829
rs77285108
rs12404911
rs73128290
All − MR Egger
All − IVW(MRE)
−2
−1
0
1
MR effect size for
'Gut microbiota abundance (genus Catenibacterium id.2153)' on 'Proteasome subunit alpha type−1'

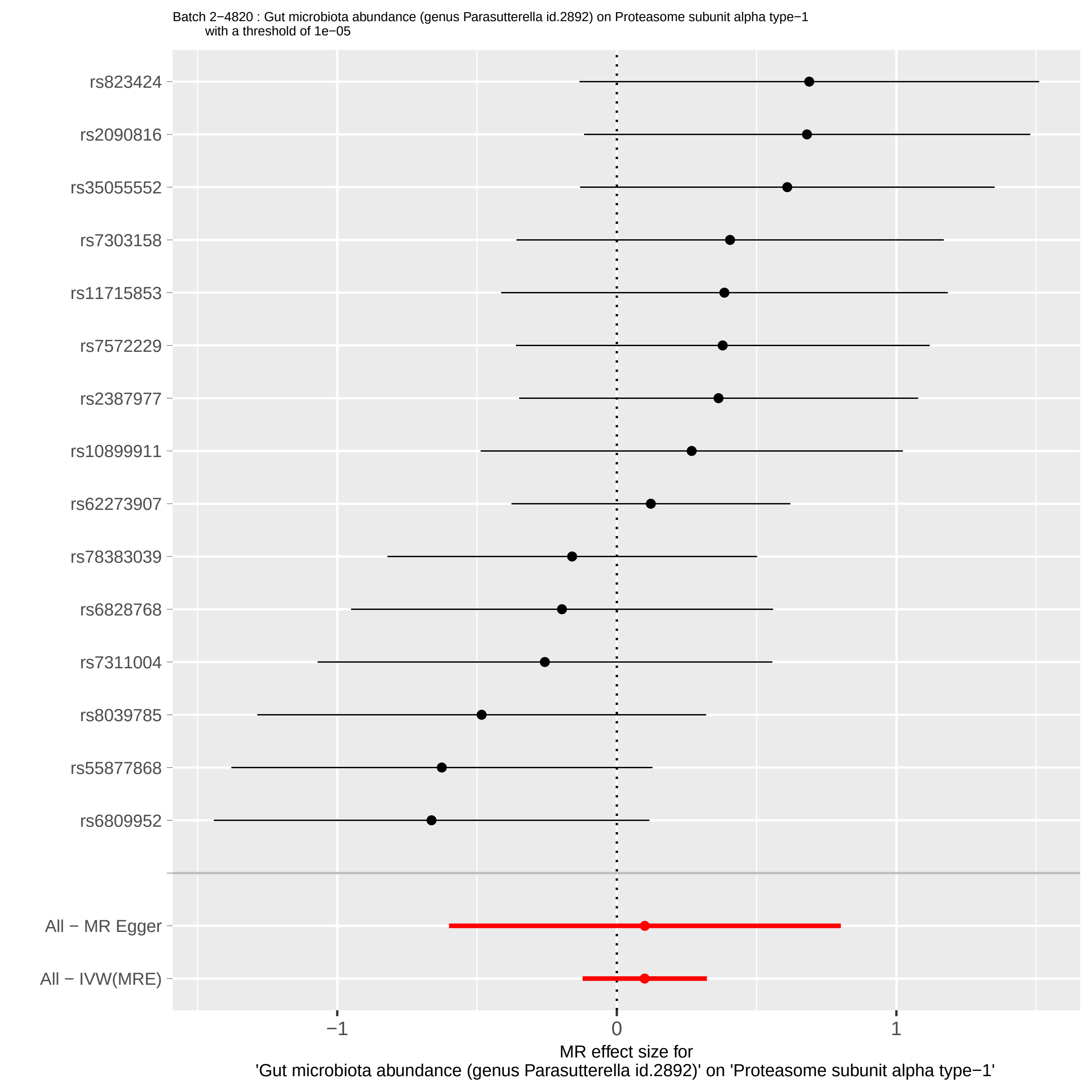
Batch 2−4820 : Gut microbiota abundance (genus Parasutterella id.2892) on Proteasome subunit alpha type−1
with a threshold of 1e−05
rs823424
rs2090816
rs35055552
rs7303158
rs11715853
rs7572229
rs2387977
rs10899911
rs62273907
rs78383039
rs6828768
rs7311004
rs8039785
rs55877868
rs6809952
All − MR Egger
All − IVW(MRE)
−1
0
1
MR effect size for
'Gut microbiota abundance (genus Parasutterella id.2892)' on 'Proteasome subunit alpha type−1'

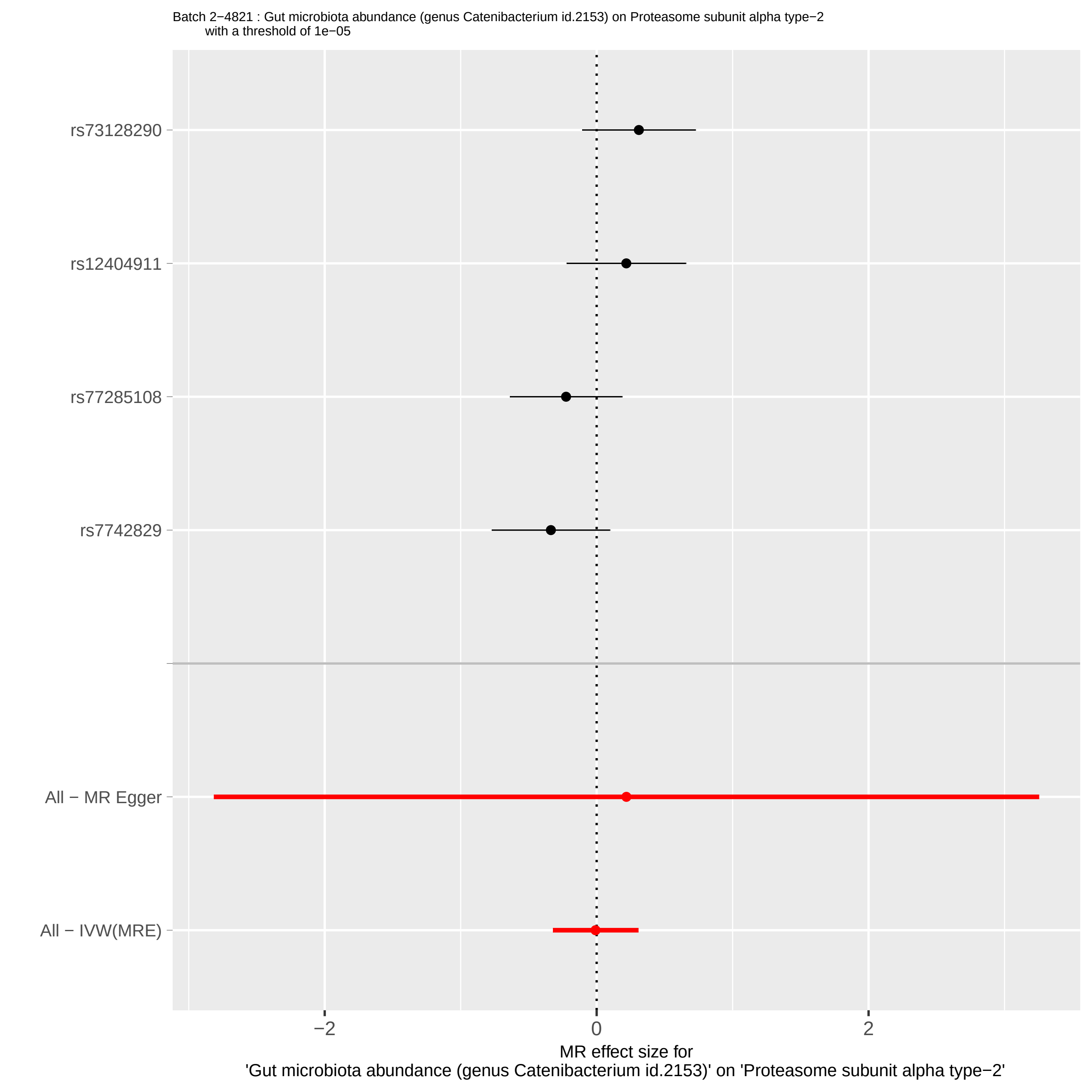
Batch 2−4821 : Gut microbiota abundance (genus Catenibacterium id.2153) on Proteasome subunit alpha type−2
with a threshold of 1e−05
rs73128290
rs12404911
rs77285108
rs7742829
All − MR Egger
All − IVW(MRE)
−2
0
2
MR effect size for
'Gut microbiota abundance (genus Catenibacterium id.2153)' on 'Proteasome subunit alpha type−2'

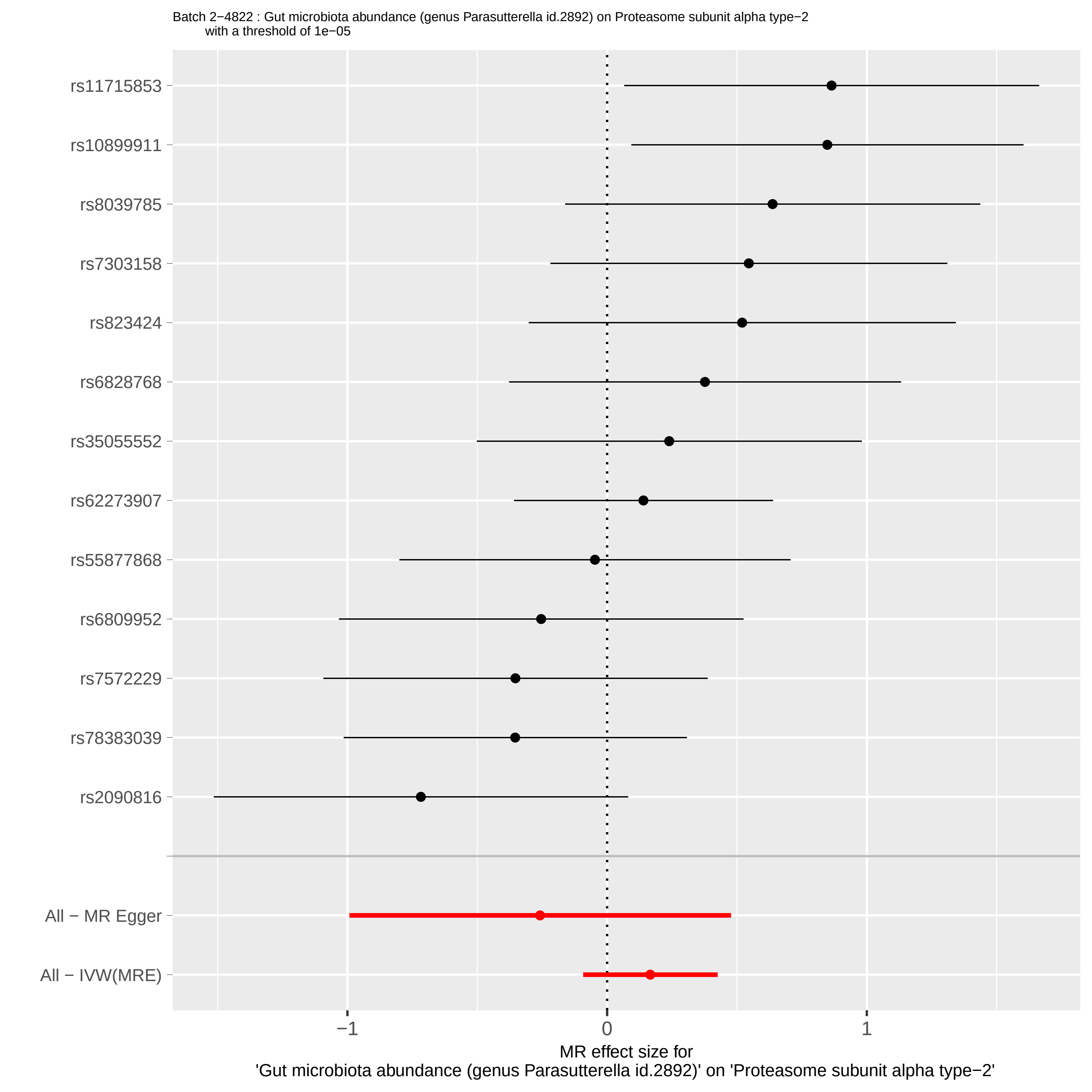

Batch 2−4822 : Gut microbiota abundance (genus Parasutterella id.2892) on Proteasome subunit alpha type−2
with a threshold of 1e−05
rs11715853
rs10899911
rs8039785
rs7303158
rs823424
rs6828768
rs35055552
rs62273907
rs55877868
rs6809952
rs7572229
rs78383039
rs2090816
All − MR Egger
All − IVW(MRE)
−1
0
1
MR effect size for
'Gut microbiota abundance (genus Parasutterella id.2892)' on 'Proteasome subunit alpha type−2'

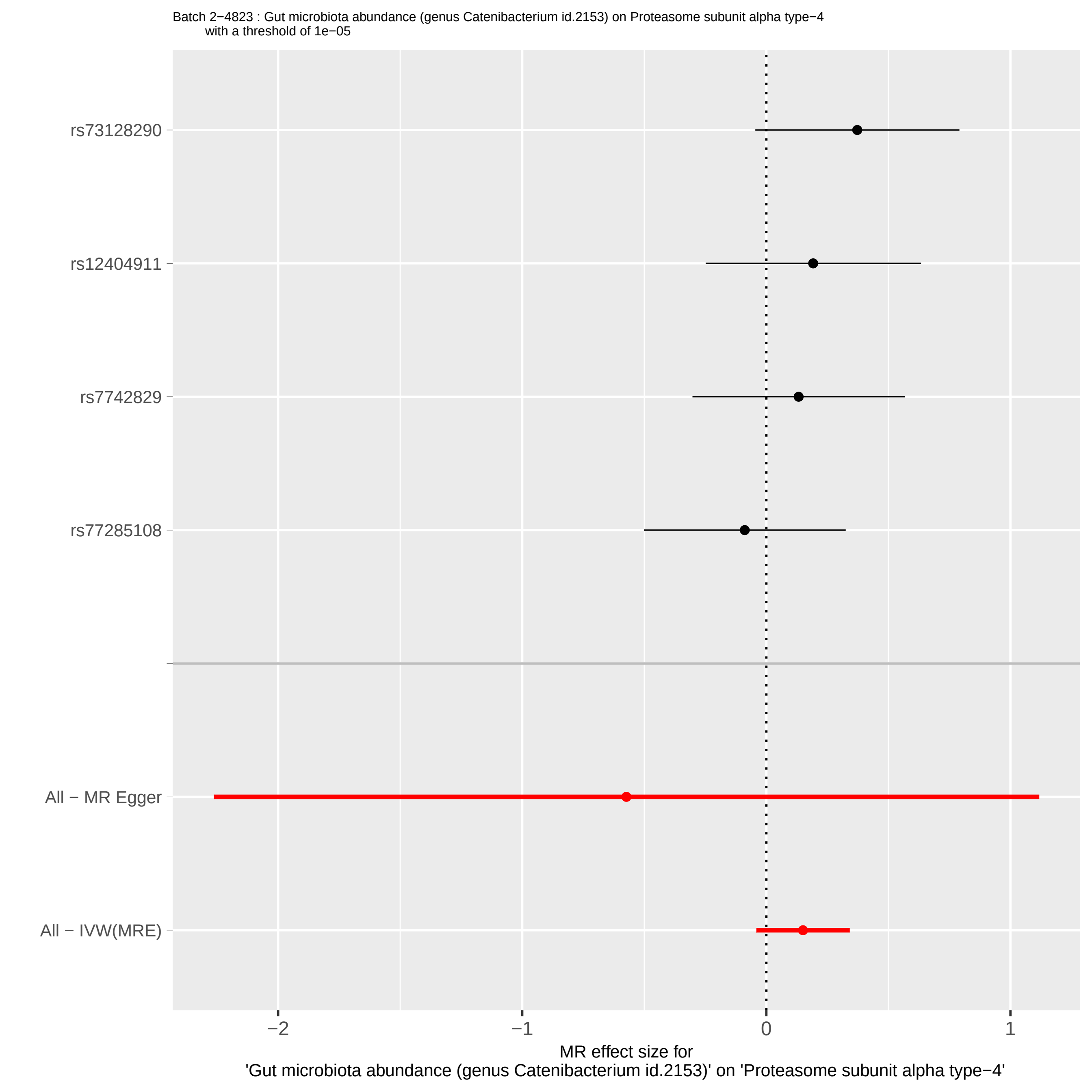

Batch 2−4823 : Gut microbiota abundance (genus Catenibacterium id.2153) on Proteasome subunit alpha type−4
with a threshold of 1e−05
rs73128290
rs12404911
rs7742829
rs77285108
All − MR Egger
All − IVW(MRE)
−2
−1
0
1
MR effect size for
'Gut microbiota abundance (genus Catenibacterium id.2153)' on 'Proteasome subunit alpha type−4'

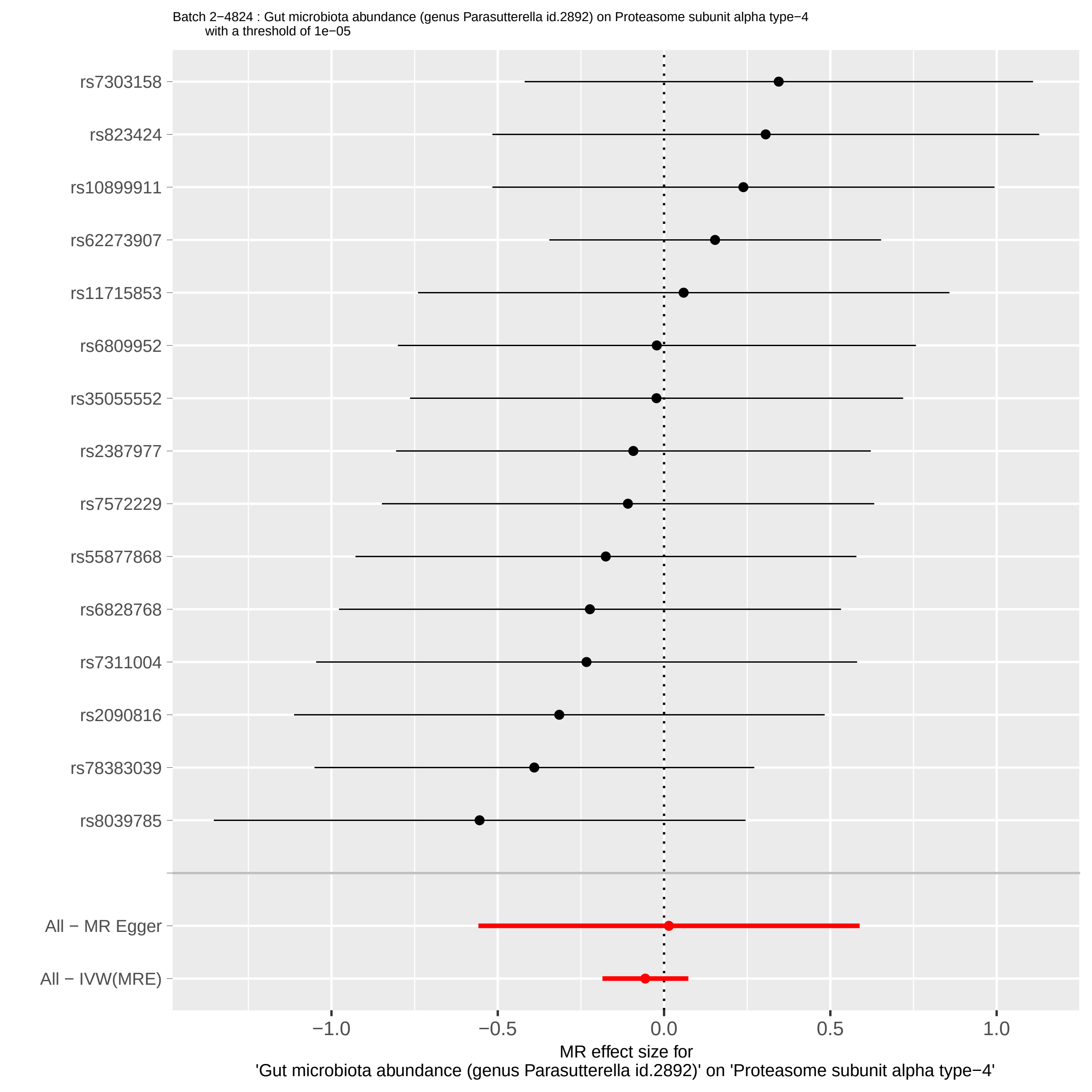

Batch 2−4824 : Gut microbiota abundance (genus Parasutterella id.2892) on Proteasome subunit alpha type−4
with a threshold of 1e−05
rs7303158
rs823424
rs10899911
rs62273907
rs11715853
rs6809952
rs35055552
rs2387977
rs7572229
rs55877868
rs6828768
rs7311004
rs2090816
rs78383039
rs8039785
All − MR Egger
All − IVW(MRE)
−1.0
−0.5
0.0
0.5
1.0
MR effect size for
'Gut microbiota abundance (genus Parasutterella id.2892)' on 'Proteasome subunit alpha type−4'

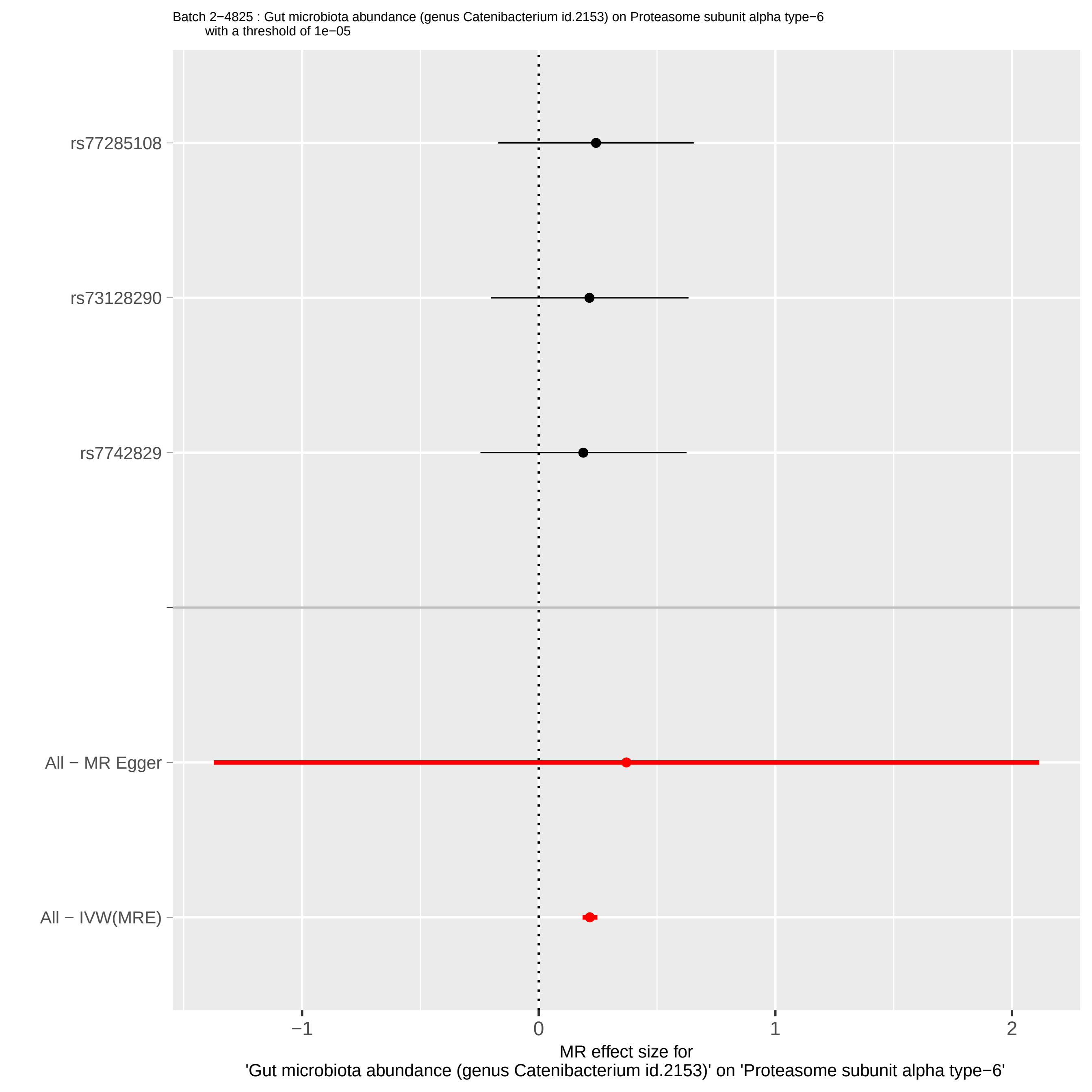
Batch 2−4825 : Gut microbiota abundance (genus Catenibacterium id.2153) on Proteasome subunit alpha type−6
with a threshold of 1e−05
rs77285108
rs73128290
rs7742829
All − MR Egger
All − IVW(MRE)
−1
0
1
2
MR effect size for
'Gut microbiota abundance (genus Catenibacterium id.2153)' on 'Proteasome subunit alpha type−6'

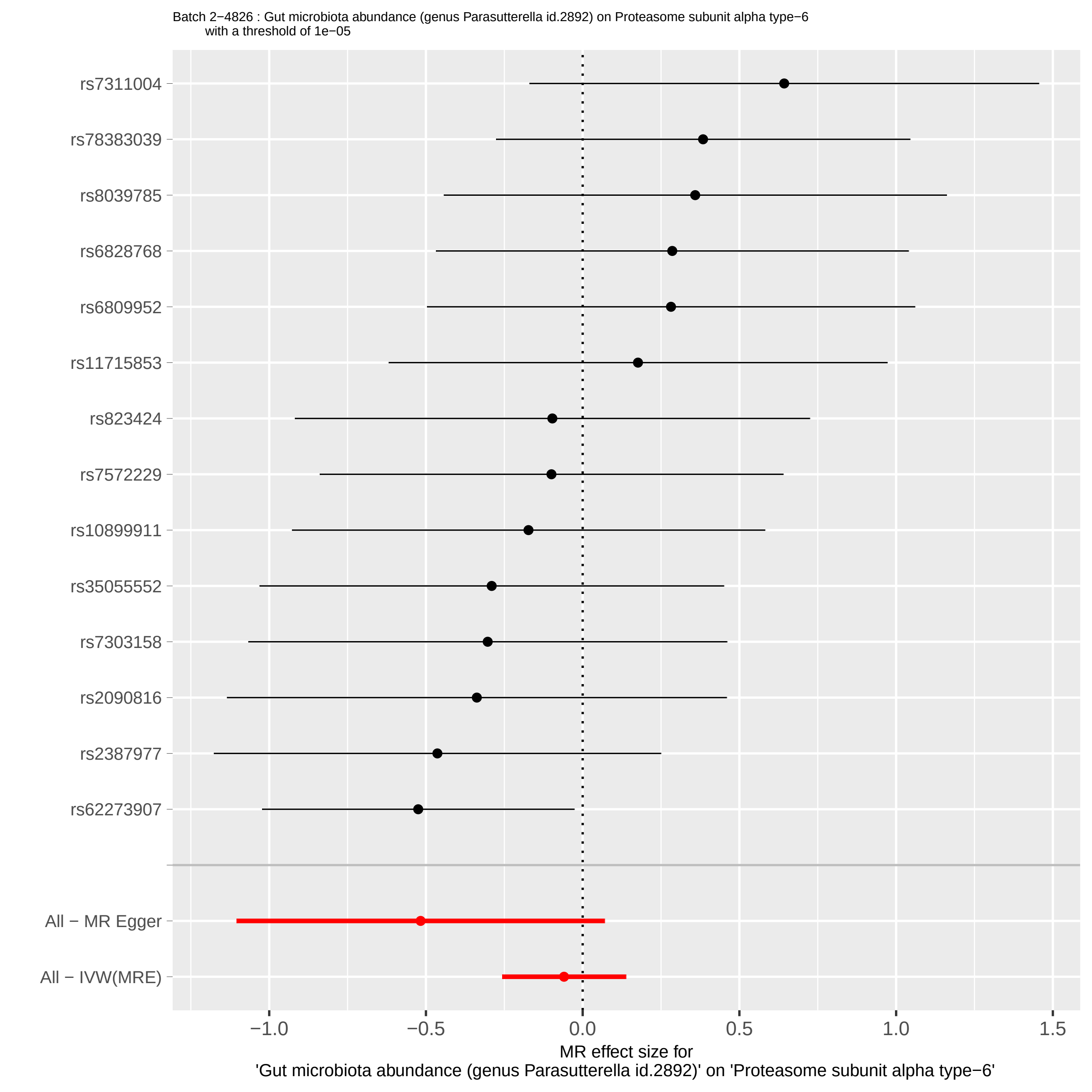
Batch 2−4826 : Gut microbiota abundance (genus Parasutterella id.2892) on Proteasome subunit alpha type−6
with a threshold of 1e−05
rs7311004
rs78383039
rs8039785
rs6828768
rs6809952
rs11715853
rs823424
rs7572229
rs10899911
rs35055552
rs7303158
rs2090816
rs2387977
rs62273907
All − MR Egger
All − IVW(MRE)
−1.0
−0.5
0.0
0.5
1.0
1.5
MR effect size for
'Gut microbiota abundance (genus Parasutterella id.2892)' on 'Proteasome subunit alpha type−6'

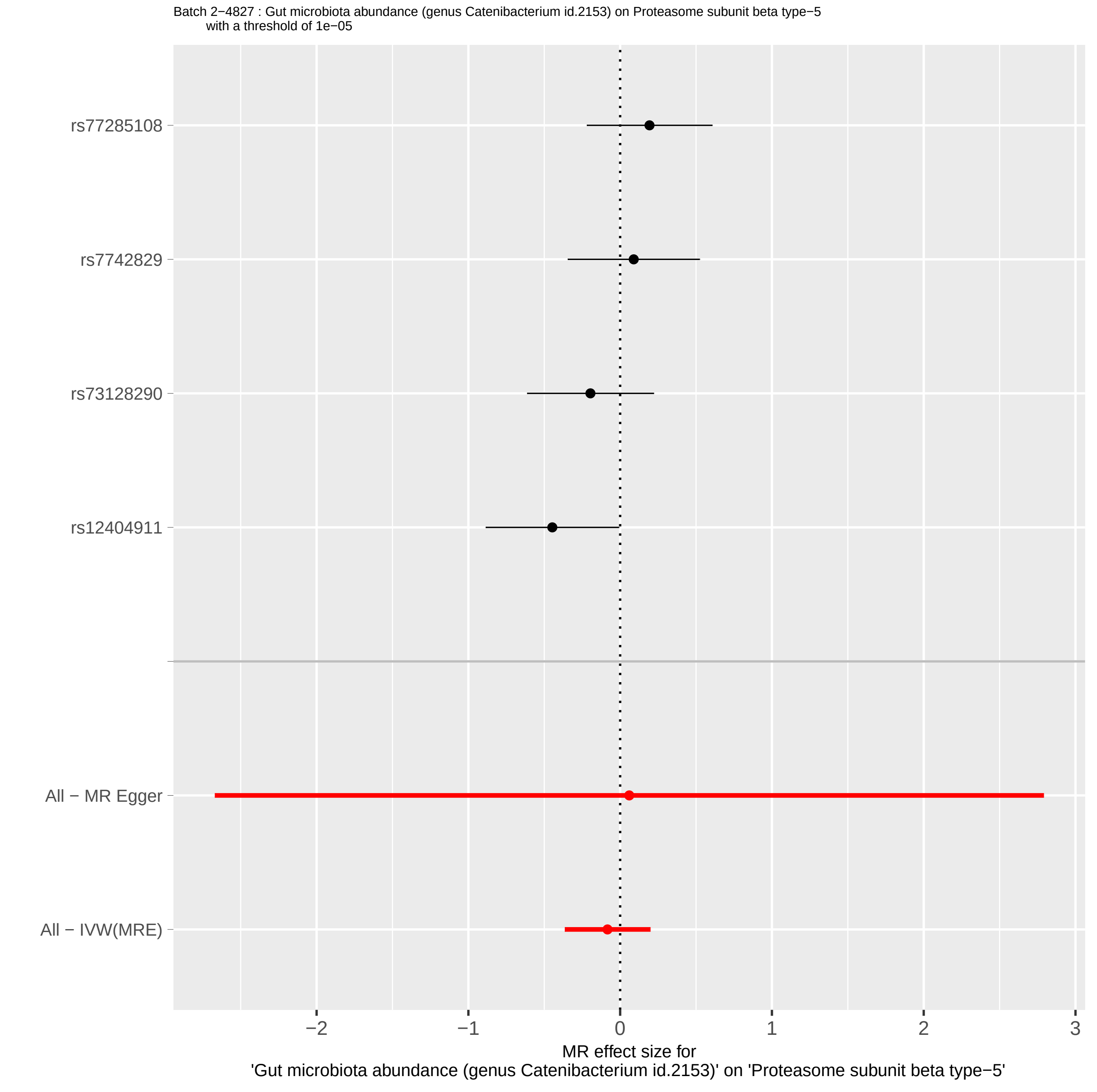
Batch 2−4827 : Gut microbiota abundance (genus Catenibacterium id.2153) on Proteasome subunit beta type−5
with a threshold of 1e−05
rs77285108
rs7742829
rs73128290
rs12404911
All − MR Egger
All − IVW(MRE)
−2
−1
0
1
2
3
MR effect size for
'Gut microbiota abundance (genus Catenibacterium id.2153)' on 'Proteasome subunit beta type−5'

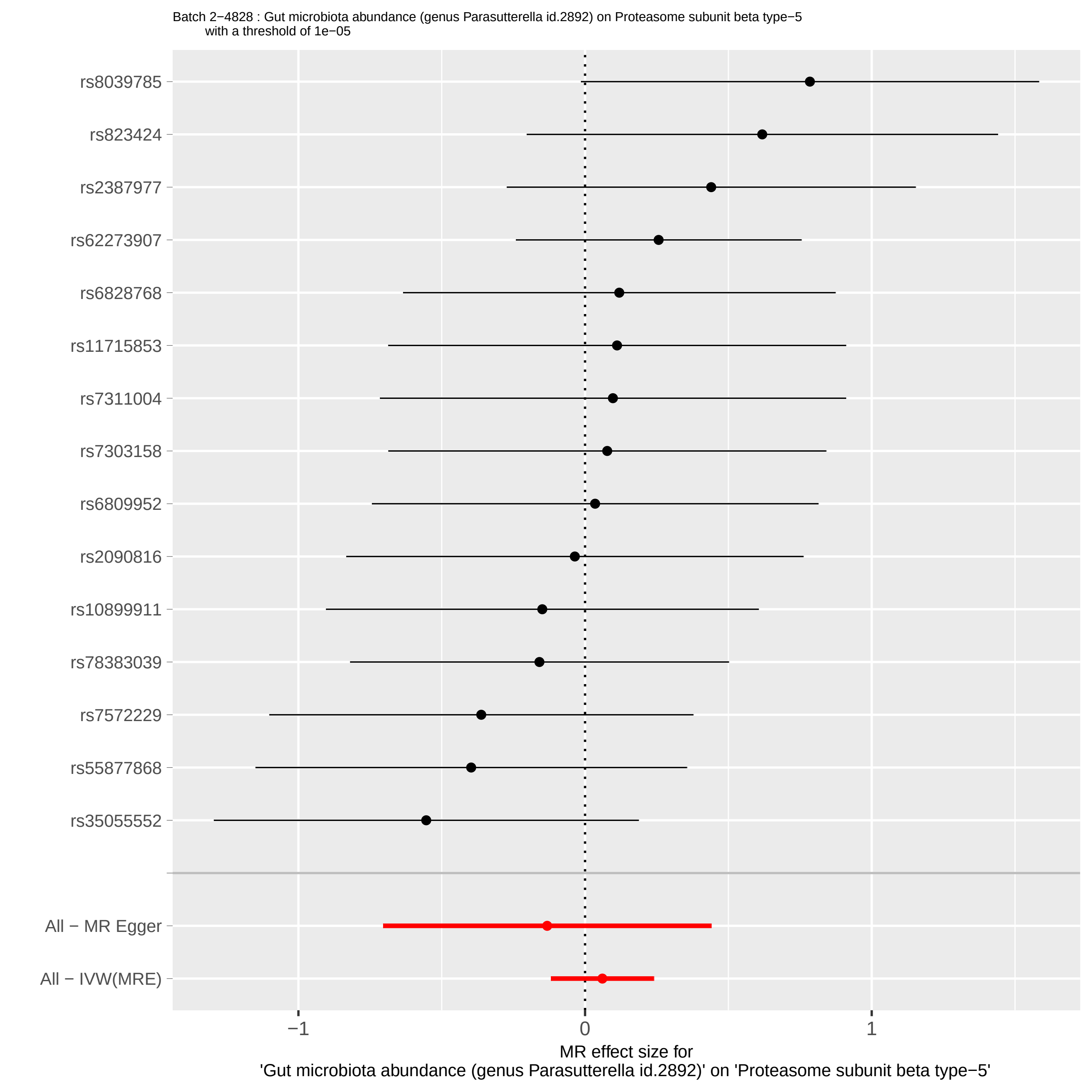

Batch 2−4828 : Gut microbiota abundance (genus Parasutterella id.2892) on Proteasome subunit beta type−5
with a threshold of 1e−05
rs8039785
rs823424
rs2387977
rs62273907
rs6828768
rs11715853
rs7311004
rs7303158
rs6809952
rs2090816
rs10899911
rs78383039
rs7572229
rs55877868
rs35055552
All − MR Egger
All − IVW(MRE)
−1
0
1
MR effect size for
'Gut microbiota abundance (genus Parasutterella id.2892)' on 'Proteasome subunit beta type−5'

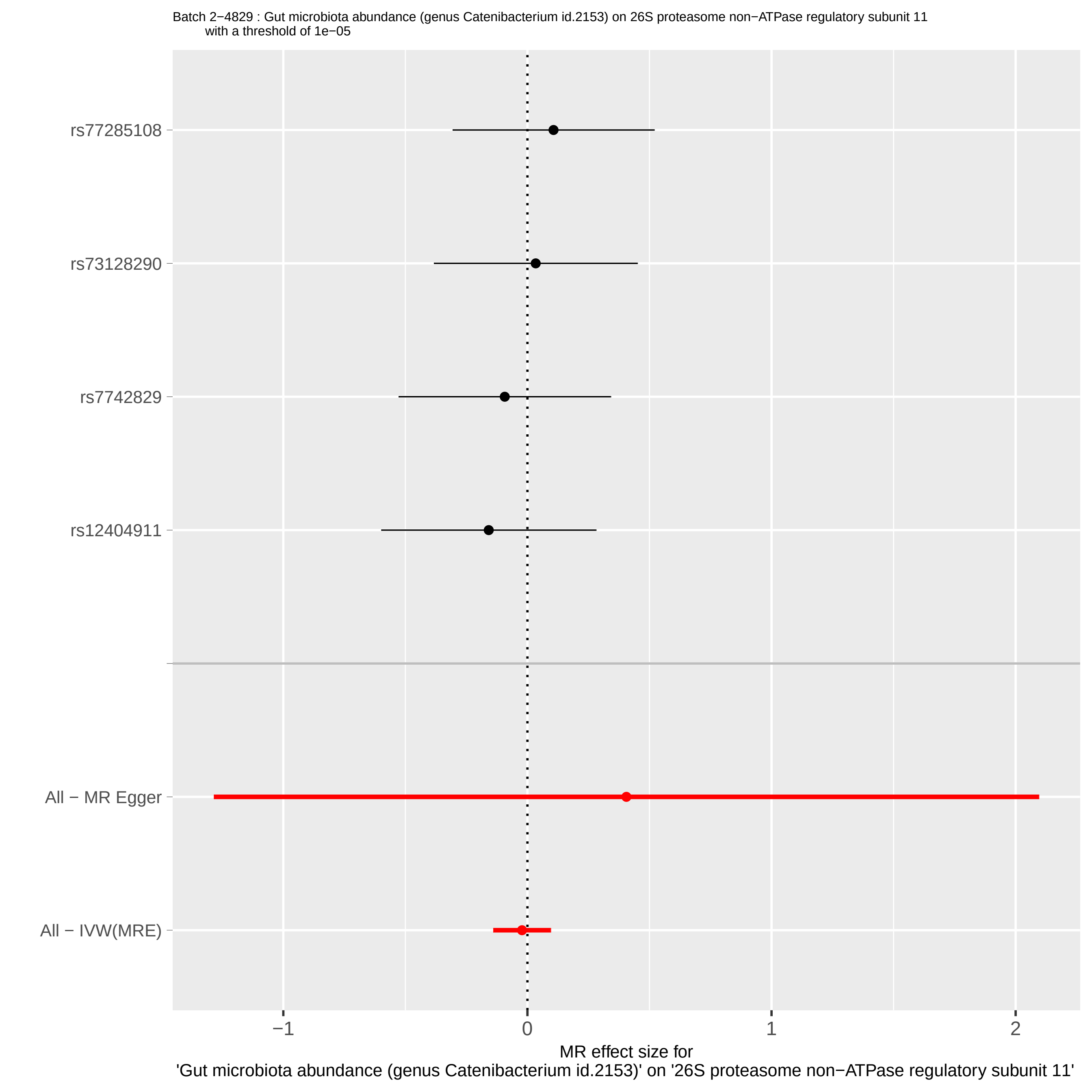

Batch 2−4829 : Gut microbiota abundance (genus Catenibacterium id.2153) on 26S proteasome non−ATPase regulatory subunit 11
with a threshold of 1e−05
rs77285108
rs73128290
rs7742829
rs12404911
All − MR Egger
All − IVW(MRE)
−1
0
1
2
MR effect size for
'Gut microbiota abundance (genus Catenibacterium id.2153)' on '26S proteasome non−ATPase regulatory subunit 11'

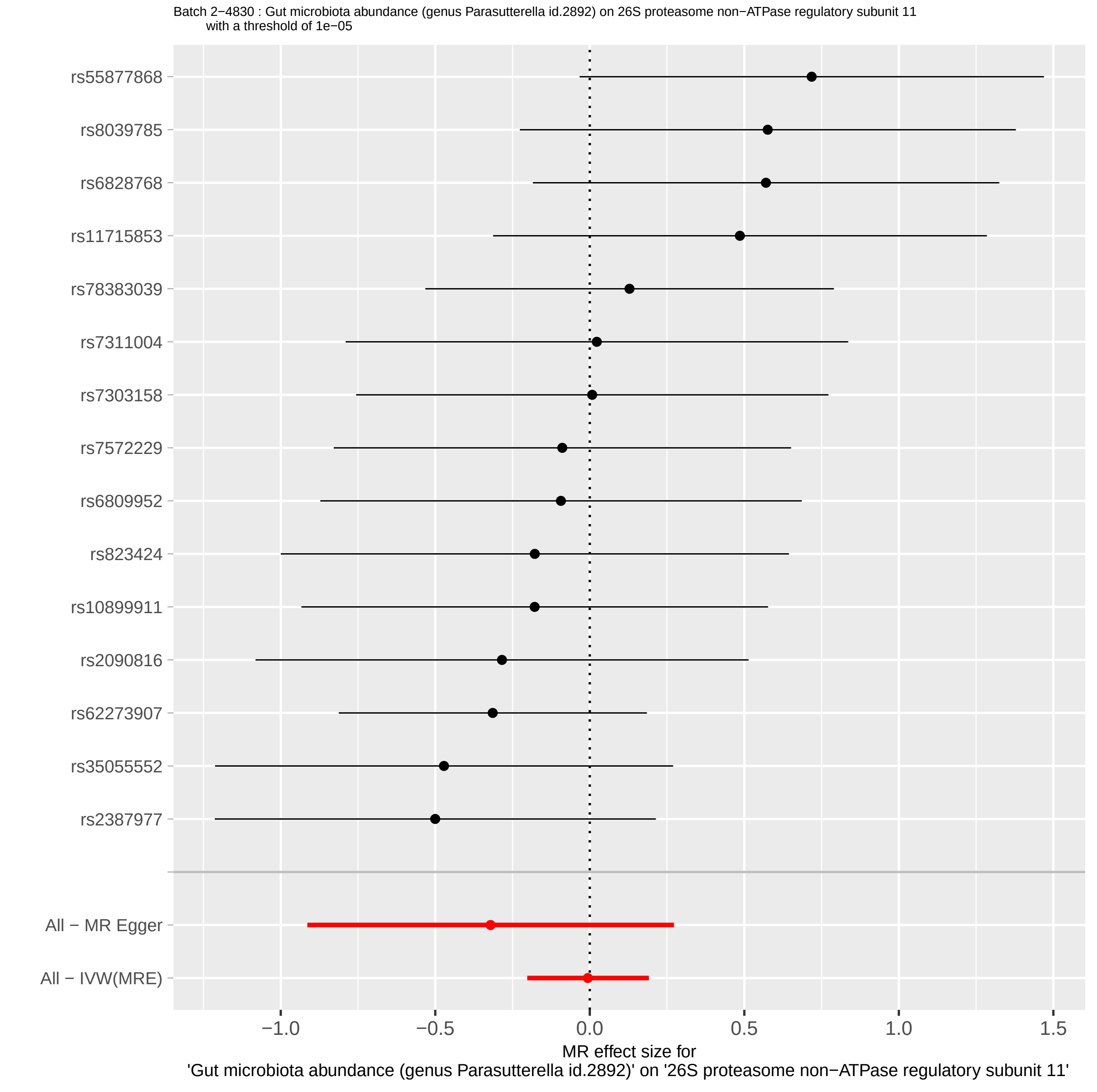

Batch 2−4830 : Gut microbiota abundance (genus Parasutterella id.2892) on 26S proteasome non−ATPase regulatory subunit 11
with a threshold of 1e−05
rs55877868
rs8039785
rs6828768
rs11715853
rs78383039
rs7311004
rs7303158
rs7572229
rs6809952
rs823424
rs10899911
rs2090816
rs62273907
rs35055552
rs2387977
All − MR Egger
All − IVW(MRE)
−1.0
−0.5
0.0
0.5
1.0
1.5
MR effect size for
'Gut microbiota abundance (genus Parasutterella id.2892)' on '26S proteasome non−ATPase regulatory subunit 11'

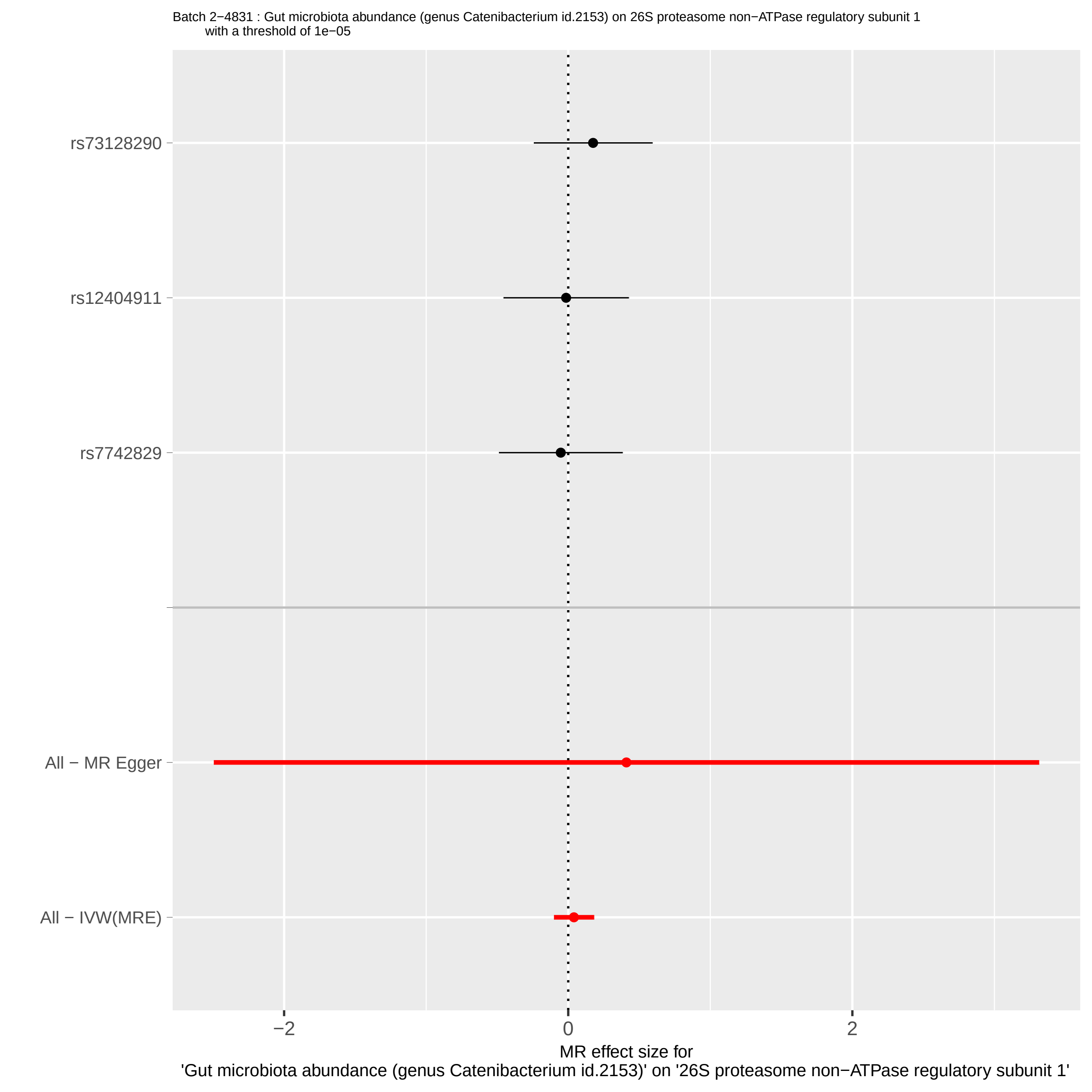

Batch 2−4831 : Gut microbiota abundance (genus Catenibacterium id.2153) on 26S proteasome non−ATPase regulatory subunit 1
with a threshold of 1e−05
rs73128290
rs12404911
rs7742829
All − MR Egger
All − IVW(MRE)
−2
0
2
MR effect size for
'Gut microbiota abundance (genus Catenibacterium id.2153)' on '26S proteasome non−ATPase regulatory subunit 1'

Batch 2−4832 : Gut microbiota abundance (genus Parasutterella id.2892) on 26S proteasome non−ATPase regulatory subunit 1
with a threshold of 1e−05

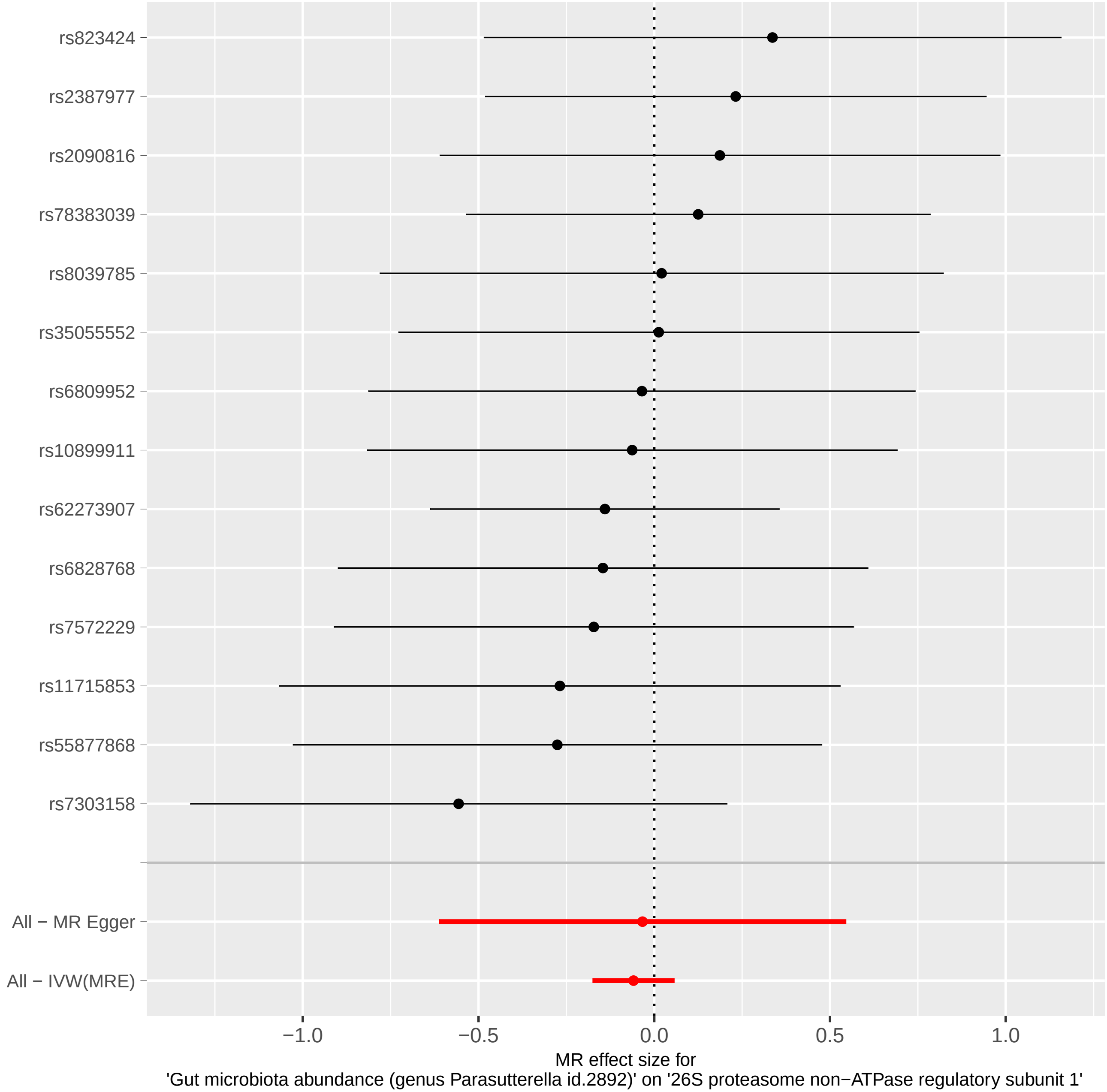

Batch 2−4833 : Gut microbiota abundance (genus Catenibacterium id.2153) on 26S proteasome non−ATPase regulatory subunit 4
with a threshold of 1e−05

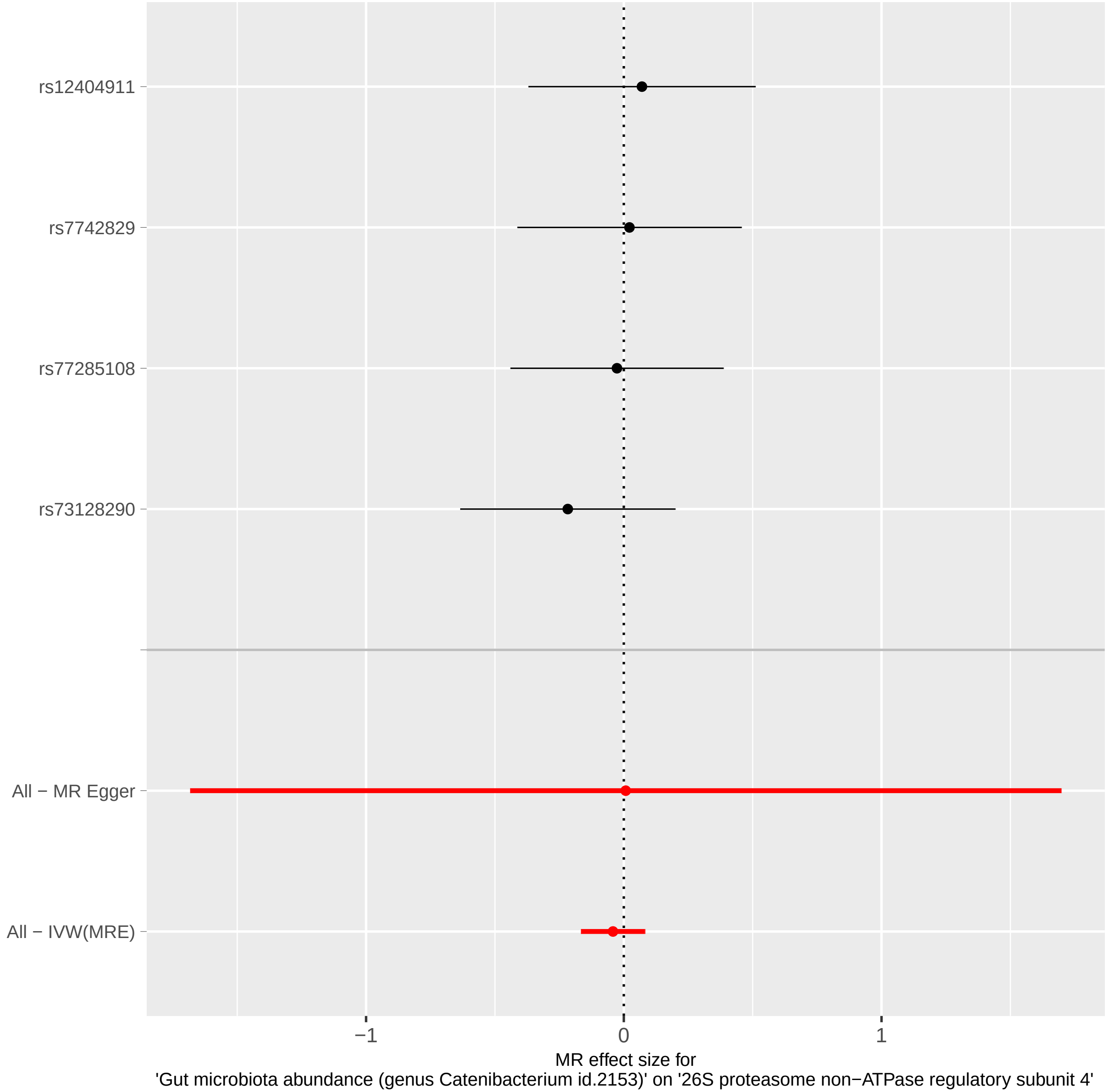

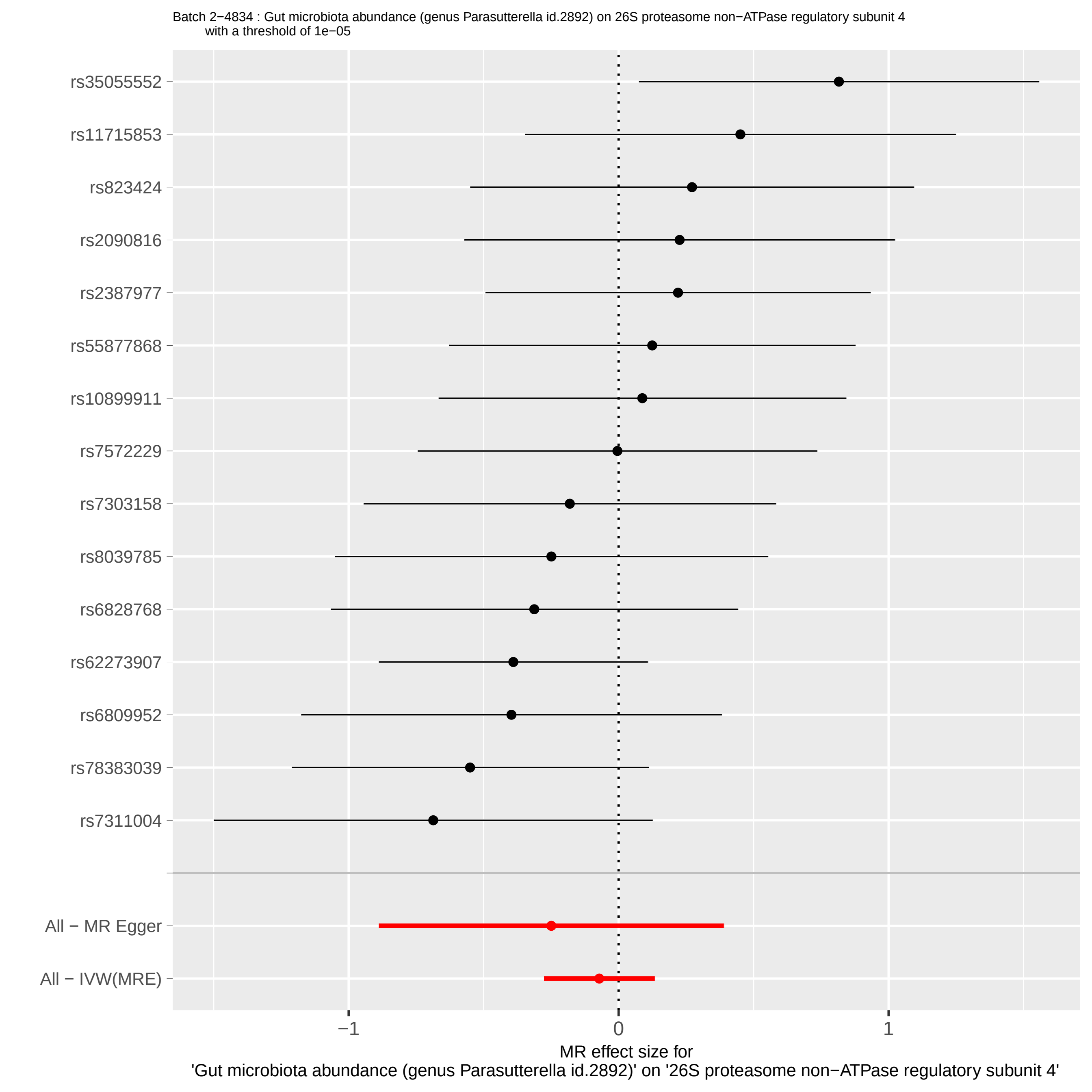

Batch 2−4834 : Gut microbiota abundance (genus Parasutterella id.2892) on 26S proteasome non−ATPase regulatory subunit 4
with a threshold of 1e−05
rs35055552
rs11715853
rs823424
rs2090816
rs2387977
rs55877868
rs10899911
rs7572229
rs7303158
rs8039785
rs6828768
rs62273907
rs6809952
rs78383039
rs7311004
All − MR Egger
All − IVW(MRE)
−1
0
1
MR effect size for
'Gut microbiota abundance (genus Parasutterella id.2892)' on '26S proteasome non−ATPase regulatory subunit 4'

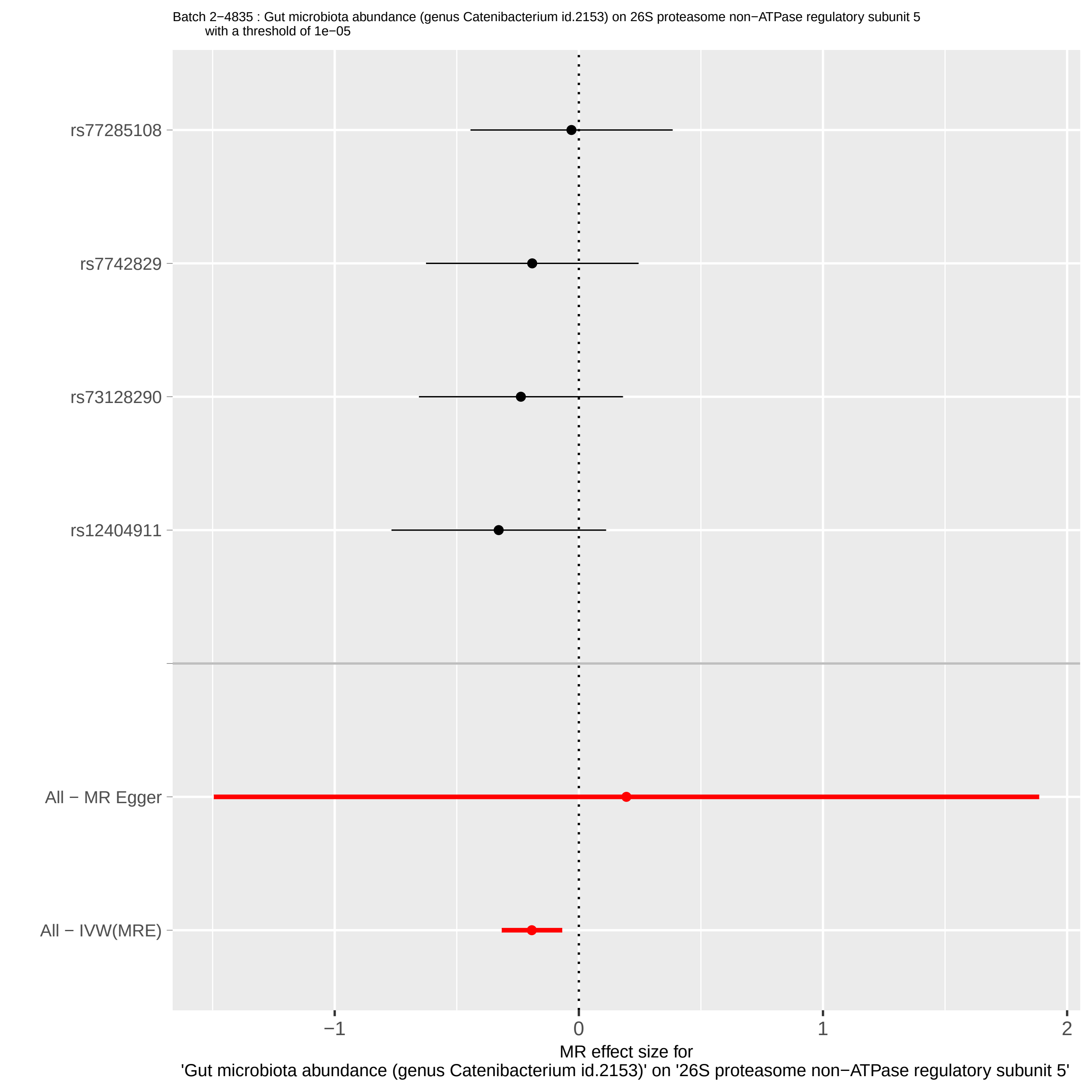
Batch 2−4835 : Gut microbiota abundance (genus Catenibacterium id.2153) on 26S proteasome non−ATPase regulatory subunit 5
with a threshold of 1e−05
rs77285108
rs7742829
rs73128290
rs12404911
All − MR Egger
All − IVW(MRE)
−1
0
1
2
MR effect size for
'Gut microbiota abundance (genus Catenibacterium id.2153)' on '26S proteasome non−ATPase regulatory subunit 5'

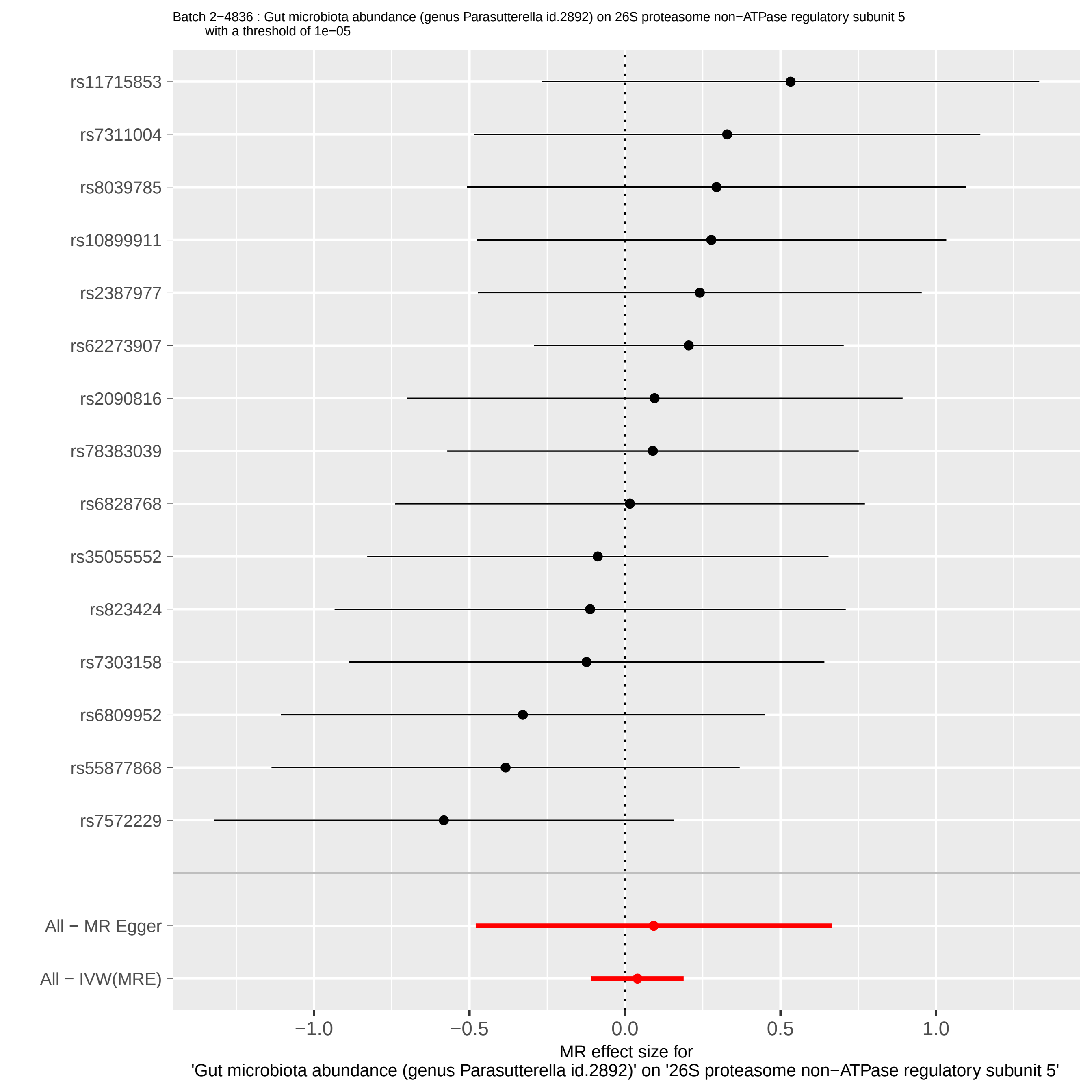

Batch 2−4836 : Gut microbiota abundance (genus Parasutterella id.2892) on 26S proteasome non−ATPase regulatory subunit 5
with a threshold of 1e−05
rs11715853
rs7311004
rs8039785
rs10899911
rs2387977
rs62273907
rs2090816
rs78383039
rs6828768
rs35055552
rs823424
rs7303158
rs6809952
rs55877868
rs7572229
All − MR Egger
All − IVW(MRE)
−1.0
−0.5
0.0
0.5
1.0
MR effect size for
'Gut microbiota abundance (genus Parasutterella id.2892)' on '26S proteasome non−ATPase regulatory subunit 5'

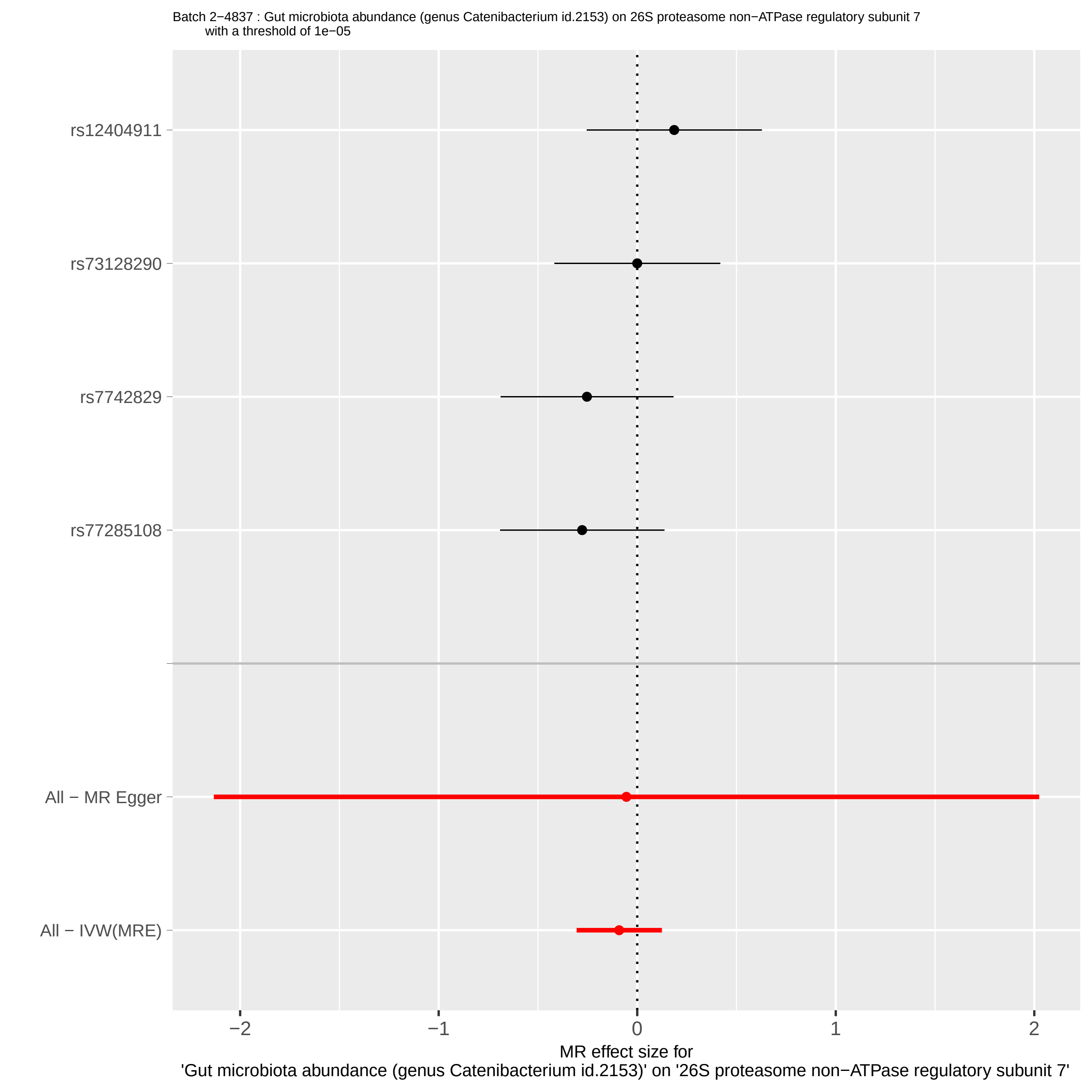
Batch 2−4837 : Gut microbiota abundance (genus Catenibacterium id.2153) on 26S proteasome non−ATPase regulatory subunit 7
with a threshold of 1e−05
rs12404911
rs73128290
rs7742829
rs77285108
All − MR Egger
All − IVW(MRE)
−2
−1
0
1
2
MR effect size for
'Gut microbiota abundance (genus Catenibacterium id.2153)' on '26S proteasome non−ATPase regulatory subunit 7'

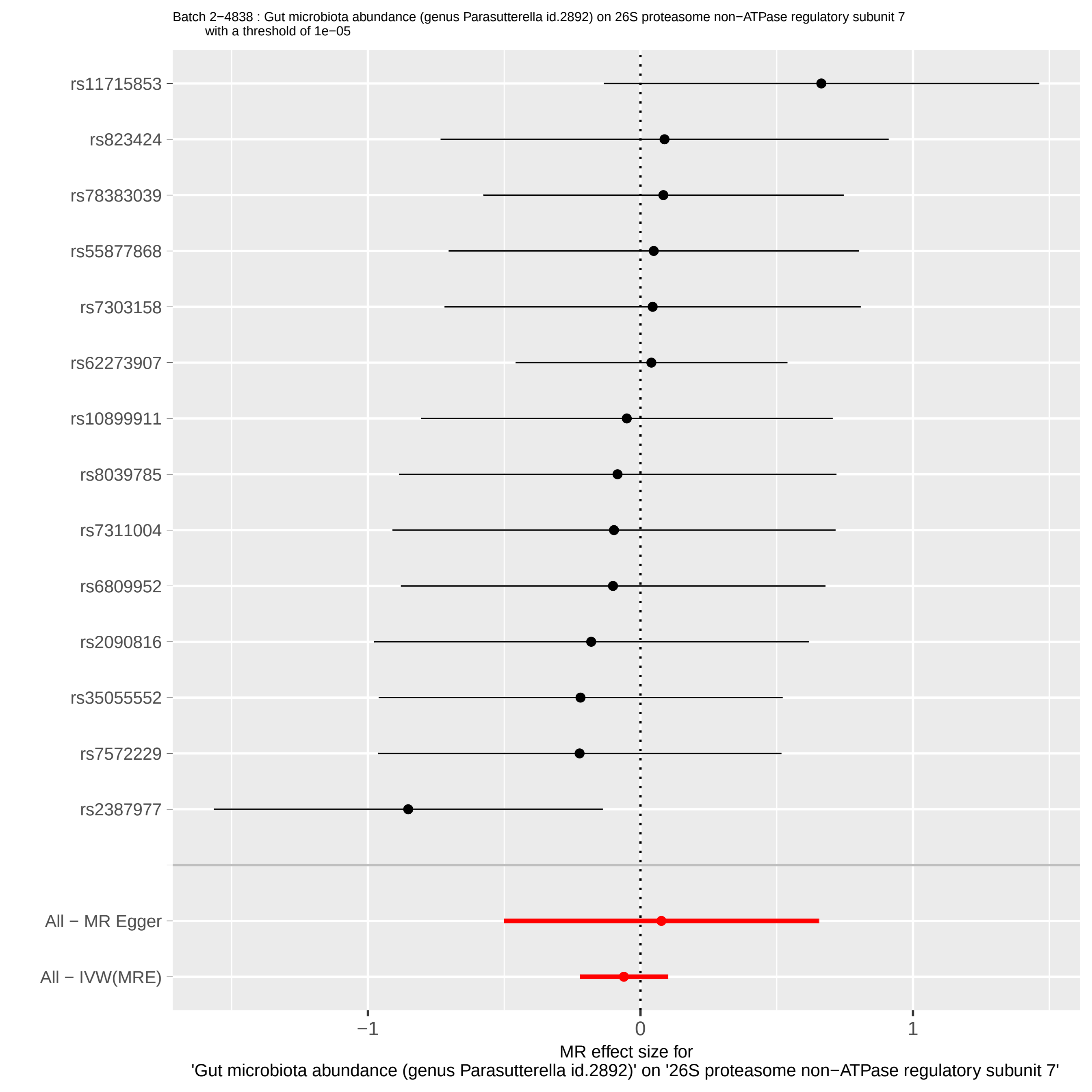
Batch 2−4838 : Gut microbiota abundance (genus Parasutterella id.2892) on 26S proteasome non−ATPase regulatory subunit 7
with a threshold of 1e−05
rs11715853
rs823424
rs78383039
rs55877868
rs7303158
rs62273907
rs10899911
rs8039785
rs7311004
rs6809952
rs2090816
rs35055552
rs7572229
rs2387977
All − MR Egger
All − IVW(MRE)
−1
0
1
MR effect size for
'Gut microbiota abundance (genus Parasutterella id.2892)' on '26S proteasome non−ATPase regulatory subunit 7'

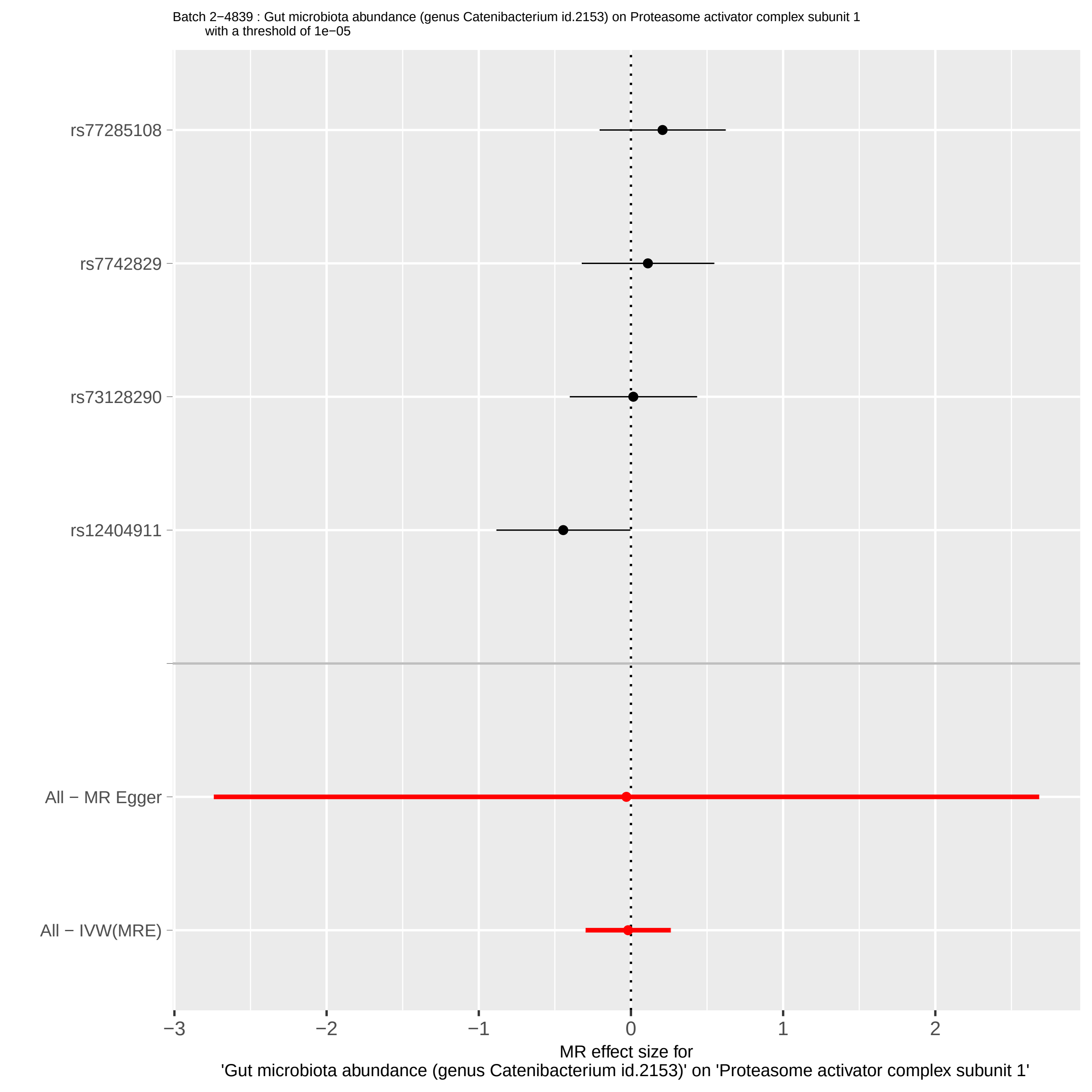
Batch 2−4839 : Gut microbiota abundance (genus Catenibacterium id.2153) on Proteasome activator complex subunit 1
with a threshold of 1e−05
rs77285108
rs7742829
rs73128290
rs12404911
All − MR Egger
All − IVW(MRE)
−3
−2
−1
0
1
2
MR effect size for
'Gut microbiota abundance (genus Catenibacterium id.2153)' on 'Proteasome activator complex subunit 1'

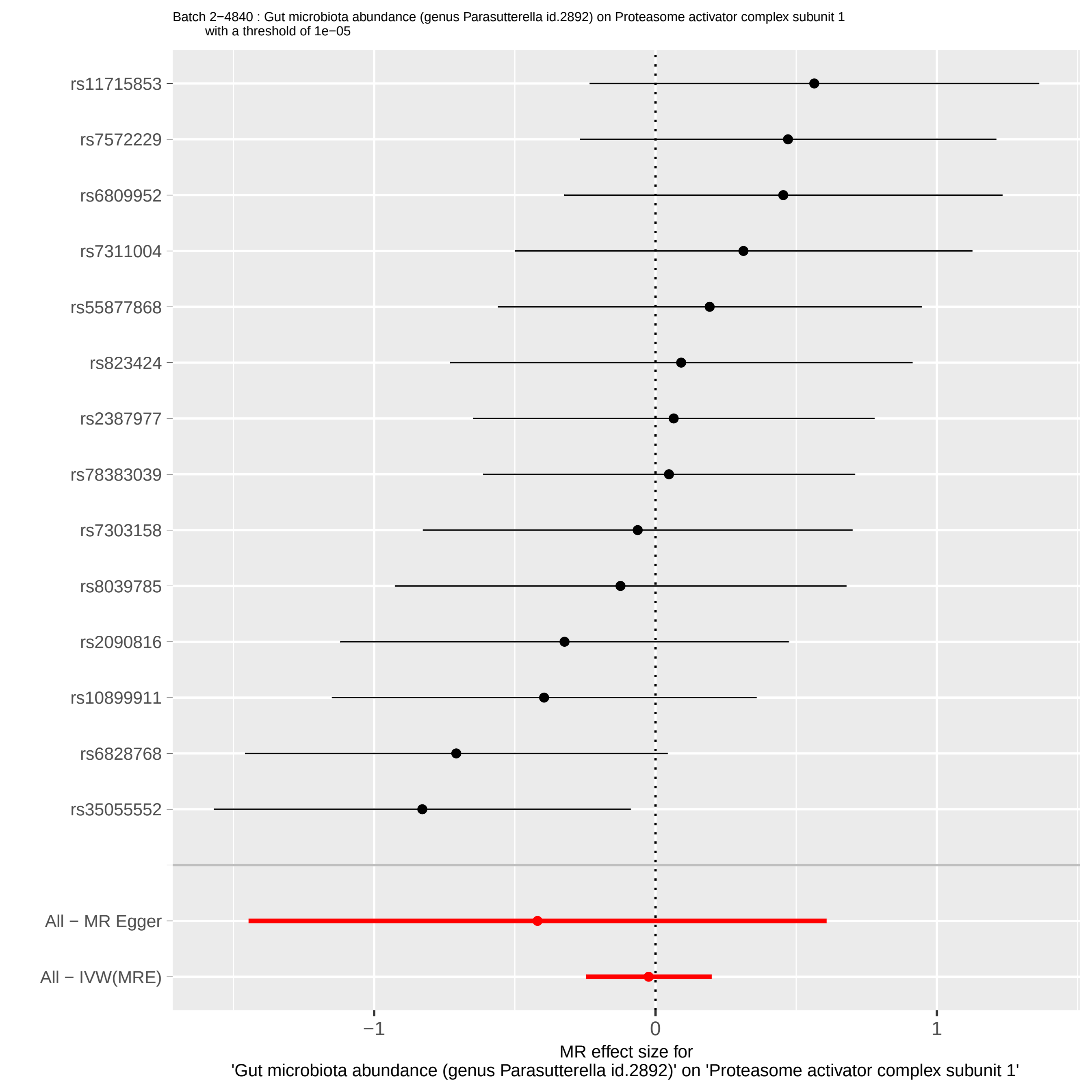
Batch 2−4840 : Gut microbiota abundance (genus Parasutterella id.2892) on Proteasome activator complex subunit 1
with a threshold of 1e−05
rs11715853
rs7572229
rs6809952
rs7311004
rs55877868
rs823424
rs2387977
rs78383039
rs7303158
rs8039785
rs2090816
rs10899911
rs6828768
rs35055552
All − MR Egger
All − IVW(MRE)
−1
0
1
MR effect size for
'Gut microbiota abundance (genus Parasutterella id.2892)' on 'Proteasome activator complex subunit 1'

Batch 2−4841 : Gut microbiota abundance (genus Catenibacterium id.2153) on Proteasome activator complex subunit 3
with a threshold of 1e−05

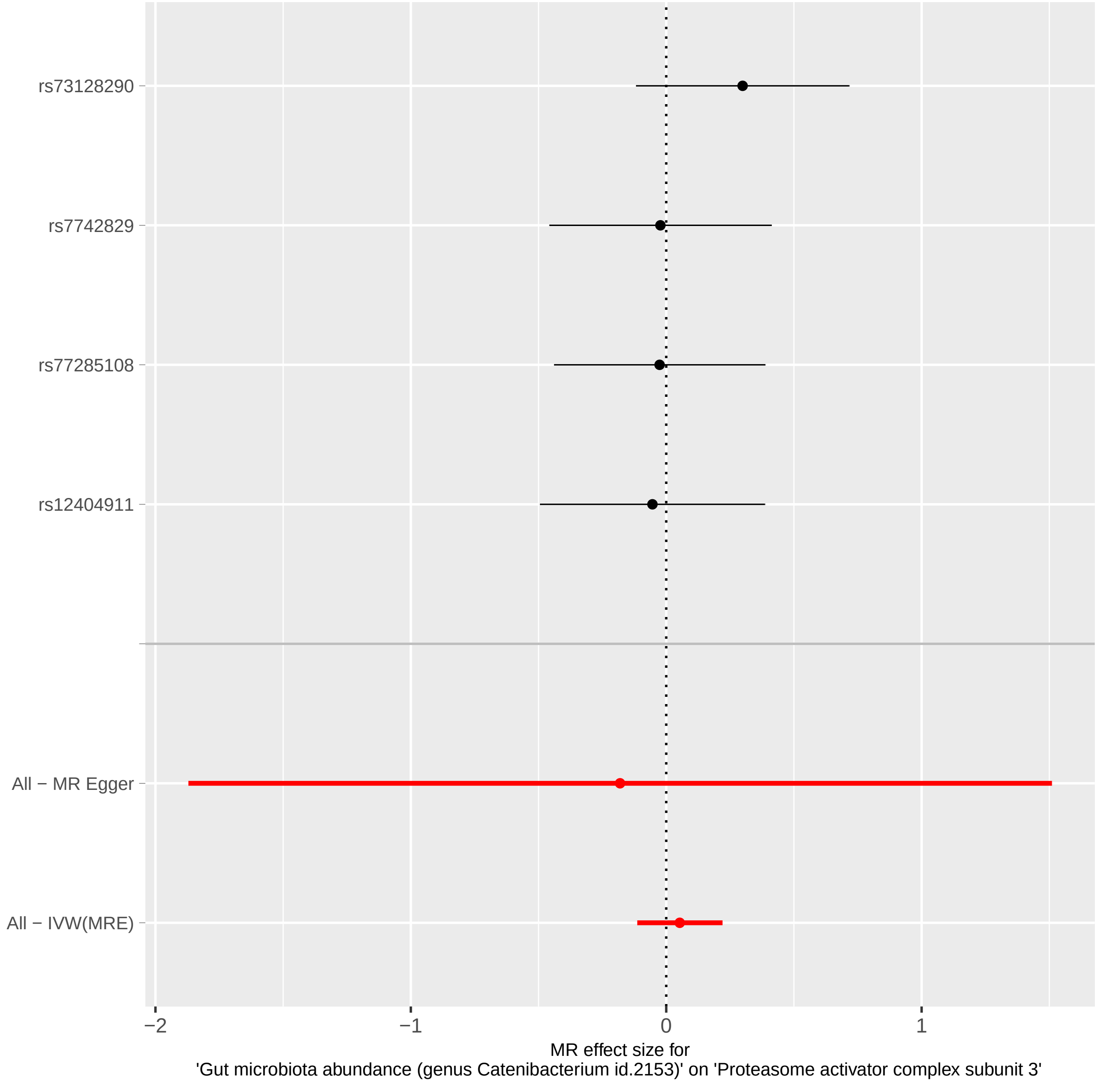

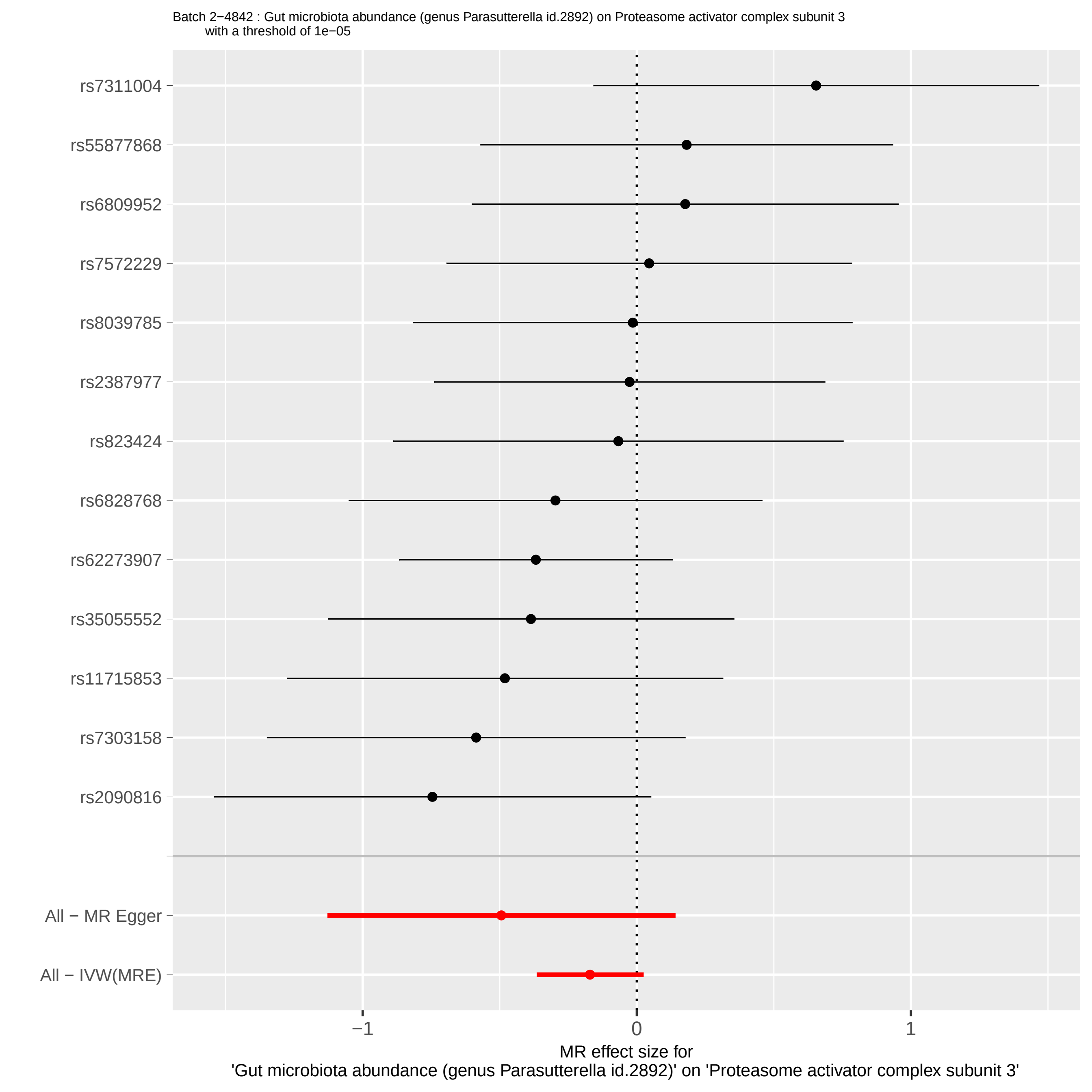
Batch 2−4842 : Gut microbiota abundance (genus Parasutterella id.2892) on Proteasome activator complex subunit 3
with a threshold of 1e−05
rs7311004
rs55877868
rs6809952
rs7572229
rs8039785
rs2387977
rs823424
rs6828768
rs62273907
rs35055552
rs11715853
rs7303158
rs2090816
All − MR Egger
All − IVW(MRE)
−1
0
1
MR effect size for
'Gut microbiota abundance (genus Parasutterella id.2892)' on 'Proteasome activator complex subunit 3'

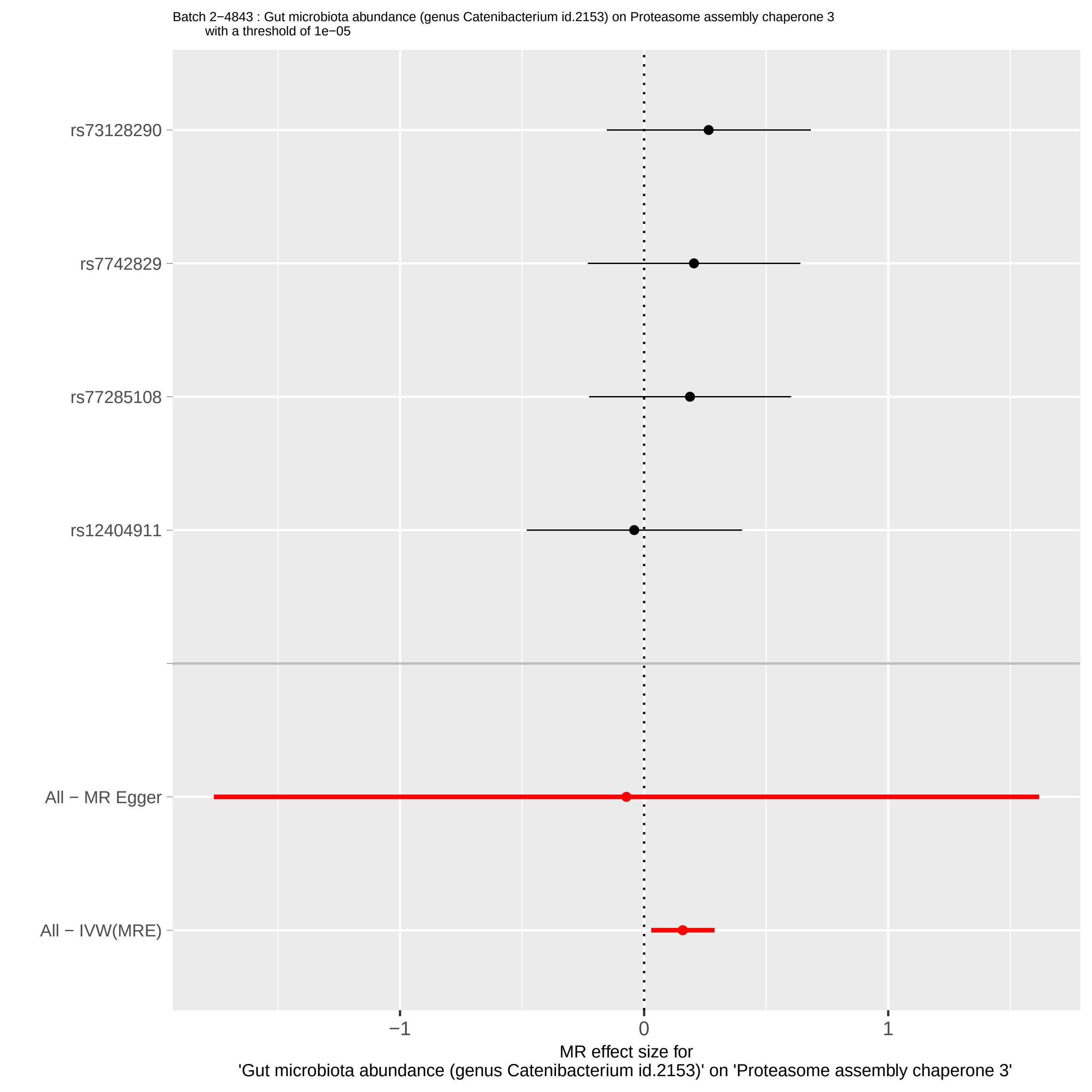

Batch 2−4843 : Gut microbiota abundance (genus Catenibacterium id.2153) on Proteasome assembly chaperone 3
with a threshold of 1e−05
rs73128290
rs7742829
rs77285108
rs12404911
All − MR Egger
All − IVW(MRE)
−1
0
1
MR effect size for
'Gut microbiota abundance (genus Catenibacterium id.2153)' on 'Proteasome assembly chaperone 3'

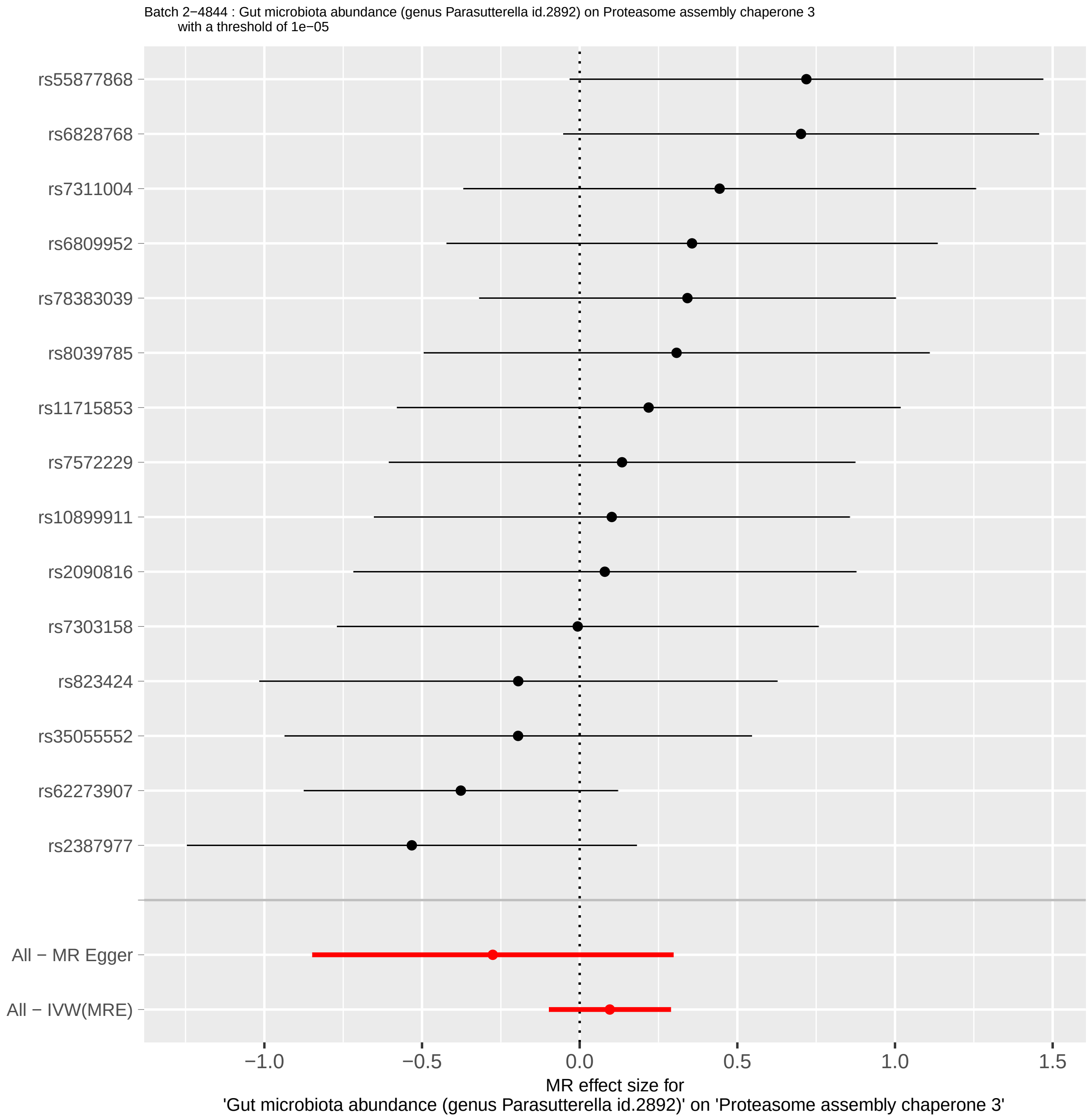
Batch 2−4844 : Gut microbiota abundance (genus Parasutterella id.2892) on Proteasome assembly chaperone 3
with a threshold of 1e−05
rs55877868
rs6828768
rs7311004
rs6809952
rs78383039
rs8039785
rs11715853
rs7572229
rs10899911
rs2090816
rs7303158
rs823424
rs35055552
rs62273907
rs2387977
All − MR Egger
All − IVW(MRE)
−1.0
−0.5
0.0
0.5
1.0
1.5
MR effect size for
'Gut microbiota abundance (genus Parasutterella id.2892)' on 'Proteasome assembly chaperone 3'

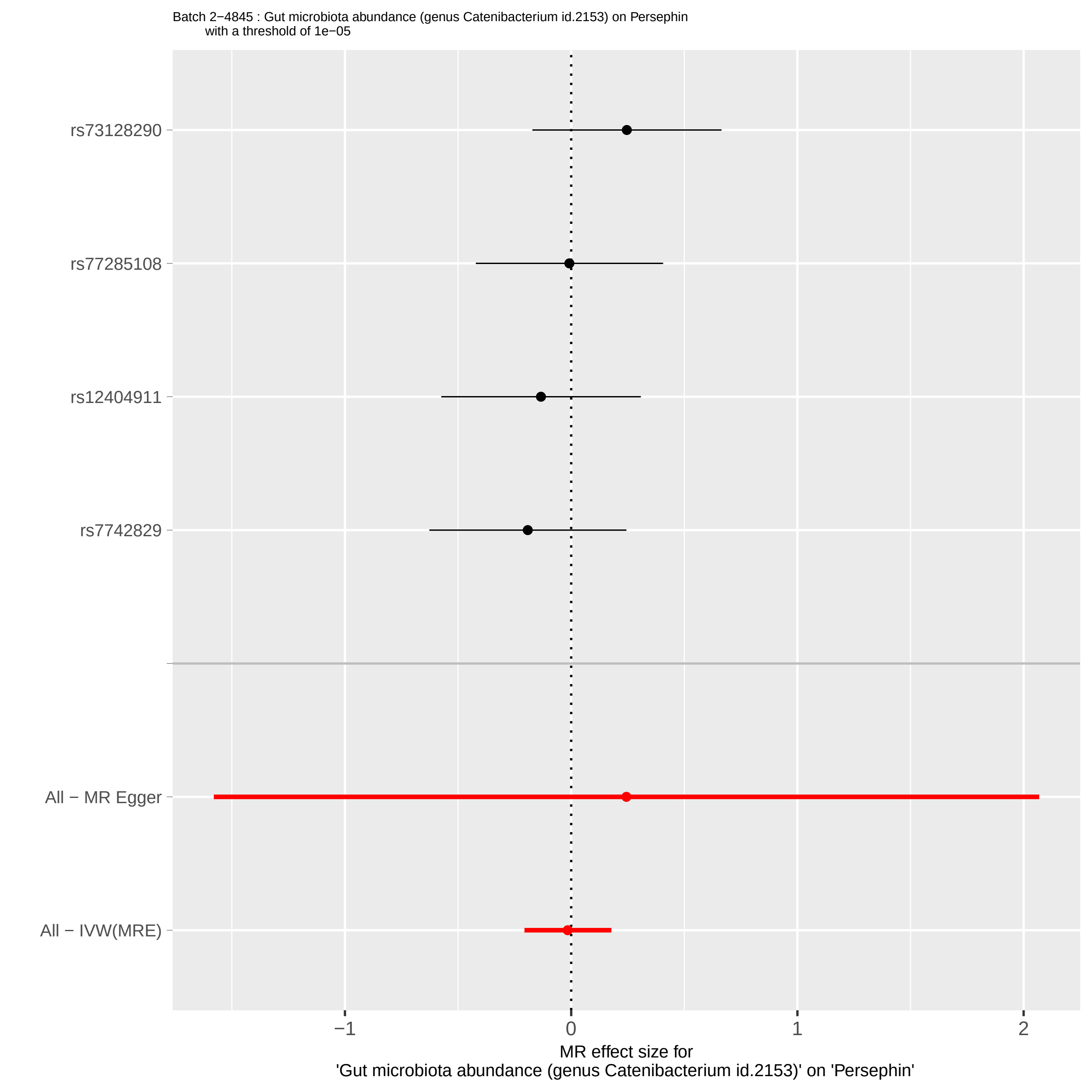

Batch 2−4845 : Gut microbiota abundance (genus Catenibacterium id.2153) on Persephin
with a threshold of 1e−05
rs73128290
rs77285108
rs12404911
rs7742829
All − MR Egger
All − IVW(MRE)
−1
0
1
2
MR effect size for
'Gut microbiota abundance (genus Catenibacterium id.2153)' on 'Persephin'

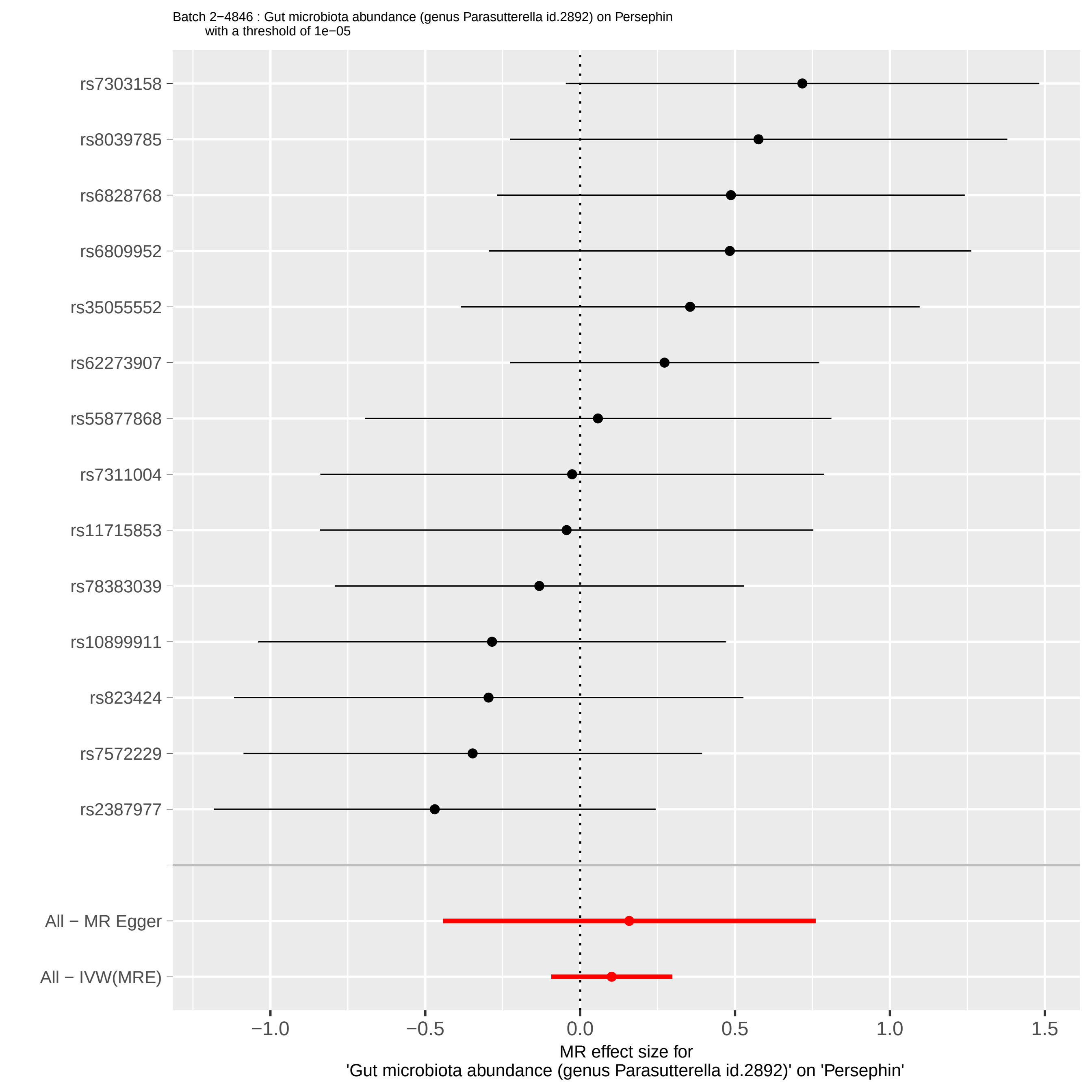

Batch 2−4846 : Gut microbiota abundance (genus Parasutterella id.2892) on Persephin
with a threshold of 1e−05
rs7303158
rs8039785
rs6828768
rs6809952
rs35055552
rs62273907
rs55877868
rs7311004
rs11715853
rs78383039
rs10899911
rs823424
rs7572229
rs2387977
All − MR Egger
All − IVW(MRE)
−1.0
−0.5
0.0
0.5
1.0
1.5
MR effect size for
'Gut microbiota abundance (genus Parasutterella id.2892)' on 'Persephin'

Batch 2−4847 : Gut microbiota abundance (genus Catenibacterium id.2153) on Patched domain−containing protein 3
with a threshold of 1e−05

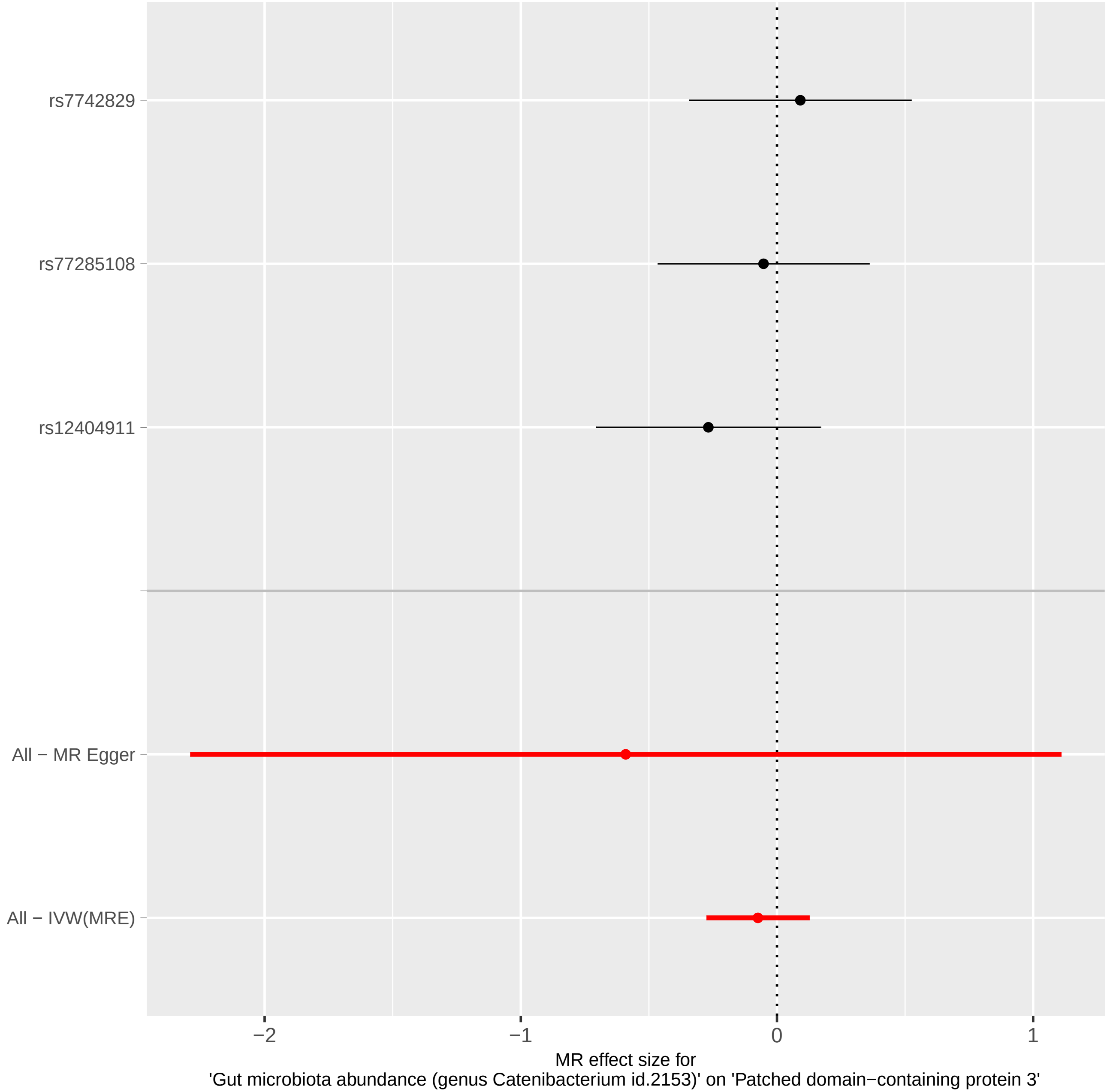

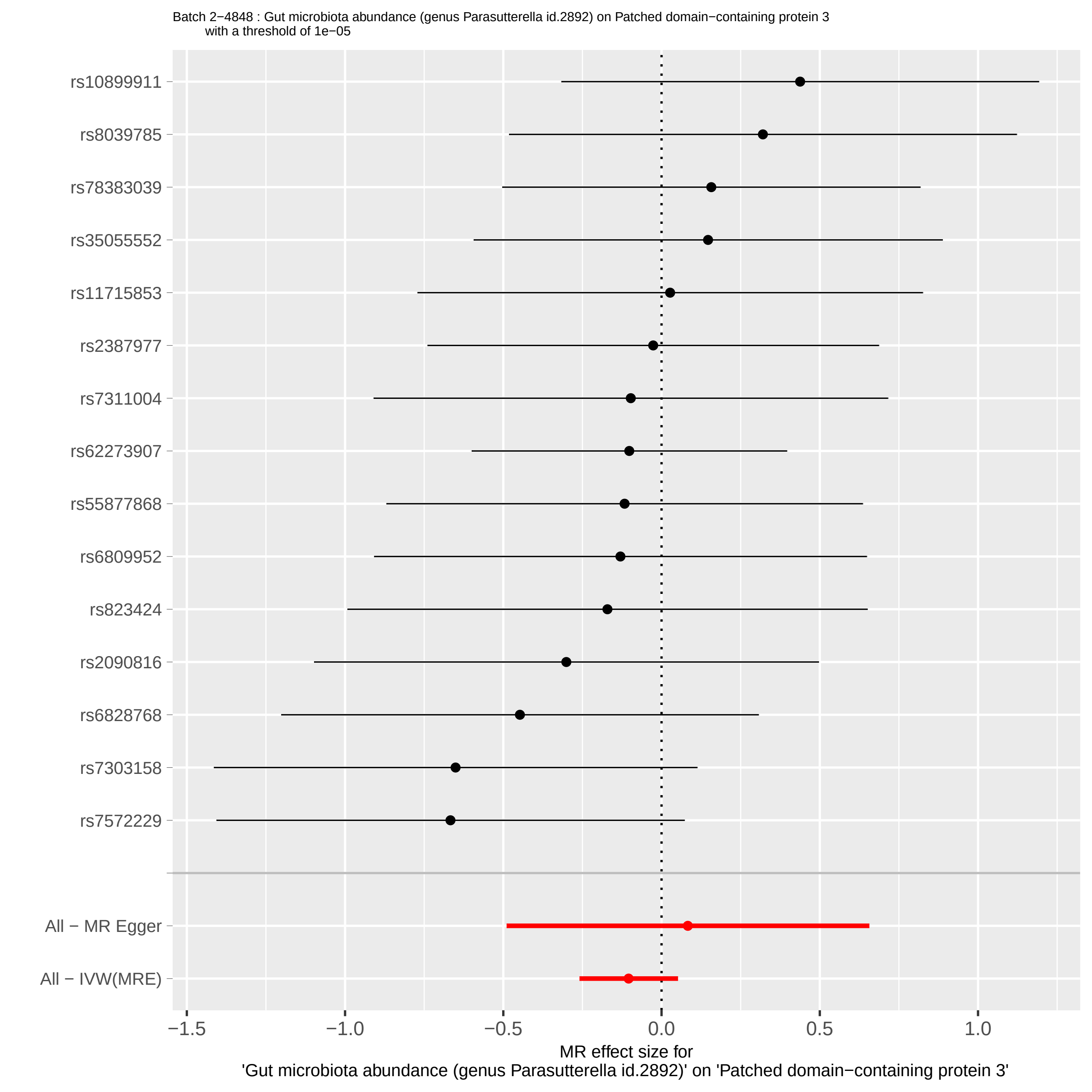
Batch 2−4848 : Gut microbiota abundance (genus Parasutterella id.2892) on Patched domain−containing protein 3
with a threshold of 1e−05
rs10899911
rs8039785
rs78383039
rs35055552
rs11715853
rs2387977
rs7311004
rs62273907
rs55877868
rs6809952
rs823424
rs2090816
rs6828768
rs7303158
rs7572229
All − MR Egger
All − IVW(MRE)
−1.5
−1.0
−0.5
0.0
0.5
1.0
MR effect size for
'Gut microbiota abundance (genus Parasutterella id.2892)' on 'Patched domain−containing protein 3'

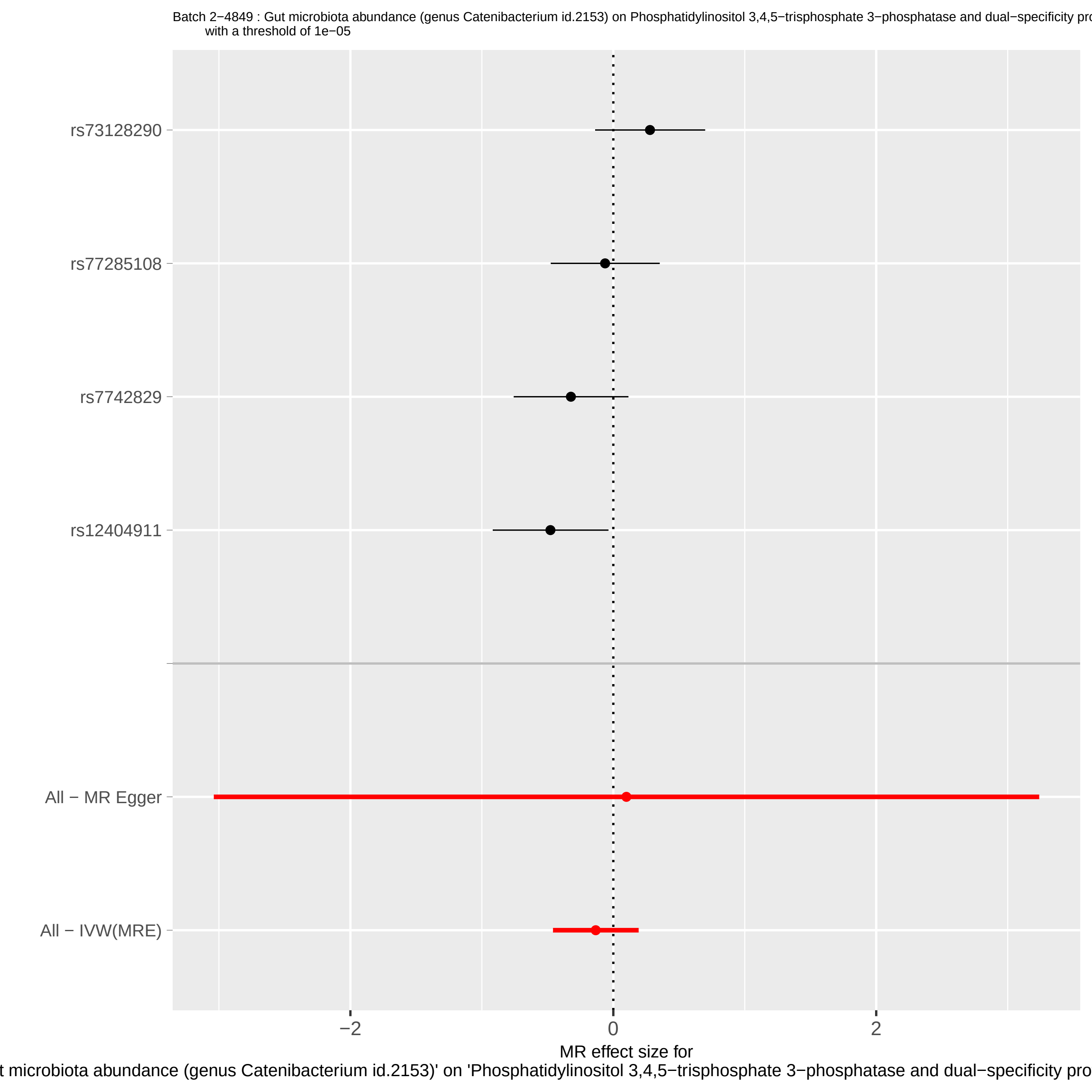
Batch 2−4849 : Gut microbiota abundance (genus Catenibacterium id.2153) on Phosphatidylinositol 3,4,5−trisphosphate 3−phosphatase and dual−specificity pro
with a threshold of 1e−05
rs73128290
rs77285108
rs7742829
rs12404911
All − MR Egger
All − IVW(MRE)
−2
0
2
MR effect size for
t microbiota abundance (genus Catenibacterium id.2153)' on 'Phosphatidylinositol 3,4,5−trisphosphate 3−phosphatase and dual−specificity pro

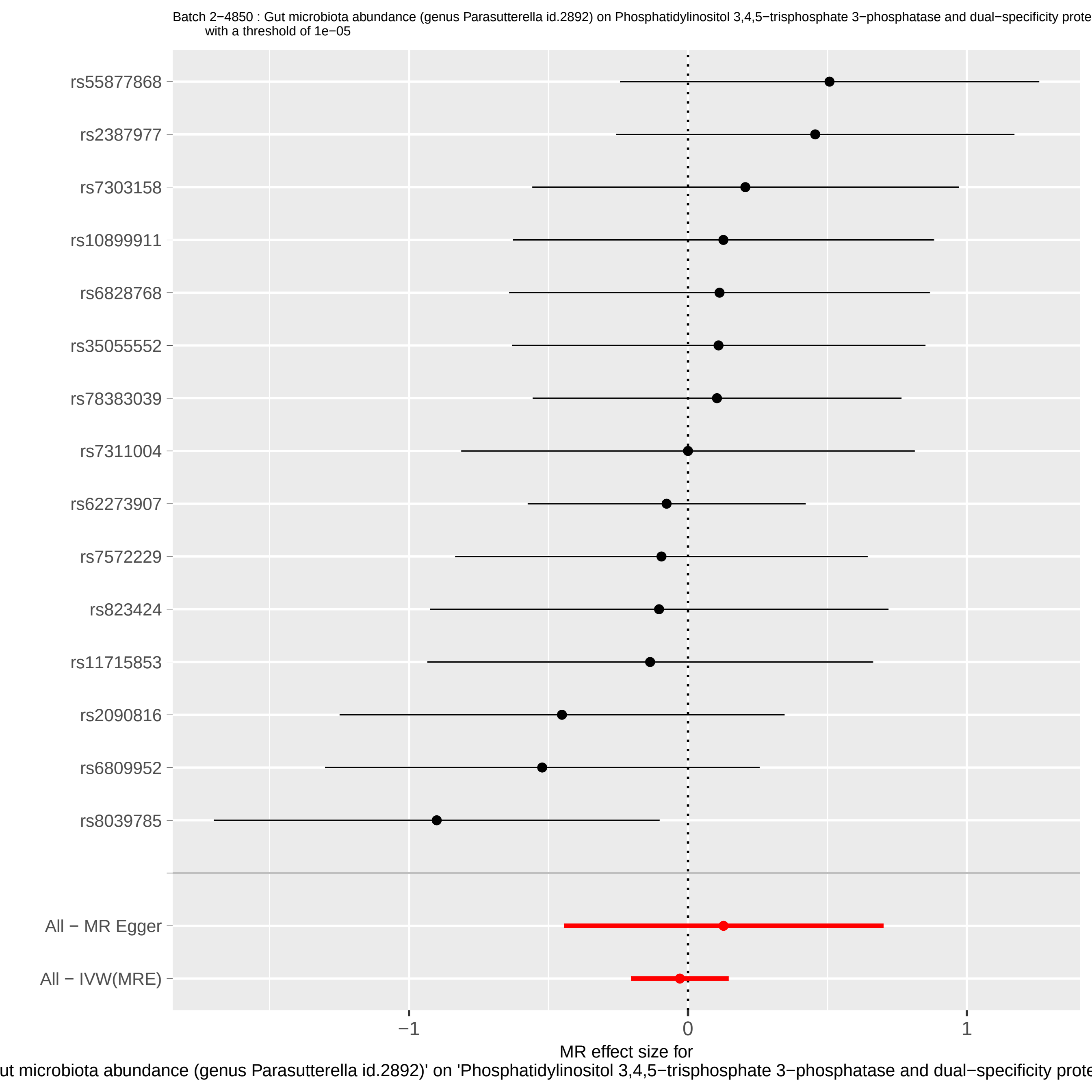

Batch 2−4850 : Gut microbiota abundance (genus Parasutterella id.2892) on Phosphatidylinositol 3,4,5−trisphosphate 3−phosphatase and dual−specificity prote
with a threshold of 1e−05
rs55877868
rs2387977
rs7303158
rs10899911
rs6828768
rs35055552
rs78383039
rs7311004
rs62273907
rs7572229
rs823424
rs11715853
rs2090816
rs6809952
rs8039785
All − MR Egger
All − IVW(MRE)
−1
0
1
MR effect size for
ut microbiota abundance (genus Parasutterella id.2892)' on 'Phosphatidylinositol 3,4,5−trisphosphate 3−phosphatase and dual−specificity prote

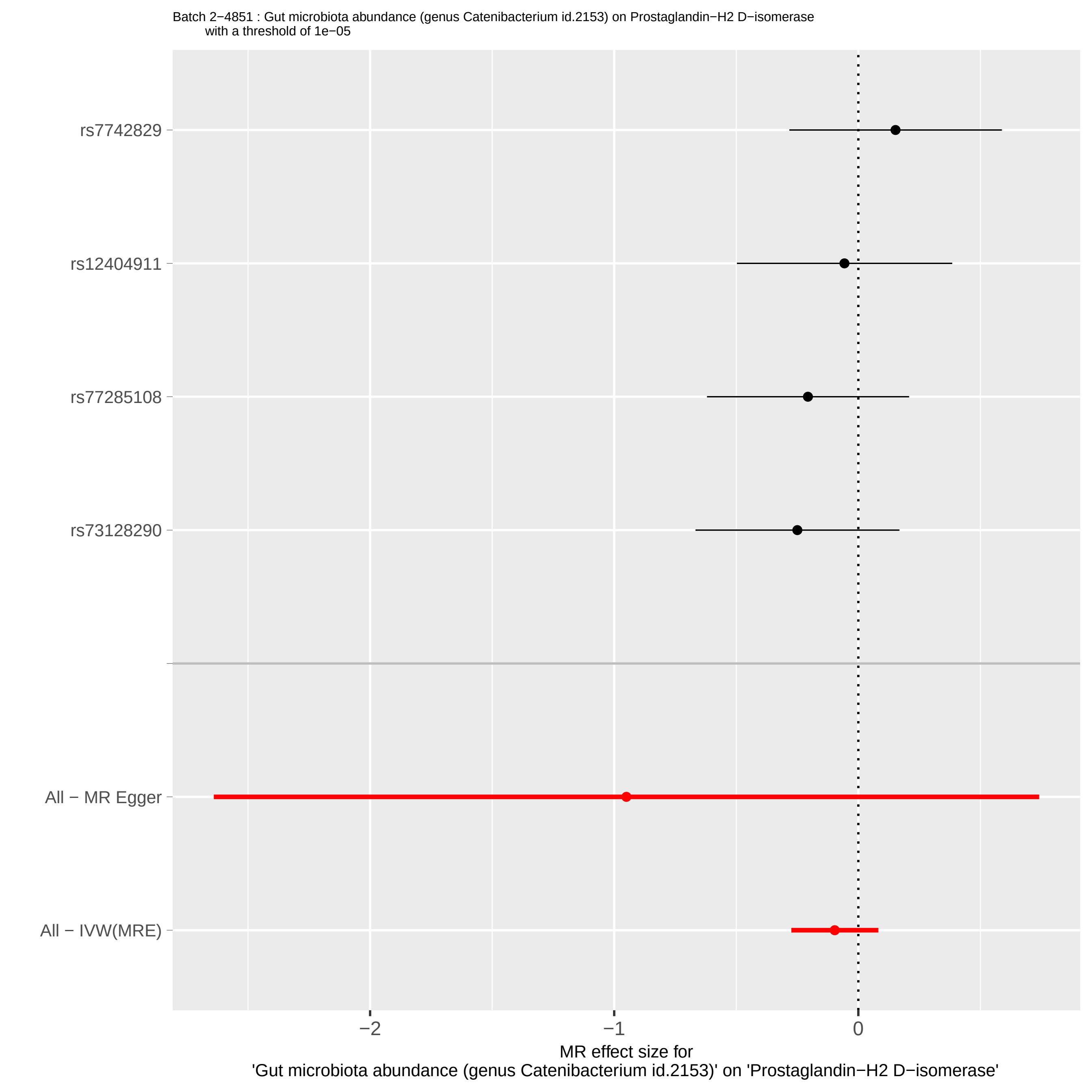

Batch 2−4851 : Gut microbiota abundance (genus Catenibacterium id.2153) on Prostaglandin−H2 D−isomerase
with a threshold of 1e−05
rs7742829
rs12404911
rs77285108
rs73128290
All − MR Egger
All − IVW(MRE)
−2
−1
0
MR effect size for
'Gut microbiota abundance (genus Catenibacterium id.2153)' on 'Prostaglandin−H2 D−isomerase'

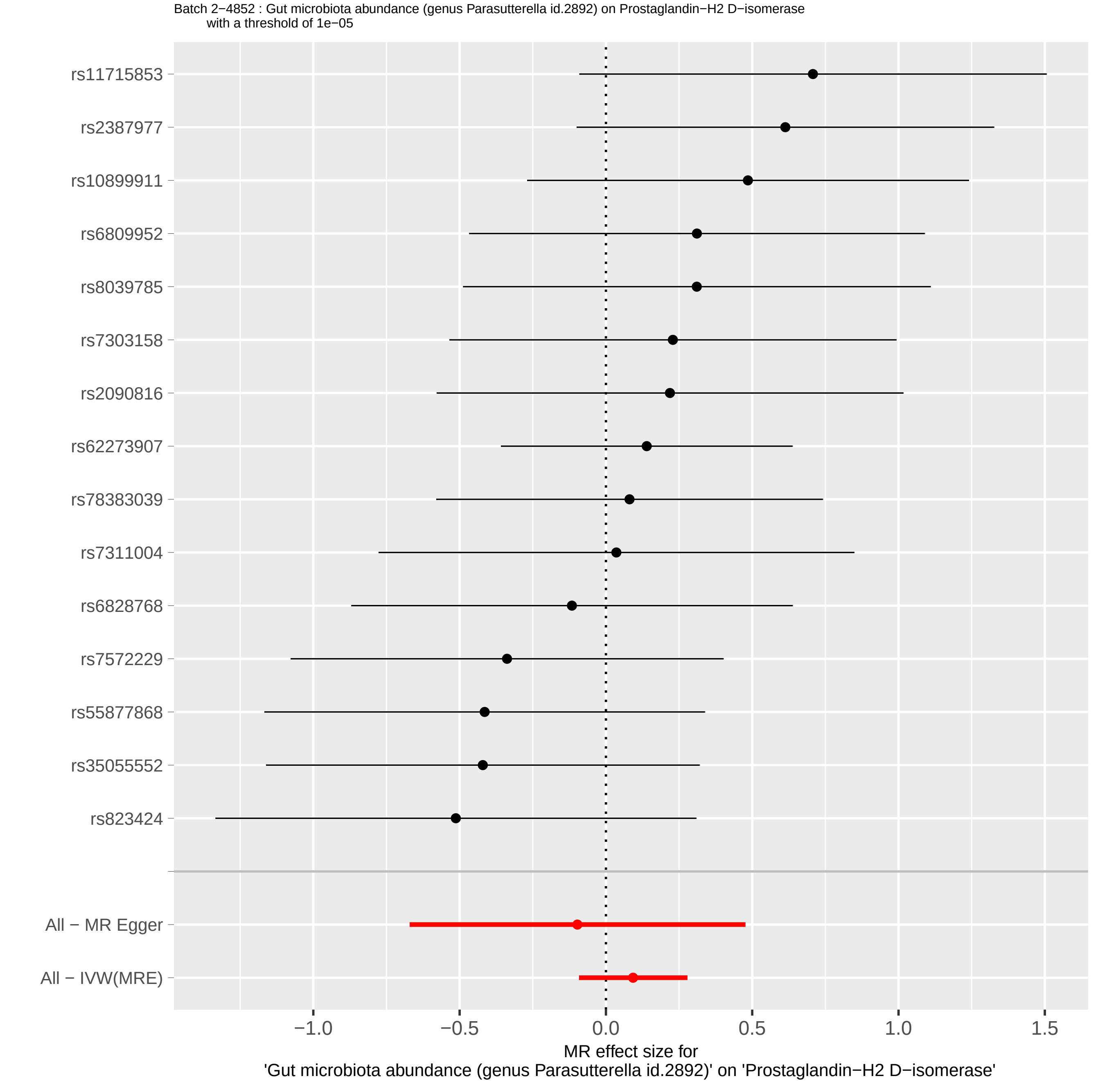
Batch 2−4852 : Gut microbiota abundance (genus Parasutterella id.2892) on Prostaglandin−H2 D−isomerase
with a threshold of 1e−05
rs11715853
rs2387977
rs10899911
rs6809952
rs8039785
rs7303158
rs2090816
rs62273907
rs78383039
rs7311004
rs6828768
rs7572229
rs55877868
rs35055552
rs823424
All − MR Egger
All − IVW(MRE)
−1.0
−0.5
0.0
0.5
1.0
1.5
MR effect size for
'Gut microbiota abundance (genus Parasutterella id.2892)' on 'Prostaglandin−H2 D−isomerase'

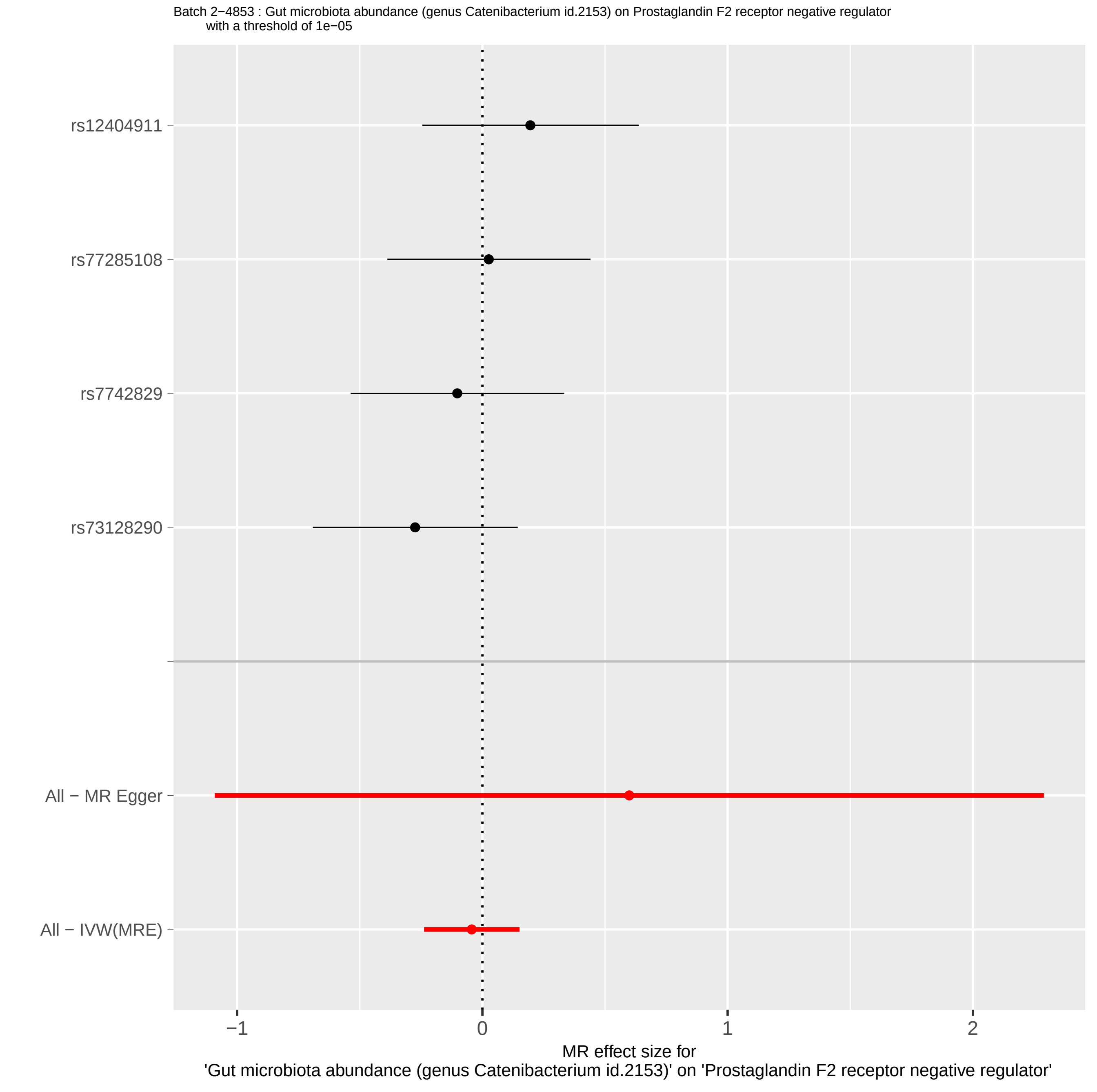

Batch 2−4853 : Gut microbiota abundance (genus Catenibacterium id.2153) on Prostaglandin F2 receptor negative regulator
with a threshold of 1e−05
rs12404911
rs77285108
rs7742829
rs73128290
All − MR Egger
All − IVW(MRE)
−1
0
1
2
MR effect size for
'Gut microbiota abundance (genus Catenibacterium id.2153)' on 'Prostaglandin F2 receptor negative regulator'

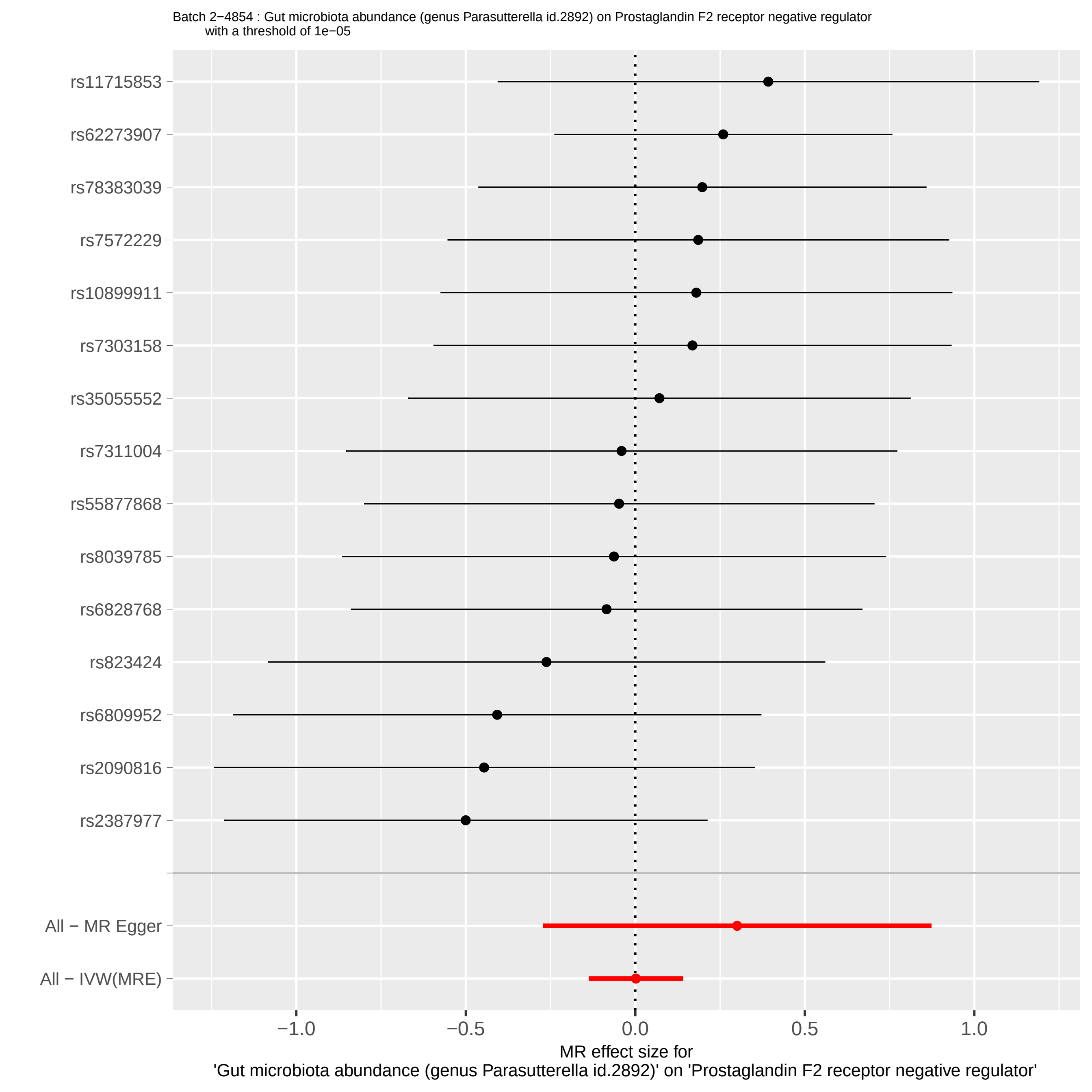

Batch 2−4854 : Gut microbiota abundance (genus Parasutterella id.2892) on Prostaglandin F2 receptor negative regulator
with a threshold of 1e−05
rs11715853
rs62273907
rs78383039
rs7572229
rs10899911
rs7303158
rs35055552
rs7311004
rs55877868
rs8039785
rs6828768
rs823424
rs6809952
rs2090816
rs2387977
All − MR Egger
All − IVW(MRE)
−1.0
−0.5
0.0
0.5
1.0
MR effect size for
'Gut microbiota abundance (genus Parasutterella id.2892)' on 'Prostaglandin F2 receptor negative regulator'

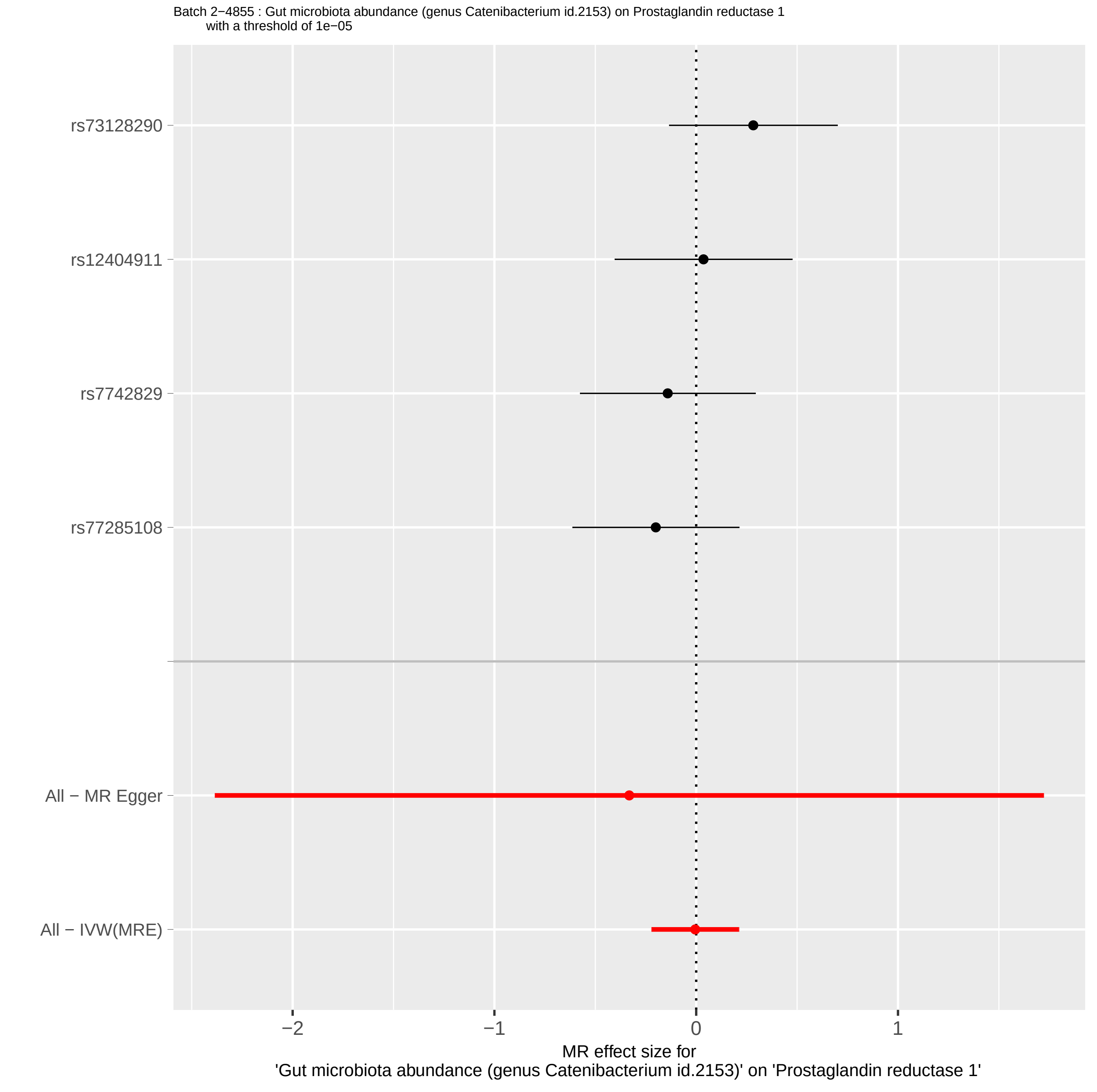

Batch 2−4855 : Gut microbiota abundance (genus Catenibacterium id.2153) on Prostaglandin reductase 1
with a threshold of 1e−05
rs73128290
rs12404911
rs7742829
rs77285108
All − MR Egger
All − IVW(MRE)
−2
−1
0
1
MR effect size for
'Gut microbiota abundance (genus Catenibacterium id.2153)' on 'Prostaglandin reductase 1'

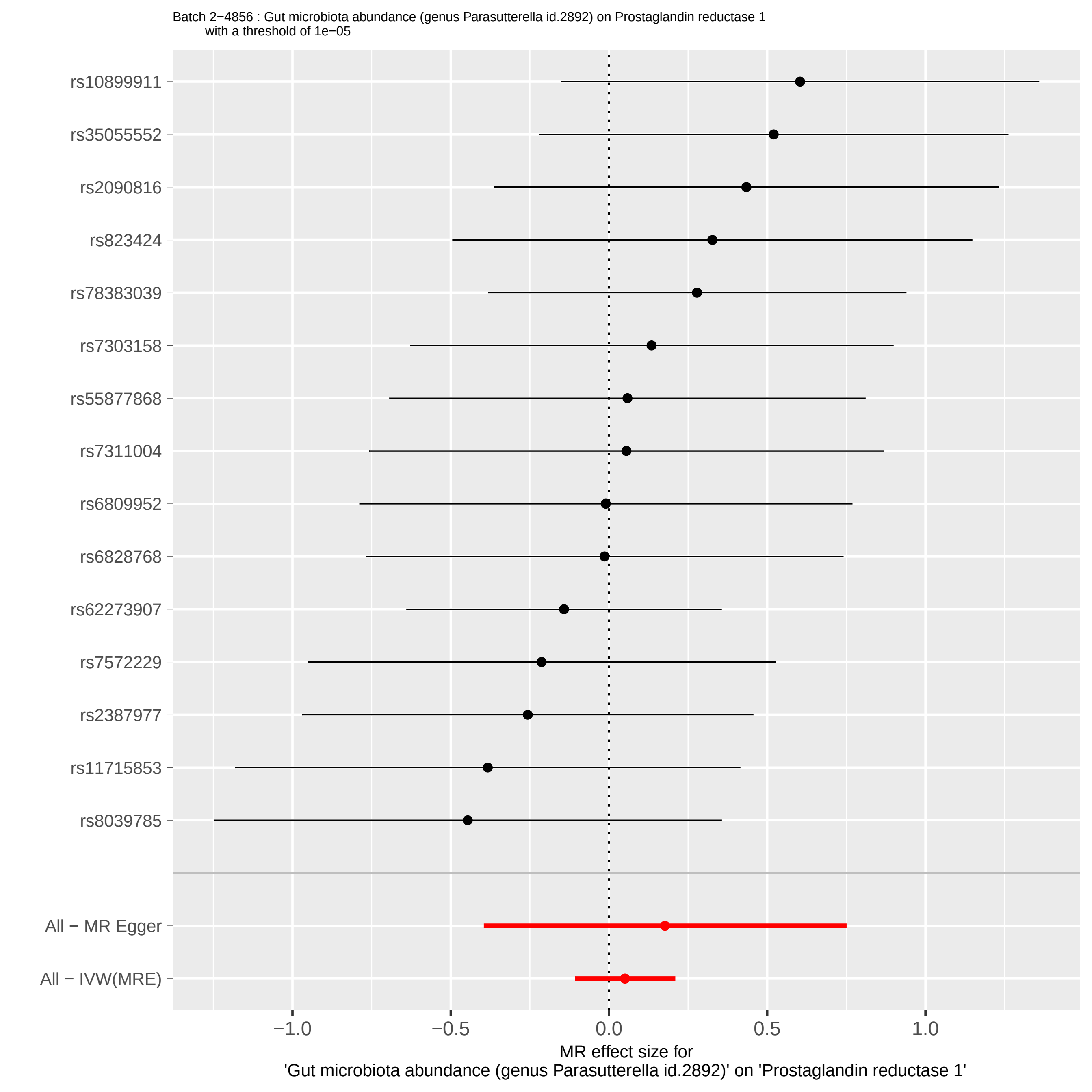
Batch 2−4856 : Gut microbiota abundance (genus Parasutterella id.2892) on Prostaglandin reductase 1
with a threshold of 1e−05
rs10899911
rs35055552
rs2090816
rs823424
rs78383039
rs7303158
rs55877868
rs7311004
rs6809952
rs6828768
rs62273907
rs7572229
rs2387977
rs11715853
rs8039785
All − MR Egger
All − IVW(MRE)
−1.0
−0.5
0.0
0.5
1.0
MR effect size for
'Gut microbiota abundance (genus Parasutterella id.2892)' on 'Prostaglandin reductase 1'

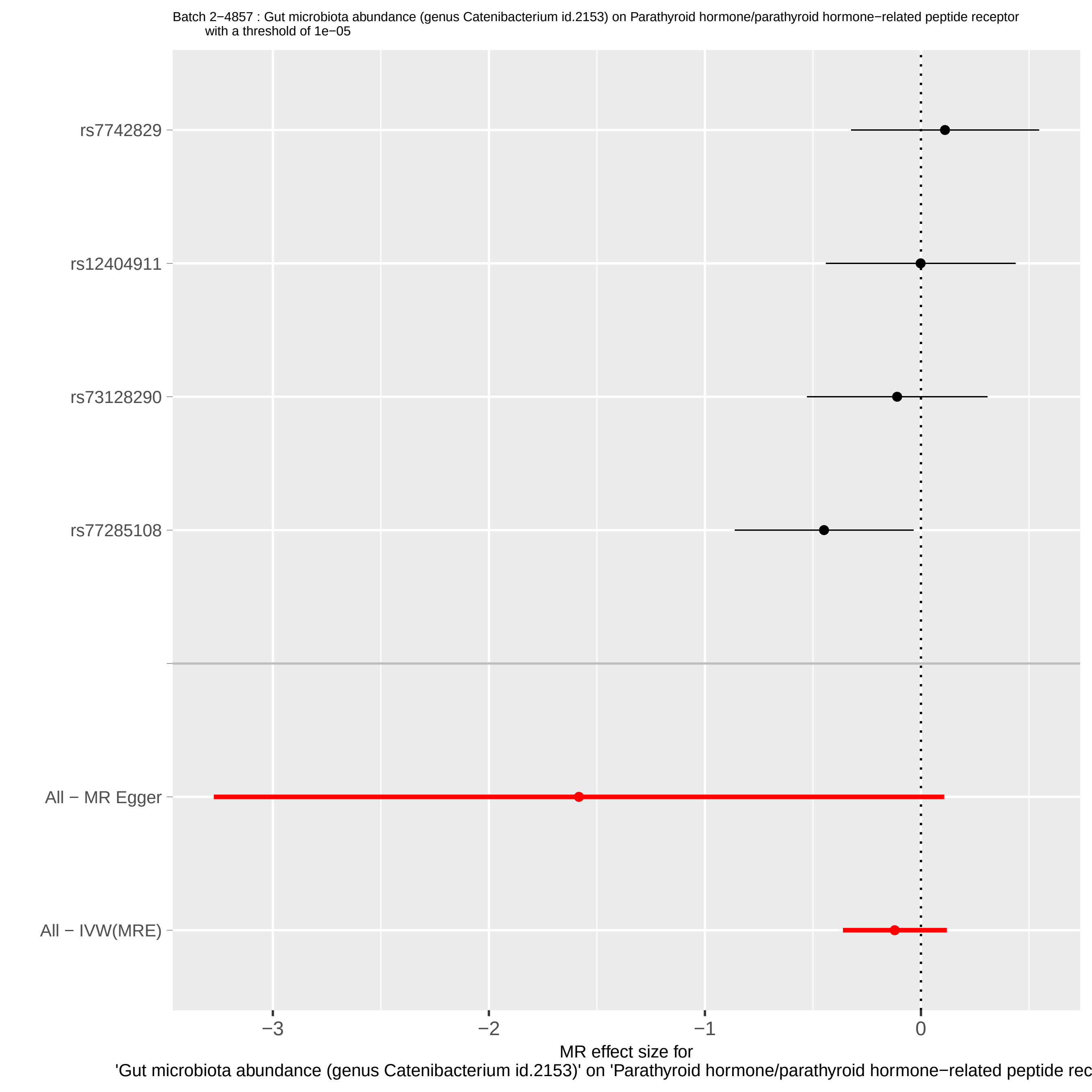

Batch 2−4857 : Gut microbiota abundance (genus Catenibacterium id.2153) on Parathyroid hormone/parathyroid hormone−related peptide receptor
with a threshold of 1e−05
rs7742829
rs12404911
rs73128290
rs77285108
All − MR Egger
All − IVW(MRE)
−3
−2
−1
0
MR effect size for
'Gut microbiota abundance (genus Catenibacterium id.2153)' on 'Parathyroid hormone/parathyroid hormone−related peptide rec

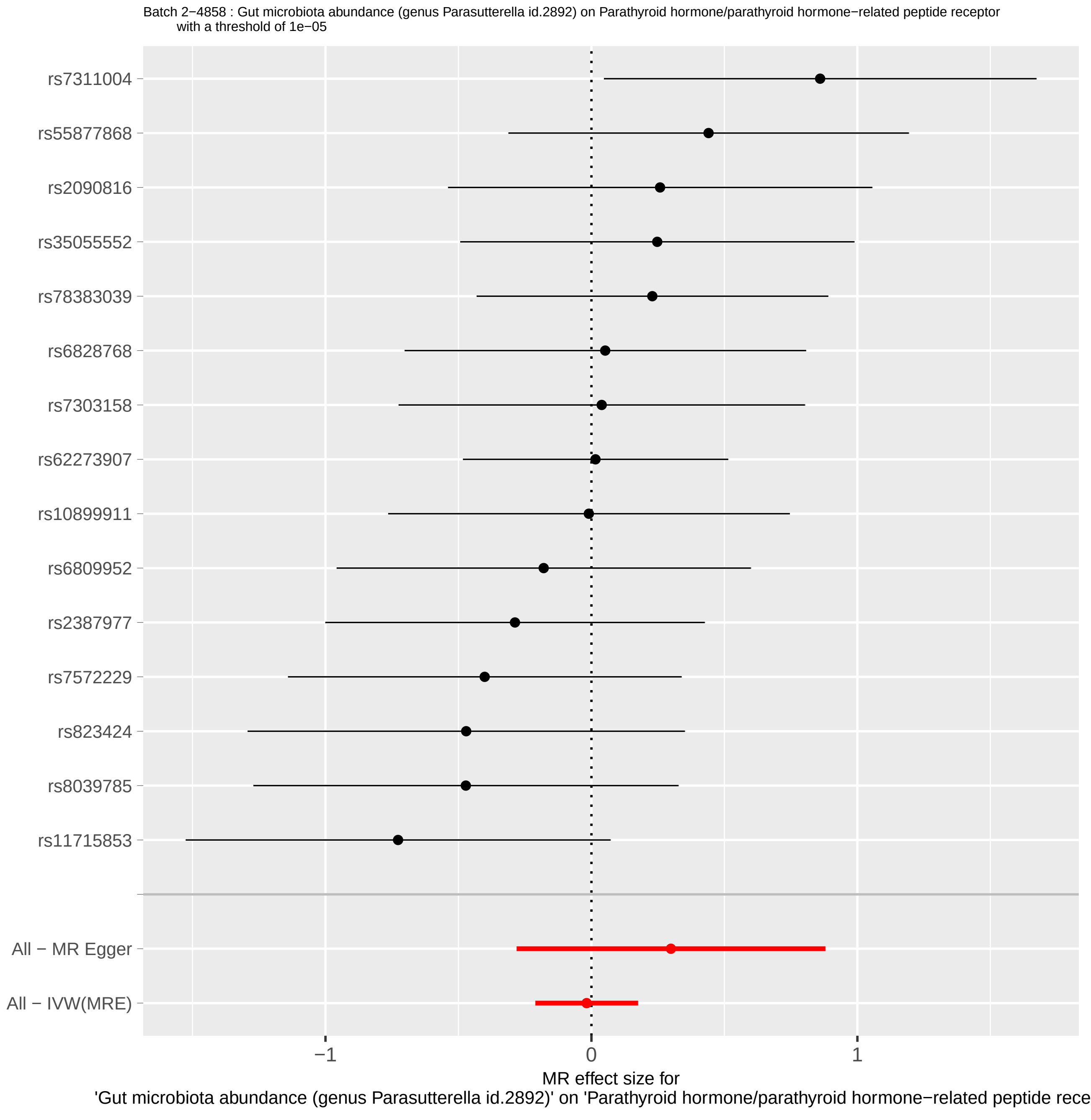

Batch 2−4858 : Gut microbiota abundance (genus Parasutterella id.2892) on Parathyroid hormone/parathyroid hormone−related peptide receptor with a threshold of 1e−05
rs7311004
rs55877868
rs2090816
rs35055552
rs78383039
rs6828768
rs7303158
rs62273907
rs10899911
rs6809952
rs2387977
rs7572229
rs823424
rs8039785
rs11715853
All − MR Egger
All − IVW(MRE)
−1
0
1
MR effect size for
'Gut microbiota abundance (genus Parasutterella id.2892)' on 'Parathyroid hormone/parathyroid hormone−related peptide rece

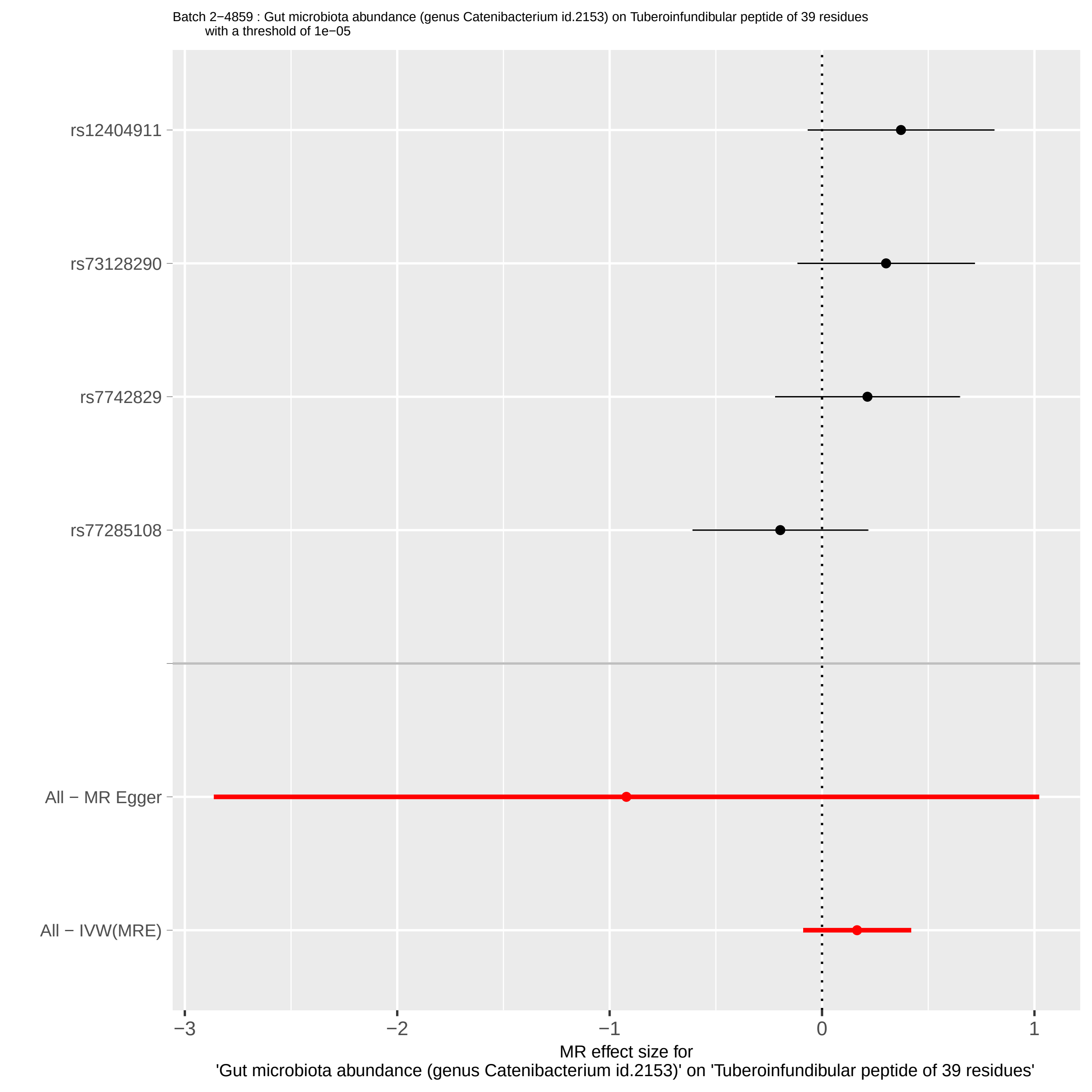
Batch 2−4859 : Gut microbiota abundance (genus Catenibacterium id.2153) on Tuberoinfundibular peptide of 39 residues
with a threshold of 1e−05
rs12404911
rs73128290
rs7742829
rs77285108
All − MR Egger
All − IVW(MRE)
−3
−2
−1
0
1
MR effect size for
'Gut microbiota abundance (genus Catenibacterium id.2153)' on 'Tuberoinfundibular peptide of 39 residues'

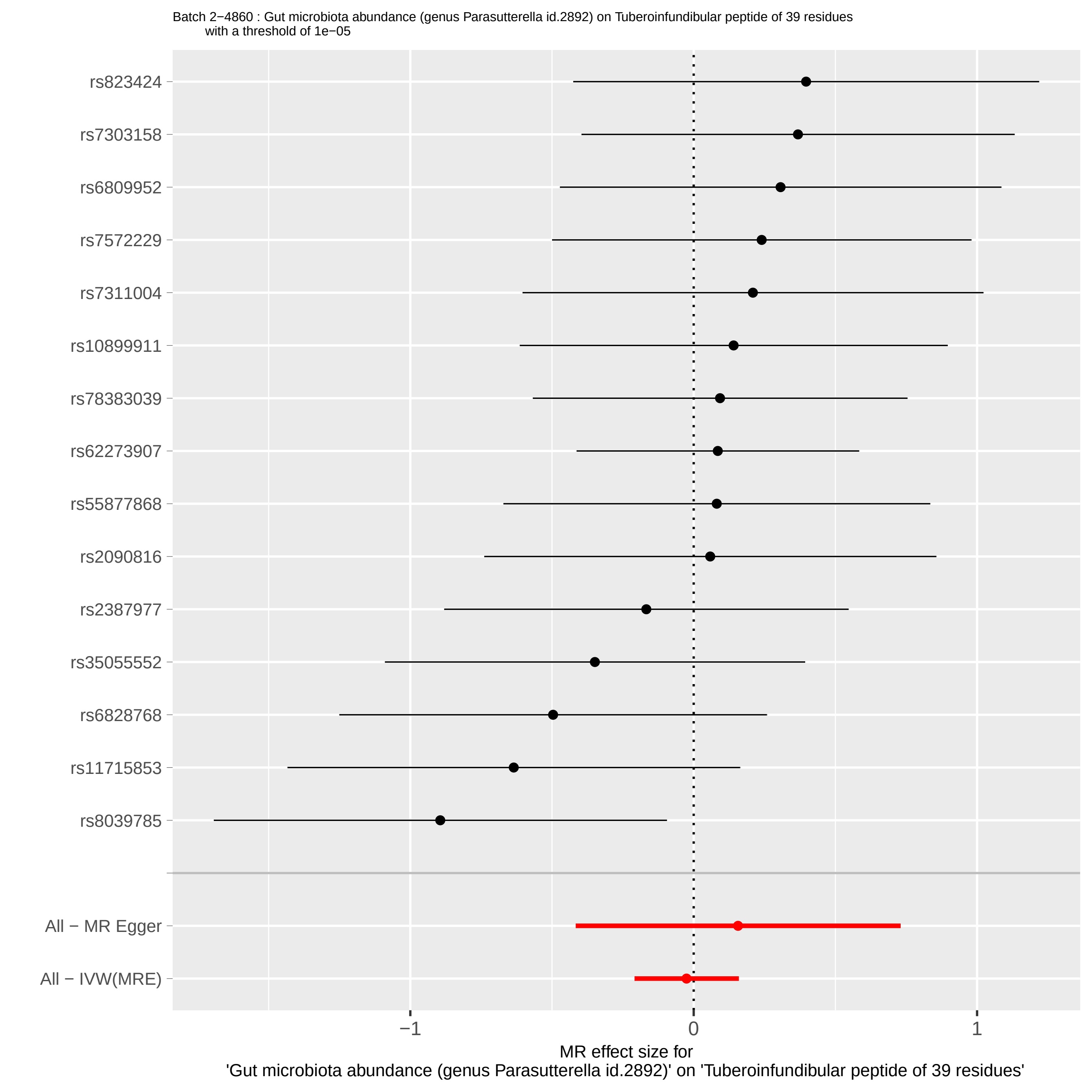
Batch 2−4860 : Gut microbiota abundance (genus Parasutterella id.2892) on Tuberoinfundibular peptide of 39 residues
with a threshold of 1e−05
rs823424
rs7303158
rs6809952
rs7572229
rs7311004
rs10899911
rs78383039
rs62273907
rs55877868
rs2090816
rs2387977
rs35055552
rs6828768
rs11715853
rs8039785
All − MR Egger
All − IVW(MRE)
−1
0
1
MR effect size for
'Gut microbiota abundance (genus Parasutterella id.2892)' on 'Tuberoinfundibular peptide of 39 residues'

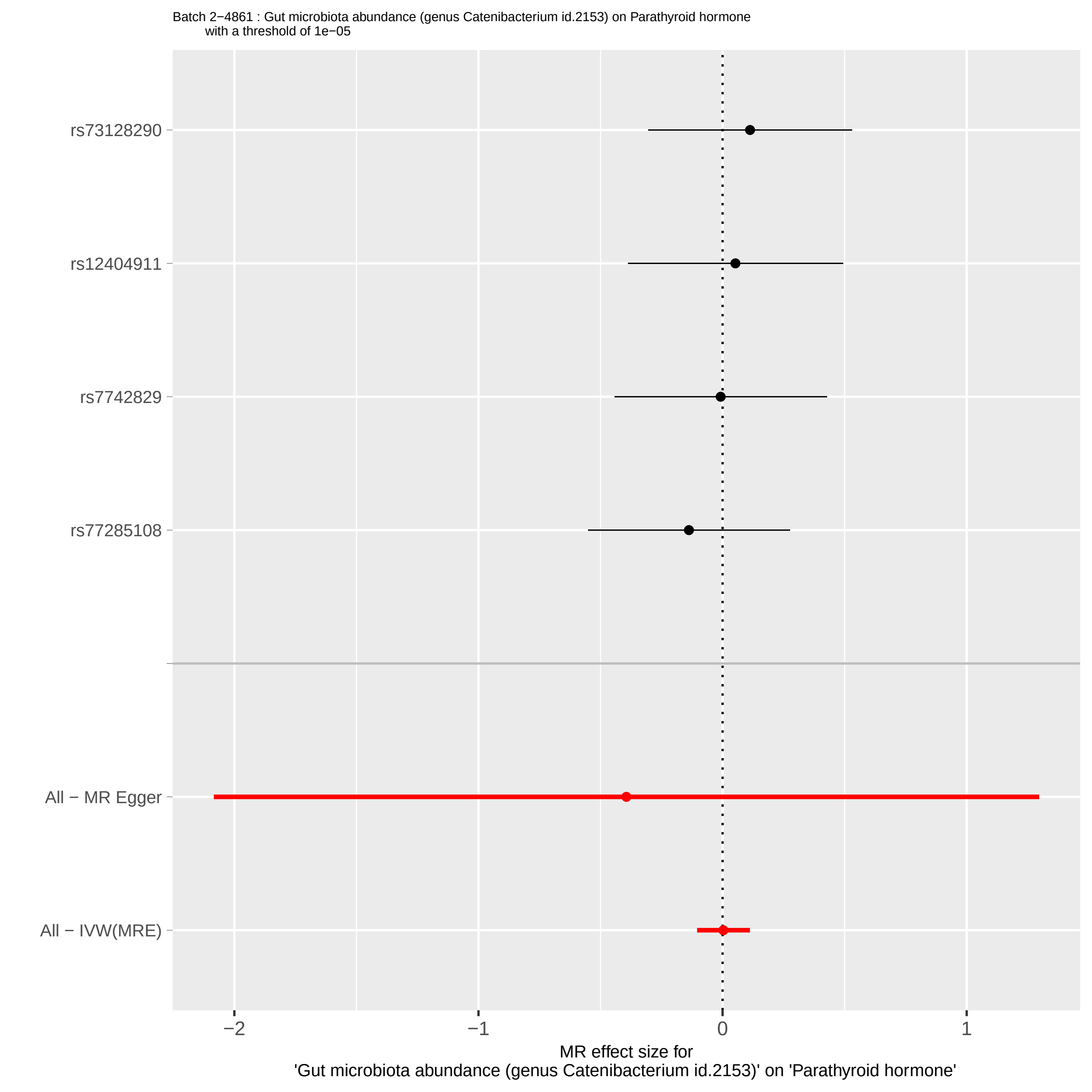

Batch 2−4861 : Gut microbiota abundance (genus Catenibacterium id.2153) on Parathyroid hormone
with a threshold of 1e−05
rs73128290
rs12404911
rs7742829
rs77285108
All − MR Egger
All − IVW(MRE)
−2
−1
0
1
MR effect size for
'Gut microbiota abundance (genus Catenibacterium id.2153)' on 'Parathyroid hormone'

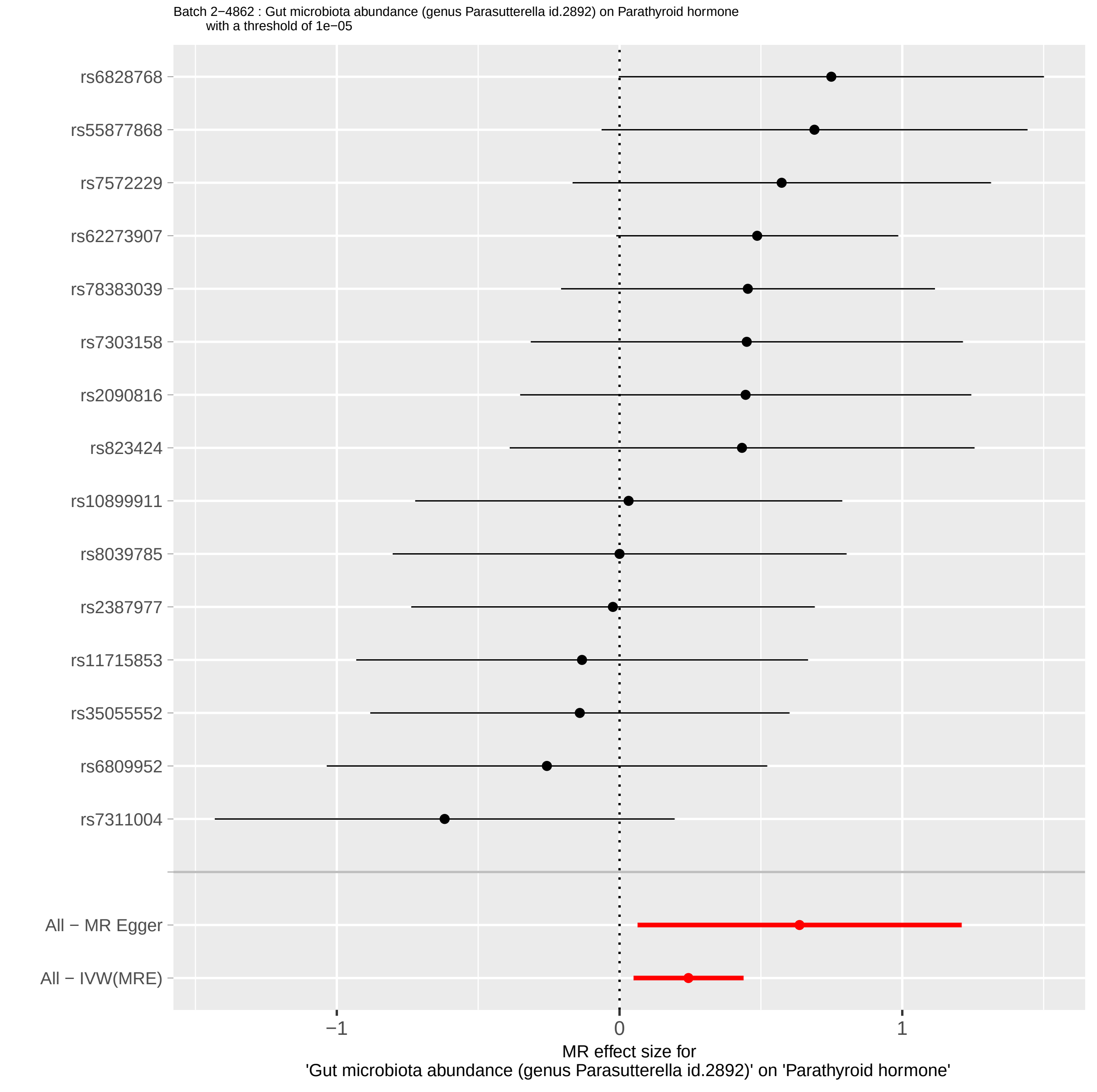

Batch 2−4862 : Gut microbiota abundance (genus Parasutterella id.2892) on Parathyroid hormone
with a threshold of 1e−05
rs6828768
rs55877868
rs7572229
rs62273907
rs78383039
rs7303158
rs2090816
rs823424
rs10899911
rs8039785
rs2387977
rs11715853
rs35055552
rs6809952
rs7311004
All − MR Egger
All − IVW(MRE)
−1
0
1
MR effect size for
'Gut microbiota abundance (genus Parasutterella id.2892)' on 'Parathyroid hormone'

Batch 2−4863 : Gut microbiota abundance (genus Catenibacterium id.2153) on Parathyroid hormone−related protein with a threshold of 1e−05

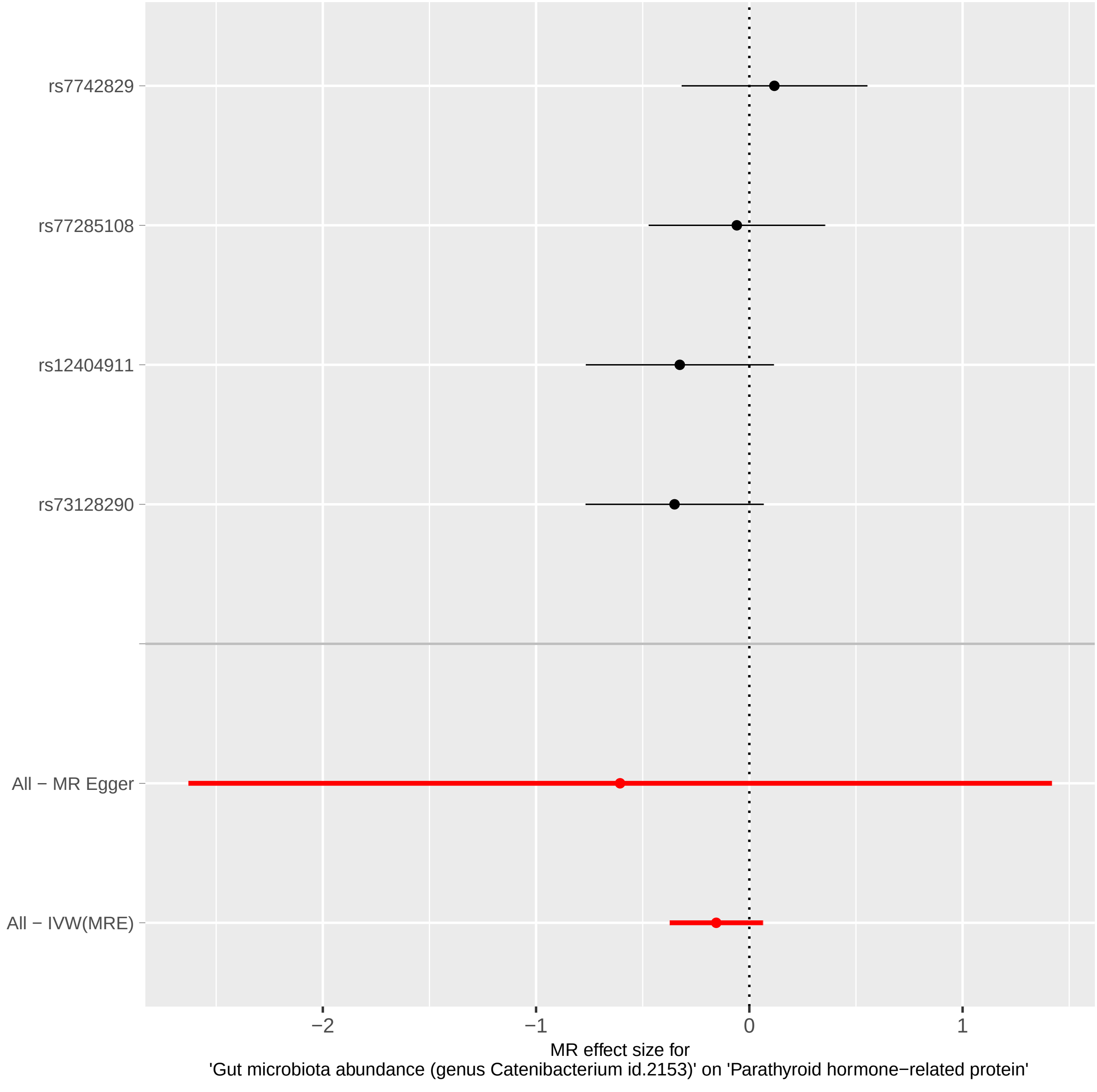

Batch 2−4864 : Gut microbiota abundance (genus Parasutterella id.2892) on Parathyroid hormone−related protein
with a threshold of 1e−05

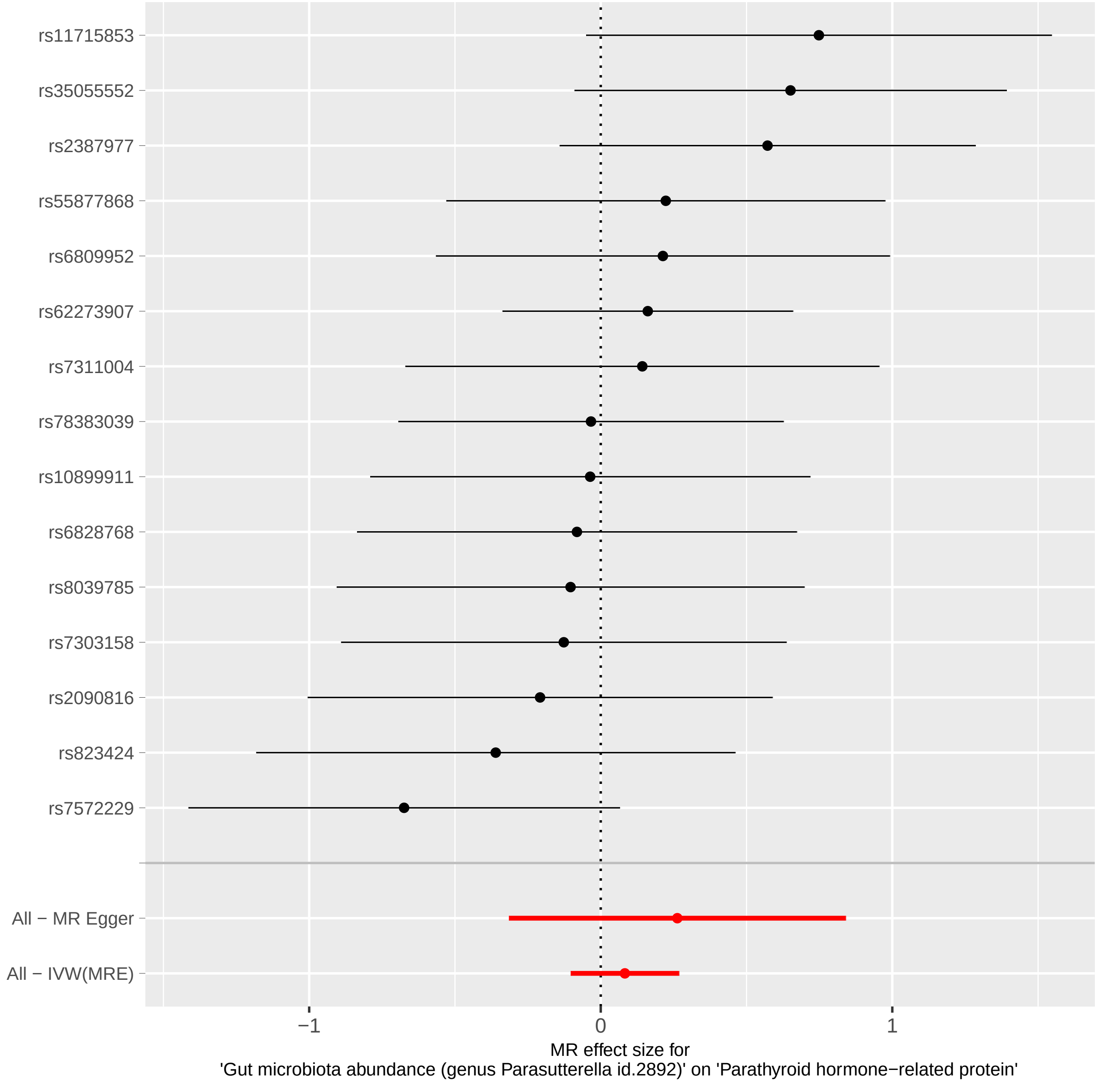

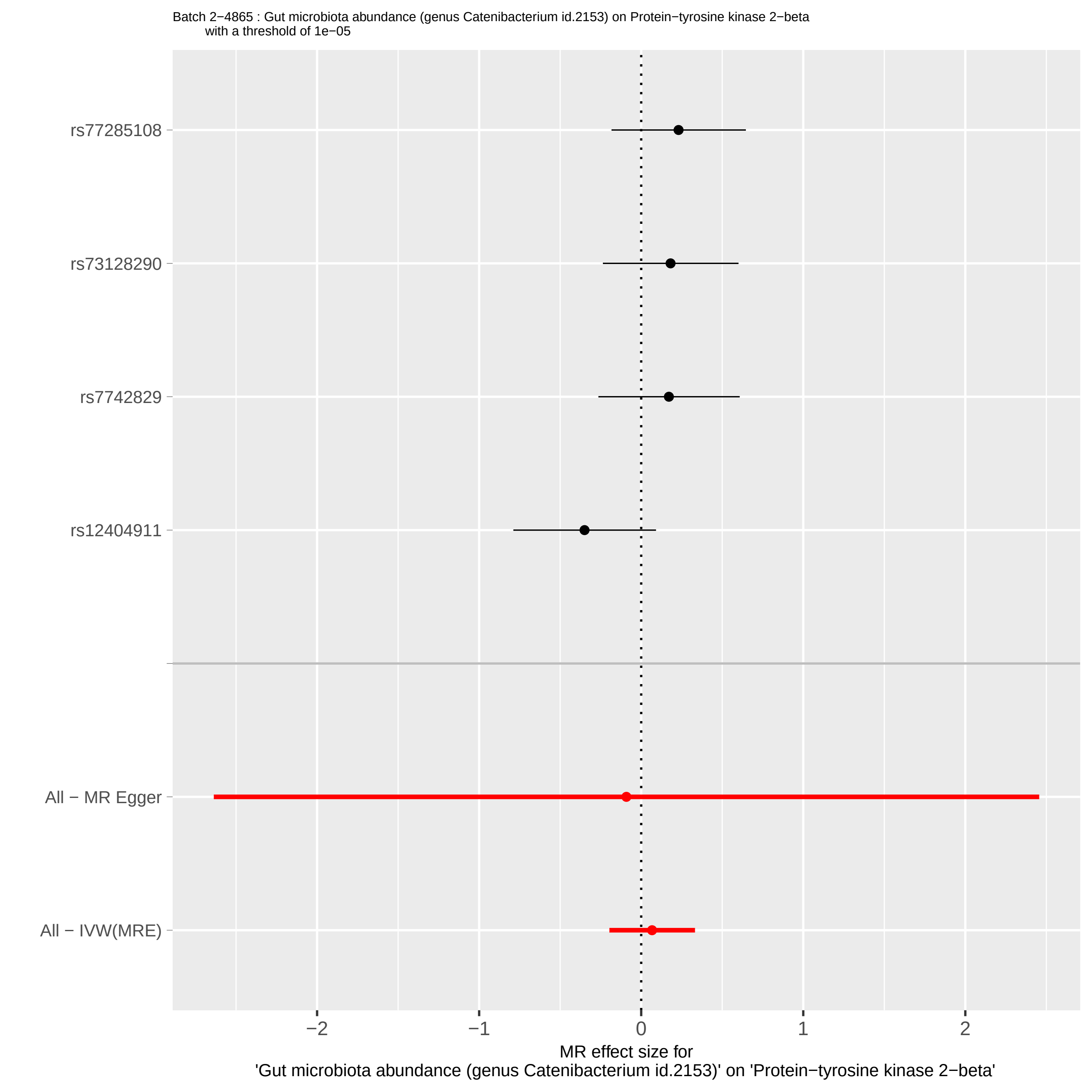
Batch 2−4865 : Gut microbiota abundance (genus Catenibacterium id.2153) on Protein−tyrosine kinase 2−beta
with a threshold of 1e−05
rs77285108
rs73128290
rs7742829
rs12404911
All − MR Egger
All − IVW(MRE)
−2
−1
0
1
2
MR effect size for
'Gut microbiota abundance (genus Catenibacterium id.2153)' on 'Protein−tyrosine kinase 2−beta'

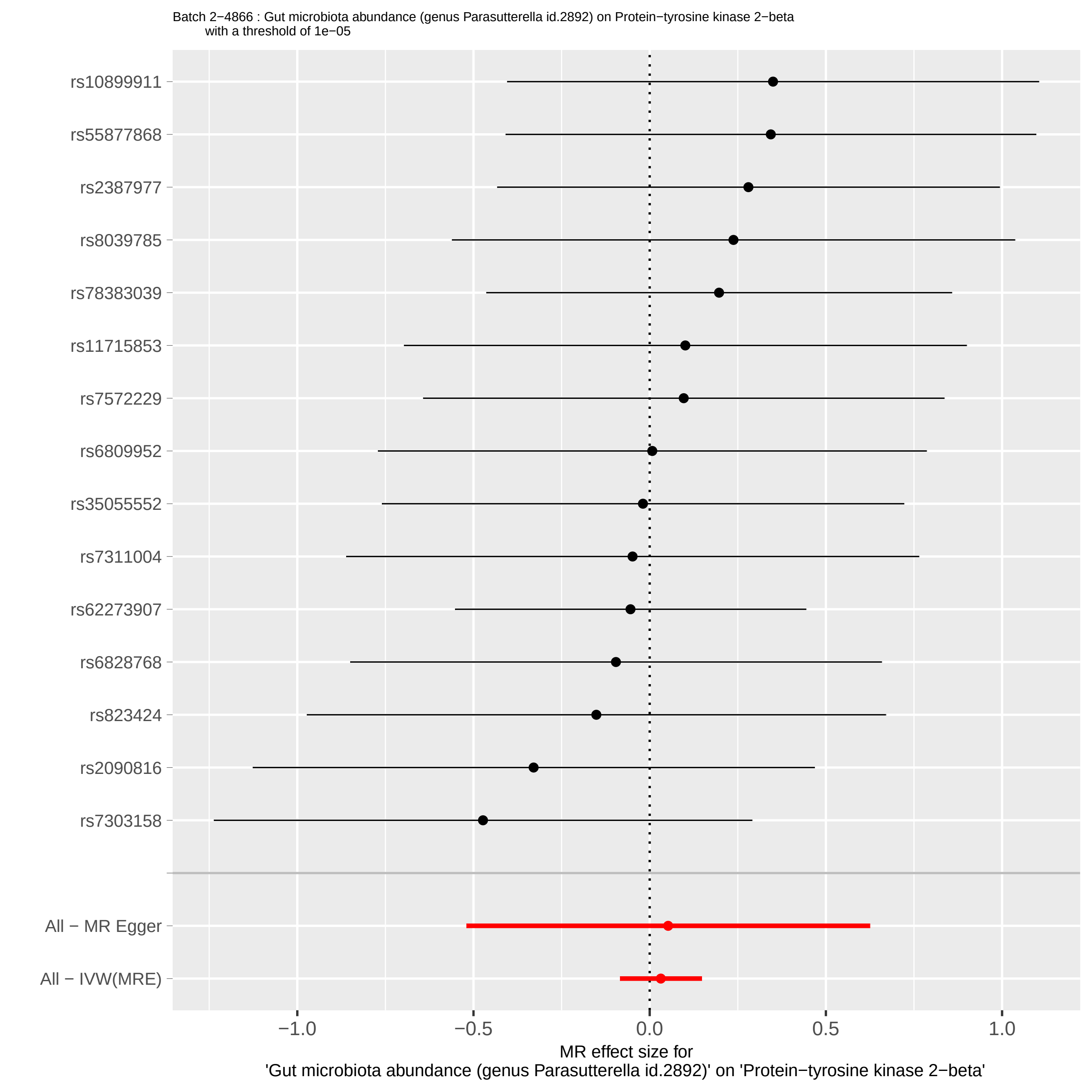

Batch 2−4866 : Gut microbiota abundance (genus Parasutterella id.2892) on Protein−tyrosine kinase 2−beta
with a threshold of 1e−05
rs10899911
rs55877868
rs2387977
rs8039785
rs78383039
rs11715853
rs7572229
rs6809952
rs35055552
rs7311004
rs62273907
rs6828768
rs823424
rs2090816
rs7303158
All − MR Egger
All − IVW(MRE)
−1.0
−0.5
0.0
0.5
1.0
MR effect size for
'Gut microbiota abundance (genus Parasutterella id.2892)' on 'Protein−tyrosine kinase 2−beta'

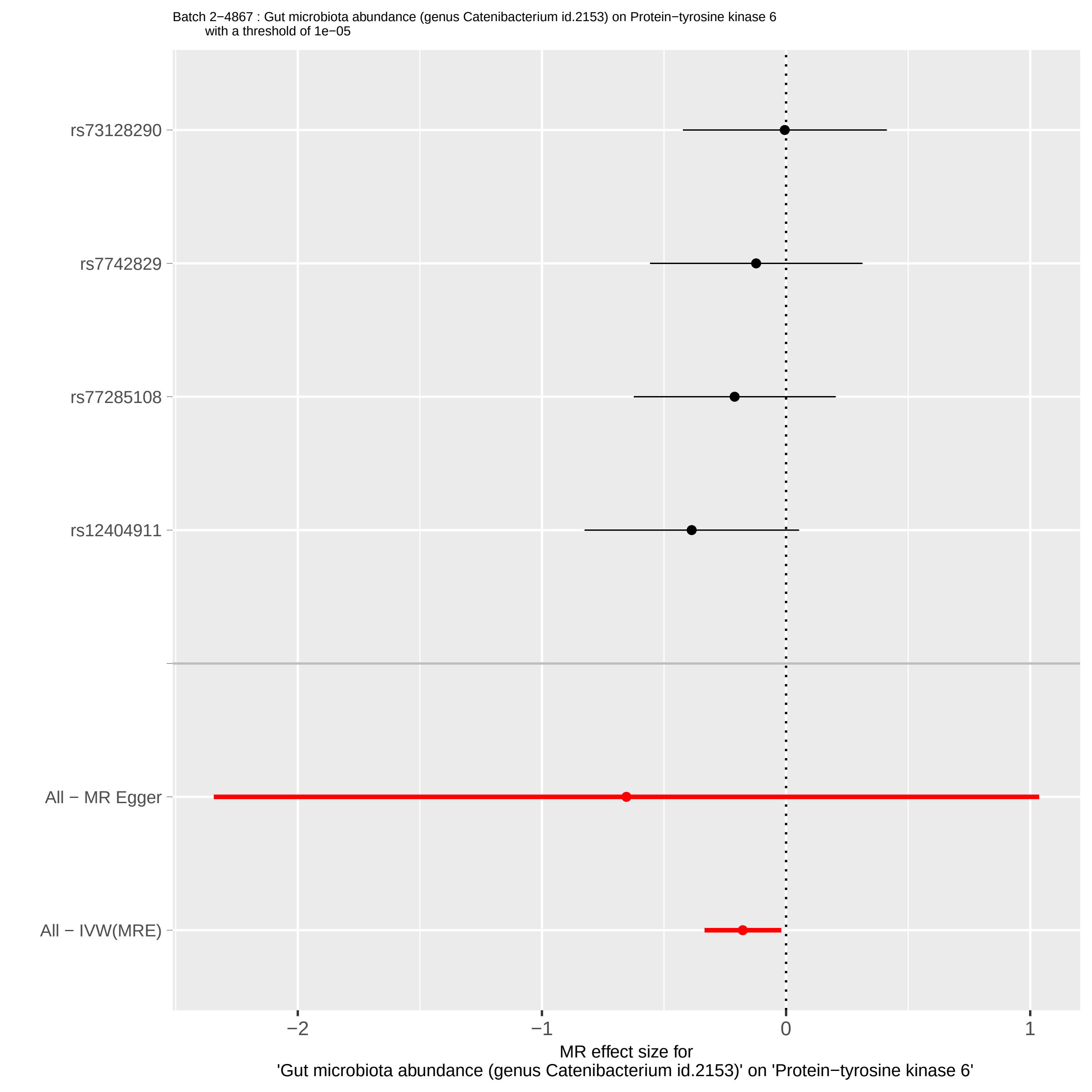

Batch 2−4867 : Gut microbiota abundance (genus Catenibacterium id.2153) on Protein−tyrosine kinase 6
with a threshold of 1e−05
rs73128290
rs7742829
rs77285108
rs12404911
All − MR Egger
All − IVW(MRE)
−2
−1
0
1
MR effect size for
'Gut microbiota abundance (genus Catenibacterium id.2153)' on 'Protein−tyrosine kinase 6'

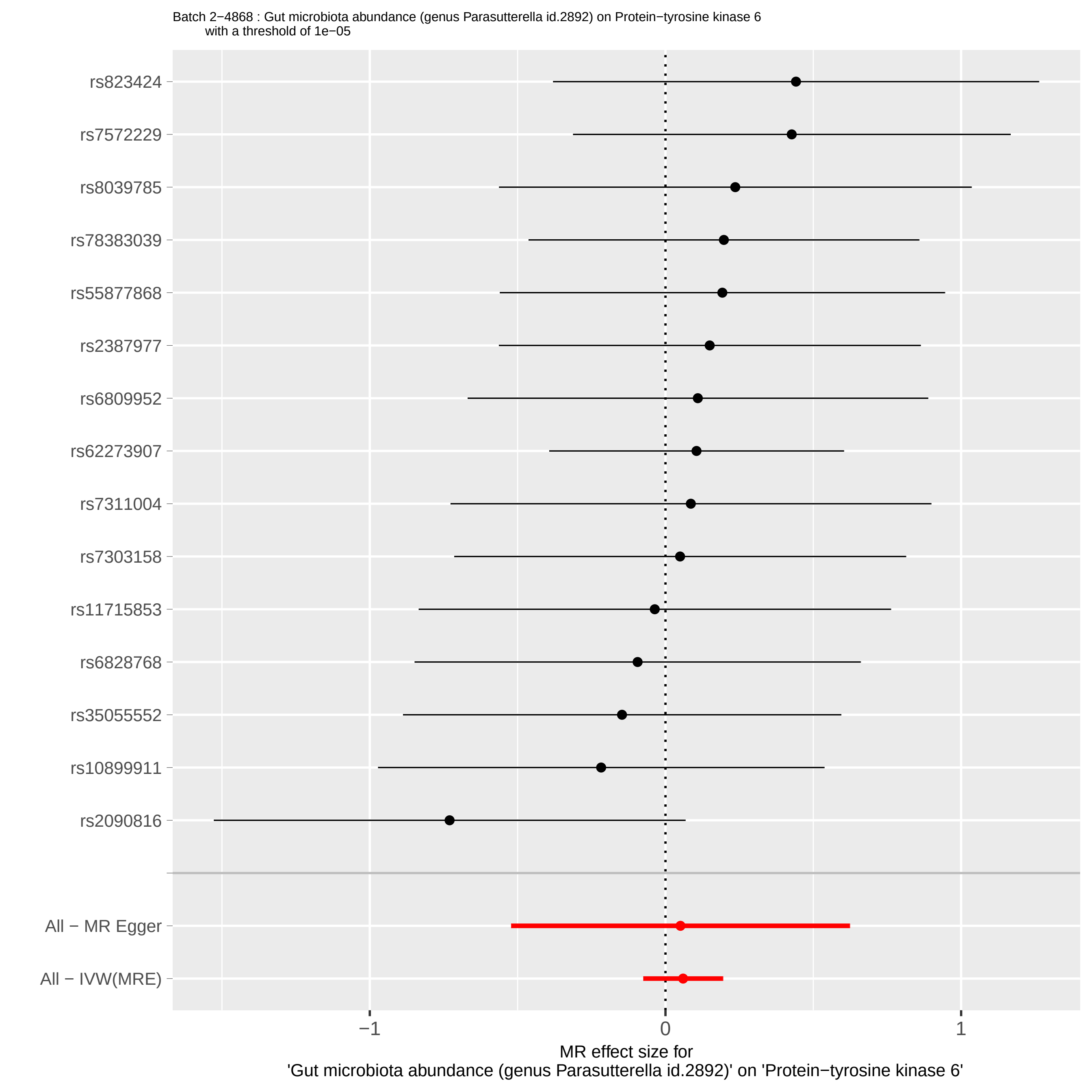

Batch 2−4868 : Gut microbiota abundance (genus Parasutterella id.2892) on Protein−tyrosine kinase 6
with a threshold of 1e−05
rs823424
rs7572229
rs8039785
rs78383039
rs55877868
rs2387977
rs6809952
rs62273907
rs7311004
rs7303158
rs11715853
rs6828768
rs35055552
rs10899911
rs2090816
All − MR Egger
All − IVW(MRE)
−1
0
1
MR effect size for
'Gut microbiota abundance (genus Parasutterella id.2892)' on 'Protein−tyrosine kinase 6'

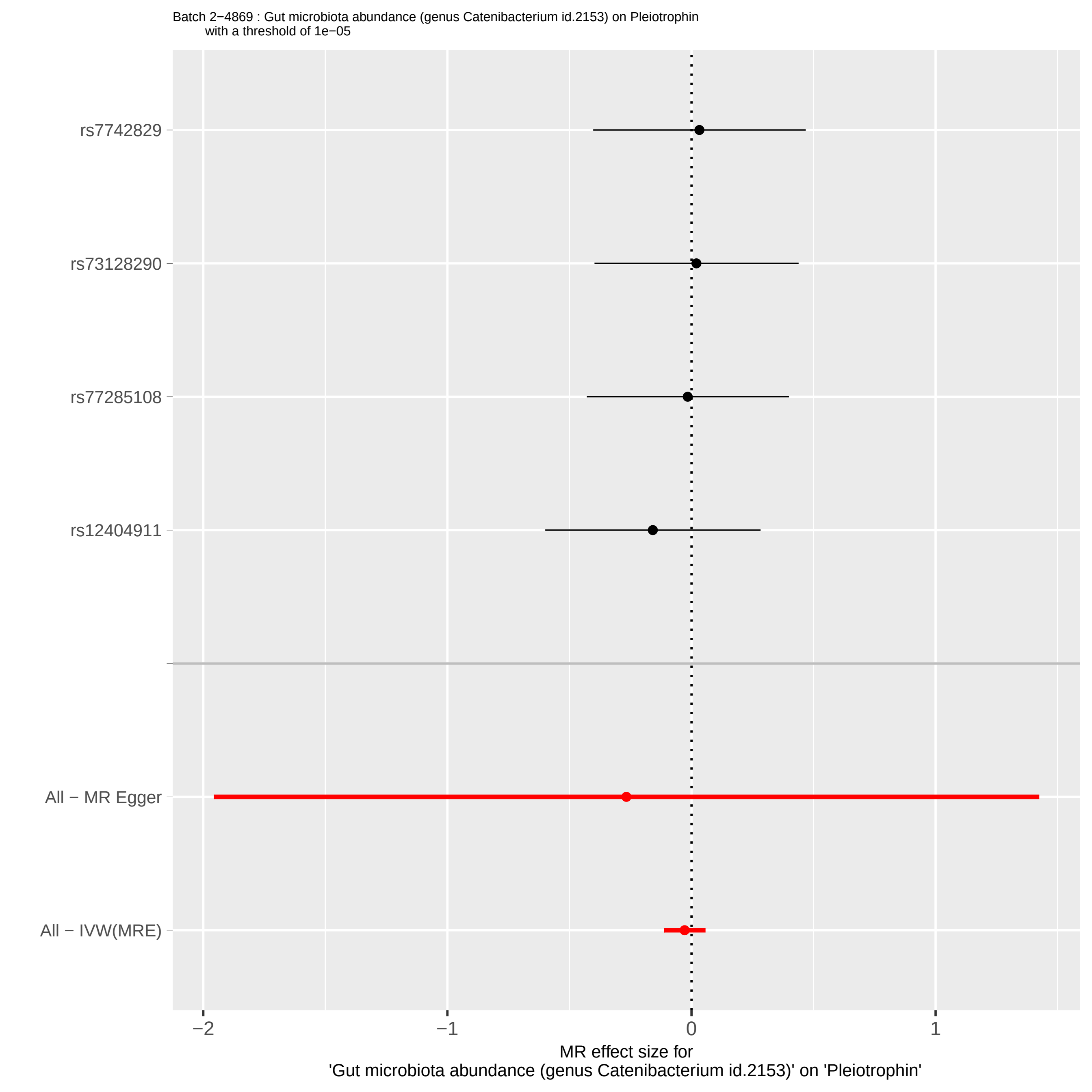
Batch 2−4869 : Gut microbiota abundance (genus Catenibacterium id.2153) on Pleiotrophin
with a threshold of 1e−05
rs7742829
rs73128290
rs77285108
rs12404911
All − MR Egger
All − IVW(MRE)
−2
−1
0
1
MR effect size for
'Gut microbiota abundance (genus Catenibacterium id.2153)' on 'Pleiotrophin'

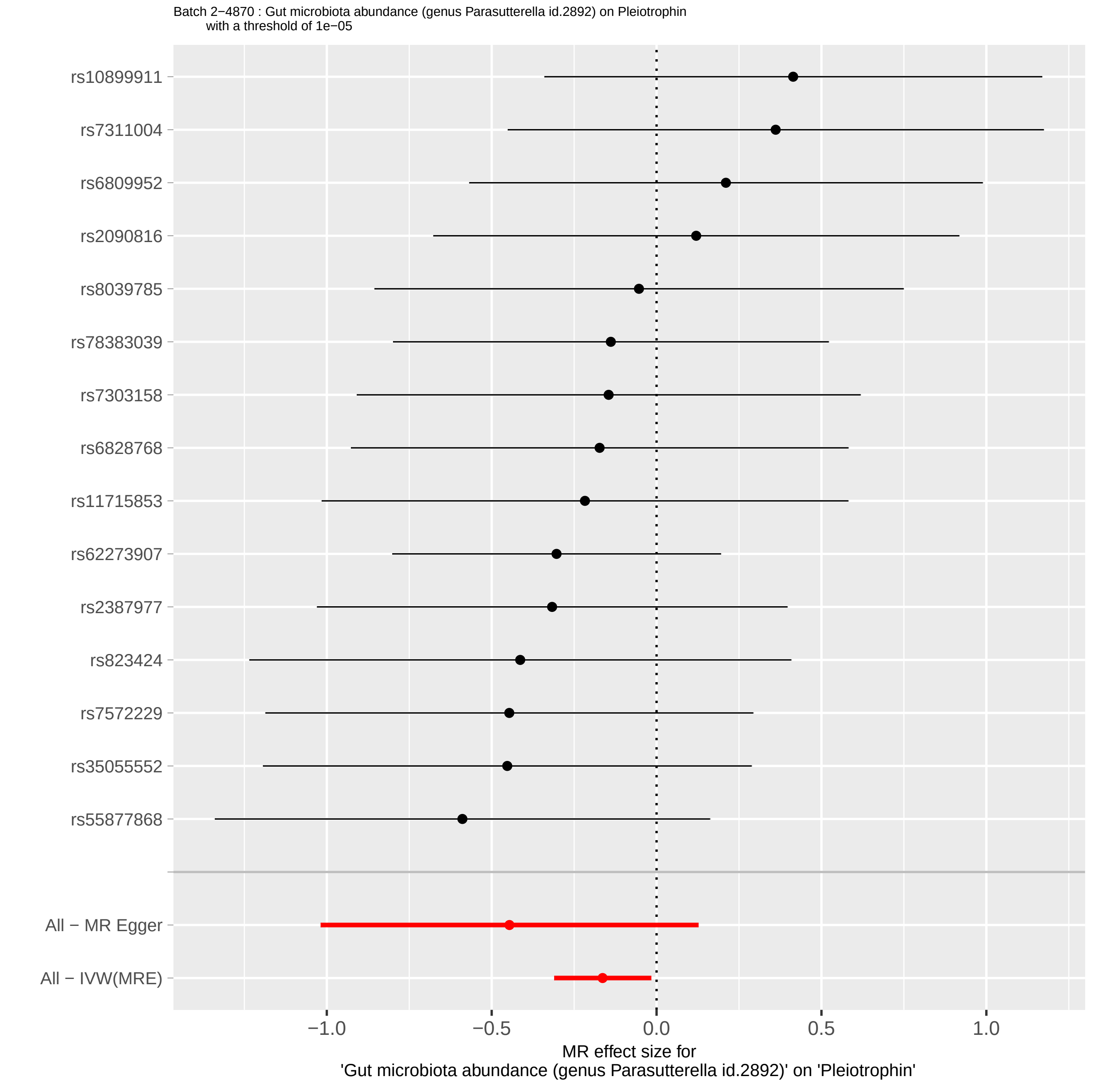
Batch 2−4870 : Gut microbiota abundance (genus Parasutterella id.2892) on Pleiotrophin
with a threshold of 1e−05
rs10899911
rs7311004
rs6809952
rs2090816
rs8039785
rs78383039
rs7303158
rs6828768
rs11715853
rs62273907
rs2387977
rs823424
rs7572229
rs35055552
rs55877868
All − MR Egger
All − IVW(MRE)
−1.0
−0.5
0.0
0.5
1.0
MR effect size for
'Gut microbiota abundance (genus Parasutterella id.2892)' on 'Pleiotrophin'

Batch 2−4871 : Gut microbiota abundance (genus Catenibacterium id.2153) on Protein tyrosine phosphatase type IVA 2
with a threshold of 1e−05

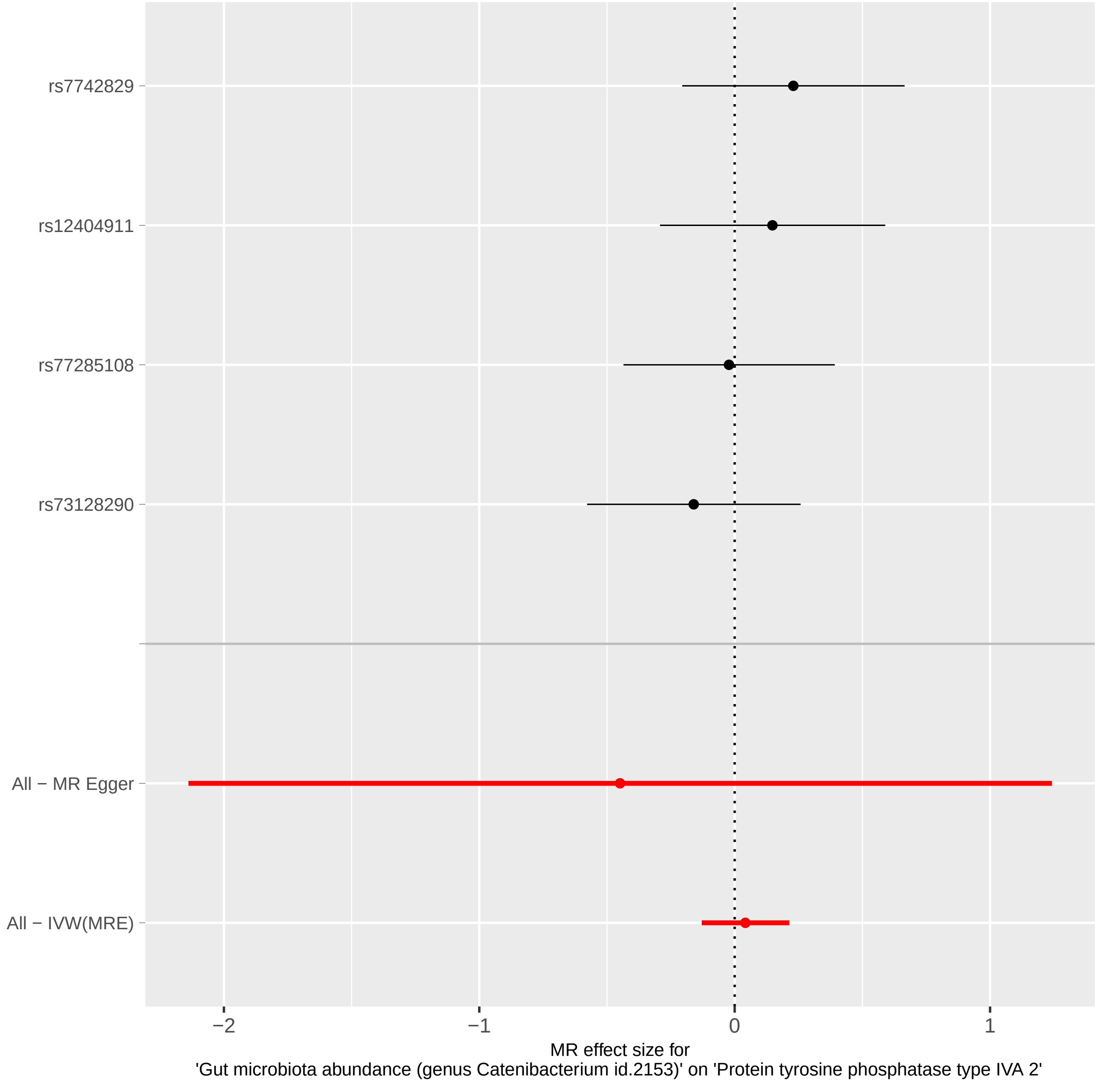

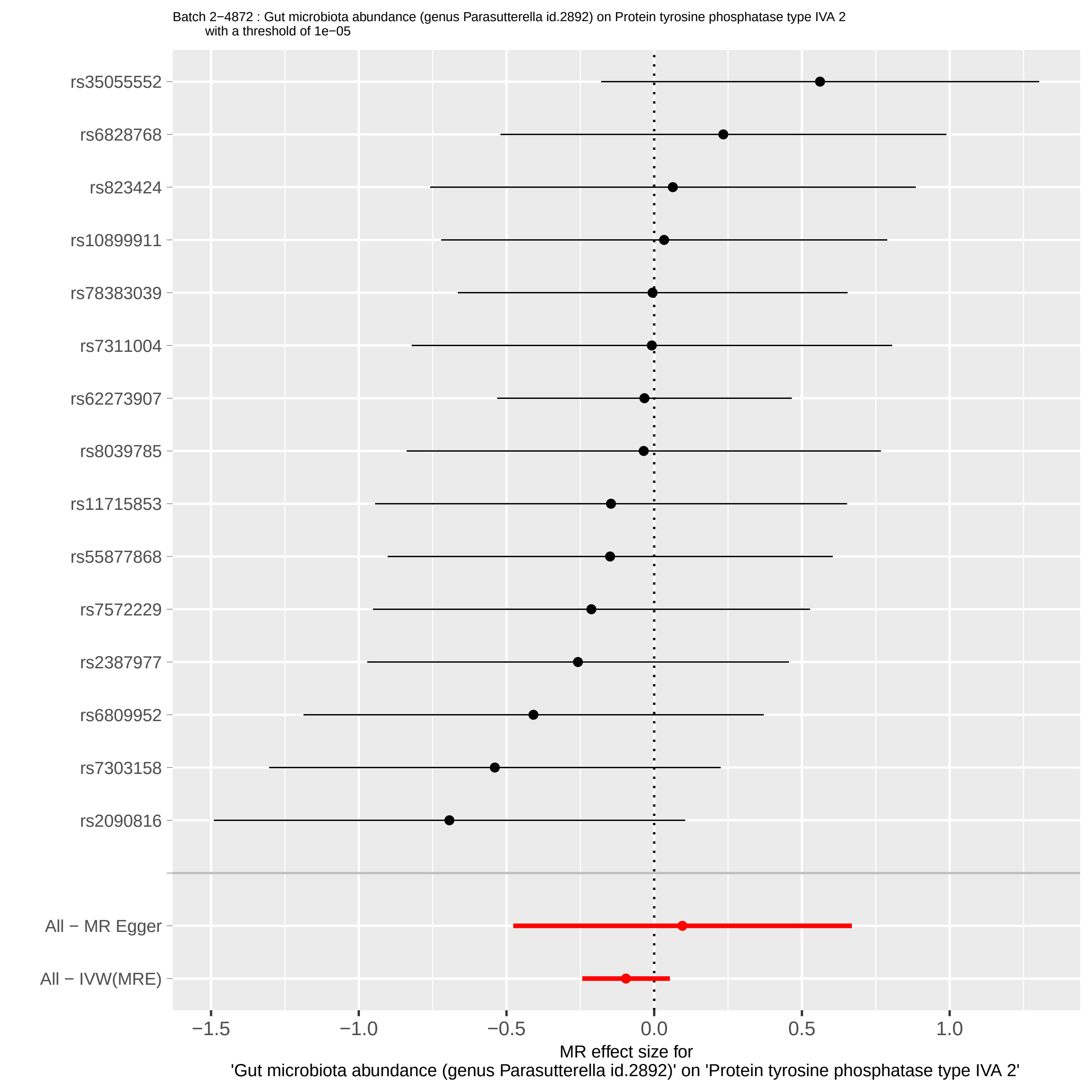

Batch 2−4872 : Gut microbiota abundance (genus Parasutterella id.2892) on Protein tyrosine phosphatase type IVA 2
with a threshold of 1e−05
rs35055552
rs6828768
rs823424
rs10899911
rs78383039
rs7311004
rs62273907
rs8039785
rs11715853
rs55877868
rs7572229
rs2387977
rs6809952
rs7303158
rs2090816
All − MR Egger
All − IVW(MRE)
−1.5
−1.0
−0.5
0.0
0.5
1.0
MR effect size for
'Gut microbiota abundance (genus Parasutterella id.2892)' on 'Protein tyrosine phosphatase type IVA 2'

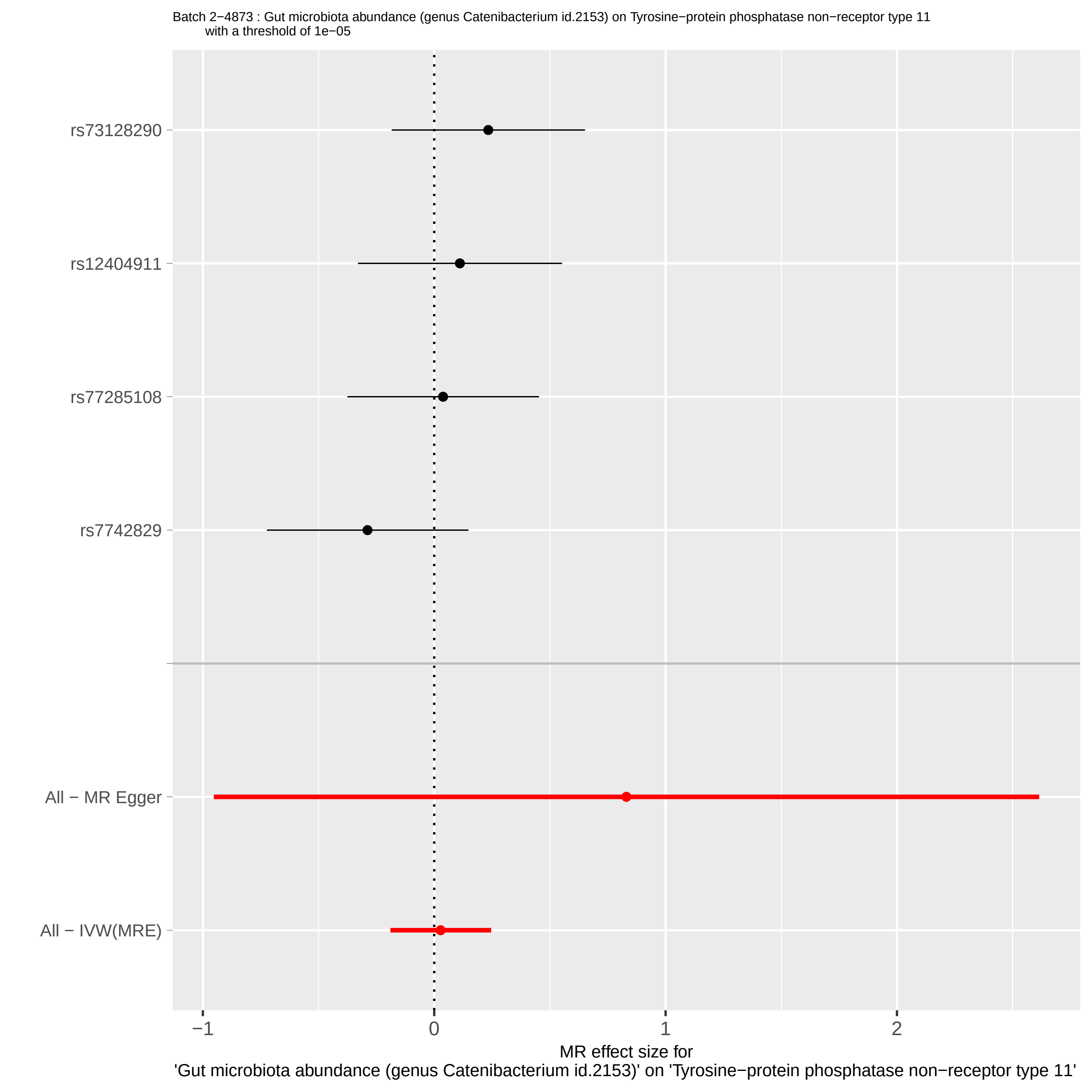
Batch 2−4873 : Gut microbiota abundance (genus Catenibacterium id.2153) on Tyrosine−protein phosphatase non−receptor type 11
with a threshold of 1e−05
rs73128290
rs12404911
rs77285108
rs7742829
All − MR Egger
All − IVW(MRE)
−1
0
1
2
MR effect size for
'Gut microbiota abundance (genus Catenibacterium id.2153)' on 'Tyrosine−protein phosphatase non−receptor type 11'

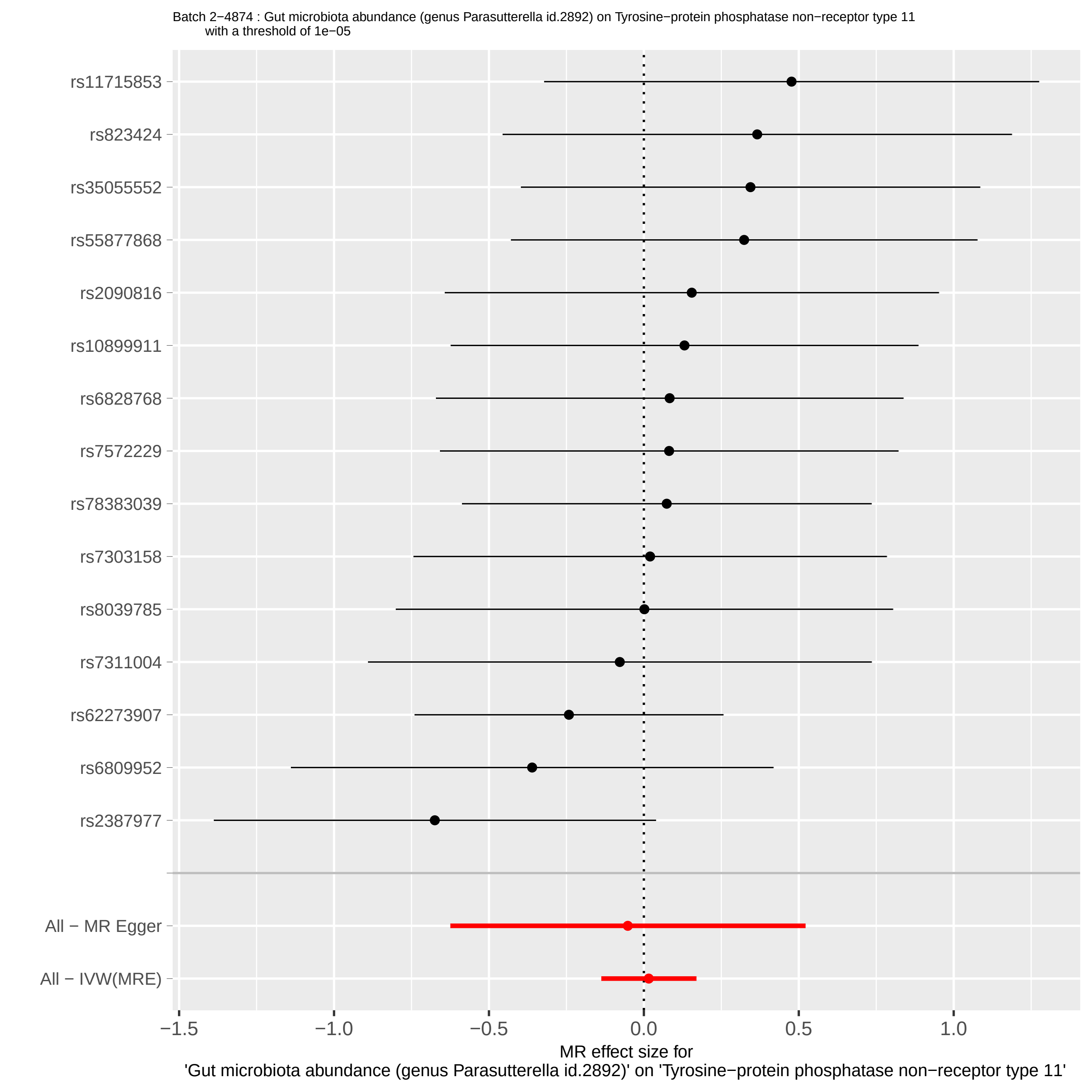

Batch 2−4874 : Gut microbiota abundance (genus Parasutterella id.2892) on Tyrosine−protein phosphatase non−receptor type 11
with a threshold of 1e−05
rs11715853
rs823424
rs35055552
rs55877868
rs2090816
rs10899911
rs6828768
rs7572229
rs78383039
rs7303158
rs8039785
rs7311004
rs62273907
rs6809952
rs2387977
All − MR Egger
All − IVW(MRE)
−1.5
−1.0
−0.5
0.0
0.5
1.0
MR effect size for
'Gut microbiota abundance (genus Parasutterella id.2892)' on 'Tyrosine−protein phosphatase non−receptor type 11'

Batch 2−4875 : Gut microbiota abundance (genus Catenibacterium id.2153) on Tyrosine−protein phosphatase non−receptor type 1
with a threshold of 1e−05

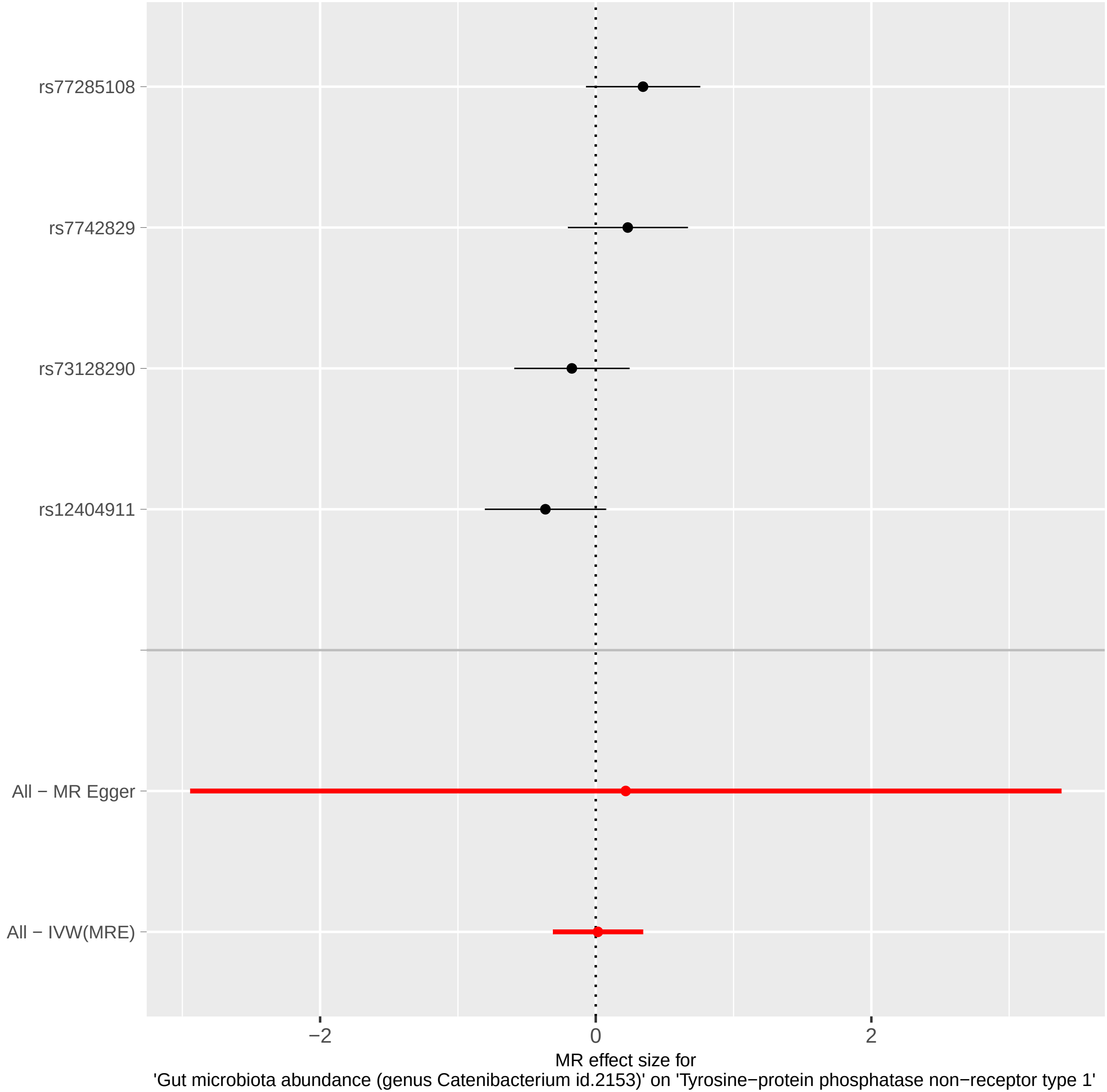

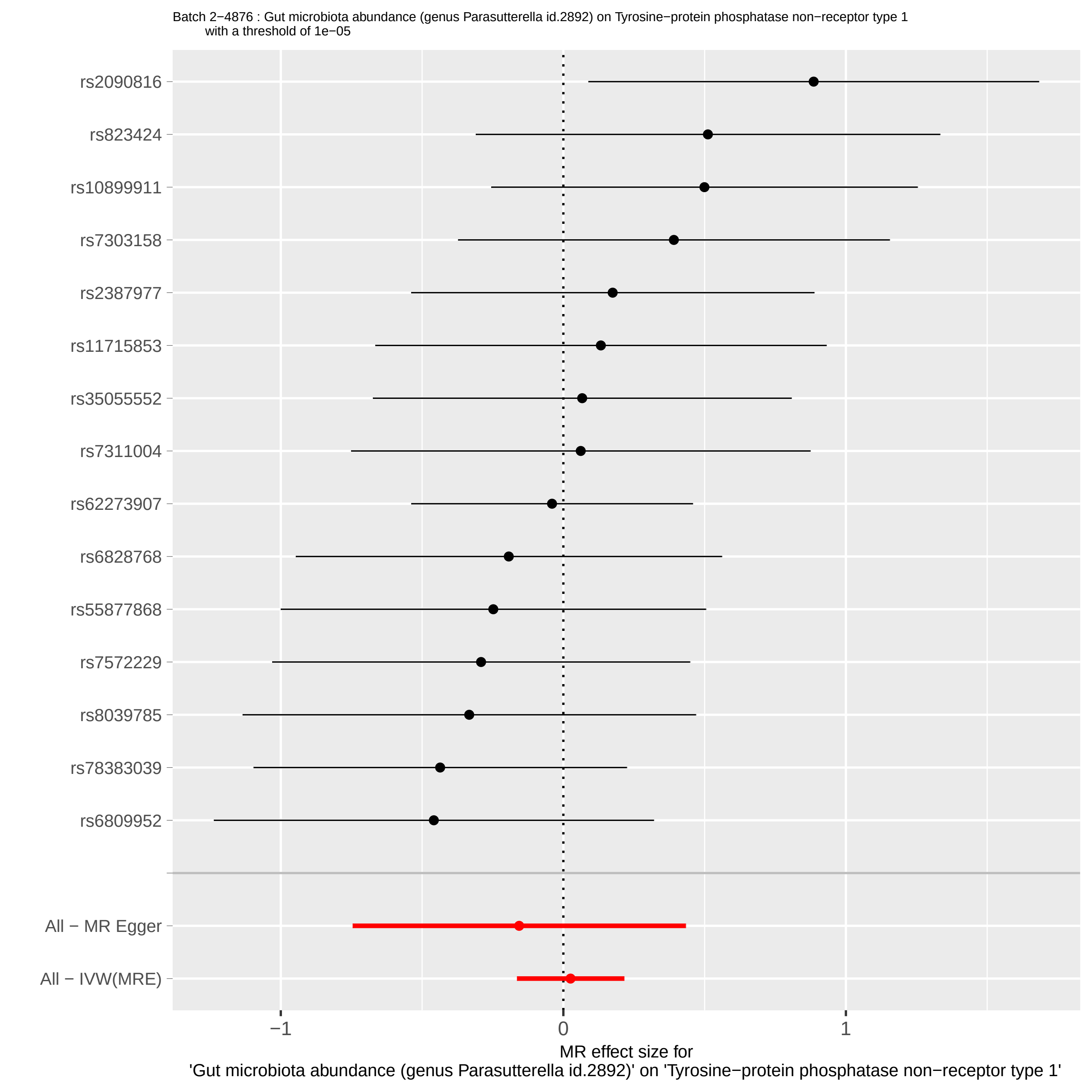
Batch 2−4876 : Gut microbiota abundance (genus Parasutterella id.2892) on Tyrosine−protein phosphatase non−receptor type 1
with a threshold of 1e−05
rs2090816
rs823424
rs10899911
rs7303158
rs2387977
rs11715853
rs35055552
rs7311004
rs62273907
rs6828768
rs55877868
rs7572229
rs8039785
rs78383039
rs6809952
All − MR Egger
All − IVW(MRE)
−1
0
1
MR effect size for
'Gut microbiota abundance (genus Parasutterella id.2892)' on 'Tyrosine−protein phosphatase non−receptor type 1'

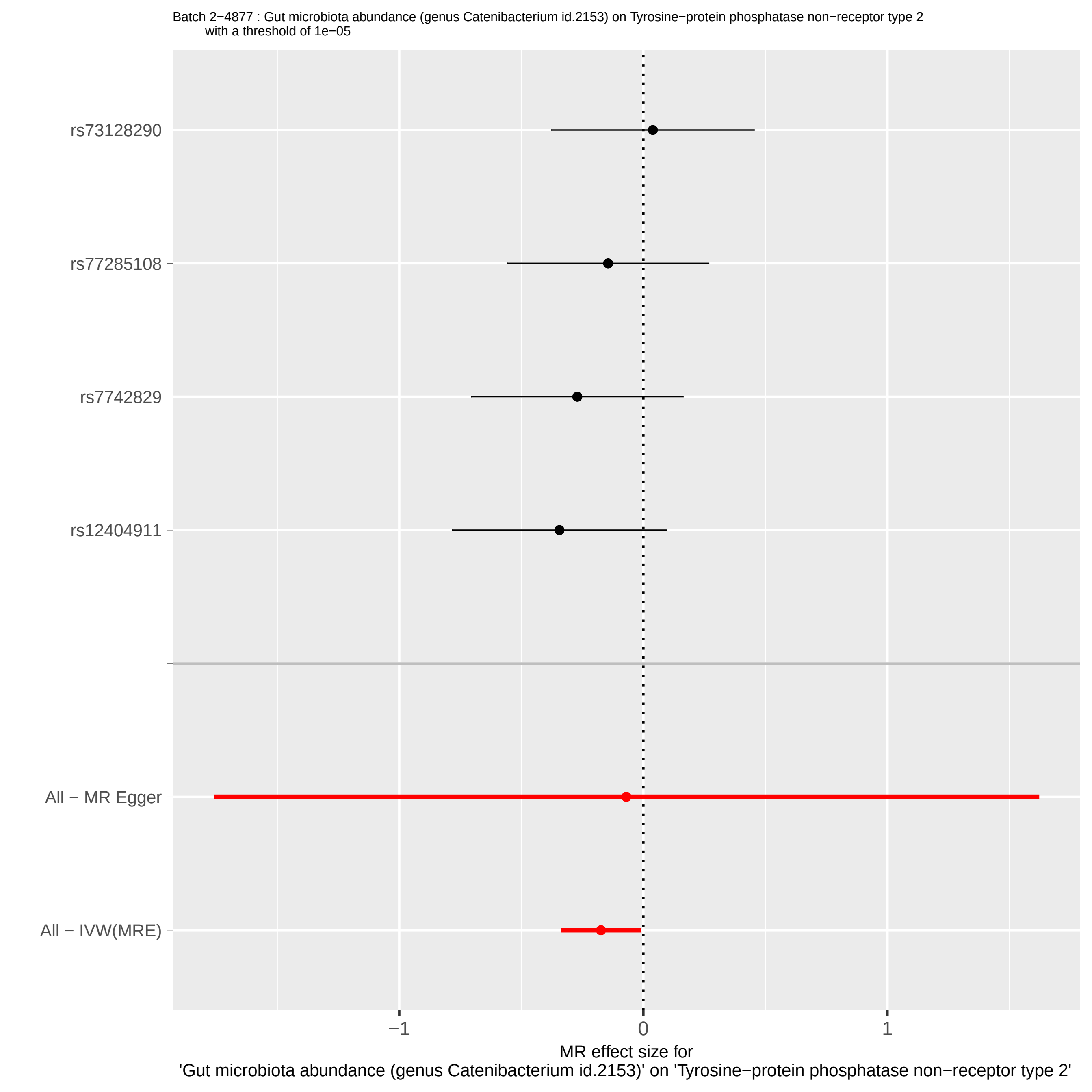

Batch 2−4877 : Gut microbiota abundance (genus Catenibacterium id.2153) on Tyrosine−protein phosphatase non−receptor type 2 with a threshold of 1e−05
rs73128290
rs77285108
rs7742829
rs12404911
All − MR Egger
All − IVW(MRE)
−1
0
1
MR effect size for
'Gut microbiota abundance (genus Catenibacterium id.2153)' on 'Tyrosine−protein phosphatase non−receptor type 2'

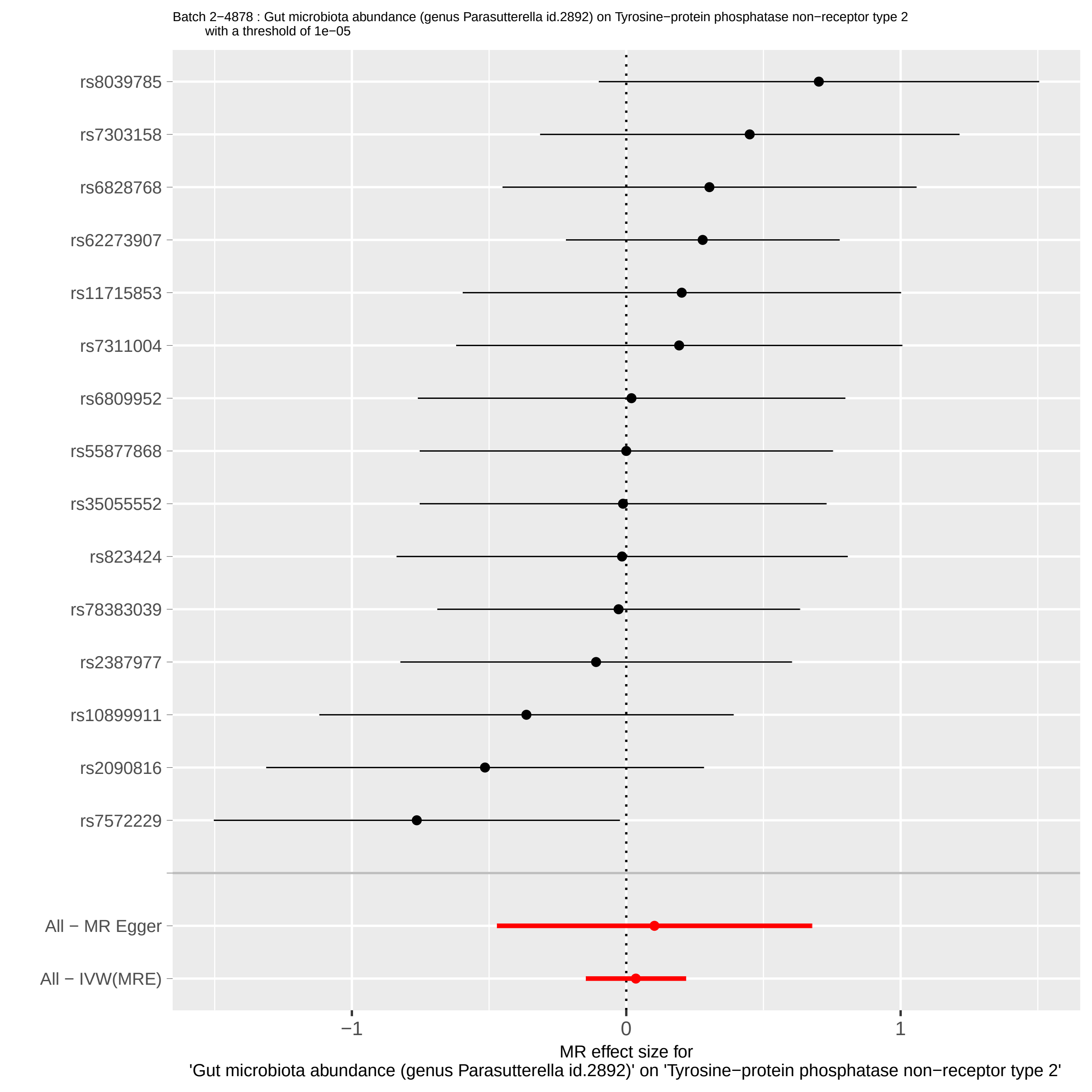

Batch 2−4878 : Gut microbiota abundance (genus Parasutterella id.2892) on Tyrosine−protein phosphatase non−receptor type 2
with a threshold of 1e−05
rs8039785
rs7303158
rs6828768
rs62273907
rs11715853
rs7311004
rs6809952
rs55877868
rs35055552
rs823424
rs78383039
rs2387977
rs10899911
rs2090816
rs7572229
All − MR Egger
All − IVW(MRE)
−1
0
1
MR effect size for
'Gut microbiota abundance (genus Parasutterella id.2892)' on 'Tyrosine−protein phosphatase non−receptor type 2'

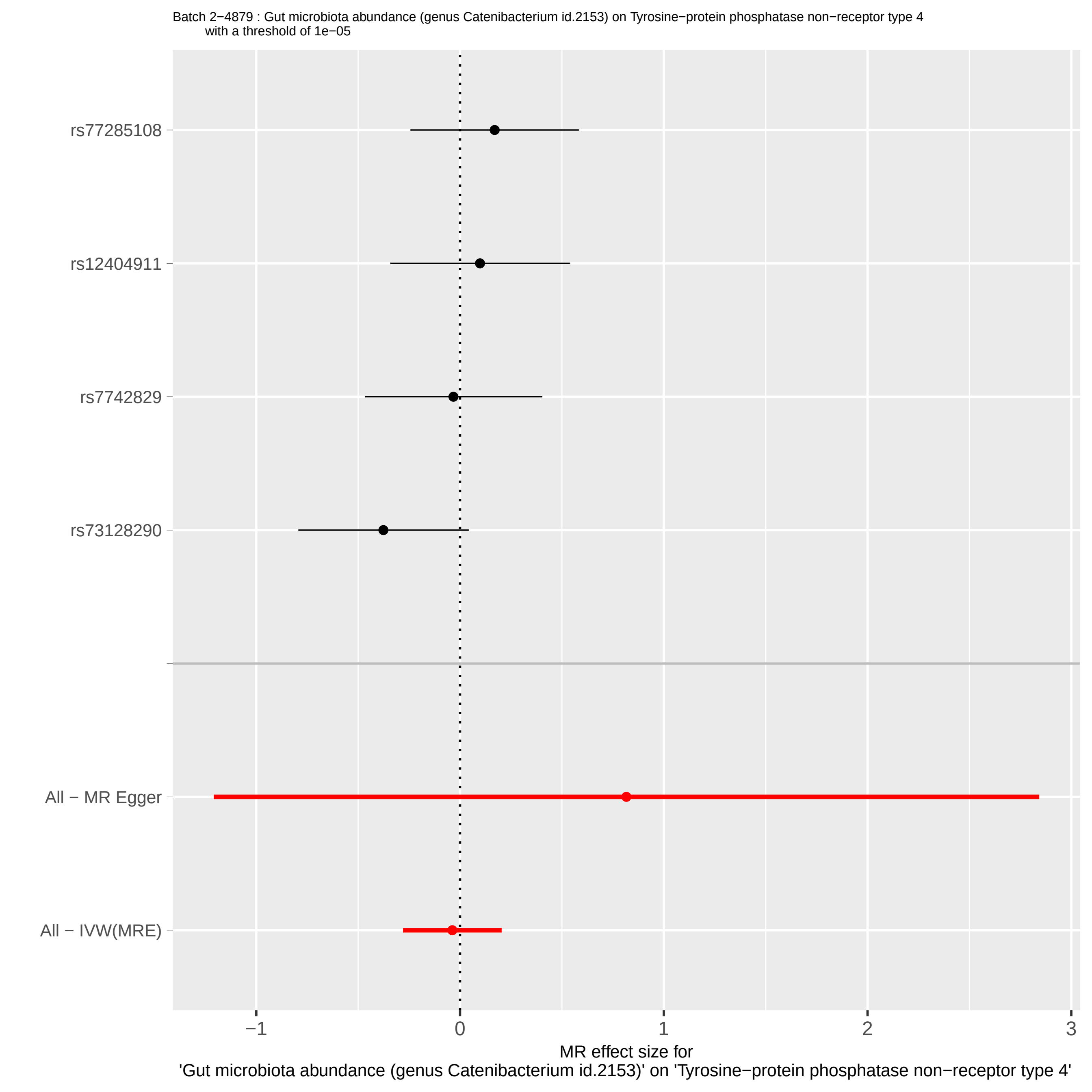
Batch 2−4879 : Gut microbiota abundance (genus Catenibacterium id.2153) on Tyrosine−protein phosphatase non−receptor type 4
with a threshold of 1e−05
rs77285108
rs12404911
rs7742829
rs73128290
All − MR Egger
All − IVW(MRE)
−1
0
1
2
3
MR effect size for
'Gut microbiota abundance (genus Catenibacterium id.2153)' on 'Tyrosine−protein phosphatase non−receptor type 4'

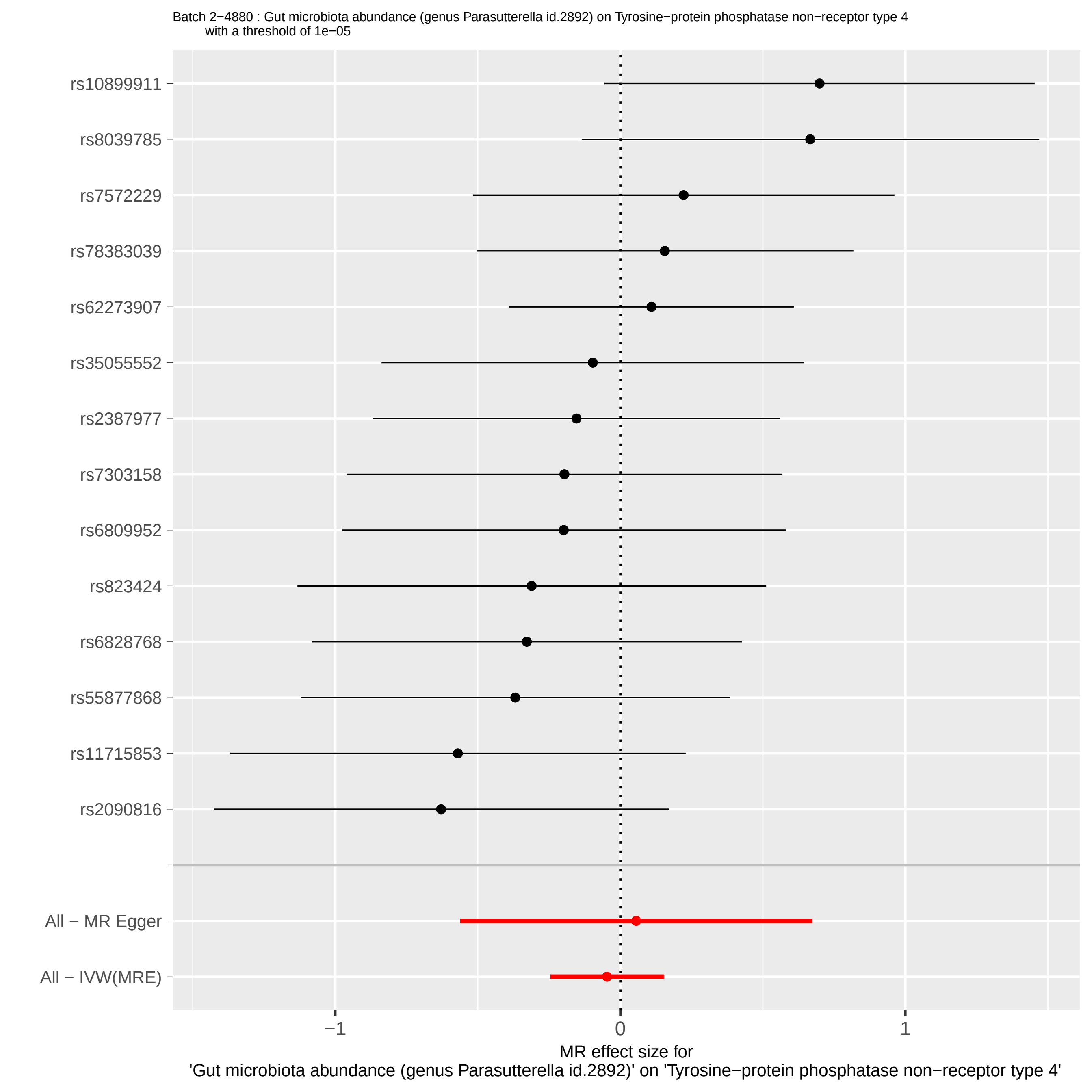

Batch 2−4880 : Gut microbiota abundance (genus Parasutterella id.2892) on Tyrosine−protein phosphatase non−receptor type 4
with a threshold of 1e−05
rs10899911
rs8039785
rs7572229
rs78383039
rs62273907
rs35055552
rs2387977
rs7303158
rs6809952
rs823424
rs6828768
rs55877868
rs11715853
rs2090816
All − MR Egger
All − IVW(MRE)
−1
0
1
MR effect size for
'Gut microbiota abundance (genus Parasutterella id.2892)' on 'Tyrosine−protein phosphatase non−receptor type 4'

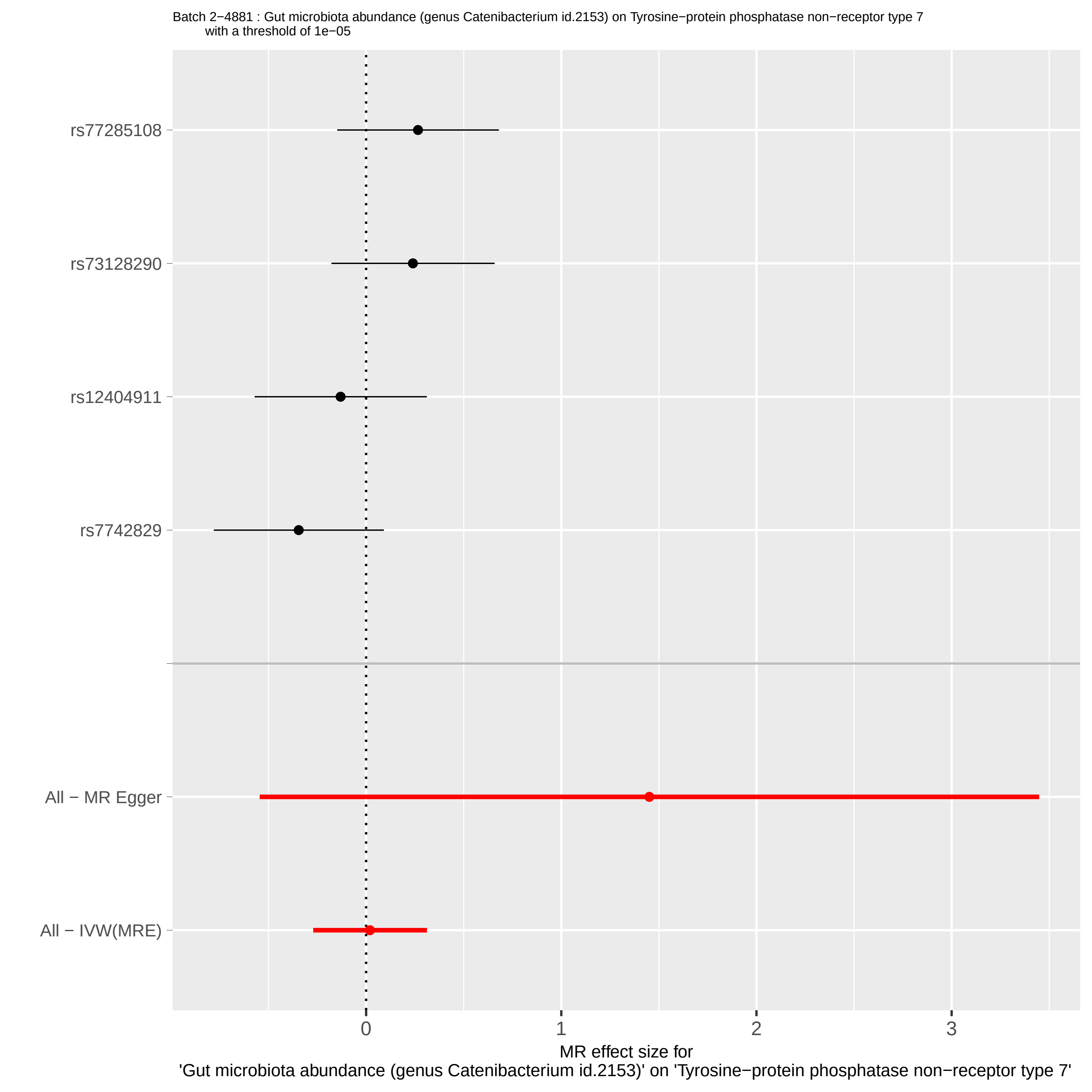
Batch 2−4881 : Gut microbiota abundance (genus Catenibacterium id.2153) on Tyrosine−protein phosphatase non−receptor type 7
with a threshold of 1e−05
rs77285108
rs73128290
rs12404911
rs7742829
All − MR Egger
All − IVW(MRE)
0
1
2
3
MR effect size for
'Gut microbiota abundance (genus Catenibacterium id.2153)' on 'Tyrosine−protein phosphatase non−receptor type 7'

Batch 2−4882 : Gut microbiota abundance (genus Parasutterella id.2892) on Tyrosine−protein phosphatase non−receptor type 7 with a threshold of 1e−05

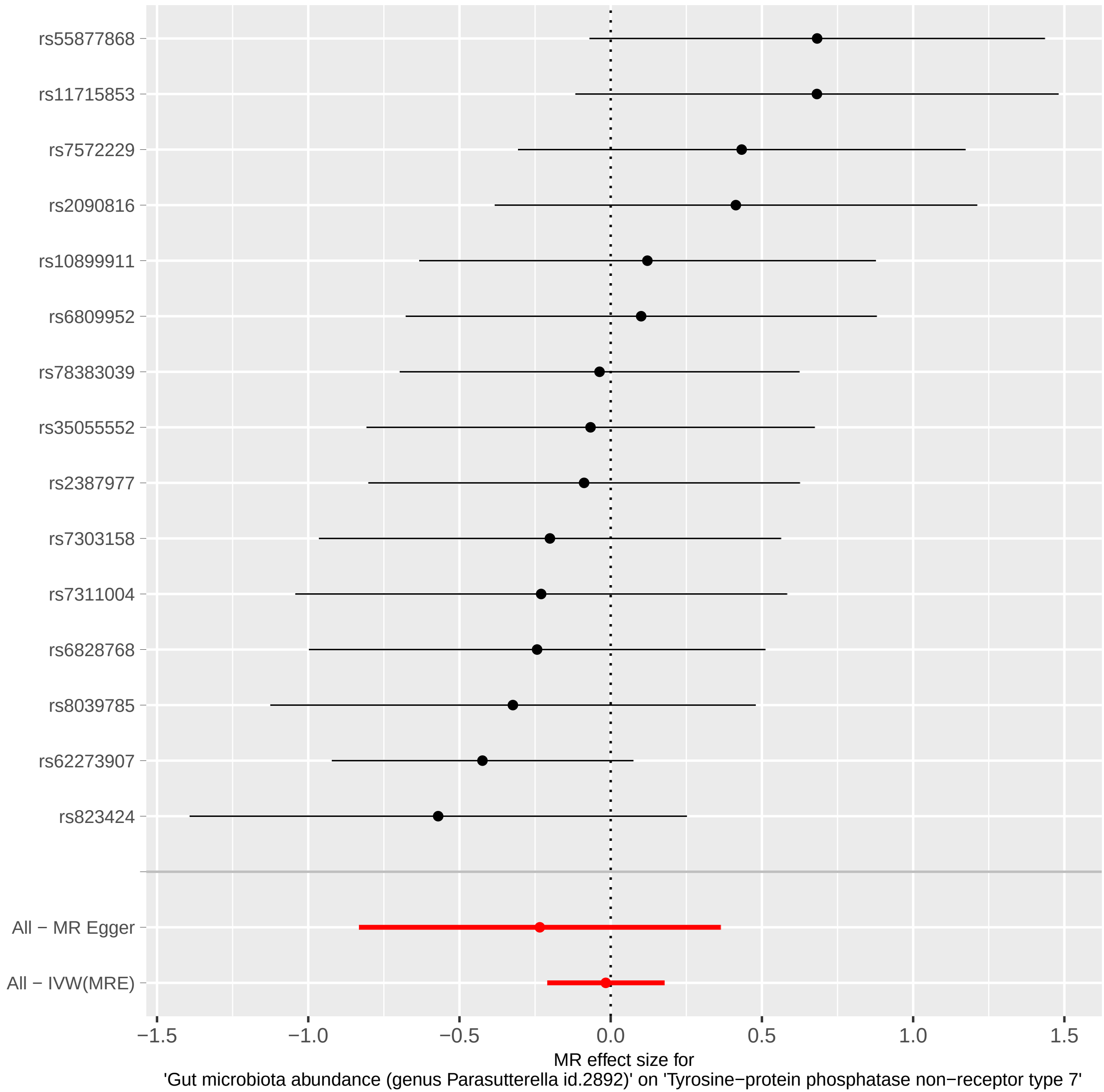

Batch 2−4883 : Gut microbiota abundance (genus Catenibacterium id.2153) on Tyrosine−protein phosphatase non−receptor type 9 with a threshold of 1e−05

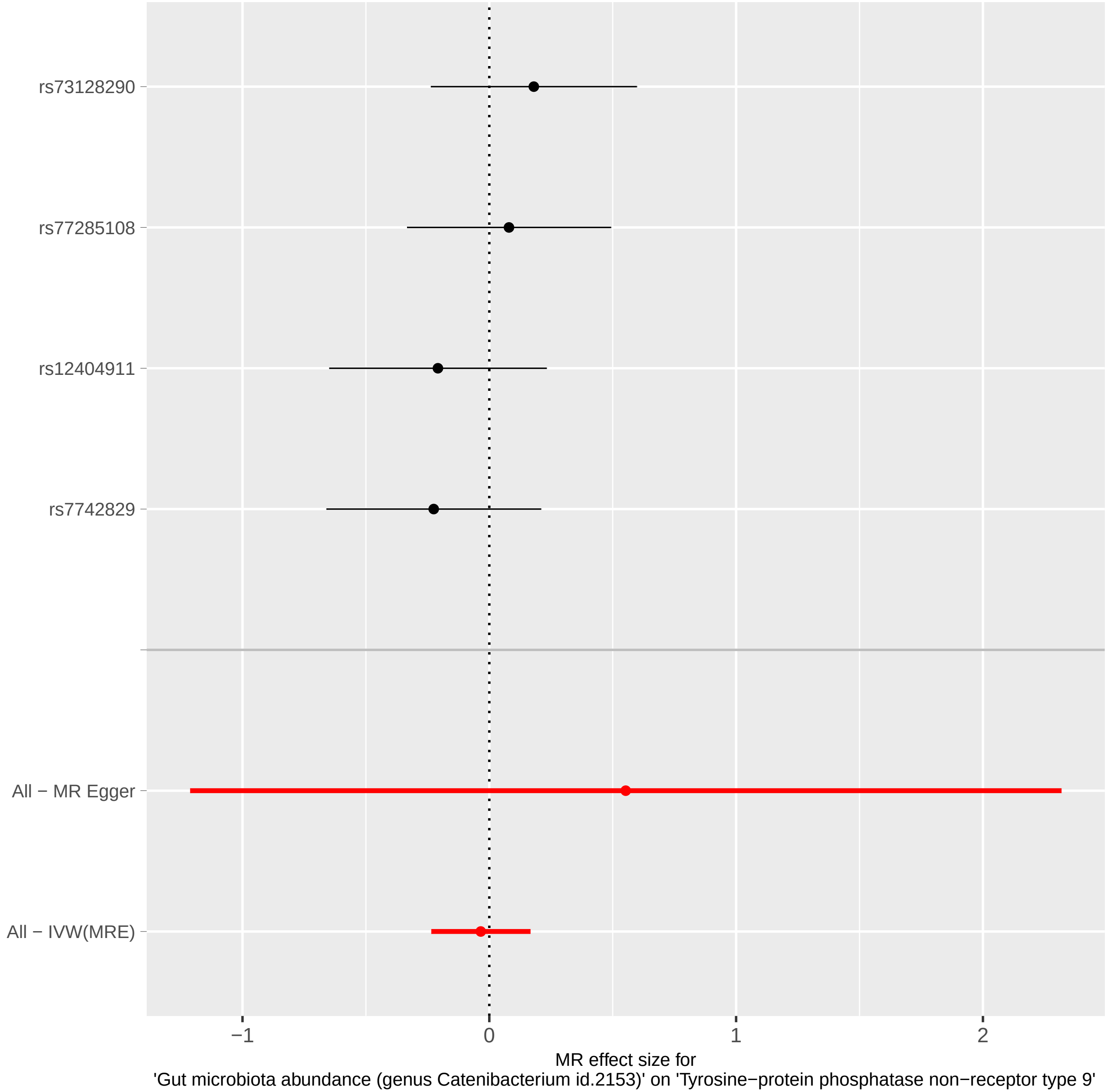

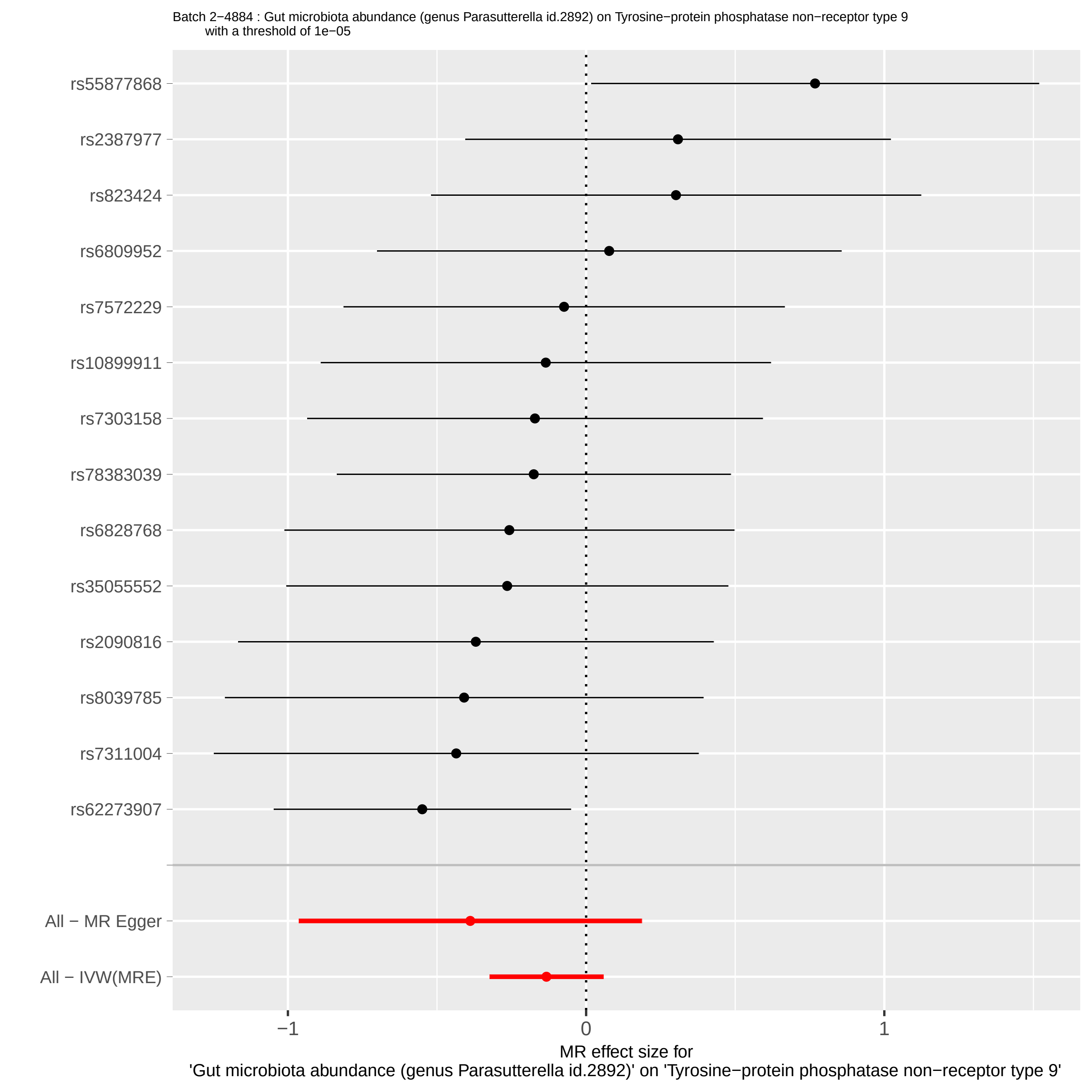

Batch 2−4884 : Gut microbiota abundance (genus Parasutterella id.2892) on Tyrosine−protein phosphatase non−receptor type 9
with a threshold of 1e−05
rs55877868
rs2387977
rs823424
rs6809952
rs7572229
rs10899911
rs7303158
rs78383039
rs6828768
rs35055552
rs2090816
rs8039785
rs7311004
rs62273907
All − MR Egger
All − IVW(MRE)
−1
0
1
MR effect size for
'Gut microbiota abundance (genus Parasutterella id.2892)' on 'Tyrosine−protein phosphatase non−receptor type 9'

Batch 2−4885 : Gut microbiota abundance (genus Catenibacterium id.2153) on Receptor−type tyrosine−protein phosphatase delta
with a threshold of 1e−05

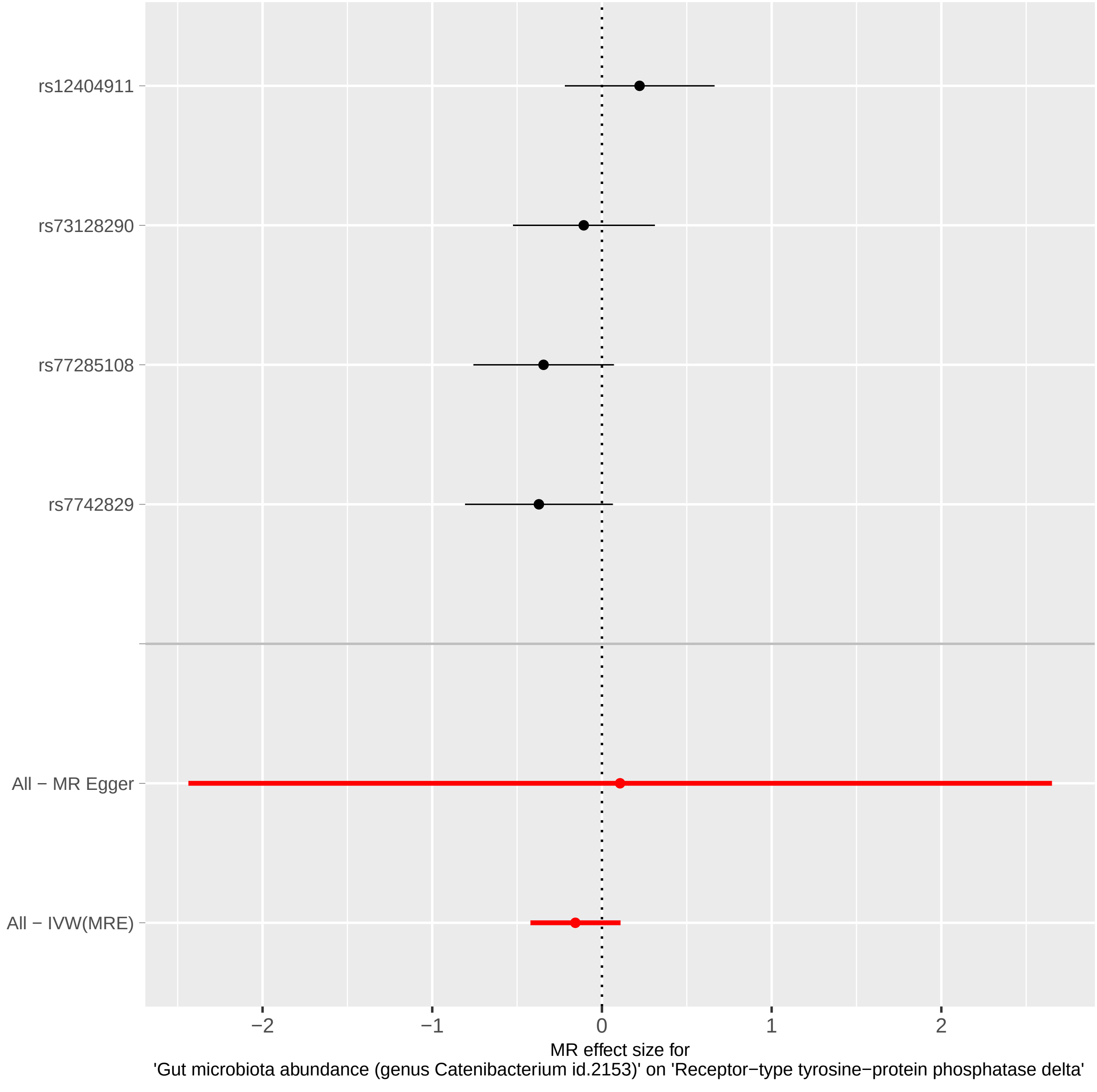

Batch 2−4886 : Gut microbiota abundance (genus Parasutterella id.2892) on Receptor−type tyrosine−protein phosphatase delta with a threshold of 1e−05

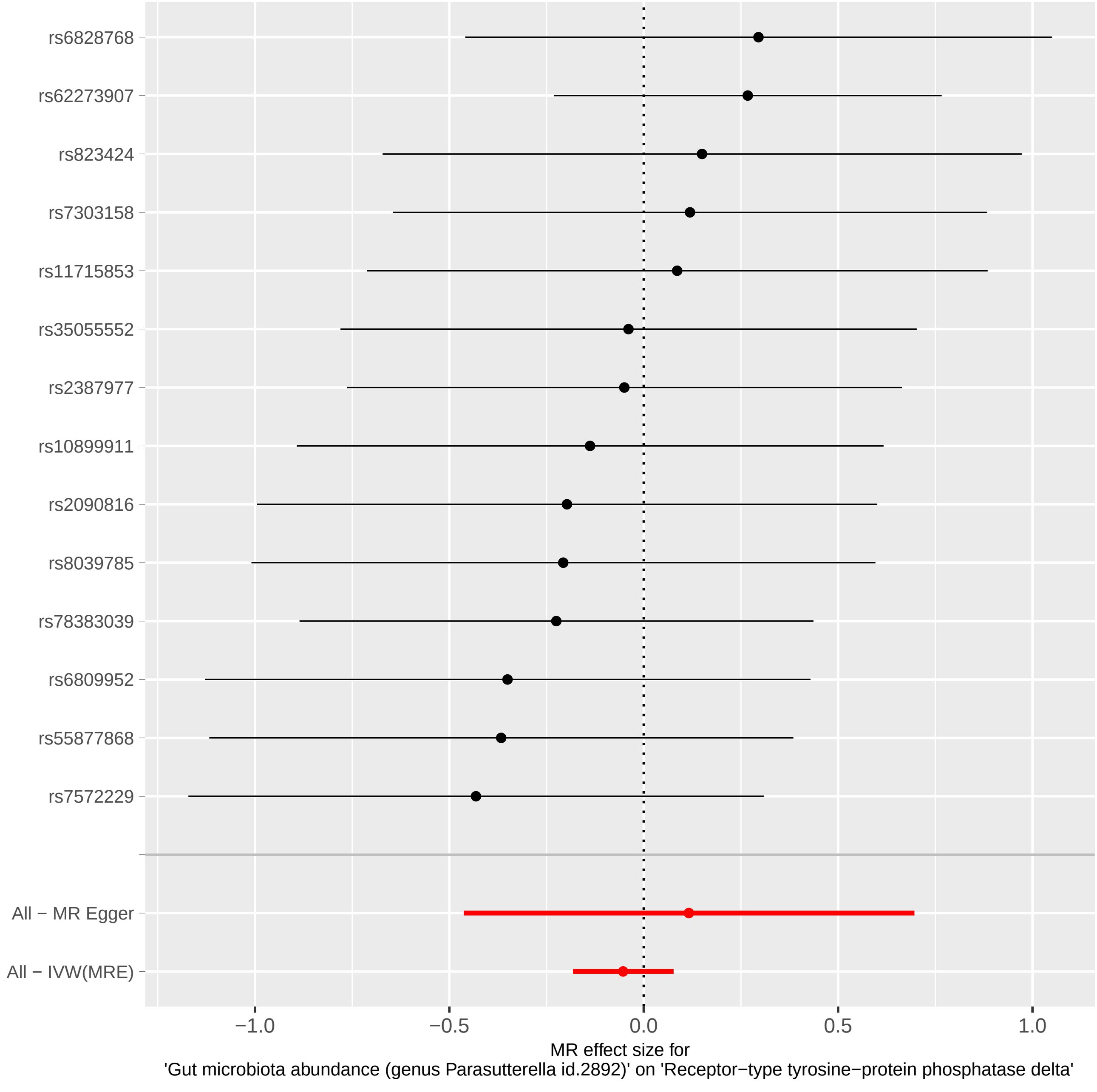

Batch 2−4887 : Gut microbiota abundance (genus Catenibacterium id.2153) on Receptor−type tyrosine−protein phosphatase H
with a threshold of 1e−05

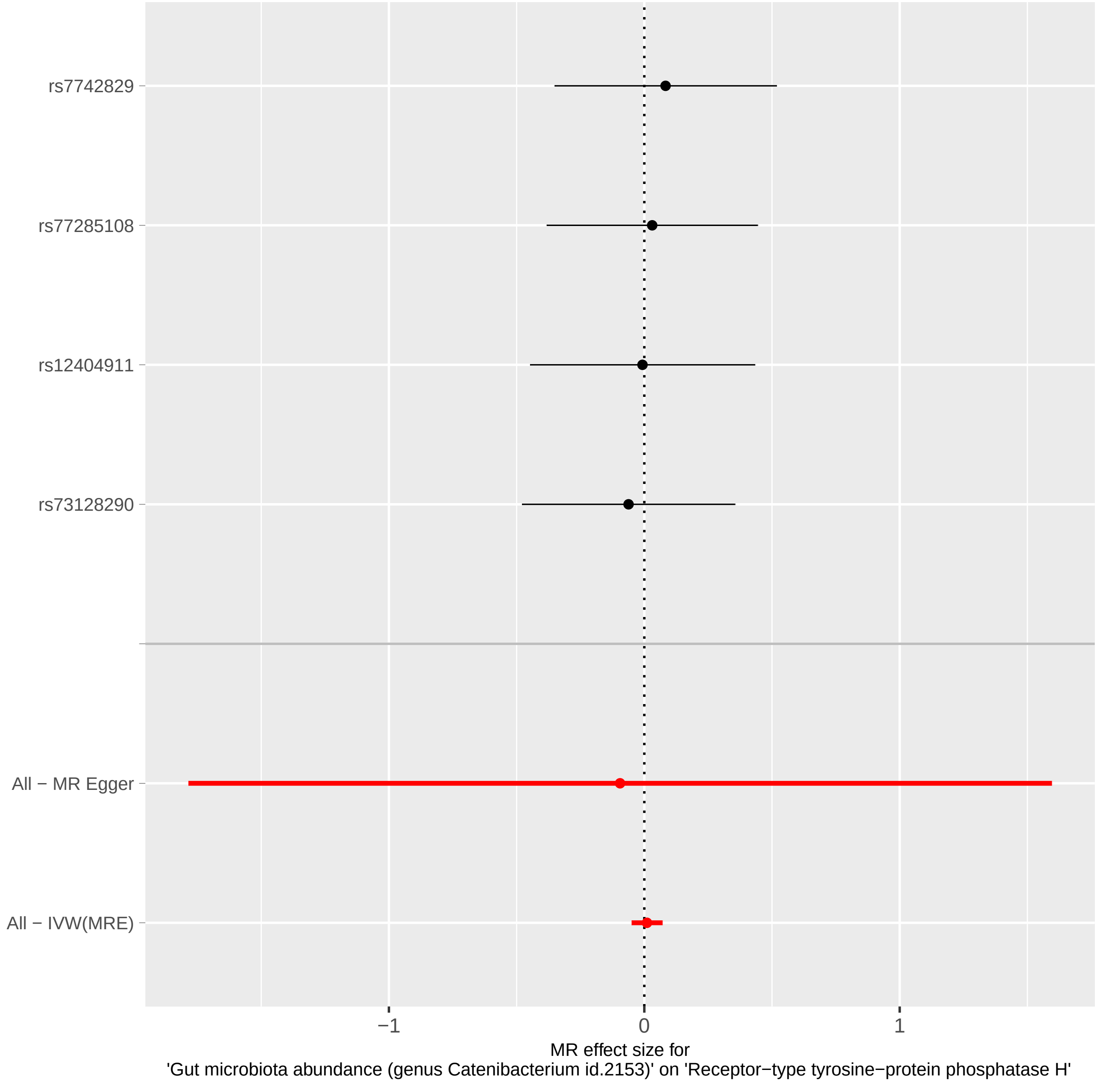

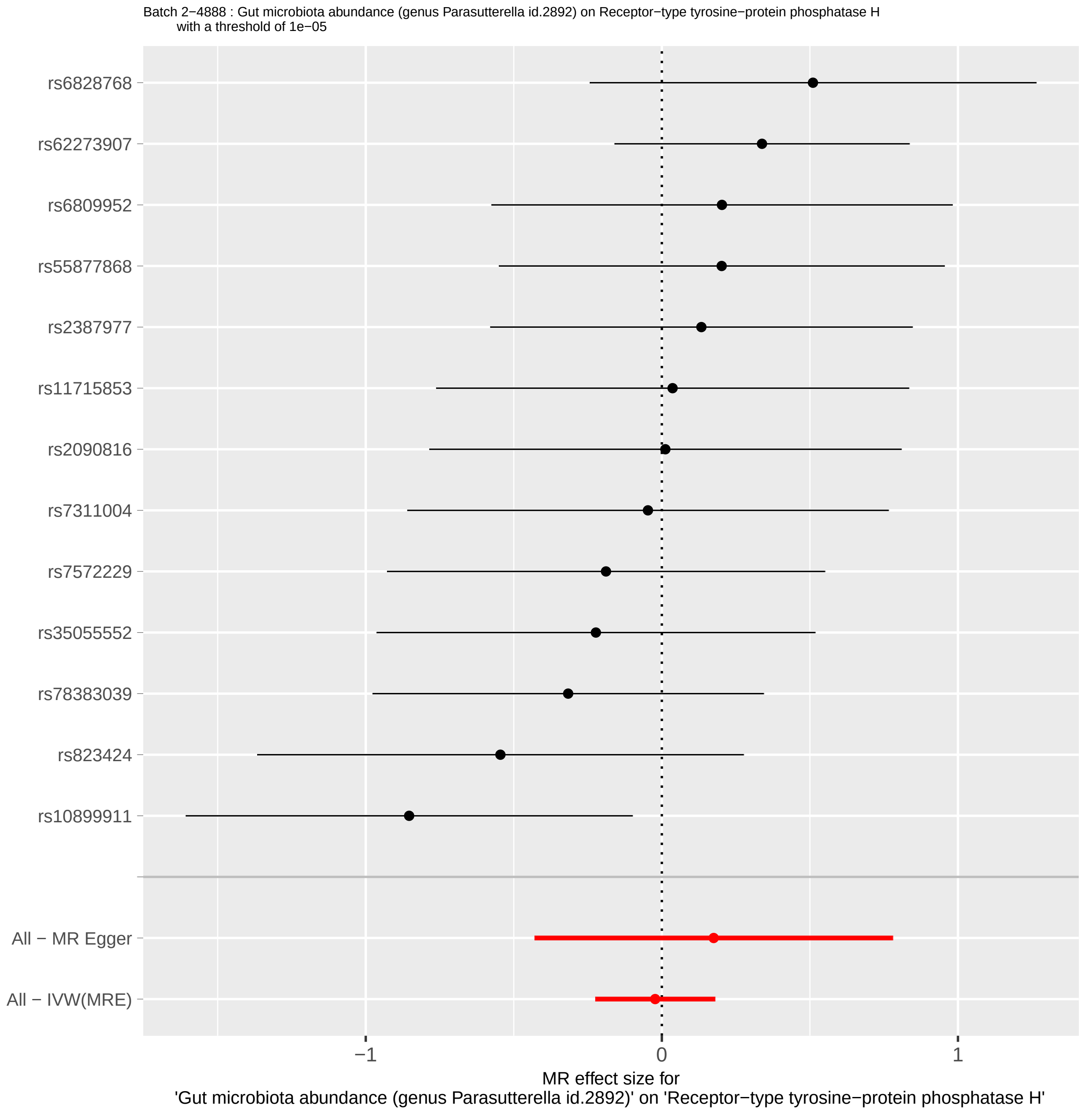
Batch 2−4888 : Gut microbiota abundance (genus Parasutterella id.2892) on Receptor−type tyrosine−protein phosphatase H
with a threshold of 1e−05
rs6828768
rs62273907
rs6809952
rs55877868
rs2387977
rs11715853
rs2090816
rs7311004
rs7572229
rs35055552
rs78383039
rs823424
rs10899911
All − MR Egger
All − IVW(MRE)
−1
0
1
MR effect size for
'Gut microbiota abundance (genus Parasutterella id.2892)' on 'Receptor−type tyrosine−protein phosphatase H'

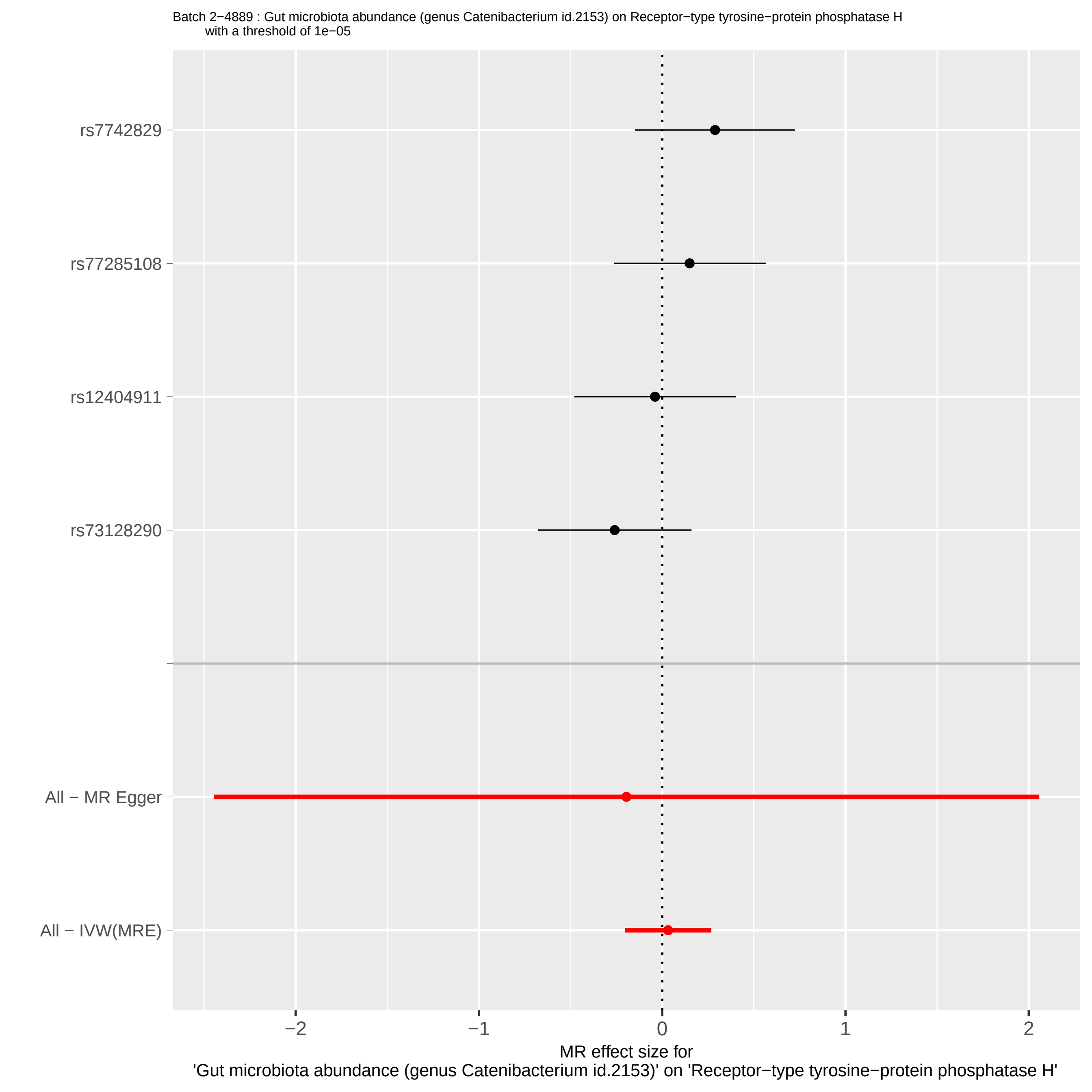

Batch 2−4889 : Gut microbiota abundance (genus Catenibacterium id.2153) on Receptor−type tyrosine−protein phosphatase H
with a threshold of 1e−05
rs7742829
rs77285108
rs12404911
rs73128290
All − MR Egger
All − IVW(MRE)
−2
−1
0
1
2
MR effect size for
'Gut microbiota abundance (genus Catenibacterium id.2153)' on 'Receptor−type tyrosine−protein phosphatase H'

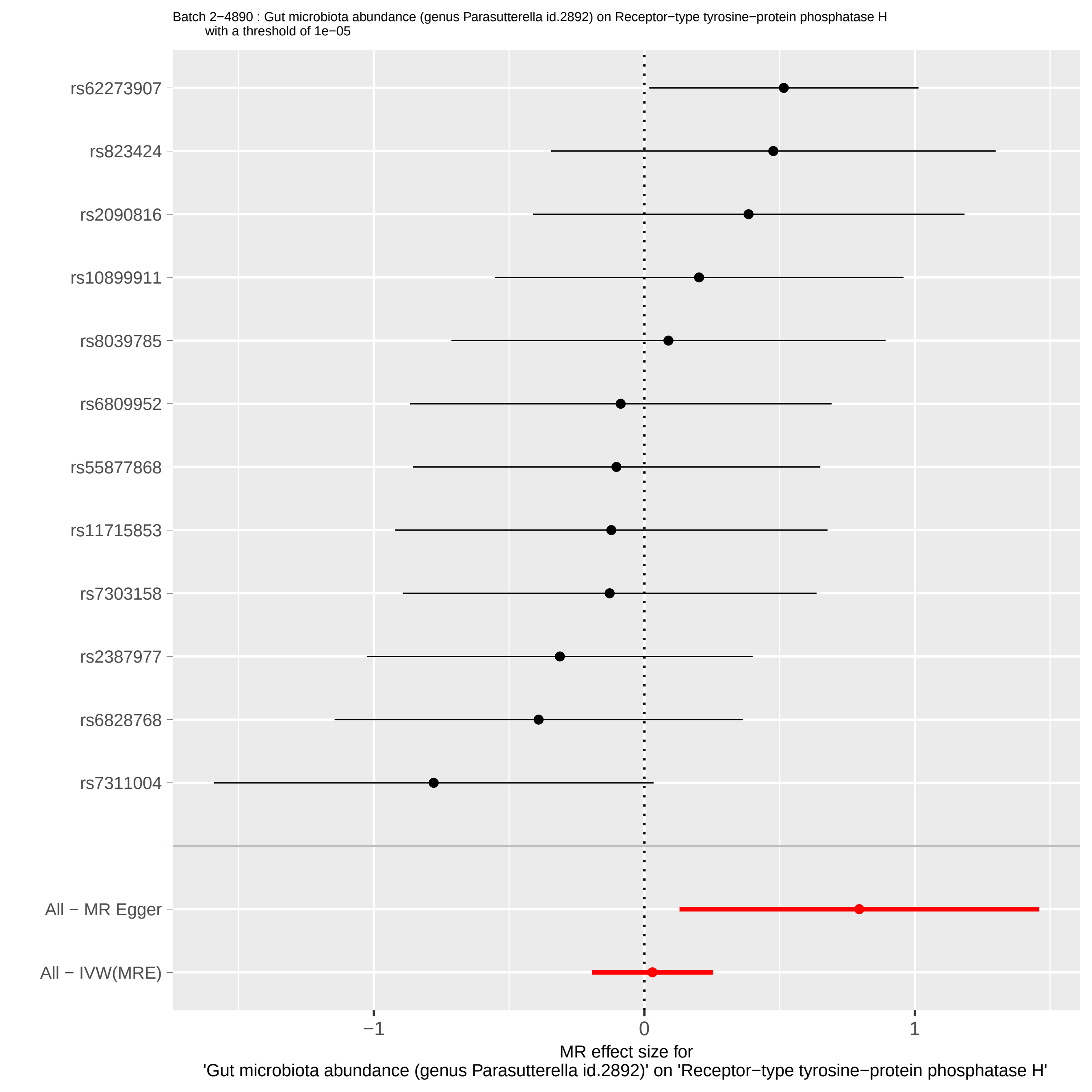

Batch 2−4890 : Gut microbiota abundance (genus Parasutterella id.2892) on Receptor−type tyrosine−protein phosphatase H
with a threshold of 1e−05
rs62273907
rs823424
rs2090816
rs10899911
rs8039785
rs6809952
rs55877868
rs11715853
rs7303158
rs2387977
rs6828768
rs7311004
All − MR Egger
All − IVW(MRE)
−1
0
1
MR effect size for
'Gut microbiota abundance (genus Parasutterella id.2892)' on 'Receptor−type tyrosine−protein phosphatase H'

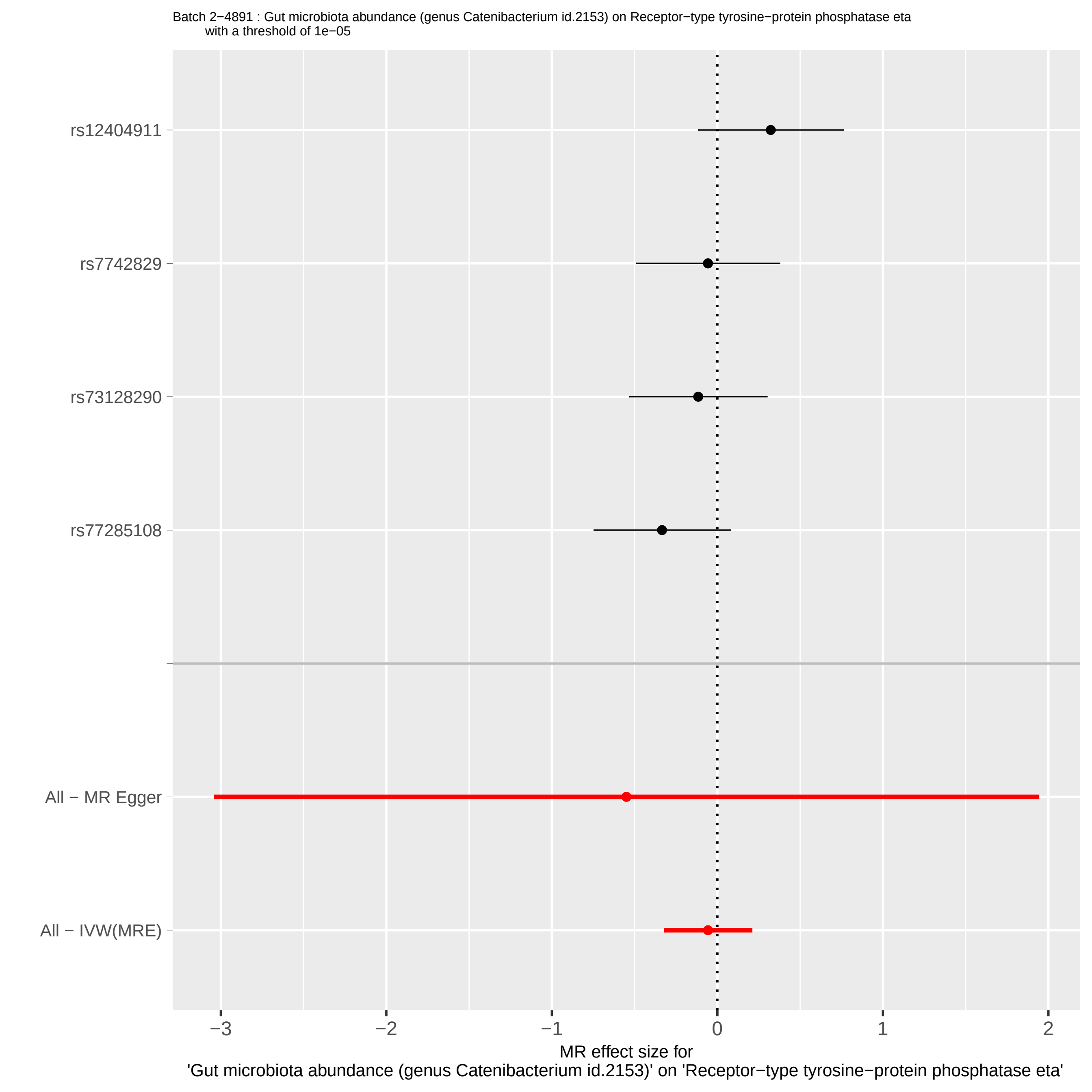

Batch 2−4891 : Gut microbiota abundance (genus Catenibacterium id.2153) on Receptor−type tyrosine−protein phosphatase eta
with a threshold of 1e−05
rs12404911
rs7742829
rs73128290
rs77285108
All − MR Egger
All − IVW(MRE)
−3
−2
−1
0
1
2
MR effect size for
'Gut microbiota abundance (genus Catenibacterium id.2153)' on 'Receptor−type tyrosine−protein phosphatase eta'

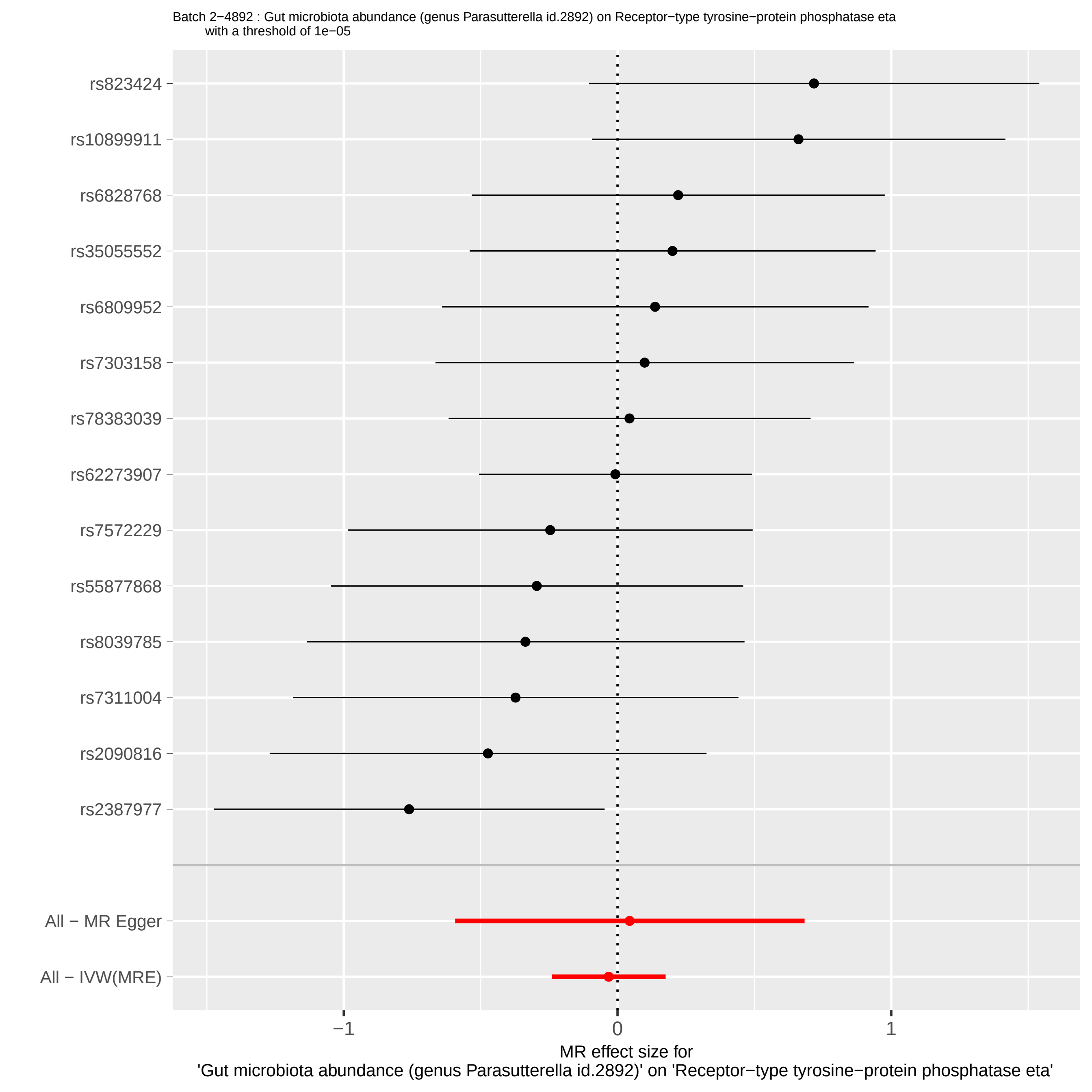

Batch 2−4892 : Gut microbiota abundance (genus Parasutterella id.2892) on Receptor−type tyrosine−protein phosphatase eta
with a threshold of 1e−05
rs823424
rs10899911
rs6828768
rs35055552
rs6809952
rs7303158
rs78383039
rs62273907
rs7572229
rs55877868
rs8039785
rs7311004
rs2090816
rs2387977
All − MR Egger
All − IVW(MRE)
−1
0
1
MR effect size for
'Gut microbiota abundance (genus Parasutterella id.2892)' on 'Receptor−type tyrosine−protein phosphatase eta'

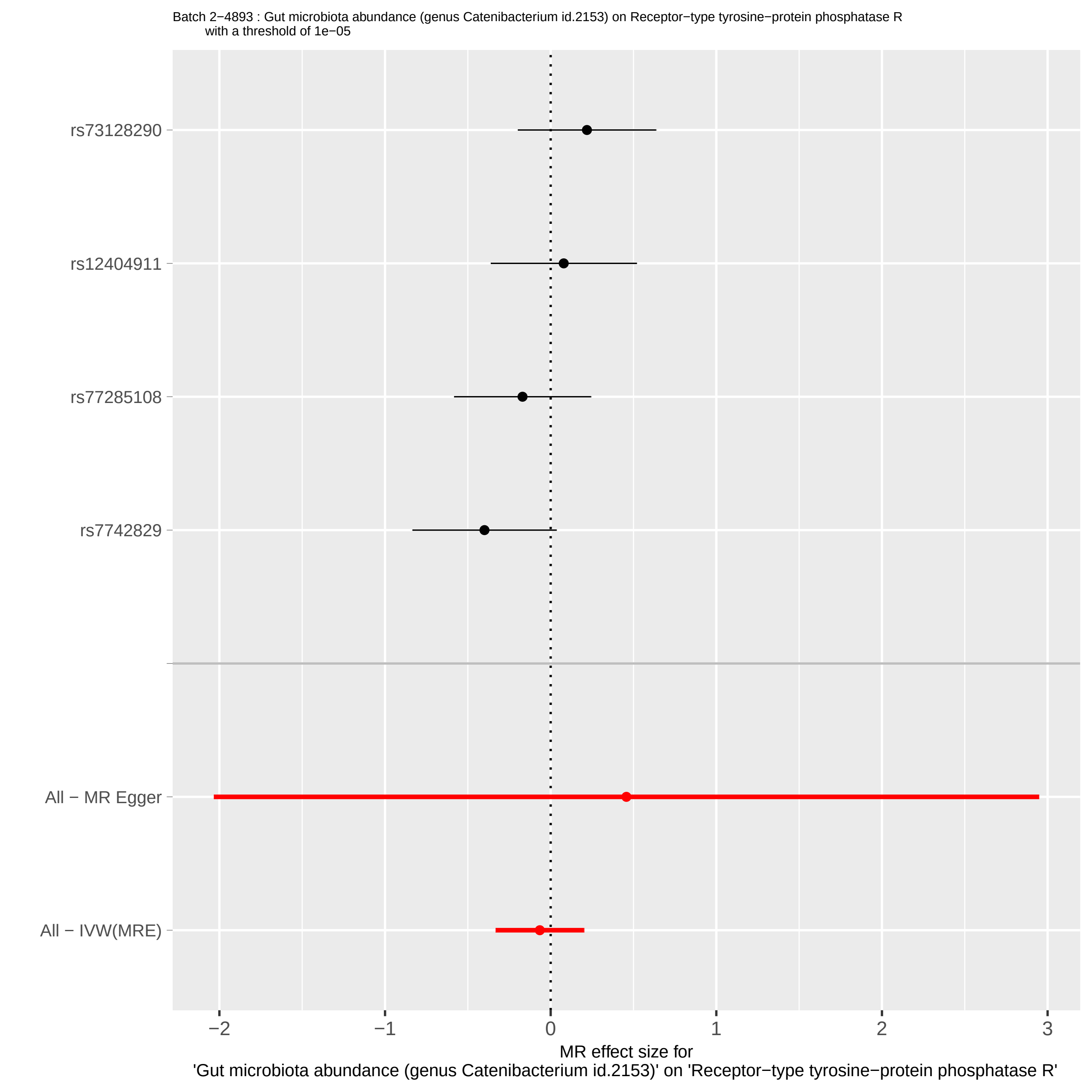
Batch 2−4893 : Gut microbiota abundance (genus Catenibacterium id.2153) on Receptor−type tyrosine−protein phosphatase R
with a threshold of 1e−05
rs73128290
rs12404911
rs77285108
rs7742829
All − MR Egger
All − IVW(MRE)
−2
−1
0
1
2
3
MR effect size for
'Gut microbiota abundance (genus Catenibacterium id.2153)' on 'Receptor−type tyrosine−protein phosphatase R'

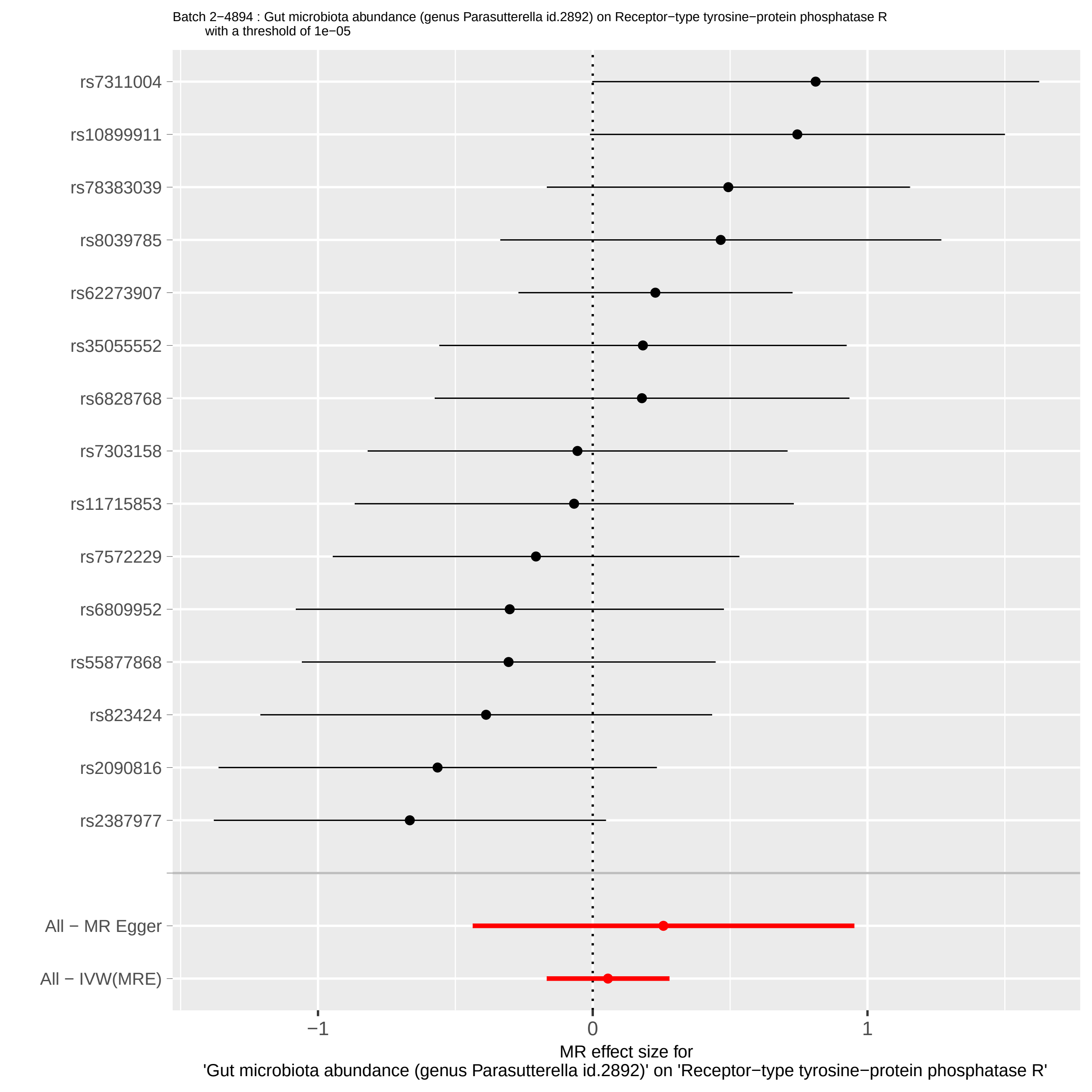

Batch 2−4894 : Gut microbiota abundance (genus Parasutterella id.2892) on Receptor−type tyrosine−protein phosphatase R
with a threshold of 1e−05
rs7311004
rs10899911
rs78383039
rs8039785
rs62273907
rs35055552
rs6828768
rs7303158
rs11715853
rs7572229
rs6809952
rs55877868
rs823424
rs2090816
rs2387977
All − MR Egger
All − IVW(MRE)
−1
0
1
MR effect size for
'Gut microbiota abundance (genus Parasutterella id.2892)' on 'Receptor−type tyrosine−protein phosphatase R'

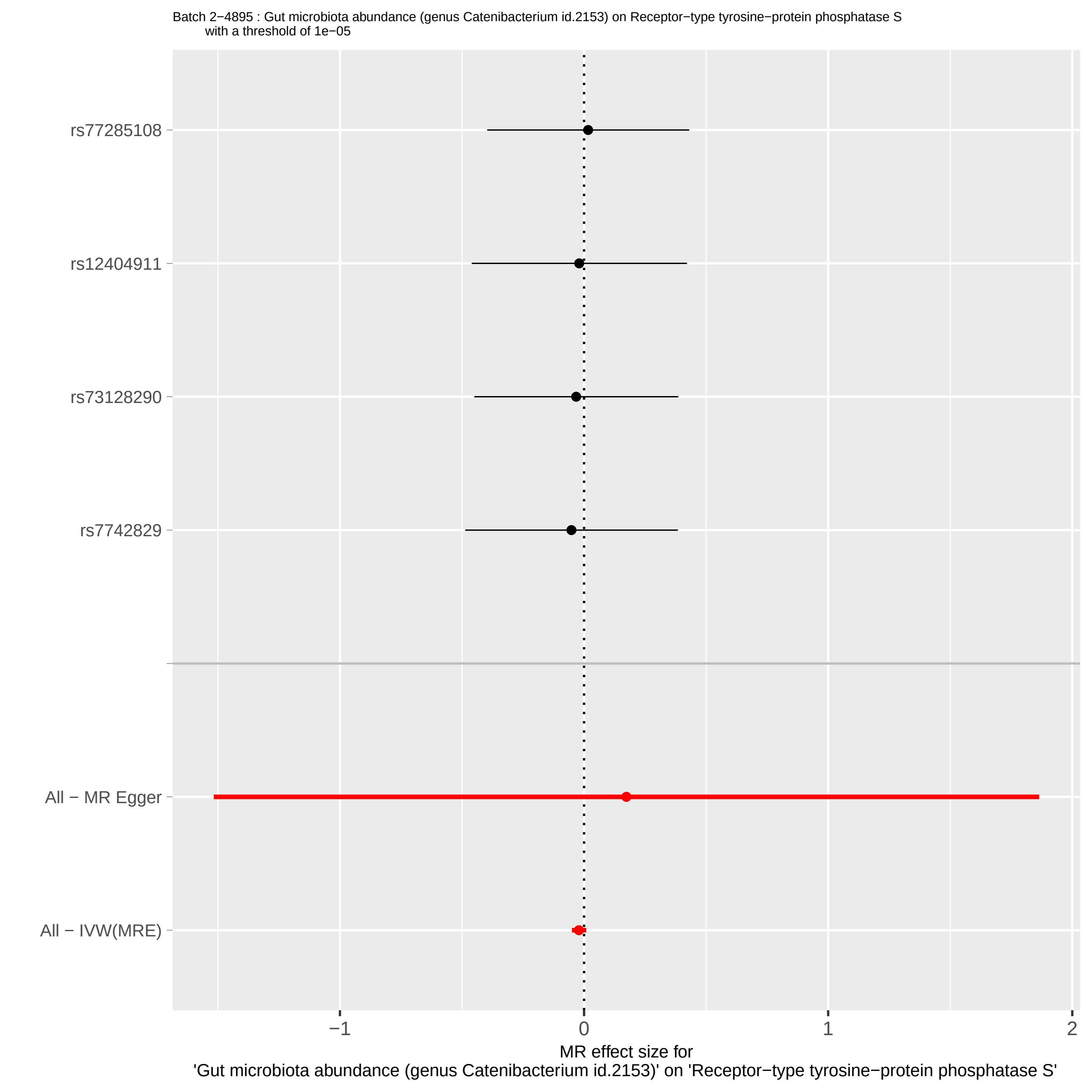
Batch 2−4895 : Gut microbiota abundance (genus Catenibacterium id.2153) on Receptor−type tyrosine−protein phosphatase S
with a threshold of 1e−05
rs77285108
rs12404911
rs73128290
rs7742829
All − MR Egger
All − IVW(MRE)
−1
0
1
2
MR effect size for
'Gut microbiota abundance (genus Catenibacterium id.2153)' on 'Receptor−type tyrosine−protein phosphatase S'

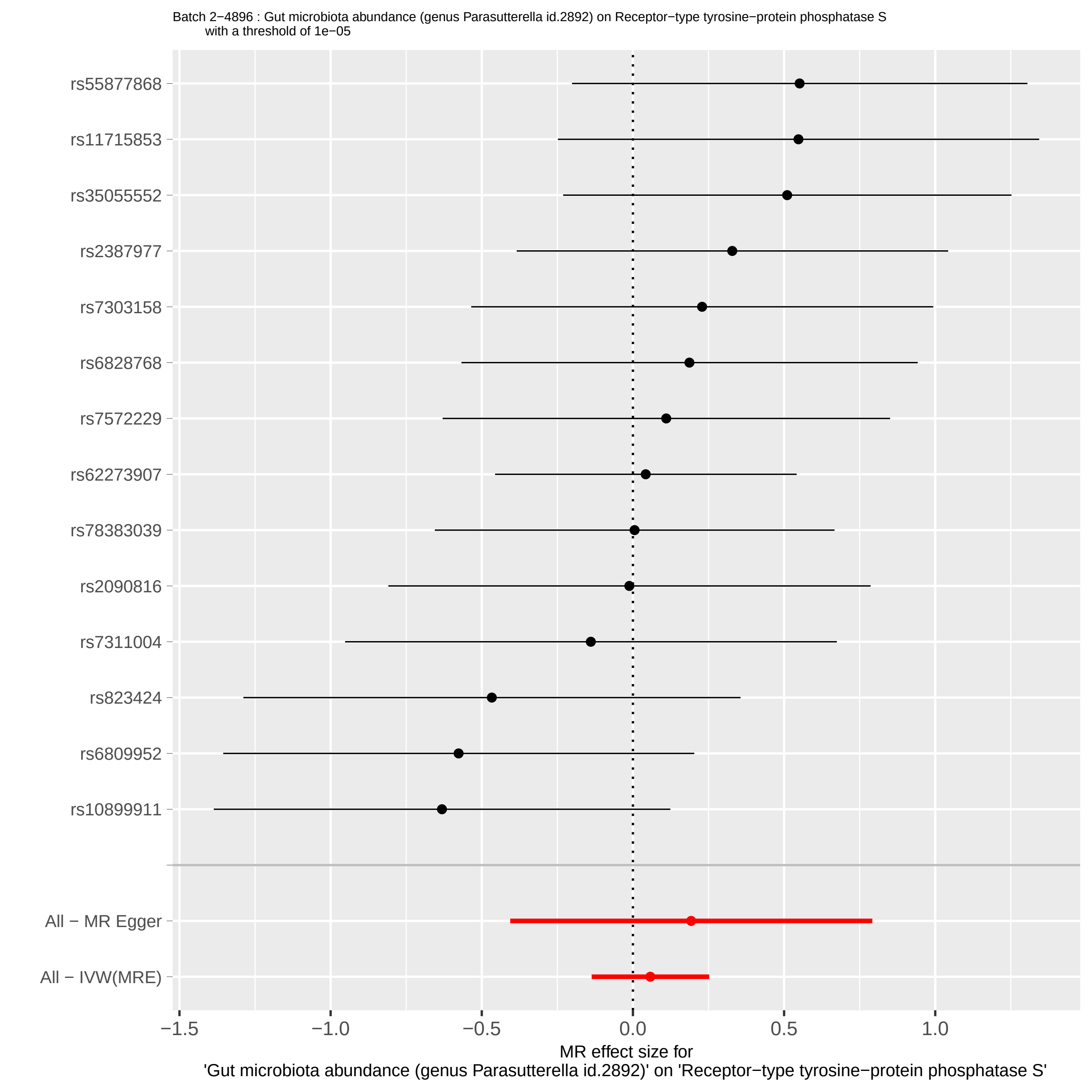

Batch 2−4896 : Gut microbiota abundance (genus Parasutterella id.2892) on Receptor−type tyrosine−protein phosphatase S
with a threshold of 1e−05
rs55877868
rs11715853
rs35055552
rs2387977
rs7303158
rs6828768
rs7572229
rs62273907
rs78383039
rs2090816
rs7311004
rs823424
rs6809952
rs10899911
All − MR Egger
All − IVW(MRE)
−1.5
−1.0
−0.5
0.0
0.5
1.0
MR effect size for
'Gut microbiota abundance (genus Parasutterella id.2892)' on 'Receptor−type tyrosine−protein phosphatase S'

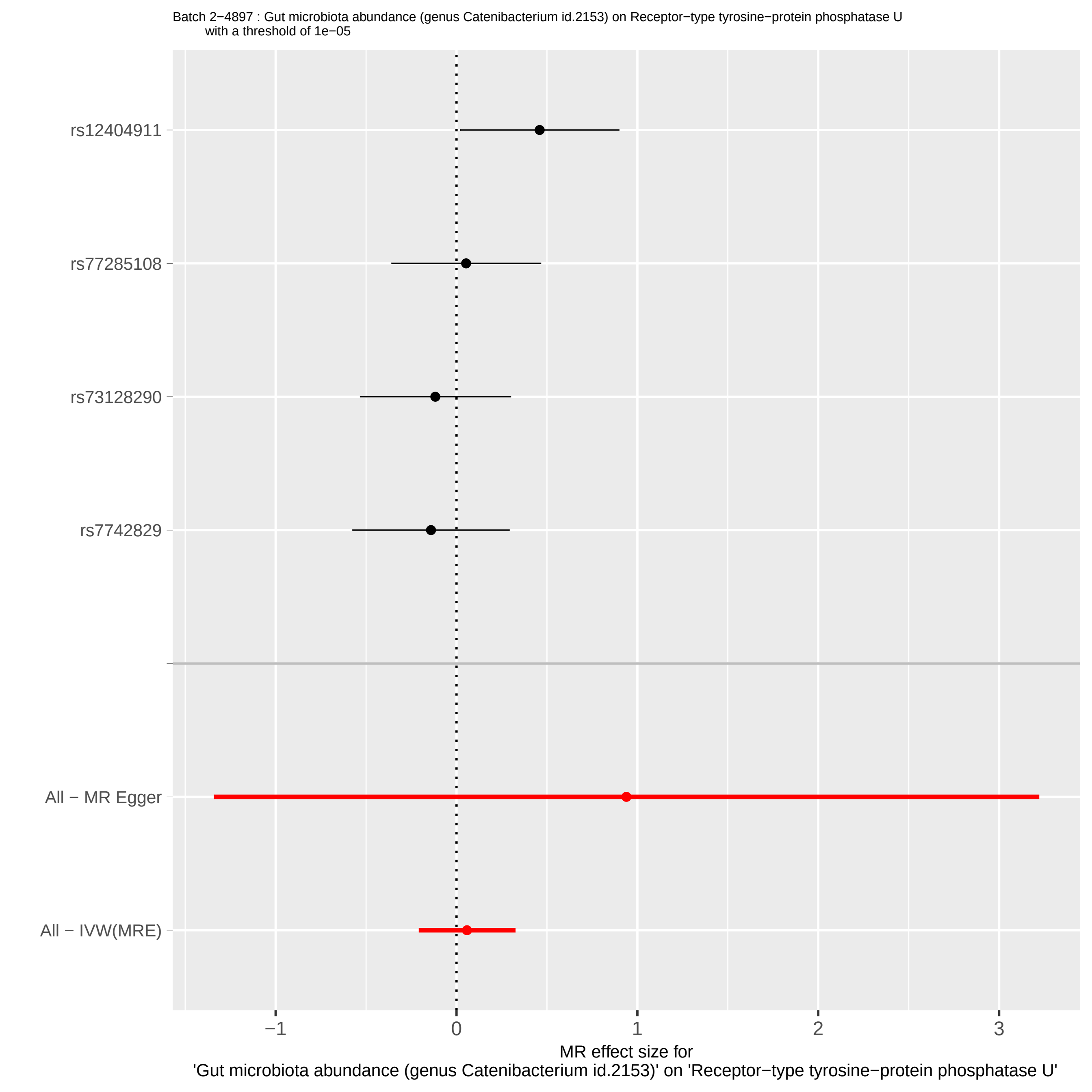
Batch 2−4897 : Gut microbiota abundance (genus Catenibacterium id.2153) on Receptor−type tyrosine−protein phosphatase U
with a threshold of 1e−05
rs12404911
rs77285108
rs73128290
rs7742829
All − MR Egger
All − IVW(MRE)
−1
0
1
2
3
MR effect size for
'Gut microbiota abundance (genus Catenibacterium id.2153)' on 'Receptor−type tyrosine−protein phosphatase U'

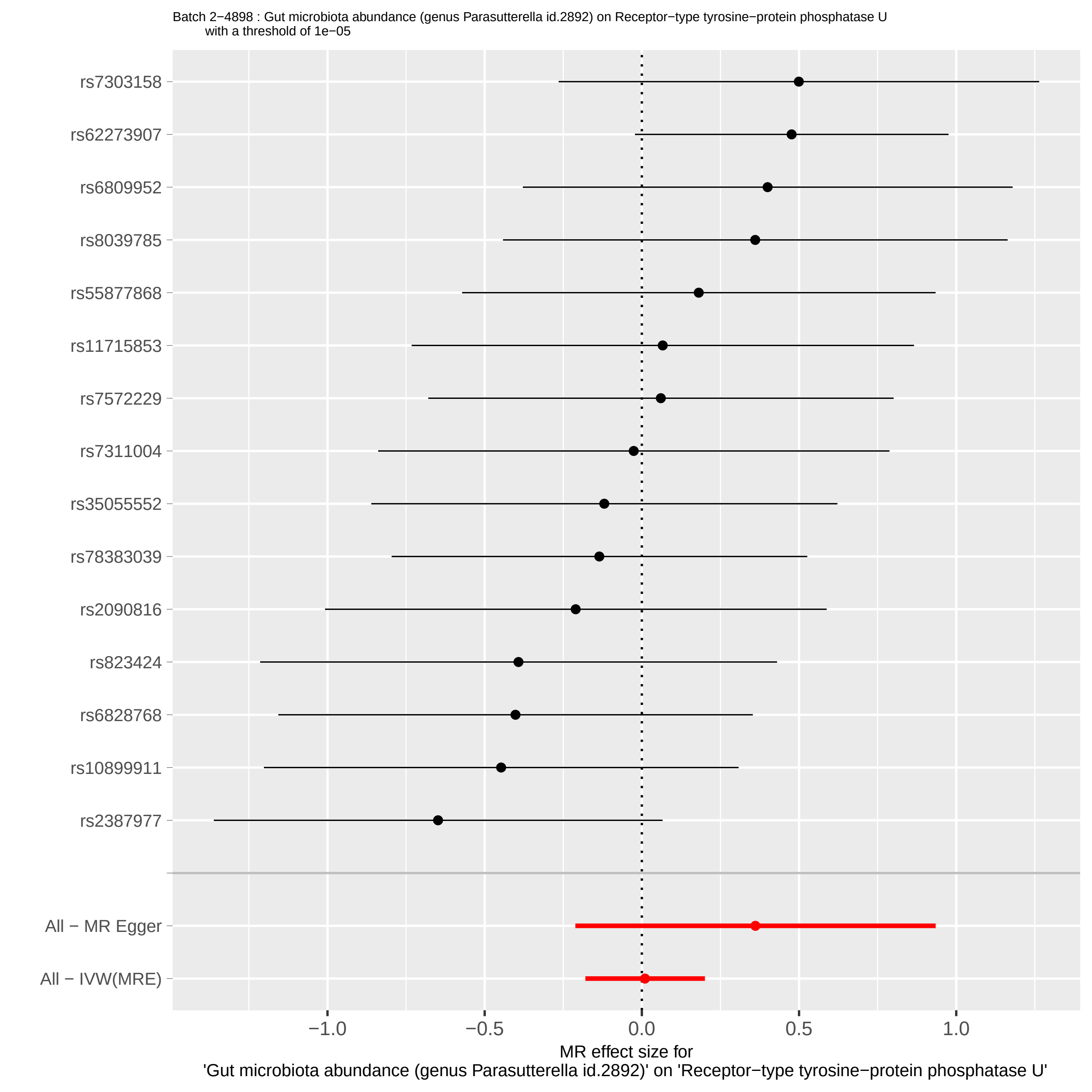

Batch 2−4898 : Gut microbiota abundance (genus Parasutterella id.2892) on Receptor−type tyrosine−protein phosphatase U
with a threshold of 1e−05
rs7303158
rs62273907
rs6809952
rs8039785
rs55877868
rs11715853
rs7572229
rs7311004
rs35055552
rs78383039
rs2090816
rs823424
rs6828768
rs10899911
rs2387977
All − MR Egger
All − IVW(MRE)
−1.0
−0.5
0.0
0.5
1.0
MR effect size for
'Gut microbiota abundance (genus Parasutterella id.2892)' on 'Receptor−type tyrosine−protein phosphatase U'

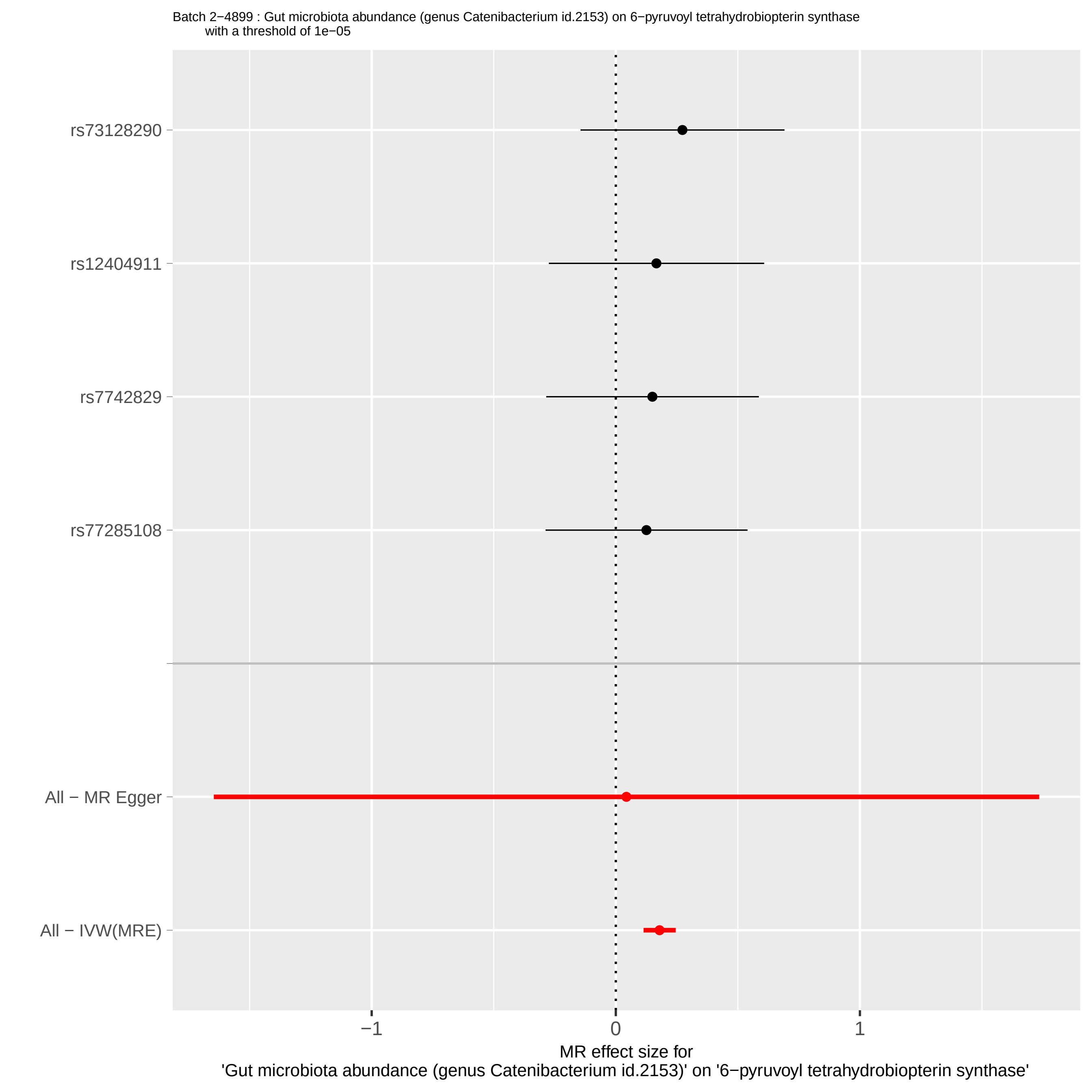
Batch 2−4899 : Gut microbiota abundance (genus Catenibacterium id.2153) on 6−pyruvoyl tetrahydrobiopterin synthase
with a threshold of 1e−05
rs73128290
rs12404911
rs7742829
rs77285108
All − MR Egger
All − IVW(MRE)
−1
0
1
MR effect size for
'Gut microbiota abundance (genus Catenibacterium id.2153)' on '6−pyruvoyl tetrahydrobiopterin synthase'

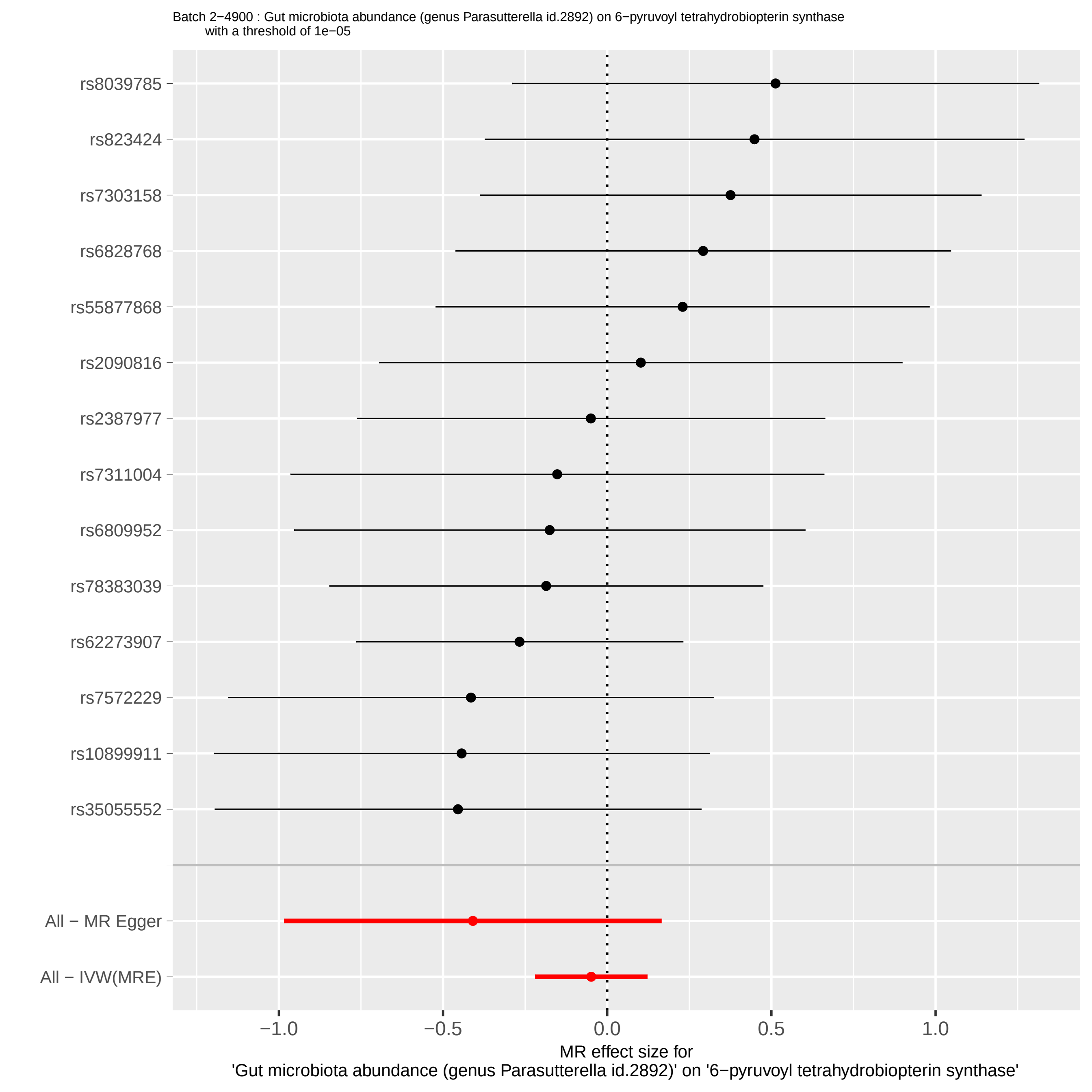

Batch 2−4900 : Gut microbiota abundance (genus Parasutterella id.2892) on 6−pyruvoyl tetrahydrobiopterin synthase
with a threshold of 1e−05
rs8039785
rs823424
rs7303158
rs6828768
rs55877868
rs2090816
rs2387977
rs7311004
rs6809952
rs78383039
rs62273907
rs7572229
rs10899911
rs35055552
All − MR Egger
All − IVW(MRE)
−1.0
−0.5
0.0
0.5
1.0
MR effect size for
'Gut microbiota abundance (genus Parasutterella id.2892)' on '6−pyruvoyl tetrahydrobiopterin synthase'

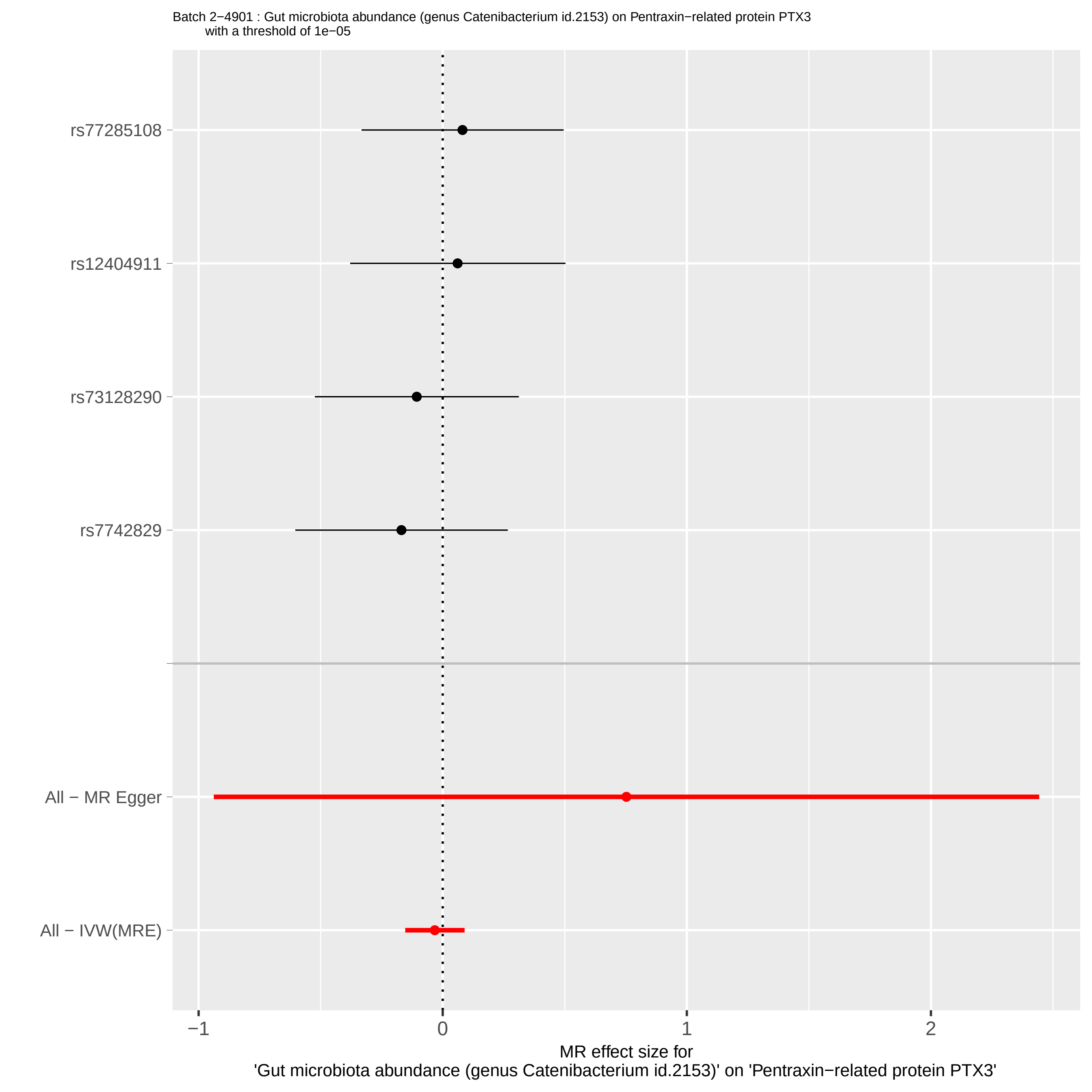
Batch 2−4901 : Gut microbiota abundance (genus Catenibacterium id.2153) on Pentraxin−related protein PTX3
with a threshold of 1e−05
rs77285108
rs12404911
rs73128290
rs7742829
All − MR Egger
All − IVW(MRE)
−1
0
1
2
MR effect size for
'Gut microbiota abundance (genus Catenibacterium id.2153)' on 'Pentraxin−related protein PTX3'

Batch 2−4902 : Gut microbiota abundance (genus Parasutterella id.2892) on Pentraxin−related protein PTX3
with a threshold of 1e−05

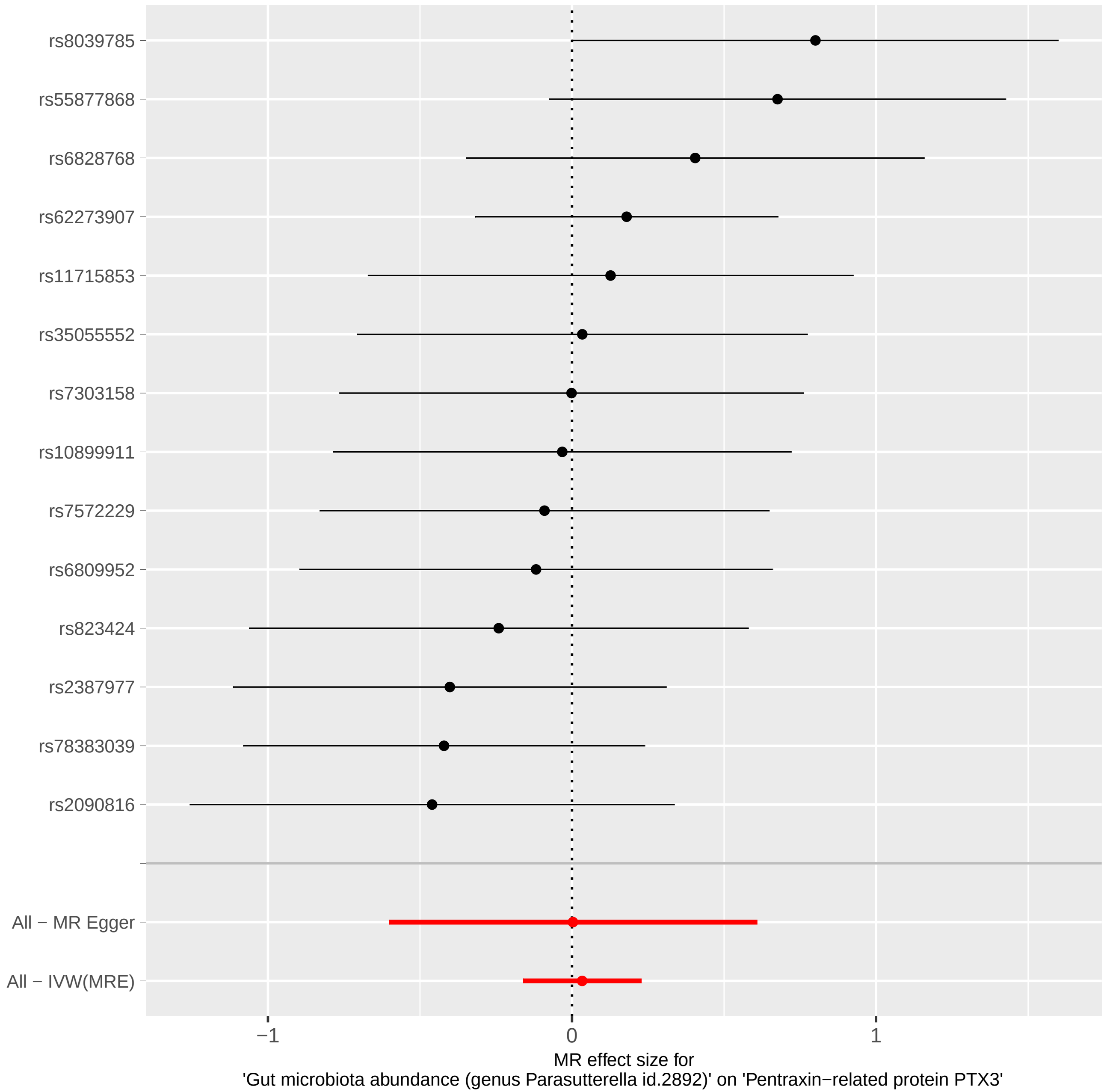

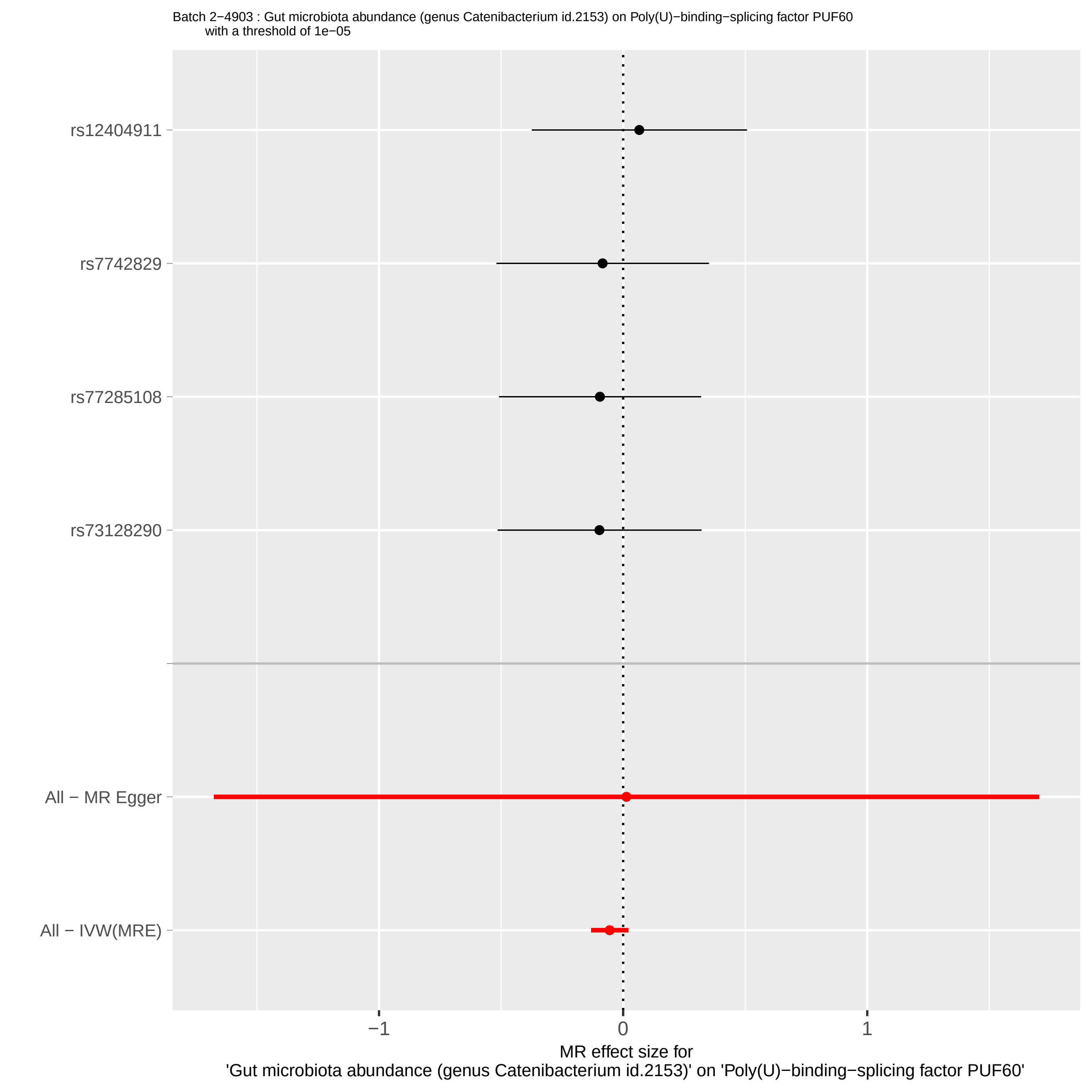
Batch 2−4903 : Gut microbiota abundance (genus Catenibacterium id.2153) on Poly(U)−binding−splicing factor PUF60
with a threshold of 1e−05
rs12404911
rs7742829
rs77285108
rs73128290
All − MR Egger
All − IVW(MRE)
−1
0
1
MR effect size for
'Gut microbiota abundance (genus Catenibacterium id.2153)' on 'Poly(U)−binding−splicing factor PUF60'

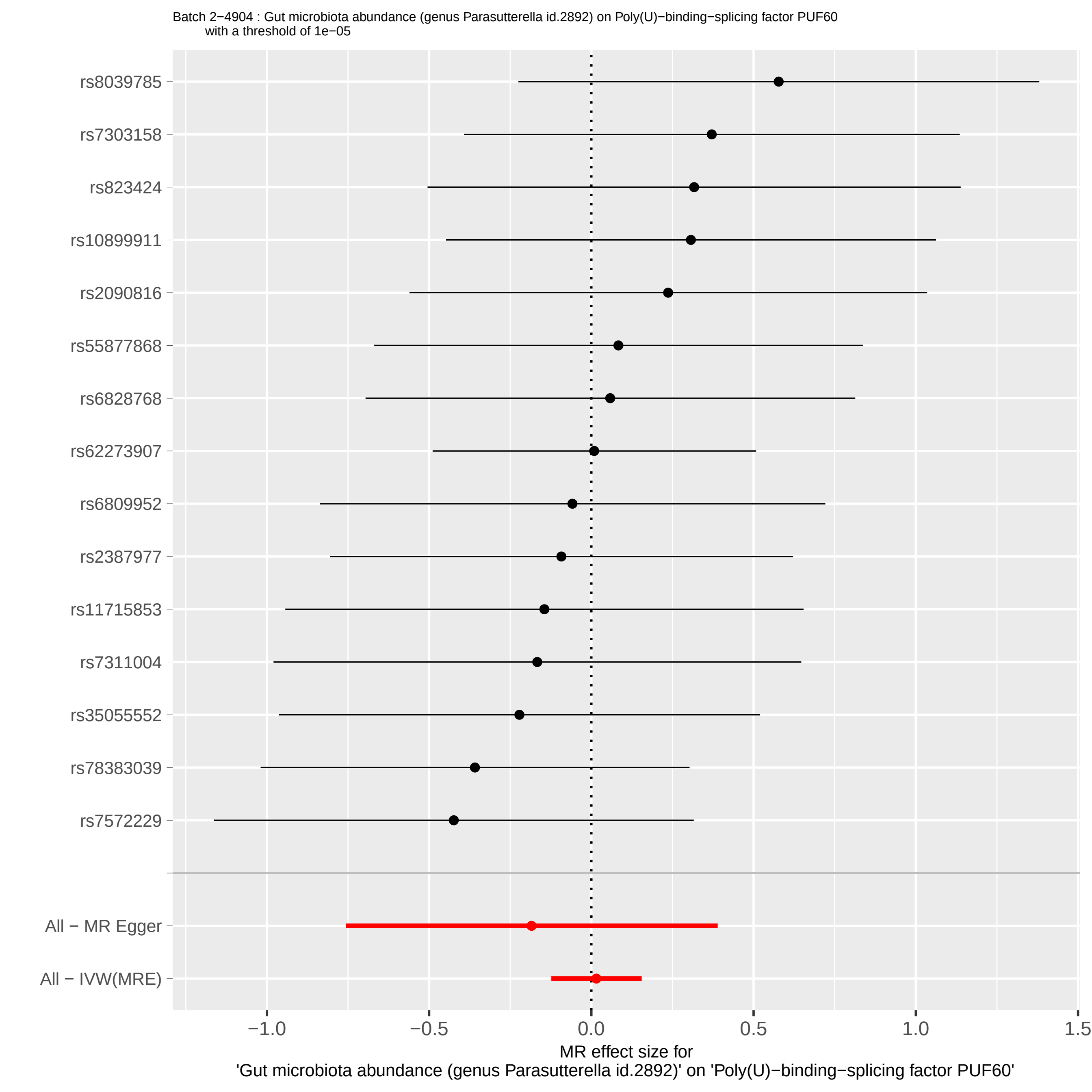

Batch 2−4904 : Gut microbiota abundance (genus Parasutterella id.2892) on Poly(U)−binding−splicing factor PUF60
with a threshold of 1e−05
rs8039785
rs7303158
rs823424
rs10899911
rs2090816
rs55877868
rs6828768
rs62273907
rs6809952
rs2387977
rs11715853
rs7311004
rs35055552
rs78383039
rs7572229
All − MR Egger
All − IVW(MRE)
−1.0
−0.5
0.0
0.5
1.0
1.5
MR effect size for
'Gut microbiota abundance (genus Parasutterella id.2892)' on 'Poly(U)−binding−splicing factor PUF60'

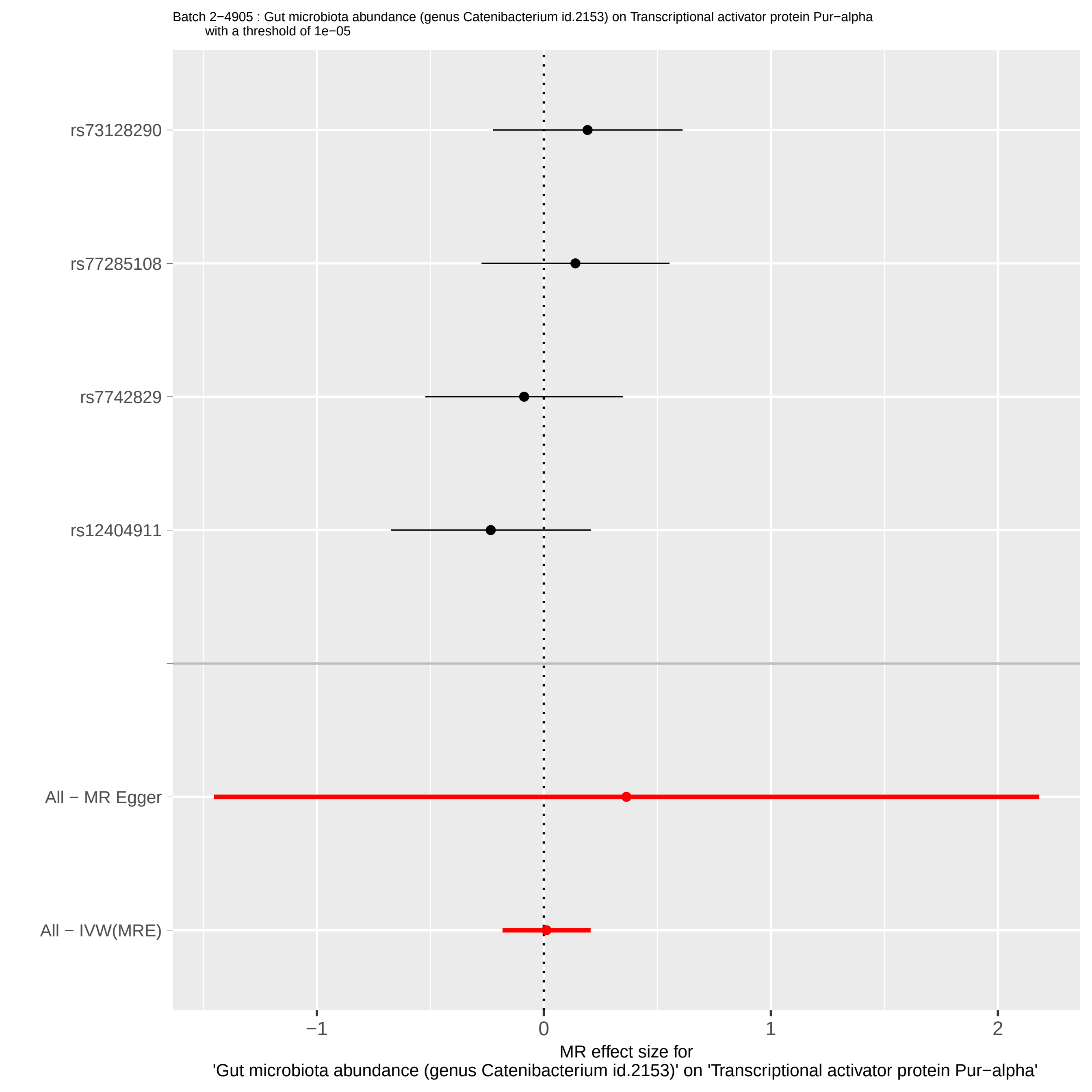

Batch 2−4905 : Gut microbiota abundance (genus Catenibacterium id.2153) on Transcriptional activator protein Pur−alpha
with a threshold of 1e−05
rs73128290
rs77285108
rs7742829
rs12404911
All − MR Egger
All − IVW(MRE)
−1
0
1
2
MR effect size for
'Gut microbiota abundance (genus Catenibacterium id.2153)' on 'Transcriptional activator protein Pur−alpha'

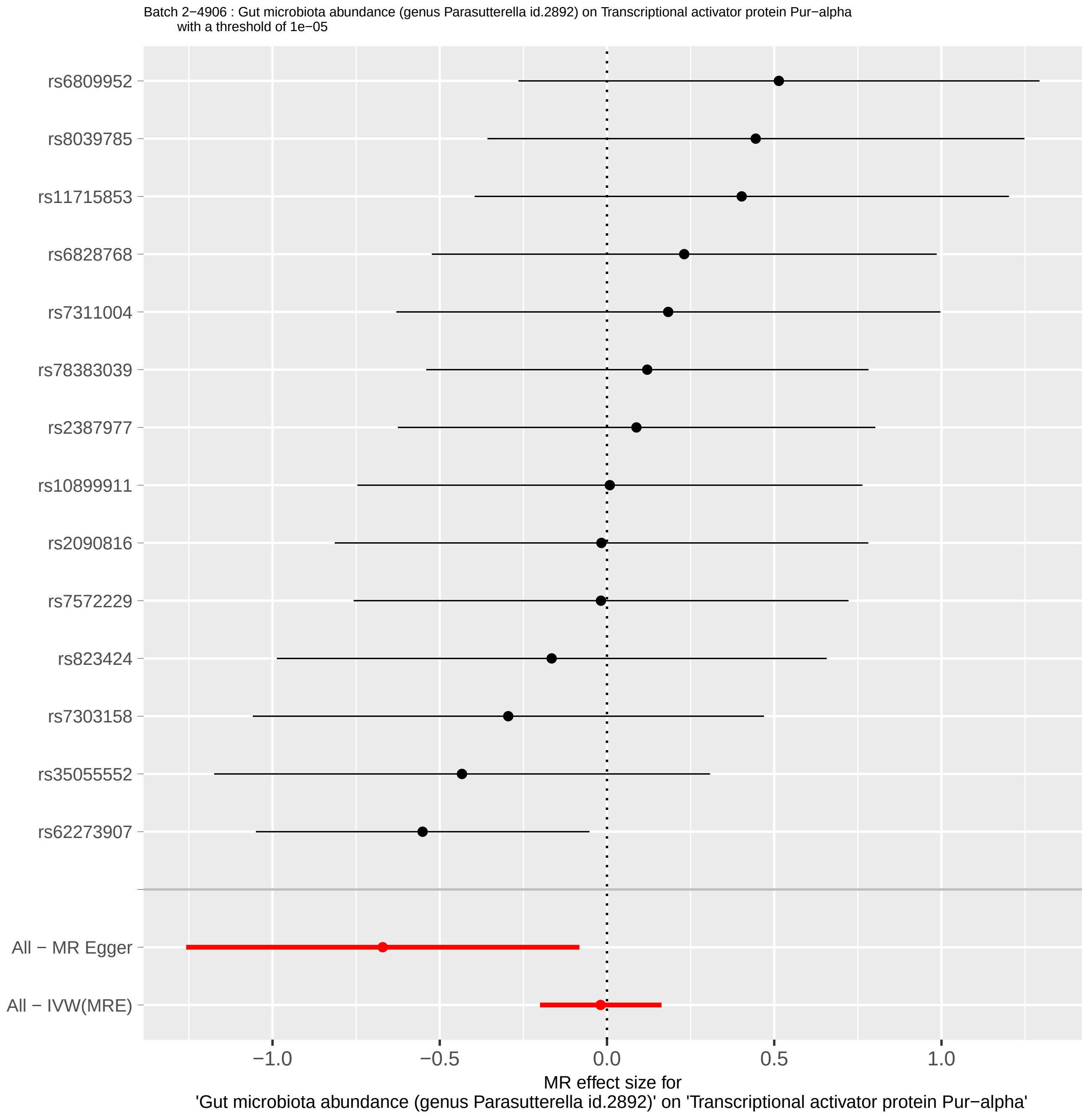
Batch 2−4906 : Gut microbiota abundance (genus Parasutterella id.2892) on Transcriptional activator protein Pur−alpha
with a threshold of 1e−05
rs6809952
rs8039785
rs11715853
rs6828768
rs7311004
rs78383039
rs2387977
rs10899911
rs2090816
rs7572229
rs823424
rs7303158
rs35055552
rs62273907
All − MR Egger
All − IVW(MRE)
−1.0
−0.5
0.0
0.5
1.0
MR effect size for
'Gut microbiota abundance (genus Parasutterella id.2892)' on 'Transcriptional activator protein Pur−alpha'

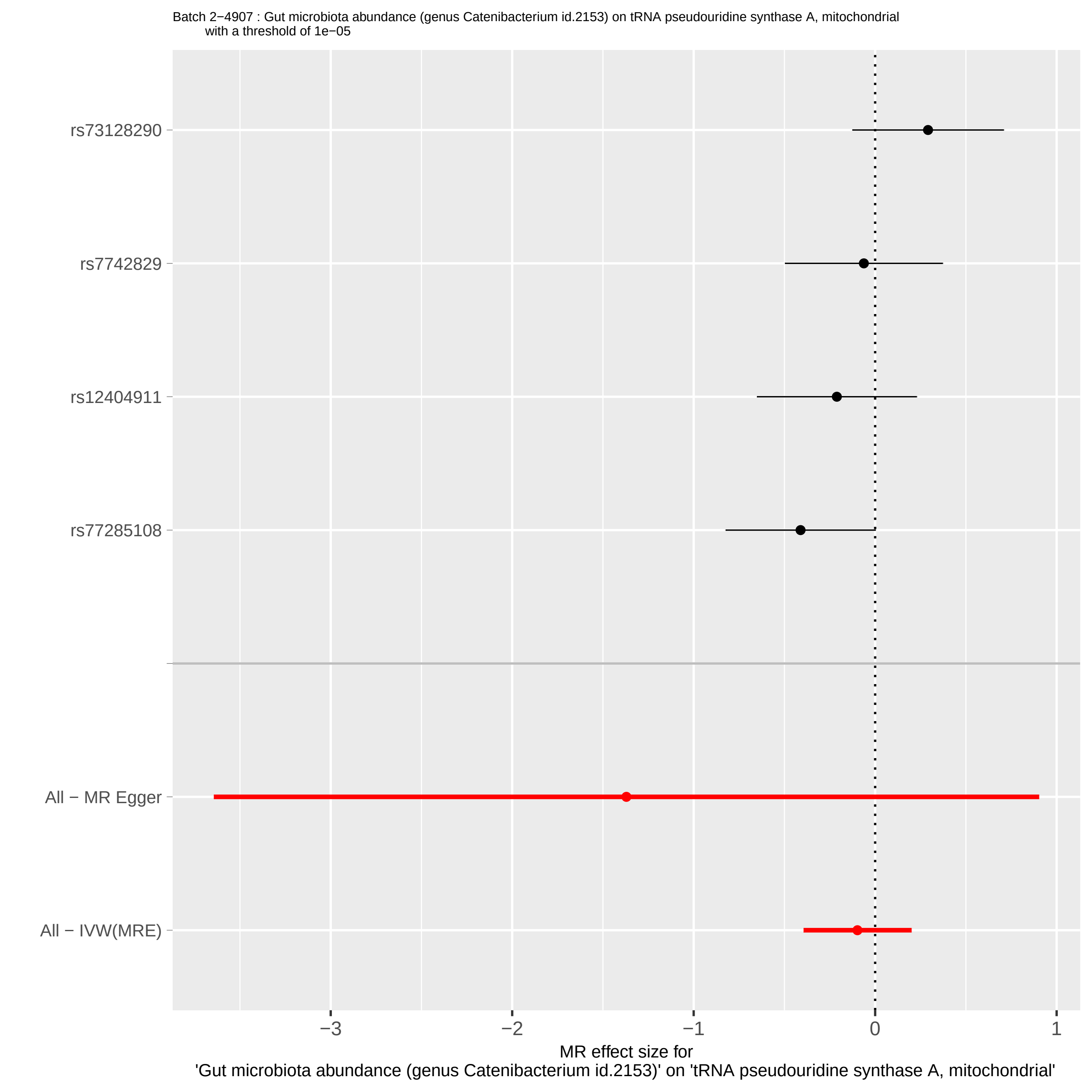

Batch 2−4907 : Gut microbiota abundance (genus Catenibacterium id.2153) on tRNA pseudouridine synthase A, mitochondrial
with a threshold of 1e−05
rs73128290
rs7742829
rs12404911
rs77285108
All − MR Egger
All − IVW(MRE)
−3
−2
−1
0
1
MR effect size for
'Gut microbiota abundance (genus Catenibacterium id.2153)' on 'tRNA pseudouridine synthase A, mitochondrial'

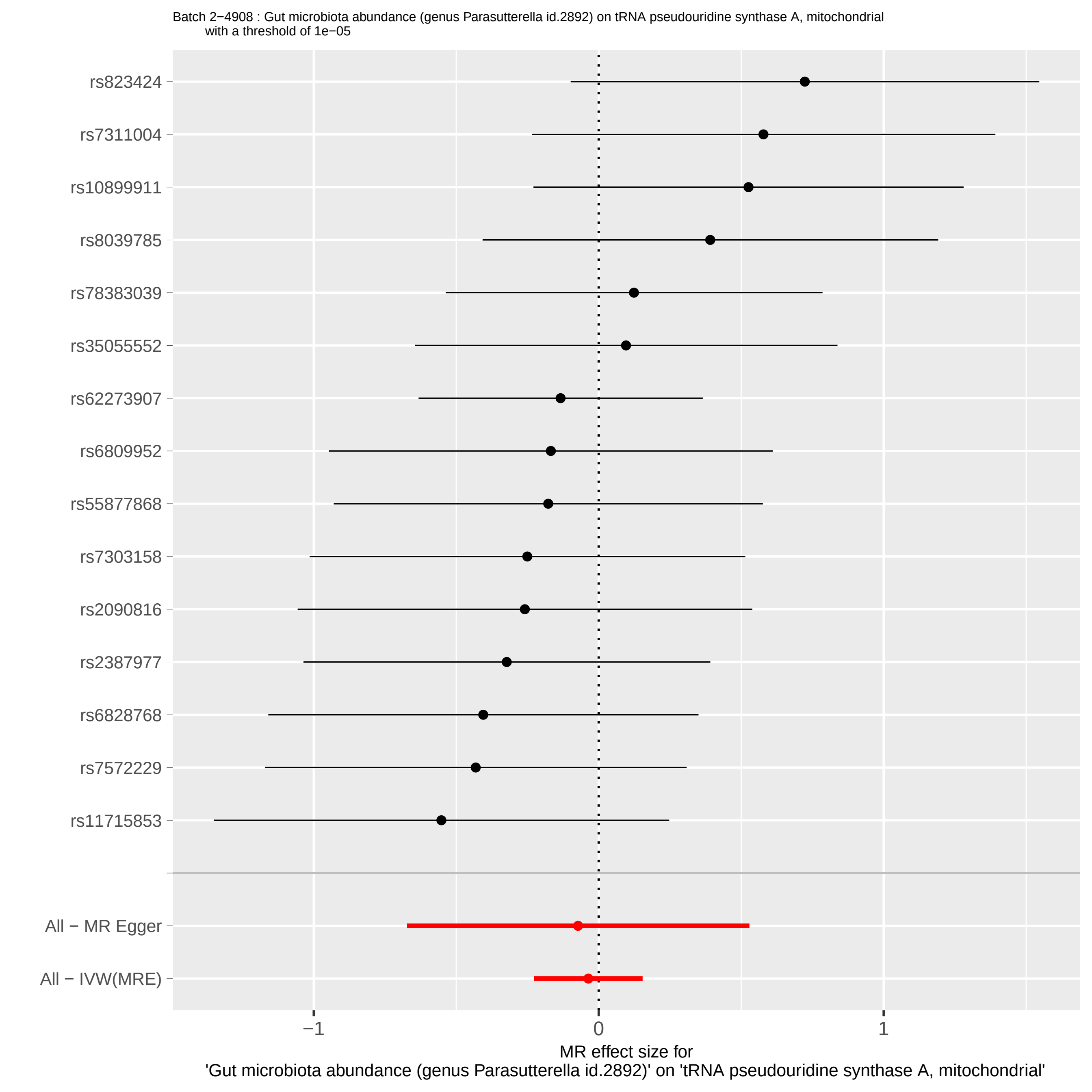

Batch 2−4908 : Gut microbiota abundance (genus Parasutterella id.2892) on tRNA pseudouridine synthase A, mitochondrial
with a threshold of 1e−05
rs823424
rs7311004
rs10899911
rs8039785
rs78383039
rs35055552
rs62273907
rs6809952
rs55877868
rs7303158
rs2090816
rs2387977
rs6828768
rs7572229
rs11715853
All − MR Egger
All − IVW(MRE)
−1
0
1
MR effect size for
'Gut microbiota abundance (genus Parasutterella id.2892)' on 'tRNA pseudouridine synthase A, mitochondrial'

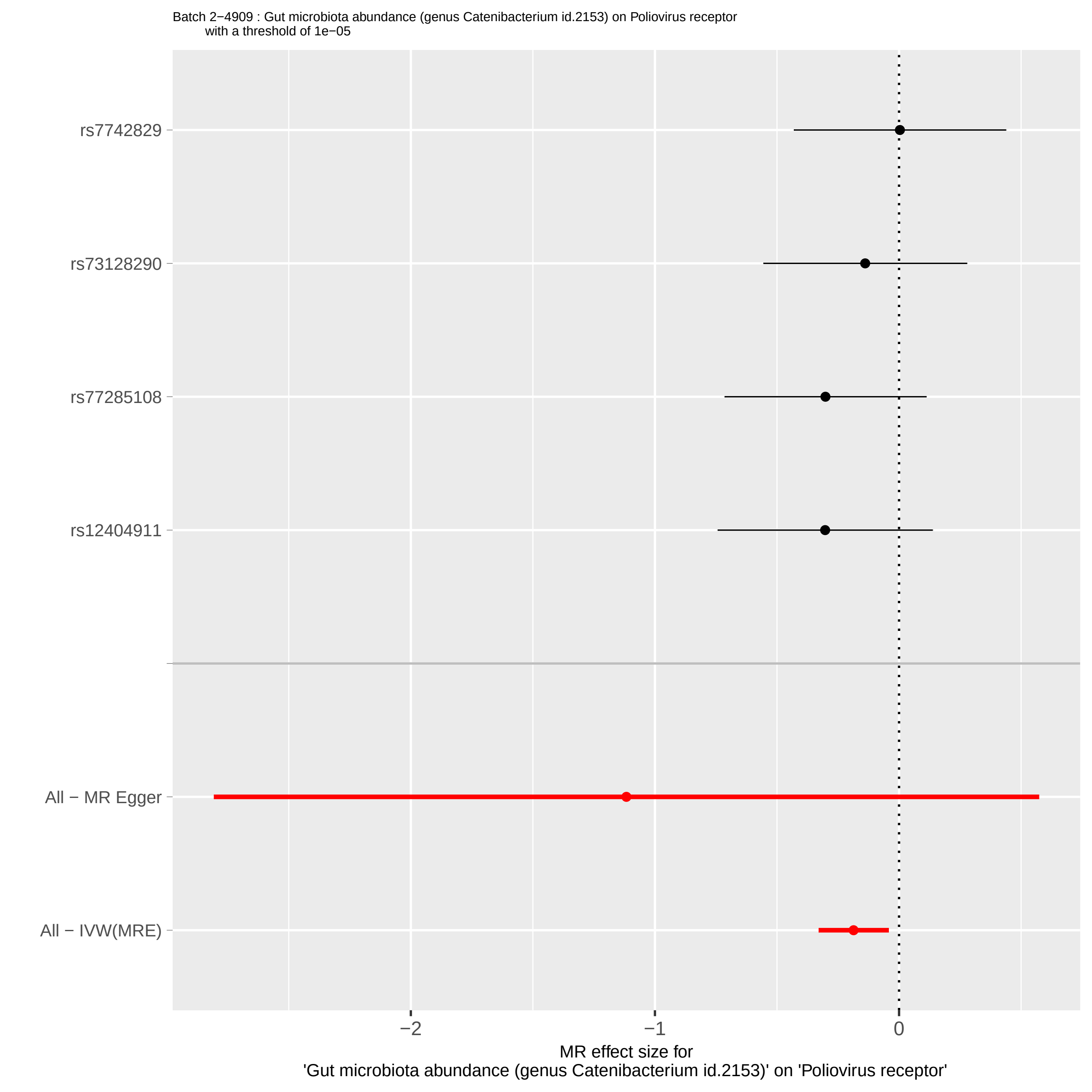

Batch 2−4909 : Gut microbiota abundance (genus Catenibacterium id.2153) on Poliovirus receptor
with a threshold of 1e−05
rs7742829
rs73128290
rs77285108
rs12404911
All − MR Egger
All − IVW(MRE)
−2
−1
0
MR effect size for
'Gut microbiota abundance (genus Catenibacterium id.2153)' on 'Poliovirus receptor'

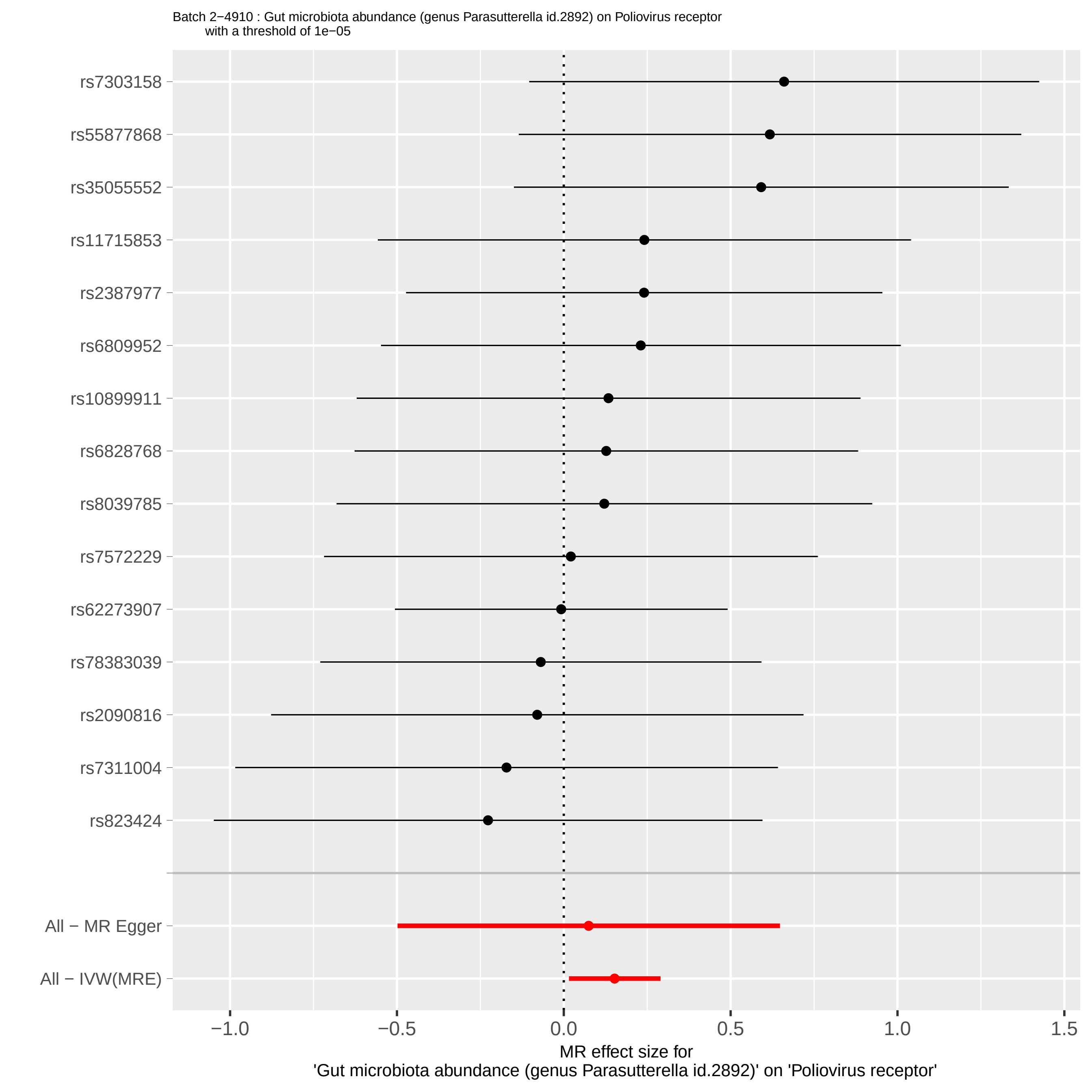

Batch 2−4910 : Gut microbiota abundance (genus Parasutterella id.2892) on Poliovirus receptor
with a threshold of 1e−05
rs7303158
rs55877868
rs35055552
rs11715853
rs2387977
rs6809952
rs10899911
rs6828768
rs8039785
rs7572229
rs62273907
rs78383039
rs2090816
rs7311004
rs823424
All − MR Egger
All − IVW(MRE)
−1.0
−0.5
0.0
0.5
1.0
1.5
MR effect size for
'Gut microbiota abundance (genus Parasutterella id.2892)' on 'Poliovirus receptor'

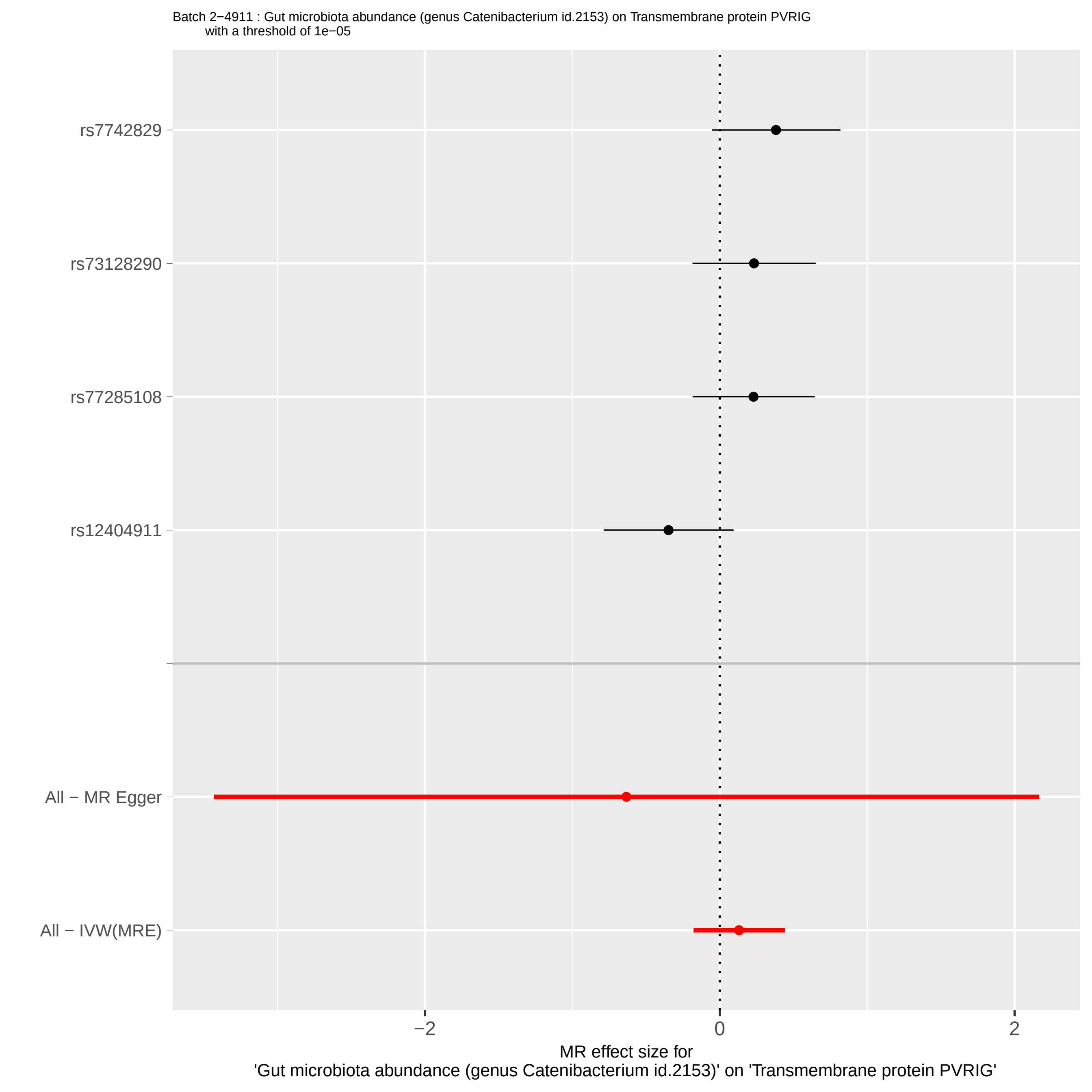

Batch 2−4911 : Gut microbiota abundance (genus Catenibacterium id.2153) on Transmembrane protein PVRIG
with a threshold of 1e−05
rs7742829
rs73128290
rs77285108
rs12404911
All − MR Egger
All − IVW(MRE)
−2
0
2
MR effect size for
'Gut microbiota abundance (genus Catenibacterium id.2153)' on 'Transmembrane protein PVRIG'

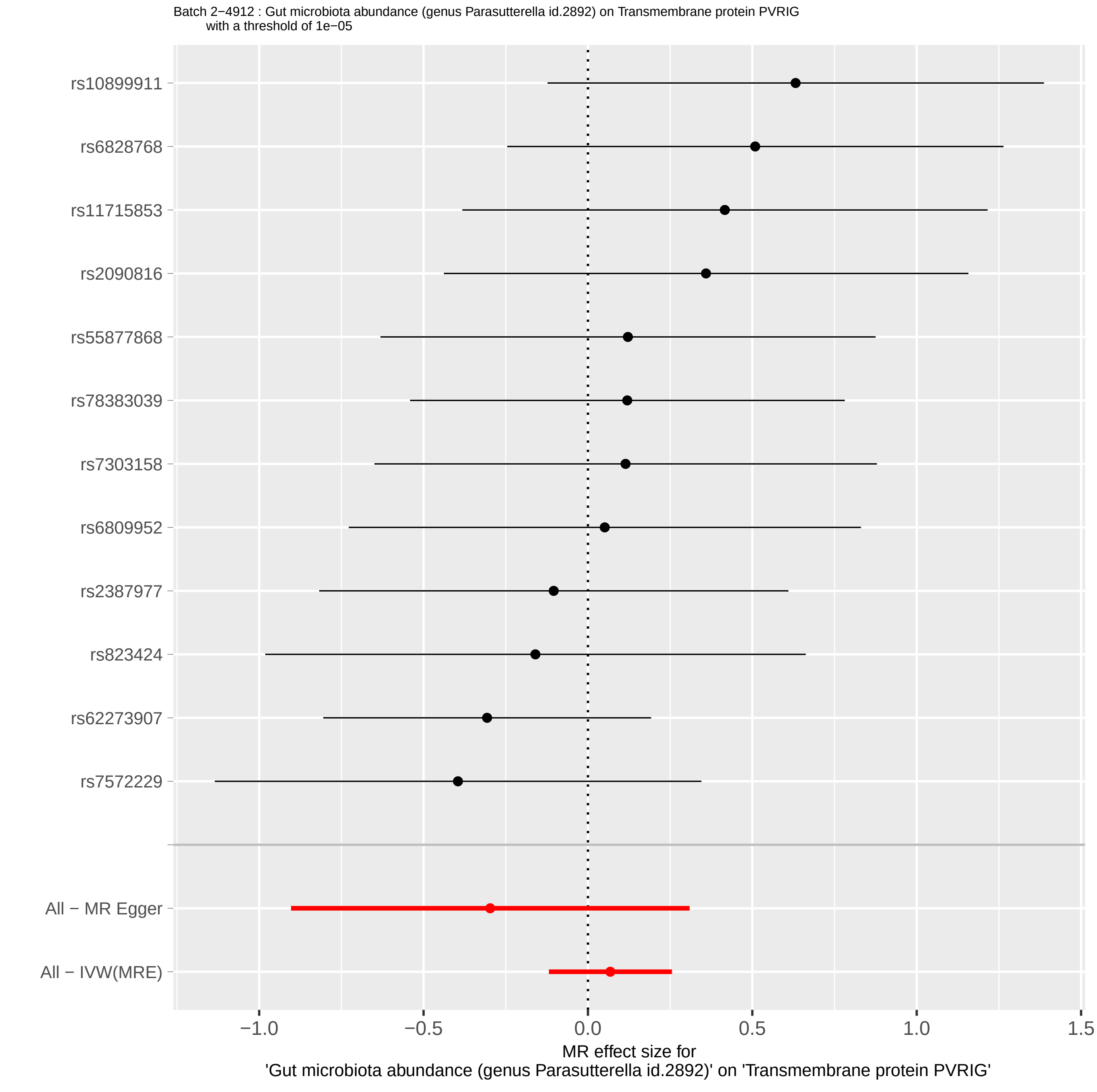

Batch 2−4912 : Gut microbiota abundance (genus Parasutterella id.2892) on Transmembrane protein PVRIG
with a threshold of 1e−05
rs10899911
rs6828768
rs11715853
rs2090816
rs55877868
rs78383039
rs7303158
rs6809952
rs2387977
rs823424
rs62273907
rs7572229
All − MR Egger
All − IVW(MRE)
−1.0
−0.5
0.0
0.5
1.0
1.5
MR effect size for
'Gut microbiota abundance (genus Parasutterella id.2892)' on 'Transmembrane protein PVRIG'

Batch 2−4913 : Gut microbiota abundance (genus Catenibacterium id.2153) on Nectin−2
with a threshold of 1e−05

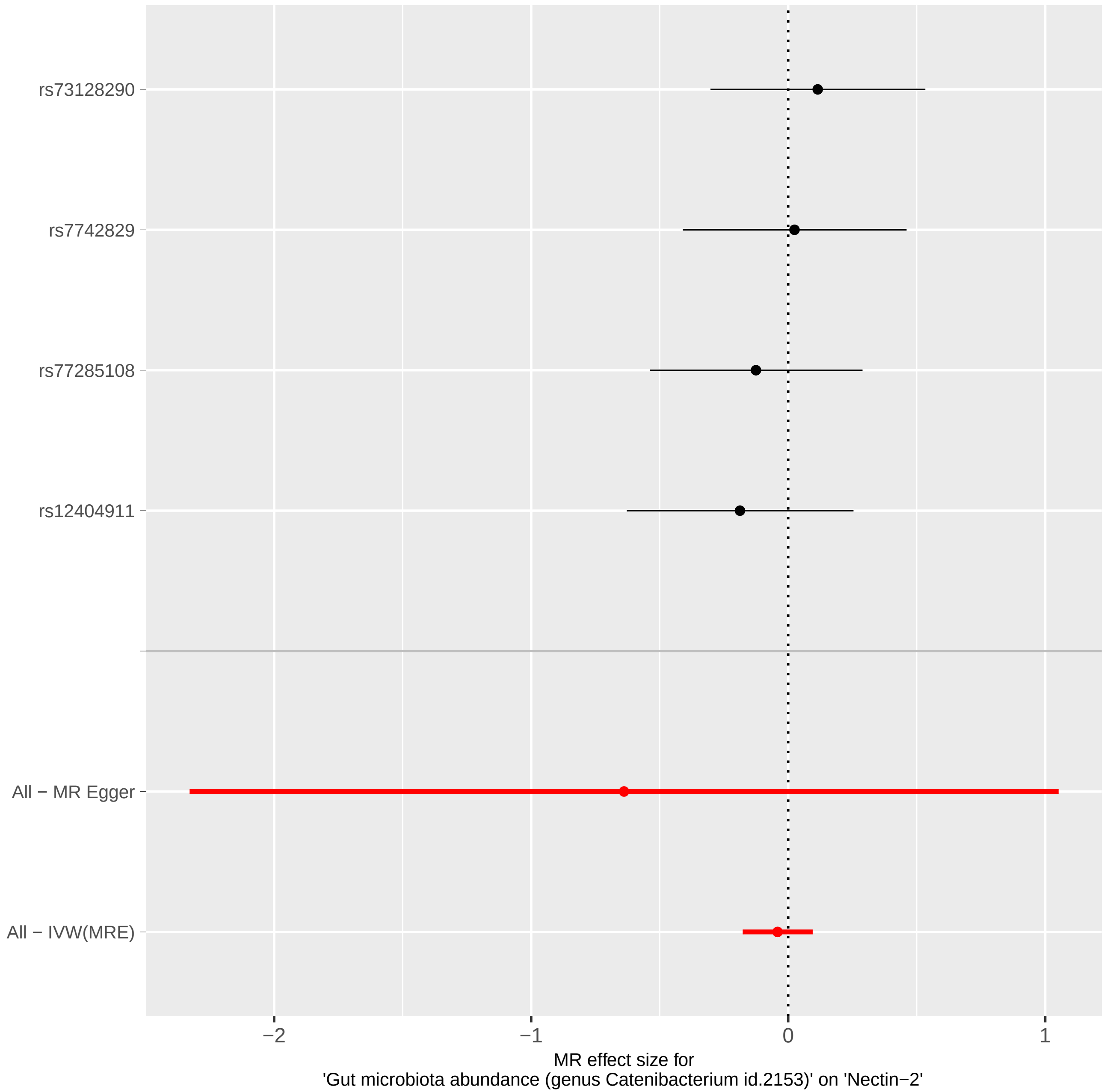

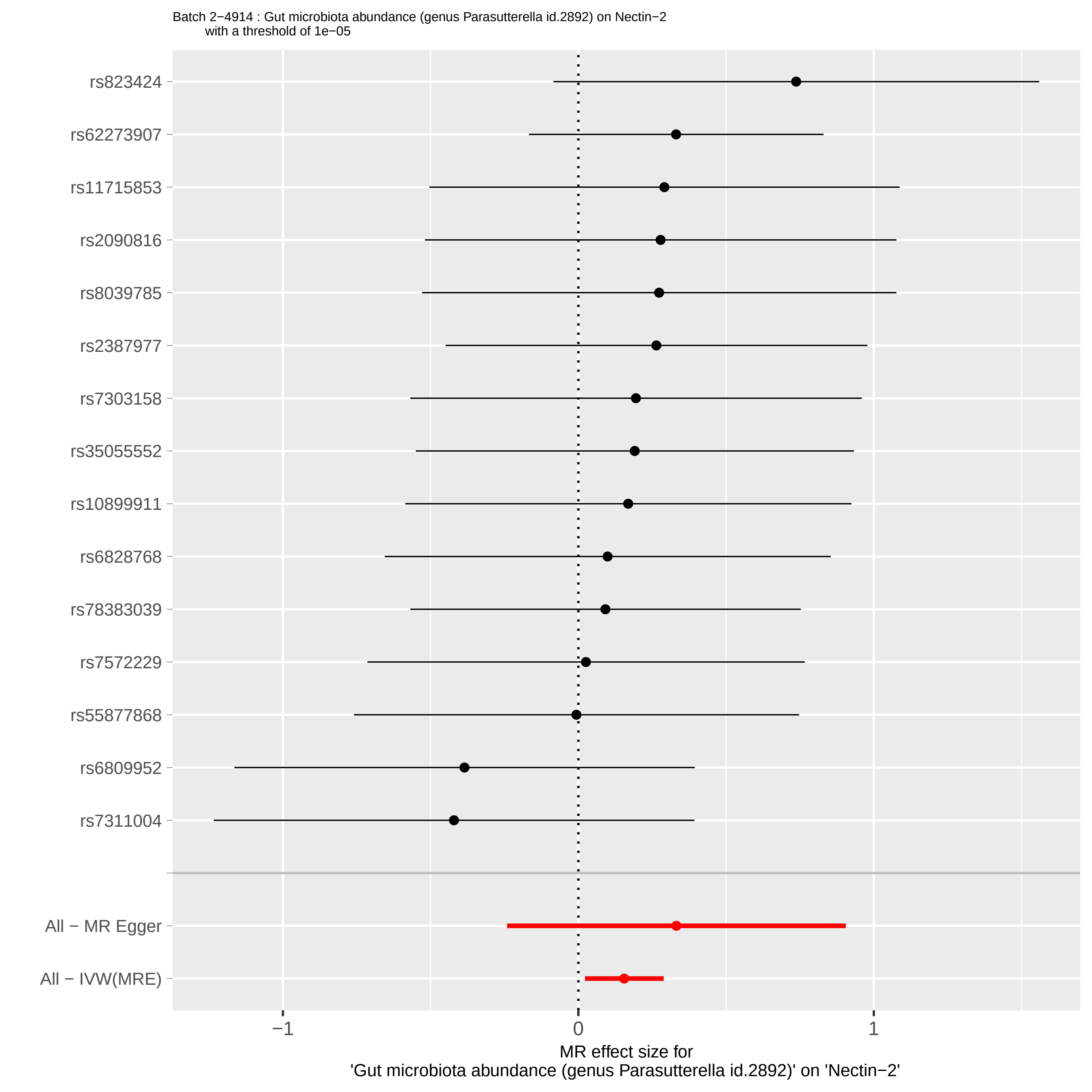
Batch 2−4914 : Gut microbiota abundance (genus Parasutterella id.2892) on Nectin−2
with a threshold of 1e−05
rs823424
rs62273907
rs11715853
rs2090816
rs8039785
rs2387977
rs7303158
rs35055552
rs10899911
rs6828768
rs78383039
rs7572229
rs55877868
rs6809952
rs7311004
All − MR Egger
All − IVW(MRE)
−1
0
1
MR effect size for
'Gut microbiota abundance (genus Parasutterella id.2892)' on 'Nectin−2'

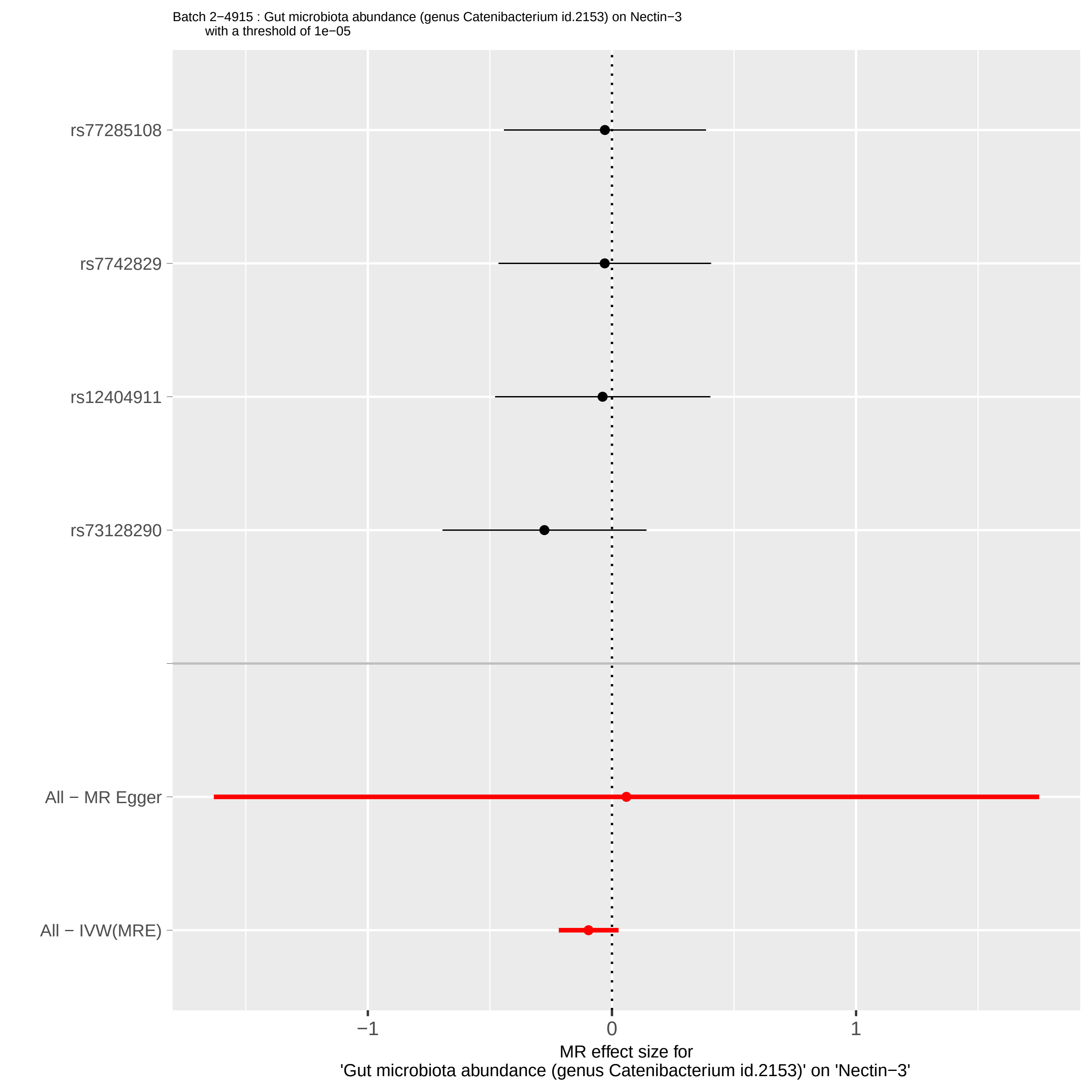

Batch 2−4915 : Gut microbiota abundance (genus Catenibacterium id.2153) on Nectin−3
with a threshold of 1e−05
rs77285108
rs7742829
rs12404911
rs73128290
All − MR Egger
All − IVW(MRE)
−1
0
1
MR effect size for
'Gut microbiota abundance (genus Catenibacterium id.2153)' on 'Nectin−3'

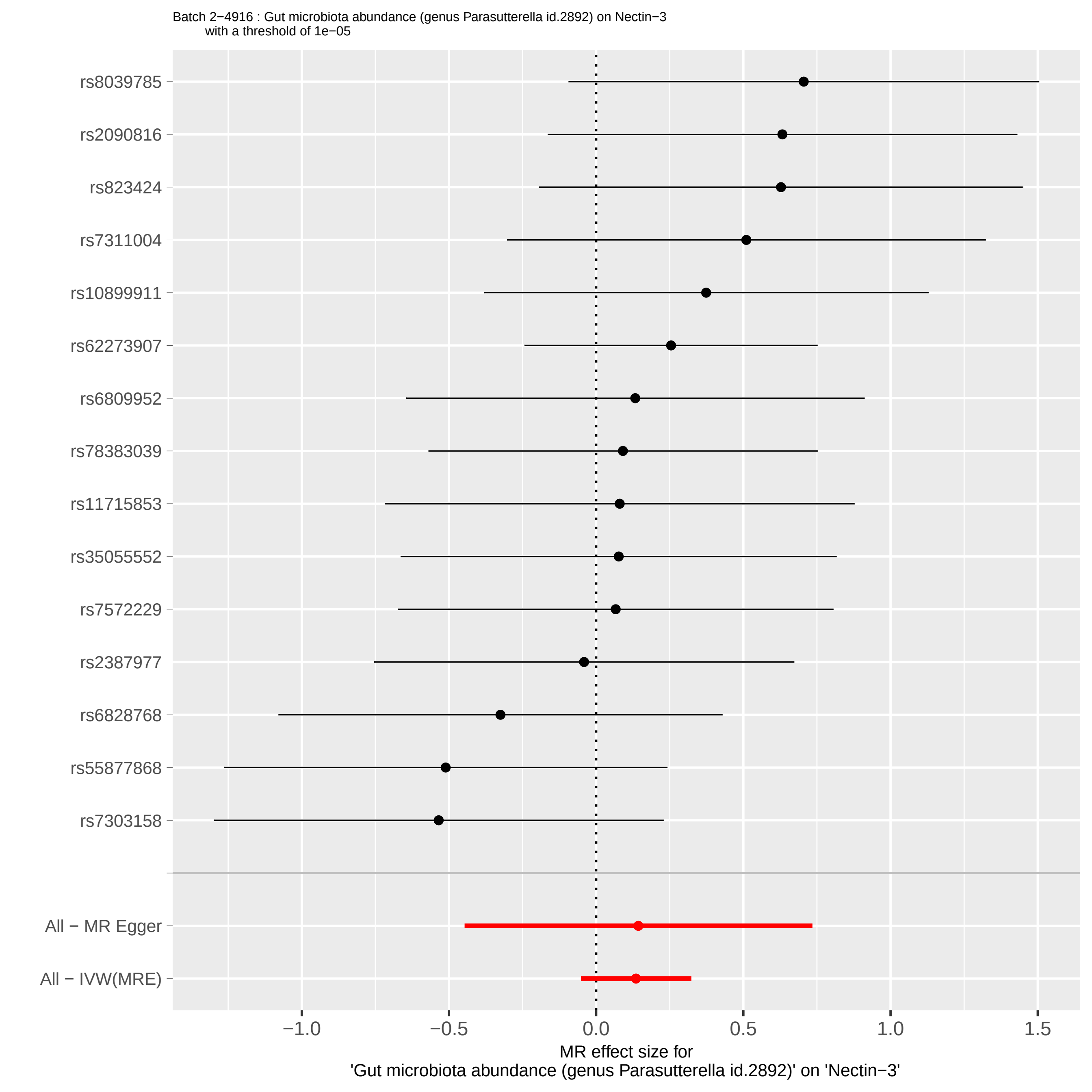

Batch 2−4916 : Gut microbiota abundance (genus Parasutterella id.2892) on Nectin−3
with a threshold of 1e−05
rs8039785
rs2090816
rs823424
rs7311004
rs10899911
rs62273907
rs6809952
rs78383039
rs11715853
rs35055552
rs7572229
rs2387977
rs6828768
rs55877868
rs7303158
All − MR Egger
All − IVW(MRE)
−1.0
−0.5
0.0
0.5
1.0
1.5
MR effect size for
'Gut microbiota abundance (genus Parasutterella id.2892)' on 'Nectin−3'

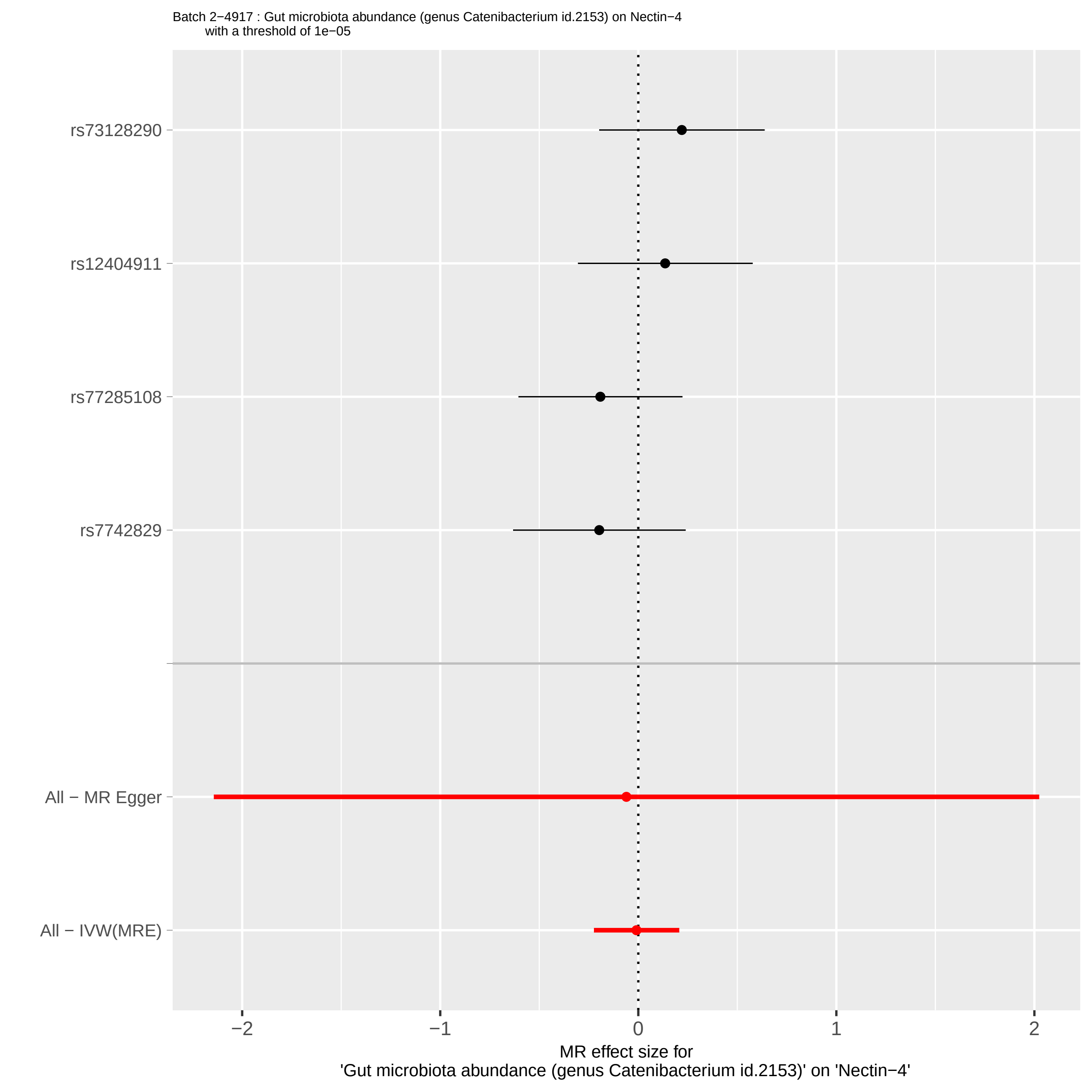

Batch 2−4917 : Gut microbiota abundance (genus Catenibacterium id.2153) on Nectin−4
with a threshold of 1e−05
rs73128290
rs12404911
rs77285108
rs7742829
All − MR Egger
All − IVW(MRE)
−2
−1
0
1
2
MR effect size for
'Gut microbiota abundance (genus Catenibacterium id.2153)' on 'Nectin−4'

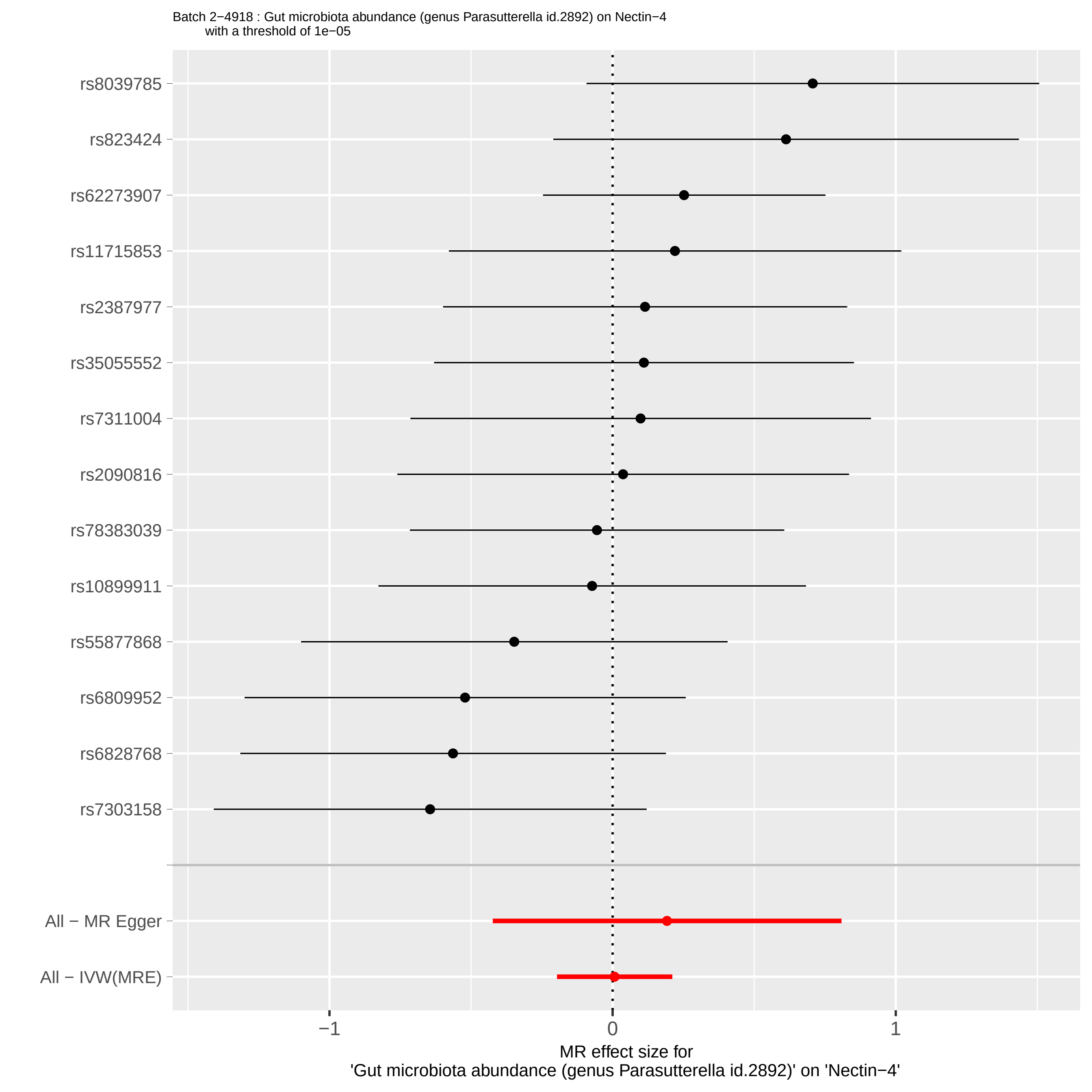

Batch 2−4918 : Gut microbiota abundance (genus Parasutterella id.2892) on Nectin−4
with a threshold of 1e−05
rs8039785
rs823424
rs62273907
rs11715853
rs2387977
rs35055552
rs7311004
rs2090816
rs78383039
rs10899911
rs55877868
rs6809952
rs6828768
rs7303158
All − MR Egger
All − IVW(MRE)
−1
0
1
MR effect size for
'Gut microbiota abundance (genus Parasutterella id.2892)' on 'Nectin−4'

Batch 2−4919 : Gut microbiota abundance (genus Catenibacterium id.2153) on Peroxidasin−like protein
with a threshold of 1e−05

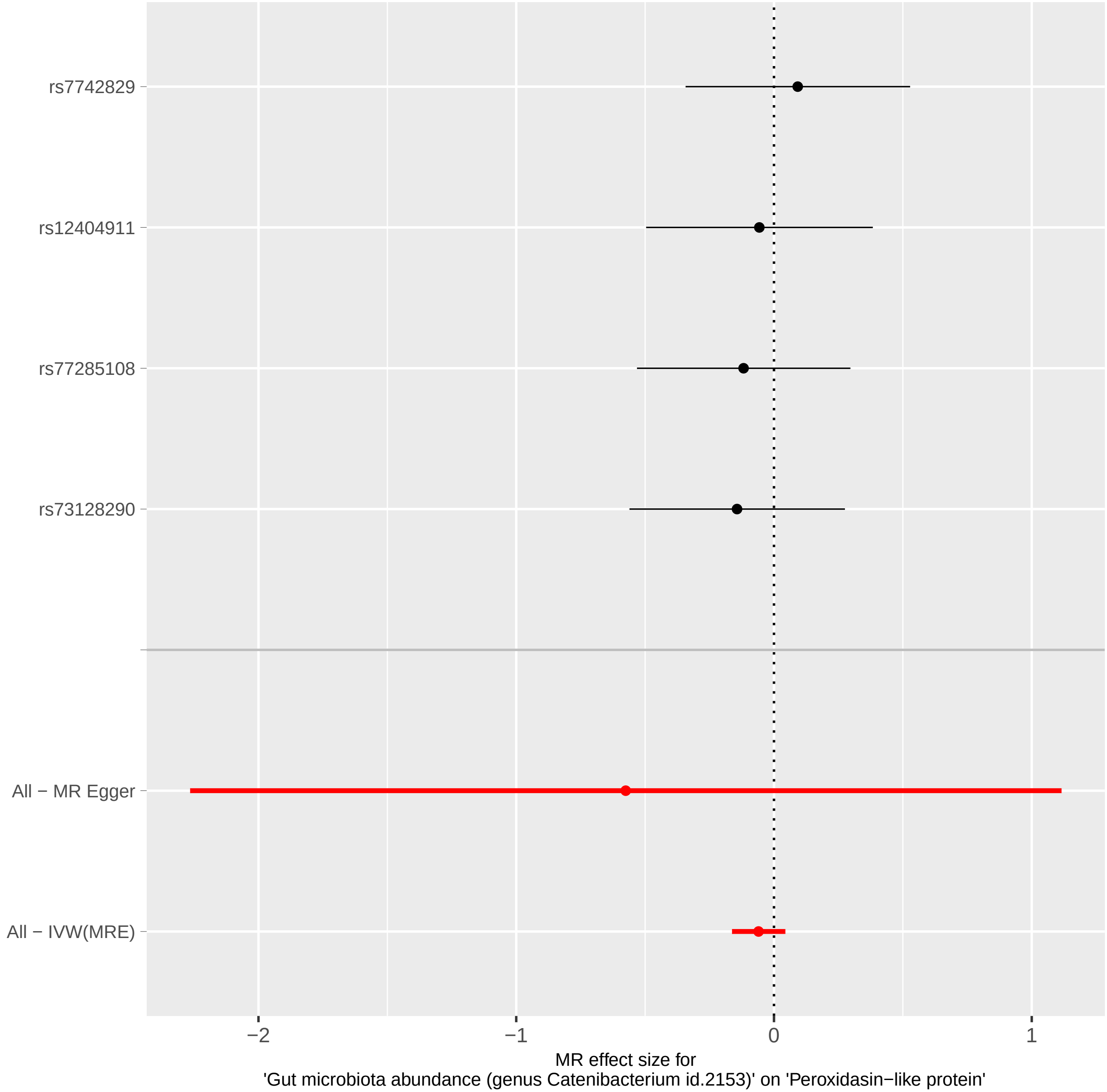

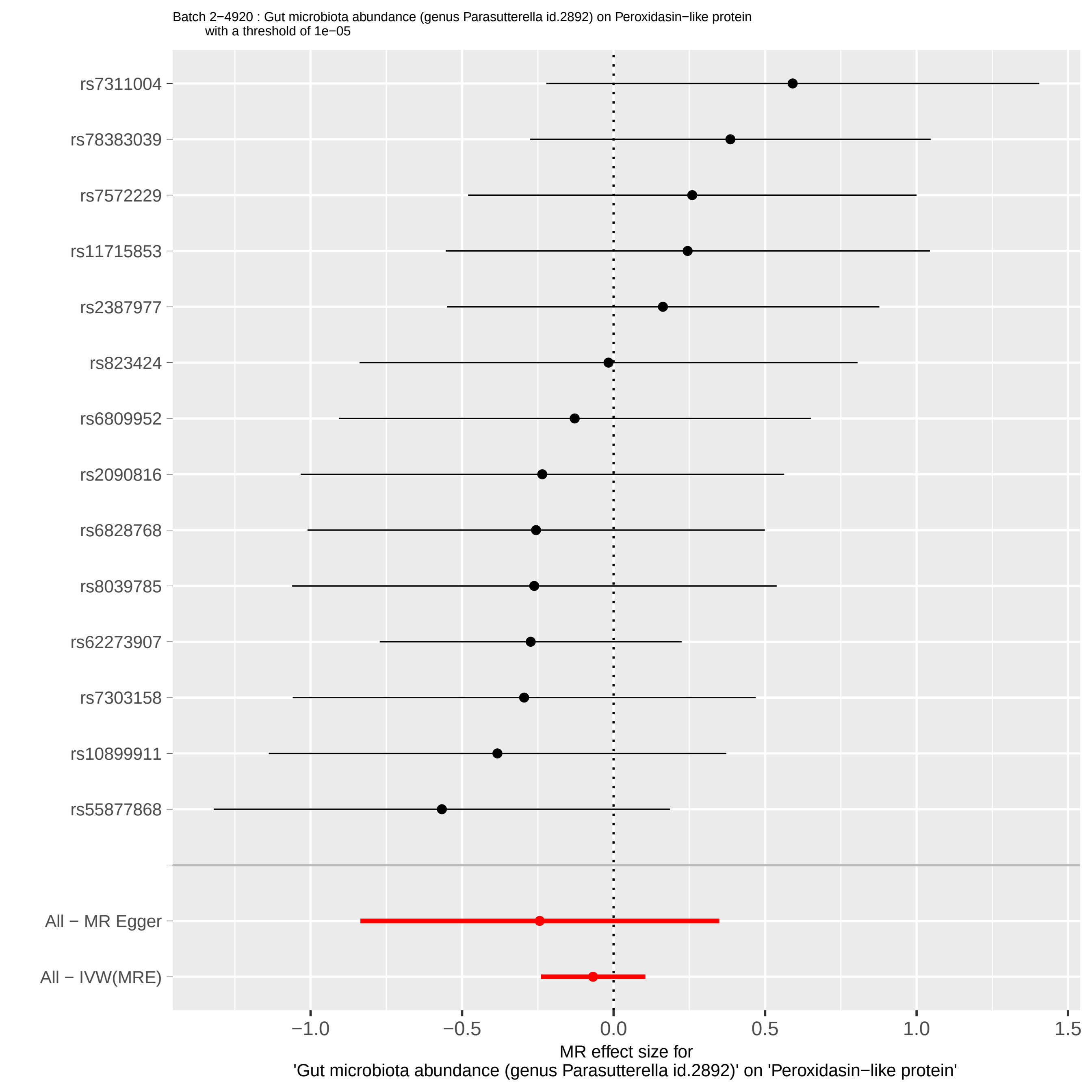

Batch 2−4920 : Gut microbiota abundance (genus Parasutterella id.2892) on Peroxidasin−like protein
with a threshold of 1e−05
rs7311004
rs78383039
rs7572229
rs11715853
rs2387977
rs823424
rs6809952
rs2090816
rs6828768
rs8039785
rs62273907
rs7303158
rs10899911
rs55877868
All − MR Egger
All − IVW(MRE)
−1.0
−0.5
0.0
0.5
1.0
1.5
MR effect size for
'Gut microbiota abundance (genus Parasutterella id.2892)' on 'Peroxidasin−like protein'

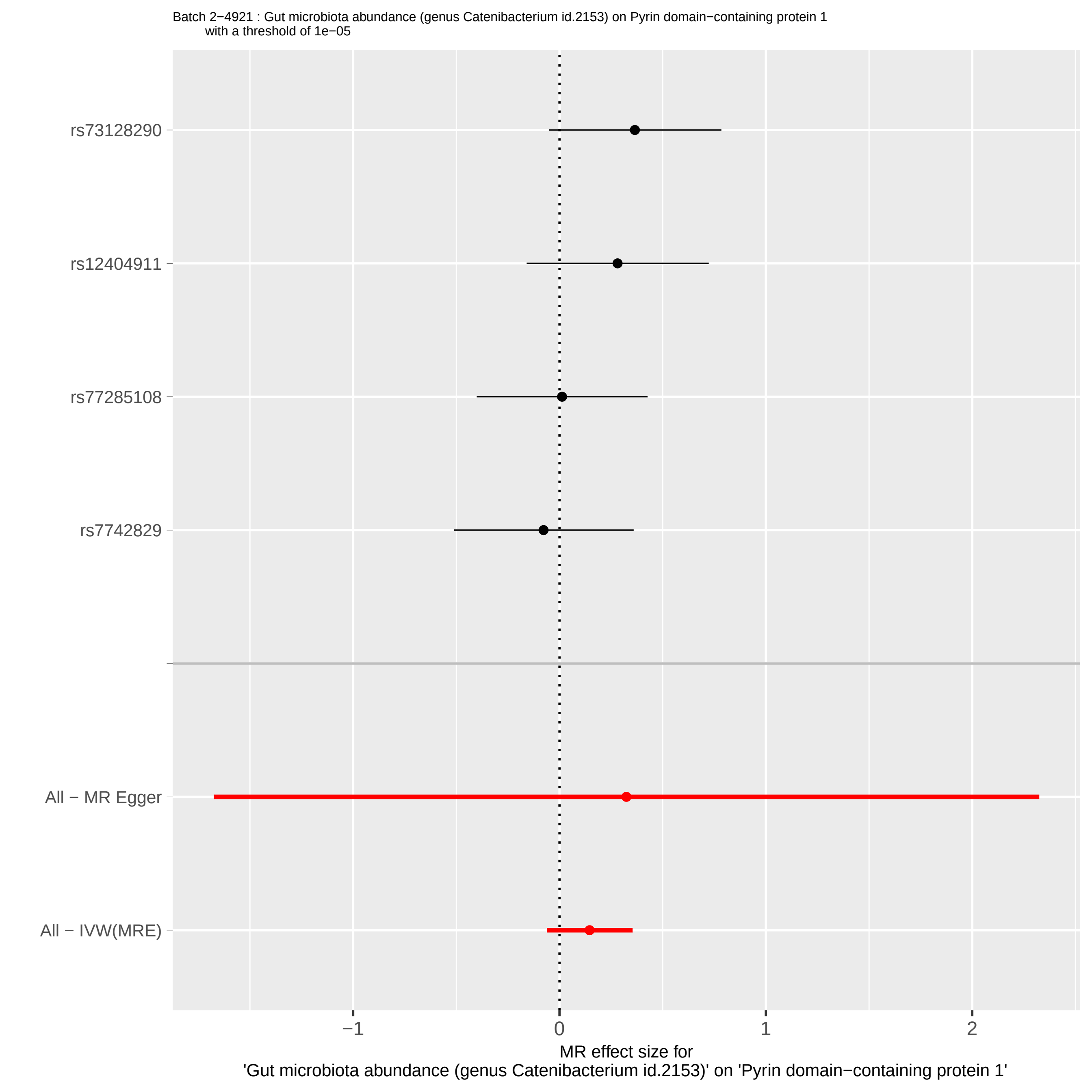

Batch 2−4921 : Gut microbiota abundance (genus Catenibacterium id.2153) on Pyrin domain−containing protein 1
with a threshold of 1e−05
rs73128290
rs12404911
rs77285108
rs7742829
All − MR Egger
All − IVW(MRE)
−1
0
1
2
MR effect size for
'Gut microbiota abundance (genus Catenibacterium id.2153)' on 'Pyrin domain−containing protein 1'

Batch 2−4922 : Gut microbiota abundance (genus Parasutterella id.2892) on Pyrin domain−containing protein 1
with a threshold of 1e−05

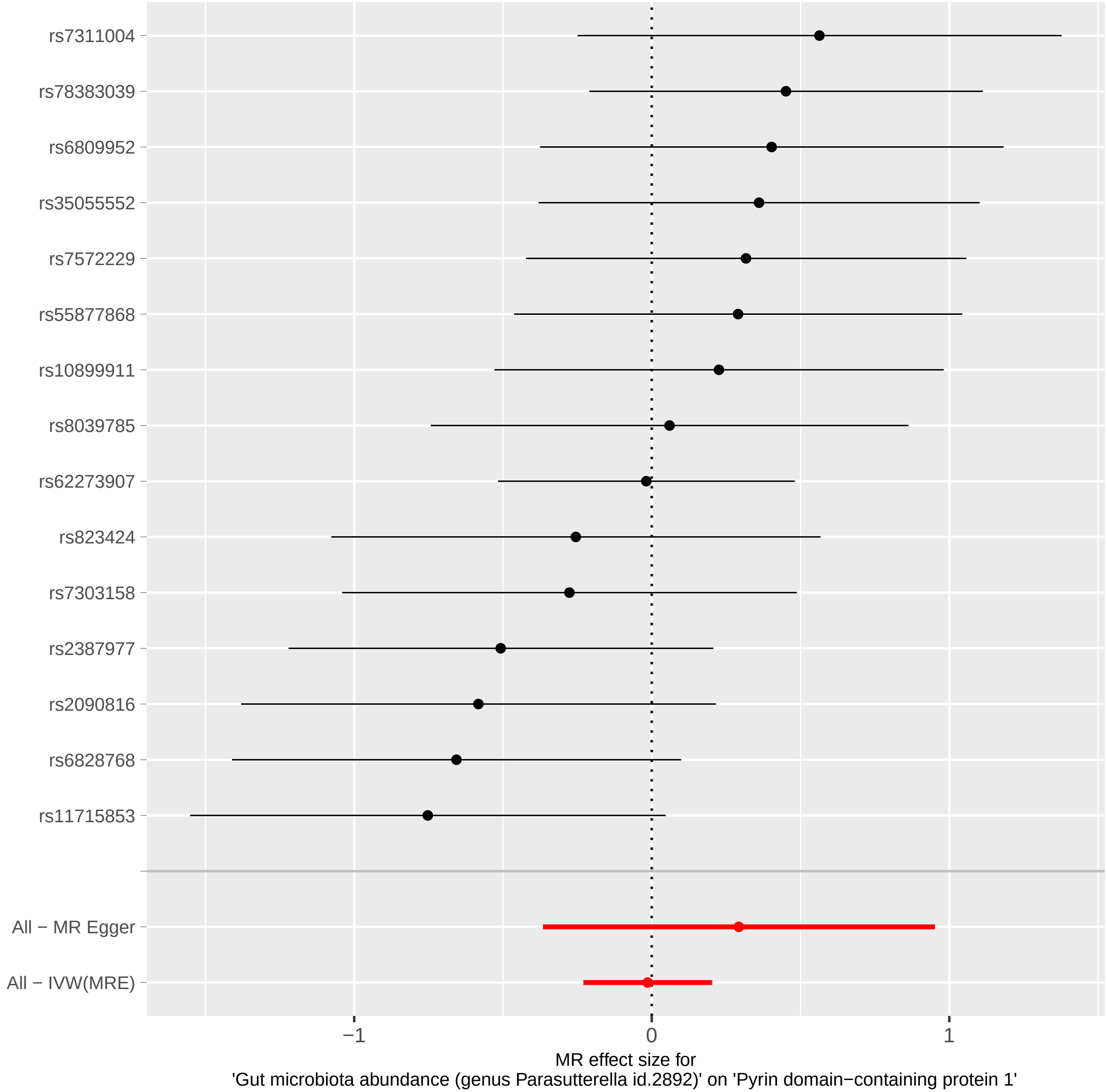

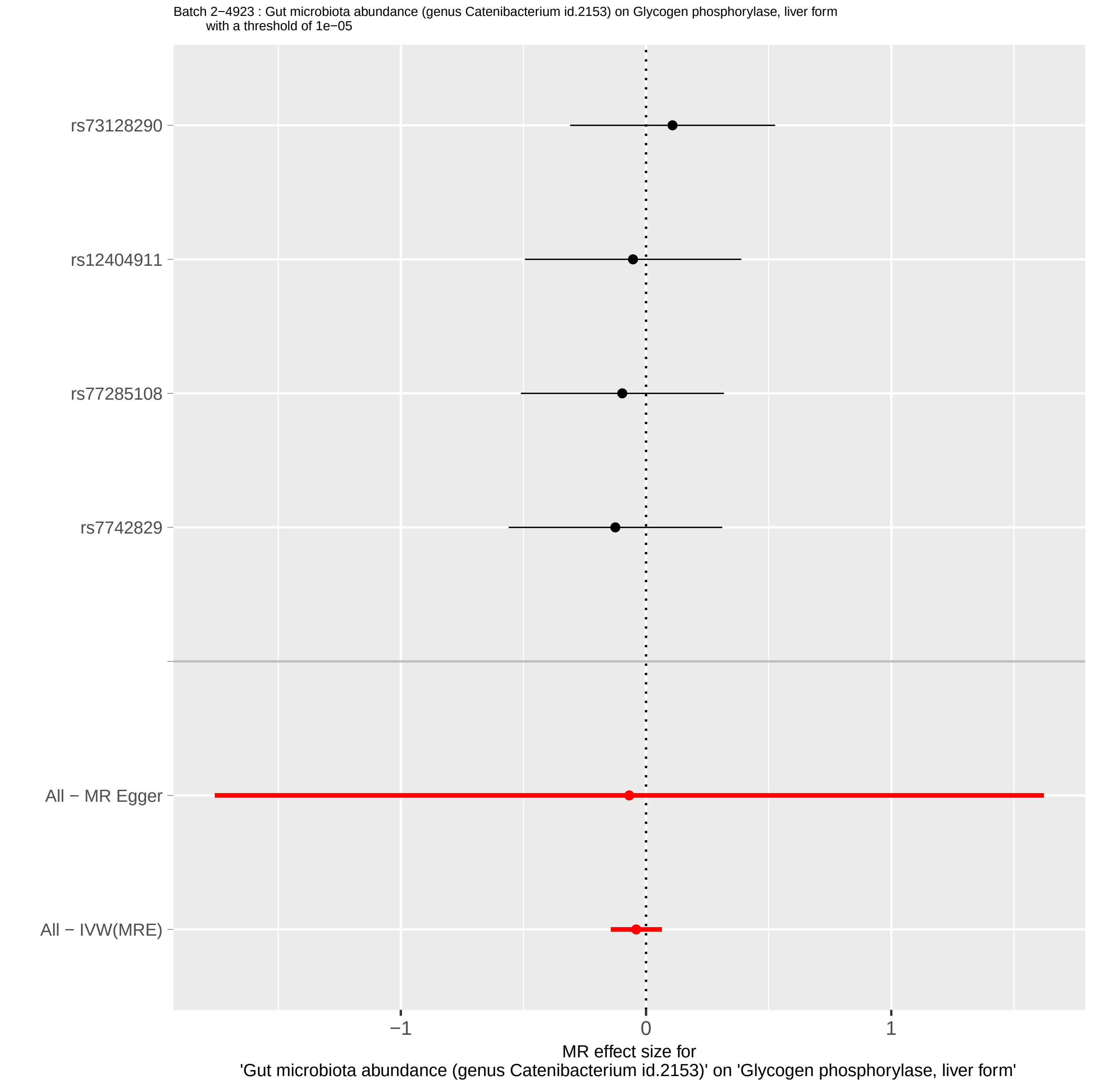

Batch 2−4923 : Gut microbiota abundance (genus Catenibacterium id.2153) on Glycogen phosphorylase, liver form
with a threshold of 1e−05
rs73128290
rs12404911
rs77285108
rs7742829
All − MR Egger
All − IVW(MRE)
−1
0
1
MR effect size for
'Gut microbiota abundance (genus Catenibacterium id.2153)' on 'Glycogen phosphorylase, liver form'

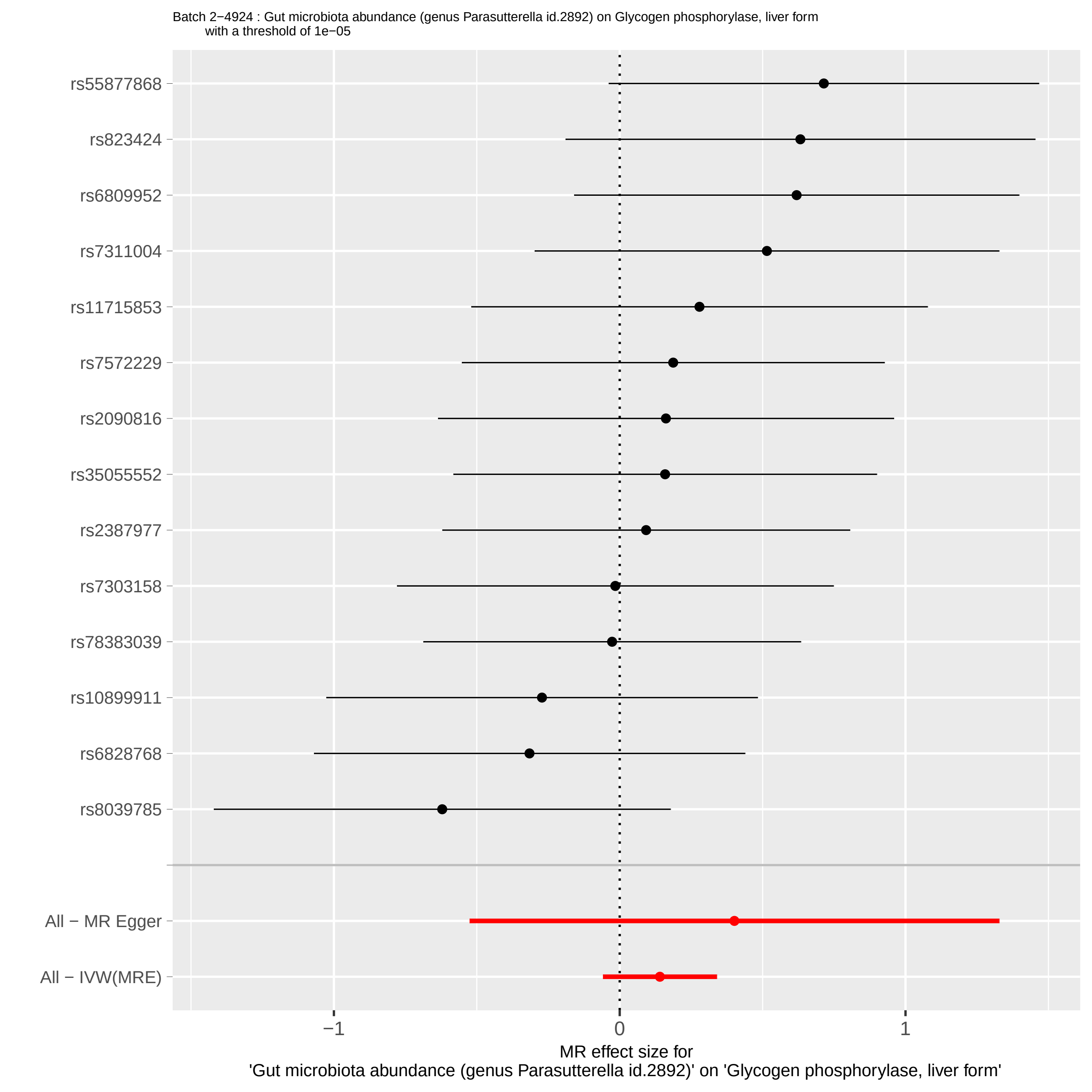

Batch 2−4924 : Gut microbiota abundance (genus Parasutterella id.2892) on Glycogen phosphorylase, liver form
with a threshold of 1e−05
rs55877868
rs823424
rs6809952
rs7311004
rs11715853
rs7572229
rs2090816
rs35055552
rs2387977
rs7303158
rs78383039
rs10899911
rs6828768
rs8039785
All − MR Egger
All − IVW(MRE)
−1
0
1
MR effect size for
'Gut microbiota abundance (genus Parasutterella id.2892)' on 'Glycogen phosphorylase, liver form'

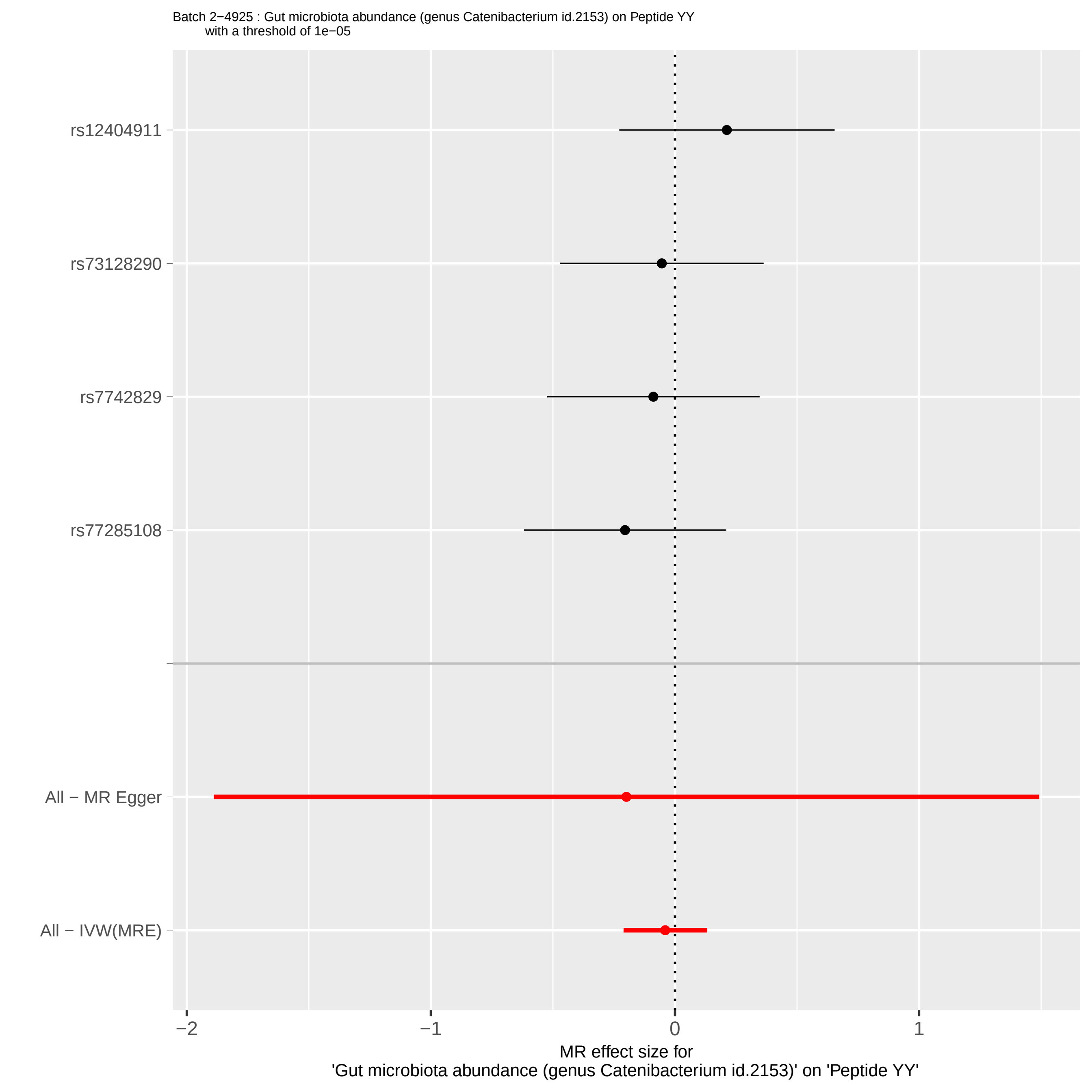

Batch 2−4925 : Gut microbiota abundance (genus Catenibacterium id.2153) on Peptide YY
with a threshold of 1e−05
rs12404911
rs73128290
rs7742829
rs77285108
All − MR Egger
All − IVW(MRE)
−2
−1
0
1
MR effect size for
'Gut microbiota abundance (genus Catenibacterium id.2153)' on 'Peptide YY'

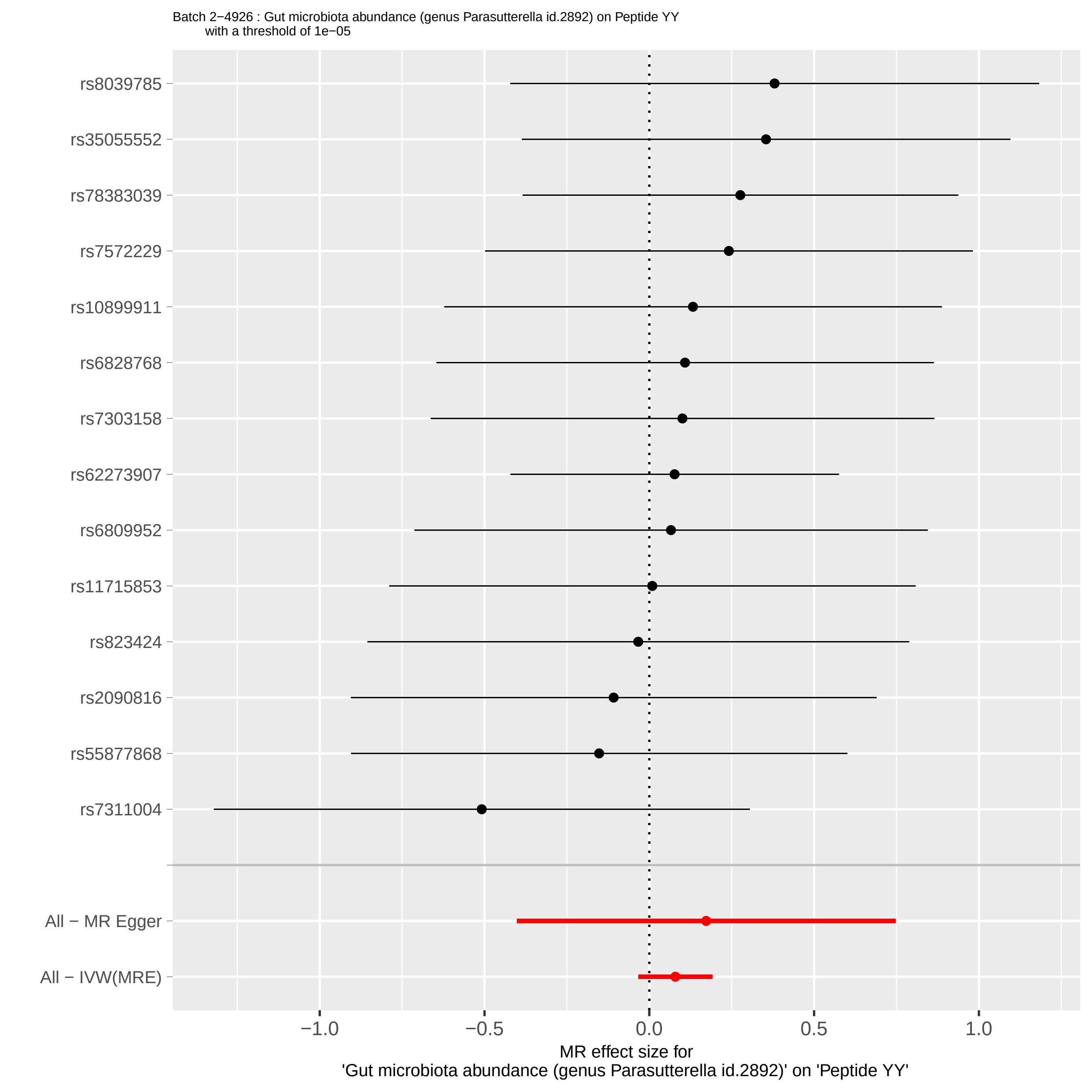

Batch 2−4926 : Gut microbiota abundance (genus Parasutterella id.2892) on Peptide YY
with a threshold of 1e−05
rs8039785
rs35055552
rs78383039
rs7572229
rs10899911
rs6828768
rs7303158
rs62273907
rs6809952
rs11715853
rs823424
rs2090816
rs55877868
rs7311004
All − MR Egger
All − IVW(MRE)
−1.0
−0.5
0.0
0.5
1.0
MR effect size for
'Gut microbiota abundance (genus Parasutterella id.2892)' on 'Peptide YY'

Batch 2−4927 : Gut microbiota abundance (genus Catenibacterium id.2153) on Dihydropteridine reductase
with a threshold of 1e−05

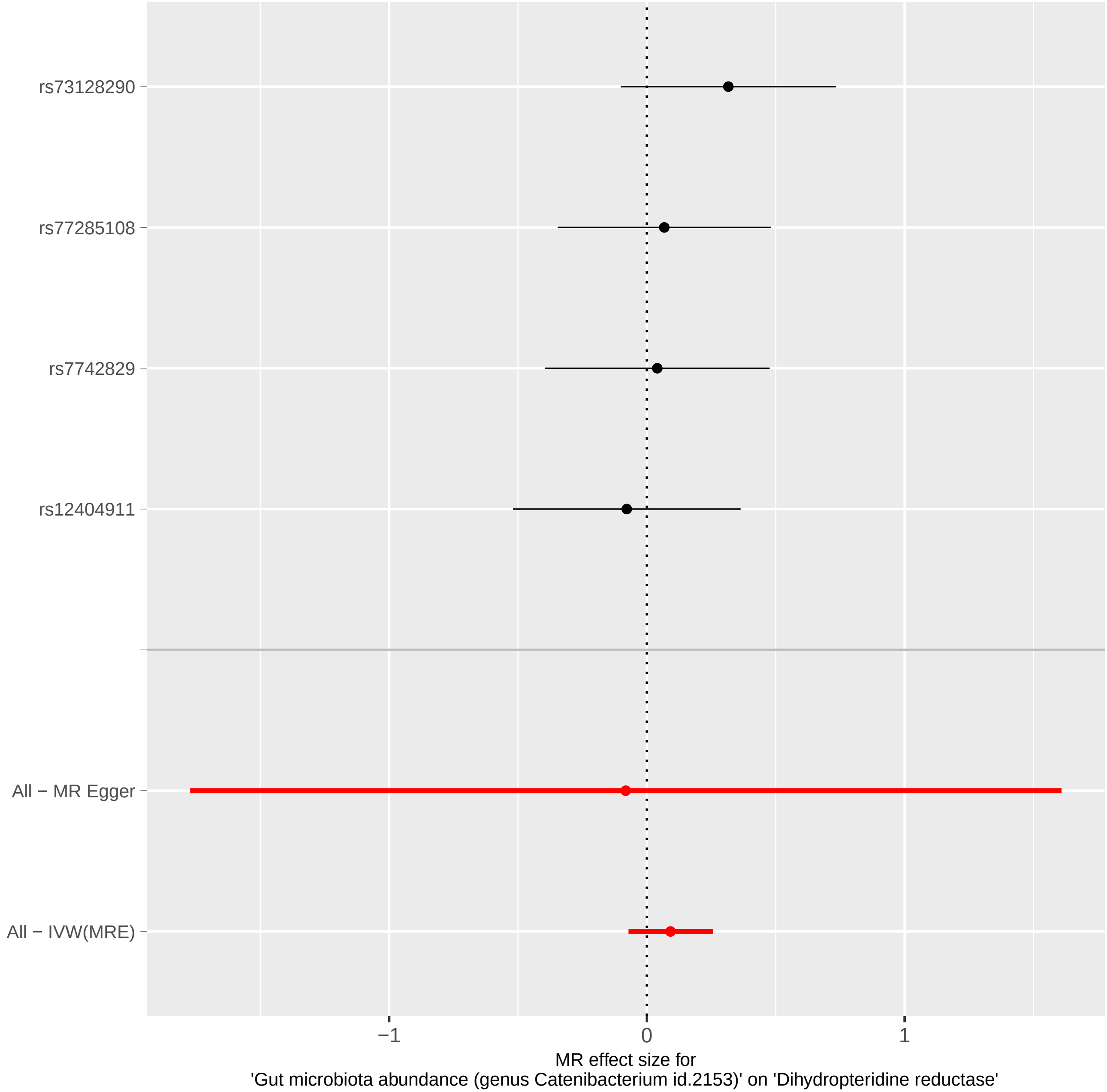

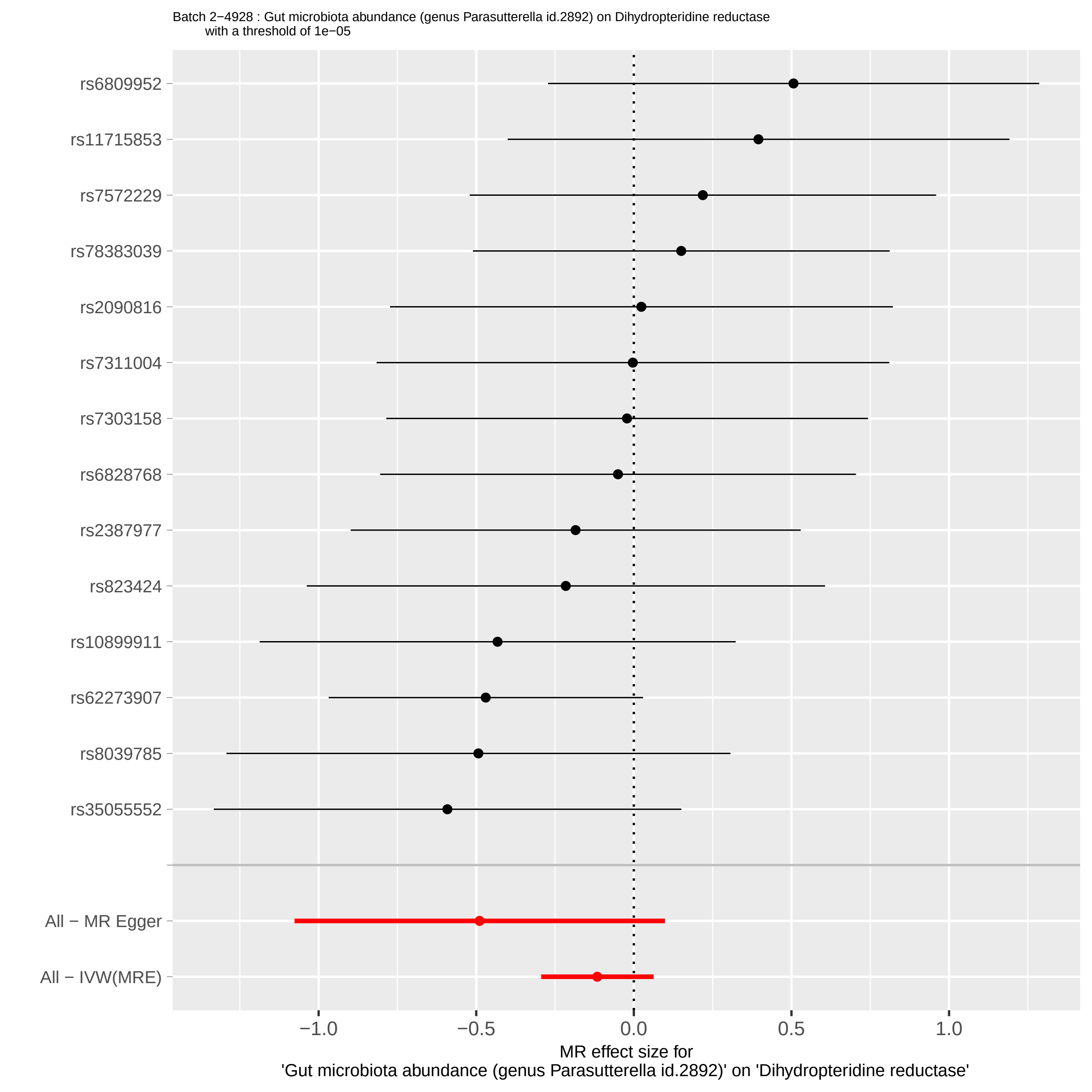

Batch 2−4928 : Gut microbiota abundance (genus Parasutterella id.2892) on Dihydropteridine reductase
with a threshold of 1e−05
rs6809952
rs11715853
rs7572229
rs78383039
rs2090816
rs7311004
rs7303158
rs6828768
rs2387977
rs823424
rs10899911
rs62273907
rs8039785
rs35055552
All − MR Egger
All − IVW(MRE)
−1.0
−0.5
0.0
0.5
1.0
MR effect size for
'Gut microbiota abundance (genus Parasutterella id.2892)' on 'Dihydropteridine reductase'

Batch 2−4929 : Gut microbiota abundance (genus Catenibacterium id.2153) on Glutaminyl−peptide cyclotransferase
with a threshold of 1e−05

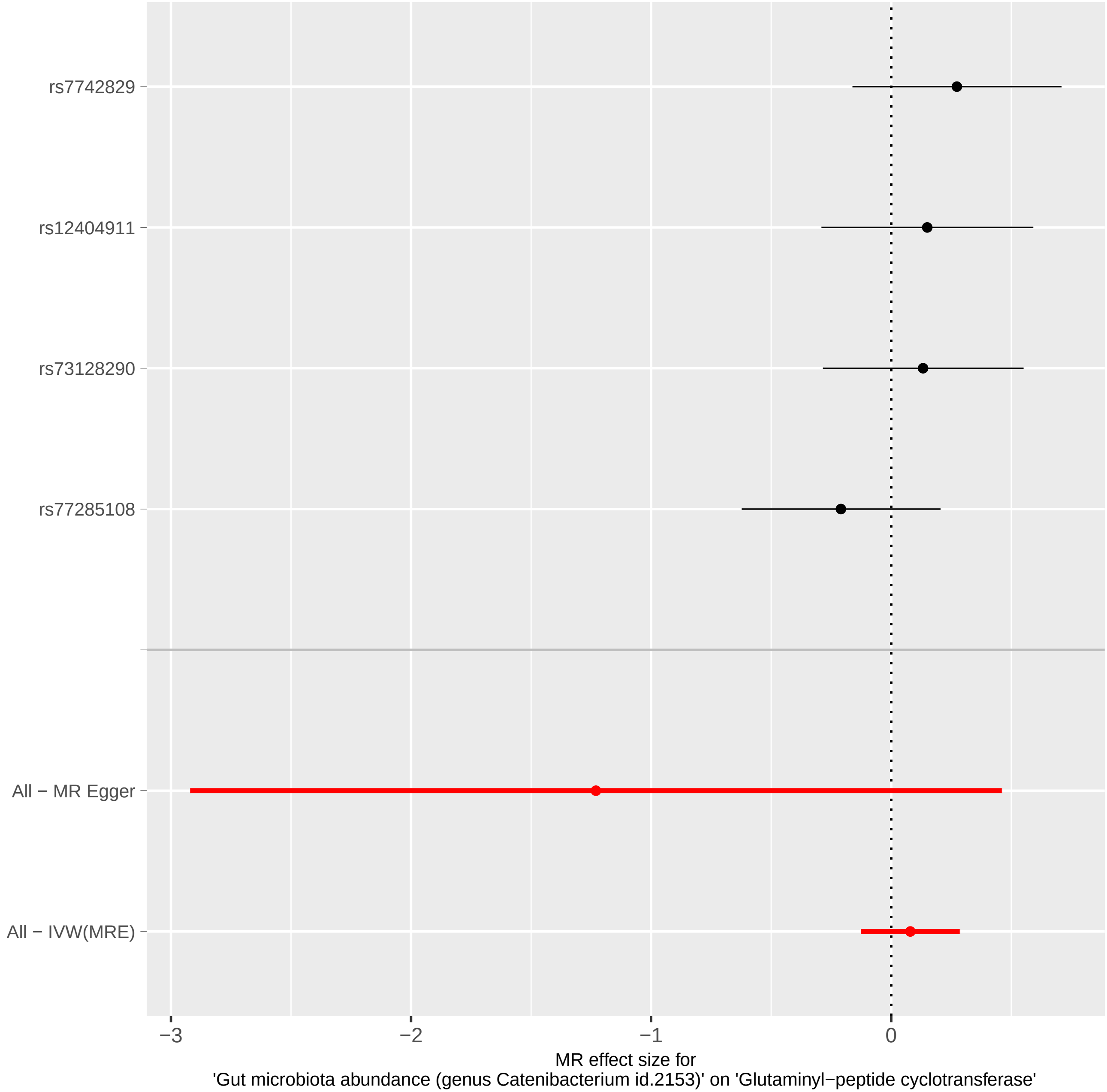

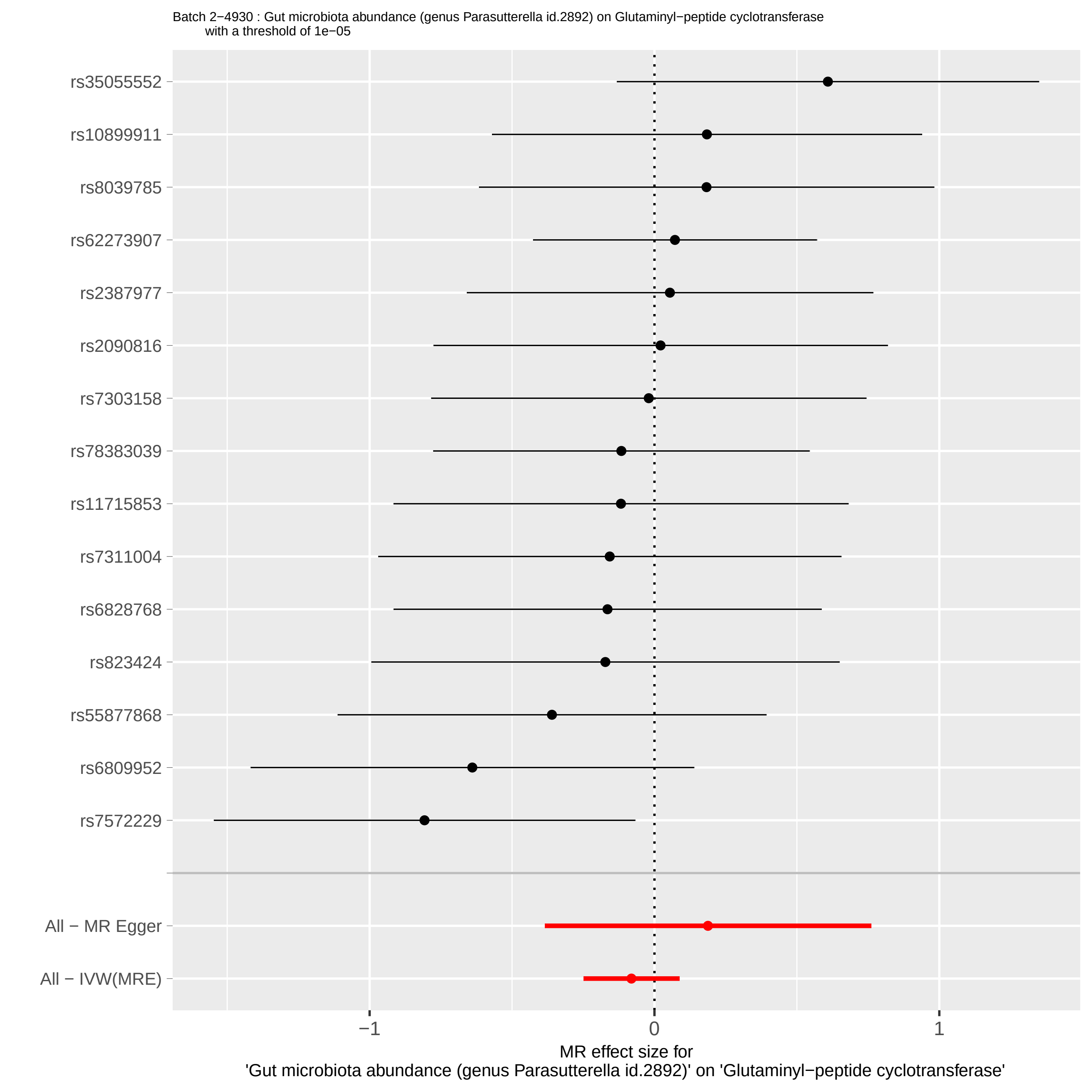

Batch 2−4930 : Gut microbiota abundance (genus Parasutterella id.2892) on Glutaminyl−peptide cyclotransferase
with a threshold of 1e−05
rs35055552
rs10899911
rs8039785
rs62273907
rs2387977
rs2090816
rs7303158
rs78383039
rs11715853
rs7311004
rs6828768
rs823424
rs55877868
rs6809952
rs7572229
All − MR Egger
All − IVW(MRE)
−1
0
1
MR effect size for
'Gut microbiota abundance (genus Parasutterella id.2892)' on 'Glutaminyl−peptide cyclotransferase'

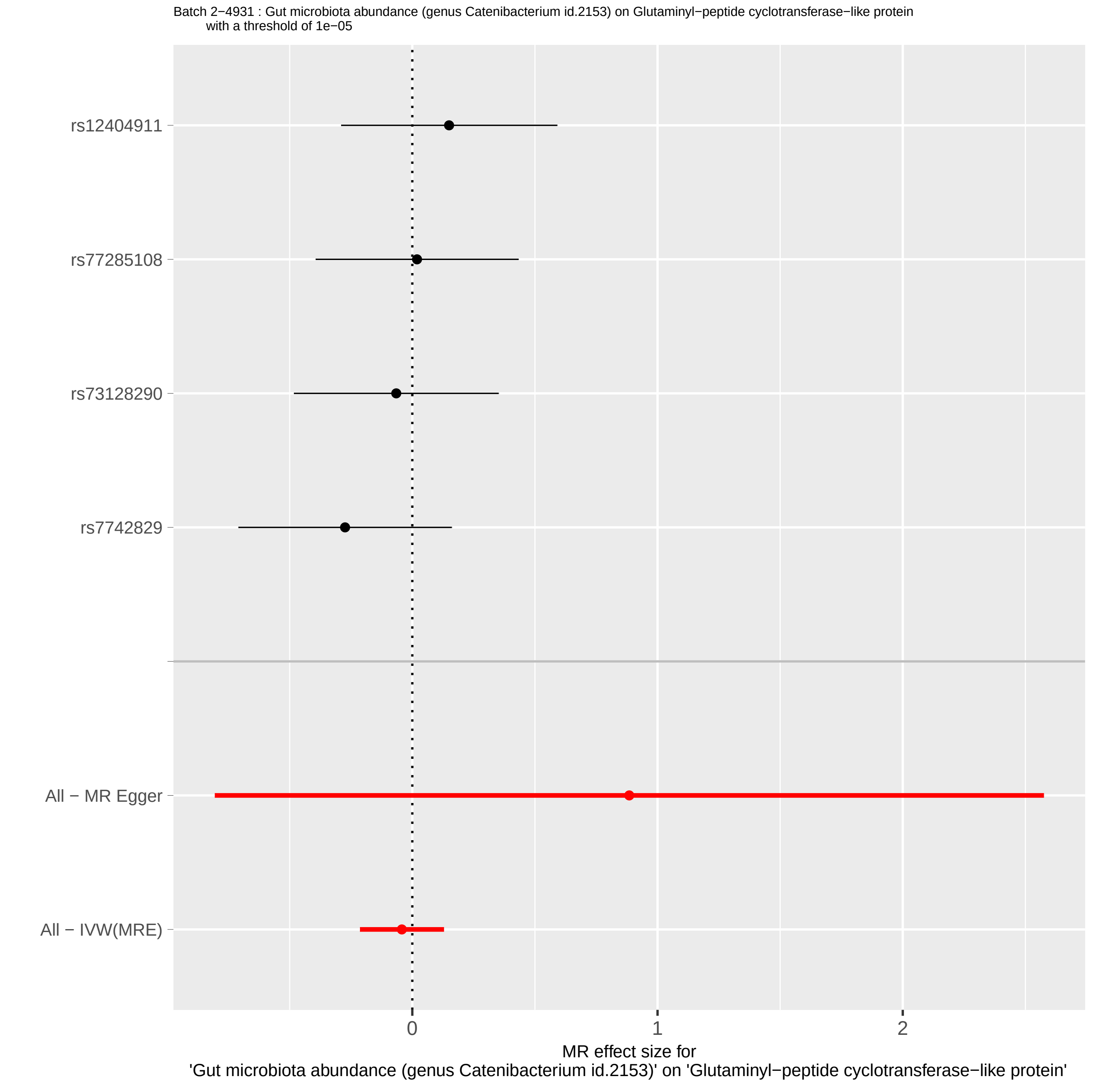

Batch 2−4931 : Gut microbiota abundance (genus Catenibacterium id.2153) on Glutaminyl−peptide cyclotransferase−like protein
with a threshold of 1e−05
rs12404911
rs77285108
rs73128290
rs7742829
All − MR Egger
All − IVW(MRE)
0
1
2
MR effect size for
'Gut microbiota abundance (genus Catenibacterium id.2153)' on 'Glutaminyl−peptide cyclotransferase−like protein'

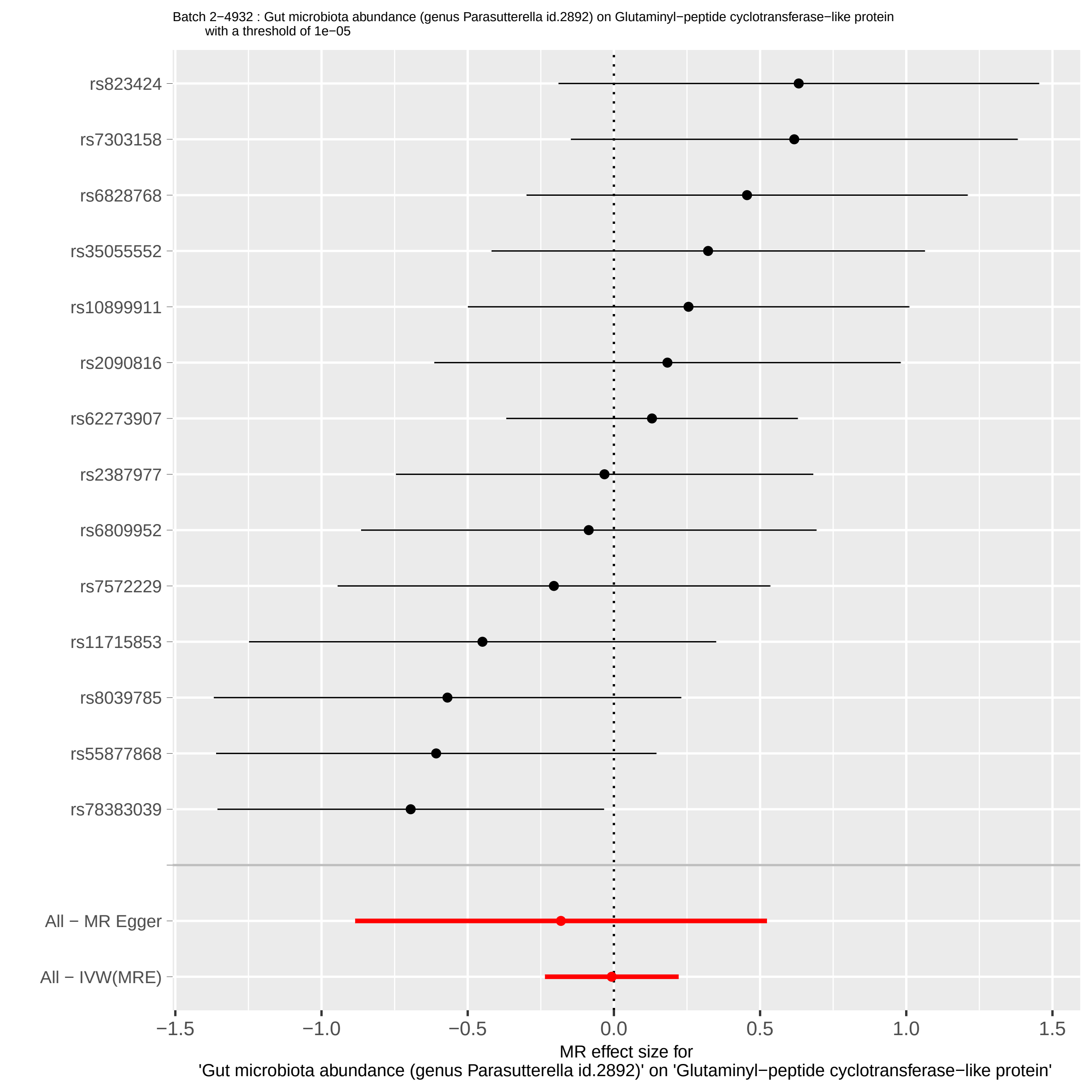

Batch 2−4932 : Gut microbiota abundance (genus Parasutterella id.2892) on Glutaminyl−peptide cyclotransferase−like protein
with a threshold of 1e−05
rs823424
rs7303158
rs6828768
rs35055552
rs10899911
rs2090816
rs62273907
rs2387977
rs6809952
rs7572229
rs11715853
rs8039785
rs55877868
rs78383039
All − MR Egger
All − IVW(MRE)
−1.5
−1.0
−0.5
0.0
0.5
1.0
1.5
MR effect size for
'Gut microbiota abundance (genus Parasutterella id.2892)' on 'Glutaminyl−peptide cyclotransferase−like protein'

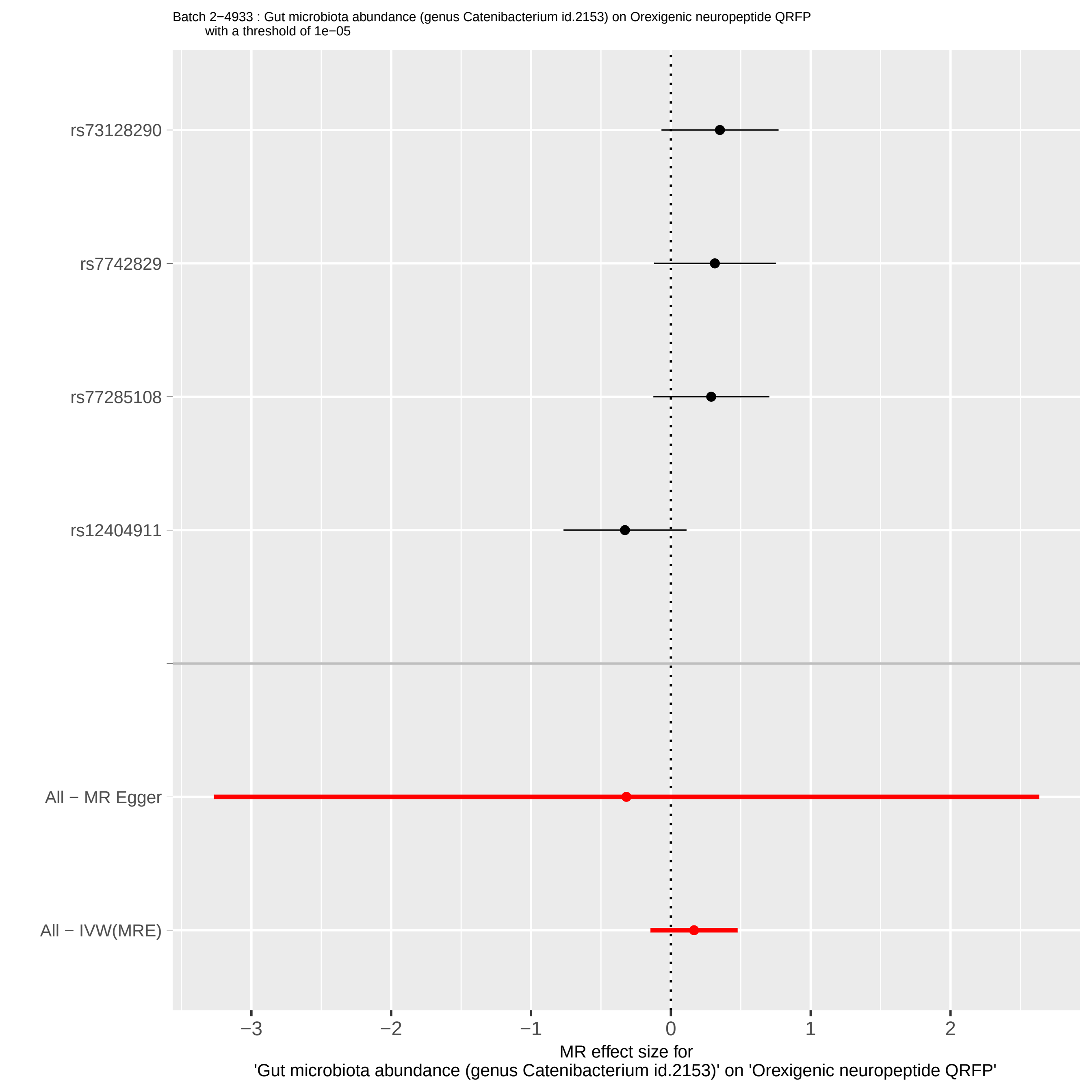
Batch 2−4933 : Gut microbiota abundance (genus Catenibacterium id.2153) on Orexigenic neuropeptide QRFP
with a threshold of 1e−05
rs73128290
rs7742829
rs77285108
rs12404911
All − MR Egger
All − IVW(MRE)
−3
−2
−1
0
1
2
MR effect size for
'Gut microbiota abundance (genus Catenibacterium id.2153)' on 'Orexigenic neuropeptide QRFP'

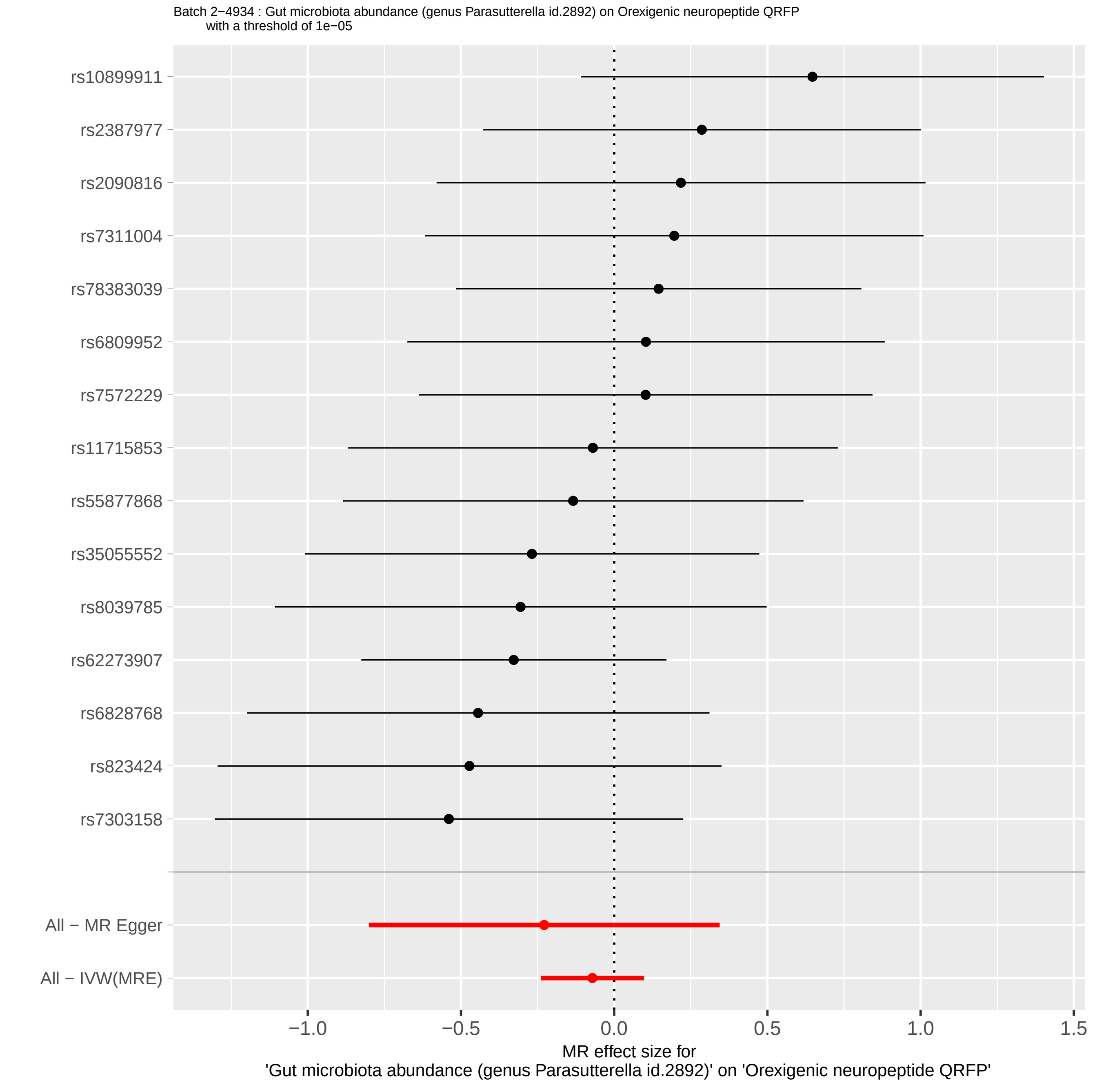

Batch 2−4934 : Gut microbiota abundance (genus Parasutterella id.2892) on Orexigenic neuropeptide QRFP
with a threshold of 1e−05
rs10899911
rs2387977
rs2090816
rs7311004
rs78383039
rs6809952
rs7572229
rs11715853
rs55877868
rs35055552
rs8039785
rs62273907
rs6828768
rs823424
rs7303158
All − MR Egger
All − IVW(MRE)
−1.0
−0.5
0.0
0.5
1.0
1.5
MR effect size for
'Gut microbiota abundance (genus Parasutterella id.2892)' on 'Orexigenic neuropeptide QRFP'

Batch 2−4935 : Gut microbiota abundance (genus Catenibacterium id.2153) on Sulfhydryl oxidase 1
with a threshold of 1e−05

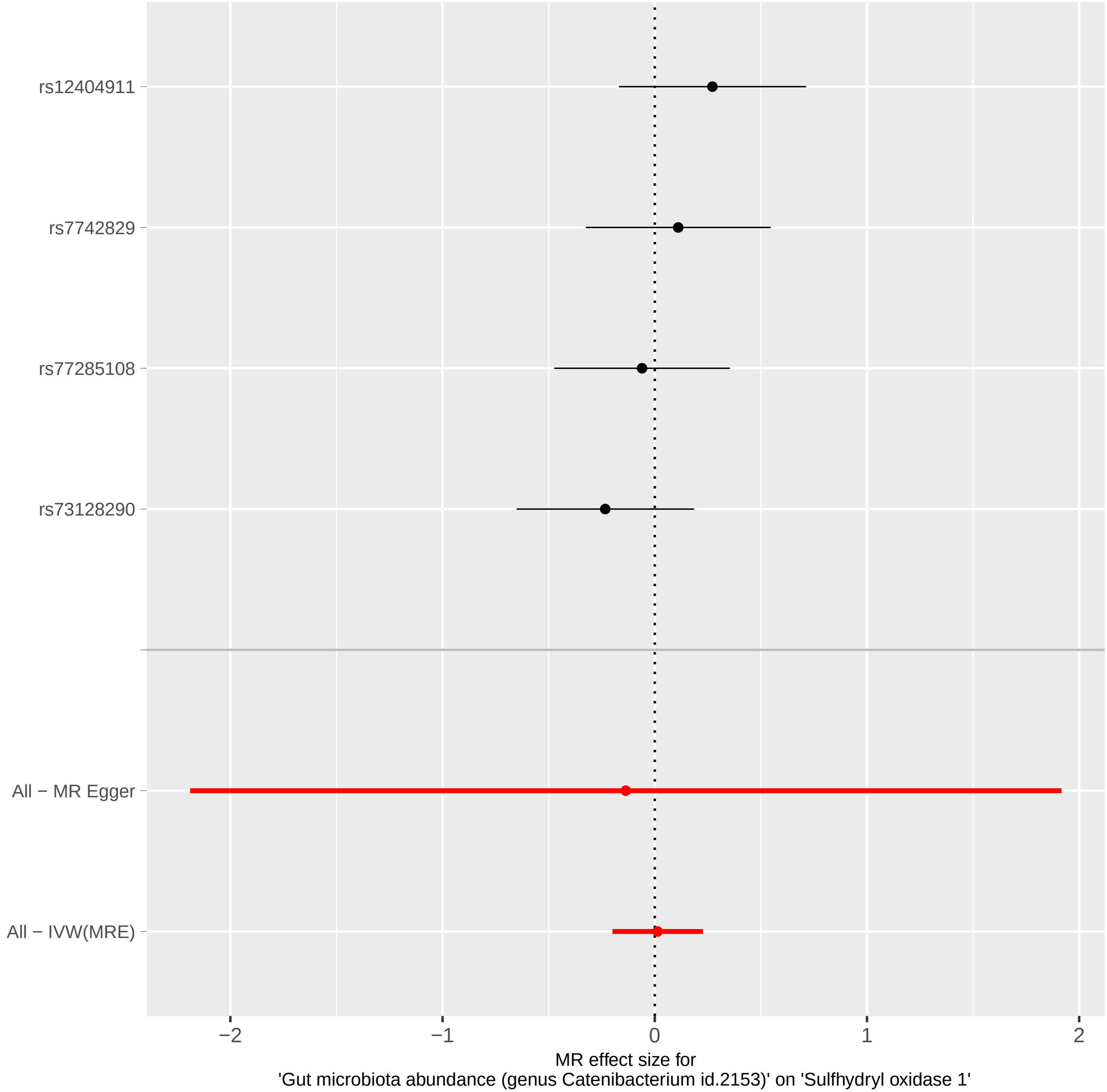

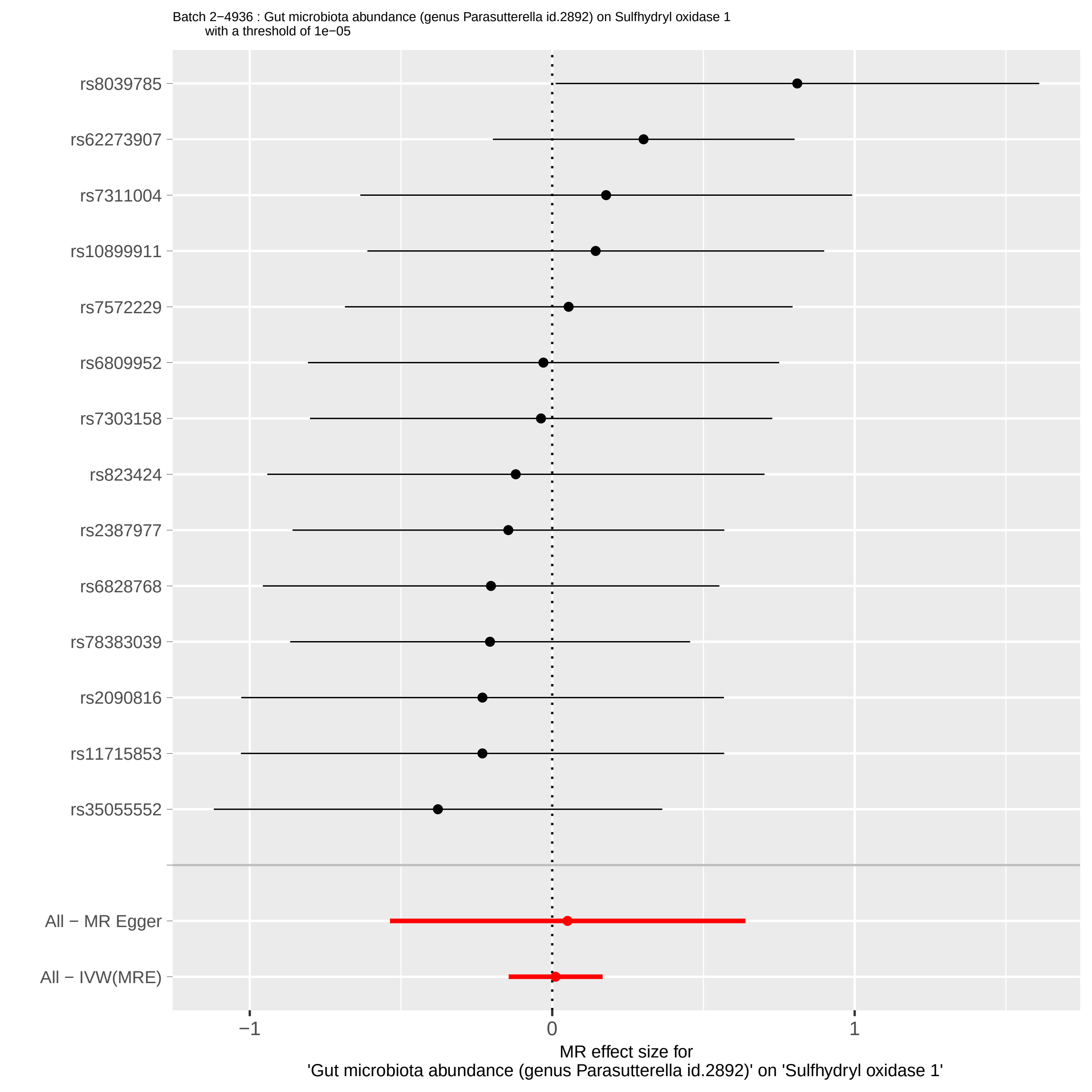
Batch 2−4936 : Gut microbiota abundance (genus Parasutterella id.2892) on Sulfhydryl oxidase 1
with a threshold of 1e−05
rs8039785
rs62273907
rs7311004
rs10899911
rs7572229
rs6809952
rs7303158
rs823424
rs2387977
rs6828768
rs78383039
rs2090816
rs11715853
rs35055552
All − MR Egger
All − IVW(MRE)
−1
0
1
MR effect size for
'Gut microbiota abundance (genus Parasutterella id.2892)' on 'Sulfhydryl oxidase 1'

Batch 2−4937 : Gut microbiota abundance (genus Catenibacterium id.2153) on Sulfhydryl oxidase 1
with a threshold of 1e−05

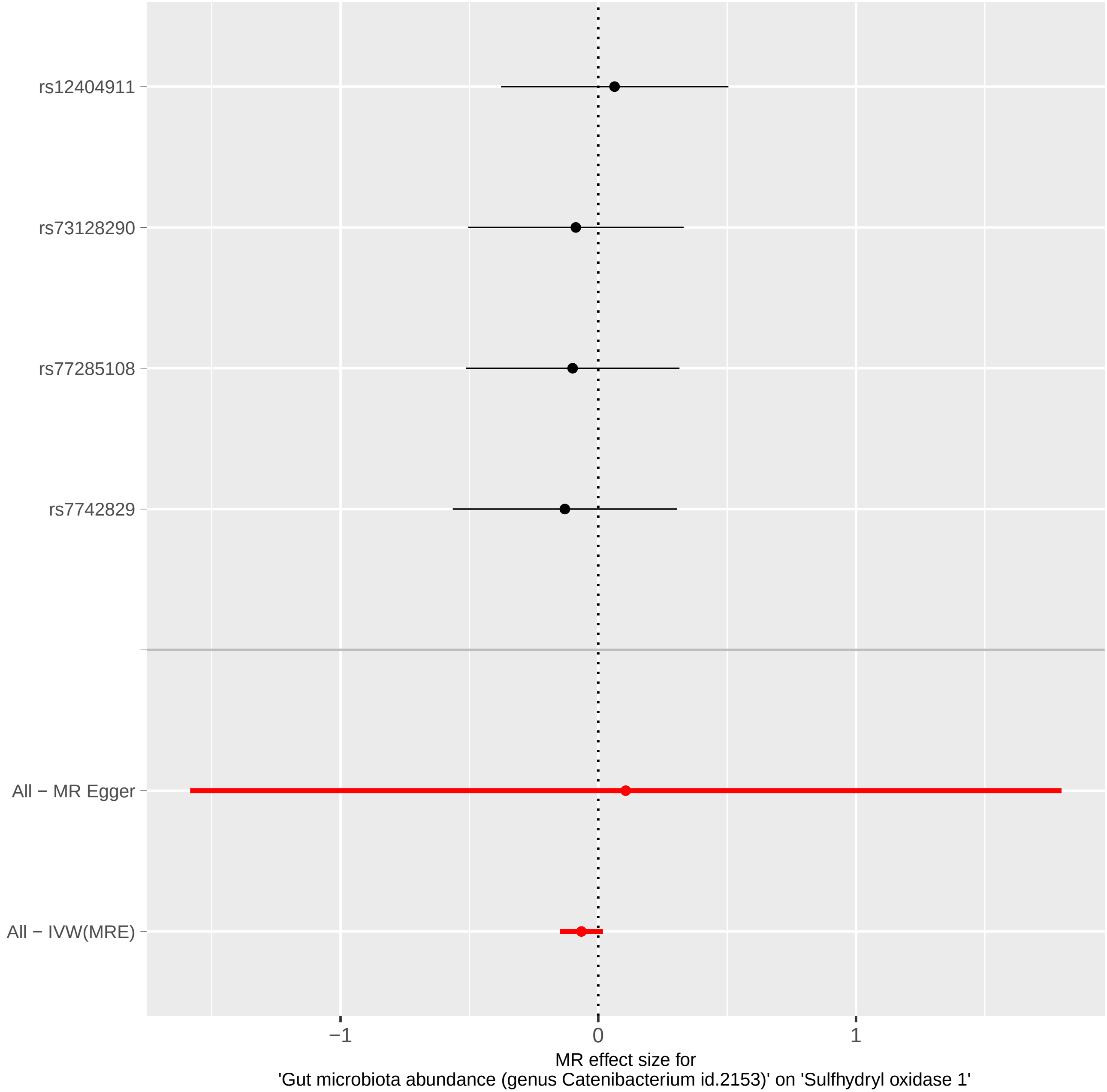

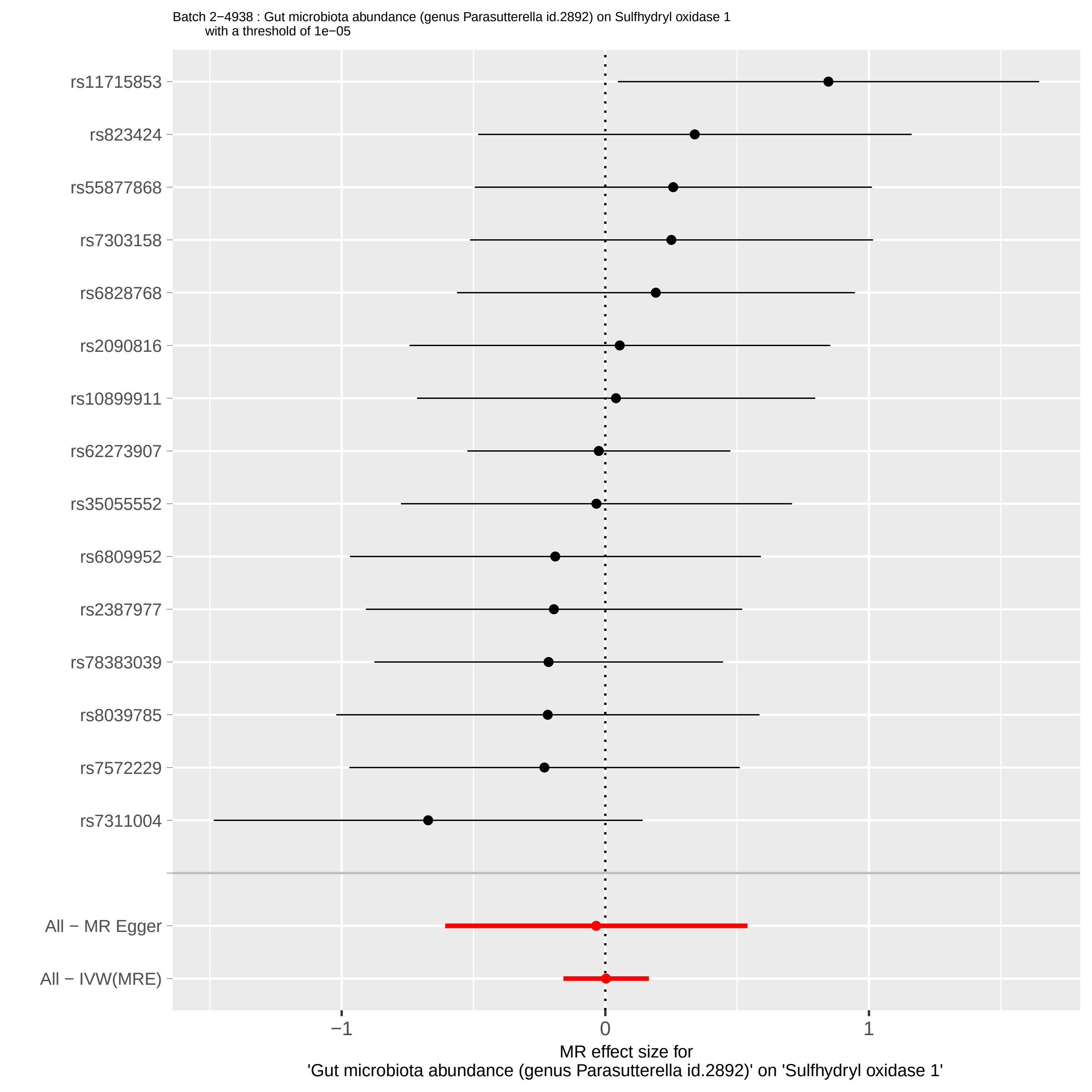

Batch 2−4938 : Gut microbiota abundance (genus Parasutterella id.2892) on Sulfhydryl oxidase 1
with a threshold of 1e−05
rs11715853
rs823424
rs55877868
rs7303158
rs6828768
rs2090816
rs10899911
rs62273907
rs35055552
rs6809952
rs2387977
rs78383039
rs8039785
rs7572229
rs7311004
All − MR Egger
All − IVW(MRE)
−1
0
1
MR effect size for
'Gut microbiota abundance (genus Parasutterella id.2892)' on 'Sulfhydryl oxidase 1'

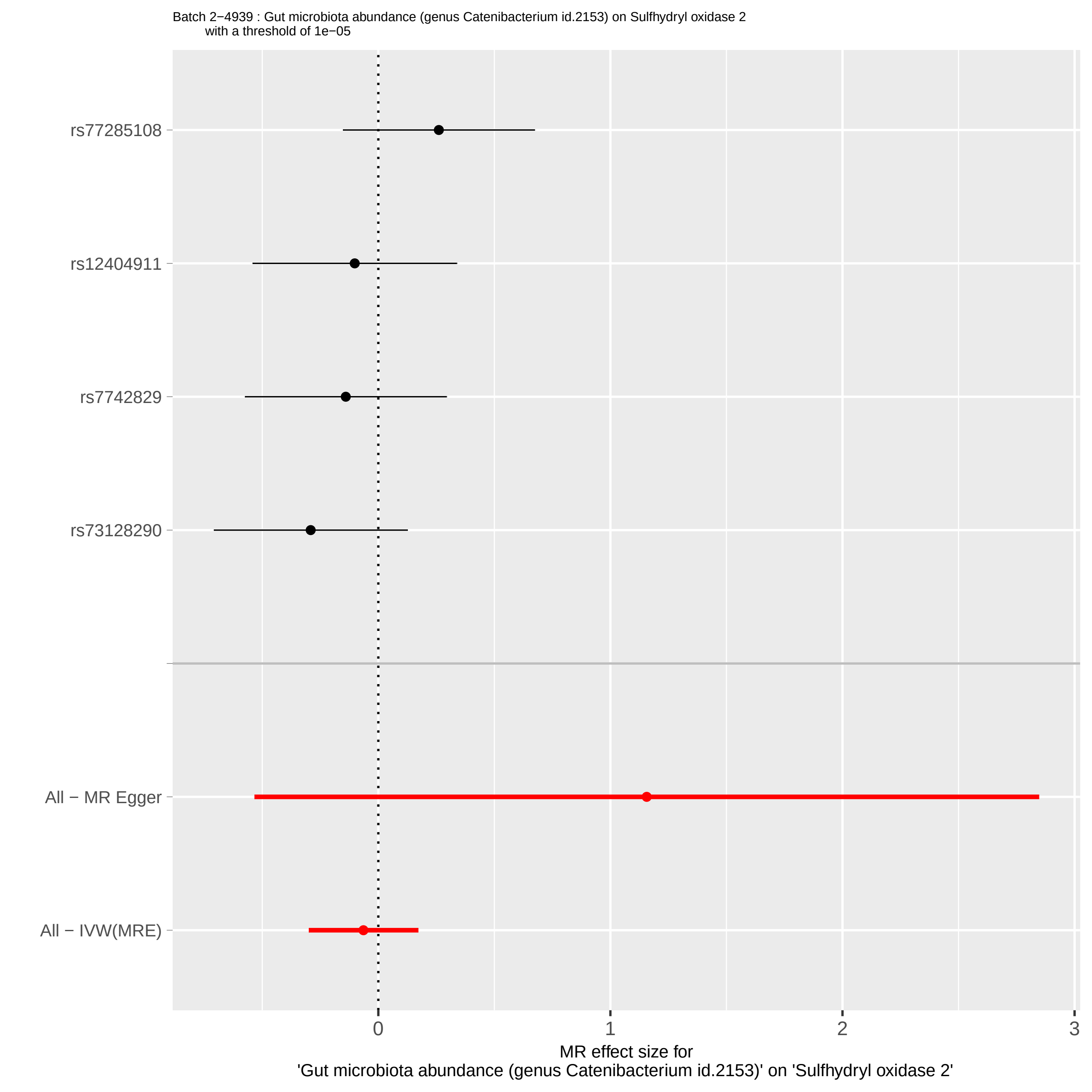
Batch 2−4939 : Gut microbiota abundance (genus Catenibacterium id.2153) on Sulfhydryl oxidase 2
with a threshold of 1e−05
rs77285108
rs12404911
rs7742829
rs73128290
All − MR Egger
All − IVW(MRE)
0
1
2
3
MR effect size for
'Gut microbiota abundance (genus Catenibacterium id.2153)' on 'Sulfhydryl oxidase 2'

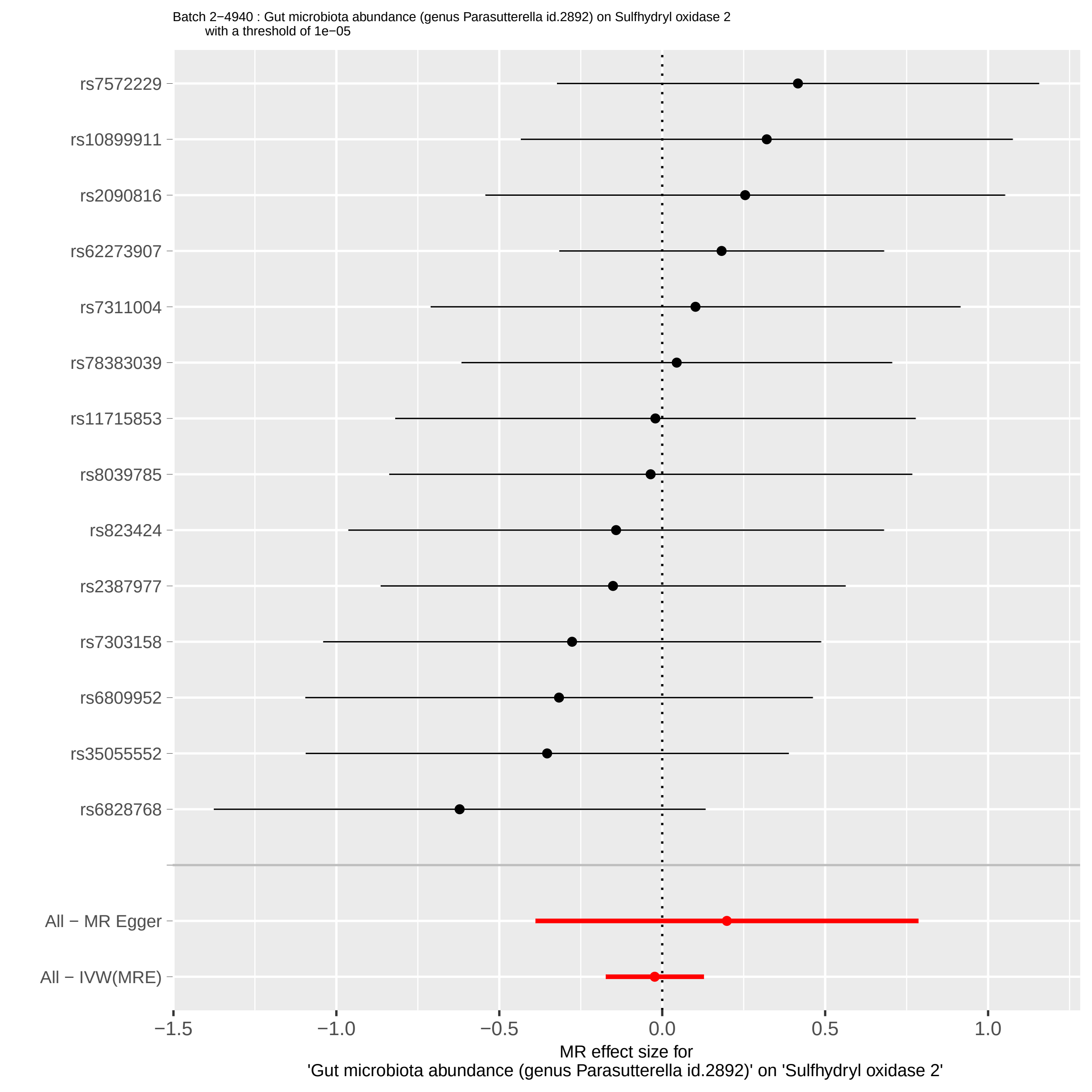

Batch 2−4940 : Gut microbiota abundance (genus Parasutterella id.2892) on Sulfhydryl oxidase 2
with a threshold of 1e−05
rs7572229
rs10899911
rs2090816
rs62273907
rs7311004
rs78383039
rs11715853
rs8039785
rs823424
rs2387977
rs7303158
rs6809952
rs35055552
rs6828768
All − MR Egger
All − IVW(MRE)
−1.5
−1.0
−0.5
0.0
0.5
1.0
MR effect size for
'Gut microbiota abundance (genus Parasutterella id.2892)' on 'Sulfhydryl oxidase 2'

Batch 2−4941 : Gut microbiota abundance (genus Catenibacterium id.2153) on Ras−related protein Rab−14
with a threshold of 1e−05

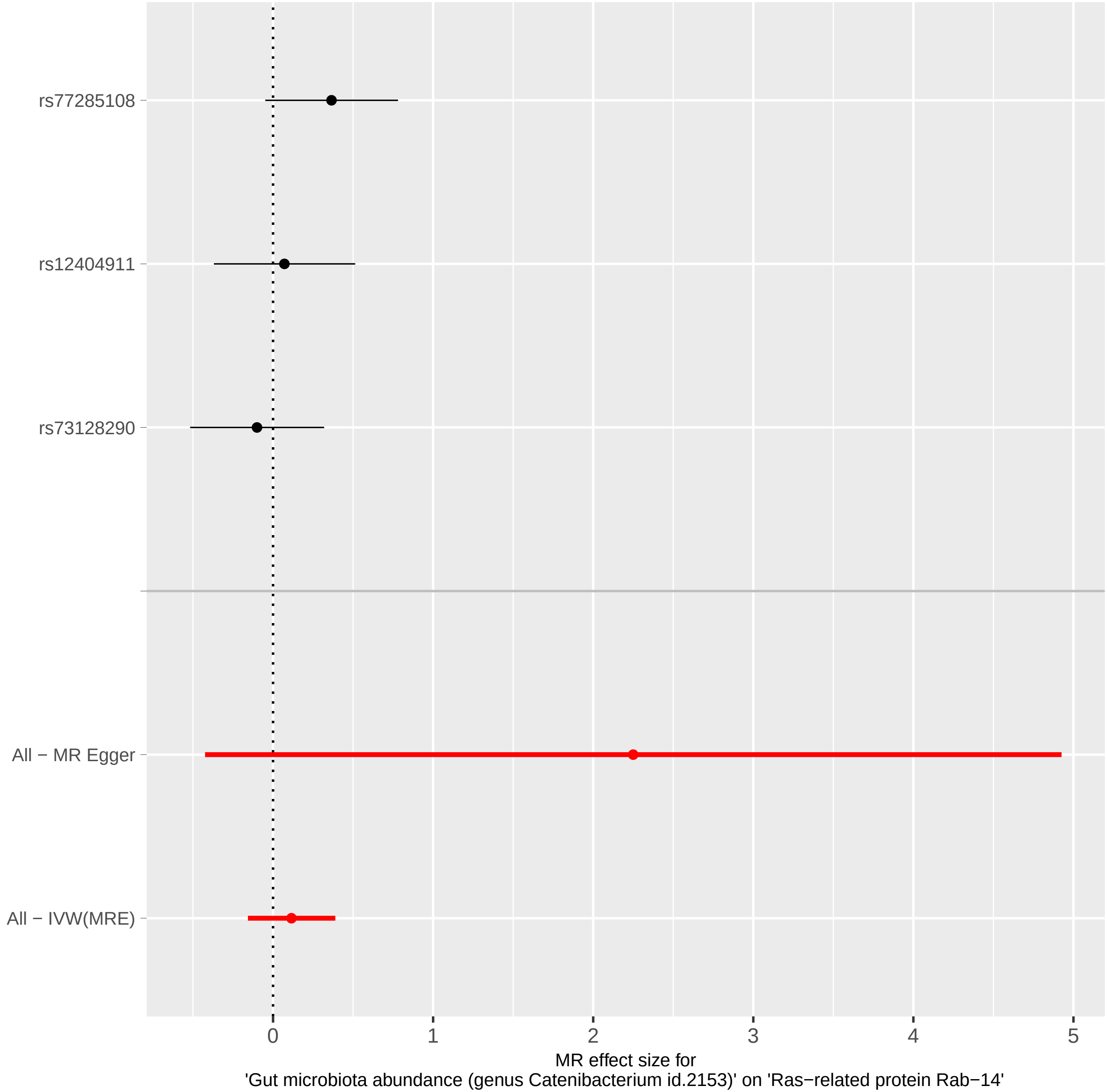

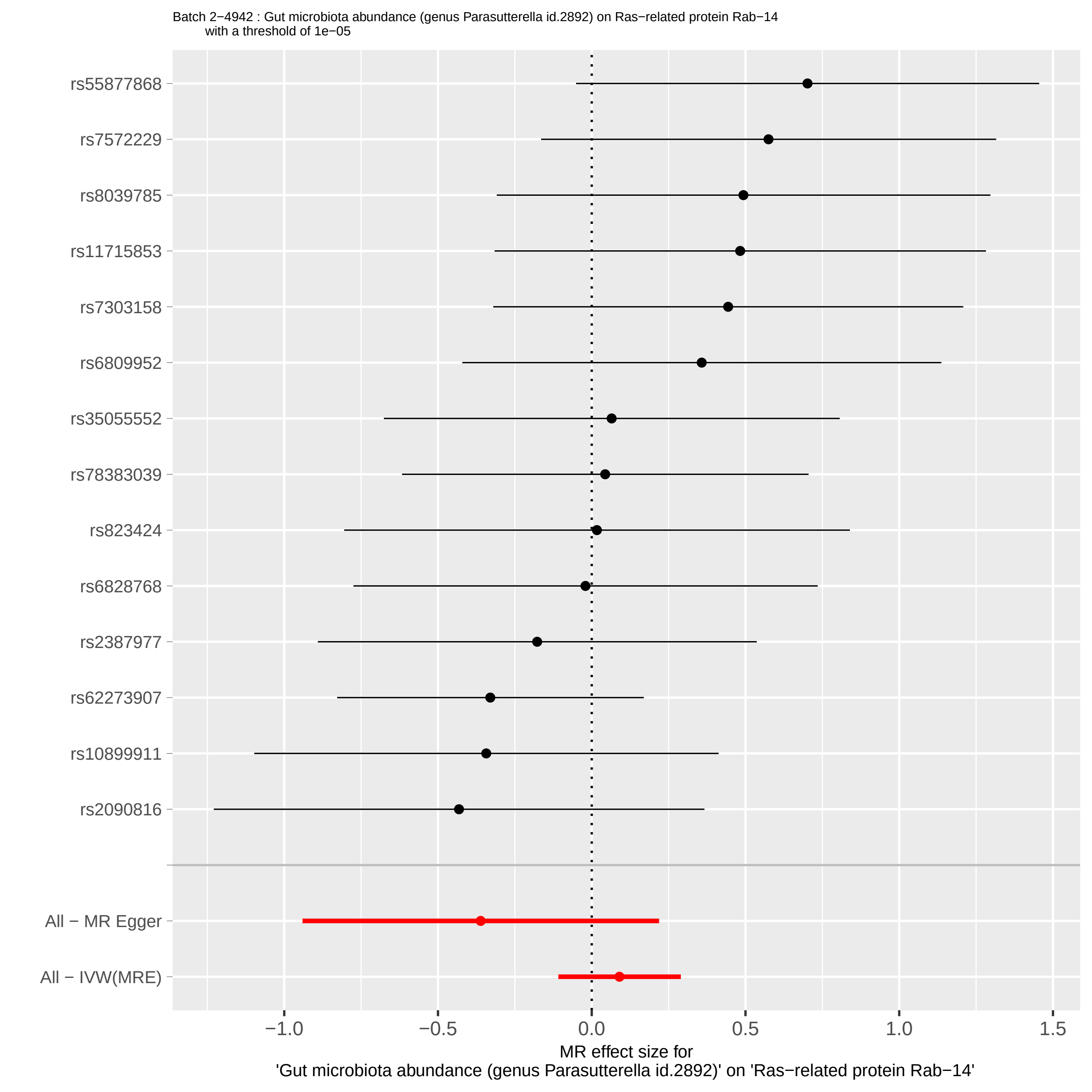
Batch 2−4942 : Gut microbiota abundance (genus Parasutterella id.2892) on Ras−related protein Rab−14
with a threshold of 1e−05
rs55877868
rs7572229
rs8039785
rs11715853
rs7303158
rs6809952
rs35055552
rs78383039
rs823424
rs6828768
rs2387977
rs62273907
rs10899911
rs2090816
All − MR Egger
All − IVW(MRE)
−1.0
−0.5
0.0
0.5
1.0
1.5
MR effect size for
'Gut microbiota abundance (genus Parasutterella id.2892)' on 'Ras−related protein Rab−14'

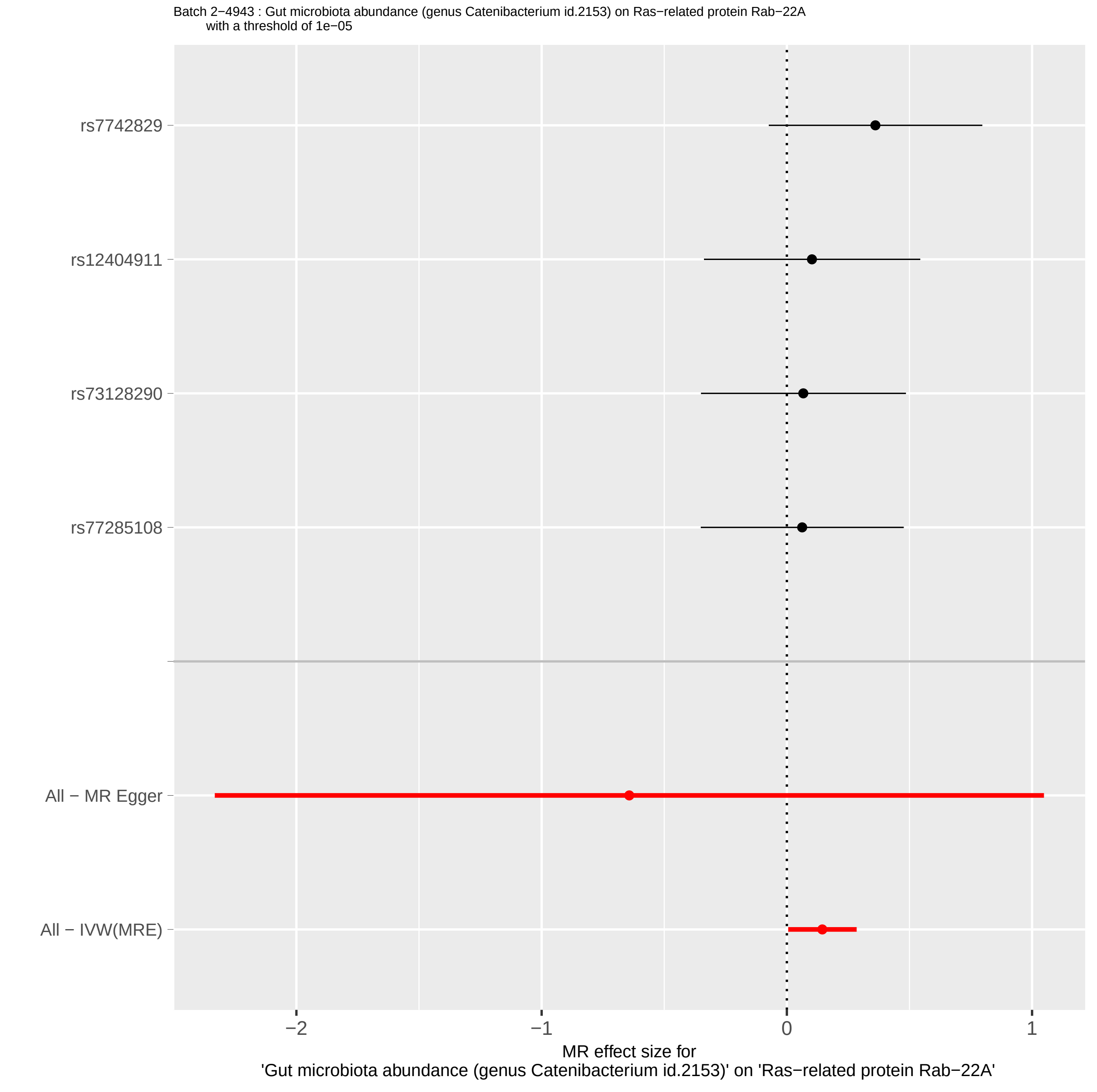

Batch 2−4943 : Gut microbiota abundance (genus Catenibacterium id.2153) on Ras−related protein Rab−22A
with a threshold of 1e−05
rs7742829
rs12404911
rs73128290
rs77285108
All − MR Egger
All − IVW(MRE)
−2
−1
0
1
MR effect size for
'Gut microbiota abundance (genus Catenibacterium id.2153)' on 'Ras−related protein Rab−22A'

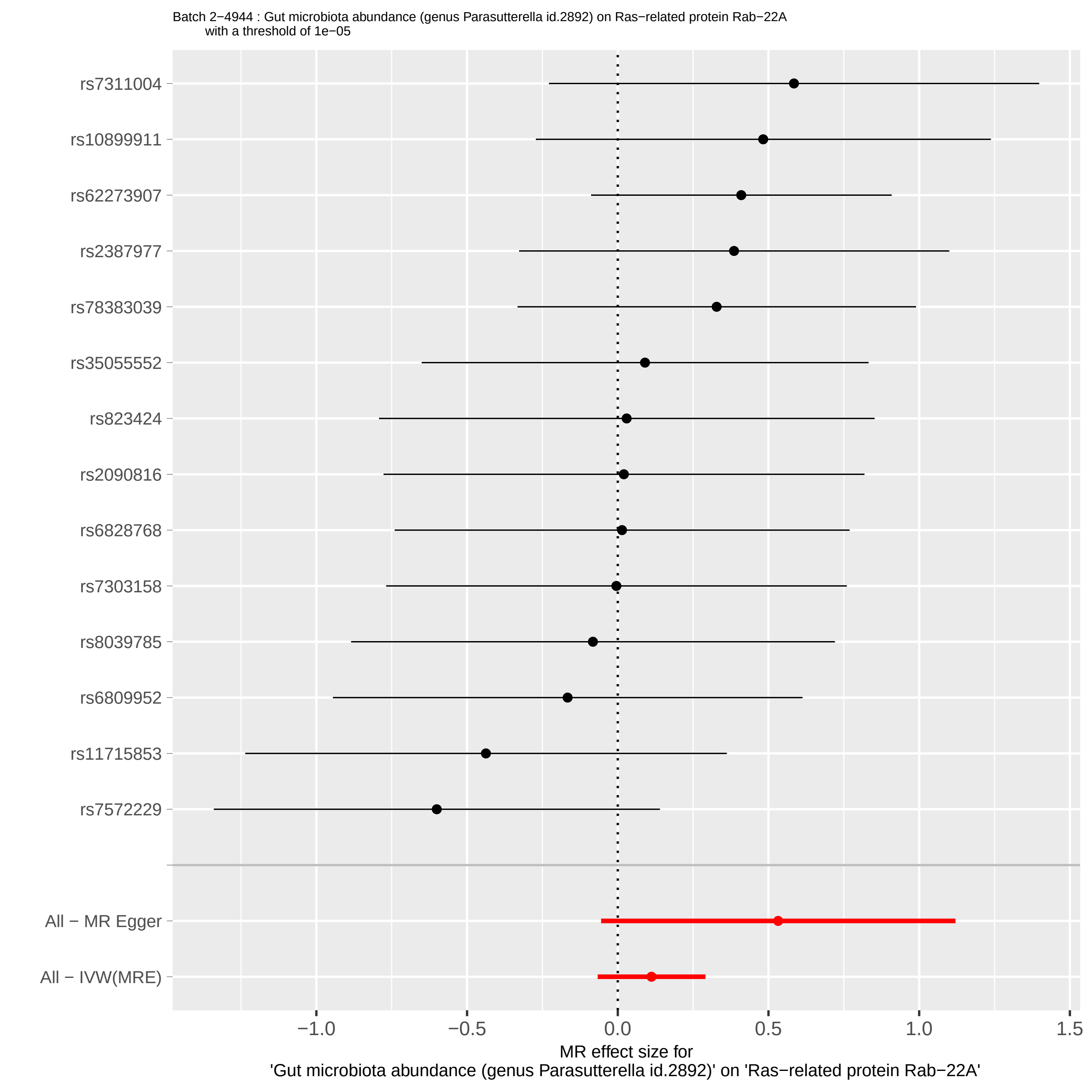

Batch 2−4944 : Gut microbiota abundance (genus Parasutterella id.2892) on Ras−related protein Rab−22A
with a threshold of 1e−05
rs7311004
rs10899911
rs62273907
rs2387977
rs78383039
rs35055552
rs823424
rs2090816
rs6828768
rs7303158
rs8039785
rs6809952
rs11715853
rs7572229
All − MR Egger
All − IVW(MRE)
−1.0
−0.5
0.0
0.5
1.0
1.5
MR effect size for
'Gut microbiota abundance (genus Parasutterella id.2892)' on 'Ras−related protein Rab−22A'

Batch 2−4945 : Gut microbiota abundance (genus Catenibacterium id.2153) on Ras−related protein Rab−26
with a threshold of 1e−05

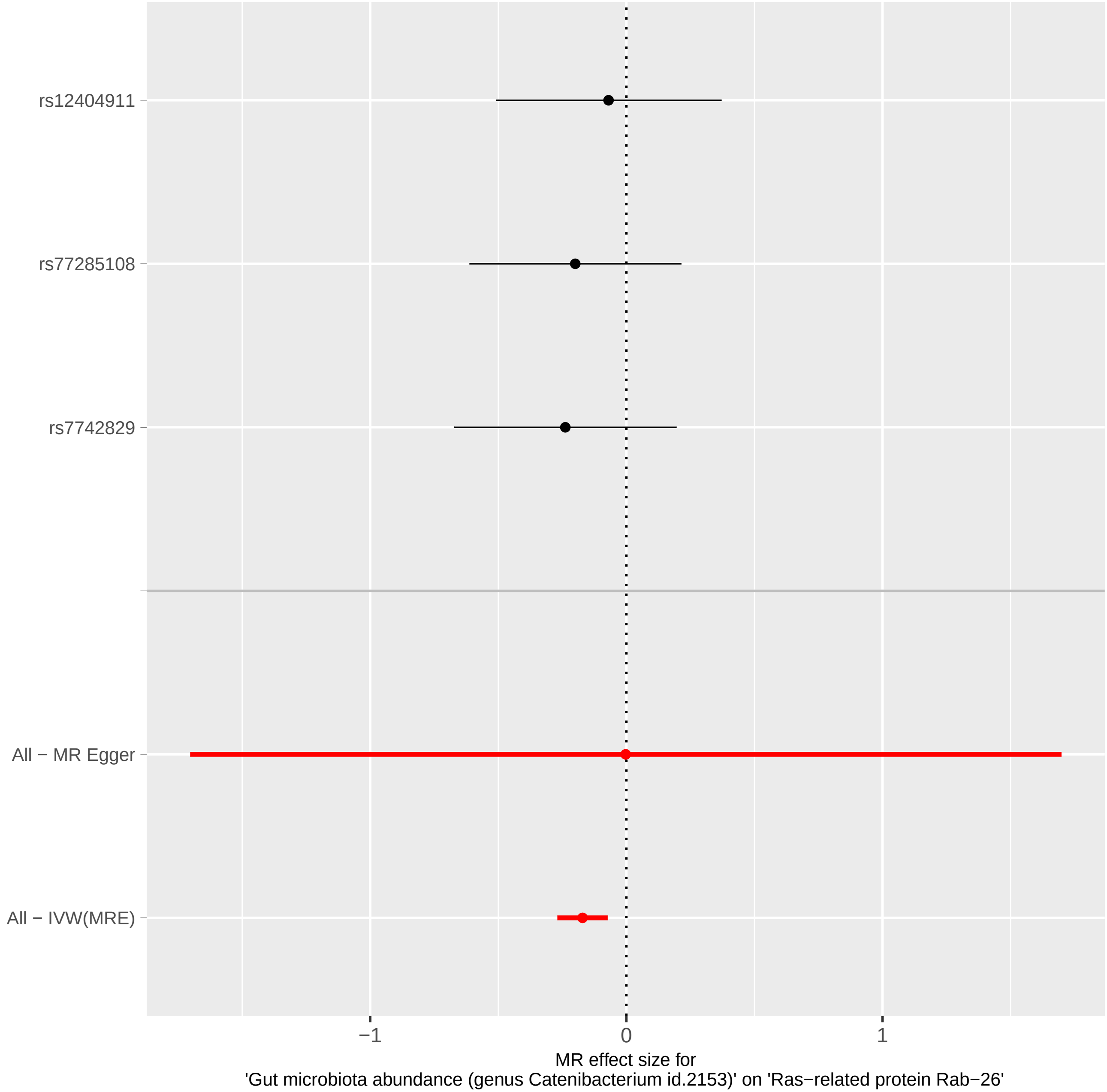

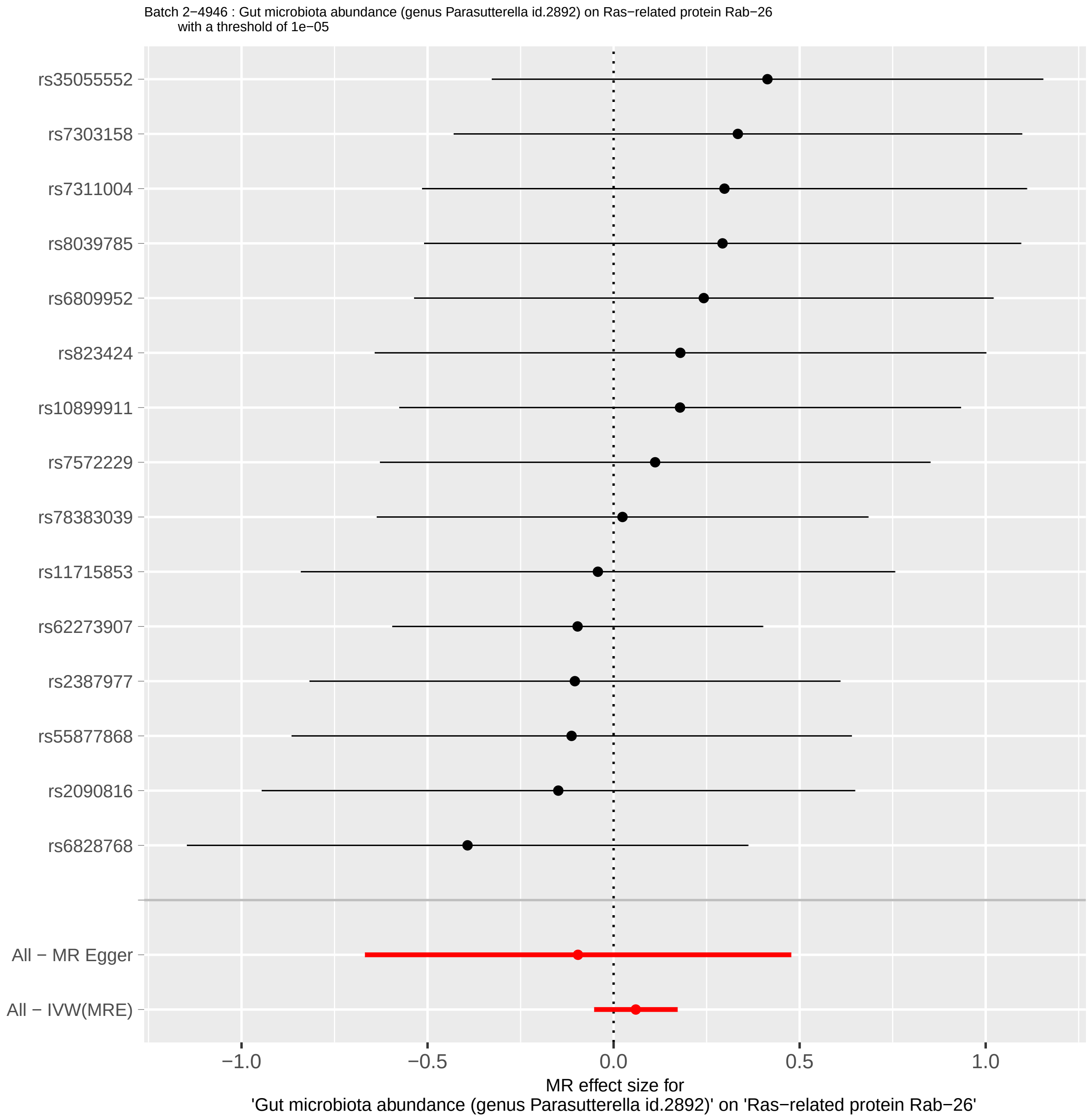
Batch 2−4946 : Gut microbiota abundance (genus Parasutterella id.2892) on Ras−related protein Rab−26
with a threshold of 1e−05
rs35055552
rs7303158
rs7311004
rs8039785
rs6809952
rs823424
rs10899911
rs7572229
rs78383039
rs11715853
rs62273907
rs2387977
rs55877868
rs2090816
rs6828768
All − MR Egger
All − IVW(MRE)
−1.0
−0.5
0.0
0.5
1.0
MR effect size for
'Gut microbiota abundance (genus Parasutterella id.2892)' on 'Ras−related protein Rab−26'

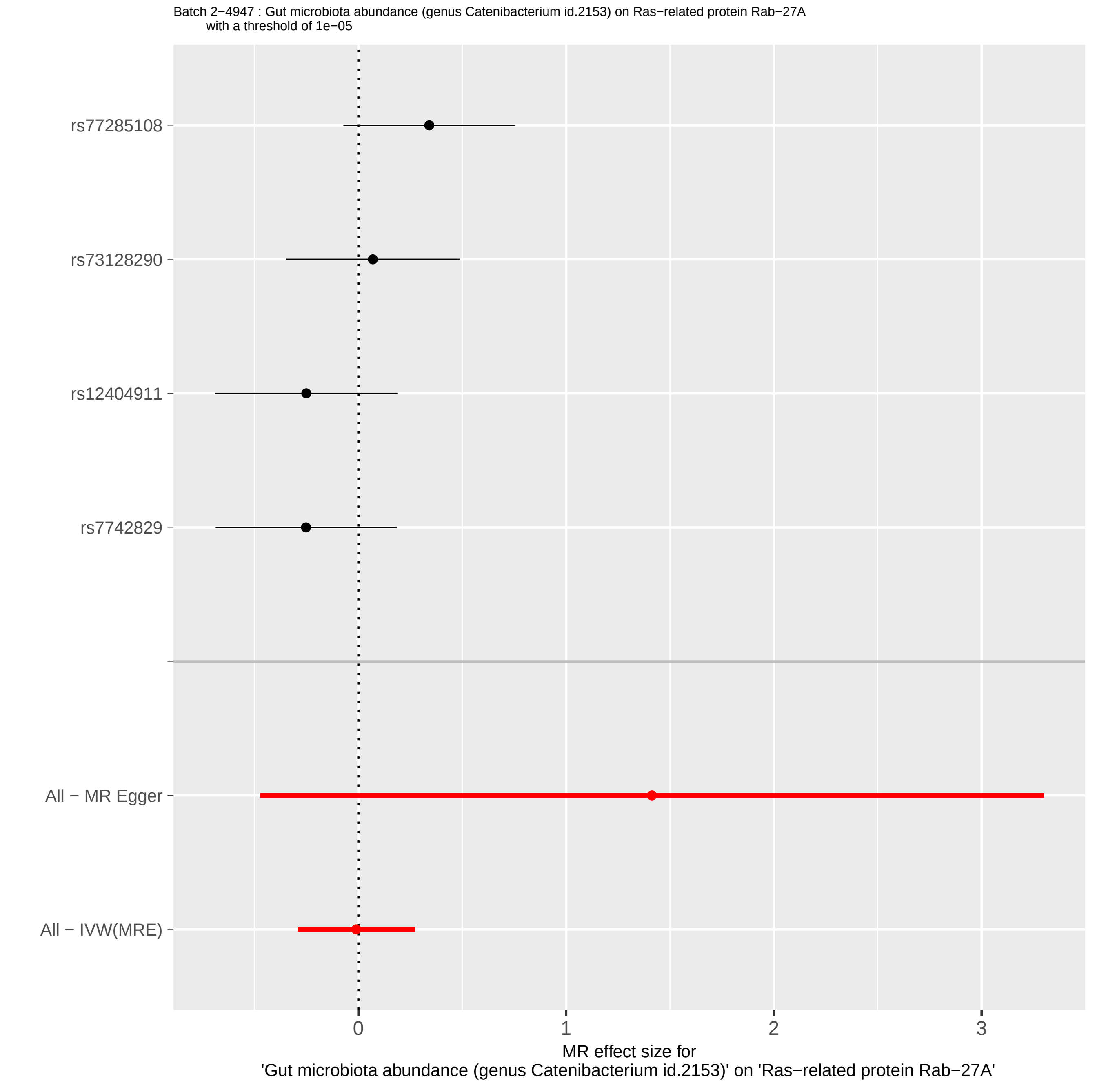

Batch 2−4947 : Gut microbiota abundance (genus Catenibacterium id.2153) on Ras−related protein Rab−27A
with a threshold of 1e−05
rs77285108
rs73128290
rs12404911
rs7742829
All − MR Egger
All − IVW(MRE)
0
1
2
3
MR effect size for
'Gut microbiota abundance (genus Catenibacterium id.2153)' on 'Ras−related protein Rab−27A'

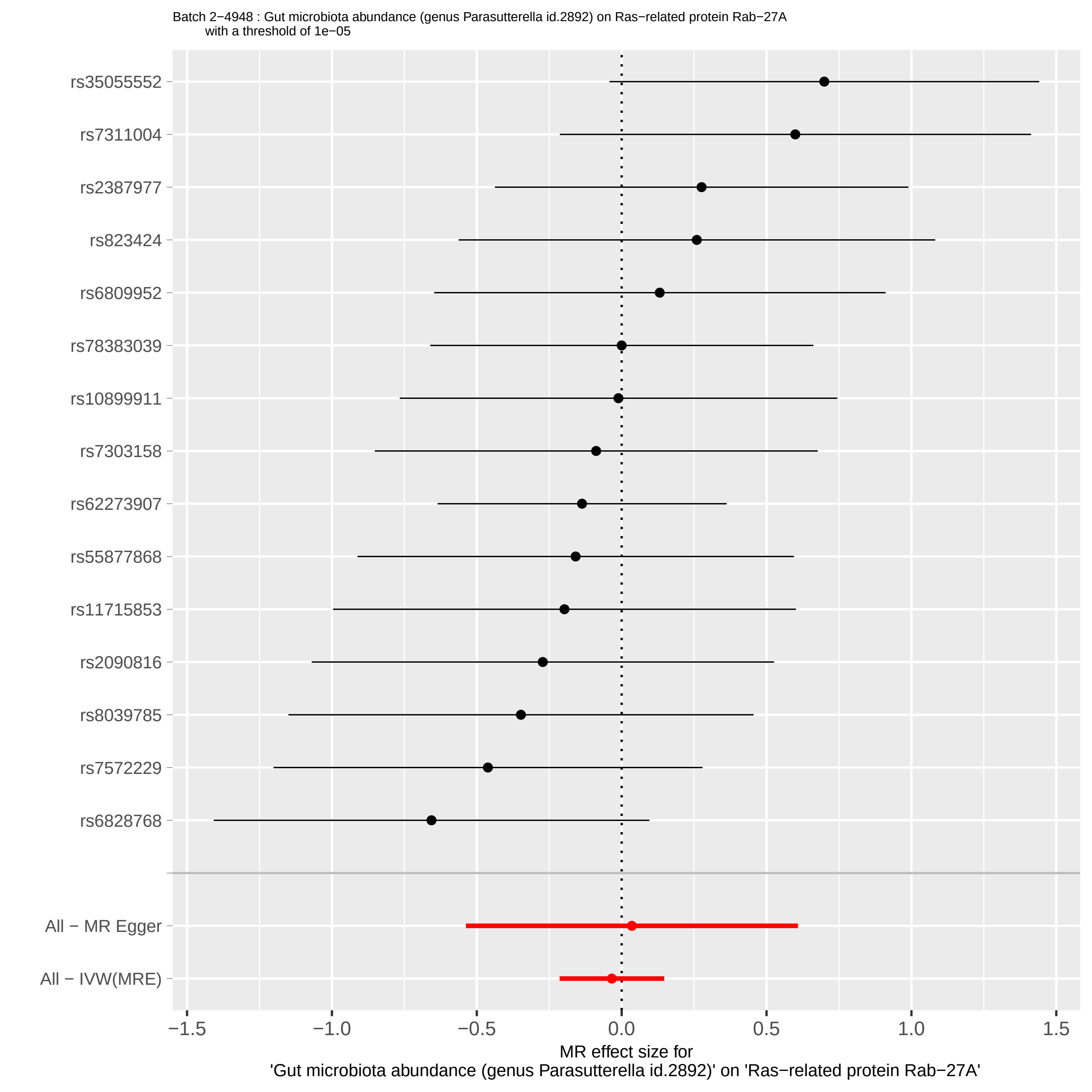

Batch 2−4948 : Gut microbiota abundance (genus Parasutterella id.2892) on Ras−related protein Rab−27A
with a threshold of 1e−05
rs35055552
rs7311004
rs2387977
rs823424
rs6809952
rs78383039
rs10899911
rs7303158
rs62273907
rs55877868
rs11715853
rs2090816
rs8039785
rs7572229
rs6828768
All − MR Egger
All − IVW(MRE)
−1.5
−1.0
−0.5
0.0
0.5
1.0
1.5
MR effect size for
'Gut microbiota abundance (genus Parasutterella id.2892)' on 'Ras−related protein Rab−27A'

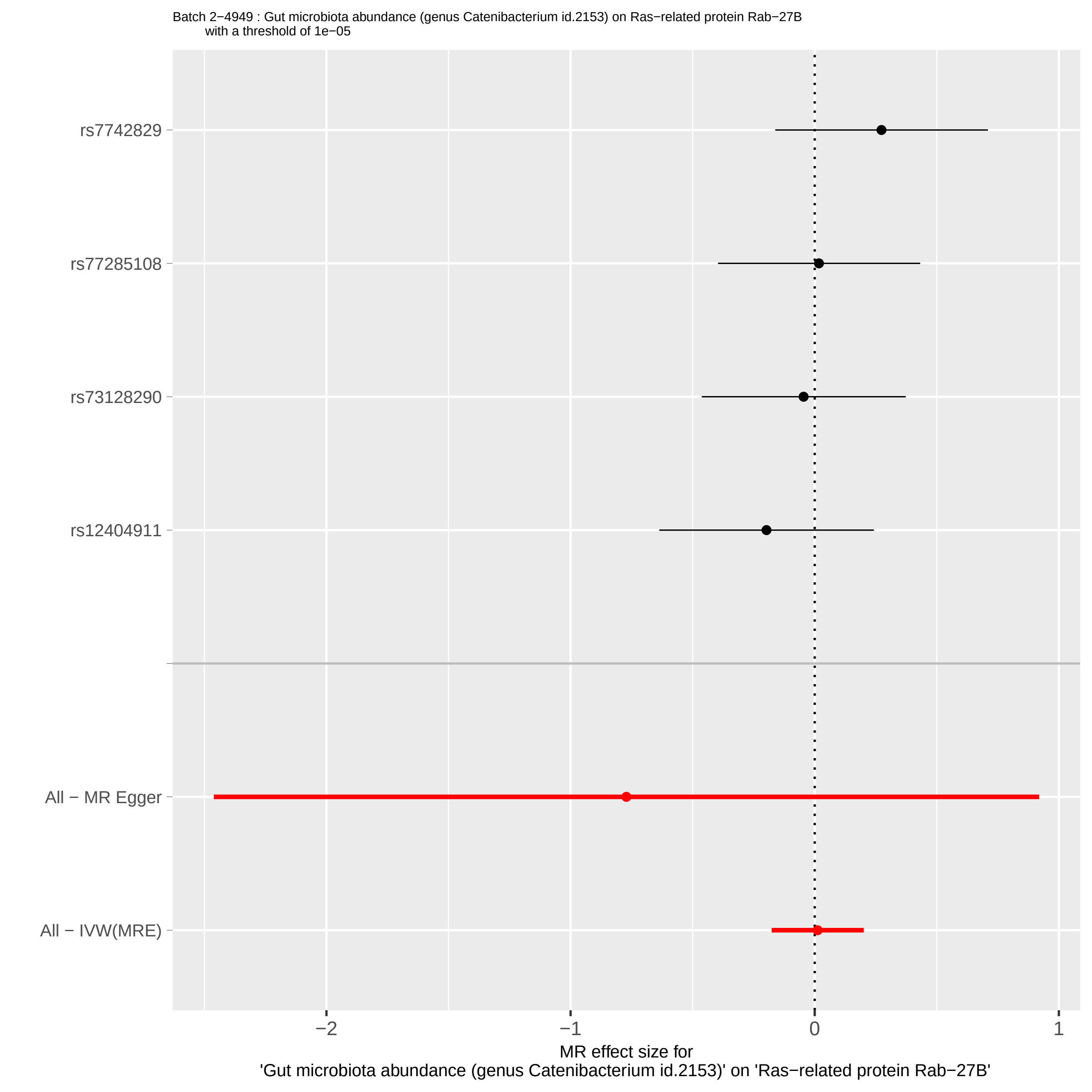

Batch 2−4949 : Gut microbiota abundance (genus Catenibacterium id.2153) on Ras−related protein Rab−27B
with a threshold of 1e−05
rs7742829
rs77285108
rs73128290
rs12404911
All − MR Egger
All − IVW(MRE)
−2
−1
0
1
MR effect size for
'Gut microbiota abundance (genus Catenibacterium id.2153)' on 'Ras−related protein Rab−27B'

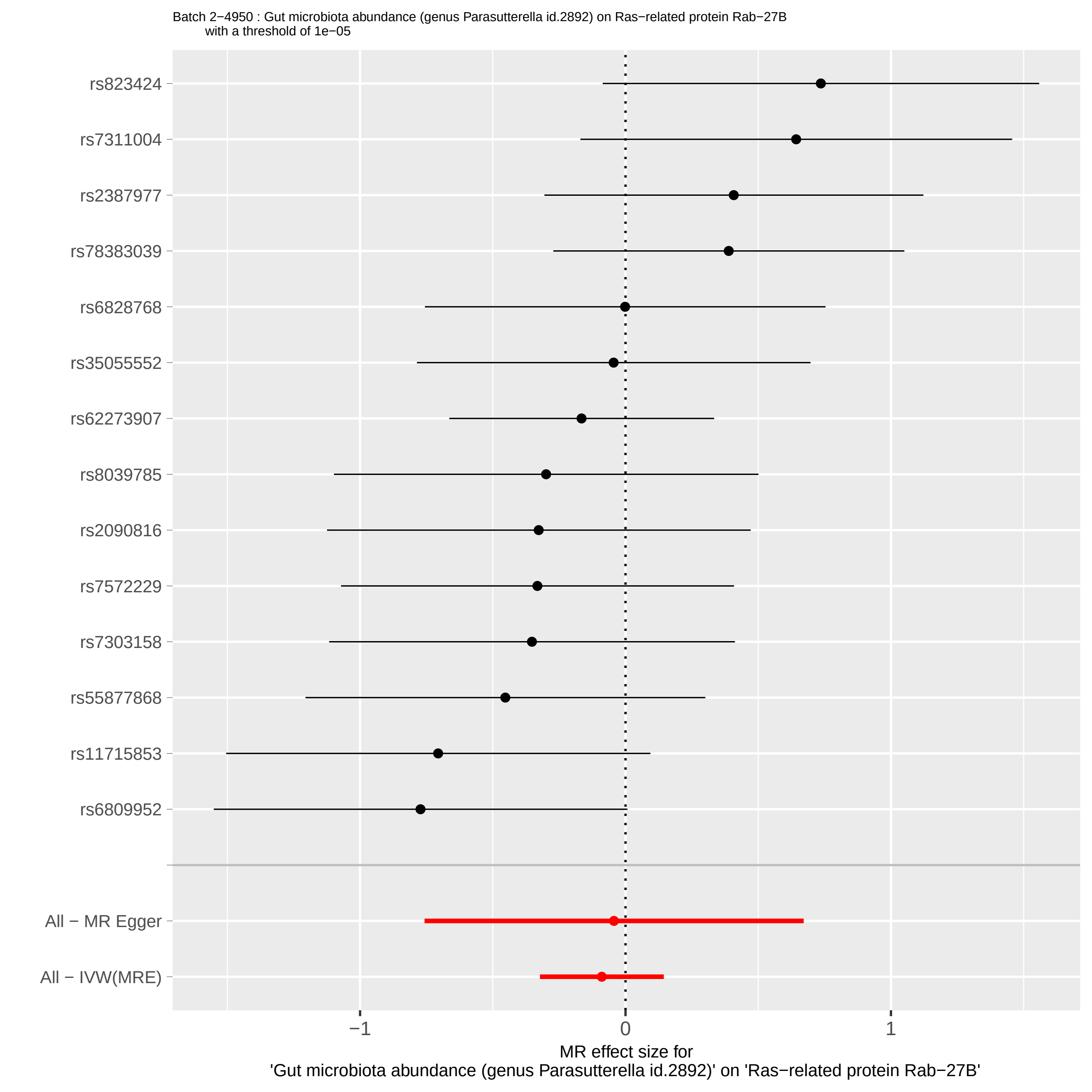
Batch 2−4950 : Gut microbiota abundance (genus Parasutterella id.2892) on Ras−related protein Rab−27B
with a threshold of 1e−05
rs823424
rs7311004
rs2387977
rs78383039
rs6828768
rs35055552
rs62273907
rs8039785
rs2090816
rs7572229
rs7303158
rs55877868
rs11715853
rs6809952
All − MR Egger
All − IVW(MRE)
−1
0
1
MR effect size for
'Gut microbiota abundance (genus Parasutterella id.2892)' on 'Ras−related protein Rab−27B'

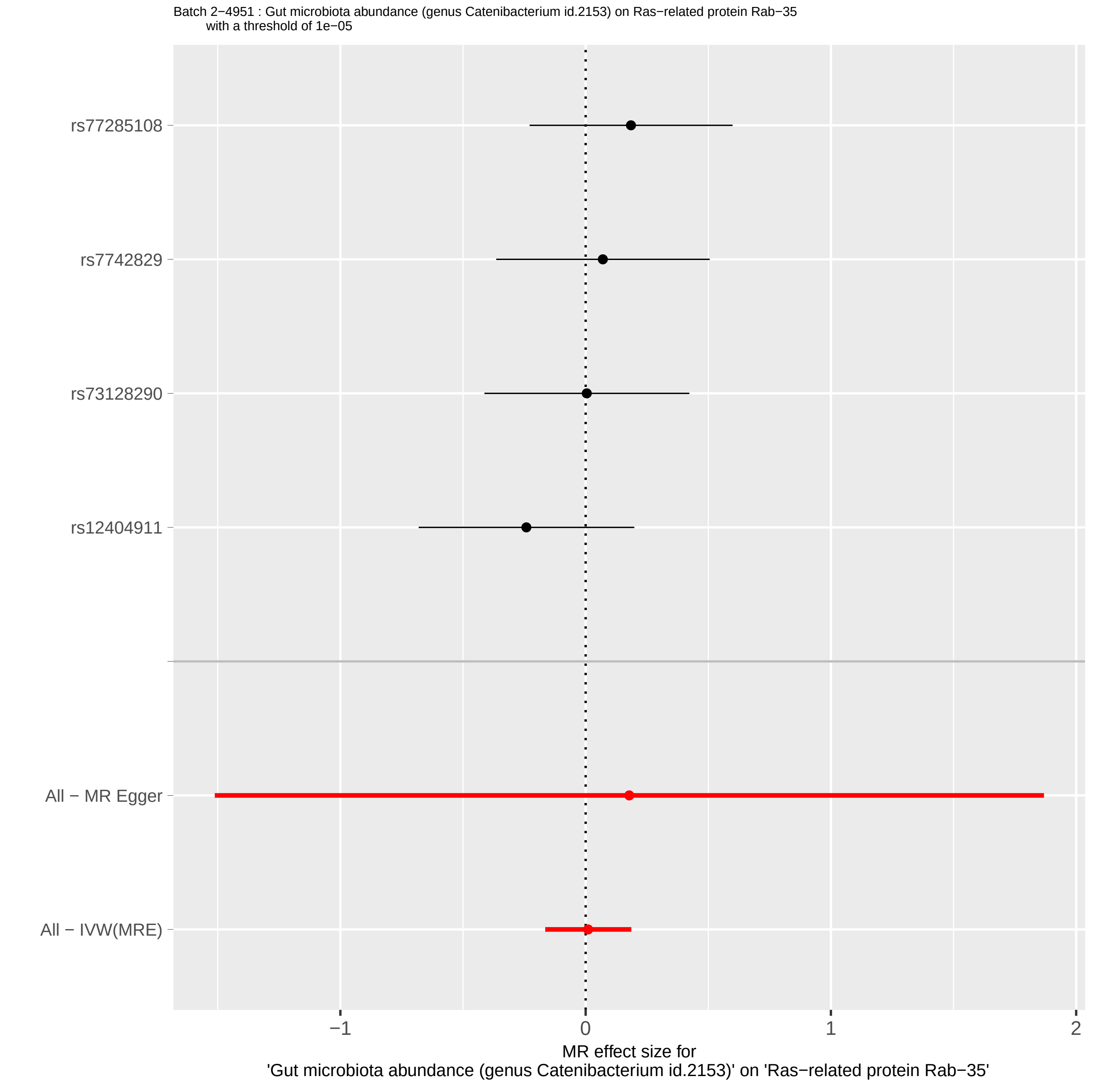

Batch 2−4951 : Gut microbiota abundance (genus Catenibacterium id.2153) on Ras−related protein Rab−35
with a threshold of 1e−05
rs77285108
rs7742829
rs73128290
rs12404911
All − MR Egger
All − IVW(MRE)
−1
0
1
2
MR effect size for
'Gut microbiota abundance (genus Catenibacterium id.2153)' on 'Ras−related protein Rab−35'

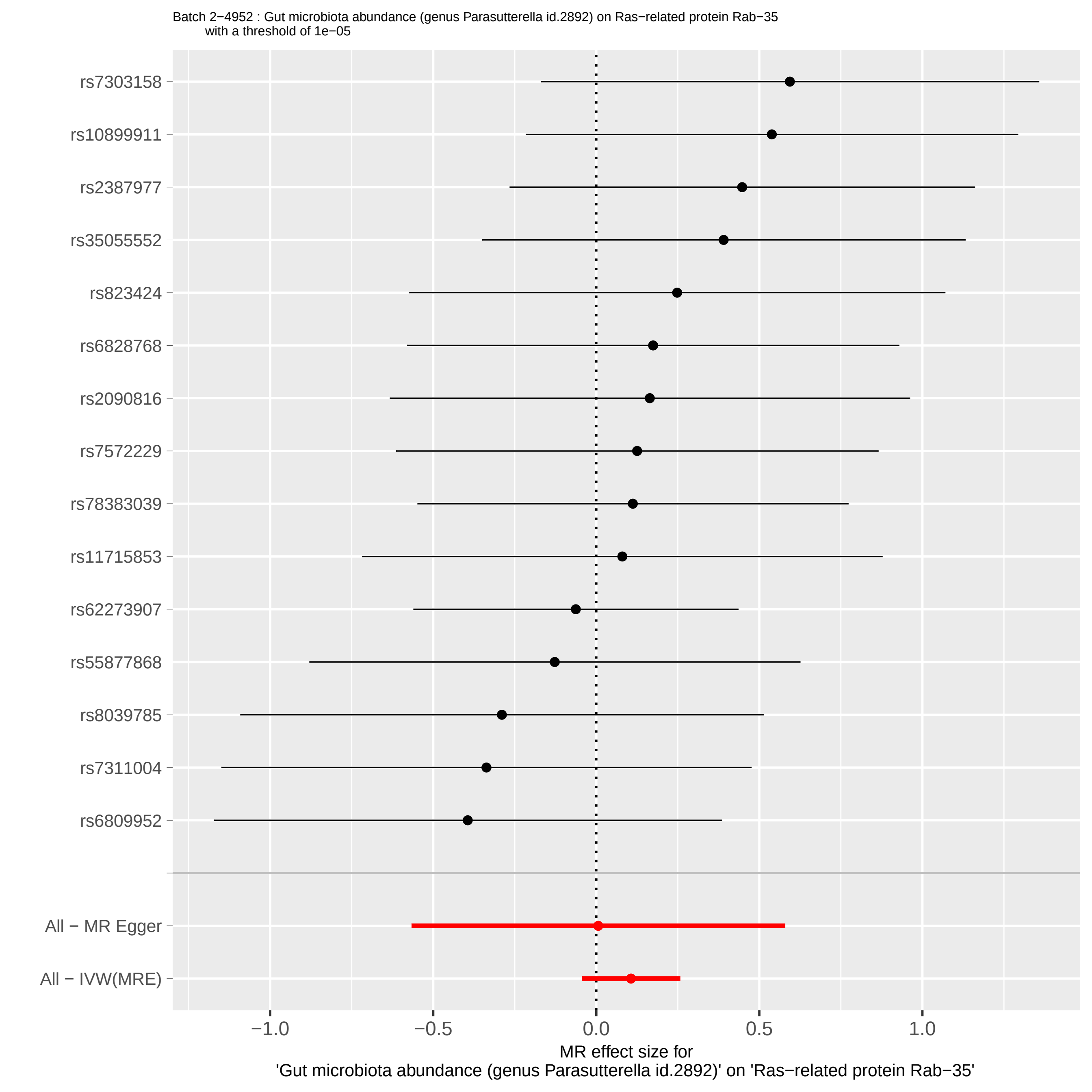
Batch 2−4952 : Gut microbiota abundance (genus Parasutterella id.2892) on Ras−related protein Rab−35
with a threshold of 1e−05
rs7303158
rs10899911
rs2387977
rs35055552
rs823424
rs6828768
rs2090816
rs7572229
rs78383039
rs11715853
rs62273907
rs55877868
rs8039785
rs7311004
rs6809952
All − MR Egger
All − IVW(MRE)
−1.0
−0.5
0.0
0.5
1.0
MR effect size for
'Gut microbiota abundance (genus Parasutterella id.2892)' on 'Ras−related protein Rab−35'

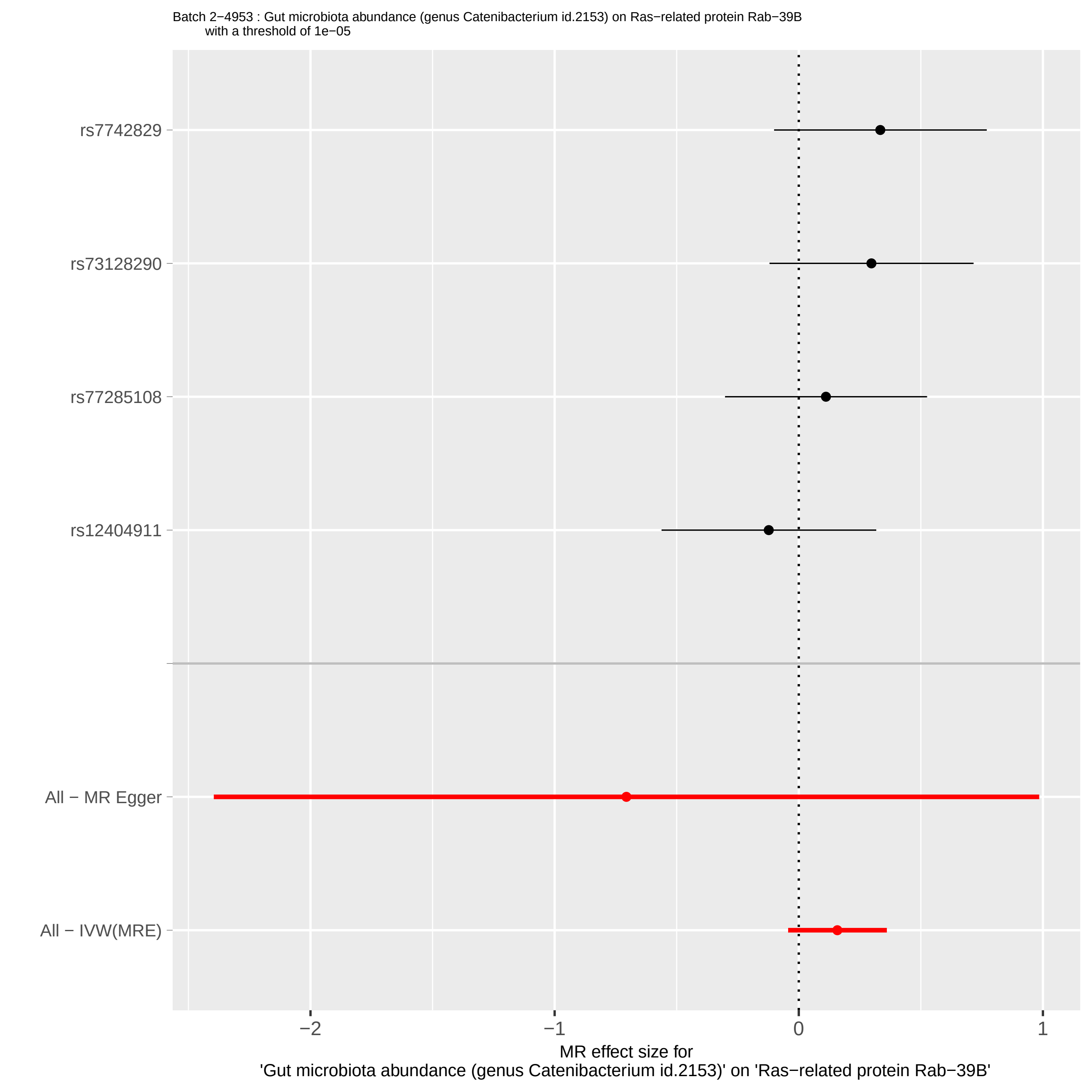
Batch 2−4953 : Gut microbiota abundance (genus Catenibacterium id.2153) on Ras−related protein Rab−39B
with a threshold of 1e−05
rs7742829
rs73128290
rs77285108
rs12404911
All − MR Egger
All − IVW(MRE)
−2
−1
0
1
MR effect size for
'Gut microbiota abundance (genus Catenibacterium id.2153)' on 'Ras−related protein Rab−39B'

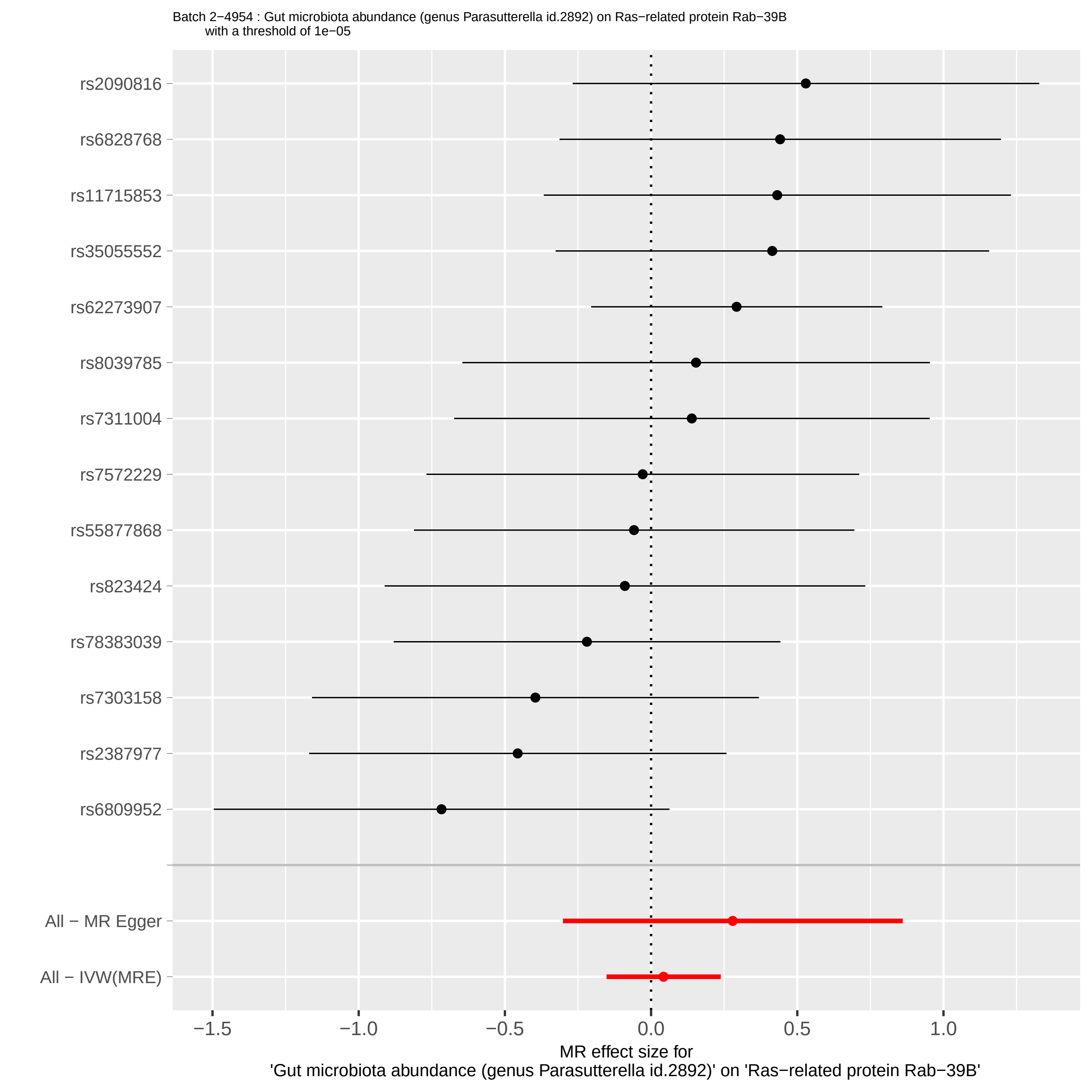
Batch 2−4954 : Gut microbiota abundance (genus Parasutterella id.2892) on Ras−related protein Rab−39B
with a threshold of 1e−05
rs2090816
rs6828768
rs11715853
rs35055552
rs62273907
rs8039785
rs7311004
rs7572229
rs55877868
rs823424
rs78383039
rs7303158
rs2387977
rs6809952
All − MR Egger
All − IVW(MRE)
−1.5
−1.0
−0.5
0.0
0.5
1.0
MR effect size for
'Gut microbiota abundance (genus Parasutterella id.2892)' on 'Ras−related protein Rab−39B'

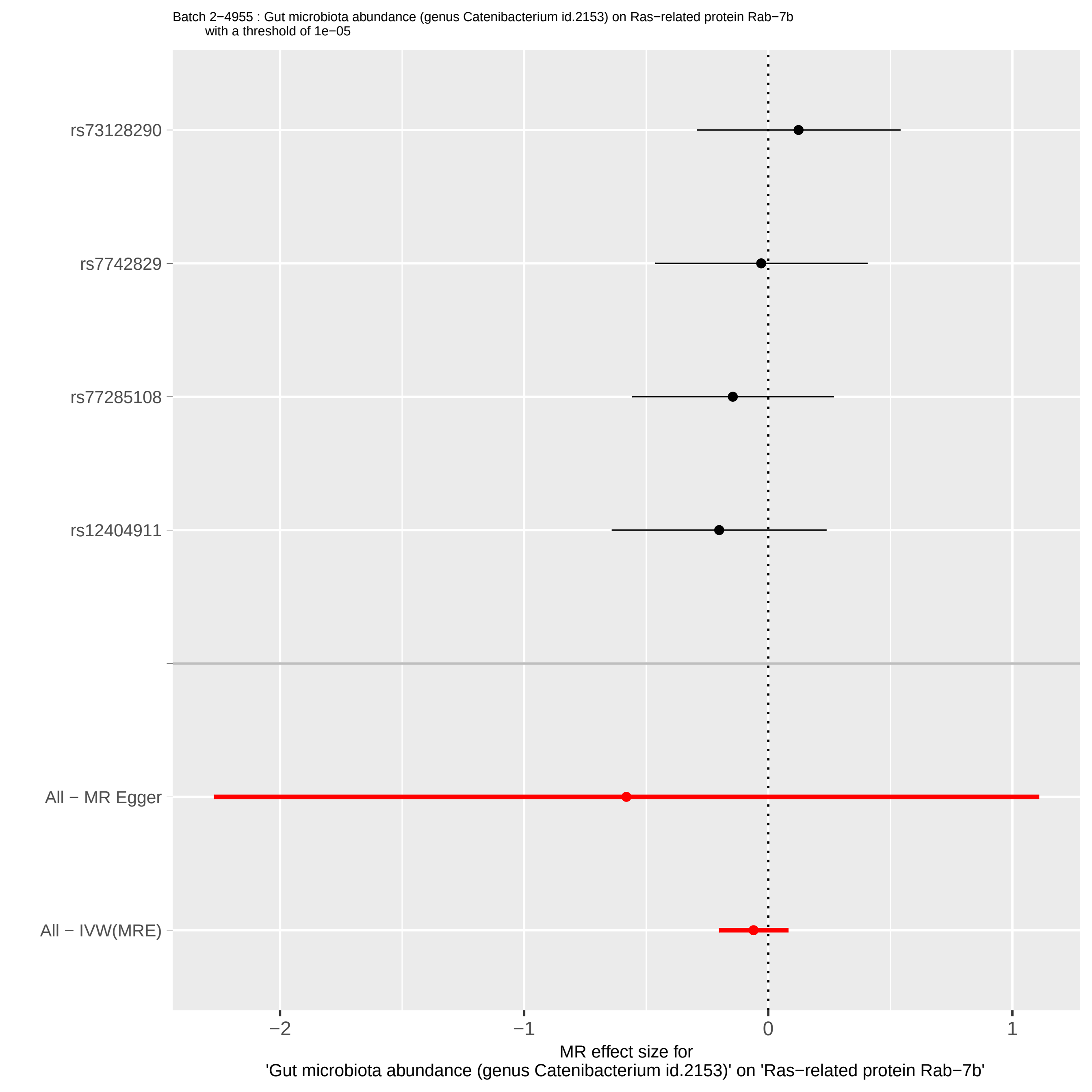
Batch 2−4955 : Gut microbiota abundance (genus Catenibacterium id.2153) on Ras−related protein Rab−7b
with a threshold of 1e−05
rs73128290
rs7742829
rs77285108
rs12404911
All − MR Egger
All − IVW(MRE)
−2
−1
0
1
MR effect size for
'Gut microbiota abundance (genus Catenibacterium id.2153)' on 'Ras−related protein Rab−7b'

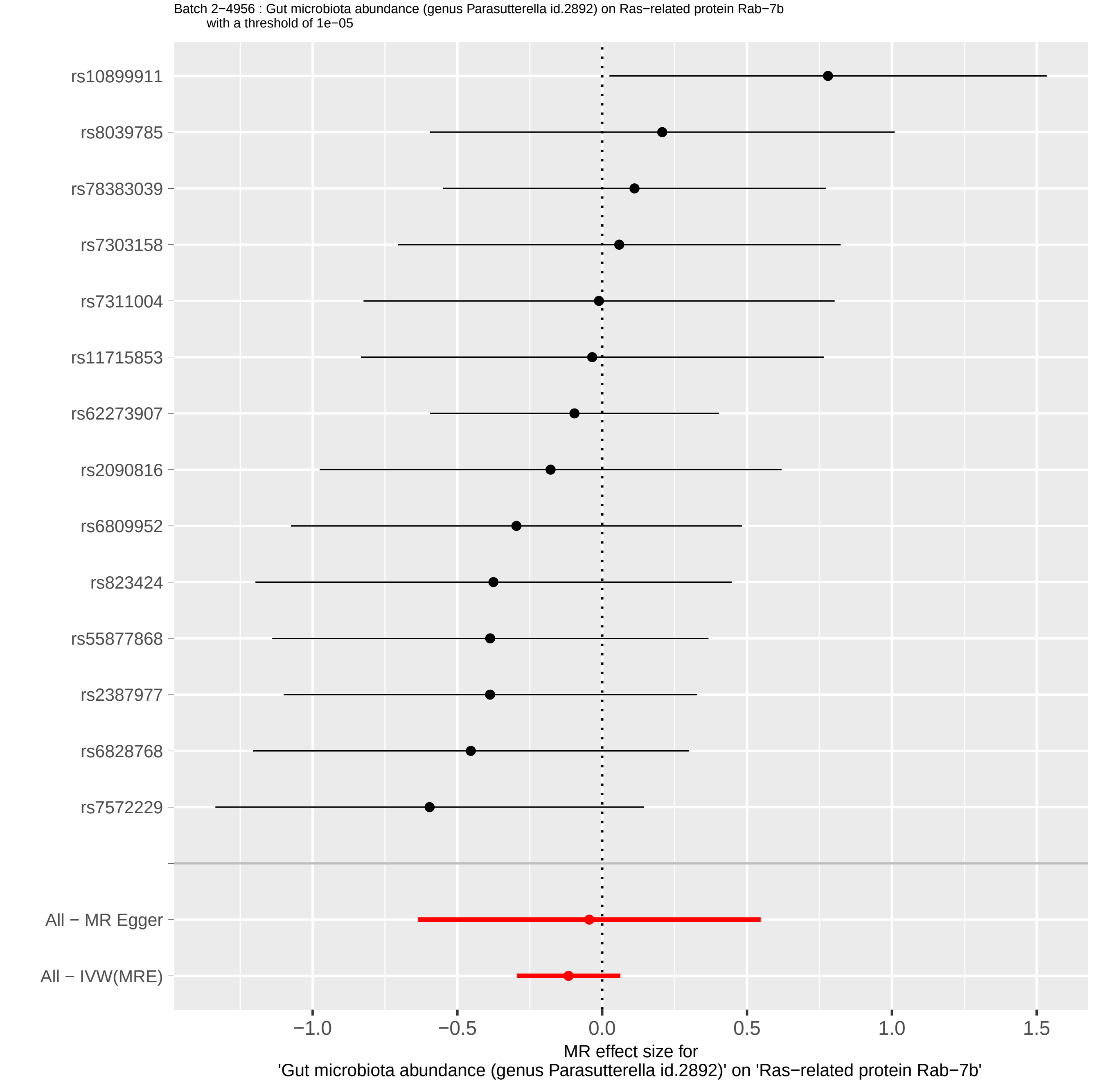

Batch 2−4956 : Gut microbiota abundance (genus Parasutterella id.2892) on Ras−related protein Rab−7b
with a threshold of 1e−05
rs10899911
rs8039785
rs78383039
rs7303158
rs7311004
rs11715853
rs62273907
rs2090816
rs6809952
rs823424
rs55877868
rs2387977
rs6828768
rs7572229
All − MR Egger
All − IVW(MRE)
−1.0
−0.5
0.0
0.5
1.0
1.5
MR effect size for
'Gut microbiota abundance (genus Parasutterella id.2892)' on 'Ras−related protein Rab−7b'

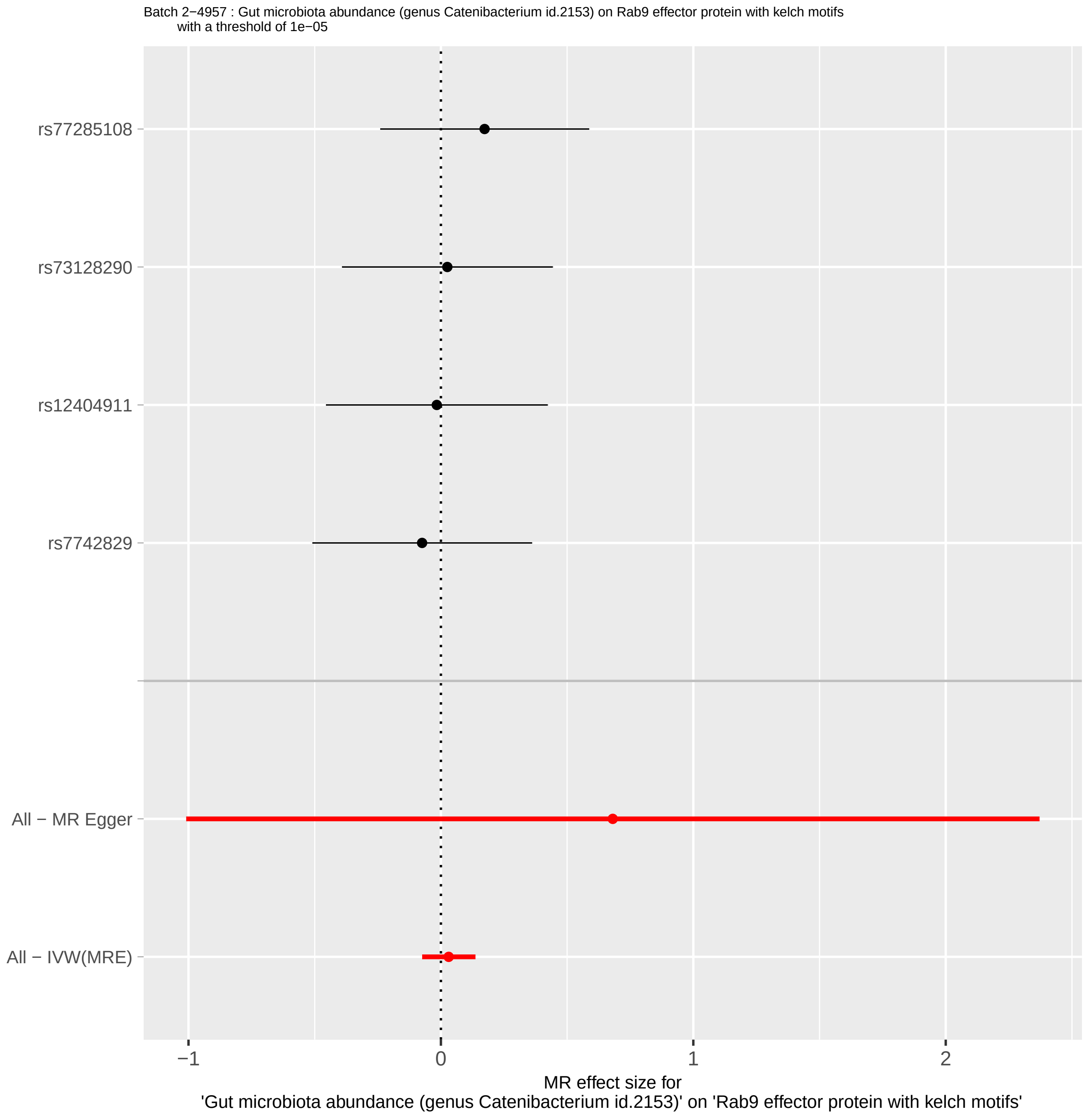
Batch 2−4957 : Gut microbiota abundance (genus Catenibacterium id.2153) on Rab9 effector protein with kelch motifs
with a threshold of 1e−05
rs77285108
rs73128290
rs12404911
rs7742829
All − MR Egger
All − IVW(MRE)
−1
0
1
2
MR effect size for
'Gut microbiota abundance (genus Catenibacterium id.2153)' on 'Rab9 effector protein with kelch motifs'

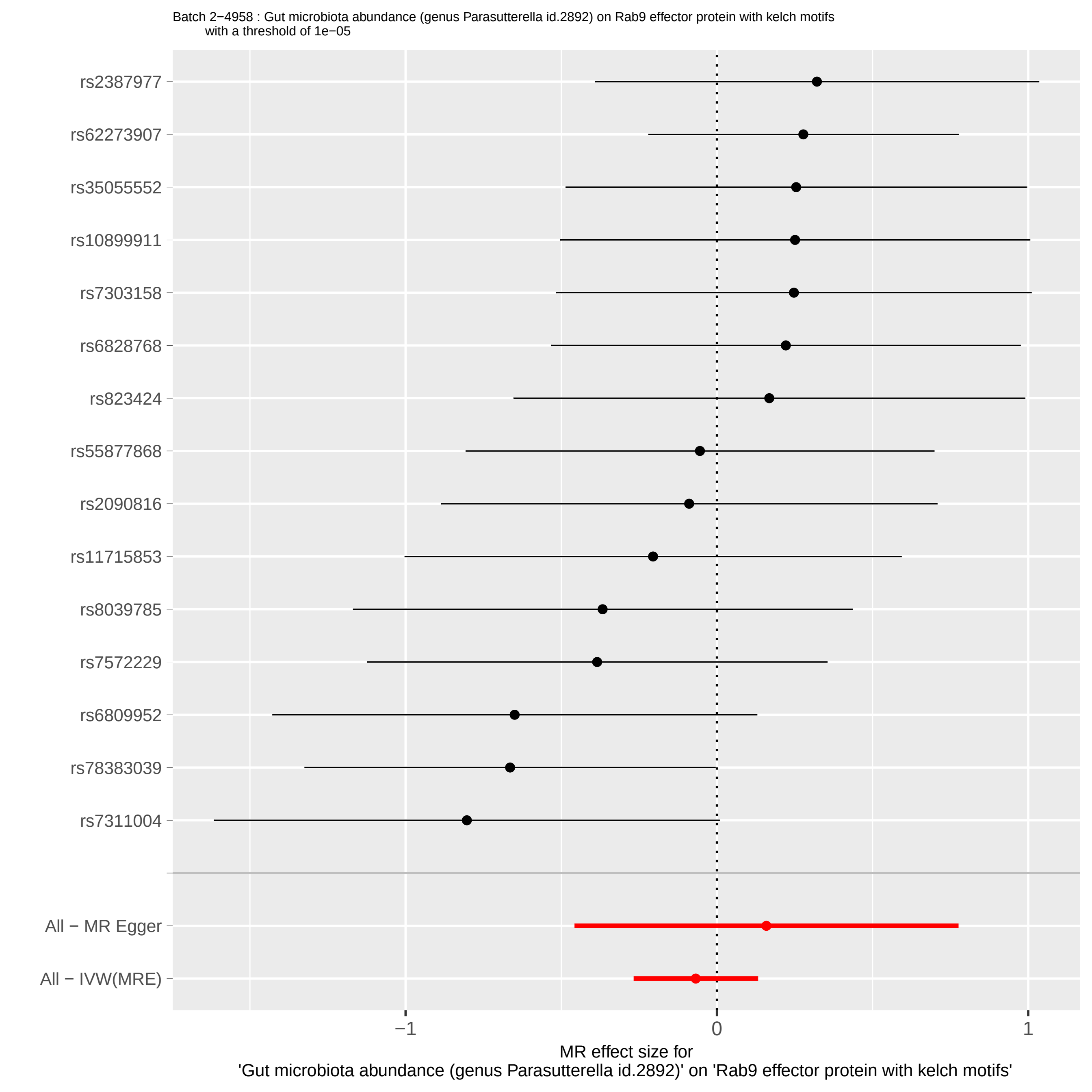

Batch 2−4958 : Gut microbiota abundance (genus Parasutterella id.2892) on Rab9 effector protein with kelch motifs
with a threshold of 1e−05
rs2387977
rs62273907
rs35055552
rs10899911
rs7303158
rs6828768
rs823424
rs55877868
rs2090816
rs11715853
rs8039785
rs7572229
rs6809952
rs78383039
rs7311004
All − MR Egger
All − IVW(MRE)
−1
0
1
MR effect size for
'Gut microbiota abundance (genus Parasutterella id.2892)' on 'Rab9 effector protein with kelch motifs'

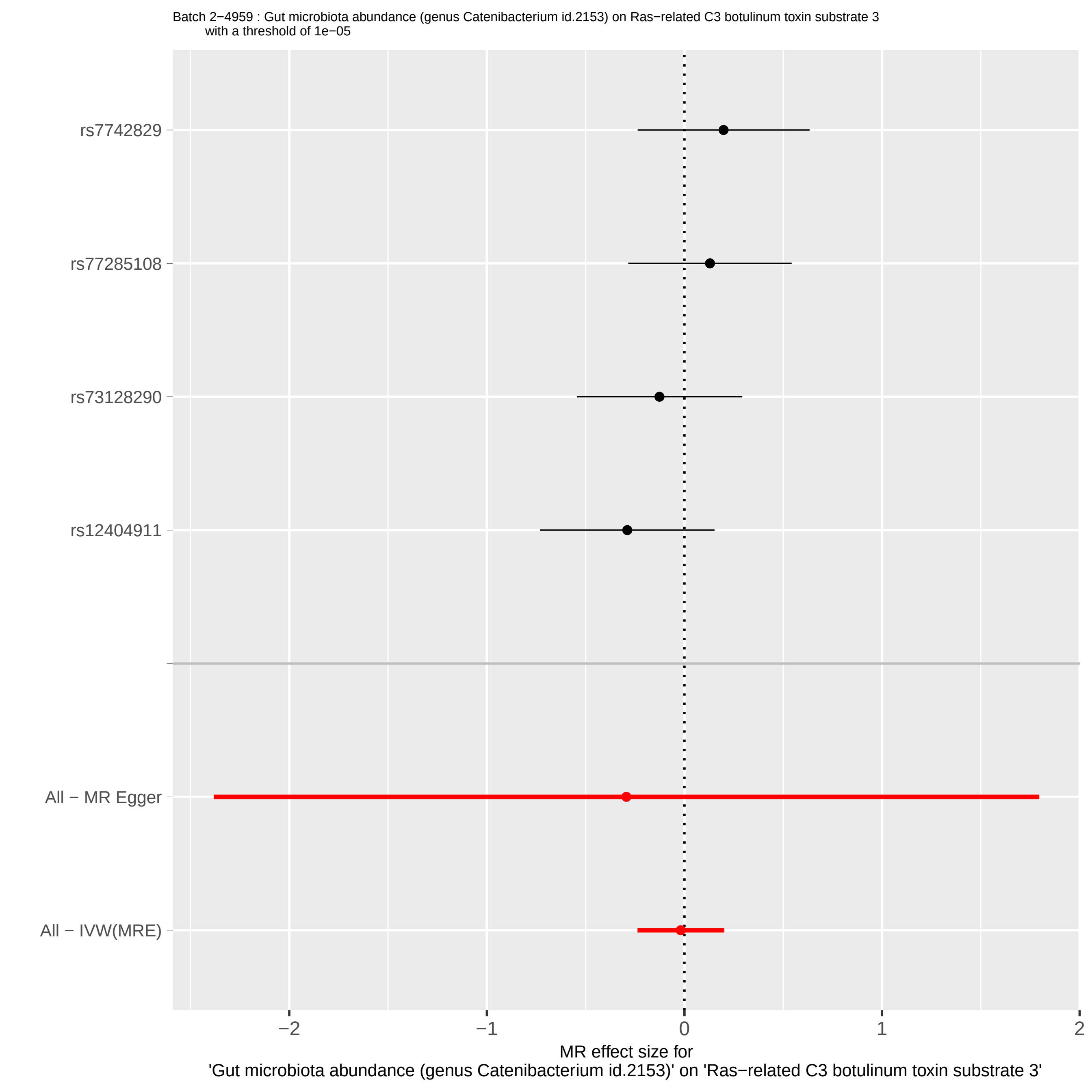

Batch 2−4959 : Gut microbiota abundance (genus Catenibacterium id.2153) on Ras−related C3 botulinum toxin substrate 3
with a threshold of 1e−05
rs7742829
rs77285108
rs73128290
rs12404911
All − MR Egger
All − IVW(MRE)
−2
−1
0
1
2
MR effect size for
'Gut microbiota abundance (genus Catenibacterium id.2153)' on 'Ras−related C3 botulinum toxin substrate 3'

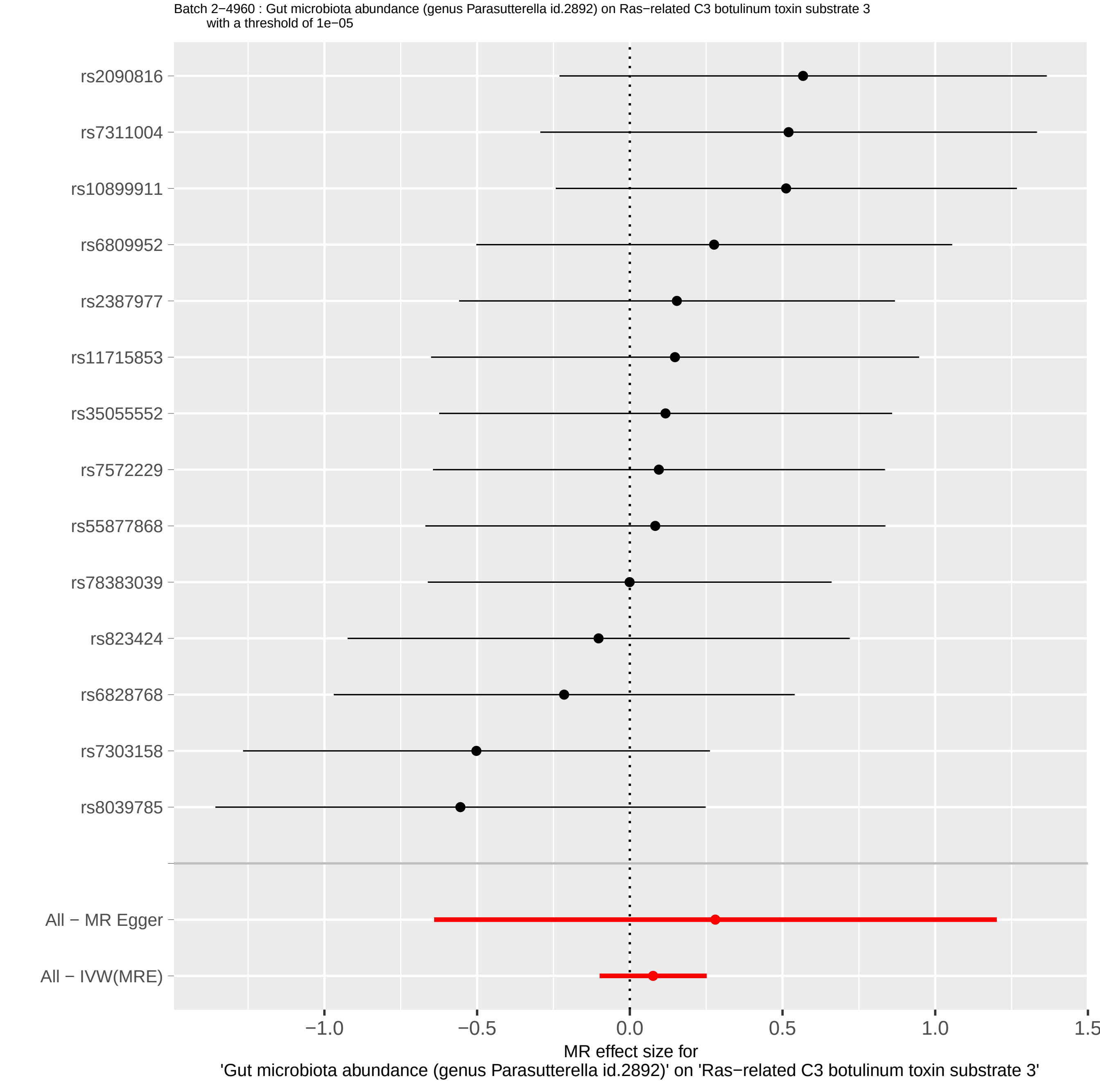
Batch 2−4960 : Gut microbiota abundance (genus Parasutterella id.2892) on Ras−related C3 botulinum toxin substrate 3
with a threshold of 1e−05
rs2090816
rs7311004
rs10899911
rs6809952
rs2387977
rs11715853
rs35055552
rs7572229
rs55877868
rs78383039
rs823424
rs6828768
rs7303158
rs8039785
All − MR Egger
All − IVW(MRE)
−1.0
−0.5
0.0
0.5
1.0
1.5
MR effect size for
'Gut microbiota abundance (genus Parasutterella id.2892)' on 'Ras−related C3 botulinum toxin substrate 3'

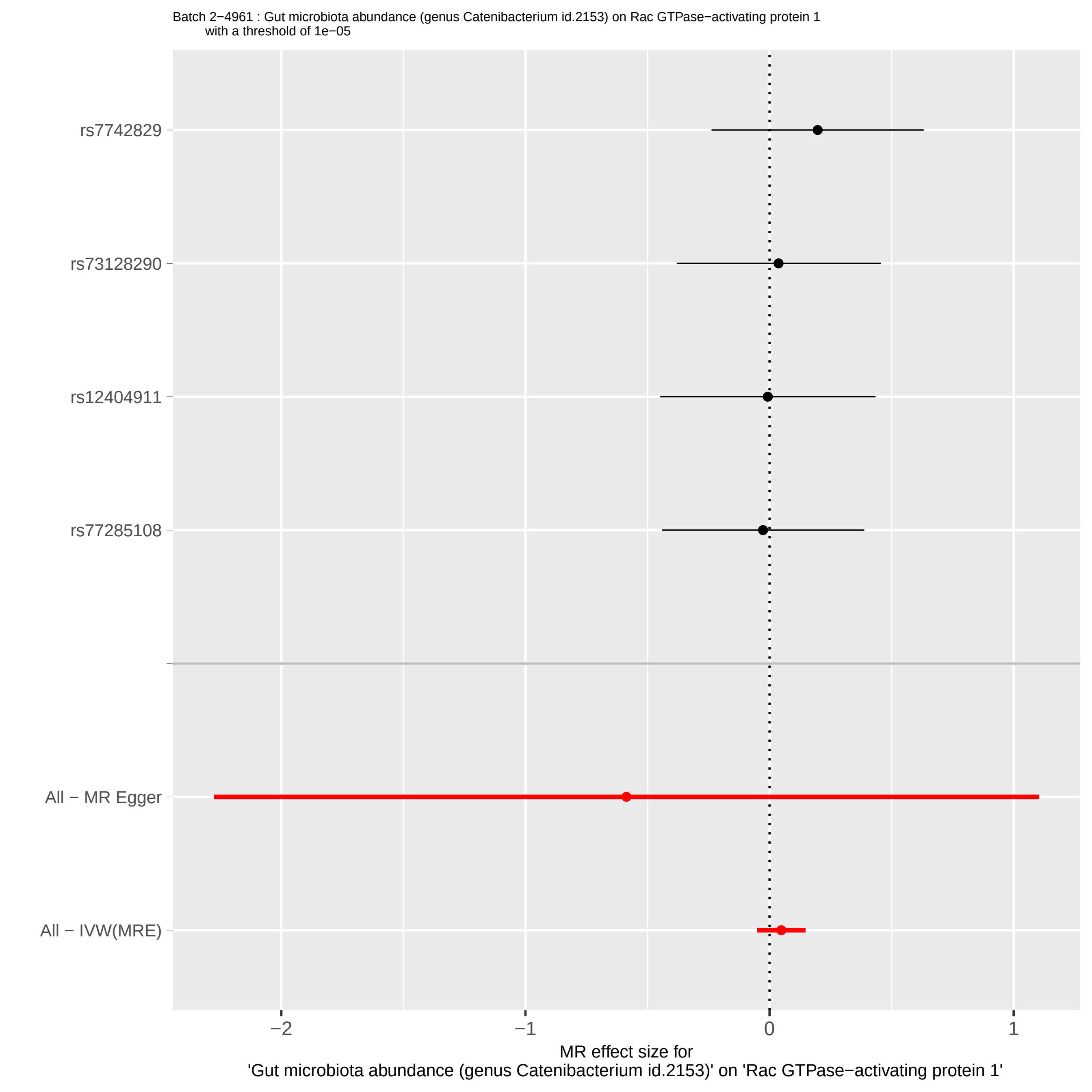
Batch 2−4961 : Gut microbiota abundance (genus Catenibacterium id.2153) on Rac GTPase−activating protein 1
with a threshold of 1e−05
rs7742829
rs73128290
rs12404911
rs77285108
All − MR Egger
All − IVW(MRE)
−2
−1
0
1
MR effect size for
'Gut microbiota abundance (genus Catenibacterium id.2153)' on 'Rac GTPase−activating protein 1'

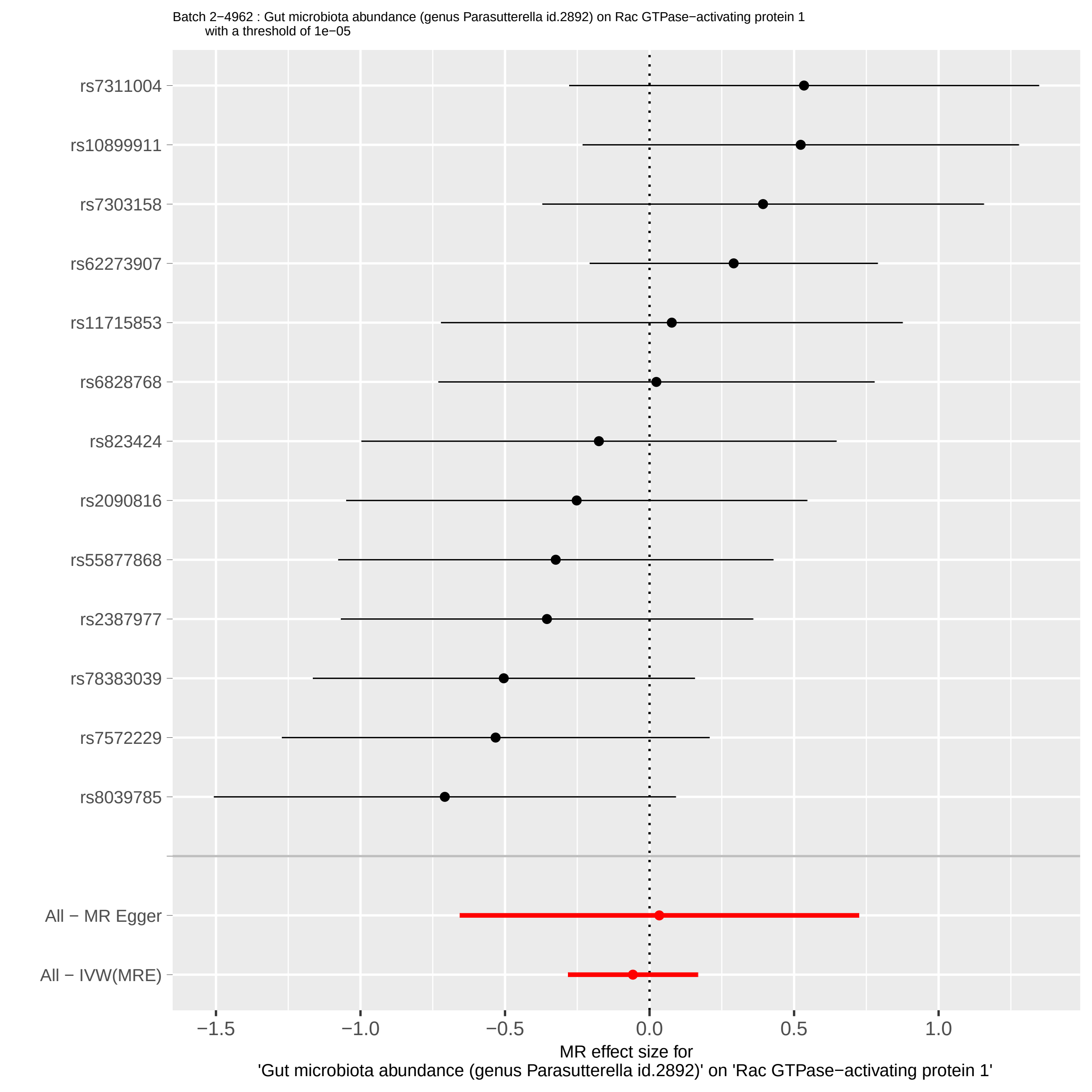

Batch 2−4962 : Gut microbiota abundance (genus Parasutterella id.2892) on Rac GTPase−activating protein 1
with a threshold of 1e−05
rs7311004
rs10899911
rs7303158
rs62273907
rs11715853
rs6828768
rs823424
rs2090816
rs55877868
rs2387977
rs78383039
rs7572229
rs8039785
All − MR Egger
All − IVW(MRE)
−1.5
−1.0
−0.5
0.0
0.5
1.0
MR effect size for
'Gut microbiota abundance (genus Parasutterella id.2892)' on 'Rac GTPase−activating protein 1'

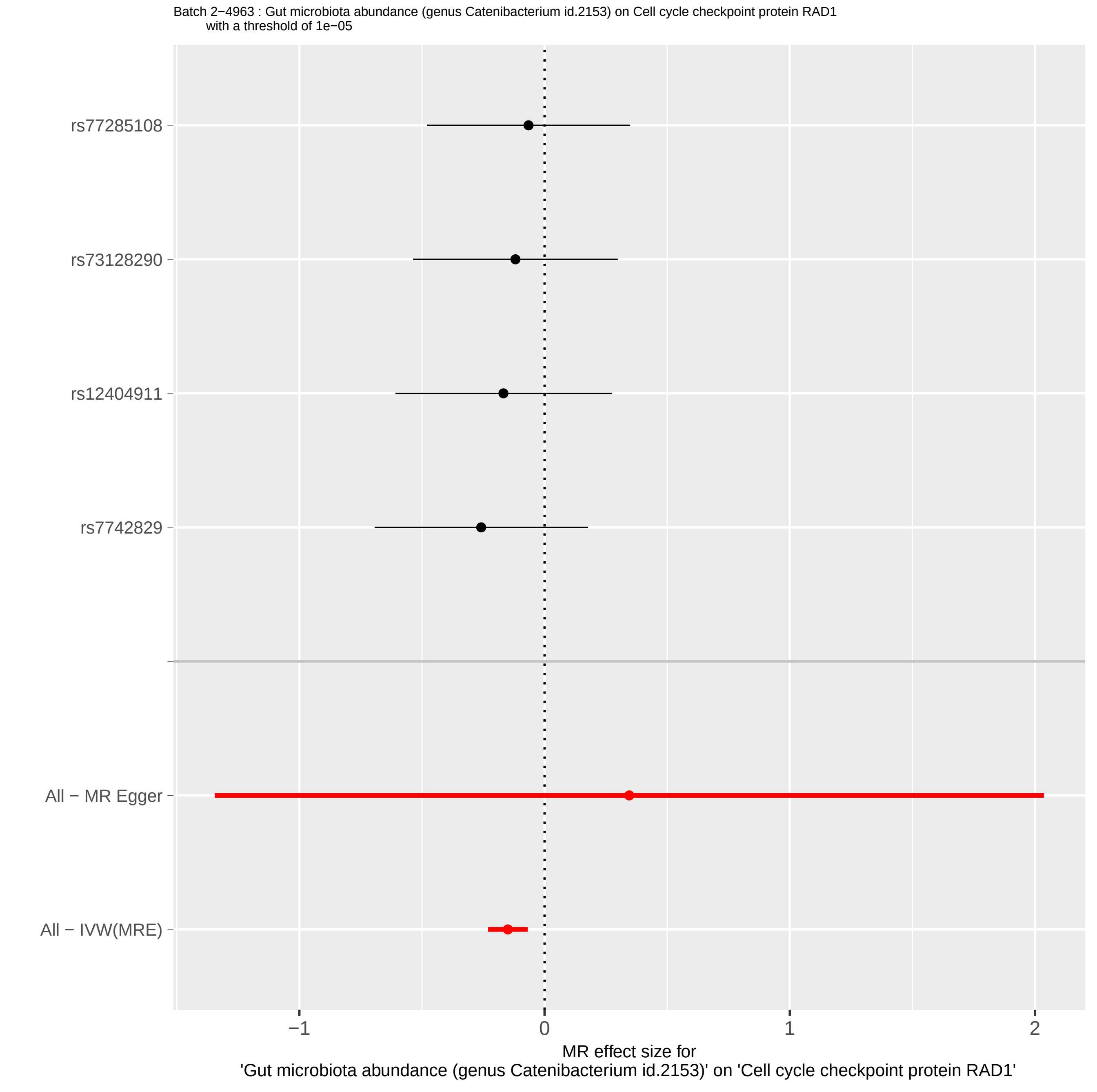
Batch 2−4963 : Gut microbiota abundance (genus Catenibacterium id.2153) on Cell cycle checkpoint protein RAD1
with a threshold of 1e−05
rs77285108
rs73128290
rs12404911
rs7742829
All − MR Egger
All − IVW(MRE)
−1
0
1
2
MR effect size for
'Gut microbiota abundance (genus Catenibacterium id.2153)' on 'Cell cycle checkpoint protein RAD1'

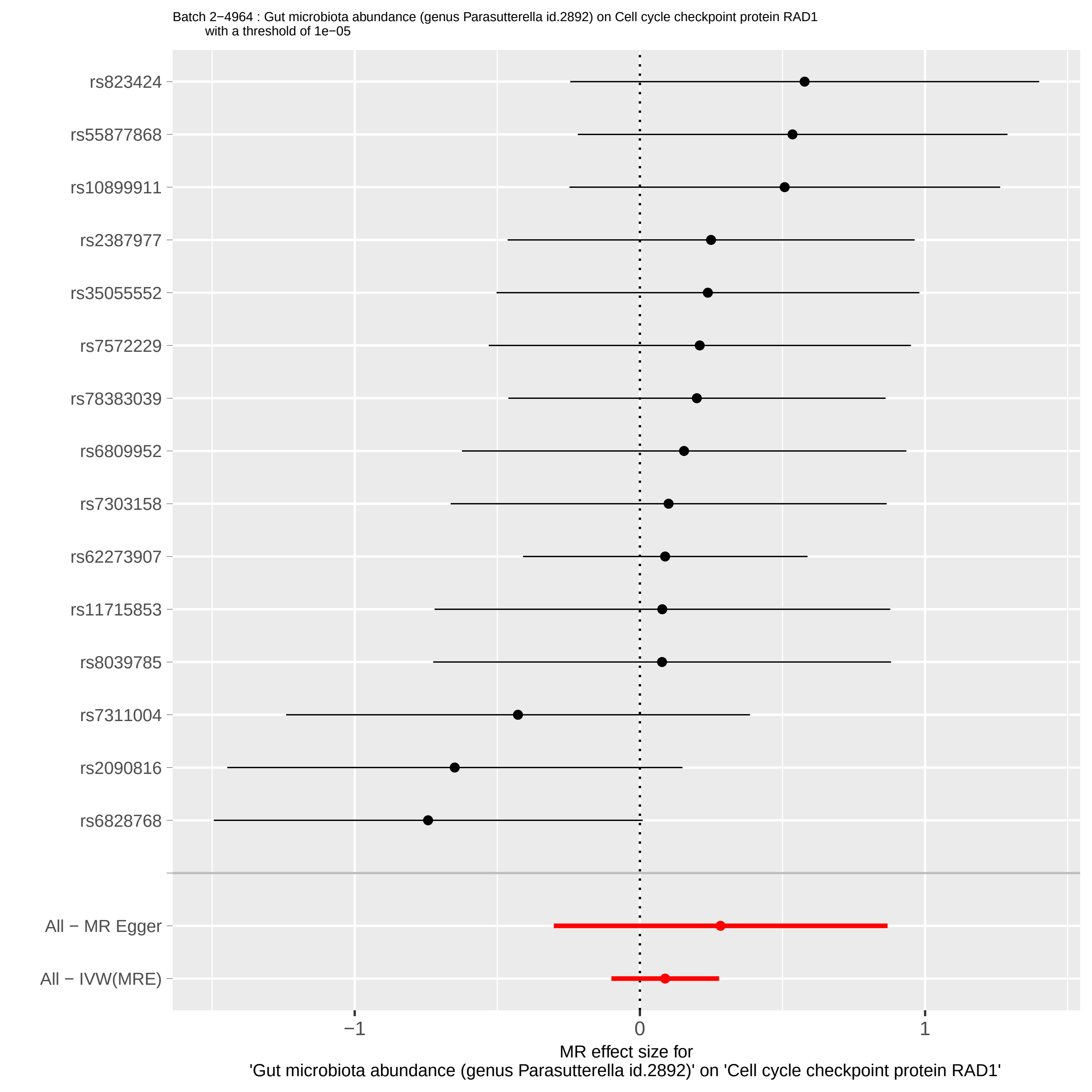
Batch 2−4964 : Gut microbiota abundance (genus Parasutterella id.2892) on Cell cycle checkpoint protein RAD1
with a threshold of 1e−05
rs823424
rs55877868
rs10899911
rs2387977
rs35055552
rs7572229
rs78383039
rs6809952
rs7303158
rs62273907
rs11715853
rs8039785
rs7311004
rs2090816
rs6828768
All − MR Egger
All − IVW(MRE)
−1
0
1
MR effect size for
'Gut microbiota abundance (genus Parasutterella id.2892)' on 'Cell cycle checkpoint protein RAD1'

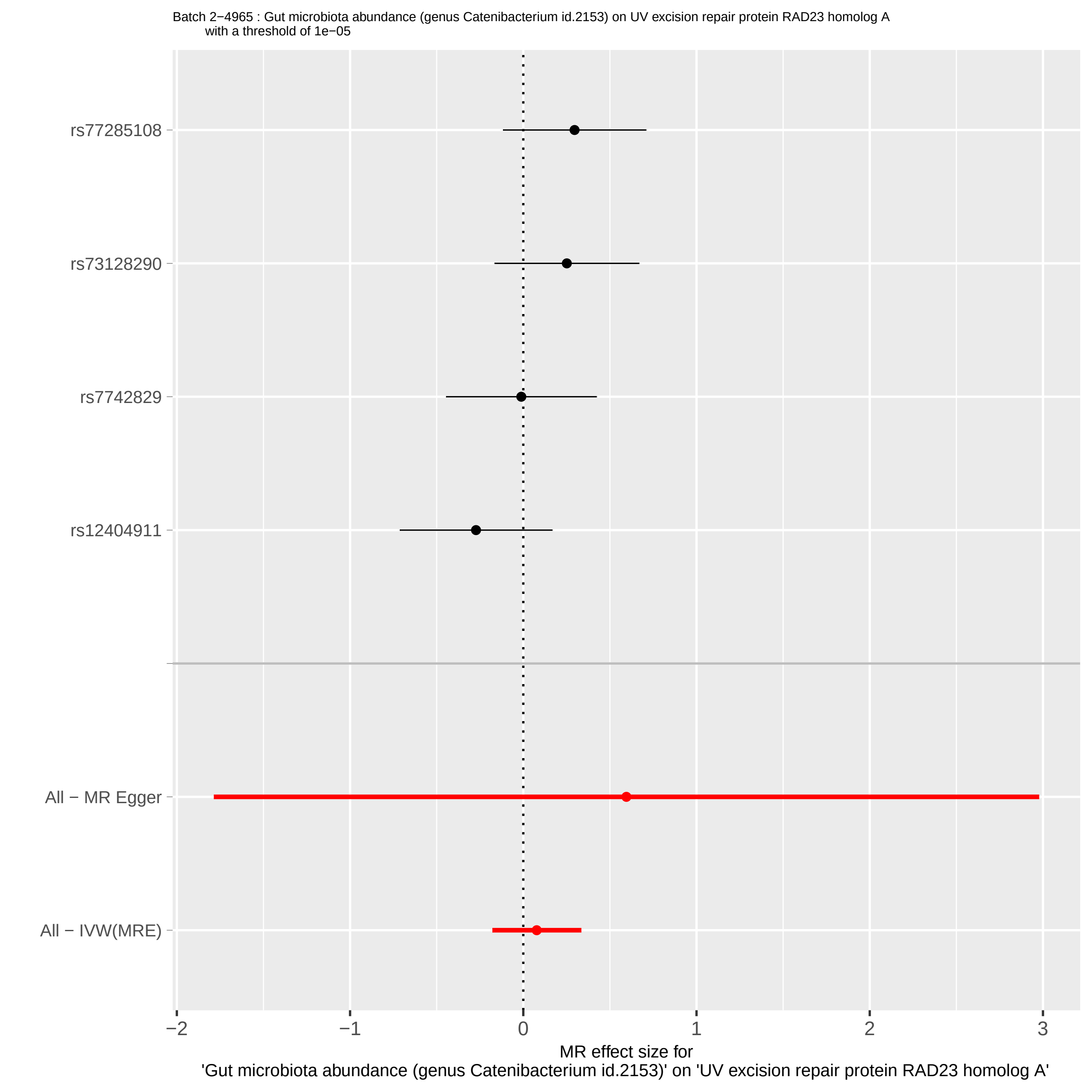
Batch 2−4965 : Gut microbiota abundance (genus Catenibacterium id.2153) on UV excision repair protein RAD23 homolog A
with a threshold of 1e−05
rs77285108
rs73128290
rs7742829
rs12404911
All − MR Egger
All − IVW(MRE)
−2
−1
0
1
2
3
MR effect size for
'Gut microbiota abundance (genus Catenibacterium id.2153)' on 'UV excision repair protein RAD23 homolog A'

Batch 2−4966 : Gut microbiota abundance (genus Parasutterella id.2892) on UV excision repair protein RAD23 homolog A
with a threshold of 1e−05

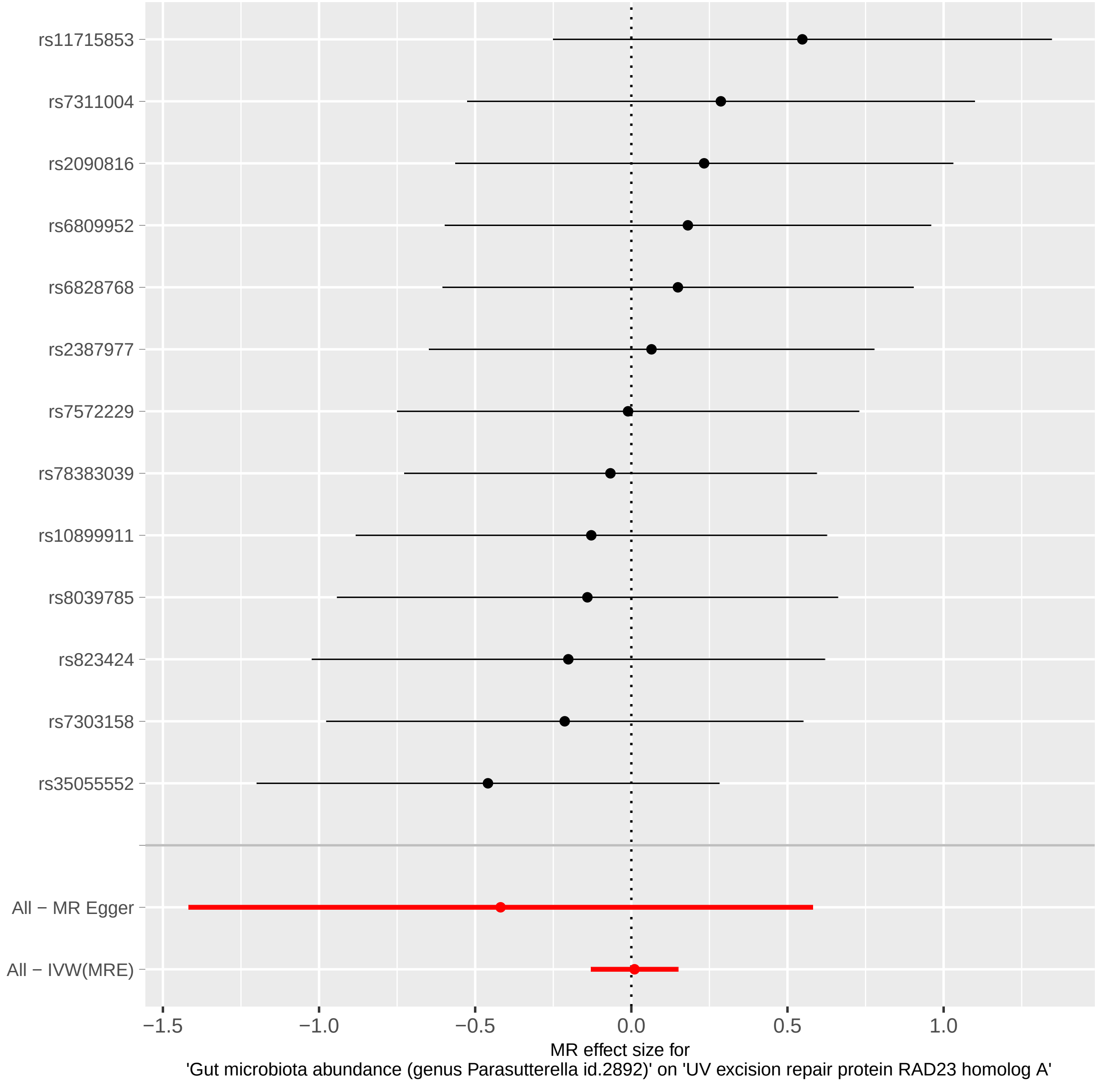

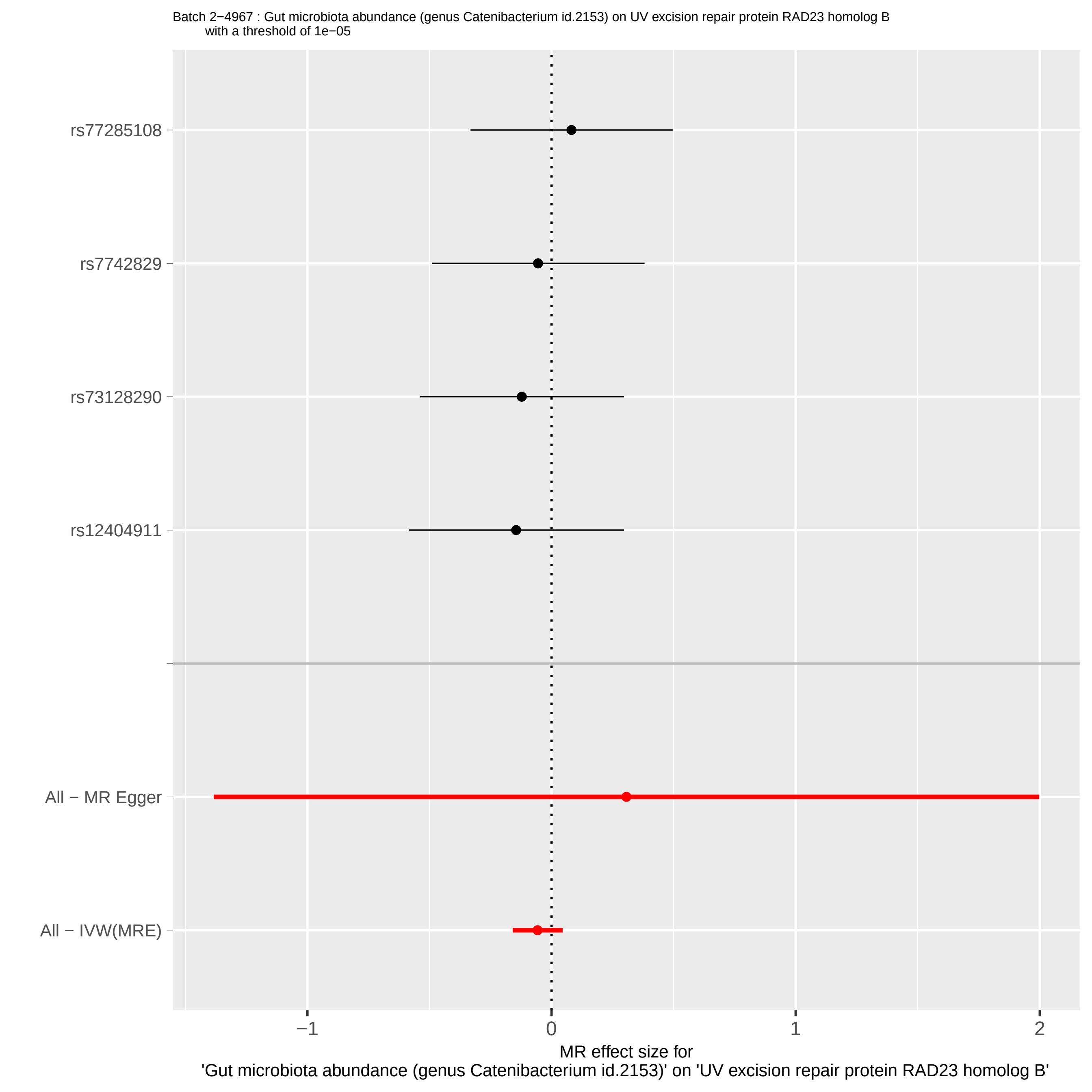

Batch 2−4967 : Gut microbiota abundance (genus Catenibacterium id.2153) on UV excision repair protein RAD23 homolog B
with a threshold of 1e−05
rs77285108
rs7742829
rs73128290
rs12404911
All − MR Egger
All − IVW(MRE)
−1
0
1
2
MR effect size for
'Gut microbiota abundance (genus Catenibacterium id.2153)' on 'UV excision repair protein RAD23 homolog B'

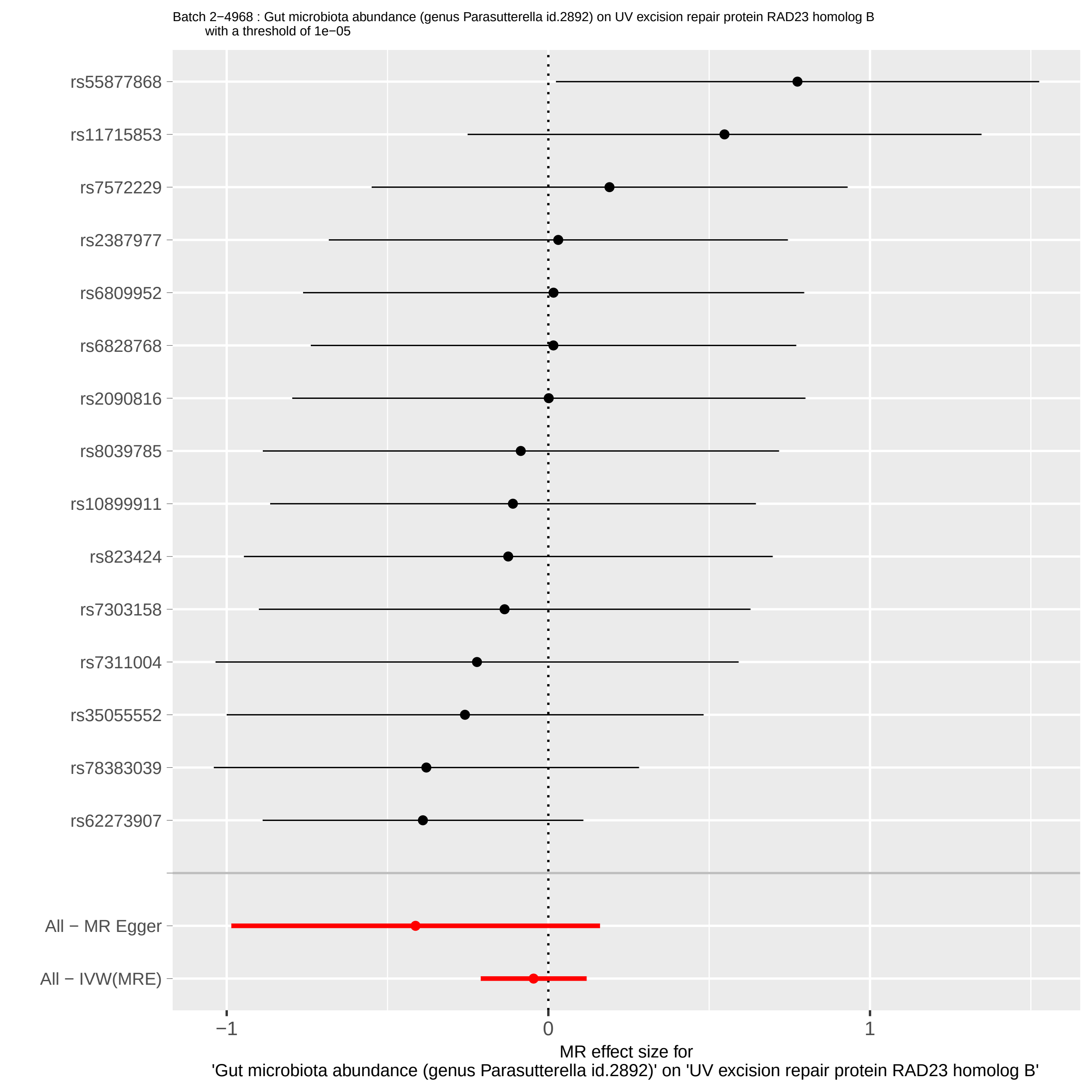
Batch 2−4968 : Gut microbiota abundance (genus Parasutterella id.2892) on UV excision repair protein RAD23 homolog B
with a threshold of 1e−05
rs55877868
rs11715853
rs7572229
rs2387977
rs6809952
rs6828768
rs2090816
rs8039785
rs10899911
rs823424
rs7303158
rs7311004
rs35055552
rs78383039
rs62273907
All − MR Egger
All − IVW(MRE)
−1
0
1
MR effect size for
'Gut microbiota abundance (genus Parasutterella id.2892)' on 'UV excision repair protein RAD23 homolog B'

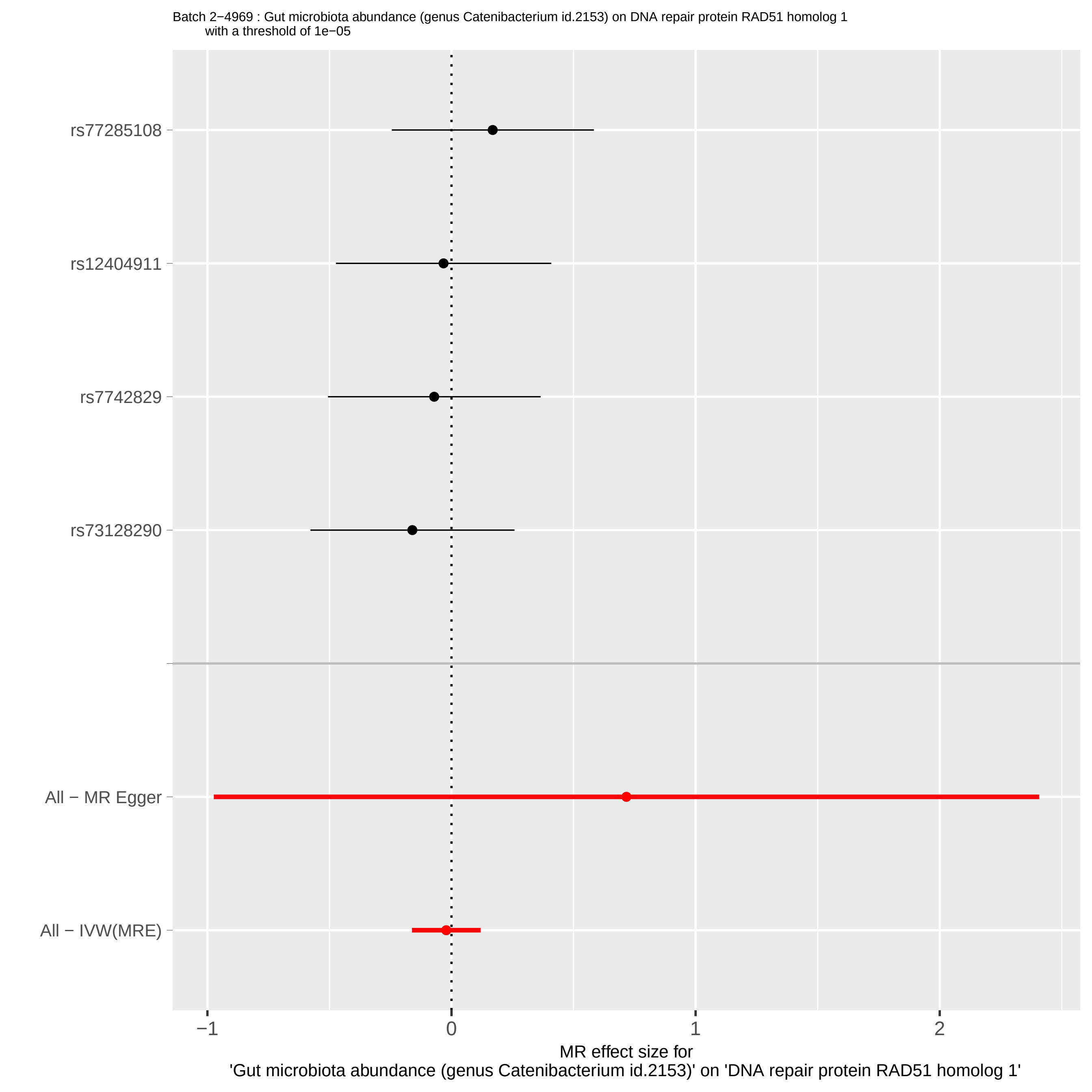

Batch 2−4969 : Gut microbiota abundance (genus Catenibacterium id.2153) on DNA repair protein RAD51 homolog 1
with a threshold of 1e−05
rs77285108
rs12404911
rs7742829
rs73128290
All − MR Egger
All − IVW(MRE)
−1
0
1
2
MR effect size for
'Gut microbiota abundance (genus Catenibacterium id.2153)' on 'DNA repair protein RAD51 homolog 1'

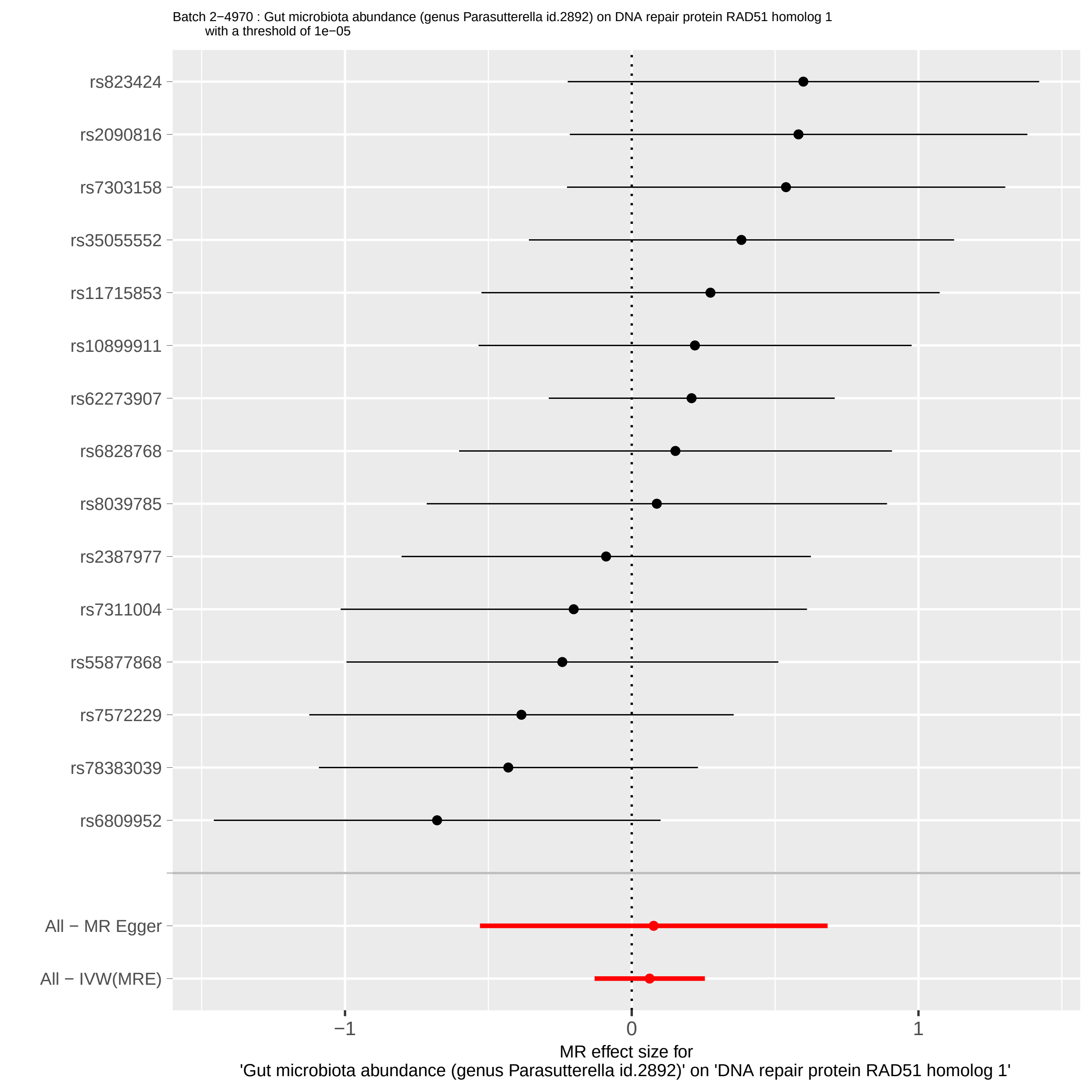

Batch 2−4970 : Gut microbiota abundance (genus Parasutterella id.2892) on DNA repair protein RAD51 homolog 1
with a threshold of 1e−05
rs823424
rs2090816
rs7303158
rs35055552
rs11715853
rs10899911
rs62273907
rs6828768
rs8039785
rs2387977
rs7311004
rs55877868
rs7572229
rs78383039
rs6809952
All − MR Egger
All − IVW(MRE)
−1
0
1
MR effect size for
'Gut microbiota abundance (genus Parasutterella id.2892)' on 'DNA repair protein RAD51 homolog 1'

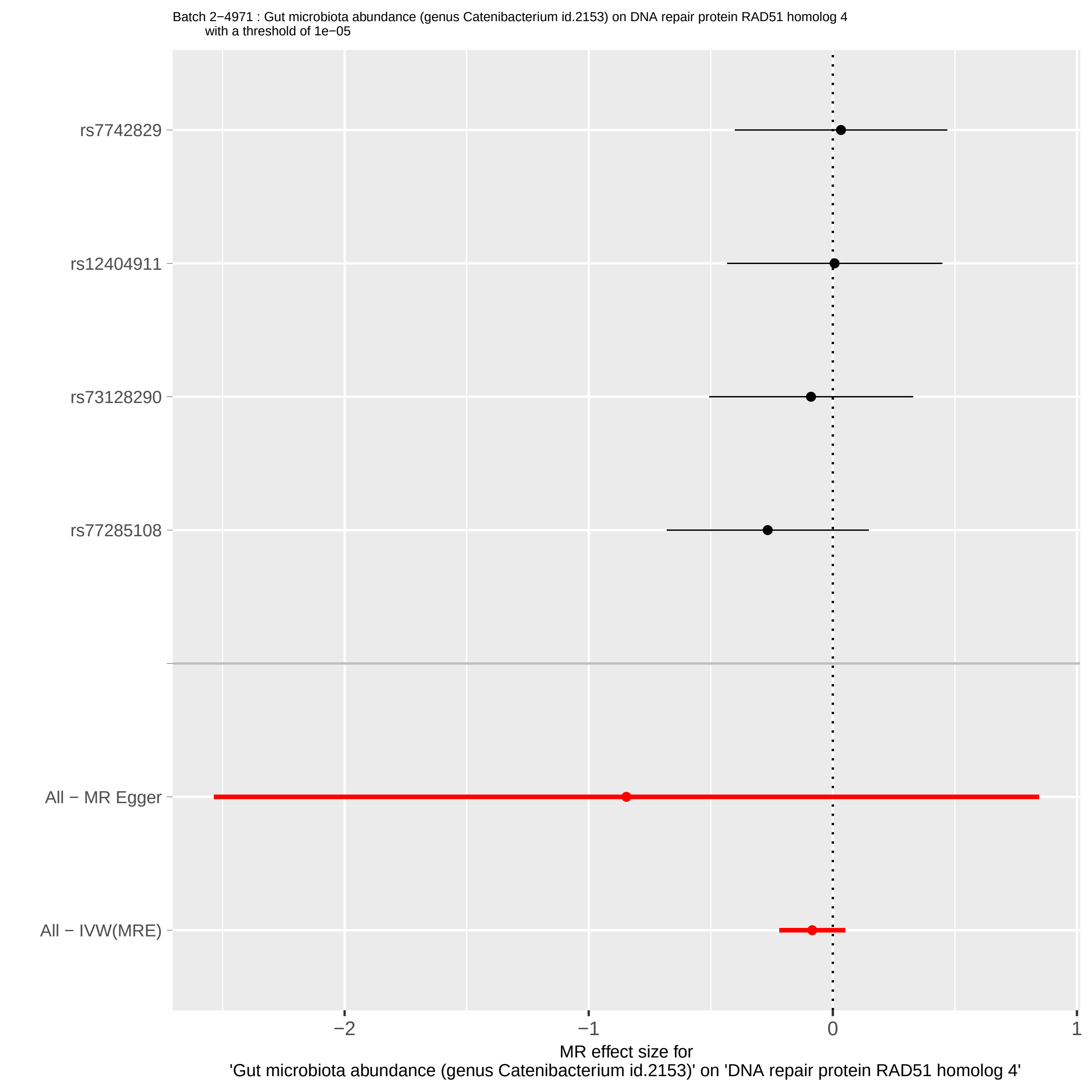
Batch 2−4971 : Gut microbiota abundance (genus Catenibacterium id.2153) on DNA repair protein RAD51 homolog 4
with a threshold of 1e−05
rs7742829
rs12404911
rs73128290
rs77285108
All − MR Egger
All − IVW(MRE)
−2
−1
0
1
MR effect size for
'Gut microbiota abundance (genus Catenibacterium id.2153)' on 'DNA repair protein RAD51 homolog 4'

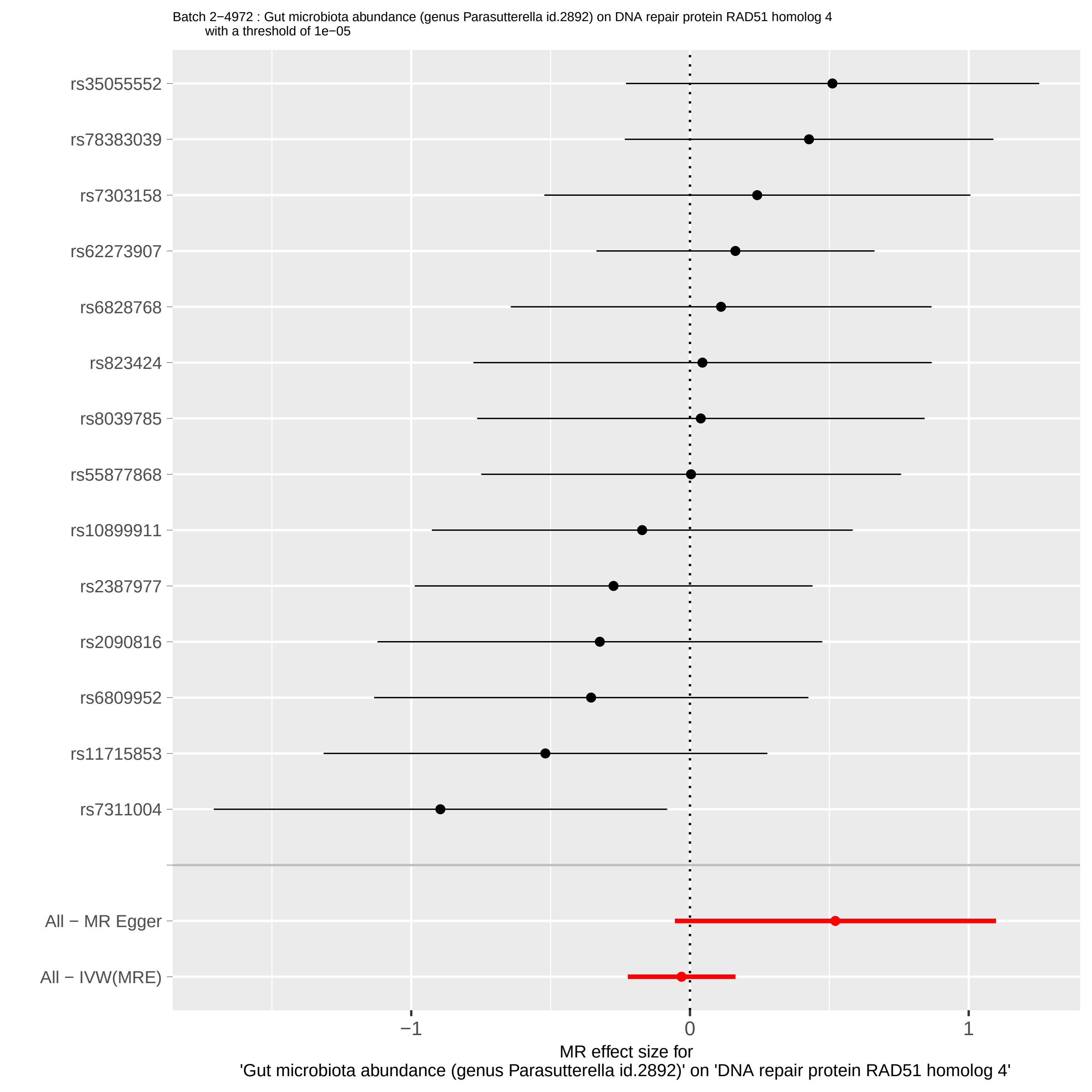
Batch 2−4972 : Gut microbiota abundance (genus Parasutterella id.2892) on DNA repair protein RAD51 homolog 4
with a threshold of 1e−05
rs35055552
rs78383039
rs7303158
rs62273907
rs6828768
rs823424
rs8039785
rs55877868
rs10899911
rs2387977
rs2090816
rs6809952
rs11715853
rs7311004
All − MR Egger
All − IVW(MRE)
−1
0
1
MR effect size for
'Gut microbiota abundance (genus Parasutterella id.2892)' on 'DNA repair protein RAD51 homolog 4'

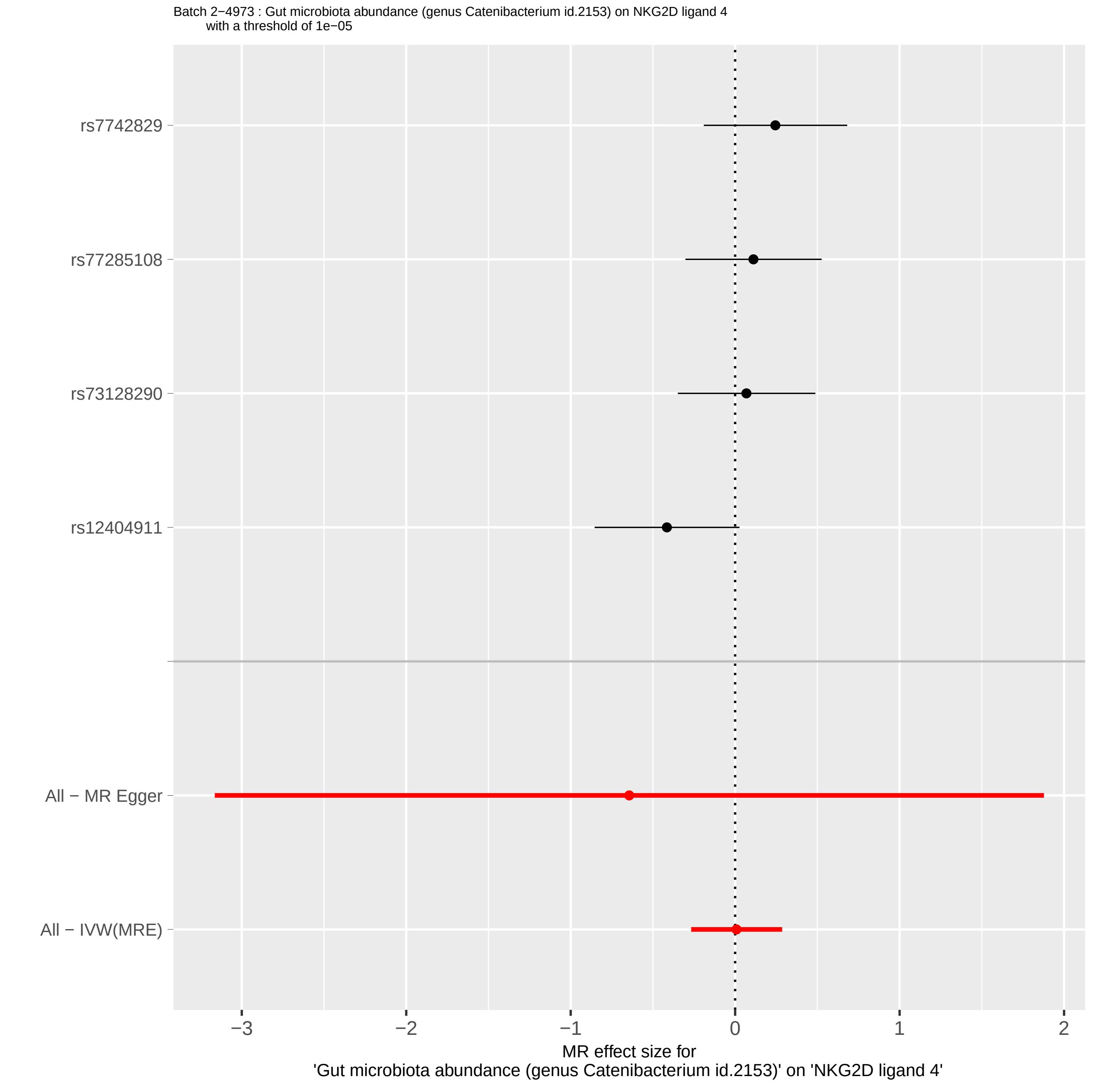

Batch 2−4973 : Gut microbiota abundance (genus Catenibacterium id.2153) on NKG2D ligand 4
with a threshold of 1e−05
rs7742829
rs77285108
rs73128290
rs12404911
All − MR Egger
All − IVW(MRE)
−3
−2
−1
0
1
2
MR effect size for
'Gut microbiota abundance (genus Catenibacterium id.2153)' on 'NKG2D ligand 4'

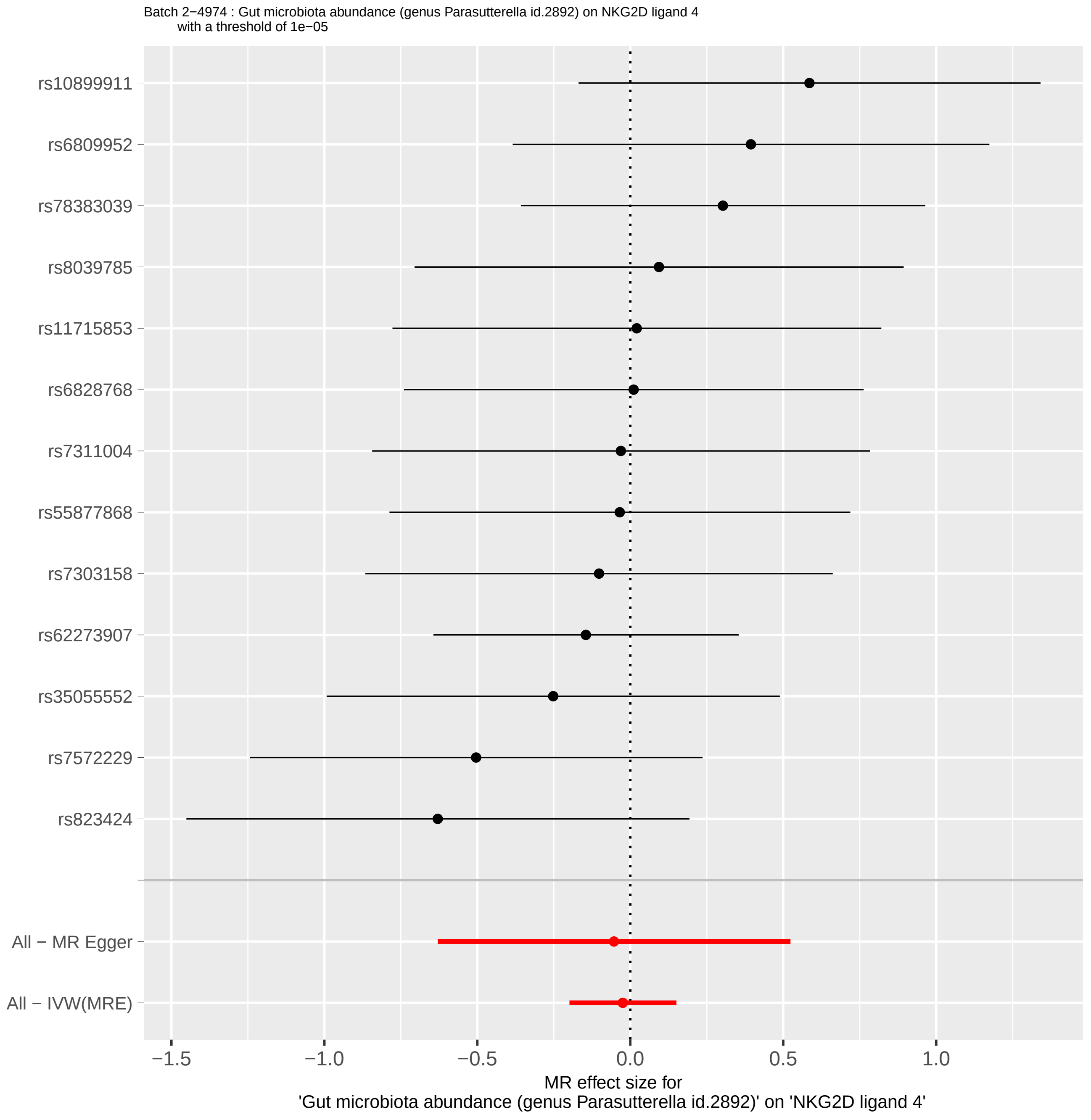

Batch 2−4974 : Gut microbiota abundance (genus Parasutterella id.2892) on NKG2D ligand 4
with a threshold of 1e−05
rs10899911
rs6809952
rs78383039
rs8039785
rs11715853
rs6828768
rs7311004
rs55877868
rs7303158
rs62273907
rs35055552
rs7572229
rs823424
All − MR Egger
All − IVW(MRE)
−1.5
−1.0
−0.5
0.0
0.5
1.0
MR effect size for
'Gut microbiota abundance (genus Parasutterella id.2892)' on 'NKG2D ligand 4'

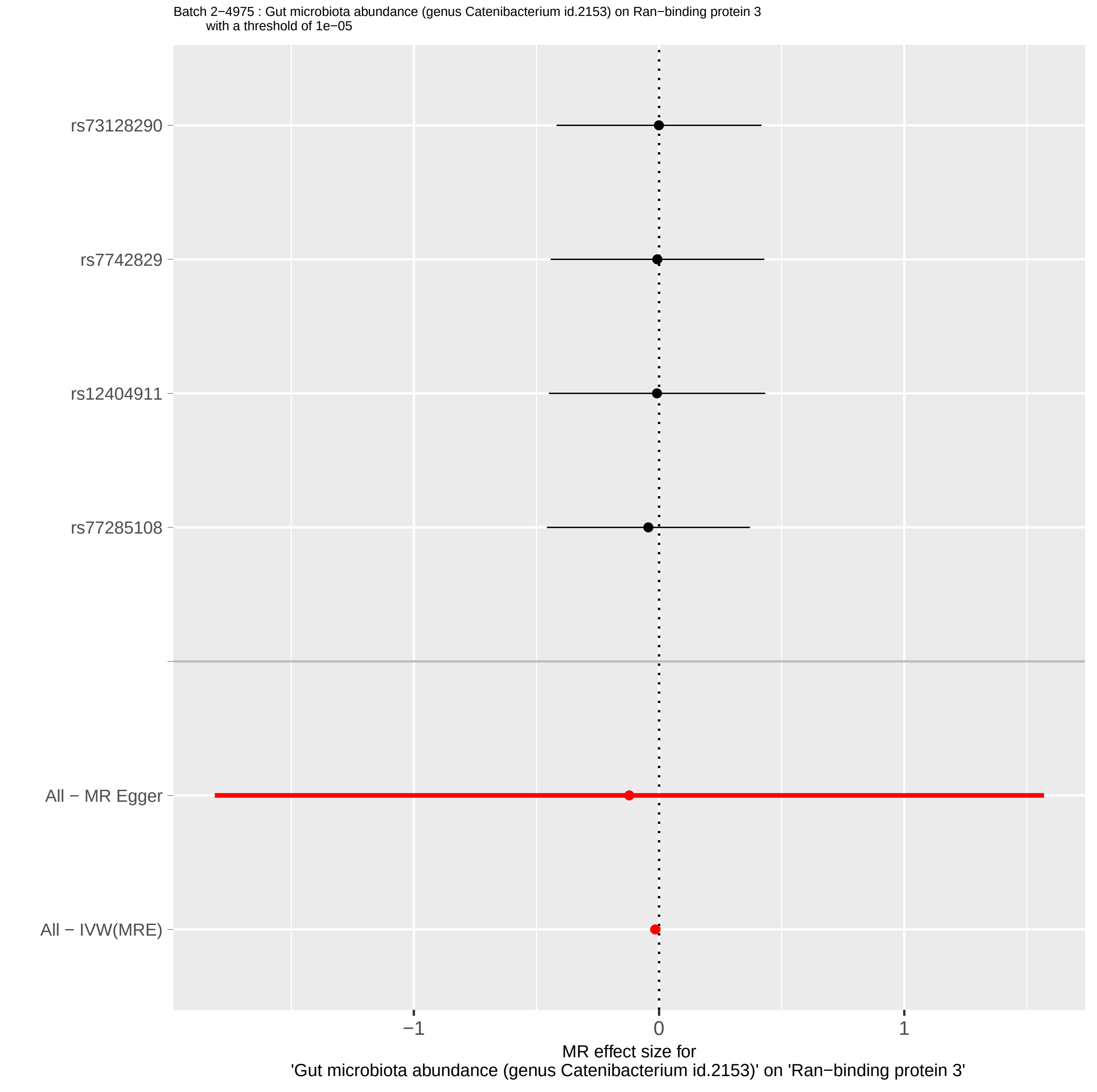
Batch 2−4975 : Gut microbiota abundance (genus Catenibacterium id.2153) on Ran−binding protein 3
with a threshold of 1e−05
rs73128290
rs7742829
rs12404911
rs77285108
All − MR Egger
All − IVW(MRE)
−1
0
1
MR effect size for
'Gut microbiota abundance (genus Catenibacterium id.2153)' on 'Ran−binding protein 3'

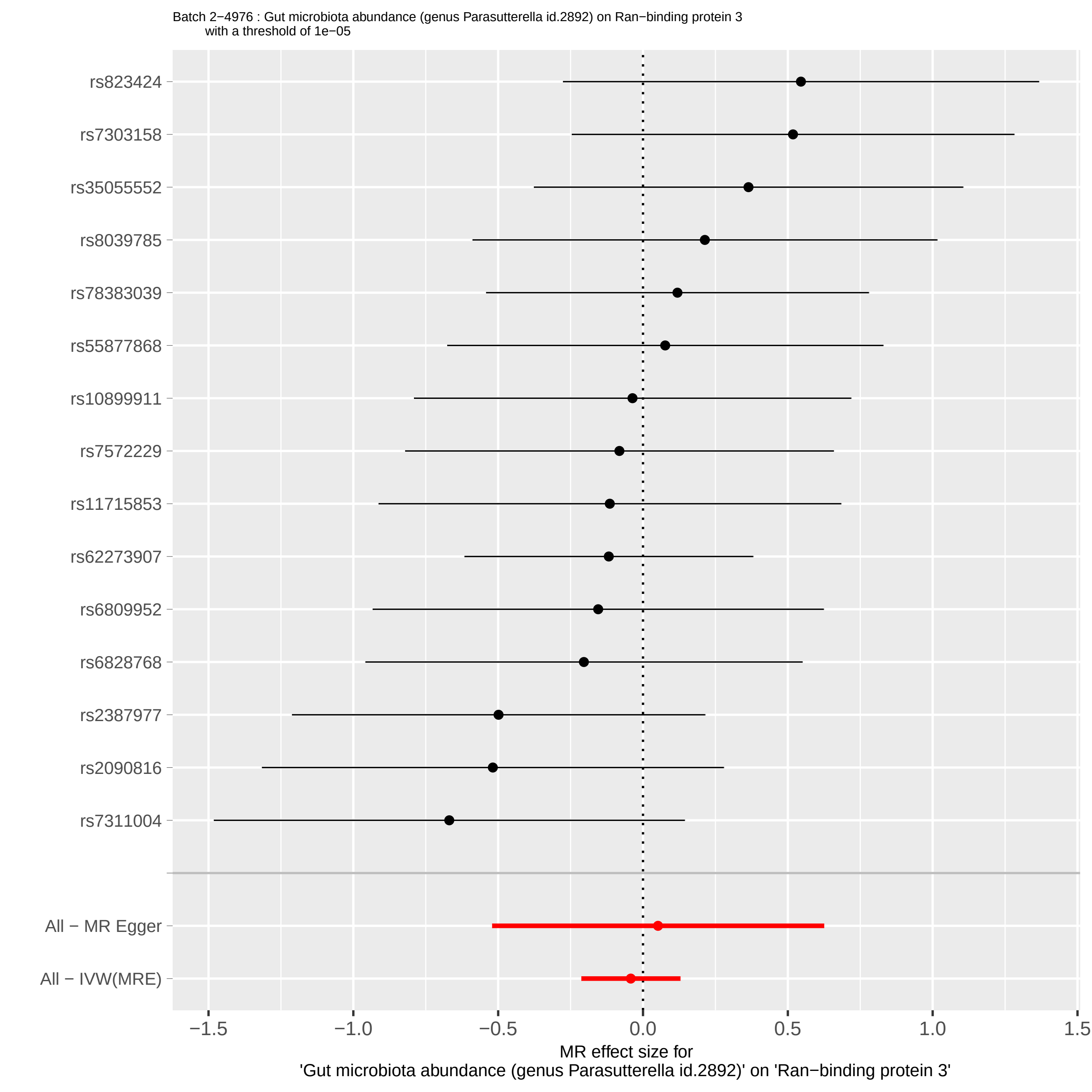

Batch 2−4976 : Gut microbiota abundance (genus Parasutterella id.2892) on Ran−binding protein 3
with a threshold of 1e−05
rs823424
rs7303158
rs35055552
rs8039785
rs78383039
rs55877868
rs10899911
rs7572229
rs11715853
rs62273907
rs6809952
rs6828768
rs2387977
rs2090816
rs7311004
All − MR Egger
All − IVW(MRE)
−1.5
−1.0
−0.5
0.0
0.5
1.0
1.5
MR effect size for
'Gut microbiota abundance (genus Parasutterella id.2892)' on 'Ran−binding protein 3'

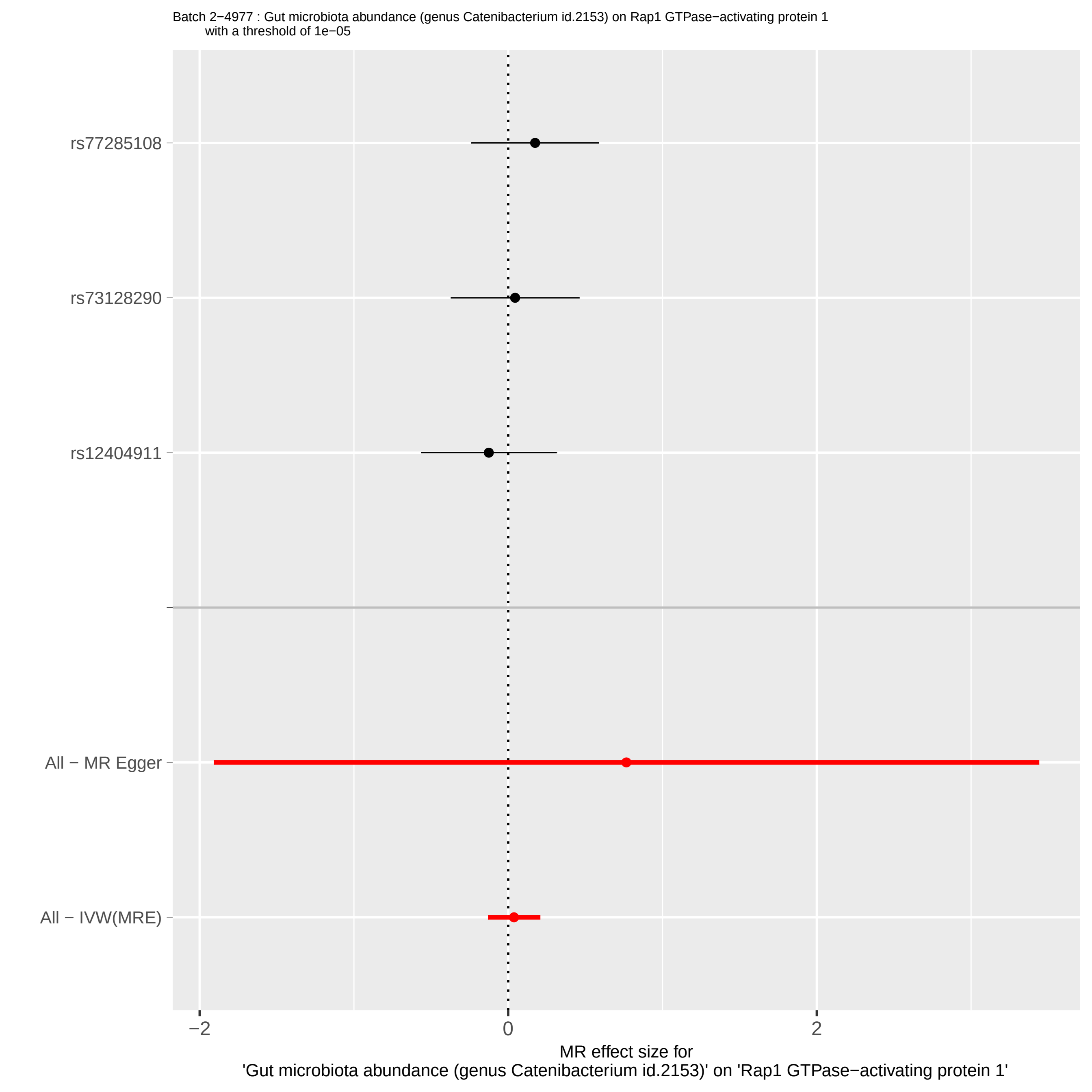

Batch 2−4977 : Gut microbiota abundance (genus Catenibacterium id.2153) on Rap1 GTPase−activating protein 1
with a threshold of 1e−05
rs77285108
rs73128290
rs12404911
All − MR Egger
All − IVW(MRE)
−2
0
2
MR effect size for
'Gut microbiota abundance (genus Catenibacterium id.2153)' on 'Rap1 GTPase−activating protein 1'

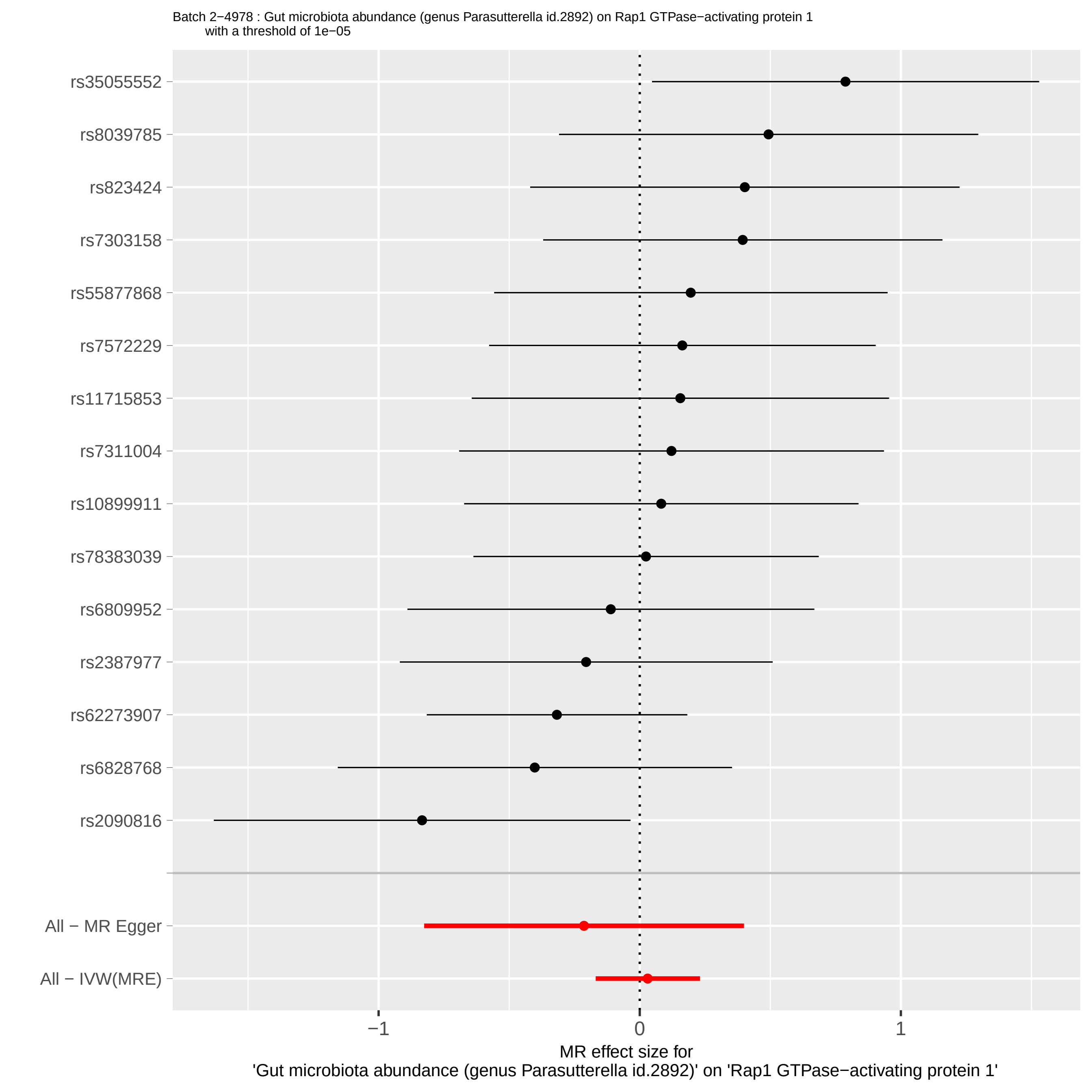

Batch 2−4978 : Gut microbiota abundance (genus Parasutterella id.2892) on Rap1 GTPase−activating protein 1
with a threshold of 1e−05
rs35055552
rs8039785
rs823424
rs7303158
rs55877868
rs7572229
rs11715853
rs7311004
rs10899911
rs78383039
rs6809952
rs2387977
rs62273907
rs6828768
rs2090816
All − MR Egger
All − IVW(MRE)
−1
0
1
MR effect size for
'Gut microbiota abundance (genus Parasutterella id.2892)' on 'Rap1 GTPase−activating protein 1'

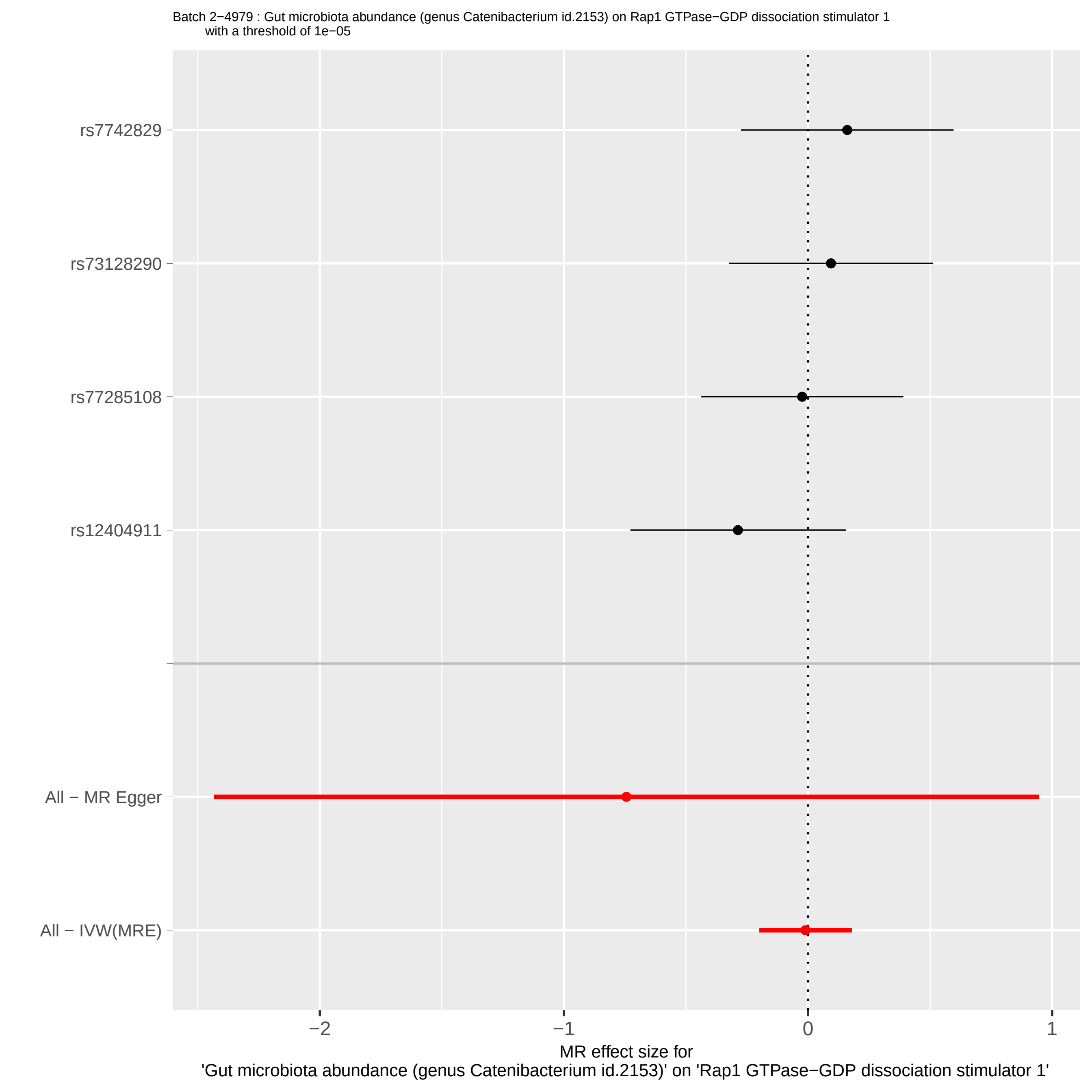

Batch 2−4979 : Gut microbiota abundance (genus Catenibacterium id.2153) on Rap1 GTPase−GDP dissociation stimulator 1
with a threshold of 1e−05
rs7742829
rs73128290
rs77285108
rs12404911
All − MR Egger
All − IVW(MRE)
−2
−1
0
1
MR effect size for
'Gut microbiota abundance (genus Catenibacterium id.2153)' on 'Rap1 GTPase−GDP dissociation stimulator 1'

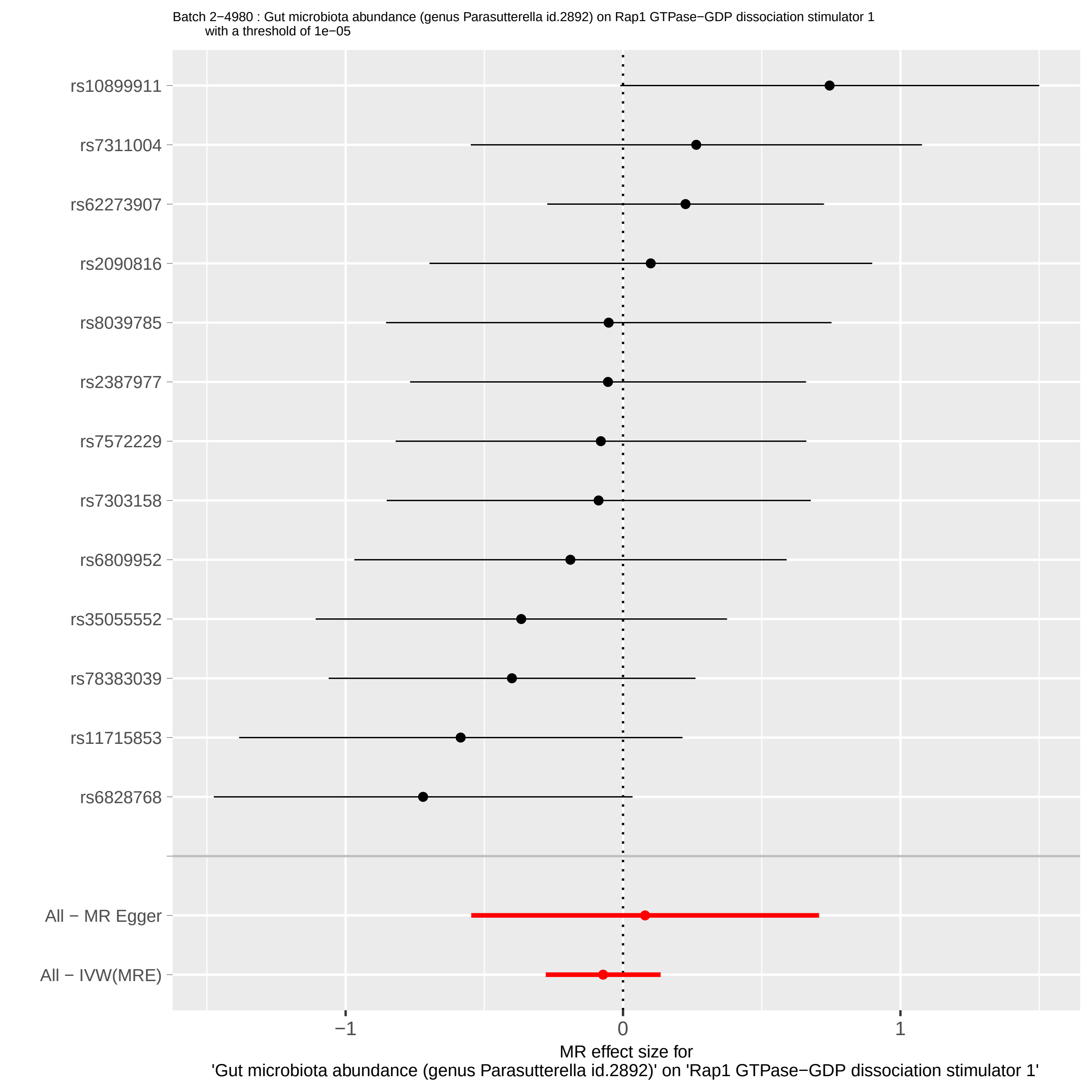

Batch 2−4980 : Gut microbiota abundance (genus Parasutterella id.2892) on Rap1 GTPase−GDP dissociation stimulator 1
with a threshold of 1e−05
rs10899911
rs7311004
rs62273907
rs2090816
rs8039785
rs2387977
rs7572229
rs7303158
rs6809952
rs35055552
rs78383039
rs11715853
rs6828768
All − MR Egger
All − IVW(MRE)
−1
0
1
MR effect size for
'Gut microbiota abundance (genus Parasutterella id.2892)' on 'Rap1 GTPase−GDP dissociation stimulator 1'

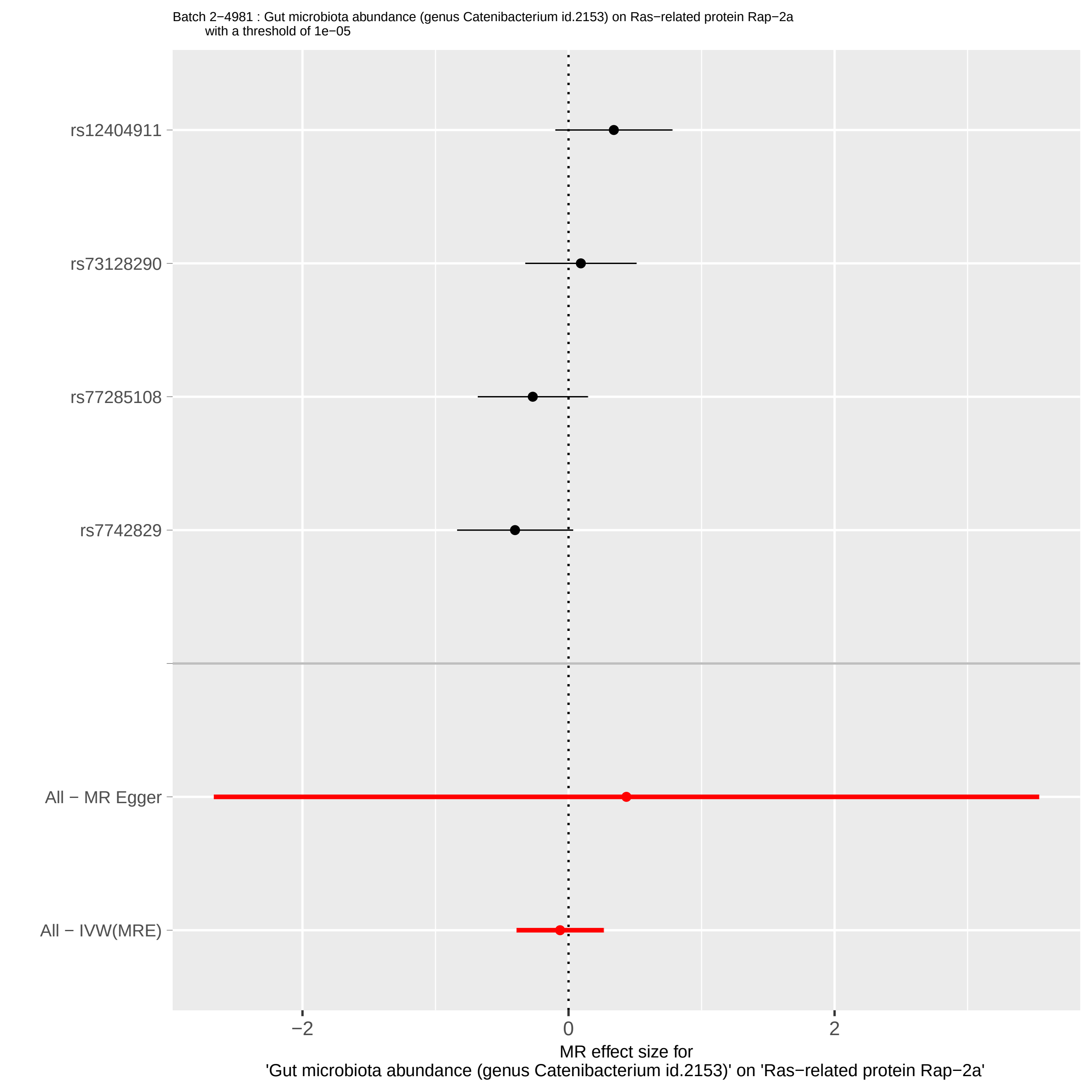
Batch 2−4981 : Gut microbiota abundance (genus Catenibacterium id.2153) on Ras−related protein Rap−2a
with a threshold of 1e−05
rs12404911
rs73128290
rs77285108
rs7742829
All − MR Egger
All − IVW(MRE)
−2
0
2
MR effect size for
'Gut microbiota abundance (genus Catenibacterium id.2153)' on 'Ras−related protein Rap−2a'

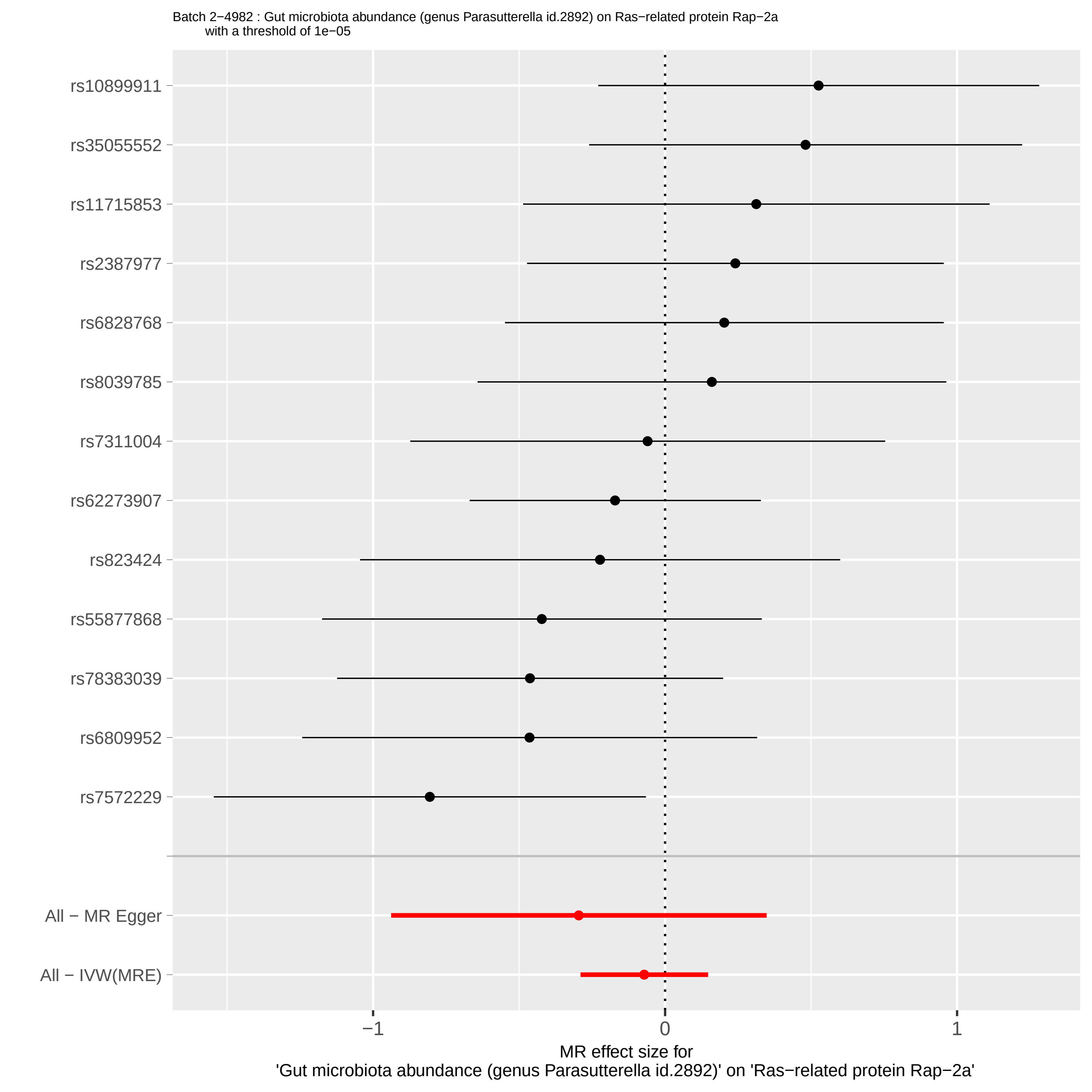

Batch 2−4982 : Gut microbiota abundance (genus Parasutterella id.2892) on Ras−related protein Rap−2a
with a threshold of 1e−05
rs10899911
rs35055552
rs11715853
rs2387977
rs6828768
rs8039785
rs7311004
rs62273907
rs823424
rs55877868
rs78383039
rs6809952
rs7572229
All − MR Egger
All − IVW(MRE)
−1
0
1
MR effect size for
'Gut microbiota abundance (genus Parasutterella id.2892)' on 'Ras−related protein Rap−2a'

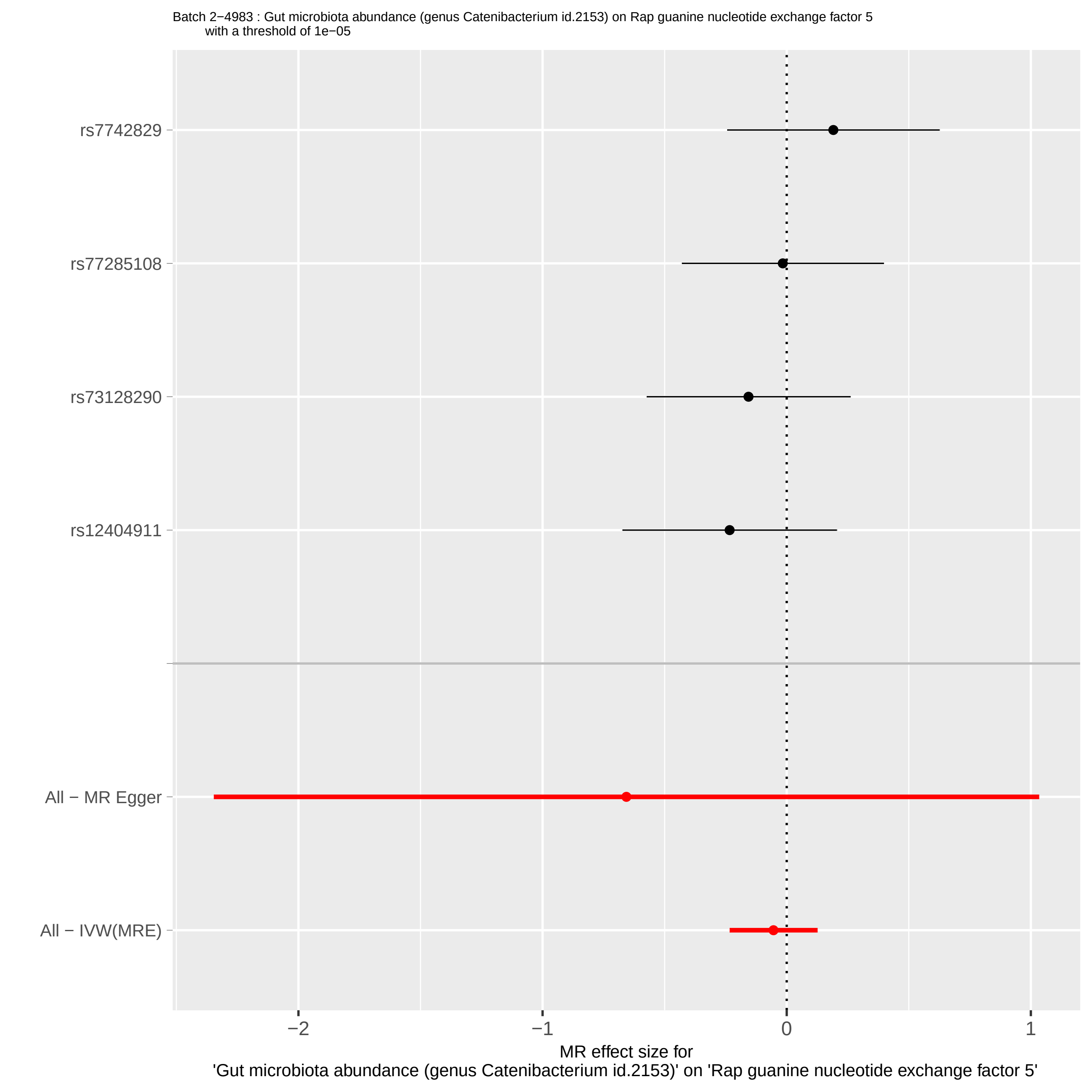
Batch 2−4983 : Gut microbiota abundance (genus Catenibacterium id.2153) on Rap guanine nucleotide exchange factor 5
with a threshold of 1e−05
rs7742829
rs77285108
rs73128290
rs12404911
All − MR Egger
All − IVW(MRE)
−2
−1
0
1
MR effect size for
'Gut microbiota abundance (genus Catenibacterium id.2153)' on 'Rap guanine nucleotide exchange factor 5'

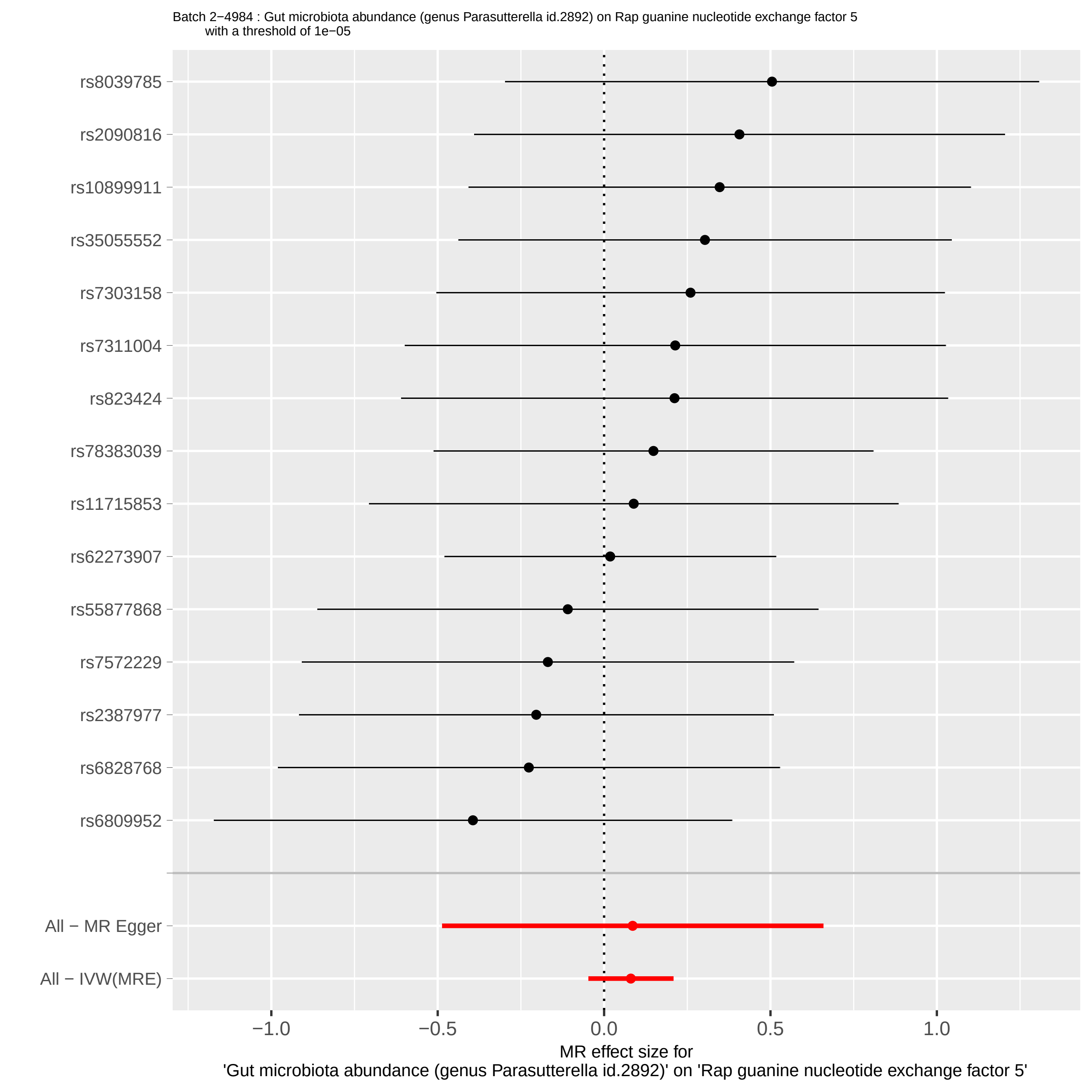

Batch 2−4984 : Gut microbiota abundance (genus Parasutterella id.2892) on Rap guanine nucleotide exchange factor 5
with a threshold of 1e−05
rs8039785
rs2090816
rs10899911
rs35055552
rs7303158
rs7311004
rs823424
rs78383039
rs11715853
rs62273907
rs55877868
rs7572229
rs2387977
rs6828768
rs6809952
All − MR Egger
All − IVW(MRE)
−1.0
−0.5
0.0
0.5
1.0
MR effect size for
'Gut microbiota abundance (genus Parasutterella id.2892)' on 'Rap guanine nucleotide exchange factor 5'

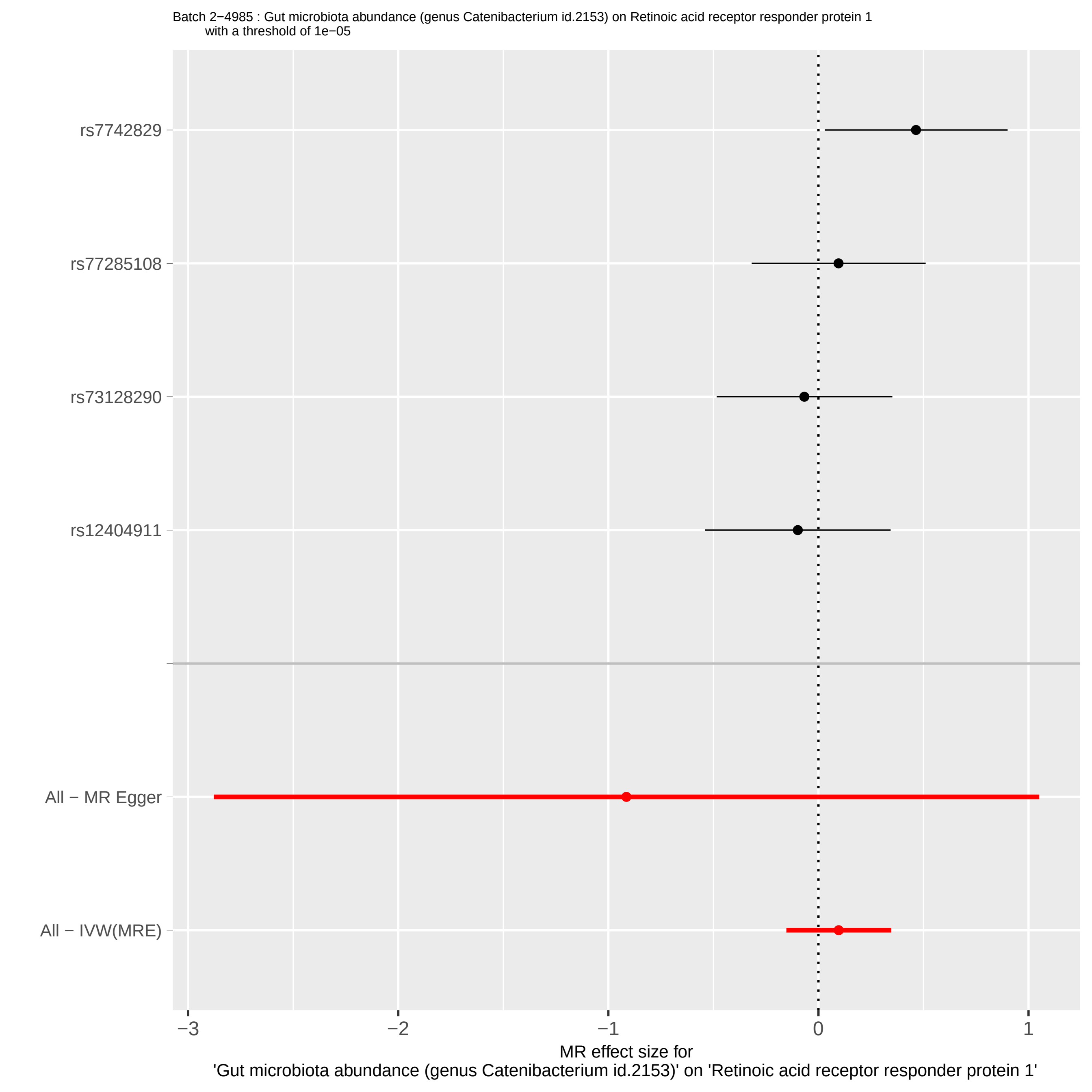
Batch 2−4985 : Gut microbiota abundance (genus Catenibacterium id.2153) on Retinoic acid receptor responder protein 1
with a threshold of 1e−05
rs7742829
rs77285108
rs73128290
rs12404911
All − MR Egger
All − IVW(MRE)
−3
−2
−1
0
1
MR effect size for
'Gut microbiota abundance (genus Catenibacterium id.2153)' on 'Retinoic acid receptor responder protein 1'

Batch 2−4986 : Gut microbiota abundance (genus Parasutterella id.2892) on Retinoic acid receptor responder protein 1
with a threshold of 1e−05

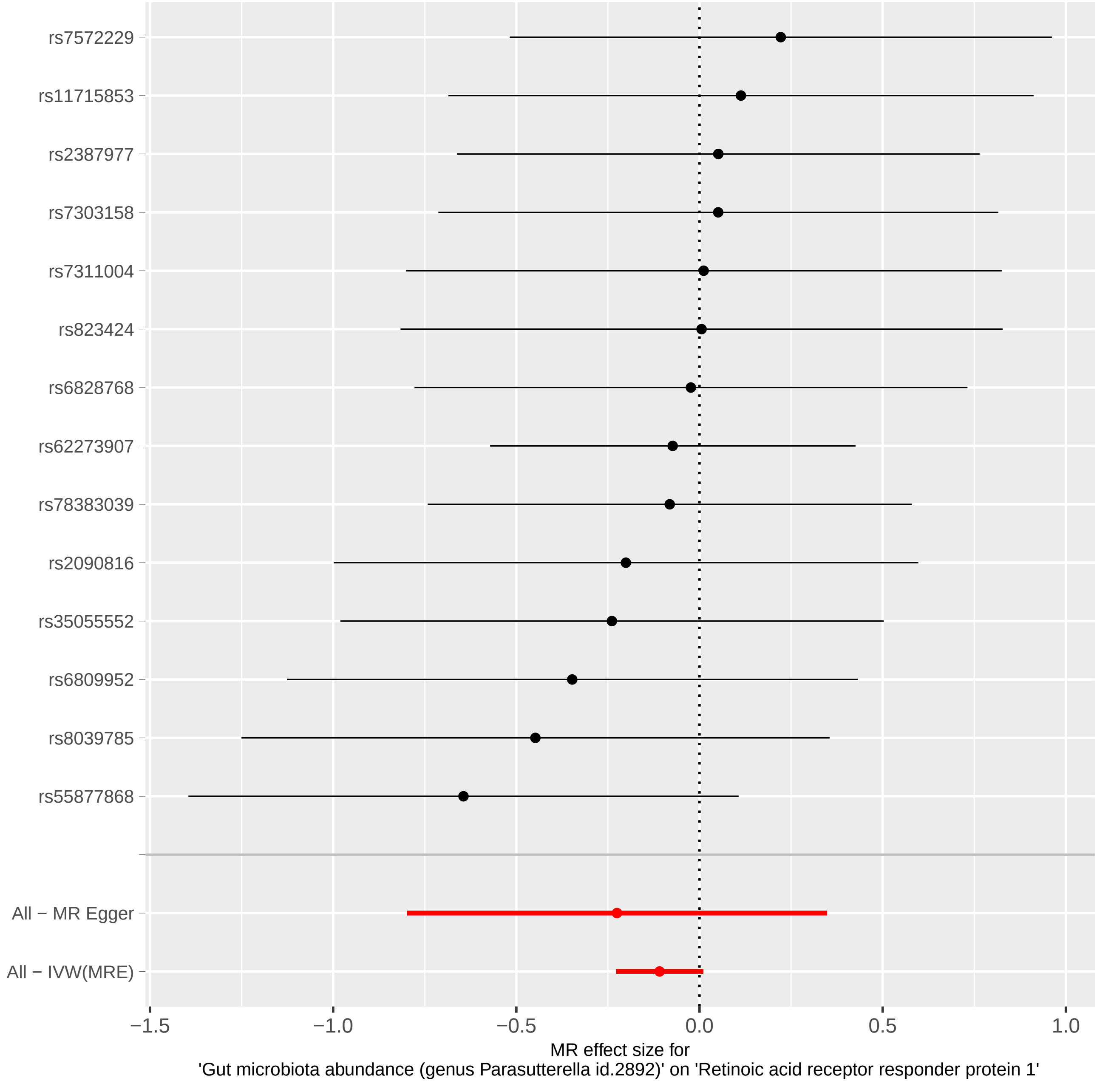

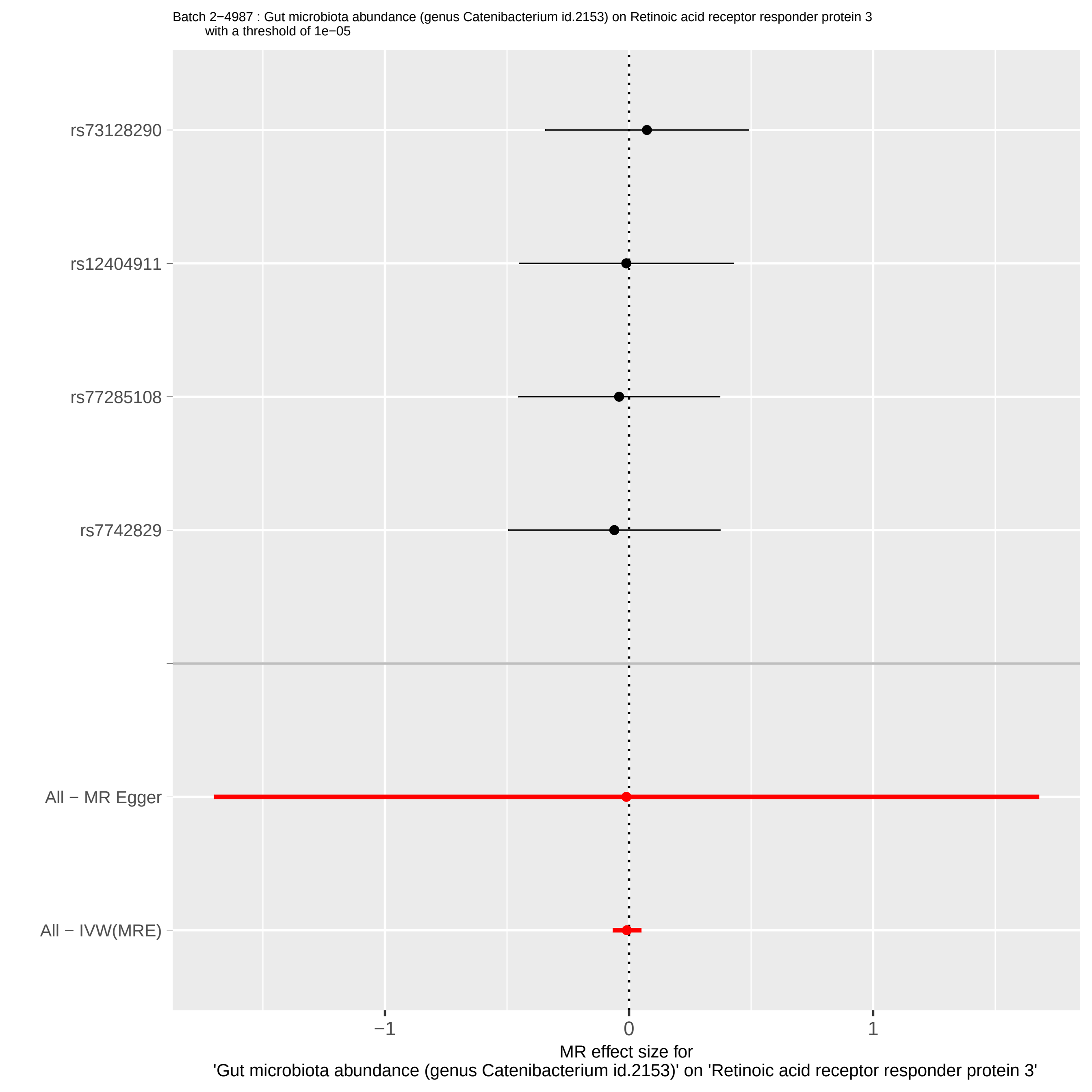
Batch 2−4987 : Gut microbiota abundance (genus Catenibacterium id.2153) on Retinoic acid receptor responder protein 3
with a threshold of 1e−05
rs73128290
rs12404911
rs77285108
rs7742829
All − MR Egger
All − IVW(MRE)
−1
0
1
MR effect size for
'Gut microbiota abundance (genus Catenibacterium id.2153)' on 'Retinoic acid receptor responder protein 3'

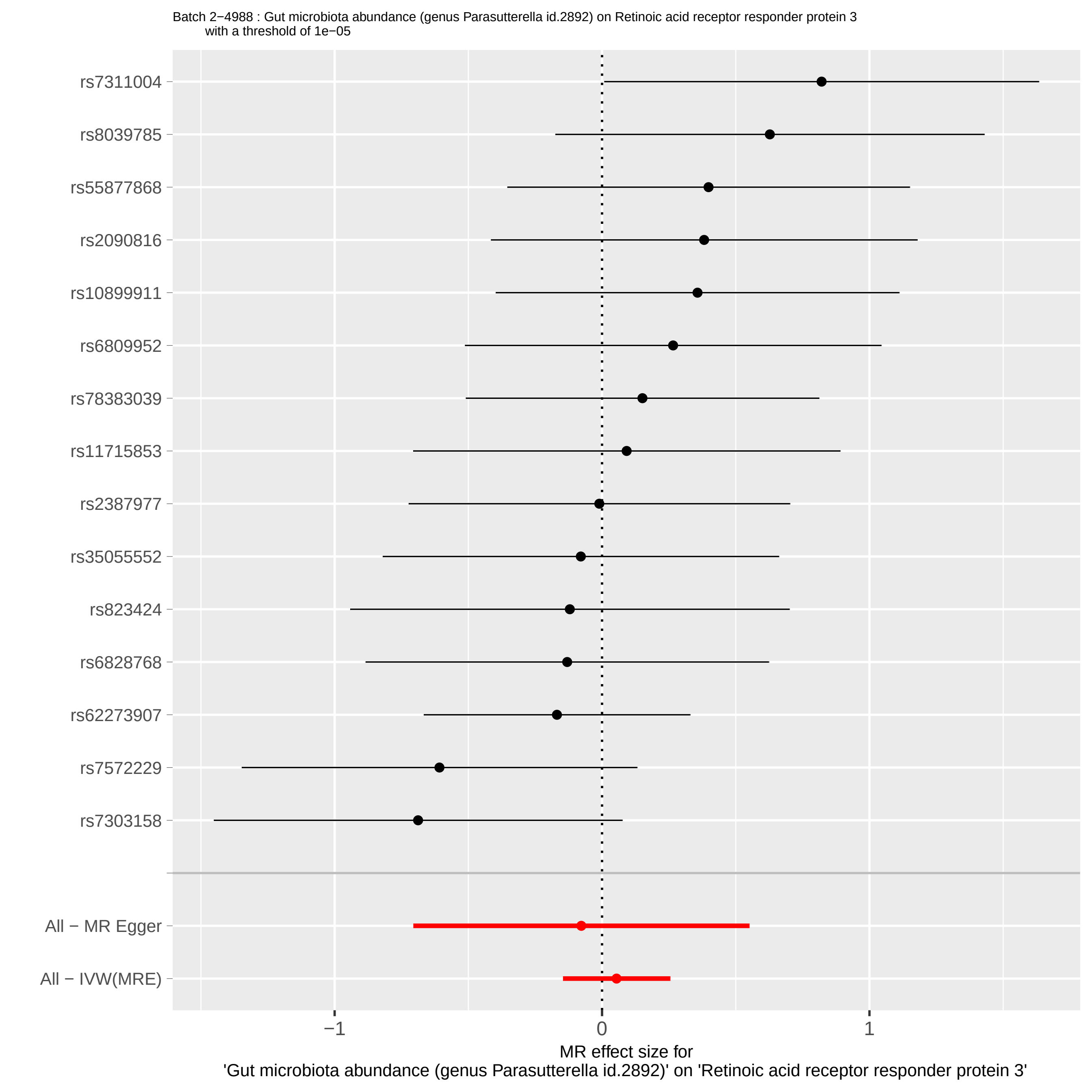

Batch 2−4988 : Gut microbiota abundance (genus Parasutterella id.2892) on Retinoic acid receptor responder protein 3
with a threshold of 1e−05
rs7311004
rs8039785
rs55877868
rs2090816
rs10899911
rs6809952
rs78383039
rs11715853
rs2387977
rs35055552
rs823424
rs6828768
rs62273907
rs7572229
rs7303158
All − MR Egger
All − IVW(MRE)
−1
0
1
MR effect size for
'Gut microbiota abundance (genus Parasutterella id.2892)' on 'Retinoic acid receptor responder protein 3'

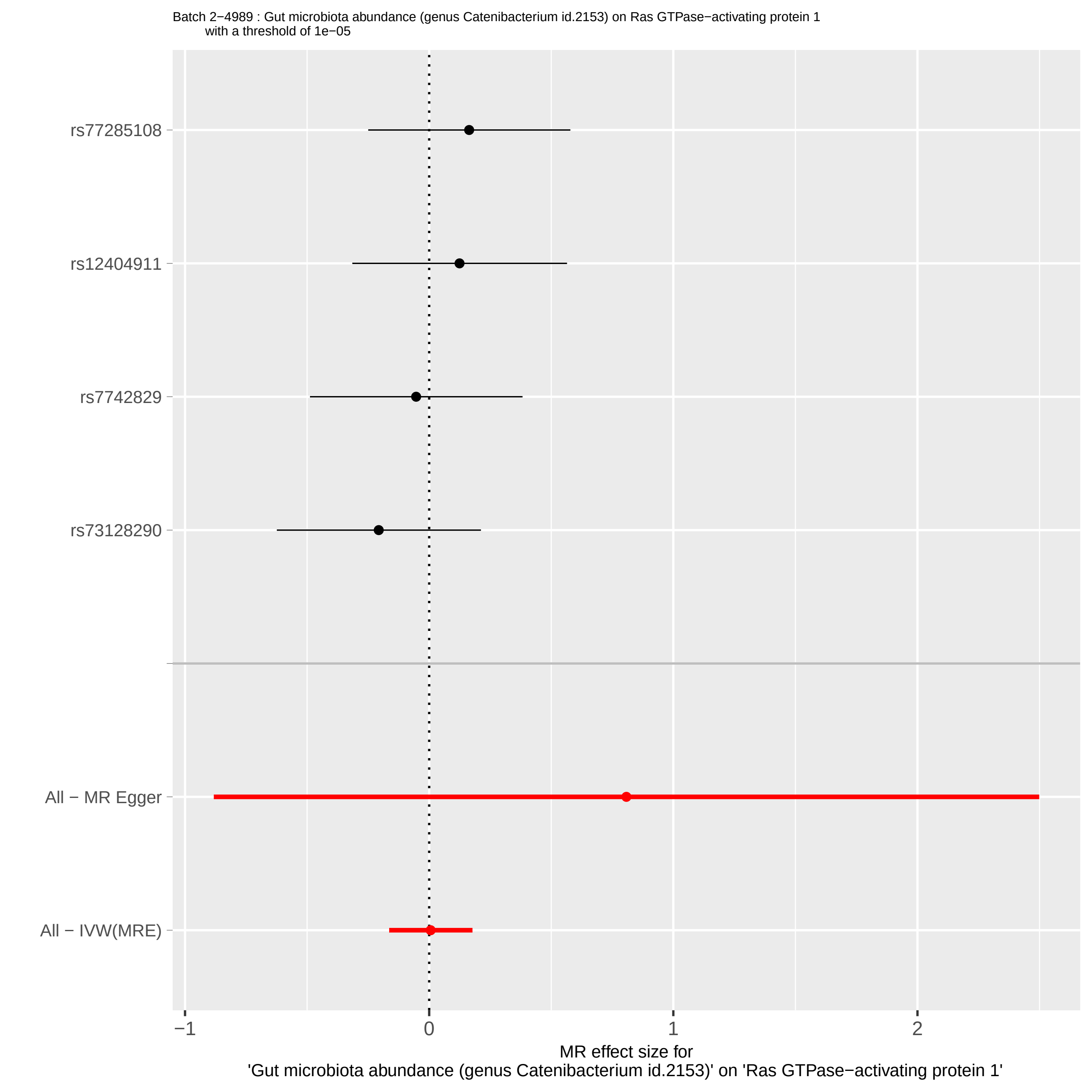

Batch 2−4989 : Gut microbiota abundance (genus Catenibacterium id.2153) on Ras GTPase−activating protein 1
with a threshold of 1e−05
rs77285108
rs12404911
rs7742829
rs73128290
All − MR Egger
All − IVW(MRE)
−1
0
1
2
MR effect size for
'Gut microbiota abundance (genus Catenibacterium id.2153)' on 'Ras GTPase−activating protein 1'

Batch 2−4990 : Gut microbiota abundance (genus Parasutterella id.2892) on Ras GTPase−activating protein 1
with a threshold of 1e−05

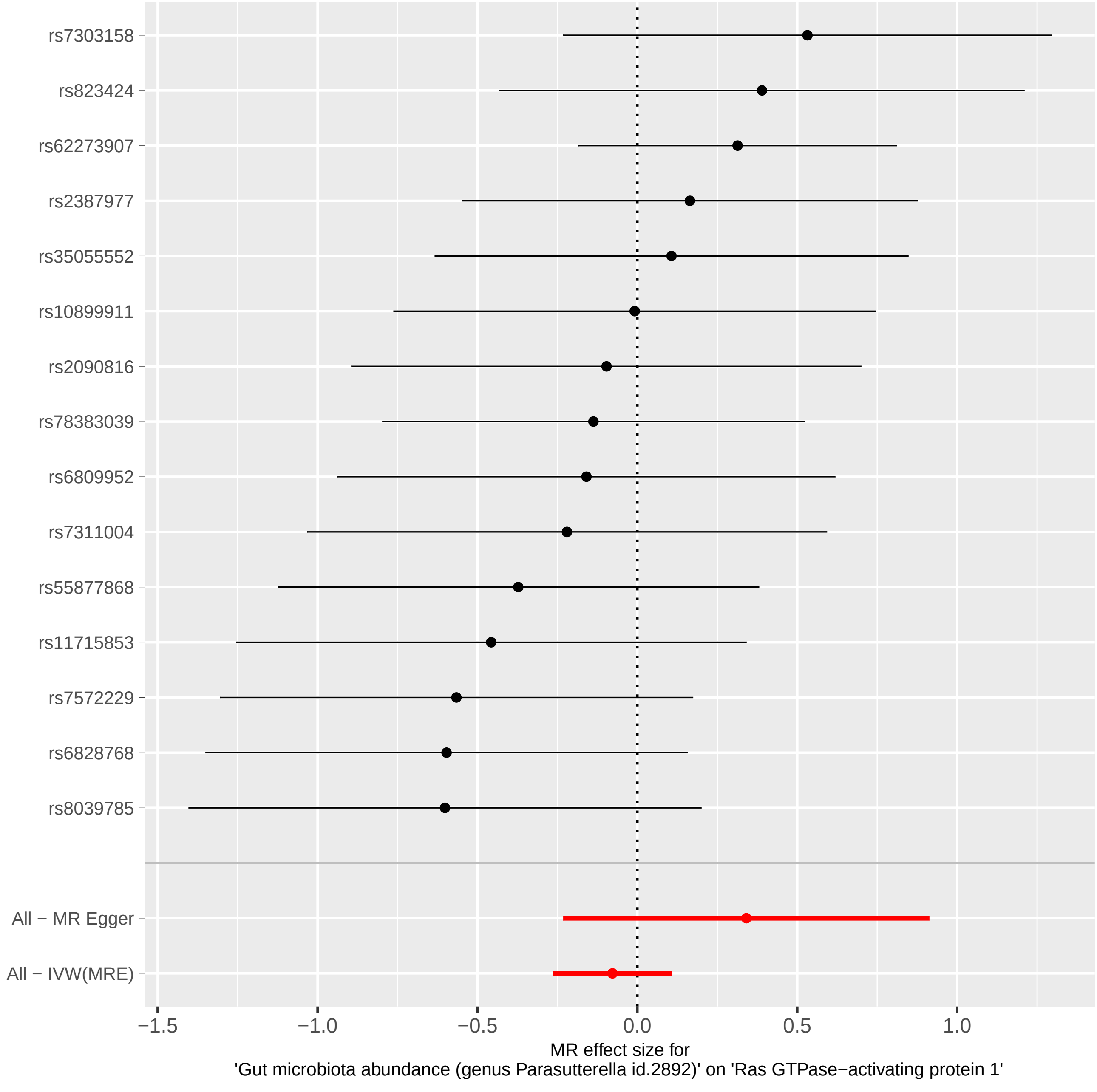

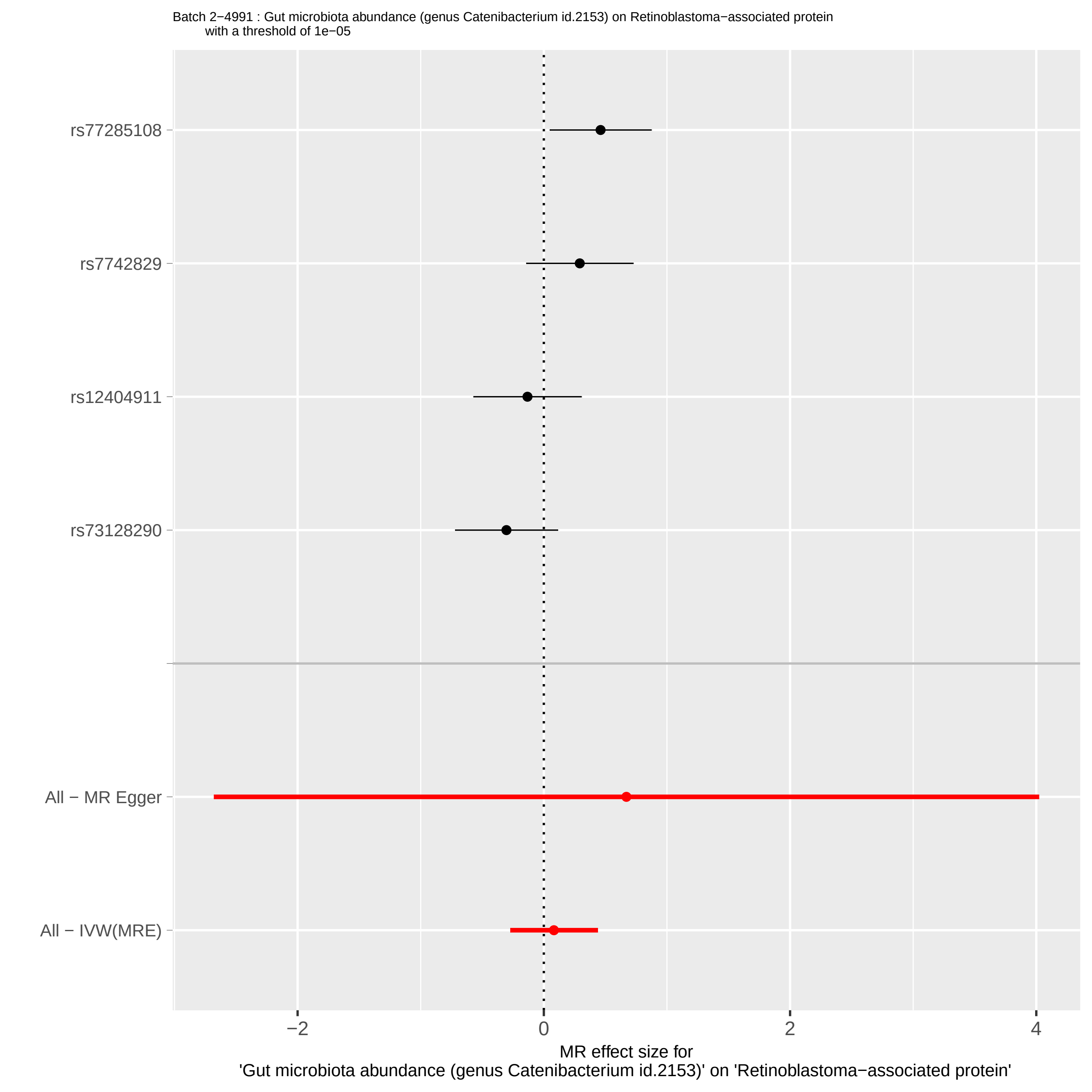
Batch 2−4991 : Gut microbiota abundance (genus Catenibacterium id.2153) on Retinoblastoma−associated protein
with a threshold of 1e−05
rs77285108
rs7742829
rs12404911
rs73128290
All − MR Egger
All − IVW(MRE)
−2
0
2
4
MR effect size for
'Gut microbiota abundance (genus Catenibacterium id.2153)' on 'Retinoblastoma−associated protein'

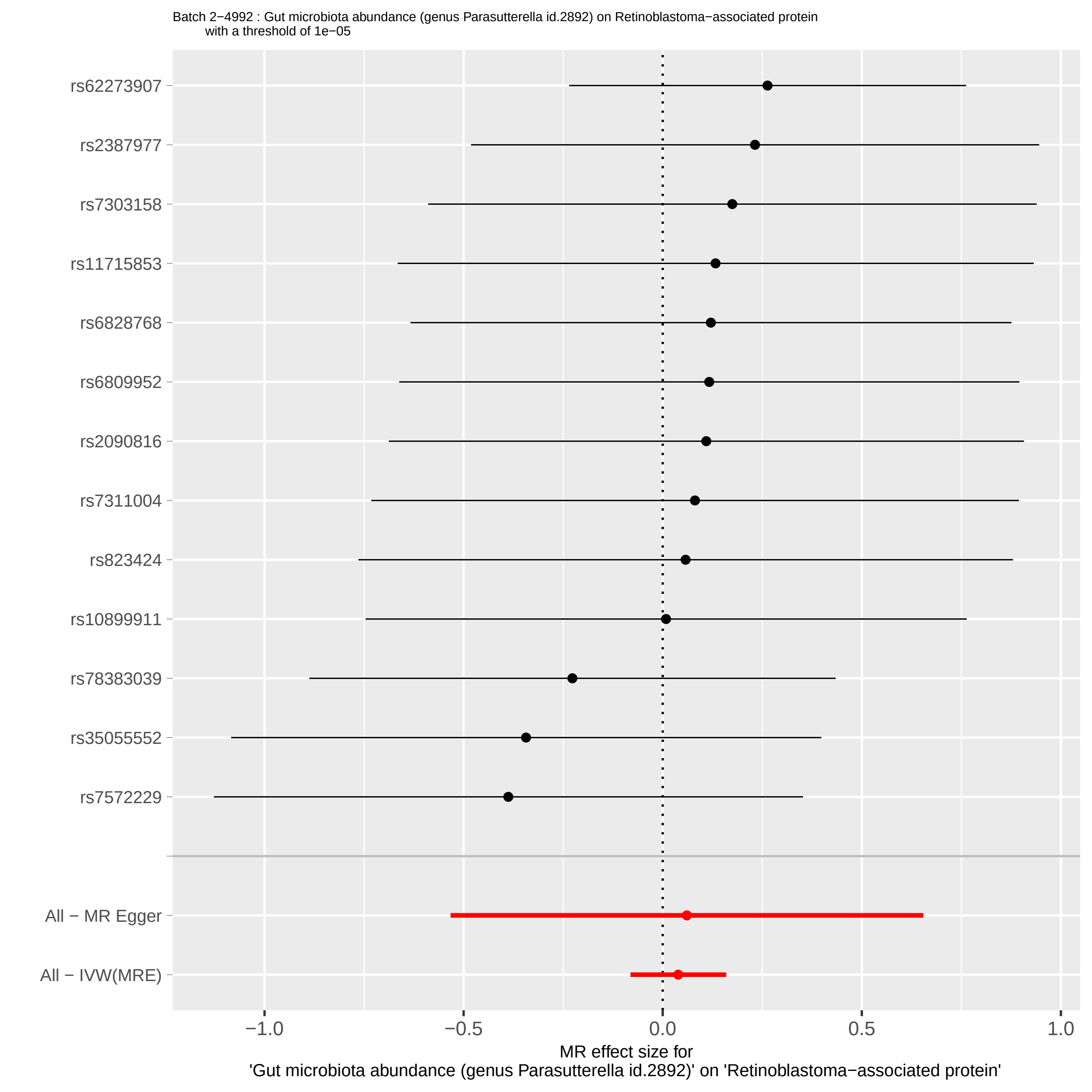
Batch 2−4992 : Gut microbiota abundance (genus Parasutterella id.2892) on Retinoblastoma−associated protein
with a threshold of 1e−05
rs62273907
rs2387977
rs7303158
rs11715853
rs6828768
rs6809952
rs2090816
rs7311004
rs823424
rs10899911
rs78383039
rs35055552
rs7572229
All − MR Egger
All − IVW(MRE)
−1.0
−0.5
0.0
0.5
1.0
MR effect size for
'Gut microbiota abundance (genus Parasutterella id.2892)' on 'Retinoblastoma−associated protein'

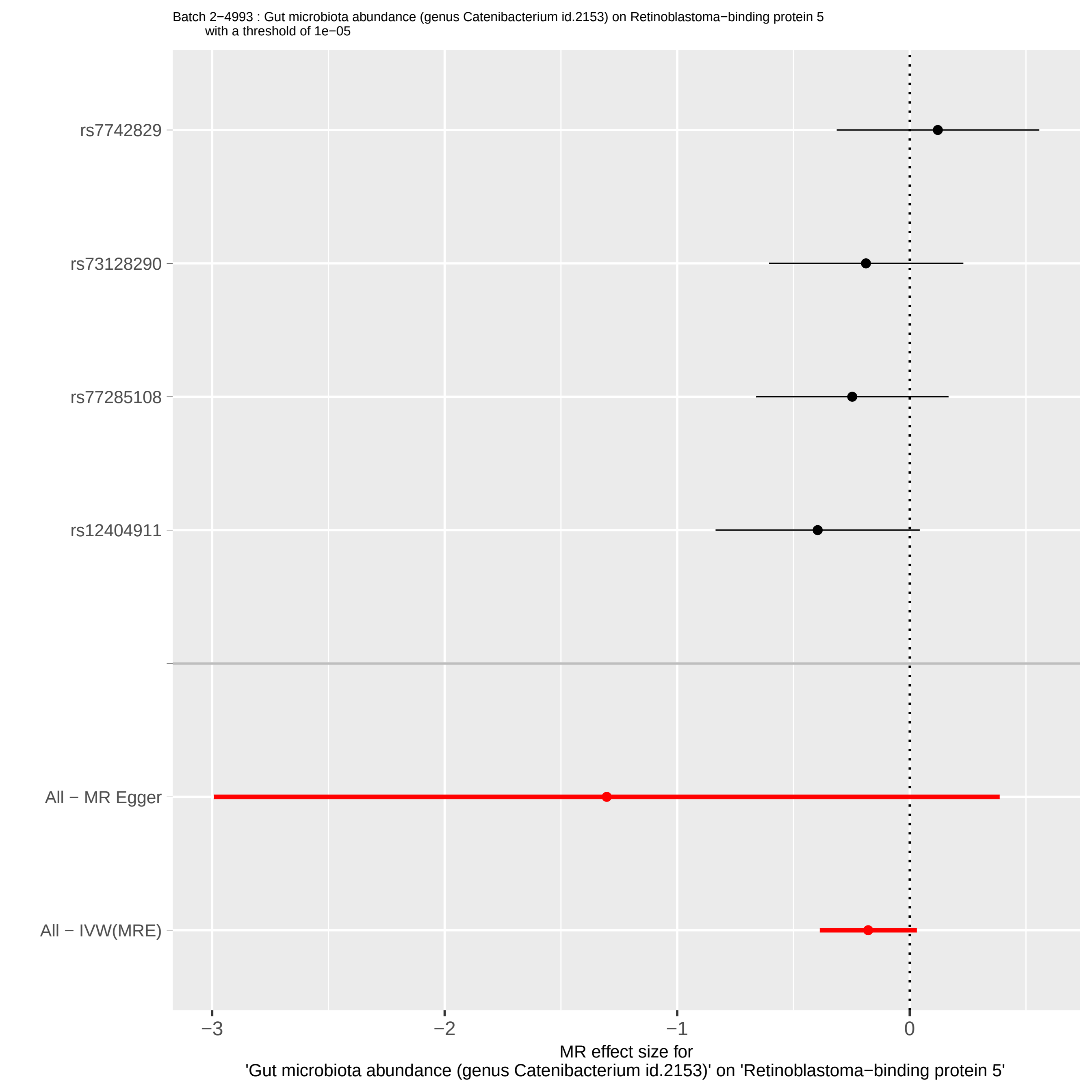

Batch 2−4993 : Gut microbiota abundance (genus Catenibacterium id.2153) on Retinoblastoma−binding protein 5
with a threshold of 1e−05
rs7742829
rs73128290
rs77285108
rs12404911
All − MR Egger
All − IVW(MRE)
−3
−2
−1
0
MR effect size for
'Gut microbiota abundance (genus Catenibacterium id.2153)' on 'Retinoblastoma−binding protein 5'

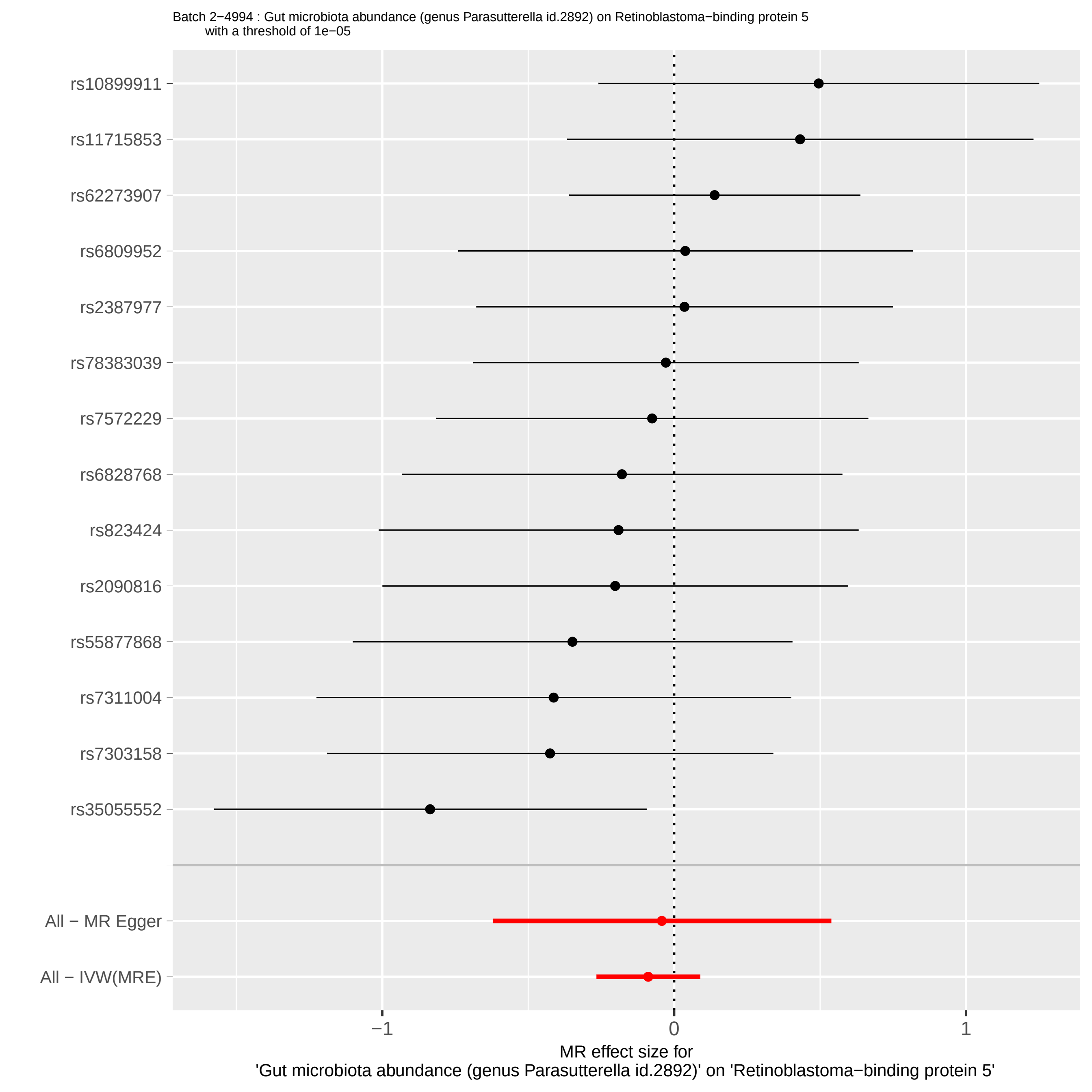

Batch 2−4994 : Gut microbiota abundance (genus Parasutterella id.2892) on Retinoblastoma−binding protein 5
with a threshold of 1e−05
rs10899911
rs11715853
rs62273907
rs6809952
rs2387977
rs78383039
rs7572229
rs6828768
rs823424
rs2090816
rs55877868
rs7311004
rs7303158
rs35055552
All − MR Egger
All − IVW(MRE)
−1
0
1
MR effect size for
'Gut microbiota abundance (genus Parasutterella id.2892)' on 'Retinoblastoma−binding protein 5'

Batch 2−4995 : Gut microbiota abundance (genus Catenibacterium id.2153) on E3 ubiquitin−protein ligase RBBP6
with a threshold of 1e−05

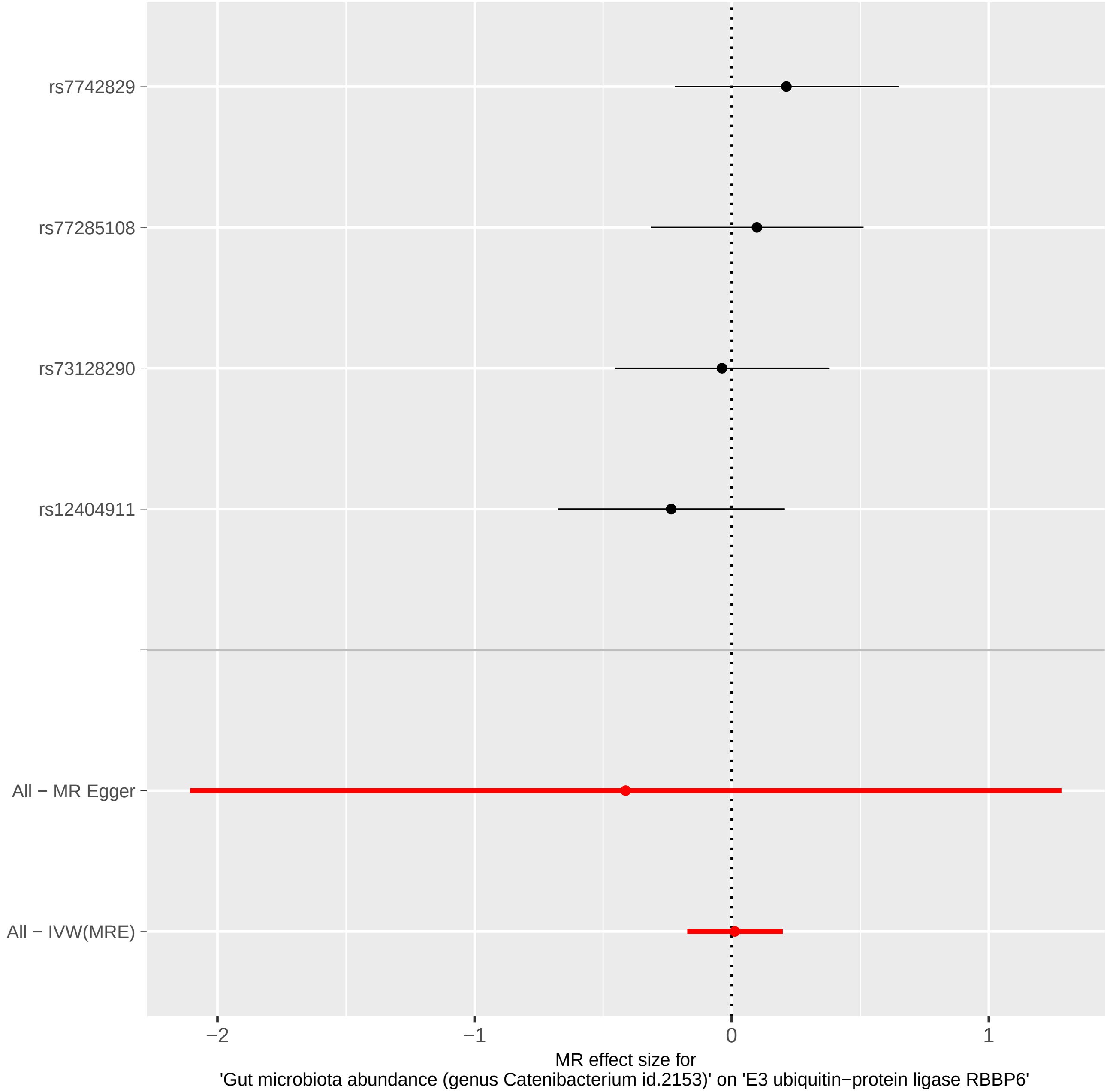

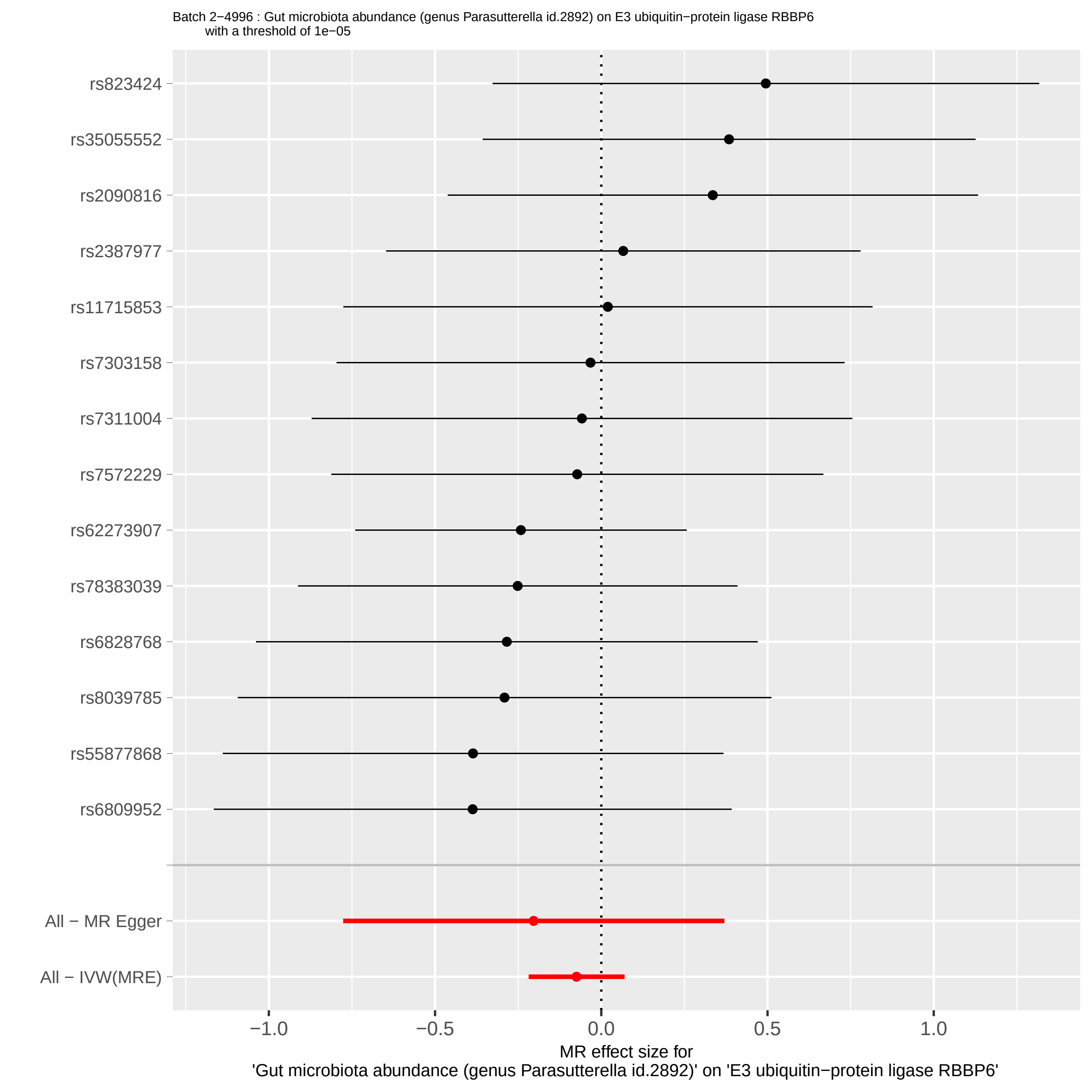
Batch 2−4996 : Gut microbiota abundance (genus Parasutterella id.2892) on E3 ubiquitin−protein ligase RBBP6
with a threshold of 1e−05
rs823424
rs35055552
rs2090816
rs2387977
rs11715853
rs7303158
rs7311004
rs7572229
rs62273907
rs78383039
rs6828768
rs8039785
rs55877868
rs6809952
All − MR Egger
All − IVW(MRE)
−1.0
−0.5
0.0
0.5
1.0
MR effect size for
'Gut microbiota abundance (genus Parasutterella id.2892)' on 'E3 ubiquitin−protein ligase RBBP6'

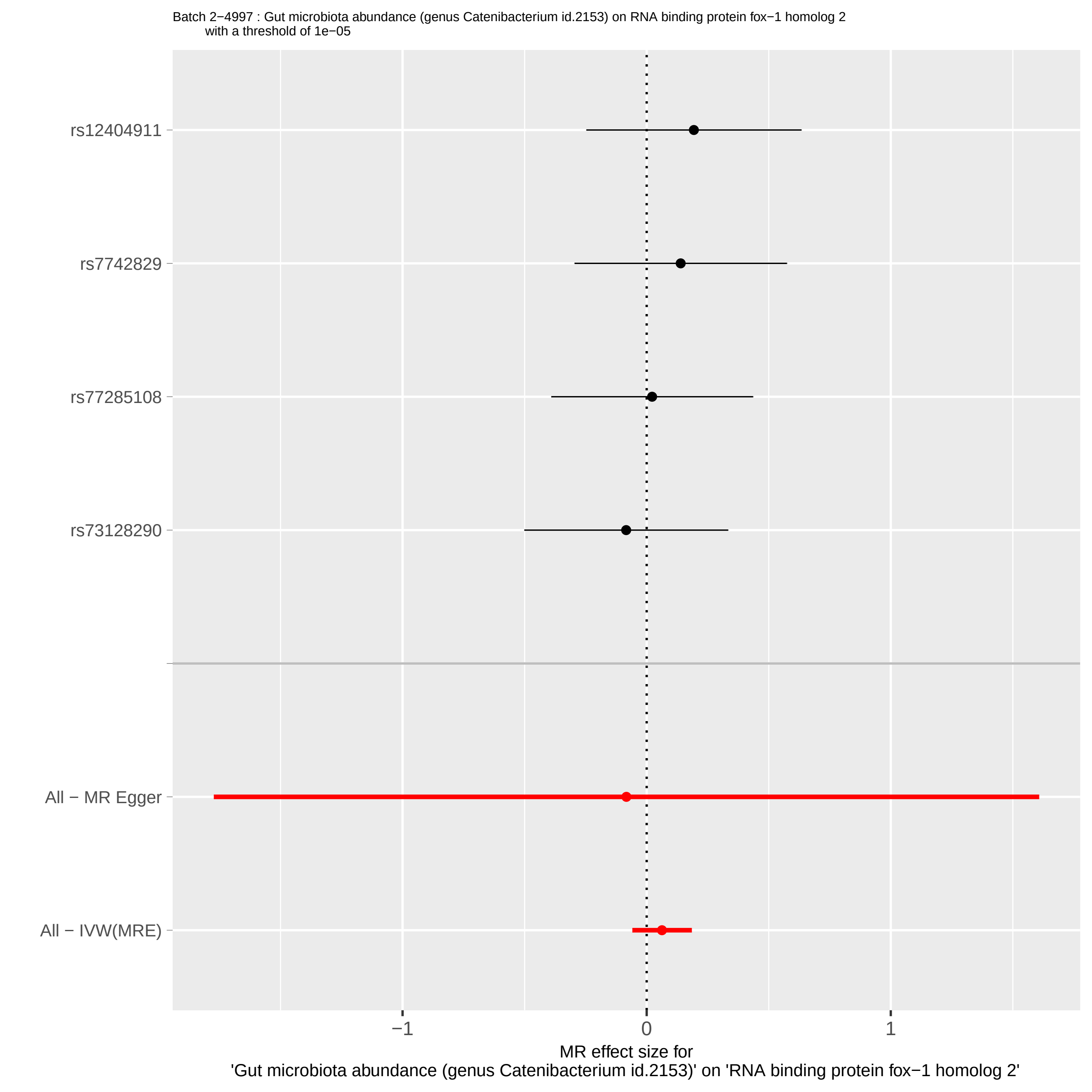

Batch 2−4997 : Gut microbiota abundance (genus Catenibacterium id.2153) on RNA binding protein fox−1 homolog 2
with a threshold of 1e−05
rs12404911
rs7742829
rs77285108
rs73128290
All − MR Egger
All − IVW(MRE)
−1
0
1
MR effect size for
'Gut microbiota abundance (genus Catenibacterium id.2153)' on 'RNA binding protein fox−1 homolog 2'

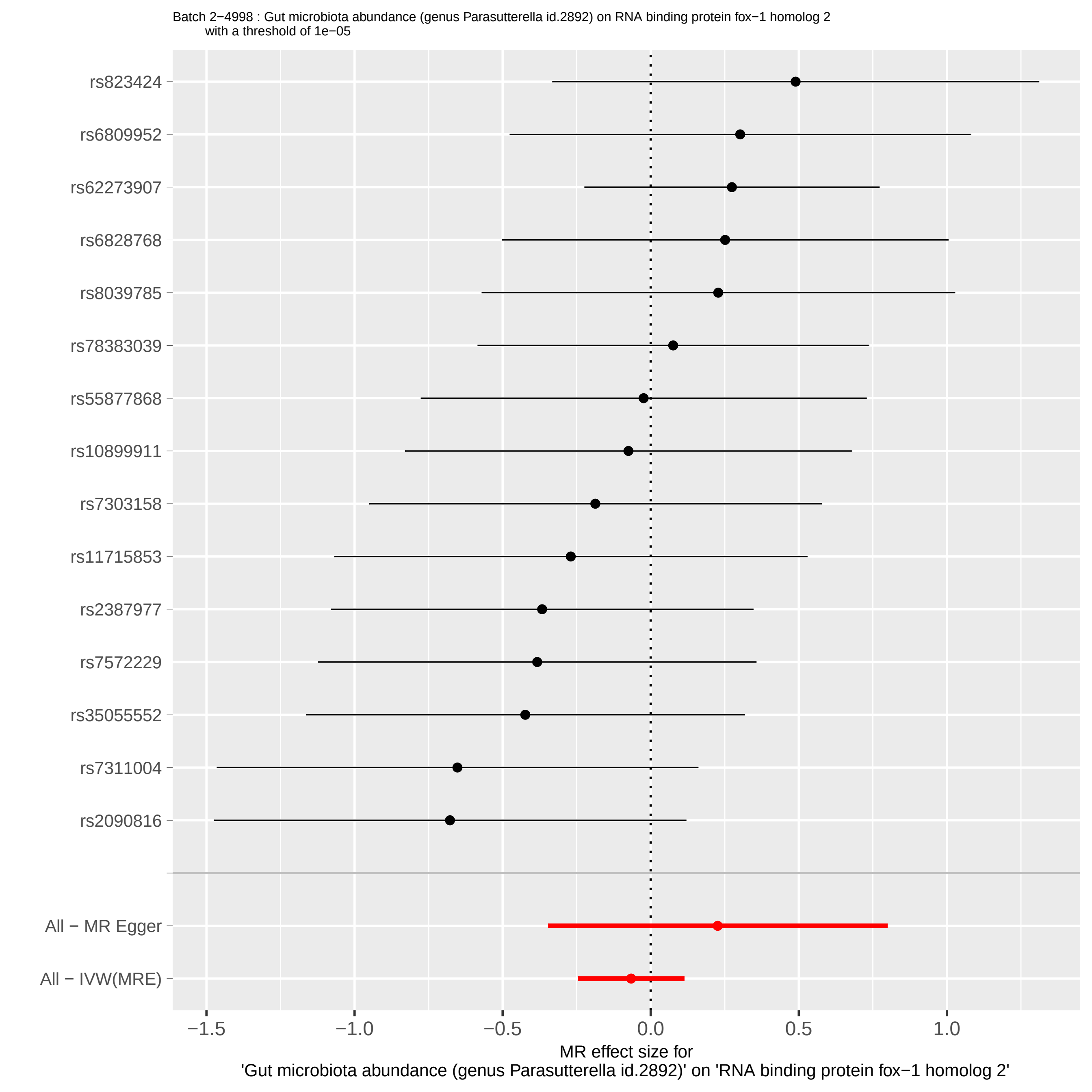

Batch 2−4998 : Gut microbiota abundance (genus Parasutterella id.2892) on RNA binding protein fox−1 homolog 2
with a threshold of 1e−05
rs823424
rs6809952
rs62273907
rs6828768
rs8039785
rs78383039
rs55877868
rs10899911
rs7303158
rs11715853
rs2387977
rs7572229
rs35055552
rs7311004
rs2090816
All − MR Egger
All − IVW(MRE)
−1.5
−1.0
−0.5
0.0
0.5
1.0
MR effect size for
'Gut microbiota abundance (genus Parasutterella id.2892)' on 'RNA binding protein fox−1 homolog 2'

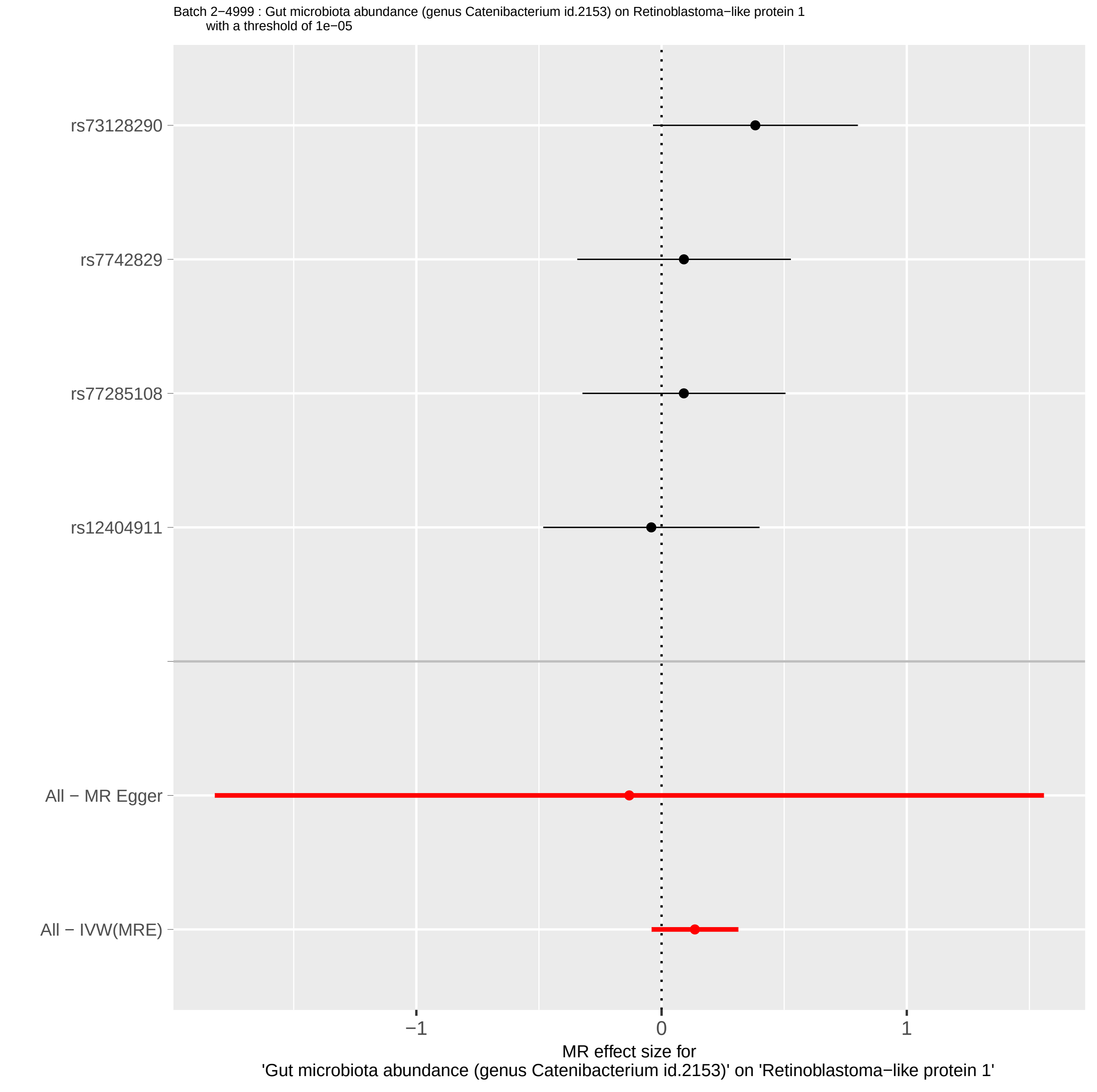

Batch 2−4999 : Gut microbiota abundance (genus Catenibacterium id.2153) on Retinoblastoma−like protein 1
with a threshold of 1e−05
rs73128290
rs7742829
rs77285108
rs12404911
All − MR Egger
All − IVW(MRE)
−1
0
1
MR effect size for
'Gut microbiota abundance (genus Catenibacterium id.2153)' on 'Retinoblastoma−like protein 1'

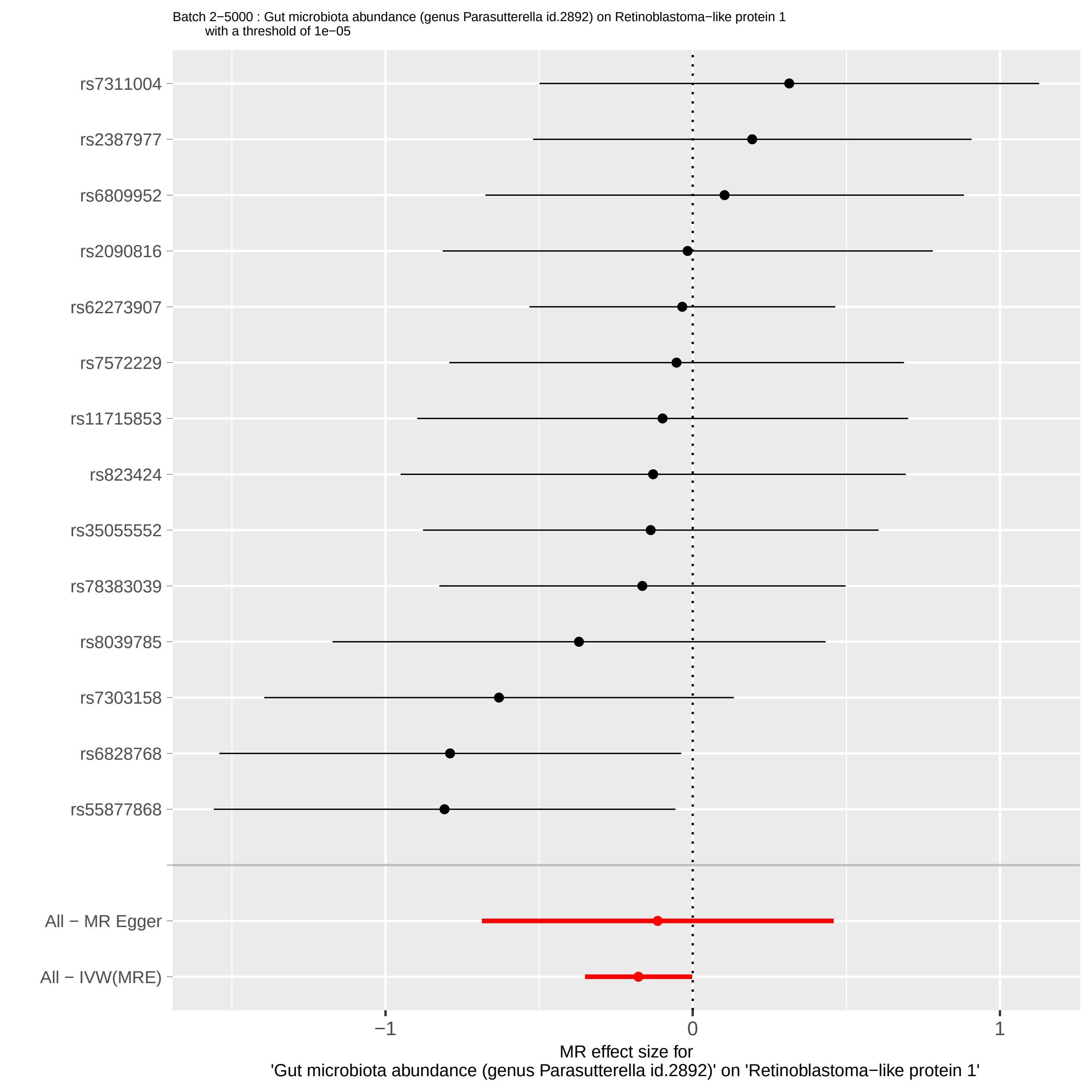

Batch 2−5000 : Gut microbiota abundance (genus Parasutterella id.2892) on Retinoblastoma−like protein 1
with a threshold of 1e−05
rs7311004
rs2387977
rs6809952
rs2090816
rs62273907
rs7572229
rs11715853
rs823424
rs35055552
rs78383039
rs8039785
rs7303158
rs6828768
rs55877868
All − MR Egger
All − IVW(MRE)
−1
0
1
MR effect size for
'Gut microbiota abundance (genus Parasutterella id.2892)' on 'Retinoblastoma−like protein 1'

Batch 2−5001 : Gut microbiota abundance (genus Catenibacterium id.2153) on Probable RNA−binding protein 19
with a threshold of 1e−05

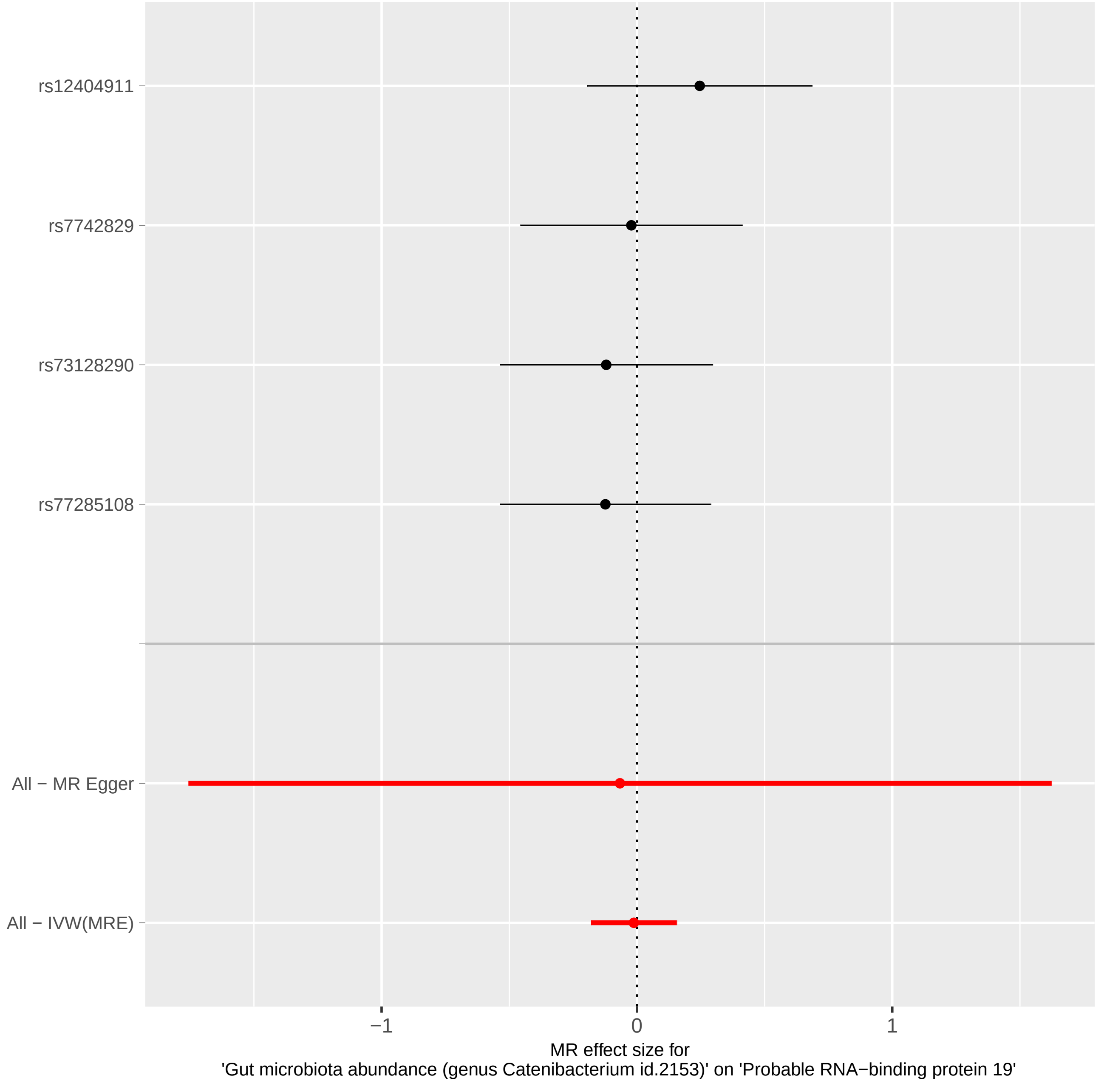

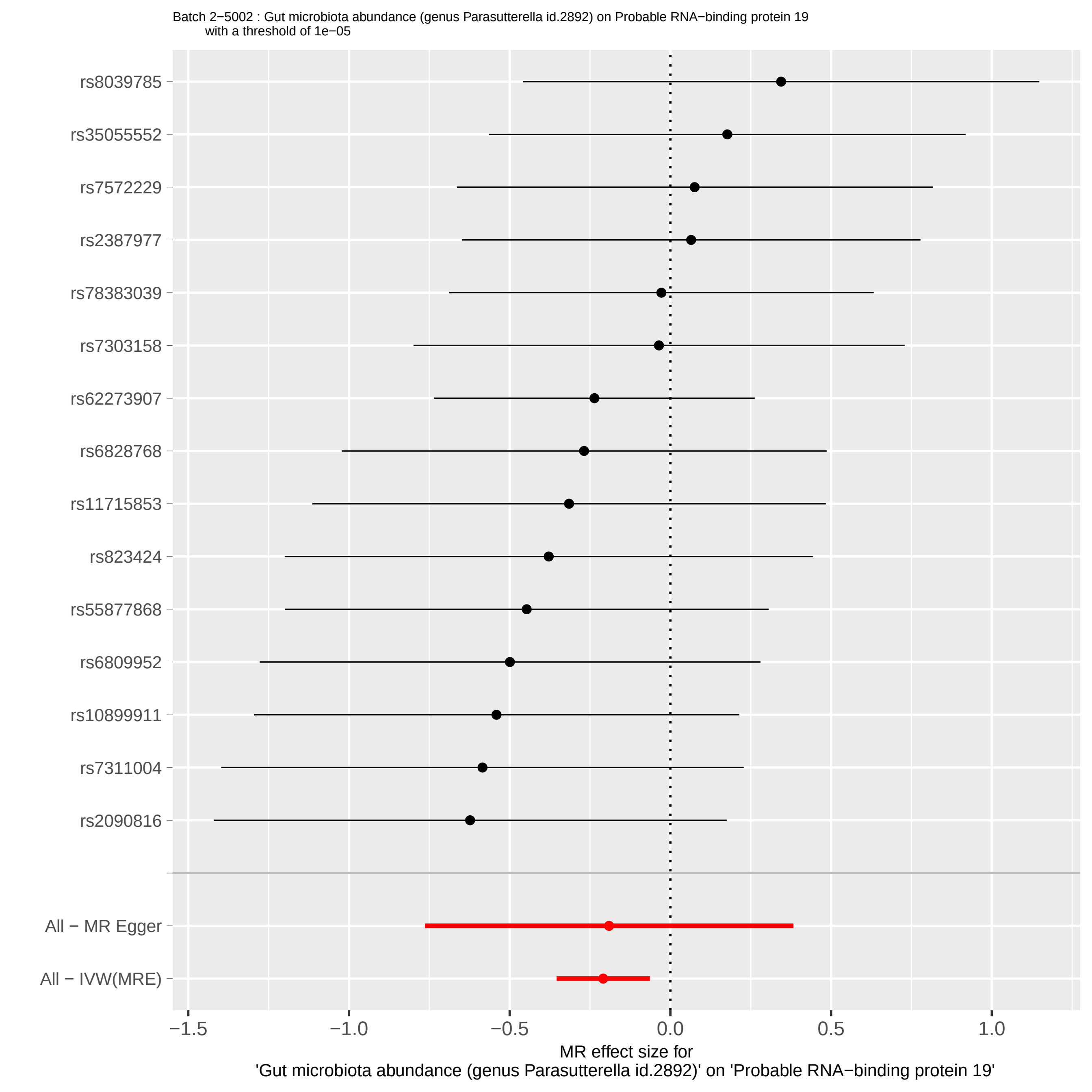
Batch 2−5002 : Gut microbiota abundance (genus Parasutterella id.2892) on Probable RNA−binding protein 19
with a threshold of 1e−05
rs8039785
rs35055552
rs7572229
rs2387977
rs78383039
rs7303158
rs62273907
rs6828768
rs11715853
rs823424
rs55877868
rs6809952
rs10899911
rs7311004
rs2090816
All − MR Egger
All − IVW(MRE)
−1.5
−1.0
−0.5
0.0
0.5
1.0
MR effect size for
'Gut microbiota abundance (genus Parasutterella id.2892)' on 'Probable RNA−binding protein 19'

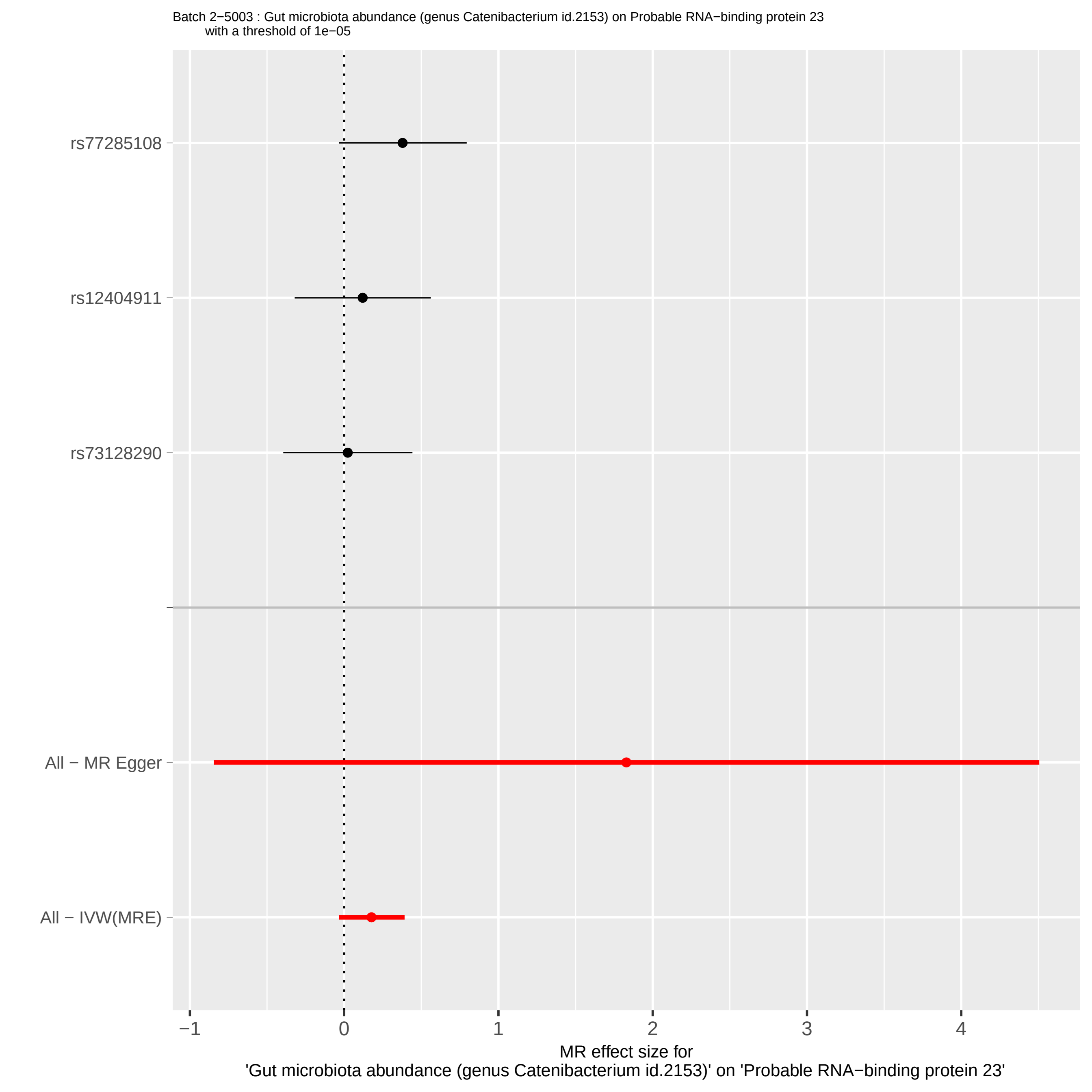

Batch 2−5003 : Gut microbiota abundance (genus Catenibacterium id.2153) on Probable RNA−binding protein 23
with a threshold of 1e−05
rs77285108
rs12404911
rs73128290
All − MR Egger
All − IVW(MRE)
−1
0
1
2
3
4
MR effect size for
'Gut microbiota abundance (genus Catenibacterium id.2153)' on 'Probable RNA−binding protein 23'

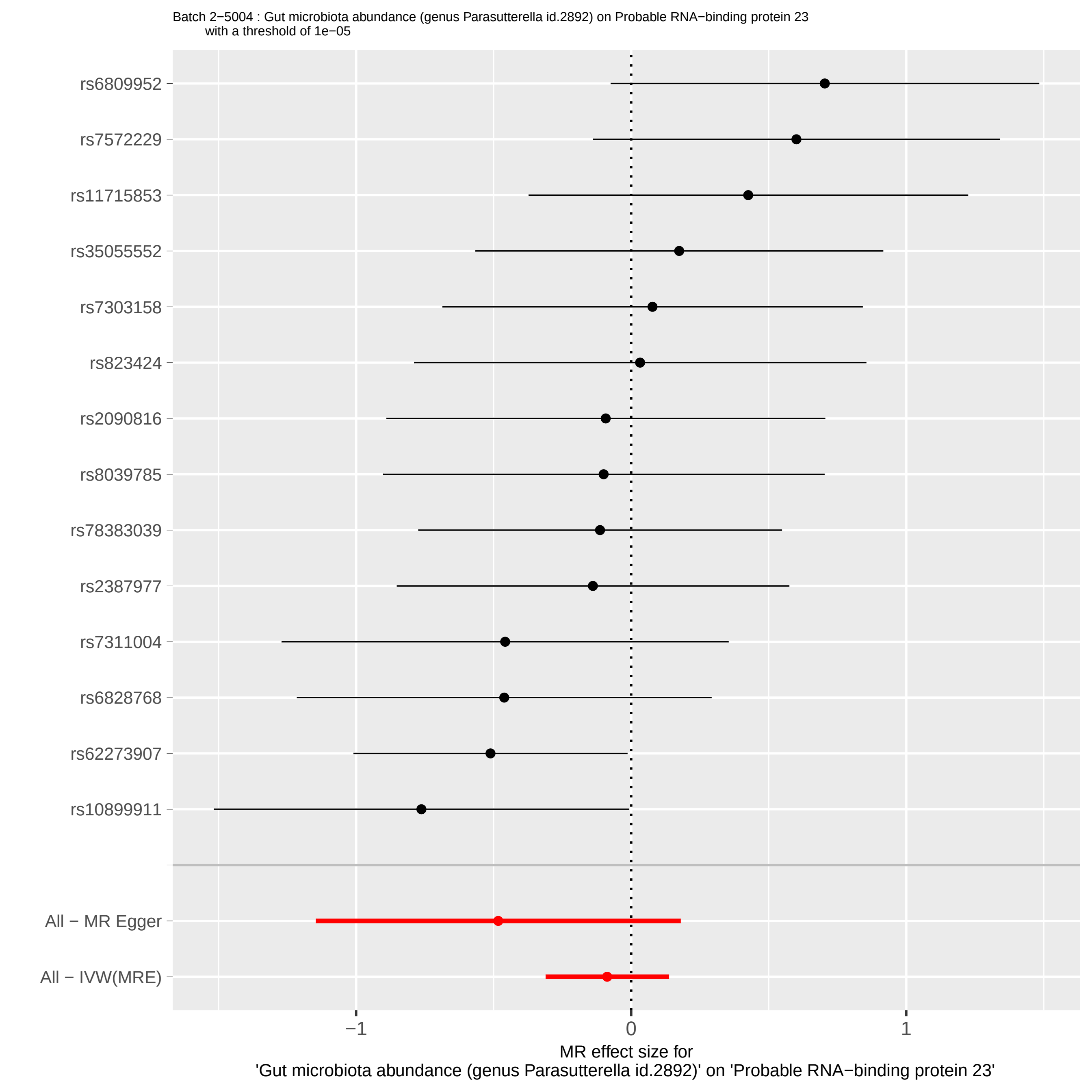
Batch 2−5004 : Gut microbiota abundance (genus Parasutterella id.2892) on Probable RNA−binding protein 23
with a threshold of 1e−05
rs6809952
rs7572229
rs11715853
rs35055552
rs7303158
rs823424
rs2090816
rs8039785
rs78383039
rs2387977
rs7311004
rs6828768
rs62273907
rs10899911
All − MR Egger
All − IVW(MRE)
−1
0
1
MR effect size for
'Gut microbiota abundance (genus Parasutterella id.2892)' on 'Probable RNA−binding protein 23'

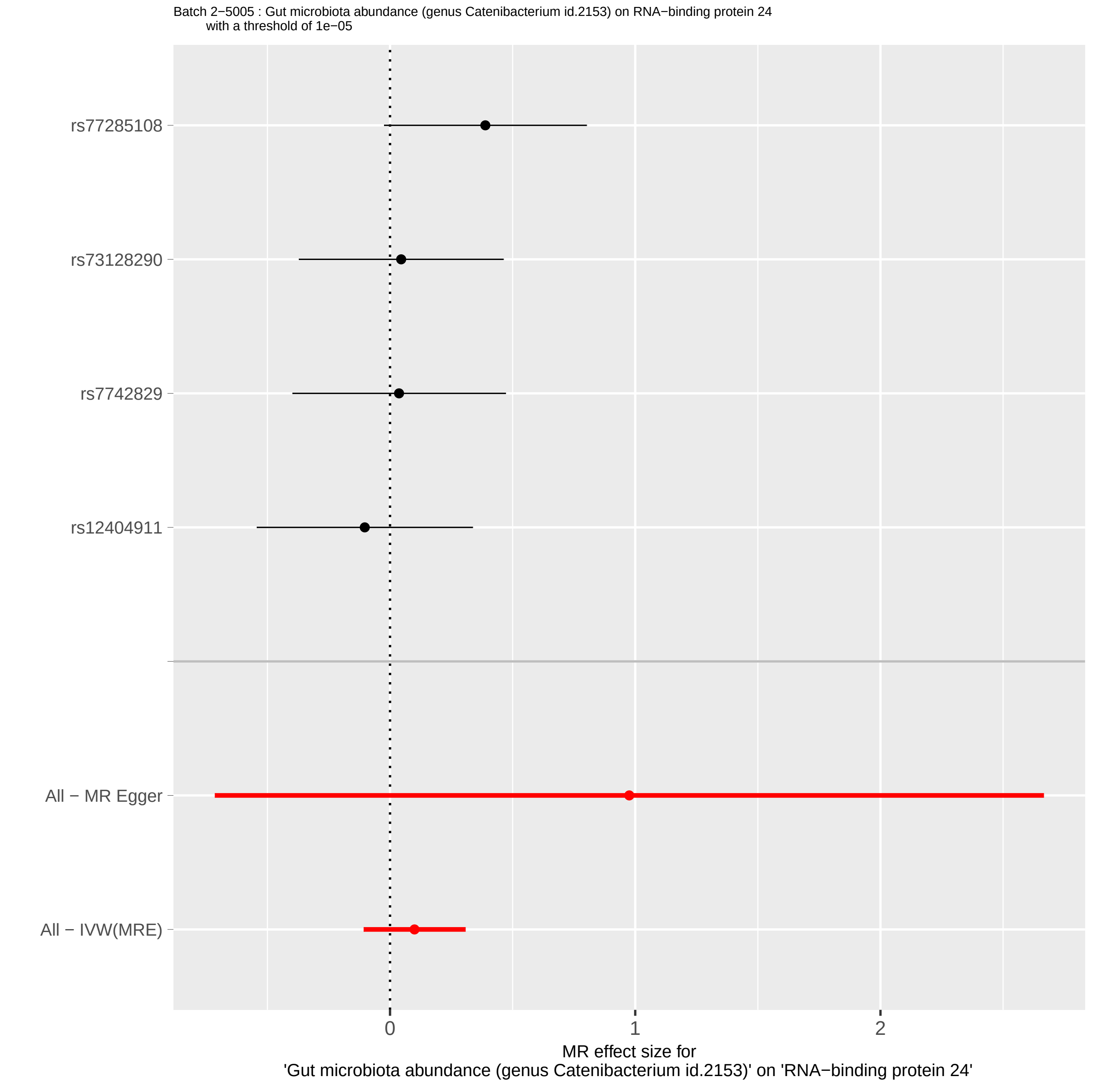
Batch 2−5005 : Gut microbiota abundance (genus Catenibacterium id.2153) on RNA−binding protein 24
with a threshold of 1e−05
rs77285108
rs73128290
rs7742829
rs12404911
All − MR Egger
All − IVW(MRE)
0
1
2
MR effect size for
'Gut microbiota abundance (genus Catenibacterium id.2153)' on 'RNA−binding protein 24'

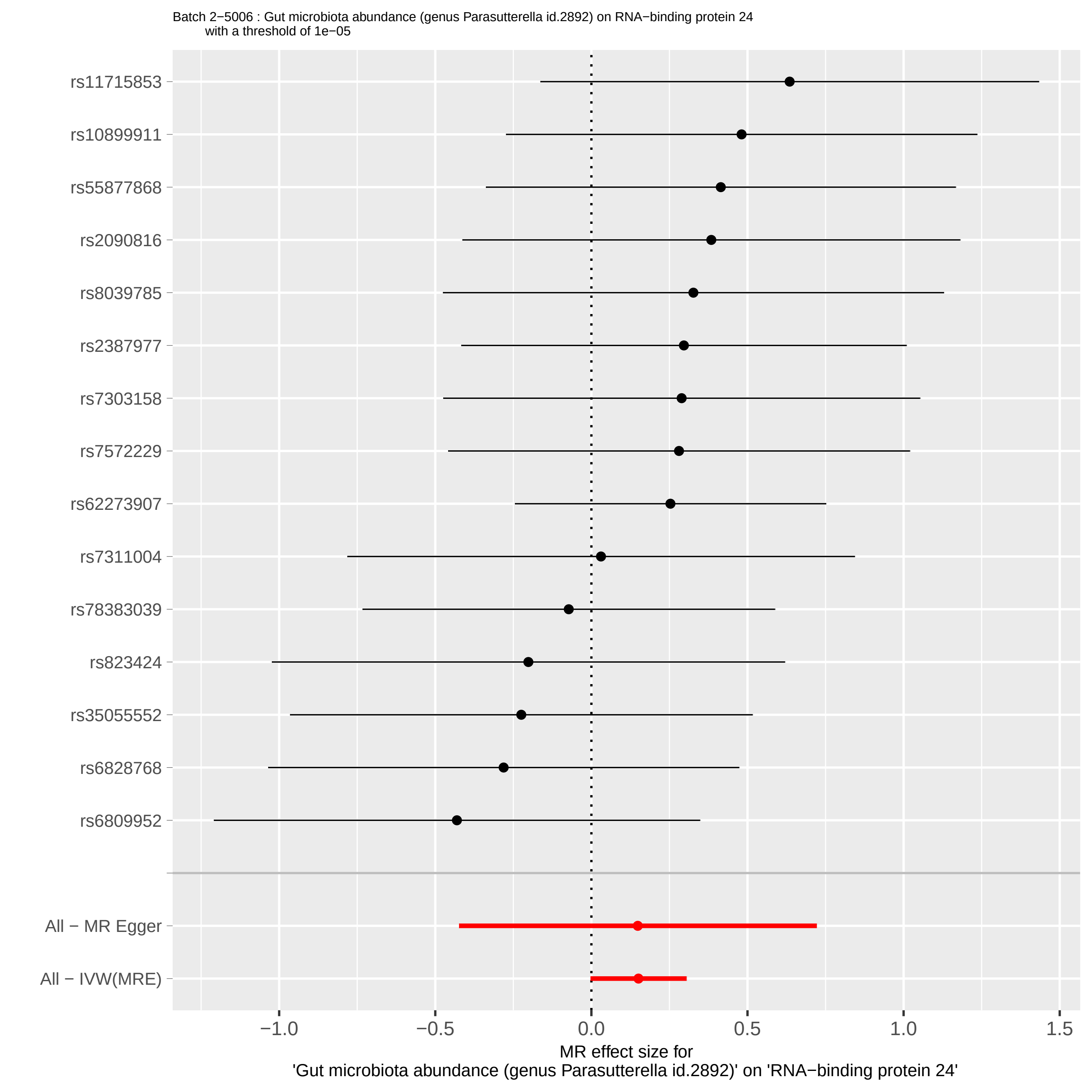

Batch 2−5006 : Gut microbiota abundance (genus Parasutterella id.2892) on RNA−binding protein 24
with a threshold of 1e−05
rs11715853
rs10899911
rs55877868
rs2090816
rs8039785
rs2387977
rs7303158
rs7572229
rs62273907
rs7311004
rs78383039
rs823424
rs35055552
rs6828768
rs6809952
All − MR Egger
All − IVW(MRE)
−1.0
−0.5
0.0
0.5
1.0
1.5
MR effect size for
'Gut microbiota abundance (genus Parasutterella id.2892)' on 'RNA−binding protein 24'

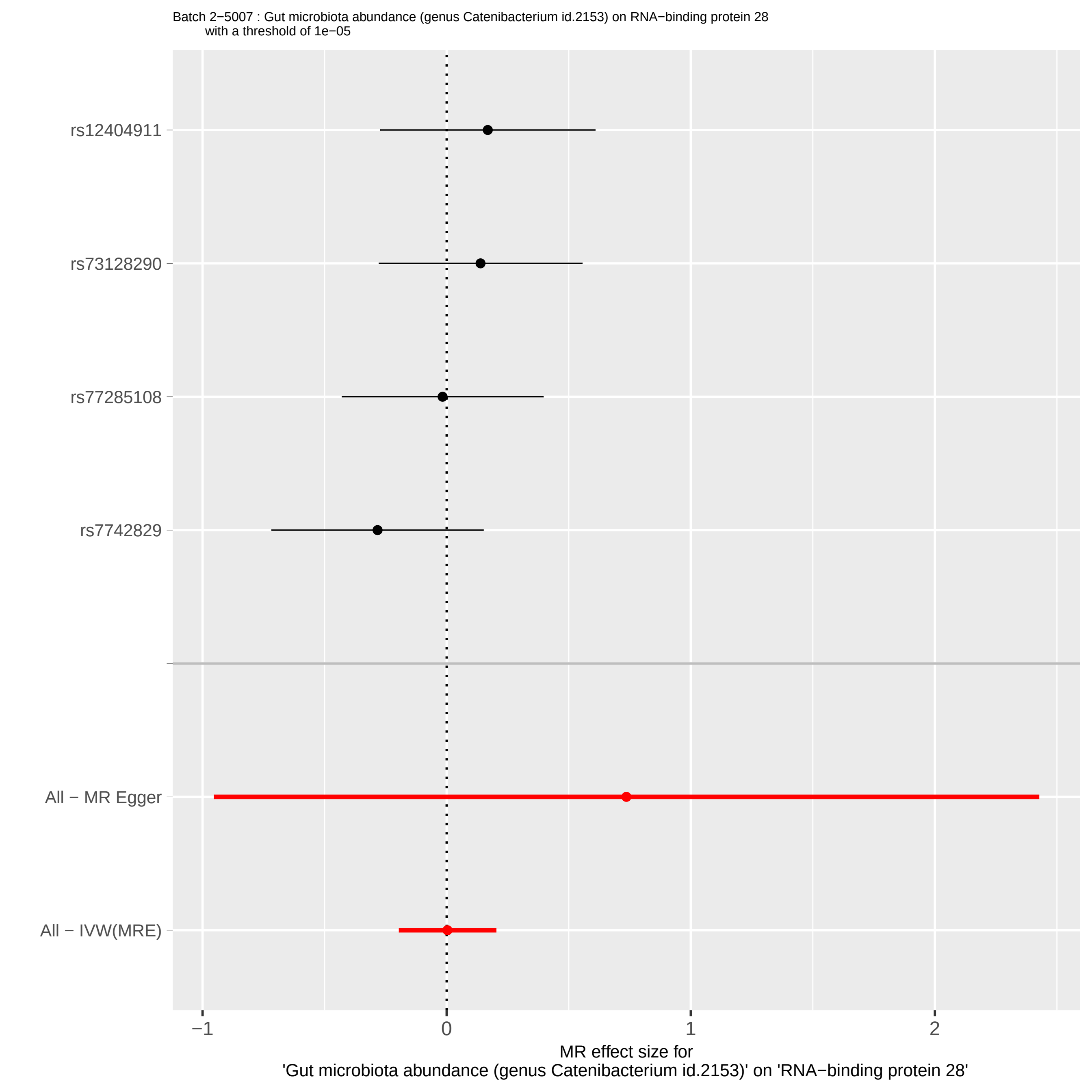

Batch 2−5007 : Gut microbiota abundance (genus Catenibacterium id.2153) on RNA−binding protein 28
with a threshold of 1e−05
rs12404911
rs73128290
rs77285108
rs7742829
All − MR Egger
All − IVW(MRE)
−1
0
1
2
MR effect size for
'Gut microbiota abundance (genus Catenibacterium id.2153)' on 'RNA−binding protein 28'

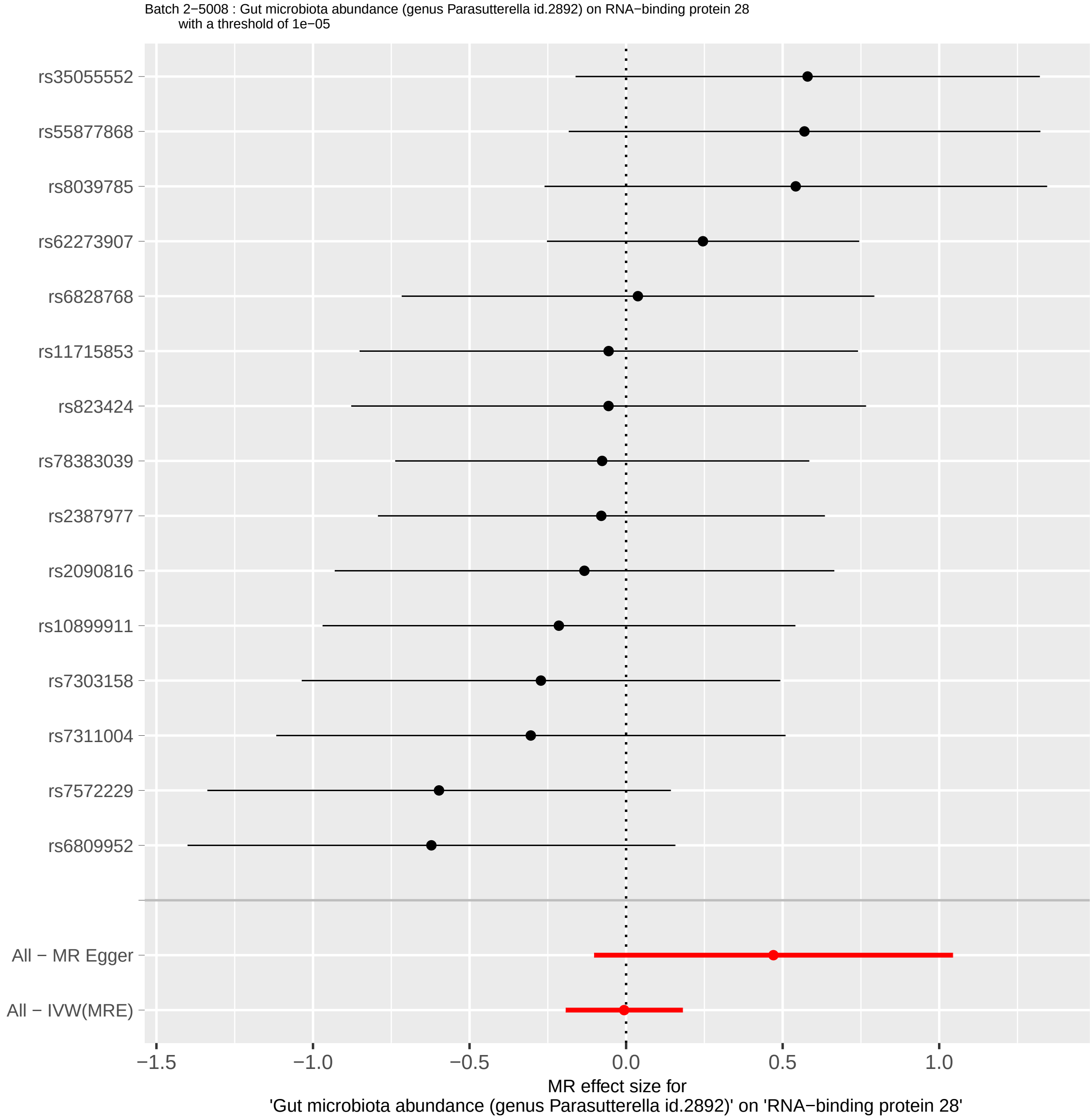
Batch 2−5008 : Gut microbiota abundance (genus Parasutterella id.2892) on RNA−binding protein 28
with a threshold of 1e−05
rs35055552
rs55877868
rs8039785
rs62273907
rs6828768
rs11715853
rs823424
rs78383039
rs2387977
rs2090816
rs10899911
rs7303158
rs7311004
rs7572229
rs6809952
All − MR Egger
All − IVW(MRE)
−1.5
−1.0
−0.5
0.0
0.5
1.0
MR effect size for
'Gut microbiota abundance (genus Parasutterella id.2892)' on 'RNA−binding protein 28'

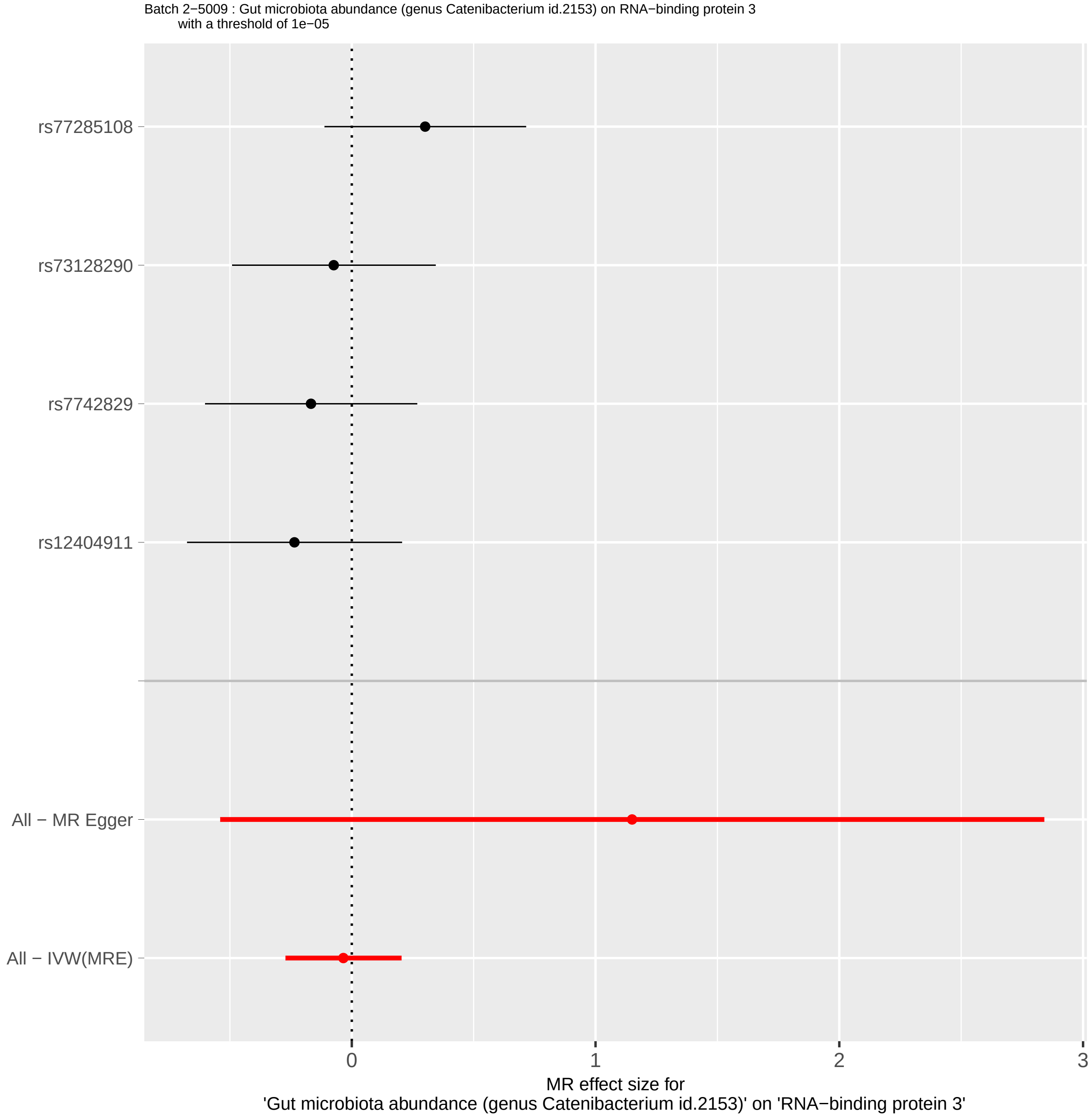

Batch 2−5009 : Gut microbiota abundance (genus Catenibacterium id.2153) on RNA−binding protein 3
with a threshold of 1e−05
rs77285108
rs73128290
rs7742829
rs12404911
All − MR Egger
All − IVW(MRE)
0
1
2
3
MR effect size for
'Gut microbiota abundance (genus Catenibacterium id.2153)' on 'RNA−binding protein 3'

Batch 2−5010 : Gut microbiota abundance (genus Parasutterella id.2892) on RNA−binding protein 3
with a threshold of 1e−05

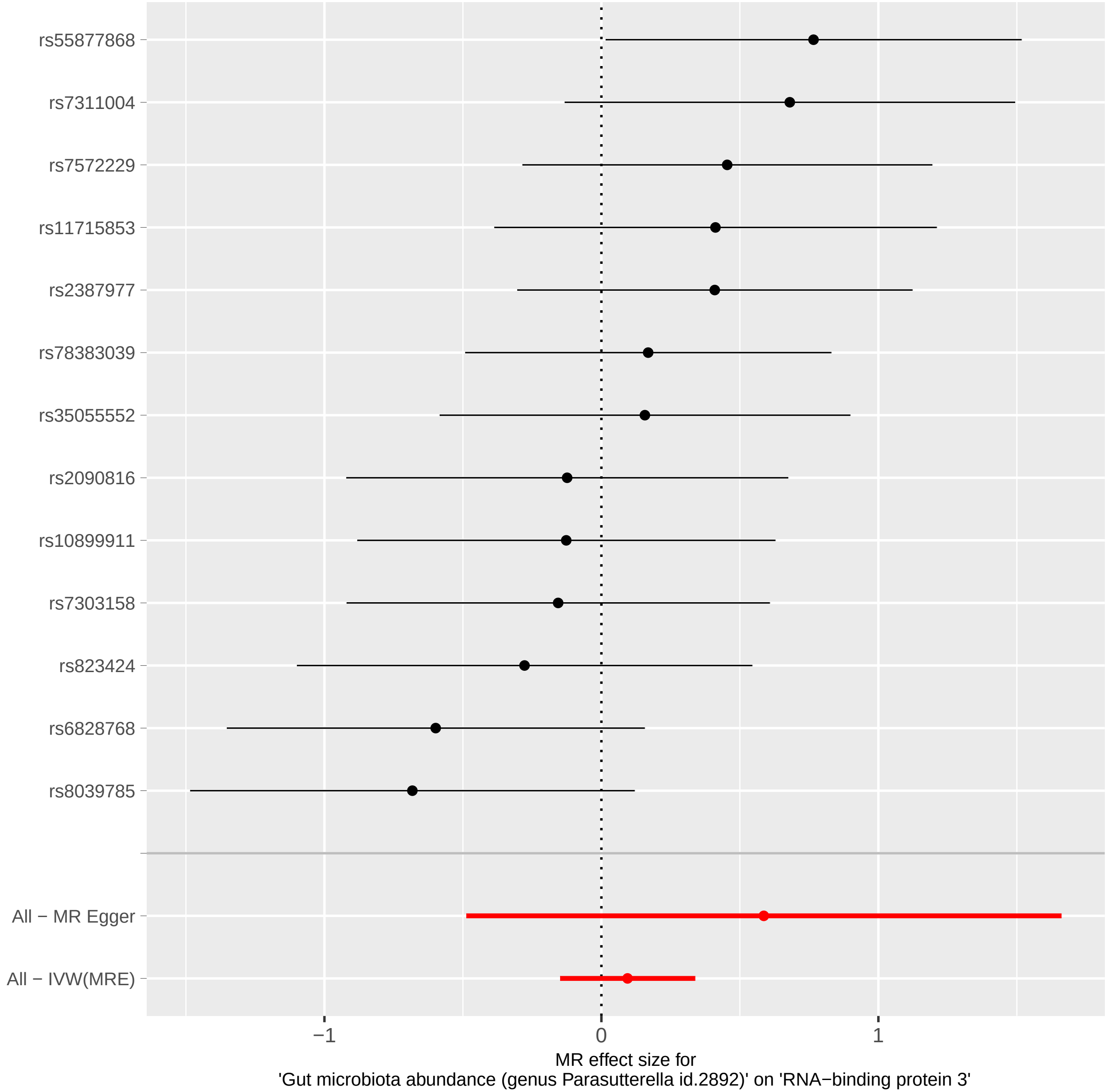

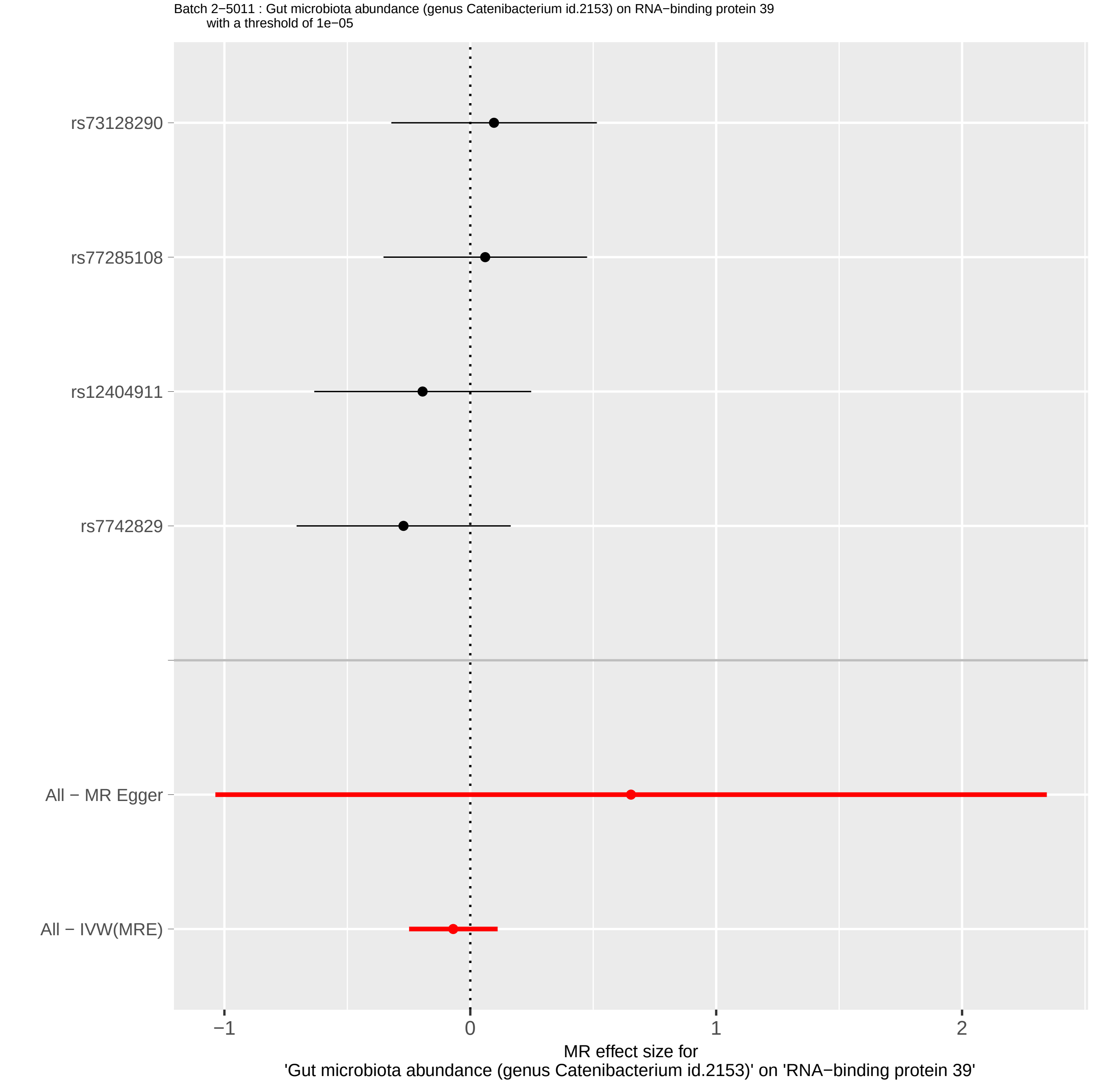

Batch 2−5011 : Gut microbiota abundance (genus Catenibacterium id.2153) on RNA−binding protein 39
with a threshold of 1e−05
rs73128290
rs77285108
rs12404911
rs7742829
All − MR Egger
All − IVW(MRE)
−1
0
1
2
MR effect size for
'Gut microbiota abundance (genus Catenibacterium id.2153)' on 'RNA−binding protein 39'

Batch 2−5012 : Gut microbiota abundance (genus Parasutterella id.2892) on RNA−binding protein 39
with a threshold of 1e−05

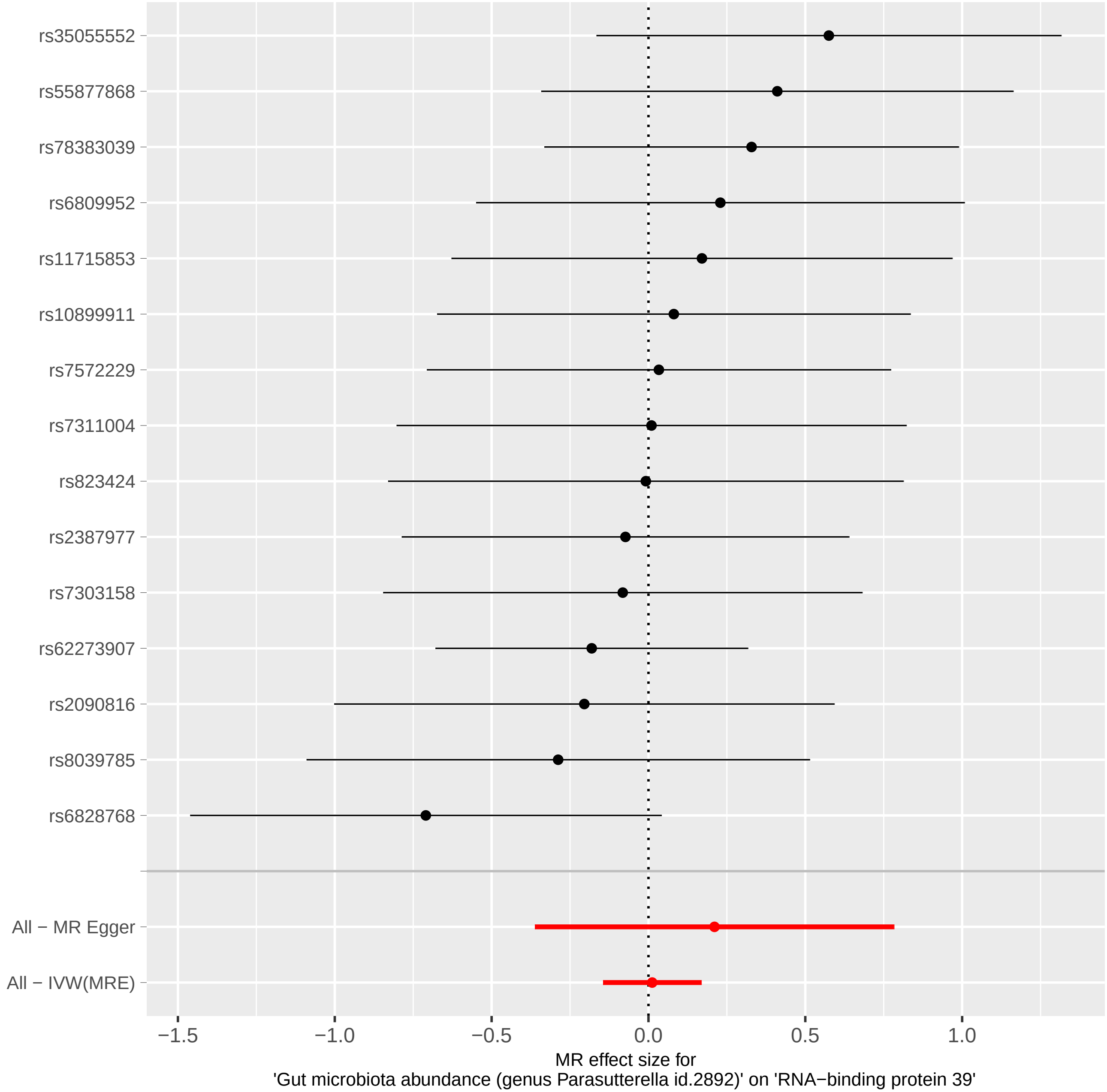

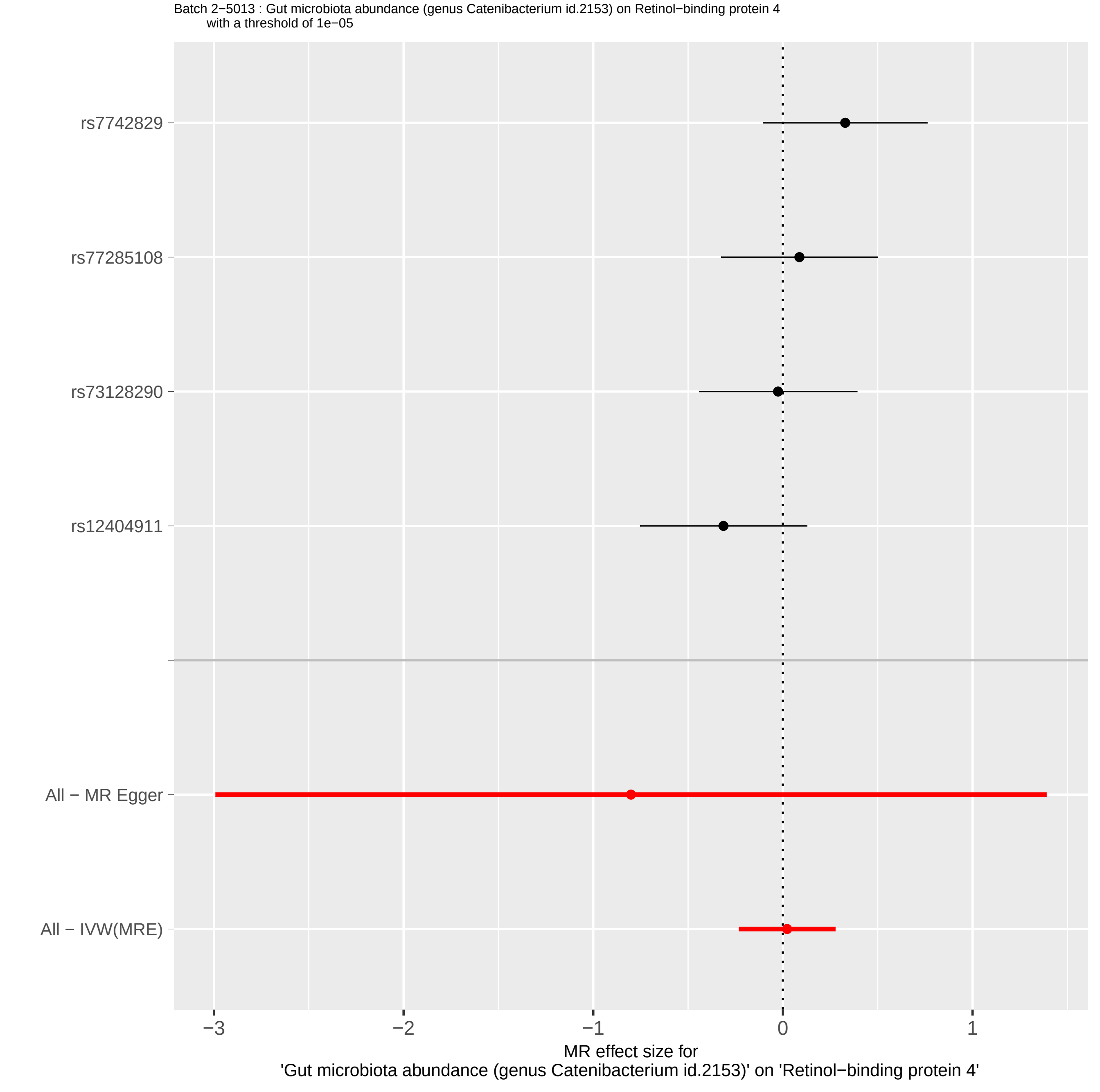
Batch 2−5013 : Gut microbiota abundance (genus Catenibacterium id.2153) on Retinol−binding protein 4
with a threshold of 1e−05
rs7742829
rs77285108
rs73128290
rs12404911
All − MR Egger
All − IVW(MRE)
−3
−2
−1
0
1
MR effect size for
'Gut microbiota abundance (genus Catenibacterium id.2153)' on 'Retinol−binding protein 4'

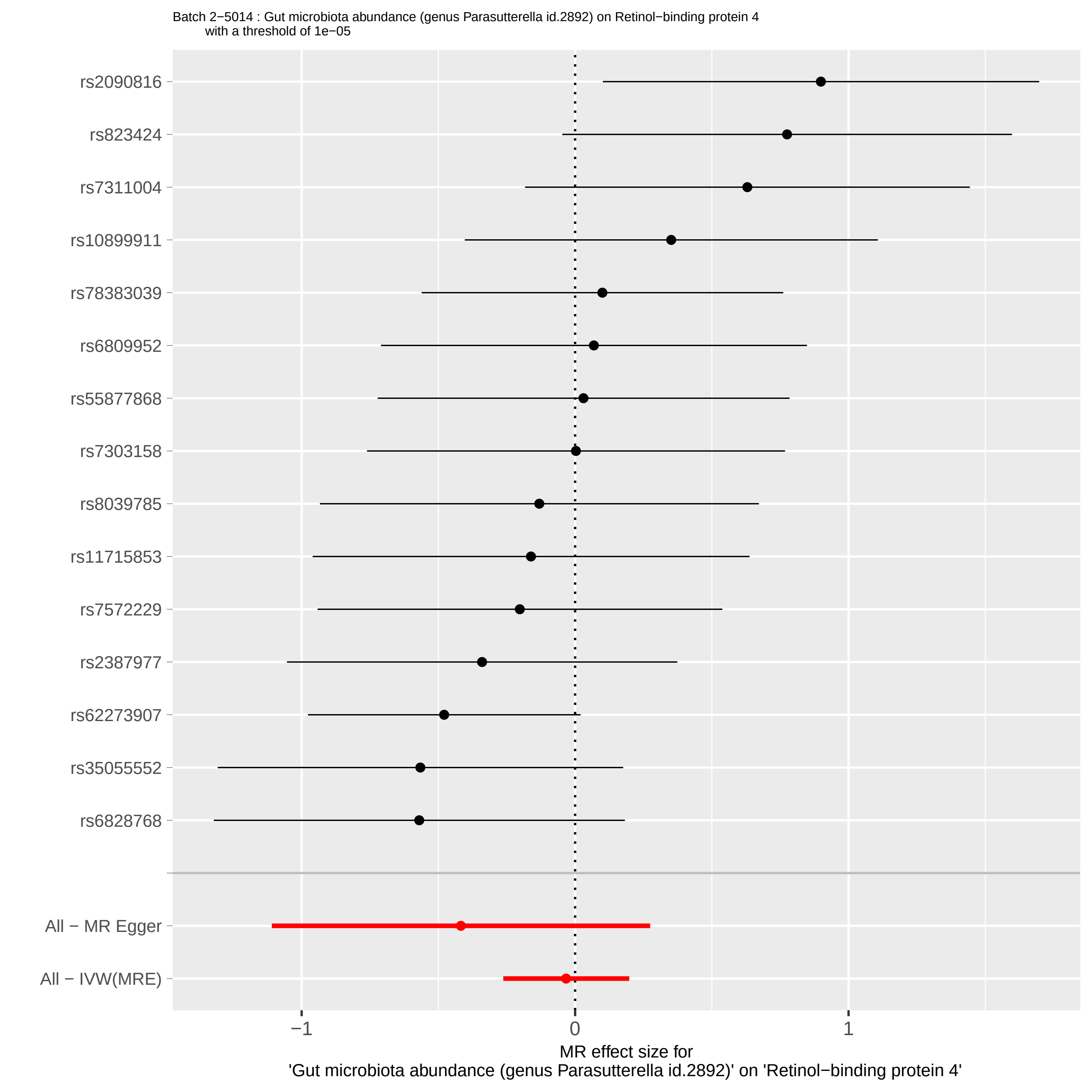
Batch 2−5014 : Gut microbiota abundance (genus Parasutterella id.2892) on Retinol−binding protein 4
with a threshold of 1e−05
rs2090816
rs823424
rs7311004
rs10899911
rs78383039
rs6809952
rs55877868
rs7303158
rs8039785
rs11715853
rs7572229
rs2387977
rs62273907
rs35055552
rs6828768
All − MR Egger
All − IVW(MRE)
−1
0
1
MR effect size for
'Gut microbiota abundance (genus Parasutterella id.2892)' on 'Retinol−binding protein 4'

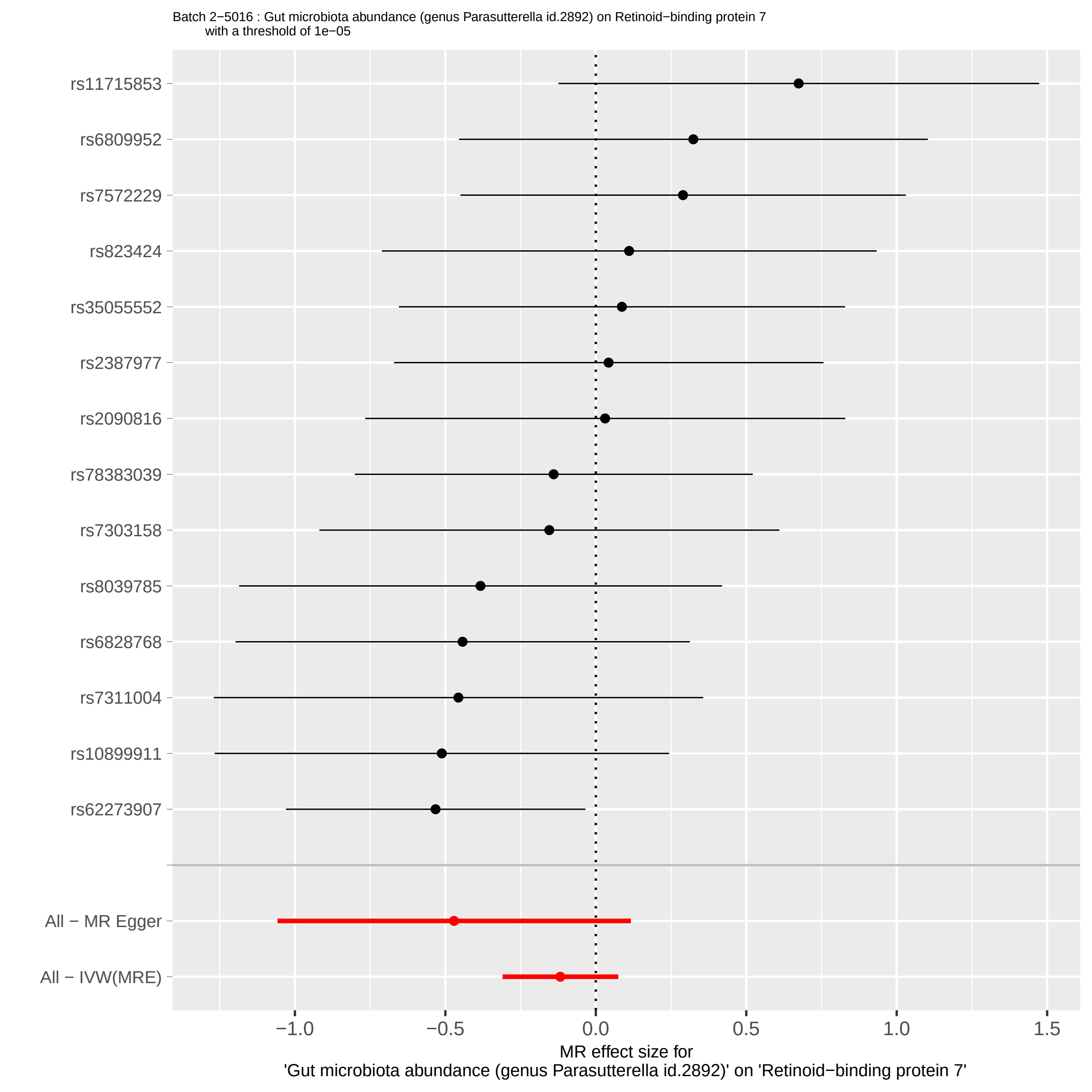

Batch 2−5016 : Gut microbiota abundance (genus Parasutterella id.2892) on Retinoid−binding protein 7
with a threshold of 1e−05
rs11715853
rs6809952
rs7572229
rs823424
rs35055552
rs2387977
rs2090816
rs78383039
rs7303158
rs8039785
rs6828768
rs7311004
rs10899911
rs62273907
All − MR Egger
All − IVW(MRE)
−1.0
−0.5
0.0
0.5
1.0
1.5
MR effect size for
'Gut microbiota abundance (genus Parasutterella id.2892)' on 'Retinoid−binding protein 7'

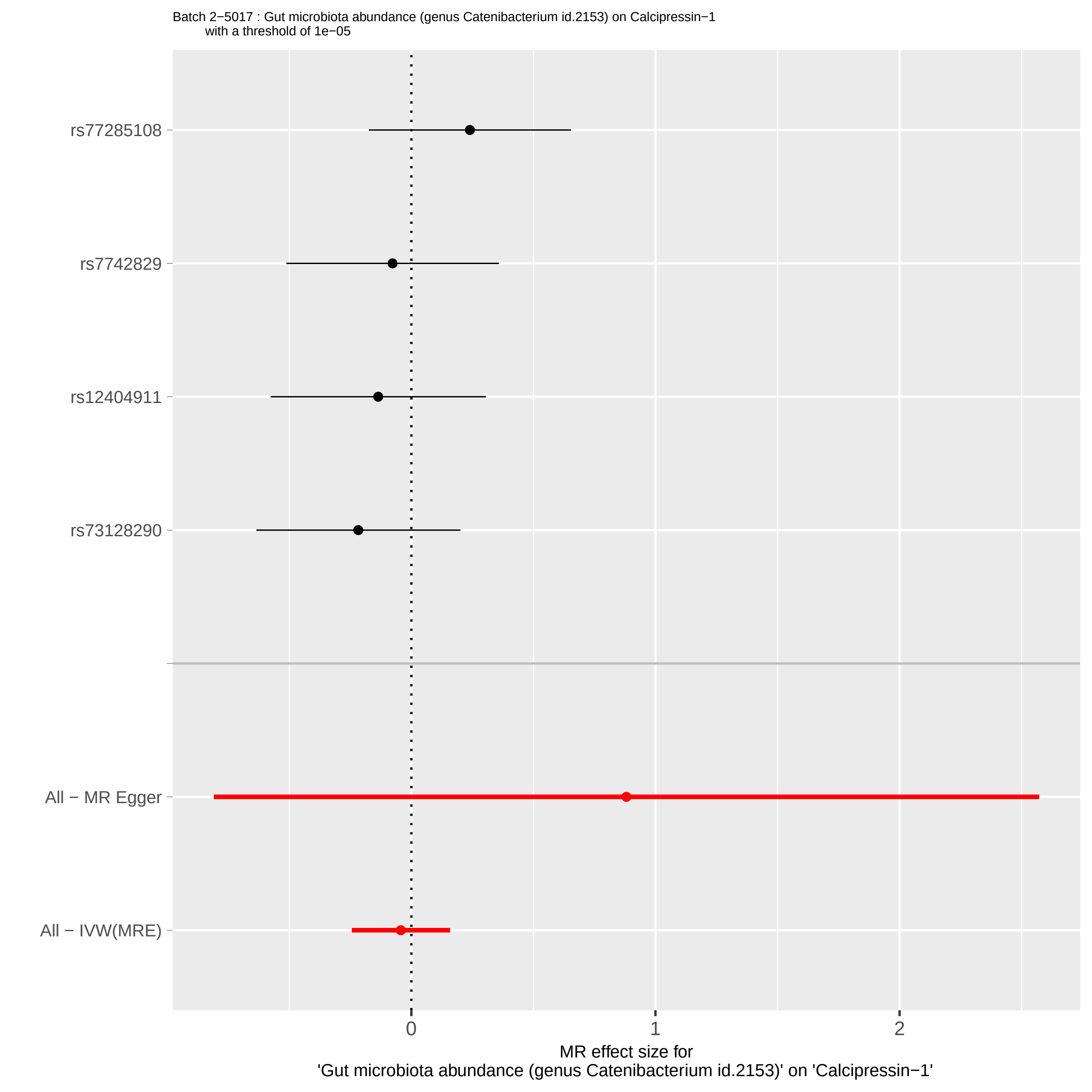
Batch 2−5017 : Gut microbiota abundance (genus Catenibacterium id.2153) on Calcipressin−1
with a threshold of 1e−05
rs77285108
rs7742829
rs12404911
rs73128290
All − MR Egger
All − IVW(MRE)
0
1
2
MR effect size for
'Gut microbiota abundance (genus Catenibacterium id.2153)' on 'Calcipressin−1'

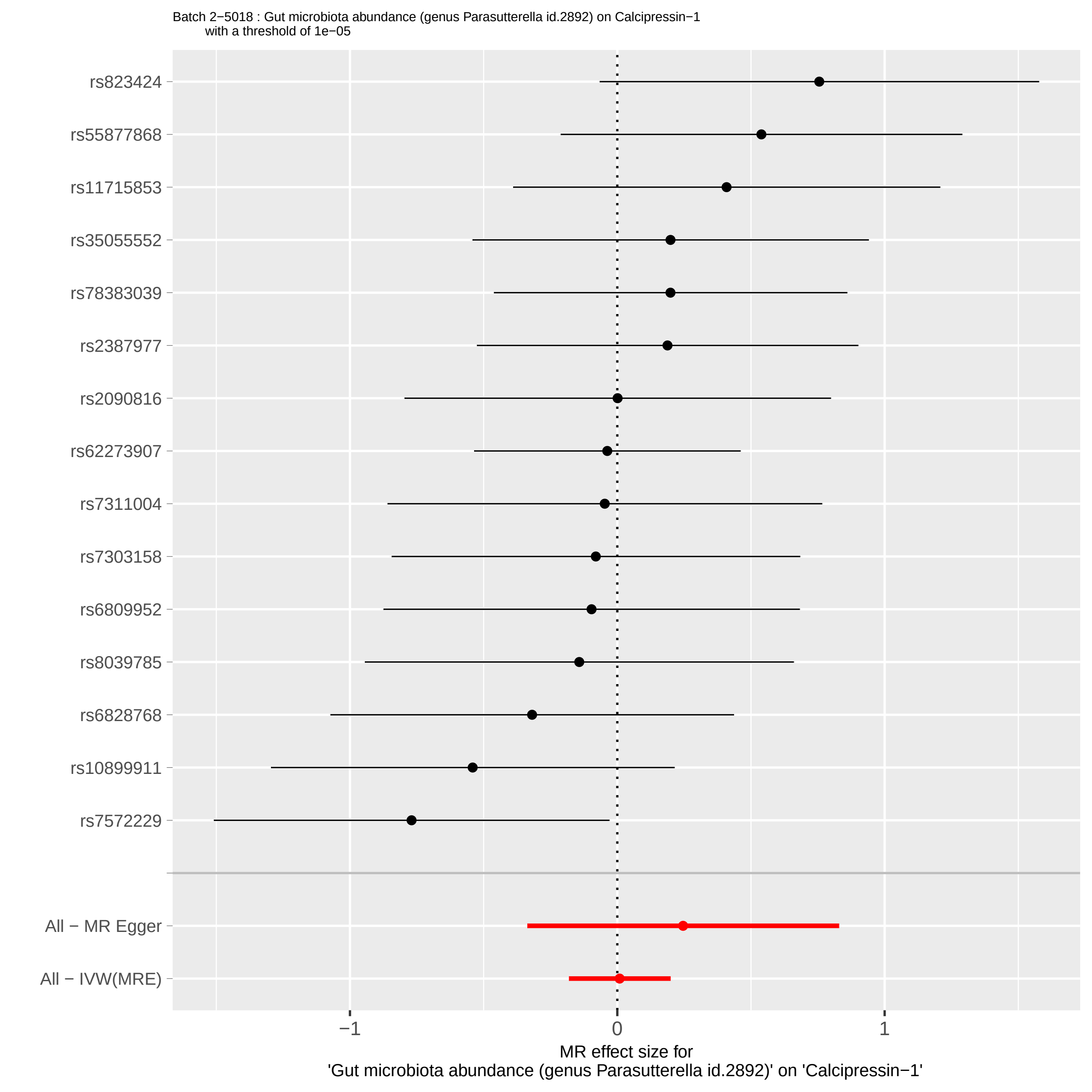

Batch 2−5018 : Gut microbiota abundance (genus Parasutterella id.2892) on Calcipressin−1
with a threshold of 1e−05
rs823424
rs55877868
rs11715853
rs35055552
rs78383039
rs2387977
rs2090816
rs62273907
rs7311004
rs7303158
rs6809952
rs8039785
rs6828768
rs10899911
rs7572229
All − MR Egger
All − IVW(MRE)
−1
0
1
MR effect size for
'Gut microbiota abundance (genus Parasutterella id.2892)' on 'Calcipressin−1'

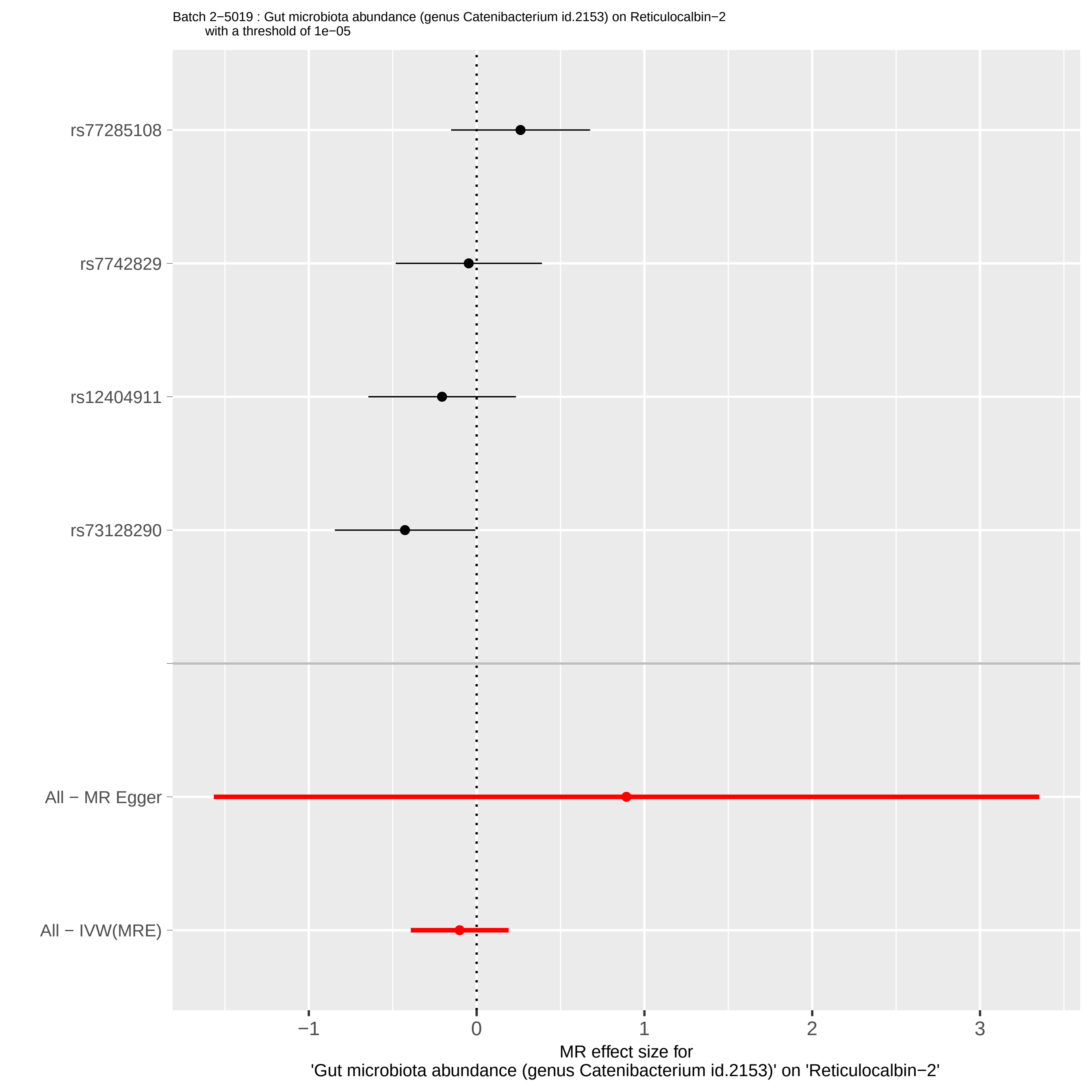

Batch 2−5019 : Gut microbiota abundance (genus Catenibacterium id.2153) on Reticulocalbin−2
with a threshold of 1e−05
rs77285108
rs7742829
rs12404911
rs73128290
All − MR Egger
All − IVW(MRE)
−1
0
1
2
3
MR effect size for
'Gut microbiota abundance (genus Catenibacterium id.2153)' on 'Reticulocalbin−2'

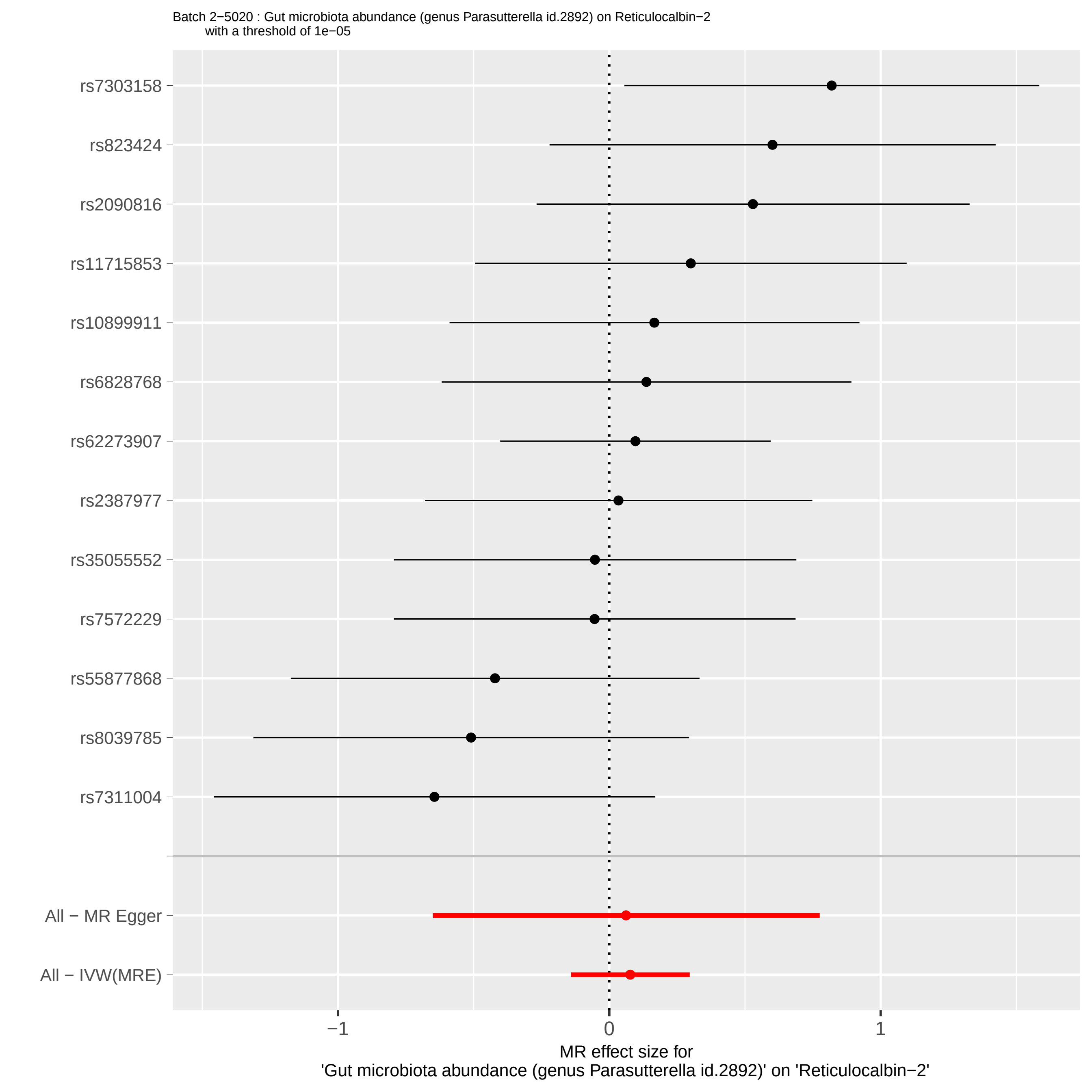

Batch 2−5020 : Gut microbiota abundance (genus Parasutterella id.2892) on Reticulocalbin−2
with a threshold of 1e−05
rs7303158
rs823424
rs2090816
rs11715853
rs10899911
rs6828768
rs62273907
rs2387977
rs35055552
rs7572229
rs55877868
rs8039785
rs7311004
All − MR Egger
All − IVW(MRE)
−1
0
1
MR effect size for
'Gut microbiota abundance (genus Parasutterella id.2892)' on 'Reticulocalbin−2'

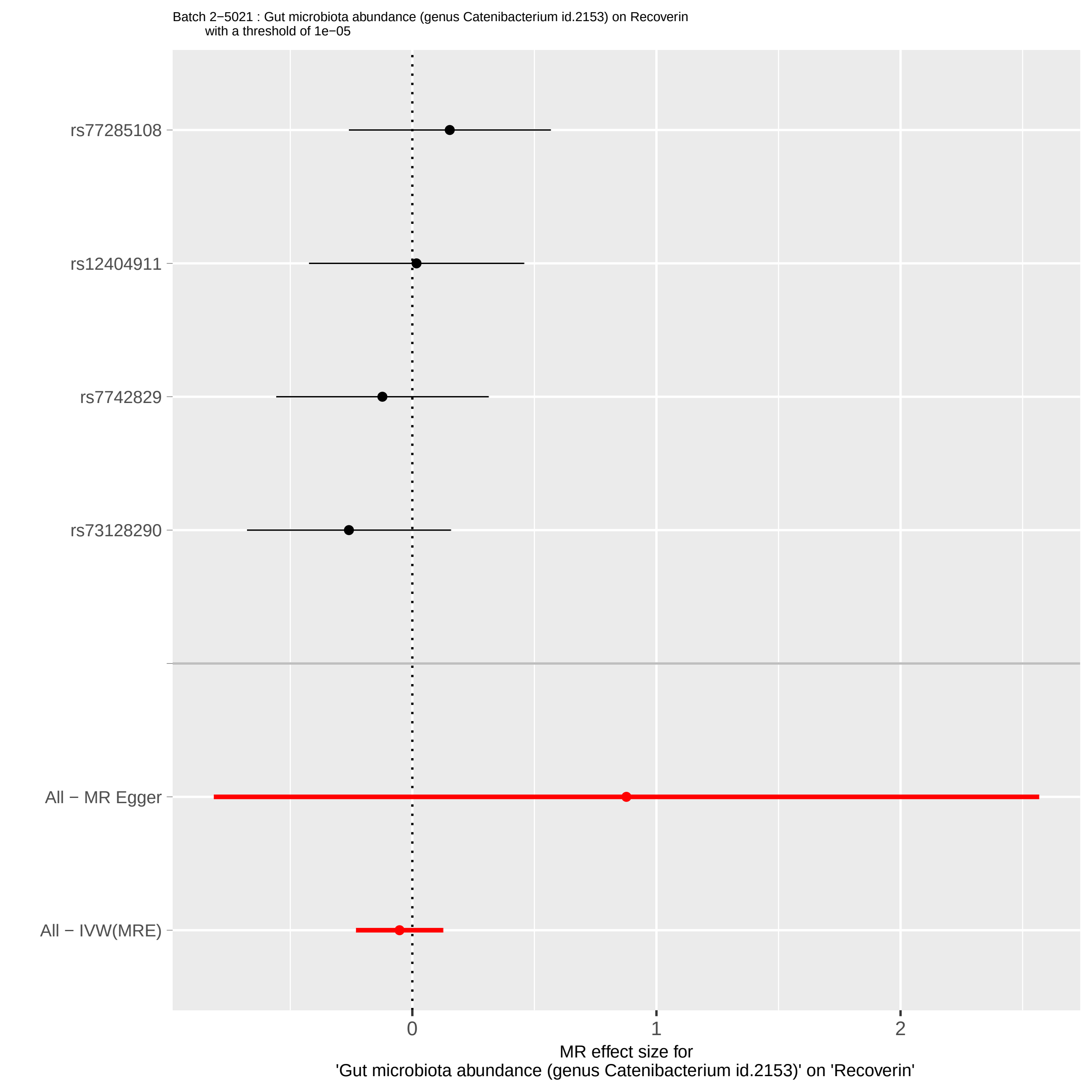
Batch 2−5021 : Gut microbiota abundance (genus Catenibacterium id.2153) on Recoverin
with a threshold of 1e−05
rs77285108
rs12404911
rs7742829
rs73128290
All − MR Egger
All − IVW(MRE)
0
1
2
MR effect size for
'Gut microbiota abundance (genus Catenibacterium id.2153)' on 'Recoverin'

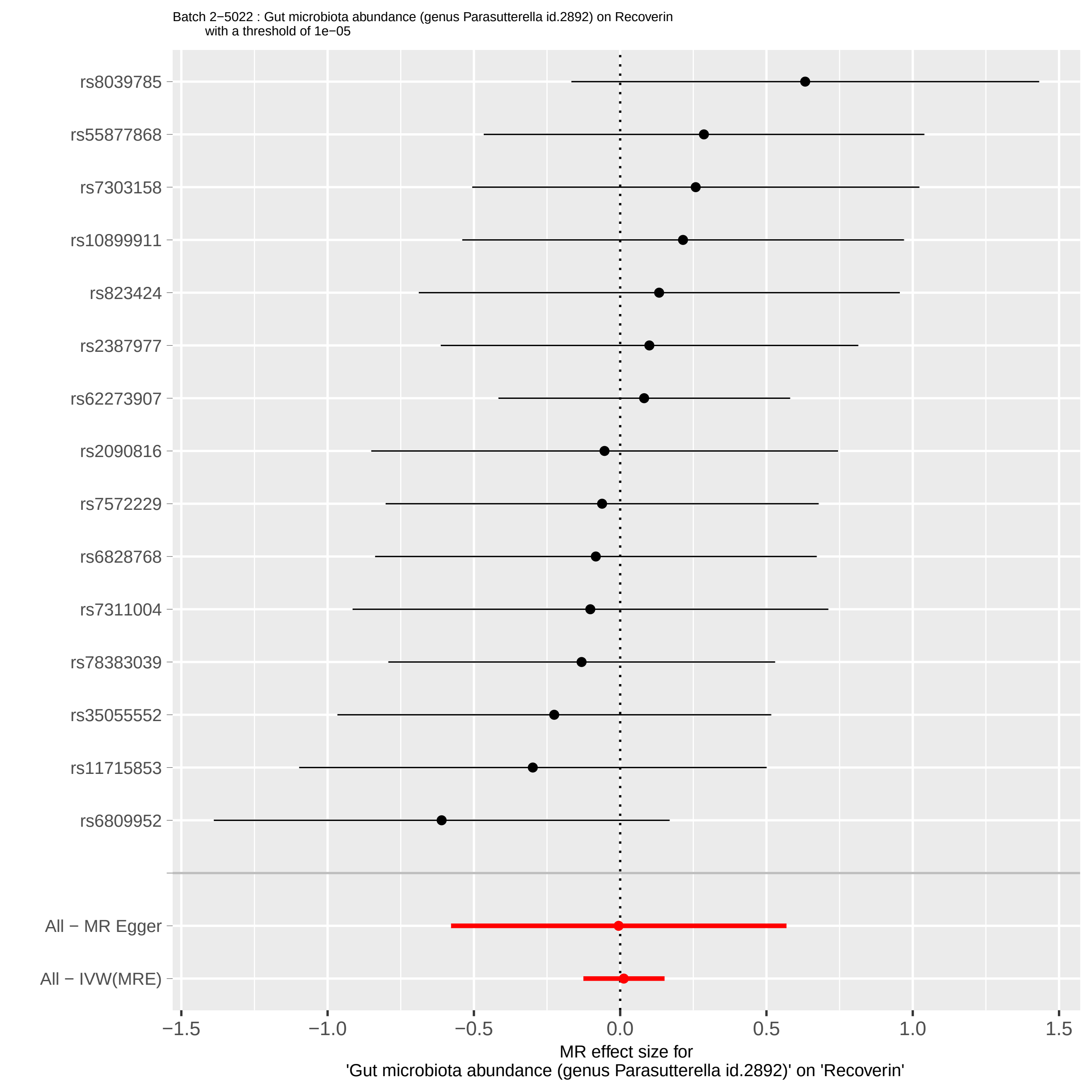

Batch 2−5022 : Gut microbiota abundance (genus Parasutterella id.2892) on Recoverin
with a threshold of 1e−05
rs8039785
rs55877868
rs7303158
rs10899911
rs823424
rs2387977
rs62273907
rs2090816
rs7572229
rs6828768
rs7311004
rs78383039
rs35055552
rs11715853
rs6809952
All − MR Egger
All − IVW(MRE)
−1.5
−1.0
−0.5
0.0
0.5
1.0
1.5
MR effect size for
'Gut microbiota abundance (genus Parasutterella id.2892)' on 'Recoverin'

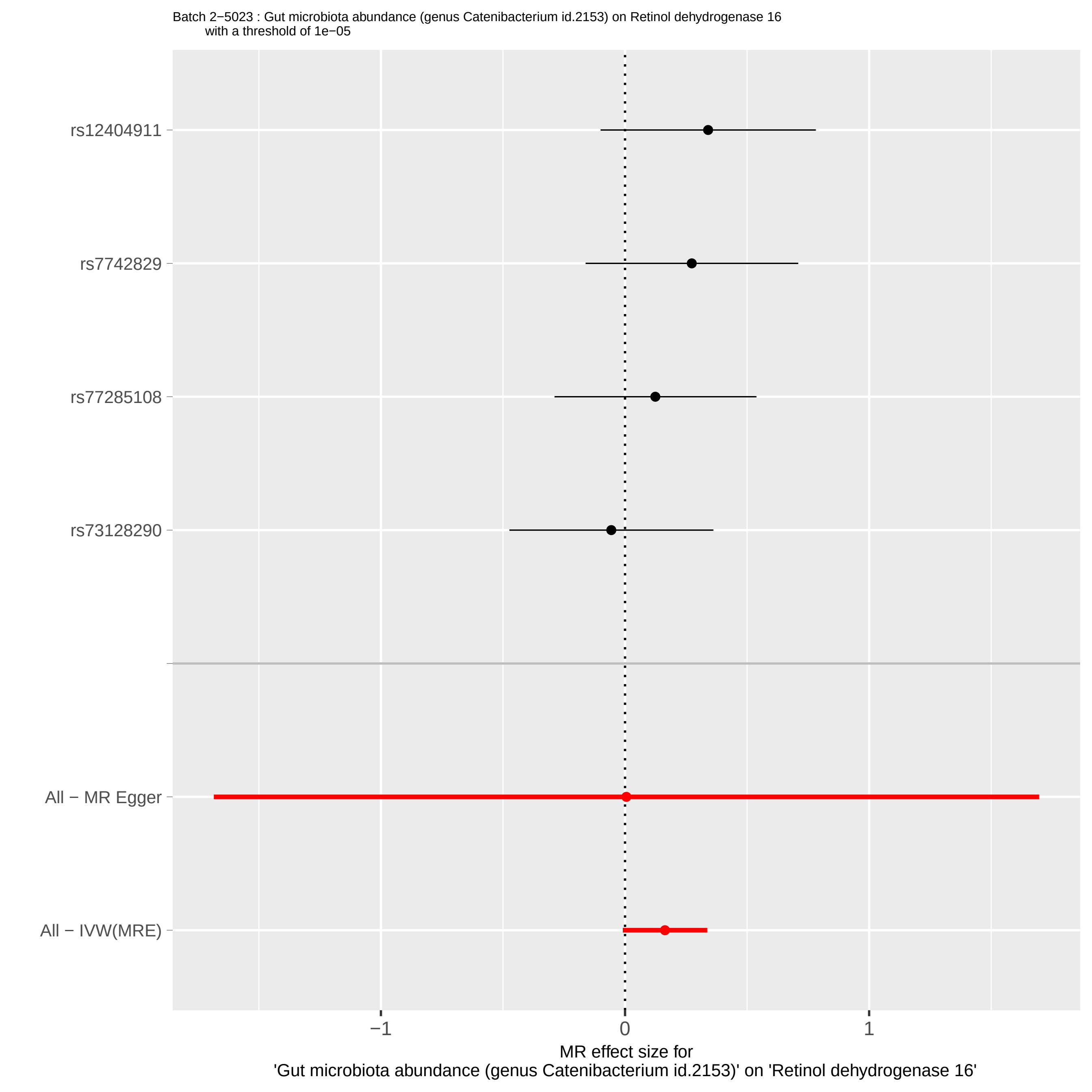
Batch 2−5023 : Gut microbiota abundance (genus Catenibacterium id.2153) on Retinol dehydrogenase 16
with a threshold of 1e−05
rs12404911
rs7742829
rs77285108
rs73128290
All − MR Egger
All − IVW(MRE)
−1
0
1
MR effect size for
'Gut microbiota abundance (genus Catenibacterium id.2153)' on 'Retinol dehydrogenase 16'

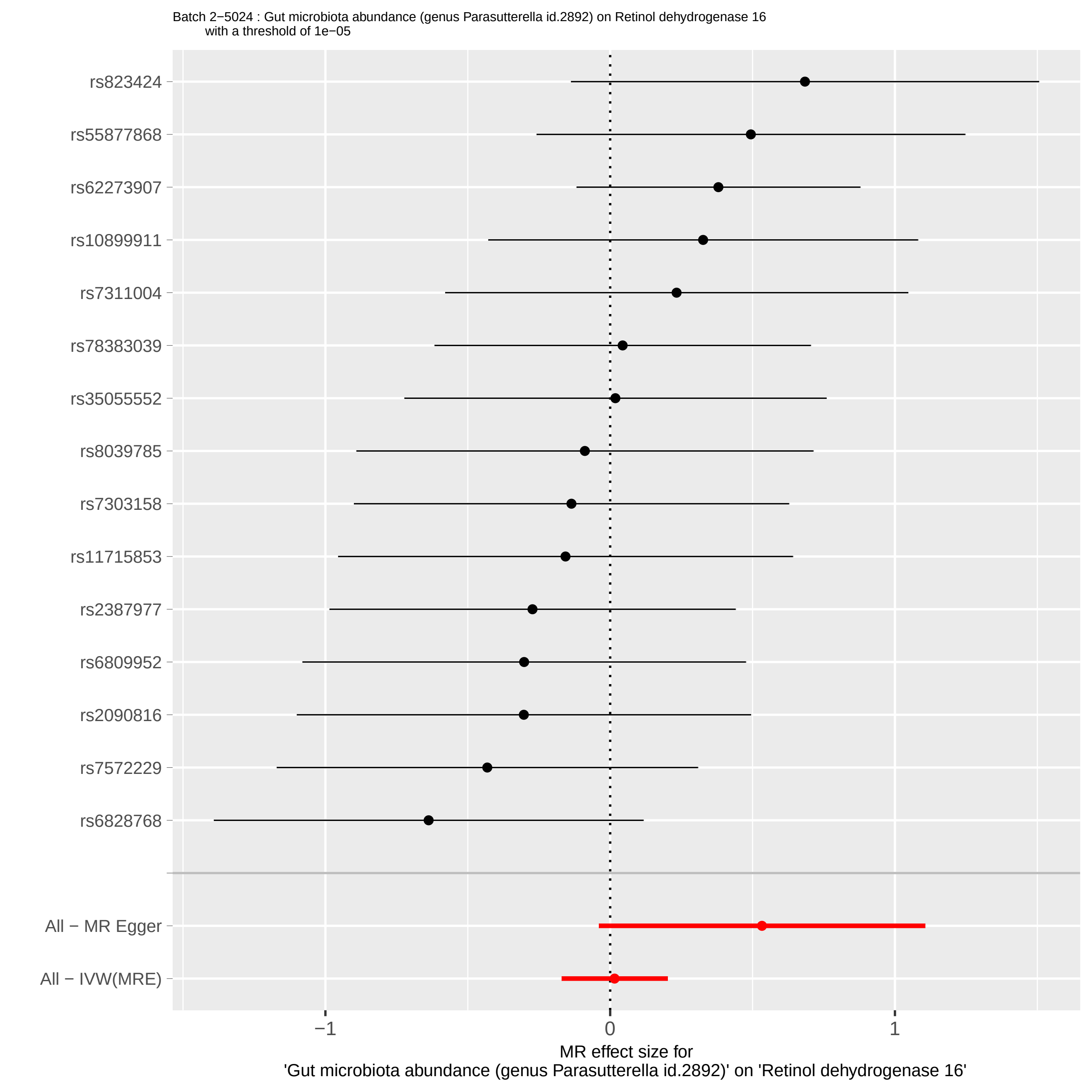

Batch 2−5024 : Gut microbiota abundance (genus Parasutterella id.2892) on Retinol dehydrogenase 16
with a threshold of 1e−05
rs823424
rs55877868
rs62273907
rs10899911
rs7311004
rs78383039
rs35055552
rs8039785
rs7303158
rs11715853
rs2387977
rs6809952
rs2090816
rs7572229
rs6828768
All − MR Egger
All − IVW(MRE)
−1
0
1
MR effect size for
'Gut microbiota abundance (genus Parasutterella id.2892)' on 'Retinol dehydrogenase 16'

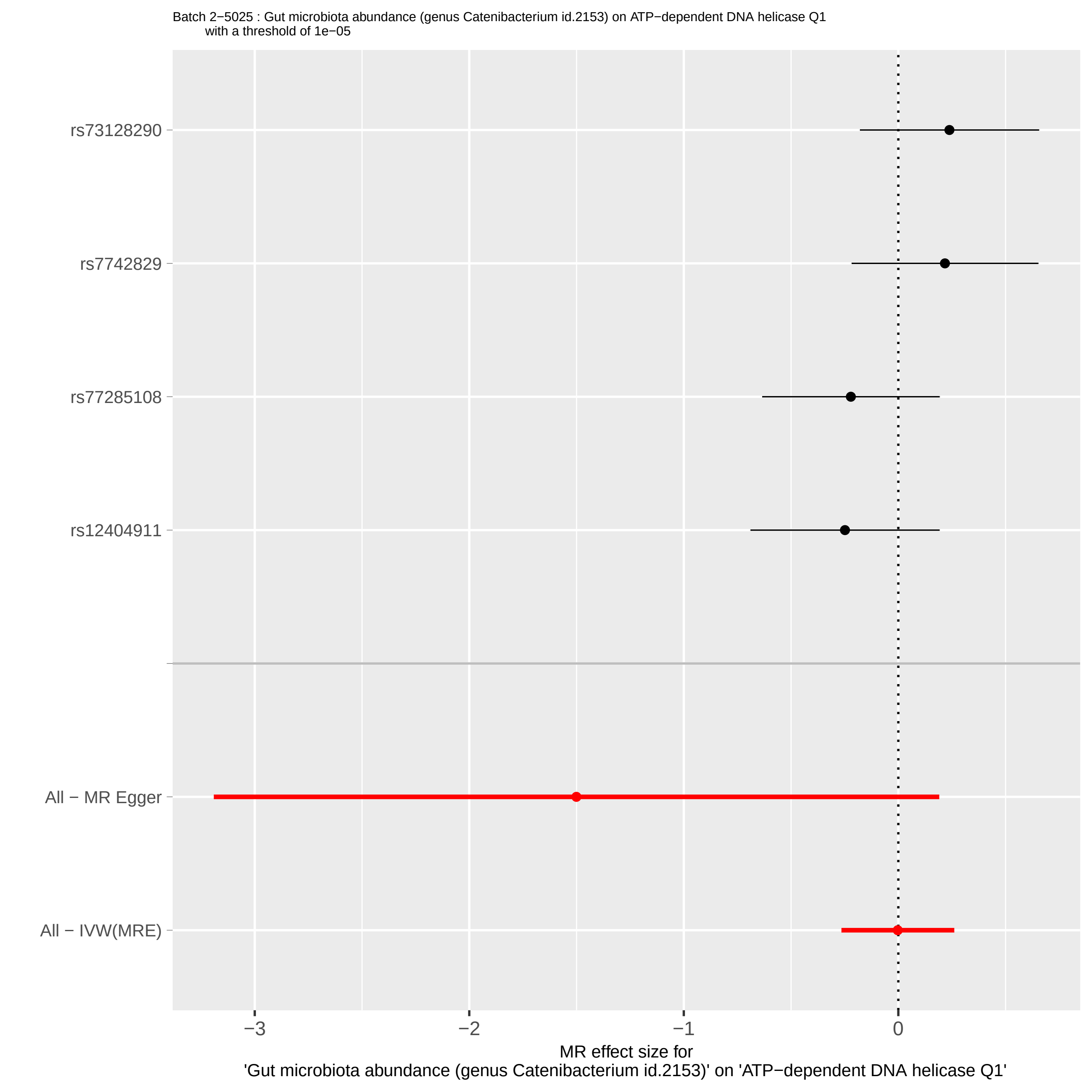
Batch 2−5025 : Gut microbiota abundance (genus Catenibacterium id.2153) on ATP−dependent DNA helicase Q1
with a threshold of 1e−05
rs73128290
rs7742829
rs77285108
rs12404911
All − MR Egger
All − IVW(MRE)
−3
−2
−1
0
MR effect size for
'Gut microbiota abundance (genus Catenibacterium id.2153)' on 'ATP−dependent DNA helicase Q1'

Batch 2−5026 : Gut microbiota abundance (genus Parasutterella id.2892) on ATP−dependent DNA helicase Q1
with a threshold of 1e−05

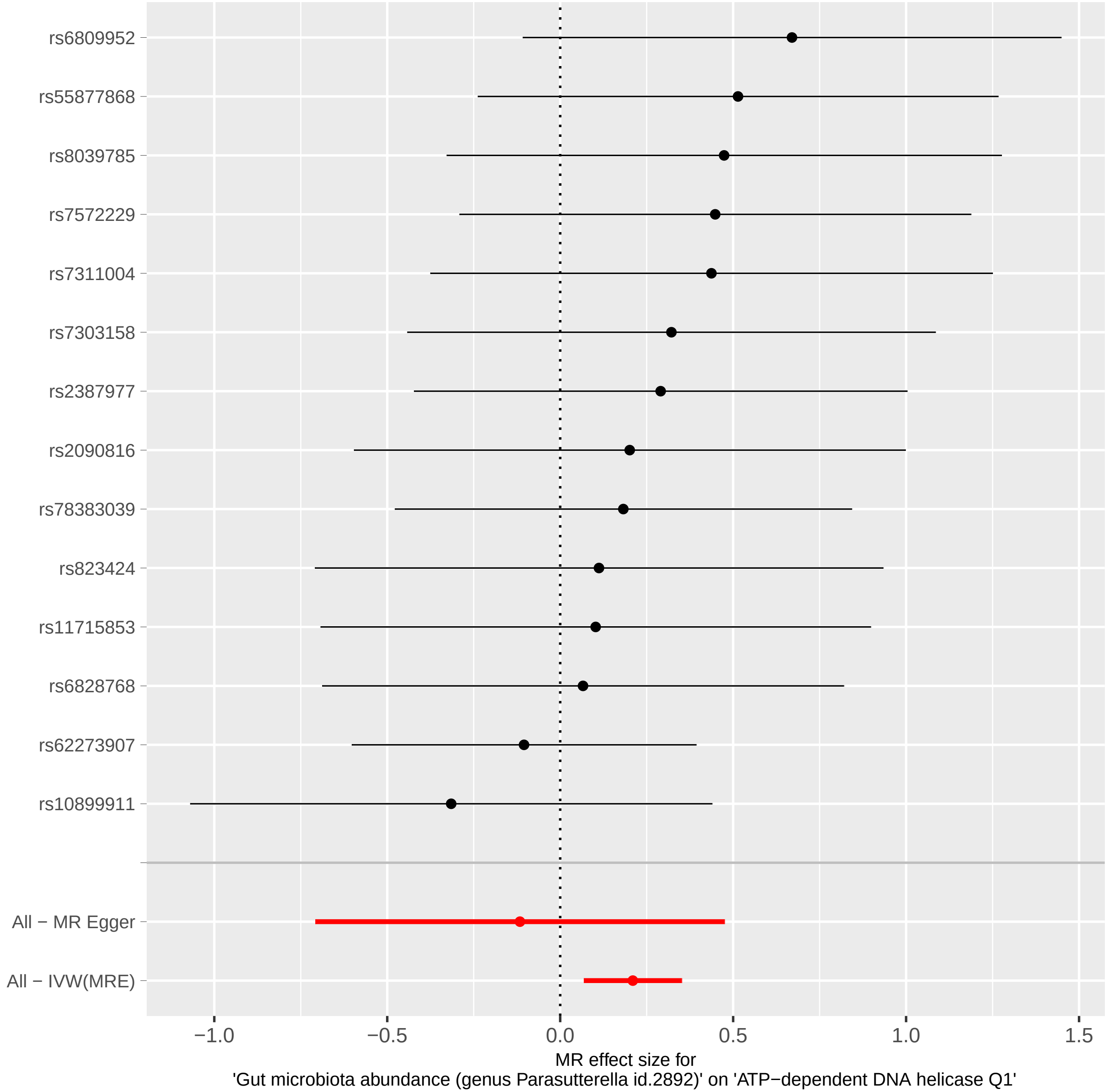

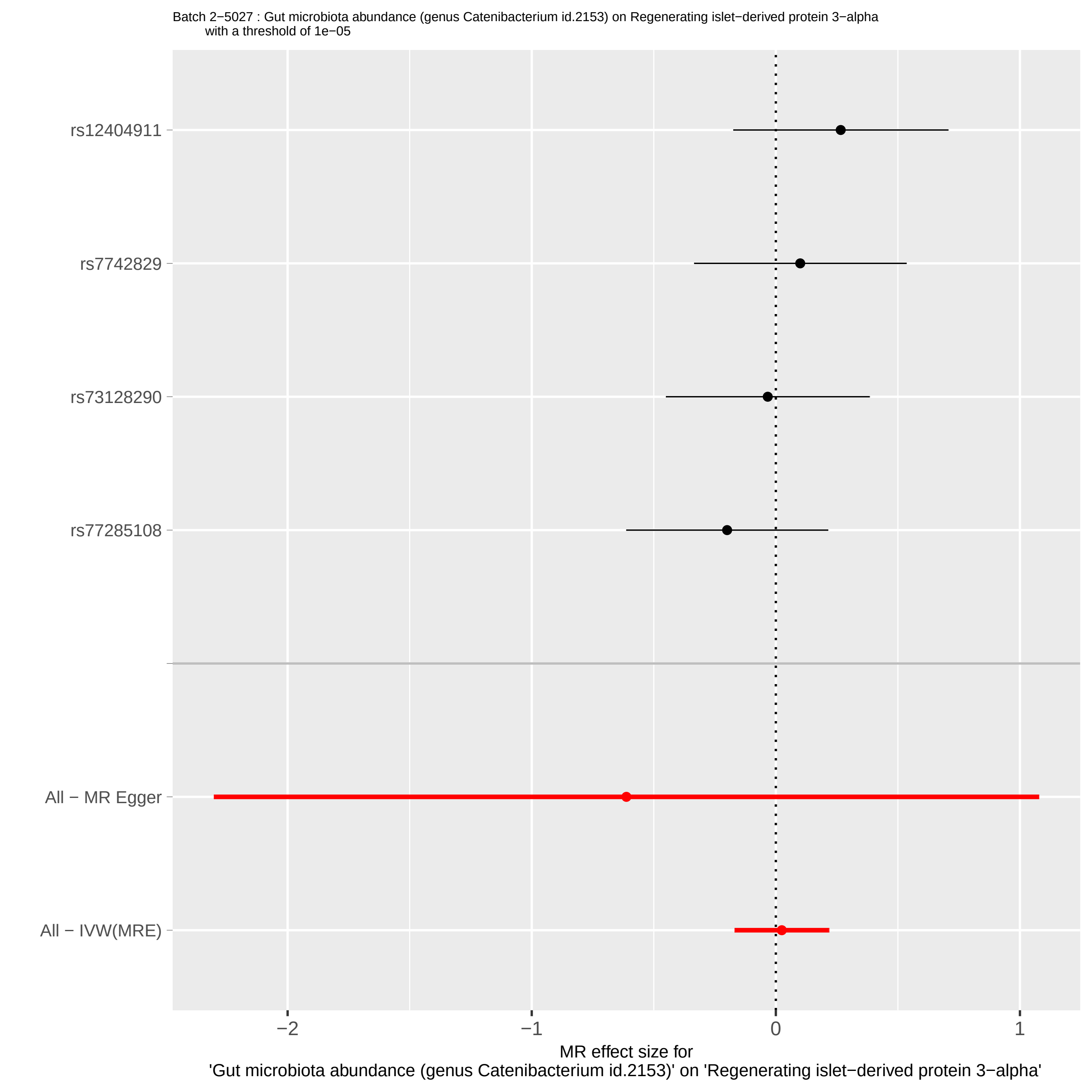

Batch 2−5027 : Gut microbiota abundance (genus Catenibacterium id.2153) on Regenerating islet−derived protein 3−alpha
with a threshold of 1e−05
rs12404911
rs7742829
rs73128290
rs77285108
All − MR Egger
All − IVW(MRE)
−2
−1
0
1
MR effect size for
'Gut microbiota abundance (genus Catenibacterium id.2153)' on 'Regenerating islet−derived protein 3−alpha'

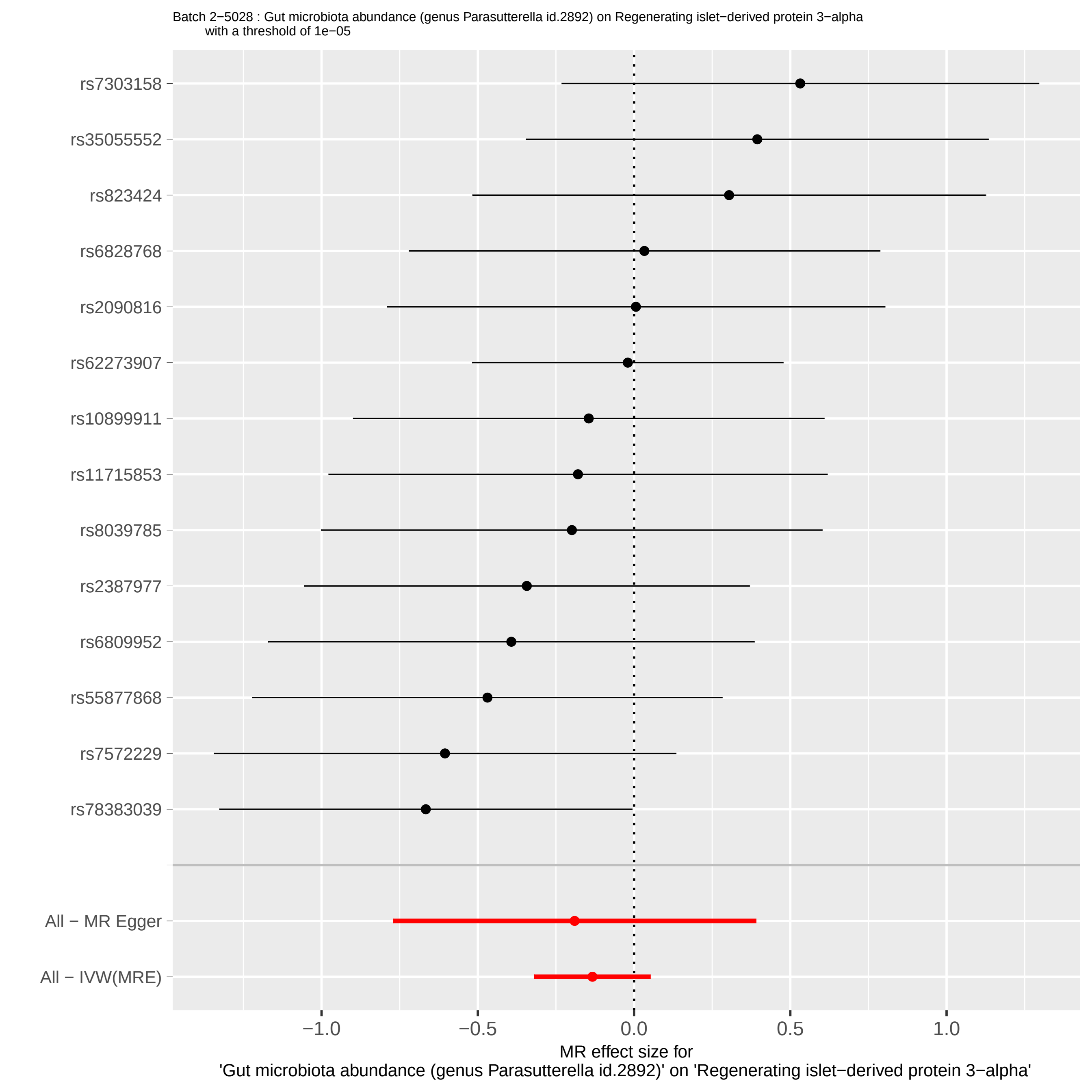
Batch 2−5028 : Gut microbiota abundance (genus Parasutterella id.2892) on Regenerating islet−derived protein 3−alpha
with a threshold of 1e−05
rs7303158
rs35055552
rs823424
rs6828768
rs2090816
rs62273907
rs10899911
rs11715853
rs8039785
rs2387977
rs6809952
rs55877868
rs7572229
rs78383039
All − MR Egger
All − IVW(MRE)
−1.0
−0.5
0.0
0.5
1.0
MR effect size for
'Gut microbiota abundance (genus Parasutterella id.2892)' on 'Regenerating islet−derived protein 3−alpha'

Batch 2−5029 : Gut microbiota abundance (genus Catenibacterium id.2153) on Regenerating islet−derived protein 4
with a threshold of 1e−05

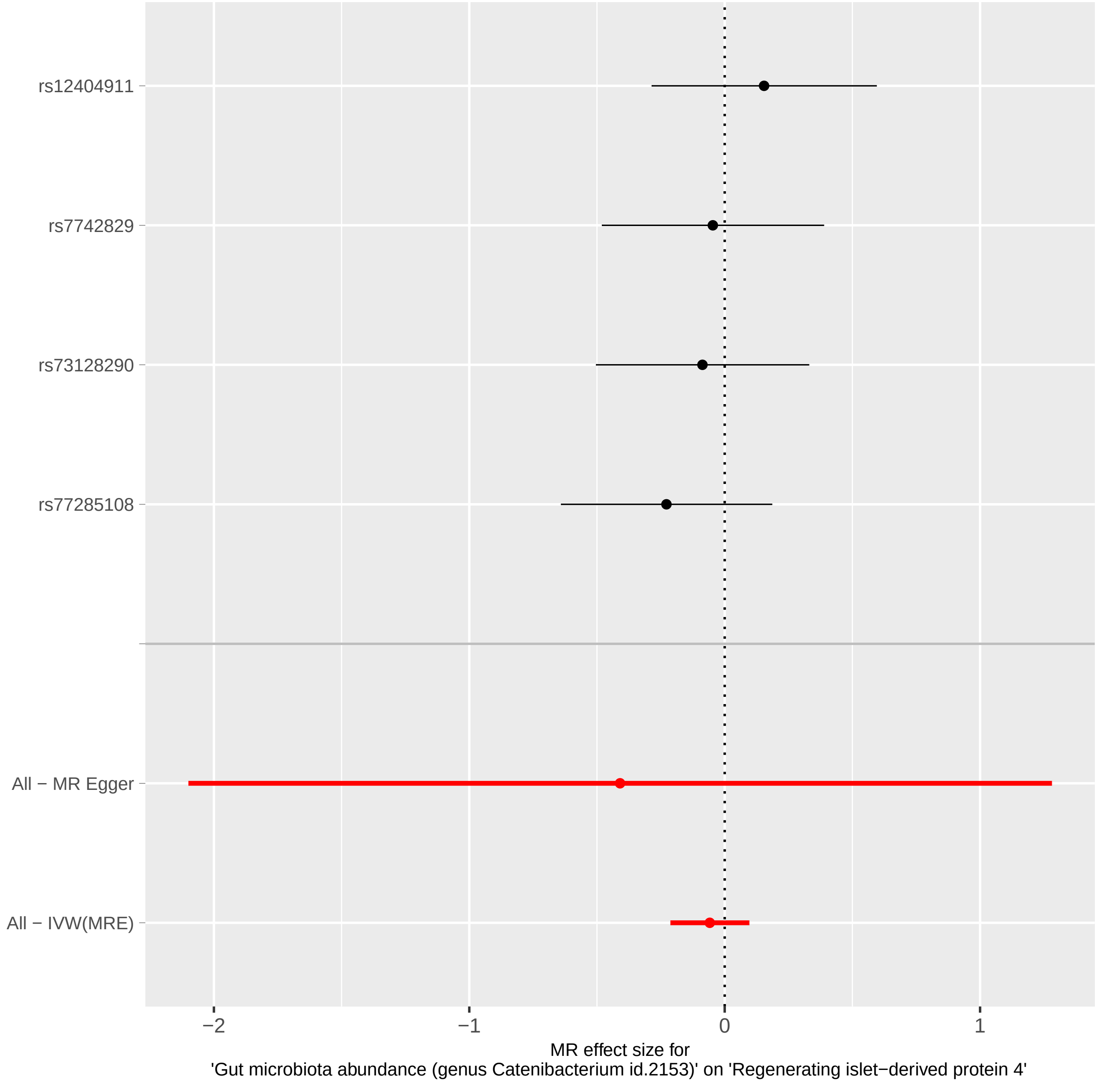

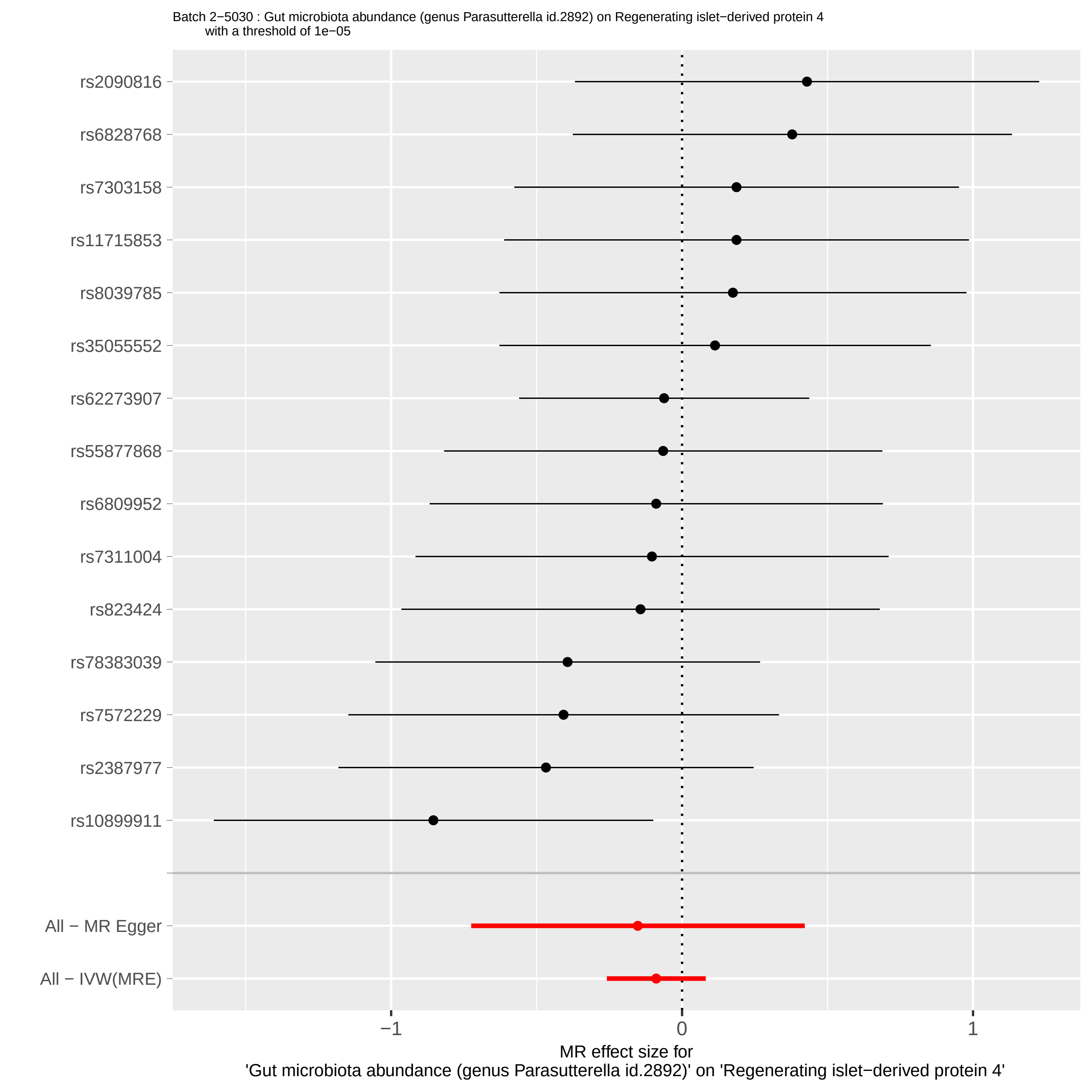

Batch 2−5030 : Gut microbiota abundance (genus Parasutterella id.2892) on Regenerating islet−derived protein 4
with a threshold of 1e−05
rs2090816
rs6828768
rs7303158
rs11715853
rs8039785
rs35055552
rs62273907
rs55877868
rs6809952
rs7311004
rs823424
rs78383039
rs7572229
rs2387977
rs10899911
All − MR Egger
All − IVW(MRE)
−1
0
1
MR effect size for
'Gut microbiota abundance (genus Parasutterella id.2892)' on 'Regenerating islet−derived protein 4'

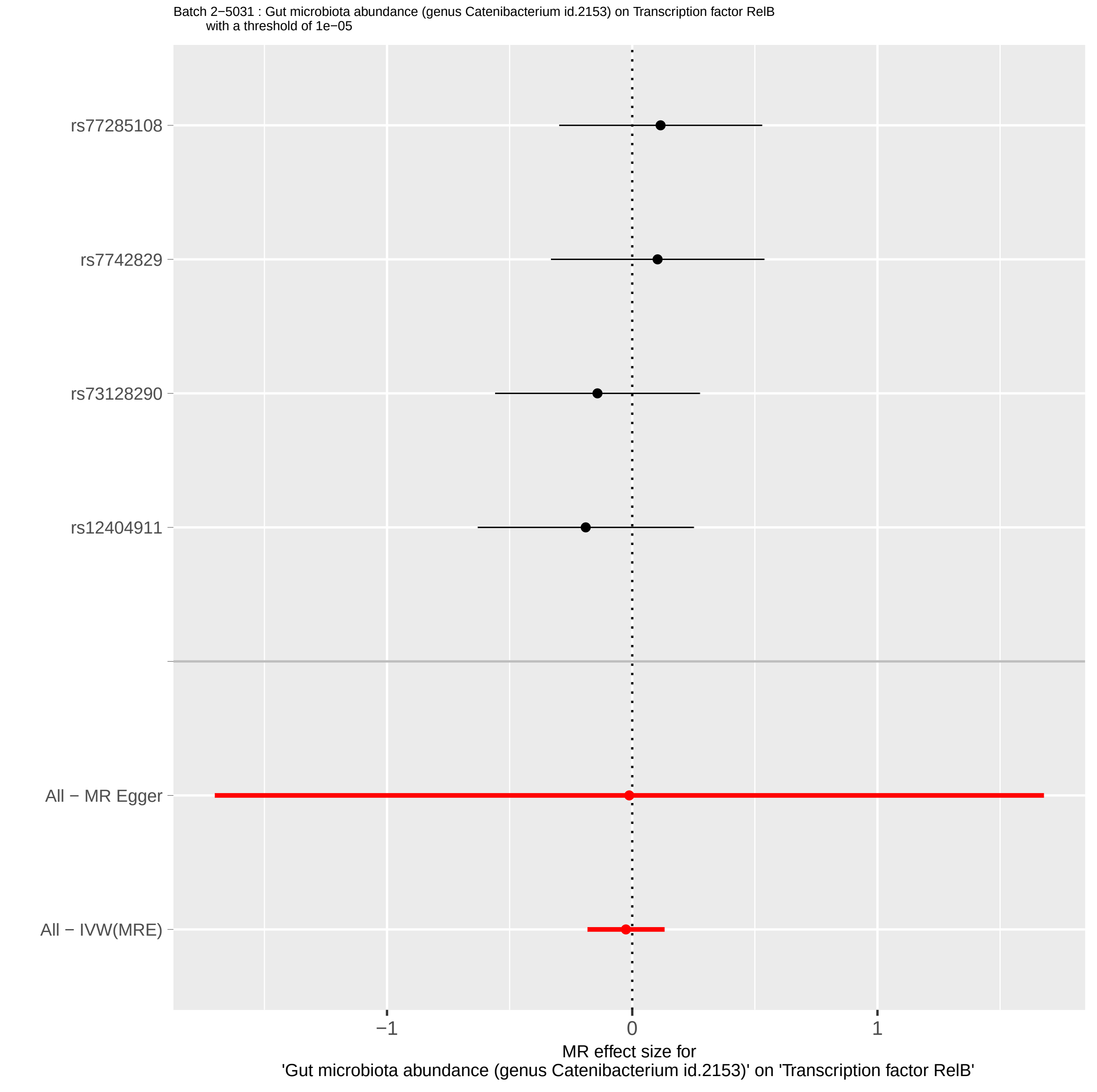

Batch 2−5031 : Gut microbiota abundance (genus Catenibacterium id.2153) on Transcription factor RelB
with a threshold of 1e−05
rs77285108
rs7742829
rs73128290
rs12404911
All − MR Egger
All − IVW(MRE)
−1
0
1
MR effect size for
'Gut microbiota abundance (genus Catenibacterium id.2153)' on 'Transcription factor RelB'

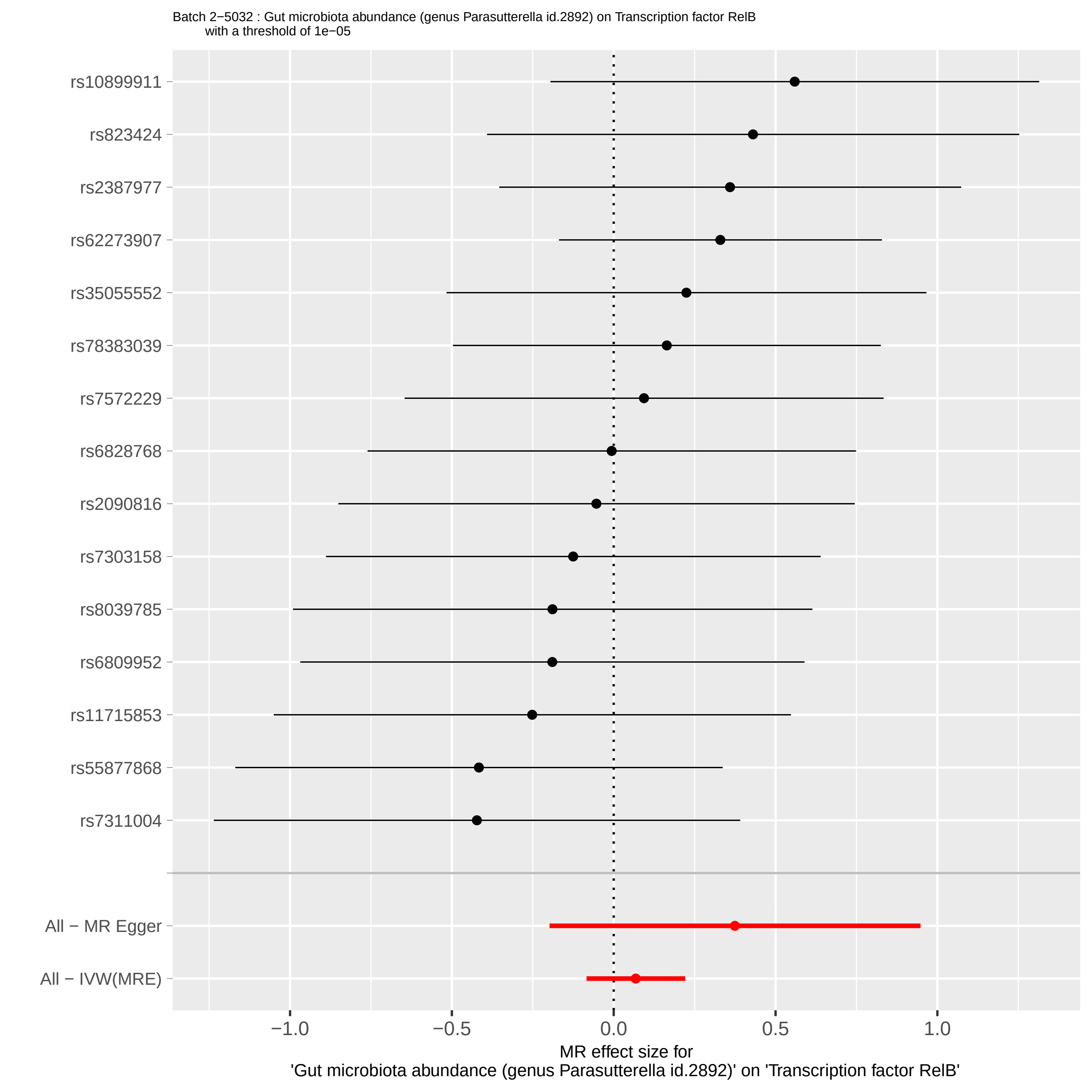

Batch 2−5032 : Gut microbiota abundance (genus Parasutterella id.2892) on Transcription factor RelB
with a threshold of 1e−05
rs10899911
rs823424
rs2387977
rs62273907
rs35055552
rs78383039
rs7572229
rs6828768
rs2090816
rs7303158
rs8039785
rs6809952
rs11715853
rs55877868
rs7311004
All − MR Egger
All − IVW(MRE)
−1.0
−0.5
0.0
0.5
1.0
MR effect size for
'Gut microbiota abundance (genus Parasutterella id.2892)' on 'Transcription factor RelB'

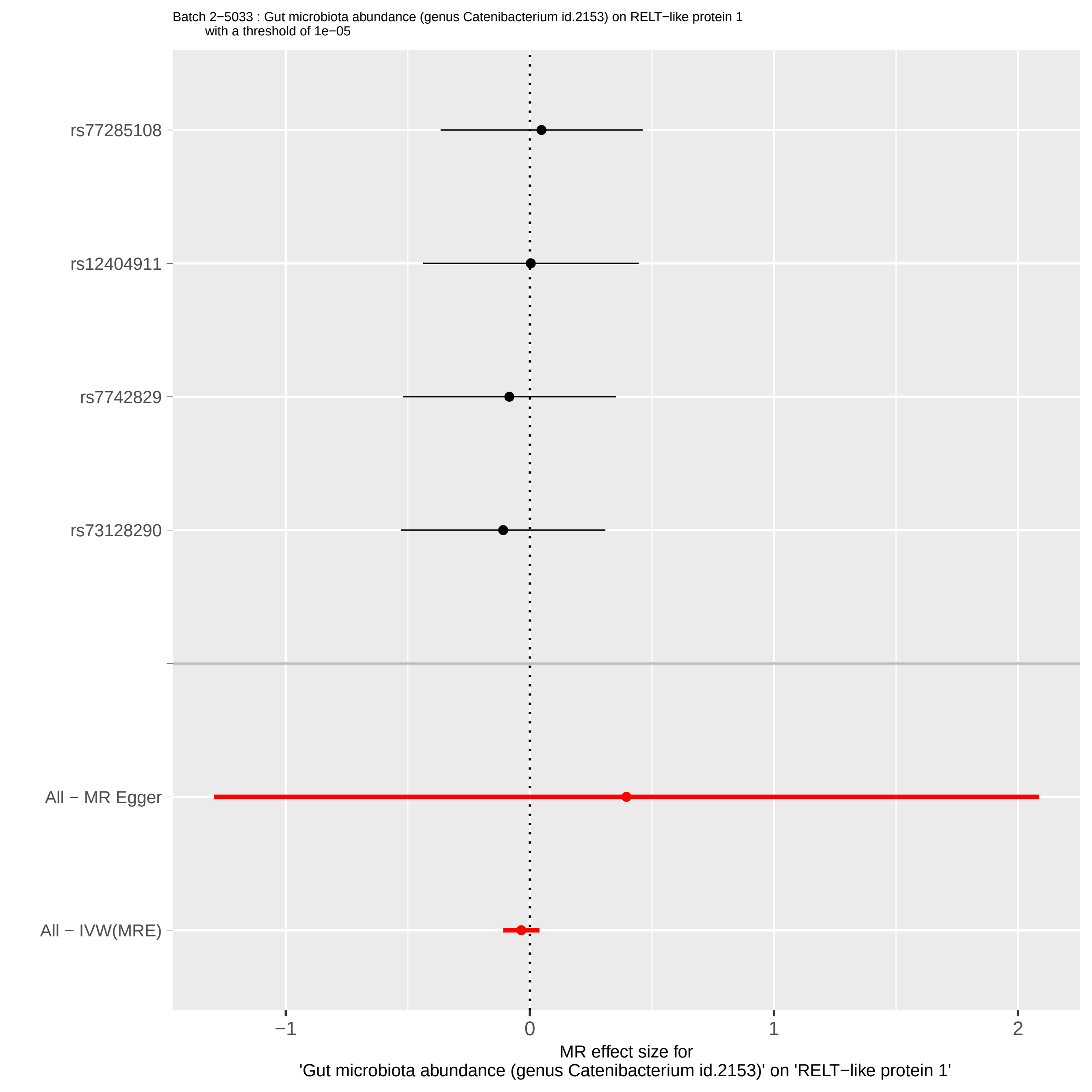
Batch 2−5033 : Gut microbiota abundance (genus Catenibacterium id.2153) on RELT−like protein 1
with a threshold of 1e−05
rs77285108
rs12404911
rs7742829
rs73128290
All − MR Egger
All − IVW(MRE)
−1
0
1
2
MR effect size for
'Gut microbiota abundance (genus Catenibacterium id.2153)' on 'RELT−like protein 1'

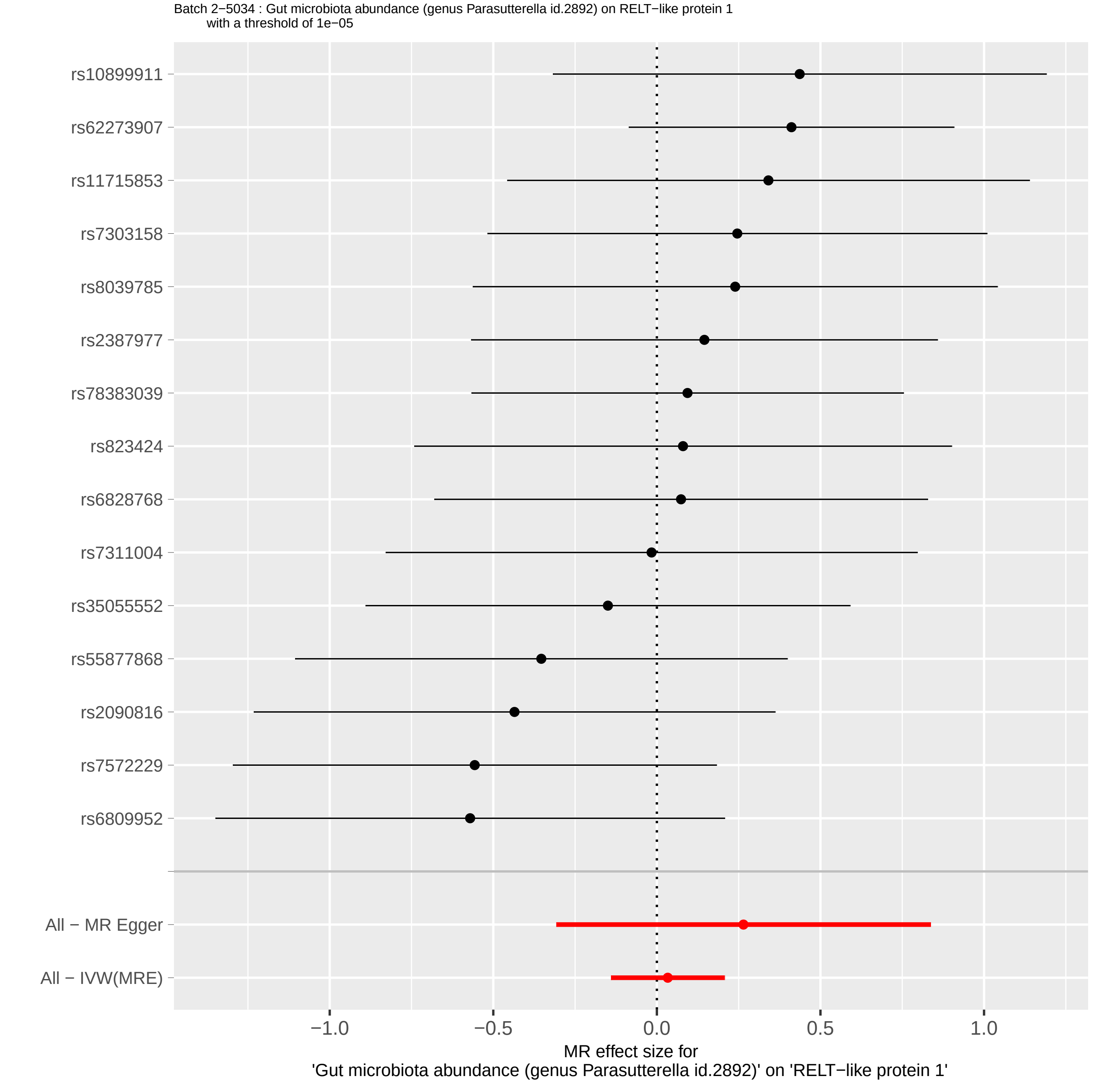

Batch 2−5034 : Gut microbiota abundance (genus Parasutterella id.2892) on RELT−like protein 1
with a threshold of 1e−05
rs10899911
rs62273907
rs11715853
rs7303158
rs8039785
rs2387977
rs78383039
rs823424
rs6828768
rs7311004
rs35055552
rs55877868
rs2090816
rs7572229
rs6809952
All − MR Egger
All − IVW(MRE)
−1.0
−0.5
0.0
0.5
1.0
MR effect size for
'Gut microbiota abundance (genus Parasutterella id.2892)' on 'RELT−like protein 1'

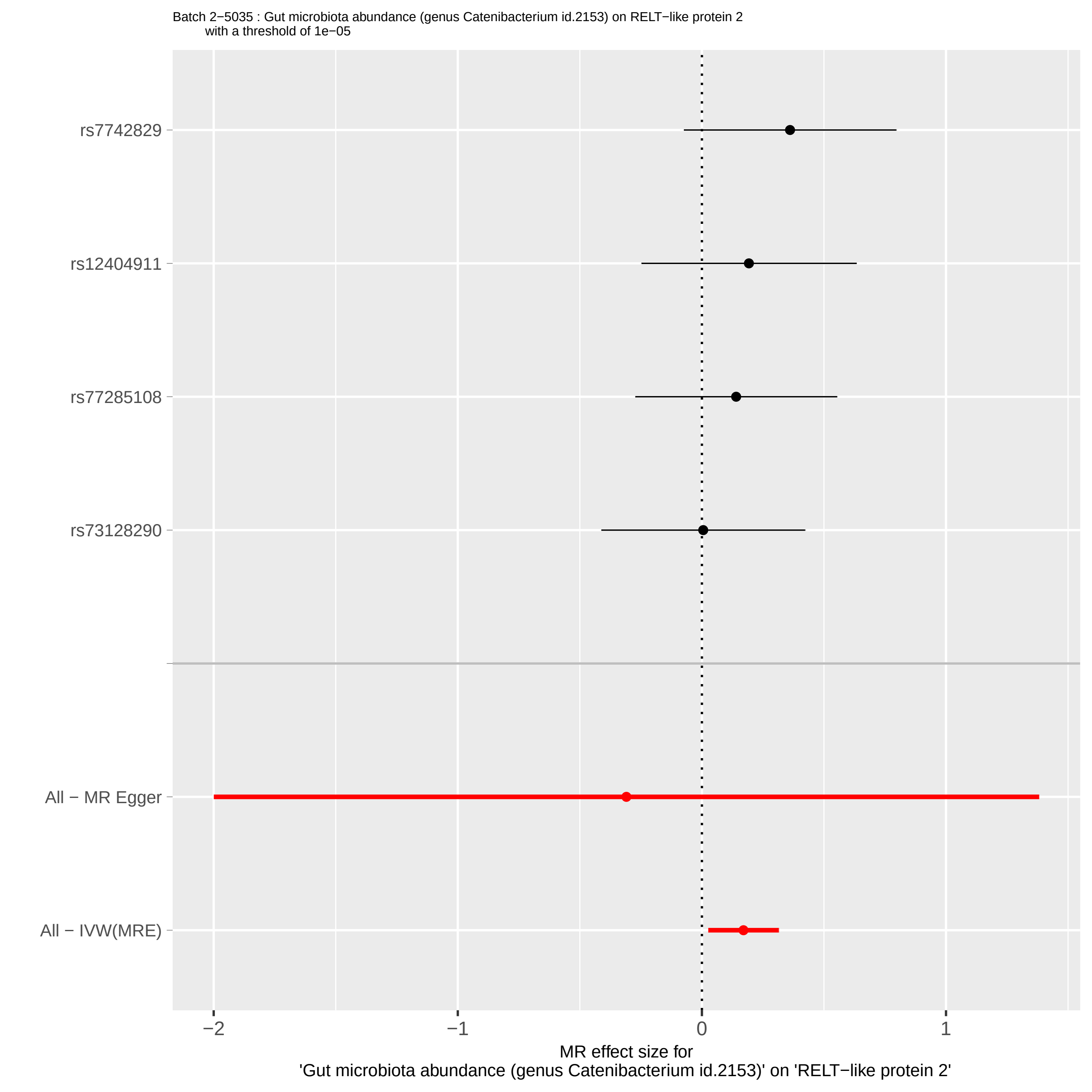

Batch 2−5035 : Gut microbiota abundance (genus Catenibacterium id.2153) on RELT−like protein 2
with a threshold of 1e−05
rs7742829
rs12404911
rs77285108
rs73128290
All − MR Egger
All − IVW(MRE)
−2
−1
0
1
MR effect size for
'Gut microbiota abundance (genus Catenibacterium id.2153)' on 'RELT−like protein 2'

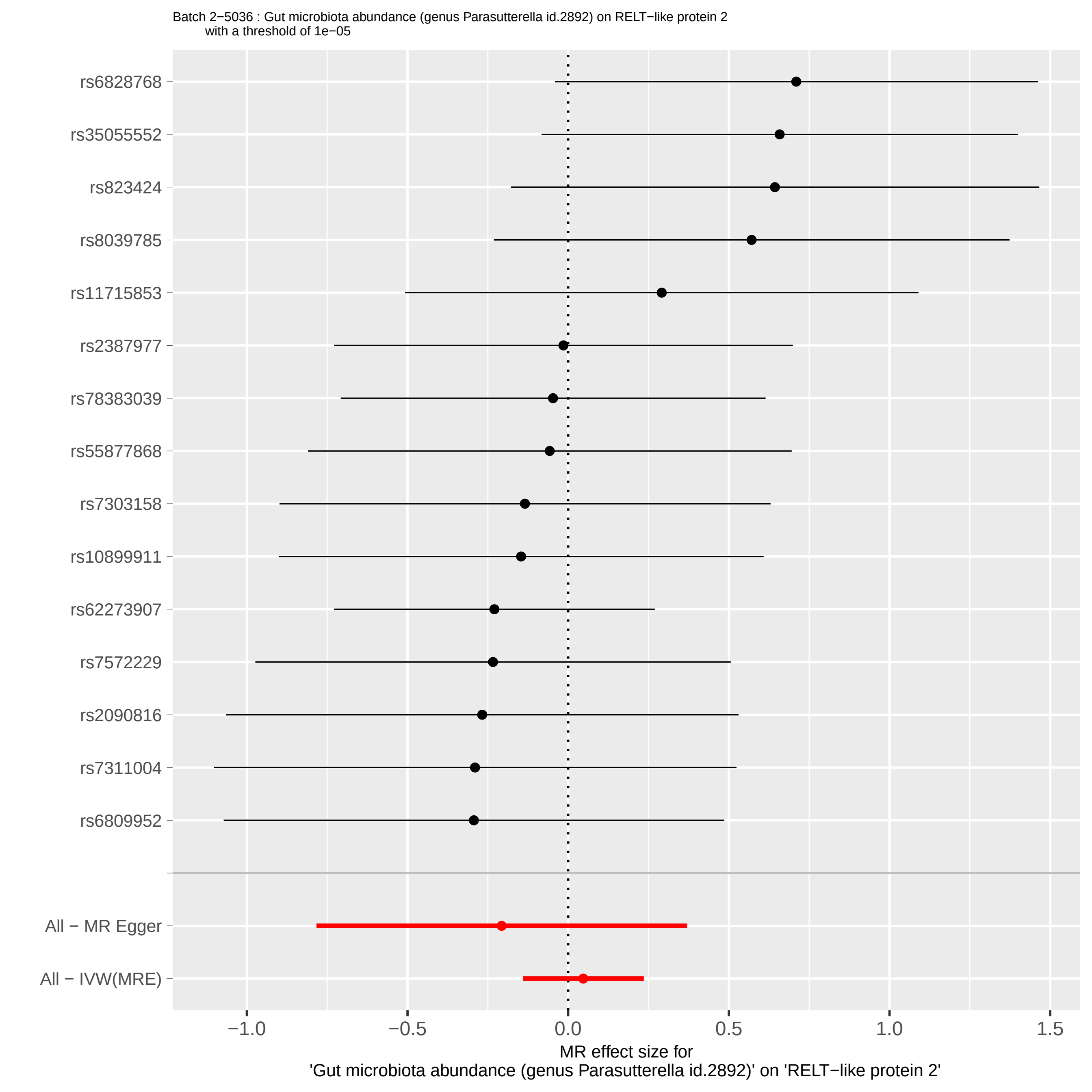
Batch 2−5036 : Gut microbiota abundance (genus Parasutterella id.2892) on RELT−like protein 2
with a threshold of 1e−05
rs6828768
rs35055552
rs823424
rs8039785
rs11715853
rs2387977
rs78383039
rs55877868
rs7303158
rs10899911
rs62273907
rs7572229
rs2090816
rs7311004
rs6809952
All − MR Egger
All − IVW(MRE)
−1.0
−0.5
0.0
0.5
1.0
1.5
MR effect size for
'Gut microbiota abundance (genus Parasutterella id.2892)' on 'RELT−like protein 2'

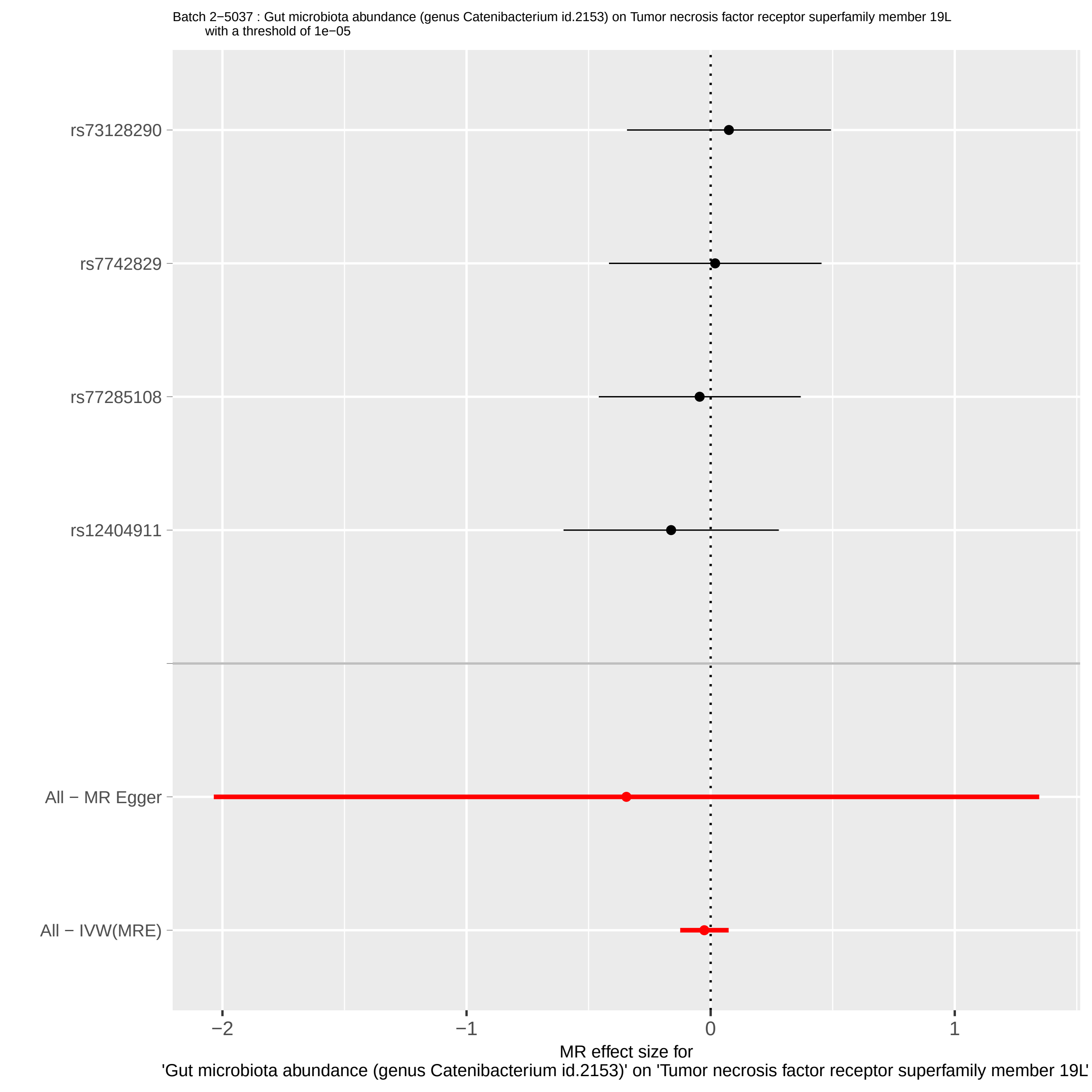
Batch 2−5037 : Gut microbiota abundance (genus Catenibacterium id.2153) on Tumor necrosis factor receptor superfamily member 19L
with a threshold of 1e−05
rs73128290
rs7742829
rs77285108
rs12404911
All − MR Egger
All − IVW(MRE)
−2
−1
0
1
MR effect size for
'Gut microbiota abundance (genus Catenibacterium id.2153)' on 'Tumor necrosis factor receptor superfamily member 19L'

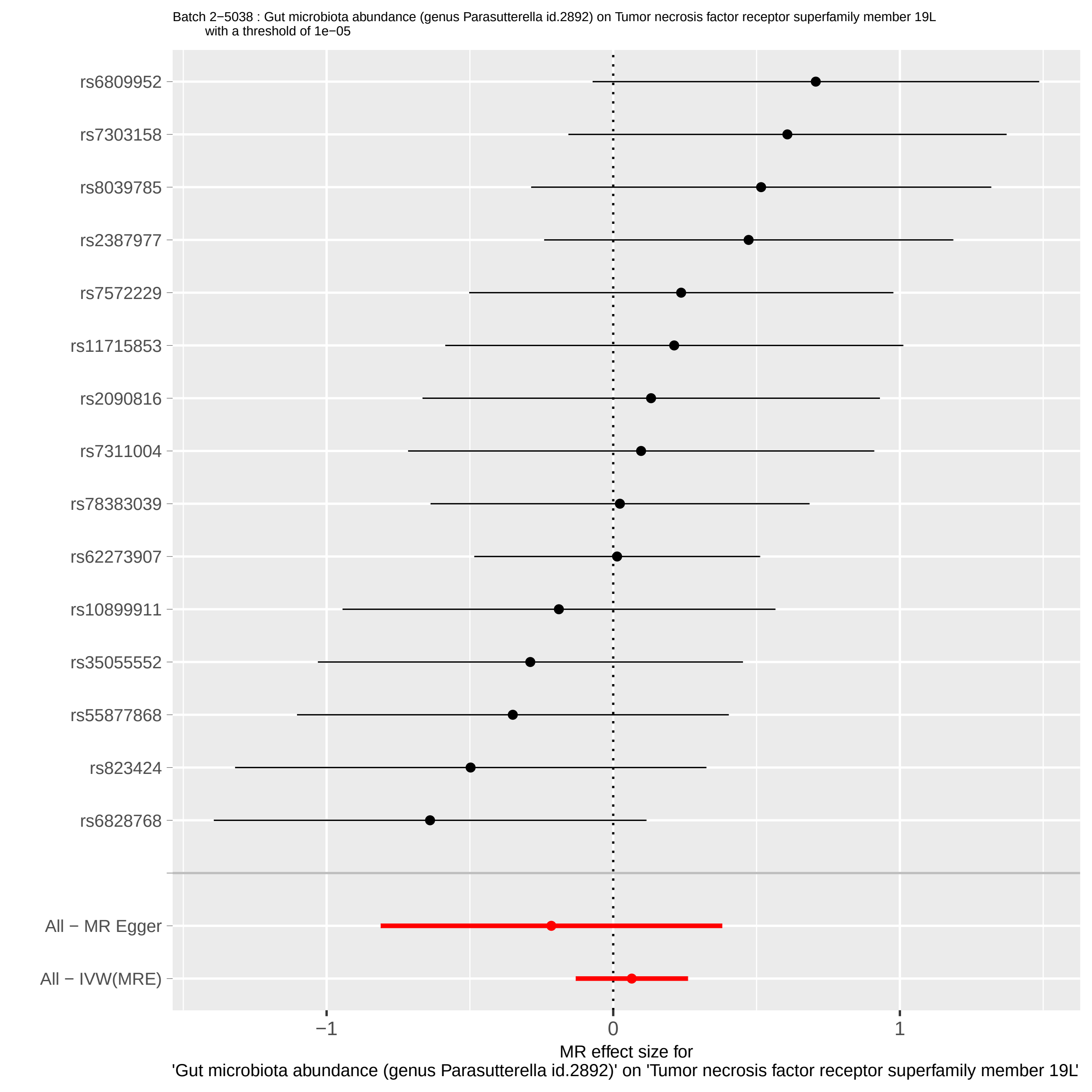
Batch 2−5038 : Gut microbiota abundance (genus Parasutterella id.2892) on Tumor necrosis factor receptor superfamily member 19L
with a threshold of 1e−05
rs6809952
rs7303158
rs8039785
rs2387977
rs7572229
rs11715853
rs2090816
rs7311004
rs78383039
rs62273907
rs10899911
rs35055552
rs55877868
rs823424
rs6828768
All − MR Egger
All − IVW(MRE)
−1
0
1
MR effect size for
'Gut microbiota abundance (genus Parasutterella id.2892)' on 'Tumor necrosis factor receptor superfamily member 19L'

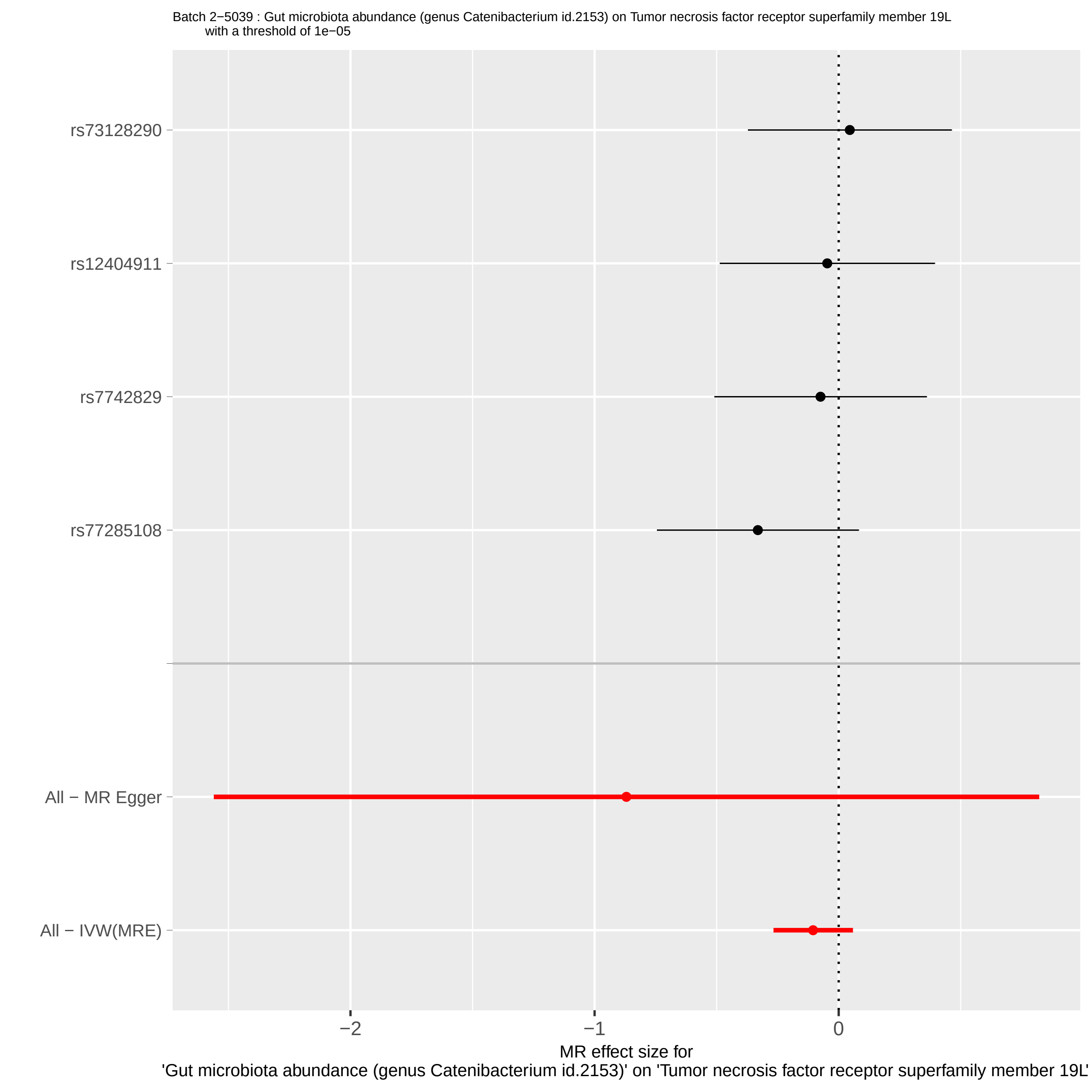
Batch 2−5039 : Gut microbiota abundance (genus Catenibacterium id.2153) on Tumor necrosis factor receptor superfamily member 19L with a threshold of 1e−05
rs73128290
rs12404911
rs7742829
rs77285108
All − MR Egger
All − IVW(MRE)
−2
−1
0
MR effect size for
'Gut microbiota abundance (genus Catenibacterium id.2153)' on 'Tumor necrosis factor receptor superfamily member 19L'

Batch 2−5040 : Gut microbiota abundance (genus Parasutterella id.2892) on Tumor necrosis factor receptor superfamily member 19L
with a threshold of 1e−05

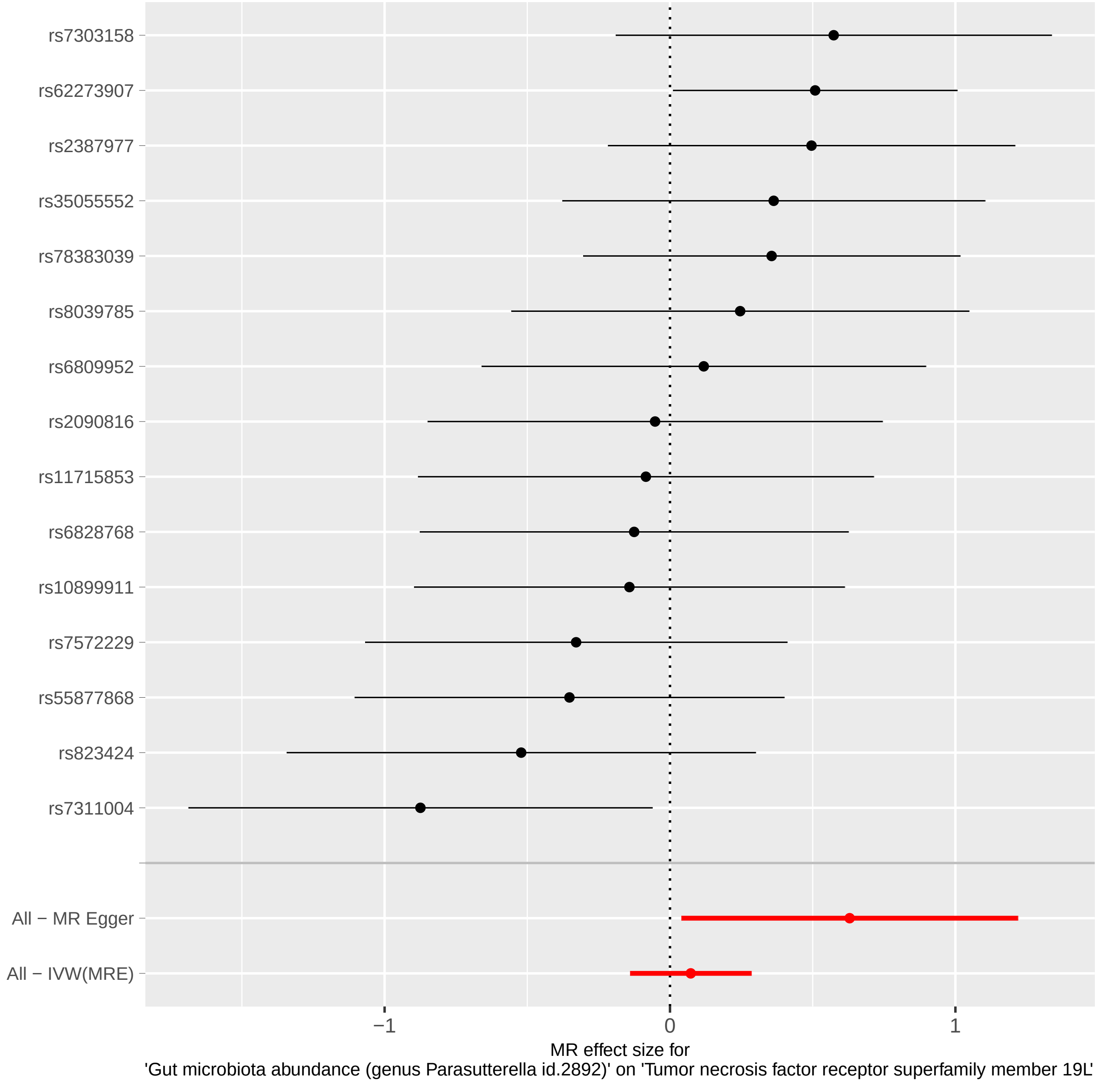

Batch 2−5041 : Gut microbiota abundance (genus Catenibacterium id.2153) on Renin
with a threshold of 1e−05

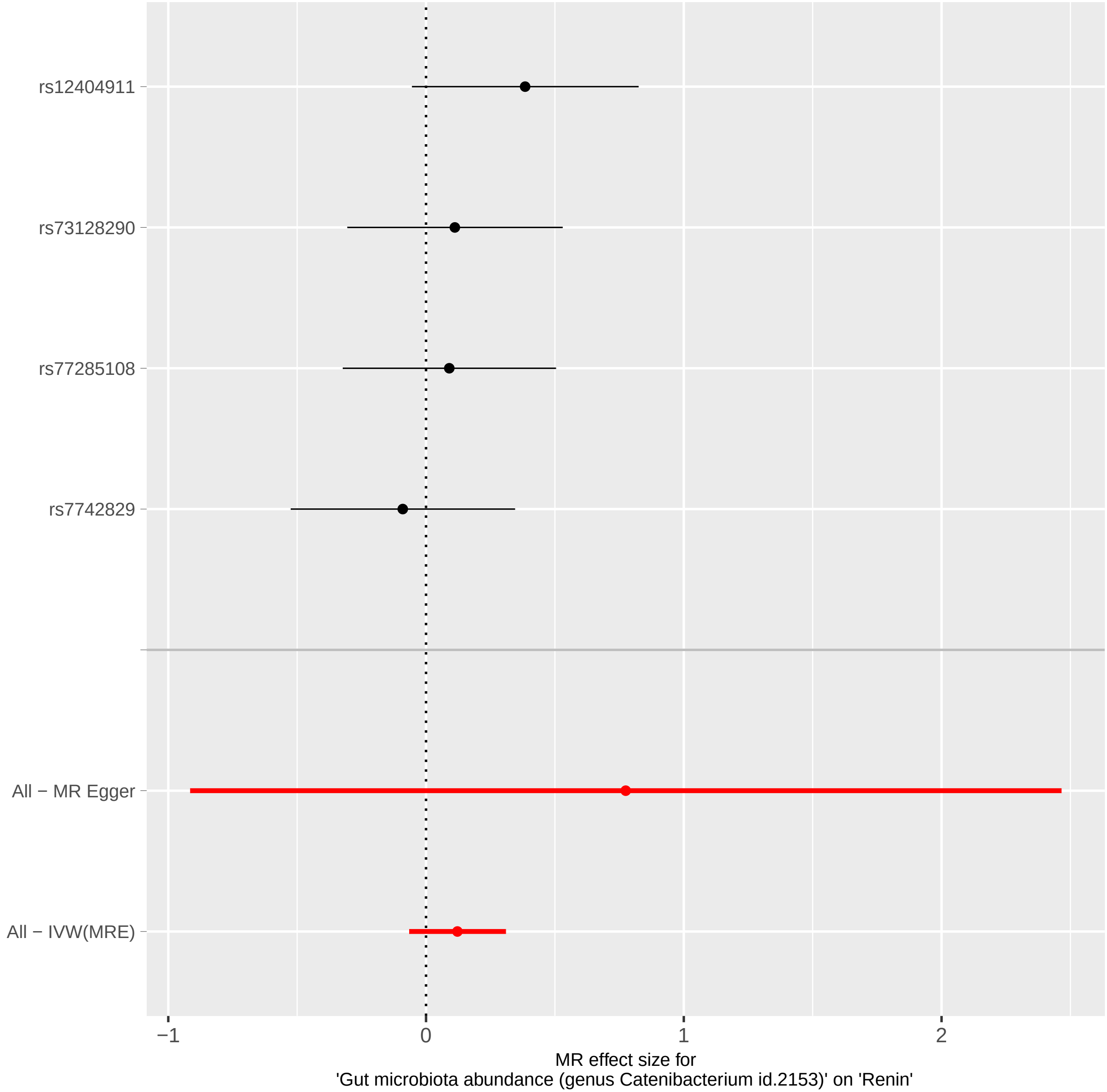

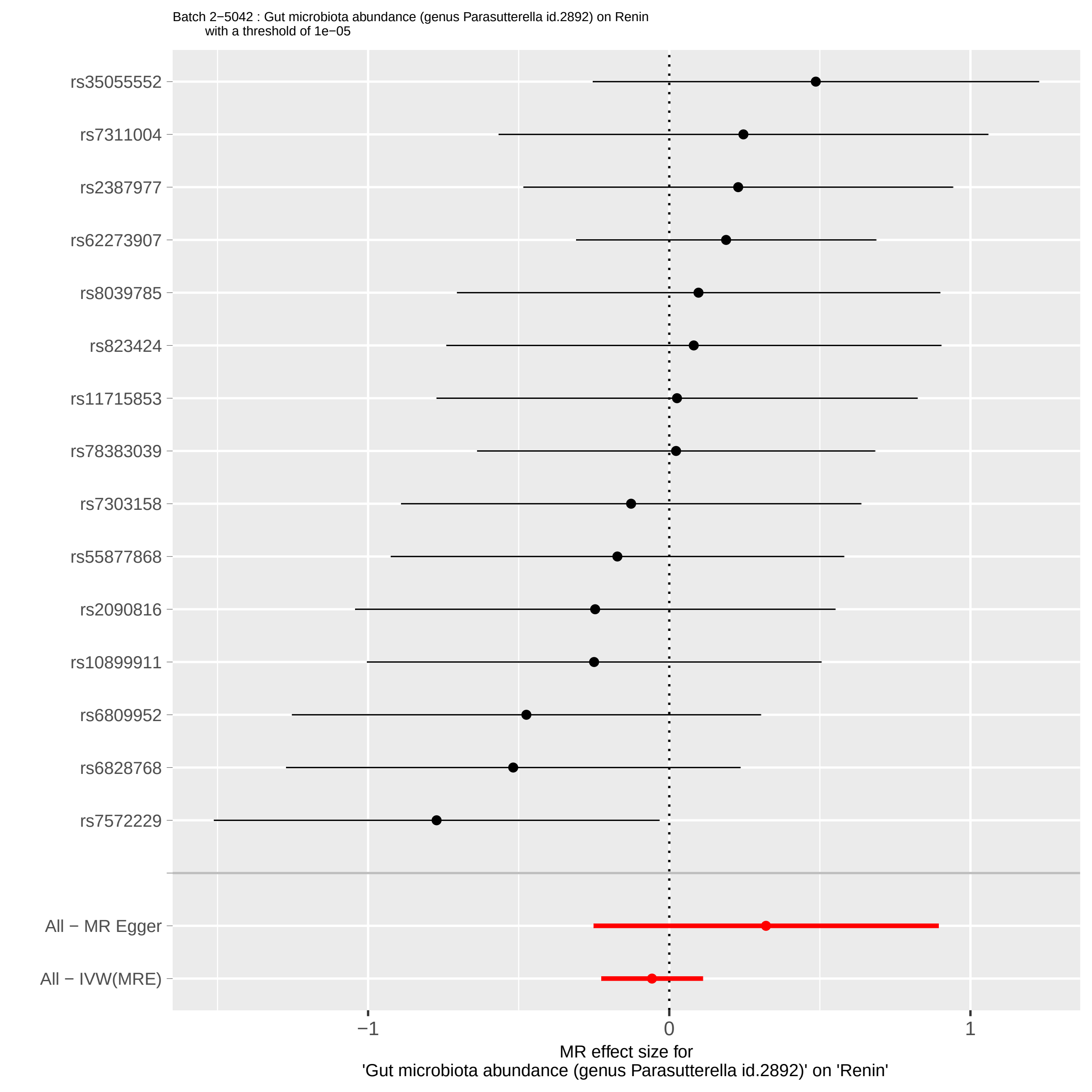

Batch 2−5042 : Gut microbiota abundance (genus Parasutterella id.2892) on Renin
with a threshold of 1e−05
rs35055552
rs7311004
rs2387977
rs62273907
rs8039785
rs823424
rs11715853
rs78383039
rs7303158
rs55877868
rs2090816
rs10899911
rs6809952
rs6828768
rs7572229
All − MR Egger
All − IVW(MRE)
−1
0
1
MR effect size for
'Gut microbiota abundance (genus Parasutterella id.2892)' on 'Renin'

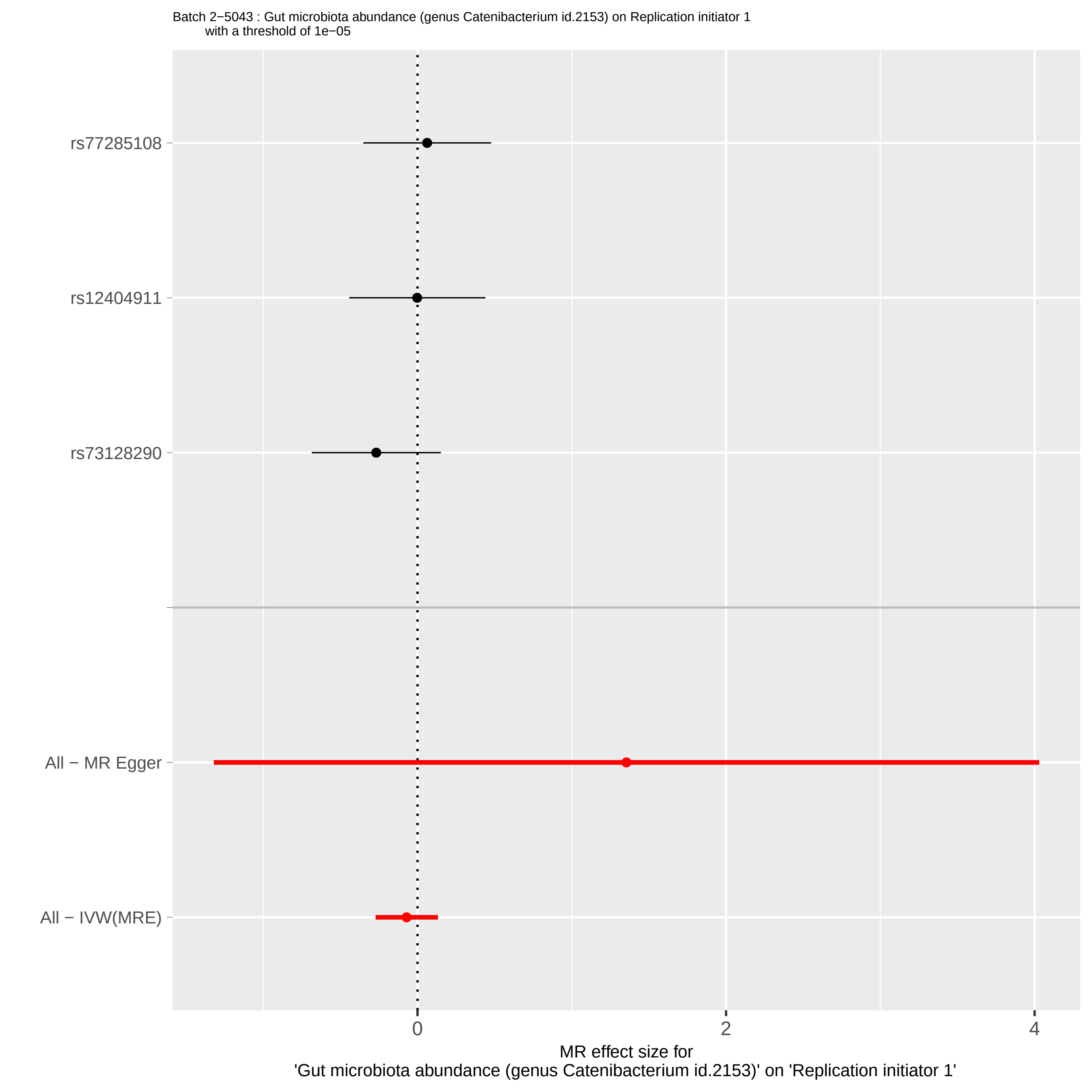
Batch 2−5043 : Gut microbiota abundance (genus Catenibacterium id.2153) on Replication initiator 1
with a threshold of 1e−05
rs77285108
rs12404911
rs73128290
All − MR Egger
All − IVW(MRE)
0
2
4
MR effect size for
'Gut microbiota abundance (genus Catenibacterium id.2153)' on 'Replication initiator 1'

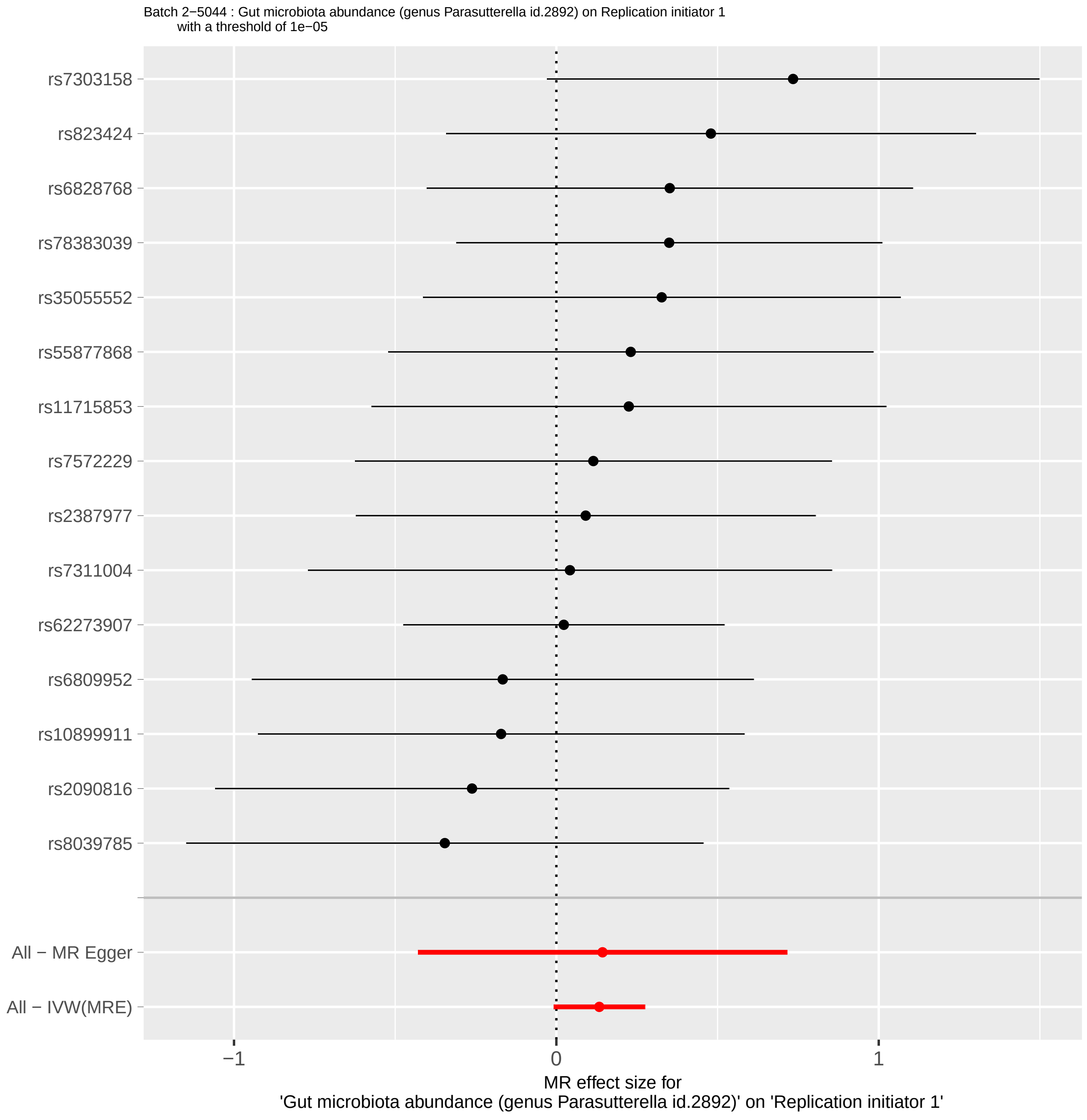

Batch 2−5044 : Gut microbiota abundance (genus Parasutterella id.2892) on Replication initiator 1
with a threshold of 1e−05
rs7303158
rs823424
rs6828768
rs78383039
rs35055552
rs55877868
rs11715853
rs7572229
rs2387977
rs7311004
rs62273907
rs6809952
rs10899911
rs2090816
rs8039785
All − MR Egger
All − IVW(MRE)
−1
0
1
MR effect size for
'Gut microbiota abundance (genus Parasutterella id.2892)' on 'Replication initiator 1'

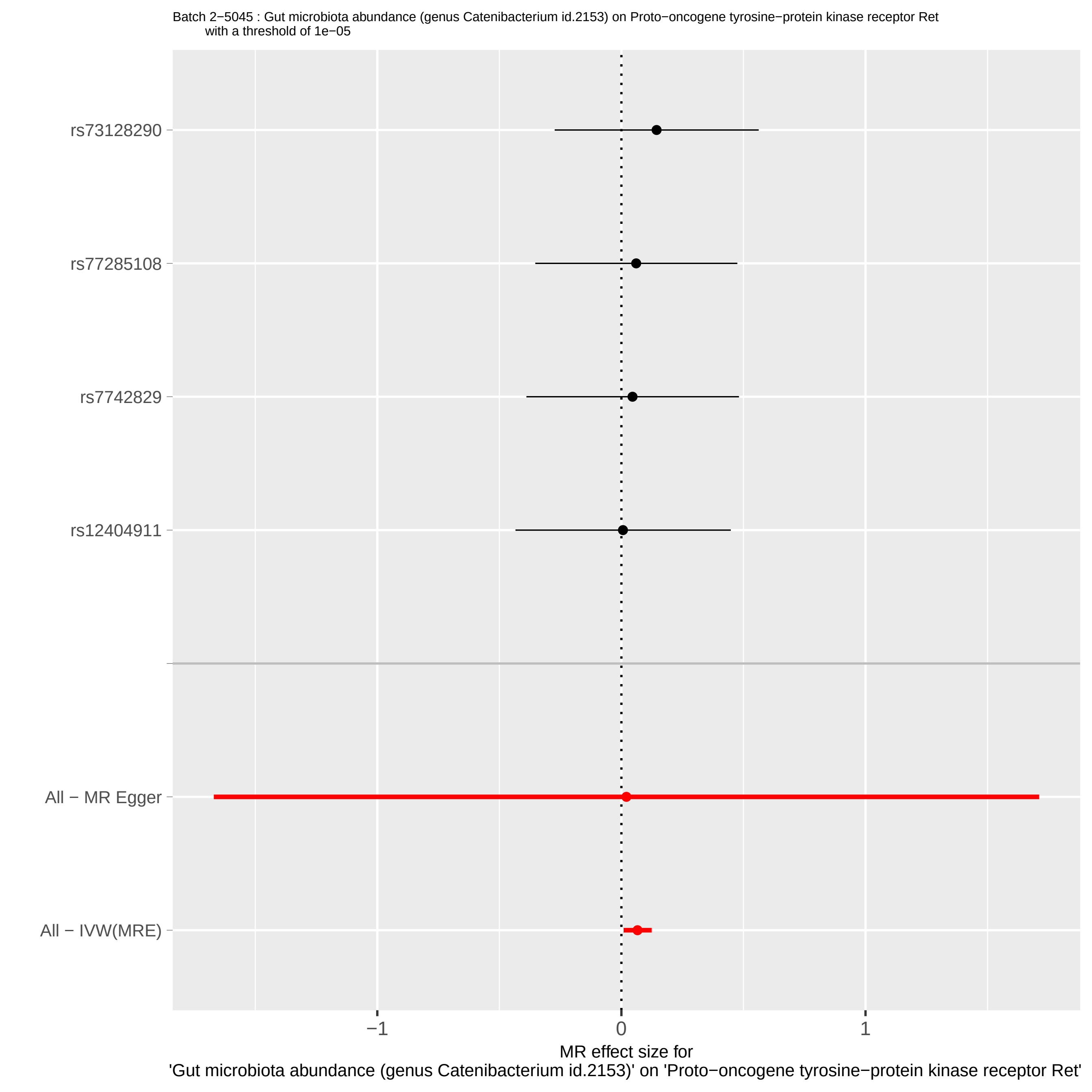

Batch 2−5045 : Gut microbiota abundance (genus Catenibacterium id.2153) on Proto−oncogene tyrosine−protein kinase receptor Ret
with a threshold of 1e−05
rs73128290
rs77285108
rs7742829
rs12404911
All − MR Egger
All − IVW(MRE)
−1
0
1
MR effect size for
'Gut microbiota abundance (genus Catenibacterium id.2153)' on 'Proto−oncogene tyrosine−protein kinase receptor Ret'

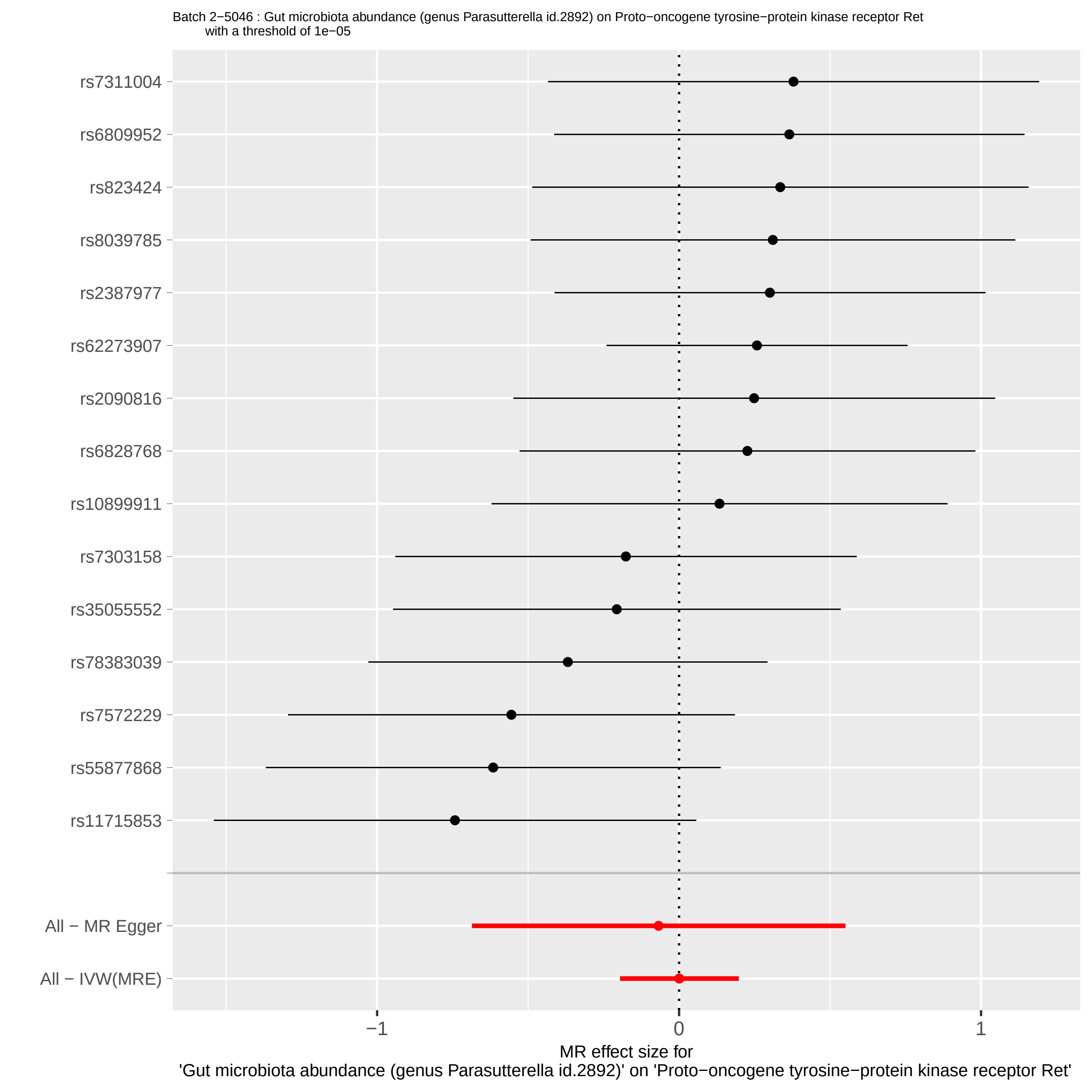

Batch 2−5046 : Gut microbiota abundance (genus Parasutterella id.2892) on Proto−oncogene tyrosine−protein kinase receptor Ret
with a threshold of 1e−05
rs7311004
rs6809952
rs823424
rs8039785
rs2387977
rs62273907
rs2090816
rs6828768
rs10899911
rs7303158
rs35055552
rs78383039
rs7572229
rs55877868
rs11715853
All − MR Egger
All − IVW(MRE)
−1
0
1
MR effect size for
'Gut microbiota abundance (genus Parasutterella id.2892)' on 'Proto−oncogene tyrosine−protein kinase receptor Ret'

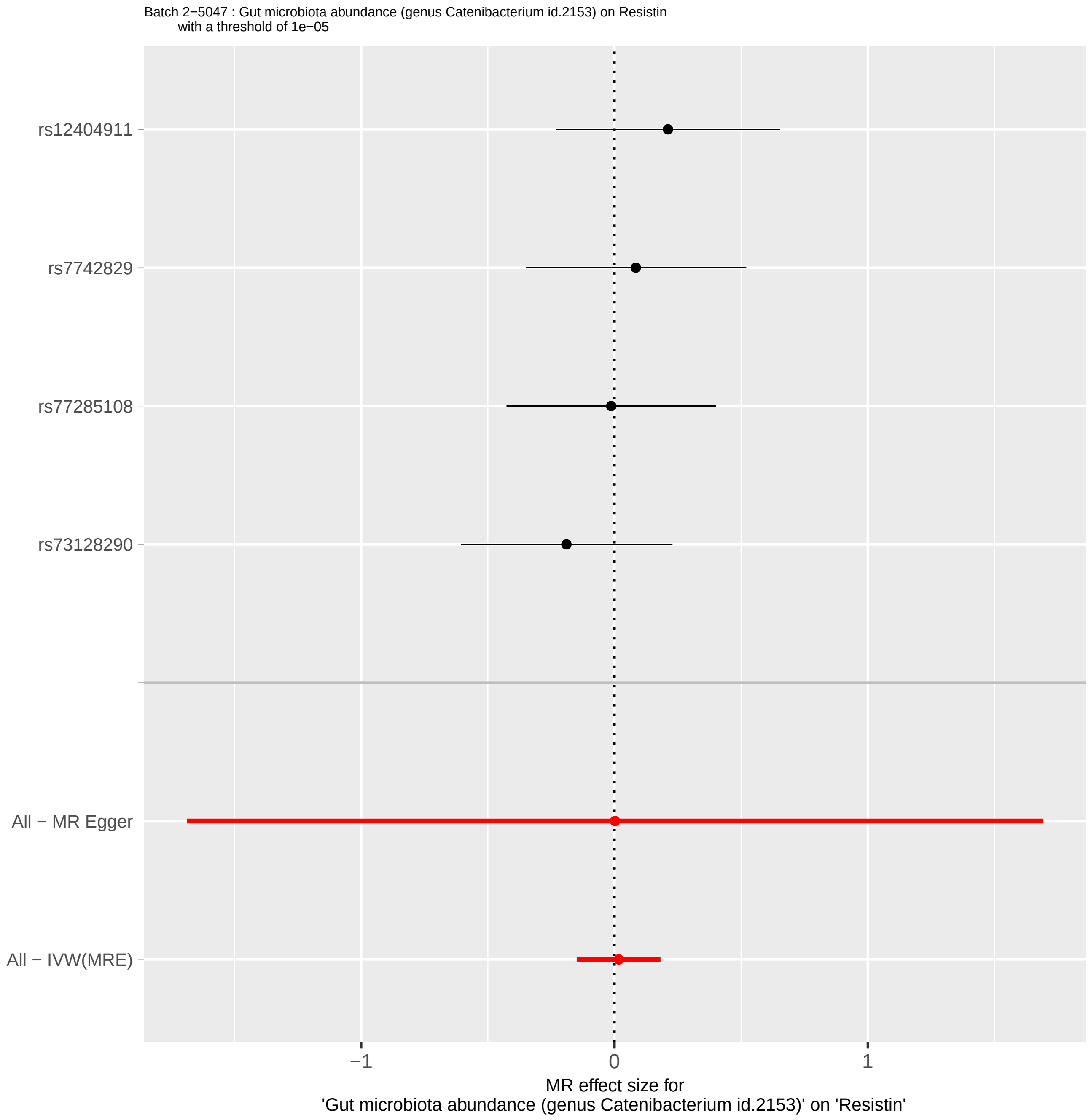

Batch 2−5047 : Gut microbiota abundance (genus Catenibacterium id.2153) on Resistin
with a threshold of 1e−05
rs12404911
rs7742829
rs77285108
rs73128290
All − MR Egger
All − IVW(MRE)
−1
0
1
MR effect size for
'Gut microbiota abundance (genus Catenibacterium id.2153)' on 'Resistin'

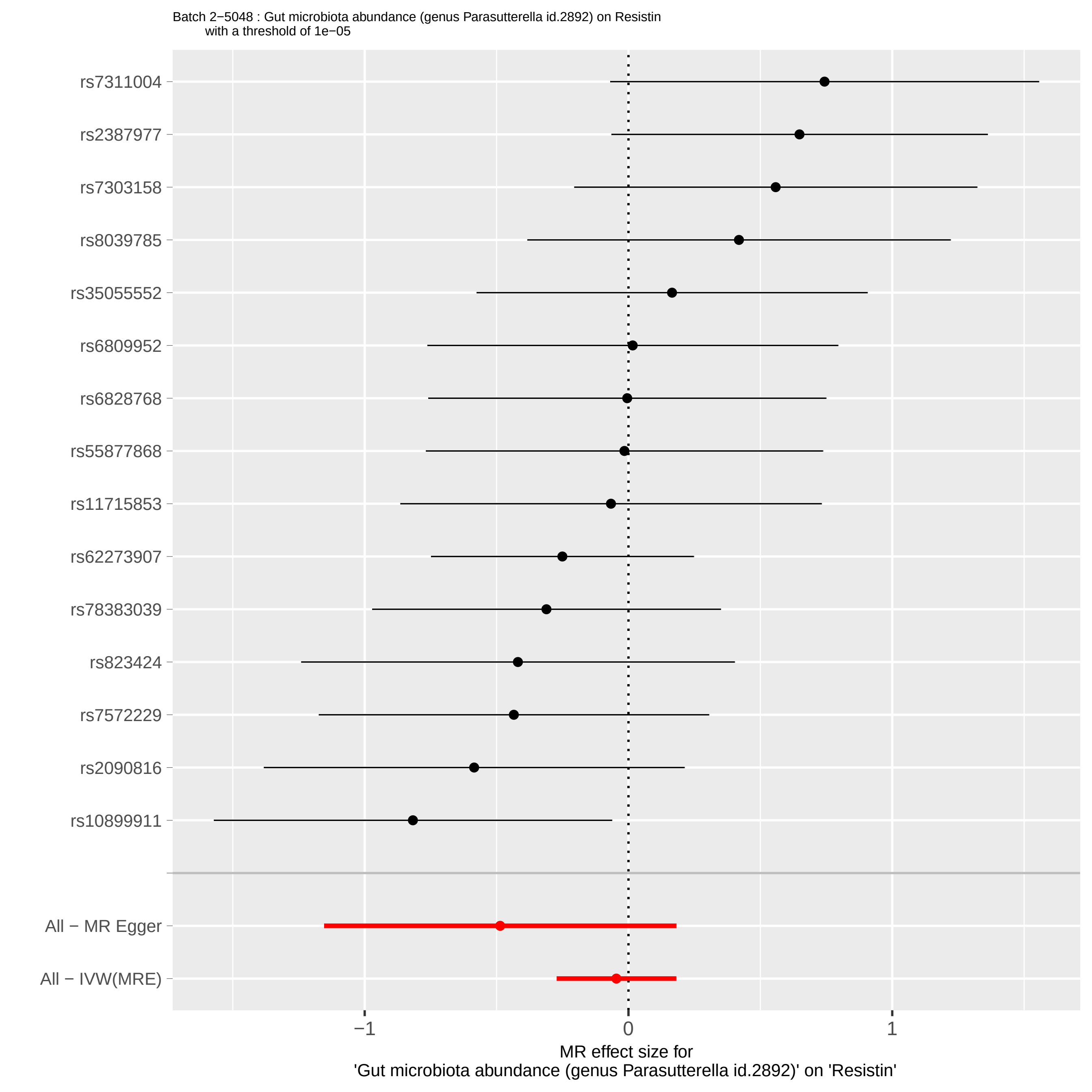

Batch 2−5048 : Gut microbiota abundance (genus Parasutterella id.2892) on Resistin
with a threshold of 1e−05
rs7311004
rs2387977
rs7303158
rs8039785
rs35055552
rs6809952
rs6828768
rs55877868
rs11715853
rs62273907
rs78383039
rs823424
rs7572229
rs2090816
rs10899911
All − MR Egger
All − IVW(MRE)
−1
0
1
MR effect size for
'Gut microbiota abundance (genus Parasutterella id.2892)' on 'Resistin'

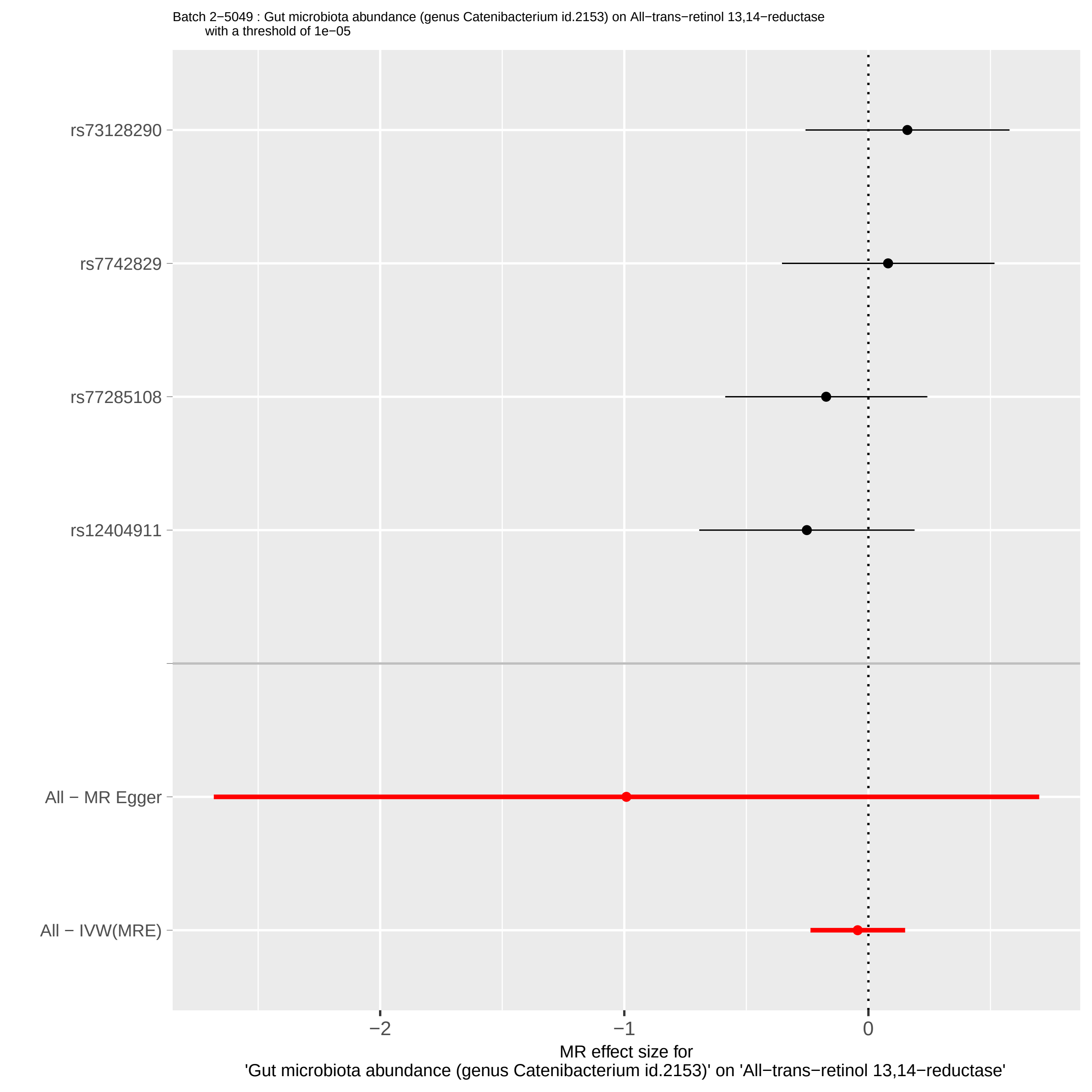

Batch 2−5049 : Gut microbiota abundance (genus Catenibacterium id.2153) on All−trans−retinol 13,14−reductase
with a threshold of 1e−05
rs73128290
rs7742829
rs77285108
rs12404911
All − MR Egger
All − IVW(MRE)
−2
−1
0
MR effect size for
'Gut microbiota abundance (genus Catenibacterium id.2153)' on 'All−trans−retinol 13,14−reductase'

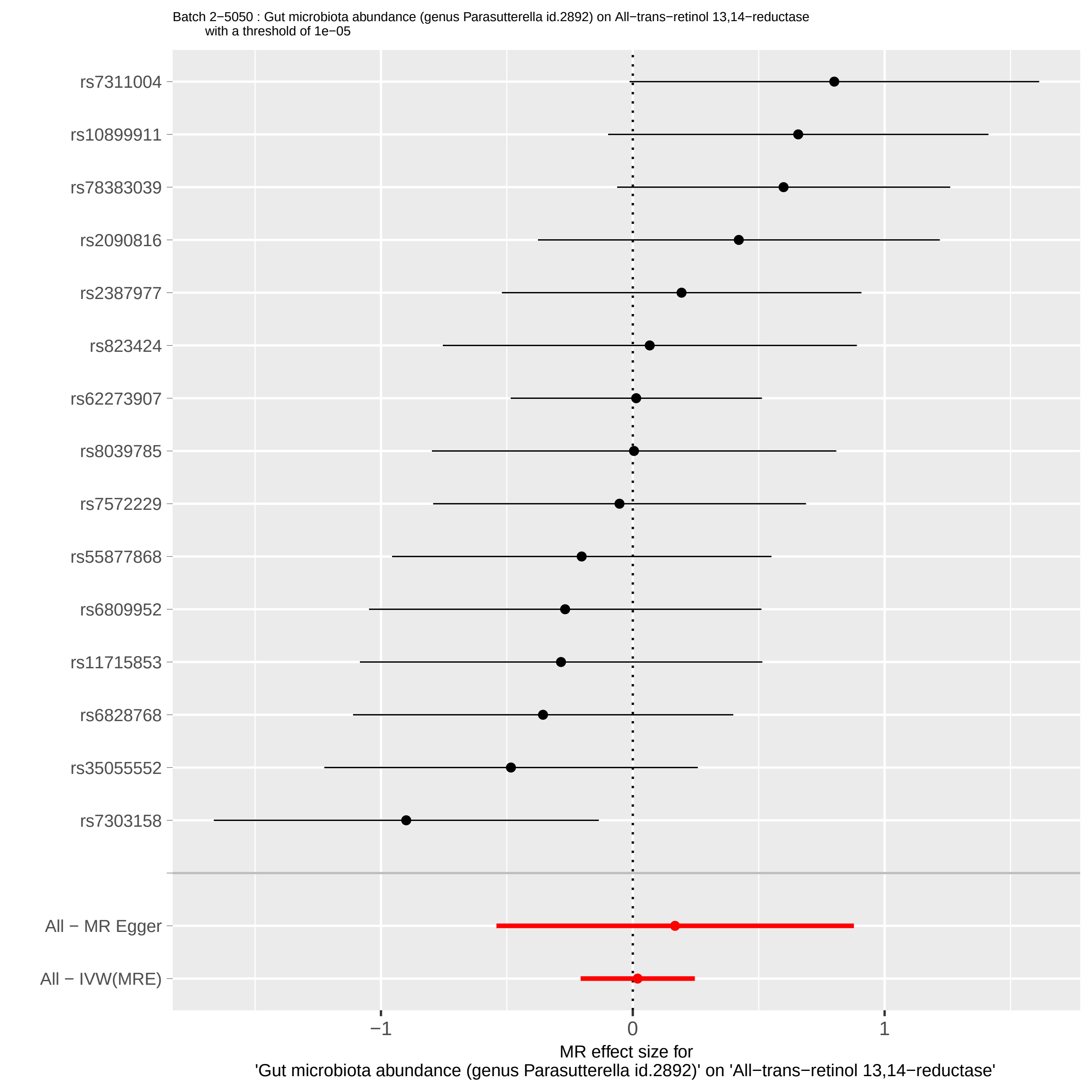

Batch 2−5050 : Gut microbiota abundance (genus Parasutterella id.2892) on All−trans−retinol 13,14−reductase
with a threshold of 1e−05
rs7311004
rs10899911
rs78383039
rs2090816
rs2387977
rs823424
rs62273907
rs8039785
rs7572229
rs55877868
rs6809952
rs11715853
rs6828768
rs35055552
rs7303158
All − MR Egger
All − IVW(MRE)
−1
0
1
MR effect size for
'Gut microbiota abundance (genus Parasutterella id.2892)' on 'All−trans−retinol 13,14−reductase'

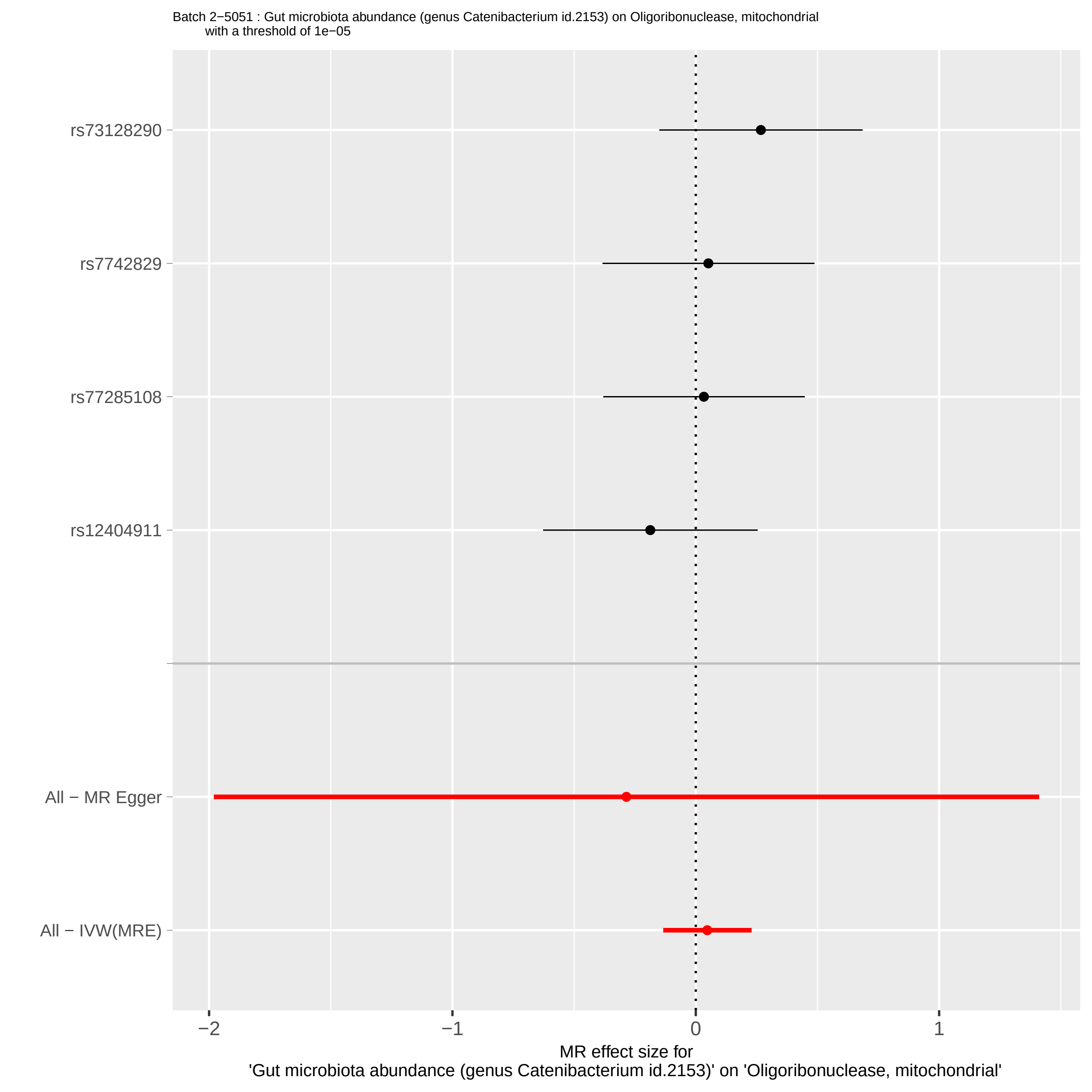

Batch 2−5051 : Gut microbiota abundance (genus Catenibacterium id.2153) on Oligoribonuclease, mitochondrial
with a threshold of 1e−05
rs73128290
rs7742829
rs77285108
rs12404911
All − MR Egger
All − IVW(MRE)
−2
−1
0
1
MR effect size for
'Gut microbiota abundance (genus Catenibacterium id.2153)' on 'Oligoribonuclease, mitochondrial'

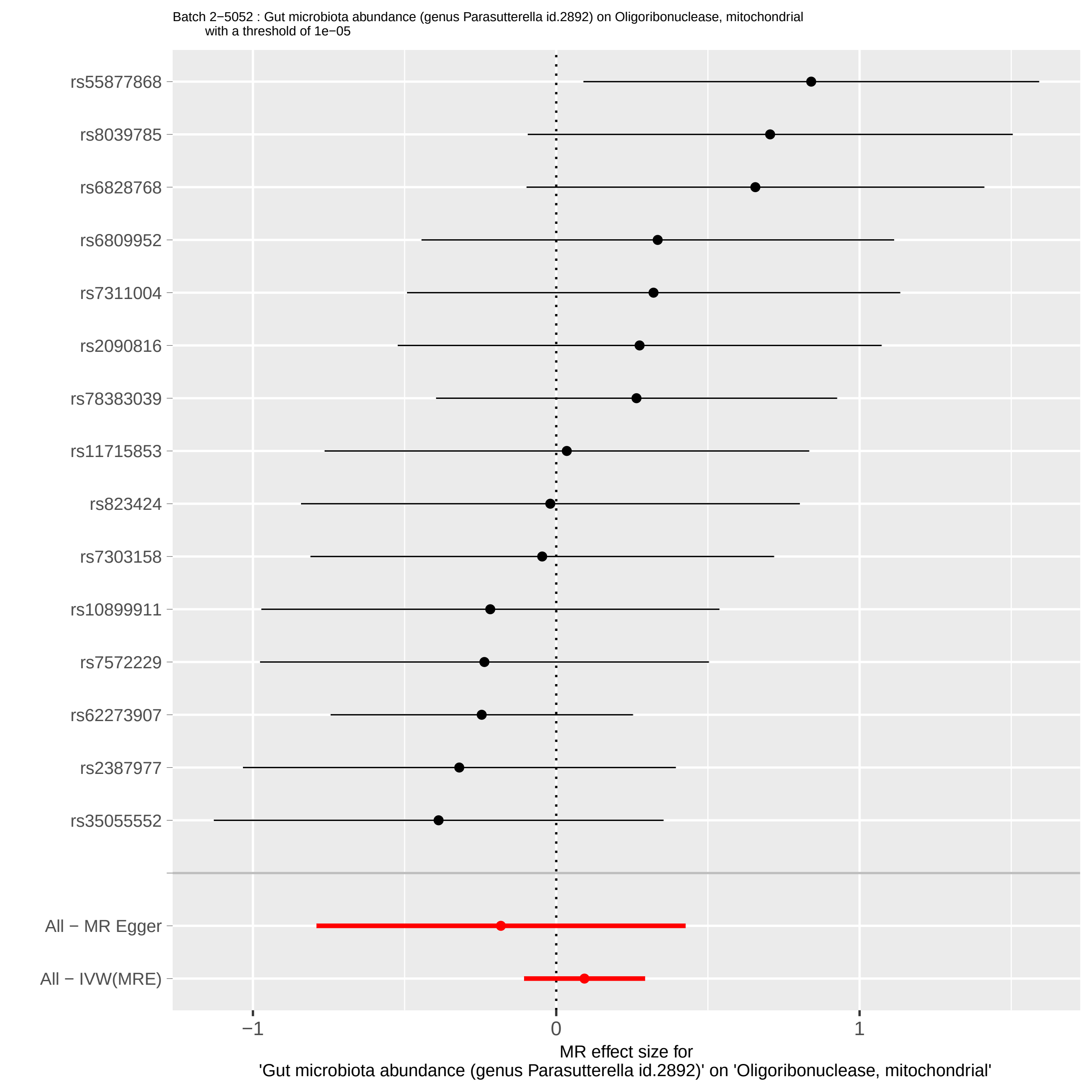
Batch 2−5052 : Gut microbiota abundance (genus Parasutterella id.2892) on Oligoribonuclease, mitochondrial
with a threshold of 1e−05
rs55877868
rs8039785
rs6828768
rs6809952
rs7311004
rs2090816
rs78383039
rs11715853
rs823424
rs7303158
rs10899911
rs7572229
rs62273907
rs2387977
rs35055552
All − MR Egger
All − IVW(MRE)
−1
0
1
MR effect size for
'Gut microbiota abundance (genus Parasutterella id.2892)' on 'Oligoribonuclease, mitochondrial'

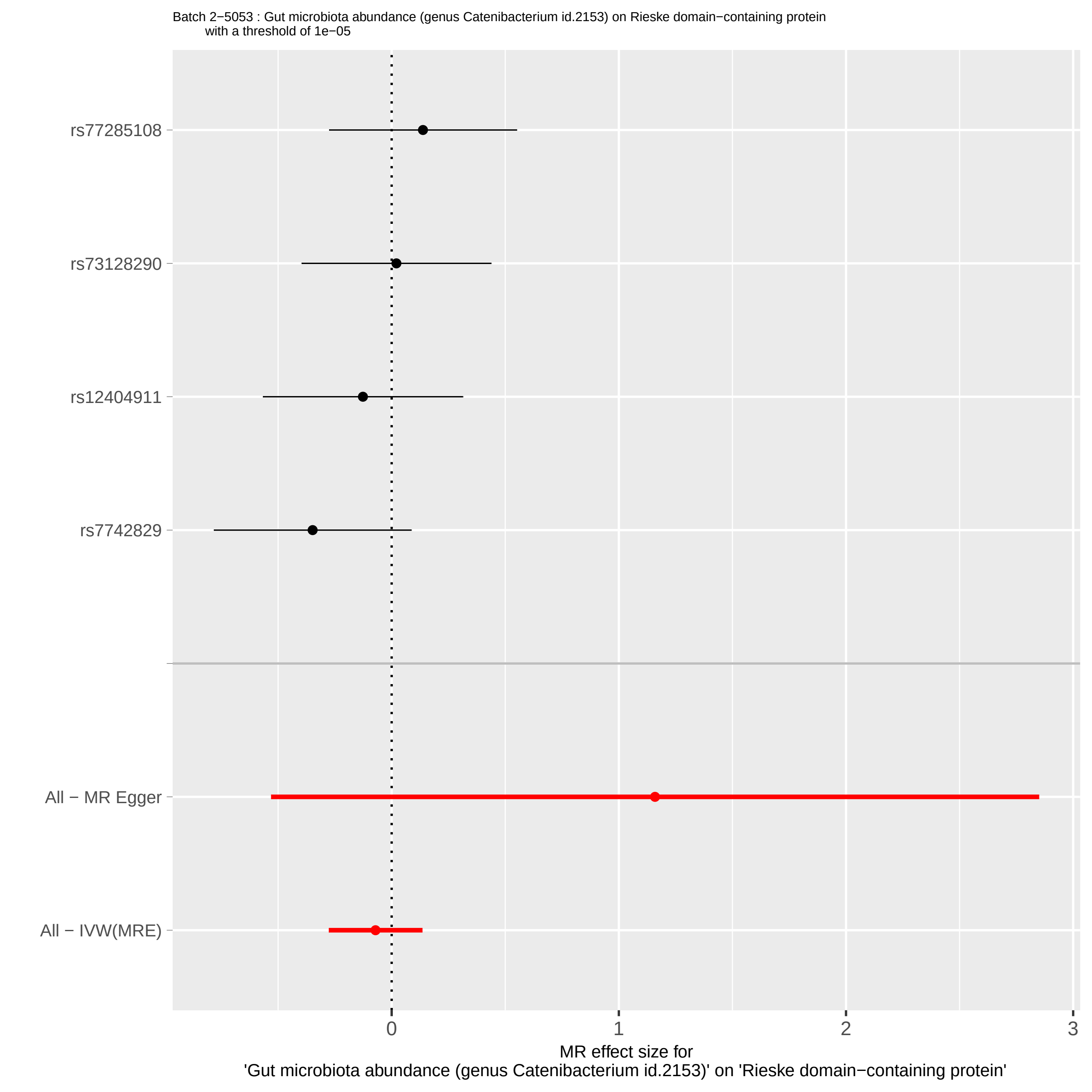

Batch 2−5053 : Gut microbiota abundance (genus Catenibacterium id.2153) on Rieske domain−containing protein
with a threshold of 1e−05
rs77285108
rs73128290
rs12404911
rs7742829
All − MR Egger
All − IVW(MRE)
0
1
2
3
MR effect size for
'Gut microbiota abundance (genus Catenibacterium id.2153)' on 'Rieske domain−containing protein'

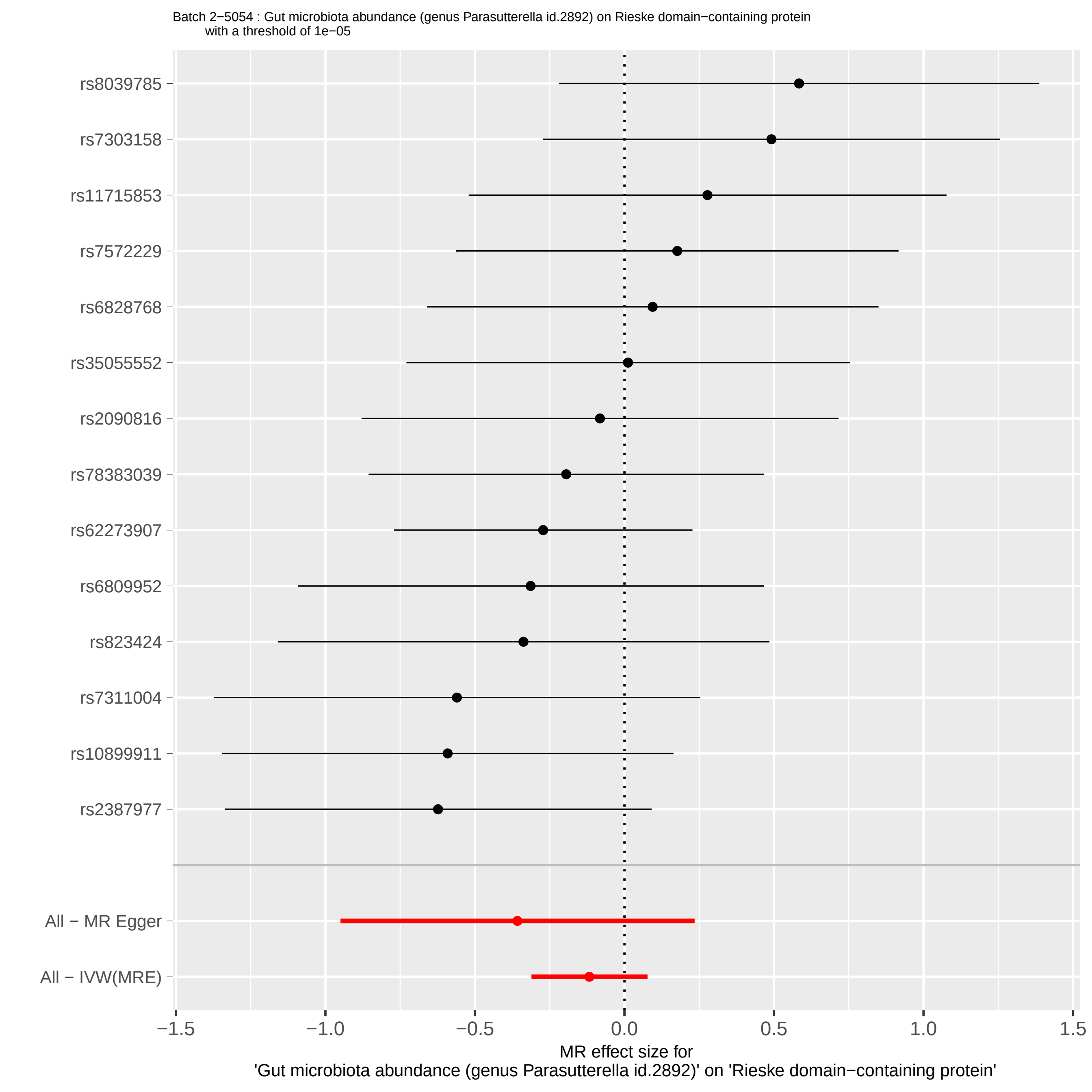

Batch 2−5054 : Gut microbiota abundance (genus Parasutterella id.2892) on Rieske domain−containing protein
with a threshold of 1e−05
rs8039785
rs7303158
rs11715853
rs7572229
rs6828768
rs35055552
rs2090816
rs78383039
rs62273907
rs6809952
rs823424
rs7311004
rs10899911
rs2387977
All − MR Egger
All − IVW(MRE)
−1.5
−1.0
−0.5
0.0
0.5
1.0
1.5
MR effect size for
'Gut microbiota abundance (genus Parasutterella id.2892)' on 'Rieske domain−containing protein'

Batch 2−5055 : Gut microbiota abundance (genus Catenibacterium id.2153) on E3 ubiquitin−protein ligase rififylin
with a threshold of 1e−05

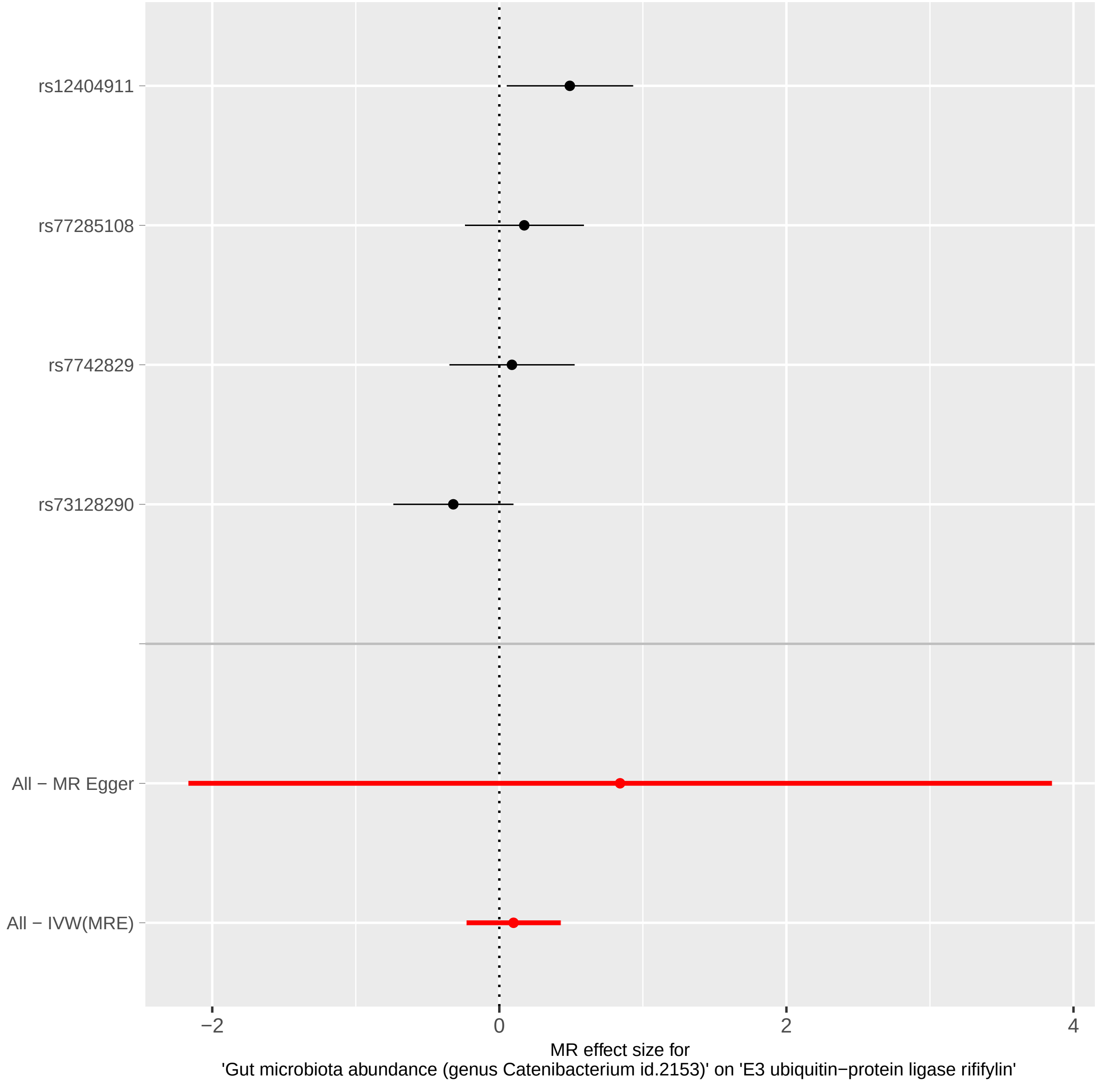

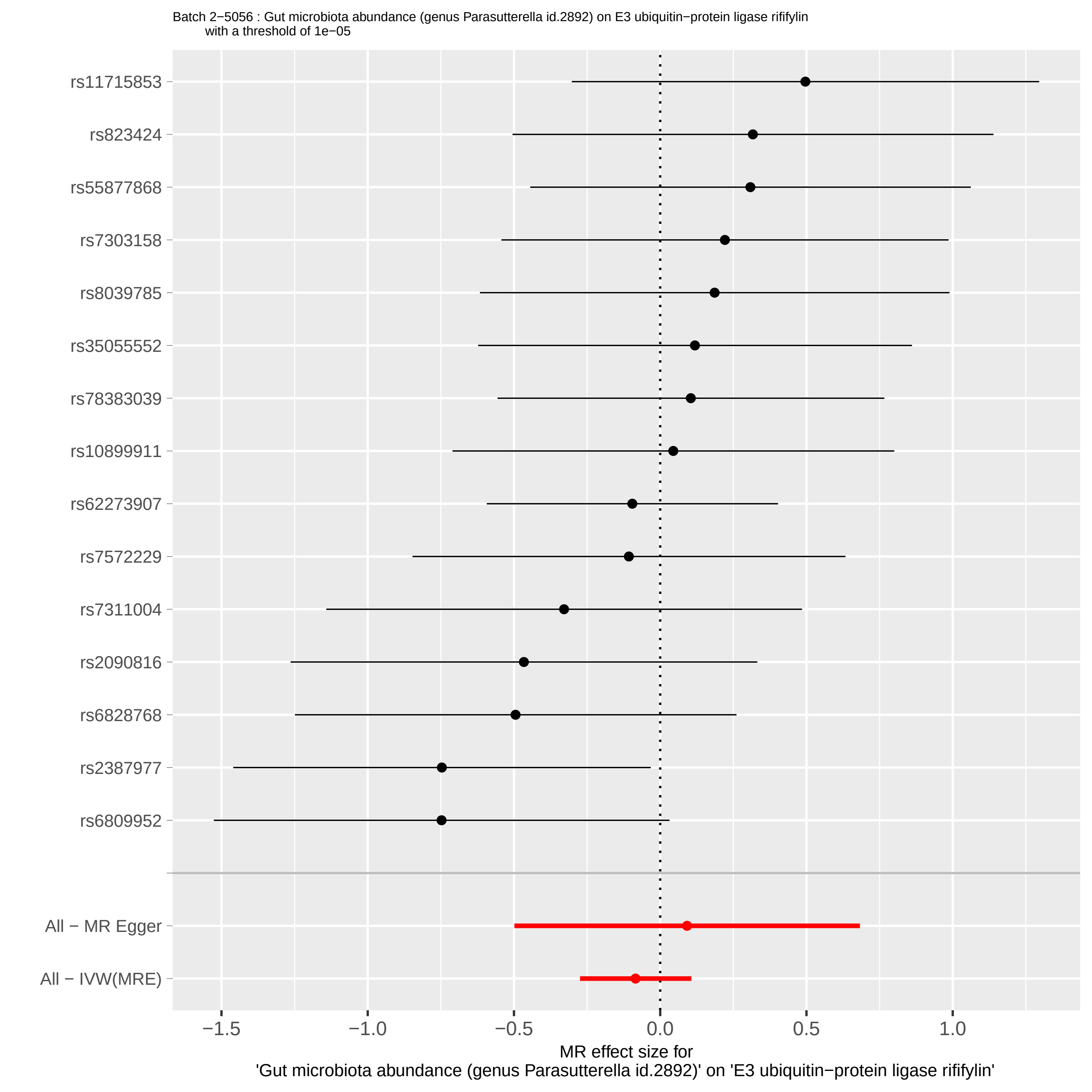
Batch 2−5056 : Gut microbiota abundance (genus Parasutterella id.2892) on E3 ubiquitin−protein ligase rififylin
with a threshold of 1e−05
rs11715853
rs823424
rs55877868
rs7303158
rs8039785
rs35055552
rs78383039
rs10899911
rs62273907
rs7572229
rs7311004
rs2090816
rs6828768
rs2387977
rs6809952
All − MR Egger
All − IVW(MRE)
−1.5
−1.0
−0.5
0.0
0.5
1.0
MR effect size for
'Gut microbiota abundance (genus Parasutterella id.2892)' on 'E3 ubiquitin−protein ligase rififylin'

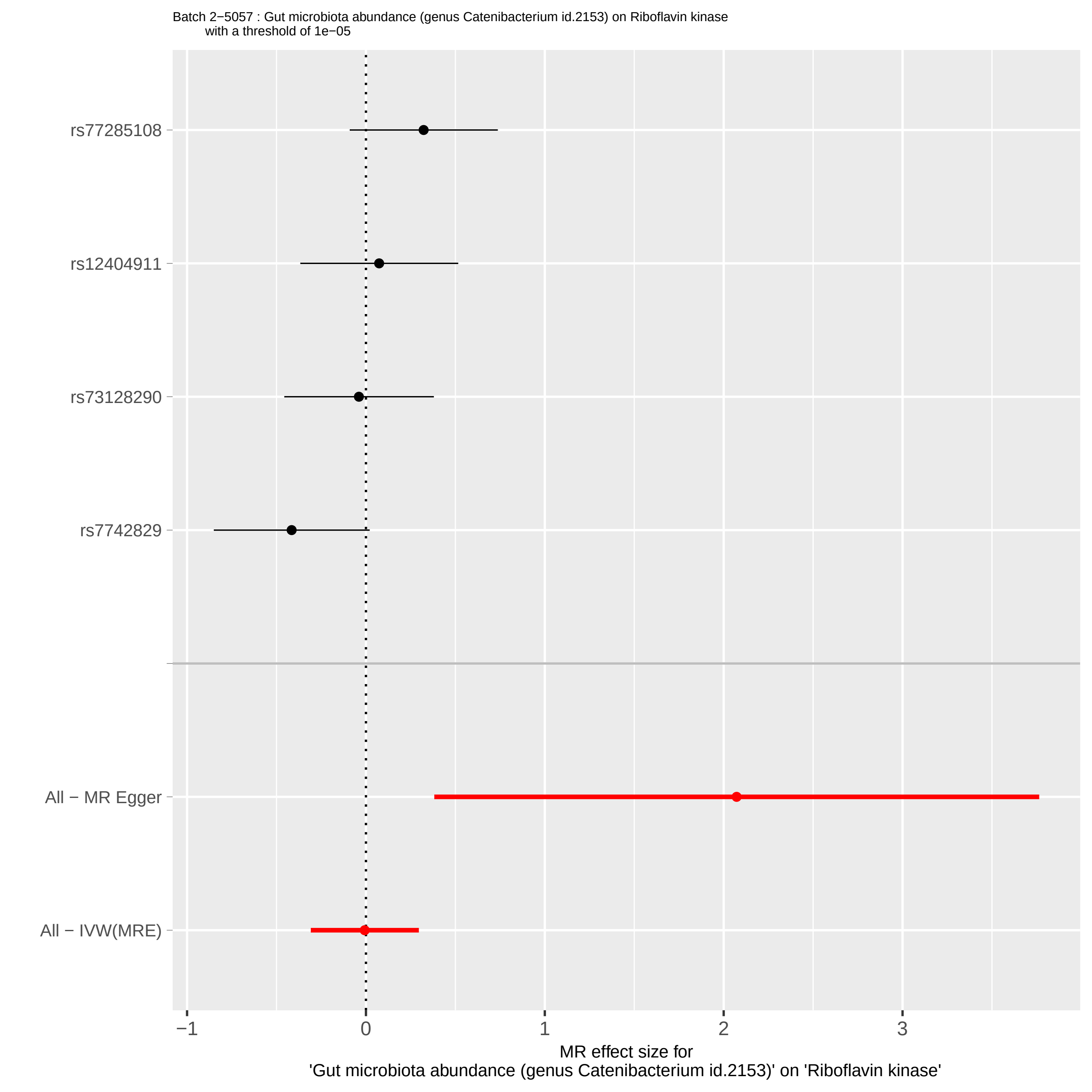

Batch 2−5057 : Gut microbiota abundance (genus Catenibacterium id.2153) on Riboflavin kinase
with a threshold of 1e−05
rs77285108
rs12404911
rs73128290
rs7742829
All − MR Egger
All − IVW(MRE)
−1
0
1
2
3
MR effect size for
'Gut microbiota abundance (genus Catenibacterium id.2153)' on 'Riboflavin kinase'

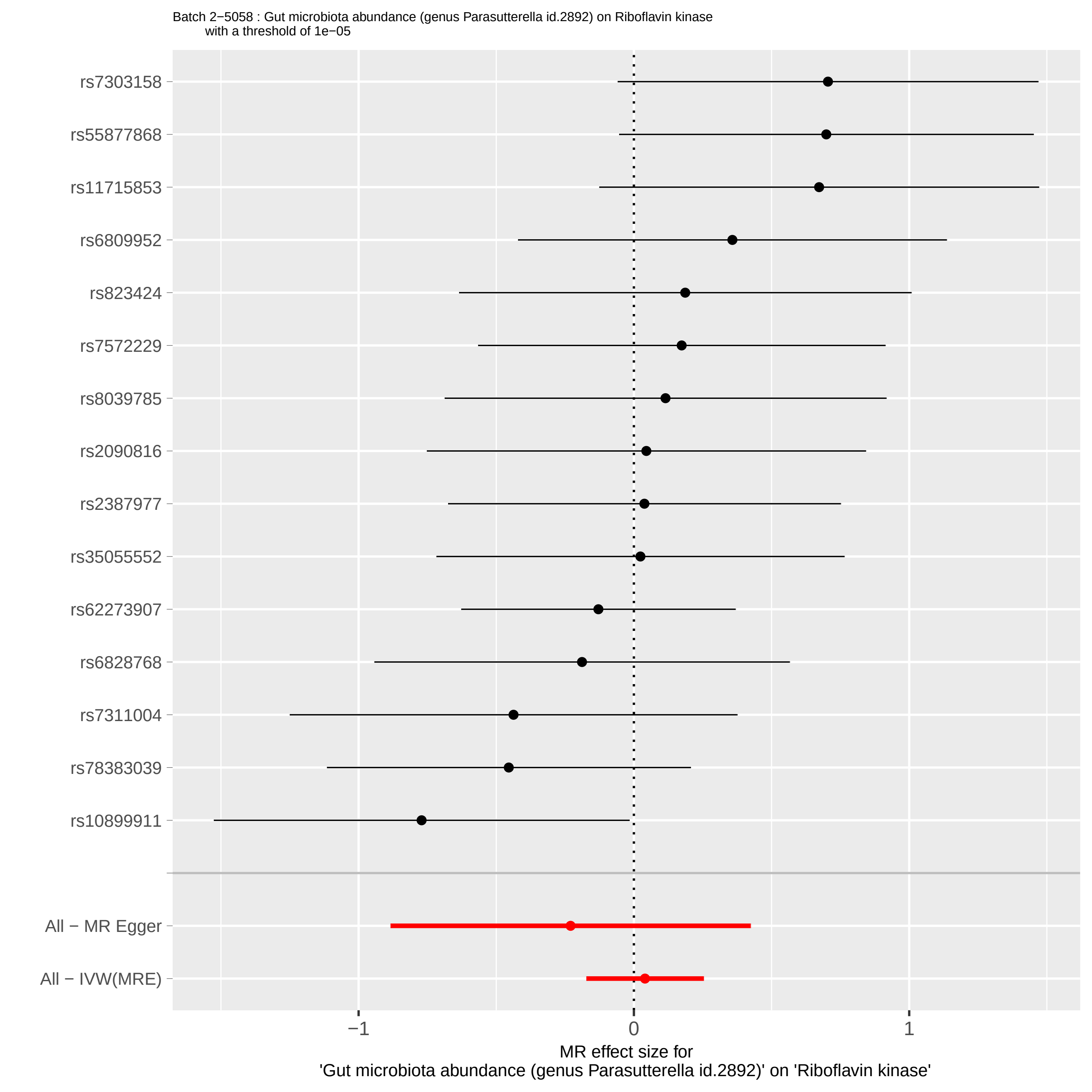
Batch 2−5058 : Gut microbiota abundance (genus Parasutterella id.2892) on Riboflavin kinase
with a threshold of 1e−05
rs7303158
rs55877868
rs11715853
rs6809952
rs823424
rs7572229
rs8039785
rs2090816
rs2387977
rs35055552
rs62273907
rs6828768
rs7311004
rs78383039
rs10899911
All − MR Egger
All − IVW(MRE)
−1
0
1
MR effect size for
'Gut microbiota abundance (genus Parasutterella id.2892)' on 'Riboflavin kinase'

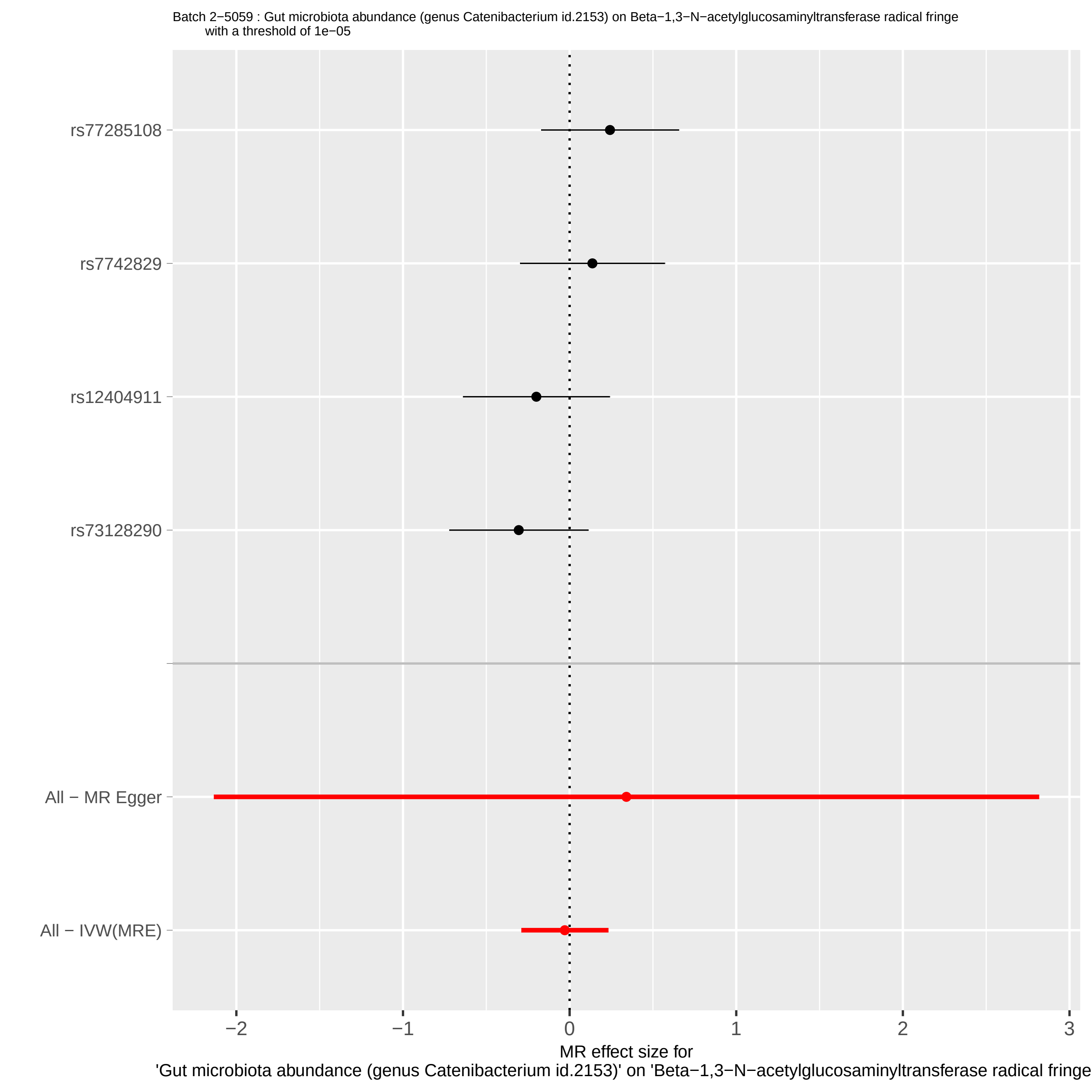
Batch 2−5059 : Gut microbiota abundance (genus Catenibacterium id.2153) on Beta−1,3−N−acetylglucosaminyltransferase radical fringe
with a threshold of 1e−05
rs77285108
rs7742829
rs12404911
rs73128290
All − MR Egger
All − IVW(MRE)
−2
−1
0
1
2
3
MR effect size for
'Gut microbiota abundance (genus Catenibacterium id.2153)' on 'Beta−1,3−N−acetylglucosaminyltransferase radical fringe'

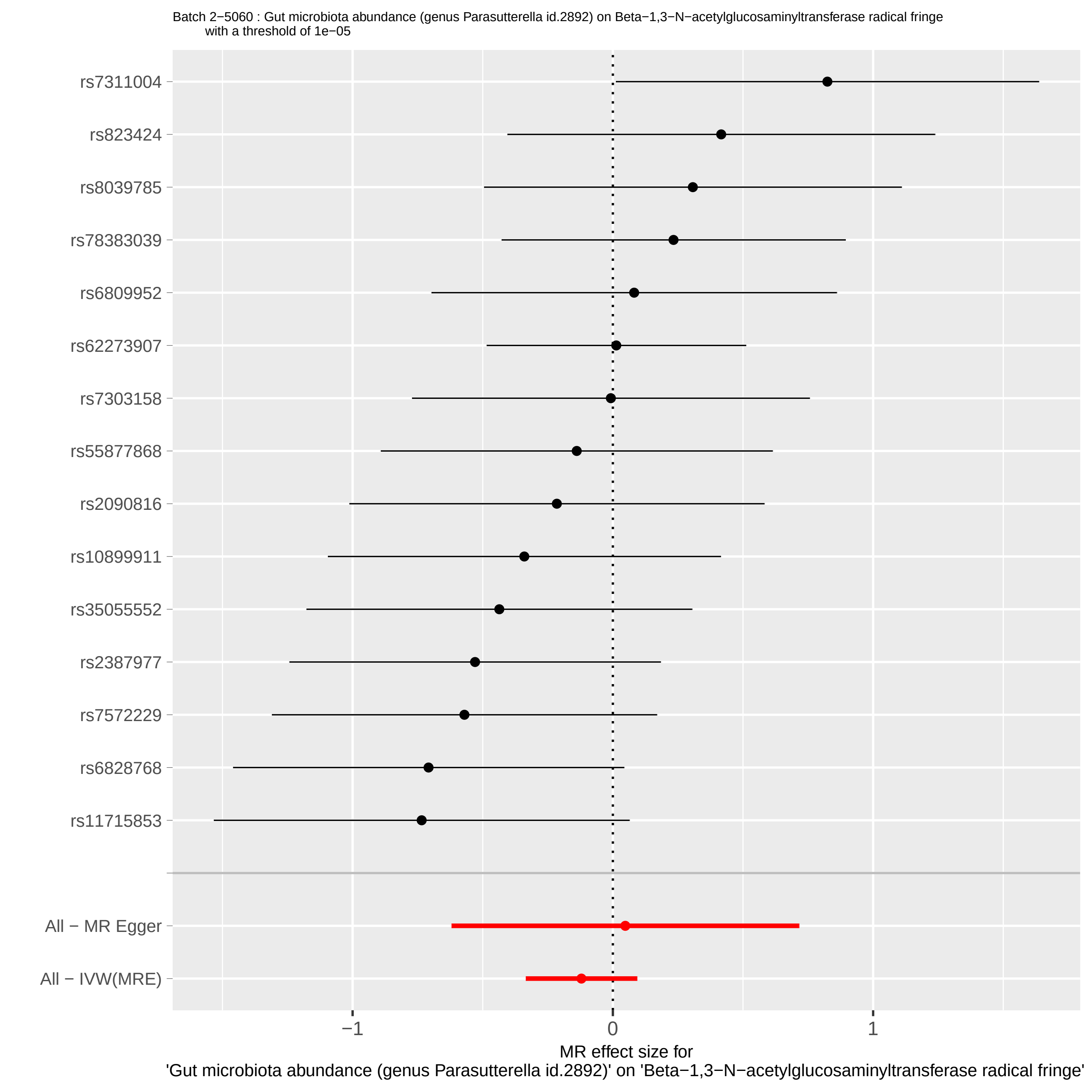

Batch 2−5060 : Gut microbiota abundance (genus Parasutterella id.2892) on Beta−1,3−N−acetylglucosaminyltransferase radical fringe
with a threshold of 1e−05
rs7311004
rs823424
rs8039785
rs78383039
rs6809952
rs62273907
rs7303158
rs55877868
rs2090816
rs10899911
rs35055552
rs2387977
rs7572229
rs6828768
rs11715853
All − MR Egger
All − IVW(MRE)
−1
0
1
MR effect size for
'Gut microbiota abundance (genus Parasutterella id.2892)' on 'Beta−1,3−N−acetylglucosaminyltransferase radical fringe'

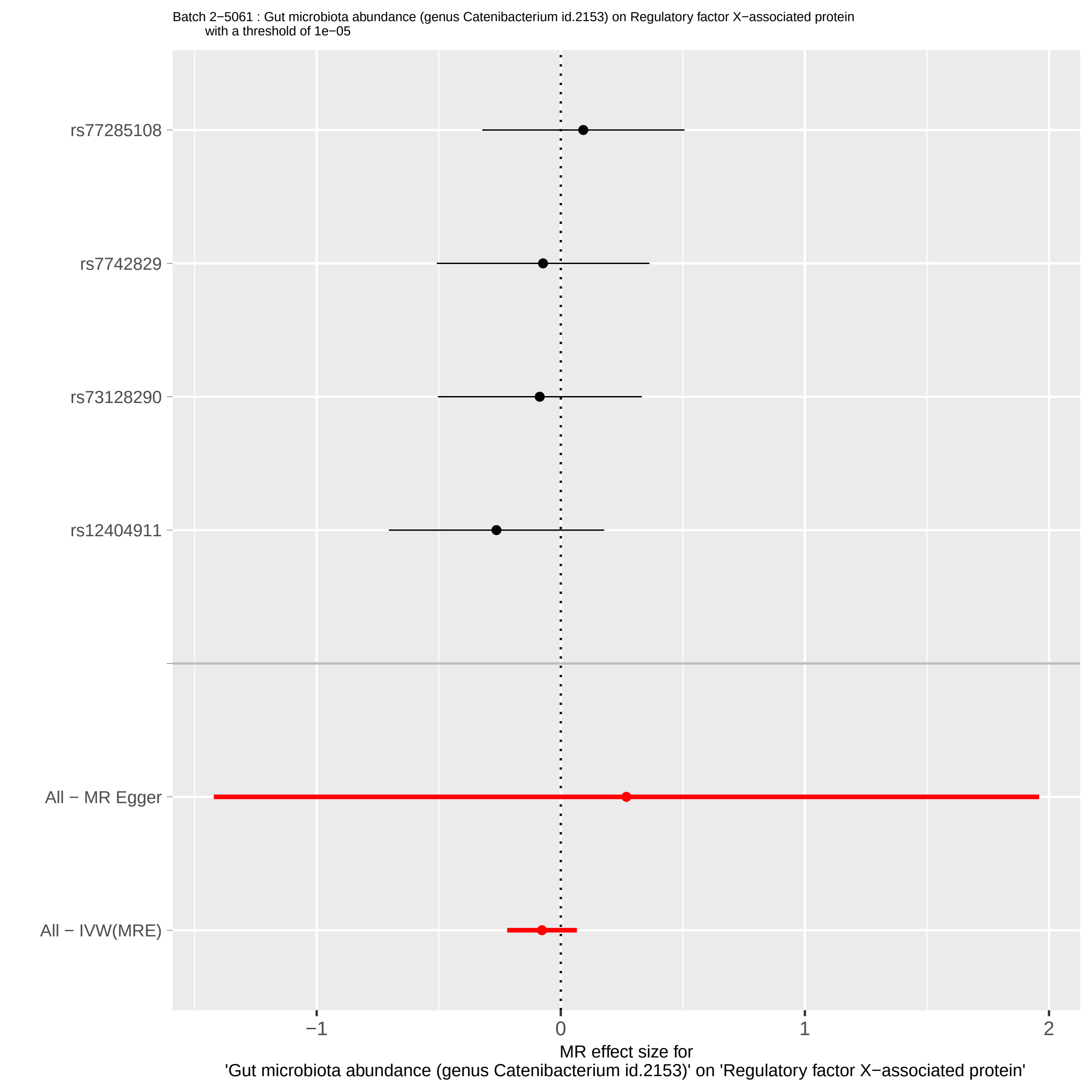
Batch 2−5061 : Gut microbiota abundance (genus Catenibacterium id.2153) on Regulatory factor X−associated protein
with a threshold of 1e−05
rs77285108
rs7742829
rs73128290
rs12404911
All − MR Egger
All − IVW(MRE)
−1
0
1
2
MR effect size for
'Gut microbiota abundance (genus Catenibacterium id.2153)' on 'Regulatory factor X−associated protein'

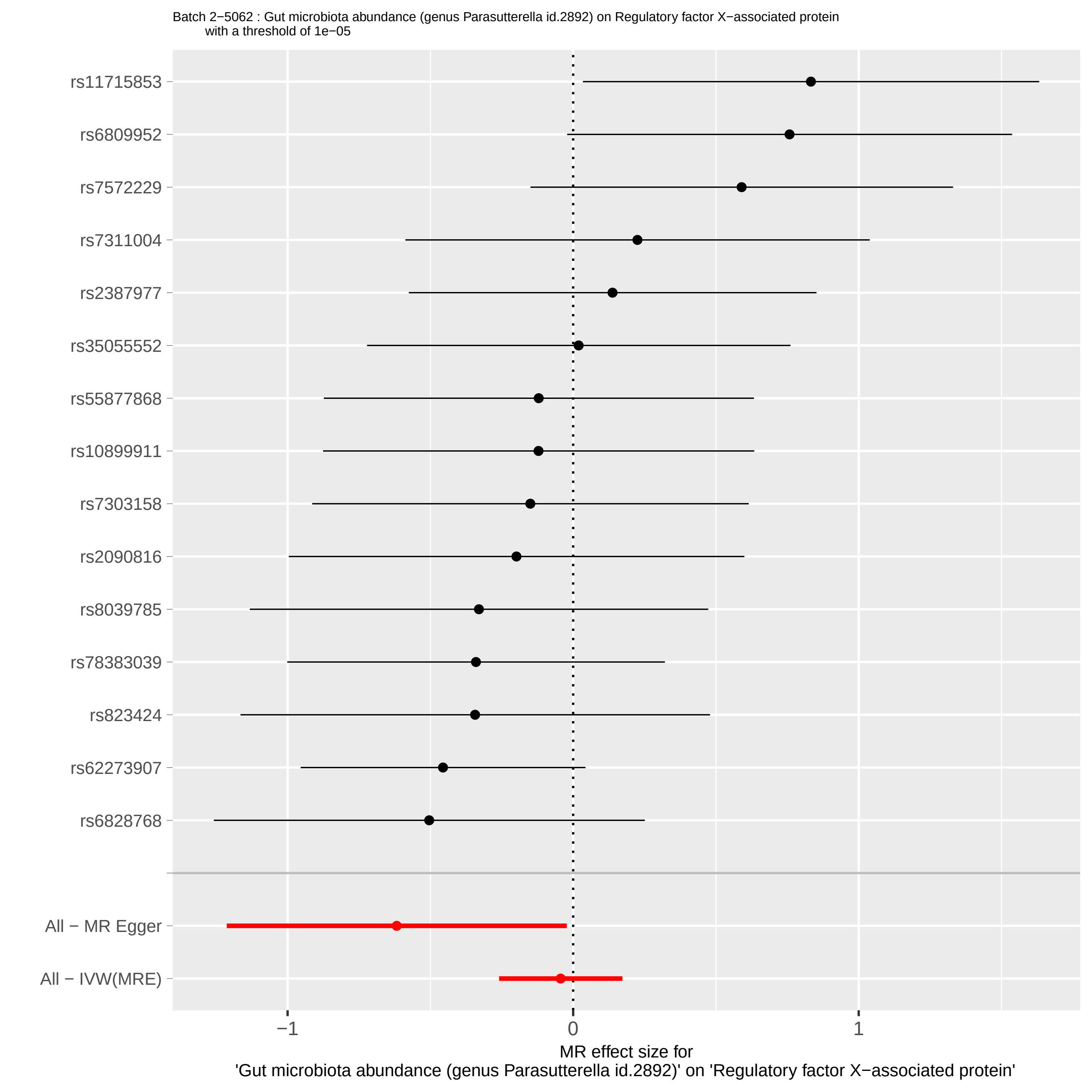

Batch 2−5062 : Gut microbiota abundance (genus Parasutterella id.2892) on Regulatory factor X−associated protein
with a threshold of 1e−05
rs11715853
rs6809952
rs7572229
rs7311004
rs2387977
rs35055552
rs55877868
rs10899911
rs7303158
rs2090816
rs8039785
rs78383039
rs823424
rs62273907
rs6828768
All − MR Egger
All − IVW(MRE)
−1
0
1
MR effect size for
'Gut microbiota abundance (genus Parasutterella id.2892)' on 'Regulatory factor X−associated protein'

Batch 2−5063 : Gut microbiota abundance (genus Catenibacterium id.2153) on Repulsive guidance molecule A
with a threshold of 1e−05

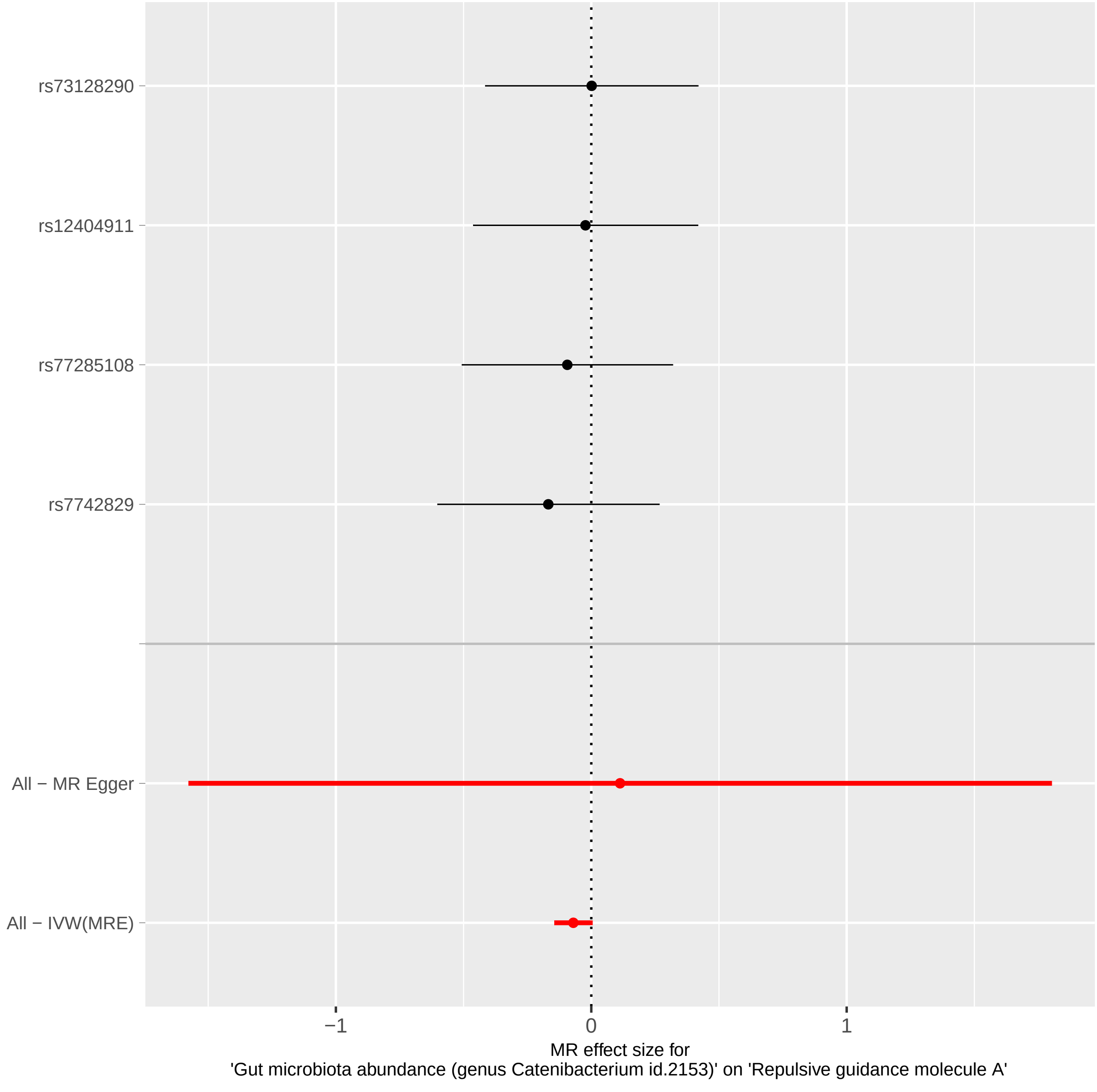

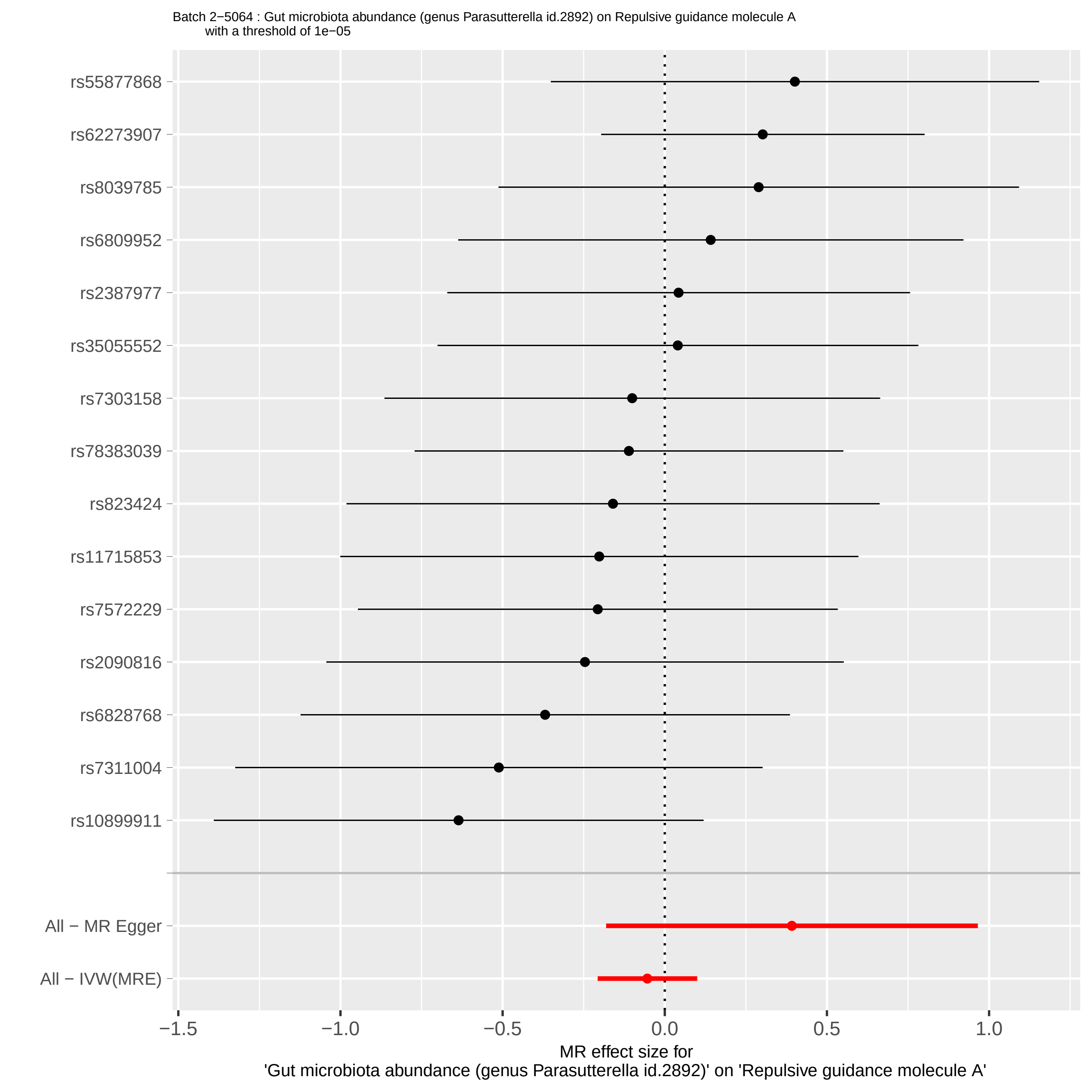

Batch 2−5064 : Gut microbiota abundance (genus Parasutterella id.2892) on Repulsive guidance molecule A
with a threshold of 1e−05
rs55877868
rs62273907
rs8039785
rs6809952
rs2387977
rs35055552
rs7303158
rs78383039
rs823424
rs11715853
rs7572229
rs2090816
rs6828768
rs7311004
rs10899911
All − MR Egger
All − IVW(MRE)
−1.5
−1.0
−0.5
0.0
0.5
1.0
MR effect size for
'Gut microbiota abundance (genus Parasutterella id.2892)' on 'Repulsive guidance molecule A'

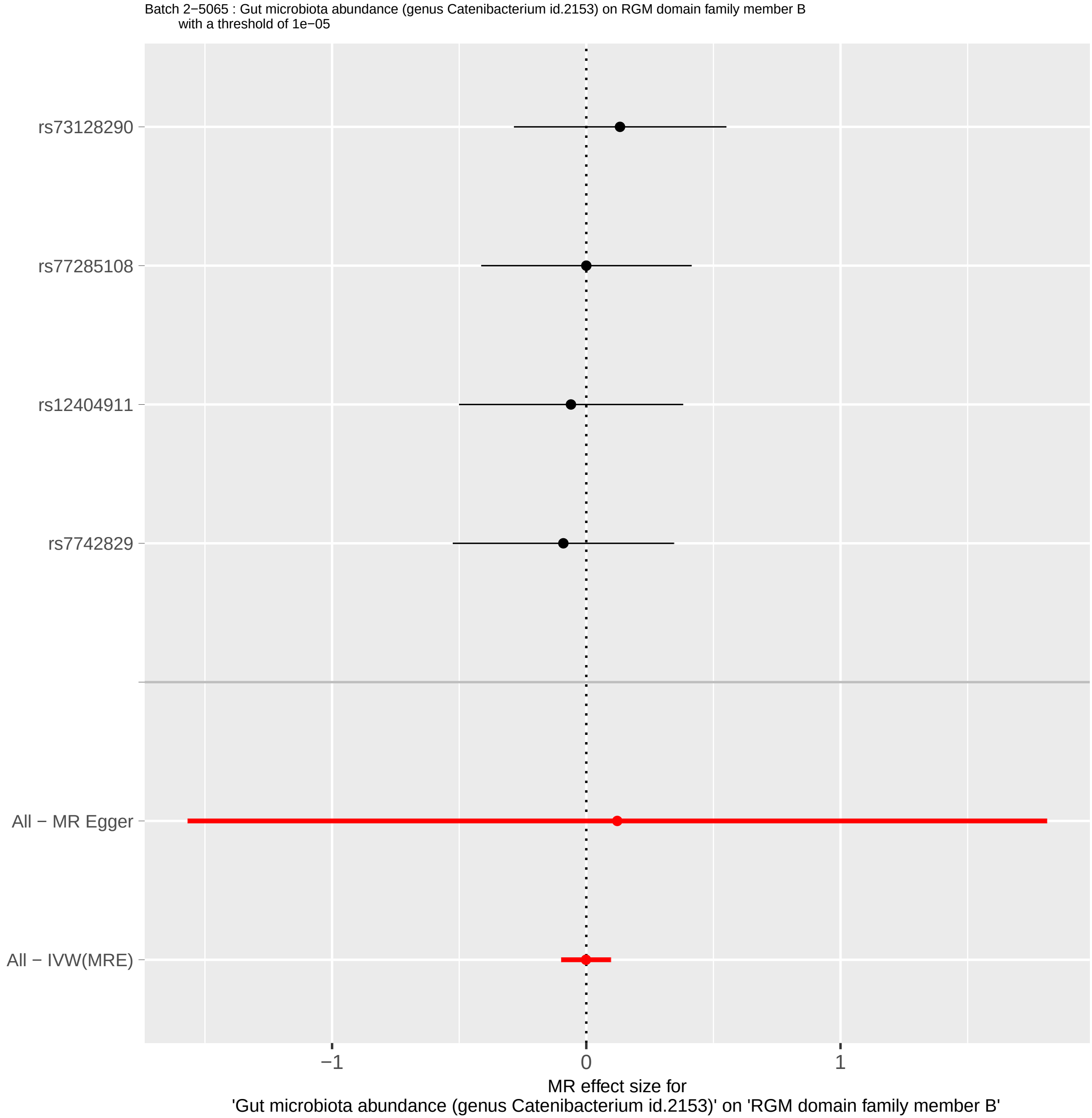
Batch 2−5065 : Gut microbiota abundance (genus Catenibacterium id.2153) on RGM domain family member B
with a threshold of 1e−05
rs73128290
rs77285108
rs12404911
rs7742829
All − MR Egger
All − IVW(MRE)
−1
0
1
MR effect size for
'Gut microbiota abundance (genus Catenibacterium id.2153)' on 'RGM domain family member B'

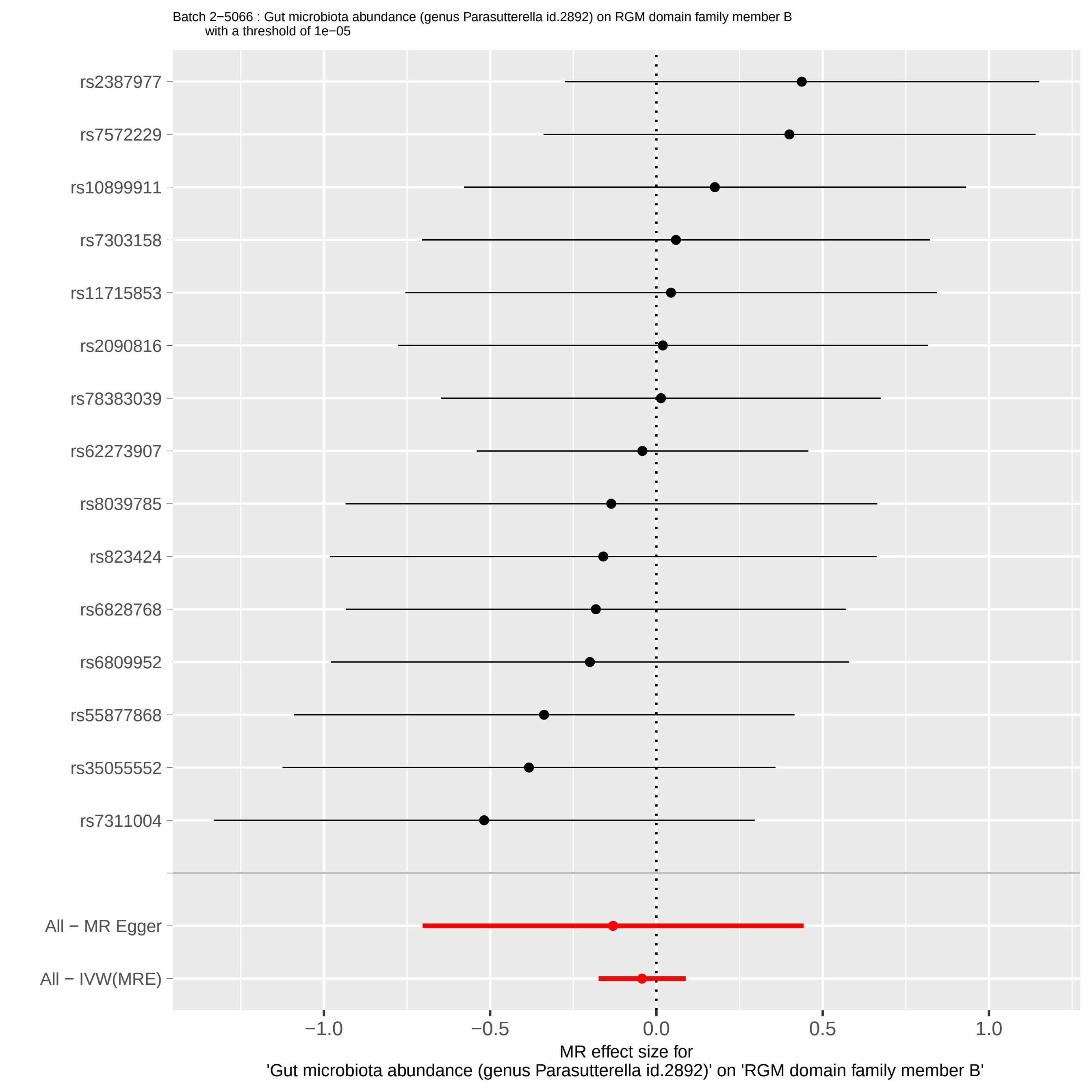

Batch 2−5066 : Gut microbiota abundance (genus Parasutterella id.2892) on RGM domain family member B
with a threshold of 1e−05
rs2387977
rs7572229
rs10899911
rs7303158
rs11715853
rs2090816
rs78383039
rs62273907
rs8039785
rs823424
rs6828768
rs6809952
rs55877868
rs35055552
rs7311004
All − MR Egger
All − IVW(MRE)
−1.0
−0.5
0.0
0.5
1.0
MR effect size for
'Gut microbiota abundance (genus Parasutterella id.2892)' on 'RGM domain family member B'

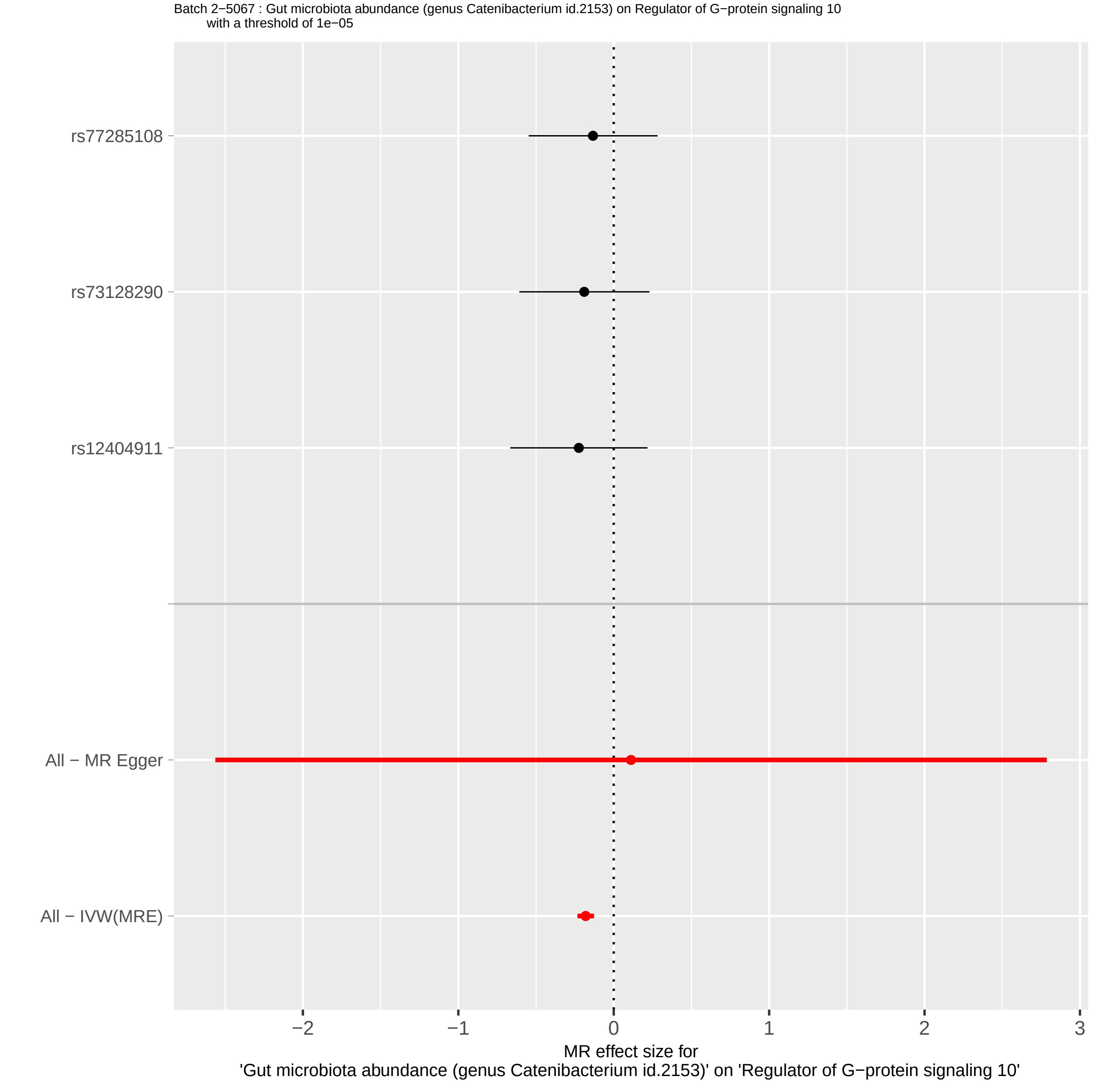

Batch 2−5067 : Gut microbiota abundance (genus Catenibacterium id.2153) on Regulator of G−protein signaling 10
with a threshold of 1e−05
rs77285108
rs73128290
rs12404911
All − MR Egger
All − IVW(MRE)
−2
−1
0
1
2
3
MR effect size for
'Gut microbiota abundance (genus Catenibacterium id.2153)' on 'Regulator of G−protein signaling 10'

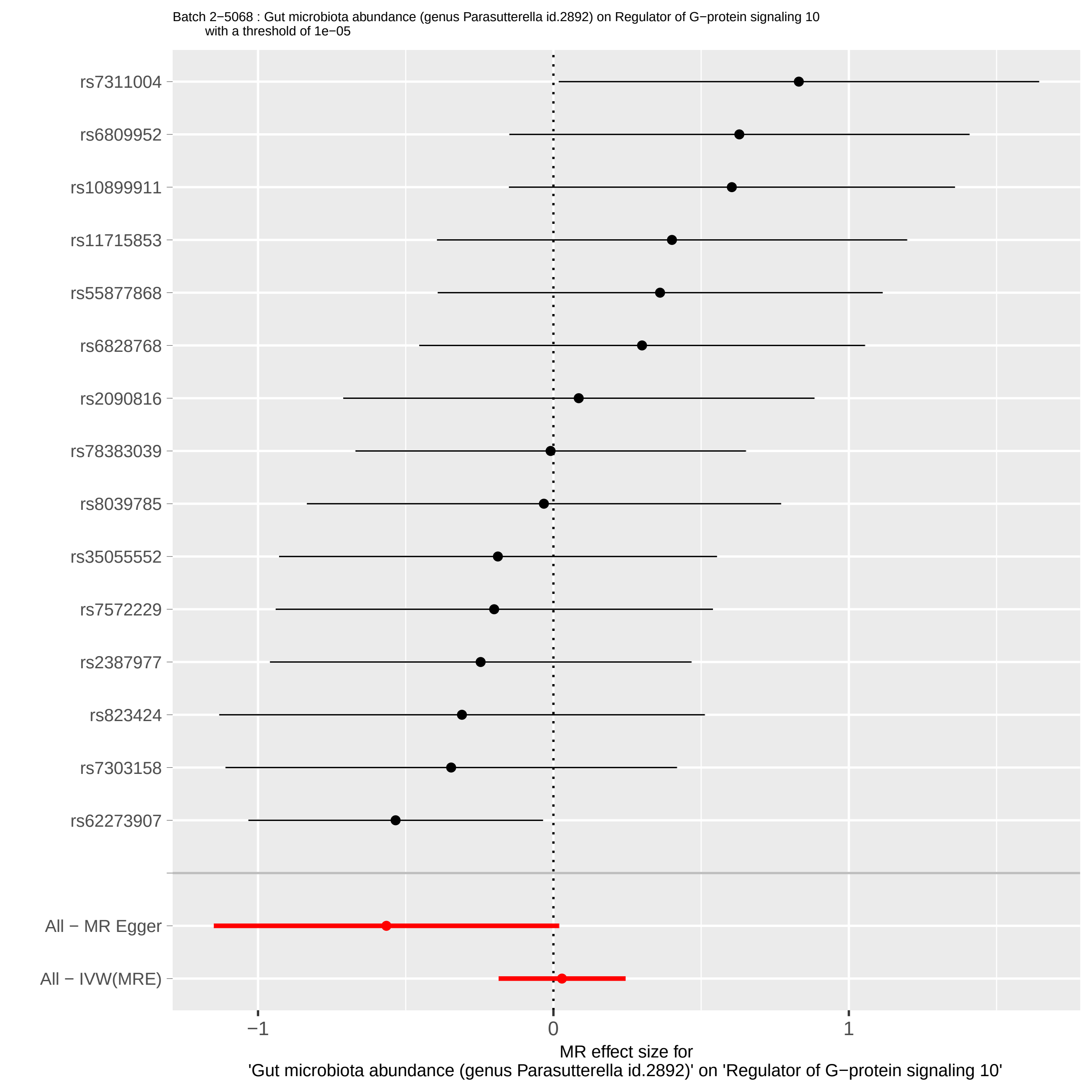

Batch 2−5068 : Gut microbiota abundance (genus Parasutterella id.2892) on Regulator of G−protein signaling 10
with a threshold of 1e−05
rs7311004
rs6809952
rs10899911
rs11715853
rs55877868
rs6828768
rs2090816
rs78383039
rs8039785
rs35055552
rs7572229
rs2387977
rs823424
rs7303158
rs62273907
All − MR Egger
All − IVW(MRE)
−1
0
1
MR effect size for
'Gut microbiota abundance (genus Parasutterella id.2892)' on 'Regulator of G−protein signaling 10'

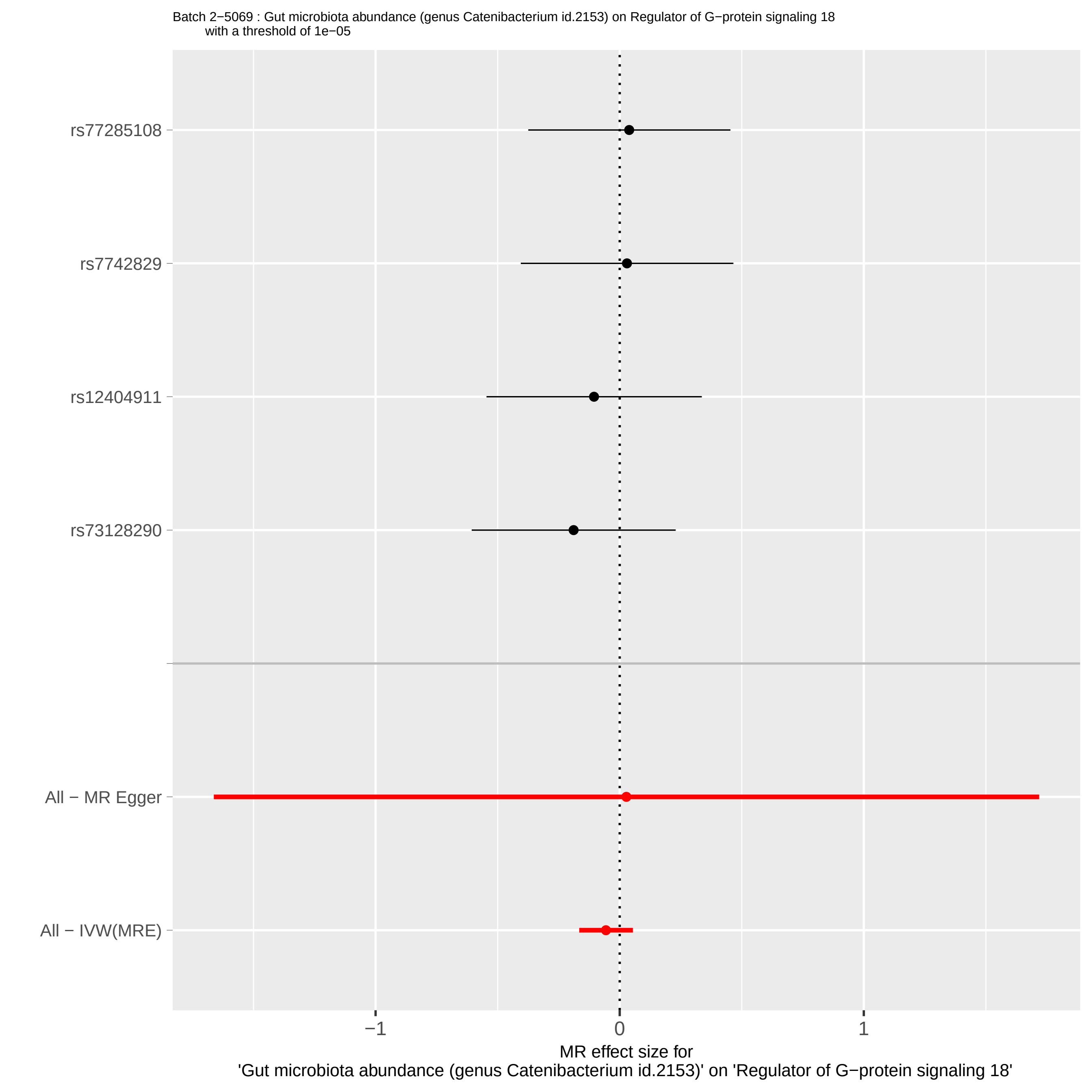

Batch 2−5069 : Gut microbiota abundance (genus Catenibacterium id.2153) on Regulator of G−protein signaling 18
with a threshold of 1e−05
rs77285108
rs7742829
rs12404911
rs73128290
All − MR Egger
All − IVW(MRE)
−1
0
1
MR effect size for
'Gut microbiota abundance (genus Catenibacterium id.2153)' on 'Regulator of G−protein signaling 18'

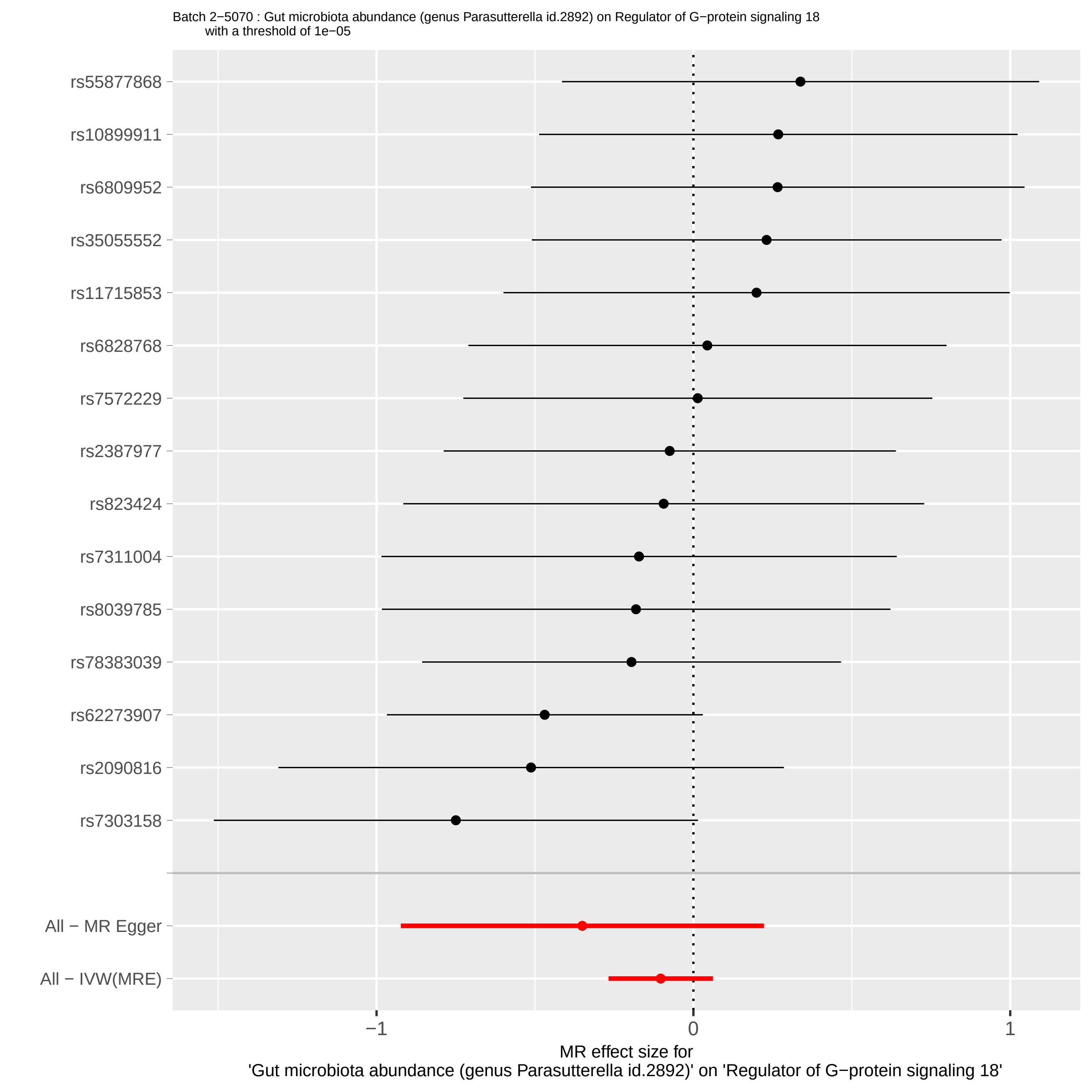

Batch 2−5070 : Gut microbiota abundance (genus Parasutterella id.2892) on Regulator of G−protein signaling 18
with a threshold of 1e−05
rs55877868
rs10899911
rs6809952
rs35055552
rs11715853
rs6828768
rs7572229
rs2387977
rs823424
rs7311004
rs8039785
rs78383039
rs62273907
rs2090816
rs7303158
All − MR Egger
All − IVW(MRE)
−1
0
1
MR effect size for
'Gut microbiota abundance (genus Parasutterella id.2892)' on 'Regulator of G−protein signaling 18'

Batch 2−5071 : Gut microbiota abundance (genus Catenibacterium id.2153) on Regulator of G−protein signaling 19 with a threshold of 1e−05

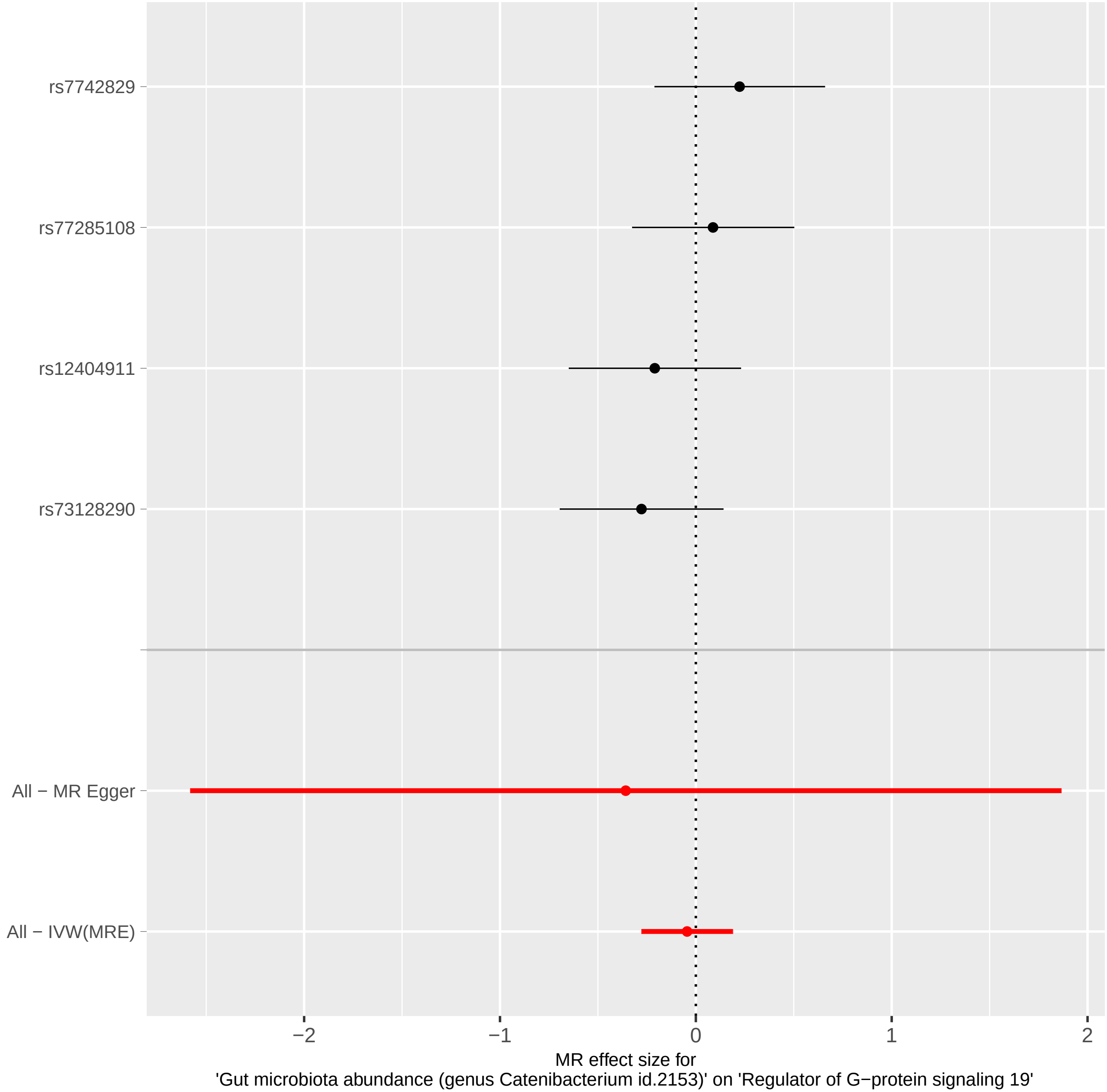

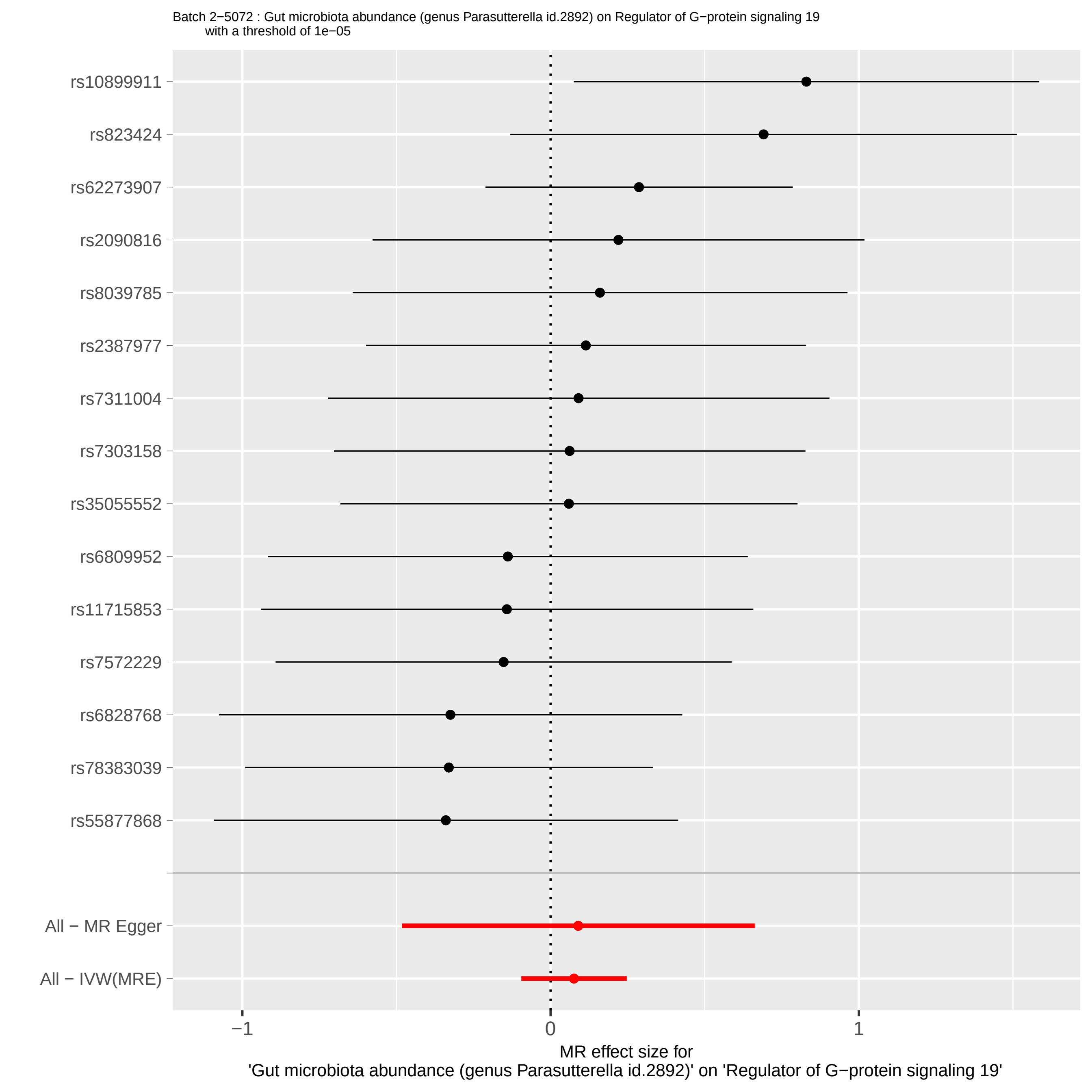

Batch 2−5072 : Gut microbiota abundance (genus Parasutterella id.2892) on Regulator of G−protein signaling 19
with a threshold of 1e−05
rs10899911
rs823424
rs62273907
rs2090816
rs8039785
rs2387977
rs7311004
rs7303158
rs35055552
rs6809952
rs11715853
rs7572229
rs6828768
rs78383039
rs55877868
All − MR Egger
All − IVW(MRE)
−1
0
1
MR effect size for
'Gut microbiota abundance (genus Parasutterella id.2892)' on 'Regulator of G−protein signaling 19'

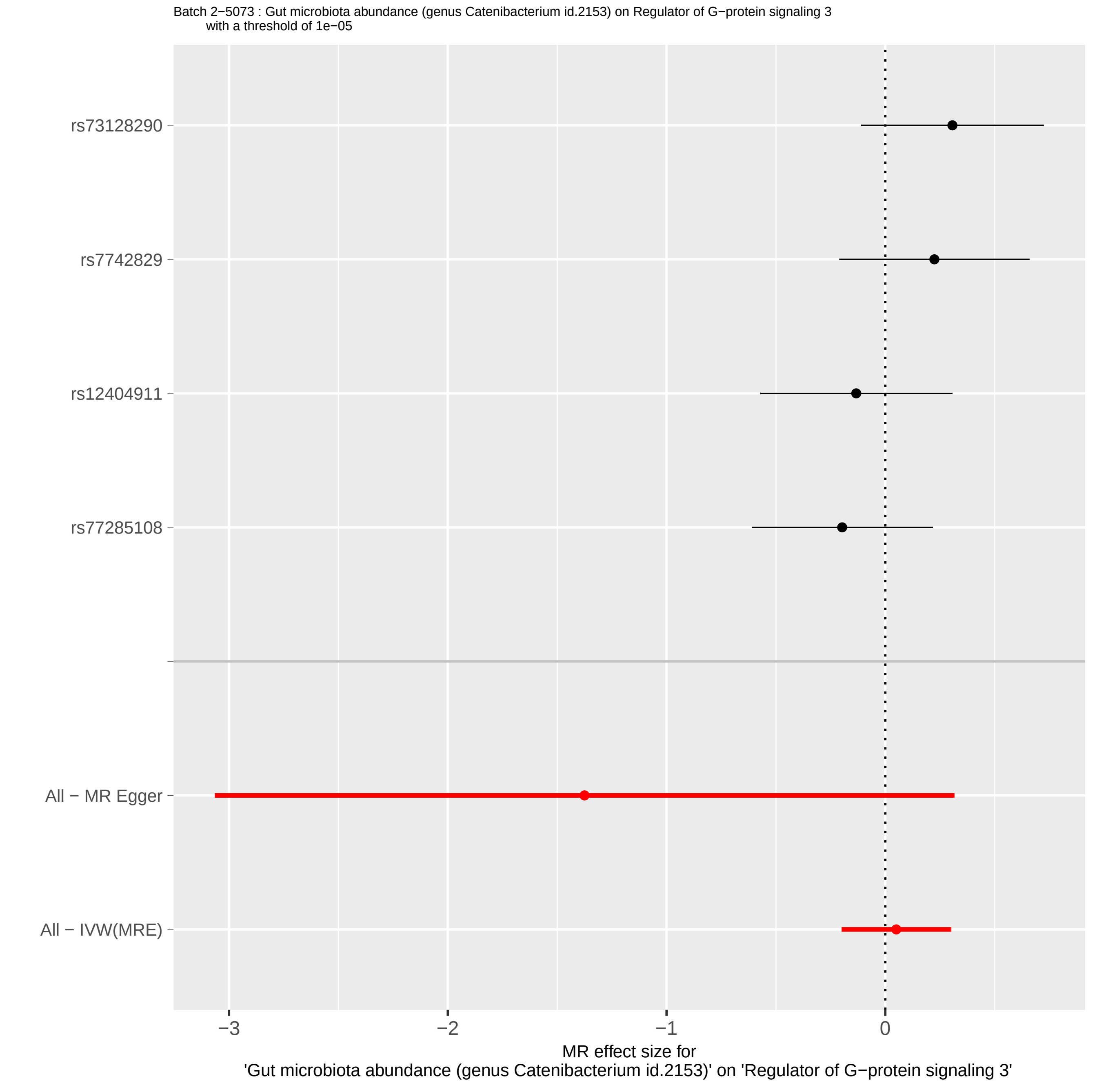
Batch 2−5073 : Gut microbiota abundance (genus Catenibacterium id.2153) on Regulator of G−protein signaling 3
with a threshold of 1e−05
rs73128290
rs7742829
rs12404911
rs77285108
All − MR Egger
All − IVW(MRE)
−3
−2
−1
0
MR effect size for
'Gut microbiota abundance (genus Catenibacterium id.2153)' on 'Regulator of G−protein signaling 3'

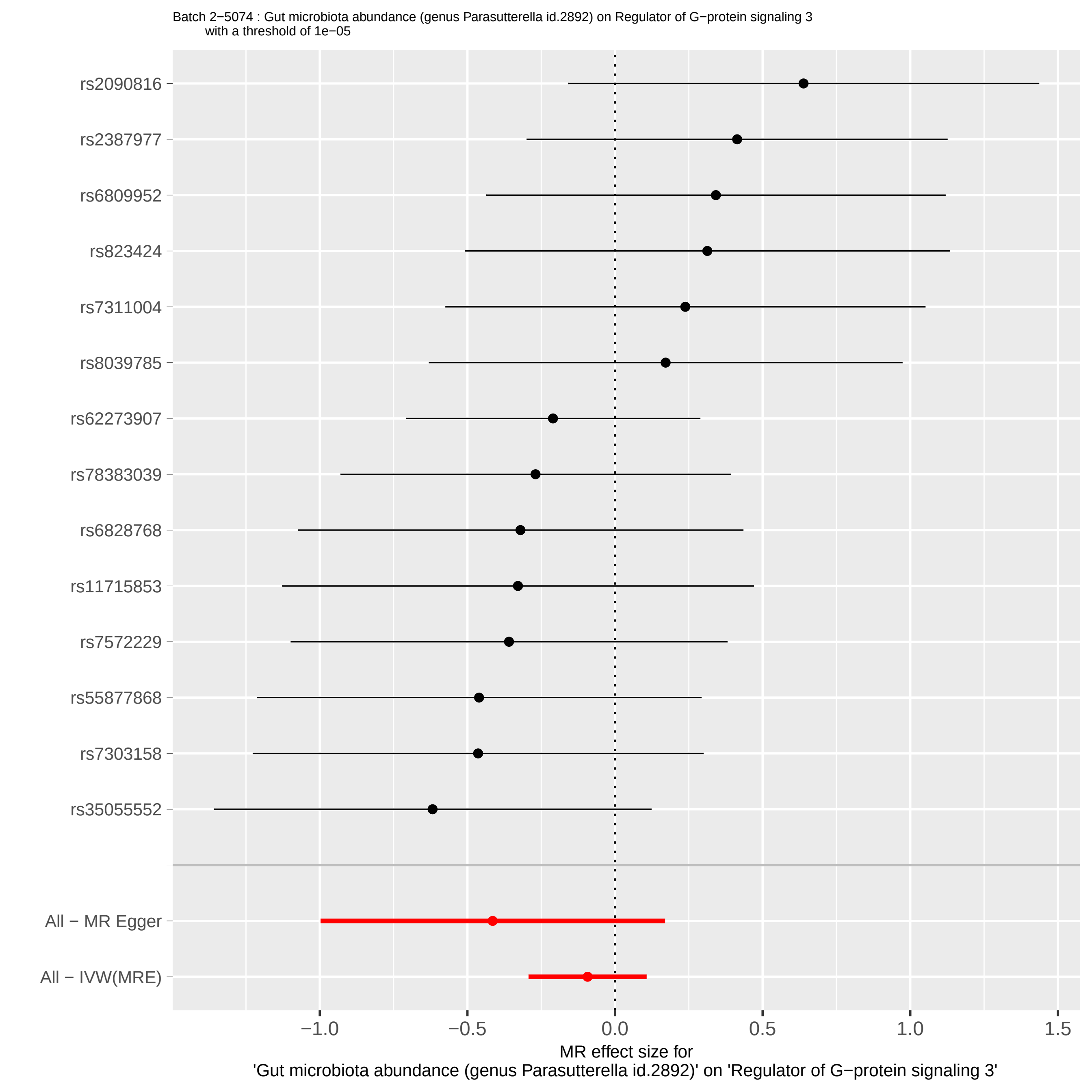
Batch 2−5074 : Gut microbiota abundance (genus Parasutterella id.2892) on Regulator of G−protein signaling 3
with a threshold of 1e−05
rs2090816
rs2387977
rs6809952
rs823424
rs7311004
rs8039785
rs62273907
rs78383039
rs6828768
rs11715853
rs7572229
rs55877868
rs7303158
rs35055552
All − MR Egger
All − IVW(MRE)
−1.0
−0.5
0.0
0.5
1.0
1.5
MR effect size for
'Gut microbiota abundance (genus Parasutterella id.2892)' on 'Regulator of G−protein signaling 3'

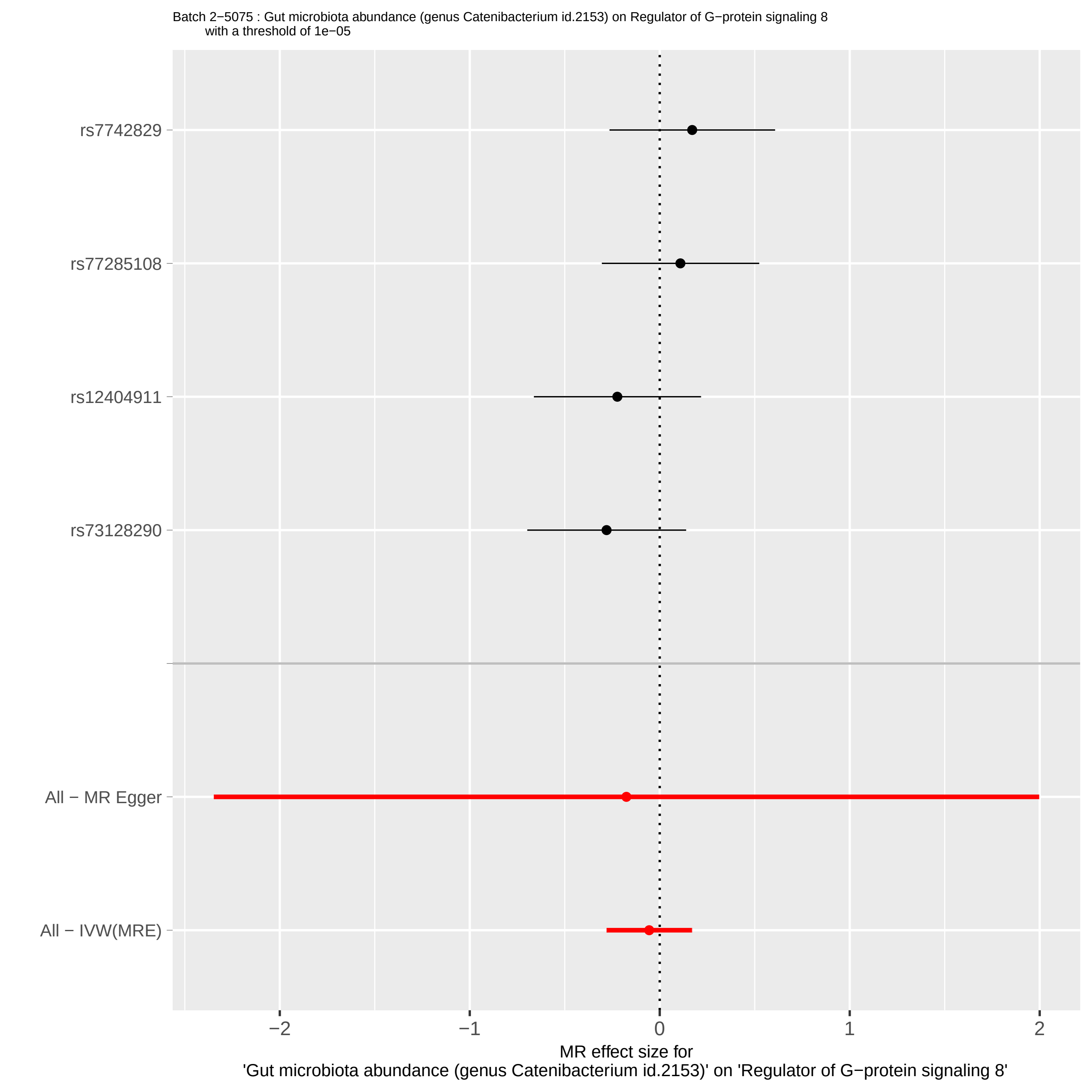
Batch 2−5075 : Gut microbiota abundance (genus Catenibacterium id.2153) on Regulator of G−protein signaling 8
with a threshold of 1e−05
rs7742829
rs77285108
rs12404911
rs73128290
All − MR Egger
All − IVW(MRE)
−2
−1
0
1
2
MR effect size for
'Gut microbiota abundance (genus Catenibacterium id.2153)' on 'Regulator of G−protein signaling 8'

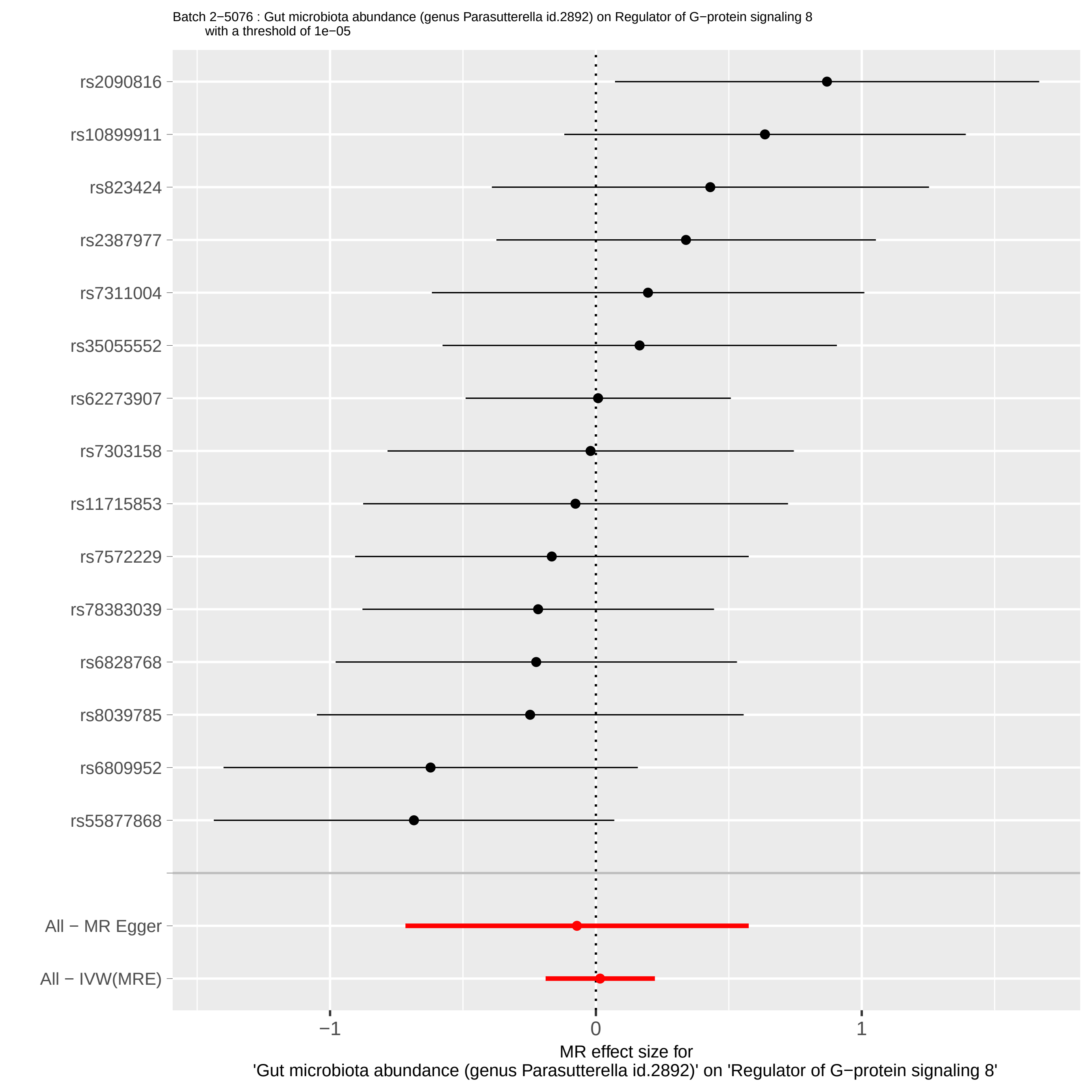

Batch 2−5076 : Gut microbiota abundance (genus Parasutterella id.2892) on Regulator of G−protein signaling 8
with a threshold of 1e−05
rs2090816
rs10899911
rs823424
rs2387977
rs7311004
rs35055552
rs62273907
rs7303158
rs11715853
rs7572229
rs78383039
rs6828768
rs8039785
rs6809952
rs55877868
All − MR Egger
All − IVW(MRE)
−1
0
1
MR effect size for
'Gut microbiota abundance (genus Parasutterella id.2892)' on 'Regulator of G−protein signaling 8'

Batch 2−5077 : Gut microbiota abundance (genus Catenibacterium id.2153) on Transforming protein RhoA
with a threshold of 1e−05

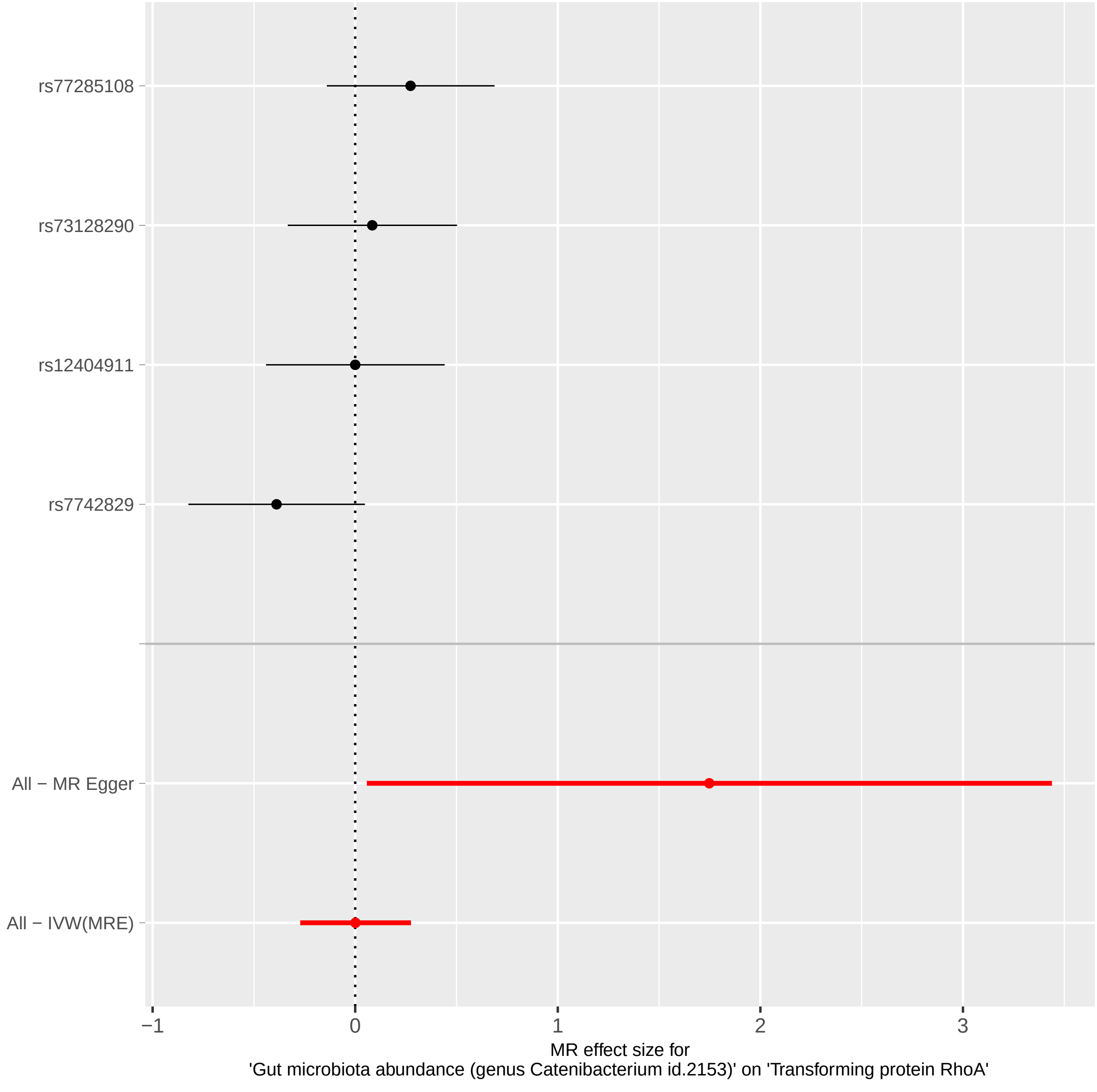

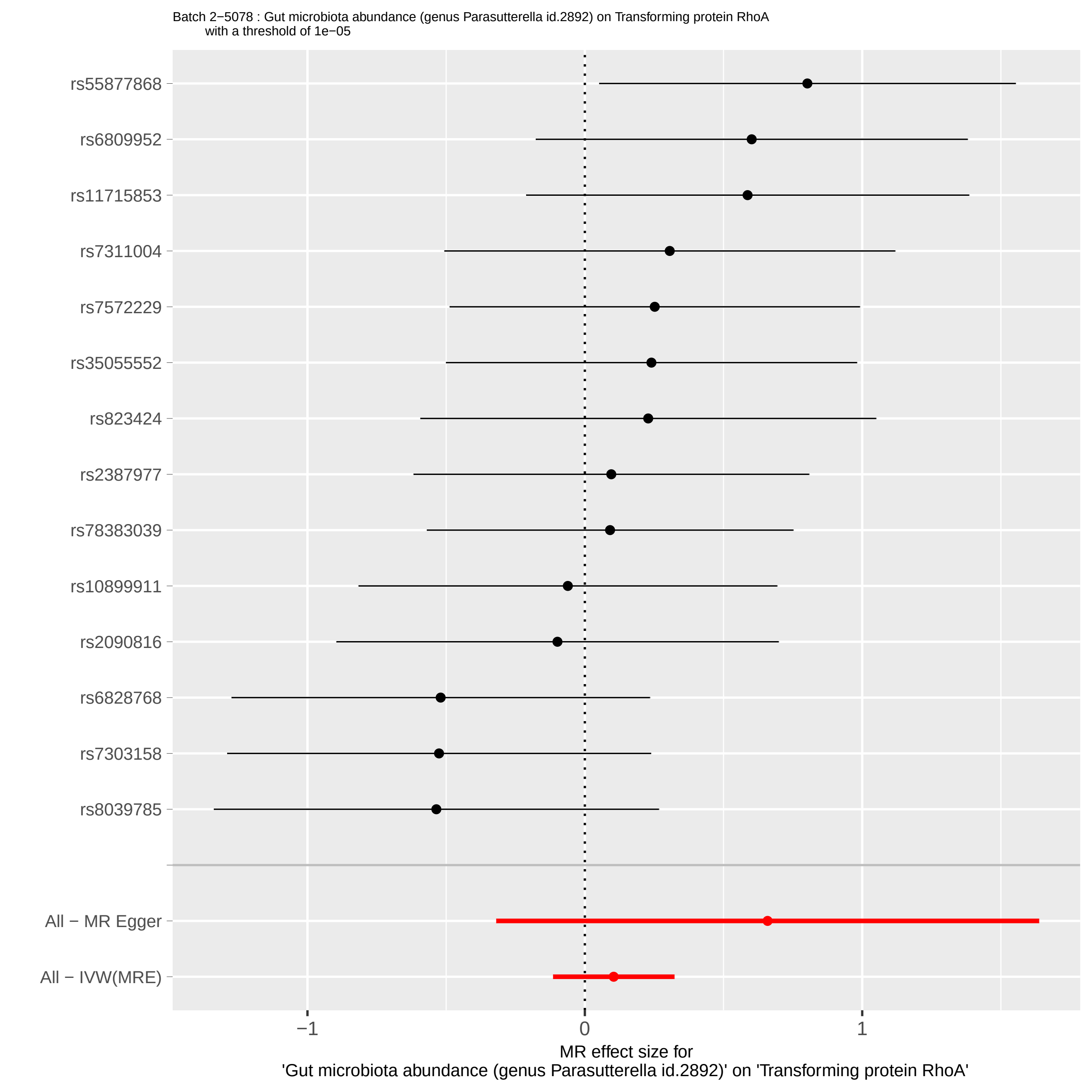
Batch 2−5078 : Gut microbiota abundance (genus Parasutterella id.2892) on Transforming protein RhoA
with a threshold of 1e−05
rs55877868
rs6809952
rs11715853
rs7311004
rs7572229
rs35055552
rs823424
rs2387977
rs78383039
rs10899911
rs2090816
rs6828768
rs7303158
rs8039785
All − MR Egger
All − IVW(MRE)
−1
0
1
MR effect size for
'Gut microbiota abundance (genus Parasutterella id.2892)' on 'Transforming protein RhoA'

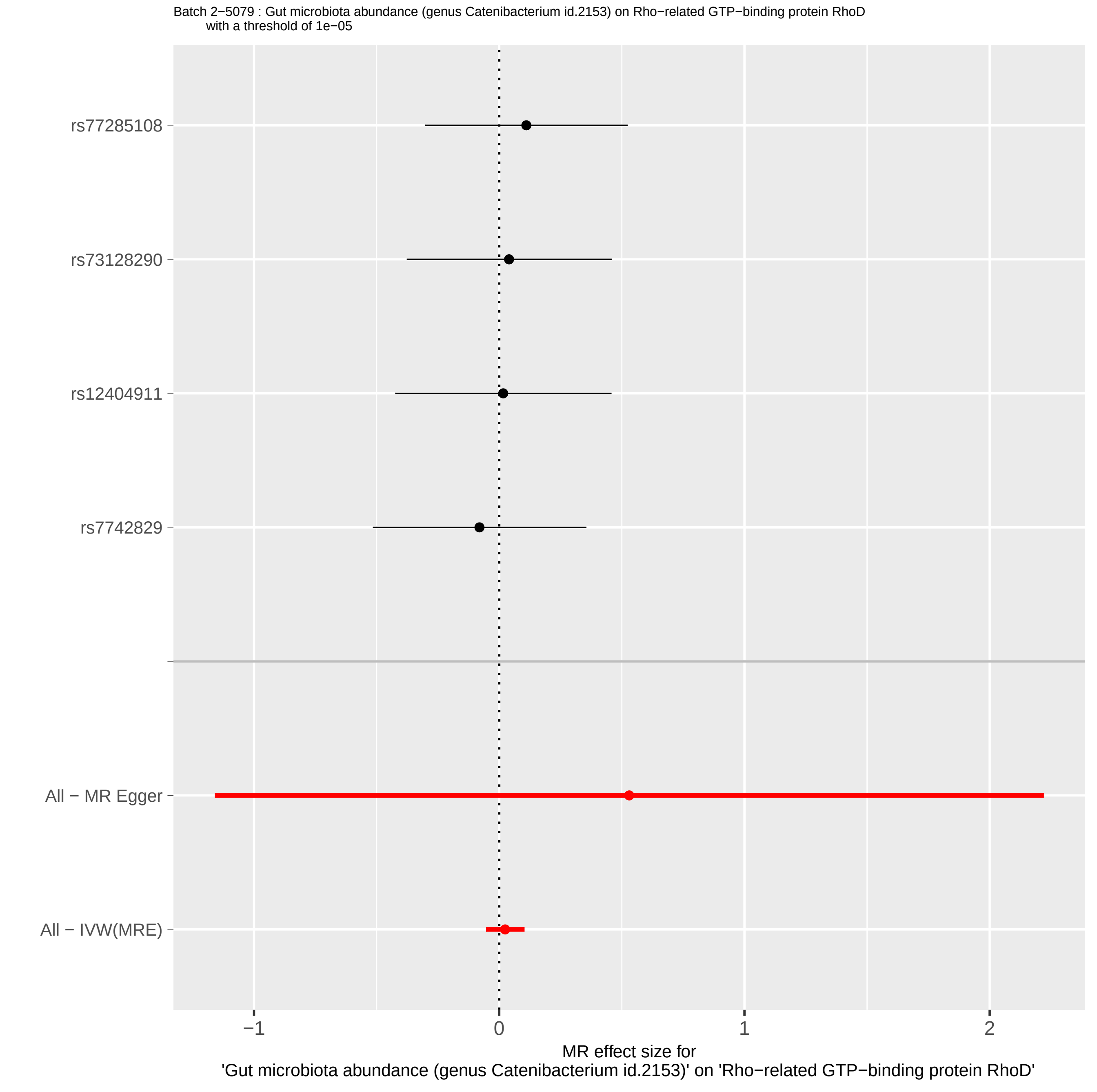
Batch 2−5079 : Gut microbiota abundance (genus Catenibacterium id.2153) on Rho−related GTP−binding protein RhoD
with a threshold of 1e−05
rs77285108
rs73128290
rs12404911
rs7742829
All − MR Egger
All − IVW(MRE)
−1
0
1
2
MR effect size for
'Gut microbiota abundance (genus Catenibacterium id.2153)' on 'Rho−related GTP−binding protein RhoD'

Batch 2−5080 : Gut microbiota abundance (genus Parasutterella id.2892) on Rho−related GTP−binding protein RhoD
with a threshold of 1e−05

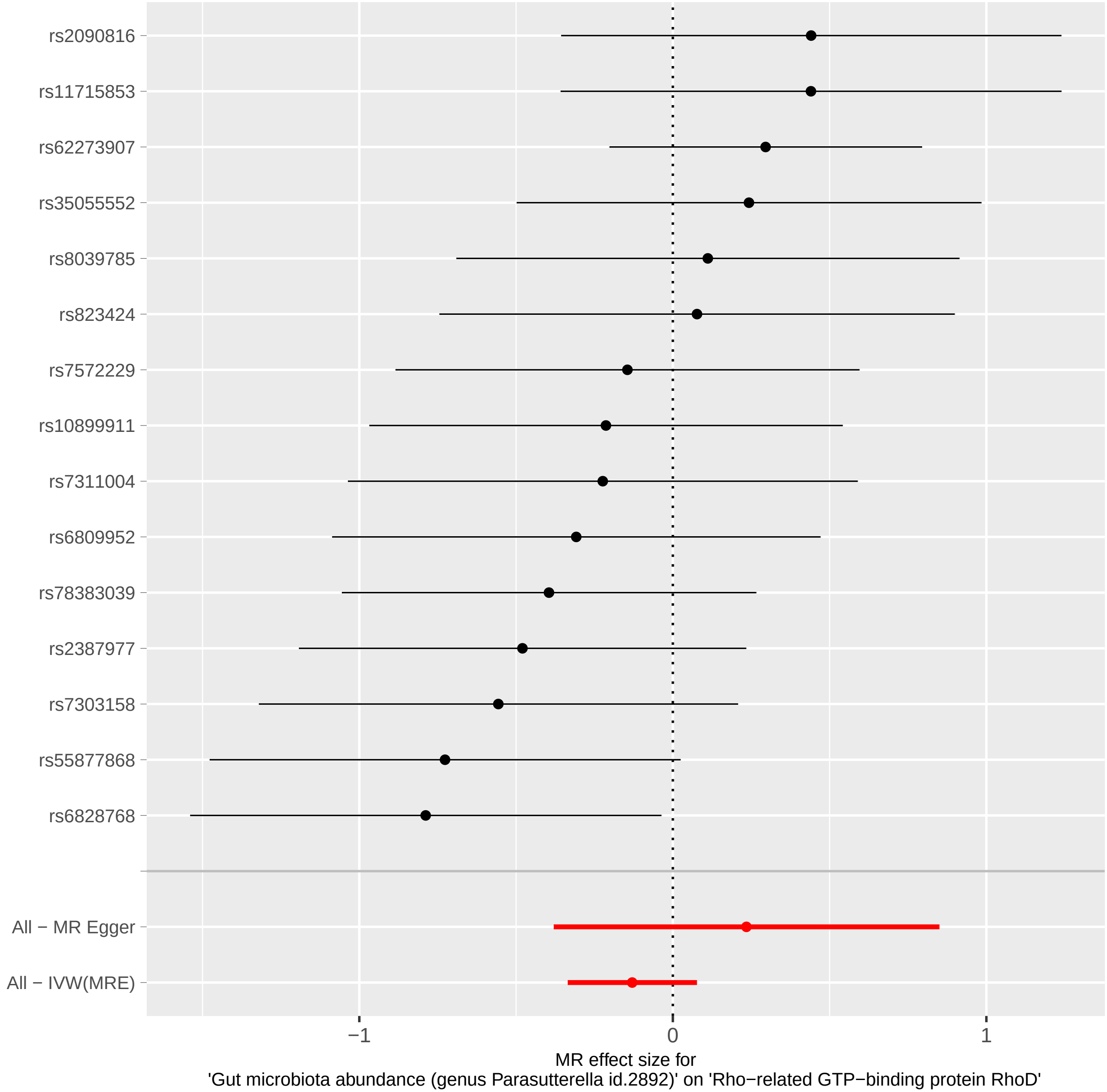

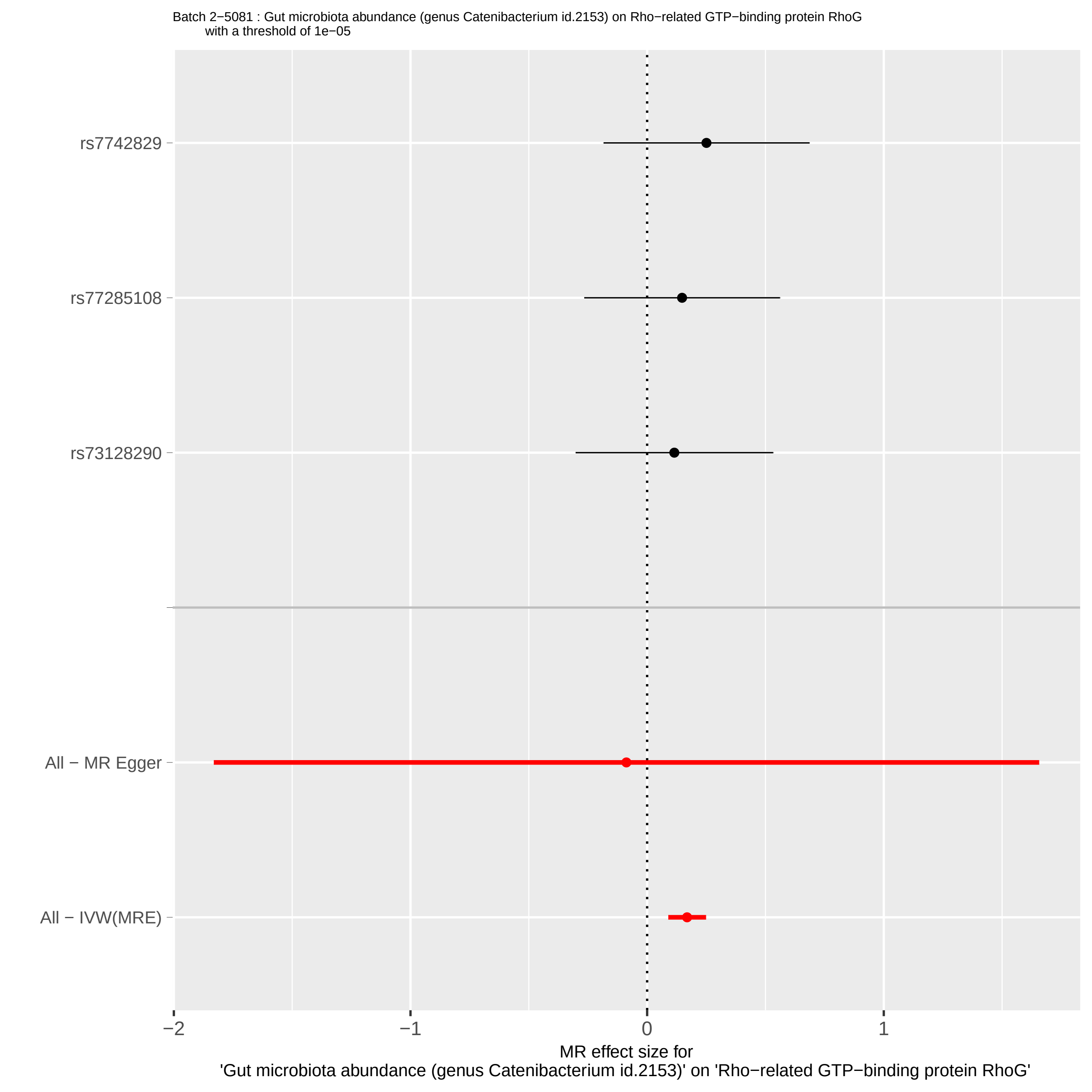
Batch 2−5081 : Gut microbiota abundance (genus Catenibacterium id.2153) on Rho−related GTP−binding protein RhoG
with a threshold of 1e−05
rs7742829
rs77285108
rs73128290
All − MR Egger
All − IVW(MRE)
−2
−1
0
1
MR effect size for
'Gut microbiota abundance (genus Catenibacterium id.2153)' on 'Rho−related GTP−binding protein RhoG'

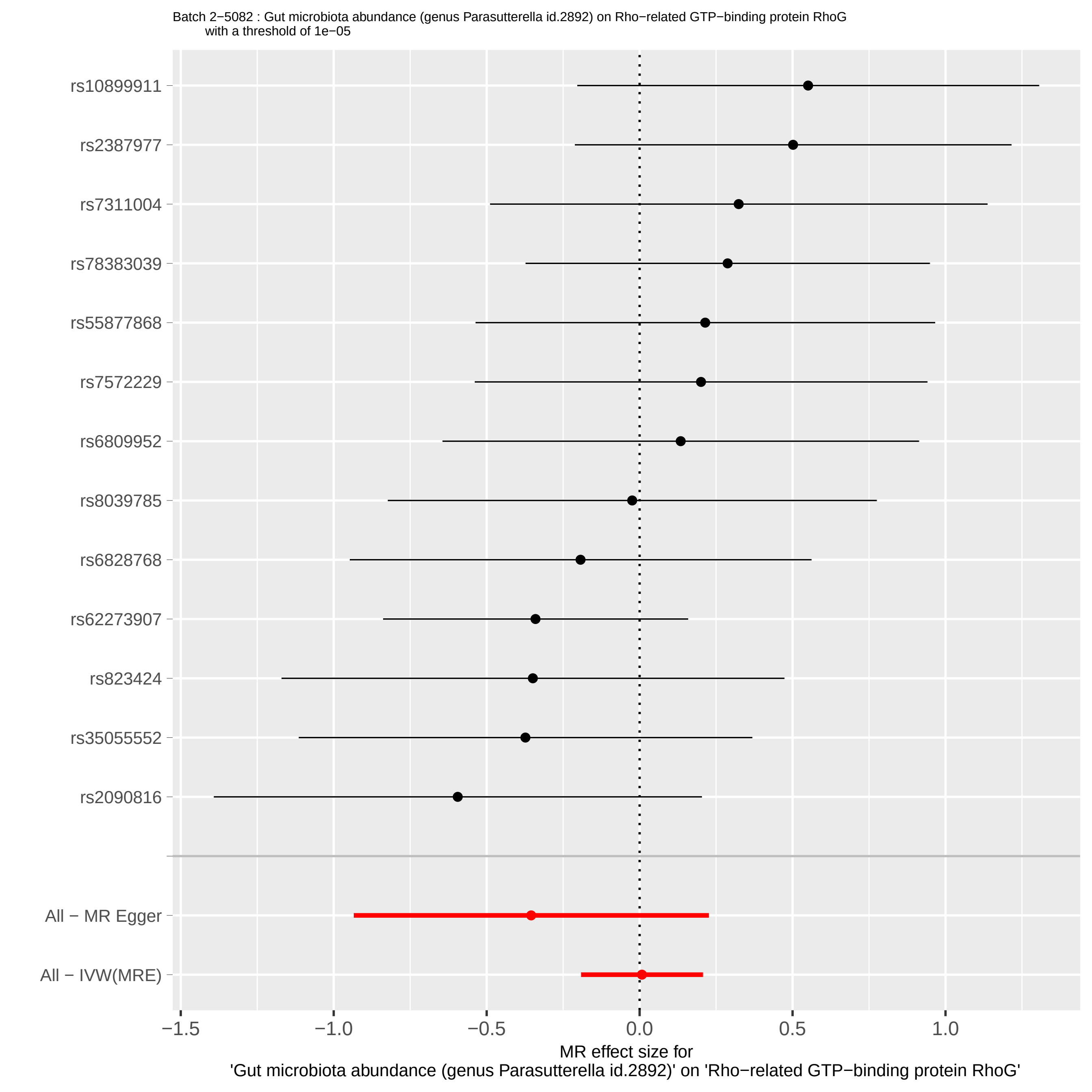

Batch 2−5082 : Gut microbiota abundance (genus Parasutterella id.2892) on Rho−related GTP−binding protein RhoG
with a threshold of 1e−05
rs10899911
rs2387977
rs7311004
rs78383039
rs55877868
rs7572229
rs6809952
rs8039785
rs6828768
rs62273907
rs823424
rs35055552
rs2090816
All − MR Egger
All − IVW(MRE)
−1.5
−1.0
−0.5
0.0
0.5
1.0
MR effect size for
'Gut microbiota abundance (genus Parasutterella id.2892)' on 'Rho−related GTP−binding protein RhoG'

Batch 2−5083 : Gut microbiota abundance (genus Catenibacterium id.2153) on Rhophilin−2
with a threshold of 1e−05

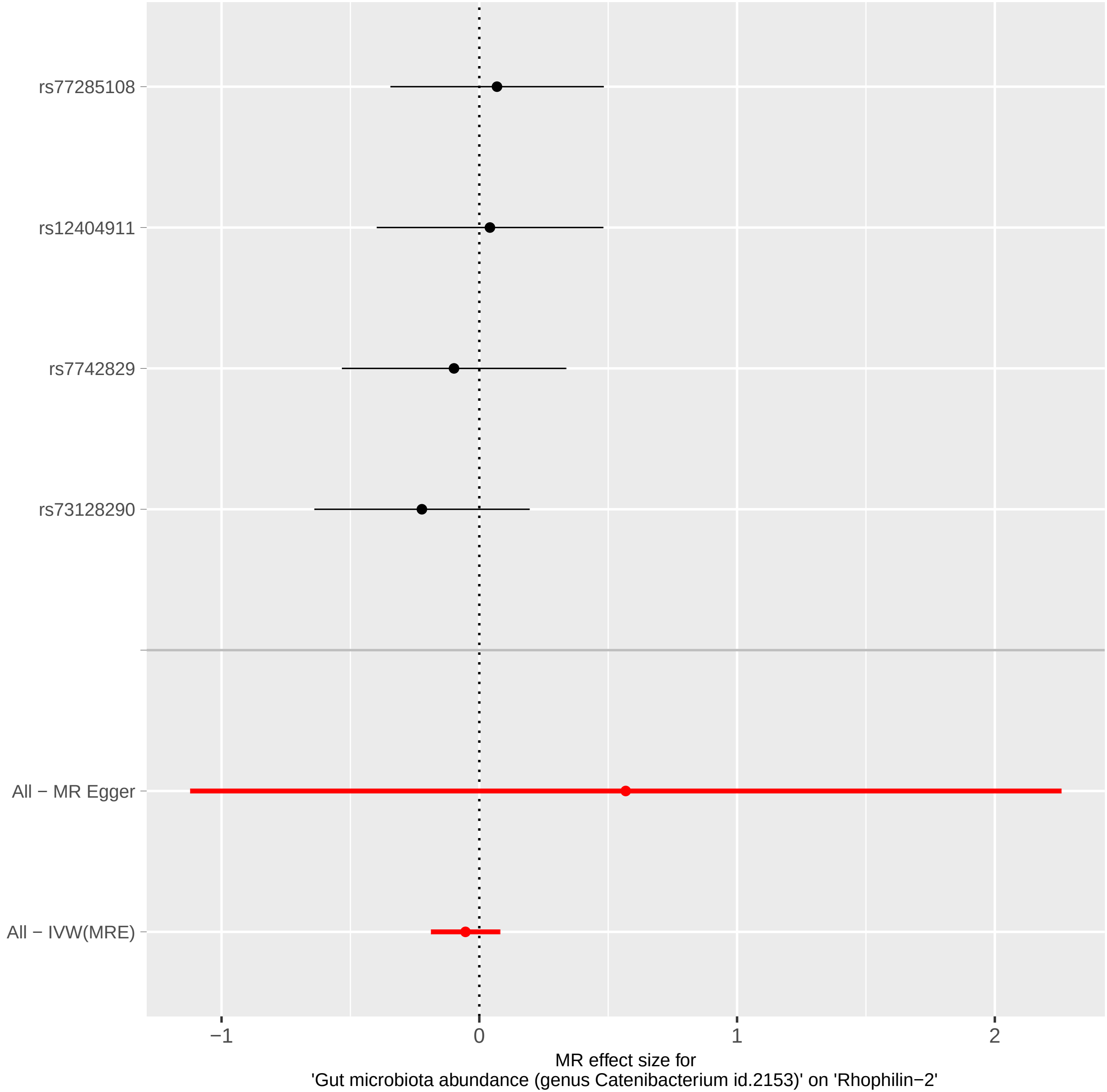

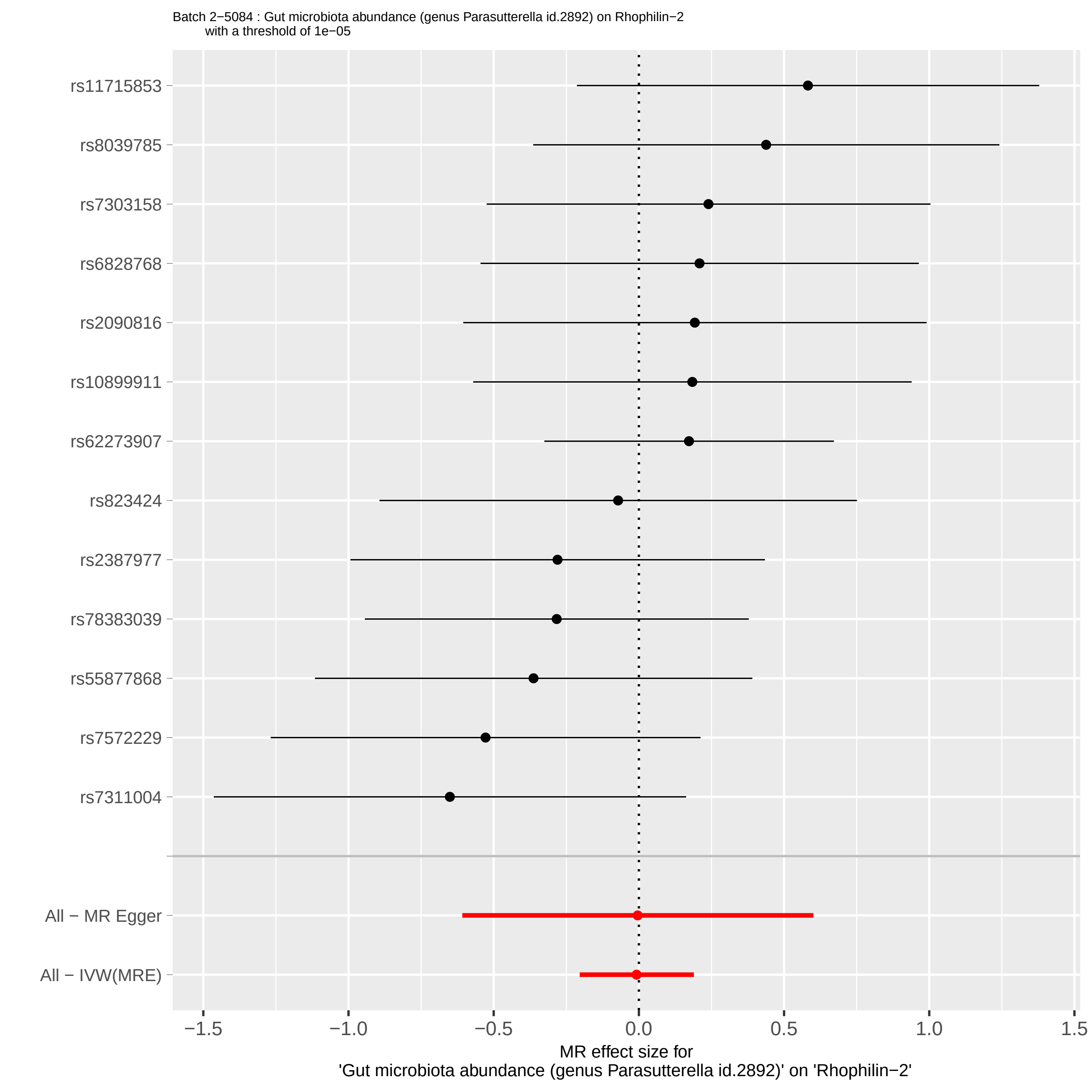
Batch 2−5084 : Gut microbiota abundance (genus Parasutterella id.2892) on Rhophilin−2
with a threshold of 1e−05
rs11715853
rs8039785
rs7303158
rs6828768
rs2090816
rs10899911
rs62273907
rs823424
rs2387977
rs78383039
rs55877868
rs7572229
rs7311004
All − MR Egger
All − IVW(MRE)
−1.5
−1.0
−0.5
0.0
0.5
1.0
1.5
MR effect size for
'Gut microbiota abundance (genus Parasutterella id.2892)' on 'Rhophilin−2'

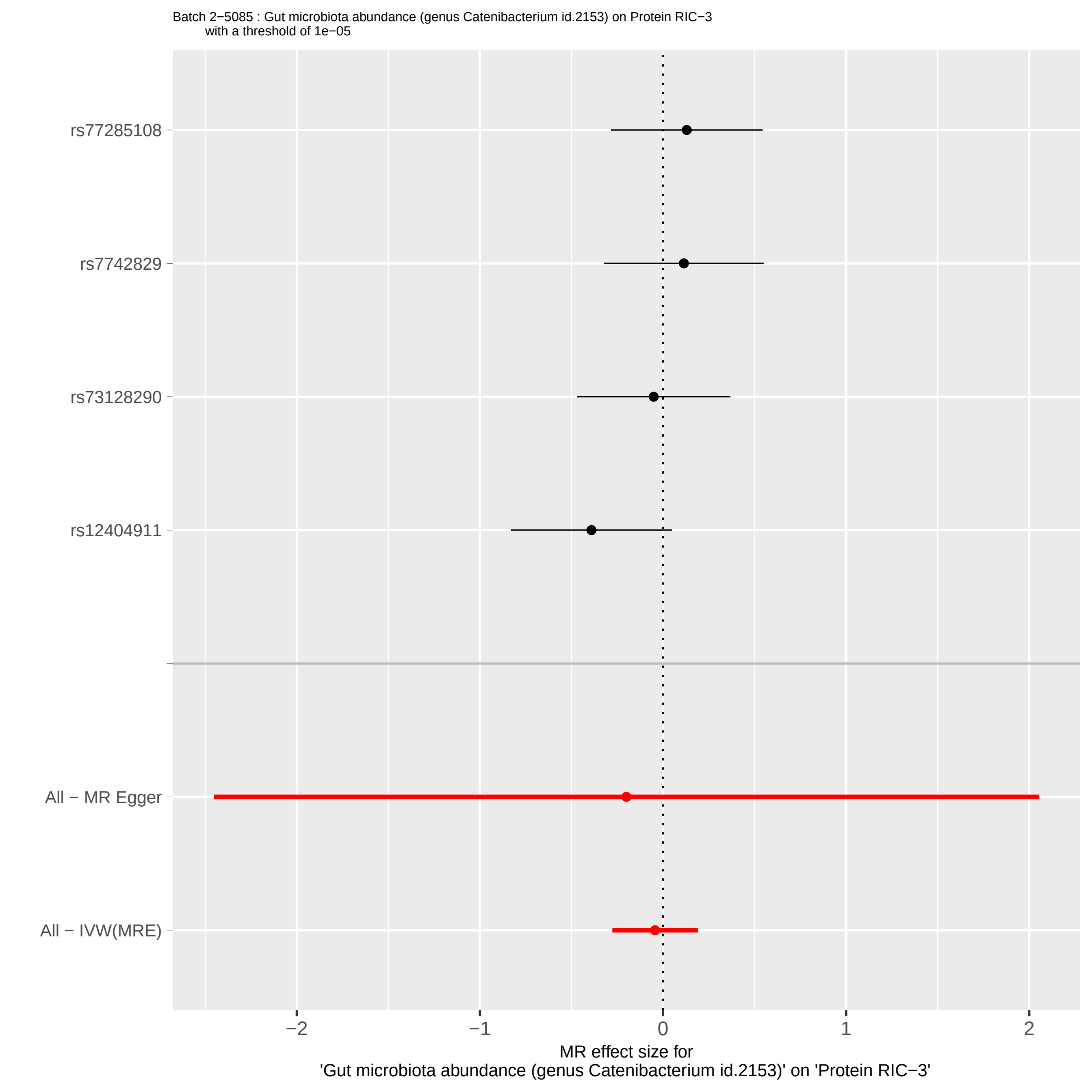

Batch 2−5085 : Gut microbiota abundance (genus Catenibacterium id.2153) on Protein RIC−3
with a threshold of 1e−05
rs77285108
rs7742829
rs73128290
rs12404911
All − MR Egger
All − IVW(MRE)
−2
−1
0
1
2
MR effect size for
'Gut microbiota abundance (genus Catenibacterium id.2153)' on 'Protein RIC−3'

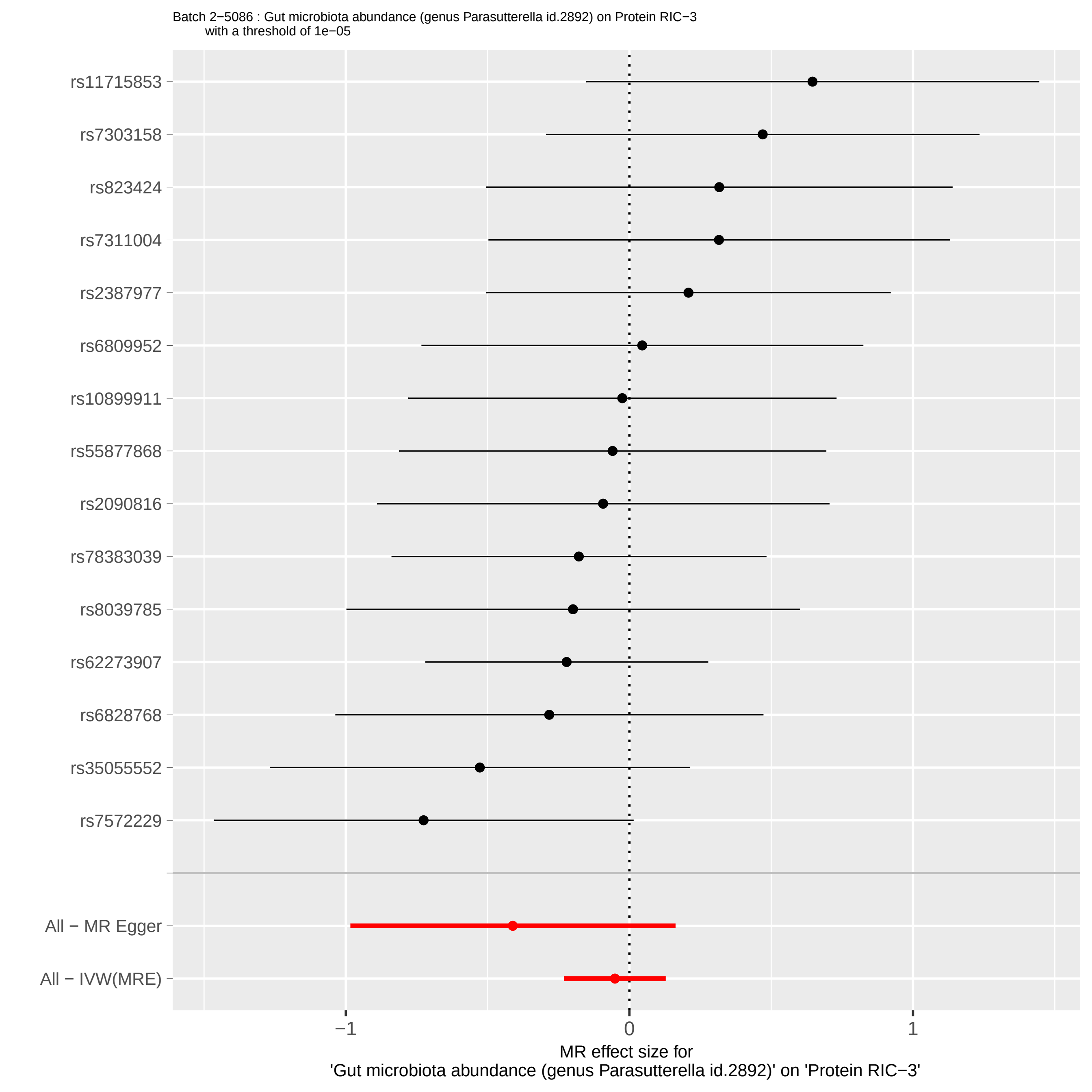

Batch 2−5086 : Gut microbiota abundance (genus Parasutterella id.2892) on Protein RIC−3
with a threshold of 1e−05
rs11715853
rs7303158
rs823424
rs7311004
rs2387977
rs6809952
rs10899911
rs55877868
rs2090816
rs78383039
rs8039785
rs62273907
rs6828768
rs35055552
rs7572229
All − MR Egger
All − IVW(MRE)
−1
0
1
MR effect size for
'Gut microbiota abundance (genus Parasutterella id.2892)' on 'Protein RIC−3'

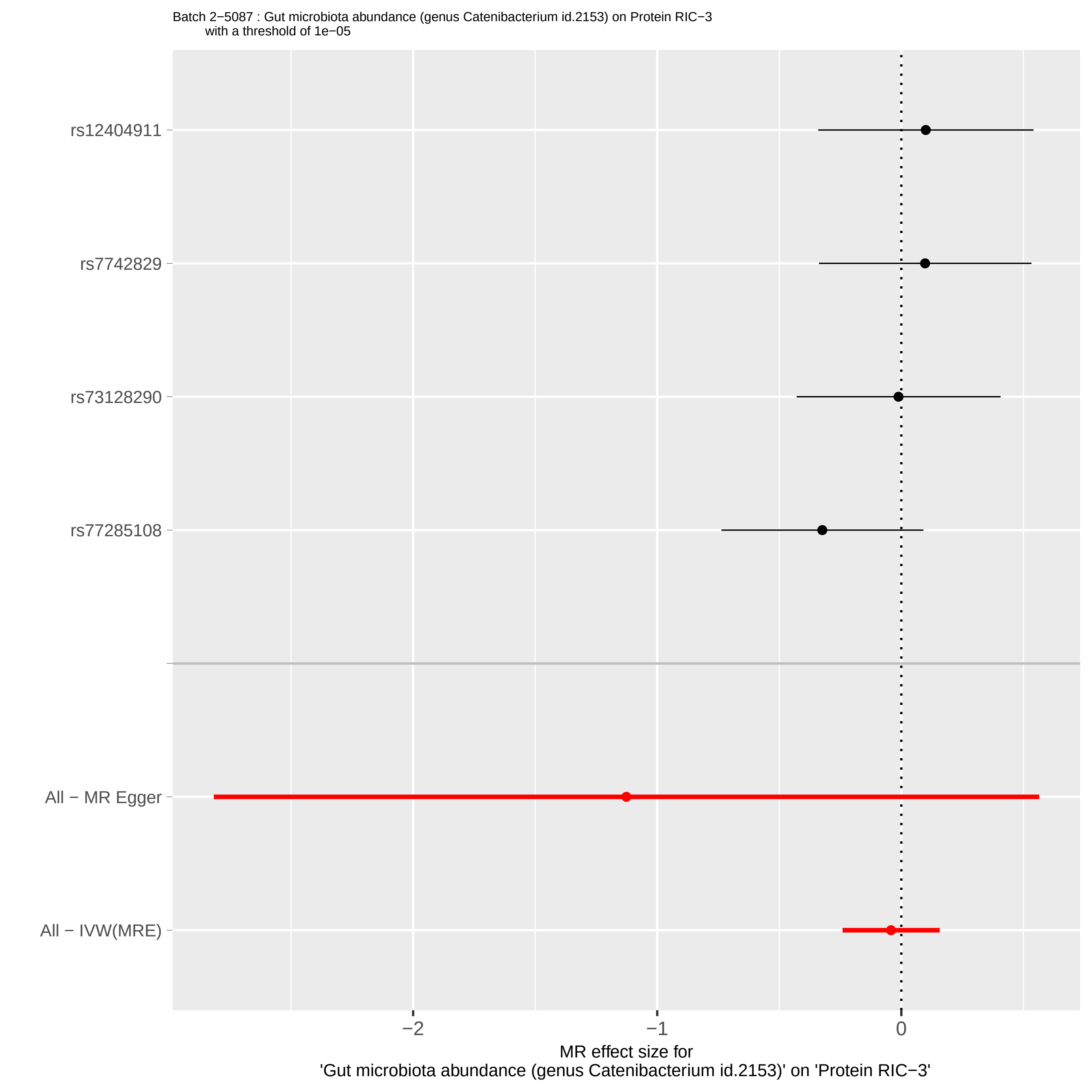

Batch 2−5087 : Gut microbiota abundance (genus Catenibacterium id.2153) on Protein RIC−3
with a threshold of 1e−05
rs12404911
rs7742829
rs73128290
rs77285108
All − MR Egger
All − IVW(MRE)
−2
−1
0
MR effect size for
'Gut microbiota abundance (genus Catenibacterium id.2153)' on 'Protein RIC−3'

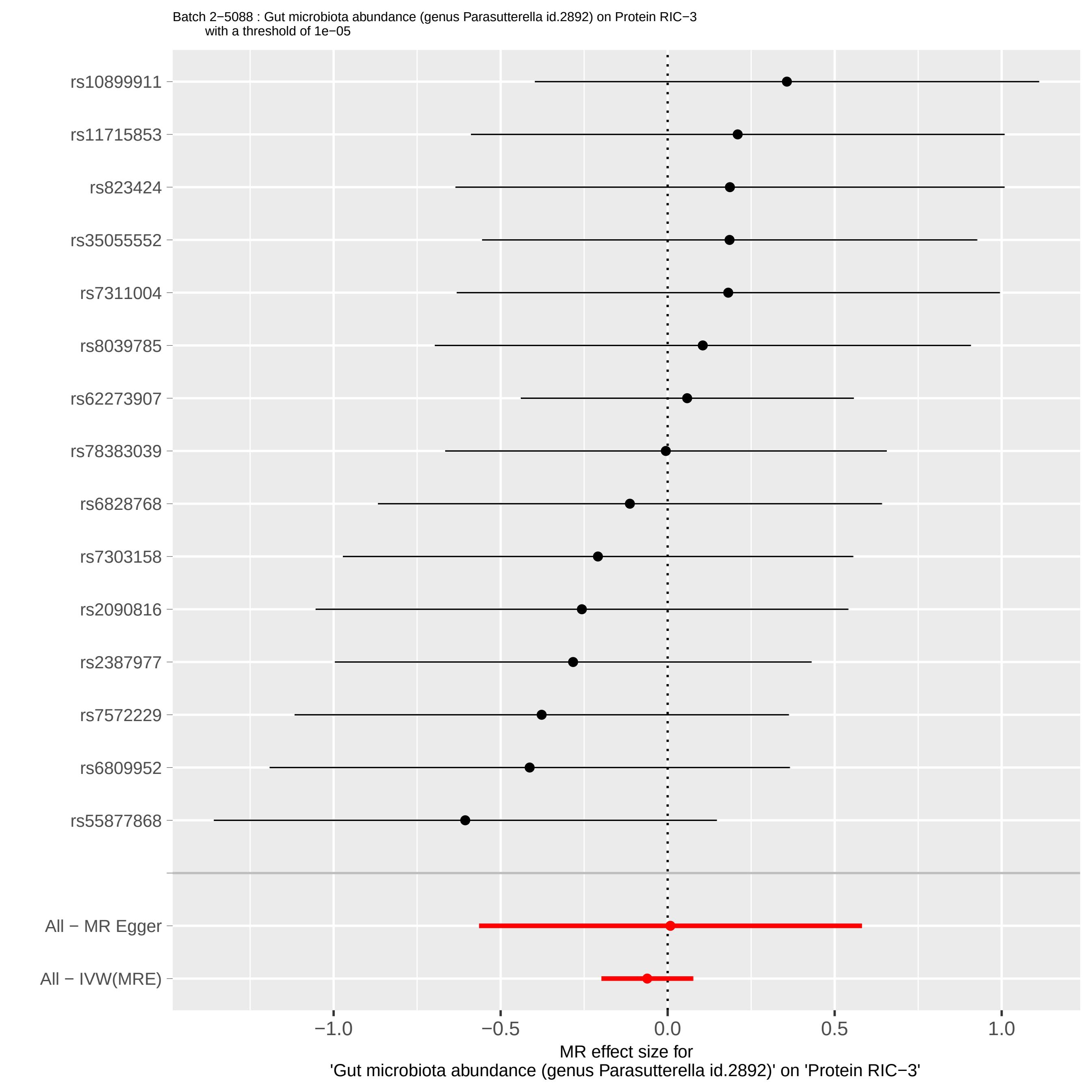

Batch 2−5088 : Gut microbiota abundance (genus Parasutterella id.2892) on Protein RIC−3
with a threshold of 1e−05
rs10899911
rs11715853
rs823424
rs35055552
rs7311004
rs8039785
rs62273907
rs78383039
rs6828768
rs7303158
rs2090816
rs2387977
rs7572229
rs6809952
rs55877868
All − MR Egger
All − IVW(MRE)
−1.0
−0.5
0.0
0.5
1.0
MR effect size for
'Gut microbiota abundance (genus Parasutterella id.2892)' on 'Protein RIC−3'

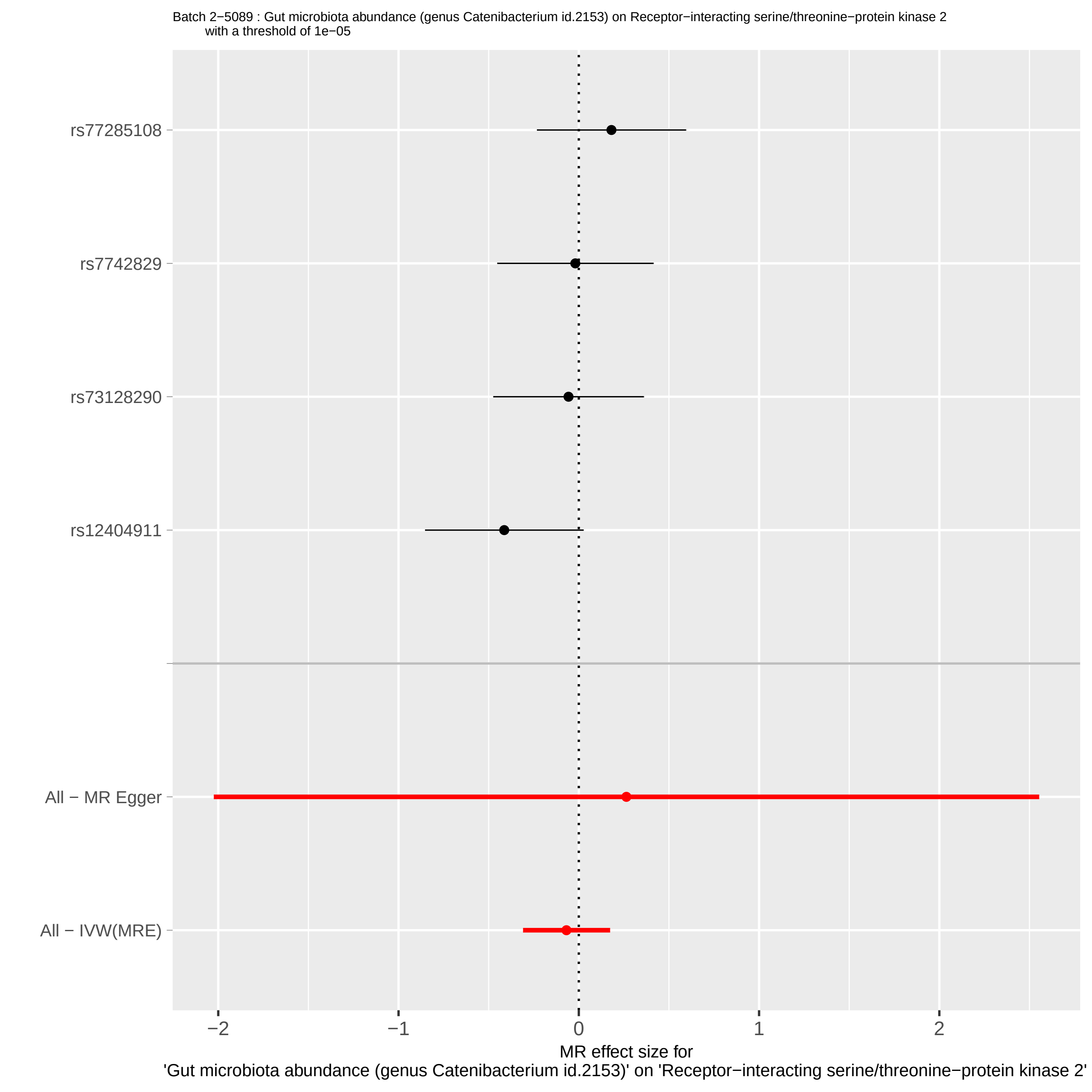
Batch 2−5089 : Gut microbiota abundance (genus Catenibacterium id.2153) on Receptor−interacting serine/threonine−protein kinase 2
with a threshold of 1e−05
rs77285108
rs7742829
rs73128290
rs12404911
All − MR Egger
All − IVW(MRE)
−2
−1
0
1
2
MR effect size for
'Gut microbiota abundance (genus Catenibacterium id.2153)' on 'Receptor−interacting serine/threonine−protein kinase 2'

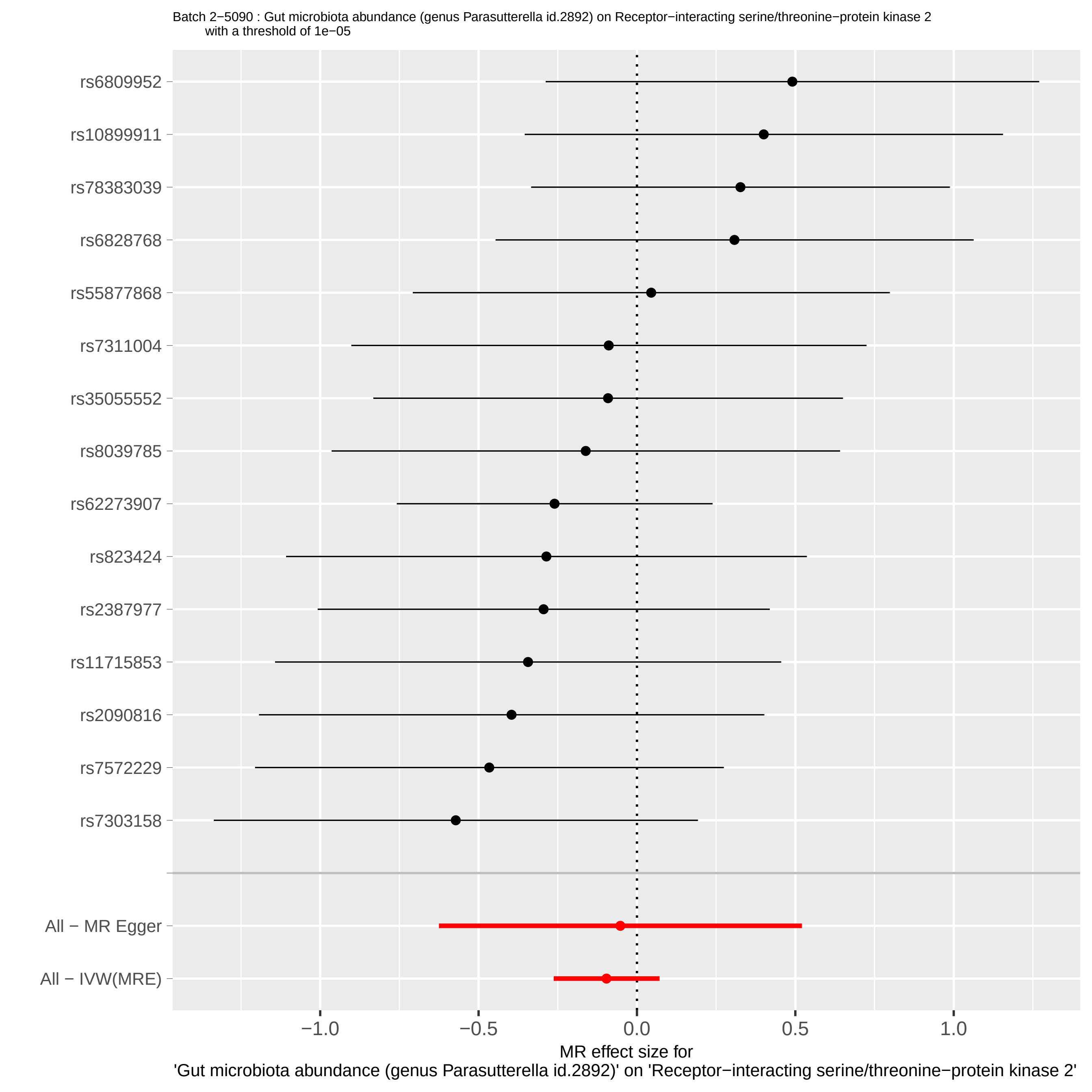

Batch 2−5090 : Gut microbiota abundance (genus Parasutterella id.2892) on Receptor−interacting serine/threonine−protein kinase 2
with a threshold of 1e−05
rs6809952
rs10899911
rs78383039
rs6828768
rs55877868
rs7311004
rs35055552
rs8039785
rs62273907
rs823424
rs2387977
rs11715853
rs2090816
rs7572229
rs7303158
All − MR Egger
All − IVW(MRE)
−1.0
−0.5
0.0
0.5
1.0
MR effect size for
'Gut microbiota abundance (genus Parasutterella id.2892)' on 'Receptor−interacting serine/threonine−protein kinase 2'

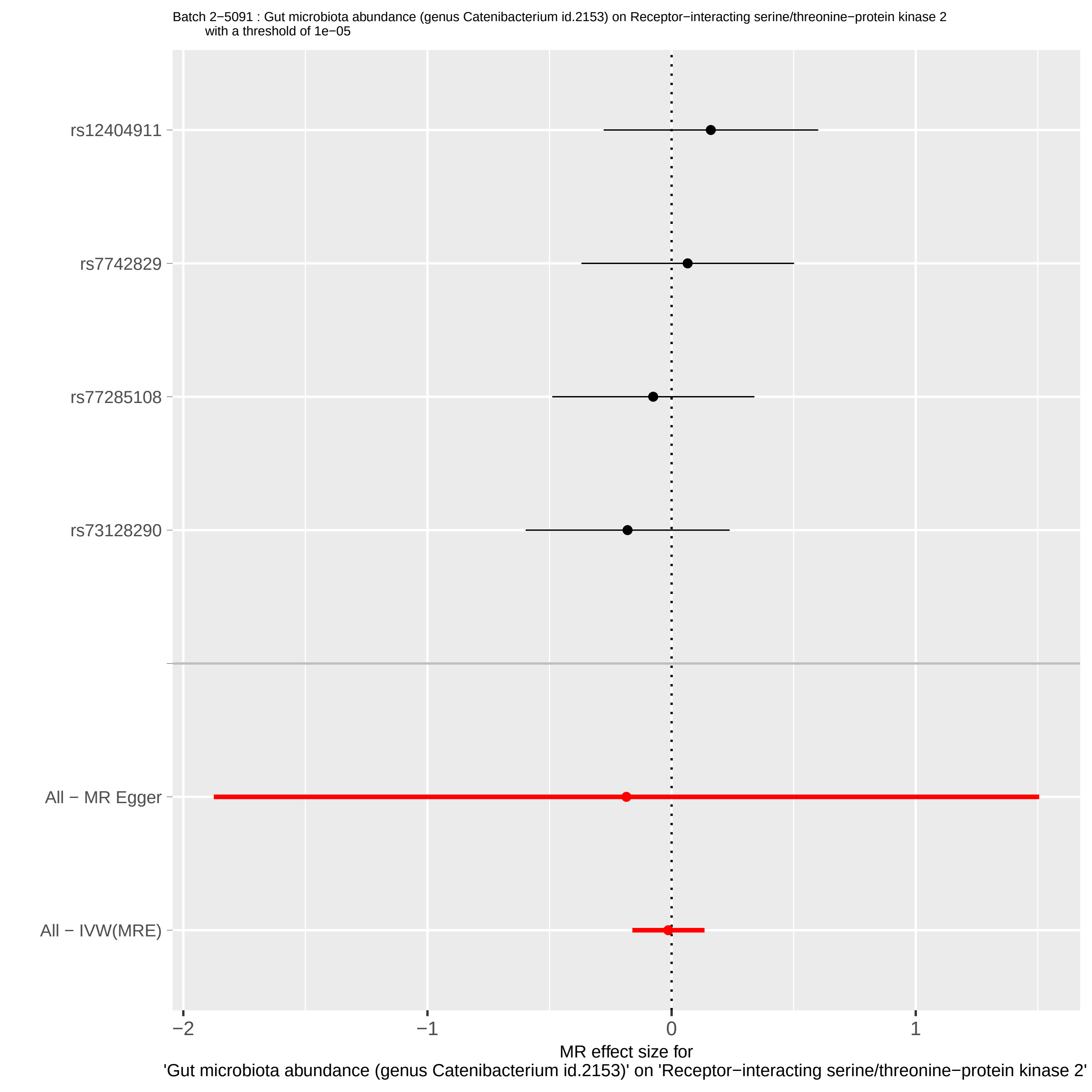

Batch 2−5091 : Gut microbiota abundance (genus Catenibacterium id.2153) on Receptor−interacting serine/threonine−protein kinase 2
with a threshold of 1e−05
rs12404911
rs7742829
rs77285108
rs73128290
All − MR Egger
All − IVW(MRE)
−2
−1
0
1
MR effect size for
'Gut microbiota abundance (genus Catenibacterium id.2153)' on 'Receptor−interacting serine/threonine−protein kinase 2'

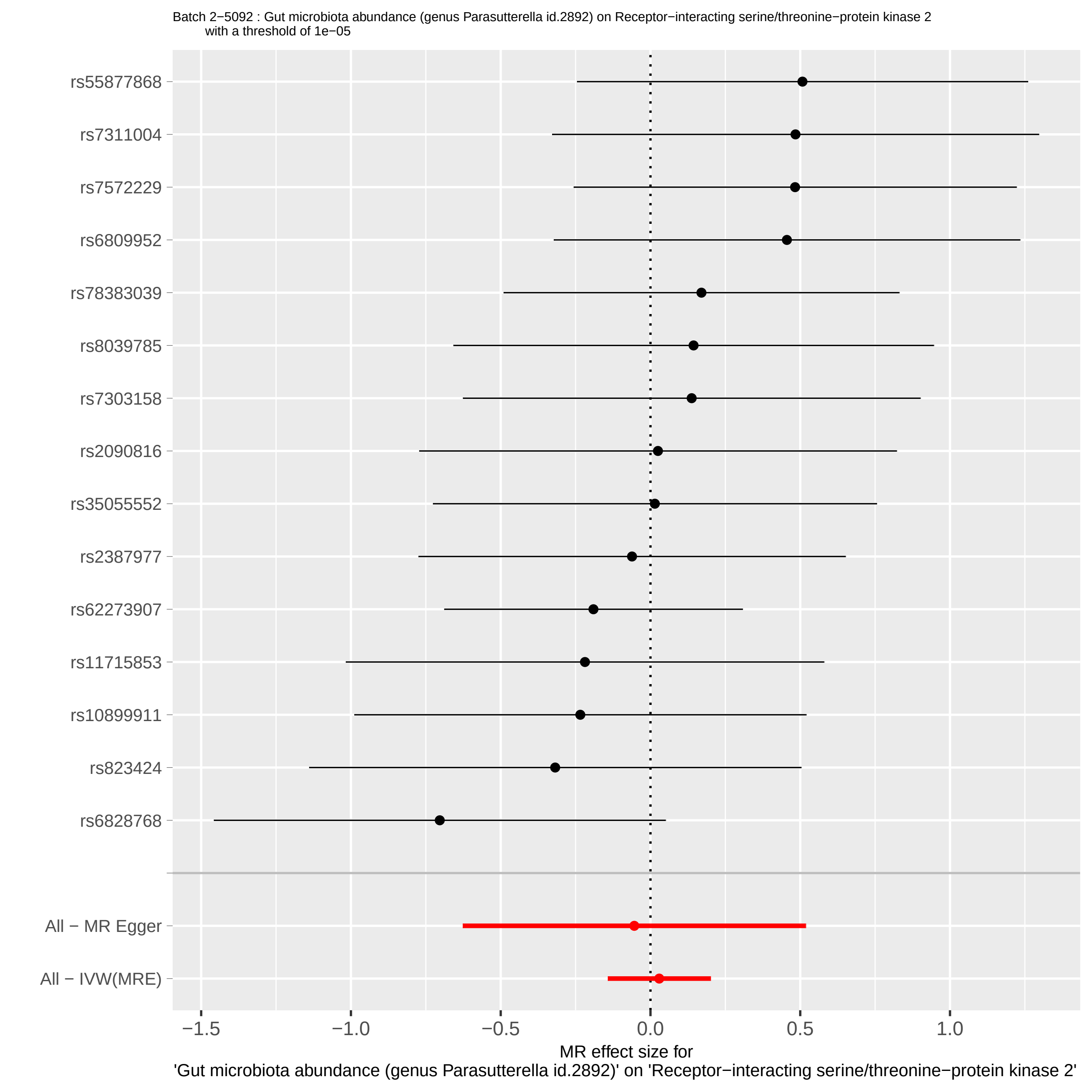

Batch 2−5092 : Gut microbiota abundance (genus Parasutterella id.2892) on Receptor−interacting serine/threonine−protein kinase 2
with a threshold of 1e−05
rs55877868
rs7311004
rs7572229
rs6809952
rs78383039
rs8039785
rs7303158
rs2090816
rs35055552
rs2387977
rs62273907
rs11715853
rs10899911
rs823424
rs6828768
All − MR Egger
All − IVW(MRE)
−1.5
−1.0
−0.5
0.0
0.5
1.0
MR effect size for
'Gut microbiota abundance (genus Parasutterella id.2892)' on 'Receptor−interacting serine/threonine−protein kinase 2'

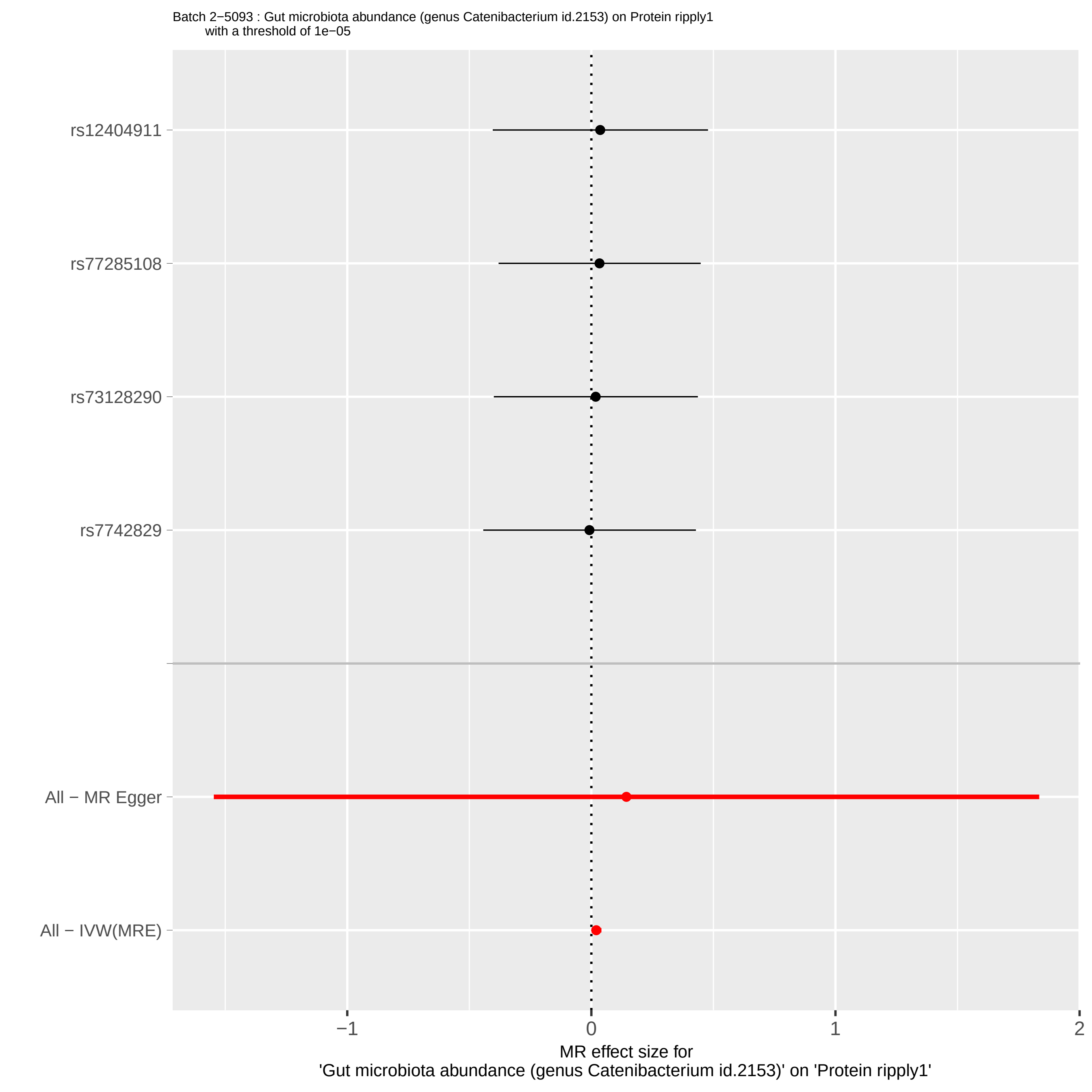

Batch 2−5093 : Gut microbiota abundance (genus Catenibacterium id.2153) on Protein ripply1
with a threshold of 1e−05
rs12404911
rs77285108
rs73128290
rs7742829
All − MR Egger
All − IVW(MRE)
−1
0
1
2
MR effect size for
'Gut microbiota abundance (genus Catenibacterium id.2153)' on 'Protein ripply1'

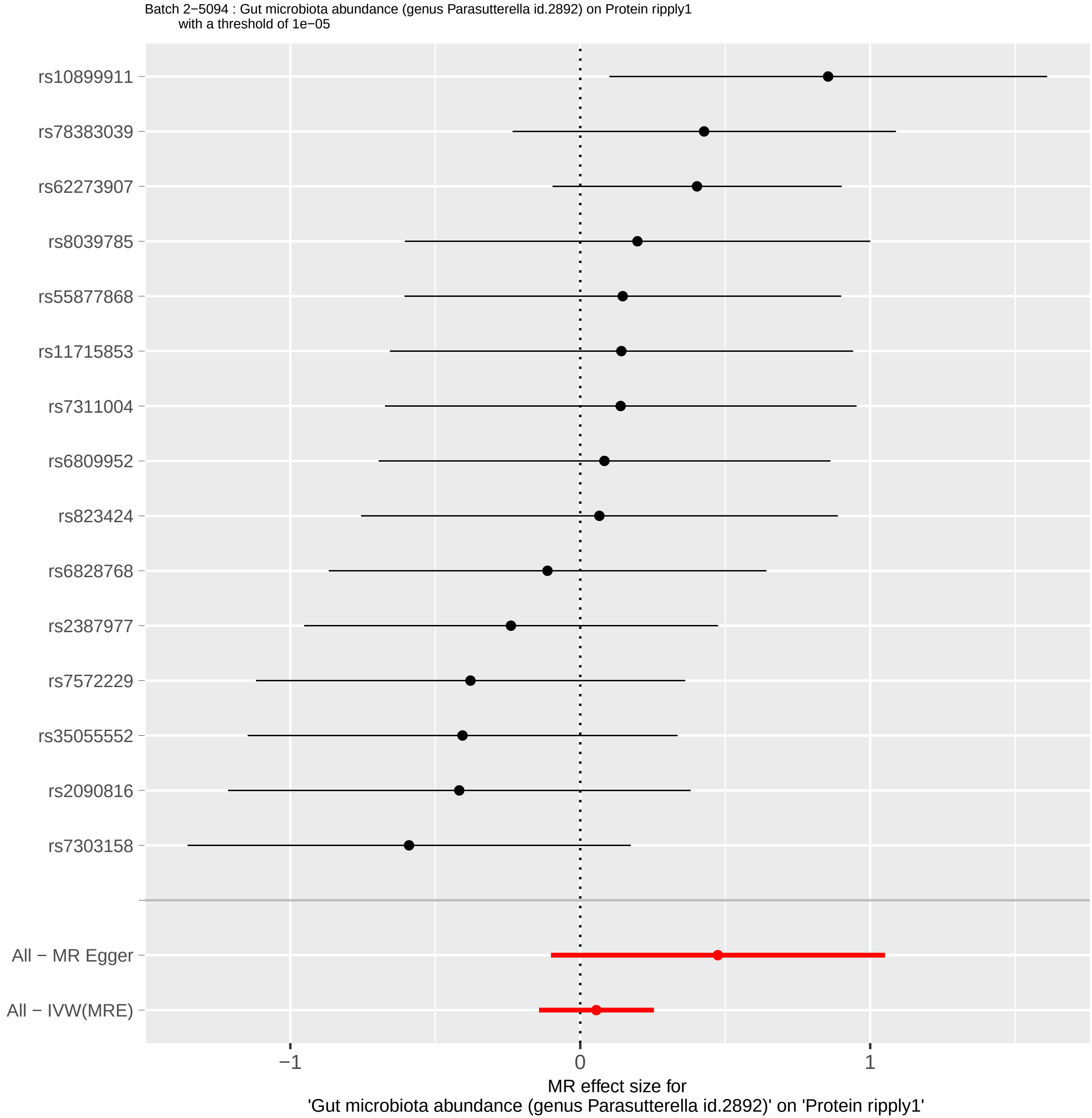

Batch 2−5094 : Gut microbiota abundance (genus Parasutterella id.2892) on Protein ripply1
with a threshold of 1e−05
rs10899911
rs78383039
rs62273907
rs8039785
rs55877868
rs11715853
rs7311004
rs6809952
rs823424
rs6828768
rs2387977
rs7572229
rs35055552
rs2090816
rs7303158
All − MR Egger
All − IVW(MRE)
−1
0
1
MR effect size for
'Gut microbiota abundance (genus Parasutterella id.2892)' on 'Protein ripply1'

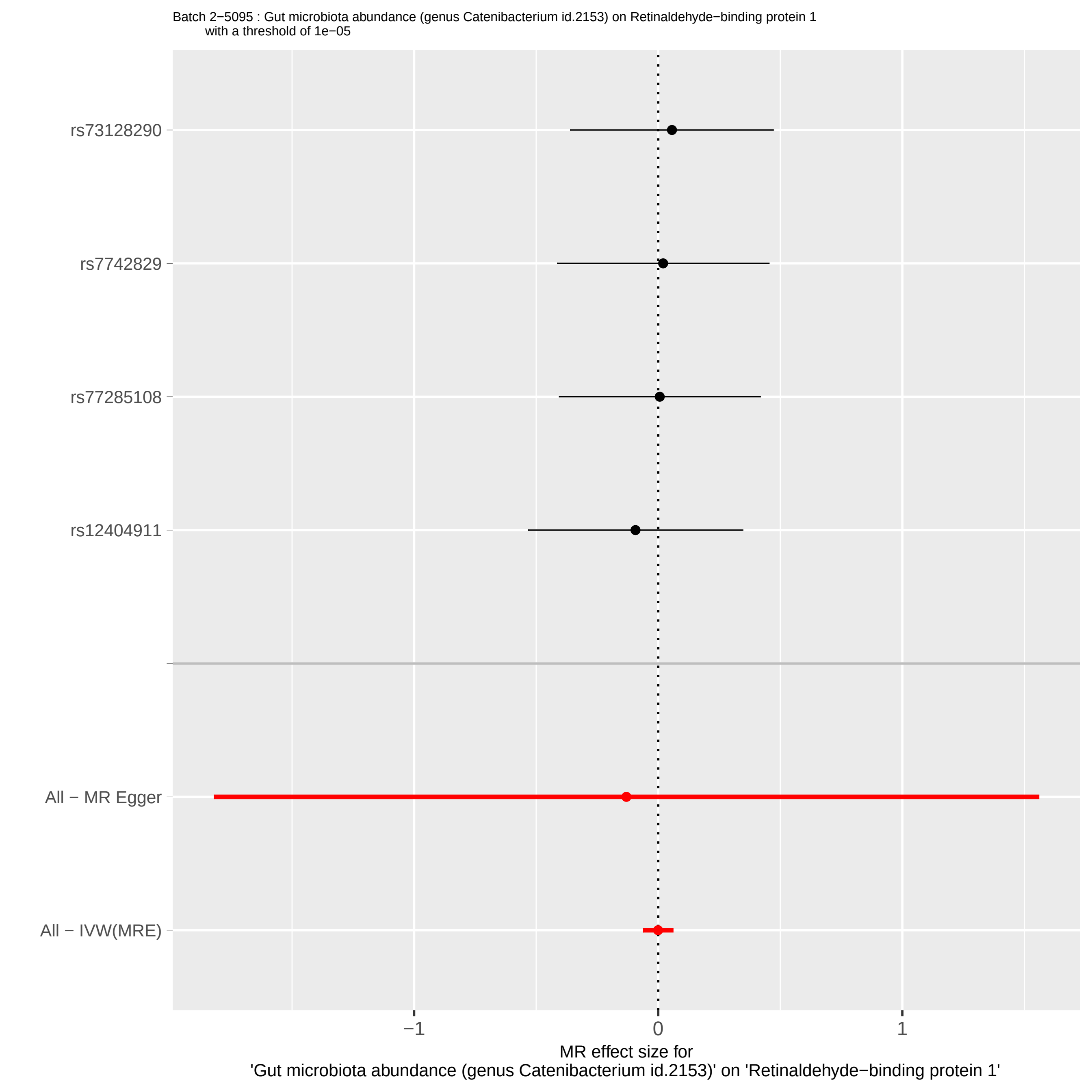
Batch 2−5095 : Gut microbiota abundance (genus Catenibacterium id.2153) on Retinaldehyde−binding protein 1
with a threshold of 1e−05
rs73128290
rs7742829
rs77285108
rs12404911
All − MR Egger
All − IVW(MRE)
−1
0
1
MR effect size for
'Gut microbiota abundance (genus Catenibacterium id.2153)' on 'Retinaldehyde−binding protein 1'

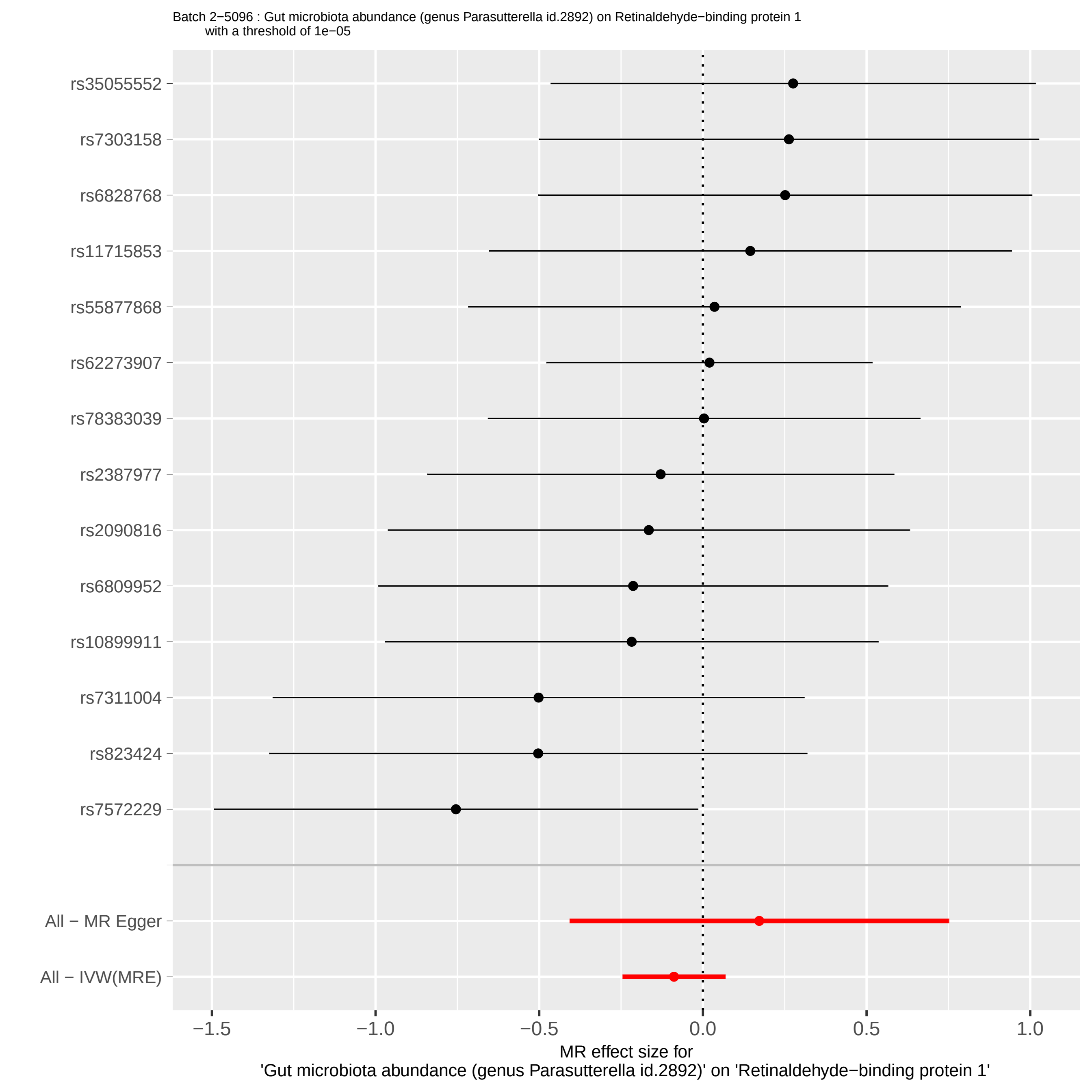

Batch 2−5096 : Gut microbiota abundance (genus Parasutterella id.2892) on Retinaldehyde−binding protein 1
with a threshold of 1e−05
rs35055552
rs7303158
rs6828768
rs11715853
rs55877868
rs62273907
rs78383039
rs2387977
rs2090816
rs6809952
rs10899911
rs7311004
rs823424
rs7572229
All − MR Egger
All − IVW(MRE)
−1.5
−1.0
−0.5
0.0
0.5
1.0
MR effect size for
'Gut microbiota abundance (genus Parasutterella id.2892)' on 'Retinaldehyde−binding protein 1'

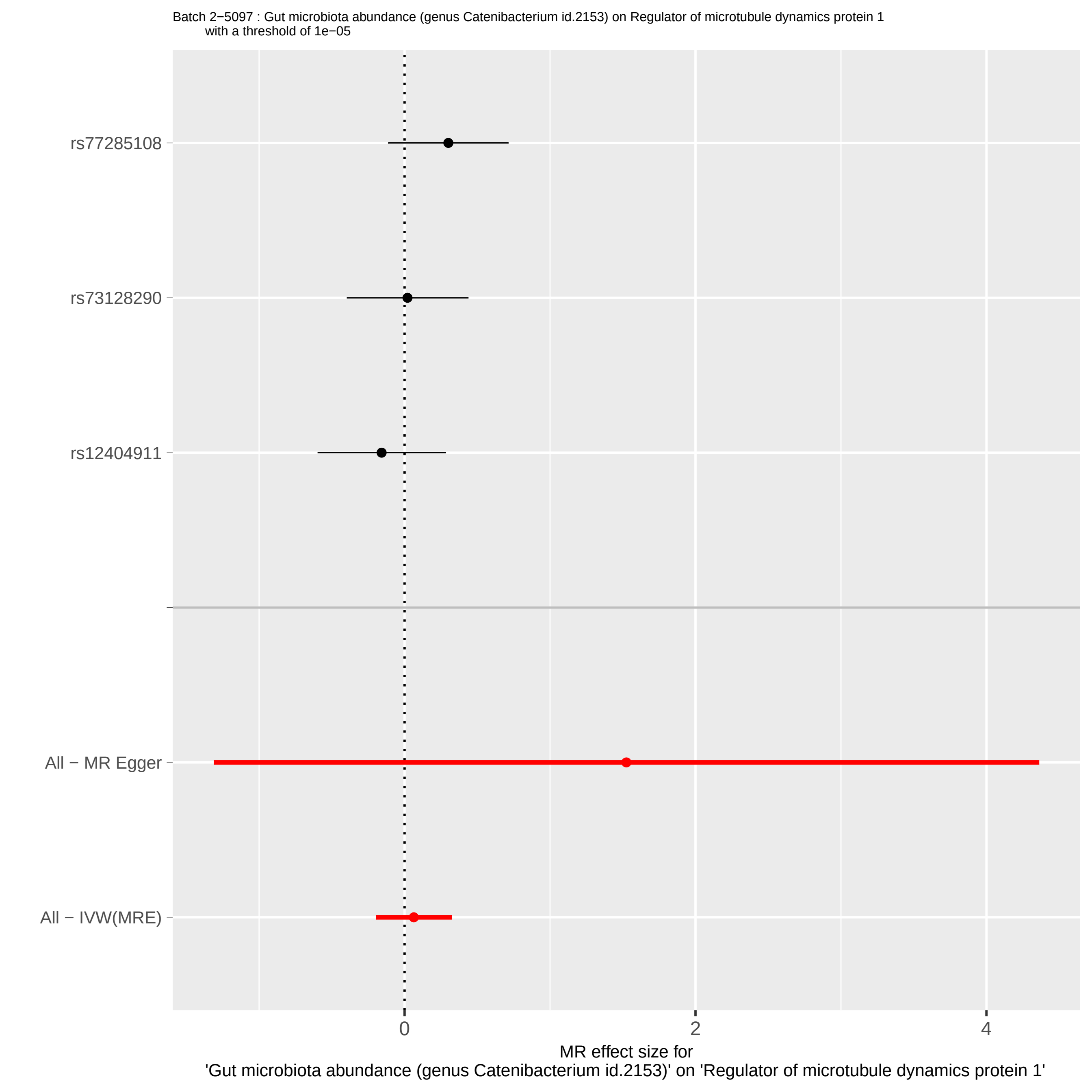

Batch 2−5097 : Gut microbiota abundance (genus Catenibacterium id.2153) on Regulator of microtubule dynamics protein 1
with a threshold of 1e−05
rs77285108
rs73128290
rs12404911
All − MR Egger
All − IVW(MRE)
0
2
4
MR effect size for
'Gut microbiota abundance (genus Catenibacterium id.2153)' on 'Regulator of microtubule dynamics protein 1'

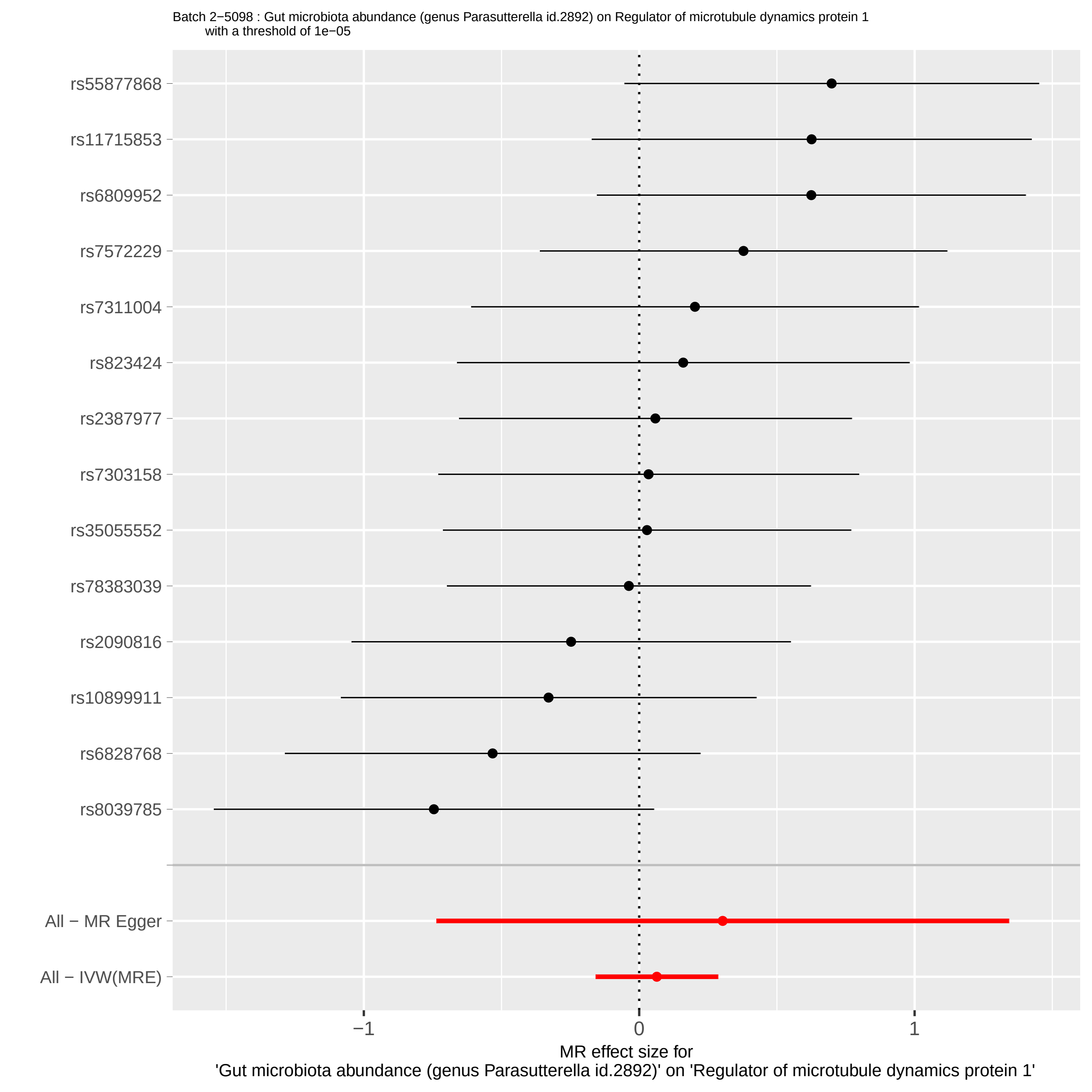
Batch 2−5098 : Gut microbiota abundance (genus Parasutterella id.2892) on Regulator of microtubule dynamics protein 1
with a threshold of 1e−05
rs55877868
rs11715853
rs6809952
rs7572229
rs7311004
rs823424
rs2387977
rs7303158
rs35055552
rs78383039
rs2090816
rs10899911
rs6828768
rs8039785
All − MR Egger
All − IVW(MRE)
−1
0
1
MR effect size for
'Gut microbiota abundance (genus Parasutterella id.2892)' on 'Regulator of microtubule dynamics protein 1'

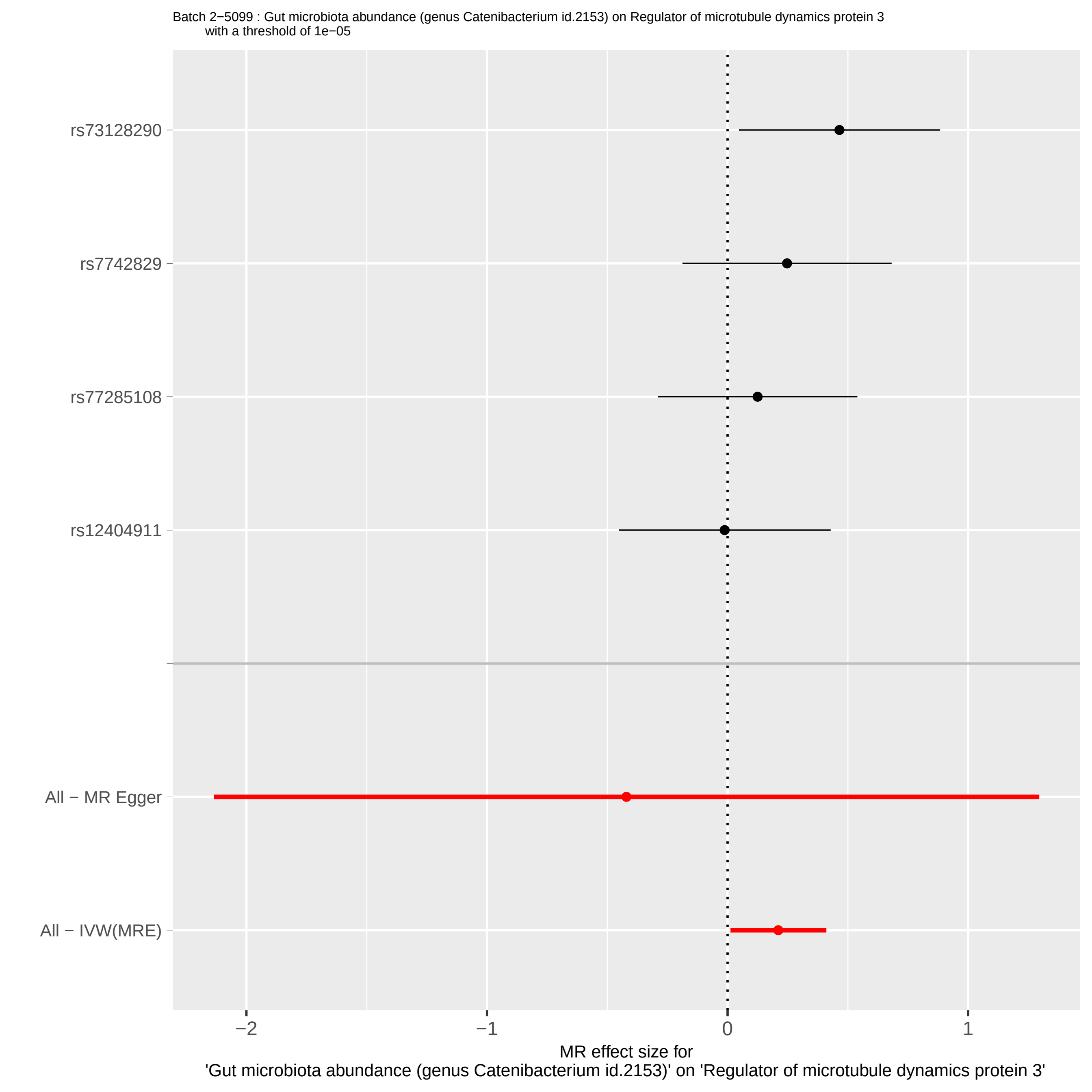
Batch 2−5099 : Gut microbiota abundance (genus Catenibacterium id.2153) on Regulator of microtubule dynamics protein 3
with a threshold of 1e−05
rs73128290
rs7742829
rs77285108
rs12404911
All − MR Egger
All − IVW(MRE)
−2
−1
0
1
MR effect size for
'Gut microbiota abundance (genus Catenibacterium id.2153)' on 'Regulator of microtubule dynamics protein 3'

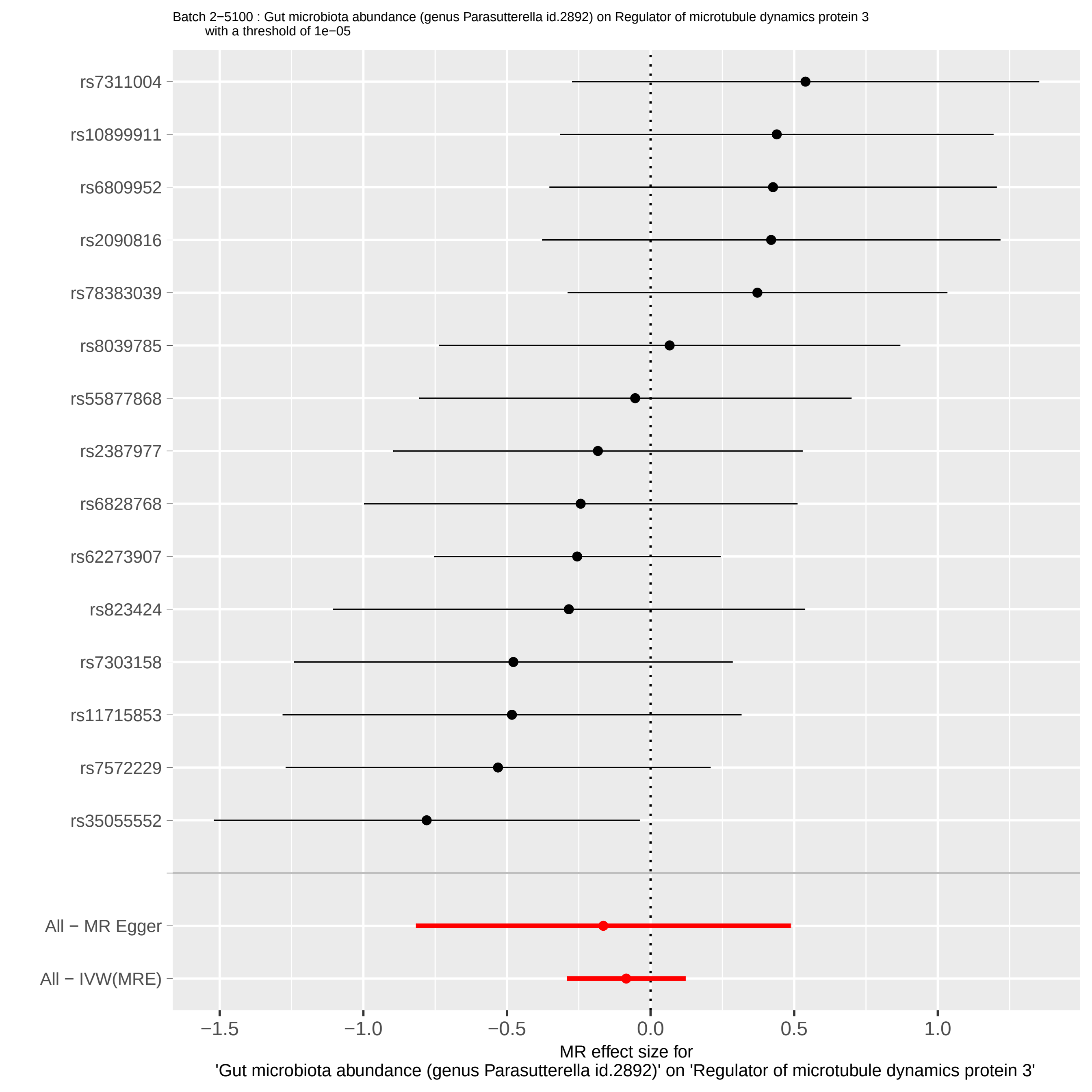
Batch 2−5100 : Gut microbiota abundance (genus Parasutterella id.2892) on Regulator of microtubule dynamics protein 3
with a threshold of 1e−05
rs7311004
rs10899911
rs6809952
rs2090816
rs78383039
rs8039785
rs55877868
rs2387977
rs6828768
rs62273907
rs823424
rs7303158
rs11715853
rs7572229
rs35055552
All − MR Egger
All − IVW(MRE)
−1.5
−1.0
−0.5
0.0
0.5
1.0
MR effect size for
'Gut microbiota abundance (genus Parasutterella id.2892)' on 'Regulator of microtubule dynamics protein 3'

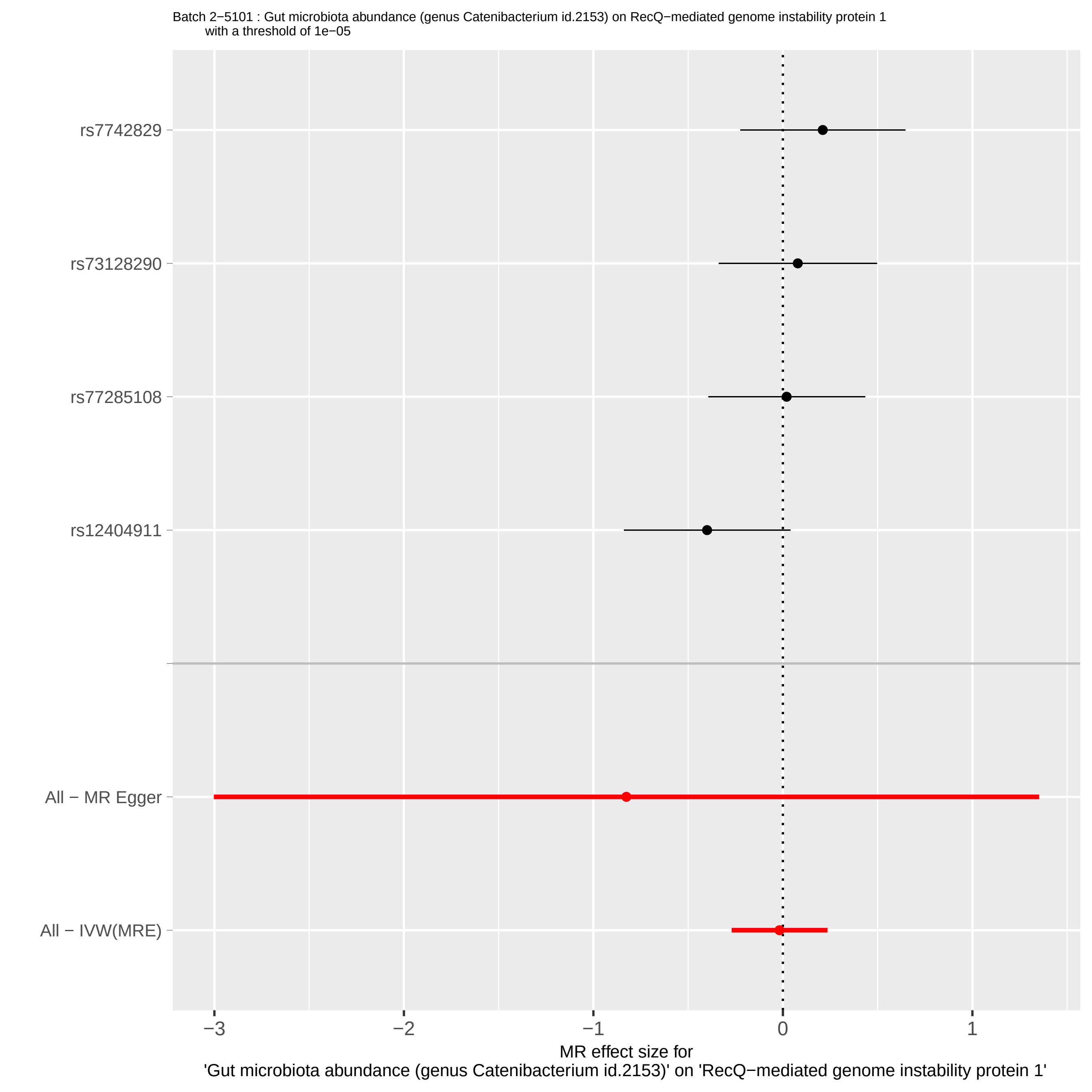

Batch 2−5101 : Gut microbiota abundance (genus Catenibacterium id.2153) on RecQ−mediated genome instability protein 1
with a threshold of 1e−05
rs7742829
rs73128290
rs77285108
rs12404911
All − MR Egger
All − IVW(MRE)
−3
−2
−1
0
1
MR effect size for
'Gut microbiota abundance (genus Catenibacterium id.2153)' on 'RecQ−mediated genome instability protein 1'

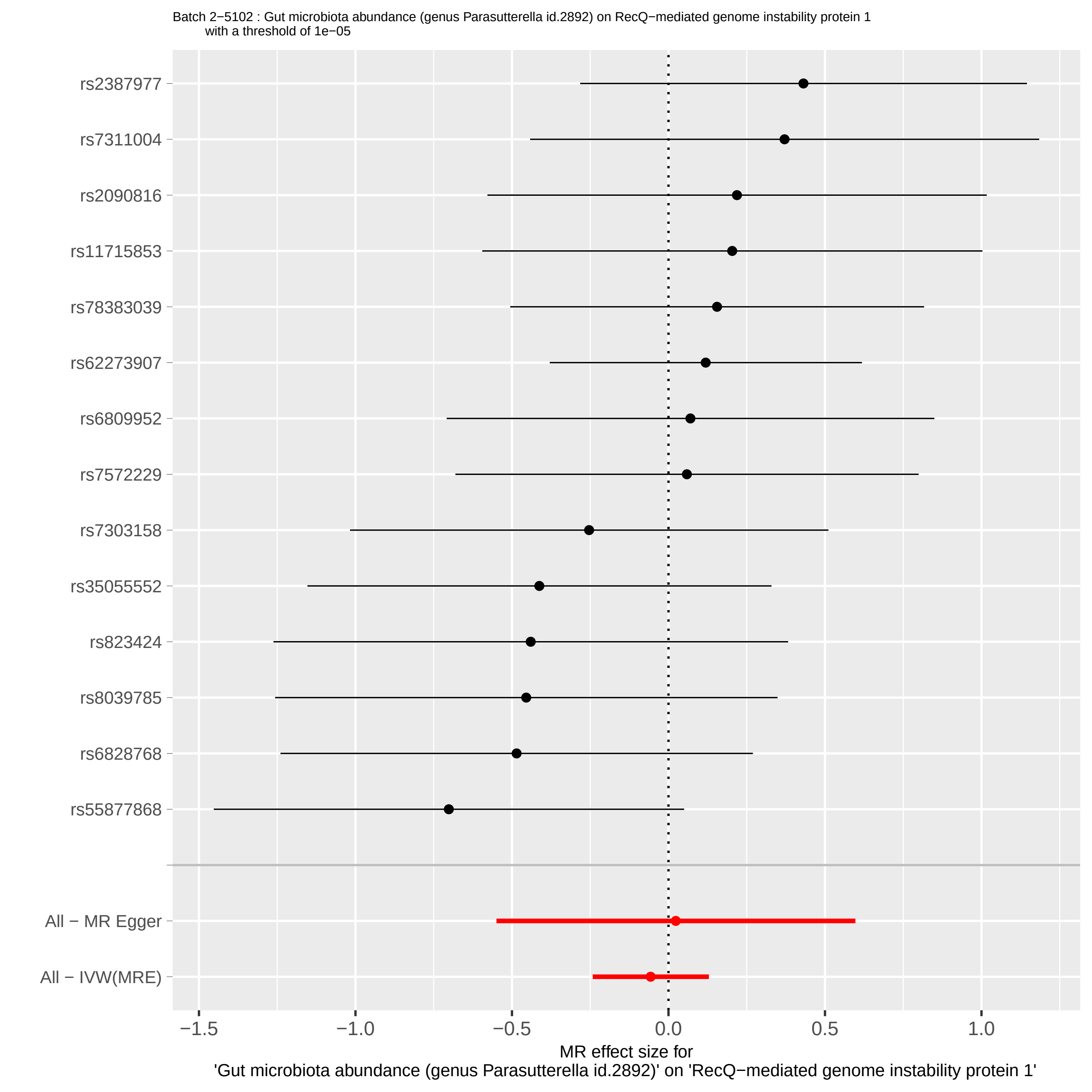
Batch 2−5102 : Gut microbiota abundance (genus Parasutterella id.2892) on RecQ−mediated genome instability protein 1
with a threshold of 1e−05
rs2387977
rs7311004
rs2090816
rs11715853
rs78383039
rs62273907
rs6809952
rs7572229
rs7303158
rs35055552
rs823424
rs8039785
rs6828768
rs55877868
All − MR Egger
All − IVW(MRE)
−1.5
−1.0
−0.5
0.0
0.5
1.0
MR effect size for
'Gut microbiota abundance (genus Parasutterella id.2892)' on 'RecQ−mediated genome instability protein 1'

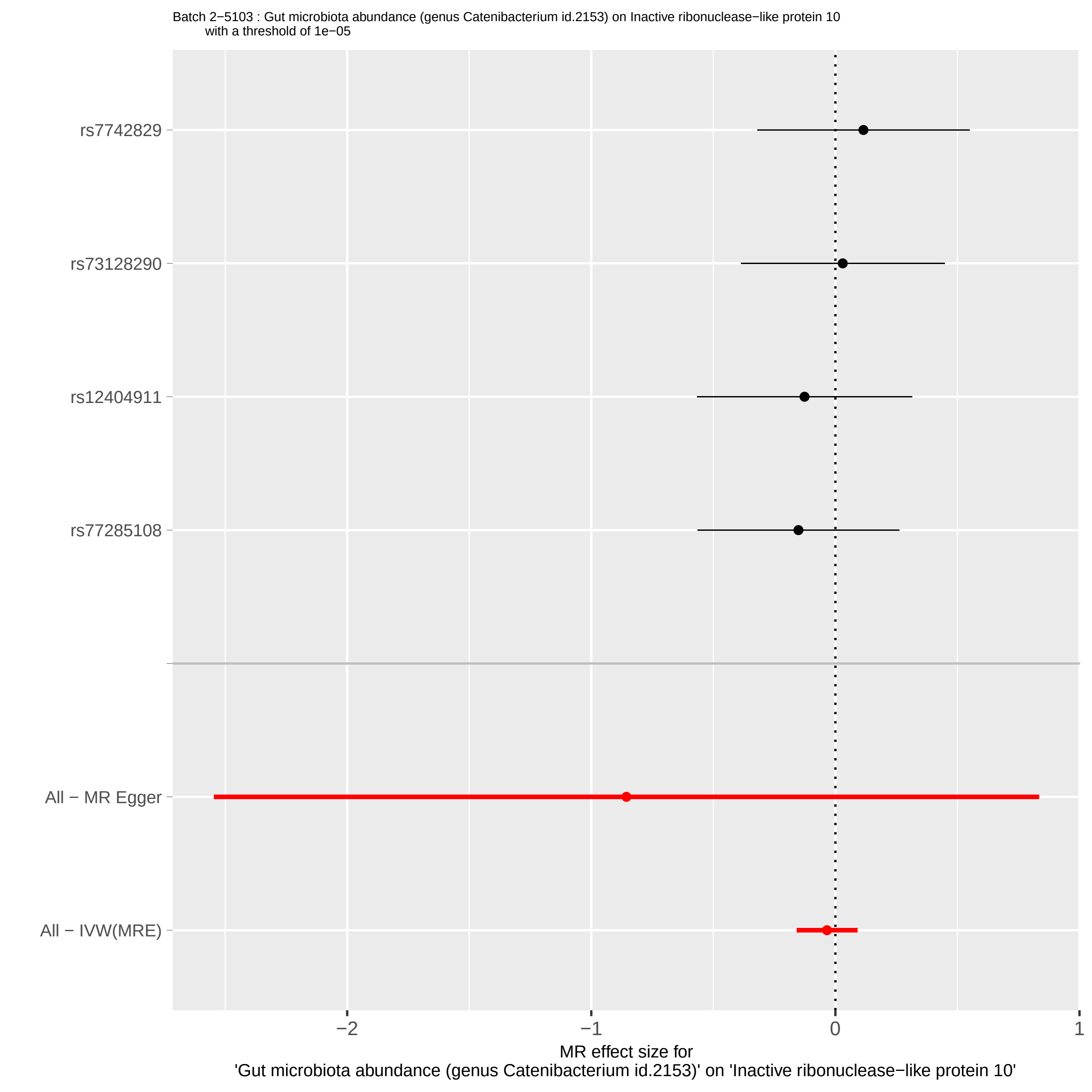
Batch 2−5103 : Gut microbiota abundance (genus Catenibacterium id.2153) on Inactive ribonuclease−like protein 10
with a threshold of 1e−05
rs7742829
rs73128290
rs12404911
rs77285108
All − MR Egger
All − IVW(MRE)
−2
−1
0
1
MR effect size for
'Gut microbiota abundance (genus Catenibacterium id.2153)' on 'Inactive ribonuclease−like protein 10'

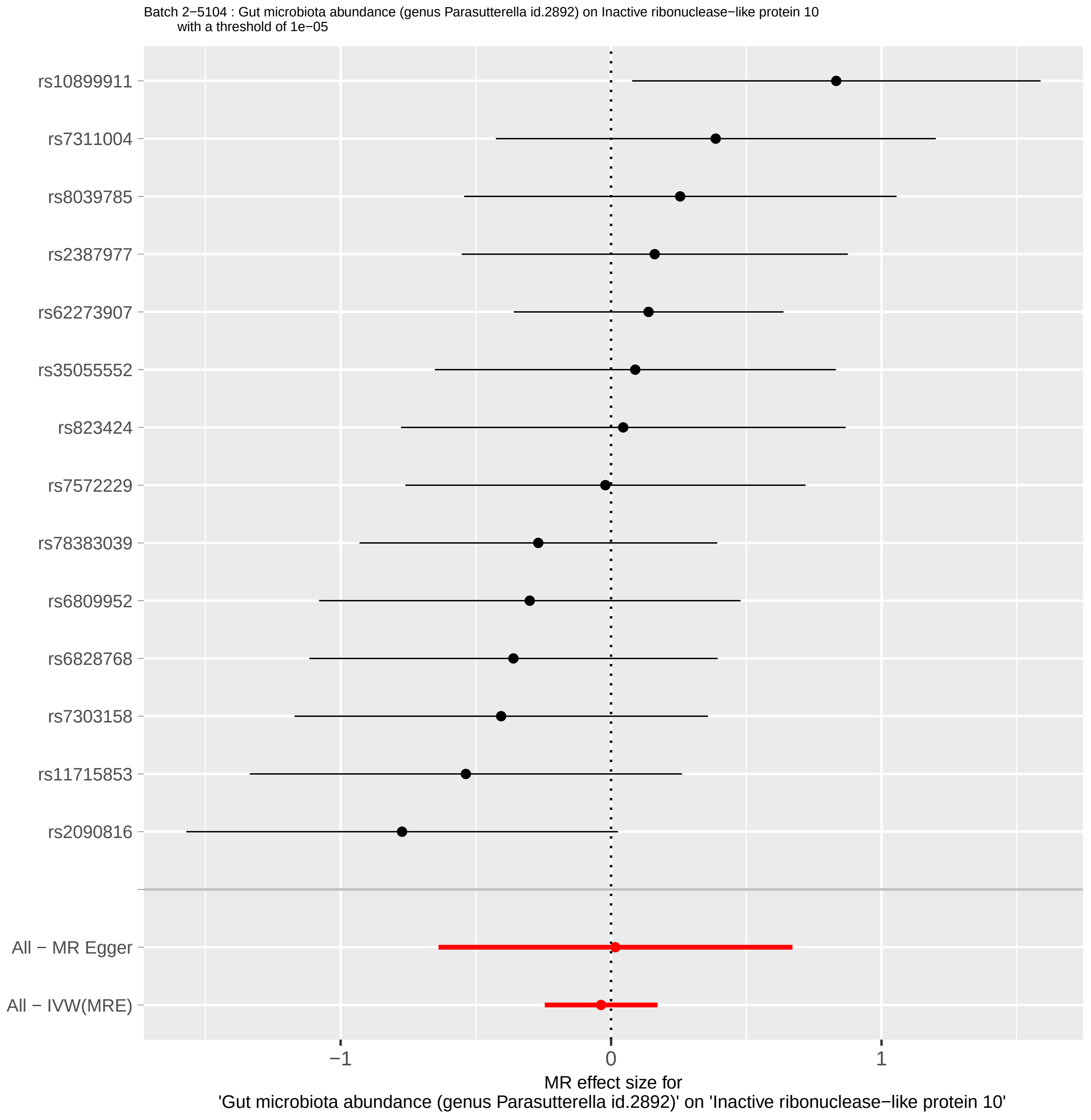
Batch 2−5104 : Gut microbiota abundance (genus Parasutterella id.2892) on Inactive ribonuclease−like protein 10
with a threshold of 1e−05
rs10899911
rs7311004
rs8039785
rs2387977
rs62273907
rs35055552
rs823424
rs7572229
rs78383039
rs6809952
rs6828768
rs7303158
rs11715853
rs2090816
All − MR Egger
All − IVW(MRE)
−1
0
1
MR effect size for
'Gut microbiota abundance (genus Parasutterella id.2892)' on 'Inactive ribonuclease−like protein 10'

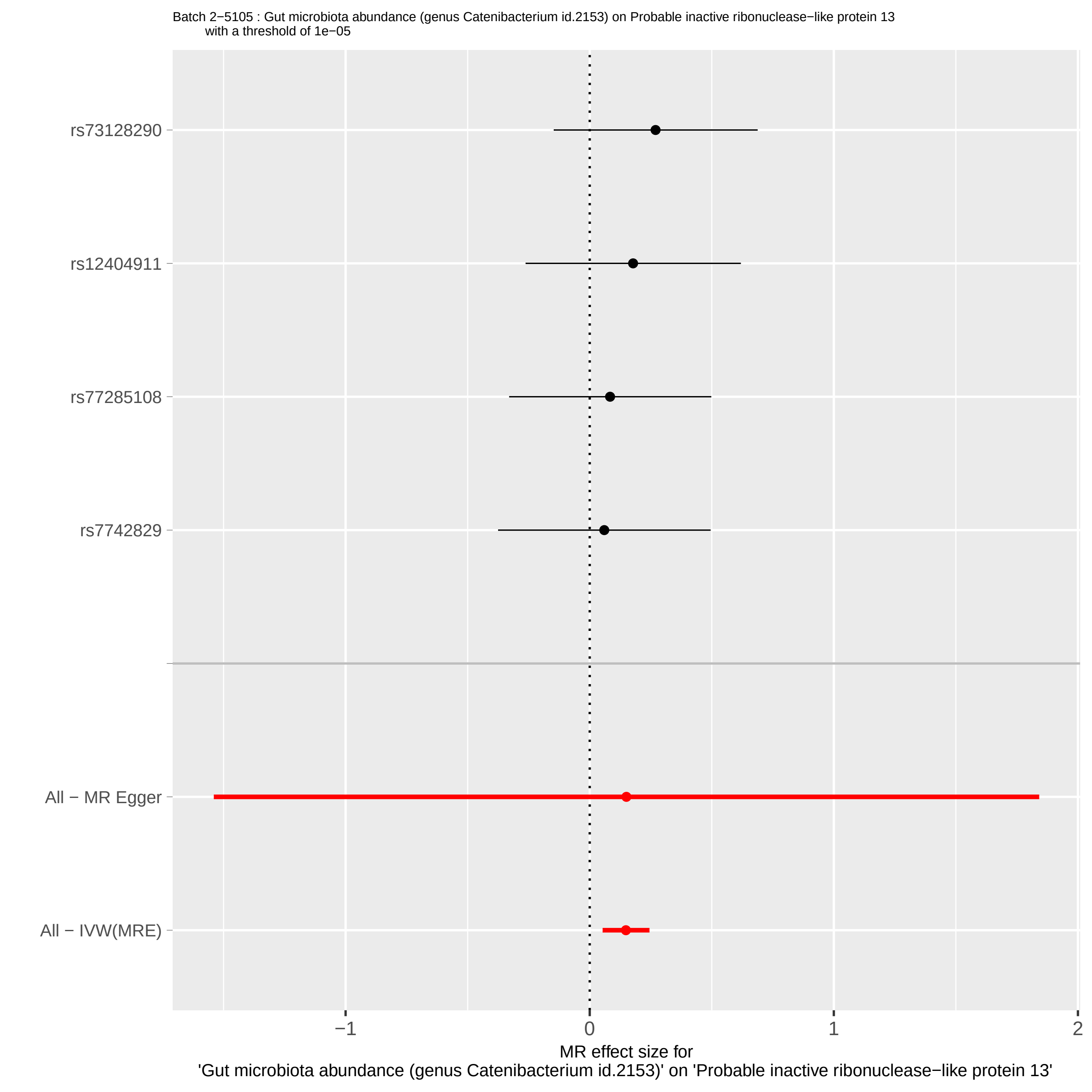

Batch 2−5105 : Gut microbiota abundance (genus Catenibacterium id.2153) on Probable inactive ribonuclease−like protein 13
with a threshold of 1e−05
rs73128290
rs12404911
rs77285108
rs7742829
All − MR Egger
All − IVW(MRE)
−1
0
1
2
MR effect size for
'Gut microbiota abundance (genus Catenibacterium id.2153)' on 'Probable inactive ribonuclease−like protein 13'

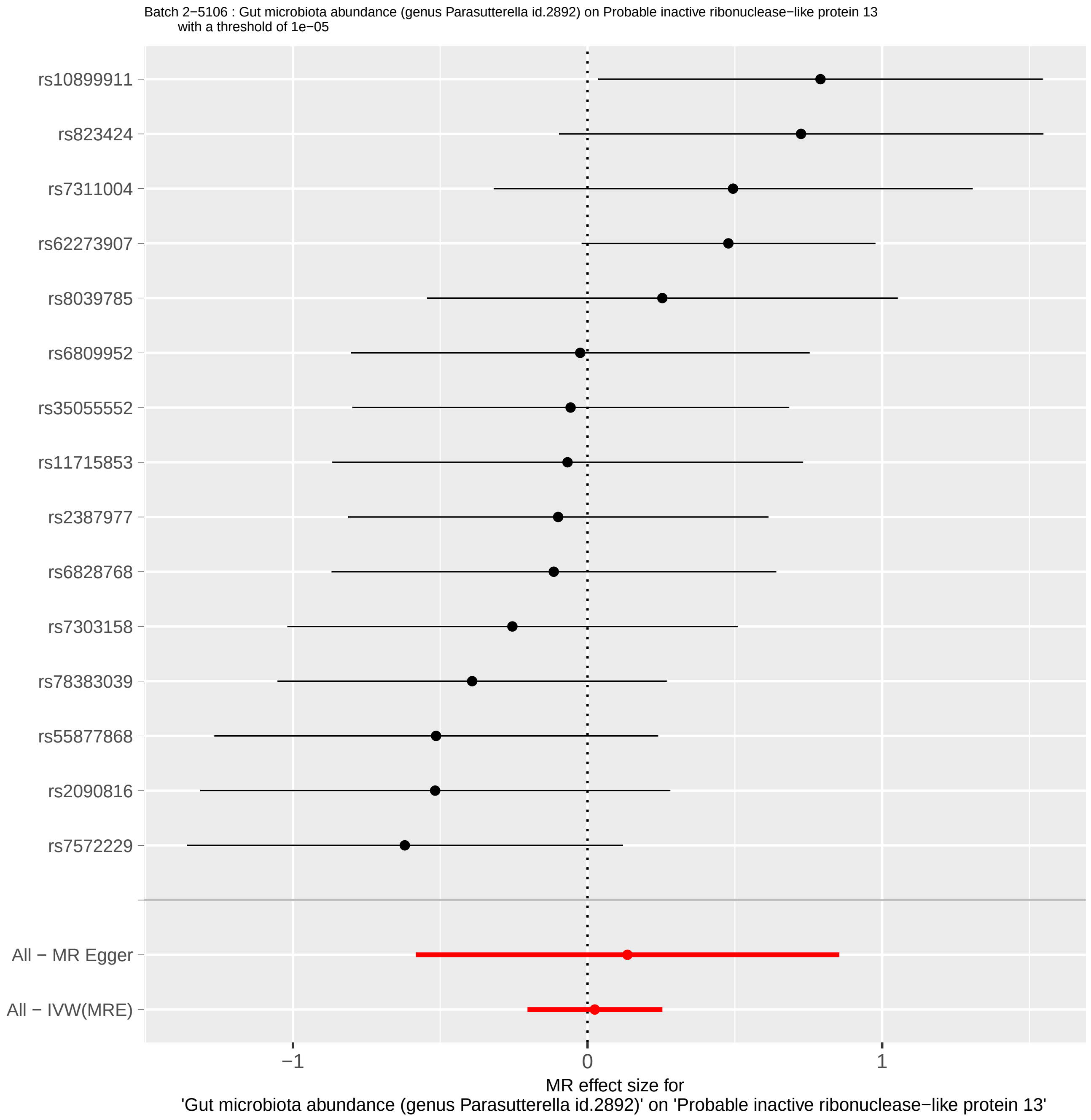

Batch 2−5106 : Gut microbiota abundance (genus Parasutterella id.2892) on Probable inactive ribonuclease−like protein 13
with a threshold of 1e−05
rs10899911
rs823424
rs7311004
rs62273907
rs8039785
rs6809952
rs35055552
rs11715853
rs2387977
rs6828768
rs7303158
rs78383039
rs55877868
rs2090816
rs7572229
All − MR Egger
All − IVW(MRE)
−1
0
1
MR effect size for
'Gut microbiota abundance (genus Parasutterella id.2892)' on 'Probable inactive ribonuclease−like protein 13'

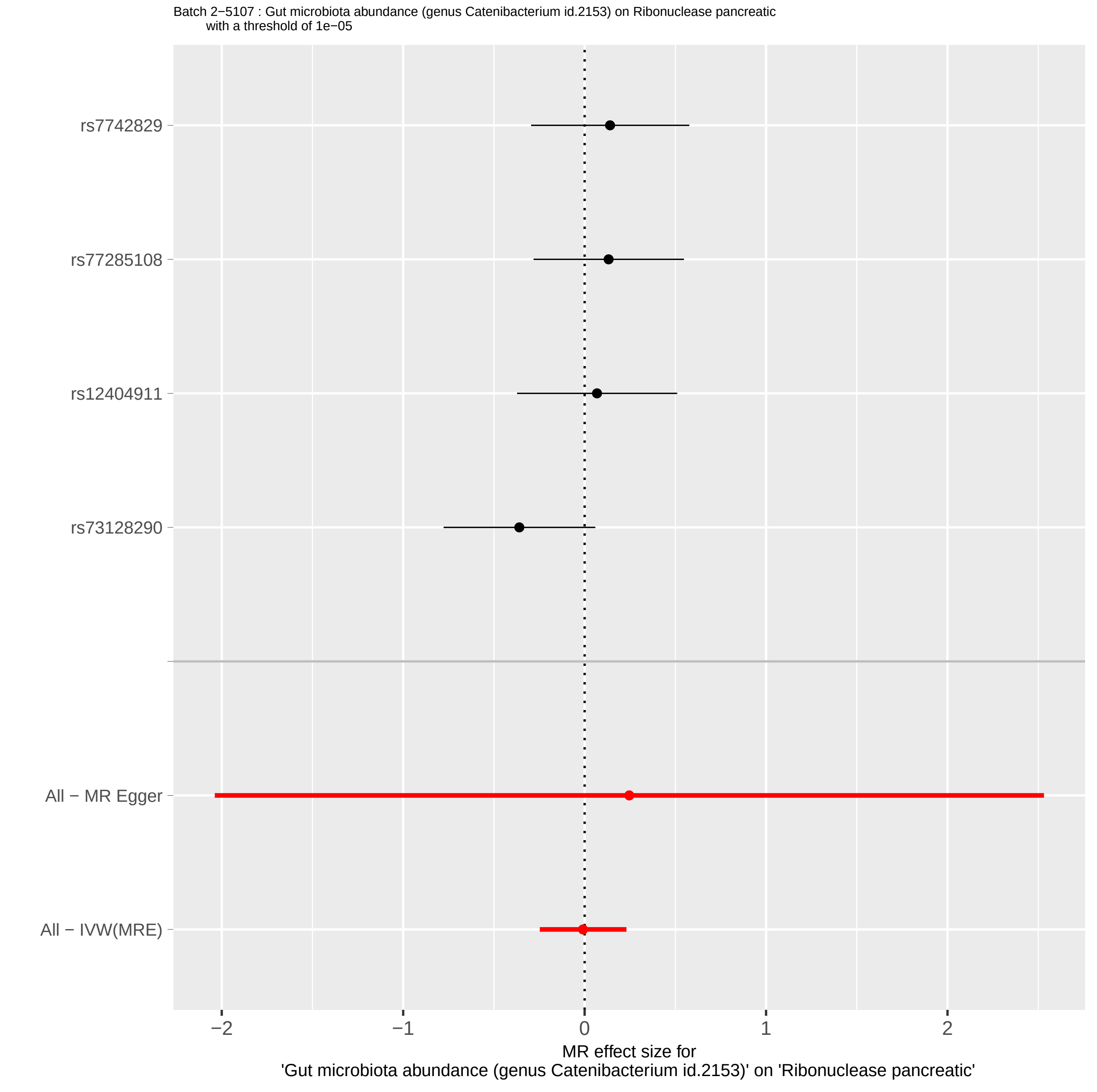
Batch 2−5107 : Gut microbiota abundance (genus Catenibacterium id.2153) on Ribonuclease pancreatic
with a threshold of 1e−05
rs7742829
rs77285108
rs12404911
rs73128290
All − MR Egger
All − IVW(MRE)
−2
−1
0
1
2
MR effect size for
'Gut microbiota abundance (genus Catenibacterium id.2153)' on 'Ribonuclease pancreatic'

Batch 2−5108 : Gut microbiota abundance (genus Parasutterella id.2892) on Ribonuclease pancreatic
with a threshold of 1e−05

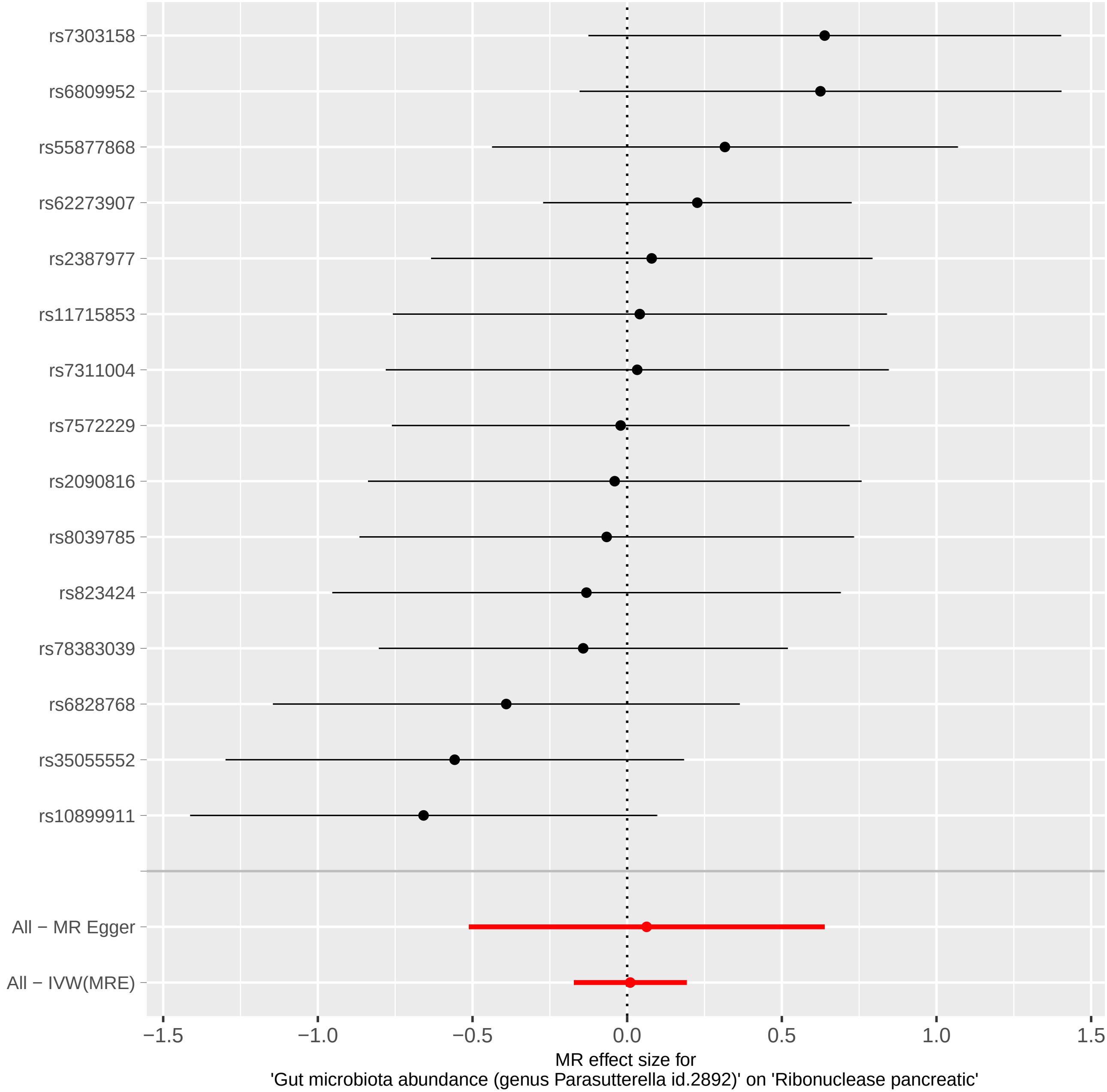

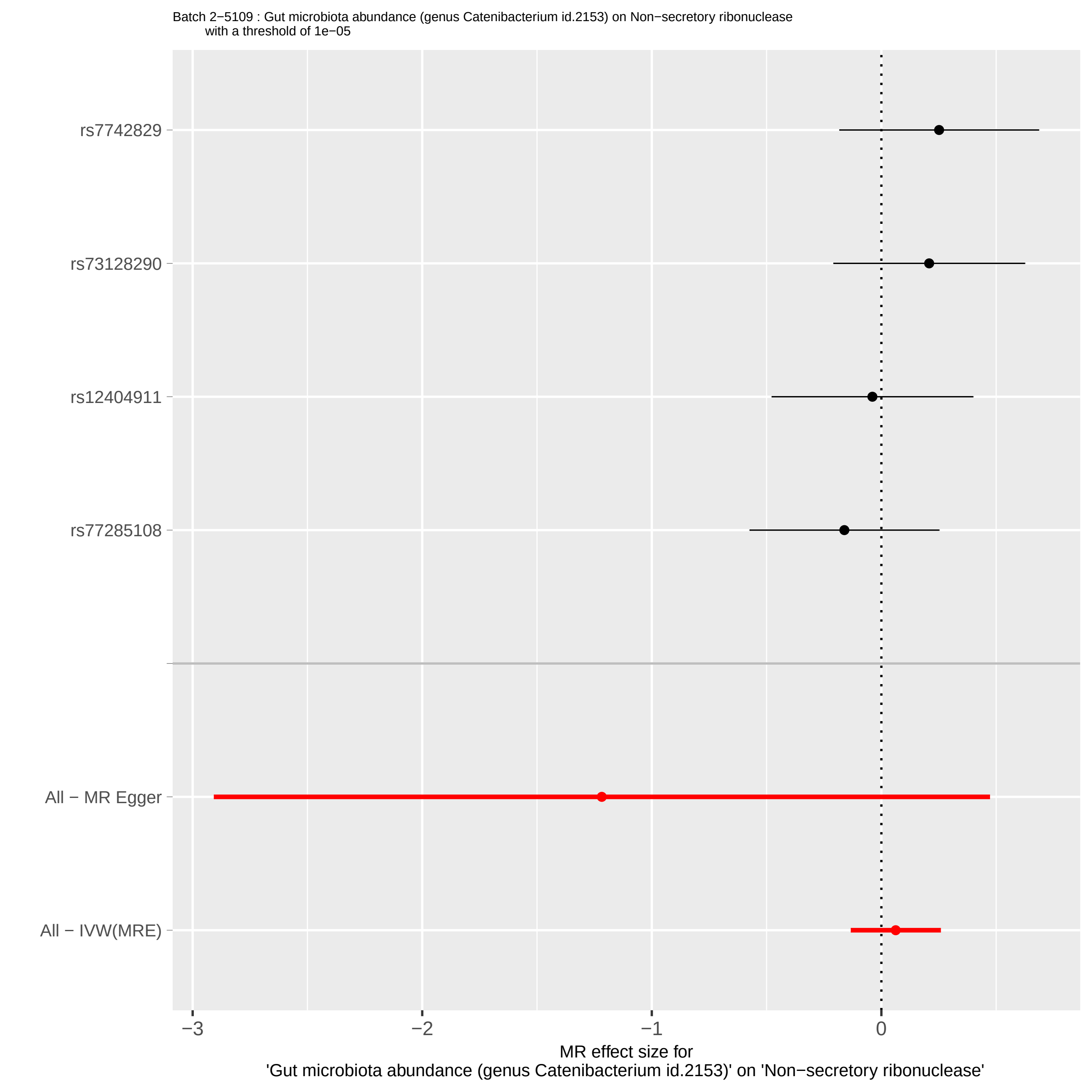

Batch 2−5109 : Gut microbiota abundance (genus Catenibacterium id.2153) on Non−secretory ribonuclease
with a threshold of 1e−05
rs7742829
rs73128290
rs12404911
rs77285108
All − MR Egger
All − IVW(MRE)
−3
−2
−1
0
MR effect size for
'Gut microbiota abundance (genus Catenibacterium id.2153)' on 'Non−secretory ribonuclease'

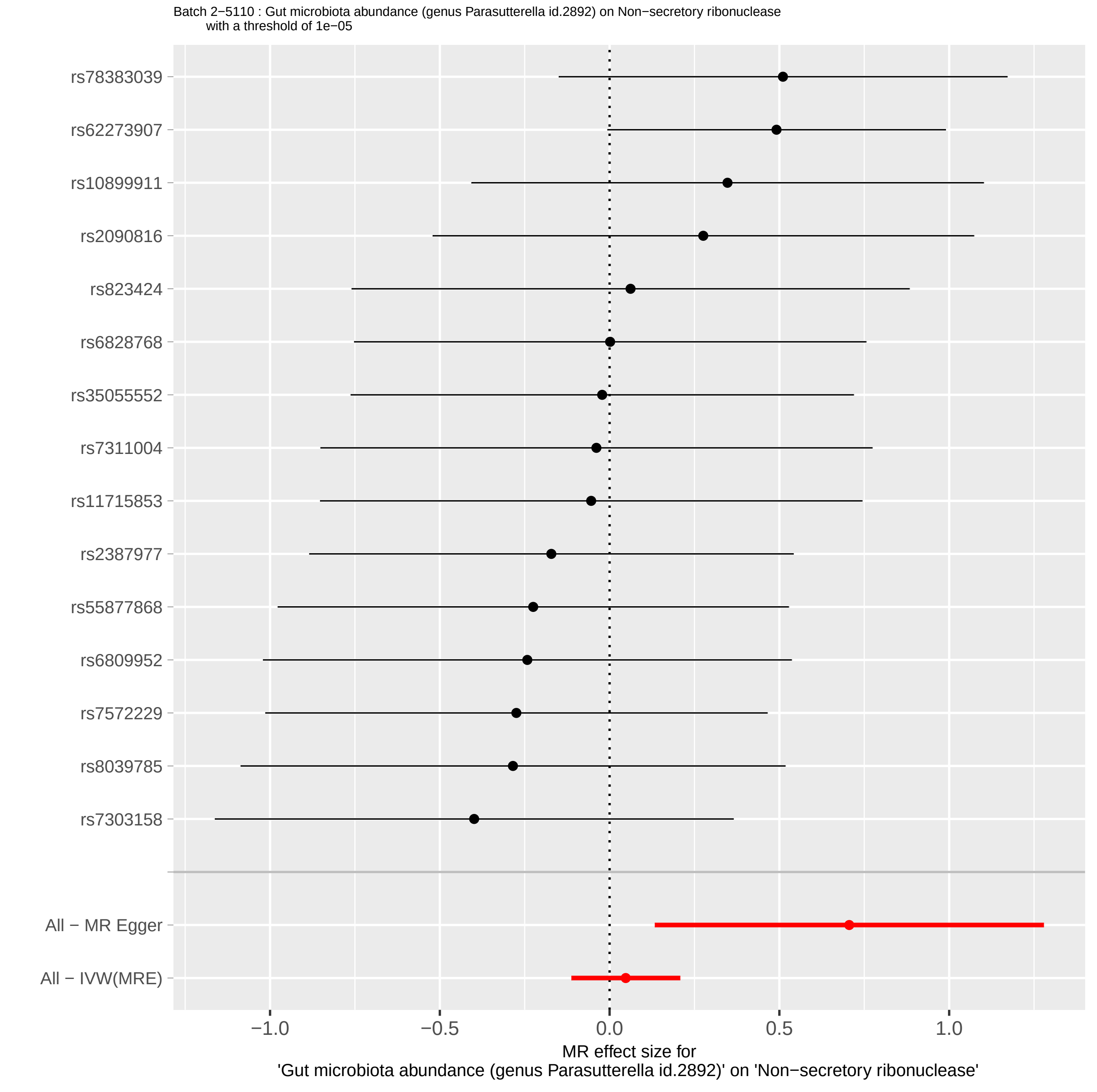

Batch 2−5110 : Gut microbiota abundance (genus Parasutterella id.2892) on Non−secretory ribonuclease
with a threshold of 1e−05
rs78383039
rs62273907
rs10899911
rs2090816
rs823424
rs6828768
rs35055552
rs7311004
rs11715853
rs2387977
rs55877868
rs6809952
rs7572229
rs8039785
rs7303158
All − MR Egger
All − IVW(MRE)
−1.0
−0.5
0.0
0.5
1.0
MR effect size for
'Gut microbiota abundance (genus Parasutterella id.2892)' on 'Non−secretory ribonuclease'

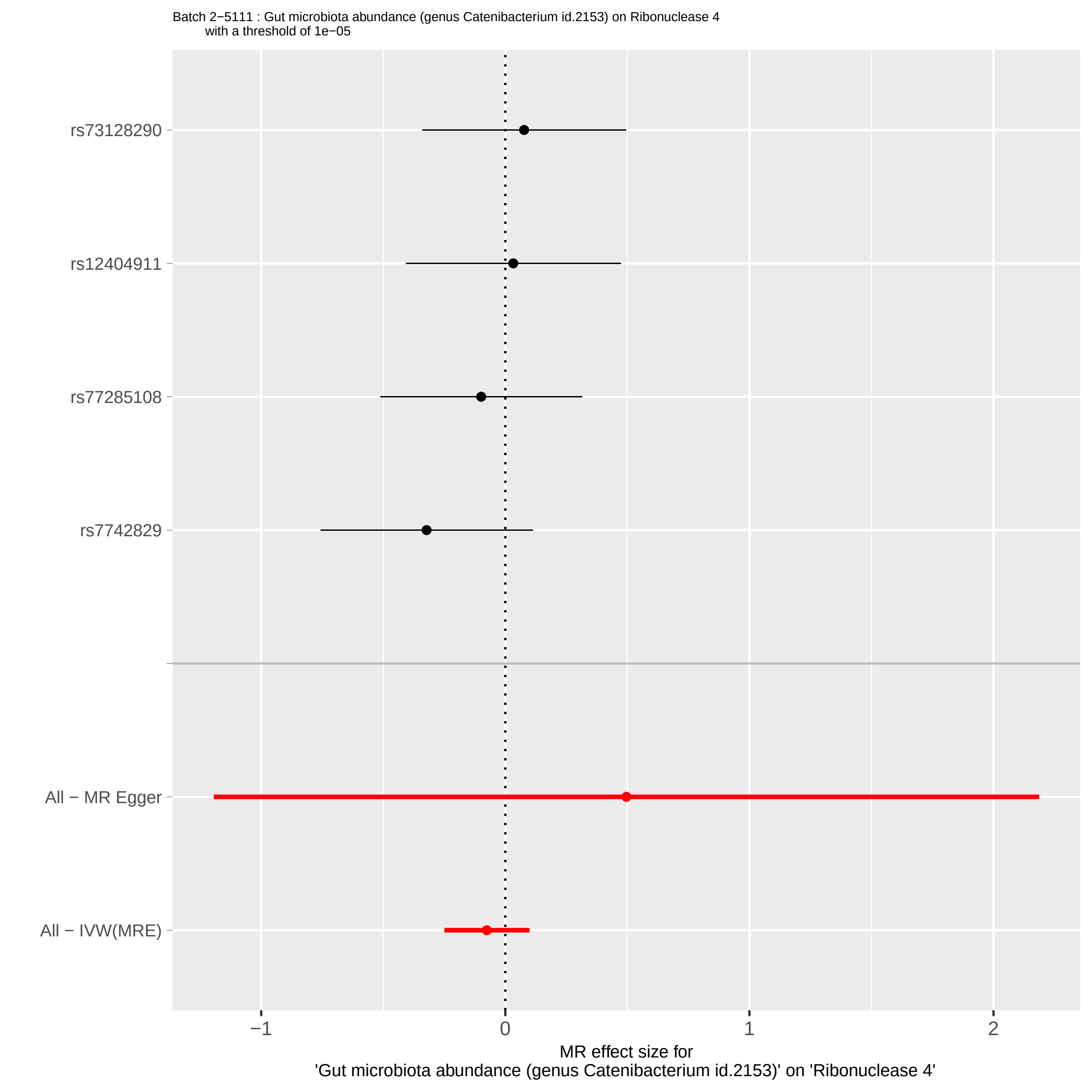

Batch 2−5111 : Gut microbiota abundance (genus Catenibacterium id.2153) on Ribonuclease 4
with a threshold of 1e−05
rs73128290
rs12404911
rs77285108
rs7742829
All − MR Egger
All − IVW(MRE)
−1
0
1
2
MR effect size for
'Gut microbiota abundance (genus Catenibacterium id.2153)' on 'Ribonuclease 4'

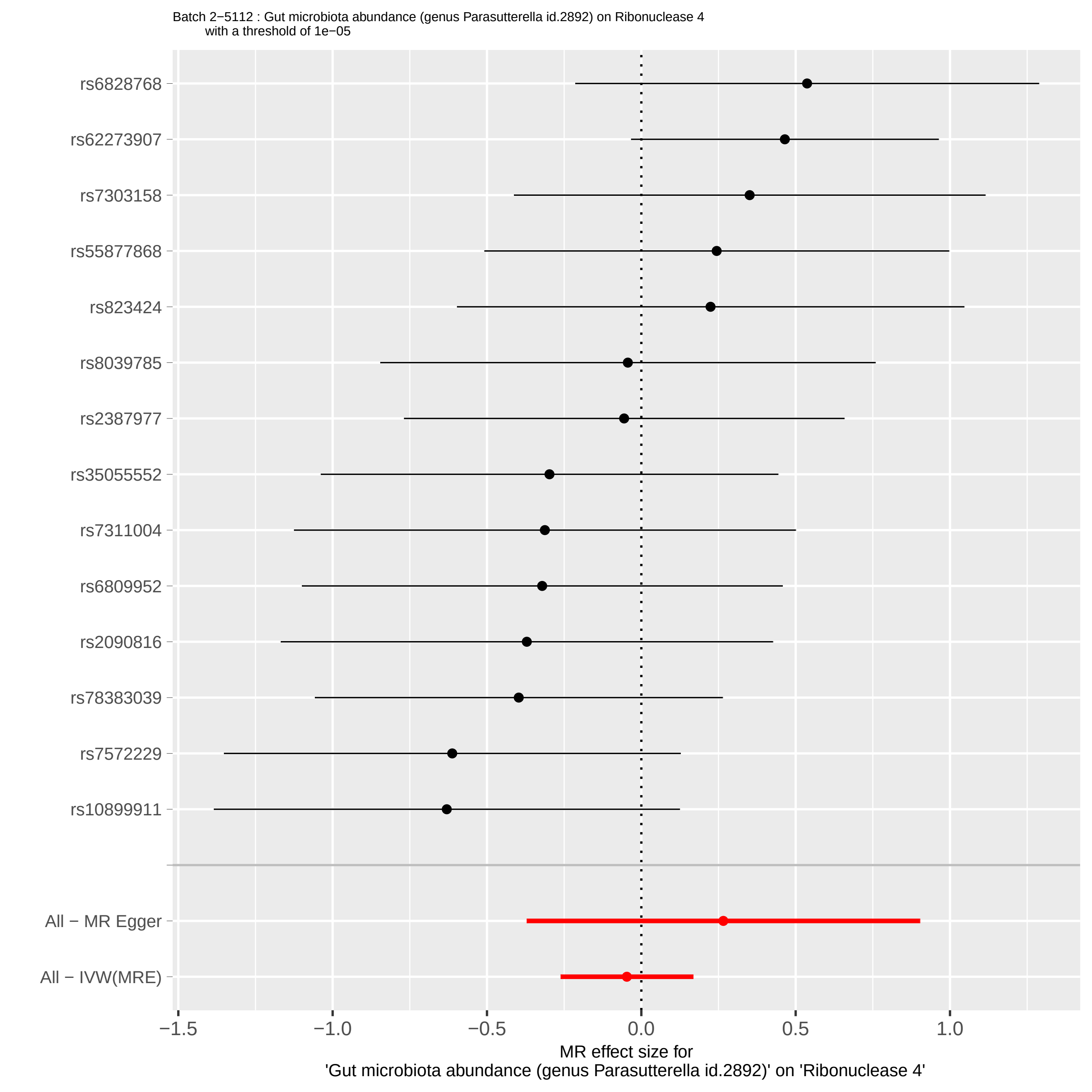

Batch 2−5112 : Gut microbiota abundance (genus Parasutterella id.2892) on Ribonuclease 4
with a threshold of 1e−05
rs6828768
rs62273907
rs7303158
rs55877868
rs823424
rs8039785
rs2387977
rs35055552
rs7311004
rs6809952
rs2090816
rs78383039
rs7572229
rs10899911
All − MR Egger
All − IVW(MRE)
−1.5
−1.0
−0.5
0.0
0.5
1.0
MR effect size for
'Gut microbiota abundance (genus Parasutterella id.2892)' on 'Ribonuclease 4'

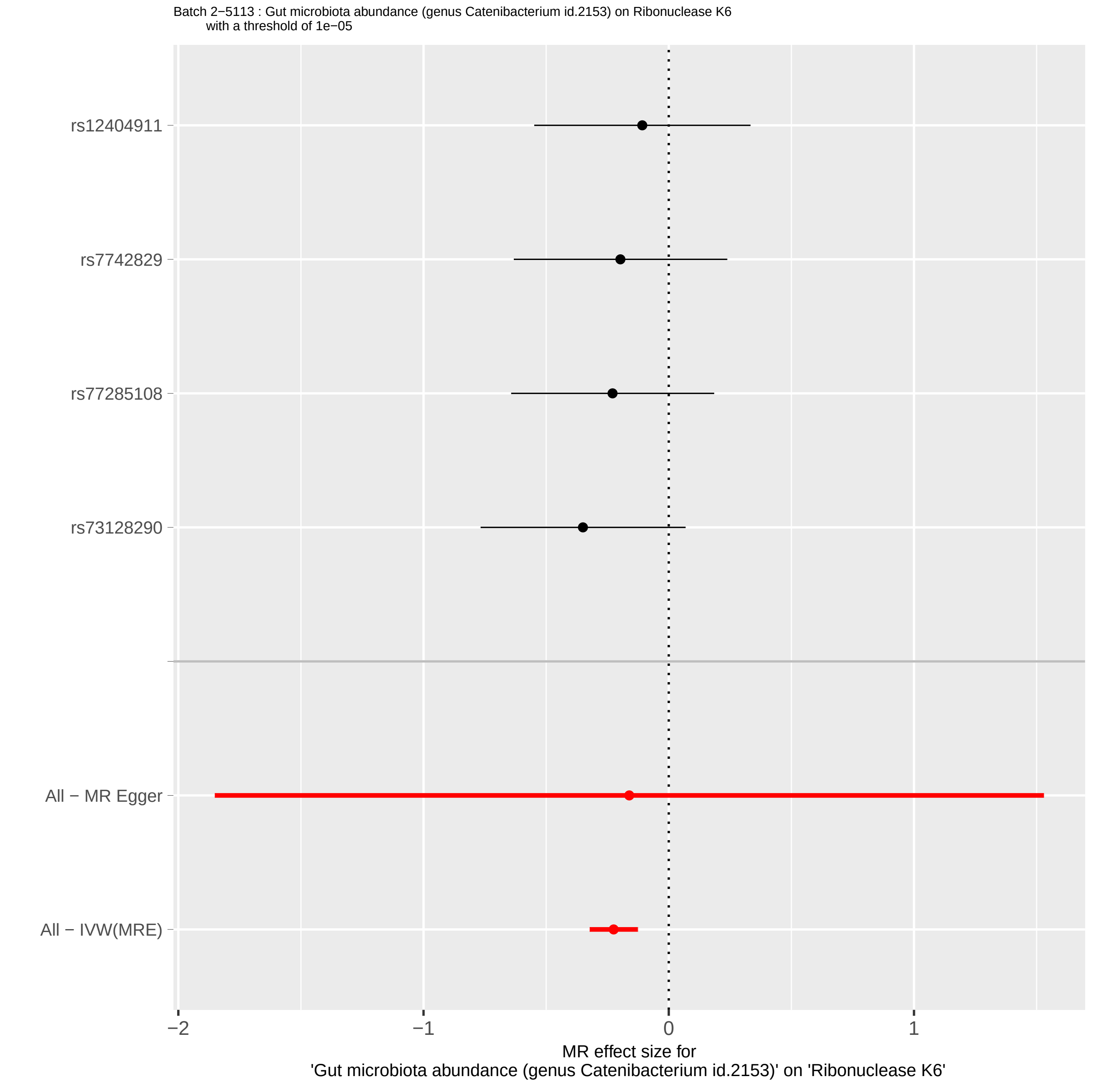
Batch 2−5113 : Gut microbiota abundance (genus Catenibacterium id.2153) on Ribonuclease K6
with a threshold of 1e−05
rs12404911
rs7742829
rs77285108
rs73128290
All − MR Egger
All − IVW(MRE)
−2
−1
0
1
MR effect size for
'Gut microbiota abundance (genus Catenibacterium id.2153)' on 'Ribonuclease K6'

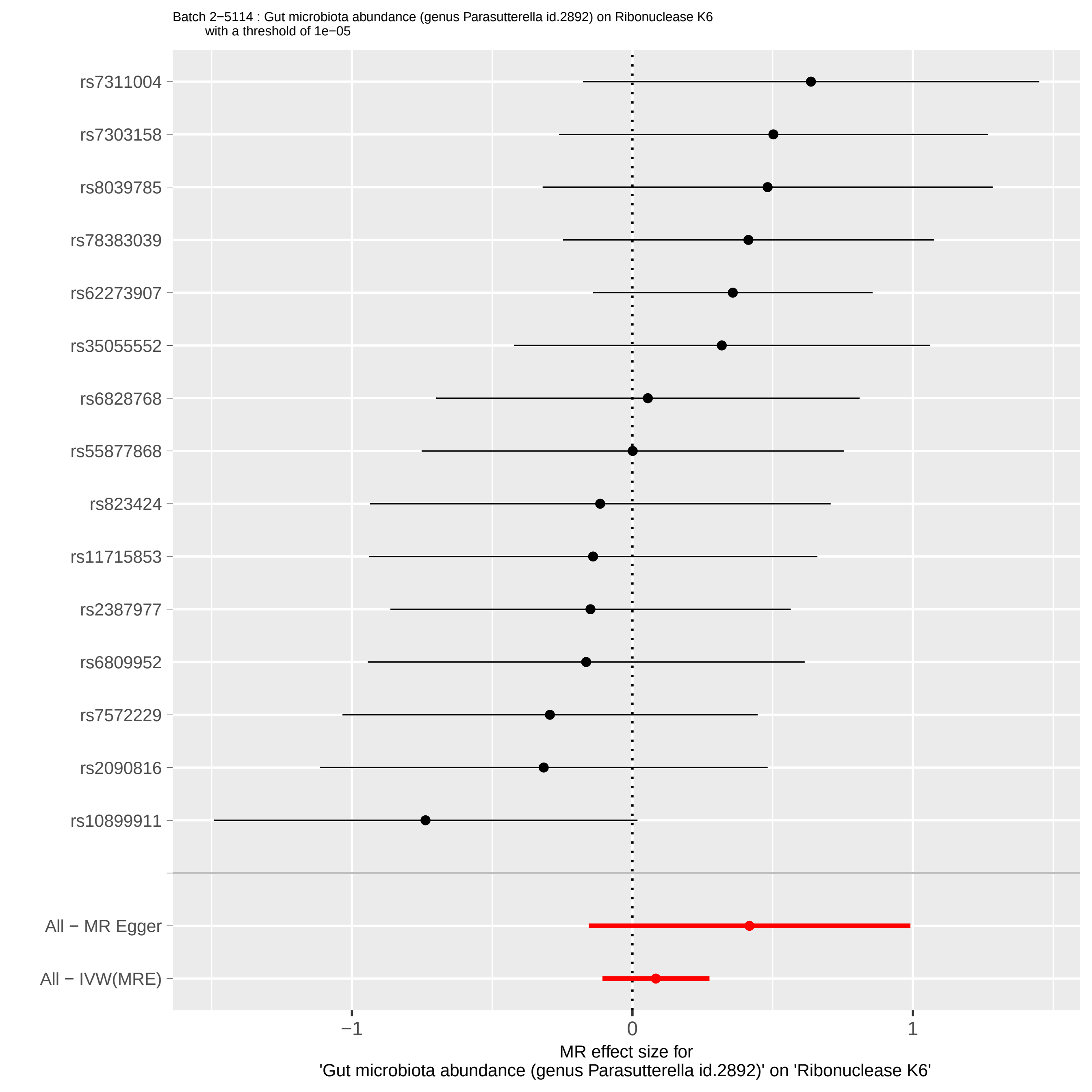

Batch 2−5114 : Gut microbiota abundance (genus Parasutterella id.2892) on Ribonuclease K6
with a threshold of 1e−05
rs7311004
rs7303158
rs8039785
rs78383039
rs62273907
rs35055552
rs6828768
rs55877868
rs823424
rs11715853
rs2387977
rs6809952
rs7572229
rs2090816
rs10899911
All − MR Egger
All − IVW(MRE)
−1
0
1
MR effect size for
'Gut microbiota abundance (genus Parasutterella id.2892)' on 'Ribonuclease K6'

Batch 2−5115 : Gut microbiota abundance (genus Catenibacterium id.2153) on Ribonuclease H1
with a threshold of 1e−05

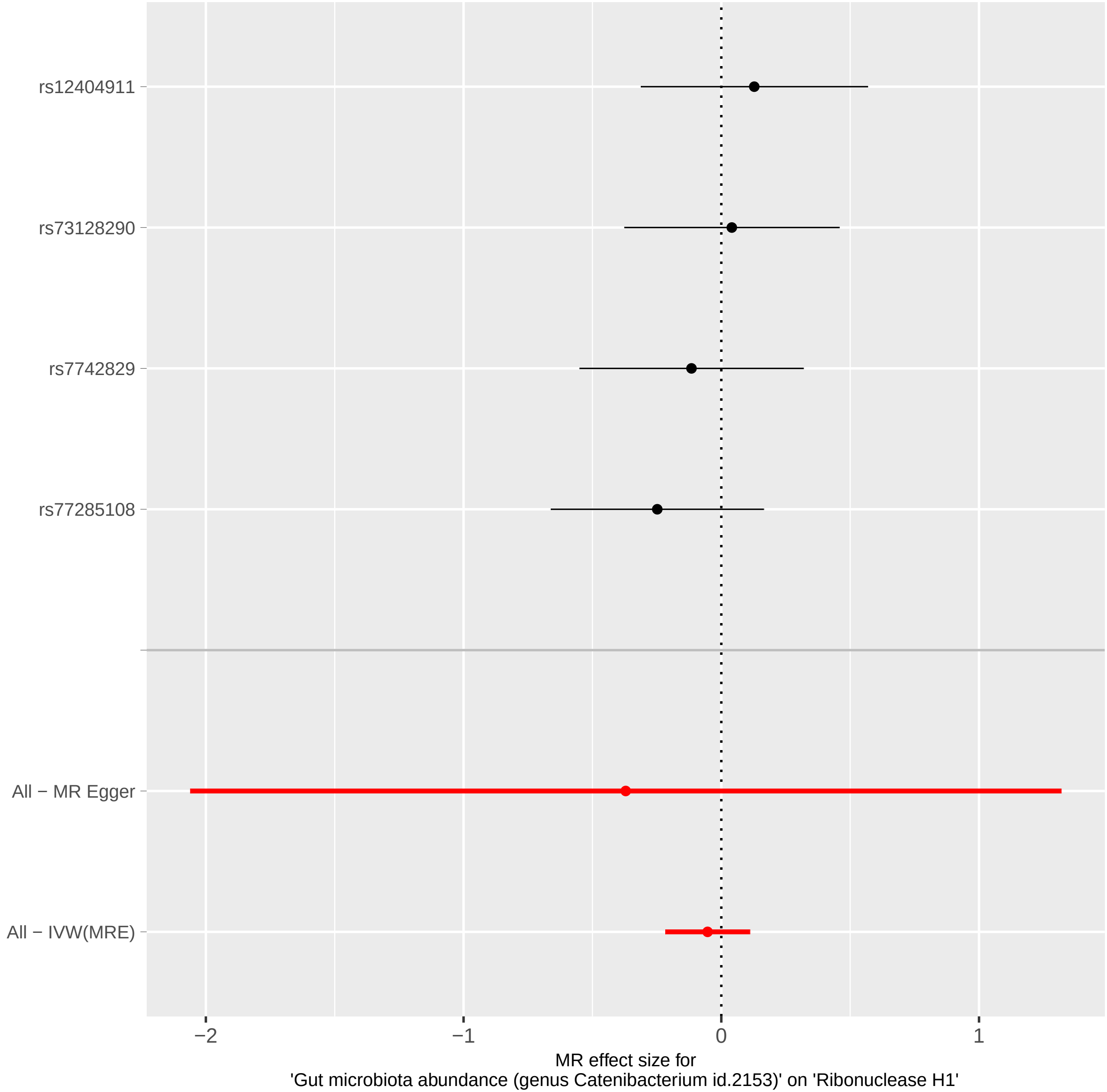

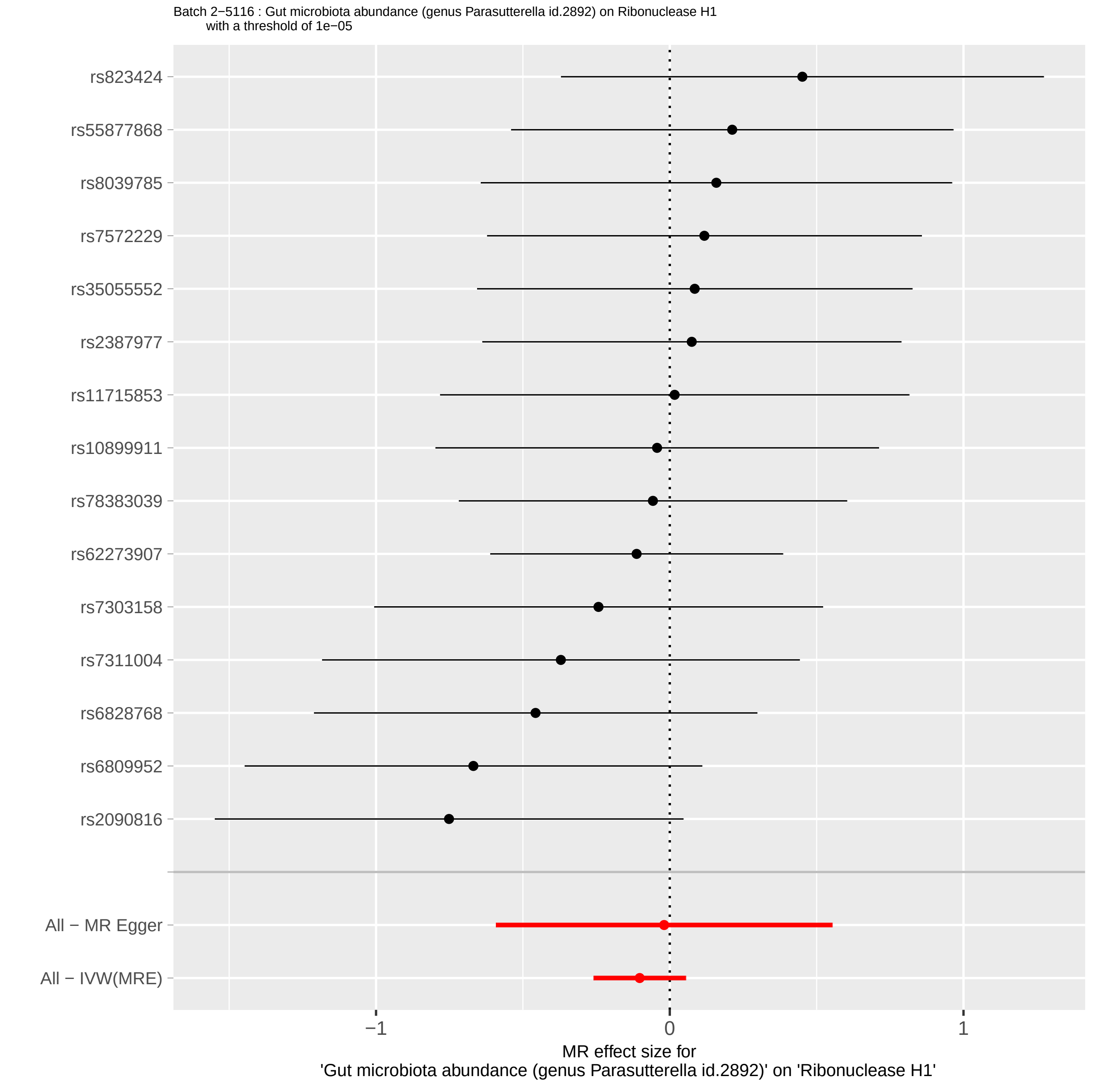

Batch 2−5116 : Gut microbiota abundance (genus Parasutterella id.2892) on Ribonuclease H1
with a threshold of 1e−05
rs823424
rs55877868
rs8039785
rs7572229
rs35055552
rs2387977
rs11715853
rs10899911
rs78383039
rs62273907
rs7303158
rs7311004
rs6828768
rs6809952
rs2090816
All − MR Egger
All − IVW(MRE)
−1
0
1
MR effect size for
'Gut microbiota abundance (genus Parasutterella id.2892)' on 'Ribonuclease H1'

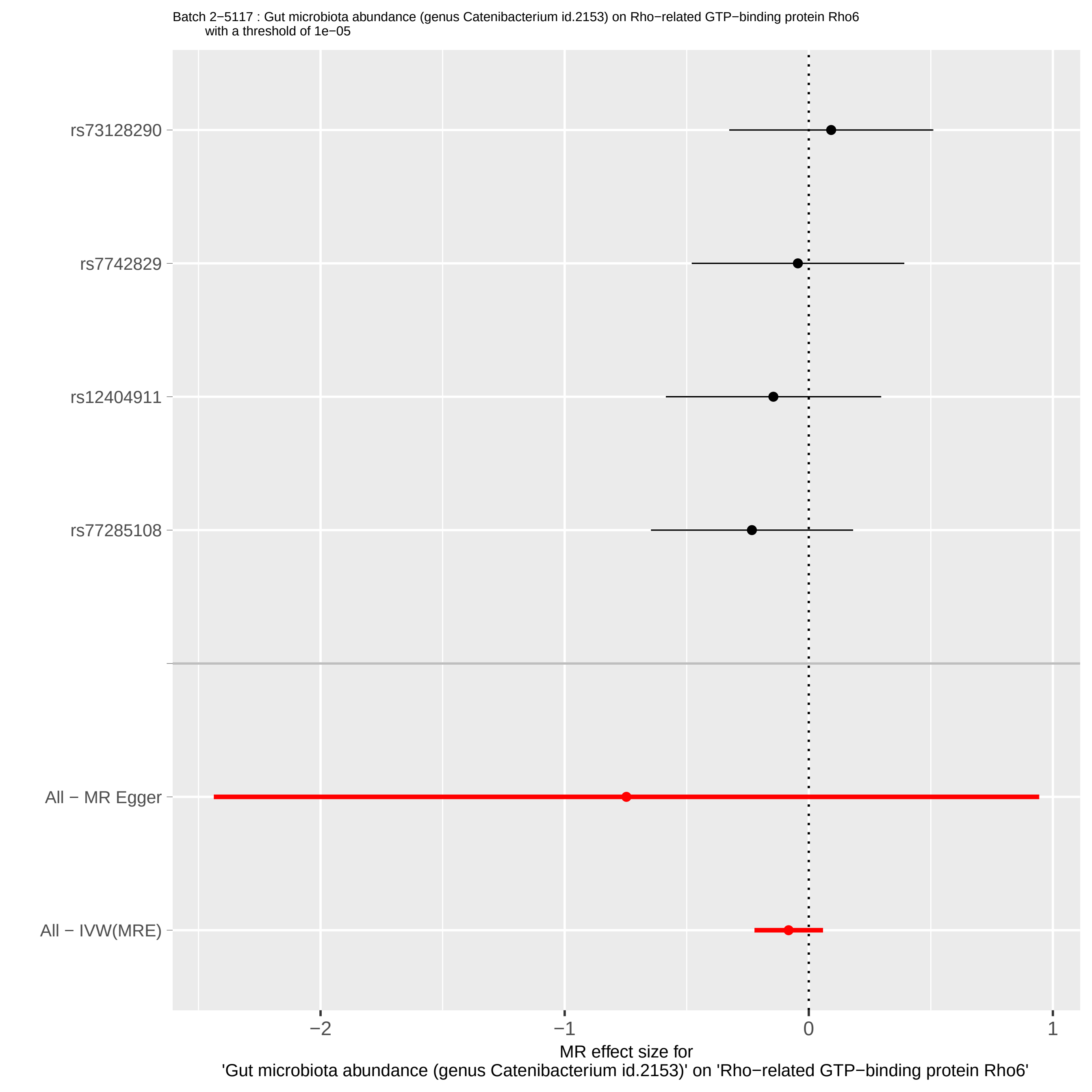

Batch 2−5117 : Gut microbiota abundance (genus Catenibacterium id.2153) on Rho−related GTP−binding protein Rho6
with a threshold of 1e−05
rs73128290
rs7742829
rs12404911
rs77285108
All − MR Egger
All − IVW(MRE)
−2
−1
0
1
MR effect size for
'Gut microbiota abundance (genus Catenibacterium id.2153)' on 'Rho−related GTP−binding protein Rho6'

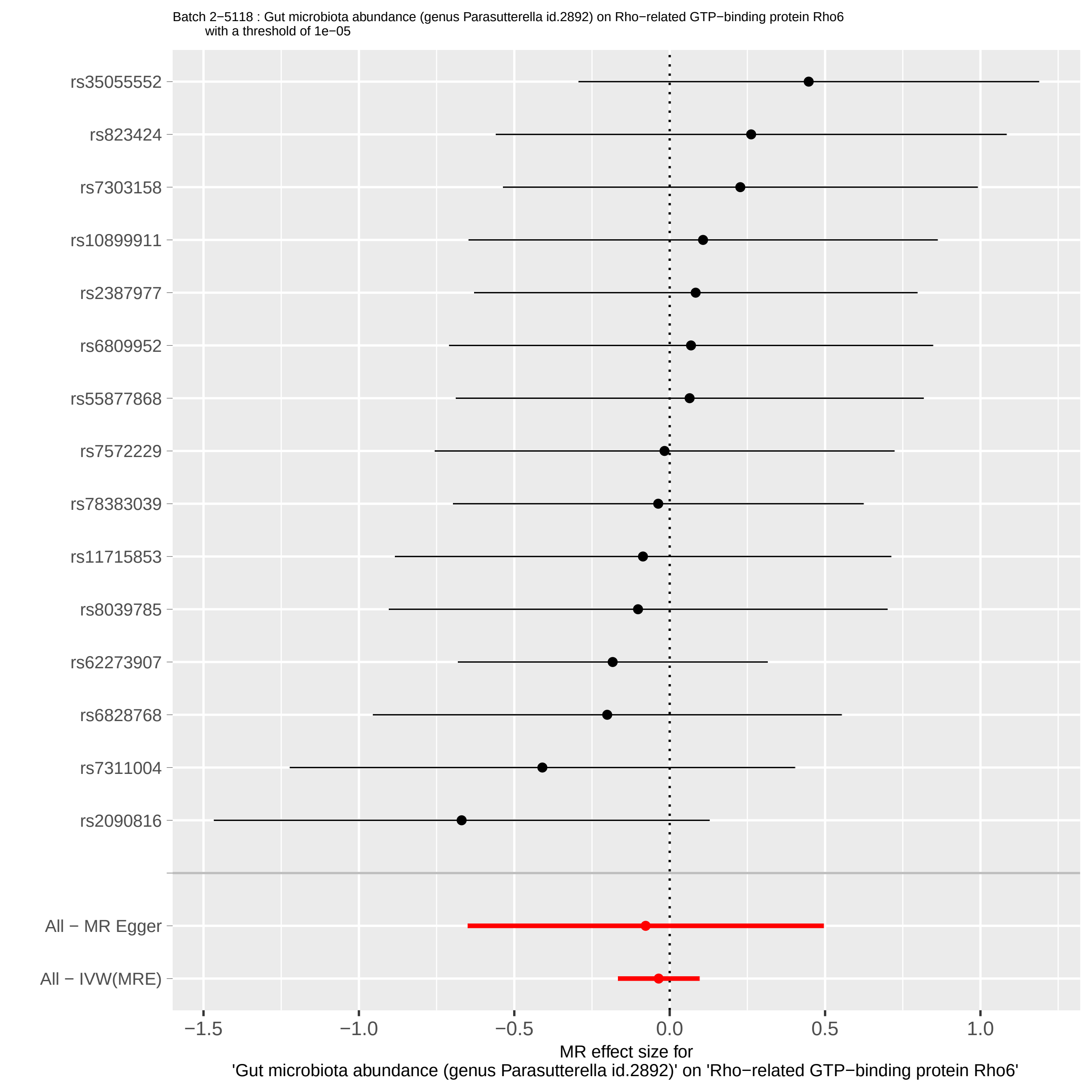
Batch 2−5118 : Gut microbiota abundance (genus Parasutterella id.2892) on Rho−related GTP−binding protein Rho6
with a threshold of 1e−05
rs35055552
rs823424
rs7303158
rs10899911
rs2387977
rs6809952
rs55877868
rs7572229
rs78383039
rs11715853
rs8039785
rs62273907
rs6828768
rs7311004
rs2090816
All − MR Egger
All − IVW(MRE)
−1.5
−1.0
−0.5
0.0
0.5
1.0
MR effect size for
'Gut microbiota abundance (genus Parasutterella id.2892)' on 'Rho−related GTP−binding protein Rho6'

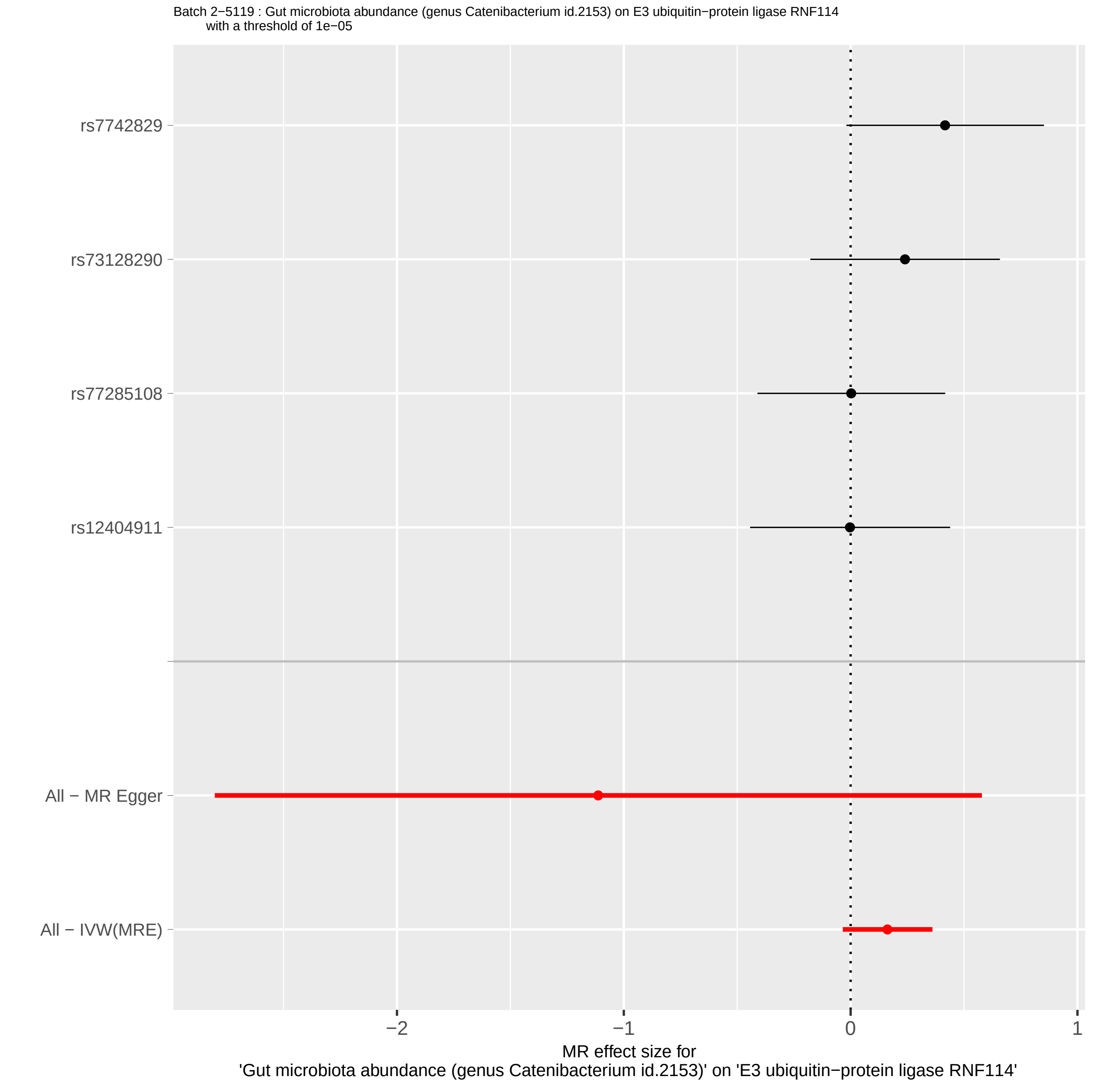

Batch 2−5119 : Gut microbiota abundance (genus Catenibacterium id.2153) on E3 ubiquitin−protein ligase RNF114
with a threshold of 1e−05
rs7742829
rs73128290
rs77285108
rs12404911
All − MR Egger
All − IVW(MRE)
−2
−1
0
1
MR effect size for
'Gut microbiota abundance (genus Catenibacterium id.2153)' on 'E3 ubiquitin−protein ligase RNF114'

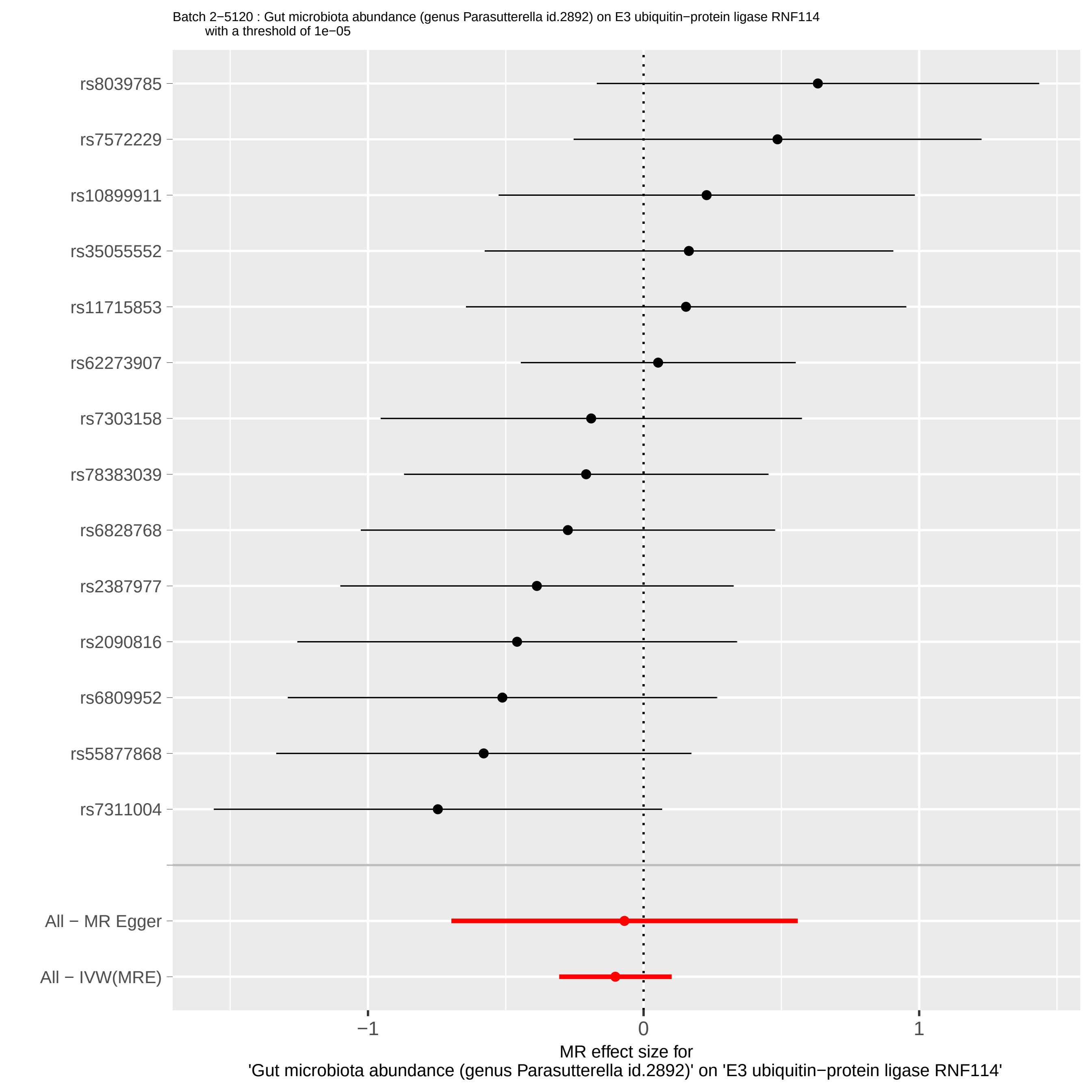
Batch 2−5120 : Gut microbiota abundance (genus Parasutterella id.2892) on E3 ubiquitin−protein ligase RNF114
with a threshold of 1e−05
rs8039785
rs7572229
rs10899911
rs35055552
rs11715853
rs62273907
rs7303158
rs78383039
rs6828768
rs2387977
rs2090816
rs6809952
rs55877868
rs7311004
All − MR Egger
All − IVW(MRE)
−1
0
1
MR effect size for
'Gut microbiota abundance (genus Parasutterella id.2892)' on 'E3 ubiquitin−protein ligase RNF114'

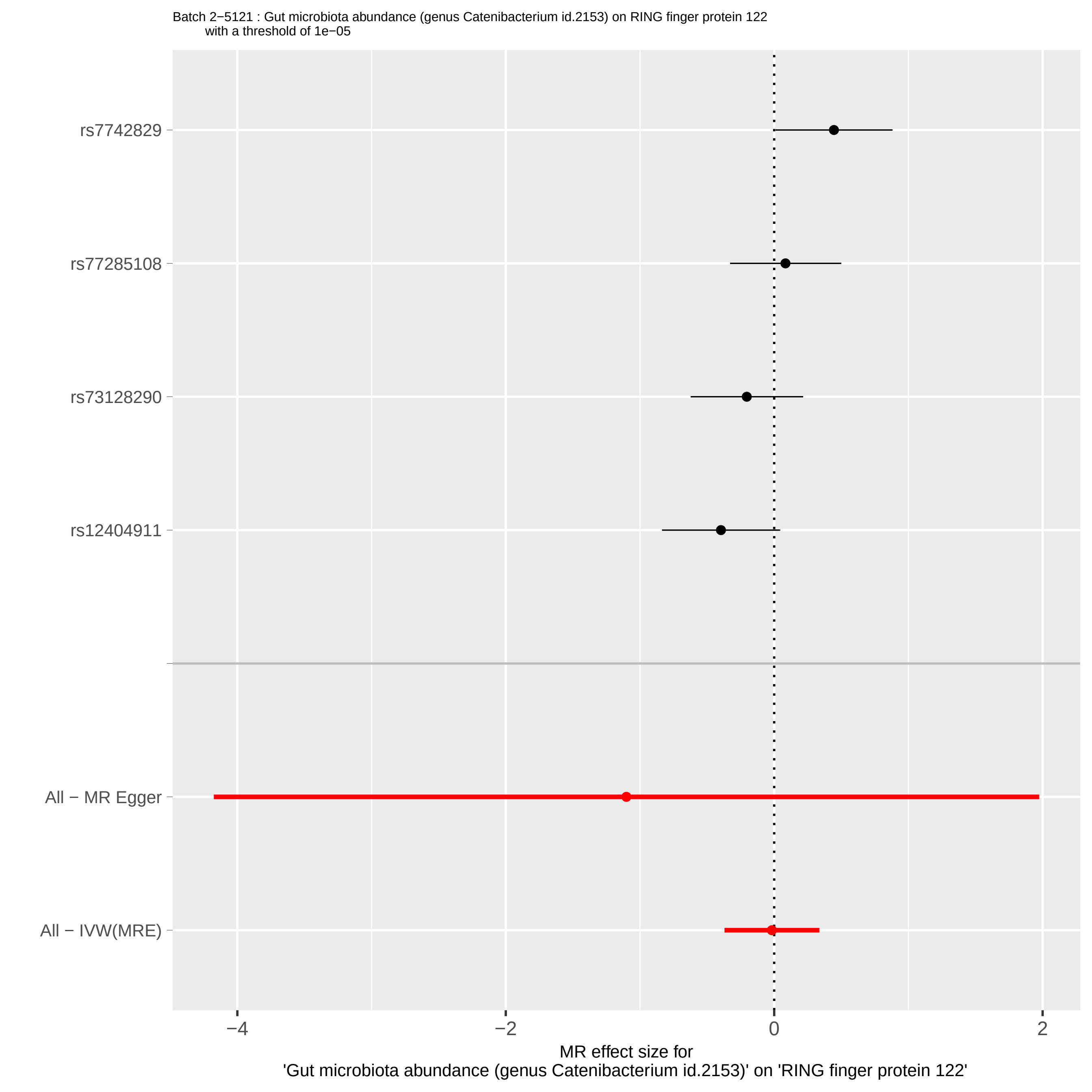
Batch 2−5121 : Gut microbiota abundance (genus Catenibacterium id.2153) on RING finger protein 122
with a threshold of 1e−05
rs7742829
rs77285108
rs73128290
rs12404911
All − MR Egger
All − IVW(MRE)
−4
−2
0
2
MR effect size for
'Gut microbiota abundance (genus Catenibacterium id.2153)' on 'RING finger protein 122'

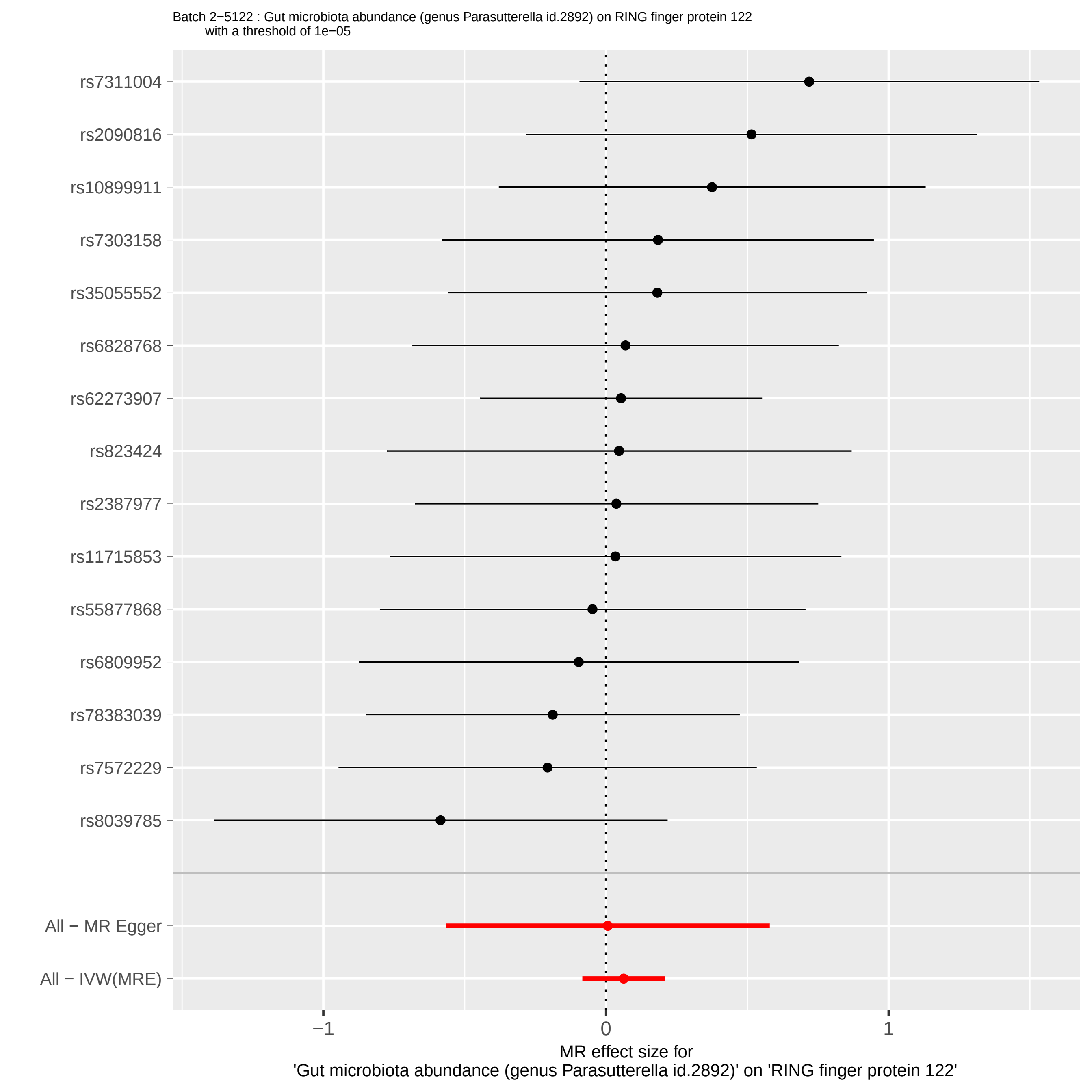
Batch 2−5122 : Gut microbiota abundance (genus Parasutterella id.2892) on RING finger protein 122
with a threshold of 1e−05
rs7311004
rs2090816
rs10899911
rs7303158
rs35055552
rs6828768
rs62273907
rs823424
rs2387977
rs11715853
rs55877868
rs6809952
rs78383039
rs7572229
rs8039785
All − MR Egger
All − IVW(MRE)
−1
0
1
MR effect size for
'Gut microbiota abundance (genus Parasutterella id.2892)' on 'RING finger protein 122'

Batch 2−5123 : Gut microbiota abundance (genus Catenibacterium id.2153) on E3 ubiquitin−protein ligase RNF128
with a threshold of 1e−05

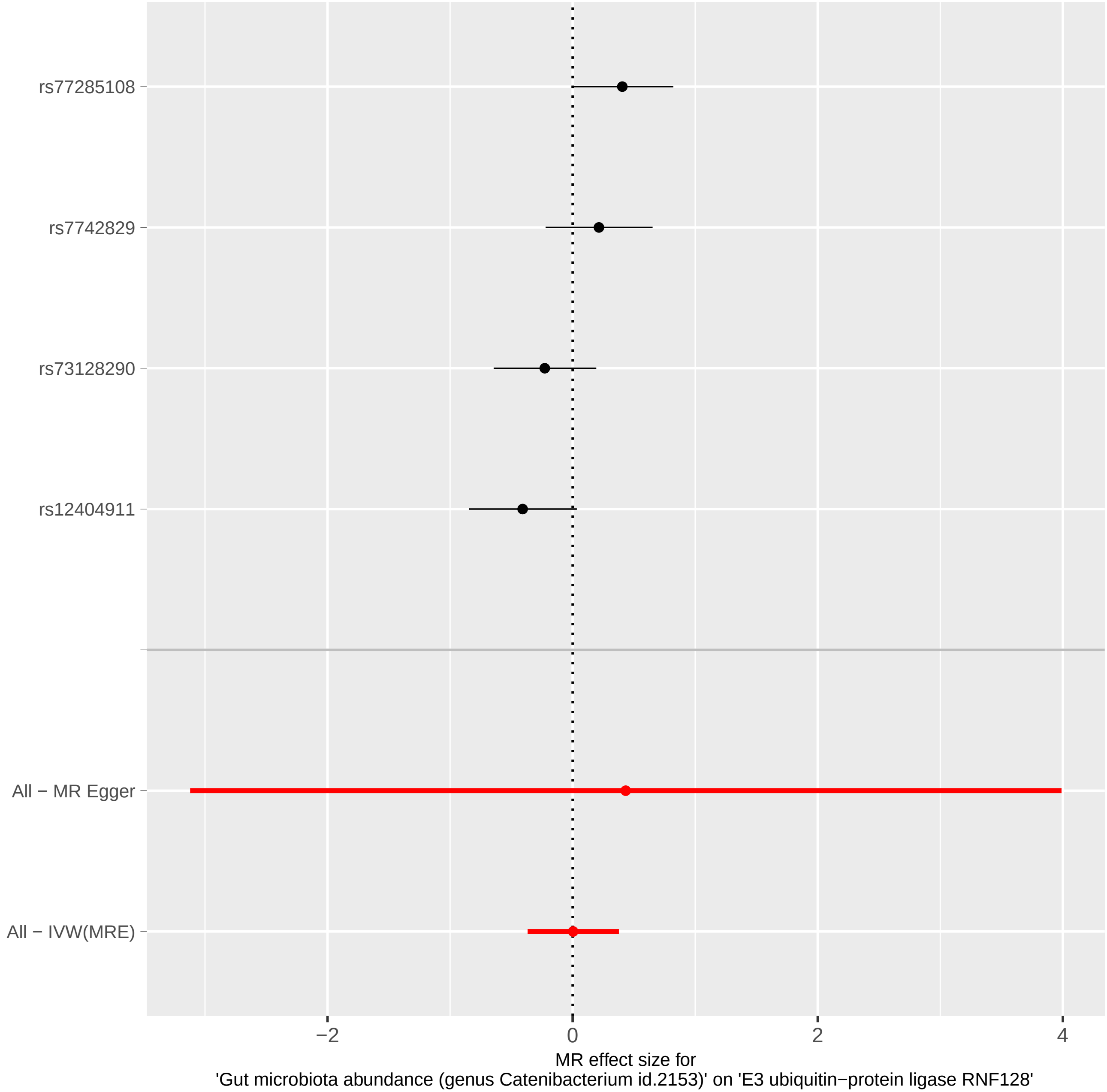

Batch 2−5124 : Gut microbiota abundance (genus Parasutterella id.2892) on E3 ubiquitin−protein ligase RNF128
with a threshold of 1e−05

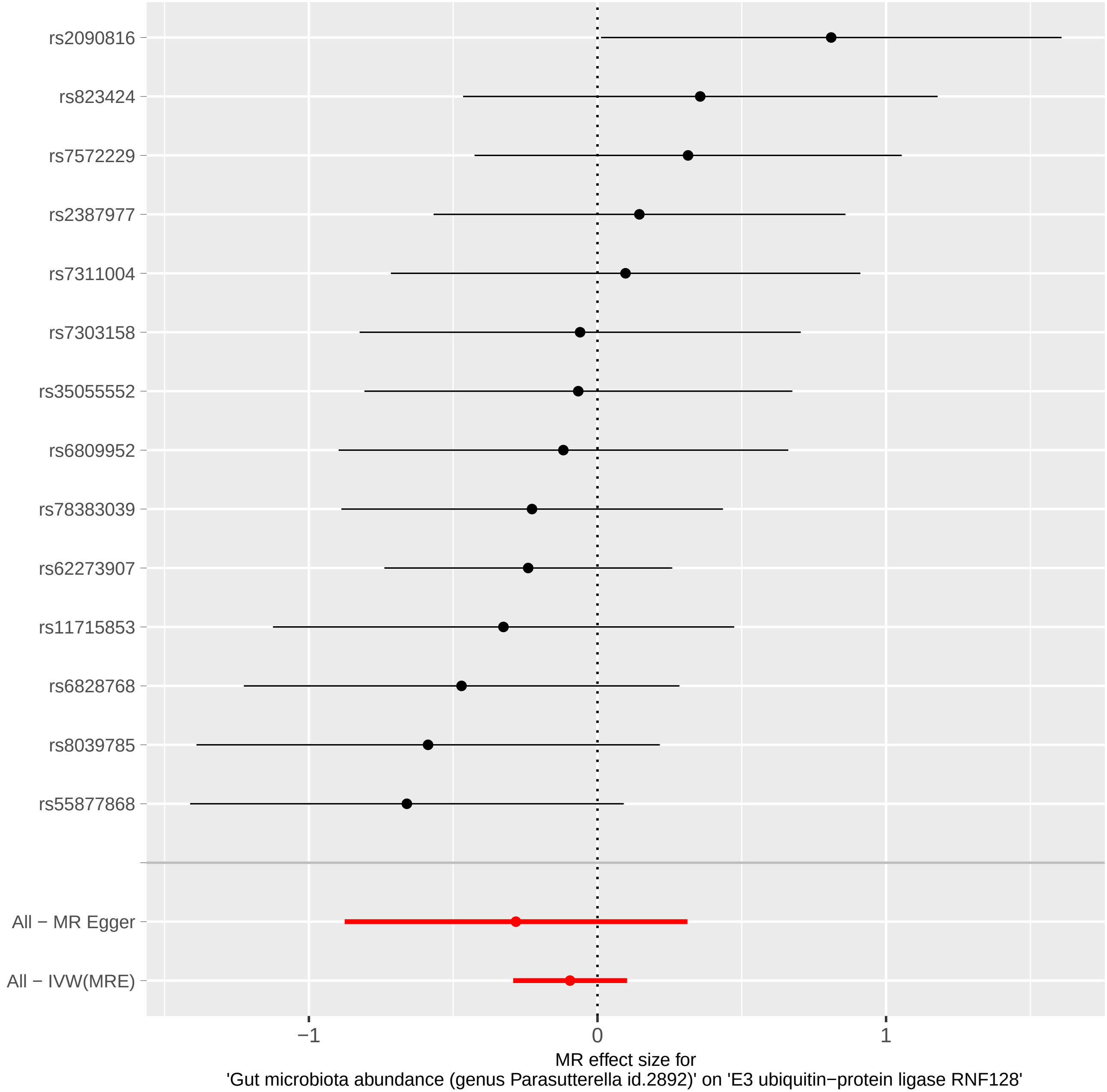

Batch 2−5125 : Gut microbiota abundance (genus Catenibacterium id.2153) on E3 ubiquitin−protein ligase RNF13
with a threshold of 1e−05

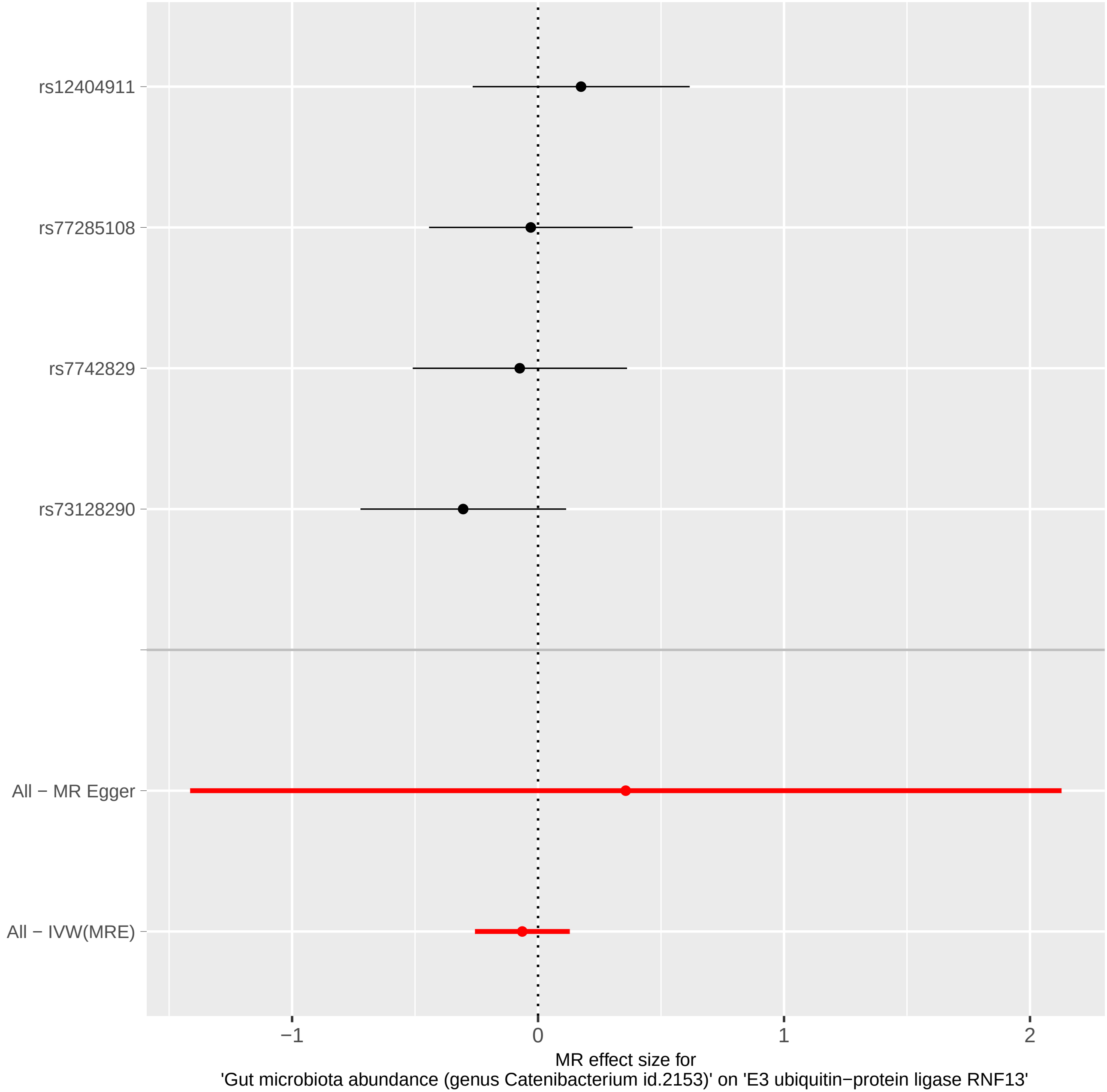

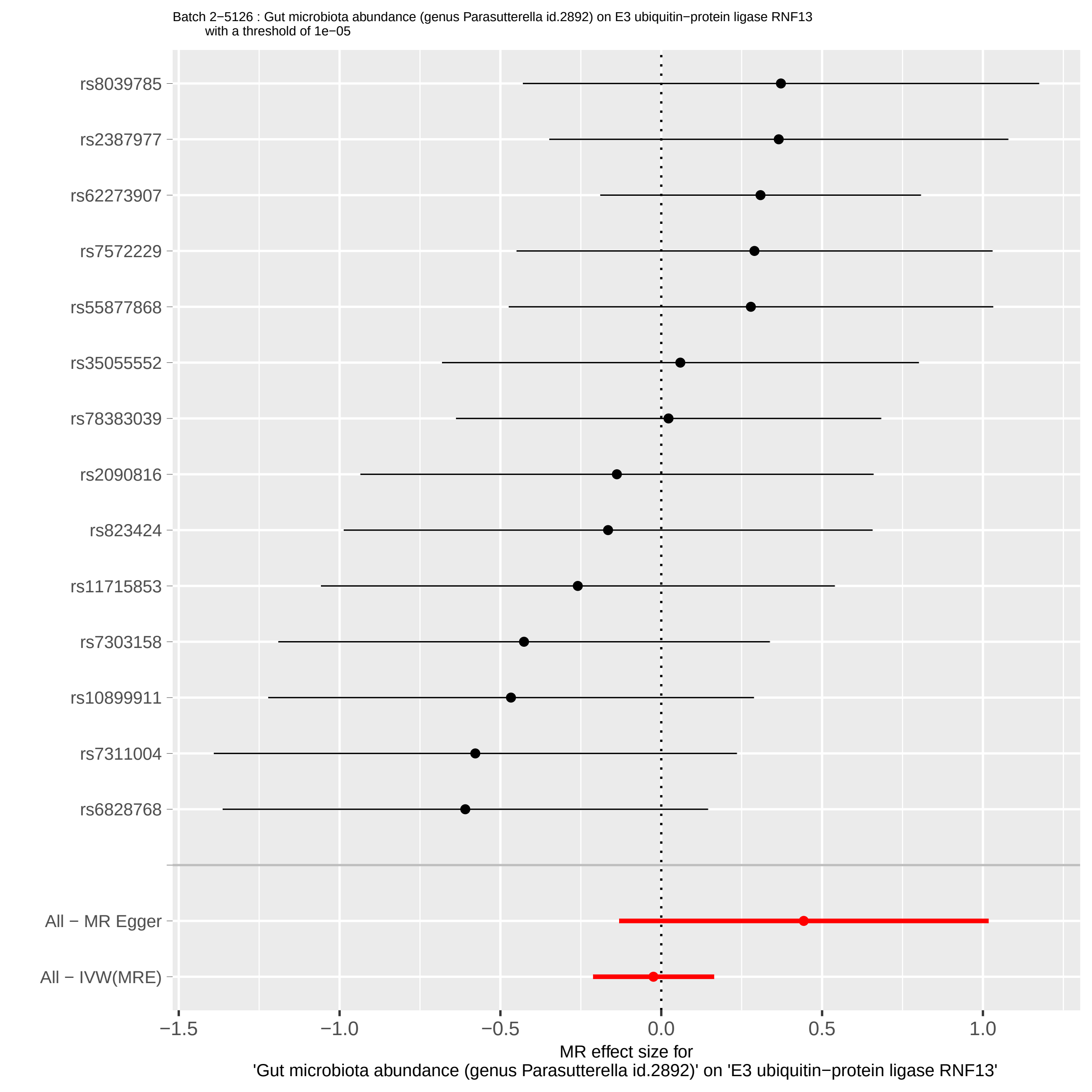

Batch 2−5126 : Gut microbiota abundance (genus Parasutterella id.2892) on E3 ubiquitin−protein ligase RNF13
with a threshold of 1e−05
rs8039785
rs2387977
rs62273907
rs7572229
rs55877868
rs35055552
rs78383039
rs2090816
rs823424
rs11715853
rs7303158
rs10899911
rs7311004
rs6828768
All − MR Egger
All − IVW(MRE)
−1.5
−1.0
−0.5
0.0
0.5
1.0
MR effect size for
'Gut microbiota abundance (genus Parasutterella id.2892)' on 'E3 ubiquitin−protein ligase RNF13'

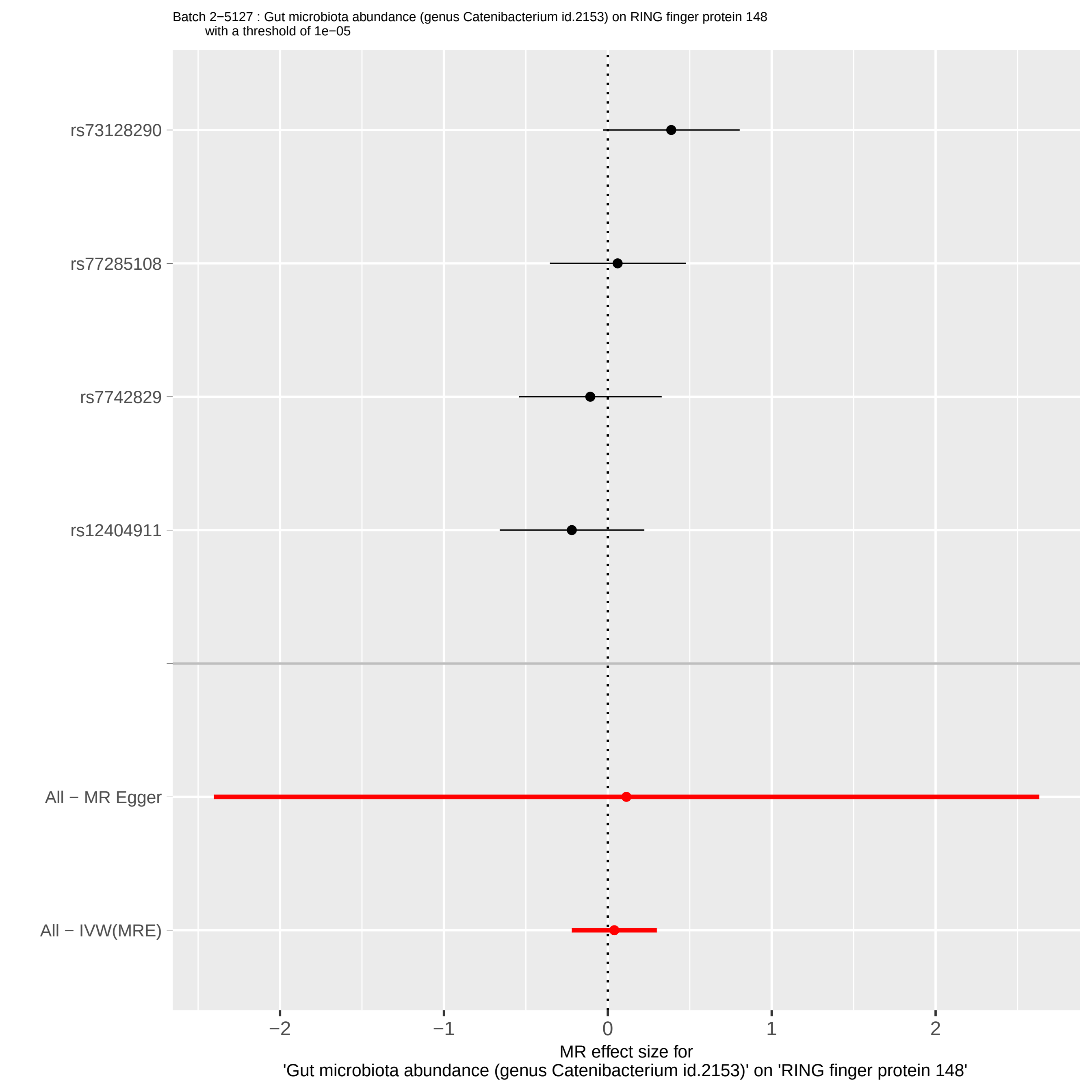

Batch 2−5127 : Gut microbiota abundance (genus Catenibacterium id.2153) on RING finger protein 148
with a threshold of 1e−05
rs73128290
rs77285108
rs7742829
rs12404911
All − MR Egger
All − IVW(MRE)
−2
−1
0
1
2
MR effect size for
'Gut microbiota abundance (genus Catenibacterium id.2153)' on 'RING finger protein 148'

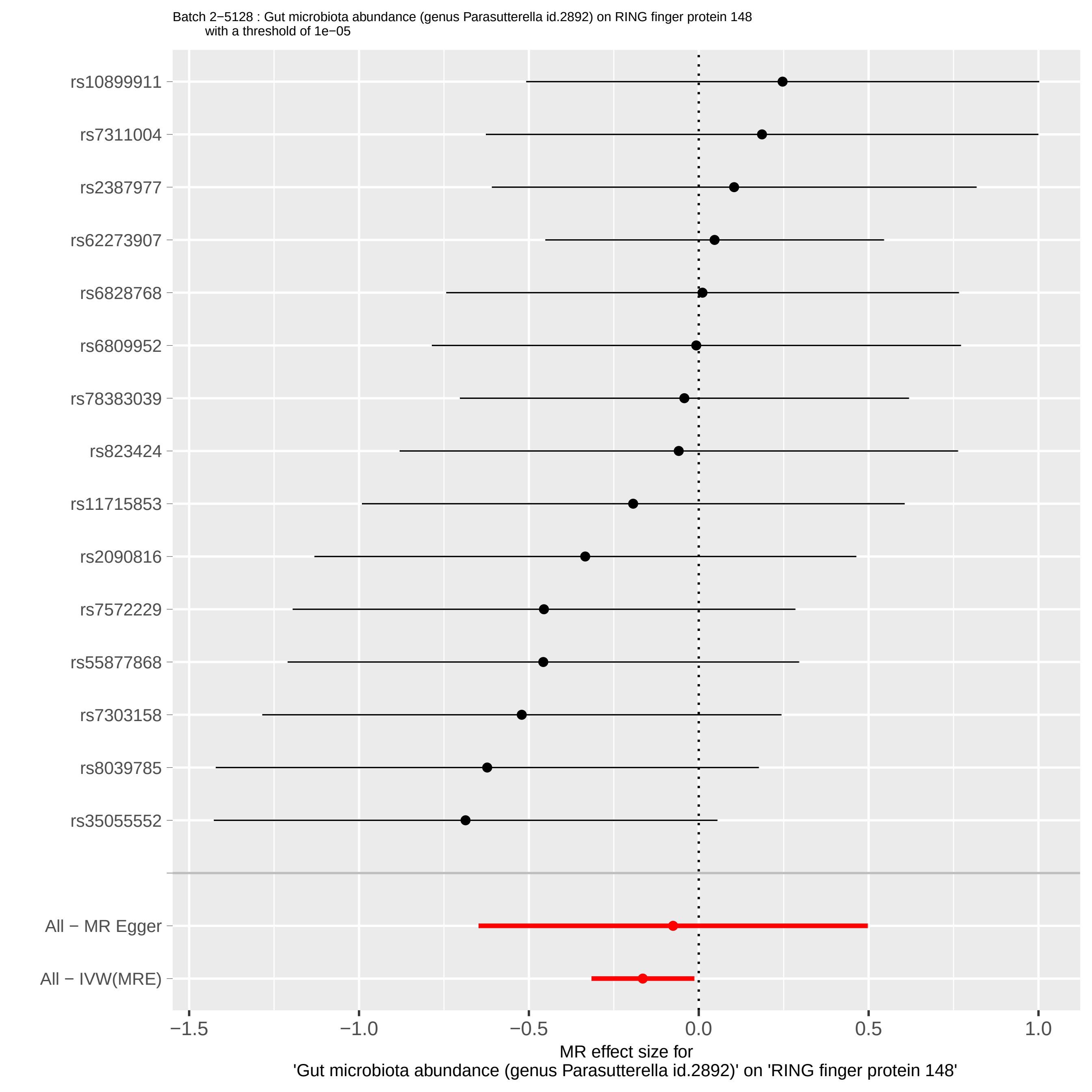

Batch 2−5128 : Gut microbiota abundance (genus Parasutterella id.2892) on RING finger protein 148
with a threshold of 1e−05
rs10899911
rs7311004
rs2387977
rs62273907
rs6828768
rs6809952
rs78383039
rs823424
rs11715853
rs2090816
rs7572229
rs55877868
rs7303158
rs8039785
rs35055552
All − MR Egger
All − IVW(MRE)
−1.5
−1.0
−0.5
0.0
0.5
1.0
MR effect size for
'Gut microbiota abundance (genus Parasutterella id.2892)' on 'RING finger protein 148'

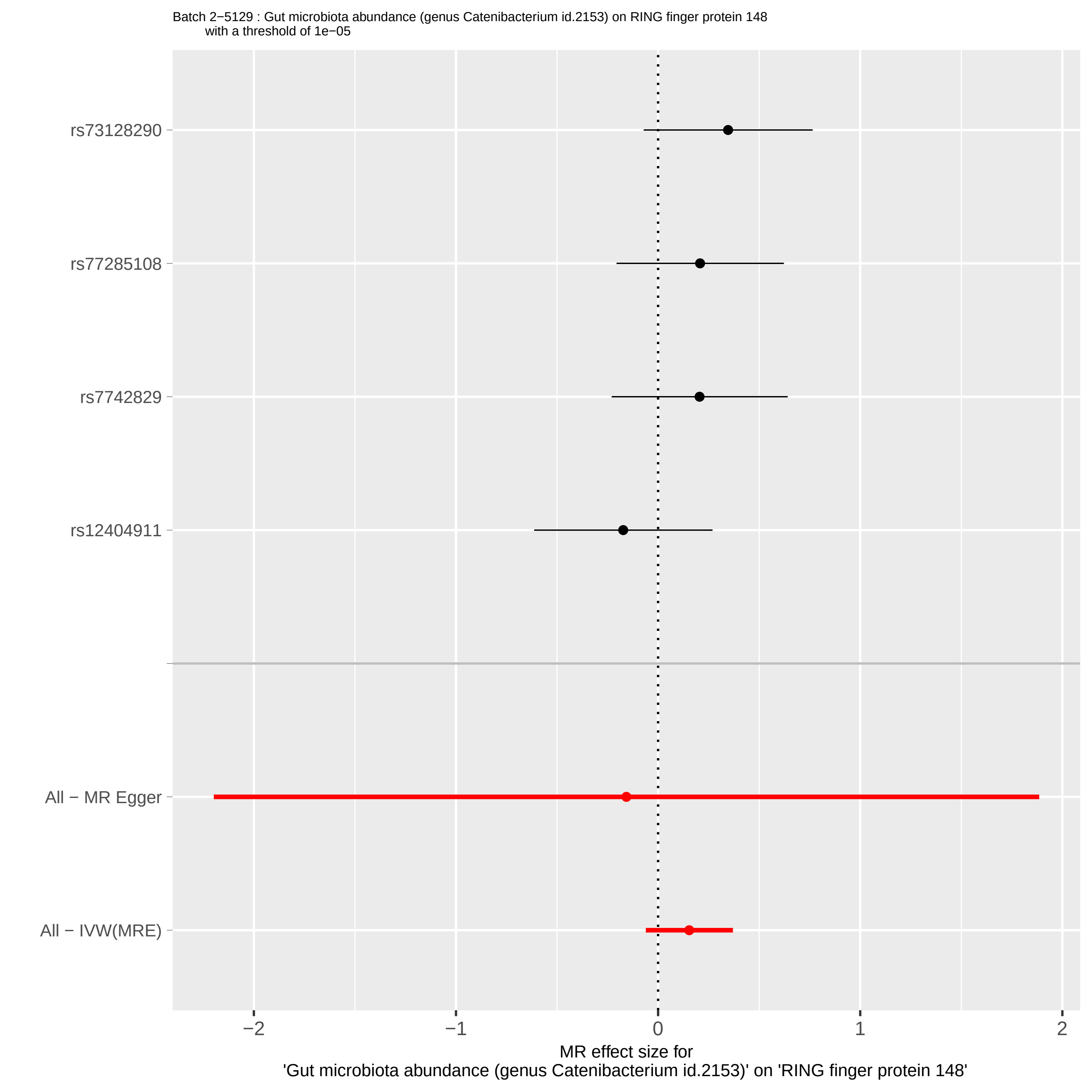
Batch 2−5129 : Gut microbiota abundance (genus Catenibacterium id.2153) on RING finger protein 148
with a threshold of 1e−05
rs73128290
rs77285108
rs7742829
rs12404911
All − MR Egger
All − IVW(MRE)
−2
−1
0
1
2
MR effect size for
'Gut microbiota abundance (genus Catenibacterium id.2153)' on 'RING finger protein 148'

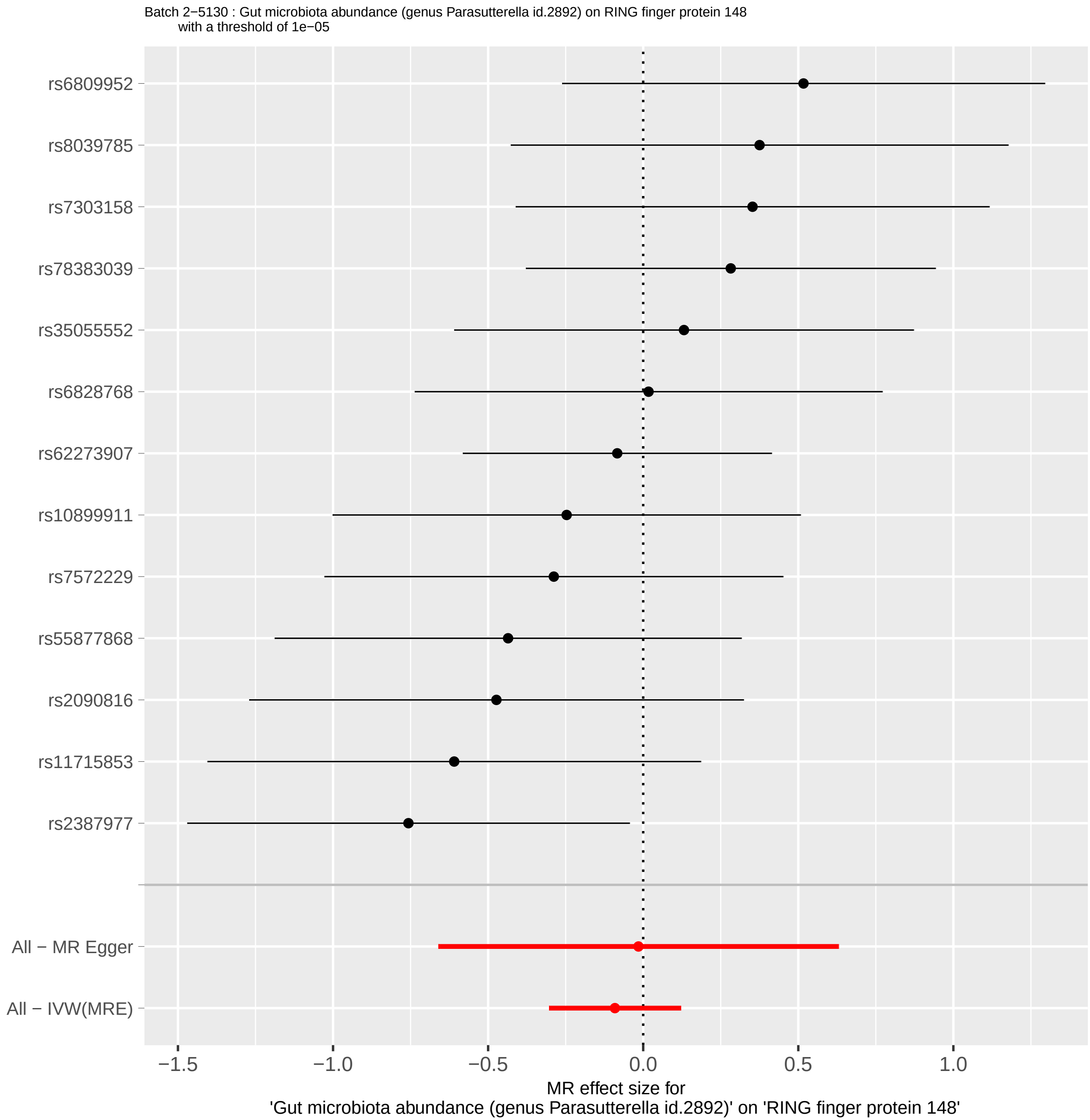
Batch 2−5130 : Gut microbiota abundance (genus Parasutterella id.2892) on RING finger protein 148
with a threshold of 1e−05
rs6809952
rs8039785
rs7303158
rs78383039
rs35055552
rs6828768
rs62273907
rs10899911
rs7572229
rs55877868
rs2090816
rs11715853
rs2387977
All − MR Egger
All − IVW(MRE)
−1.5
−1.0
−0.5
0.0
0.5
1.0
MR effect size for
'Gut microbiota abundance (genus Parasutterella id.2892)' on 'RING finger protein 148'

Batch 2−5131 : Gut microbiota abundance (genus Catenibacterium id.2153) on E3 ubiquitin−protein ligase RNF149
with a threshold of 1e−05

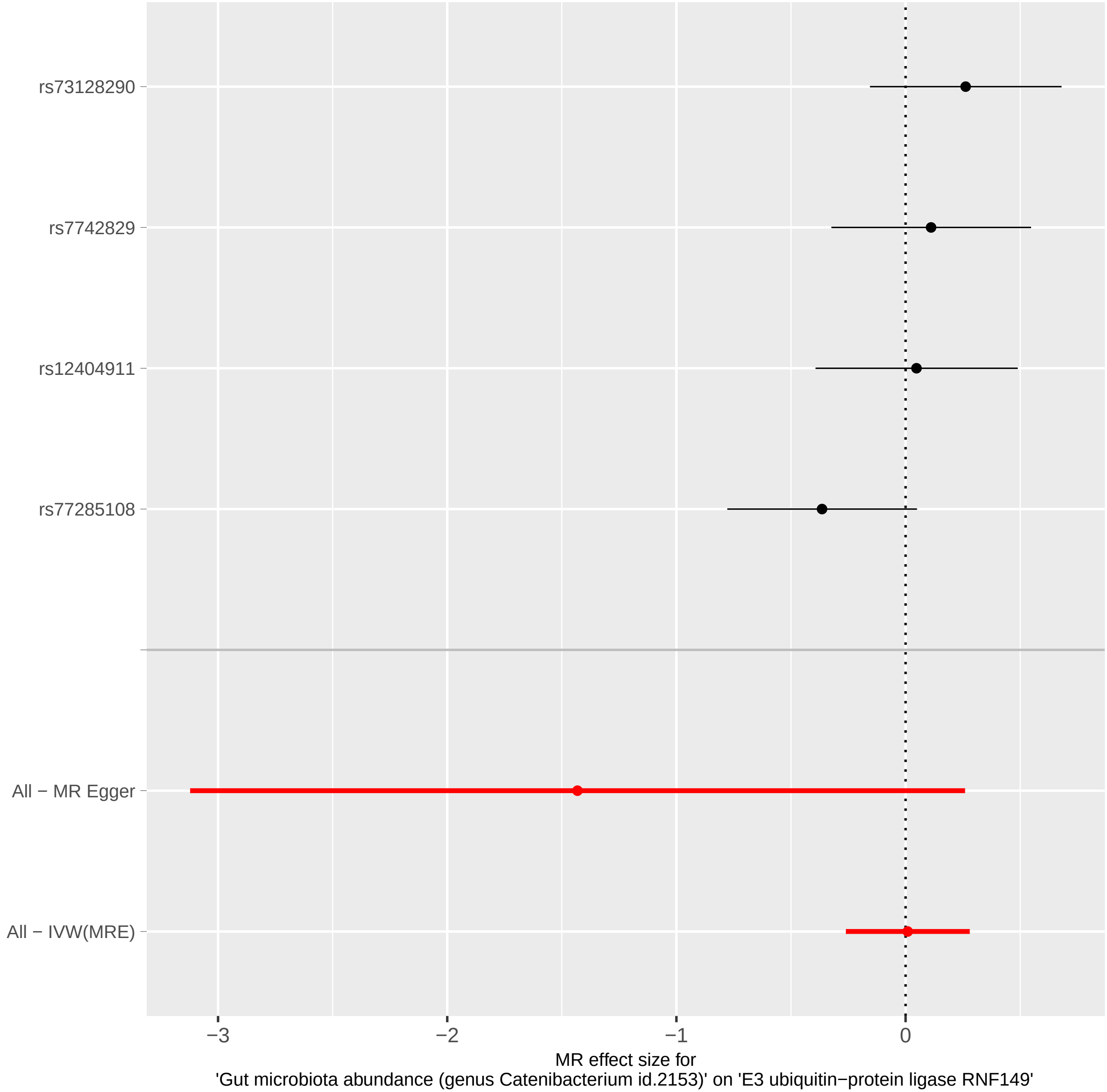

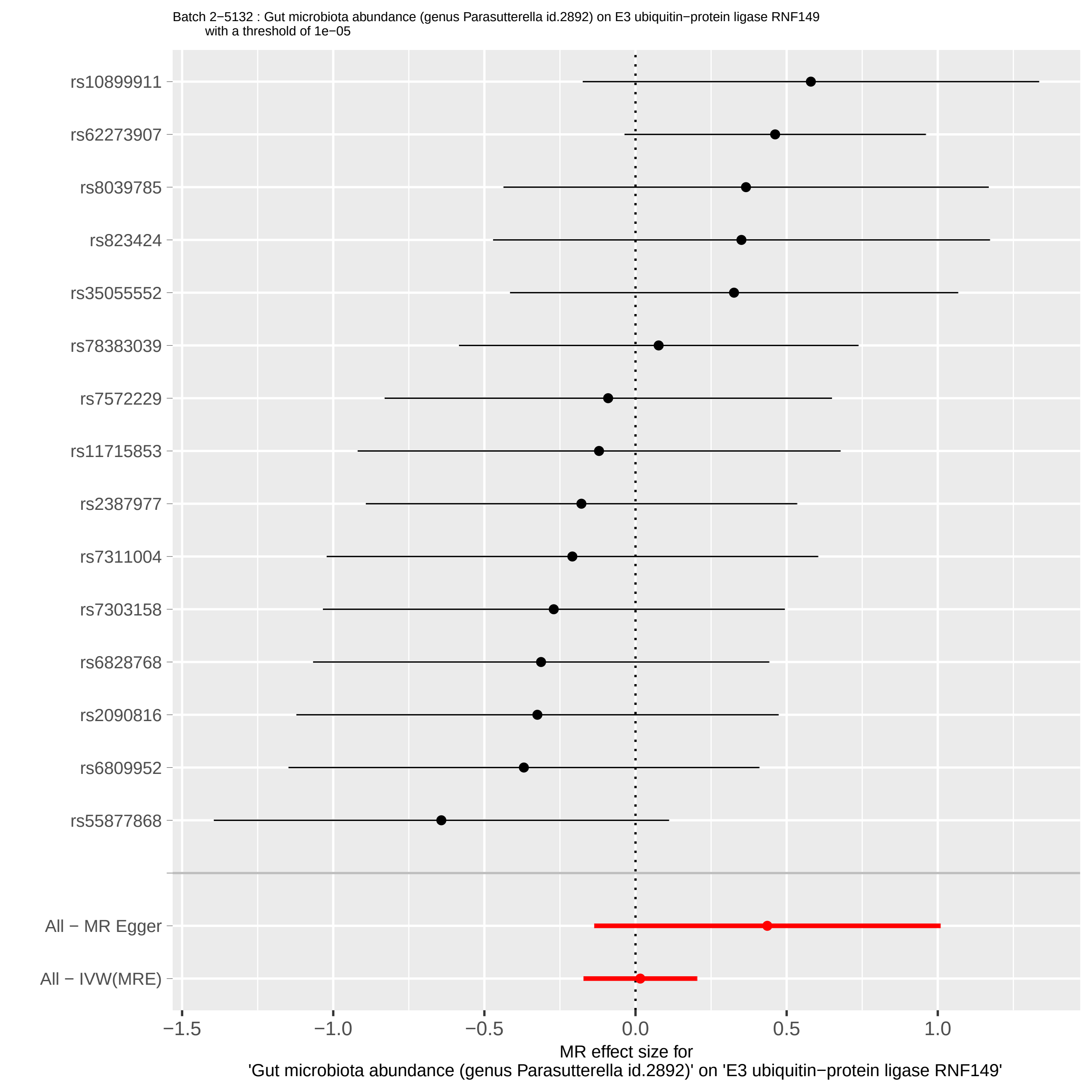

Batch 2−5132 : Gut microbiota abundance (genus Parasutterella id.2892) on E3 ubiquitin−protein ligase RNF149
with a threshold of 1e−05
rs10899911
rs62273907
rs8039785
rs823424
rs35055552
rs78383039
rs7572229
rs11715853
rs2387977
rs7311004
rs7303158
rs6828768
rs2090816
rs6809952
rs55877868
All − MR Egger
All − IVW(MRE)
−1.5
−1.0
−0.5
0.0
0.5
1.0
MR effect size for
'Gut microbiota abundance (genus Parasutterella id.2892)' on 'E3 ubiquitin−protein ligase RNF149'

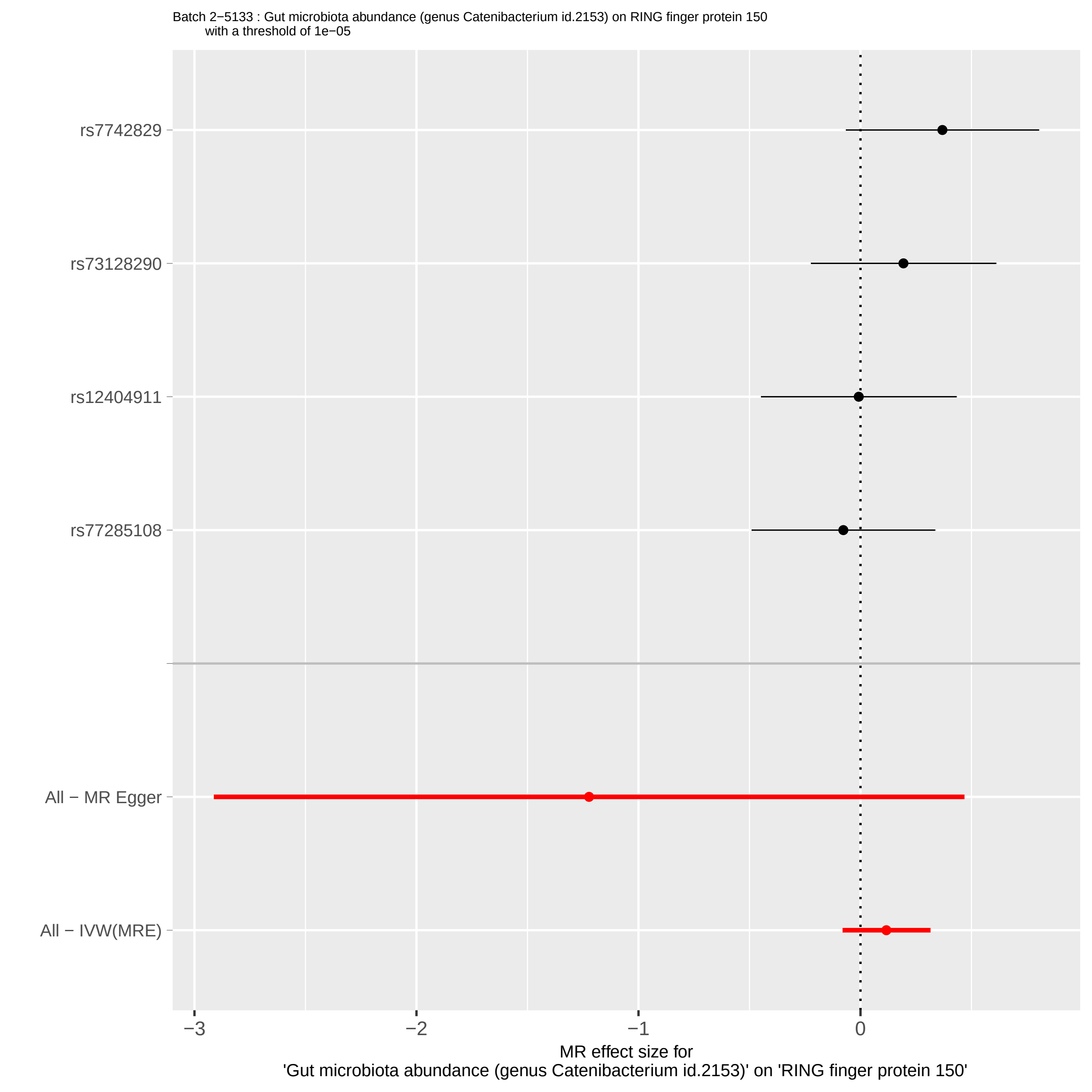
Batch 2−5133 : Gut microbiota abundance (genus Catenibacterium id.2153) on RING finger protein 150
with a threshold of 1e−05
rs7742829
rs73128290
rs12404911
rs77285108
All − MR Egger
All − IVW(MRE)
−3
−2
−1
0
MR effect size for
'Gut microbiota abundance (genus Catenibacterium id.2153)' on 'RING finger protein 150'

Batch 2−5134 : Gut microbiota abundance (genus Parasutterella id.2892) on RING finger protein 150
with a threshold of 1e−05

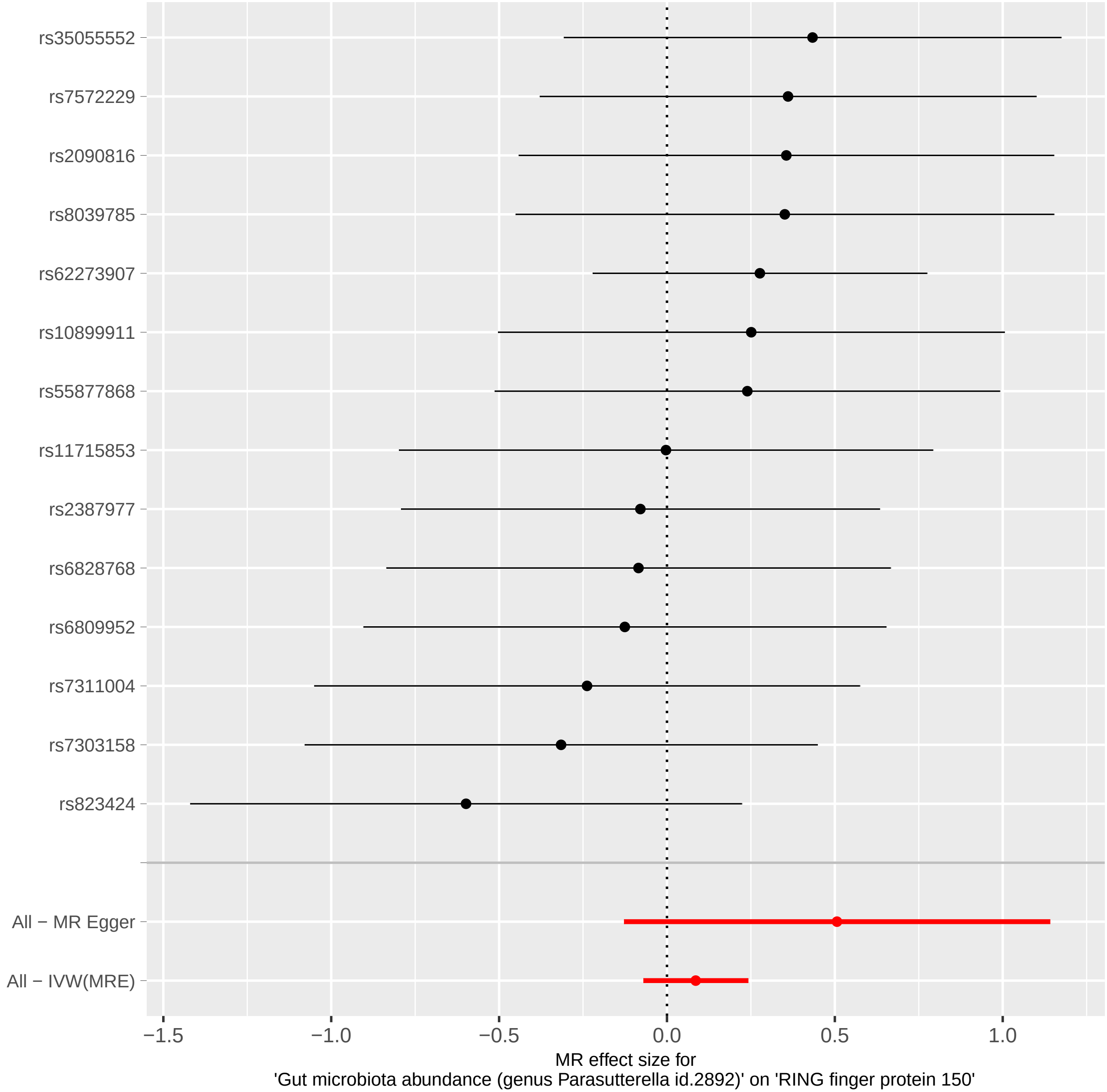

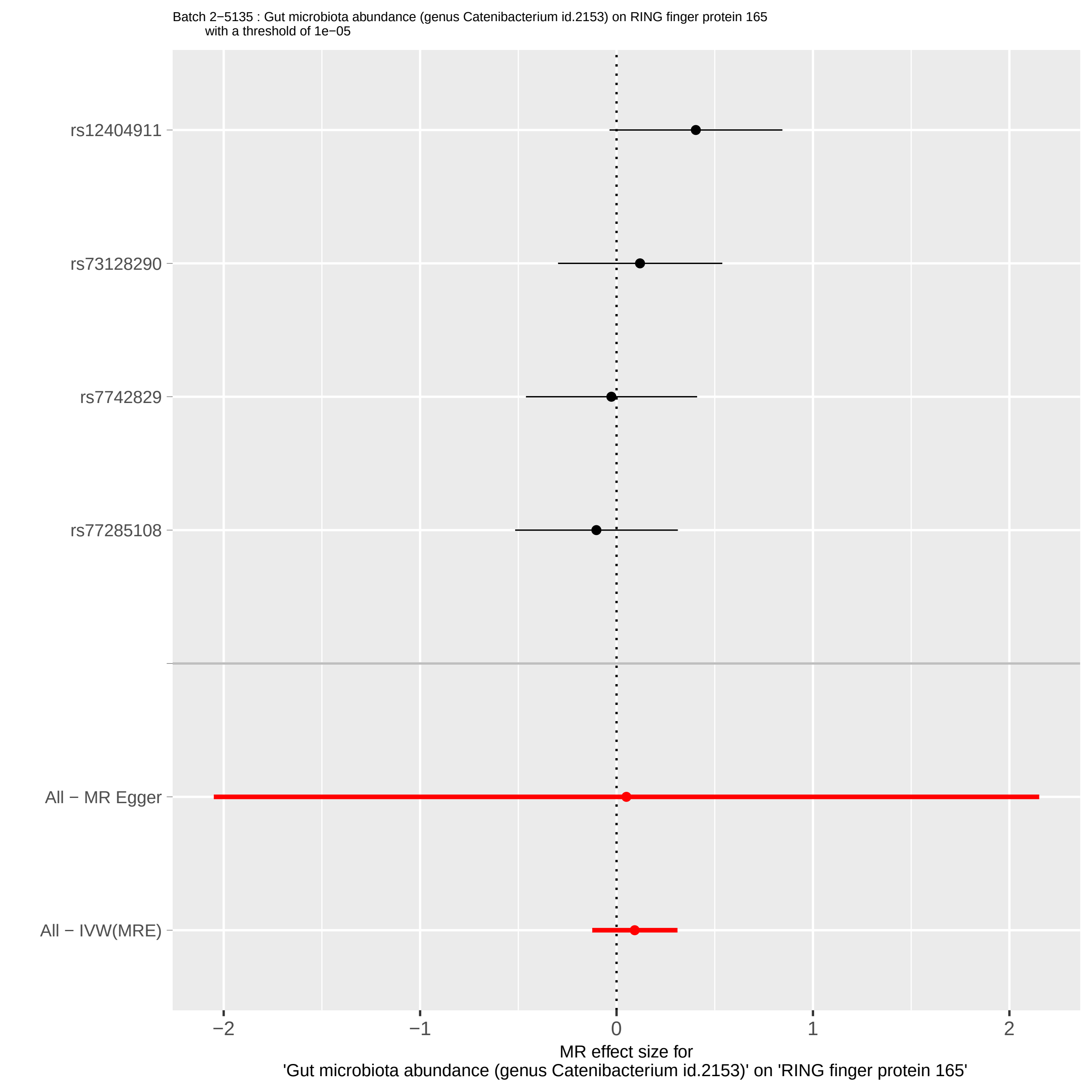

Batch 2−5135 : Gut microbiota abundance (genus Catenibacterium id.2153) on RING finger protein 165
with a threshold of 1e−05
rs12404911
rs73128290
rs7742829
rs77285108
All − MR Egger
All − IVW(MRE)
−2
−1
0
1
2
MR effect size for
'Gut microbiota abundance (genus Catenibacterium id.2153)' on 'RING finger protein 165'

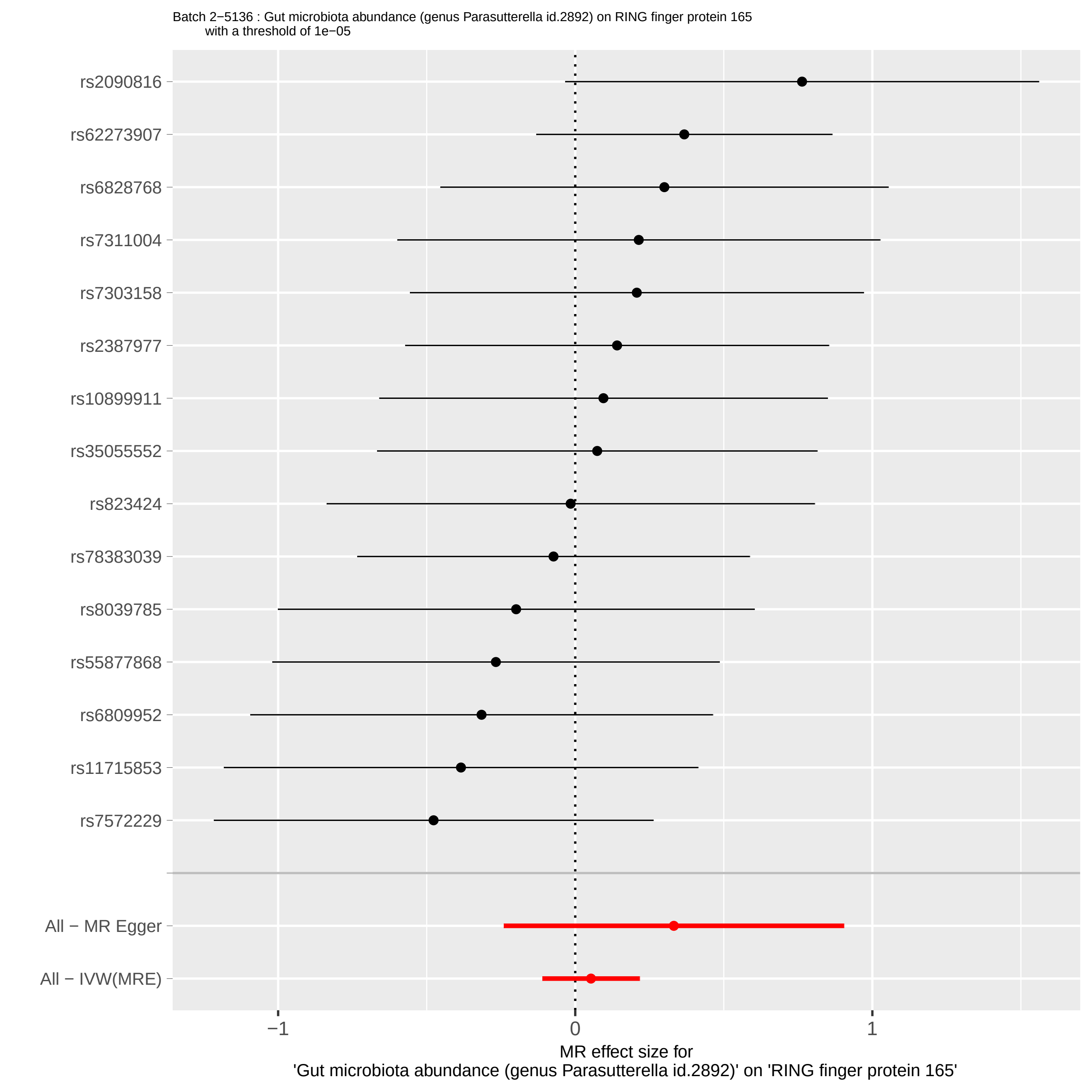

Batch 2−5136 : Gut microbiota abundance (genus Parasutterella id.2892) on RING finger protein 165
with a threshold of 1e−05
rs2090816
rs62273907
rs6828768
rs7311004
rs7303158
rs2387977
rs10899911
rs35055552
rs823424
rs78383039
rs8039785
rs55877868
rs6809952
rs11715853
rs7572229
All − MR Egger
All − IVW(MRE)
−1
0
1
MR effect size for
'Gut microbiota abundance (genus Parasutterella id.2892)' on 'RING finger protein 165'

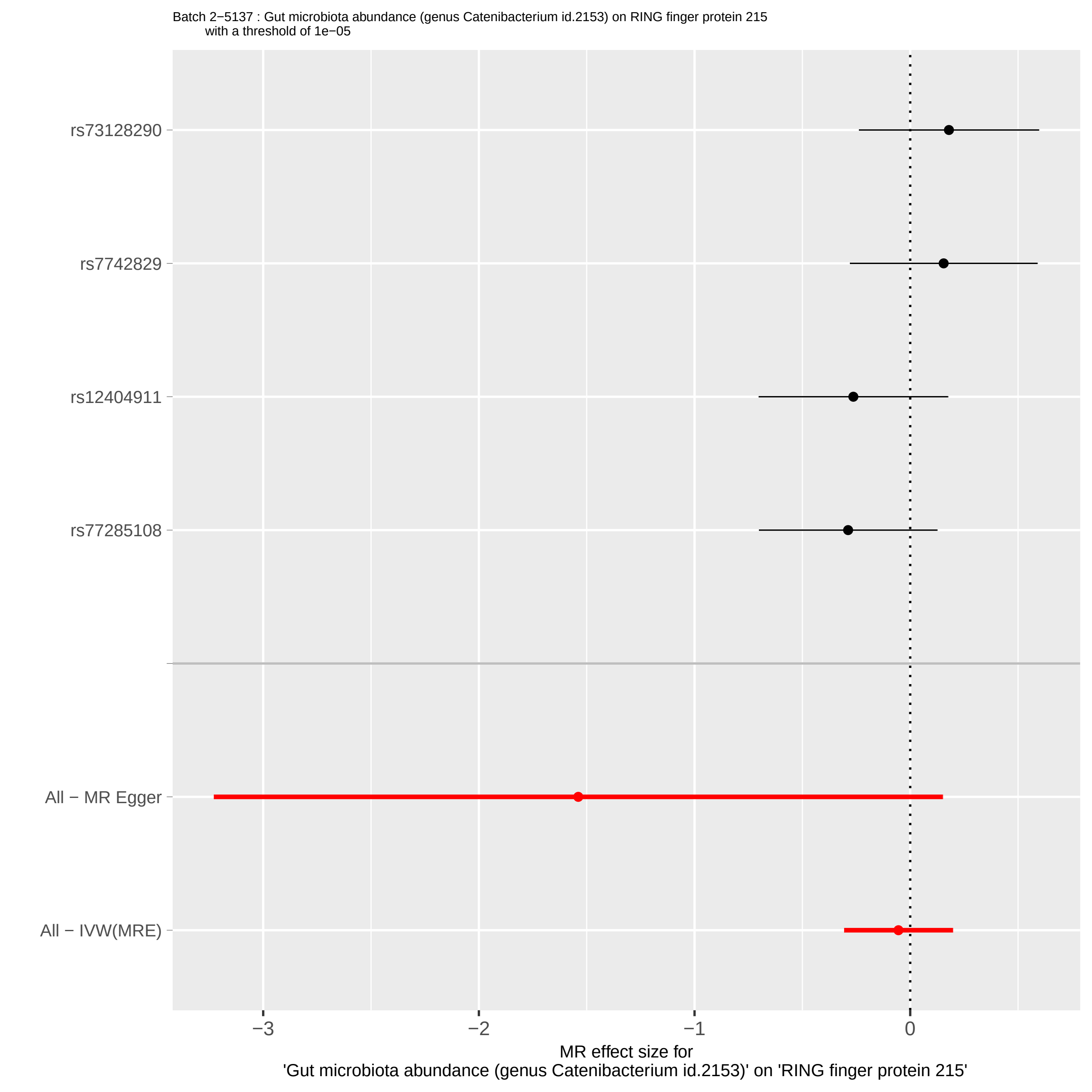
Batch 2−5137 : Gut microbiota abundance (genus Catenibacterium id.2153) on RING finger protein 215
with a threshold of 1e−05
rs73128290
rs7742829
rs12404911
rs77285108
All − MR Egger
All − IVW(MRE)
−3
−2
−1
0
MR effect size for
'Gut microbiota abundance (genus Catenibacterium id.2153)' on 'RING finger protein 215'

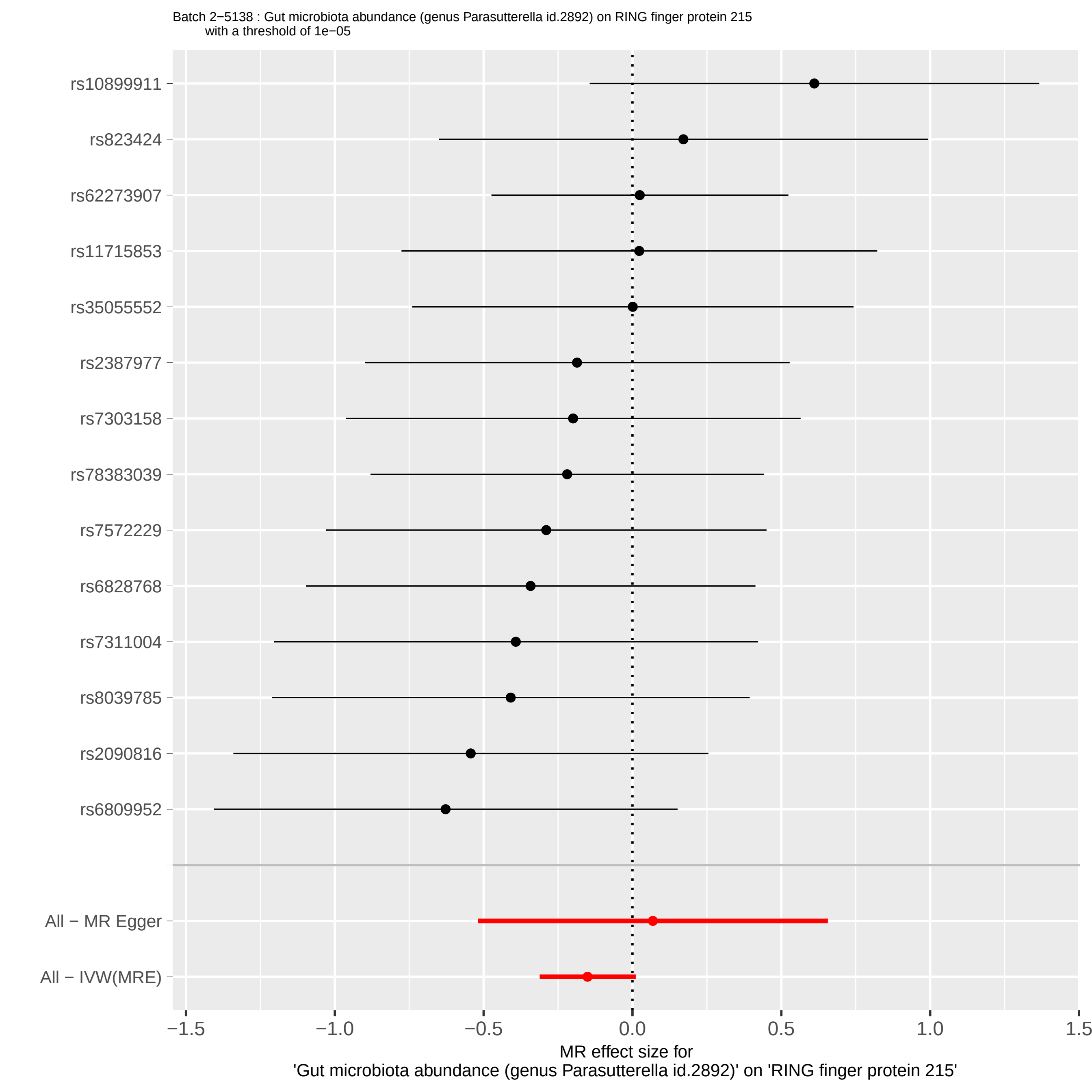

Batch 2−5138 : Gut microbiota abundance (genus Parasutterella id.2892) on RING finger protein 215
with a threshold of 1e−05
rs10899911
rs823424
rs62273907
rs11715853
rs35055552
rs2387977
rs7303158
rs78383039
rs7572229
rs6828768
rs7311004
rs8039785
rs2090816
rs6809952
All − MR Egger
All − IVW(MRE)
−1.5
−1.0
−0.5
0.0
0.5
1.0
1.5
MR effect size for
'Gut microbiota abundance (genus Parasutterella id.2892)' on 'RING finger protein 215'

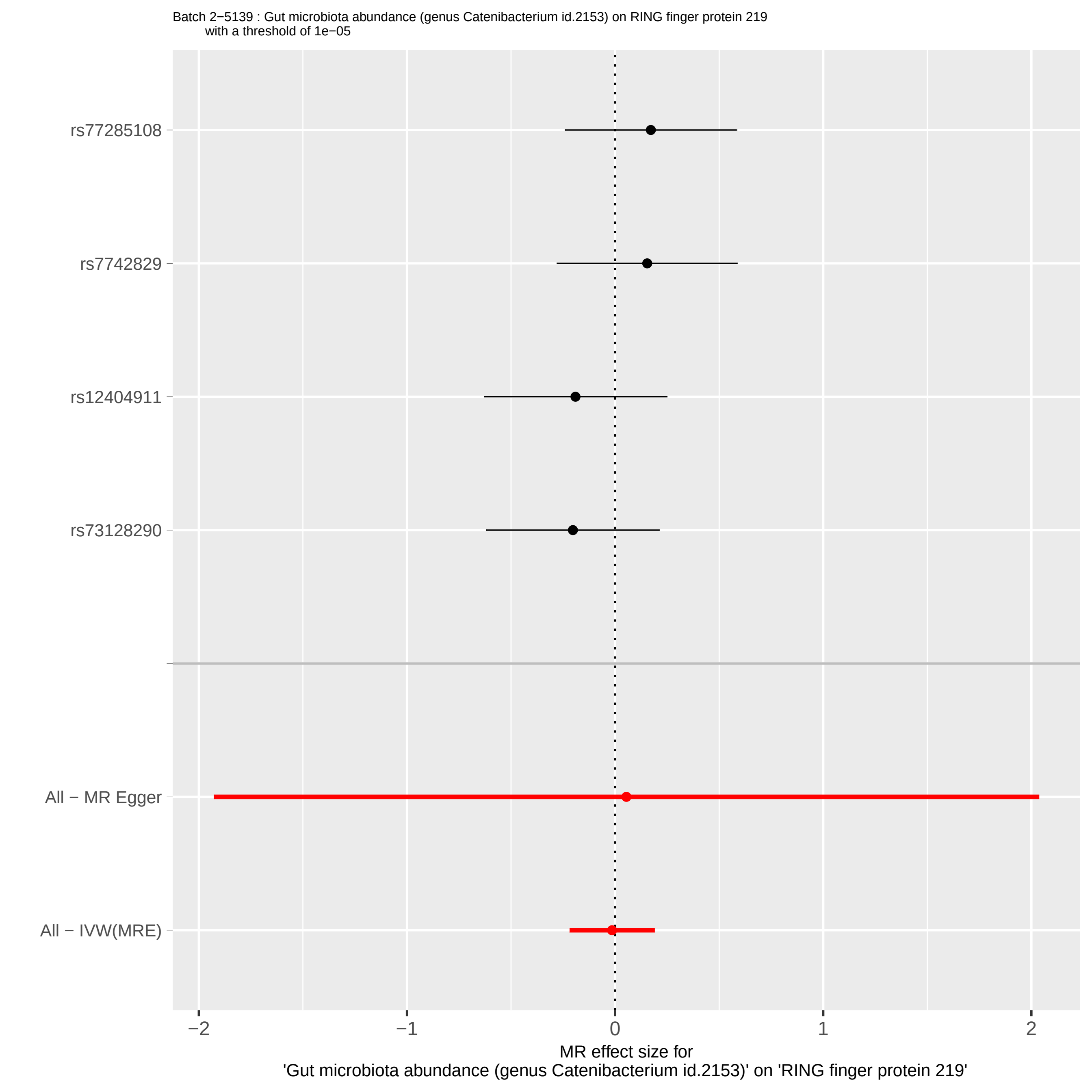
Batch 2−5139 : Gut microbiota abundance (genus Catenibacterium id.2153) on RING finger protein 219
with a threshold of 1e−05
rs77285108
rs7742829
rs12404911
rs73128290
All − MR Egger
All − IVW(MRE)
−2
−1
0
1
2
MR effect size for
'Gut microbiota abundance (genus Catenibacterium id.2153)' on 'RING finger protein 219'

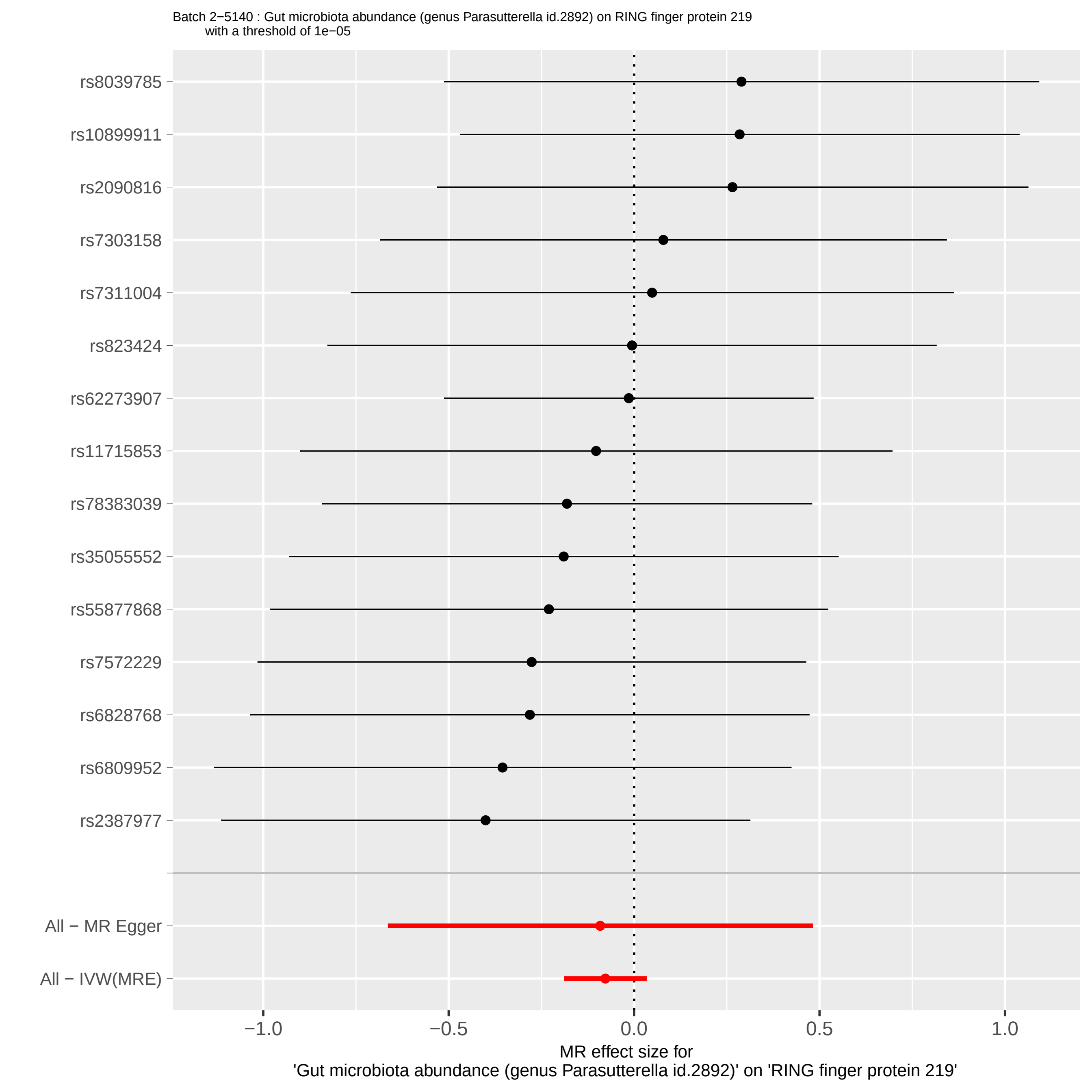

Batch 2−5140 : Gut microbiota abundance (genus Parasutterella id.2892) on RING finger protein 219
with a threshold of 1e−05
rs8039785
rs10899911
rs2090816
rs7303158
rs7311004
rs823424
rs62273907
rs11715853
rs78383039
rs35055552
rs55877868
rs7572229
rs6828768
rs6809952
rs2387977
All − MR Egger
All − IVW(MRE)
−1.0
−0.5
0.0
0.5
1.0
MR effect size for
'Gut microbiota abundance (genus Parasutterella id.2892)' on 'RING finger protein 219'

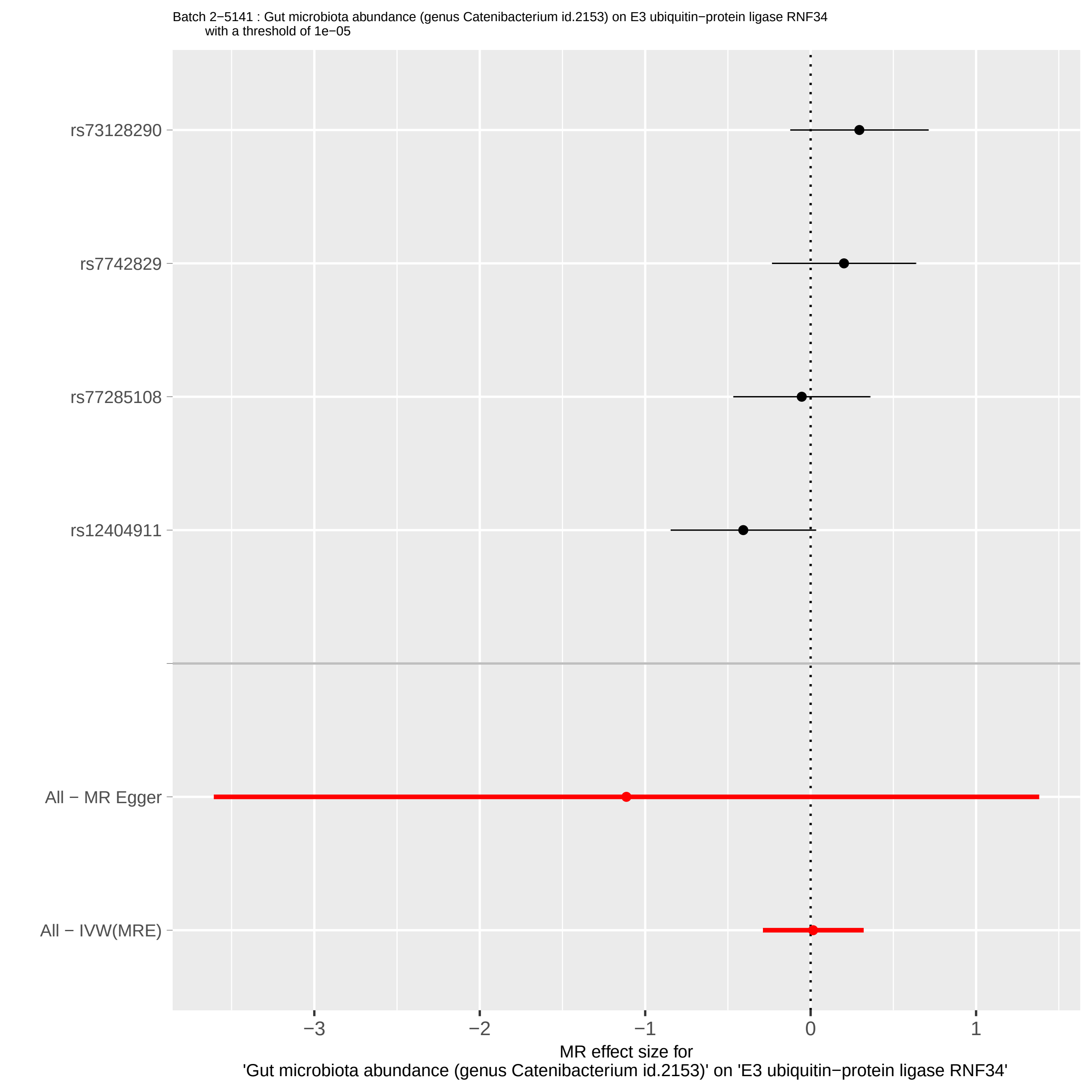

Batch 2−5141 : Gut microbiota abundance (genus Catenibacterium id.2153) on E3 ubiquitin−protein ligase RNF34
with a threshold of 1e−05
rs73128290
rs7742829
rs77285108
rs12404911
All − MR Egger
All − IVW(MRE)
−3
−2
−1
0
1
MR effect size for
'Gut microbiota abundance (genus Catenibacterium id.2153)' on 'E3 ubiquitin−protein ligase RNF34'

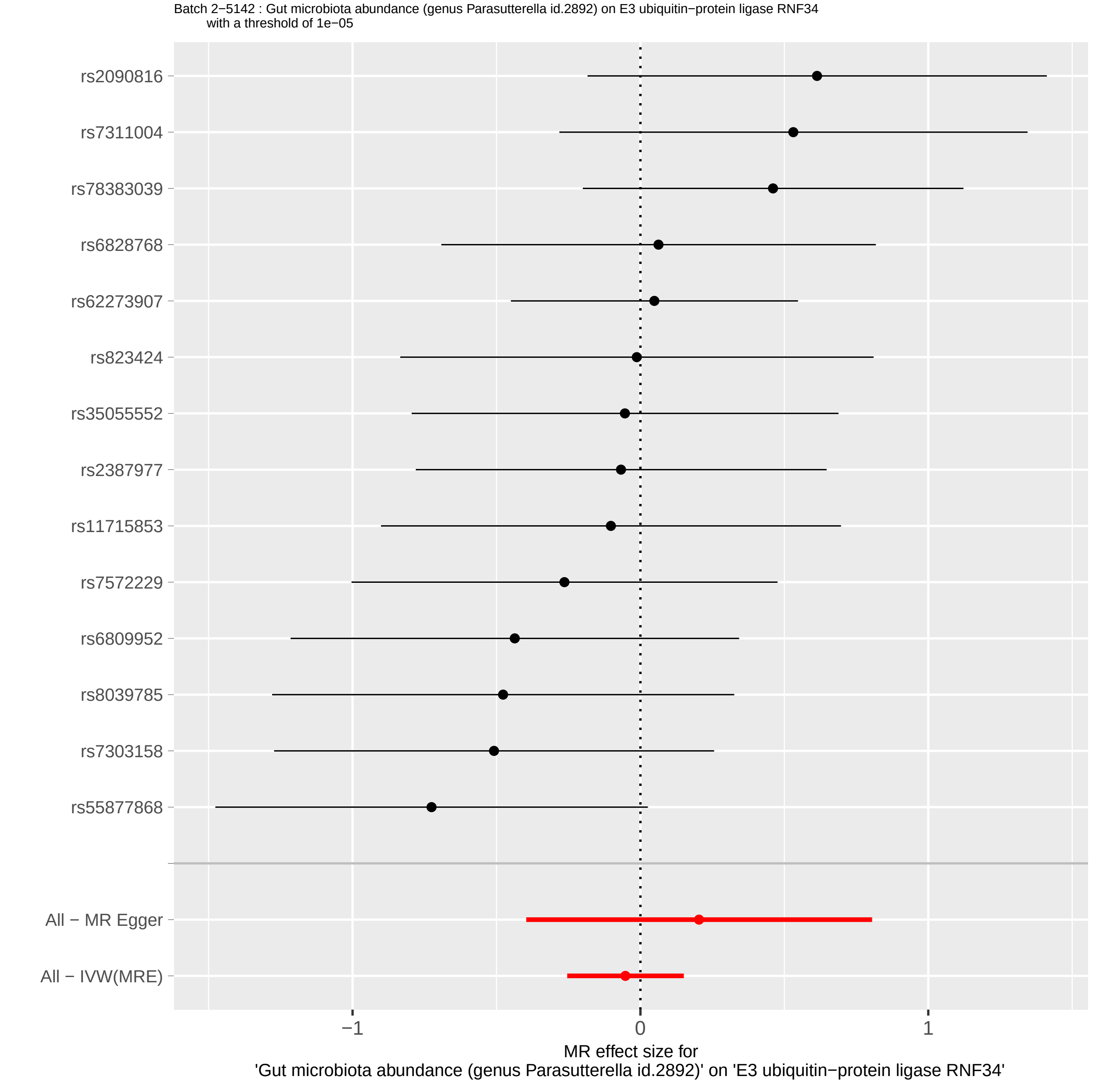
Batch 2−5142 : Gut microbiota abundance (genus Parasutterella id.2892) on E3 ubiquitin−protein ligase RNF34
with a threshold of 1e−05
rs2090816
rs7311004
rs78383039
rs6828768
rs62273907
rs823424
rs35055552
rs2387977
rs11715853
rs7572229
rs6809952
rs8039785
rs7303158
rs55877868
All − MR Egger
All − IVW(MRE)
−1
0
1
MR effect size for
'Gut microbiota abundance (genus Parasutterella id.2892)' on 'E3 ubiquitin−protein ligase RNF34'

Batch 2−5143 : Gut microbiota abundance (genus Catenibacterium id.2153) on E3 ubiquitin−protein ligase RNF43
with a threshold of 1e−05

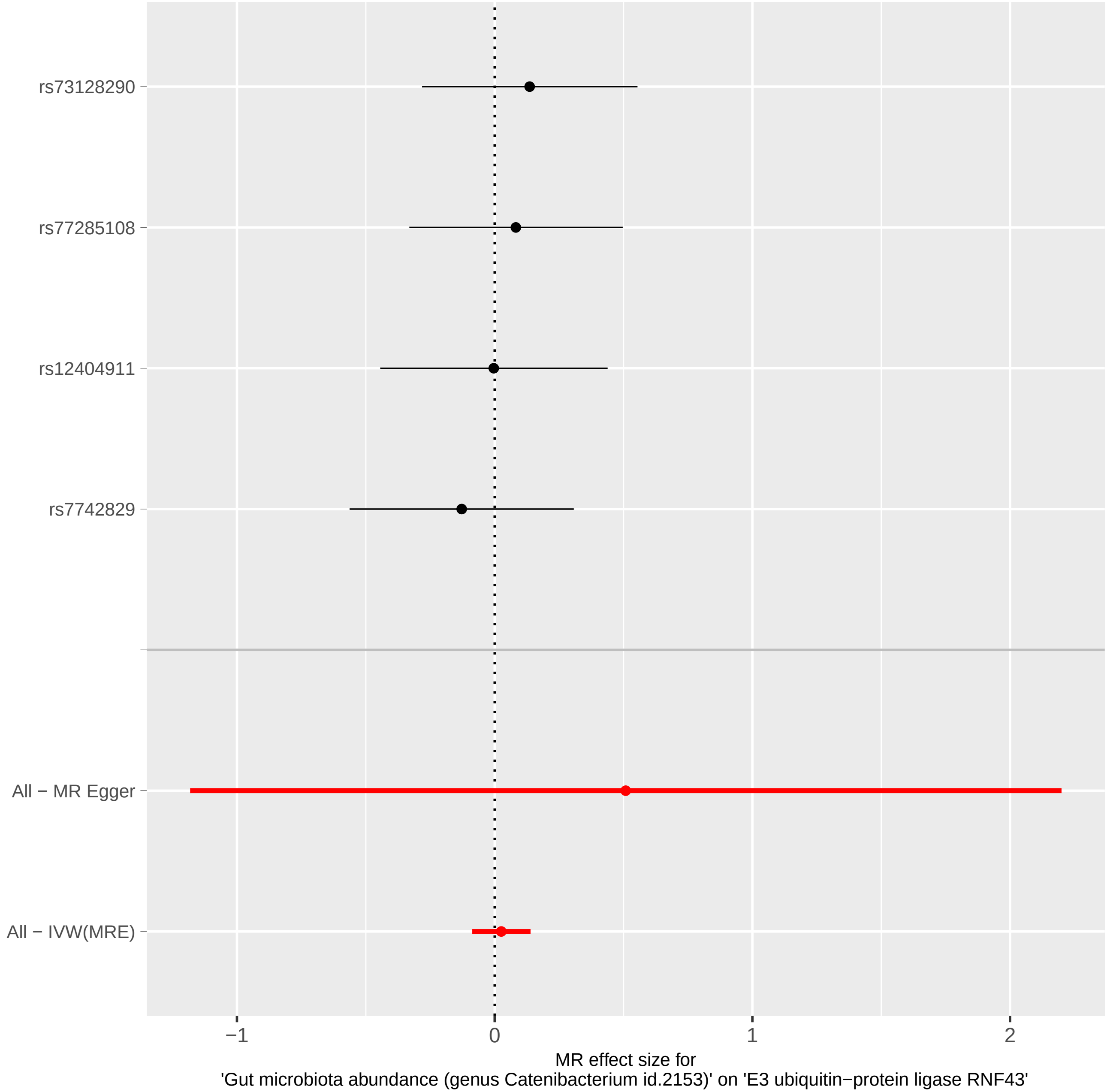

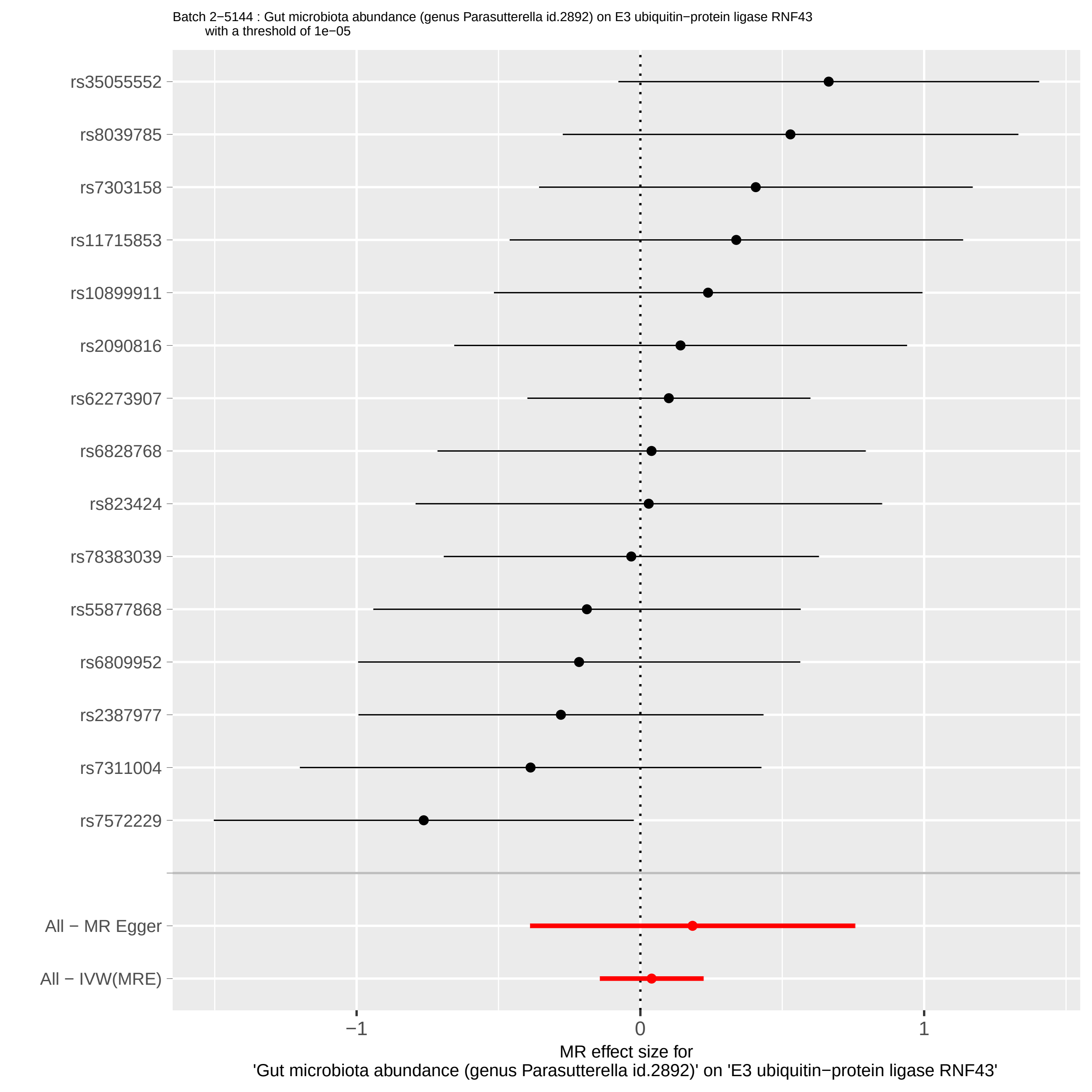

Batch 2−5144 : Gut microbiota abundance (genus Parasutterella id.2892) on E3 ubiquitin−protein ligase RNF43
with a threshold of 1e−05
rs35055552
rs8039785
rs7303158
rs11715853
rs10899911
rs2090816
rs62273907
rs6828768
rs823424
rs78383039
rs55877868
rs6809952
rs2387977
rs7311004
rs7572229
All − MR Egger
All − IVW(MRE)
−1
0
1
MR effect size for
'Gut microbiota abundance (genus Parasutterella id.2892)' on 'E3 ubiquitin−protein ligase RNF43'

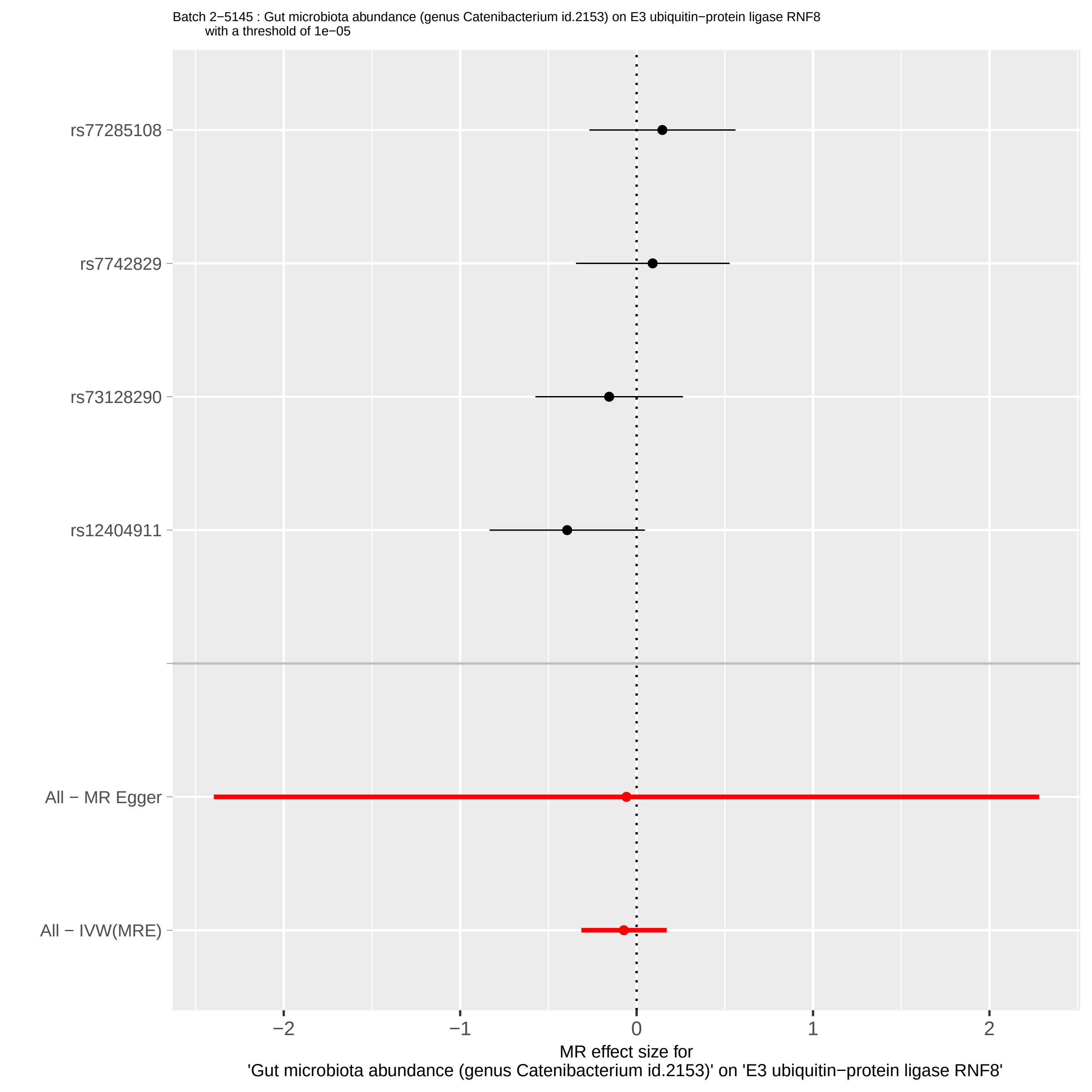

Batch 2−5145 : Gut microbiota abundance (genus Catenibacterium id.2153) on E3 ubiquitin−protein ligase RNF8
with a threshold of 1e−05
rs77285108
rs7742829
rs73128290
rs12404911
All − MR Egger
All − IVW(MRE)
−2
−1
0
1
2
MR effect size for
'Gut microbiota abundance (genus Catenibacterium id.2153)' on 'E3 ubiquitin−protein ligase RNF8'

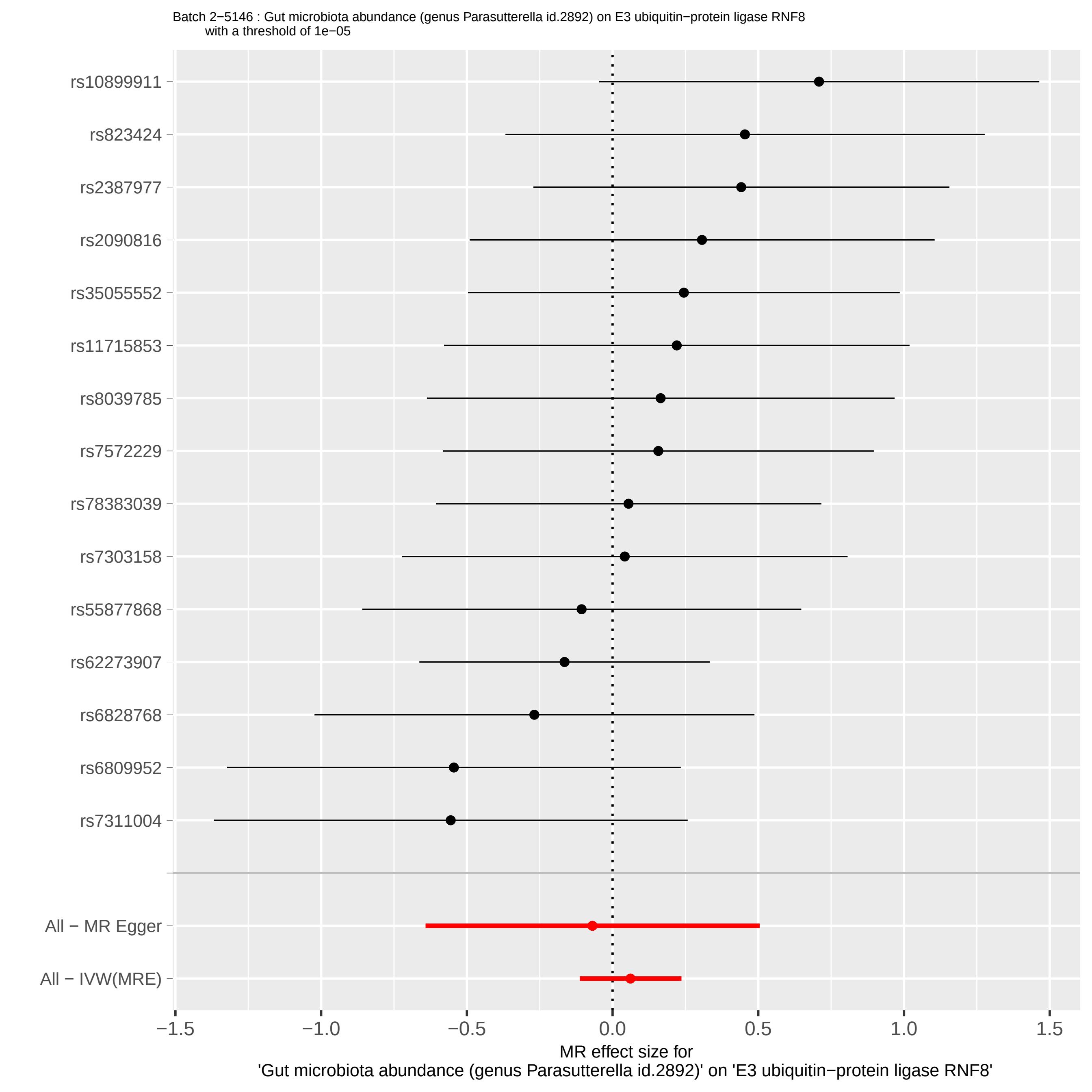
Batch 2−5146 : Gut microbiota abundance (genus Parasutterella id.2892) on E3 ubiquitin−protein ligase RNF8
with a threshold of 1e−05
rs10899911
rs823424
rs2387977
rs2090816
rs35055552
rs11715853
rs8039785
rs7572229
rs78383039
rs7303158
rs55877868
rs62273907
rs6828768
rs6809952
rs7311004
All − MR Egger
All − IVW(MRE)
−1.5
−1.0
−0.5
0.0
0.5
1.0
1.5
MR effect size for
'Gut microbiota abundance (genus Parasutterella id.2892)' on 'E3 ubiquitin−protein ligase RNF8'

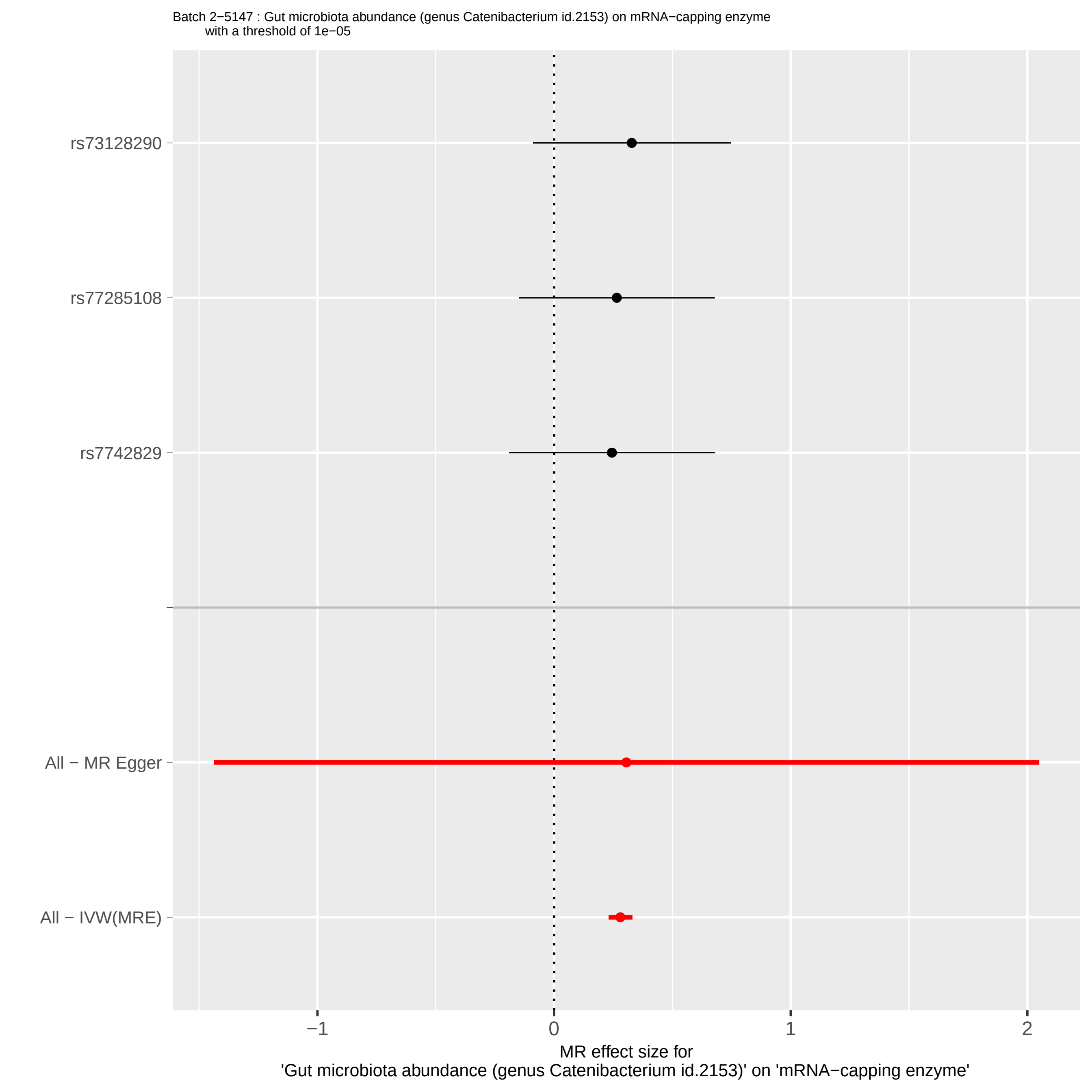
Batch 2−5147 : Gut microbiota abundance (genus Catenibacterium id.2153) on mRNA−capping enzyme
with a threshold of 1e−05
rs73128290
rs77285108
rs7742829
All − MR Egger
All − IVW(MRE)
−1
0
1
2
MR effect size for
'Gut microbiota abundance (genus Catenibacterium id.2153)' on 'mRNA−capping enzyme'

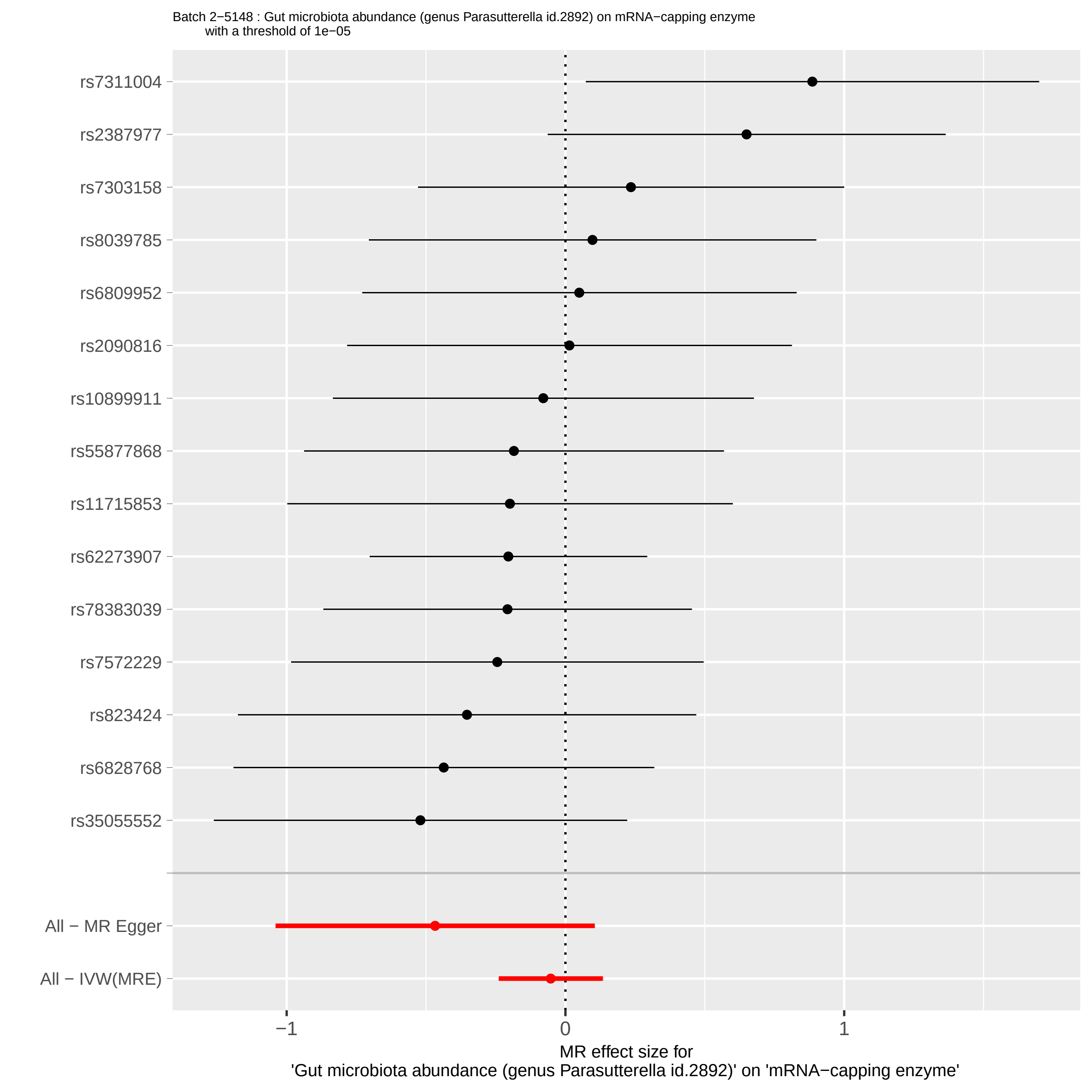
Batch 2−5148 : Gut microbiota abundance (genus Parasutterella id.2892) on mRNA−capping enzyme
with a threshold of 1e−05
rs7311004
rs2387977
rs7303158
rs8039785
rs6809952
rs2090816
rs10899911
rs55877868
rs11715853
rs62273907
rs78383039
rs7572229
rs823424
rs6828768
rs35055552
All − MR Egger
All − IVW(MRE)
−1
0
1
MR effect size for
'Gut microbiota abundance (genus Parasutterella id.2892)' on 'mRNA−capping enzyme'

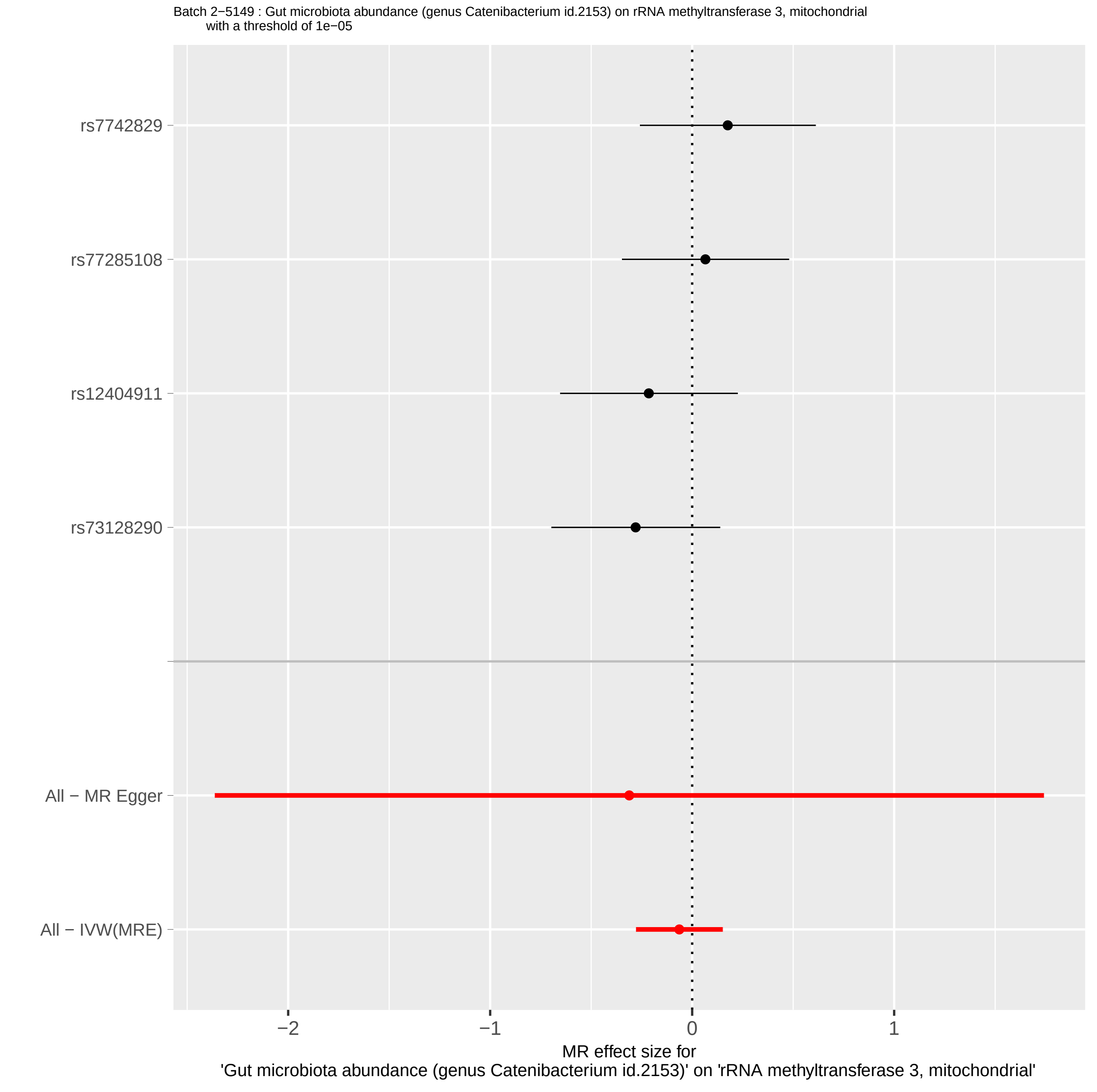

Batch 2−5149 : Gut microbiota abundance (genus Catenibacterium id.2153) on rRNA methyltransferase 3, mitochondrial with a threshold of 1e−05
rs7742829
rs77285108
rs12404911
rs73128290
All − MR Egger
All − IVW(MRE)
−2
−1
0
1
MR effect size for
'Gut microbiota abundance (genus Catenibacterium id.2153)' on 'rRNA methyltransferase 3, mitochondrial'

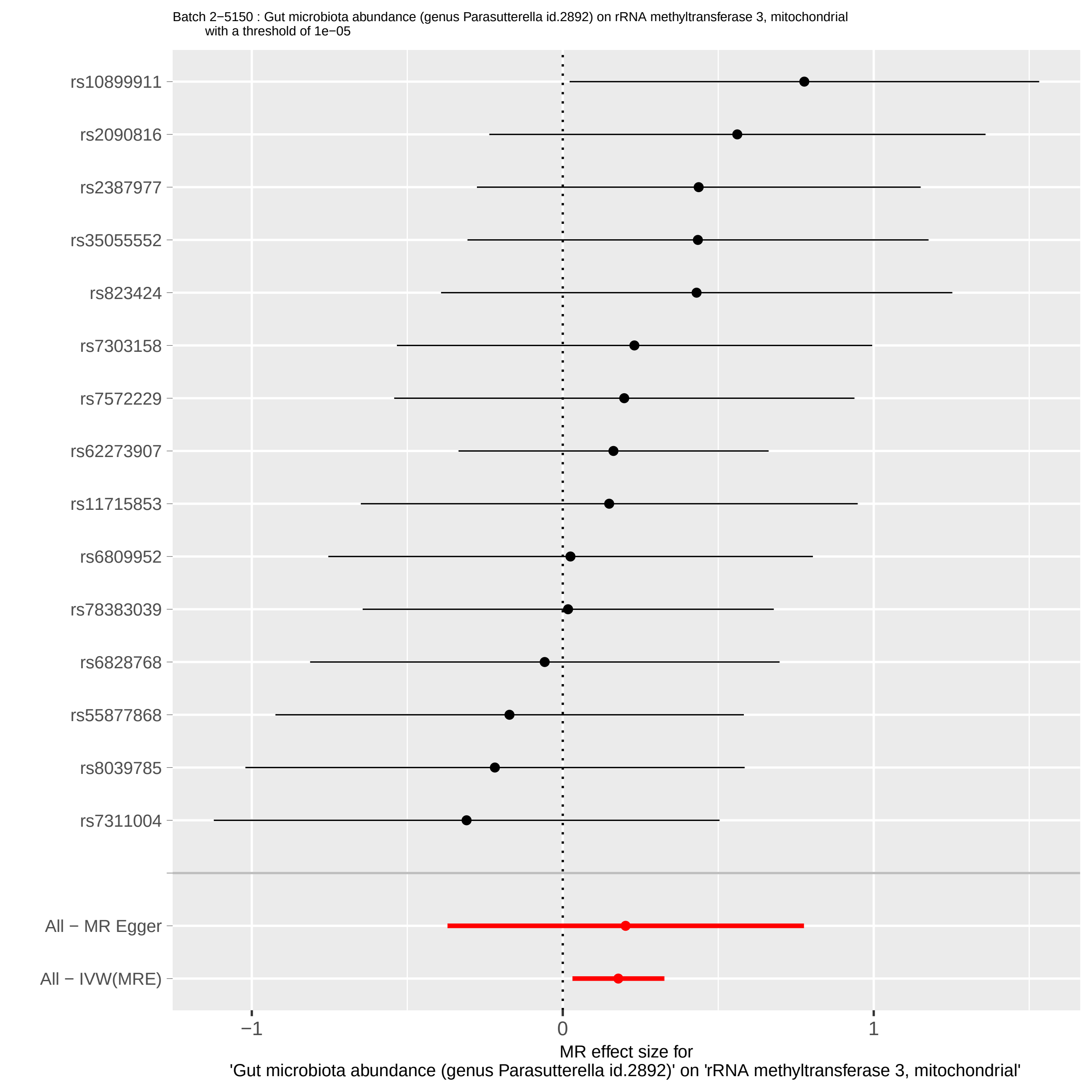
Batch 2−5150 : Gut microbiota abundance (genus Parasutterella id.2892) on rRNA methyltransferase 3, mitochondrial
with a threshold of 1e−05
rs10899911
rs2090816
rs2387977
rs35055552
rs823424
rs7303158
rs7572229
rs62273907
rs11715853
rs6809952
rs78383039
rs6828768
rs55877868
rs8039785
rs7311004
All − MR Egger
All − IVW(MRE)
−1
0
1
MR effect size for
'Gut microbiota abundance (genus Parasutterella id.2892)' on 'rRNA methyltransferase 3, mitochondrial'

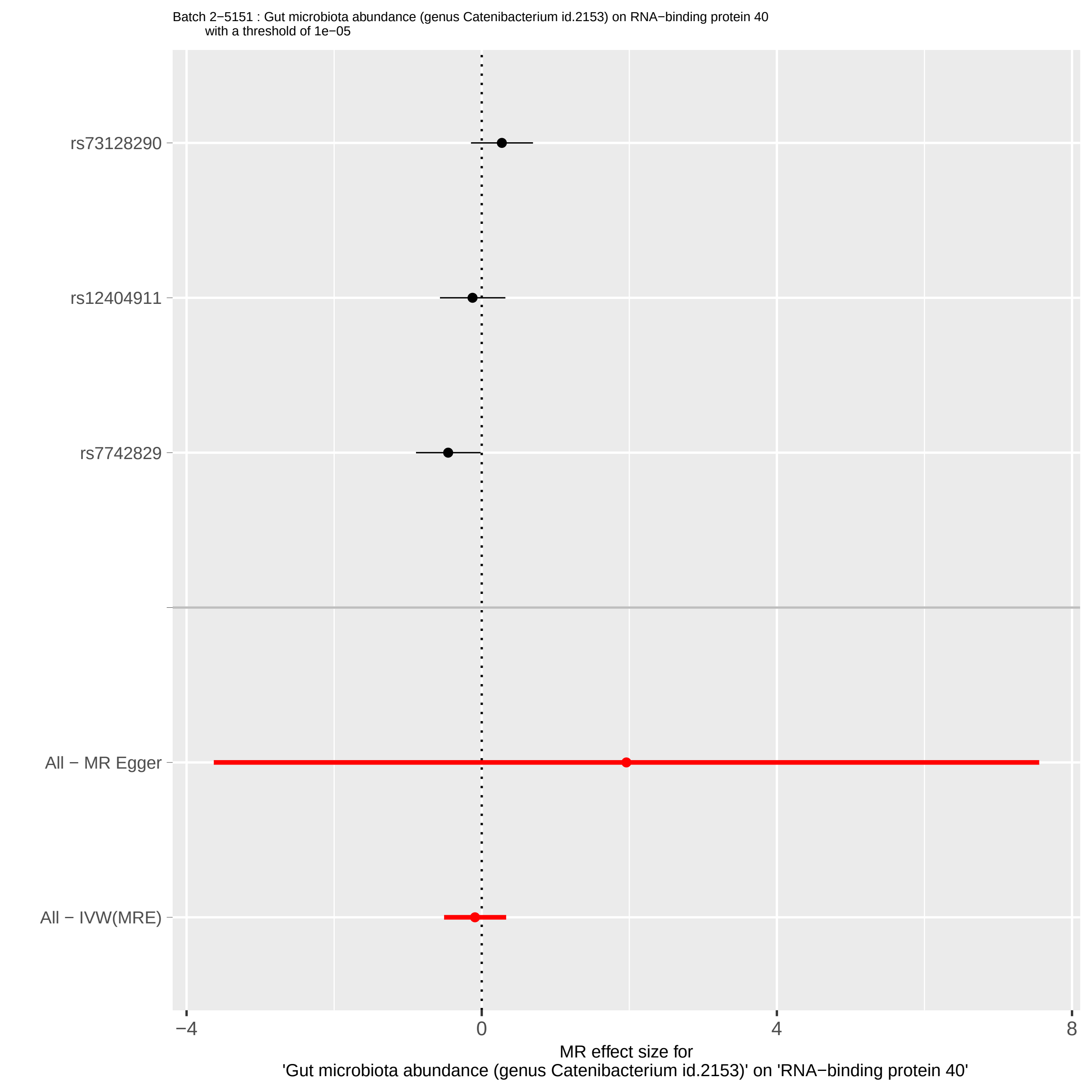

Batch 2−5151 : Gut microbiota abundance (genus Catenibacterium id.2153) on RNA−binding protein 40
with a threshold of 1e−05
rs73128290
rs12404911
rs7742829
All − MR Egger
All − IVW(MRE)
−4
0
4
8
MR effect size for
'Gut microbiota abundance (genus Catenibacterium id.2153)' on 'RNA−binding protein 40'

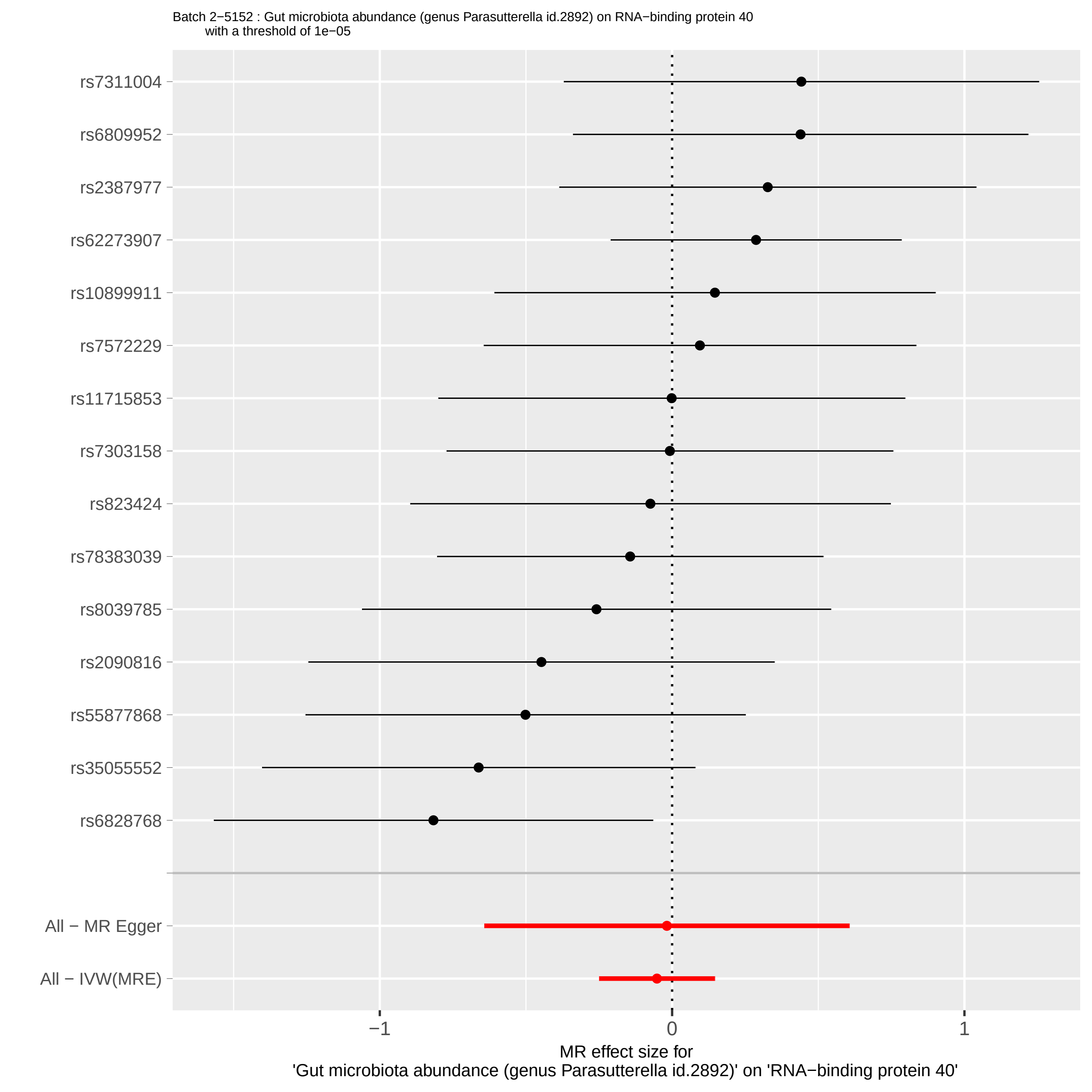

Batch 2−5152 : Gut microbiota abundance (genus Parasutterella id.2892) on RNA−binding protein 40
with a threshold of 1e−05
rs7311004
rs6809952
rs2387977
rs62273907
rs10899911
rs7572229
rs11715853
rs7303158
rs823424
rs78383039
rs8039785
rs2090816
rs55877868
rs35055552
rs6828768
All − MR Egger
All − IVW(MRE)
−1
0
1
MR effect size for
'Gut microbiota abundance (genus Parasutterella id.2892)' on 'RNA−binding protein 40'

Batch 2−5153 : Gut microbiota abundance (genus Catenibacterium id.2153) on Roundabout homolog 1
with a threshold of 1e−05

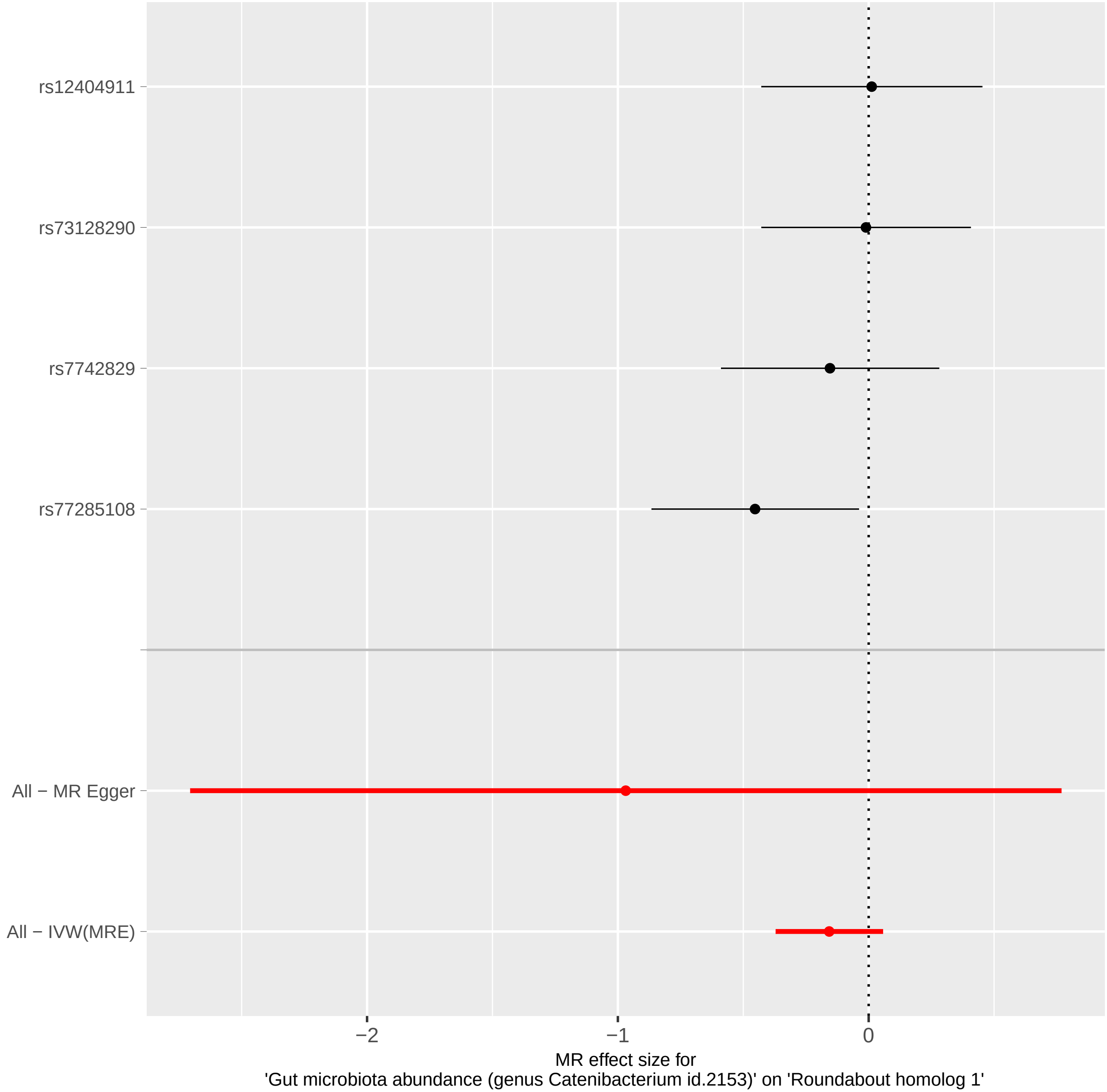

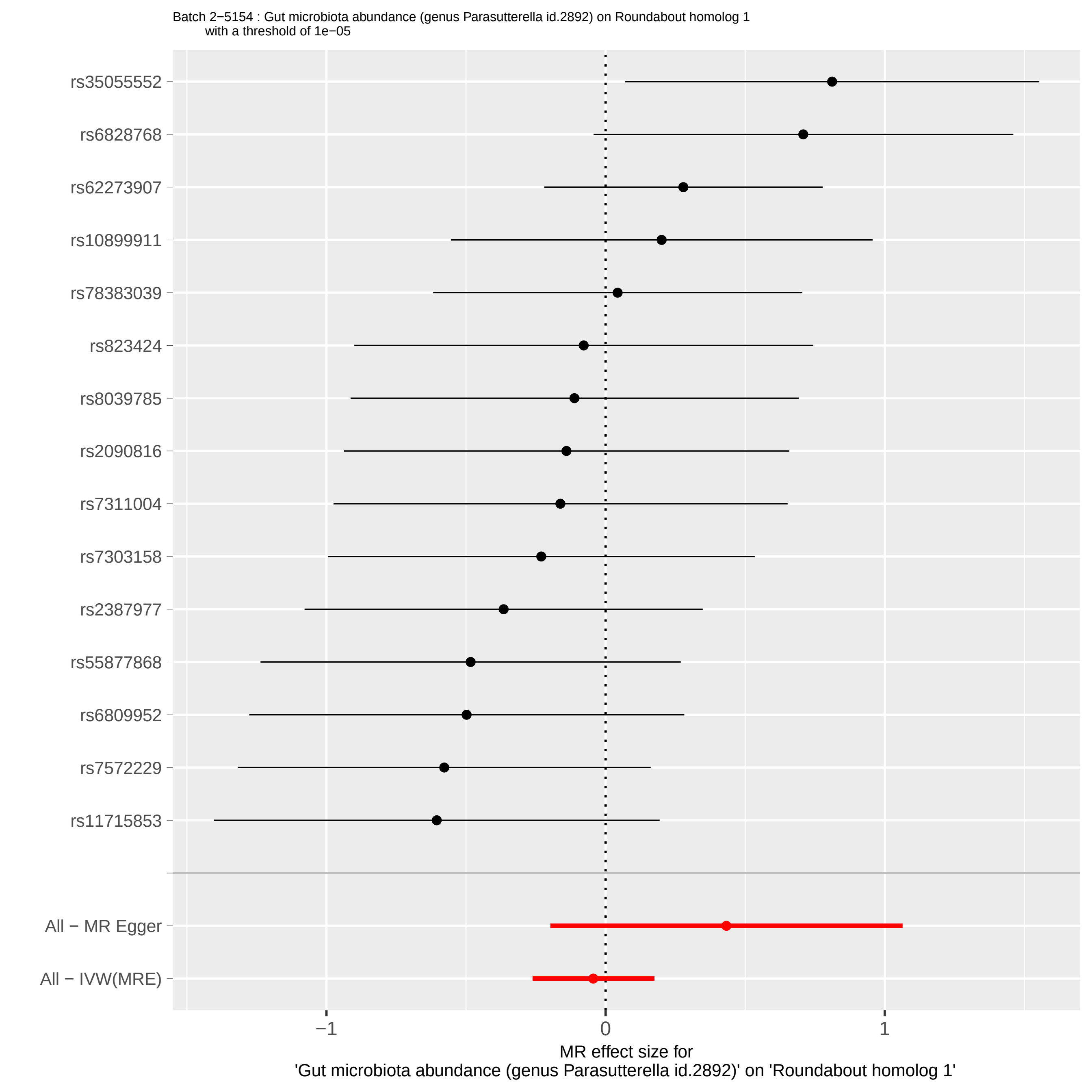

Batch 2−5154 : Gut microbiota abundance (genus Parasutterella id.2892) on Roundabout homolog 1 with a threshold of 1e−05
rs35055552
rs6828768
rs62273907
rs10899911
rs78383039
rs823424
rs8039785
rs2090816
rs7311004
rs7303158
rs2387977
rs55877868
rs6809952
rs7572229
rs11715853
All − MR Egger
All − IVW(MRE)
−1
0
1
MR effect size for
'Gut microbiota abundance (genus Parasutterella id.2892)' on 'Roundabout homolog 1'

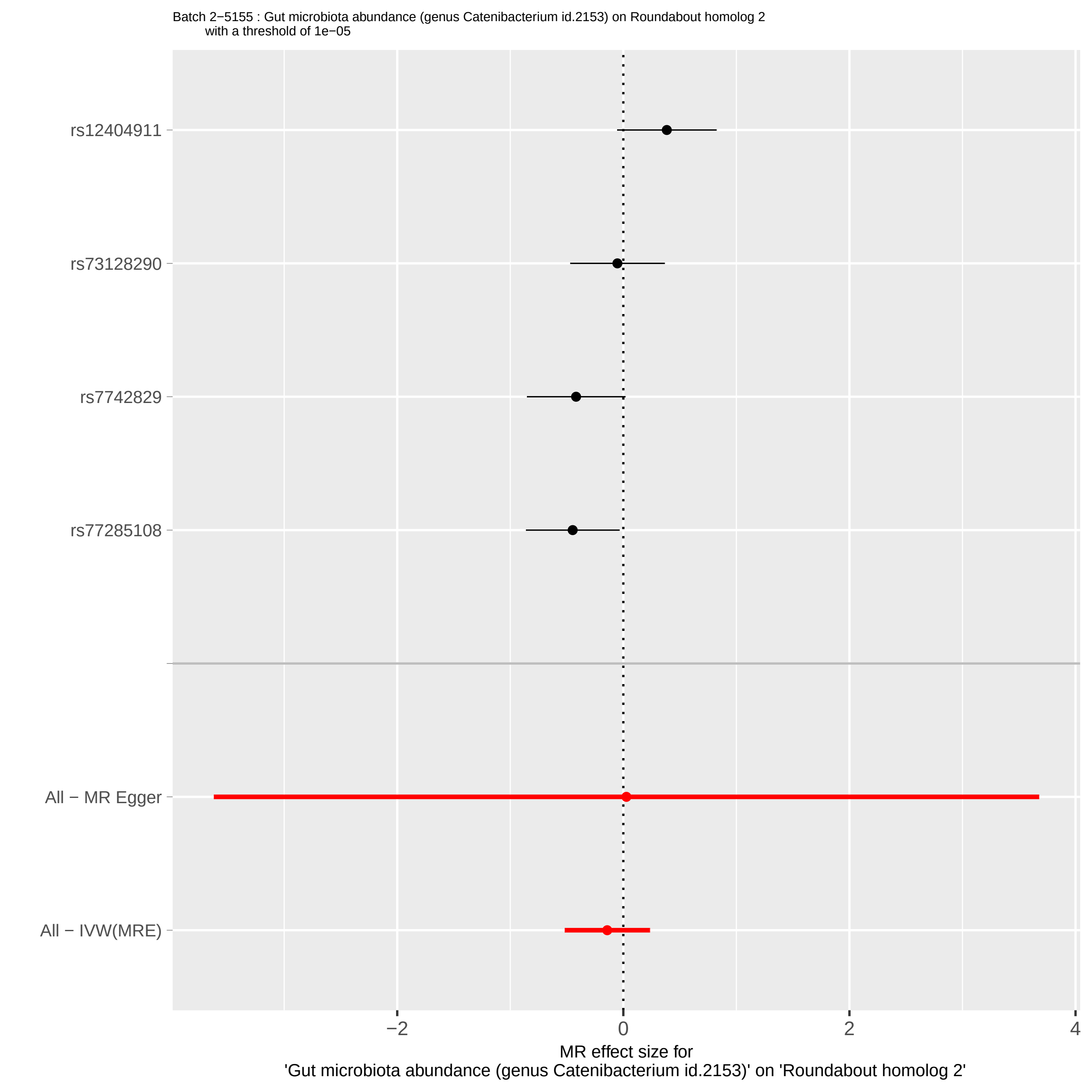
Batch 2−5155 : Gut microbiota abundance (genus Catenibacterium id.2153) on Roundabout homolog 2
with a threshold of 1e−05
rs12404911
rs73128290
rs7742829
rs77285108
All − MR Egger
All − IVW(MRE)
−2
0
2
4
MR effect size for
'Gut microbiota abundance (genus Catenibacterium id.2153)' on 'Roundabout homolog 2'

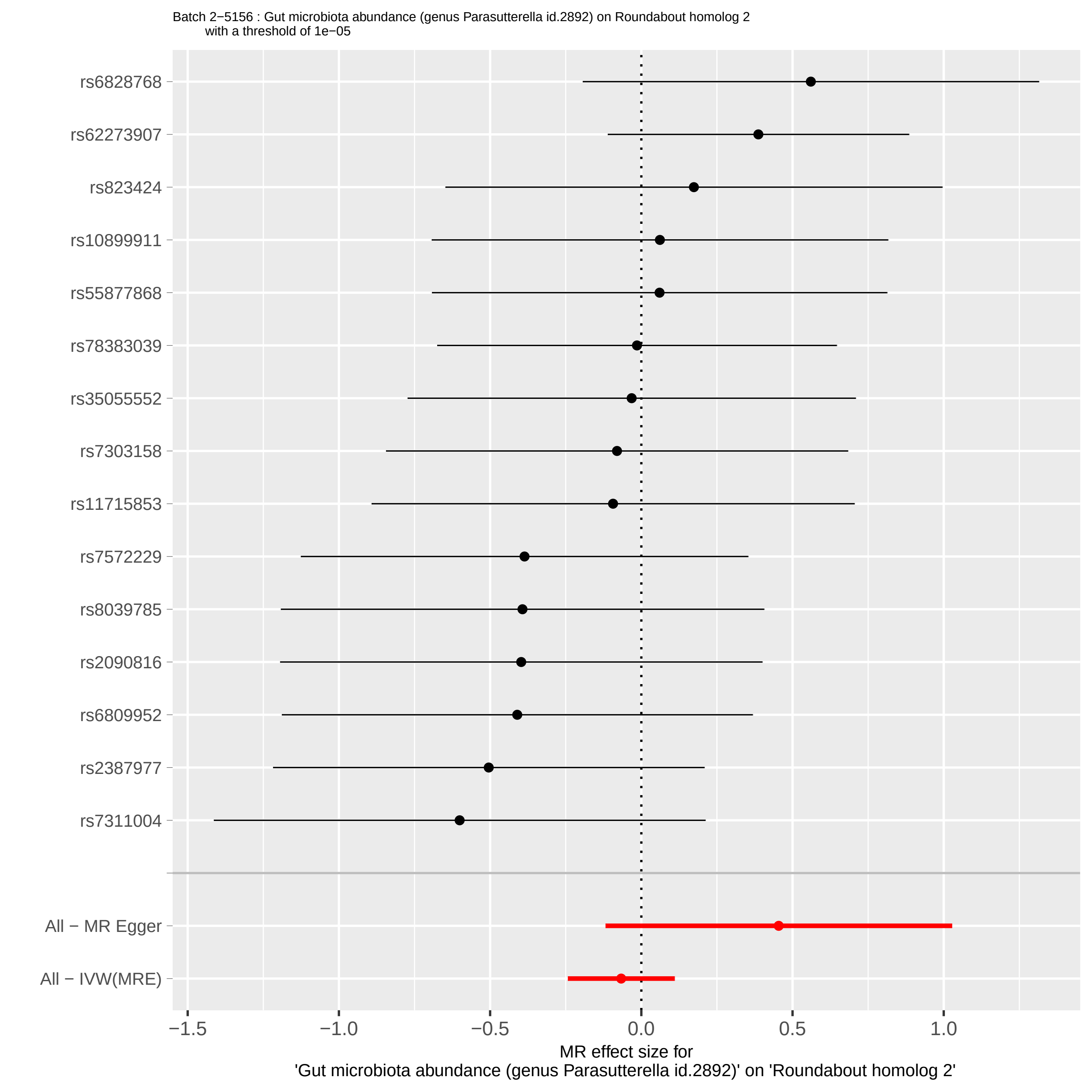

Batch 2−5156 : Gut microbiota abundance (genus Parasutterella id.2892) on Roundabout homolog 2
with a threshold of 1e−05
rs6828768
rs62273907
rs823424
rs10899911
rs55877868
rs78383039
rs35055552
rs7303158
rs11715853
rs7572229
rs8039785
rs2090816
rs6809952
rs2387977
rs7311004
All − MR Egger
All − IVW(MRE)
−1.5
−1.0
−0.5
0.0
0.5
1.0
MR effect size for
'Gut microbiota abundance (genus Parasutterella id.2892)' on 'Roundabout homolog 2'

Batch 2−5157 : Gut microbiota abundance (genus Catenibacterium id.2153) on Roundabout homolog 3
with a threshold of 1e−05

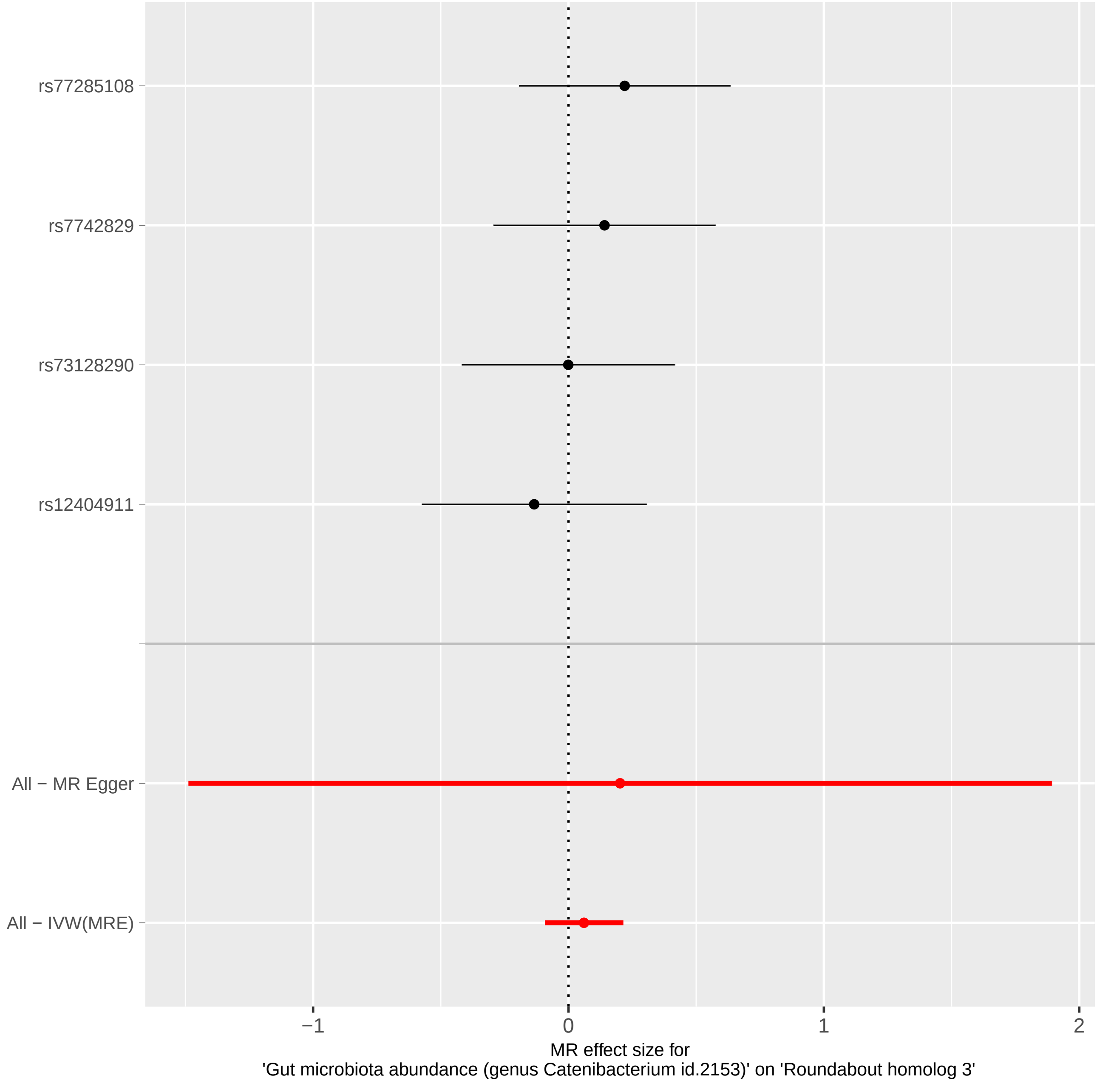

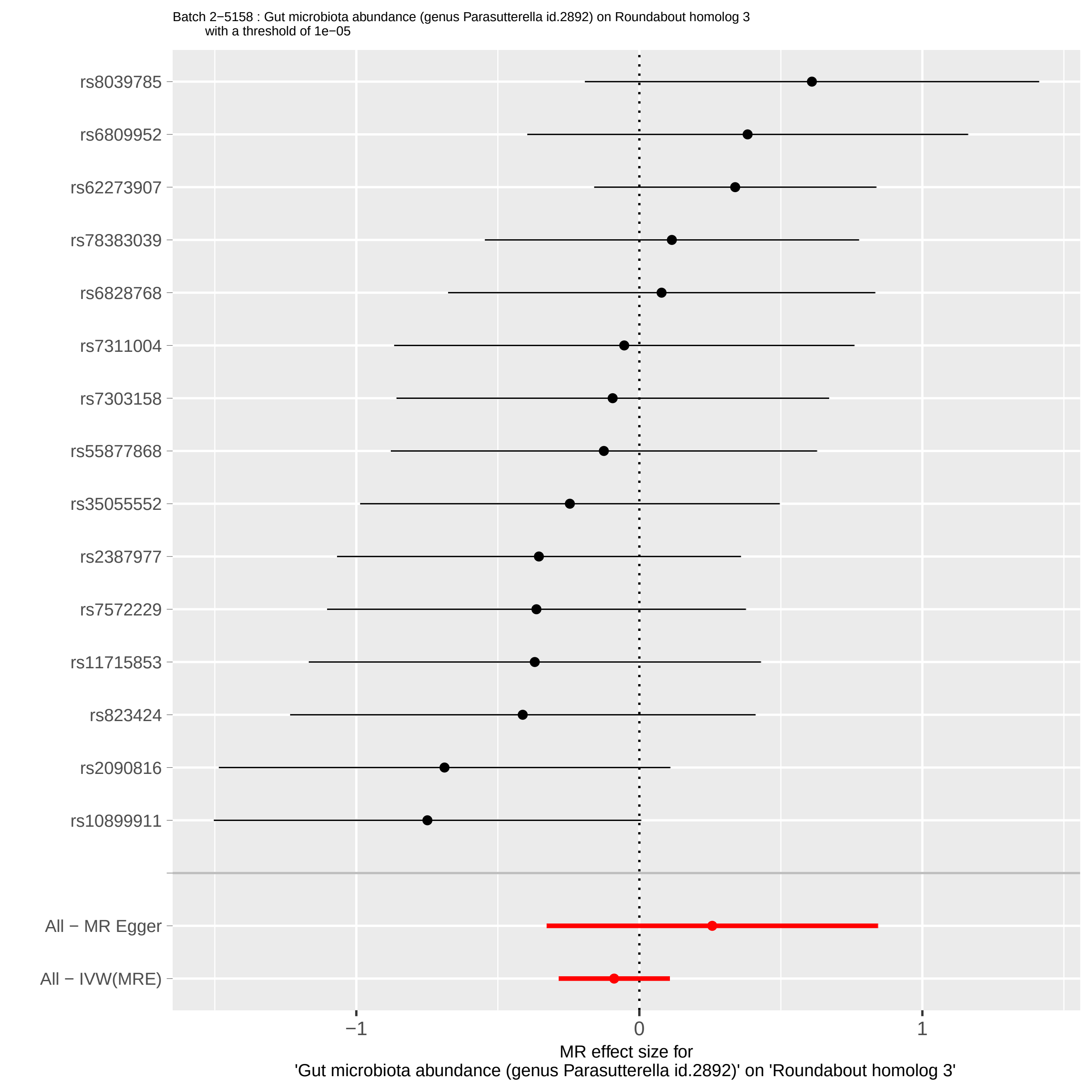

Batch 2−5158 : Gut microbiota abundance (genus Parasutterella id.2892) on Roundabout homolog 3
with a threshold of 1e−05
rs8039785
rs6809952
rs62273907
rs78383039
rs6828768
rs7311004
rs7303158
rs55877868
rs35055552
rs2387977
rs7572229
rs11715853
rs823424
rs2090816
rs10899911
All − MR Egger
All − IVW(MRE)
−1
0
1
MR effect size for
'Gut microbiota abundance (genus Parasutterella id.2892)' on 'Roundabout homolog 3'

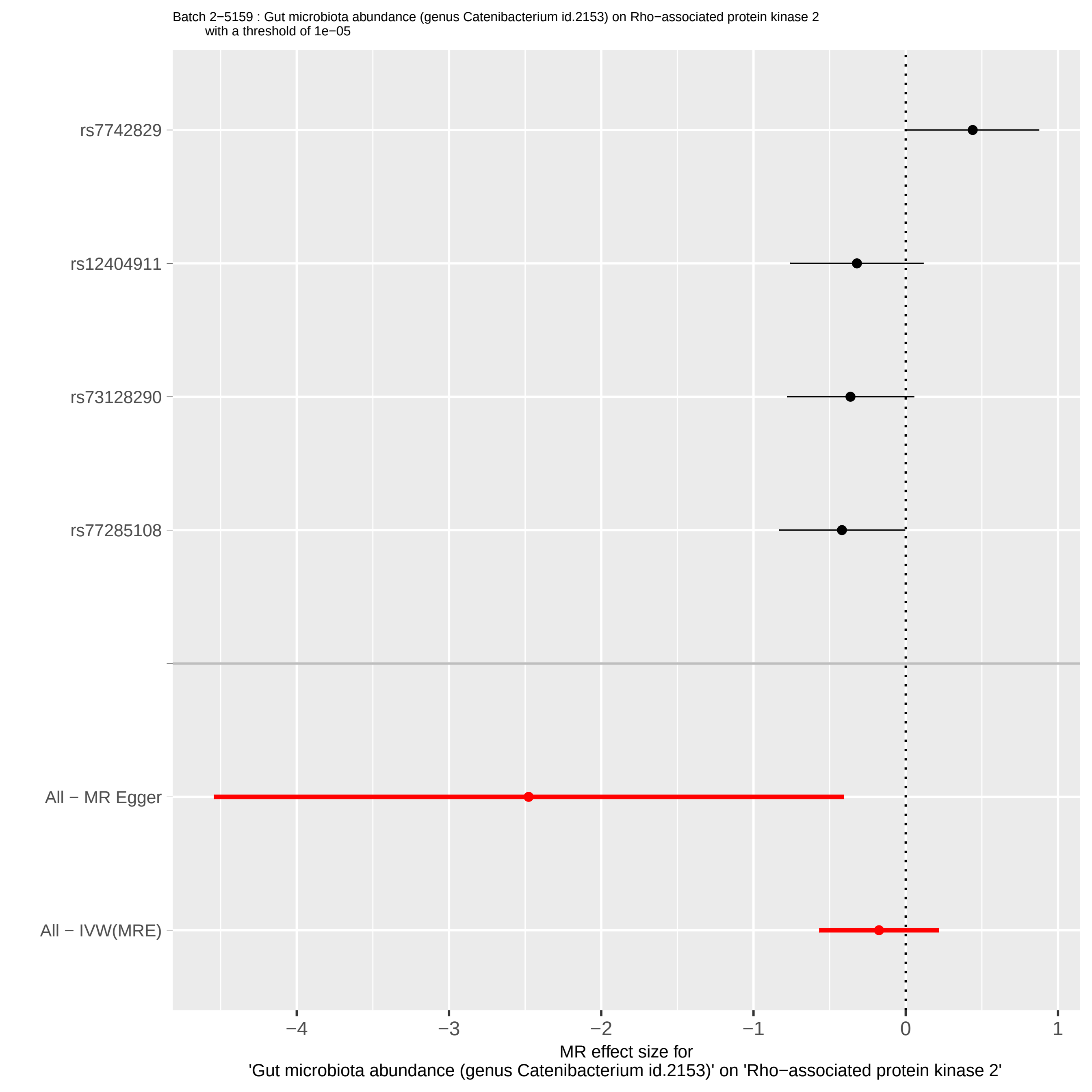

Batch 2−5159 : Gut microbiota abundance (genus Catenibacterium id.2153) on Rho−associated protein kinase 2
with a threshold of 1e−05
rs7742829
rs12404911
rs73128290
rs77285108
All − MR Egger
All − IVW(MRE)
−4
−3
−2
−1
0
1
MR effect size for
'Gut microbiota abundance (genus Catenibacterium id.2153)' on 'Rho−associated protein kinase 2'

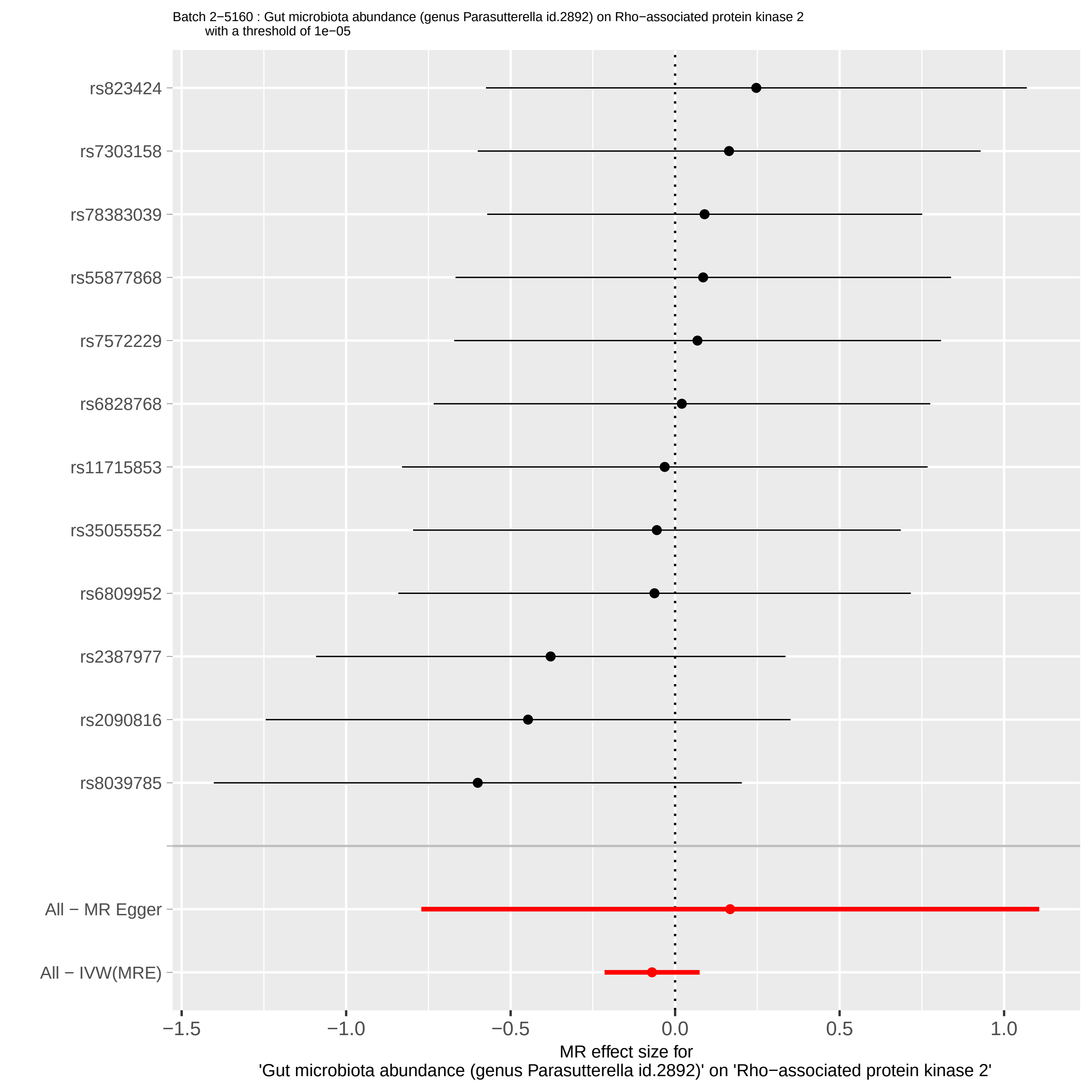

Batch 2−5160 : Gut microbiota abundance (genus Parasutterella id.2892) on Rho−associated protein kinase 2
with a threshold of 1e−05
rs823424
rs7303158
rs78383039
rs55877868
rs7572229
rs6828768
rs11715853
rs35055552
rs6809952
rs2387977
rs2090816
rs8039785
All − MR Egger
All − IVW(MRE)
−1.5
−1.0
−0.5
0.0
0.5
1.0
MR effect size for
'Gut microbiota abundance (genus Parasutterella id.2892)' on 'Rho−associated protein kinase 2'

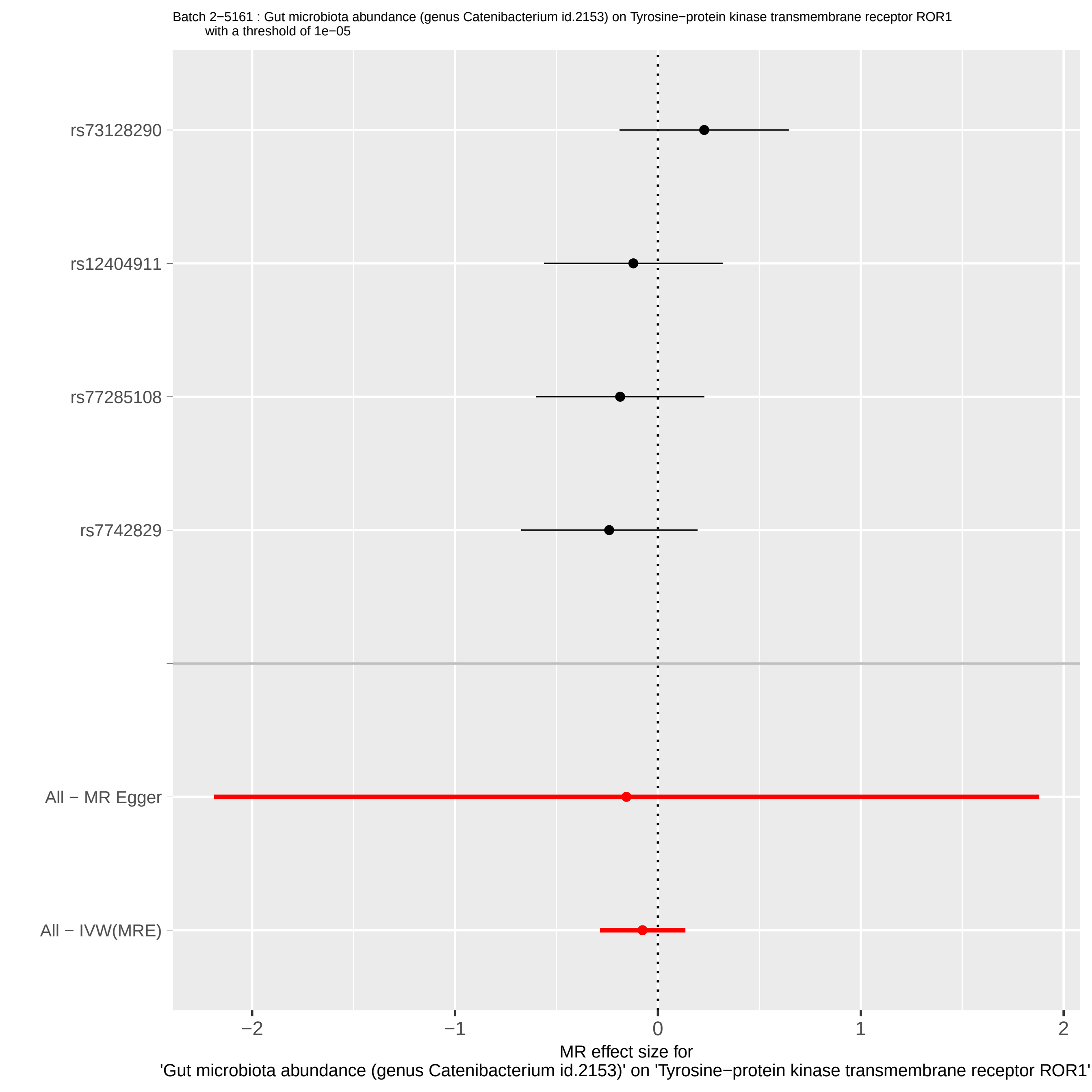

Batch 2−5161 : Gut microbiota abundance (genus Catenibacterium id.2153) on Tyrosine−protein kinase transmembrane receptor ROR1
with a threshold of 1e−05
rs73128290
rs12404911
rs77285108
rs7742829
All − MR Egger
All − IVW(MRE)
−2
−1
0
1
2
MR effect size for
'Gut microbiota abundance (genus Catenibacterium id.2153)' on 'Tyrosine−protein kinase transmembrane receptor ROR1'

Batch 2−5162 : Gut microbiota abundance (genus Parasutterella id.2892) on Tyrosine−protein kinase transmembrane receptor ROR1
with a threshold of 1e−05

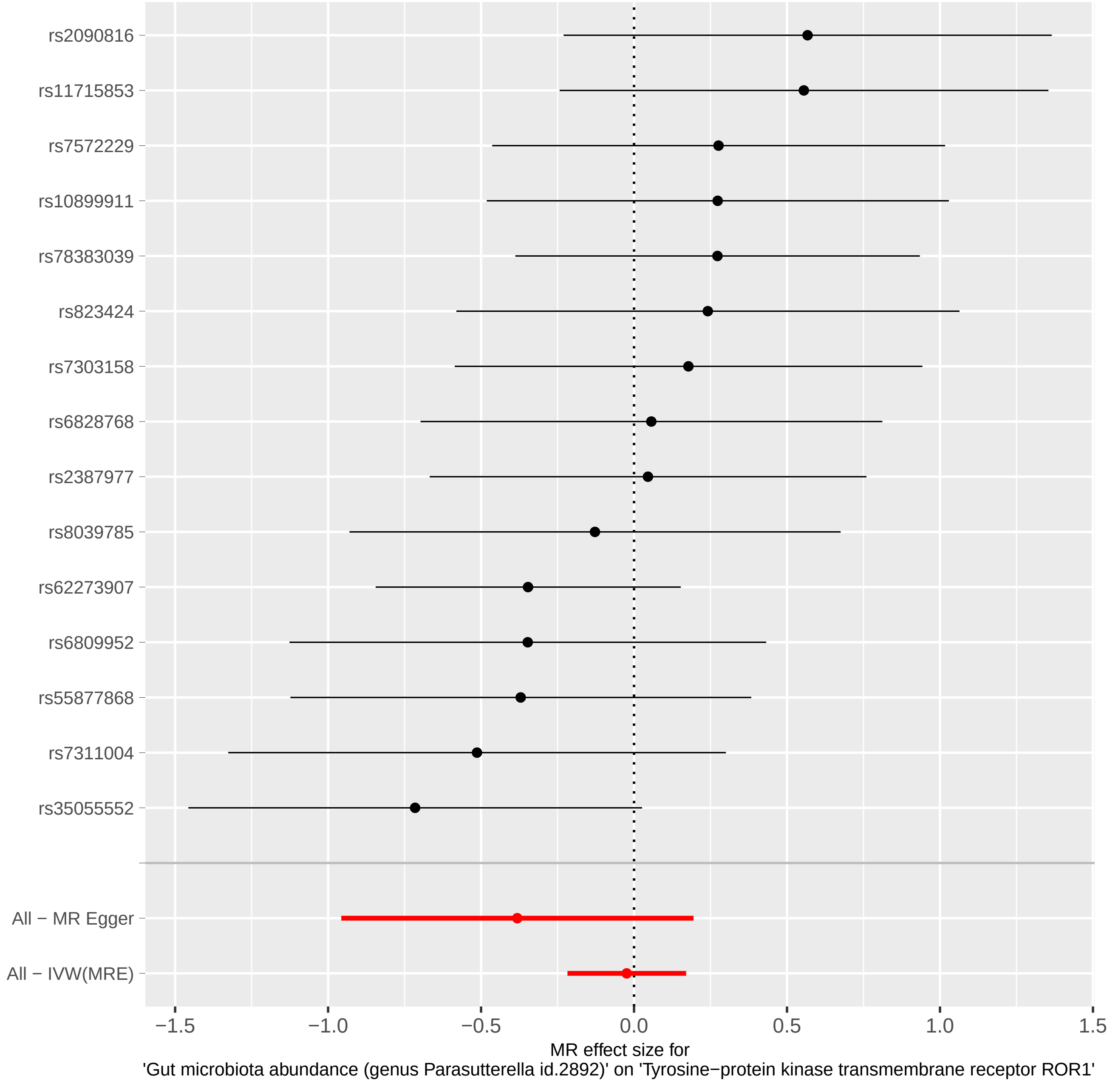

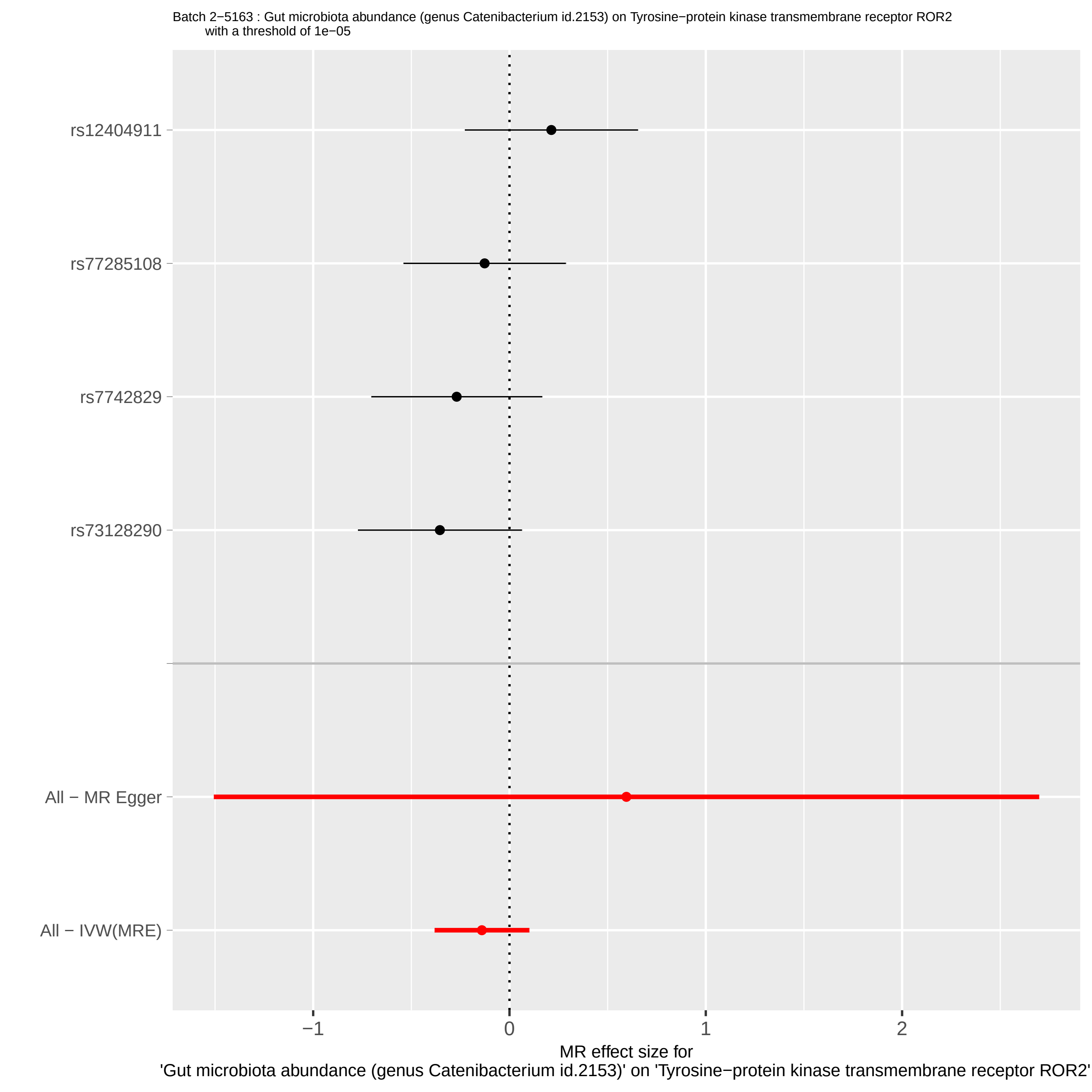
Batch 2−5163 : Gut microbiota abundance (genus Catenibacterium id.2153) on Tyrosine−protein kinase transmembrane receptor ROR2 with a threshold of 1e−05
rs12404911
rs77285108
rs7742829
rs73128290
All − MR Egger
All − IVW(MRE)
−1
0
1
2
MR effect size for
'Gut microbiota abundance (genus Catenibacterium id.2153)' on 'Tyrosine−protein kinase transmembrane receptor ROR2'

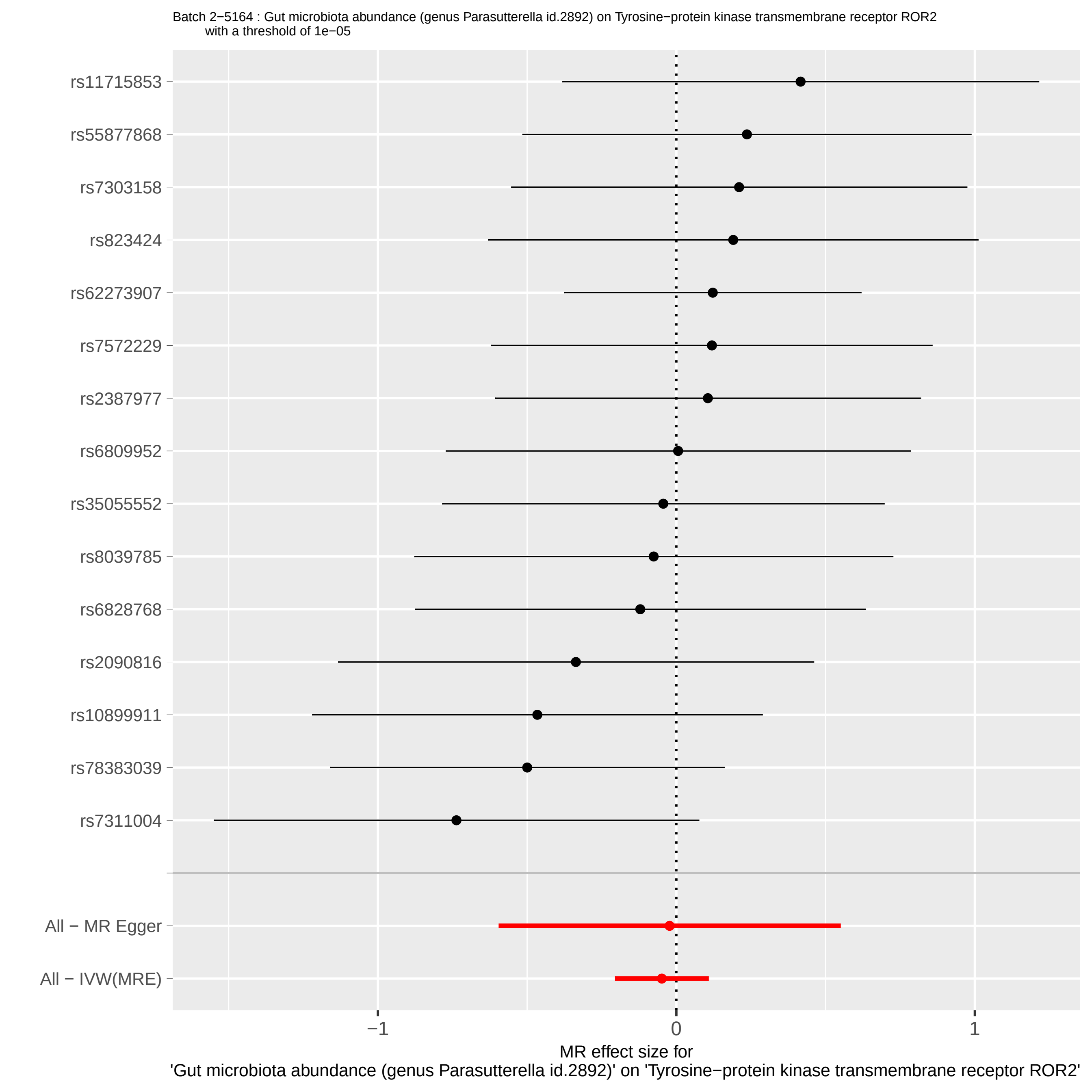

Batch 2−5164 : Gut microbiota abundance (genus Parasutterella id.2892) on Tyrosine−protein kinase transmembrane receptor ROR2
with a threshold of 1e−05
rs11715853
rs55877868
rs7303158
rs823424
rs62273907
rs7572229
rs2387977
rs6809952
rs35055552
rs8039785
rs6828768
rs2090816
rs10899911
rs78383039
rs7311004
All − MR Egger
All − IVW(MRE)
−1
0
1
MR effect size for
'Gut microbiota abundance (genus Parasutterella id.2892)' on 'Tyrosine−protein kinase transmembrane receptor ROR2'

Batch 2−5165 : Gut microbiota abundance (genus Catenibacterium id.2153) on Nuclear receptor ROR−beta
with a threshold of 1e−05

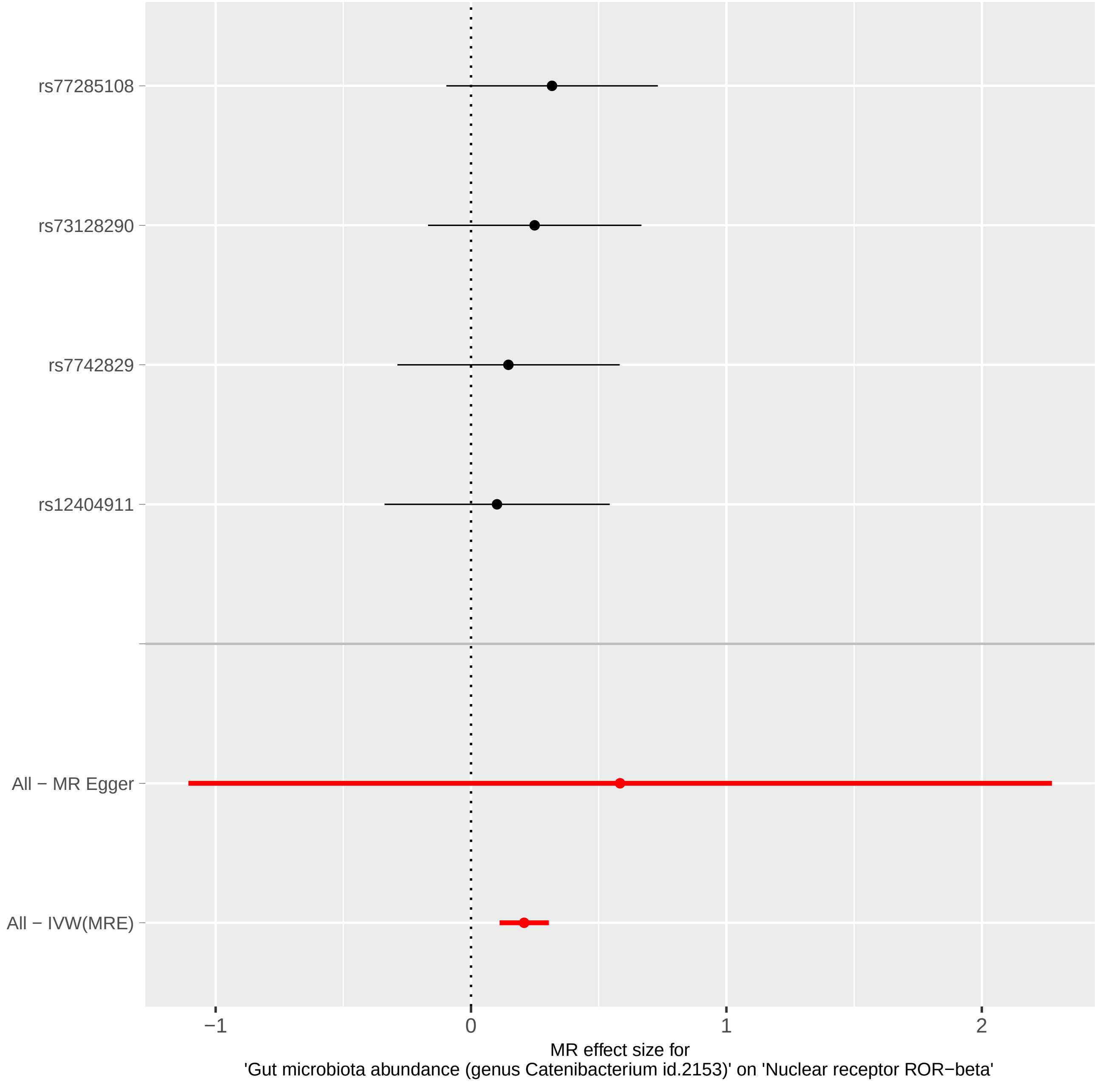

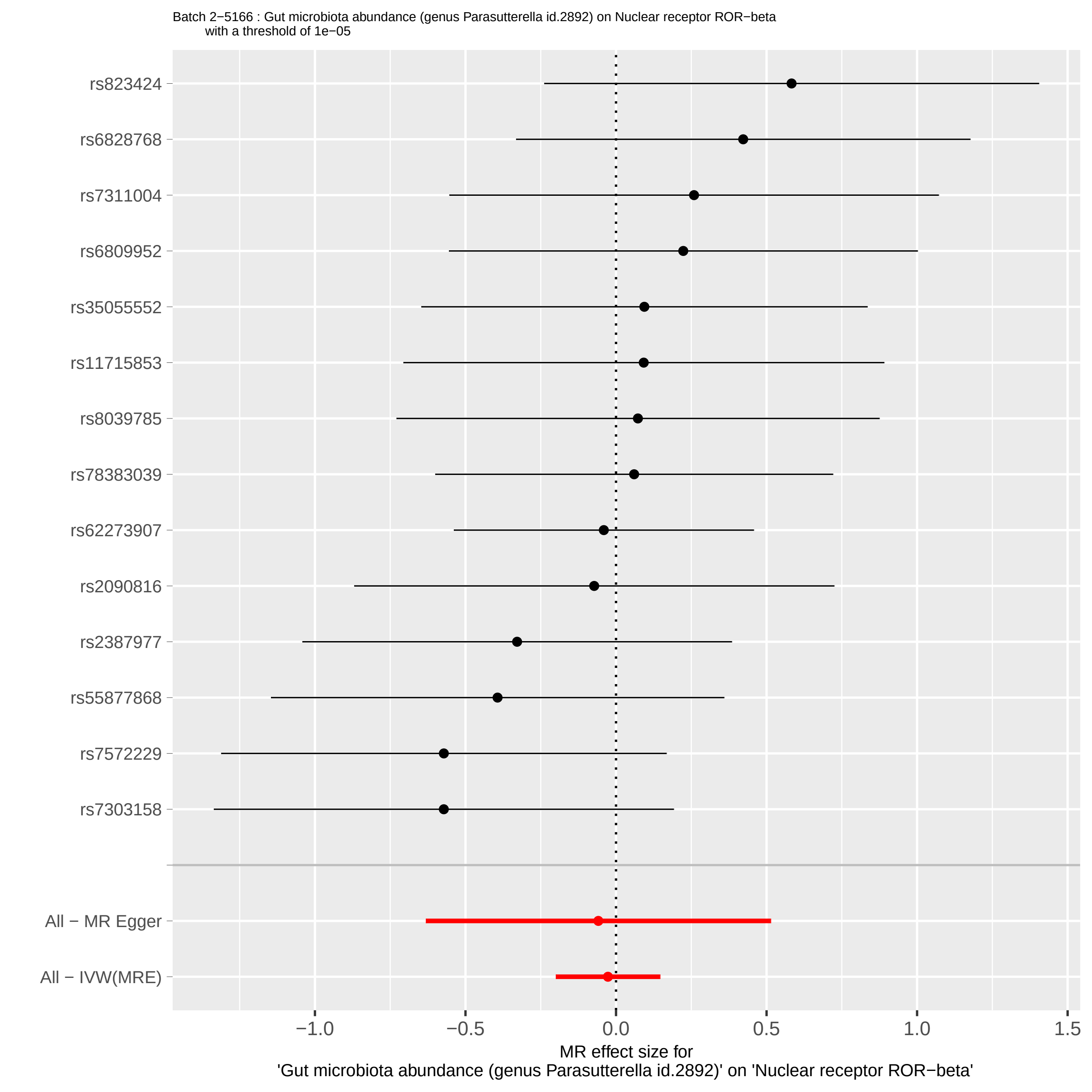

Batch 2−5166 : Gut microbiota abundance (genus Parasutterella id.2892) on Nuclear receptor ROR−beta with a threshold of 1e−05
rs823424
rs6828768
rs7311004
rs6809952
rs35055552
rs11715853
rs8039785
rs78383039
rs62273907
rs2090816
rs2387977
rs55877868
rs7572229
rs7303158
All − MR Egger
All − IVW(MRE)
−1.0
−0.5
0.0
0.5
1.0
1.5
MR effect size for
'Gut microbiota abundance (genus Parasutterella id.2892)' on 'Nuclear receptor ROR−beta'

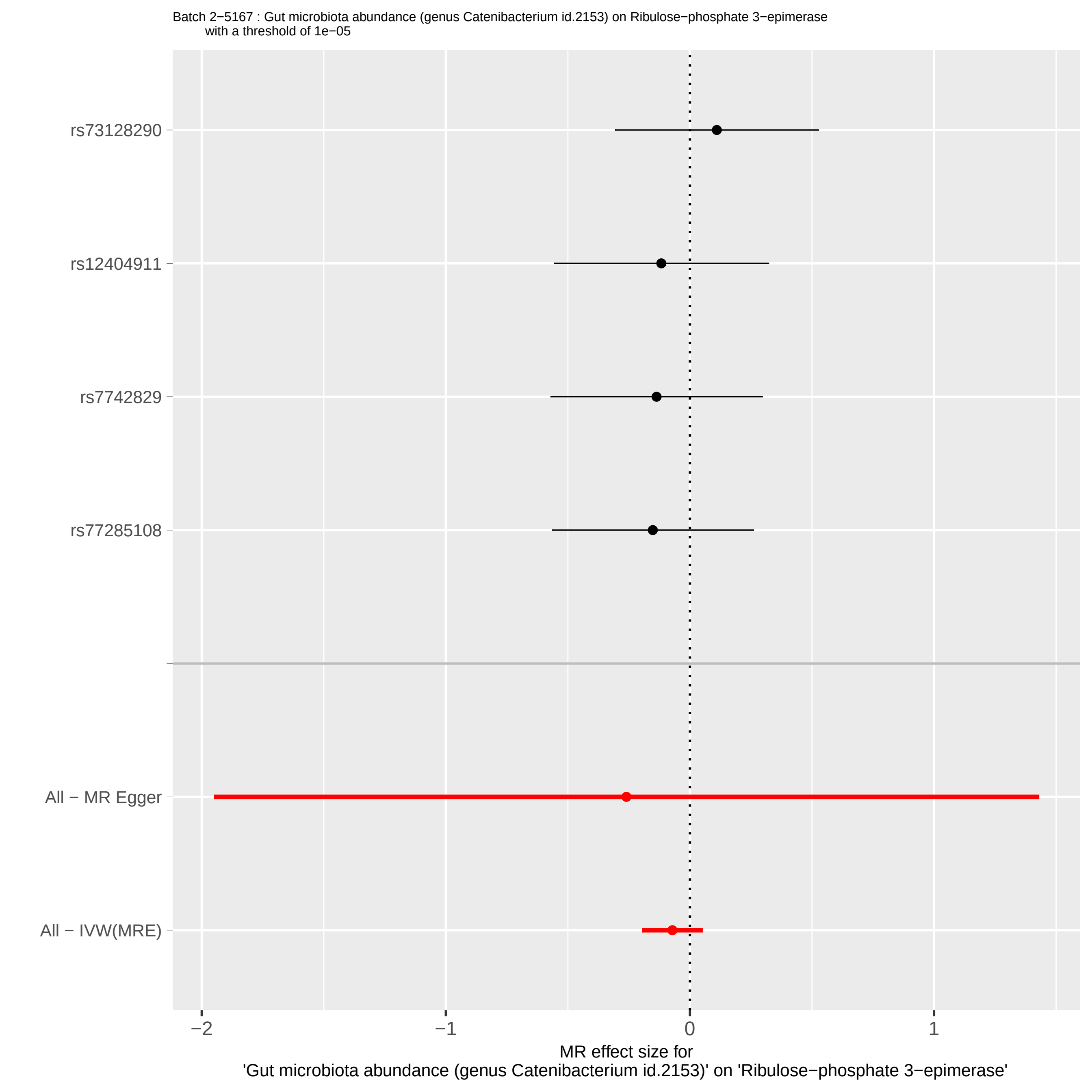
Batch 2−5167 : Gut microbiota abundance (genus Catenibacterium id.2153) on Ribulose−phosphate 3−epimerase
with a threshold of 1e−05
rs73128290
rs12404911
rs7742829
rs77285108
All − MR Egger
All − IVW(MRE)
−2
−1
0
1
MR effect size for
'Gut microbiota abundance (genus Catenibacterium id.2153)' on 'Ribulose−phosphate 3−epimerase'

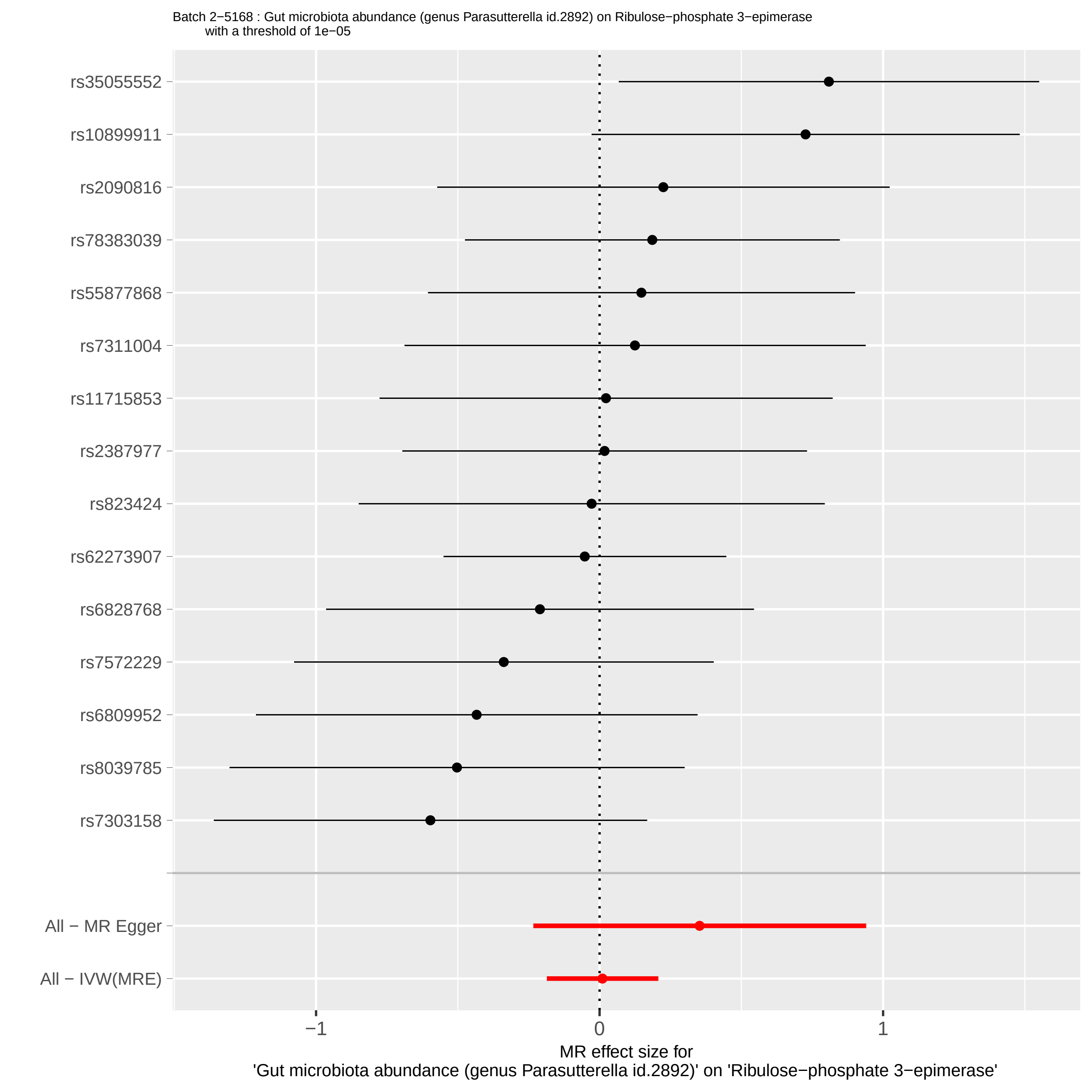
Batch 2−5168 : Gut microbiota abundance (genus Parasutterella id.2892) on Ribulose−phosphate 3−epimerase
with a threshold of 1e−05
rs35055552
rs10899911
rs2090816
rs78383039
rs55877868
rs7311004
rs11715853
rs2387977
rs823424
rs62273907
rs6828768
rs7572229
rs6809952
rs8039785
rs7303158
All − MR Egger
All − IVW(MRE)
−1
0
1
MR effect size for
'Gut microbiota abundance (genus Parasutterella id.2892)' on 'Ribulose−phosphate 3−epimerase'

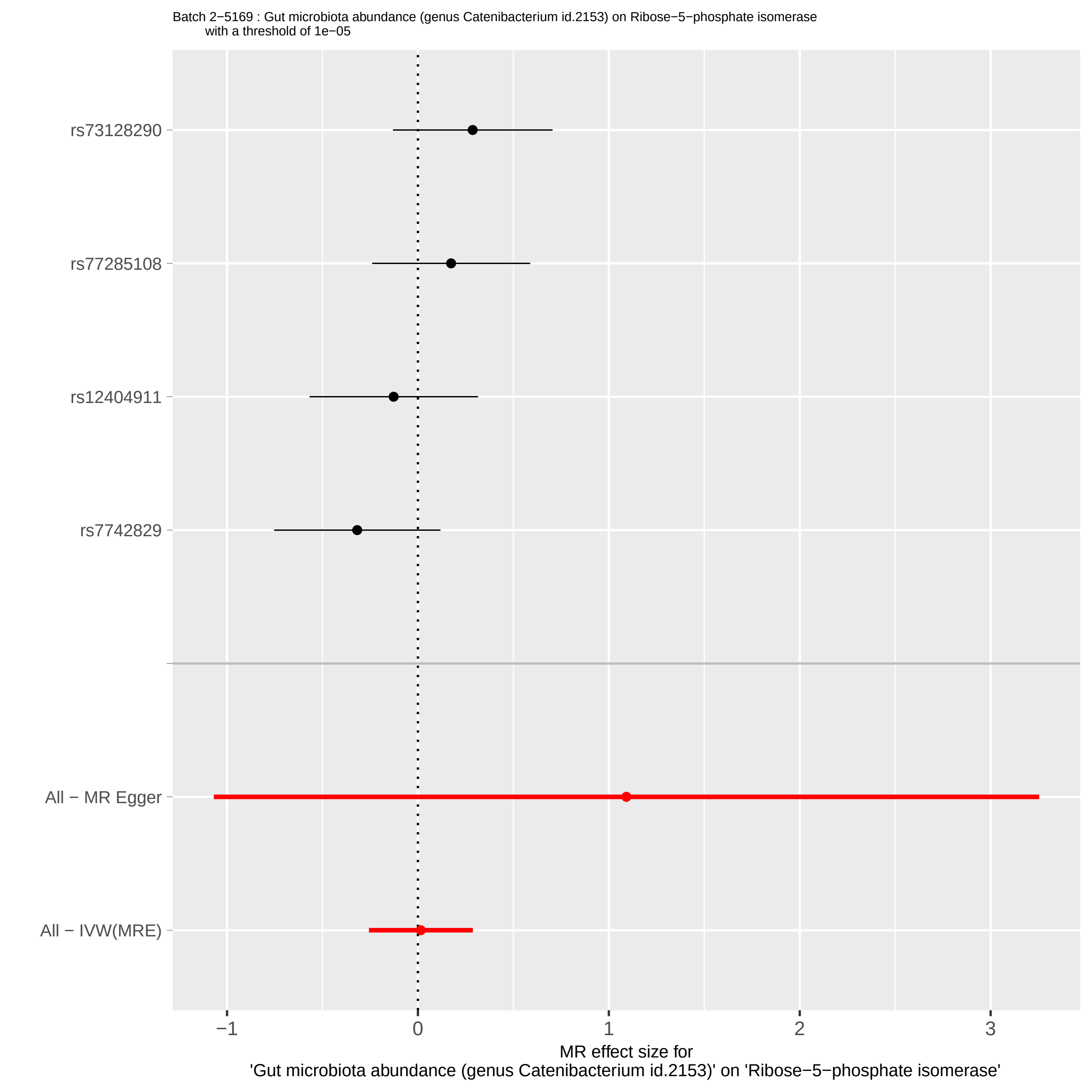

Batch 2−5169 : Gut microbiota abundance (genus Catenibacterium id.2153) on Ribose−5−phosphate isomerase
with a threshold of 1e−05
rs73128290
rs77285108
rs12404911
rs7742829
All − MR Egger
All − IVW(MRE)
−1
0
1
2
3
MR effect size for
'Gut microbiota abundance (genus Catenibacterium id.2153)' on 'Ribose−5−phosphate isomerase'

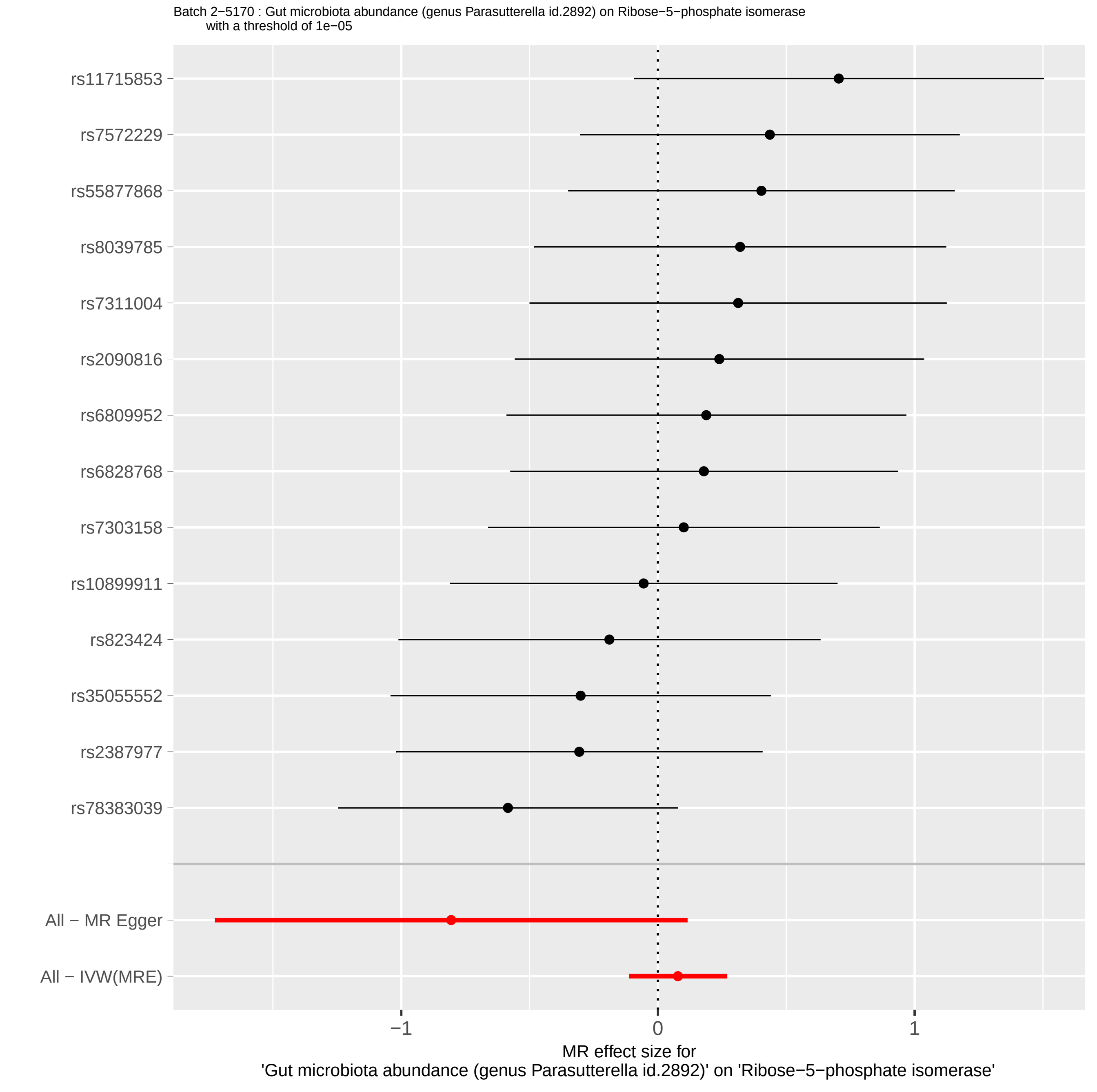

Batch 2−5170 : Gut microbiota abundance (genus Parasutterella id.2892) on Ribose−5−phosphate isomerase
with a threshold of 1e−05
rs11715853
rs7572229
rs55877868
rs8039785
rs7311004
rs2090816
rs6809952
rs6828768
rs7303158
rs10899911
rs823424
rs35055552
rs2387977
rs78383039
All − MR Egger
All − IVW(MRE)
−1
0
1
MR effect size for
'Gut microbiota abundance (genus Parasutterella id.2892)' on 'Ribose−5−phosphate isomerase'

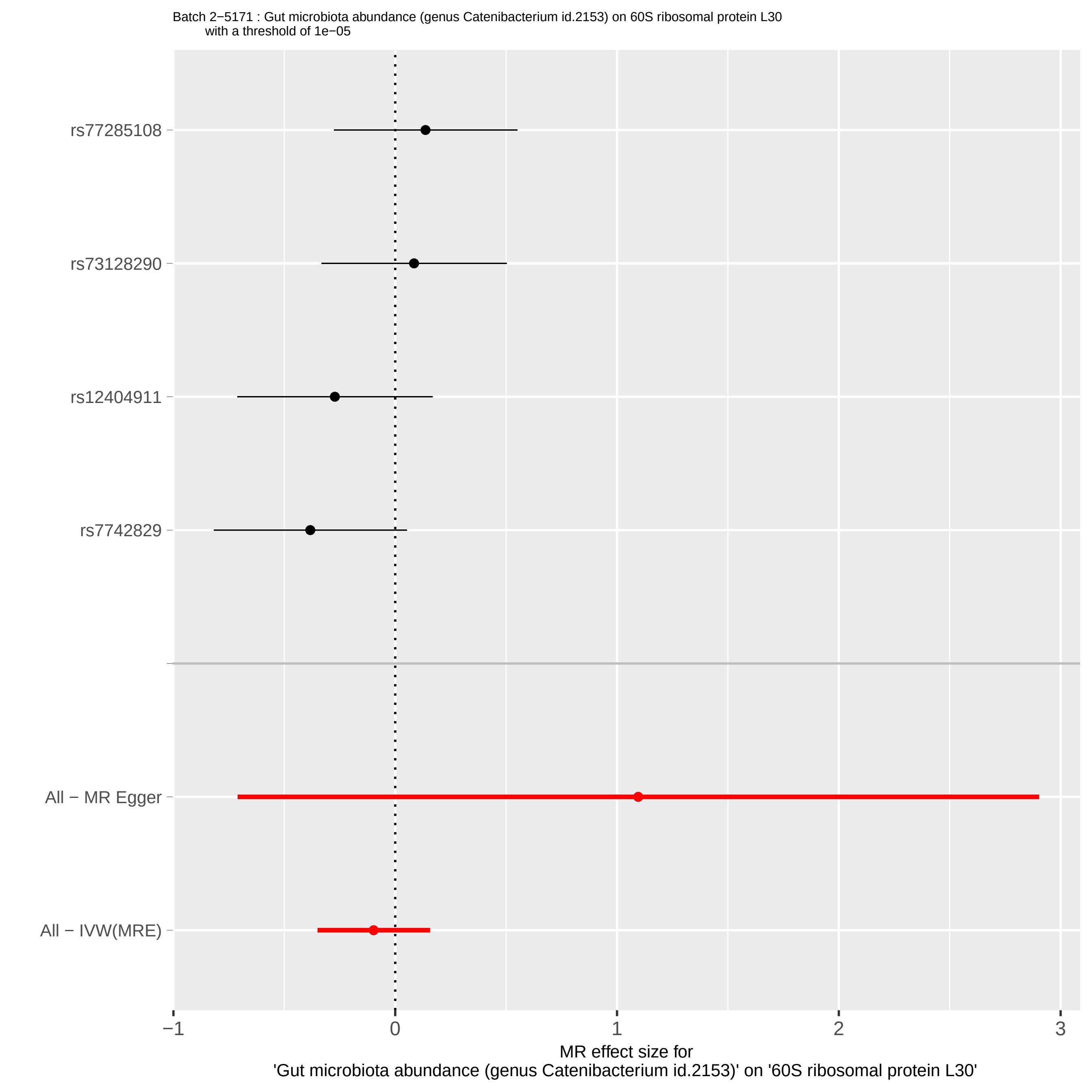

Batch 2−5171 : Gut microbiota abundance (genus Catenibacterium id.2153) on 60S ribosomal protein L30
with a threshold of 1e−05
rs77285108
rs73128290
rs12404911
rs7742829
All − MR Egger
All − IVW(MRE)
−1
0
1
2
3
MR effect size for
'Gut microbiota abundance (genus Catenibacterium id.2153)' on '60S ribosomal protein L30'

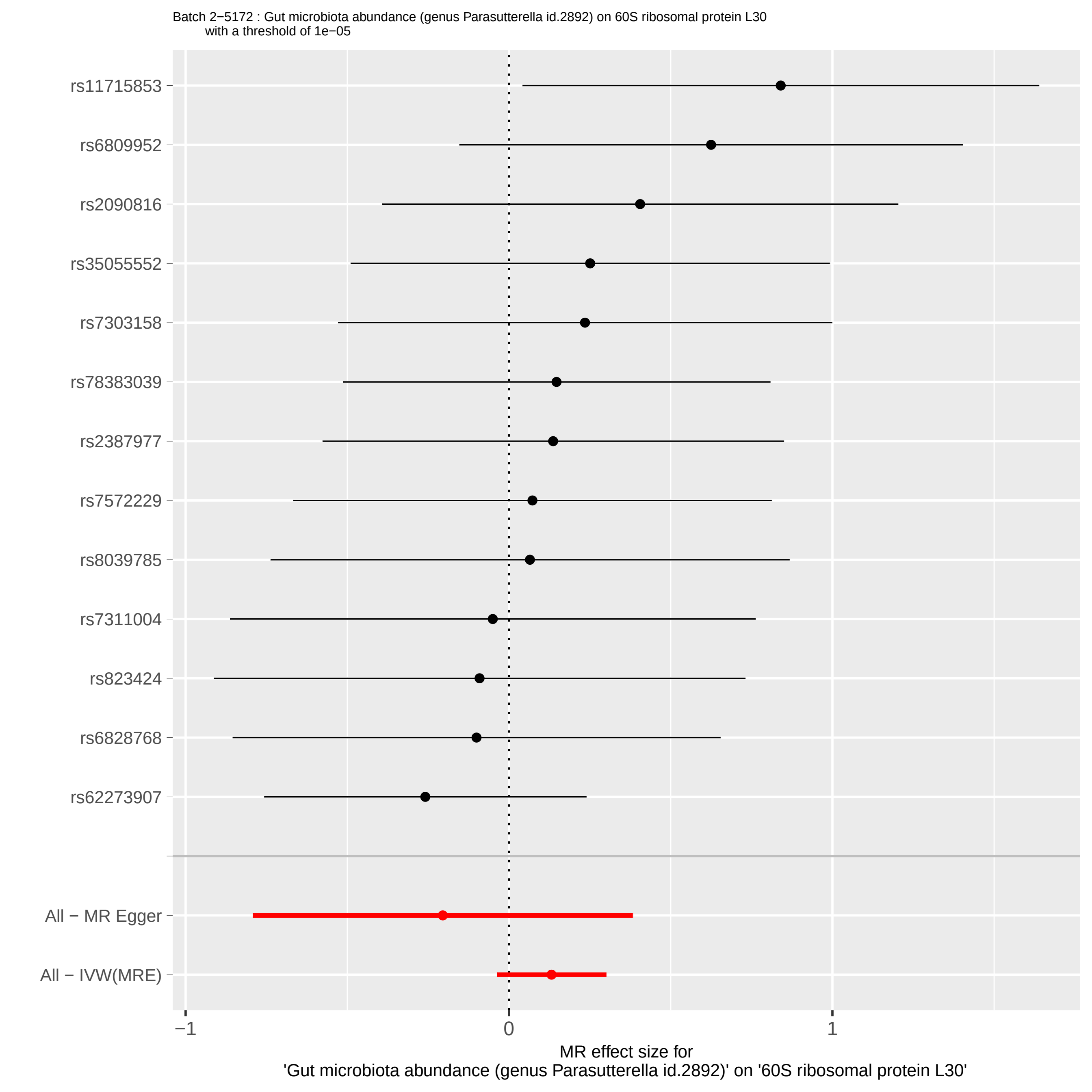

Batch 2−5172 : Gut microbiota abundance (genus Parasutterella id.2892) on 60S ribosomal protein L30
with a threshold of 1e−05
rs11715853
rs6809952
rs2090816
rs35055552
rs7303158
rs78383039
rs2387977
rs7572229
rs8039785
rs7311004
rs823424
rs6828768
rs62273907
All − MR Egger
All − IVW(MRE)
−1
0
1
MR effect size for
'Gut microbiota abundance (genus Parasutterella id.2892)' on '60S ribosomal protein L30'

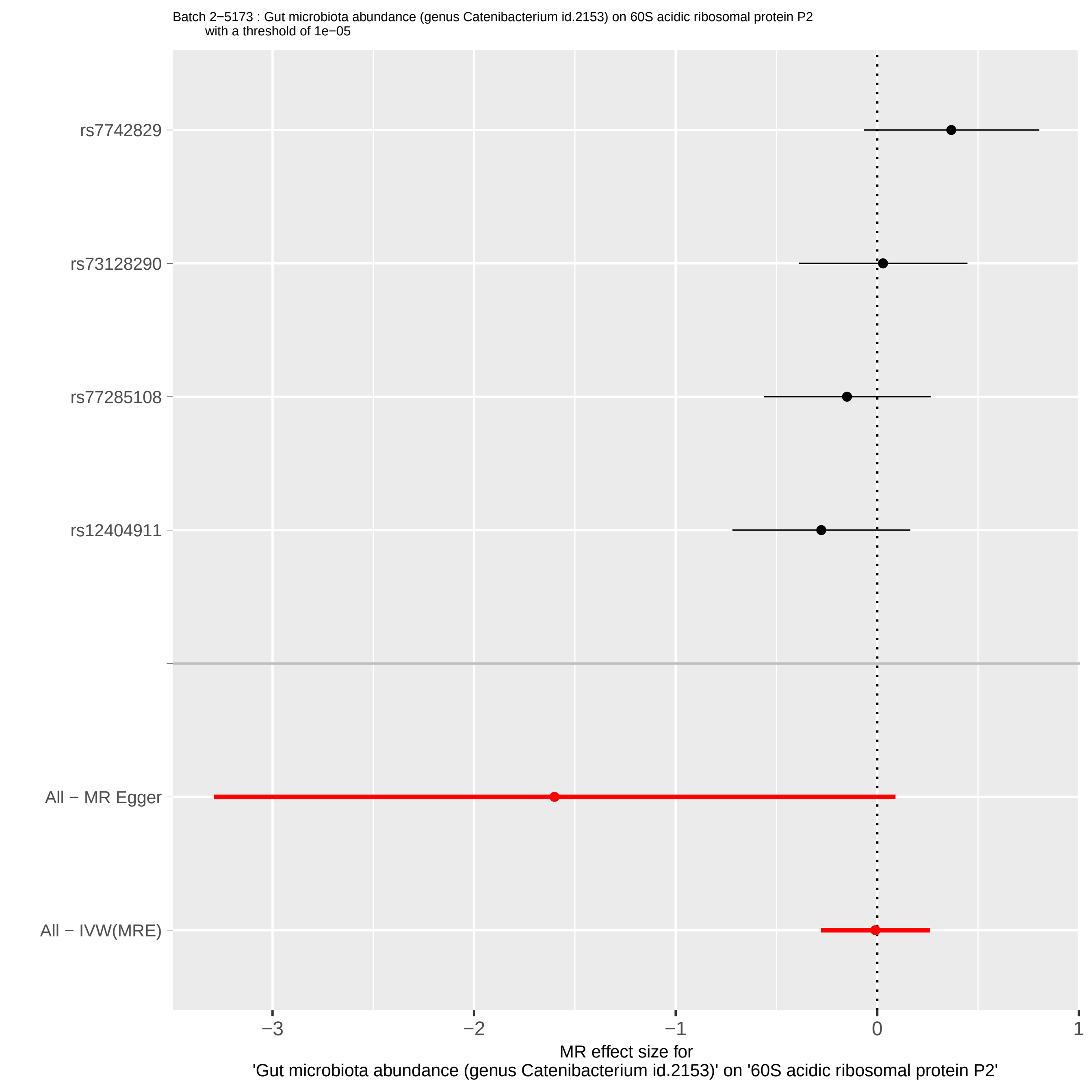
Batch 2−5173 : Gut microbiota abundance (genus Catenibacterium id.2153) on 60S acidic ribosomal protein P2
with a threshold of 1e−05
rs7742829
rs73128290
rs77285108
rs12404911
All − MR Egger
All − IVW(MRE)
−3
−2
−1
0
1
MR effect size for
'Gut microbiota abundance (genus Catenibacterium id.2153)' on '60S acidic ribosomal protein P2'

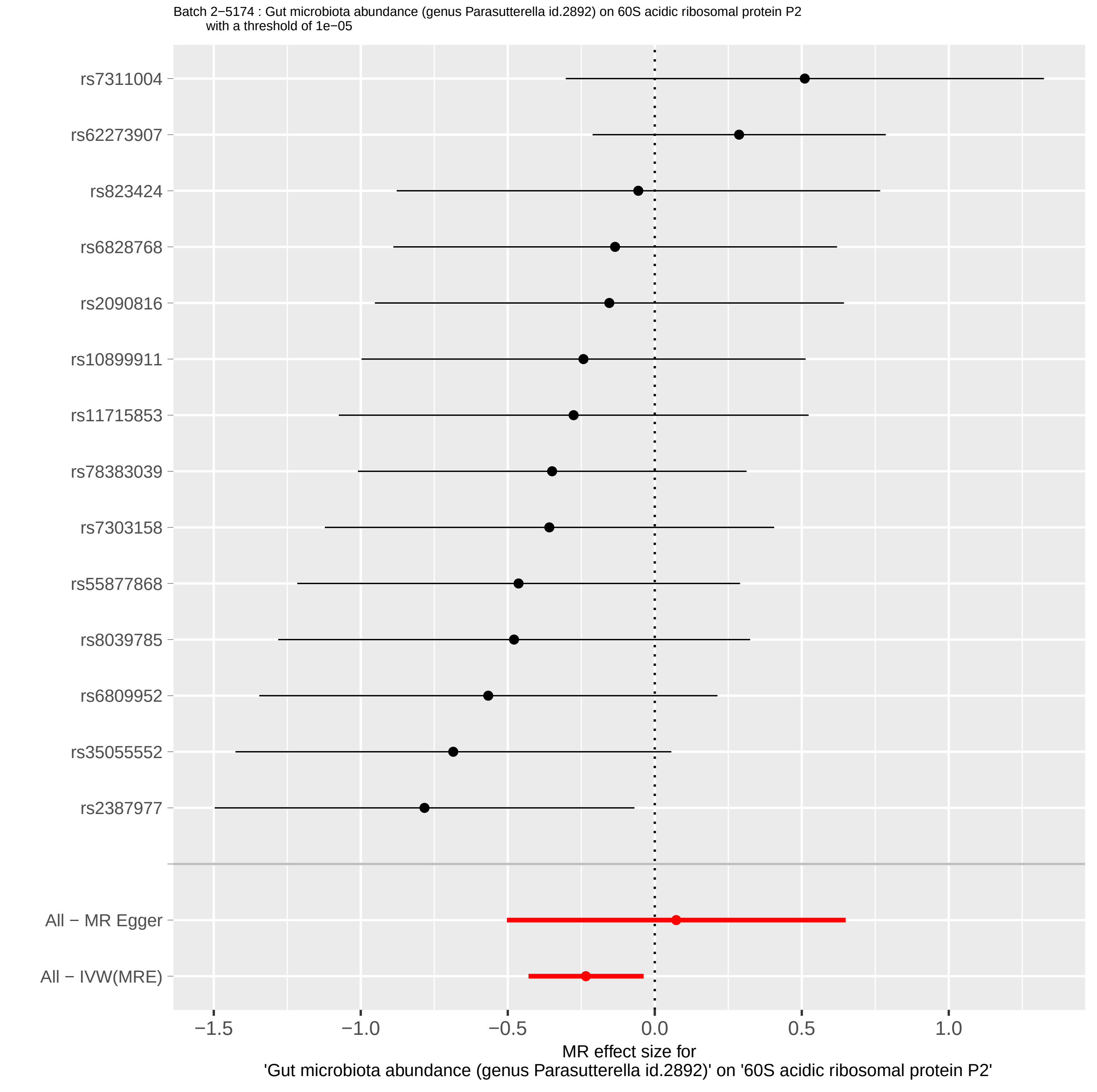

Batch 2−5174 : Gut microbiota abundance (genus Parasutterella id.2892) on 60S acidic ribosomal protein P2
with a threshold of 1e−05
rs7311004
rs62273907
rs823424
rs6828768
rs2090816
rs10899911
rs11715853
rs78383039
rs7303158
rs55877868
rs8039785
rs6809952
rs35055552
rs2387977
All − MR Egger
All − IVW(MRE)
−1.5
−1.0
−0.5
0.0
0.5
1.0
MR effect size for
'Gut microbiota abundance (genus Parasutterella id.2892)' on '60S acidic ribosomal protein P2'

Batch 2−5175 : Gut microbiota abundance (genus Catenibacterium id.2153) on Dolichyl−diphosphooligosaccharide−−protein glycosyltransferase subunit 1
with a threshold of 1e−05

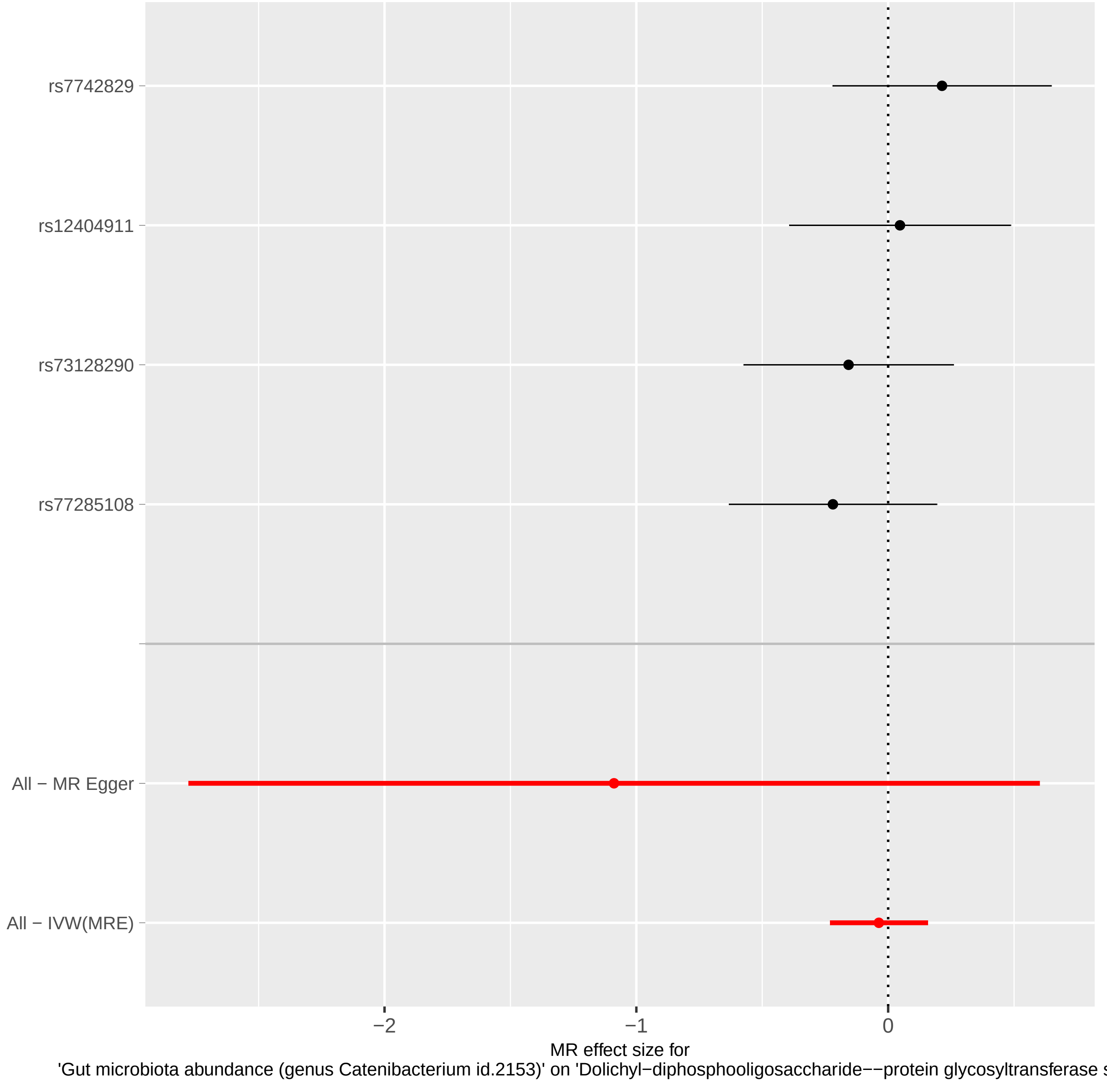

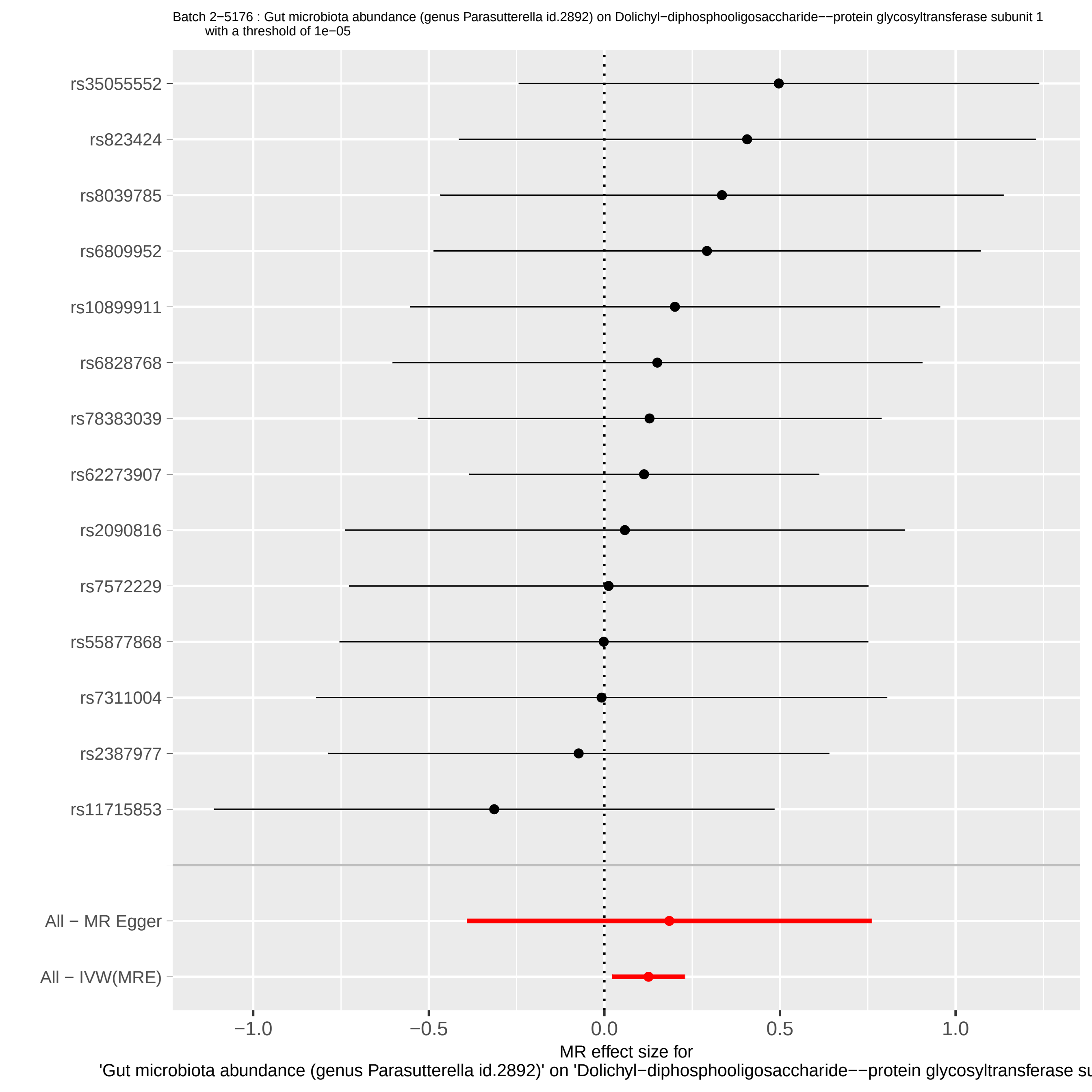

Batch 2−5176 : Gut microbiota abundance (genus Parasutterella id.2892) on Dolichyl−diphosphooligosaccharide−−protein glycosyltransferase subunit 1 with a threshold of 1e−05
rs35055552
rs823424
rs8039785
rs6809952
rs10899911
rs6828768
rs78383039
rs62273907
rs2090816
rs7572229
rs55877868
rs7311004
rs2387977
rs11715853
All − MR Egger
All − IVW(MRE)
−1.0
−0.5
0.0
0.5
1.0
MR effect size for
'Gut microbiota abundance (genus Parasutterella id.2892)' on 'Dolichyl−diphosphooligosaccharide−−protein glycosyltransferase su

Batch 2−5177 : Gut microbiota abundance (genus Catenibacterium id.2153) on Dolichyl−diphosphooligosaccharide−−protein glycosyltransferase subunit 1 with a threshold of 1e−05

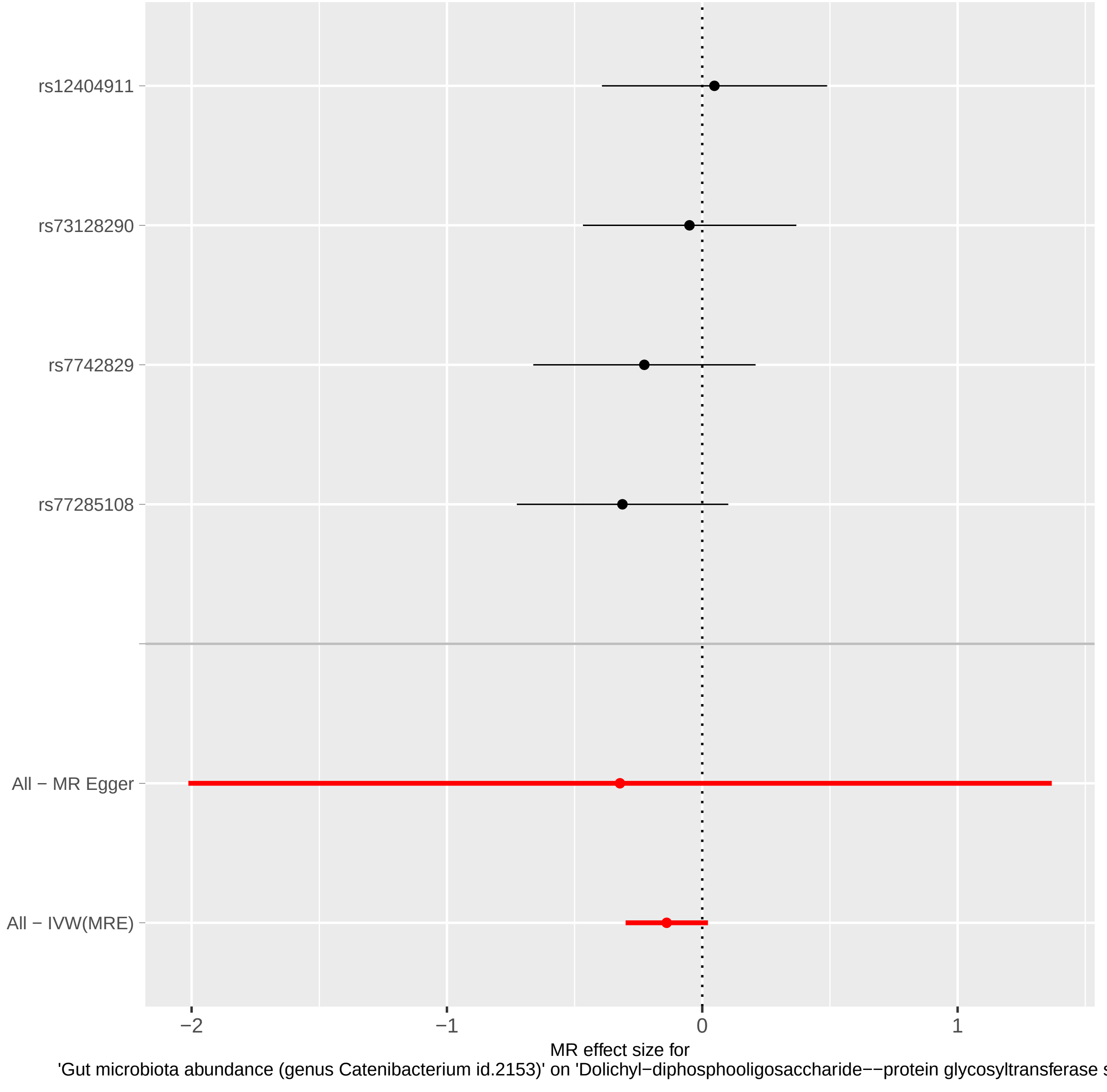

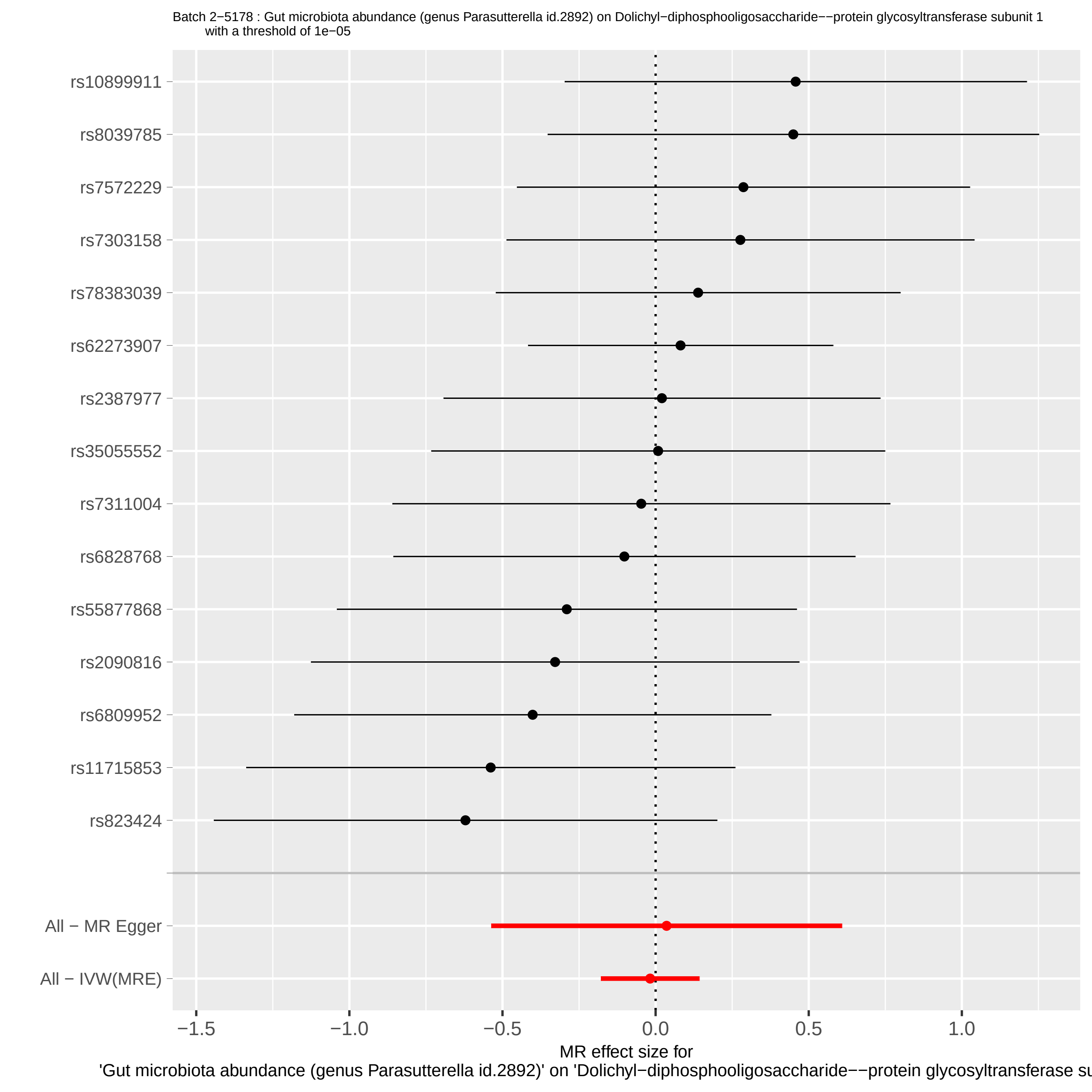

Batch 2−5178 : Gut microbiota abundance (genus Parasutterella id.2892) on Dolichyl−diphosphooligosaccharide−−protein glycosyltransferase subunit 1
with a threshold of 1e−05
rs10899911
rs8039785
rs7572229
rs7303158
rs78383039
rs62273907
rs2387977
rs35055552
rs7311004
rs6828768
rs55877868
rs2090816
rs6809952
rs11715853
rs823424
All − MR Egger
All − IVW(MRE)
−1.5
−1.0
−0.5
0.0
0.5
1.0
MR effect size for
'Gut microbiota abundance (genus Parasutterella id.2892)' on 'Dolichyl−diphosphooligosaccharide−−protein glycosyltransferase su

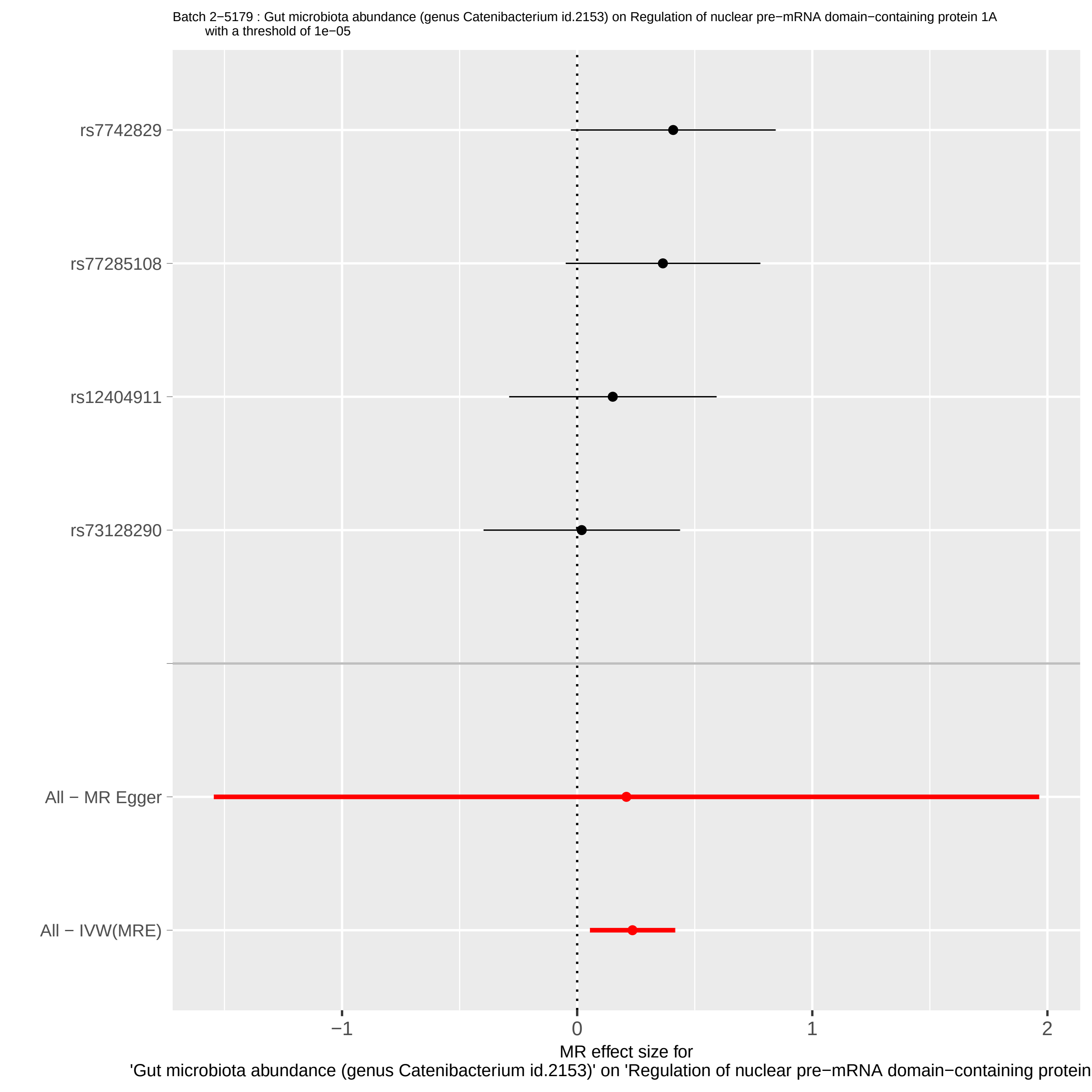
Batch 2−5179 : Gut microbiota abundance (genus Catenibacterium id.2153) on Regulation of nuclear pre−mRNA domain−containing protein 1A
with a threshold of 1e−05
rs7742829
rs77285108
rs12404911
rs73128290
All − MR Egger
All − IVW(MRE)
−1
0
1
2
MR effect size for
'Gut microbiota abundance (genus Catenibacterium id.2153)' on 'Regulation of nuclear pre−mRNA domain−containing protein

Batch 2−5180 : Gut microbiota abundance (genus Parasutterella id.2892) on Regulation of nuclear pre−mRNA domain−containing protein 1A
with a threshold of 1e−05

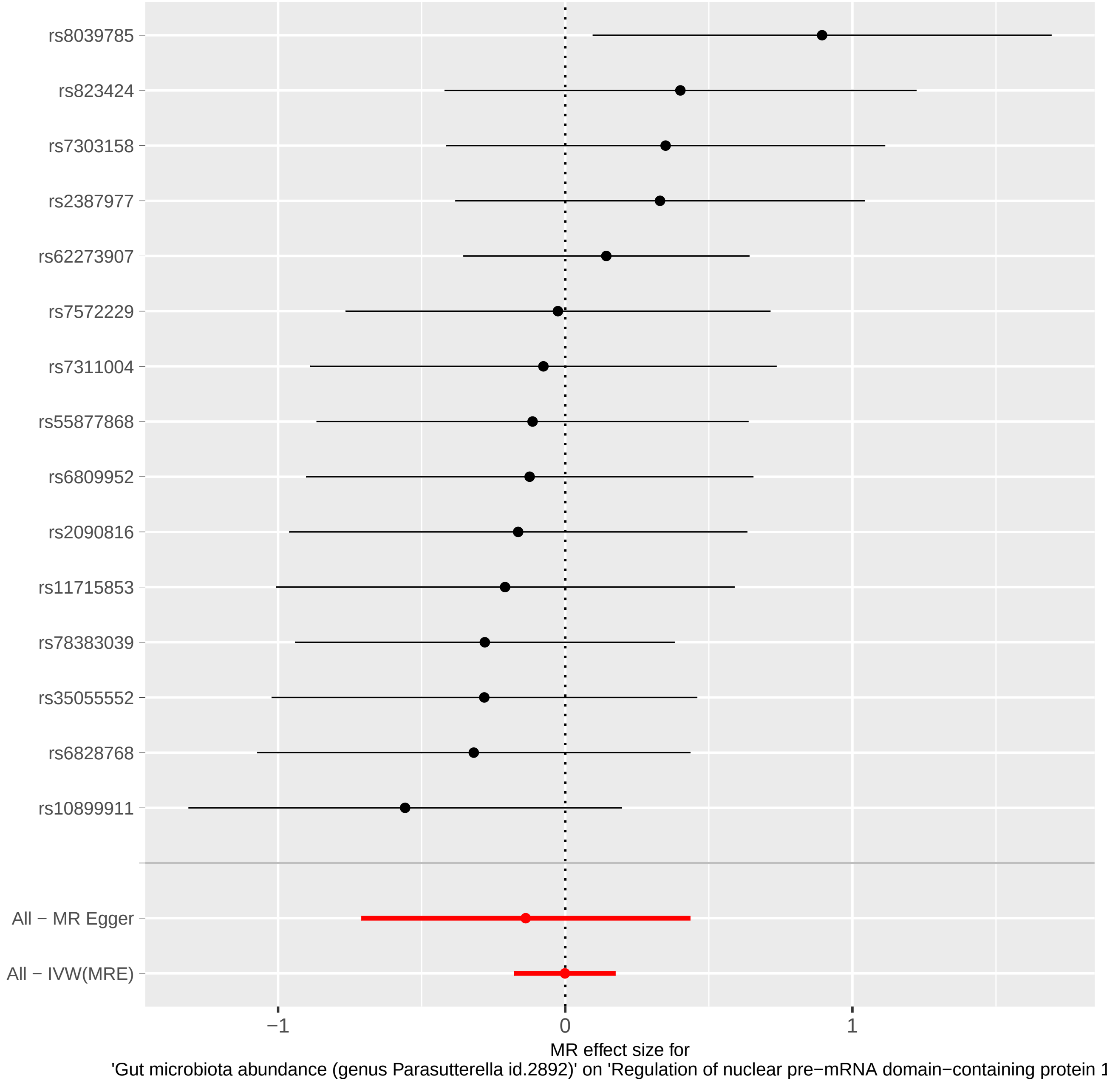

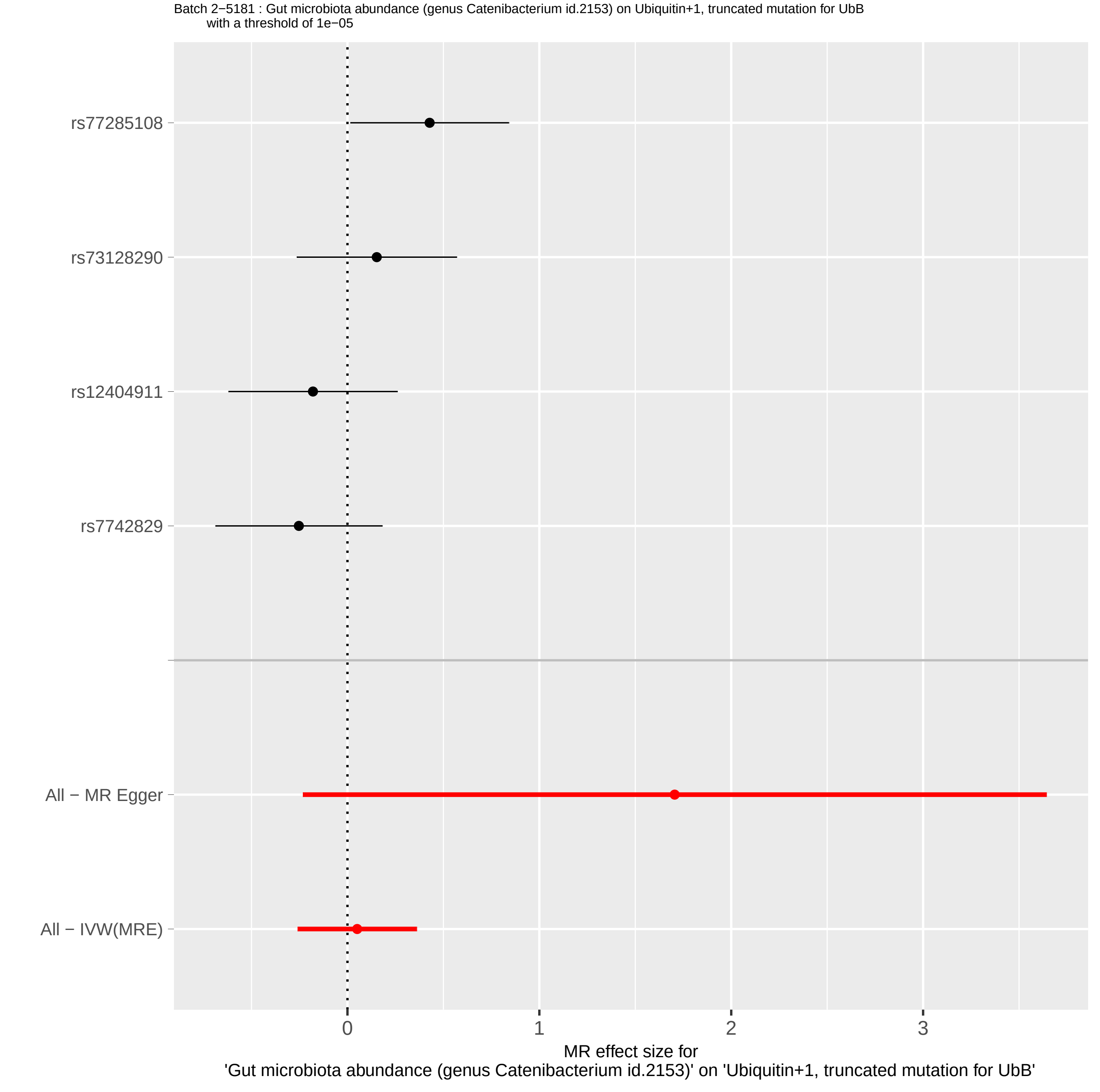

Batch 2−5181 : Gut microbiota abundance (genus Catenibacterium id.2153) on Ubiquitin+1, truncated mutation for UbB
with a threshold of 1e−05
rs77285108
rs73128290
rs12404911
rs7742829
All − MR Egger
All − IVW(MRE)
0
1
2
3
MR effect size for
'Gut microbiota abundance (genus Catenibacterium id.2153)' on 'Ubiquitin+1, truncated mutation for UbB'

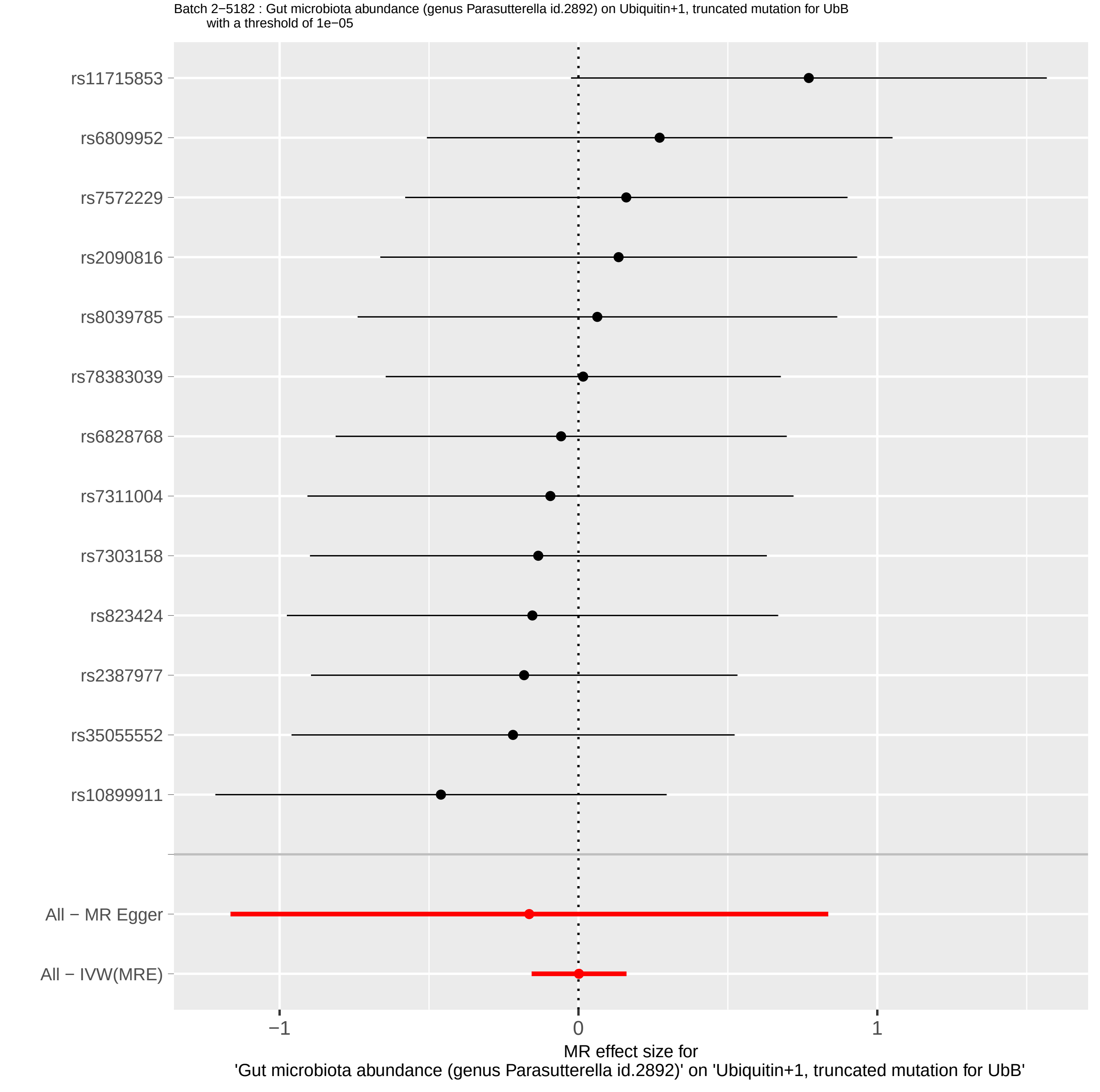
Batch 2−5182 : Gut microbiota abundance (genus Parasutterella id.2892) on Ubiquitin+1, truncated mutation for UbB
with a threshold of 1e−05
rs11715853
rs6809952
rs7572229
rs2090816
rs8039785
rs78383039
rs6828768
rs7311004
rs7303158
rs823424
rs2387977
rs35055552
rs10899911
All − MR Egger
All − IVW(MRE)
−1
0
1
MR effect size for
'Gut microbiota abundance (genus Parasutterella id.2892)' on 'Ubiquitin+1, truncated mutation for UbB'

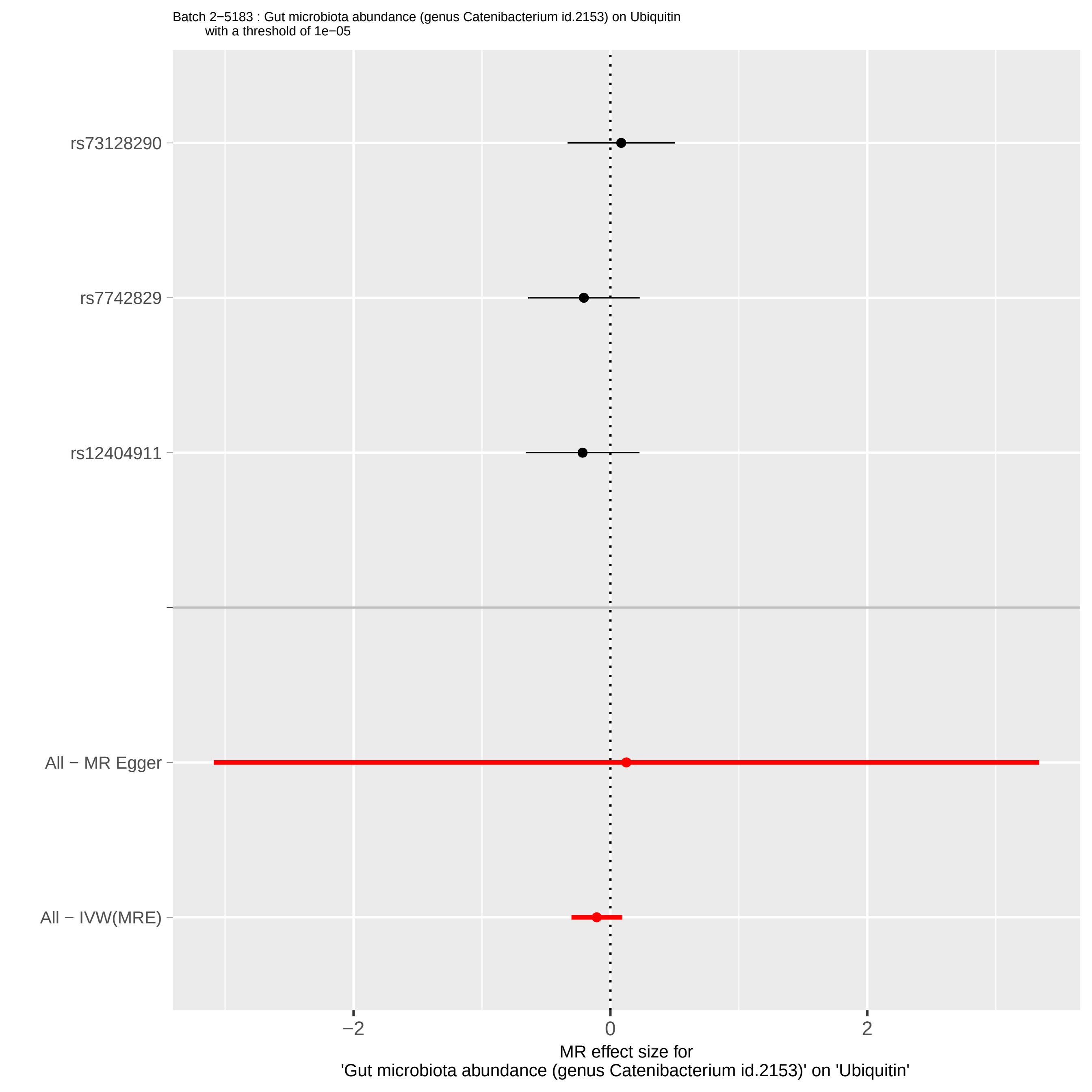

Batch 2−5183 : Gut microbiota abundance (genus Catenibacterium id.2153) on Ubiquitin
with a threshold of 1e−05
rs73128290
rs7742829
rs12404911
All − MR Egger
All − IVW(MRE)
−2
0
2
MR effect size for
'Gut microbiota abundance (genus Catenibacterium id.2153)' on 'Ubiquitin'

Batch 2−5184 : Gut microbiota abundance (genus Parasutterella id.2892) on Ubiquitin
with a threshold of 1e−05

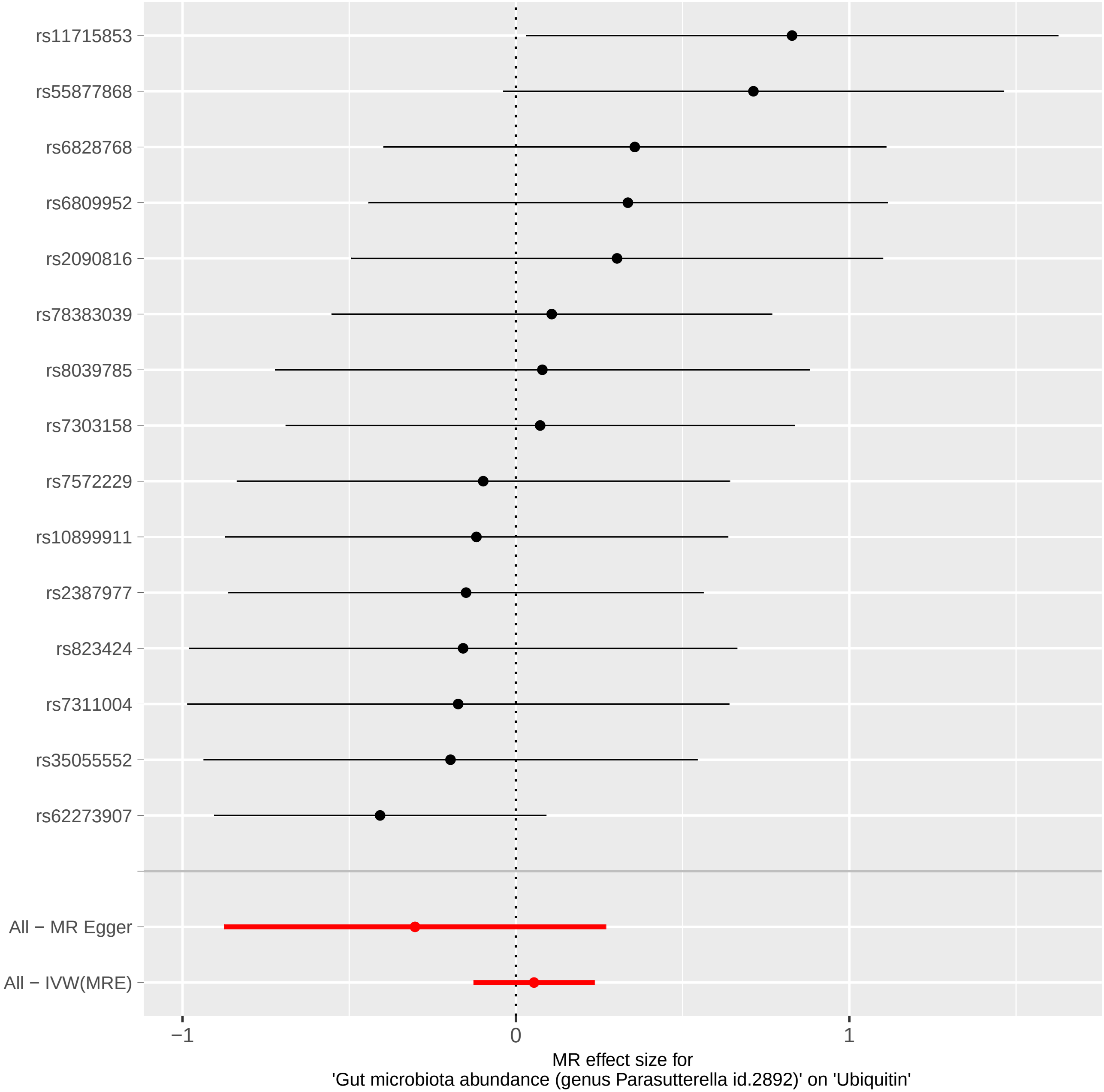

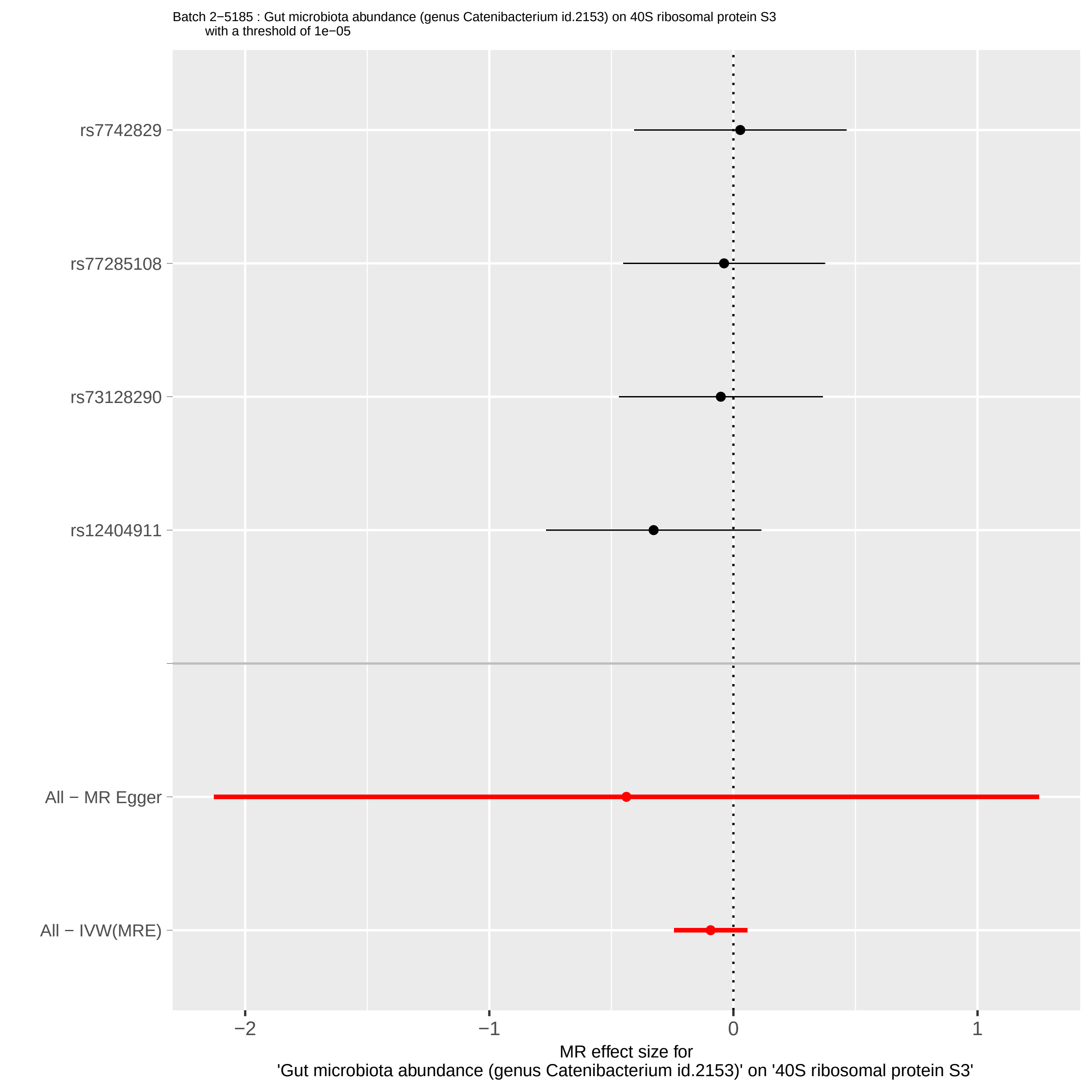
Batch 2−5185 : Gut microbiota abundance (genus Catenibacterium id.2153) on 40S ribosomal protein S3
with a threshold of 1e−05
rs7742829
rs77285108
rs73128290
rs12404911
All − MR Egger
All − IVW(MRE)
−2
−1
0
1
MR effect size for
'Gut microbiota abundance (genus Catenibacterium id.2153)' on '40S ribosomal protein S3'

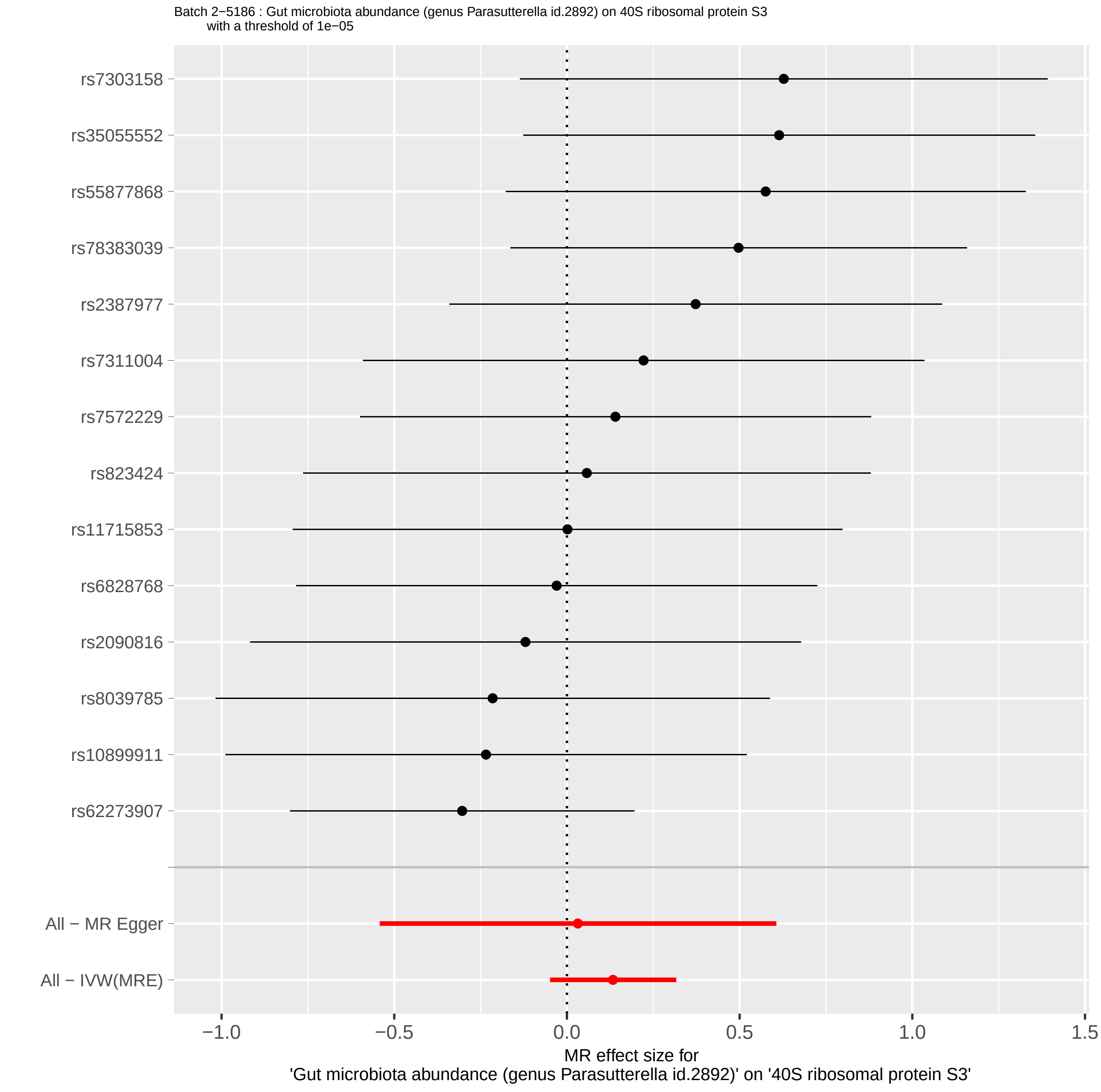

Batch 2−5186 : Gut microbiota abundance (genus Parasutterella id.2892) on 40S ribosomal protein S3
with a threshold of 1e−05
rs7303158
rs35055552
rs55877868
rs78383039
rs2387977
rs7311004
rs7572229
rs823424
rs11715853
rs6828768
rs2090816
rs8039785
rs10899911
rs62273907
All − MR Egger
All − IVW(MRE)
−1.0
−0.5
0.0
0.5
1.0
1.5
MR effect size for
'Gut microbiota abundance (genus Parasutterella id.2892)' on '40S ribosomal protein S3'

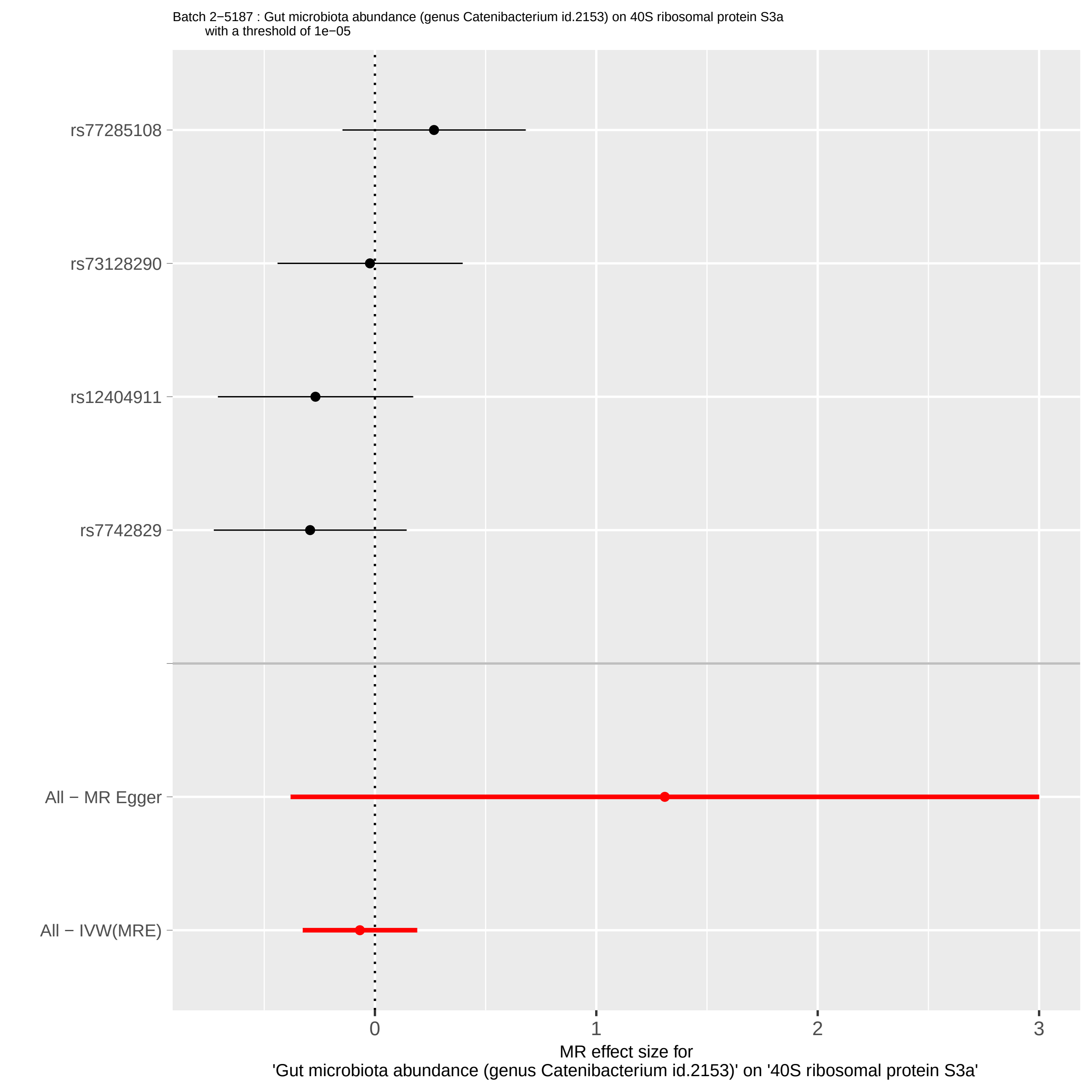
Batch 2−5187 : Gut microbiota abundance (genus Catenibacterium id.2153) on 40S ribosomal protein S3a
with a threshold of 1e−05
rs77285108
rs73128290
rs12404911
rs7742829
All − MR Egger
All − IVW(MRE)
0
1
2
3
MR effect size for
'Gut microbiota abundance (genus Catenibacterium id.2153)' on '40S ribosomal protein S3a'

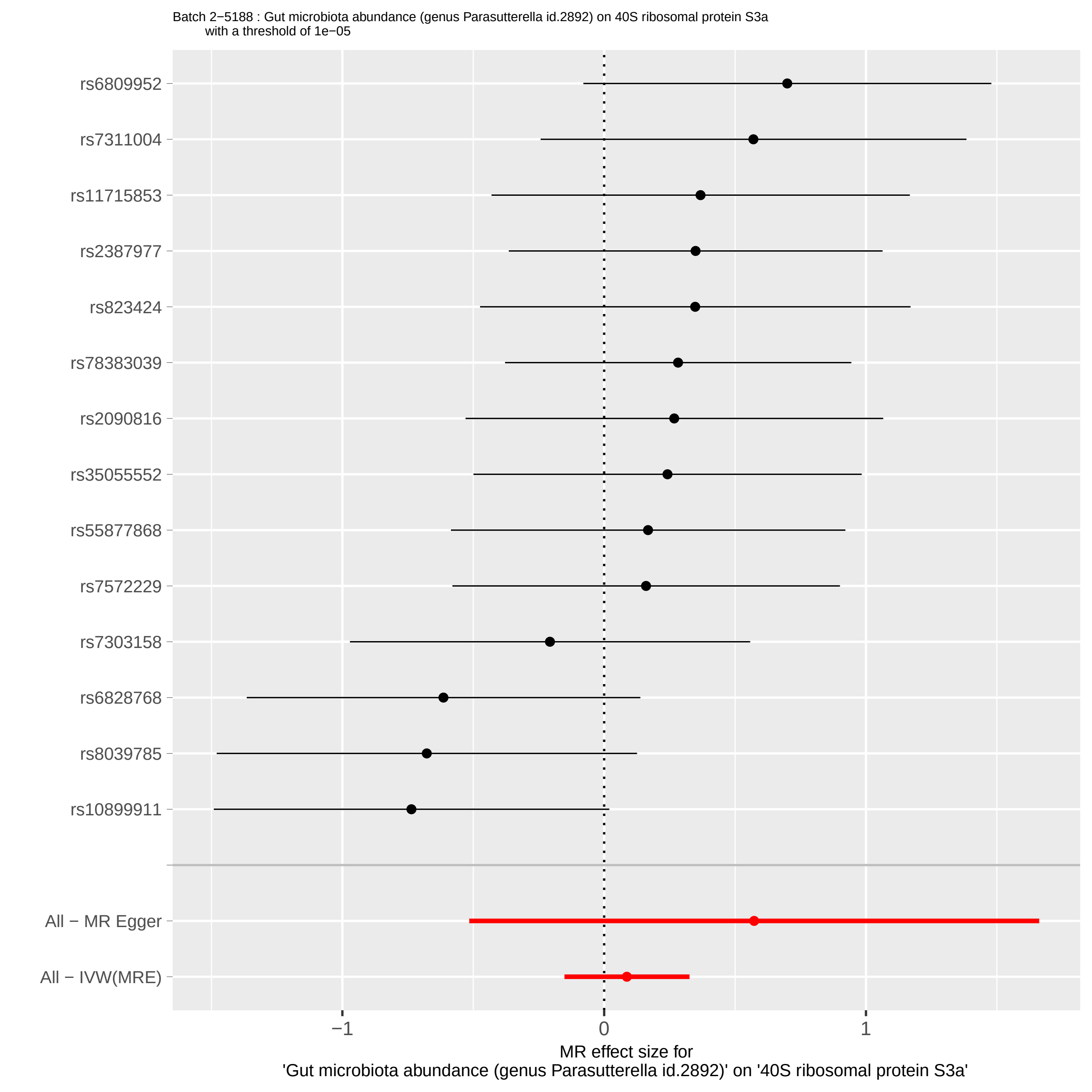
Batch 2−5188 : Gut microbiota abundance (genus Parasutterella id.2892) on 40S ribosomal protein S3a
with a threshold of 1e−05
rs6809952
rs7311004
rs11715853
rs2387977
rs823424
rs78383039
rs2090816
rs35055552
rs55877868
rs7572229
rs7303158
rs6828768
rs8039785
rs10899911
All − MR Egger
All − IVW(MRE)
−1
0
1
MR effect size for
'Gut microbiota abundance (genus Parasutterella id.2892)' on '40S ribosomal protein S3a'

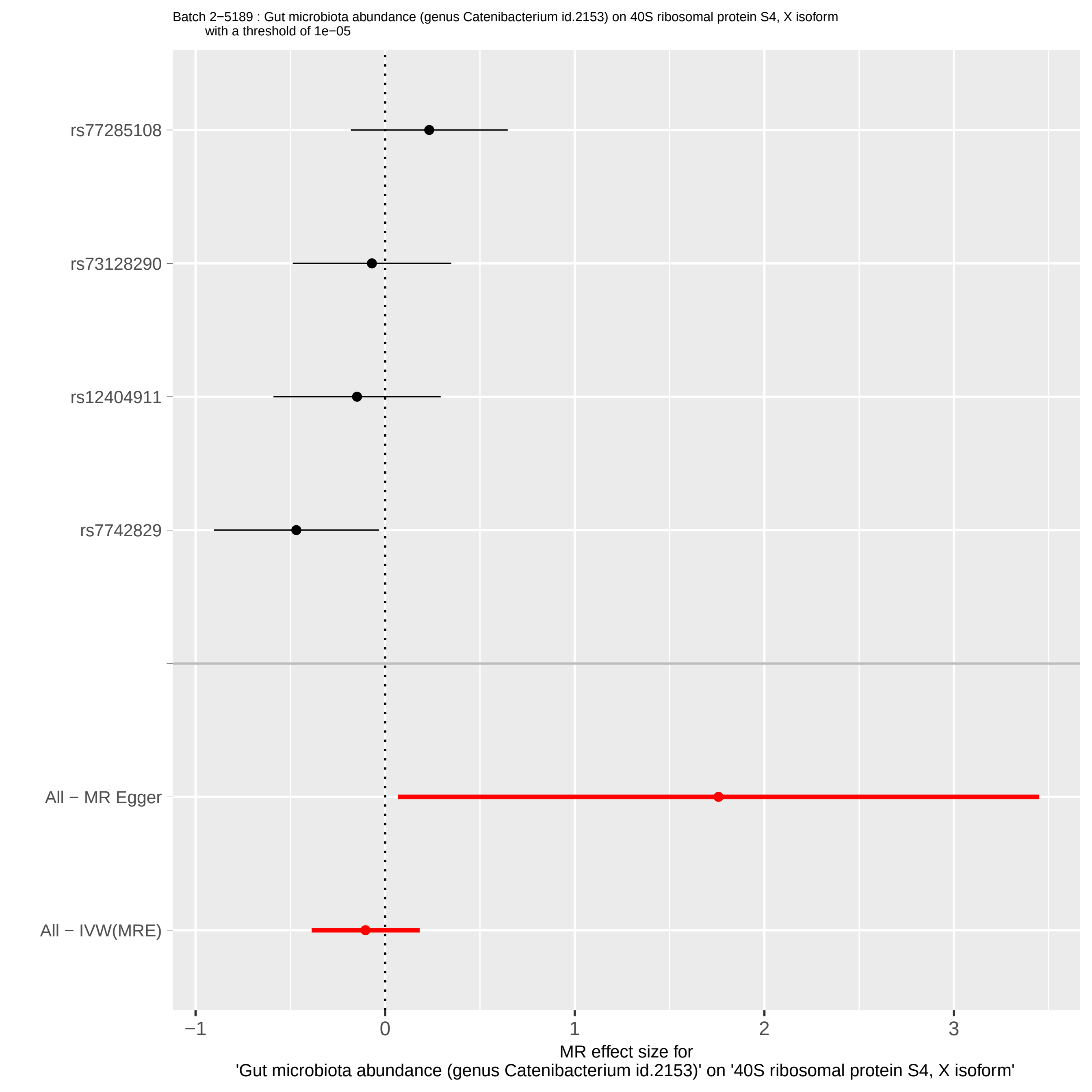
Batch 2−5189 : Gut microbiota abundance (genus Catenibacterium id.2153) on 40S ribosomal protein S4, X isoform
with a threshold of 1e−05
rs77285108
rs73128290
rs12404911
rs7742829
All − MR Egger
All − IVW(MRE)
−1
0
1
2
3
MR effect size for
'Gut microbiota abundance (genus Catenibacterium id.2153)' on '40S ribosomal protein S4, X isoform'

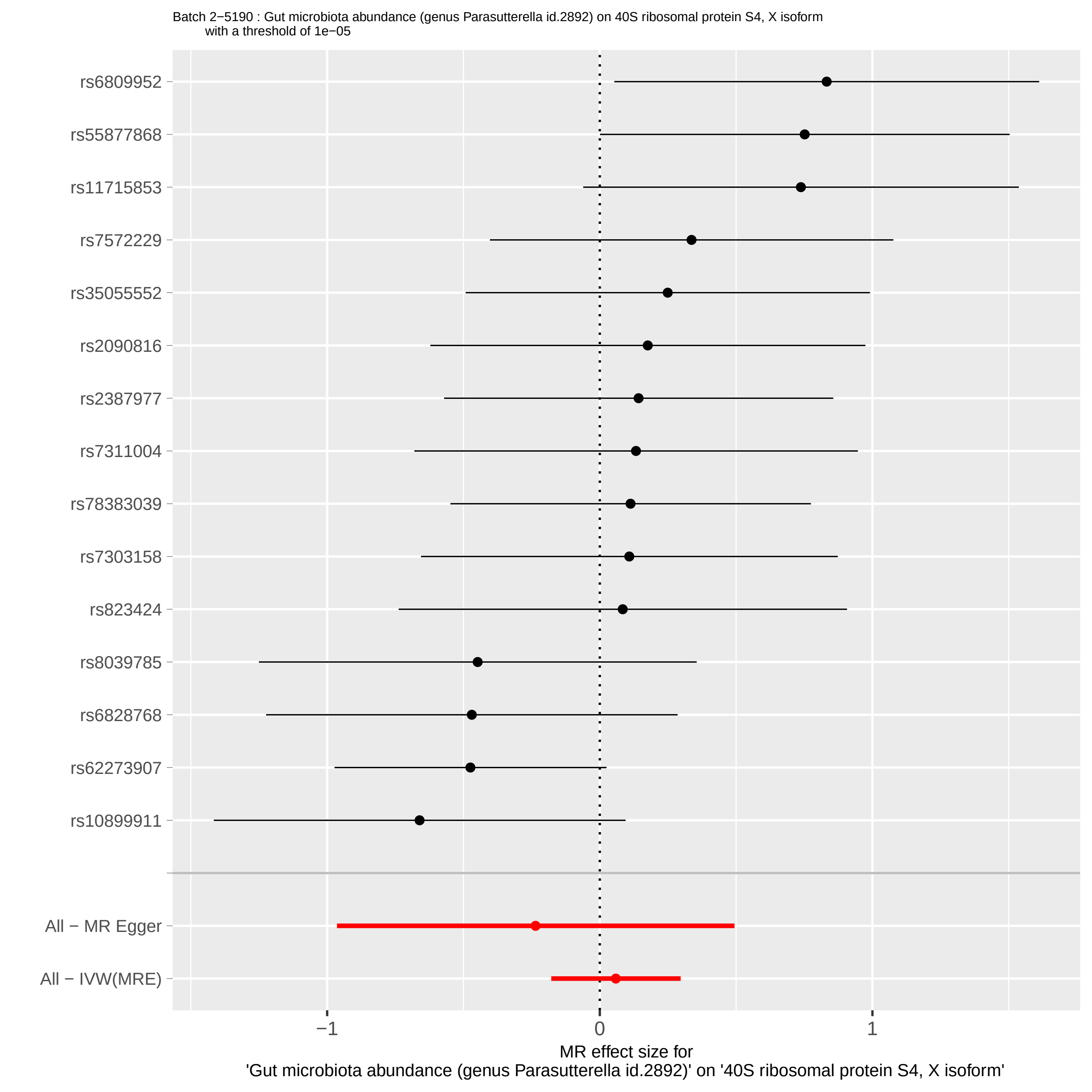

Batch 2−5190 : Gut microbiota abundance (genus Parasutterella id.2892) on 40S ribosomal protein S4, X isoform
with a threshold of 1e−05
rs6809952
rs55877868
rs11715853
rs7572229
rs35055552
rs2090816
rs2387977
rs7311004
rs78383039
rs7303158
rs823424
rs8039785
rs6828768
rs62273907
rs10899911
All − MR Egger
All − IVW(MRE)
−1
0
1
MR effect size for
'Gut microbiota abundance (genus Parasutterella id.2892)' on '40S ribosomal protein S4, X isoform'

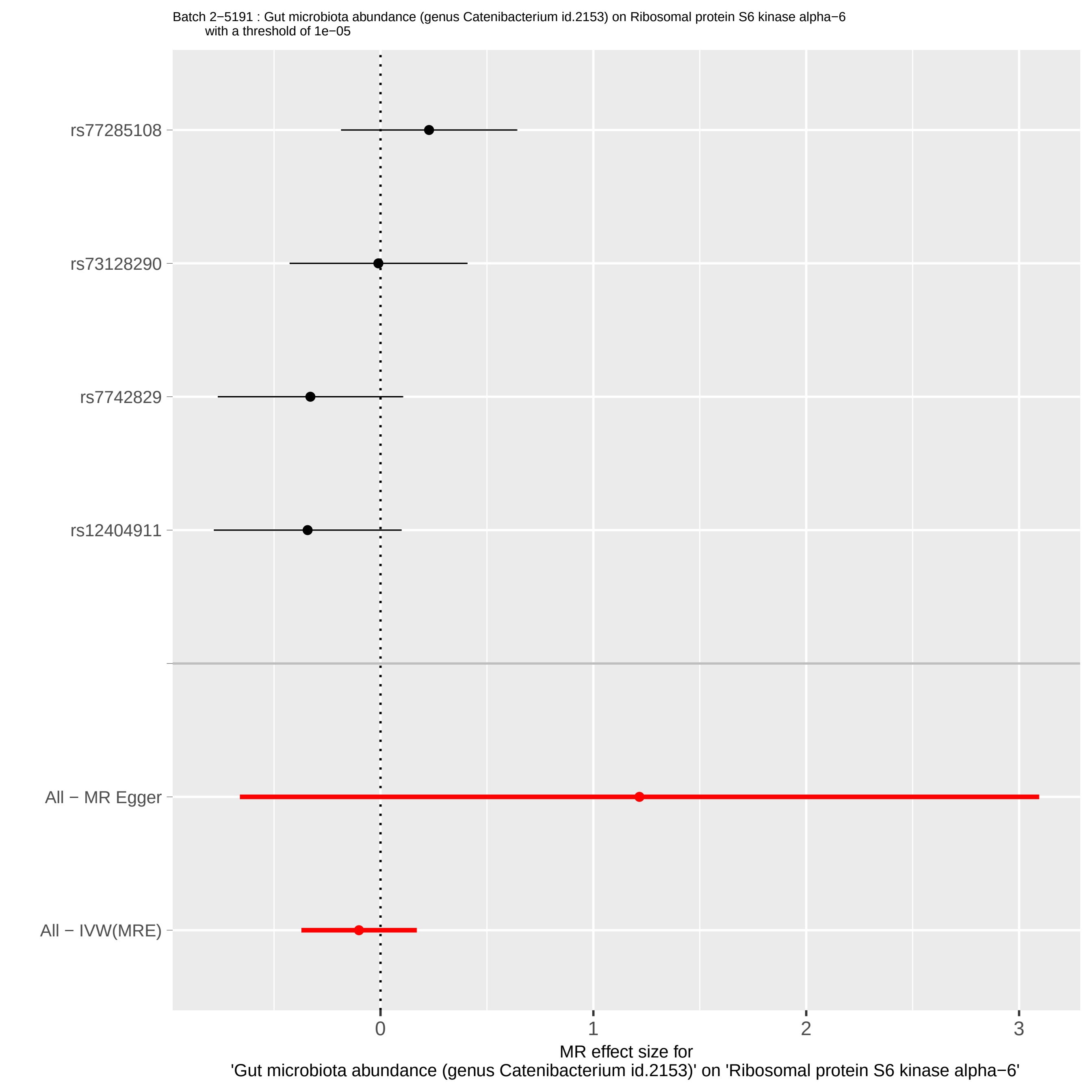

Batch 2−5191 : Gut microbiota abundance (genus Catenibacterium id.2153) on Ribosomal protein S6 kinase alpha−6
with a threshold of 1e−05
rs77285108
rs73128290
rs7742829
rs12404911
All − MR Egger
All − IVW(MRE)
0
1
2
3
MR effect size for
'Gut microbiota abundance (genus Catenibacterium id.2153)' on 'Ribosomal protein S6 kinase alpha−6'

Batch 2−5192 : Gut microbiota abundance (genus Parasutterella id.2892) on Ribosomal protein S6 kinase alpha−6
with a threshold of 1e−05

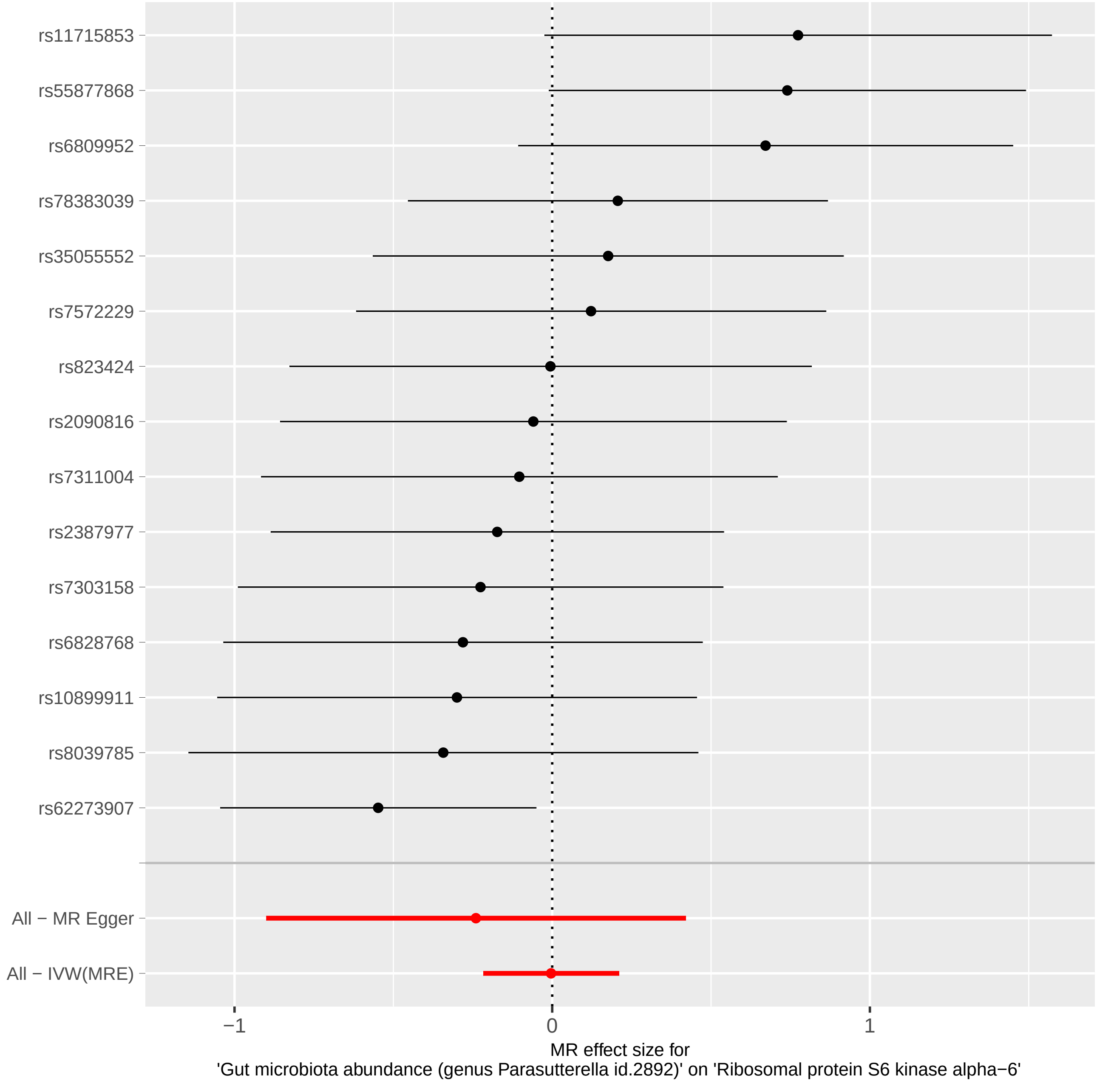

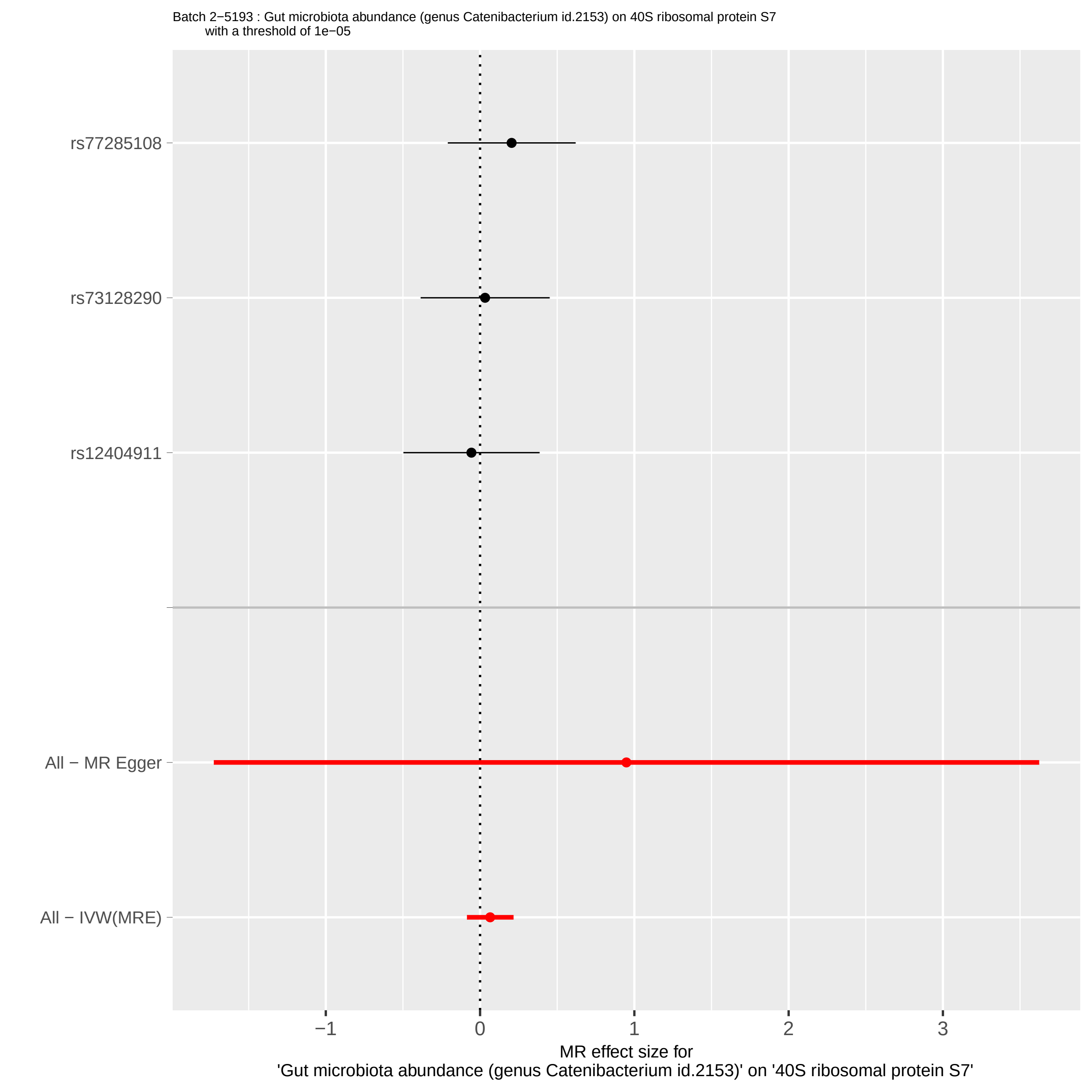
Batch 2−5193 : Gut microbiota abundance (genus Catenibacterium id.2153) on 40S ribosomal protein S7
with a threshold of 1e−05
rs77285108
rs73128290
rs12404911
All − MR Egger
All − IVW(MRE)
−1
0
1
2
3
MR effect size for
'Gut microbiota abundance (genus Catenibacterium id.2153)' on '40S ribosomal protein S7'

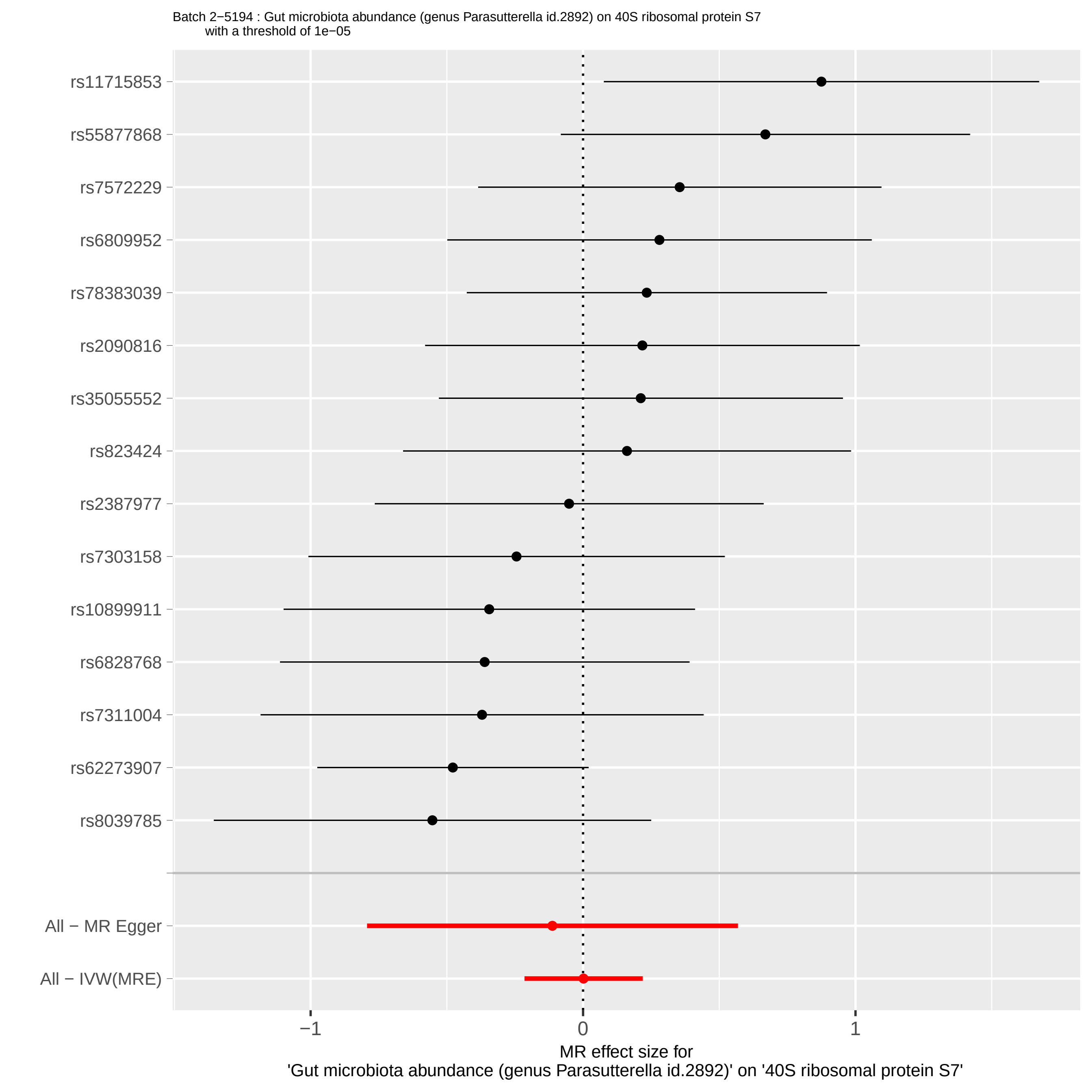
Batch 2−5194 : Gut microbiota abundance (genus Parasutterella id.2892) on 40S ribosomal protein S7
with a threshold of 1e−05
rs11715853
rs55877868
rs7572229
rs6809952
rs78383039
rs2090816
rs35055552
rs823424
rs2387977
rs7303158
rs10899911
rs6828768
rs7311004
rs62273907
rs8039785
All − MR Egger
All − IVW(MRE)
−1
0
1
MR effect size for
'Gut microbiota abundance (genus Parasutterella id.2892)' on '40S ribosomal protein S7'

Batch 2−5195 : Gut microbiota abundance (genus Catenibacterium id.2153) on 40S ribosomal protein SA
with a threshold of 1e−05

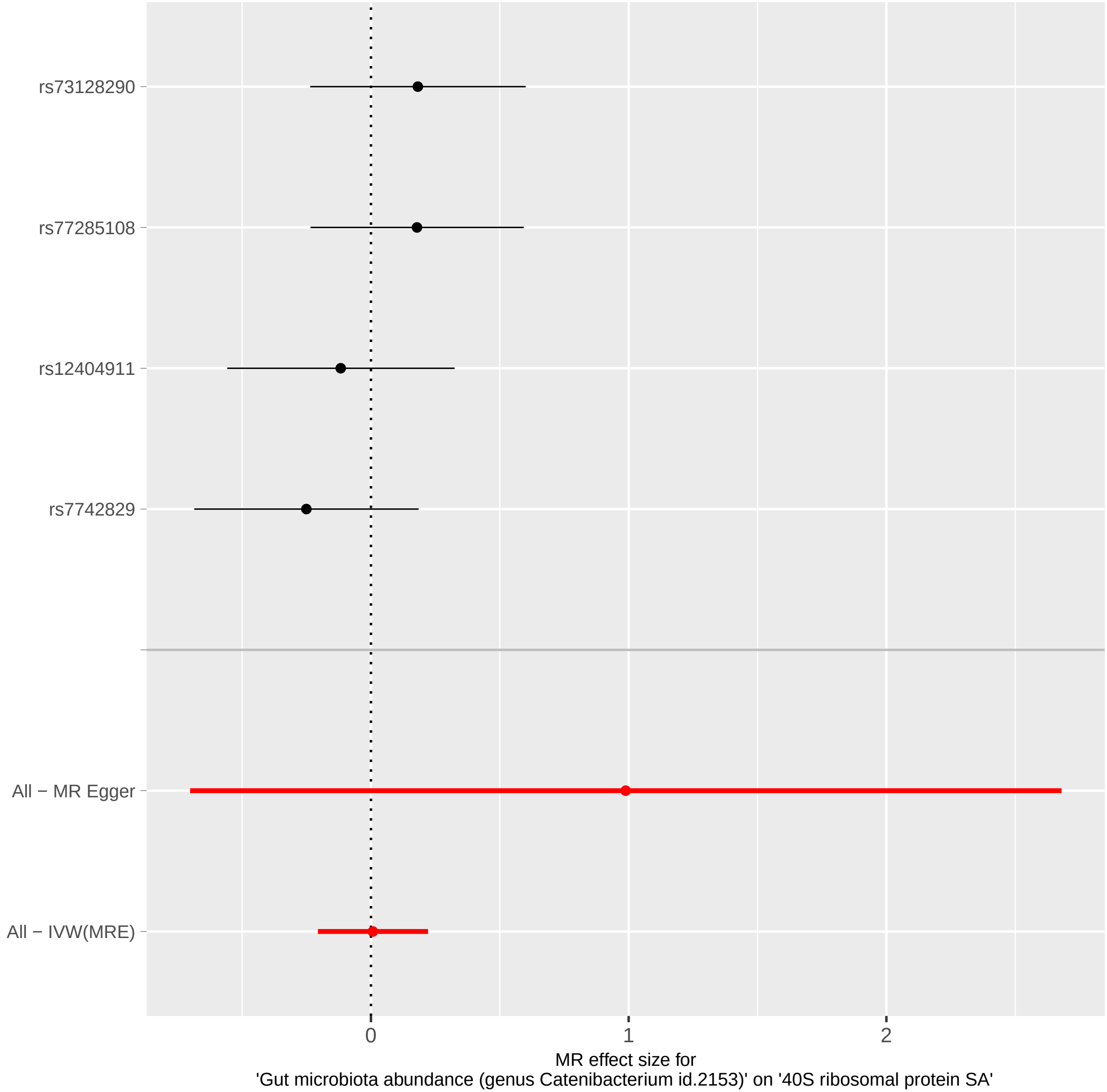

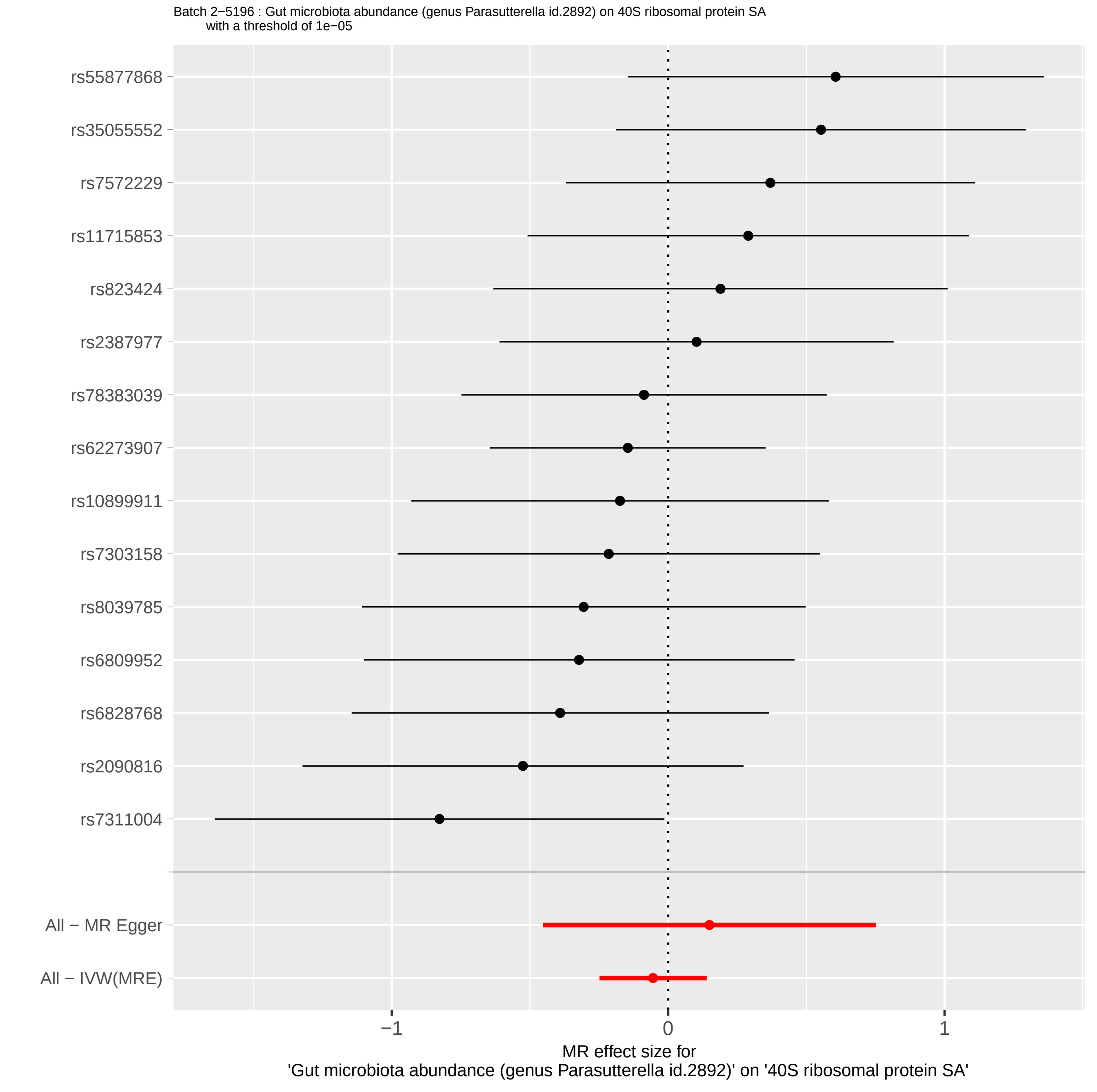

Batch 2−5196 : Gut microbiota abundance (genus Parasutterella id.2892) on 40S ribosomal protein SA
with a threshold of 1e−05
rs55877868
rs35055552
rs7572229
rs11715853
rs823424
rs2387977
rs78383039
rs62273907
rs10899911
rs7303158
rs8039785
rs6809952
rs6828768
rs2090816
rs7311004
All − MR Egger
All − IVW(MRE)
−1
0
1
MR effect size for
'Gut microbiota abundance (genus Parasutterella id.2892)' on '40S ribosomal protein SA'

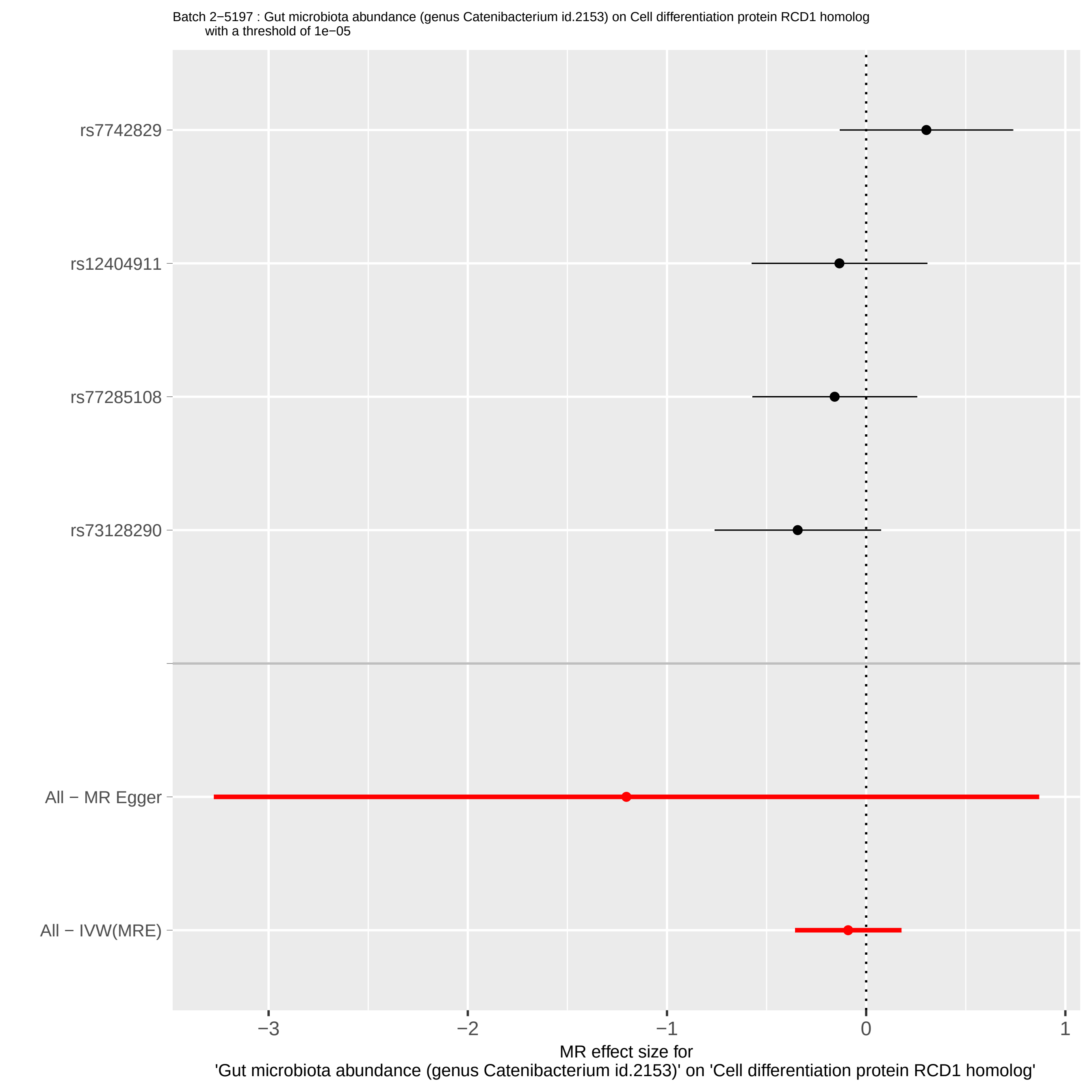
Batch 2−5197 : Gut microbiota abundance (genus Catenibacterium id.2153) on Cell differentiation protein RCD1 homolog
with a threshold of 1e−05
rs7742829
rs12404911
rs77285108
rs73128290
All − MR Egger
All − IVW(MRE)
−3
−2
−1
0
1
MR effect size for
'Gut microbiota abundance (genus Catenibacterium id.2153)' on 'Cell differentiation protein RCD1 homolog'

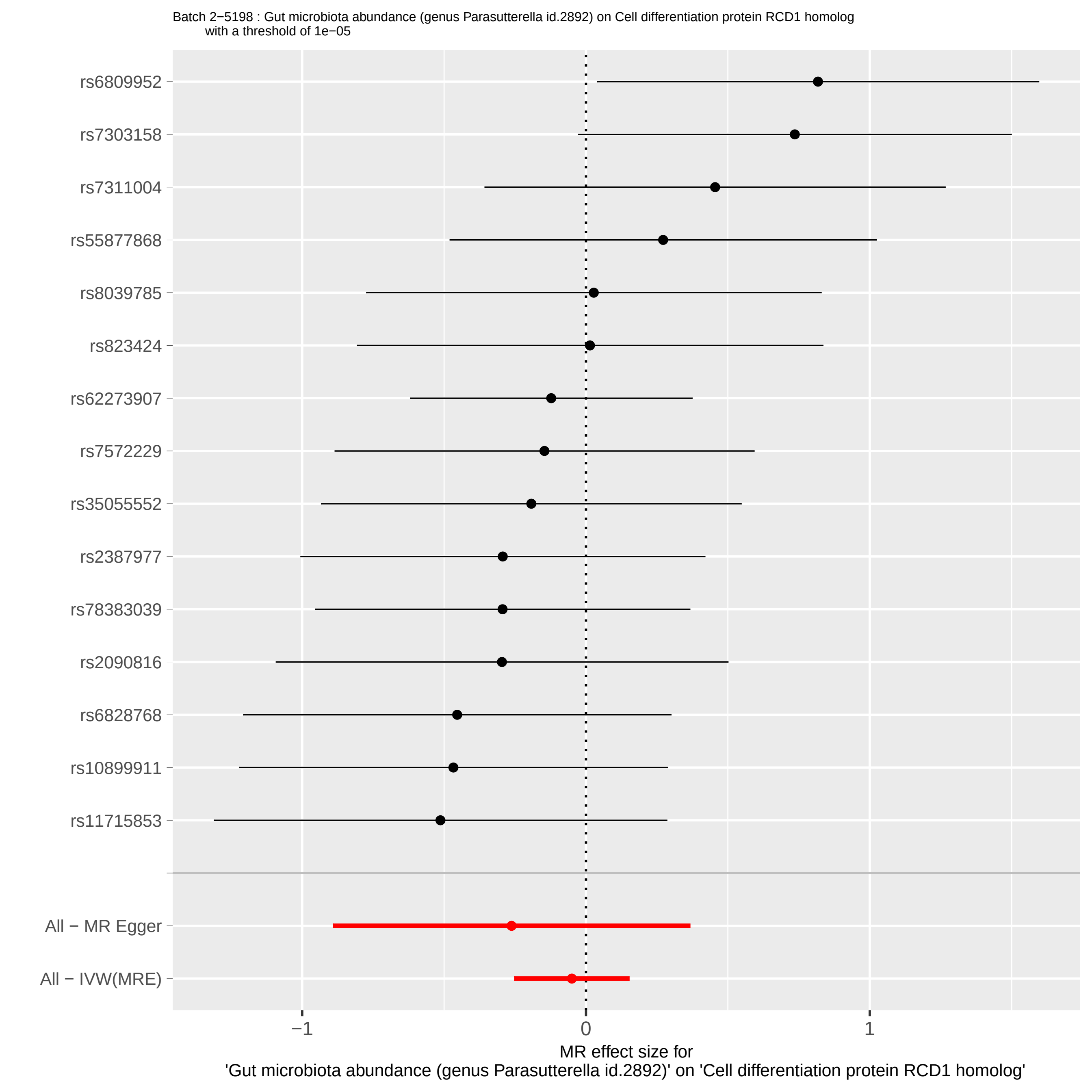

Batch 2−5198 : Gut microbiota abundance (genus Parasutterella id.2892) on Cell differentiation protein RCD1 homolog
with a threshold of 1e−05
rs6809952
rs7303158
rs7311004
rs55877868
rs8039785
rs823424
rs62273907
rs7572229
rs35055552
rs2387977
rs78383039
rs2090816
rs6828768
rs10899911
rs11715853
All − MR Egger
All − IVW(MRE)
−1
0
1
MR effect size for
'Gut microbiota abundance (genus Parasutterella id.2892)' on 'Cell differentiation protein RCD1 homolog'

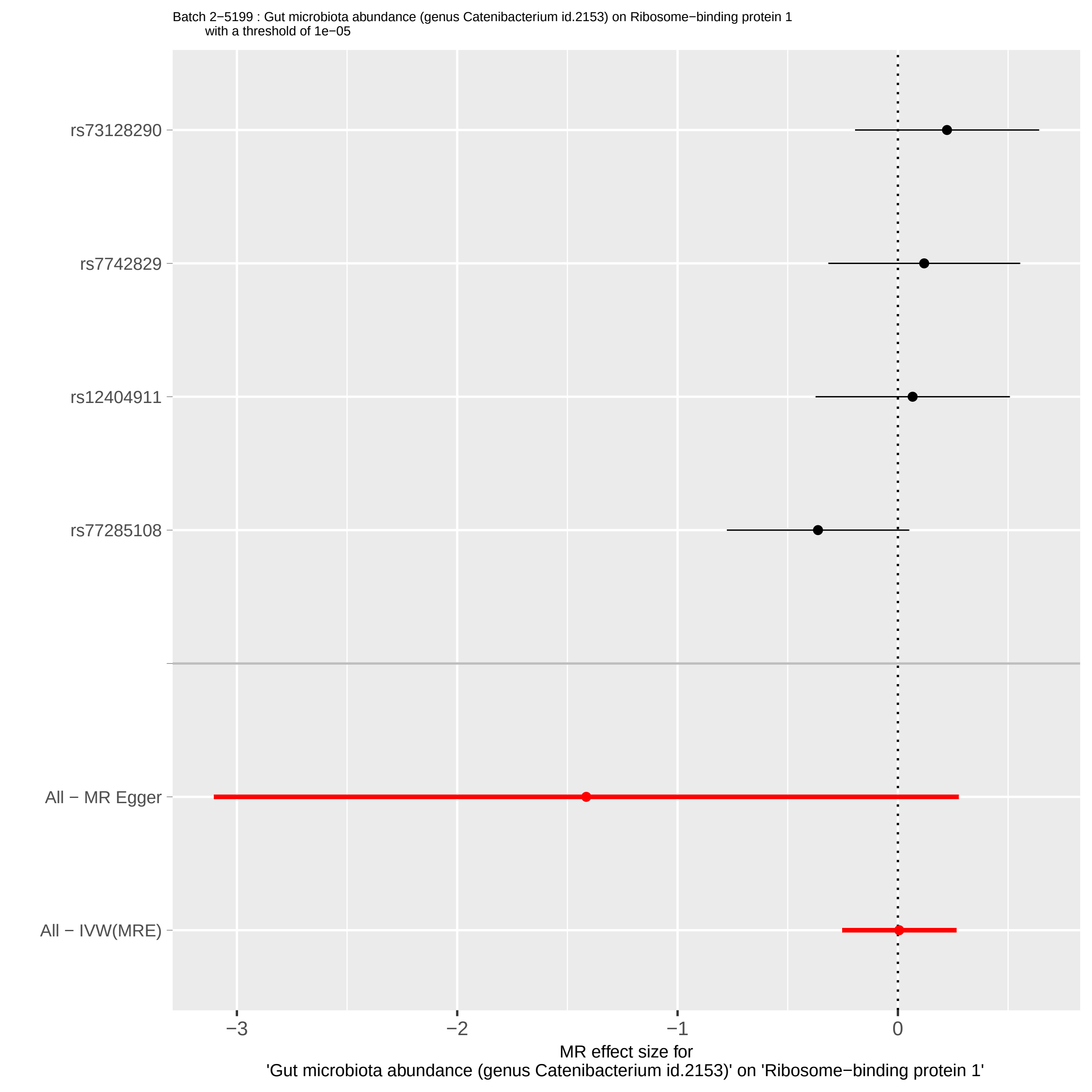

Batch 2−5199 : Gut microbiota abundance (genus Catenibacterium id.2153) on Ribosome−binding protein 1
with a threshold of 1e−05
rs73128290
rs7742829
rs12404911
rs77285108
All − MR Egger
All − IVW(MRE)
−3
−2
−1
0
MR effect size for
'Gut microbiota abundance (genus Catenibacterium id.2153)' on 'Ribosome−binding protein 1'

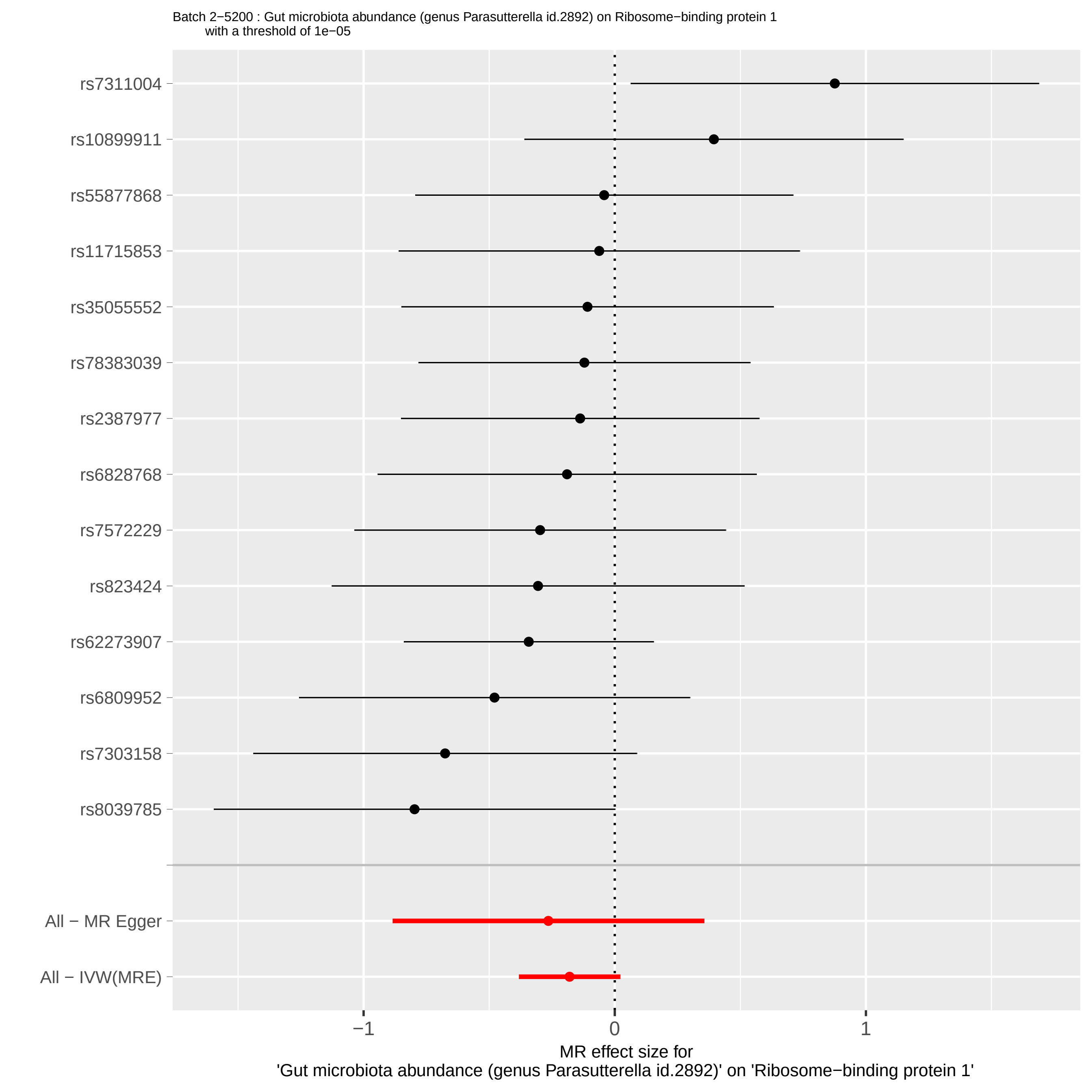
Batch 2−5200 : Gut microbiota abundance (genus Parasutterella id.2892) on Ribosome−binding protein 1
with a threshold of 1e−05
rs7311004
rs10899911
rs55877868
rs11715853
rs35055552
rs78383039
rs2387977
rs6828768
rs7572229
rs823424
rs62273907
rs6809952
rs7303158
rs8039785
All − MR Egger
All − IVW(MRE)
−1
0
1
MR effect size for
'Gut microbiota abundance (genus Parasutterella id.2892)' on 'Ribosome−binding protein 1'

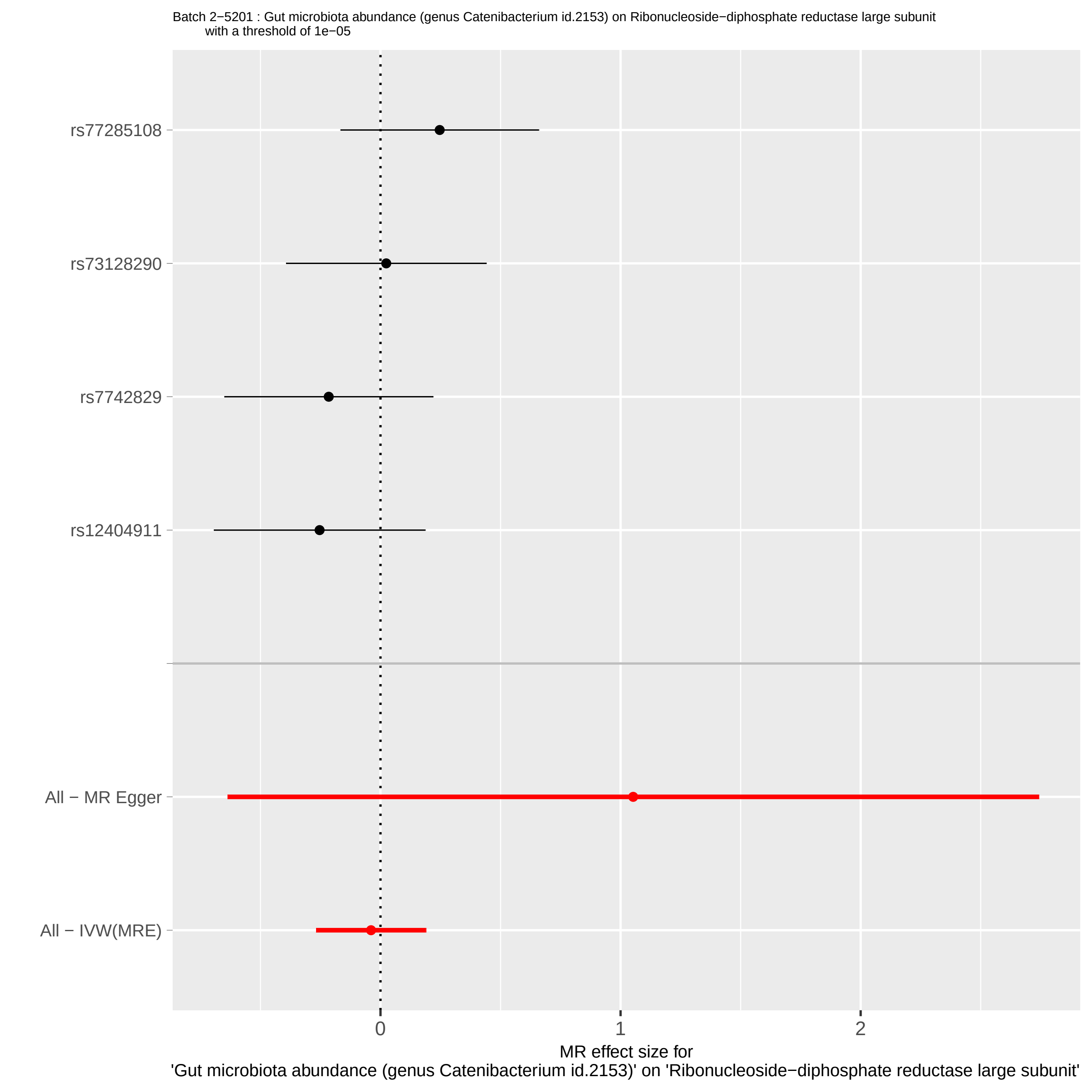
Batch 2−5201 : Gut microbiota abundance (genus Catenibacterium id.2153) on Ribonucleoside−diphosphate reductase large subunit
with a threshold of 1e−05
rs77285108
rs73128290
rs7742829
rs12404911
All − MR Egger
All − IVW(MRE)
0
1
2
MR effect size for
'Gut microbiota abundance (genus Catenibacterium id.2153)' on 'Ribonucleoside−diphosphate reductase large subunit'

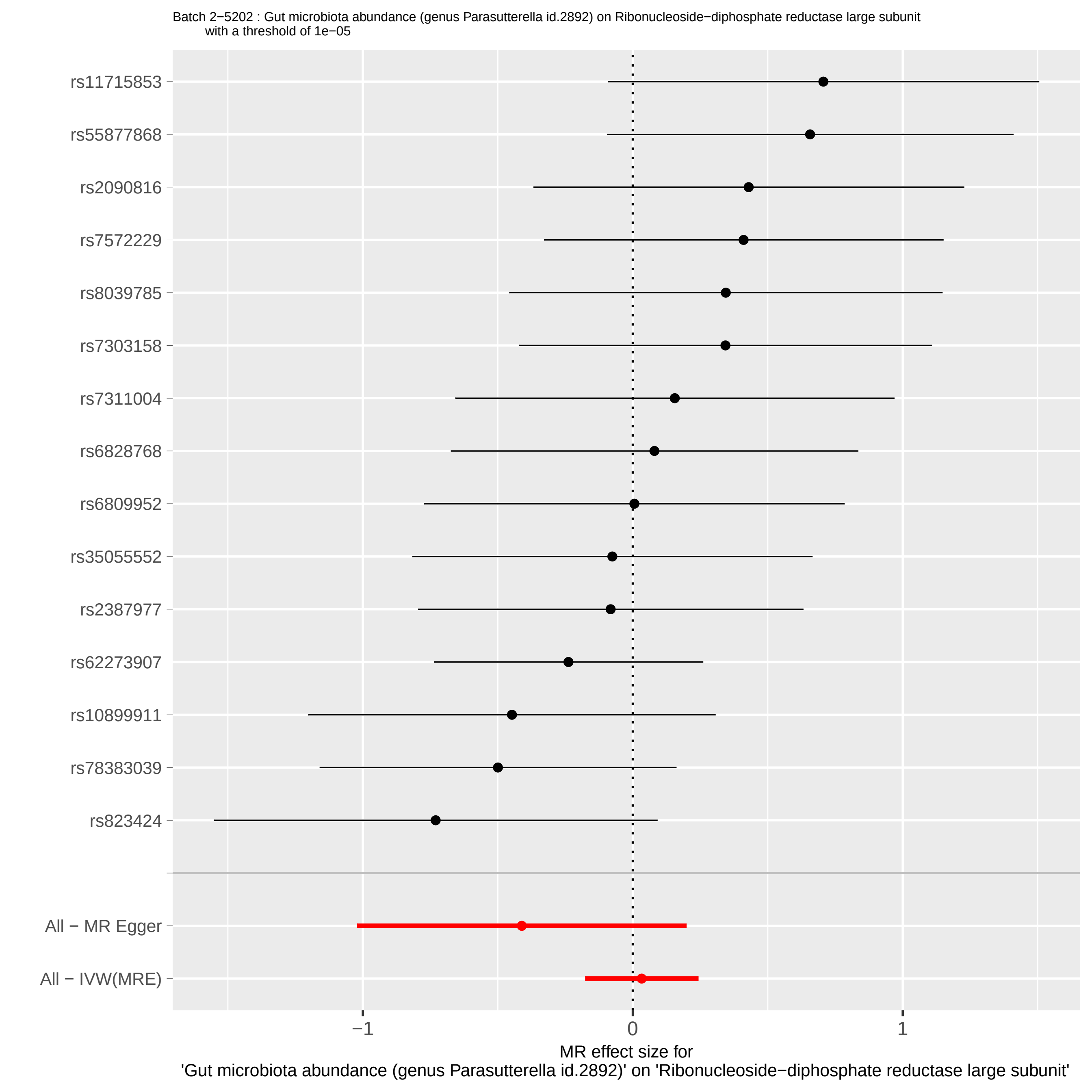

Batch 2−5202 : Gut microbiota abundance (genus Parasutterella id.2892) on Ribonucleoside−diphosphate reductase large subunit
with a threshold of 1e−05
rs11715853
rs55877868
rs2090816
rs7572229
rs8039785
rs7303158
rs7311004
rs6828768
rs6809952
rs35055552
rs2387977
rs62273907
rs10899911
rs78383039
rs823424
All − MR Egger
All − IVW(MRE)
−1
0
1
MR effect size for
'Gut microbiota abundance (genus Parasutterella id.2892)' on 'Ribonucleoside−diphosphate reductase large subunit'

Batch 2−5203 : Gut microbiota abundance (genus Catenibacterium id.2153) on Ribonucleoside−diphosphate reductase subunit M2 B
with a threshold of 1e−05

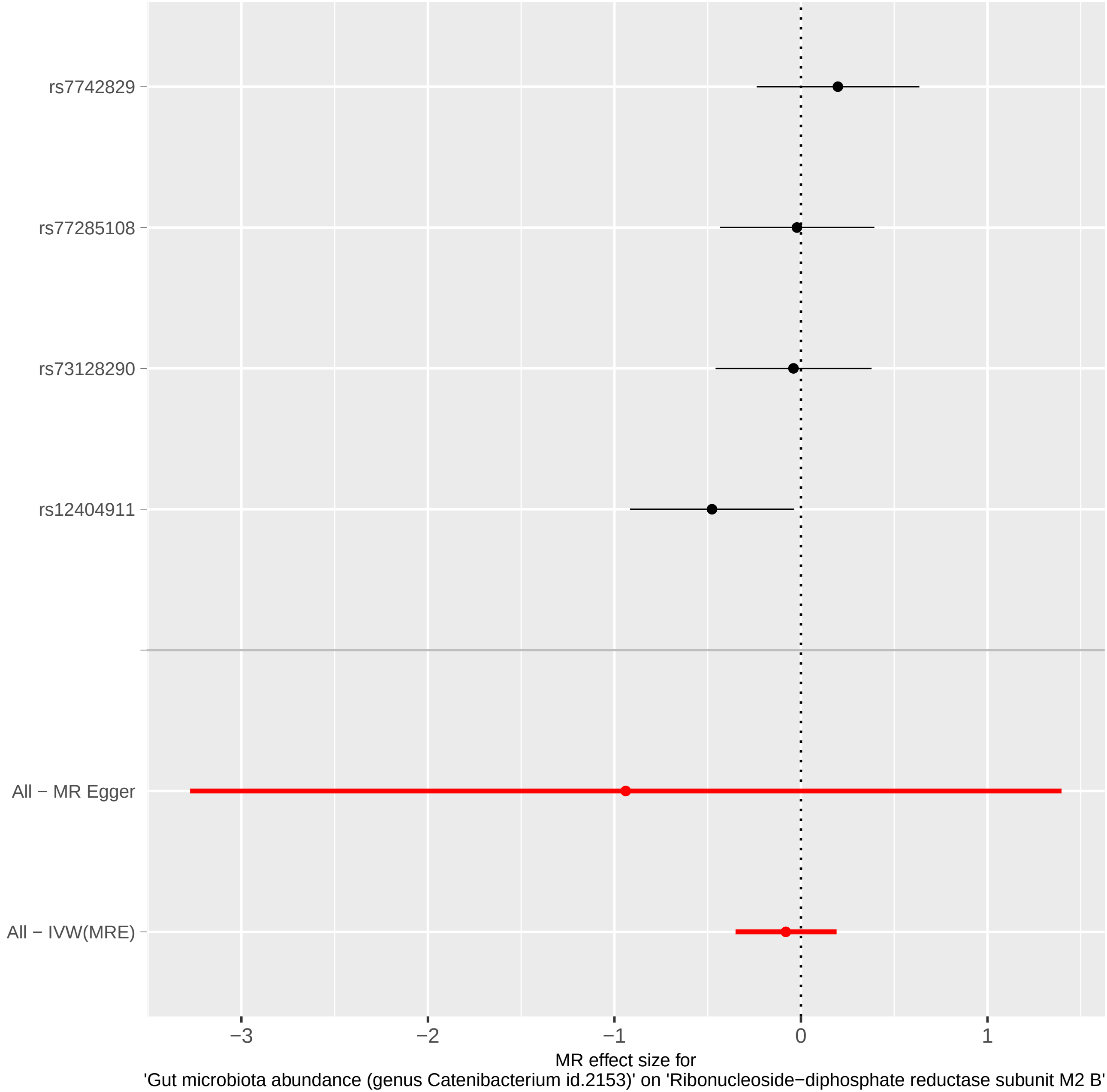

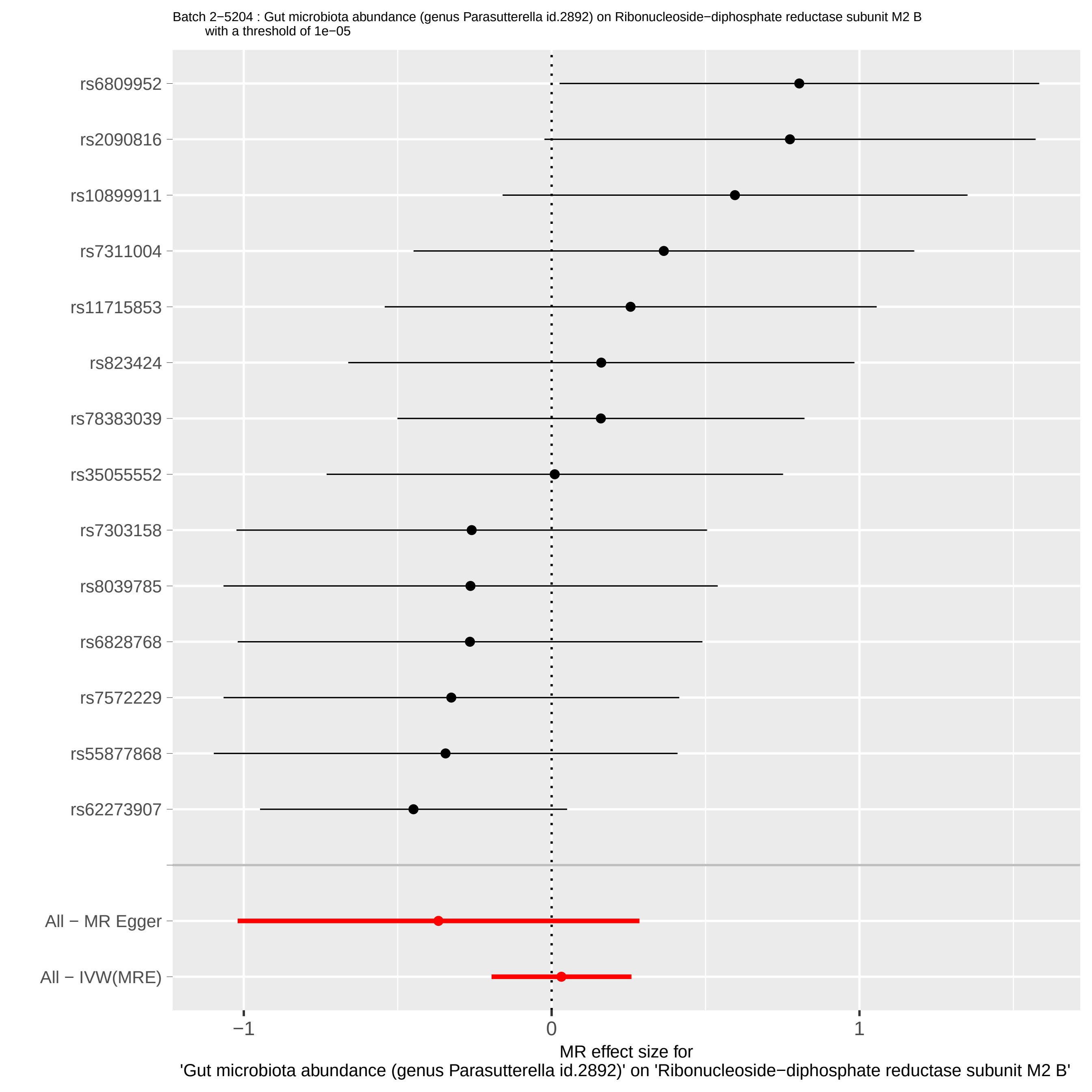
Batch 2−5204 : Gut microbiota abundance (genus Parasutterella id.2892) on Ribonucleoside−diphosphate reductase subunit M2 B
with a threshold of 1e−05
rs6809952
rs2090816
rs10899911
rs7311004
rs11715853
rs823424
rs78383039
rs35055552
rs7303158
rs8039785
rs6828768
rs7572229
rs55877868
rs62273907
All − MR Egger
All − IVW(MRE)
−1
0
1
MR effect size for
'Gut microbiota abundance (genus Parasutterella id.2892)' on 'Ribonucleoside−diphosphate reductase subunit M2 B'

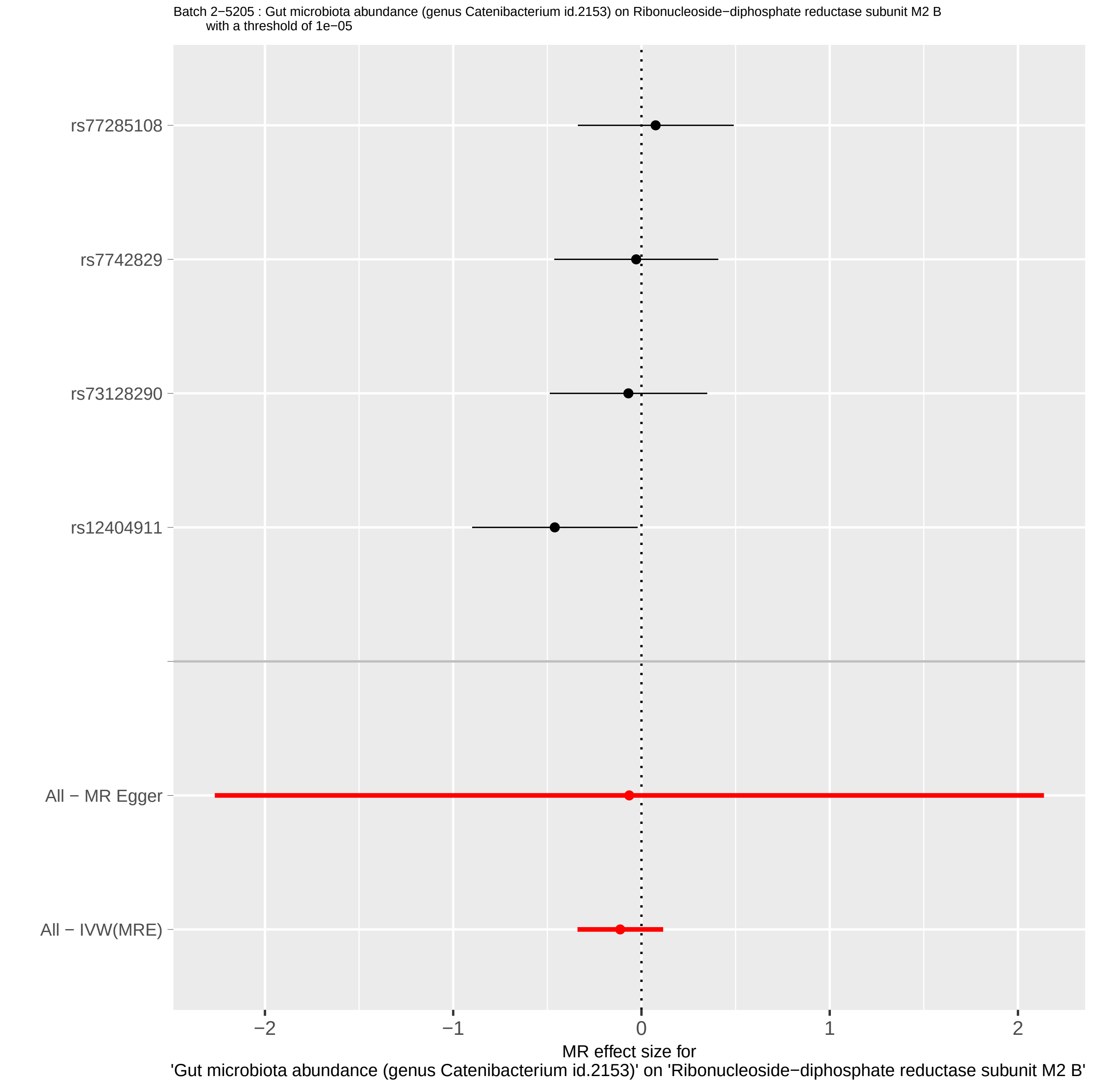
Batch 2−5205 : Gut microbiota abundance (genus Catenibacterium id.2153) on Ribonucleoside−diphosphate reductase subunit M2 B
with a threshold of 1e−05
rs77285108
rs7742829
rs73128290
rs12404911
All − MR Egger
All − IVW(MRE)
−2
−1
0
1
2
MR effect size for
'Gut microbiota abundance (genus Catenibacterium id.2153)' on 'Ribonucleoside−diphosphate reductase subunit M2 B'

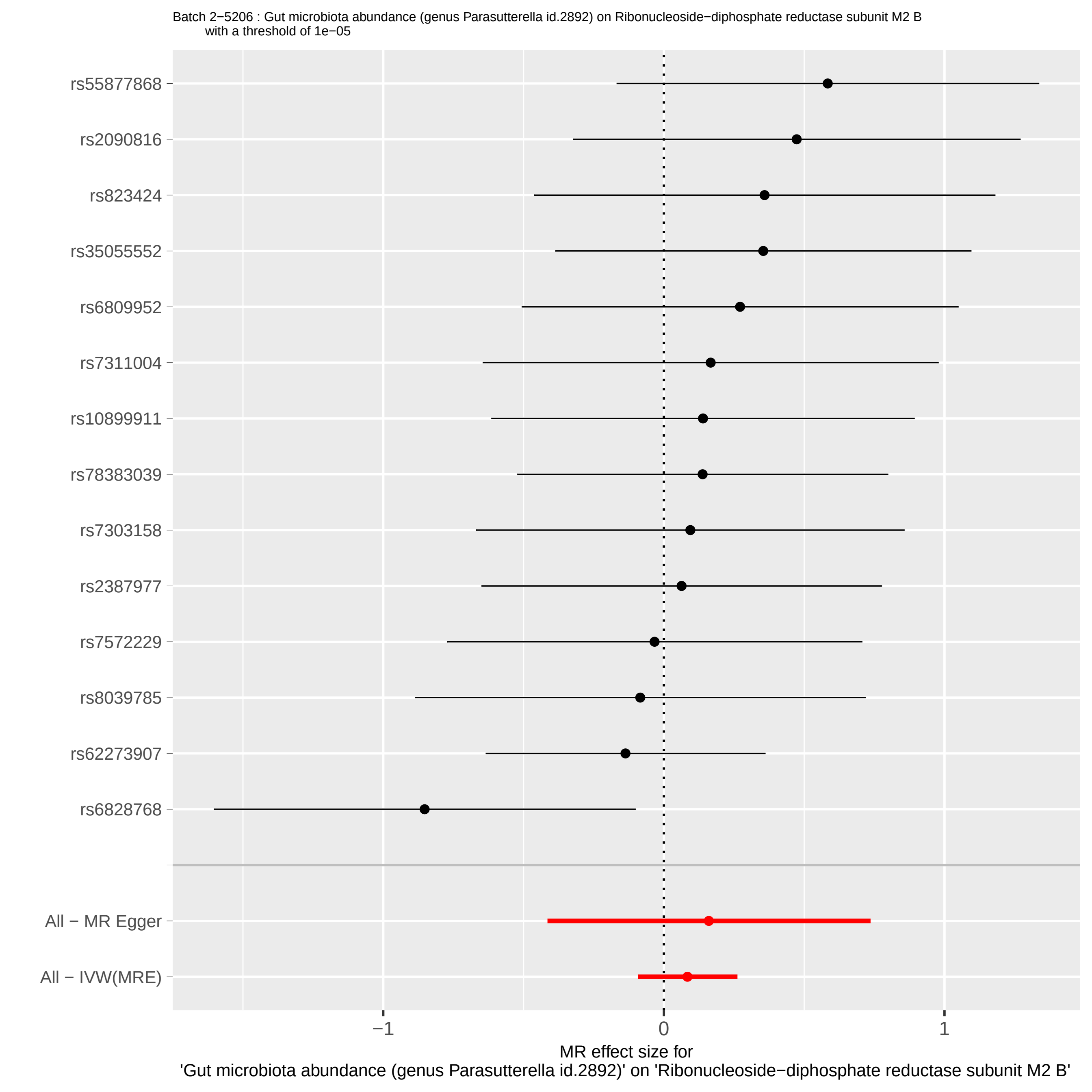

Batch 2−5206 : Gut microbiota abundance (genus Parasutterella id.2892) on Ribonucleoside−diphosphate reductase subunit M2 B
with a threshold of 1e−05
rs55877868
rs2090816
rs823424
rs35055552
rs6809952
rs7311004
rs10899911
rs78383039
rs7303158
rs2387977
rs7572229
rs8039785
rs62273907
rs6828768
All − MR Egger
All − IVW(MRE)
−1
0
1
MR effect size for
'Gut microbiota abundance (genus Parasutterella id.2892)' on 'Ribonucleoside−diphosphate reductase subunit M2 B'

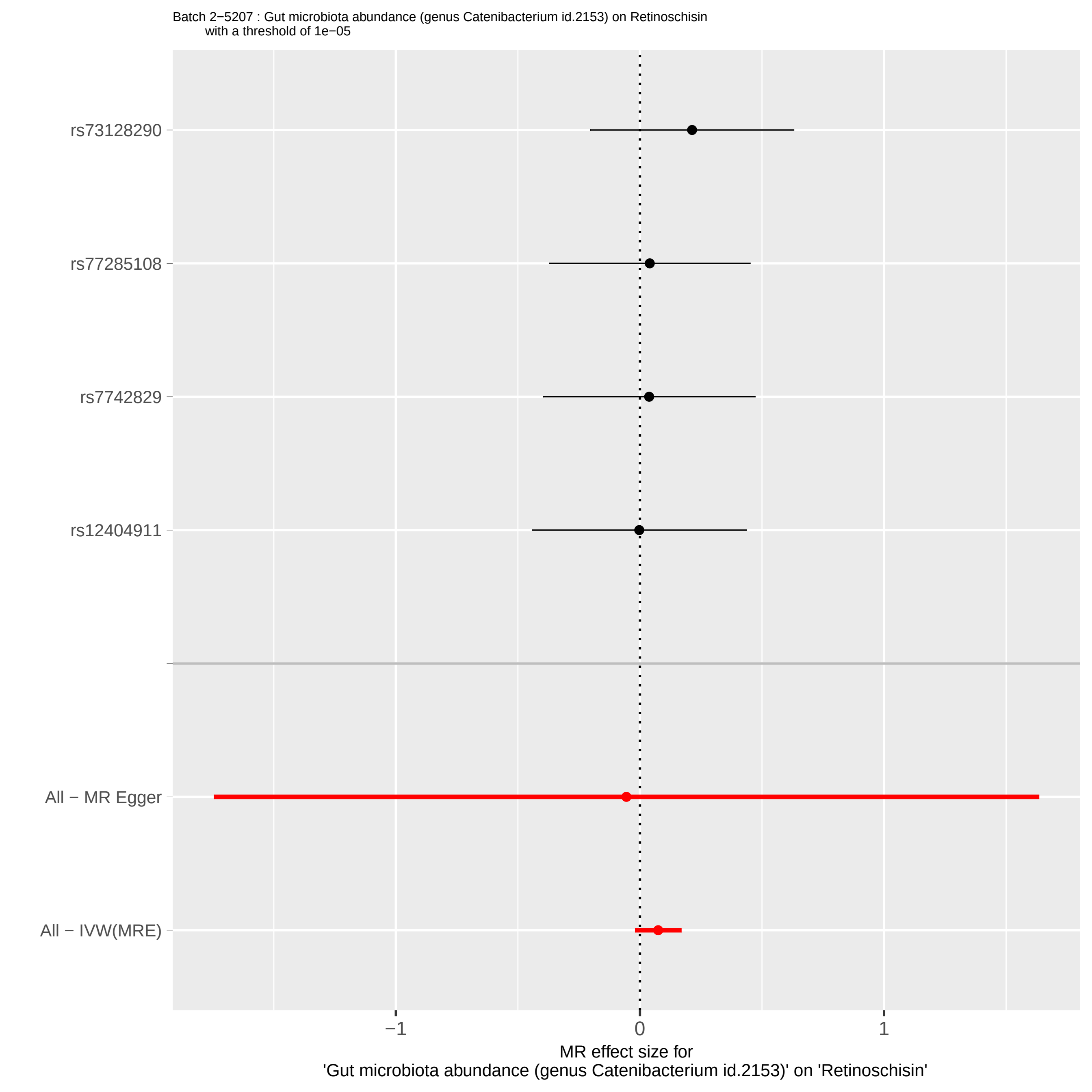
Batch 2−5207 : Gut microbiota abundance (genus Catenibacterium id.2153) on Retinoschisin
with a threshold of 1e−05
rs73128290
rs77285108
rs7742829
rs12404911
All − MR Egger
All − IVW(MRE)
−1
0
1
MR effect size for
'Gut microbiota abundance (genus Catenibacterium id.2153)' on 'Retinoschisin'

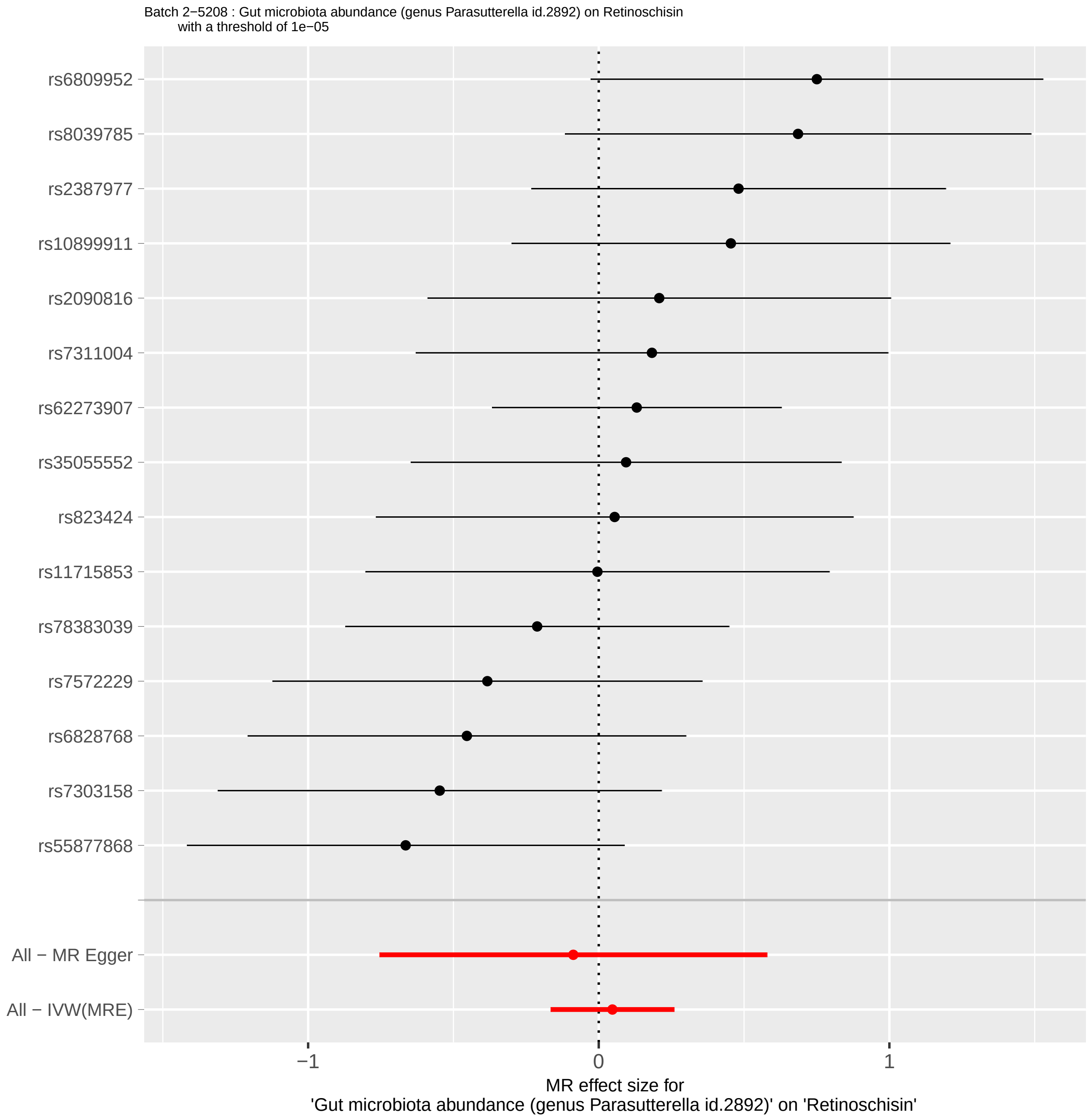

Batch 2−5208 : Gut microbiota abundance (genus Parasutterella id.2892) on Retinoschisin
with a threshold of 1e−05
rs6809952
rs8039785
rs2387977
rs10899911
rs2090816
rs7311004
rs62273907
rs35055552
rs823424
rs11715853
rs78383039
rs7572229
rs6828768
rs7303158
rs55877868
All − MR Egger
All − IVW(MRE)
−1
0
1
MR effect size for
'Gut microbiota abundance (genus Parasutterella id.2892)' on 'Retinoschisin'

Batch 2−5209 : Gut microbiota abundance (genus Catenibacterium id.2153) on R−spondin−3
with a threshold of 1e−05

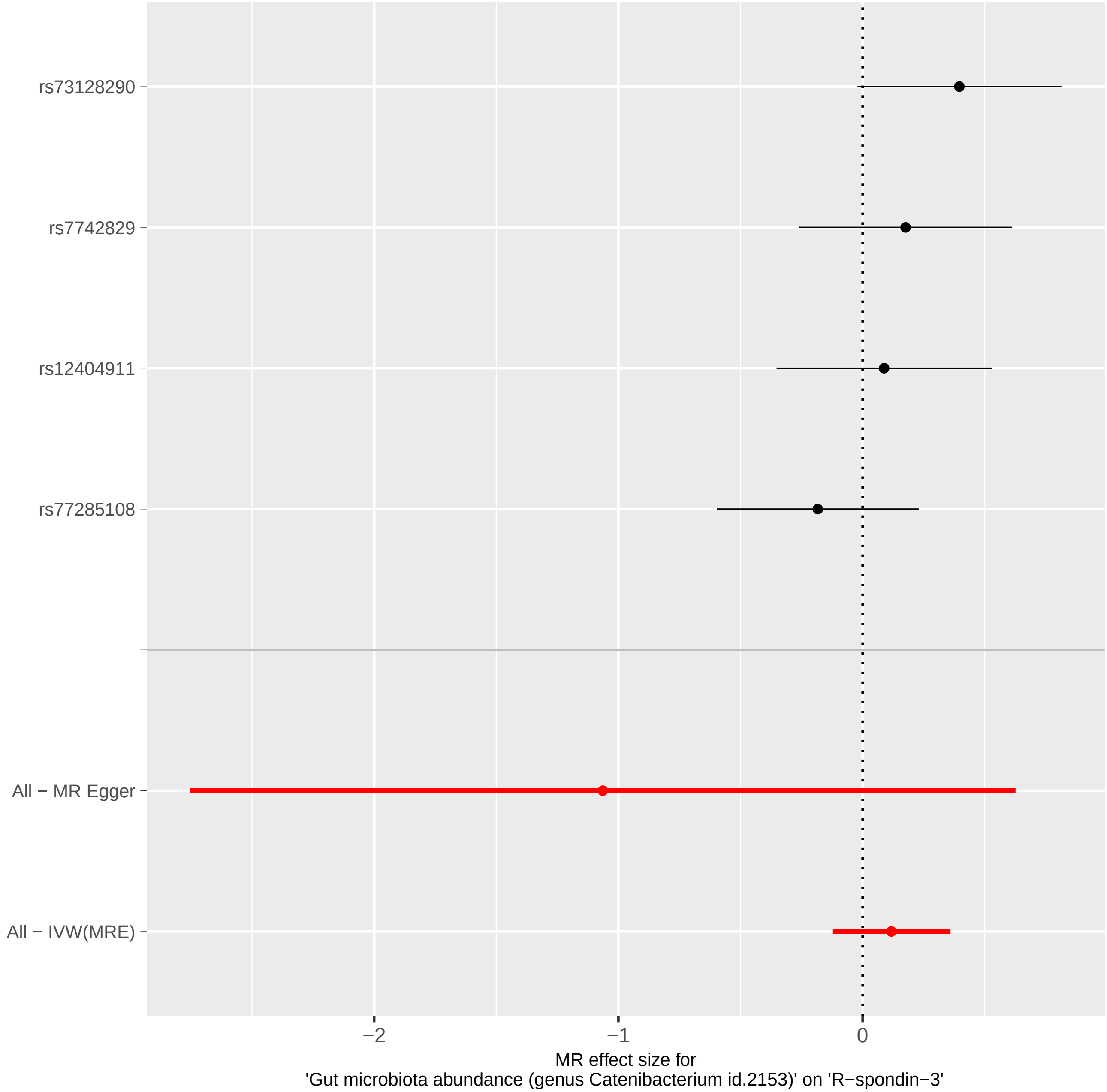

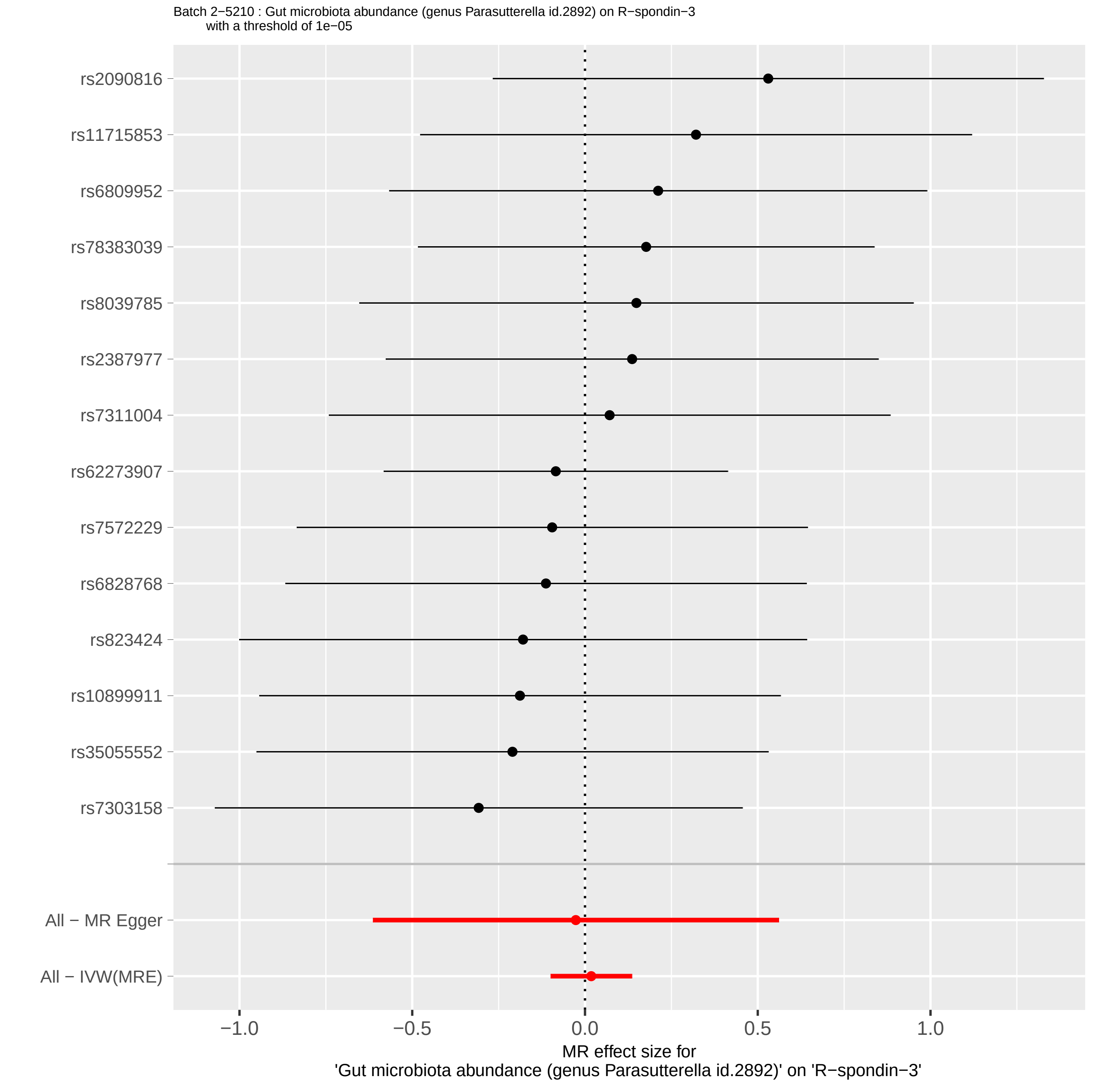

Batch 2−5210 : Gut microbiota abundance (genus Parasutterella id.2892) on R−spondin−3
with a threshold of 1e−05
rs2090816
rs11715853
rs6809952
rs78383039
rs8039785
rs2387977
rs7311004
rs62273907
rs7572229
rs6828768
rs823424
rs10899911
rs35055552
rs7303158
All − MR Egger
All − IVW(MRE)
−1.0
−0.5
0.0
0.5
1.0
MR effect size for
'Gut microbiota abundance (genus Parasutterella id.2892)' on 'R−spondin−3'

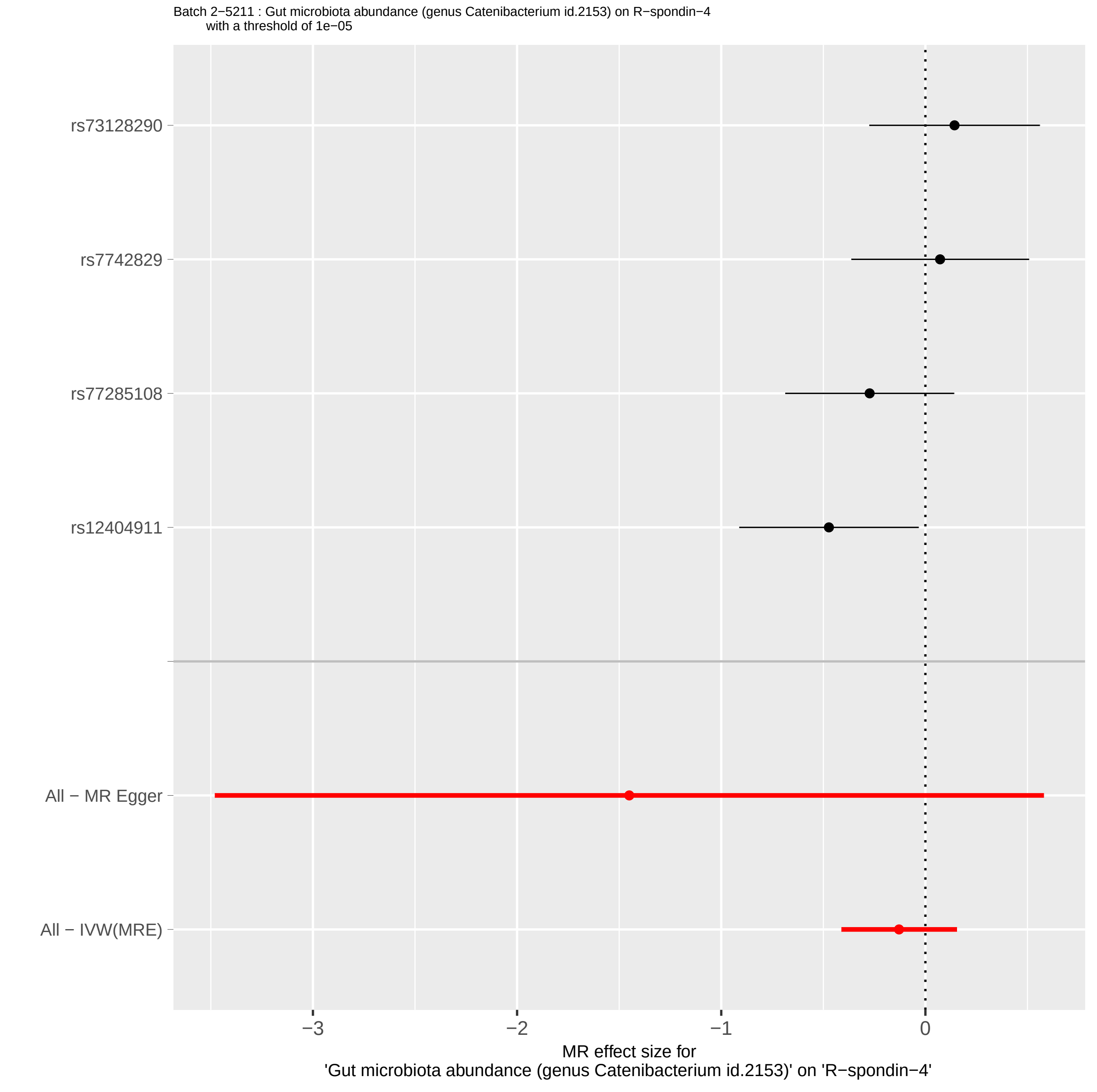
Batch 2−5211 : Gut microbiota abundance (genus Catenibacterium id.2153) on R−spondin−4
with a threshold of 1e−05
rs73128290
rs7742829
rs77285108
rs12404911
All − MR Egger
All − IVW(MRE)
−3
−2
−1
0
MR effect size for
'Gut microbiota abundance (genus Catenibacterium id.2153)' on 'R−spondin−4'

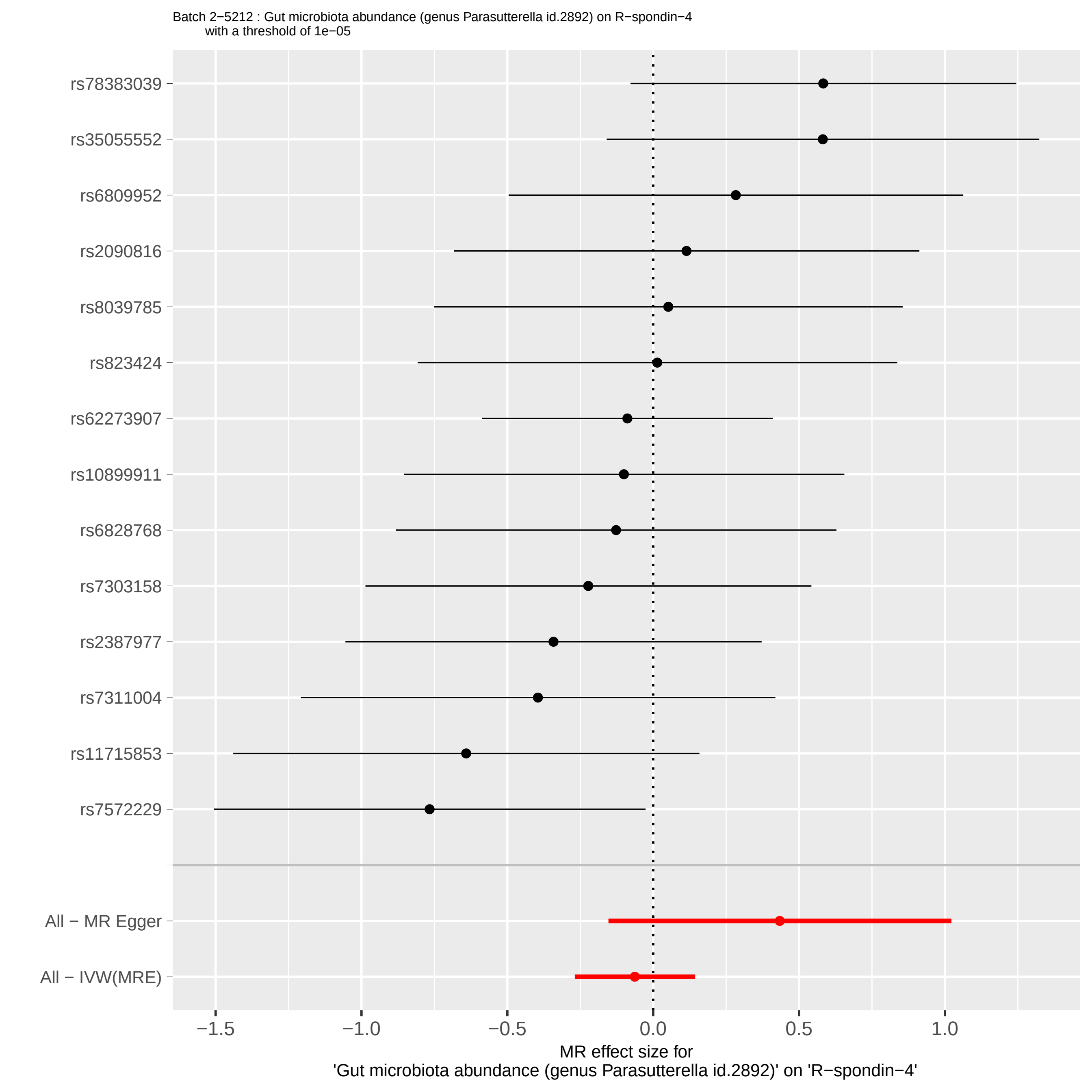

Batch 2−5212 : Gut microbiota abundance (genus Parasutterella id.2892) on R−spondin−4
with a threshold of 1e−05
rs78383039
rs35055552
rs6809952
rs2090816
rs8039785
rs823424
rs62273907
rs10899911
rs6828768
rs7303158
rs2387977
rs7311004
rs11715853
rs7572229
All − MR Egger
All − IVW(MRE)
−1.5
−1.0
−0.5
0.0
0.5
1.0
MR effect size for
'Gut microbiota abundance (genus Parasutterella id.2892)' on 'R−spondin−4'

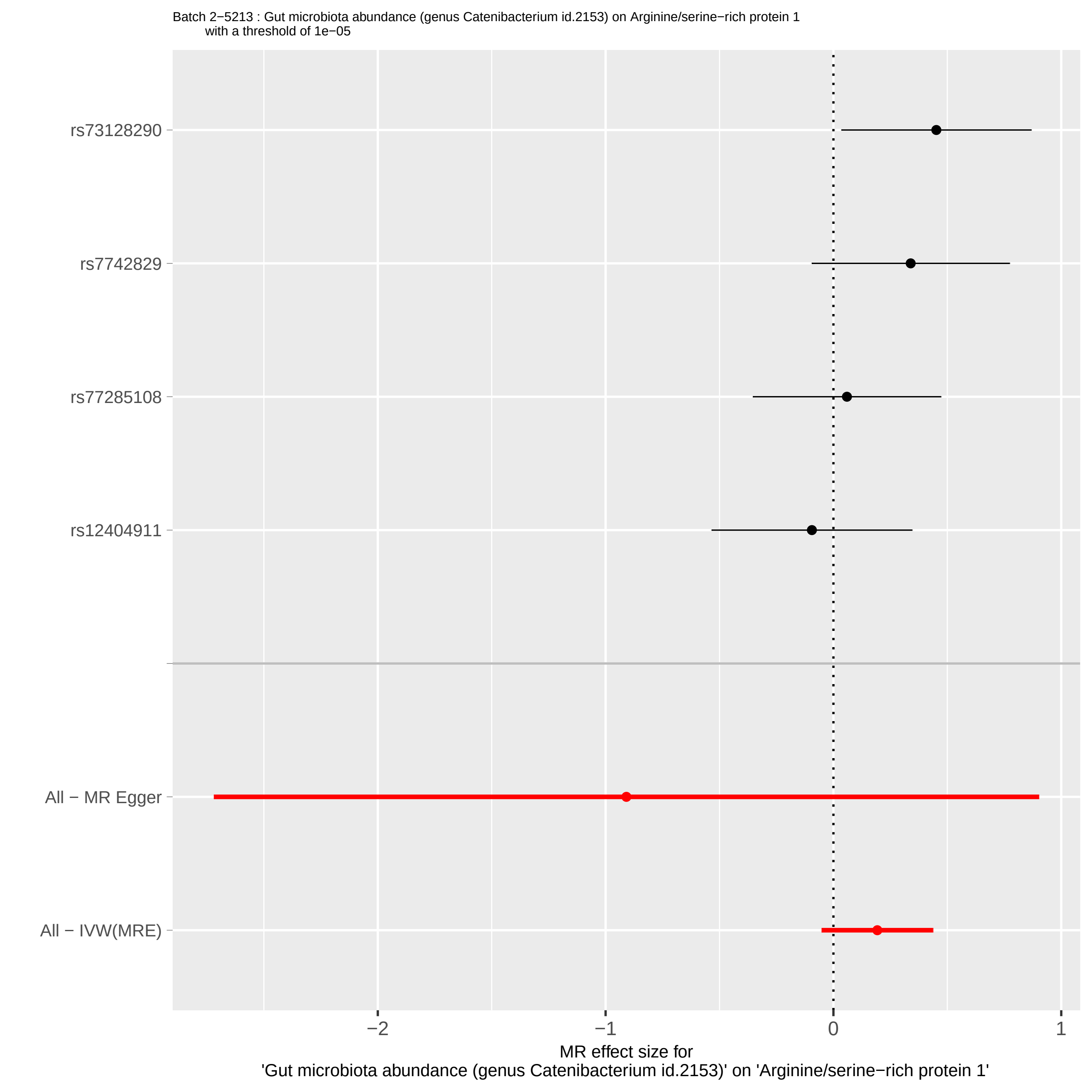
Batch 2−5213 : Gut microbiota abundance (genus Catenibacterium id.2153) on Arginine/serine−rich protein 1
with a threshold of 1e−05
rs73128290
rs7742829
rs77285108
rs12404911
All − MR Egger
All − IVW(MRE)
−2
−1
0
1
MR effect size for
'Gut microbiota abundance (genus Catenibacterium id.2153)' on 'Arginine/serine−rich protein 1'

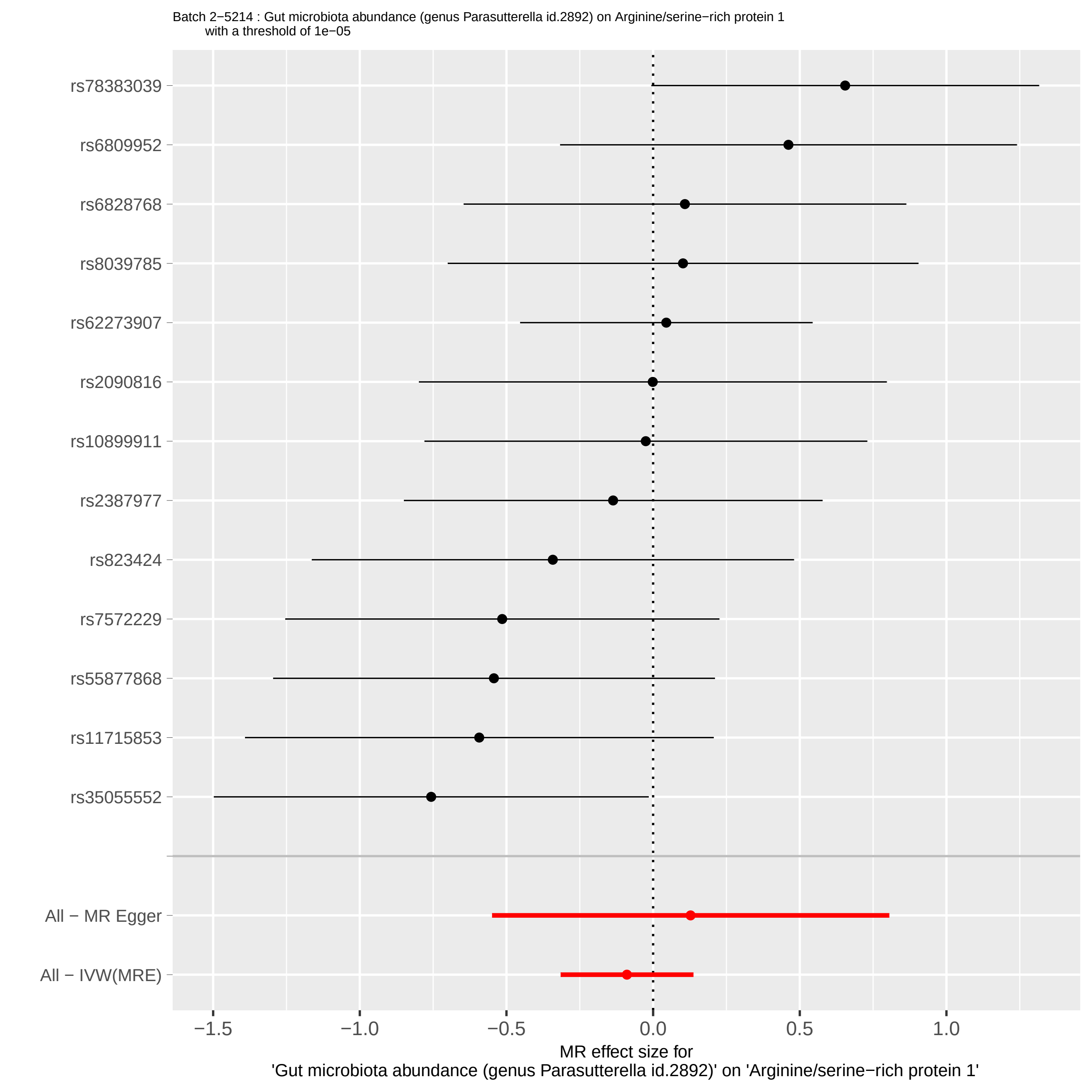

Batch 2−5214 : Gut microbiota abundance (genus Parasutterella id.2892) on Arginine/serine−rich protein 1
with a threshold of 1e−05
rs78383039
rs6809952
rs6828768
rs8039785
rs62273907
rs2090816
rs10899911
rs2387977
rs823424
rs7572229
rs55877868
rs11715853
rs35055552
All − MR Egger
All − IVW(MRE)
−1.5
−1.0
−0.5
0.0
0.5
1.0
MR effect size for
'Gut microbiota abundance (genus Parasutterella id.2892)' on 'Arginine/serine−rich protein 1'

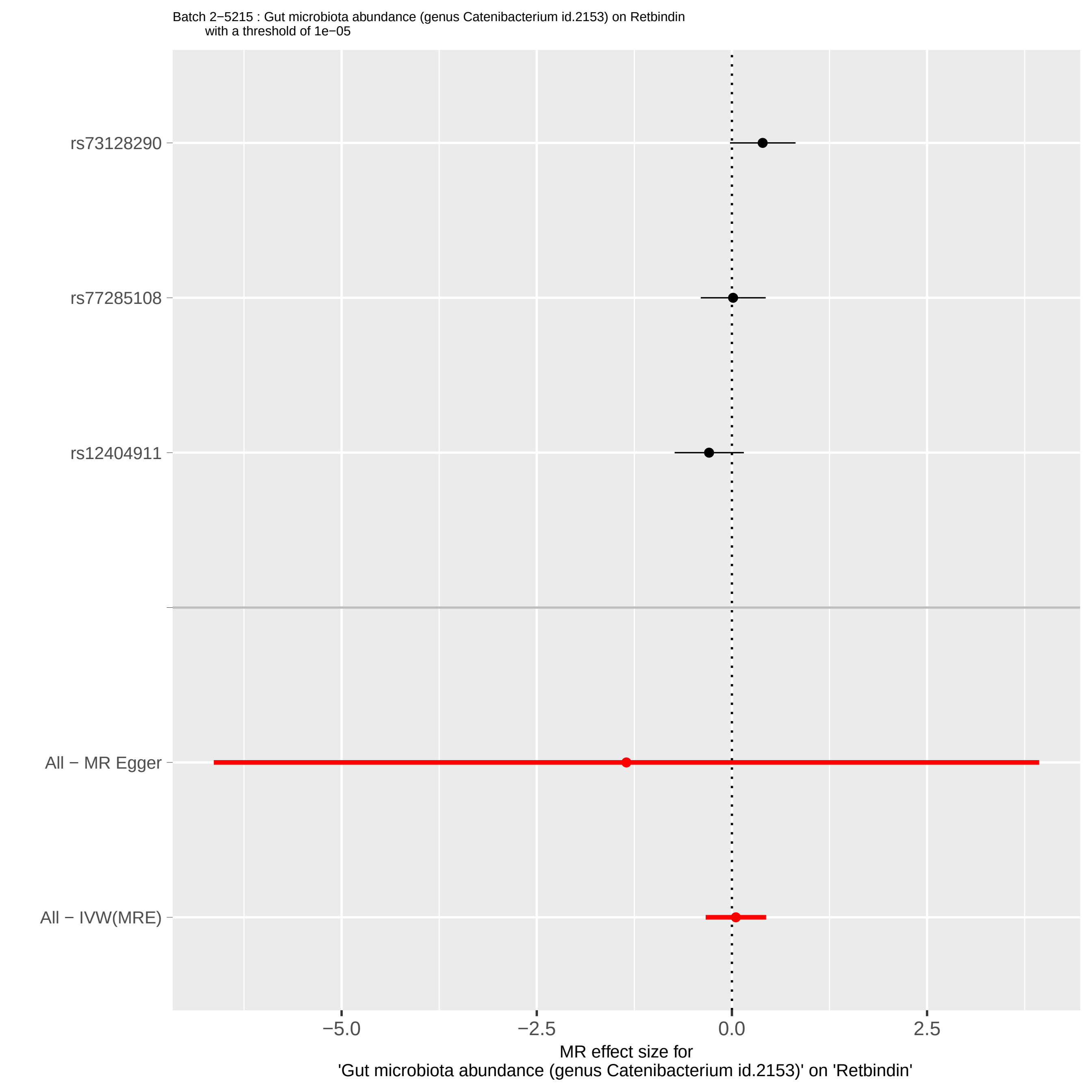

Batch 2−5215 : Gut microbiota abundance (genus Catenibacterium id.2153) on Retbindin
with a threshold of 1e−05
rs73128290
rs77285108
rs12404911
All − MR Egger
All − IVW(MRE)
−5.0
−2.5
0.0
2.5
MR effect size for
'Gut microbiota abundance (genus Catenibacterium id.2153)' on 'Retbindin'

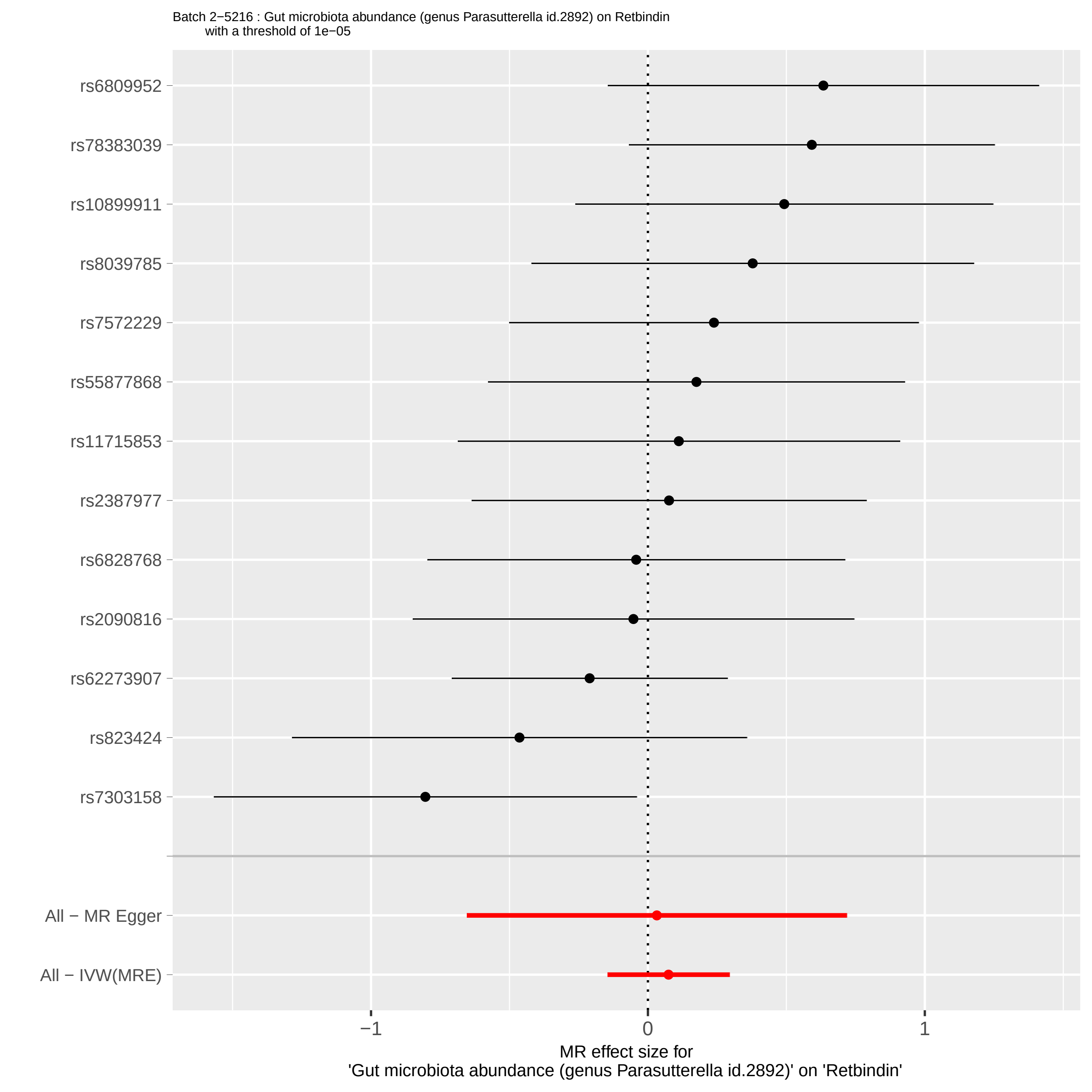
Batch 2−5216 : Gut microbiota abundance (genus Parasutterella id.2892) on Retbindin
with a threshold of 1e−05
rs6809952
rs78383039
rs10899911
rs8039785
rs7572229
rs55877868
rs11715853
rs2387977
rs6828768
rs2090816
rs62273907
rs823424
rs7303158
All − MR Egger
All − IVW(MRE)
−1
0
1
MR effect size for
'Gut microbiota abundance (genus Parasutterella id.2892)' on 'Retbindin'

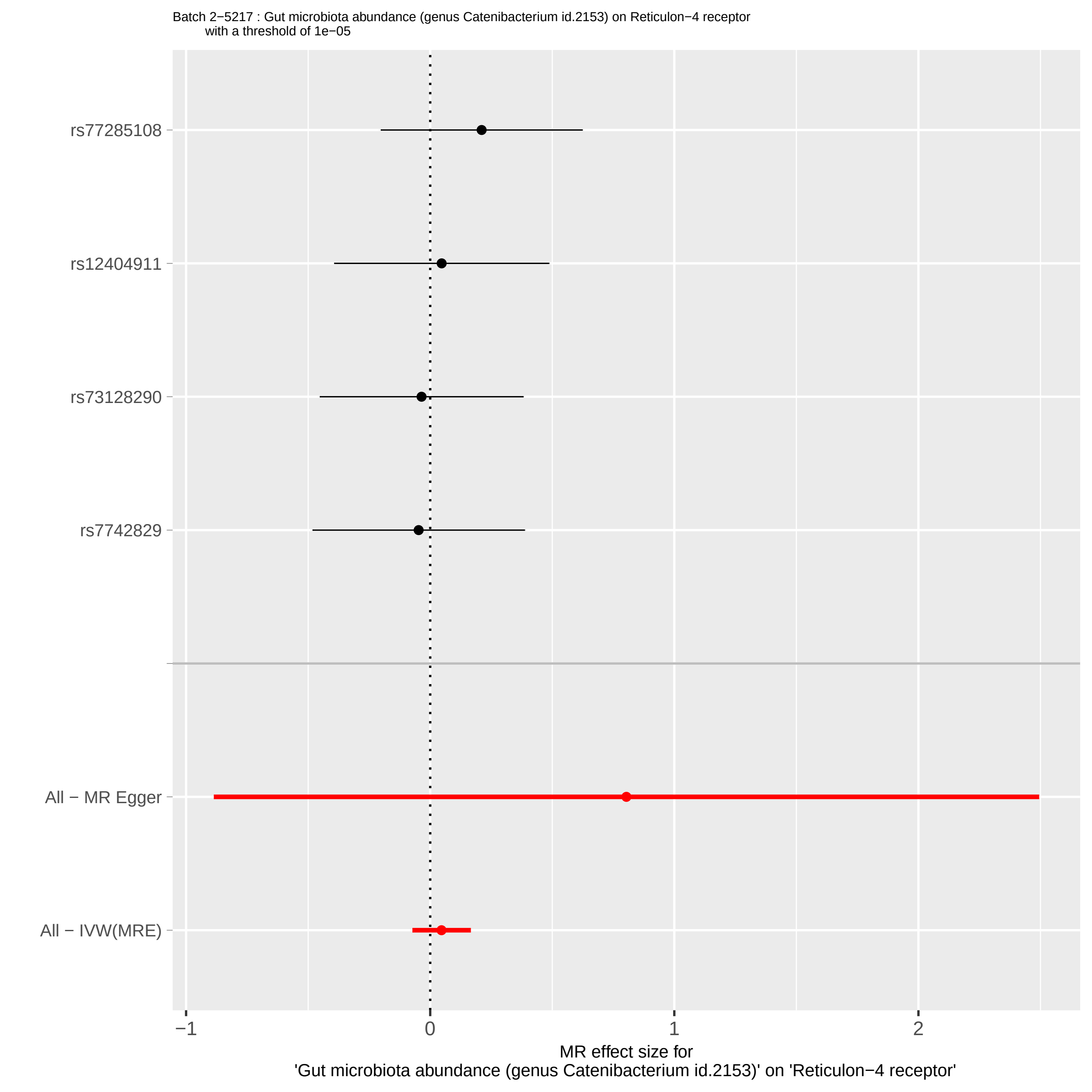
Batch 2−5217 : Gut microbiota abundance (genus Catenibacterium id.2153) on Reticulon−4 receptor
with a threshold of 1e−05
rs77285108
rs12404911
rs73128290
rs7742829
All − MR Egger
All − IVW(MRE)
−1
0
1
2
MR effect size for
'Gut microbiota abundance (genus Catenibacterium id.2153)' on 'Reticulon−4 receptor'

Batch 2−5218 : Gut microbiota abundance (genus Parasutterella id.2892) on Reticulon−4 receptor
with a threshold of 1e−05

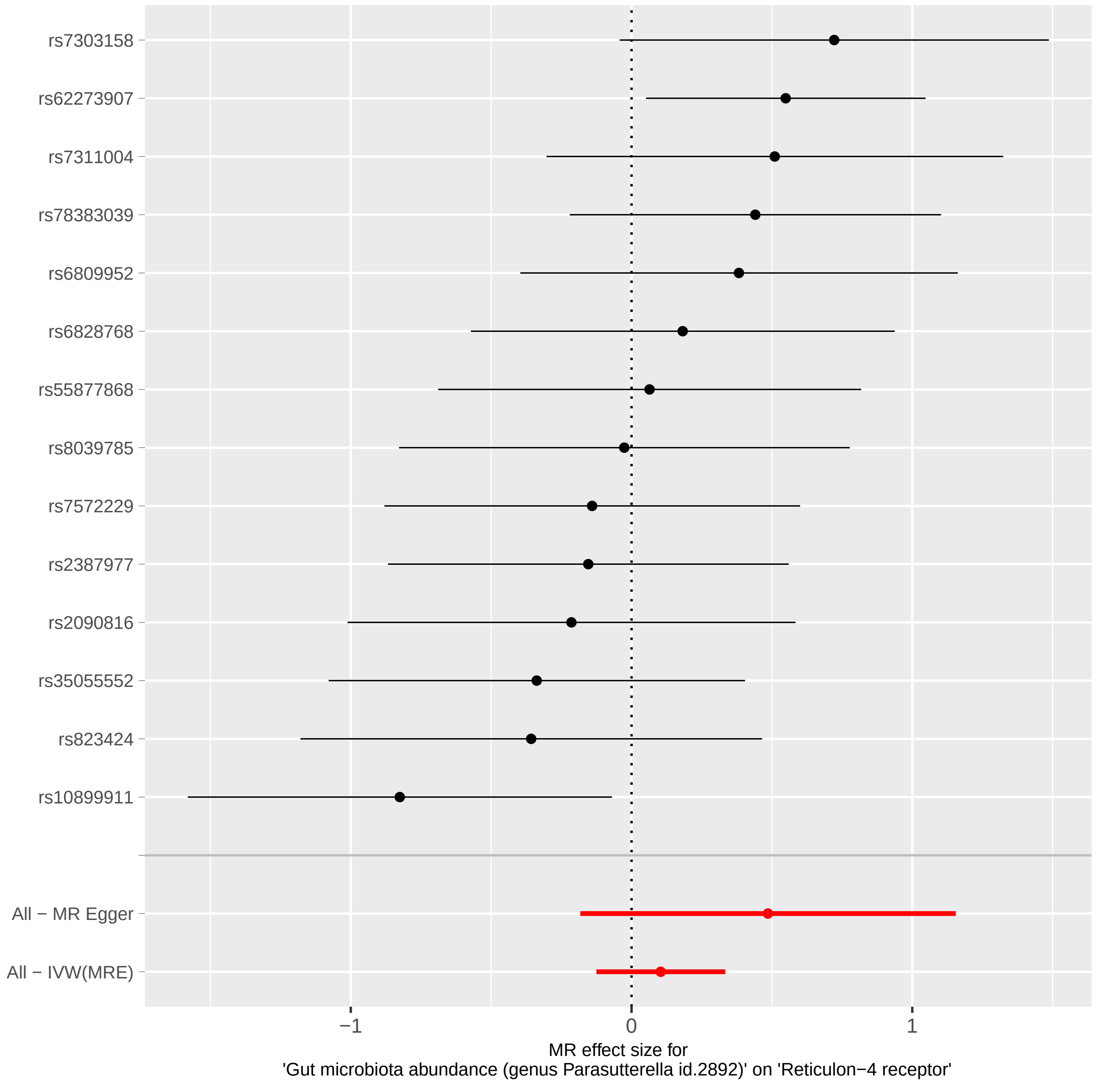

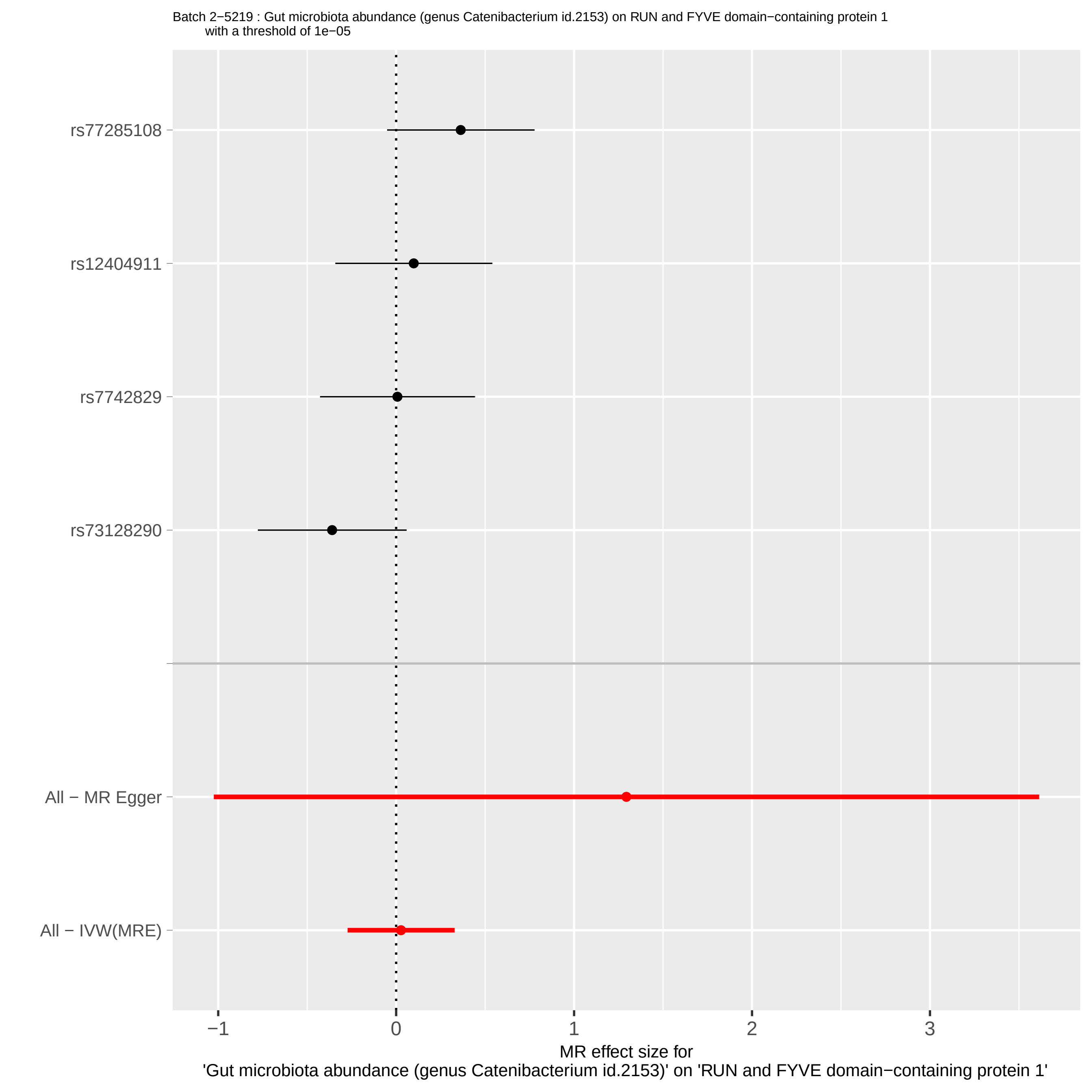
Batch 2−5219 : Gut microbiota abundance (genus Catenibacterium id.2153) on RUN and FYVE domain−containing protein 1
with a threshold of 1e−05
rs77285108
rs12404911
rs7742829
rs73128290
All − MR Egger
All − IVW(MRE)
−1
0
1
2
3
MR effect size for
'Gut microbiota abundance (genus Catenibacterium id.2153)' on 'RUN and FYVE domain−containing protein 1'

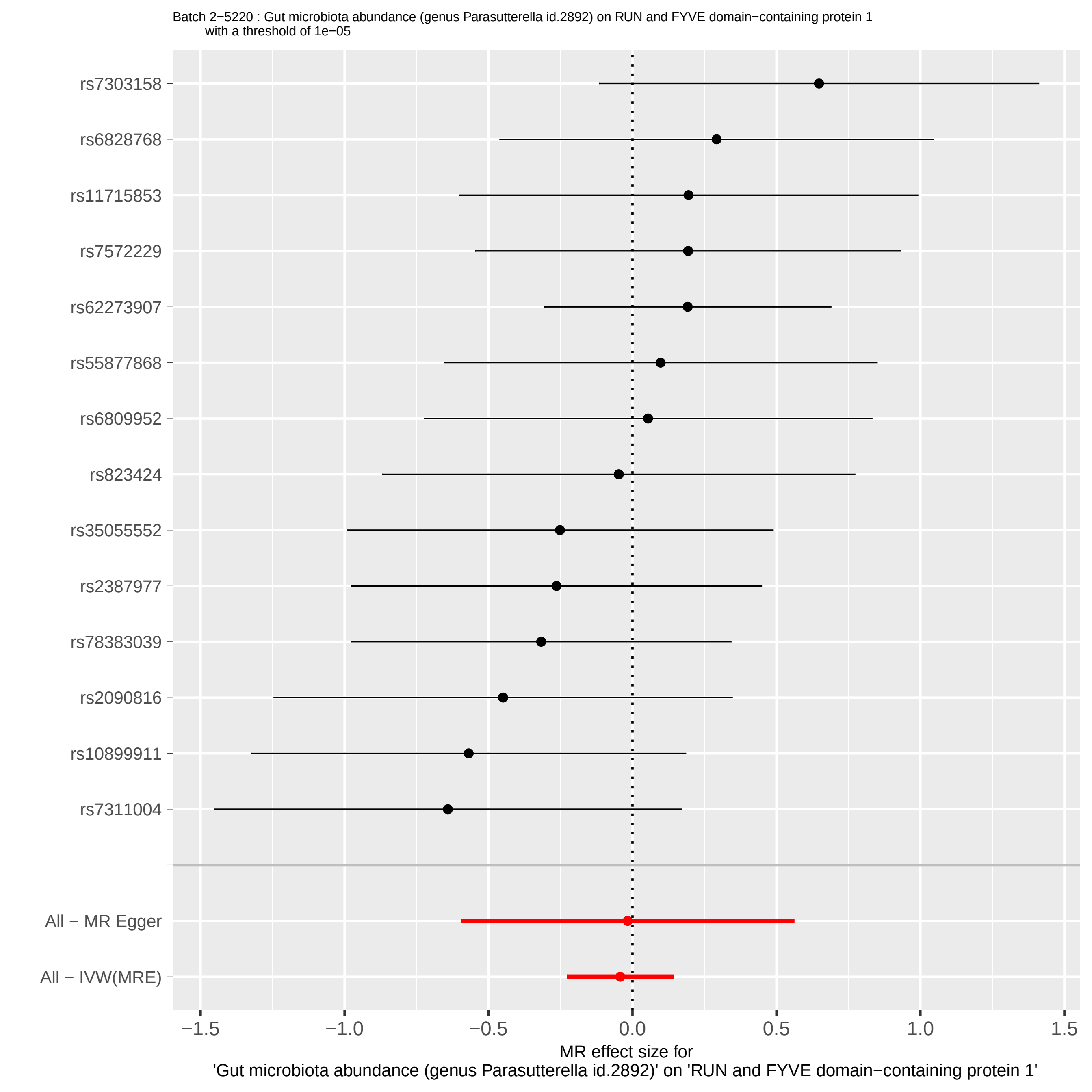

Batch 2−5220 : Gut microbiota abundance (genus Parasutterella id.2892) on RUN and FYVE domain−containing protein 1
with a threshold of 1e−05
rs7303158
rs6828768
rs11715853
rs7572229
rs62273907
rs55877868
rs6809952
rs823424
rs35055552
rs2387977
rs78383039
rs2090816
rs10899911
rs7311004
All − MR Egger
All − IVW(MRE)
−1.5
−1.0
−0.5
0.0
0.5
1.0
1.5
MR effect size for
'Gut microbiota abundance (genus Parasutterella id.2892)' on 'RUN and FYVE domain−containing protein 1'

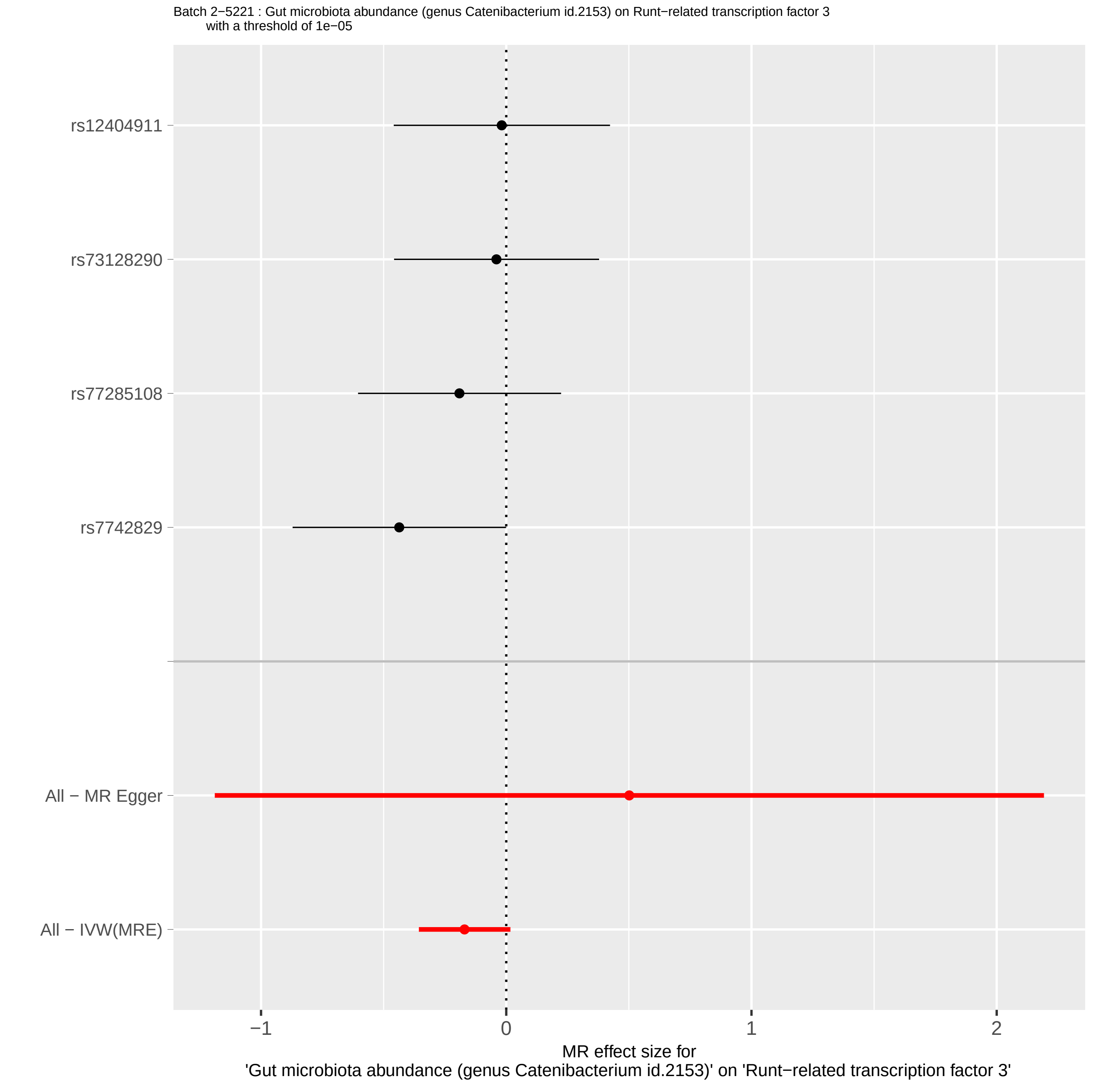
Batch 2−5221 : Gut microbiota abundance (genus Catenibacterium id.2153) on Runt−related transcription factor 3
with a threshold of 1e−05
rs12404911
rs73128290
rs77285108
rs7742829
All − MR Egger
All − IVW(MRE)
−1
0
1
2
MR effect size for
'Gut microbiota abundance (genus Catenibacterium id.2153)' on 'Runt−related transcription factor 3'

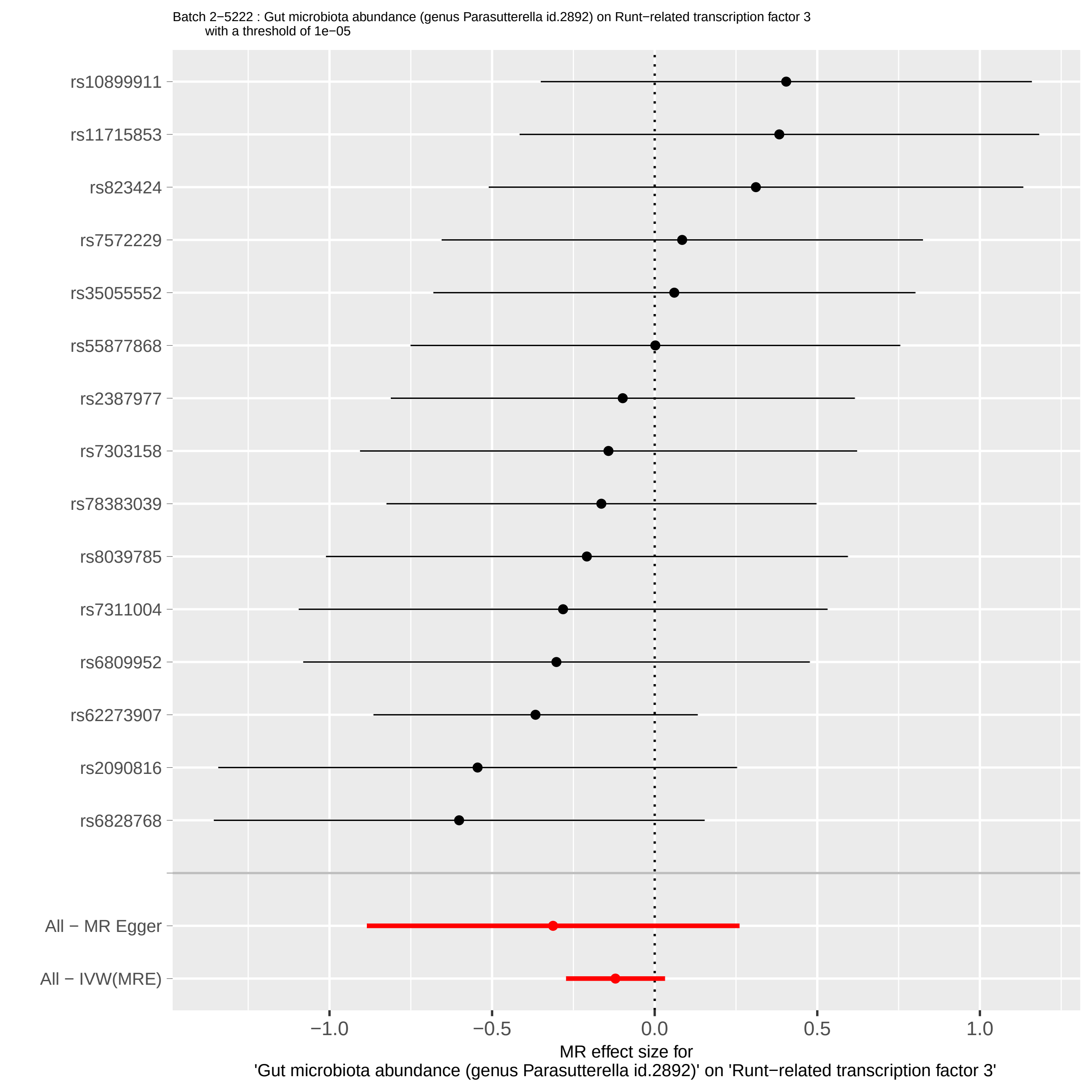

Batch 2−5222 : Gut microbiota abundance (genus Parasutterella id.2892) on Runt−related transcription factor 3
with a threshold of 1e−05
rs10899911
rs11715853
rs823424
rs7572229
rs35055552
rs55877868
rs2387977
rs7303158
rs78383039
rs8039785
rs7311004
rs6809952
rs62273907
rs2090816
rs6828768
All − MR Egger
All − IVW(MRE)
−1.0
−0.5
0.0
0.5
1.0
MR effect size for
'Gut microbiota abundance (genus Parasutterella id.2892)' on 'Runt−related transcription factor 3'

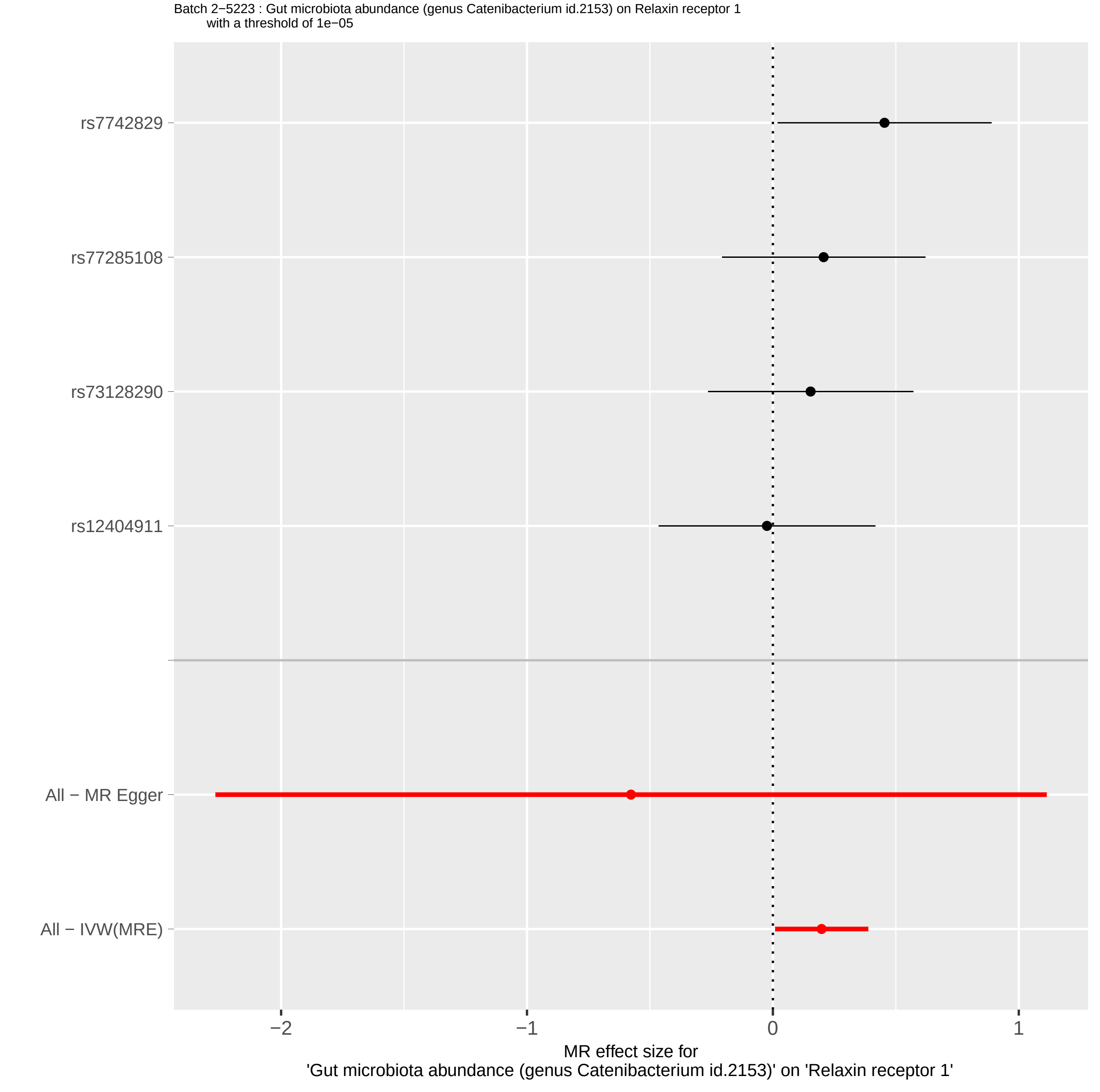
Batch 2−5223 : Gut microbiota abundance (genus Catenibacterium id.2153) on Relaxin receptor 1
with a threshold of 1e−05
rs7742829
rs77285108
rs73128290
rs12404911
All − MR Egger
All − IVW(MRE)
−2
−1
0
1
MR effect size for
'Gut microbiota abundance (genus Catenibacterium id.2153)' on 'Relaxin receptor 1'

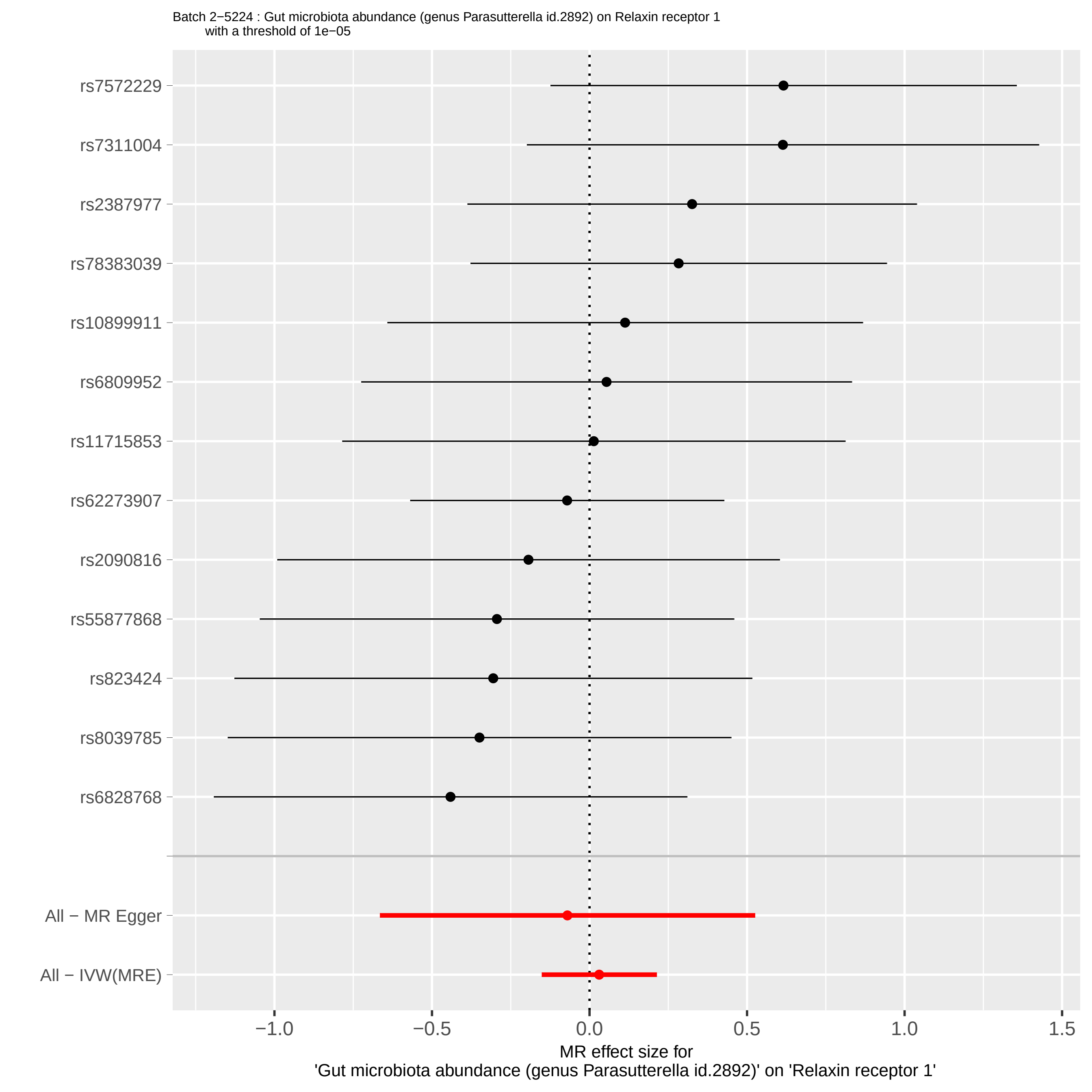

Batch 2−5224 : Gut microbiota abundance (genus Parasutterella id.2892) on Relaxin receptor 1
with a threshold of 1e−05
rs7572229
rs7311004
rs2387977
rs78383039
rs10899911
rs6809952
rs11715853
rs62273907
rs2090816
rs55877868
rs823424
rs8039785
rs6828768
All − MR Egger
All − IVW(MRE)
−1.0
−0.5
0.0
0.5
1.0
1.5
MR effect size for
'Gut microbiota abundance (genus Parasutterella id.2892)' on 'Relaxin receptor 1'

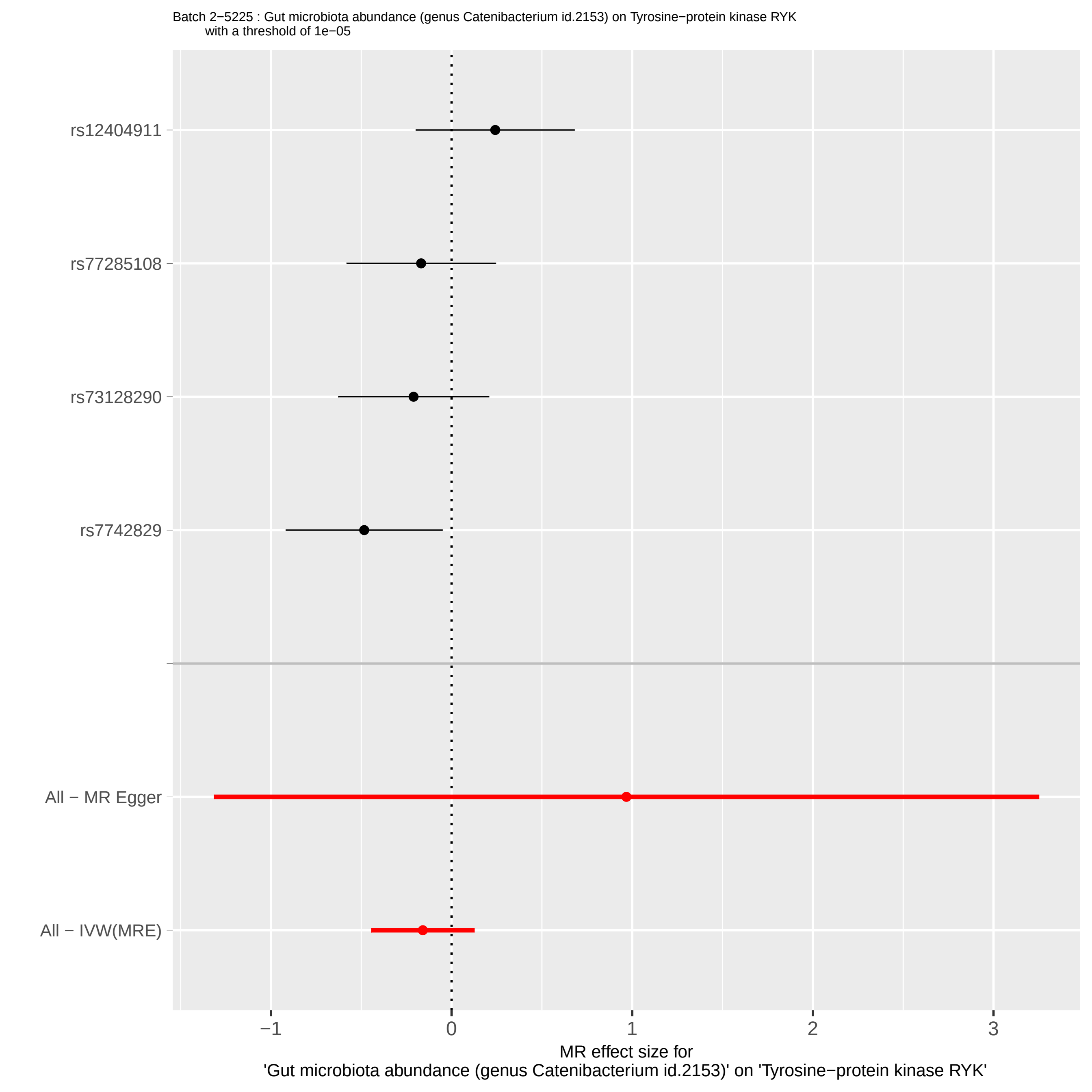

Batch 2−5225 : Gut microbiota abundance (genus Catenibacterium id.2153) on Tyrosine−protein kinase RYK
with a threshold of 1e−05
rs12404911
rs77285108
rs73128290
rs7742829
All − MR Egger
All − IVW(MRE)
−1
0
1
2
3
MR effect size for
'Gut microbiota abundance (genus Catenibacterium id.2153)' on 'Tyrosine−protein kinase RYK'

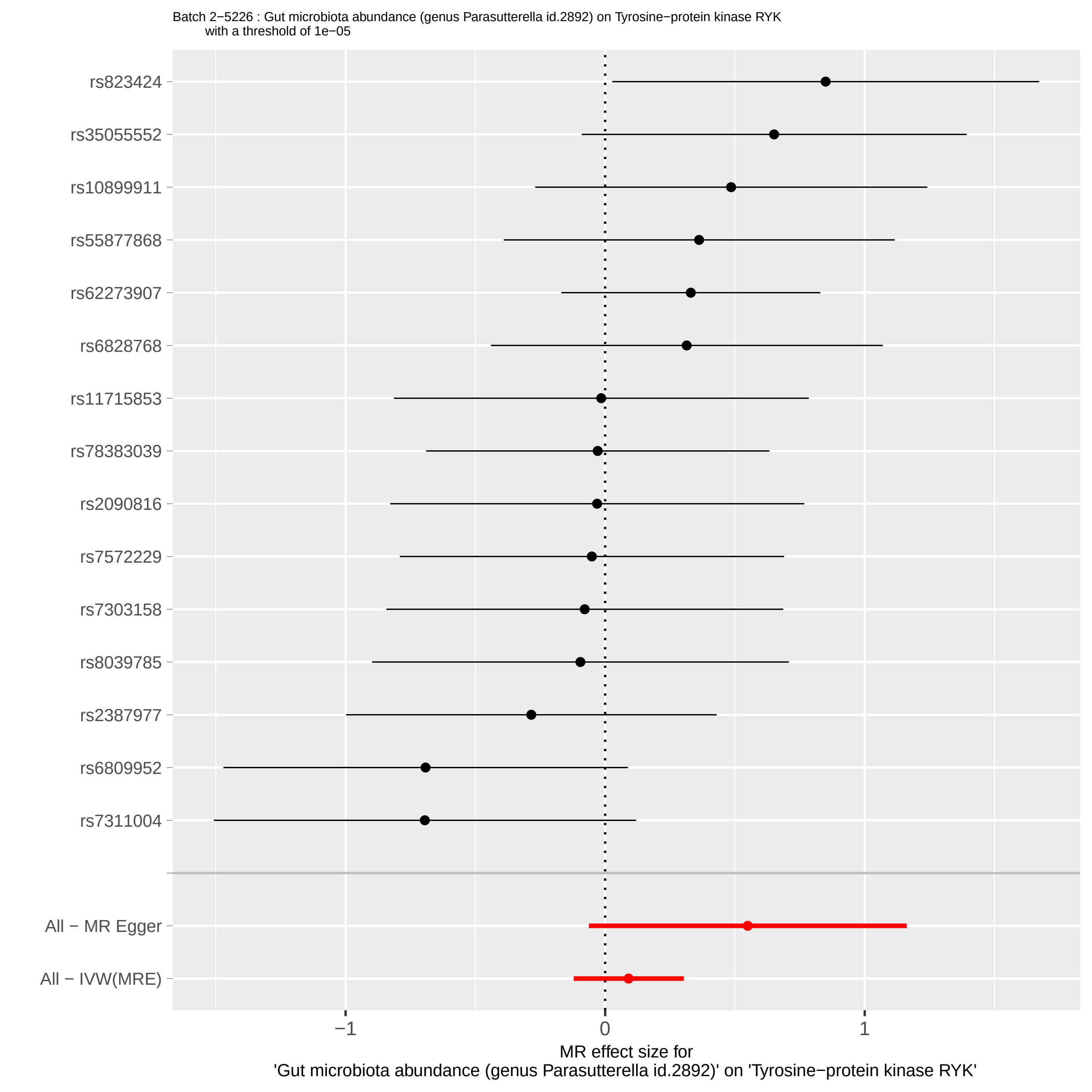
Batch 2−5226 : Gut microbiota abundance (genus Parasutterella id.2892) on Tyrosine−protein kinase RYK
with a threshold of 1e−05
rs823424
rs35055552
rs10899911
rs55877868
rs62273907
rs6828768
rs11715853
rs78383039
rs2090816
rs7572229
rs7303158
rs8039785
rs2387977
rs6809952
rs7311004
All − MR Egger
All − IVW(MRE)
−1
0
1
MR effect size for
'Gut microbiota abundance (genus Parasutterella id.2892)' on 'Tyrosine−protein kinase RYK'

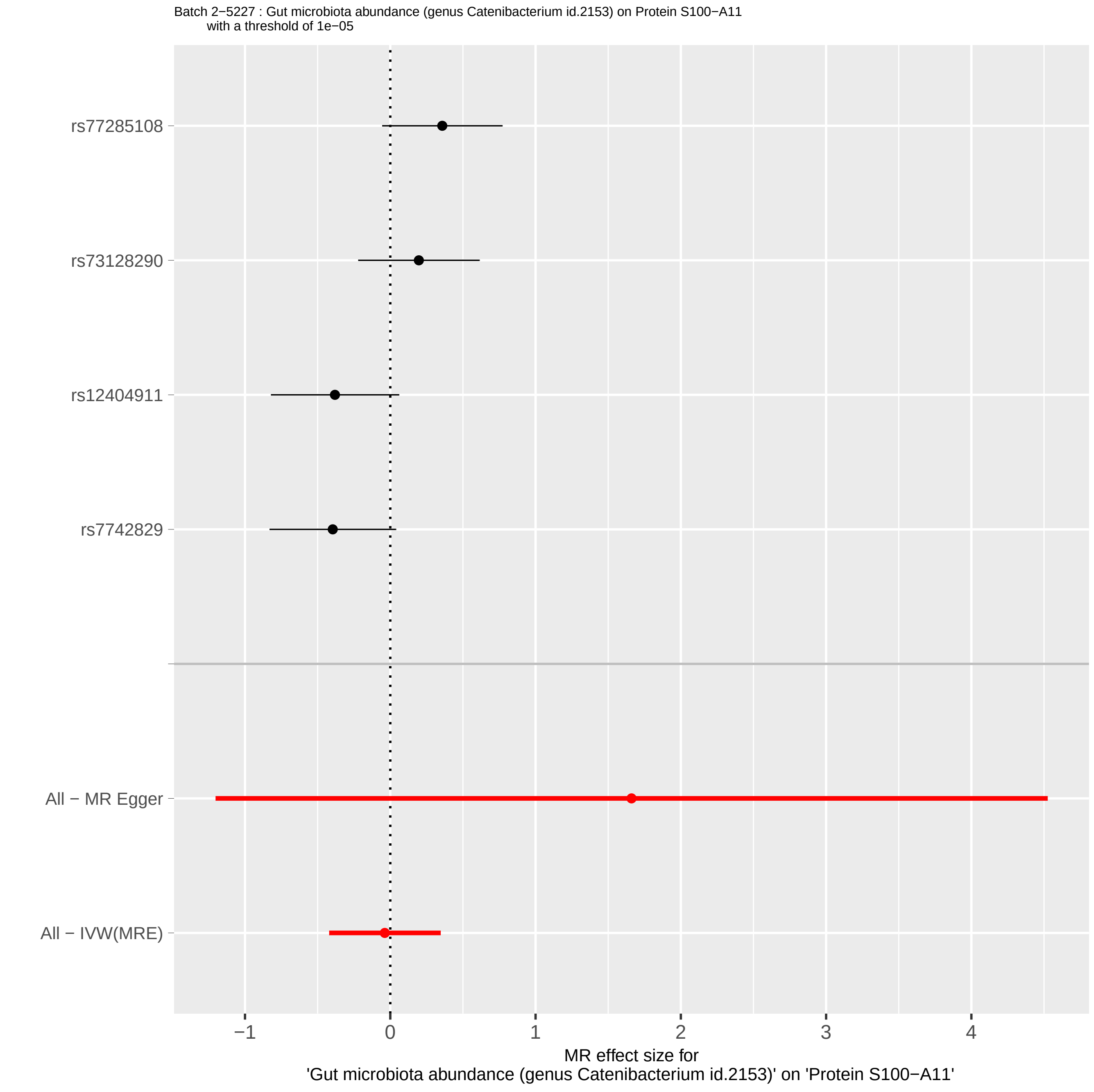

Batch 2−5227 : Gut microbiota abundance (genus Catenibacterium id.2153) on Protein S100−A11
with a threshold of 1e−05
rs77285108
rs73128290
rs12404911
rs7742829
All − MR Egger
All − IVW(MRE)
−1
0
1
2
3
4
MR effect size for
'Gut microbiota abundance (genus Catenibacterium id.2153)' on 'Protein S100−A11'

Batch 2−5228 : Gut microbiota abundance (genus Parasutterella id.2892) on Protein S100−A11
with a threshold of 1e−05

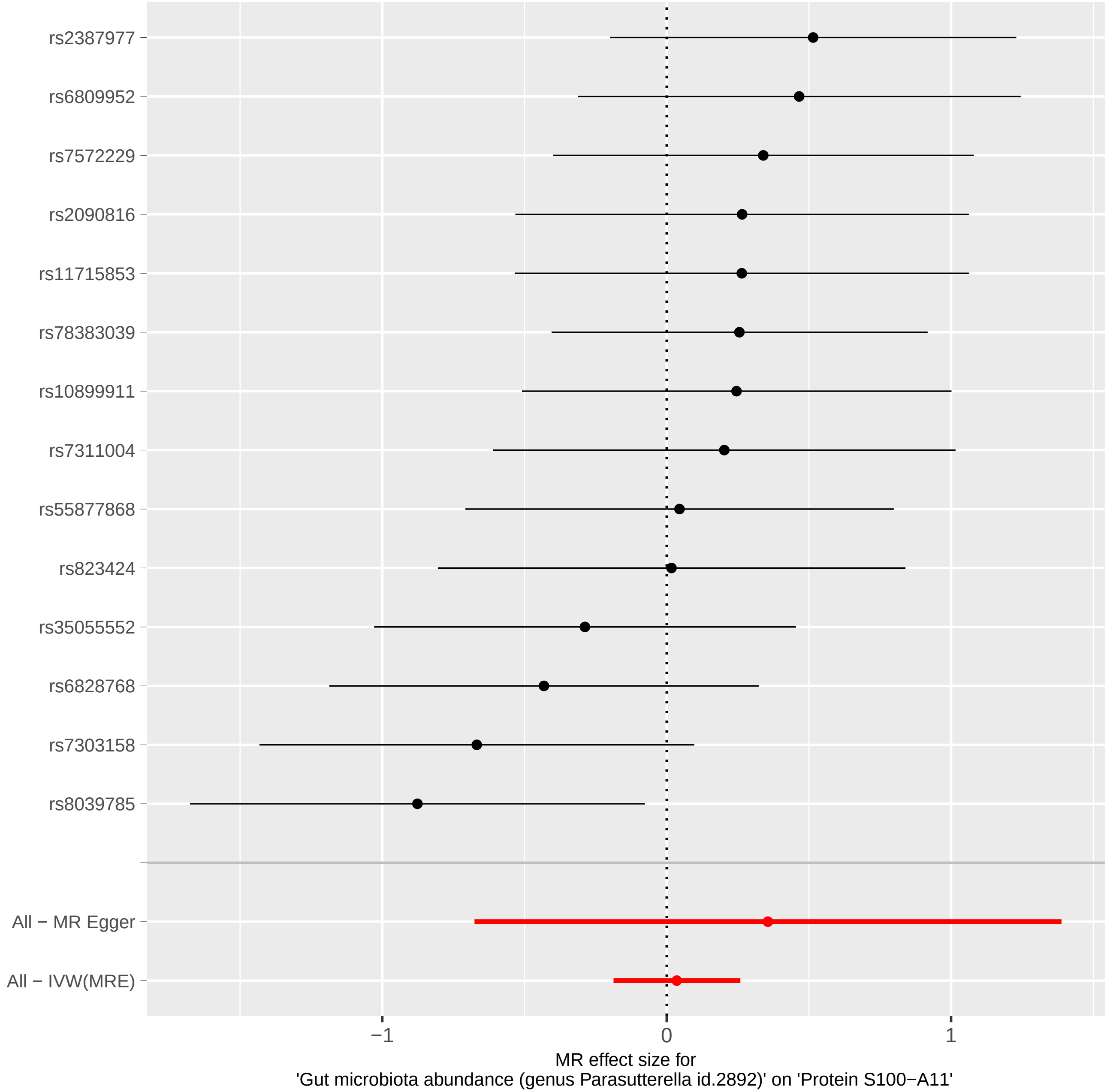

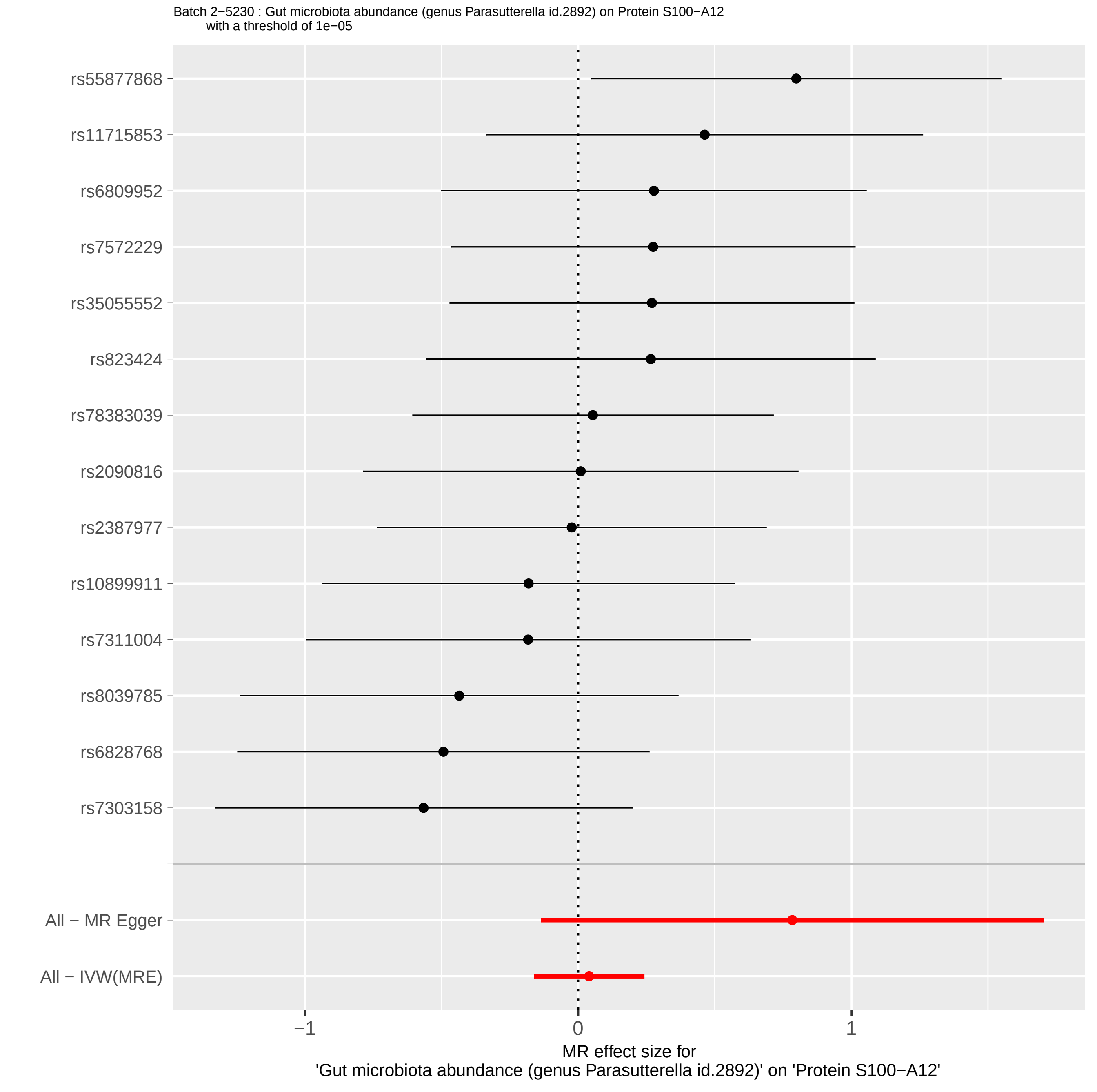

Batch 2−5230 : Gut microbiota abundance (genus Parasutterella id.2892) on Protein S100−A12
with a threshold of 1e−05
rs55877868
rs11715853
rs6809952
rs7572229
rs35055552
rs823424
rs78383039
rs2090816
rs2387977
rs10899911
rs7311004
rs8039785
rs6828768
rs7303158
All − MR Egger
All − IVW(MRE)
−1
0
1
MR effect size for
'Gut microbiota abundance (genus Parasutterella id.2892)' on 'Protein S100−A12'

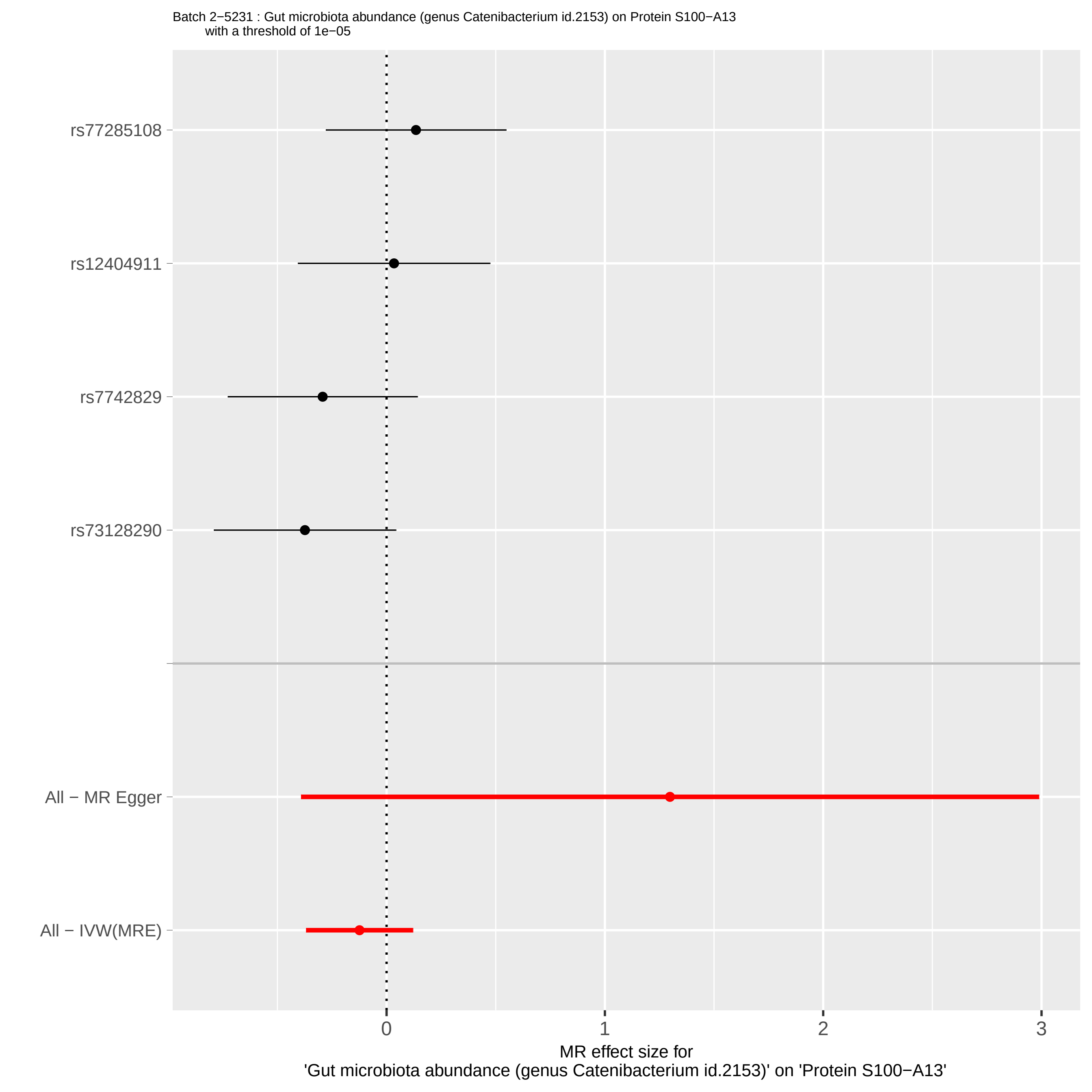

Batch 2−5231 : Gut microbiota abundance (genus Catenibacterium id.2153) on Protein S100−A13
with a threshold of 1e−05
rs77285108
rs12404911
rs7742829
rs73128290
All − MR Egger
All − IVW(MRE)
0
1
2
3
MR effect size for
'Gut microbiota abundance (genus Catenibacterium id.2153)' on 'Protein S100−A13'

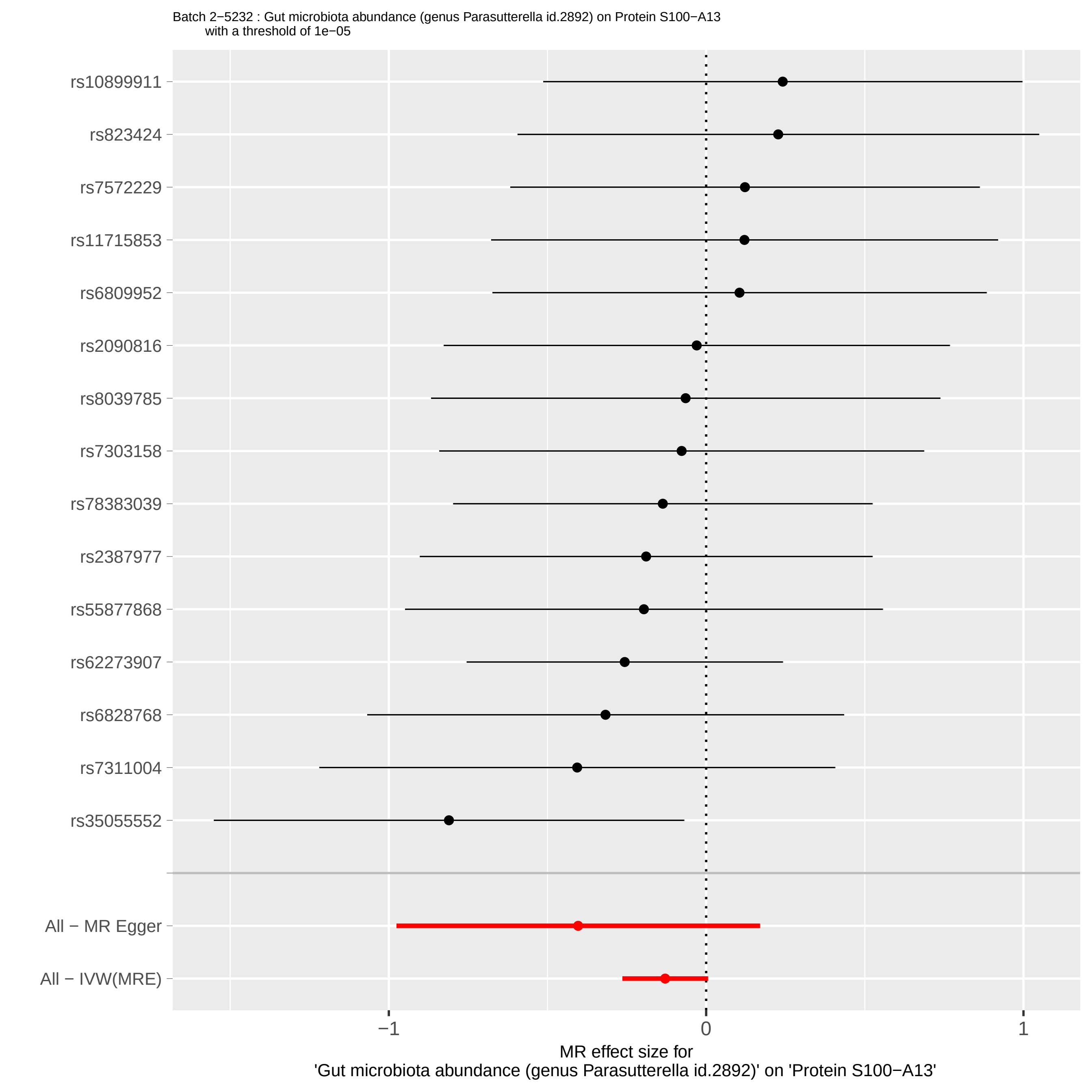

Batch 2−5232 : Gut microbiota abundance (genus Parasutterella id.2892) on Protein S100−A13
with a threshold of 1e−05
rs10899911
rs823424
rs7572229
rs11715853
rs6809952
rs2090816
rs8039785
rs7303158
rs78383039
rs2387977
rs55877868
rs62273907
rs6828768
rs7311004
rs35055552
All − MR Egger
All − IVW(MRE)
−1
0
1
MR effect size for
'Gut microbiota abundance (genus Parasutterella id.2892)' on 'Protein S100−A13'

Batch 2−5233 : Gut microbiota abundance (genus Catenibacterium id.2153) on Protein S100−A2
with a threshold of 1e−05

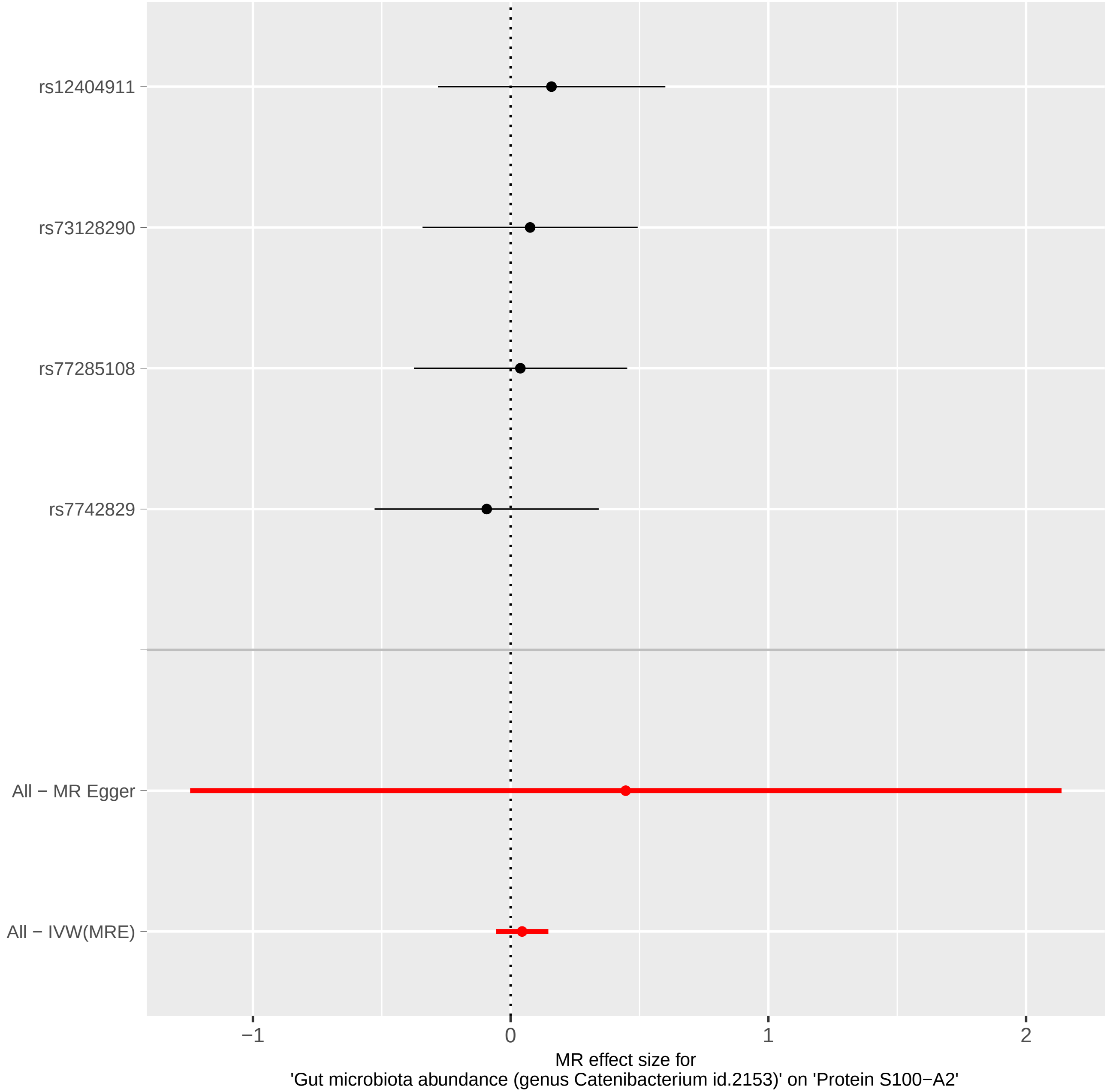

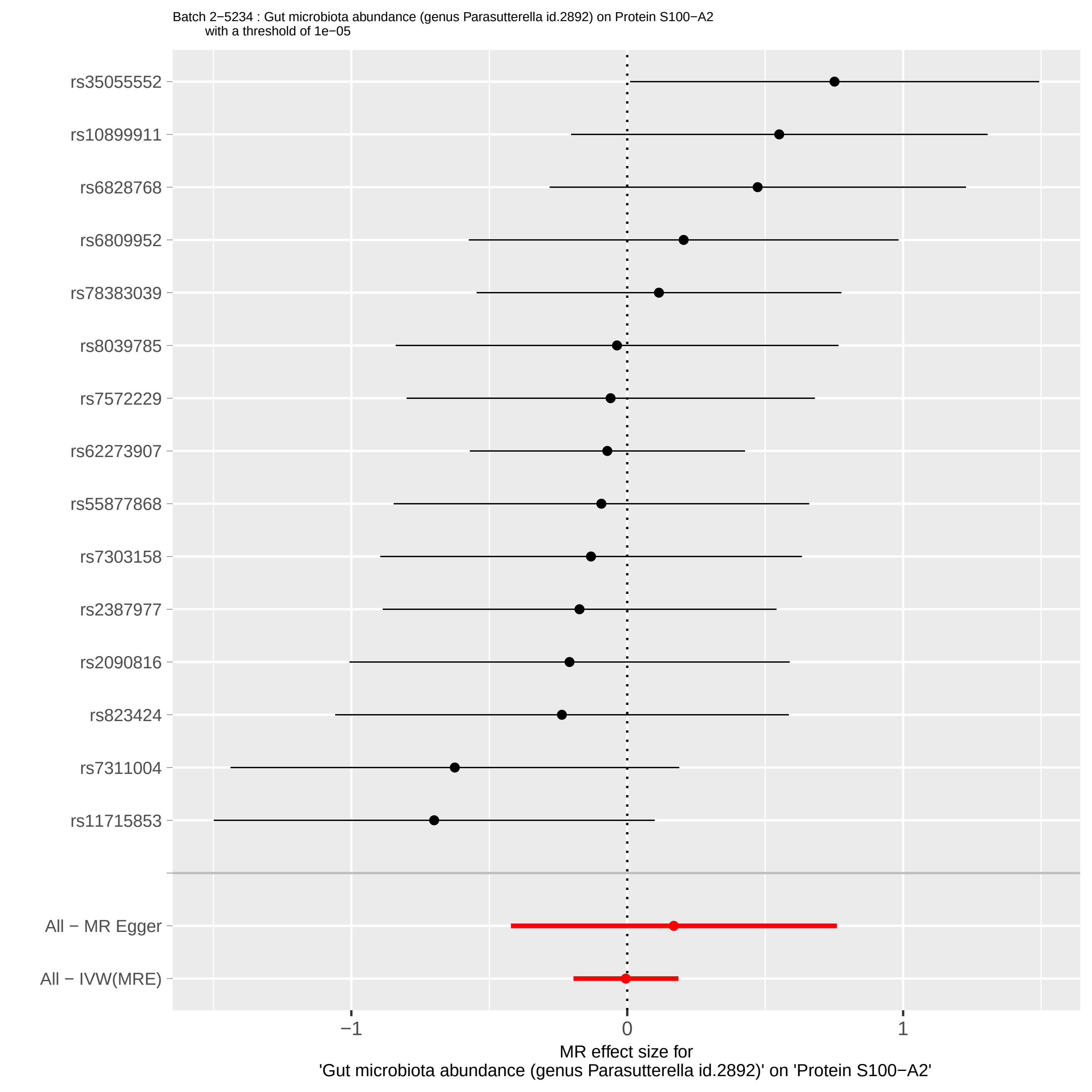

Batch 2−5234 : Gut microbiota abundance (genus Parasutterella id.2892) on Protein S100−A2
with a threshold of 1e−05
rs35055552
rs10899911
rs6828768
rs6809952
rs78383039
rs8039785
rs7572229
rs62273907
rs55877868
rs7303158
rs2387977
rs2090816
rs823424
rs7311004
rs11715853
All − MR Egger
All − IVW(MRE)
−1
0
1
MR effect size for
'Gut microbiota abundance (genus Parasutterella id.2892)' on 'Protein S100−A2'

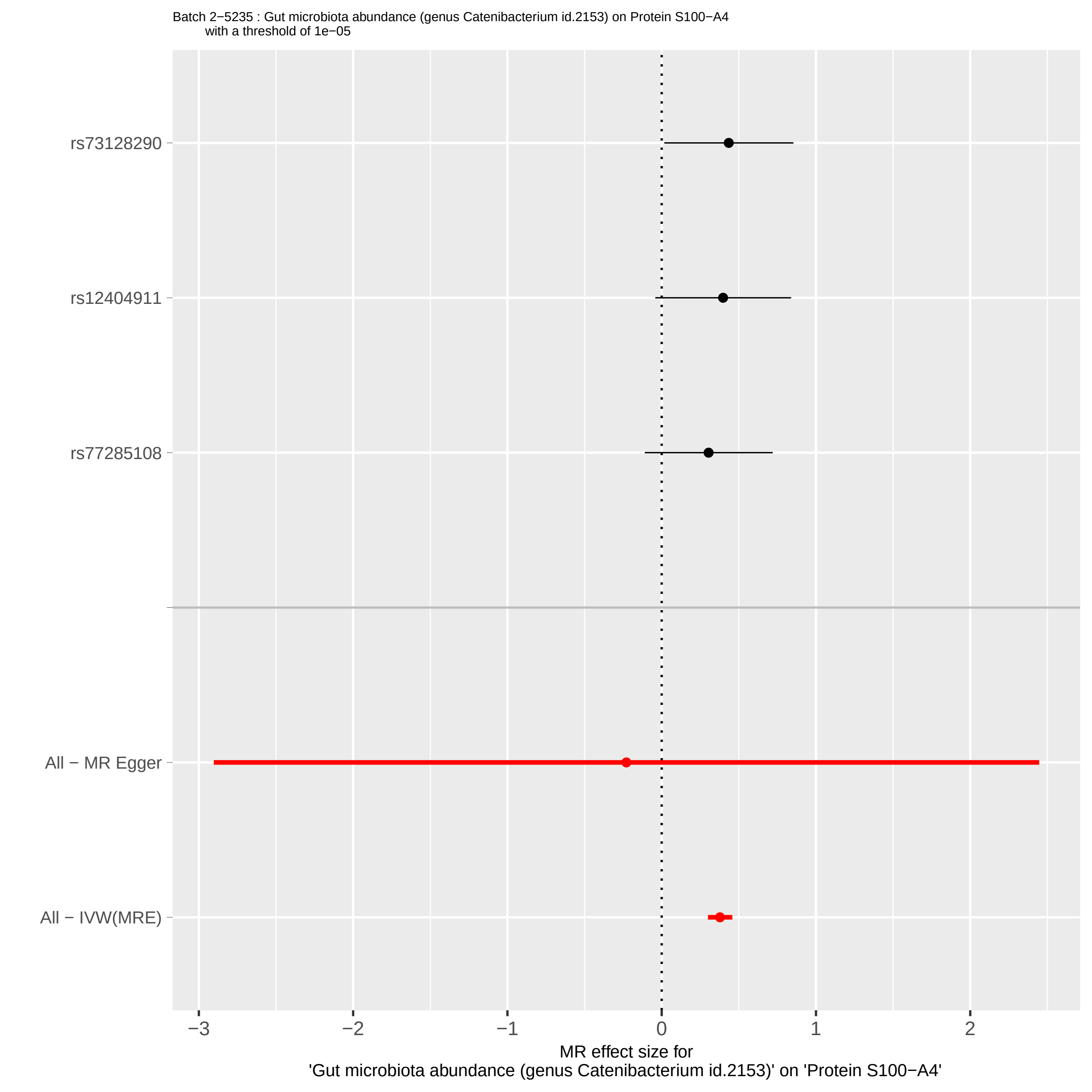

Batch 2−5235 : Gut microbiota abundance (genus Catenibacterium id.2153) on Protein S100−A4
with a threshold of 1e−05
rs73128290
rs12404911
rs77285108
All − MR Egger
All − IVW(MRE)
−3
−2
−1
0
1
2
MR effect size for
'Gut microbiota abundance (genus Catenibacterium id.2153)' on 'Protein S100−A4'

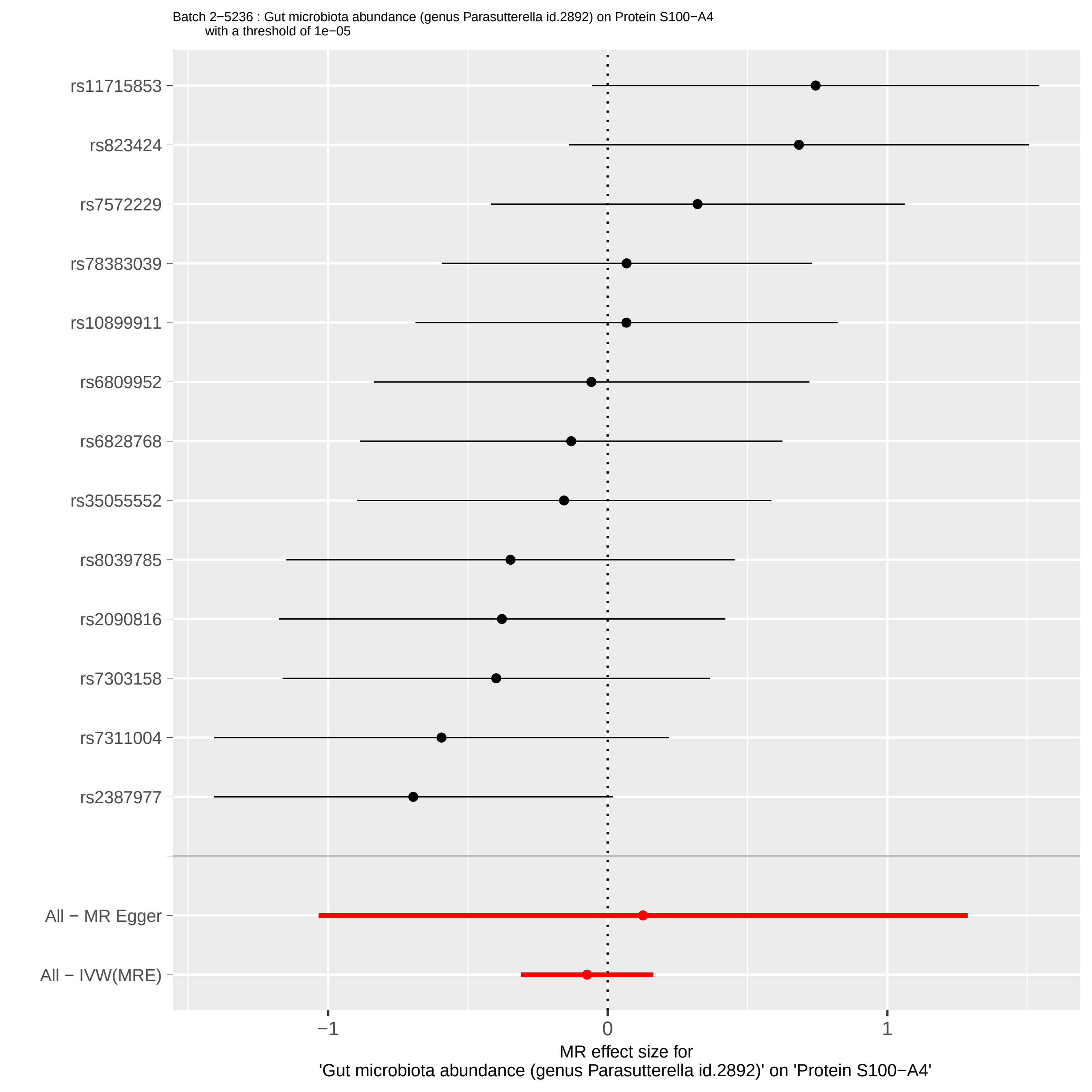

Batch 2−5236 : Gut microbiota abundance (genus Parasutterella id.2892) on Protein S100−A4
with a threshold of 1e−05
rs11715853
rs823424
rs7572229
rs78383039
rs10899911
rs6809952
rs6828768
rs35055552
rs8039785
rs2090816
rs7303158
rs7311004
rs2387977
All − MR Egger
All − IVW(MRE)
−1
0
1
MR effect size for
'Gut microbiota abundance (genus Parasutterella id.2892)' on 'Protein S100−A4'

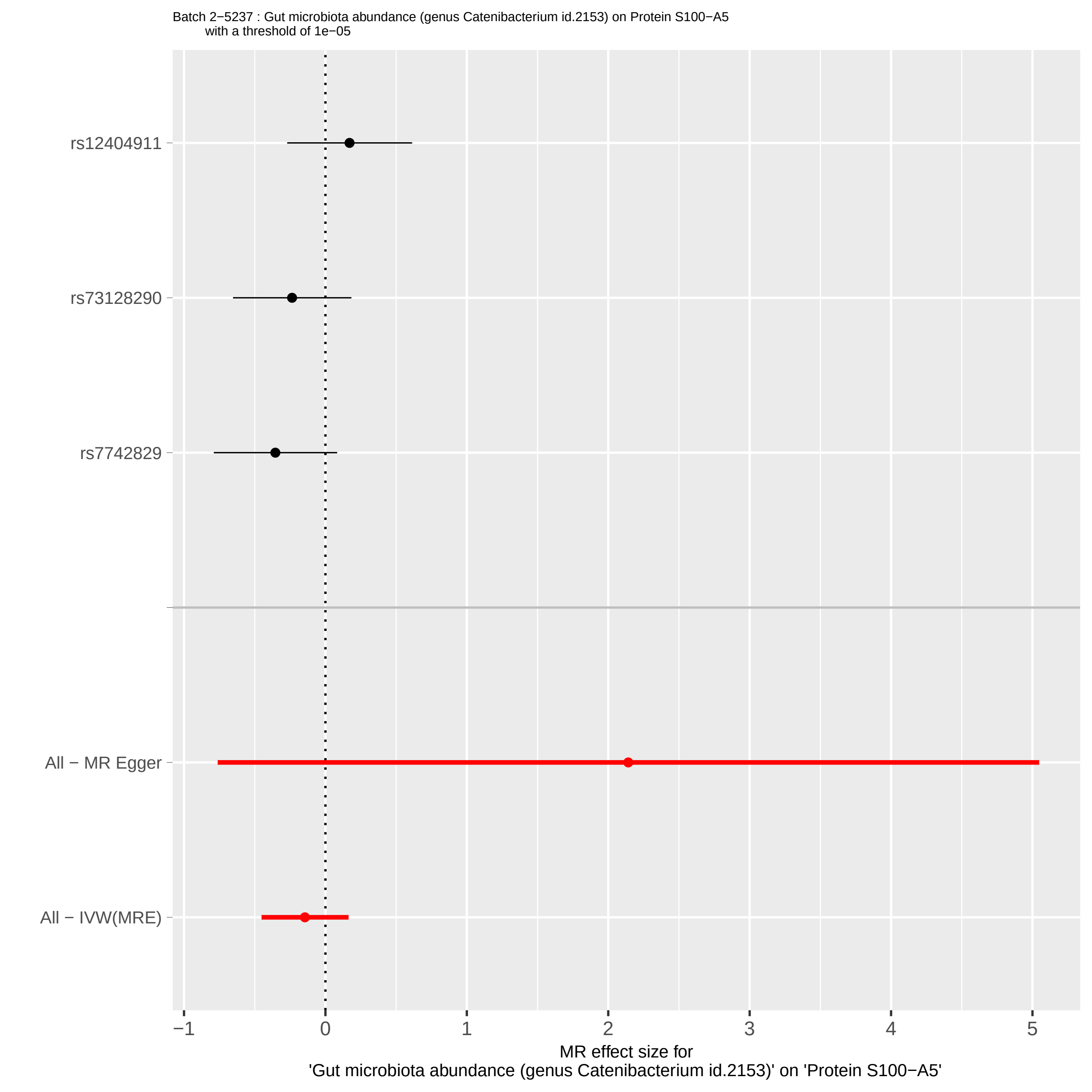

Batch 2−5237 : Gut microbiota abundance (genus Catenibacterium id.2153) on Protein S100−A5
with a threshold of 1e−05
rs12404911
rs73128290
rs7742829
All − MR Egger
All − IVW(MRE)
−1
0
1
2
3
4
5
MR effect size for
'Gut microbiota abundance (genus Catenibacterium id.2153)' on 'Protein S100−A5'

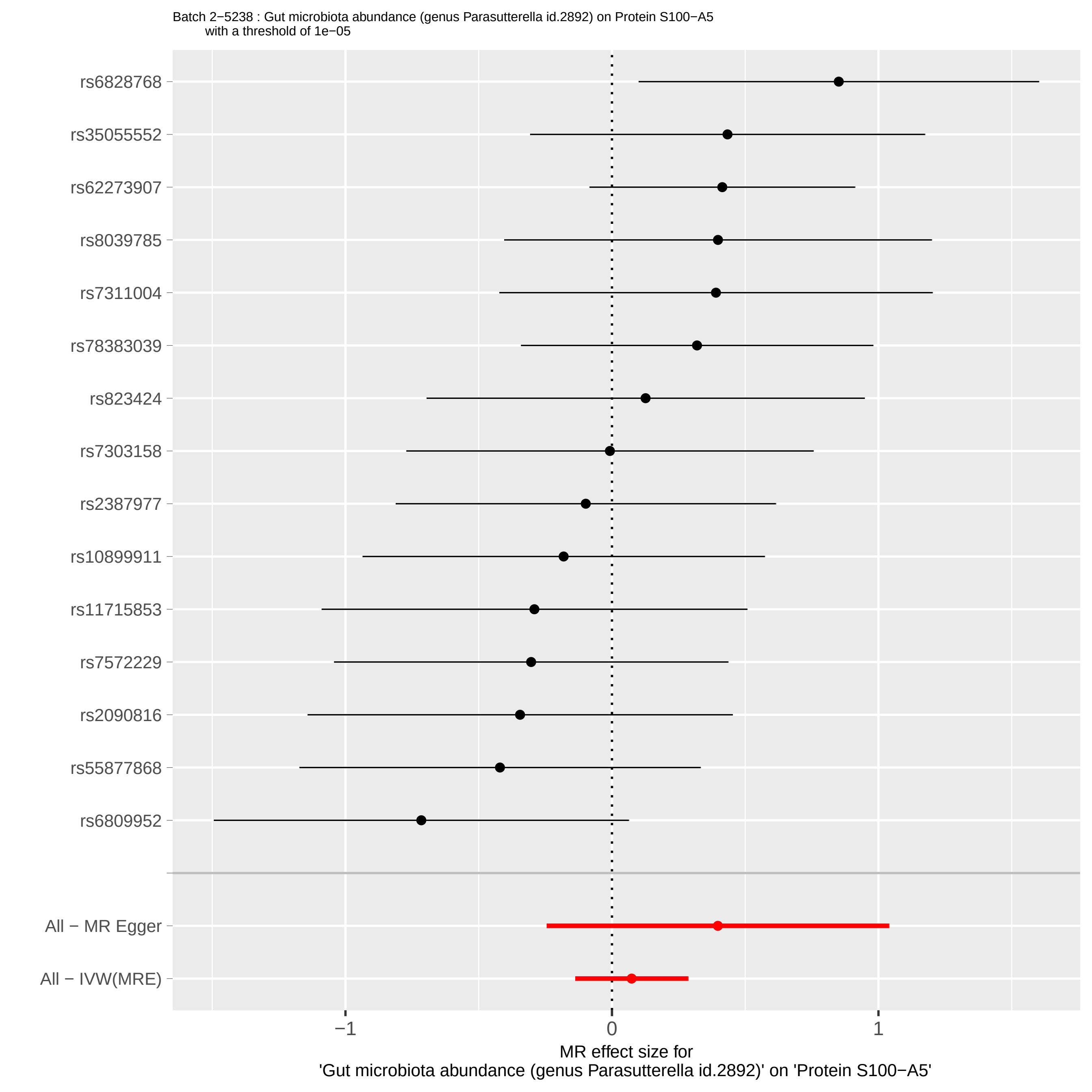

Batch 2−5238 : Gut microbiota abundance (genus Parasutterella id.2892) on Protein S100−A5
with a threshold of 1e−05
rs6828768
rs35055552
rs62273907
rs8039785
rs7311004
rs78383039
rs823424
rs7303158
rs2387977
rs10899911
rs11715853
rs7572229
rs2090816
rs55877868
rs6809952
All − MR Egger
All − IVW(MRE)
−1
0
1
MR effect size for
'Gut microbiota abundance (genus Parasutterella id.2892)' on 'Protein S100−A5'

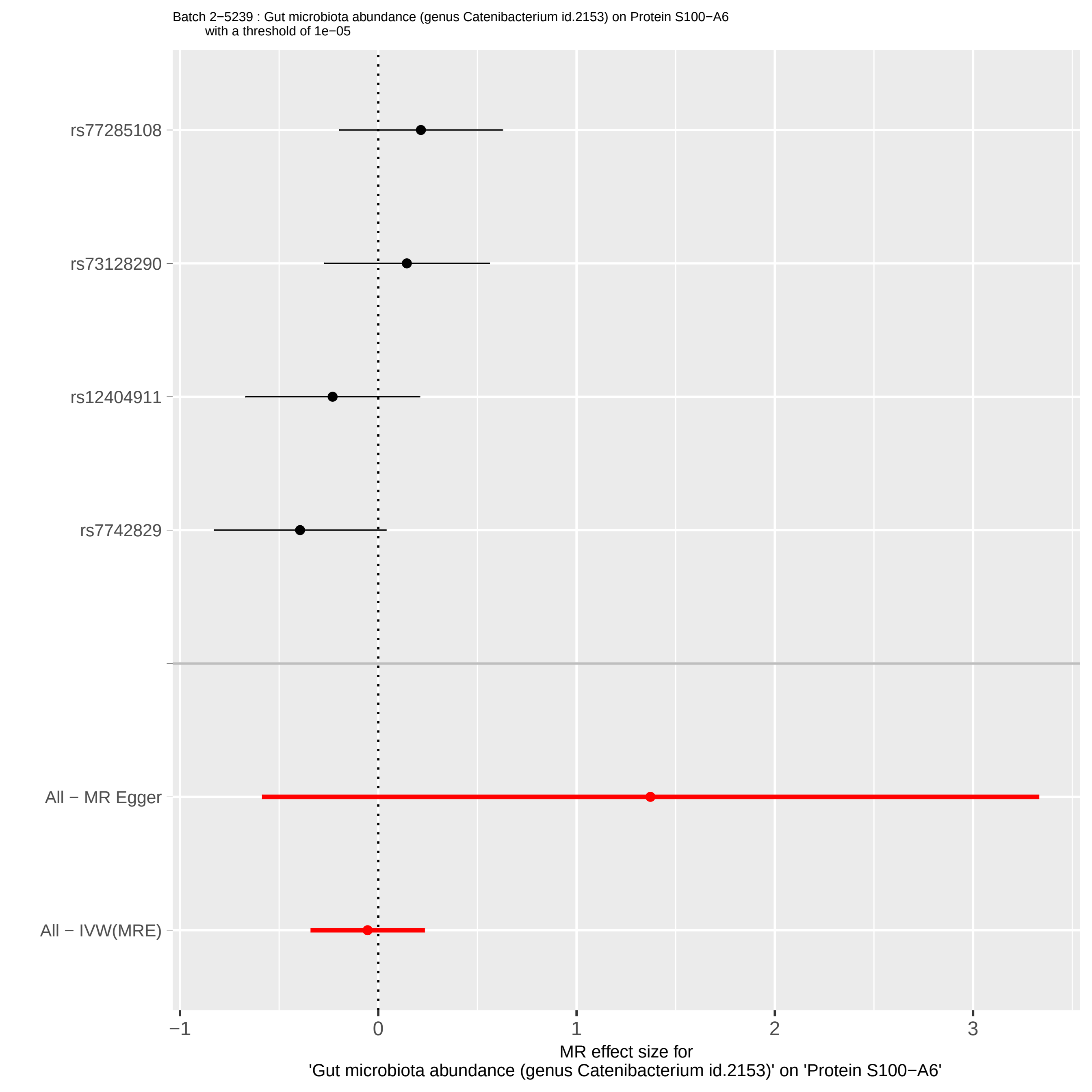

Batch 2−5239 : Gut microbiota abundance (genus Catenibacterium id.2153) on Protein S100−A6
with a threshold of 1e−05
rs77285108
rs73128290
rs12404911
rs7742829
All − MR Egger
All − IVW(MRE)
−1
0
1
2
3
MR effect size for
'Gut microbiota abundance (genus Catenibacterium id.2153)' on 'Protein S100−A6'

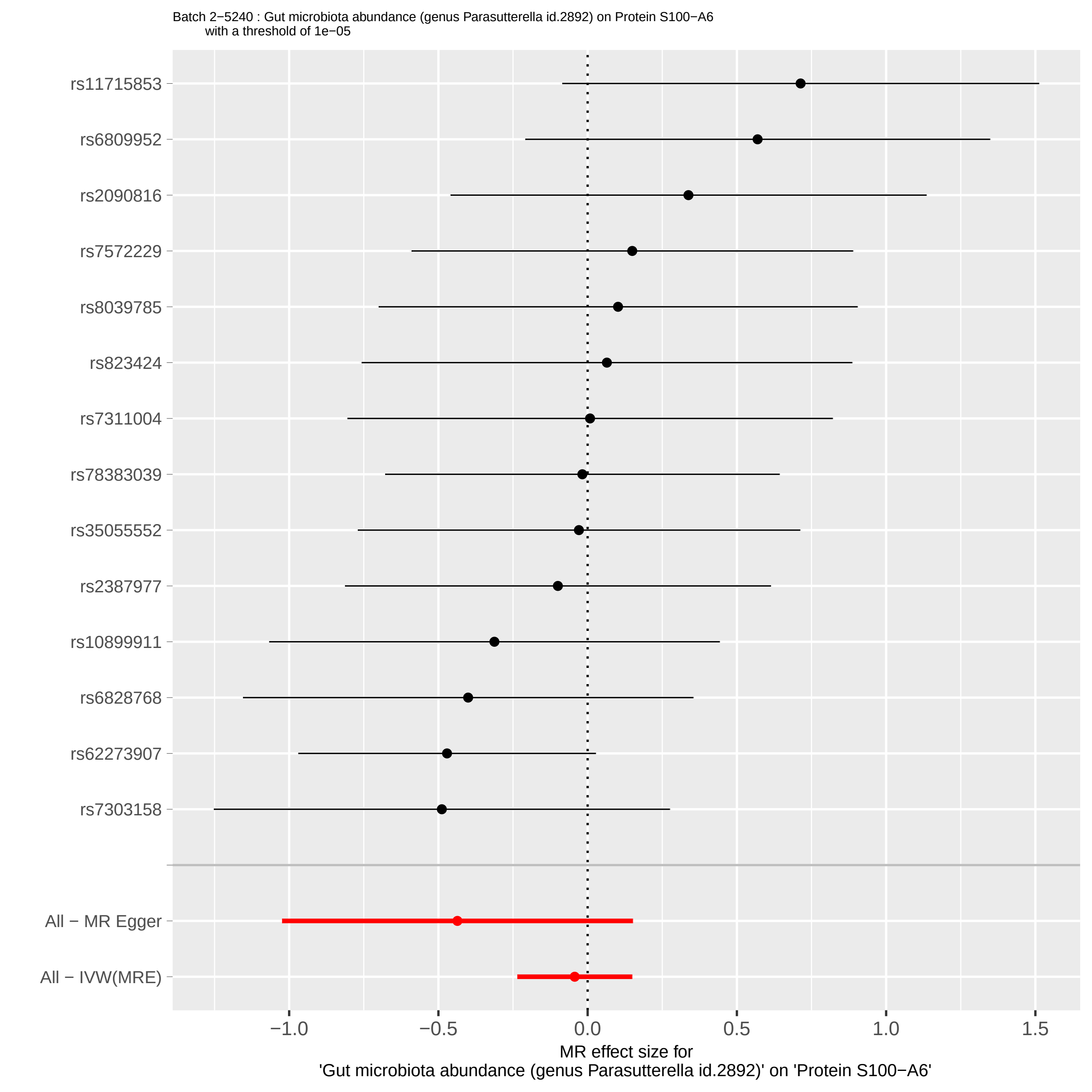

Batch 2−5240 : Gut microbiota abundance (genus Parasutterella id.2892) on Protein S100−A6
with a threshold of 1e−05
rs11715853
rs6809952
rs2090816
rs7572229
rs8039785
rs823424
rs7311004
rs78383039
rs35055552
rs2387977
rs10899911
rs6828768
rs62273907
rs7303158
All − MR Egger
All − IVW(MRE)
−1.0
−0.5
0.0
0.5
1.0
1.5
MR effect size for
'Gut microbiota abundance (genus Parasutterella id.2892)' on 'Protein S100−A6'

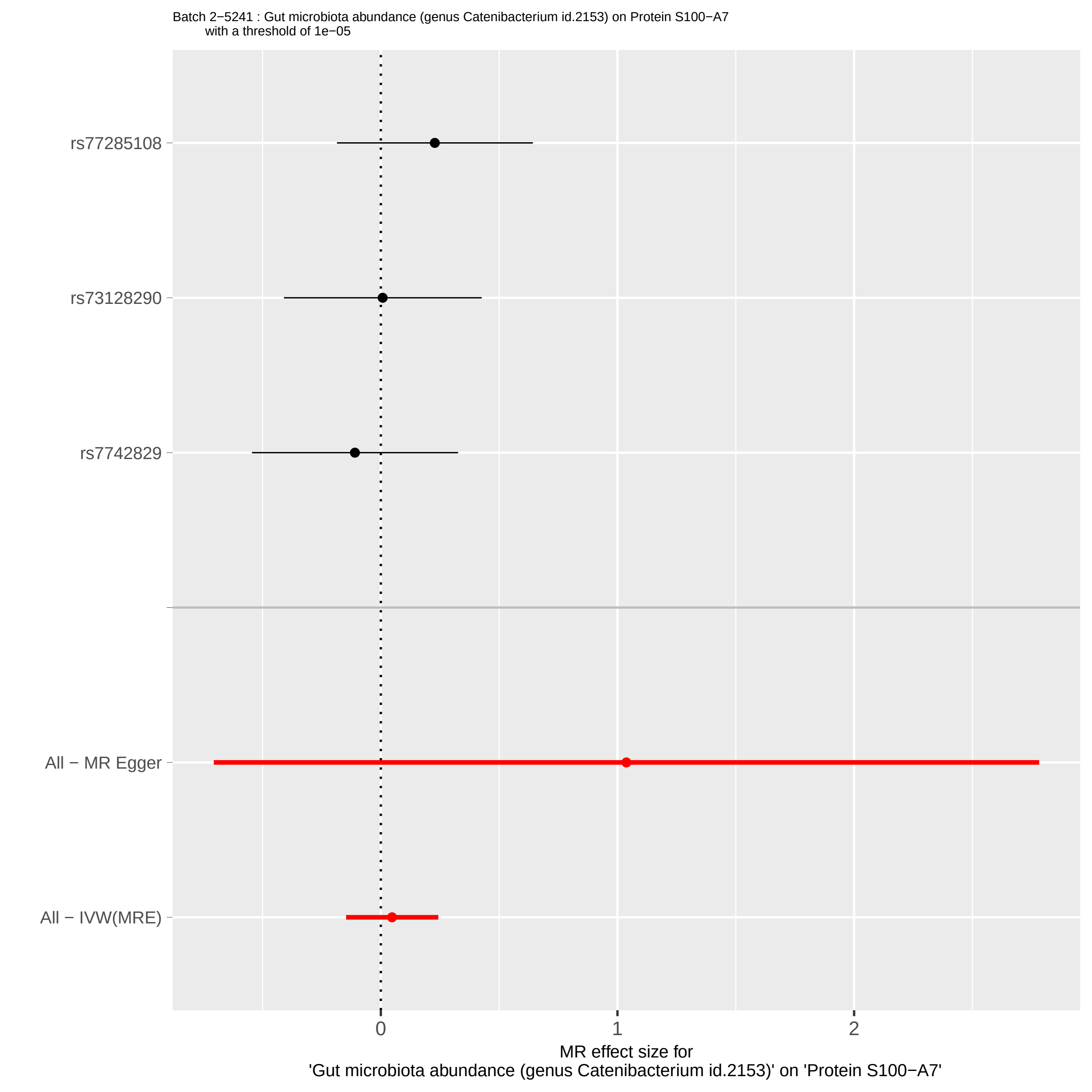
Batch 2−5241 : Gut microbiota abundance (genus Catenibacterium id.2153) on Protein S100−A7
with a threshold of 1e−05
rs77285108
rs73128290
rs7742829
All − MR Egger
All − IVW(MRE)
0
1
2
MR effect size for
'Gut microbiota abundance (genus Catenibacterium id.2153)' on 'Protein S100−A7'

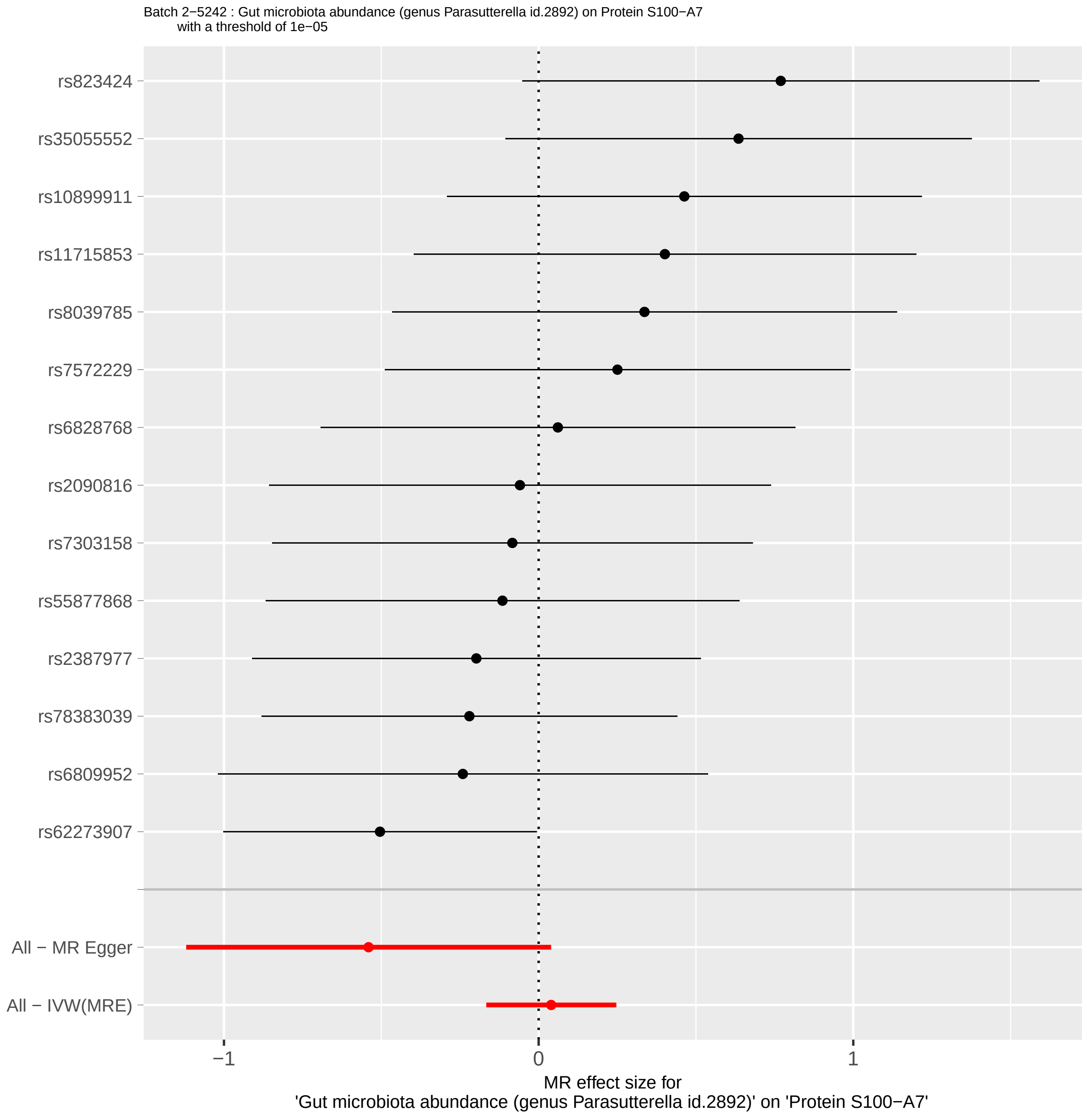
Batch 2−5242 : Gut microbiota abundance (genus Parasutterella id.2892) on Protein S100−A7
with a threshold of 1e−05
rs823424
rs35055552
rs10899911
rs11715853
rs8039785
rs7572229
rs6828768
rs2090816
rs7303158
rs55877868
rs2387977
rs78383039
rs6809952
rs62273907
All − MR Egger
All − IVW(MRE)
−1
0
1
MR effect size for
'Gut microbiota abundance (genus Parasutterella id.2892)' on 'Protein S100−A7'

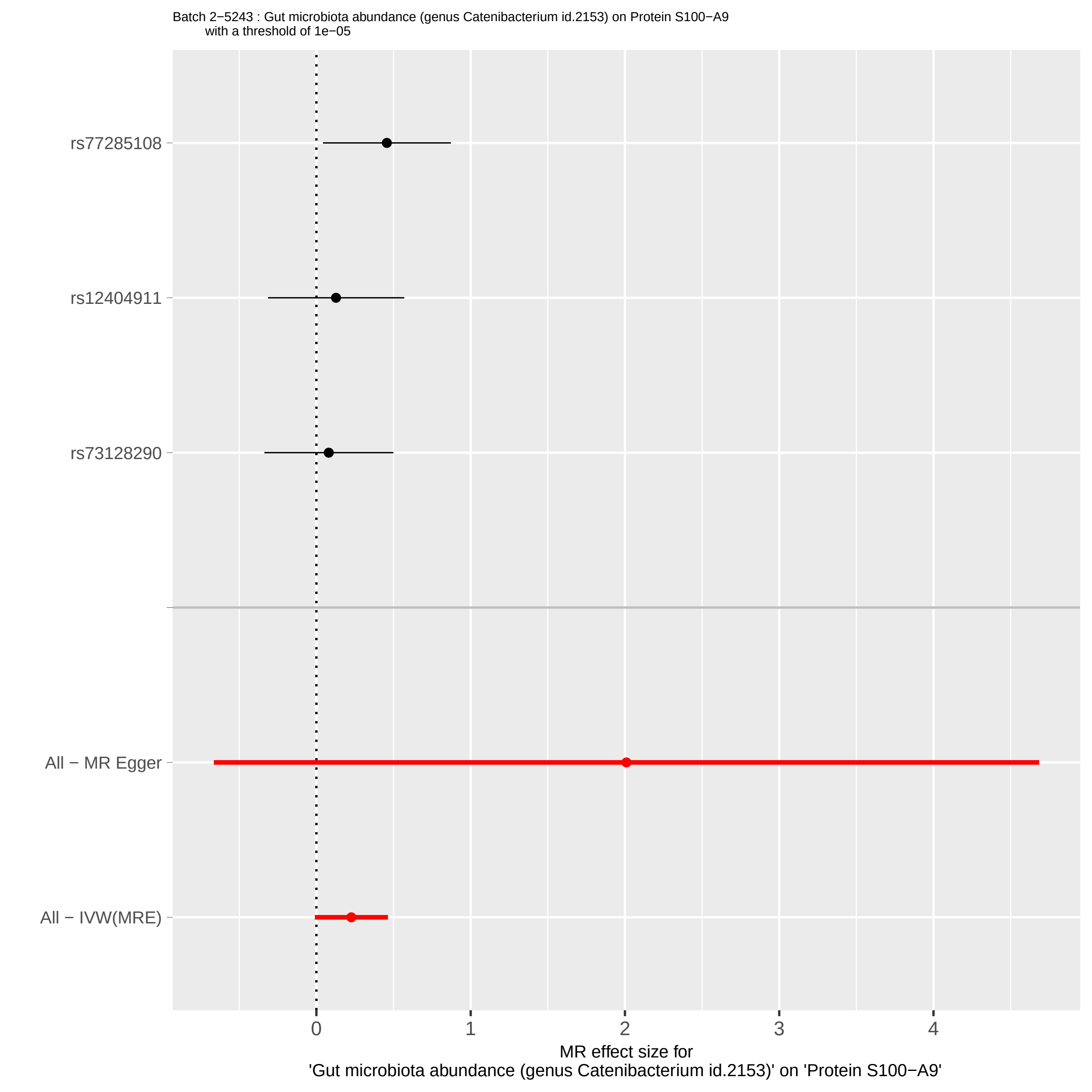
Batch 2−5243 : Gut microbiota abundance (genus Catenibacterium id.2153) on Protein S100−A9
with a threshold of 1e−05
rs77285108
rs12404911
rs73128290
All − MR Egger
All − IVW(MRE)
0
1
2
3
4
MR effect size for
'Gut microbiota abundance (genus Catenibacterium id.2153)' on 'Protein S100−A9'

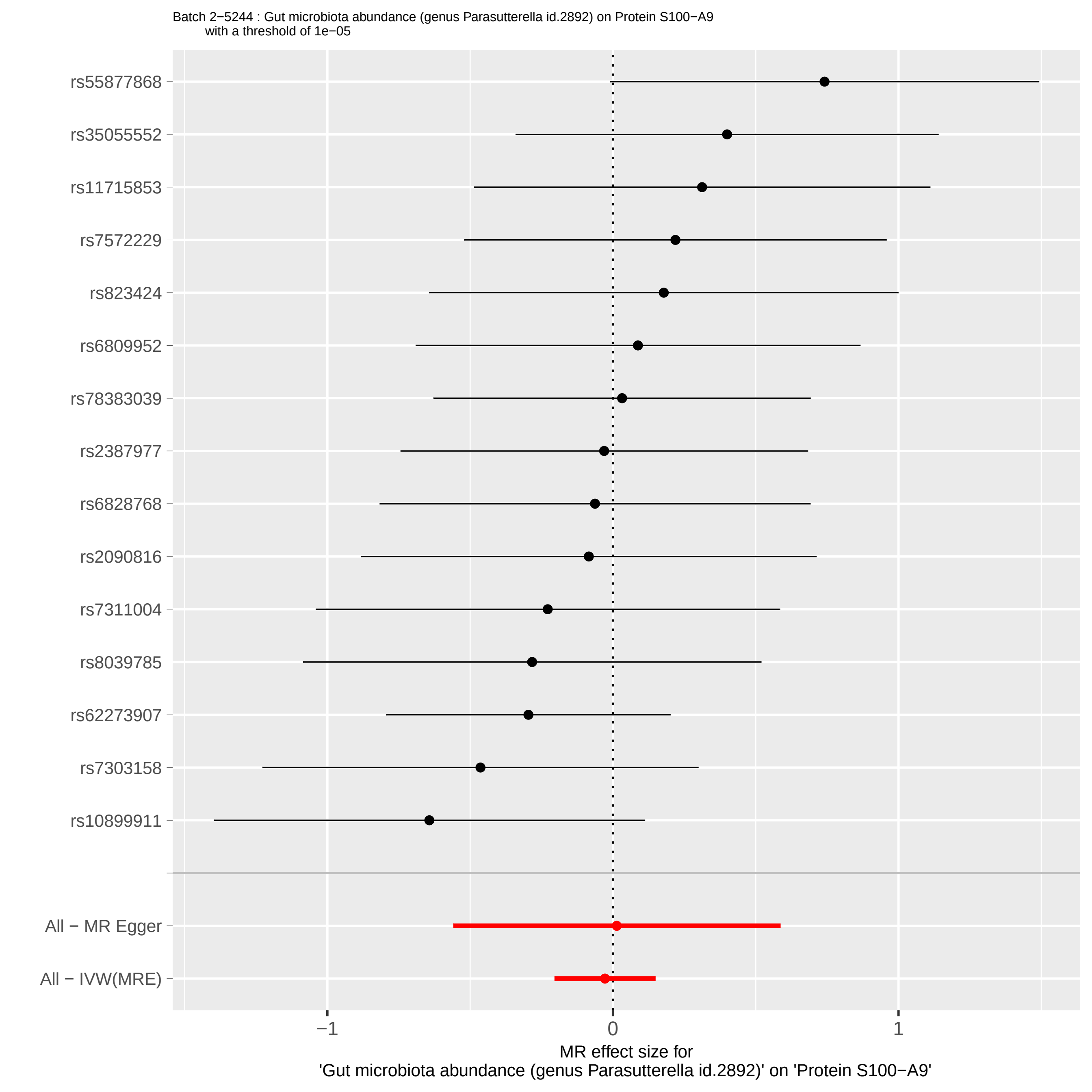

Batch 2−5244 : Gut microbiota abundance (genus Parasutterella id.2892) on Protein S100−A9
with a threshold of 1e−05
rs55877868
rs35055552
rs11715853
rs7572229
rs823424
rs6809952
rs78383039
rs2387977
rs6828768
rs2090816
rs7311004
rs8039785
rs62273907
rs7303158
rs10899911
All − MR Egger
All − IVW(MRE)
−1
0
1
MR effect size for
'Gut microbiota abundance (genus Parasutterella id.2892)' on 'Protein S100−A9'

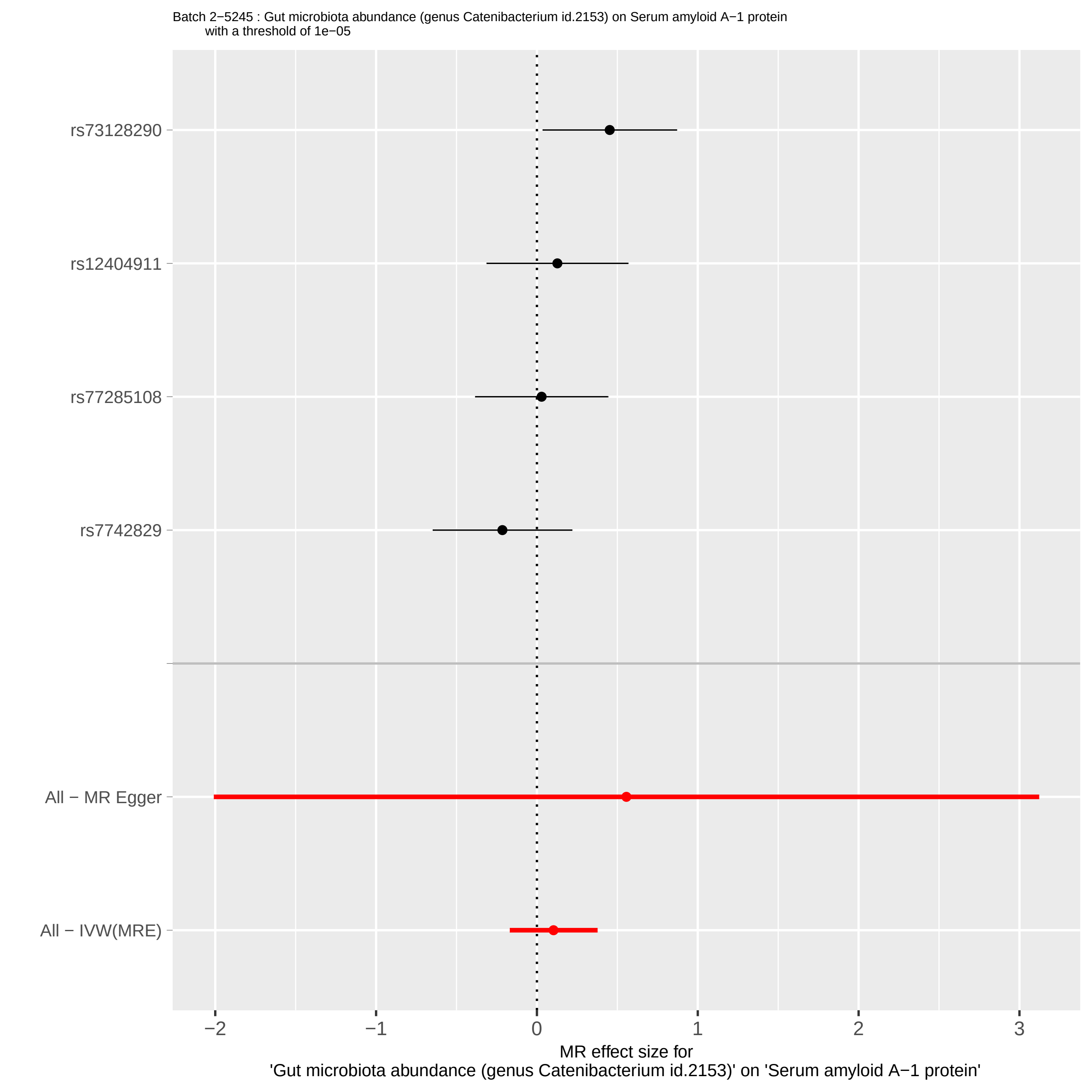
Batch 2−5245 : Gut microbiota abundance (genus Catenibacterium id.2153) on Serum amyloid A−1 protein
with a threshold of 1e−05
rs73128290
rs12404911
rs77285108
rs7742829
All − MR Egger
All − IVW(MRE)
−2
−1
0
1
2
3
MR effect size for
'Gut microbiota abundance (genus Catenibacterium id.2153)' on 'Serum amyloid A−1 protein'

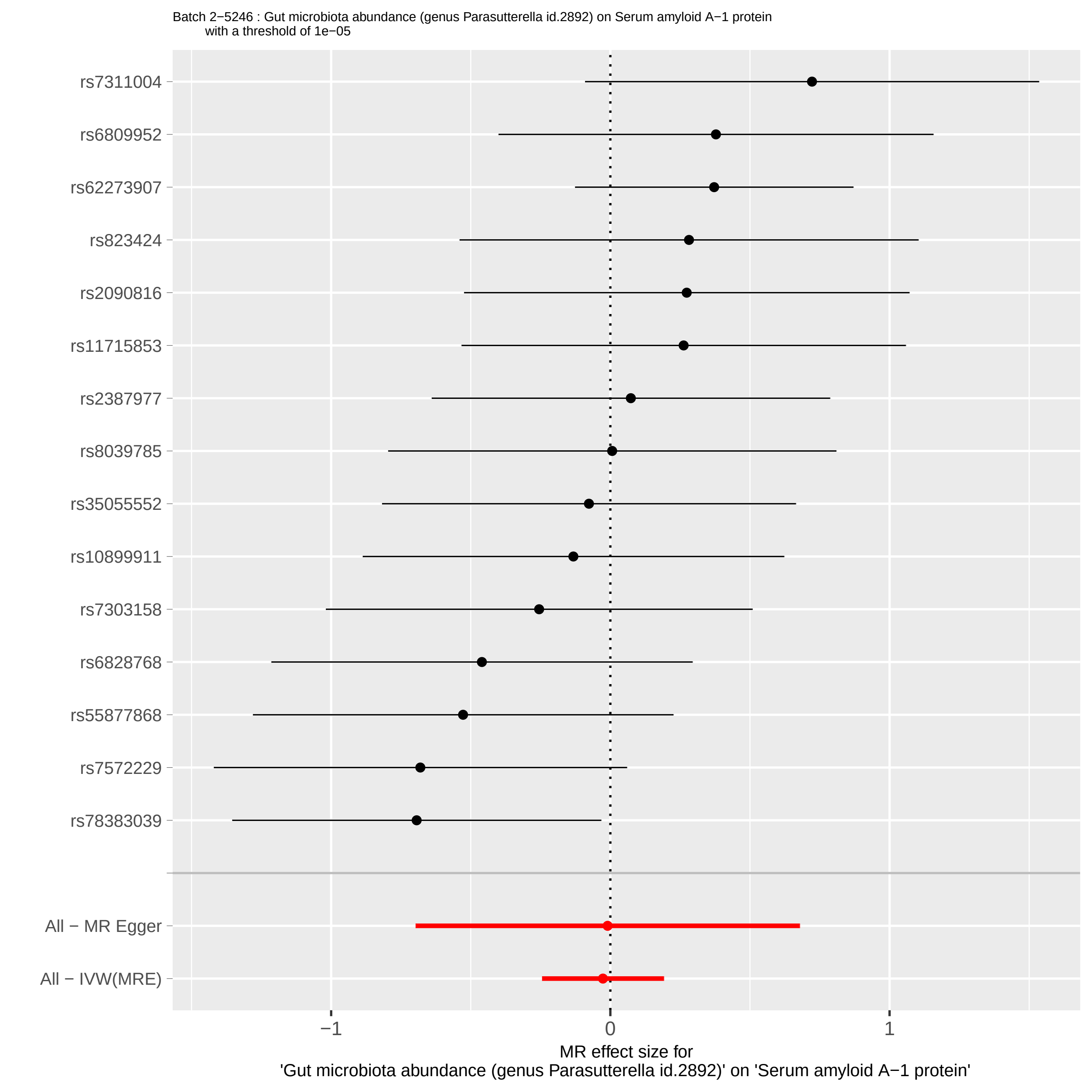
Batch 2−5246 : Gut microbiota abundance (genus Parasutterella id.2892) on Serum amyloid A−1 protein
with a threshold of 1e−05
rs7311004
rs6809952
rs62273907
rs823424
rs2090816
rs11715853
rs2387977
rs8039785
rs35055552
rs10899911
rs7303158
rs6828768
rs55877868
rs7572229
rs78383039
All − MR Egger
All − IVW(MRE)
−1
0
1
MR effect size for
'Gut microbiota abundance (genus Parasutterella id.2892)' on 'Serum amyloid A−1 protein'

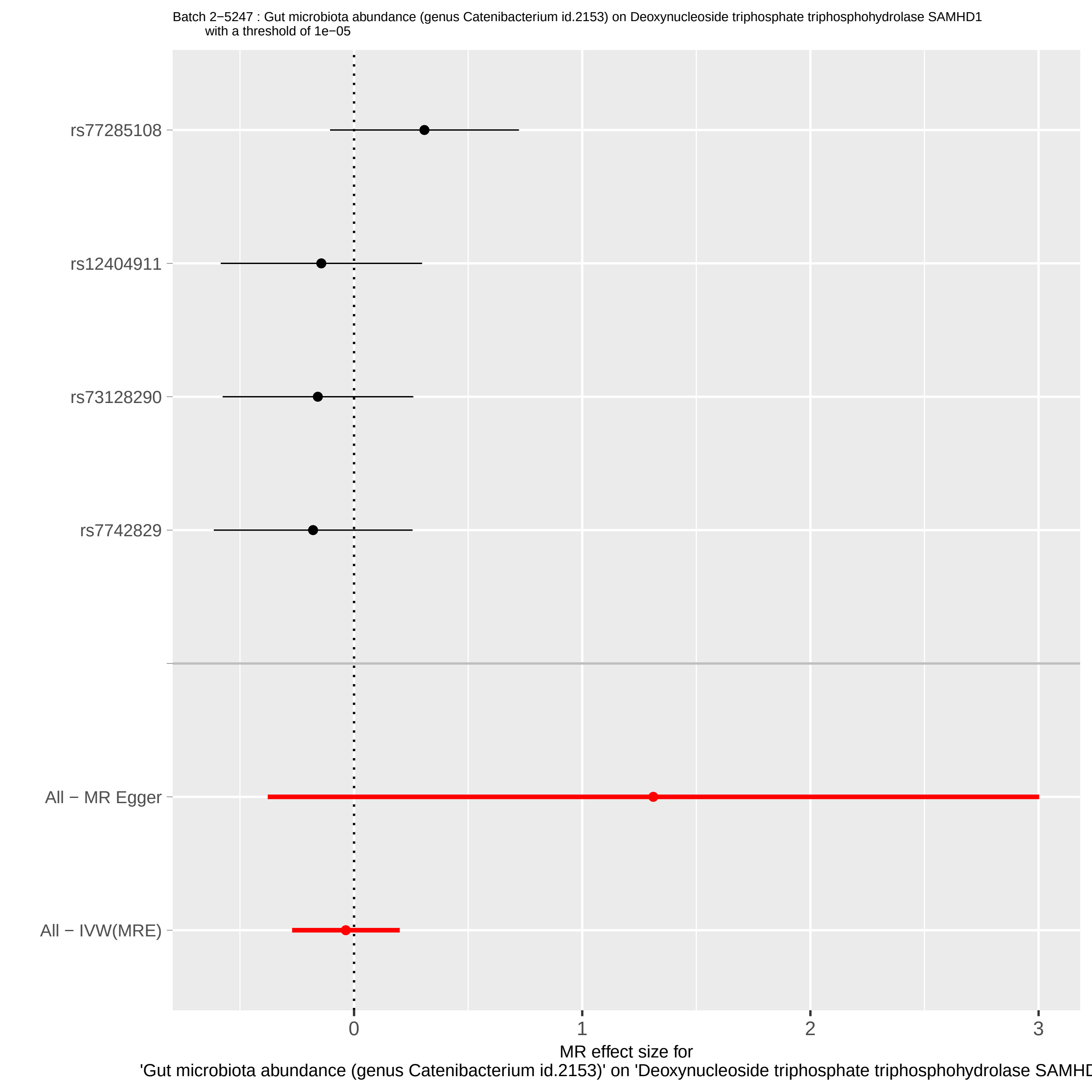

Batch 2−5247 : Gut microbiota abundance (genus Catenibacterium id.2153) on Deoxynucleoside triphosphate triphosphohydrolase SAMHD1
with a threshold of 1e−05
rs77285108
rs12404911
rs73128290
rs7742829
All − MR Egger
All − IVW(MRE)
0
1
2
3
MR effect size for
'Gut microbiota abundance (genus Catenibacterium id.2153)' on 'Deoxynucleoside triphosphate triphosphohydrolase SAMHD

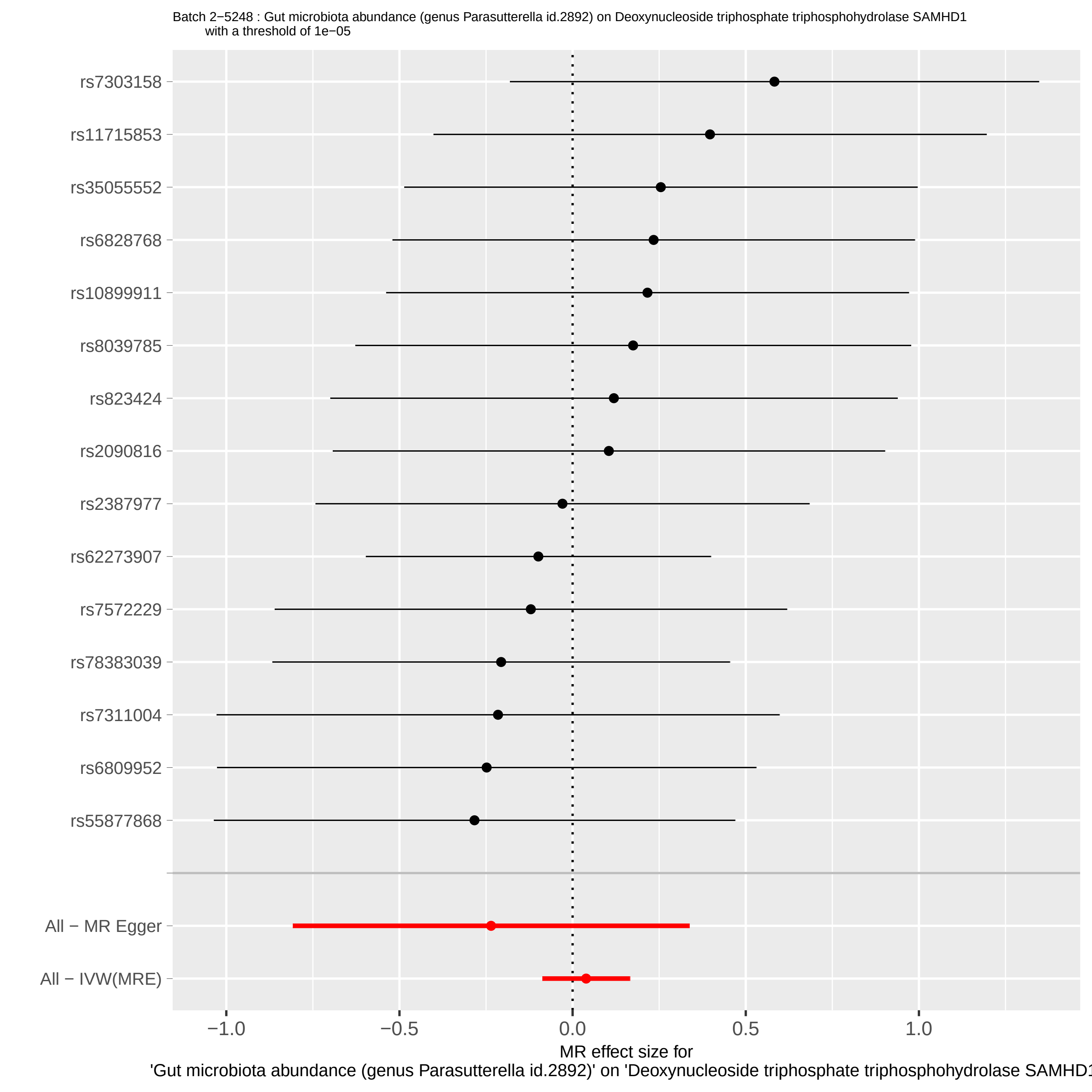

Batch 2−5248 : Gut microbiota abundance (genus Parasutterella id.2892) on Deoxynucleoside triphosphate triphosphohydrolase SAMHD1
with a threshold of 1e−05
rs7303158
rs11715853
rs35055552
rs6828768
rs10899911
rs8039785
rs823424
rs2090816
rs2387977
rs62273907
rs7572229
rs78383039
rs7311004
rs6809952
rs55877868
All − MR Egger
All − IVW(MRE)
−1.0
−0.5
0.0
0.5
1.0
MR effect size for
'Gut microbiota abundance (genus Parasutterella id.2892)' on 'Deoxynucleoside triphosphate triphosphohydrolase SAMHD1

Batch 2−5249 : Gut microbiota abundance (genus Catenibacterium id.2153) on Histone deacetylase complex subunit SAP18
with a threshold of 1e−05

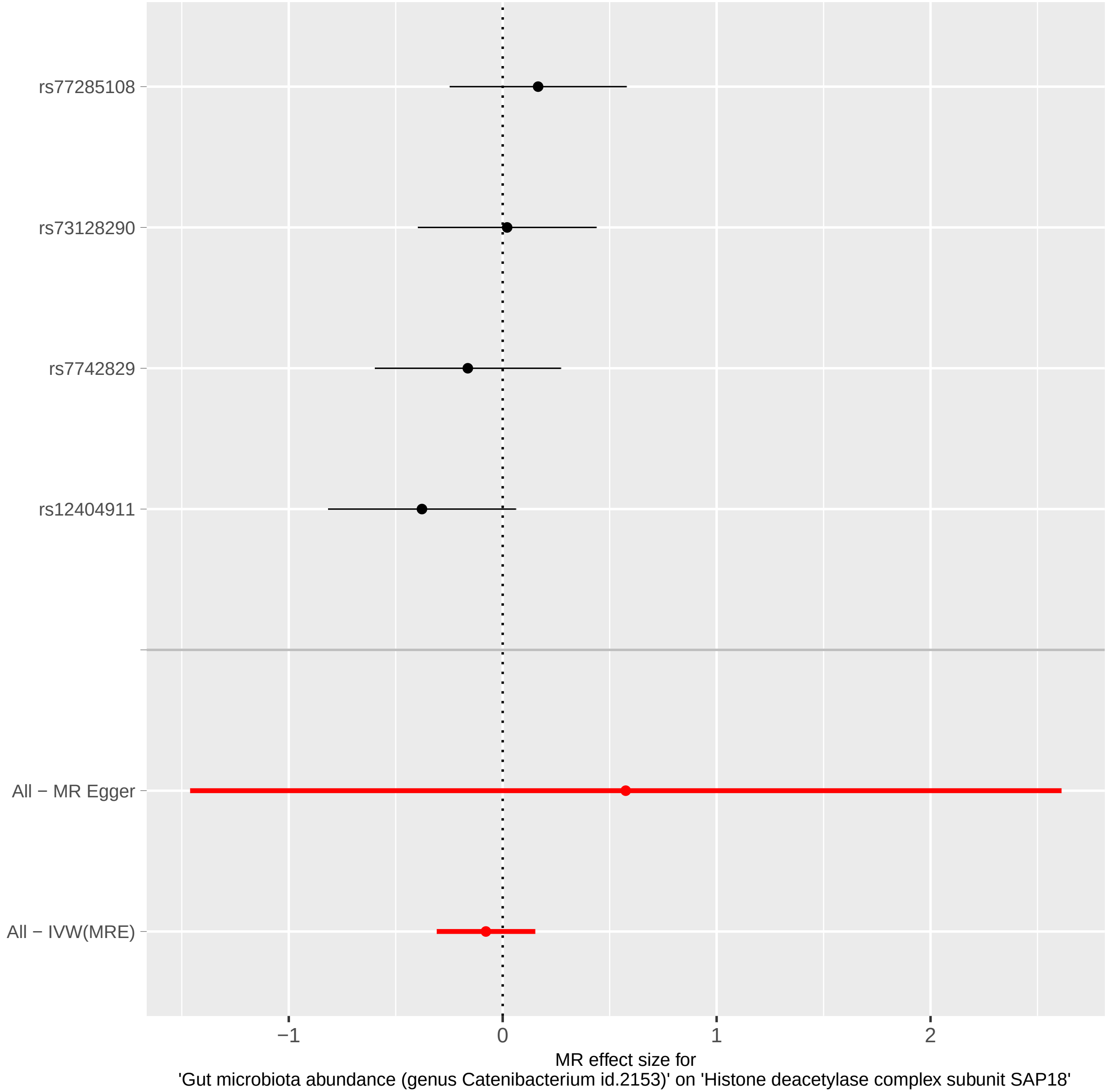

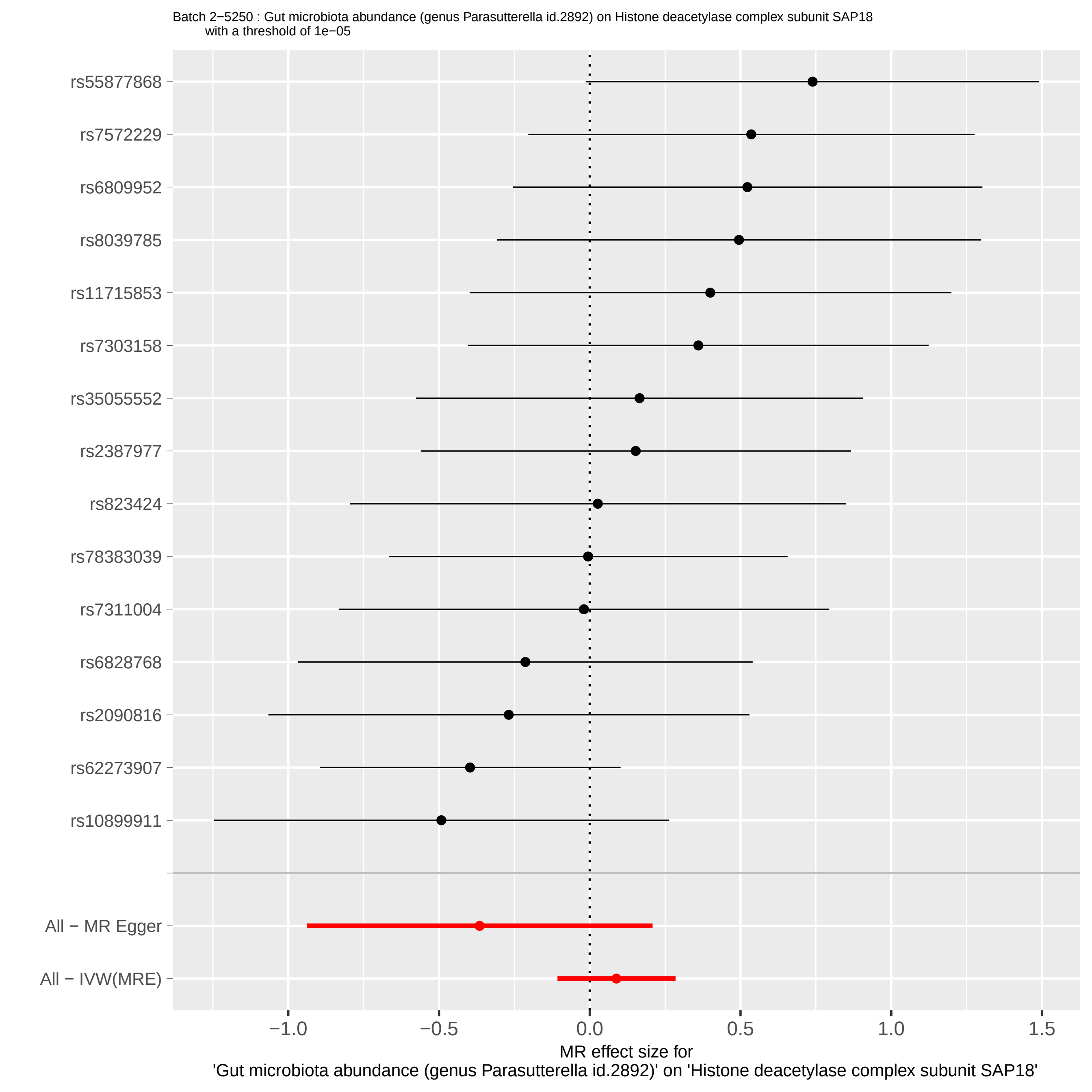

Batch 2−5250 : Gut microbiota abundance (genus Parasutterella id.2892) on Histone deacetylase complex subunit SAP18
with a threshold of 1e−05
rs55877868
rs7572229
rs6809952
rs8039785
rs11715853
rs7303158
rs35055552
rs2387977
rs823424
rs78383039
rs7311004
rs6828768
rs2090816
rs62273907
rs10899911
All − MR Egger
All − IVW(MRE)
−1.0
−0.5
0.0
0.5
1.0
1.5
MR effect size for
'Gut microbiota abundance (genus Parasutterella id.2892)' on 'Histone deacetylase complex subunit SAP18'

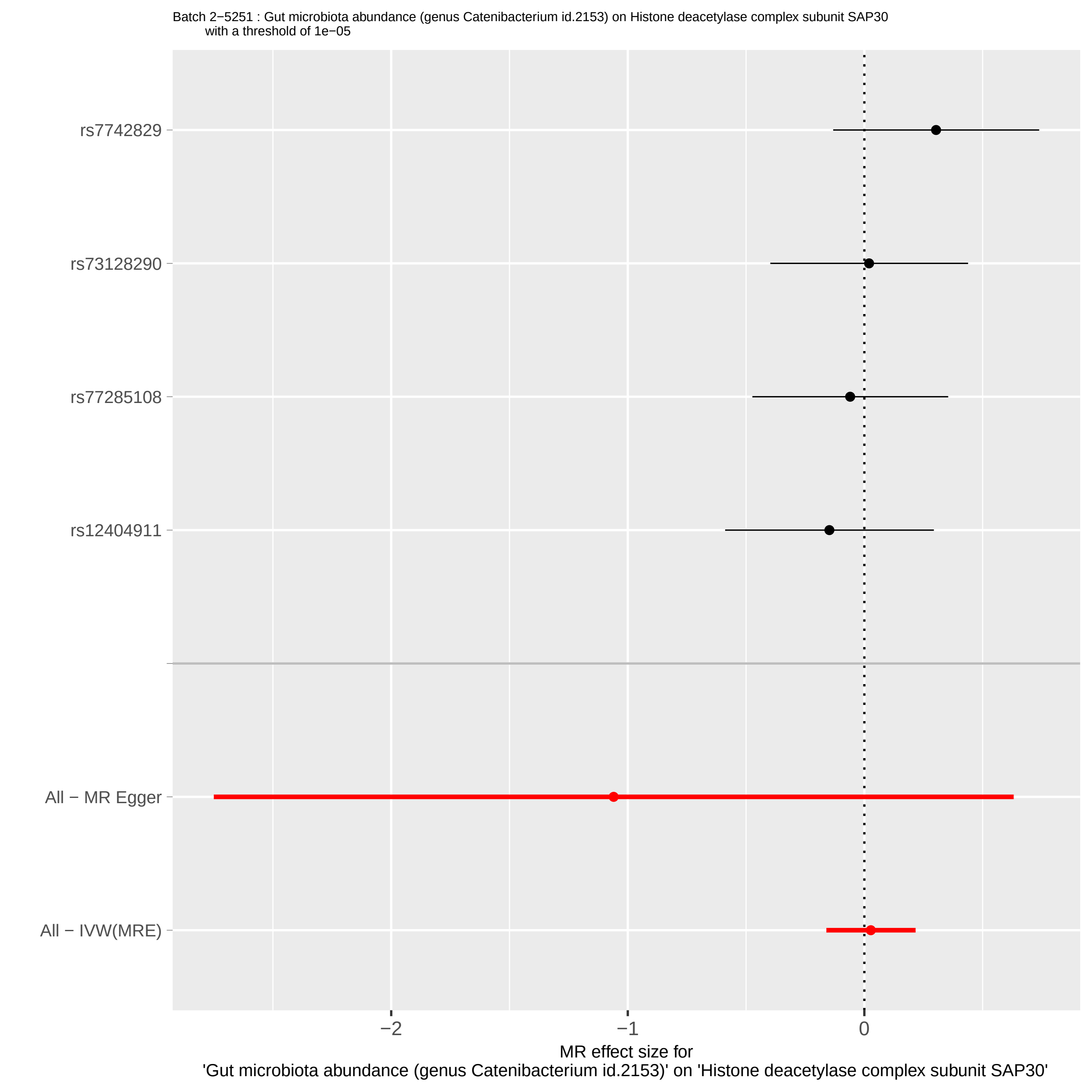

Batch 2−5251 : Gut microbiota abundance (genus Catenibacterium id.2153) on Histone deacetylase complex subunit SAP30
with a threshold of 1e−05
rs7742829
rs73128290
rs77285108
rs12404911
All − MR Egger
All − IVW(MRE)
−2
−1
0
MR effect size for
'Gut microbiota abundance (genus Catenibacterium id.2153)' on 'Histone deacetylase complex subunit SAP30'

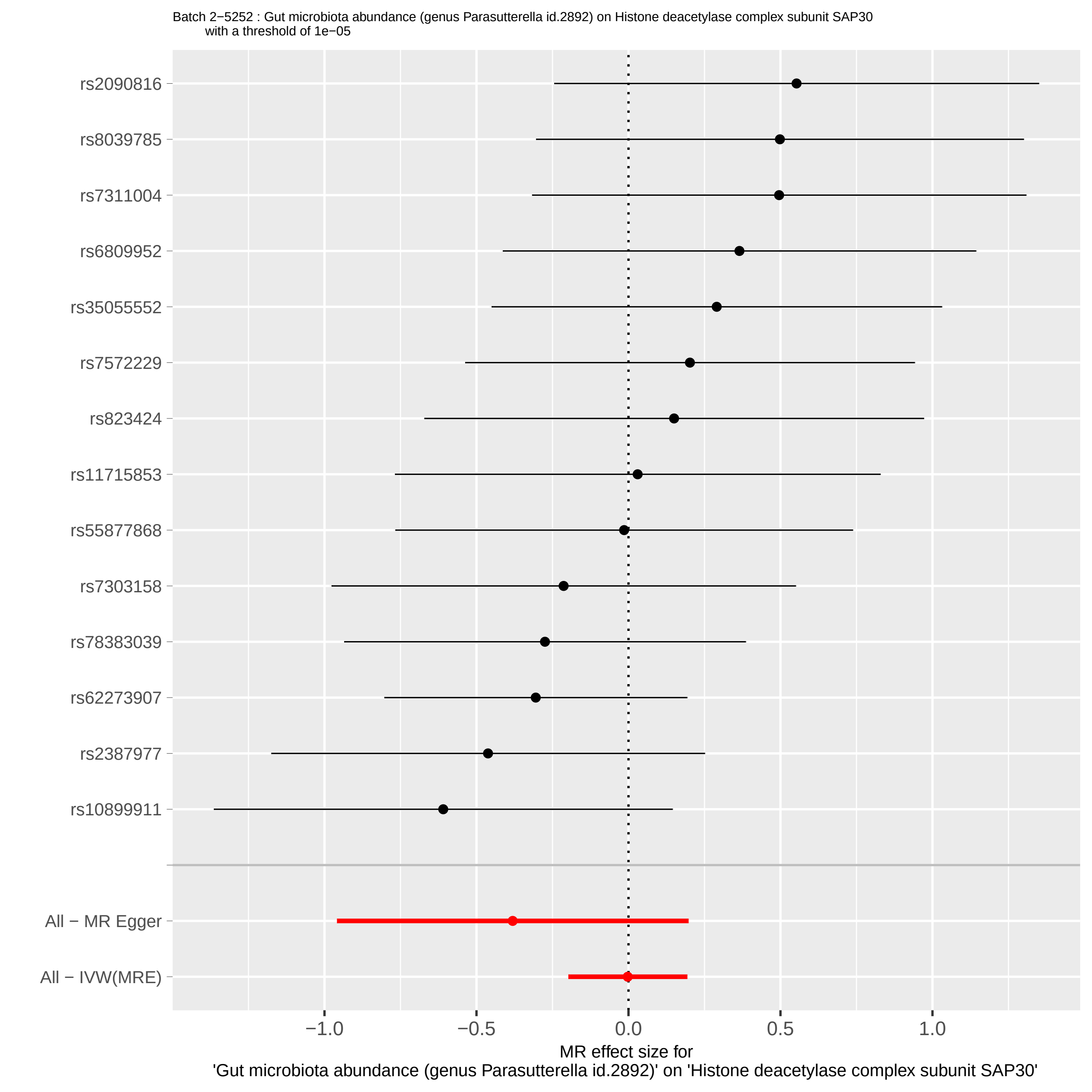

Batch 2−5252 : Gut microbiota abundance (genus Parasutterella id.2892) on Histone deacetylase complex subunit SAP30
with a threshold of 1e−05
rs2090816
rs8039785
rs7311004
rs6809952
rs35055552
rs7572229
rs823424
rs11715853
rs55877868
rs7303158
rs78383039
rs62273907
rs2387977
rs10899911
All − MR Egger
All − IVW(MRE)
−1.0
−0.5
0.0
0.5
1.0
MR effect size for
'Gut microbiota abundance (genus Parasutterella id.2892)' on 'Histone deacetylase complex subunit SAP30'

Batch 2−5253 : Gut microbiota abundance (genus Catenibacterium id.2153) on Serine−−tRNA ligase, mitochondrial
with a threshold of 1e−05

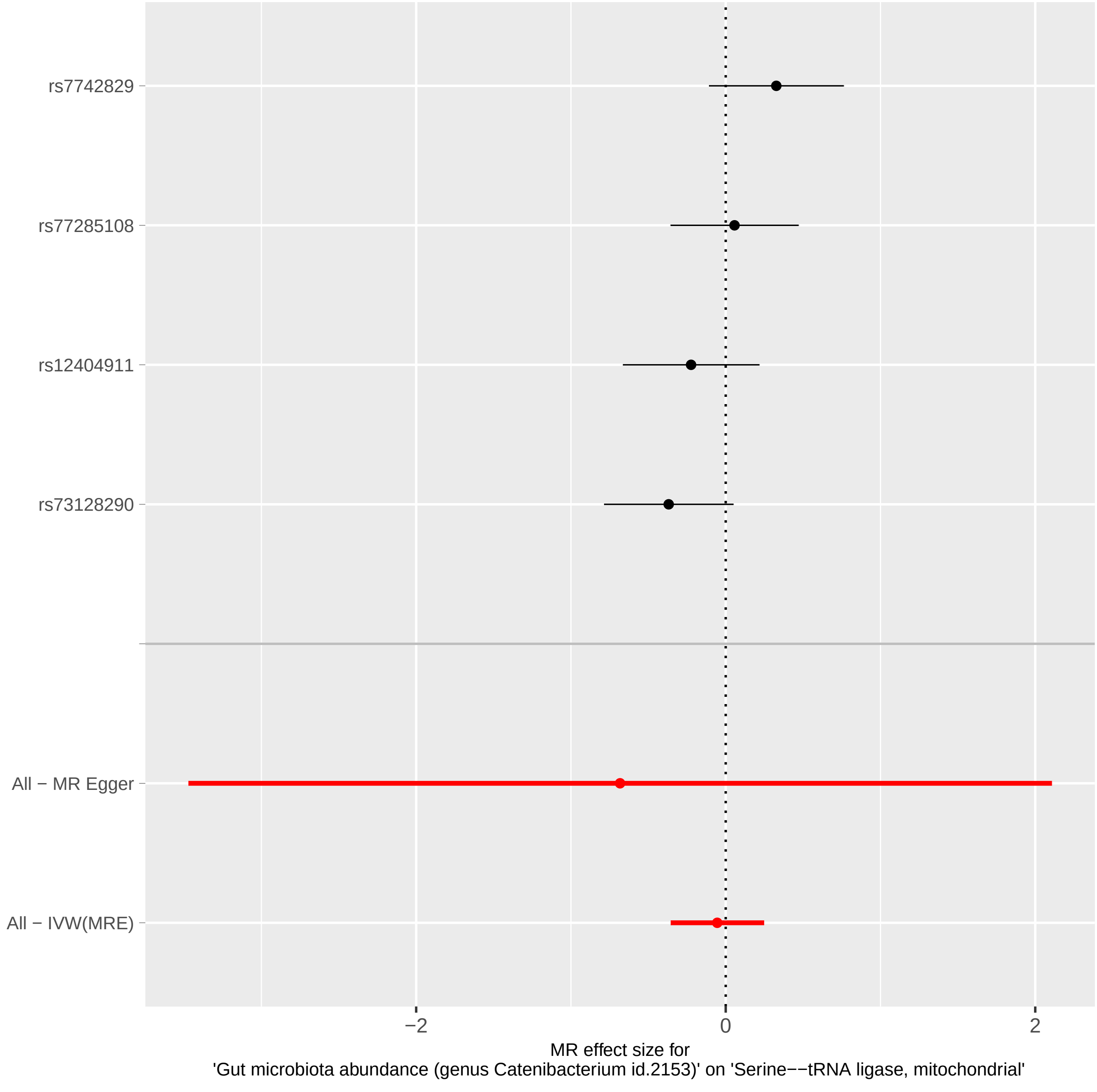

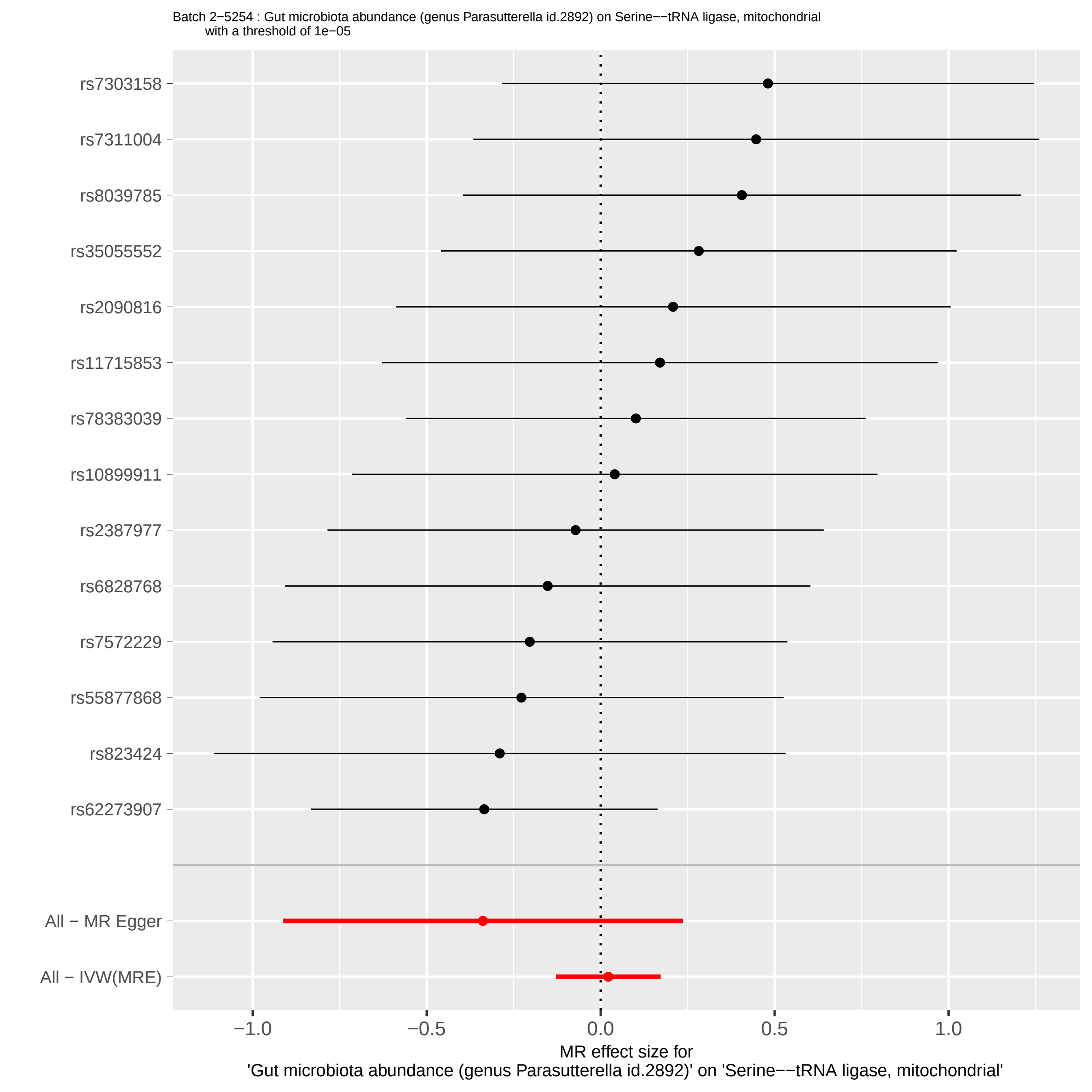
Batch 2−5254 : Gut microbiota abundance (genus Parasutterella id.2892) on Serine−−tRNA ligase, mitochondrial
with a threshold of 1e−05
rs7303158
rs7311004
rs8039785
rs35055552
rs2090816
rs11715853
rs78383039
rs10899911
rs2387977
rs6828768
rs7572229
rs55877868
rs823424
rs62273907
All − MR Egger
All − IVW(MRE)
−1.0
−0.5
0.0
0.5
1.0
MR effect size for
'Gut microbiota abundance (genus Parasutterella id.2892)' on 'Serine−−tRNA ligase, mitochondrial'

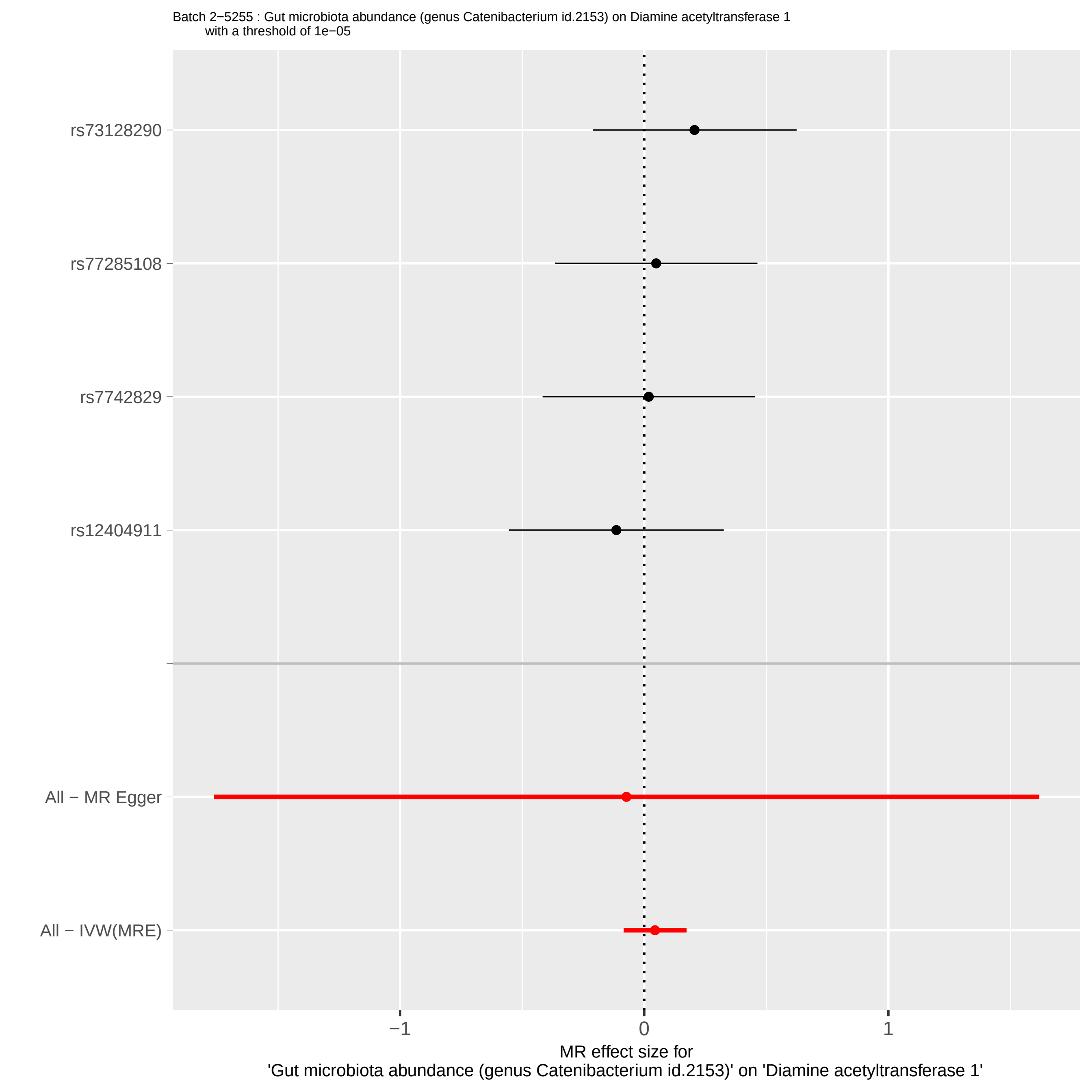
Batch 2−5255 : Gut microbiota abundance (genus Catenibacterium id.2153) on Diamine acetyltransferase 1
with a threshold of 1e−05
rs73128290
rs77285108
rs7742829
rs12404911
All − MR Egger
All − IVW(MRE)
−1
0
1
MR effect size for
'Gut microbiota abundance (genus Catenibacterium id.2153)' on 'Diamine acetyltransferase 1'

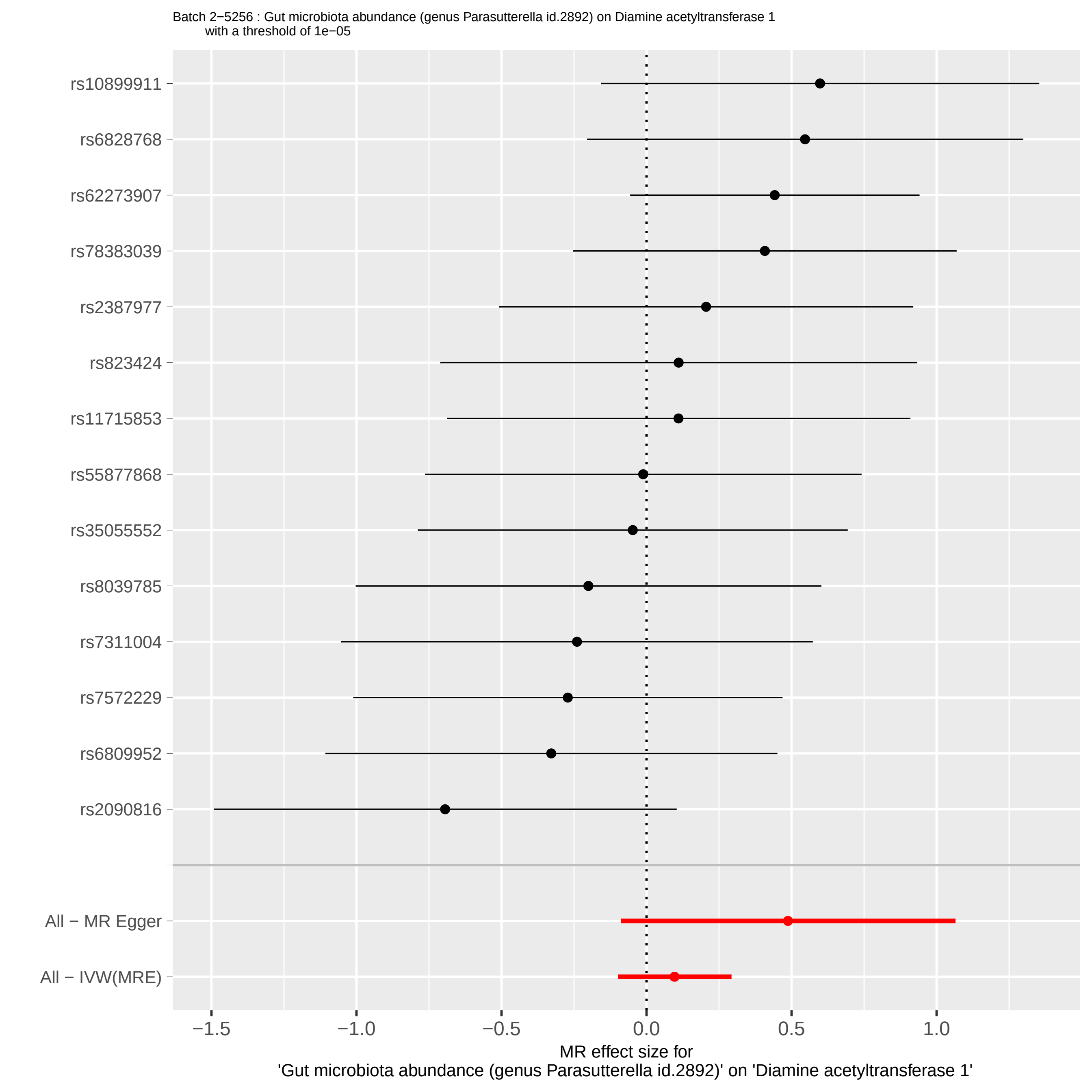

Batch 2−5256 : Gut microbiota abundance (genus Parasutterella id.2892) on Diamine acetyltransferase 1
with a threshold of 1e−05
rs10899911
rs6828768
rs62273907
rs78383039
rs2387977
rs823424
rs11715853
rs55877868
rs35055552
rs8039785
rs7311004
rs7572229
rs6809952
rs2090816
All − MR Egger
All − IVW(MRE)
−1.5
−1.0
−0.5
0.0
0.5
1.0
MR effect size for
'Gut microbiota abundance (genus Parasutterella id.2892)' on 'Diamine acetyltransferase 1'

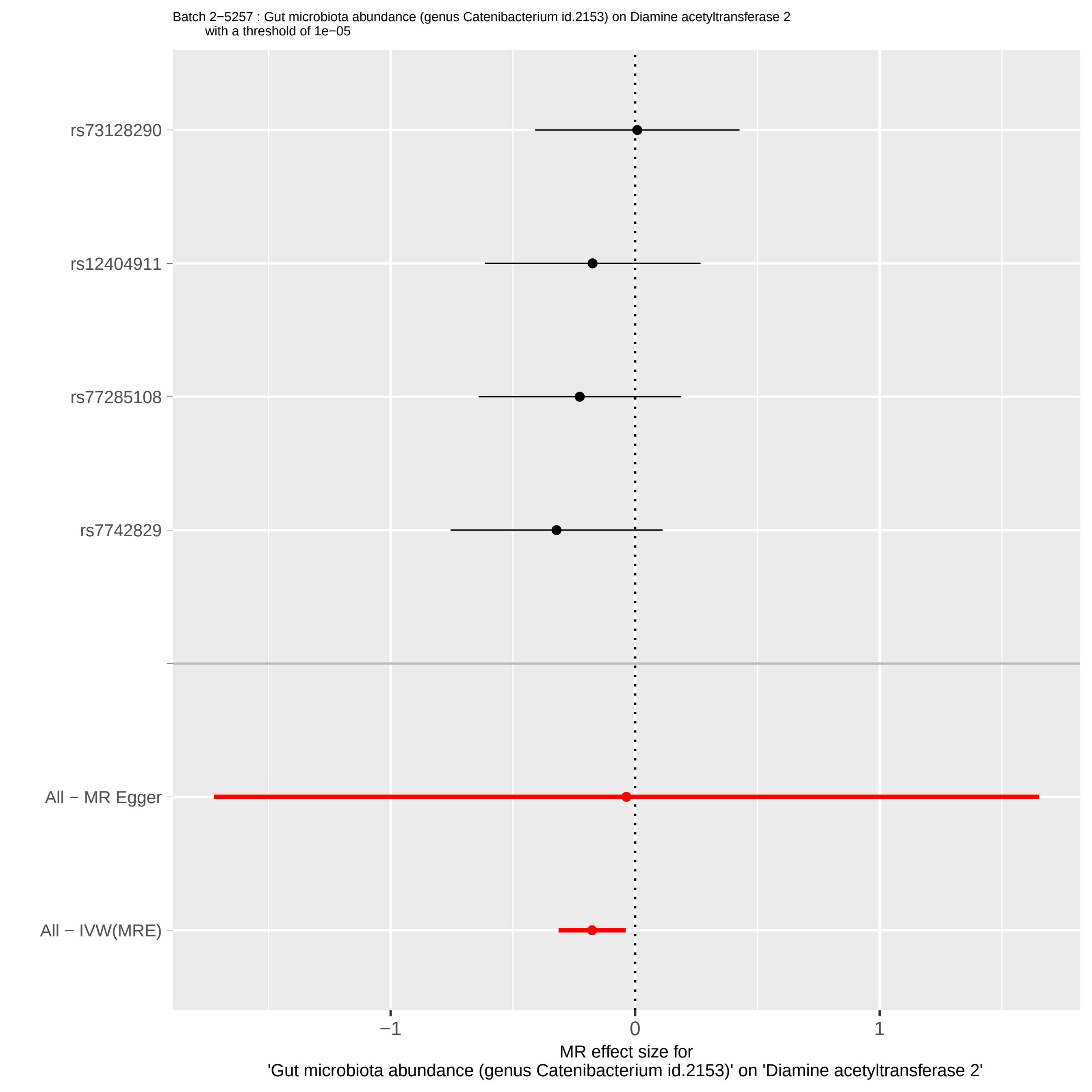

Batch 2−5257 : Gut microbiota abundance (genus Catenibacterium id.2153) on Diamine acetyltransferase 2
with a threshold of 1e−05
rs73128290
rs12404911
rs77285108
rs7742829
All − MR Egger
All − IVW(MRE)
−1
0
1
MR effect size for
'Gut microbiota abundance (genus Catenibacterium id.2153)' on 'Diamine acetyltransferase 2'

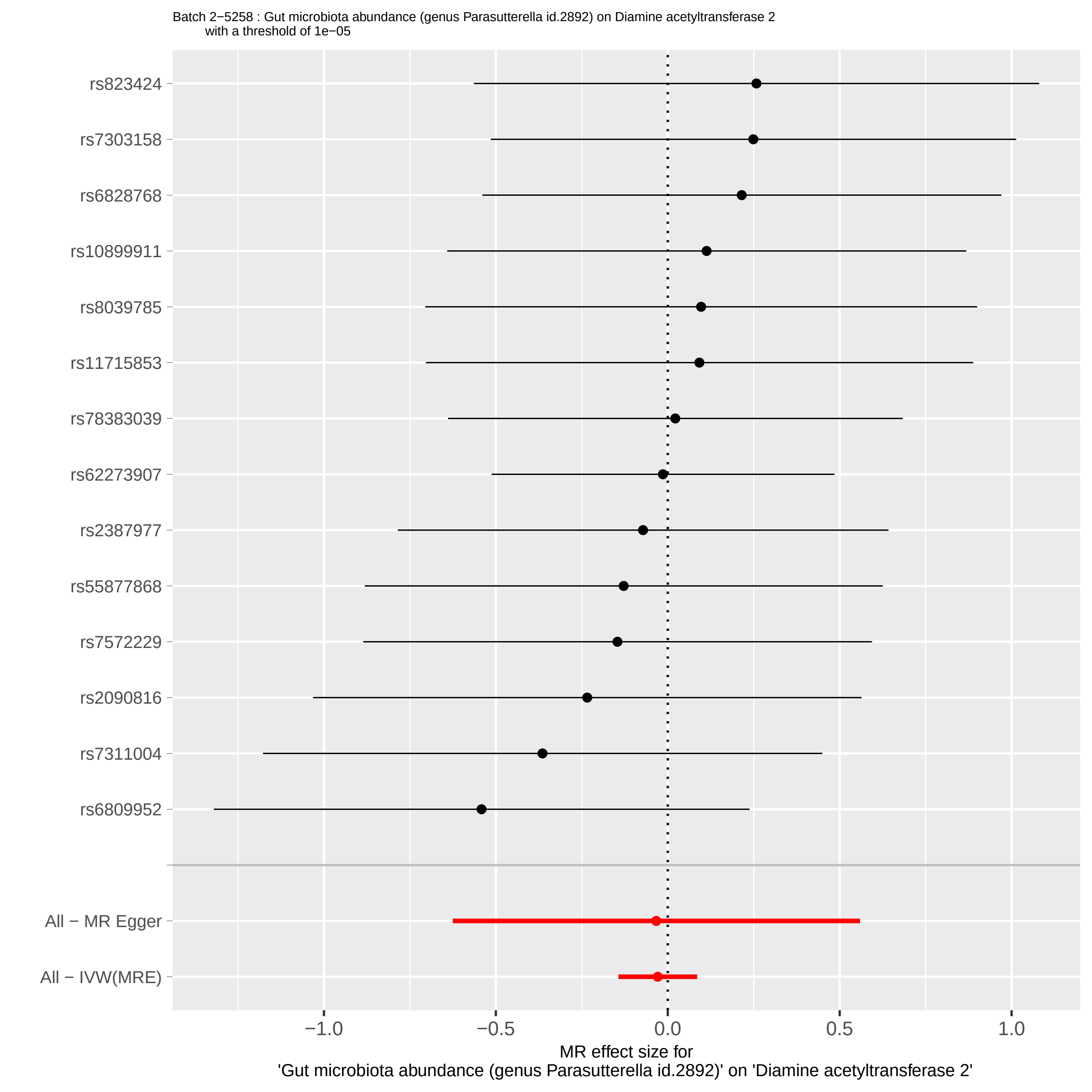

Batch 2−5258 : Gut microbiota abundance (genus Parasutterella id.2892) on Diamine acetyltransferase 2
with a threshold of 1e−05
rs823424
rs7303158
rs6828768
rs10899911
rs8039785
rs11715853
rs78383039
rs62273907
rs2387977
rs55877868
rs7572229
rs2090816
rs7311004
rs6809952
All − MR Egger
All − IVW(MRE)
−1.0
−0.5
0.0
0.5
1.0
MR effect size for
'Gut microbiota abundance (genus Parasutterella id.2892)' on 'Diamine acetyltransferase 2'

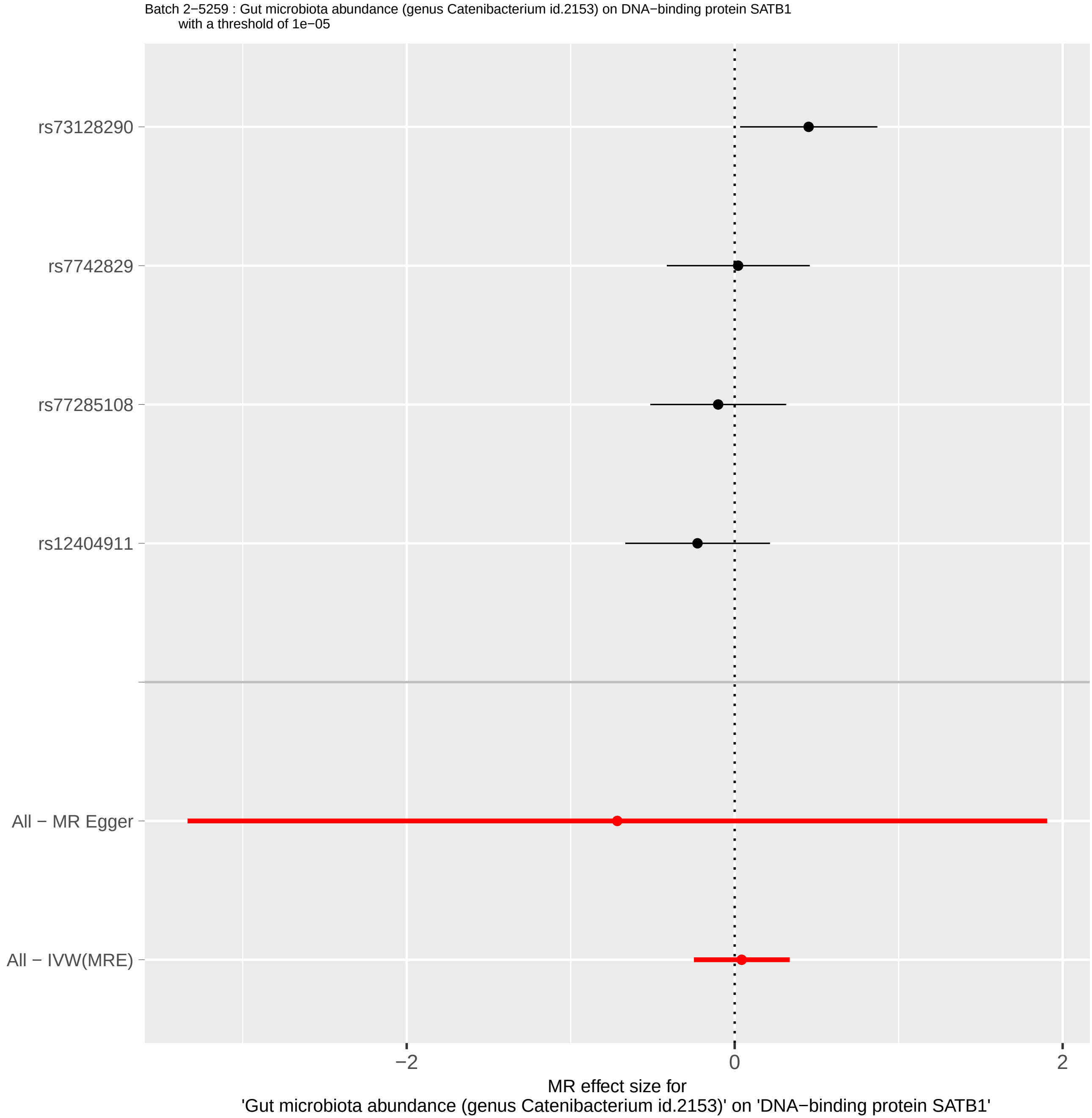

Batch 2−5259 : Gut microbiota abundance (genus Catenibacterium id.2153) on DNA−binding protein SATB1
with a threshold of 1e−05
rs73128290
rs7742829
rs77285108
rs12404911
All − MR Egger
All − IVW(MRE)
−2
0
2
MR effect size for
'Gut microbiota abundance (genus Catenibacterium id.2153)' on 'DNA−binding protein SATB1'

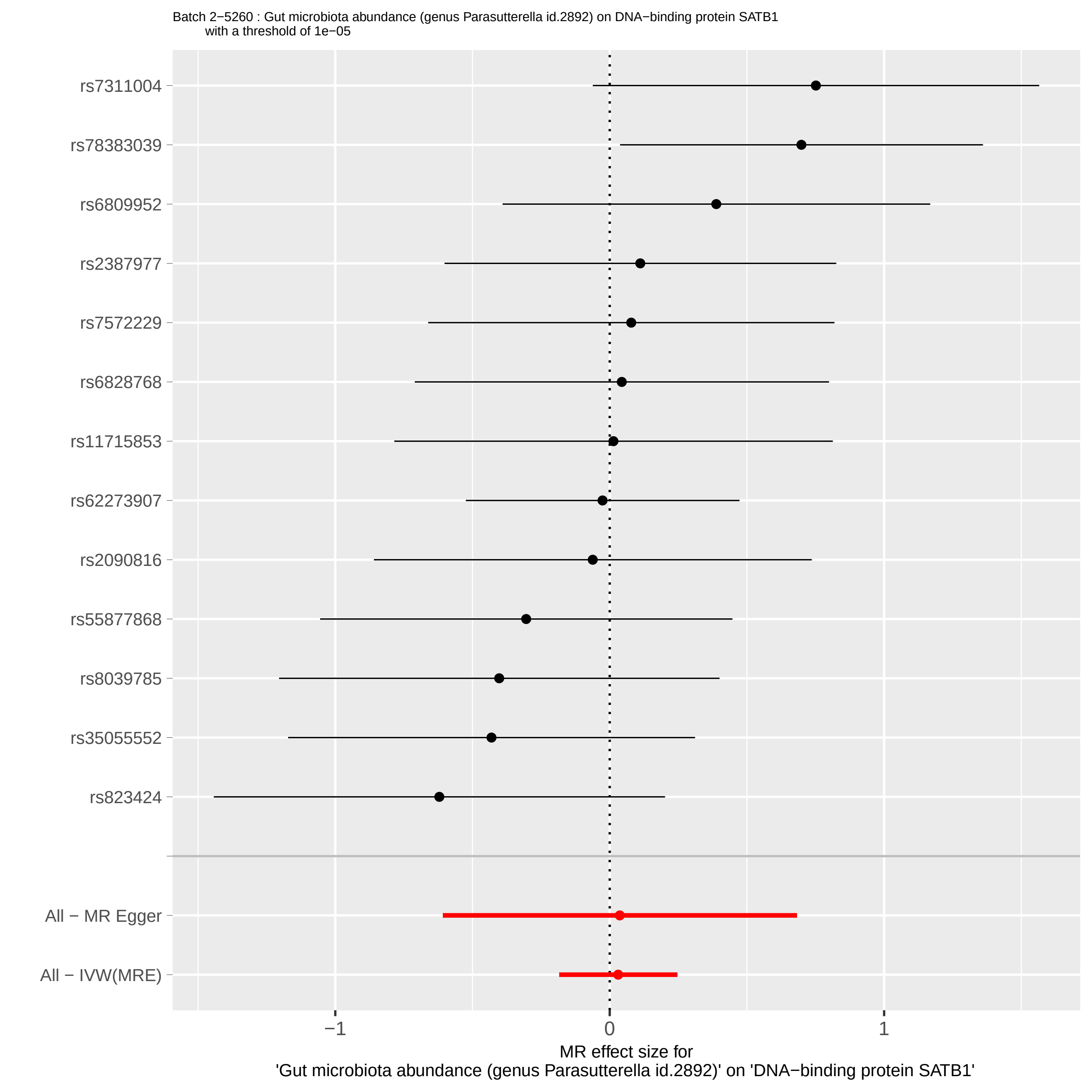
Batch 2−5260 : Gut microbiota abundance (genus Parasutterella id.2892) on DNA−binding protein SATB1
with a threshold of 1e−05
rs7311004
rs78383039
rs6809952
rs2387977
rs7572229
rs6828768
rs11715853
rs62273907
rs2090816
rs55877868
rs8039785
rs35055552
rs823424
All − MR Egger
All − IVW(MRE)
−1
0
1
MR effect size for
'Gut microbiota abundance (genus Parasutterella id.2892)' on 'DNA−binding protein SATB1'

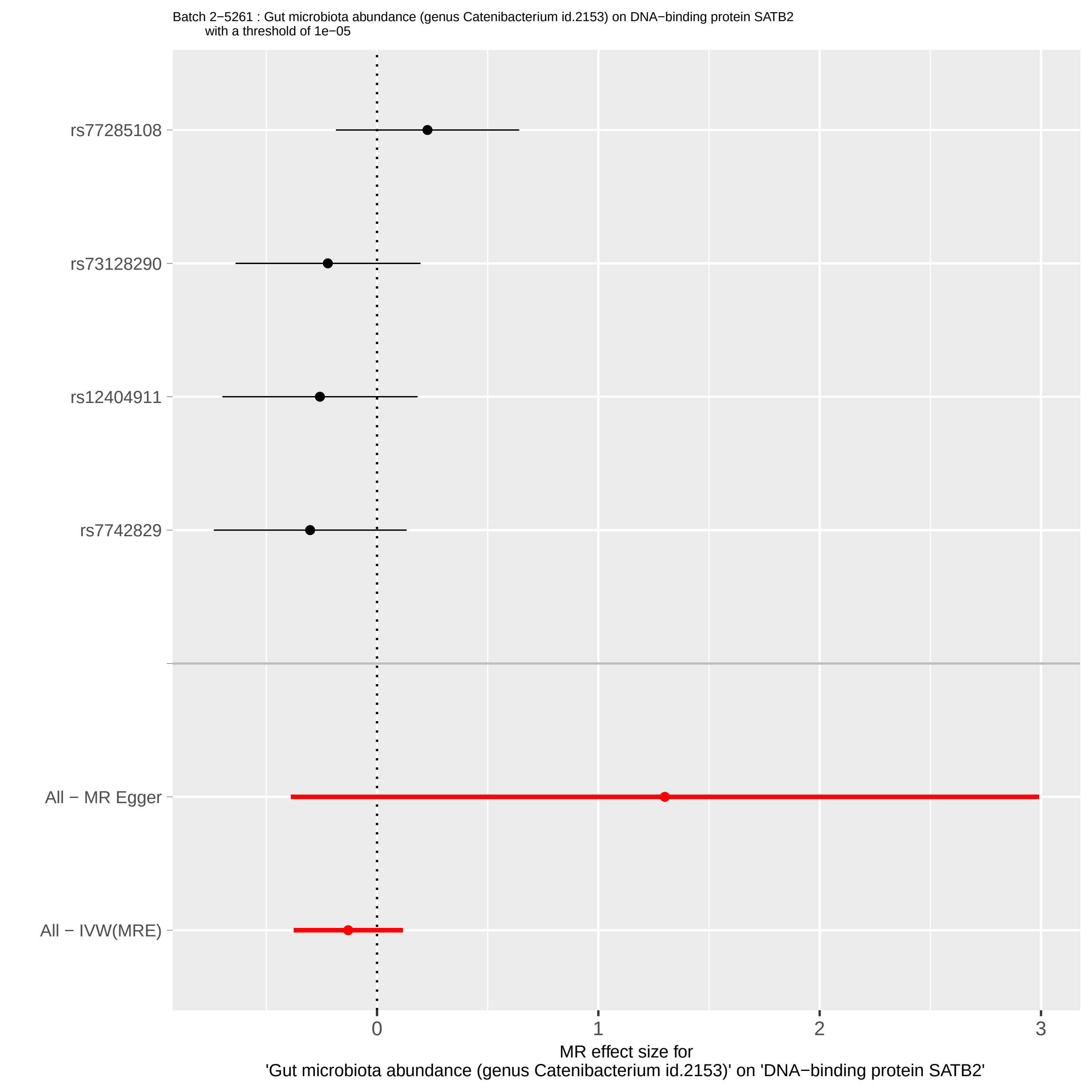
Batch 2−5261 : Gut microbiota abundance (genus Catenibacterium id.2153) on DNA−binding protein SATB2
with a threshold of 1e−05
rs77285108
rs73128290
rs12404911
rs7742829
All − MR Egger
All − IVW(MRE)
0
1
2
3
MR effect size for
'Gut microbiota abundance (genus Catenibacterium id.2153)' on 'DNA−binding protein SATB2'

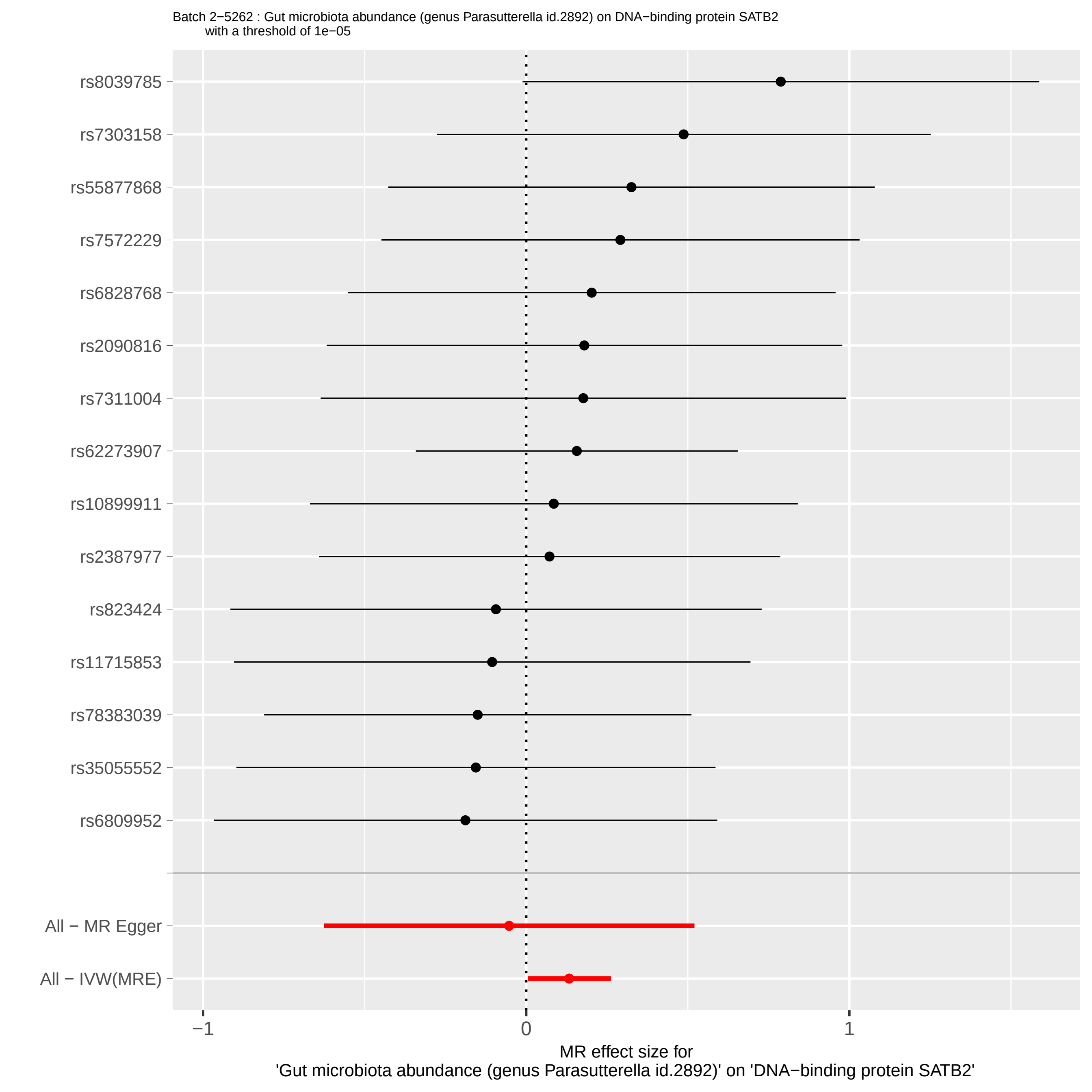

Batch 2−5262 : Gut microbiota abundance (genus Parasutterella id.2892) on DNA−binding protein SATB2
with a threshold of 1e−05
rs8039785
rs7303158
rs55877868
rs7572229
rs6828768
rs2090816
rs7311004
rs62273907
rs10899911
rs2387977
rs823424
rs11715853
rs78383039
rs35055552
rs6809952
All − MR Egger
All − IVW(MRE)
−1
0
1
MR effect size for
'Gut microbiota abundance (genus Parasutterella id.2892)' on 'DNA−binding protein SATB2'

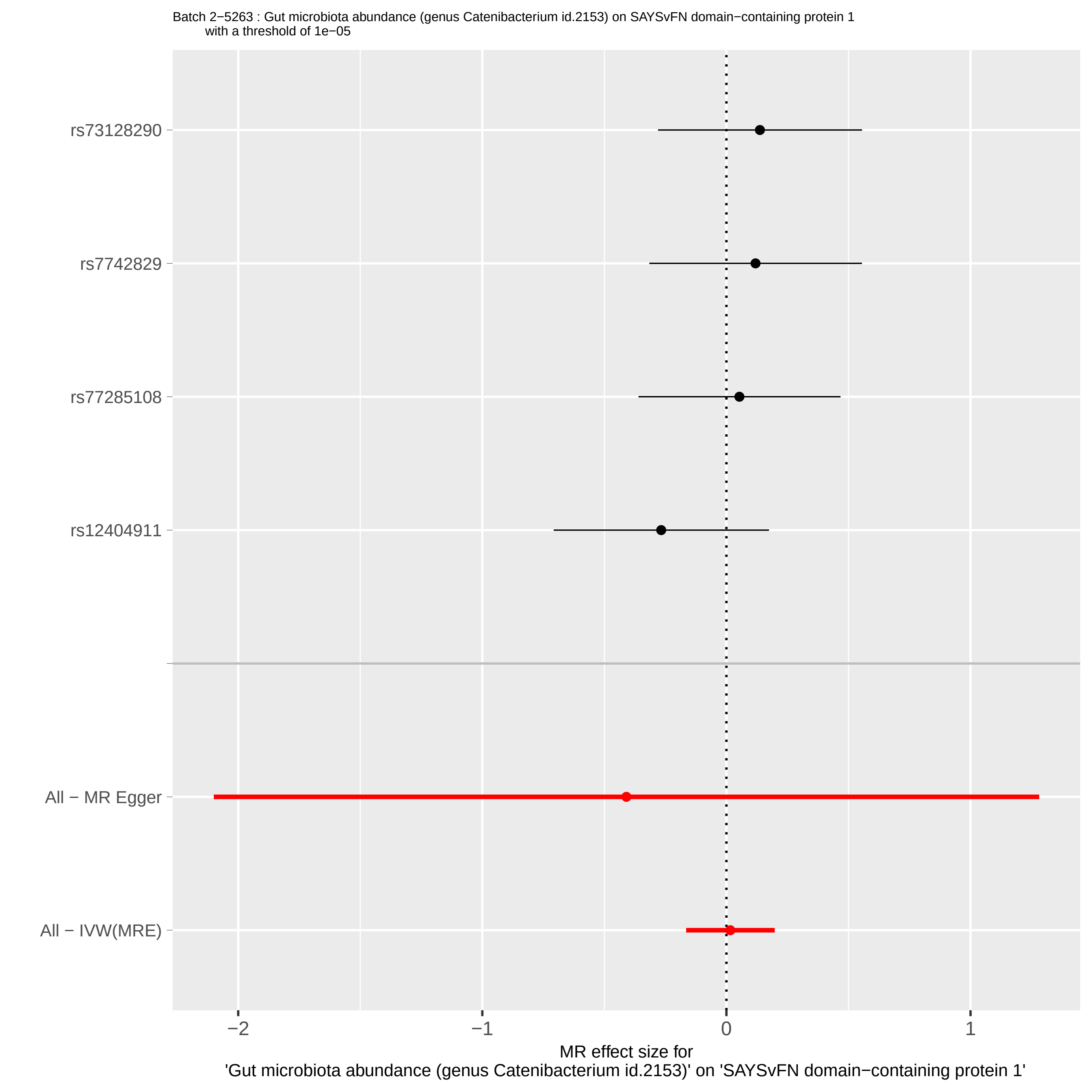
Batch 2−5263 : Gut microbiota abundance (genus Catenibacterium id.2153) on SAYSvFN domain−containing protein 1
with a threshold of 1e−05
rs73128290
rs7742829
rs77285108
rs12404911
All − MR Egger
All − IVW(MRE)
−2
−1
0
1
MR effect size for
'Gut microbiota abundance (genus Catenibacterium id.2153)' on 'SAYSvFN domain−containing protein 1'

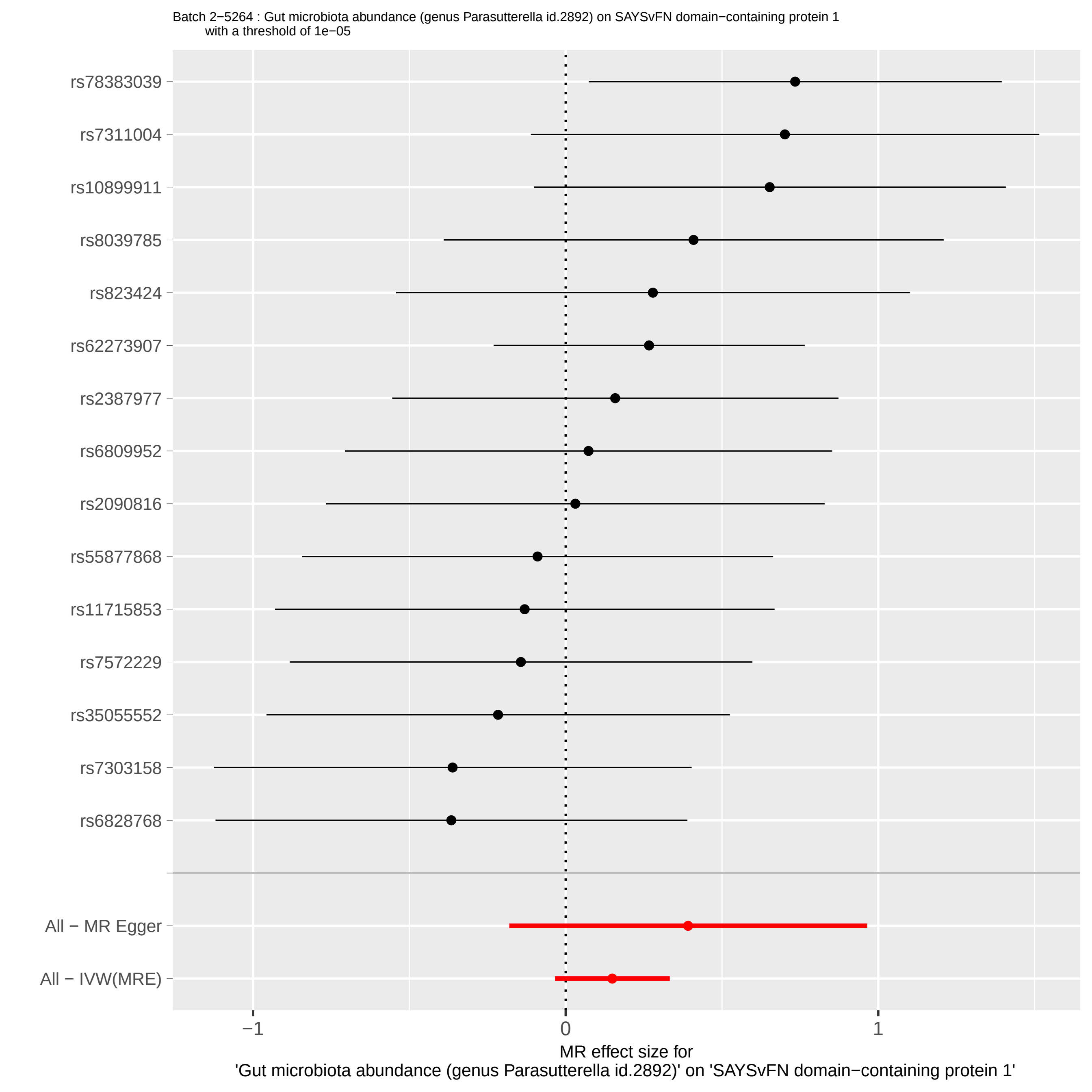

Batch 2−5264 : Gut microbiota abundance (genus Parasutterella id.2892) on SAYSvFN domain−containing protein 1
with a threshold of 1e−05
rs78383039
rs7311004
rs10899911
rs8039785
rs823424
rs62273907
rs2387977
rs6809952
rs2090816
rs55877868
rs11715853
rs7572229
rs35055552
rs7303158
rs6828768
All − MR Egger
All − IVW(MRE)
−1
0
1
MR effect size for
'Gut microbiota abundance (genus Parasutterella id.2892)' on 'SAYSvFN domain−containing protein 1'

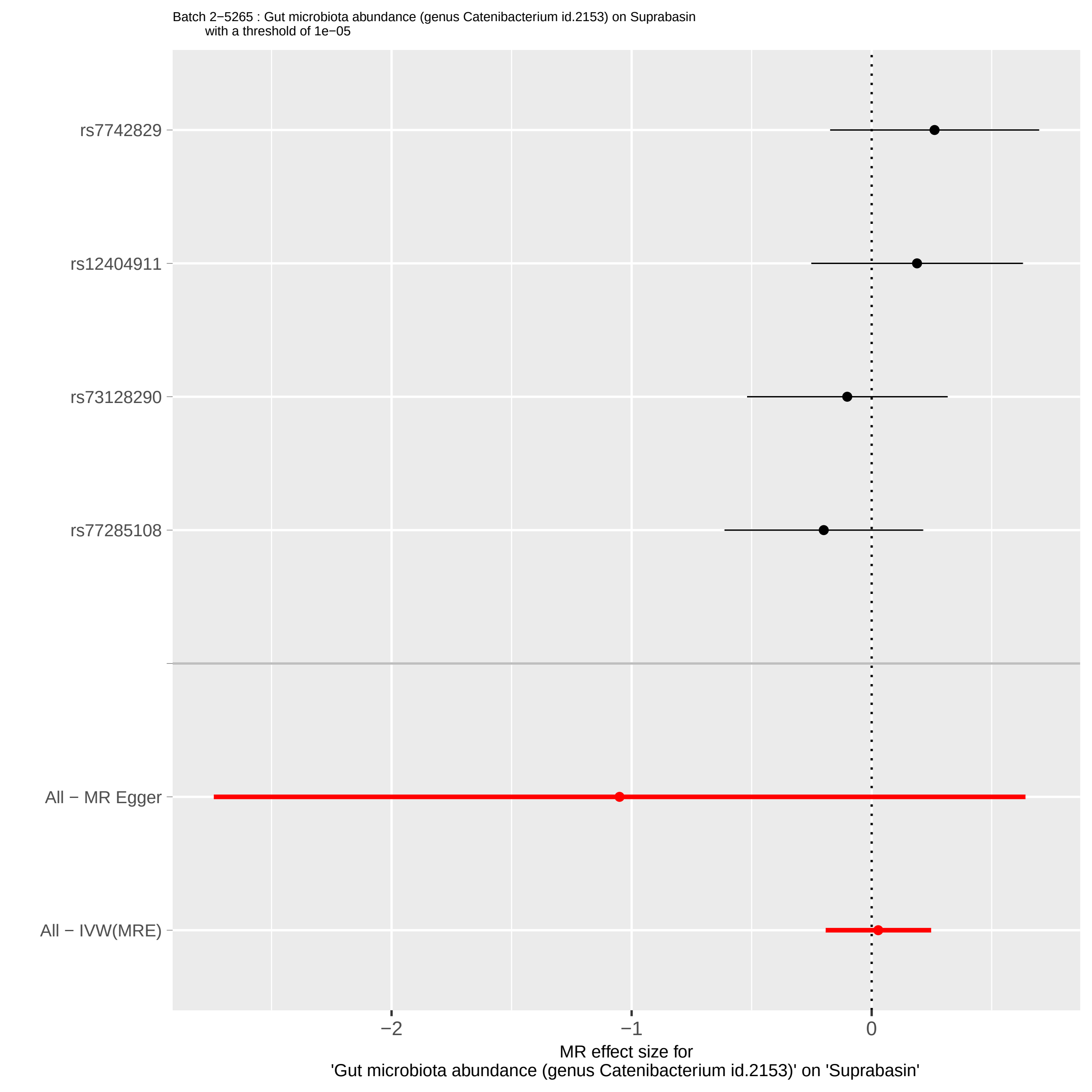

Batch 2−5265 : Gut microbiota abundance (genus Catenibacterium id.2153) on Suprabasin
with a threshold of 1e−05
rs7742829
rs12404911
rs73128290
rs77285108
All − MR Egger
All − IVW(MRE)
−2
−1
0
MR effect size for
'Gut microbiota abundance (genus Catenibacterium id.2153)' on 'Suprabasin'

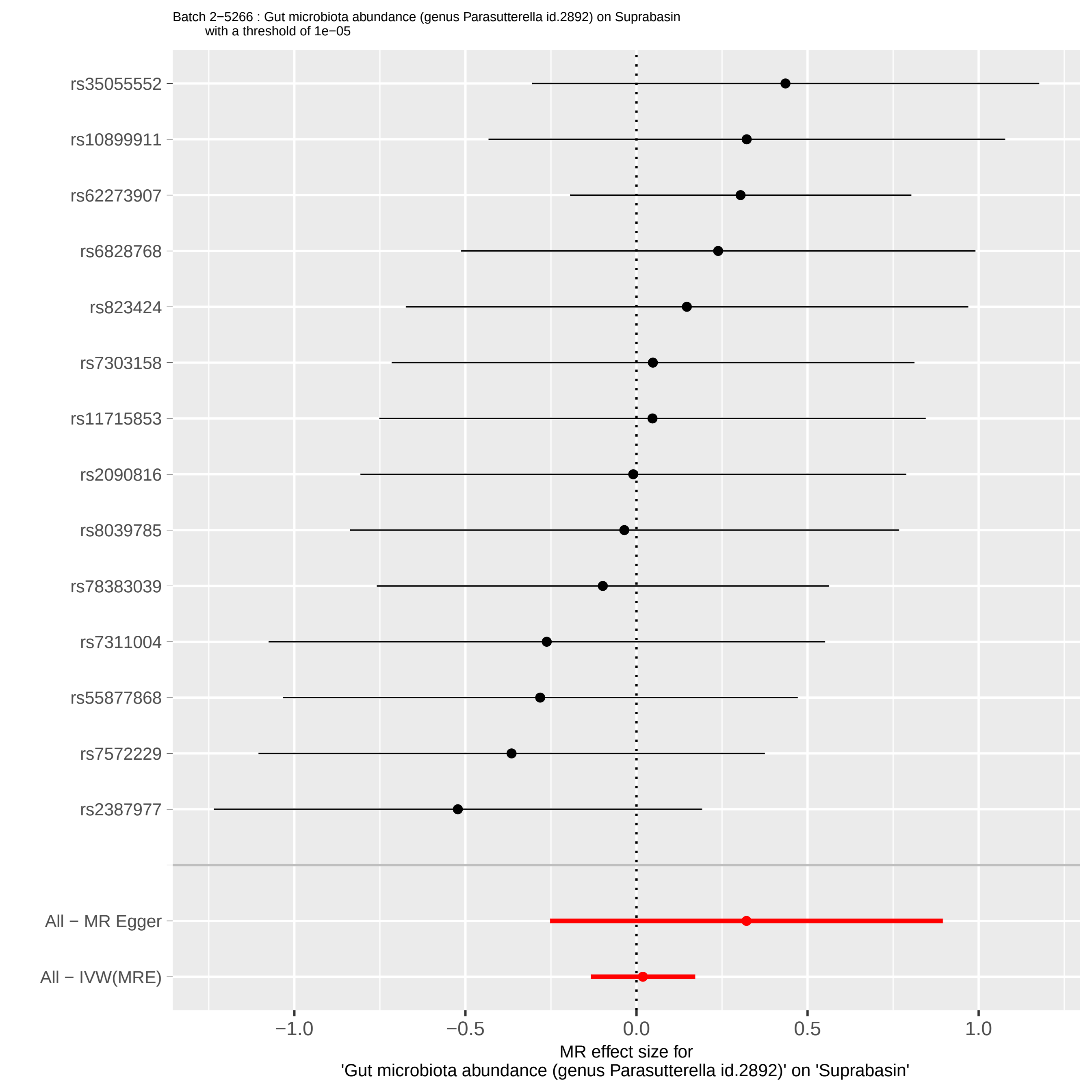

Batch 2−5266 : Gut microbiota abundance (genus Parasutterella id.2892) on Suprabasin
with a threshold of 1e−05
rs35055552
rs10899911
rs62273907
rs6828768
rs823424
rs7303158
rs11715853
rs2090816
rs8039785
rs78383039
rs7311004
rs55877868
rs7572229
rs2387977
All − MR Egger
All − IVW(MRE)
−1.0
−0.5
0.0
0.5
1.0
MR effect size for
'Gut microbiota abundance (genus Parasutterella id.2892)' on 'Suprabasin'

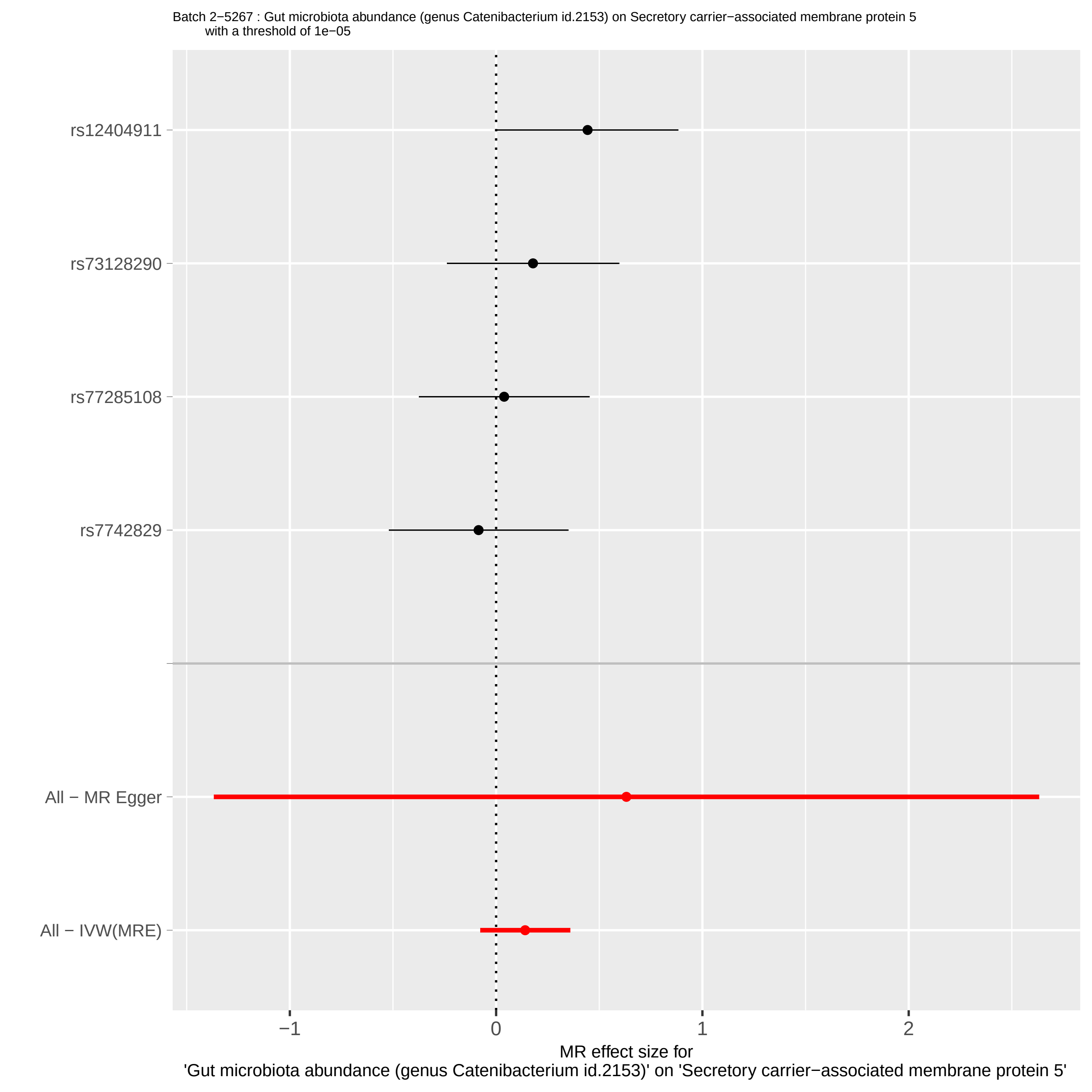
Batch 2−5267 : Gut microbiota abundance (genus Catenibacterium id.2153) on Secretory carrier−associated membrane protein 5
with a threshold of 1e−05
rs12404911
rs73128290
rs77285108
rs7742829
All − MR Egger
All − IVW(MRE)
−1
0
1
2
MR effect size for
'Gut microbiota abundance (genus Catenibacterium id.2153)' on 'Secretory carrier−associated membrane protein 5'

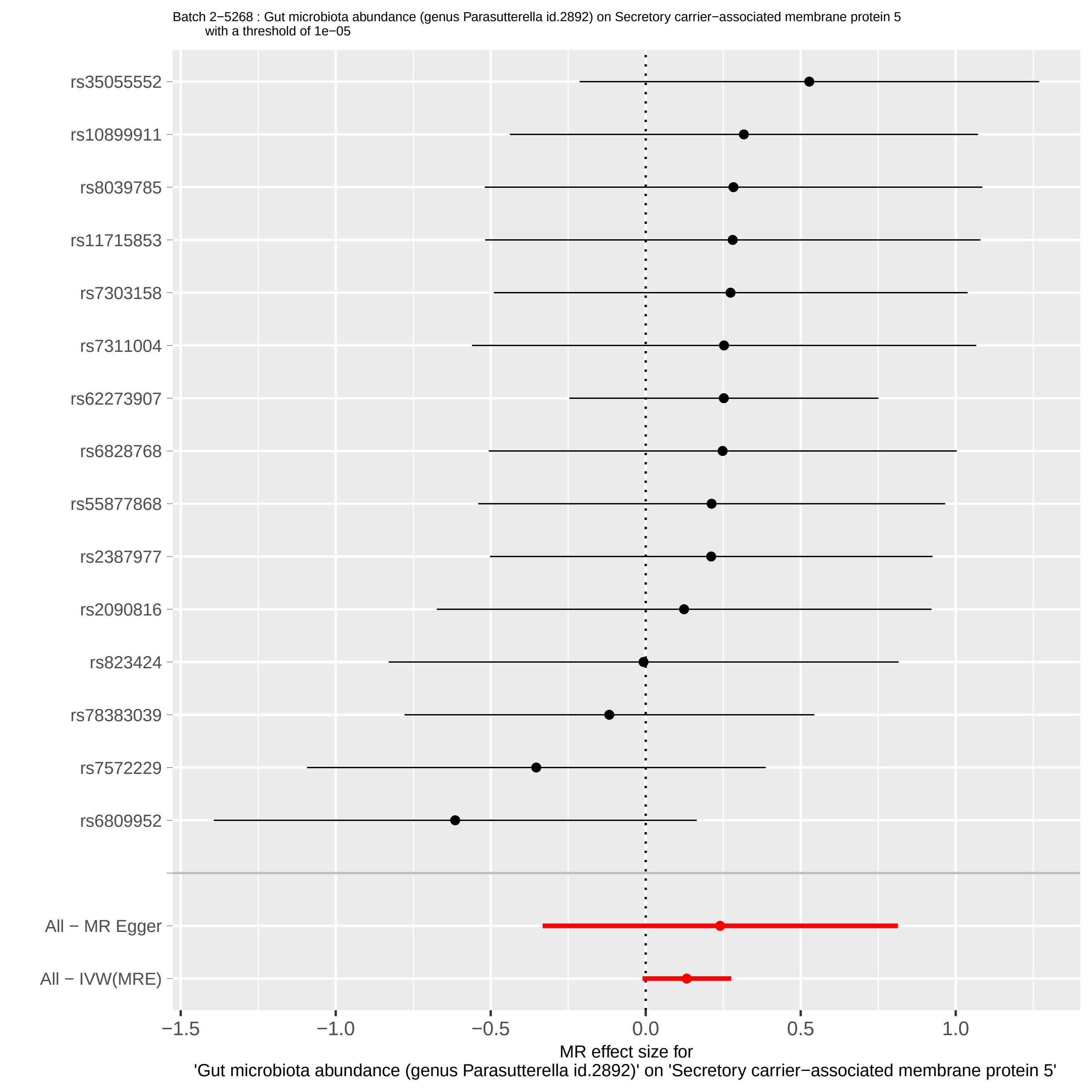
Batch 2−5268 : Gut microbiota abundance (genus Parasutterella id.2892) on Secretory carrier−associated membrane protein 5
with a threshold of 1e−05
rs35055552
rs10899911
rs8039785
rs11715853
rs7303158
rs7311004
rs62273907
rs6828768
rs55877868
rs2387977
rs2090816
rs823424
rs78383039
rs7572229
rs6809952
All − MR Egger
All − IVW(MRE)
−1.5
−1.0
−0.5
0.0
0.5
1.0
MR effect size for
'Gut microbiota abundance (genus Parasutterella id.2892)' on 'Secretory carrier−associated membrane protein 5'

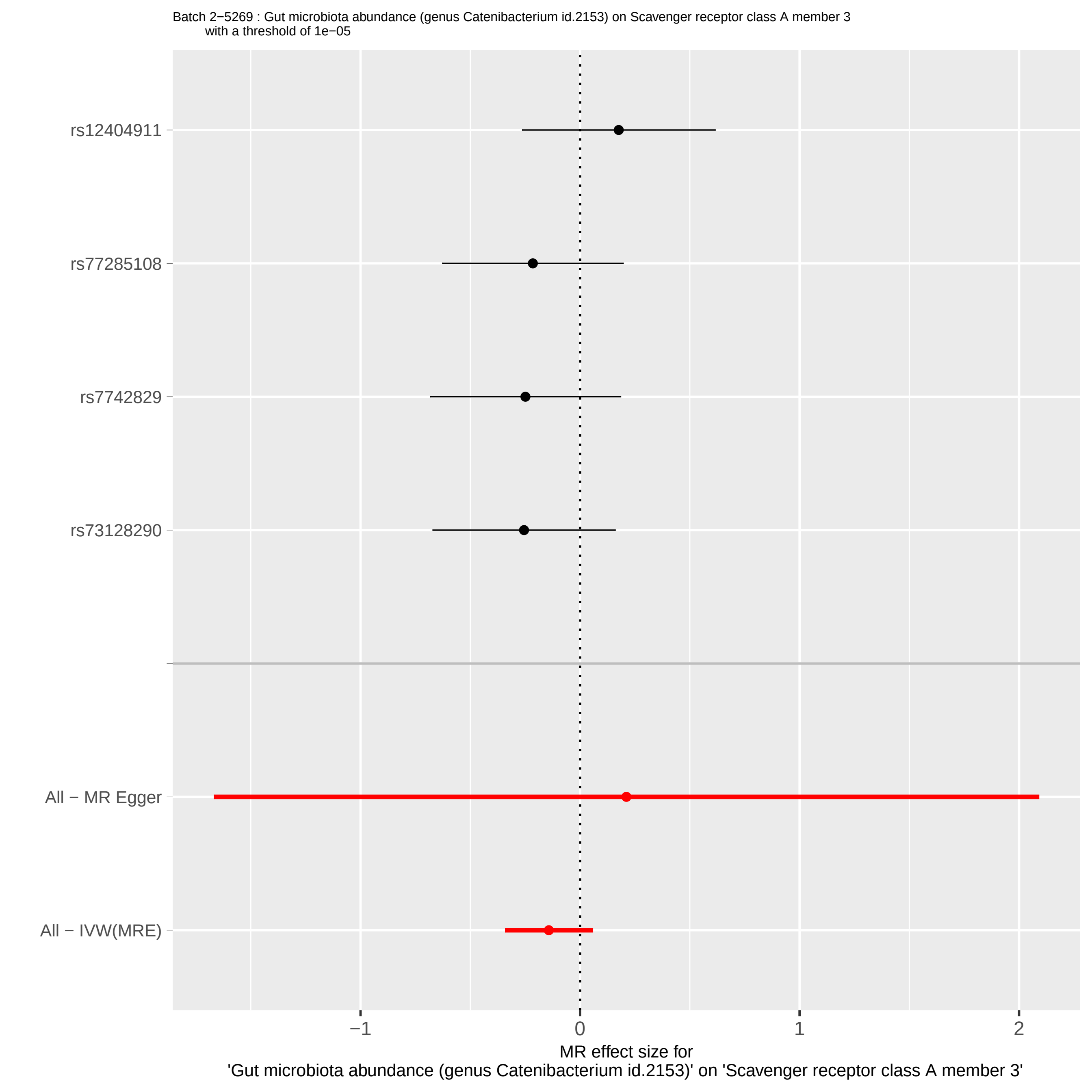

Batch 2−5269 : Gut microbiota abundance (genus Catenibacterium id.2153) on Scavenger receptor class A member 3
with a threshold of 1e−05
rs12404911
rs77285108
rs7742829
rs73128290
All − MR Egger
All − IVW(MRE)
−1
0
1
2
MR effect size for
'Gut microbiota abundance (genus Catenibacterium id.2153)' on 'Scavenger receptor class A member 3'

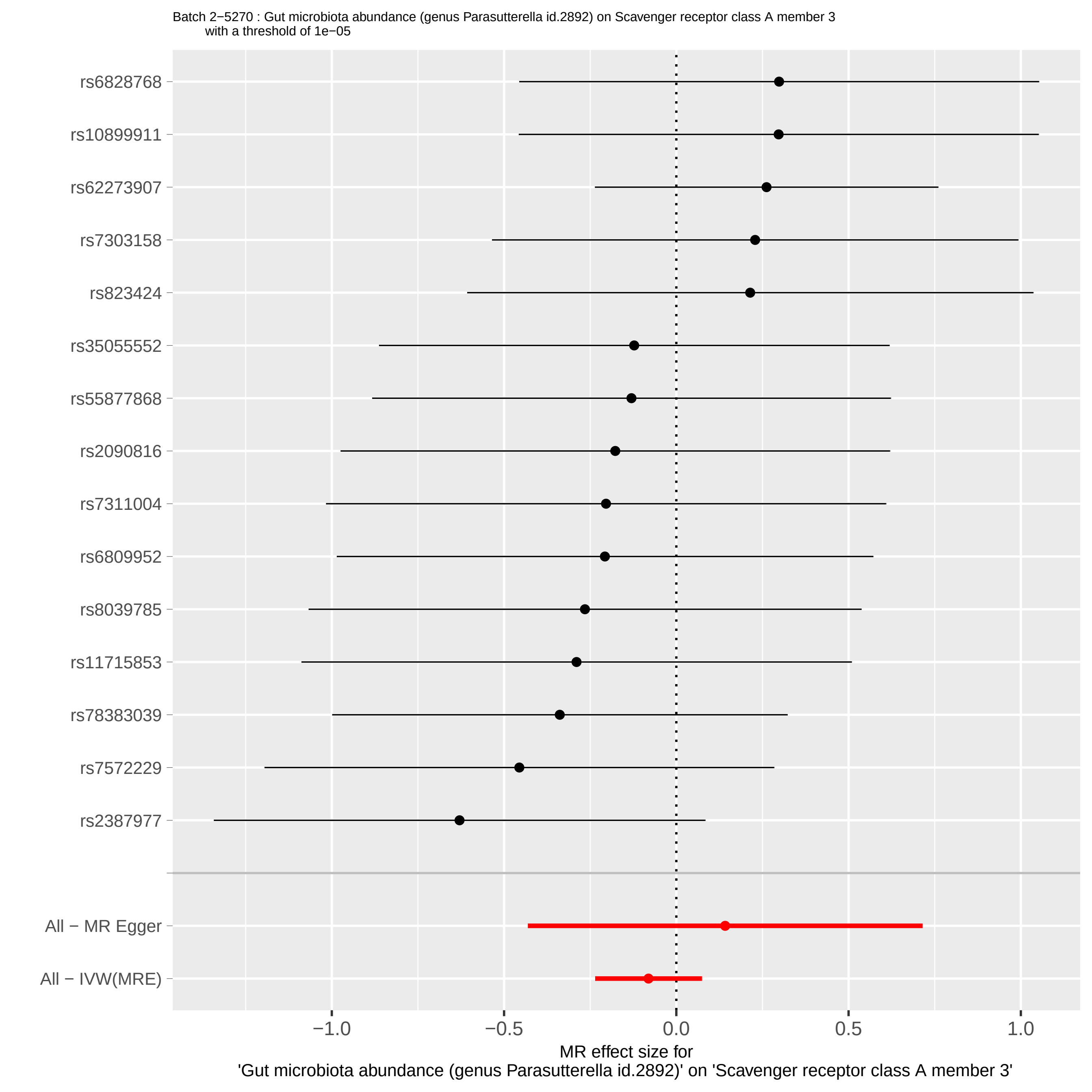

Batch 2−5270 : Gut microbiota abundance (genus Parasutterella id.2892) on Scavenger receptor class A member 3
with a threshold of 1e−05
rs6828768
rs10899911
rs62273907
rs7303158
rs823424
rs35055552
rs55877868
rs2090816
rs7311004
rs6809952
rs8039785
rs11715853
rs78383039
rs7572229
rs2387977
All − MR Egger
All − IVW(MRE)
−1.0
−0.5
0.0
0.5
1.0
MR effect size for
'Gut microbiota abundance (genus Parasutterella id.2892)' on 'Scavenger receptor class A member 3'

Batch 2−5271 : Gut microbiota abundance (genus Catenibacterium id.2153) on Scavenger receptor class A member 3
with a threshold of 1e−05

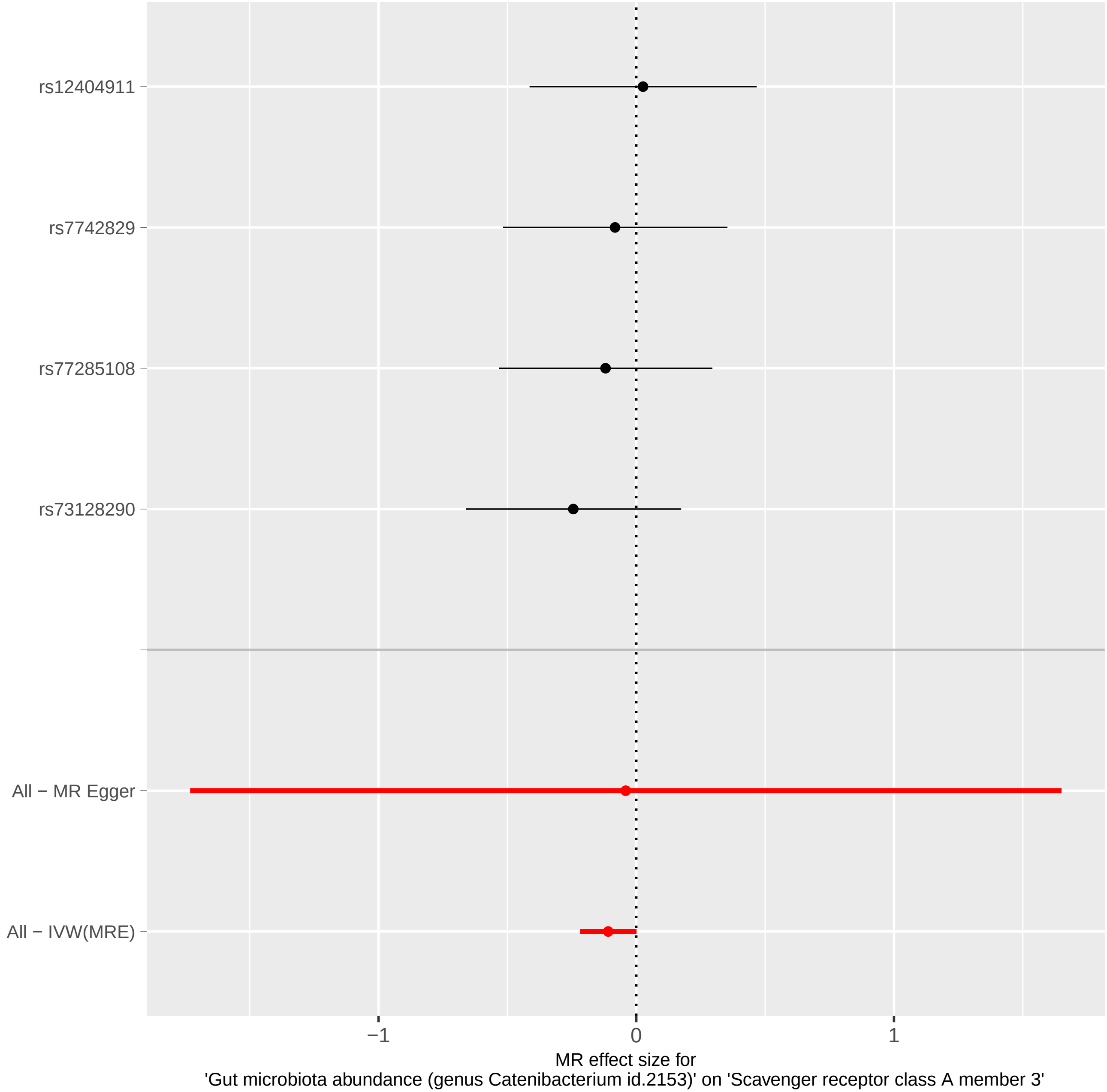

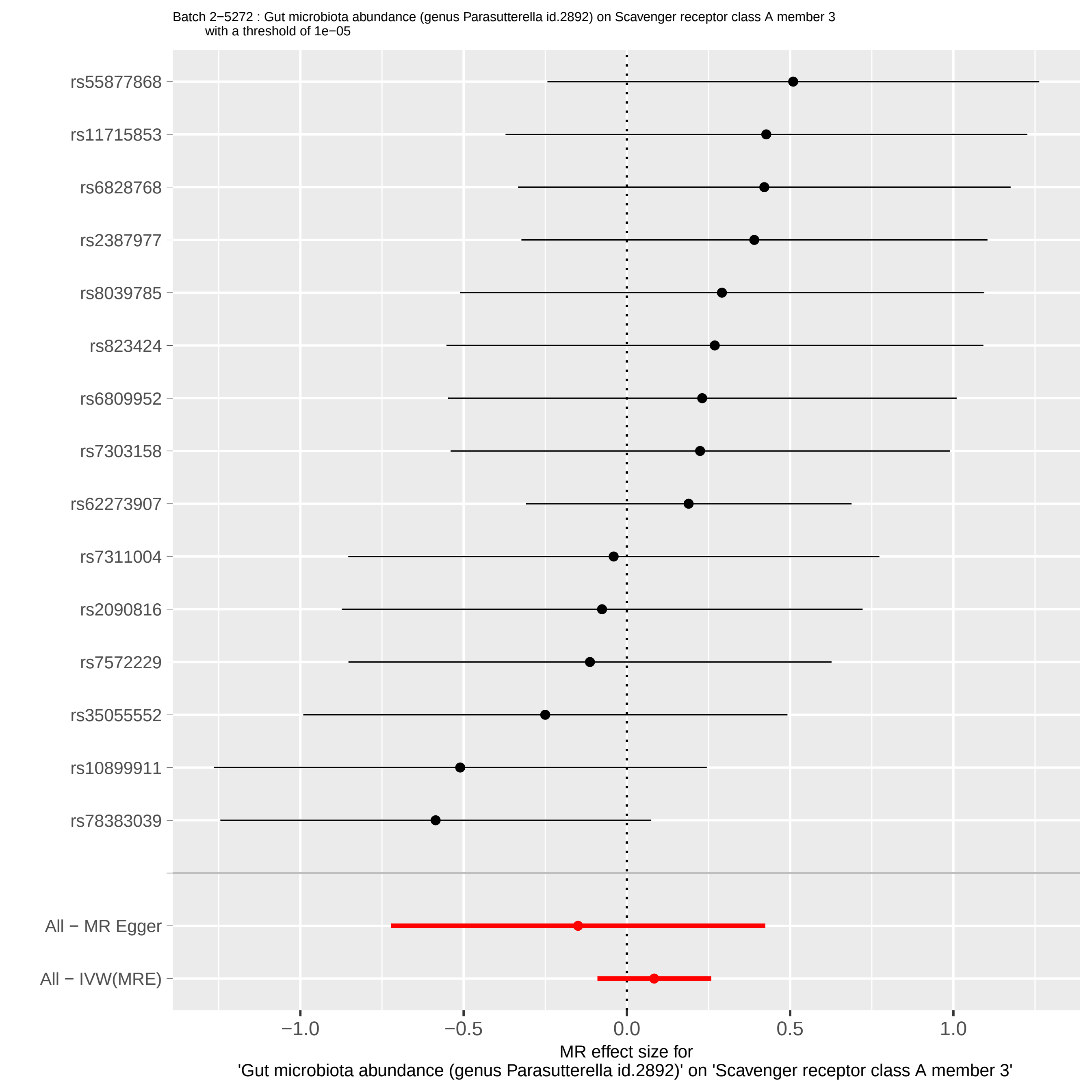

Batch 2−5272 : Gut microbiota abundance (genus Parasutterella id.2892) on Scavenger receptor class A member 3
with a threshold of 1e−05
rs55877868
rs11715853
rs6828768
rs2387977
rs8039785
rs823424
rs6809952
rs7303158
rs62273907
rs7311004
rs2090816
rs7572229
rs35055552
rs10899911
rs78383039
All − MR Egger
All − IVW(MRE)
−1.0
−0.5
0.0
0.5
1.0
MR effect size for
'Gut microbiota abundance (genus Parasutterella id.2892)' on 'Scavenger receptor class A member 3'

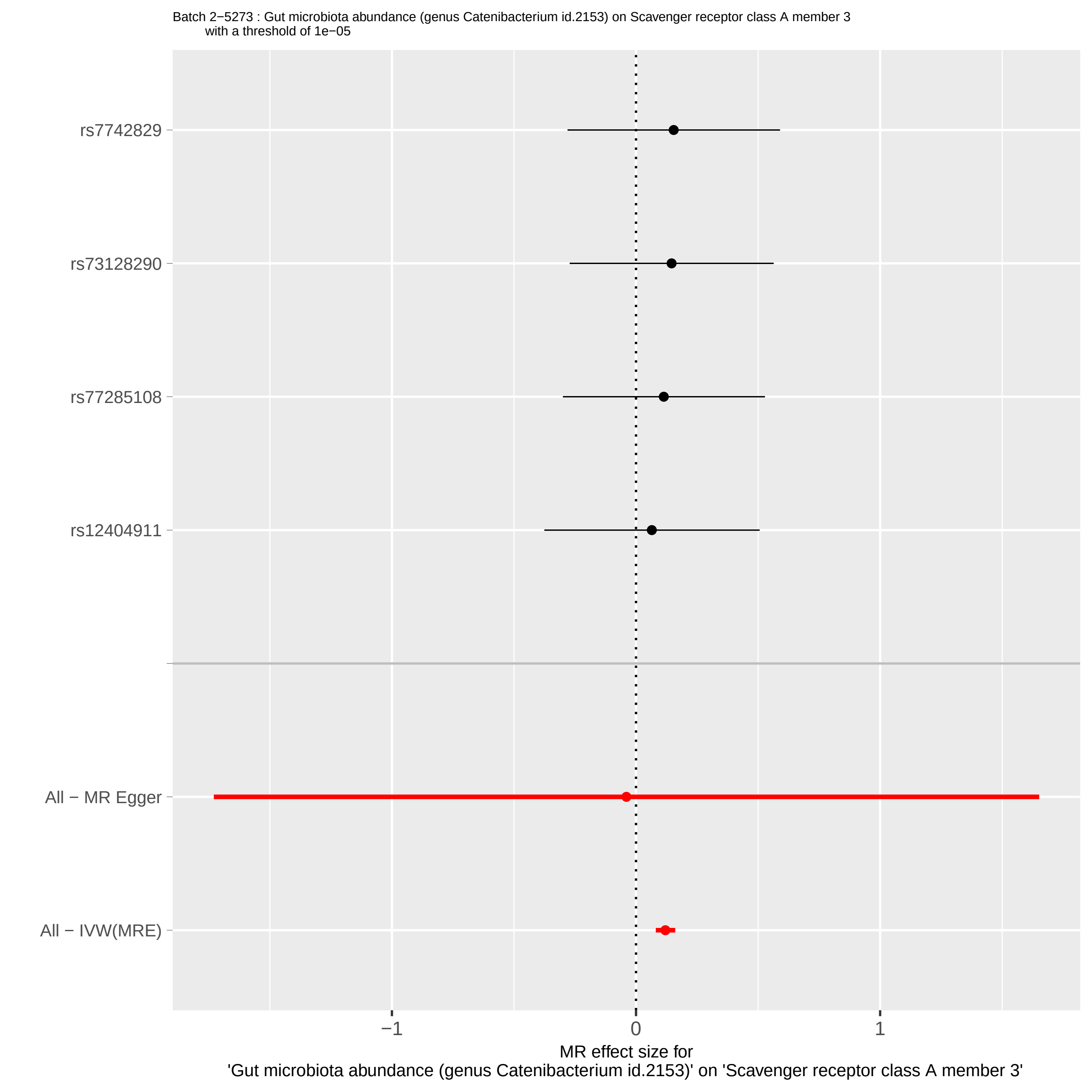

Batch 2−5273 : Gut microbiota abundance (genus Catenibacterium id.2153) on Scavenger receptor class A member 3
with a threshold of 1e−05
rs7742829
rs73128290
rs77285108
rs12404911
All − MR Egger
All − IVW(MRE)
−1
0
1
MR effect size for
'Gut microbiota abundance (genus Catenibacterium id.2153)' on 'Scavenger receptor class A member 3'

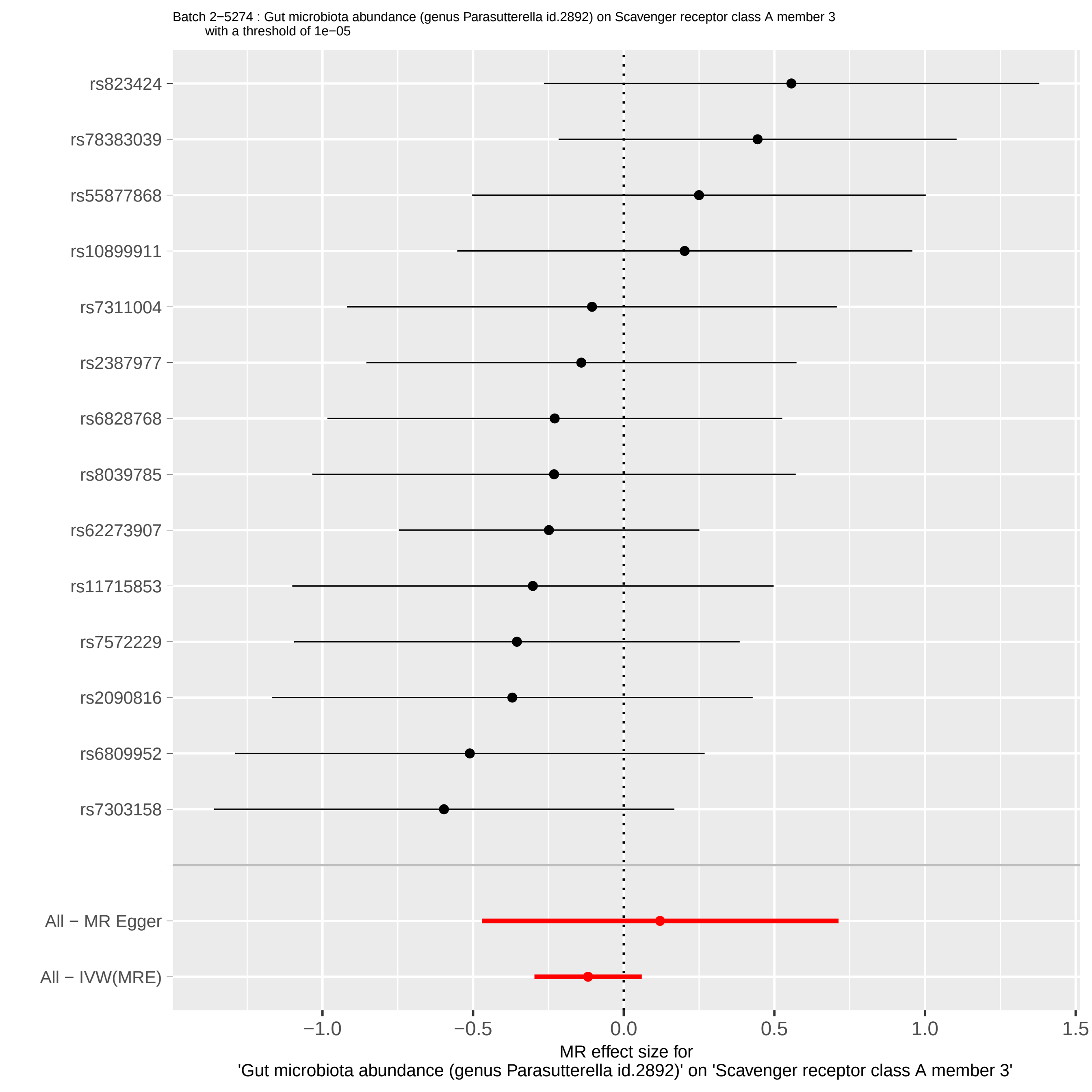

Batch 2−5274 : Gut microbiota abundance (genus Parasutterella id.2892) on Scavenger receptor class A member 3
with a threshold of 1e−05
rs823424
rs78383039
rs55877868
rs10899911
rs7311004
rs2387977
rs6828768
rs8039785
rs62273907
rs11715853
rs7572229
rs2090816
rs6809952
rs7303158
All − MR Egger
All − IVW(MRE)
−1.0
−0.5
0.0
0.5
1.0
1.5
MR effect size for
'Gut microbiota abundance (genus Parasutterella id.2892)' on 'Scavenger receptor class A member 3'

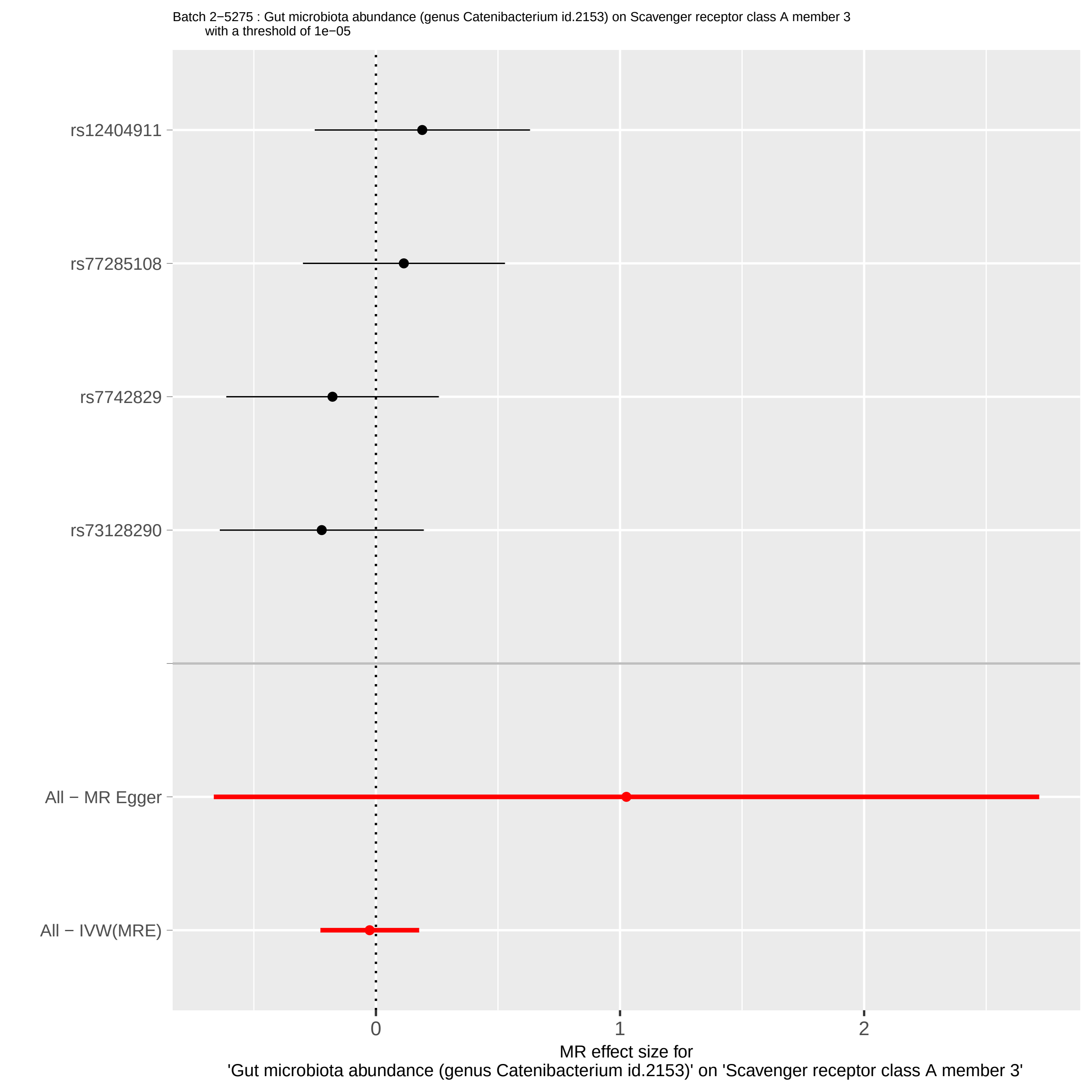

Batch 2−5275 : Gut microbiota abundance (genus Catenibacterium id.2153) on Scavenger receptor class A member 3
with a threshold of 1e−05
rs12404911
rs77285108
rs7742829
rs73128290
All − MR Egger
All − IVW(MRE)
0
1
2
MR effect size for
'Gut microbiota abundance (genus Catenibacterium id.2153)' on 'Scavenger receptor class A member 3'

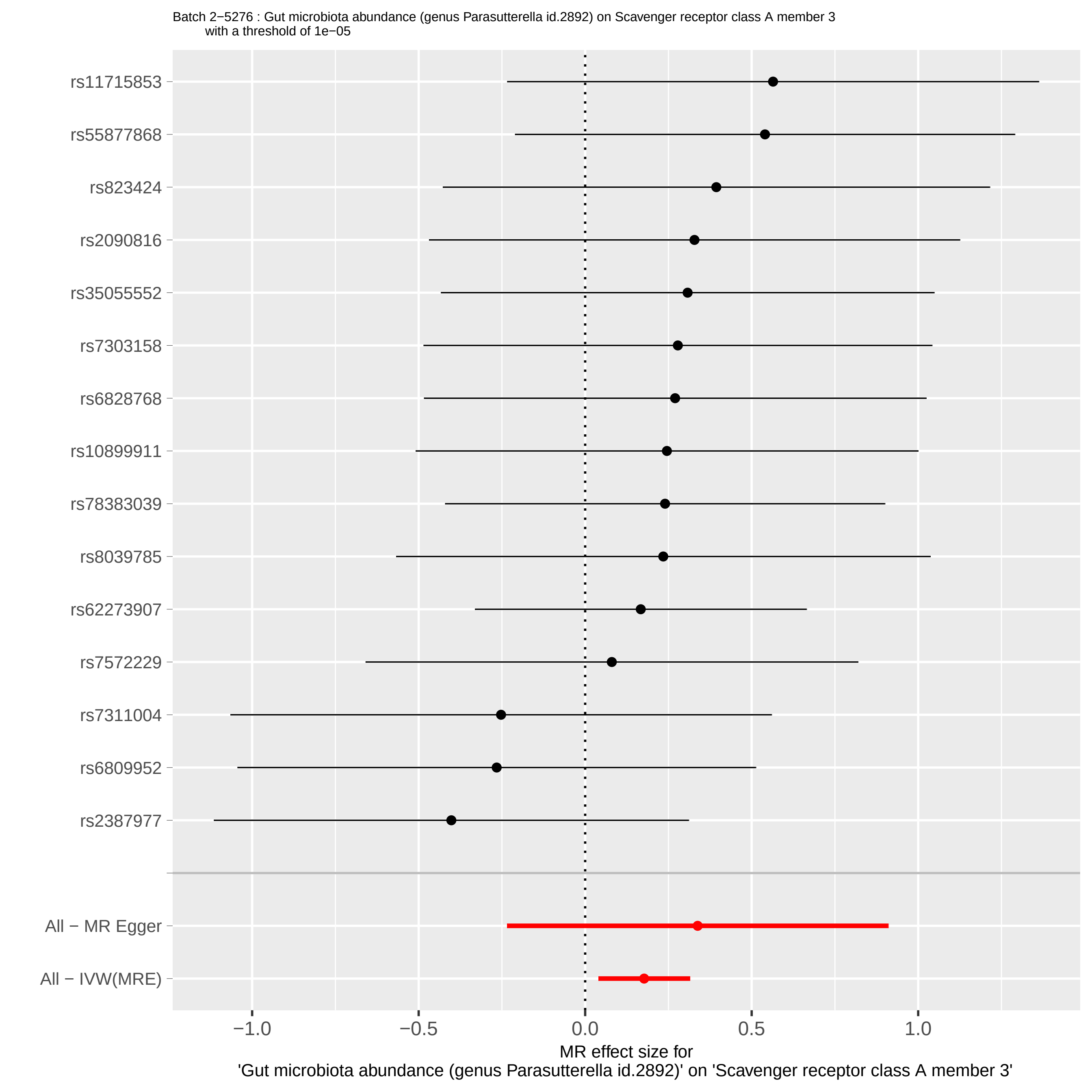

Batch 2−5276 : Gut microbiota abundance (genus Parasutterella id.2892) on Scavenger receptor class A member 3
with a threshold of 1e−05
rs11715853
rs55877868
rs823424
rs2090816
rs35055552
rs7303158
rs6828768
rs10899911
rs78383039
rs8039785
rs62273907
rs7572229
rs7311004
rs6809952
rs2387977
All − MR Egger
All − IVW(MRE)
−1.0
−0.5
0.0
0.5
1.0
MR effect size for
'Gut microbiota abundance (genus Parasutterella id.2892)' on 'Scavenger receptor class A member 3'

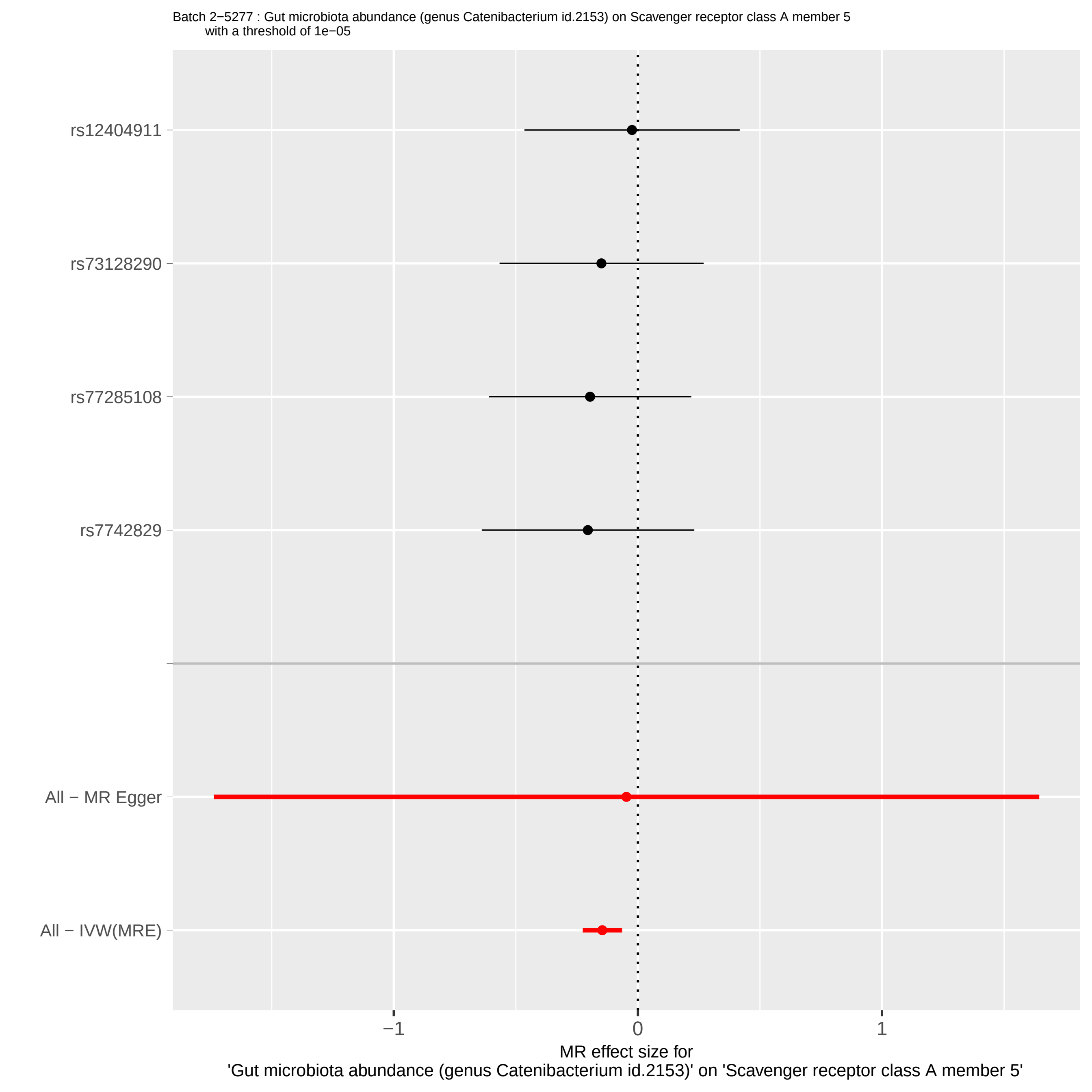
Batch 2−5277 : Gut microbiota abundance (genus Catenibacterium id.2153) on Scavenger receptor class A member 5
with a threshold of 1e−05
rs12404911
rs73128290
rs77285108
rs7742829
All − MR Egger
All − IVW(MRE)
−1
0
1
MR effect size for
'Gut microbiota abundance (genus Catenibacterium id.2153)' on 'Scavenger receptor class A member 5'

Batch 2−5278 : Gut microbiota abundance (genus Parasutterella id.2892) on Scavenger receptor class A member 5
with a threshold of 1e−05

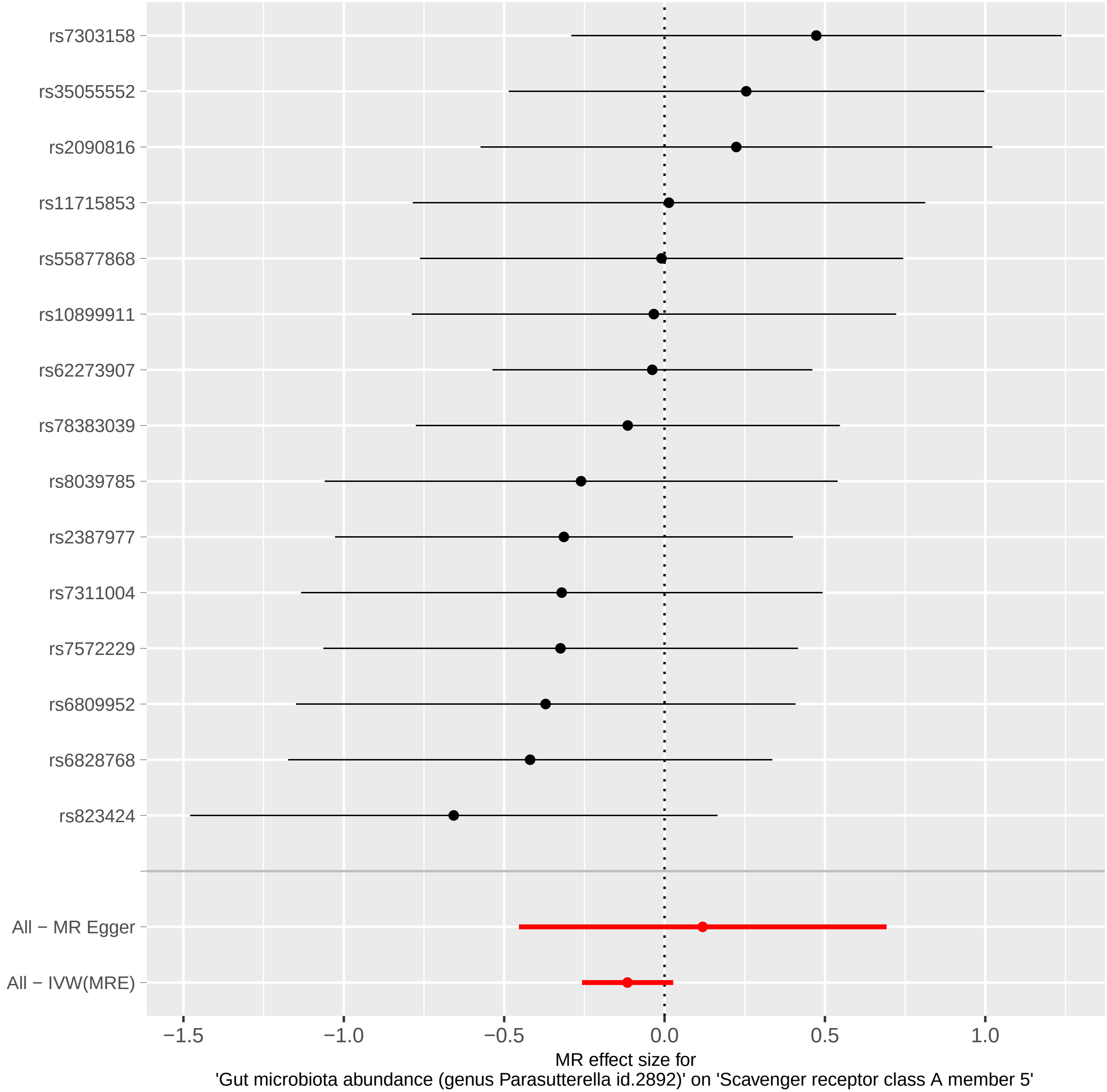

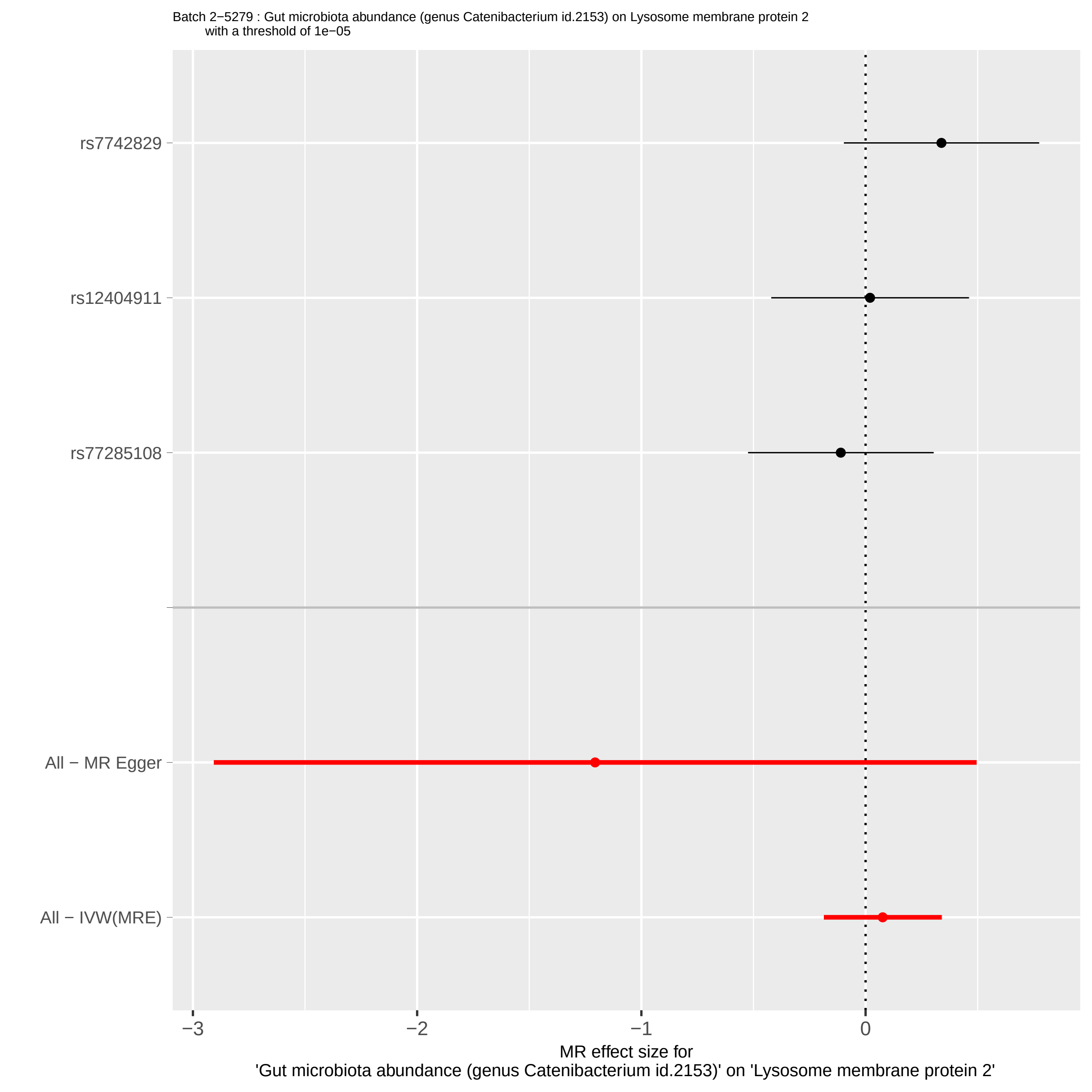

Batch 2−5279 : Gut microbiota abundance (genus Catenibacterium id.2153) on Lysosome membrane protein 2
with a threshold of 1e−05
rs7742829
rs12404911
rs77285108
All − MR Egger
All − IVW(MRE)
−3
−2
−1
0
MR effect size for
'Gut microbiota abundance (genus Catenibacterium id.2153)' on 'Lysosome membrane protein 2'

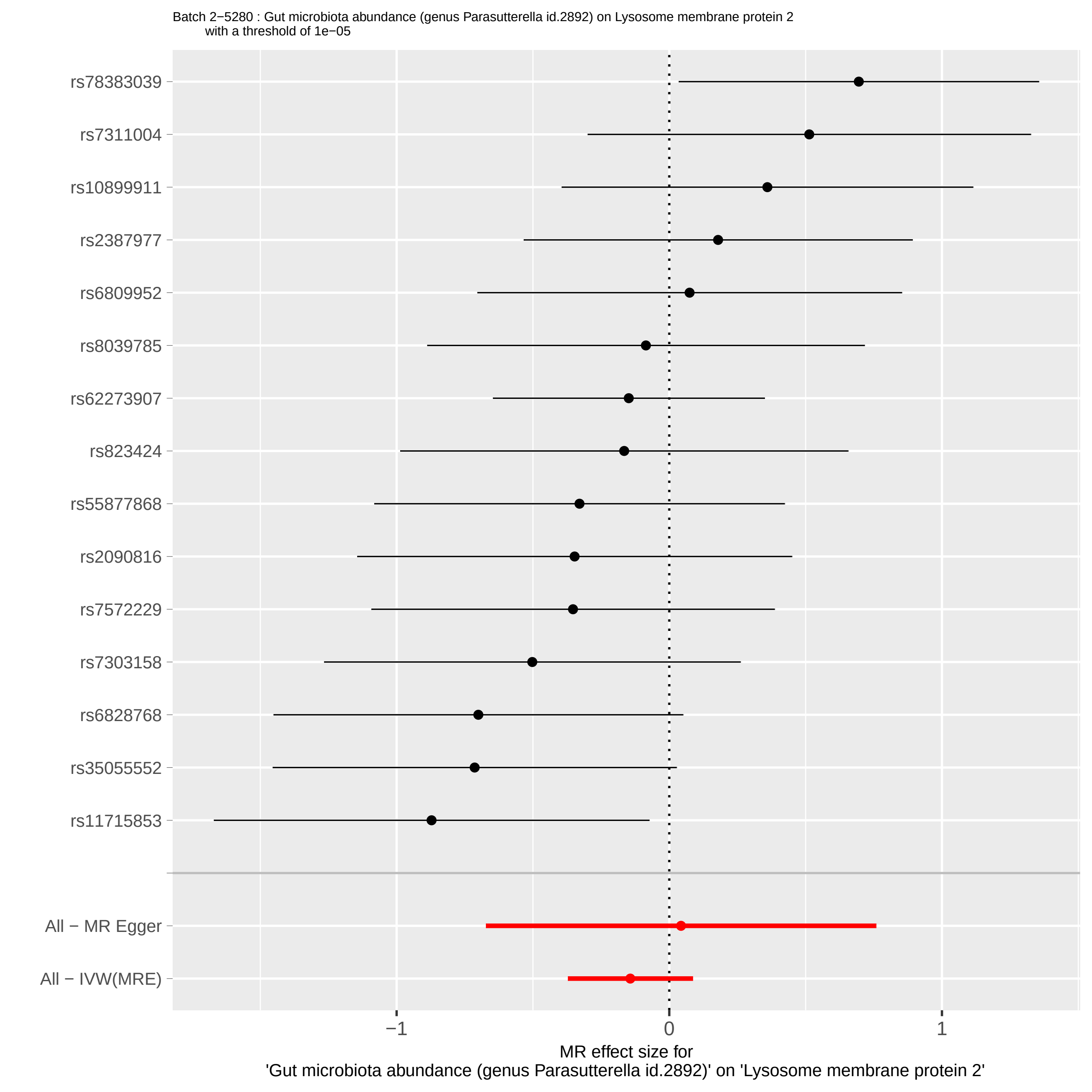
Batch 2−5280 : Gut microbiota abundance (genus Parasutterella id.2892) on Lysosome membrane protein 2
with a threshold of 1e−05
rs78383039
rs7311004
rs10899911
rs2387977
rs6809952
rs8039785
rs62273907
rs823424
rs55877868
rs2090816
rs7572229
rs7303158
rs6828768
rs35055552
rs11715853
All − MR Egger
All − IVW(MRE)
−1
0
1
MR effect size for
'Gut microbiota abundance (genus Parasutterella id.2892)' on 'Lysosome membrane protein 2'

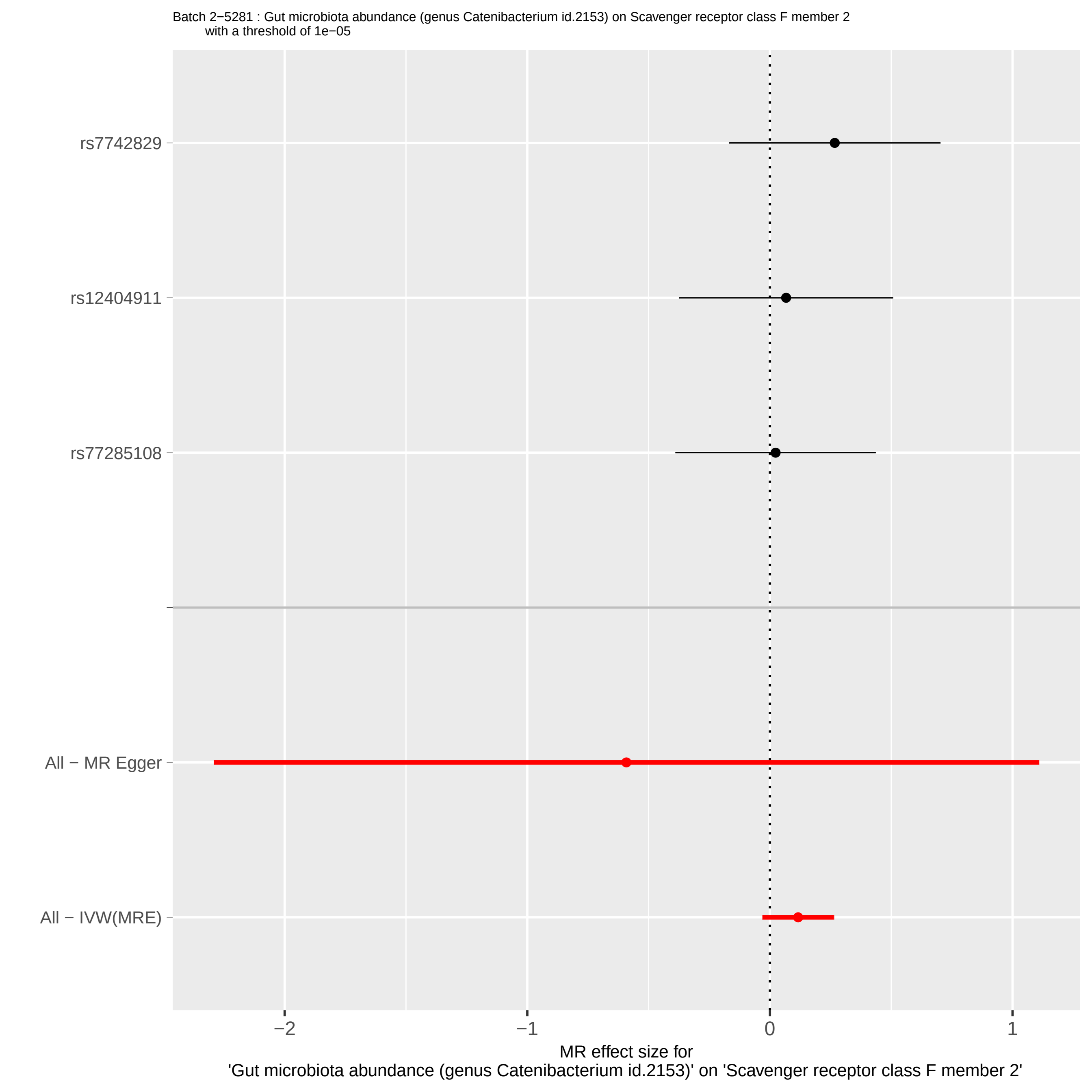

Batch 2−5281 : Gut microbiota abundance (genus Catenibacterium id.2153) on Scavenger receptor class F member 2
with a threshold of 1e−05
rs7742829
rs12404911
rs77285108
All − MR Egger
All − IVW(MRE)
−2
−1
0
1
MR effect size for
'Gut microbiota abundance (genus Catenibacterium id.2153)' on 'Scavenger receptor class F member 2'

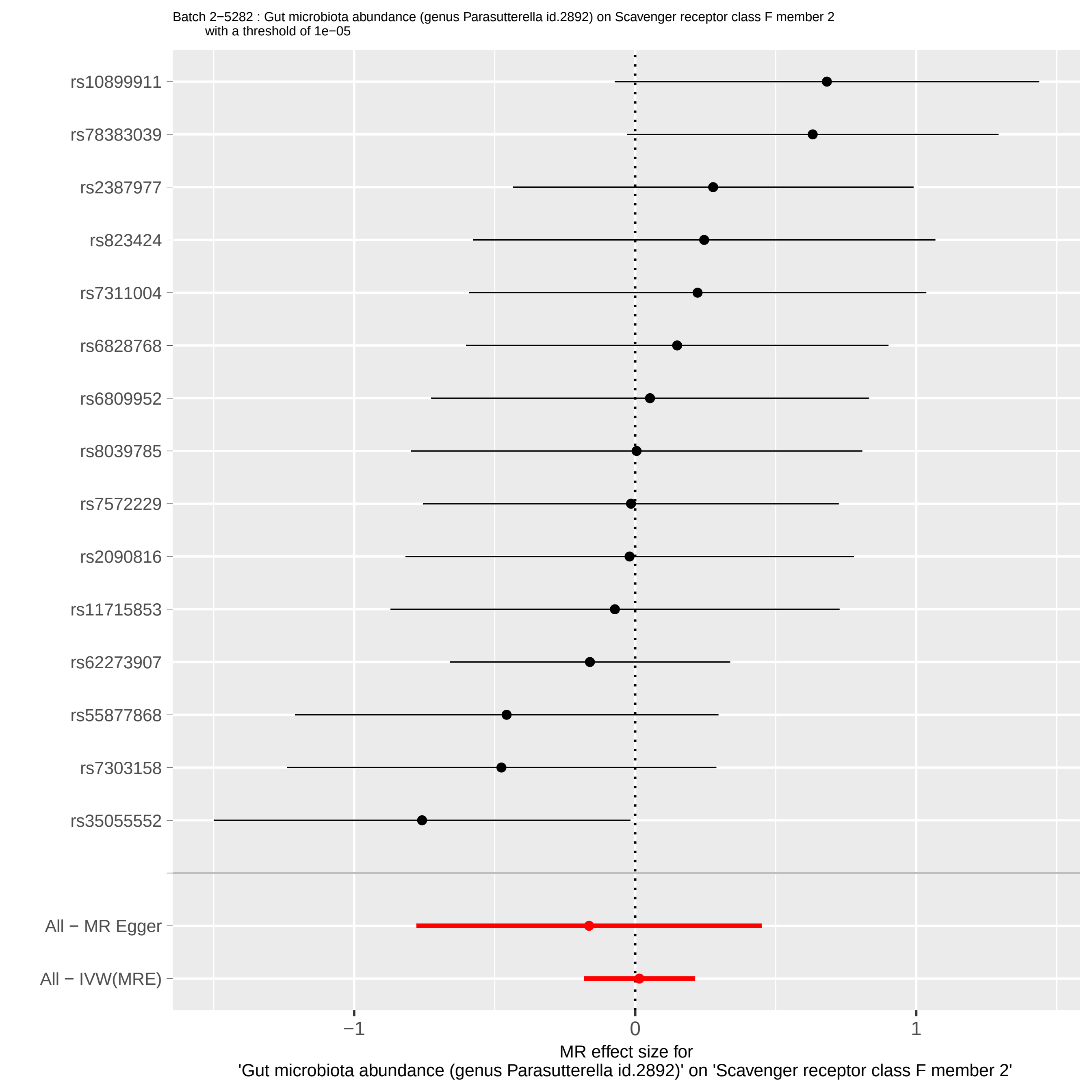
Batch 2−5282 : Gut microbiota abundance (genus Parasutterella id.2892) on Scavenger receptor class F member 2
with a threshold of 1e−05
rs10899911
rs78383039
rs2387977
rs823424
rs7311004
rs6828768
rs6809952
rs8039785
rs7572229
rs2090816
rs11715853
rs62273907
rs55877868
rs7303158
rs35055552
All − MR Egger
All − IVW(MRE)
−1
0
1
MR effect size for
'Gut microbiota abundance (genus Parasutterella id.2892)' on 'Scavenger receptor class F member 2'

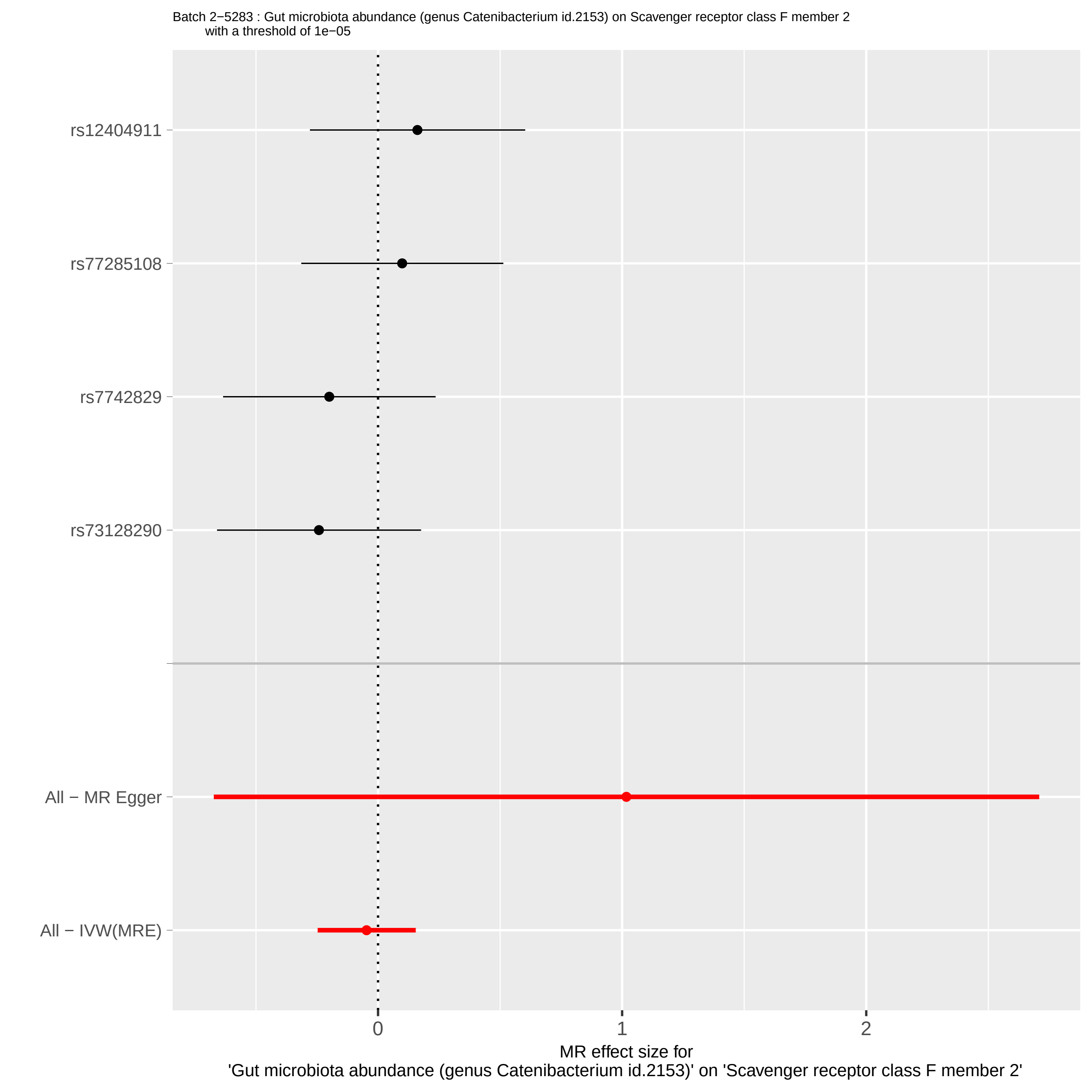

Batch 2−5283 : Gut microbiota abundance (genus Catenibacterium id.2153) on Scavenger receptor class F member 2
with a threshold of 1e−05
rs12404911
rs77285108
rs7742829
rs73128290
All − MR Egger
All − IVW(MRE)
0
1
2
MR effect size for
'Gut microbiota abundance (genus Catenibacterium id.2153)' on 'Scavenger receptor class F member 2'

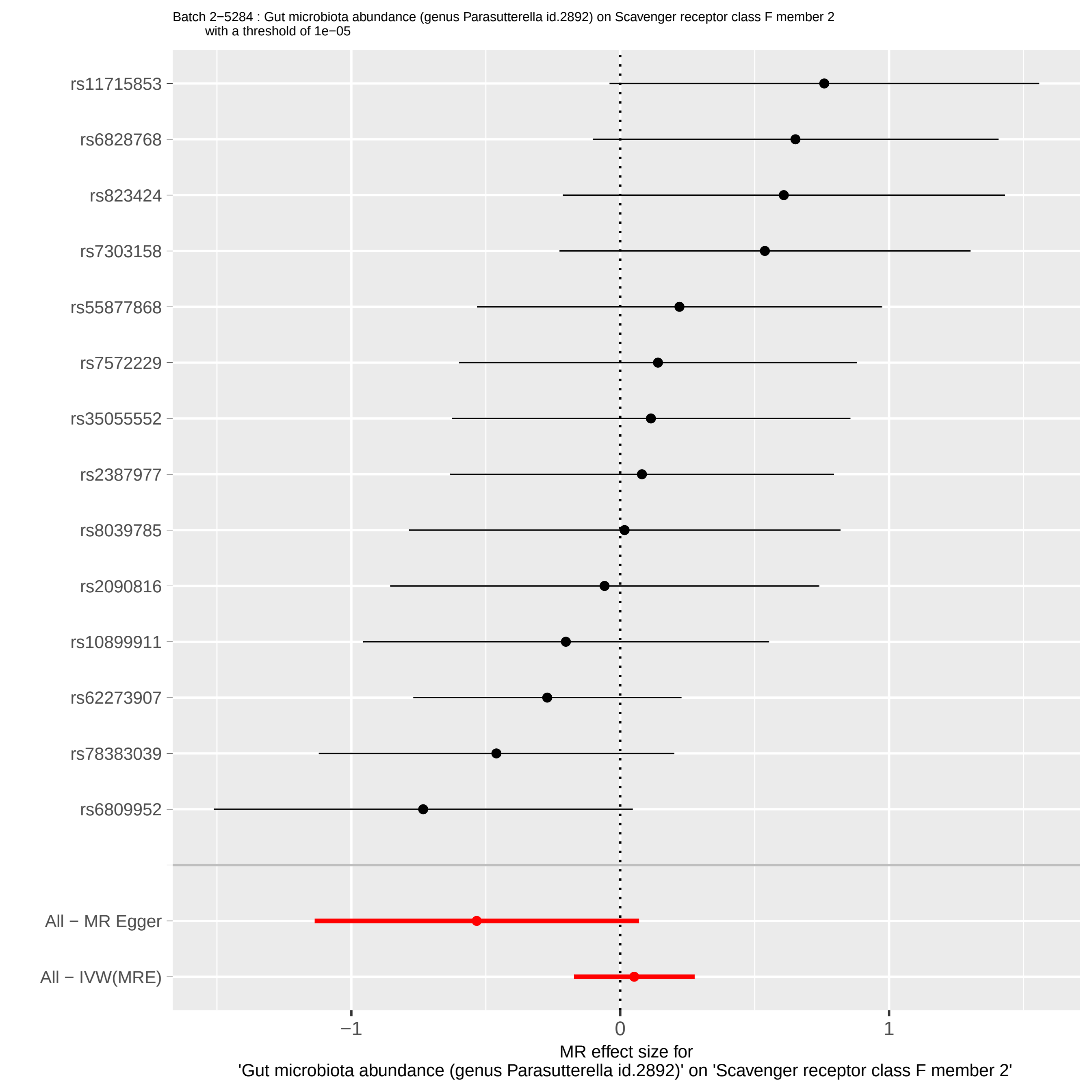
Batch 2−5284 : Gut microbiota abundance (genus Parasutterella id.2892) on Scavenger receptor class F member 2
with a threshold of 1e−05
rs11715853
rs6828768
rs823424
rs7303158
rs55877868
rs7572229
rs35055552
rs2387977
rs8039785
rs2090816
rs10899911
rs62273907
rs78383039
rs6809952
All − MR Egger
All − IVW(MRE)
−1
0
1
MR effect size for
'Gut microbiota abundance (genus Parasutterella id.2892)' on 'Scavenger receptor class F member 2'

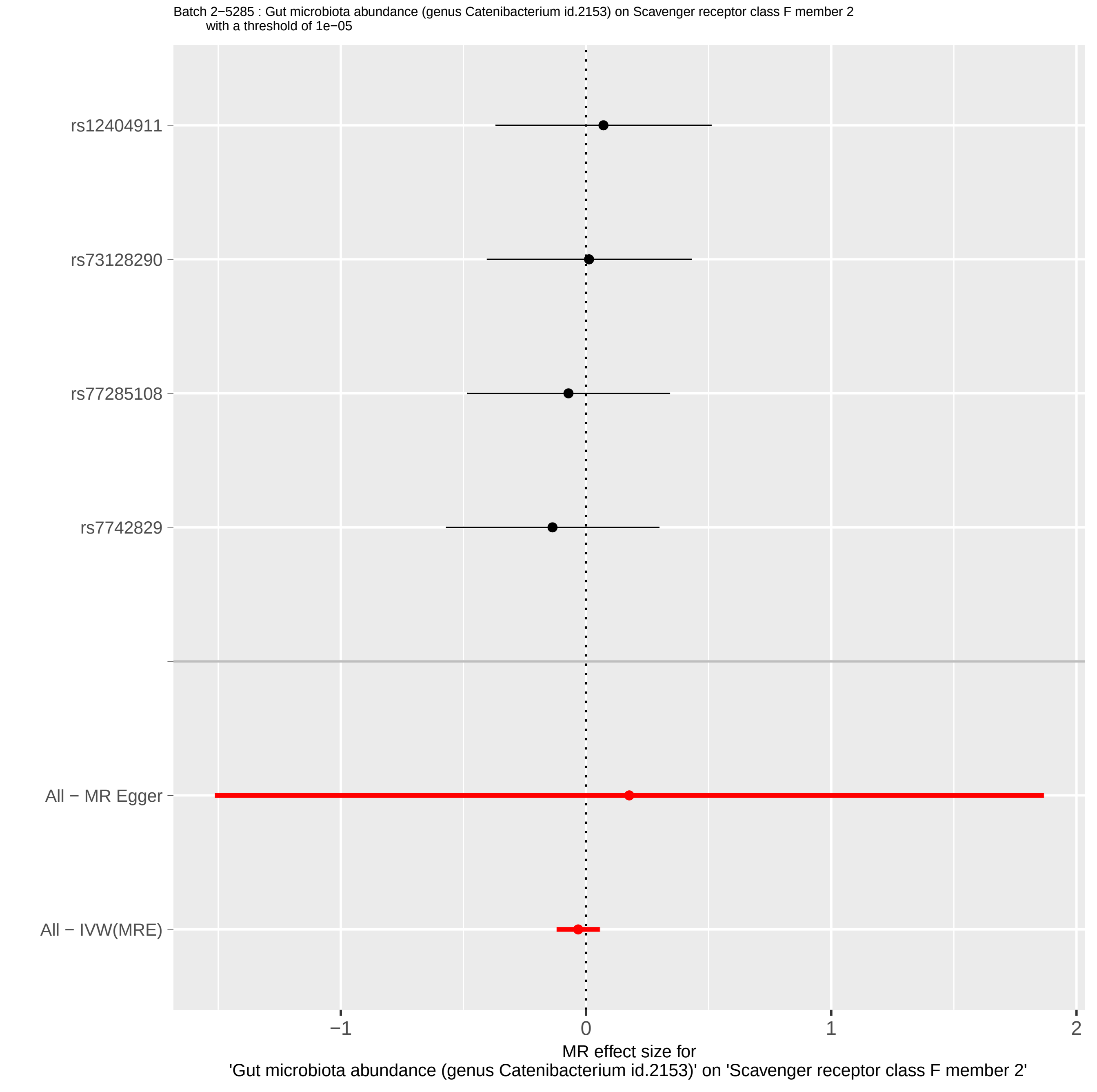
Batch 2−5285 : Gut microbiota abundance (genus Catenibacterium id.2153) on Scavenger receptor class F member 2
with a threshold of 1e−05
rs12404911
rs73128290
rs77285108
rs7742829
All − MR Egger
All − IVW(MRE)
−1
0
1
2
MR effect size for
'Gut microbiota abundance (genus Catenibacterium id.2153)' on 'Scavenger receptor class F member 2'

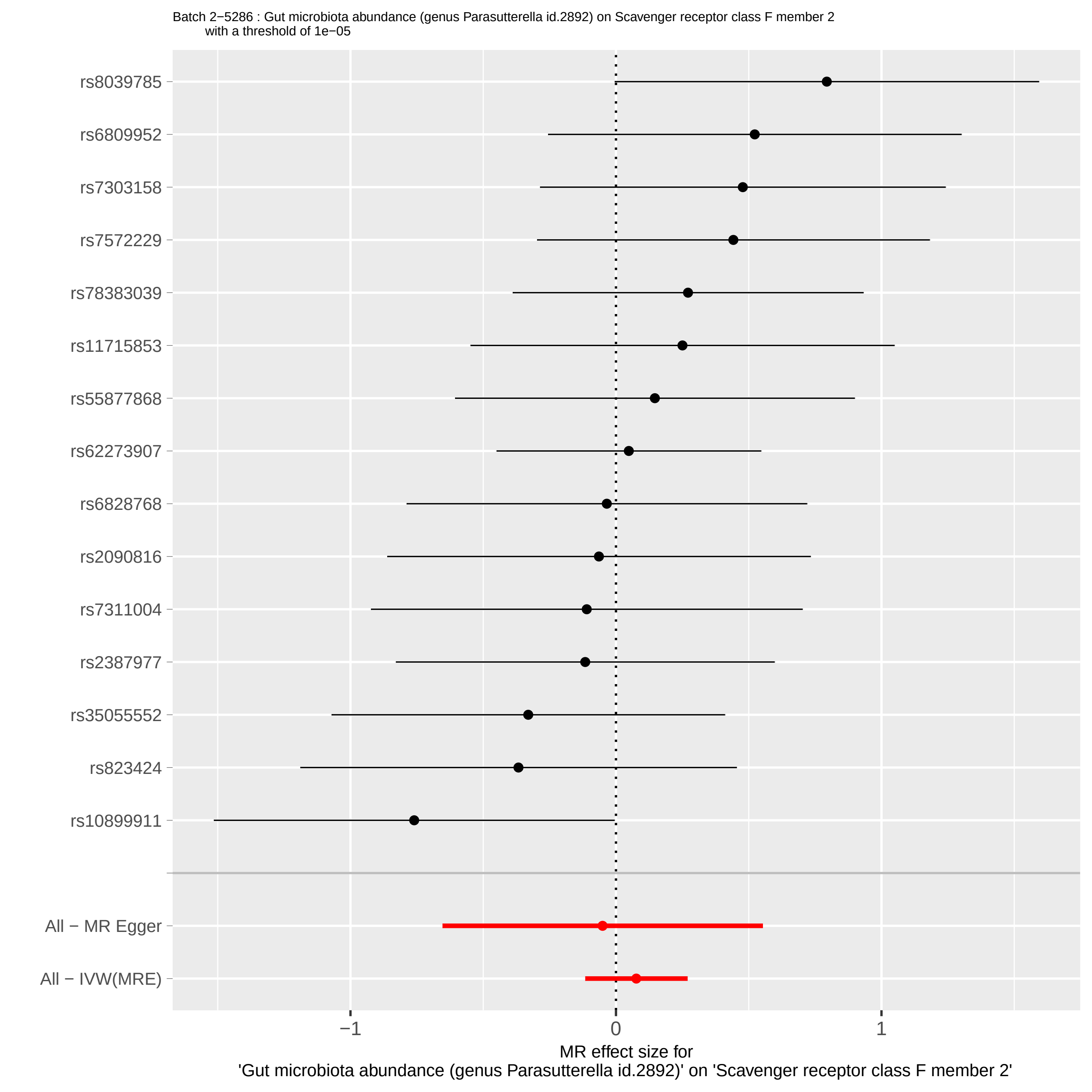

Batch 2−5286 : Gut microbiota abundance (genus Parasutterella id.2892) on Scavenger receptor class F member 2
with a threshold of 1e−05
rs8039785
rs6809952
rs7303158
rs7572229
rs78383039
rs11715853
rs55877868
rs62273907
rs6828768
rs2090816
rs7311004
rs2387977
rs35055552
rs823424
rs10899911
All − MR Egger
All − IVW(MRE)
−1
0
1
MR effect size for
'Gut microbiota abundance (genus Parasutterella id.2892)' on 'Scavenger receptor class F member 2'

Batch 2−5287 : Gut microbiota abundance (genus Catenibacterium id.2153) on Secretogranin−2
with a threshold of 1e−05

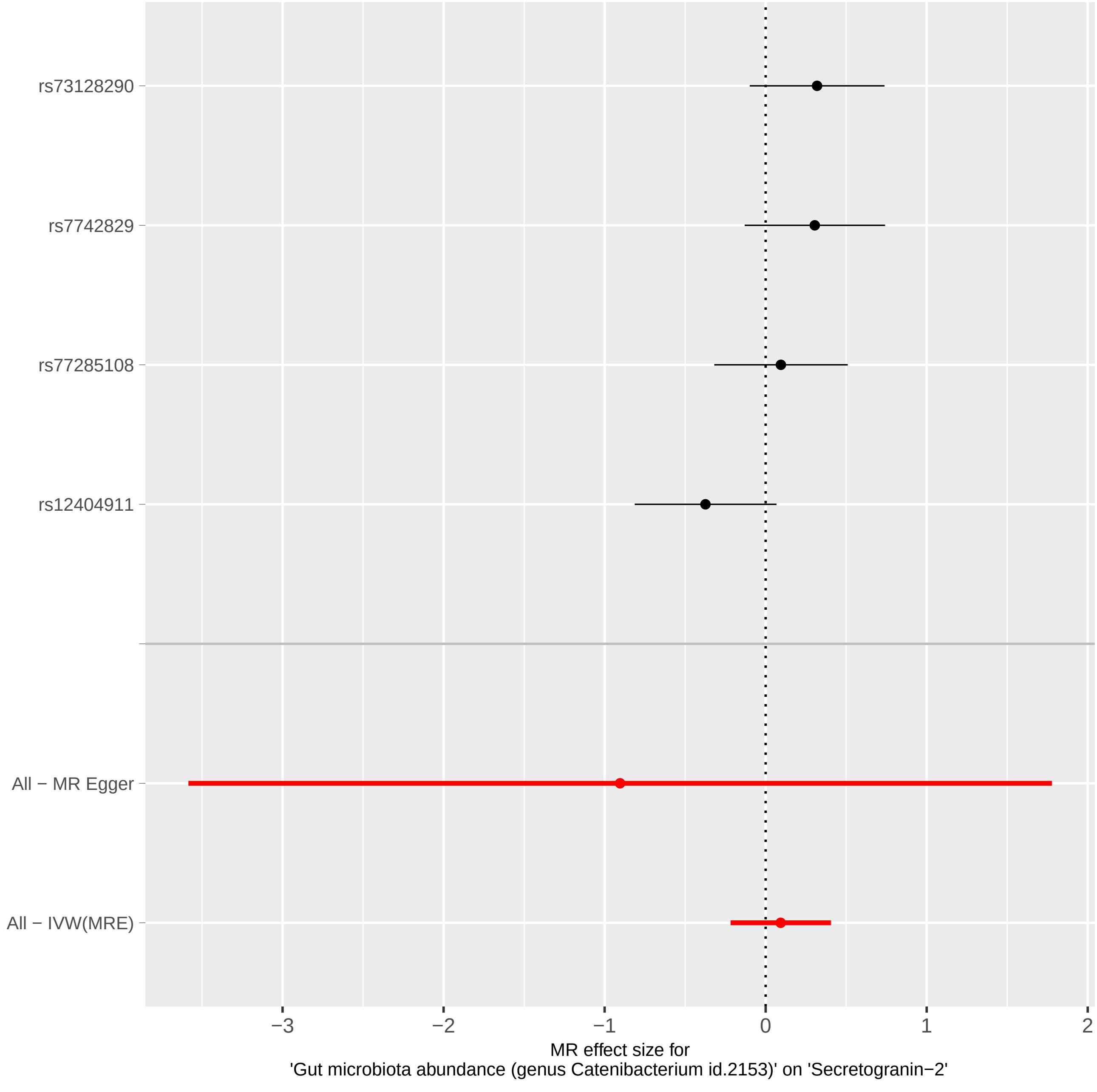

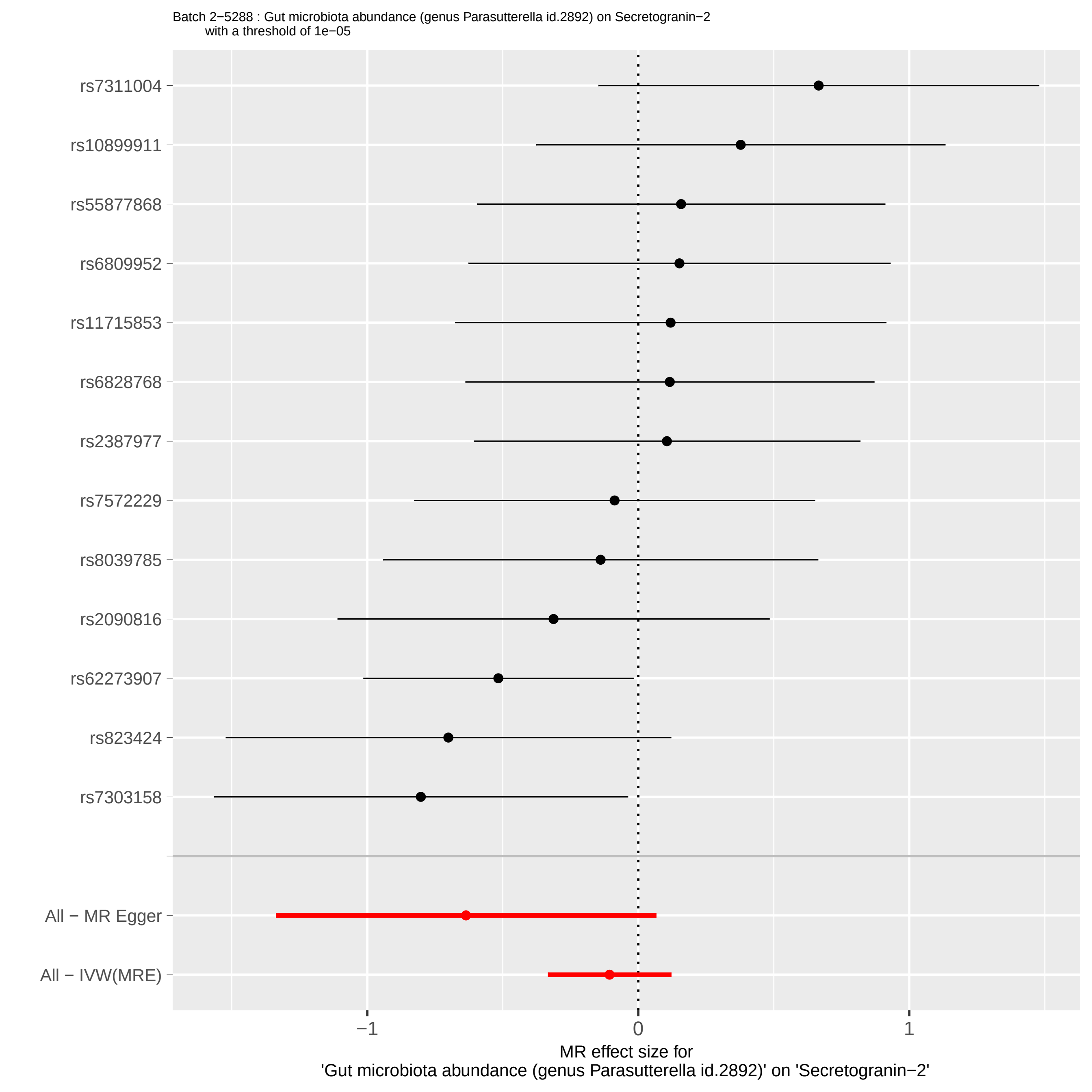
Batch 2−5288 : Gut microbiota abundance (genus Parasutterella id.2892) on Secretogranin−2
with a threshold of 1e−05
rs7311004
rs10899911
rs55877868
rs6809952
rs11715853
rs6828768
rs2387977
rs7572229
rs8039785
rs2090816
rs62273907
rs823424
rs7303158
All − MR Egger
All − IVW(MRE)
−1
0
1
MR effect size for
'Gut microbiota abundance (genus Parasutterella id.2892)' on 'Secretogranin−2'

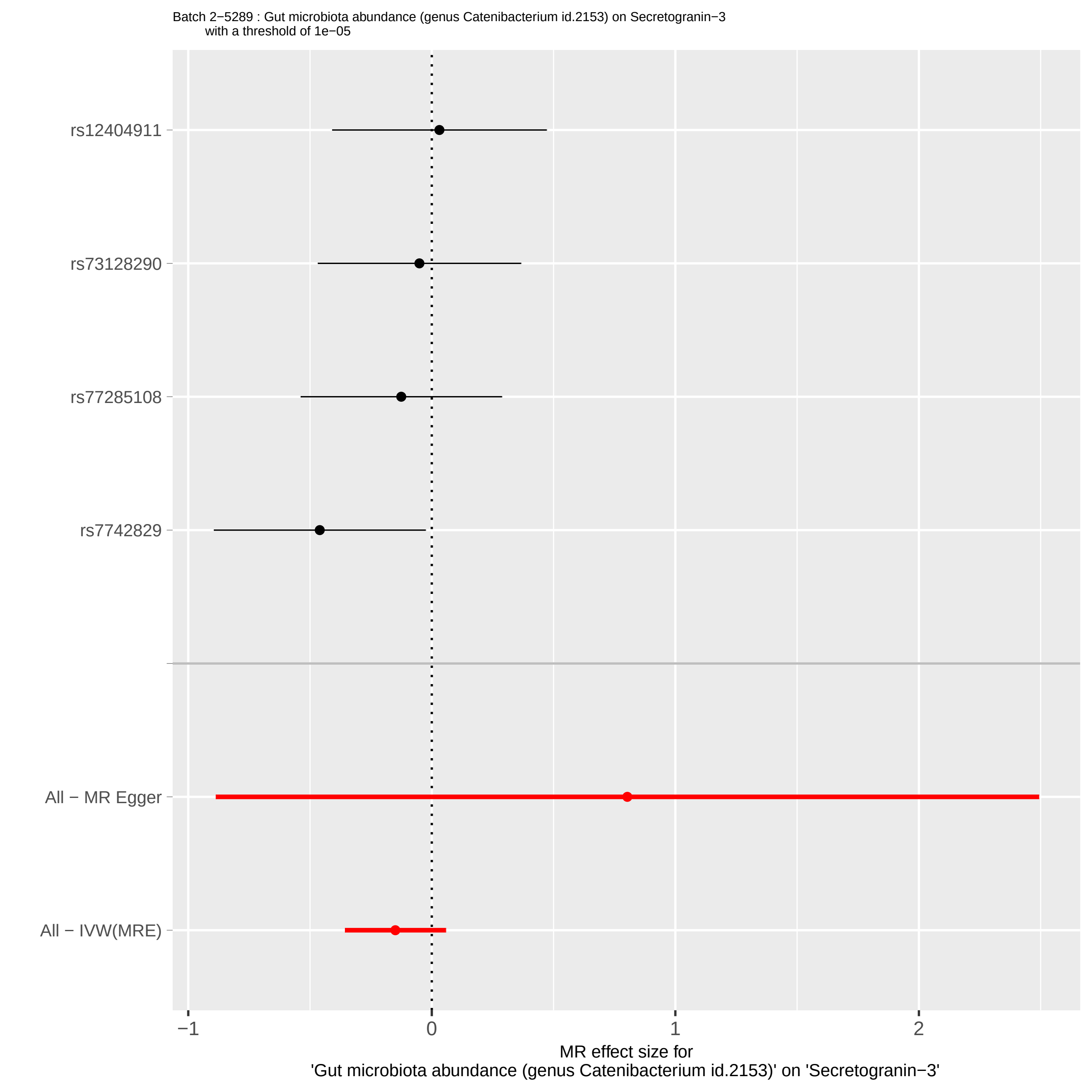

Batch 2−5289 : Gut microbiota abundance (genus Catenibacterium id.2153) on Secretogranin−3
with a threshold of 1e−05
rs12404911
rs73128290
rs77285108
rs7742829
All − MR Egger
All − IVW(MRE)
−1
0
1
2
MR effect size for
'Gut microbiota abundance (genus Catenibacterium id.2153)' on 'Secretogranin−3'

Batch 2−5290 : Gut microbiota abundance (genus Parasutterella id.2892) on Secretogranin−3
with a threshold of 1e−05

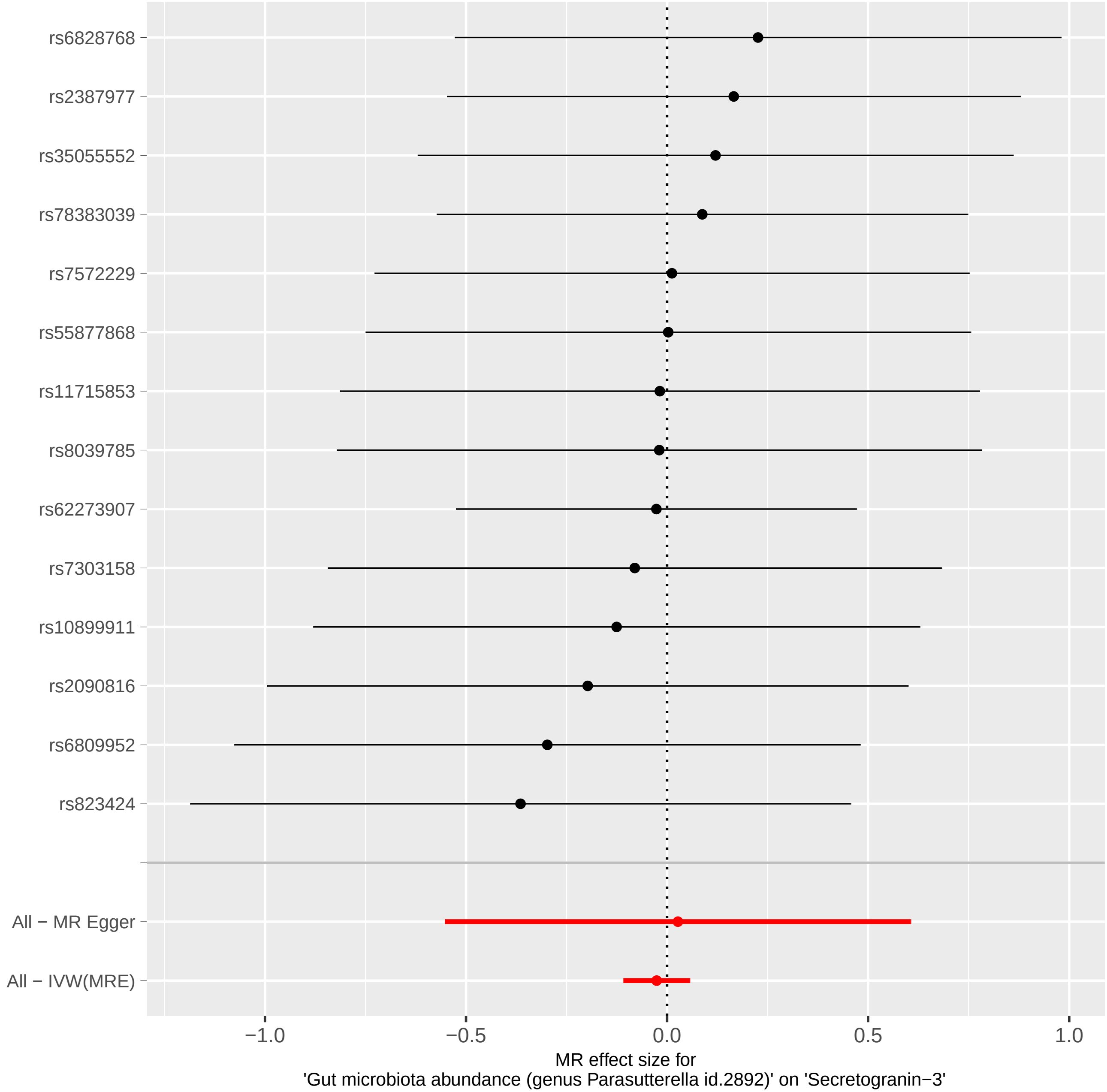

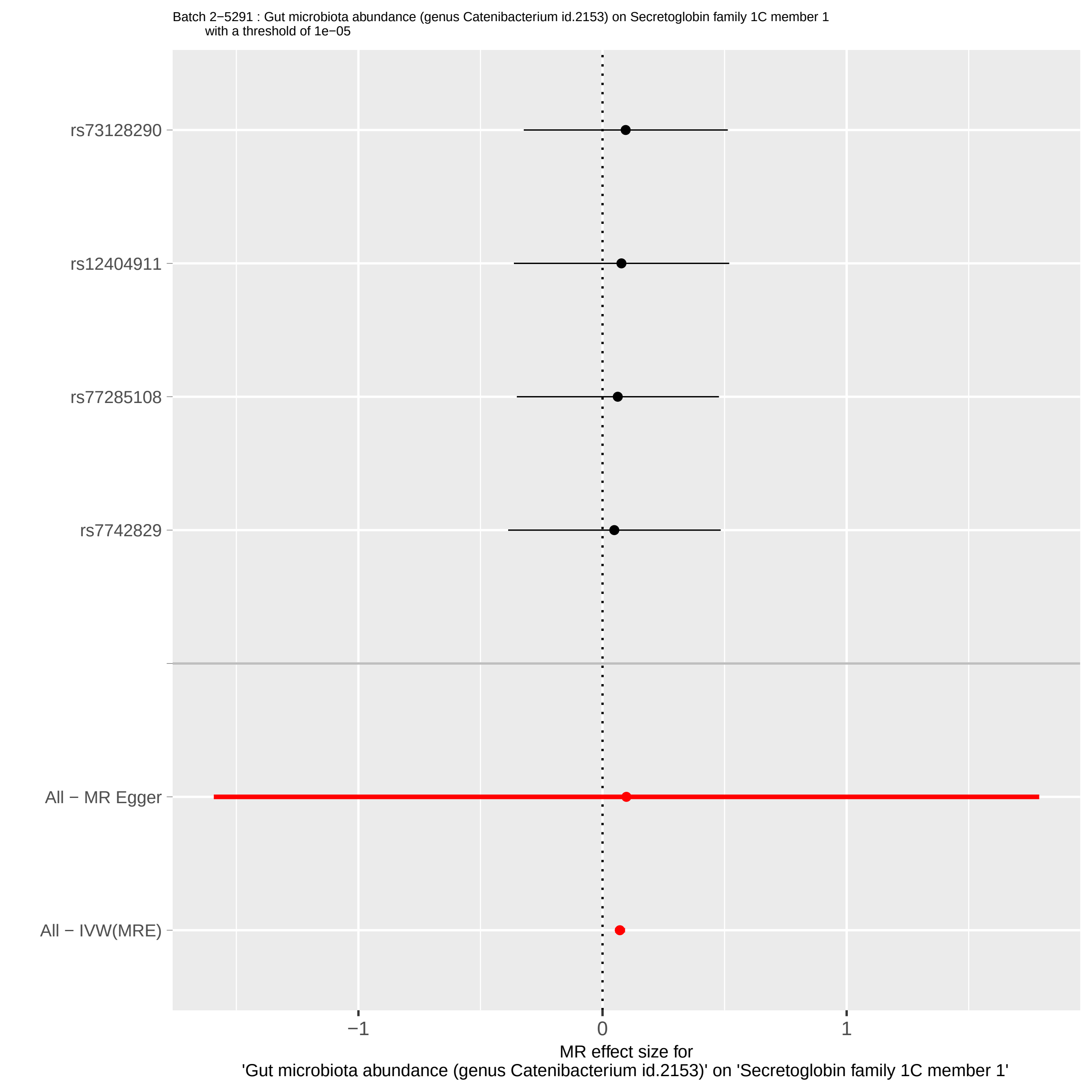
Batch 2−5291 : Gut microbiota abundance (genus Catenibacterium id.2153) on Secretoglobin family 1C member 1
with a threshold of 1e−05
rs73128290
rs12404911
rs77285108
rs7742829
All − MR Egger
All − IVW(MRE)
−1
0
1
MR effect size for
'Gut microbiota abundance (genus Catenibacterium id.2153)' on 'Secretoglobin family 1C member 1'

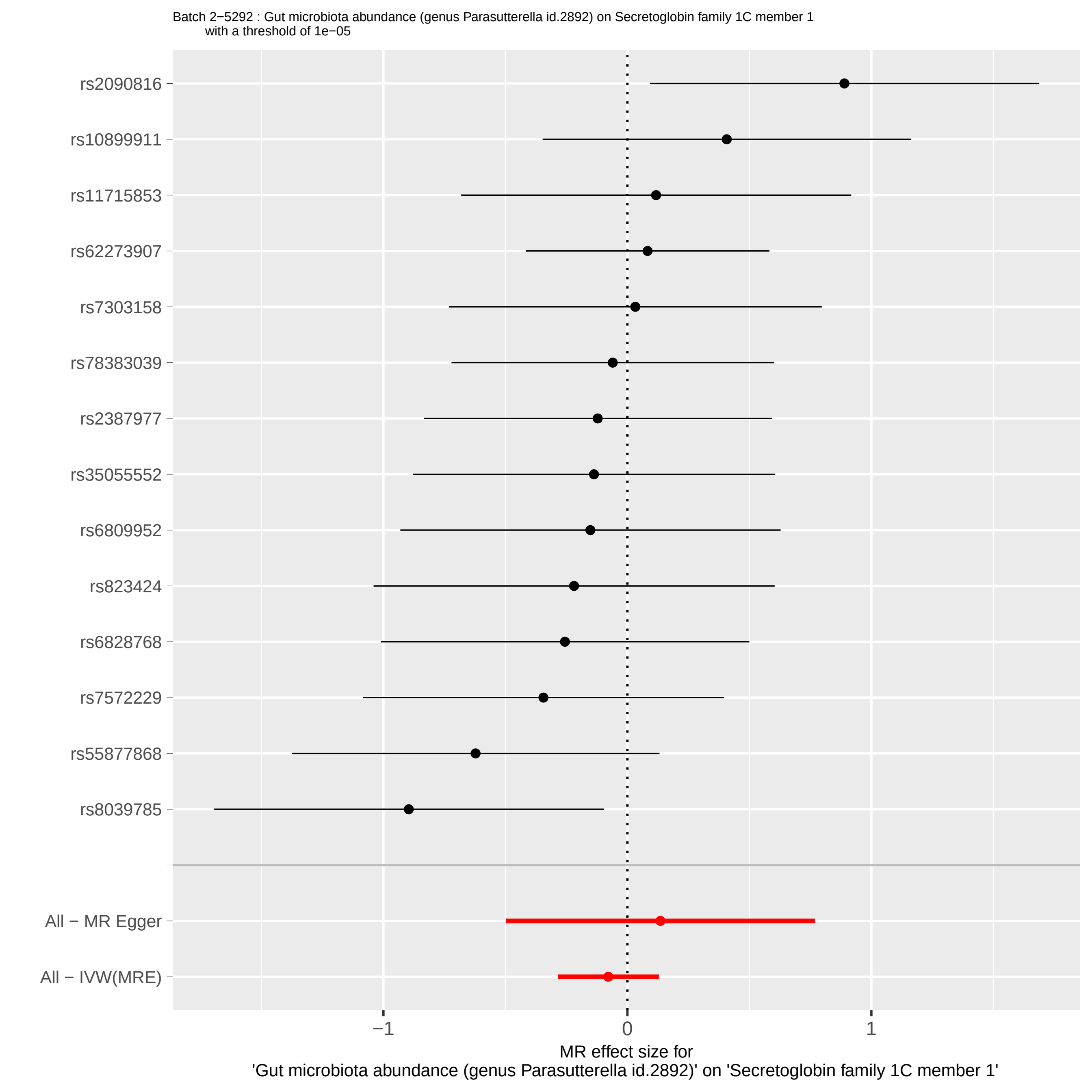
Batch 2−5292 : Gut microbiota abundance (genus Parasutterella id.2892) on Secretoglobin family 1C member 1
with a threshold of 1e−05
rs2090816
rs10899911
rs11715853
rs62273907
rs7303158
rs78383039
rs2387977
rs35055552
rs6809952
rs823424
rs6828768
rs7572229
rs55877868
rs8039785
All − MR Egger
All − IVW(MRE)
−1
0
1
MR effect size for
'Gut microbiota abundance (genus Parasutterella id.2892)' on 'Secretoglobin family 1C member 1'

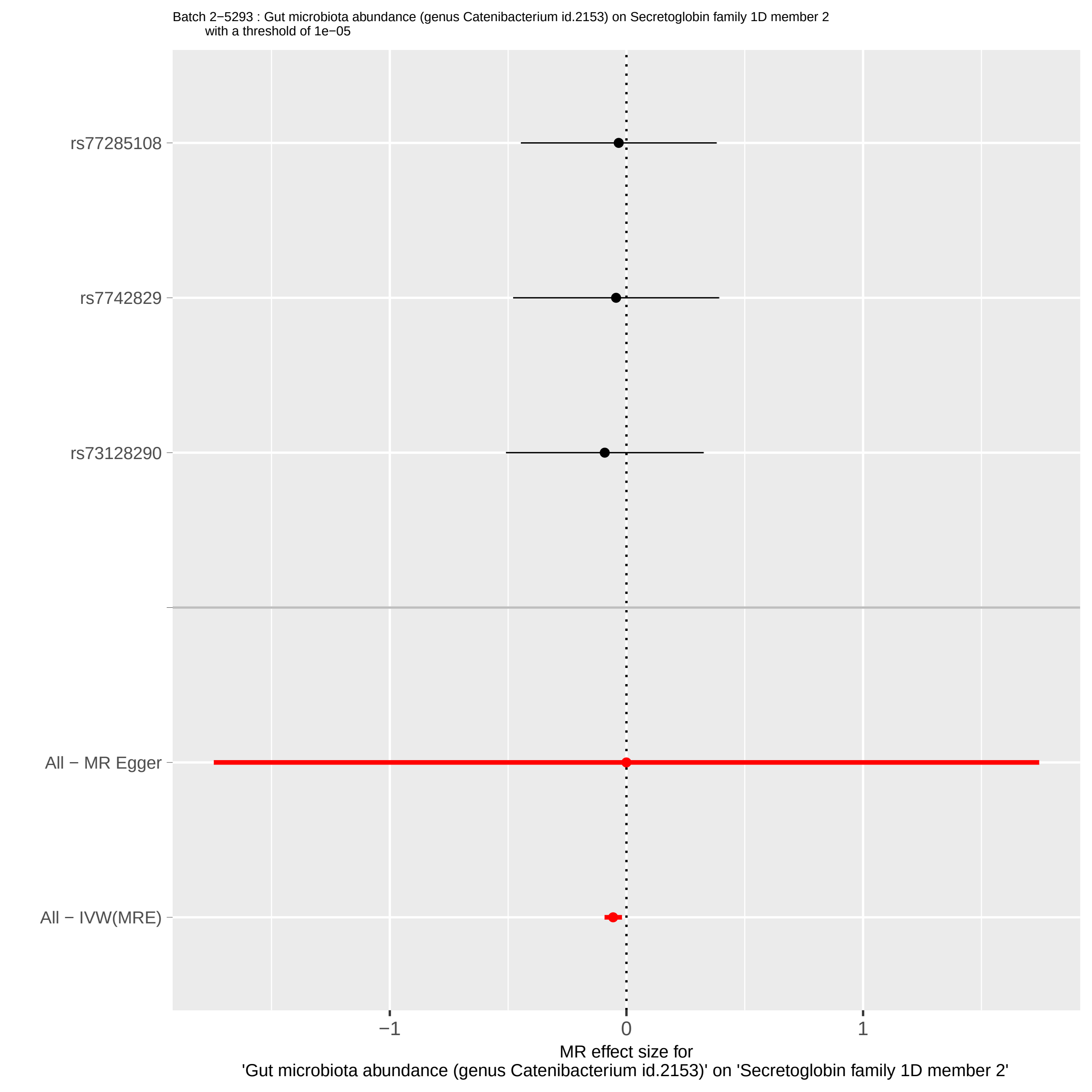

Batch 2−5293 : Gut microbiota abundance (genus Catenibacterium id.2153) on Secretoglobin family 1D member 2
with a threshold of 1e−05
rs77285108
rs7742829
rs73128290
All − MR Egger
All − IVW(MRE)
−1
0
1
MR effect size for
'Gut microbiota abundance (genus Catenibacterium id.2153)' on 'Secretoglobin family 1D member 2'

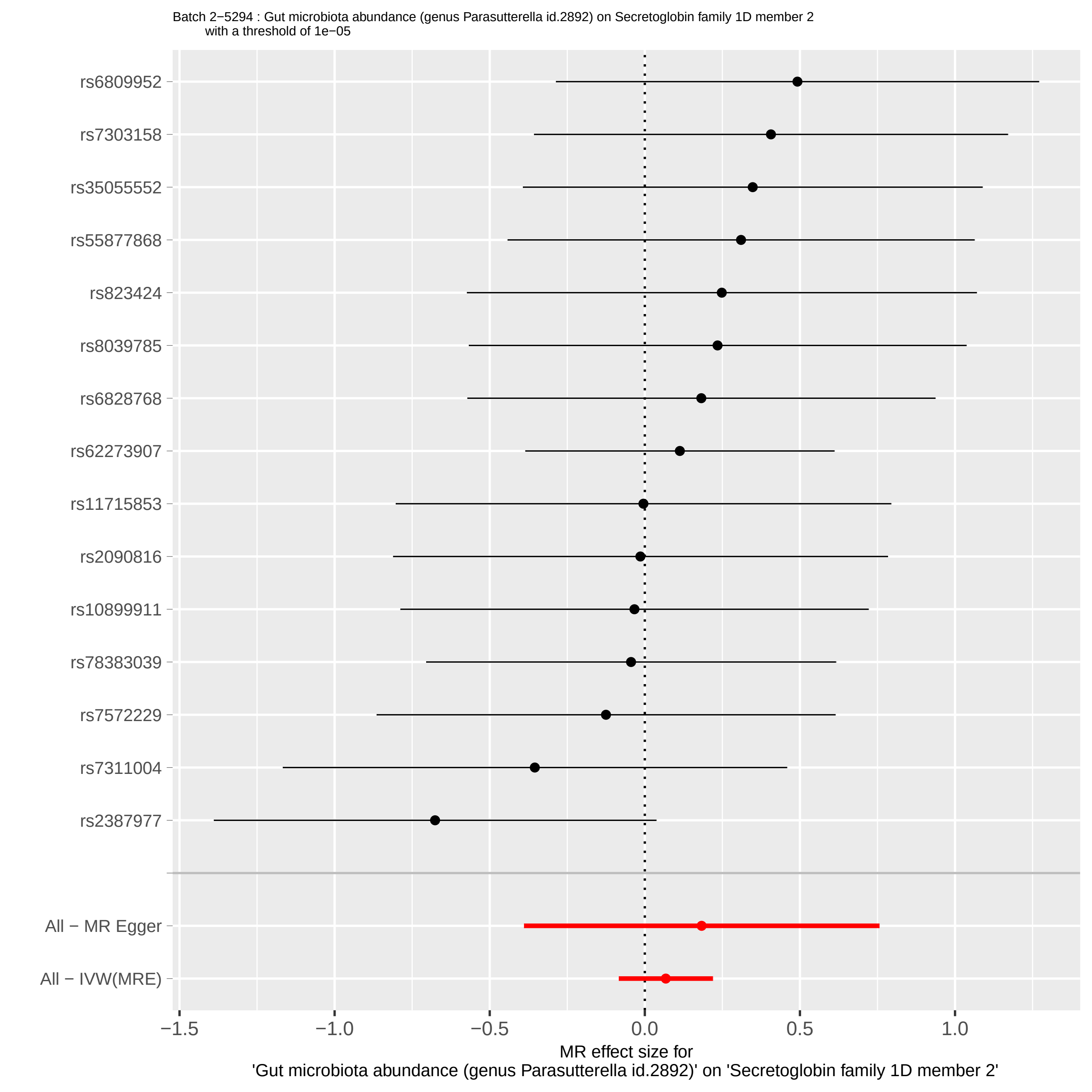
Batch 2−5294 : Gut microbiota abundance (genus Parasutterella id.2892) on Secretoglobin family 1D member 2
with a threshold of 1e−05
rs6809952
rs7303158
rs35055552
rs55877868
rs823424
rs8039785
rs6828768
rs62273907
rs11715853
rs2090816
rs10899911
rs78383039
rs7572229
rs7311004
rs2387977
All − MR Egger
All − IVW(MRE)
−1.5
−1.0
−0.5
0.0
0.5
1.0
MR effect size for
'Gut microbiota abundance (genus Parasutterella id.2892)' on 'Secretoglobin family 1D member 2'

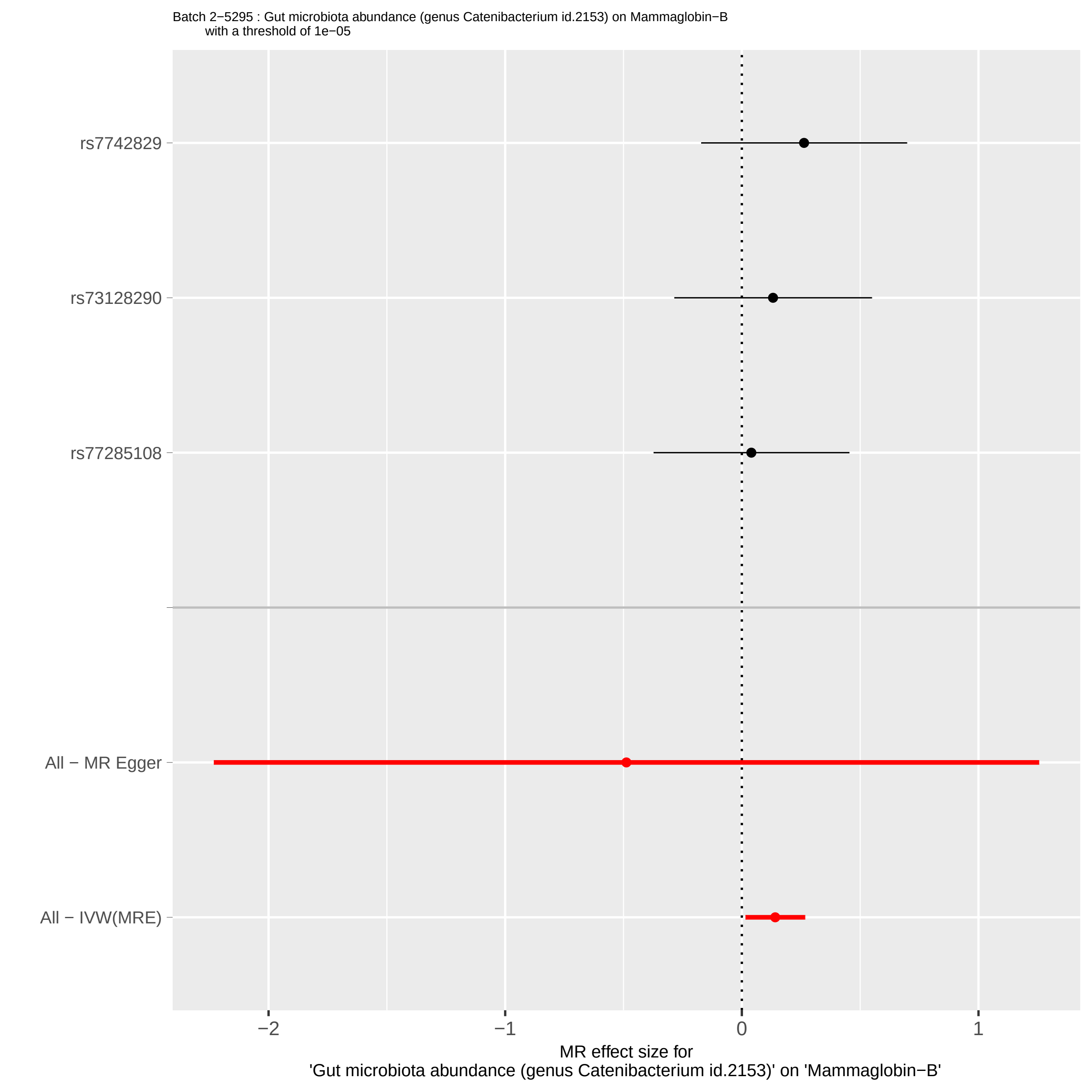
Batch 2−5295 : Gut microbiota abundance (genus Catenibacterium id.2153) on Mammaglobin−B
with a threshold of 1e−05
rs7742829
rs73128290
rs77285108
All − MR Egger
All − IVW(MRE)
−2
−1
0
1
MR effect size for
'Gut microbiota abundance (genus Catenibacterium id.2153)' on 'Mammaglobin−B'

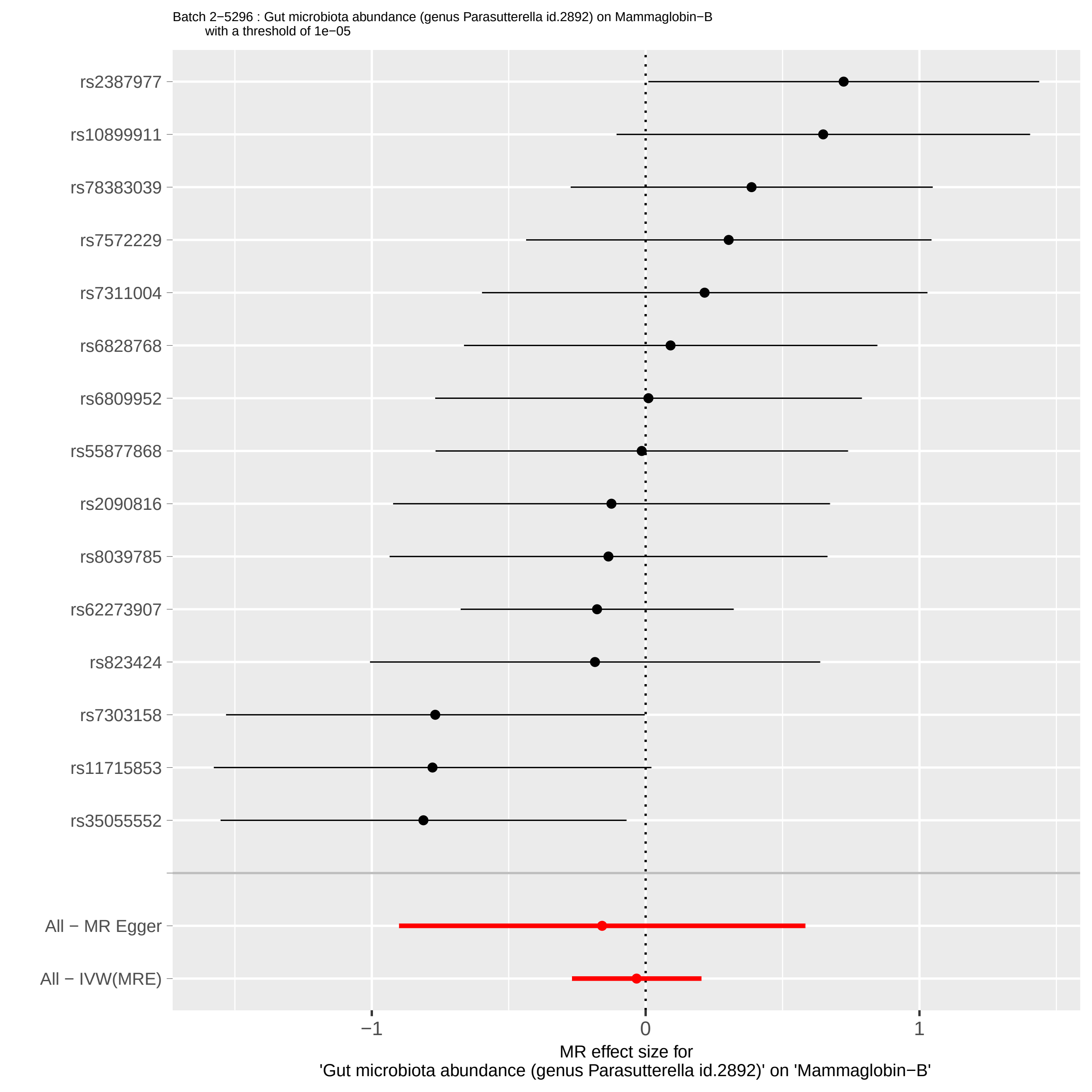

Batch 2−5296 : Gut microbiota abundance (genus Parasutterella id.2892) on Mammaglobin−B
with a threshold of 1e−05
rs2387977
rs10899911
rs78383039
rs7572229
rs7311004
rs6828768
rs6809952
rs55877868
rs2090816
rs8039785
rs62273907
rs823424
rs7303158
rs11715853
rs35055552
All − MR Egger
All − IVW(MRE)
−1
0
1
MR effect size for
'Gut microbiota abundance (genus Parasutterella id.2892)' on 'Mammaglobin−B'

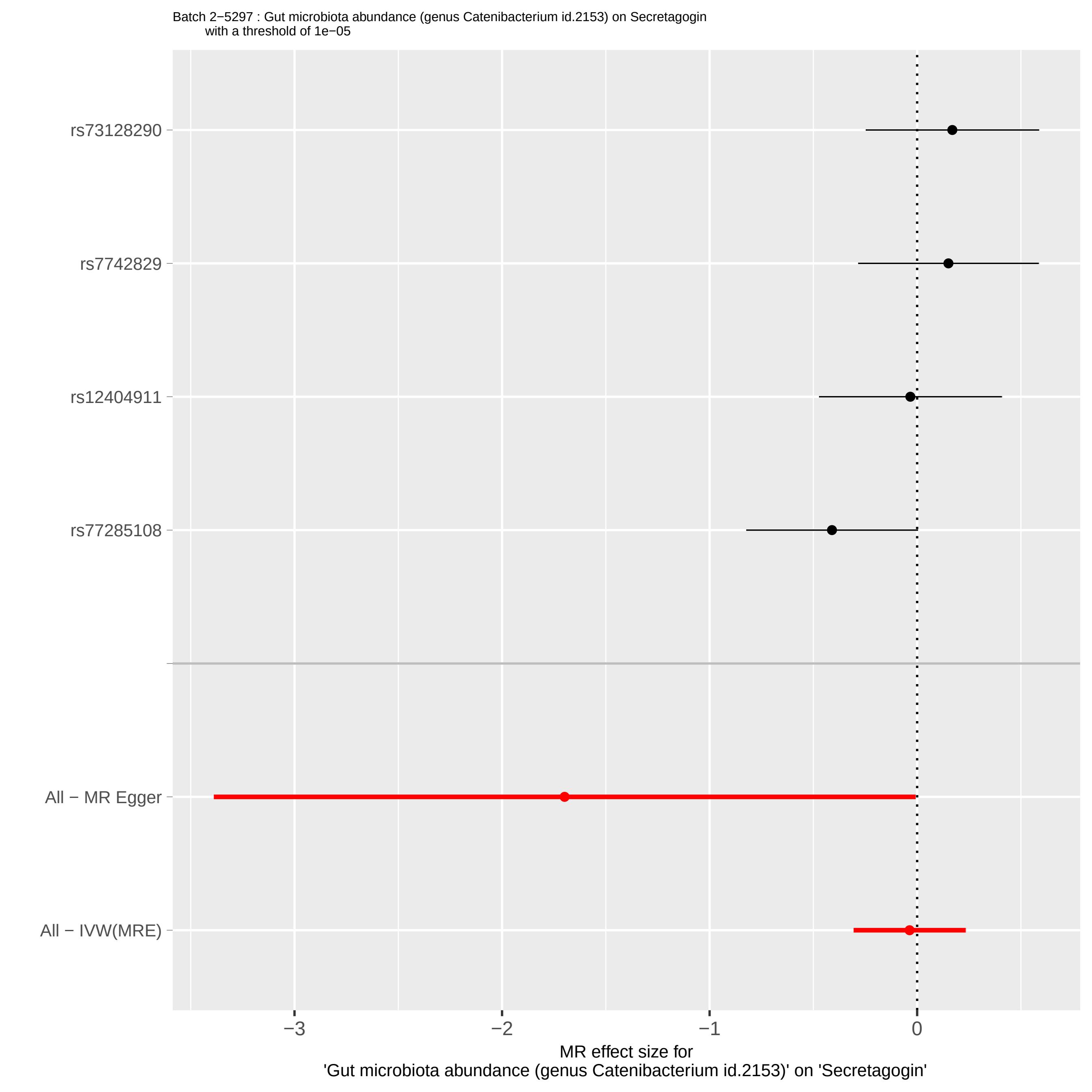
Batch 2−5297 : Gut microbiota abundance (genus Catenibacterium id.2153) on Secretagogin
with a threshold of 1e−05
rs73128290
rs7742829
rs12404911
rs77285108
All − MR Egger
All − IVW(MRE)
−3
−2
−1
0
MR effect size for
'Gut microbiota abundance (genus Catenibacterium id.2153)' on 'Secretagogin'

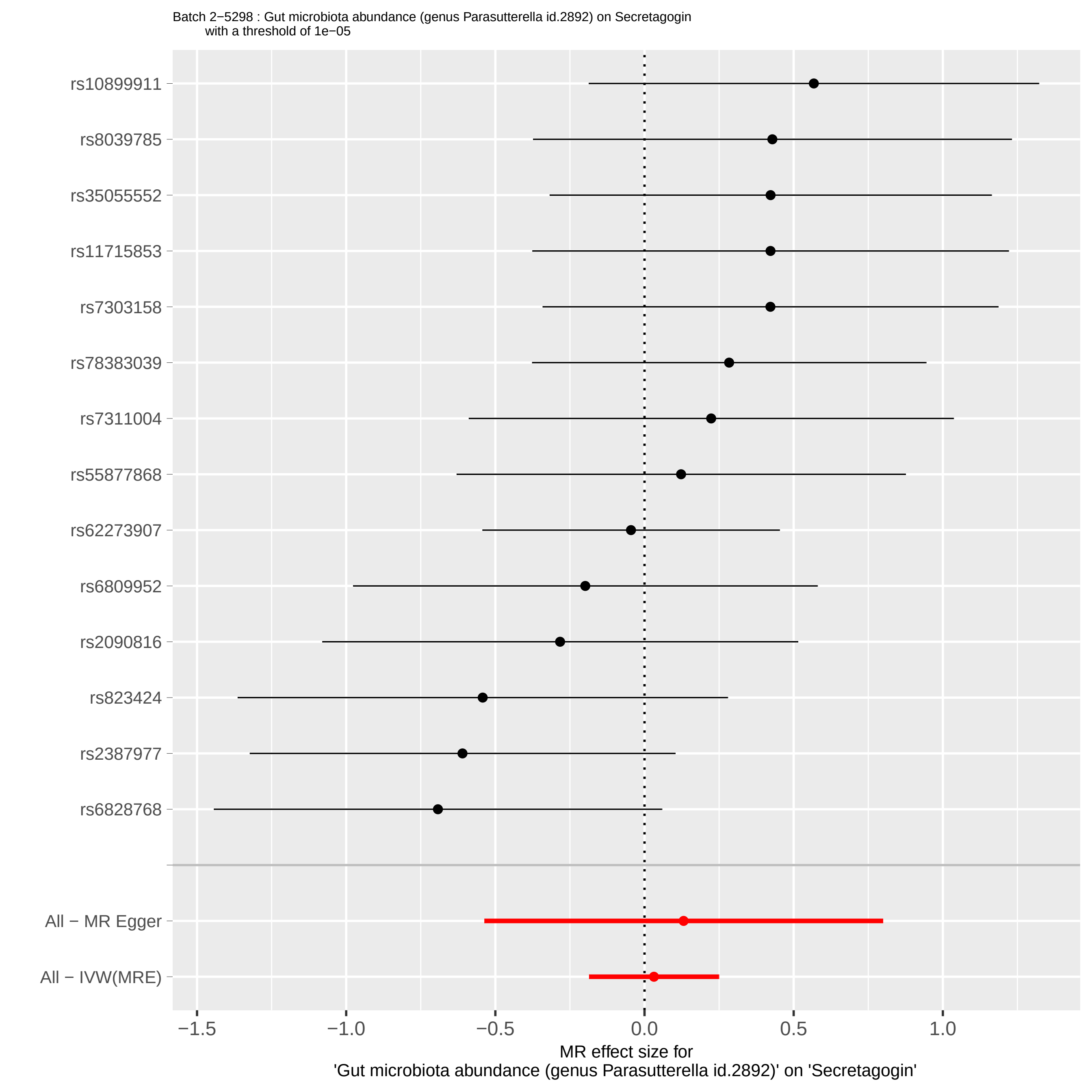
Batch 2−5298 : Gut microbiota abundance (genus Parasutterella id.2892) on Secretagogin
with a threshold of 1e−05
rs10899911
rs8039785
rs35055552
rs11715853
rs7303158
rs78383039
rs7311004
rs55877868
rs62273907
rs6809952
rs2090816
rs823424
rs2387977
rs6828768
All − MR Egger
All − IVW(MRE)
−1.5
−1.0
−0.5
0.0
0.5
1.0
MR effect size for
'Gut microbiota abundance (genus Parasutterella id.2892)' on 'Secretagogin'

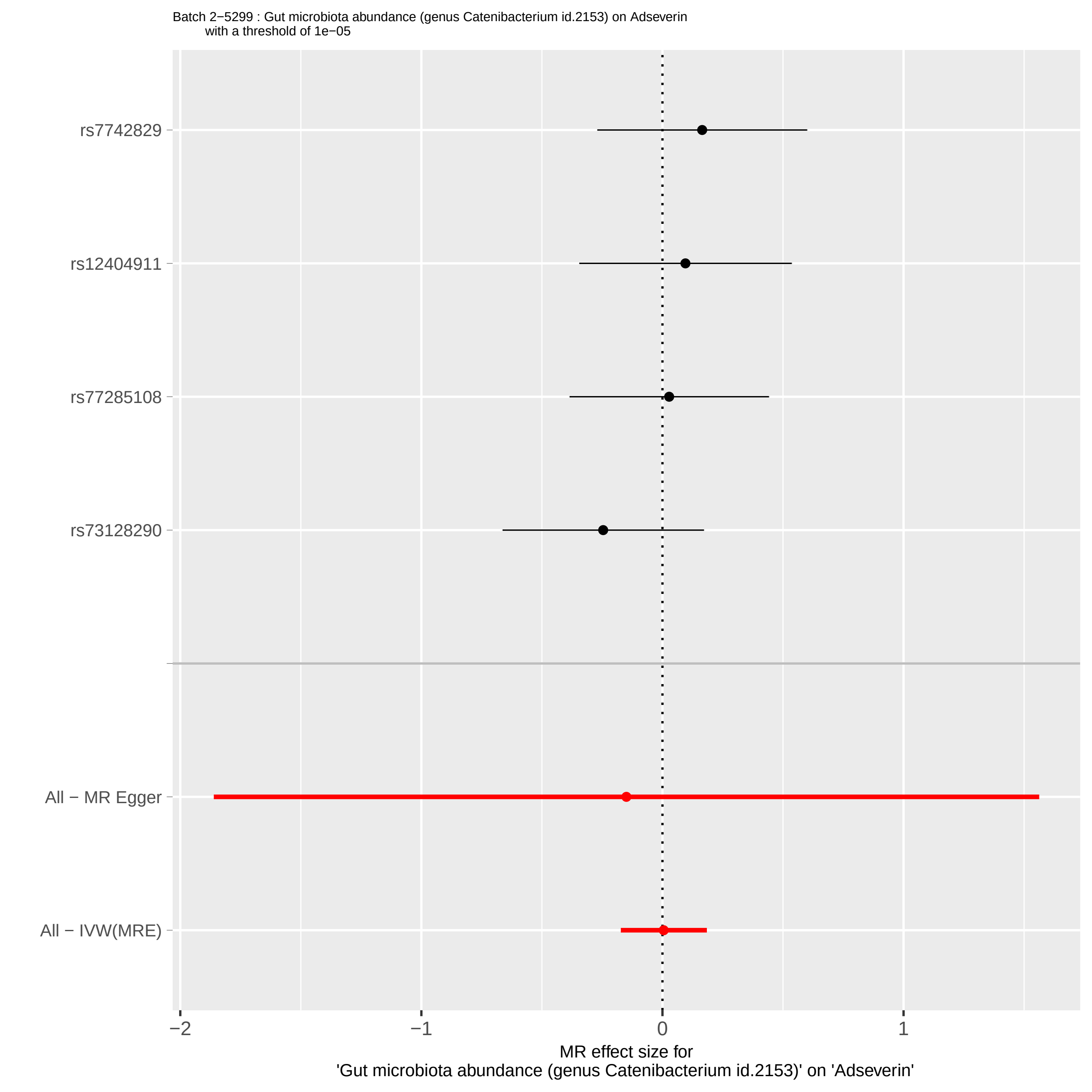
Batch 2−5299 : Gut microbiota abundance (genus Catenibacterium id.2153) on Adseverin
with a threshold of 1e−05
rs7742829
rs12404911
rs77285108
rs73128290
All − MR Egger
All − IVW(MRE)
−2
−1
0
1
MR effect size for
'Gut microbiota abundance (genus Catenibacterium id.2153)' on 'Adseverin'

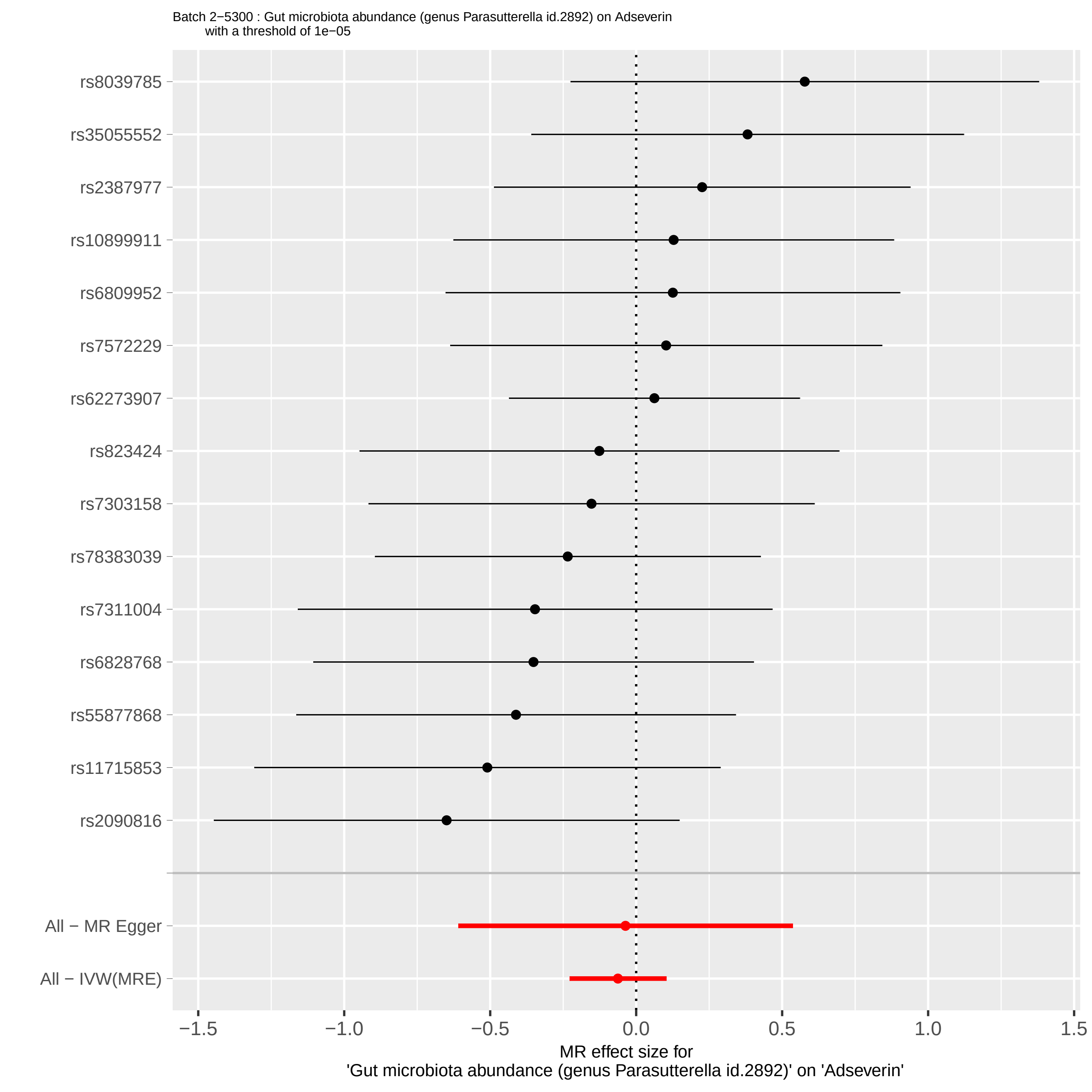

Batch 2−5300 : Gut microbiota abundance (genus Parasutterella id.2892) on Adseverin
with a threshold of 1e−05
rs8039785
rs35055552
rs2387977
rs10899911
rs6809952
rs7572229
rs62273907
rs823424
rs7303158
rs78383039
rs7311004
rs6828768
rs55877868
rs11715853
rs2090816
All − MR Egger
All − IVW(MRE)
−1.5
−1.0
−0.5
0.0
0.5
1.0
1.5
MR effect size for
'Gut microbiota abundance (genus Parasutterella id.2892)' on 'Adseverin'

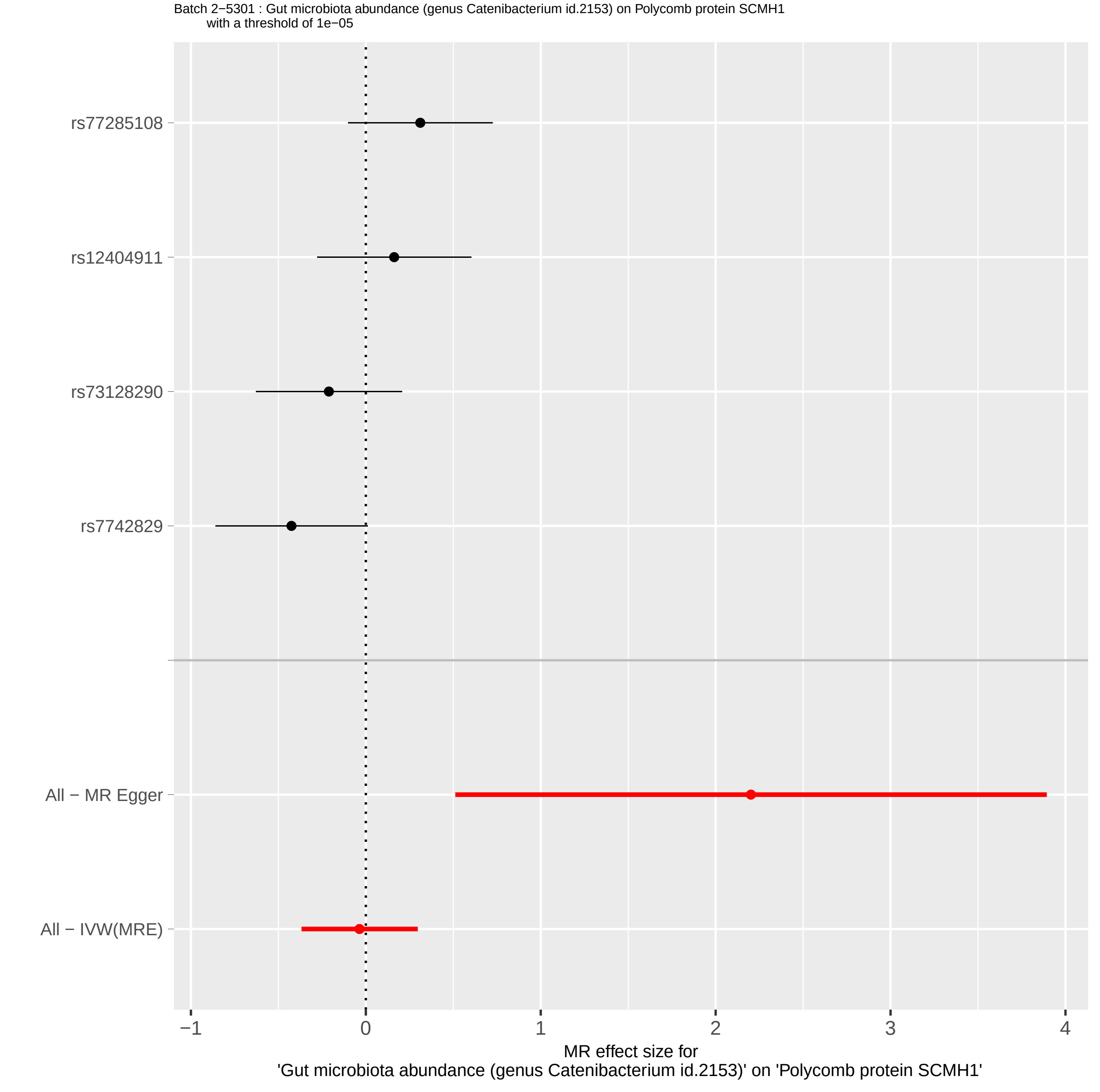
Batch 2−5301 : Gut microbiota abundance (genus Catenibacterium id.2153) on Polycomb protein SCMH1
with a threshold of 1e−05
rs77285108
rs12404911
rs73128290
rs7742829
All − MR Egger
All − IVW(MRE)
−1
0
1
2
3
4
MR effect size for
'Gut microbiota abundance (genus Catenibacterium id.2153)' on 'Polycomb protein SCMH1'

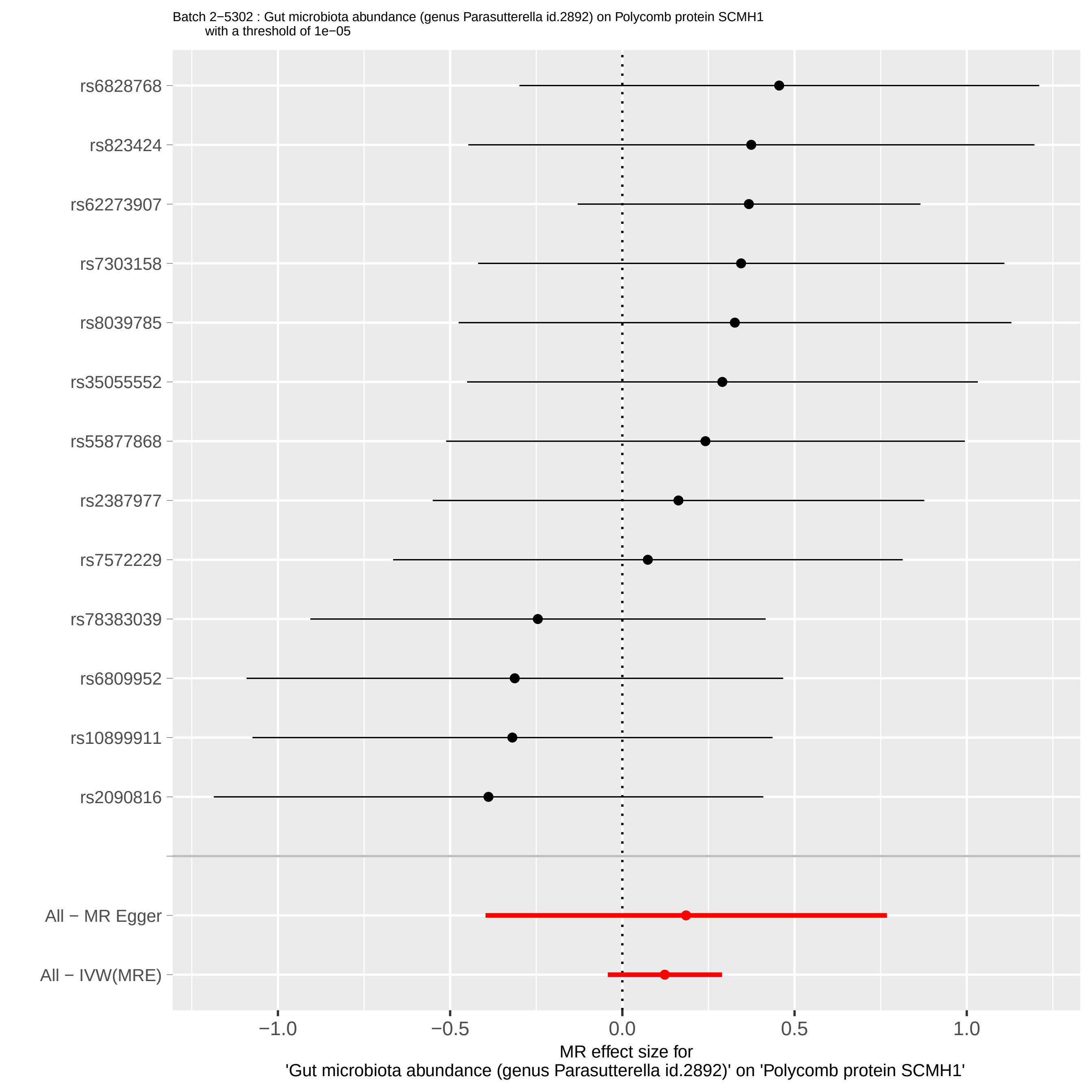
Batch 2−5302 : Gut microbiota abundance (genus Parasutterella id.2892) on Polycomb protein SCMH1
with a threshold of 1e−05
rs6828768
rs823424
rs62273907
rs7303158
rs8039785
rs35055552
rs55877868
rs2387977
rs7572229
rs78383039
rs6809952
rs10899911
rs2090816
All − MR Egger
All − IVW(MRE)
−1.0
−0.5
0.0
0.5
1.0
MR effect size for
'Gut microbiota abundance (genus Parasutterella id.2892)' on 'Polycomb protein SCMH1'

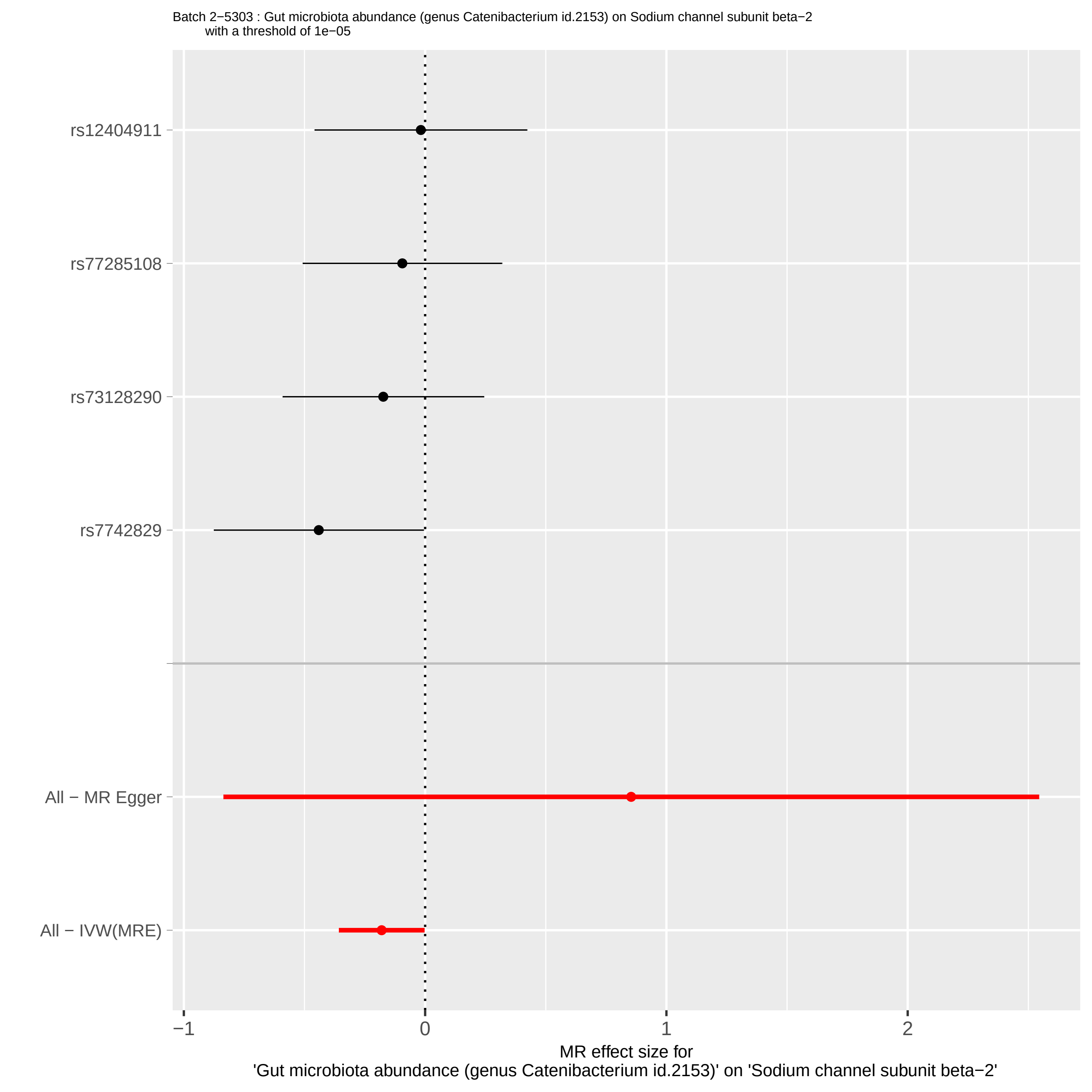

Batch 2−5303 : Gut microbiota abundance (genus Catenibacterium id.2153) on Sodium channel subunit beta−2
with a threshold of 1e−05
rs12404911
rs77285108
rs73128290
rs7742829
All − MR Egger
All − IVW(MRE)
−1
0
1
2
MR effect size for
'Gut microbiota abundance (genus Catenibacterium id.2153)' on 'Sodium channel subunit beta−2'

Batch 2−5304 : Gut microbiota abundance (genus Parasutterella id.2892) on Sodium channel subunit beta−2
with a threshold of 1e−05

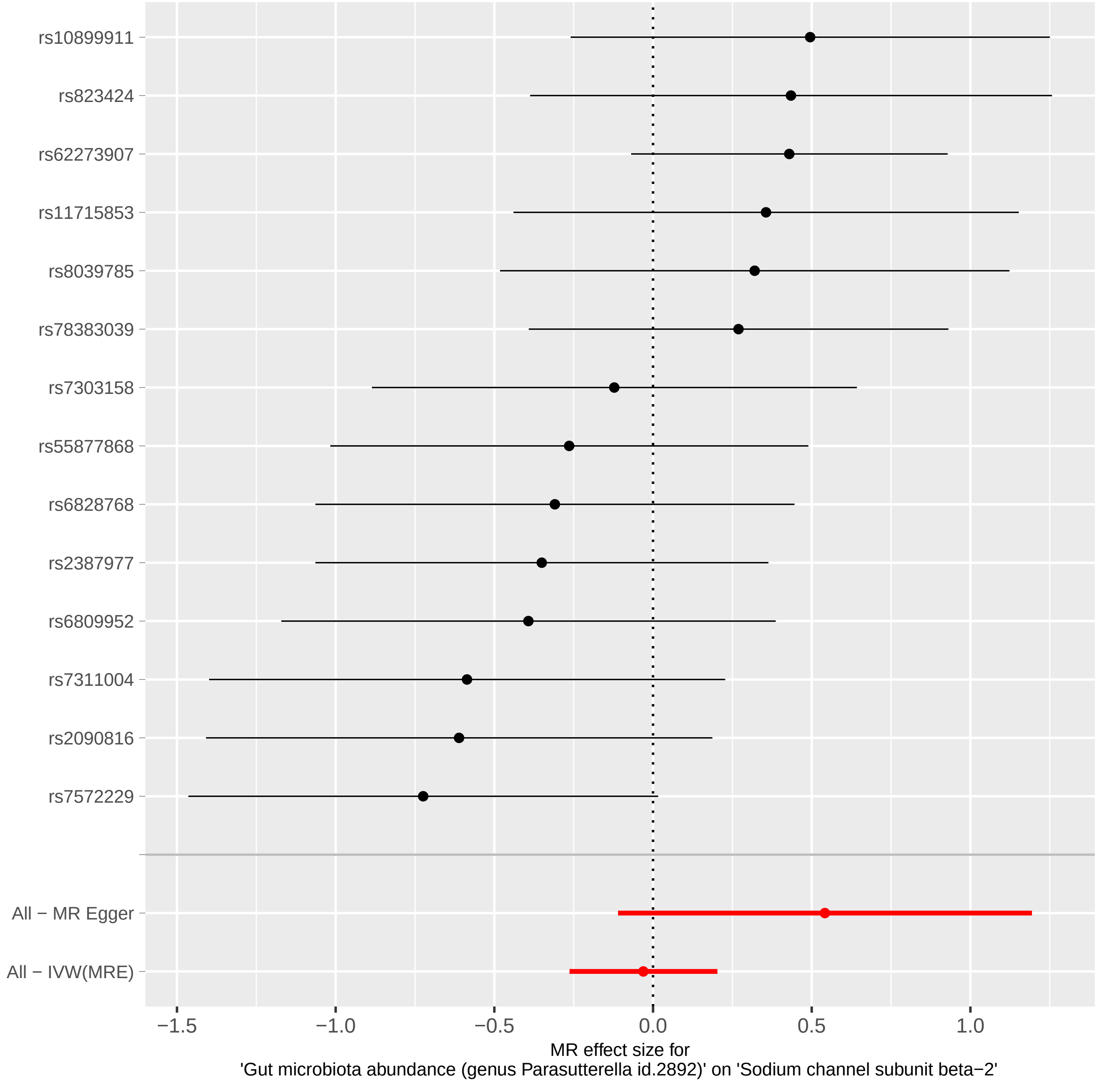

Batch 2−5305 : Gut microbiota abundance (genus Catenibacterium id.2153) on Protein SCO1 homolog, mitochondrial
with a threshold of 1e−05

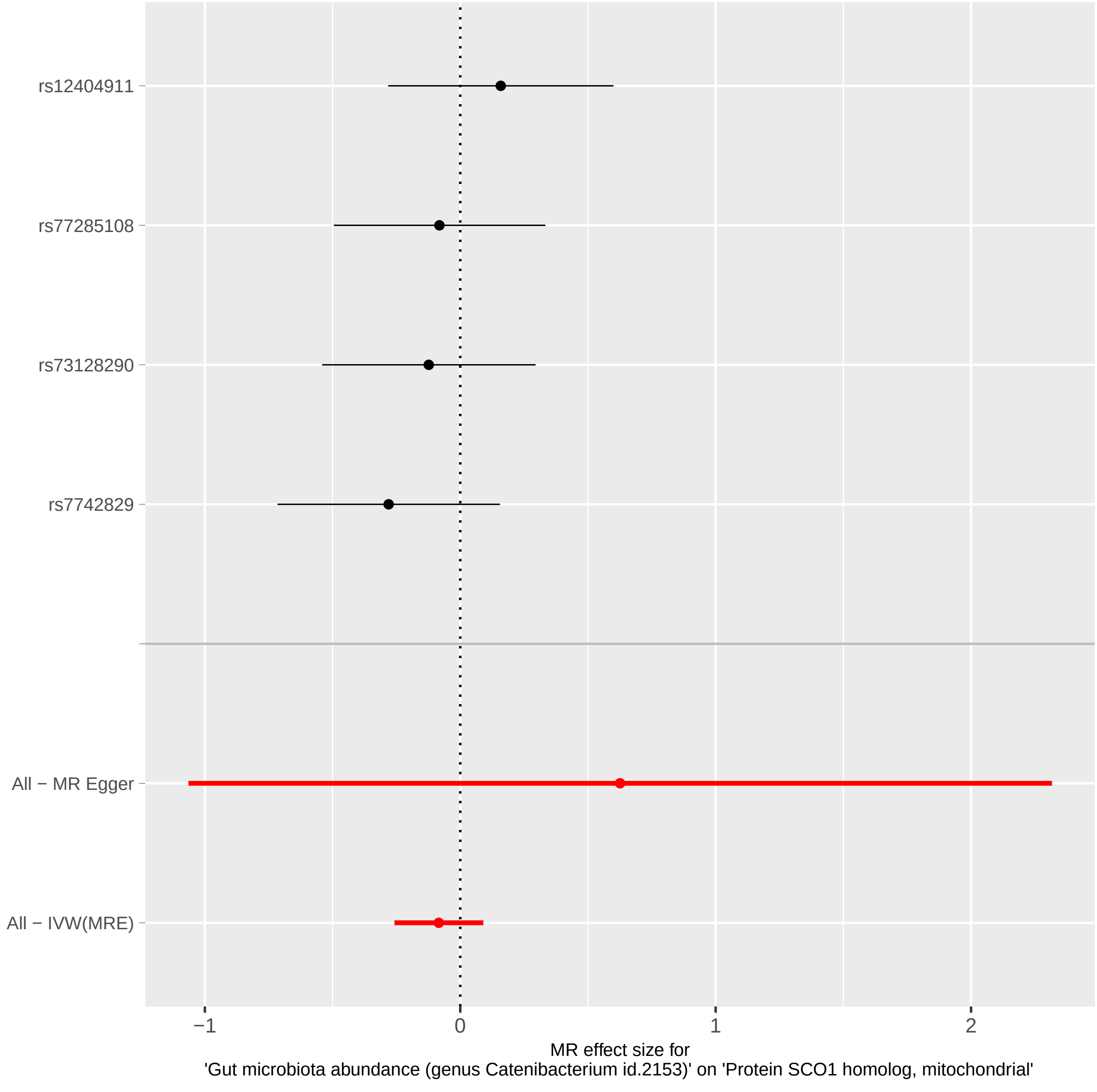

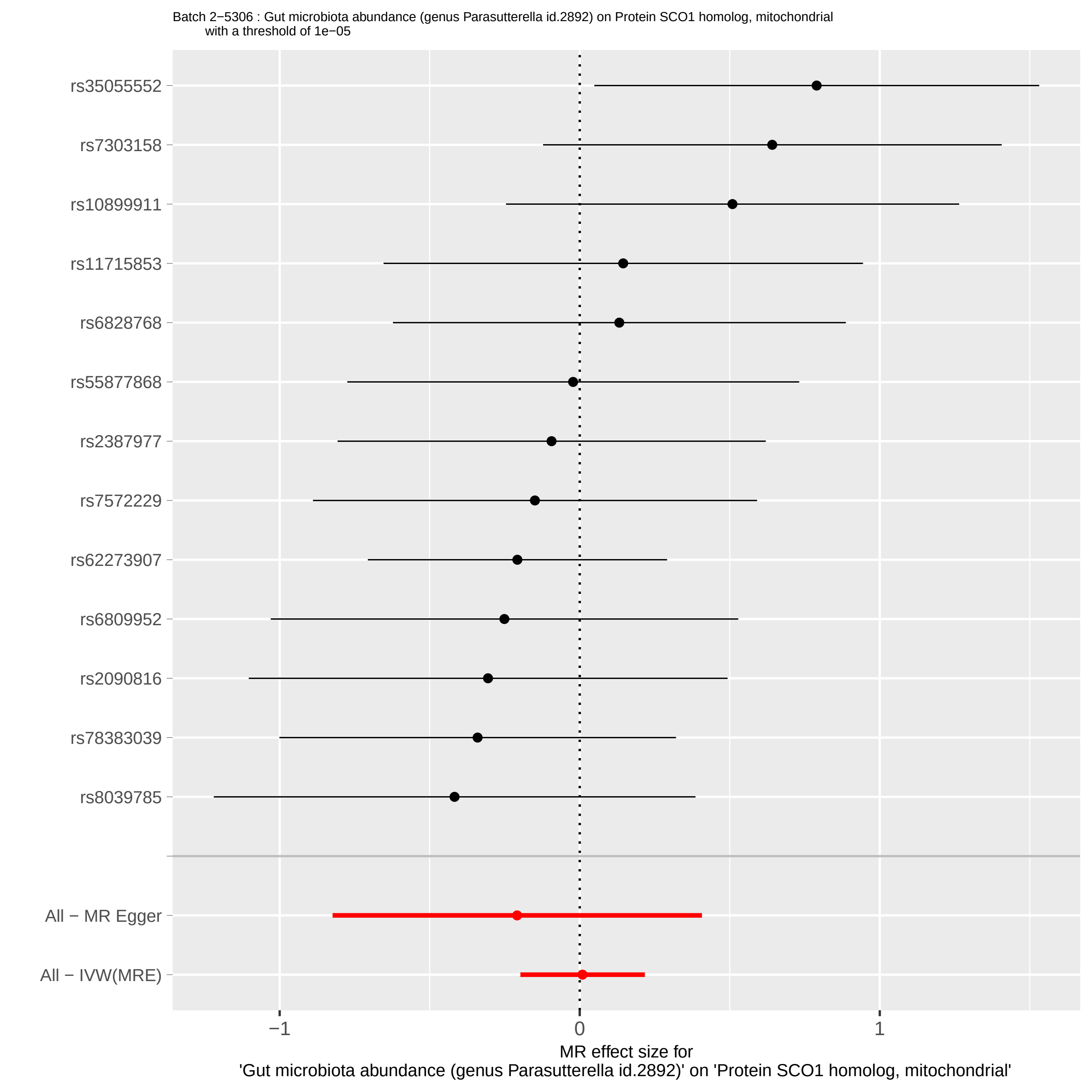
Batch 2−5306 : Gut microbiota abundance (genus Parasutterella id.2892) on Protein SCO1 homolog, mitochondrial
with a threshold of 1e−05
rs35055552
rs7303158
rs10899911
rs11715853
rs6828768
rs55877868
rs2387977
rs7572229
rs62273907
rs6809952
rs2090816
rs78383039
rs8039785
All − MR Egger
All − IVW(MRE)
−1
0
1
MR effect size for
'Gut microbiota abundance (genus Parasutterella id.2892)' on 'Protein SCO1 homolog, mitochondrial'

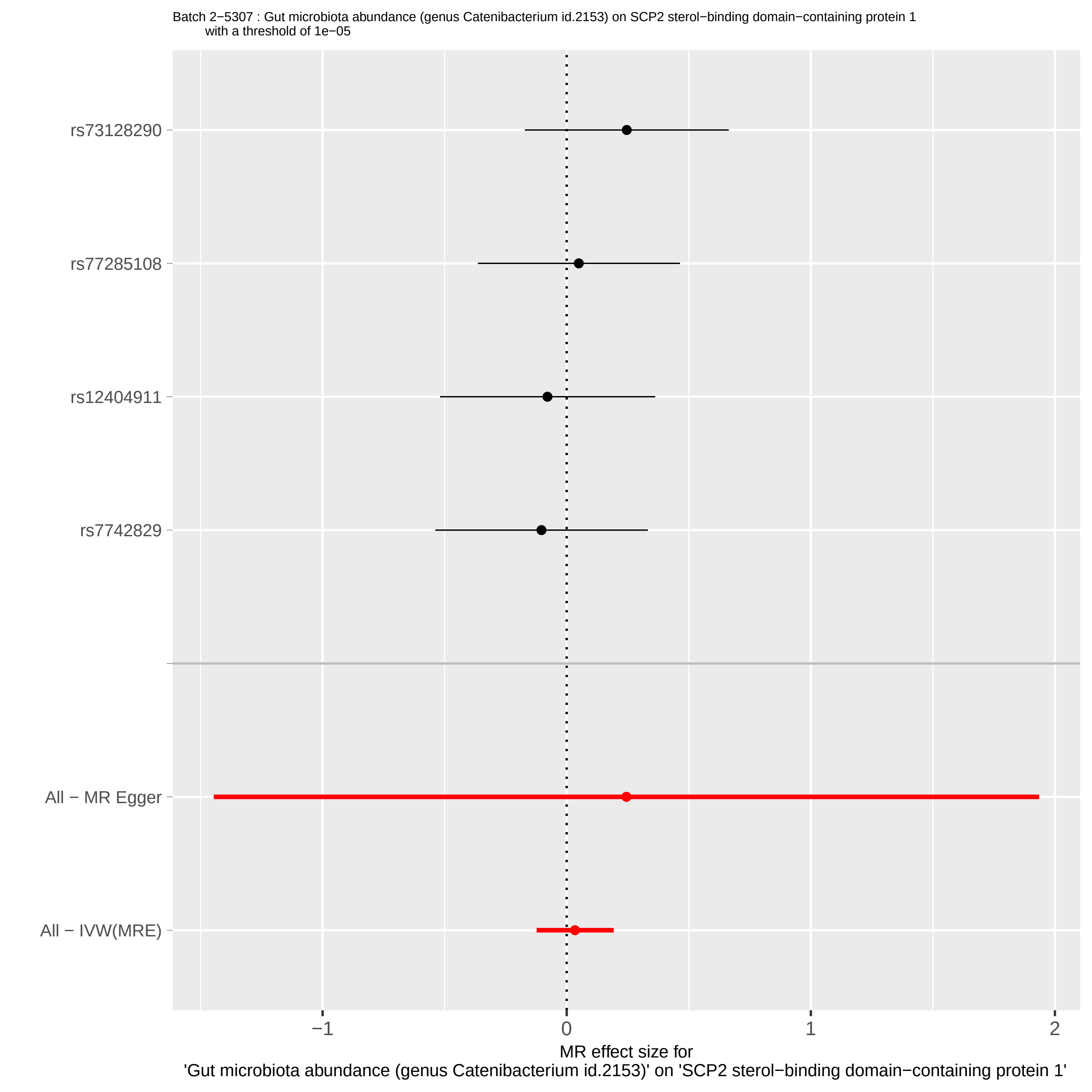

Batch 2−5307 : Gut microbiota abundance (genus Catenibacterium id.2153) on SCP2 sterol−binding domain−containing protein 1
with a threshold of 1e−05
rs73128290
rs77285108
rs12404911
rs7742829
All − MR Egger
All − IVW(MRE)
−1
0
1
2
MR effect size for
'Gut microbiota abundance (genus Catenibacterium id.2153)' on 'SCP2 sterol−binding domain−containing protein 1'

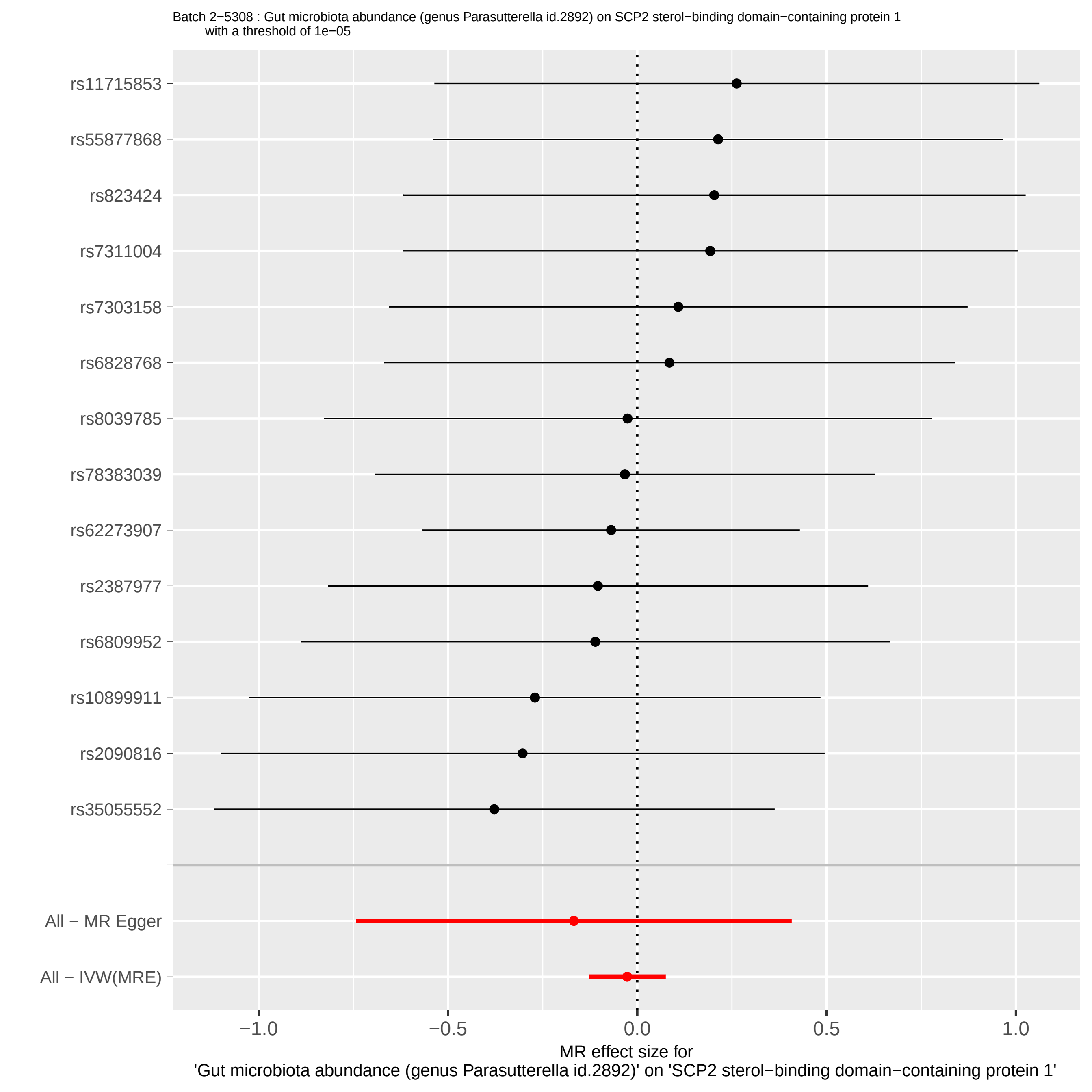
Batch 2−5308 : Gut microbiota abundance (genus Parasutterella id.2892) on SCP2 sterol−binding domain−containing protein 1
with a threshold of 1e−05
rs11715853
rs55877868
rs823424
rs7311004
rs7303158
rs6828768
rs8039785
rs78383039
rs62273907
rs2387977
rs6809952
rs10899911
rs2090816
rs35055552
All − MR Egger
All − IVW(MRE)
−1.0
−0.5
0.0
0.5
1.0
MR effect size for
'Gut microbiota abundance (genus Parasutterella id.2892)' on 'SCP2 sterol−binding domain−containing protein 1'

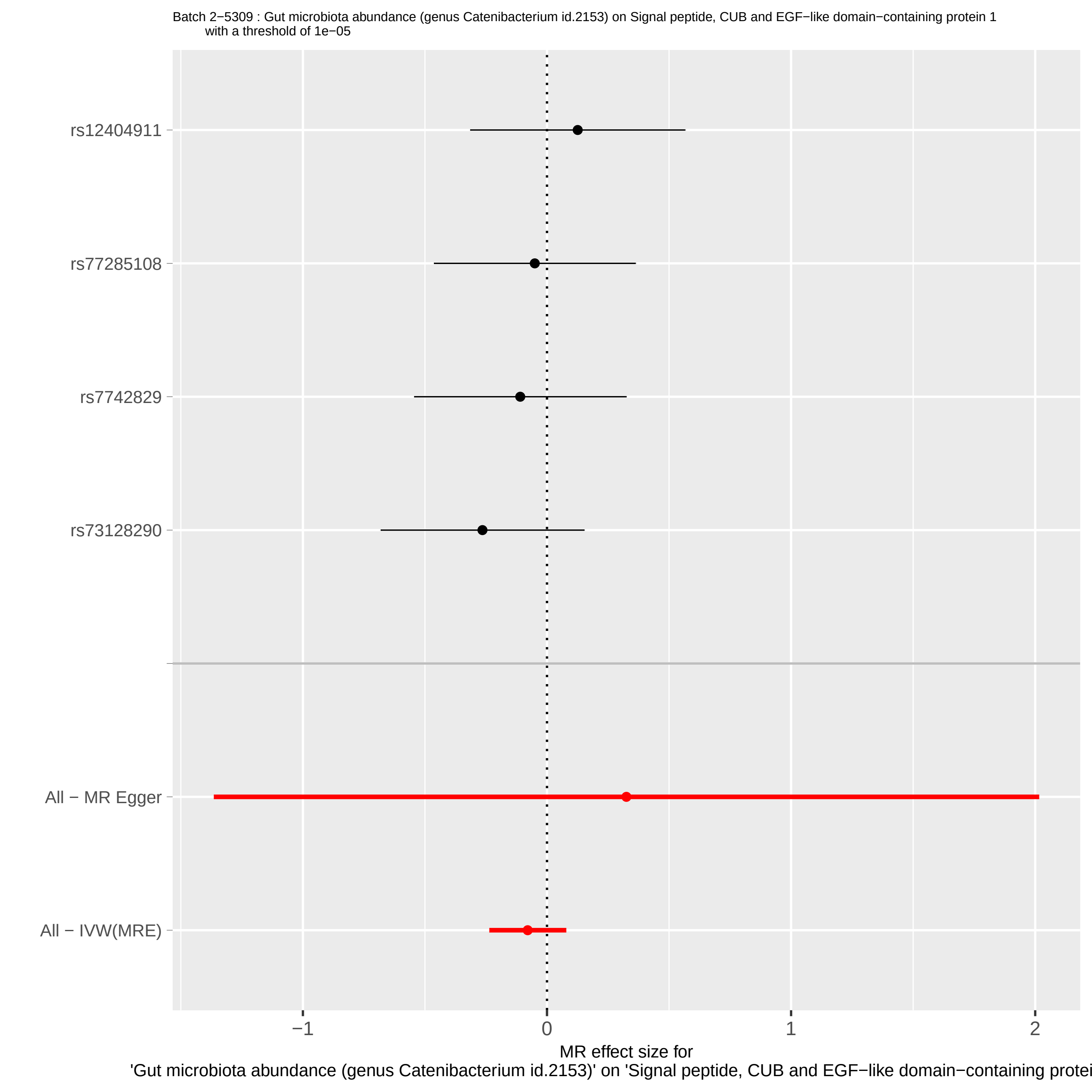
Batch 2−5309 : Gut microbiota abundance (genus Catenibacterium id.2153) on Signal peptide, CUB and EGF−like domain−containing protein 1
with a threshold of 1e−05
rs12404911
rs77285108
rs7742829
rs73128290
All − MR Egger
All − IVW(MRE)
−1
0
1
2
MR effect size for
'Gut microbiota abundance (genus Catenibacterium id.2153)' on 'Signal peptide, CUB and EGF−like domain−containing protei

Batch 2−5310 : Gut microbiota abundance (genus Parasutterella id.2892) on Signal peptide, CUB and EGF−like domain−containing protein 1 with a threshold of 1e−05

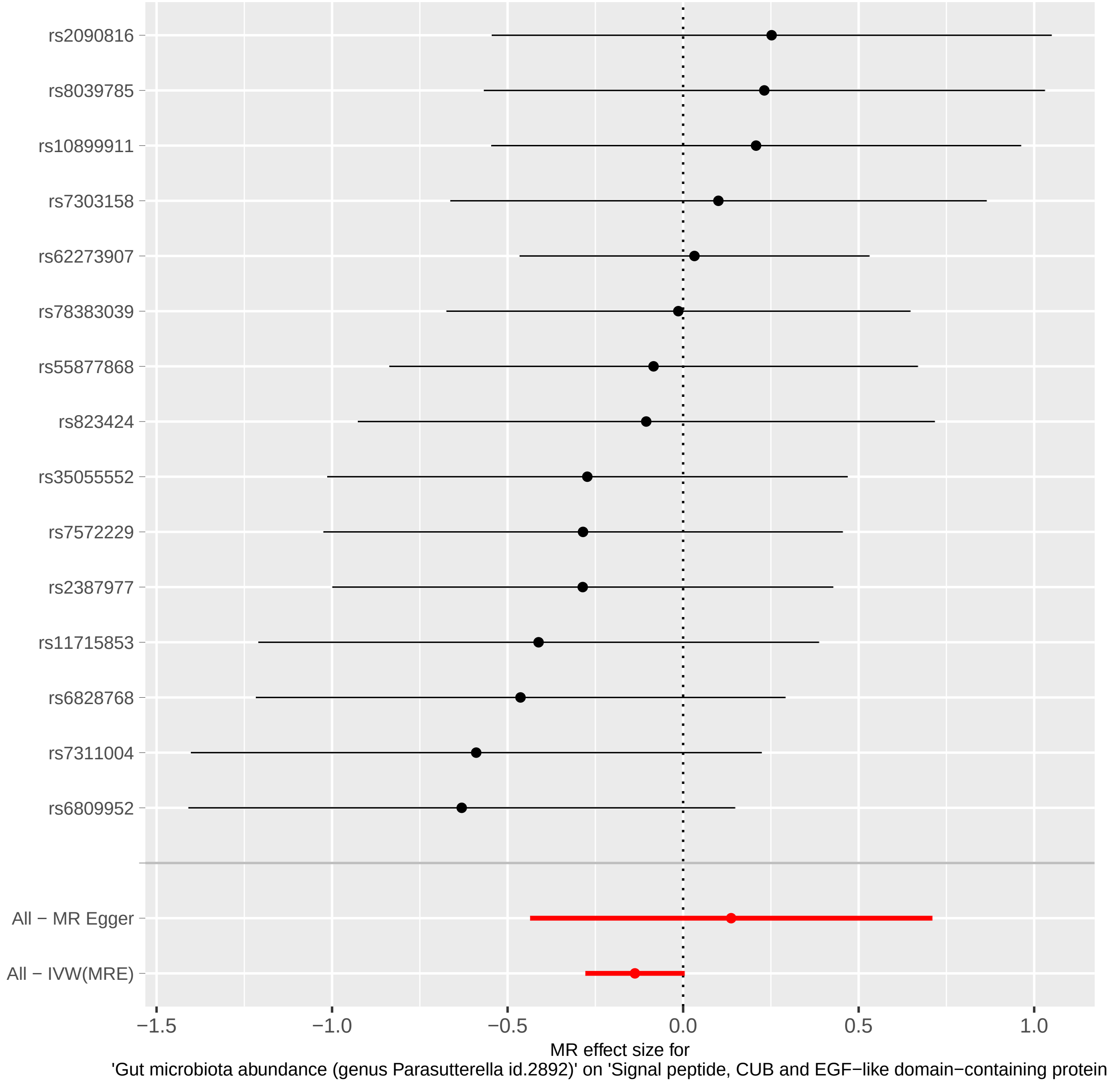

Batch 2−5311 : Gut microbiota abundance (genus Catenibacterium id.2153) on Stromal cell−derived factor 2−like protein 1
with a threshold of 1e−05

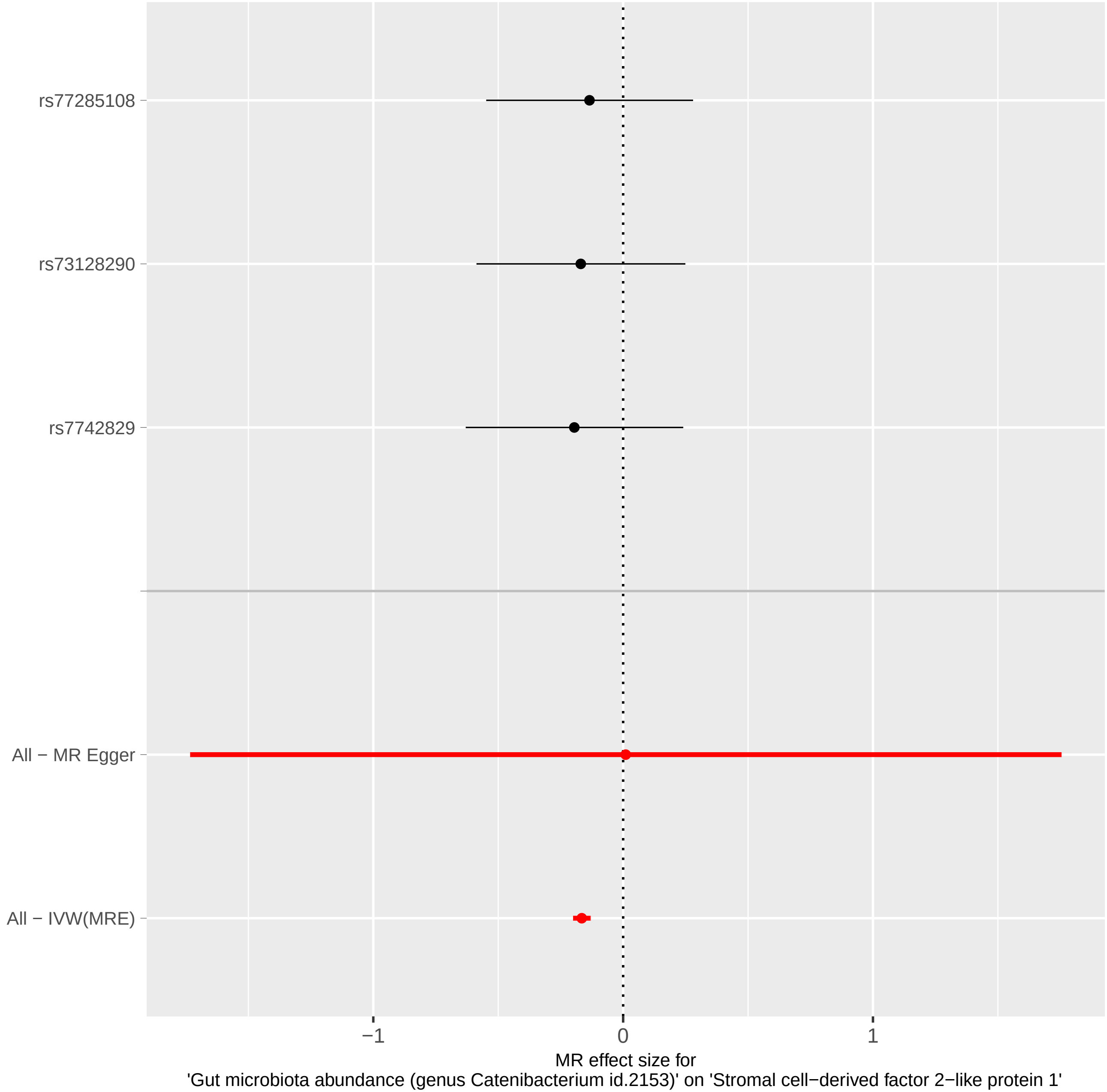

Batch 2−5312 : Gut microbiota abundance (genus Parasutterella id.2892) on Stromal cell−derived factor 2−like protein 1
with a threshold of 1e−05

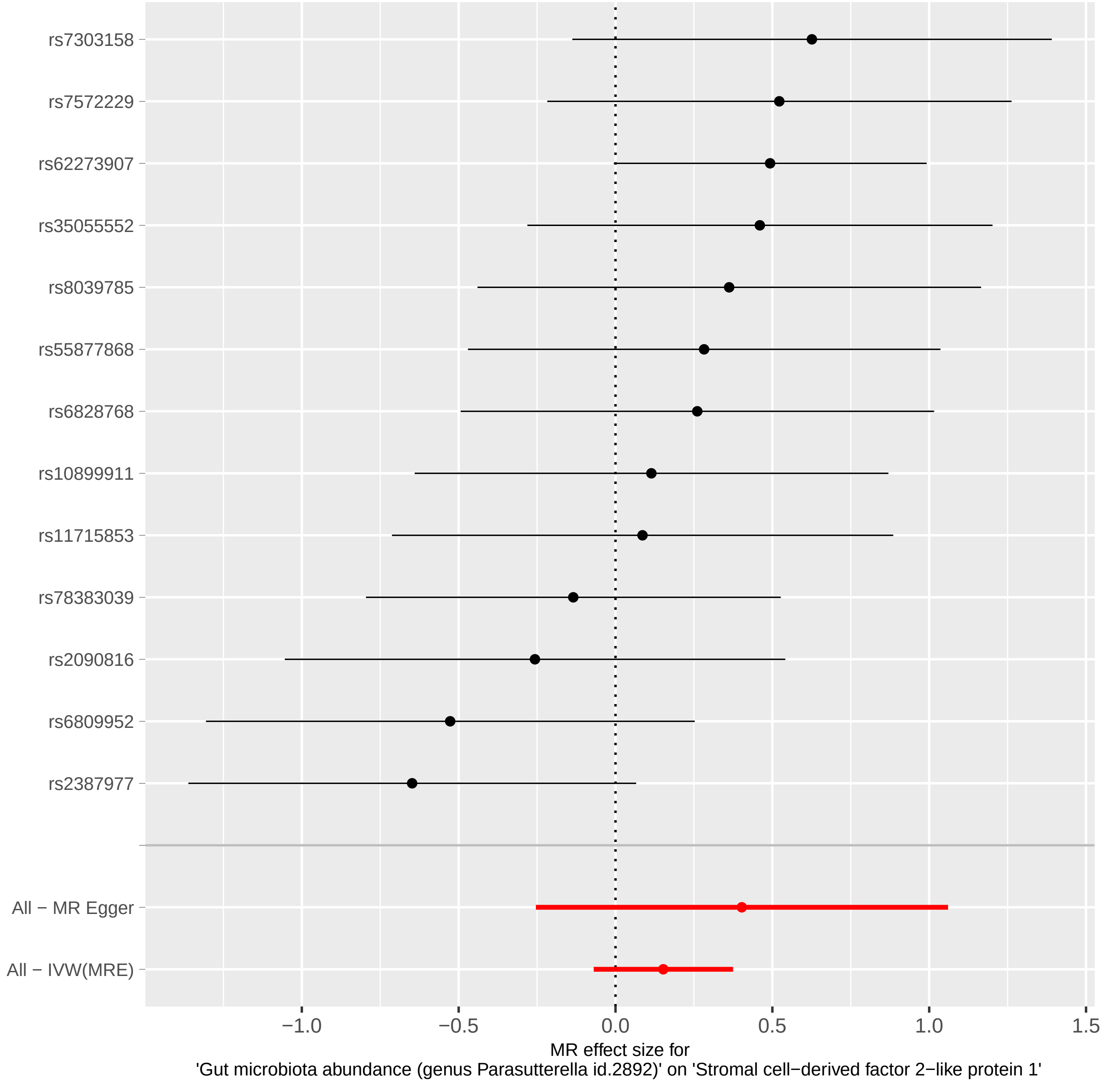

Batch 2−5313 : Gut microbiota abundance (genus Catenibacterium id.2153) on Succinate dehydrogenase assembly factor 2, mitochondrial with a threshold of 1e−05

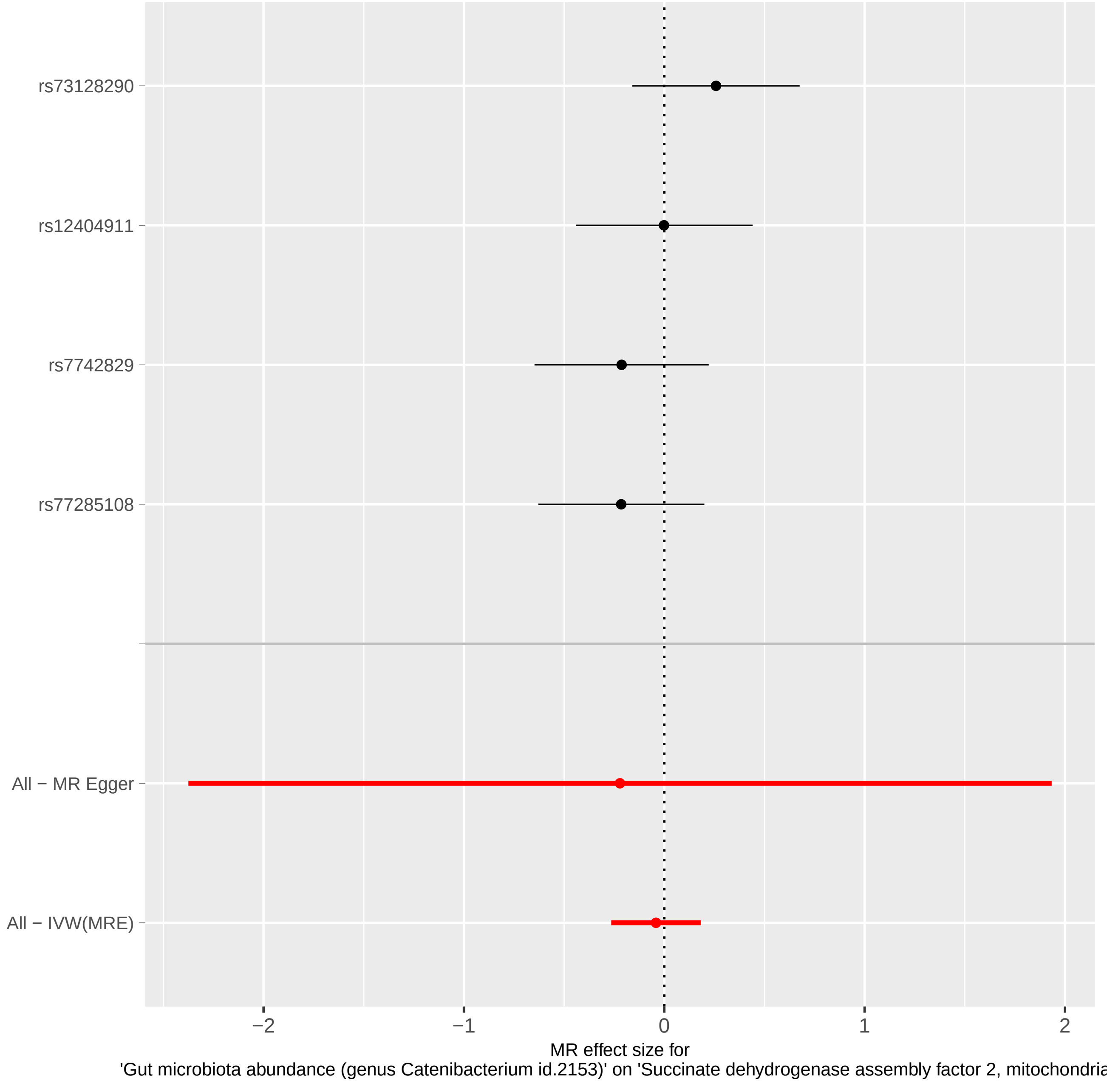

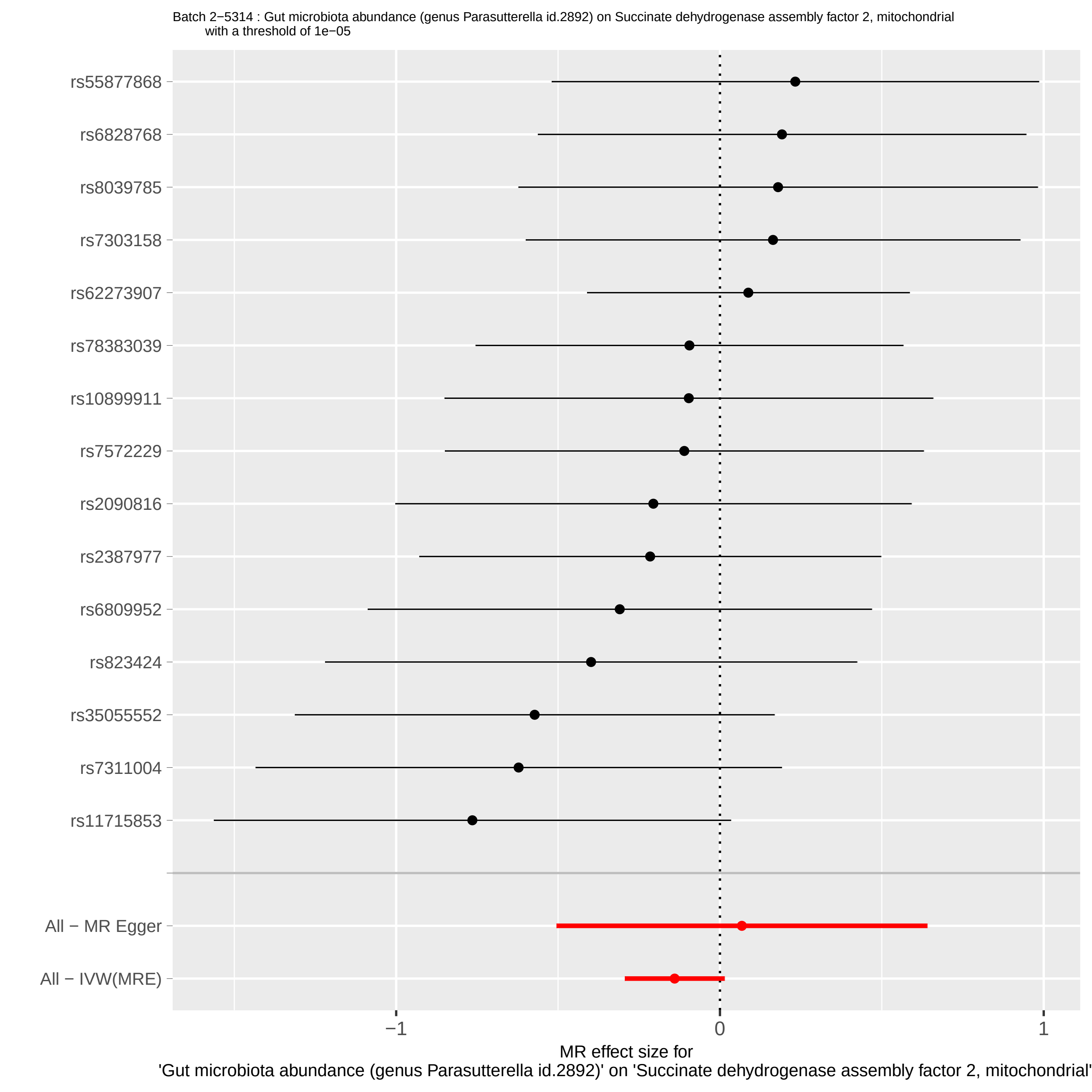

Batch 2−5314 : Gut microbiota abundance (genus Parasutterella id.2892) on Succinate dehydrogenase assembly factor 2, mitochondrial
with a threshold of 1e−05
rs55877868
rs6828768
rs8039785
rs7303158
rs62273907
rs78383039
rs10899911
rs7572229
rs2090816
rs2387977
rs6809952
rs823424
rs35055552
rs7311004
rs11715853
All − MR Egger
All − IVW(MRE)
−1
0
1
MR effect size for
'Gut microbiota abundance (genus Parasutterella id.2892)' on 'Succinate dehydrogenase assembly factor 2, mitochondrial'

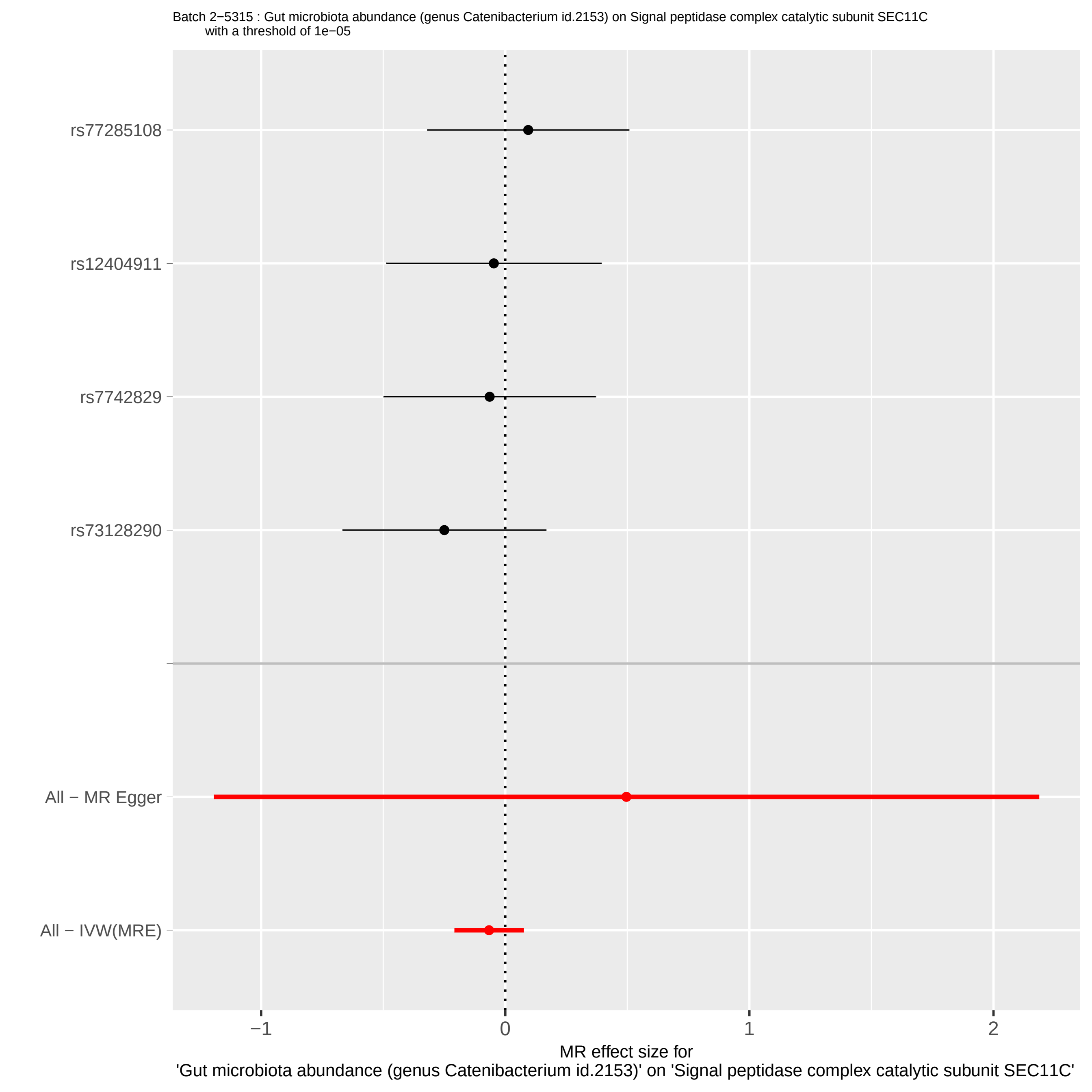

Batch 2−5315 : Gut microbiota abundance (genus Catenibacterium id.2153) on Signal peptidase complex catalytic subunit SEC11C
with a threshold of 1e−05
rs77285108
rs12404911
rs7742829
rs73128290
All − MR Egger
All − IVW(MRE)
−1
0
1
2
MR effect size for
'Gut microbiota abundance (genus Catenibacterium id.2153)' on 'Signal peptidase complex catalytic subunit SEC11C'

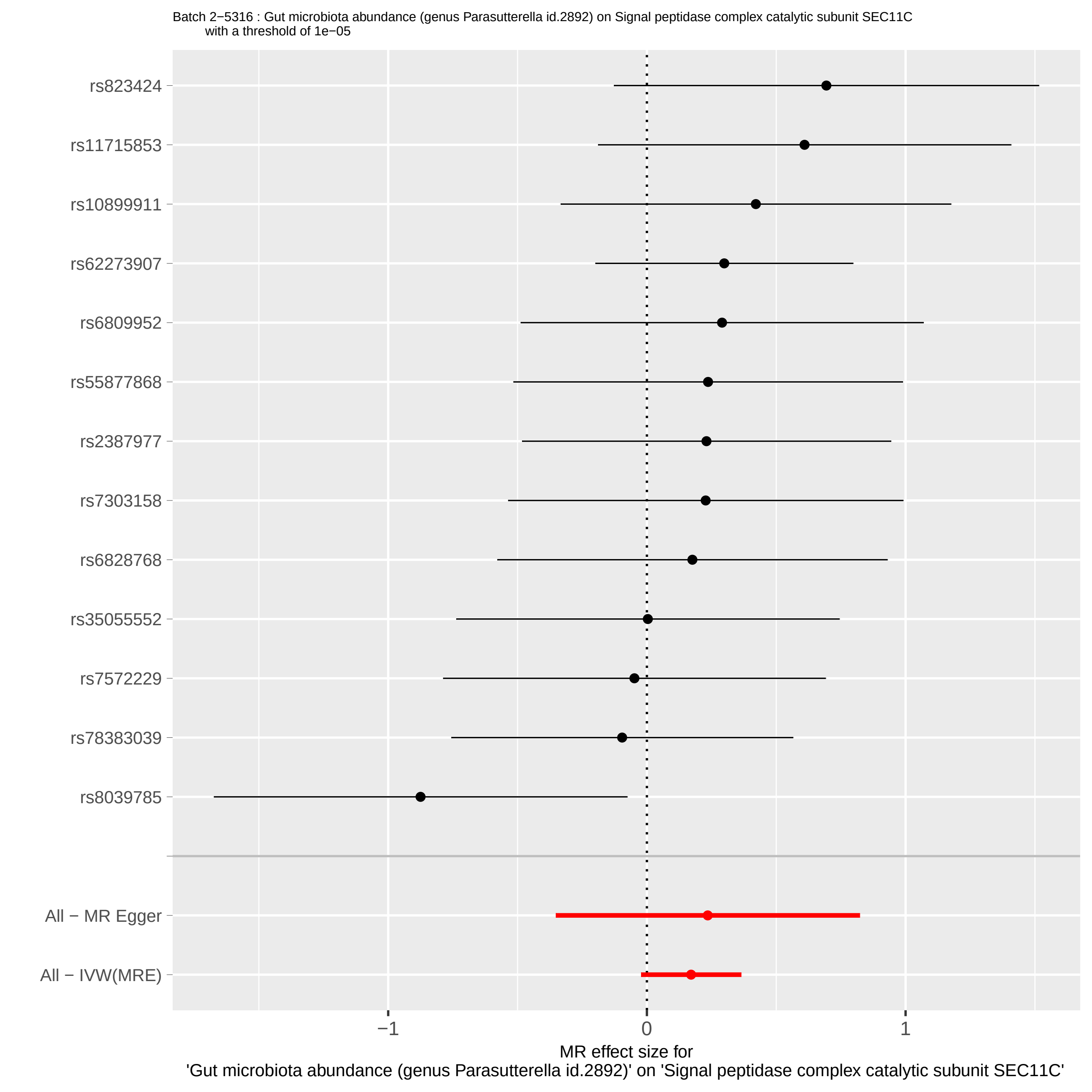

Batch 2−5316 : Gut microbiota abundance (genus Parasutterella id.2892) on Signal peptidase complex catalytic subunit SEC11C
with a threshold of 1e−05
rs823424
rs11715853
rs10899911
rs62273907
rs6809952
rs55877868
rs2387977
rs7303158
rs6828768
rs35055552
rs7572229
rs78383039
rs8039785
All − MR Egger
All − IVW(MRE)
−1
0
1
MR effect size for
'Gut microbiota abundance (genus Parasutterella id.2892)' on 'Signal peptidase complex catalytic subunit SEC11C'

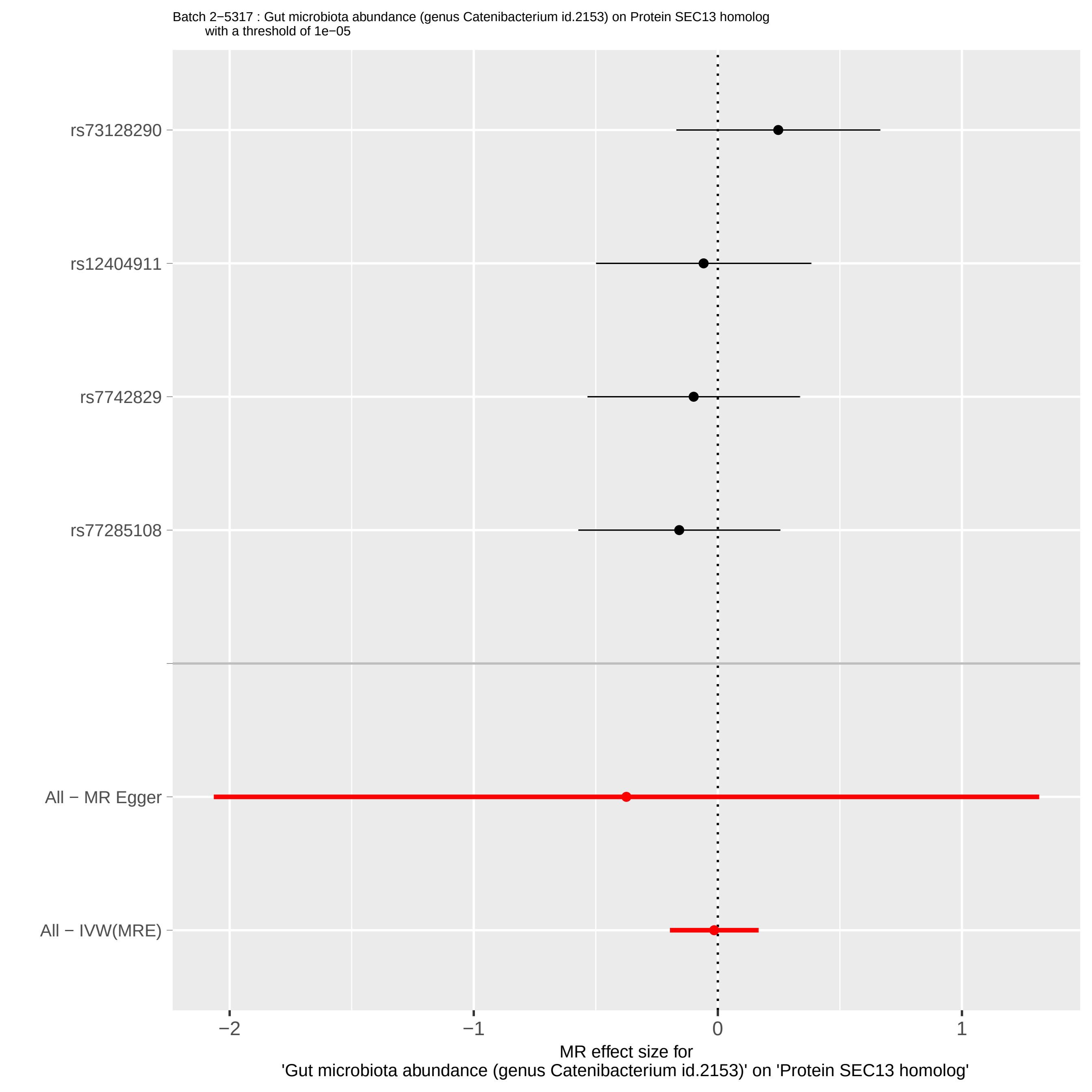

Batch 2−5317 : Gut microbiota abundance (genus Catenibacterium id.2153) on Protein SEC13 homolog
with a threshold of 1e−05
rs73128290
rs12404911
rs7742829
rs77285108
All − MR Egger
All − IVW(MRE)
−2
−1
0
1
MR effect size for
'Gut microbiota abundance (genus Catenibacterium id.2153)' on 'Protein SEC13 homolog'

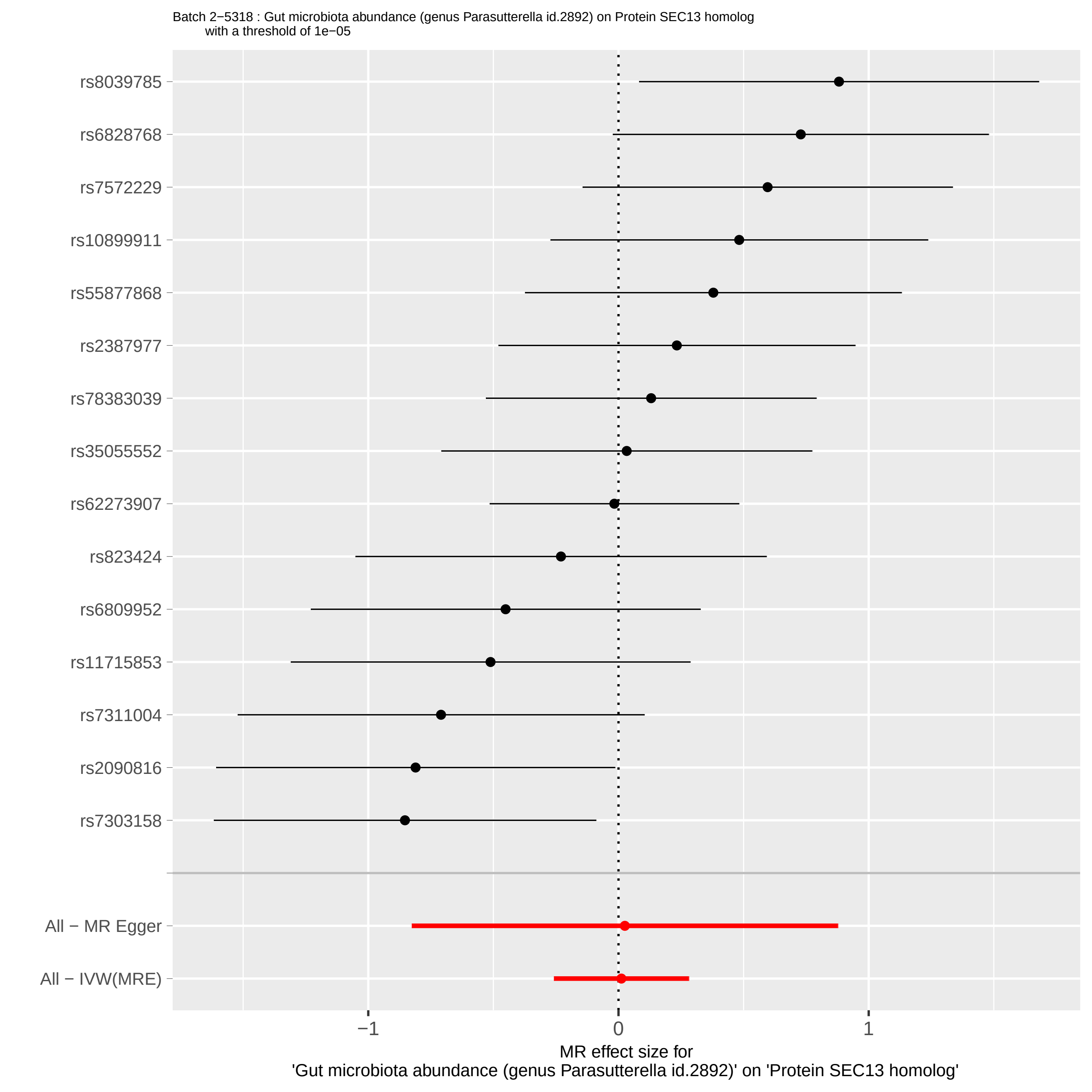

Batch 2−5318 : Gut microbiota abundance (genus Parasutterella id.2892) on Protein SEC13 homolog
with a threshold of 1e−05
rs8039785
rs6828768
rs7572229
rs10899911
rs55877868
rs2387977
rs78383039
rs35055552
rs62273907
rs823424
rs6809952
rs11715853
rs7311004
rs2090816
rs7303158
All − MR Egger
All − IVW(MRE)
−1
0
1
MR effect size for
'Gut microbiota abundance (genus Parasutterella id.2892)' on 'Protein SEC13 homolog'

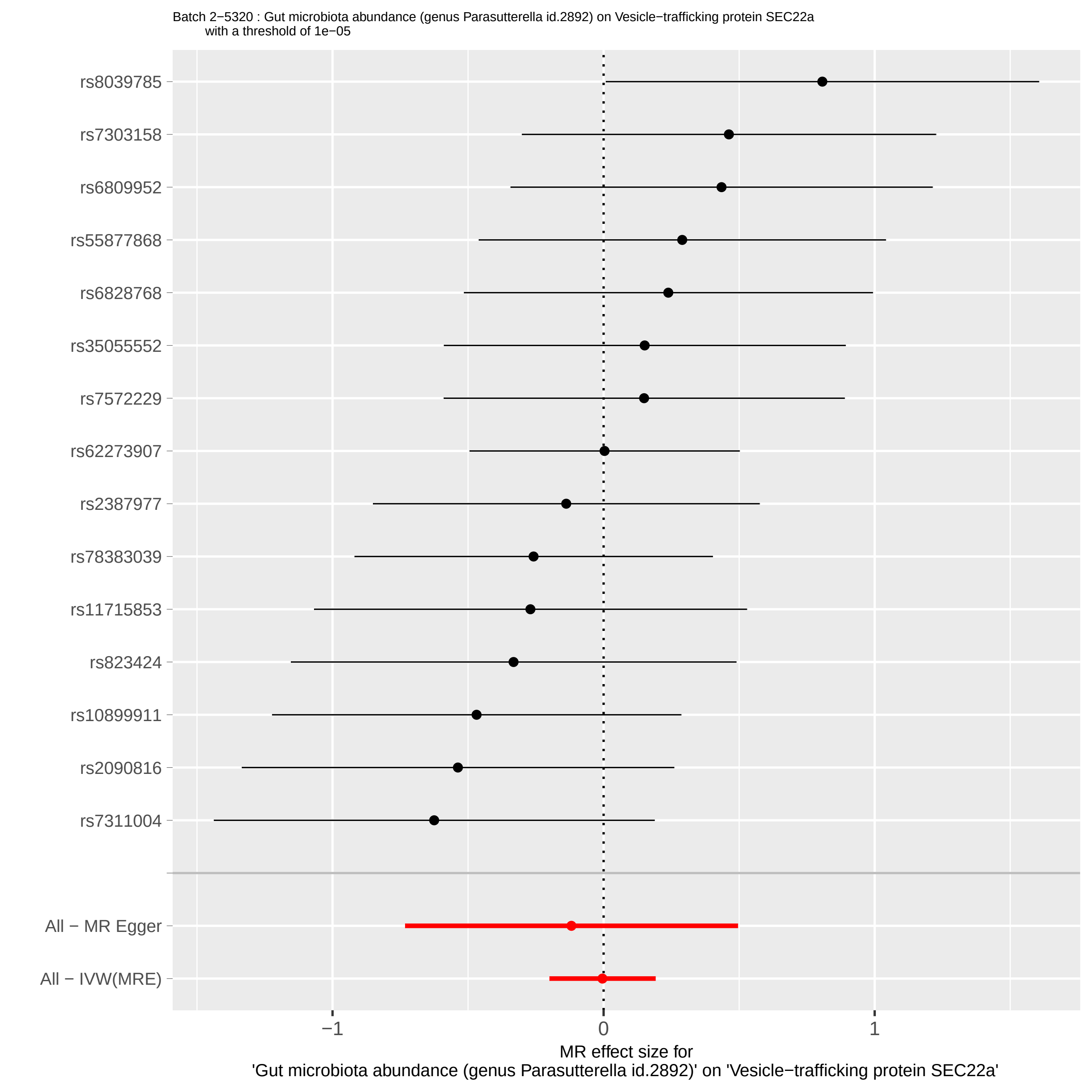

Batch 2−5320 : Gut microbiota abundance (genus Parasutterella id.2892) on Vesicle−trafficking protein SEC22a
with a threshold of 1e−05
rs8039785
rs7303158
rs6809952
rs55877868
rs6828768
rs35055552
rs7572229
rs62273907
rs2387977
rs78383039
rs11715853
rs823424
rs10899911
rs2090816
rs7311004
All − MR Egger
All − IVW(MRE)
−1
0
1
MR effect size for
'Gut microbiota abundance (genus Parasutterella id.2892)' on 'Vesicle−trafficking protein SEC22a'

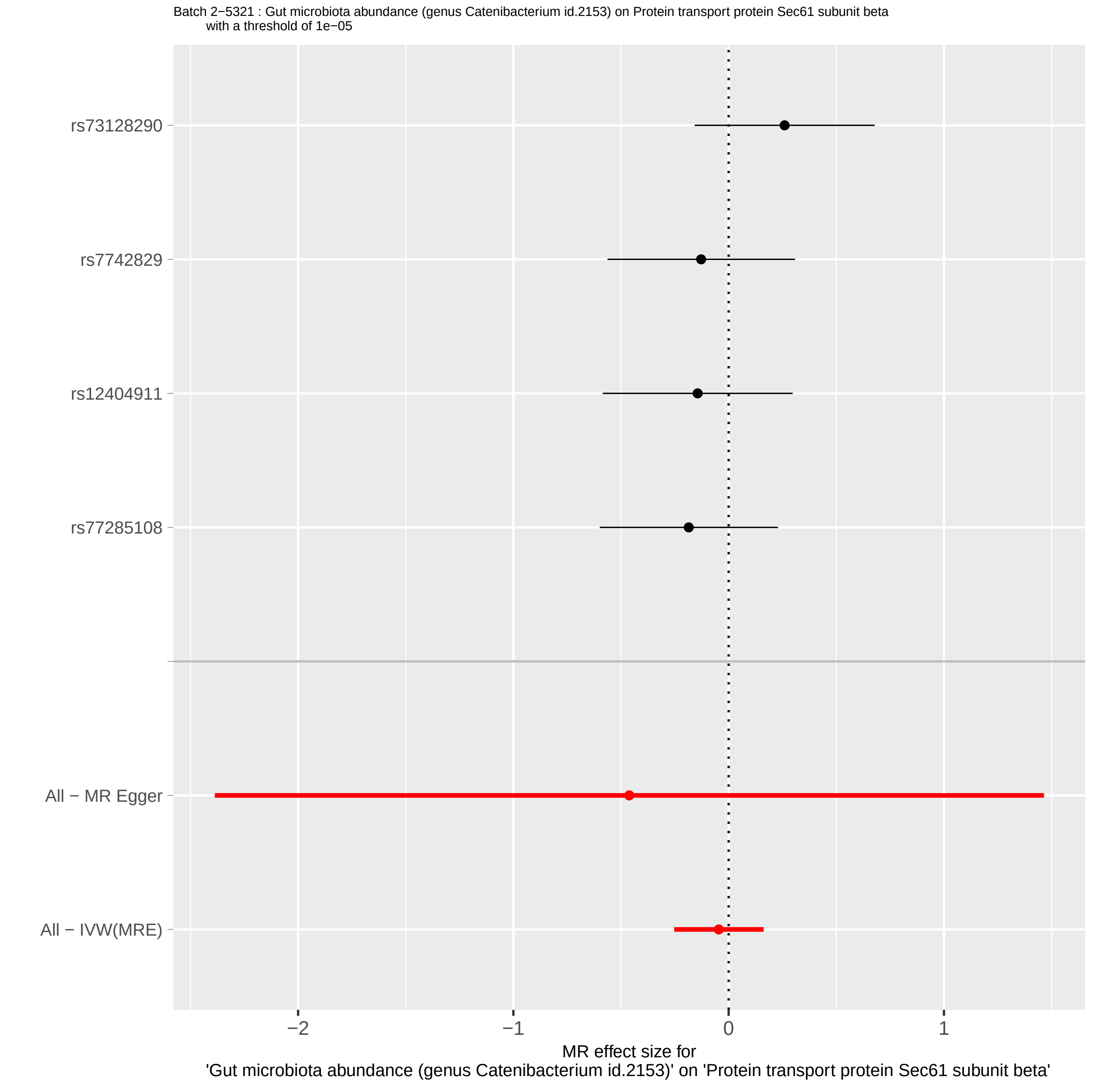

Batch 2−5321 : Gut microbiota abundance (genus Catenibacterium id.2153) on Protein transport protein Sec61 subunit beta
with a threshold of 1e−05
rs73128290
rs7742829
rs12404911
rs77285108
All − MR Egger
All − IVW(MRE)
−2
−1
0
1
MR effect size for
'Gut microbiota abundance (genus Catenibacterium id.2153)' on 'Protein transport protein Sec61 subunit beta'

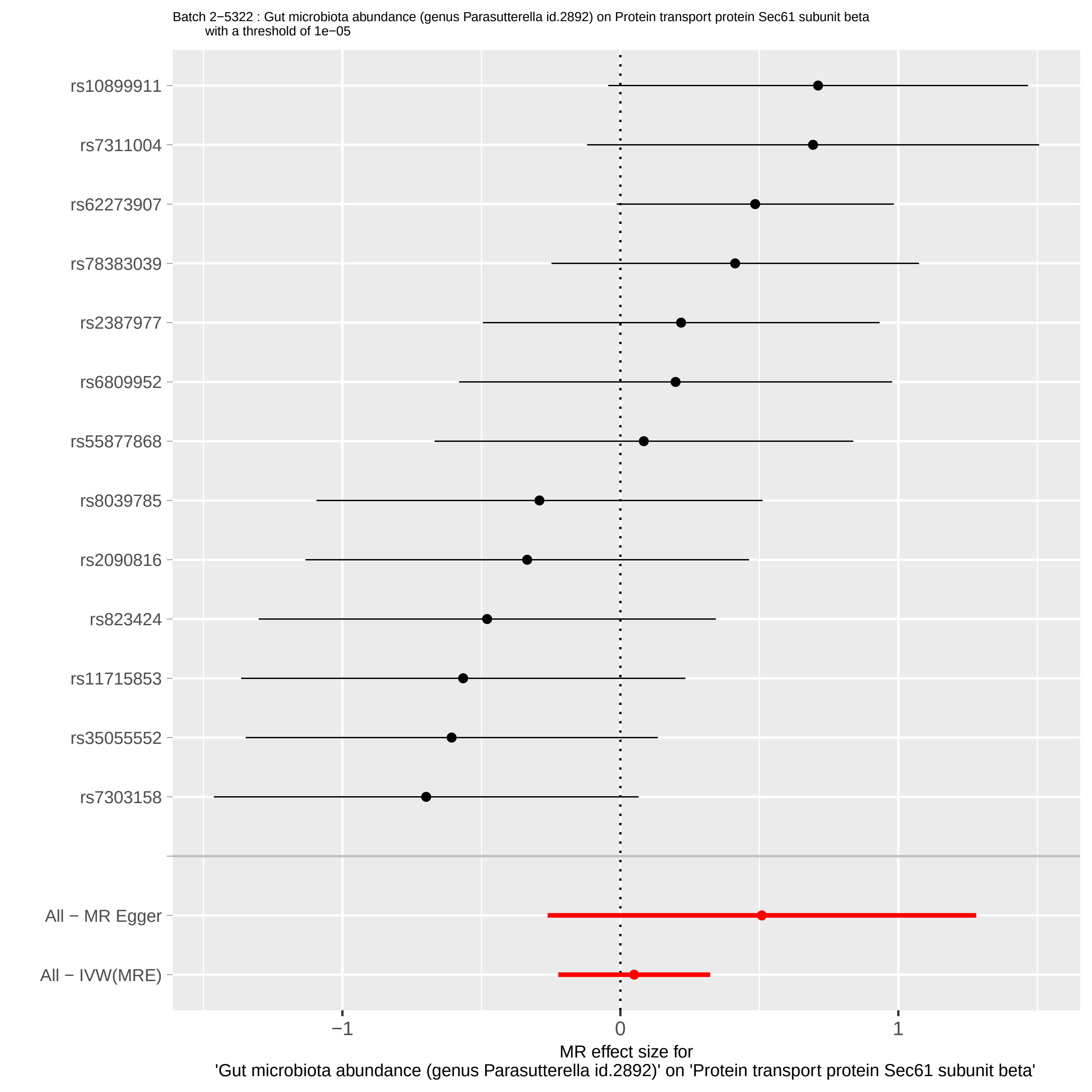

Batch 2−5322 : Gut microbiota abundance (genus Parasutterella id.2892) on Protein transport protein Sec61 subunit beta
with a threshold of 1e−05
rs10899911
rs7311004
rs62273907
rs78383039
rs2387977
rs6809952
rs55877868
rs8039785
rs2090816
rs823424
rs11715853
rs35055552
rs7303158
All − MR Egger
All − IVW(MRE)
−1
0
1
MR effect size for
'Gut microbiota abundance (genus Parasutterella id.2892)' on 'Protein transport protein Sec61 subunit beta'

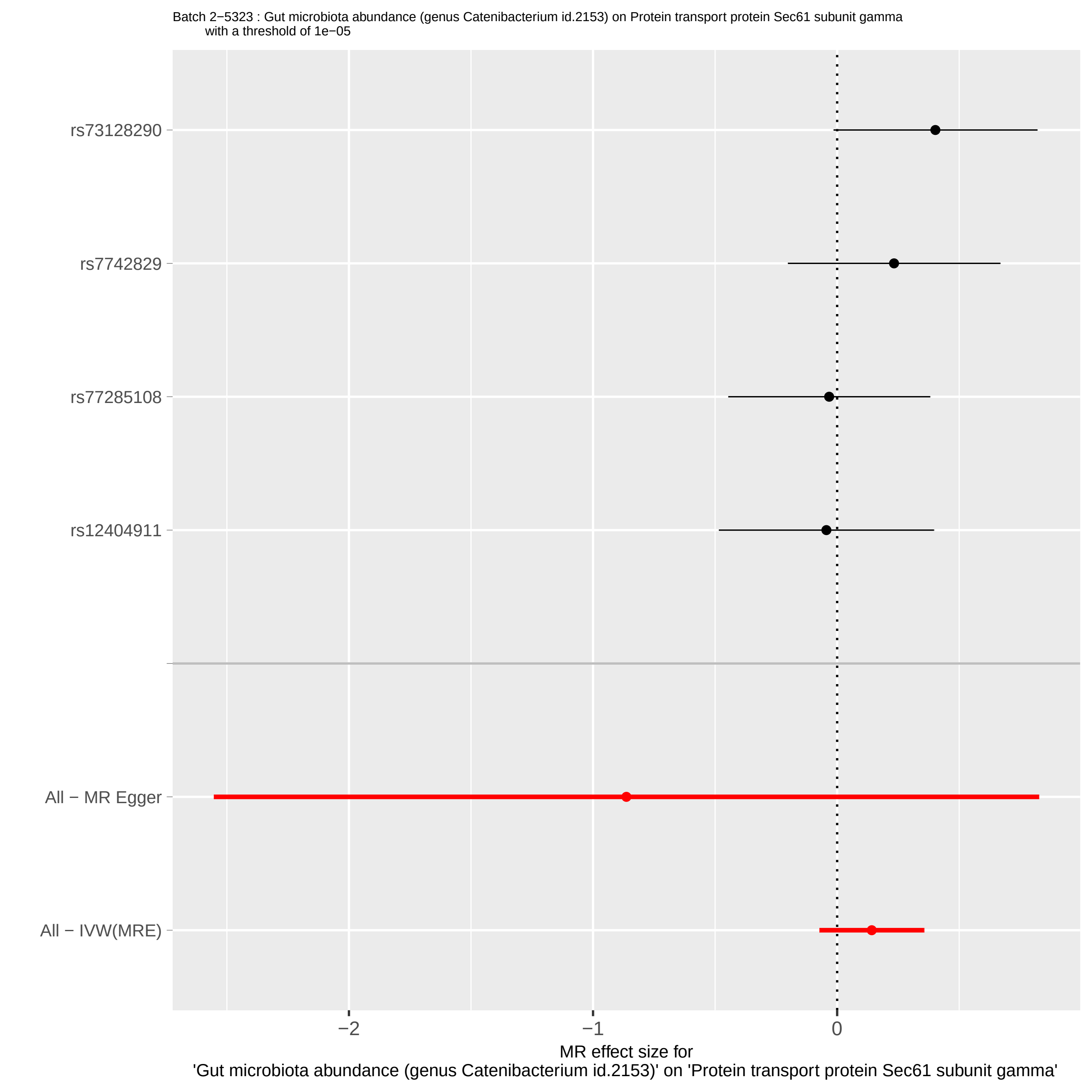
Batch 2−5323 : Gut microbiota abundance (genus Catenibacterium id.2153) on Protein transport protein Sec61 subunit gamma
with a threshold of 1e−05
rs73128290
rs7742829
rs77285108
rs12404911
All − MR Egger
All − IVW(MRE)
−2
−1
0
MR effect size for
'Gut microbiota abundance (genus Catenibacterium id.2153)' on 'Protein transport protein Sec61 subunit gamma'

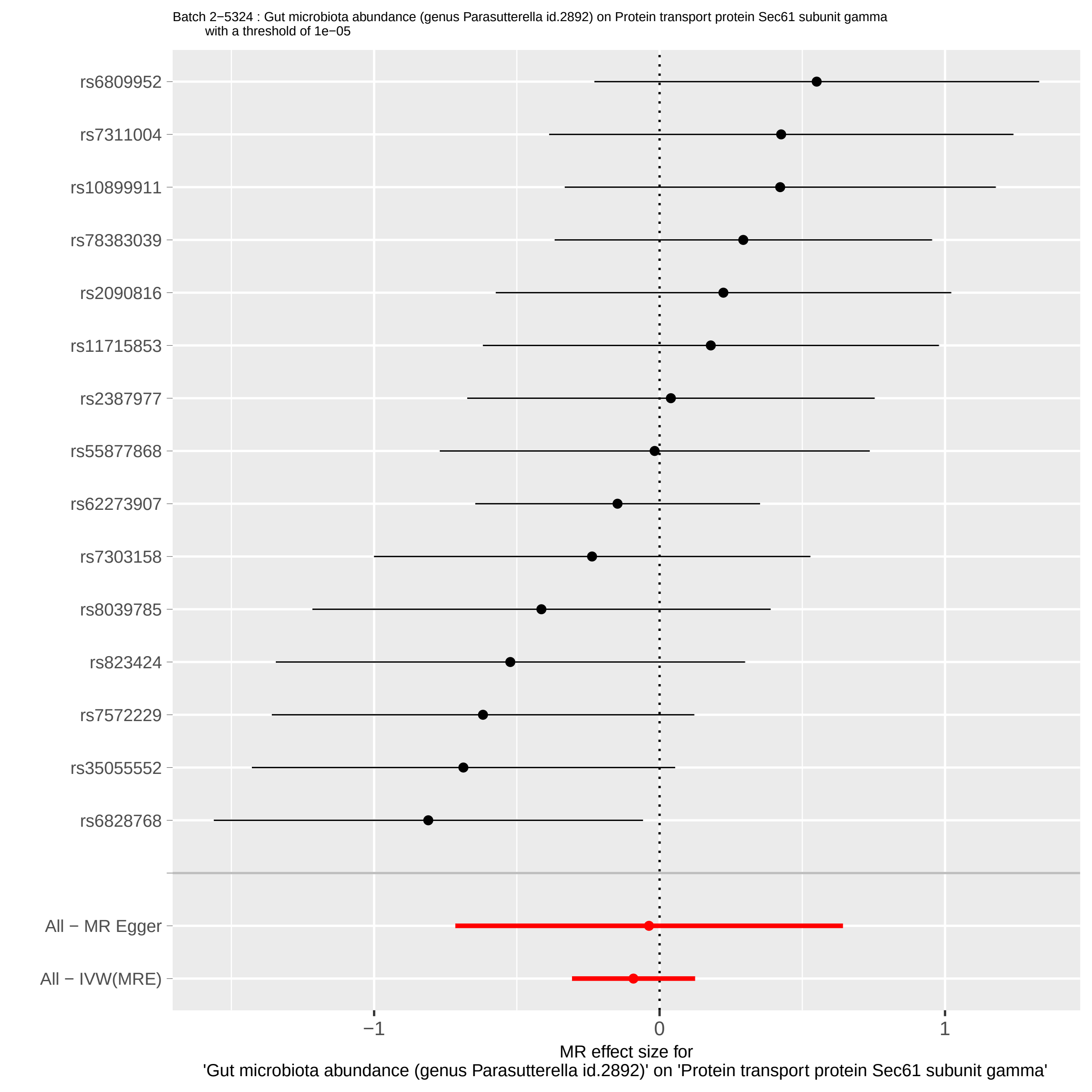
Batch 2−5324 : Gut microbiota abundance (genus Parasutterella id.2892) on Protein transport protein Sec61 subunit gamma
with a threshold of 1e−05
rs6809952
rs7311004
rs10899911
rs78383039
rs2090816
rs11715853
rs2387977
rs55877868
rs62273907
rs7303158
rs8039785
rs823424
rs7572229
rs35055552
rs6828768
All − MR Egger
All − IVW(MRE)
−1
0
1
MR effect size for
'Gut microbiota abundance (genus Parasutterella id.2892)' on 'Protein transport protein Sec61 subunit gamma'

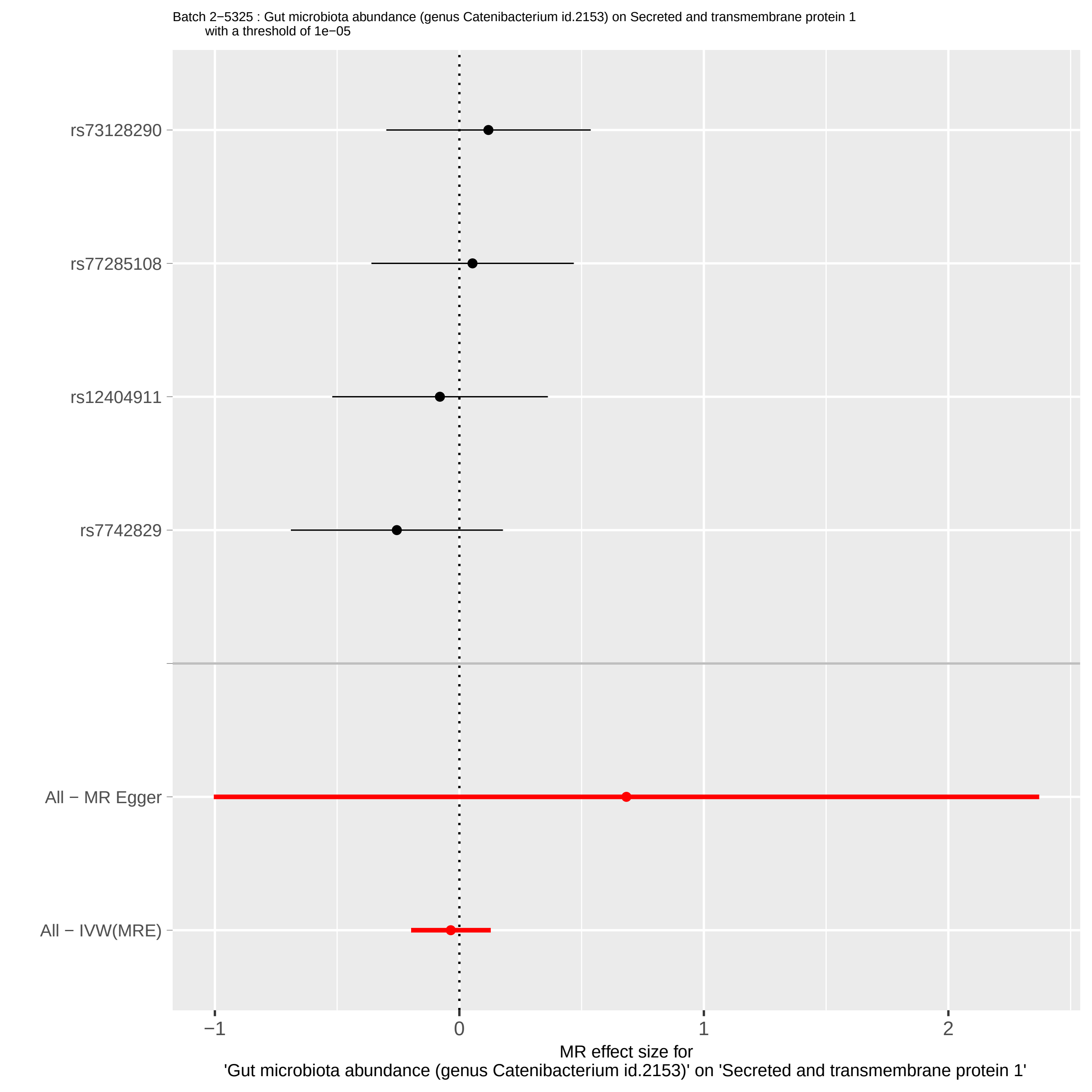
Batch 2−5325 : Gut microbiota abundance (genus Catenibacterium id.2153) on Secreted and transmembrane protein 1
with a threshold of 1e−05
rs73128290
rs77285108
rs12404911
rs7742829
All − MR Egger
All − IVW(MRE)
−1
0
1
2
MR effect size for
'Gut microbiota abundance (genus Catenibacterium id.2153)' on 'Secreted and transmembrane protein 1'

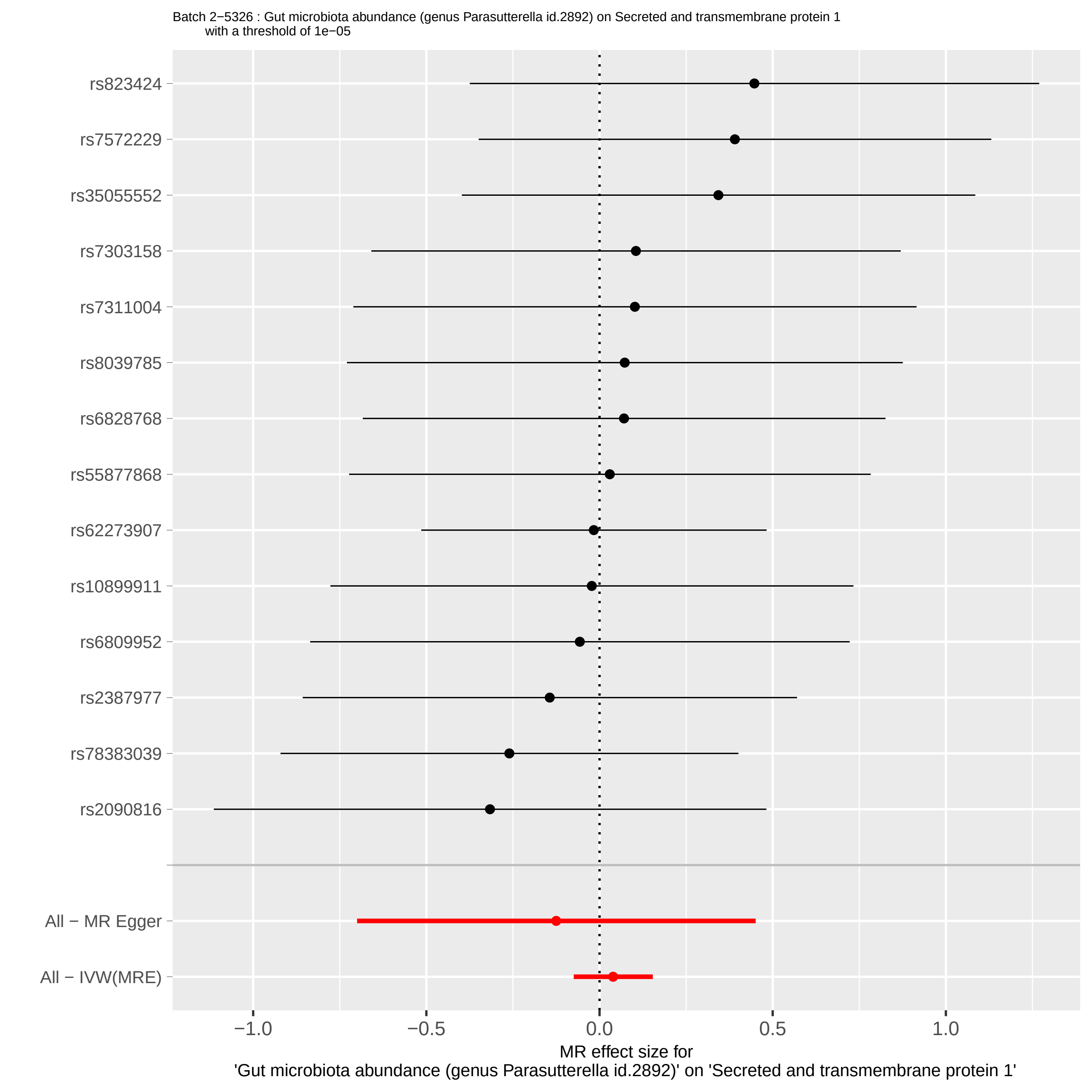

Batch 2−5326 : Gut microbiota abundance (genus Parasutterella id.2892) on Secreted and transmembrane protein 1
with a threshold of 1e−05
rs823424
rs7572229
rs35055552
rs7303158
rs7311004
rs8039785
rs6828768
rs55877868
rs62273907
rs10899911
rs6809952
rs2387977
rs78383039
rs2090816
All − MR Egger
All − IVW(MRE)
−1.0
−0.5
0.0
0.5
1.0
MR effect size for
'Gut microbiota abundance (genus Parasutterella id.2892)' on 'Secreted and transmembrane protein 1'

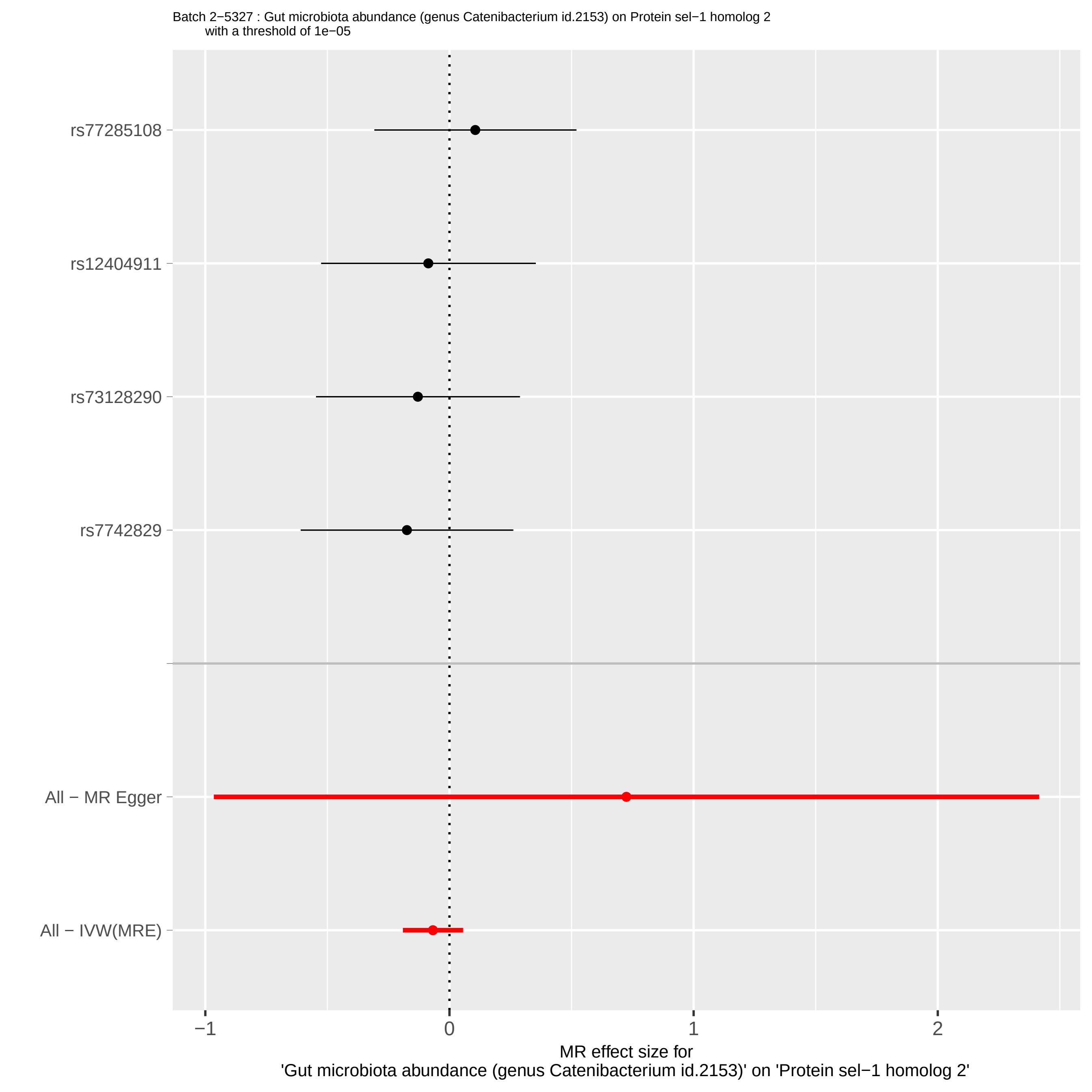

Batch 2−5327 : Gut microbiota abundance (genus Catenibacterium id.2153) on Protein sel−1 homolog 2
with a threshold of 1e−05
rs77285108
rs12404911
rs73128290
rs7742829
All − MR Egger
All − IVW(MRE)
−1
0
1
2
MR effect size for
'Gut microbiota abundance (genus Catenibacterium id.2153)' on 'Protein sel−1 homolog 2'

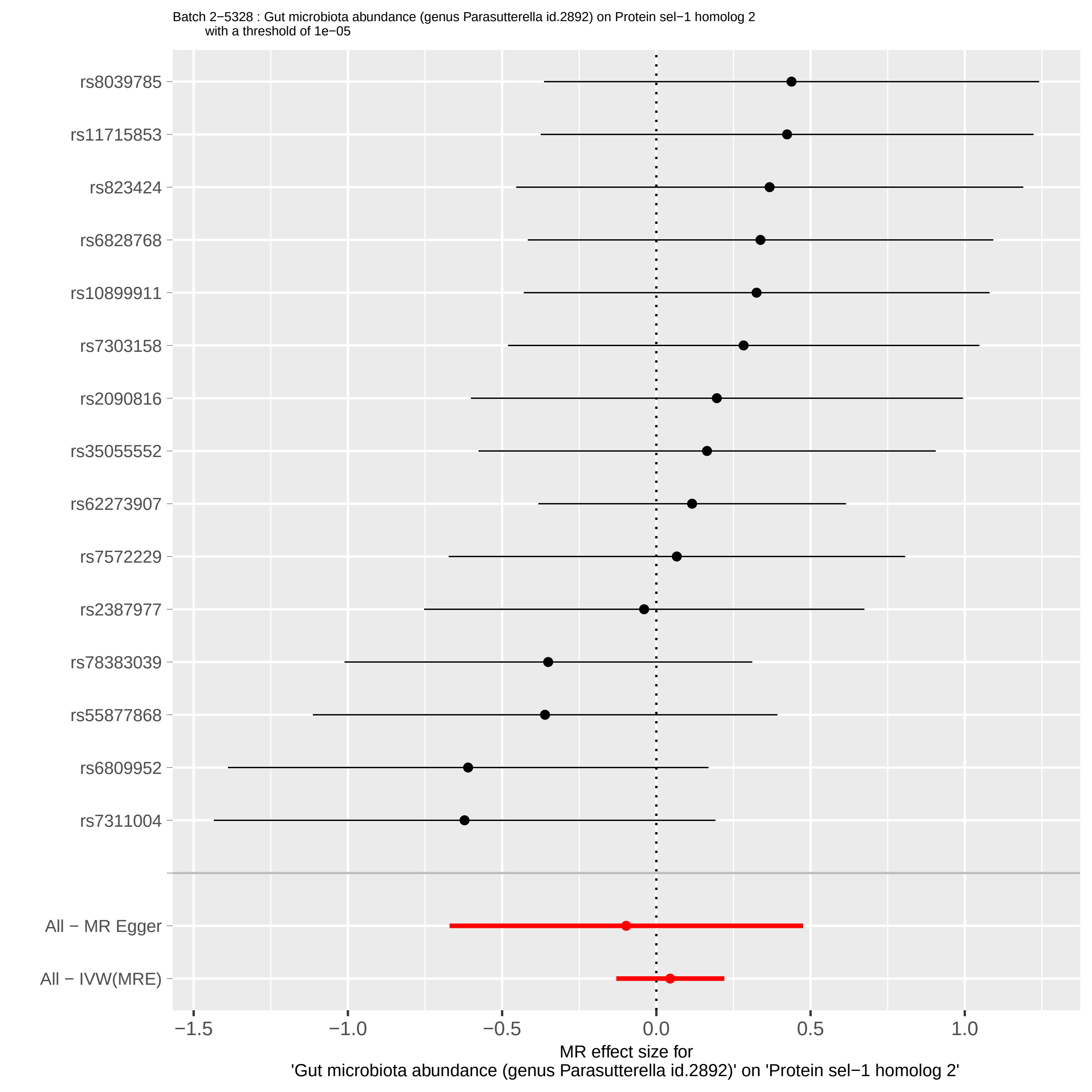

Batch 2−5328 : Gut microbiota abundance (genus Parasutterella id.2892) on Protein sel−1 homolog 2
with a threshold of 1e−05
rs8039785
rs11715853
rs823424
rs6828768
rs10899911
rs7303158
rs2090816
rs35055552
rs62273907
rs7572229
rs2387977
rs78383039
rs55877868
rs6809952
rs7311004
All − MR Egger
All − IVW(MRE)
−1.5
−1.0
−0.5
0.0
0.5
1.0
MR effect size for
'Gut microbiota abundance (genus Parasutterella id.2892)' on 'Protein sel−1 homolog 2'

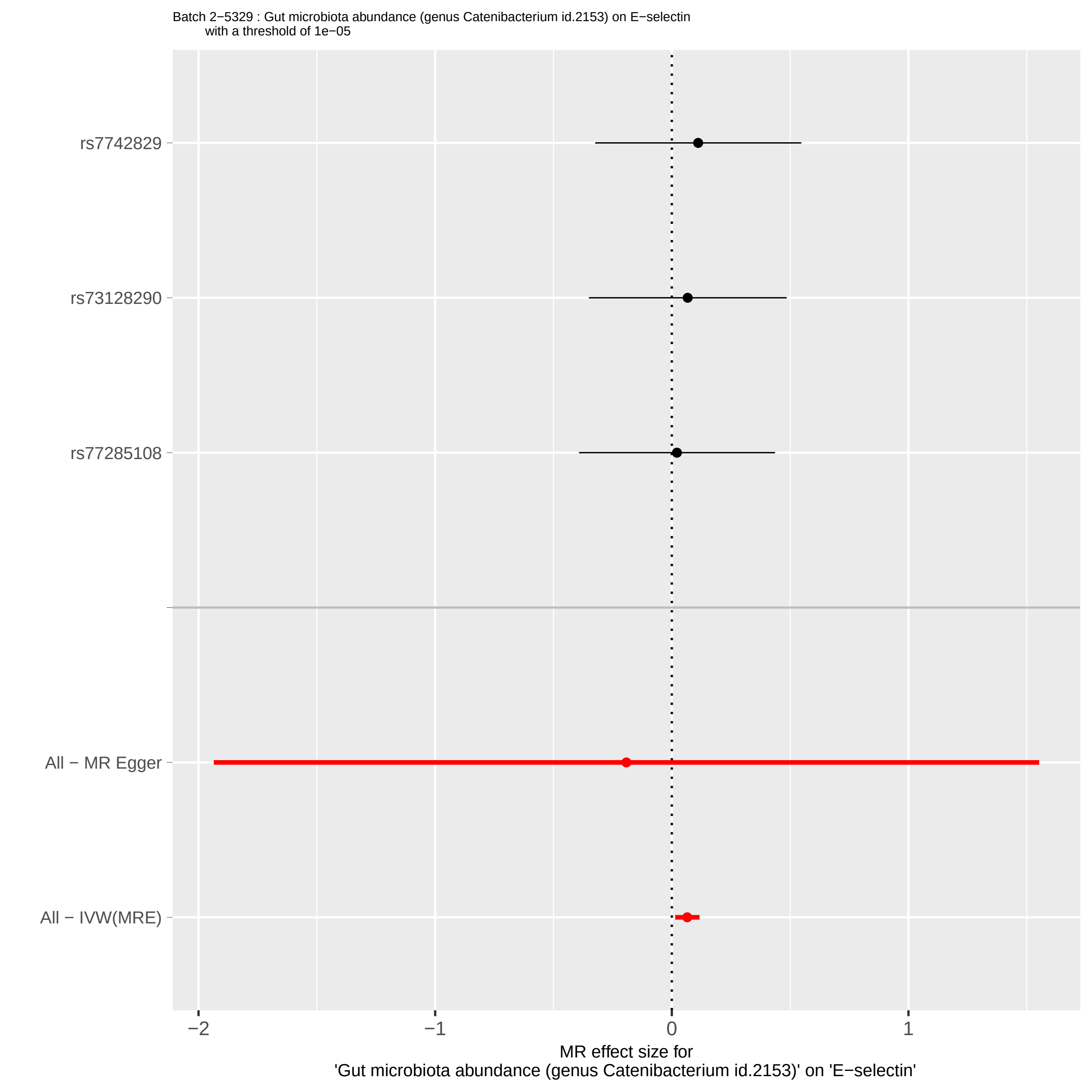
Batch 2−5329 : Gut microbiota abundance (genus Catenibacterium id.2153) on E−selectin
with a threshold of 1e−05
rs7742829
rs73128290
rs77285108
All − MR Egger
All − IVW(MRE)
−2
−1
0
1
MR effect size for
'Gut microbiota abundance (genus Catenibacterium id.2153)' on 'E−selectin'

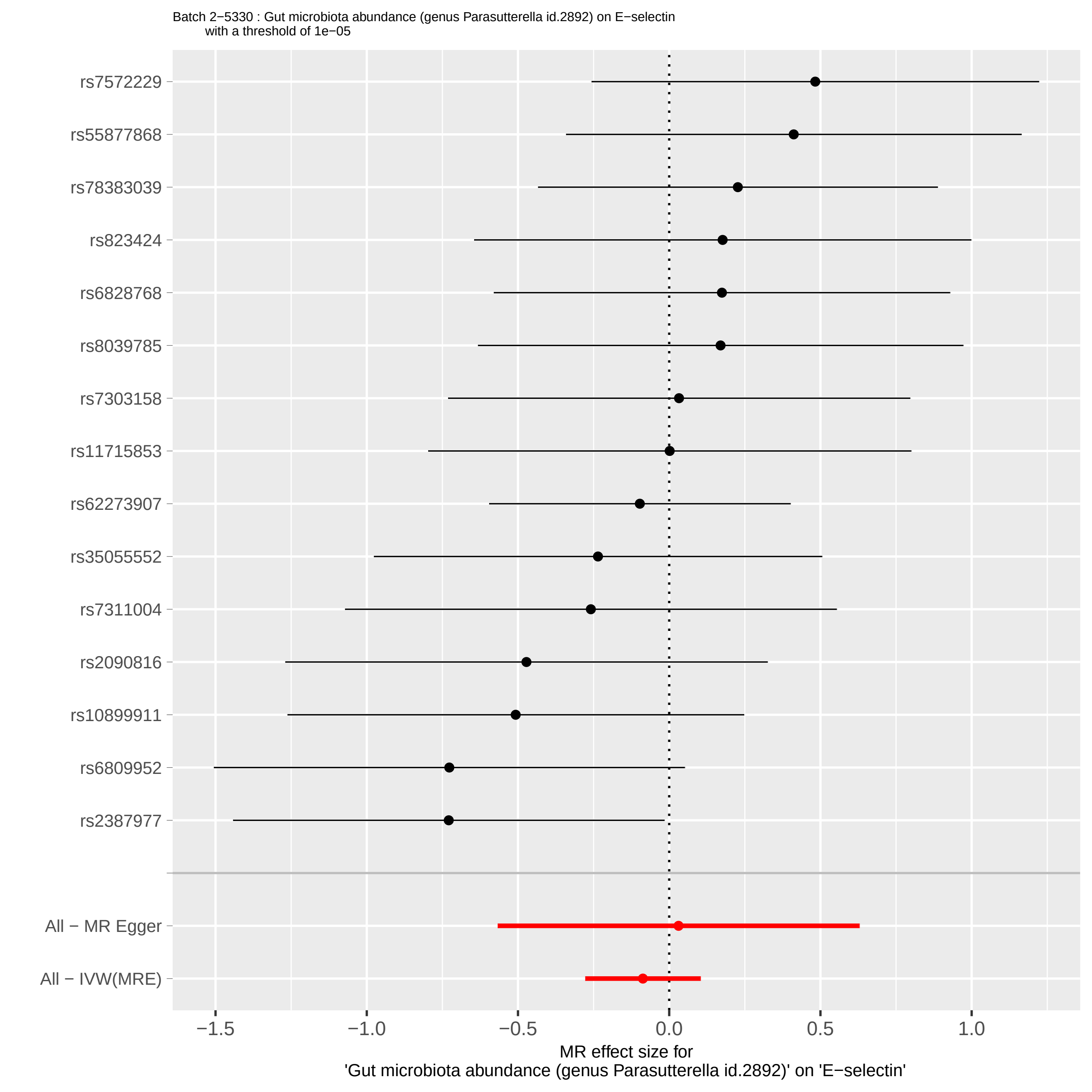
Batch 2−5330 : Gut microbiota abundance (genus Parasutterella id.2892) on E−selectin
with a threshold of 1e−05
rs7572229
rs55877868
rs78383039
rs823424
rs6828768
rs8039785
rs7303158
rs11715853
rs62273907
rs35055552
rs7311004
rs2090816
rs10899911
rs6809952
rs2387977
All − MR Egger
All − IVW(MRE)
−1.5
−1.0
−0.5
0.0
0.5
1.0
MR effect size for
'Gut microbiota abundance (genus Parasutterella id.2892)' on 'E−selectin'

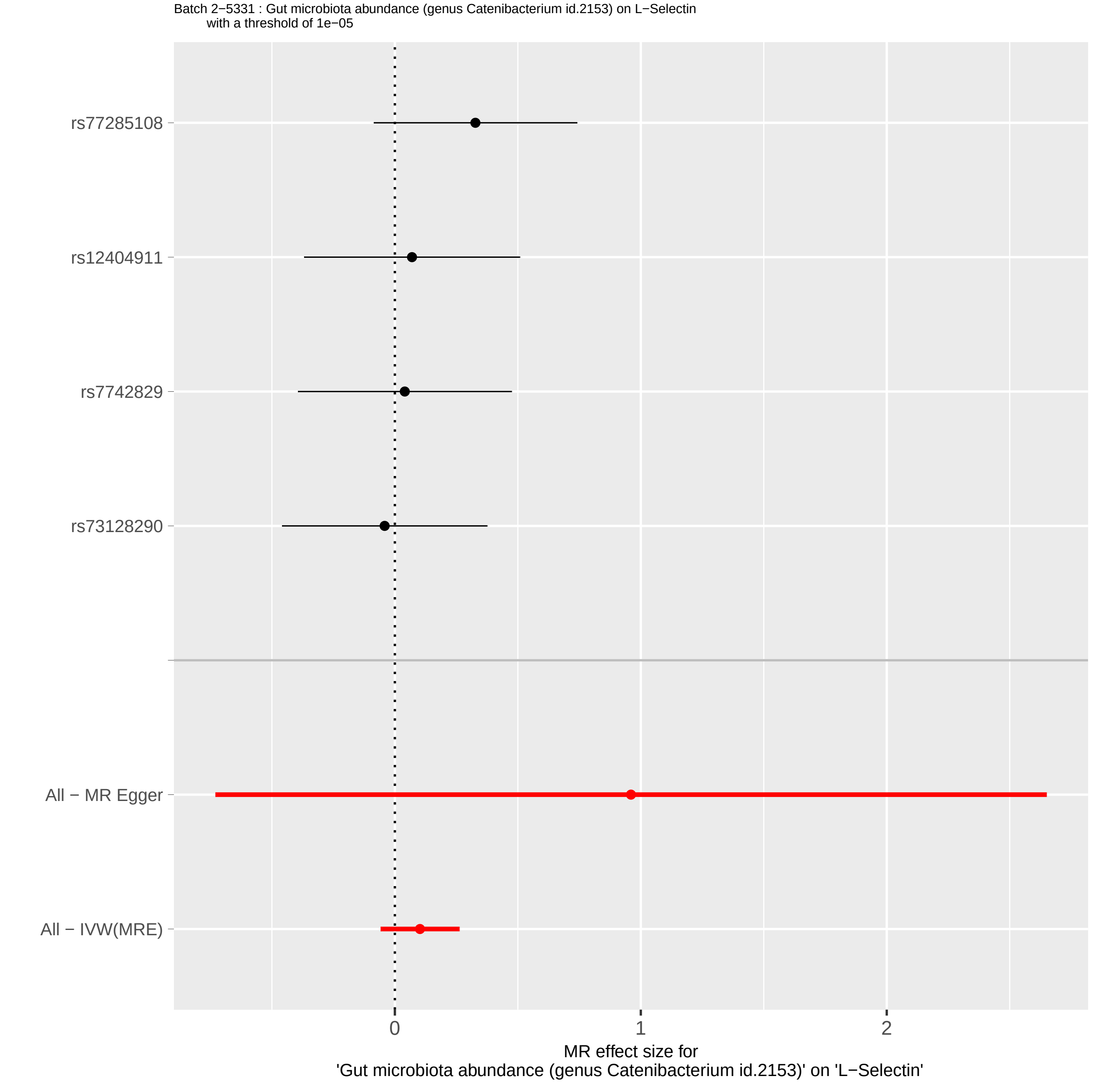

Batch 2−5331 : Gut microbiota abundance (genus Catenibacterium id.2153) on L−Selectin
with a threshold of 1e−05
rs77285108
rs12404911
rs7742829
rs73128290
All − MR Egger
All − IVW(MRE)
0
1
2
MR effect size for
'Gut microbiota abundance (genus Catenibacterium id.2153)' on 'L−Selectin'

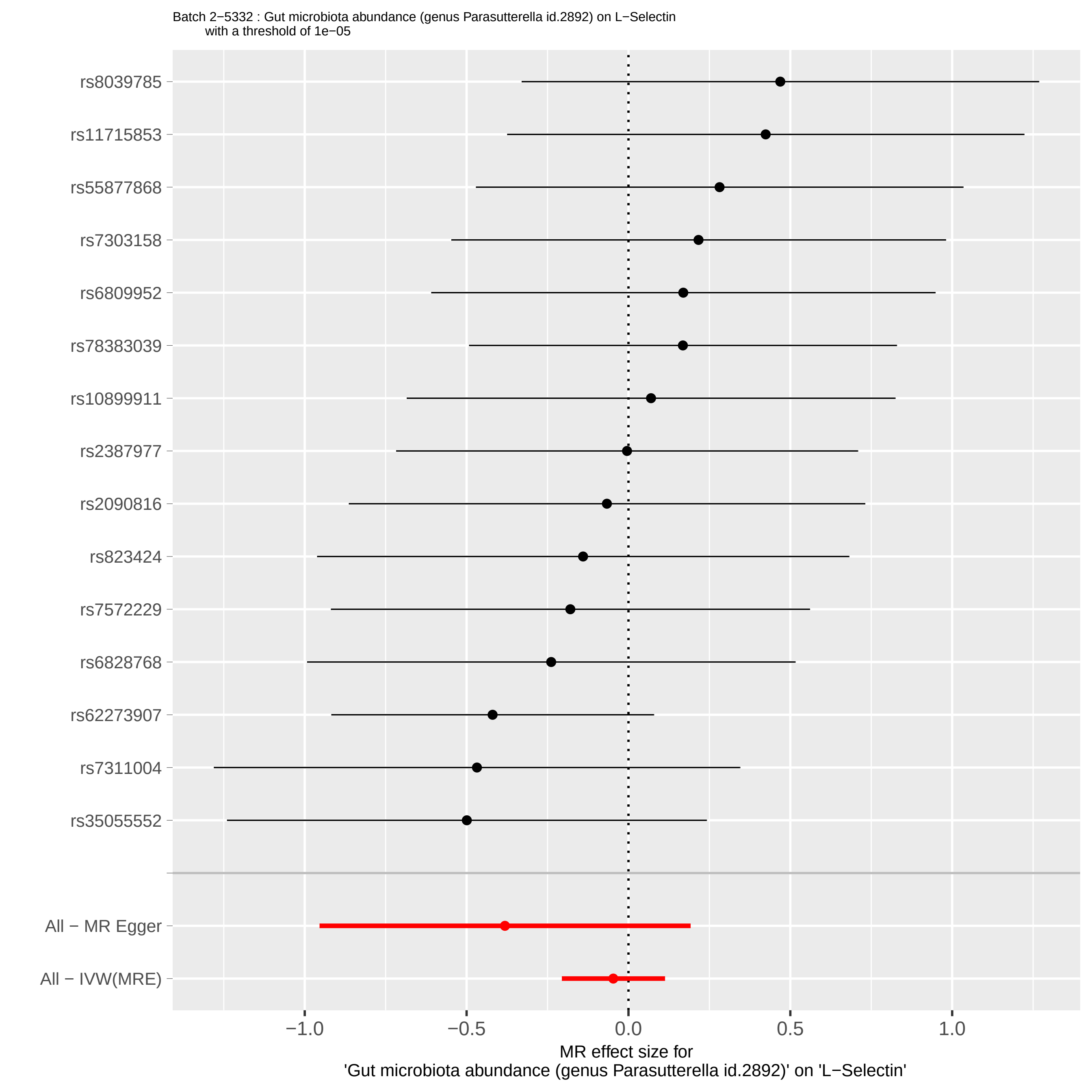

Batch 2−5332 : Gut microbiota abundance (genus Parasutterella id.2892) on L−Selectin
with a threshold of 1e−05
rs8039785
rs11715853
rs55877868
rs7303158
rs6809952
rs78383039
rs10899911
rs2387977
rs2090816
rs823424
rs7572229
rs6828768
rs62273907
rs7311004
rs35055552
All − MR Egger
All − IVW(MRE)
−1.0
−0.5
0.0
0.5
1.0
MR effect size for
'Gut microbiota abundance (genus Parasutterella id.2892)' on 'L−Selectin'

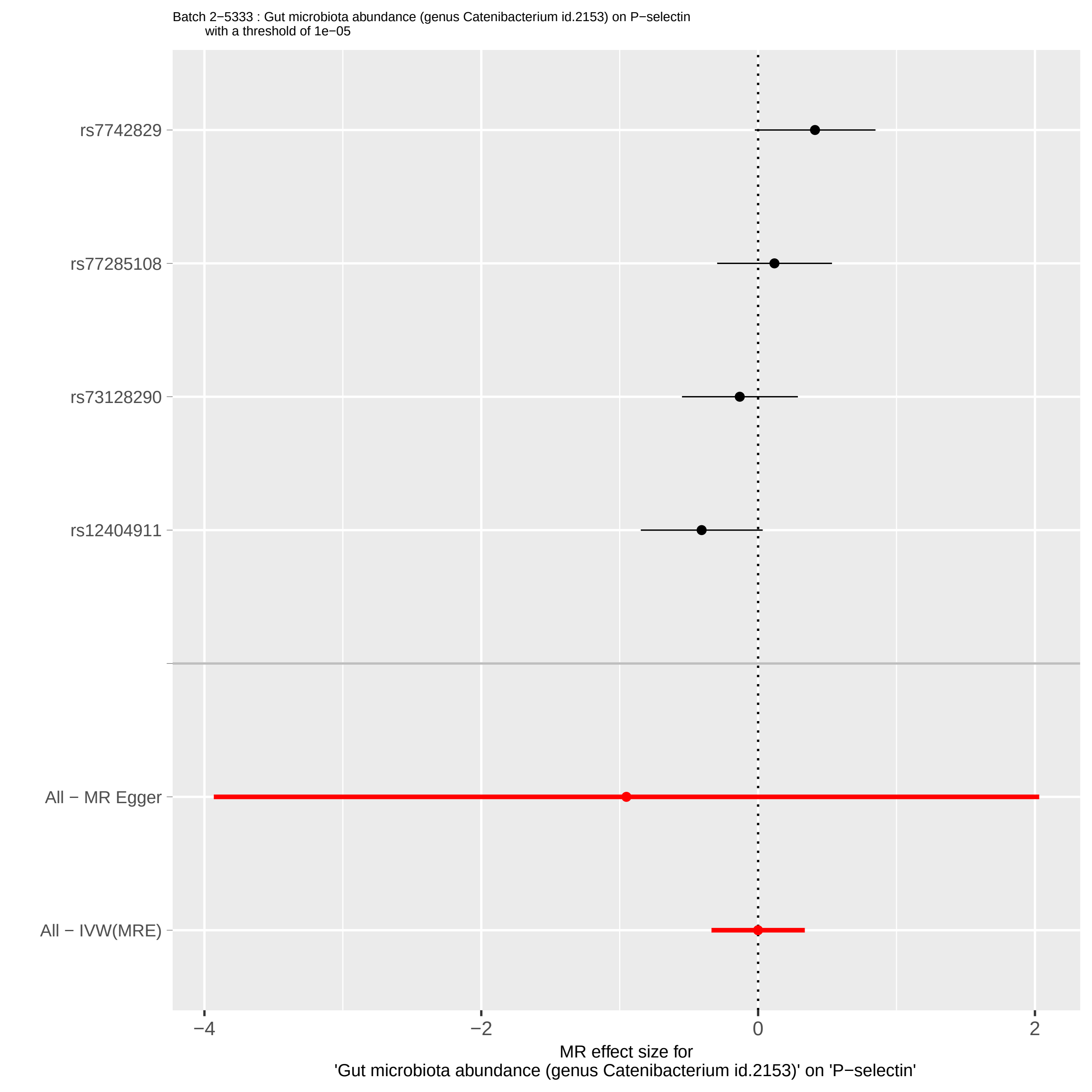

Batch 2−5333 : Gut microbiota abundance (genus Catenibacterium id.2153) on P−selectin
with a threshold of 1e−05
rs7742829
rs77285108
rs73128290
rs12404911
All − MR Egger
All − IVW(MRE)
−4
−2
0
2
MR effect size for
'Gut microbiota abundance (genus Catenibacterium id.2153)' on 'P−selectin'

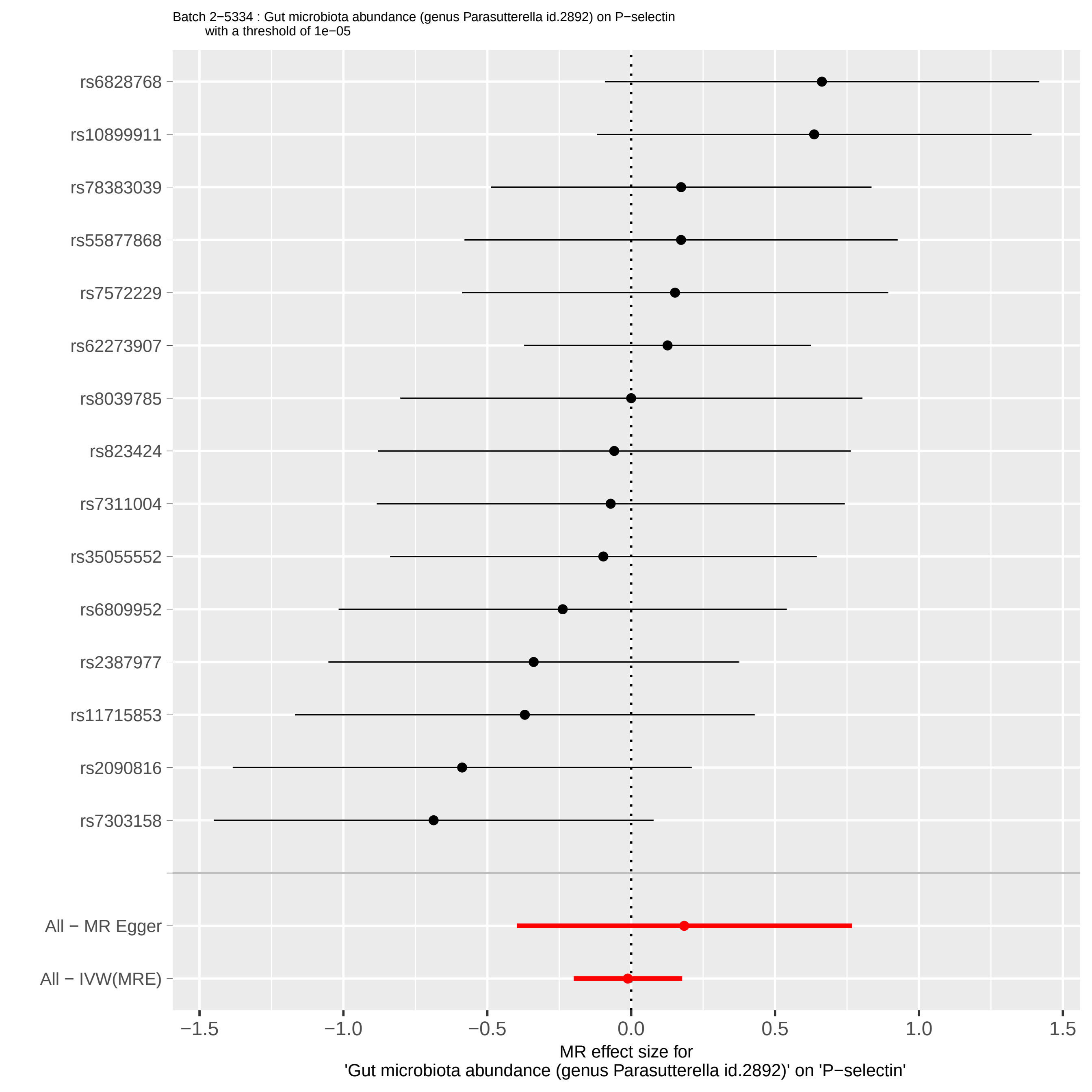
Batch 2−5334 : Gut microbiota abundance (genus Parasutterella id.2892) on P−selectin
with a threshold of 1e−05
rs6828768
rs10899911
rs78383039
rs55877868
rs7572229
rs62273907
rs8039785
rs823424
rs7311004
rs35055552
rs6809952
rs2387977
rs11715853
rs2090816
rs7303158
All − MR Egger
All − IVW(MRE)
−1.5
−1.0
−0.5
0.0
0.5
1.0
1.5
MR effect size for
'Gut microbiota abundance (genus Parasutterella id.2892)' on 'P−selectin'

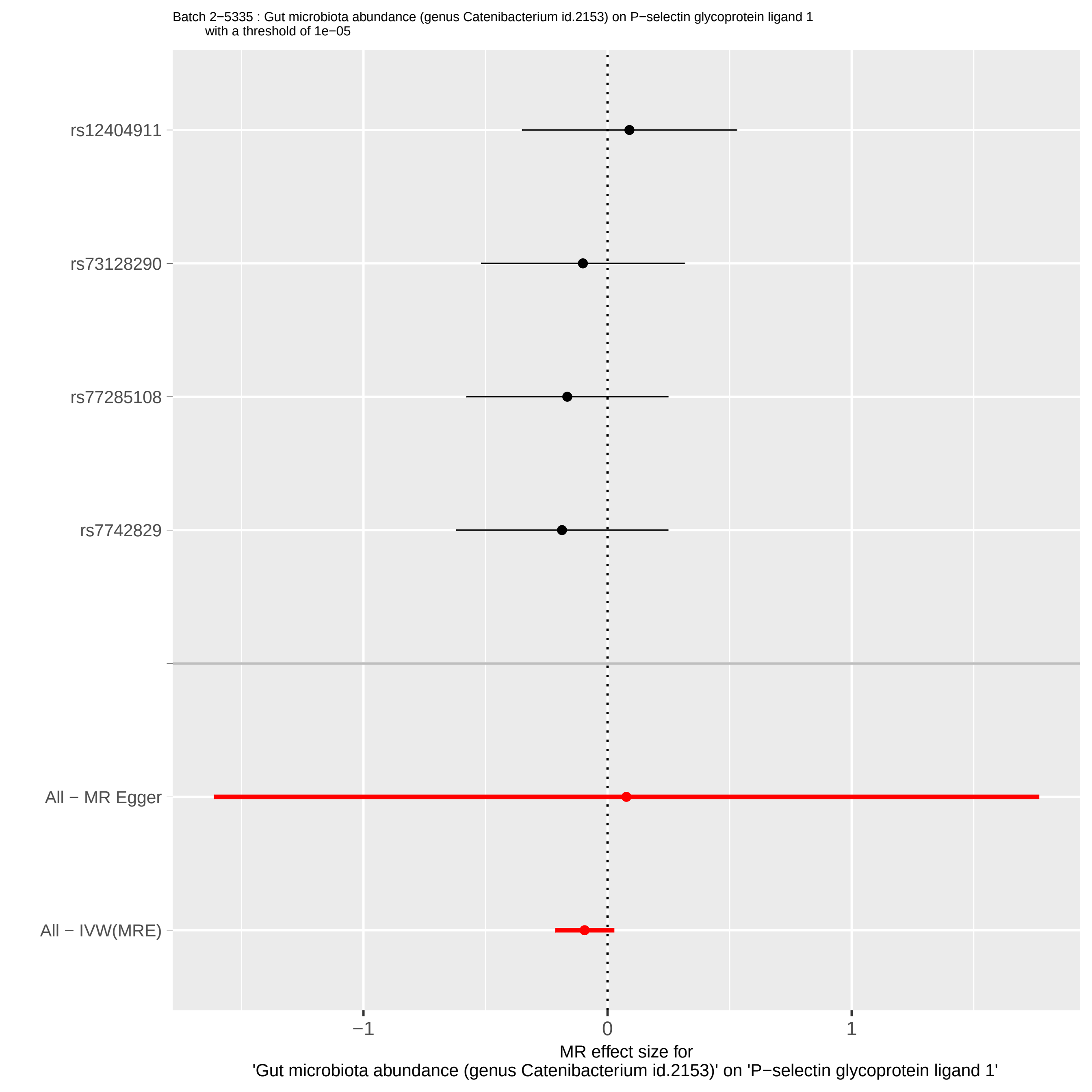

Batch 2−5335 : Gut microbiota abundance (genus Catenibacterium id.2153) on P−selectin glycoprotein ligand 1
with a threshold of 1e−05
rs12404911
rs73128290
rs77285108
rs7742829
All − MR Egger
All − IVW(MRE)
−1
0
1
MR effect size for
'Gut microbiota abundance (genus Catenibacterium id.2153)' on 'P−selectin glycoprotein ligand 1'

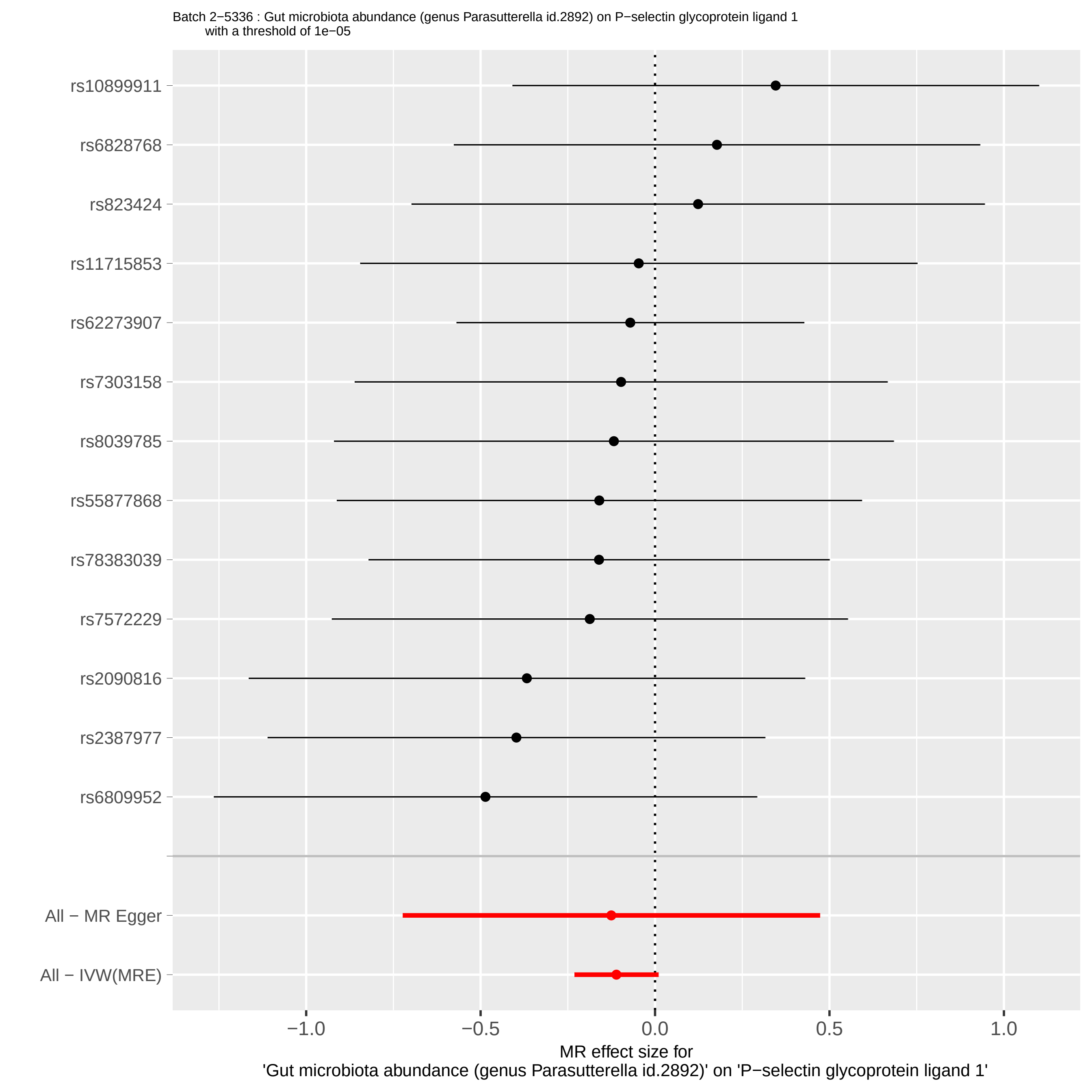

Batch 2−5336 : Gut microbiota abundance (genus Parasutterella id.2892) on P−selectin glycoprotein ligand 1
with a threshold of 1e−05
rs10899911
rs6828768
rs823424
rs11715853
rs62273907
rs7303158
rs8039785
rs55877868
rs78383039
rs7572229
rs2090816
rs2387977
rs6809952
All − MR Egger
All − IVW(MRE)
−1.0
−0.5
0.0
0.5
1.0
MR effect size for
'Gut microbiota abundance (genus Parasutterella id.2892)' on 'P−selectin glycoprotein ligand 1'

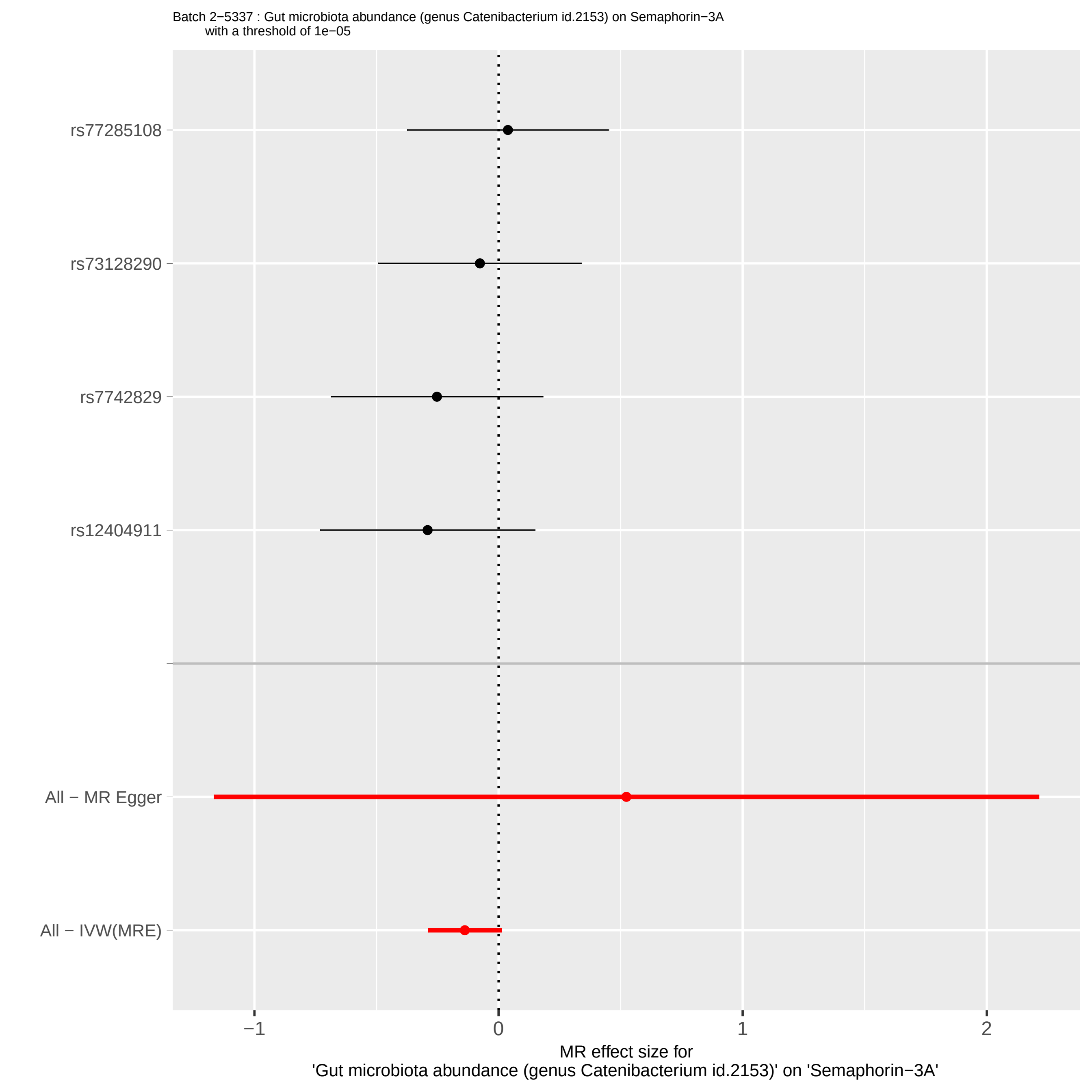
Batch 2−5337 : Gut microbiota abundance (genus Catenibacterium id.2153) on Semaphorin−3A
with a threshold of 1e−05
rs77285108
rs73128290
rs7742829
rs12404911
All − MR Egger
All − IVW(MRE)
−1
0
1
2
MR effect size for
'Gut microbiota abundance (genus Catenibacterium id.2153)' on 'Semaphorin−3A'

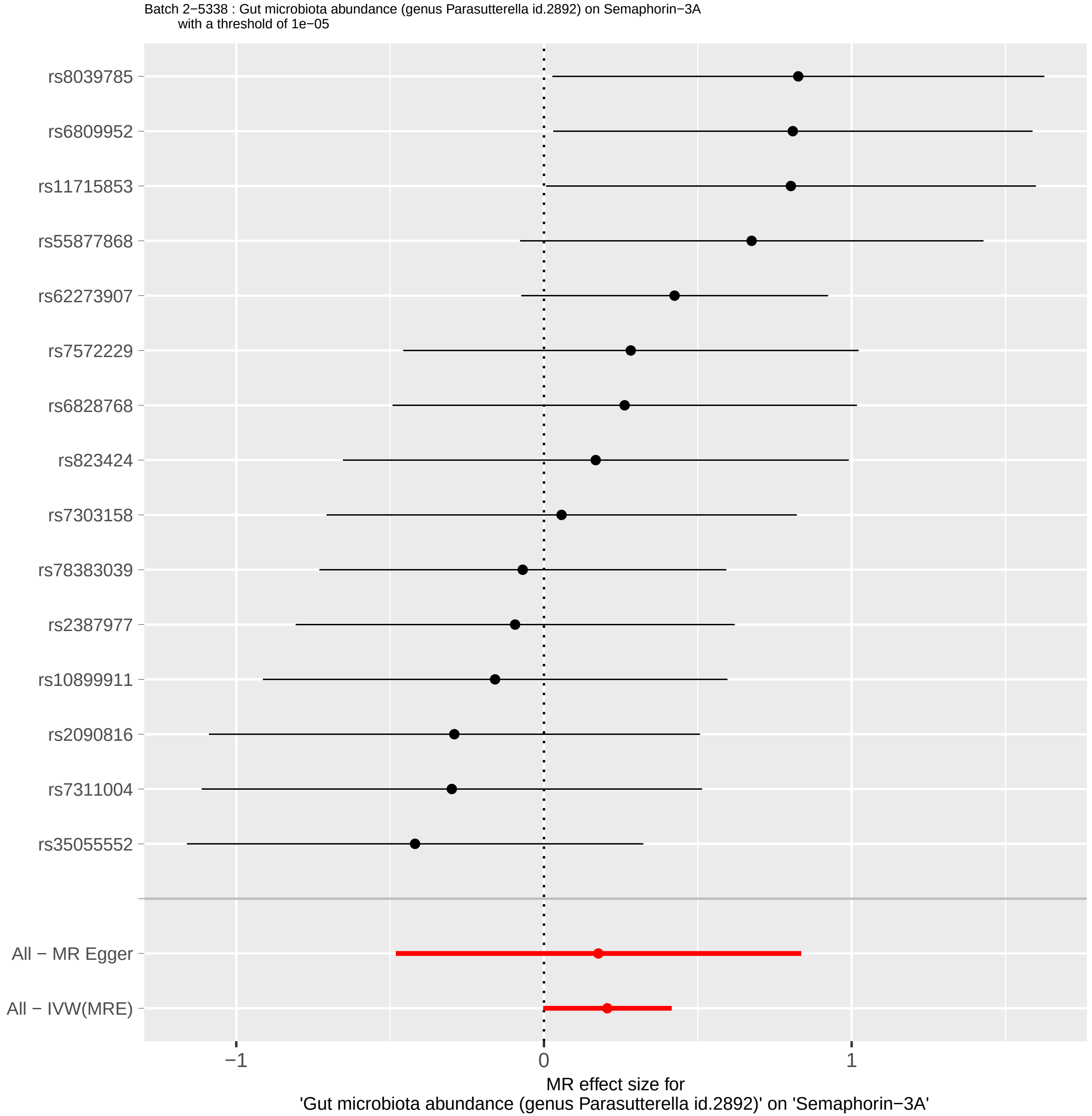

Batch 2−5338 : Gut microbiota abundance (genus Parasutterella id.2892) on Semaphorin−3A
with a threshold of 1e−05
rs8039785
rs6809952
rs11715853
rs55877868
rs62273907
rs7572229
rs6828768
rs823424
rs7303158
rs78383039
rs2387977
rs10899911
rs2090816
rs7311004
rs35055552
All − MR Egger
All − IVW(MRE)
−1
0
1
MR effect size for
'Gut microbiota abundance (genus Parasutterella id.2892)' on 'Semaphorin−3A'

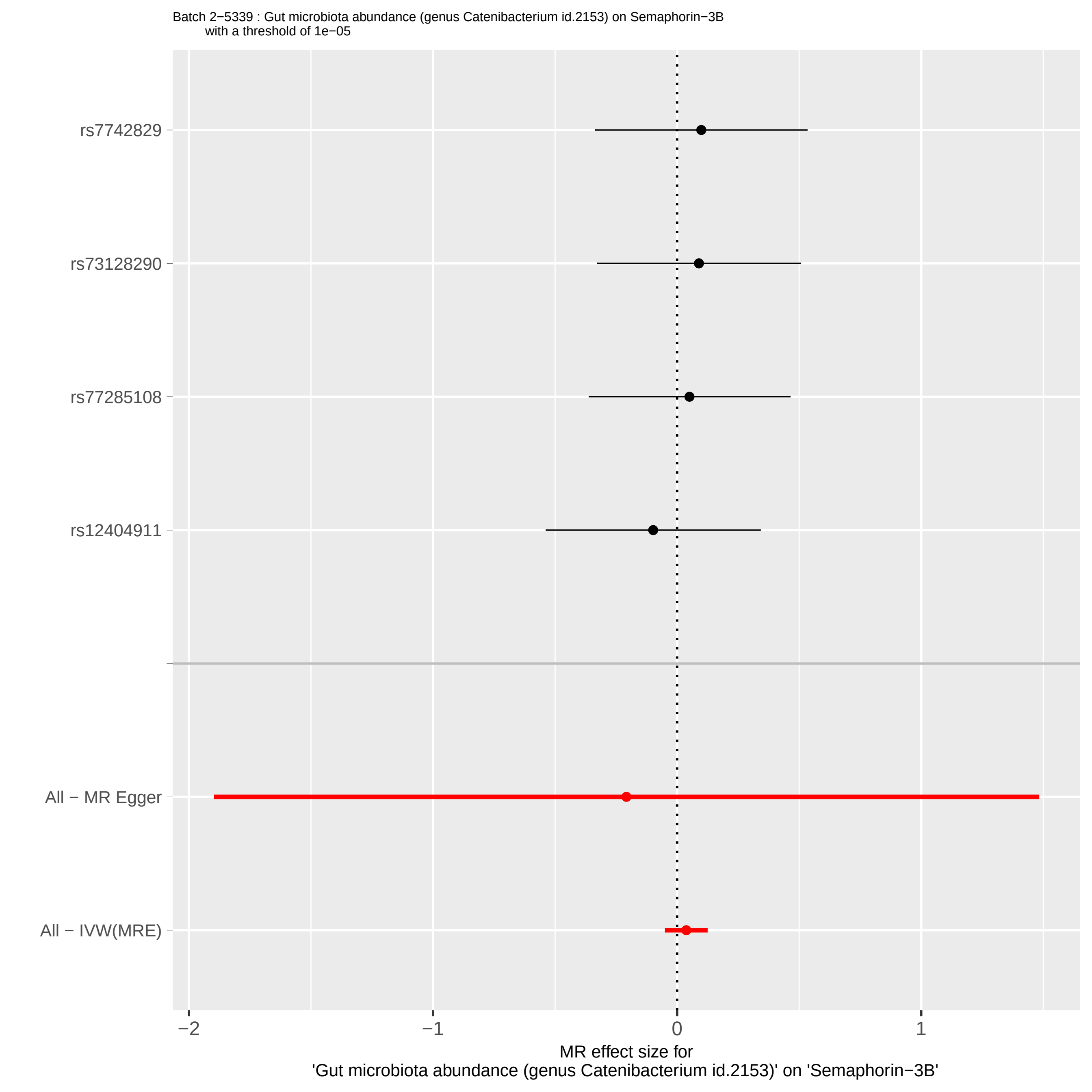
Batch 2−5339 : Gut microbiota abundance (genus Catenibacterium id.2153) on Semaphorin−3B
with a threshold of 1e−05
rs7742829
rs73128290
rs77285108
rs12404911
All − MR Egger
All − IVW(MRE)
−2
−1
0
1
MR effect size for
'Gut microbiota abundance (genus Catenibacterium id.2153)' on 'Semaphorin−3B'

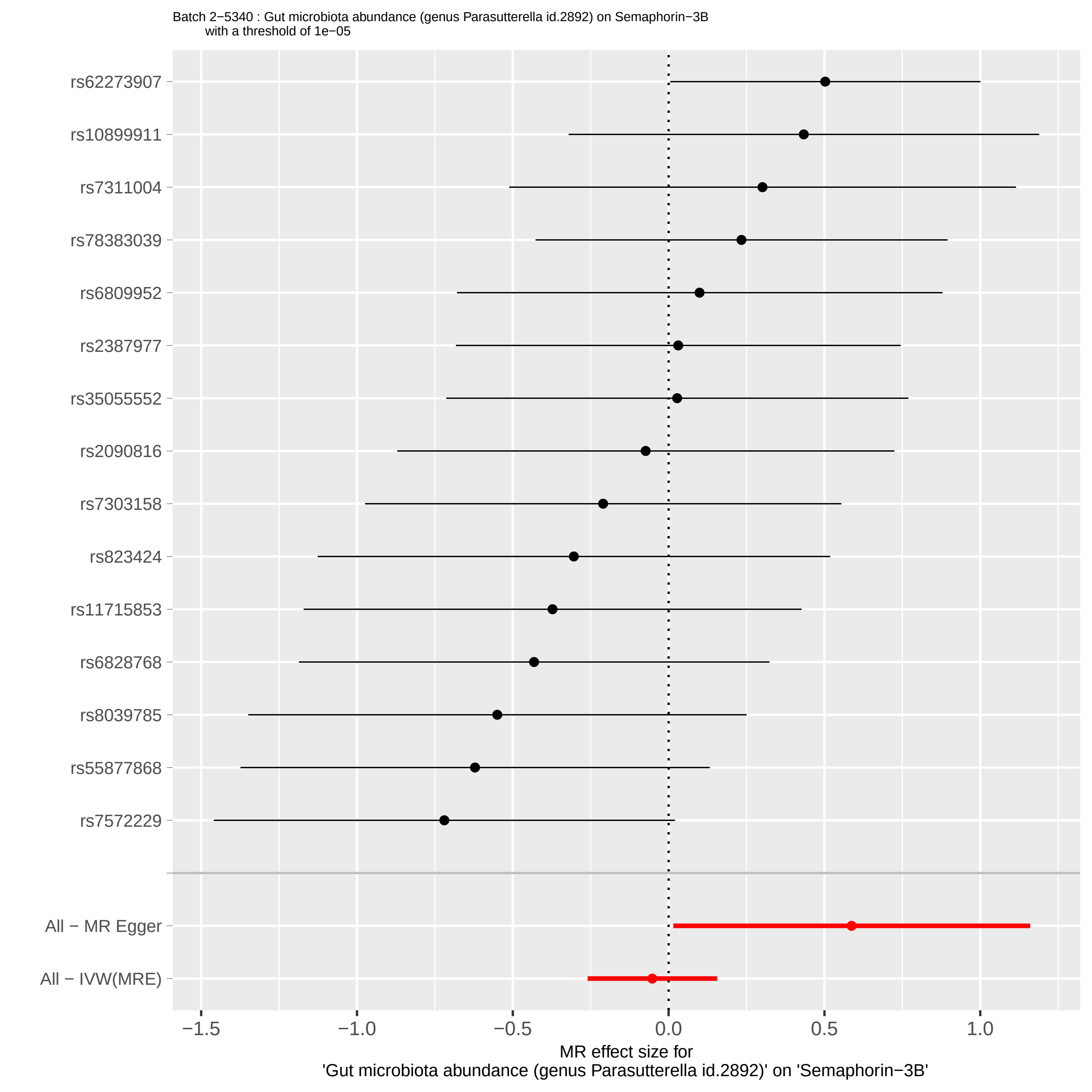

Batch 2−5340 : Gut microbiota abundance (genus Parasutterella id.2892) on Semaphorin−3B
with a threshold of 1e−05
rs62273907
rs10899911
rs7311004
rs78383039
rs6809952
rs2387977
rs35055552
rs2090816
rs7303158
rs823424
rs11715853
rs6828768
rs8039785
rs55877868
rs7572229
All − MR Egger
All − IVW(MRE)
−1.5
−1.0
−0.5
0.0
0.5
1.0
MR effect size for
'Gut microbiota abundance (genus Parasutterella id.2892)' on 'Semaphorin−3B'

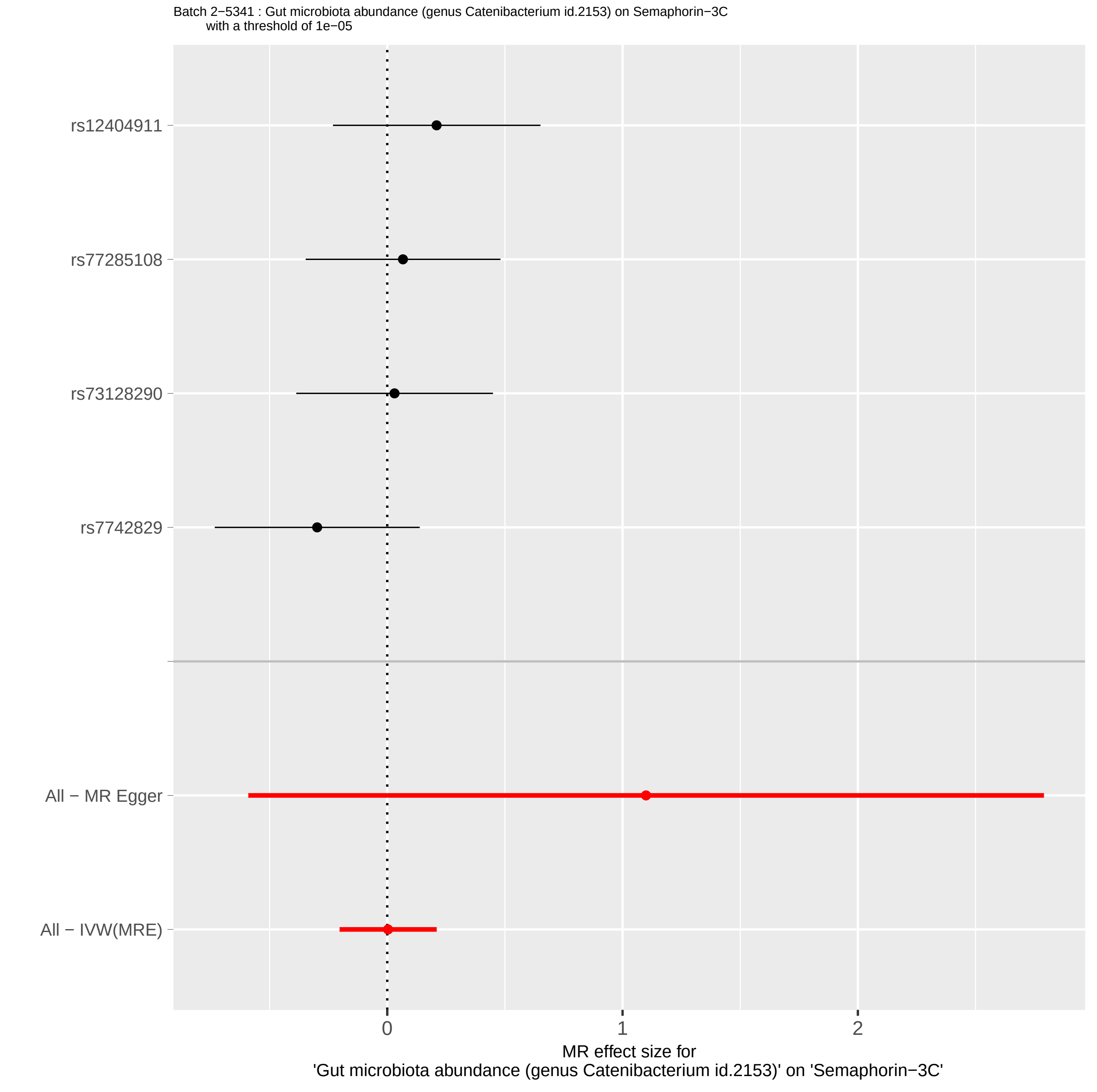

Batch 2−5341 : Gut microbiota abundance (genus Catenibacterium id.2153) on Semaphorin−3C
with a threshold of 1e−05
rs12404911
rs77285108
rs73128290
rs7742829
All − MR Egger
All − IVW(MRE)
0
1
2
MR effect size for
'Gut microbiota abundance (genus Catenibacterium id.2153)' on 'Semaphorin−3C'

Batch 2−5342 : Gut microbiota abundance (genus Parasutterella id.2892) on Semaphorin−3C
with a threshold of 1e−05

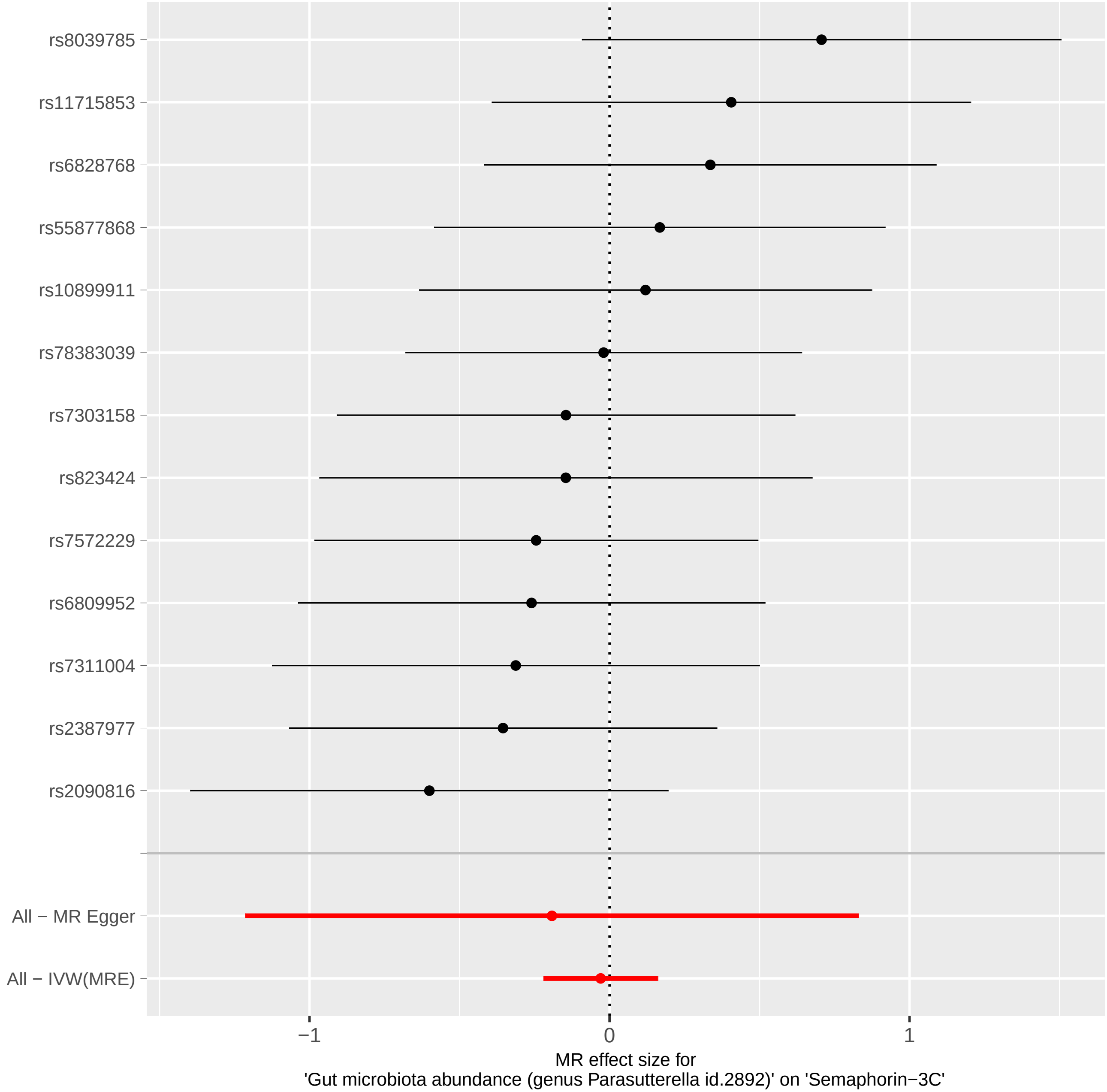

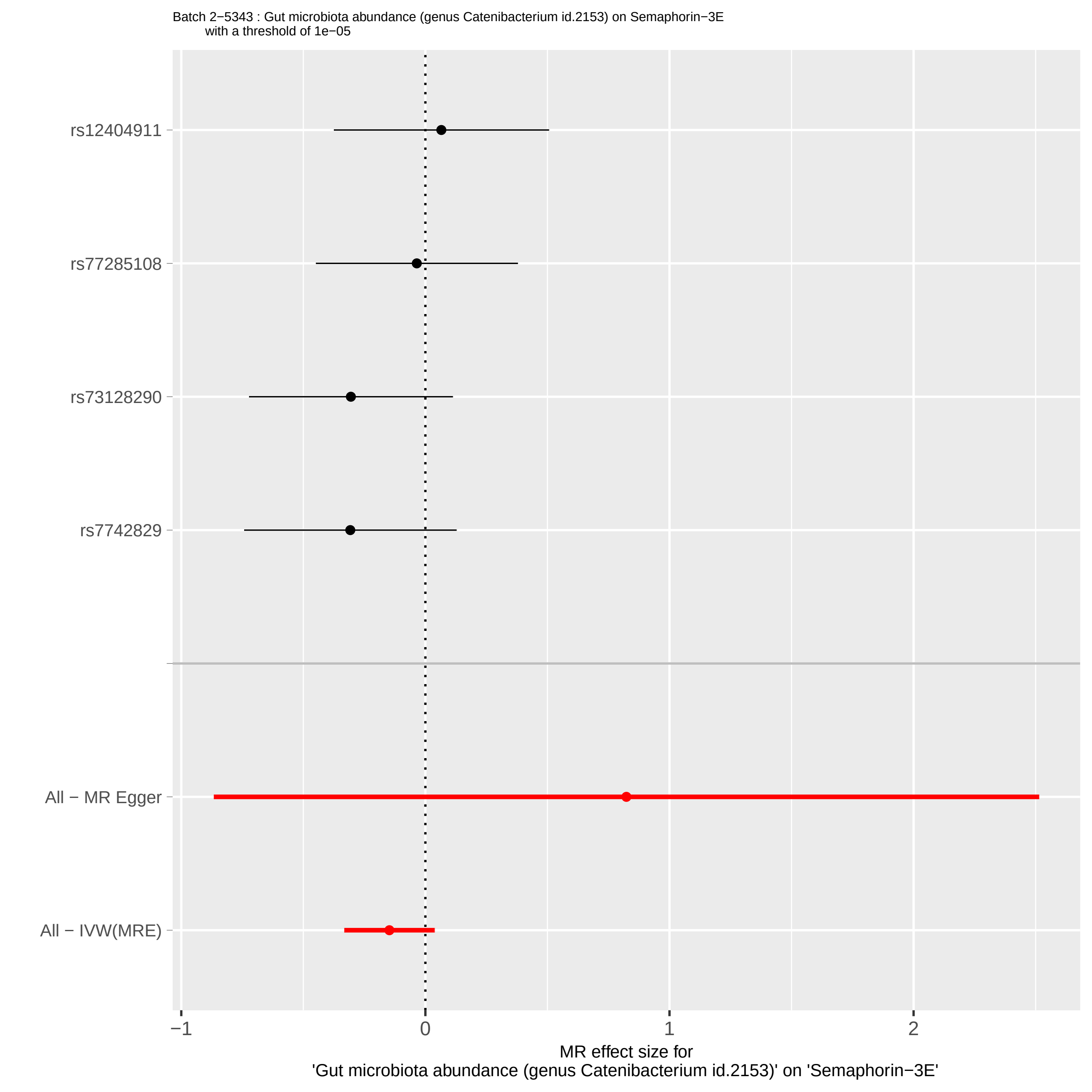

Batch 2−5343 : Gut microbiota abundance (genus Catenibacterium id.2153) on Semaphorin−3E
with a threshold of 1e−05
rs12404911
rs77285108
rs73128290
rs7742829
All − MR Egger
All − IVW(MRE)
−1
0
1
2
MR effect size for
'Gut microbiota abundance (genus Catenibacterium id.2153)' on 'Semaphorin−3E'

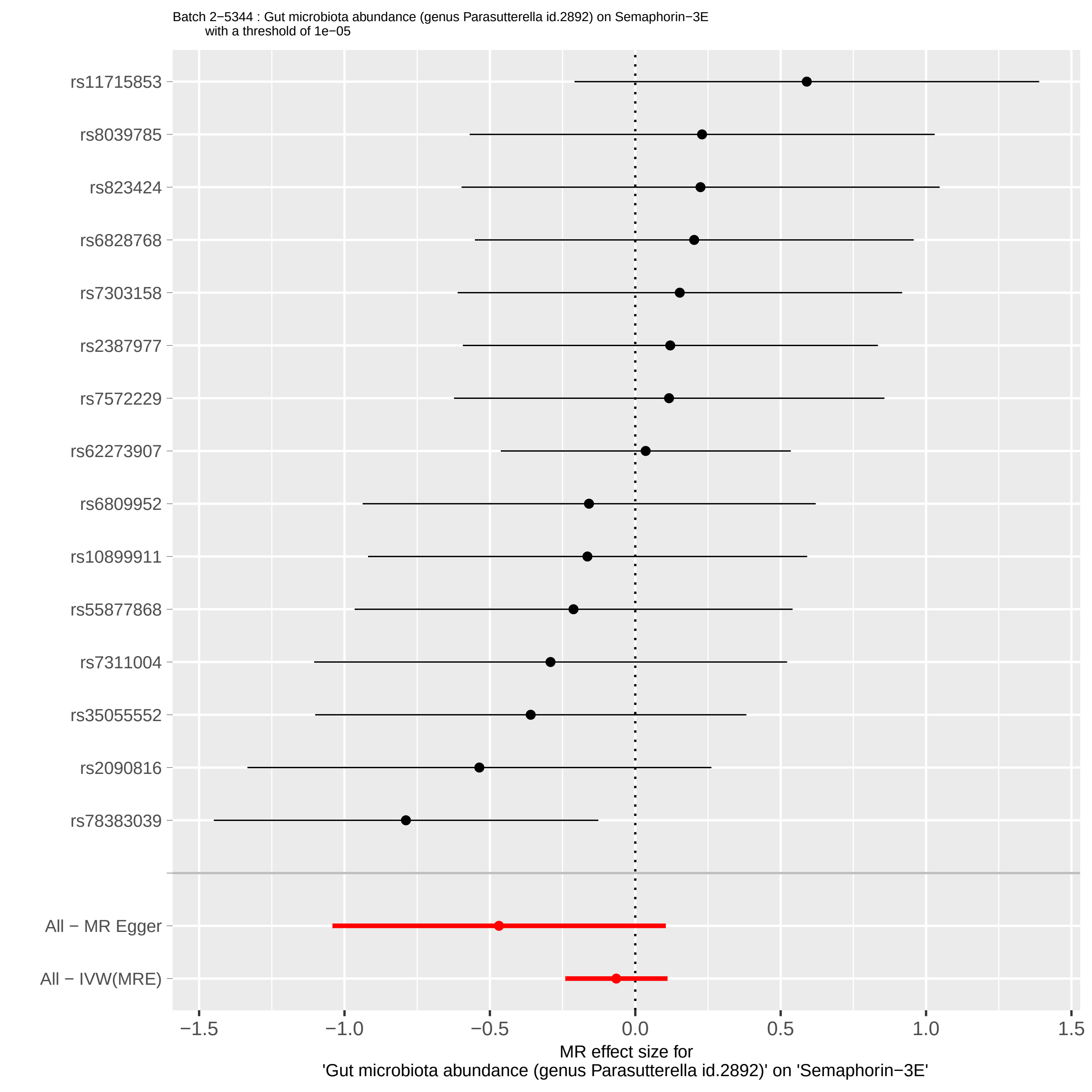

Batch 2−5344 : Gut microbiota abundance (genus Parasutterella id.2892) on Semaphorin−3E
with a threshold of 1e−05
rs11715853
rs8039785
rs823424
rs6828768
rs7303158
rs2387977
rs7572229
rs62273907
rs6809952
rs10899911
rs55877868
rs7311004
rs35055552
rs2090816
rs78383039
All − MR Egger
All − IVW(MRE)
−1.5
−1.0
−0.5
0.0
0.5
1.0
1.5
MR effect size for
'Gut microbiota abundance (genus Parasutterella id.2892)' on 'Semaphorin−3E'

Batch 2−5345 : Gut microbiota abundance (genus Catenibacterium id.2153) on Semaphorin−3G
with a threshold of 1e−05

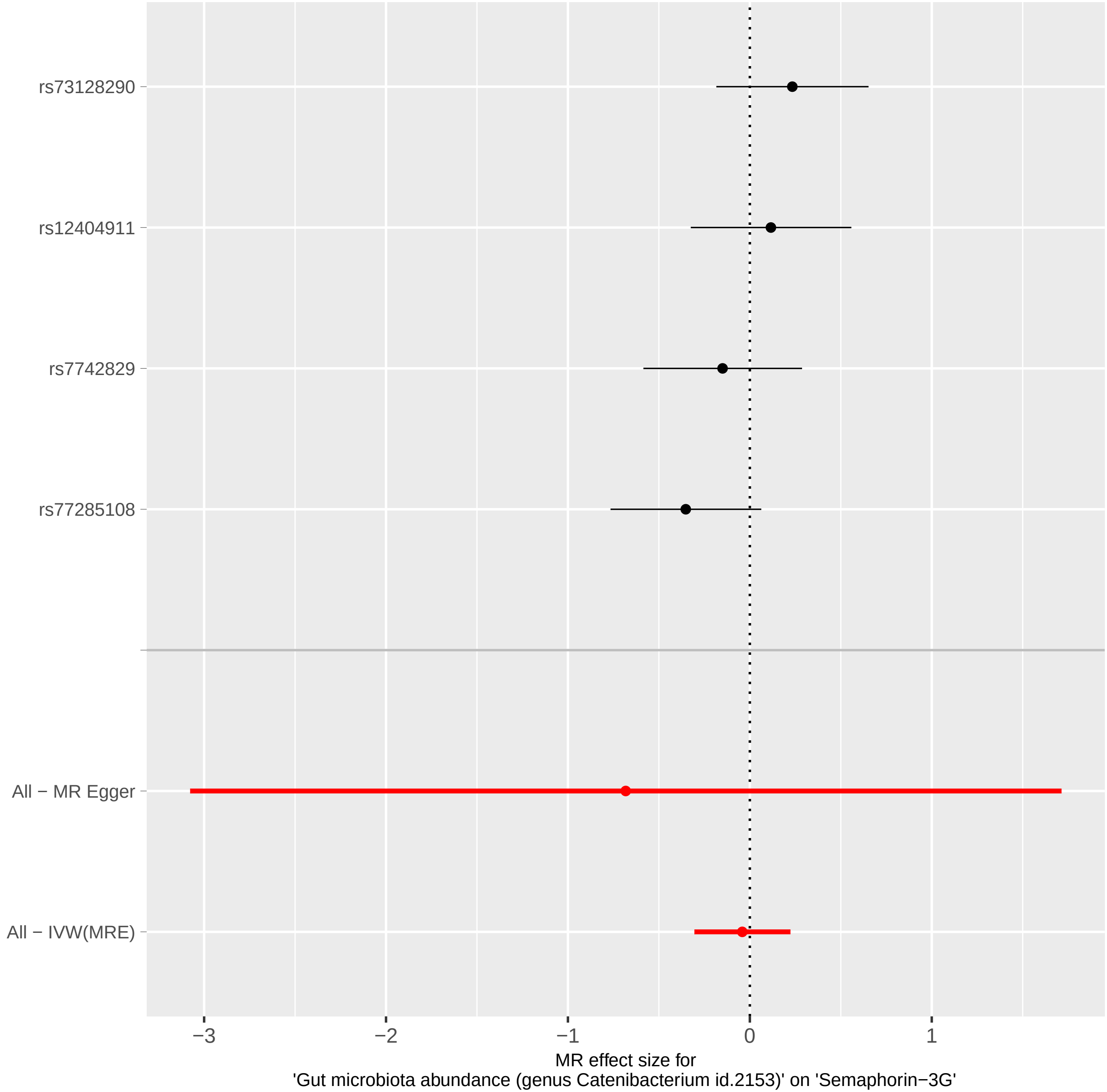

Batch 2−5346 : Gut microbiota abundance (genus Parasutterella id.2892) on Semaphorin−3G
with a threshold of 1e−05

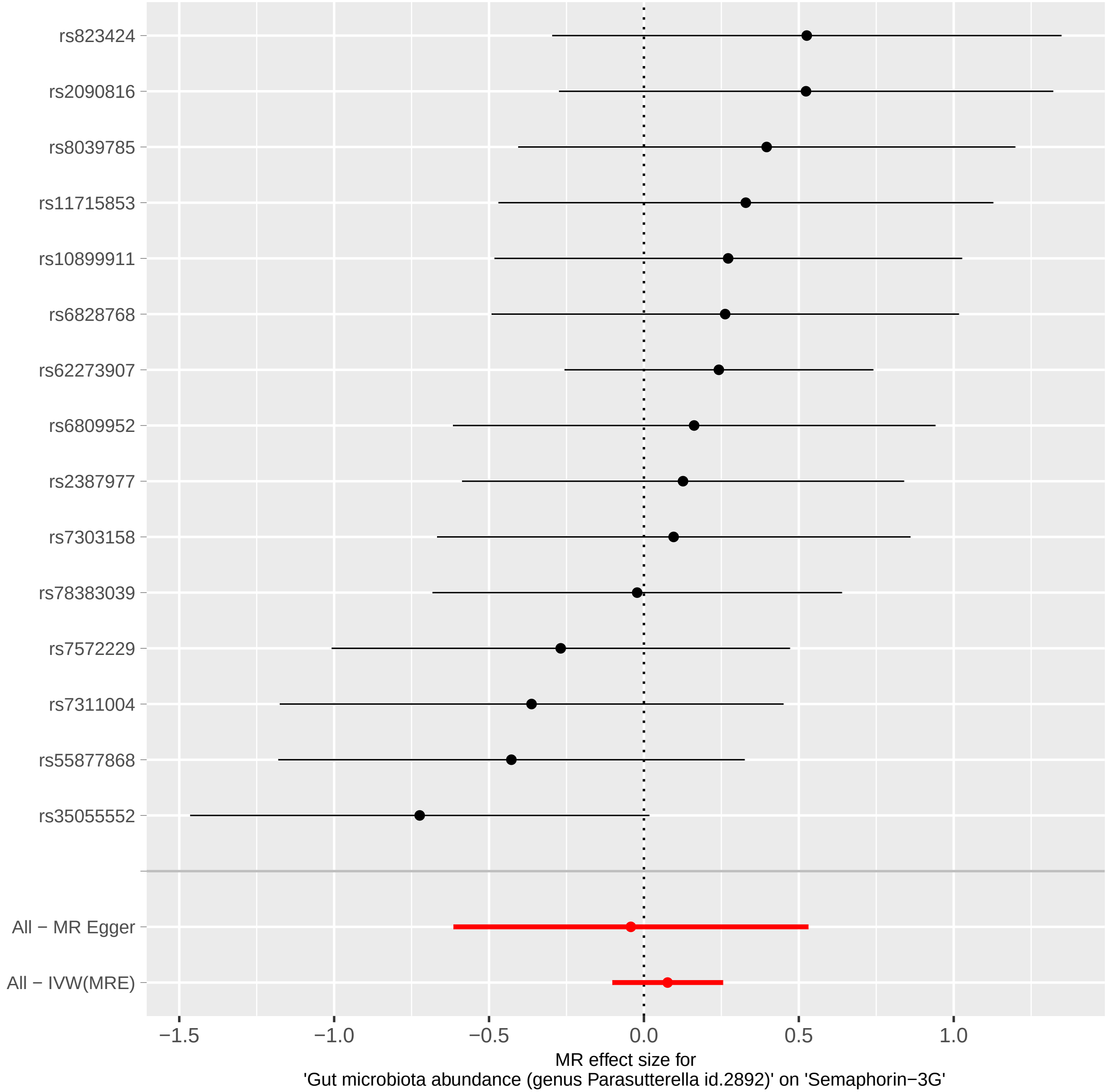

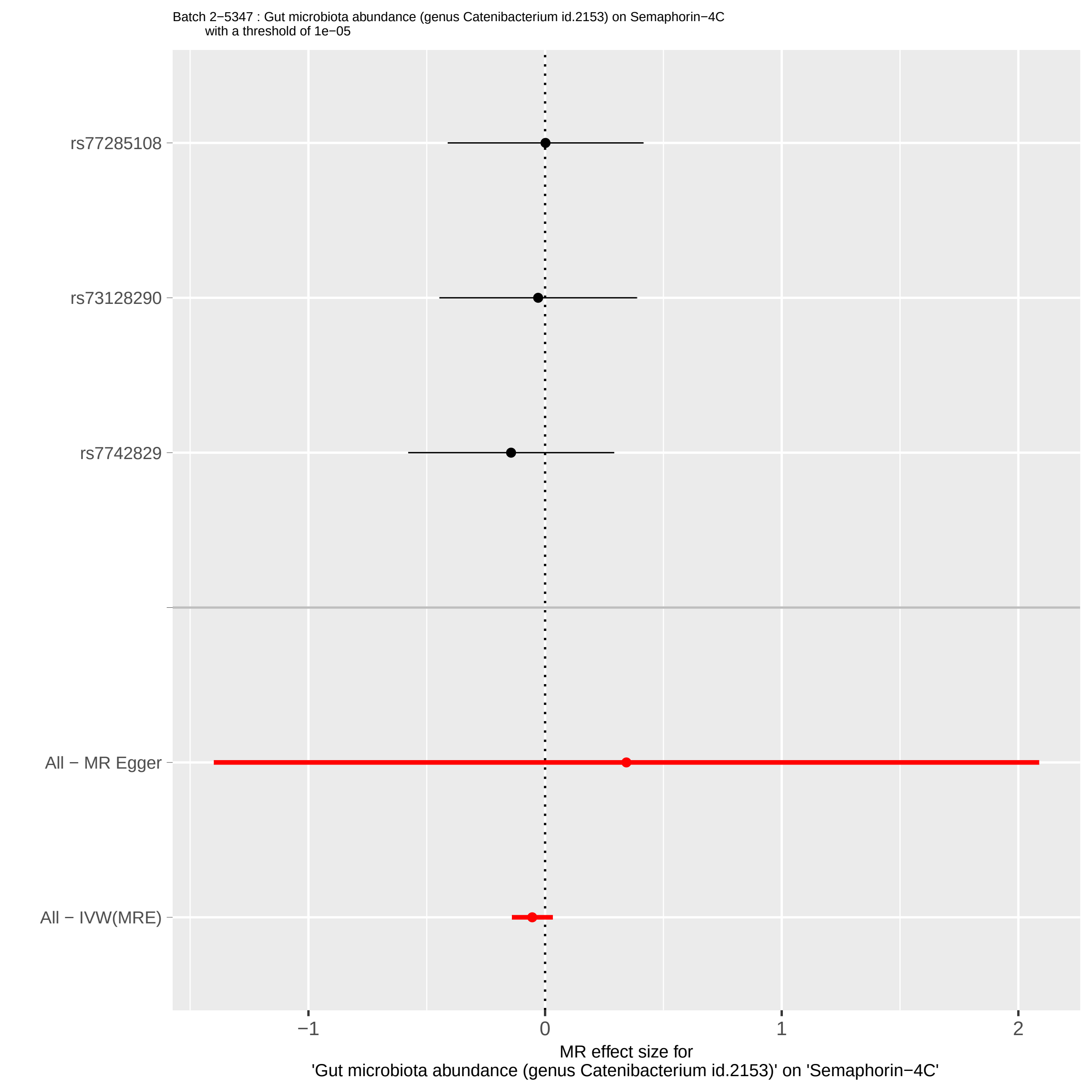
Batch 2−5347 : Gut microbiota abundance (genus Catenibacterium id.2153) on Semaphorin−4C
with a threshold of 1e−05
rs77285108
rs73128290
rs7742829
All − MR Egger
All − IVW(MRE)
−1
0
1
2
MR effect size for
'Gut microbiota abundance (genus Catenibacterium id.2153)' on 'Semaphorin−4C'

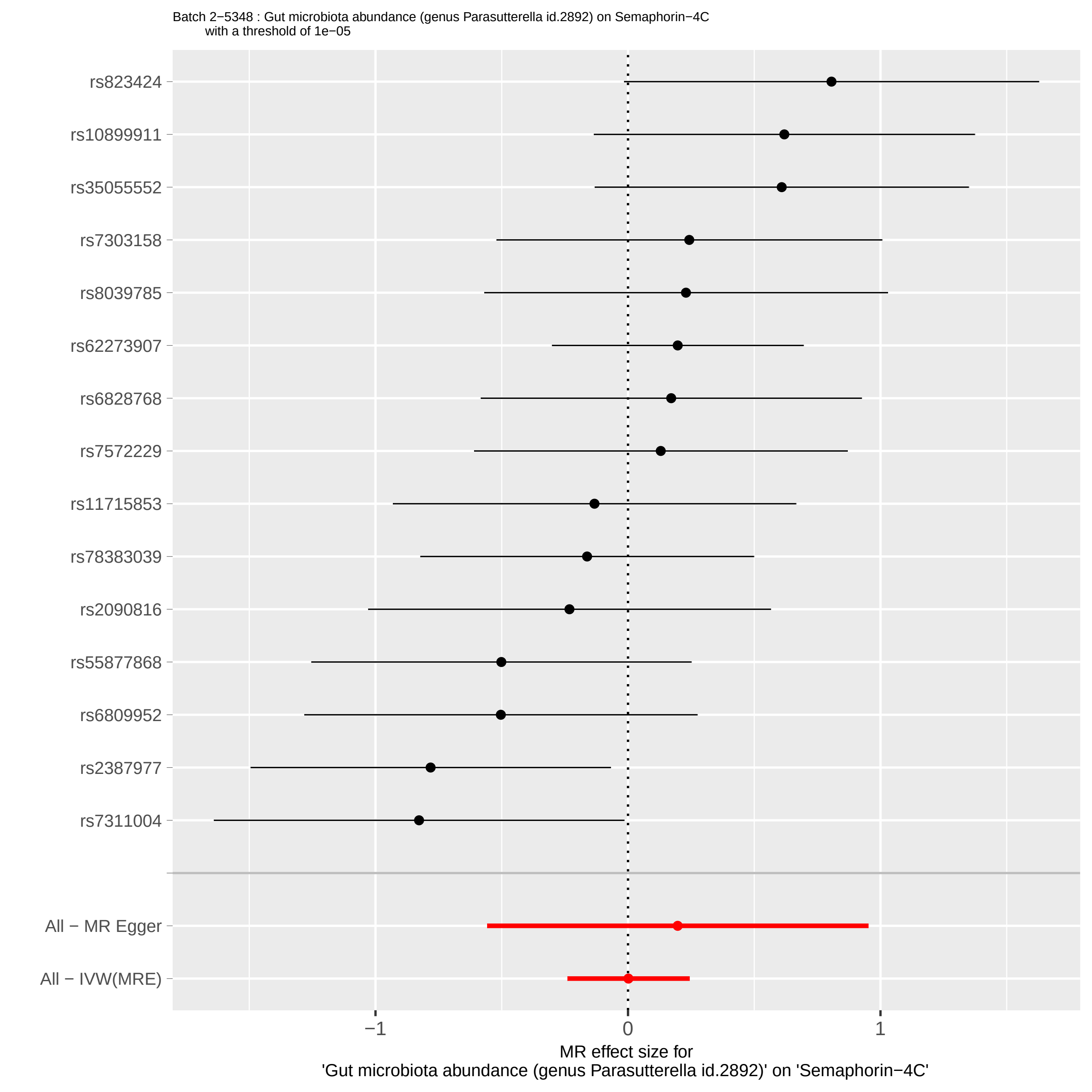
Batch 2−5348 : Gut microbiota abundance (genus Parasutterella id.2892) on Semaphorin−4C
with a threshold of 1e−05
rs823424
rs10899911
rs35055552
rs7303158
rs8039785
rs62273907
rs6828768
rs7572229
rs11715853
rs78383039
rs2090816
rs55877868
rs6809952
rs2387977
rs7311004
All − MR Egger
All − IVW(MRE)
−1
0
1
MR effect size for
'Gut microbiota abundance (genus Parasutterella id.2892)' on 'Semaphorin−4C'

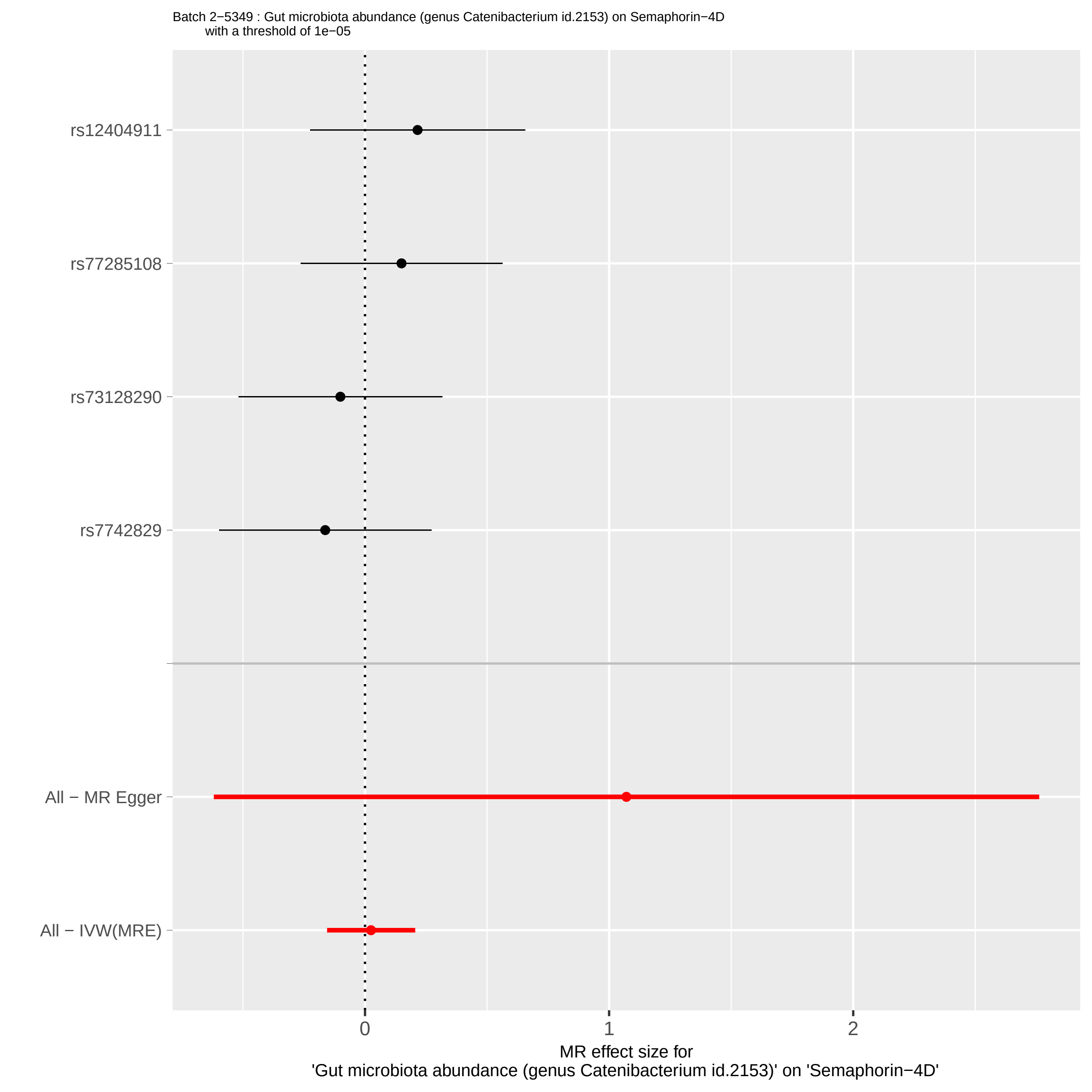

Batch 2−5349 : Gut microbiota abundance (genus Catenibacterium id.2153) on Semaphorin−4D
with a threshold of 1e−05
rs12404911
rs77285108
rs73128290
rs7742829
All − MR Egger
All − IVW(MRE)
0
1
2
MR effect size for
'Gut microbiota abundance (genus Catenibacterium id.2153)' on 'Semaphorin−4D'

Batch 2−5350 : Gut microbiota abundance (genus Parasutterella id.2892) on Semaphorin−4D
with a threshold of 1e−05

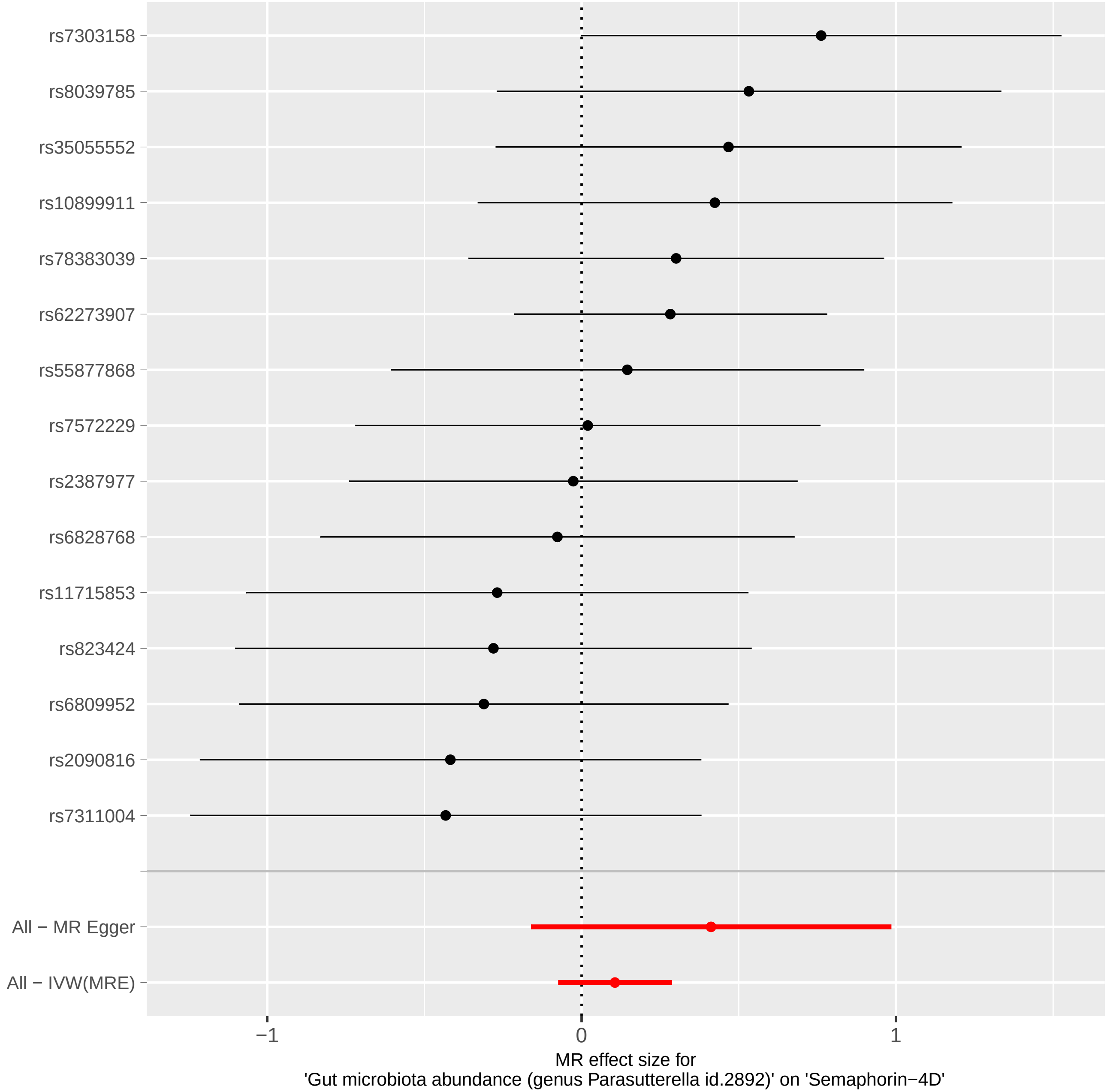

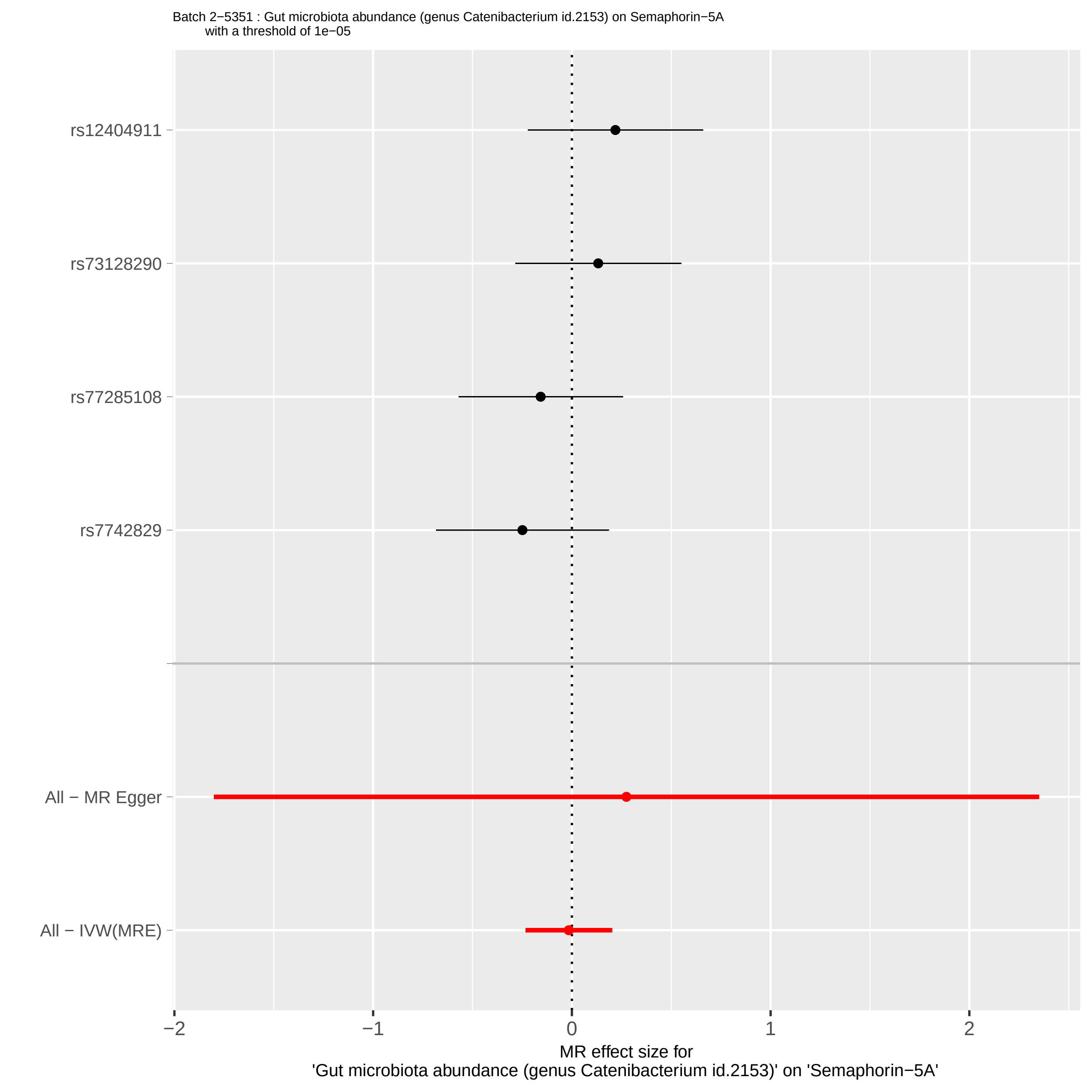

Batch 2−5351 : Gut microbiota abundance (genus Catenibacterium id.2153) on Semaphorin−5A
with a threshold of 1e−05
rs12404911
rs73128290
rs77285108
rs7742829
All − MR Egger
All − IVW(MRE)
−2
−1
0
1
2
MR effect size for
'Gut microbiota abundance (genus Catenibacterium id.2153)' on 'Semaphorin−5A'

Batch 2−5352 : Gut microbiota abundance (genus Parasutterella id.2892) on Semaphorin−5A
with a threshold of 1e−05

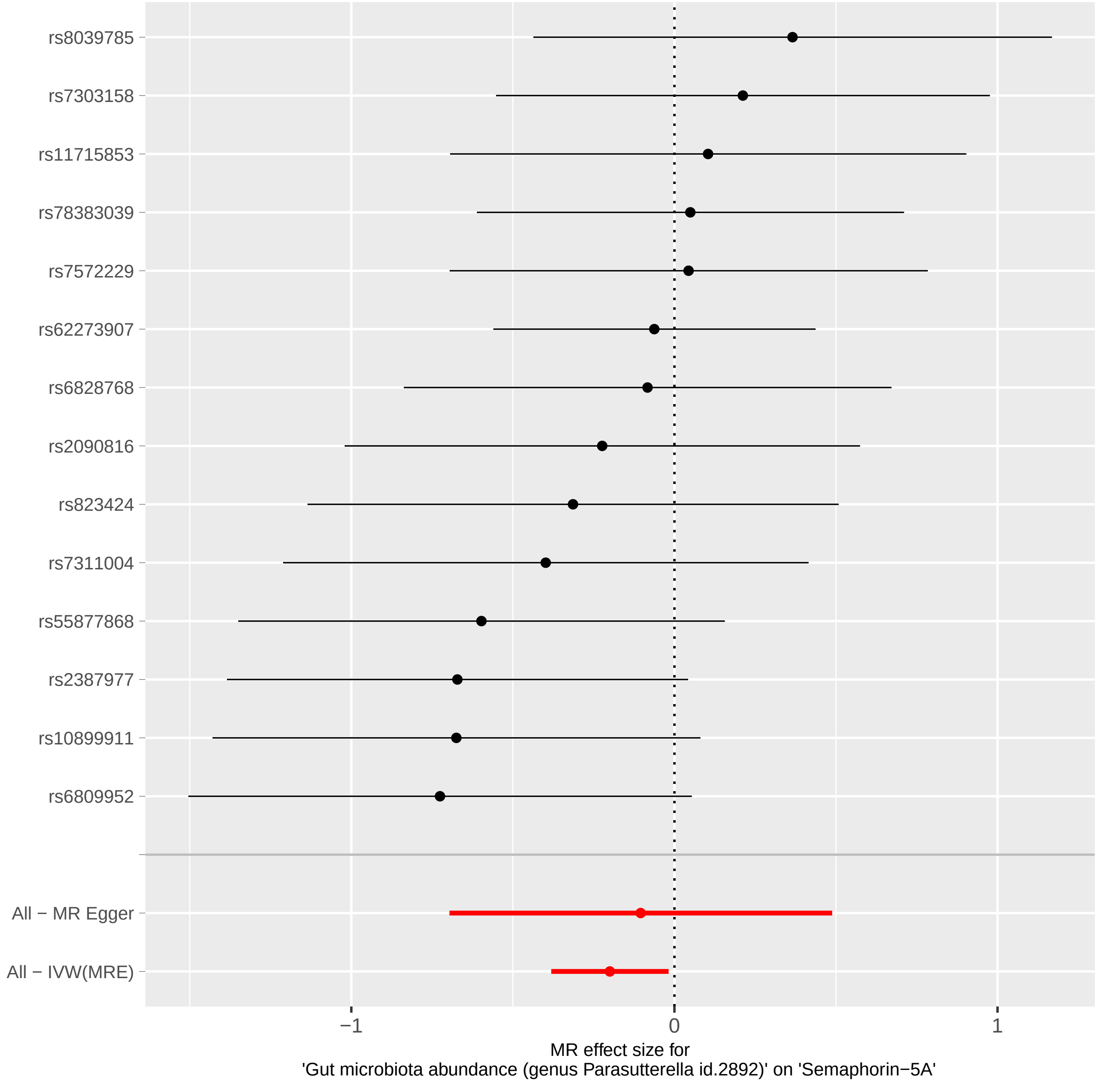

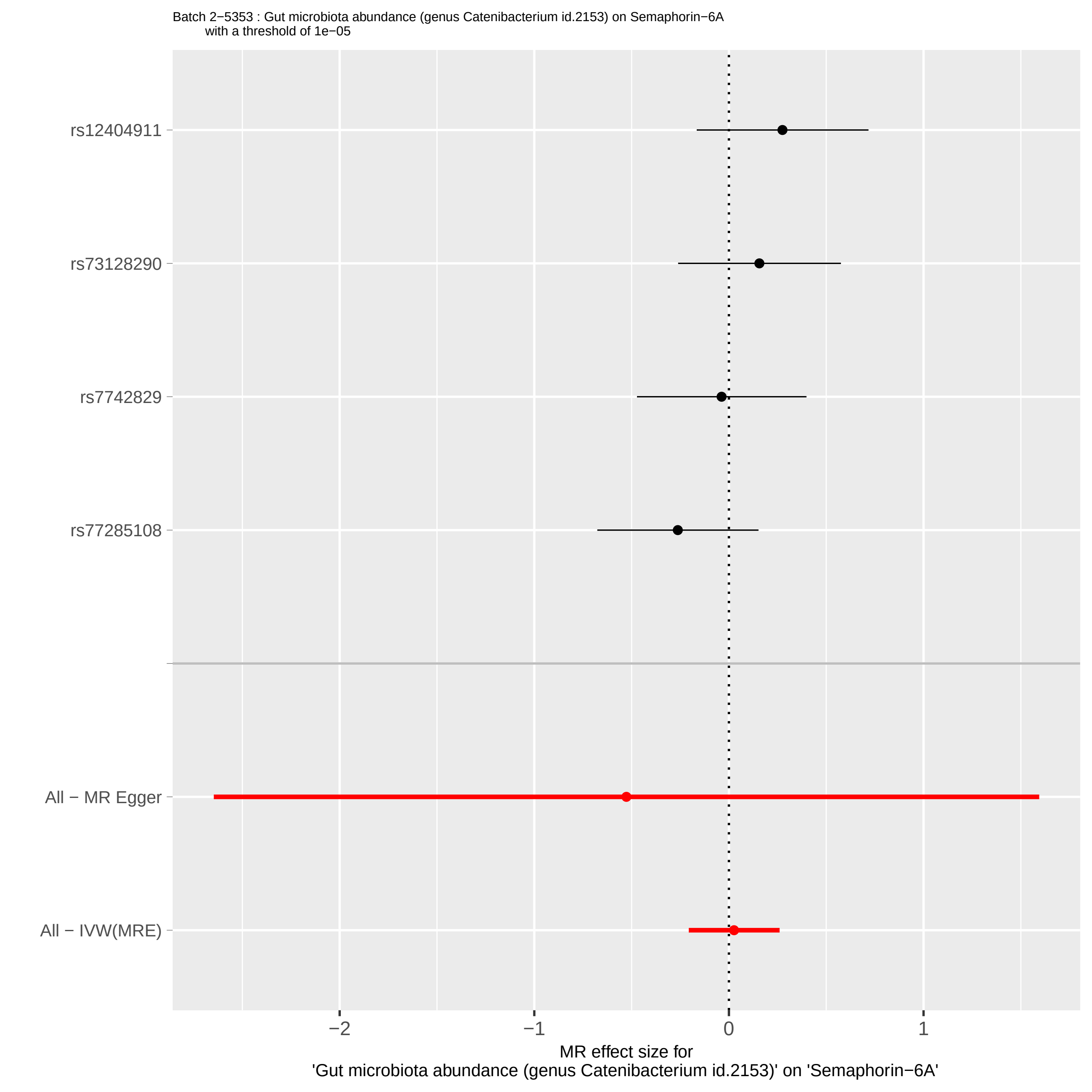

Batch 2−5353 : Gut microbiota abundance (genus Catenibacterium id.2153) on Semaphorin−6A
with a threshold of 1e−05
rs12404911
rs73128290
rs7742829
rs77285108
All − MR Egger
All − IVW(MRE)
−2
−1
0
1
MR effect size for
'Gut microbiota abundance (genus Catenibacterium id.2153)' on 'Semaphorin−6A'

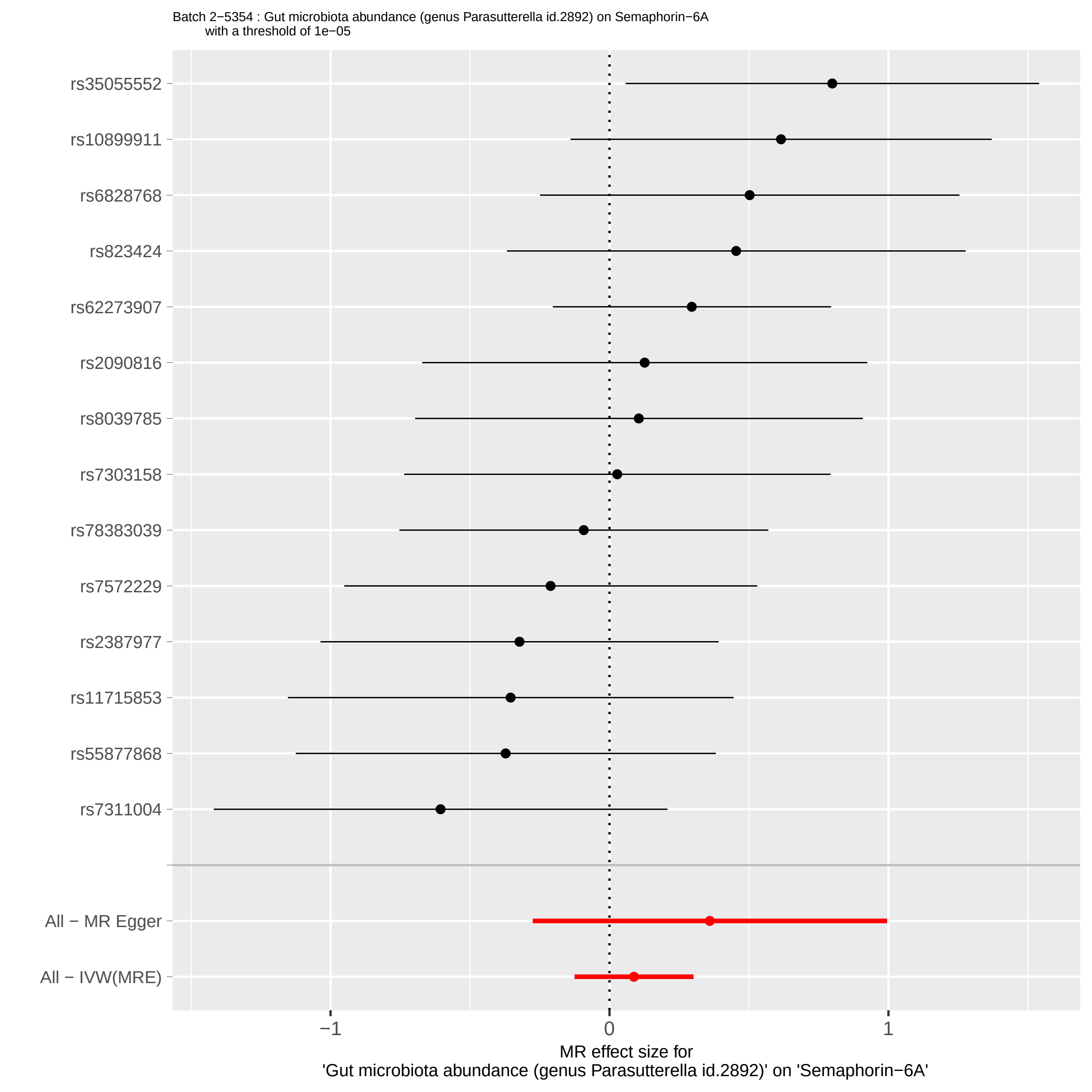

Batch 2−5354 : Gut microbiota abundance (genus Parasutterella id.2892) on Semaphorin−6A
with a threshold of 1e−05
rs35055552
rs10899911
rs6828768
rs823424
rs62273907
rs2090816
rs8039785
rs7303158
rs78383039
rs7572229
rs2387977
rs11715853
rs55877868
rs7311004
All − MR Egger
All − IVW(MRE)
−1
0
1
MR effect size for
'Gut microbiota abundance (genus Parasutterella id.2892)' on 'Semaphorin−6A'

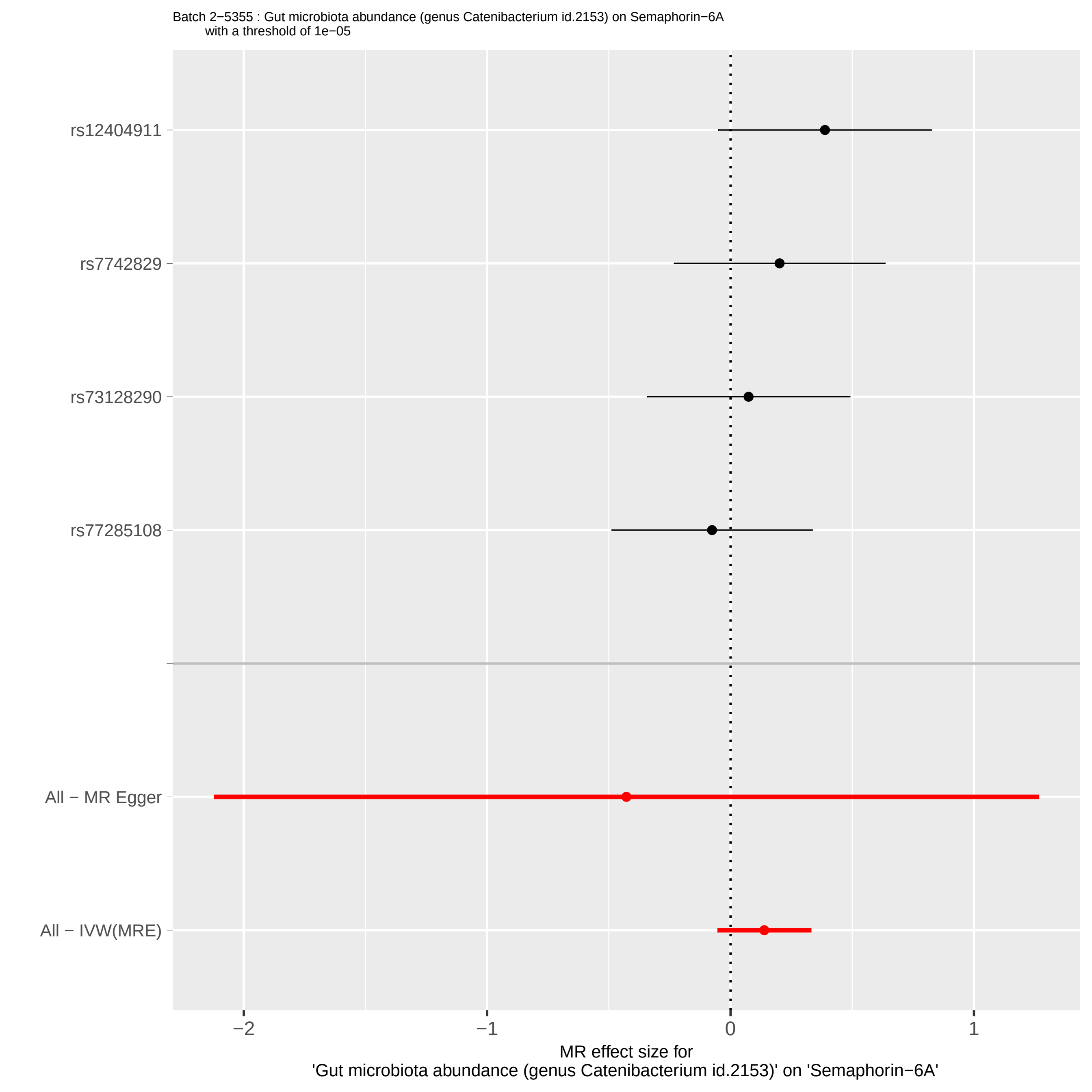
Batch 2−5355 : Gut microbiota abundance (genus Catenibacterium id.2153) on Semaphorin−6A
with a threshold of 1e−05
rs12404911
rs7742829
rs73128290
rs77285108
All − MR Egger
All − IVW(MRE)
−2
−1
0
1
MR effect size for
'Gut microbiota abundance (genus Catenibacterium id.2153)' on 'Semaphorin−6A'

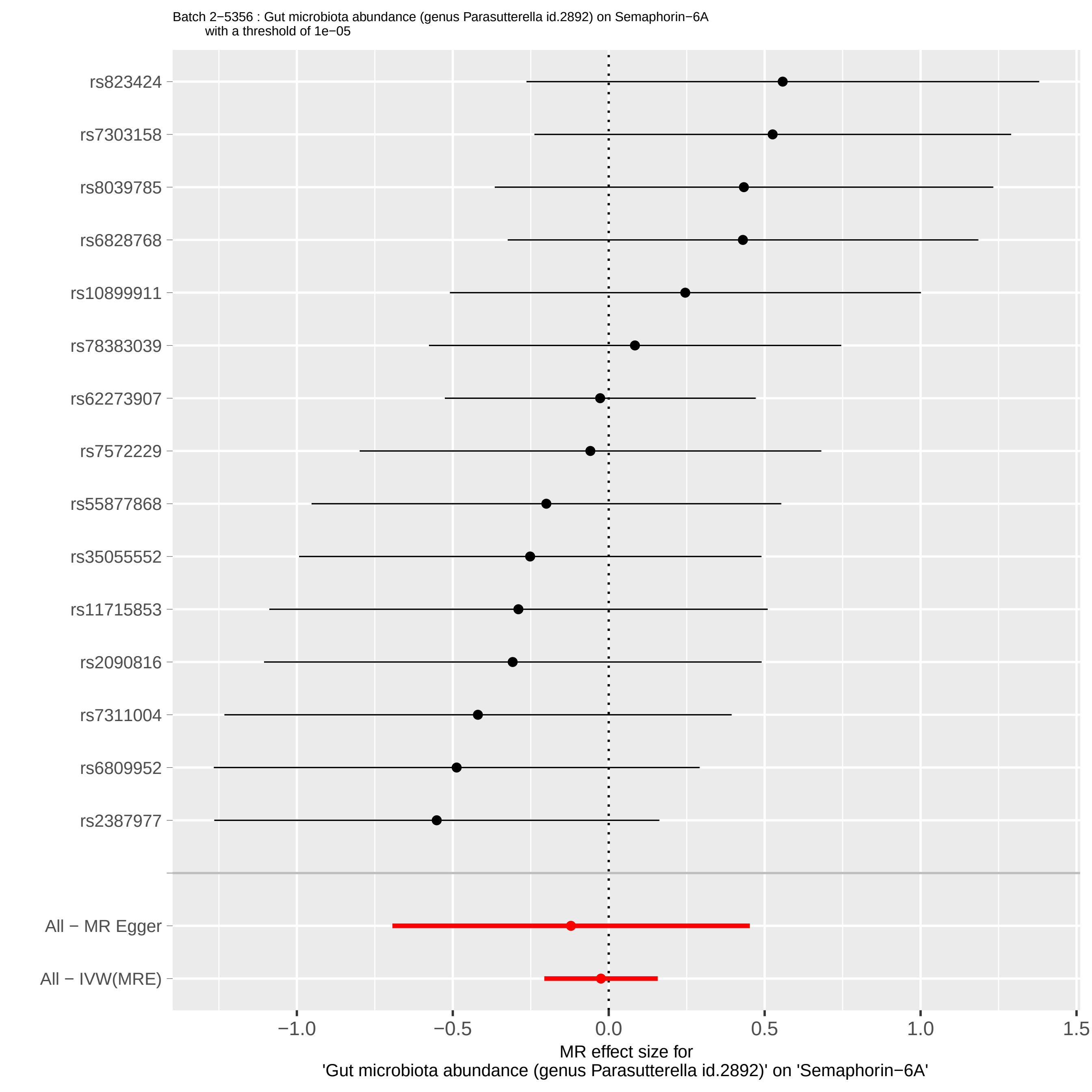
Batch 2−5356 : Gut microbiota abundance (genus Parasutterella id.2892) on Semaphorin−6A
with a threshold of 1e−05
rs823424
rs7303158
rs8039785
rs6828768
rs10899911
rs78383039
rs62273907
rs7572229
rs55877868
rs35055552
rs11715853
rs2090816
rs7311004
rs6809952
rs2387977
All − MR Egger
All − IVW(MRE)
−1.0
−0.5
0.0
0.5
1.0
1.5
MR effect size for
'Gut microbiota abundance (genus Parasutterella id.2892)' on 'Semaphorin−6A'

Batch 2−5357 : Gut microbiota abundance (genus Catenibacterium id.2153) on Semaphorin−6C
with a threshold of 1e−05

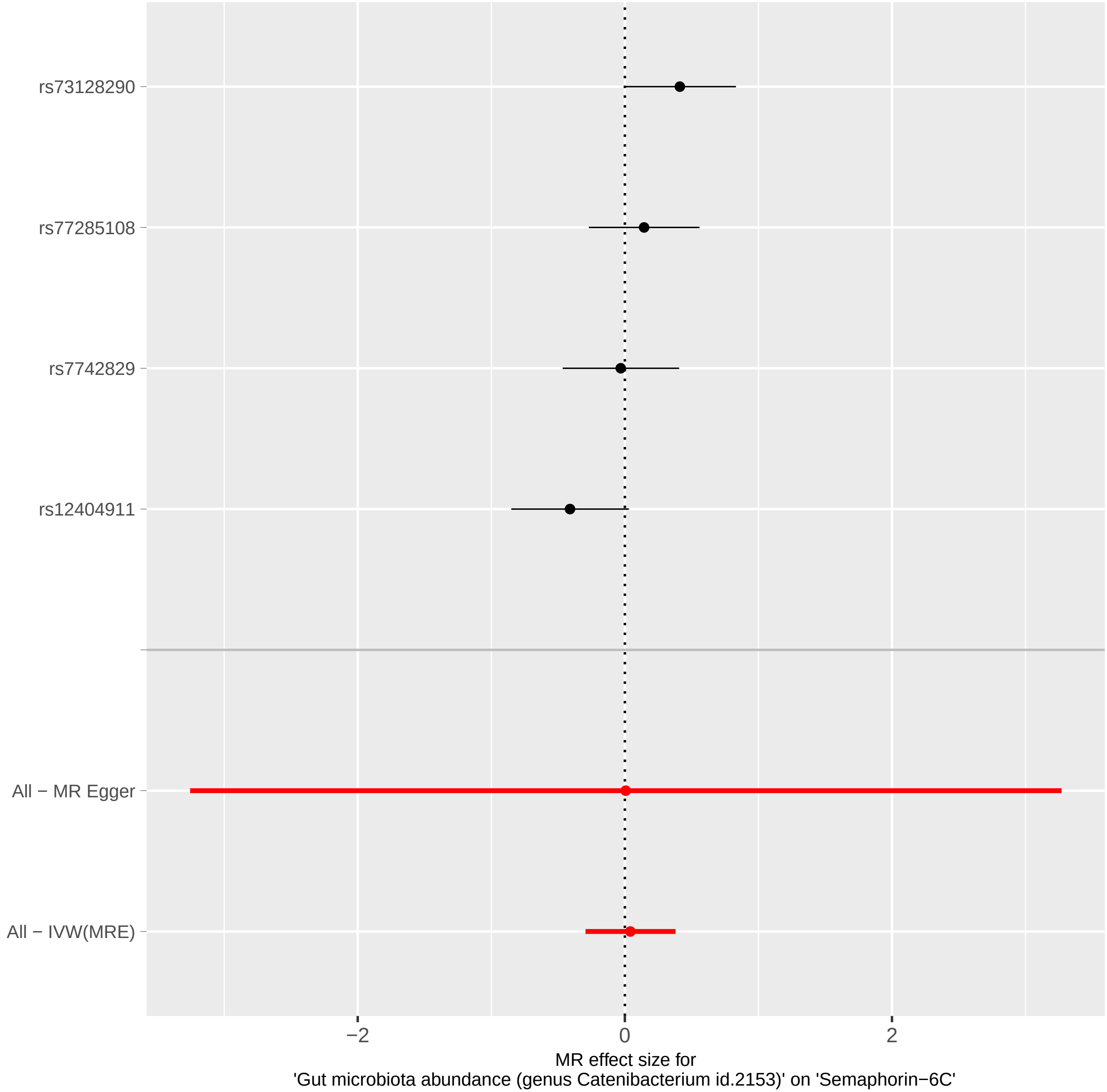

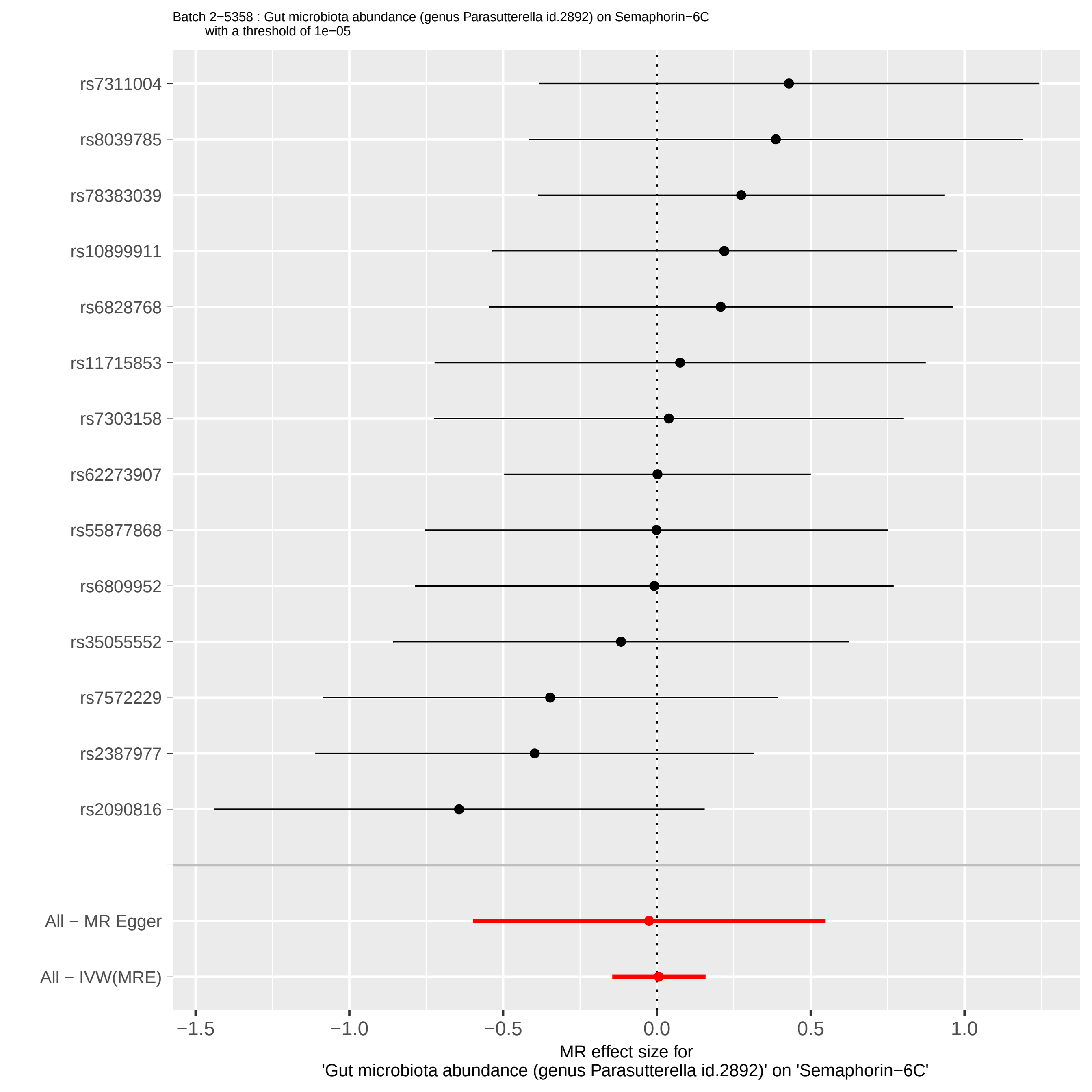
Batch 2−5358 : Gut microbiota abundance (genus Parasutterella id.2892) on Semaphorin−6C
with a threshold of 1e−05
rs7311004
rs8039785
rs78383039
rs10899911
rs6828768
rs11715853
rs7303158
rs62273907
rs55877868
rs6809952
rs35055552
rs7572229
rs2387977
rs2090816
All − MR Egger
All − IVW(MRE)
−1.5
−1.0
−0.5
0.0
0.5
1.0
MR effect size for
'Gut microbiota abundance (genus Parasutterella id.2892)' on 'Semaphorin−6C'

Batch 2−5359 : Gut microbiota abundance (genus Catenibacterium id.2153) on Semaphorin−7A
with a threshold of 1e−05

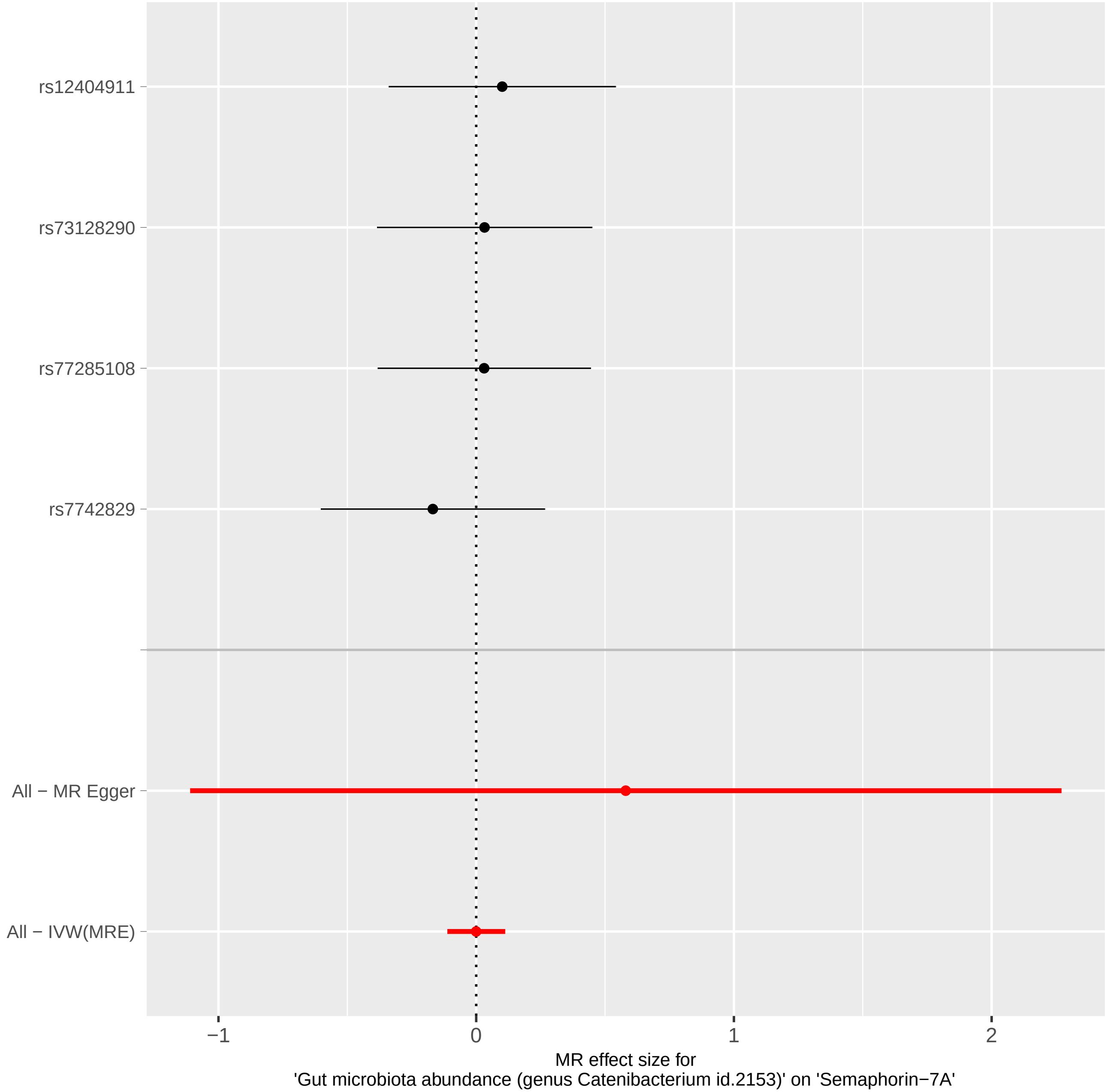

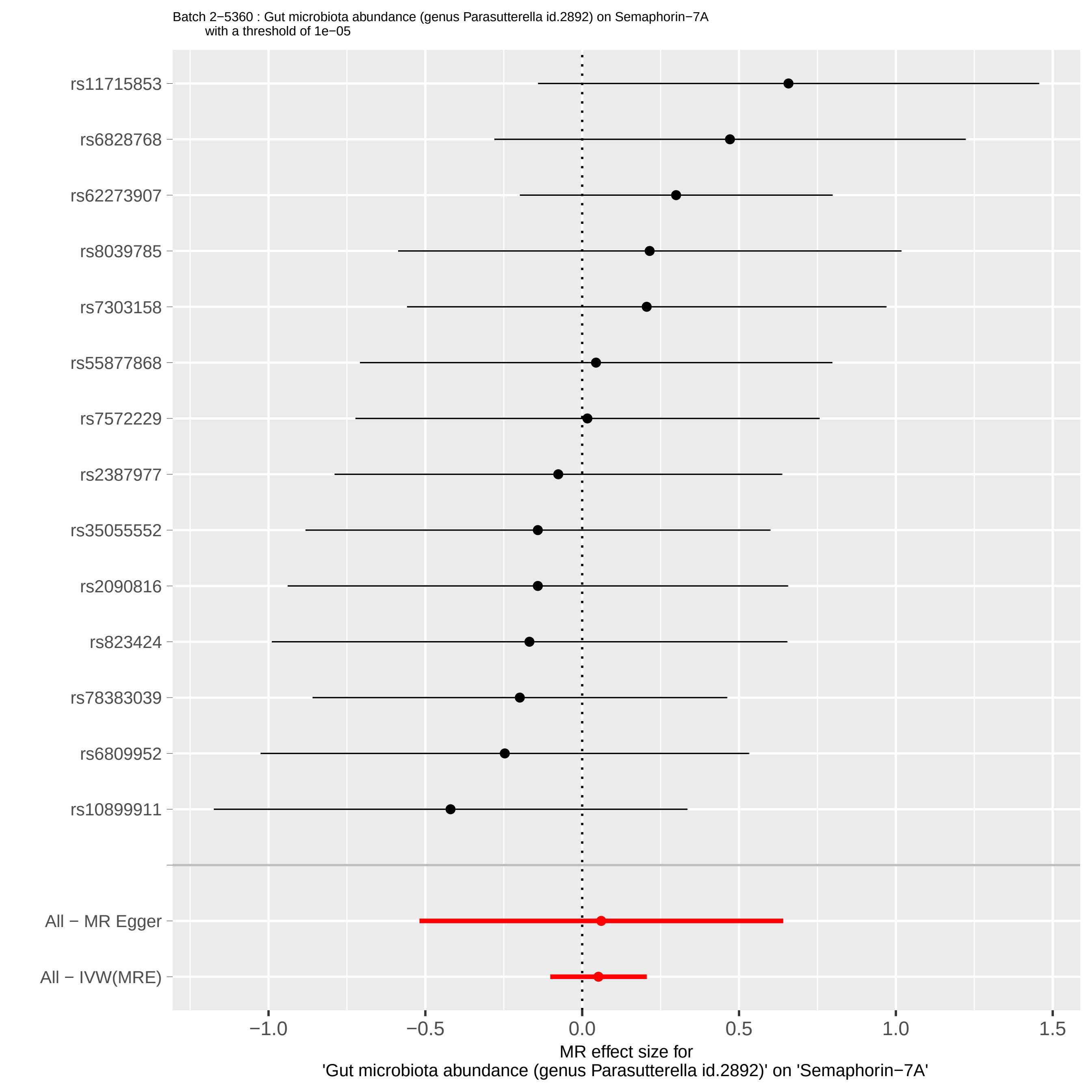
Batch 2−5360 : Gut microbiota abundance (genus Parasutterella id.2892) on Semaphorin−7A
with a threshold of 1e−05
rs11715853
rs6828768
rs62273907
rs8039785
rs7303158
rs55877868
rs7572229
rs2387977
rs35055552
rs2090816
rs823424
rs78383039
rs6809952
rs10899911
All − MR Egger
All − IVW(MRE)
−1.0
−0.5
0.0
0.5
1.0
1.5
MR effect size for
'Gut microbiota abundance (genus Parasutterella id.2892)' on 'Semaphorin−7A'

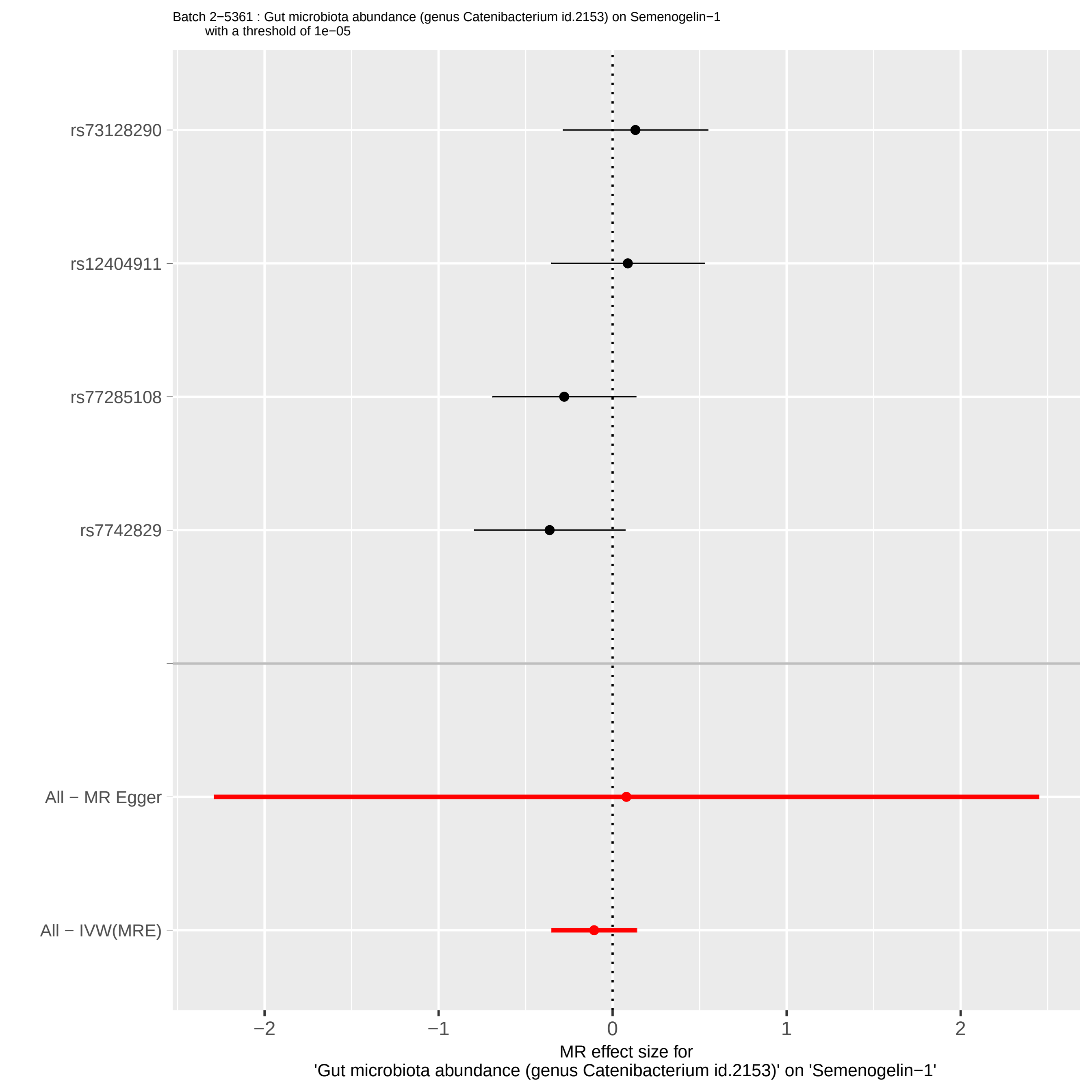
Batch 2−5361 : Gut microbiota abundance (genus Catenibacterium id.2153) on Semenogelin−1
with a threshold of 1e−05
rs73128290
rs12404911
rs77285108
rs7742829
All − MR Egger
All − IVW(MRE)
−2
−1
0
1
2
MR effect size for
'Gut microbiota abundance (genus Catenibacterium id.2153)' on 'Semenogelin−1'

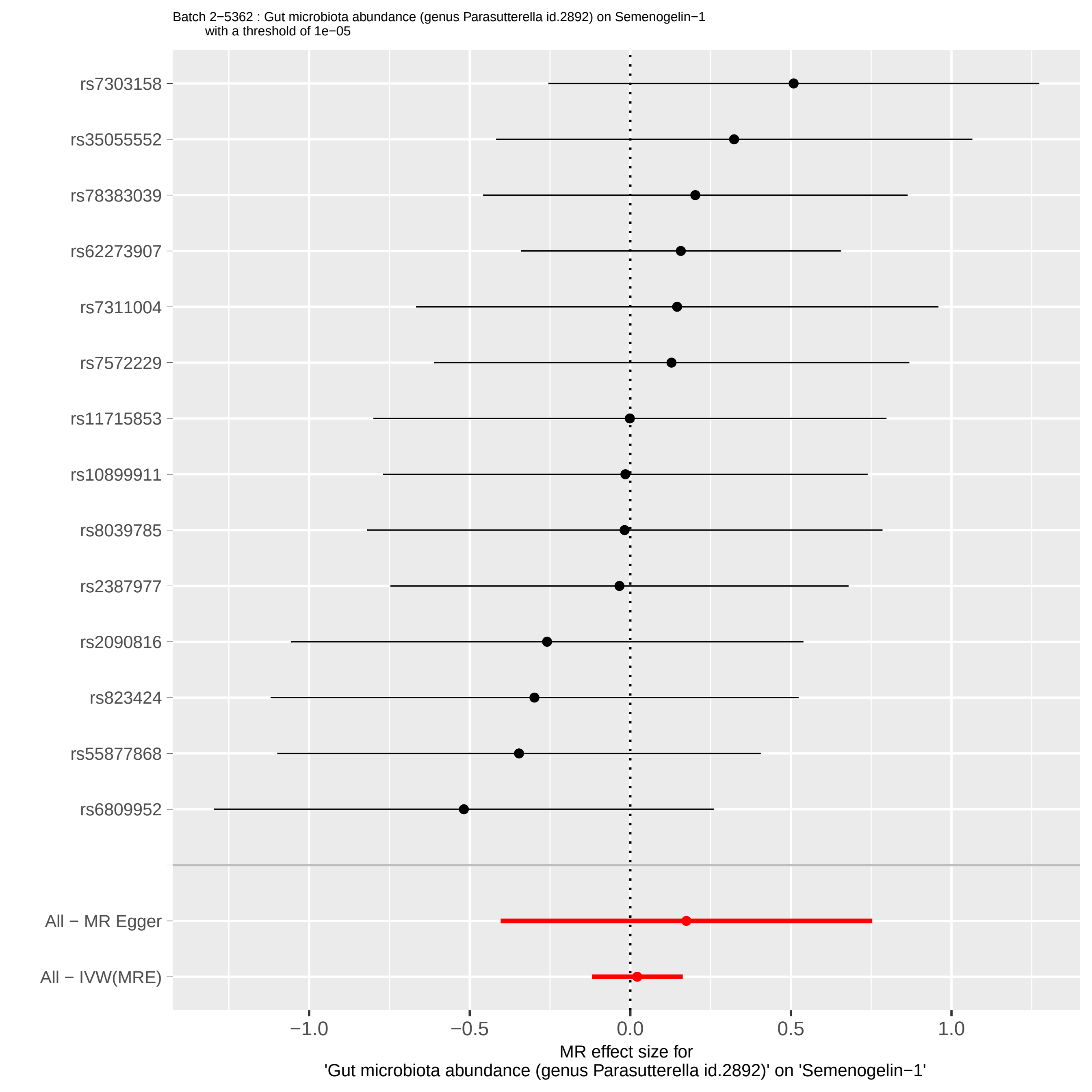

Batch 2−5362 : Gut microbiota abundance (genus Parasutterella id.2892) on Semenogelin−1
with a threshold of 1e−05
rs7303158
rs35055552
rs78383039
rs62273907
rs7311004
rs7572229
rs11715853
rs10899911
rs8039785
rs2387977
rs2090816
rs823424
rs55877868
rs6809952
All − MR Egger
All − IVW(MRE)
−1.0
−0.5
0.0
0.5
1.0
MR effect size for
'Gut microbiota abundance (genus Parasutterella id.2892)' on 'Semenogelin−1'

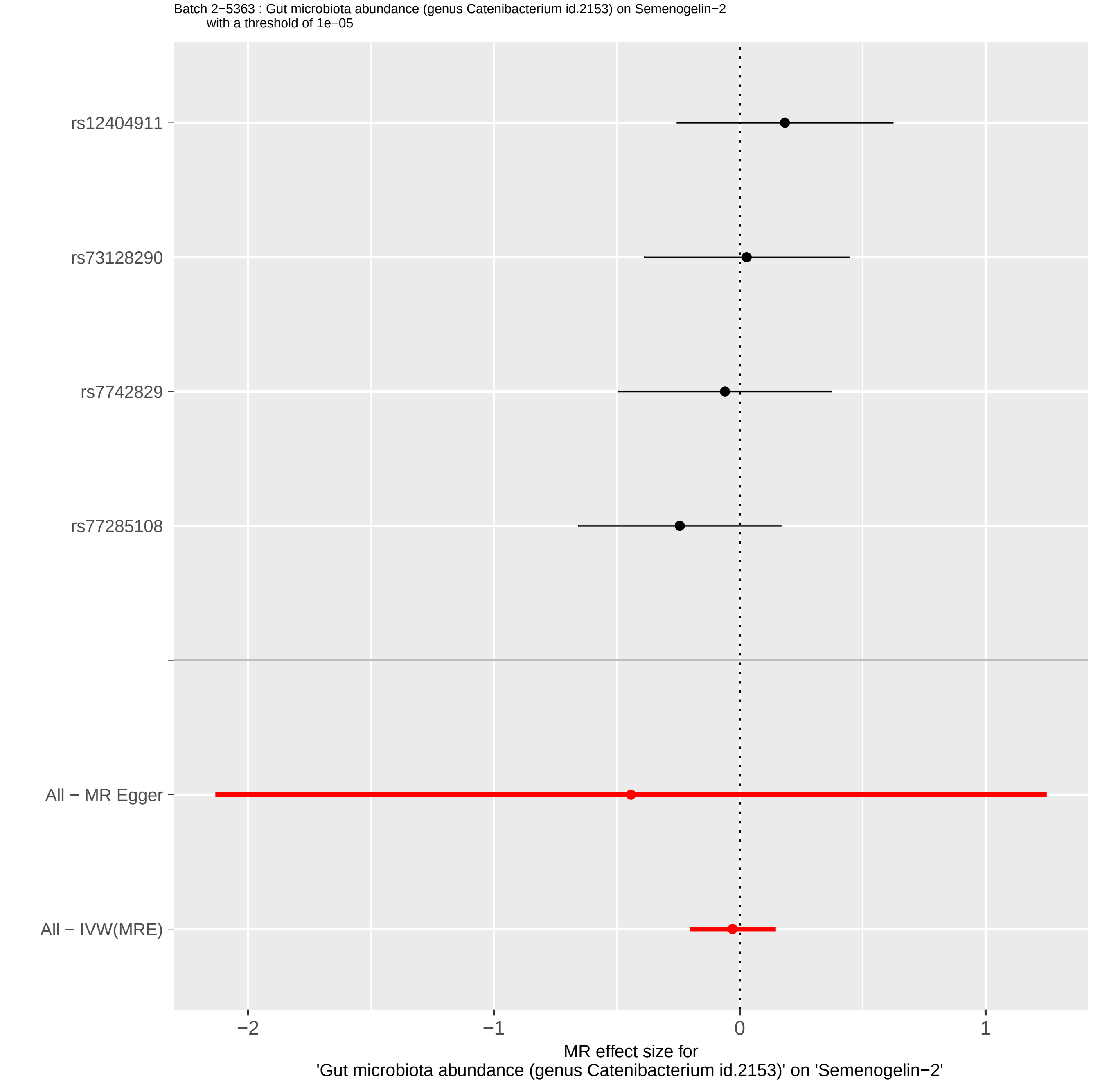

Batch 2−5363 : Gut microbiota abundance (genus Catenibacterium id.2153) on Semenogelin−2
with a threshold of 1e−05
rs12404911
rs73128290
rs7742829
rs77285108
All − MR Egger
All − IVW(MRE)
−2
−1
0
1
MR effect size for
'Gut microbiota abundance (genus Catenibacterium id.2153)' on 'Semenogelin−2'

Batch 2−5364 : Gut microbiota abundance (genus Parasutterella id.2892) on Semenogelin−2
with a threshold of 1e−05

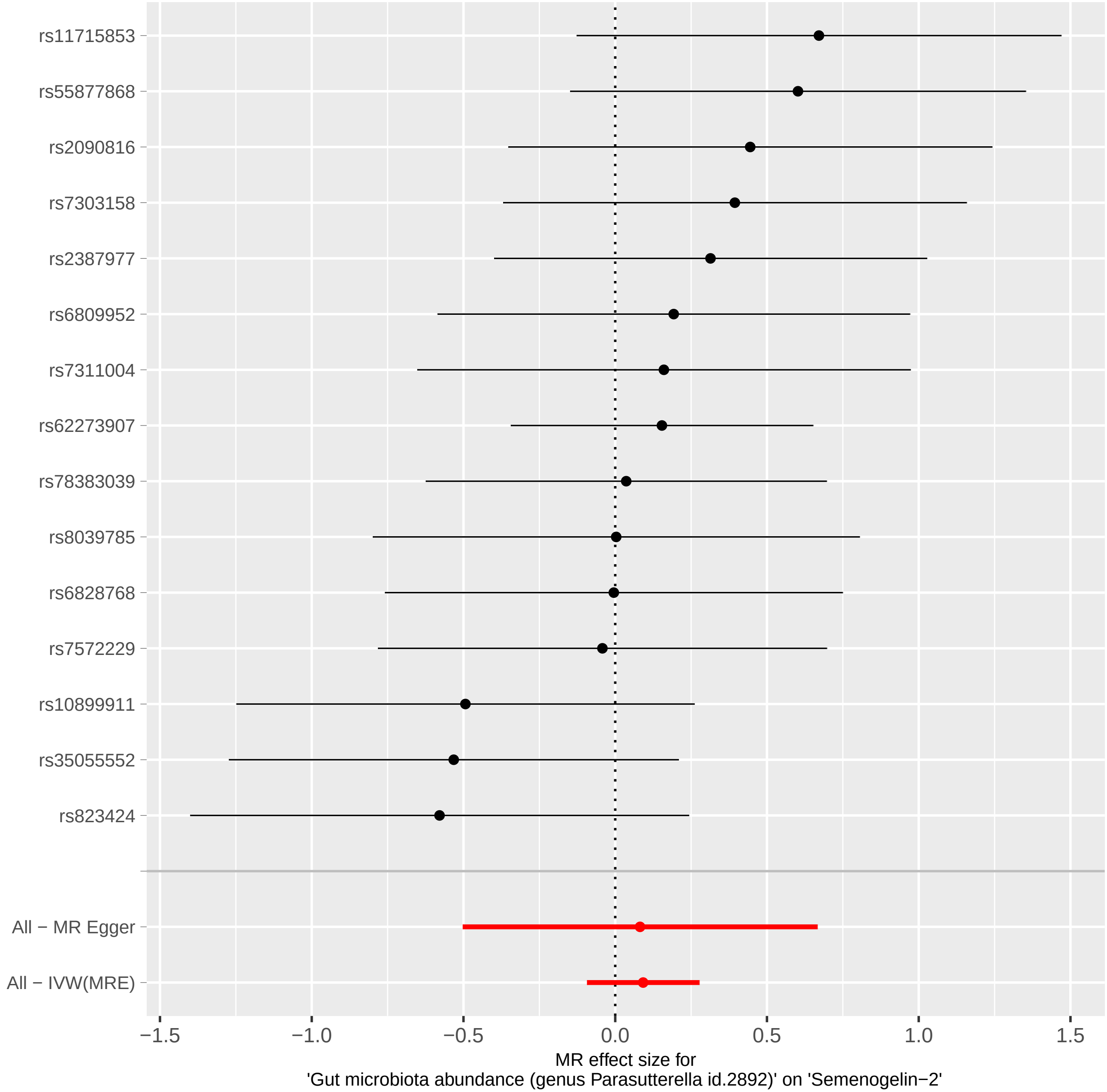

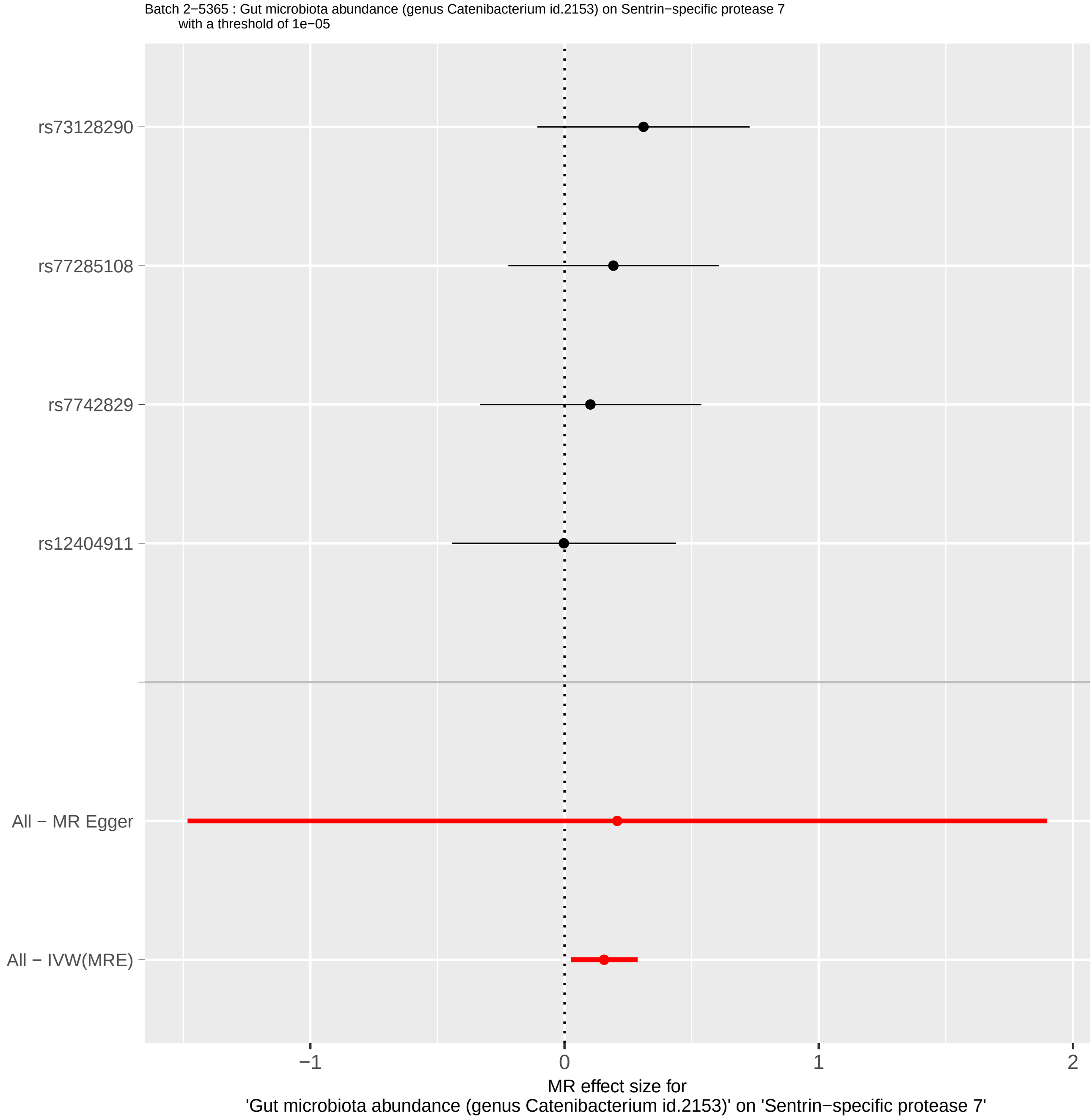

Batch 2−5365 : Gut microbiota abundance (genus Catenibacterium id.2153) on Sentrin−specific protease 7
with a threshold of 1e−05
rs73128290
rs77285108
rs7742829
rs12404911
All − MR Egger
All − IVW(MRE)
−1
0
1
2
MR effect size for
'Gut microbiota abundance (genus Catenibacterium id.2153)' on 'Sentrin−specific protease 7'

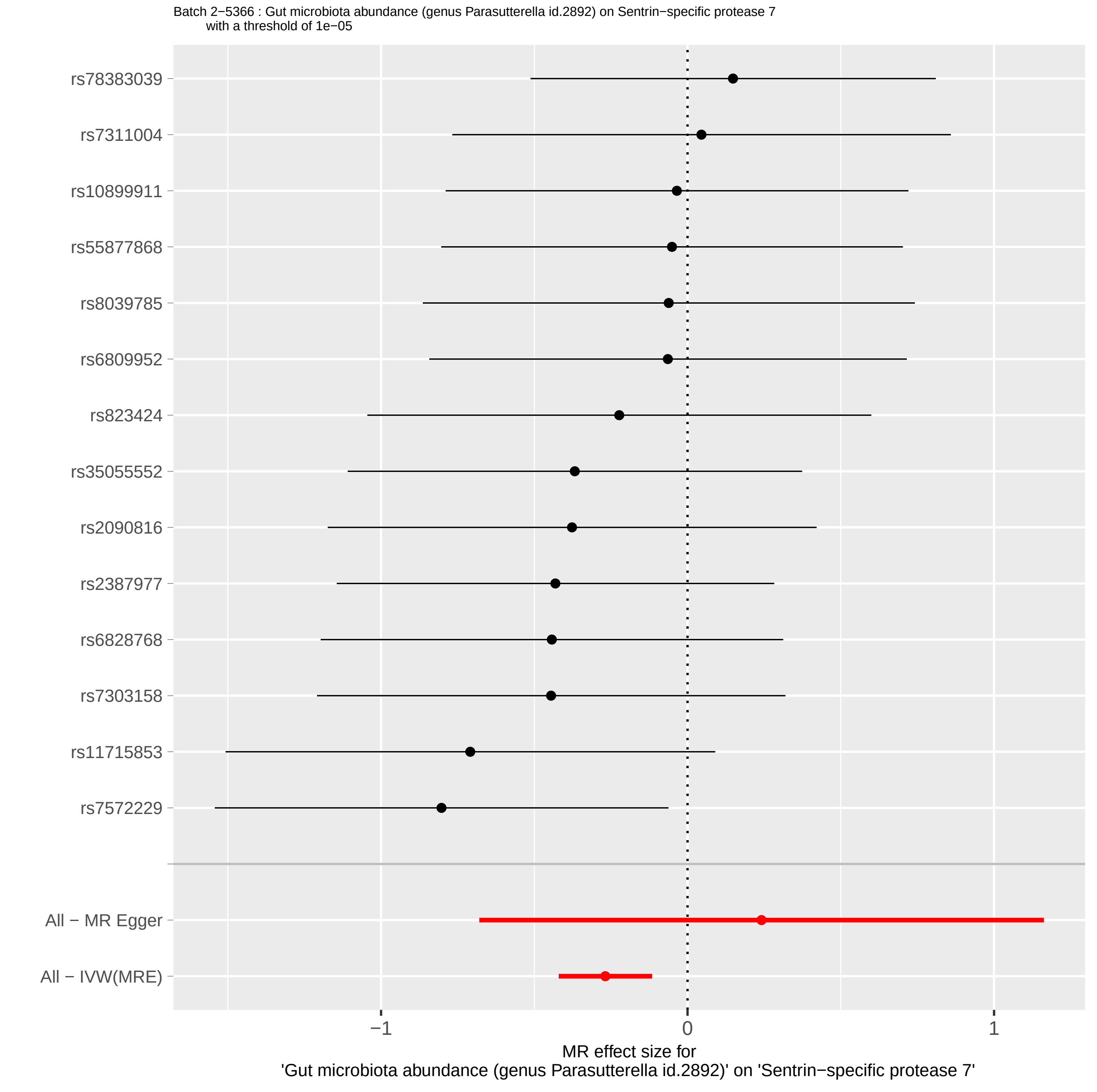

Batch 2−5366 : Gut microbiota abundance (genus Parasutterella id.2892) on Sentrin−specific protease 7
with a threshold of 1e−05
rs78383039
rs7311004
rs10899911
rs55877868
rs8039785
rs6809952
rs823424
rs35055552
rs2090816
rs2387977
rs6828768
rs7303158
rs11715853
rs7572229
All − MR Egger
All − IVW(MRE)
−1
0
1
MR effect size for
'Gut microbiota abundance (genus Parasutterella id.2892)' on 'Sentrin−specific protease 7'

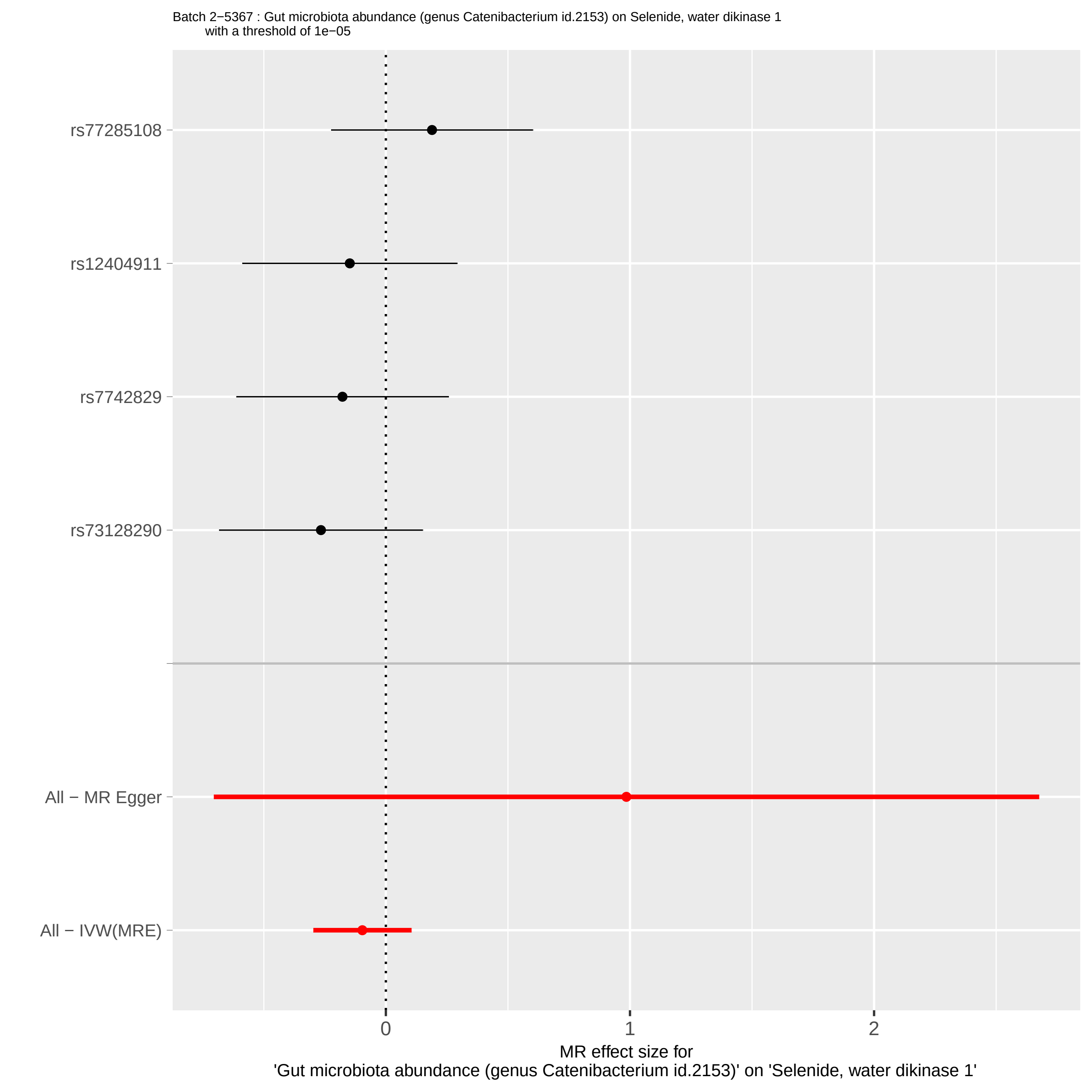
Batch 2−5367 : Gut microbiota abundance (genus Catenibacterium id.2153) on Selenide, water dikinase 1
with a threshold of 1e−05
rs77285108
rs12404911
rs7742829
rs73128290
All − MR Egger
All − IVW(MRE)
0
1
2
MR effect size for
'Gut microbiota abundance (genus Catenibacterium id.2153)' on 'Selenide, water dikinase 1'

Batch 2−5368 : Gut microbiota abundance (genus Parasutterella id.2892) on Selenide, water dikinase 1
with a threshold of 1e−05

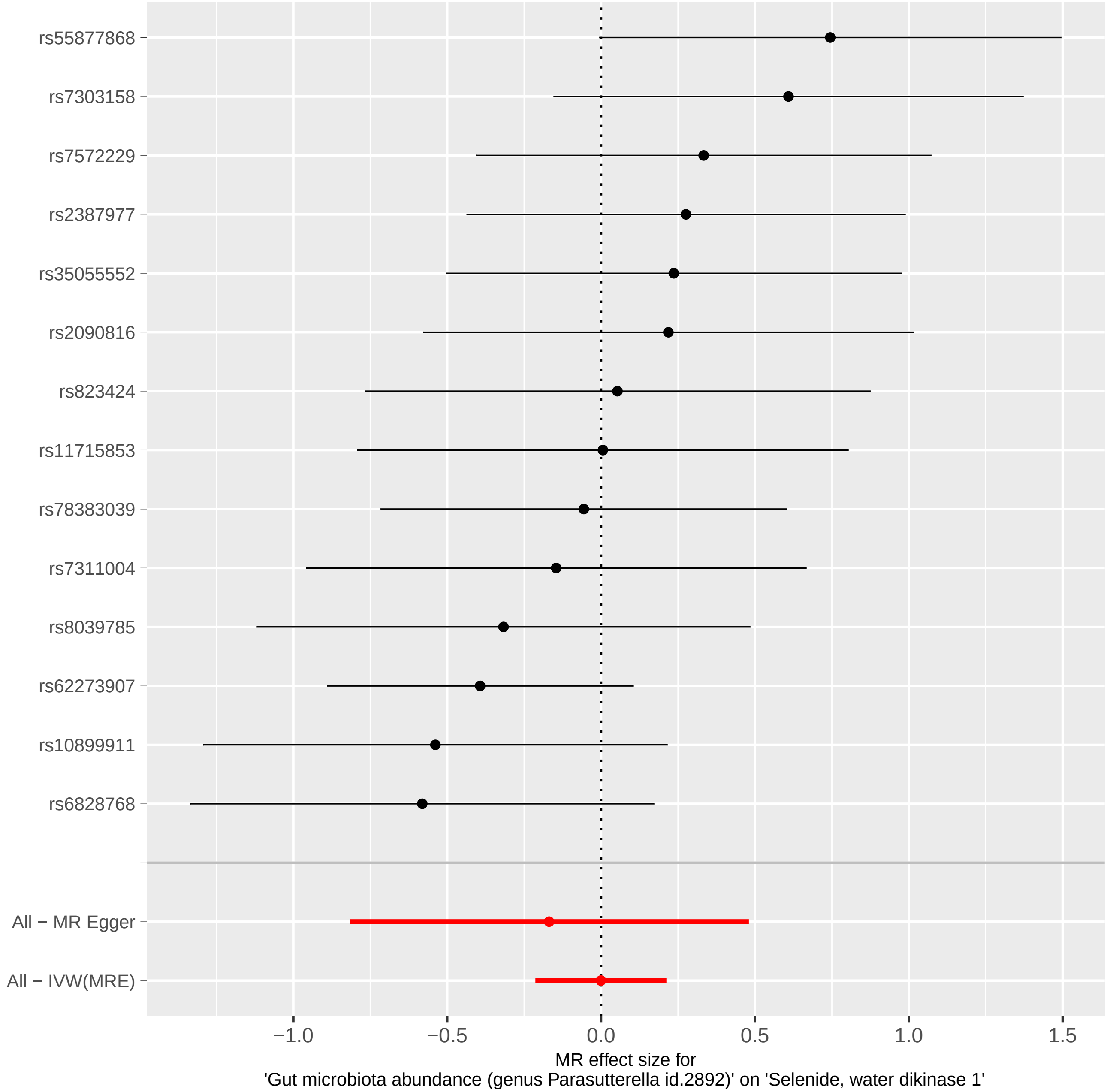

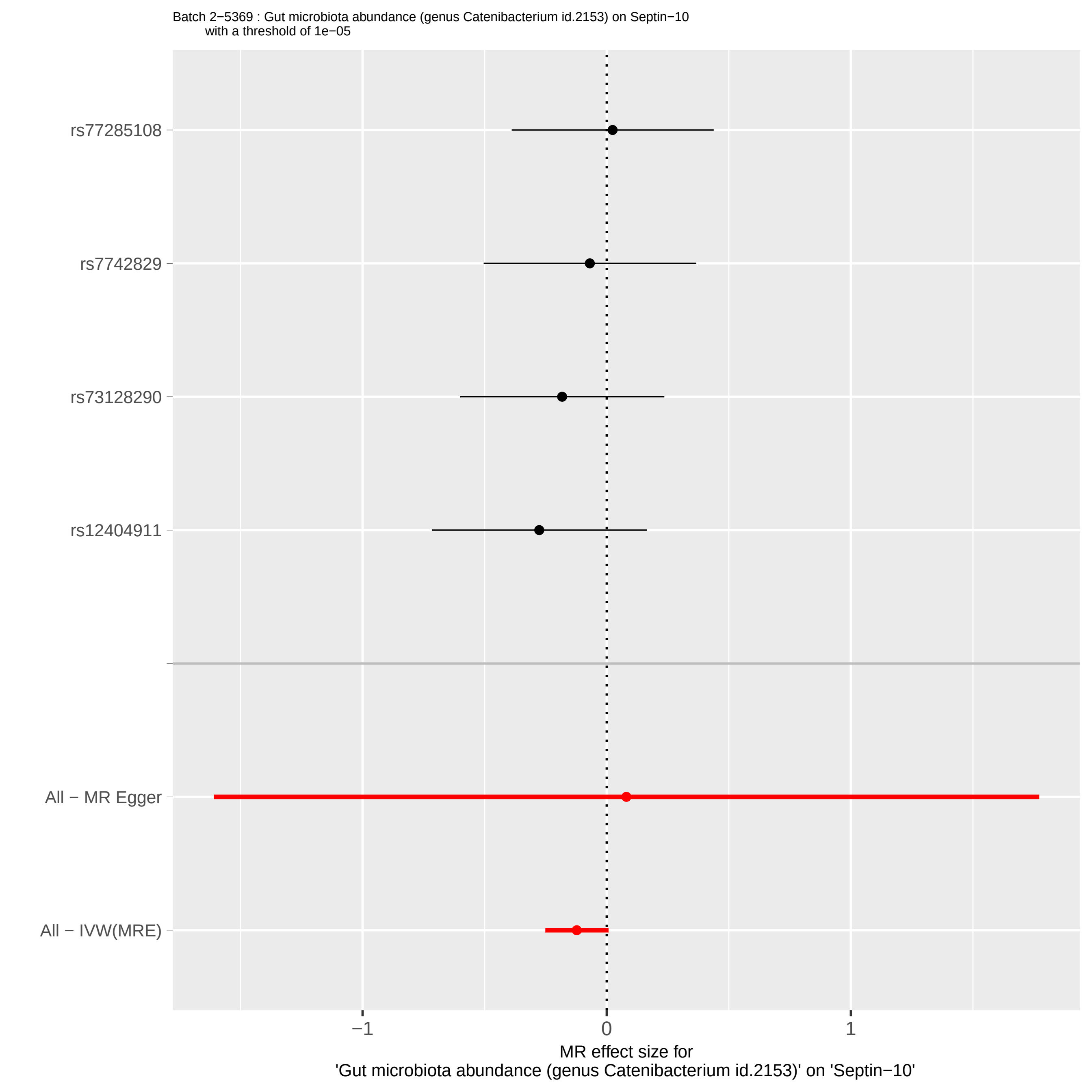

Batch 2−5369 : Gut microbiota abundance (genus Catenibacterium id.2153) on Septin−10
with a threshold of 1e−05
rs77285108
rs7742829
rs73128290
rs12404911
All − MR Egger
All − IVW(MRE)
−1
0
1
MR effect size for
'Gut microbiota abundance (genus Catenibacterium id.2153)' on 'Septin−10'

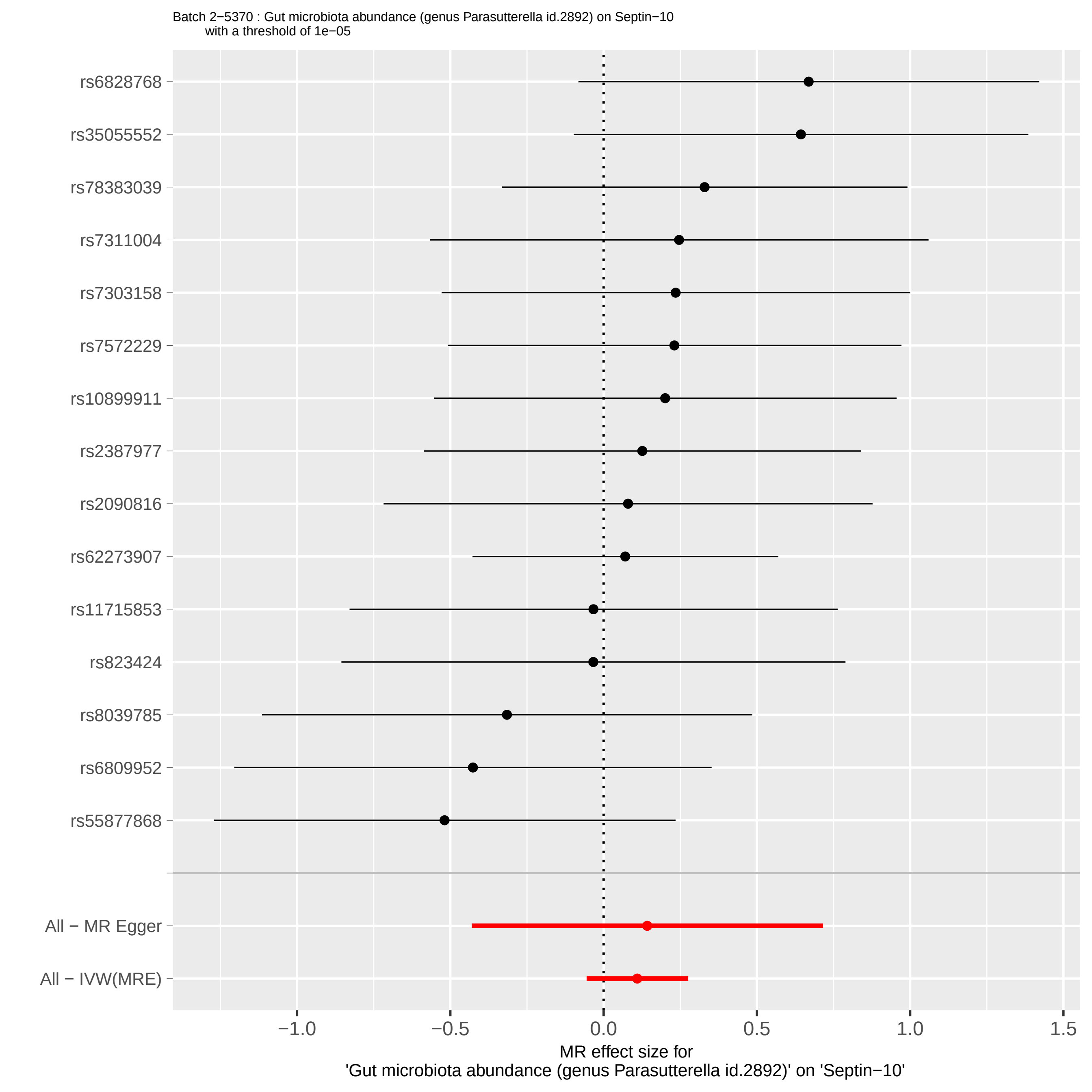
Batch 2−5370 : Gut microbiota abundance (genus Parasutterella id.2892) on Septin−10
with a threshold of 1e−05
rs6828768
rs35055552
rs78383039
rs7311004
rs7303158
rs7572229
rs10899911
rs2387977
rs2090816
rs62273907
rs11715853
rs823424
rs8039785
rs6809952
rs55877868
All − MR Egger
All − IVW(MRE)
−1.0
−0.5
0.0
0.5
1.0
1.5
MR effect size for
'Gut microbiota abundance (genus Parasutterella id.2892)' on 'Septin−10'

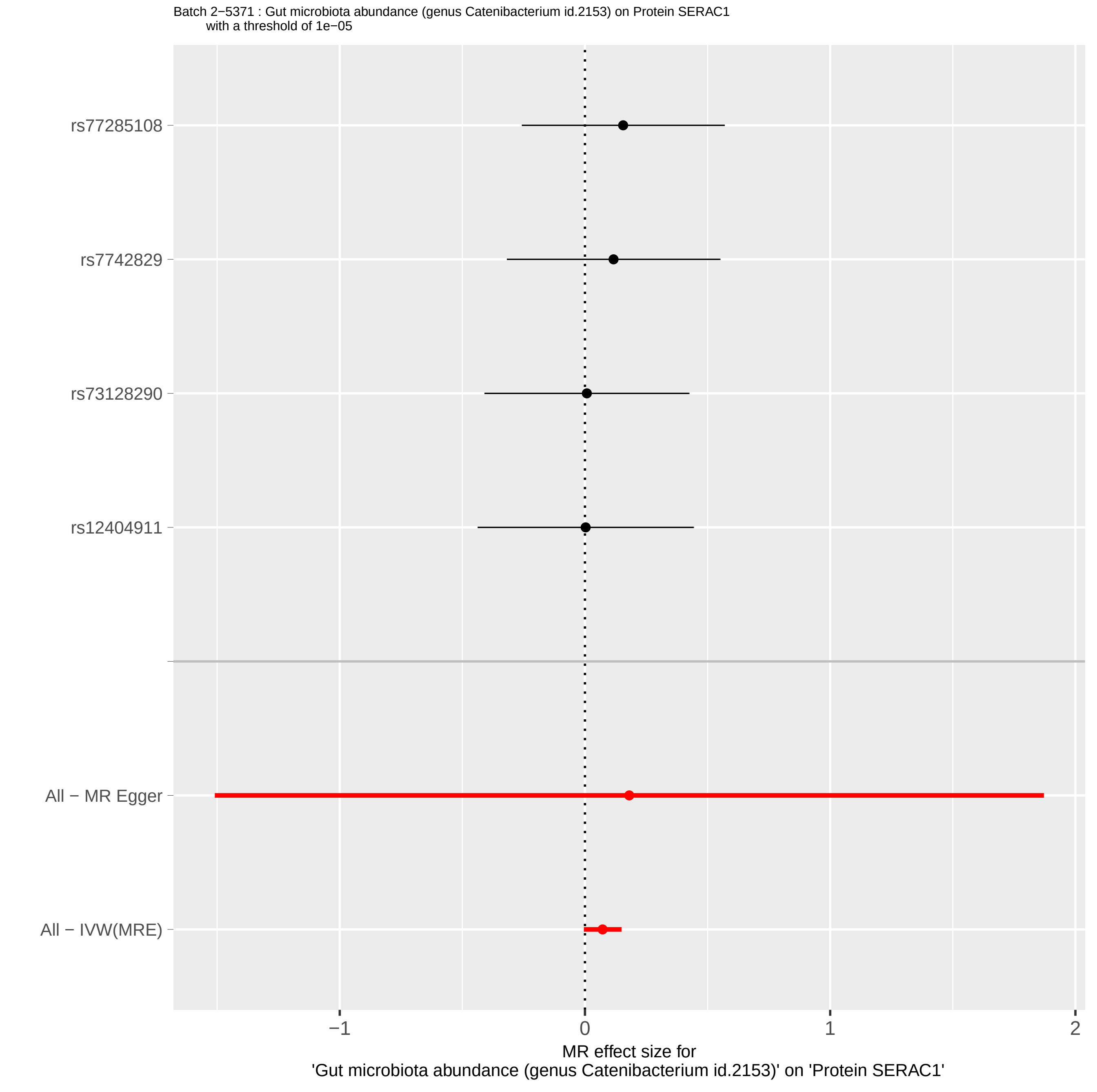

Batch 2−5371 : Gut microbiota abundance (genus Catenibacterium id.2153) on Protein SERAC1
with a threshold of 1e−05
rs77285108
rs7742829
rs73128290
rs12404911
All − MR Egger
All − IVW(MRE)
−1
0
1
2
MR effect size for
'Gut microbiota abundance (genus Catenibacterium id.2153)' on 'Protein SERAC1'

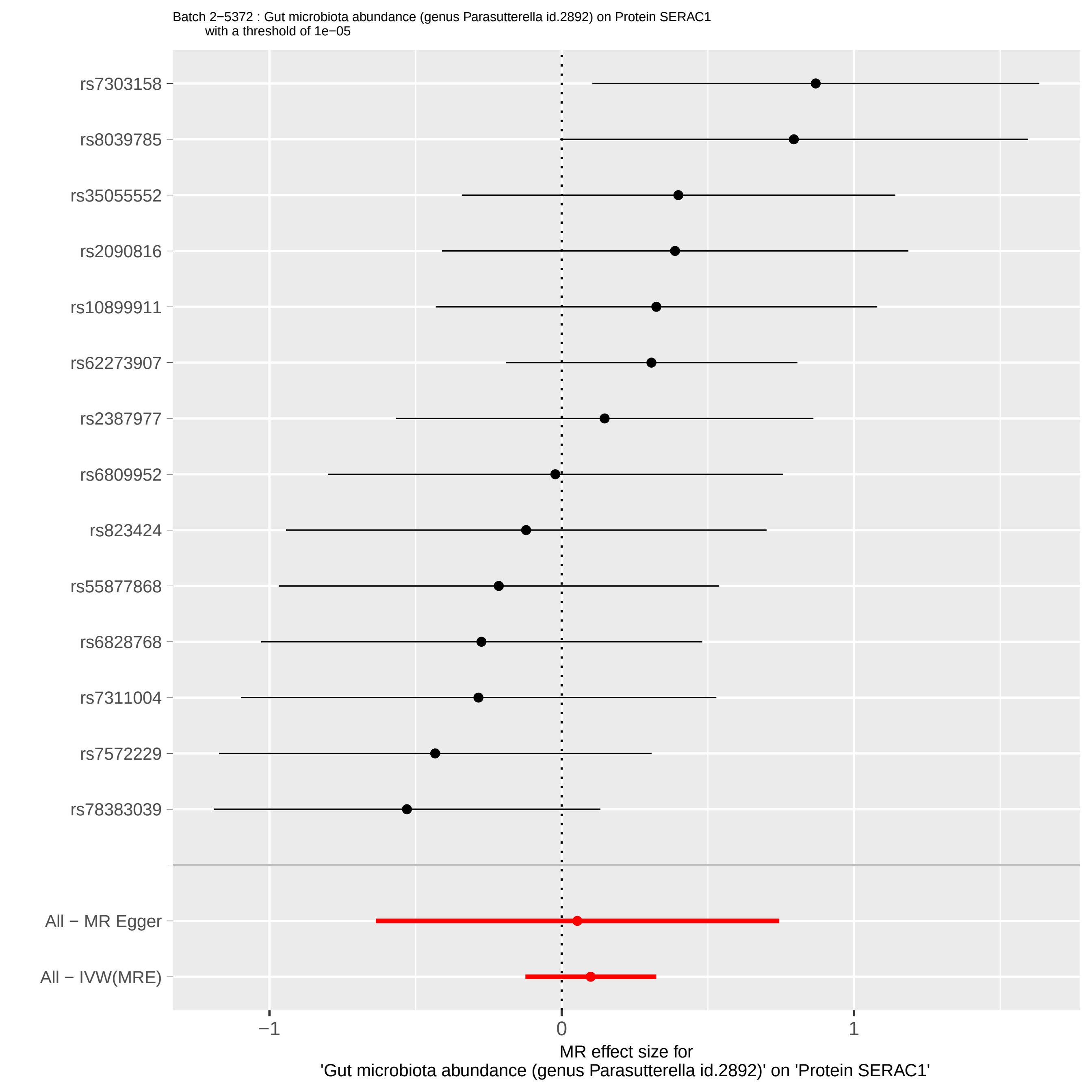
Batch 2−5372 : Gut microbiota abundance (genus Parasutterella id.2892) on Protein SERAC1
with a threshold of 1e−05
rs7303158
rs8039785
rs35055552
rs2090816
rs10899911
rs62273907
rs2387977
rs6809952
rs823424
rs55877868
rs6828768
rs7311004
rs7572229
rs78383039
All − MR Egger
All − IVW(MRE)
−1
0
1
MR effect size for
'Gut microbiota abundance (genus Parasutterella id.2892)' on 'Protein SERAC1'

Batch 2−5374 : Gut microbiota abundance (genus Parasutterella id.2892) on Small EDRK−rich factor 1 with a threshold of 1e−05

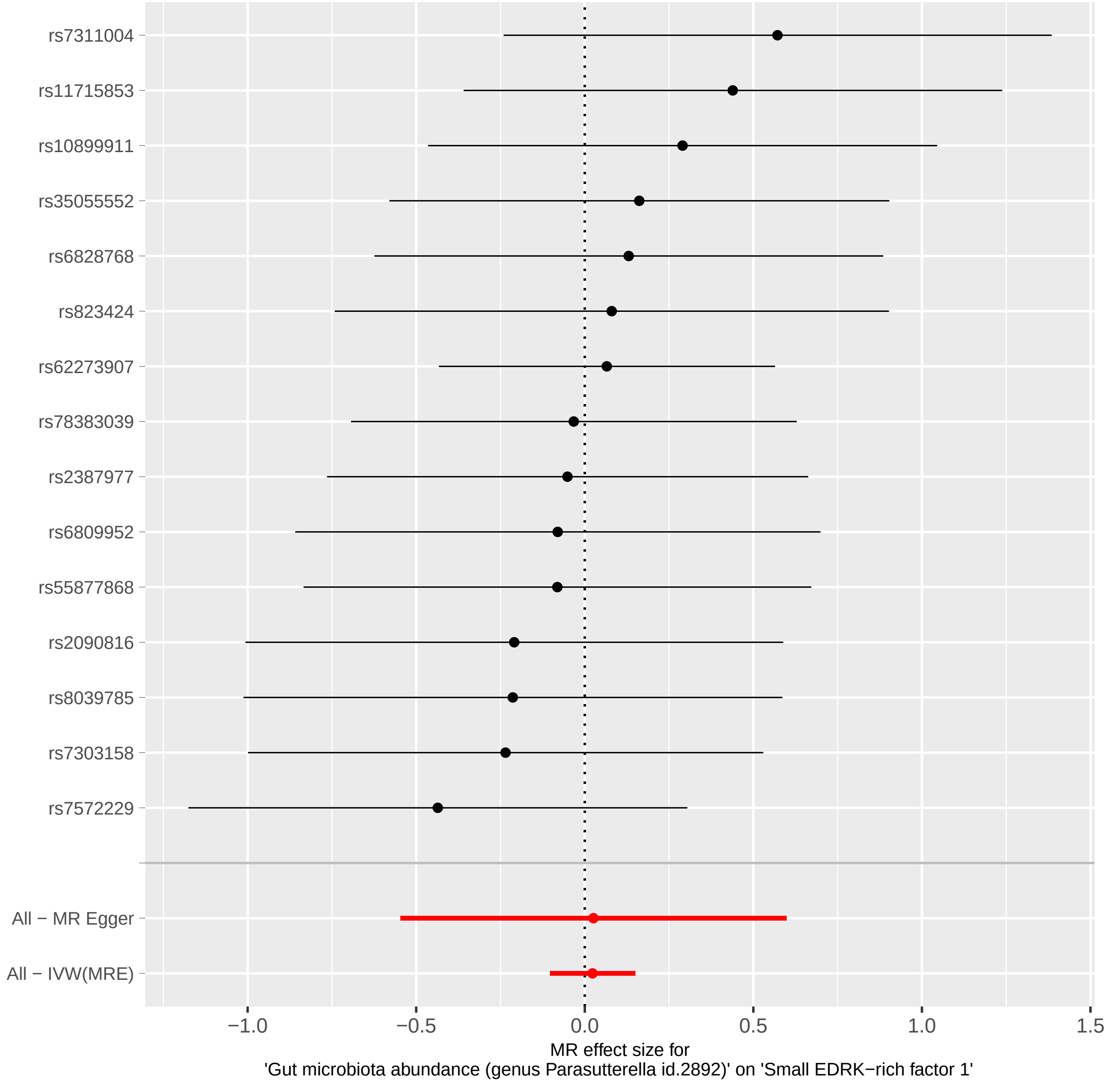

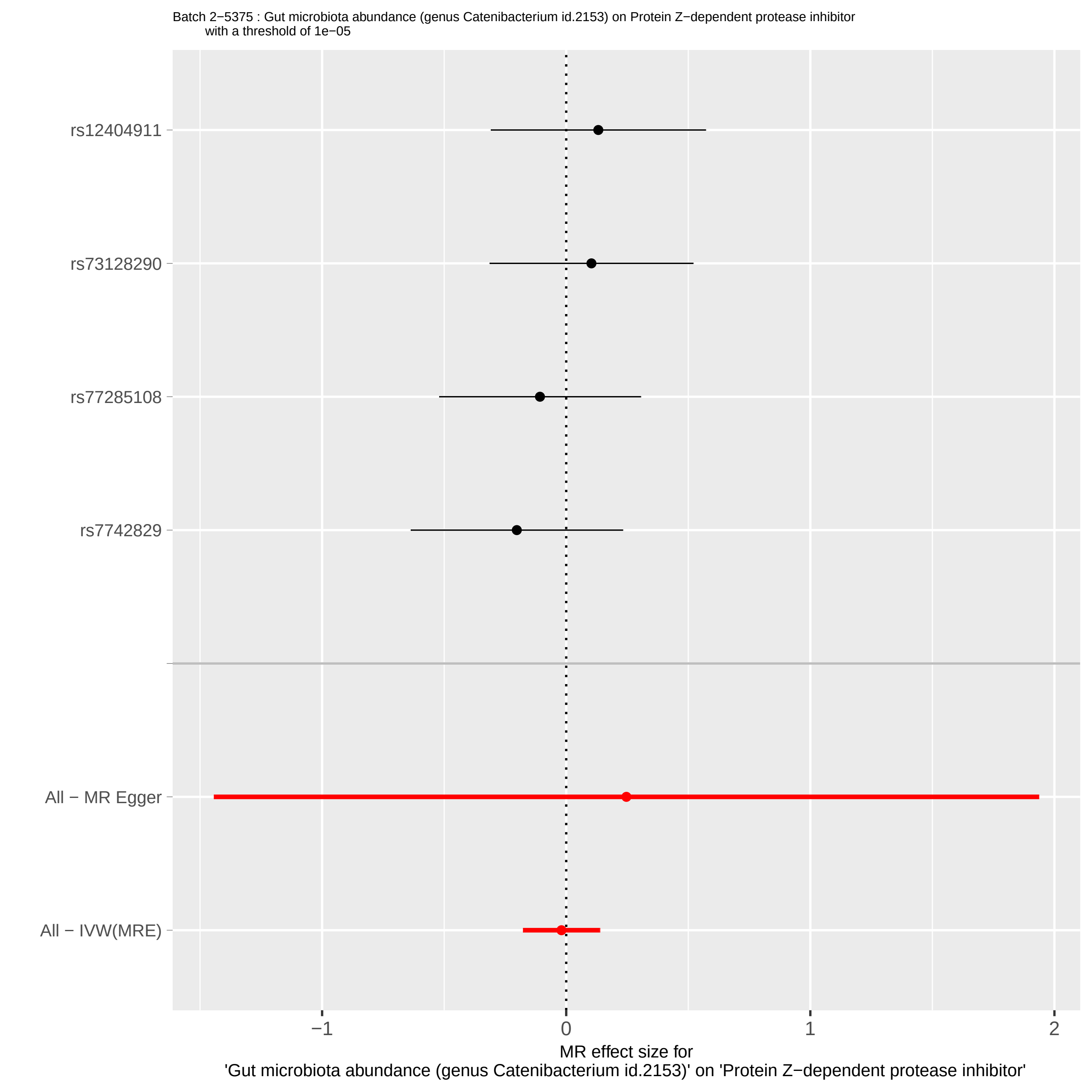

Batch 2−5375 : Gut microbiota abundance (genus Catenibacterium id.2153) on Protein Z−dependent protease inhibitor
with a threshold of 1e−05
rs12404911
rs73128290
rs77285108
rs7742829
All − MR Egger
All − IVW(MRE)
−1
0
1
2
MR effect size for
'Gut microbiota abundance (genus Catenibacterium id.2153)' on 'Protein Z−dependent protease inhibitor'

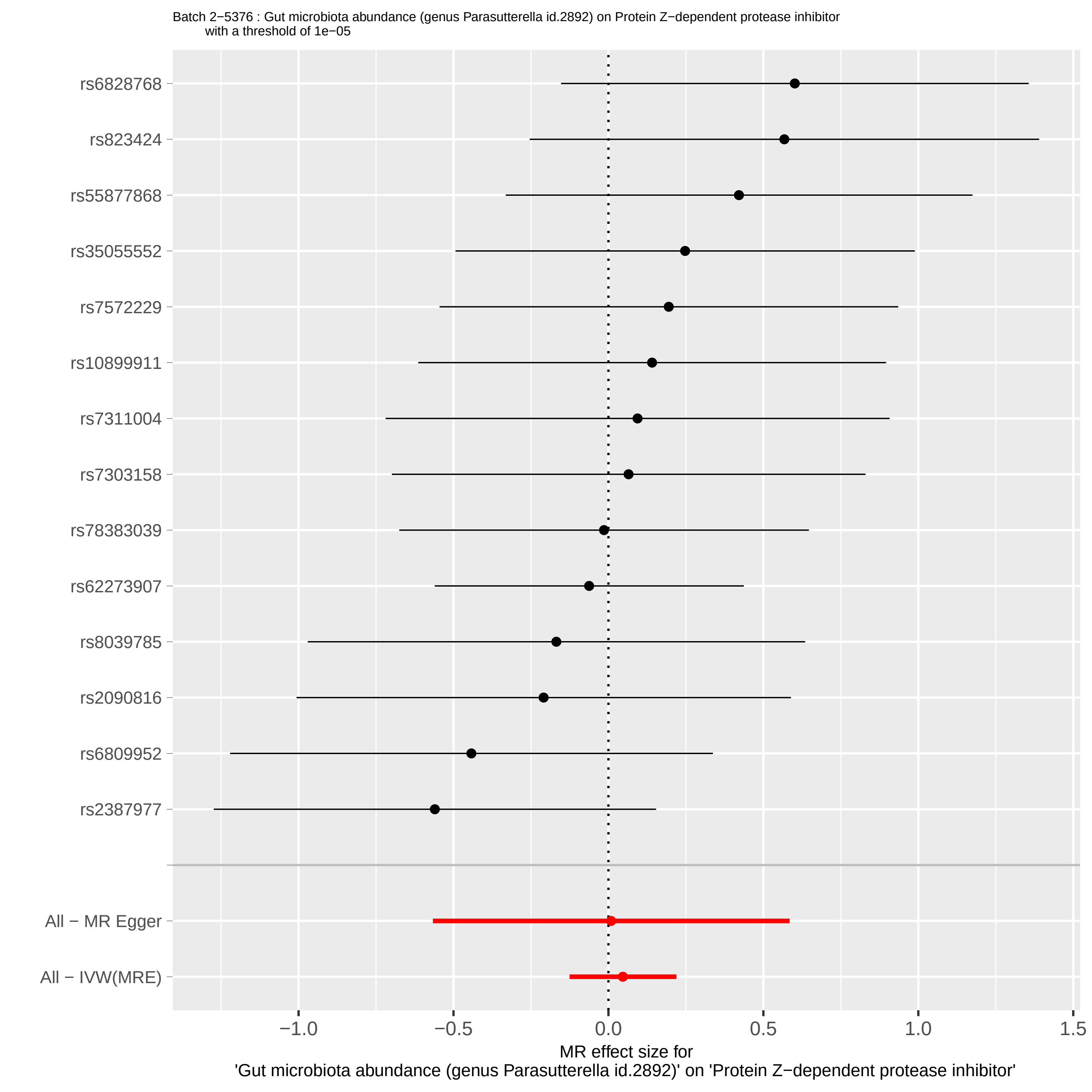
Batch 2−5376 : Gut microbiota abundance (genus Parasutterella id.2892) on Protein Z−dependent protease inhibitor
with a threshold of 1e−05
rs6828768
rs823424
rs55877868
rs35055552
rs7572229
rs10899911
rs7311004
rs7303158
rs78383039
rs62273907
rs8039785
rs2090816
rs6809952
rs2387977
All − MR Egger
All − IVW(MRE)
−1.0
−0.5
0.0
0.5
1.0
1.5
MR effect size for
'Gut microbiota abundance (genus Parasutterella id.2892)' on 'Protein Z−dependent protease inhibitor'

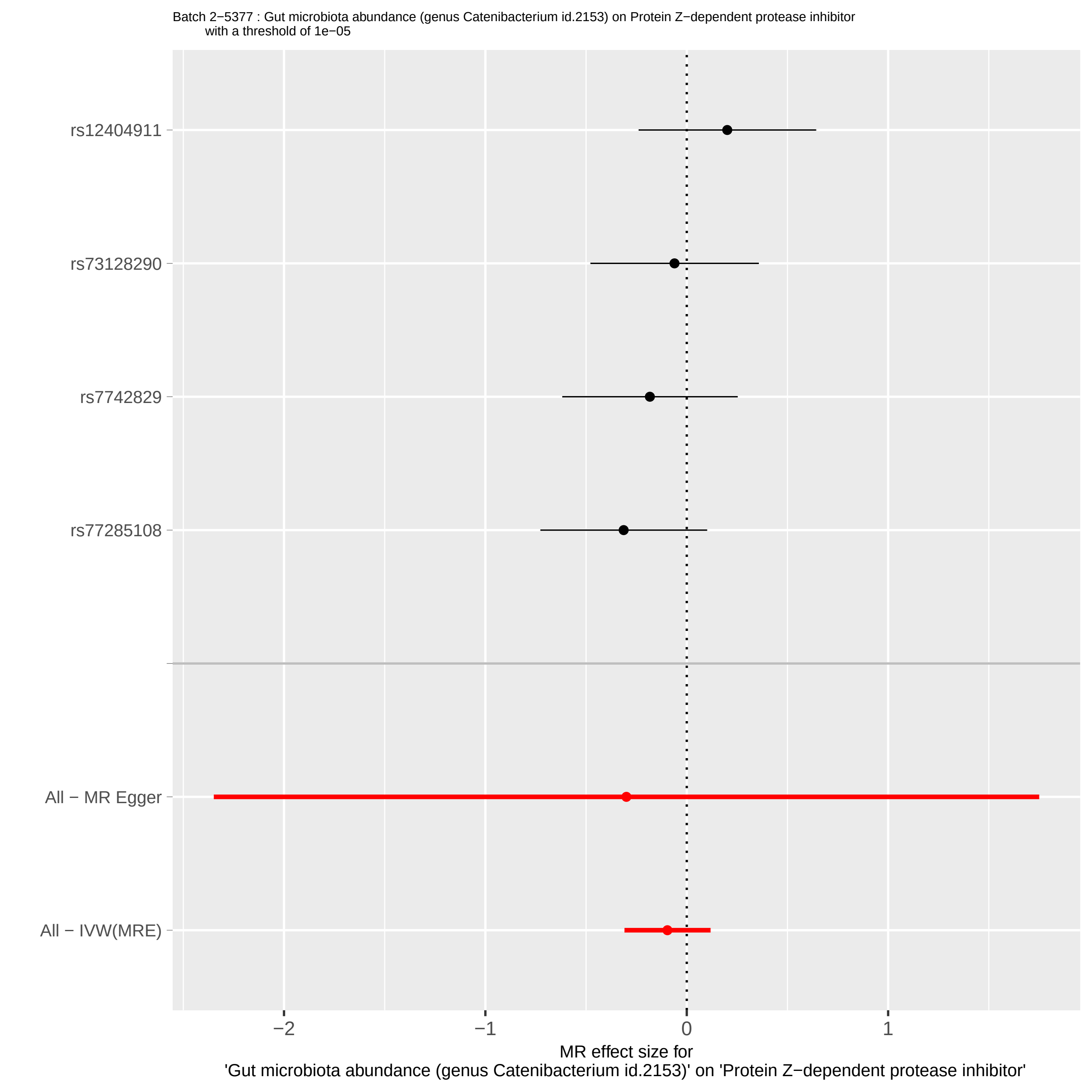
Batch 2−5377 : Gut microbiota abundance (genus Catenibacterium id.2153) on Protein Z−dependent protease inhibitor
with a threshold of 1e−05
rs12404911
rs73128290
rs7742829
rs77285108
All − MR Egger
All − IVW(MRE)
−2
−1
0
1
MR effect size for
'Gut microbiota abundance (genus Catenibacterium id.2153)' on 'Protein Z−dependent protease inhibitor'

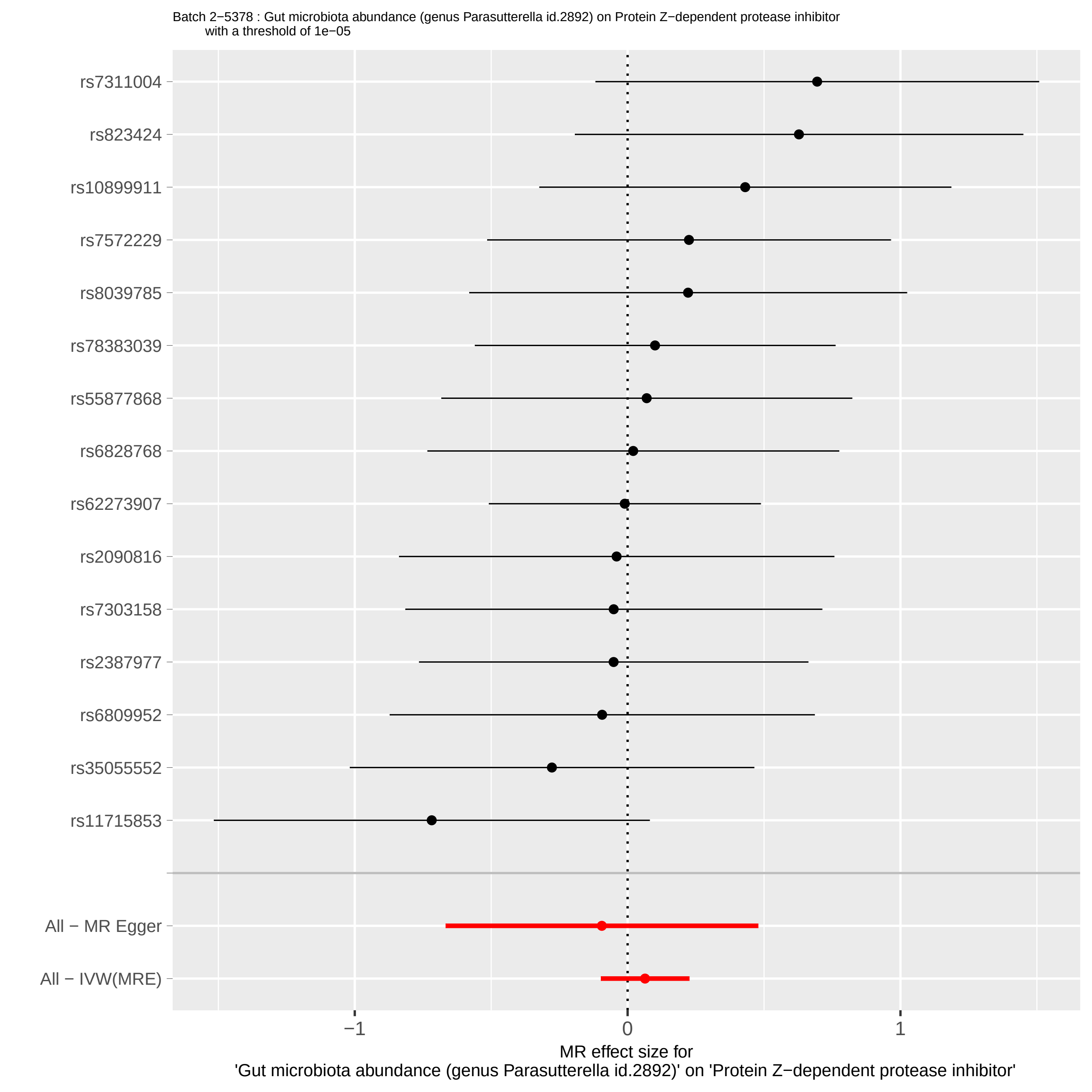

Batch 2−5378 : Gut microbiota abundance (genus Parasutterella id.2892) on Protein Z−dependent protease inhibitor
with a threshold of 1e−05
rs7311004
rs823424
rs10899911
rs7572229
rs8039785
rs78383039
rs55877868
rs6828768
rs62273907
rs2090816
rs7303158
rs2387977
rs6809952
rs35055552
rs11715853
All − MR Egger
All − IVW(MRE)
−1
0
1
MR effect size for
'Gut microbiota abundance (genus Parasutterella id.2892)' on 'Protein Z−dependent protease inhibitor'

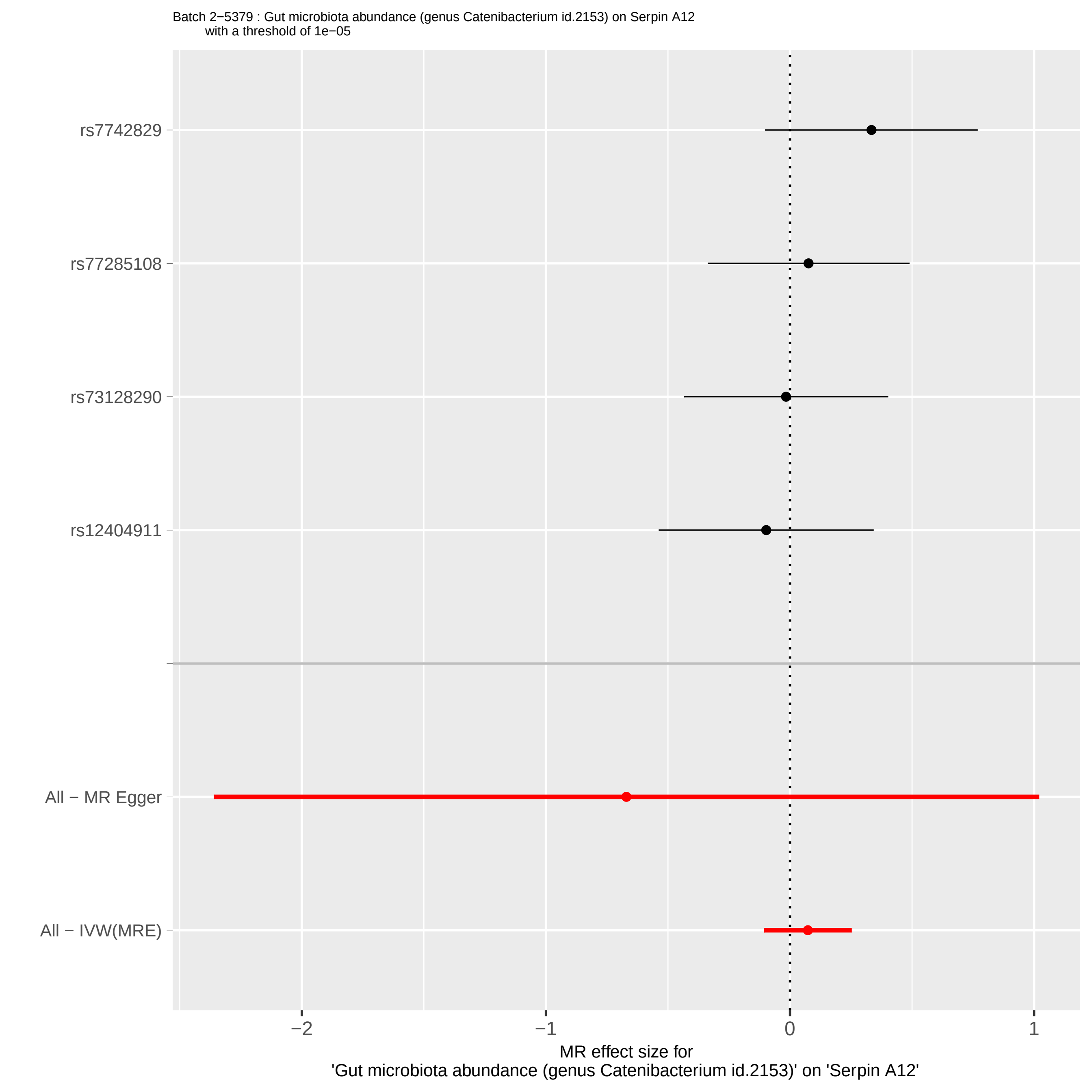

Batch 2−5379 : Gut microbiota abundance (genus Catenibacterium id.2153) on Serpin A12
with a threshold of 1e−05
rs7742829
rs77285108
rs73128290
rs12404911
All − MR Egger
All − IVW(MRE)
−2
−1
0
1
MR effect size for
'Gut microbiota abundance (genus Catenibacterium id.2153)' on 'Serpin A12'

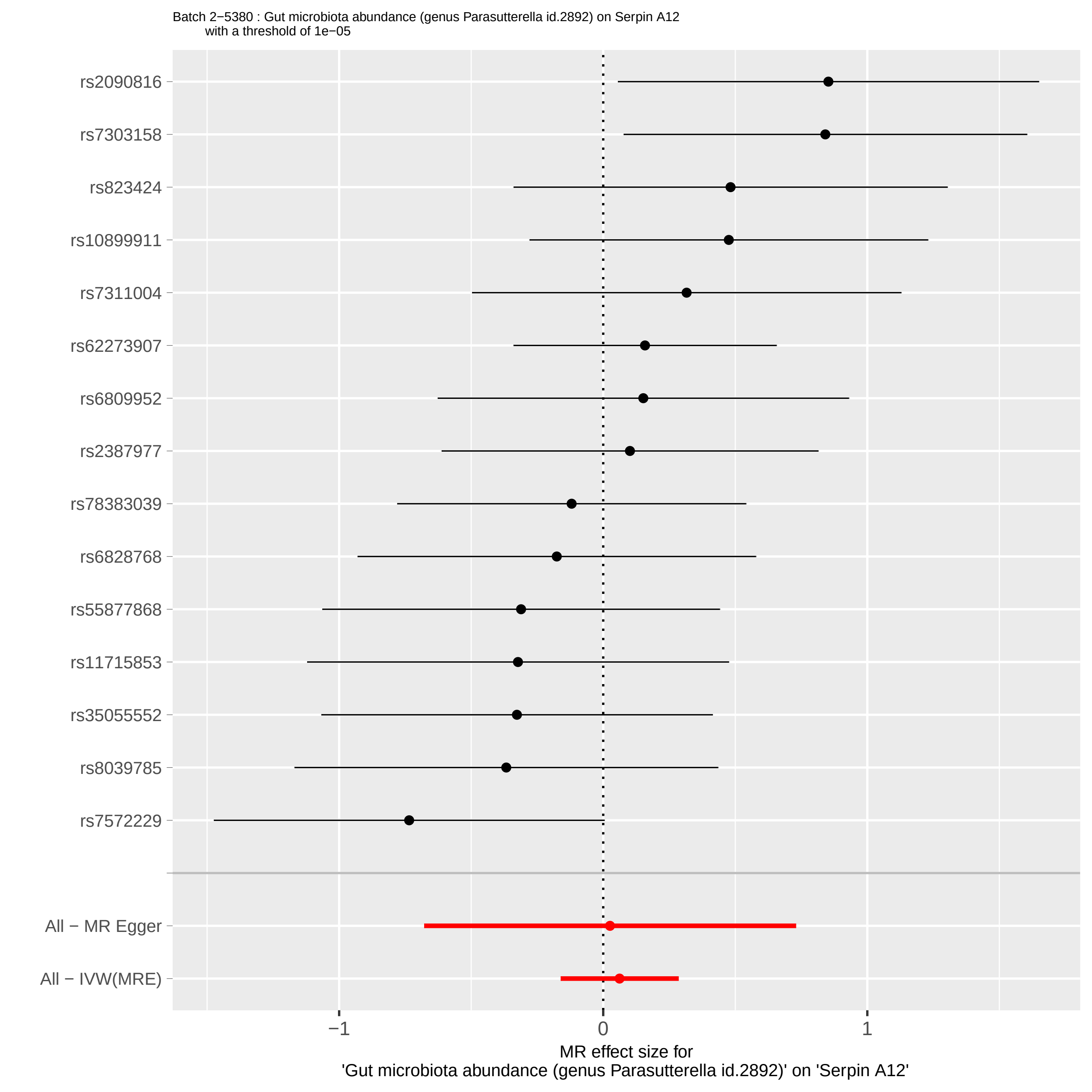
Batch 2−5380 : Gut microbiota abundance (genus Parasutterella id.2892) on Serpin A12
with a threshold of 1e−05
rs2090816
rs7303158
rs823424
rs10899911
rs7311004
rs62273907
rs6809952
rs2387977
rs78383039
rs6828768
rs55877868
rs11715853
rs35055552
rs8039785
rs7572229
All − MR Egger
All − IVW(MRE)
−1
0
1
MR effect size for
'Gut microbiota abundance (genus Parasutterella id.2892)' on 'Serpin A12'

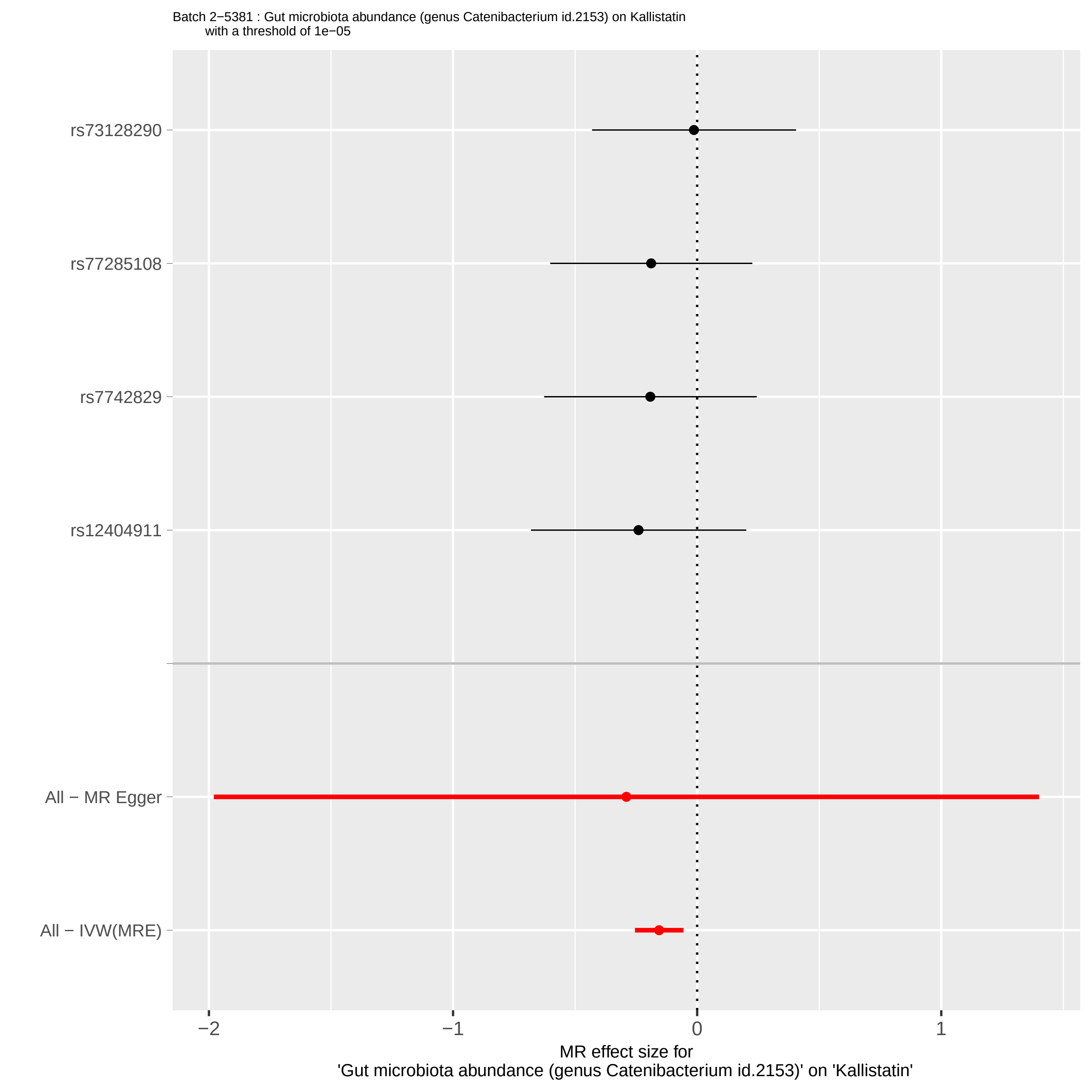
Batch 2−5381 : Gut microbiota abundance (genus Catenibacterium id.2153) on Kallistatin
with a threshold of 1e−05
rs73128290
rs77285108
rs7742829
rs12404911
All − MR Egger
All − IVW(MRE)
−2
−1
0
1
MR effect size for
'Gut microbiota abundance (genus Catenibacterium id.2153)' on 'Kallistatin'

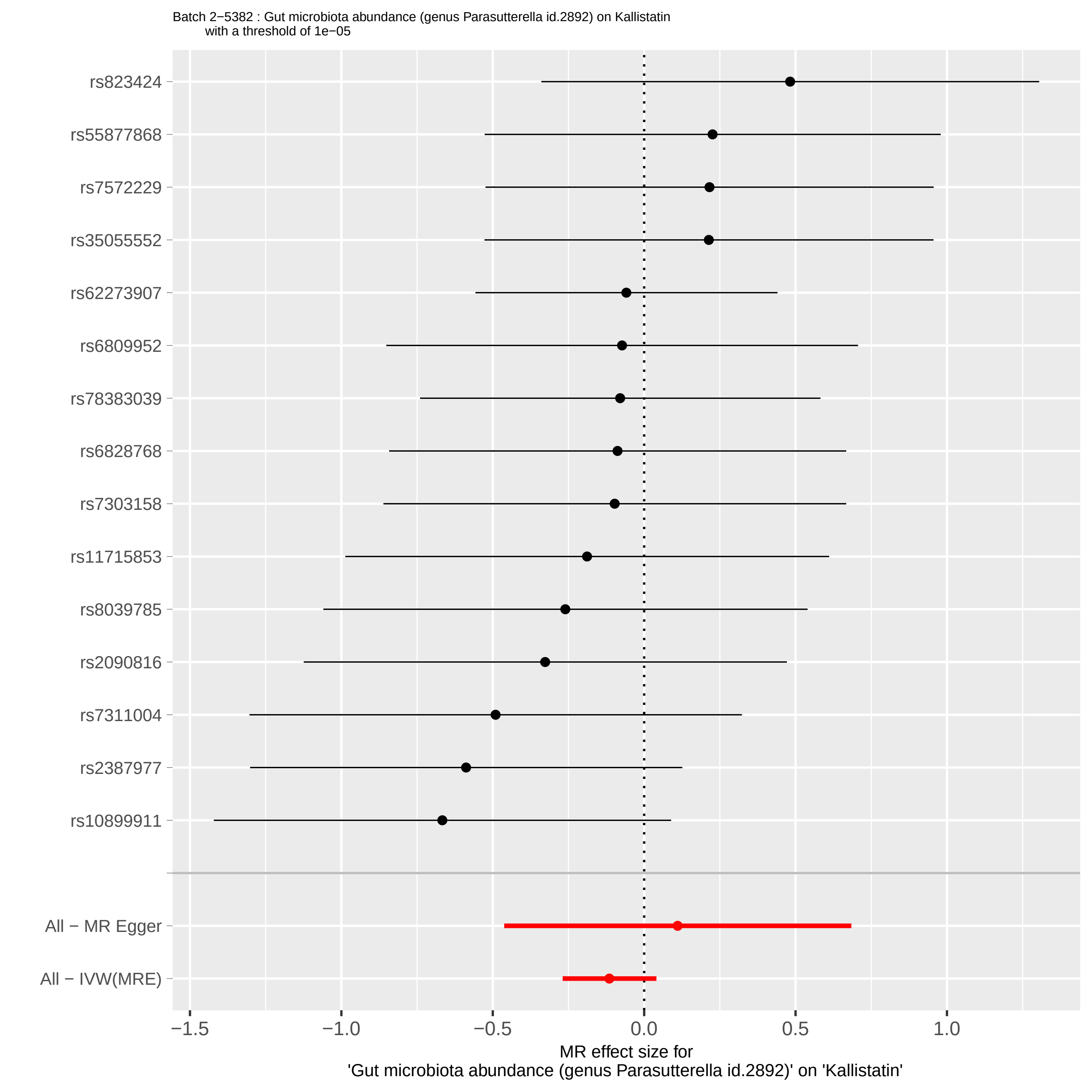

Batch 2−5382 : Gut microbiota abundance (genus Parasutterella id.2892) on Kallistatin
with a threshold of 1e−05
rs823424
rs55877868
rs7572229
rs35055552
rs62273907
rs6809952
rs78383039
rs6828768
rs7303158
rs11715853
rs8039785
rs2090816
rs7311004
rs2387977
rs10899911
All − MR Egger
All − IVW(MRE)
−1.5
−1.0
−0.5
0.0
0.5
1.0
MR effect size for
'Gut microbiota abundance (genus Parasutterella id.2892)' on 'Kallistatin'

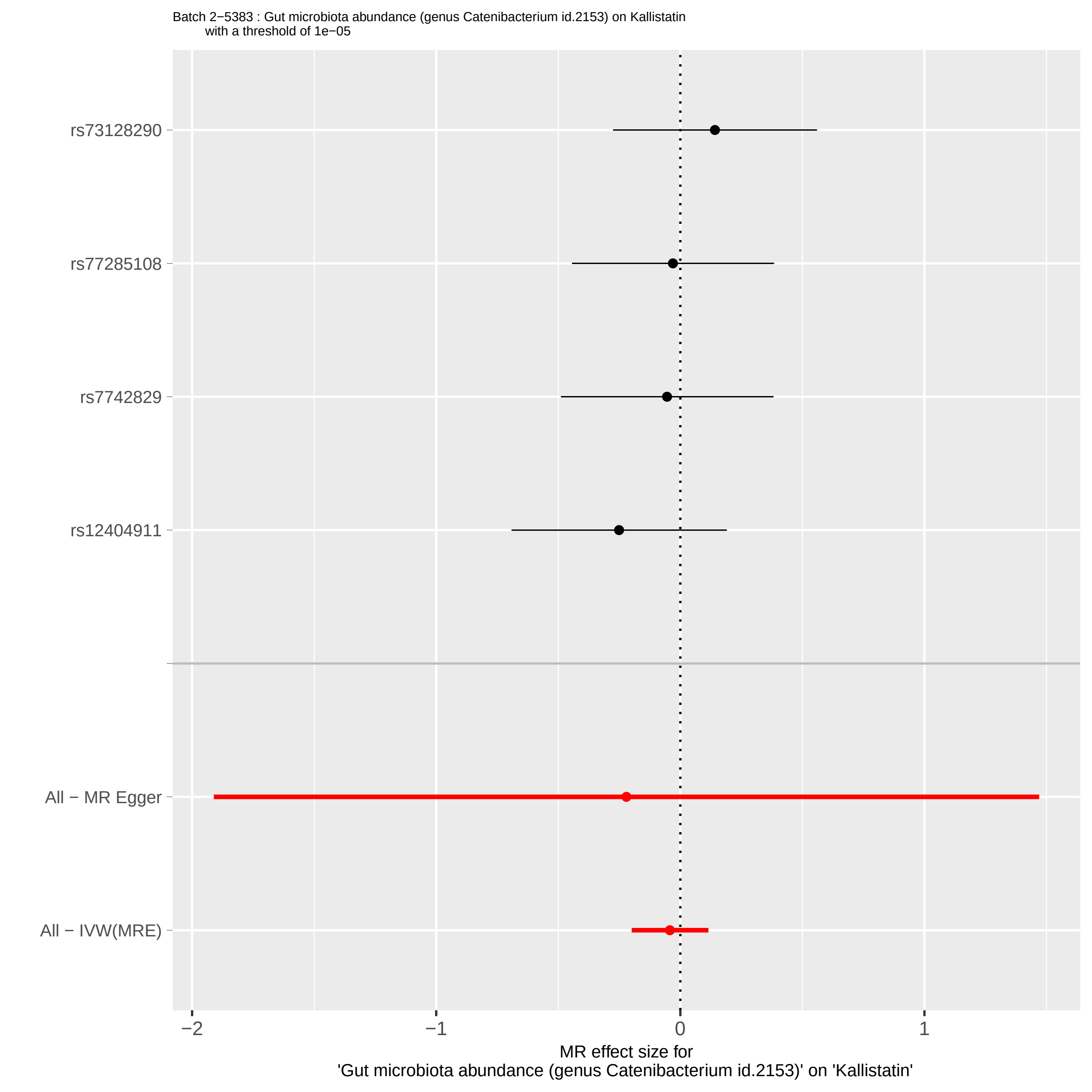

Batch 2−5383 : Gut microbiota abundance (genus Catenibacterium id.2153) on Kallistatin
with a threshold of 1e−05
rs73128290
rs77285108
rs7742829
rs12404911
All − MR Egger
All − IVW(MRE)
−2
−1
0
1
MR effect size for
'Gut microbiota abundance (genus Catenibacterium id.2153)' on 'Kallistatin'

Batch 2−5384 : Gut microbiota abundance (genus Parasutterella id.2892) on Kallistatin
with a threshold of 1e−05

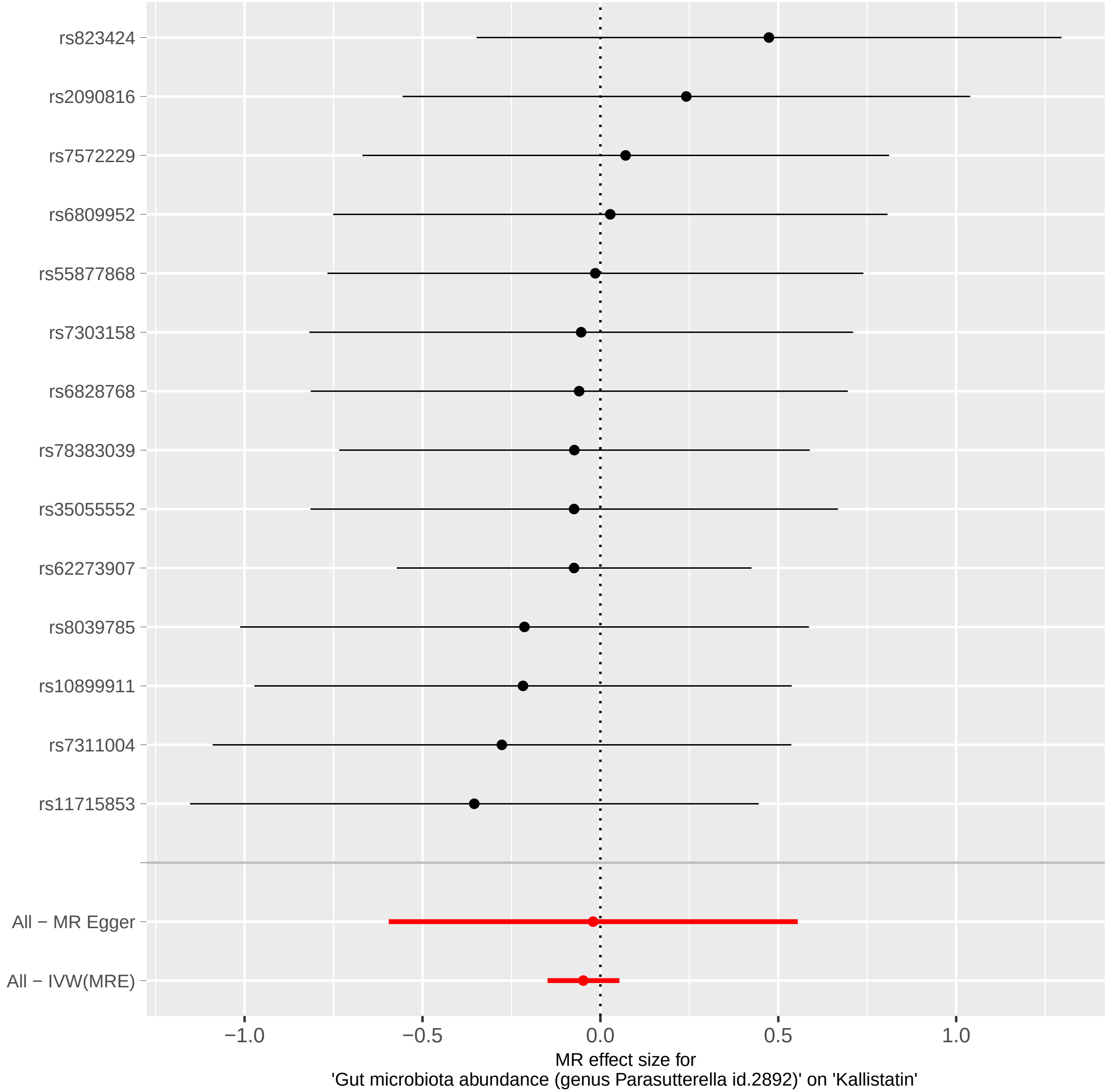

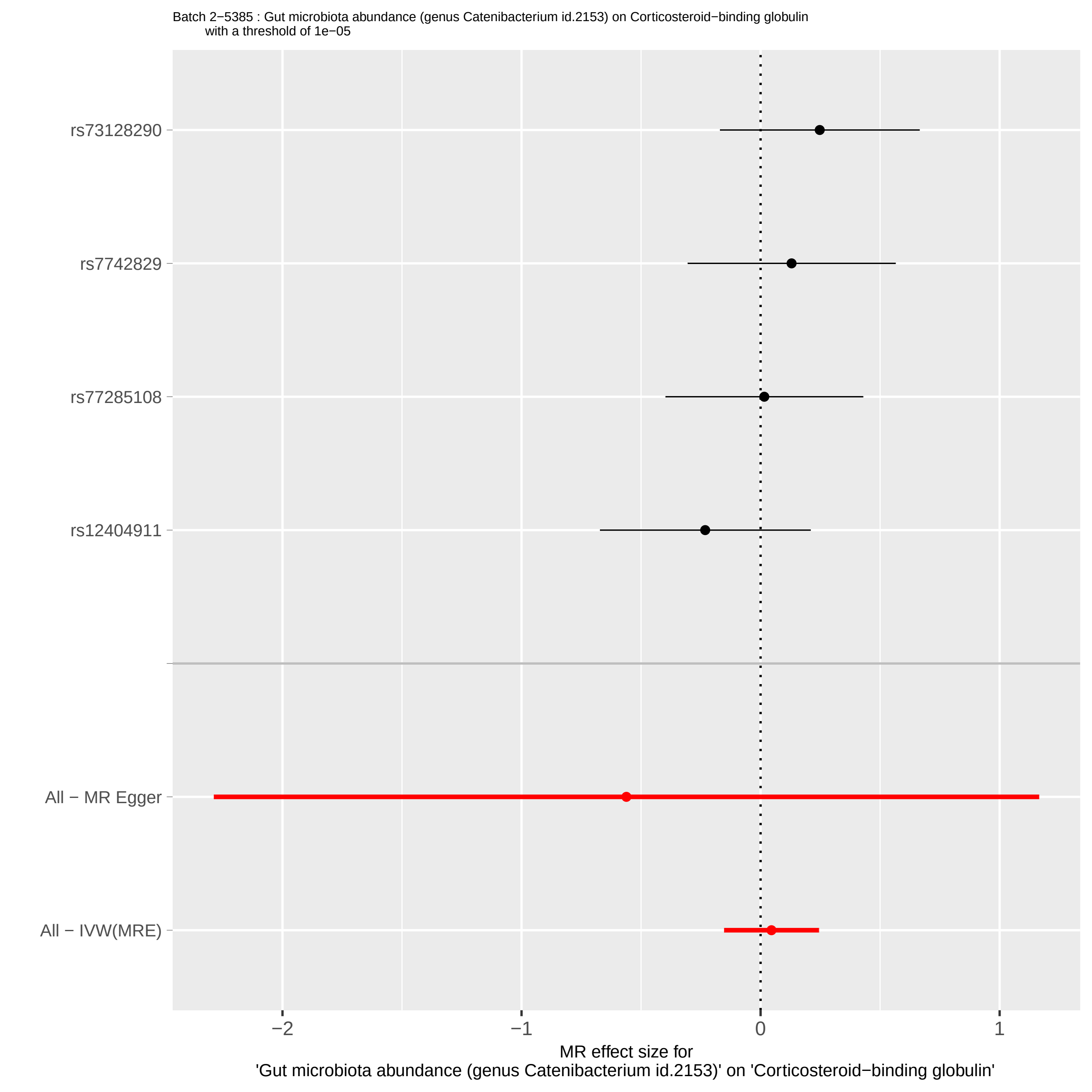
Batch 2−5385 : Gut microbiota abundance (genus Catenibacterium id.2153) on Corticosteroid−binding globulin
with a threshold of 1e−05
rs73128290
rs7742829
rs77285108
rs12404911
All − MR Egger
All − IVW(MRE)
−2
−1
0
1
MR effect size for
'Gut microbiota abundance (genus Catenibacterium id.2153)' on 'Corticosteroid−binding globulin'

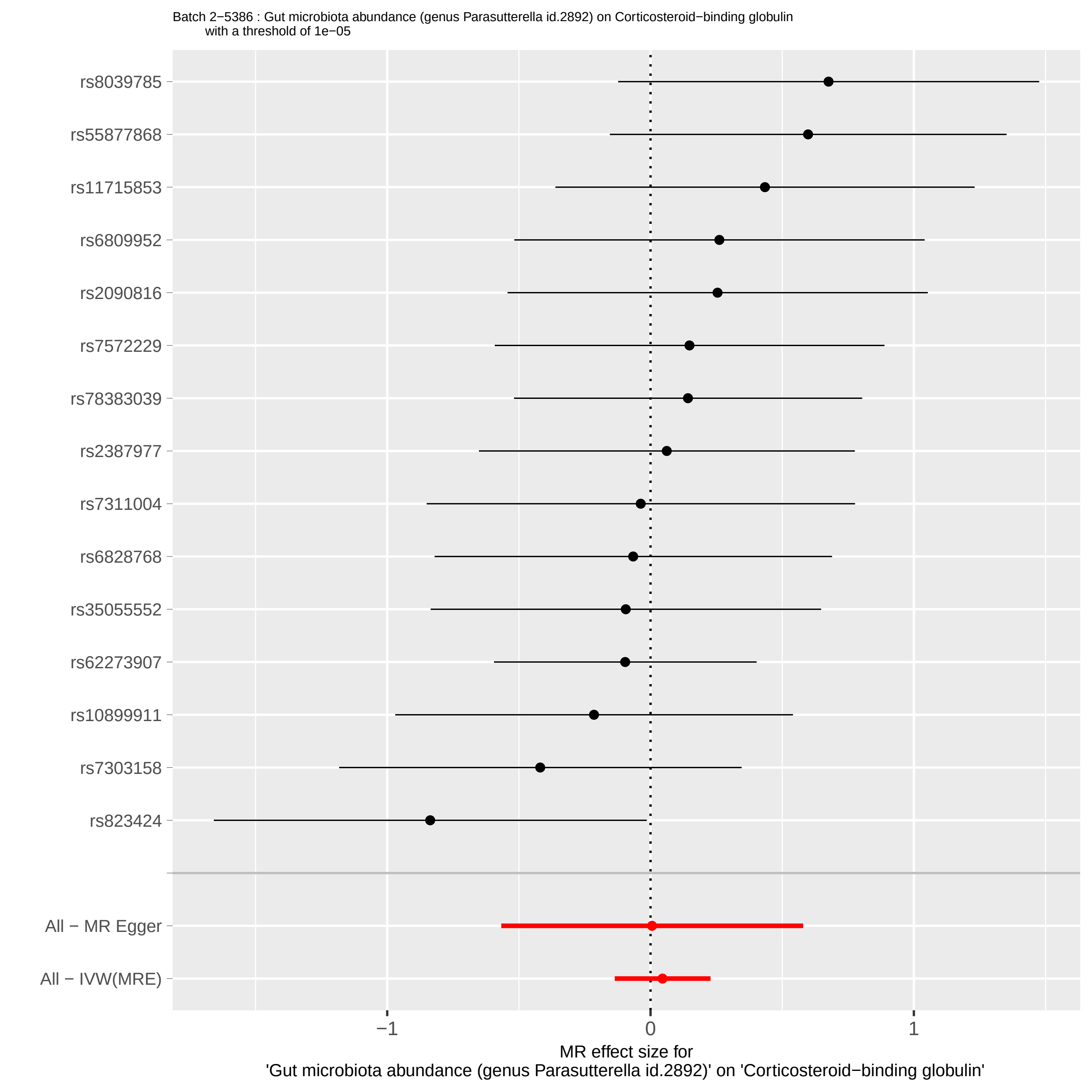
Batch 2−5386 : Gut microbiota abundance (genus Parasutterella id.2892) on Corticosteroid−binding globulin
with a threshold of 1e−05
rs8039785
rs55877868
rs11715853
rs6809952
rs2090816
rs7572229
rs78383039
rs2387977
rs7311004
rs6828768
rs35055552
rs62273907
rs10899911
rs7303158
rs823424
All − MR Egger
All − IVW(MRE)
−1
0
1
MR effect size for
'Gut microbiota abundance (genus Parasutterella id.2892)' on 'Corticosteroid−binding globulin'

Batch 2−5387 : Gut microbiota abundance (genus Catenibacterium id.2153) on Serpin A9
with a threshold of 1e−05

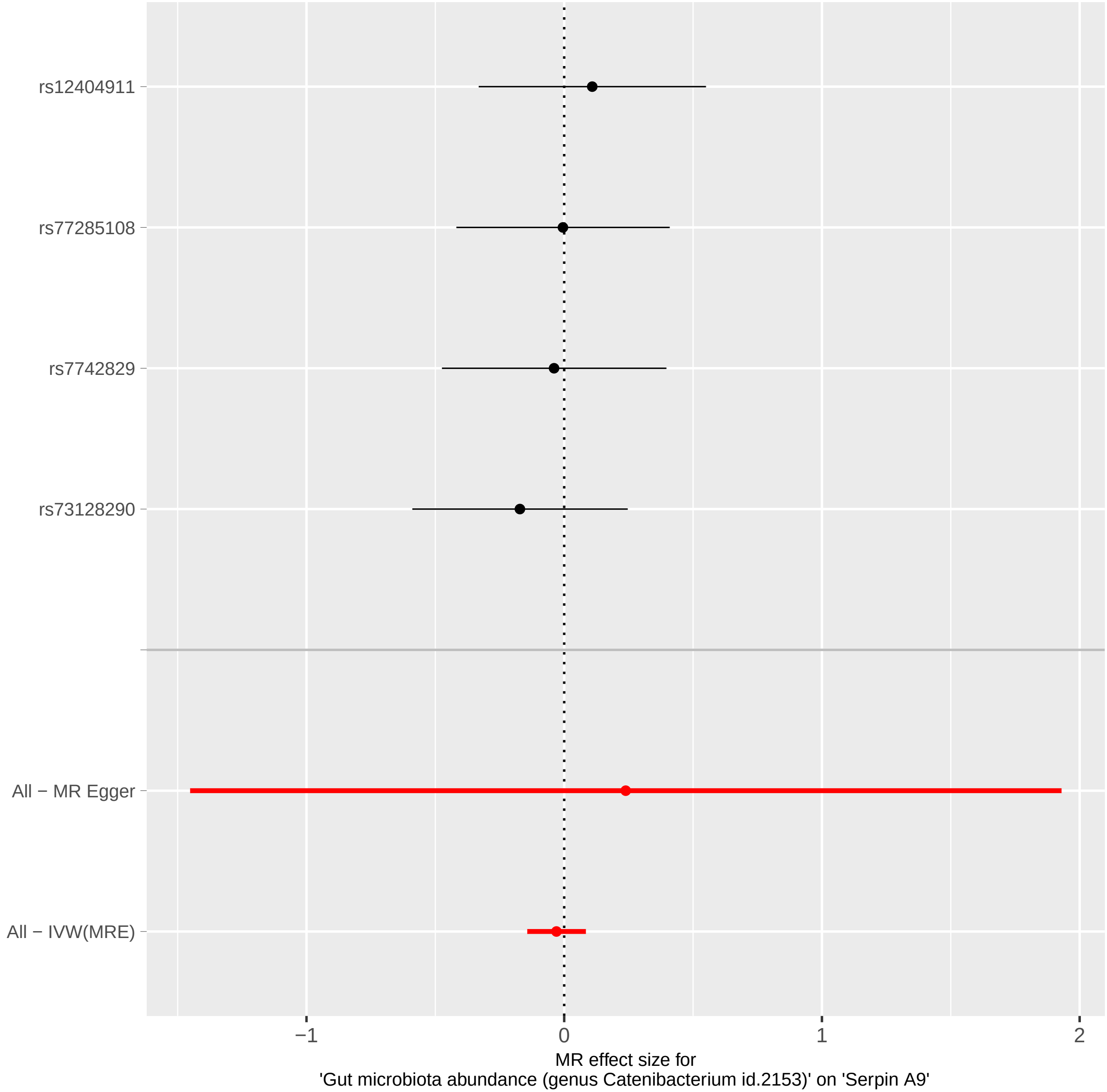

Batch 2−5388 : Gut microbiota abundance (genus Parasutterella id.2892) on Serpin A9
with a threshold of 1e−05

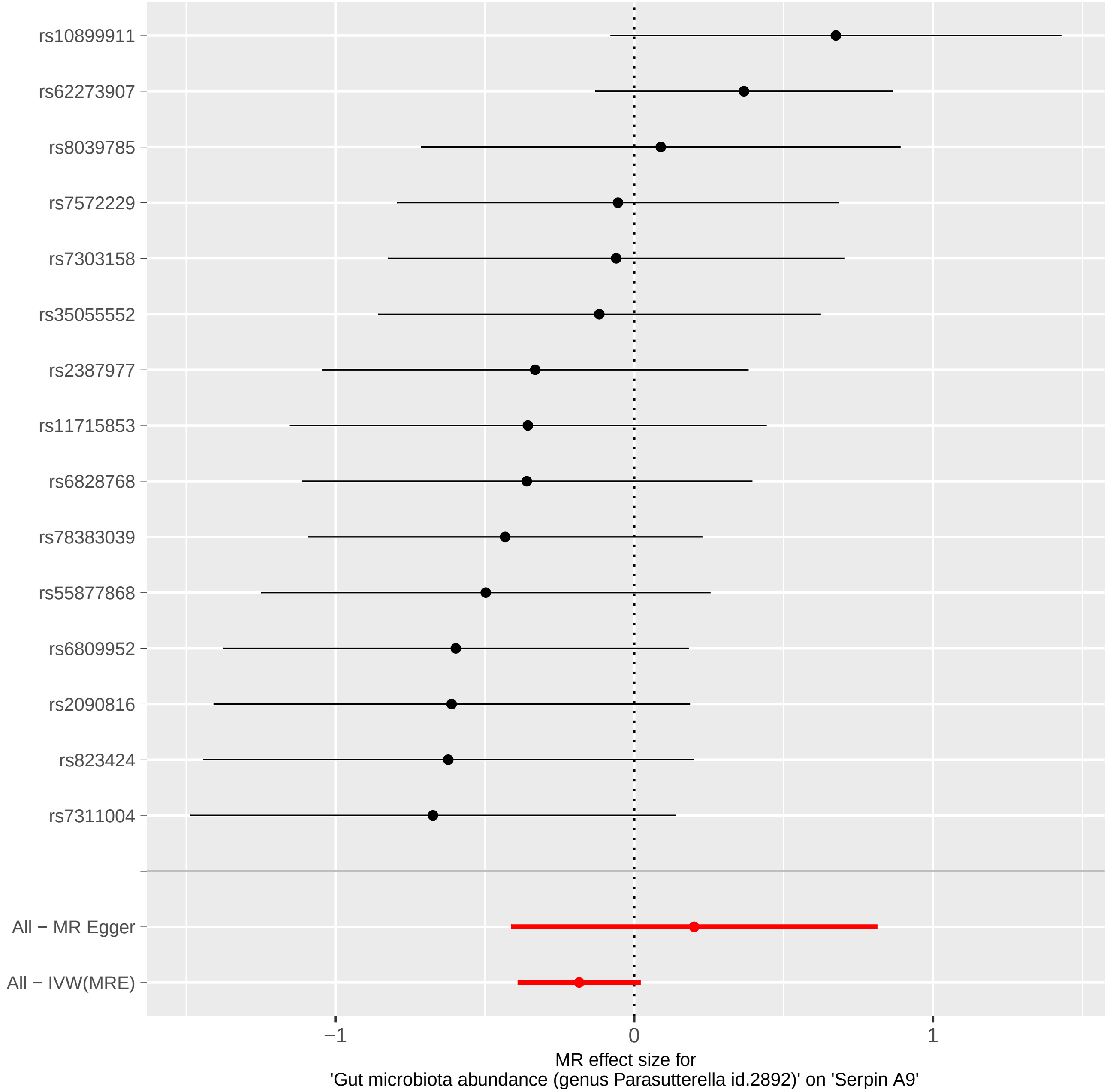

Batch 2−5389 : Gut microbiota abundance (genus Catenibacterium id.2153) on Antithrombin−III
with a threshold of 1e−05

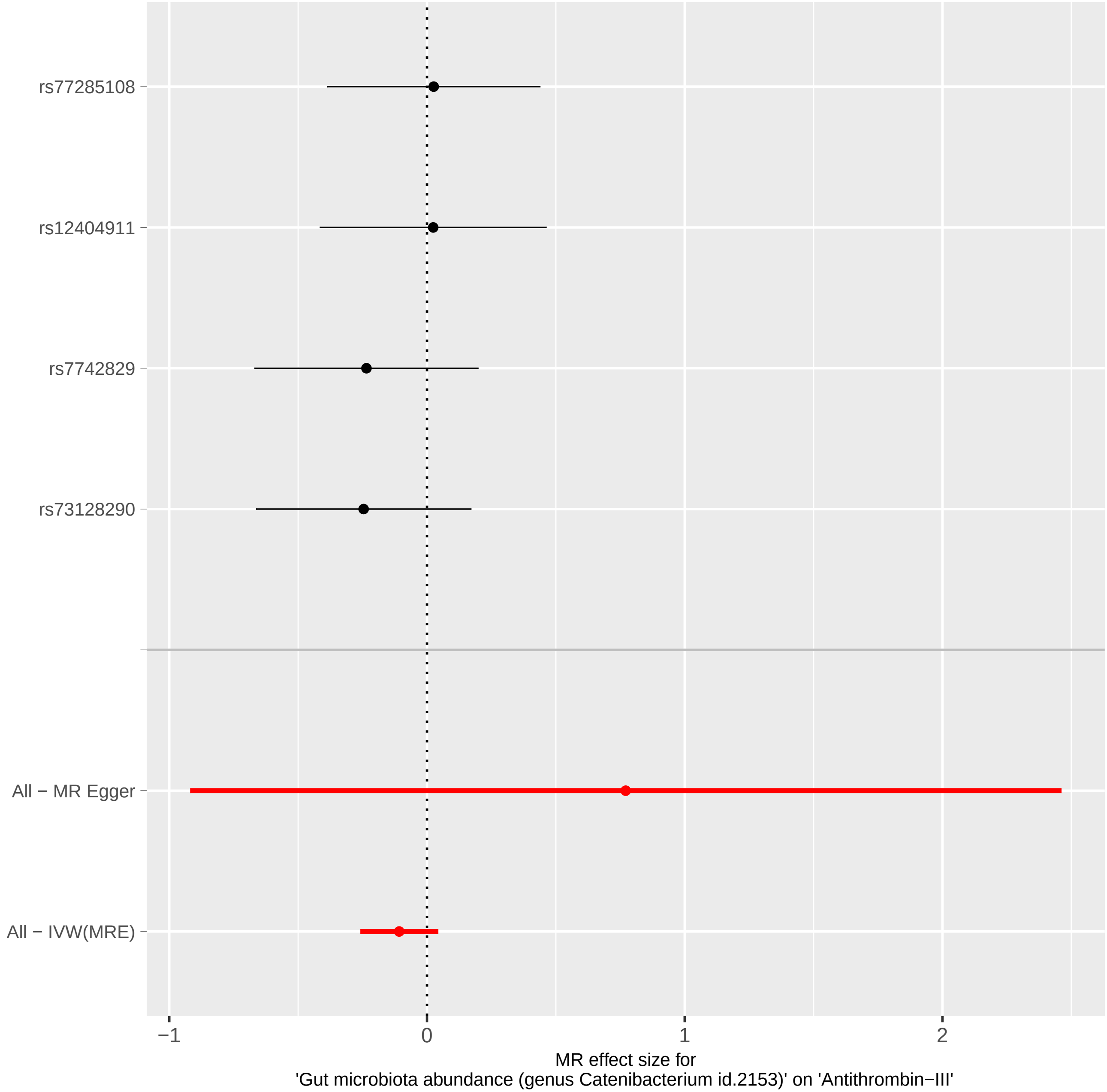

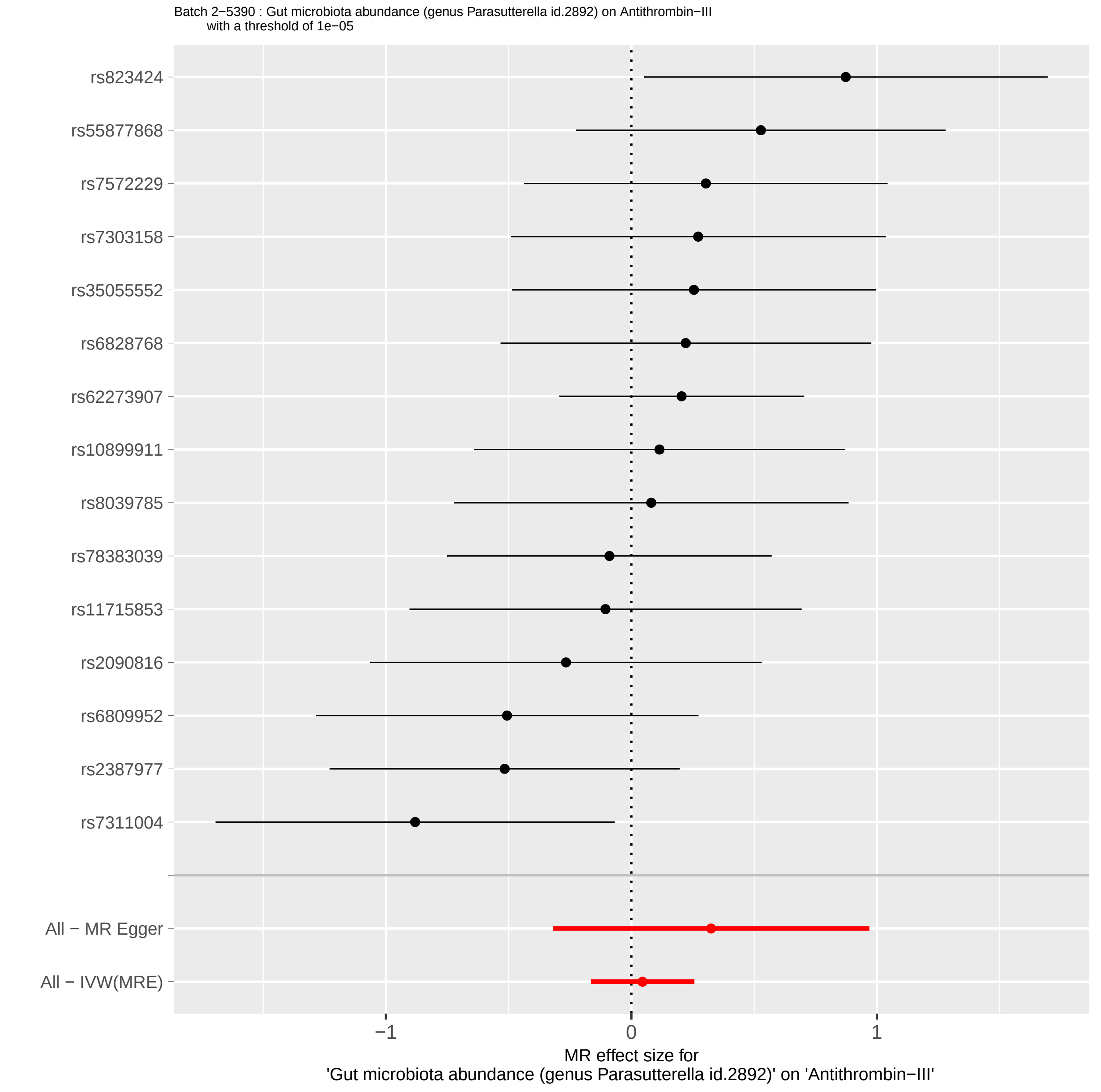

Batch 2−5390 : Gut microbiota abundance (genus Parasutterella id.2892) on Antithrombin−III
with a threshold of 1e−05
rs823424
rs55877868
rs7572229
rs7303158
rs35055552
rs6828768
rs62273907
rs10899911
rs8039785
rs78383039
rs11715853
rs2090816
rs6809952
rs2387977
rs7311004
All − MR Egger
All − IVW(MRE)
−1
0
1
MR effect size for
'Gut microbiota abundance (genus Parasutterella id.2892)' on 'Antithrombin−III'

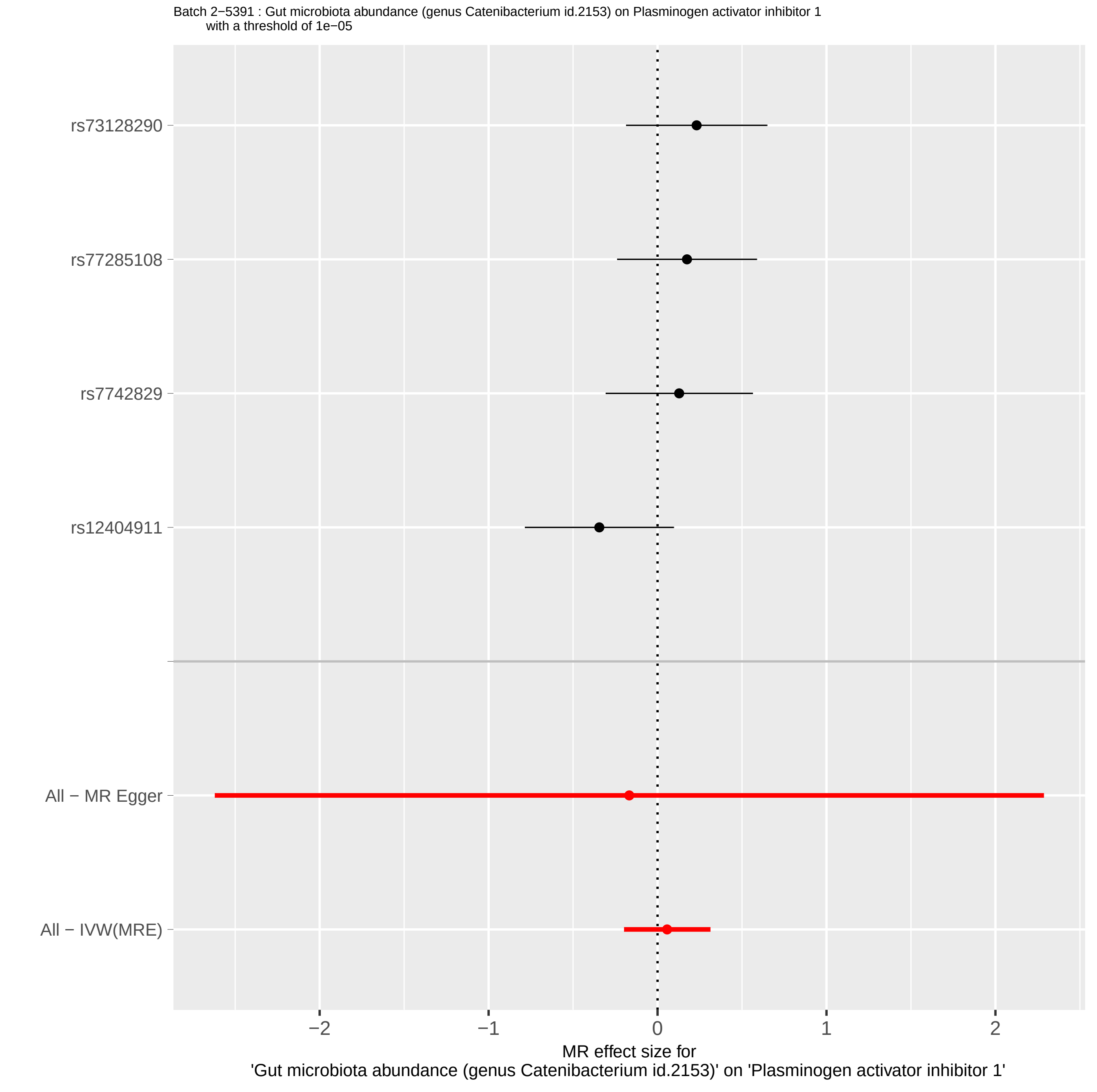
Batch 2−5391 : Gut microbiota abundance (genus Catenibacterium id.2153) on Plasminogen activator inhibitor 1
with a threshold of 1e−05
rs73128290
rs77285108
rs7742829
rs12404911
All − MR Egger
All − IVW(MRE)
−2
−1
0
1
2
MR effect size for
'Gut microbiota abundance (genus Catenibacterium id.2153)' on 'Plasminogen activator inhibitor 1'

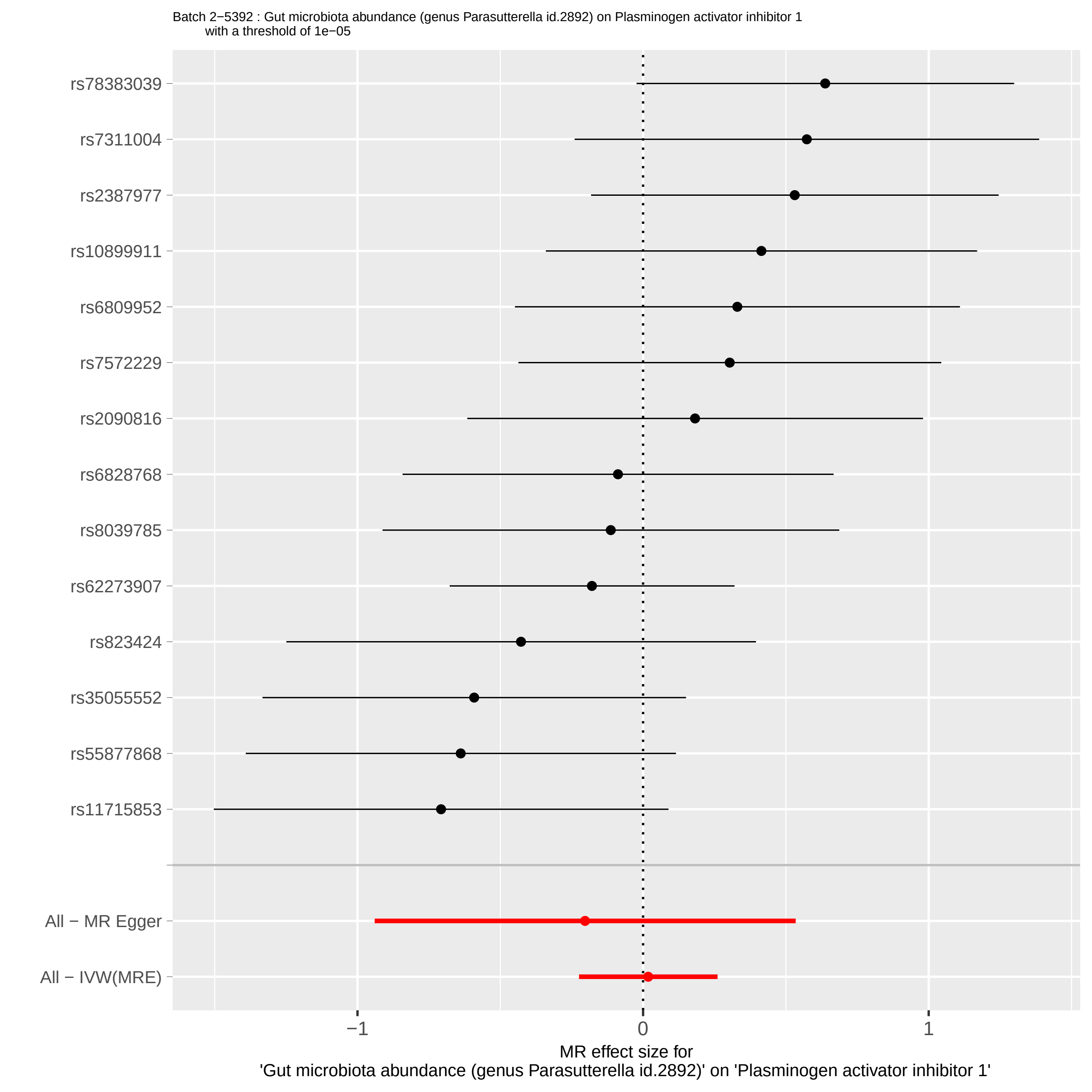
Batch 2−5392 : Gut microbiota abundance (genus Parasutterella id.2892) on Plasminogen activator inhibitor 1
with a threshold of 1e−05
rs78383039
rs7311004
rs2387977
rs10899911
rs6809952
rs7572229
rs2090816
rs6828768
rs8039785
rs62273907
rs823424
rs35055552
rs55877868
rs11715853
All − MR Egger
All − IVW(MRE)
−1
0
1
MR effect size for
'Gut microbiota abundance (genus Parasutterella id.2892)' on 'Plasminogen activator inhibitor 1'

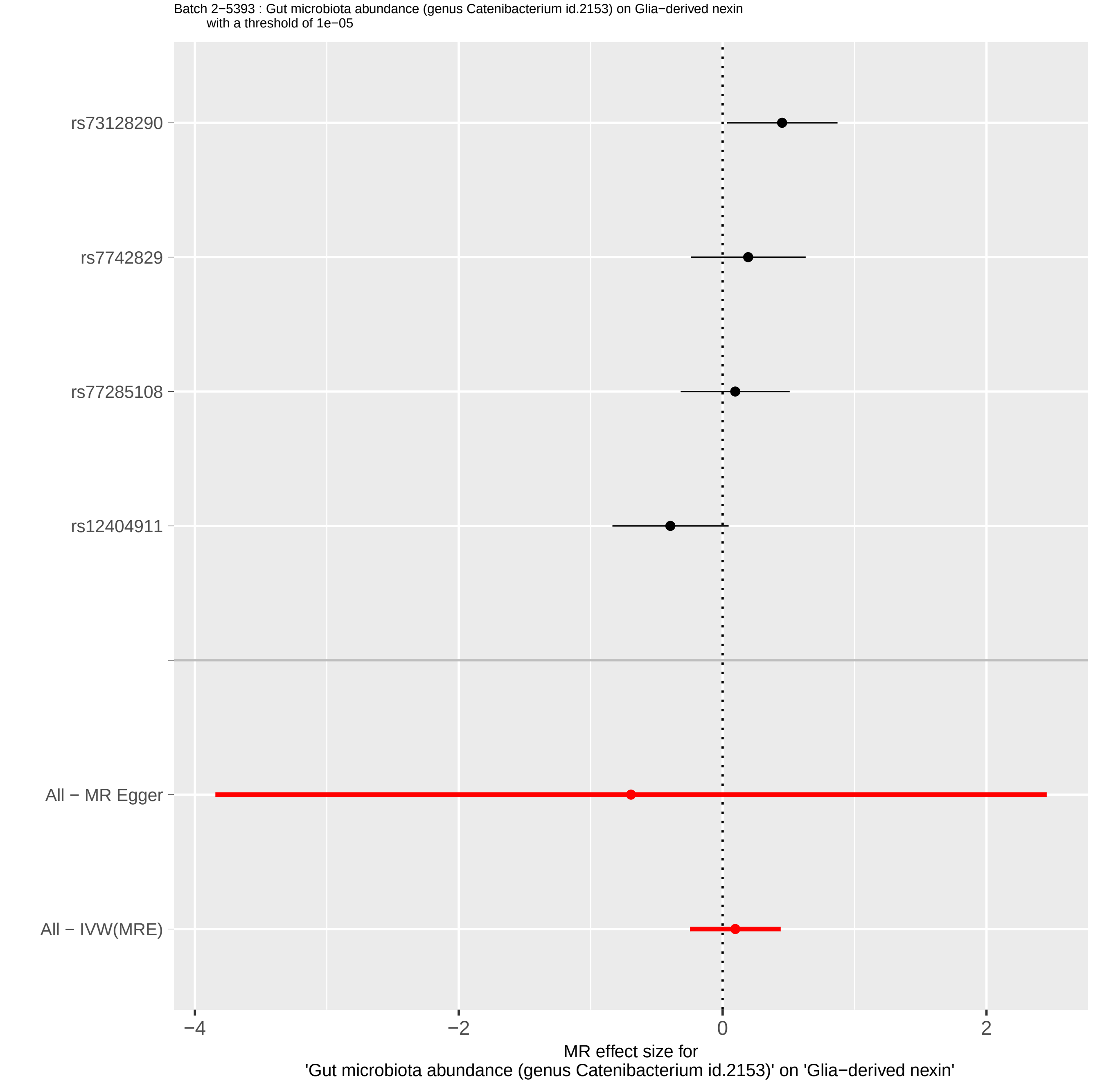

Batch 2−5393 : Gut microbiota abundance (genus Catenibacterium id.2153) on Glia−derived nexin
with a threshold of 1e−05
rs73128290
rs7742829
rs77285108
rs12404911
All − MR Egger
All − IVW(MRE)
−4
−2
0
2
MR effect size for
'Gut microbiota abundance (genus Catenibacterium id.2153)' on 'Glia−derived nexin'

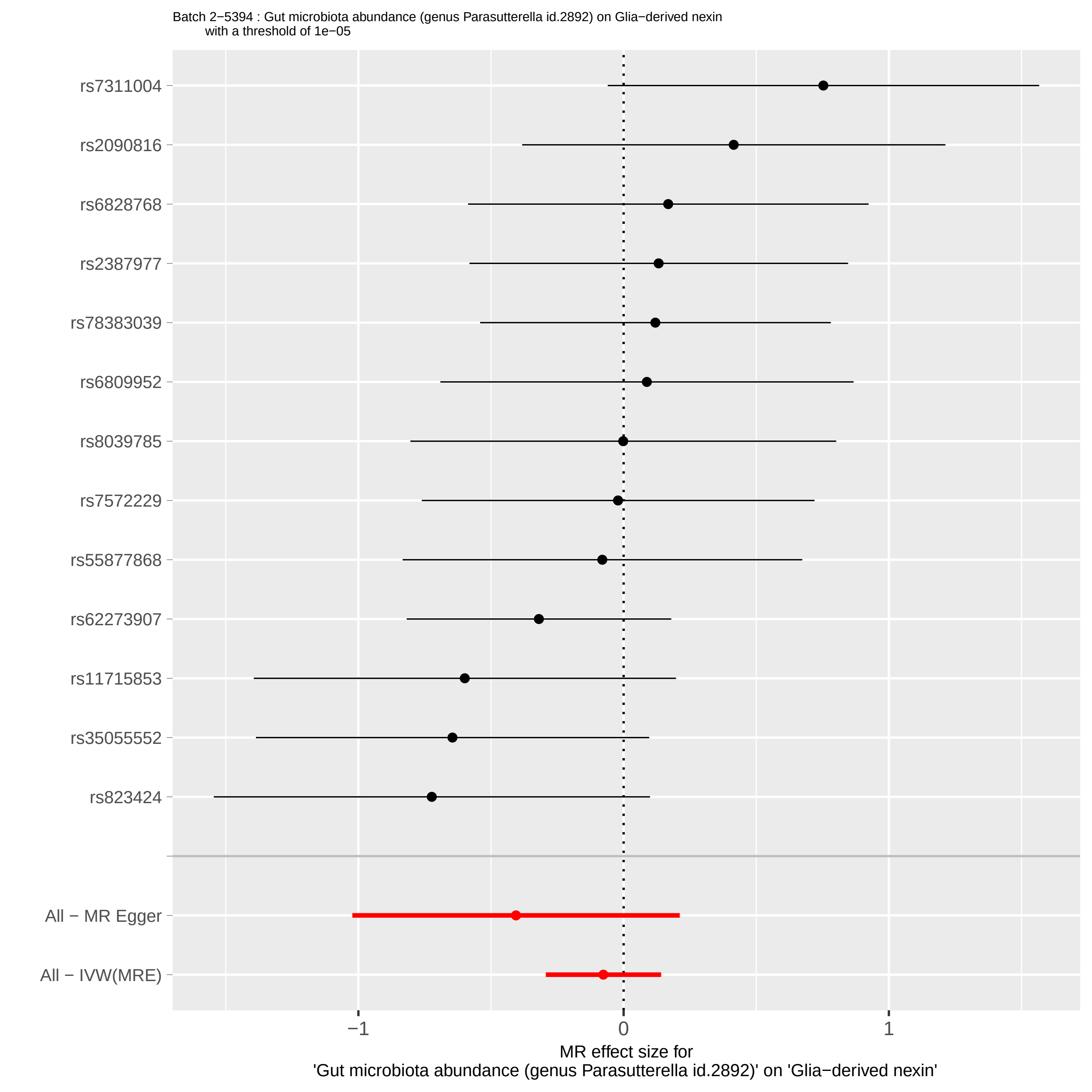
Batch 2−5394 : Gut microbiota abundance (genus Parasutterella id.2892) on Glia−derived nexin
with a threshold of 1e−05
rs7311004
rs2090816
rs6828768
rs2387977
rs78383039
rs6809952
rs8039785
rs7572229
rs55877868
rs62273907
rs11715853
rs35055552
rs823424
All − MR Egger
All − IVW(MRE)
−1
0
1
MR effect size for
'Gut microbiota abundance (genus Parasutterella id.2892)' on 'Glia−derived nexin'

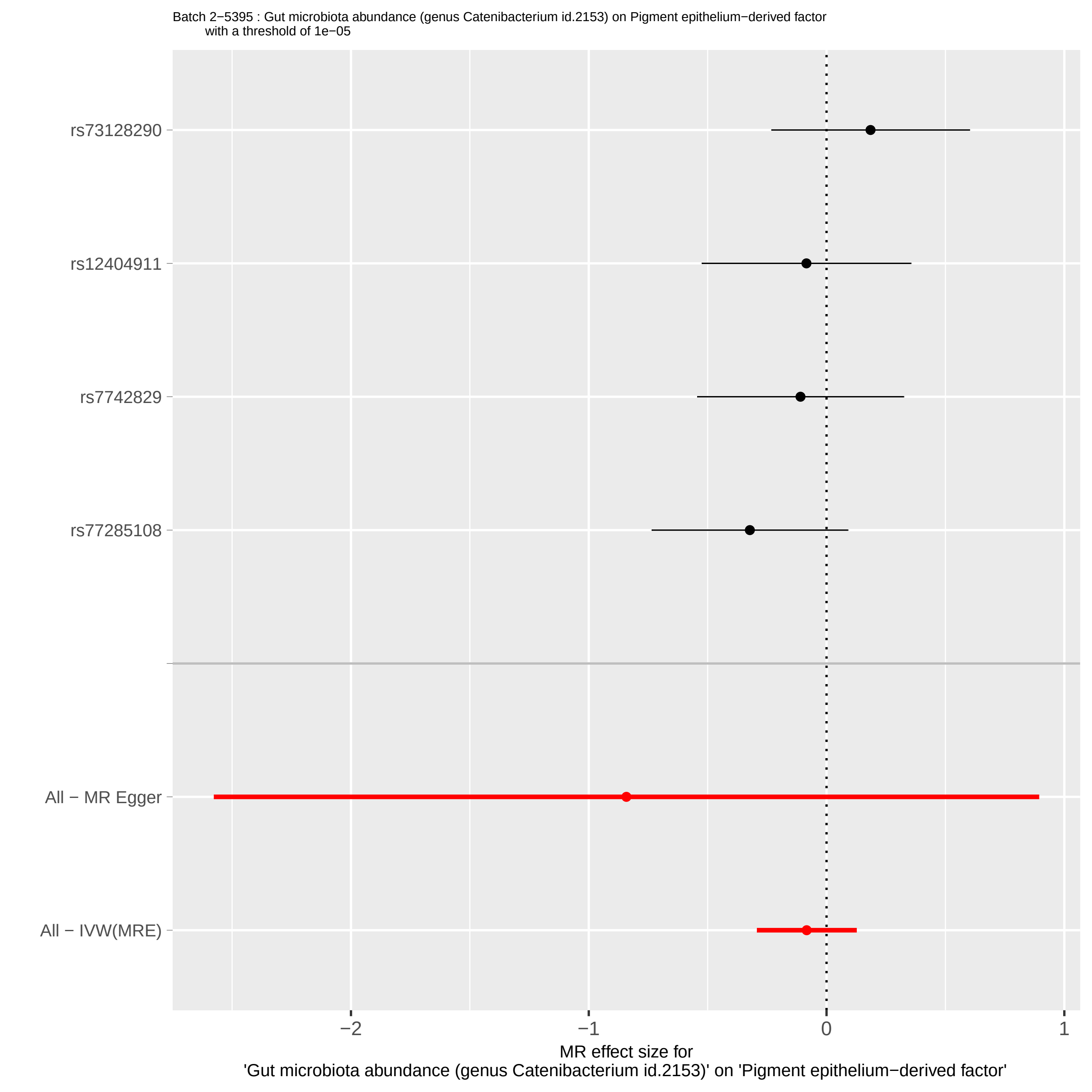
Batch 2−5395 : Gut microbiota abundance (genus Catenibacterium id.2153) on Pigment epithelium−derived factor
with a threshold of 1e−05
rs73128290
rs12404911
rs7742829
rs77285108
All − MR Egger
All − IVW(MRE)
−2
−1
0
1
MR effect size for
'Gut microbiota abundance (genus Catenibacterium id.2153)' on 'Pigment epithelium−derived factor'

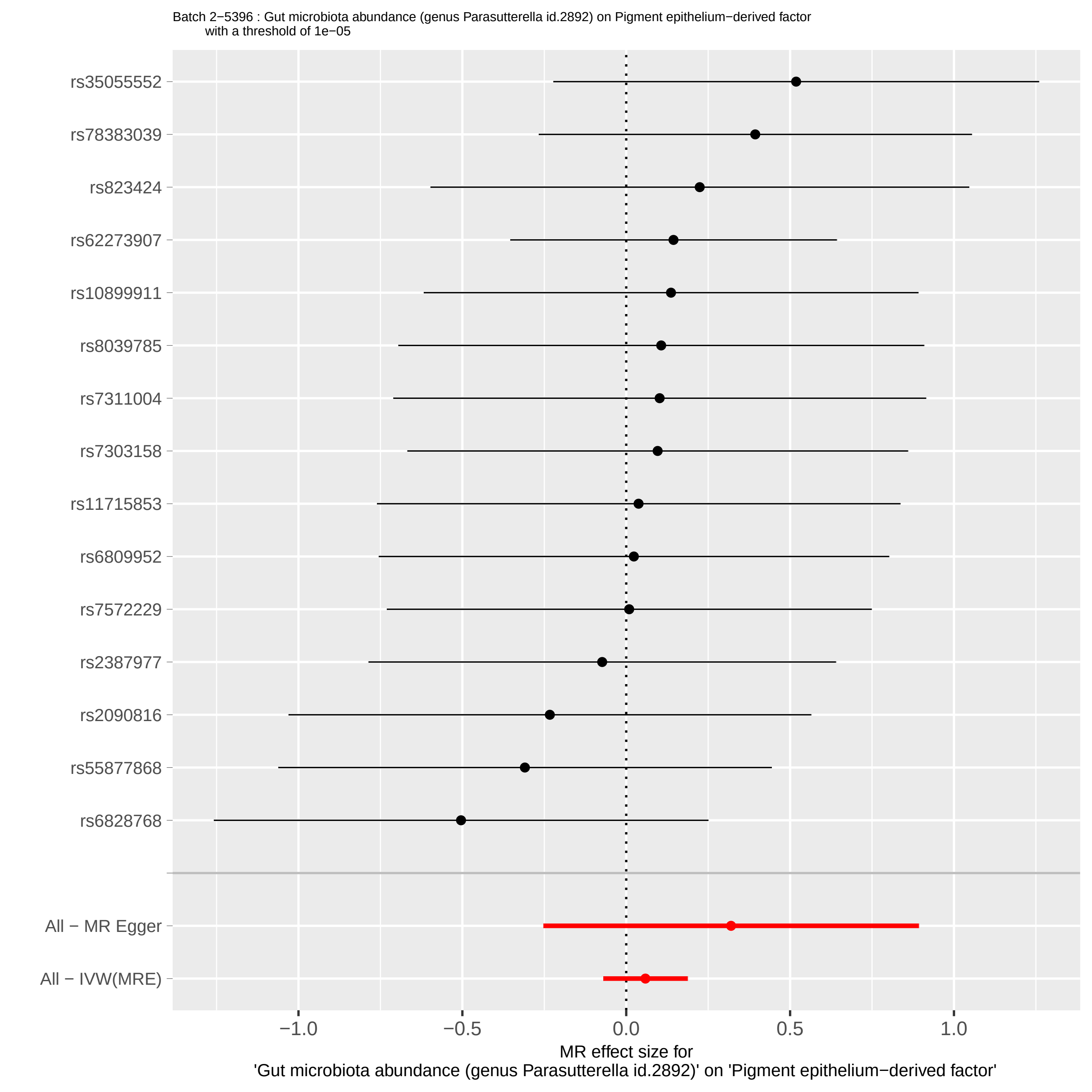
Batch 2−5396 : Gut microbiota abundance (genus Parasutterella id.2892) on Pigment epithelium−derived factor
with a threshold of 1e−05
rs35055552
rs78383039
rs823424
rs62273907
rs10899911
rs8039785
rs7311004
rs7303158
rs11715853
rs6809952
rs7572229
rs2387977
rs2090816
rs55877868
rs6828768
All − MR Egger
All − IVW(MRE)
−1.0
−0.5
0.0
0.5
1.0
MR effect size for
'Gut microbiota abundance (genus Parasutterella id.2892)' on 'Pigment epithelium−derived factor'

Batch 2−5397 : Gut microbiota abundance (genus Catenibacterium id.2153) on Pigment epithelium−derived factor
with a threshold of 1e−05

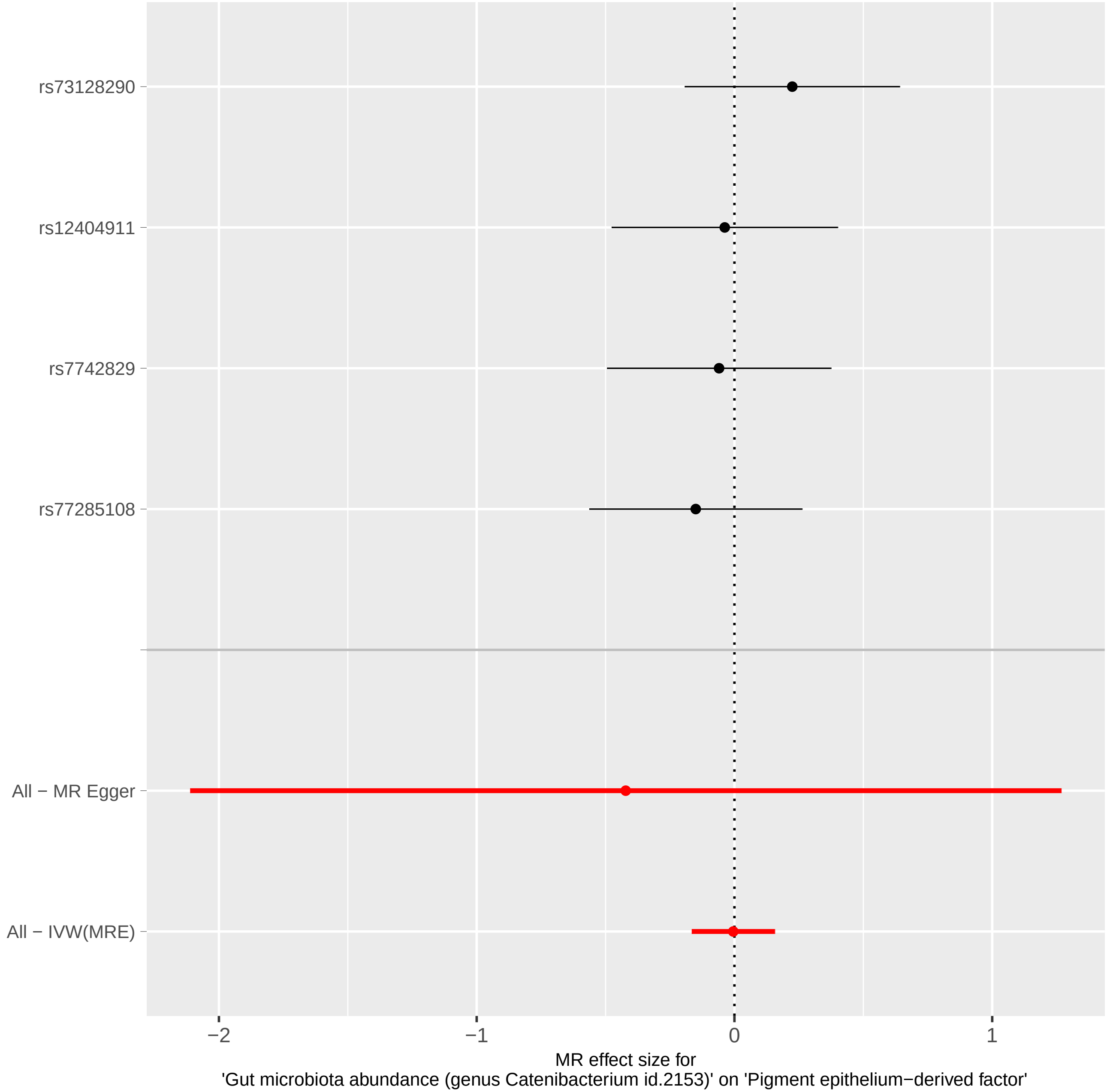

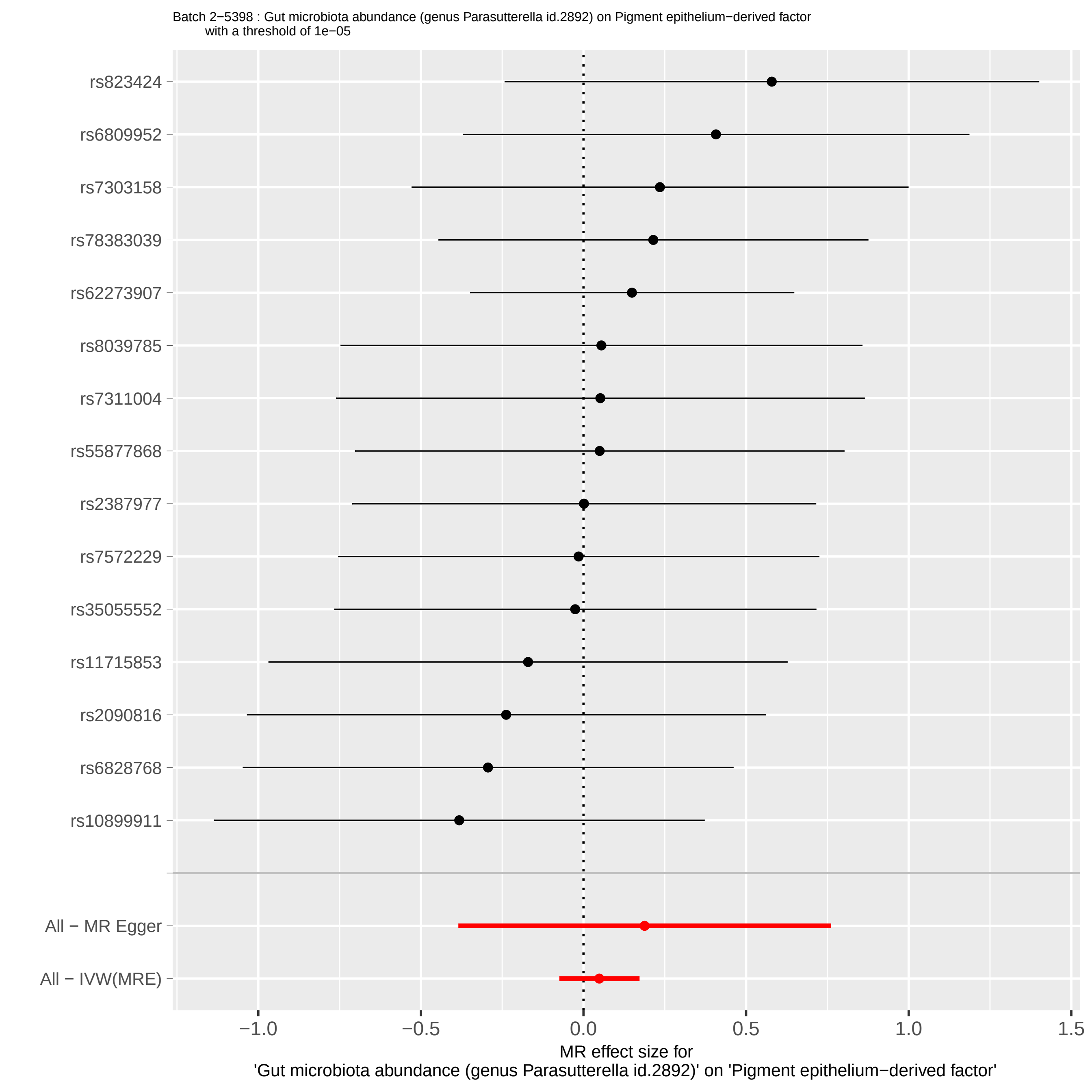

Batch 2−5398 : Gut microbiota abundance (genus Parasutterella id.2892) on Pigment epithelium−derived factor
with a threshold of 1e−05
rs823424
rs6809952
rs7303158
rs78383039
rs62273907
rs8039785
rs7311004
rs55877868
rs2387977
rs7572229
rs35055552
rs11715853
rs2090816
rs6828768
rs10899911
All − MR Egger
All − IVW(MRE)
−1.0
−0.5
0.0
0.5
1.0
1.5
MR effect size for
'Gut microbiota abundance (genus Parasutterella id.2892)' on 'Pigment epithelium−derived factor'

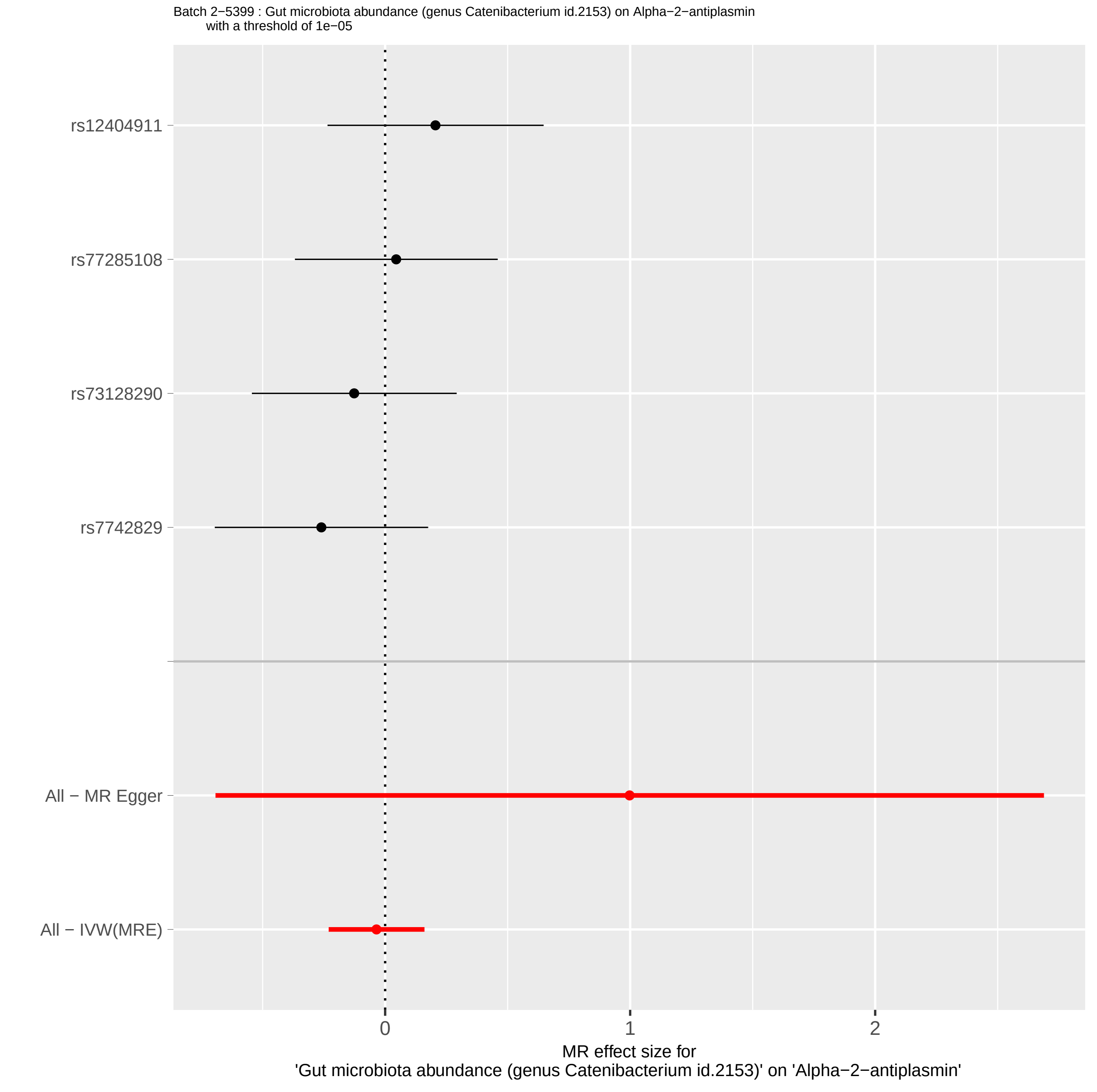

Batch 2−5399 : Gut microbiota abundance (genus Catenibacterium id.2153) on Alpha−2−antiplasmin
with a threshold of 1e−05
rs12404911
rs77285108
rs73128290
rs7742829
All − MR Egger
All − IVW(MRE)
0
1
2
MR effect size for
'Gut microbiota abundance (genus Catenibacterium id.2153)' on 'Alpha−2−antiplasmin'

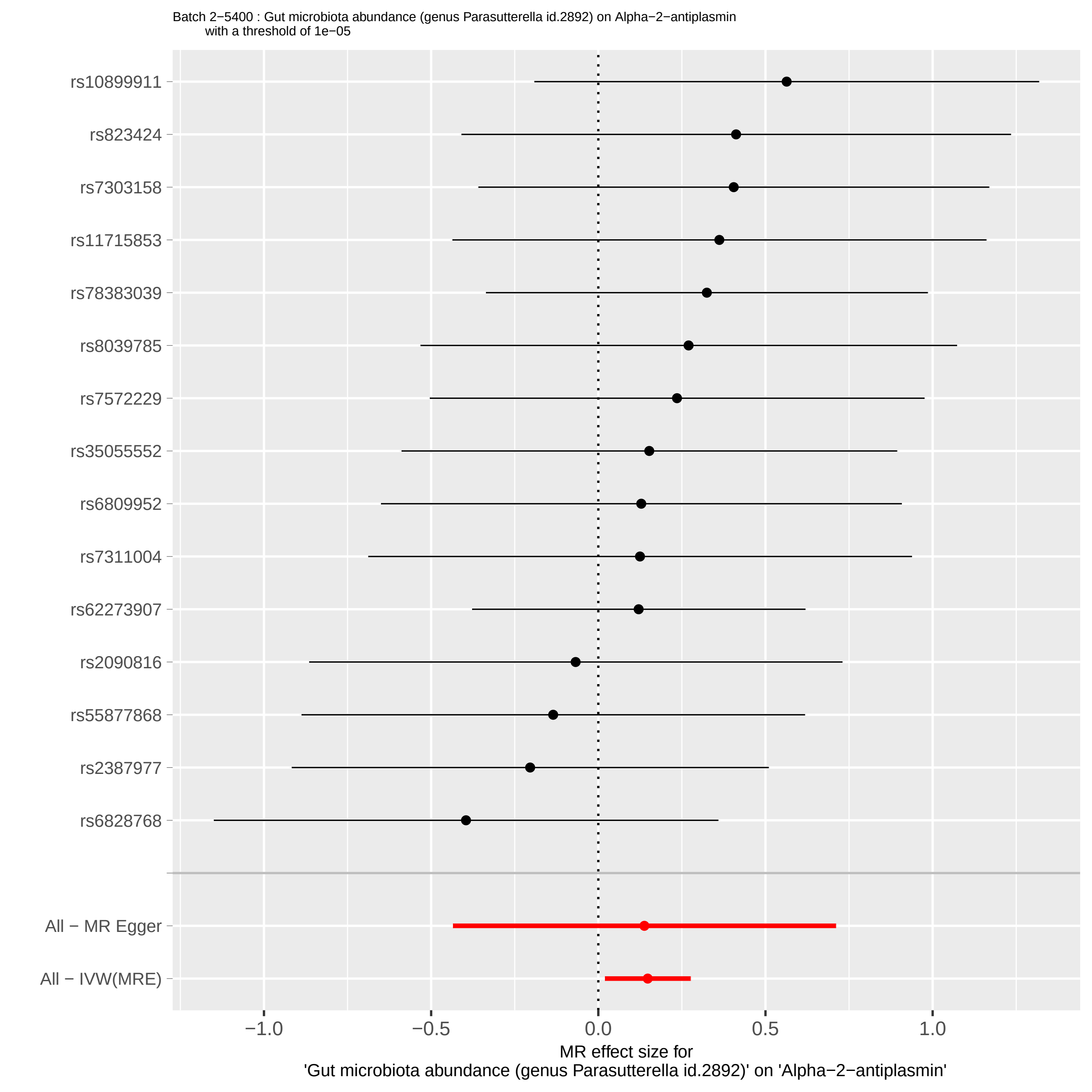
Batch 2−5400 : Gut microbiota abundance (genus Parasutterella id.2892) on Alpha−2−antiplasmin
with a threshold of 1e−05
rs10899911
rs823424
rs7303158
rs11715853
rs78383039
rs8039785
rs7572229
rs35055552
rs6809952
rs7311004
rs62273907
rs2090816
rs55877868
rs2387977
rs6828768
All − MR Egger
All − IVW(MRE)
−1.0
−0.5
0.0
0.5
1.0
MR effect size for
'Gut microbiota abundance (genus Parasutterella id.2892)' on 'Alpha−2−antiplasmin'

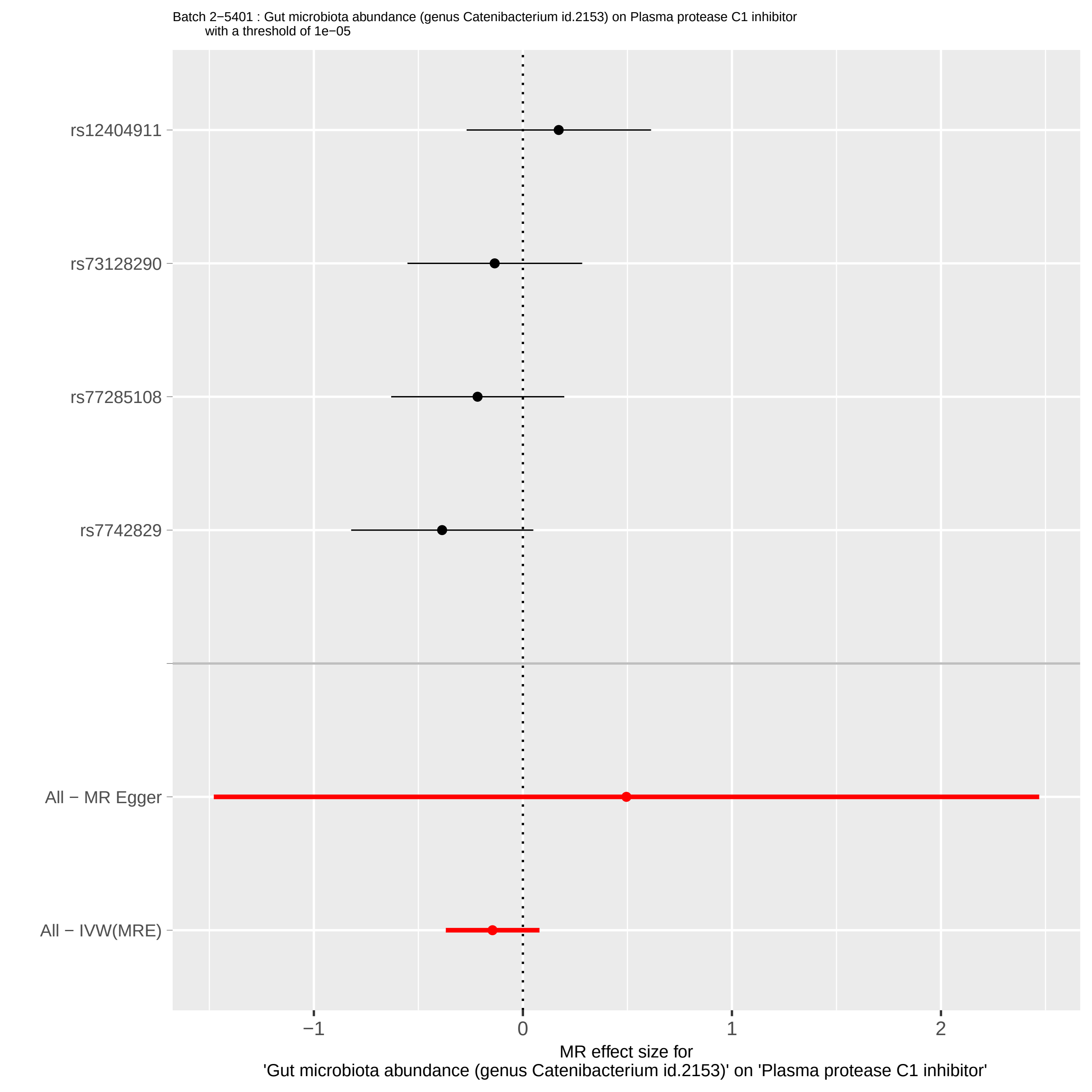
Batch 2−5401 : Gut microbiota abundance (genus Catenibacterium id.2153) on Plasma protease C1 inhibitor
with a threshold of 1e−05
rs12404911
rs73128290
rs77285108
rs7742829
All − MR Egger
All − IVW(MRE)
−1
0
1
2
MR effect size for
'Gut microbiota abundance (genus Catenibacterium id.2153)' on 'Plasma protease C1 inhibitor'

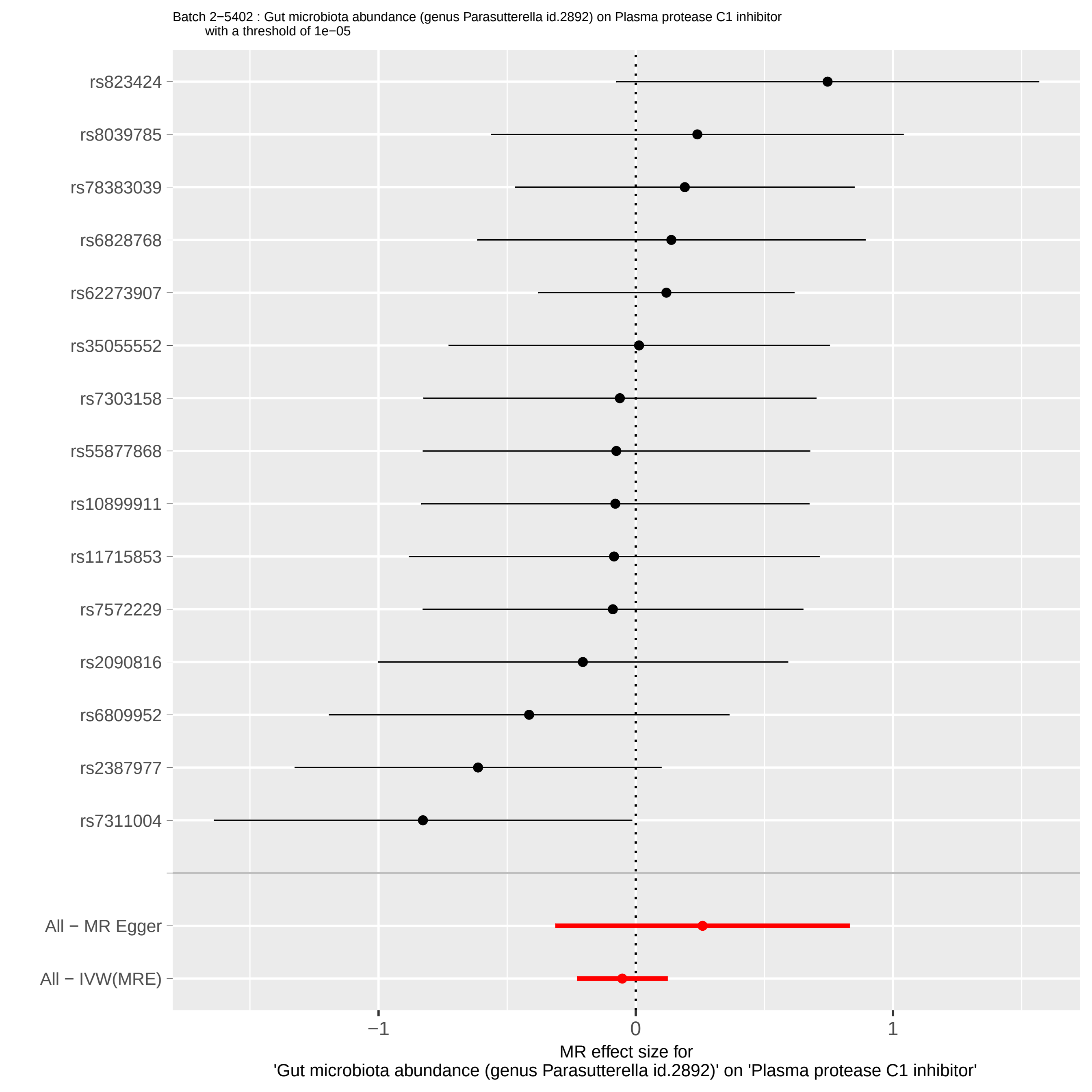

Batch 2−5402 : Gut microbiota abundance (genus Parasutterella id.2892) on Plasma protease C1 inhibitor
with a threshold of 1e−05
rs823424
rs8039785
rs78383039
rs6828768
rs62273907
rs35055552
rs7303158
rs55877868
rs10899911
rs11715853
rs7572229
rs2090816
rs6809952
rs2387977
rs7311004
All − MR Egger
All − IVW(MRE)
−1
0
1
MR effect size for
'Gut microbiota abundance (genus Parasutterella id.2892)' on 'Plasma protease C1 inhibitor'

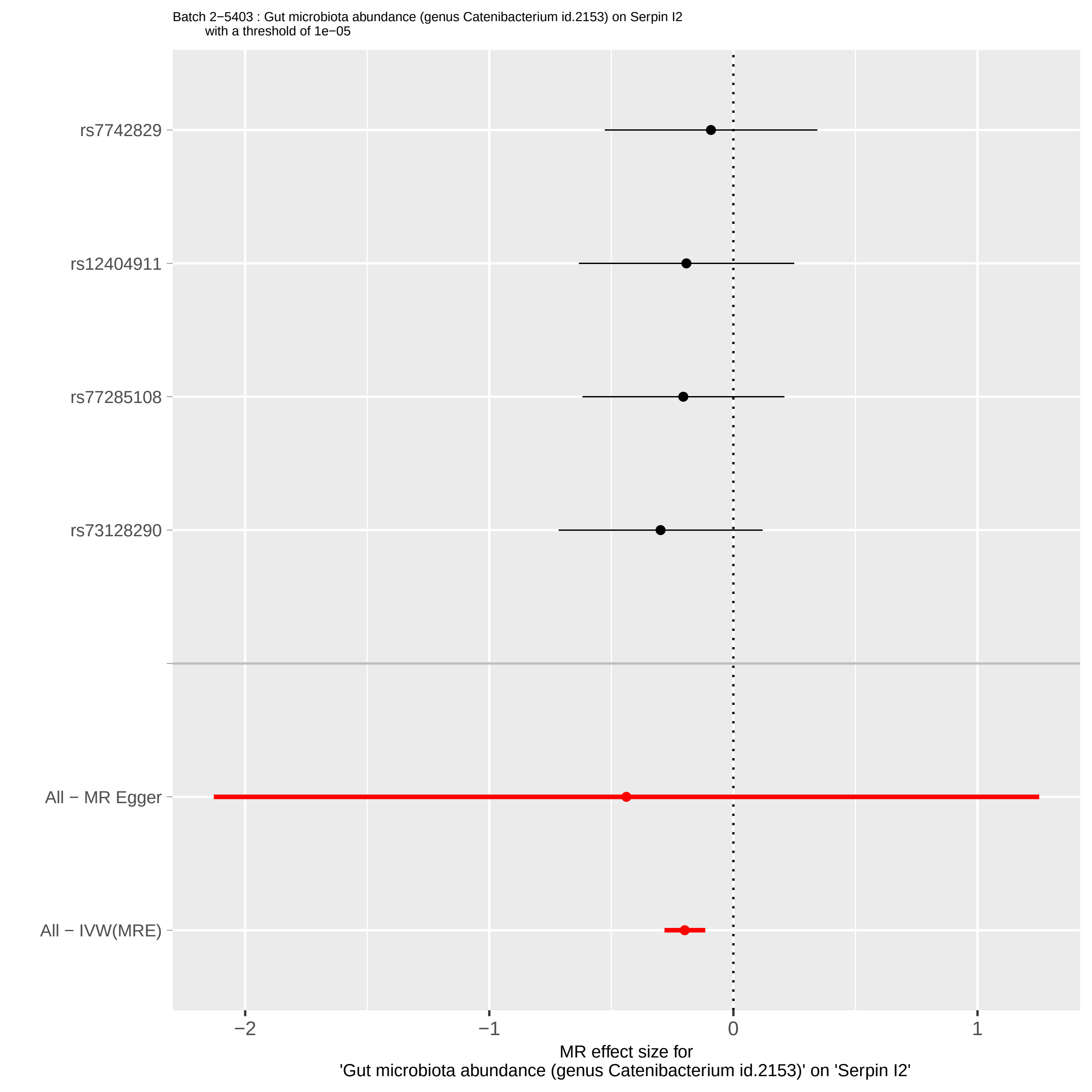

Batch 2−5403 : Gut microbiota abundance (genus Catenibacterium id.2153) on Serpin I2
with a threshold of 1e−05
rs7742829
rs12404911
rs77285108
rs73128290
All − MR Egger
All − IVW(MRE)
−2
−1
0
1
MR effect size for
'Gut microbiota abundance (genus Catenibacterium id.2153)' on 'Serpin I2'

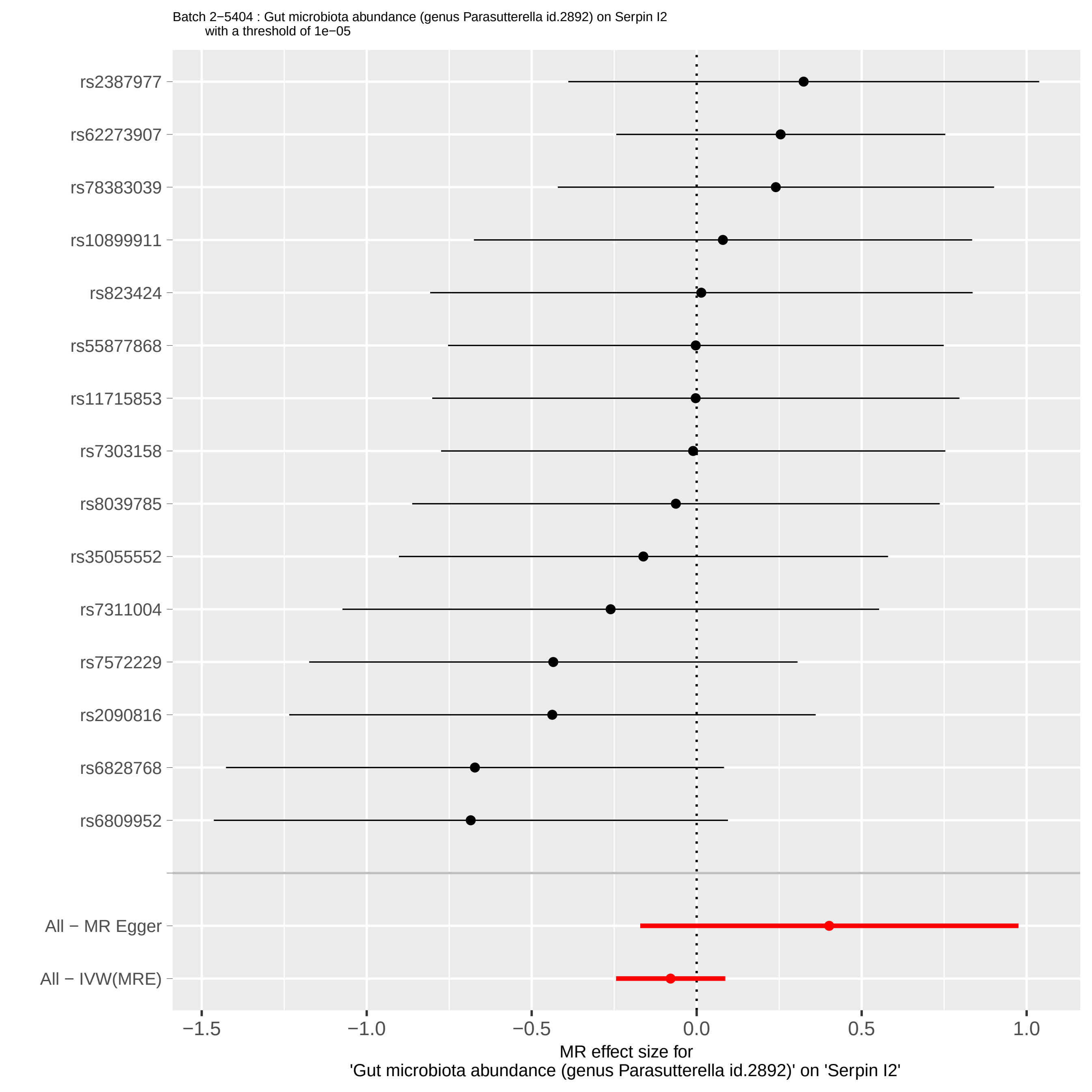
Batch 2−5404 : Gut microbiota abundance (genus Parasutterella id.2892) on Serpin I2
with a threshold of 1e−05
rs2387977
rs62273907
rs78383039
rs10899911
rs823424
rs55877868
rs11715853
rs7303158
rs8039785
rs35055552
rs7311004
rs7572229
rs2090816
rs6828768
rs6809952
All − MR Egger
All − IVW(MRE)
−1.5
−1.0
−0.5
0.0
0.5
1.0
MR effect size for
'Gut microbiota abundance (genus Parasutterella id.2892)' on 'Serpin I2'

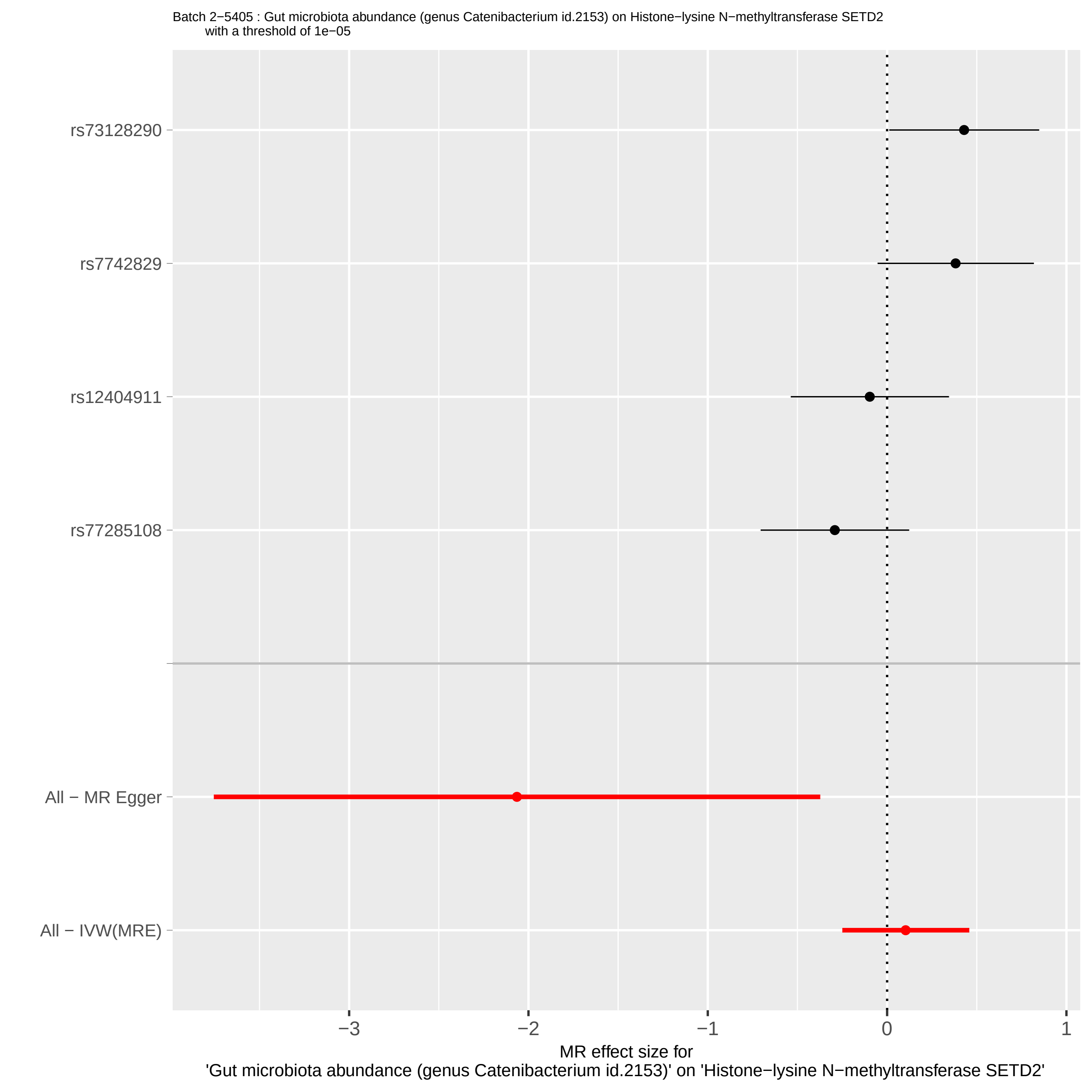
Batch 2−5405 : Gut microbiota abundance (genus Catenibacterium id.2153) on Histone−lysine N−methyltransferase SETD2
with a threshold of 1e−05
rs73128290
rs7742829
rs12404911
rs77285108
All − MR Egger
All − IVW(MRE)
−3
−2
−1
0
1
MR effect size for
'Gut microbiota abundance (genus Catenibacterium id.2153)' on 'Histone−lysine N−methyltransferase SETD2'

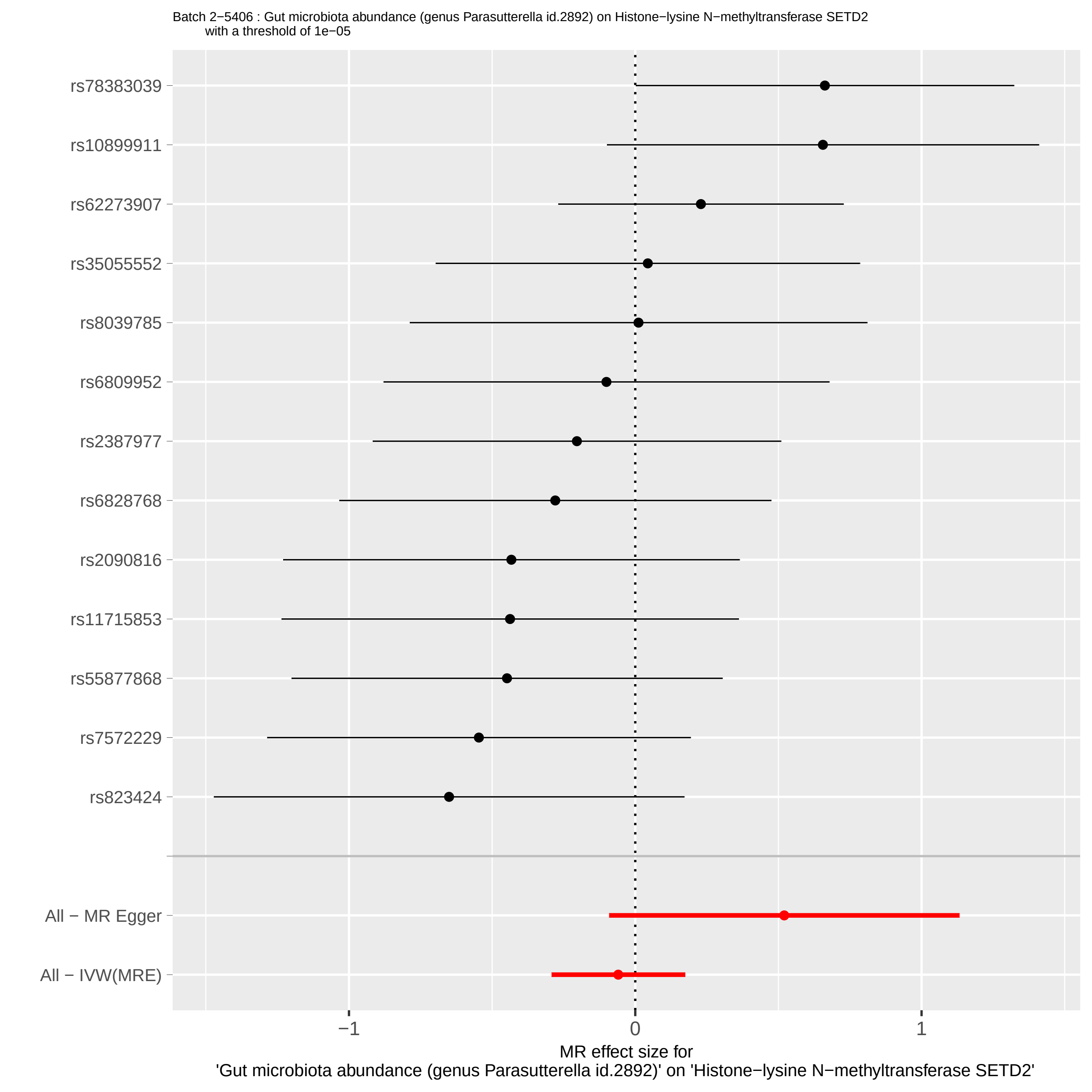

Batch 2−5406 : Gut microbiota abundance (genus Parasutterella id.2892) on Histone−lysine N−methyltransferase SETD2
with a threshold of 1e−05
rs78383039
rs10899911
rs62273907
rs35055552
rs8039785
rs6809952
rs2387977
rs6828768
rs2090816
rs11715853
rs55877868
rs7572229
rs823424
All − MR Egger
All − IVW(MRE)
−1
0
1
MR effect size for
'Gut microbiota abundance (genus Parasutterella id.2892)' on 'Histone−lysine N−methyltransferase SETD2'

Batch 2−5407 : Gut microbiota abundance (genus Catenibacterium id.2153) on Histone−lysine N−methyltransferase SETMAR
with a threshold of 1e−05

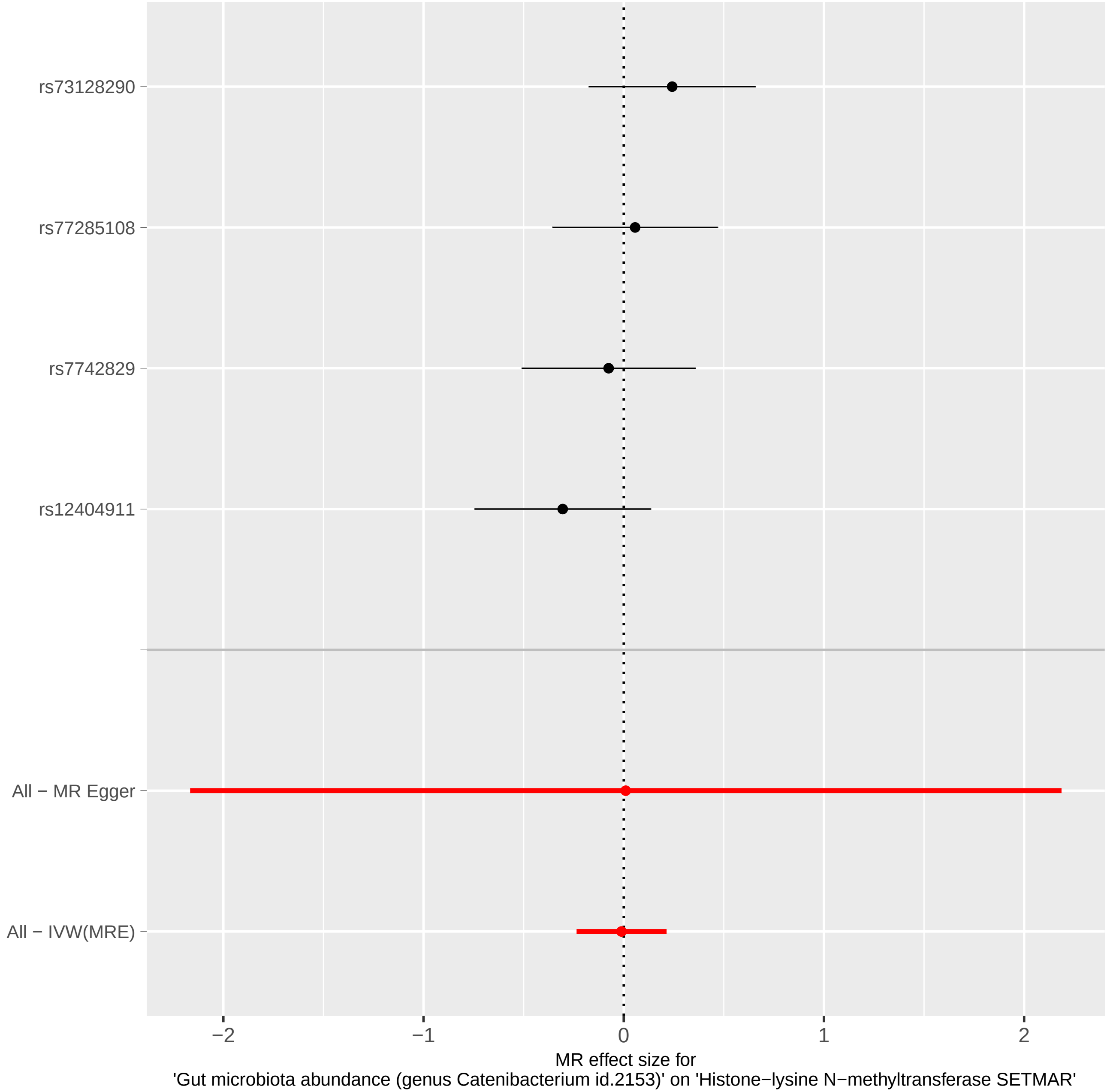

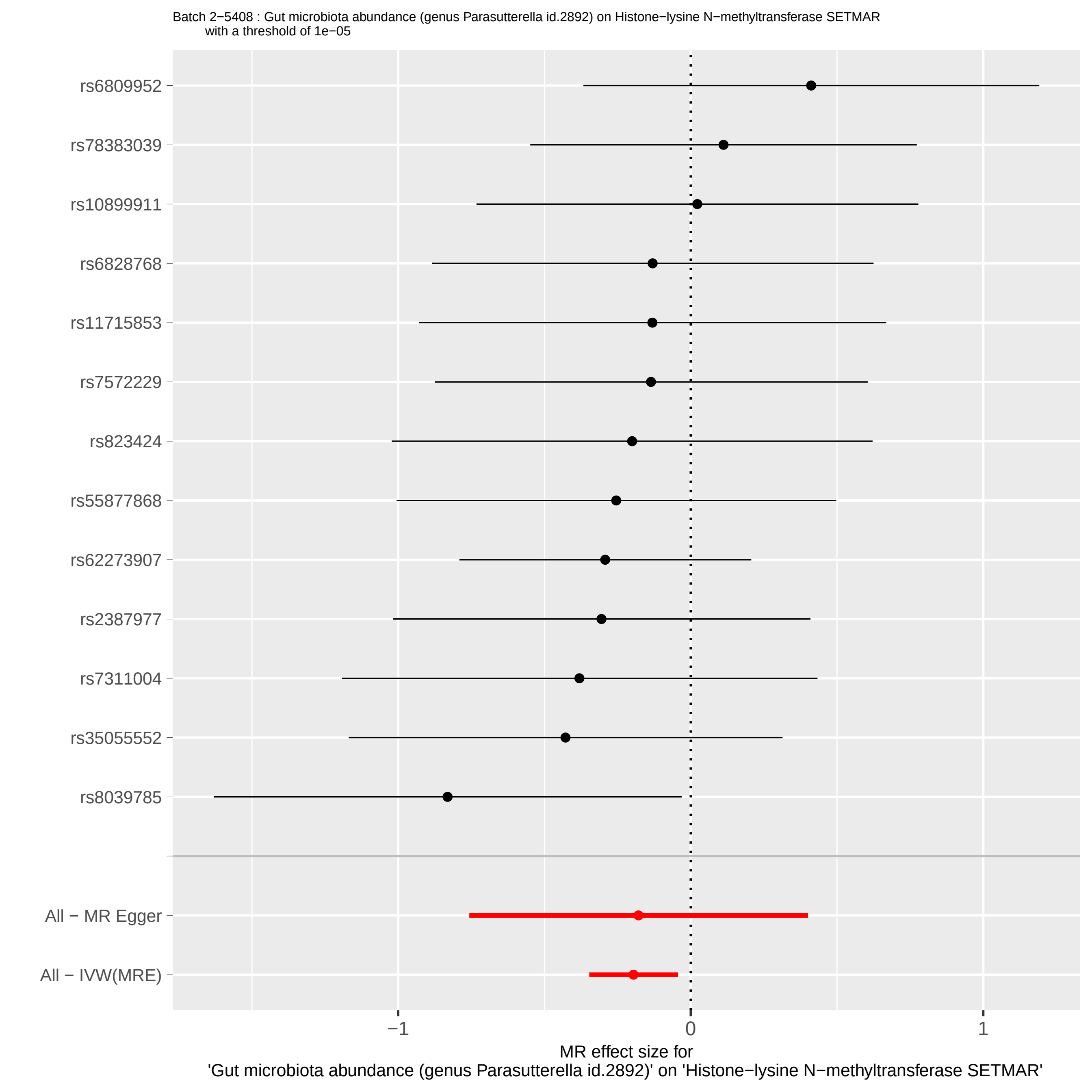

Batch 2−5408 : Gut microbiota abundance (genus Parasutterella id.2892) on Histone−lysine N−methyltransferase SETMAR
with a threshold of 1e−05
rs6809952
rs78383039
rs10899911
rs6828768
rs11715853
rs7572229
rs823424
rs55877868
rs62273907
rs2387977
rs7311004
rs35055552
rs8039785
All − MR Egger
All − IVW(MRE)
−1
0
1
MR effect size for
'Gut microbiota abundance (genus Parasutterella id.2892)' on 'Histone−lysine N−methyltransferase SETMAR'

Batch 2−5409 : Gut microbiota abundance (genus Catenibacterium id.2153) on Seizure 6−like protein 2
with a threshold of 1e−05

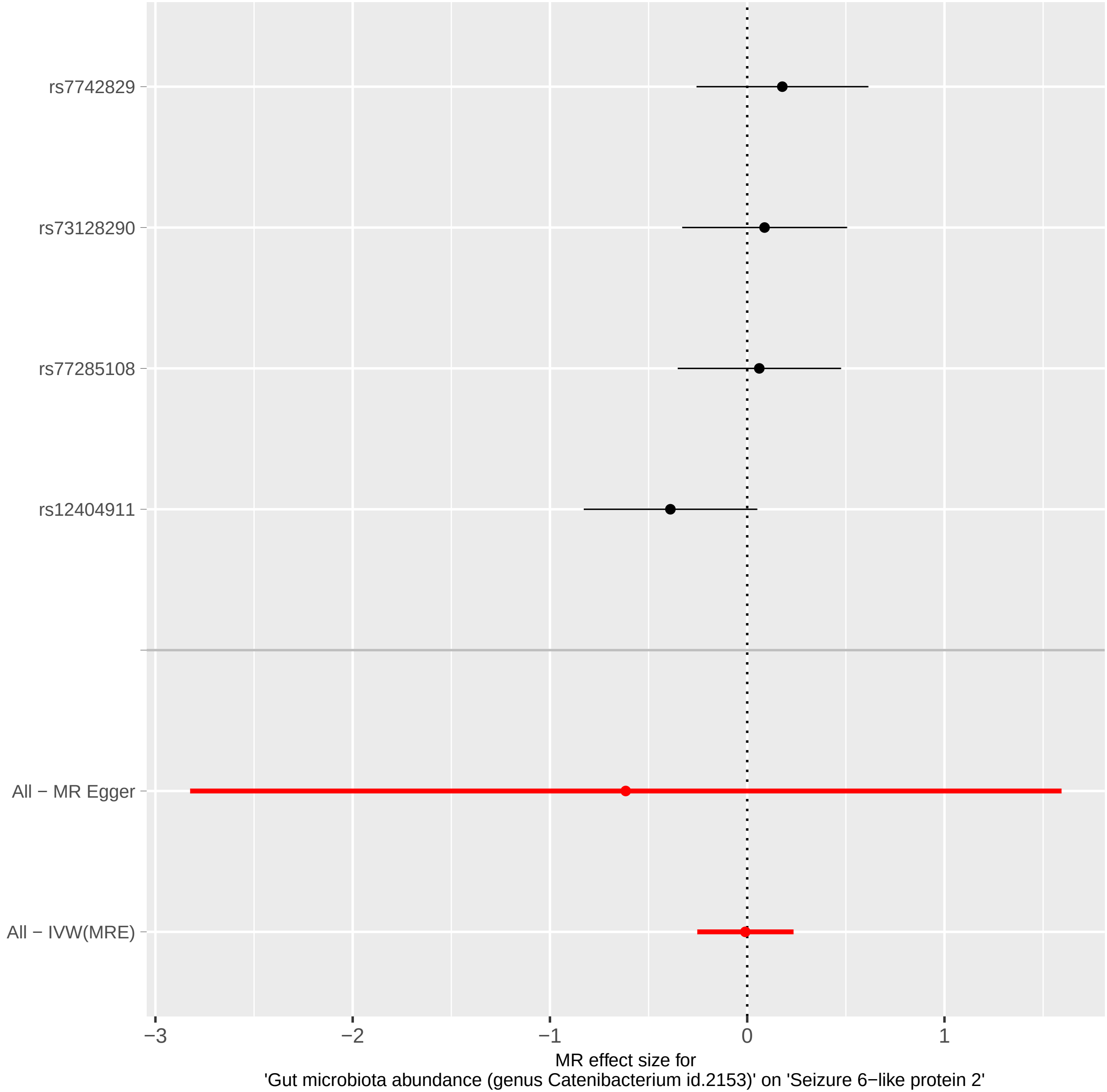

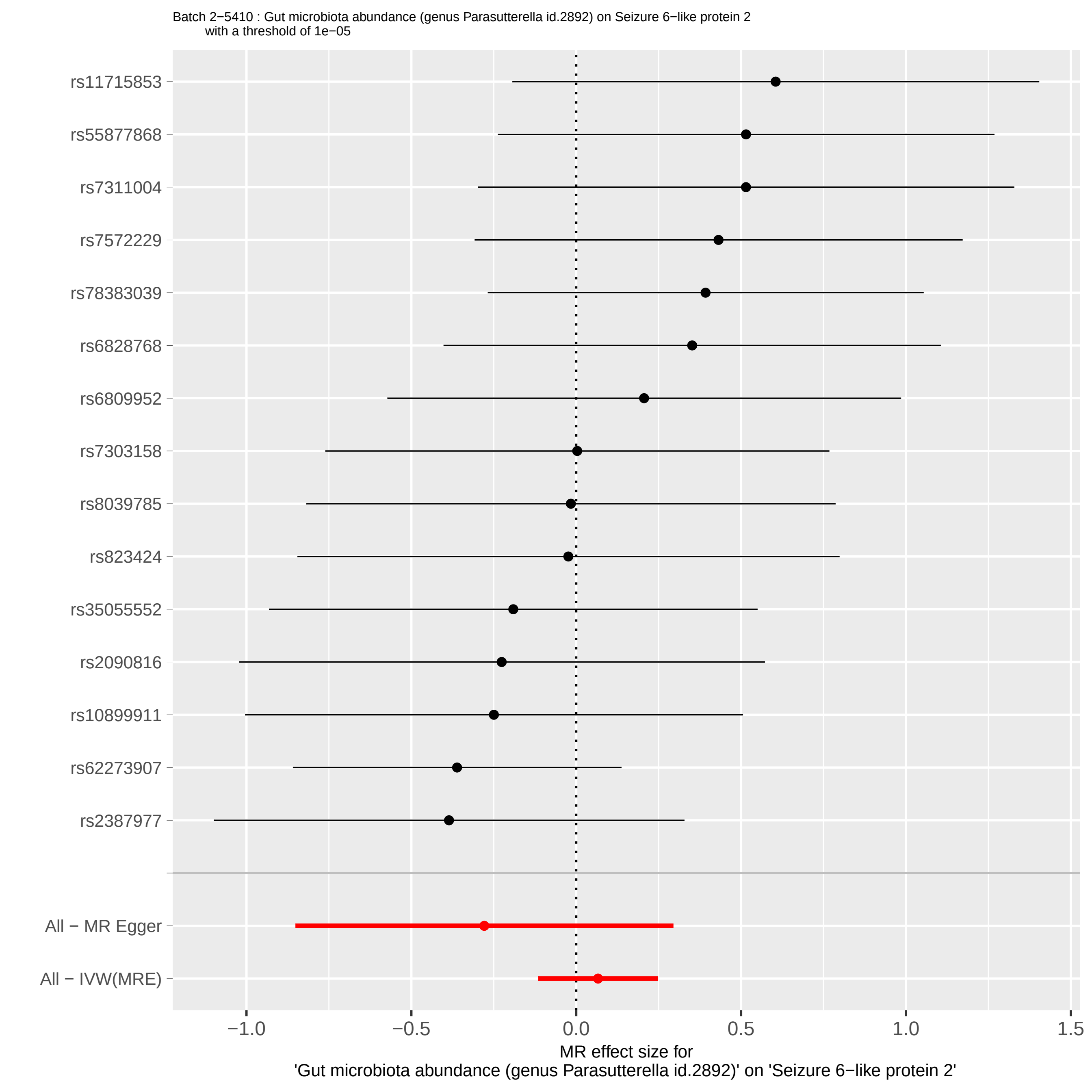

Batch 2−5410 : Gut microbiota abundance (genus Parasutterella id.2892) on Seizure 6−like protein 2
with a threshold of 1e−05
rs11715853
rs55877868
rs7311004
rs7572229
rs78383039
rs6828768
rs6809952
rs7303158
rs8039785
rs823424
rs35055552
rs2090816
rs10899911
rs62273907
rs2387977
All − MR Egger
All − IVW(MRE)
−1.0
−0.5
0.0
0.5
1.0
1.5
MR effect size for
'Gut microbiota abundance (genus Parasutterella id.2892)' on 'Seizure 6−like protein 2'

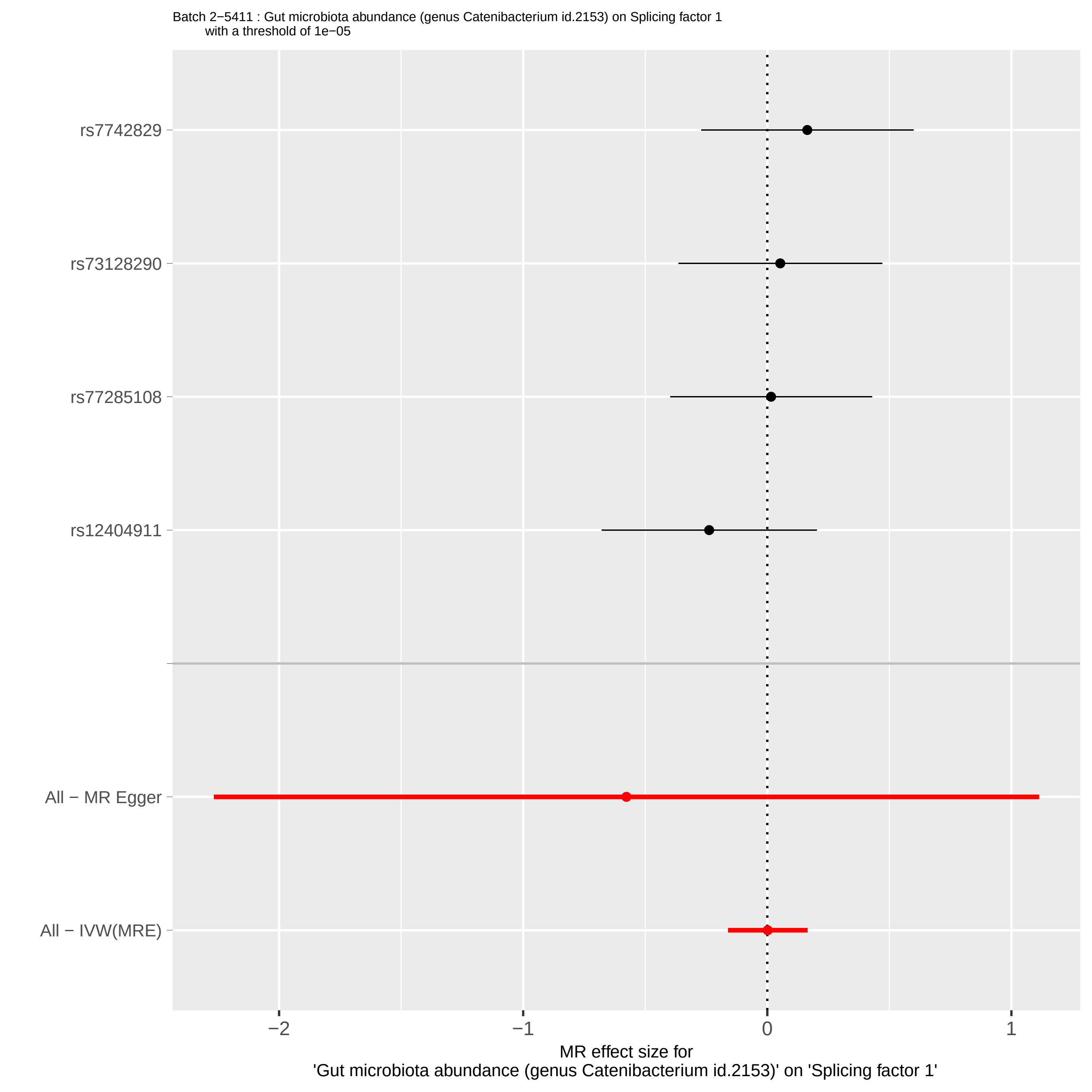
Batch 2−5411 : Gut microbiota abundance (genus Catenibacterium id.2153) on Splicing factor 1
with a threshold of 1e−05
rs7742829
rs73128290
rs77285108
rs12404911
All − MR Egger
All − IVW(MRE)
−2
−1
0
1
MR effect size for
'Gut microbiota abundance (genus Catenibacterium id.2153)' on 'Splicing factor 1'

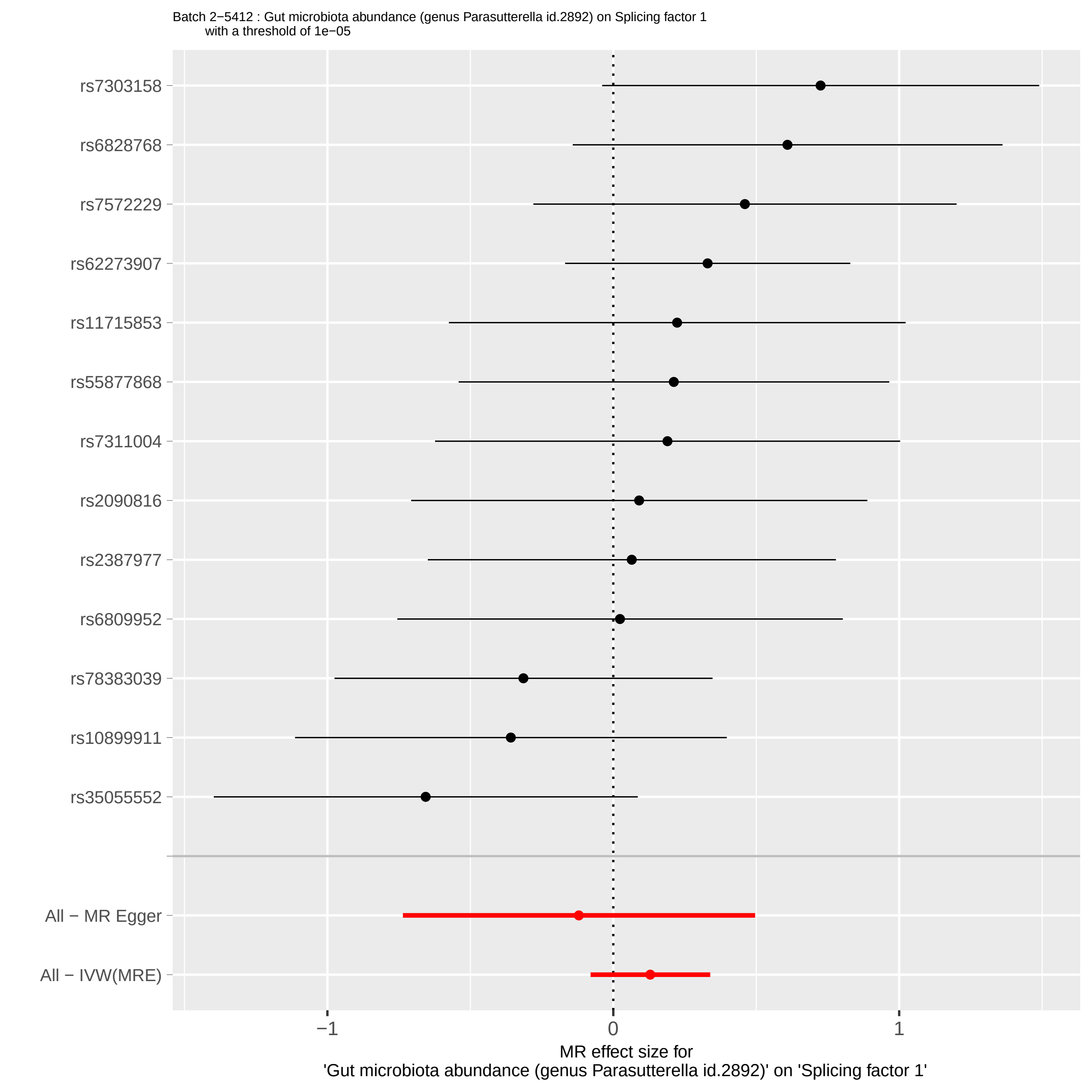
Batch 2−5412 : Gut microbiota abundance (genus Parasutterella id.2892) on Splicing factor 1
with a threshold of 1e−05
rs7303158
rs6828768
rs7572229
rs62273907
rs11715853
rs55877868
rs7311004
rs2090816
rs2387977
rs6809952
rs78383039
rs10899911
rs35055552
All − MR Egger
All − IVW(MRE)
−1
0
1
MR effect size for
'Gut microbiota abundance (genus Parasutterella id.2892)' on 'Splicing factor 1'

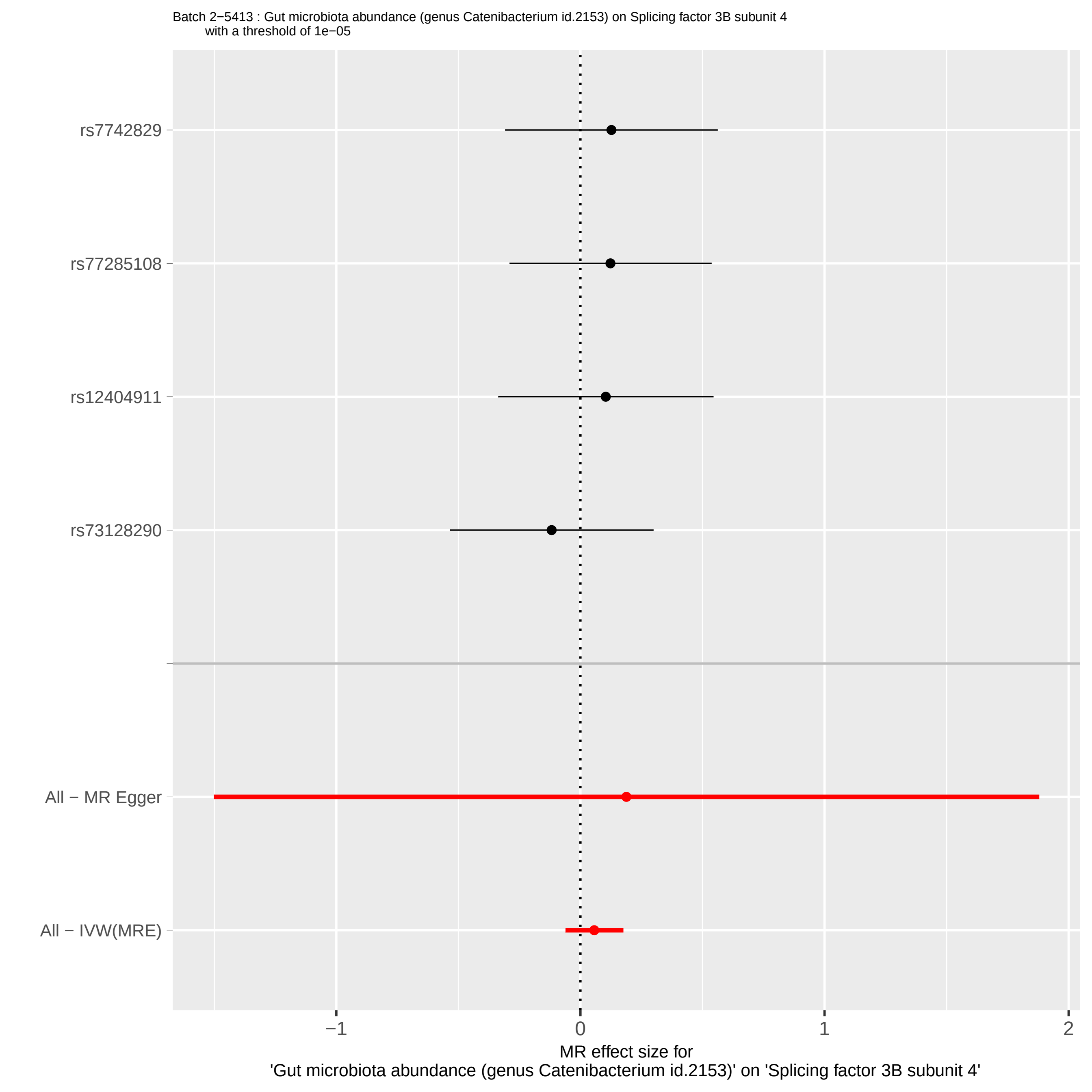

Batch 2−5413 : Gut microbiota abundance (genus Catenibacterium id.2153) on Splicing factor 3B subunit 4
with a threshold of 1e−05
rs7742829
rs77285108
rs12404911
rs73128290
All − MR Egger
All − IVW(MRE)
−1
0
1
2
MR effect size for
'Gut microbiota abundance (genus Catenibacterium id.2153)' on 'Splicing factor 3B subunit 4'

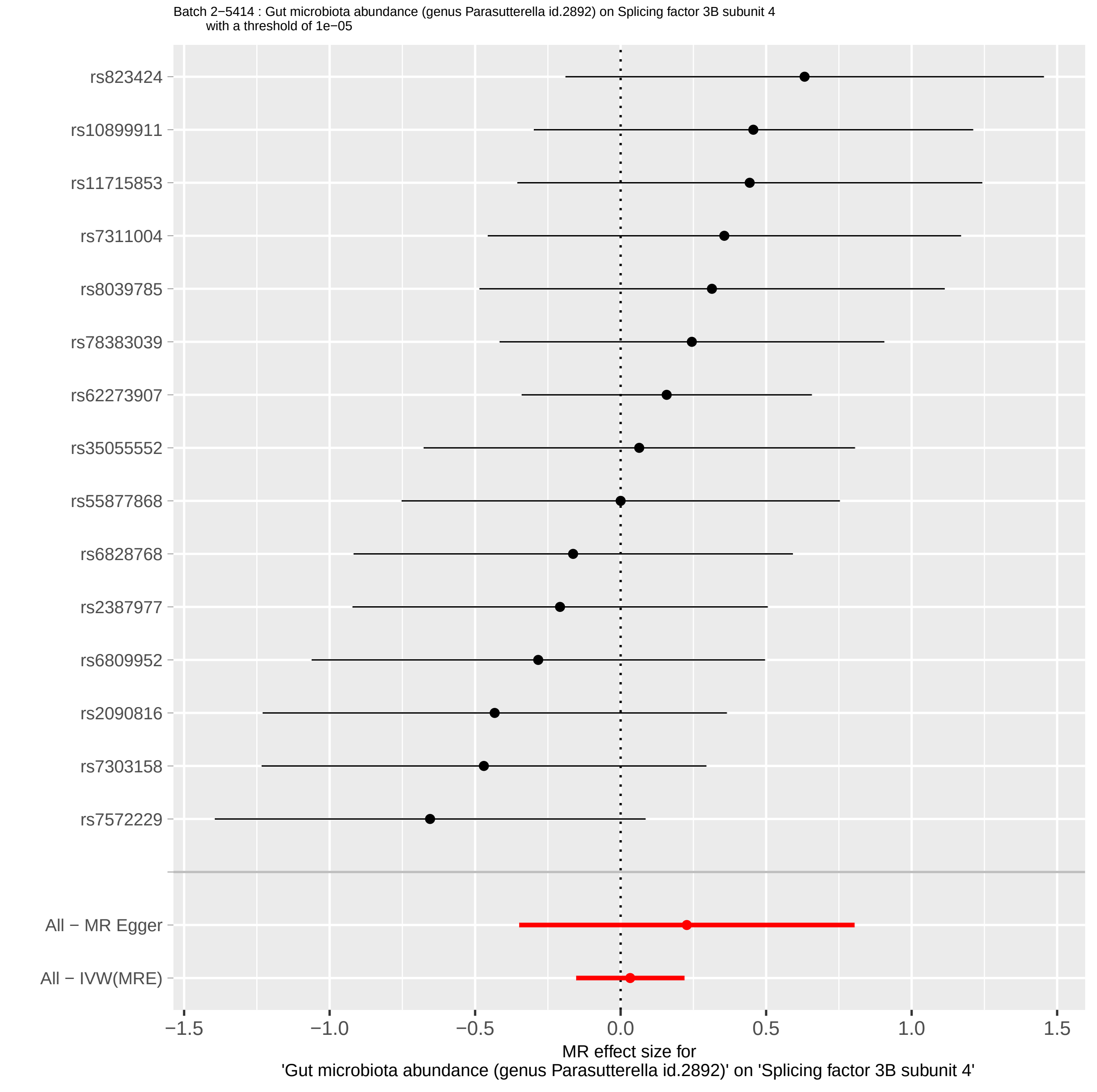

Batch 2−5414 : Gut microbiota abundance (genus Parasutterella id.2892) on Splicing factor 3B subunit 4
with a threshold of 1e−05
rs823424
rs10899911
rs11715853
rs7311004
rs8039785
rs78383039
rs62273907
rs35055552
rs55877868
rs6828768
rs2387977
rs6809952
rs2090816
rs7303158
rs7572229
All − MR Egger
All − IVW(MRE)
−1.5
−1.0
−0.5
0.0
0.5
1.0
1.5
MR effect size for
'Gut microbiota abundance (genus Parasutterella id.2892)' on 'Splicing factor 3B subunit 4'

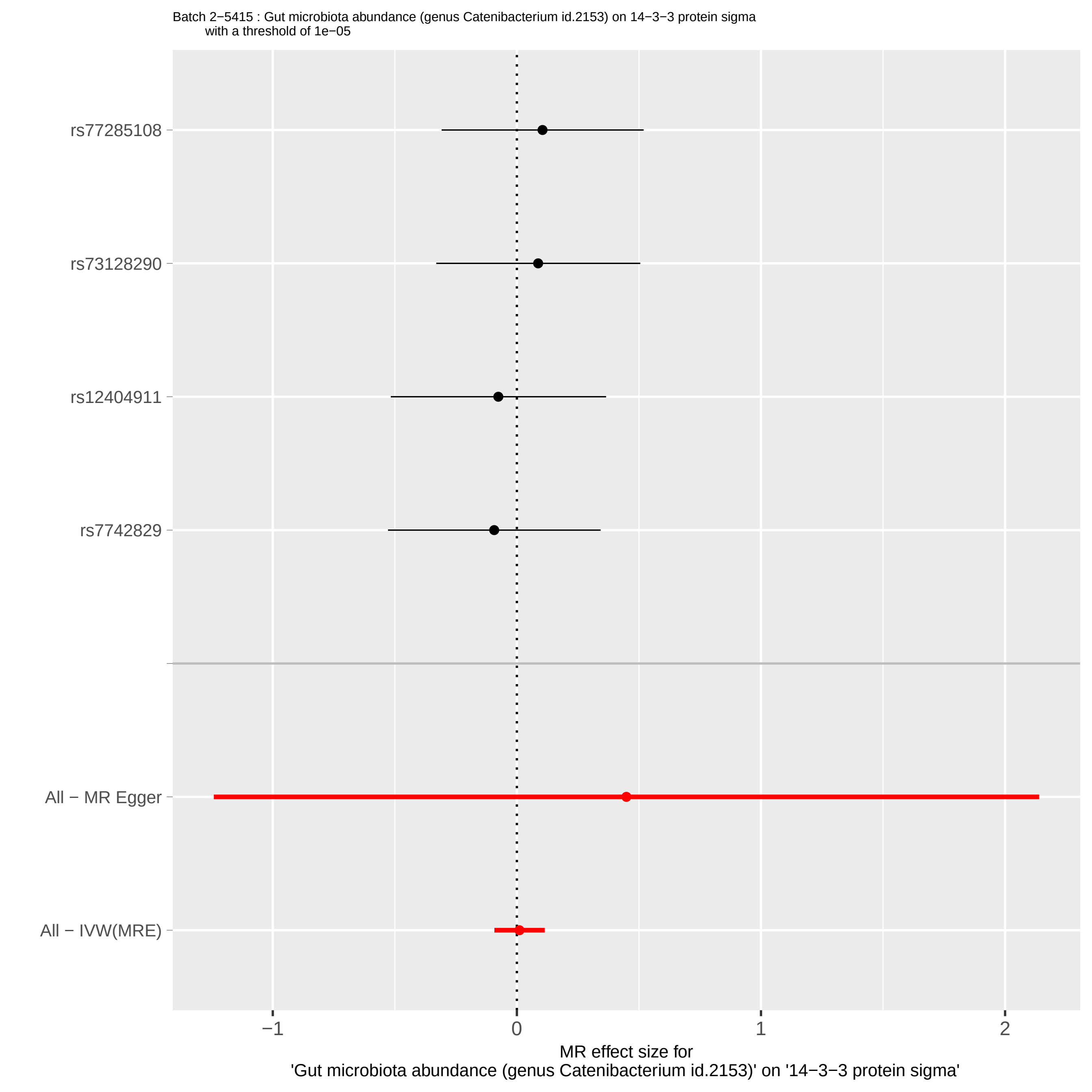

Batch 2−5415 : Gut microbiota abundance (genus Catenibacterium id.2153) on 14−3−3 protein sigma
with a threshold of 1e−05
rs77285108
rs73128290
rs12404911
rs7742829
All − MR Egger
All − IVW(MRE)
−1
0
1
2
MR effect size for
'Gut microbiota abundance (genus Catenibacterium id.2153)' on '14−3−3 protein sigma'

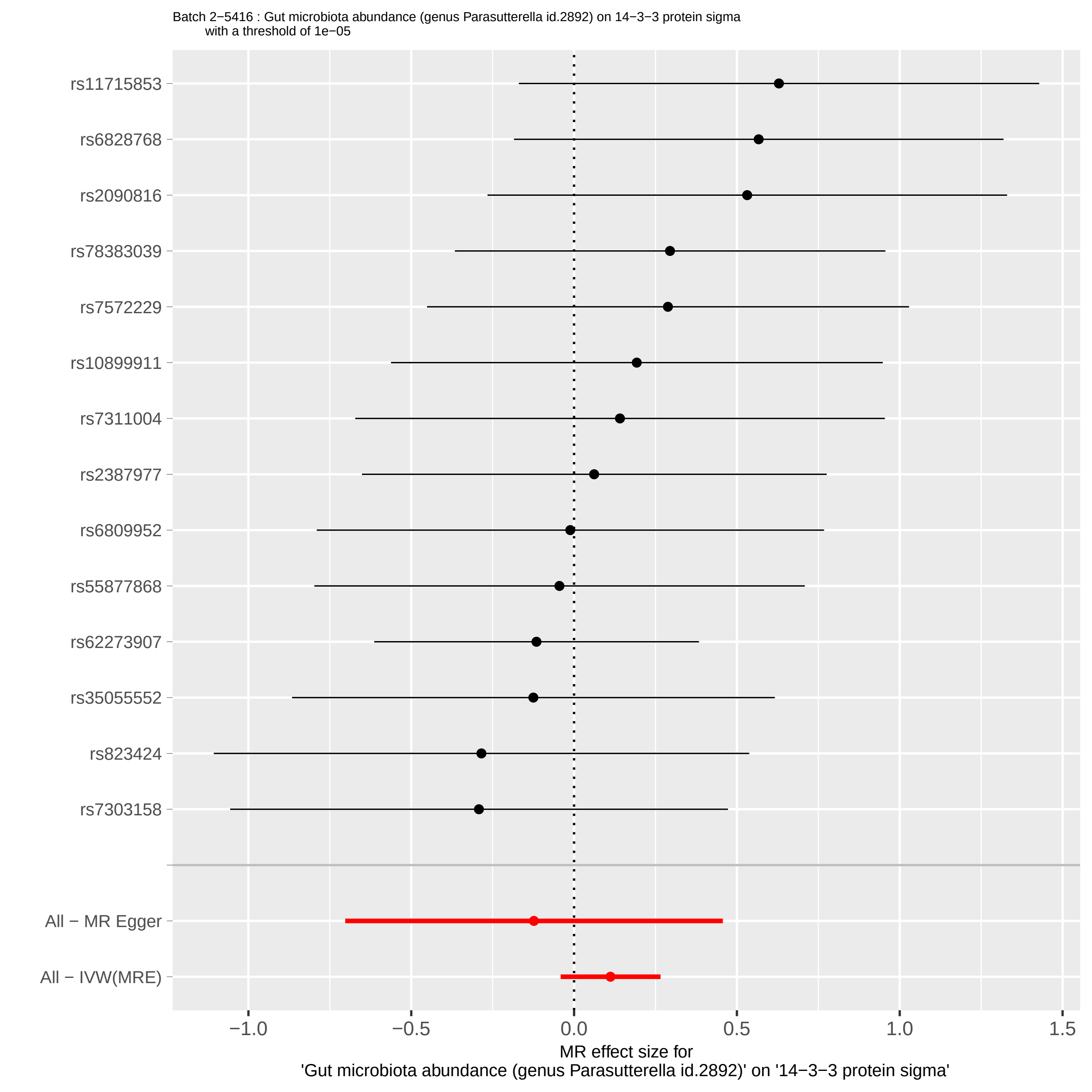

Batch 2−5416 : Gut microbiota abundance (genus Parasutterella id.2892) on 14−3−3 protein sigma
with a threshold of 1e−05
rs11715853
rs6828768
rs2090816
rs78383039
rs7572229
rs10899911
rs7311004
rs2387977
rs6809952
rs55877868
rs62273907
rs35055552
rs823424
rs7303158
All − MR Egger
All − IVW(MRE)
−1.0
−0.5
0.0
0.5
1.0
1.5
MR effect size for
'Gut microbiota abundance (genus Parasutterella id.2892)' on '14−3−3 protein sigma'

Batch 2−5417 : Gut microbiota abundance (genus Catenibacterium id.2153) on Secreted frizzled−related protein 1
with a threshold of 1e−05

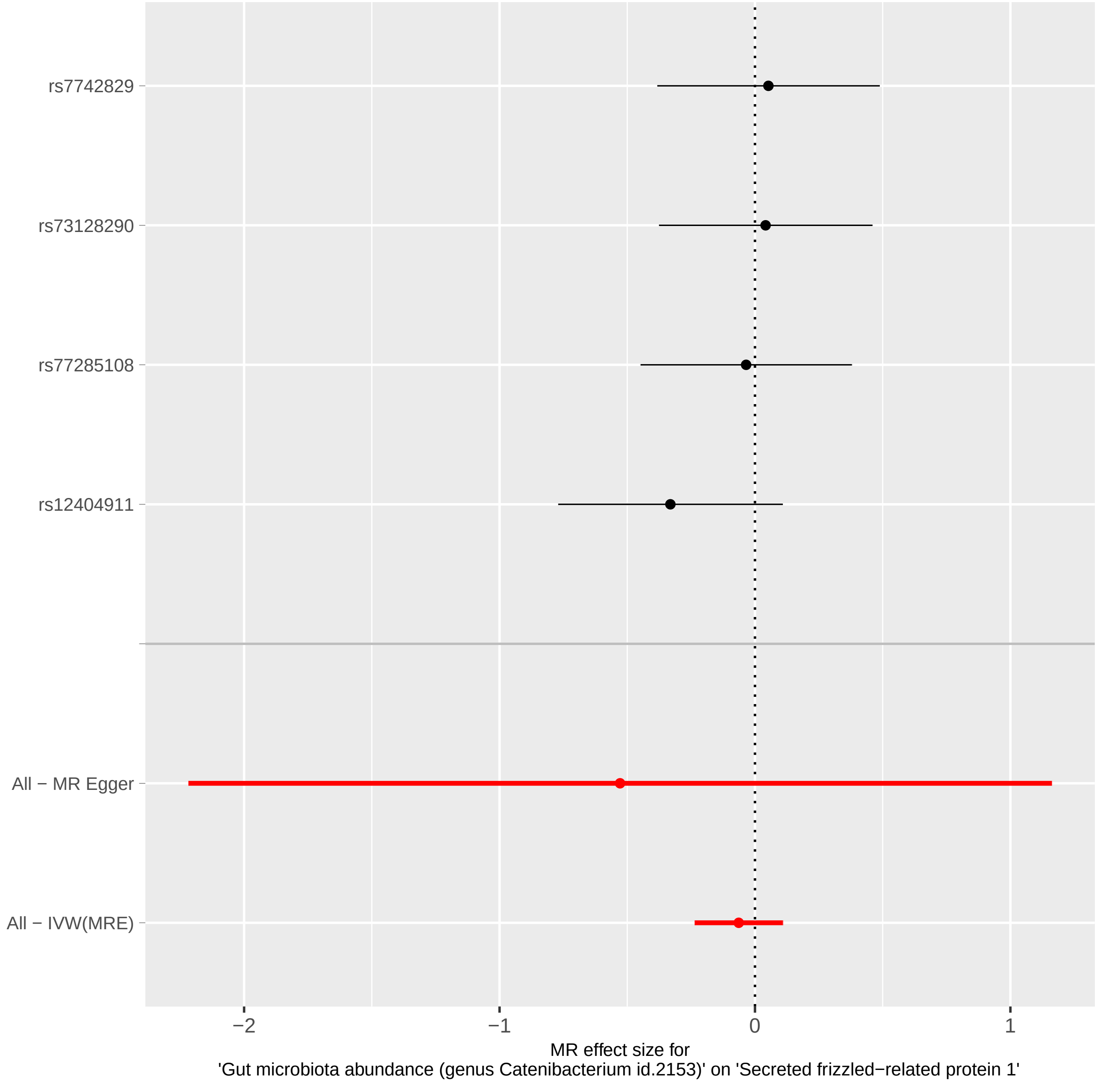

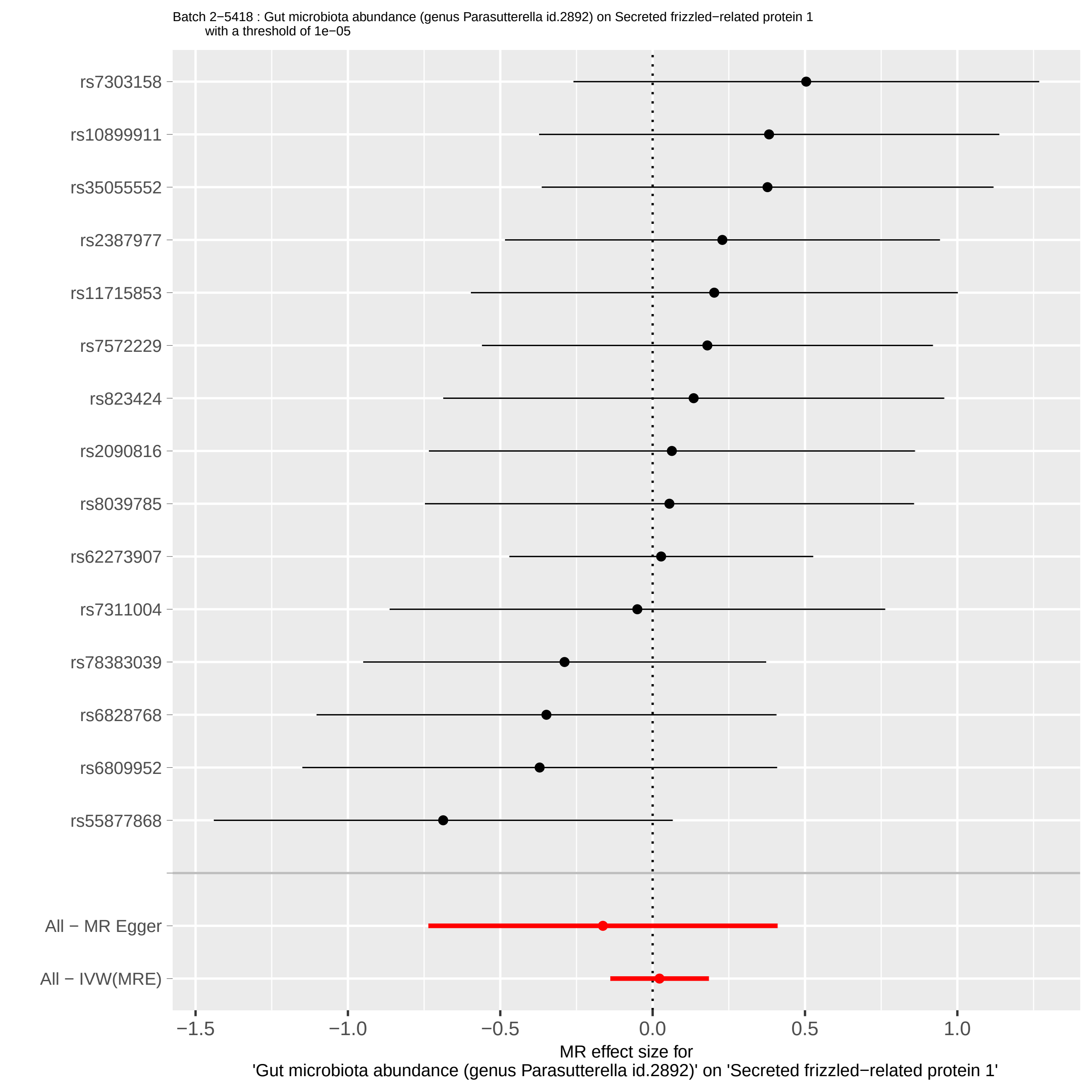

Batch 2−5418 : Gut microbiota abundance (genus Parasutterella id.2892) on Secreted frizzled−related protein 1
with a threshold of 1e−05
rs7303158
rs10899911
rs35055552
rs2387977
rs11715853
rs7572229
rs823424
rs2090816
rs8039785
rs62273907
rs7311004
rs78383039
rs6828768
rs6809952
rs55877868
All − MR Egger
All − IVW(MRE)
−1.5
−1.0
−0.5
0.0
0.5
1.0
MR effect size for
'Gut microbiota abundance (genus Parasutterella id.2892)' on 'Secreted frizzled−related protein 1'

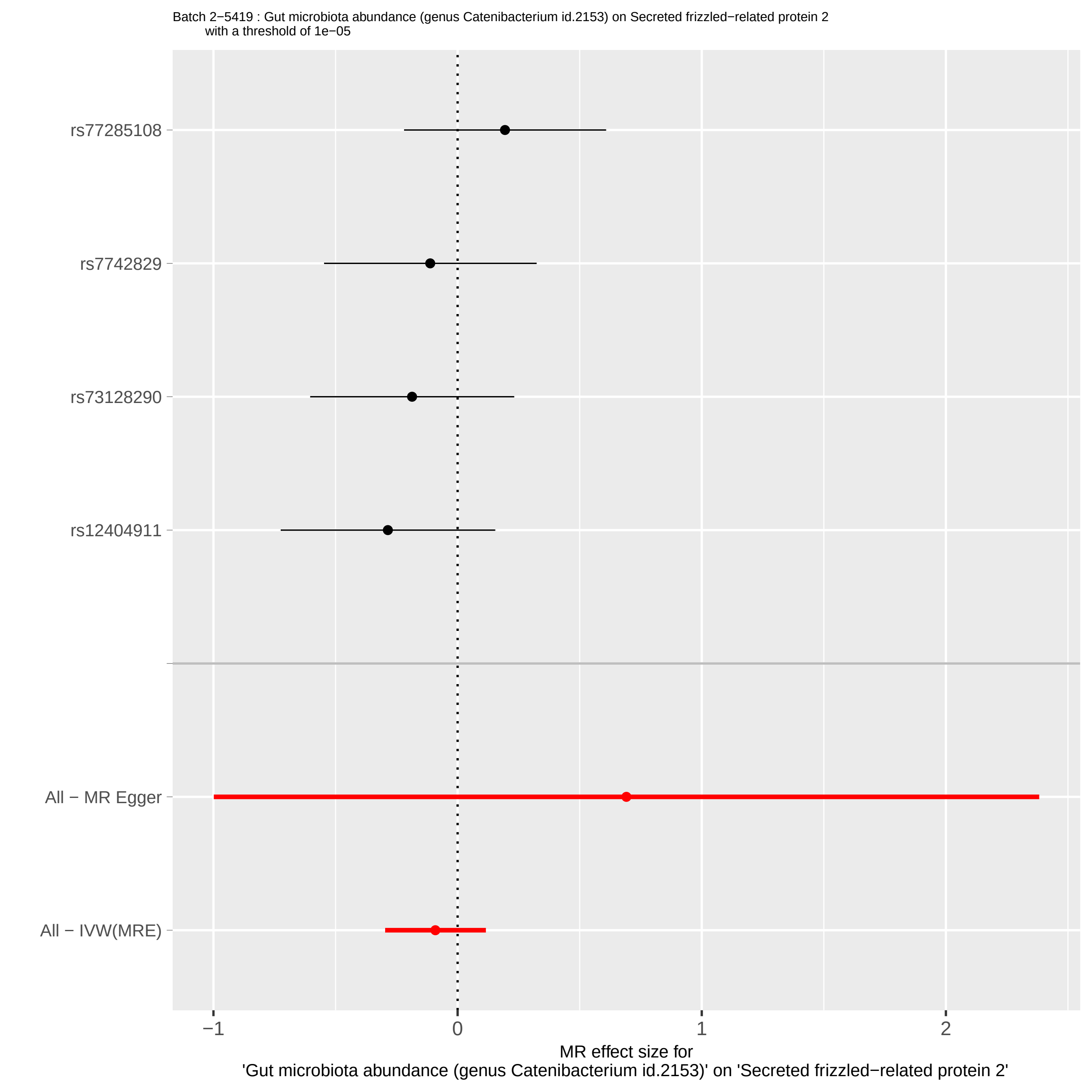

Batch 2−5419 : Gut microbiota abundance (genus Catenibacterium id.2153) on Secreted frizzled−related protein 2
with a threshold of 1e−05
rs77285108
rs7742829
rs73128290
rs12404911
All − MR Egger
All − IVW(MRE)
−1
0
1
2
MR effect size for
'Gut microbiota abundance (genus Catenibacterium id.2153)' on 'Secreted frizzled−related protein 2'

Batch 2−5420 : Gut microbiota abundance (genus Parasutterella id.2892) on Secreted frizzled−related protein 2
with a threshold of 1e−05

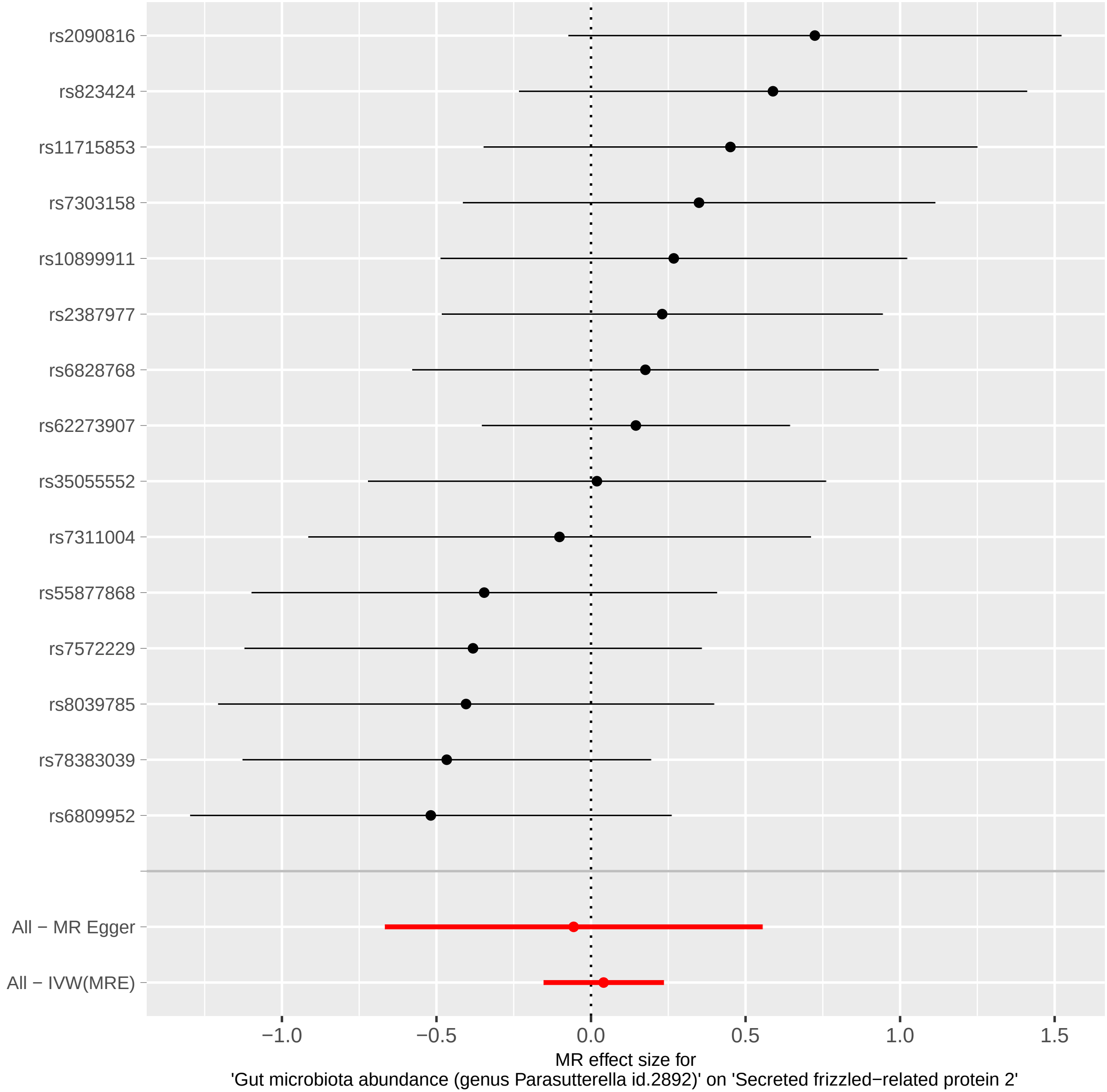

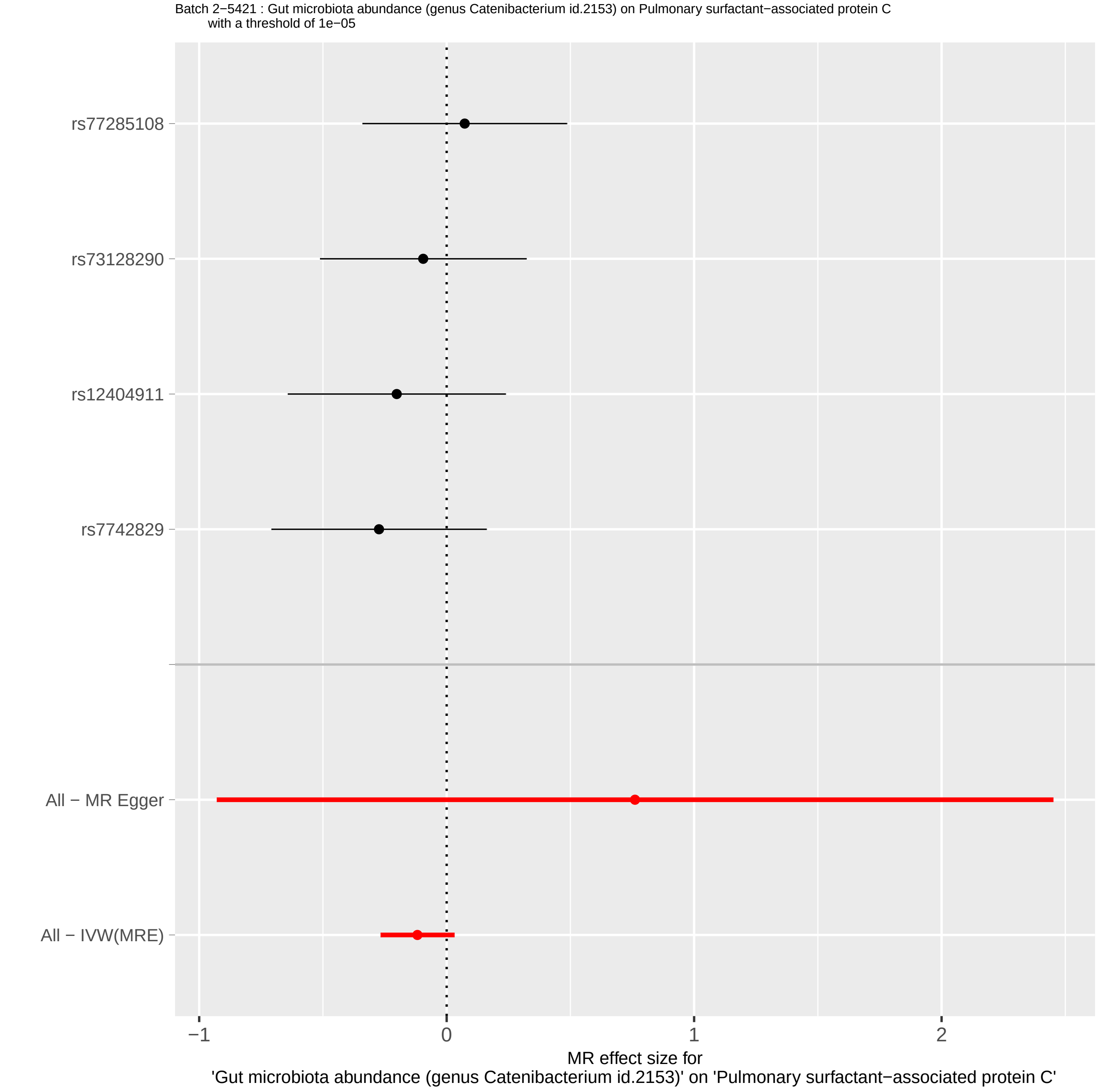
Batch 2−5421 : Gut microbiota abundance (genus Catenibacterium id.2153) on Pulmonary surfactant−associated protein C
with a threshold of 1e−05
rs77285108
rs73128290
rs12404911
rs7742829
All − MR Egger
All − IVW(MRE)
−1
0
1
2
MR effect size for
'Gut microbiota abundance (genus Catenibacterium id.2153)' on 'Pulmonary surfactant−associated protein C'

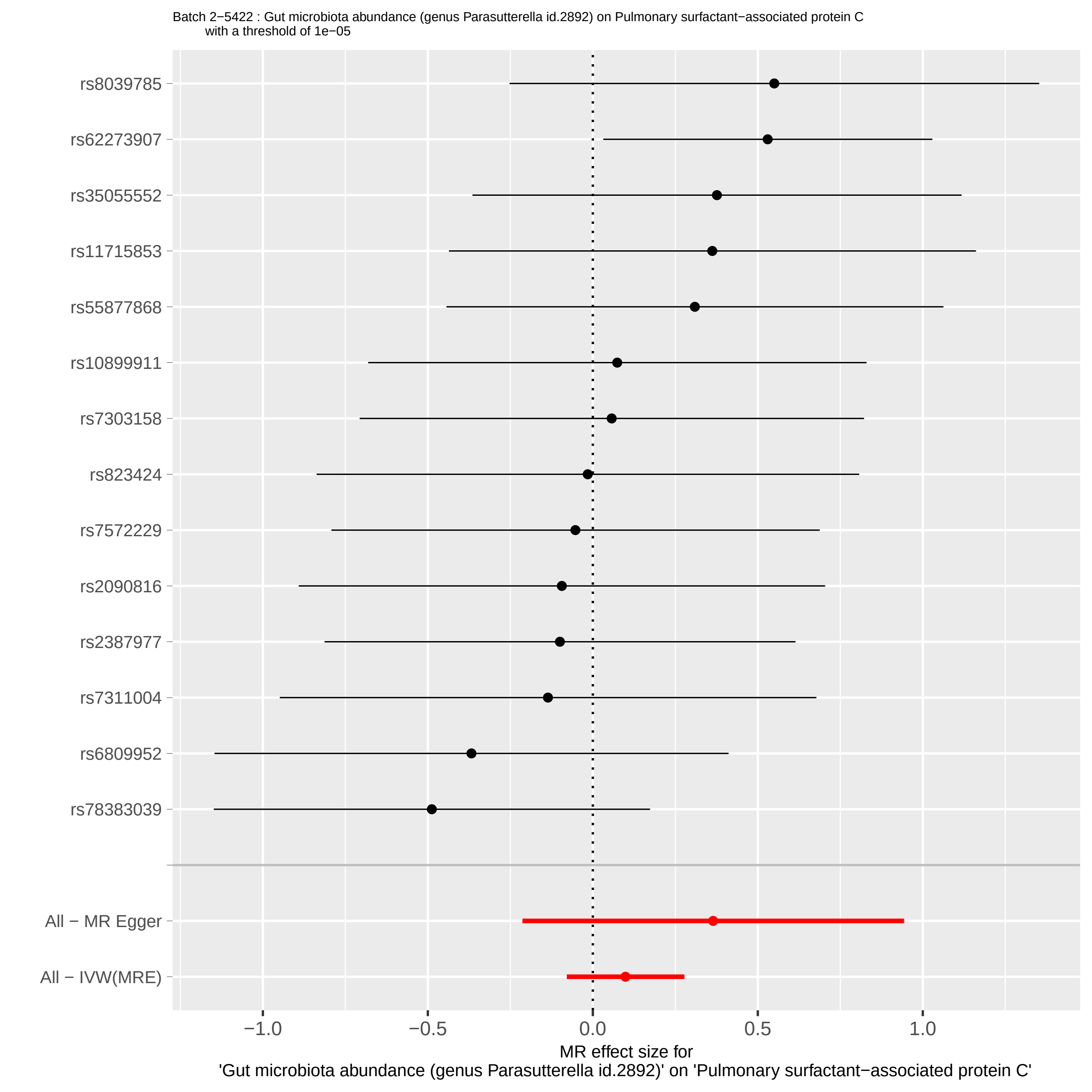

Batch 2−5422 : Gut microbiota abundance (genus Parasutterella id.2892) on Pulmonary surfactant−associated protein C
with a threshold of 1e−05
rs8039785
rs62273907
rs35055552
rs11715853
rs55877868
rs10899911
rs7303158
rs823424
rs7572229
rs2090816
rs2387977
rs7311004
rs6809952
rs78383039
All − MR Egger
All − IVW(MRE)
−1.0
−0.5
0.0
0.5
1.0
MR effect size for
'Gut microbiota abundance (genus Parasutterella id.2892)' on 'Pulmonary surfactant−associated protein C'

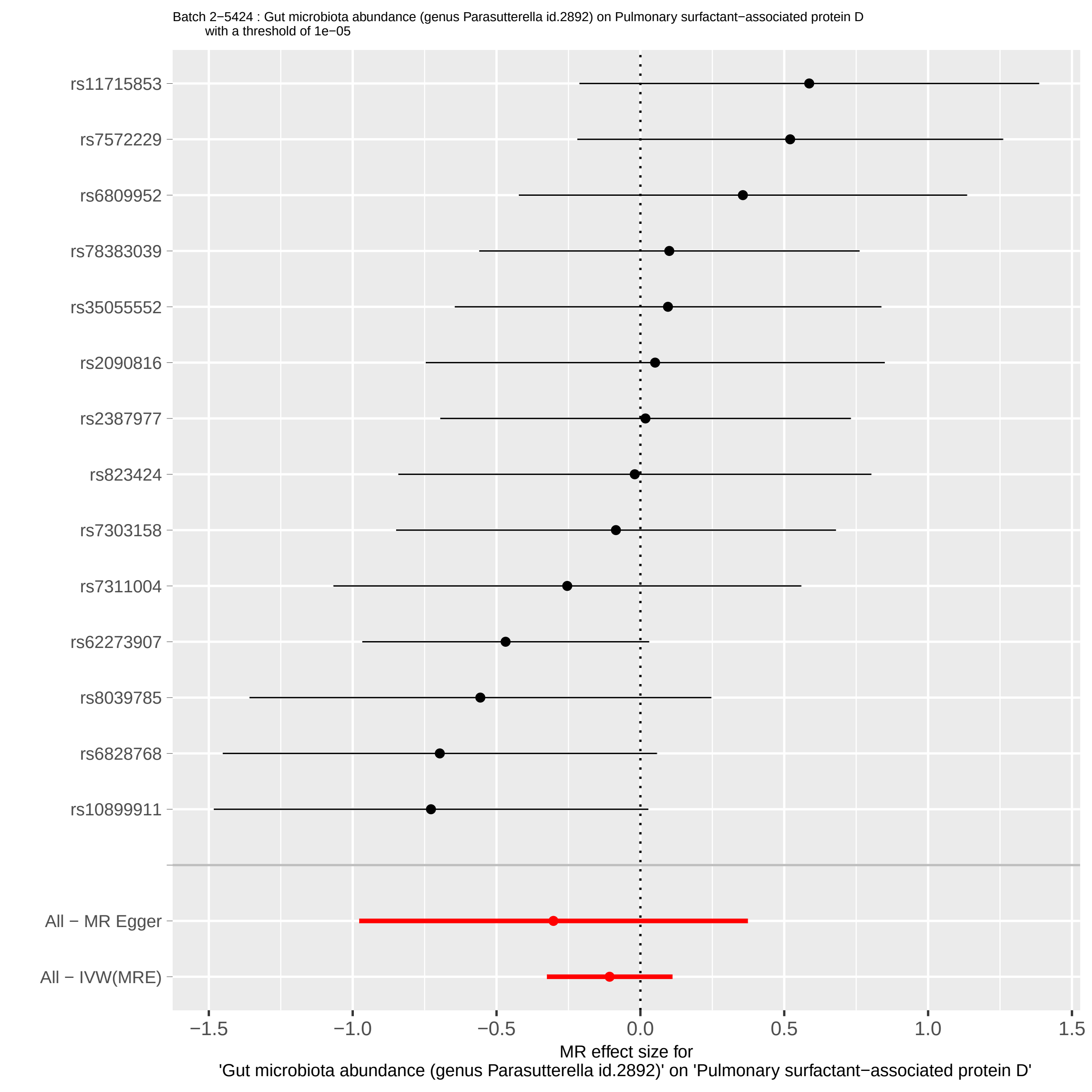

Batch 2−5424 : Gut microbiota abundance (genus Parasutterella id.2892) on Pulmonary surfactant−associated protein D
with a threshold of 1e−05
rs11715853
rs7572229
rs6809952
rs78383039
rs35055552
rs2090816
rs2387977
rs823424
rs7303158
rs7311004
rs62273907
rs8039785
rs6828768
rs10899911
All − MR Egger
All − IVW(MRE)
−1.5
−1.0
−0.5
0.0
0.5
1.0
1.5
MR effect size for
'Gut microbiota abundance (genus Parasutterella id.2892)' on 'Pulmonary surfactant−associated protein D'

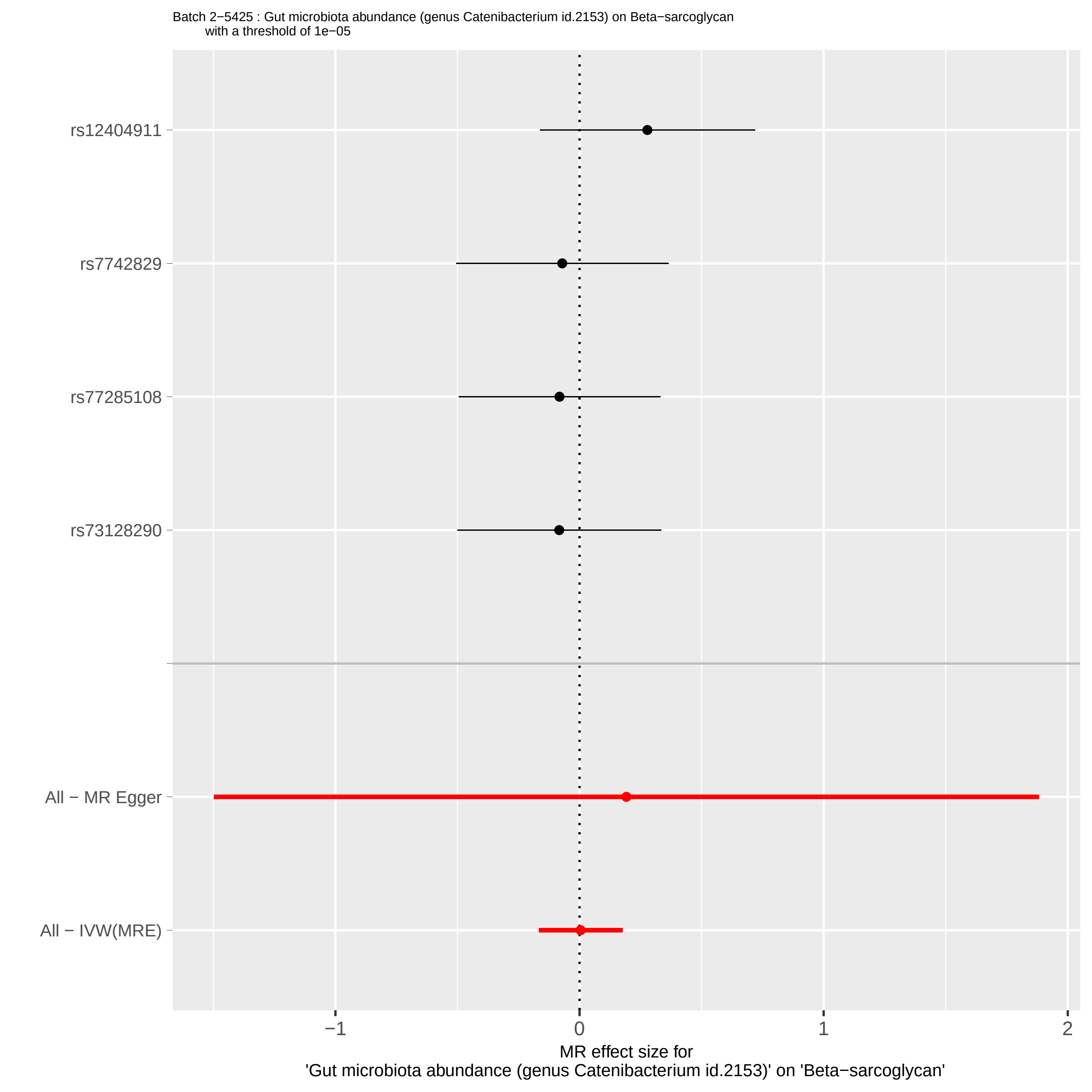

Batch 2−5425 : Gut microbiota abundance (genus Catenibacterium id.2153) on Beta−sarcoglycan
with a threshold of 1e−05
rs12404911
rs7742829
rs77285108
rs73128290
All − MR Egger
All − IVW(MRE)
−1
0
1
2
MR effect size for
'Gut microbiota abundance (genus Catenibacterium id.2153)' on 'Beta−sarcoglycan'

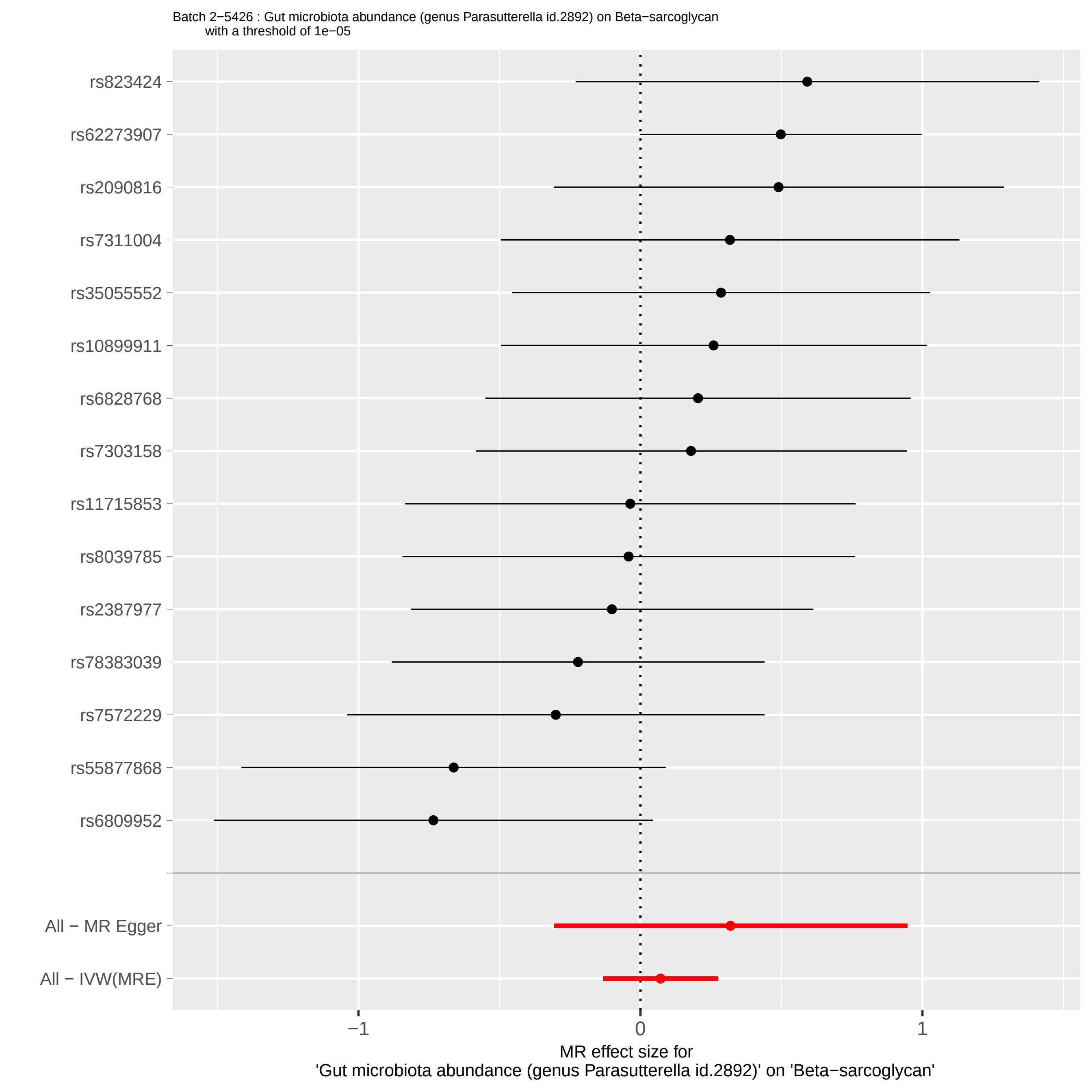
Batch 2−5426 : Gut microbiota abundance (genus Parasutterella id.2892) on Beta−sarcoglycan
with a threshold of 1e−05
rs823424
rs62273907
rs2090816
rs7311004
rs35055552
rs10899911
rs6828768
rs7303158
rs11715853
rs8039785
rs2387977
rs78383039
rs7572229
rs55877868
rs6809952
All − MR Egger
All − IVW(MRE)
−1
0
1
MR effect size for
'Gut microbiota abundance (genus Parasutterella id.2892)' on 'Beta−sarcoglycan'

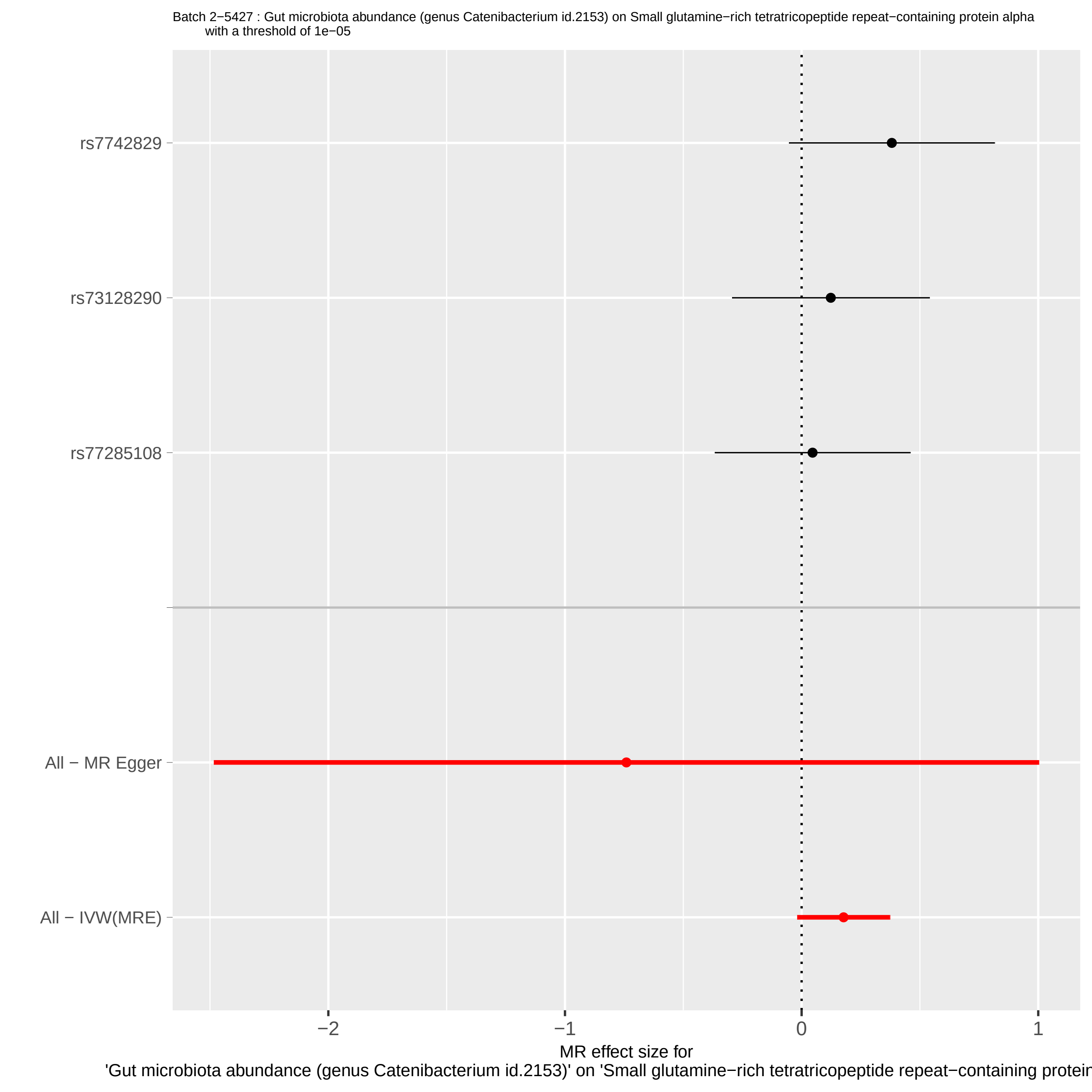
Batch 2−5427 : Gut microbiota abundance (genus Catenibacterium id.2153) on Small glutamine−rich tetratricopeptide repeat−containing protein alpha
with a threshold of 1e−05
rs7742829
rs73128290
rs77285108
All − MR Egger
All − IVW(MRE)
−2
−1
0
1
MR effect size for
'Gut microbiota abundance (genus Catenibacterium id.2153)' on 'Small glutamine−rich tetratricopeptide repeat−containing protein

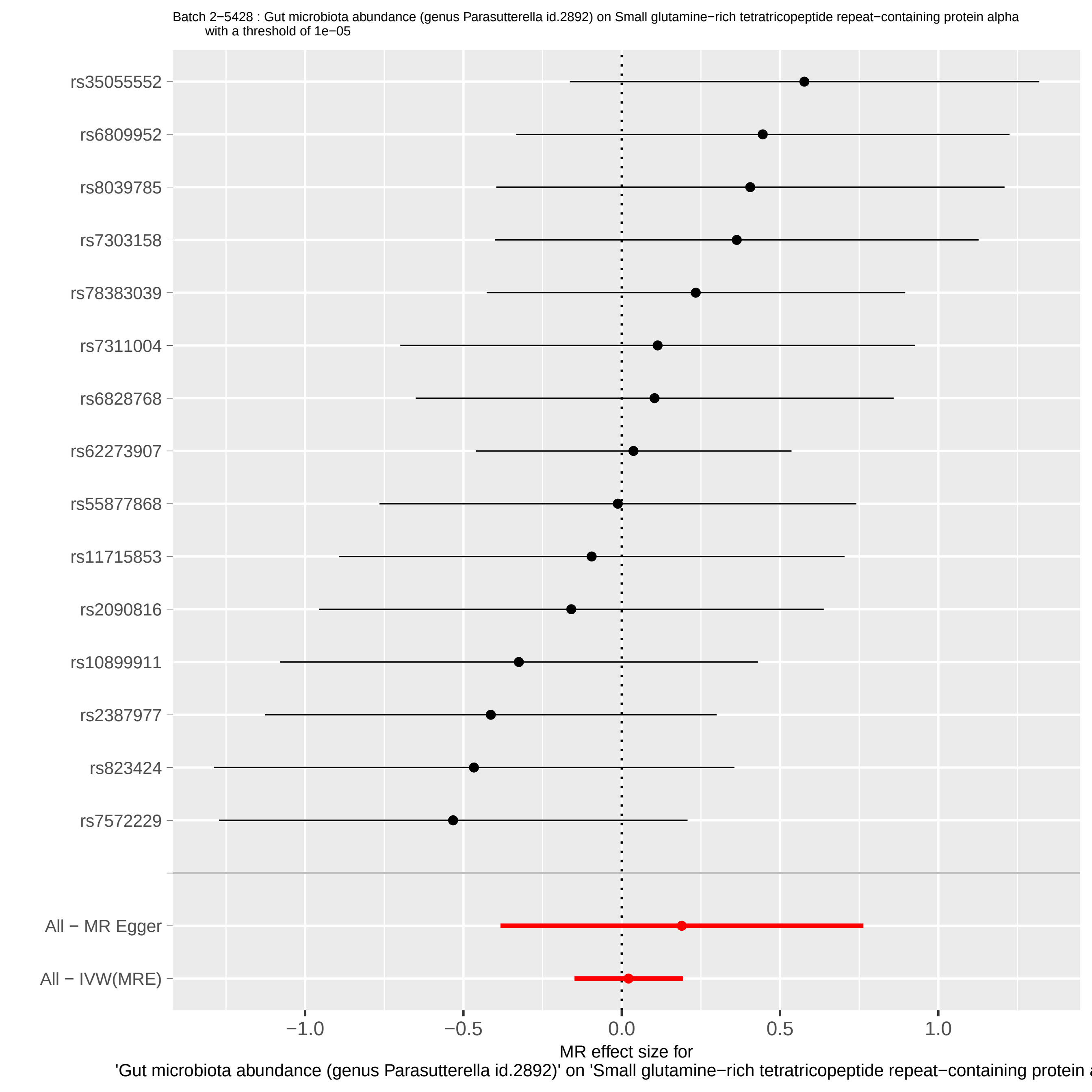
Batch 2−5428 : Gut microbiota abundance (genus Parasutterella id.2892) on Small glutamine−rich tetratricopeptide repeat−containing protein alpha
with a threshold of 1e−05
rs35055552
rs6809952
rs8039785
rs7303158
rs78383039
rs7311004
rs6828768
rs62273907
rs55877868
rs11715853
rs2090816
rs10899911
rs2387977
rs823424
rs7572229
All − MR Egger
All − IVW(MRE)
−1.0
−0.5
0.0
0.5
1.0
MR effect size for
'Gut microbiota abundance (genus Parasutterella id.2892)' on 'Small glutamine−rich tetratricopeptide repeat−containing protein a

Batch 2−5429 : Gut microbiota abundance (genus Catenibacterium id.2153) on SH2 domain−containing protein 1A
with a threshold of 1e−05

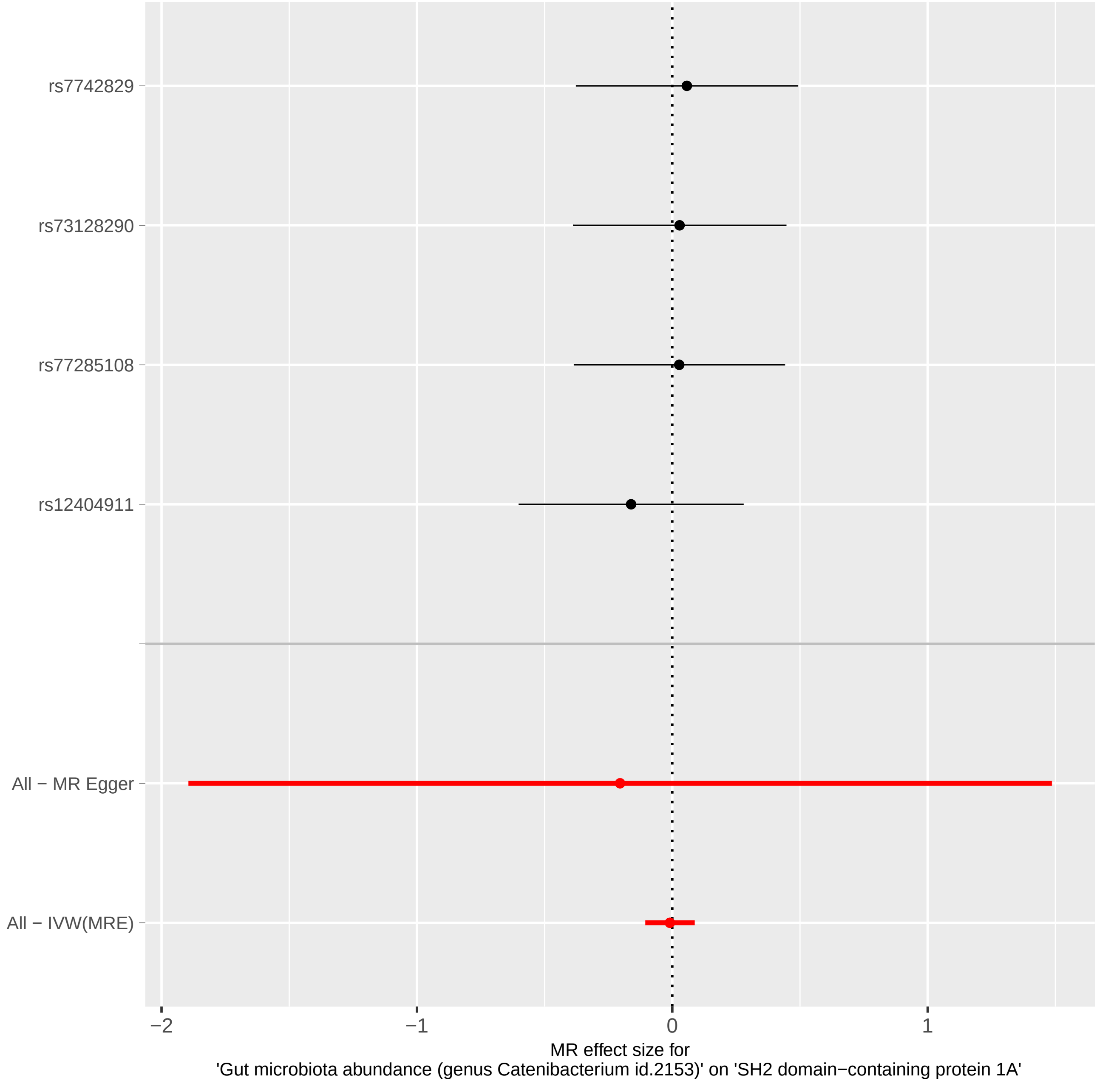

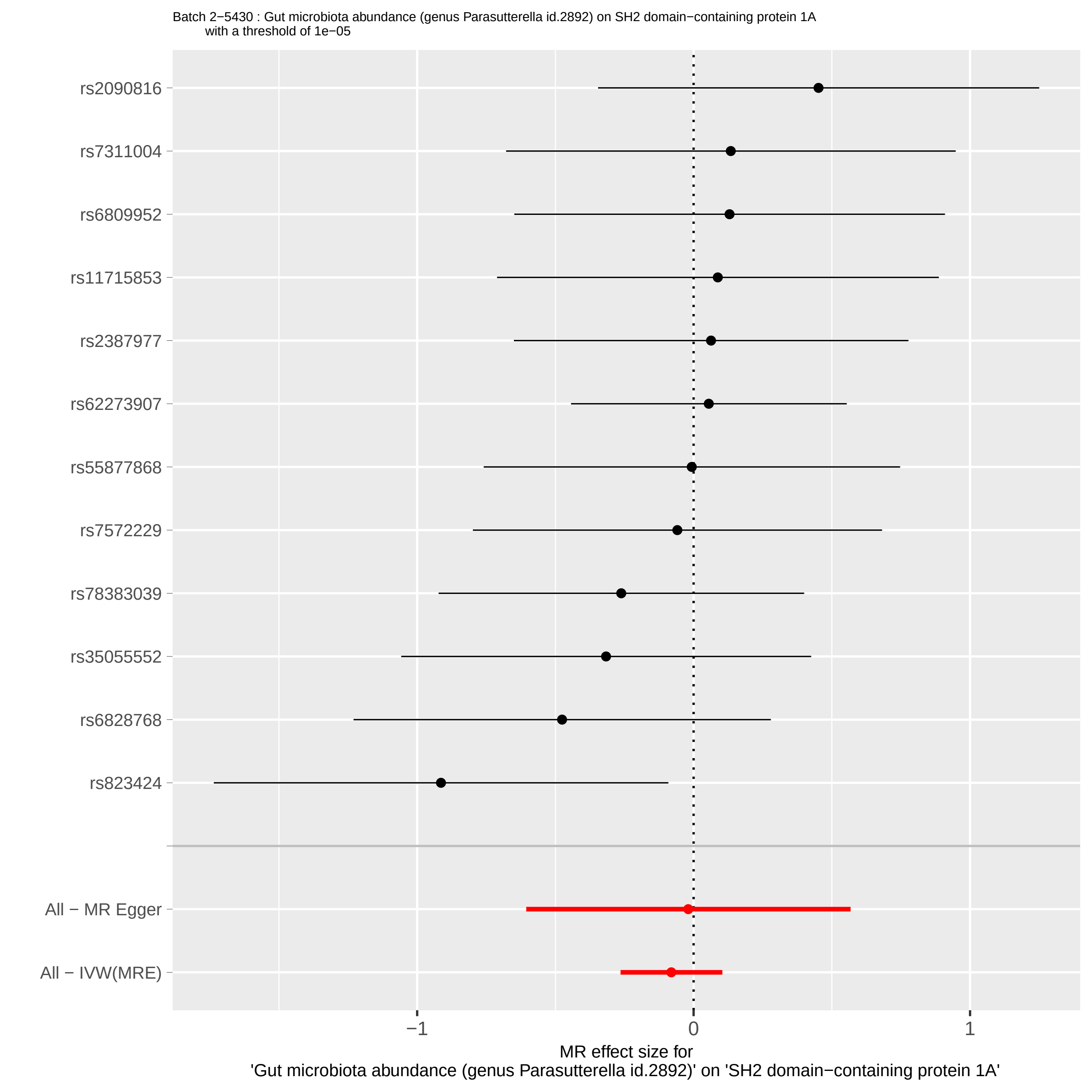

Batch 2−5430 : Gut microbiota abundance (genus Parasutterella id.2892) on SH2 domain−containing protein 1A
with a threshold of 1e−05
rs2090816
rs7311004
rs6809952
rs11715853
rs2387977
rs62273907
rs55877868
rs7572229
rs78383039
rs35055552
rs6828768
rs823424
All − MR Egger
All − IVW(MRE)
−1
0
1
MR effect size for
'Gut microbiota abundance (genus Parasutterella id.2892)' on 'SH2 domain−containing protein 1A'

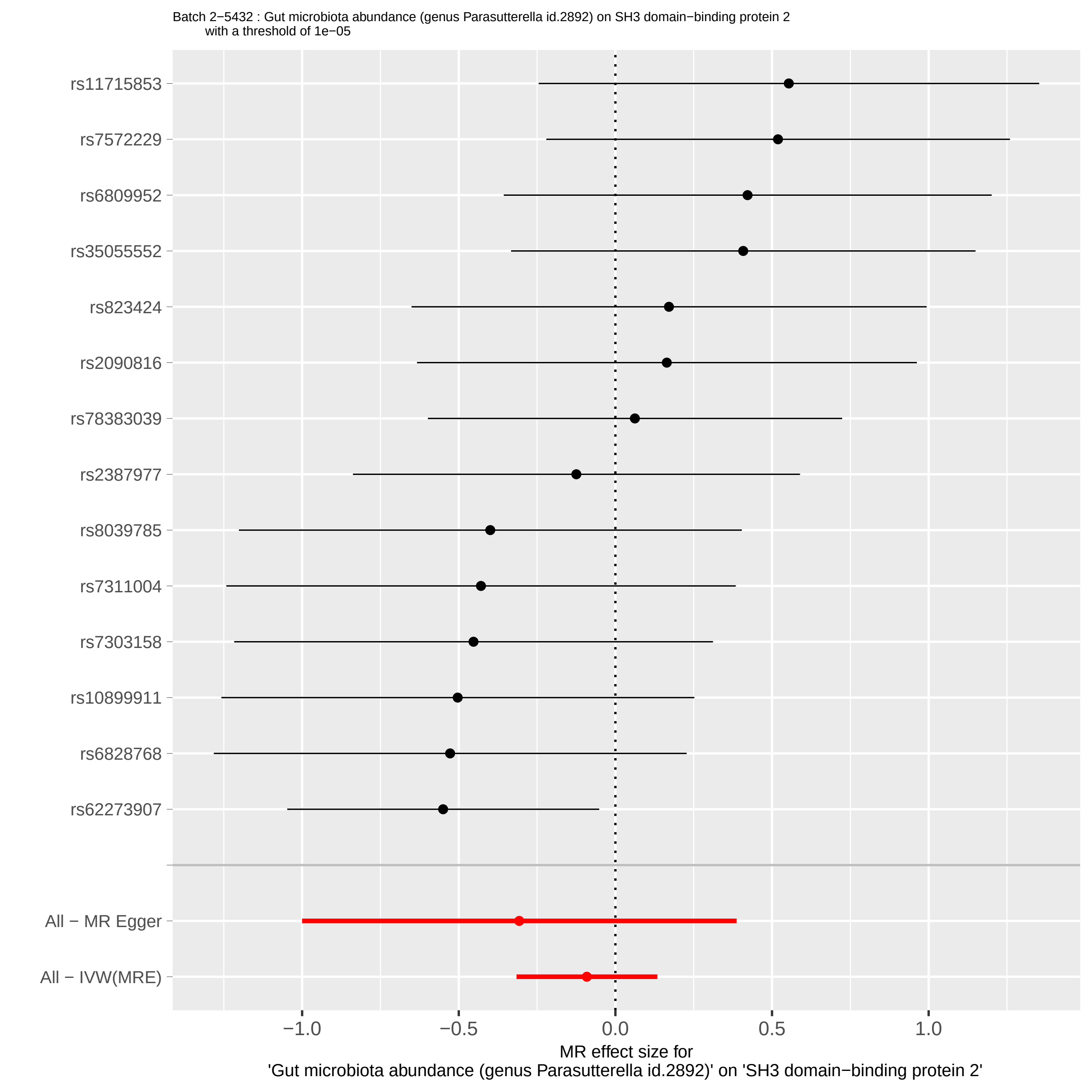

Batch 2−5432 : Gut microbiota abundance (genus Parasutterella id.2892) on SH3 domain−binding protein 2
with a threshold of 1e−05
rs11715853
rs7572229
rs6809952
rs35055552
rs823424
rs2090816
rs78383039
rs2387977
rs8039785
rs7311004
rs7303158
rs10899911
rs6828768
rs62273907
All − MR Egger
All − IVW(MRE)
−1.0
−0.5
0.0
0.5
1.0
MR effect size for
'Gut microbiota abundance (genus Parasutterella id.2892)' on 'SH3 domain−binding protein 2'

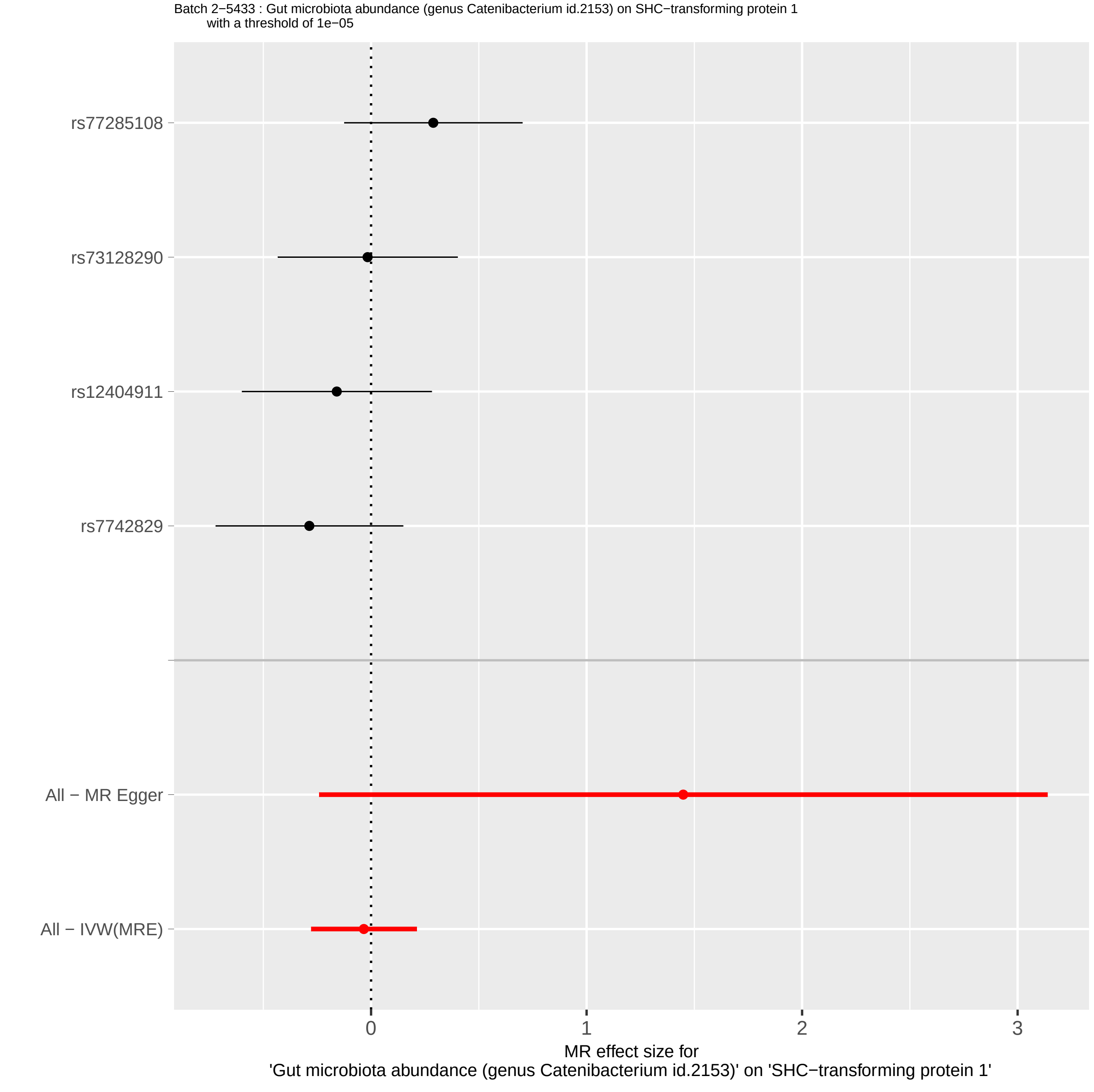

Batch 2−5433 : Gut microbiota abundance (genus Catenibacterium id.2153) on SHC−transforming protein 1
with a threshold of 1e−05
rs77285108
rs73128290
rs12404911
rs7742829
All − MR Egger
All − IVW(MRE)
0
1
2
3
MR effect size for
'Gut microbiota abundance (genus Catenibacterium id.2153)' on 'SHC−transforming protein 1'

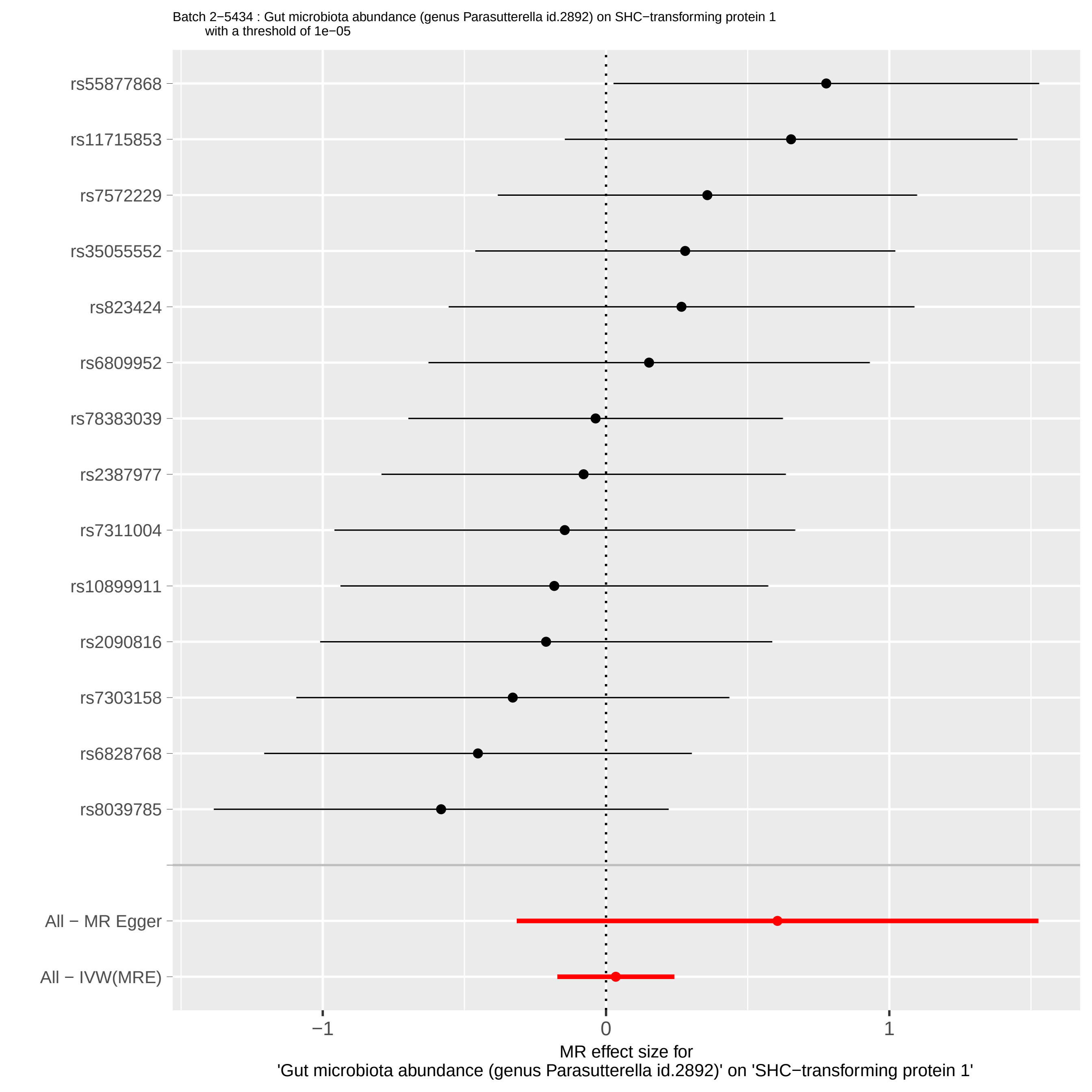
Batch 2−5434 : Gut microbiota abundance (genus Parasutterella id.2892) on SHC−transforming protein 1
with a threshold of 1e−05
rs55877868
rs11715853
rs7572229
rs35055552
rs823424
rs6809952
rs78383039
rs2387977
rs7311004
rs10899911
rs2090816
rs7303158
rs6828768
rs8039785
All − MR Egger
All − IVW(MRE)
−1
0
1
MR effect size for
'Gut microbiota abundance (genus Parasutterella id.2892)' on 'SHC−transforming protein 1'

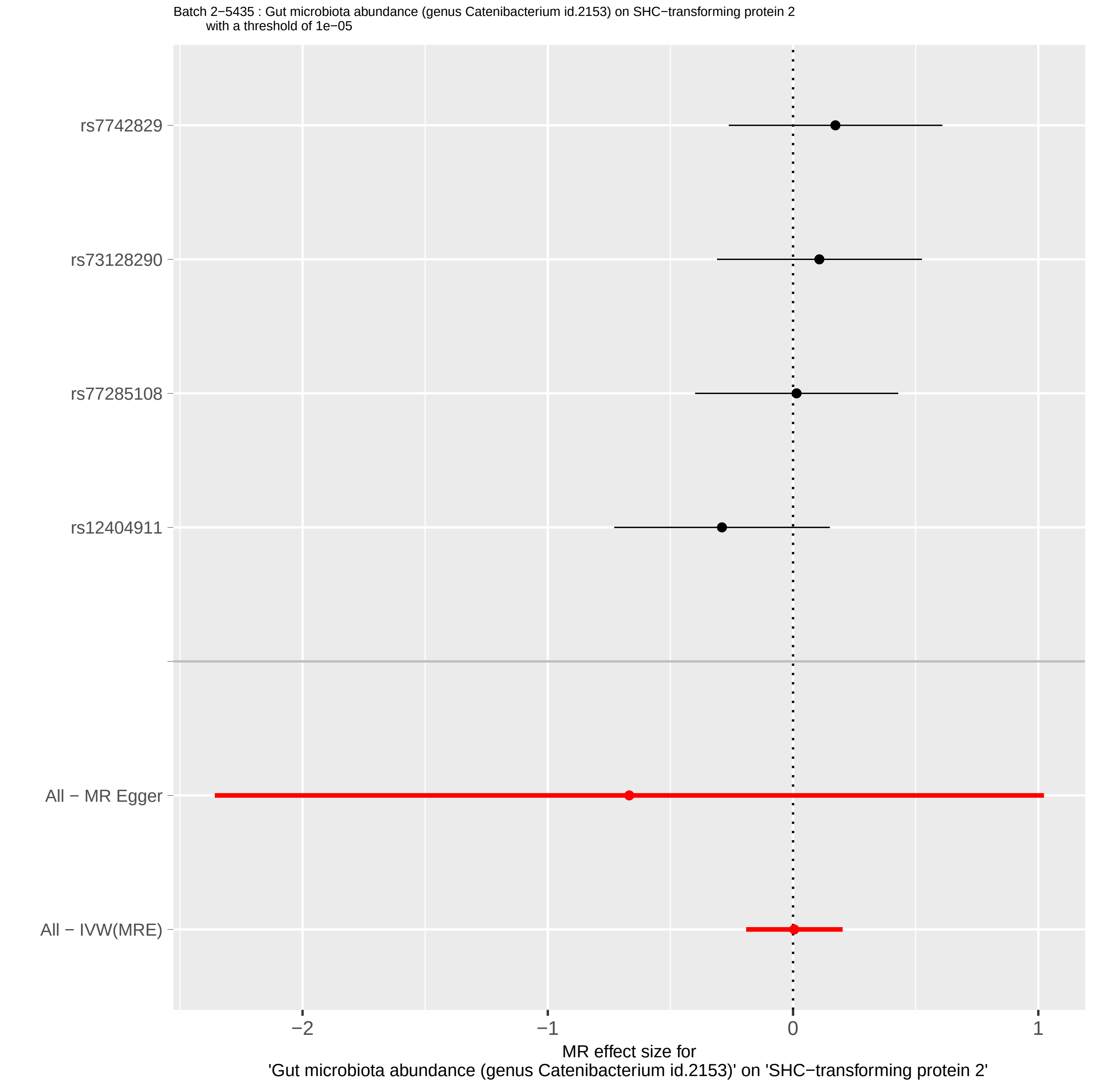

Batch 2−5435 : Gut microbiota abundance (genus Catenibacterium id.2153) on SHC−transforming protein 2
with a threshold of 1e−05
rs7742829
rs73128290
rs77285108
rs12404911
All − MR Egger
All − IVW(MRE)
−2
−1
0
1
MR effect size for
'Gut microbiota abundance (genus Catenibacterium id.2153)' on 'SHC−transforming protein 2'

Batch 2−5436 : Gut microbiota abundance (genus Parasutterella id.2892) on SHC−transforming protein 2
with a threshold of 1e−05

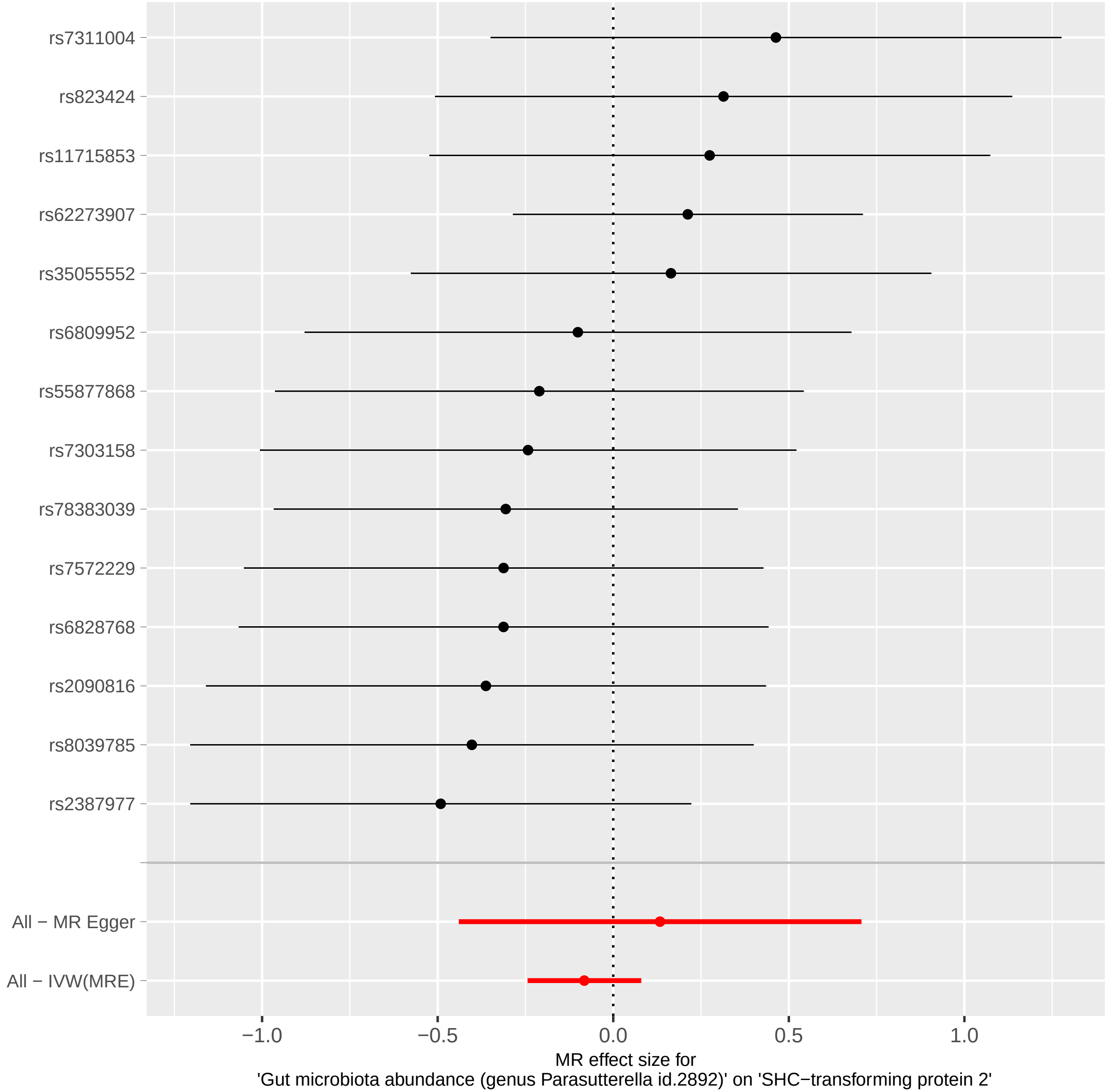

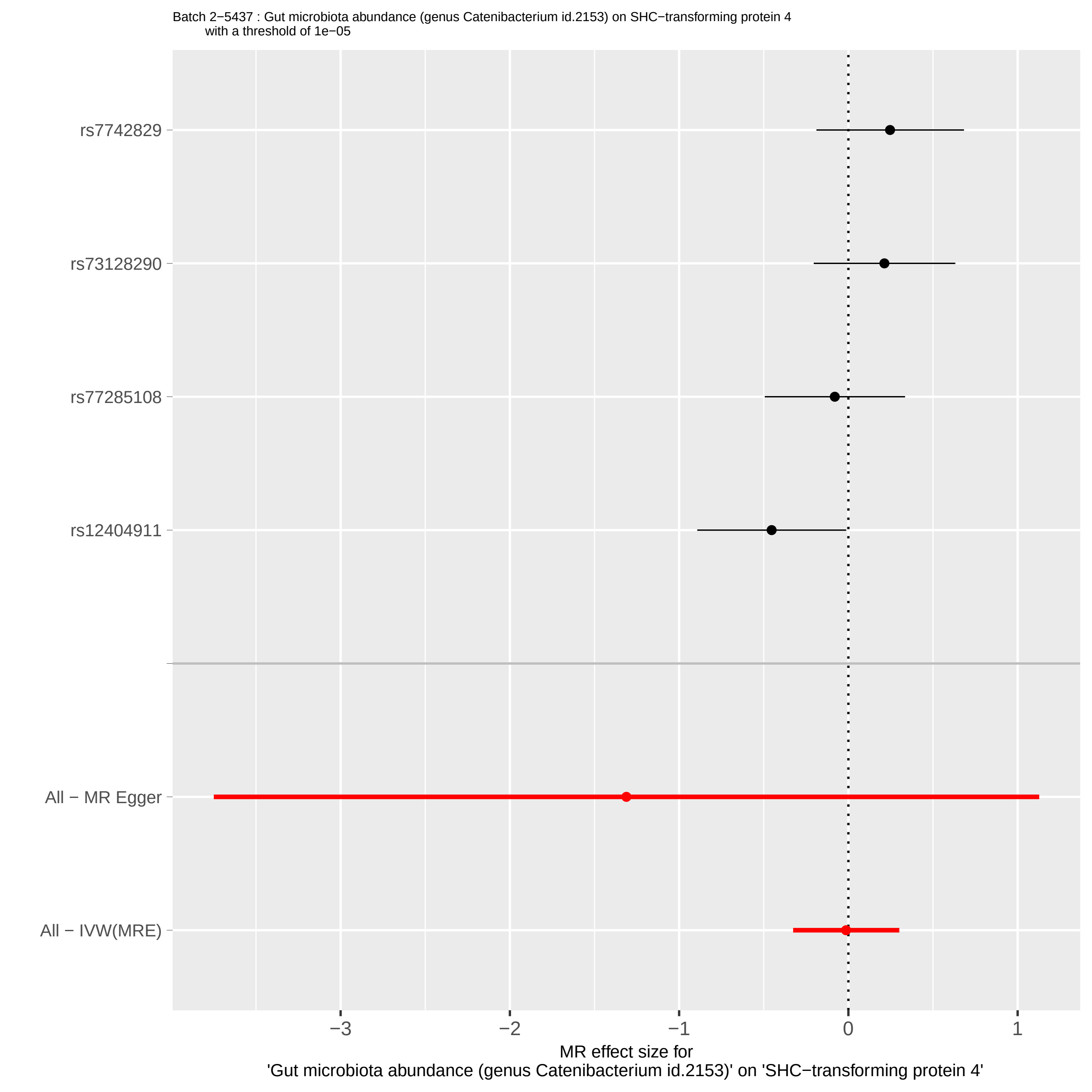
Batch 2−5437 : Gut microbiota abundance (genus Catenibacterium id.2153) on SHC−transforming protein 4
with a threshold of 1e−05
rs7742829
rs73128290
rs77285108
rs12404911
All − MR Egger
All − IVW(MRE)
−3
−2
−1
0
1
MR effect size for
'Gut microbiota abundance (genus Catenibacterium id.2153)' on 'SHC−transforming protein 4'

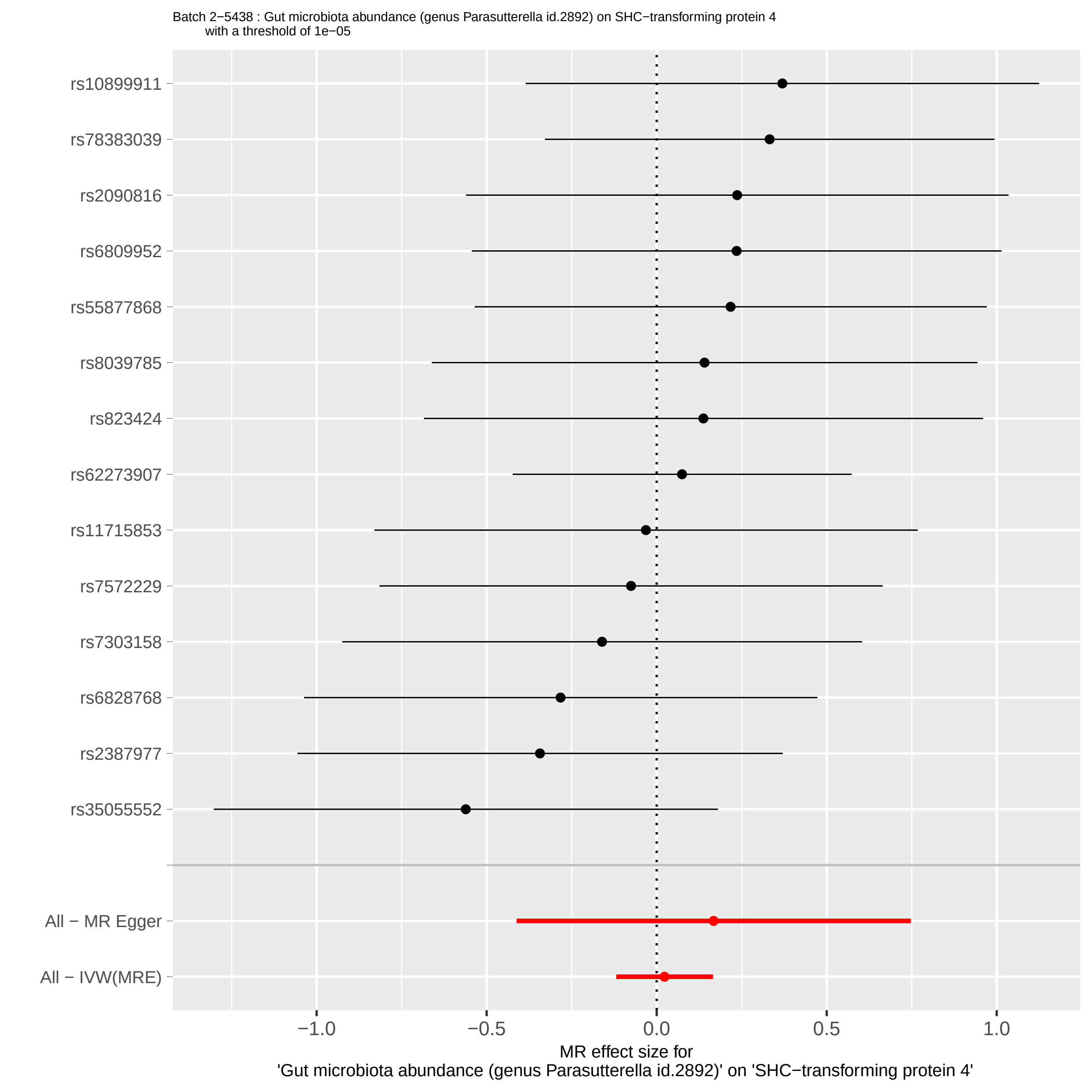
Batch 2−5438 : Gut microbiota abundance (genus Parasutterella id.2892) on SHC−transforming protein 4
with a threshold of 1e−05
rs10899911
rs78383039
rs2090816
rs6809952
rs55877868
rs8039785
rs823424
rs62273907
rs11715853
rs7572229
rs7303158
rs6828768
rs2387977
rs35055552
All − MR Egger
All − IVW(MRE)
−1.0
−0.5
0.0
0.5
1.0
MR effect size for
'Gut microbiota abundance (genus Parasutterella id.2892)' on 'SHC−transforming protein 4'

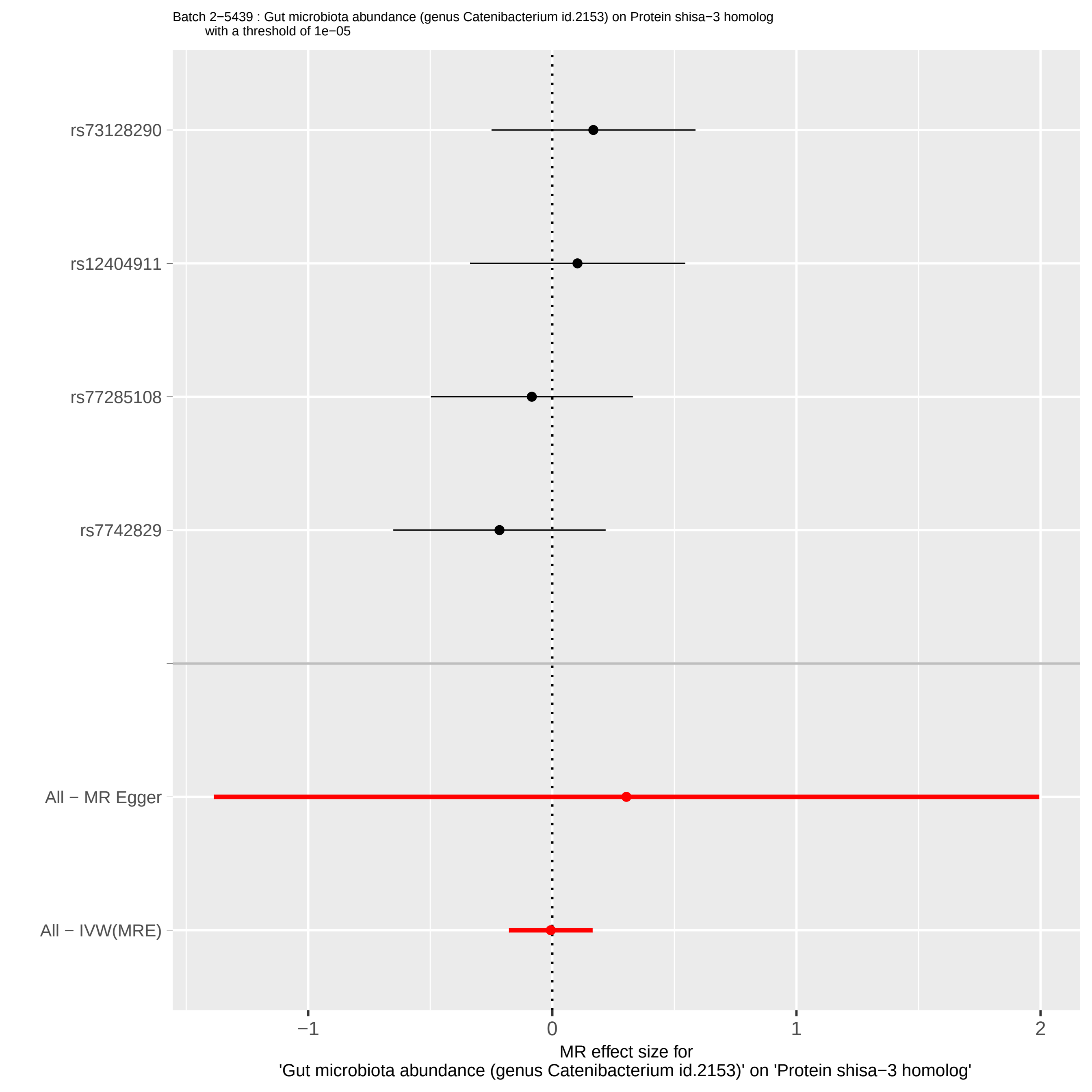

Batch 2−5439 : Gut microbiota abundance (genus Catenibacterium id.2153) on Protein shisa−3 homolog
with a threshold of 1e−05
rs73128290
rs12404911
rs77285108
rs7742829
All − MR Egger
All − IVW(MRE)
−1
0
1
2
MR effect size for
'Gut microbiota abundance (genus Catenibacterium id.2153)' on 'Protein shisa−3 homolog'

Batch 2−5440 : Gut microbiota abundance (genus Parasutterella id.2892) on Protein shisa−3 homolog
with a threshold of 1e−05

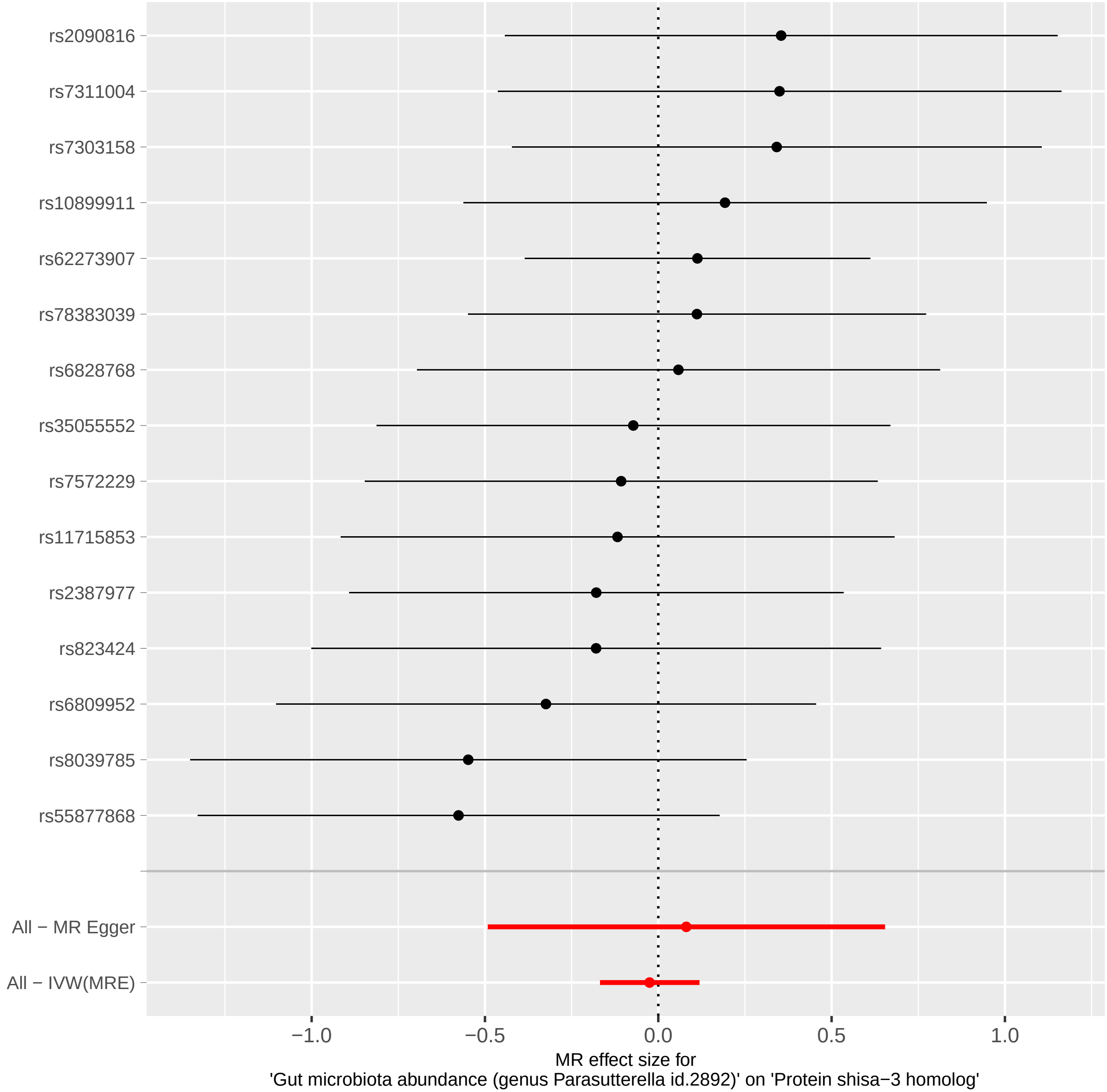

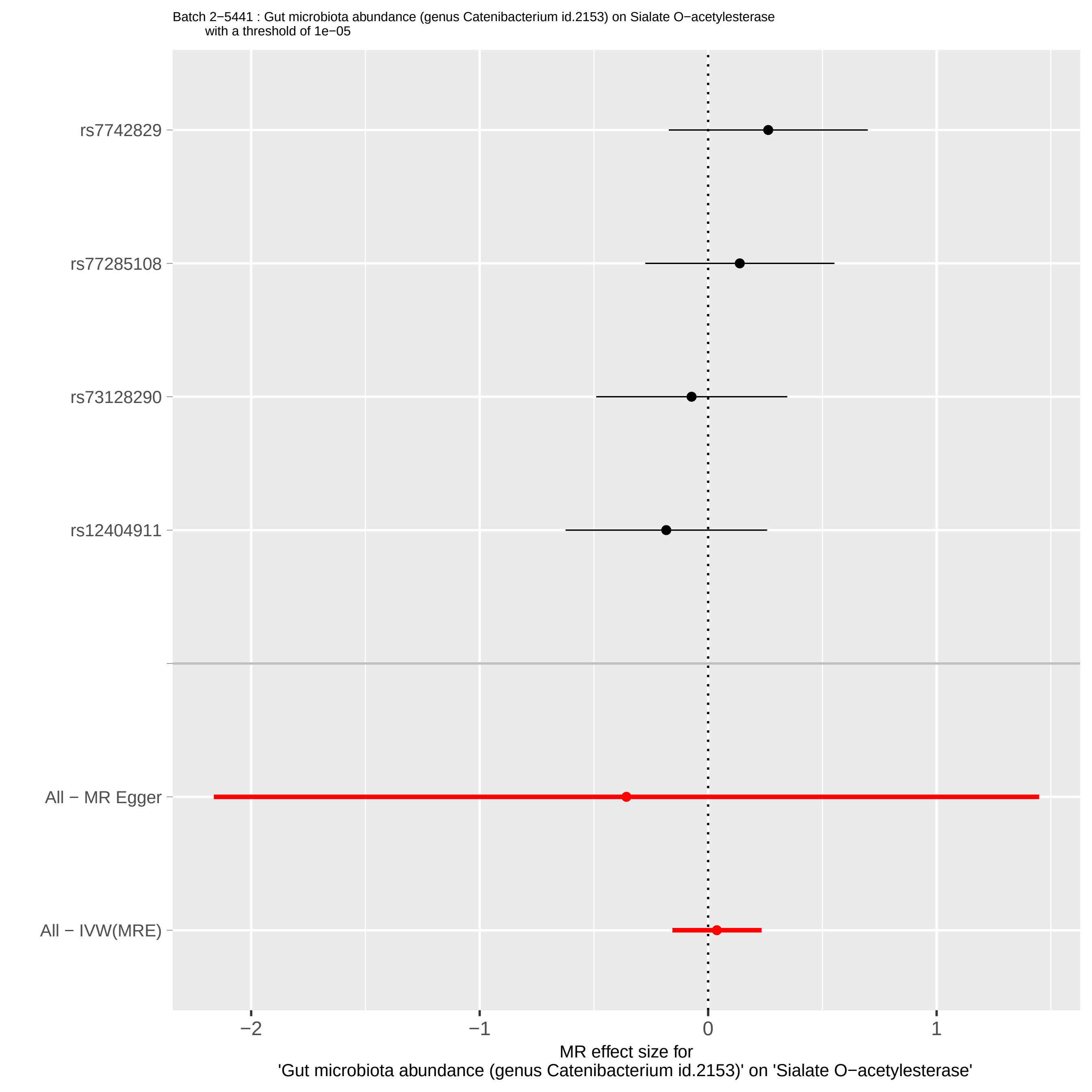

Batch 2−5441 : Gut microbiota abundance (genus Catenibacterium id.2153) on Sialate O−acetylesterase
with a threshold of 1e−05
rs7742829
rs77285108
rs73128290
rs12404911
All − MR Egger
All − IVW(MRE)
−2
−1
0
1
MR effect size for
'Gut microbiota abundance (genus Catenibacterium id.2153)' on 'Sialate O−acetylesterase'

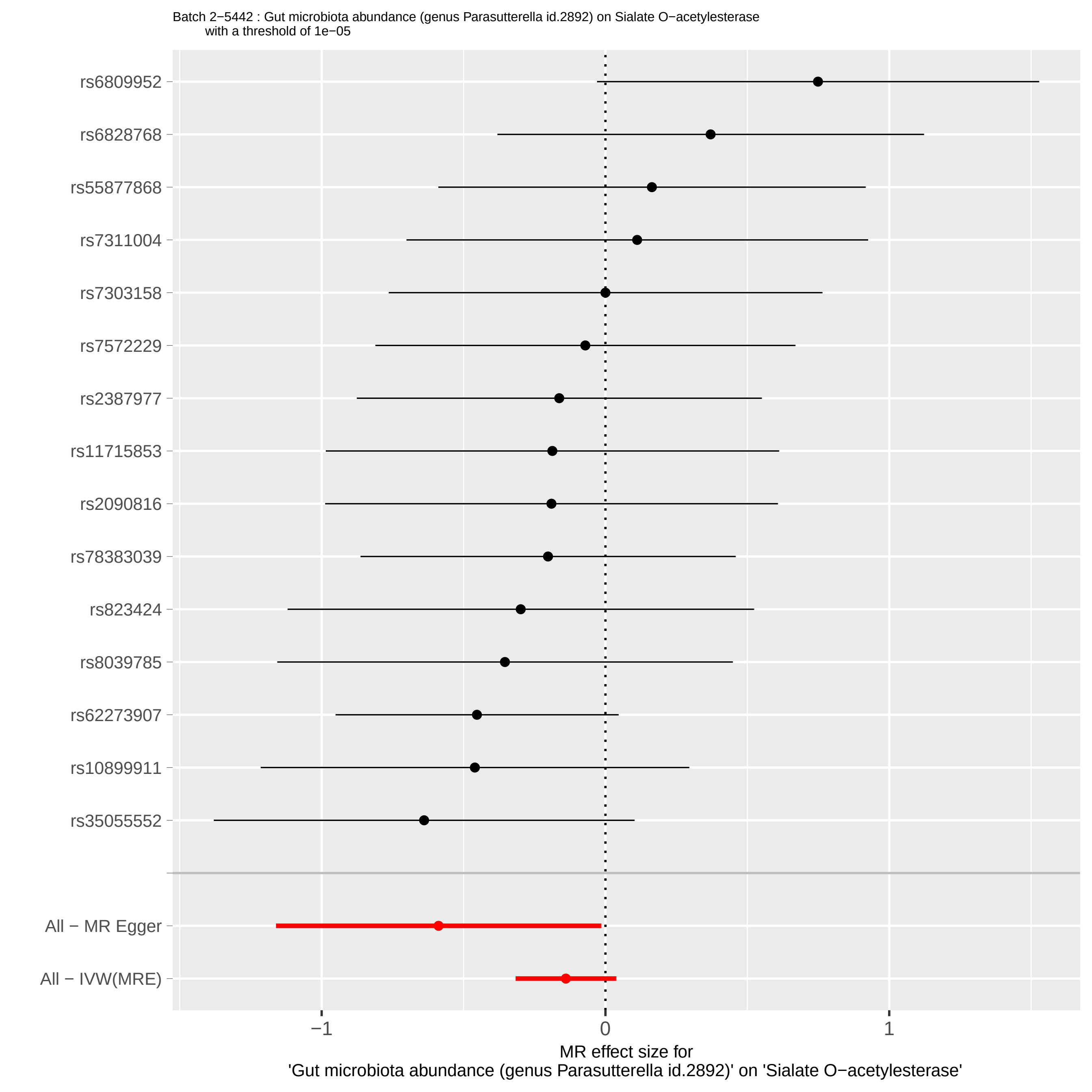

Batch 2−5442 : Gut microbiota abundance (genus Parasutterella id.2892) on Sialate O−acetylesterase
with a threshold of 1e−05
rs6809952
rs6828768
rs55877868
rs7311004
rs7303158
rs7572229
rs2387977
rs11715853
rs2090816
rs78383039
rs823424
rs8039785
rs62273907
rs10899911
rs35055552
All − MR Egger
All − IVW(MRE)
−1
0
1
MR effect size for
'Gut microbiota abundance (genus Parasutterella id.2892)' on 'Sialate O−acetylesterase'

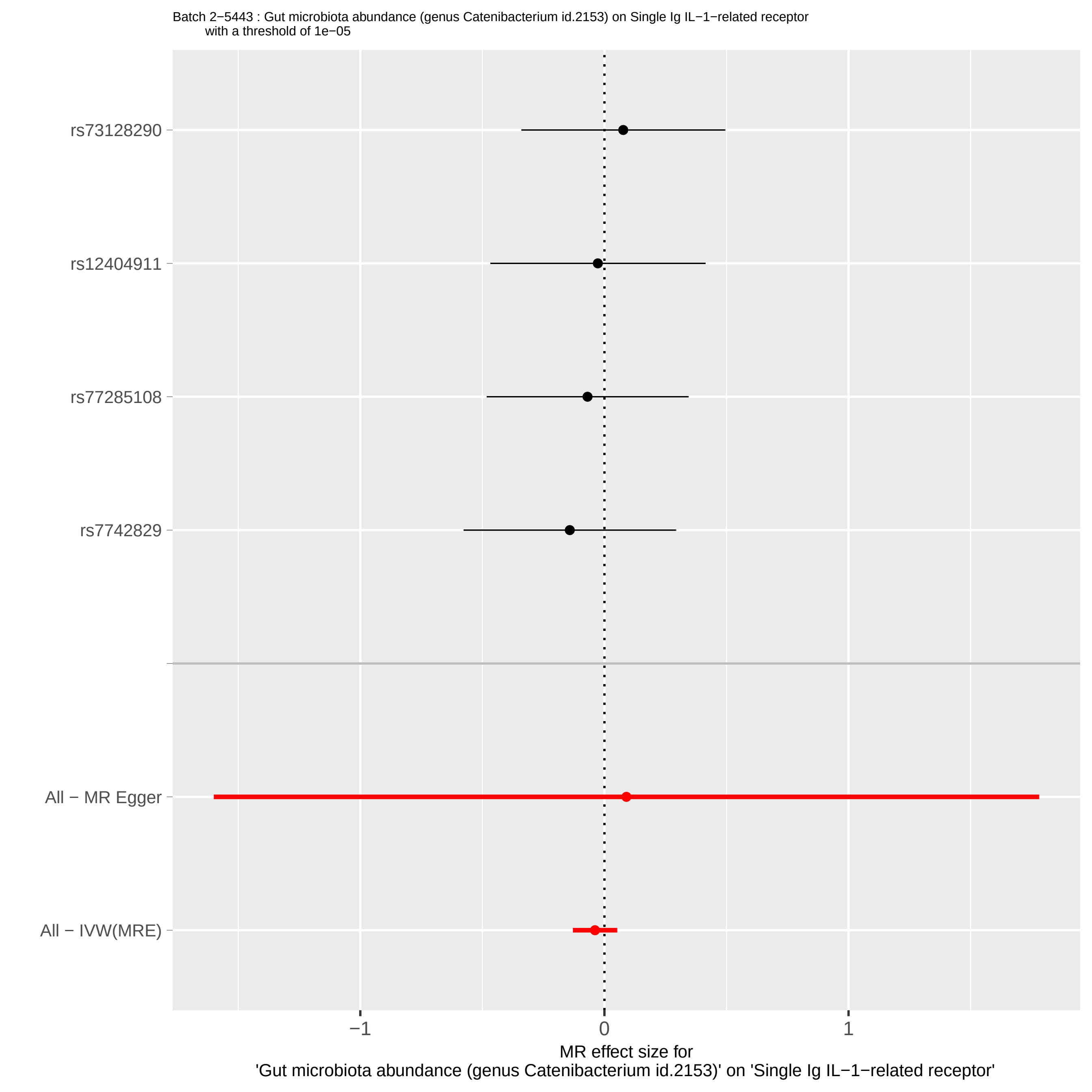

Batch 2−5443 : Gut microbiota abundance (genus Catenibacterium id.2153) on Single Ig IL−1−related receptor
with a threshold of 1e−05
rs73128290
rs12404911
rs77285108
rs7742829
All − MR Egger
All − IVW(MRE)
−1
0
1
MR effect size for
'Gut microbiota abundance (genus Catenibacterium id.2153)' on 'Single Ig IL−1−related receptor'

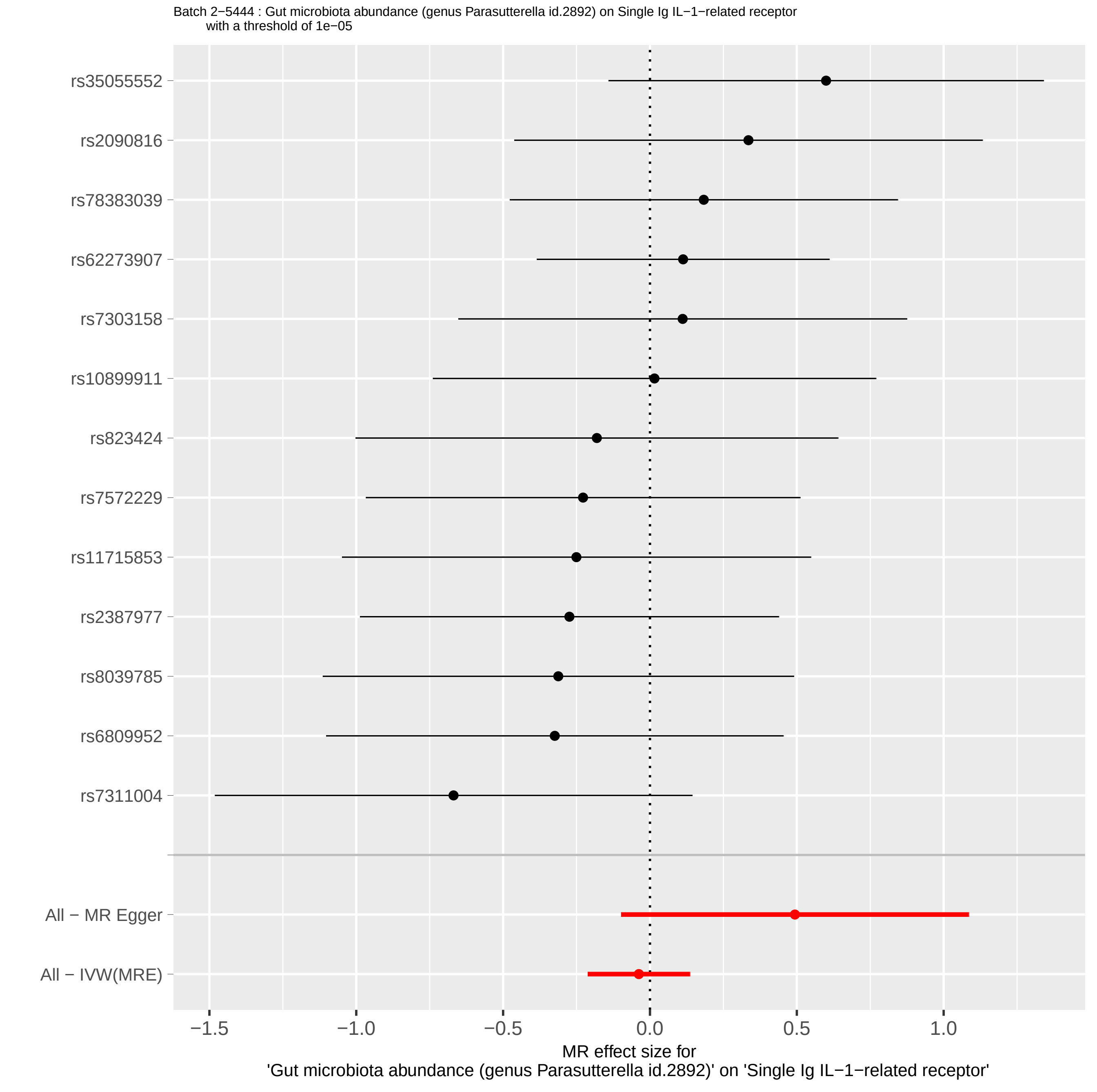

Batch 2−5444 : Gut microbiota abundance (genus Parasutterella id.2892) on Single Ig IL−1−related receptor
with a threshold of 1e−05
rs35055552
rs2090816
rs78383039
rs62273907
rs7303158
rs10899911
rs823424
rs7572229
rs11715853
rs2387977
rs8039785
rs6809952
rs7311004
All − MR Egger
All − IVW(MRE)
−1.5
−1.0
−0.5
0.0
0.5
1.0
MR effect size for
'Gut microbiota abundance (genus Parasutterella id.2892)' on 'Single Ig IL−1−related receptor'

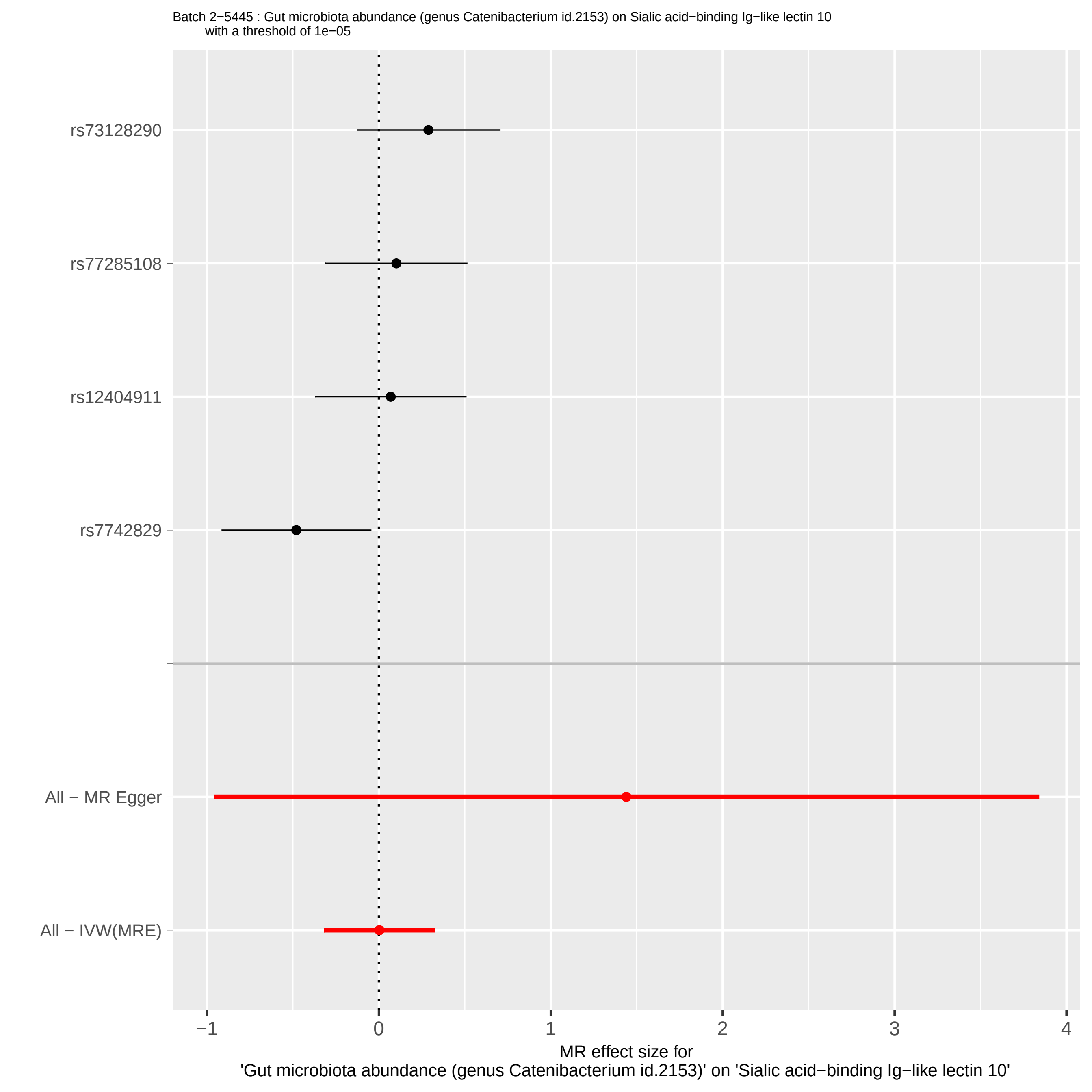
Batch 2−5445 : Gut microbiota abundance (genus Catenibacterium id.2153) on Sialic acid−binding Ig−like lectin 10
with a threshold of 1e−05
rs73128290
rs77285108
rs12404911
rs7742829
All − MR Egger
All − IVW(MRE)
−1
0
1
2
3
4
MR effect size for
'Gut microbiota abundance (genus Catenibacterium id.2153)' on 'Sialic acid−binding Ig−like lectin 10'

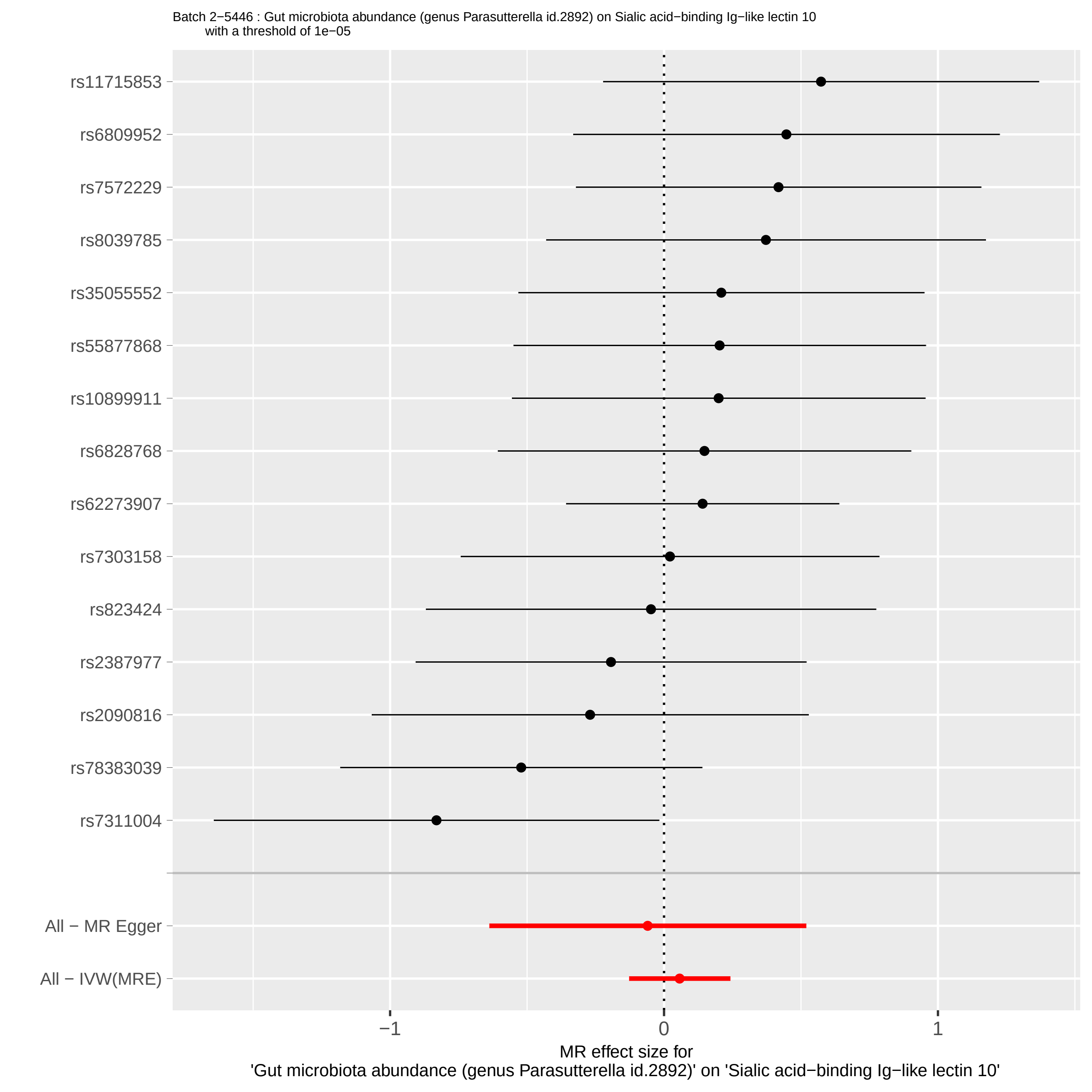
Batch 2−5446 : Gut microbiota abundance (genus Parasutterella id.2892) on Sialic acid−binding Ig−like lectin 10
with a threshold of 1e−05
rs11715853
rs6809952
rs7572229
rs8039785
rs35055552
rs55877868
rs10899911
rs6828768
rs62273907
rs7303158
rs823424
rs2387977
rs2090816
rs78383039
rs7311004
All − MR Egger
All − IVW(MRE)
−1
0
1
MR effect size for
'Gut microbiota abundance (genus Parasutterella id.2892)' on 'Sialic acid−binding Ig−like lectin 10'

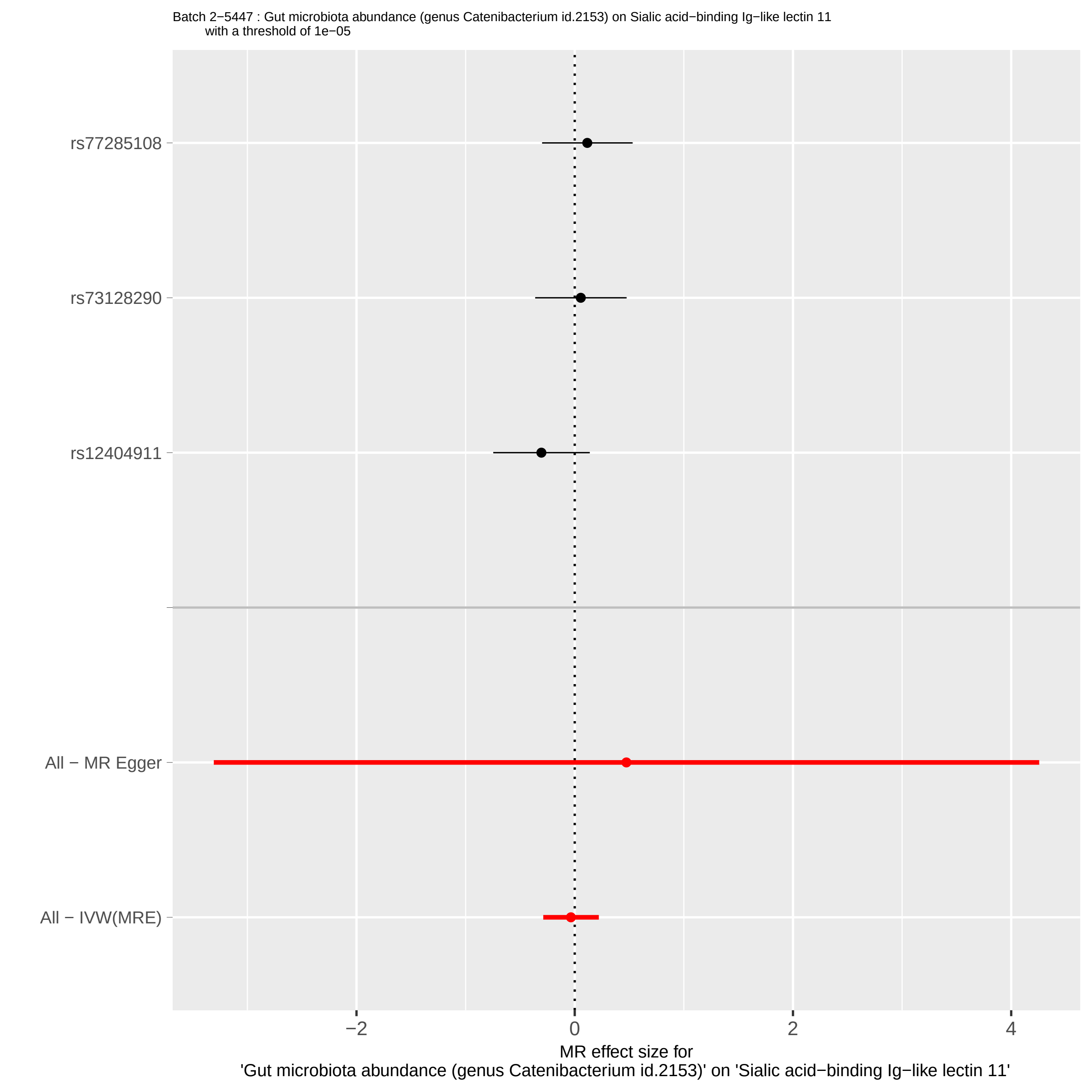

Batch 2−5447 : Gut microbiota abundance (genus Catenibacterium id.2153) on Sialic acid−binding Ig−like lectin 11
with a threshold of 1e−05
rs77285108
rs73128290
rs12404911
All − MR Egger
All − IVW(MRE)
−2
0
2
4
MR effect size for
'Gut microbiota abundance (genus Catenibacterium id.2153)' on 'Sialic acid−binding Ig−like lectin 11'

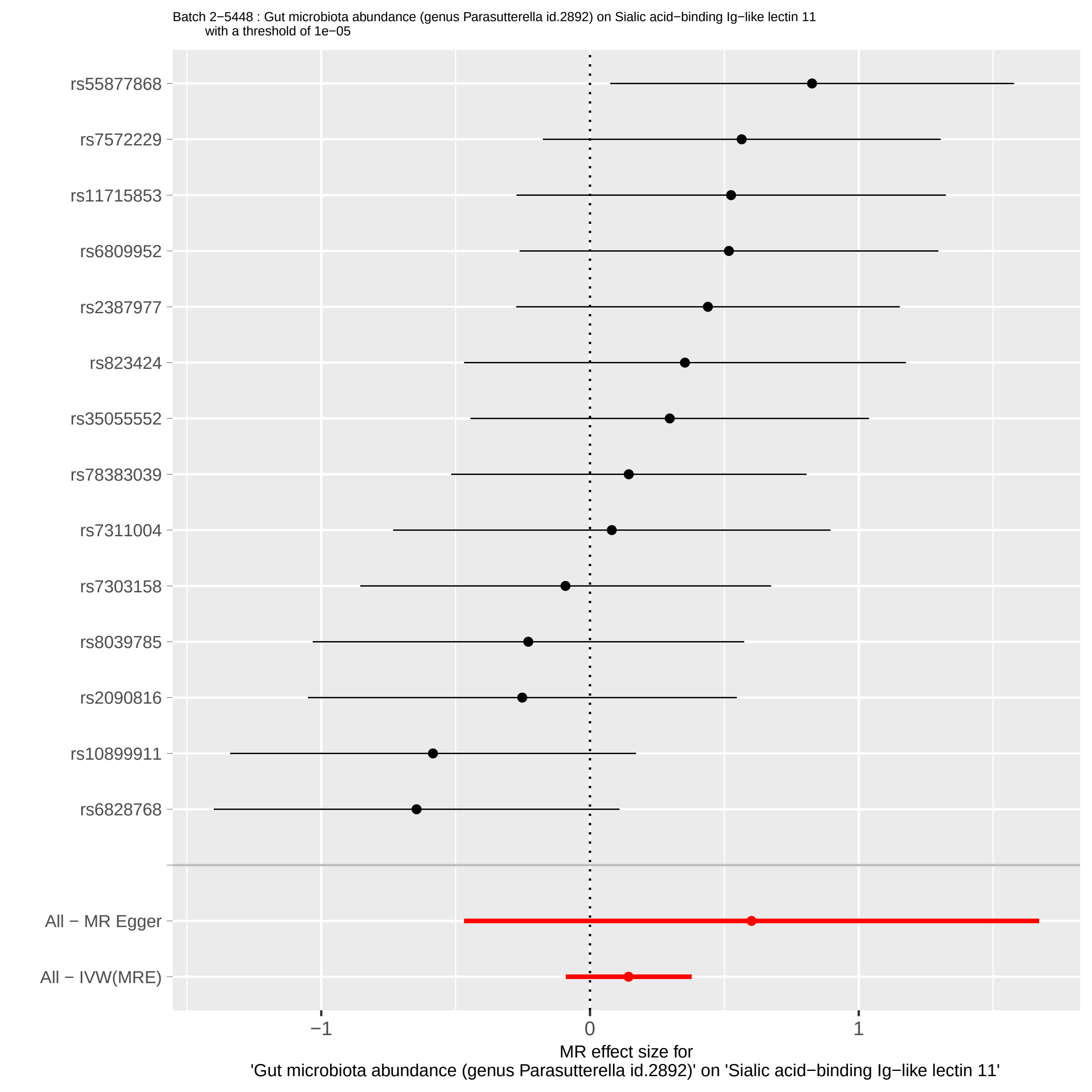

Batch 2−5448 : Gut microbiota abundance (genus Parasutterella id.2892) on Sialic acid−binding Ig−like lectin 11
with a threshold of 1e−05
rs55877868
rs7572229
rs11715853
rs6809952
rs2387977
rs823424
rs35055552
rs78383039
rs7311004
rs7303158
rs8039785
rs2090816
rs10899911
rs6828768
All − MR Egger
All − IVW(MRE)
−1
0
1
MR effect size for
'Gut microbiota abundance (genus Parasutterella id.2892)' on 'Sialic acid−binding Ig−like lectin 11'

Batch 2−5449 : Gut microbiota abundance (genus Catenibacterium id.2153) on Sialic acid−binding Ig−like lectin 12
with a threshold of 1e−05

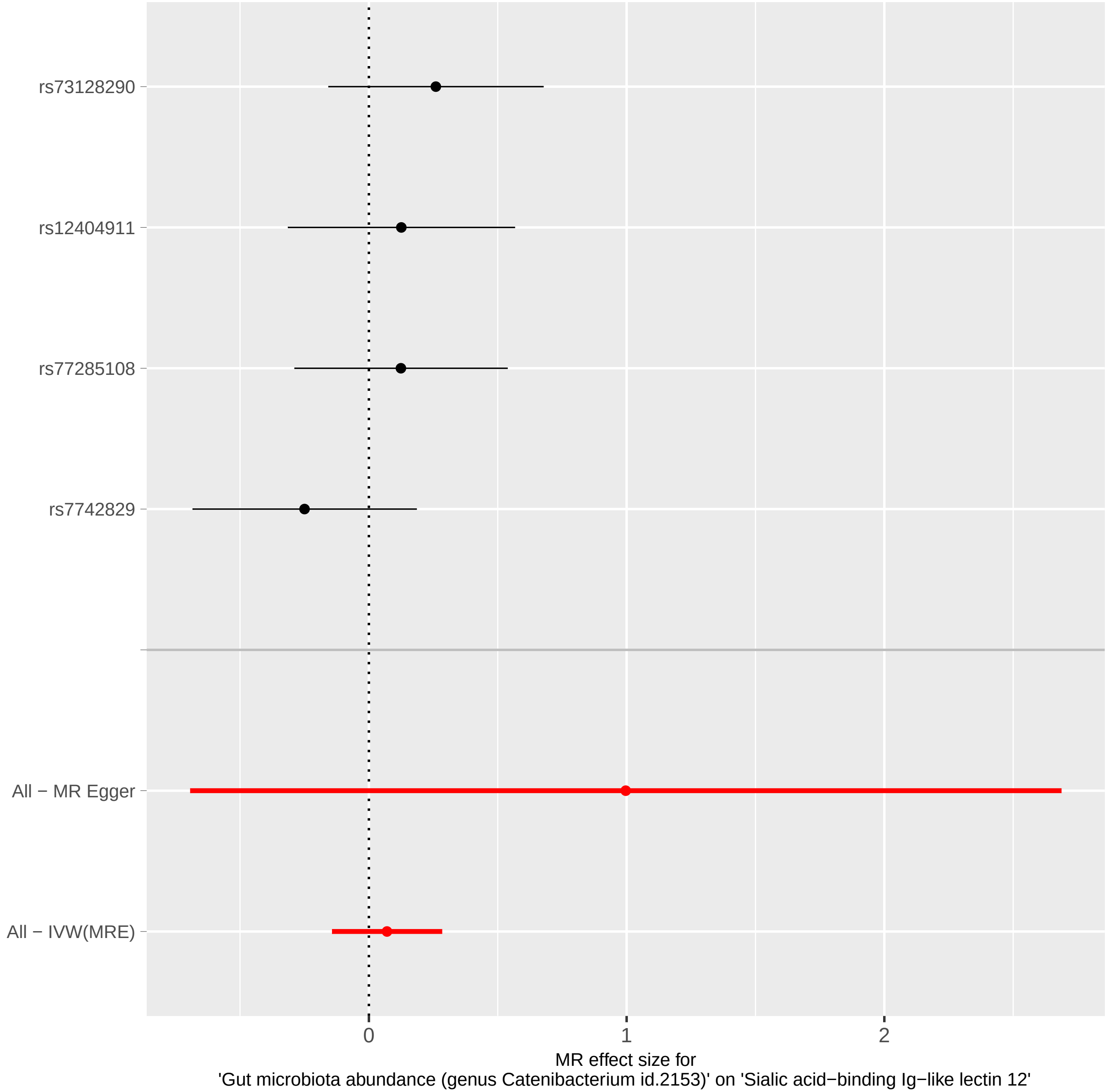

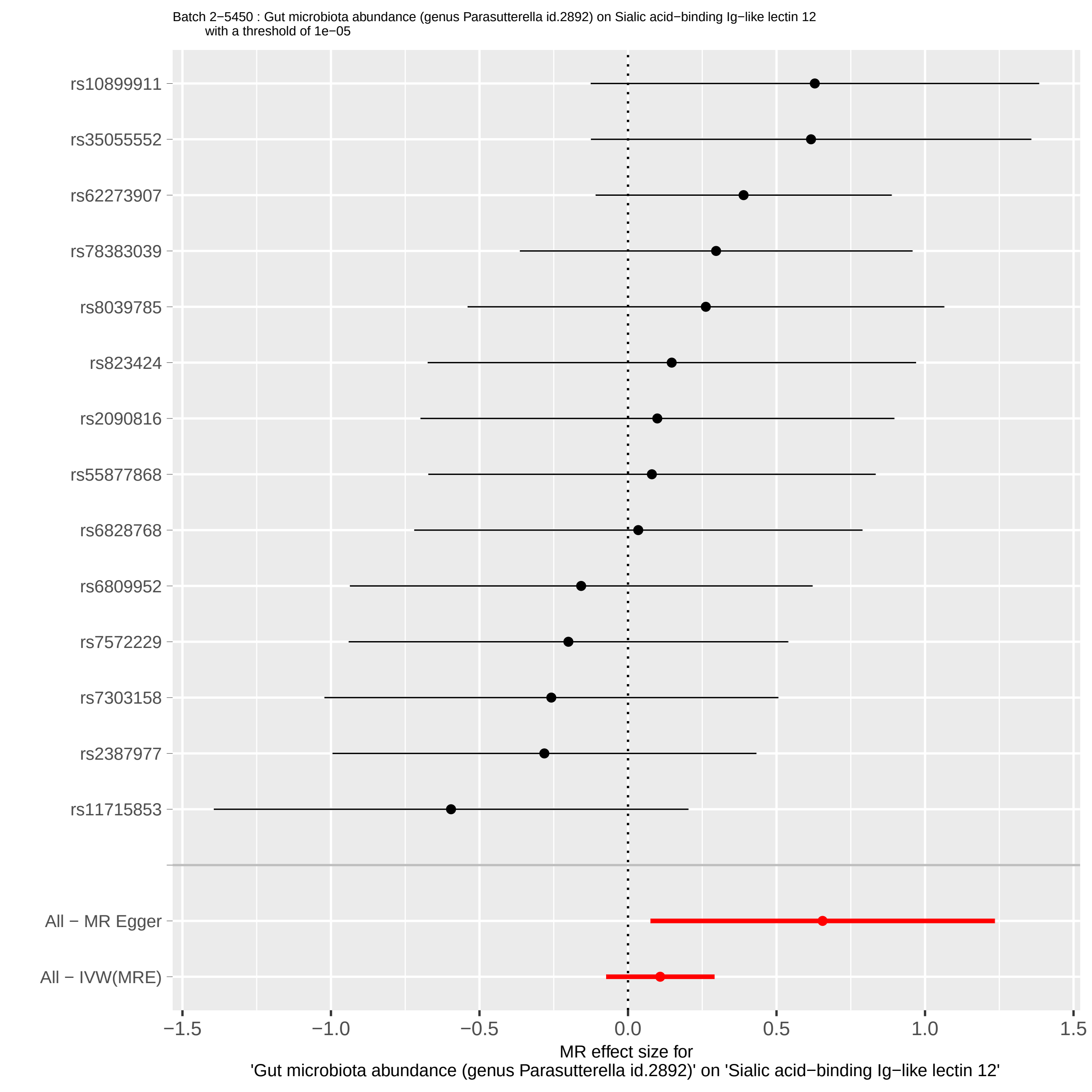

Batch 2−5450 : Gut microbiota abundance (genus Parasutterella id.2892) on Sialic acid−binding Ig−like lectin 12
with a threshold of 1e−05
rs10899911
rs35055552
rs62273907
rs78383039
rs8039785
rs823424
rs2090816
rs55877868
rs6828768
rs6809952
rs7572229
rs7303158
rs2387977
rs11715853
All − MR Egger
All − IVW(MRE)
−1.5
−1.0
−0.5
0.0
0.5
1.0
1.5
MR effect size for
'Gut microbiota abundance (genus Parasutterella id.2892)' on 'Sialic acid−binding Ig−like lectin 12'

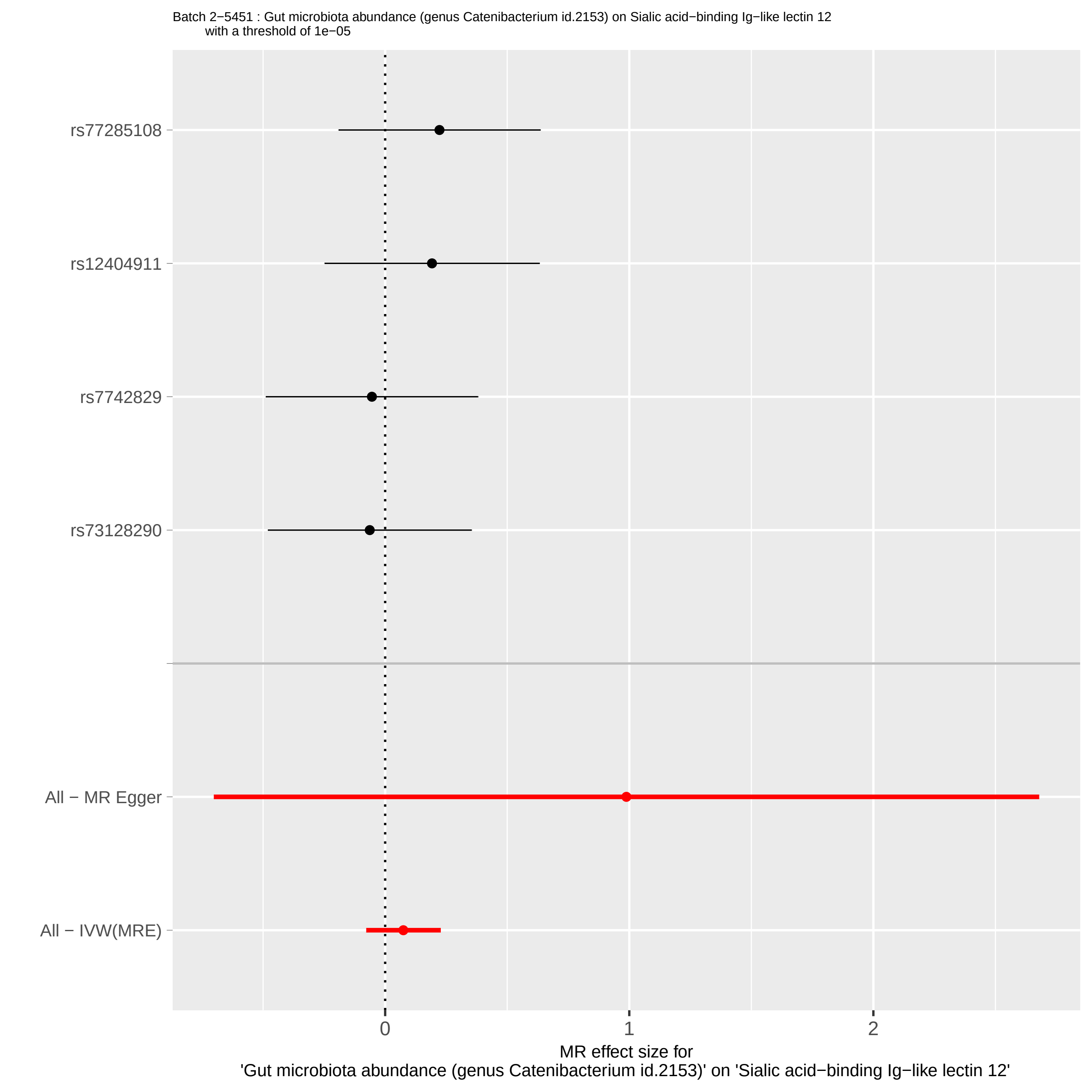

Batch 2−5451 : Gut microbiota abundance (genus Catenibacterium id.2153) on Sialic acid−binding Ig−like lectin 12
with a threshold of 1e−05
rs77285108
rs12404911
rs7742829
rs73128290
All − MR Egger
All − IVW(MRE)
0
1
2
MR effect size for
'Gut microbiota abundance (genus Catenibacterium id.2153)' on 'Sialic acid−binding Ig−like lectin 12'

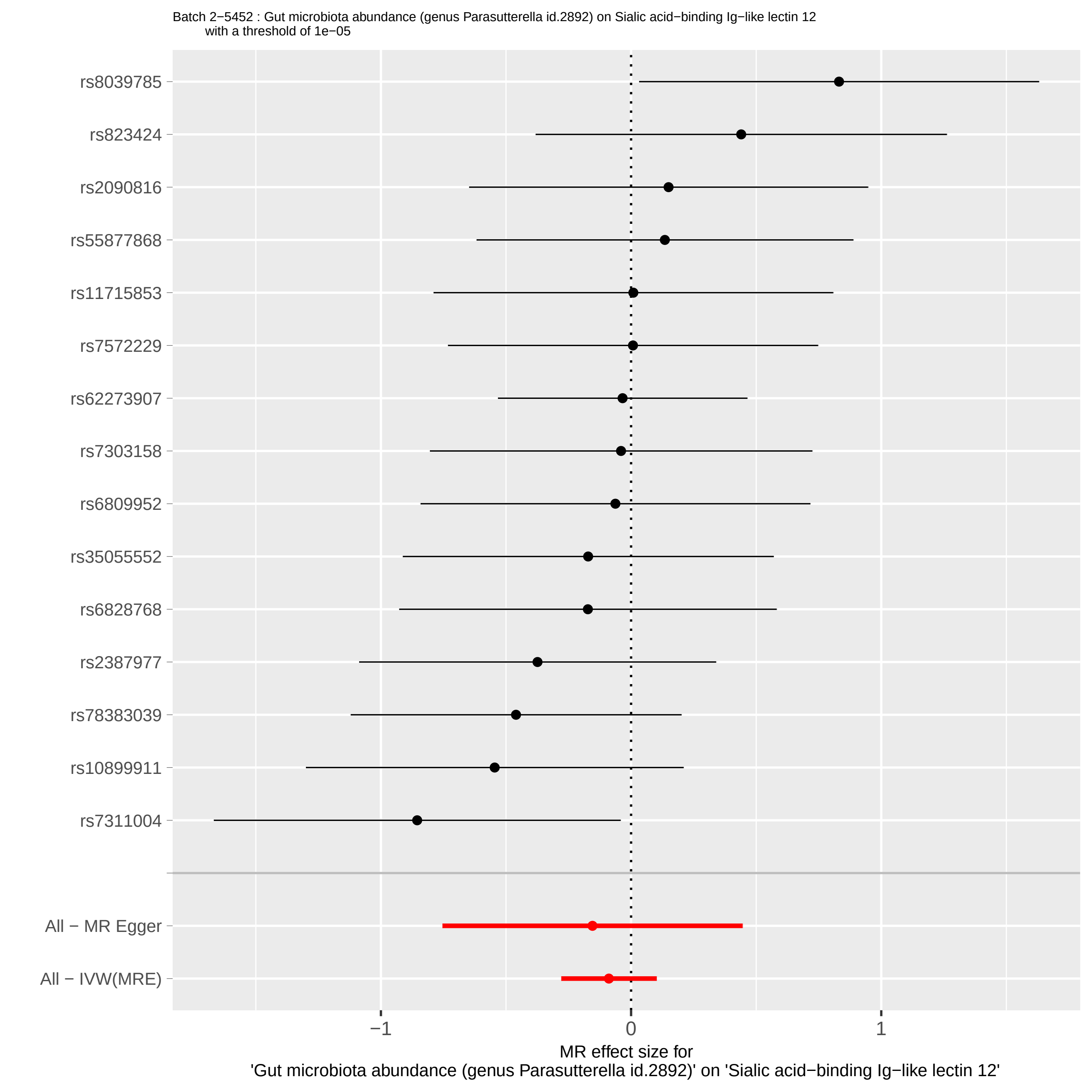
Batch 2−5452 : Gut microbiota abundance (genus Parasutterella id.2892) on Sialic acid−binding Ig−like lectin 12
with a threshold of 1e−05
rs8039785
rs823424
rs2090816
rs55877868
rs11715853
rs7572229
rs62273907
rs7303158
rs6809952
rs35055552
rs6828768
rs2387977
rs78383039
rs10899911
rs7311004
All − MR Egger
All − IVW(MRE)
−1
0
1
MR effect size for
'Gut microbiota abundance (genus Parasutterella id.2892)' on 'Sialic acid−binding Ig−like lectin 12'

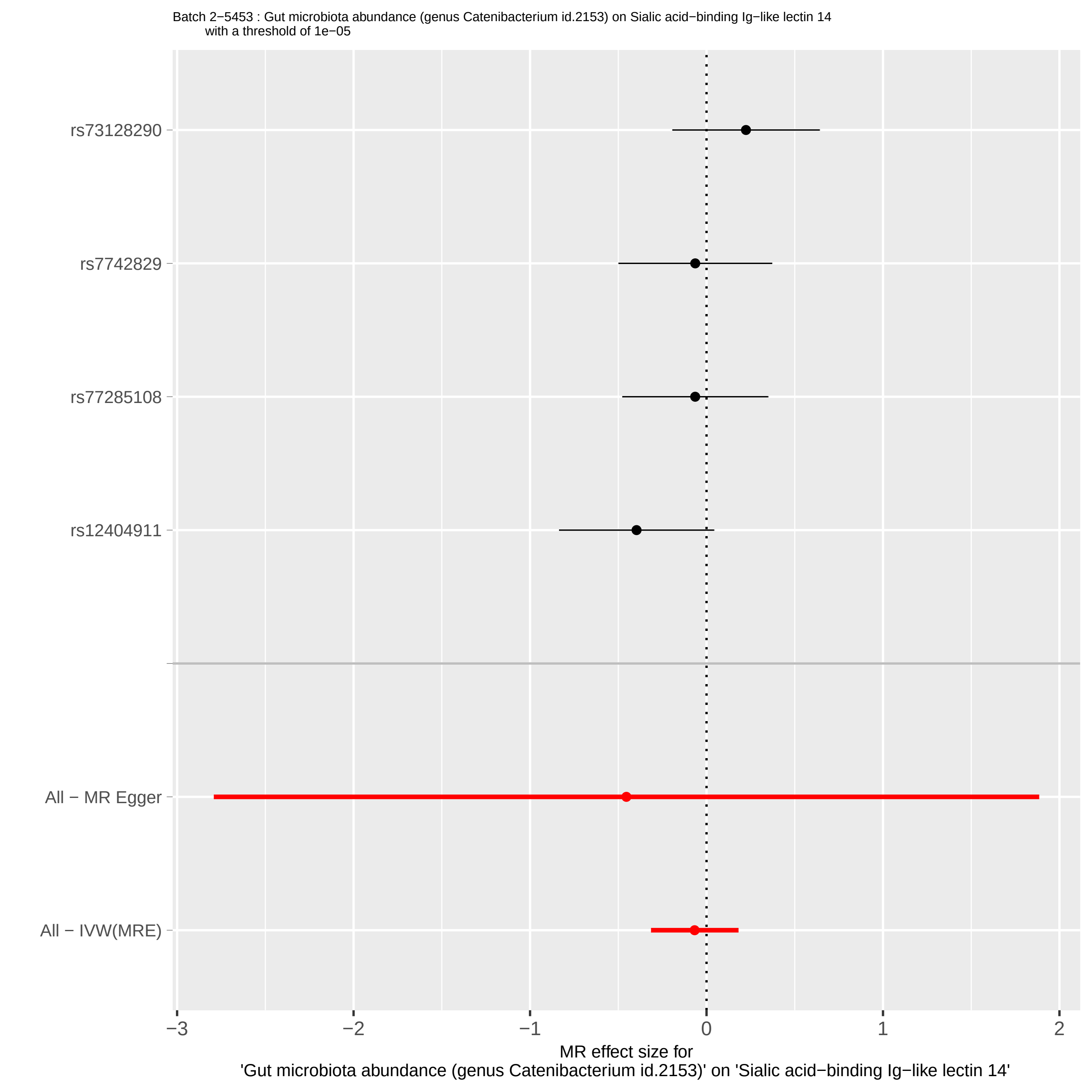

Batch 2−5453 : Gut microbiota abundance (genus Catenibacterium id.2153) on Sialic acid−binding Ig−like lectin 14
with a threshold of 1e−05
rs73128290
rs7742829
rs77285108
rs12404911
All − MR Egger
All − IVW(MRE)
−3
−2
−1
0
1
2
MR effect size for
'Gut microbiota abundance (genus Catenibacterium id.2153)' on 'Sialic acid−binding Ig−like lectin 14'

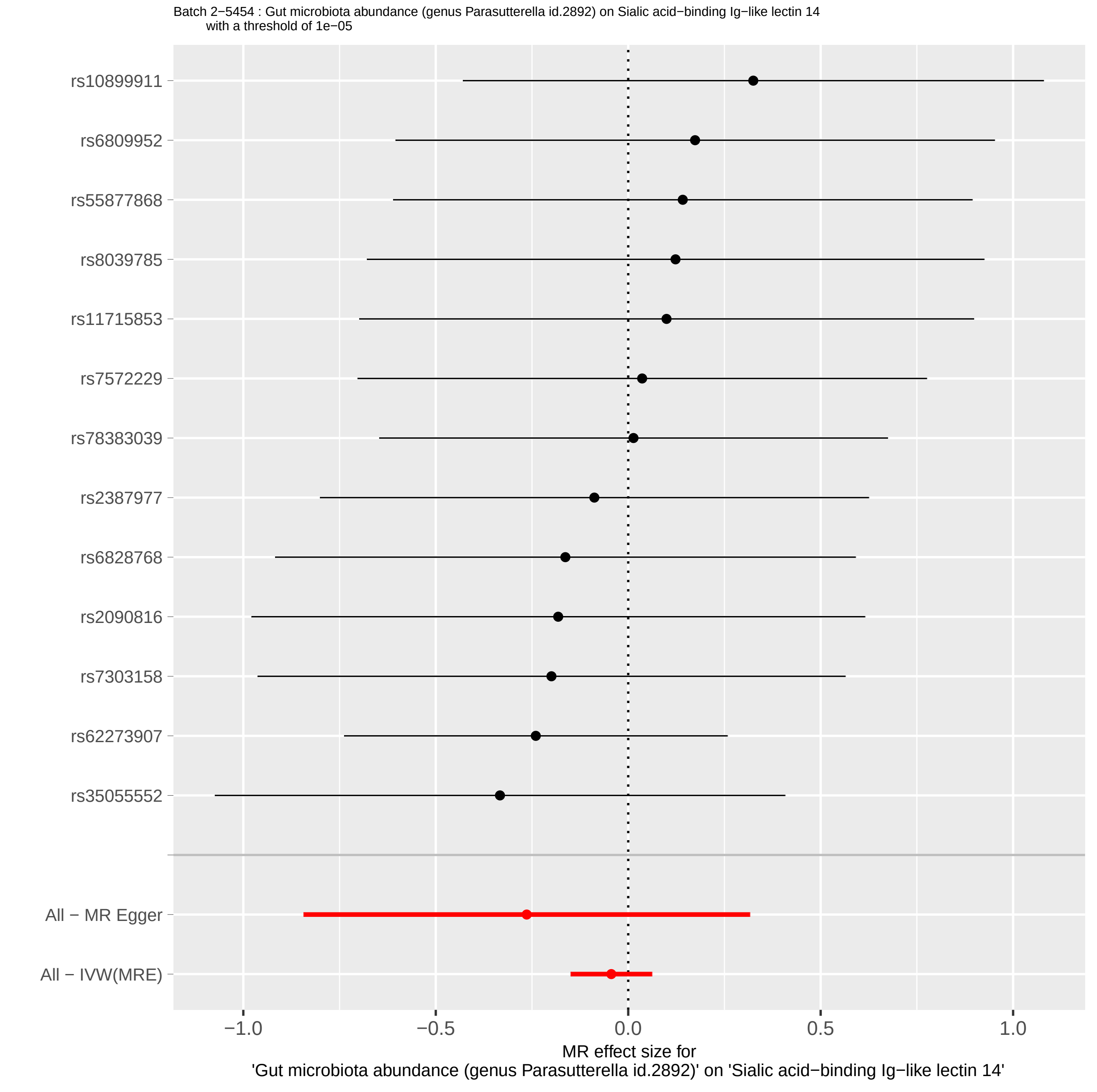
Batch 2−5454 : Gut microbiota abundance (genus Parasutterella id.2892) on Sialic acid−binding Ig−like lectin 14
with a threshold of 1e−05
rs10899911
rs6809952
rs55877868
rs8039785
rs11715853
rs7572229
rs78383039
rs2387977
rs6828768
rs2090816
rs7303158
rs62273907
rs35055552
All − MR Egger
All − IVW(MRE)
−1.0
−0.5
0.0
0.5
1.0
MR effect size for
'Gut microbiota abundance (genus Parasutterella id.2892)' on 'Sialic acid−binding Ig−like lectin 14'

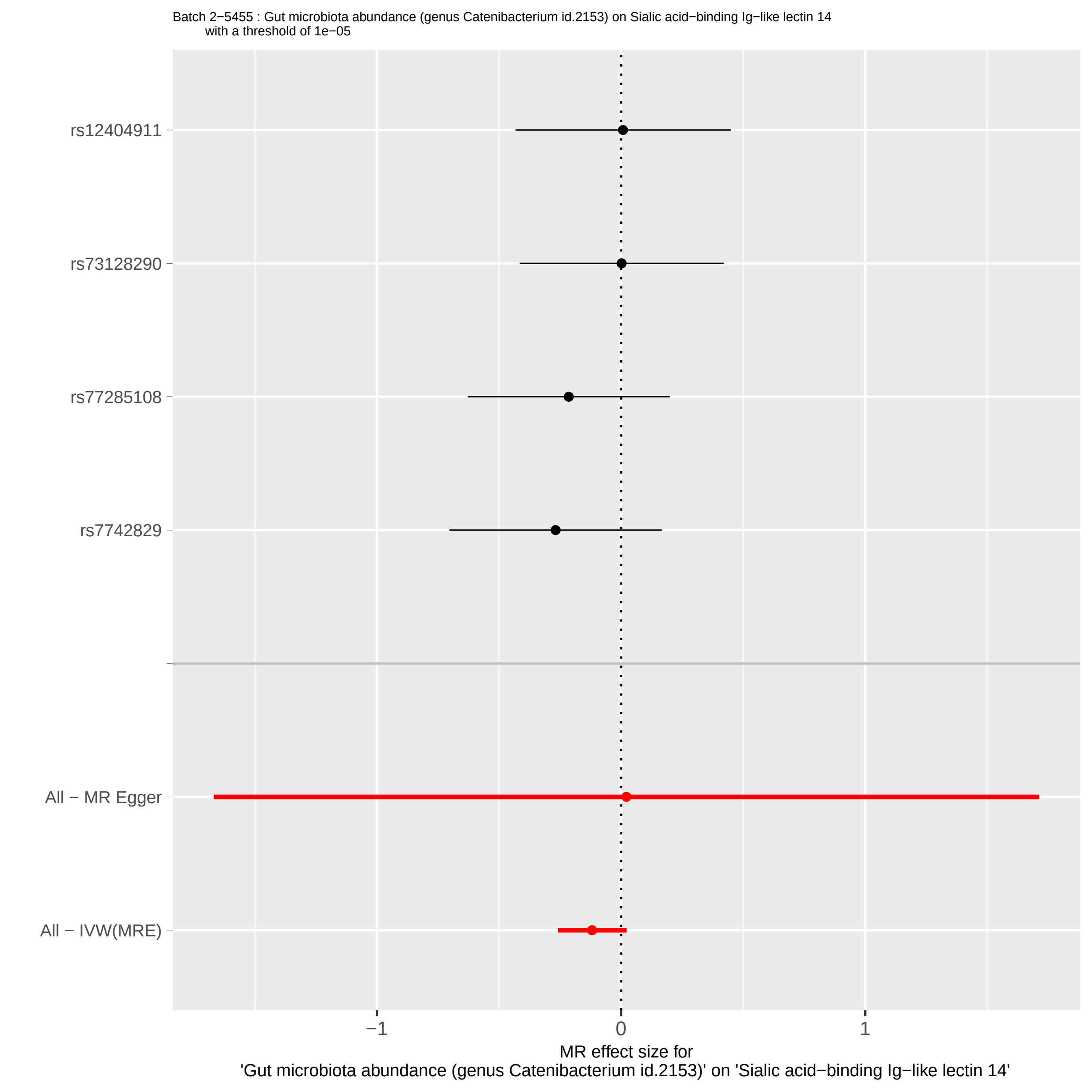
Batch 2−5455 : Gut microbiota abundance (genus Catenibacterium id.2153) on Sialic acid−binding Ig−like lectin 14
with a threshold of 1e−05
rs12404911
rs73128290
rs77285108
rs7742829
All − MR Egger
All − IVW(MRE)
−1
0
1
MR effect size for
'Gut microbiota abundance (genus Catenibacterium id.2153)' on 'Sialic acid−binding Ig−like lectin 14'

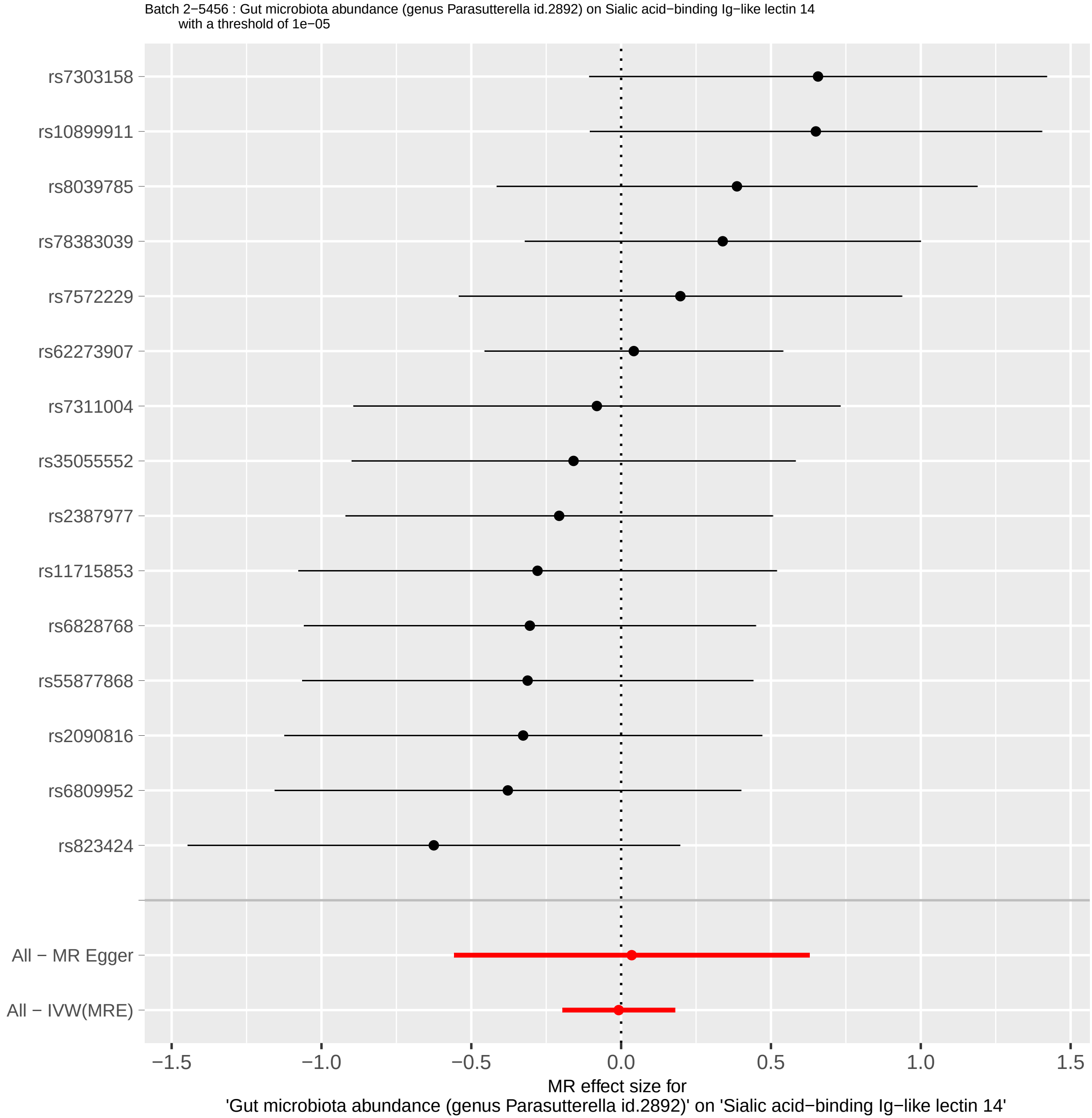

Batch 2−5456 : Gut microbiota abundance (genus Parasutterella id.2892) on Sialic acid−binding Ig−like lectin 14
with a threshold of 1e−05
rs7303158
rs10899911
rs8039785
rs78383039
rs7572229
rs62273907
rs7311004
rs35055552
rs2387977
rs11715853
rs6828768
rs55877868
rs2090816
rs6809952
rs823424
All − MR Egger
All − IVW(MRE)
−1.5
−1.0
−0.5
0.0
0.5
1.0
1.5
MR effect size for
'Gut microbiota abundance (genus Parasutterella id.2892)' on 'Sialic acid−binding Ig−like lectin 14'

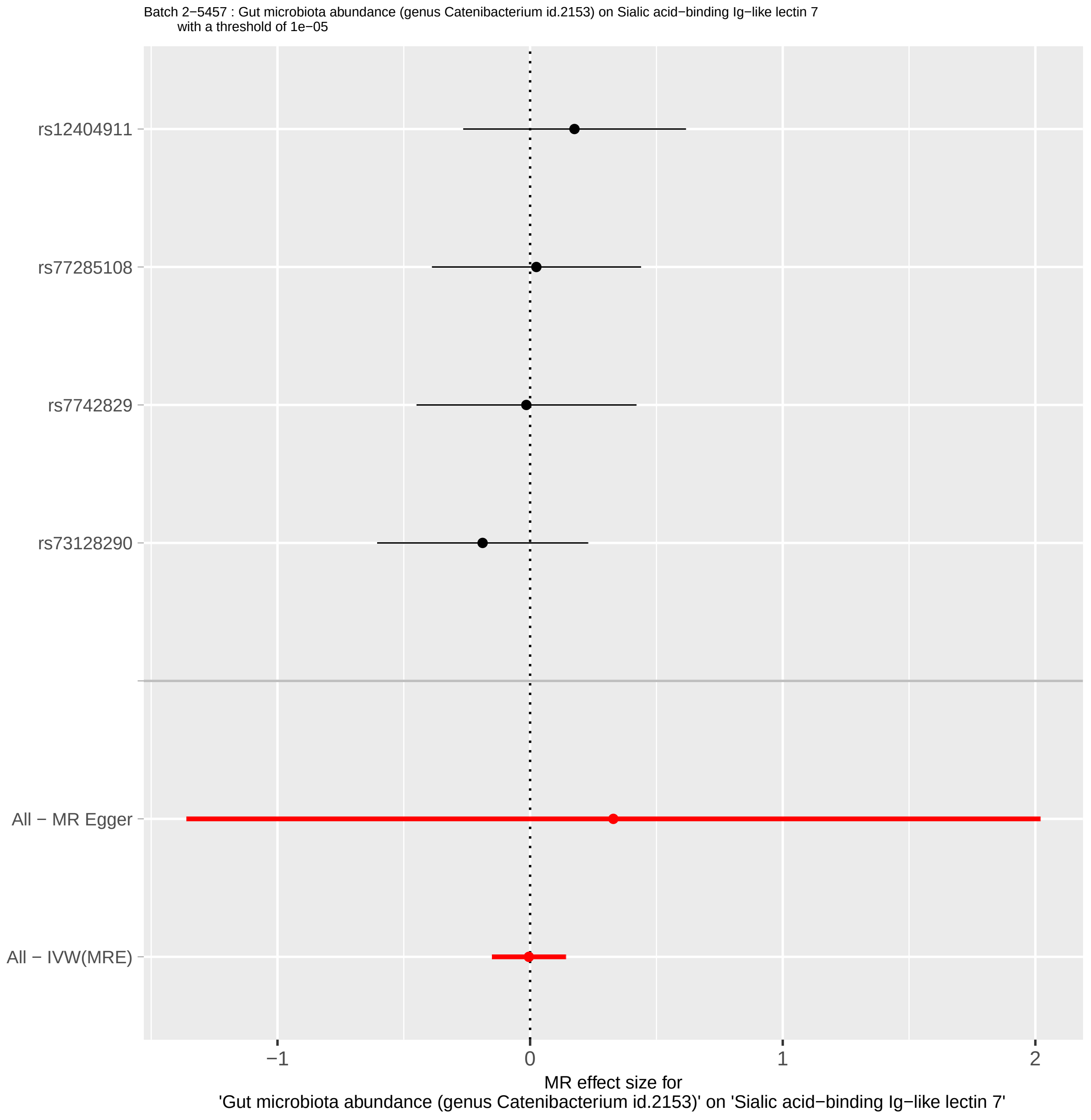

Batch 2−5457 : Gut microbiota abundance (genus Catenibacterium id.2153) on Sialic acid−binding Ig−like lectin 7
with a threshold of 1e−05
rs12404911
rs77285108
rs7742829
rs73128290
All − MR Egger
All − IVW(MRE)
−1
0
1
2
MR effect size for
'Gut microbiota abundance (genus Catenibacterium id.2153)' on 'Sialic acid−binding Ig−like lectin 7'

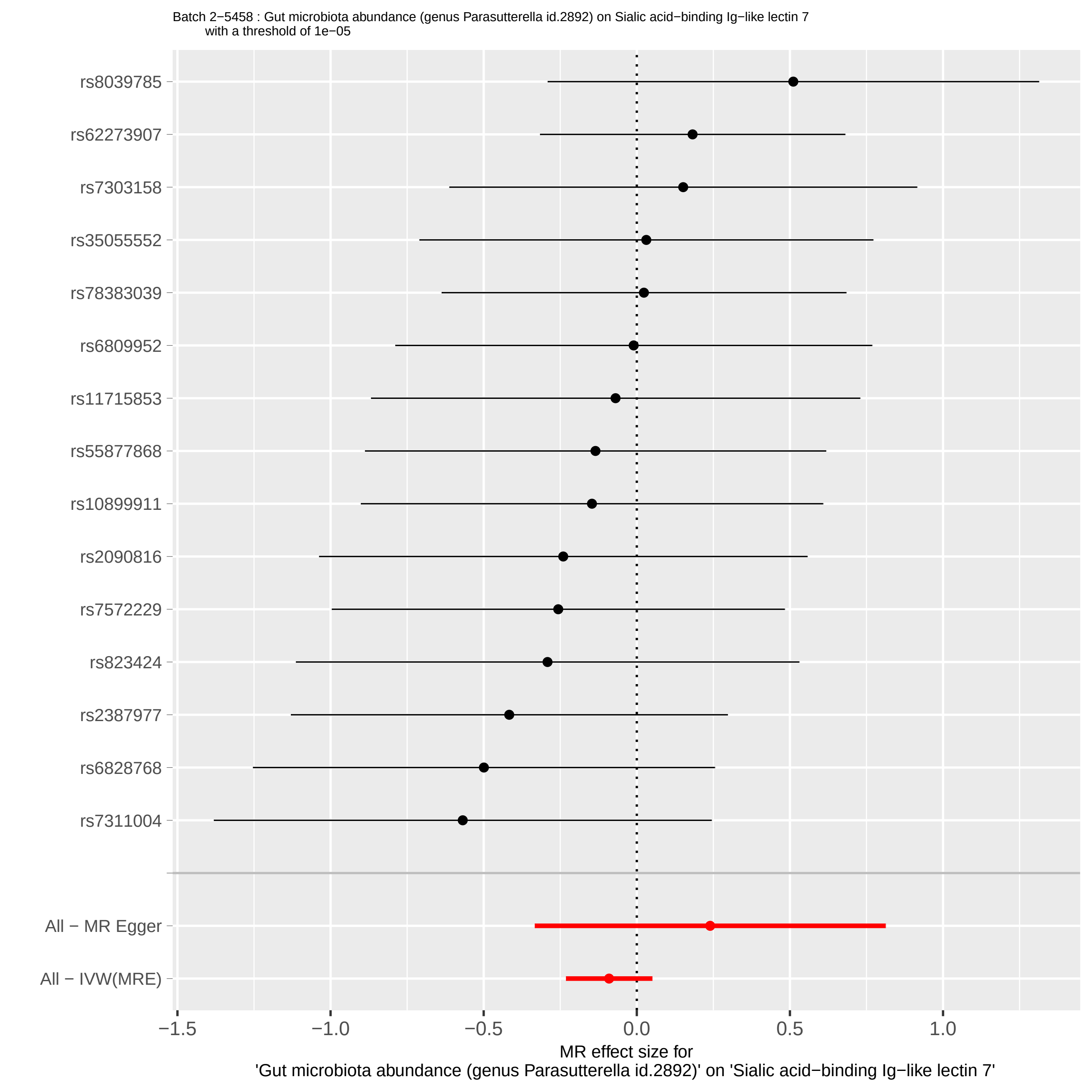
Batch 2−5458 : Gut microbiota abundance (genus Parasutterella id.2892) on Sialic acid−binding Ig−like lectin 7
with a threshold of 1e−05
rs8039785
rs62273907
rs7303158
rs35055552
rs78383039
rs6809952
rs11715853
rs55877868
rs10899911
rs2090816
rs7572229
rs823424
rs2387977
rs6828768
rs7311004
All − MR Egger
All − IVW(MRE)
−1.5
−1.0
−0.5
0.0
0.5
1.0
MR effect size for
'Gut microbiota abundance (genus Parasutterella id.2892)' on 'Sialic acid−binding Ig−like lectin 7'

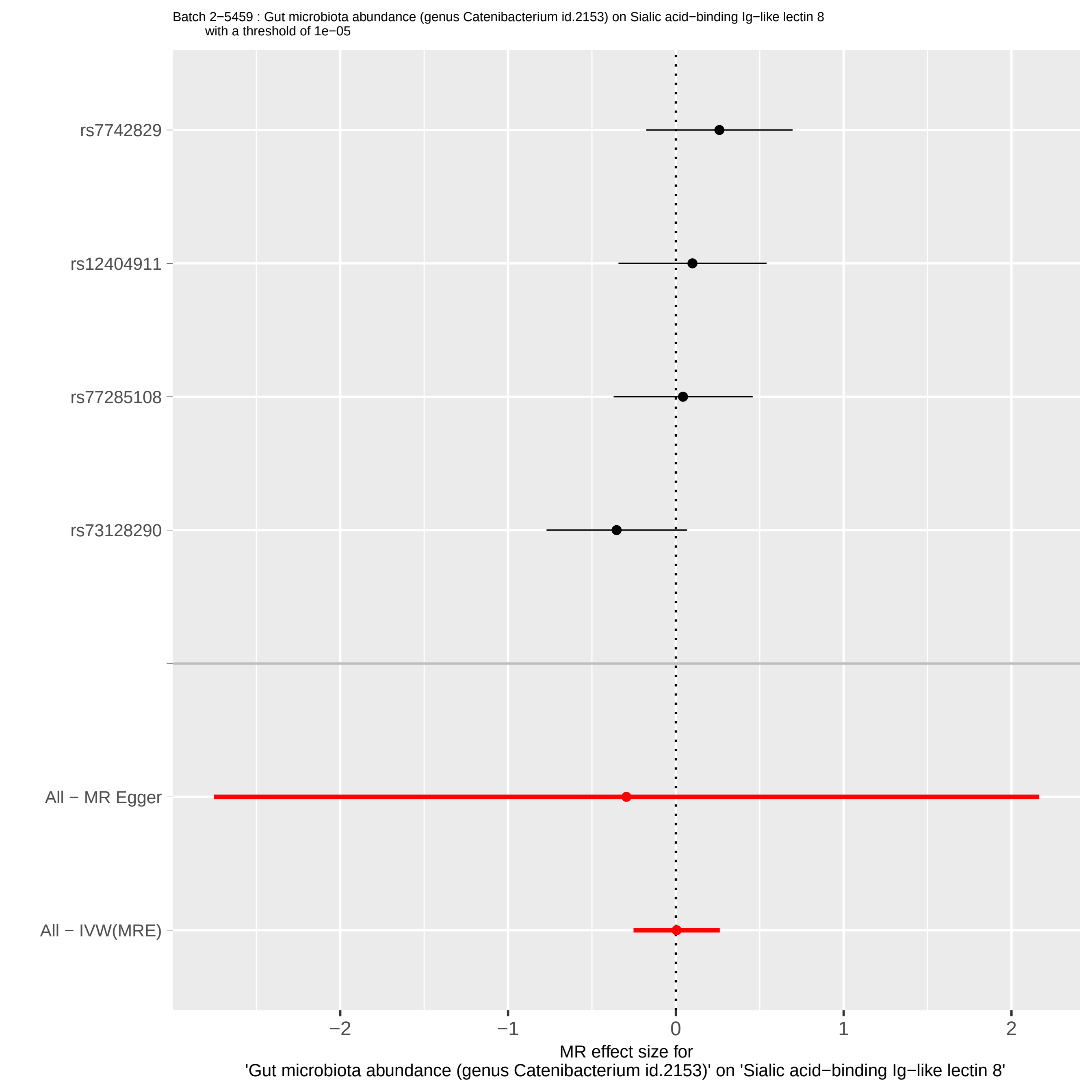
Batch 2−5459 : Gut microbiota abundance (genus Catenibacterium id.2153) on Sialic acid−binding Ig−like lectin 8
with a threshold of 1e−05
rs7742829
rs12404911
rs77285108
rs73128290
All − MR Egger
All − IVW(MRE)
−2
−1
0
1
2
MR effect size for
'Gut microbiota abundance (genus Catenibacterium id.2153)' on 'Sialic acid−binding Ig−like lectin 8'

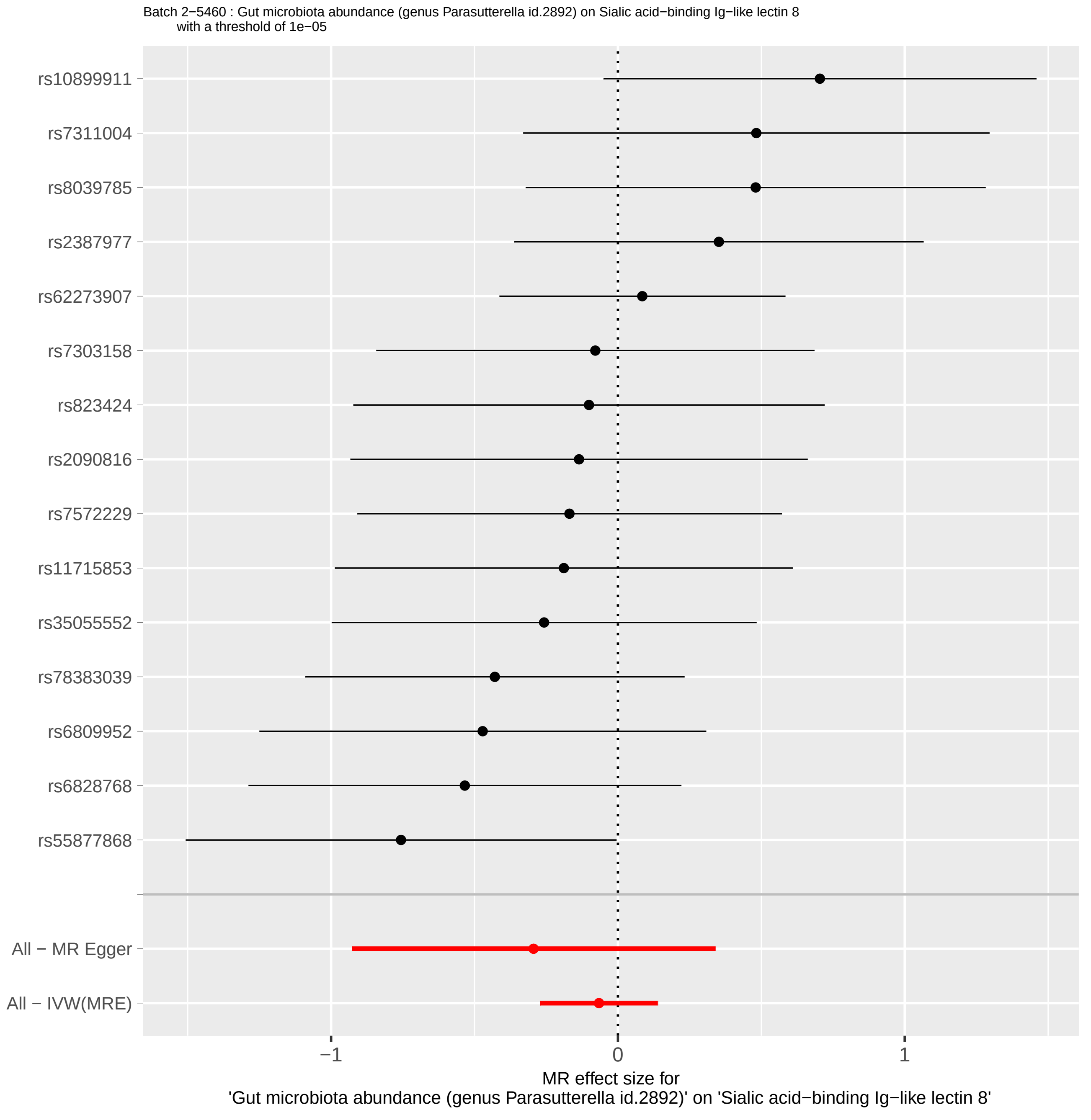
Batch 2−5460 : Gut microbiota abundance (genus Parasutterella id.2892) on Sialic acid−binding Ig−like lectin 8
with a threshold of 1e−05
rs10899911
rs7311004
rs8039785
rs2387977
rs62273907
rs7303158
rs823424
rs2090816
rs7572229
rs11715853
rs35055552
rs78383039
rs6809952
rs6828768
rs55877868
All − MR Egger
All − IVW(MRE)
−1
0
1
MR effect size for
'Gut microbiota abundance (genus Parasutterella id.2892)' on 'Sialic acid−binding Ig−like lectin 8'

Batch 2−5461 : Gut microbiota abundance (genus Catenibacterium id.2153) on Sialic acid−binding Ig−like lectin 9 with a threshold of 1e−05

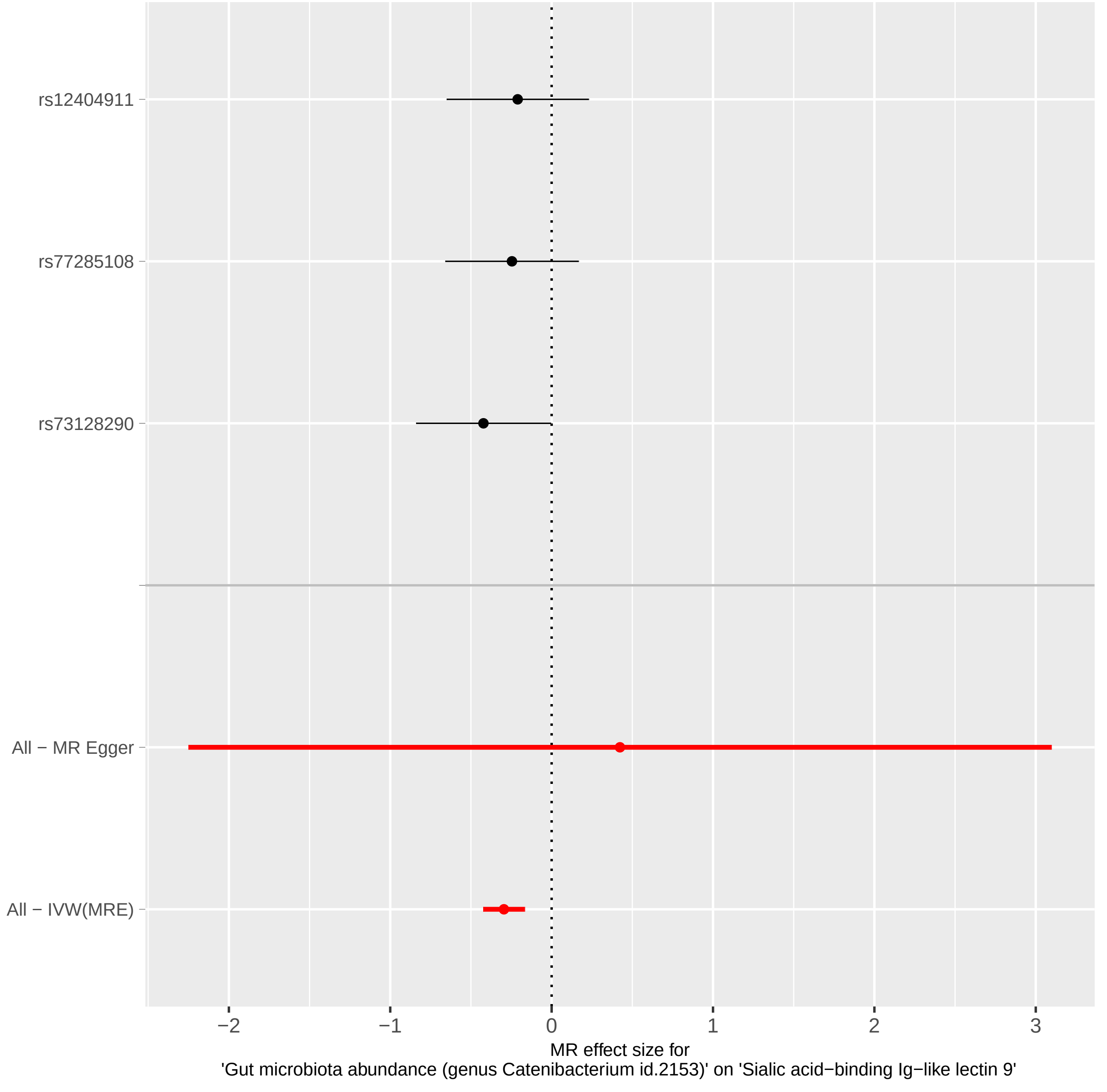

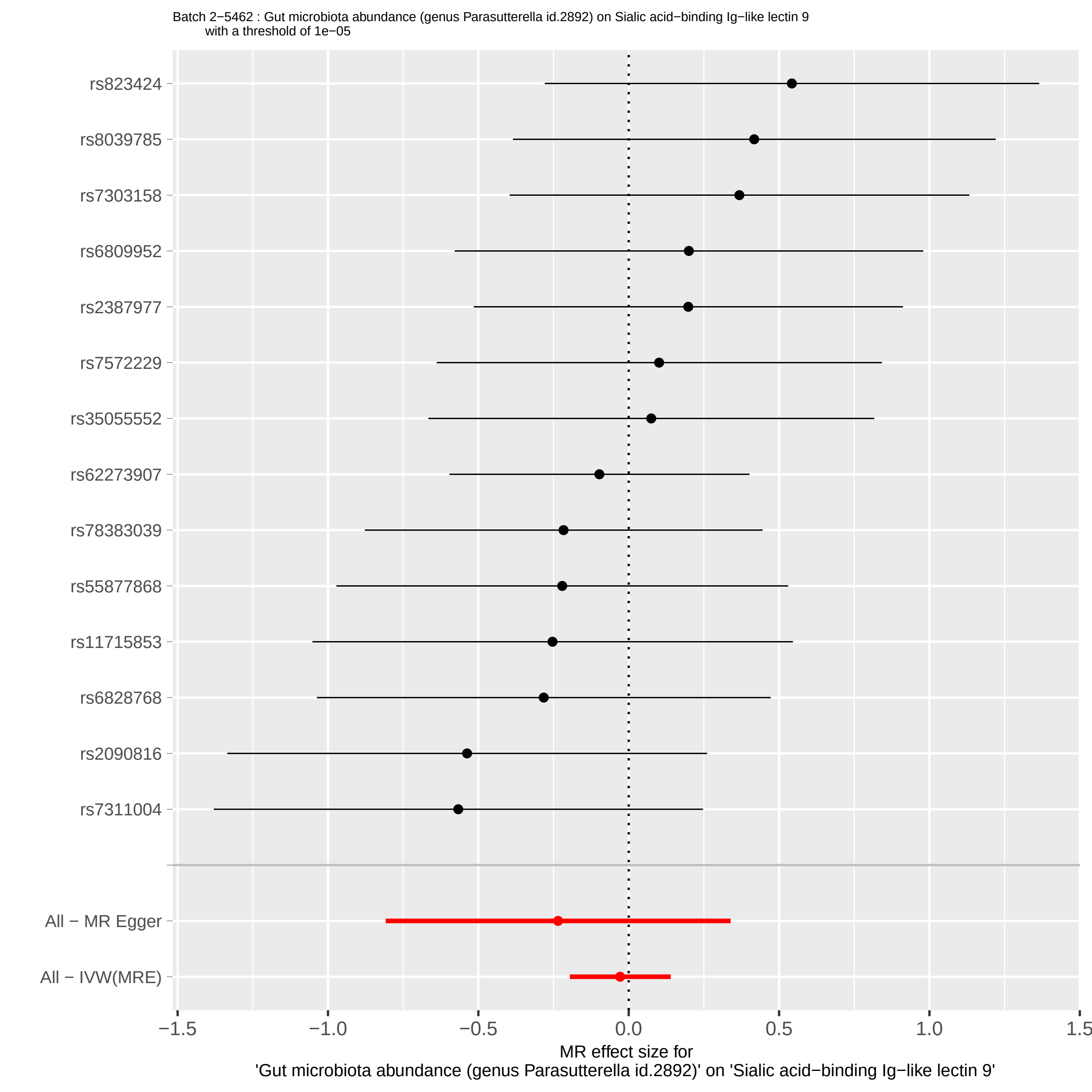
Batch 2−5462 : Gut microbiota abundance (genus Parasutterella id.2892) on Sialic acid−binding Ig−like lectin 9
with a threshold of 1e−05
rs823424
rs8039785
rs7303158
rs6809952
rs2387977
rs7572229
rs35055552
rs62273907
rs78383039
rs55877868
rs11715853
rs6828768
rs2090816
rs7311004
All − MR Egger
All − IVW(MRE)
−1.5
−1.0
−0.5
0.0
0.5
1.0
1.5
MR effect size for
'Gut microbiota abundance (genus Parasutterella id.2892)' on 'Sialic acid−binding Ig−like lectin 9'

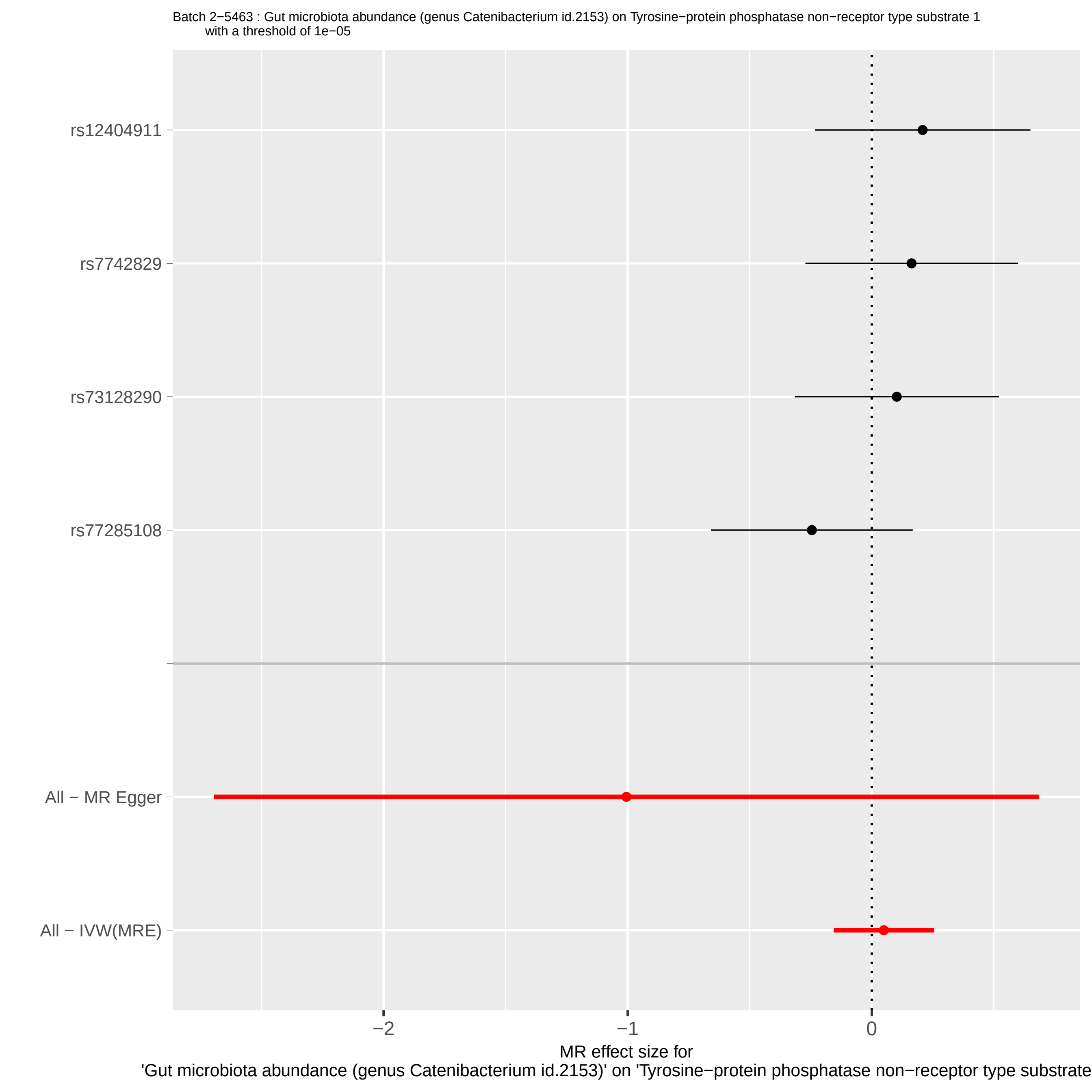
Batch 2−5463 : Gut microbiota abundance (genus Catenibacterium id.2153) on Tyrosine−protein phosphatase non−receptor type substrate 1
with a threshold of 1e−05
rs12404911
rs7742829
rs73128290
rs77285108
All − MR Egger
All − IVW(MRE)
−2
−1
0
MR effect size for
'Gut microbiota abundance (genus Catenibacterium id.2153)' on 'Tyrosine−protein phosphatase non−receptor type substrate

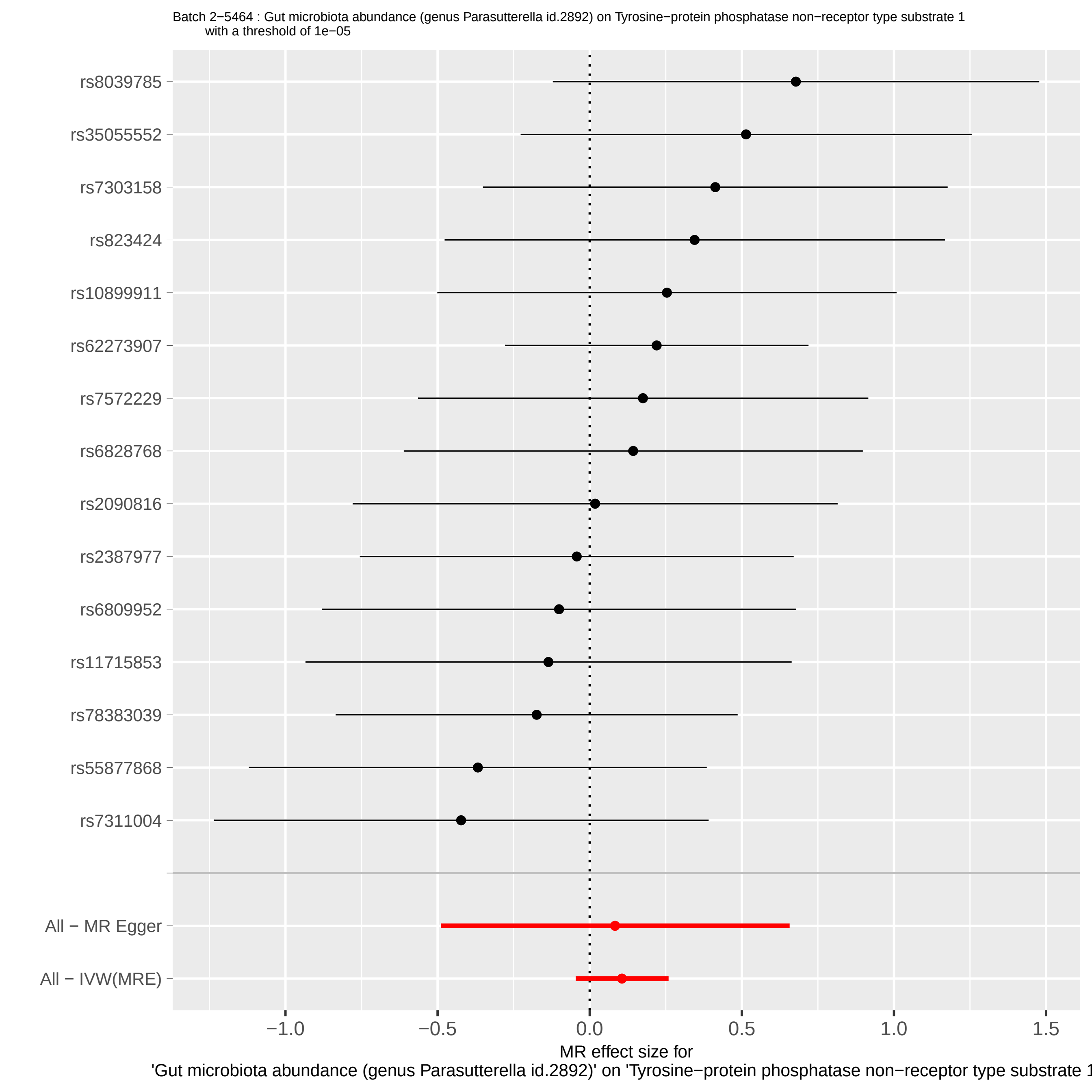

Batch 2−5464 : Gut microbiota abundance (genus Parasutterella id.2892) on Tyrosine−protein phosphatase non−receptor type substrate 1
with a threshold of 1e−05
rs8039785
rs35055552
rs7303158
rs823424
rs10899911
rs62273907
rs7572229
rs6828768
rs2090816
rs2387977
rs6809952
rs11715853
rs78383039
rs55877868
rs7311004
All − MR Egger
All − IVW(MRE)
−1.0
−0.5
0.0
0.5
1.0
1.5
MR effect size for
'Gut microbiota abundance (genus Parasutterella id.2892)' on 'Tyrosine−protein phosphatase non−receptor type substrate 1

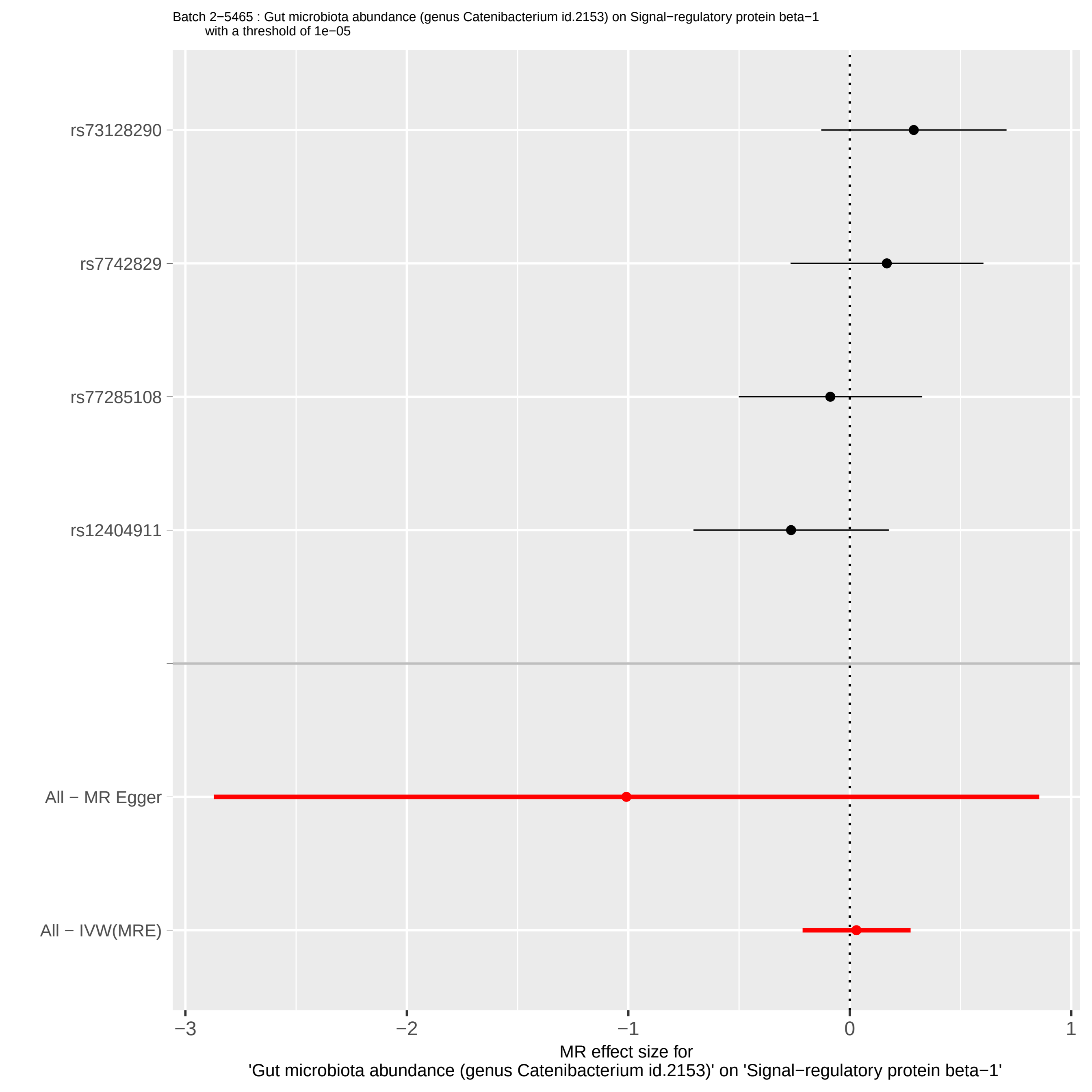

Batch 2−5465 : Gut microbiota abundance (genus Catenibacterium id.2153) on Signal−regulatory protein beta−1
with a threshold of 1e−05
rs73128290
rs7742829
rs77285108
rs12404911
All − MR Egger
All − IVW(MRE)
−3
−2
−1
0
1
MR effect size for
'Gut microbiota abundance (genus Catenibacterium id.2153)' on 'Signal−regulatory protein beta−1'

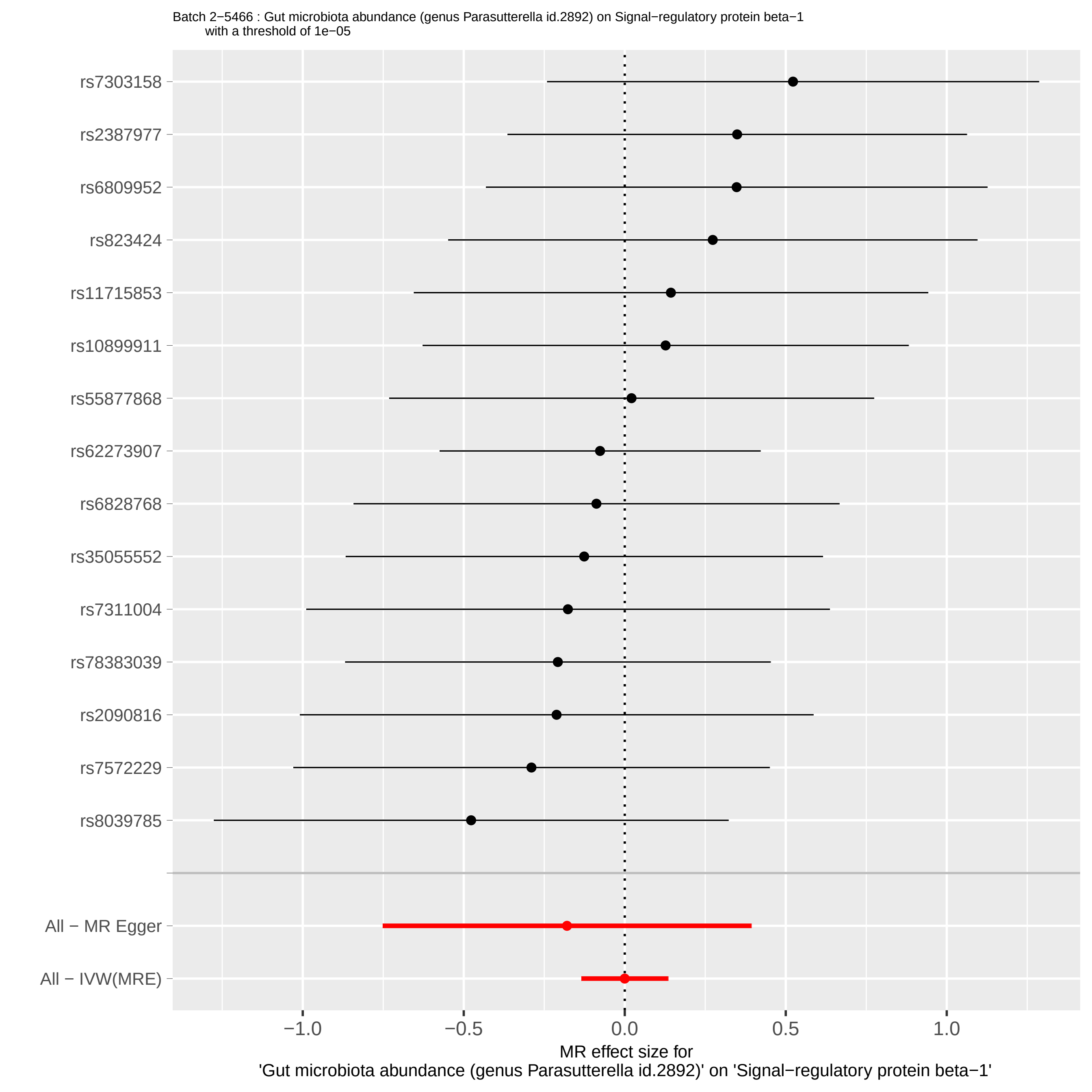
Batch 2−5466 : Gut microbiota abundance (genus Parasutterella id.2892) on Signal−regulatory protein beta−1
with a threshold of 1e−05
rs7303158
rs2387977
rs6809952
rs823424
rs11715853
rs10899911
rs55877868
rs62273907
rs6828768
rs35055552
rs7311004
rs78383039
rs2090816
rs7572229
rs8039785
All − MR Egger
All − IVW(MRE)
−1.0
−0.5
0.0
0.5
1.0
MR effect size for
'Gut microbiota abundance (genus Parasutterella id.2892)' on 'Signal−regulatory protein beta−1'

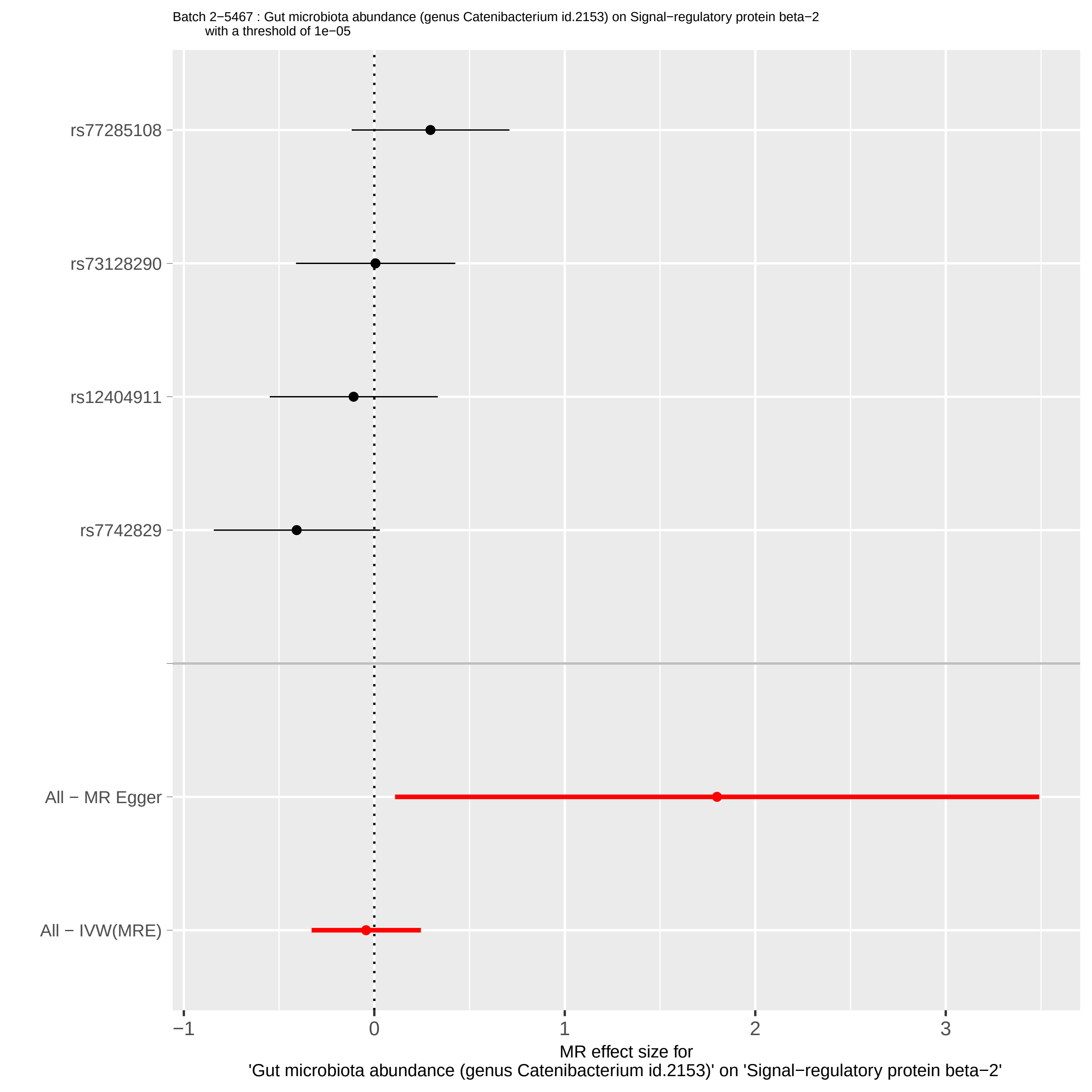

Batch 2−5467 : Gut microbiota abundance (genus Catenibacterium id.2153) on Signal−regulatory protein beta−2
with a threshold of 1e−05
rs77285108
rs73128290
rs12404911
rs7742829
All − MR Egger
All − IVW(MRE)
−1
0
1
2
3
MR effect size for
'Gut microbiota abundance (genus Catenibacterium id.2153)' on 'Signal−regulatory protein beta−2'

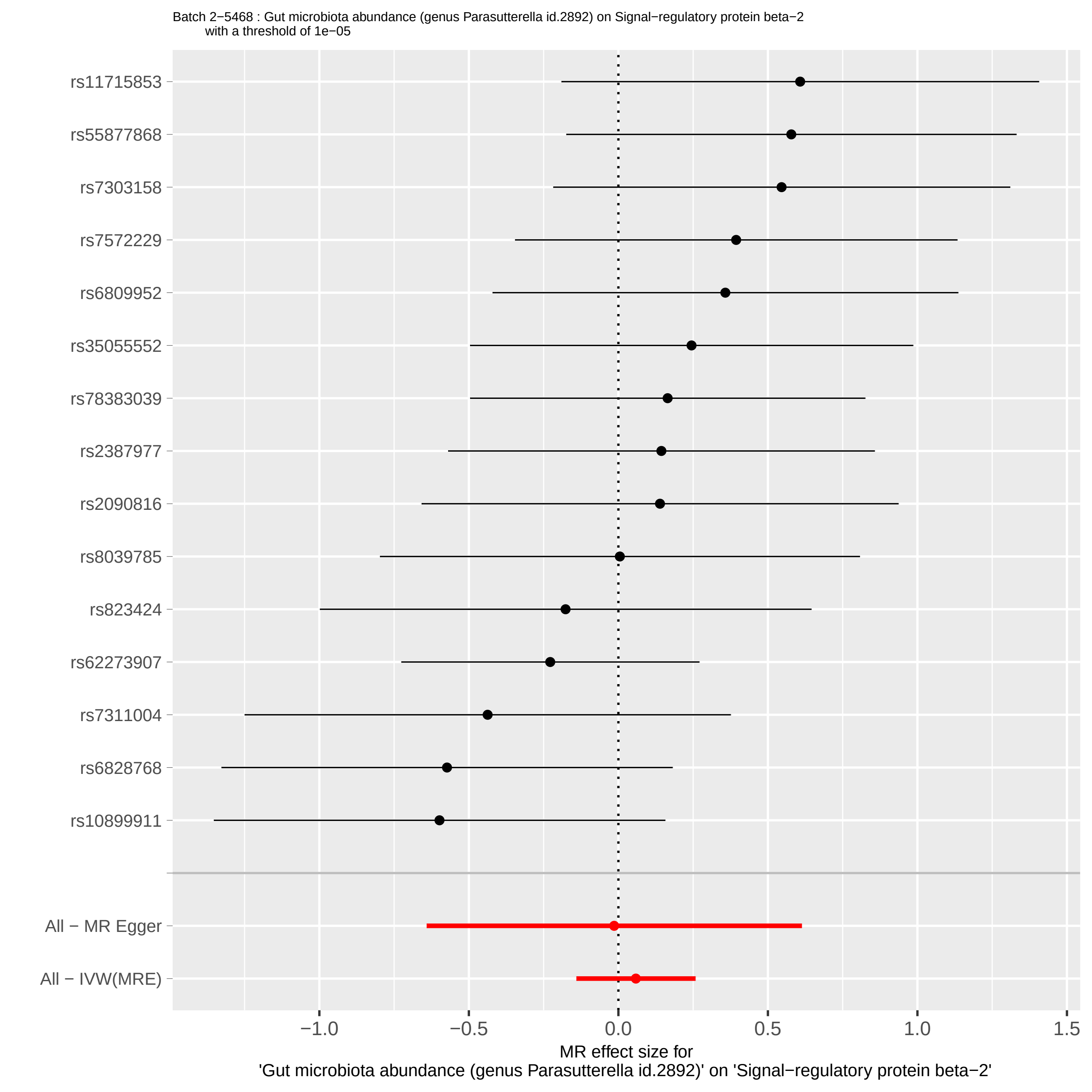

Batch 2−5468 : Gut microbiota abundance (genus Parasutterella id.2892) on Signal−regulatory protein beta−2
with a threshold of 1e−05
rs11715853
rs55877868
rs7303158
rs7572229
rs6809952
rs35055552
rs78383039
rs2387977
rs2090816
rs8039785
rs823424
rs62273907
rs7311004
rs6828768
rs10899911
All − MR Egger
All − IVW(MRE)
−1.0
−0.5
0.0
0.5
1.0
1.5
MR effect size for
'Gut microbiota abundance (genus Parasutterella id.2892)' on 'Signal−regulatory protein beta−2'

Batch 2−5469 : Gut microbiota abundance (genus Catenibacterium id.2153) on Signal−regulatory protein gamma
with a threshold of 1e−05

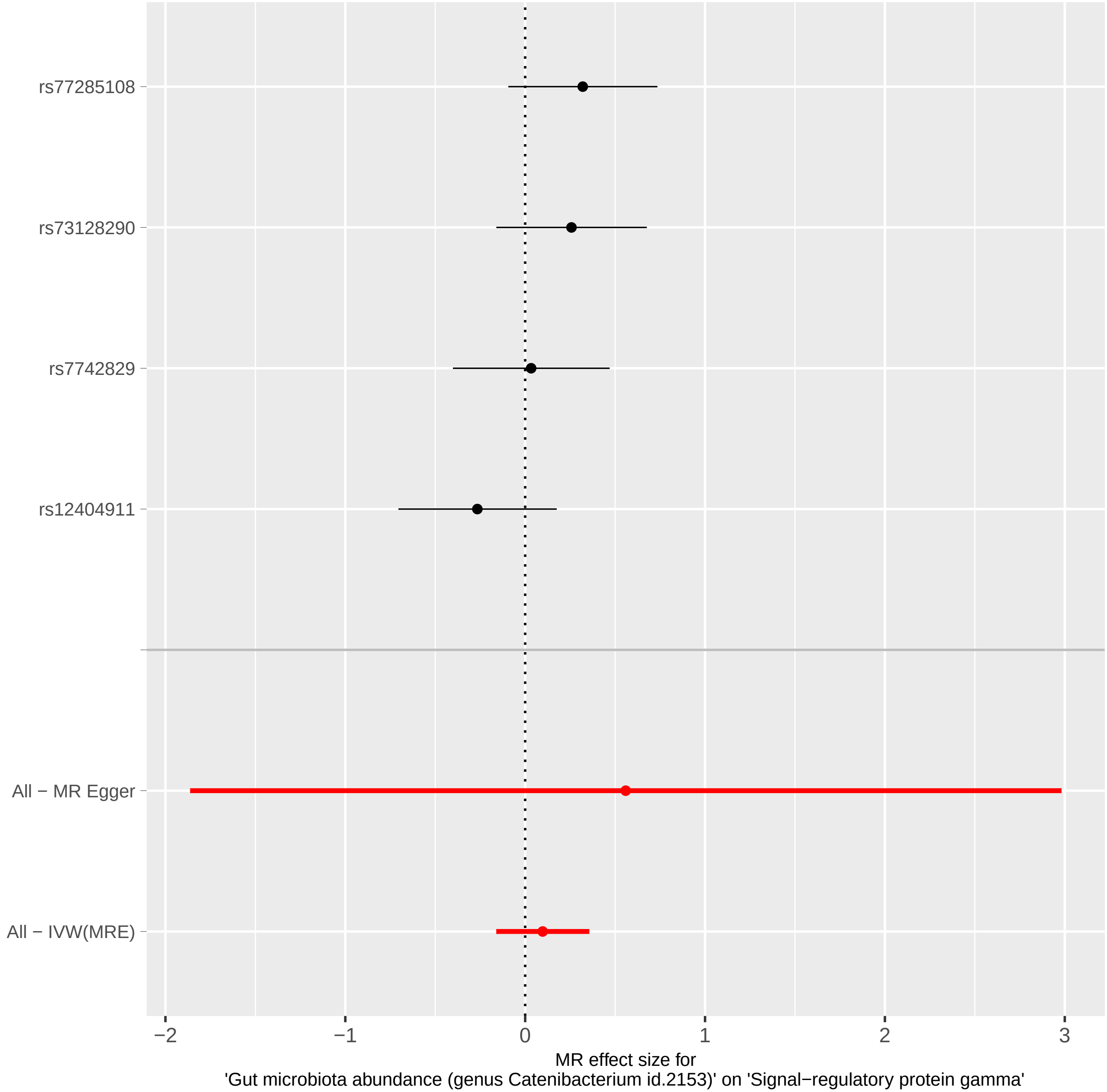

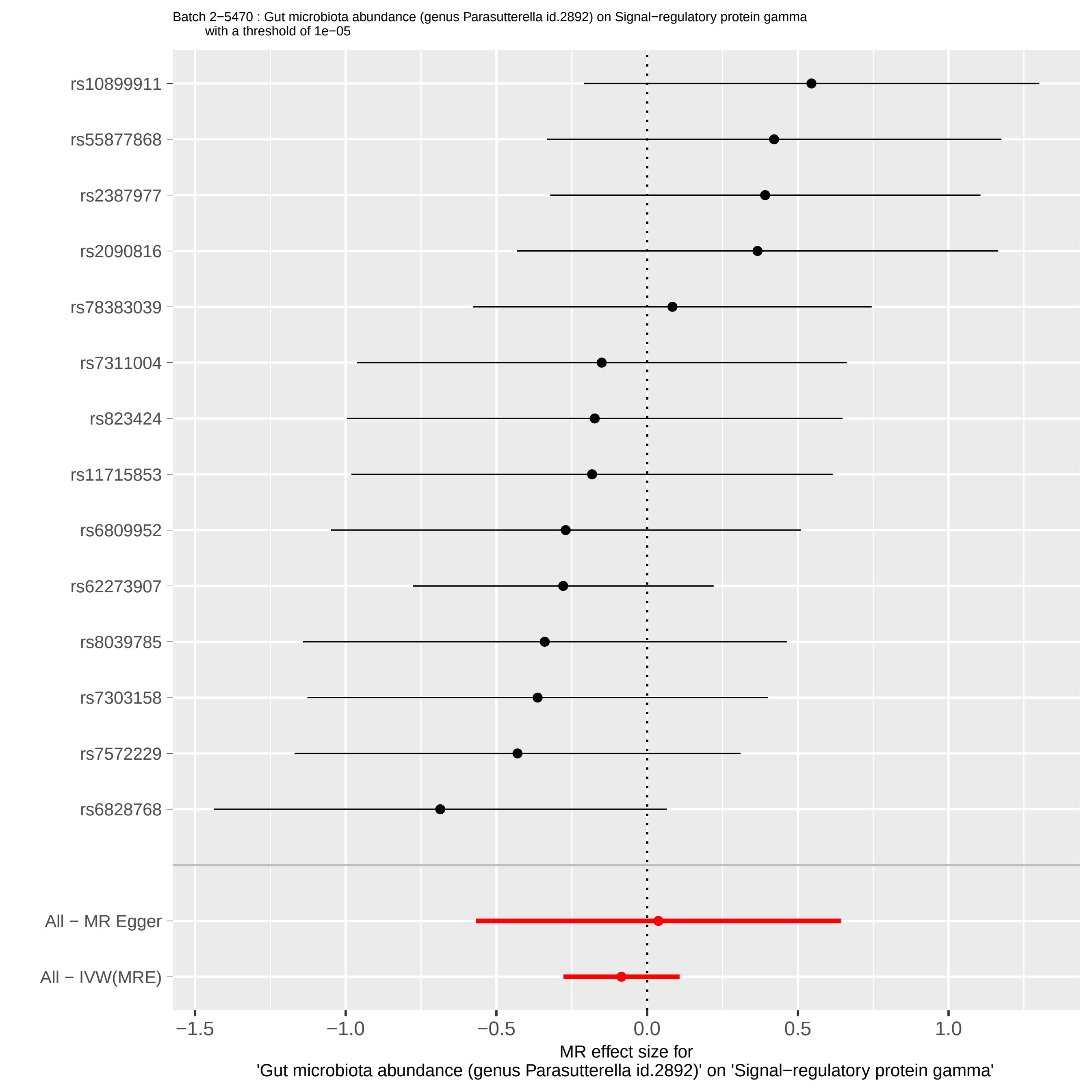

Batch 2−5470 : Gut microbiota abundance (genus Parasutterella id.2892) on Signal−regulatory protein gamma
with a threshold of 1e−05
rs10899911
rs55877868
rs2387977
rs2090816
rs78383039
rs7311004
rs823424
rs11715853
rs6809952
rs62273907
rs8039785
rs7303158
rs7572229
rs6828768
All − MR Egger
All − IVW(MRE)
−1.5
−1.0
−0.5
0.0
0.5
1.0
MR effect size for
'Gut microbiota abundance (genus Parasutterella id.2892)' on 'Signal−regulatory protein gamma'

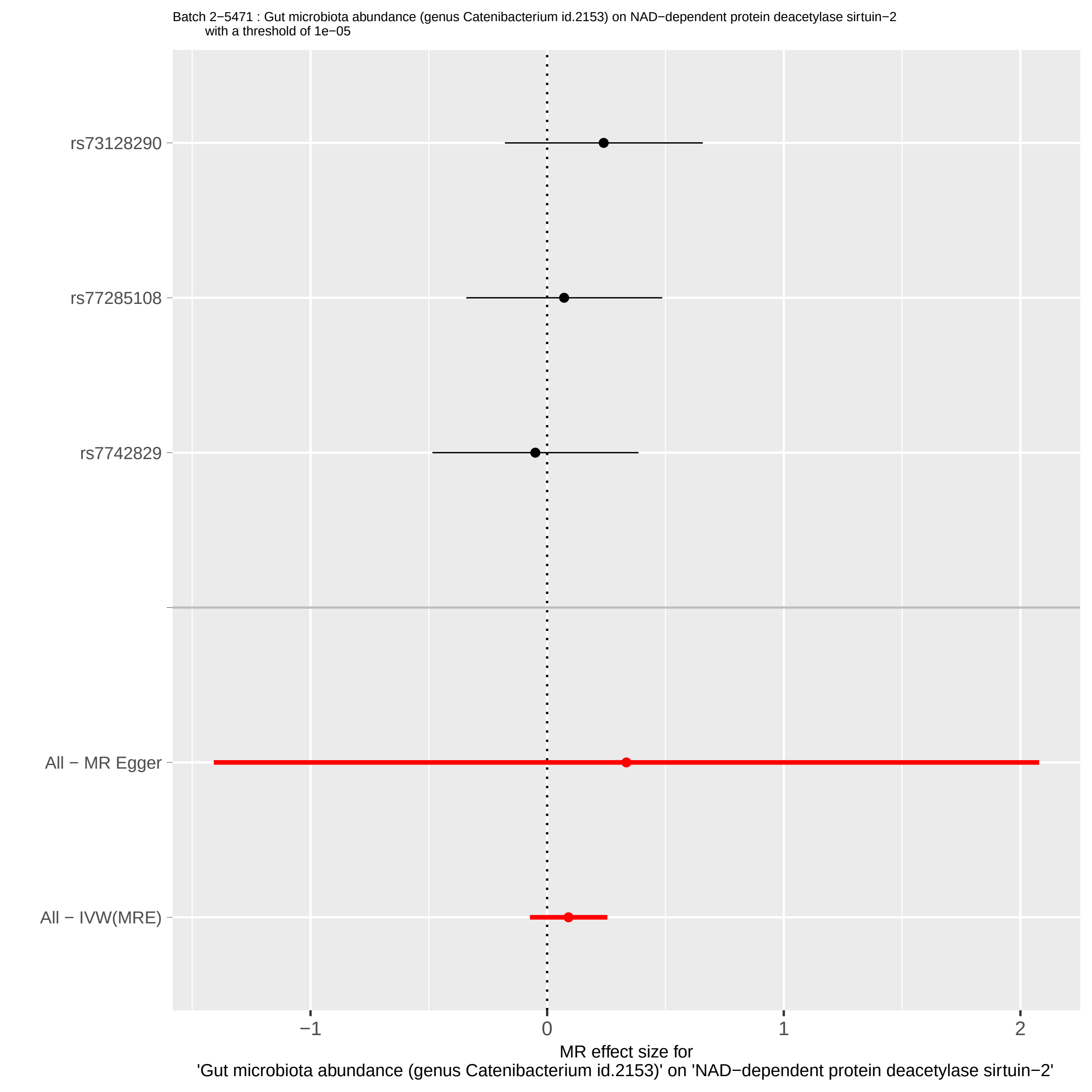

Batch 2−5471 : Gut microbiota abundance (genus Catenibacterium id.2153) on NAD−dependent protein deacetylase sirtuin−2
with a threshold of 1e−05
rs73128290
rs77285108
rs7742829
All − MR Egger
All − IVW(MRE)
−1
0
1
2
MR effect size for
'Gut microbiota abundance (genus Catenibacterium id.2153)' on 'NAD−dependent protein deacetylase sirtuin−2'

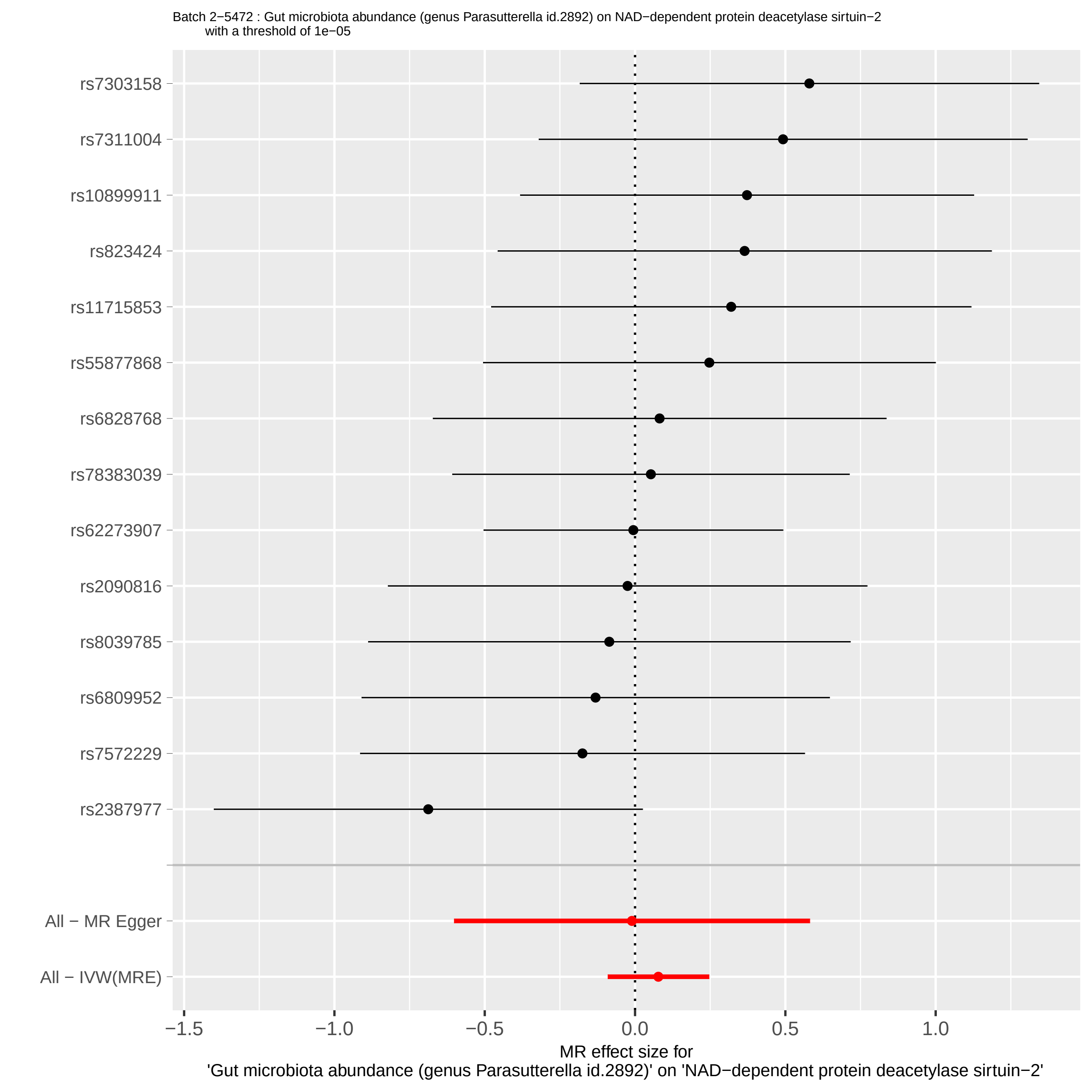
Batch 2−5472 : Gut microbiota abundance (genus Parasutterella id.2892) on NAD−dependent protein deacetylase sirtuin−2
with a threshold of 1e−05
rs7303158
rs7311004
rs10899911
rs823424
rs11715853
rs55877868
rs6828768
rs78383039
rs62273907
rs2090816
rs8039785
rs6809952
rs7572229
rs2387977
All − MR Egger
All − IVW(MRE)
−1.5
−1.0
−0.5
0.0
0.5
1.0
MR effect size for
'Gut microbiota abundance (genus Parasutterella id.2892)' on 'NAD−dependent protein deacetylase sirtuin−2'

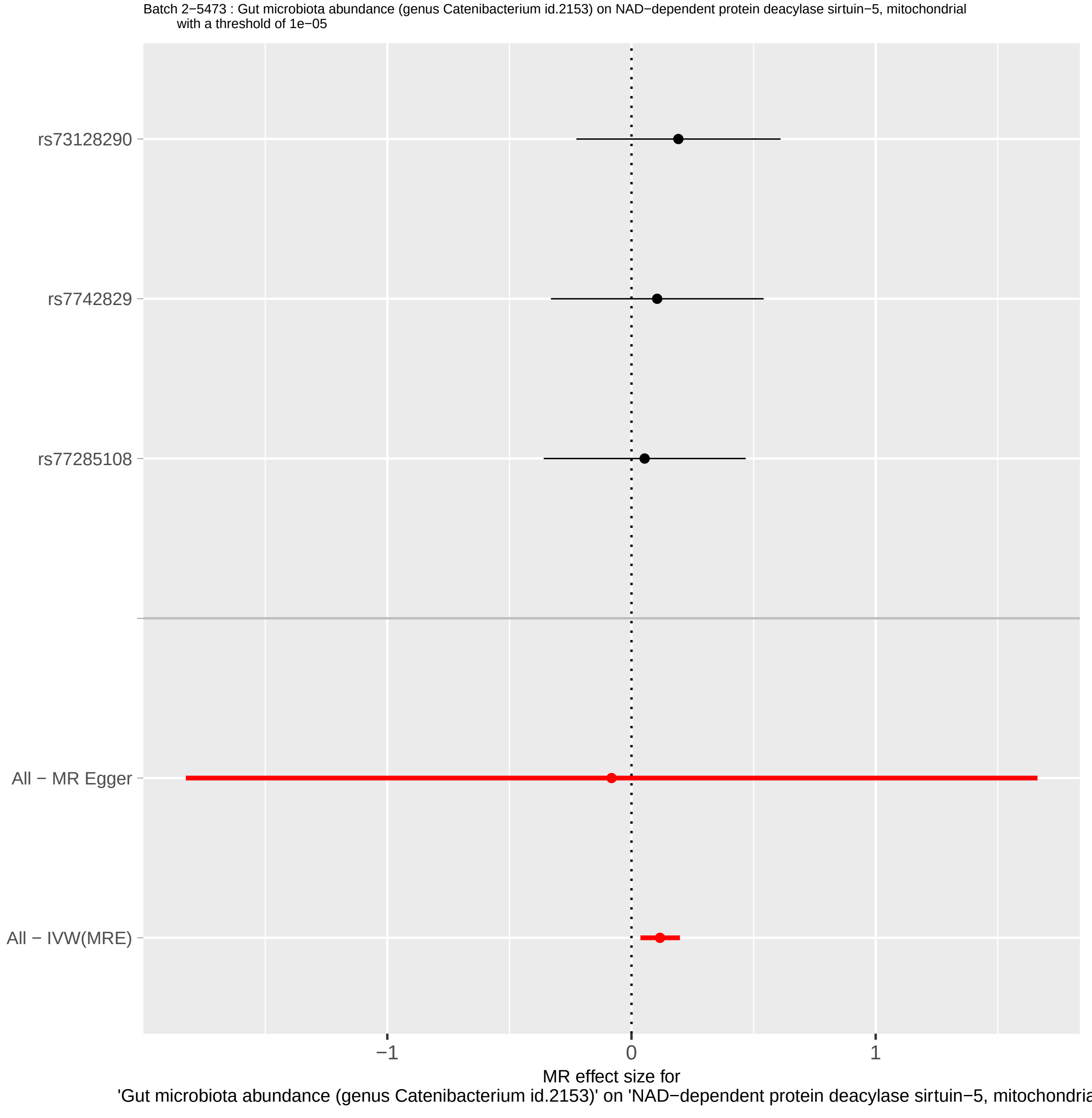

Batch 2−5473 : Gut microbiota abundance (genus Catenibacterium id.2153) on NAD−dependent protein deacylase sirtuin−5, mitochondrial
with a threshold of 1e−05
rs73128290
rs7742829
rs77285108
All − MR Egger
All − IVW(MRE)
−1
0
1
MR effect size for
'Gut microbiota abundance (genus Catenibacterium id.2153)' on 'NAD−dependent protein deacylase sirtuin−5, mitochondria

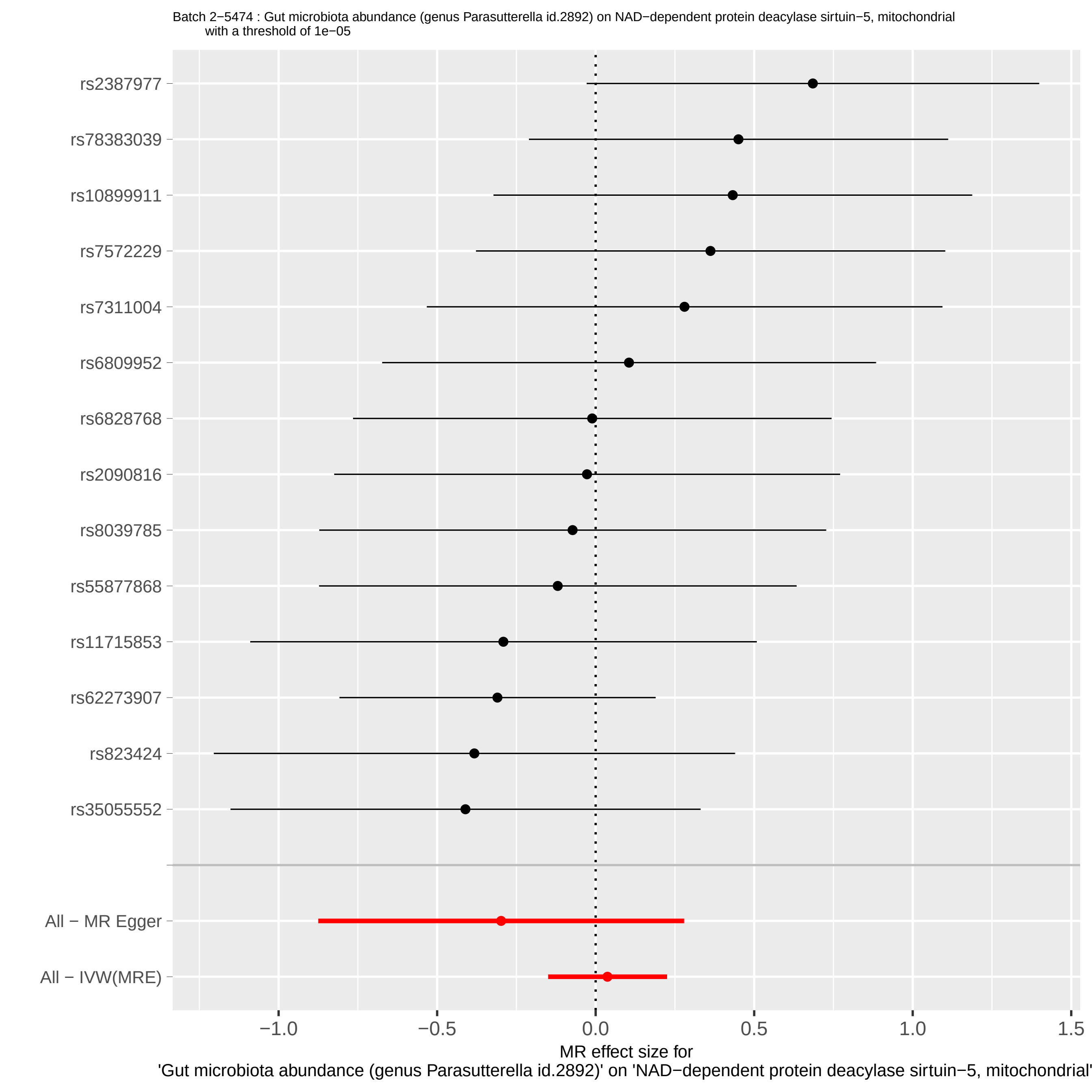

Batch 2−5474 : Gut microbiota abundance (genus Parasutterella id.2892) on NAD−dependent protein deacylase sirtuin−5, mitochondrial
with a threshold of 1e−05
rs2387977
rs78383039
rs10899911
rs7572229
rs7311004
rs6809952
rs6828768
rs2090816
rs8039785
rs55877868
rs11715853
rs62273907
rs823424
rs35055552
All − MR Egger
All − IVW(MRE)
−1.0
−0.5
0.0
0.5
1.0
1.5
MR effect size for
'Gut microbiota abundance (genus Parasutterella id.2892)' on 'NAD−dependent protein deacylase sirtuin−5, mitochondrial'

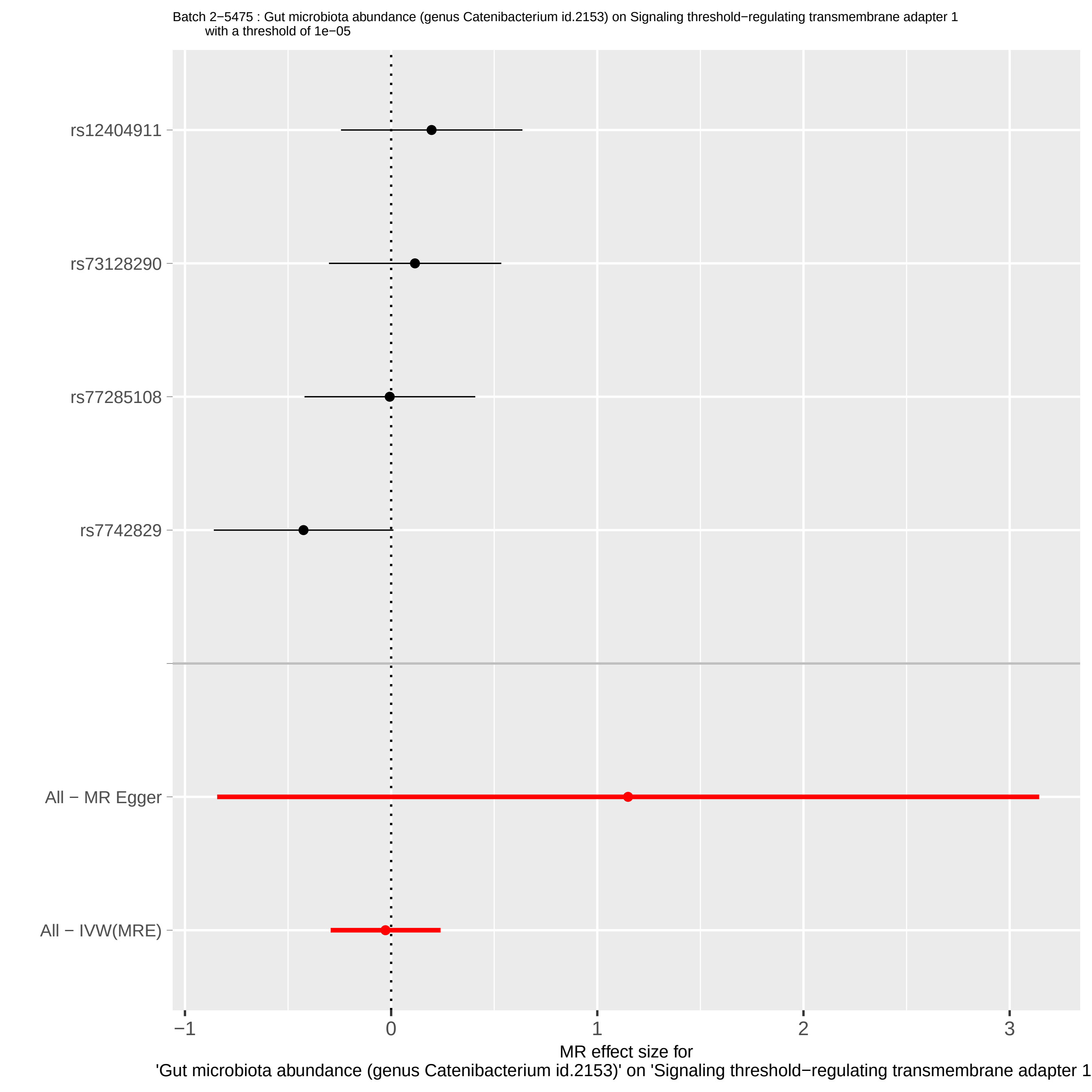

Batch 2−5475 : Gut microbiota abundance (genus Catenibacterium id.2153) on Signaling threshold−regulating transmembrane adapter 1
with a threshold of 1e−05
rs12404911
rs73128290
rs77285108
rs7742829
All − MR Egger
All − IVW(MRE)
−1
0
1
2
3
MR effect size for
'Gut microbiota abundance (genus Catenibacterium id.2153)' on 'Signaling threshold−regulating transmembrane adapter 1

Batch 2−5476 : Gut microbiota abundance (genus Parasutterella id.2892) on Signaling threshold−regulating transmembrane adapter 1 with a threshold of 1e−05

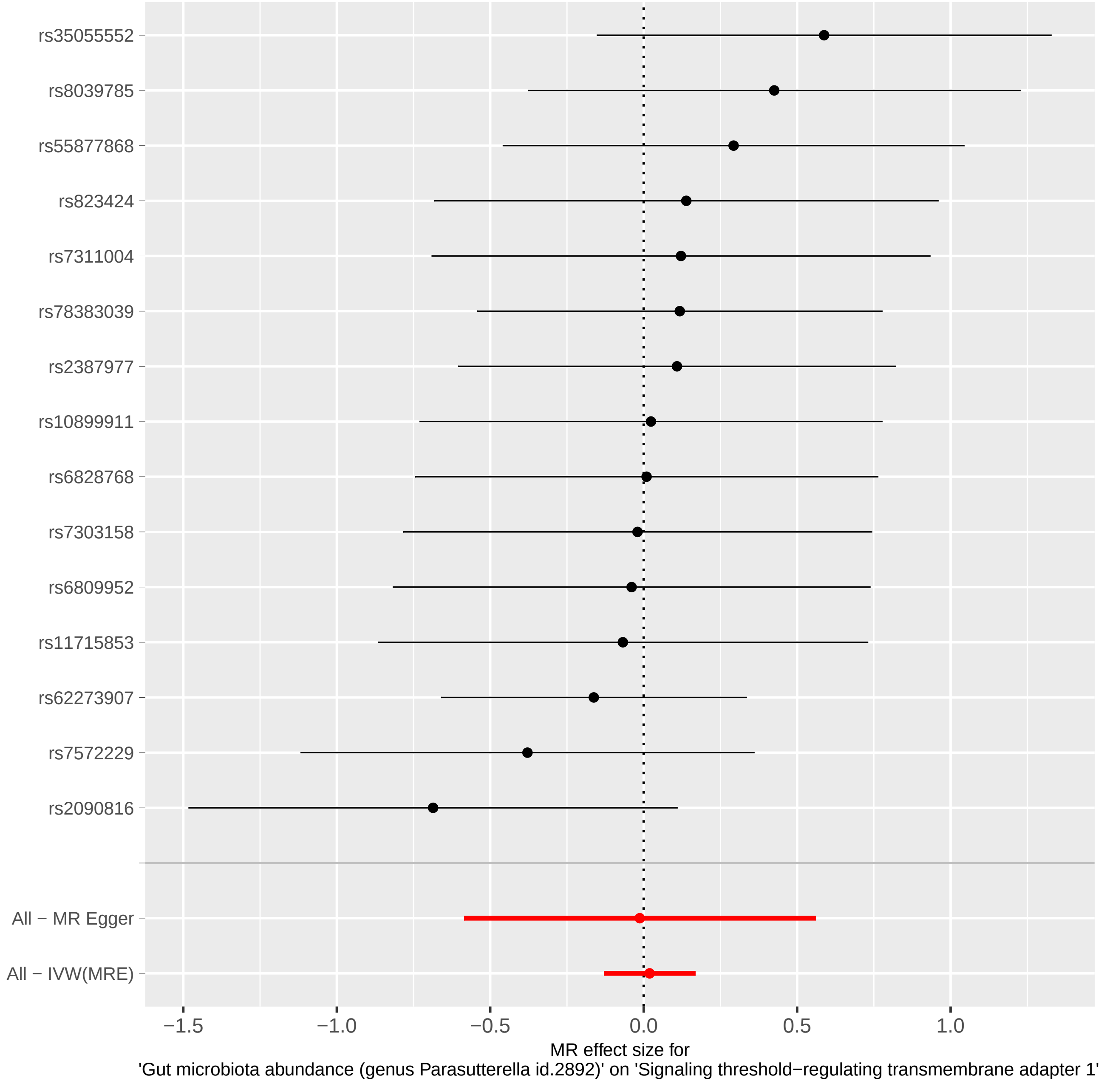

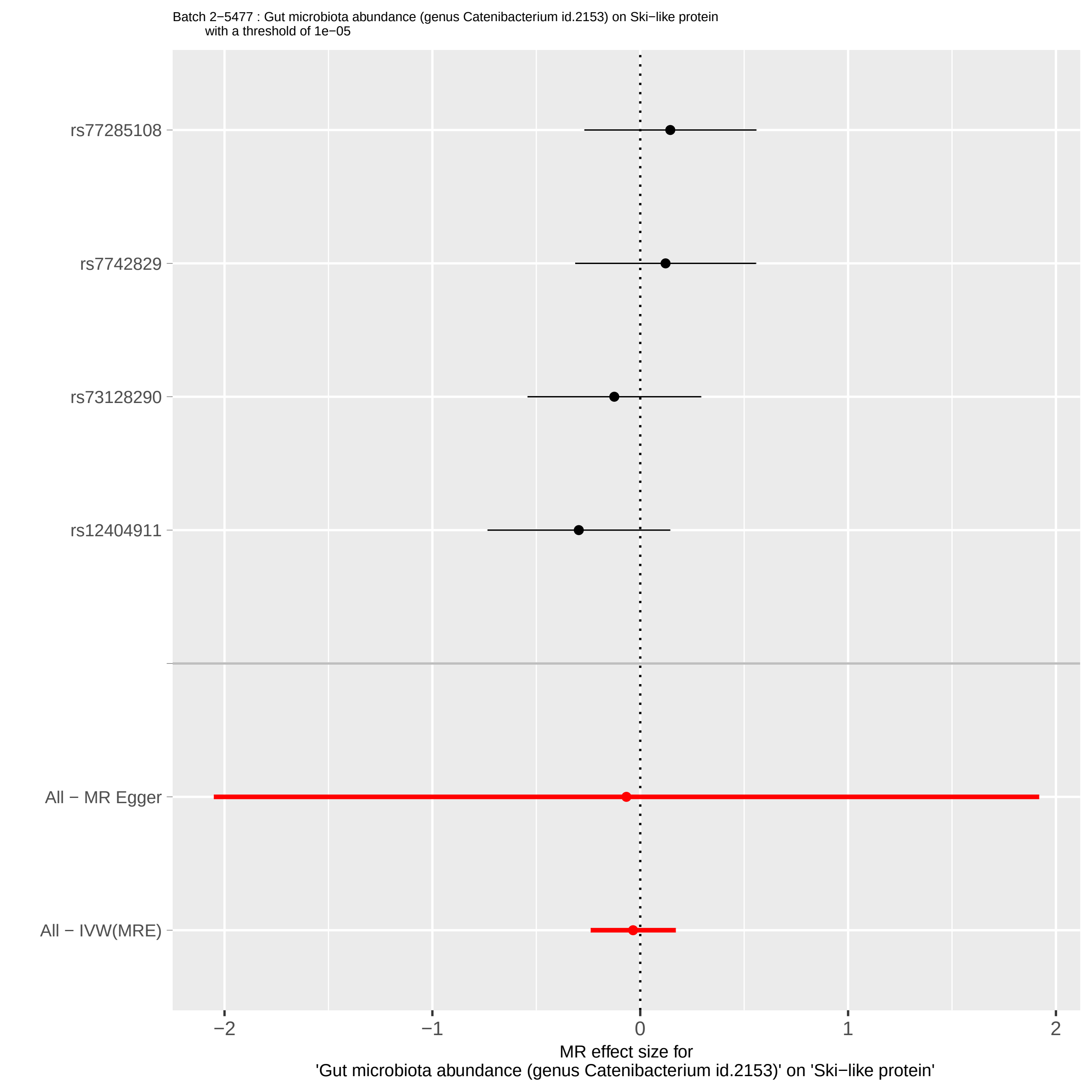
Batch 2−5477 : Gut microbiota abundance (genus Catenibacterium id.2153) on Ski−like protein
with a threshold of 1e−05
rs77285108
rs7742829
rs73128290
rs12404911
All − MR Egger
All − IVW(MRE)
−2
−1
0
1
2
MR effect size for
'Gut microbiota abundance (genus Catenibacterium id.2153)' on 'Ski−like protein'

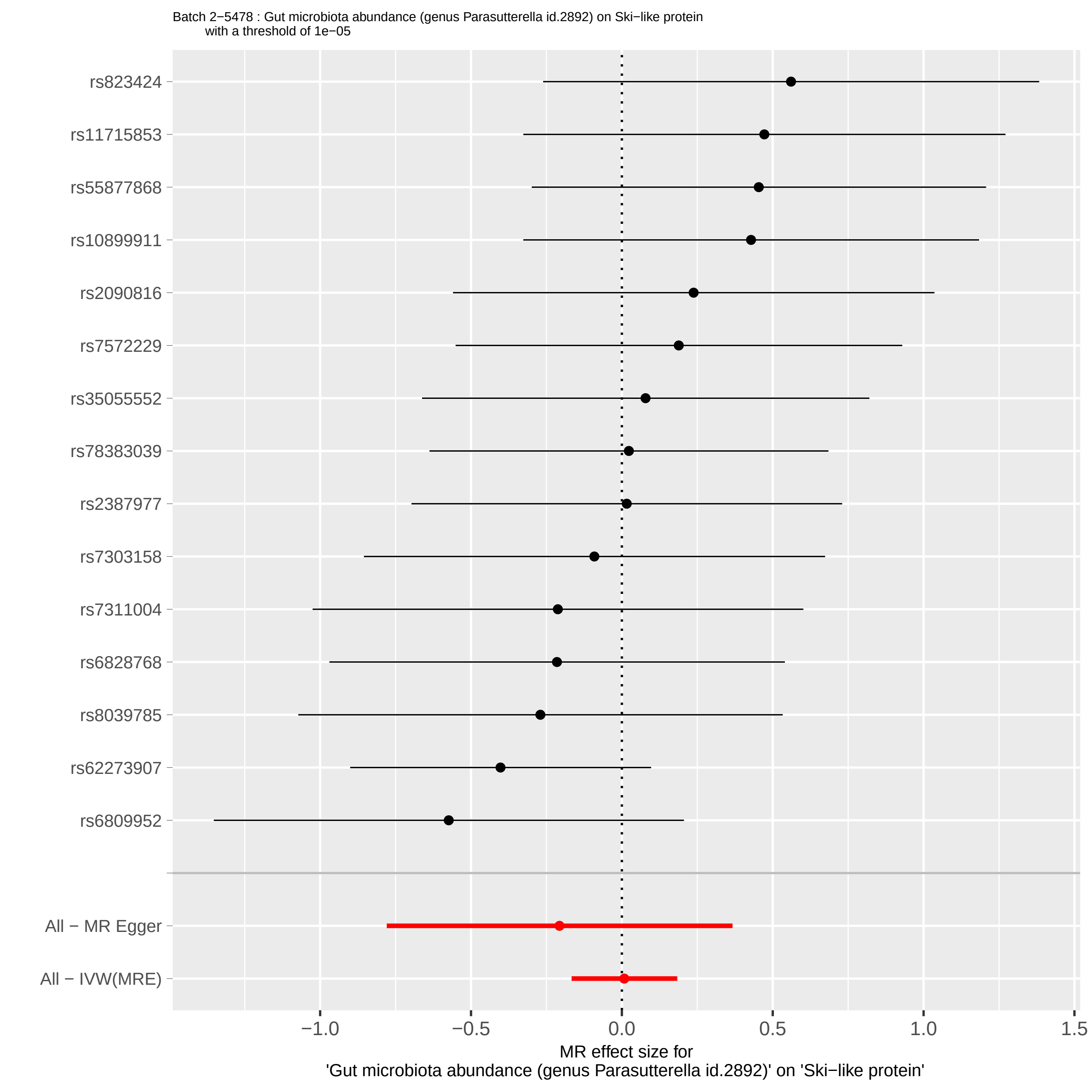

Batch 2−5478 : Gut microbiota abundance (genus Parasutterella id.2892) on Ski−like protein
with a threshold of 1e−05
rs823424
rs11715853
rs55877868
rs10899911
rs2090816
rs7572229
rs35055552
rs78383039
rs2387977
rs7303158
rs7311004
rs6828768
rs8039785
rs62273907
rs6809952
All − MR Egger
All − IVW(MRE)
−1.0
−0.5
0.0
0.5
1.0
1.5
MR effect size for
'Gut microbiota abundance (genus Parasutterella id.2892)' on 'Ski−like protein'

Batch 2−5479 : Gut microbiota abundance (genus Catenibacterium id.2153) on S−phase kinase−associated protein 1
with a threshold of 1e−05

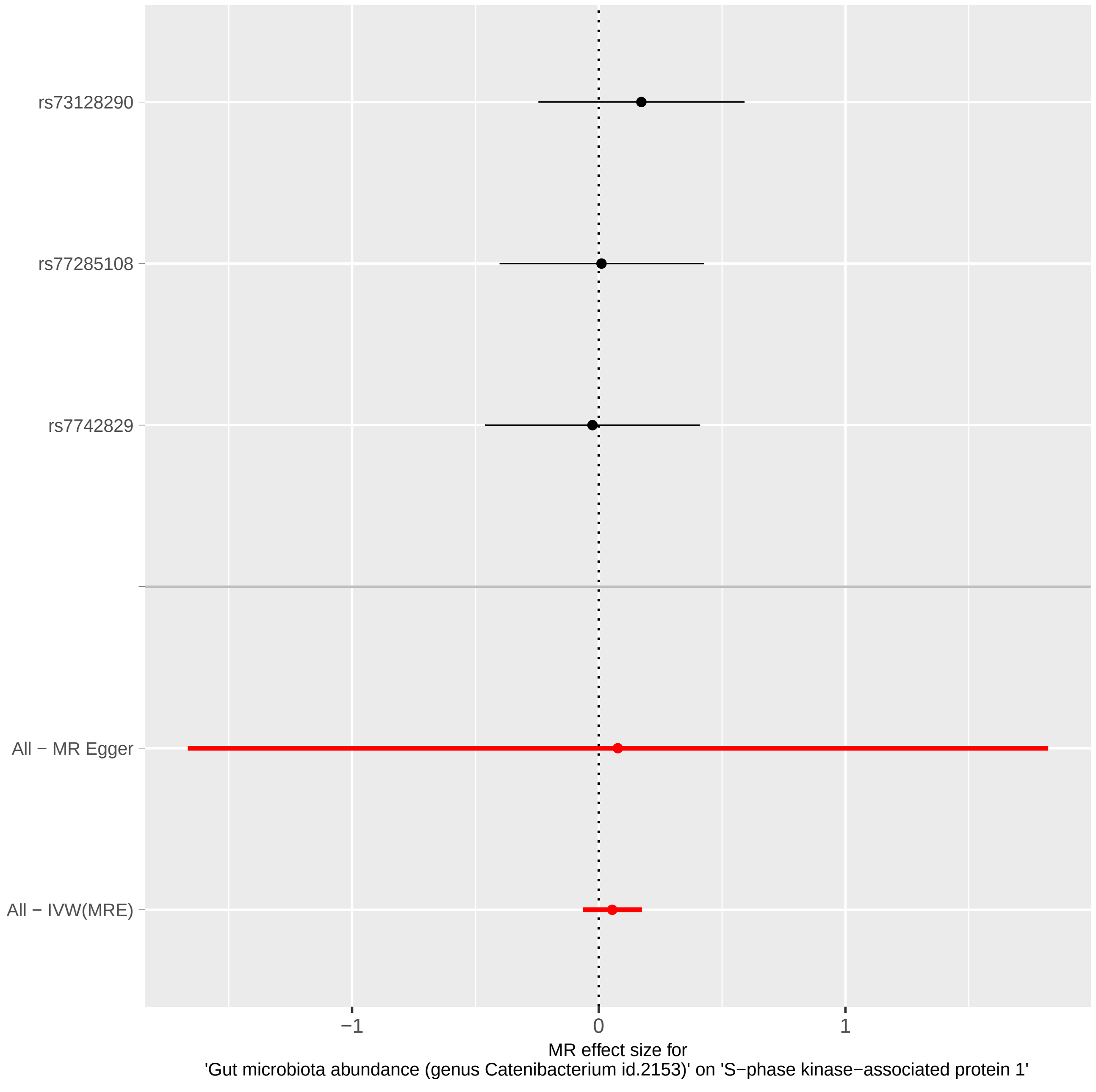

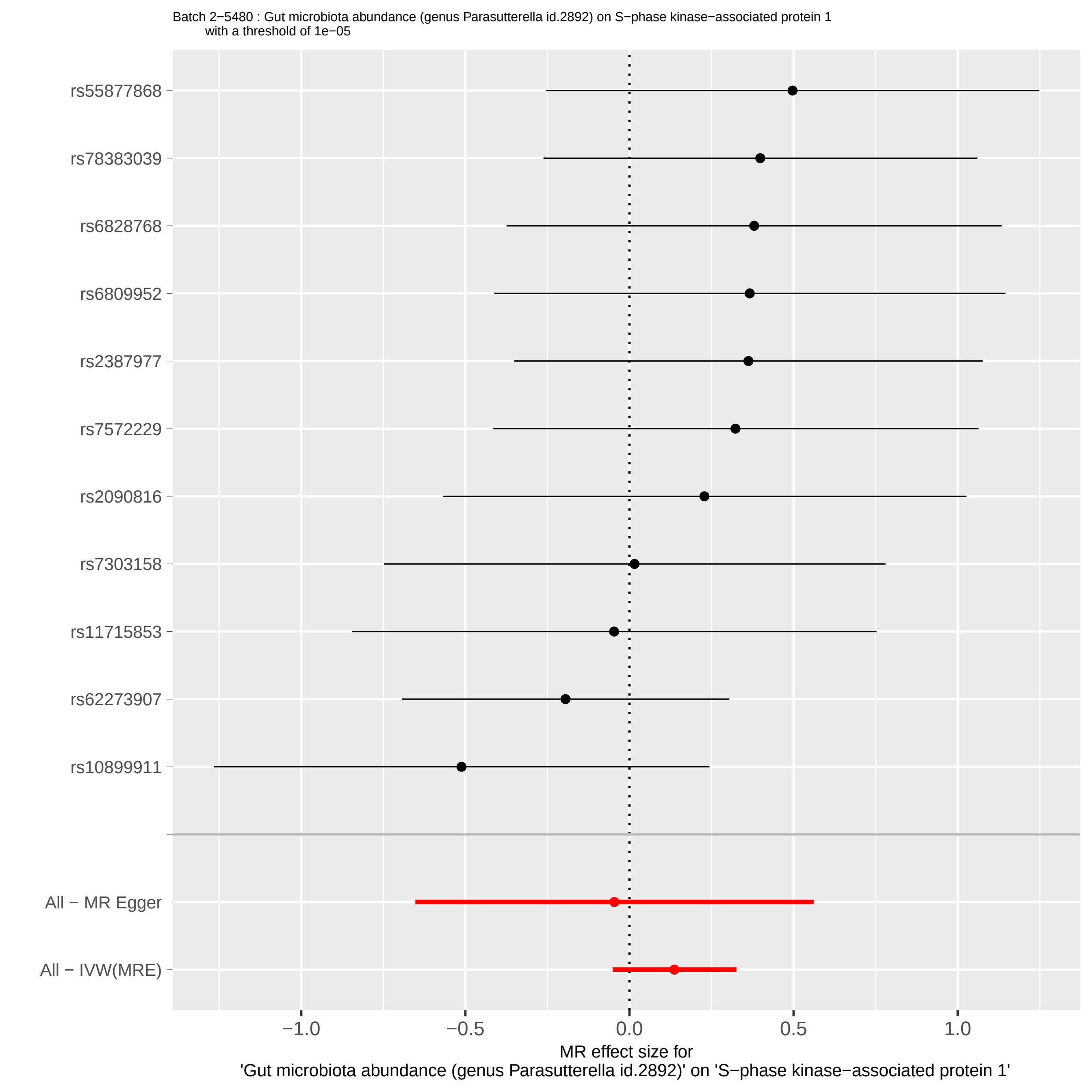

Batch 2−5480 : Gut microbiota abundance (genus Parasutterella id.2892) on S−phase kinase−associated protein 1
with a threshold of 1e−05
rs55877868
rs78383039
rs6828768
rs6809952
rs2387977
rs7572229
rs2090816
rs7303158
rs11715853
rs62273907
rs10899911
All − MR Egger
All − IVW(MRE)
−1.0
−0.5
0.0
0.5
1.0
MR effect size for
'Gut microbiota abundance (genus Parasutterella id.2892)' on 'S−phase kinase−associated protein 1'

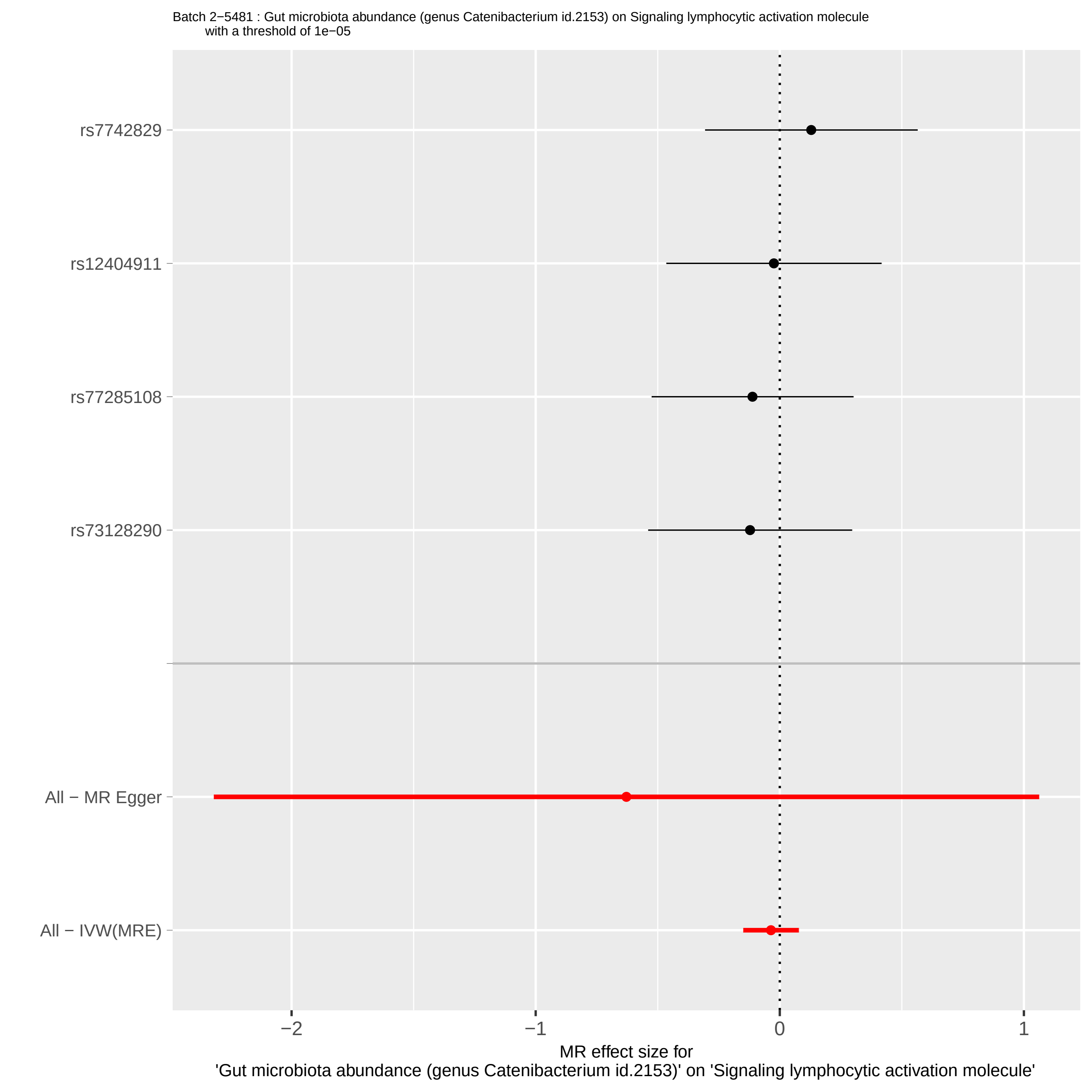

Batch 2−5481 : Gut microbiota abundance (genus Catenibacterium id.2153) on Signaling lymphocytic activation molecule
with a threshold of 1e−05
rs7742829
rs12404911
rs77285108
rs73128290
All − MR Egger
All − IVW(MRE)
−2
−1
0
1
MR effect size for
'Gut microbiota abundance (genus Catenibacterium id.2153)' on 'Signaling lymphocytic activation molecule'

Batch 2−5482 : Gut microbiota abundance (genus Parasutterella id.2892) on Signaling lymphocytic activation molecule
with a threshold of 1e−05

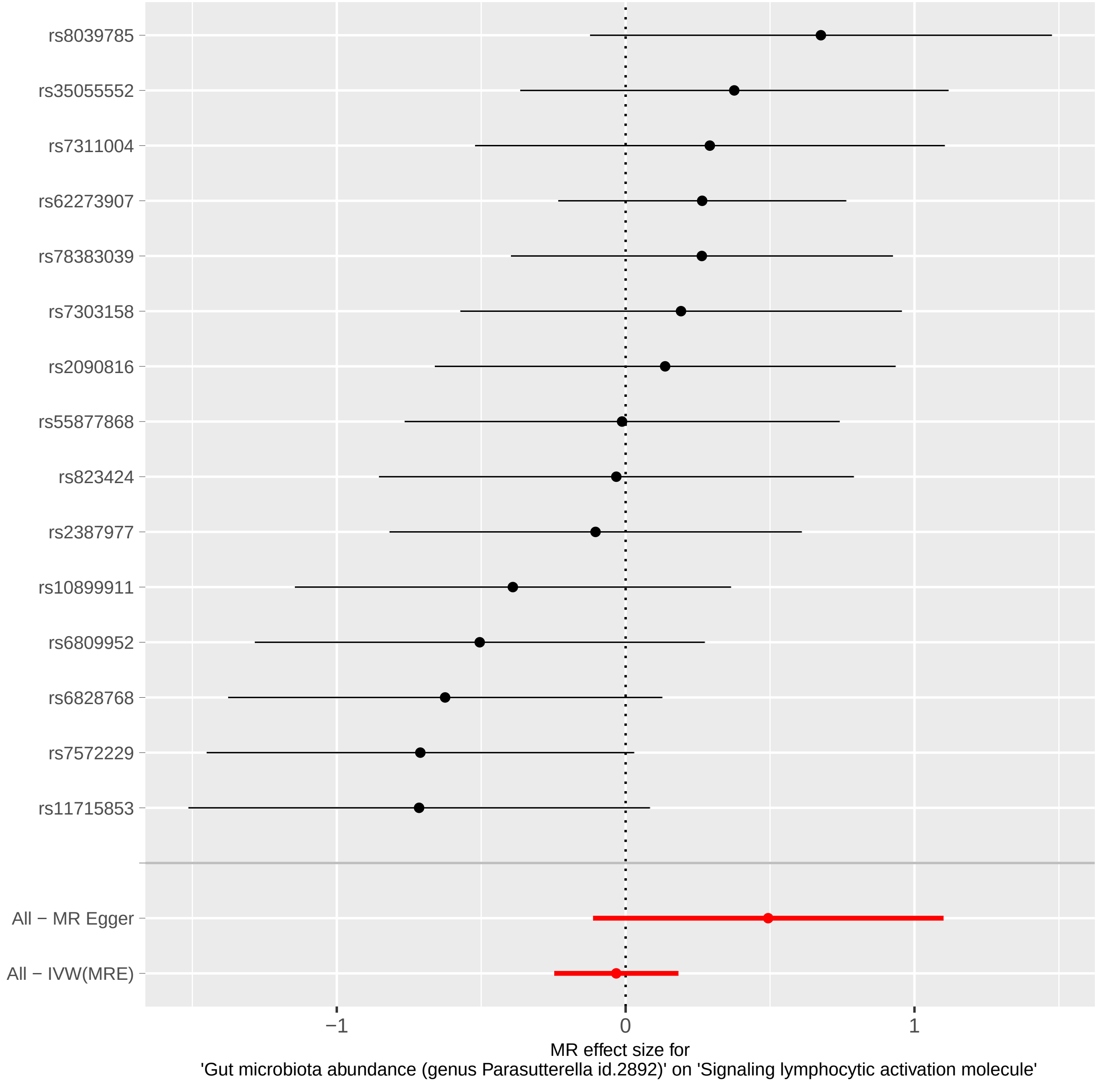

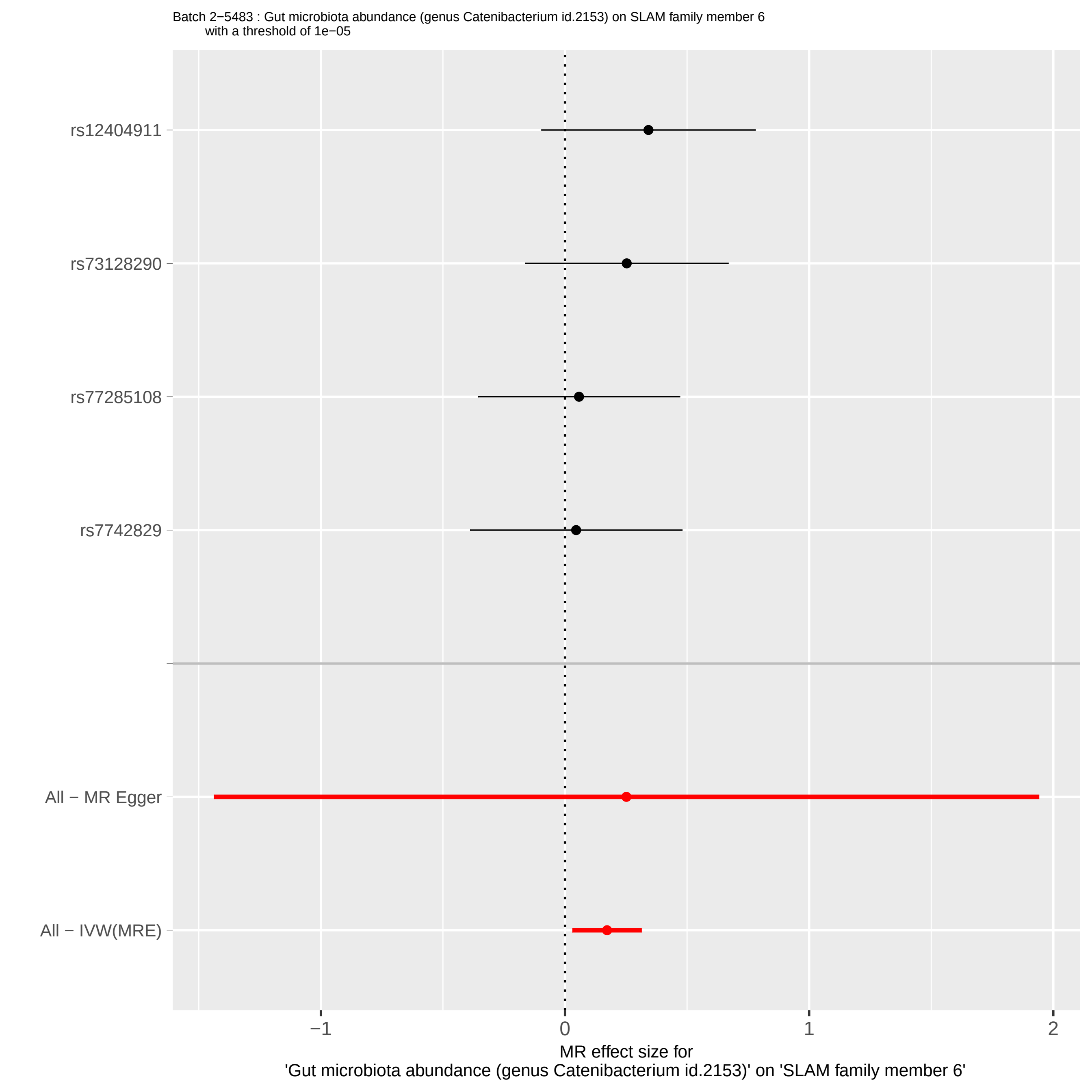

Batch 2−5483 : Gut microbiota abundance (genus Catenibacterium id.2153) on SLAM family member 6
with a threshold of 1e−05
rs12404911
rs73128290
rs77285108
rs7742829
All − MR Egger
All − IVW(MRE)
−1
0
1
2
MR effect size for
'Gut microbiota abundance (genus Catenibacterium id.2153)' on 'SLAM family member 6'

Batch 2−5484 : Gut microbiota abundance (genus Parasutterella id.2892) on SLAM family member 6
with a threshold of 1e−05

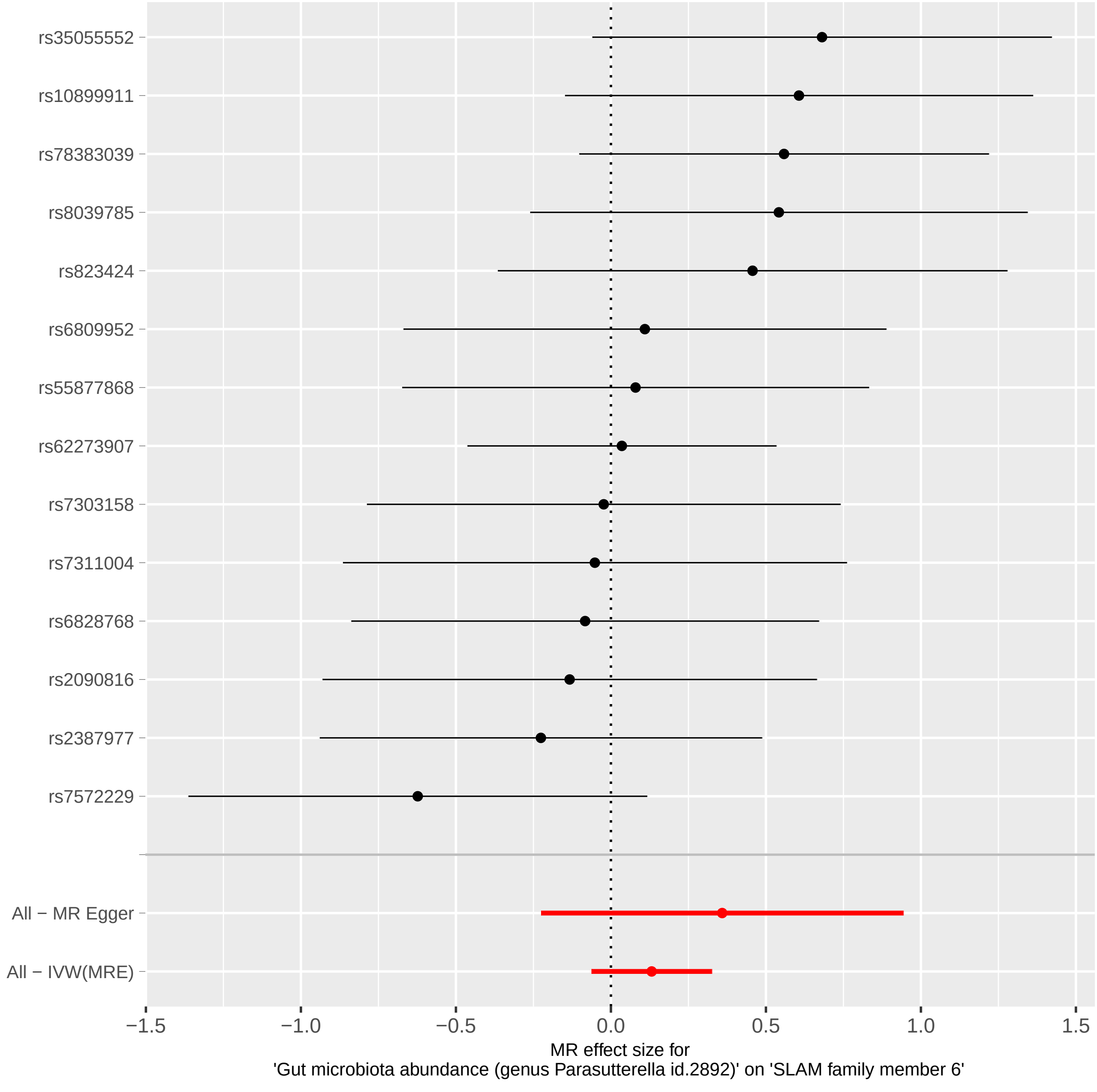

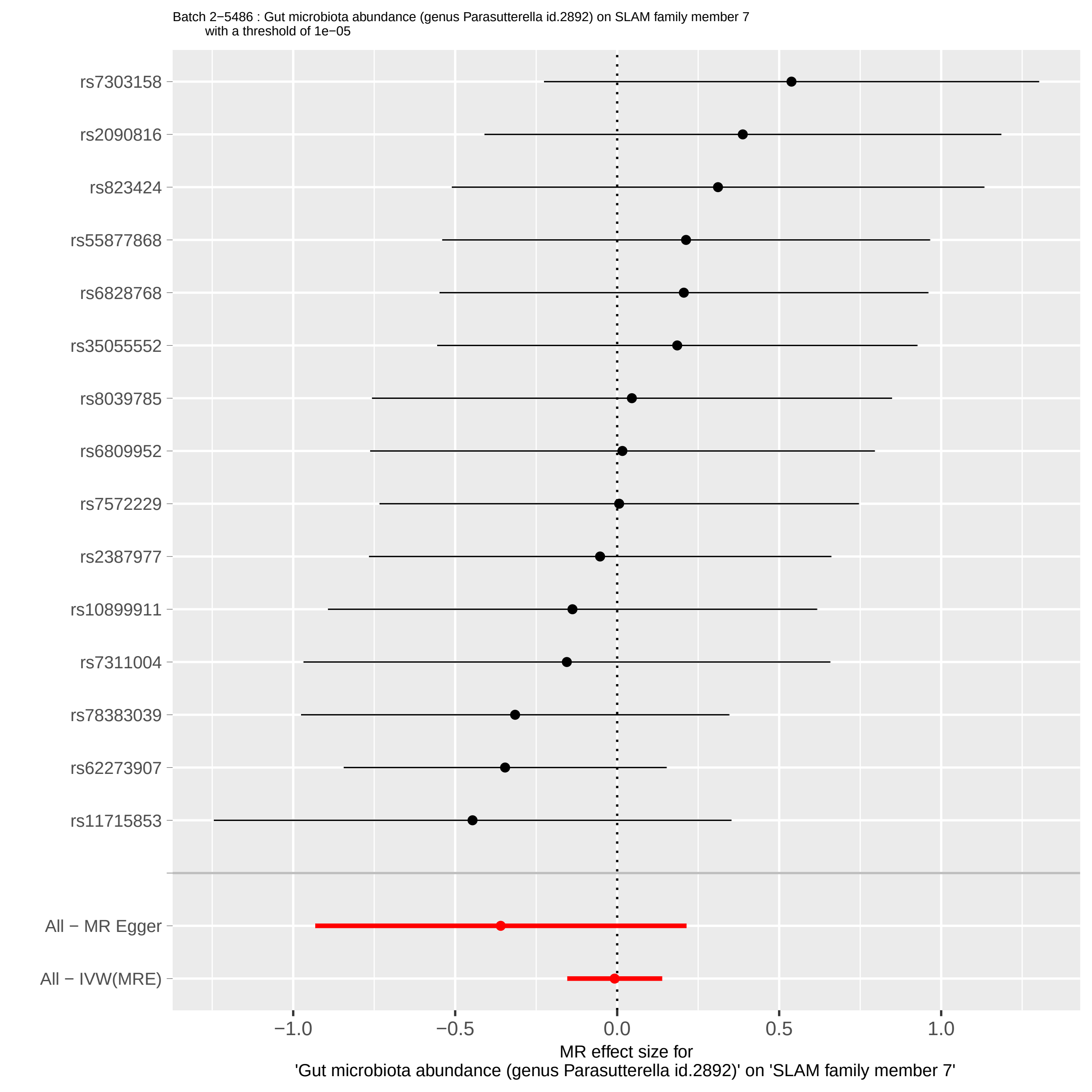

Batch 2−5486 : Gut microbiota abundance (genus Parasutterella id.2892) on SLAM family member 7
with a threshold of 1e−05
rs7303158
rs2090816
rs823424
rs55877868
rs6828768
rs35055552
rs8039785
rs6809952
rs7572229
rs2387977
rs10899911
rs7311004
rs78383039
rs62273907
rs11715853
All − MR Egger
All − IVW(MRE)
−1.0
−0.5
0.0
0.5
1.0
MR effect size for
'Gut microbiota abundance (genus Parasutterella id.2892)' on 'SLAM family member 7'

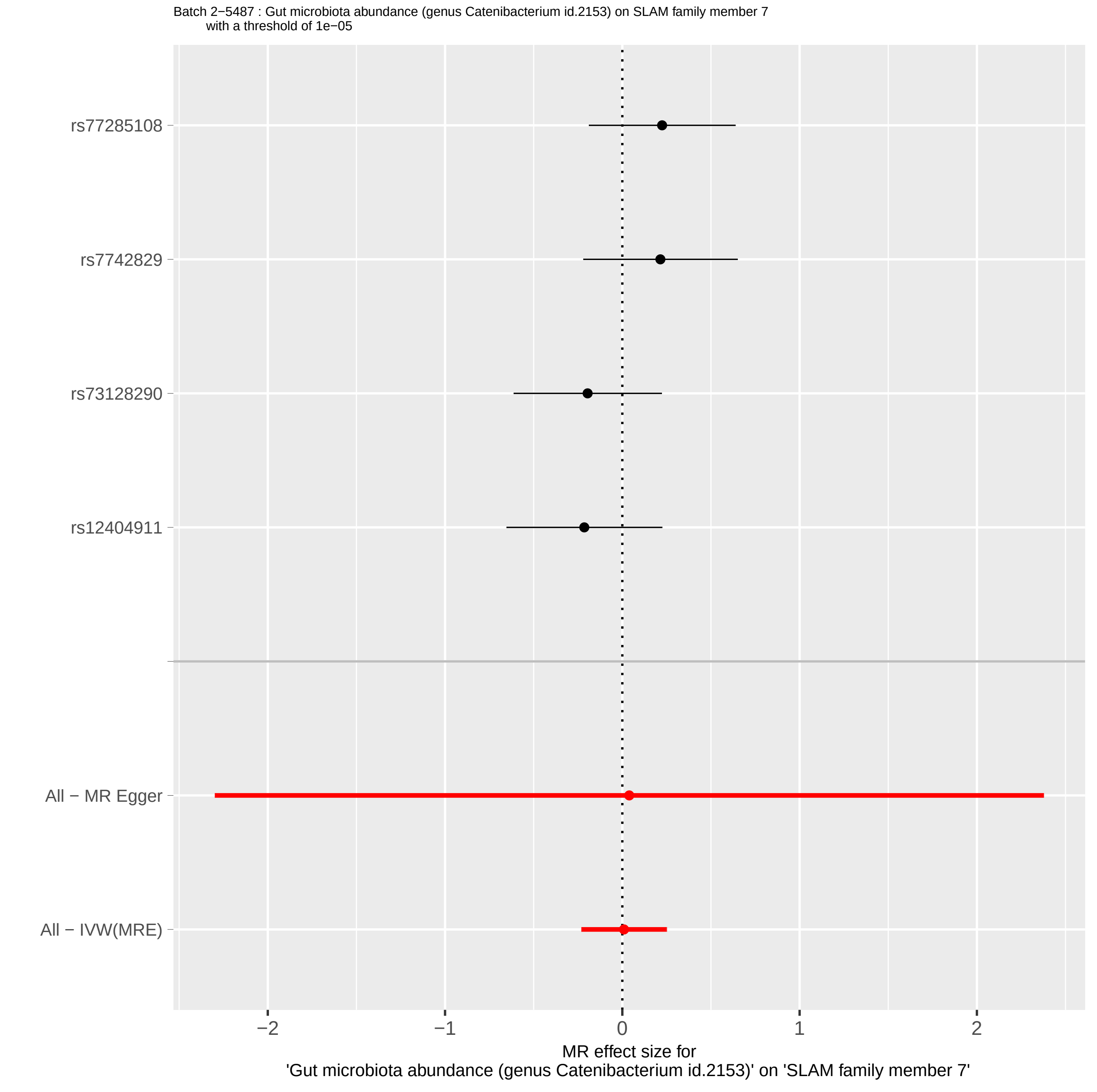

Batch 2−5487 : Gut microbiota abundance (genus Catenibacterium id.2153) on SLAM family member 7
with a threshold of 1e−05
rs77285108
rs7742829
rs73128290
rs12404911
All − MR Egger
All − IVW(MRE)
−2
−1
0
1
2
MR effect size for
'Gut microbiota abundance (genus Catenibacterium id.2153)' on 'SLAM family member 7'

Batch 2−5488 : Gut microbiota abundance (genus Parasutterella id.2892) on SLAM family member 7
with a threshold of 1e−05

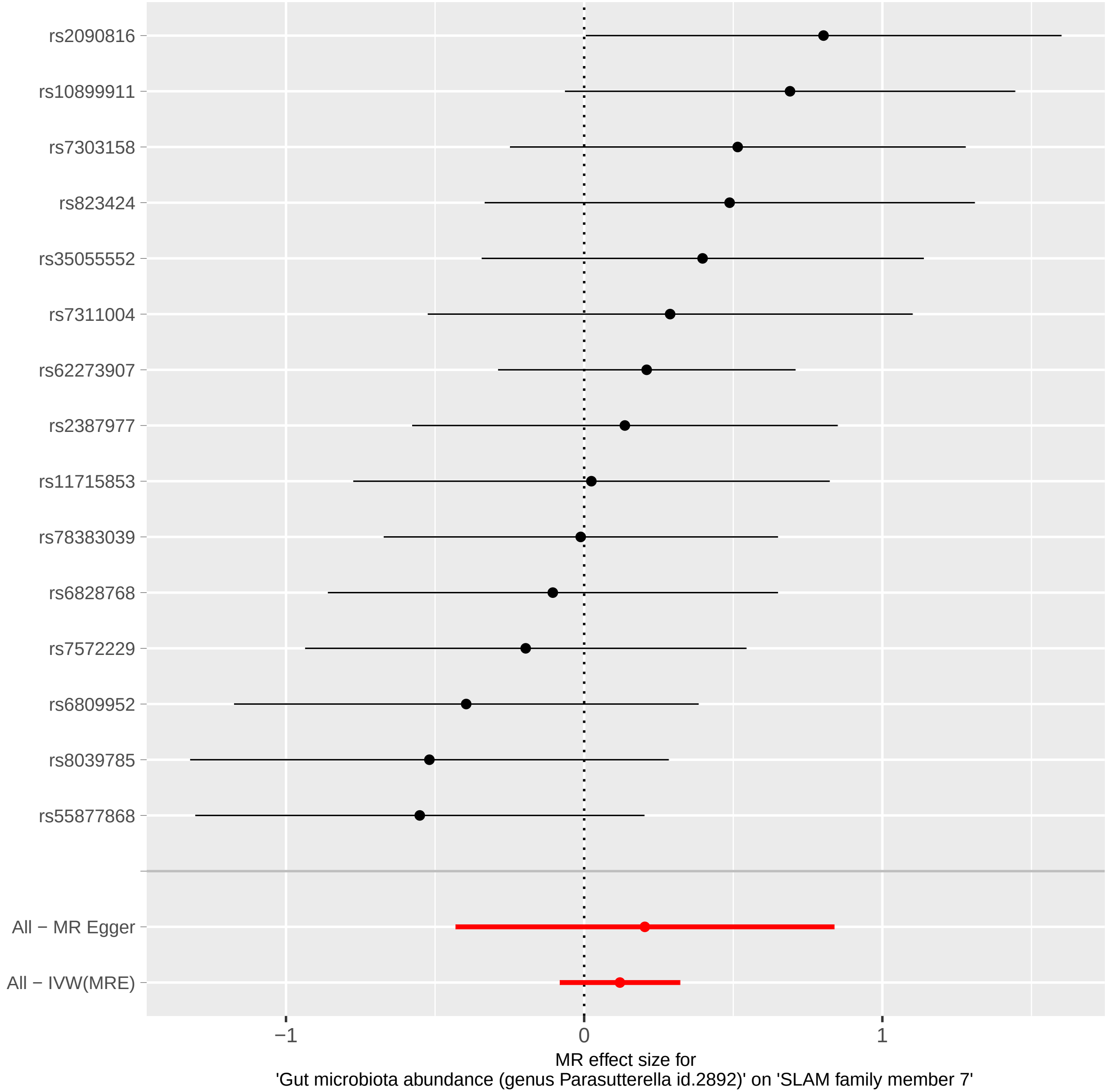

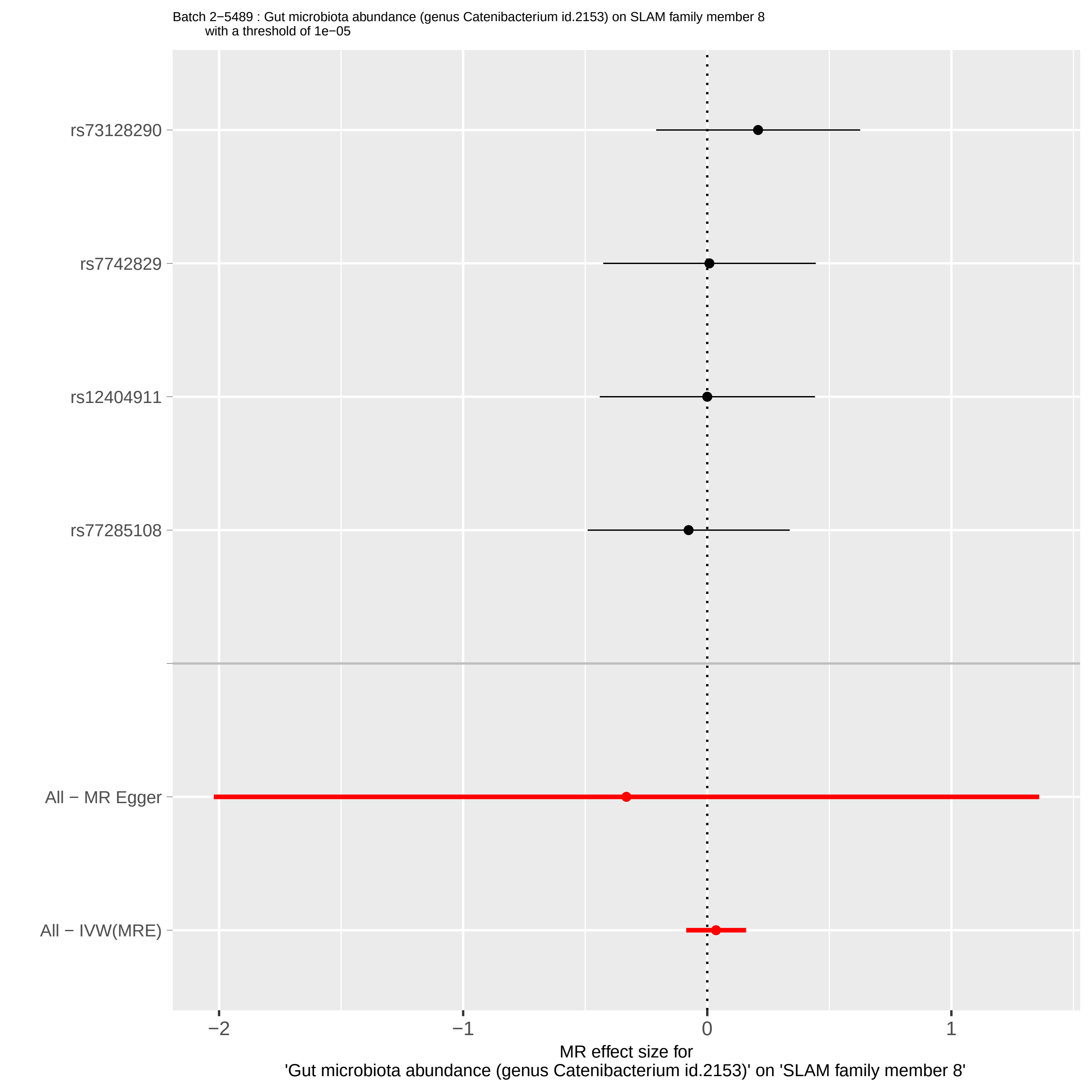

Batch 2−5489 : Gut microbiota abundance (genus Catenibacterium id.2153) on SLAM family member 8
with a threshold of 1e−05
rs73128290
rs7742829
rs12404911
rs77285108
All − MR Egger
All − IVW(MRE)
−2
−1
0
1
MR effect size for
'Gut microbiota abundance (genus Catenibacterium id.2153)' on 'SLAM family member 8'

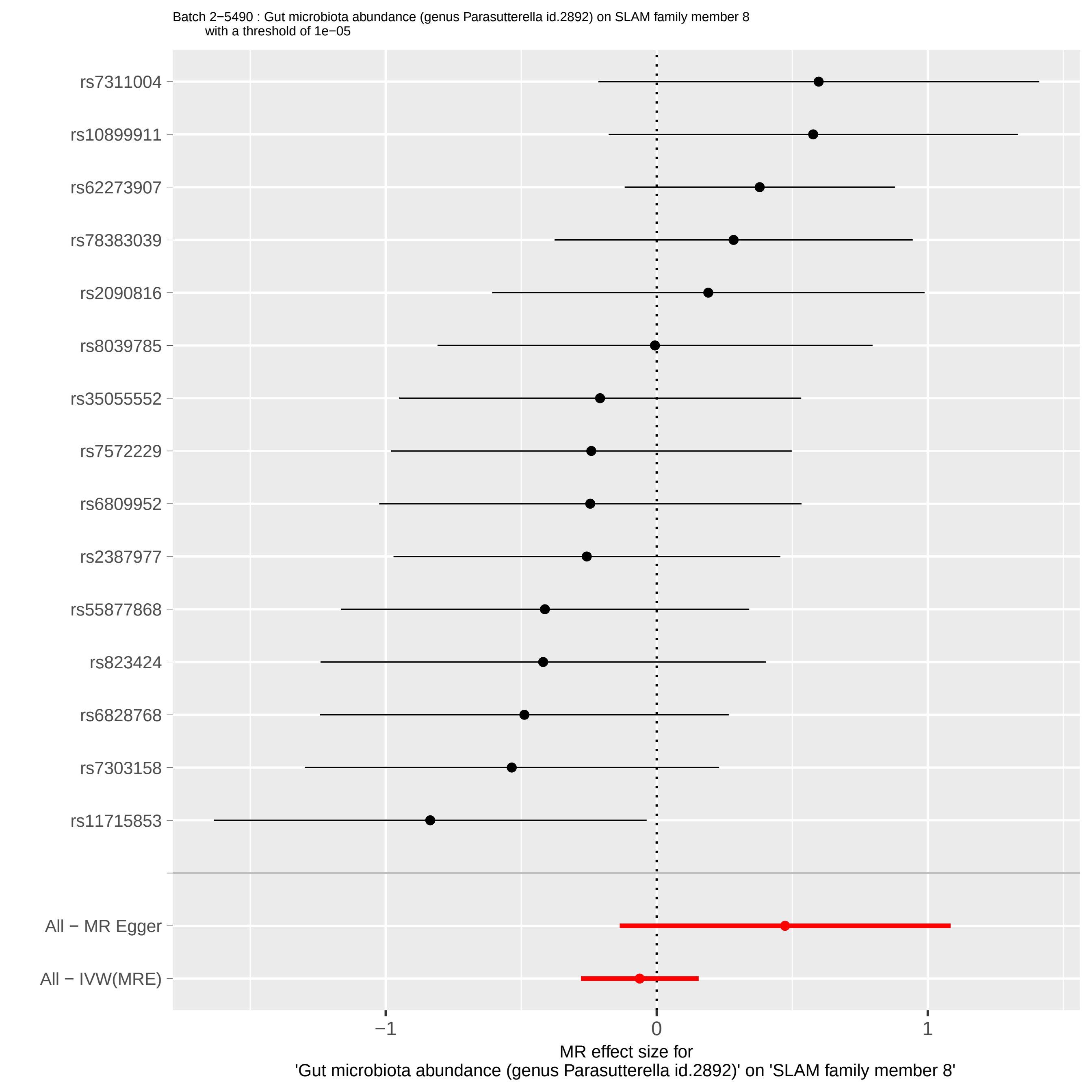

Batch 2−5490 : Gut microbiota abundance (genus Parasutterella id.2892) on SLAM family member 8
with a threshold of 1e−05
rs7311004
rs10899911
rs62273907
rs78383039
rs2090816
rs8039785
rs35055552
rs7572229
rs6809952
rs2387977
rs55877868
rs823424
rs6828768
rs7303158
rs11715853
All − MR Egger
All − IVW(MRE)
−1
0
1
MR effect size for
'Gut microbiota abundance (genus Parasutterella id.2892)' on 'SLAM family member 8'

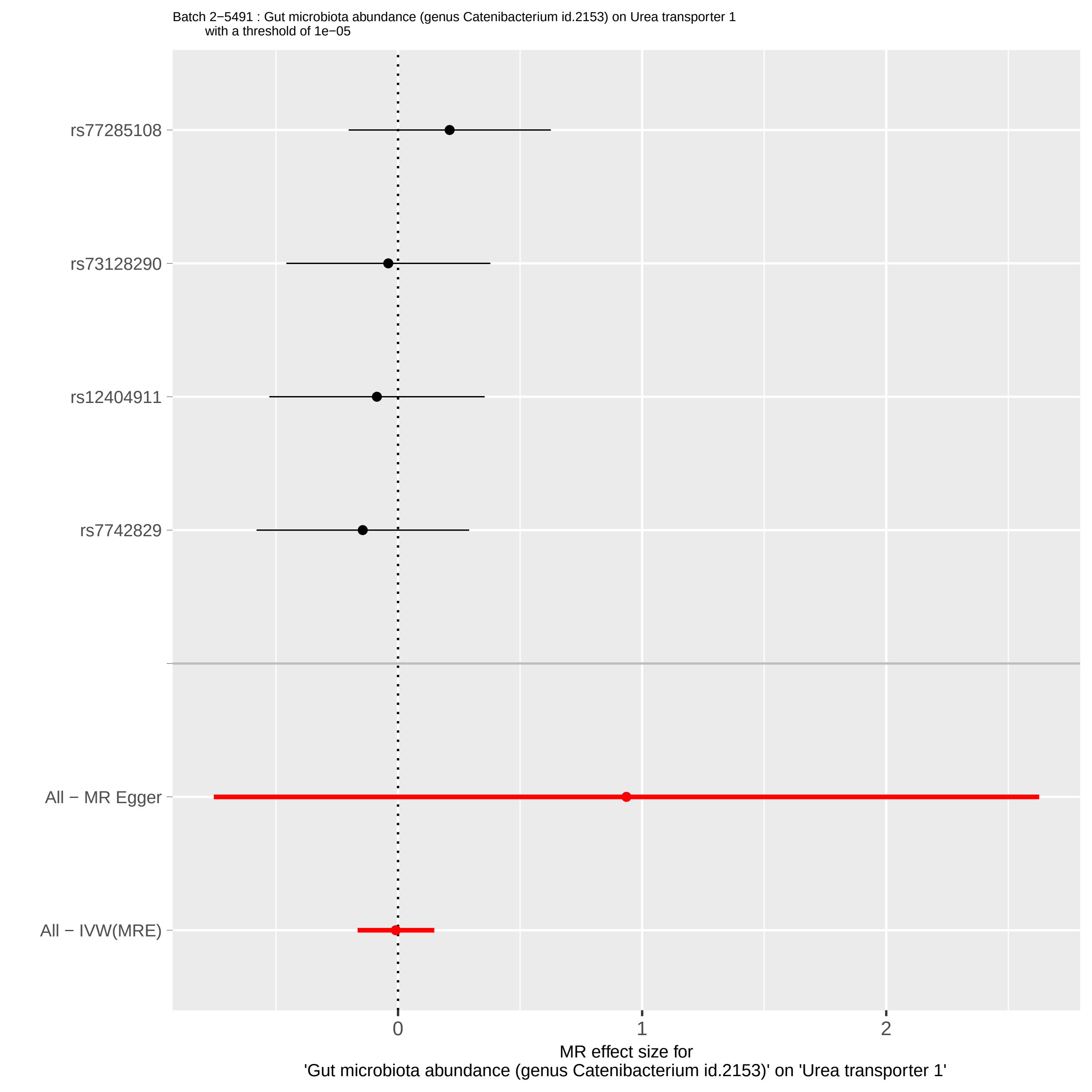

Batch 2−5491 : Gut microbiota abundance (genus Catenibacterium id.2153) on Urea transporter 1
with a threshold of 1e−05
rs77285108
rs73128290
rs12404911
rs7742829
All − MR Egger
All − IVW(MRE)
0
1
2
MR effect size for
'Gut microbiota abundance (genus Catenibacterium id.2153)' on 'Urea transporter 1'

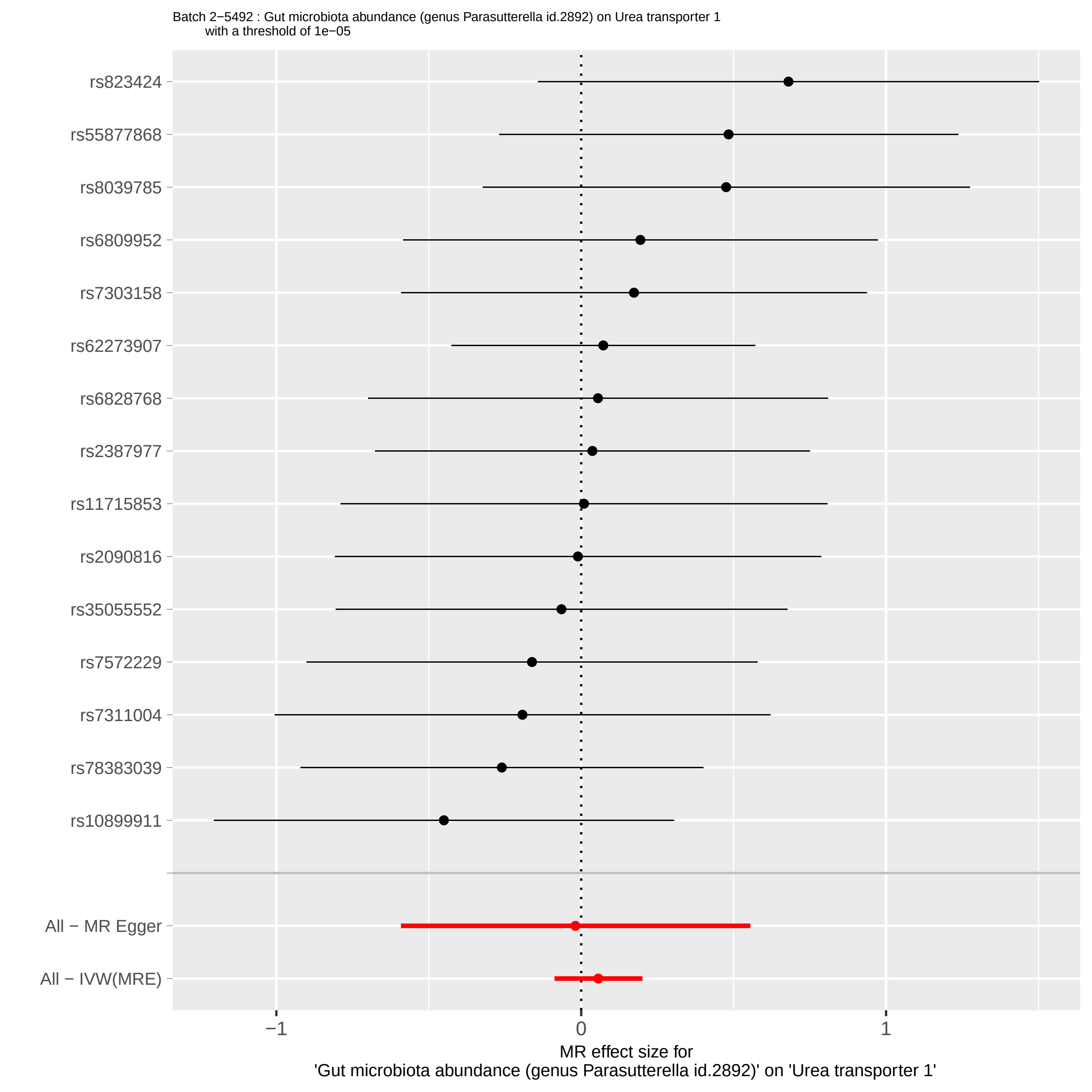

Batch 2−5492 : Gut microbiota abundance (genus Parasutterella id.2892) on Urea transporter 1
with a threshold of 1e−05
rs823424
rs55877868
rs8039785
rs6809952
rs7303158
rs62273907
rs6828768
rs2387977
rs11715853
rs2090816
rs35055552
rs7572229
rs7311004
rs78383039
rs10899911
All − MR Egger
All − IVW(MRE)
−1
0
1
MR effect size for
'Gut microbiota abundance (genus Parasutterella id.2892)' on 'Urea transporter 1'

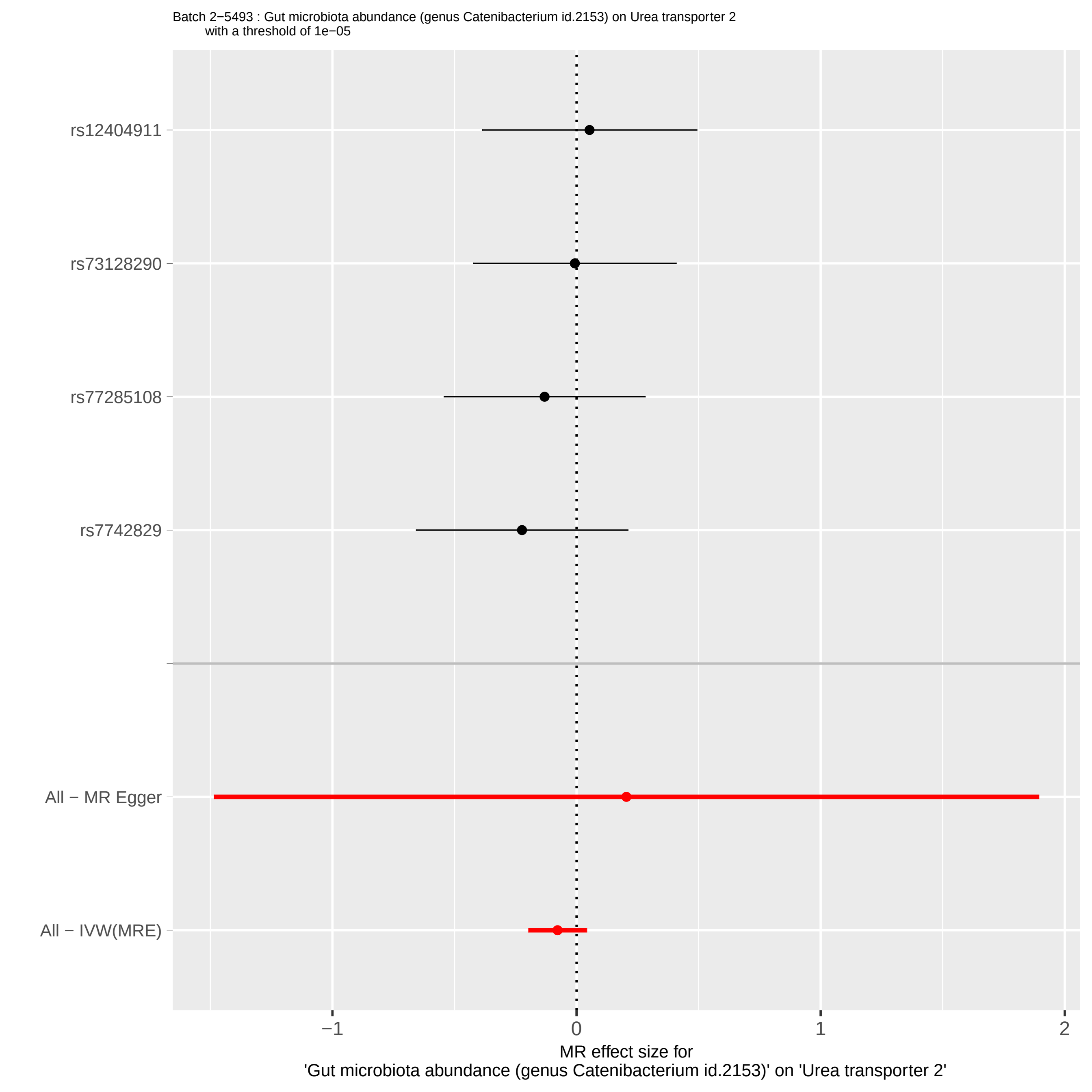

Batch 2−5493 : Gut microbiota abundance (genus Catenibacterium id.2153) on Urea transporter 2
with a threshold of 1e−05
rs12404911
rs73128290
rs77285108
rs7742829
All − MR Egger
All − IVW(MRE)
−1
0
1
2
MR effect size for
'Gut microbiota abundance (genus Catenibacterium id.2153)' on 'Urea transporter 2'

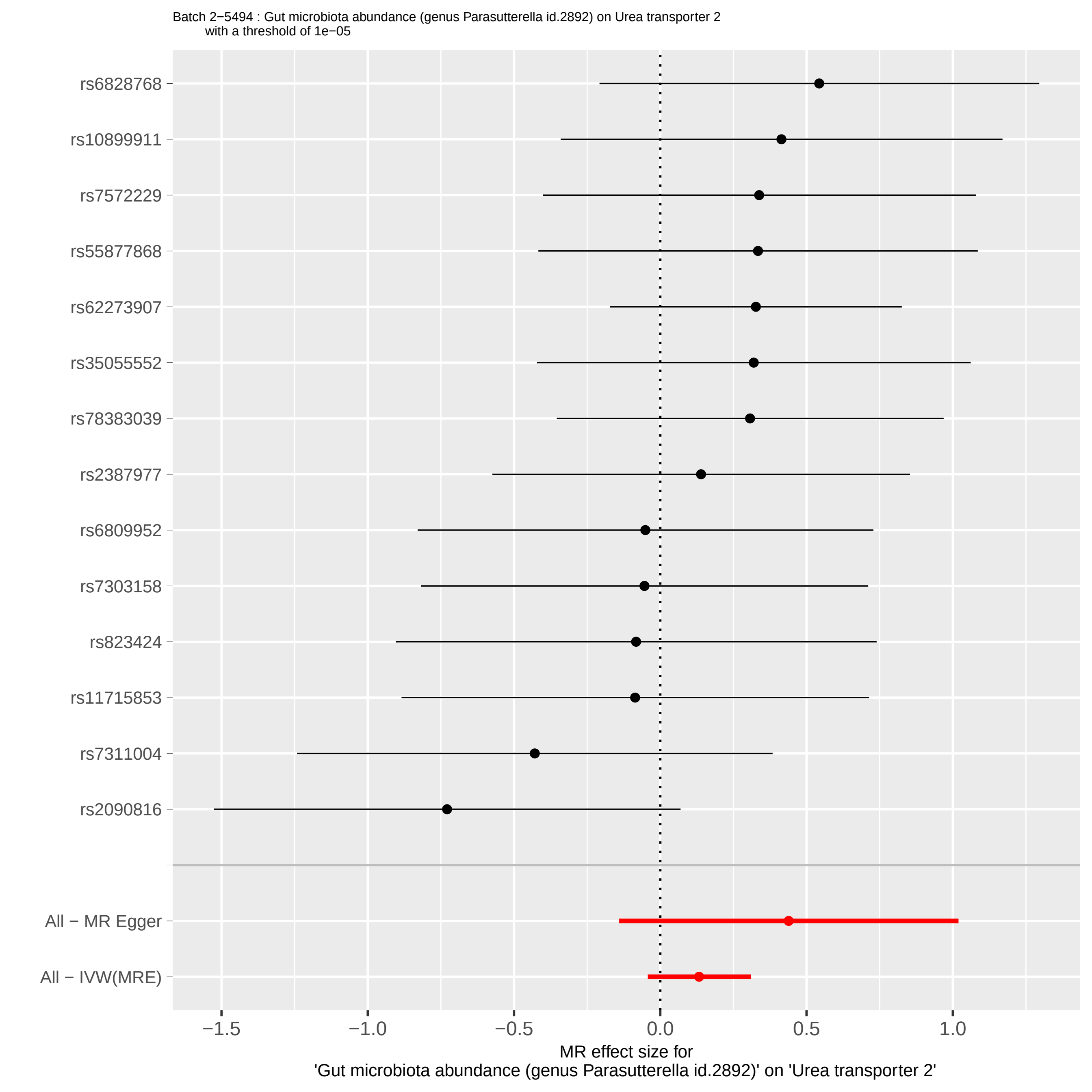
Batch 2−5494 : Gut microbiota abundance (genus Parasutterella id.2892) on Urea transporter 2
with a threshold of 1e−05
rs6828768
rs10899911
rs7572229
rs55877868
rs62273907
rs35055552
rs78383039
rs2387977
rs6809952
rs7303158
rs823424
rs11715853
rs7311004
rs2090816
All − MR Egger
All − IVW(MRE)
−1.5
−1.0
−0.5
0.0
0.5
1.0
MR effect size for
'Gut microbiota abundance (genus Parasutterella id.2892)' on 'Urea transporter 2'

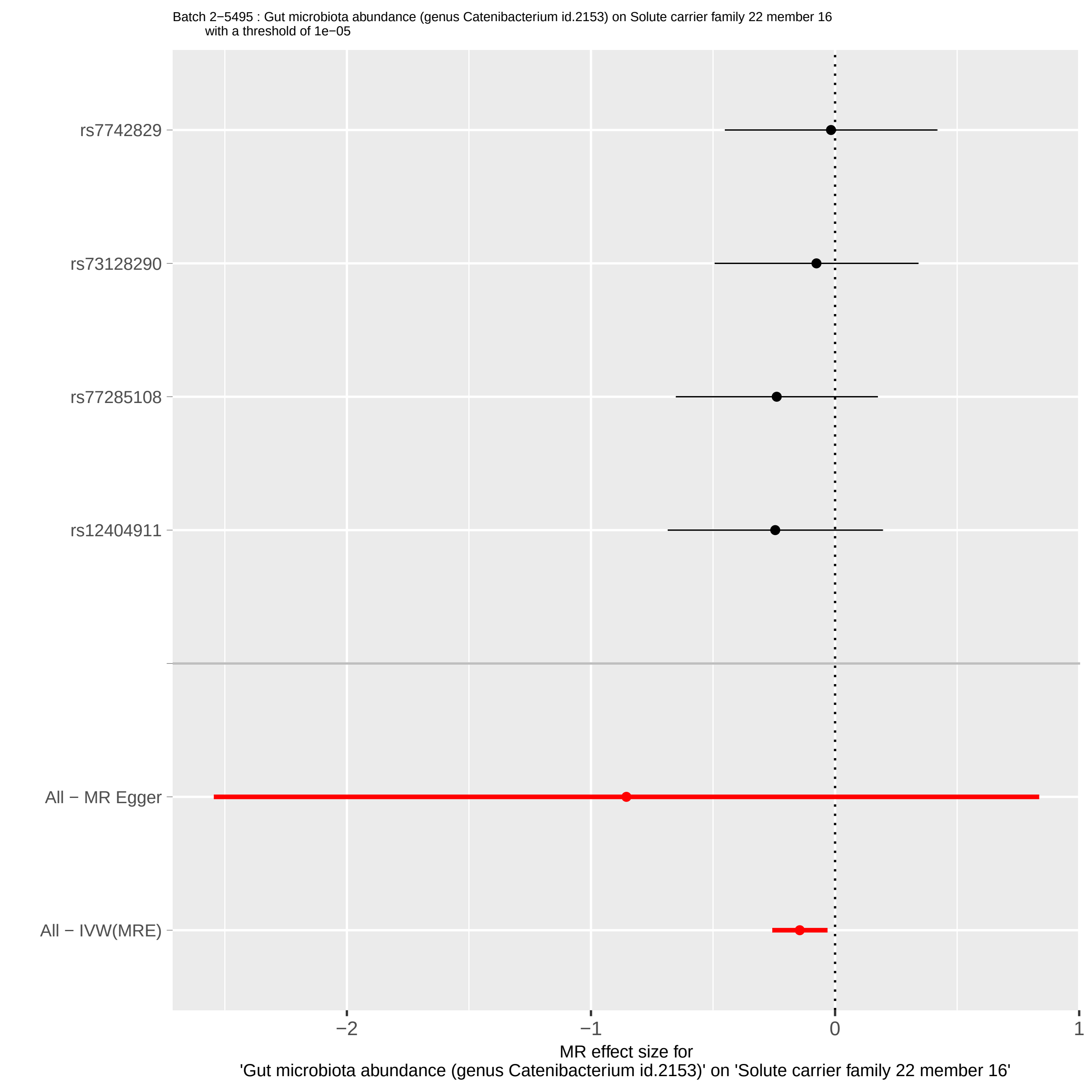

Batch 2−5495 : Gut microbiota abundance (genus Catenibacterium id.2153) on Solute carrier family 22 member 16
with a threshold of 1e−05
rs7742829
rs73128290
rs77285108
rs12404911
All − MR Egger
All − IVW(MRE)
−2
−1
0
1
MR effect size for
'Gut microbiota abundance (genus Catenibacterium id.2153)' on 'Solute carrier family 22 member 16'

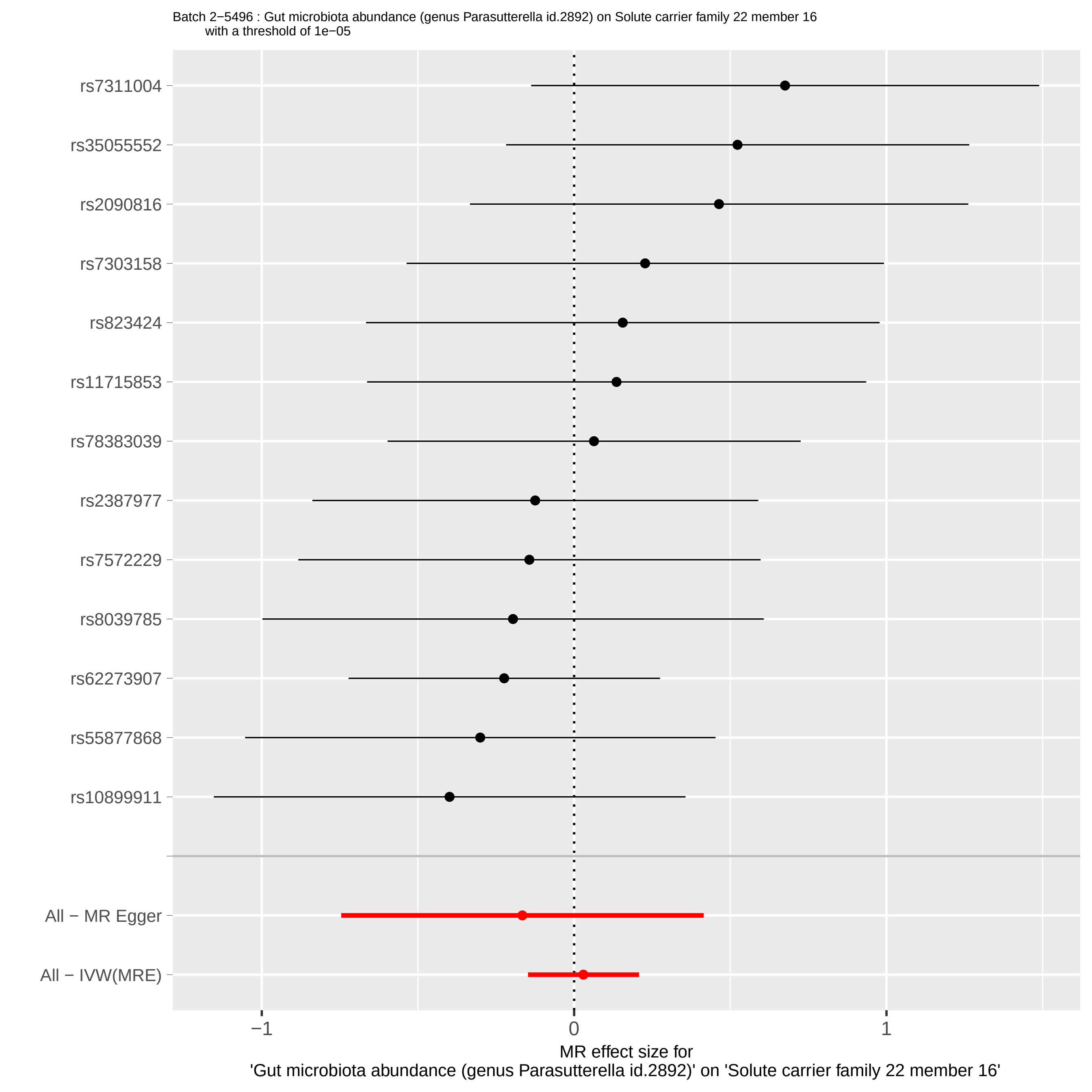

Batch 2−5496 : Gut microbiota abundance (genus Parasutterella id.2892) on Solute carrier family 22 member 16
with a threshold of 1e−05
rs7311004
rs35055552
rs2090816
rs7303158
rs823424
rs11715853
rs78383039
rs2387977
rs7572229
rs8039785
rs62273907
rs55877868
rs10899911
All − MR Egger
All − IVW(MRE)
−1
0
1
MR effect size for
'Gut microbiota abundance (genus Parasutterella id.2892)' on 'Solute carrier family 22 member 16'

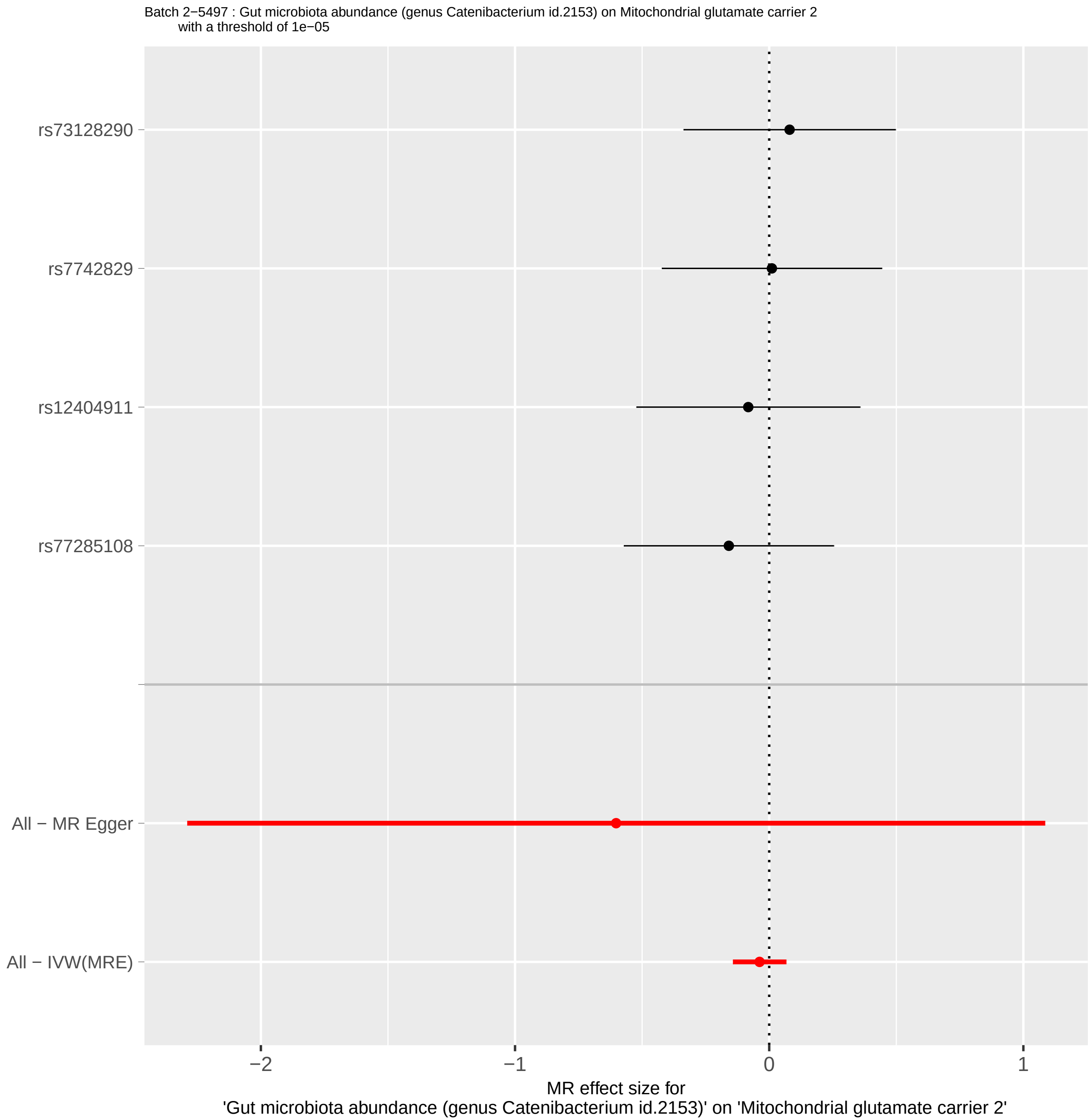
Batch 2−5497 : Gut microbiota abundance (genus Catenibacterium id.2153) on Mitochondrial glutamate carrier 2
with a threshold of 1e−05
rs73128290
rs7742829
rs12404911
rs77285108
All − MR Egger
All − IVW(MRE)
−2
−1
0
1
MR effect size for
'Gut microbiota abundance (genus Catenibacterium id.2153)' on 'Mitochondrial glutamate carrier 2'

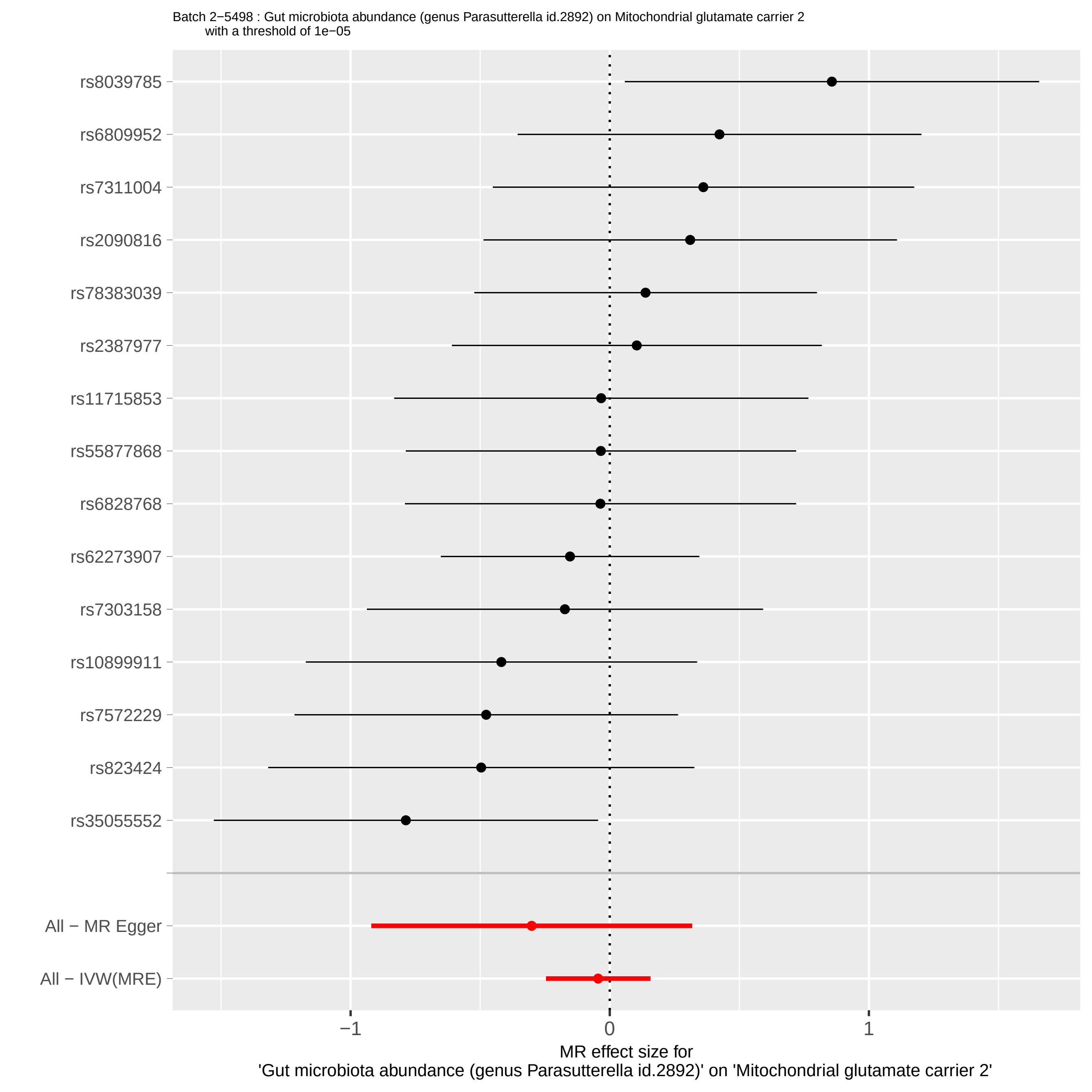

Batch 2−5498 : Gut microbiota abundance (genus Parasutterella id.2892) on Mitochondrial glutamate carrier 2
with a threshold of 1e−05
rs8039785
rs6809952
rs7311004
rs2090816
rs78383039
rs2387977
rs11715853
rs55877868
rs6828768
rs62273907
rs7303158
rs10899911
rs7572229
rs823424
rs35055552
All − MR Egger
All − IVW(MRE)
−1
0
1
MR effect size for
'Gut microbiota abundance (genus Parasutterella id.2892)' on 'Mitochondrial glutamate carrier 2'

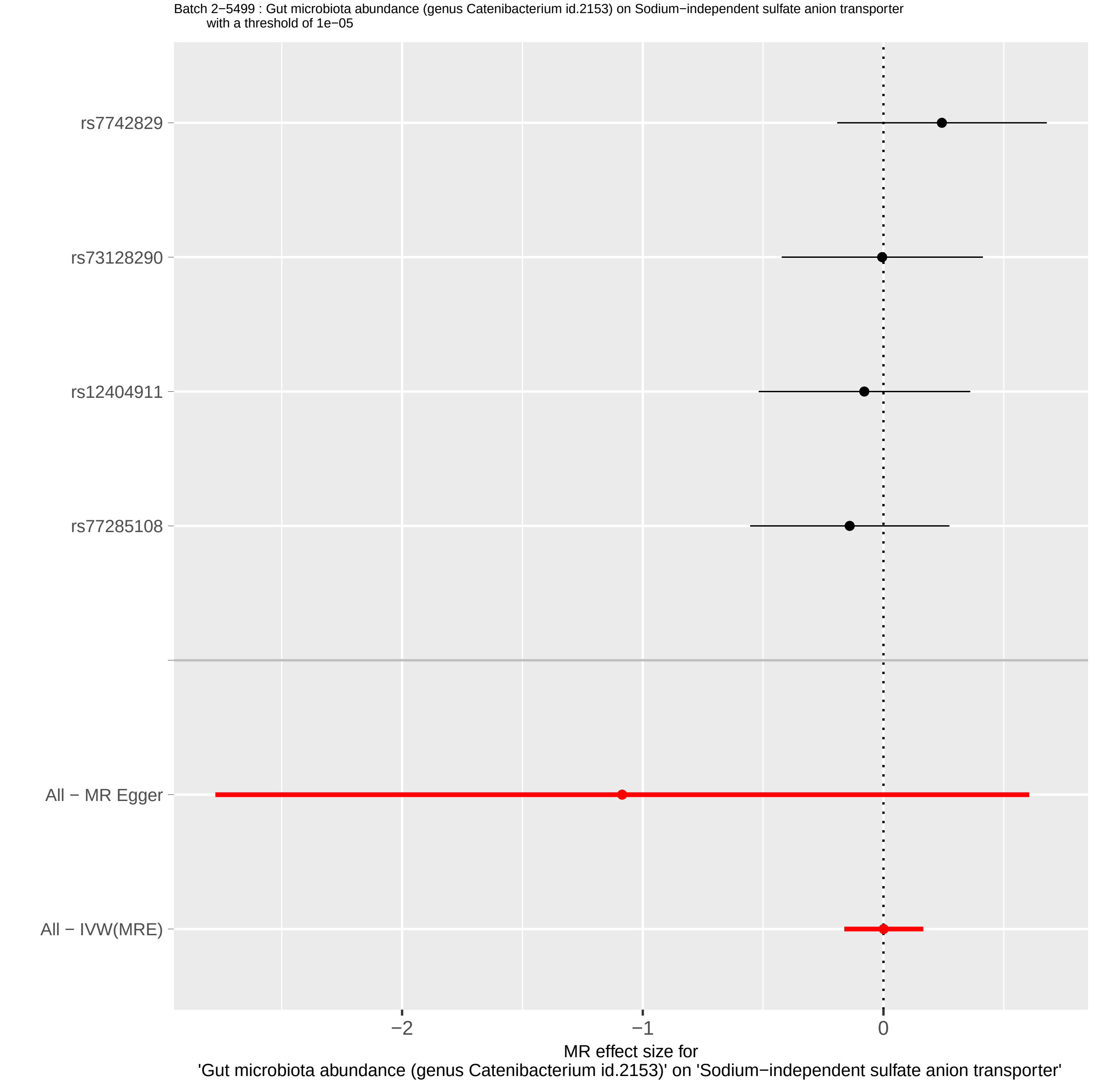

Batch 2−5499 : Gut microbiota abundance (genus Catenibacterium id.2153) on Sodium−independent sulfate anion transporter
with a threshold of 1e−05
rs7742829
rs73128290
rs12404911
rs77285108
All − MR Egger
All − IVW(MRE)
−2
−1
0
MR effect size for
'Gut microbiota abundance (genus Catenibacterium id.2153)' on 'Sodium−independent sulfate anion transporter'

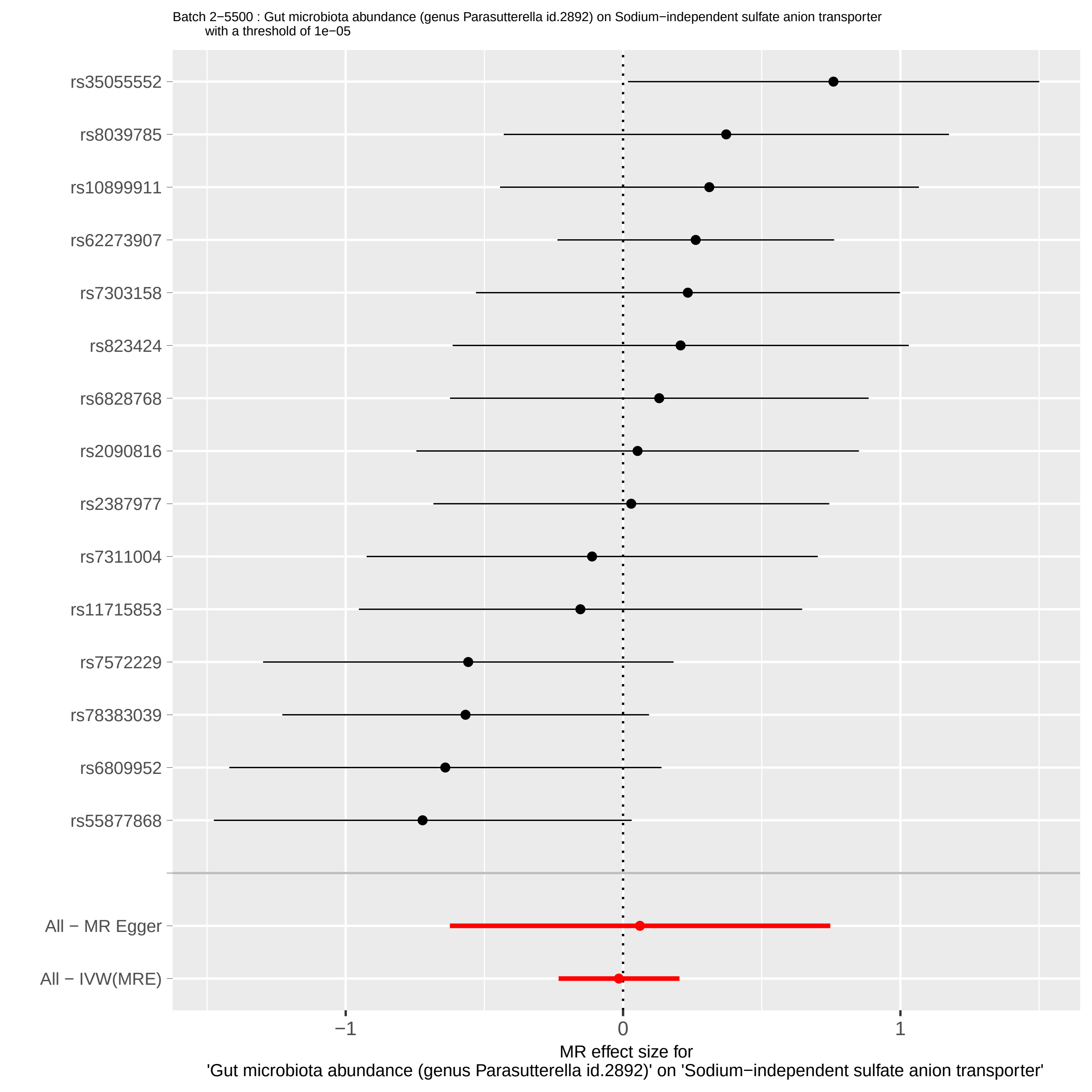

Batch 2−5500 : Gut microbiota abundance (genus Parasutterella id.2892) on Sodium−independent sulfate anion transporter
with a threshold of 1e−05
rs35055552
rs8039785
rs10899911
rs62273907
rs7303158
rs823424
rs6828768
rs2090816
rs2387977
rs7311004
rs11715853
rs7572229
rs78383039
rs6809952
rs55877868
All − MR Egger
All − IVW(MRE)
−1
0
1
MR effect size for
'Gut microbiota abundance (genus Parasutterella id.2892)' on 'Sodium−independent sulfate anion transporter'

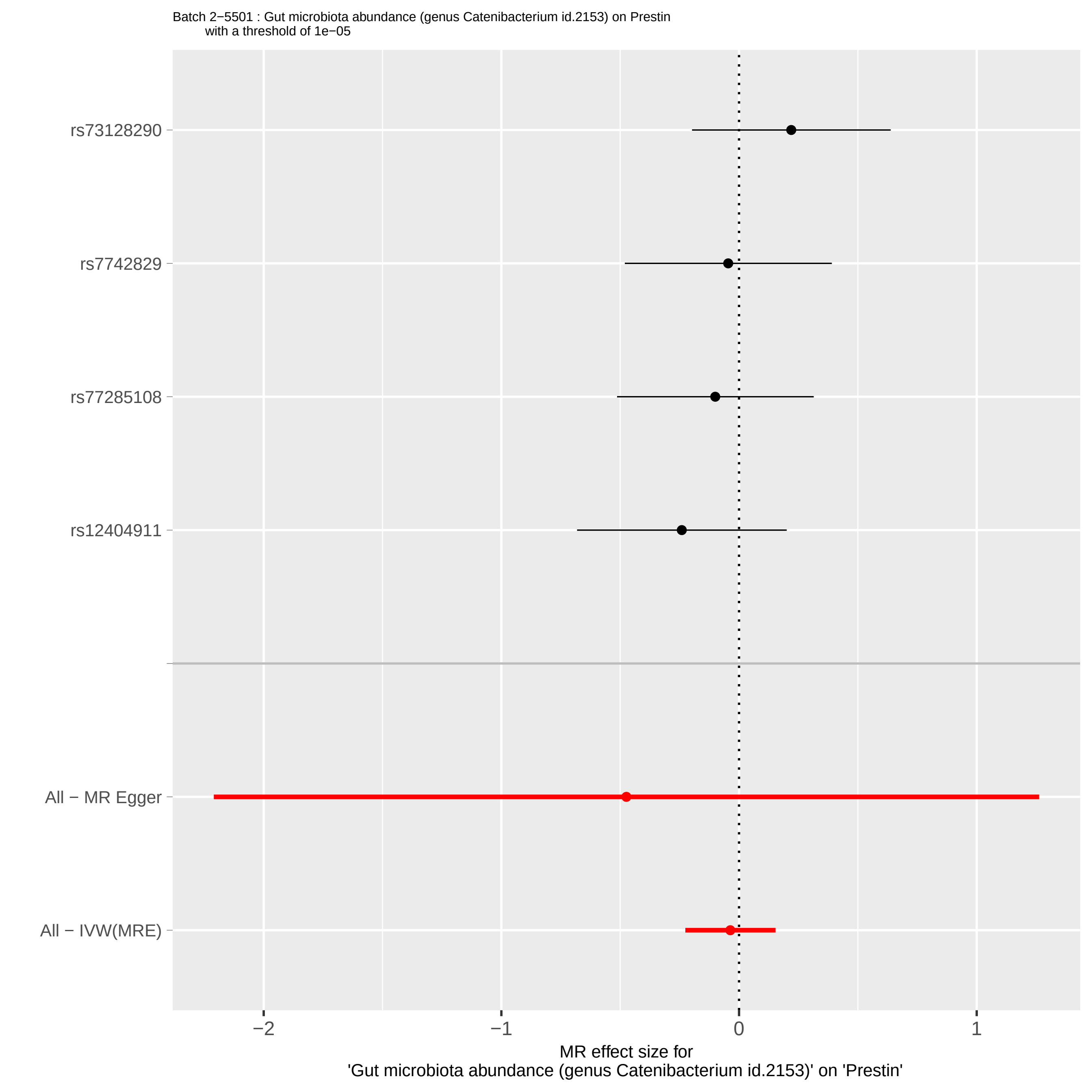
Batch 2−5501 : Gut microbiota abundance (genus Catenibacterium id.2153) on Prestin
with a threshold of 1e−05
rs73128290
rs7742829
rs77285108
rs12404911
All − MR Egger
All − IVW(MRE)
−2
−1
0
1
MR effect size for
'Gut microbiota abundance (genus Catenibacterium id.2153)' on 'Prestin'

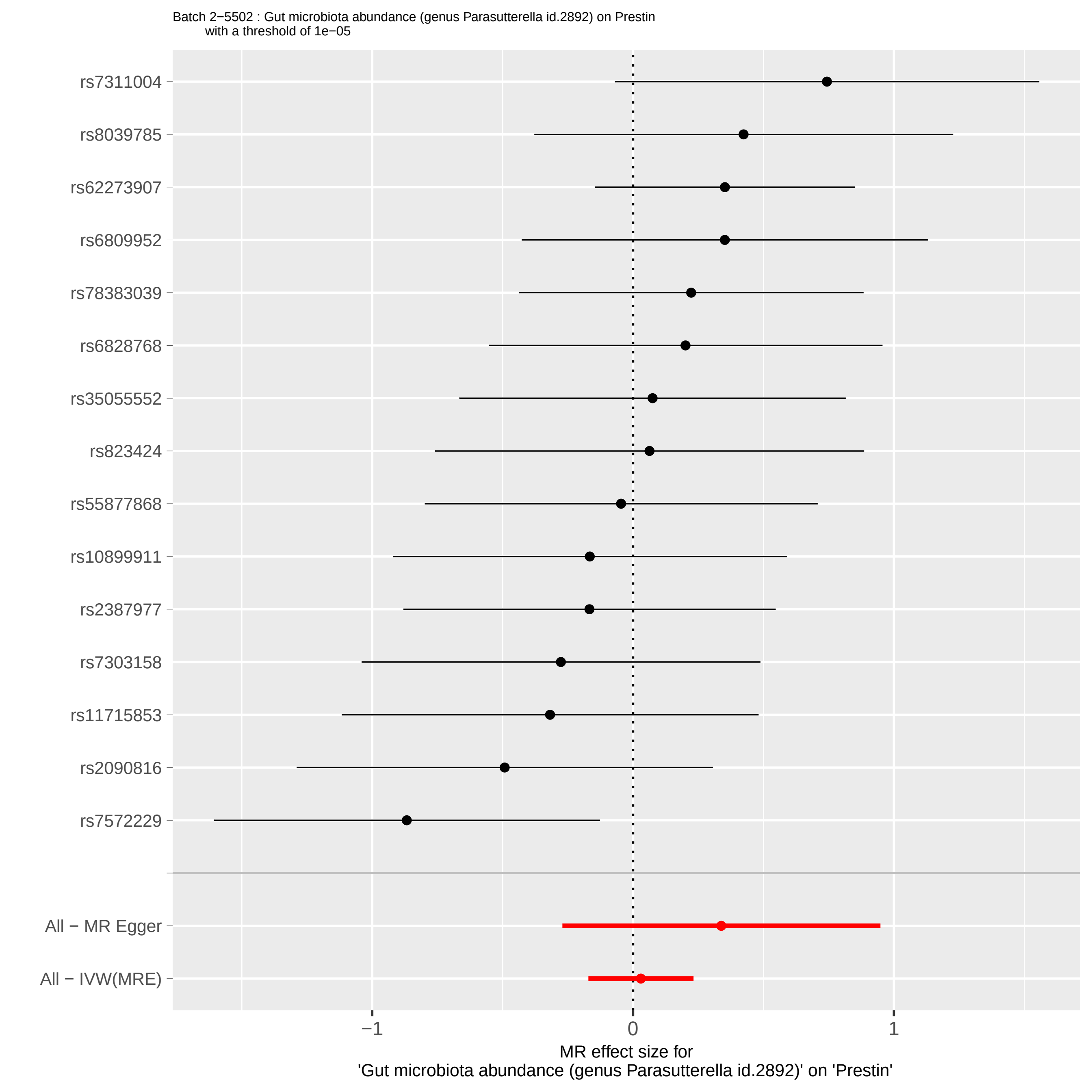

Batch 2−5502 : Gut microbiota abundance (genus Parasutterella id.2892) on Prestin
with a threshold of 1e−05
rs7311004
rs8039785
rs62273907
rs6809952
rs78383039
rs6828768
rs35055552
rs823424
rs55877868
rs10899911
rs2387977
rs7303158
rs11715853
rs2090816
rs7572229
All − MR Egger
All − IVW(MRE)
−1
0
1
MR effect size for
'Gut microbiota abundance (genus Parasutterella id.2892)' on 'Prestin'

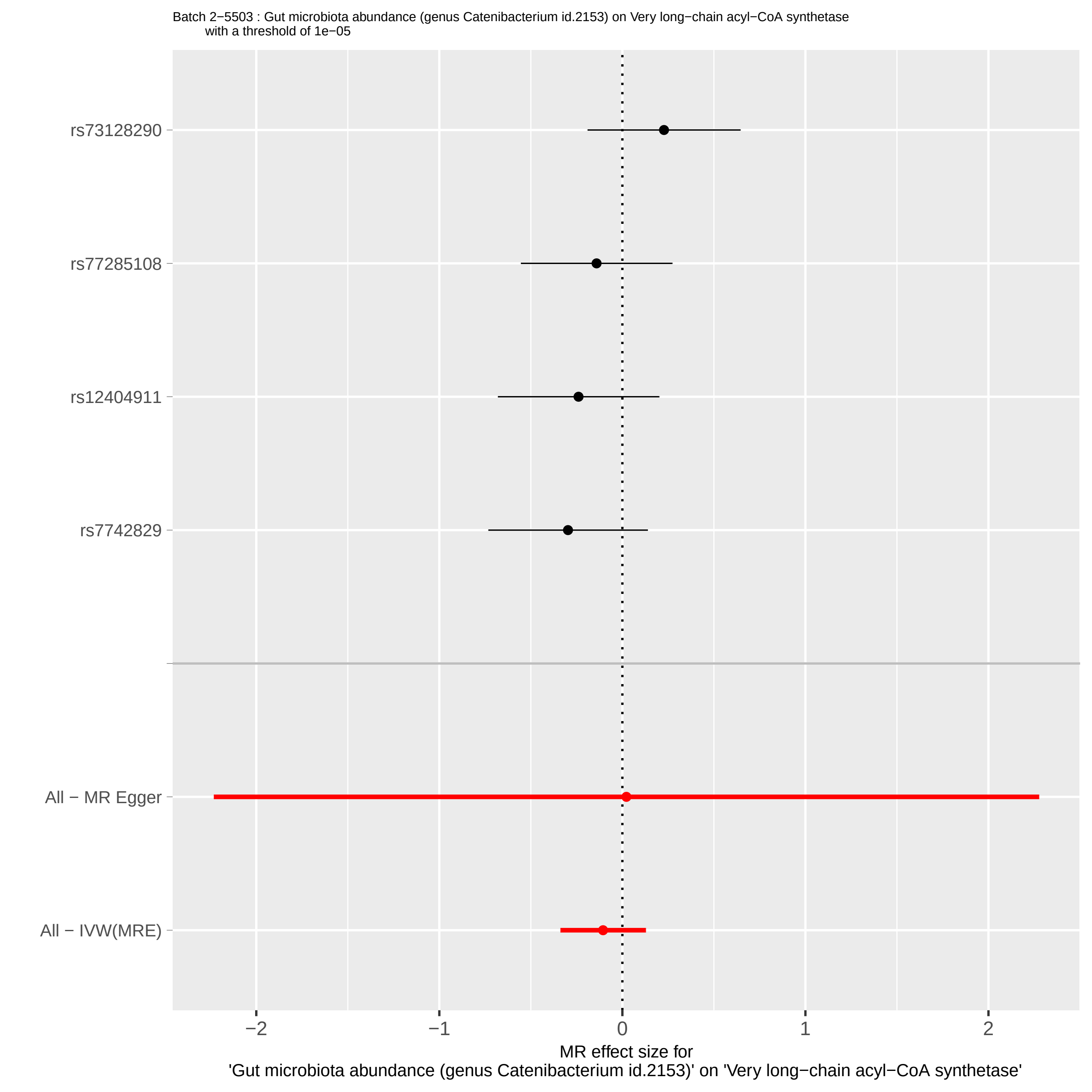
Batch 2−5503 : Gut microbiota abundance (genus Catenibacterium id.2153) on Very long−chain acyl−CoA synthetase
with a threshold of 1e−05
rs73128290
rs77285108
rs12404911
rs7742829
All − MR Egger
All − IVW(MRE)
−2
−1
0
1
2
MR effect size for
'Gut microbiota abundance (genus Catenibacterium id.2153)' on 'Very long−chain acyl−CoA synthetase'

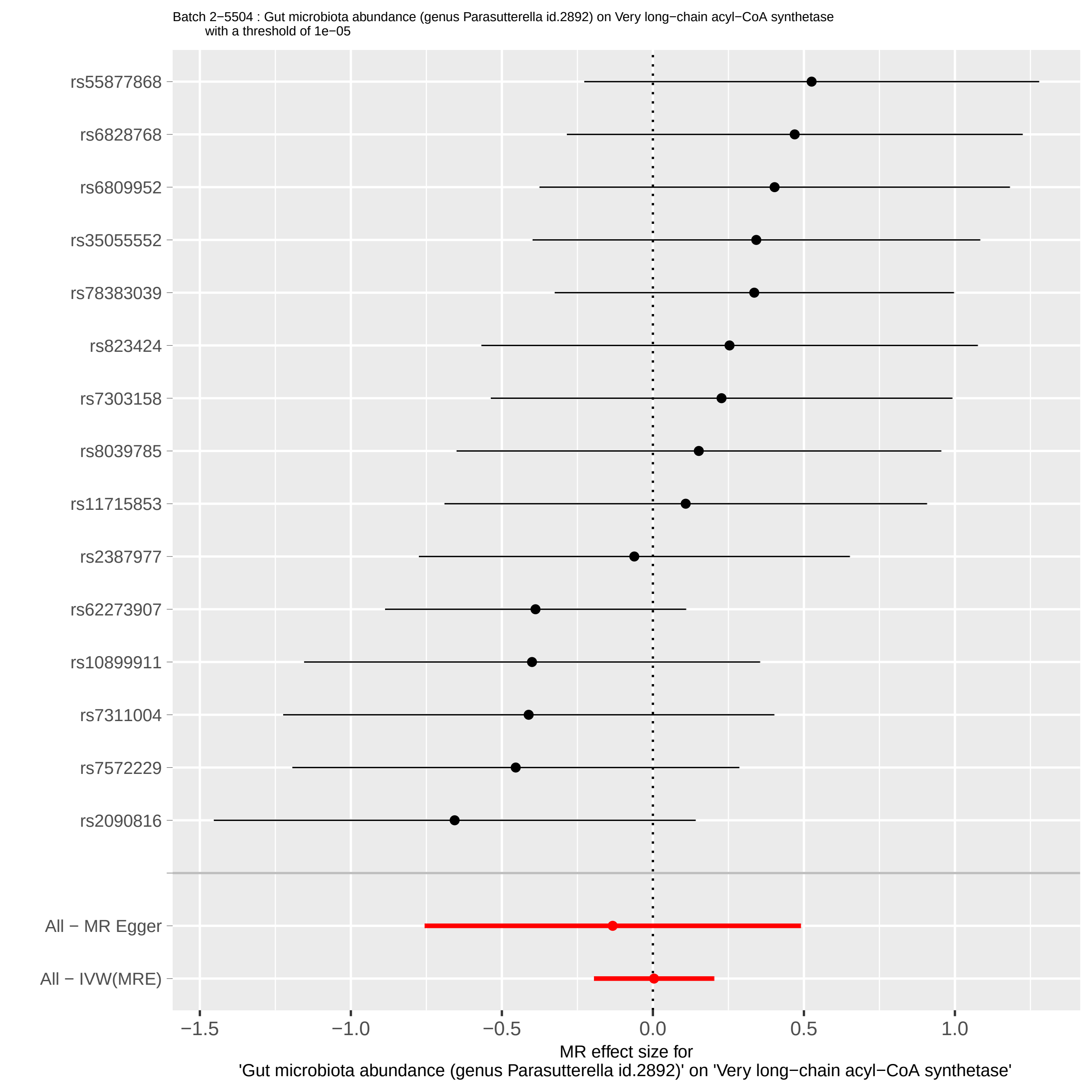

Batch 2−5504 : Gut microbiota abundance (genus Parasutterella id.2892) on Very long−chain acyl−CoA synthetase
with a threshold of 1e−05
rs55877868
rs6828768
rs6809952
rs35055552
rs78383039
rs823424
rs7303158
rs8039785
rs11715853
rs2387977
rs62273907
rs10899911
rs7311004
rs7572229
rs2090816
All − MR Egger
All − IVW(MRE)
−1.5
−1.0
−0.5
0.0
0.5
1.0
MR effect size for
'Gut microbiota abundance (genus Parasutterella id.2892)' on 'Very long−chain acyl−CoA synthetase'

Batch 2−5505 : Gut microbiota abundance (genus Catenibacterium id.2153) on Zinc transporter 3
with a threshold of 1e−05

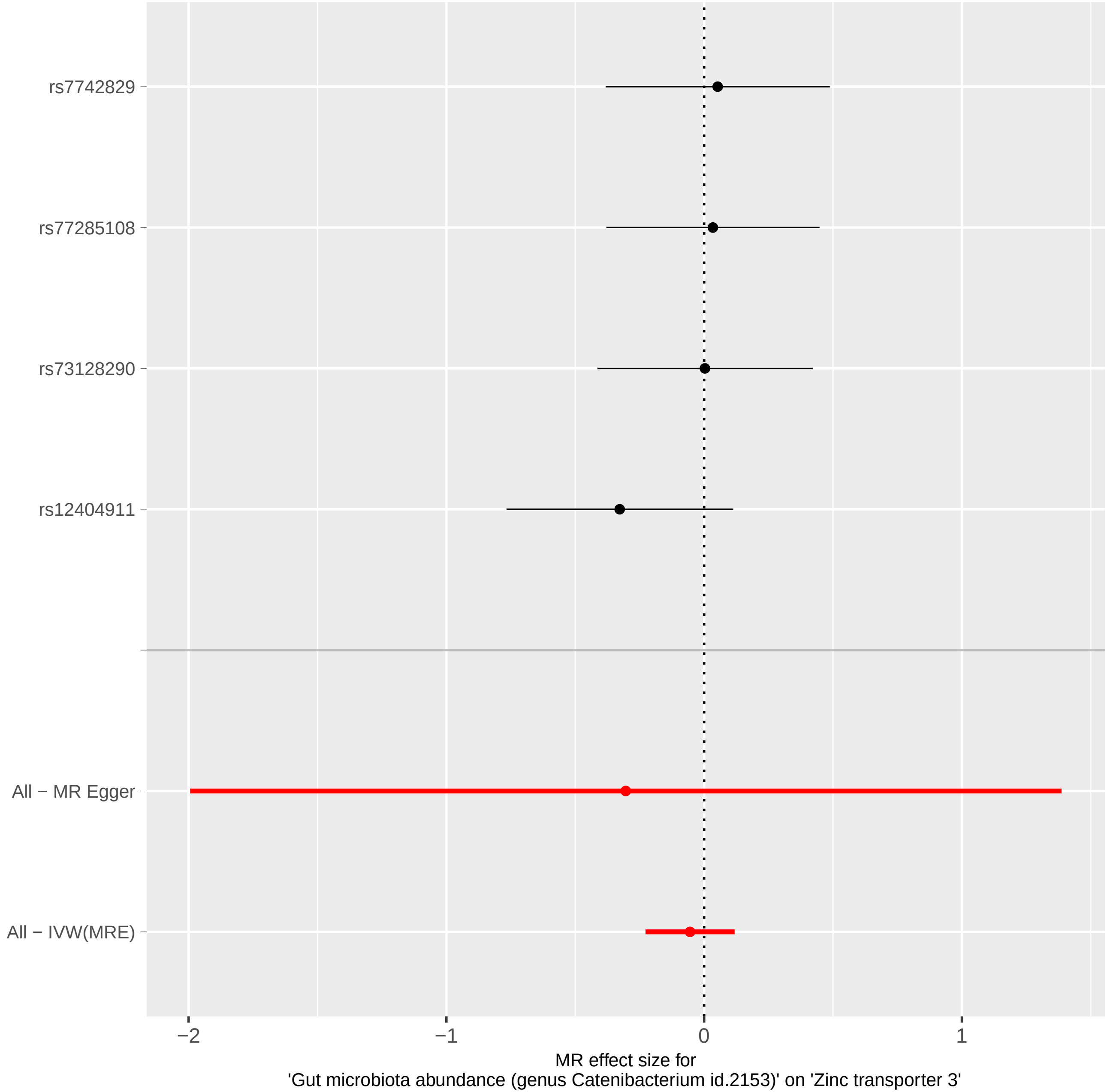

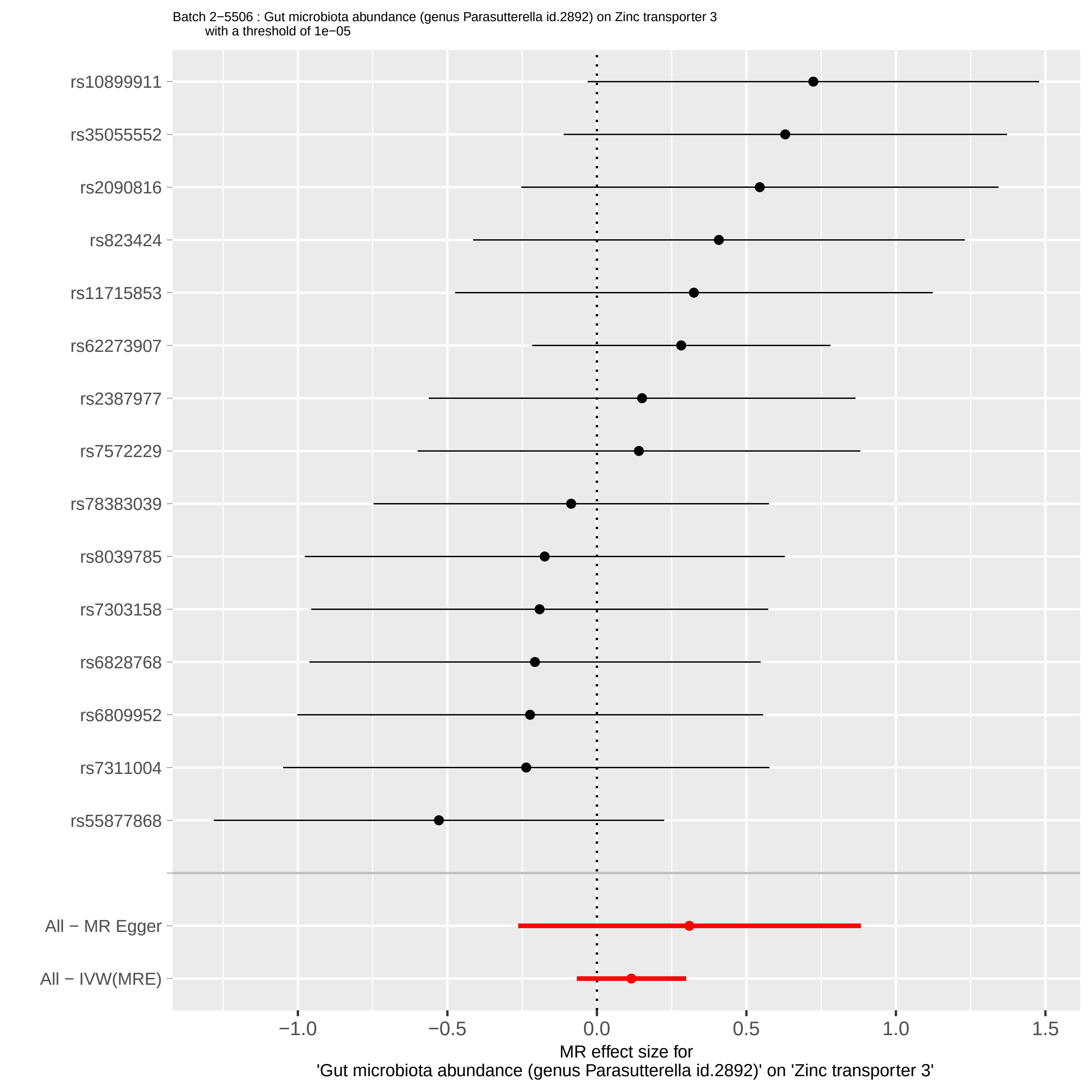

Batch 2−5506 : Gut microbiota abundance (genus Parasutterella id.2892) on Zinc transporter 3
with a threshold of 1e−05
rs10899911
rs35055552
rs2090816
rs823424
rs11715853
rs62273907
rs2387977
rs7572229
rs78383039
rs8039785
rs7303158
rs6828768
rs6809952
rs7311004
rs55877868
All − MR Egger
All − IVW(MRE)
−1.0
−0.5
0.0
0.5
1.0
1.5
MR effect size for
'Gut microbiota abundance (genus Parasutterella id.2892)' on 'Zinc transporter 3'

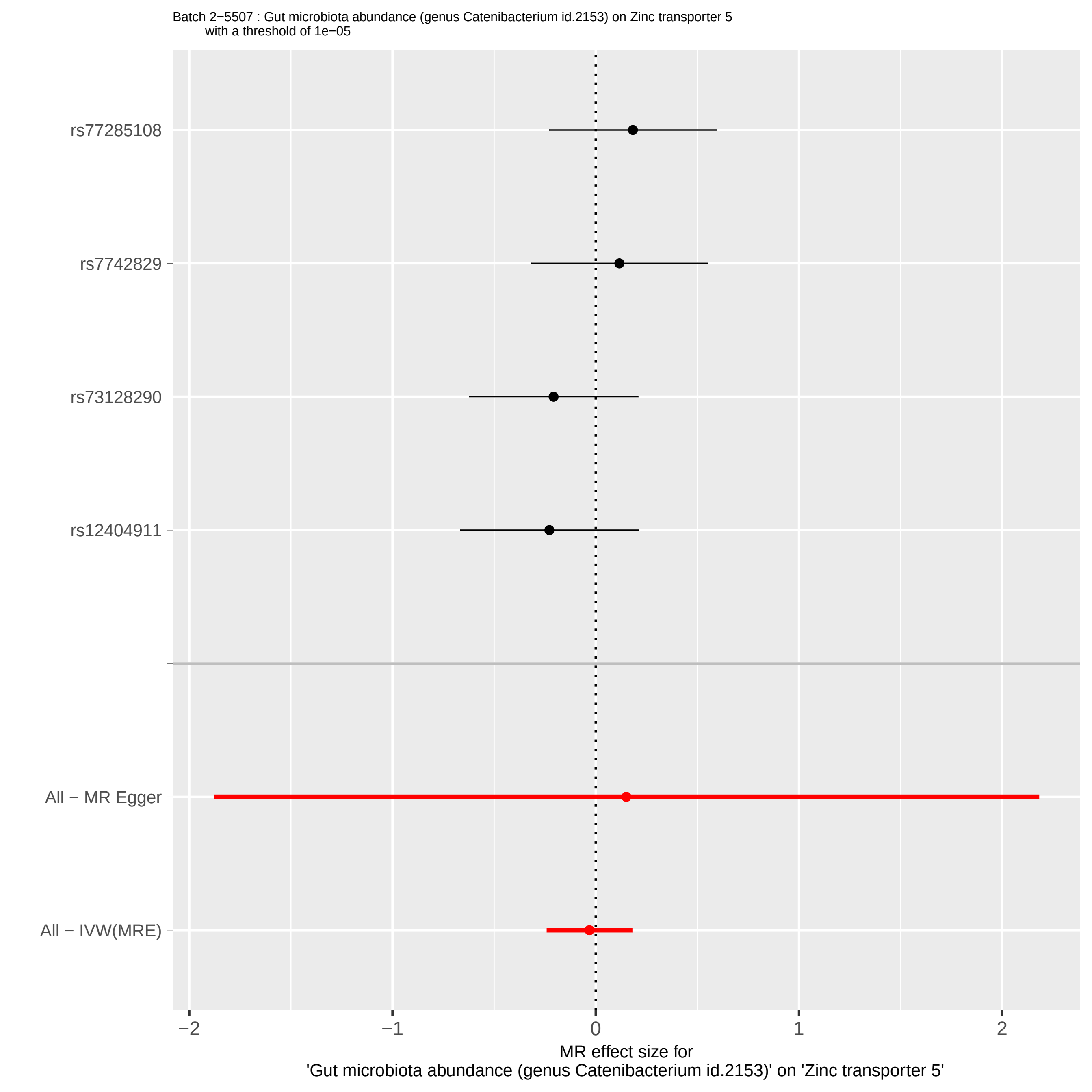
Batch 2−5507 : Gut microbiota abundance (genus Catenibacterium id.2153) on Zinc transporter 5
with a threshold of 1e−05
rs77285108
rs7742829
rs73128290
rs12404911
All − MR Egger
All − IVW(MRE)
−2
−1
0
1
2
MR effect size for
'Gut microbiota abundance (genus Catenibacterium id.2153)' on 'Zinc transporter 5'

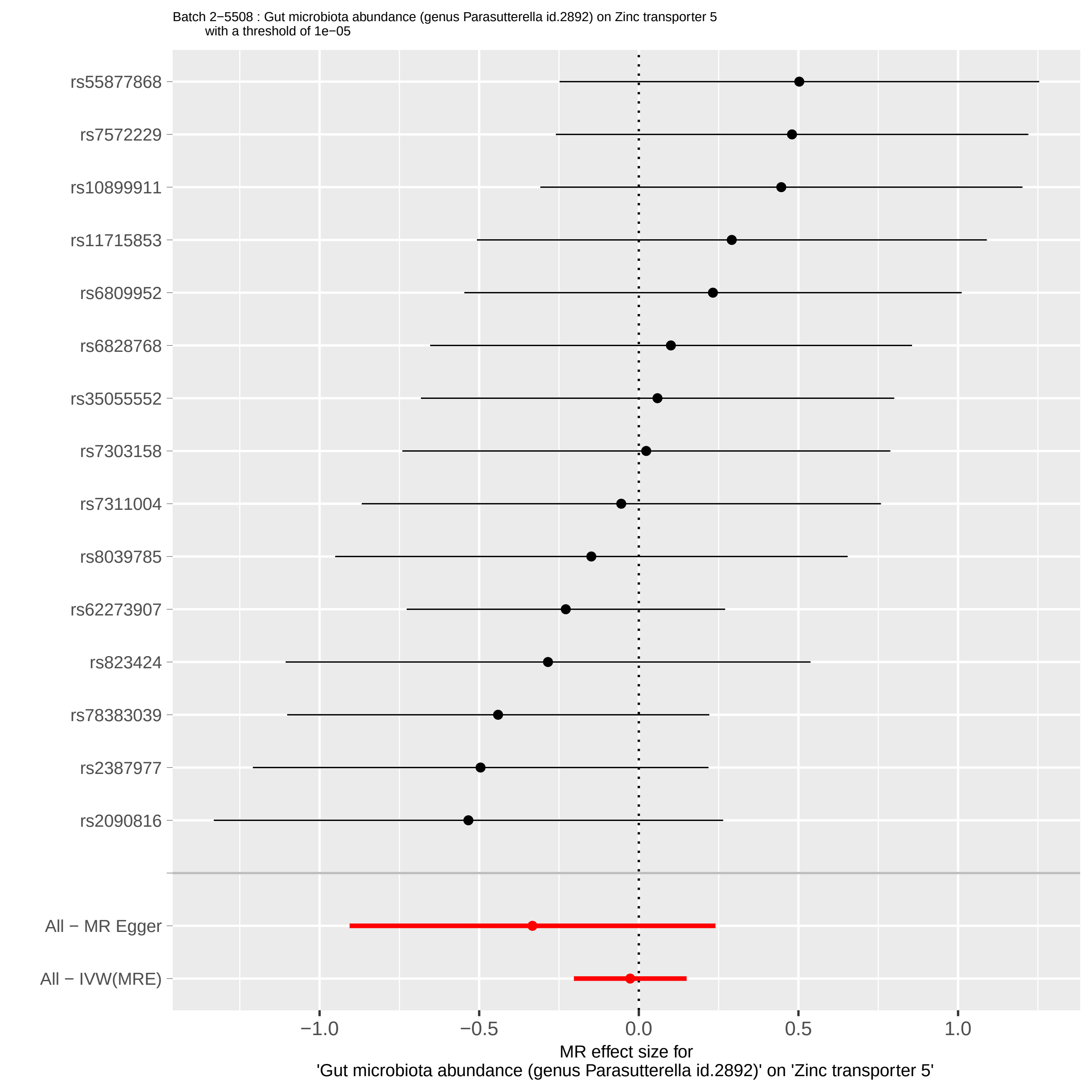
Batch 2−5508 : Gut microbiota abundance (genus Parasutterella id.2892) on Zinc transporter 5
with a threshold of 1e−05
rs55877868
rs7572229
rs10899911
rs11715853
rs6809952
rs6828768
rs35055552
rs7303158
rs7311004
rs8039785
rs62273907
rs823424
rs78383039
rs2387977
rs2090816
All − MR Egger
All − IVW(MRE)
−1.0
−0.5
0.0
0.5
1.0
MR effect size for
'Gut microbiota abundance (genus Parasutterella id.2892)' on 'Zinc transporter 5'

Batch 2−5509 : Gut microbiota abundance (genus Catenibacterium id.2153) on Adenosine 3'−phospho 5'−phosphosulfate transporter 2 with a threshold of 1e−05

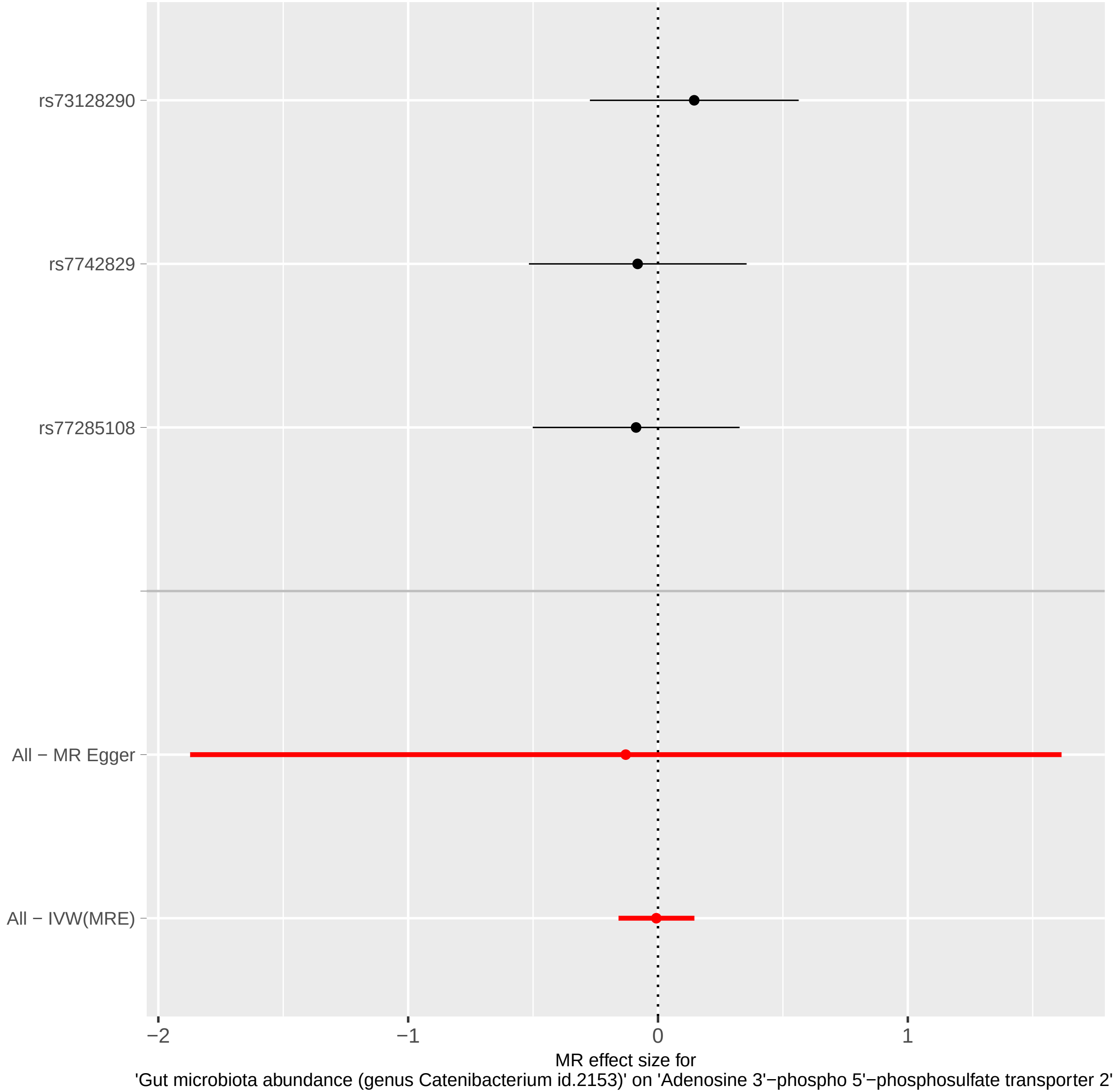

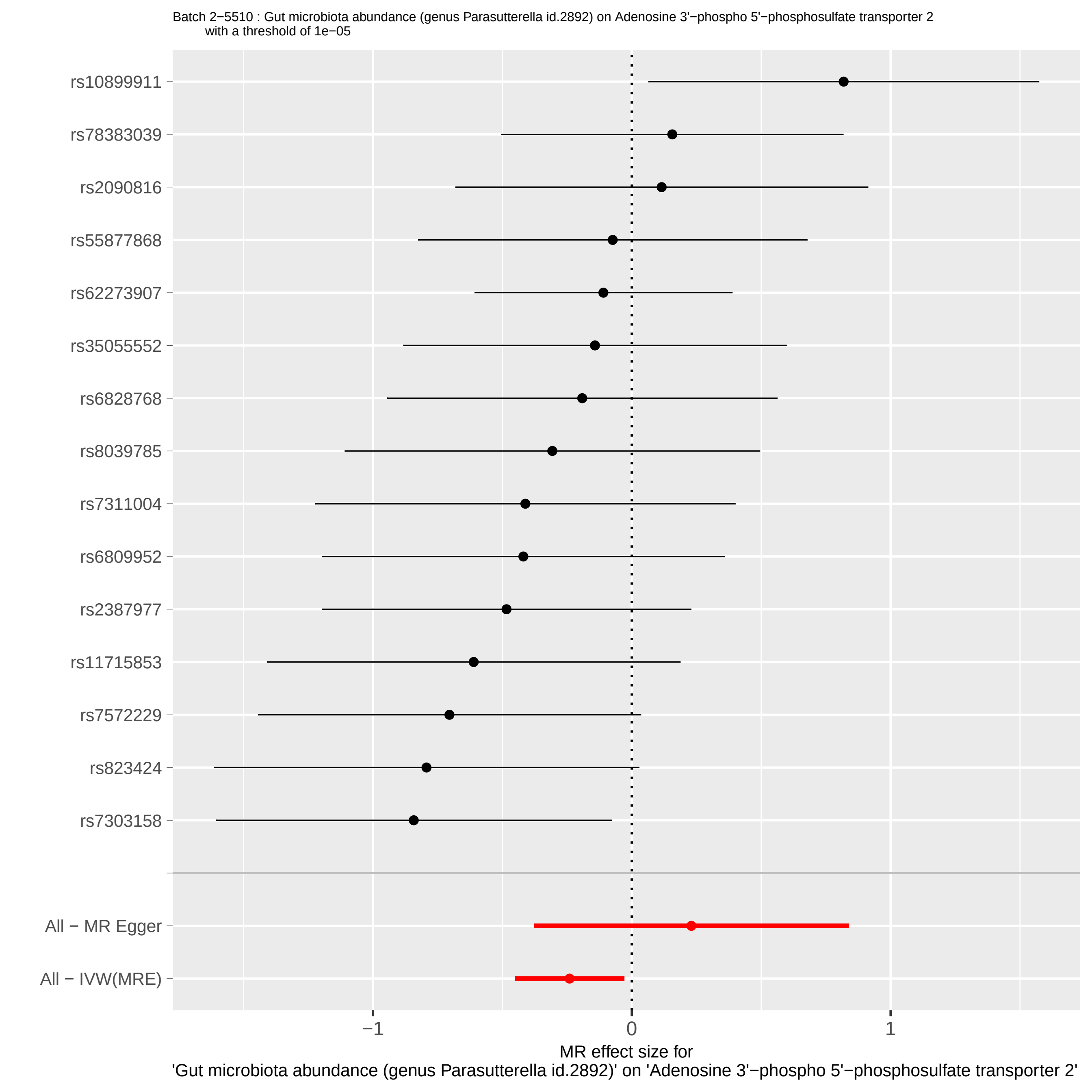

Batch 2−5510 : Gut microbiota abundance (genus Parasutterella id.2892) on Adenosine 3'−phospho 5'−phosphosulfate transporter 2
with a threshold of 1e−05
rs10899911
rs78383039
rs2090816
rs55877868
rs62273907
rs35055552
rs6828768
rs8039785
rs7311004
rs6809952
rs2387977
rs11715853
rs7572229
rs823424
rs7303158
All − MR Egger
All − IVW(MRE)
−1
0
1
MR effect size for
'Gut microbiota abundance (genus Parasutterella id.2892)' on 'Adenosine 3'−phospho 5'−phosphosulfate transporter 2'

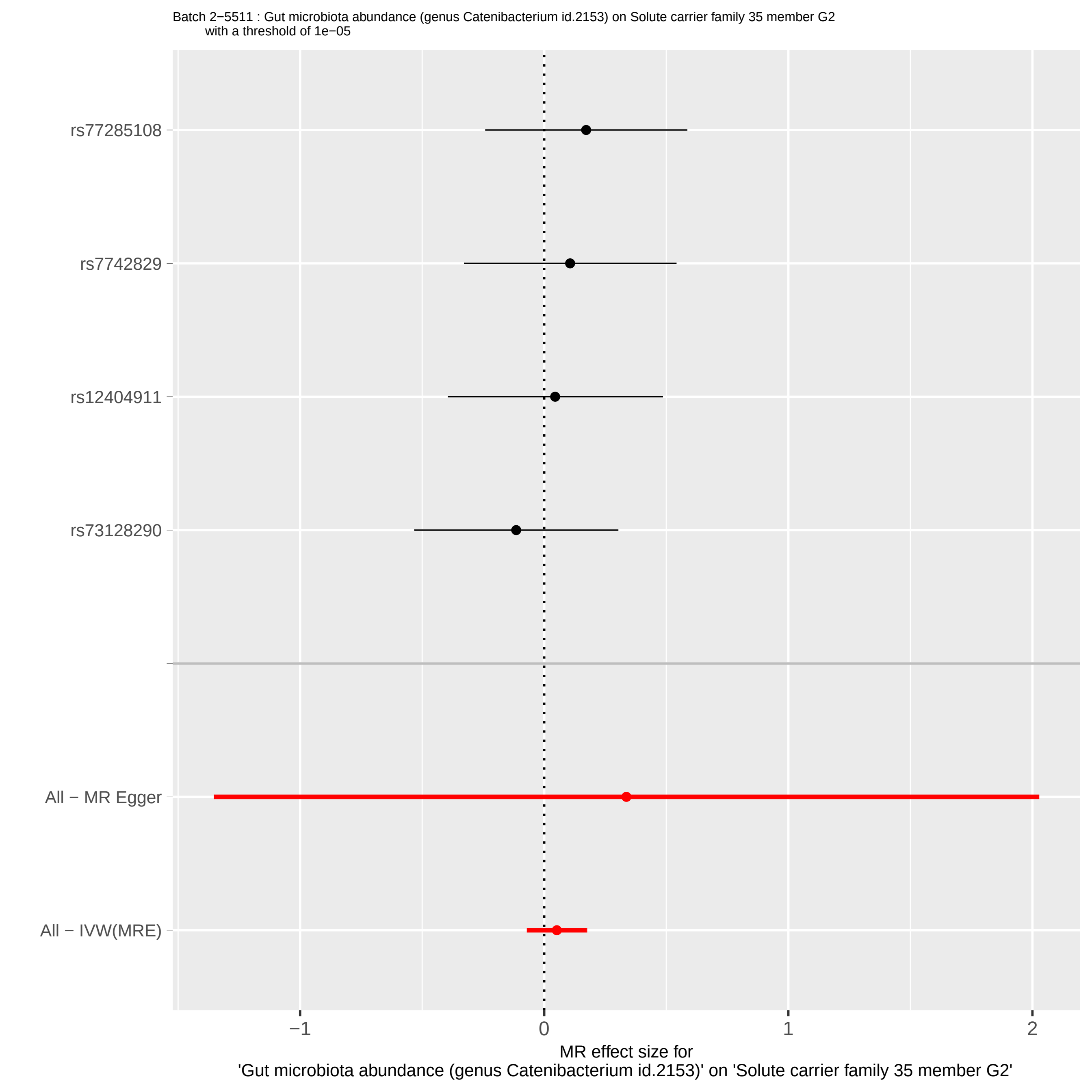

Batch 2−5511 : Gut microbiota abundance (genus Catenibacterium id.2153) on Solute carrier family 35 member G2
with a threshold of 1e−05
rs77285108
rs7742829
rs12404911
rs73128290
All − MR Egger
All − IVW(MRE)
−1
0
1
2
MR effect size for
'Gut microbiota abundance (genus Catenibacterium id.2153)' on 'Solute carrier family 35 member G2'

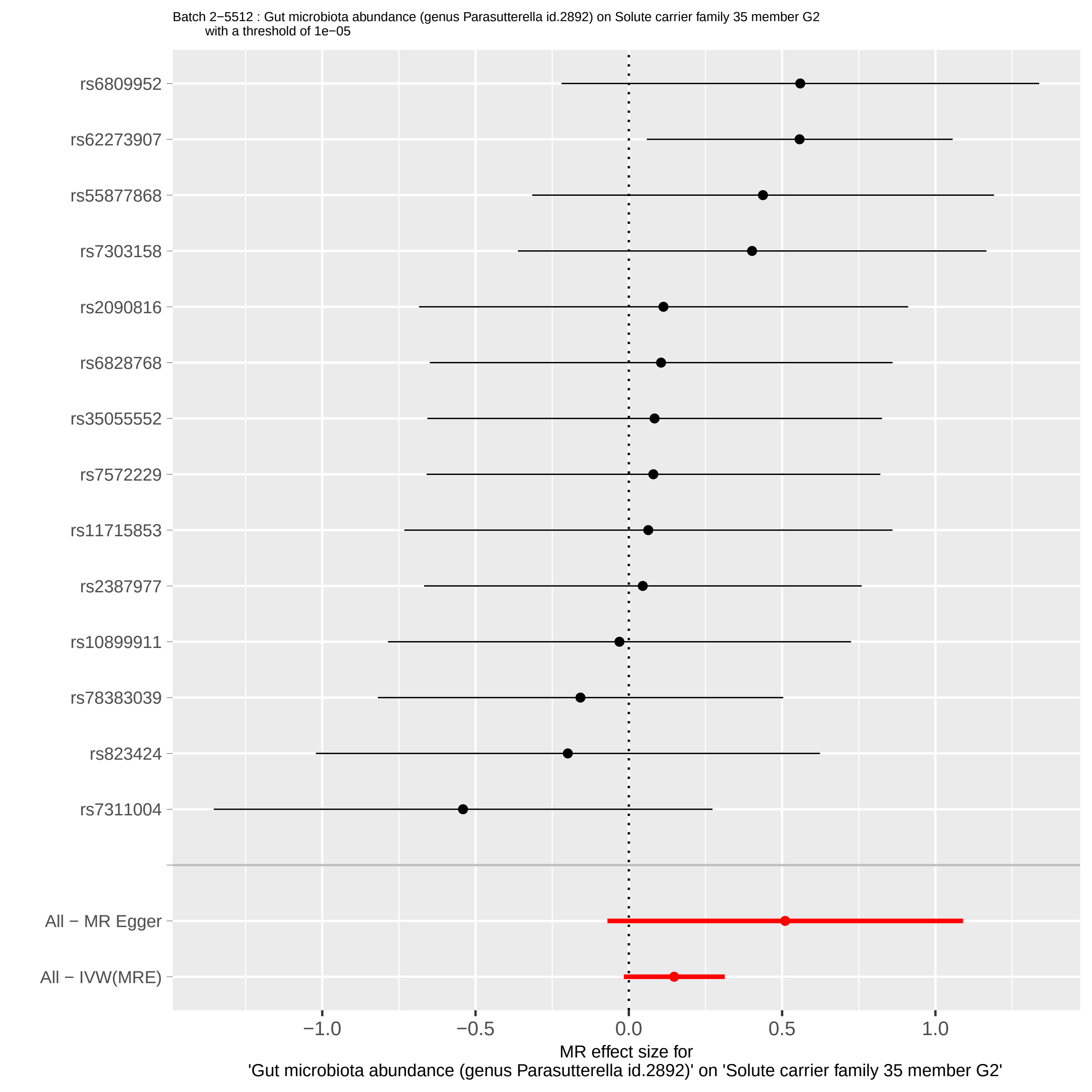

Batch 2−5512 : Gut microbiota abundance (genus Parasutterella id.2892) on Solute carrier family 35 member G2
with a threshold of 1e−05
rs6809952
rs62273907
rs55877868
rs7303158
rs2090816
rs6828768
rs35055552
rs7572229
rs11715853
rs2387977
rs10899911
rs78383039
rs823424
rs7311004
All − MR Egger
All − IVW(MRE)
−1.0
−0.5
0.0
0.5
1.0
MR effect size for
'Gut microbiota abundance (genus Parasutterella id.2892)' on 'Solute carrier family 35 member G2'

Batch 2−5513 : Gut microbiota abundance (genus Catenibacterium id.2153) on Solute carrier family 41 member 2
with a threshold of 1e−05

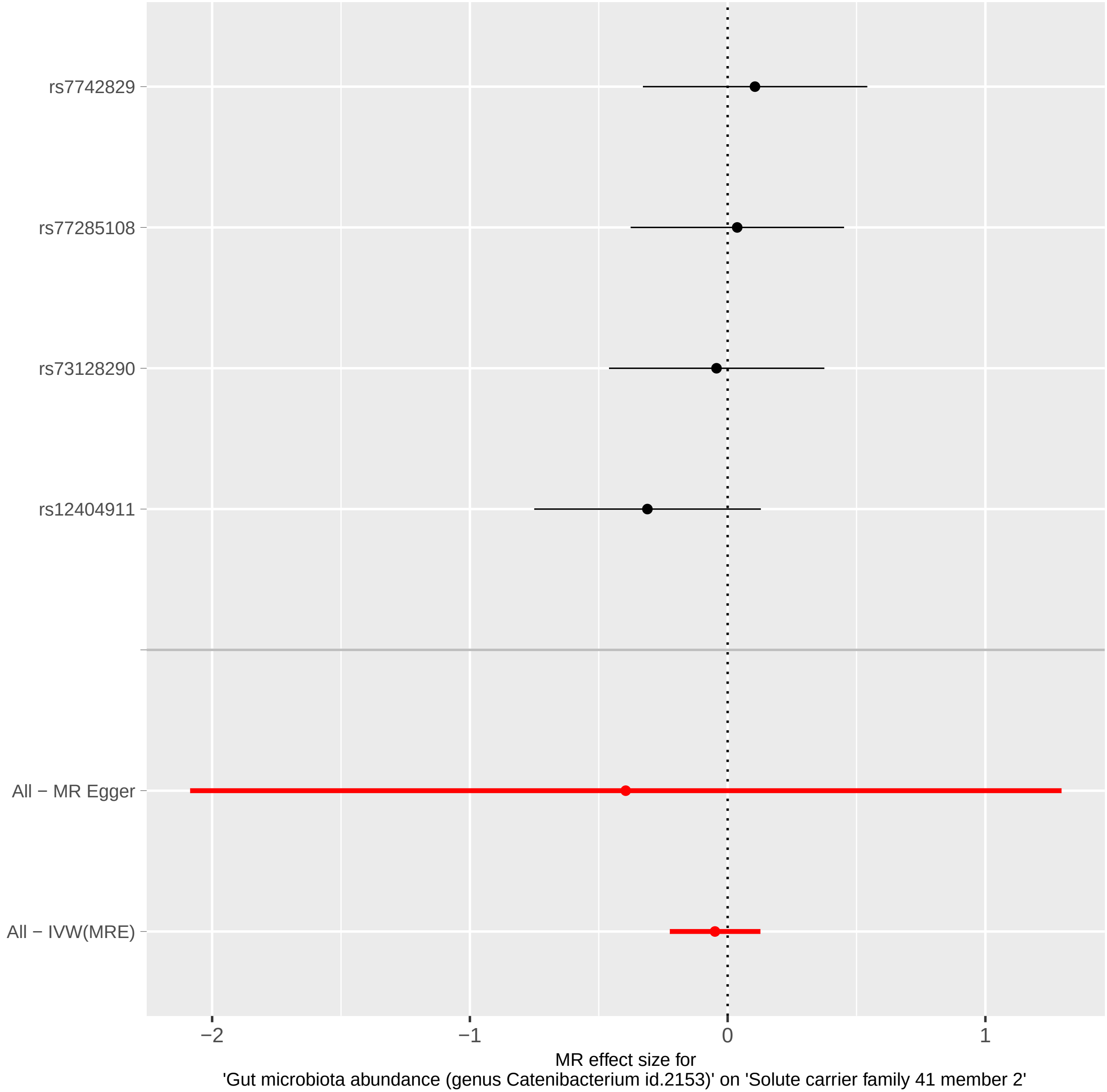

Batch 2−5514 : Gut microbiota abundance (genus Parasutterella id.2892) on Solute carrier family 41 member 2
with a threshold of 1e−05

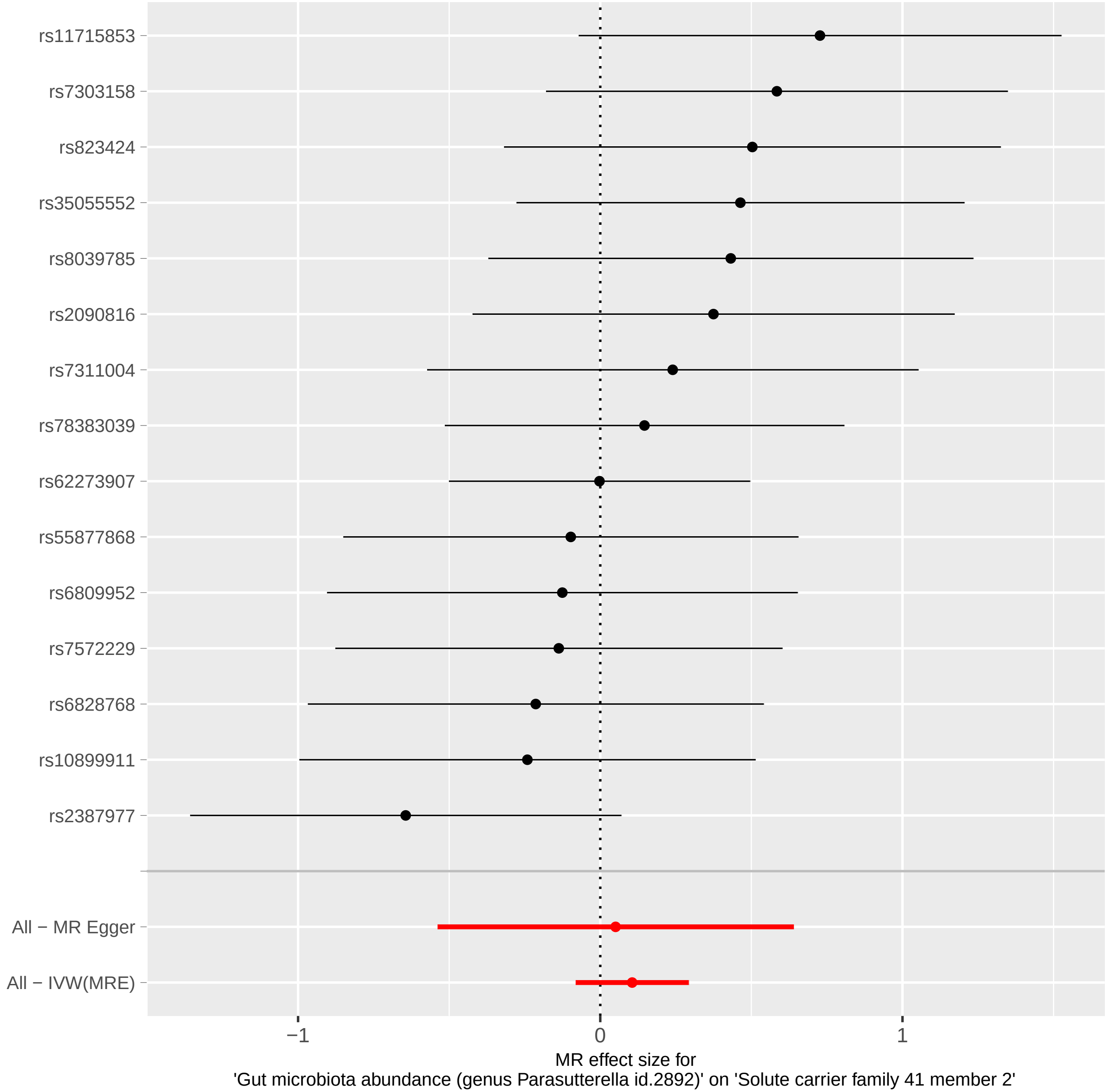

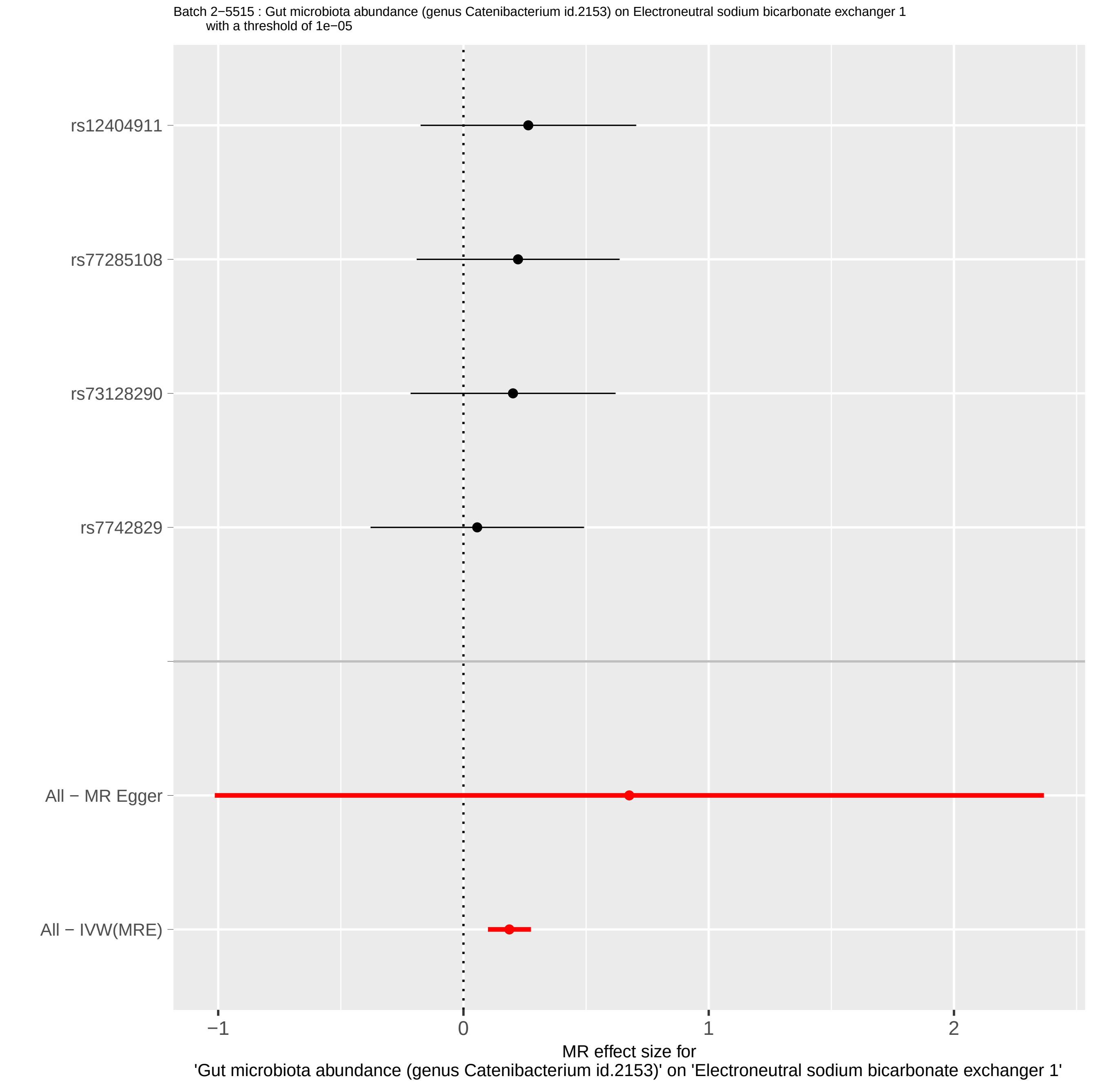

Batch 2−5515 : Gut microbiota abundance (genus Catenibacterium id.2153) on Electroneutral sodium bicarbonate exchanger 1
with a threshold of 1e−05
rs12404911
rs77285108
rs73128290
rs7742829
All − MR Egger
All − IVW(MRE)
−1
0
1
2
MR effect size for
'Gut microbiota abundance (genus Catenibacterium id.2153)' on 'Electroneutral sodium bicarbonate exchanger 1'

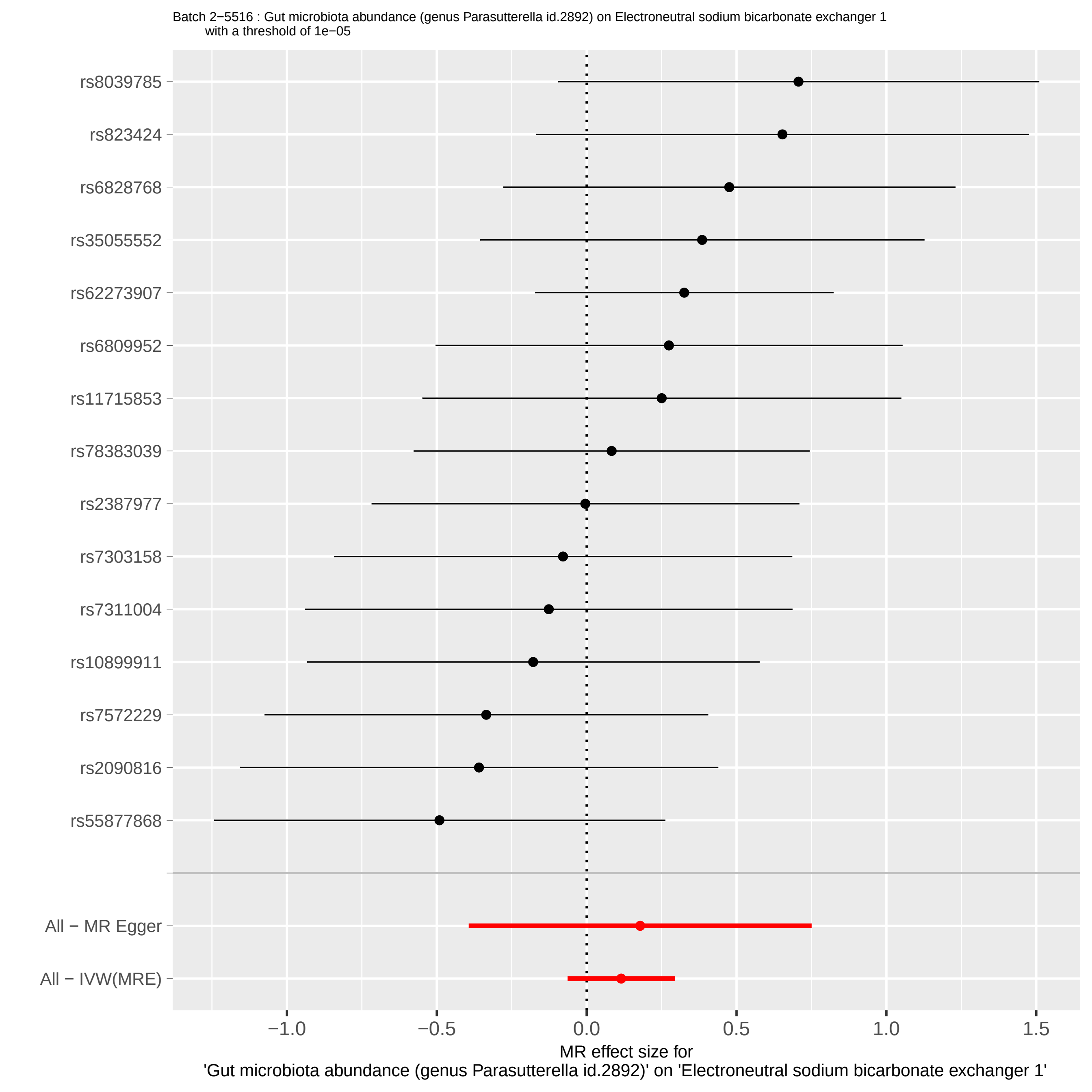

Batch 2−5516 : Gut microbiota abundance (genus Parasutterella id.2892) on Electroneutral sodium bicarbonate exchanger 1
with a threshold of 1e−05
rs8039785
rs823424
rs6828768
rs35055552
rs62273907
rs6809952
rs11715853
rs78383039
rs2387977
rs7303158
rs7311004
rs10899911
rs7572229
rs2090816
rs55877868
All − MR Egger
All − IVW(MRE)
−1.0
−0.5
0.0
0.5
1.0
1.5
MR effect size for
'Gut microbiota abundance (genus Parasutterella id.2892)' on 'Electroneutral sodium bicarbonate exchanger 1'

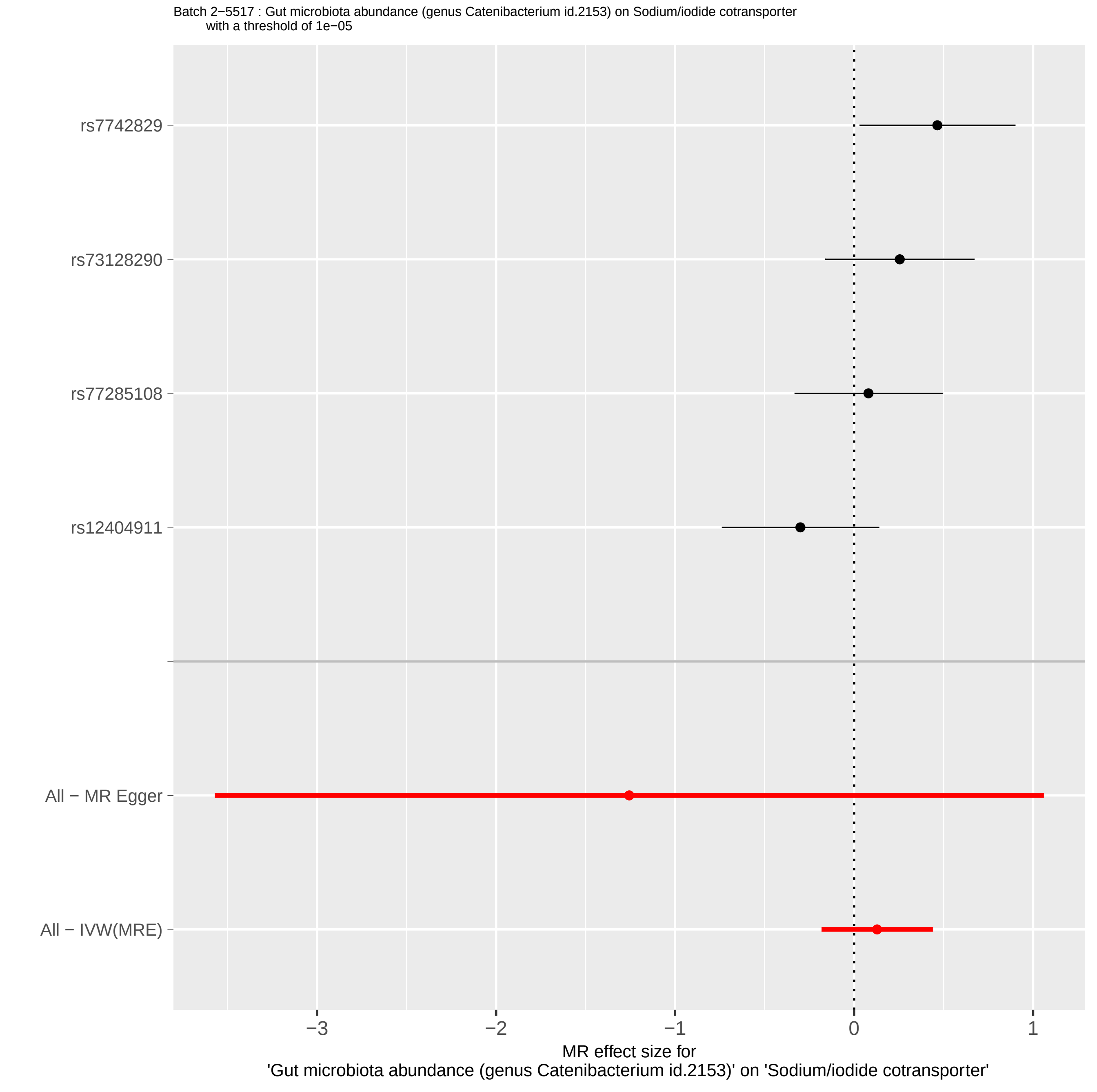

Batch 2−5517 : Gut microbiota abundance (genus Catenibacterium id.2153) on Sodium/iodide cotransporter
with a threshold of 1e−05
rs7742829
rs73128290
rs77285108
rs12404911
All − MR Egger
All − IVW(MRE)
−3
−2
−1
0
1
MR effect size for
'Gut microbiota abundance (genus Catenibacterium id.2153)' on 'Sodium/iodide cotransporter'

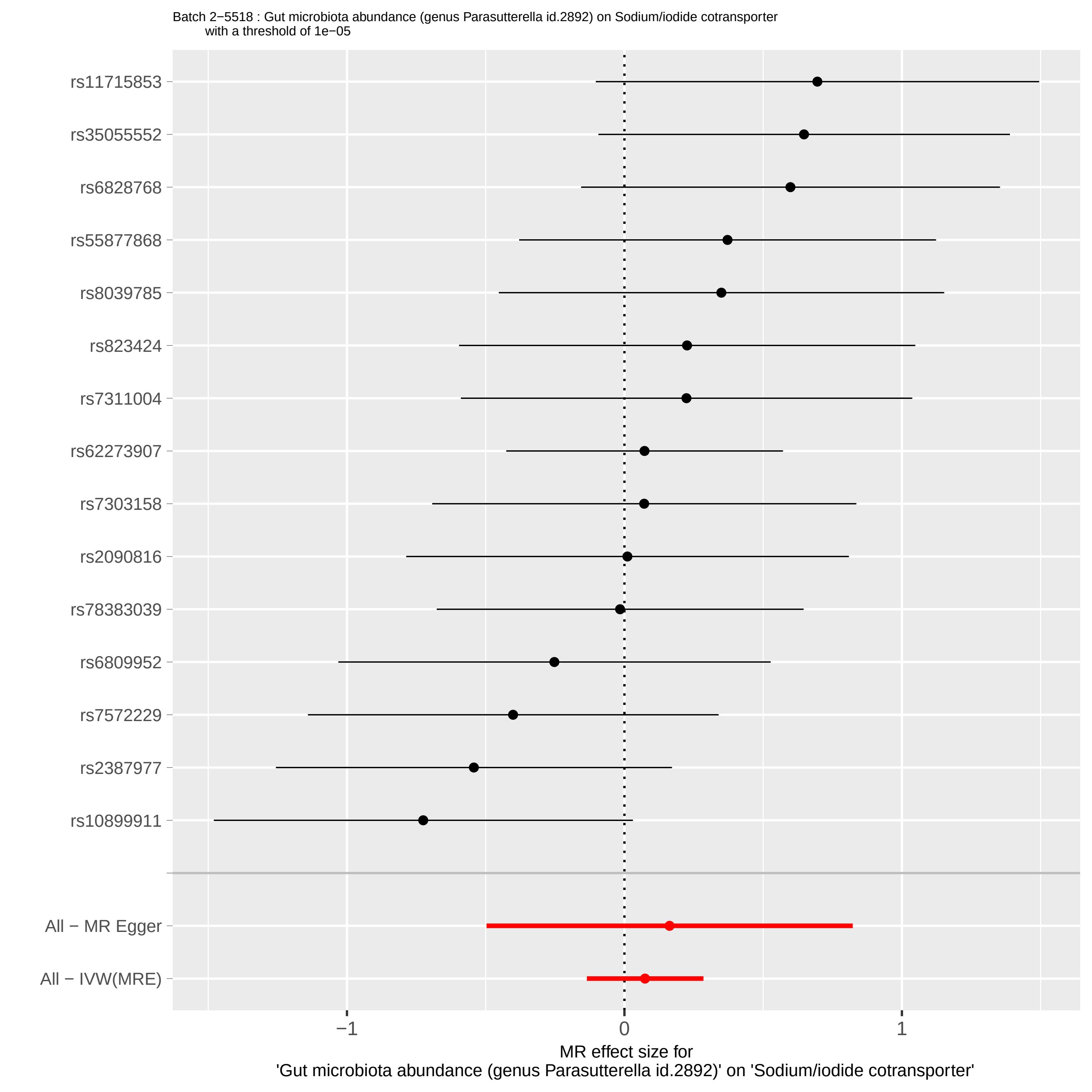
Batch 2−5518 : Gut microbiota abundance (genus Parasutterella id.2892) on Sodium/iodide cotransporter
with a threshold of 1e−05
rs11715853
rs35055552
rs6828768
rs55877868
rs8039785
rs823424
rs7311004
rs62273907
rs7303158
rs2090816
rs78383039
rs6809952
rs7572229
rs2387977
rs10899911
All − MR Egger
All − IVW(MRE)
−1
0
1
MR effect size for
'Gut microbiota abundance (genus Parasutterella id.2892)' on 'Sodium/iodide cotransporter'

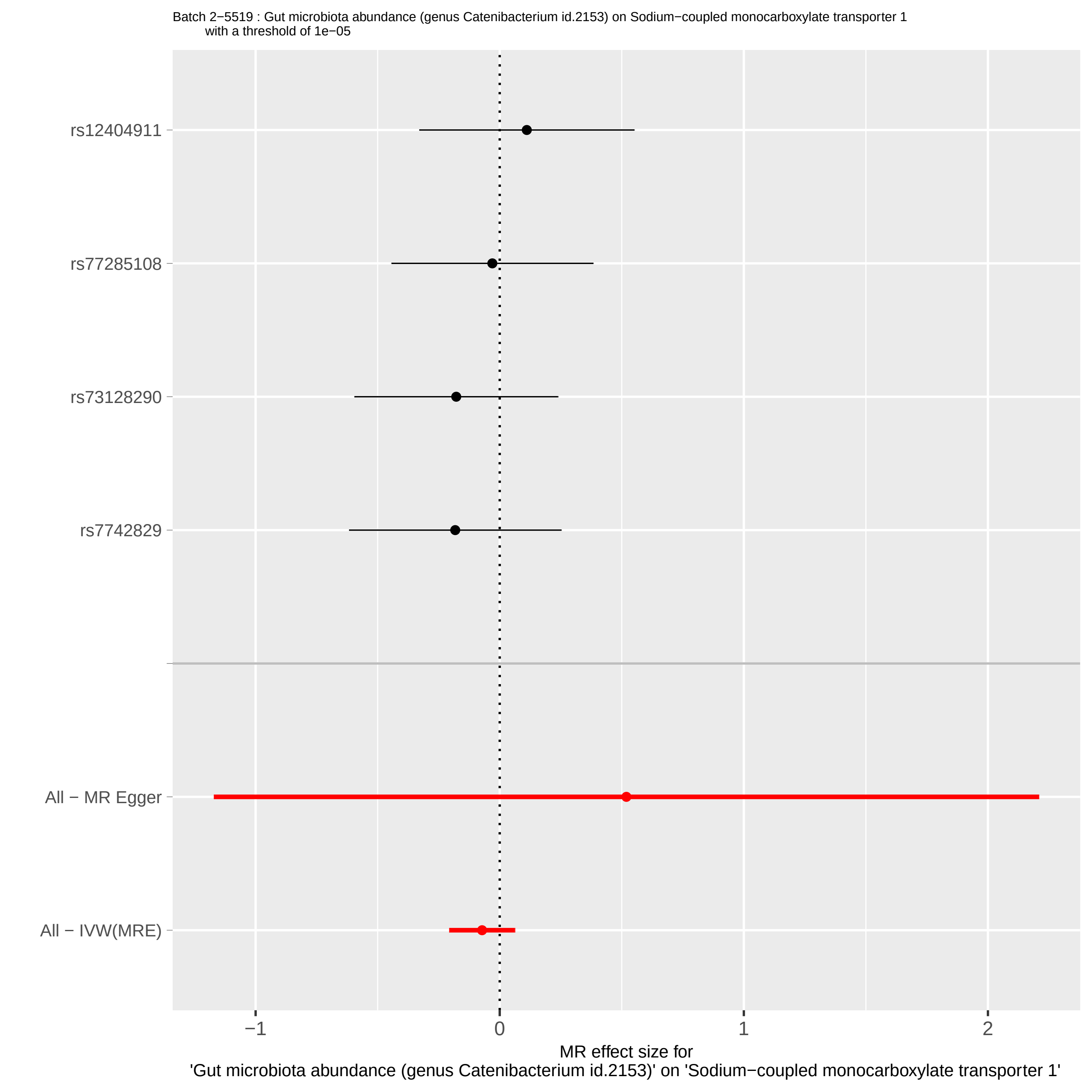

Batch 2−5519 : Gut microbiota abundance (genus Catenibacterium id.2153) on Sodium−coupled monocarboxylate transporter 1
with a threshold of 1e−05
rs12404911
rs77285108
rs73128290
rs7742829
All − MR Egger
All − IVW(MRE)
−1
0
1
2
MR effect size for
'Gut microbiota abundance (genus Catenibacterium id.2153)' on 'Sodium−coupled monocarboxylate transporter 1'

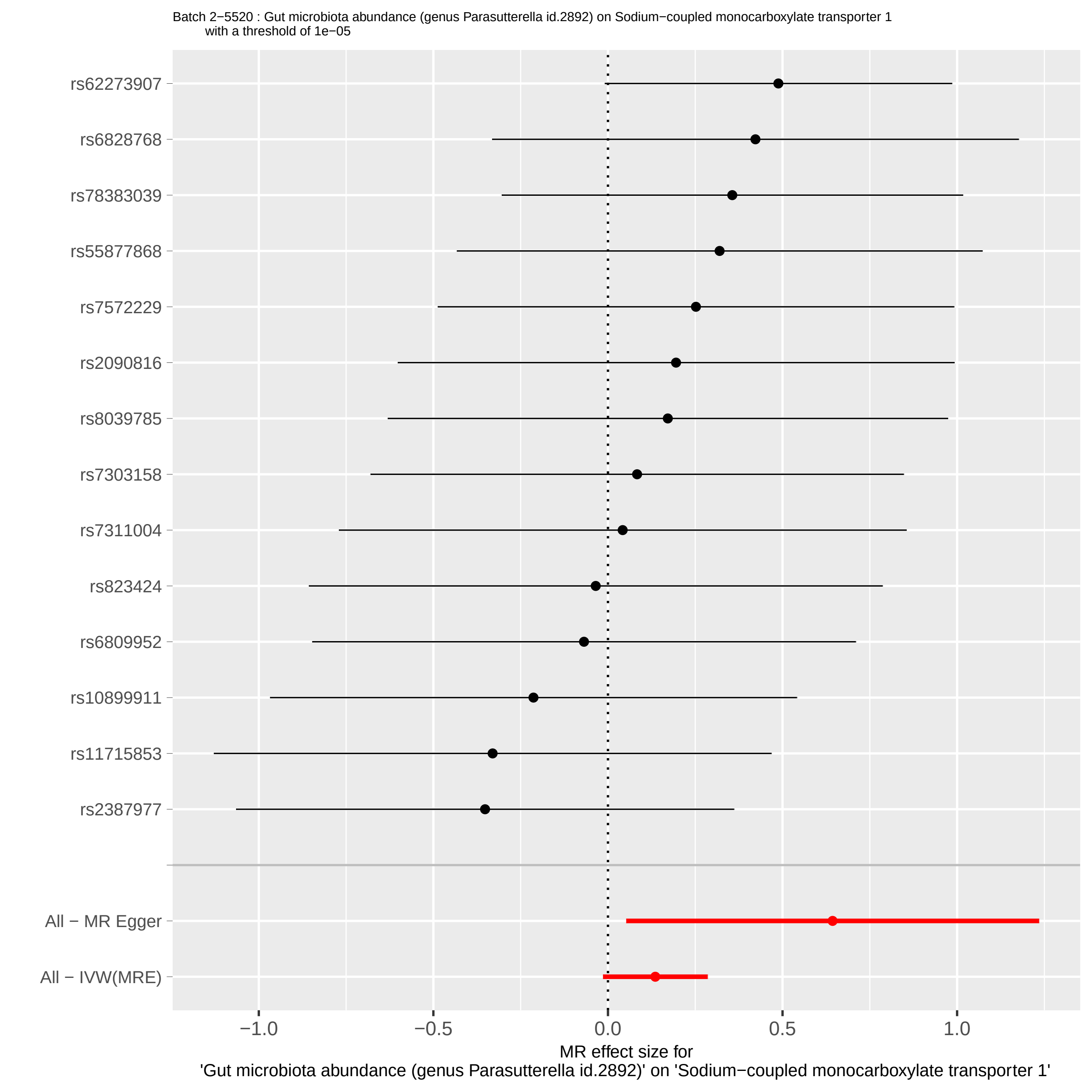

Batch 2−5520 : Gut microbiota abundance (genus Parasutterella id.2892) on Sodium−coupled monocarboxylate transporter 1
with a threshold of 1e−05
rs62273907
rs6828768
rs78383039
rs55877868
rs7572229
rs2090816
rs8039785
rs7303158
rs7311004
rs823424
rs6809952
rs10899911
rs11715853
rs2387977
All − MR Egger
All − IVW(MRE)
−1.0
−0.5
0.0
0.5
1.0
MR effect size for
'Gut microbiota abundance (genus Parasutterella id.2892)' on 'Sodium−coupled monocarboxylate transporter 1'

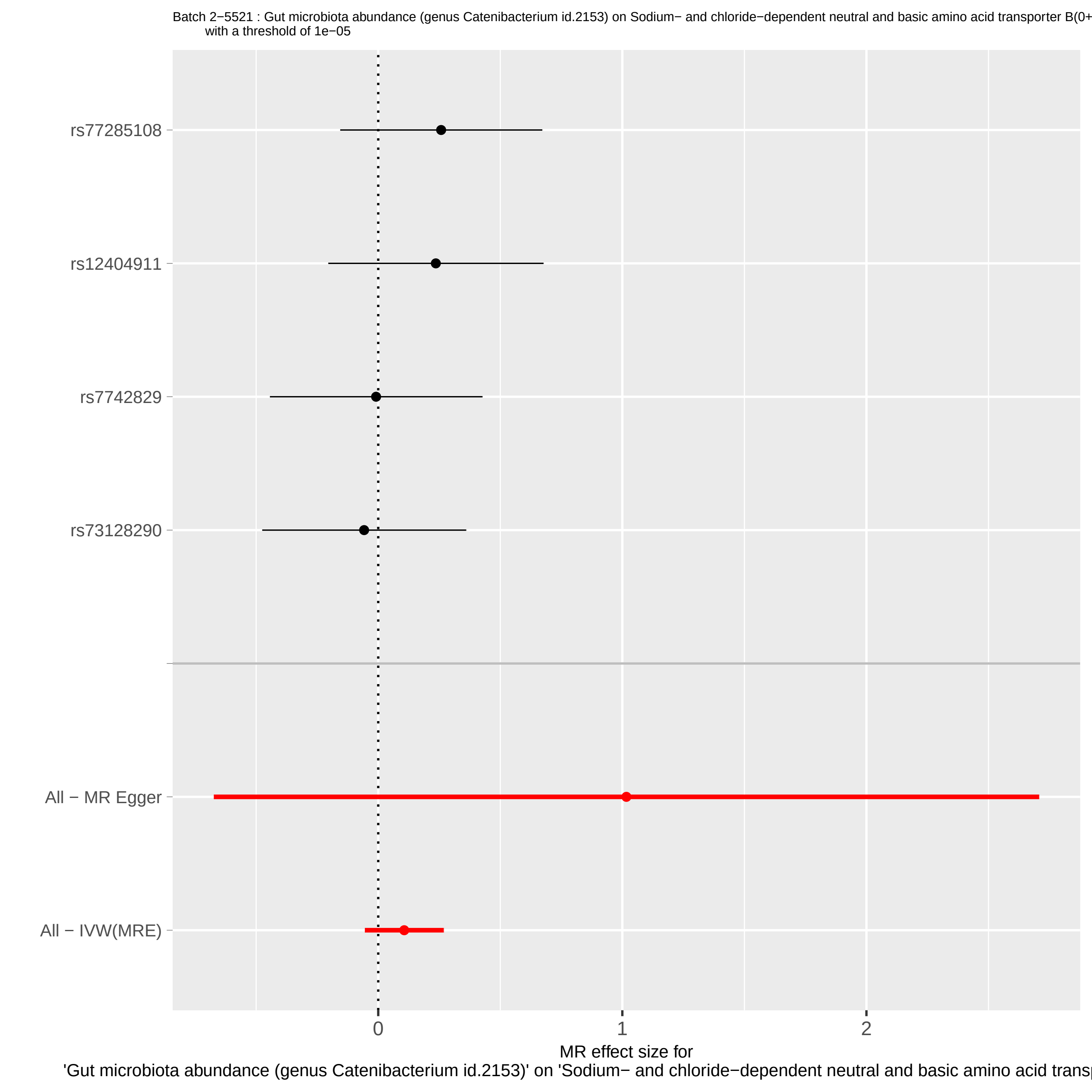

Batch 2−5521 : Gut microbiota abundance (genus Catenibacterium id.2153) on Sodium− and chloride−dependent neutral and basic amino acid transporter B(0+
with a threshold of 1e−05
rs77285108
rs12404911
rs7742829
rs73128290
All − MR Egger
All − IVW(MRE)
0
1
2
MR effect size for
'Gut microbiota abundance (genus Catenibacterium id.2153)' on 'Sodium− and chloride−dependent neutral and basic amino acid transp

Batch 2−5522 : Gut microbiota abundance (genus Parasutterella id.2892) on Sodium− and chloride−dependent neutral and basic amino acid transporter B(0+)
with a threshold of 1e−05

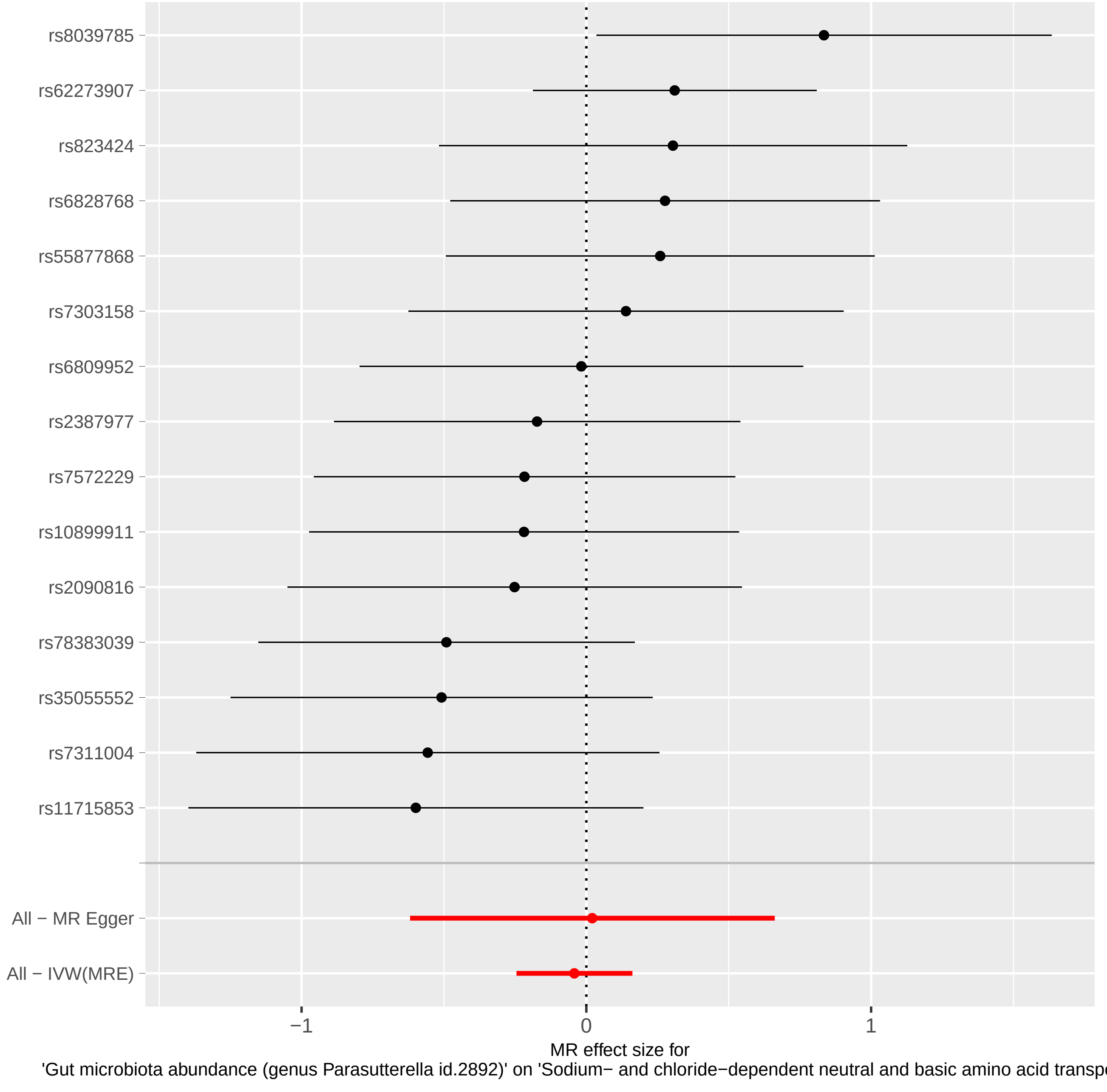

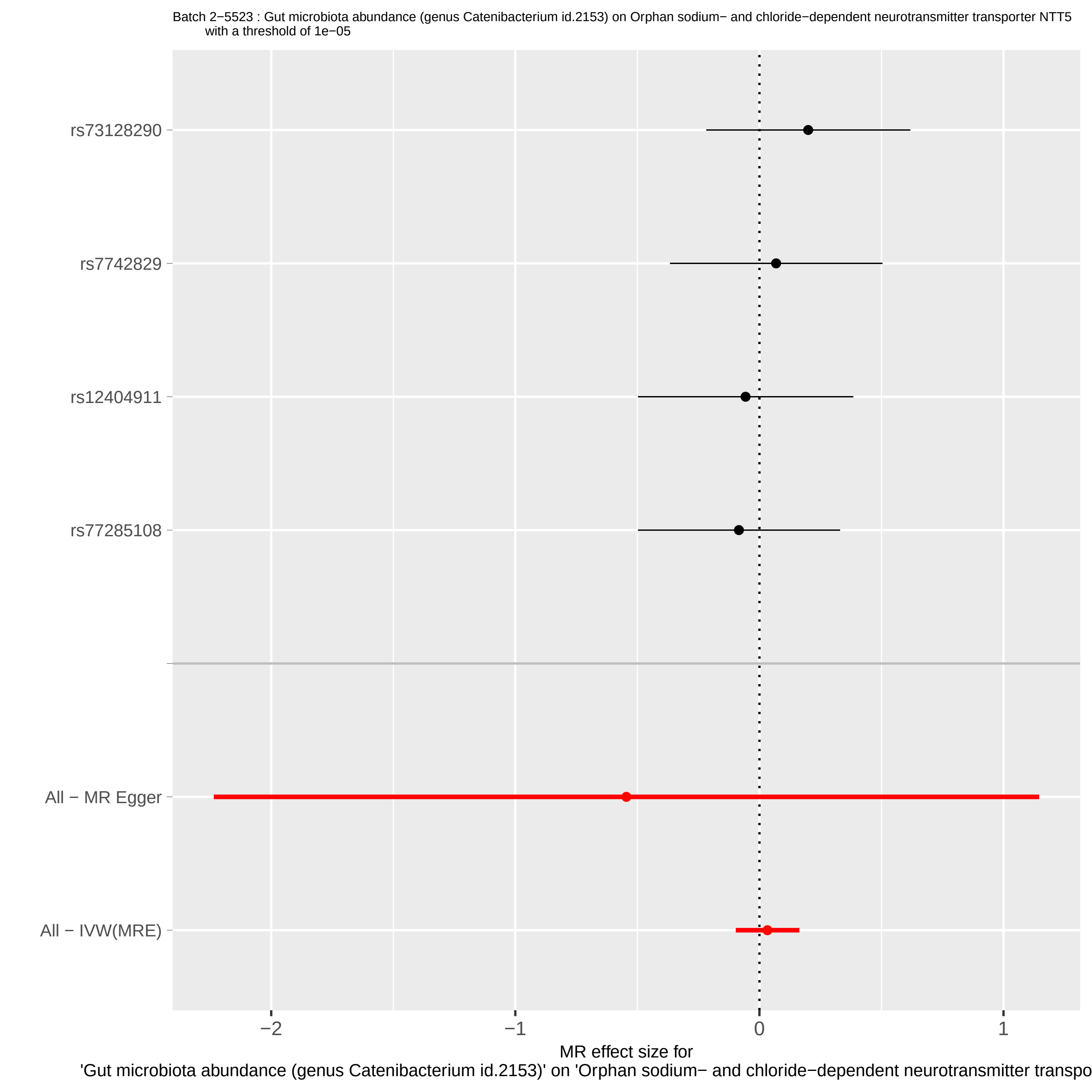
Batch 2−5523 : Gut microbiota abundance (genus Catenibacterium id.2153) on Orphan sodium− and chloride−dependent neurotransmitter transporter NTT5
with a threshold of 1e−05
rs73128290
rs7742829
rs12404911
rs77285108
All − MR Egger
All − IVW(MRE)
−2
−1
0
1
MR effect size for
'Gut microbiota abundance (genus Catenibacterium id.2153)' on 'Orphan sodium− and chloride−dependent neurotransmitter transpo

Batch 2−5524 : Gut microbiota abundance (genus Parasutterella id.2892) on Orphan sodium− and chloride−dependent neurotransmitter transporter NTT5 with a threshold of 1e−05

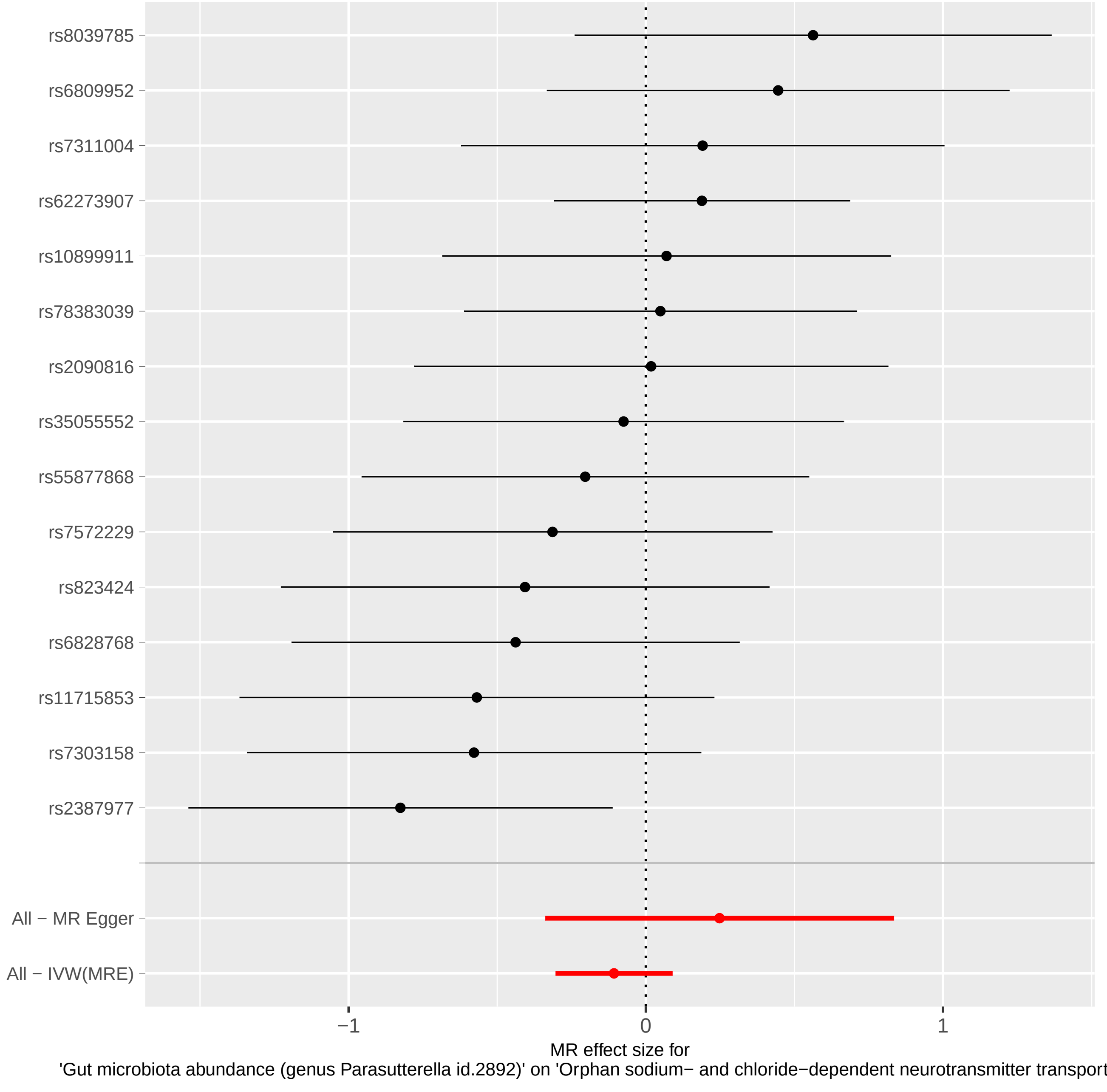

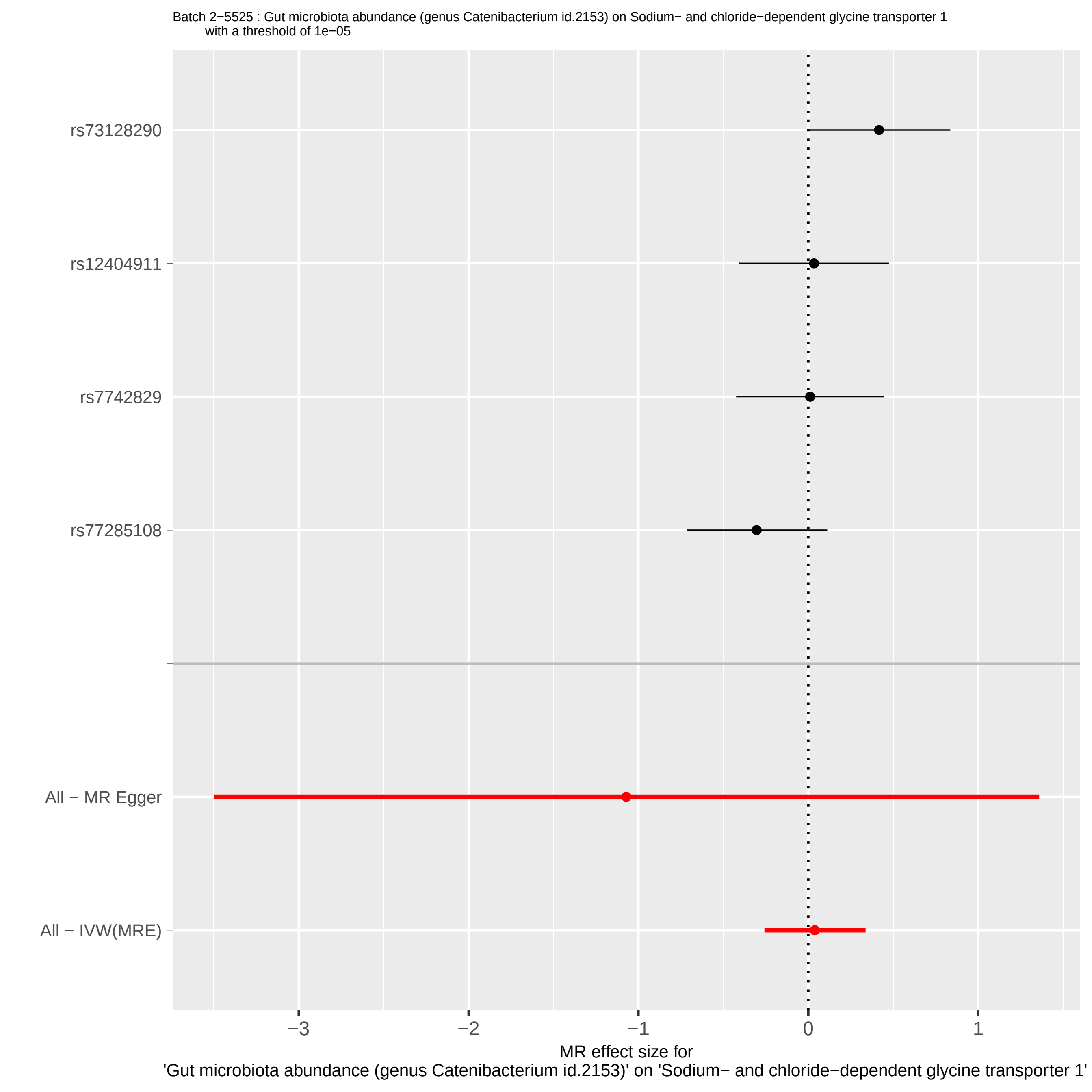

Batch 2−5525 : Gut microbiota abundance (genus Catenibacterium id.2153) on Sodium− and chloride−dependent glycine transporter 1
with a threshold of 1e−05
rs73128290
rs12404911
rs7742829
rs77285108
All − MR Egger
All − IVW(MRE)
−3
−2
−1
0
1
MR effect size for
'Gut microbiota abundance (genus Catenibacterium id.2153)' on 'Sodium− and chloride−dependent glycine transporter 1'

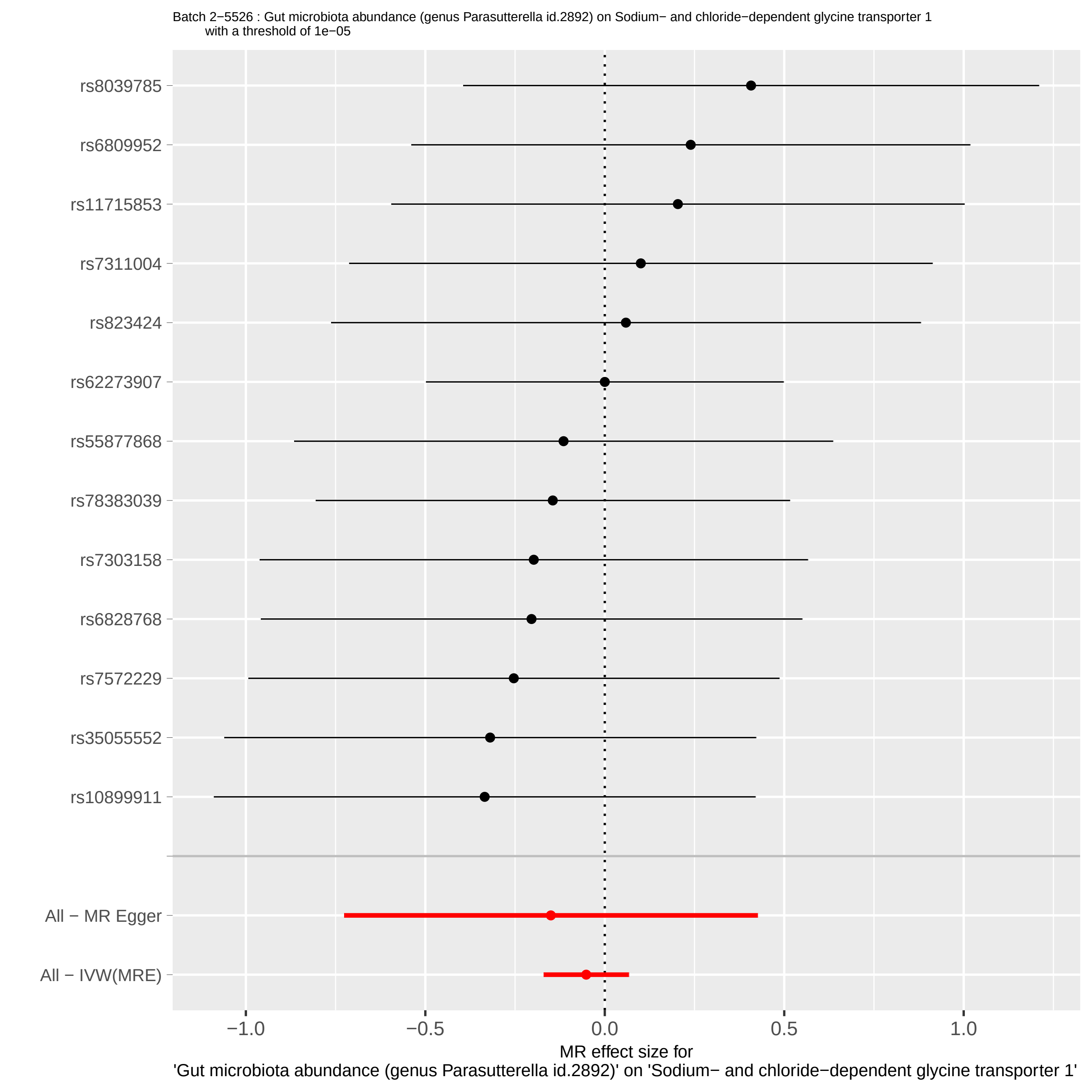

Batch 2−5526 : Gut microbiota abundance (genus Parasutterella id.2892) on Sodium− and chloride−dependent glycine transporter 1
with a threshold of 1e−05
rs8039785
rs6809952
rs11715853
rs7311004
rs823424
rs62273907
rs55877868
rs78383039
rs7303158
rs6828768
rs7572229
rs35055552
rs10899911
All − MR Egger
All − IVW(MRE)
−1.0
−0.5
0.0
0.5
1.0
MR effect size for
'Gut microbiota abundance (genus Parasutterella id.2892)' on 'Sodium− and chloride−dependent glycine transporter 1'

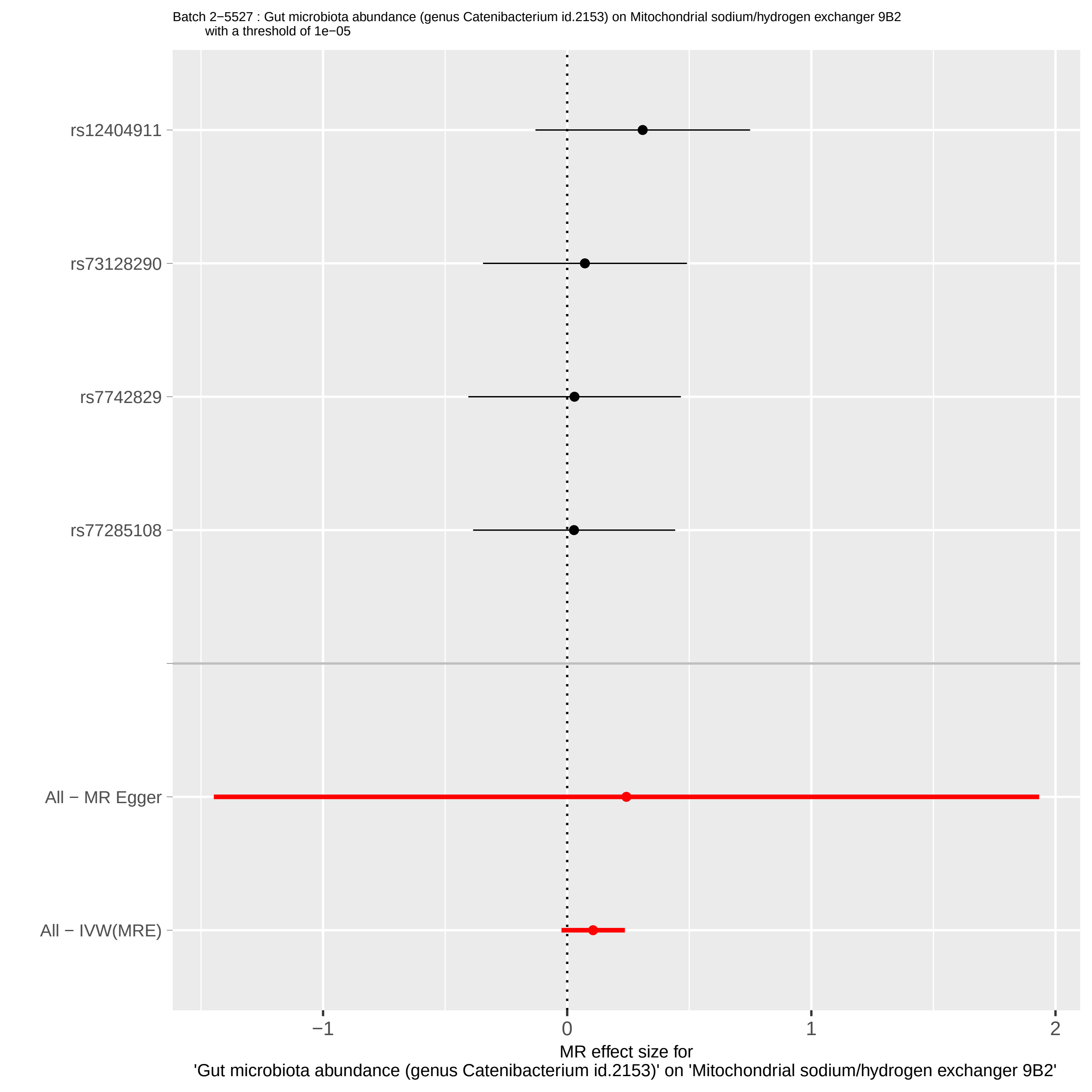

Batch 2−5527 : Gut microbiota abundance (genus Catenibacterium id.2153) on Mitochondrial sodium/hydrogen exchanger 9B2
with a threshold of 1e−05
rs12404911
rs73128290
rs7742829
rs77285108
All − MR Egger
All − IVW(MRE)
−1
0
1
2
MR effect size for
'Gut microbiota abundance (genus Catenibacterium id.2153)' on 'Mitochondrial sodium/hydrogen exchanger 9B2'

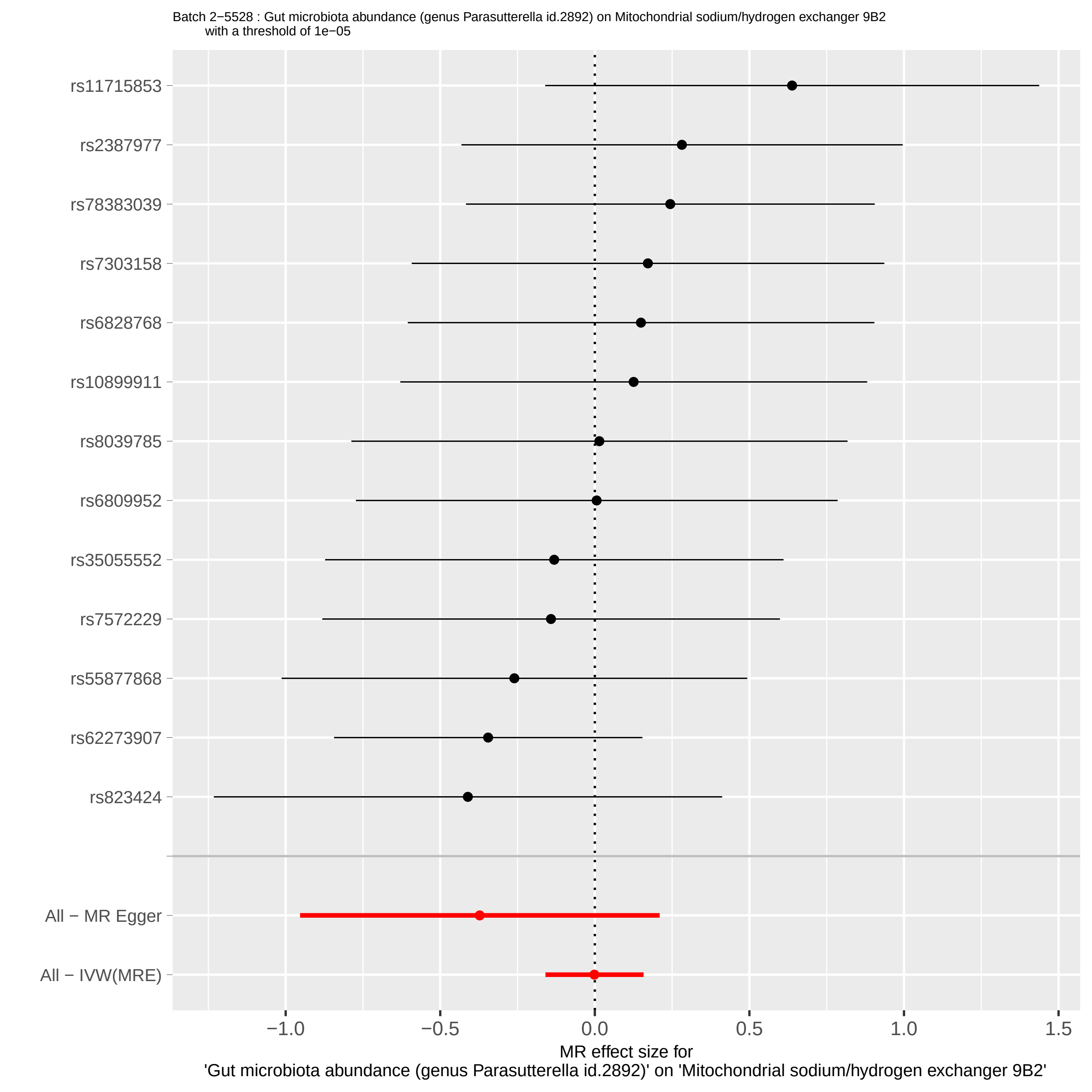
Batch 2−5528 : Gut microbiota abundance (genus Parasutterella id.2892) on Mitochondrial sodium/hydrogen exchanger 9B2
with a threshold of 1e−05
rs11715853
rs2387977
rs78383039
rs7303158
rs6828768
rs10899911
rs8039785
rs6809952
rs35055552
rs7572229
rs55877868
rs62273907
rs823424
All − MR Egger
All − IVW(MRE)
−1.0
−0.5
0.0
0.5
1.0
1.5
MR effect size for
'Gut microbiota abundance (genus Parasutterella id.2892)' on 'Mitochondrial sodium/hydrogen exchanger 9B2'

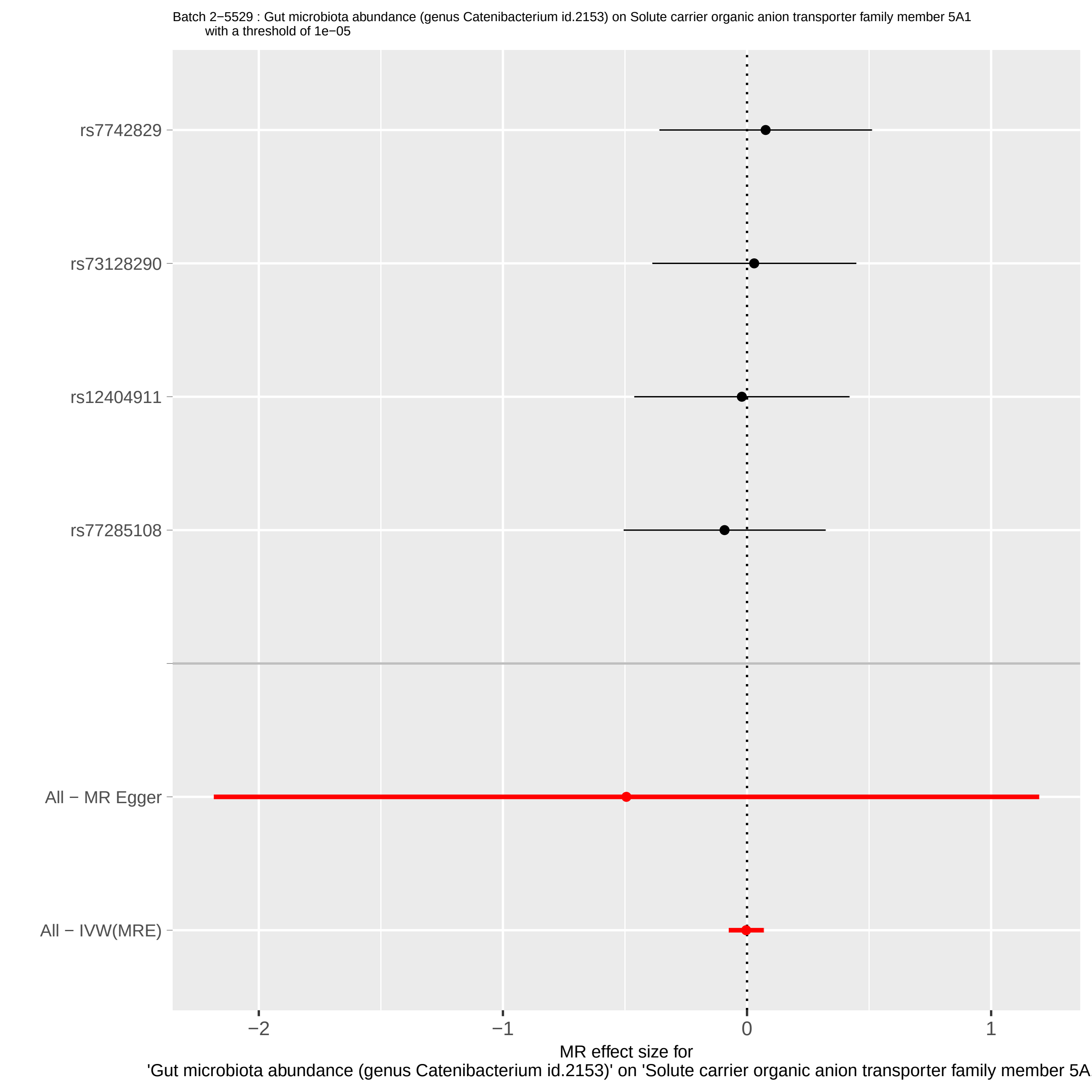
Batch 2−5529 : Gut microbiota abundance (genus Catenibacterium id.2153) on Solute carrier organic anion transporter family member 5A1
with a threshold of 1e−05
rs7742829
rs73128290
rs12404911
rs77285108
All − MR Egger
All − IVW(MRE)
−2
−1
0
1
MR effect size for
'Gut microbiota abundance (genus Catenibacterium id.2153)' on 'Solute carrier organic anion transporter family member 5A

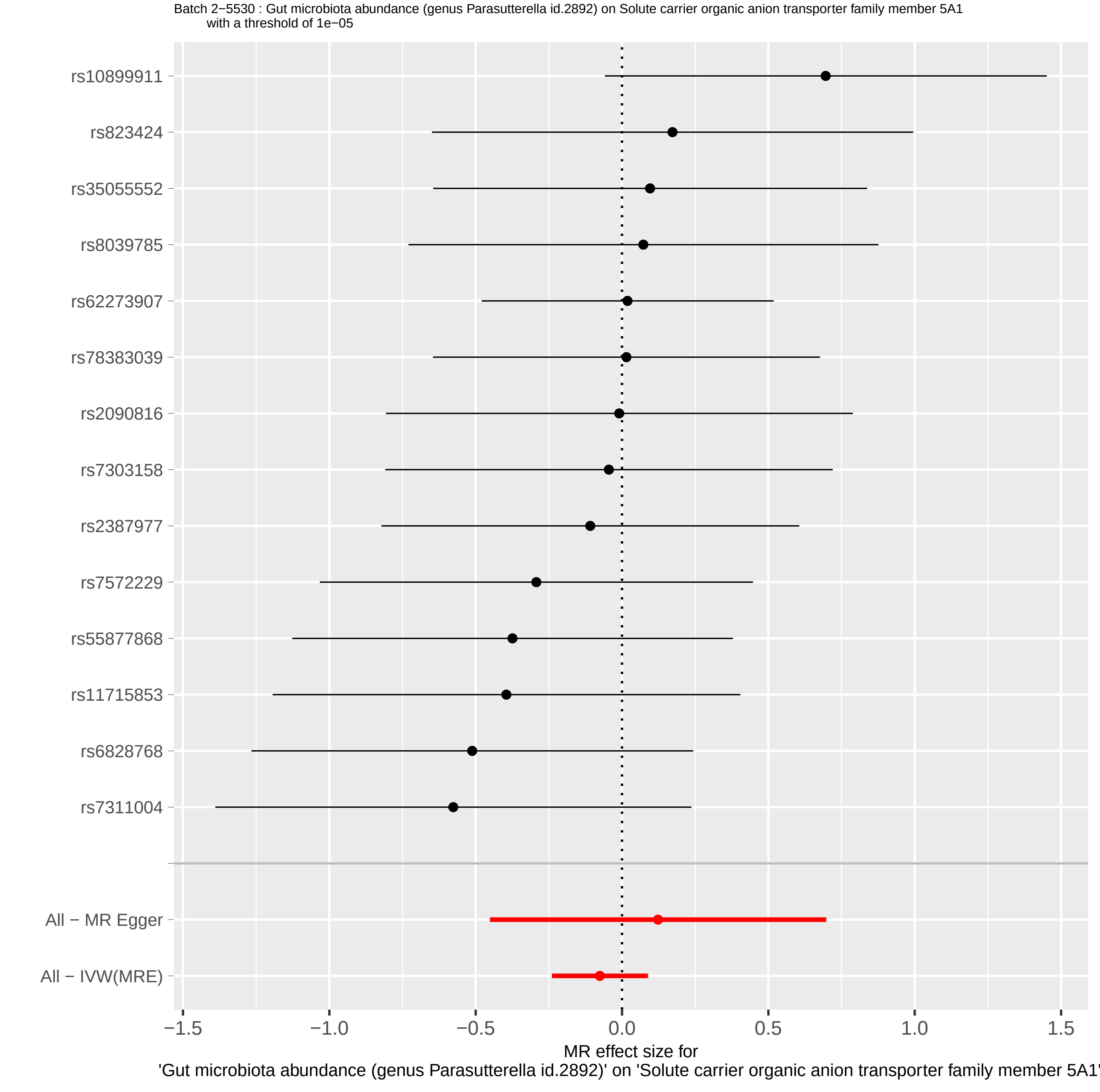
Batch 2−5530 : Gut microbiota abundance (genus Parasutterella id.2892) on Solute carrier organic anion transporter family member 5A1
with a threshold of 1e−05
rs10899911
rs823424
rs35055552
rs8039785
rs62273907
rs78383039
rs2090816
rs7303158
rs2387977
rs7572229
rs55877868
rs11715853
rs6828768
rs7311004
All − MR Egger
All − IVW(MRE)
−1.5
−1.0
−0.5
0.0
0.5
1.0
1.5
MR effect size for
'Gut microbiota abundance (genus Parasutterella id.2892)' on 'Solute carrier organic anion transporter family member 5A1'

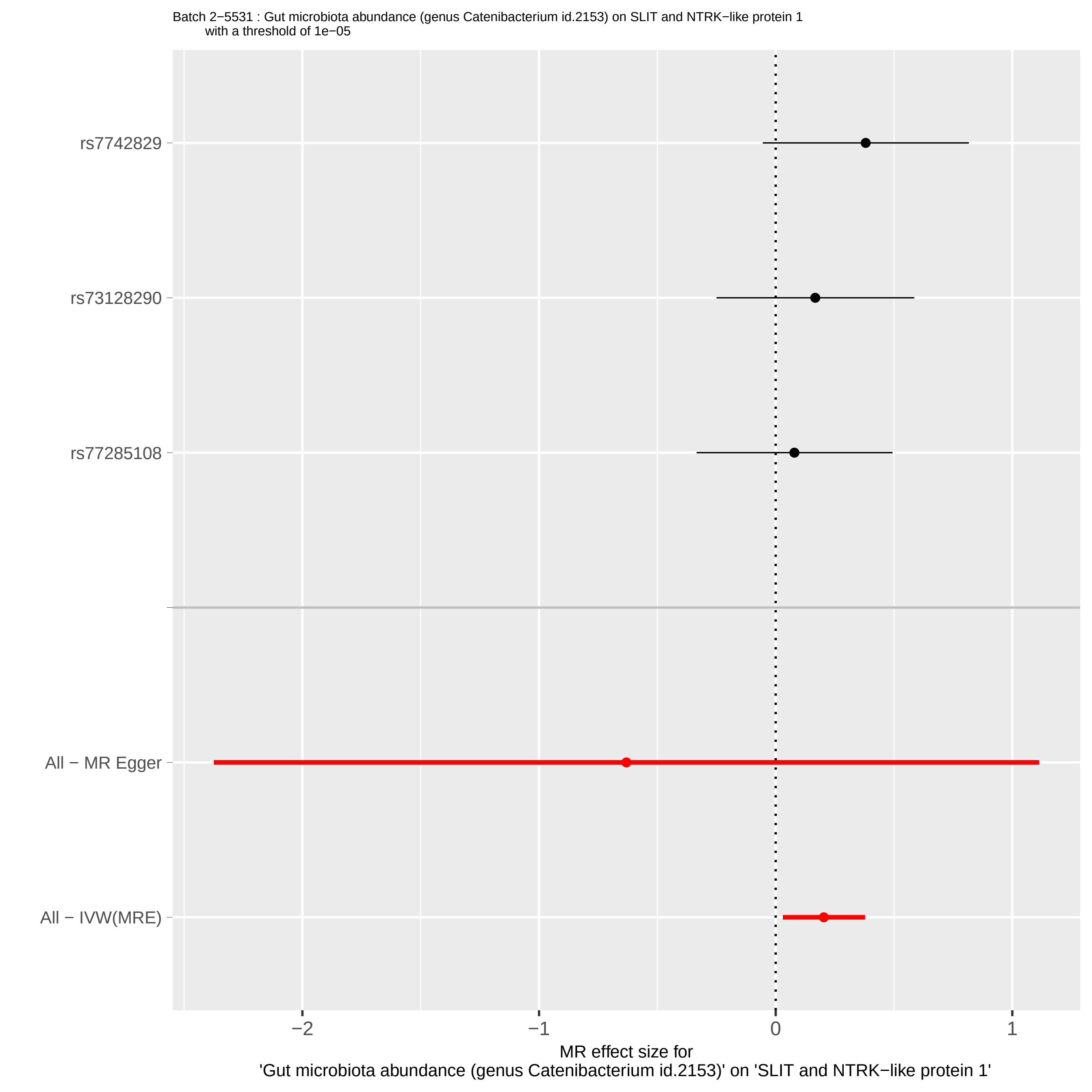

Batch 2−5531 : Gut microbiota abundance (genus Catenibacterium id.2153) on SLIT and NTRK−like protein 1
with a threshold of 1e−05
rs7742829
rs73128290
rs77285108
All − MR Egger
All − IVW(MRE)
−2
−1
0
1
MR effect size for
'Gut microbiota abundance (genus Catenibacterium id.2153)' on 'SLIT and NTRK−like protein 1'

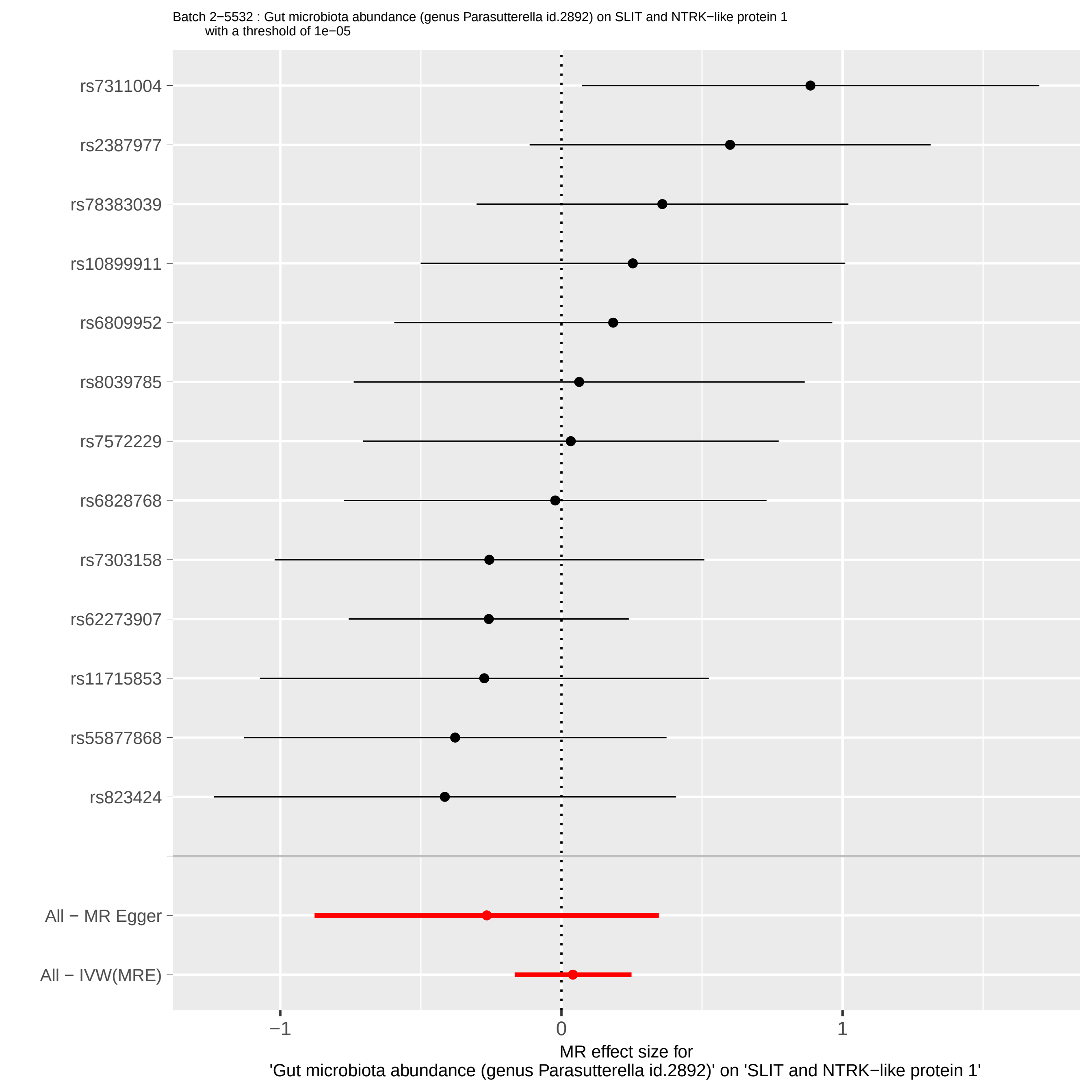

Batch 2−5532 : Gut microbiota abundance (genus Parasutterella id.2892) on SLIT and NTRK−like protein 1
with a threshold of 1e−05
rs7311004
rs2387977
rs78383039
rs10899911
rs6809952
rs8039785
rs7572229
rs6828768
rs7303158
rs62273907
rs11715853
rs55877868
rs823424
All − MR Egger
All − IVW(MRE)
−1
0
1
MR effect size for
'Gut microbiota abundance (genus Parasutterella id.2892)' on 'SLIT and NTRK−like protein 1'

Batch 2−5533 : Gut microbiota abundance (genus Catenibacterium id.2153) on SLIT and NTRK−like protein 3
with a threshold of 1e−05

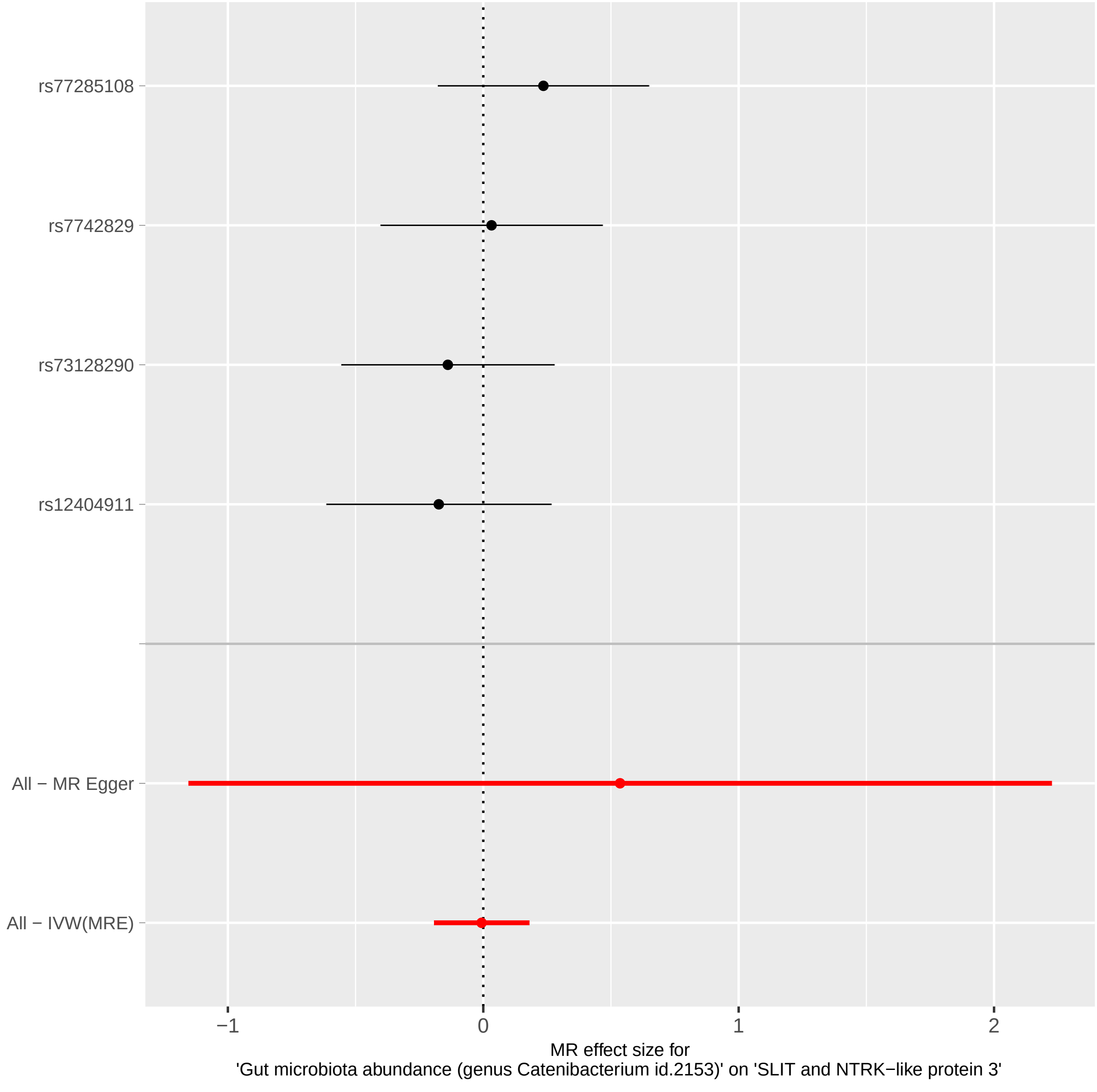

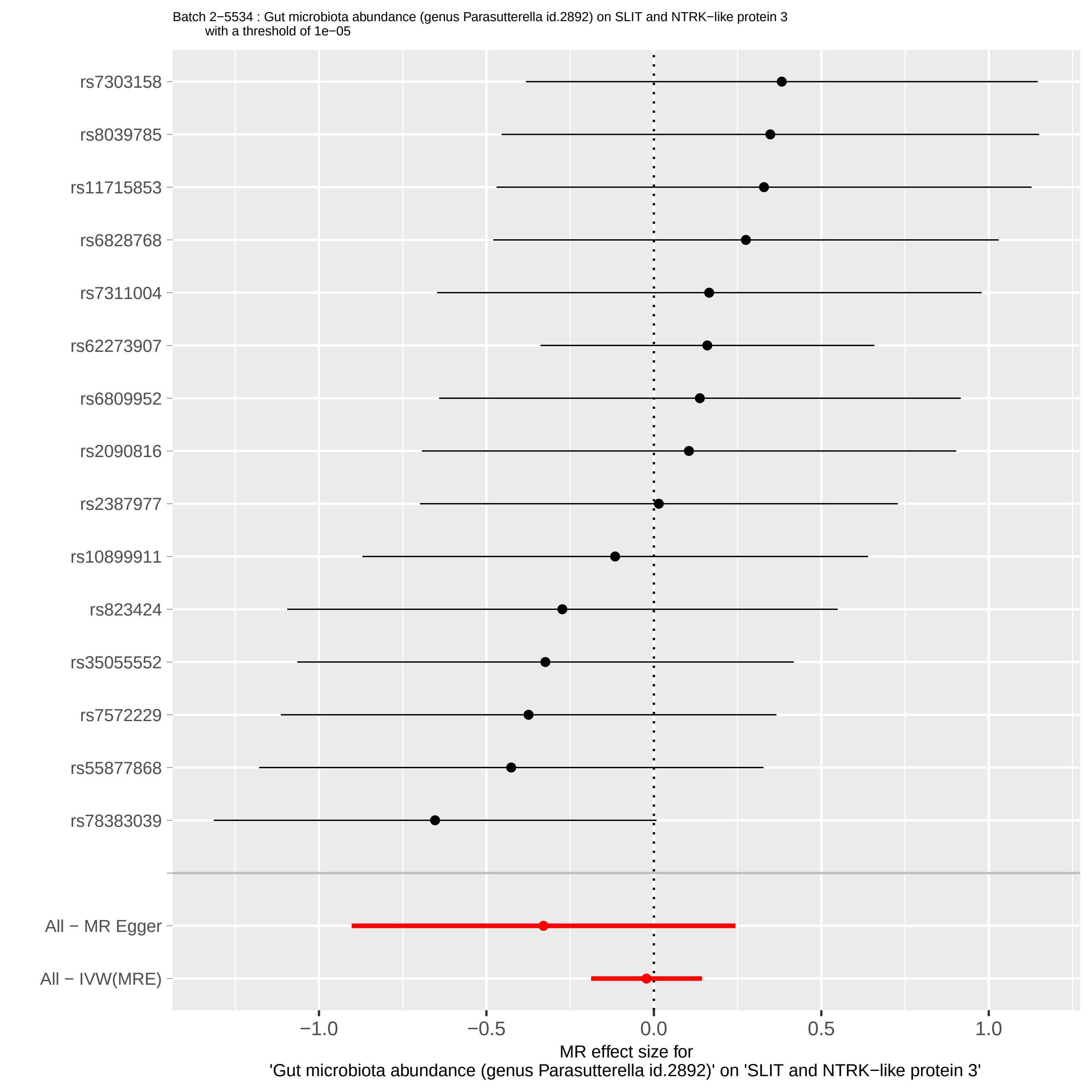
Batch 2−5534 : Gut microbiota abundance (genus Parasutterella id.2892) on SLIT and NTRK−like protein 3
with a threshold of 1e−05
rs7303158
rs8039785
rs11715853
rs6828768
rs7311004
rs62273907
rs6809952
rs2090816
rs2387977
rs10899911
rs823424
rs35055552
rs7572229
rs55877868
rs78383039
All − MR Egger
All − IVW(MRE)
−1.0
−0.5
0.0
0.5
1.0
MR effect size for
'Gut microbiota abundance (genus Parasutterella id.2892)' on 'SLIT and NTRK−like protein 3'

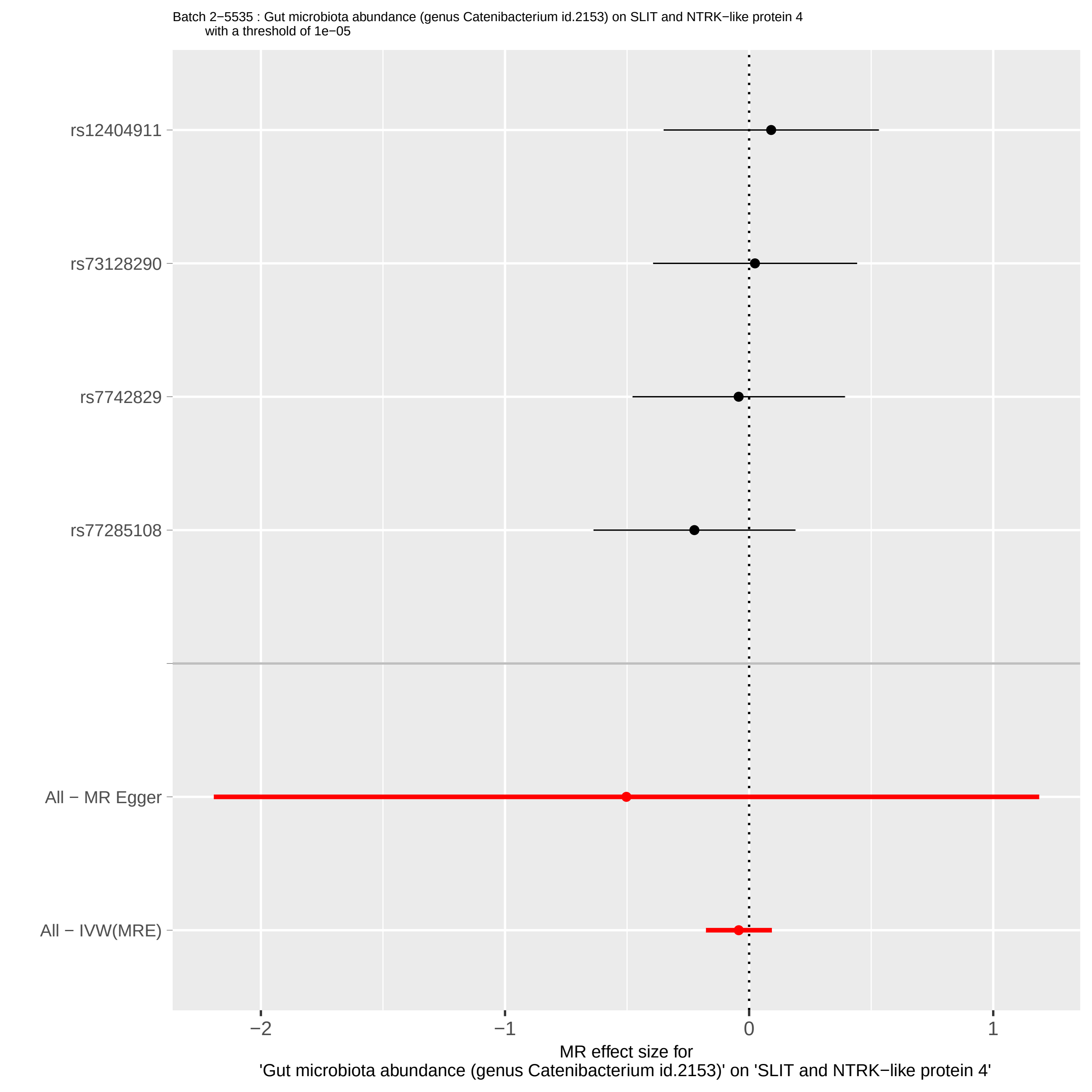

Batch 2−5535 : Gut microbiota abundance (genus Catenibacterium id.2153) on SLIT and NTRK−like protein 4
with a threshold of 1e−05
rs12404911
rs73128290
rs7742829
rs77285108
All − MR Egger
All − IVW(MRE)
−2
−1
0
1
MR effect size for
'Gut microbiota abundance (genus Catenibacterium id.2153)' on 'SLIT and NTRK−like protein 4'

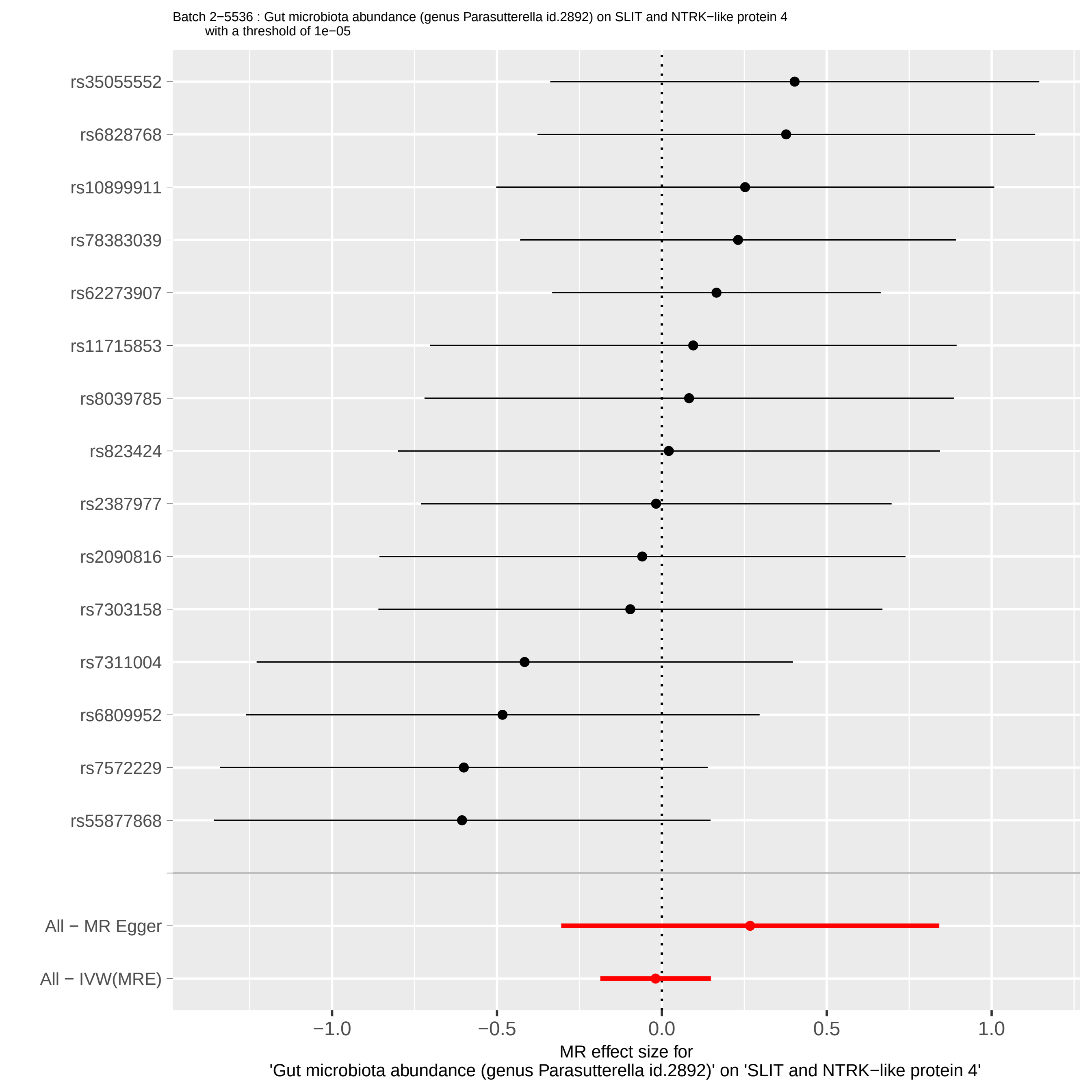
Batch 2−5536 : Gut microbiota abundance (genus Parasutterella id.2892) on SLIT and NTRK−like protein 4
with a threshold of 1e−05
rs35055552
rs6828768
rs10899911
rs78383039
rs62273907
rs11715853
rs8039785
rs823424
rs2387977
rs2090816
rs7303158
rs7311004
rs6809952
rs7572229
rs55877868
All − MR Egger
All − IVW(MRE)
−1.0
−0.5
0.0
0.5
1.0
MR effect size for
'Gut microbiota abundance (genus Parasutterella id.2892)' on 'SLIT and NTRK−like protein 4'

Batch 2−5537 : Gut microbiota abundance (genus Catenibacterium id.2153) on SLIT and NTRK−like protein 5
with a threshold of 1e−05

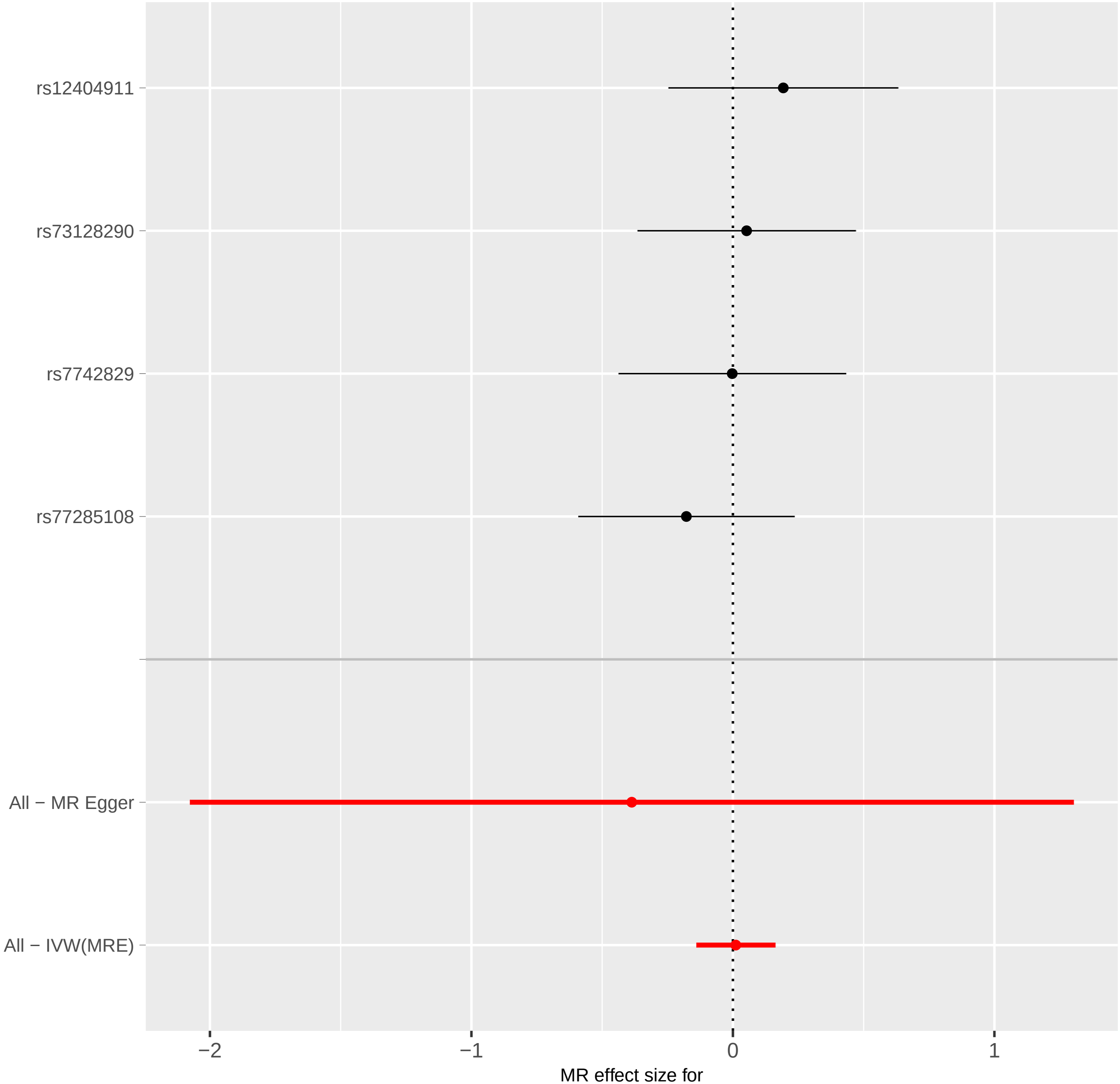

MR effect size for
'Gut microbiota abundance (genus Catenibacterium id.2153)' on 'SLIT and NTRK−like protein 5'

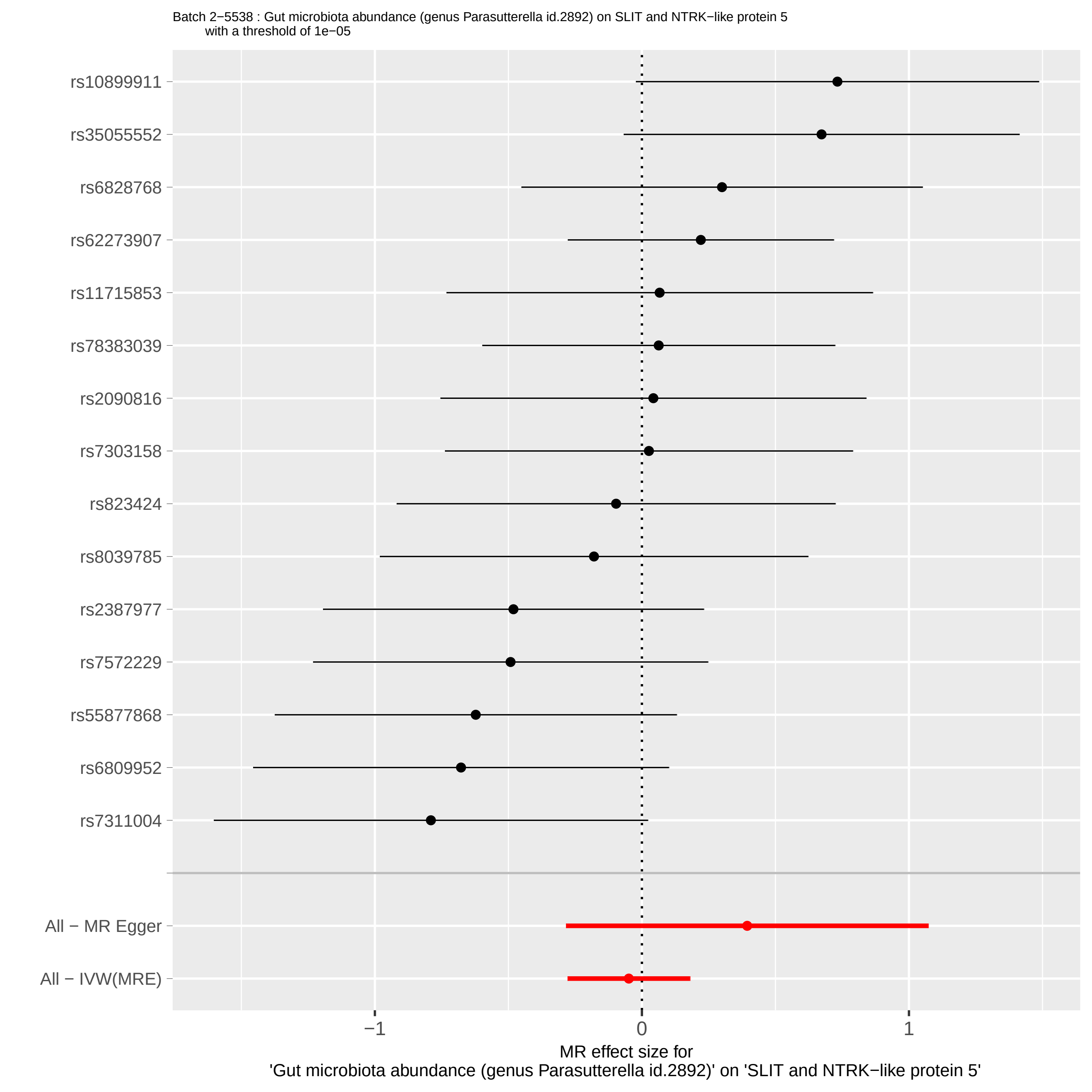

Batch 2−5538 : Gut microbiota abundance (genus Parasutterella id.2892) on SLIT and NTRK−like protein 5
with a threshold of 1e−05
rs10899911
rs35055552
rs6828768
rs62273907
rs11715853
rs78383039
rs2090816
rs7303158
rs823424
rs8039785
rs2387977
rs7572229
rs55877868
rs6809952
rs7311004
All − MR Egger
All − IVW(MRE)
−1
0
1
MR effect size for
'Gut microbiota abundance (genus Parasutterella id.2892)' on 'SLIT and NTRK−like protein 5'

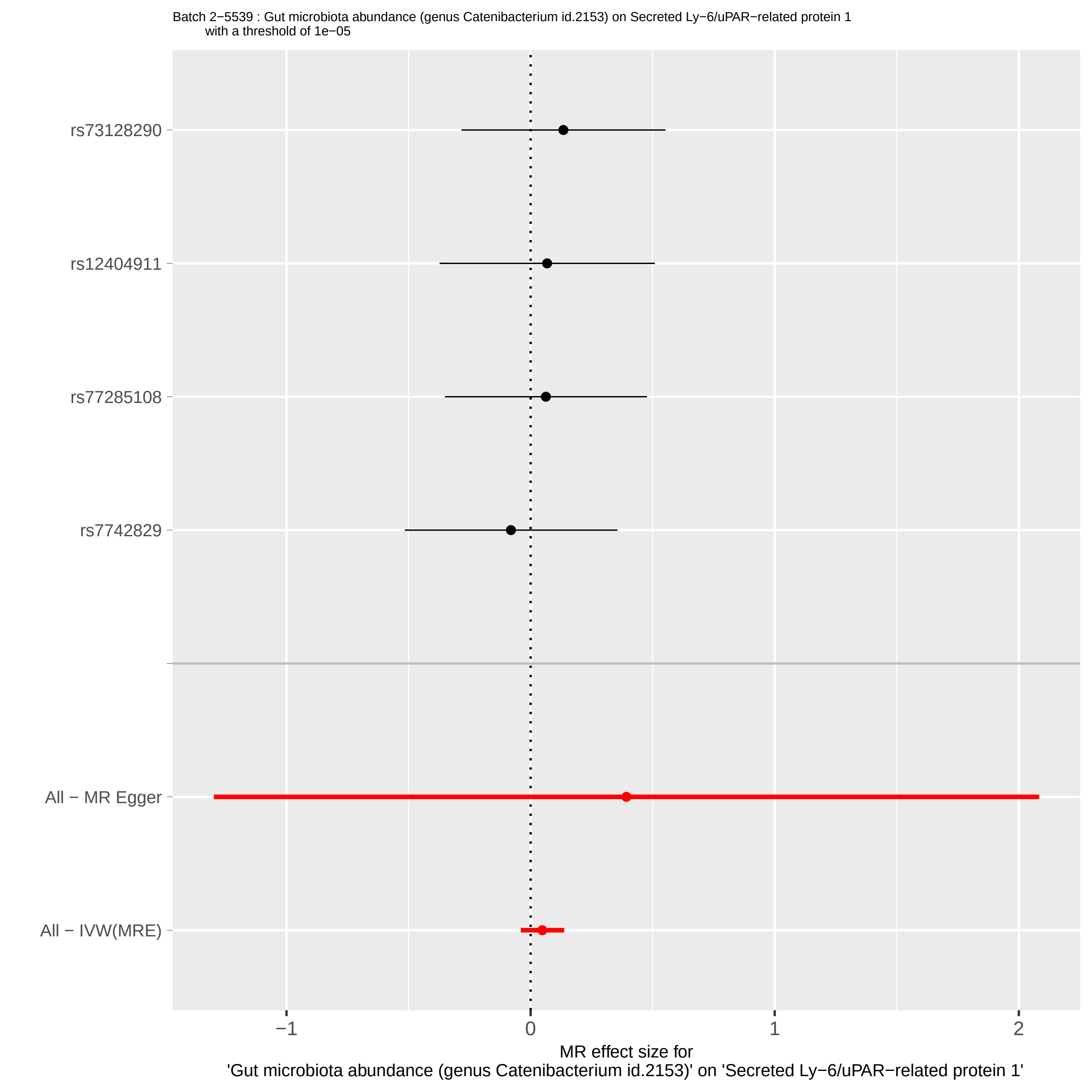

Batch 2−5539 : Gut microbiota abundance (genus Catenibacterium id.2153) on Secreted Ly−6/uPAR−related protein 1
with a threshold of 1e−05
rs73128290
rs12404911
rs77285108
rs7742829
All − MR Egger
All − IVW(MRE)
−1
0
1
2
MR effect size for
'Gut microbiota abundance (genus Catenibacterium id.2153)' on 'Secreted Ly−6/uPAR−related protein 1'

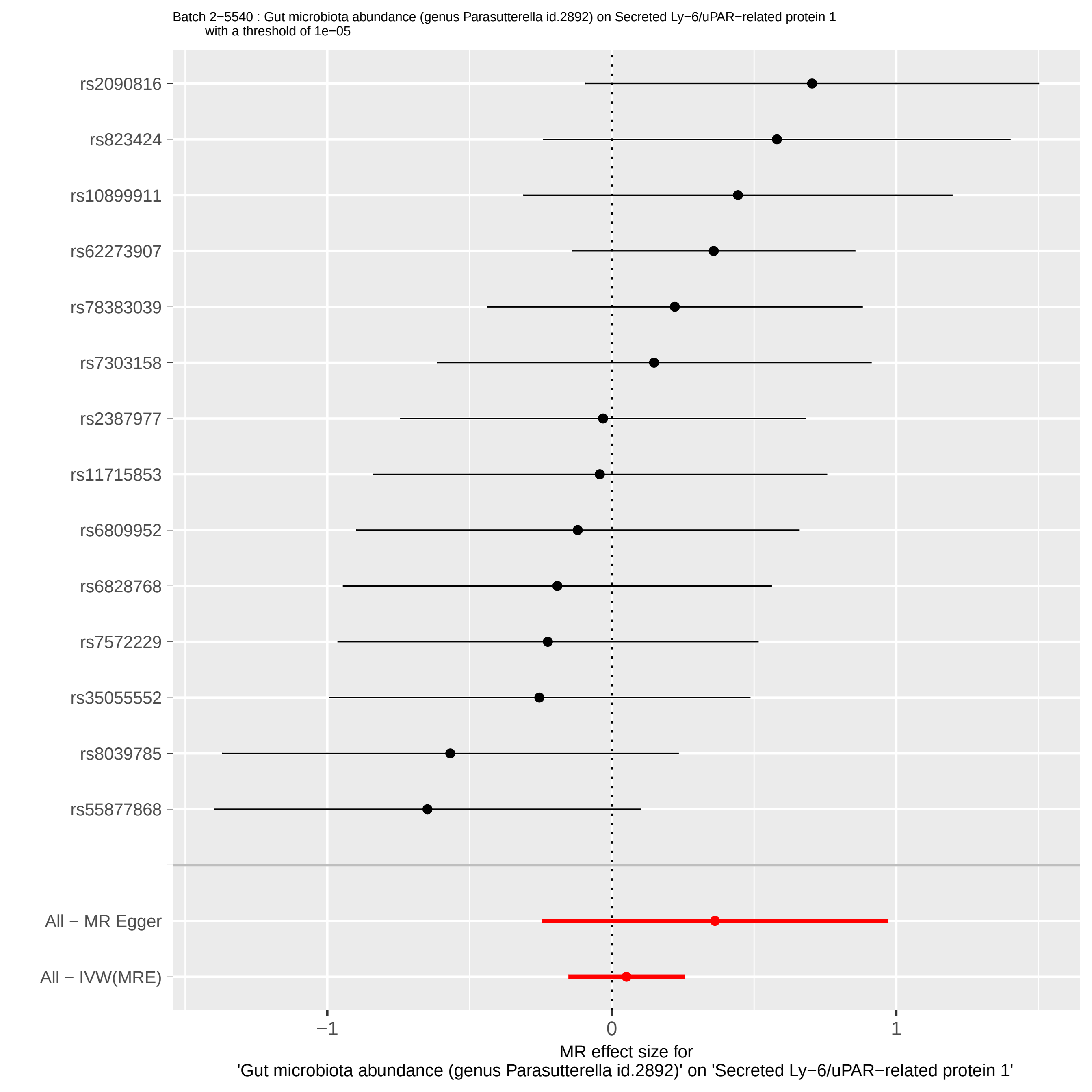
Batch 2−5540 : Gut microbiota abundance (genus Parasutterella id.2892) on Secreted Ly−6/uPAR−related protein 1
with a threshold of 1e−05
rs2090816
rs823424
rs10899911
rs62273907
rs78383039
rs7303158
rs2387977
rs11715853
rs6809952
rs6828768
rs7572229
rs35055552
rs8039785
rs55877868
All − MR Egger
All − IVW(MRE)
−1
0
1
MR effect size for
'Gut microbiota abundance (genus Parasutterella id.2892)' on 'Secreted Ly−6/uPAR−related protein 1'

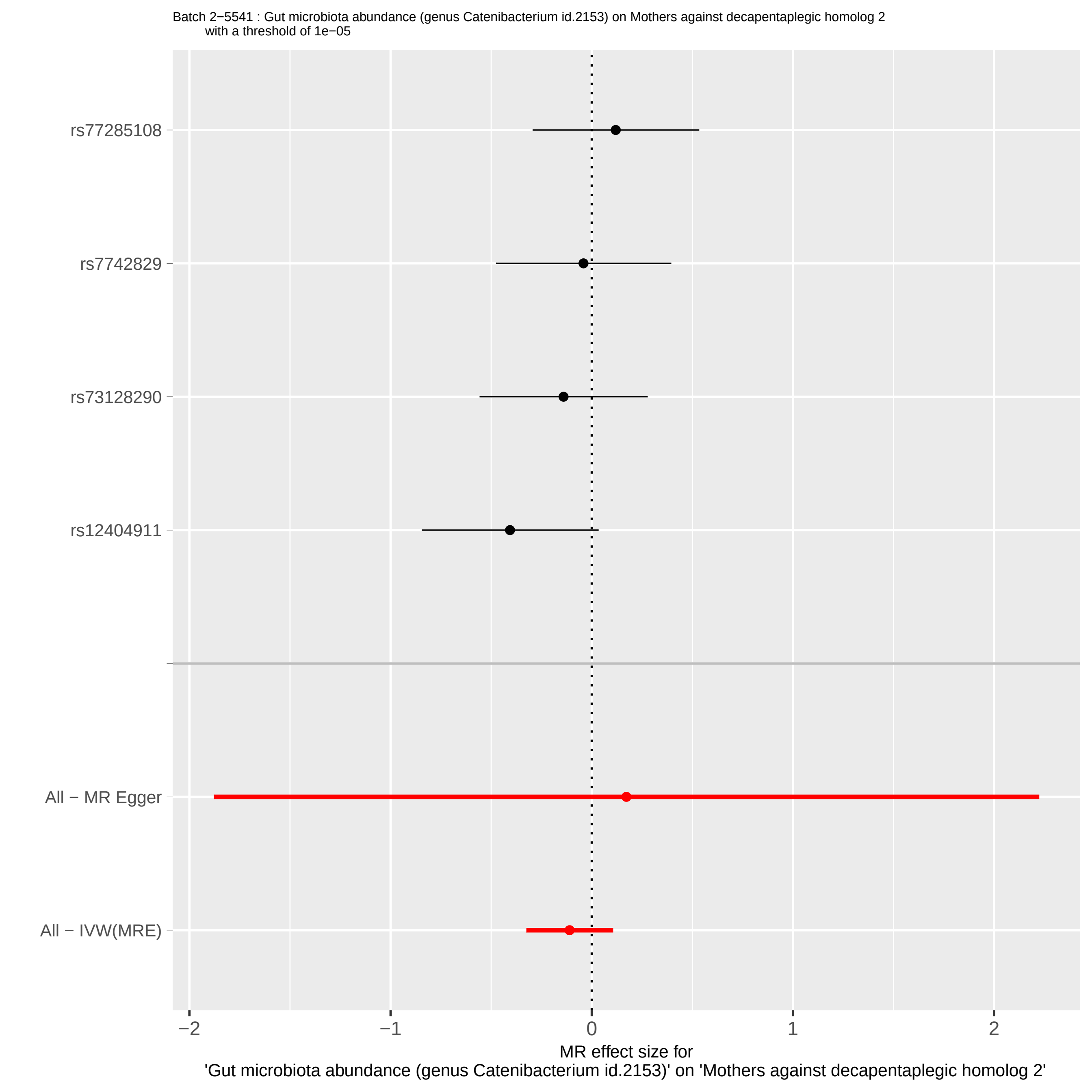
Batch 2−5541 : Gut microbiota abundance (genus Catenibacterium id.2153) on Mothers against decapentaplegic homolog 2
with a threshold of 1e−05
rs77285108
rs7742829
rs73128290
rs12404911
All − MR Egger
All − IVW(MRE)
−2
−1
0
1
2
MR effect size for
'Gut microbiota abundance (genus Catenibacterium id.2153)' on 'Mothers against decapentaplegic homolog 2'

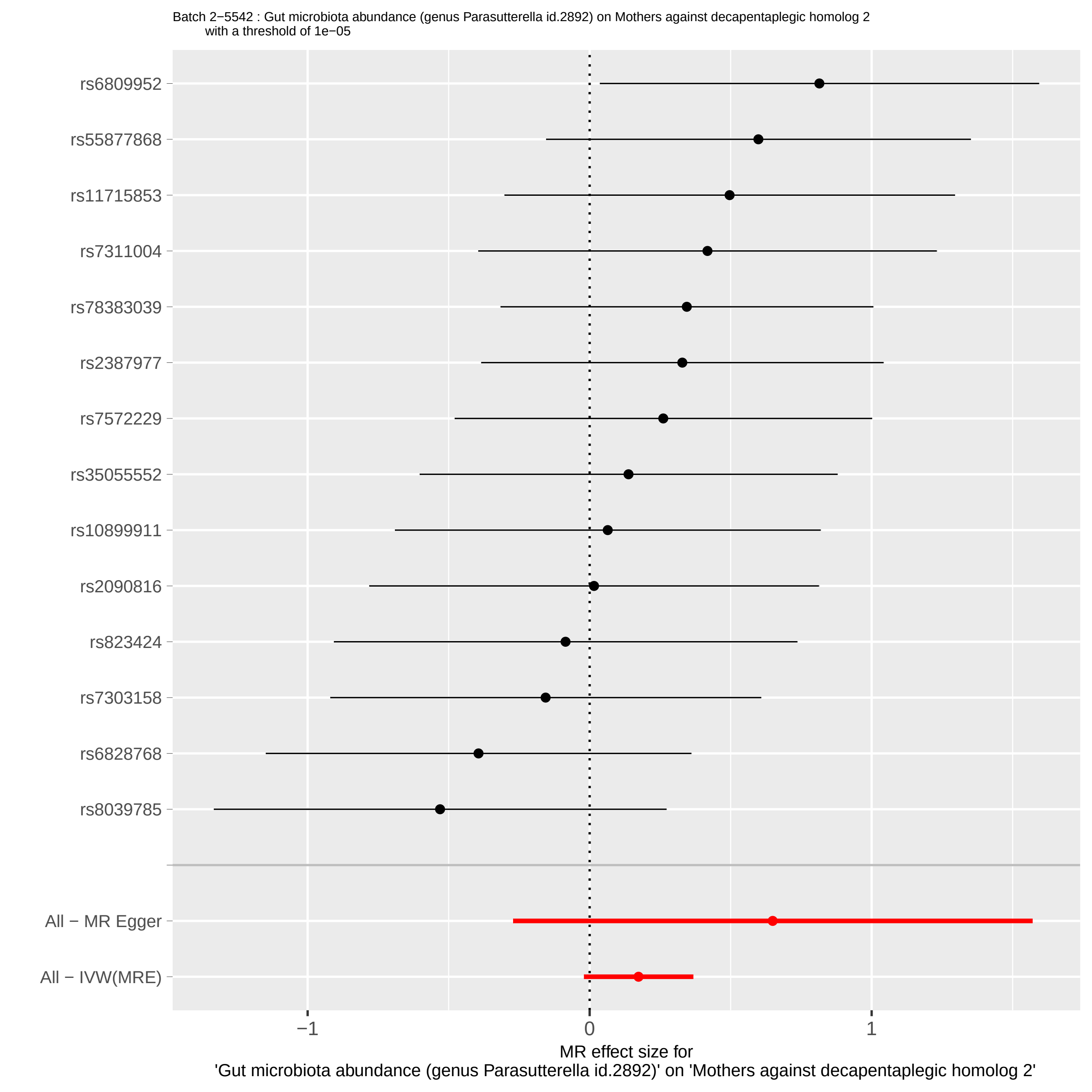

Batch 2−5542 : Gut microbiota abundance (genus Parasutterella id.2892) on Mothers against decapentaplegic homolog 2
with a threshold of 1e−05
rs6809952
rs55877868
rs11715853
rs7311004
rs78383039
rs2387977
rs7572229
rs35055552
rs10899911
rs2090816
rs823424
rs7303158
rs6828768
rs8039785
All − MR Egger
All − IVW(MRE)
−1
0
1
MR effect size for
'Gut microbiota abundance (genus Parasutterella id.2892)' on 'Mothers against decapentaplegic homolog 2'

Batch 2−5543 : Gut microbiota abundance (genus Catenibacterium id.2153) on Mothers against decapentaplegic homolog 4
with a threshold of 1e−05

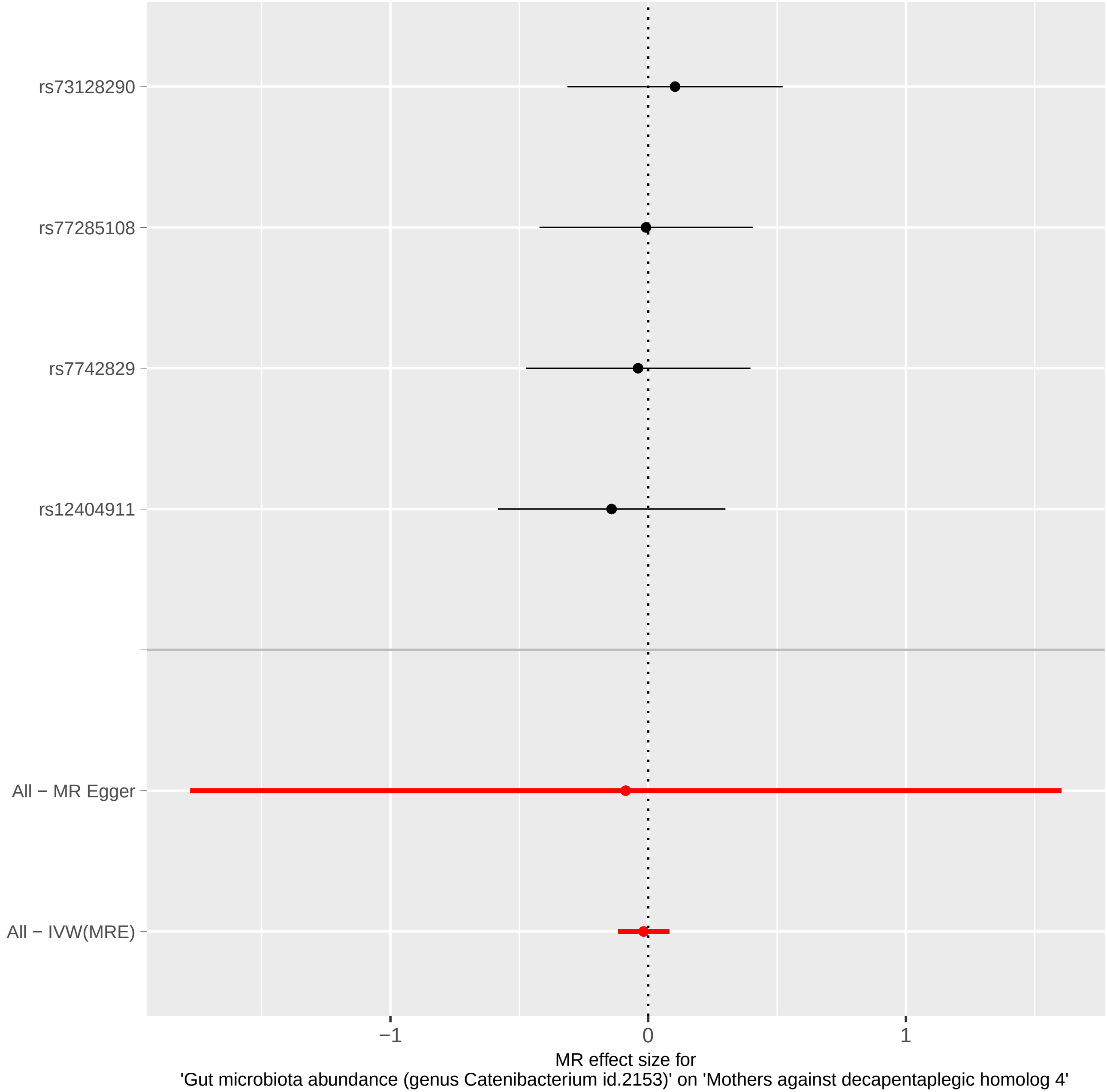

Batch 2−5544 : Gut microbiota abundance (genus Parasutterella id.2892) on Mothers against decapentaplegic homolog 4
with a threshold of 1e−05

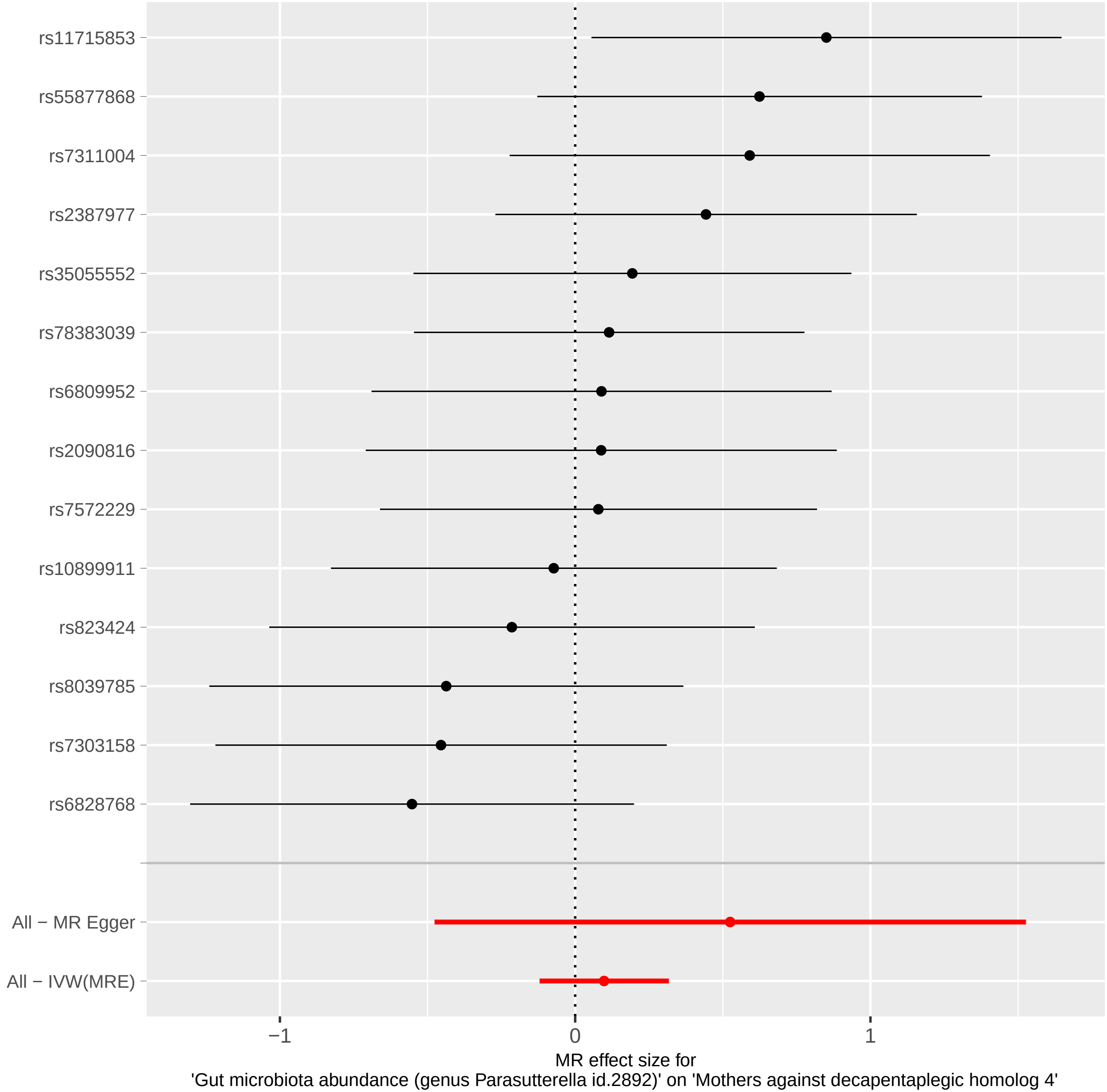

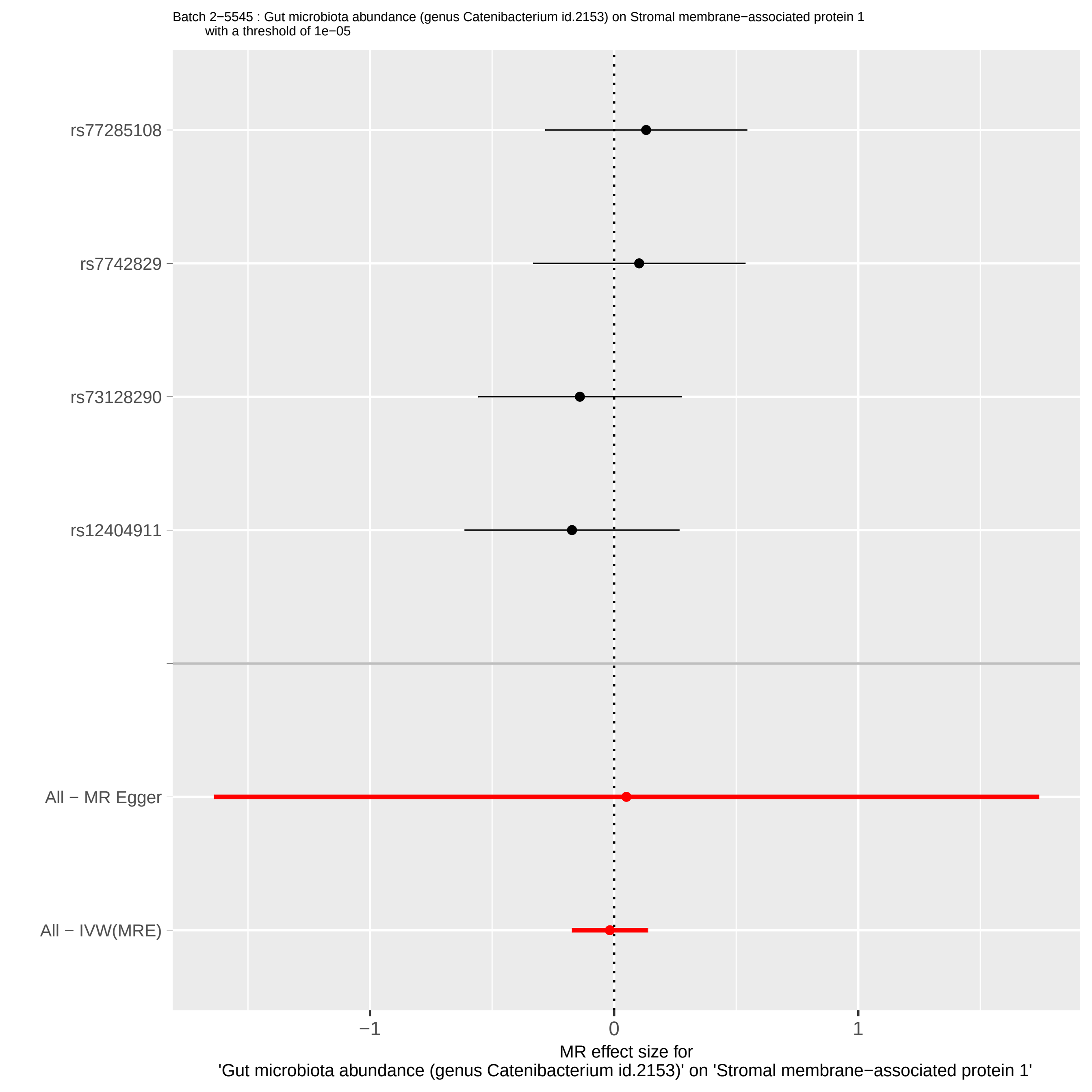

Batch 2−5545 : Gut microbiota abundance (genus Catenibacterium id.2153) on Stromal membrane−associated protein 1
with a threshold of 1e−05
rs77285108
rs7742829
rs73128290
rs12404911
All − MR Egger
All − IVW(MRE)
−1
0
1
MR effect size for
'Gut microbiota abundance (genus Catenibacterium id.2153)' on 'Stromal membrane−associated protein 1'

Batch 2−5546 : Gut microbiota abundance (genus Parasutterella id.2892) on Stromal membrane−associated protein 1
with a threshold of 1e−05

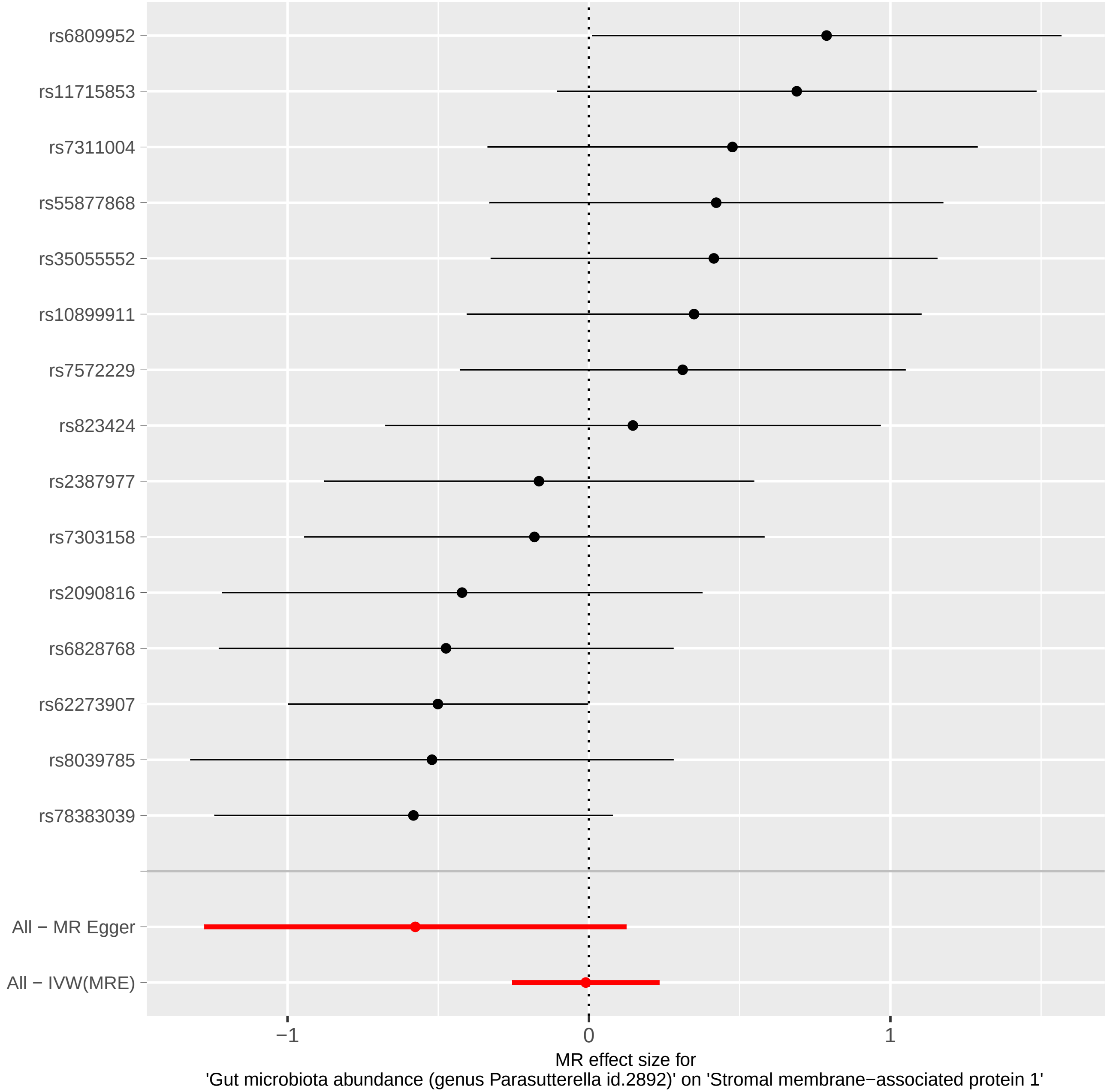

Batch 2−5547 : Gut microbiota abundance (genus Catenibacterium id.2153) on Structural maintenance of chromosomes protein 3
with a threshold of 1e−05

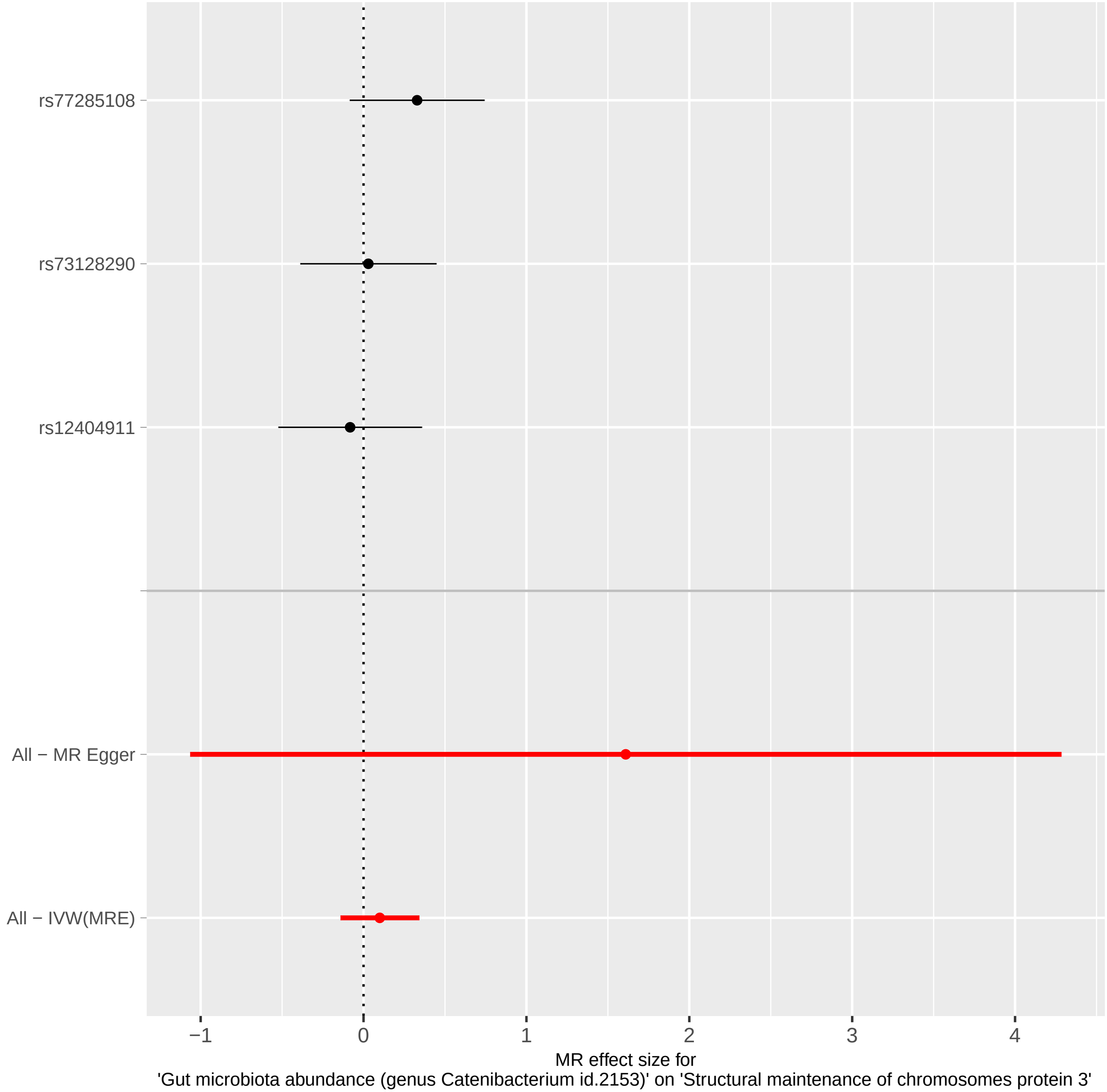

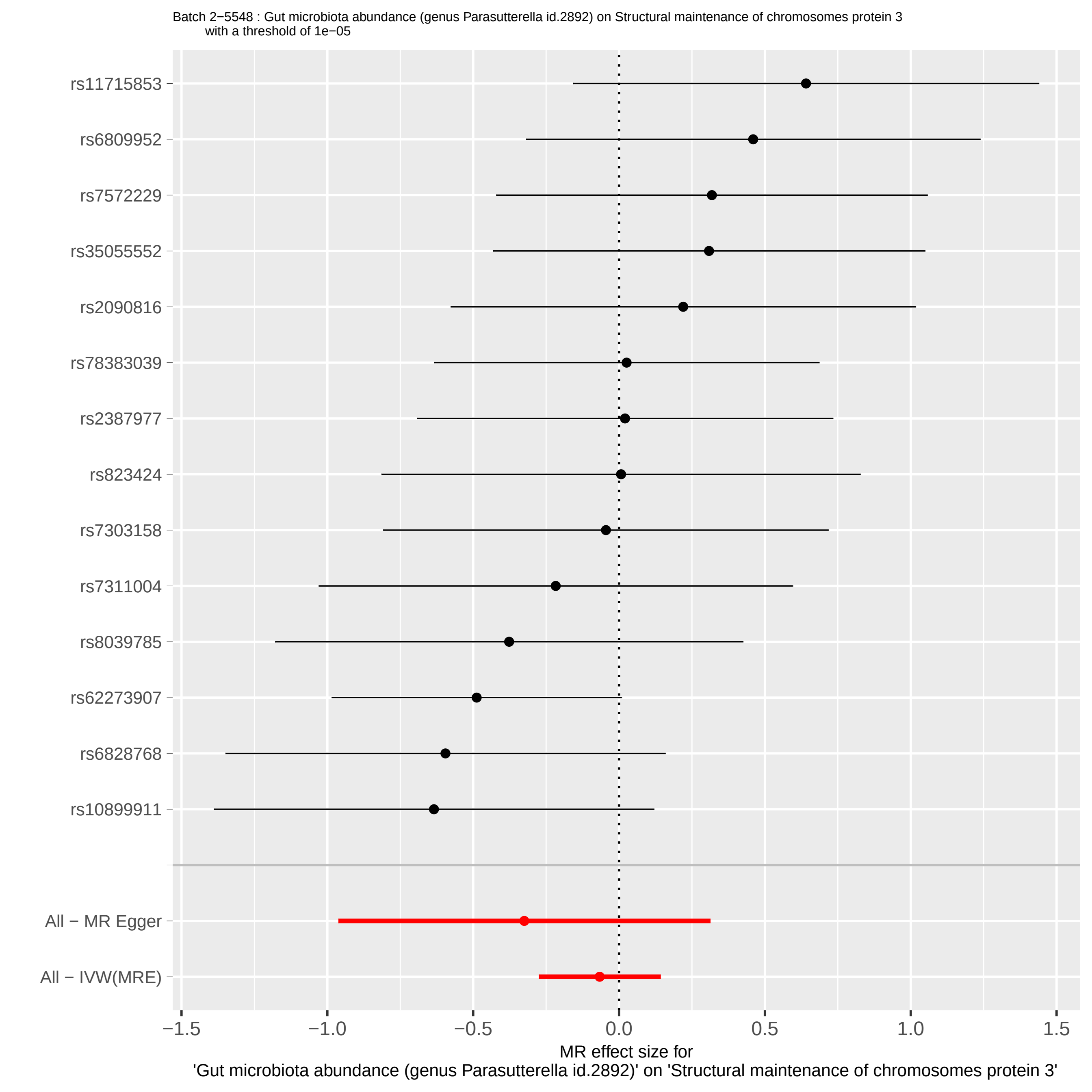
Batch 2−5548 : Gut microbiota abundance (genus Parasutterella id.2892) on Structural maintenance of chromosomes protein 3
[truncated: 415,577 more chars]
