# Supplementary material for: Investigating the role of gut microbiota in diabetic nephropathy through plasma proteome mediated analysis
Source: Sci Rep. 2025 Feb 14;15:5457. doi: 10.1038/s41598-025-90306-7 (PMC11828962; doi:10.1038/s41598-025-90306-7)
Supplement: Supplementary file 4 — Supplementary Material 4 [file 41598_2025_90306_MOESM4_ESM.pdf]

Supplementary Figure 4: Leave-one-out analyses of all MR analyses in this study

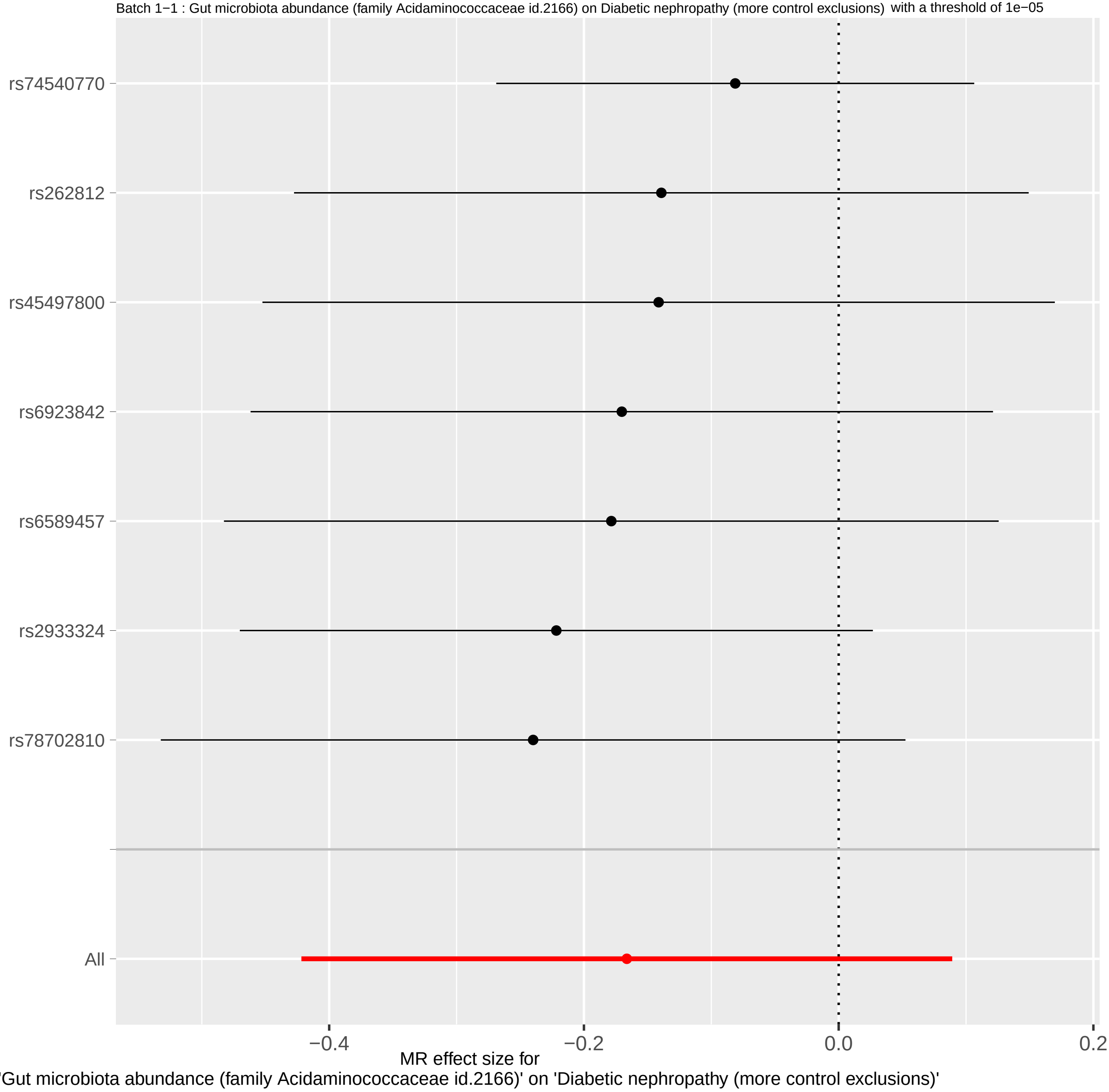

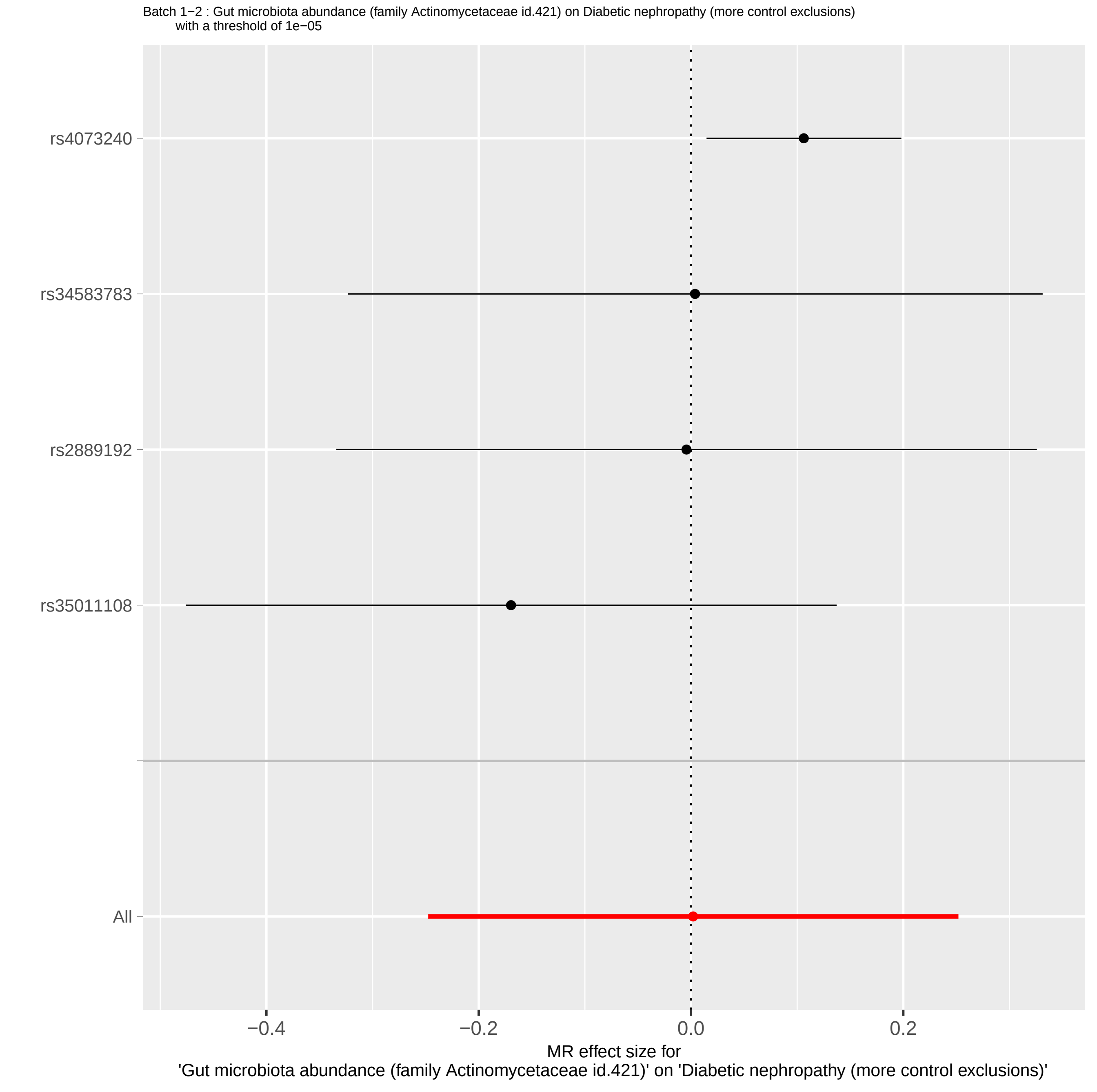

Batch 1−2 : Gut microbiota abundance (family Actinomycetaceae id.421) on Diabetic nephropathy (more control exclusions)
with a threshold of 1e−05
rs4073240
rs34583783
rs2889192
rs35011108
All
−0.4
−0.2
0.0
0.2
MR effect size for
'Gut microbiota abundance (family Actinomycetaceae id.421)' on 'Diabetic nephropathy (more control exclusions)'

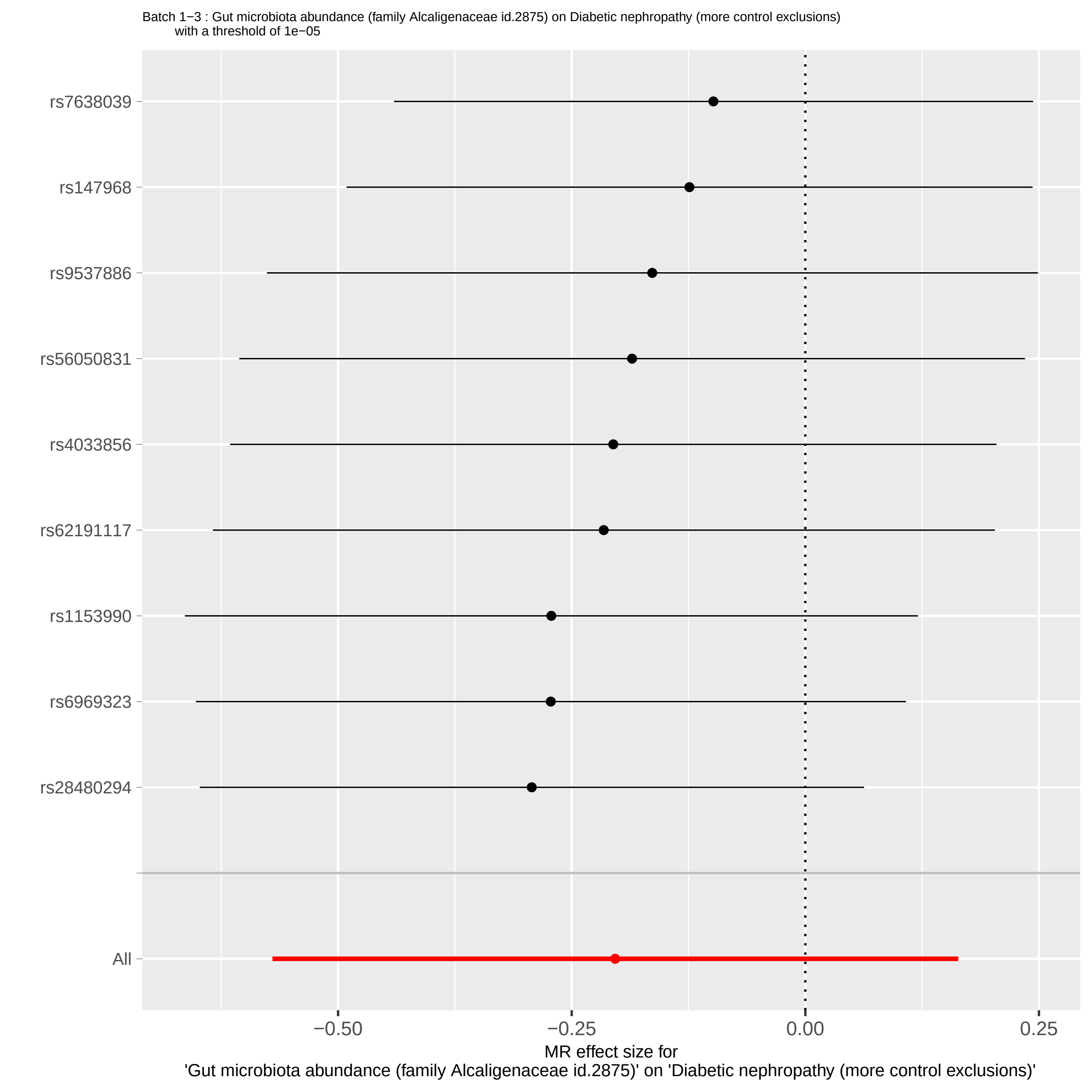
Batch 1−3 : Gut microbiota abundance (family Alcaligenaceae id.2875) on Diabetic nephropathy (more control exclusions)
with a threshold of 1e−05
rs7638039
rs147968
rs9537886
rs56050831
rs4033856
rs62191117
rs1153990
rs6969323
rs28480294
All
−0.50
−0.25
0.00
0.25
MR effect size for
'Gut microbiota abundance (family Alcaligenaceae id.2875)' on 'Diabetic nephropathy (more control exclusions)'

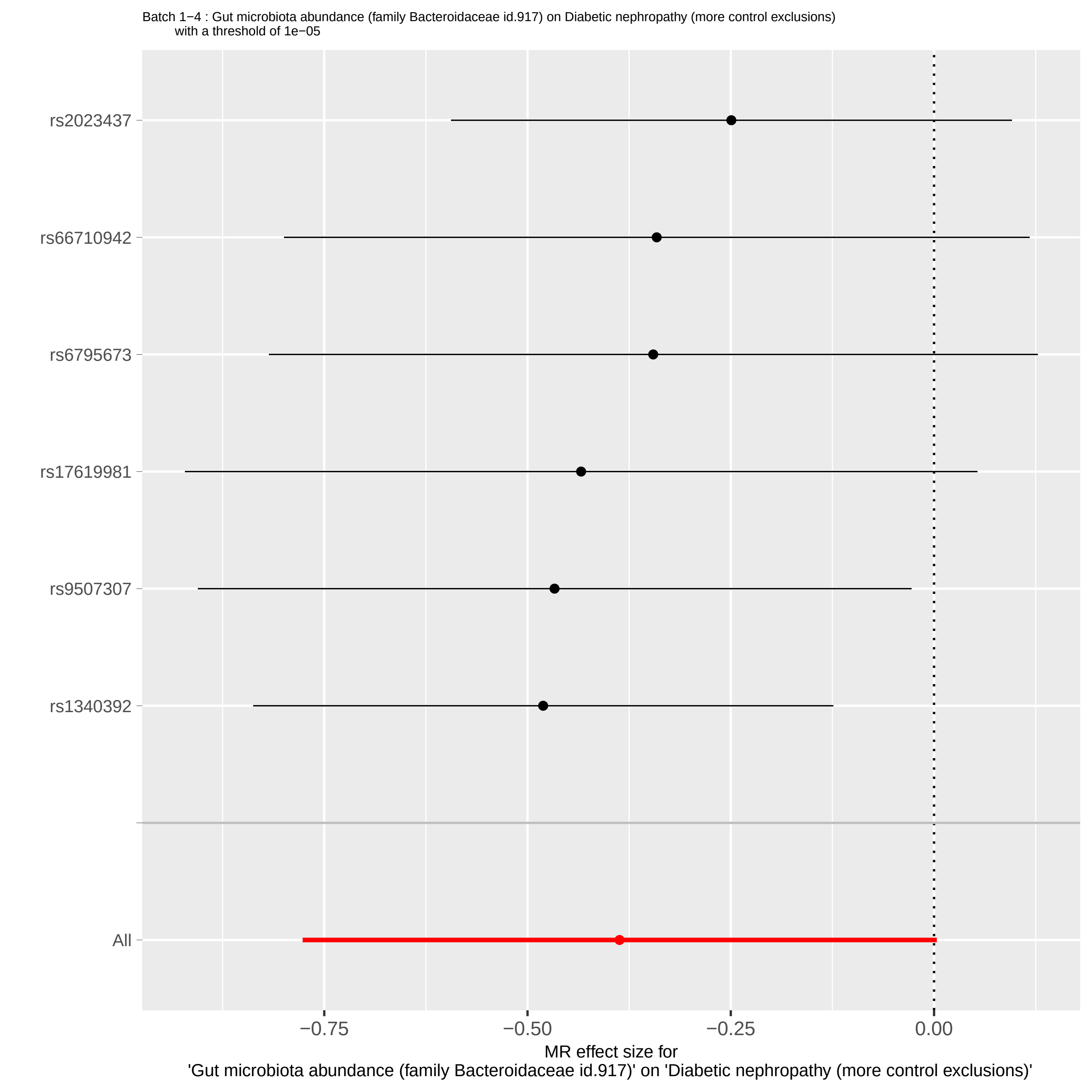
Batch 1−4 : Gut microbiota abundance (family Bacteroidaceae id.917) on Diabetic nephropathy (more control exclusions)
with a threshold of 1e−05
rs2023437
rs66710942
rs6795673
rs17619981
rs9507307
rs1340392
All
−0.75
−0.50
−0.25
0.00
MR effect size for
'Gut microbiota abundance (family Bacteroidaceae id.917)' on 'Diabetic nephropathy (more control exclusions)'

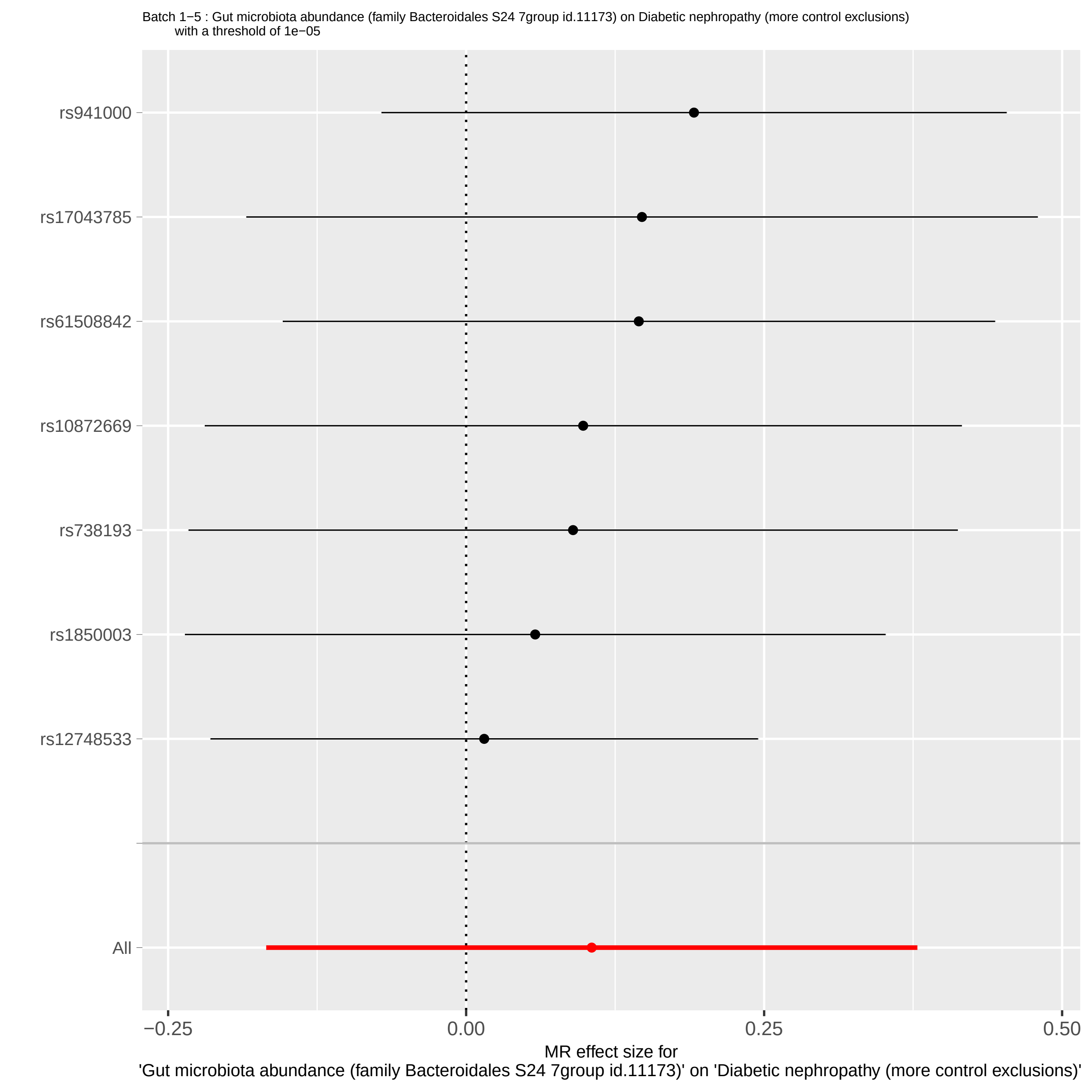
Batch 1−5 : Gut microbiota abundance (family Bacteroidales S24 7group id.11173) on Diabetic nephropathy (more control exclusions)
with a threshold of 1e−05
rs941000
rs17043785
rs61508842
rs10872669
rs738193
rs1850003
rs12748533
All
−0.25
0.00
0.25
0.50
MR effect size for
'Gut microbiota abundance (family Bacteroidales S24 7group id.11173)' on 'Diabetic nephropathy (more control exclusions)'

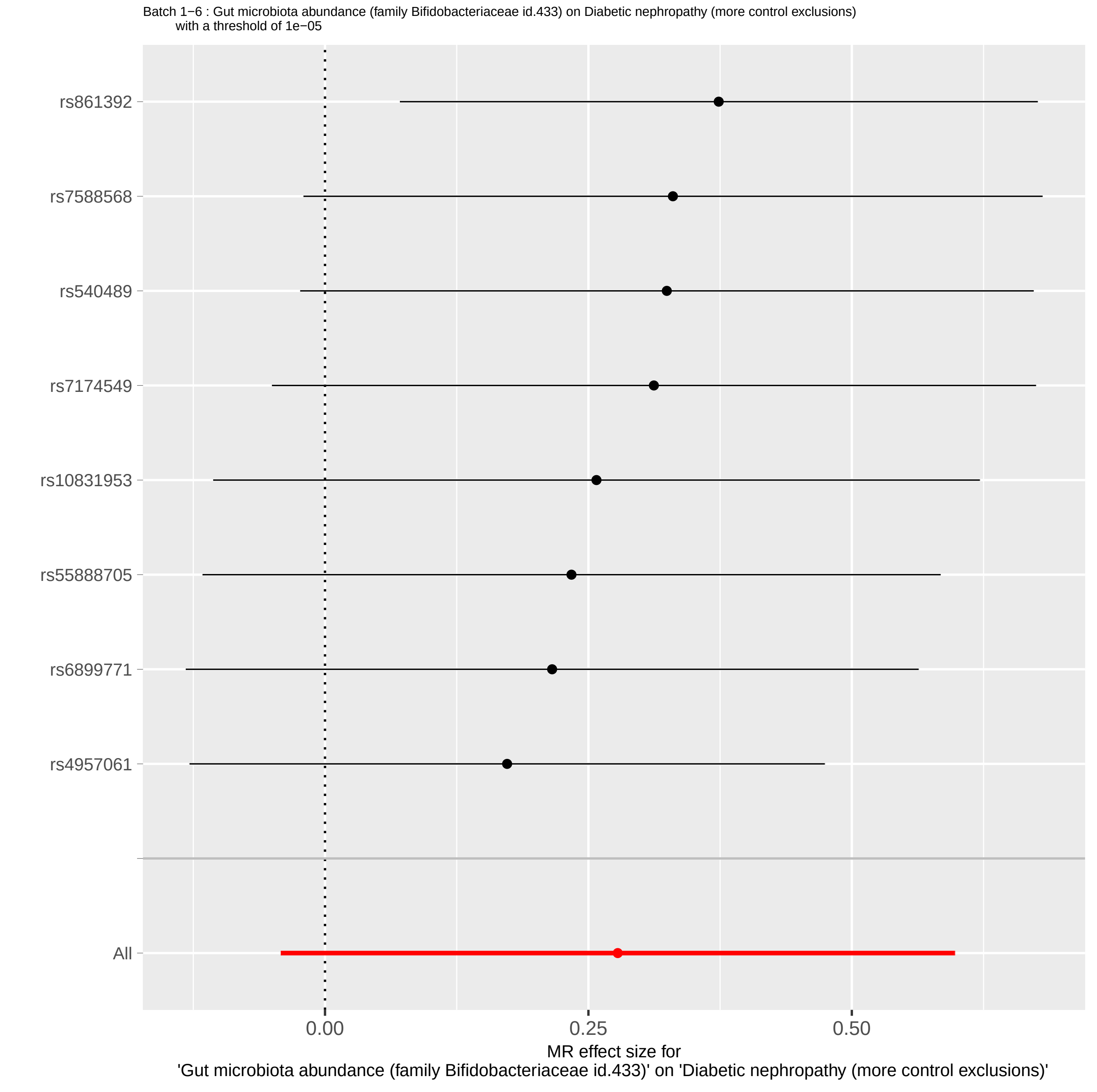

Batch 1−6 : Gut microbiota abundance (family Bifidobacteriaceae id.433) on Diabetic nephropathy (more control exclusions)
with a threshold of 1e−05
rs861392
rs7588568
rs540489
rs7174549
rs10831953
rs55888705
rs6899771
rs4957061
All
0.00
0.25
0.50
MR effect size for
'Gut microbiota abundance (family Bifidobacteriaceae id.433)' on 'Diabetic nephropathy (more control exclusions)'

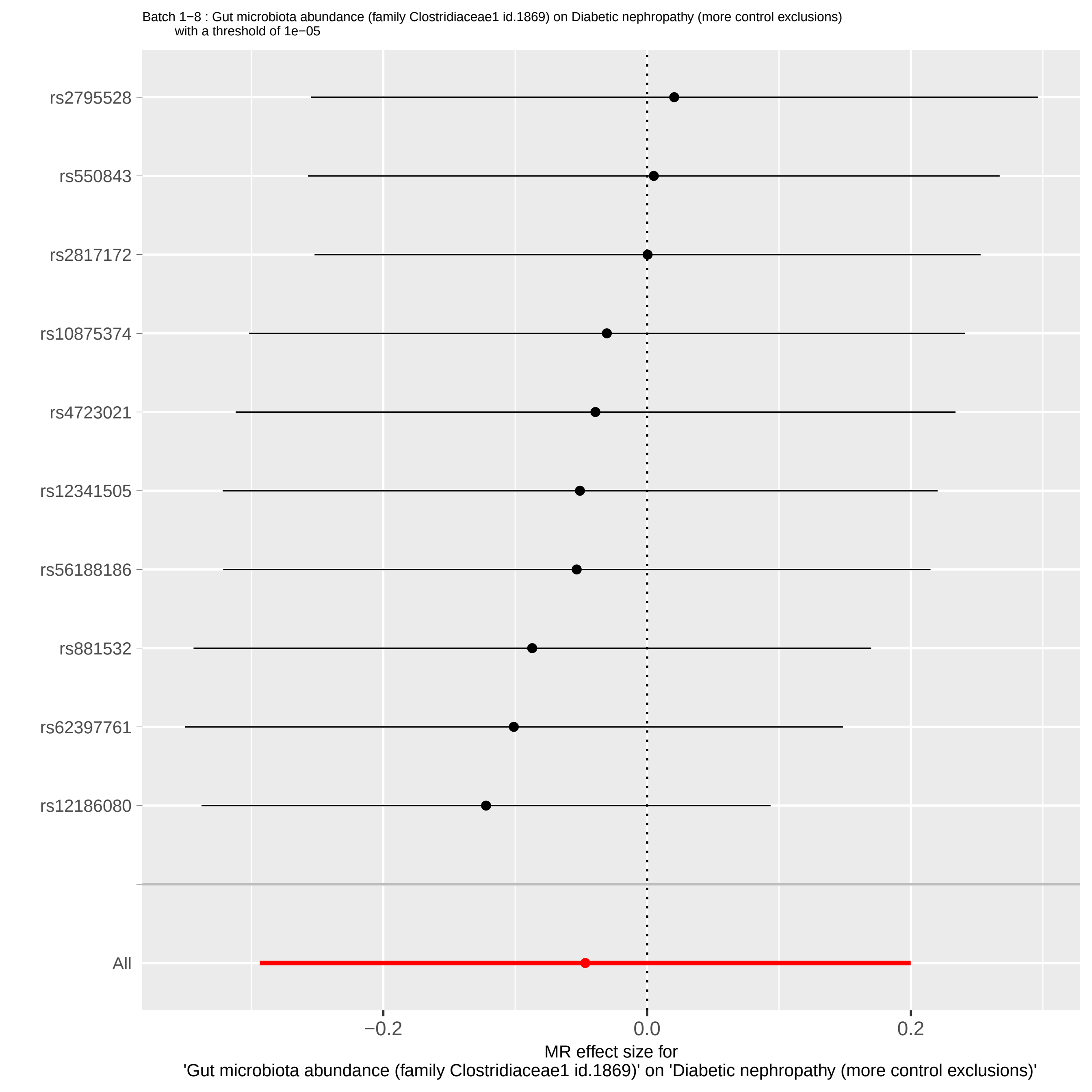

Batch 1−8 : Gut microbiota abundance (family Clostridiaceae1 id.1869) on Diabetic nephropathy (more control exclusions) with a threshold of 1e−05
rs2795528
rs550843
rs2817172
rs10875374
rs4723021
rs12341505
rs56188186
rs881532
rs62397761
rs12186080
All
−0.2
0.0
0.2
MR effect size for
'Gut microbiota abundance (family Clostridiaceae1 id.1869)' on 'Diabetic nephropathy (more control exclusions)'

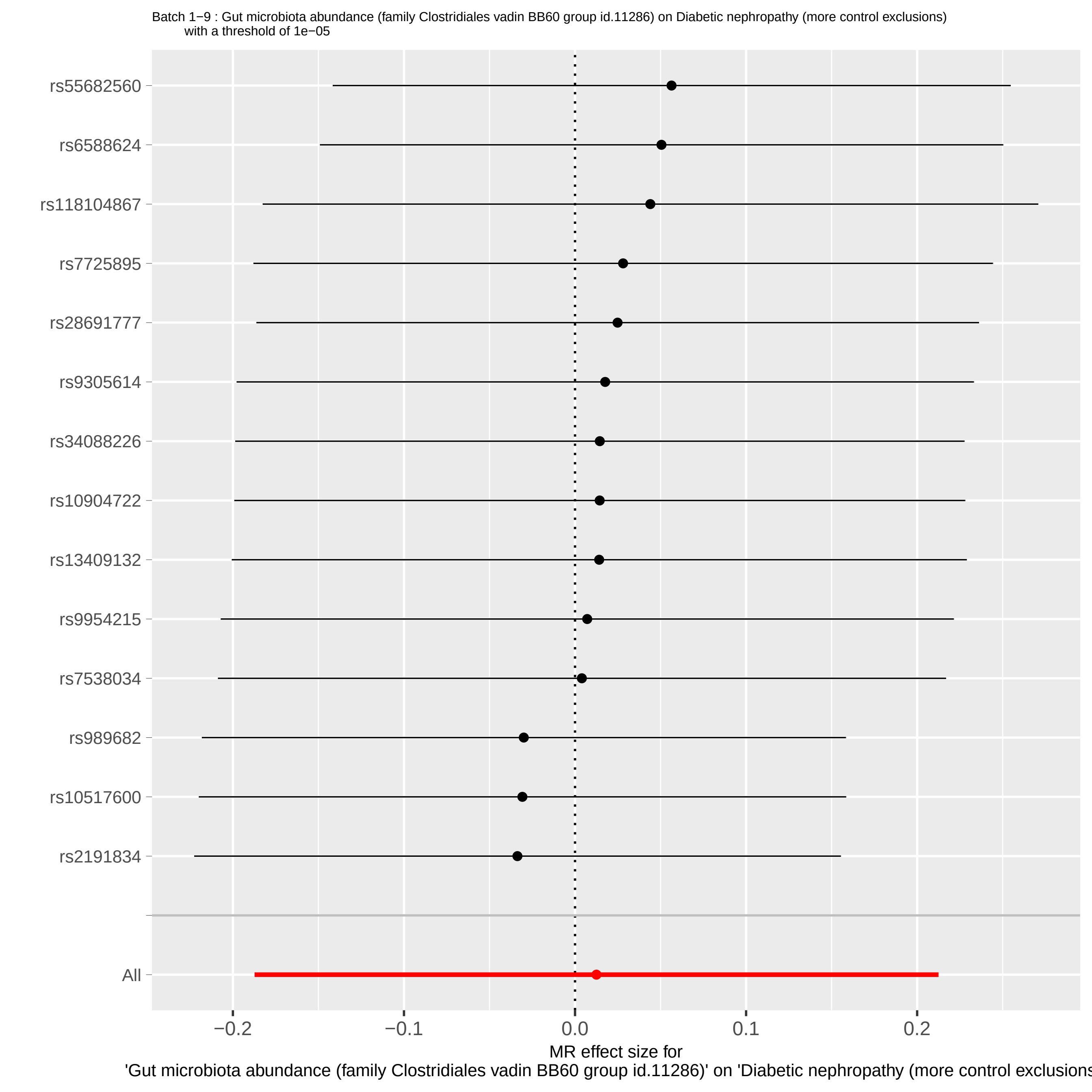
Batch 1−9 : Gut microbiota abundance (family Clostridiales vadin BB60 group id.11286) on Diabetic nephropathy (more control exclusions)
with a threshold of 1e−05
rs55682560
rs6588624
rs118104867
rs7725895
rs28691777
rs9305614
rs34088226
rs10904722
rs13409132
rs9954215
rs7538034
rs989682
rs10517600
rs2191834
All
−0.2
−0.1
0.0
0.1
0.2
MR effect size for
'Gut microbiota abundance (family Clostridiales vadin BB60 group id.11286)' on 'Diabetic nephropathy (more control exclusions

Batch 1−10 : Gut microbiota abundance (family Coriobacteriaceae id.811) on Diabetic nephropathy (more control exclusions) with a threshold of 1e−05

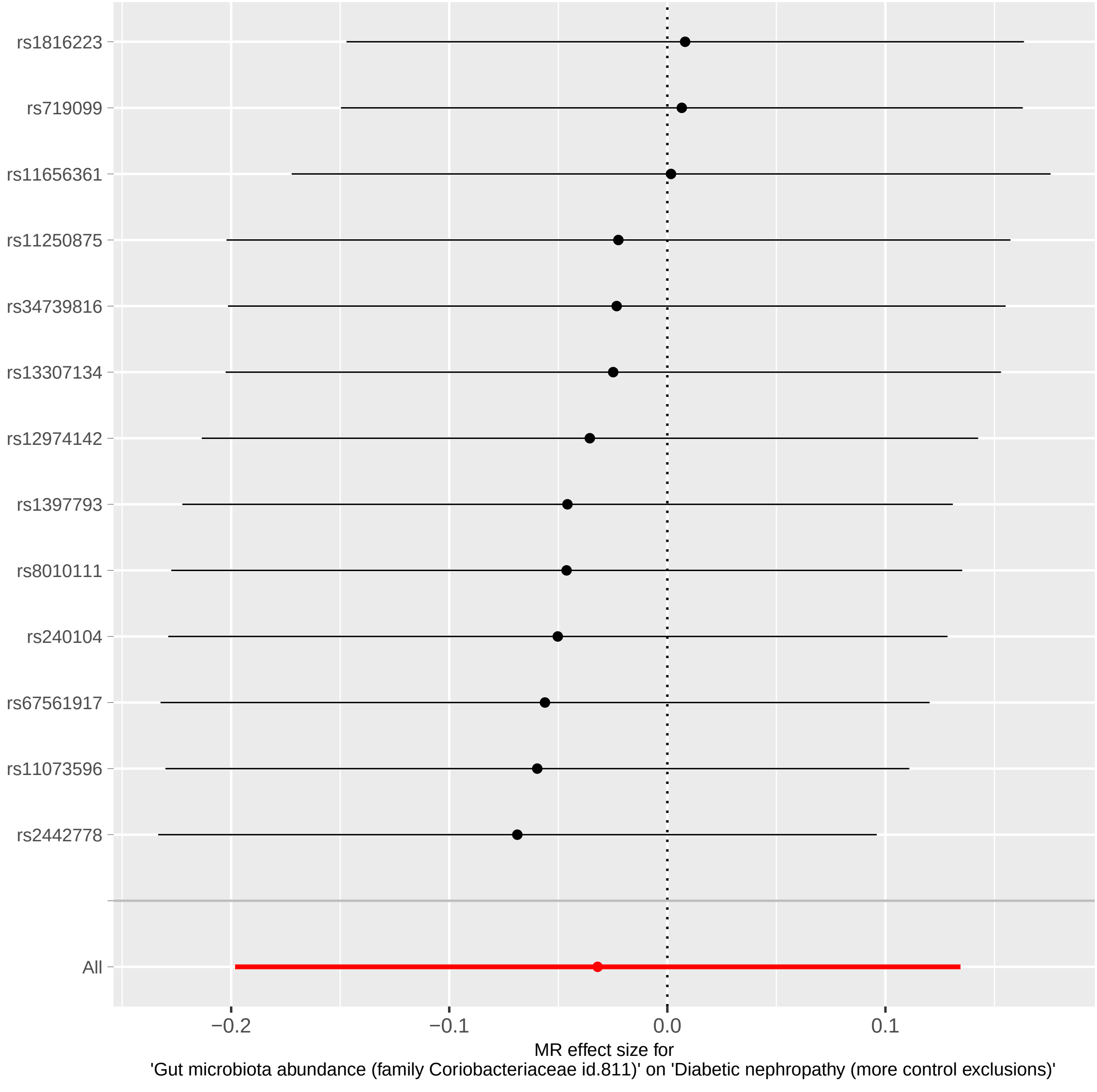

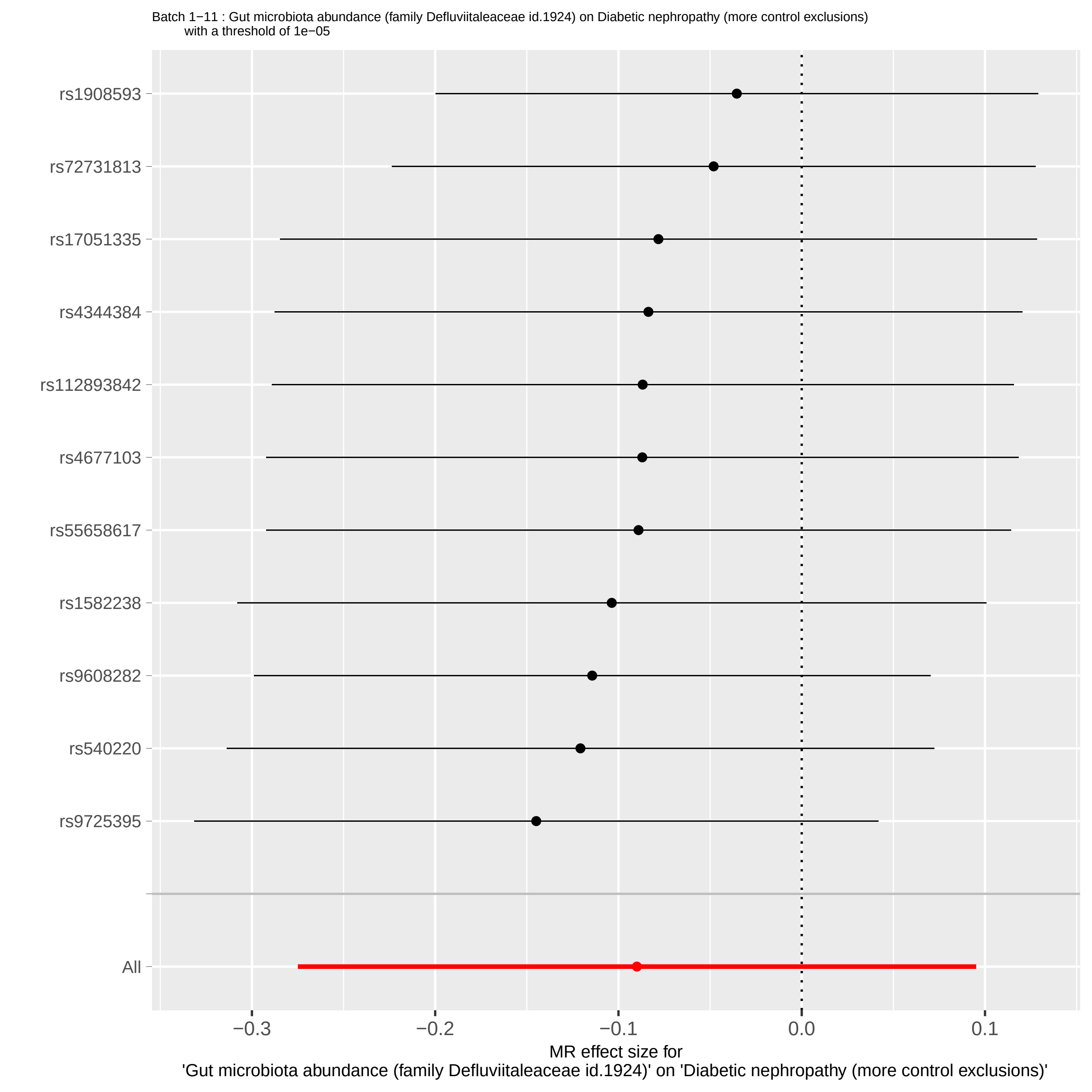
Batch 1−11 : Gut microbiota abundance (family Defluviitaleaceae id.1924) on Diabetic nephropathy (more control exclusions)
with a threshold of 1e−05
rs1908593
rs72731813
rs17051335
rs4344384
rs112893842
rs4677103
rs55658617
rs1582238
rs9608282
rs540220
rs9725395
All
−0.3
−0.2
−0.1
0.0
0.1
MR effect size for
'Gut microbiota abundance (family Defluviitaleaceae id.1924)' on 'Diabetic nephropathy (more control exclusions)'

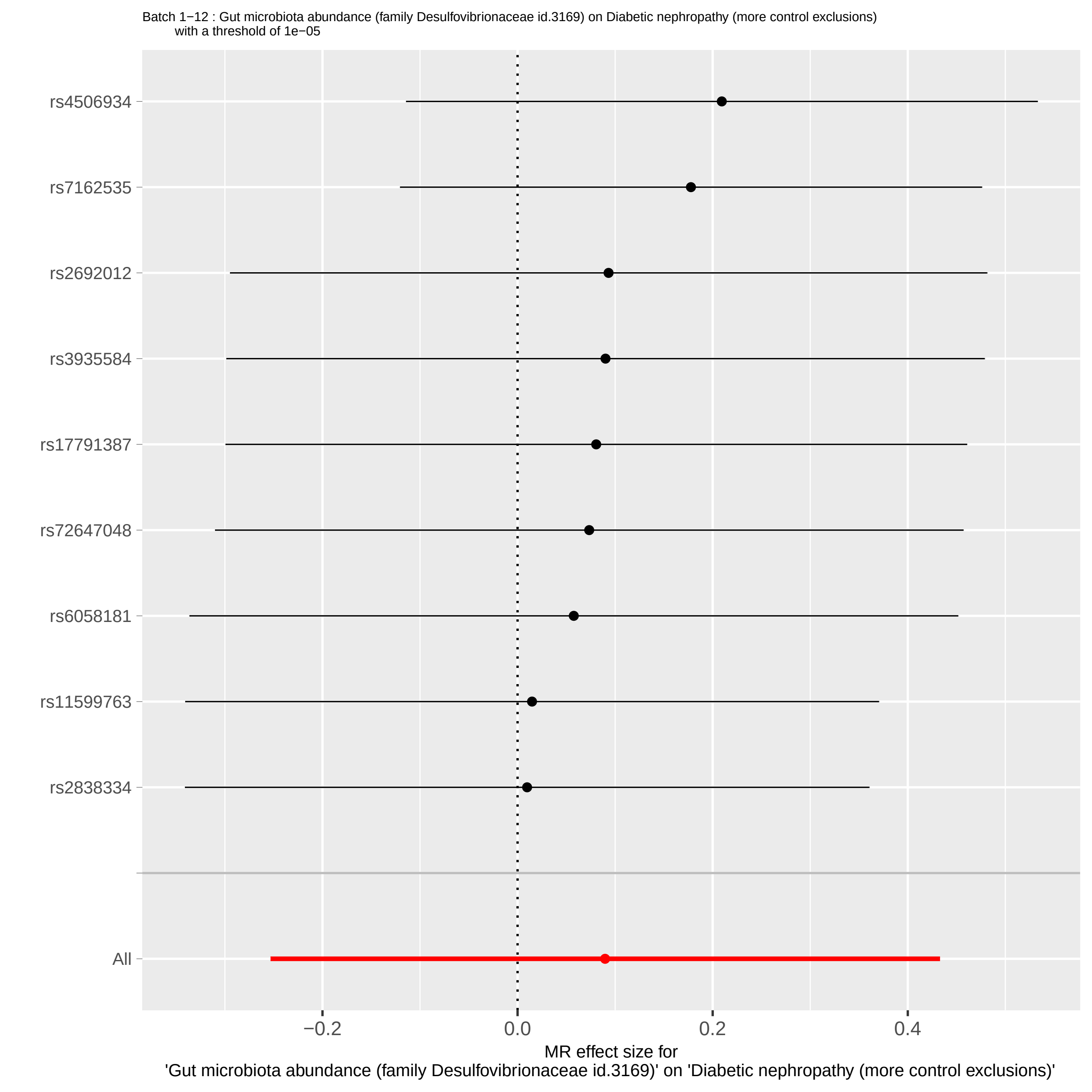

Batch 1−12 : Gut microbiota abundance (family Desulfovibrionaceae id.3169) on Diabetic nephropathy (more control exclusions)
with a threshold of 1e−05
rs4506934
rs7162535
rs2692012
rs3935584
rs17791387
rs72647048
rs6058181
rs11599763
rs2838334
All
−0.2
0.0
0.2
0.4
MR effect size for
'Gut microbiota abundance (family Desulfovibrionaceae id.3169)' on 'Diabetic nephropathy (more control exclusions)'

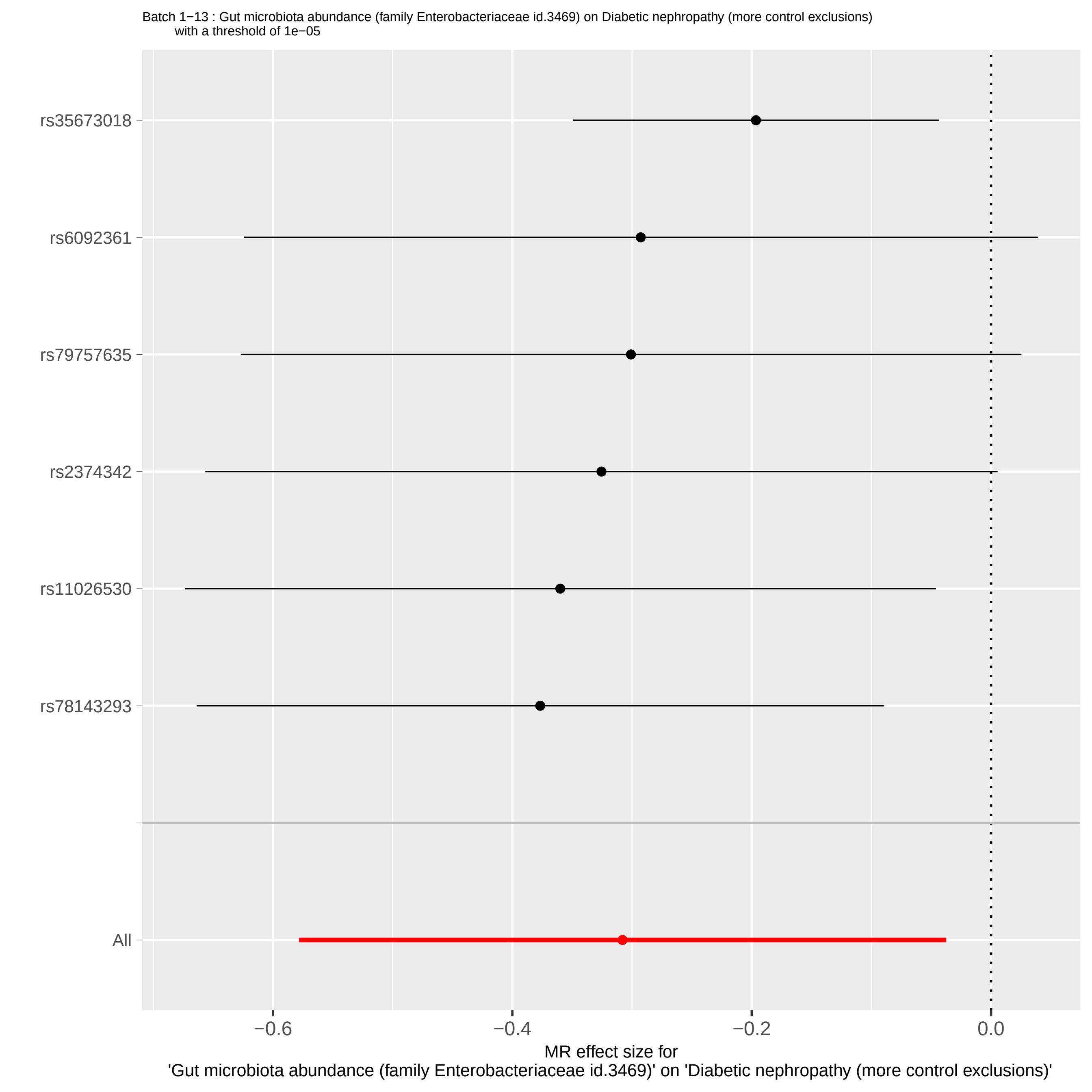
Batch 1−13 : Gut microbiota abundance (family Enterobacteriaceae id.3469) on Diabetic nephropathy (more control exclusions)
with a threshold of 1e−05
rs35673018
rs6092361
rs79757635
rs2374342
rs11026530
rs78143293
All
−0.6
−0.4
−0.2
0.0
MR effect size for
'Gut microbiota abundance (family Enterobacteriaceae id.3469)' on 'Diabetic nephropathy (more control exclusions)'

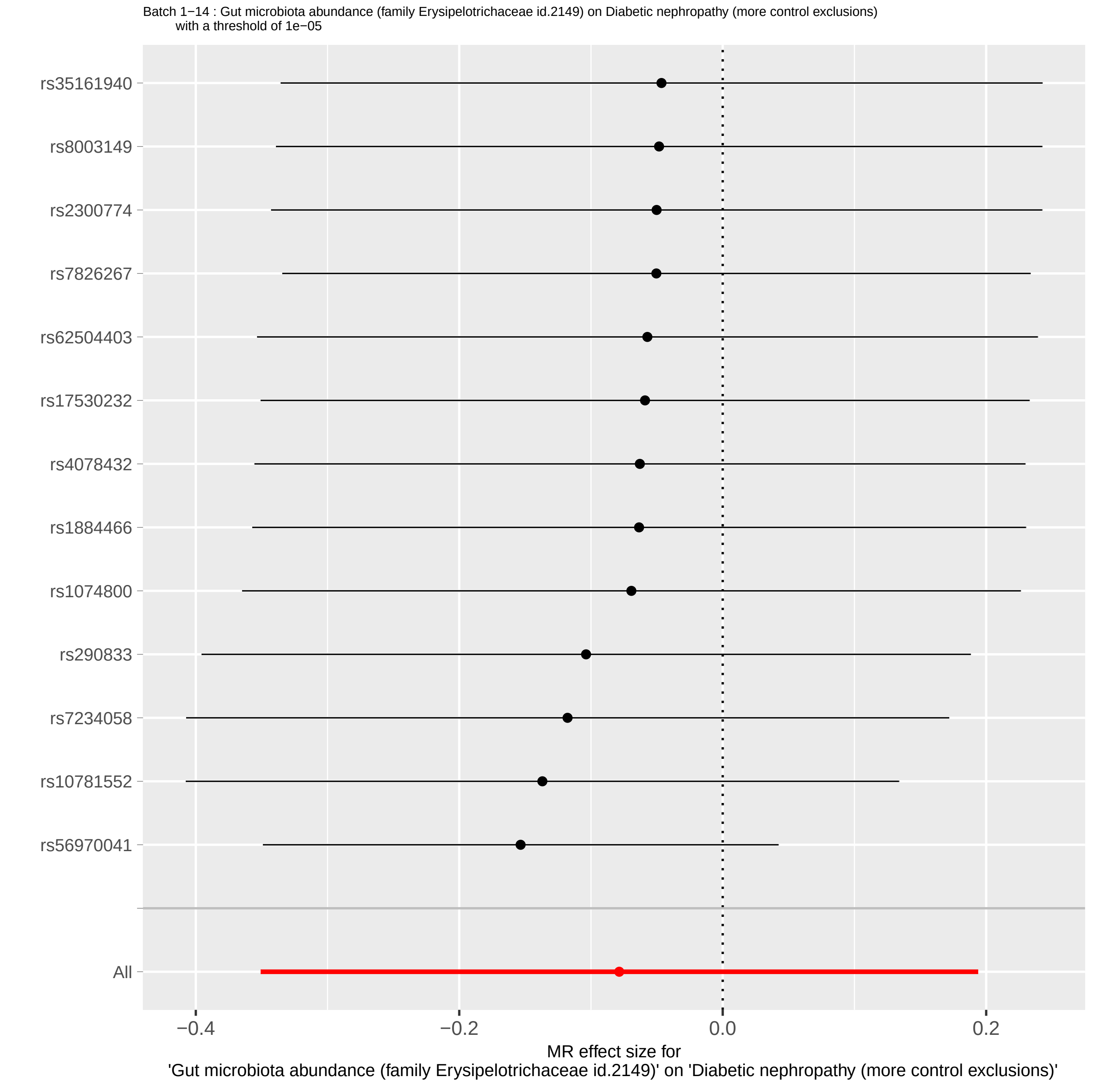

Batch 1−14 : Gut microbiota abundance (family Erysipelotrichaceae id.2149) on Diabetic nephropathy (more control exclusions)
with a threshold of 1e−05
rs35161940
rs8003149
rs2300774
rs7826267
rs62504403
rs17530232
rs4078432
rs1884466
rs1074800
rs290833
rs7234058
rs10781552
rs56970041
All
−0.4
−0.2
0.0
0.2
MR effect size for
'Gut microbiota abundance (family Erysipelotrichaceae id.2149)' on 'Diabetic nephropathy (more control exclusions)'

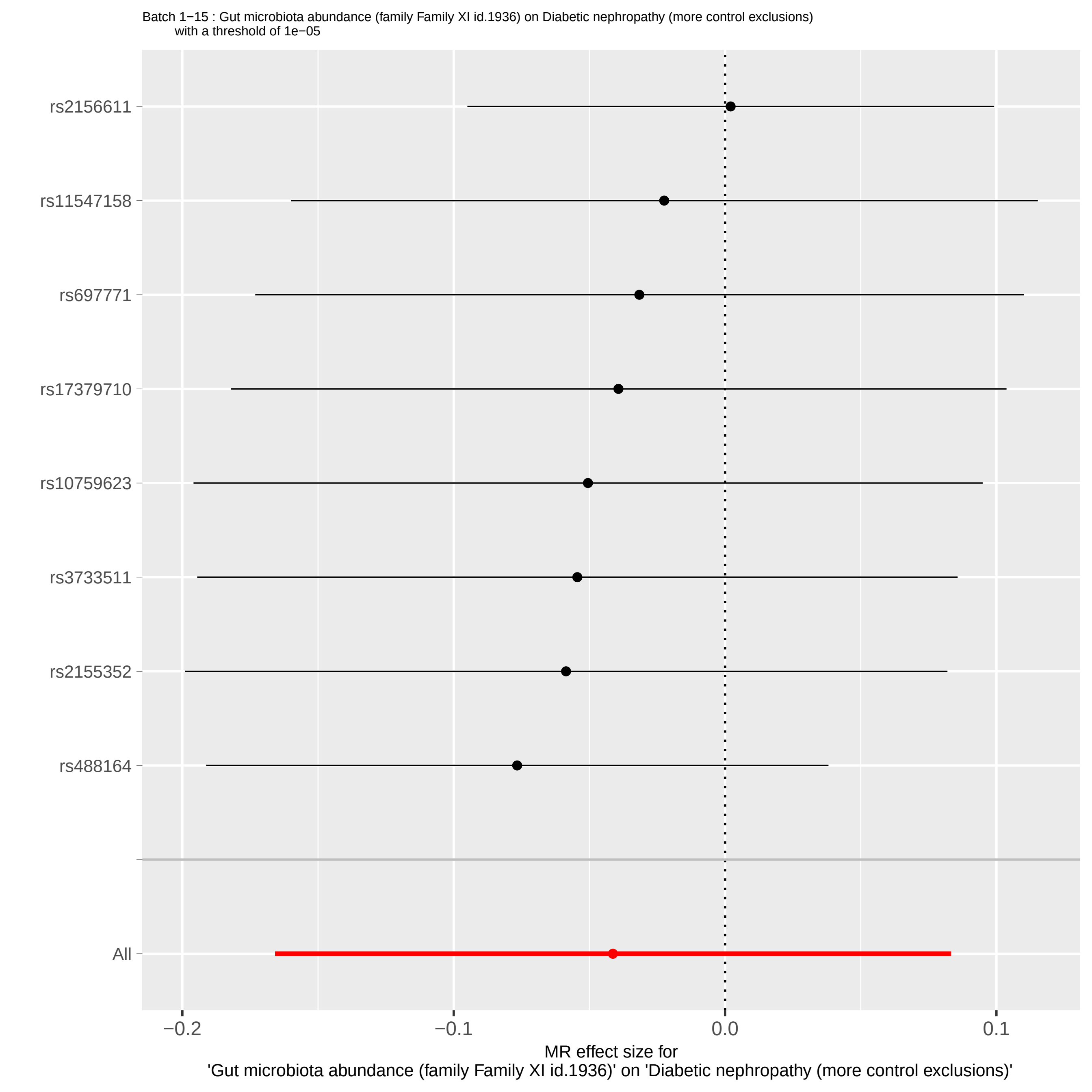

Batch 1−15 : Gut microbiota abundance (family Family XI id.1936) on Diabetic nephropathy (more control exclusions)
with a threshold of 1e−05
rs2156611
rs11547158
rs697771
rs17379710
rs10759623
rs3733511
rs2155352
rs488164
All
−0.2
−0.1
0.0
0.1
MR effect size for
'Gut microbiota abundance (family Family XI id.1936)' on 'Diabetic nephropathy (more control exclusions)'

Batch 1−16 : Gut microbiota abundance (family Family XIII id.1957) on Diabetic nephropathy (more control exclusions) with a threshold of 1e−05

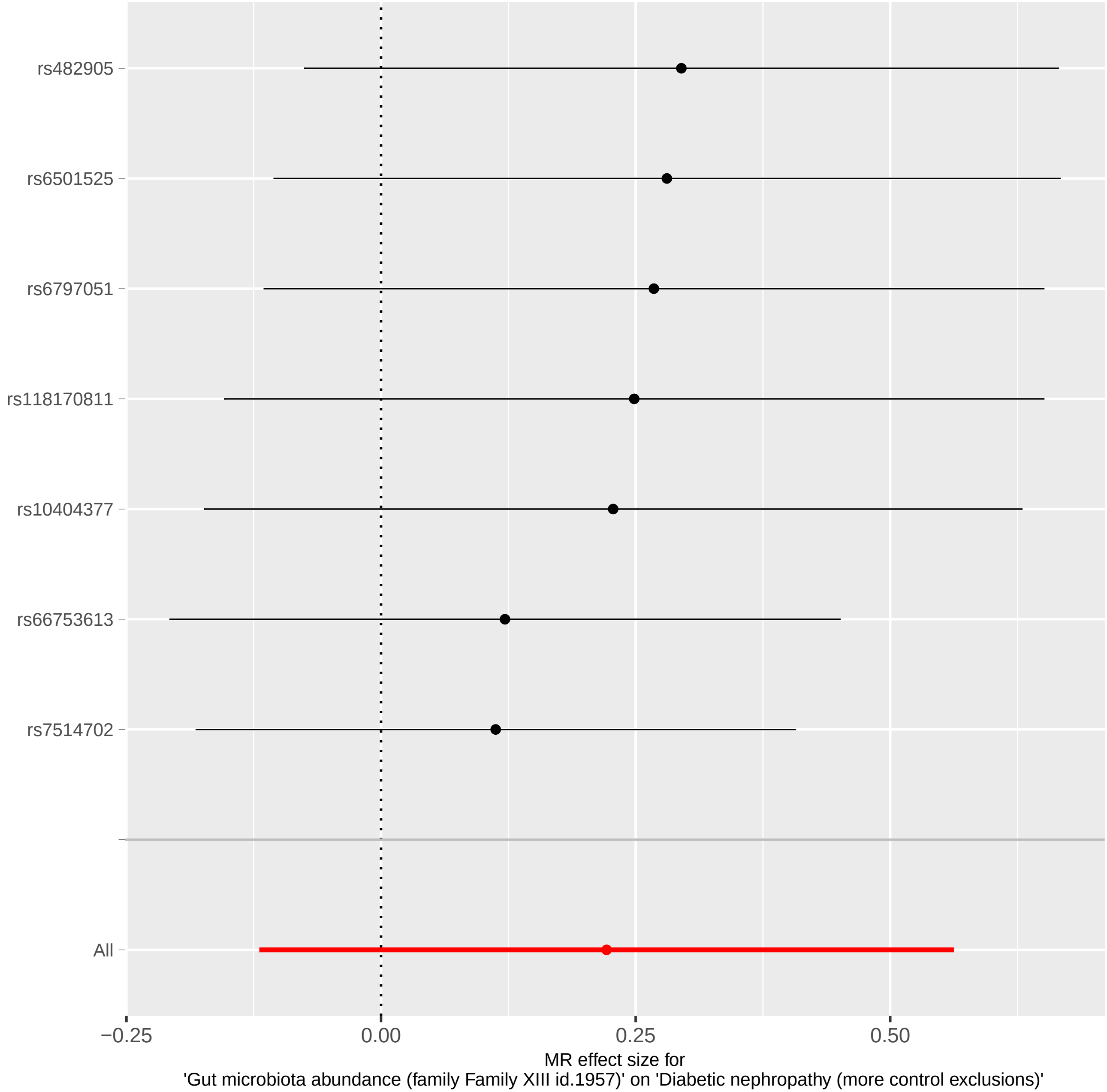

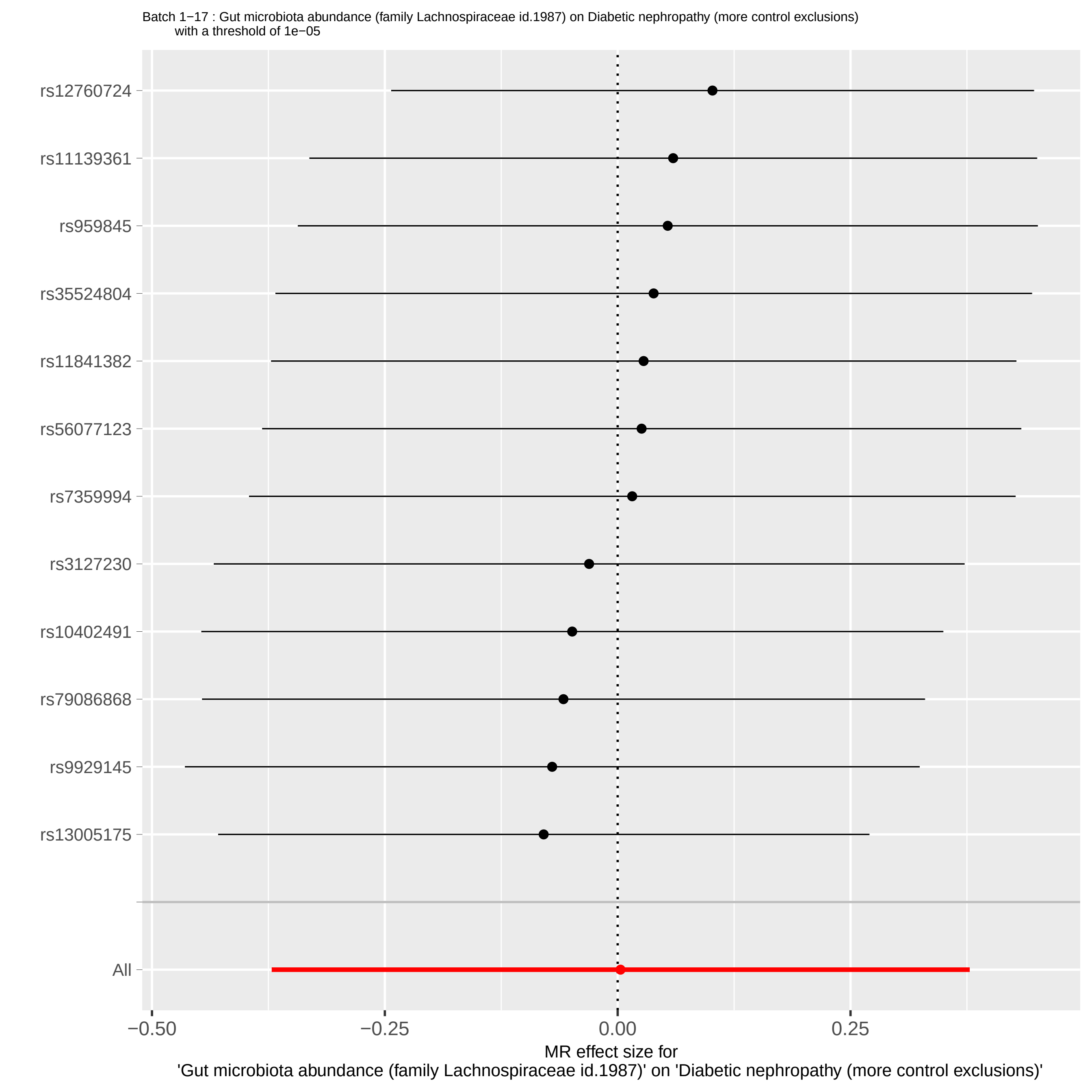

Batch 1−17 : Gut microbiota abundance (family Lachnospiraceae id.1987) on Diabetic nephropathy (more control exclusions) with a threshold of 1e−05
rs12760724
rs11139361
rs959845
rs35524804
rs11841382
rs56077123
rs7359994
rs3127230
rs10402491
rs79086868
rs9929145
rs13005175
All
−0.50
−0.25
0.00
0.25
MR effect size for
'Gut microbiota abundance (family Lachnospiraceae id.1987)' on 'Diabetic nephropathy (more control exclusions)'

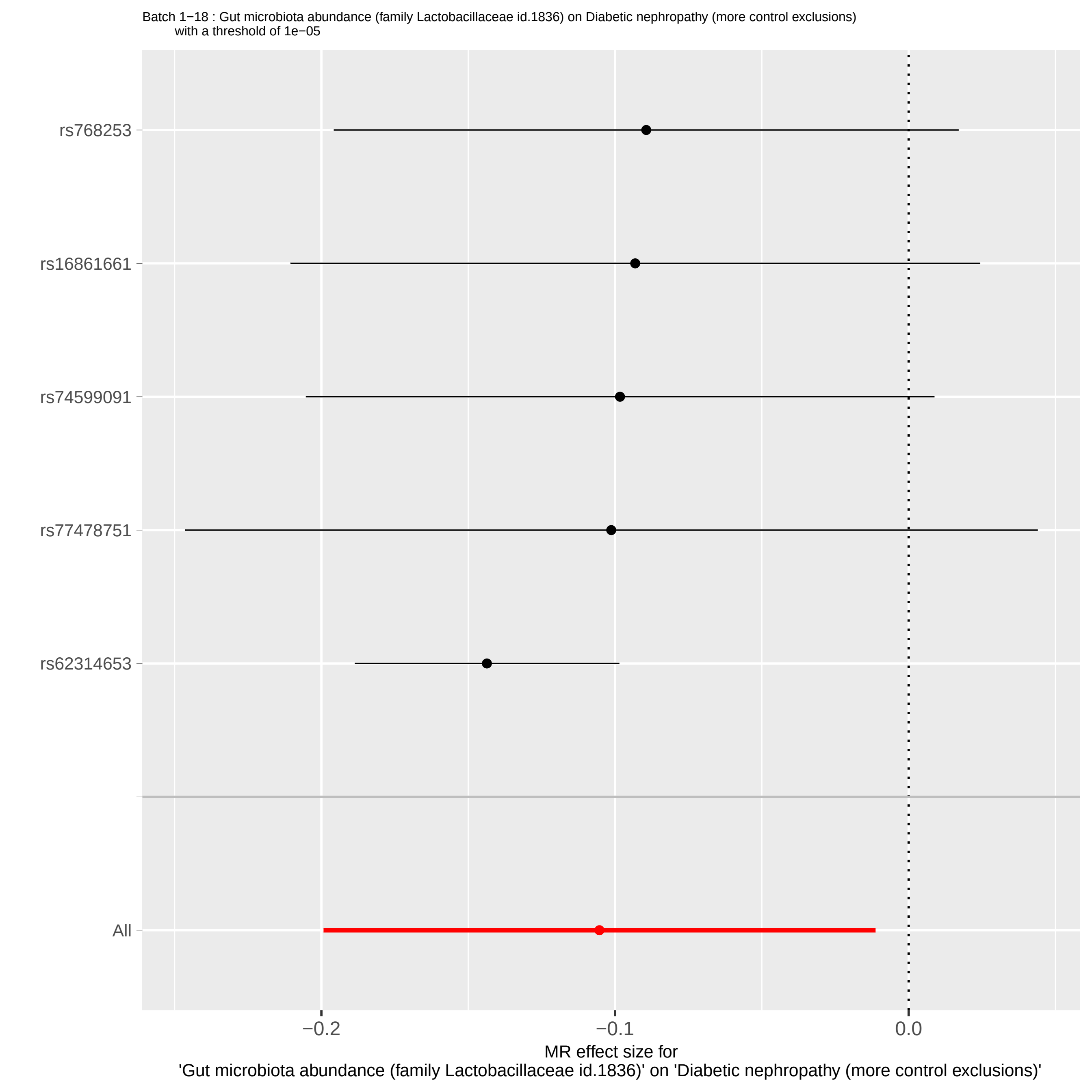
Batch 1−18 : Gut microbiota abundance (family Lactobacillaceae id.1836) on Diabetic nephropathy (more control exclusions)
with a threshold of 1e−05
rs768253
rs16861661
rs74599091
rs77478751
rs62314653
All
−0.2
−0.1
0.0
MR effect size for
'Gut microbiota abundance (family Lactobacillaceae id.1836)' on 'Diabetic nephropathy (more control exclusions)'

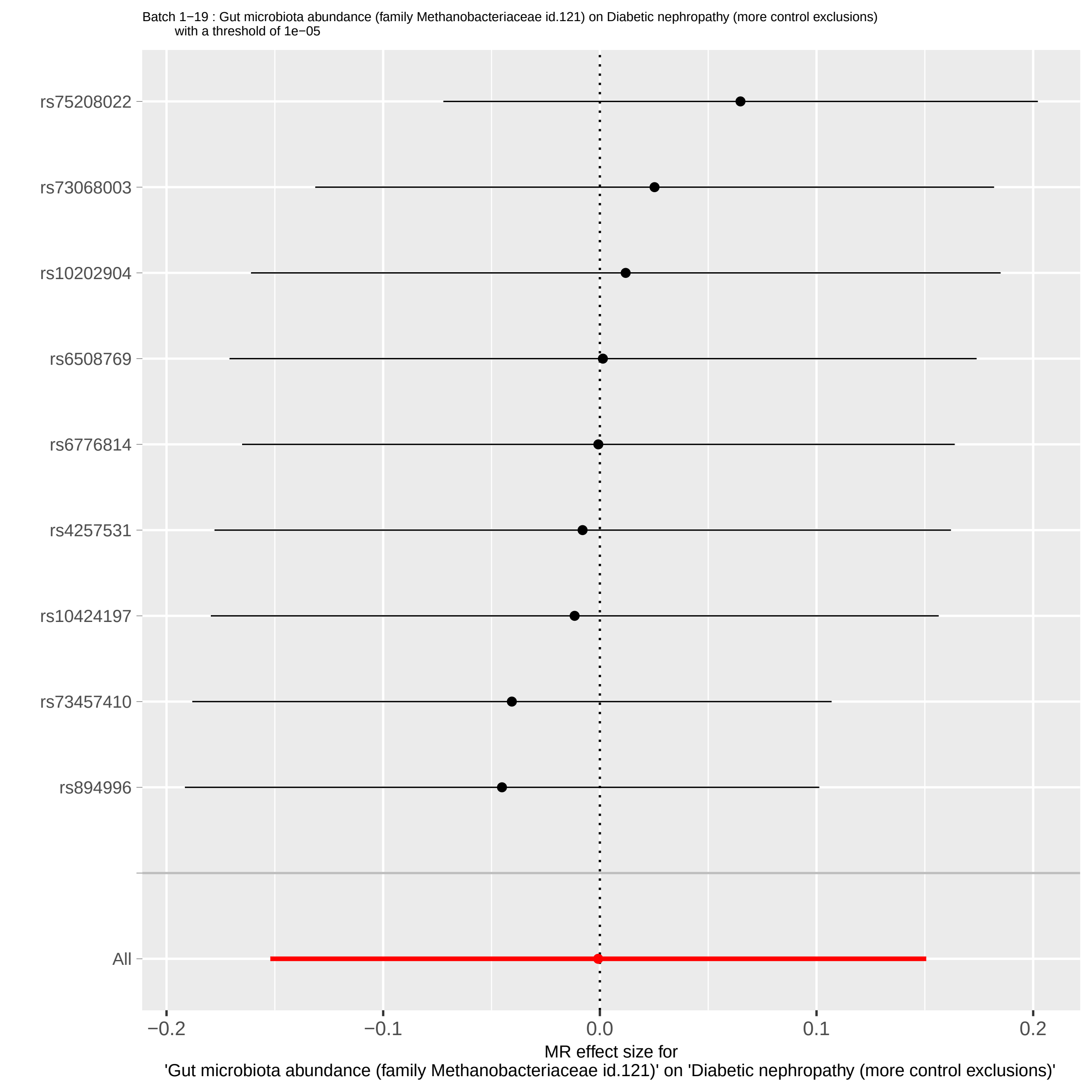

Batch 1−19 : Gut microbiota abundance (family Methanobacteriaceae id.121) on Diabetic nephropathy (more control exclusions)
with a threshold of 1e−05
rs75208022
rs73068003
rs10202904
rs6508769
rs6776814
rs4257531
rs10424197
rs73457410
rs894996
All
−0.2
−0.1
0.0
0.1
0.2
MR effect size for
'Gut microbiota abundance (family Methanobacteriaceae id.121)' on 'Diabetic nephropathy (more control exclusions)'

Batch 1−20 : Gut microbiota abundance (family Oxalobacteraceae id.2966) on Diabetic nephropathy (more control exclusions) with a threshold of 1e−05

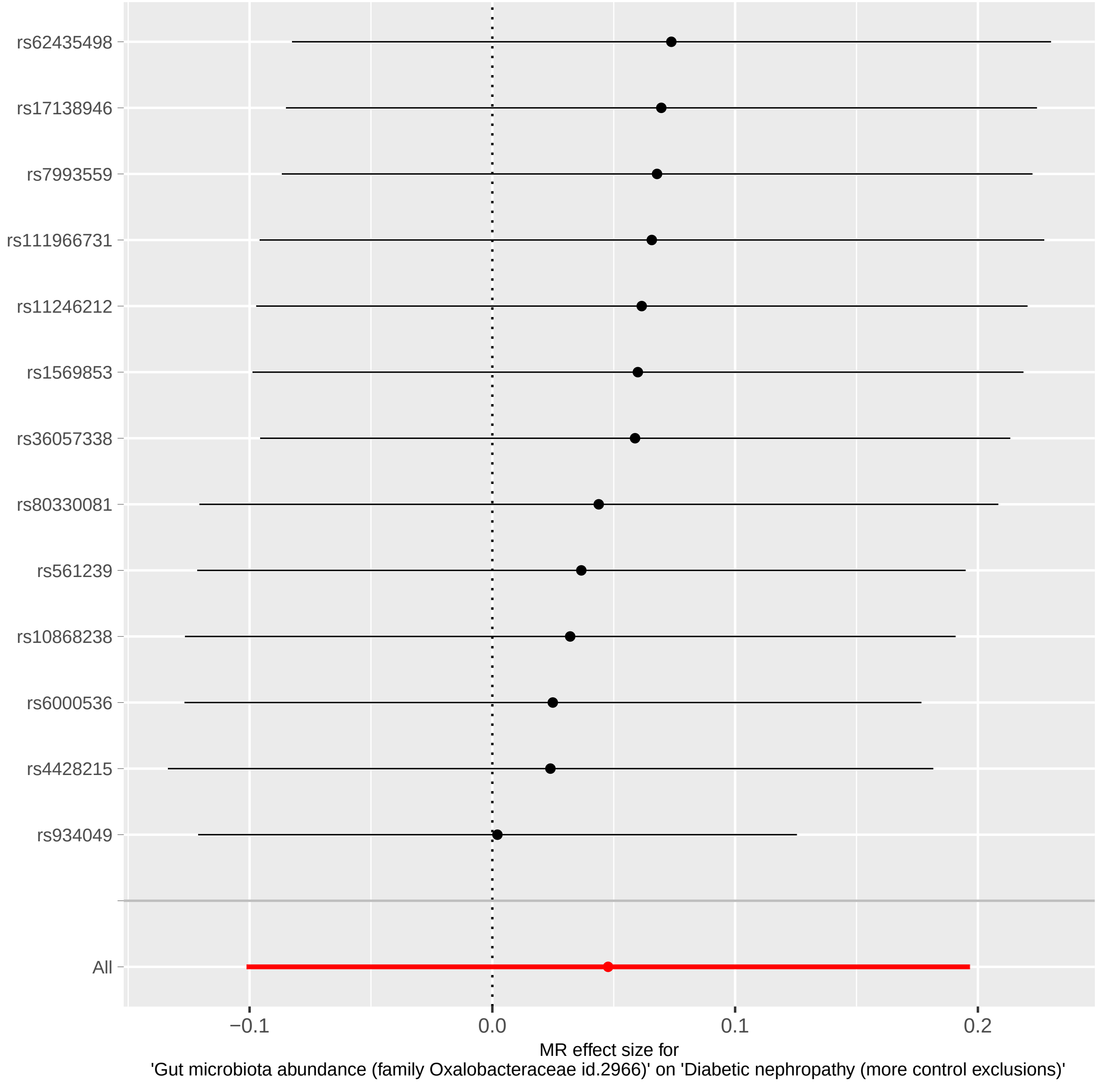

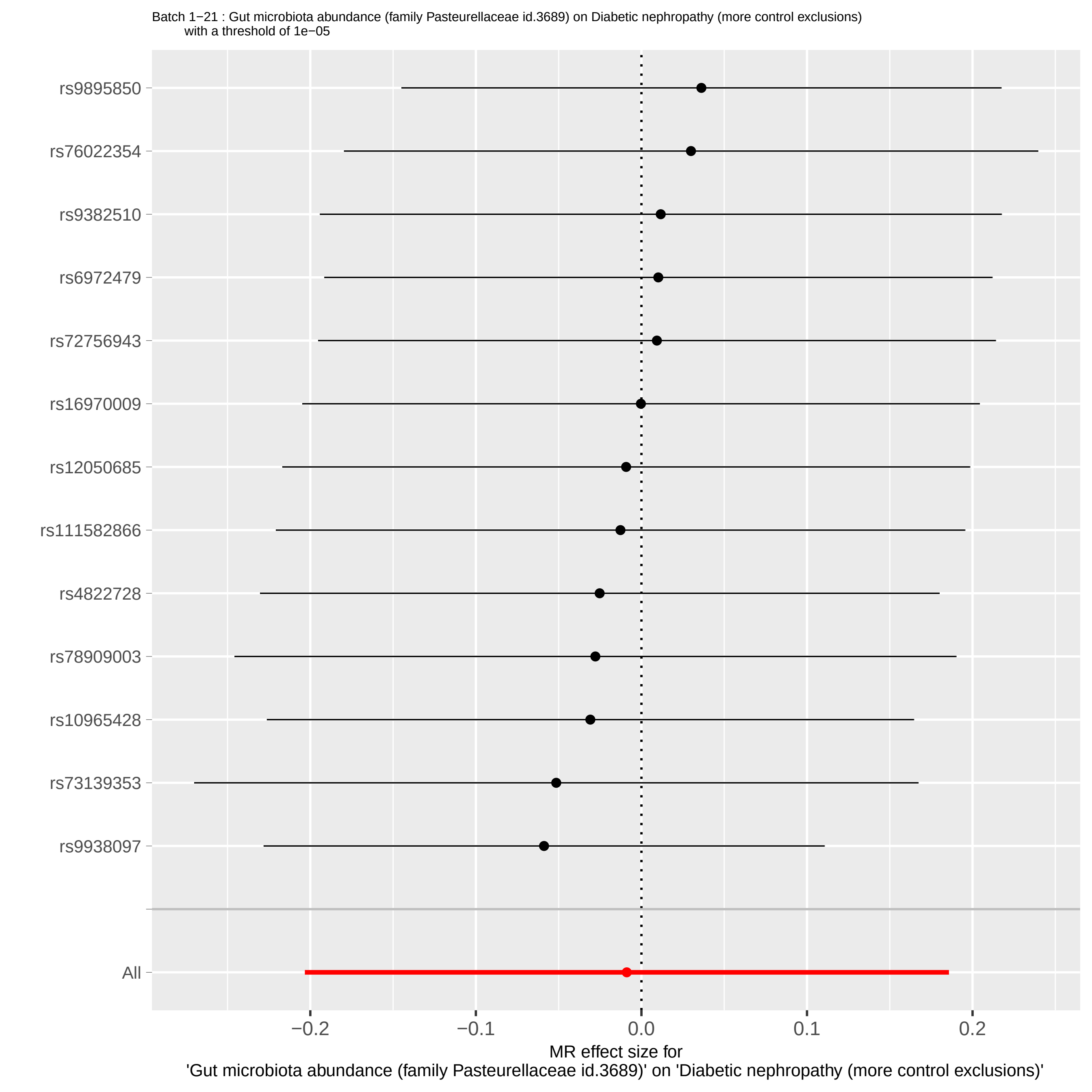

Batch 1−21 : Gut microbiota abundance (family Pasteurellaceae id.3689) on Diabetic nephropathy (more control exclusions)
with a threshold of 1e−05
rs9895850
rs76022354
rs9382510
rs6972479
rs72756943
rs16970009
rs12050685
rs111582866
rs4822728
rs78909003
rs10965428
rs73139353
rs9938097
All
−0.2
−0.1
0.0
0.1
0.2
MR effect size for
'Gut microbiota abundance (family Pasteurellaceae id.3689)' on 'Diabetic nephropathy (more control exclusions)'

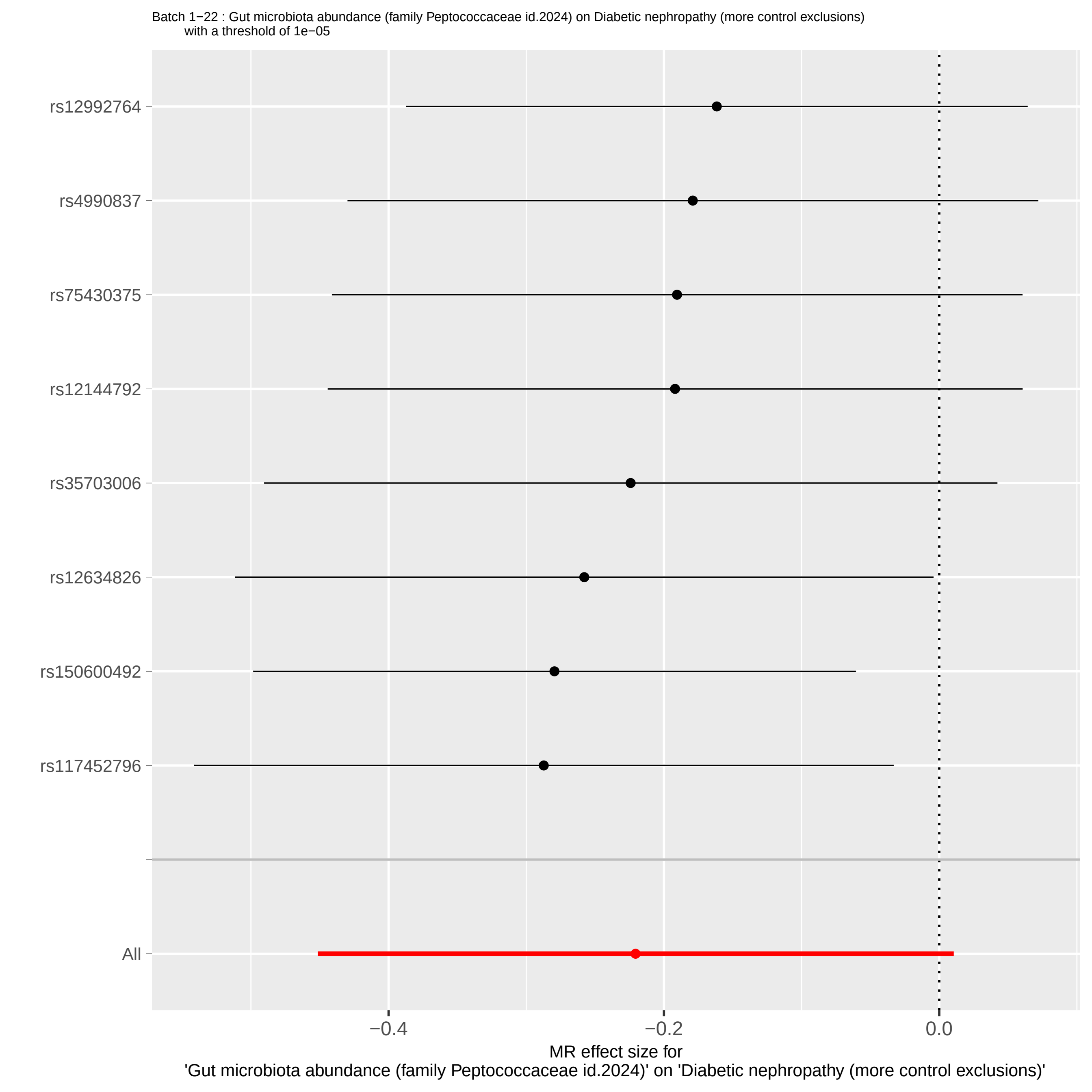

Batch 1−22 : Gut microbiota abundance (family Peptococcaceae id.2024) on Diabetic nephropathy (more control exclusions)
with a threshold of 1e−05
rs12992764
rs4990837
rs75430375
rs12144792
rs35703006
rs12634826
rs150600492
rs117452796
All
−0.4
−0.2
0.0
MR effect size for
'Gut microbiota abundance (family Peptococcaceae id.2024)' on 'Diabetic nephropathy (more control exclusions)'

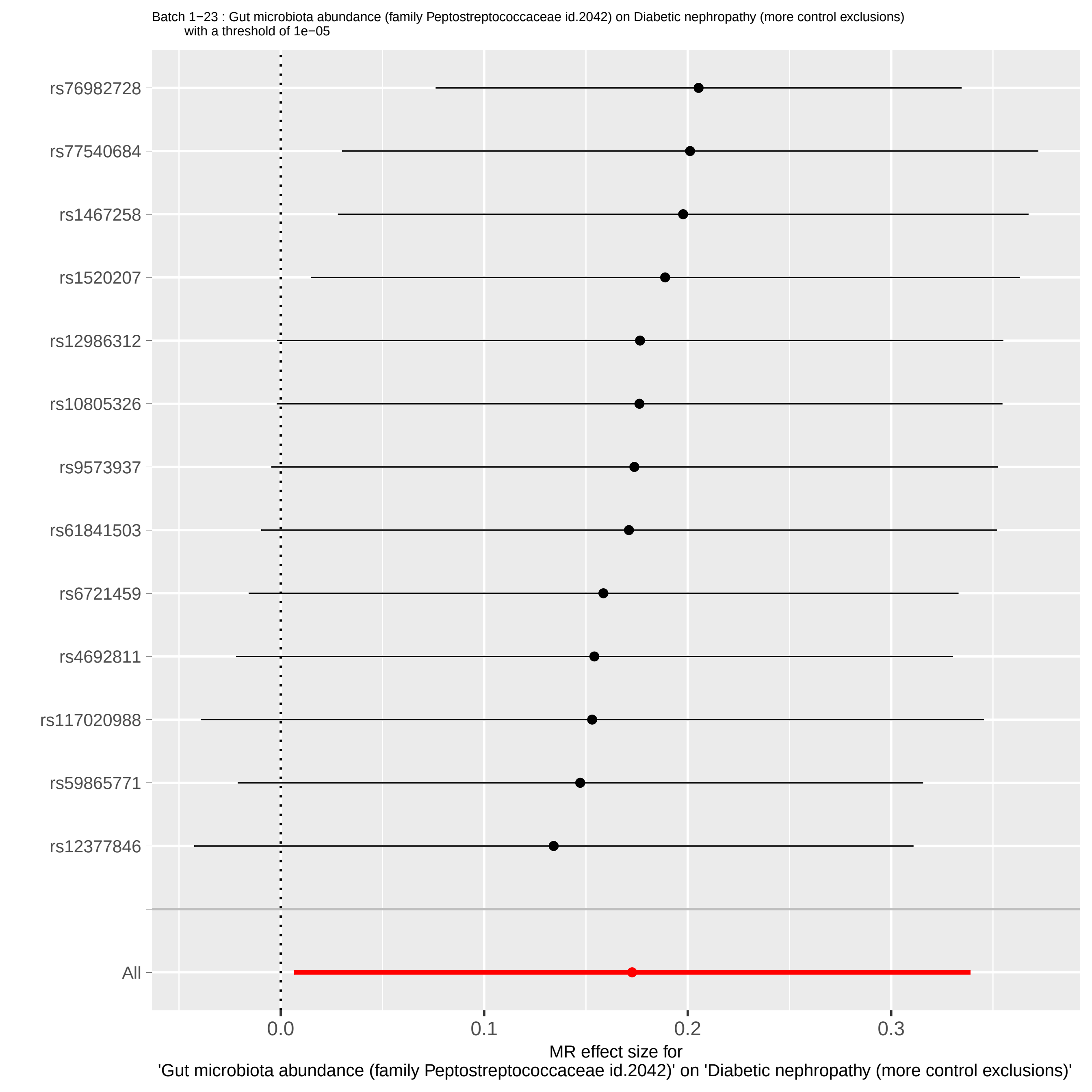
Batch 1−23 : Gut microbiota abundance (family Peptostreptococcaceae id.2042) on Diabetic nephropathy (more control exclusions)
with a threshold of 1e−05
rs76982728
rs77540684
rs1467258
rs1520207
rs12986312
rs10805326
rs9573937
rs61841503
rs6721459
rs4692811
rs117020988
rs59865771
rs12377846
All
0.0
0.1
0.2
0.3
MR effect size for
'Gut microbiota abundance (family Peptostreptococcaceae id.2042)' on 'Diabetic nephropathy (more control exclusions)'

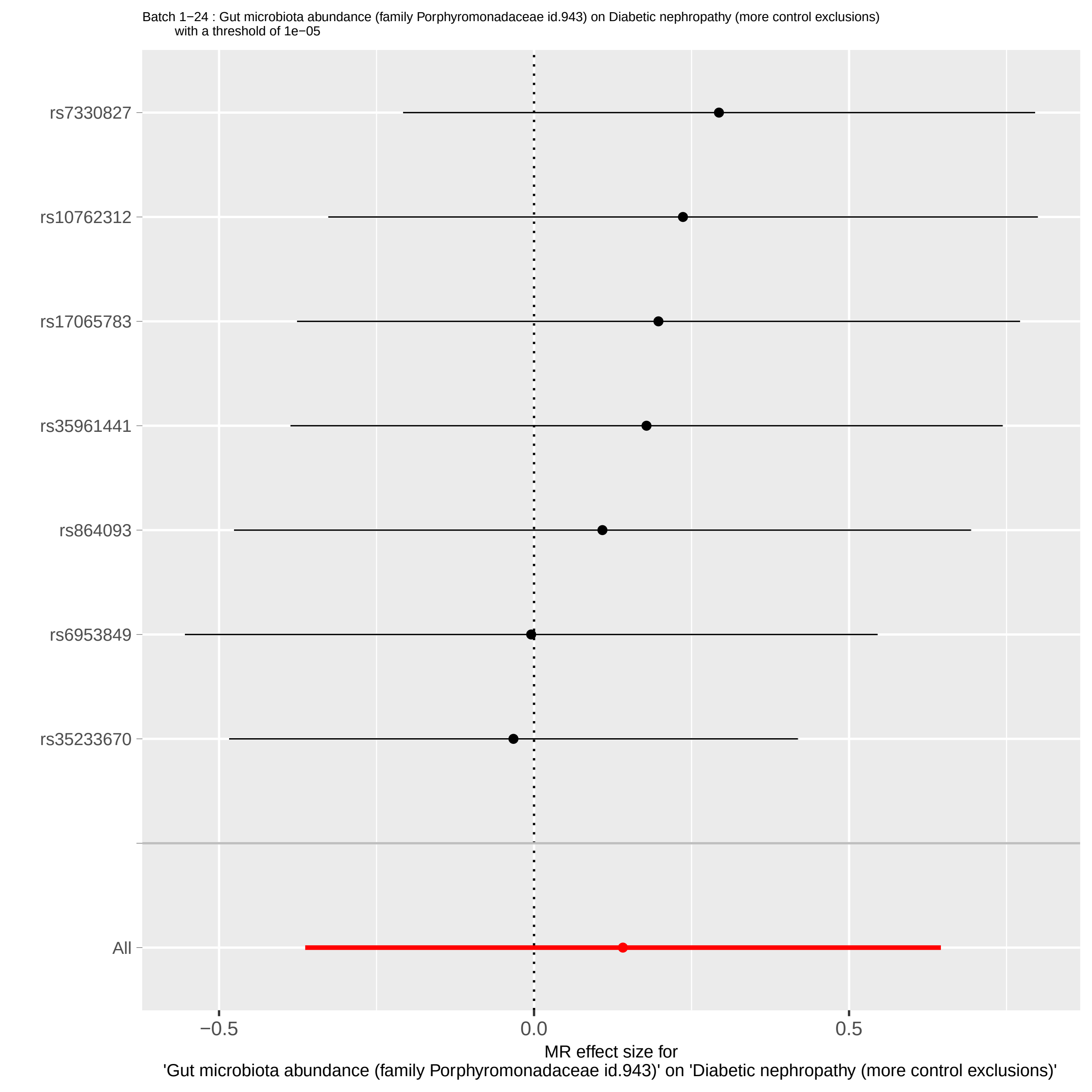
Batch 1−24 : Gut microbiota abundance (family Porphyromonadaceae id.943) on Diabetic nephropathy (more control exclusions)
with a threshold of 1e−05
rs7330827
rs10762312
rs17065783
rs35961441
rs864093
rs6953849
rs35233670
All
−0.5
0.0
0.5
MR effect size for
'Gut microbiota abundance (family Porphyromonadaceae id.943)' on 'Diabetic nephropathy (more control exclusions)'

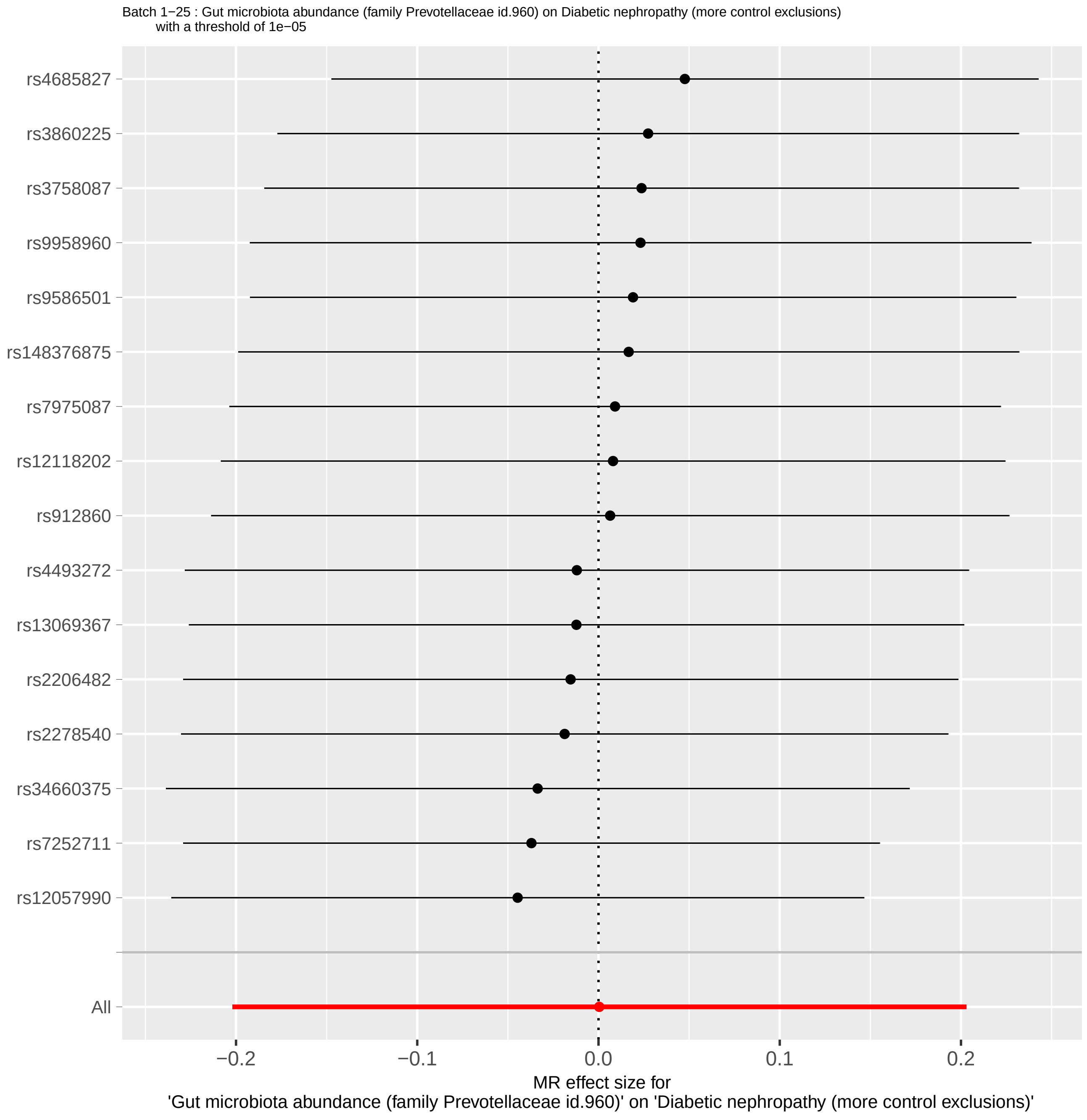
Batch 1−25 : Gut microbiota abundance (family Prevotellaceae id.960) on Diabetic nephropathy (more control exclusions)
with a threshold of 1e−05
rs4685827
rs3860225
rs3758087
rs9958960
rs9586501
rs148376875
rs7975087
rs12118202
rs912860
rs4493272
rs13069367
rs2206482
rs2278540
rs34660375
rs7252711
rs12057990
All
−0.2
−0.1
0.0
0.1
0.2
MR effect size for
'Gut microbiota abundance (family Prevotellaceae id.960)' on 'Diabetic nephropathy (more control exclusions)'

Batch 1−26 : Gut microbiota abundance (family Rhodospirillaceae id.2717) on Diabetic nephropathy (more control exclusions)
with a threshold of 1e−05

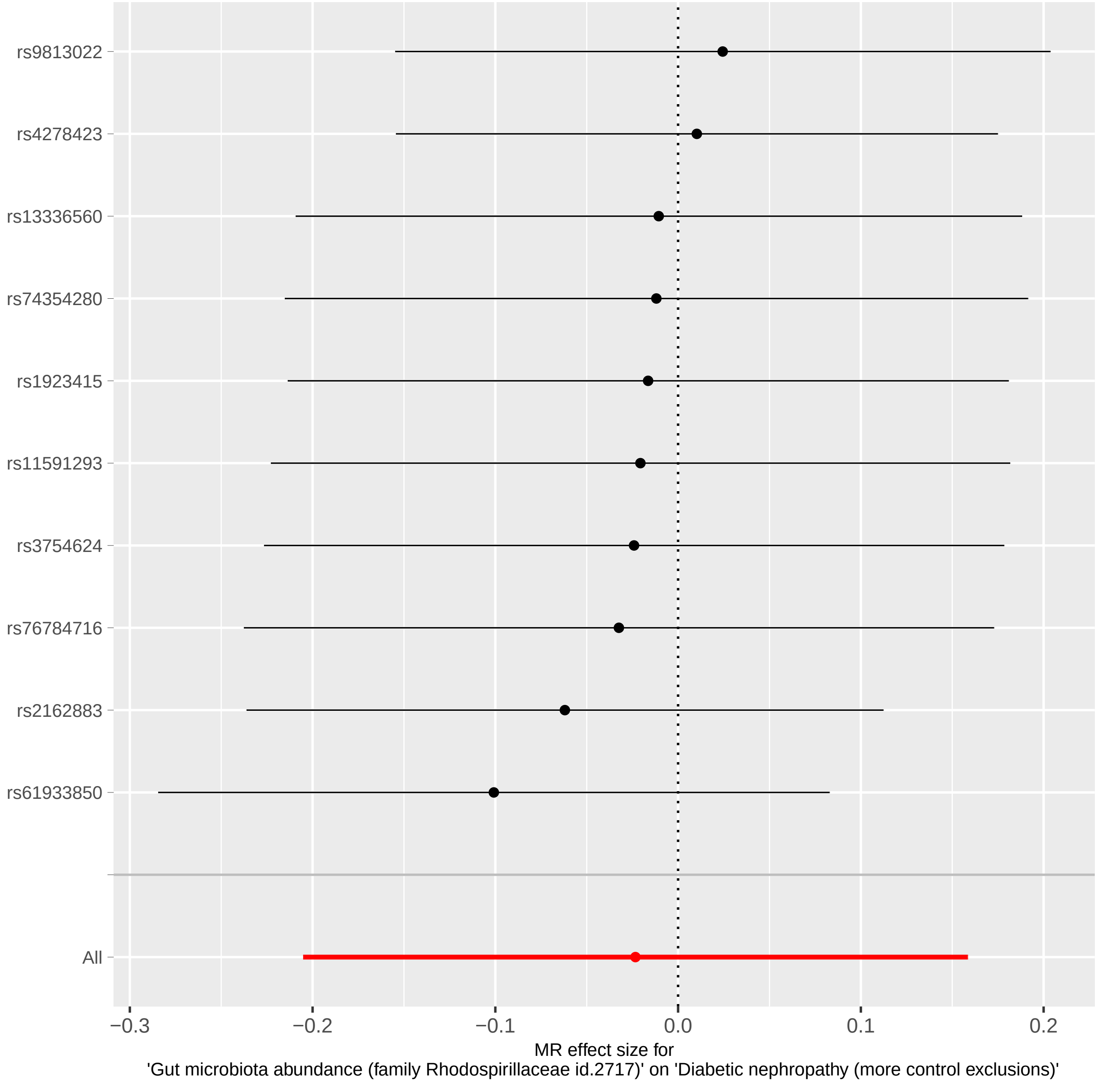

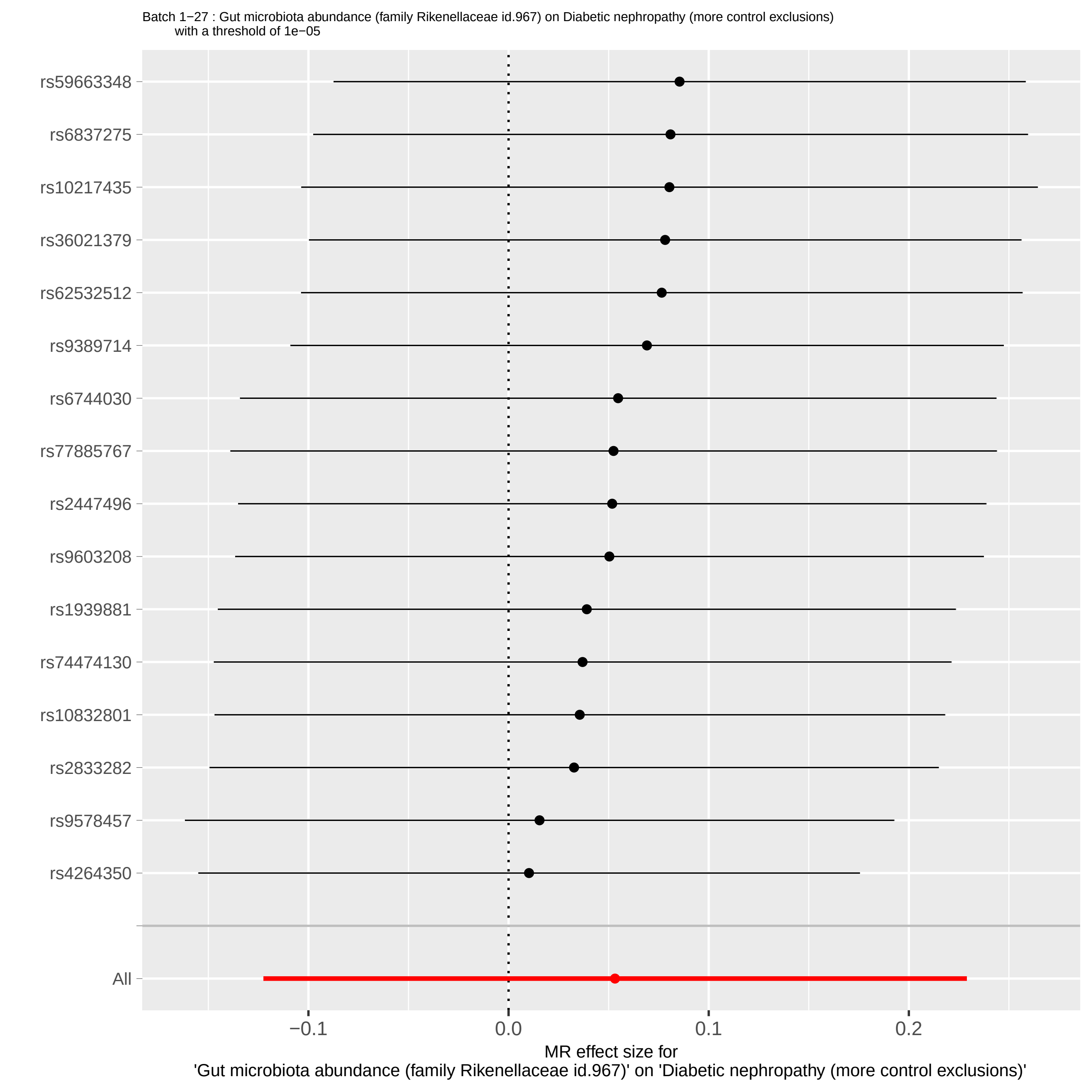
Batch 1−27 : Gut microbiota abundance (family Rikenellaceae id.967) on Diabetic nephropathy (more control exclusions) with a threshold of 1e−05
rs59663348
rs6837275
rs10217435
rs36021379
rs62532512
rs9389714
rs6744030
rs77885767
rs2447496
rs9603208
rs1939881
rs74474130
rs10832801
rs2833282
rs9578457
rs4264350
All
−0.1
0.0
0.1
0.2
MR effect size for
'Gut microbiota abundance (family Rikenellaceae id.967)' on 'Diabetic nephropathy (more control exclusions)'

Batch 1−28 : Gut microbiota abundance (family Ruminococcaceae id.2050) on Diabetic nephropathy (more control exclusions)
with a threshold of 1e−05

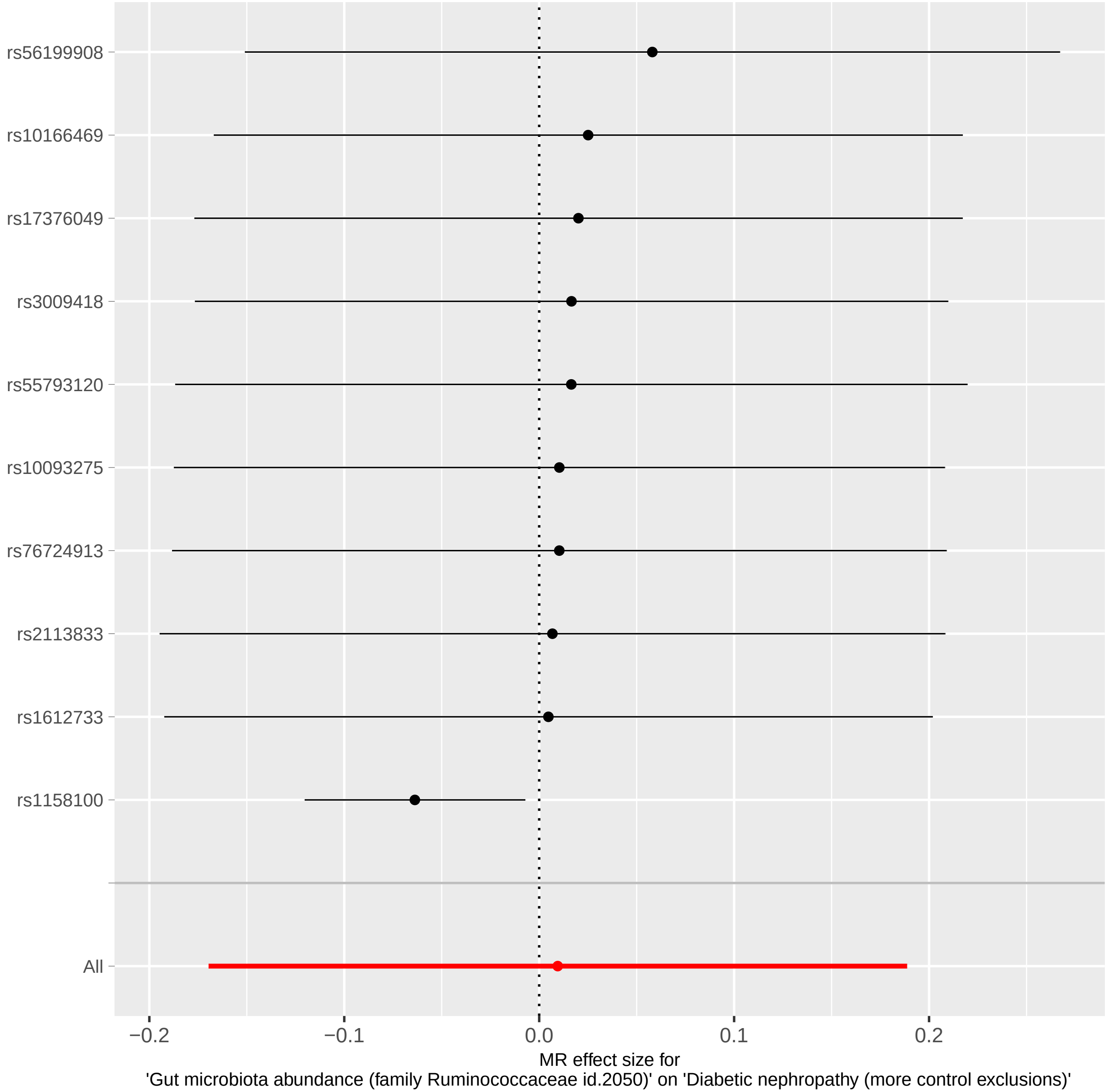

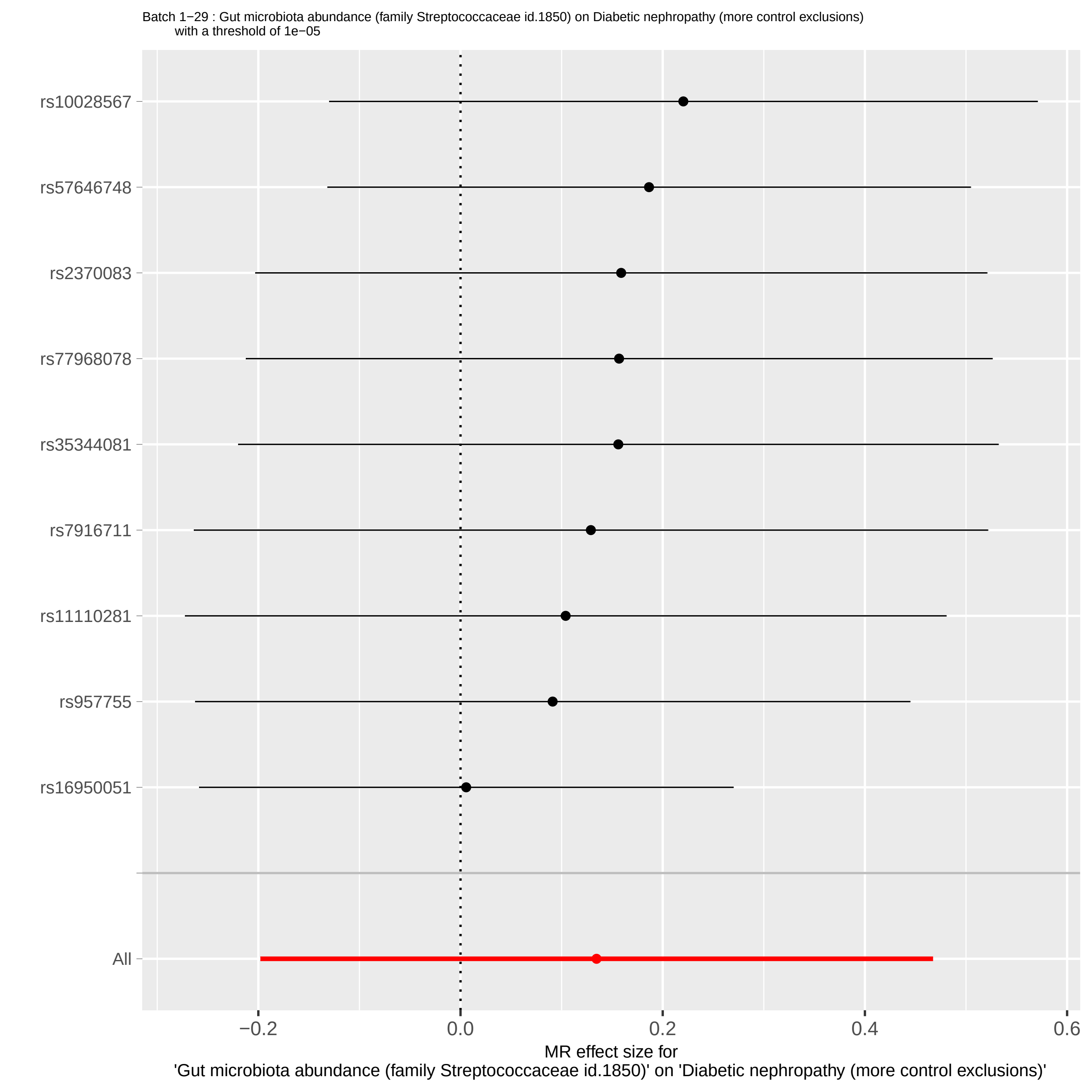
Batch 1−29 : Gut microbiota abundance (family Streptococcaceae id.1850) on Diabetic nephropathy (more control exclusions)
with a threshold of 1e−05
rs10028567
rs57646748
rs2370083
rs77968078
rs35344081
rs7916711
rs11110281
rs957755
rs16950051
All
−0.2
0.0
0.2
0.4
0.6
MR effect size for
'Gut microbiota abundance (family Streptococcaceae id.1850)' on 'Diabetic nephropathy (more control exclusions)'

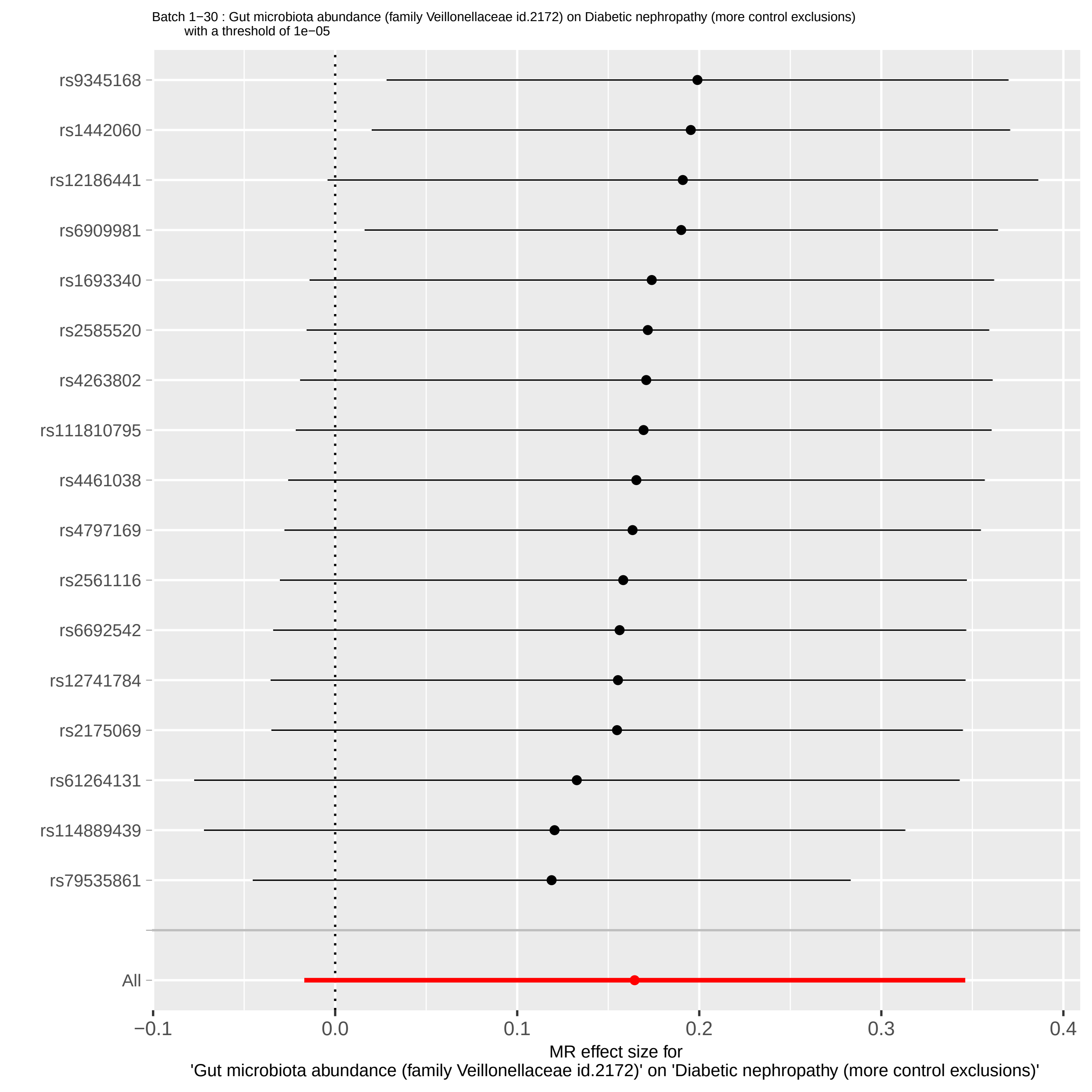
Batch 1−30 : Gut microbiota abundance (family Veillonellaceae id.2172) on Diabetic nephropathy (more control exclusions)
with a threshold of 1e−05
rs9345168
rs1442060
rs12186441
rs6909981
rs1693340
rs2585520
rs4263802
rs111810795
rs4461038
rs4797169
rs2561116
rs6692542
rs12741784
rs2175069
rs61264131
rs114889439
rs79535861
All
−0.1
0.0
0.1
0.2
0.3
0.4
MR effect size for
'Gut microbiota abundance (family Veillonellaceae id.2172)' on 'Diabetic nephropathy (more control exclusions)'

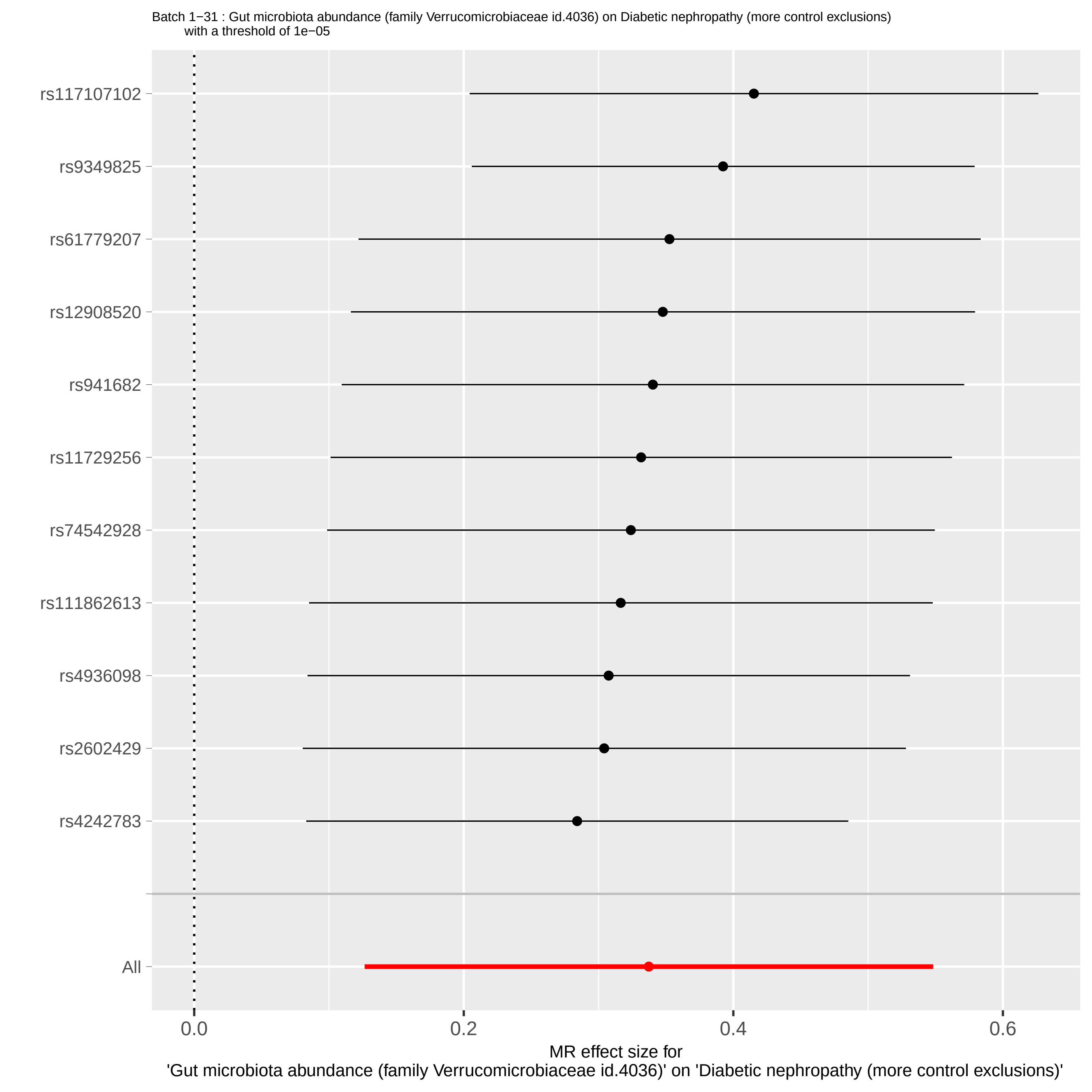
Batch 1−31 : Gut microbiota abundance (family Verrucomicrobiaceae id.4036) on Diabetic nephropathy (more control exclusions)
with a threshold of 1e−05
rs117107102
rs9349825
rs61779207
rs12908520
rs941682
rs11729256
rs74542928
rs111862613
rs4936098
rs2602429
rs4242783
All
0.0
0.2
0.4
0.6
MR effect size for
'Gut microbiota abundance (family Verrucomicrobiaceae id.4036)' on 'Diabetic nephropathy (more control exclusions)'

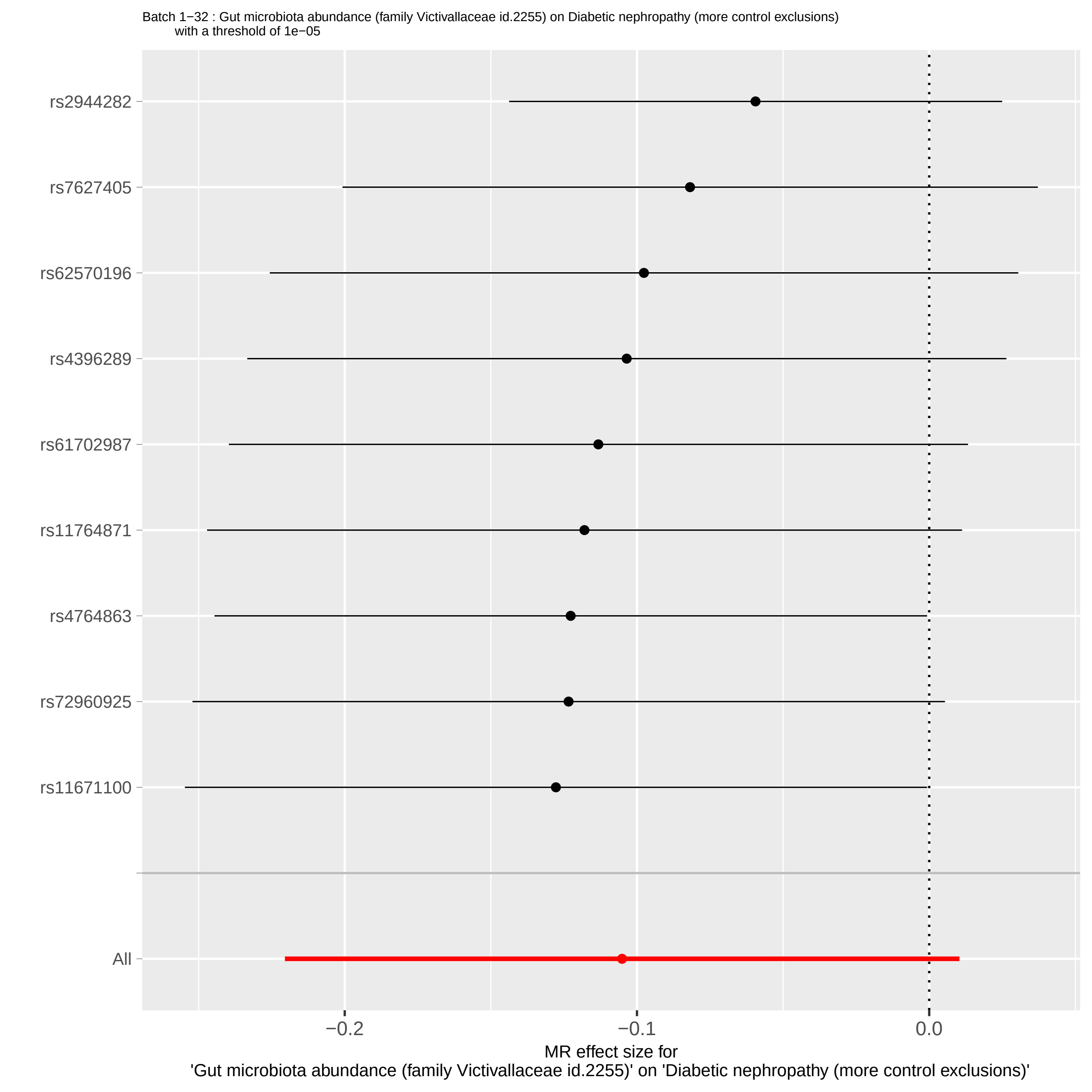
Batch 1−32 : Gut microbiota abundance (family Victivallaceae id.2255) on Diabetic nephropathy (more control exclusions)
with a threshold of 1e−05
rs2944282
rs7627405
rs62570196
rs4396289
rs61702987
rs11764871
rs4764863
rs72960925
rs11671100
All
−0.2
−0.1
0.0
MR effect size for
'Gut microbiota abundance (family Victivallaceae id.2255)' on 'Diabetic nephropathy (more control exclusions)'

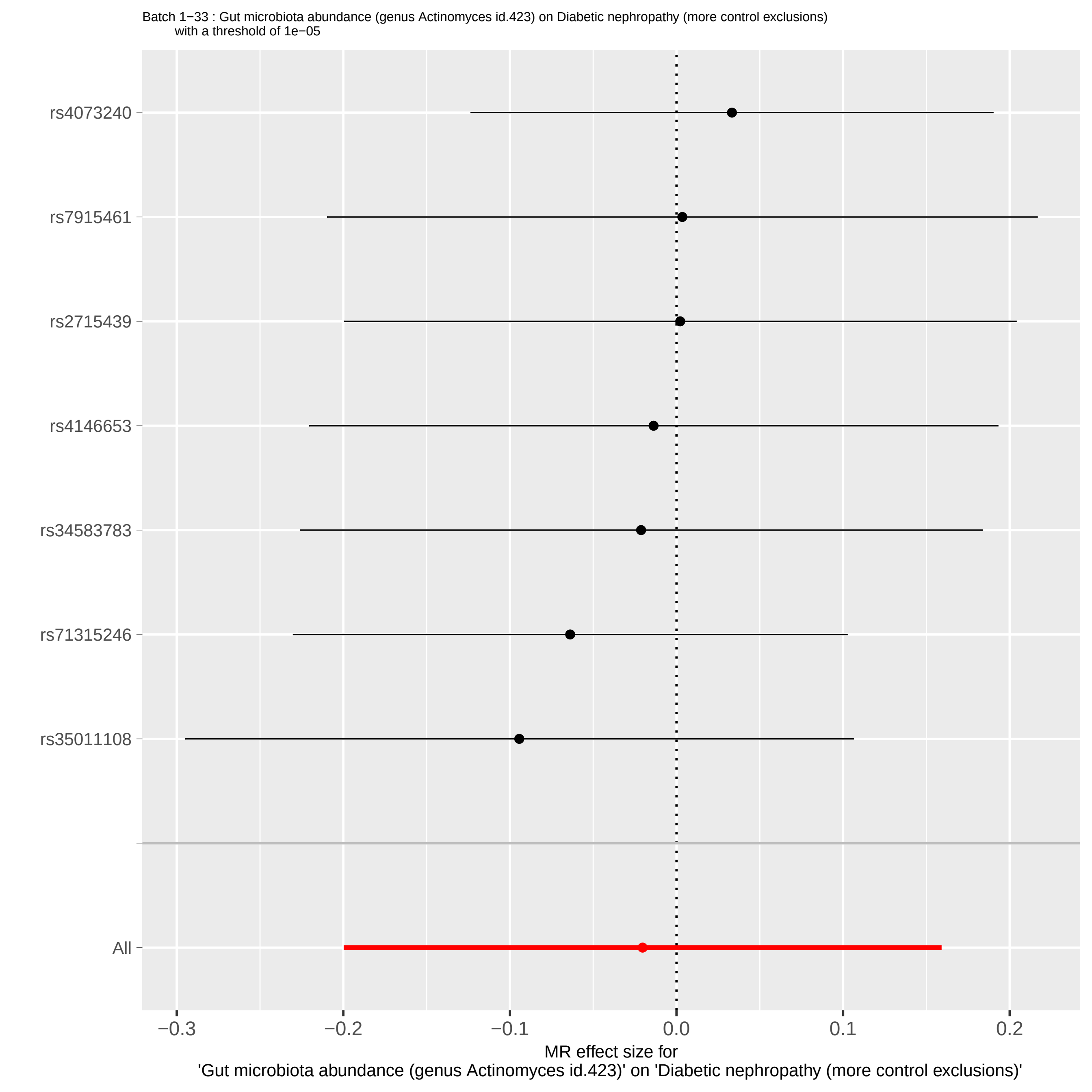

Batch 1−33 : Gut microbiota abundance (genus Actinomyces id.423) on Diabetic nephropathy (more control exclusions)
with a threshold of 1e−05
rs4073240
rs7915461
rs2715439
rs4146653
rs34583783
rs71315246
rs35011108
All
−0.3
−0.2
−0.1
0.0
0.1
0.2
MR effect size for
'Gut microbiota abundance (genus Actinomyces id.423)' on 'Diabetic nephropathy (more control exclusions)'

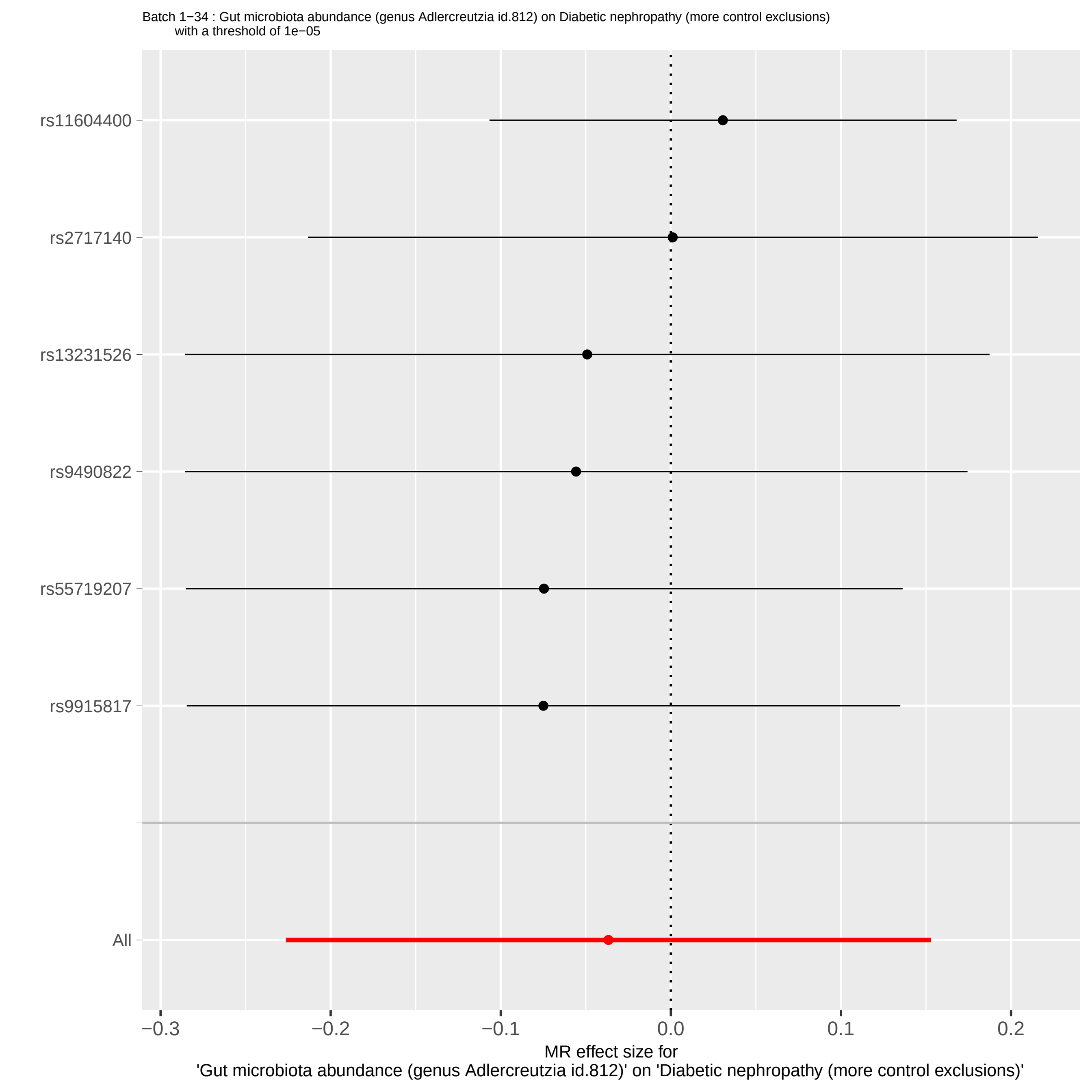

Batch 1−34 : Gut microbiota abundance (genus Adlercreutzia id.812) on Diabetic nephropathy (more control exclusions)
with a threshold of 1e−05
rs11604400
rs2717140
rs13231526
rs9490822
rs55719207
rs9915817
All
−0.3
−0.2
−0.1
0.0
0.1
0.2
MR effect size for
'Gut microbiota abundance (genus Adlercreutzia id.812)' on 'Diabetic nephropathy (more control exclusions)'

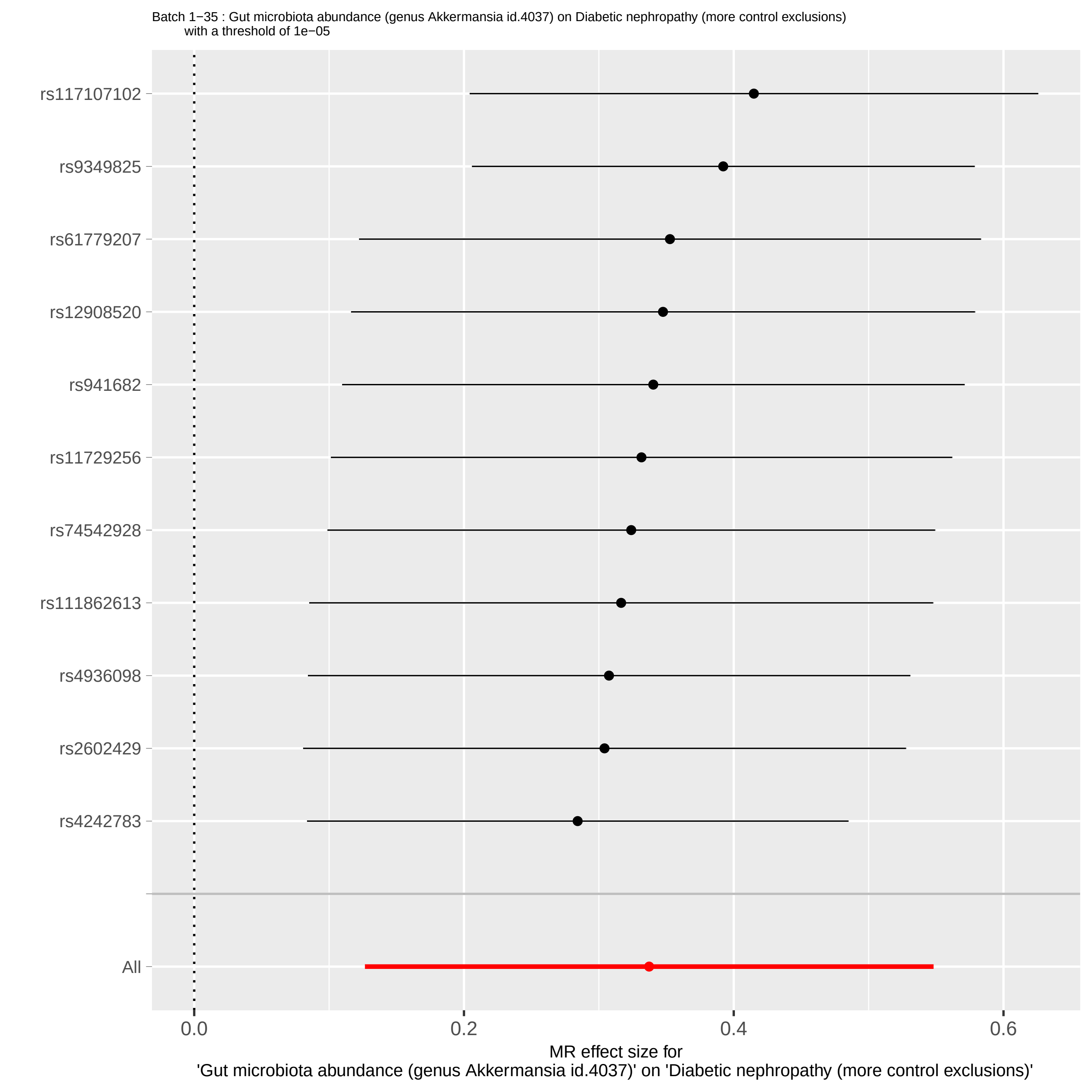

Batch 1−35 : Gut microbiota abundance (genus Akkermansia id.4037) on Diabetic nephropathy (more control exclusions)
with a threshold of 1e−05
rs117107102
rs9349825
rs61779207
rs12908520
rs941682
rs11729256
rs74542928
rs111862613
rs4936098
rs2602429
rs4242783
All
0.0
0.2
0.4
0.6
MR effect size for
'Gut microbiota abundance (genus Akkermansia id.4037)' on 'Diabetic nephropathy (more control exclusions)'

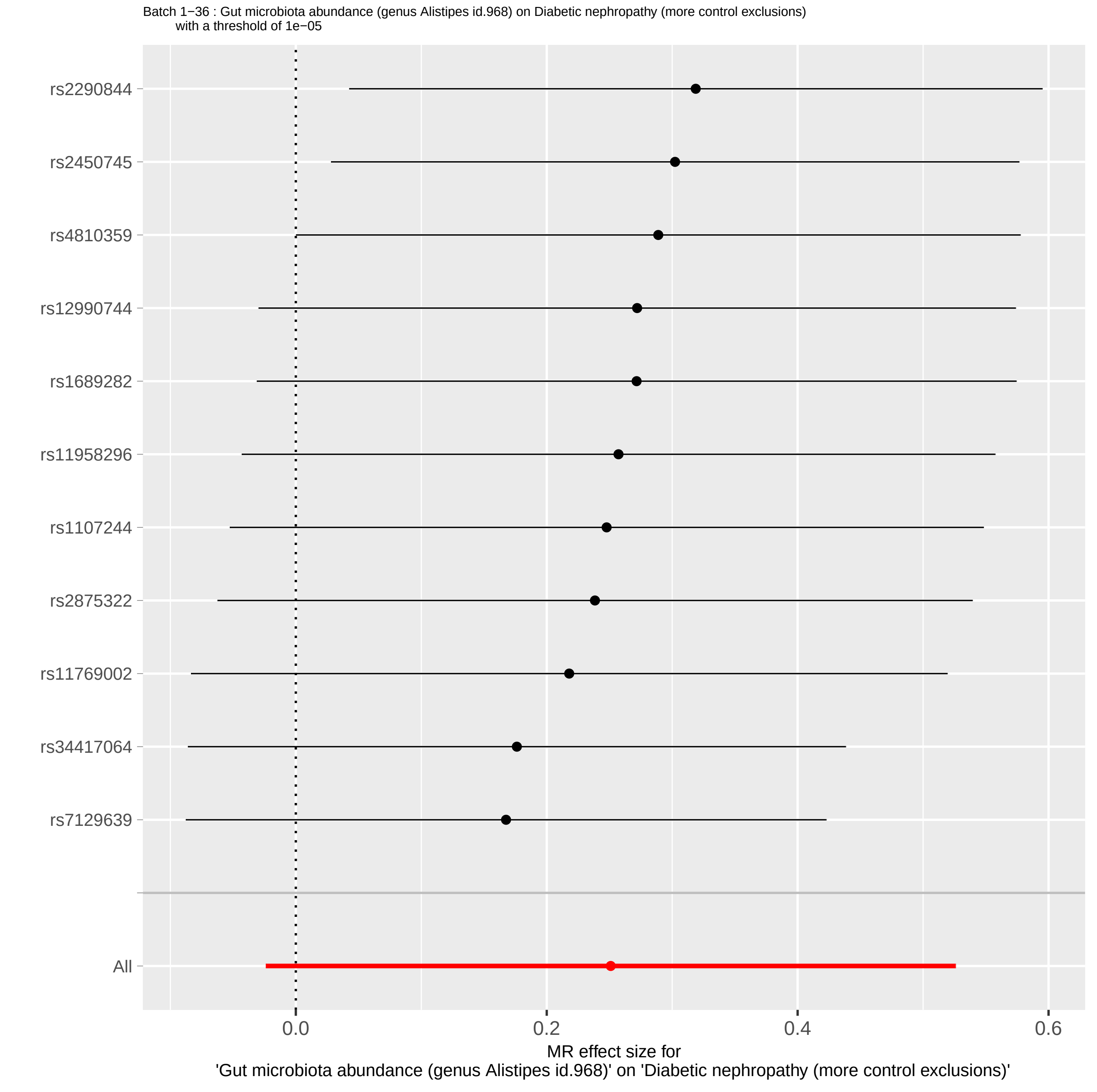
Batch 1−36 : Gut microbiota abundance (genus Alistipes id.968) on Diabetic nephropathy (more control exclusions)
with a threshold of 1e−05
rs2290844
rs2450745
rs4810359
rs12990744
rs1689282
rs11958296
rs1107244
rs2875322
rs11769002
rs34417064
rs7129639
All
0.0
0.2
0.4
0.6
MR effect size for
'Gut microbiota abundance (genus Alistipes id.968)' on 'Diabetic nephropathy (more control exclusions)'

Batch 1−37 : Gut microbiota abundance (genus Allisonella id.2174) on Diabetic nephropathy (more control exclusions) with a threshold of 1e−05

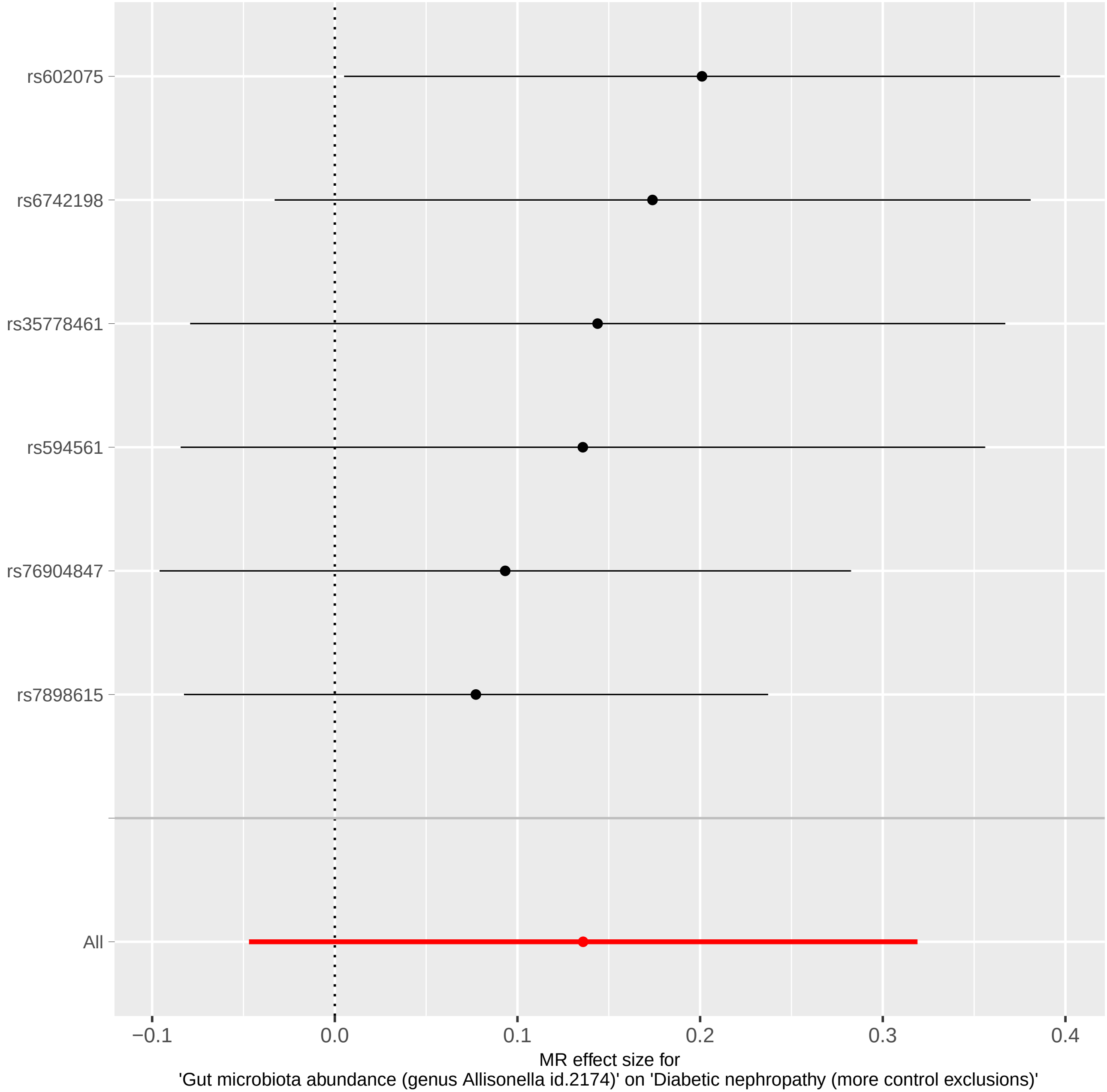

Batch 1−38 : Gut microbiota abundance (genus Alloprevotella id.961) on Diabetic nephropathy (more control exclusions) with a threshold of 1e−05

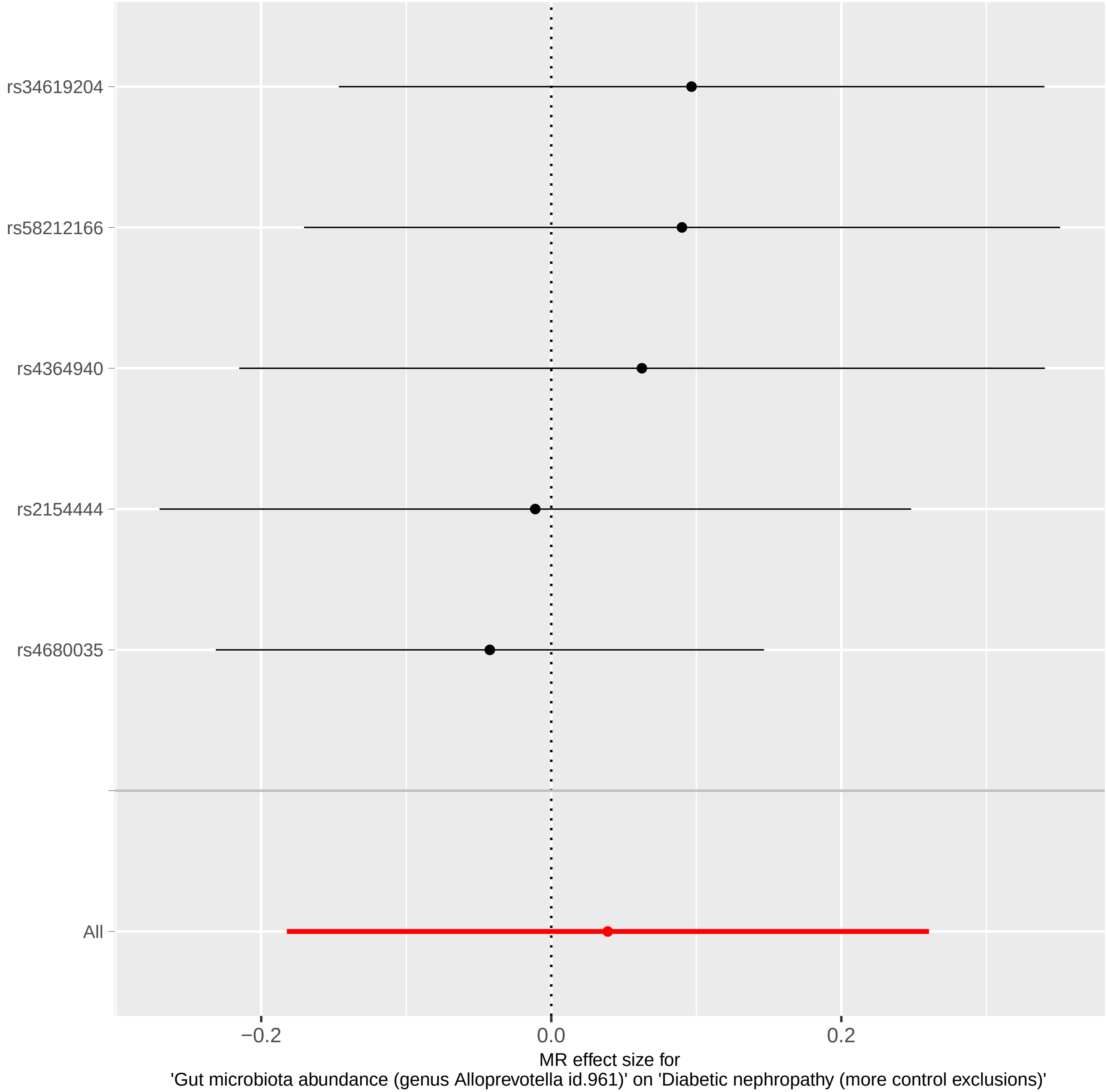

Batch 1−39 : Gut microbiota abundance (genus Anaerofilum id.2053) on Diabetic nephropathy (more control exclusions) with a threshold of 1e−05

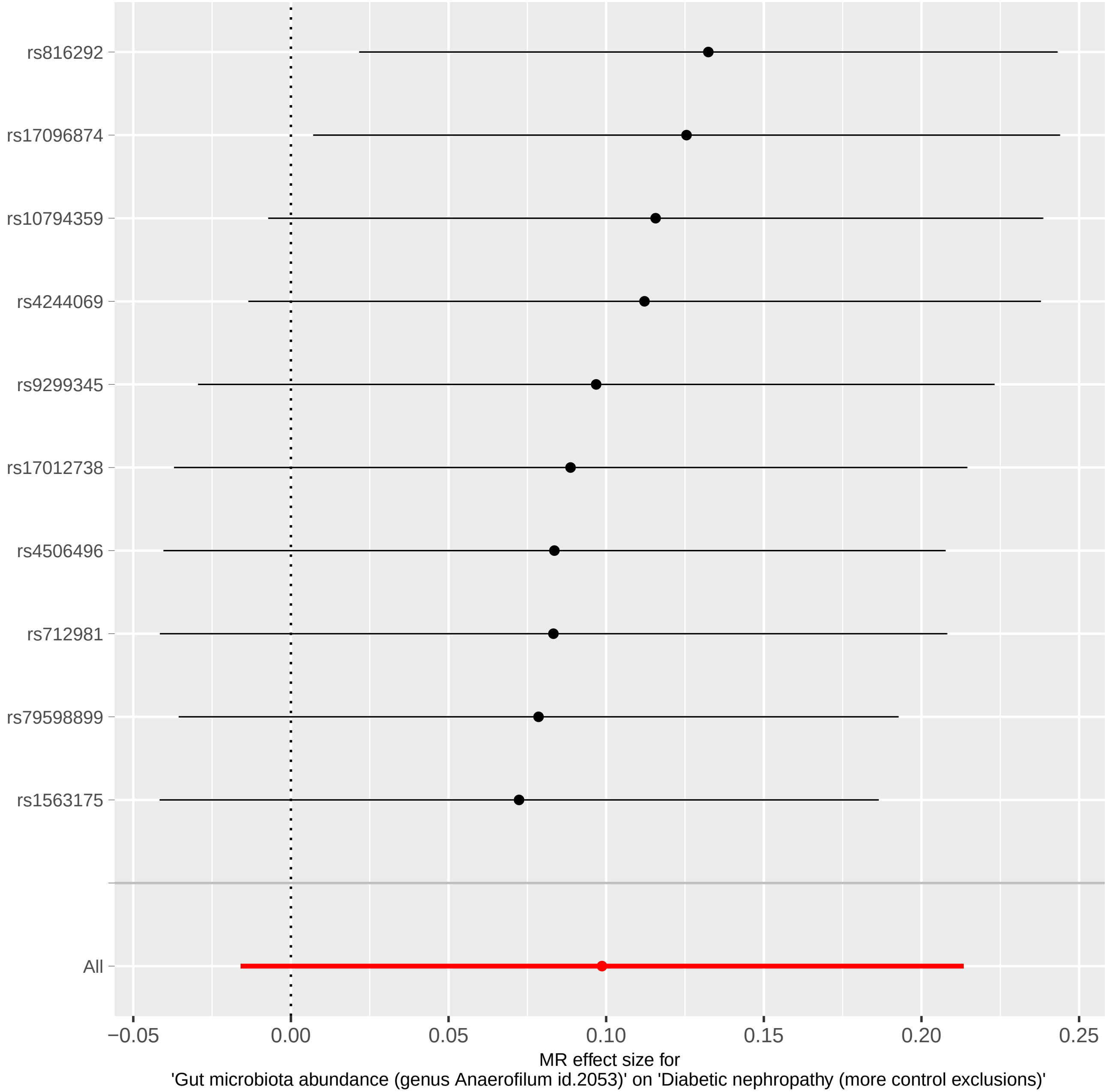

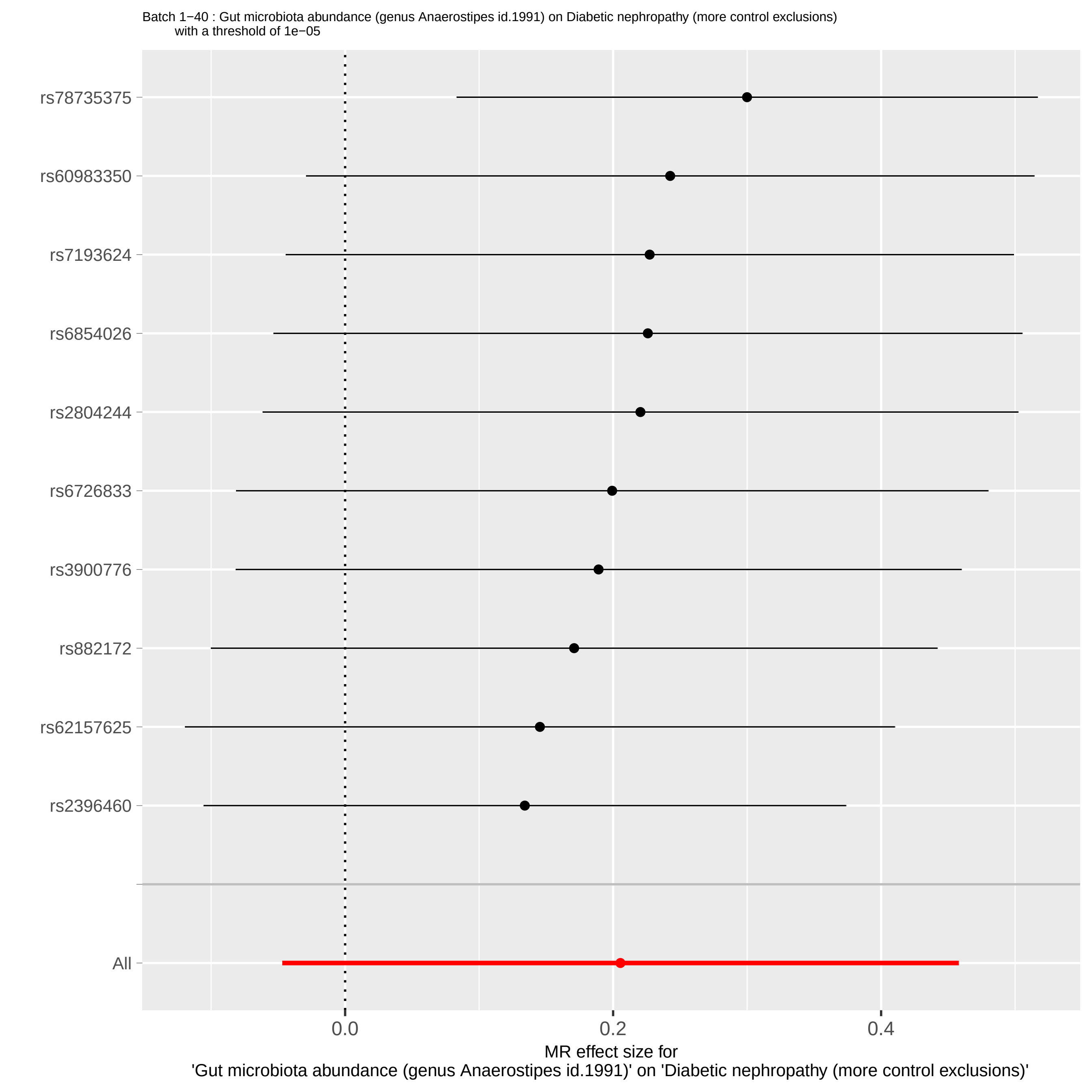

Batch 1−40 : Gut microbiota abundance (genus Anaerostipes id.1991) on Diabetic nephropathy (more control exclusions) with a threshold of 1e−05
rs78735375
rs60983350
rs7193624
rs6854026
rs2804244
rs6726833
rs3900776
rs882172
rs62157625
rs2396460
All
0.0
0.2
0.4
MR effect size for
'Gut microbiota abundance (genus Anaerostipes id.1991)' on 'Diabetic nephropathy (more control exclusions)'

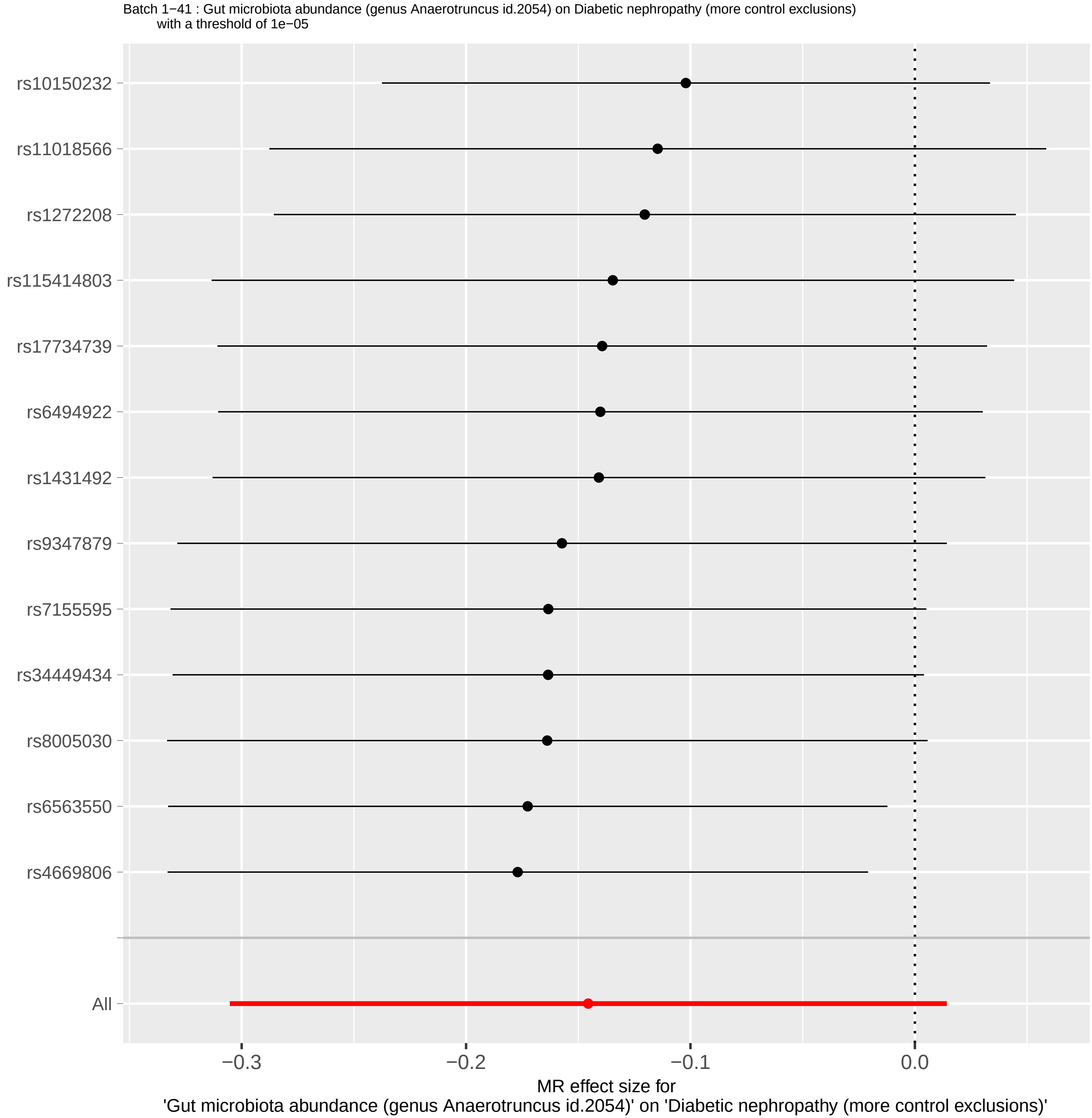
Batch 1−41 : Gut microbiota abundance (genus Anaerotruncus id.2054) on Diabetic nephropathy (more control exclusions) with a threshold of 1e−05
rs10150232
rs11018566
rs1272208
rs115414803
rs17734739
rs6494922
rs1431492
rs9347879
rs7155595
rs34449434
rs8005030
rs6563550
rs4669806
All
−0.3
−0.2
−0.1
0.0
MR effect size for
'Gut microbiota abundance (genus Anaerotruncus id.2054)' on 'Diabetic nephropathy (more control exclusions)'

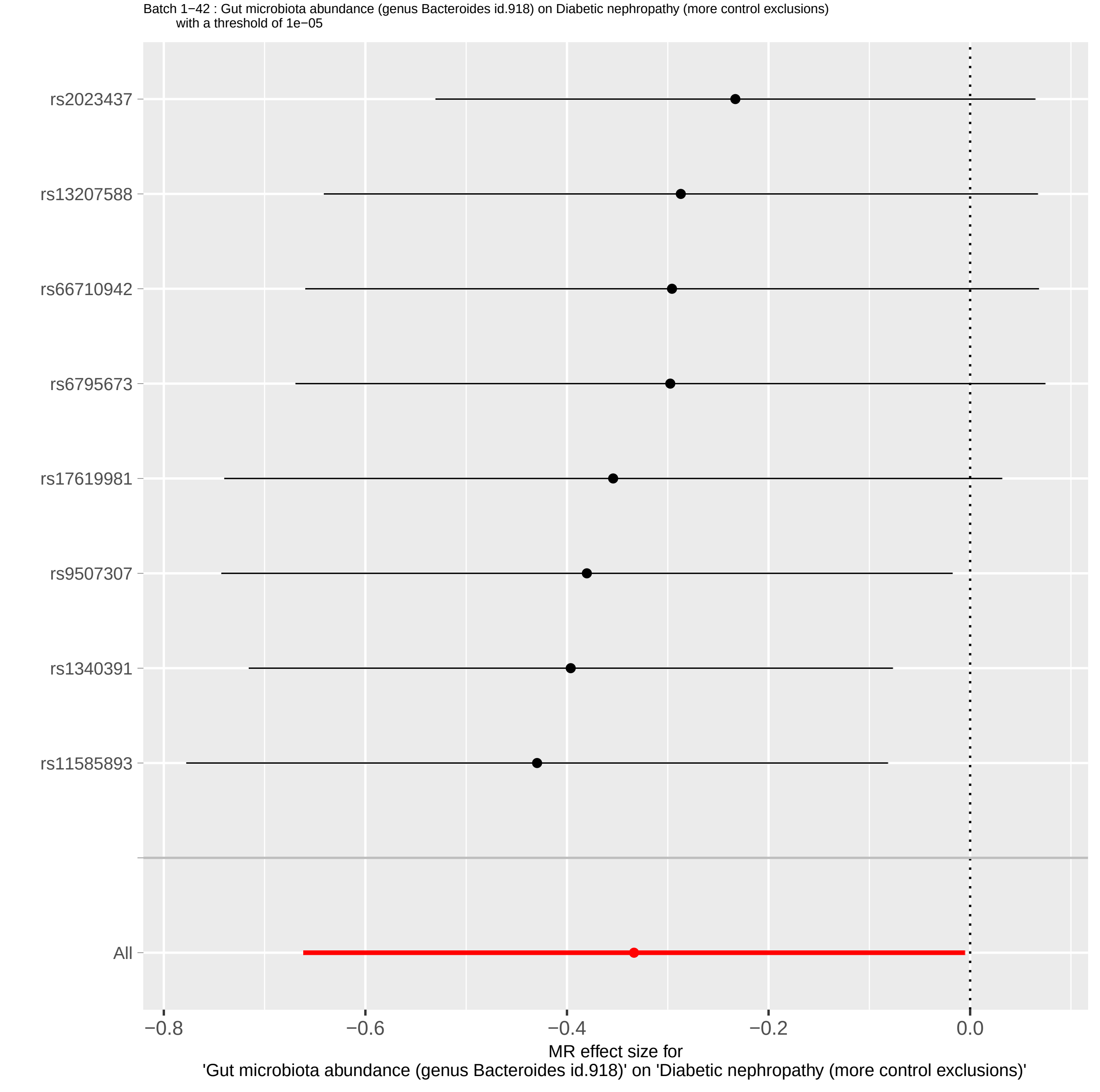

Batch 1−42 : Gut microbiota abundance (genus Bacteroides id.918) on Diabetic nephropathy (more control exclusions) with a threshold of 1e−05
rs2023437
rs13207588
rs66710942
rs6795673
rs17619981
rs9507307
rs1340391
rs11585893
All
−0.8
−0.6
−0.4
−0.2
0.0
MR effect size for
'Gut microbiota abundance (genus Bacteroides id.918)' on 'Diabetic nephropathy (more control exclusions)'

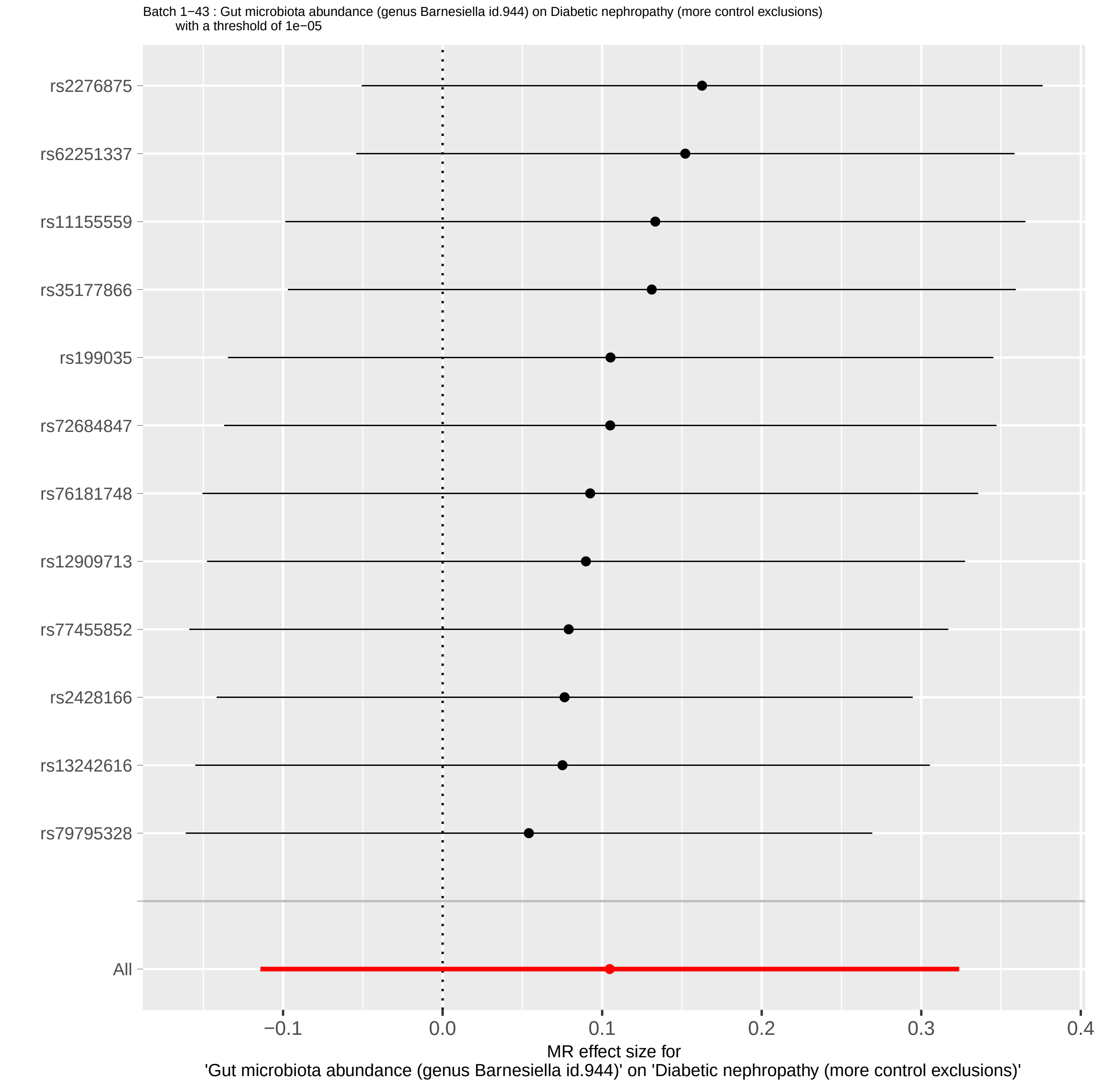

Batch 1−43 : Gut microbiota abundance (genus Barnesiella id.944) on Diabetic nephropathy (more control exclusions) with a threshold of 1e−05
rs2276875
rs62251337
rs11155559
rs35177866
rs199035
rs72684847
rs76181748
rs12909713
rs77455852
rs2428166
rs13242616
rs79795328
All
−0.1
0.0
0.1
0.2
0.3
0.4
MR effect size for
'Gut microbiota abundance (genus Barnesiella id.944)' on 'Diabetic nephropathy (more control exclusions)'

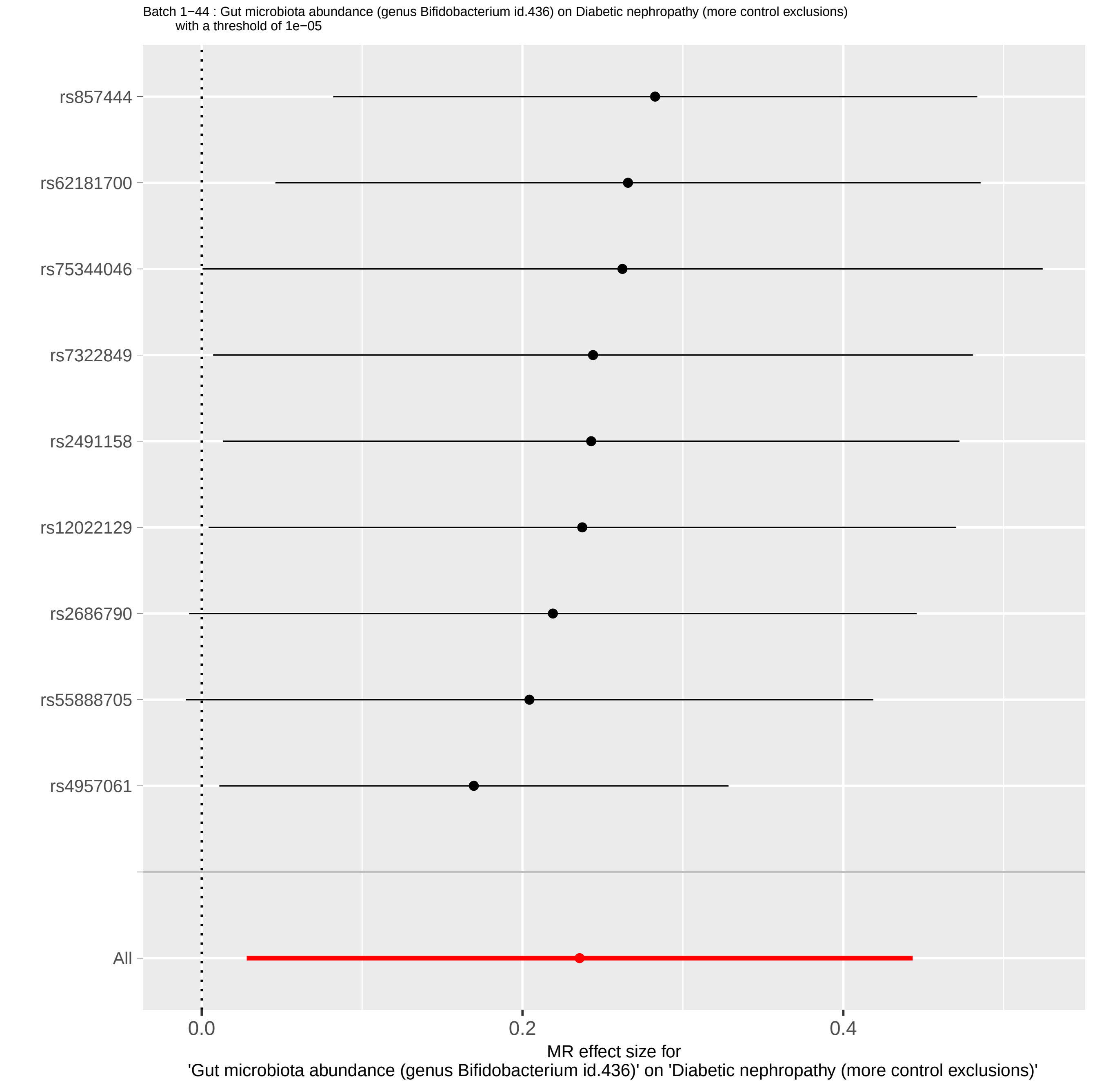
Batch 1−44 : Gut microbiota abundance (genus Bifidobacterium id.436) on Diabetic nephropathy (more control exclusions)
with a threshold of 1e−05
rs857444
rs62181700
rs75344046
rs7322849
rs2491158
rs12022129
rs2686790
rs55888705
rs4957061
All
0.0
0.2
0.4
MR effect size for
'Gut microbiota abundance (genus Bifidobacterium id.436)' on 'Diabetic nephropathy (more control exclusions)'

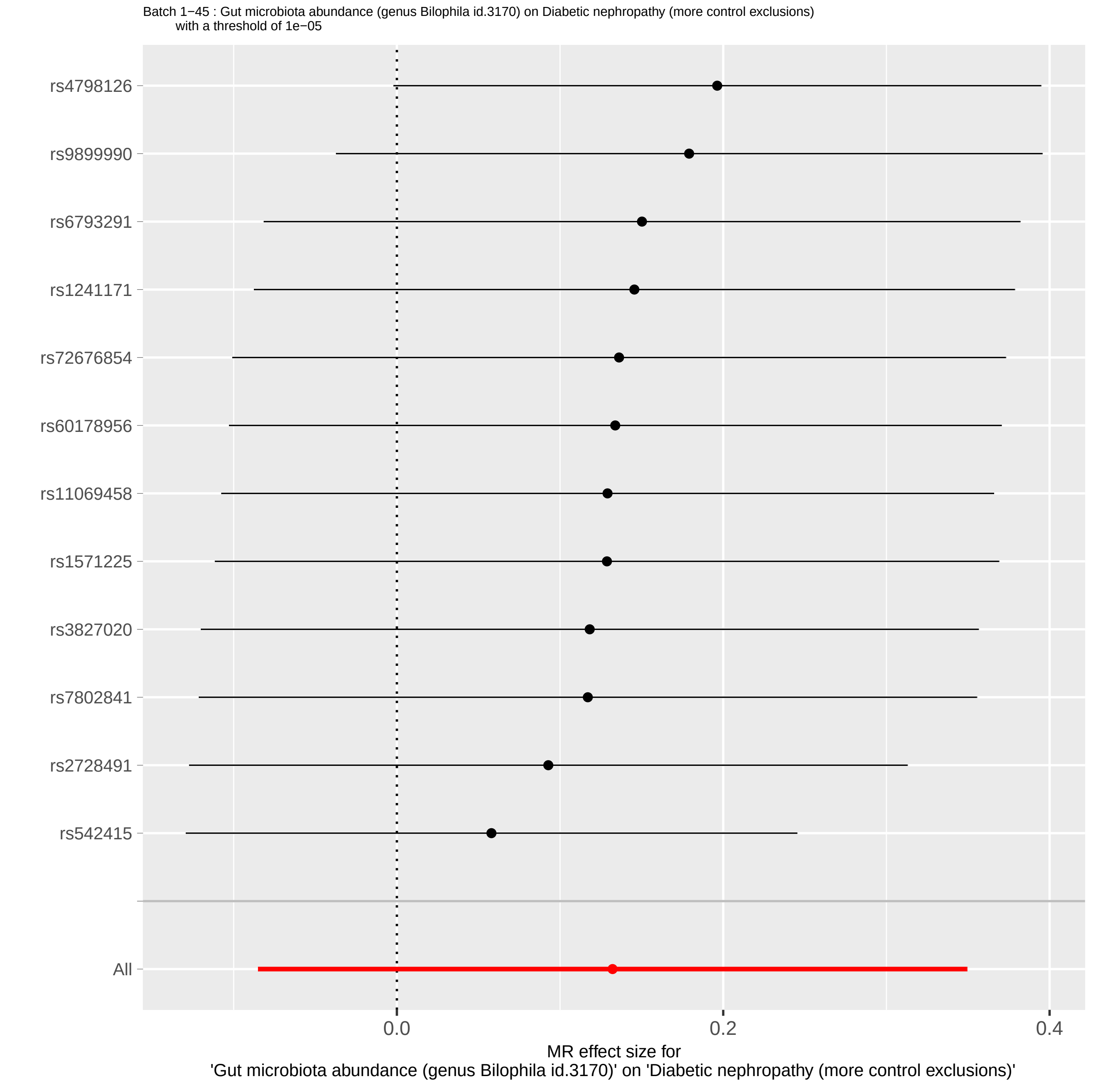
Batch 1−45 : Gut microbiota abundance (genus Bilophila id.3170) on Diabetic nephropathy (more control exclusions)
with a threshold of 1e−05
rs4798126
rs9899990
rs6793291
rs1241171
rs72676854
rs60178956
rs11069458
rs1571225
rs3827020
rs7802841
rs2728491
rs542415
All
0.0
0.2
0.4
MR effect size for
'Gut microbiota abundance (genus Bilophila id.3170)' on 'Diabetic nephropathy (more control exclusions)'

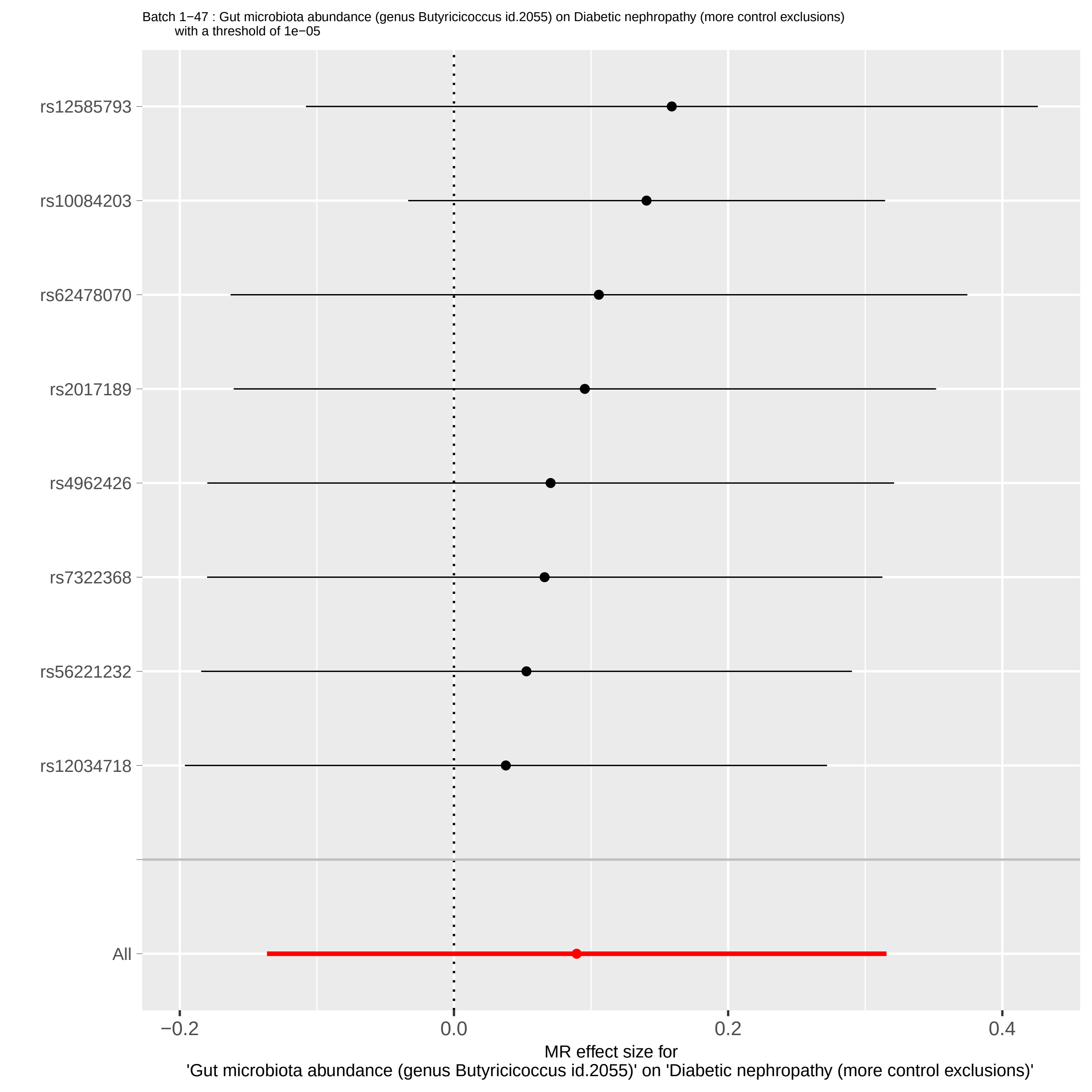

Batch 1−47 : Gut microbiota abundance (genus Butyricicoccus id.2055) on Diabetic nephropathy (more control exclusions)
with a threshold of 1e−05
rs12585793
rs10084203
rs62478070
rs2017189
rs4962426
rs7322368
rs56221232
rs12034718
All
−0.2
0.0
0.2
0.4
MR effect size for
'Gut microbiota abundance (genus Butyricicoccus id.2055)' on 'Diabetic nephropathy (more control exclusions)'

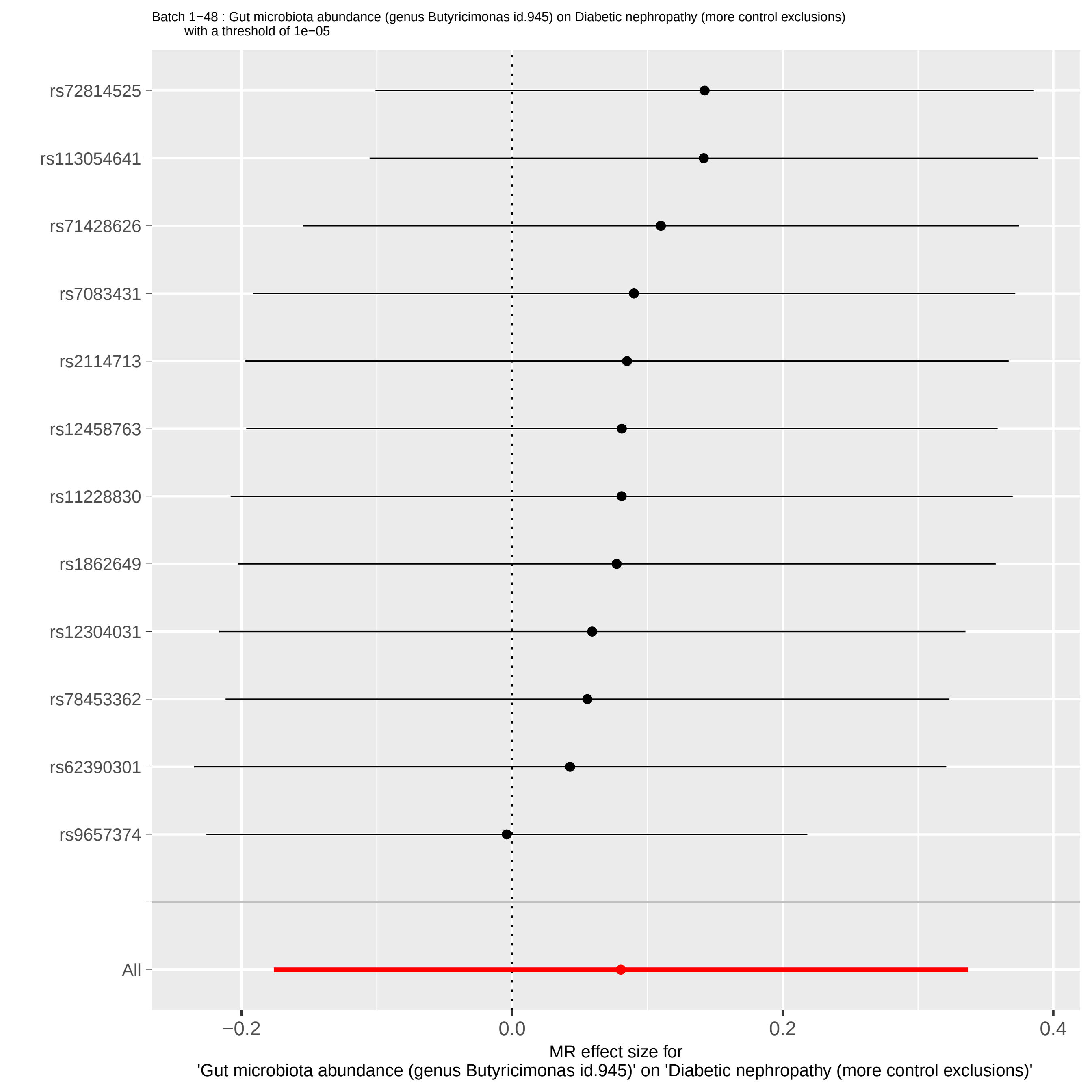

Batch 1−48 : Gut microbiota abundance (genus Butyricimonas id.945) on Diabetic nephropathy (more control exclusions)
with a threshold of 1e−05
rs72814525
rs113054641
rs71428626
rs7083431
rs2114713
rs12458763
rs11228830
rs1862649
rs12304031
rs78453362
rs62390301
rs9657374
All
−0.2
0.0
0.2
0.4
MR effect size for
'Gut microbiota abundance (genus Butyricimonas id.945)' on 'Diabetic nephropathy (more control exclusions)'

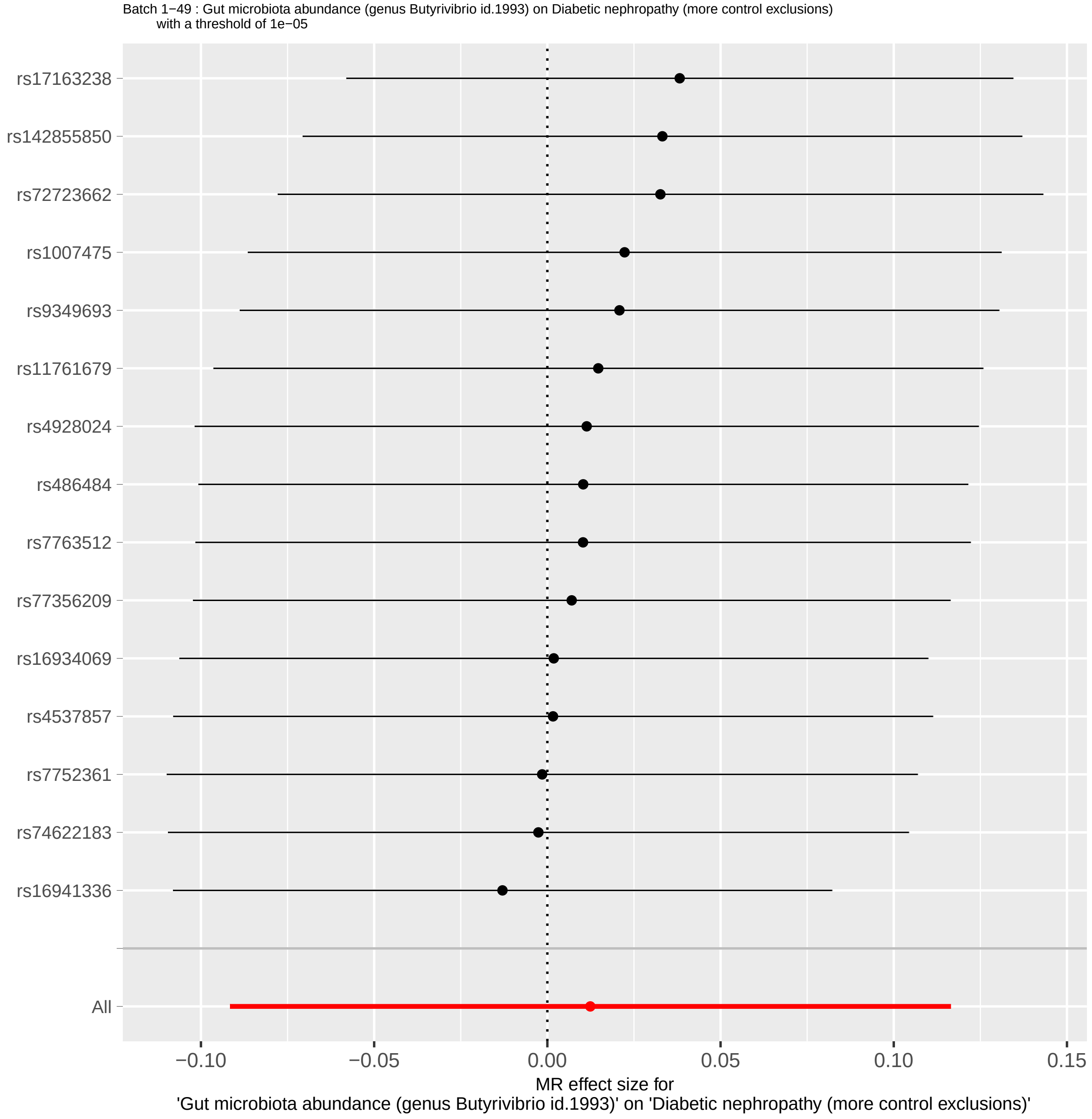

Batch 1−49 : Gut microbiota abundance (genus Butyrivibrio id.1993) on Diabetic nephropathy (more control exclusions) with a threshold of 1e−05
rs17163238
rs142855850
rs72723662
rs1007475
rs9349693
rs11761679
rs4928024
rs486484
rs7763512
rs77356209
rs16934069
rs4537857
rs7752361
rs74622183
rs16941336
All
−0.10
−0.05
0.00
0.05
0.10
0.15
MR effect size for
'Gut microbiota abundance (genus Butyrivibrio id.1993)' on 'Diabetic nephropathy (more control exclusions)'

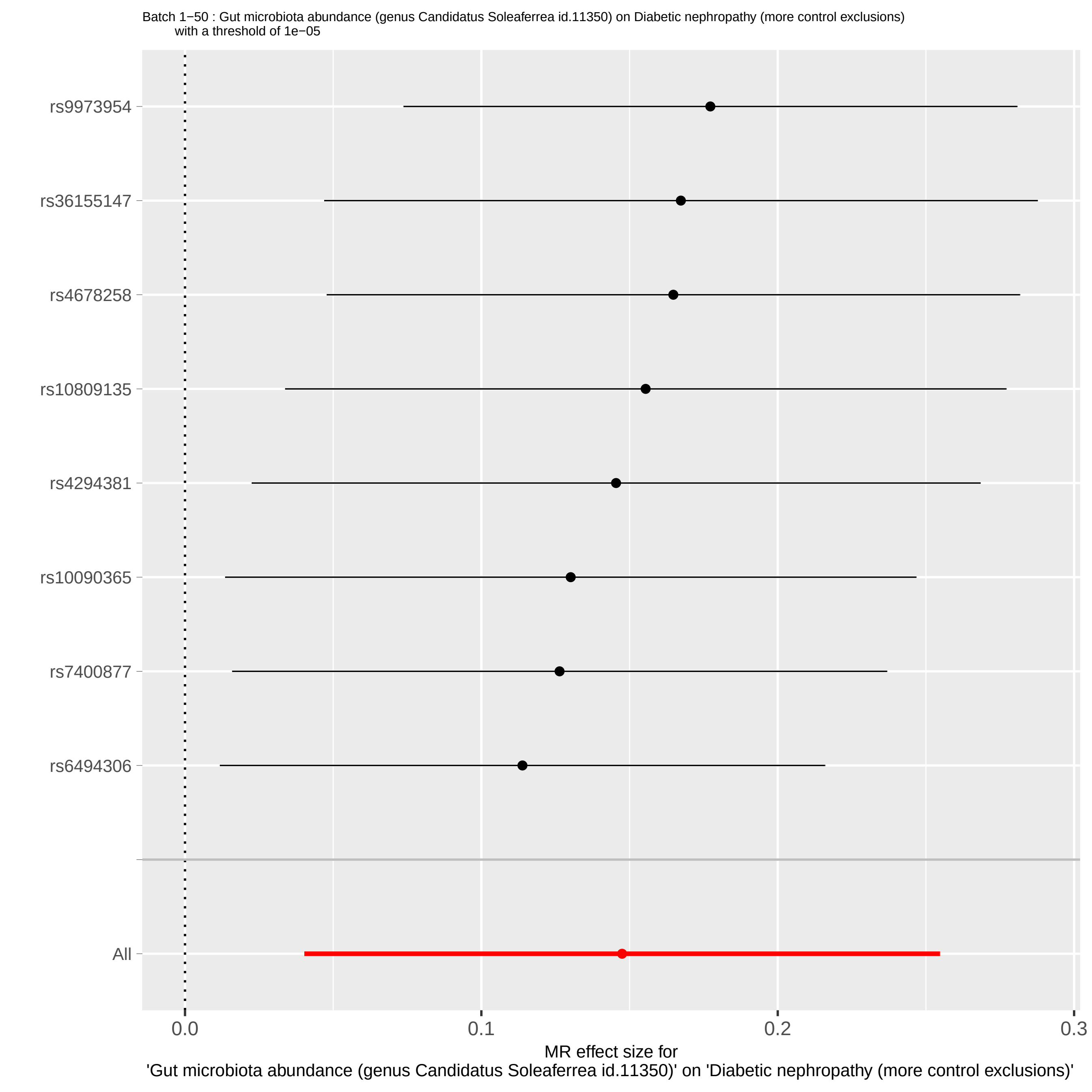

Batch 1−50 : Gut microbiota abundance (genus Candidatus Soleaferrea id.11350) on Diabetic nephropathy (more control exclusions)
with a threshold of 1e−05
rs9973954
rs36155147
rs4678258
rs10809135
rs4294381
rs10090365
rs7400877
rs6494306
All
0.0
0.1
0.2
0.3
MR effect size for
'Gut microbiota abundance (genus Candidatus Soleaferrea id.11350)' on 'Diabetic nephropathy (more control exclusions)'

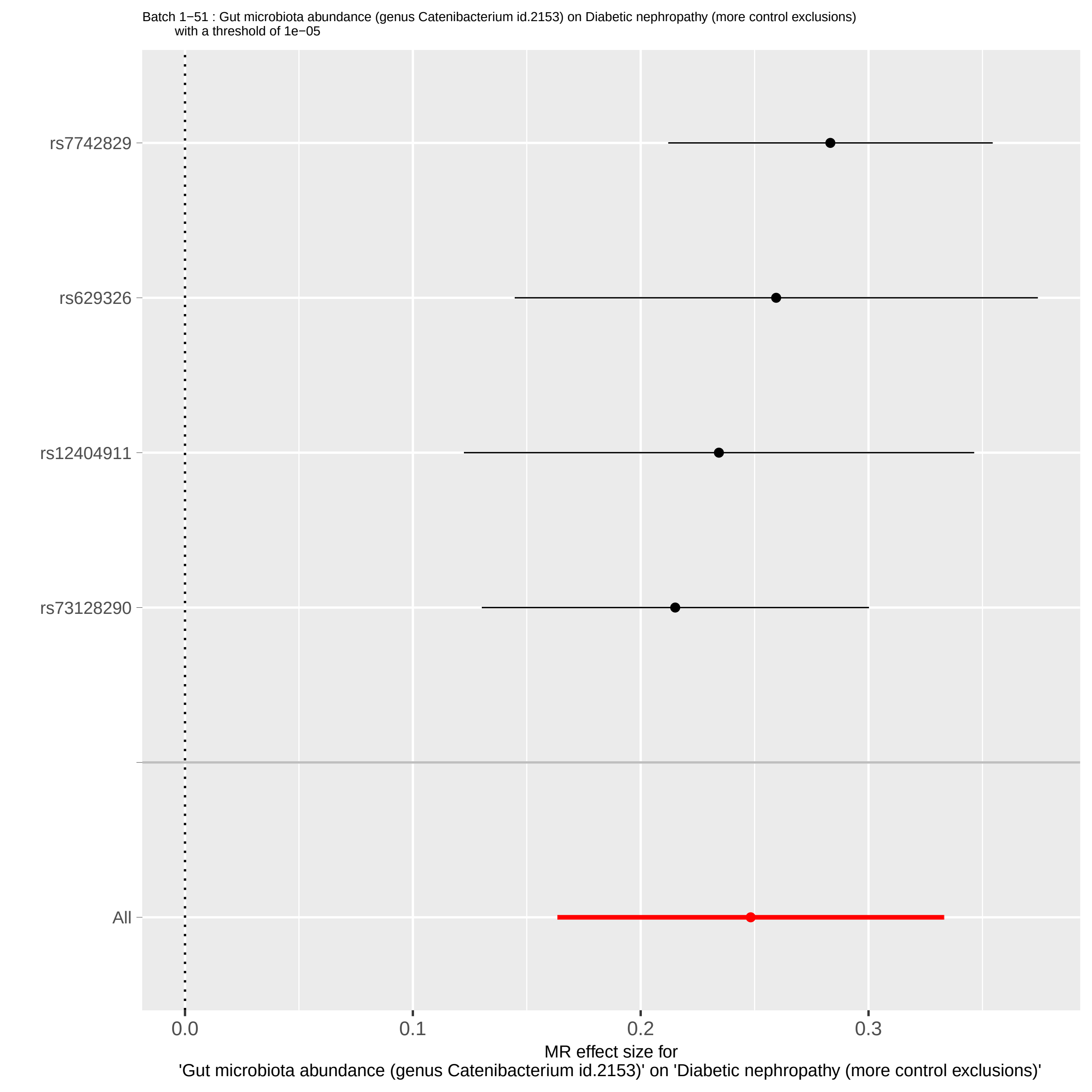
Batch 1−51 : Gut microbiota abundance (genus Catenibacterium id.2153) on Diabetic nephropathy (more control exclusions)
with a threshold of 1e−05
rs7742829
rs629326
rs12404911
rs73128290
All
0.0
0.1
0.2
0.3
MR effect size for
'Gut microbiota abundance (genus Catenibacterium id.2153)' on 'Diabetic nephropathy (more control exclusions)'

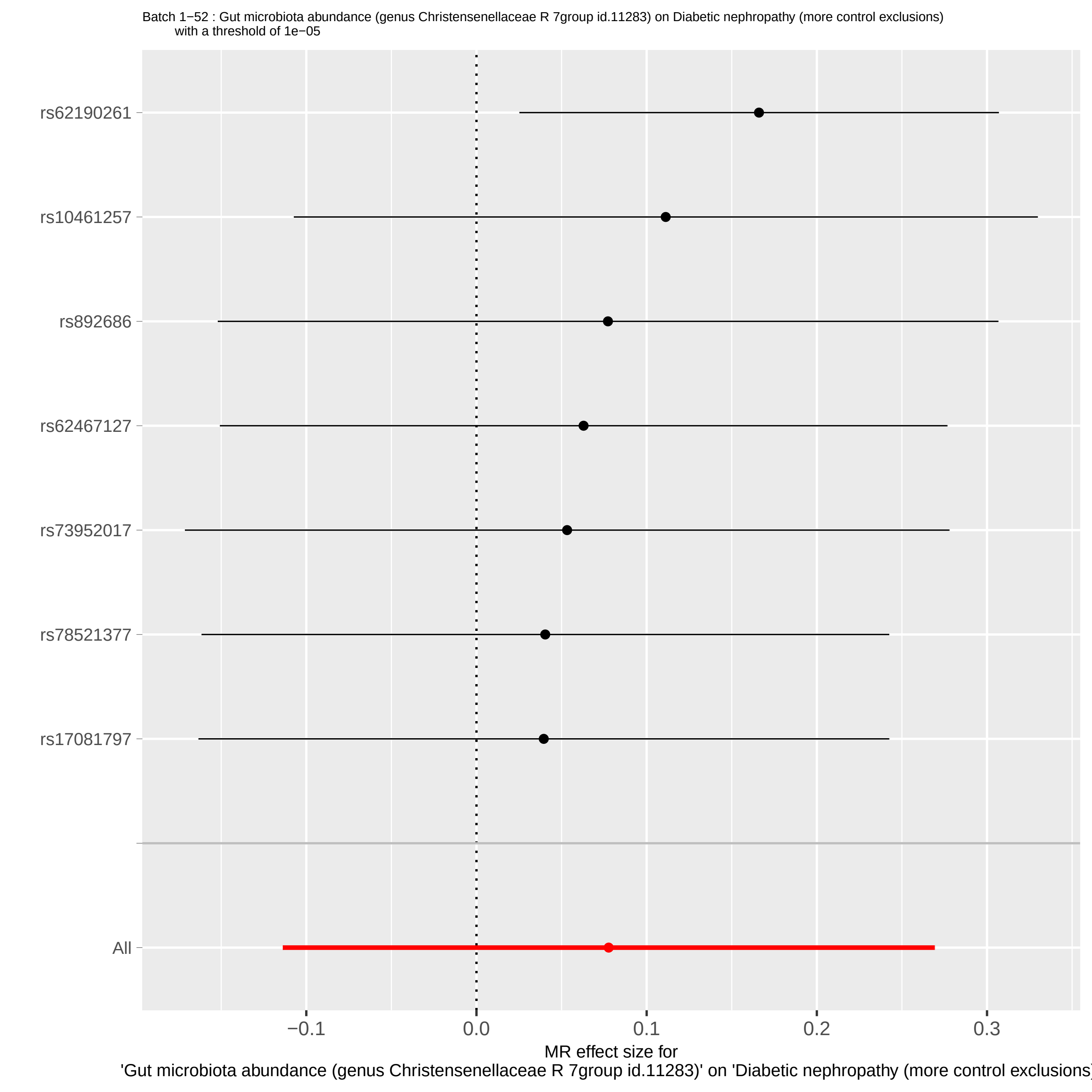

Batch 1−52 : Gut microbiota abundance (genus Christensenellaceae R 7group id.11283) on Diabetic nephropathy (more control exclusions)
with a threshold of 1e−05
rs62190261
rs10461257
rs892686
rs62467127
rs73952017
rs78521377
rs17081797
All
−0.1
0.0
0.1
0.2
0.3
MR effect size for
'Gut microbiota abundance (genus Christensenellaceae R 7group id.11283)' on 'Diabetic nephropathy (more control exclusions)'

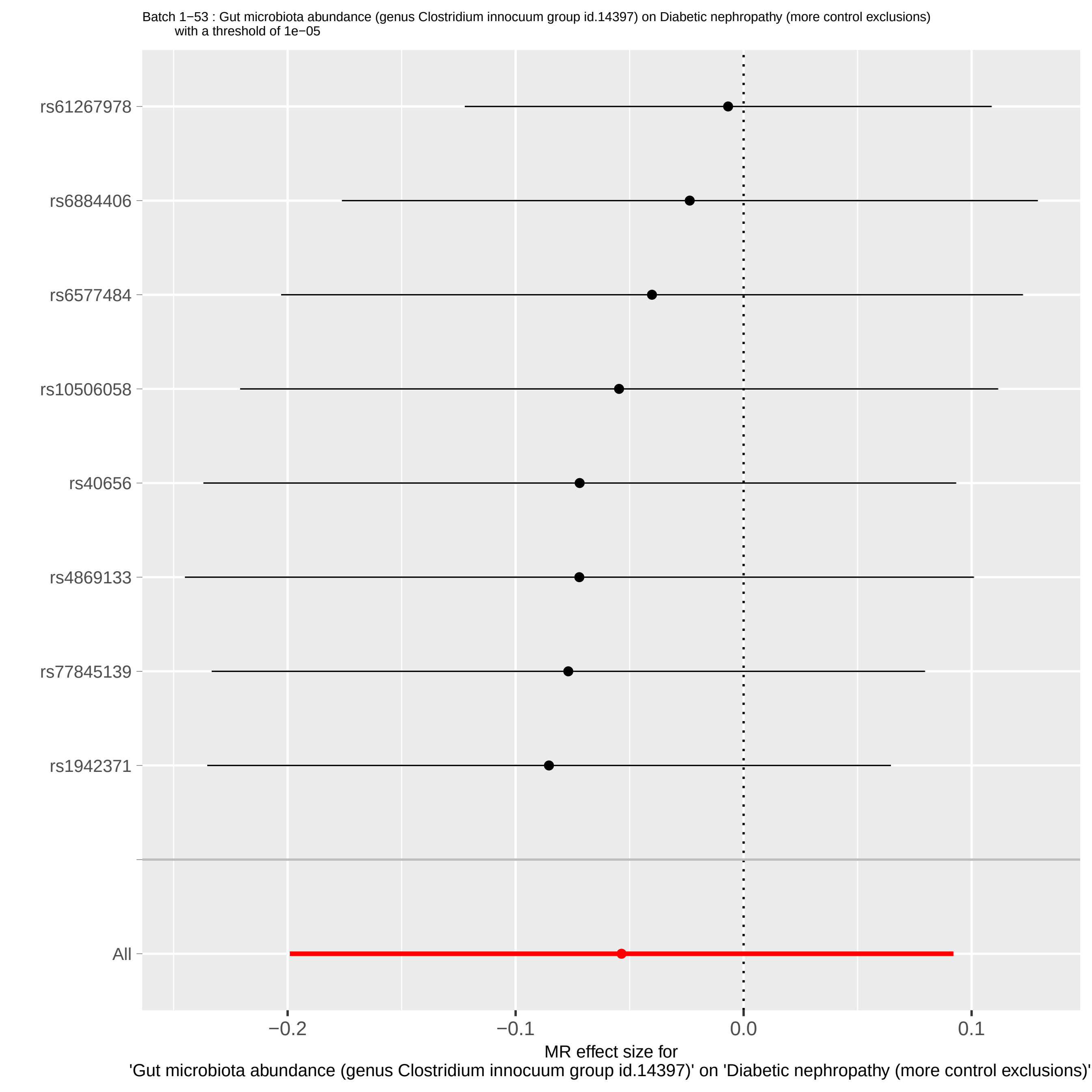

Batch 1−53 : Gut microbiota abundance (genus Clostridium innocuum group id.14397) on Diabetic nephropathy (more control exclusions) with a threshold of 1e−05
rs61267978
rs6884406
rs6577484
rs10506058
rs40656
rs4869133
rs77845139
rs1942371
All
−0.2
−0.1
0.0
0.1
MR effect size for
'Gut microbiota abundance (genus Clostridium innocuum group id.14397)' on 'Diabetic nephropathy (more control exclusions)'

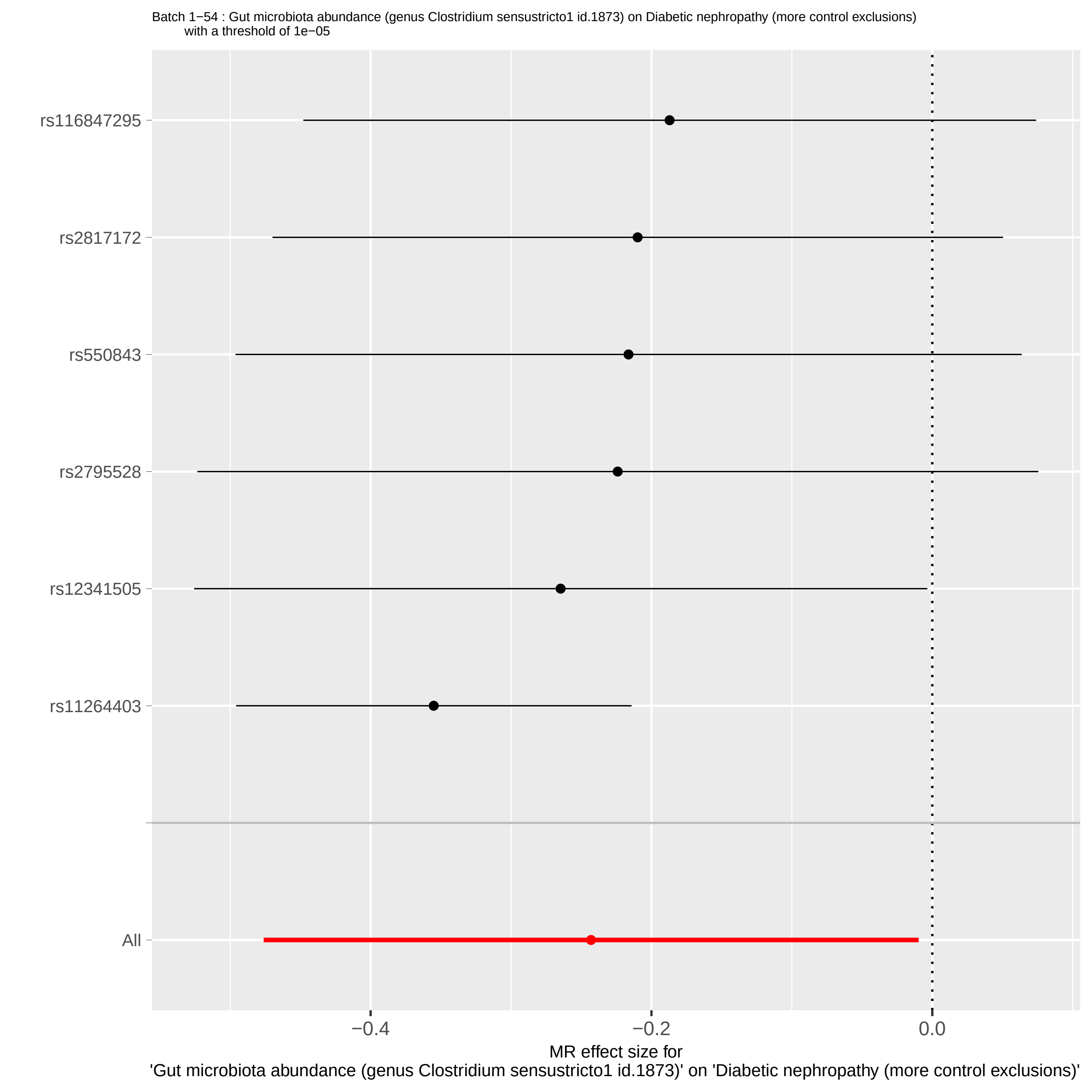

Batch 1−54 : Gut microbiota abundance (genus Clostridium sensustricto1 id.1873) on Diabetic nephropathy (more control exclusions)
with a threshold of 1e−05
rs116847295
rs2817172
rs550843
rs2795528
rs12341505
rs11264403
All
−0.4
−0.2
0.0
MR effect size for
'Gut microbiota abundance (genus Clostridium sensustricto1 id.1873)' on 'Diabetic nephropathy (more control exclusions)'

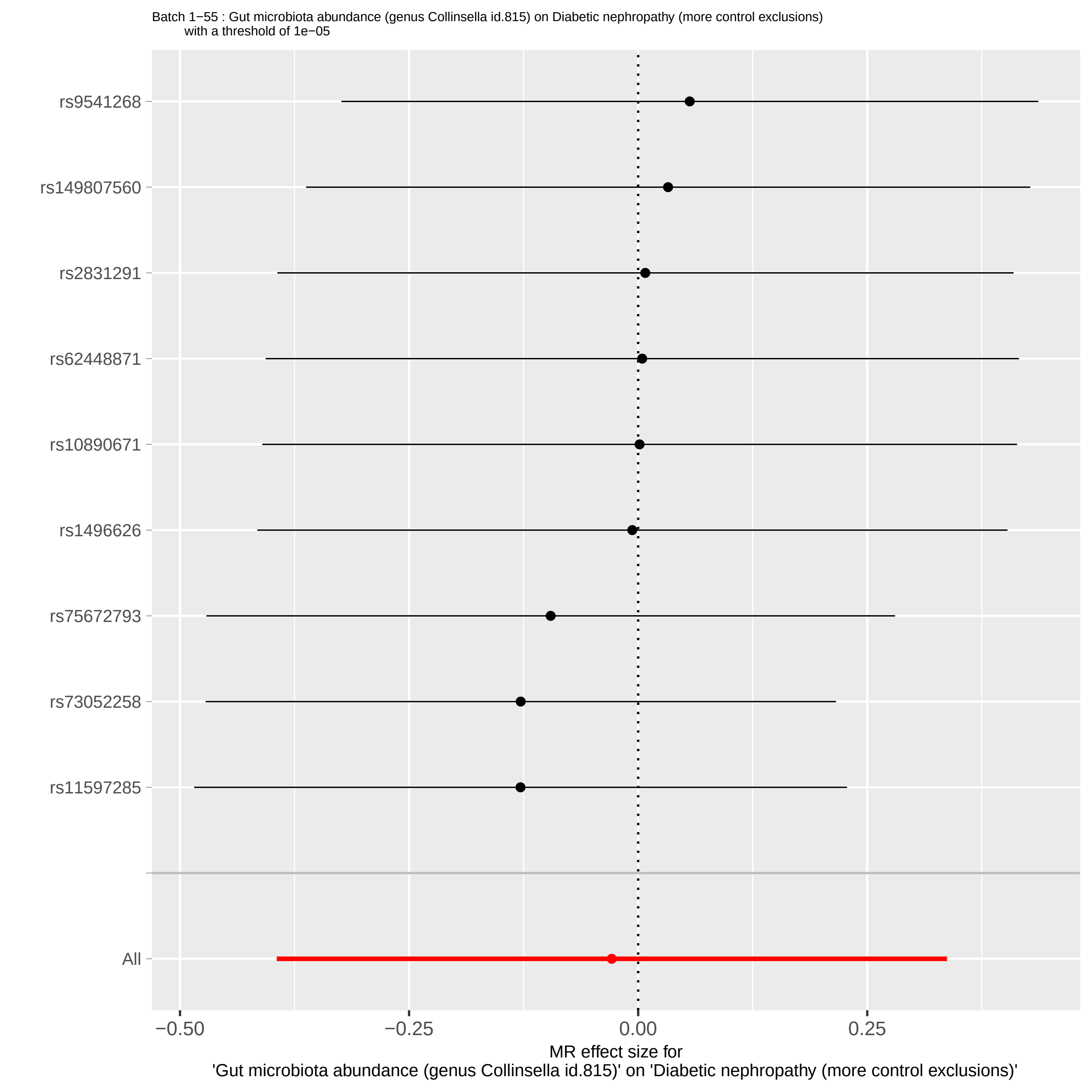
Batch 1−55 : Gut microbiota abundance (genus Collinsella id.815) on Diabetic nephropathy (more control exclusions)
with a threshold of 1e−05
rs9541268
rs149807560
rs2831291
rs62448871
rs10890671
rs1496626
rs75672793
rs73052258
rs11597285
All
−0.50
−0.25
0.00
0.25
MR effect size for
'Gut microbiota abundance (genus Collinsella id.815)' on 'Diabetic nephropathy (more control exclusions)'

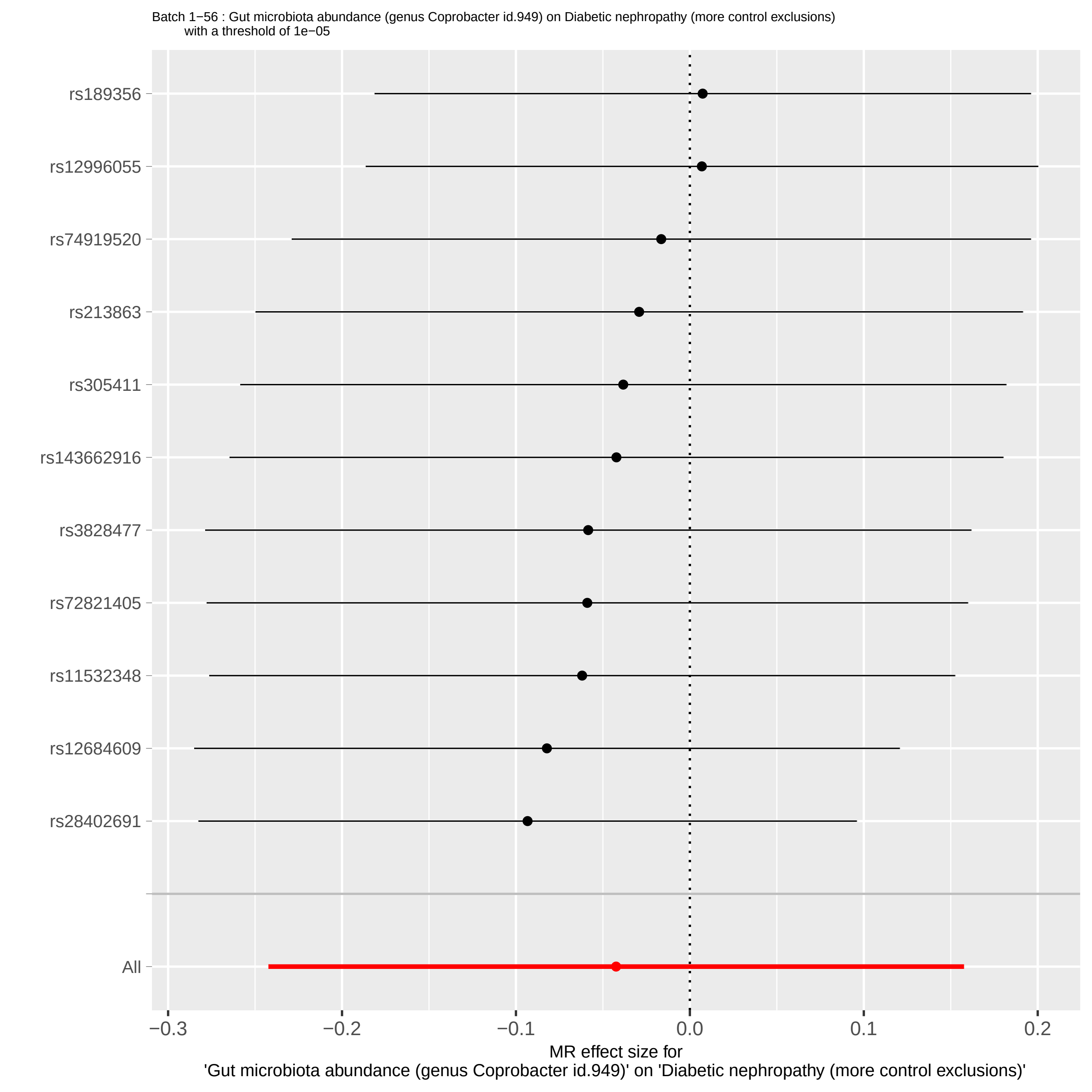

Batch 1−56 : Gut microbiota abundance (genus Coprobacter id.949) on Diabetic nephropathy (more control exclusions) with a threshold of 1e−05
rs189356
rs12996055
rs74919520
rs213863
rs305411
rs143662916
rs3828477
rs72821405
rs11532348
rs12684609
rs28402691
All
−0.3
−0.2
−0.1
0.0
0.1
0.2
MR effect size for
'Gut microbiota abundance (genus Coprobacter id.949)' on 'Diabetic nephropathy (more control exclusions)'

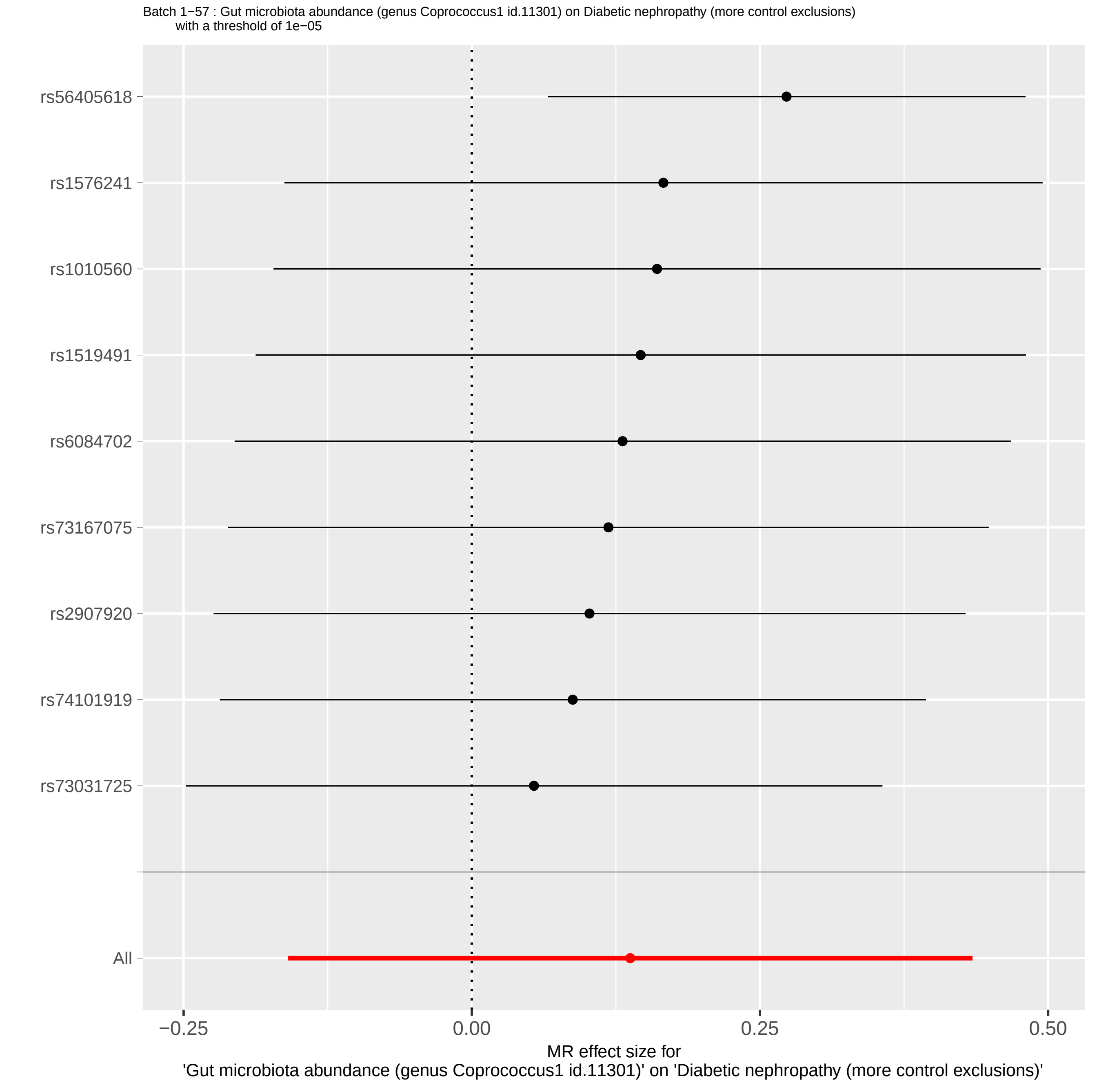

Batch 1−57 : Gut microbiota abundance (genus Coprococcus1 id.11301) on Diabetic nephropathy (more control exclusions)
with a threshold of 1e−05
rs56405618
rs1576241
rs1010560
rs1519491
rs6084702
rs73167075
rs2907920
rs74101919
rs73031725
All
−0.25
0.00
0.25
0.50
MR effect size for
'Gut microbiota abundance (genus Coprococcus1 id.11301)' on 'Diabetic nephropathy (more control exclusions)'

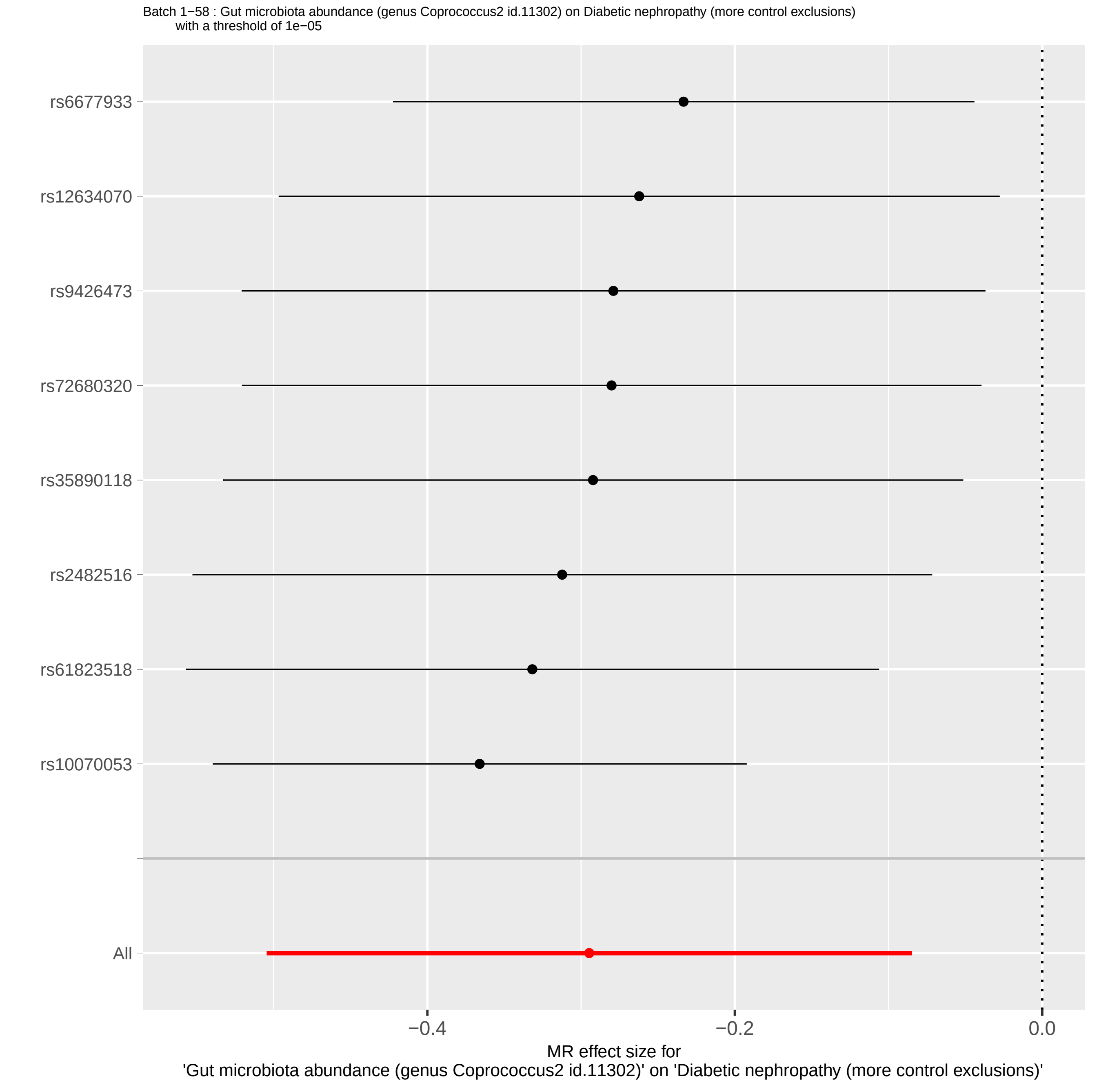

Batch 1−58 : Gut microbiota abundance (genus Coprococcus2 id.11302) on Diabetic nephropathy (more control exclusions) with a threshold of 1e−05
rs6677933
rs12634070
rs9426473
rs72680320
rs35890118
rs2482516
rs61823518
rs10070053
All
−0.4
−0.2
0.0
MR effect size for
'Gut microbiota abundance (genus Coprococcus2 id.11302)' on 'Diabetic nephropathy (more control exclusions)'

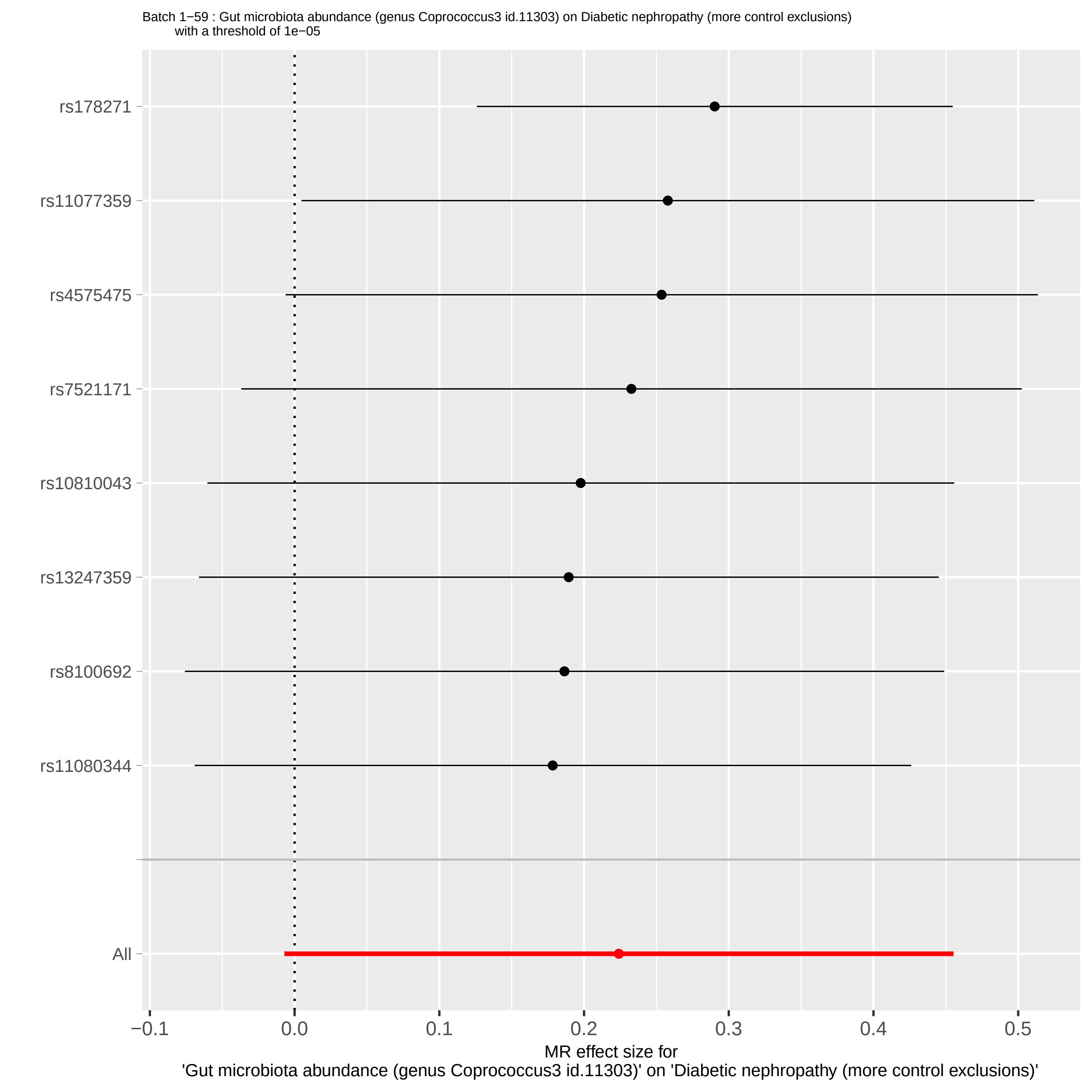

Batch 1−59 : Gut microbiota abundance (genus Coprococcus3 id.11303) on Diabetic nephropathy (more control exclusions)
with a threshold of 1e−05
rs178271
rs11077359
rs4575475
rs7521171
rs10810043
rs13247359
rs8100692
rs11080344
All
−0.1
0.0
0.1
0.2
0.3
0.4
0.5
MR effect size for
'Gut microbiota abundance (genus Coprococcus3 id.11303)' on 'Diabetic nephropathy (more control exclusions)'

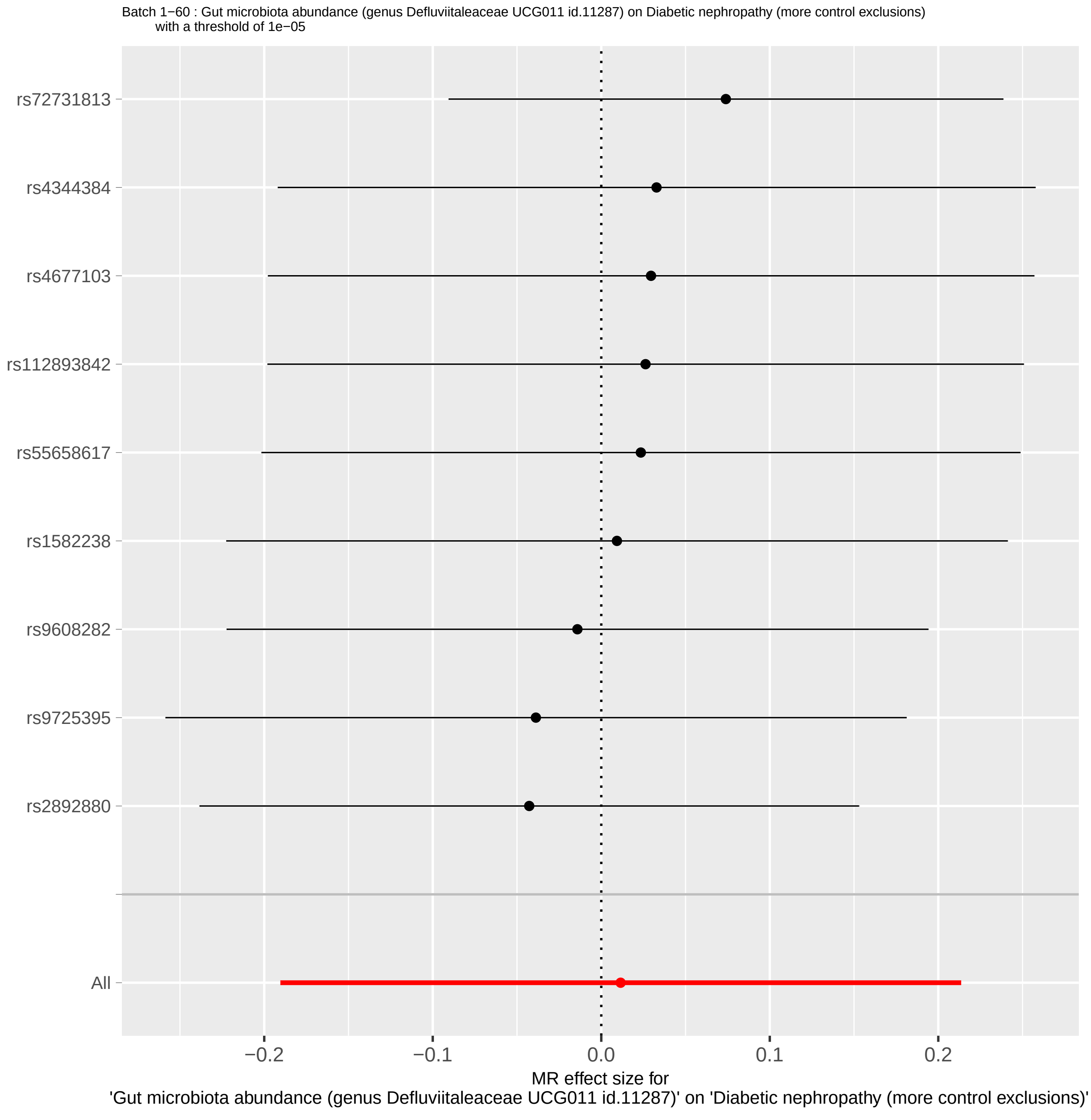
Batch 1−60 : Gut microbiota abundance (genus Defluviitaleaceae UCG011 id.11287) on Diabetic nephropathy (more control exclusions)
with a threshold of 1e−05
rs72731813
rs4344384
rs4677103
rs112893842
rs55658617
rs1582238
rs9608282
rs9725395
rs2892880
All
−0.2
−0.1
0.0
0.1
0.2
MR effect size for
'Gut microbiota abundance (genus Defluviitaleaceae UCG011 id.11287)' on 'Diabetic nephropathy (more control exclusions)'

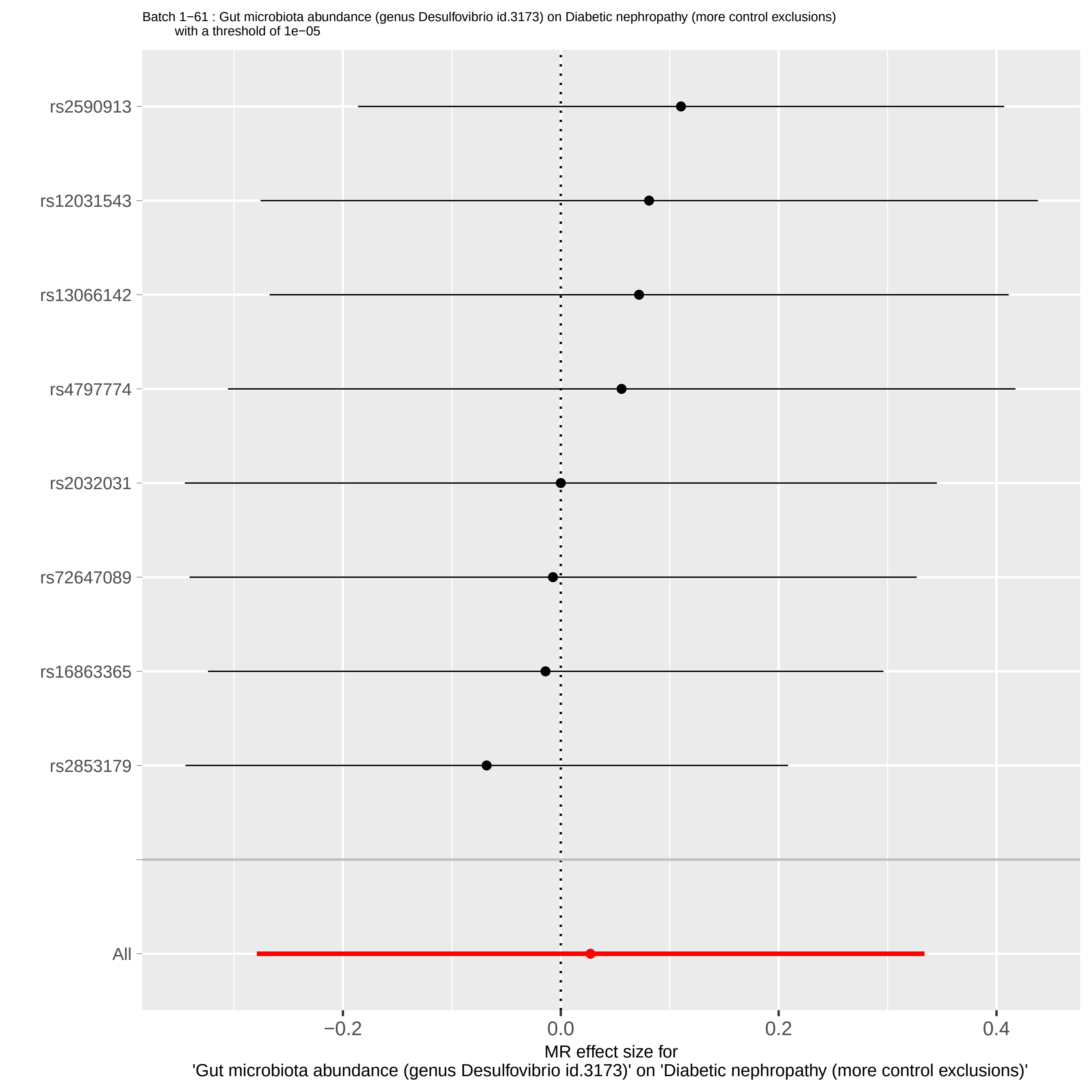
Batch 1−61 : Gut microbiota abundance (genus Desulfovibrio id.3173) on Diabetic nephropathy (more control exclusions) with a threshold of 1e−05
rs2590913
rs12031543
rs13066142
rs4797774
rs2032031
rs72647089
rs16863365
rs2853179
All
−0.2
0.0
0.2
0.4
MR effect size for
'Gut microbiota abundance (genus Desulfovibrio id.3173)' on 'Diabetic nephropathy (more control exclusions)'

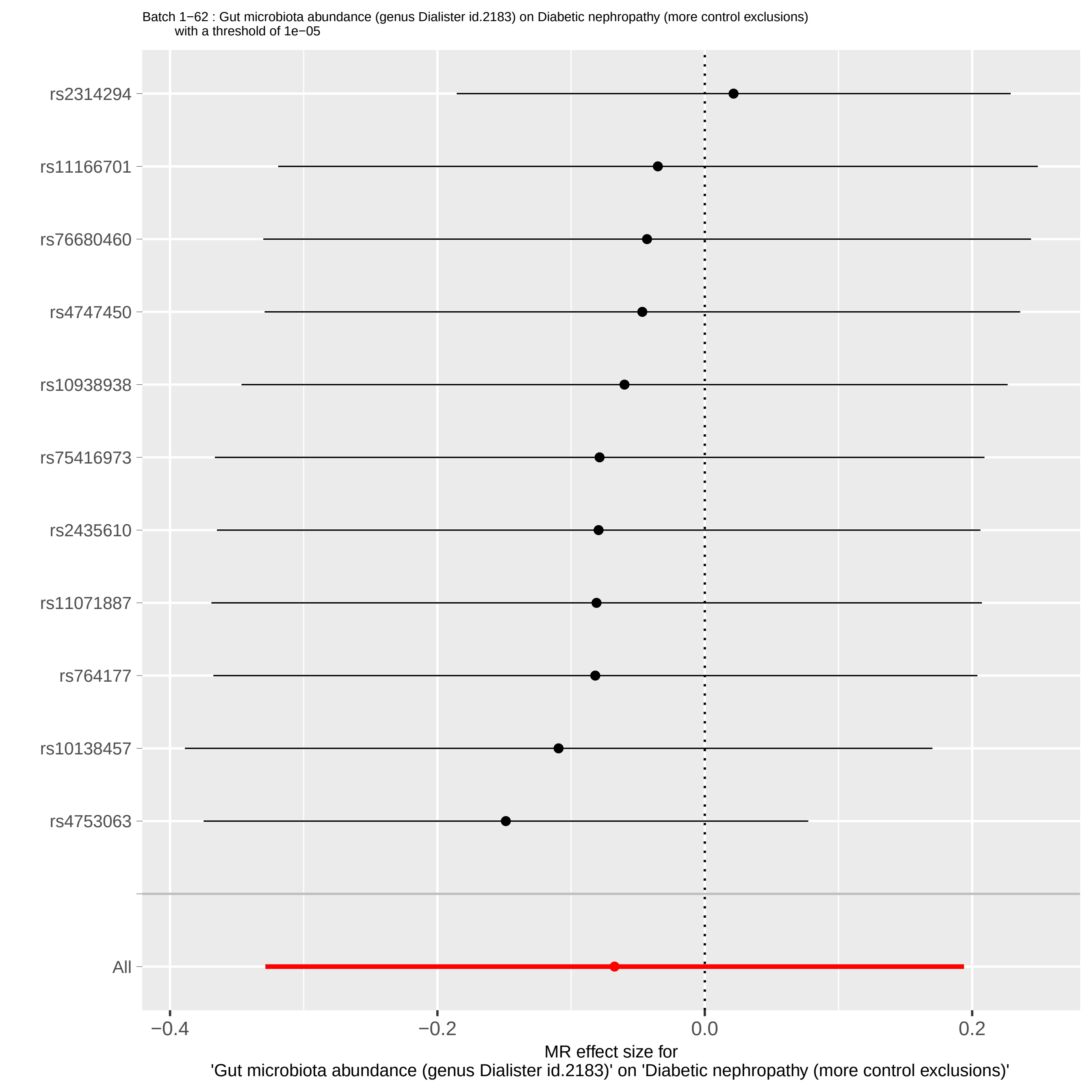
Batch 1−62 : Gut microbiota abundance (genus Dialister id.2183) on Diabetic nephropathy (more control exclusions)
with a threshold of 1e−05
rs2314294
rs11166701
rs76680460
rs4747450
rs10938938
rs75416973
rs2435610
rs11071887
rs764177
rs10138457
rs4753063
All
−0.4
−0.2
0.0
0.2
MR effect size for
'Gut microbiota abundance (genus Dialister id.2183)' on 'Diabetic nephropathy (more control exclusions)'

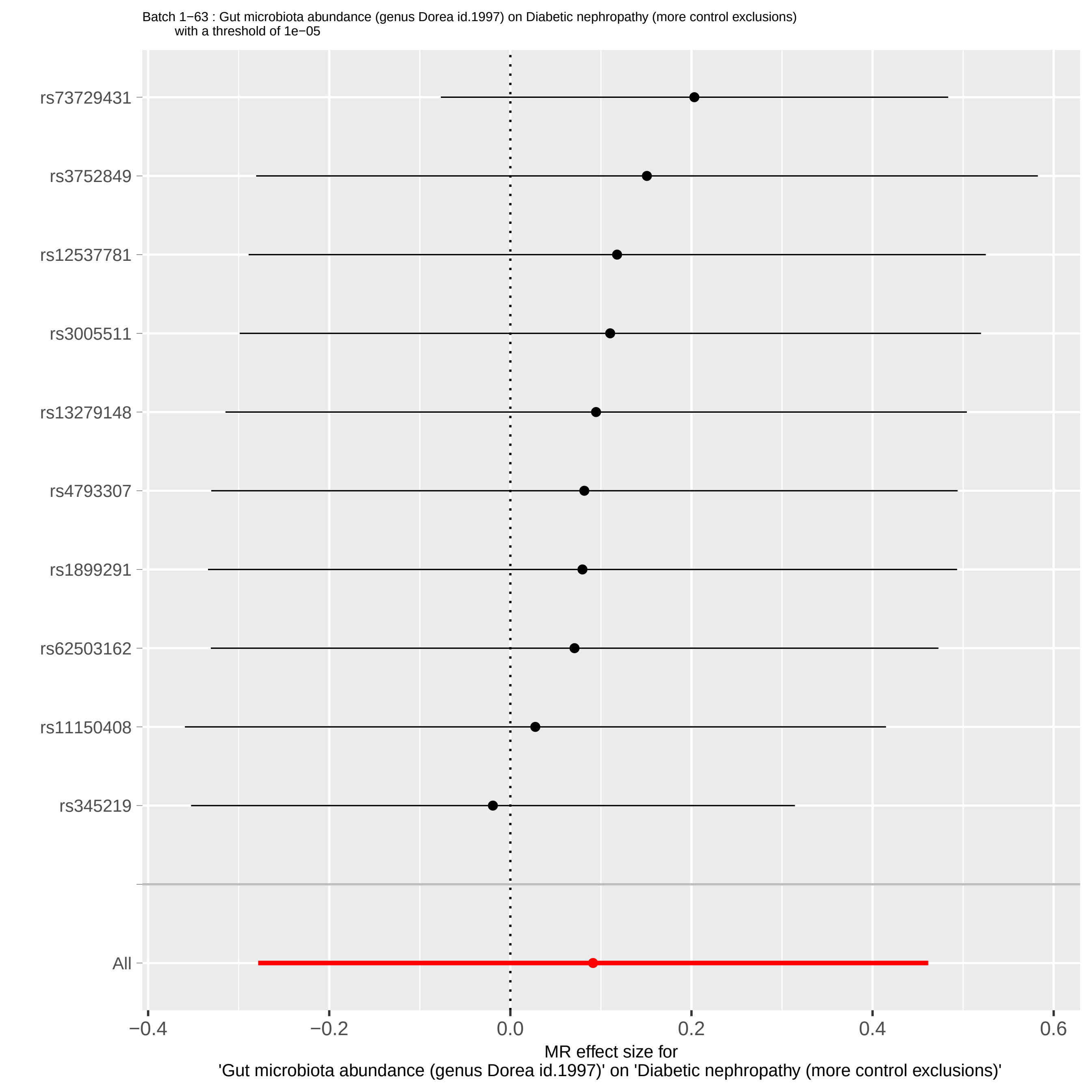

Batch 1−63 : Gut microbiota abundance (genus Dorea id.1997) on Diabetic nephropathy (more control exclusions)
with a threshold of 1e−05
rs73729431
rs3752849
rs12537781
rs3005511
rs13279148
rs4793307
rs1899291
rs62503162
rs11150408
rs345219
All
−0.4
−0.2
0.0
0.2
0.4
0.6
MR effect size for
'Gut microbiota abundance (genus Dorea id.1997)' on 'Diabetic nephropathy (more control exclusions)'

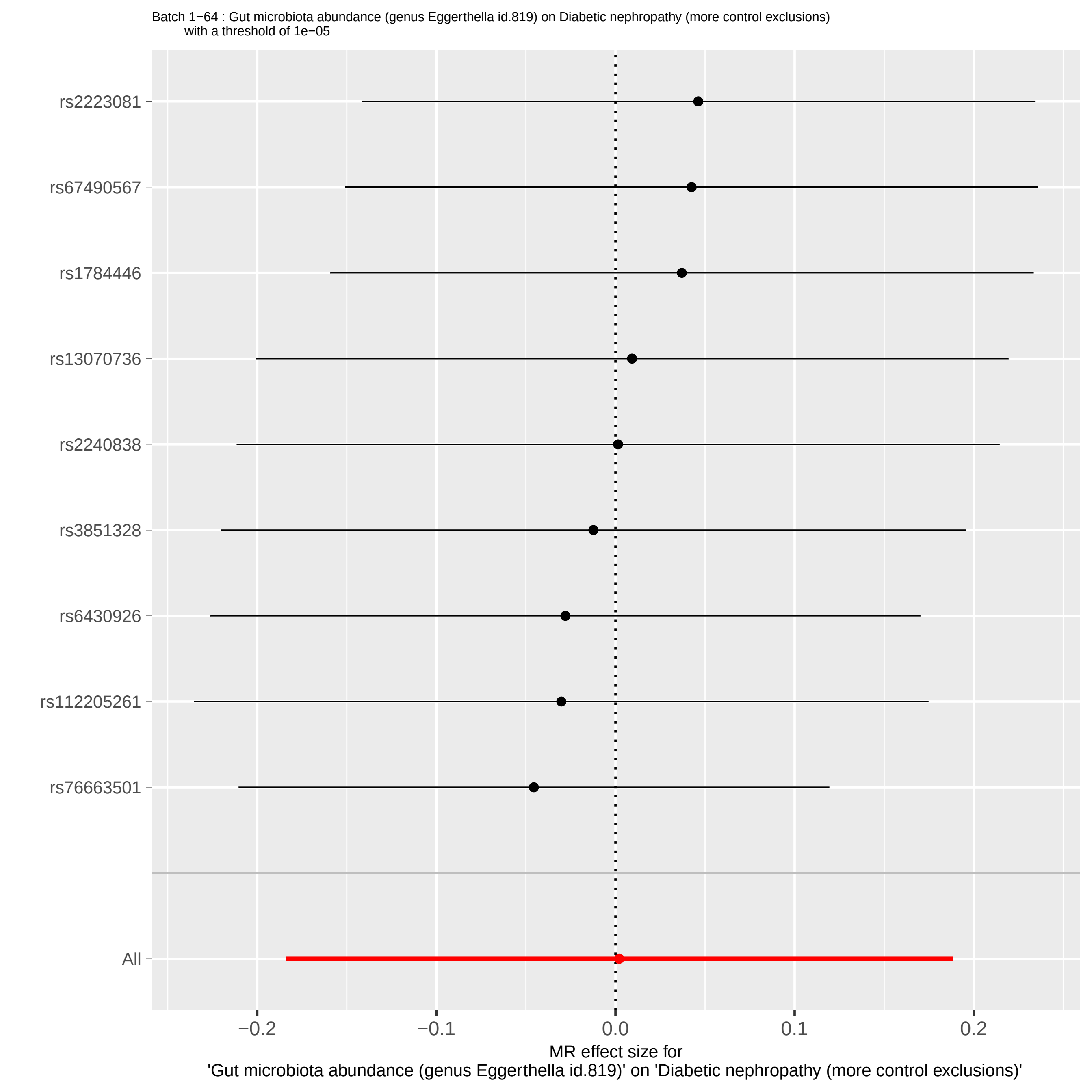

Batch 1−64 : Gut microbiota abundance (genus Eggerthella id.819) on Diabetic nephropathy (more control exclusions)
with a threshold of 1e−05
rs2223081
rs67490567
rs1784446
rs13070736
rs2240838
rs3851328
rs6430926
rs112205261
rs76663501
All
−0.2
−0.1
0.0
0.1
0.2
MR effect size for
'Gut microbiota abundance (genus Eggerthella id.819)' on 'Diabetic nephropathy (more control exclusions)'

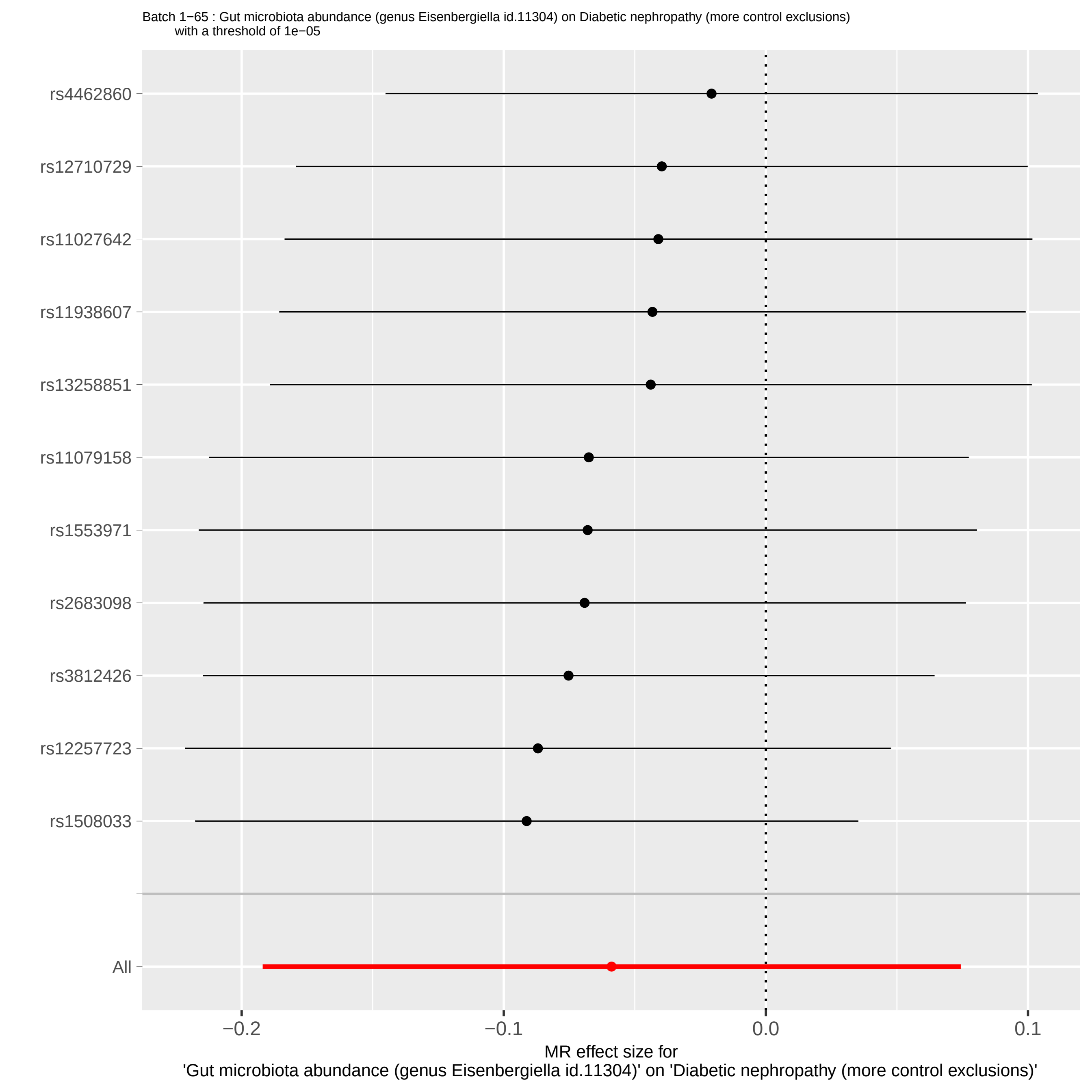
Batch 1−65 : Gut microbiota abundance (genus Eisenbergiella id.11304) on Diabetic nephropathy (more control exclusions)
with a threshold of 1e−05
rs4462860
rs12710729
rs11027642
rs11938607
rs13258851
rs11079158
rs1553971
rs2683098
rs3812426
rs12257723
rs1508033
All
−0.2
−0.1
0.0
0.1
MR effect size for
'Gut microbiota abundance (genus Eisenbergiella id.11304)' on 'Diabetic nephropathy (more control exclusions)'

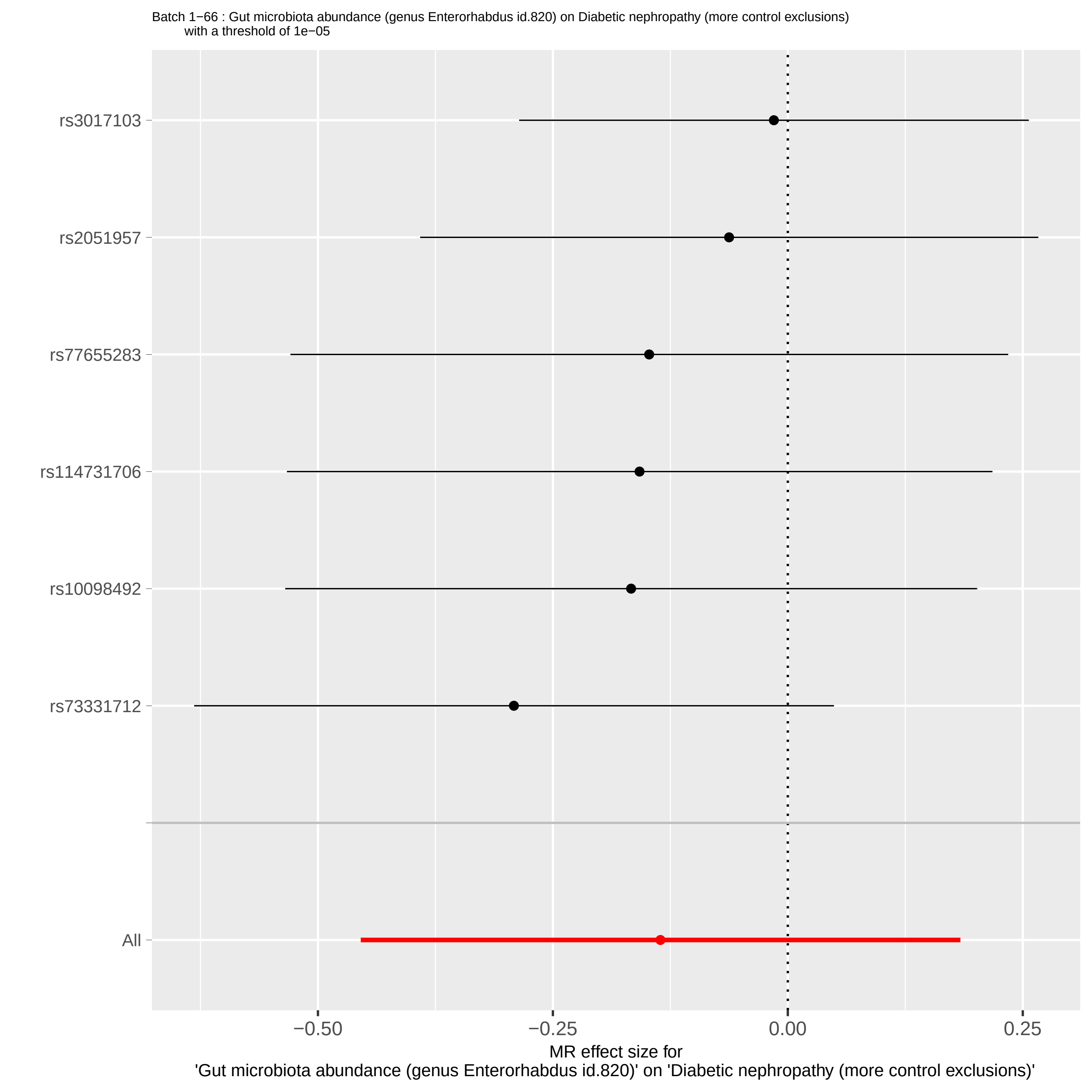
Batch 1−66 : Gut microbiota abundance (genus Enterorhabdus id.820) on Diabetic nephropathy (more control exclusions)
with a threshold of 1e−05
rs3017103
rs2051957
rs77655283
rs114731706
rs10098492
rs73331712
All
−0.50
−0.25
0.00
0.25
MR effect size for
'Gut microbiota abundance (genus Enterorhabdus id.820)' on 'Diabetic nephropathy (more control exclusions)'

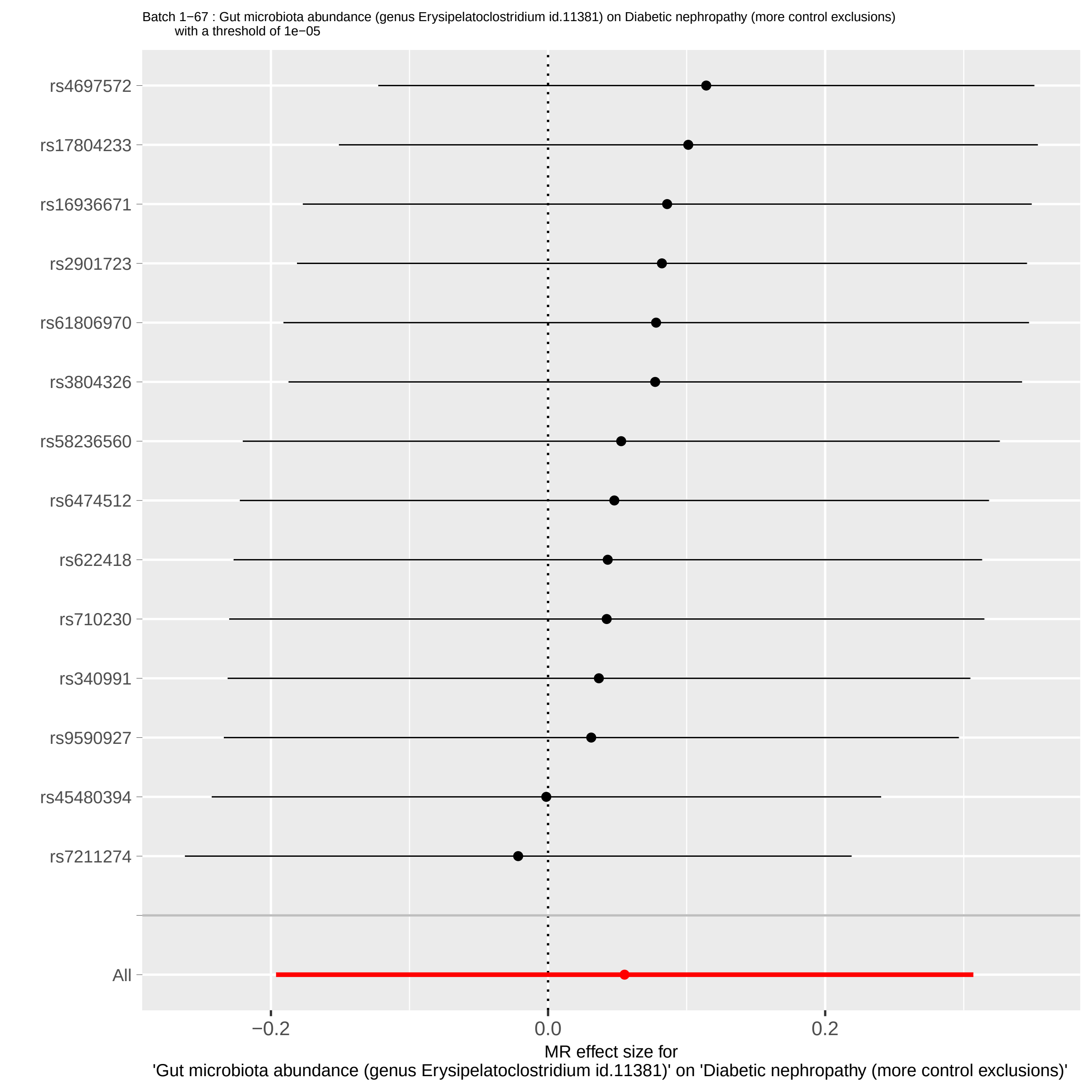

Batch 1−67 : Gut microbiota abundance (genus Erysipelatoclostridium id.11381) on Diabetic nephropathy (more control exclusions)
with a threshold of 1e−05
rs4697572
rs17804233
rs16936671
rs2901723
rs61806970
rs3804326
rs58236560
rs6474512
rs622418
rs710230
rs340991
rs9590927
rs45480394
rs7211274
All
−0.2
0.0
0.2
MR effect size for
'Gut microbiota abundance (genus Erysipelatoclostridium id.11381)' on 'Diabetic nephropathy (more control exclusions)'

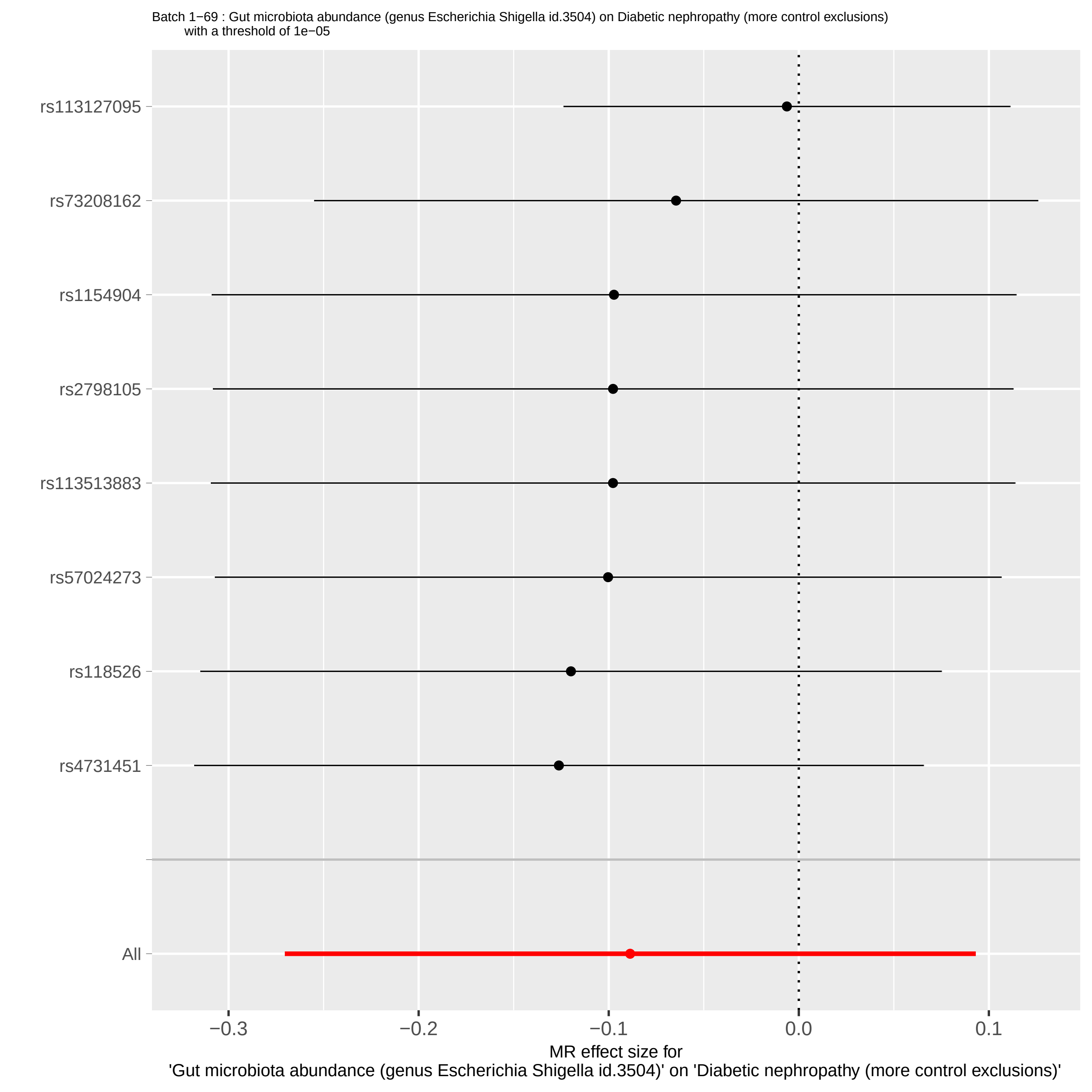
Batch 1−69 : Gut microbiota abundance (genus Escherichia Shigella id.3504) on Diabetic nephropathy (more control exclusions)
with a threshold of 1e−05
rs113127095
rs73208162
rs1154904
rs2798105
rs113513883
rs57024273
rs118526
rs4731451
All
−0.3
−0.2
−0.1
0.0
0.1
MR effect size for
'Gut microbiota abundance (genus Escherichia Shigella id.3504)' on 'Diabetic nephropathy (more control exclusions)'

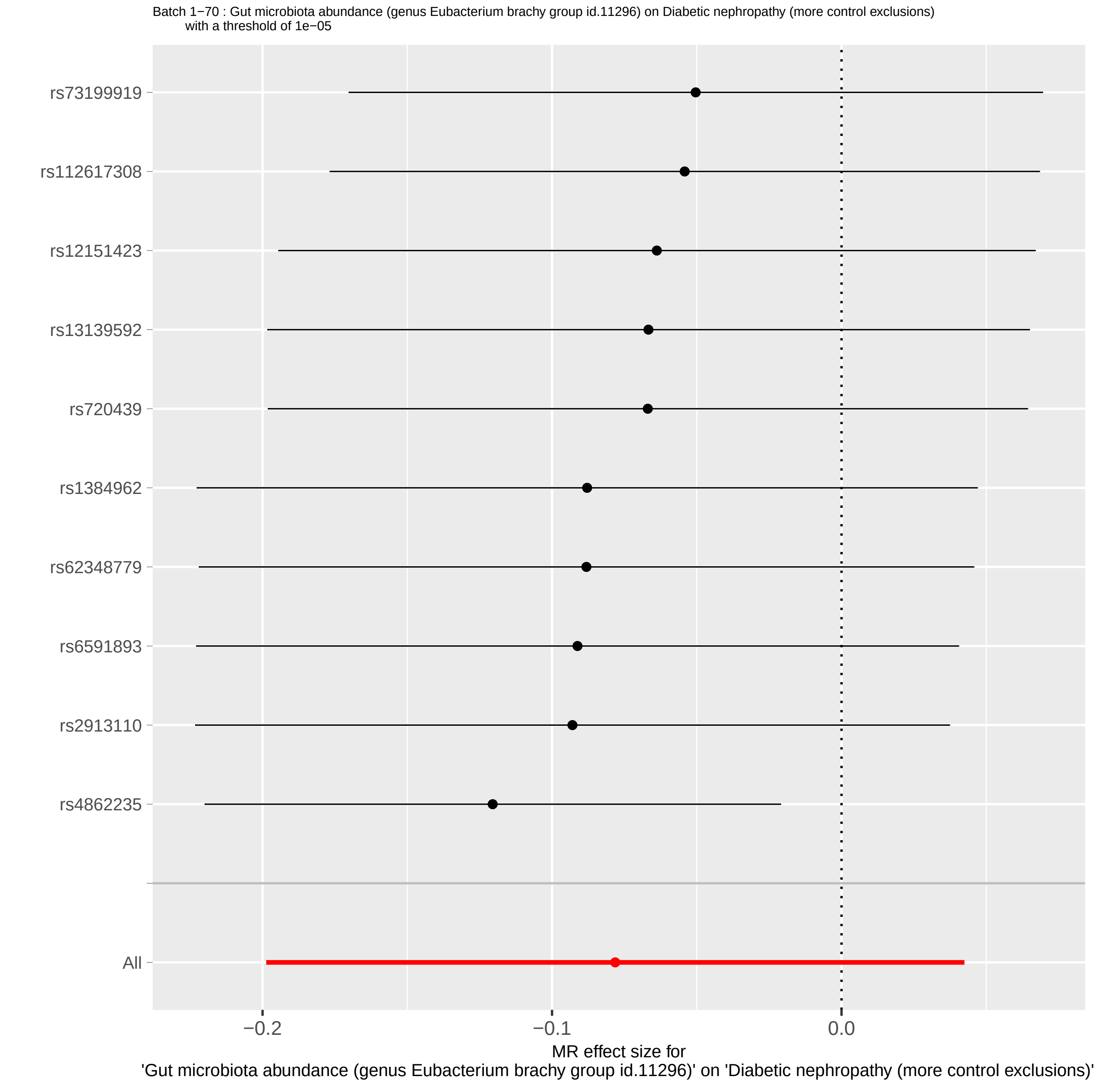
Batch 1−70 : Gut microbiota abundance (genus Eubacterium brachy group id.11296) on Diabetic nephropathy (more control exclusions) with a threshold of 1e−05
rs73199919
rs112617308
rs12151423
rs13139592
rs720439
rs1384962
rs62348779
rs6591893
rs2913110
rs4862235
All
−0.2
−0.1
0.0
MR effect size for
'Gut microbiota abundance (genus Eubacterium brachy group id.11296)' on 'Diabetic nephropathy (more control exclusions)'

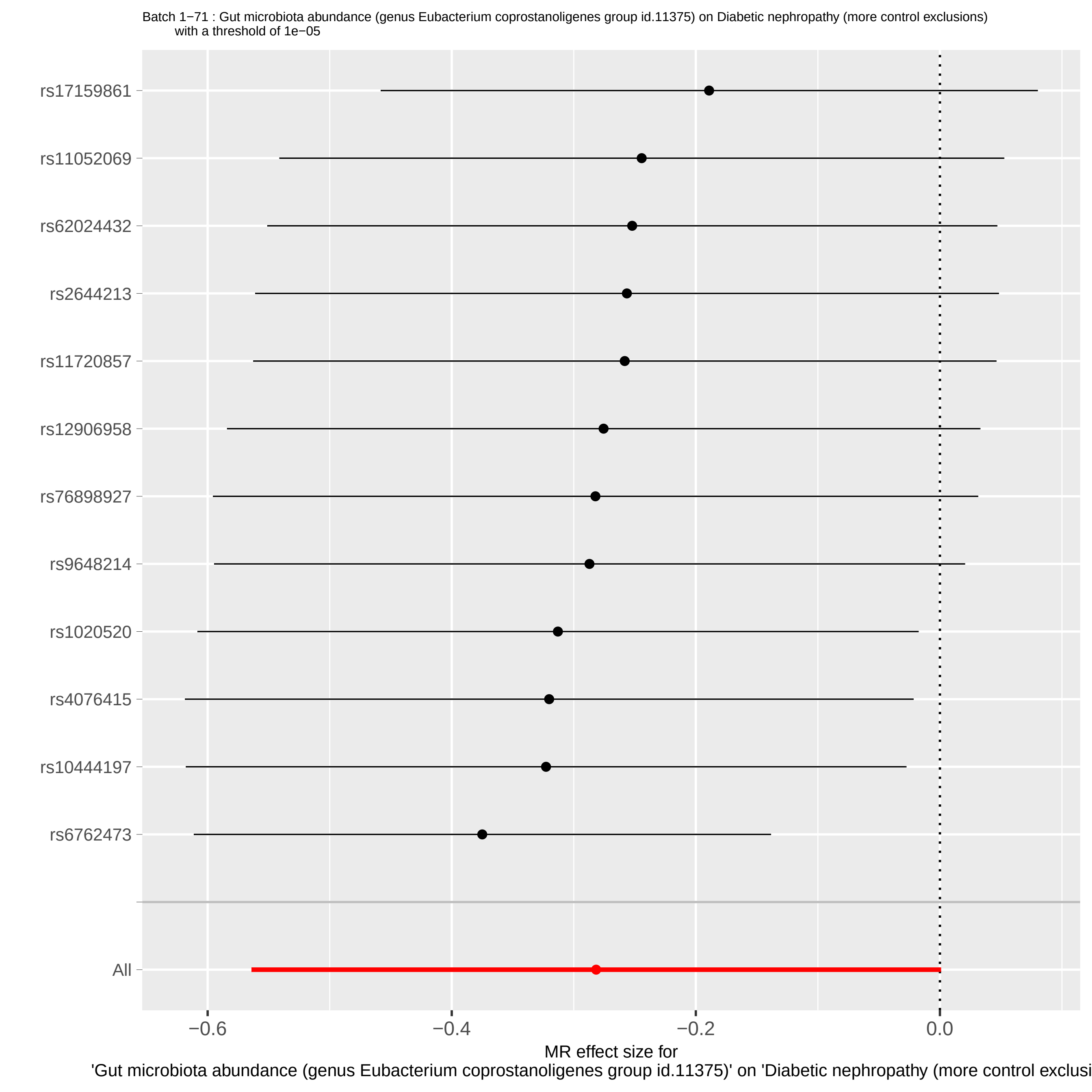

Batch 1−71 : Gut microbiota abundance (genus Eubacterium coprostanoligenes group id.11375) on Diabetic nephropathy (more control exclusions)
with a threshold of 1e−05
rs17159861
rs11052069
rs62024432
rs2644213
rs11720857
rs12906958
rs76898927
rs9648214
rs1020520
rs4076415
rs10444197
rs6762473
All
−0.6
−0.4
−0.2
0.0
MR effect size for
'Gut microbiota abundance (genus Eubacterium coprostanoligenes group id.11375)' on 'Diabetic nephropathy (more control exclusi

Batch 1−72 : Gut microbiota abundance (genus Eubacterium eligens group id.14372) on Diabetic nephropathy (more control exclusions)
with a threshold of 1e−05

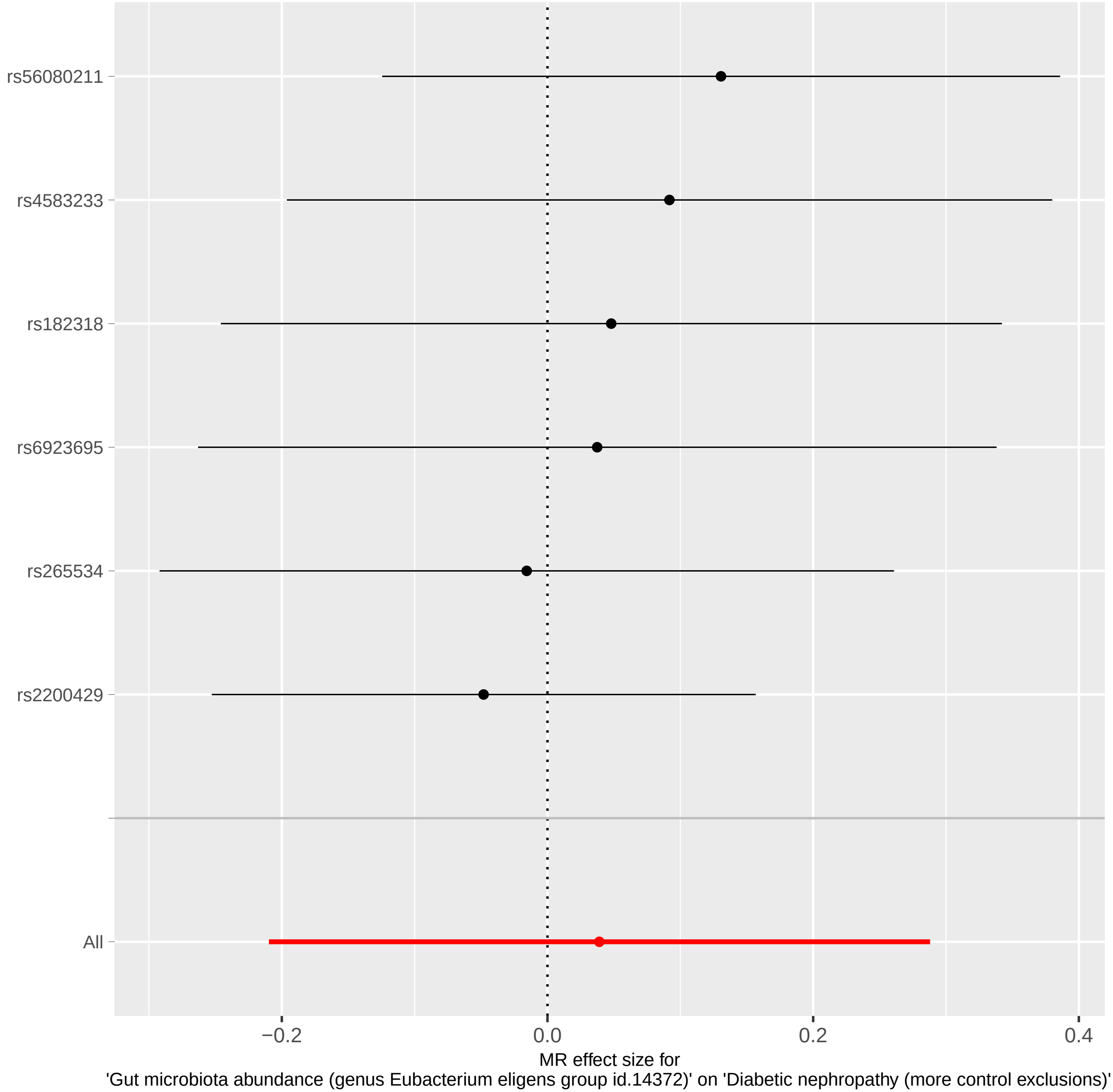

Batch 1−73 : Gut microbiota abundance (genus Eubacterium fissicatena group id.14373) on Diabetic nephropathy (more control exclusions) with a threshold of 1e−05

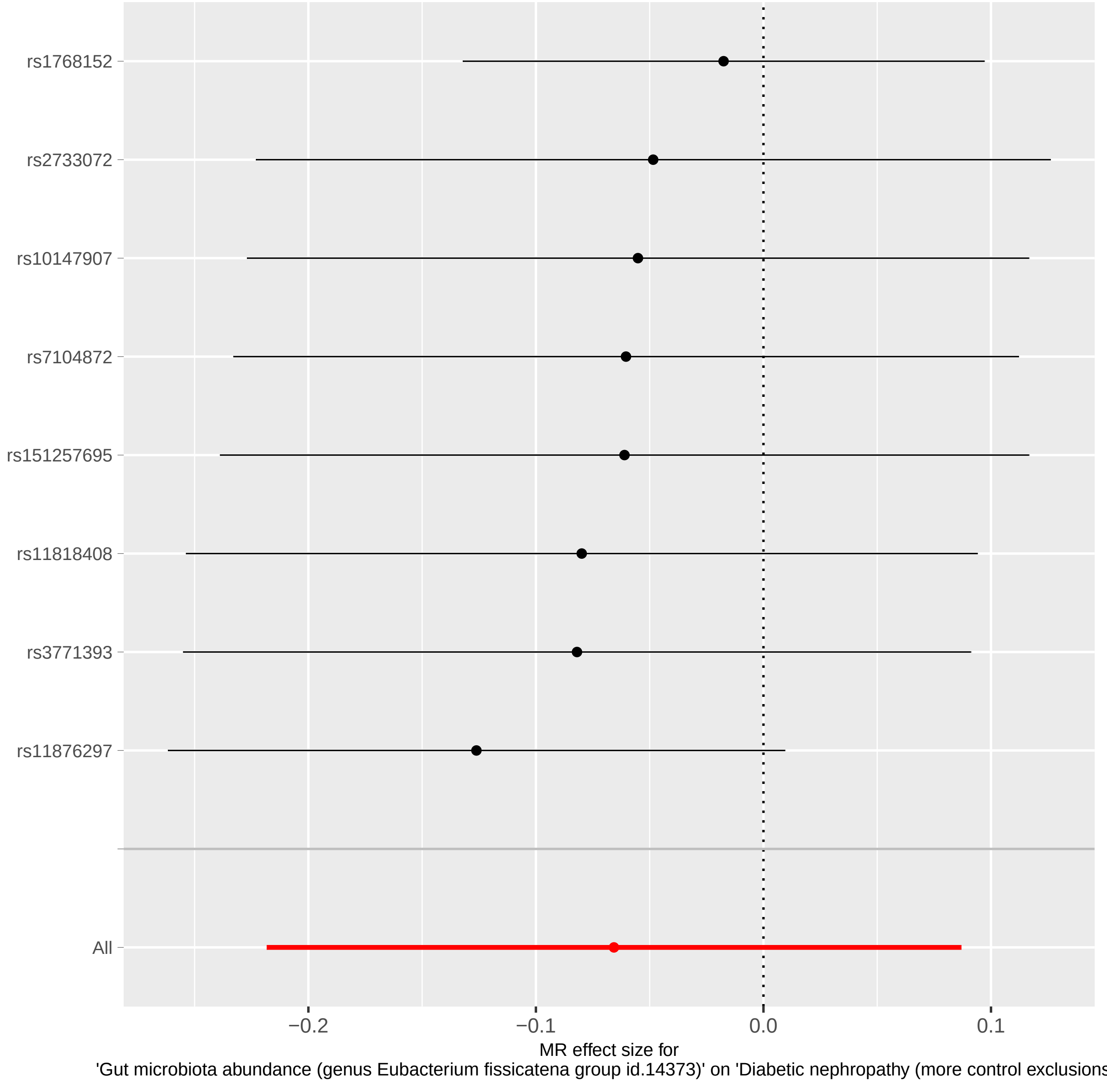

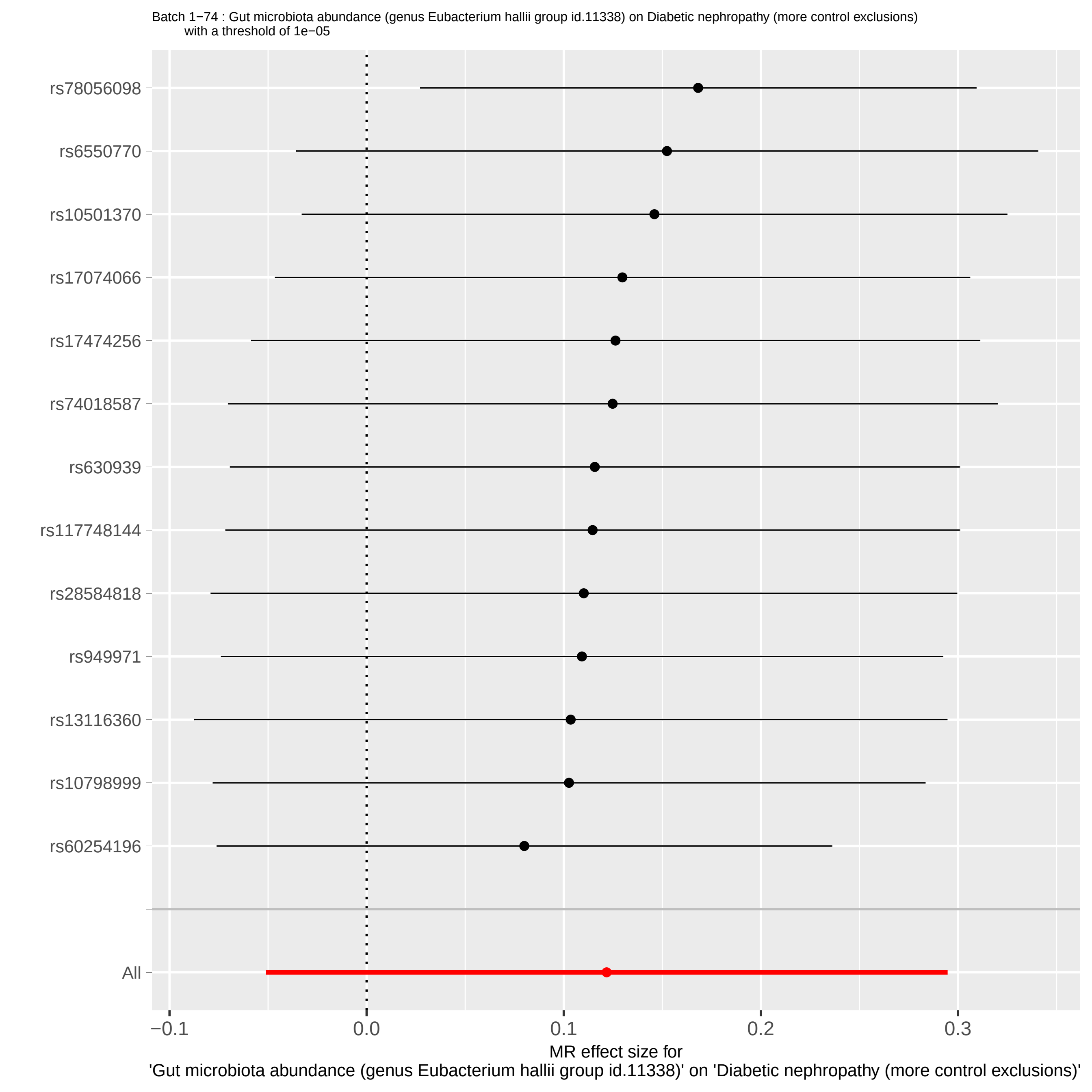

Batch 1−74 : Gut microbiota abundance (genus Eubacterium hallii group id.11338) on Diabetic nephropathy (more control exclusions) with a threshold of 1e−05
rs78056098
rs6550770
rs10501370
rs17074066
rs17474256
rs74018587
rs630939
rs117748144
rs28584818
rs949971
rs13116360
rs10798999
rs60254196
All
−0.1
0.0
0.1
0.2
0.3
MR effect size for
'Gut microbiota abundance (genus Eubacterium hallii group id.11338)' on 'Diabetic nephropathy (more control exclusions)'

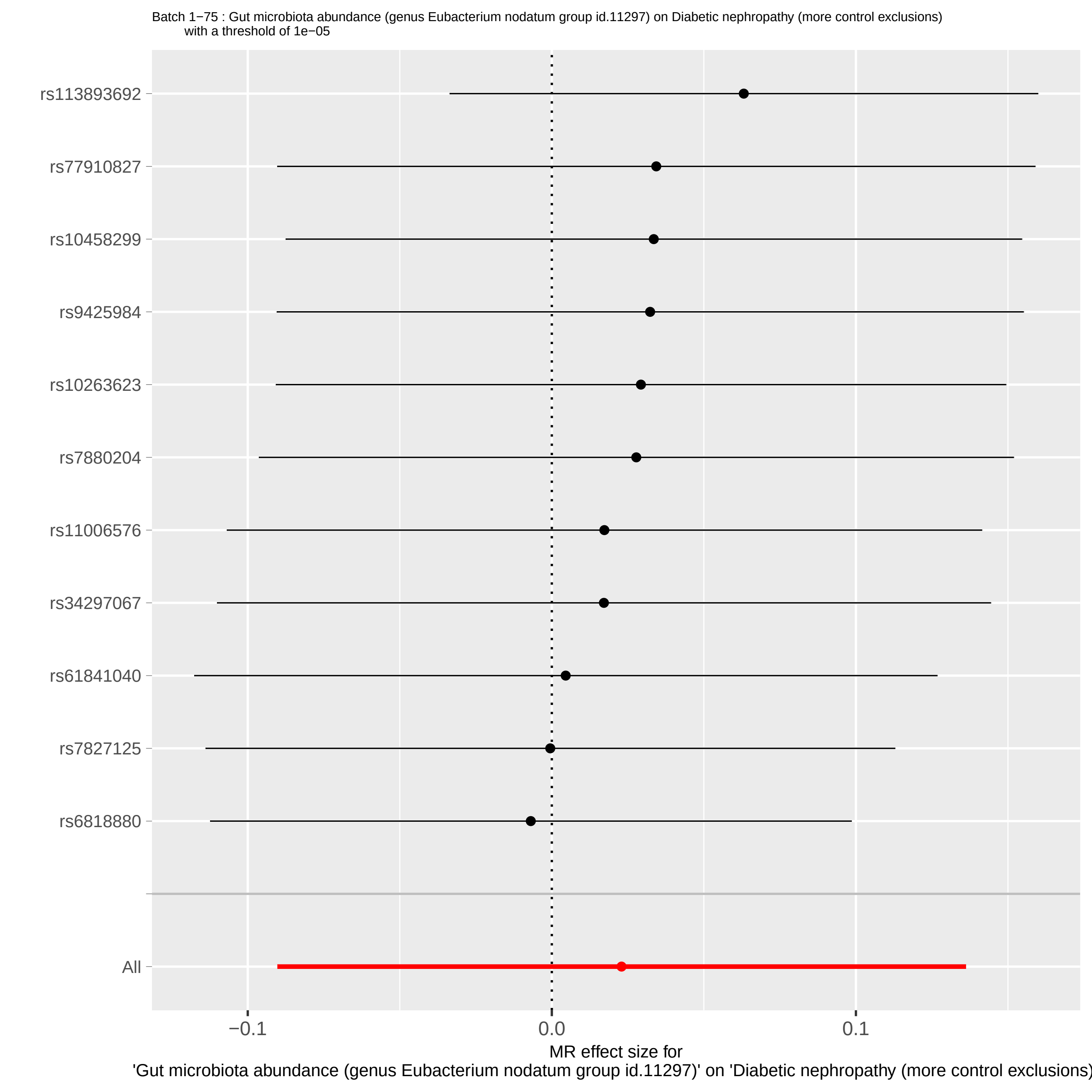

Batch 1−75 : Gut microbiota abundance (genus Eubacterium nodatum group id.11297) on Diabetic nephropathy (more control exclusions) with a threshold of 1e−05
rs113893692
rs77910827
rs10458299
rs9425984
rs10263623
rs7880204
rs11006576
rs34297067
rs61841040
rs7827125
rs6818880
All
−0.1
0.0
0.1
MR effect size for
'Gut microbiota abundance (genus Eubacterium nodatum group id.11297)' on 'Diabetic nephropathy (more control exclusions)'

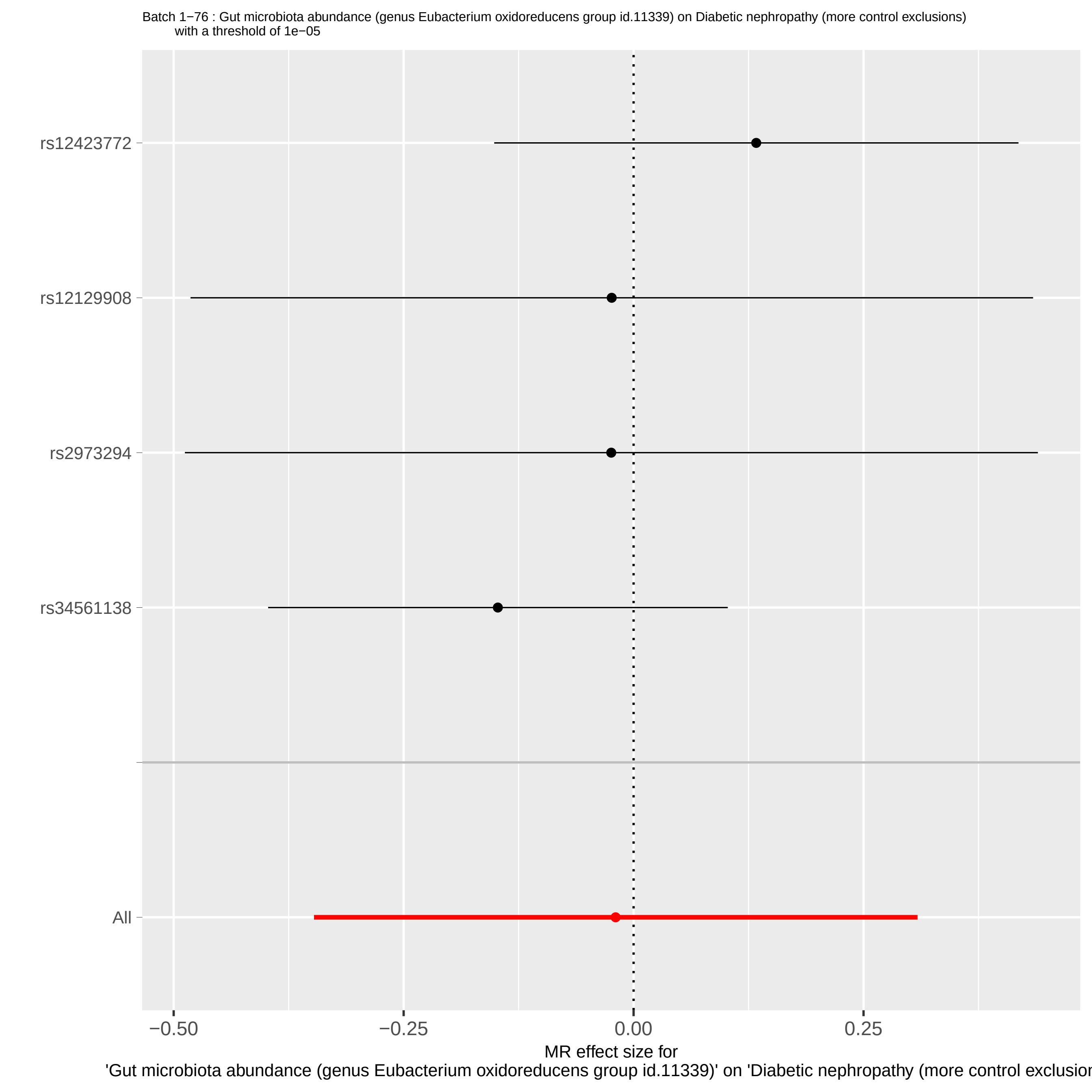

Batch 1−76 : Gut microbiota abundance (genus Eubacterium oxidoreducens group id.11339) on Diabetic nephropathy (more control exclusions)
with a threshold of 1e−05
rs12423772
rs12129908
rs2973294
rs34561138
All
−0.50
−0.25
0.00
0.25
MR effect size for
'Gut microbiota abundance (genus Eubacterium oxidoreducens group id.11339)' on 'Diabetic nephropathy (more control exclusio

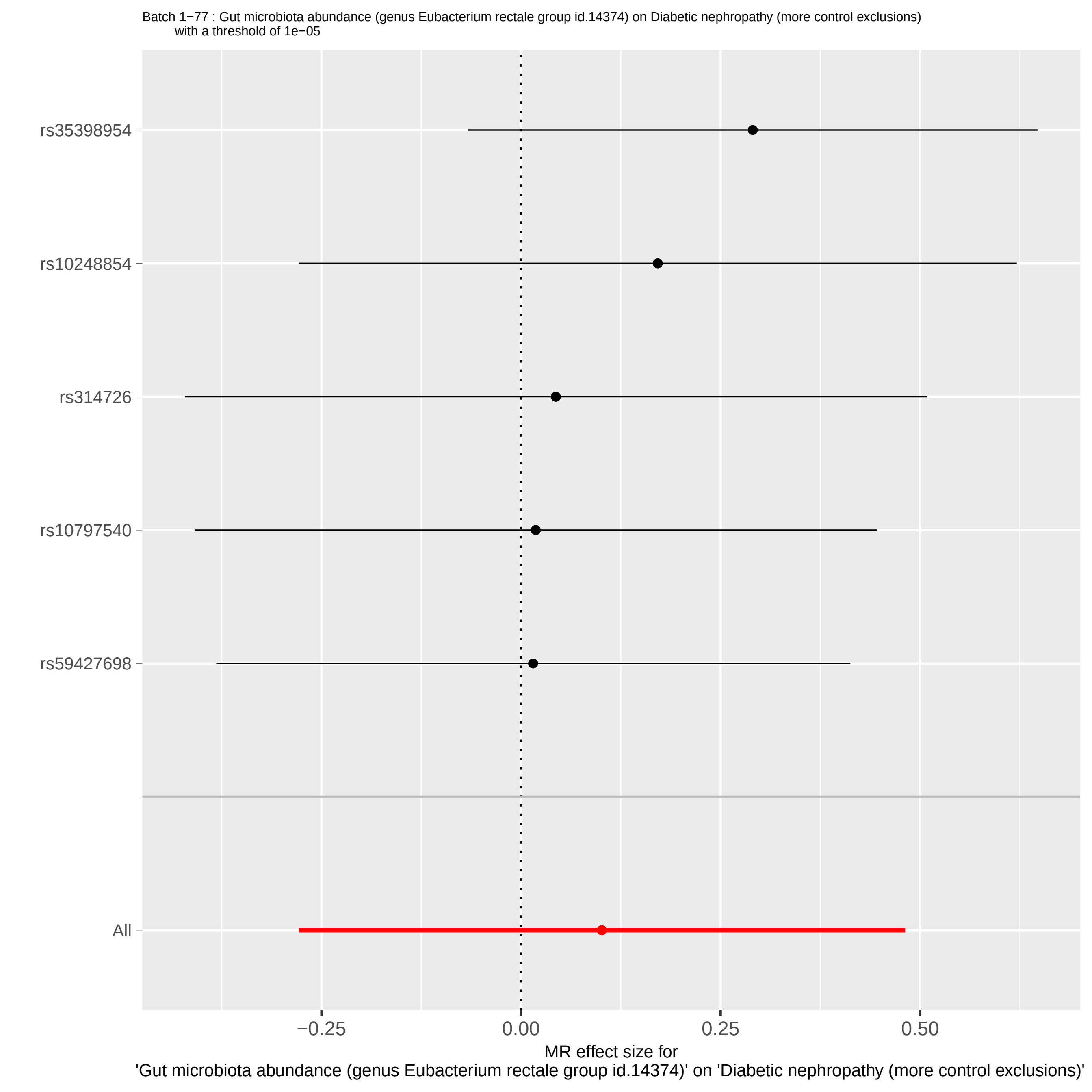

Batch 1−77 : Gut microbiota abundance (genus Eubacterium rectale group id.14374) on Diabetic nephropathy (more control exclusions) with a threshold of 1e−05
rs35398954
rs10248854
rs314726
rs10797540
rs59427698
All
−0.25
0.00
0.25
0.50
MR effect size for
'Gut microbiota abundance (genus Eubacterium rectale group id.14374)' on 'Diabetic nephropathy (more control exclusions)'

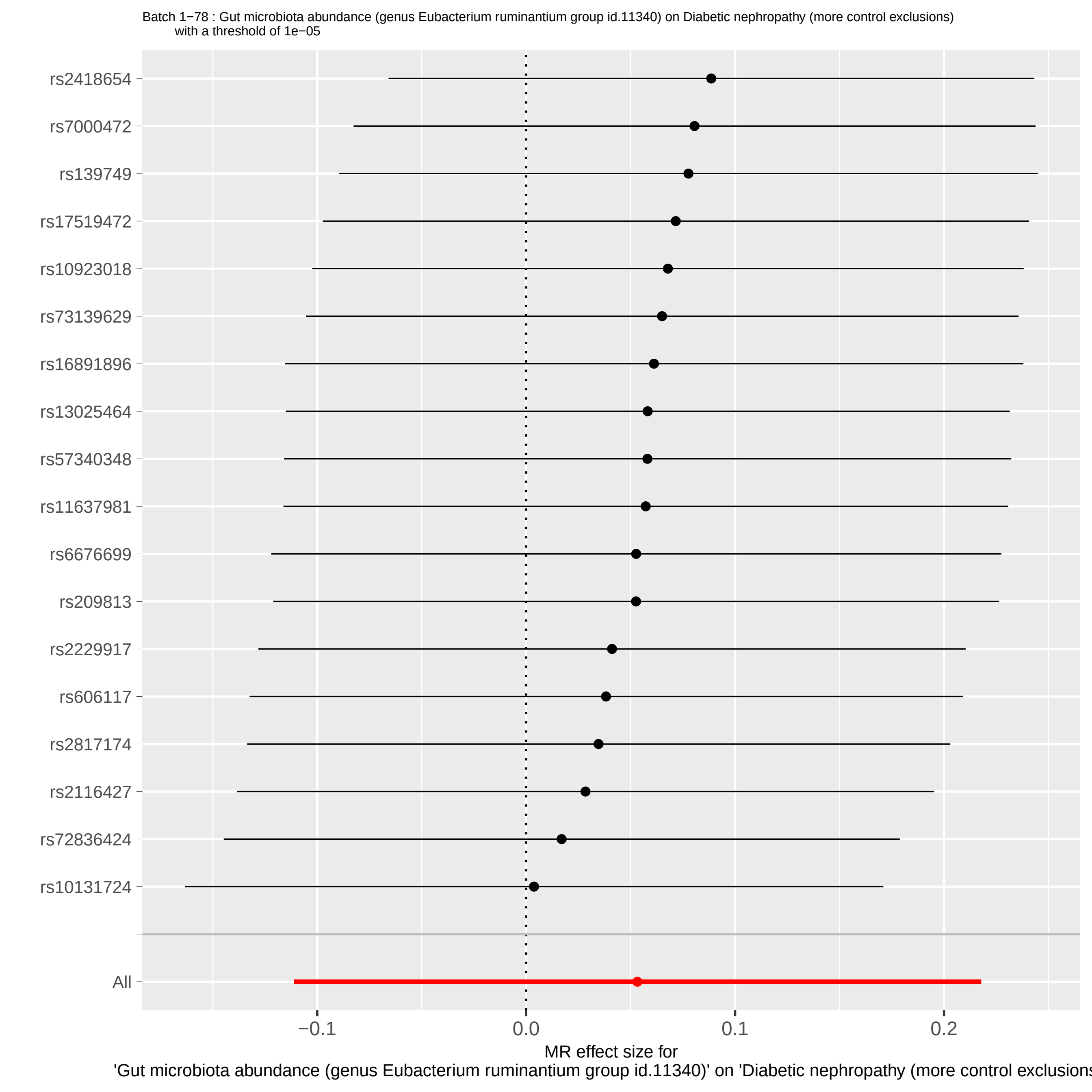

Batch 1−78 : Gut microbiota abundance (genus Eubacterium ruminantium group id.11340) on Diabetic nephropathy (more control exclusions)
with a threshold of 1e−05
rs2418654
rs7000472
rs139749
rs17519472
rs10923018
rs73139629
rs16891896
rs13025464
rs57340348
rs11637981
rs6676699
rs209813
rs2229917
rs606117
rs2817174
rs2116427
rs72836424
rs10131724
All
−0.1
0.0
0.1
0.2
MR effect size for
'Gut microbiota abundance (genus Eubacterium ruminantium group id.11340)' on 'Diabetic nephropathy (more control exclusions

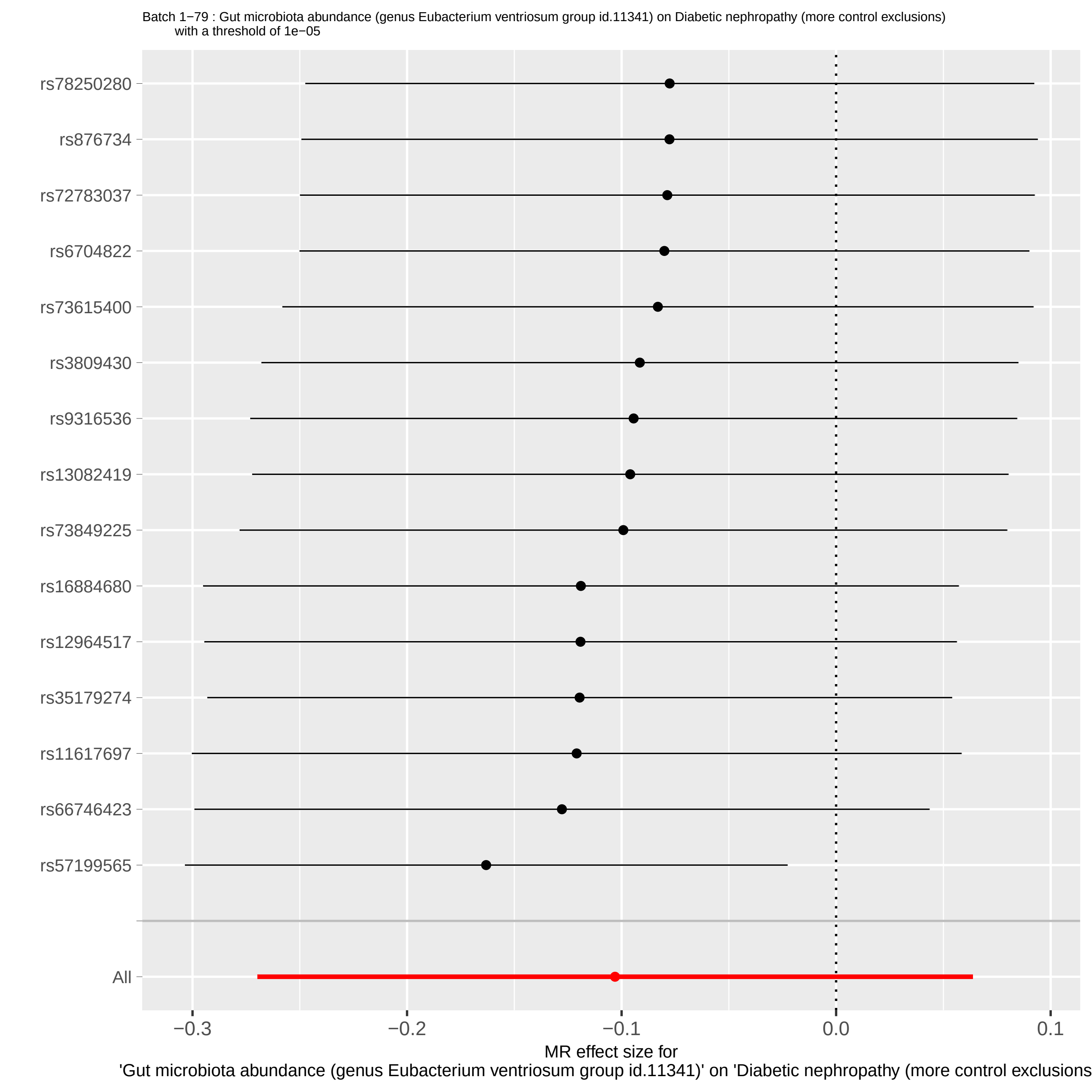

Batch 1−79 : Gut microbiota abundance (genus Eubacterium ventriosum group id.11341) on Diabetic nephropathy (more control exclusions) with a threshold of 1e−05
rs78250280
rs876734
rs72783037
rs6704822
rs73615400
rs3809430
rs9316536
rs13082419
rs73849225
rs16884680
rs12964517
rs35179274
rs11617697
rs66746423
rs57199565
All
−0.3
−0.2
−0.1
0.0
0.1
MR effect size for
'Gut microbiota abundance (genus Eubacterium ventriosum group id.11341)' on 'Diabetic nephropathy (more control exclusions

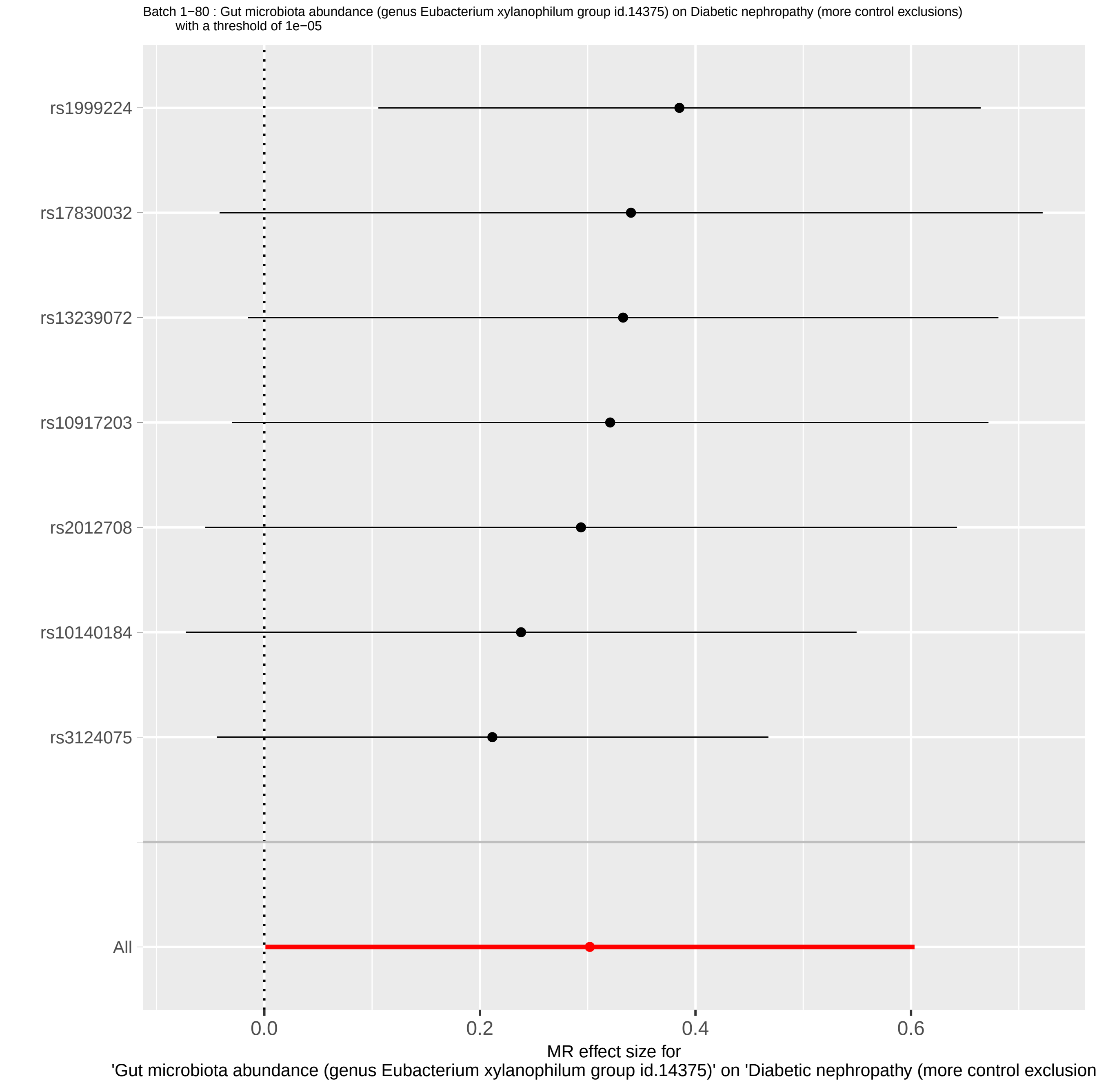

Batch 1−80 : Gut microbiota abundance (genus Eubacterium xylanophilum group id.14375) on Diabetic nephropathy (more control exclusions) with a threshold of 1e−05
rs1999224
rs17830032
rs13239072
rs10917203
rs2012708
rs10140184
rs3124075
All
0.0
0.2
0.4
0.6
MR effect size for
'Gut microbiota abundance (genus Eubacterium xylanophilum group id.14375)' on 'Diabetic nephropathy (more control exclusion

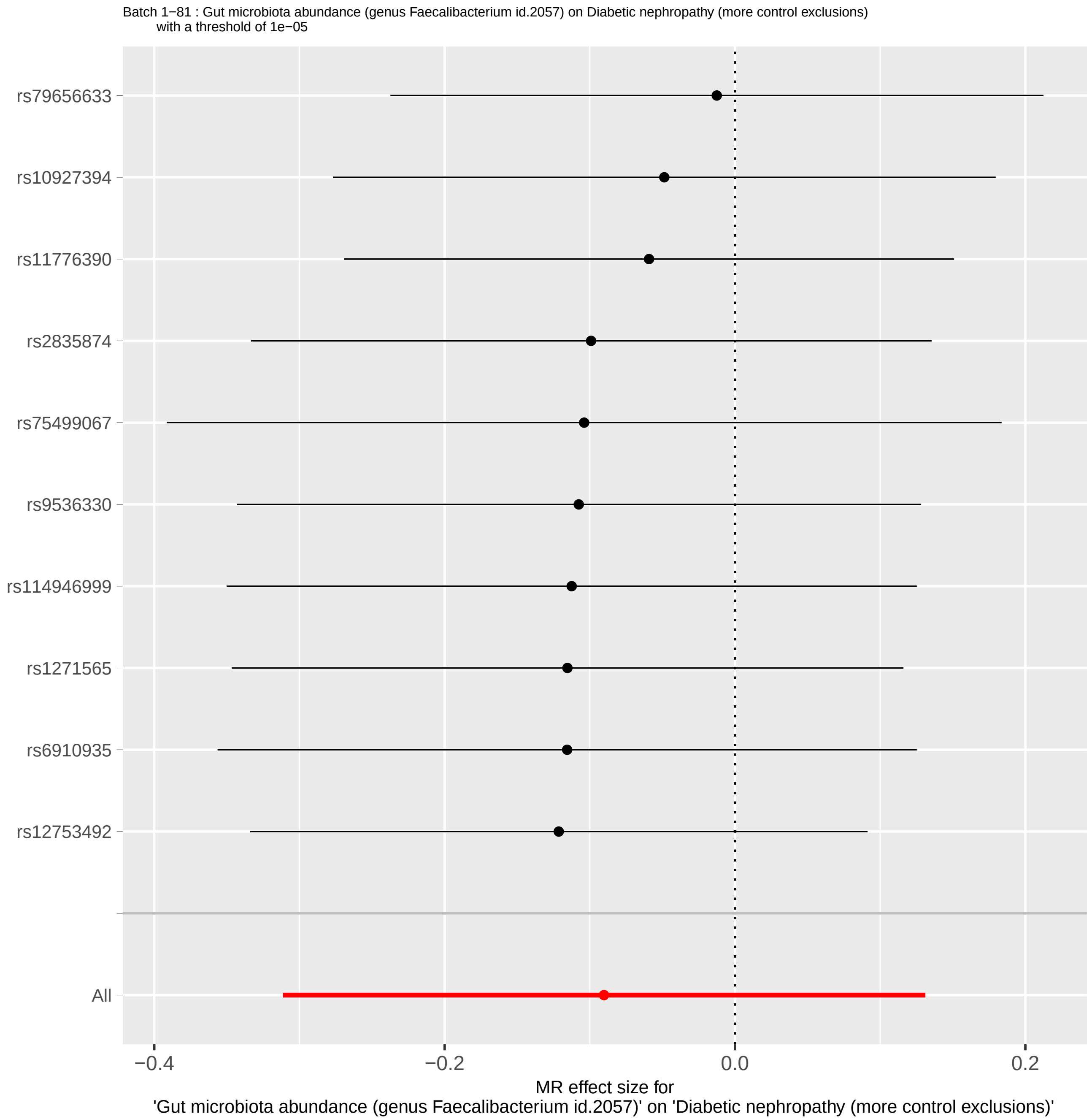
Batch 1−81 : Gut microbiota abundance (genus Faecalibacterium id.2057) on Diabetic nephropathy (more control exclusions)
with a threshold of 1e−05
rs79656633
rs10927394
rs11776390
rs2835874
rs75499067
rs9536330
rs114946999
rs1271565
rs6910935
rs12753492
All
−0.4
−0.2
0.0
0.2
MR effect size for
'Gut microbiota abundance (genus Faecalibacterium id.2057)' on 'Diabetic nephropathy (more control exclusions)'

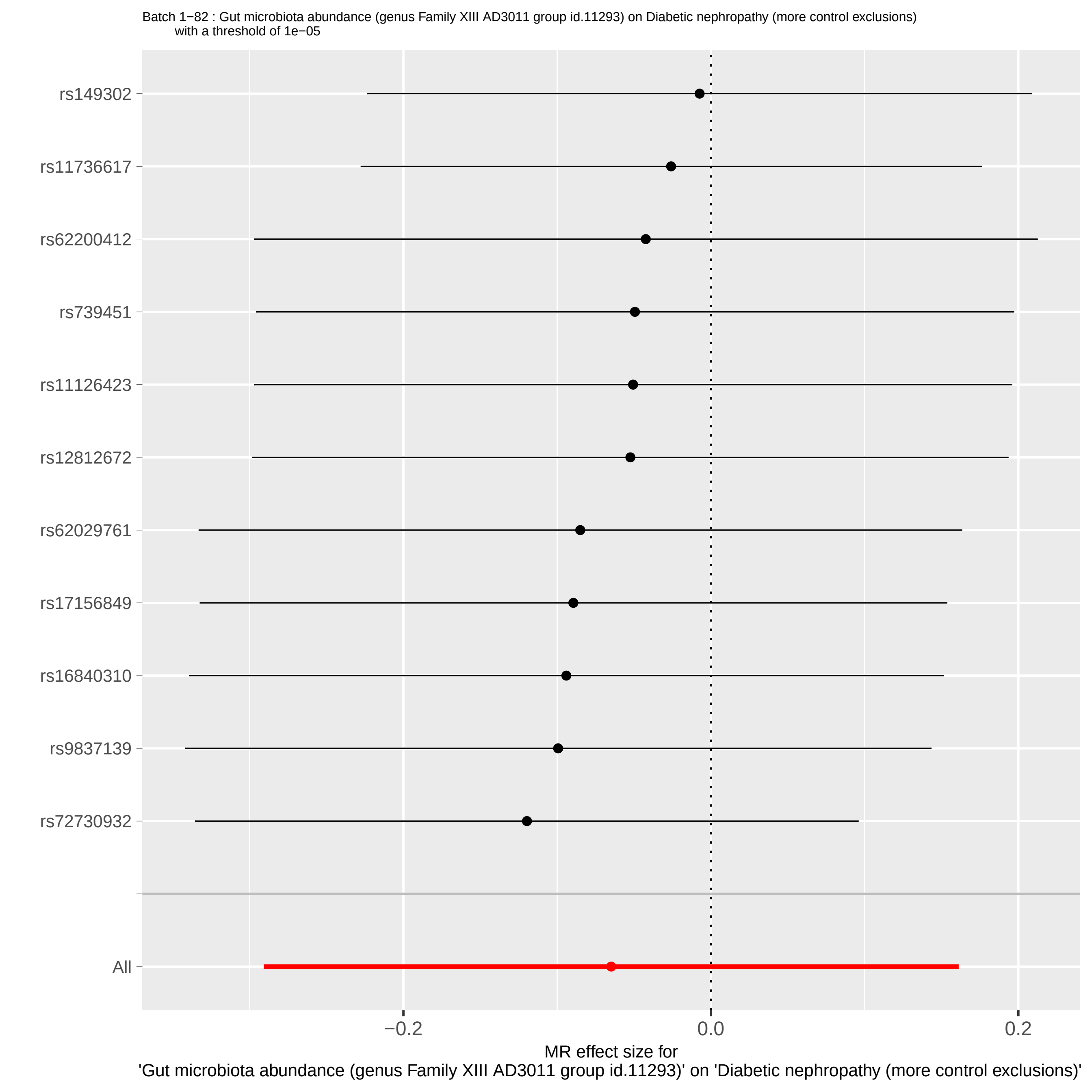

Batch 1−82 : Gut microbiota abundance (genus Family XIII AD3011 group id.11293) on Diabetic nephropathy (more control exclusions)
with a threshold of 1e−05
rs149302
rs11736617
rs62200412
rs739451
rs11126423
rs12812672
rs62029761
rs17156849
rs16840310
rs9837139
rs72730932
All
−0.2
0.0
0.2
MR effect size for
'Gut microbiota abundance (genus Family XIII AD3011 group id.11293)' on 'Diabetic nephropathy (more control exclusions)'

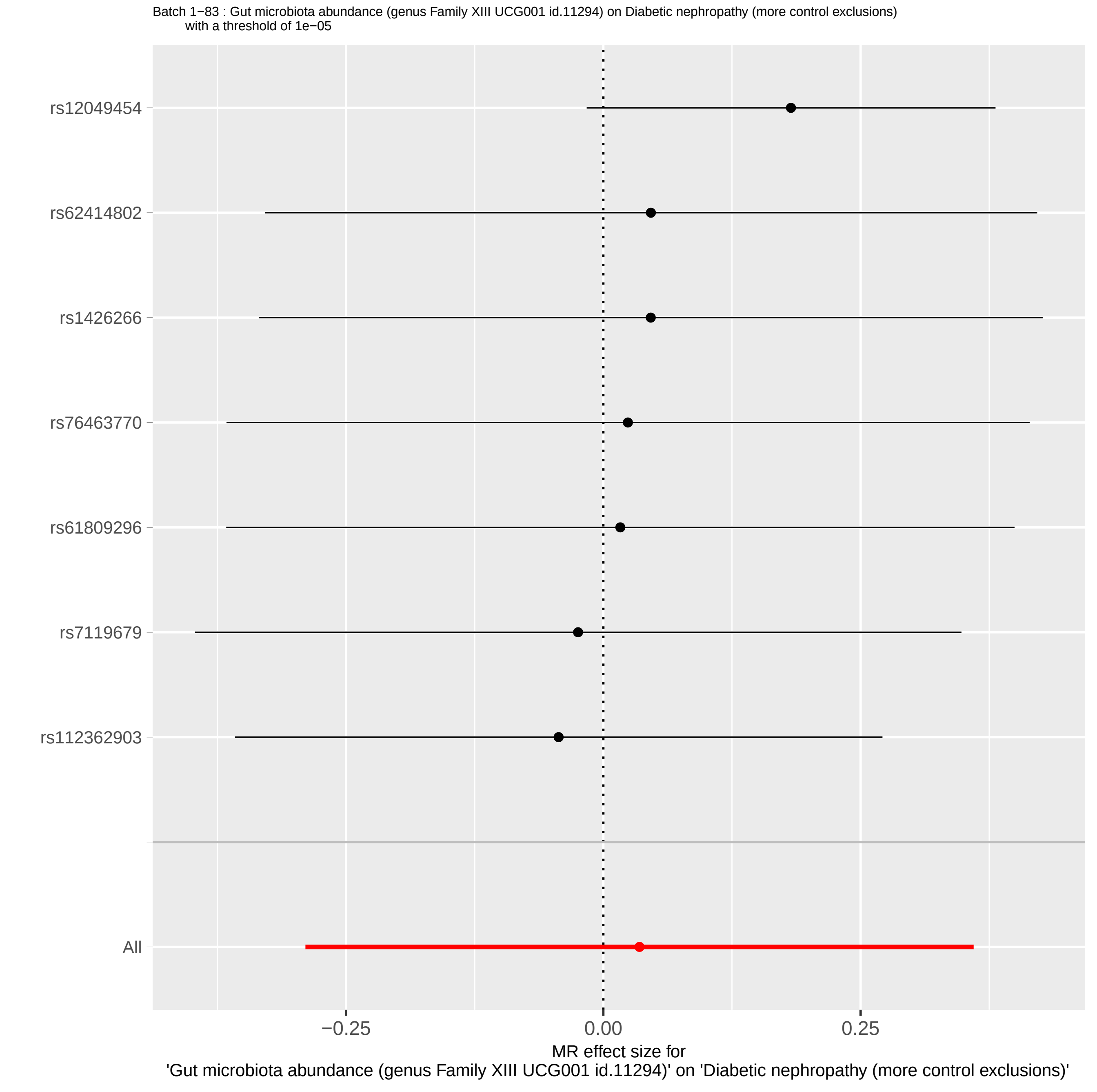
Batch 1−83 : Gut microbiota abundance (genus Family XIII UCG001 id.11294) on Diabetic nephropathy (more control exclusions)
with a threshold of 1e−05
rs12049454
rs62414802
rs1426266
rs76463770
rs61809296
rs7119679
rs112362903
All
−0.25
0.00
0.25
MR effect size for
'Gut microbiota abundance (genus Family XIII UCG001 id.11294)' on 'Diabetic nephropathy (more control exclusions)'

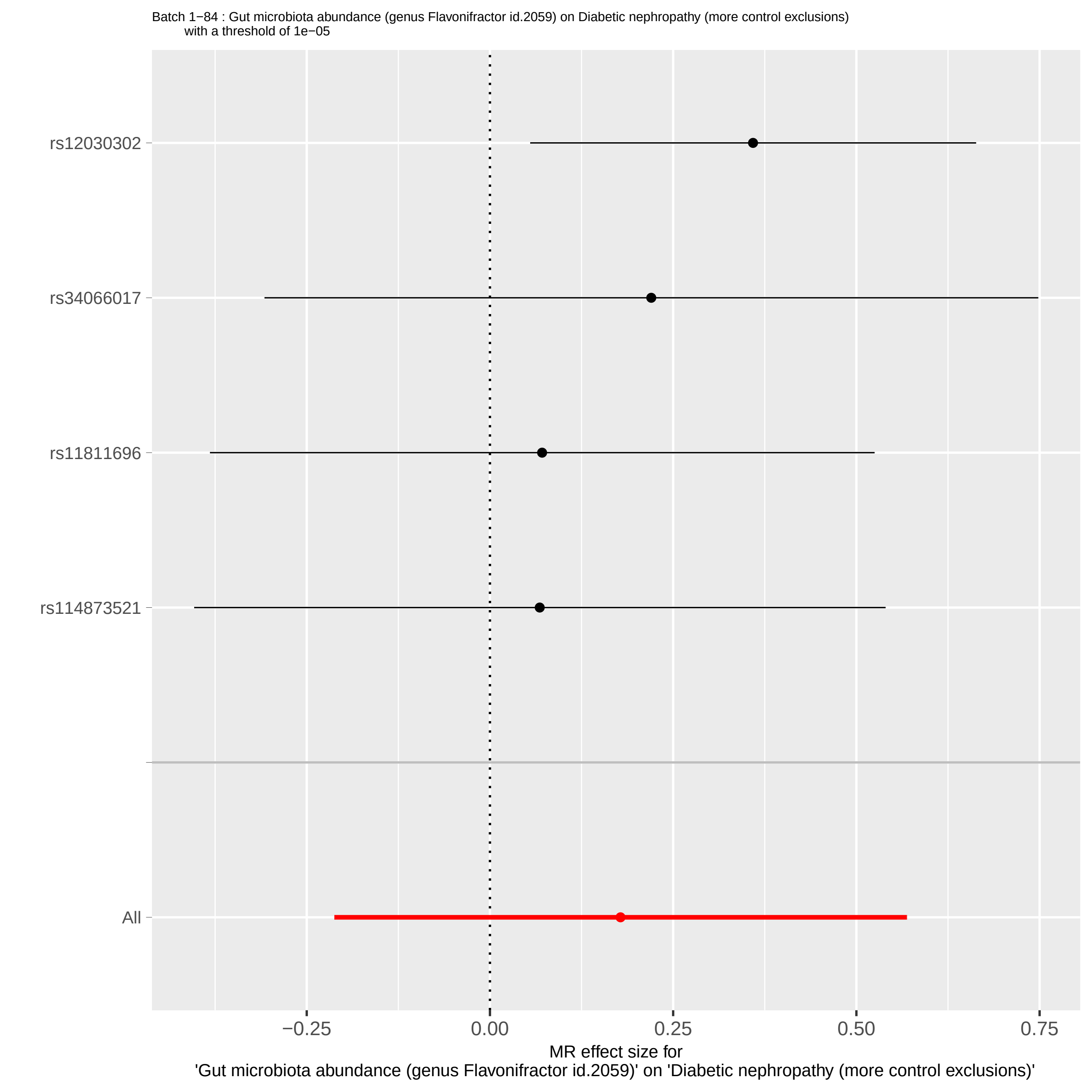
Batch 1−84 : Gut microbiota abundance (genus Flavonifractor id.2059) on Diabetic nephropathy (more control exclusions)
with a threshold of 1e−05
rs12030302
rs34066017
rs11811696
rs114873521
All
−0.25
0.00
0.25
0.50
0.75
MR effect size for
'Gut microbiota abundance (genus Flavonifractor id.2059)' on 'Diabetic nephropathy (more control exclusions)'

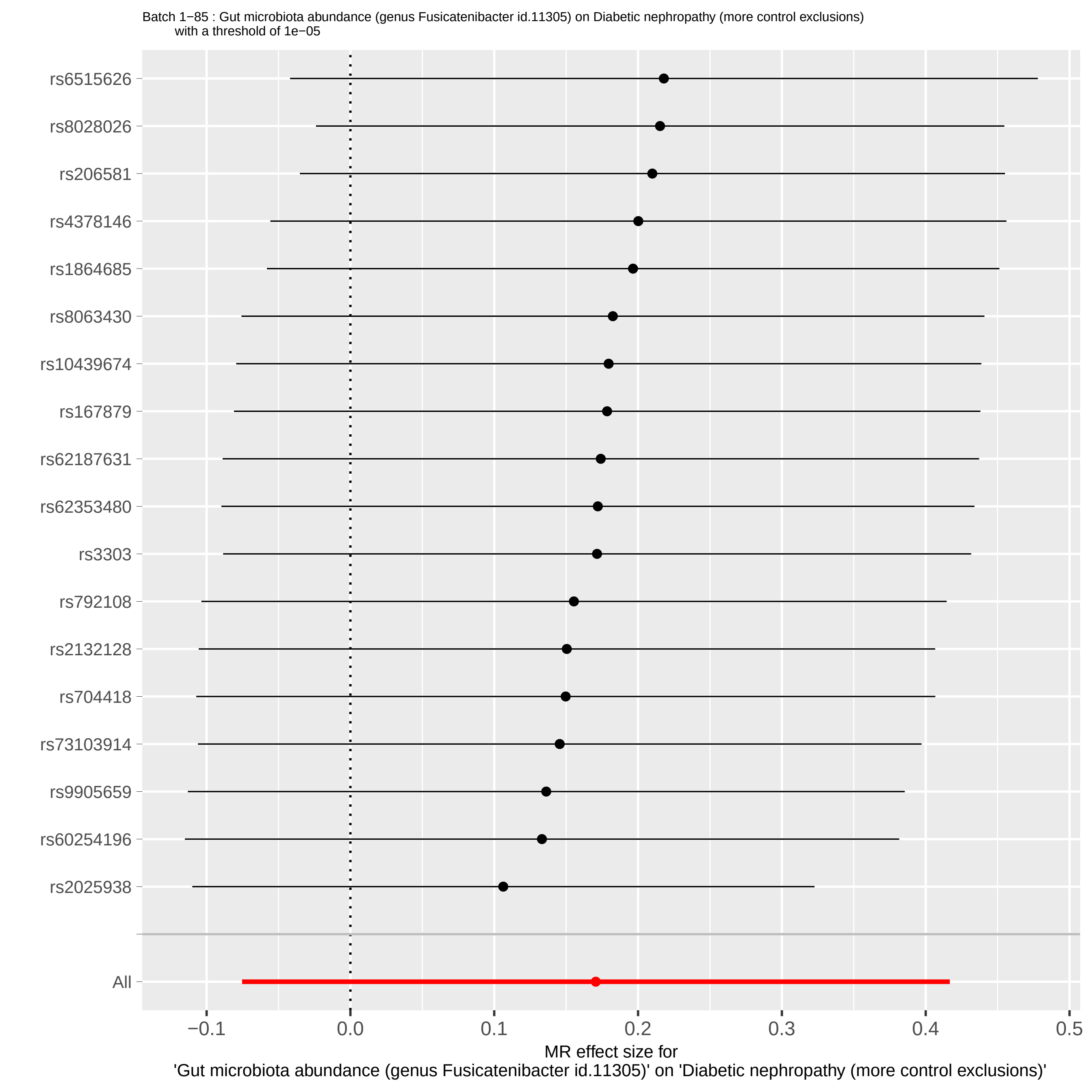
Batch 1−85 : Gut microbiota abundance (genus Fusicatenibacter id.11305) on Diabetic nephropathy (more control exclusions) with a threshold of 1e−05
rs6515626
rs8028026
rs206581
rs4378146
rs1864685
rs8063430
rs10439674
rs167879
rs62187631
rs62353480
rs3303
rs792108
rs2132128
rs704418
rs73103914
rs9905659
rs60254196
rs2025938
All
−0.1
0.0
0.1
0.2
0.3
0.4
0.5
MR effect size for
'Gut microbiota abundance (genus Fusicatenibacter id.11305)' on 'Diabetic nephropathy (more control exclusions)'

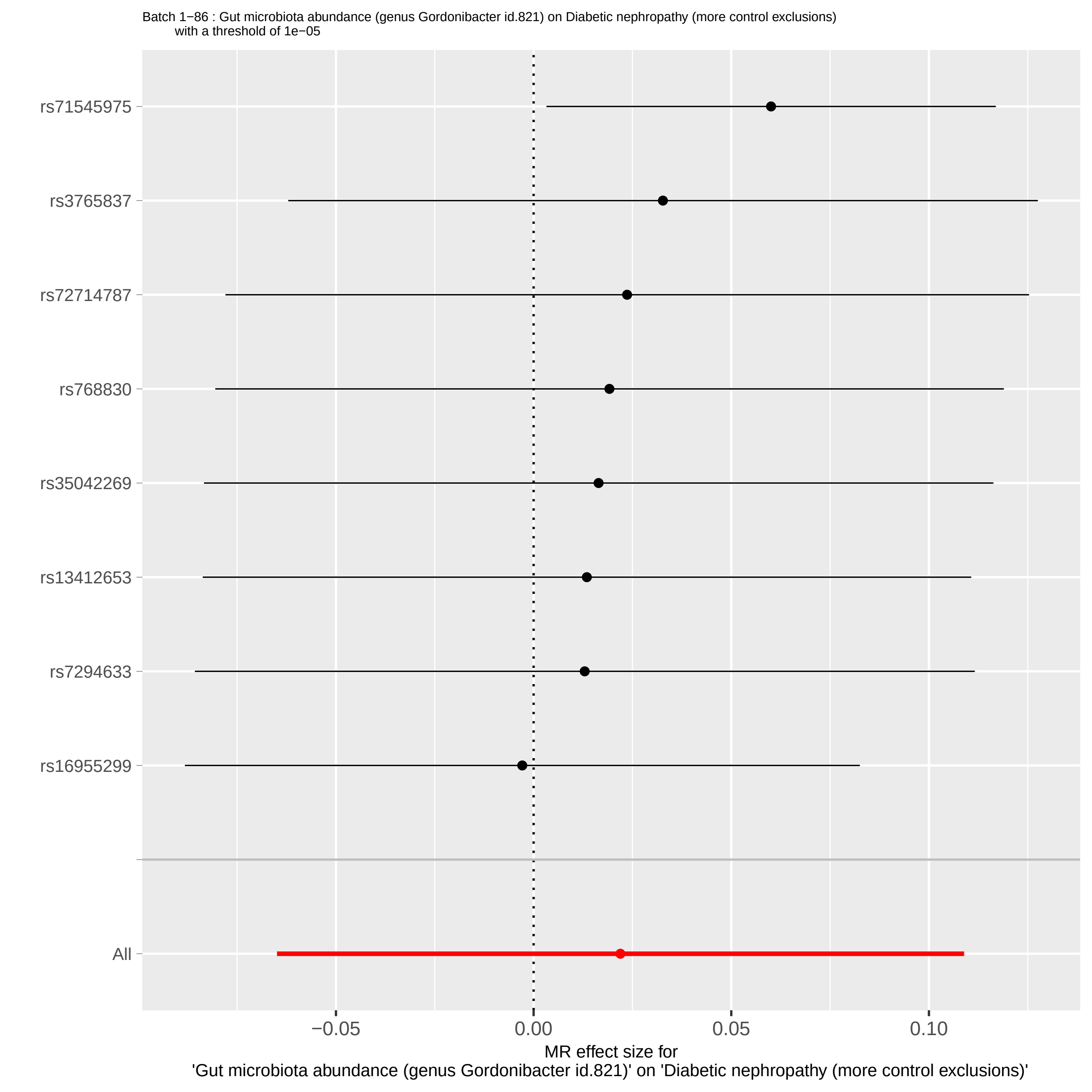
Batch 1−86 : Gut microbiota abundance (genus Gordonibacter id.821) on Diabetic nephropathy (more control exclusions)
with a threshold of 1e−05
rs71545975
rs3765837
rs72714787
rs768830
rs35042269
rs13412653
rs7294633
rs16955299
All
−0.05
0.00
0.05
0.10
MR effect size for
'Gut microbiota abundance (genus Gordonibacter id.821)' on 'Diabetic nephropathy (more control exclusions)'

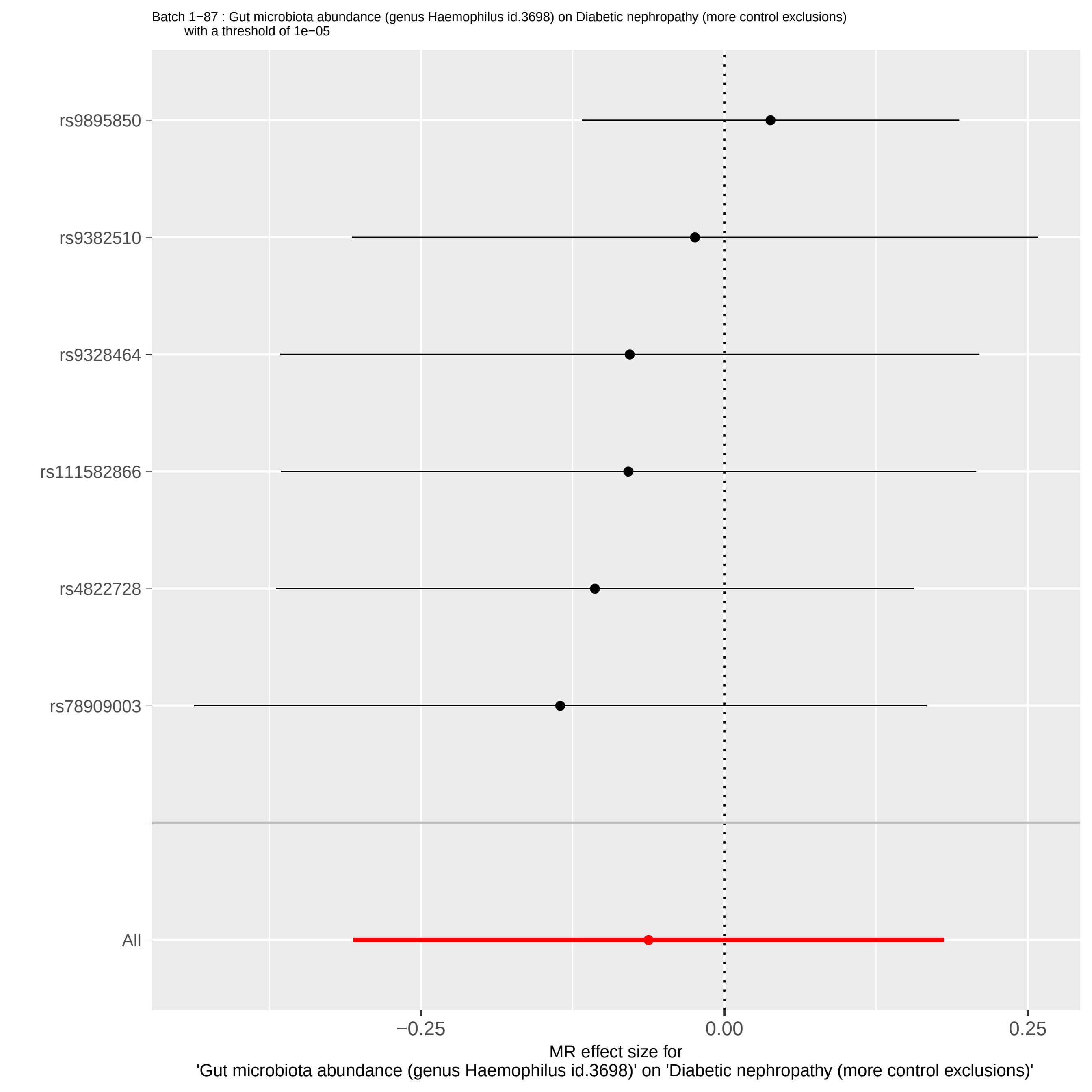
Batch 1−87 : Gut microbiota abundance (genus Haemophilus id.3698) on Diabetic nephropathy (more control exclusions)
with a threshold of 1e−05
rs9895850
rs9382510
rs9328464
rs111582866
rs4822728
rs78909003
All
−0.25
0.00
0.25
MR effect size for
'Gut microbiota abundance (genus Haemophilus id.3698)' on 'Diabetic nephropathy (more control exclusions)'

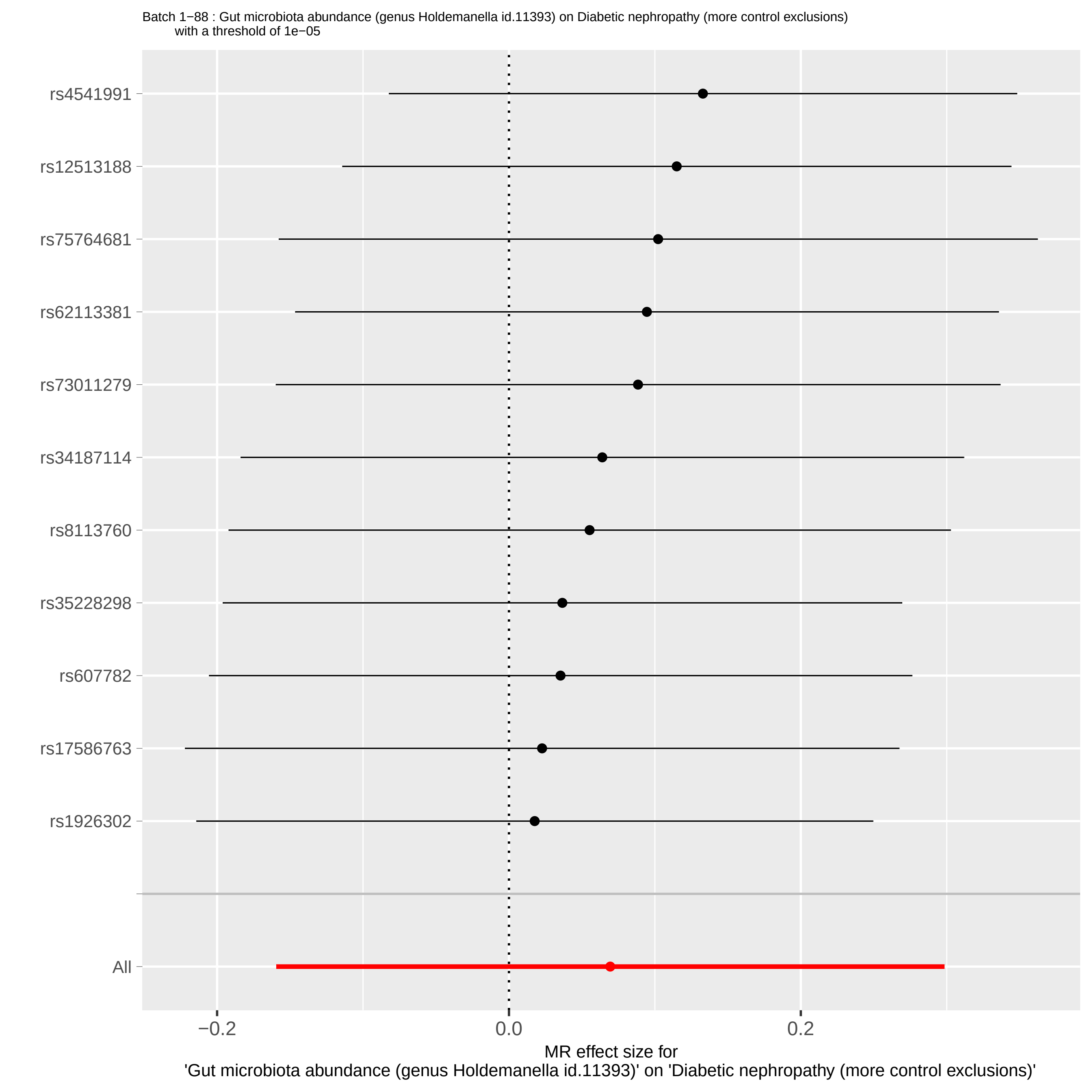
Batch 1−88 : Gut microbiota abundance (genus Holdemanella id.11393) on Diabetic nephropathy (more control exclusions) with a threshold of 1e−05
rs4541991
rs12513188
rs75764681
rs62113381
rs73011279
rs34187114
rs8113760
rs35228298
rs607782
rs17586763
rs1926302
All
−0.2
0.0
0.2
MR effect size for
'Gut microbiota abundance (genus Holdemanella id.11393)' on 'Diabetic nephropathy (more control exclusions)'

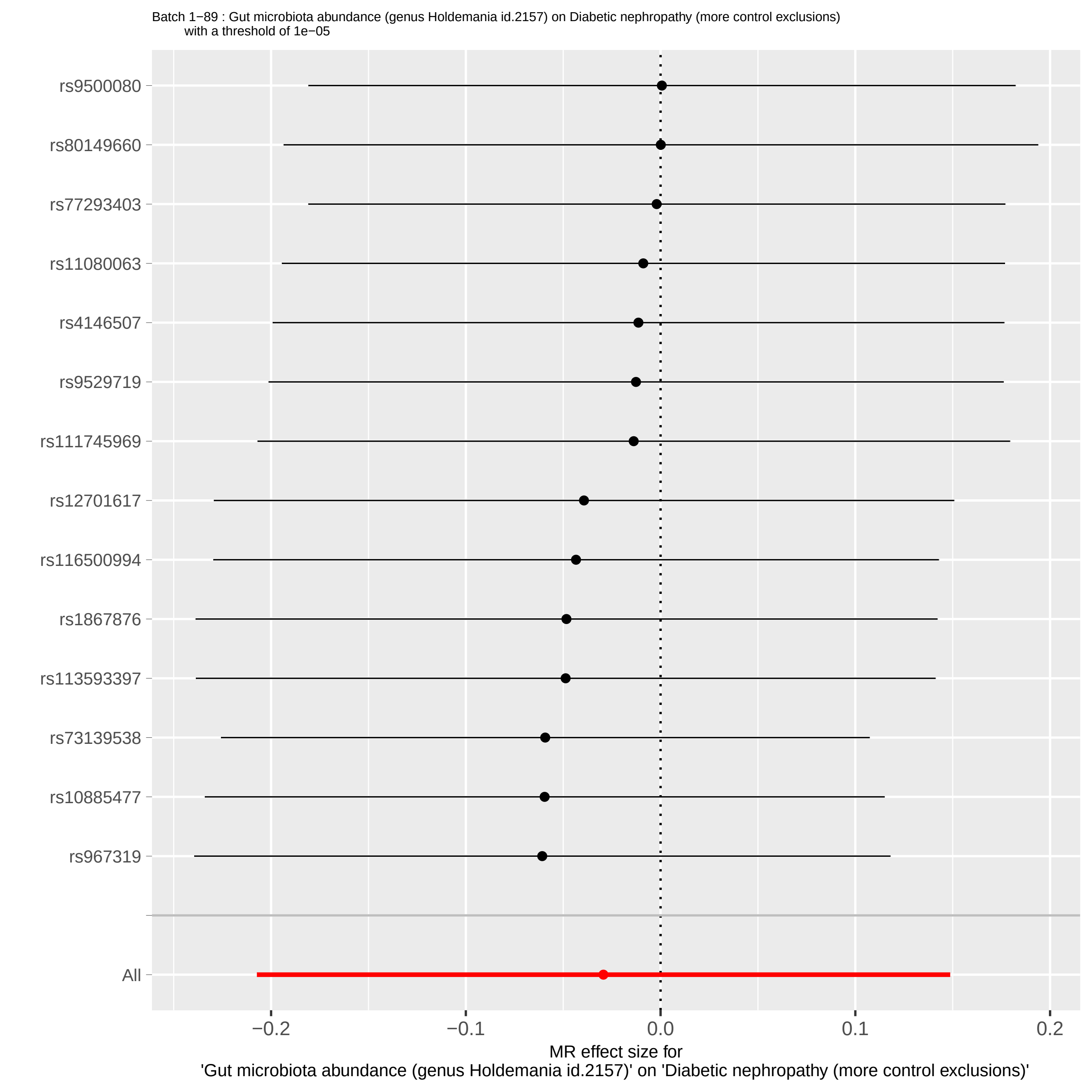
Batch 1−89 : Gut microbiota abundance (genus Holdemania id.2157) on Diabetic nephropathy (more control exclusions)
with a threshold of 1e−05
rs9500080
rs80149660
rs77293403
rs11080063
rs4146507
rs9529719
rs111745969
rs12701617
rs116500994
rs1867876
rs113593397
rs73139538
rs10885477
rs967319
All
−0.2
−0.1
0.0
0.1
0.2
MR effect size for
'Gut microbiota abundance (genus Holdemania id.2157)' on 'Diabetic nephropathy (more control exclusions)'

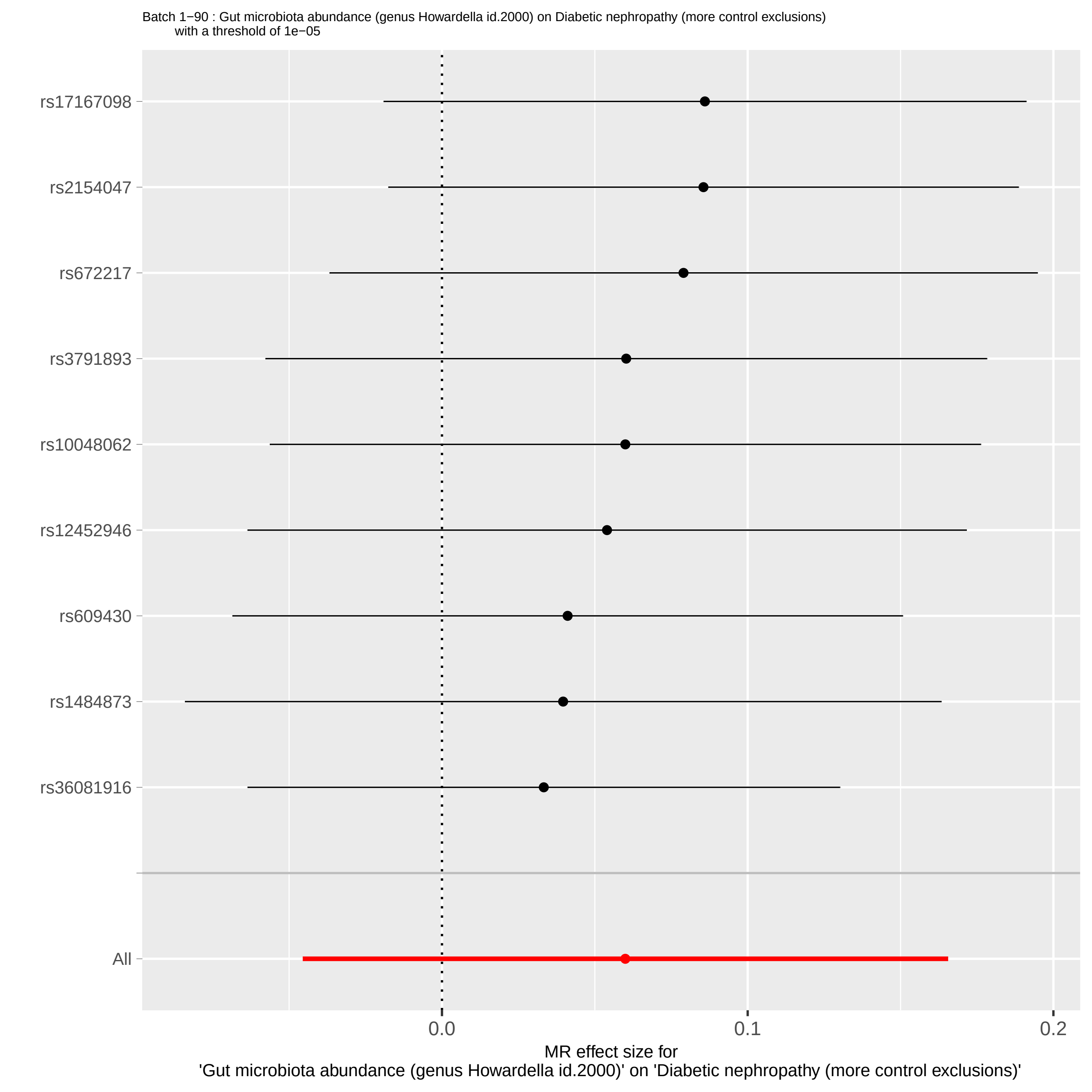
Batch 1−90 : Gut microbiota abundance (genus Howardella id.2000) on Diabetic nephropathy (more control exclusions)
with a threshold of 1e−05
rs17167098
rs2154047
rs672217
rs3791893
rs10048062
rs12452946
rs609430
rs1484873
rs36081916
All
0.0
0.1
0.2
MR effect size for
'Gut microbiota abundance (genus Howardella id.2000)' on 'Diabetic nephropathy (more control exclusions)'

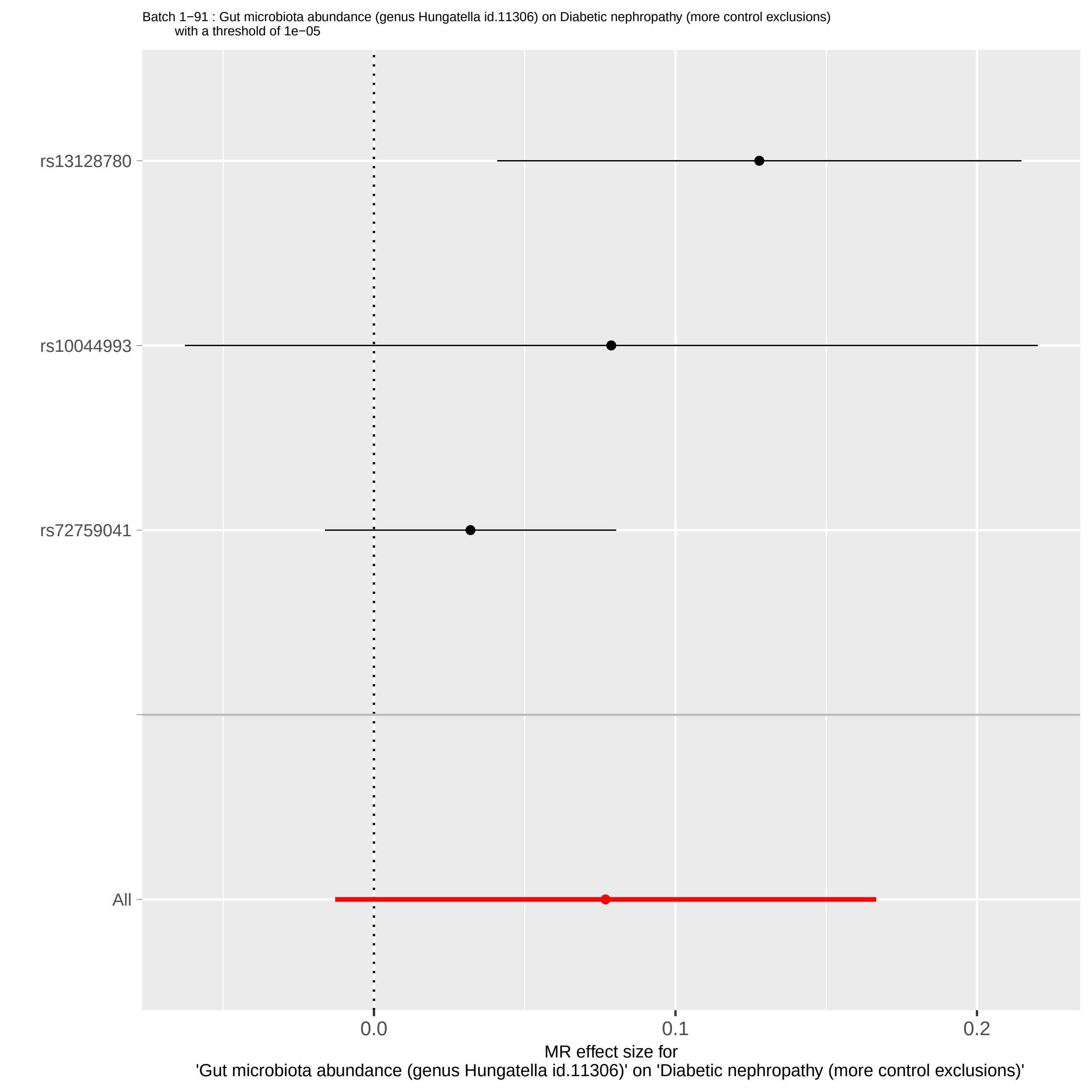
Batch 1−91 : Gut microbiota abundance (genus Hungatella id.11306) on Diabetic nephropathy (more control exclusions)
with a threshold of 1e−05
rs13128780
rs10044993
rs72759041
All
0.0
0.1
0.2
MR effect size for
'Gut microbiota abundance (genus Hungatella id.11306)' on 'Diabetic nephropathy (more control exclusions)'

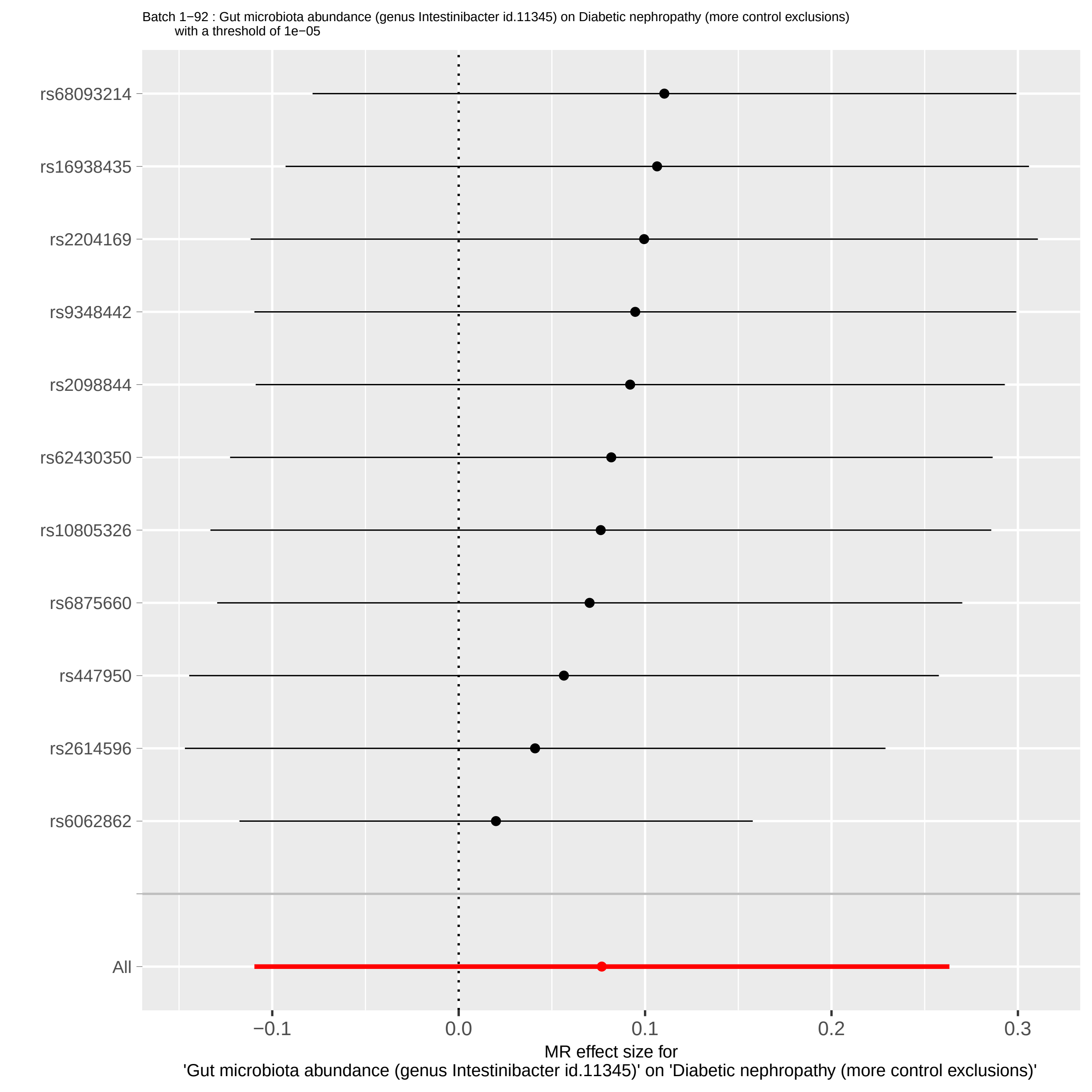

Batch 1−92 : Gut microbiota abundance (genus Intestinibacter id.11345) on Diabetic nephropathy (more control exclusions)
with a threshold of 1e−05
rs68093214
rs16938435
rs2204169
rs9348442
rs2098844
rs62430350
rs10805326
rs6875660
rs447950
rs2614596
rs6062862
All
−0.1
0.0
0.1
0.2
0.3
MR effect size for
'Gut microbiota abundance (genus Intestinibacter id.11345)' on 'Diabetic nephropathy (more control exclusions)'

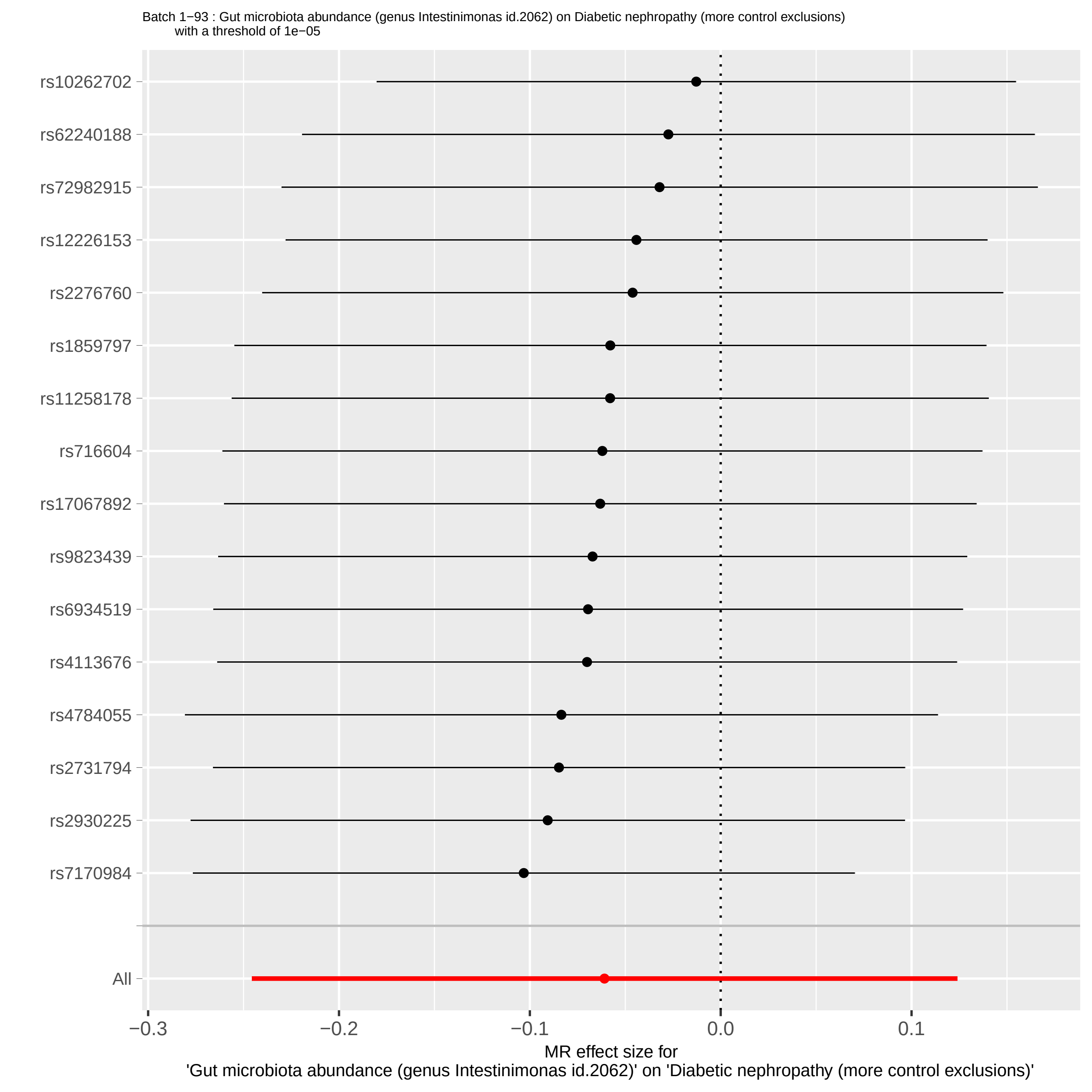

Batch 1−93 : Gut microbiota abundance (genus Intestinimonas id.2062) on Diabetic nephropathy (more control exclusions) with a threshold of 1e−05
rs10262702
rs62240188
rs72982915
rs12226153
rs2276760
rs1859797
rs11258178
rs716604
rs17067892
rs9823439
rs6934519
rs4113676
rs4784055
rs2731794
rs2930225
rs7170984
All
−0.3
−0.2
−0.1
0.0
0.1
MR effect size for
'Gut microbiota abundance (genus Intestinimonas id.2062)' on 'Diabetic nephropathy (more control exclusions)'

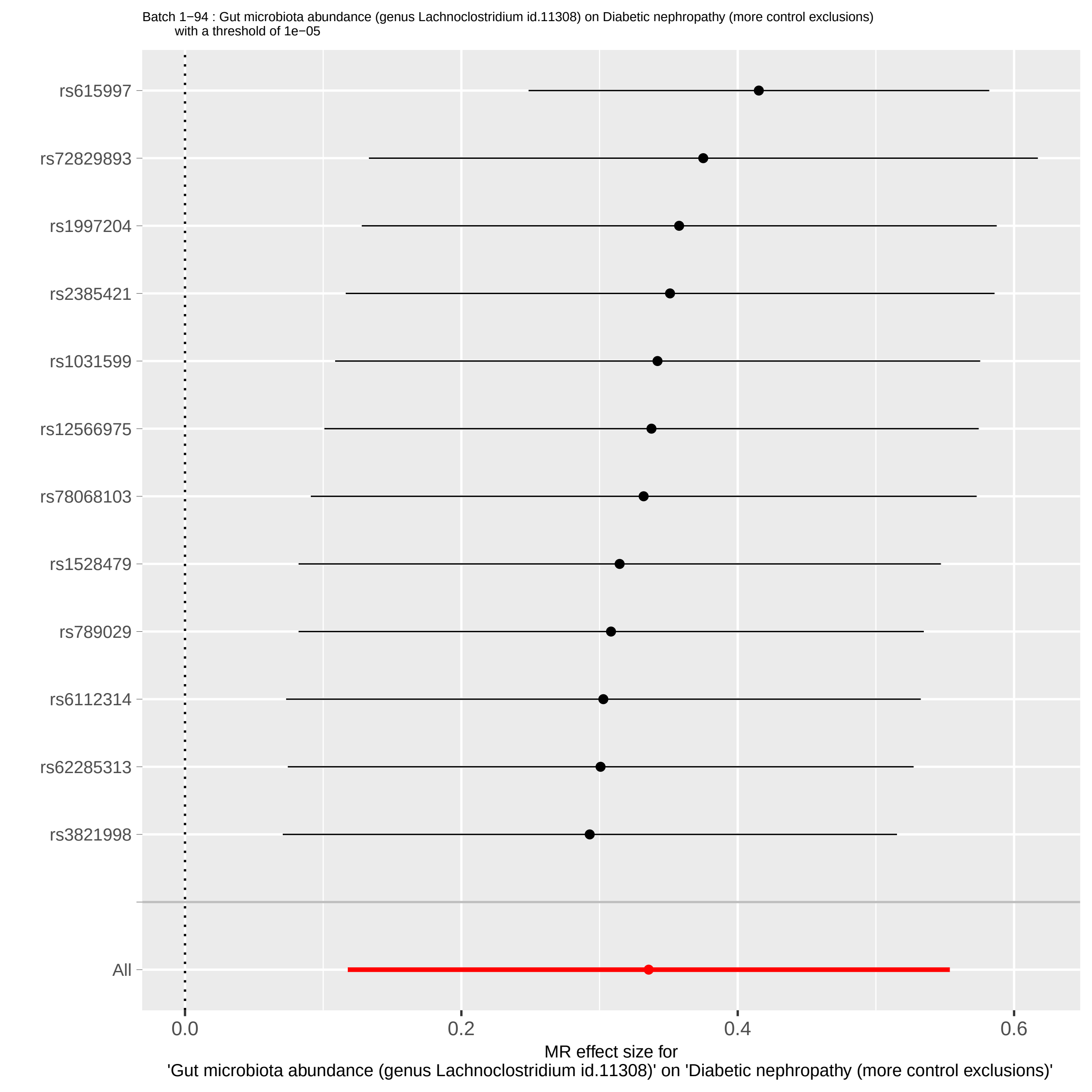

Batch 1−94 : Gut microbiota abundance (genus Lachnoclostridium id.11308) on Diabetic nephropathy (more control exclusions)
with a threshold of 1e−05
rs615997
rs72829893
rs1997204
rs2385421
rs1031599
rs12566975
rs78068103
rs1528479
rs789029
rs6112314
rs62285313
rs3821998
All
0.0
0.2
0.4
0.6
MR effect size for
'Gut microbiota abundance (genus Lachnoclostridium id.11308)' on 'Diabetic nephropathy (more control exclusions)'

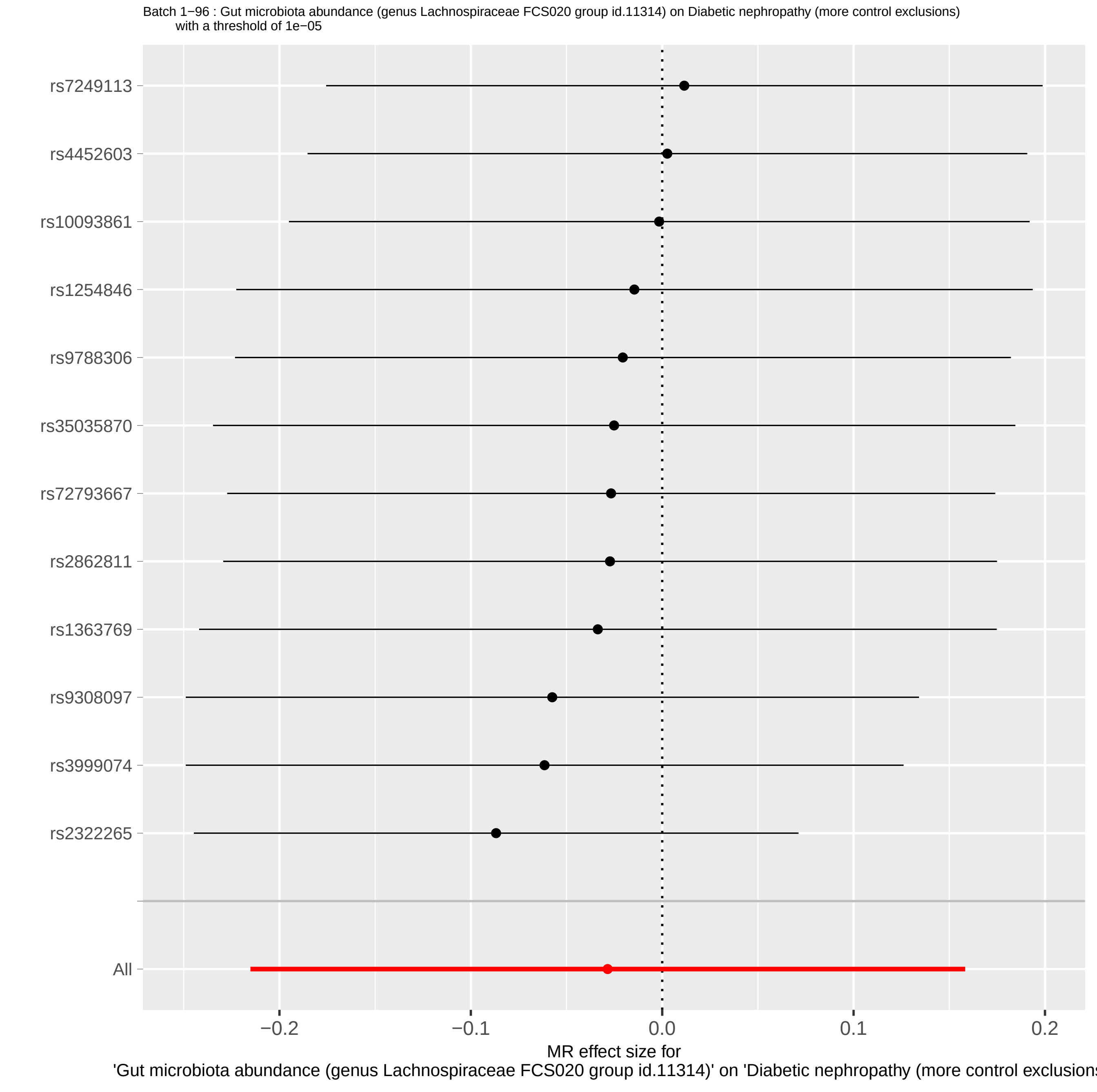

Batch 1−96 : Gut microbiota abundance (genus Lachnospiraceae FCS020 group id.11314) on Diabetic nephropathy (more control exclusions) with a threshold of 1e−05
rs7249113
rs4452603
rs10093861
rs1254846
rs9788306
rs35035870
rs72793667
rs2862811
rs1363769
rs9308097
rs3999074
rs2322265
All
−0.2
−0.1
0.0
0.1
0.2
MR effect size for
'Gut microbiota abundance (genus Lachnospiraceae FCS020 group id.11314)' on 'Diabetic nephropathy (more control exclusions

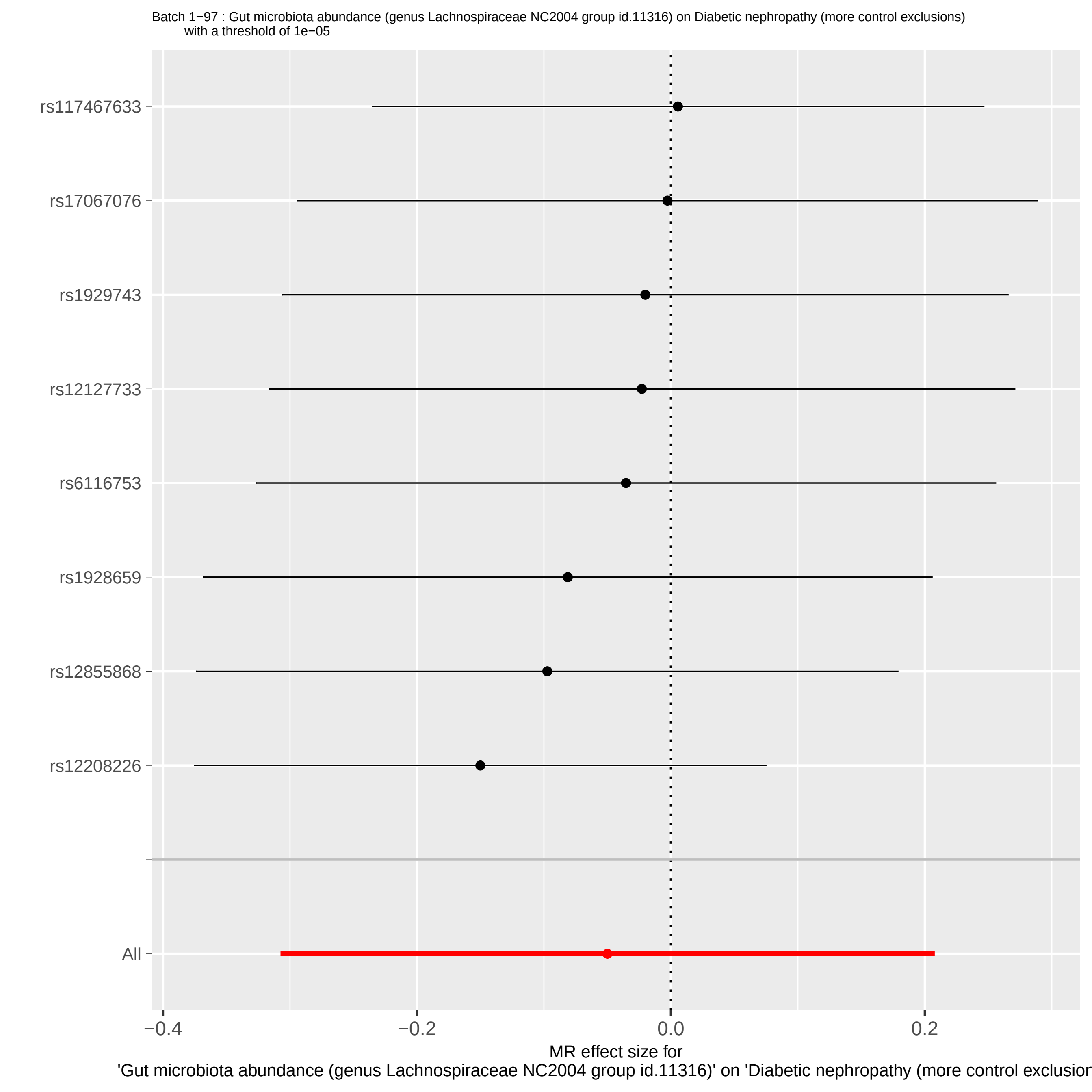
Batch 1−97 : Gut microbiota abundance (genus Lachnospiraceae NC2004 group id.11316) on Diabetic nephropathy (more control exclusions)
with a threshold of 1e−05
rs117467633
rs17067076
rs1929743
rs12127733
rs6116753
rs1928659
rs12855868
rs12208226
All
−0.4
−0.2
0.0
0.2
MR effect size for
'Gut microbiota abundance (genus Lachnospiraceae NC2004 group id.11316)' on 'Diabetic nephropathy (more control exclusion

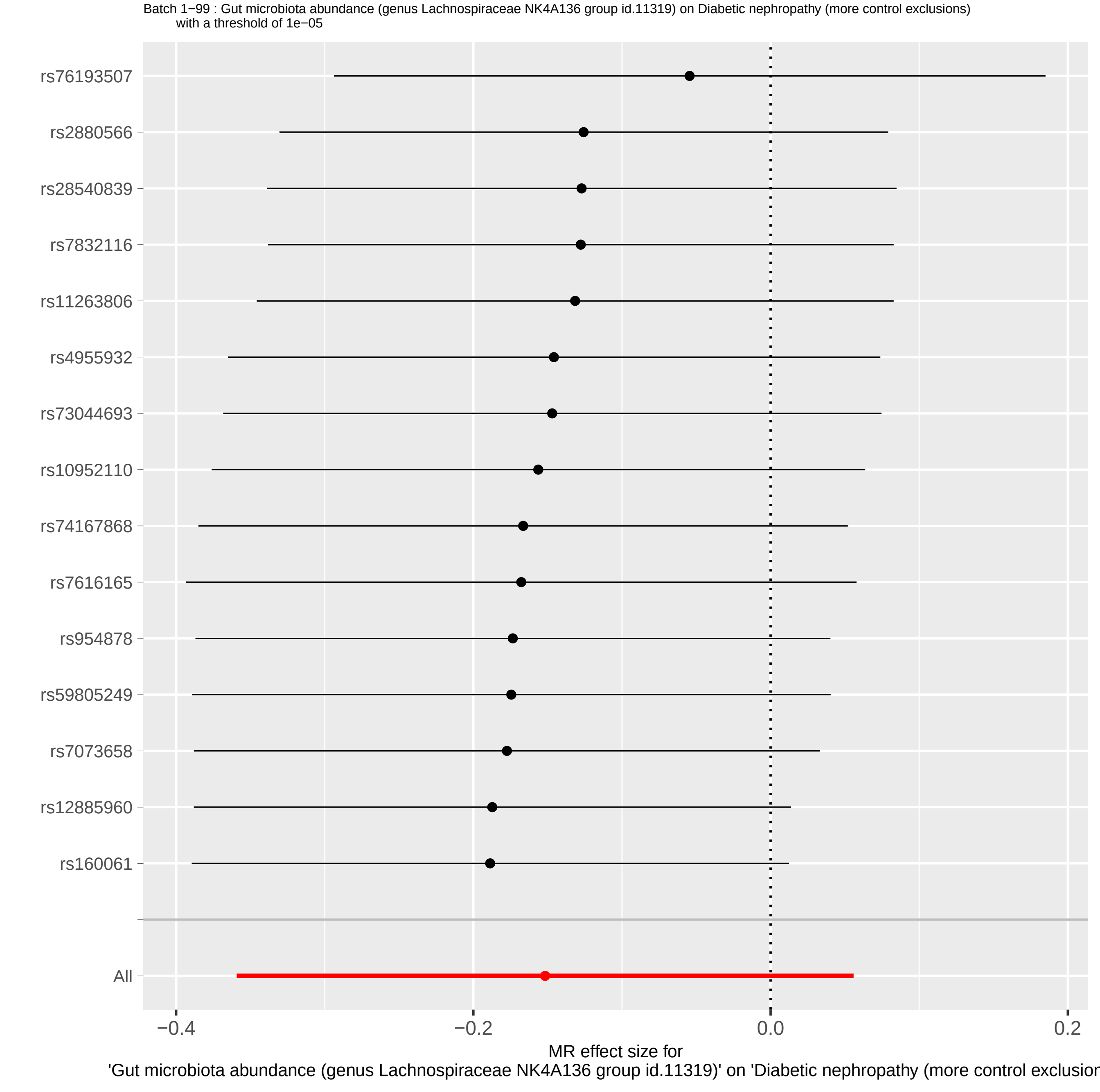
Batch 1−99 : Gut microbiota abundance (genus Lachnospiraceae NK4A136 group id.11319) on Diabetic nephropathy (more control exclusions)
with a threshold of 1e−05
rs76193507
rs2880566
rs28540839
rs7832116
rs11263806
rs4955932
rs73044693
rs10952110
rs74167868
rs7616165
rs954878
rs59805249
rs7073658
rs12885960
rs160061
All
−0.4
−0.2
0.0
0.2
MR effect size for
'Gut microbiota abundance (genus Lachnospiraceae NK4A136 group id.11319)' on 'Diabetic nephropathy (more control exclusion

Batch 1−100 : Gut microbiota abundance (genus Lachnospiraceae UCG001 id.11321) on Diabetic nephropathy (more control exclusions) with a threshold of 1e−05

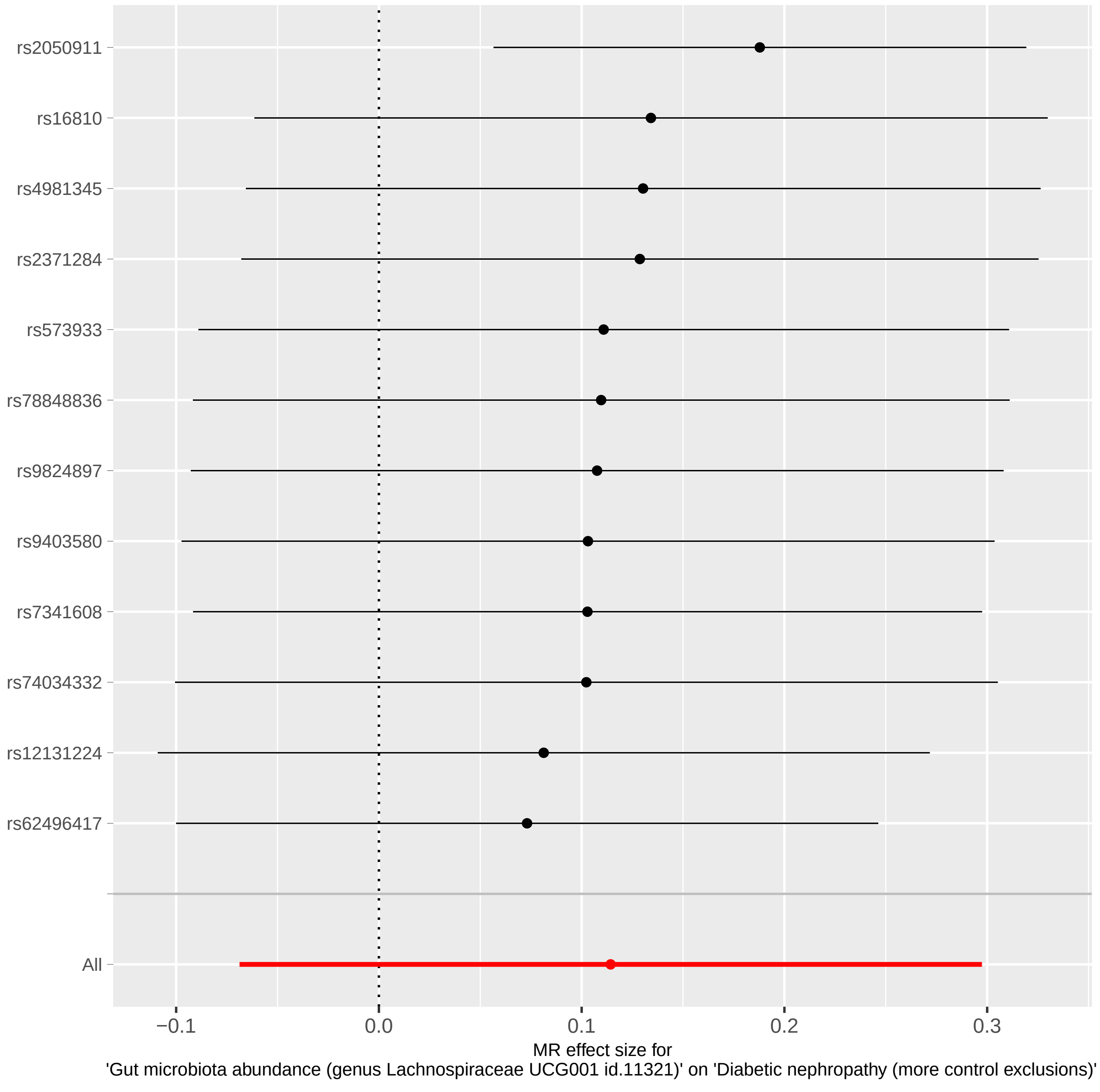

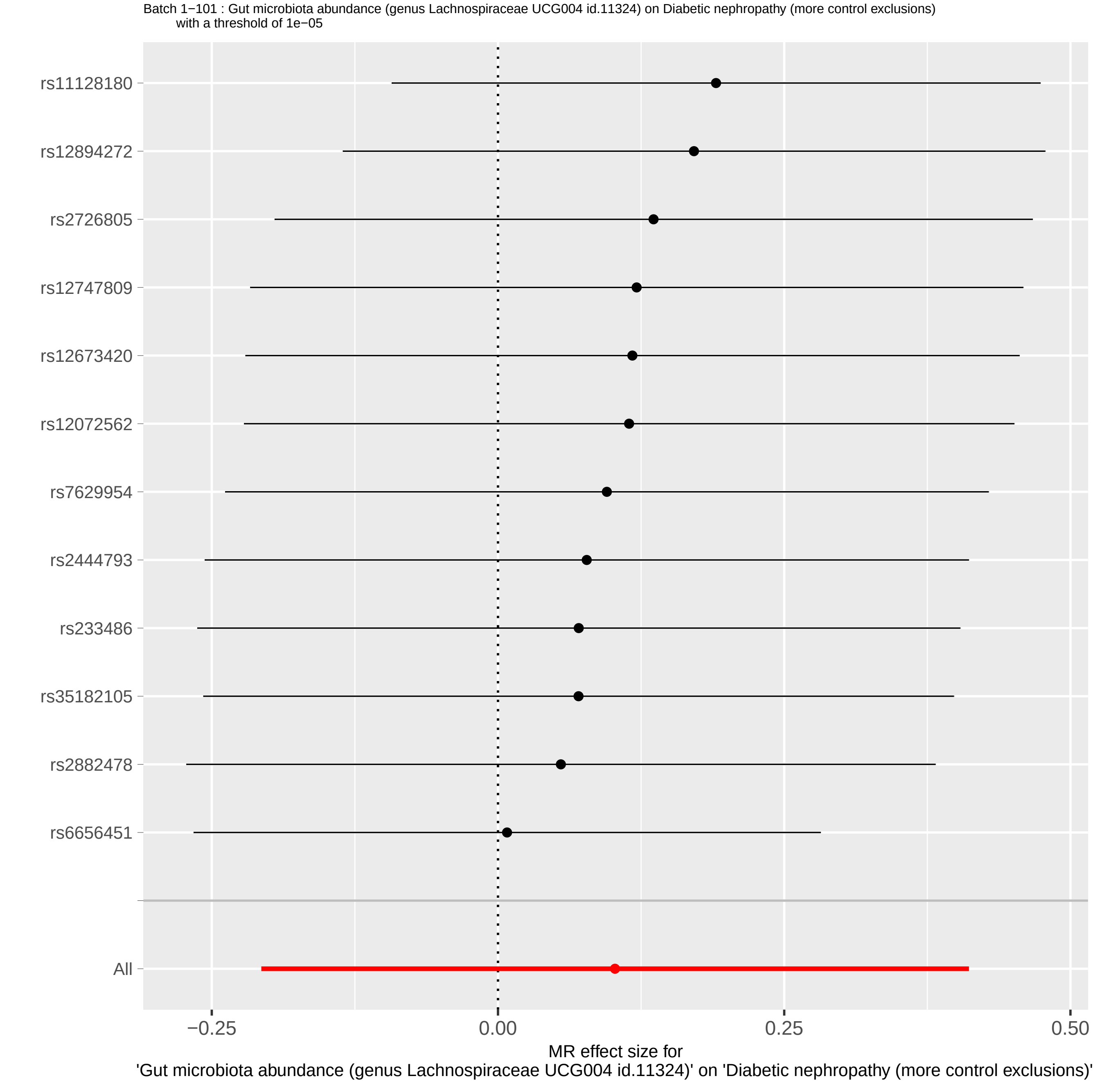
Batch 1−101 : Gut microbiota abundance (genus Lachnospiraceae UCG004 id.11324) on Diabetic nephropathy (more control exclusions)
with a threshold of 1e−05
rs11128180
rs12894272
rs2726805
rs12747809
rs12673420
rs12072562
rs7629954
rs2444793
rs233486
rs35182105
rs2882478
rs6656451
All
−0.25
0.00
0.25
0.50
MR effect size for
'Gut microbiota abundance (genus Lachnospiraceae UCG004 id.11324)' on 'Diabetic nephropathy (more control exclusions)'

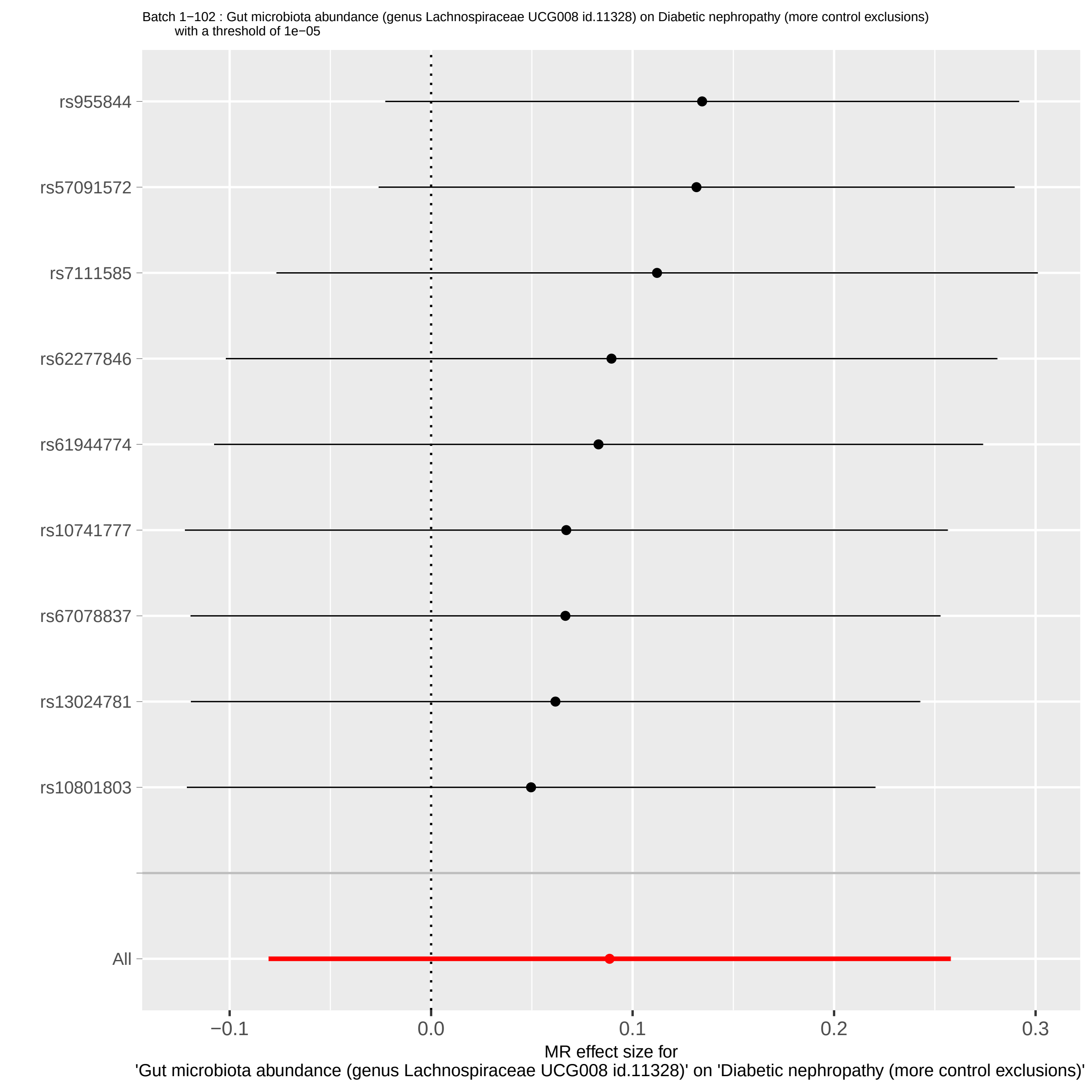
Batch 1−102 : Gut microbiota abundance (genus Lachnospiraceae UCG008 id.11328) on Diabetic nephropathy (more control exclusions) with a threshold of 1e−05
rs955844
rs57091572
rs7111585
rs62277846
rs61944774
rs10741777
rs67078837
rs13024781
rs10801803
All
−0.1
0.0
0.1
0.2
0.3
MR effect size for
'Gut microbiota abundance (genus Lachnospiraceae UCG008 id.11328)' on 'Diabetic nephropathy (more control exclusions)'

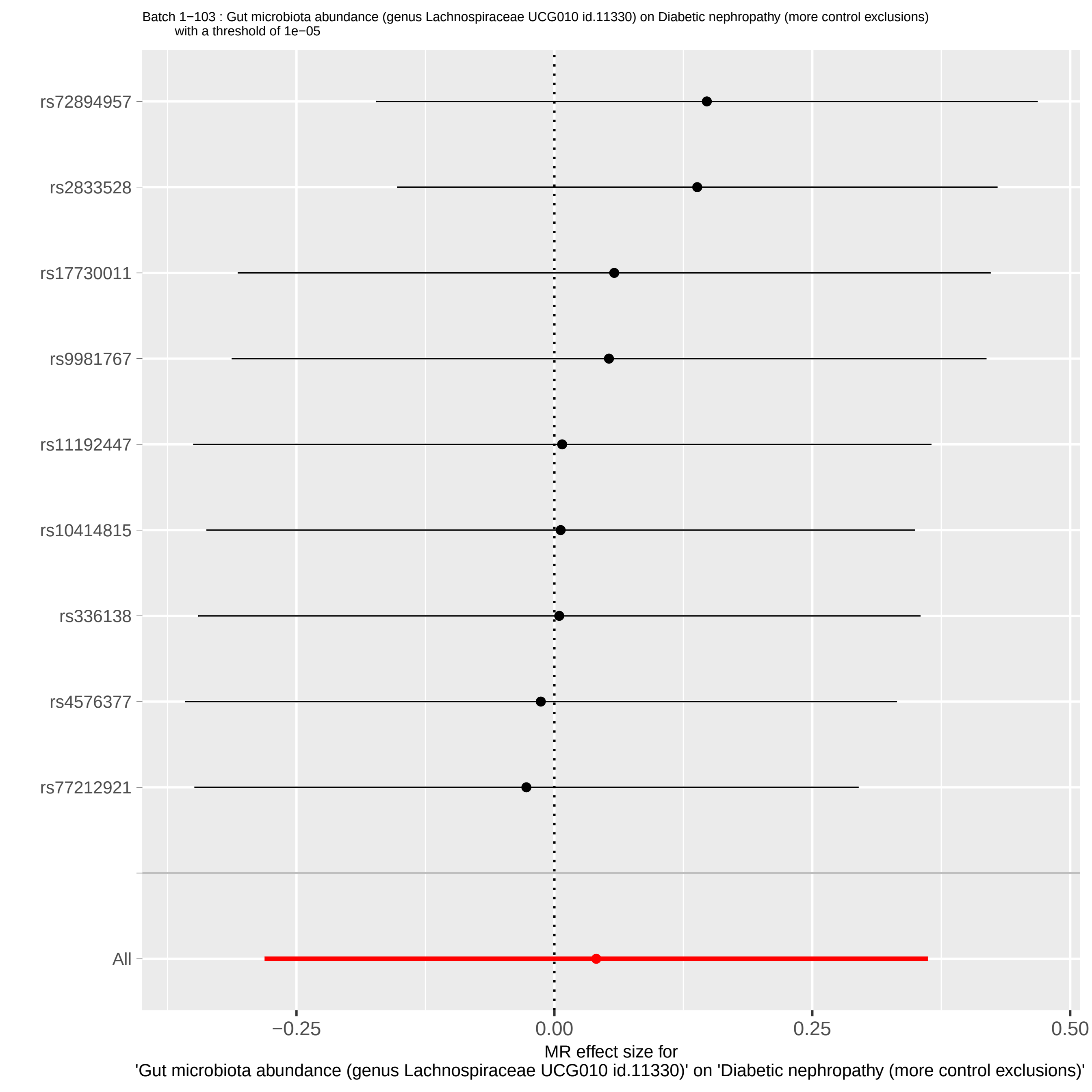

Batch 1−103 : Gut microbiota abundance (genus Lachnospiraceae UCG010 id.11330) on Diabetic nephropathy (more control exclusions)
with a threshold of 1e−05
rs72894957
rs2833528
rs17730011
rs9981767
rs11192447
rs10414815
rs336138
rs4576377
rs77212921
All
−0.25
0.00
0.25
0.50
MR effect size for
'Gut microbiota abundance (genus Lachnospiraceae UCG010 id.11330)' on 'Diabetic nephropathy (more control exclusions)'

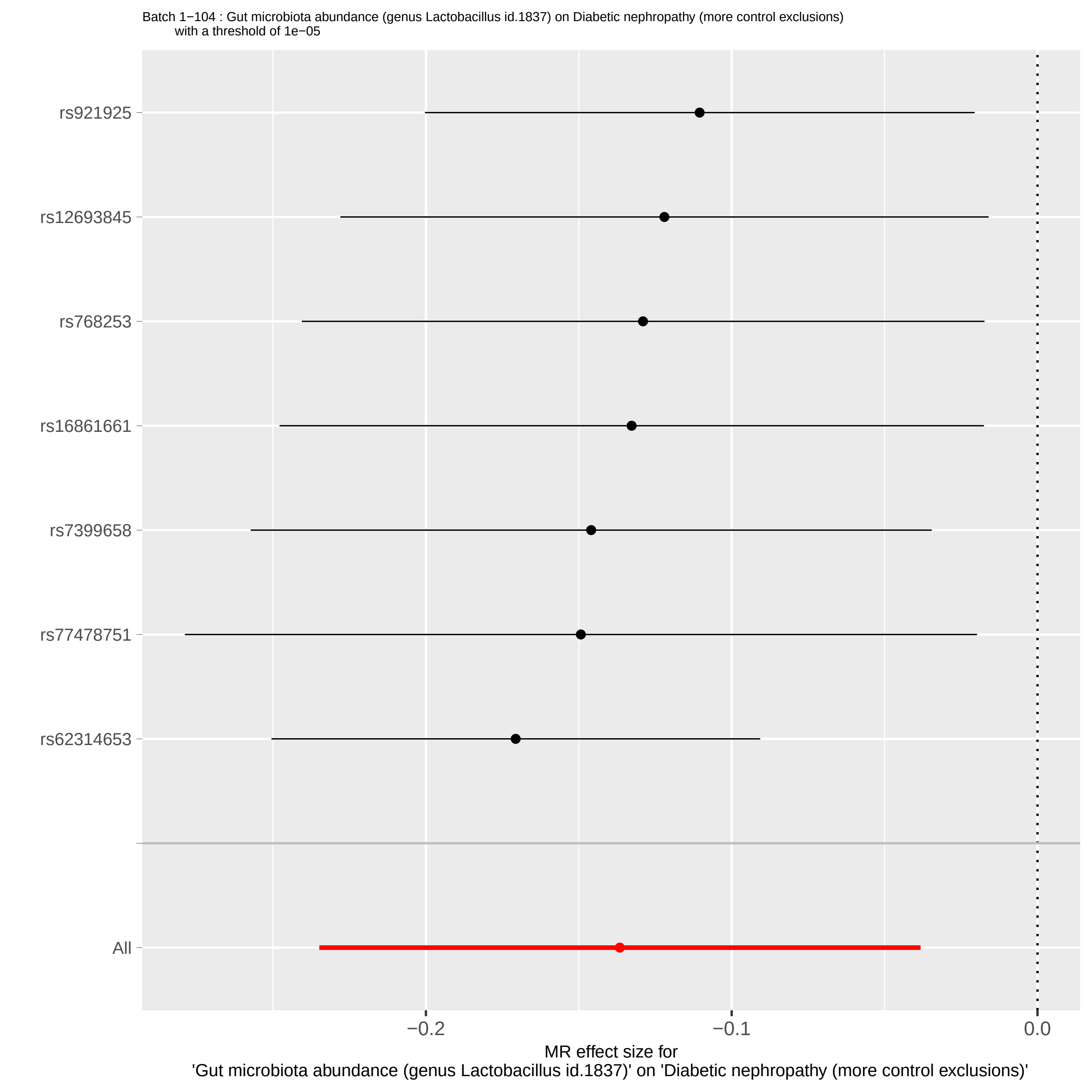
Batch 1−104 : Gut microbiota abundance (genus Lactobacillus id.1837) on Diabetic nephropathy (more control exclusions) with a threshold of 1e−05
rs921925
rs12693845
rs768253
rs16861661
rs7399658
rs77478751
rs62314653
All
−0.2
−0.1
0.0
MR effect size for
'Gut microbiota abundance (genus Lactobacillus id.1837)' on 'Diabetic nephropathy (more control exclusions)'

Batch 1−105 : Gut microbiota abundance (genus Lactococcus id.1851) on Diabetic nephropathy (more control exclusions)
with a threshold of 1e−05

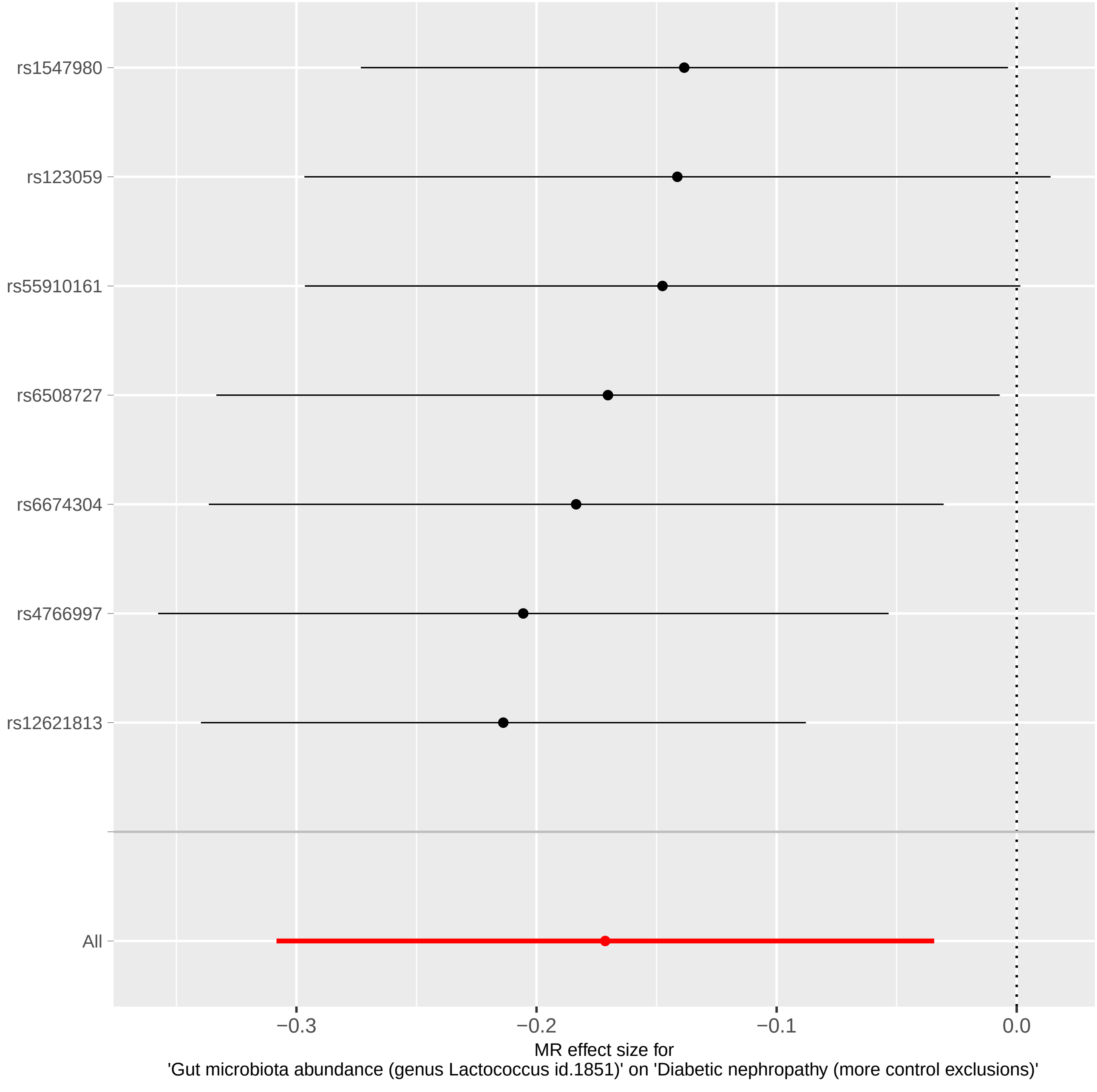

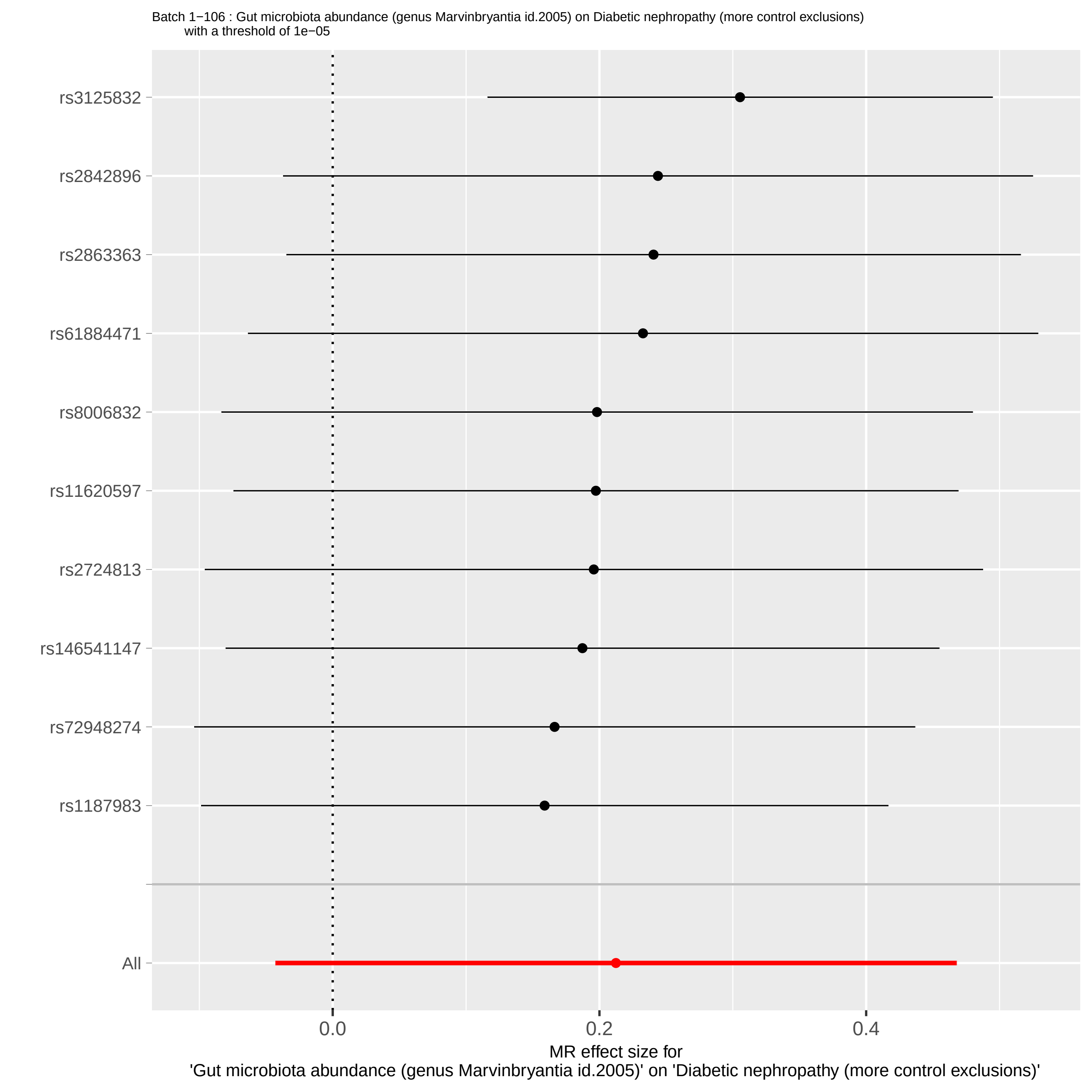
Batch 1−106 : Gut microbiota abundance (genus Marvinbryantia id.2005) on Diabetic nephropathy (more control exclusions)
with a threshold of 1e−05
rs3125832
rs2842896
rs2863363
rs61884471
rs8006832
rs11620597
rs2724813
rs146541147
rs72948274
rs1187983
All
0.0
0.2
0.4
MR effect size for
'Gut microbiota abundance (genus Marvinbryantia id.2005)' on 'Diabetic nephropathy (more control exclusions)'

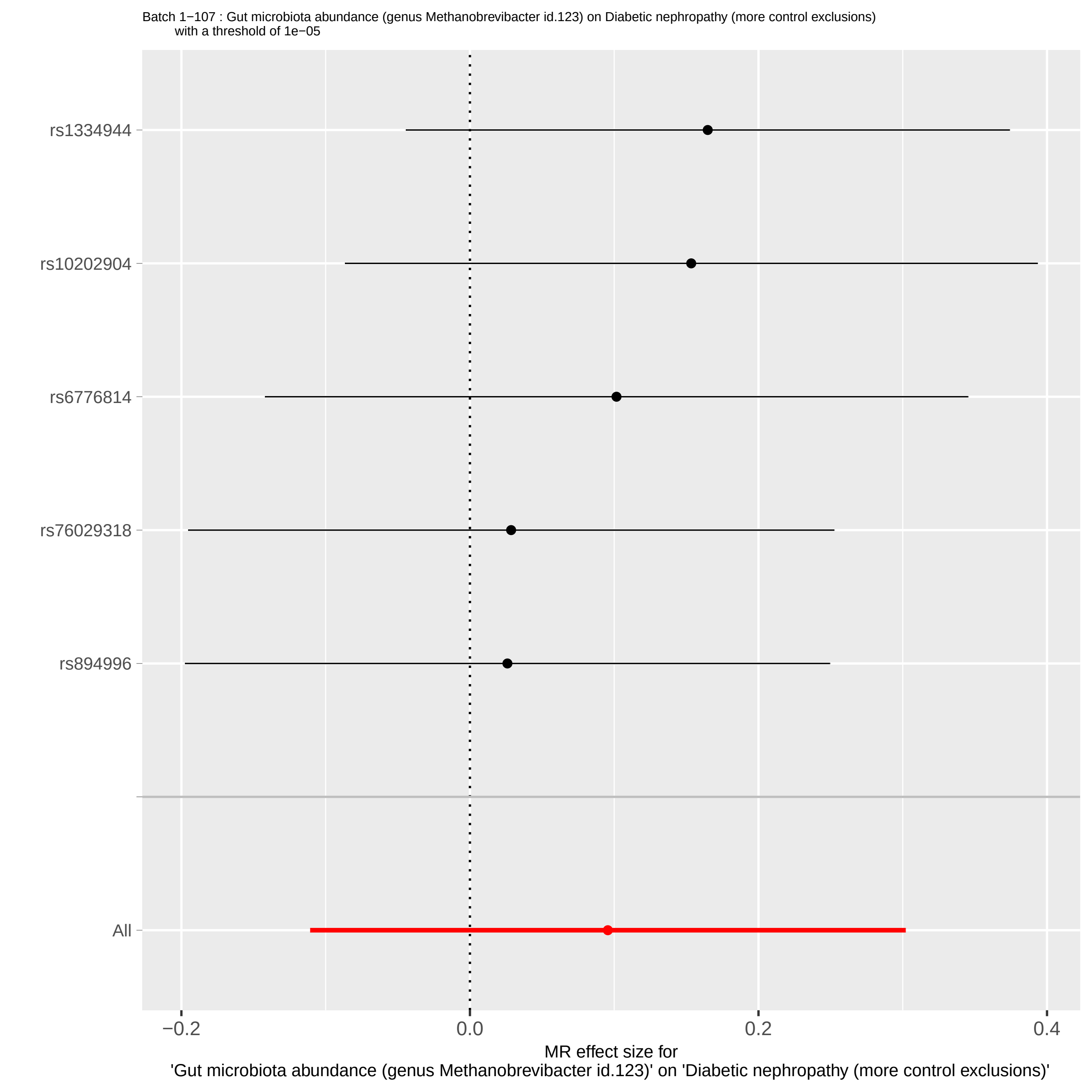
Batch 1−107 : Gut microbiota abundance (genus Methanobrevibacter id.123) on Diabetic nephropathy (more control exclusions) with a threshold of 1e−05
rs1334944
rs10202904
rs6776814
rs76029318
rs894996
All
−0.2
0.0
0.2
0.4
MR effect size for
'Gut microbiota abundance (genus Methanobrevibacter id.123)' on 'Diabetic nephropathy (more control exclusions)'

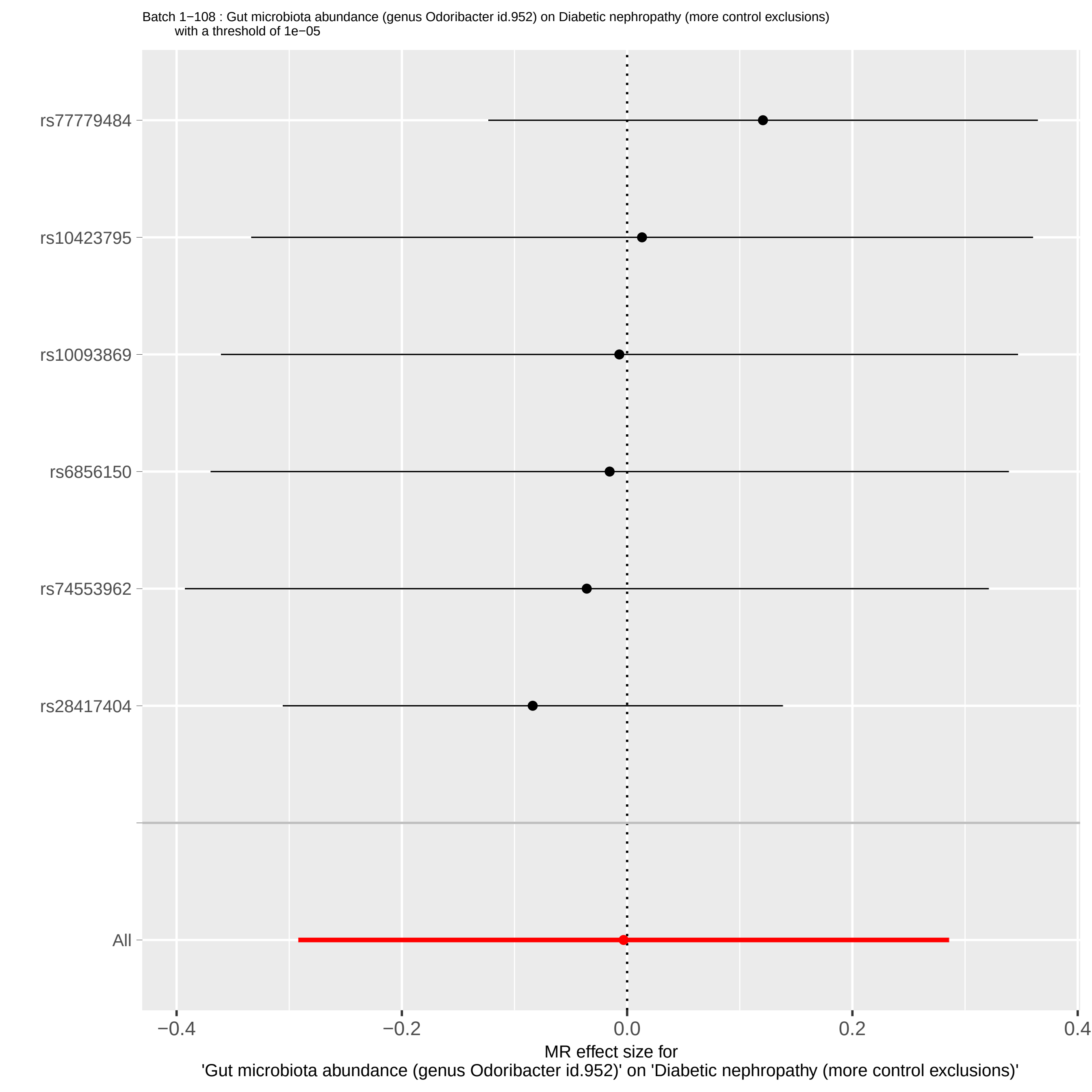

Batch 1−108 : Gut microbiota abundance (genus Odoribacter id.952) on Diabetic nephropathy (more control exclusions)
with a threshold of 1e−05
rs77779484
rs10423795
rs10093869
rs6856150
rs74553962
rs28417404
All
−0.4
−0.2
0.0
0.2
0.4
MR effect size for
'Gut microbiota abundance (genus Odoribacter id.952)' on 'Diabetic nephropathy (more control exclusions)'

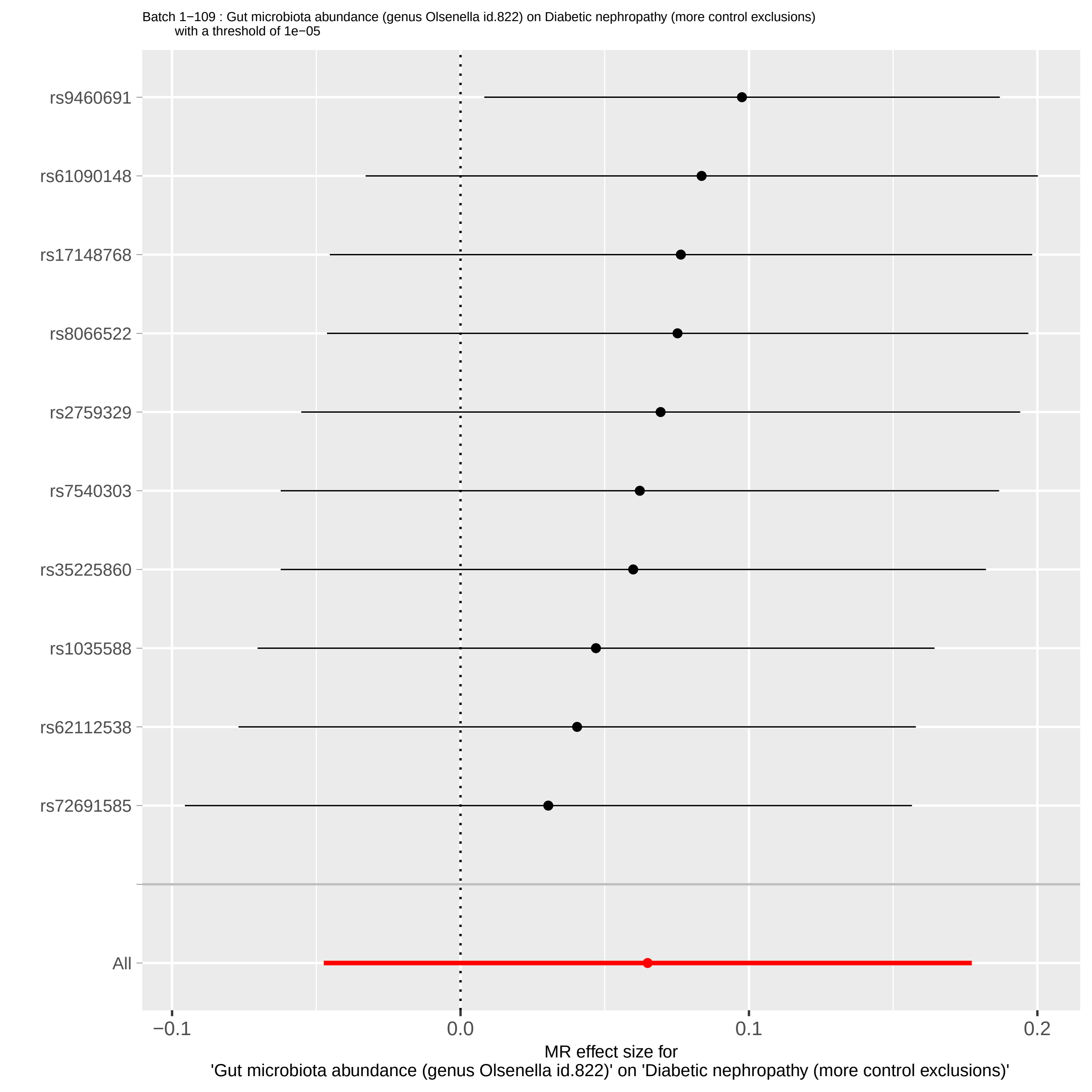
Batch 1−109 : Gut microbiota abundance (genus Olsenella id.822) on Diabetic nephropathy (more control exclusions)
with a threshold of 1e−05
rs9460691
rs61090148
rs17148768
rs8066522
rs2759329
rs7540303
rs35225860
rs1035588
rs62112538
rs72691585
All
−0.1
0.0
0.1
0.2
MR effect size for
'Gut microbiota abundance (genus Olsenella id.822)' on 'Diabetic nephropathy (more control exclusions)'

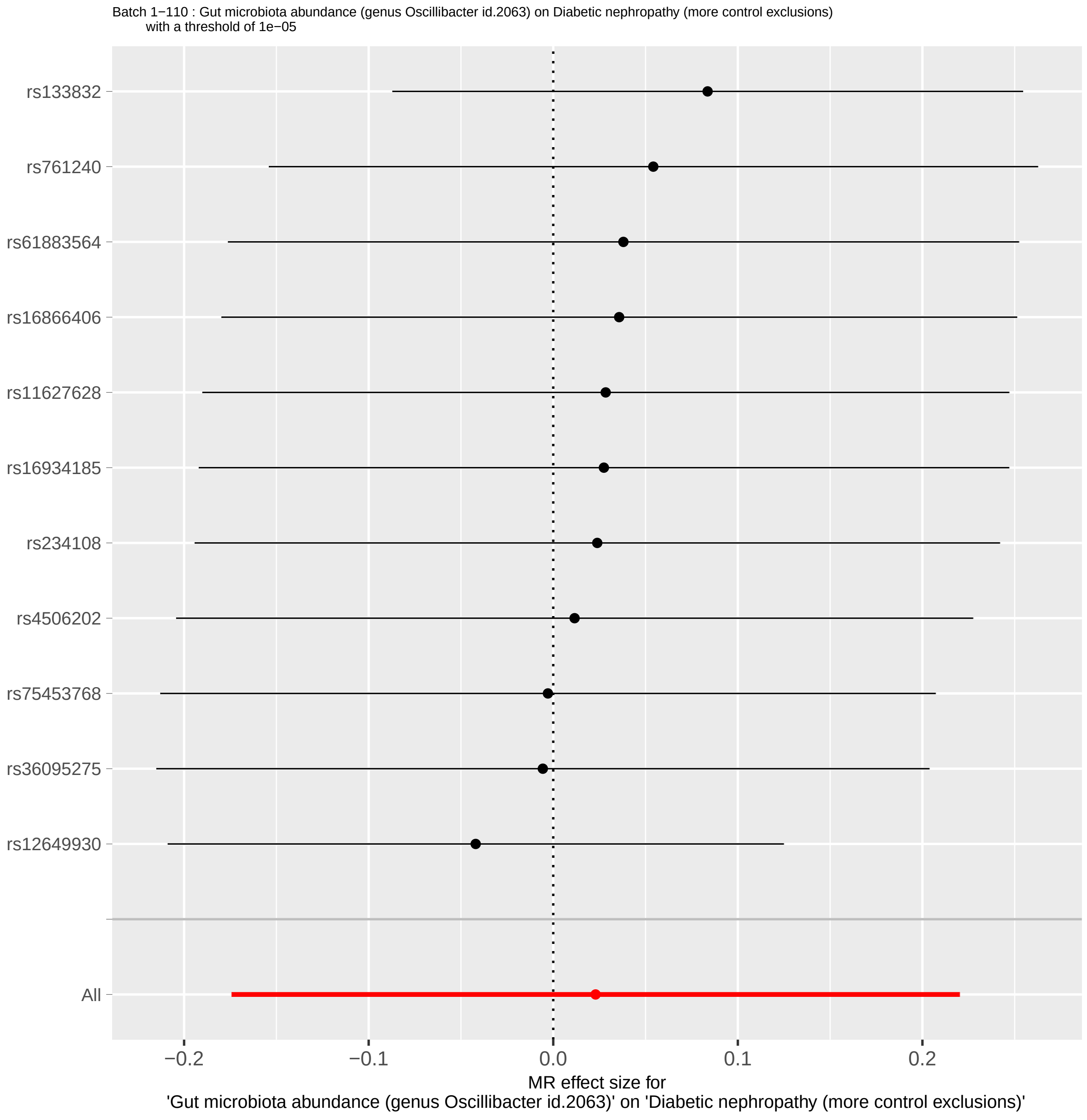

Batch 1−110 : Gut microbiota abundance (genus Oscillibacter id.2063) on Diabetic nephropathy (more control exclusions)
with a threshold of 1e−05
rs133832
rs761240
rs61883564
rs16866406
rs11627628
rs16934185
rs234108
rs4506202
rs75453768
rs36095275
rs12649930
All
−0.2
−0.1
0.0
0.1
0.2
MR effect size for
'Gut microbiota abundance (genus Oscillibacter id.2063)' on 'Diabetic nephropathy (more control exclusions)'

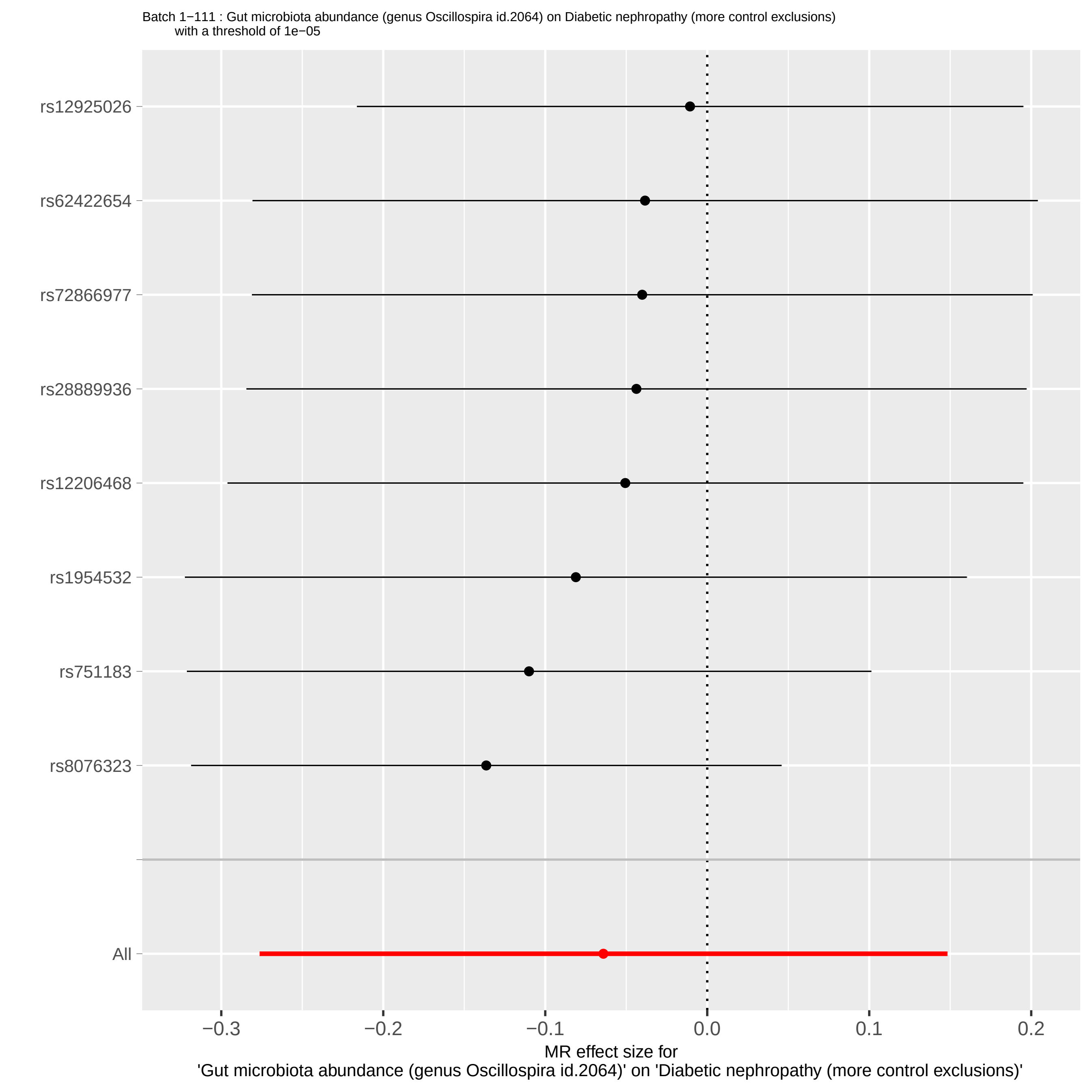

Batch 1−111 : Gut microbiota abundance (genus Oscillospira id.2064) on Diabetic nephropathy (more control exclusions)
with a threshold of 1e−05
rs12925026
rs62422654
rs72866977
rs28889936
rs12206468
rs1954532
rs751183
rs8076323
All
−0.3
−0.2
−0.1
0.0
0.1
0.2
MR effect size for
'Gut microbiota abundance (genus Oscillospira id.2064)' on 'Diabetic nephropathy (more control exclusions)'

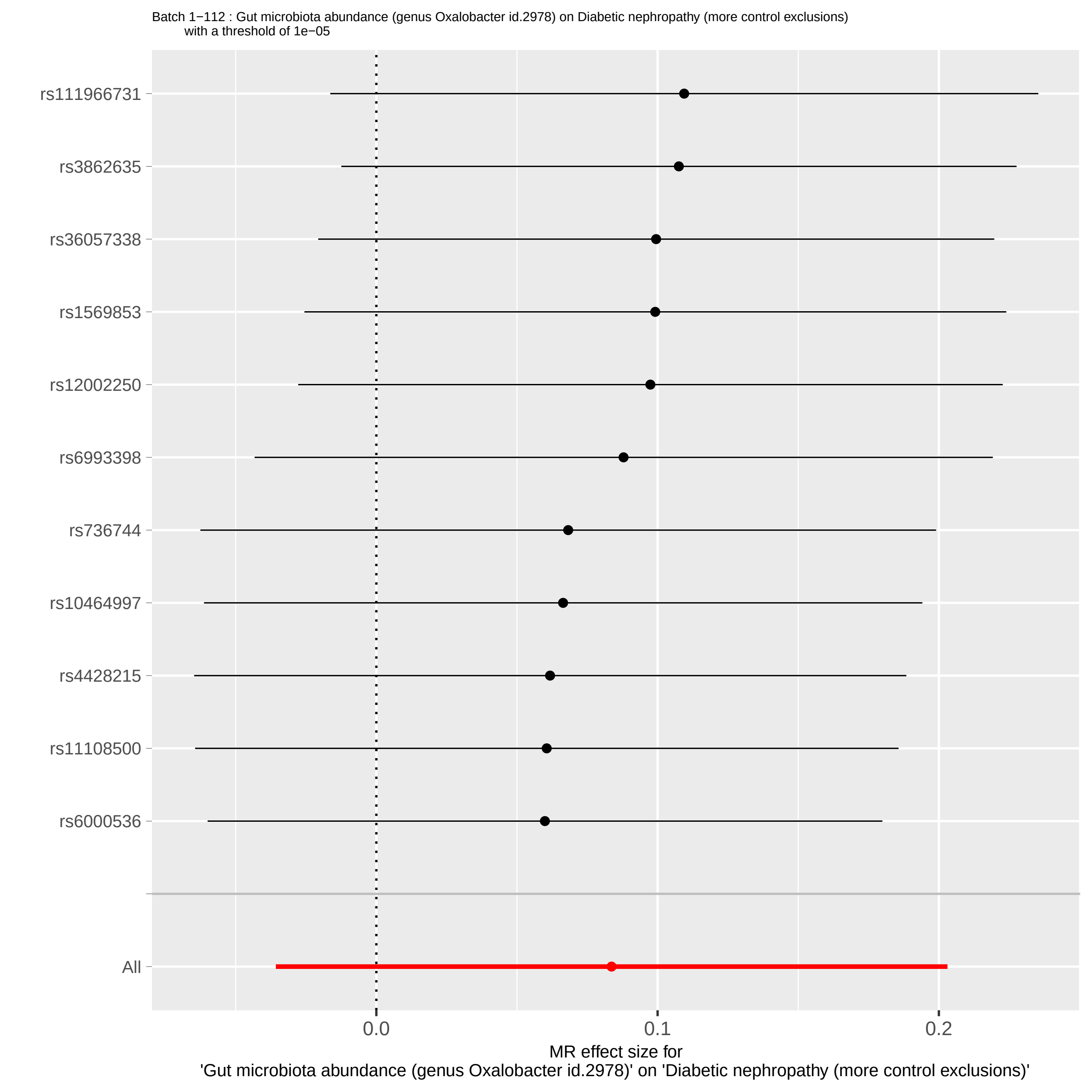

Batch 1−112 : Gut microbiota abundance (genus Oxalobacter id.2978) on Diabetic nephropathy (more control exclusions)
with a threshold of 1e−05
rs111966731
rs3862635
rs36057338
rs1569853
rs12002250
rs6993398
rs736744
rs10464997
rs4428215
rs11108500
rs6000536
All
0.0
0.1
0.2
MR effect size for
'Gut microbiota abundance (genus Oxalobacter id.2978)' on 'Diabetic nephropathy (more control exclusions)'

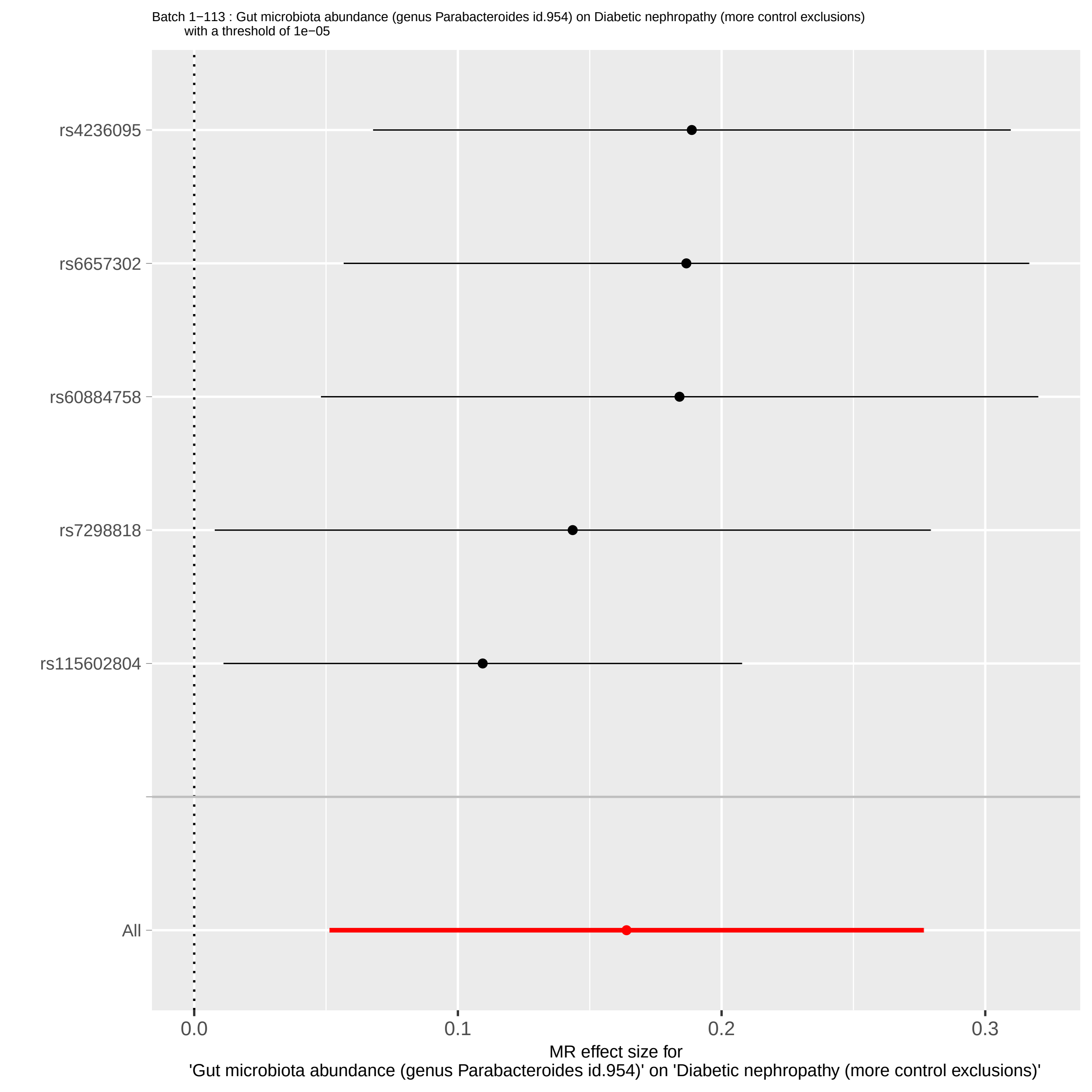
Batch 1−113 : Gut microbiota abundance (genus Parabacteroides id.954) on Diabetic nephropathy (more control exclusions)
with a threshold of 1e−05
rs4236095
rs6657302
rs60884758
rs7298818
rs115602804
All
0.0
0.1
0.2
0.3
MR effect size for
'Gut microbiota abundance (genus Parabacteroides id.954)' on 'Diabetic nephropathy (more control exclusions)'

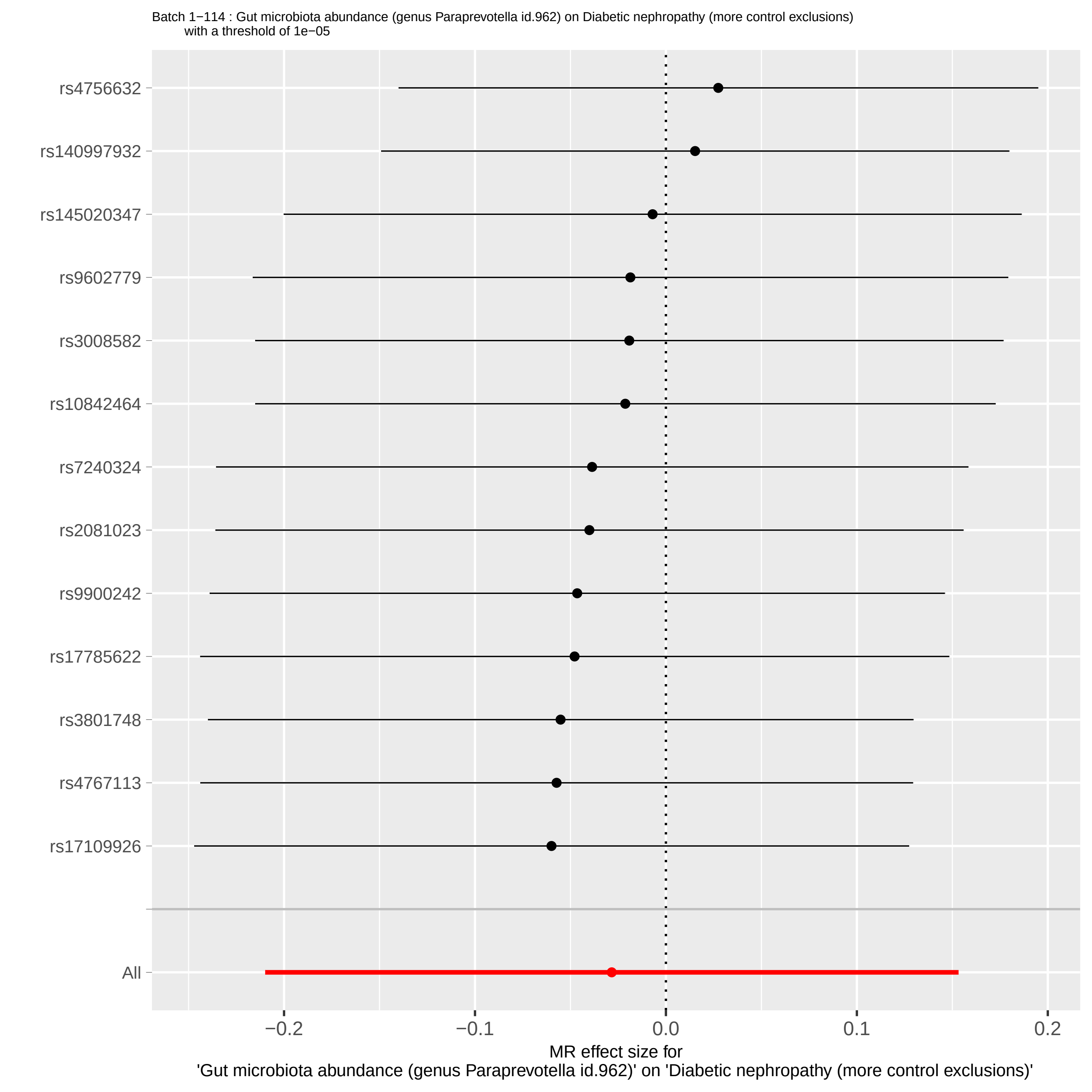
Batch 1−114 : Gut microbiota abundance (genus Paraprevotella id.962) on Diabetic nephropathy (more control exclusions)
with a threshold of 1e−05
rs4756632
rs140997932
rs145020347
rs9602779
rs3008582
rs10842464
rs7240324
rs2081023
rs9900242
rs17785622
rs3801748
rs4767113
rs17109926
All
−0.2
−0.1
0.0
0.1
0.2
MR effect size for
'Gut microbiota abundance (genus Paraprevotella id.962)' on 'Diabetic nephropathy (more control exclusions)'

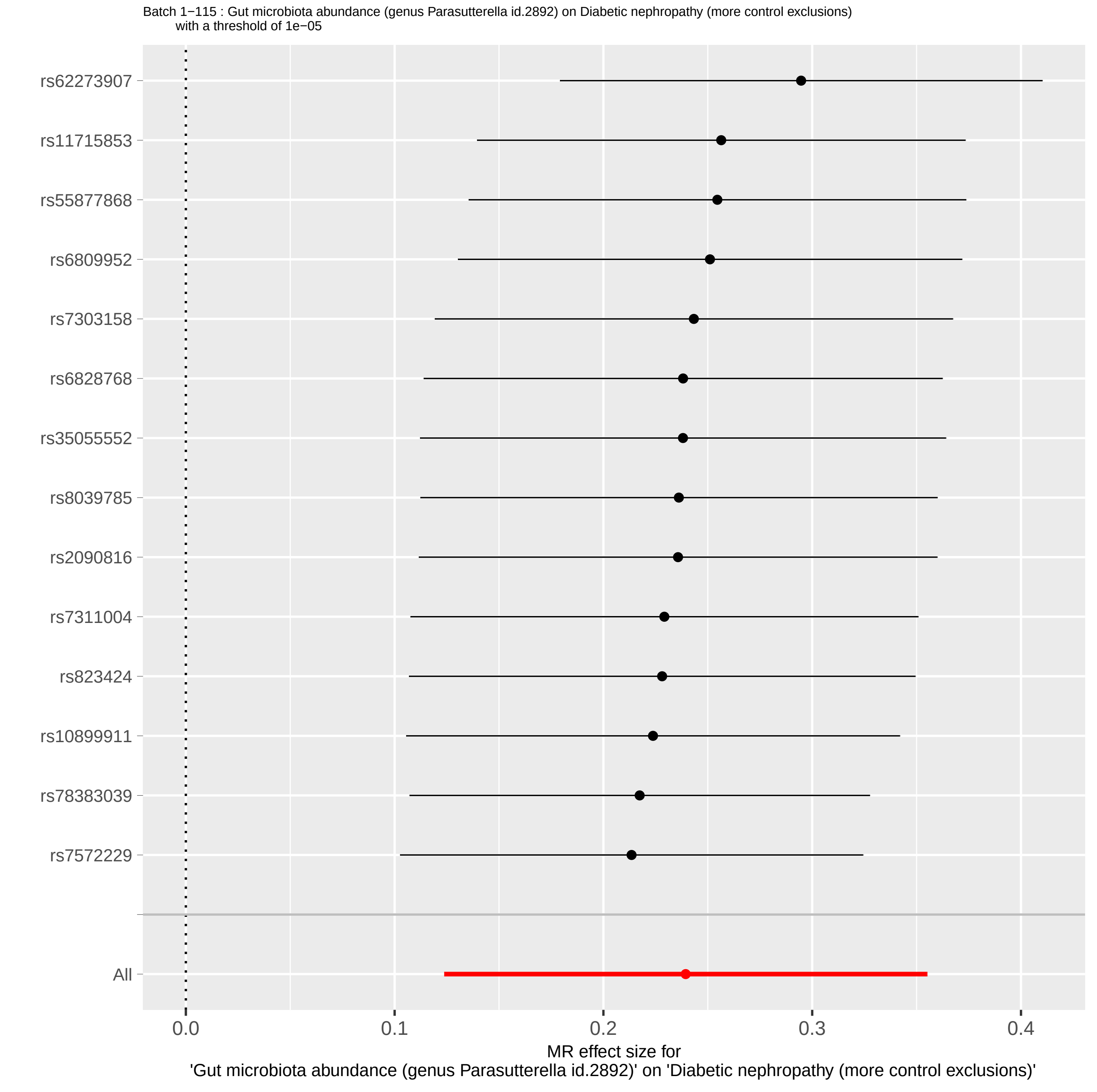
Batch 1−115 : Gut microbiota abundance (genus Parasutterella id.2892) on Diabetic nephropathy (more control exclusions)
with a threshold of 1e−05
rs62273907
rs11715853
rs55877868
rs6809952
rs7303158
rs6828768
rs35055552
rs8039785
rs2090816
rs7311004
rs823424
rs10899911
rs78383039
rs7572229
All
0.0
0.1
0.2
0.3
0.4
MR effect size for
'Gut microbiota abundance (genus Parasutterella id.2892)' on 'Diabetic nephropathy (more control exclusions)'

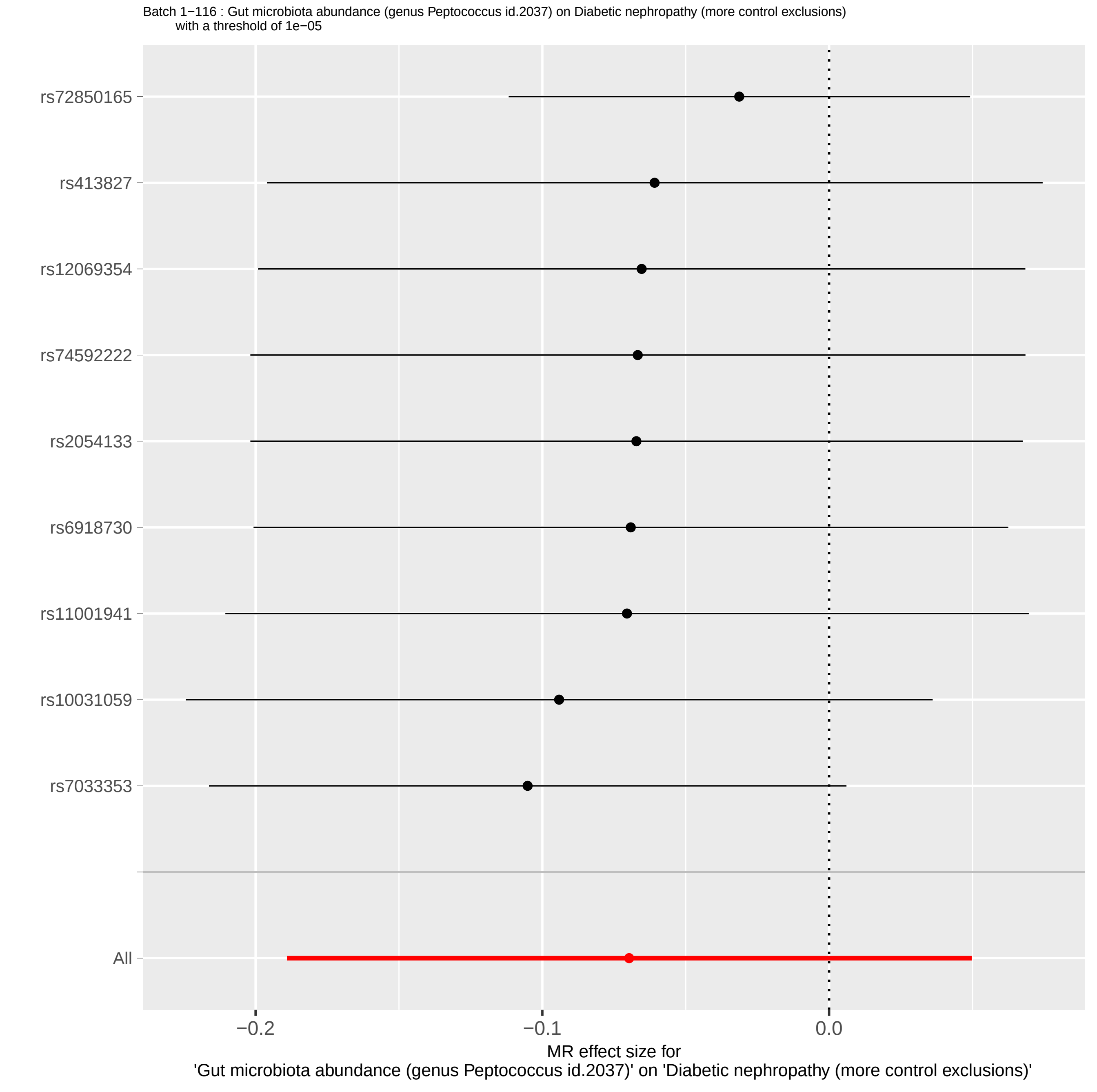

Batch 1−116 : Gut microbiota abundance (genus Peptococcus id.2037) on Diabetic nephropathy (more control exclusions)
with a threshold of 1e−05
rs72850165
rs413827
rs12069354
rs74592222
rs2054133
rs6918730
rs11001941
rs10031059
rs7033353
All
−0.2
−0.1
0.0
MR effect size for
'Gut microbiota abundance (genus Peptococcus id.2037)' on 'Diabetic nephropathy (more control exclusions)'

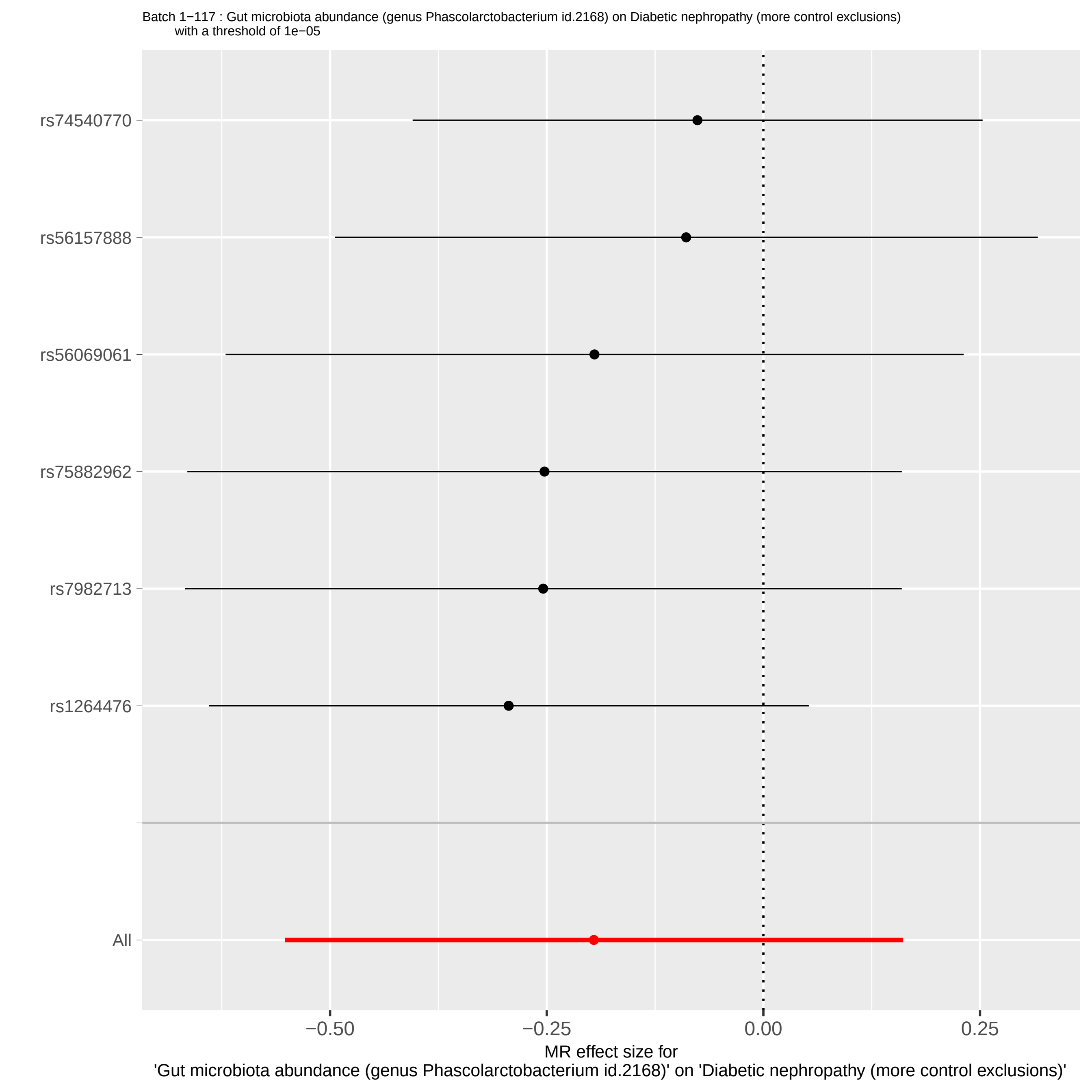

Batch 1−117 : Gut microbiota abundance (genus Phascolarctobacterium id.2168) on Diabetic nephropathy (more control exclusions)
with a threshold of 1e−05
rs74540770
rs56157888
rs56069061
rs75882962
rs7982713
rs1264476
All
−0.50
−0.25
0.00
0.25
MR effect size for
'Gut microbiota abundance (genus Phascolarctobacterium id.2168)' on 'Diabetic nephropathy (more control exclusions)'

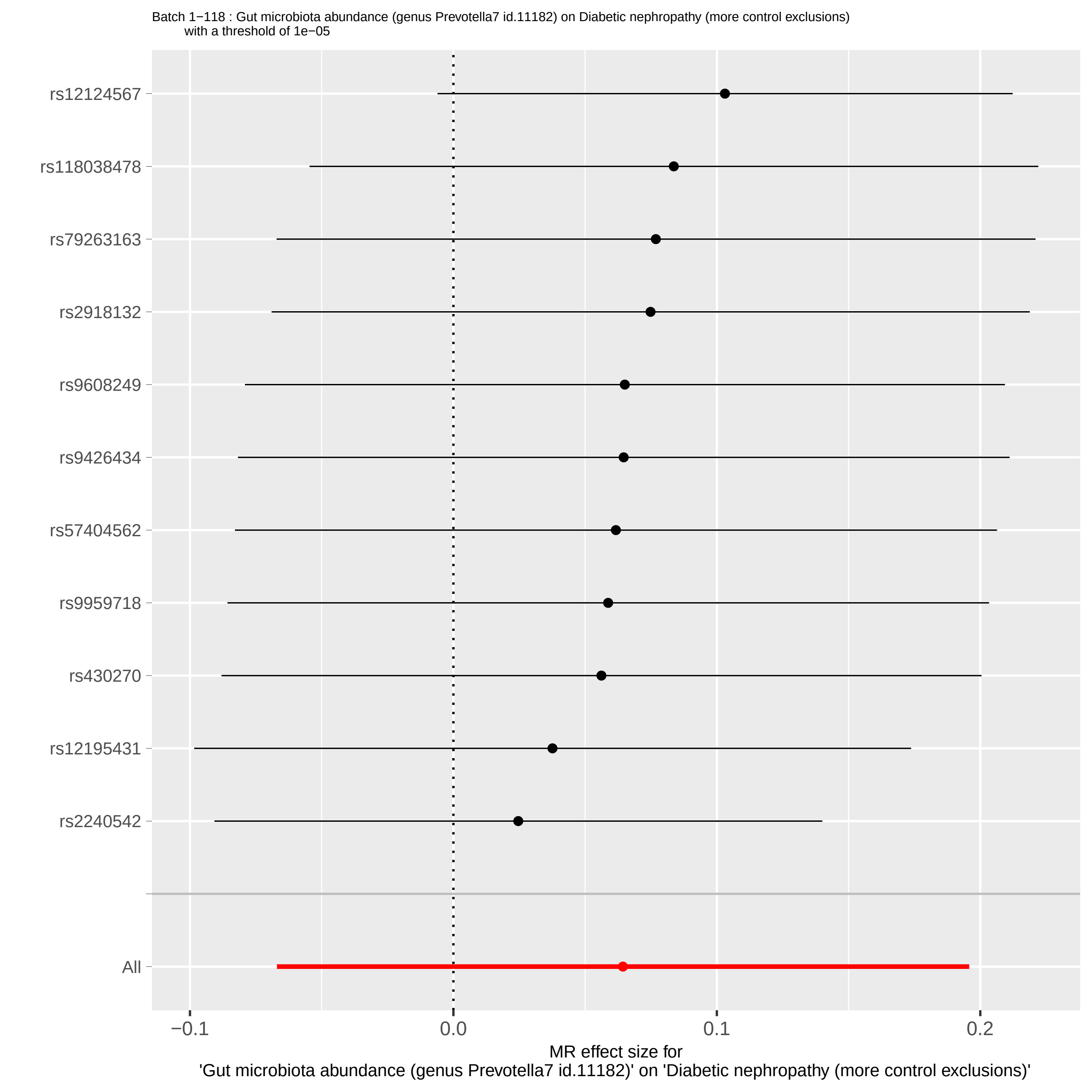
Batch 1−118 : Gut microbiota abundance (genus Prevotella7 id.11182) on Diabetic nephropathy (more control exclusions)
with a threshold of 1e−05
rs12124567
rs118038478
rs79263163
rs2918132
rs9608249
rs9426434
rs57404562
rs9959718
rs430270
rs12195431
rs2240542
All
−0.1
0.0
0.1
0.2
MR effect size for
'Gut microbiota abundance (genus Prevotella7 id.11182)' on 'Diabetic nephropathy (more control exclusions)'

Batch 1−119 : Gut microbiota abundance (genus Prevotella9 id.11183) on Diabetic nephropathy (more control exclusions)
with a threshold of 1e−05

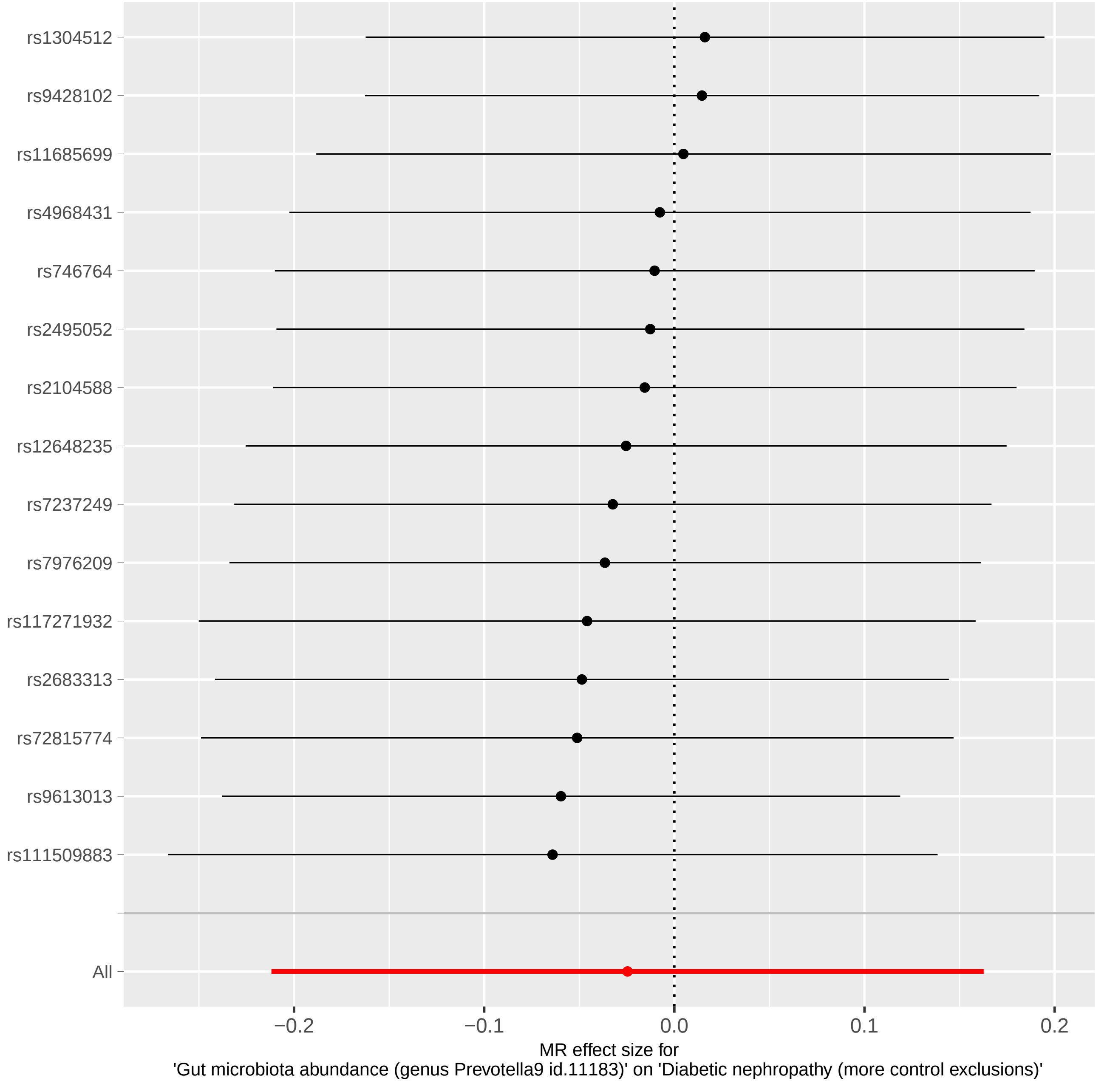

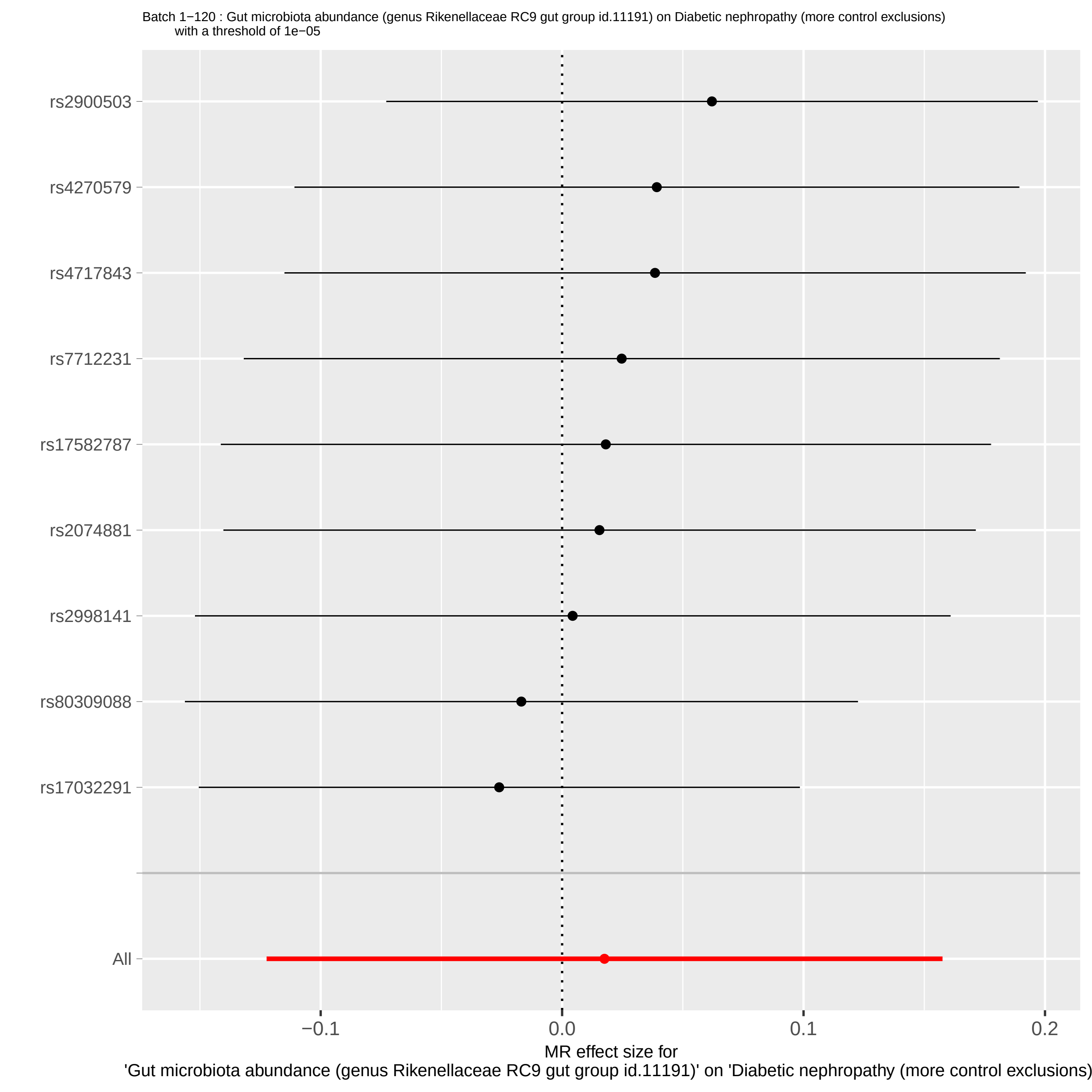

Batch 1−120 : Gut microbiota abundance (genus Rikenellaceae RC9 gut group id.11191) on Diabetic nephropathy (more control exclusions) with a threshold of 1e−05
rs2900503
rs4270579
rs4717843
rs7712231
rs17582787
rs2074881
rs2998141
rs80309088
rs17032291
All
−0.1
0.0
0.1
0.2
MR effect size for
'Gut microbiota abundance (genus Rikenellaceae RC9 gut group id.11191)' on 'Diabetic nephropathy (more control exclusions)'

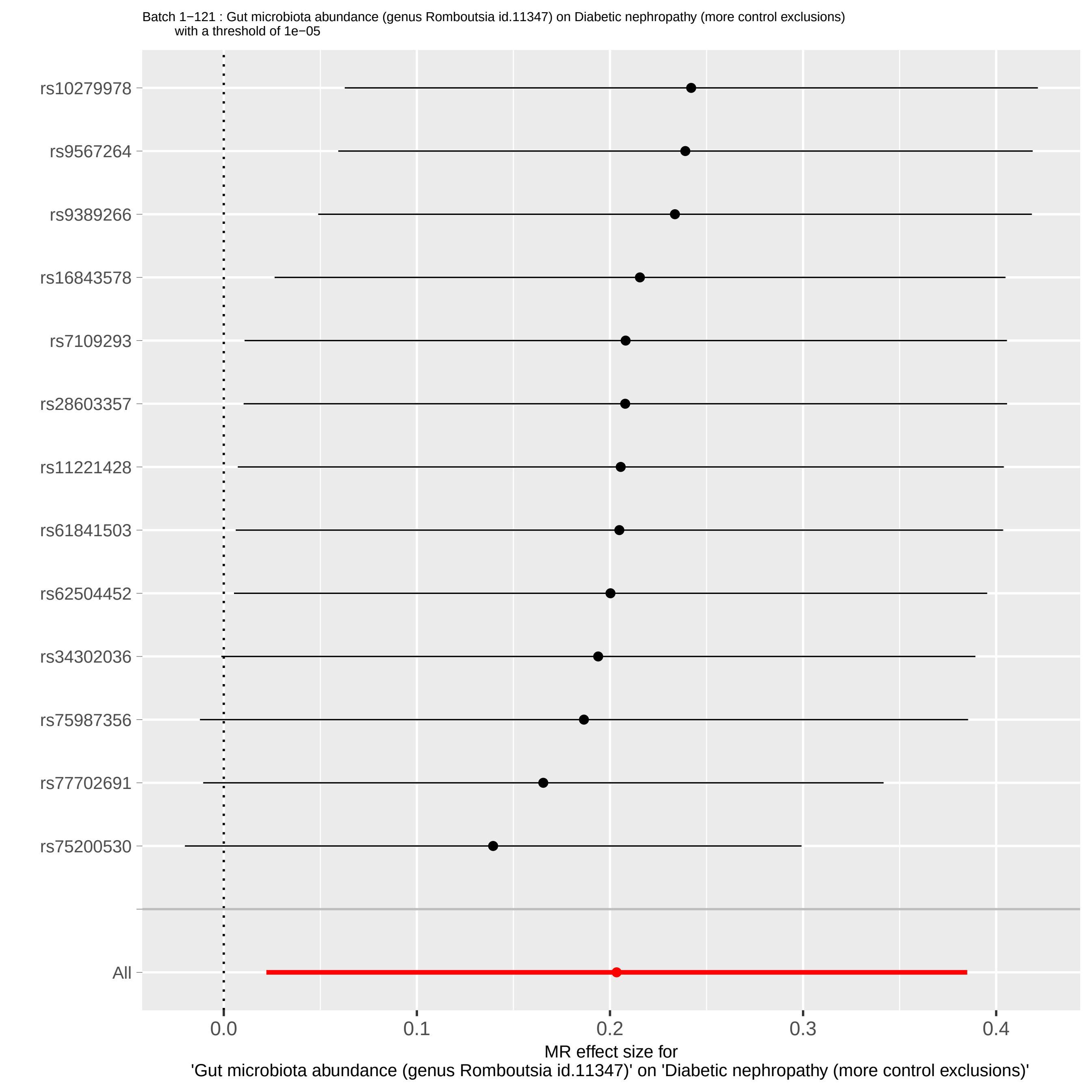
Batch 1−121 : Gut microbiota abundance (genus Romboutsia id.11347) on Diabetic nephropathy (more control exclusions)
with a threshold of 1e−05
rs10279978
rs9567264
rs9389266
rs16843578
rs7109293
rs28603357
rs11221428
rs61841503
rs62504452
rs34302036
rs75987356
rs77702691
rs75200530
All
0.0
0.1
0.2
0.3
0.4
MR effect size for
'Gut microbiota abundance (genus Romboutsia id.11347)' on 'Diabetic nephropathy (more control exclusions)'

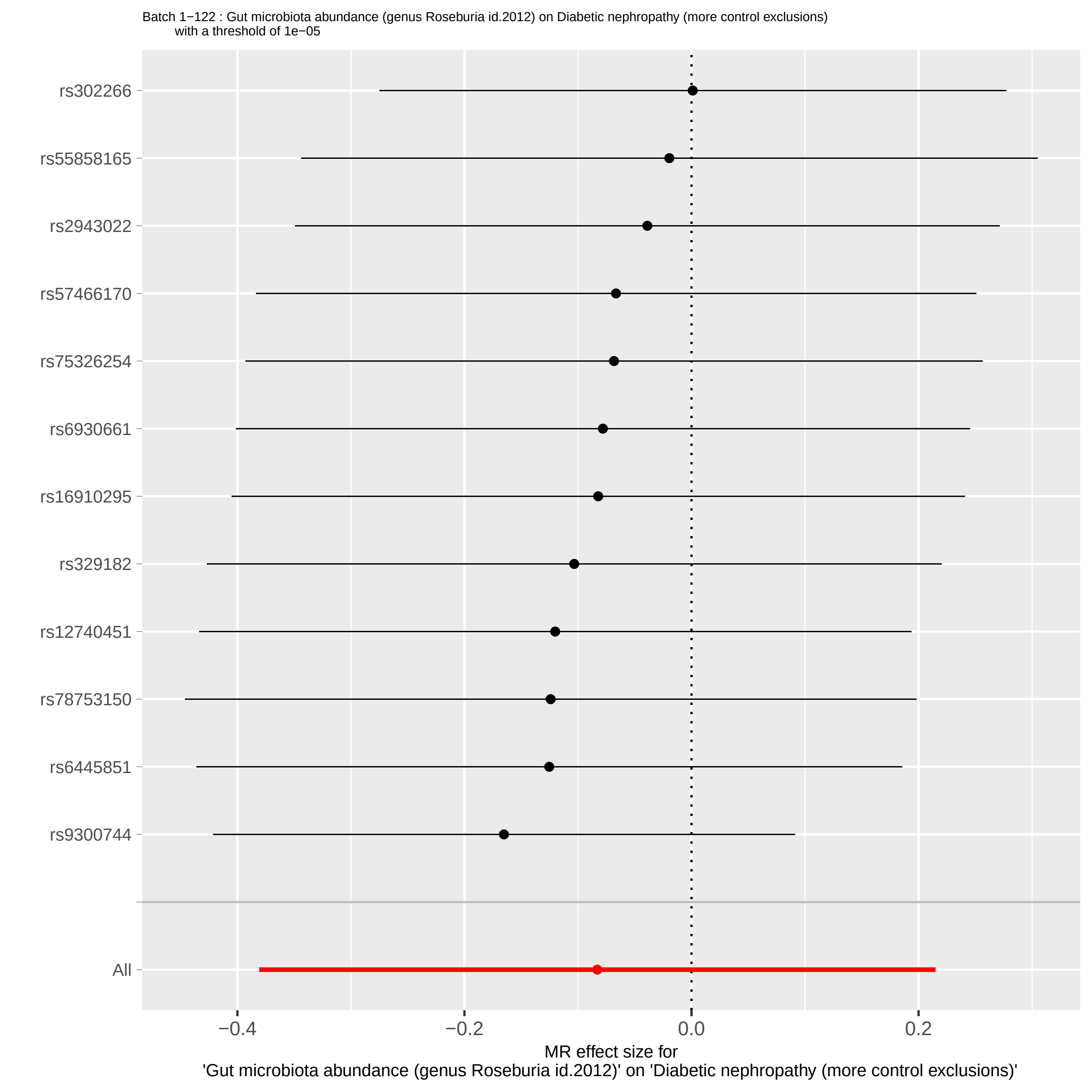
Batch 1−122 : Gut microbiota abundance (genus Roseburia id.2012) on Diabetic nephropathy (more control exclusions)
with a threshold of 1e−05
rs302266
rs55858165
rs2943022
rs57466170
rs75326254
rs6930661
rs16910295
rs329182
rs12740451
rs78753150
rs6445851
rs9300744
All
−0.4
−0.2
0.0
0.2
MR effect size for
'Gut microbiota abundance (genus Roseburia id.2012)' on 'Diabetic nephropathy (more control exclusions)'

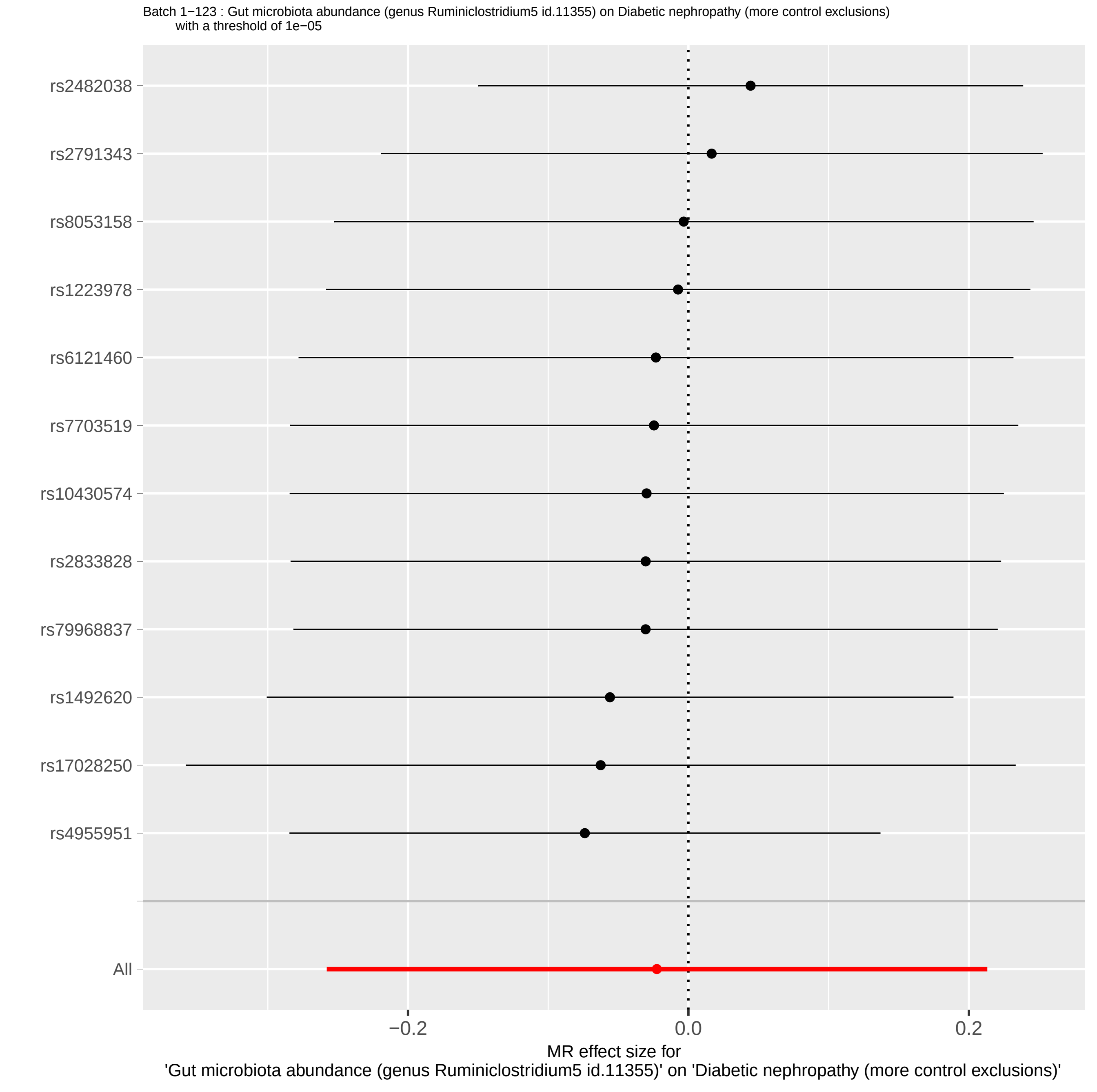

Batch 1−123 : Gut microbiota abundance (genus Ruminiclostridium5 id.11355) on Diabetic nephropathy (more control exclusions) with a threshold of 1e−05
rs2482038
rs2791343
rs8053158
rs1223978
rs6121460
rs7703519
rs10430574
rs2833828
rs79968837
rs1492620
rs17028250
rs4955951
All
−0.2
0.0
0.2
MR effect size for
'Gut microbiota abundance (genus Ruminiclostridium5 id.11355)' on 'Diabetic nephropathy (more control exclusions)'

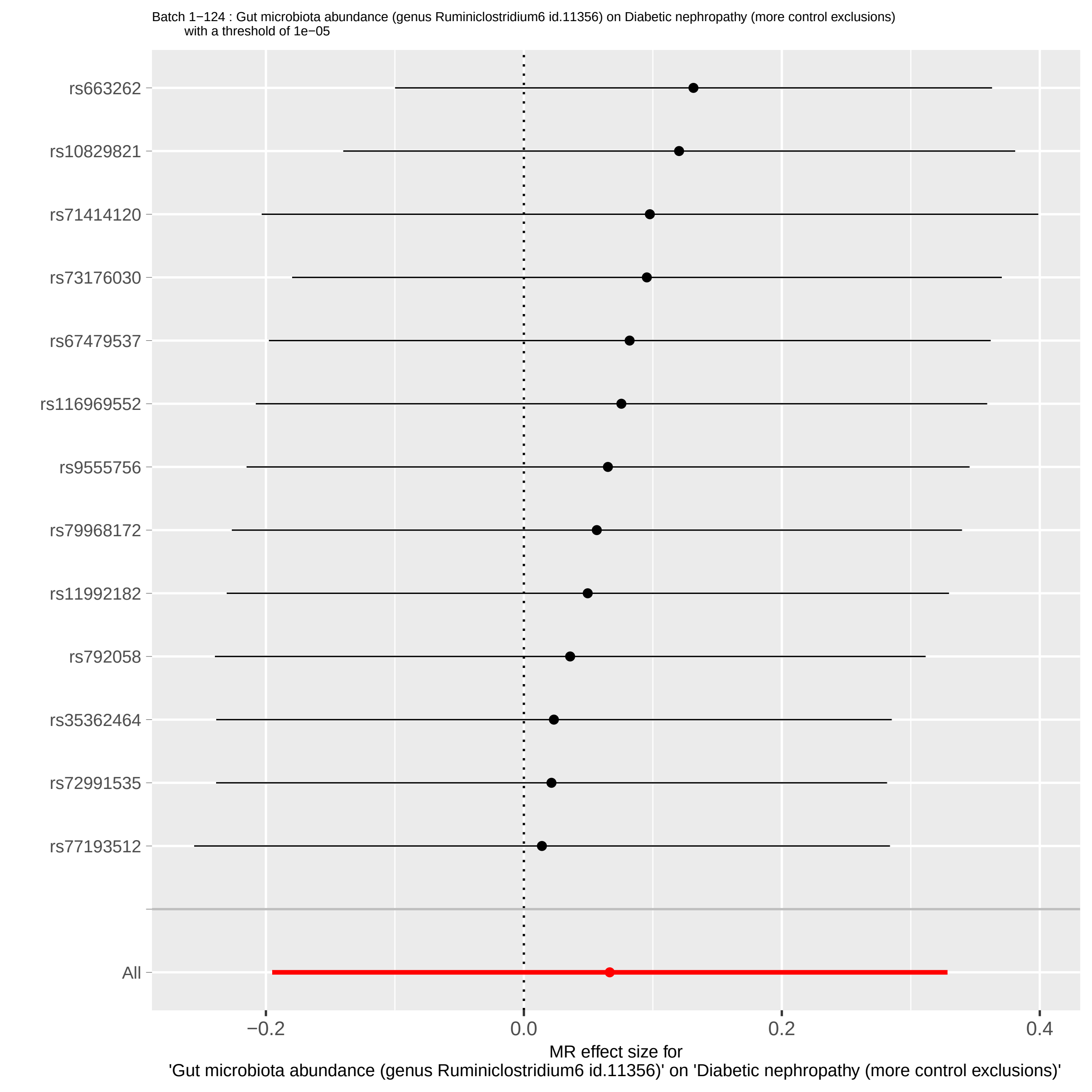

Batch 1−124 : Gut microbiota abundance (genus Ruminiclostridium6 id.11356) on Diabetic nephropathy (more control exclusions) with a threshold of 1e−05
rs663262
rs10829821
rs71414120
rs73176030
rs67479537
rs116969552
rs9555756
rs79968172
rs11992182
rs792058
rs35362464
rs72991535
rs77193512
All
−0.2
0.0
0.2
0.4
MR effect size for
'Gut microbiota abundance (genus Ruminiclostridium6 id.11356)' on 'Diabetic nephropathy (more control exclusions)'

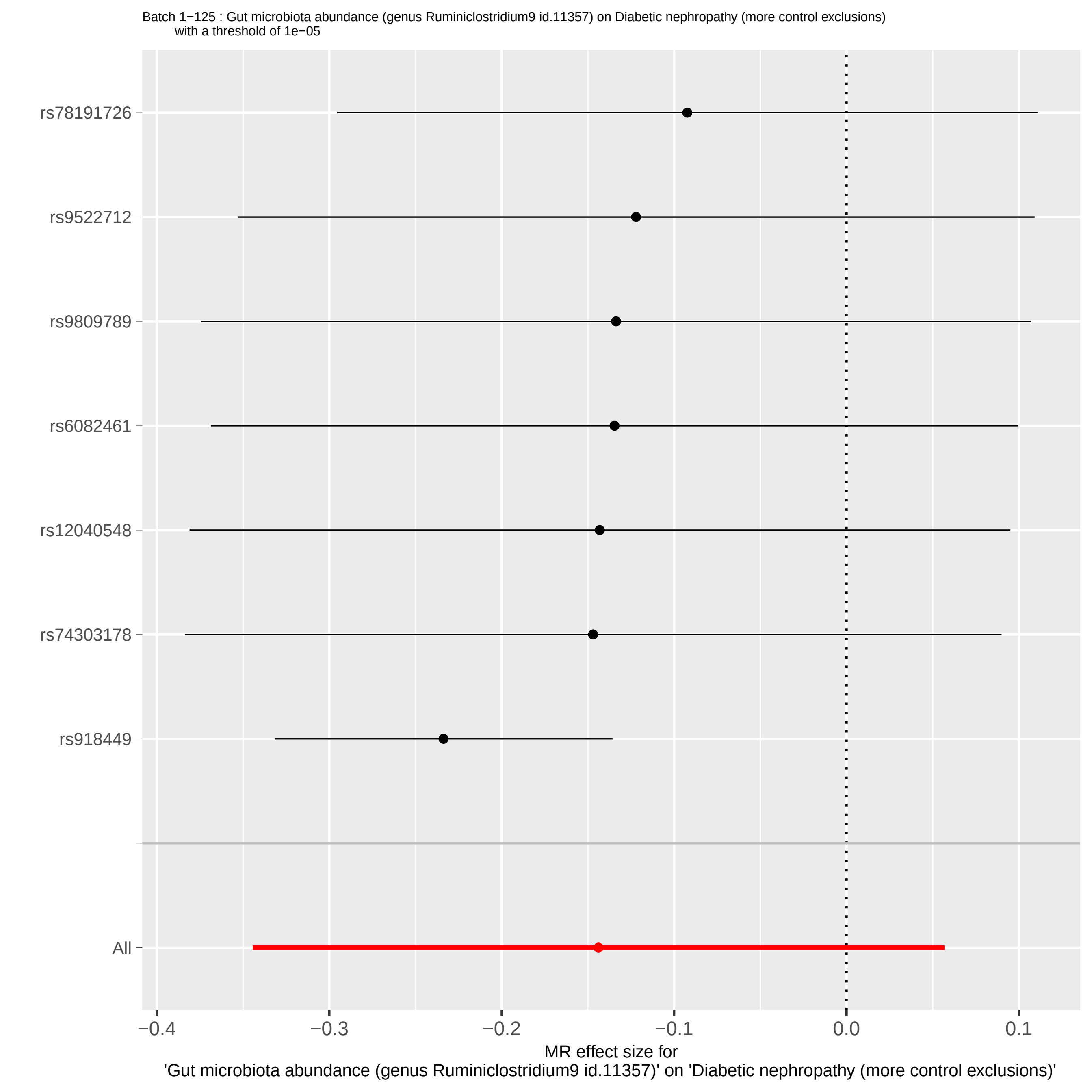
Batch 1−125 : Gut microbiota abundance (genus Ruminiclostridium9 id.11357) on Diabetic nephropathy (more control exclusions)
with a threshold of 1e−05
rs78191726
rs9522712
rs9809789
rs6082461
rs12040548
rs74303178
rs918449
All
−0.4
−0.3
−0.2
−0.1
0.0
0.1
MR effect size for
'Gut microbiota abundance (genus Ruminiclostridium9 id.11357)' on 'Diabetic nephropathy (more control exclusions)'

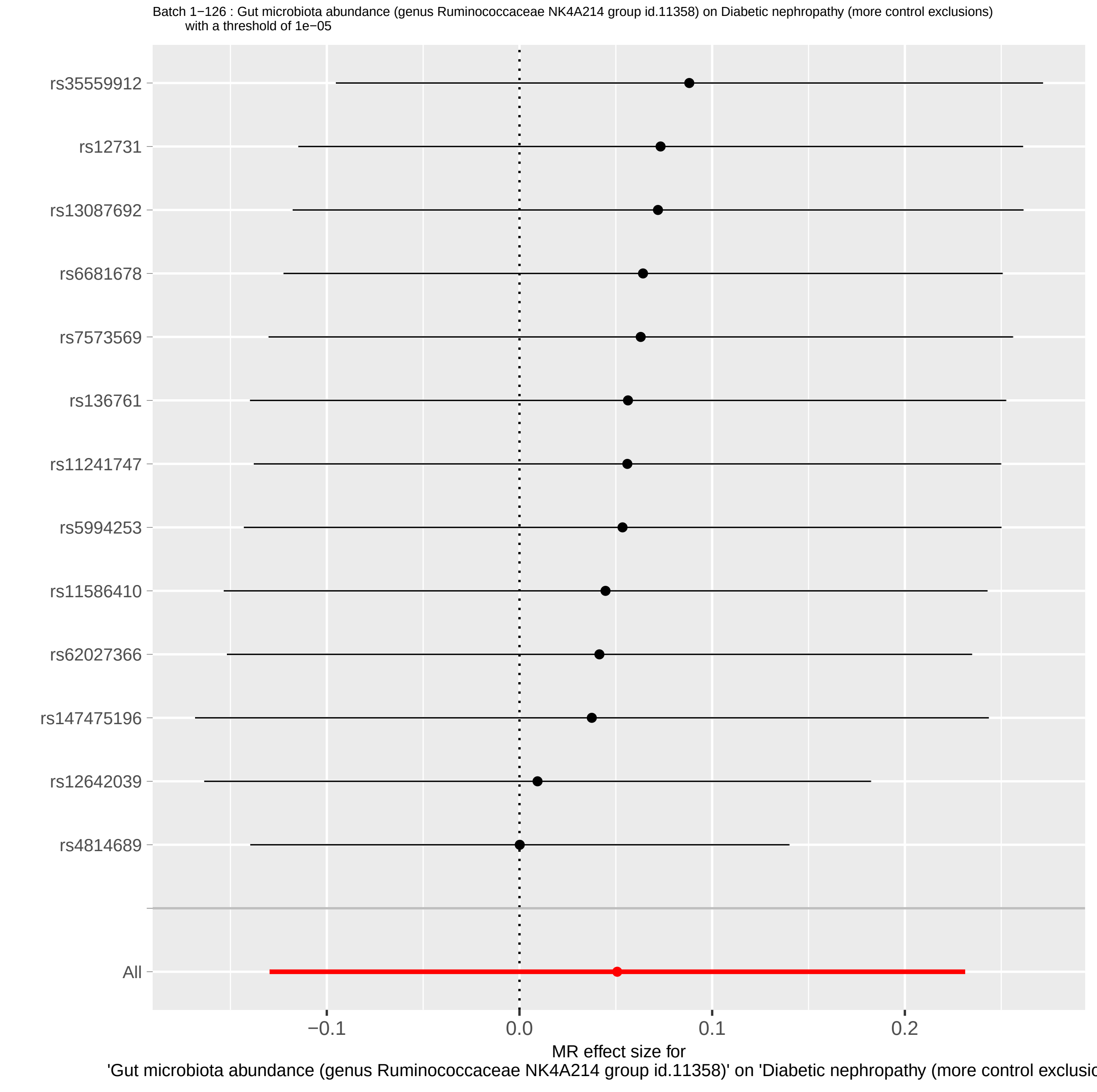
Batch 1−126 : Gut microbiota abundance (genus Ruminococcaceae NK4A214 group id.11358) on Diabetic nephropathy (more control exclusions) with a threshold of 1e−05
rs35559912
rs12731
rs13087692
rs6681678
rs7573569
rs136761
rs11241747
rs5994253
rs11586410
rs62027366
rs147475196
rs12642039
rs4814689
All
−0.1
0.0
0.1
0.2
MR effect size for
'Gut microbiota abundance (genus Ruminococcaceae NK4A214 group id.11358)' on 'Diabetic nephropathy (more control exclusio

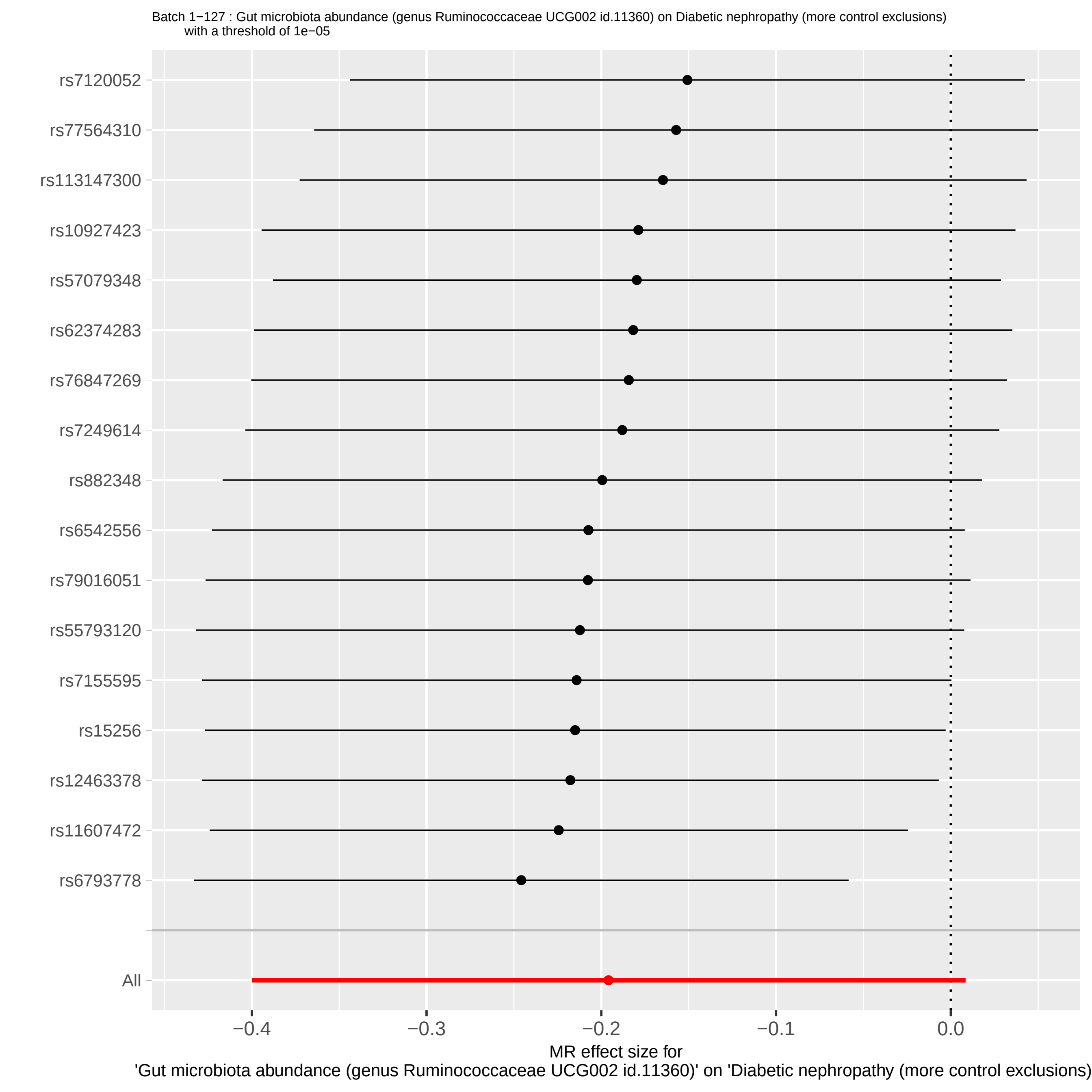
Batch 1−127 : Gut microbiota abundance (genus Ruminococcaceae UCG002 id.11360) on Diabetic nephropathy (more control exclusions)
with a threshold of 1e−05
rs7120052
rs77564310
rs113147300
rs10927423
rs57079348
rs62374283
rs76847269
rs7249614
rs882348
rs6542556
rs79016051
rs55793120
rs7155595
rs15256
rs12463378
rs11607472
rs6793778
All
−0.4
−0.3
−0.2
−0.1
0.0
MR effect size for
'Gut microbiota abundance (genus Ruminococcaceae UCG002 id.11360)' on 'Diabetic nephropathy (more control exclusions)'

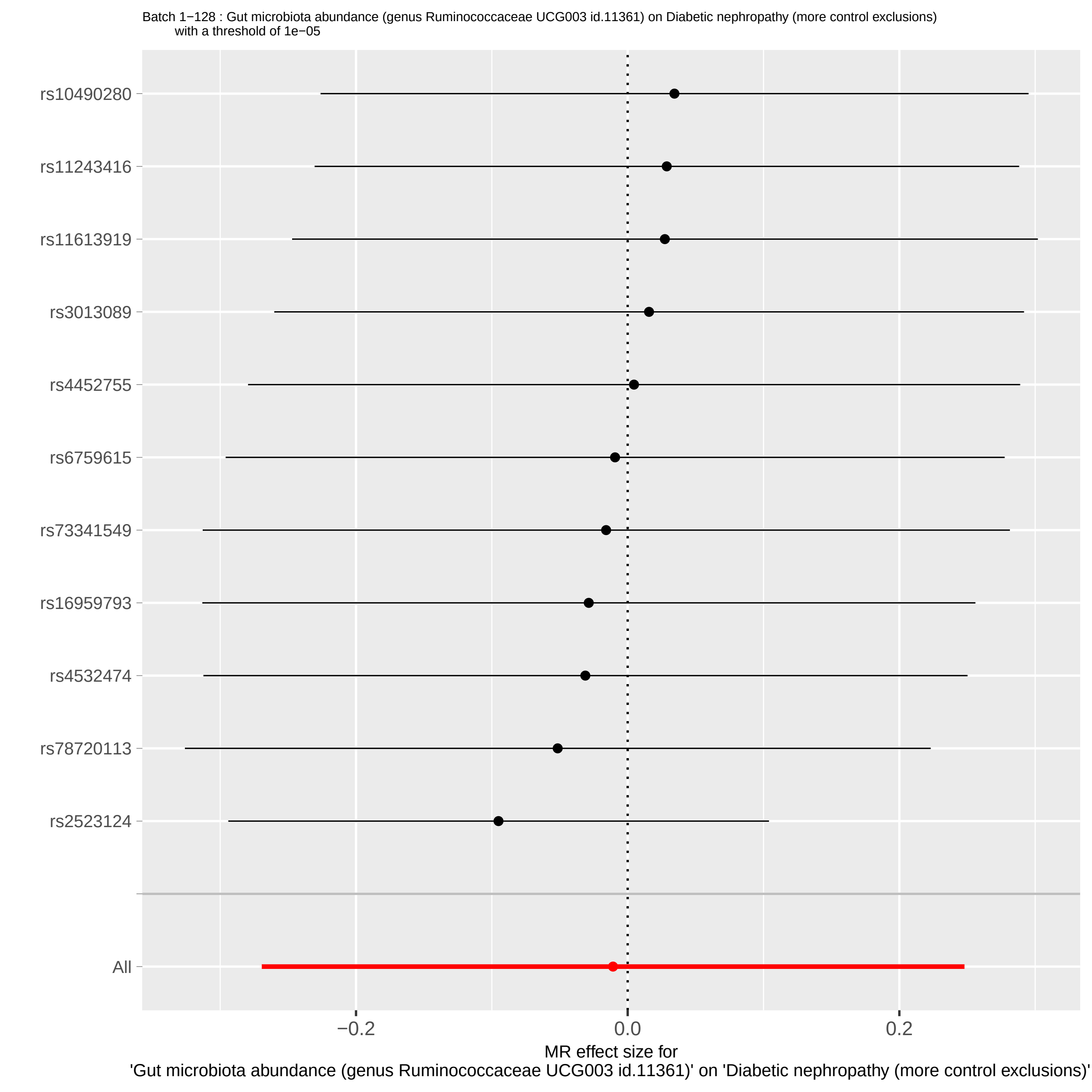

Batch 1−128 : Gut microbiota abundance (genus Ruminococcaceae UCG003 id.11361) on Diabetic nephropathy (more control exclusions)
with a threshold of 1e−05
rs10490280
rs11243416
rs11613919
rs3013089
rs4452755
rs6759615
rs73341549
rs16959793
rs4532474
rs78720113
rs2523124
All
−0.2
0.0
0.2
MR effect size for
'Gut microbiota abundance (genus Ruminococcaceae UCG003 id.11361)' on 'Diabetic nephropathy (more control exclusions)'

Batch 1−129 : Gut microbiota abundance (genus Ruminococcaceae UCG004 id.11362) on Diabetic nephropathy (more control exclusions) with a threshold of 1e−05

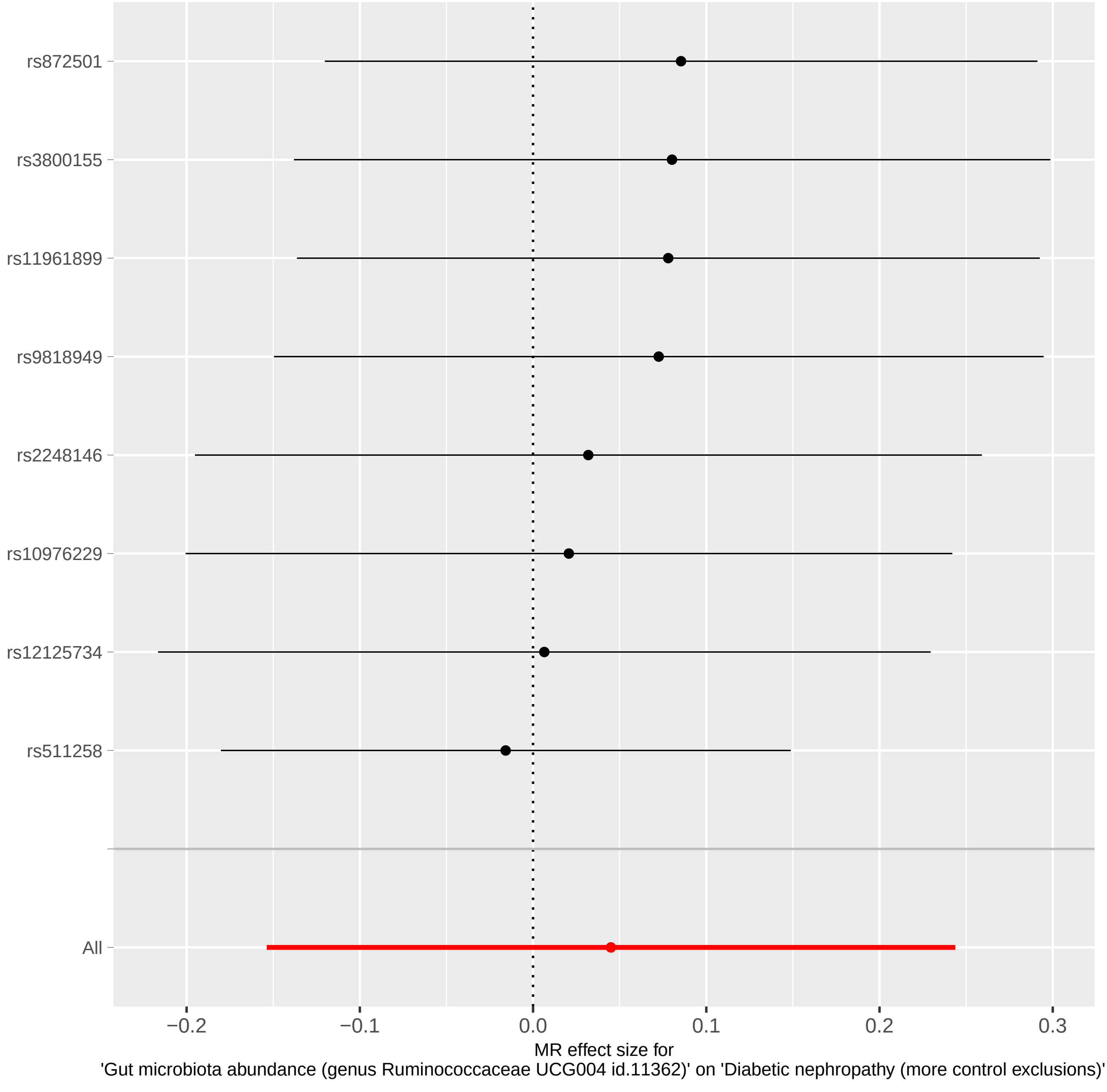

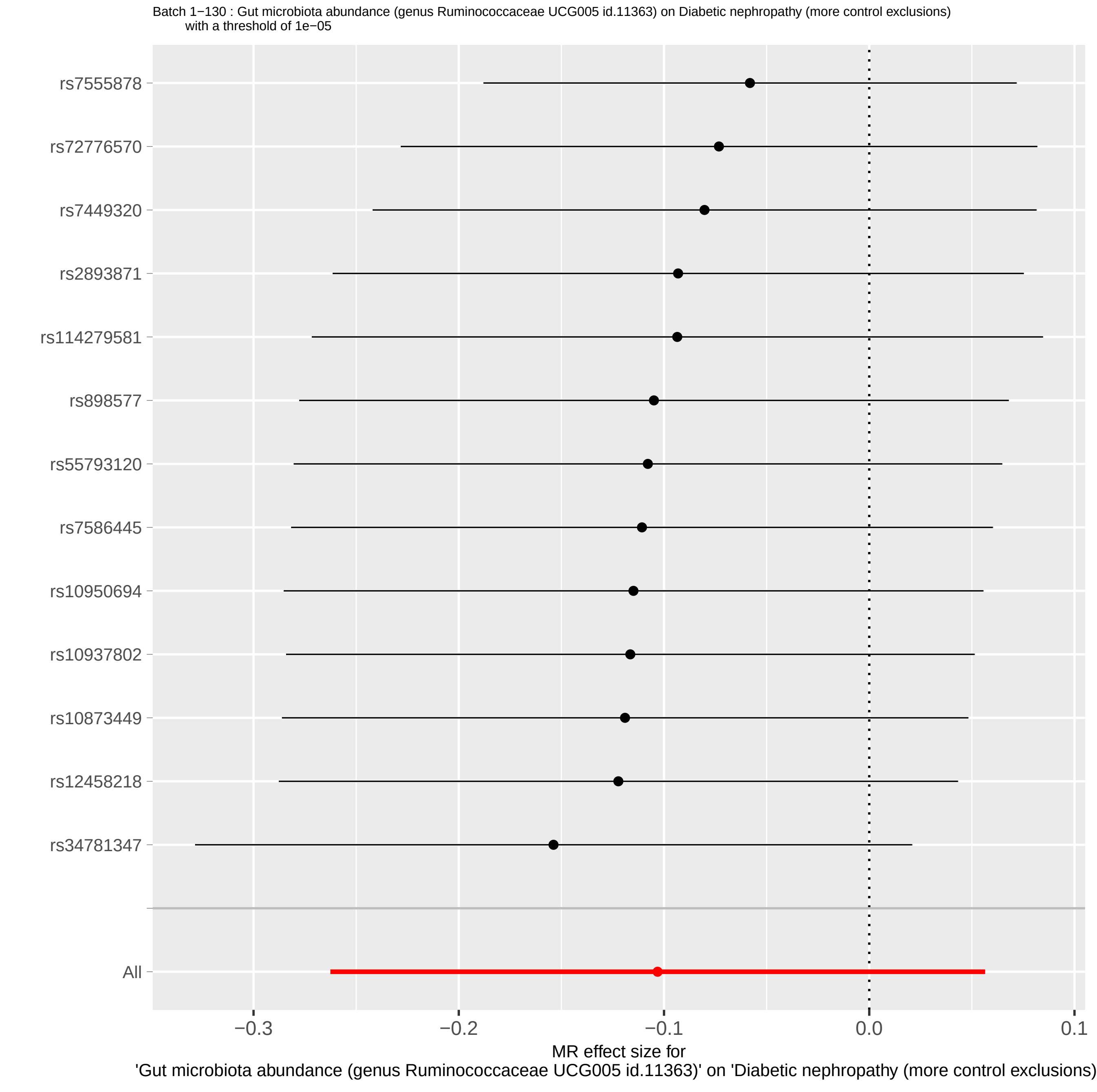
Batch 1−130 : Gut microbiota abundance (genus Ruminococcaceae UCG005 id.11363) on Diabetic nephropathy (more control exclusions) with a threshold of 1e−05
rs7555878
rs72776570
rs7449320
rs2893871
rs114279581
rs898577
rs55793120
rs7586445
rs10950694
rs10937802
rs10873449
rs12458218
rs34781347
All
−0.3
−0.2
−0.1
0.0
0.1
MR effect size for
'Gut microbiota abundance (genus Ruminococcaceae UCG005 id.11363)' on 'Diabetic nephropathy (more control exclusions)'

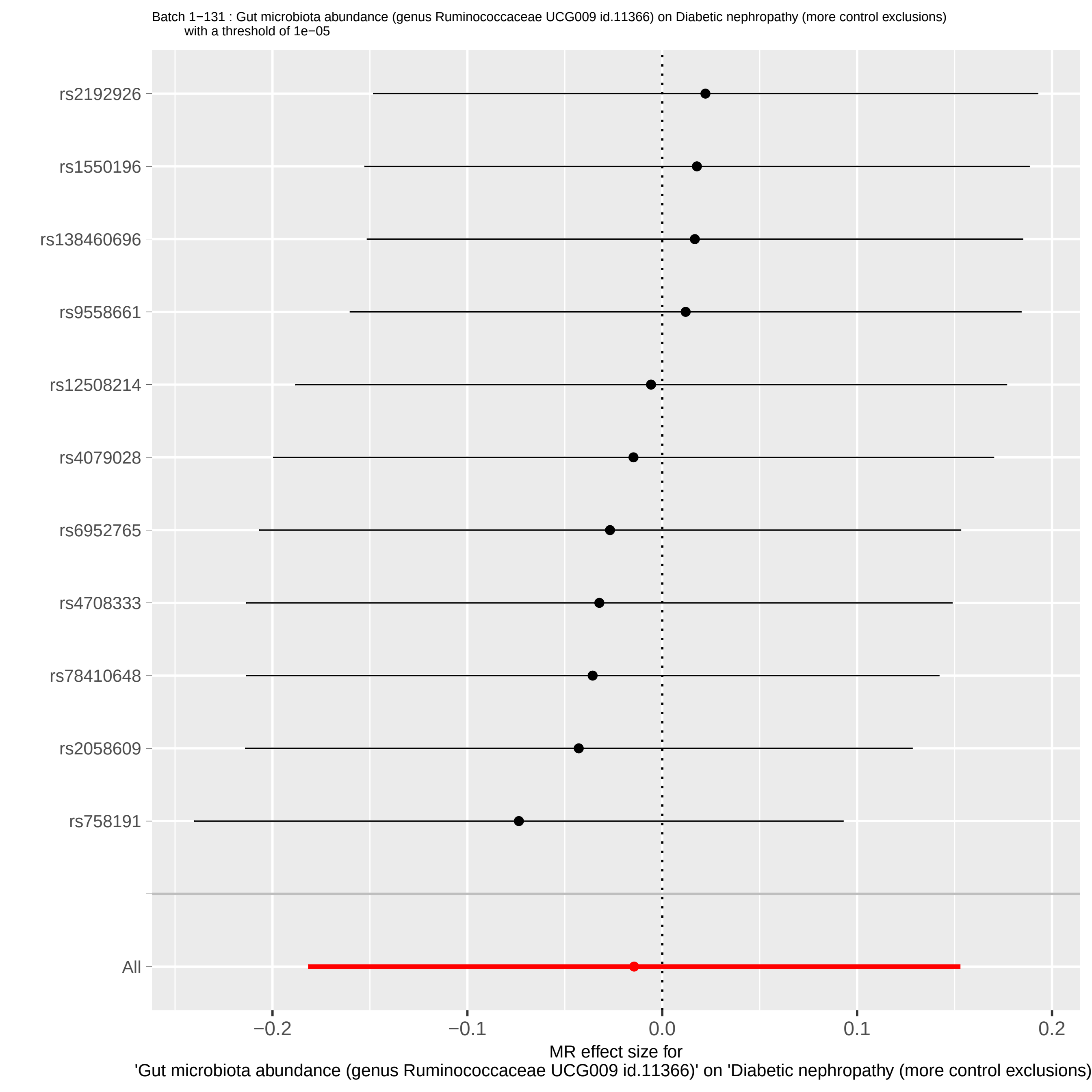
Batch 1−131 : Gut microbiota abundance (genus Ruminococcaceae UCG009 id.11366) on Diabetic nephropathy (more control exclusions) with a threshold of 1e−05
rs2192926
rs1550196
rs138460696
rs9558661
rs12508214
rs4079028
rs6952765
rs4708333
rs78410648
rs2058609
rs758191
All
−0.2
−0.1
0.0
0.1
0.2
MR effect size for
'Gut microbiota abundance (genus Ruminococcaceae UCG009 id.11366)' on 'Diabetic nephropathy (more control exclusions)'

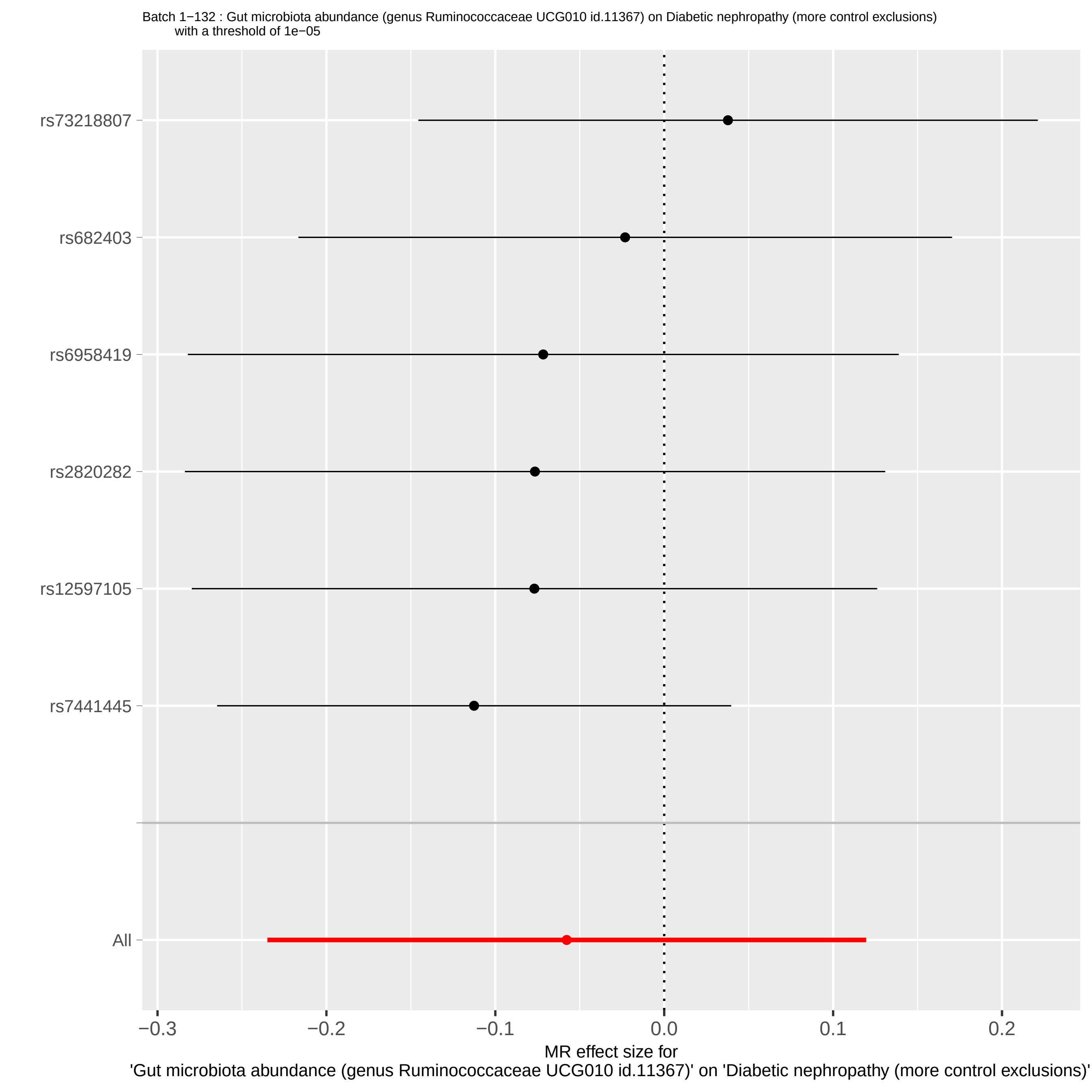

Batch 1−132 : Gut microbiota abundance (genus Ruminococcaceae UCG010 id.11367) on Diabetic nephropathy (more control exclusions)
with a threshold of 1e−05
rs73218807
rs682403
rs6958419
rs2820282
rs12597105
rs7441445
All
−0.3
−0.2
−0.1
0.0
0.1
0.2
MR effect size for
'Gut microbiota abundance (genus Ruminococcaceae UCG010 id.11367)' on 'Diabetic nephropathy (more control exclusions)'

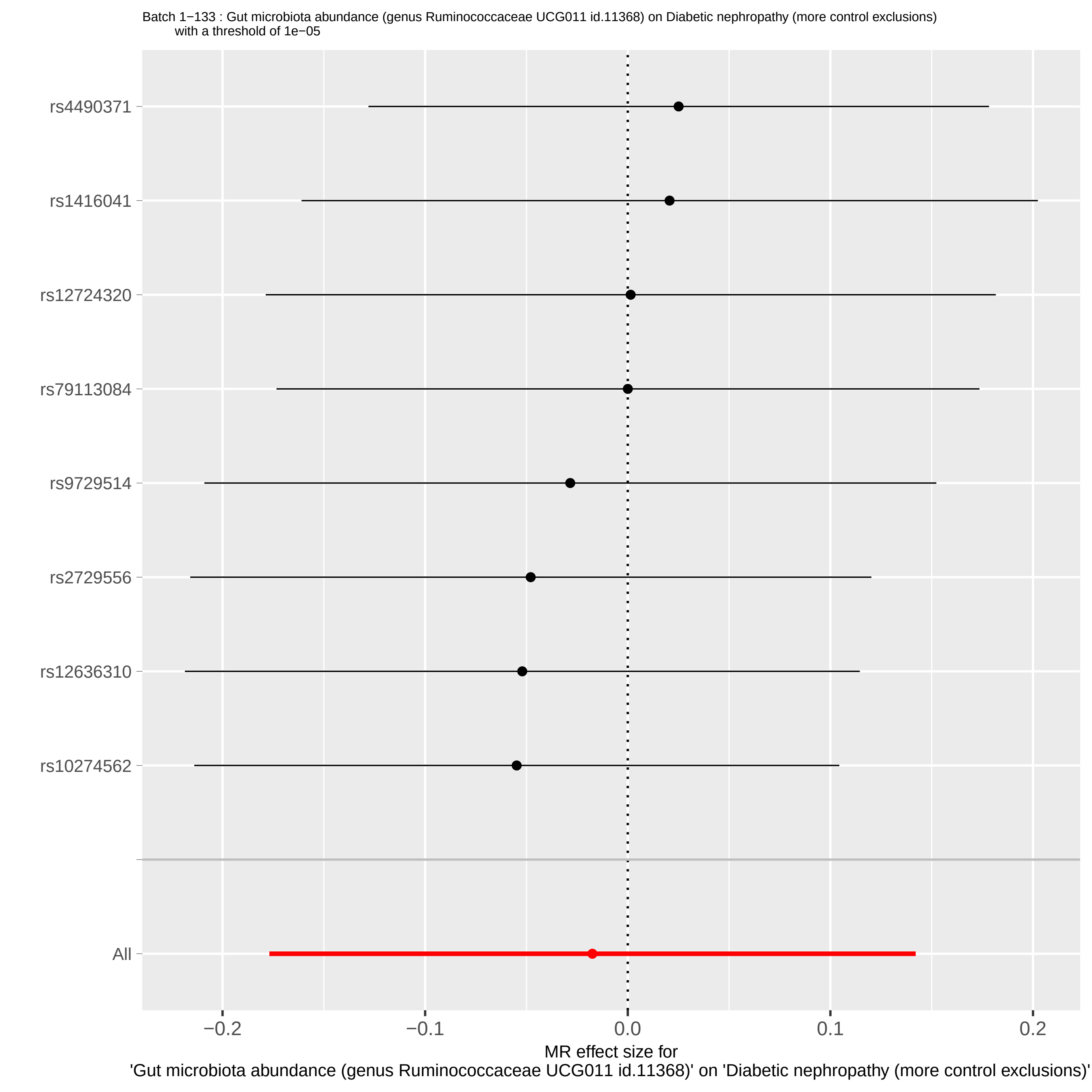

Batch 1−133 : Gut microbiota abundance (genus Ruminococcaceae UCG011 id.11368) on Diabetic nephropathy (more control exclusions)
with a threshold of 1e−05
rs4490371
rs1416041
rs12724320
rs79113084
rs9729514
rs2729556
rs12636310
rs10274562
All
−0.2
−0.1
0.0
0.1
0.2
MR effect size for
'Gut microbiota abundance (genus Ruminococcaceae UCG011 id.11368)' on 'Diabetic nephropathy (more control exclusions)'

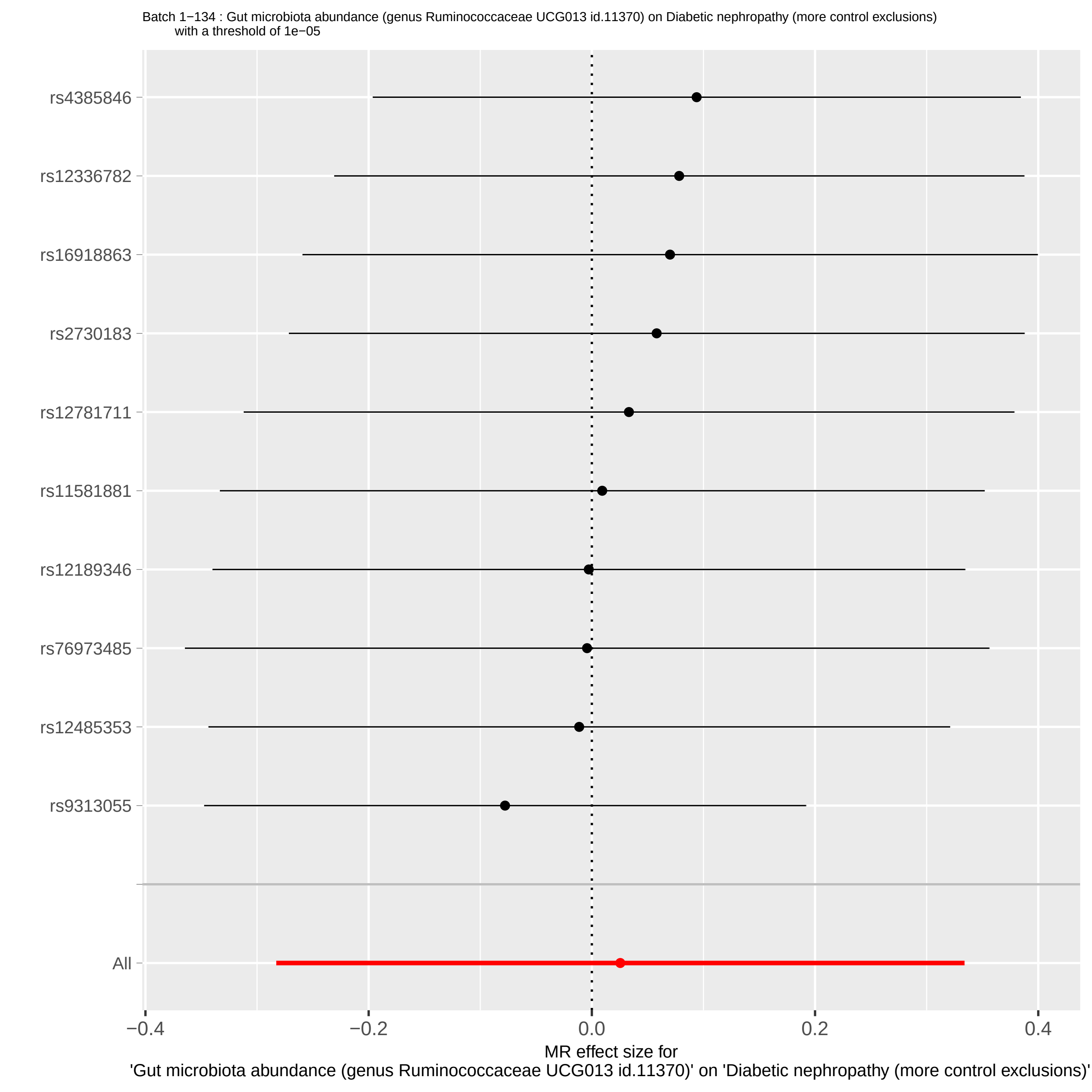
Batch 1−134 : Gut microbiota abundance (genus Ruminococcaceae UCG013 id.11370) on Diabetic nephropathy (more control exclusions)
with a threshold of 1e−05
rs4385846
rs12336782
rs16918863
rs2730183
rs12781711
rs11581881
rs12189346
rs76973485
rs12485353
rs9313055
All
−0.4
−0.2
0.0
0.2
0.4
MR effect size for
'Gut microbiota abundance (genus Ruminococcaceae UCG013 id.11370)' on 'Diabetic nephropathy (more control exclusions)'

Batch 1−135 : Gut microbiota abundance (genus Ruminococcaceae UCG014 id.11371) on Diabetic nephropathy (more control exclusions) with a threshold of 1e−05

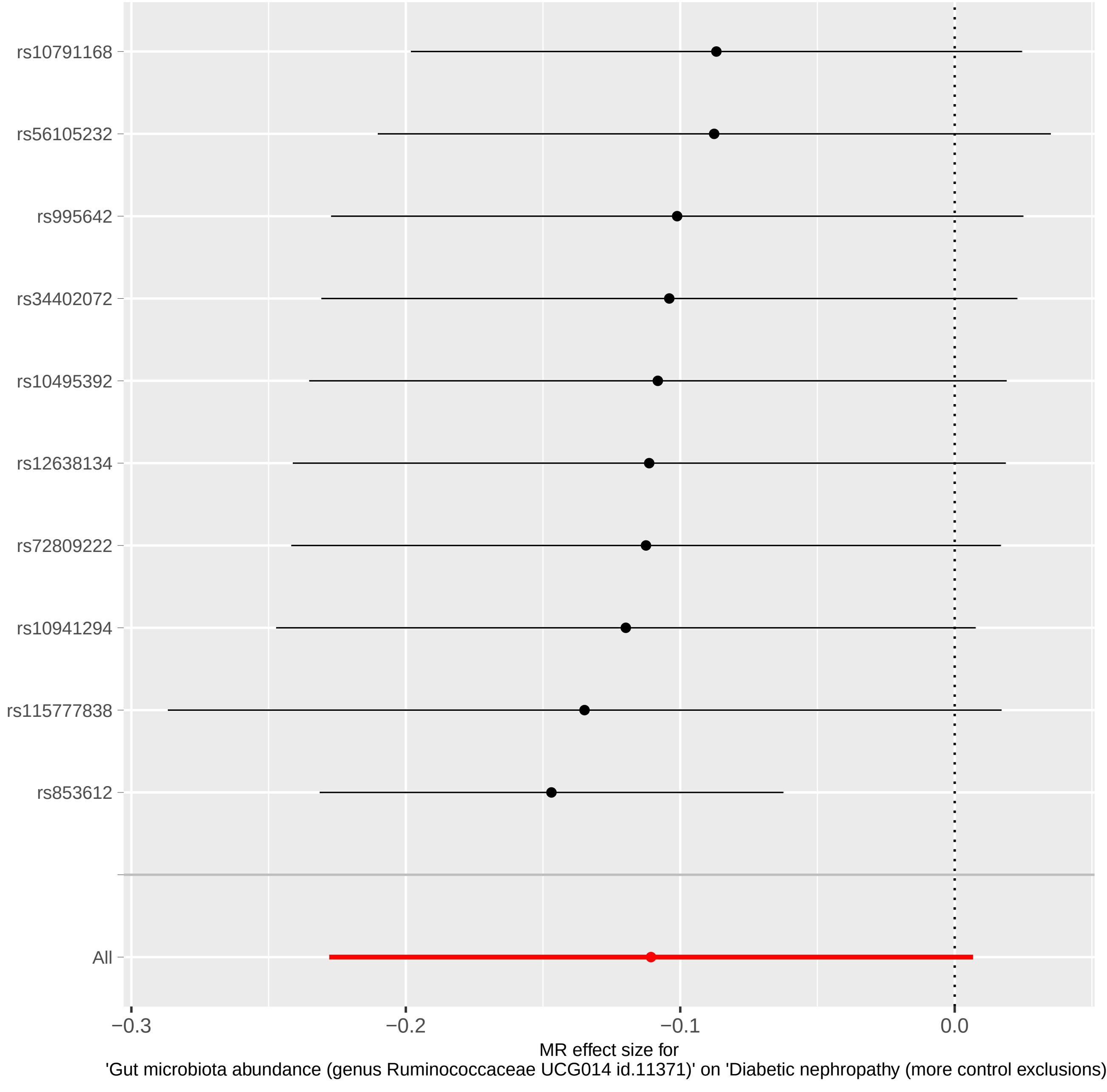

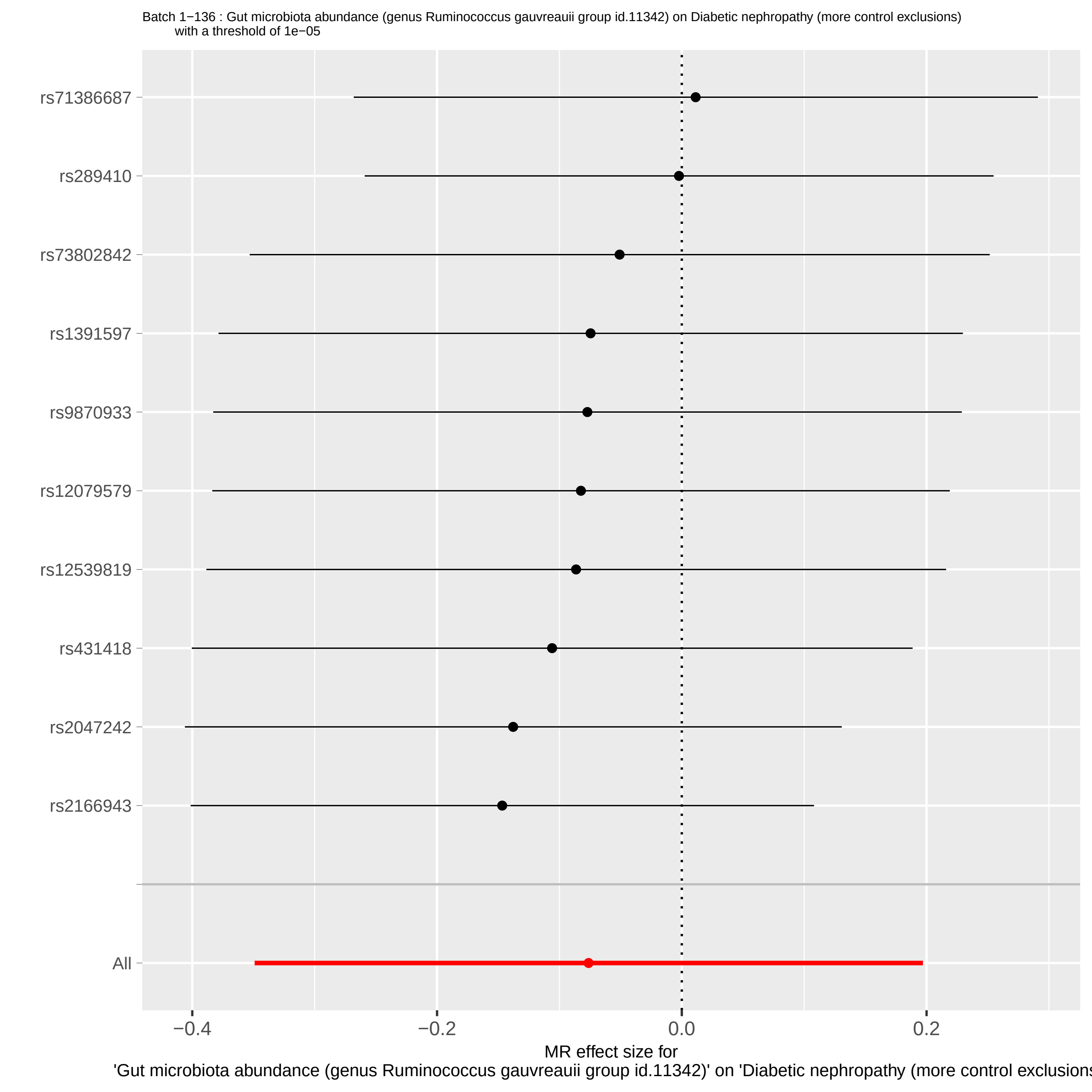

Batch 1−136 : Gut microbiota abundance (genus Ruminococcus gauvreauii group id.11342) on Diabetic nephropathy (more control exclusions)
with a threshold of 1e−05
rs71386687
rs289410
rs73802842
rs1391597
rs9870933
rs12079579
rs12539819
rs431418
rs2047242
rs2166943
All
−0.4
−0.2
0.0
0.2
MR effect size for
'Gut microbiota abundance (genus Ruminococcus gauvreauii group id.11342)' on 'Diabetic nephropathy (more control exclusions

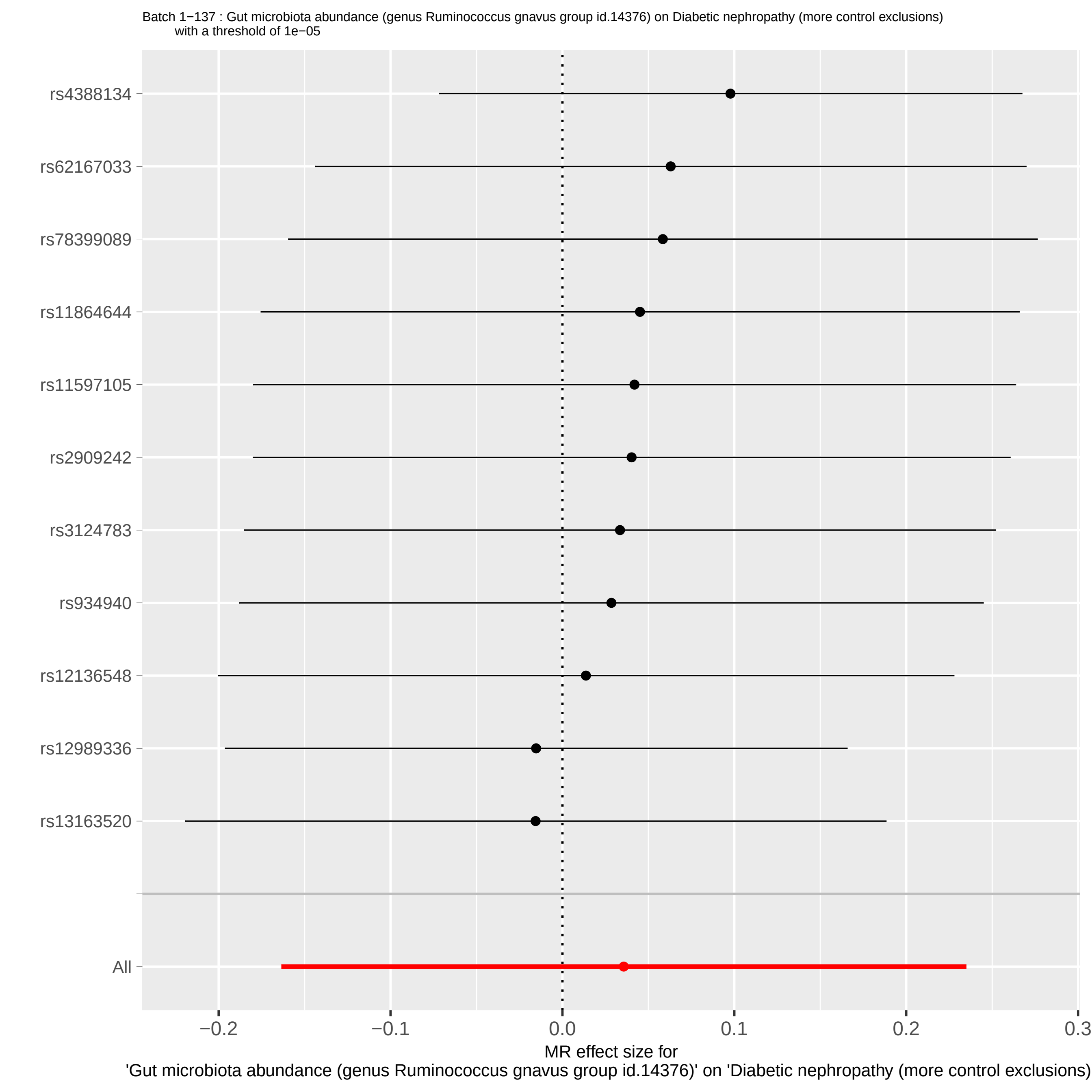
Batch 1−137 : Gut microbiota abundance (genus Ruminococcus gnavus group id.14376) on Diabetic nephropathy (more control exclusions)
with a threshold of 1e−05
rs4388134
rs62167033
rs78399089
rs11864644
rs11597105
rs2909242
rs3124783
rs934940
rs12136548
rs12989336
rs13163520
All
−0.2
−0.1
0.0
0.1
0.2
0.3
MR effect size for
'Gut microbiota abundance (genus Ruminococcus gnavus group id.14376)' on 'Diabetic nephropathy (more control exclusions)'

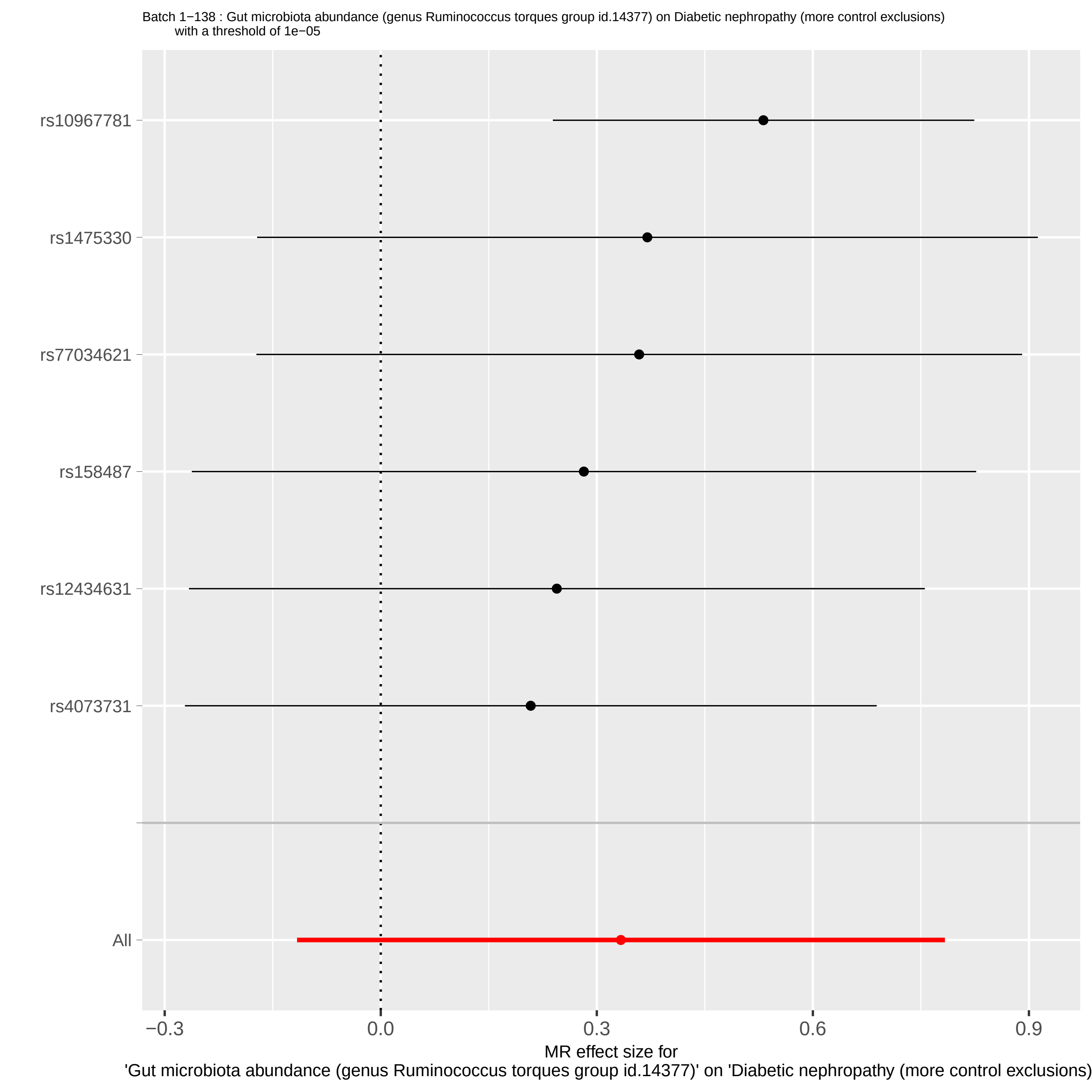

Batch 1−138 : Gut microbiota abundance (genus Ruminococcus torques group id.14377) on Diabetic nephropathy (more control exclusions) with a threshold of 1e−05
rs10967781
rs1475330
rs77034621
rs158487
rs12434631
rs4073731
All
−0.3
0.0
0.3
0.6
0.9
MR effect size for
'Gut microbiota abundance (genus Ruminococcus torques group id.14377)' on 'Diabetic nephropathy (more control exclusions)'

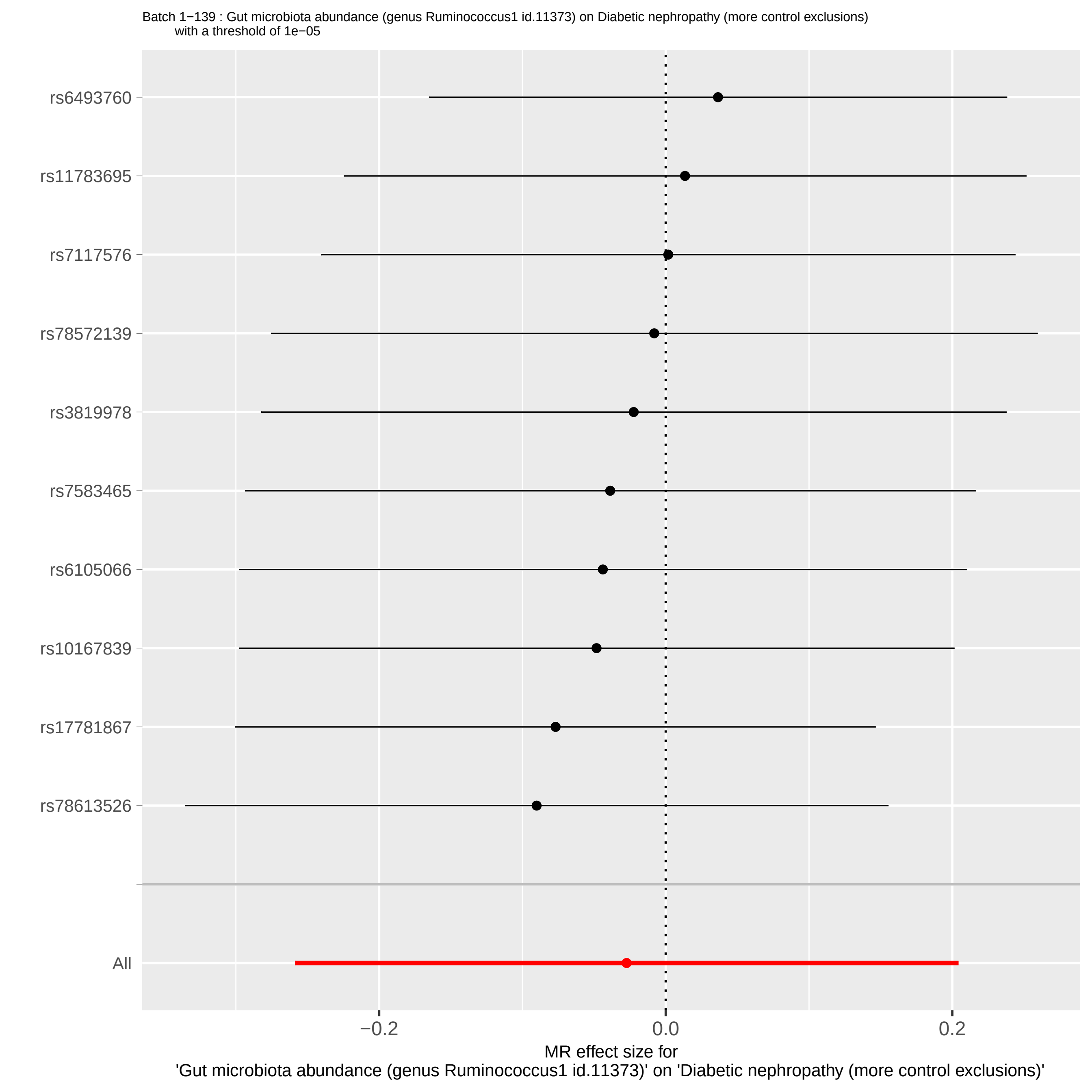
Batch 1−139 : Gut microbiota abundance (genus Ruminococcus1 id.11373) on Diabetic nephropathy (more control exclusions) with a threshold of 1e−05
rs6493760
rs11783695
rs7117576
rs78572139
rs3819978
rs7583465
rs6105066
rs10167839
rs17781867
rs78613526
All
−0.2
0.0
0.2
MR effect size for
'Gut microbiota abundance (genus Ruminococcus1 id.11373)' on 'Diabetic nephropathy (more control exclusions)'

Batch 1−140 : Gut microbiota abundance (genus Ruminococcus2 id.11374) on Diabetic nephropathy (more control exclusions) with a threshold of 1e−05

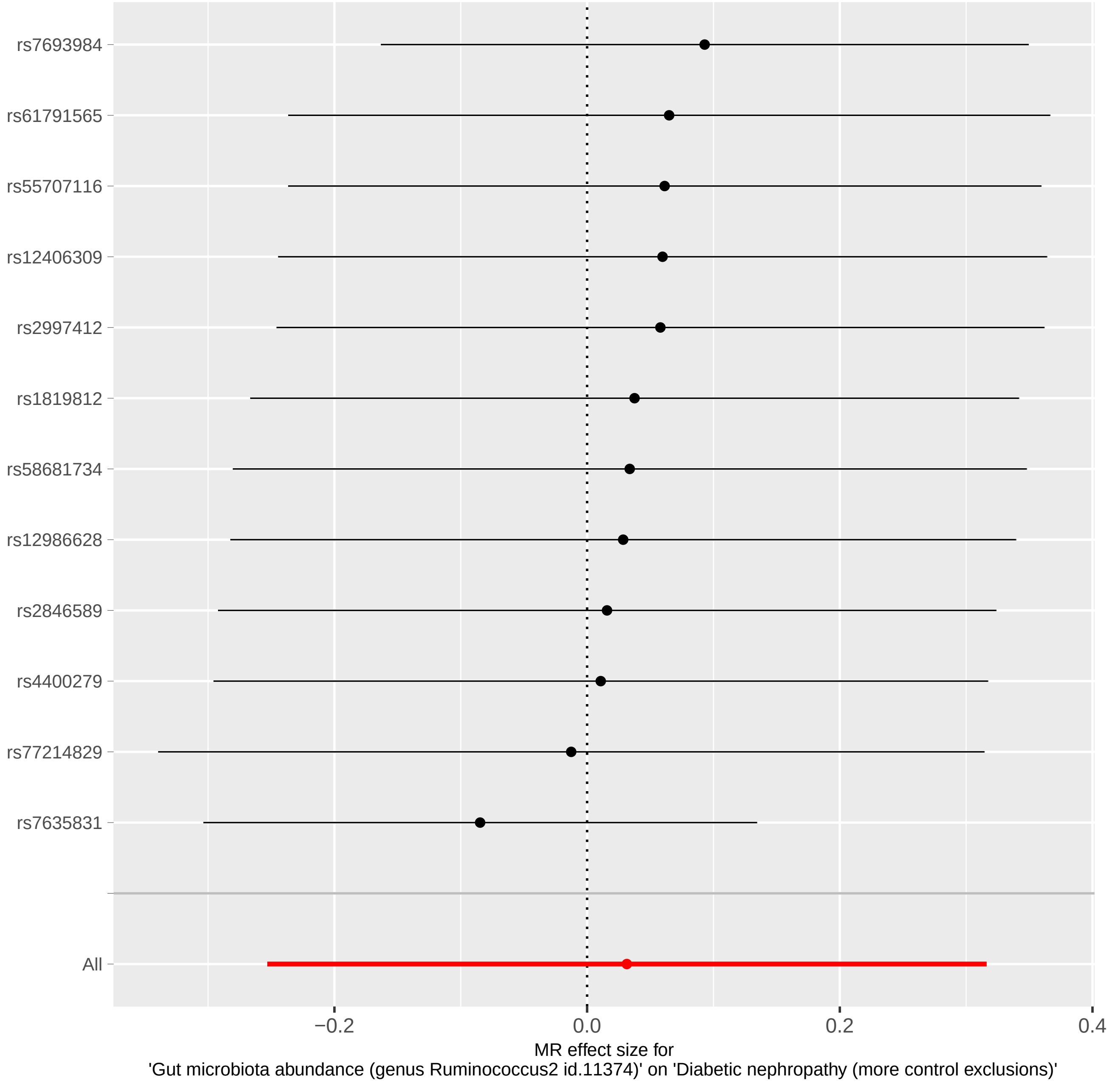

Batch 1−141 : Gut microbiota abundance (genus Sellimonas id.14369) on Diabetic nephropathy (more control exclusions)
with a threshold of 1e−05

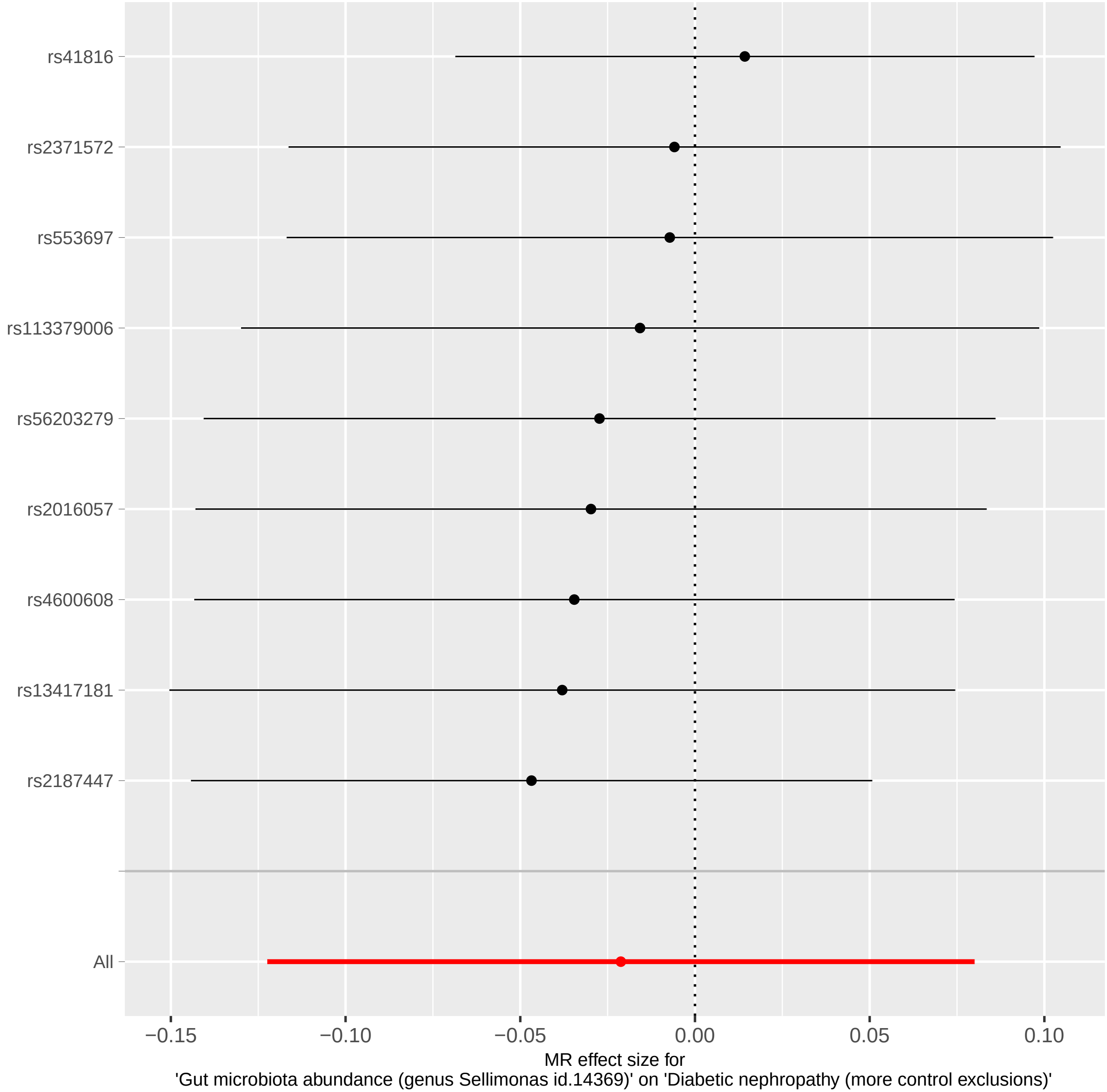

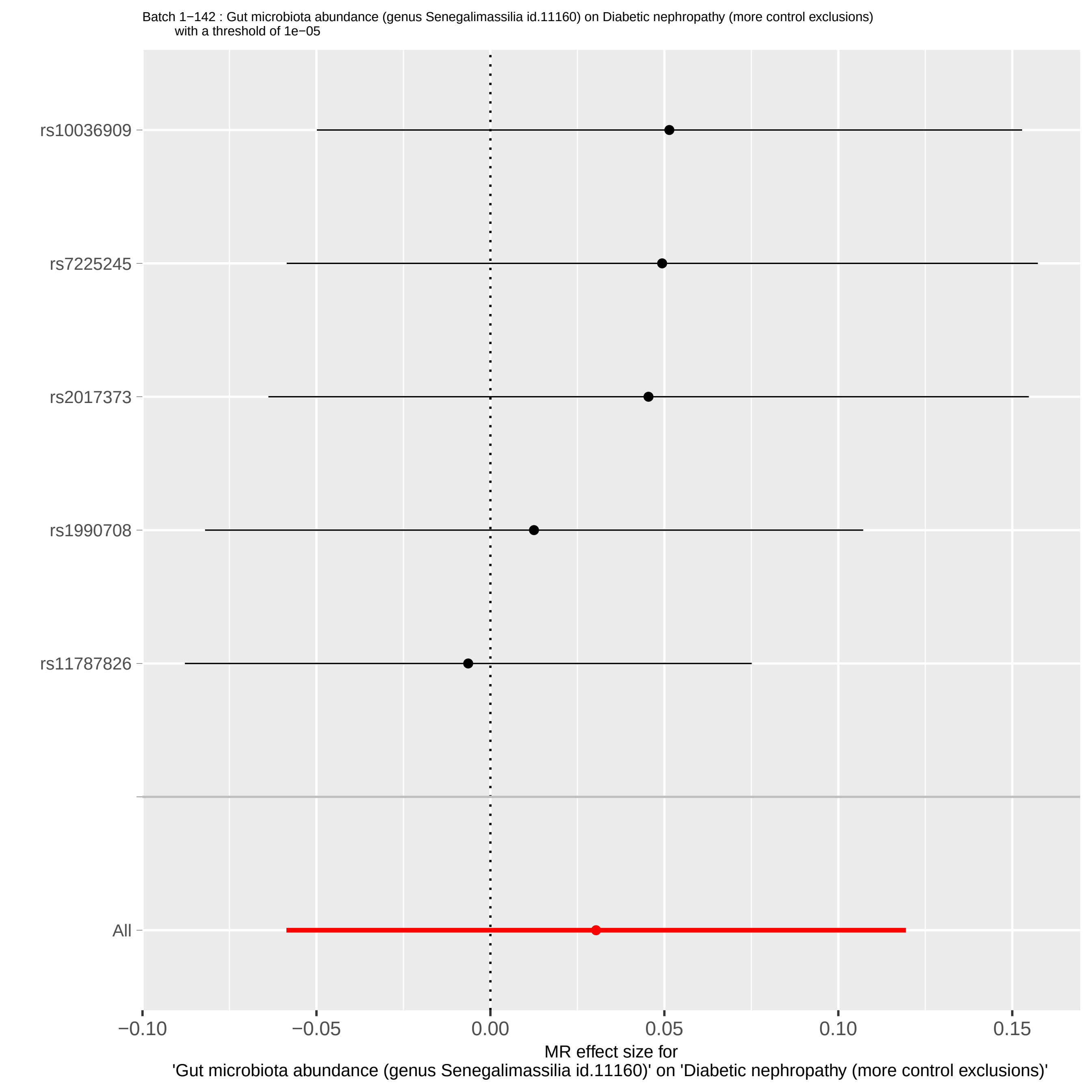

Batch 1−142 : Gut microbiota abundance (genus Senegalimassilia id.11160) on Diabetic nephropathy (more control exclusions)
with a threshold of 1e−05
rs10036909
rs7225245
rs2017373
rs1990708
rs11787826
All
−0.10
−0.05
0.00
0.05
0.10
0.15
MR effect size for
'Gut microbiota abundance (genus Senegalimassilia id.11160)' on 'Diabetic nephropathy (more control exclusions)'

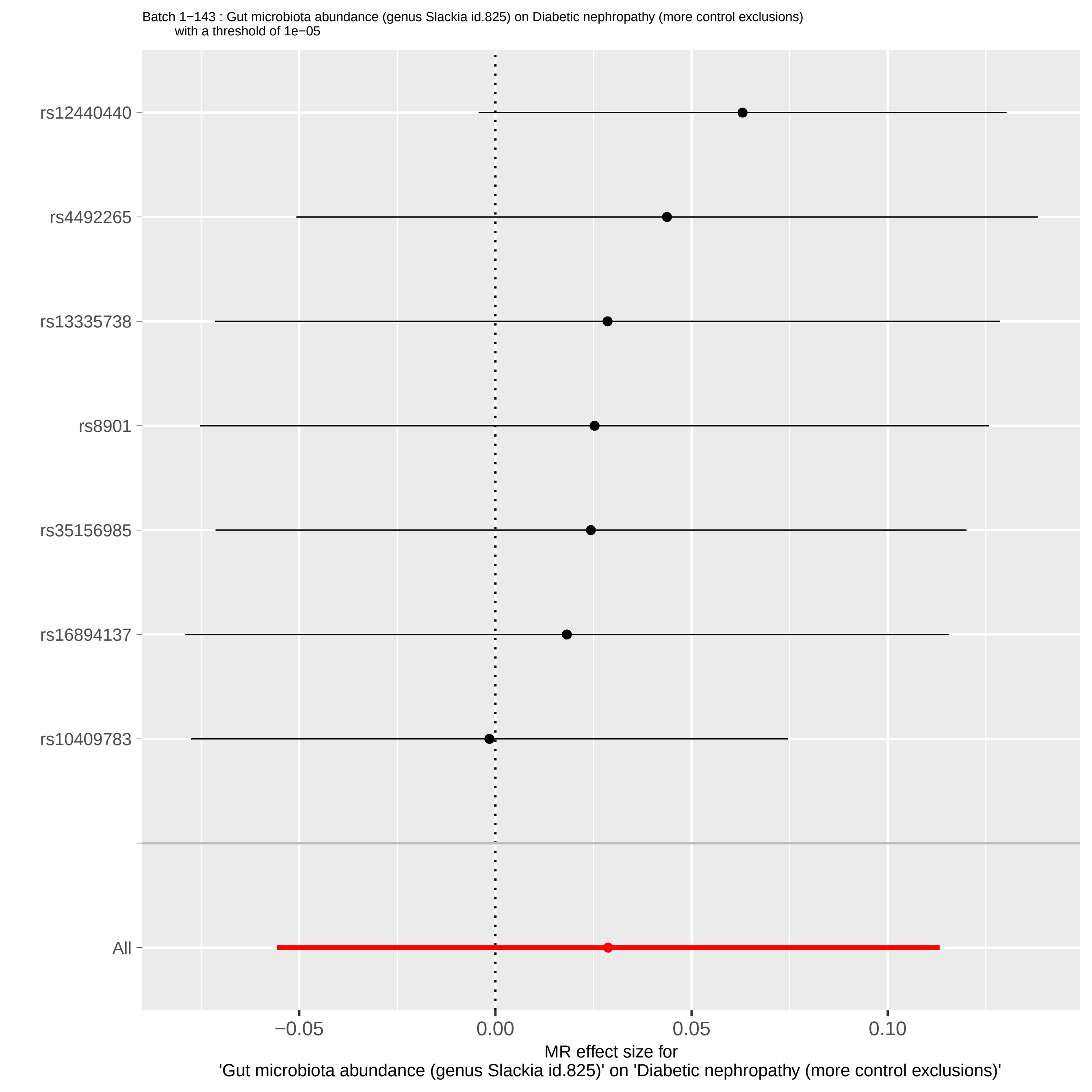
Batch 1−143 : Gut microbiota abundance (genus Slackia id.825) on Diabetic nephropathy (more control exclusions)
with a threshold of 1e−05
rs12440440
rs4492265
rs13335738
rs8901
rs35156985
rs16894137
rs10409783
All
−0.05
0.00
0.05
0.10
MR effect size for
'Gut microbiota abundance (genus Slackia id.825)' on 'Diabetic nephropathy (more control exclusions)'

Batch 1−144 : Gut microbiota abundance (genus Streptococcus id.1853) on Diabetic nephropathy (more control exclusions) with a threshold of 1e−05

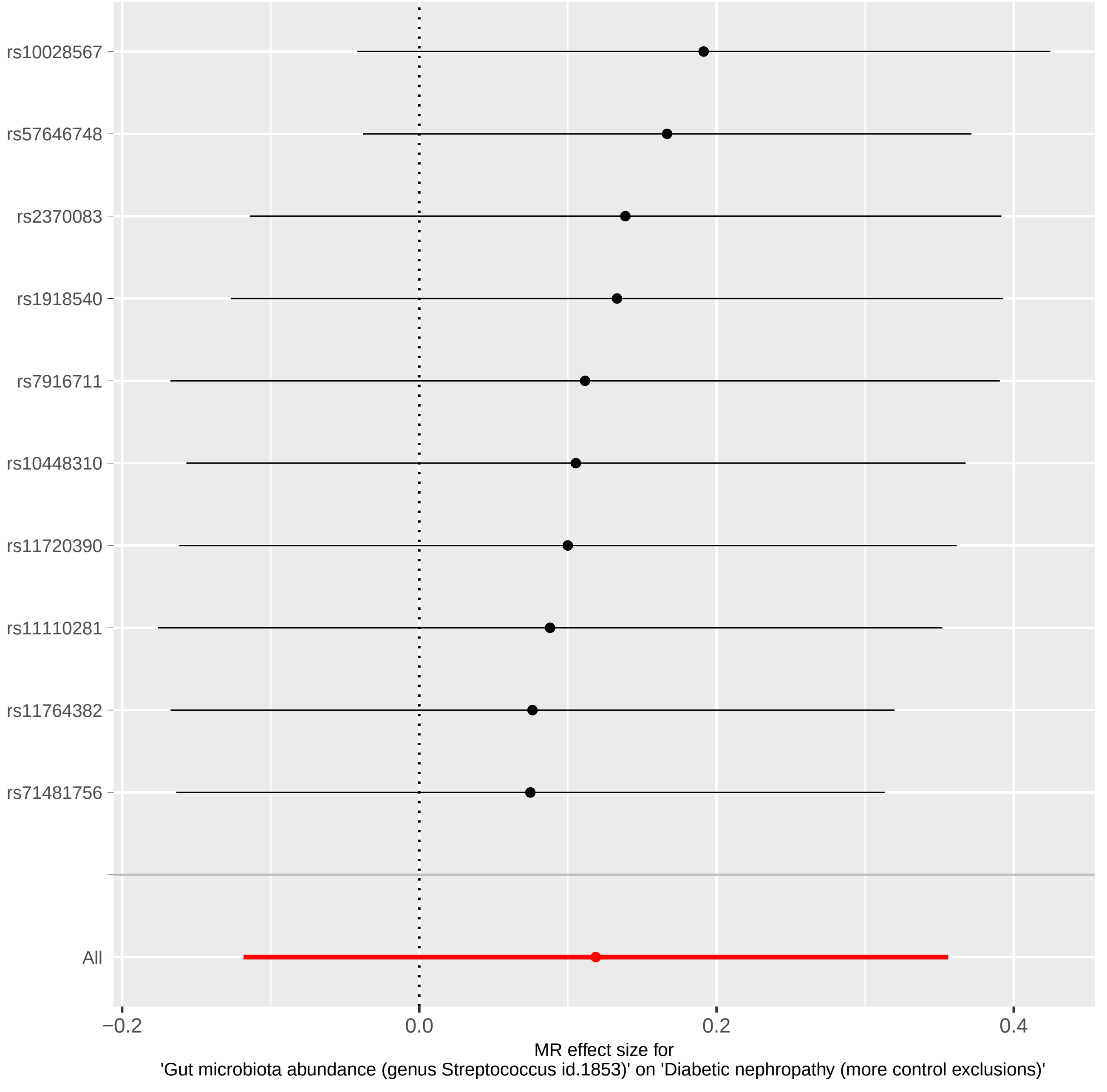

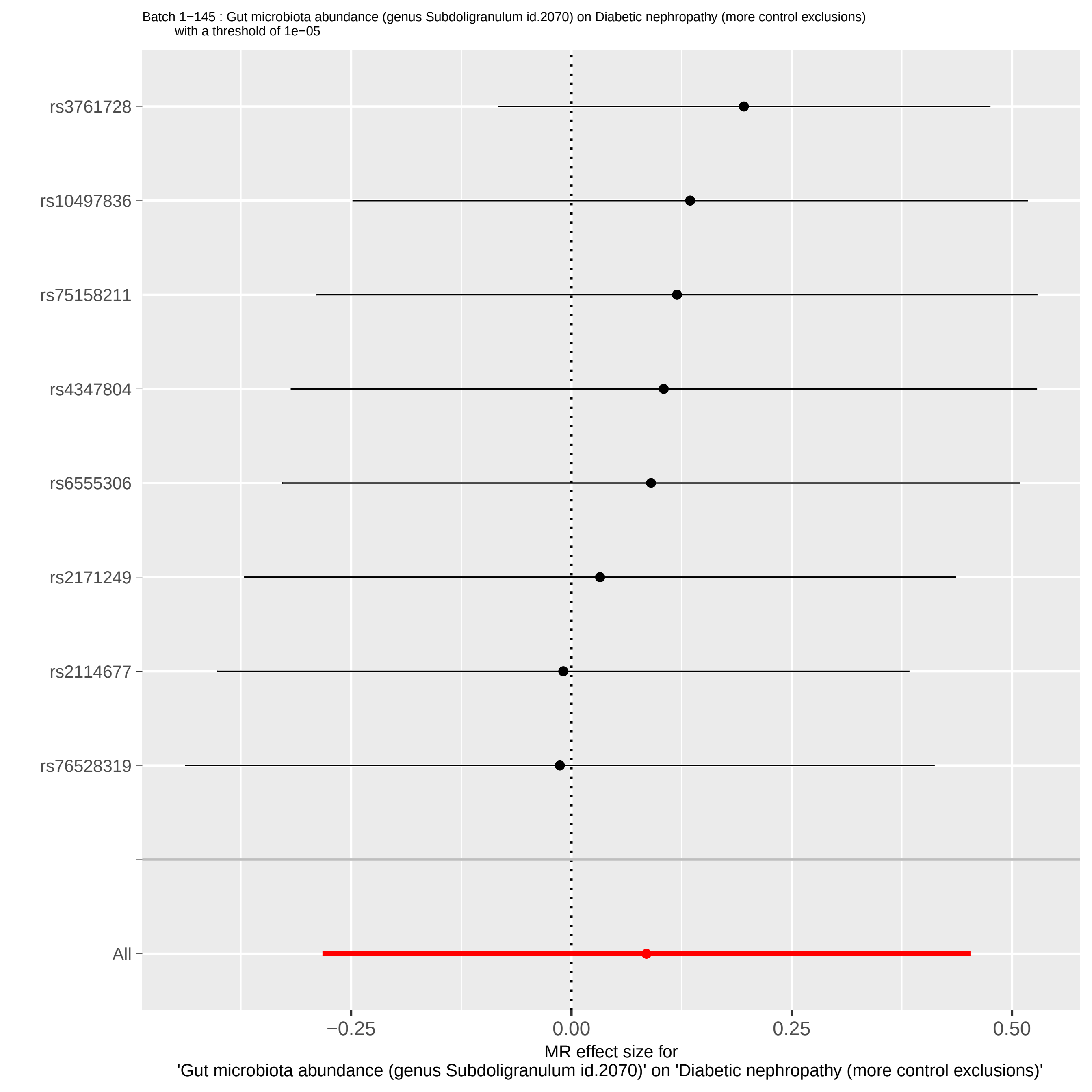

Batch 1−145 : Gut microbiota abundance (genus Subdoligranulum id.2070) on Diabetic nephropathy (more control exclusions)
with a threshold of 1e−05
rs3761728
rs10497836
rs75158211
rs4347804
rs6555306
rs2171249
rs2114677
rs76528319
All
−0.25
0.00
0.25
0.50
MR effect size for
'Gut microbiota abundance (genus Subdoligranulum id.2070)' on 'Diabetic nephropathy (more control exclusions)'

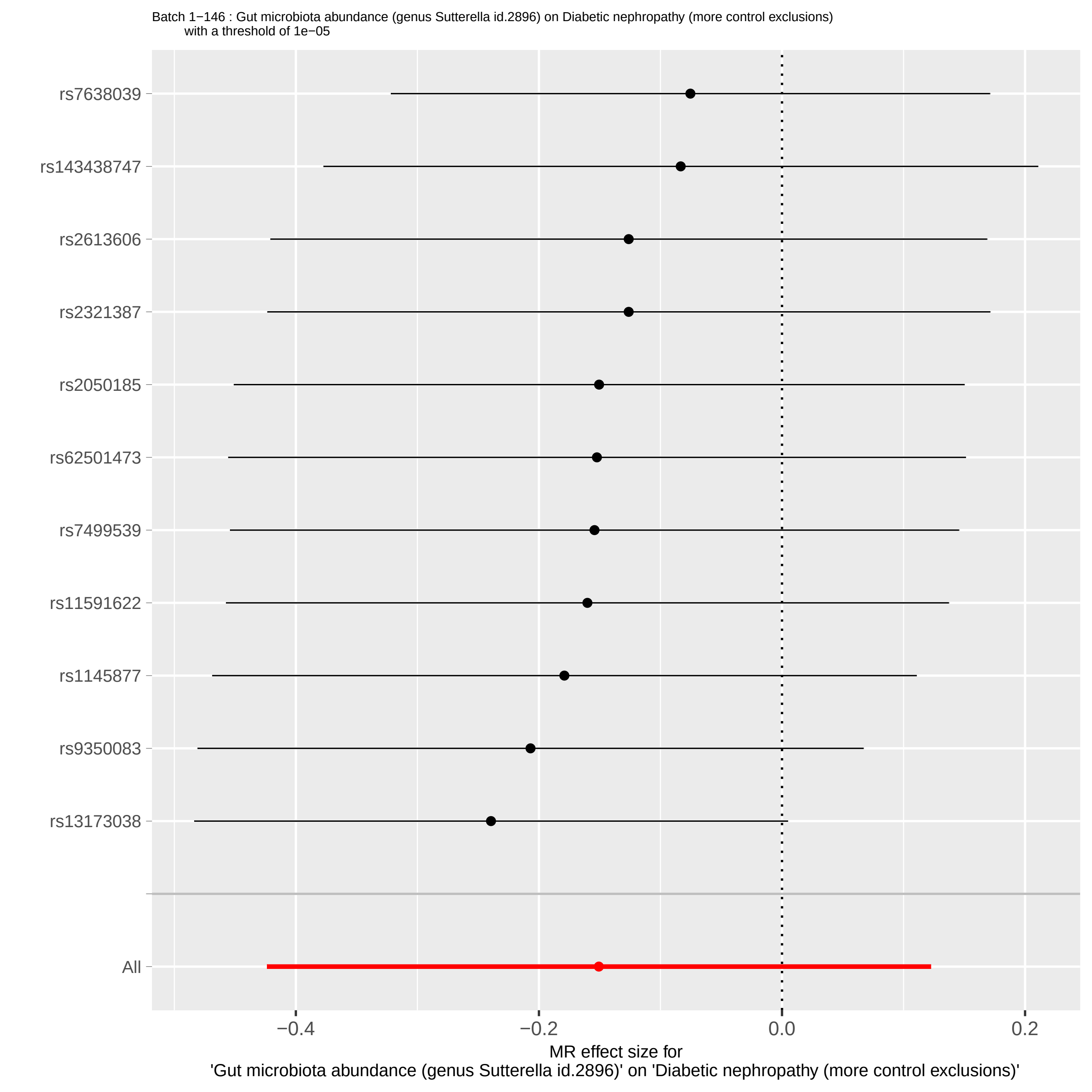

Batch 1−146 : Gut microbiota abundance (genus Sutterella id.2896) on Diabetic nephropathy (more control exclusions)
with a threshold of 1e−05
rs7638039
rs143438747
rs2613606
rs2321387
rs2050185
rs62501473
rs7499539
rs11591622
rs1145877
rs9350083
rs13173038
All
−0.4
−0.2
0.0
0.2
MR effect size for
'Gut microbiota abundance (genus Sutterella id.2896)' on 'Diabetic nephropathy (more control exclusions)'

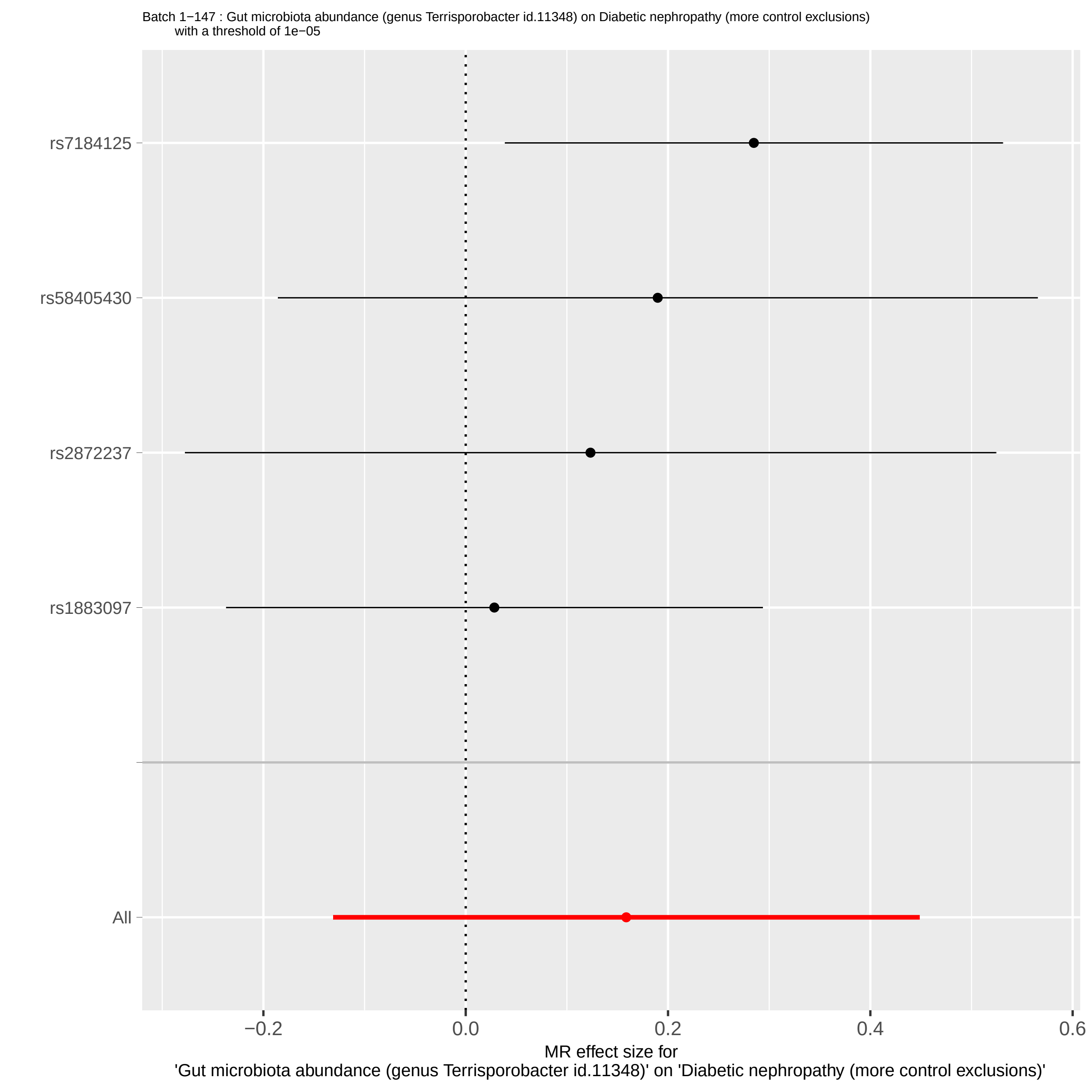
Batch 1−147 : Gut microbiota abundance (genus Terrisporobacter id.11348) on Diabetic nephropathy (more control exclusions)
with a threshold of 1e−05
rs7184125
rs58405430
rs2872237
rs1883097
All
−0.2
0.0
0.2
0.4
0.6
MR effect size for
'Gut microbiota abundance (genus Terrisporobacter id.11348)' on 'Diabetic nephropathy (more control exclusions)'

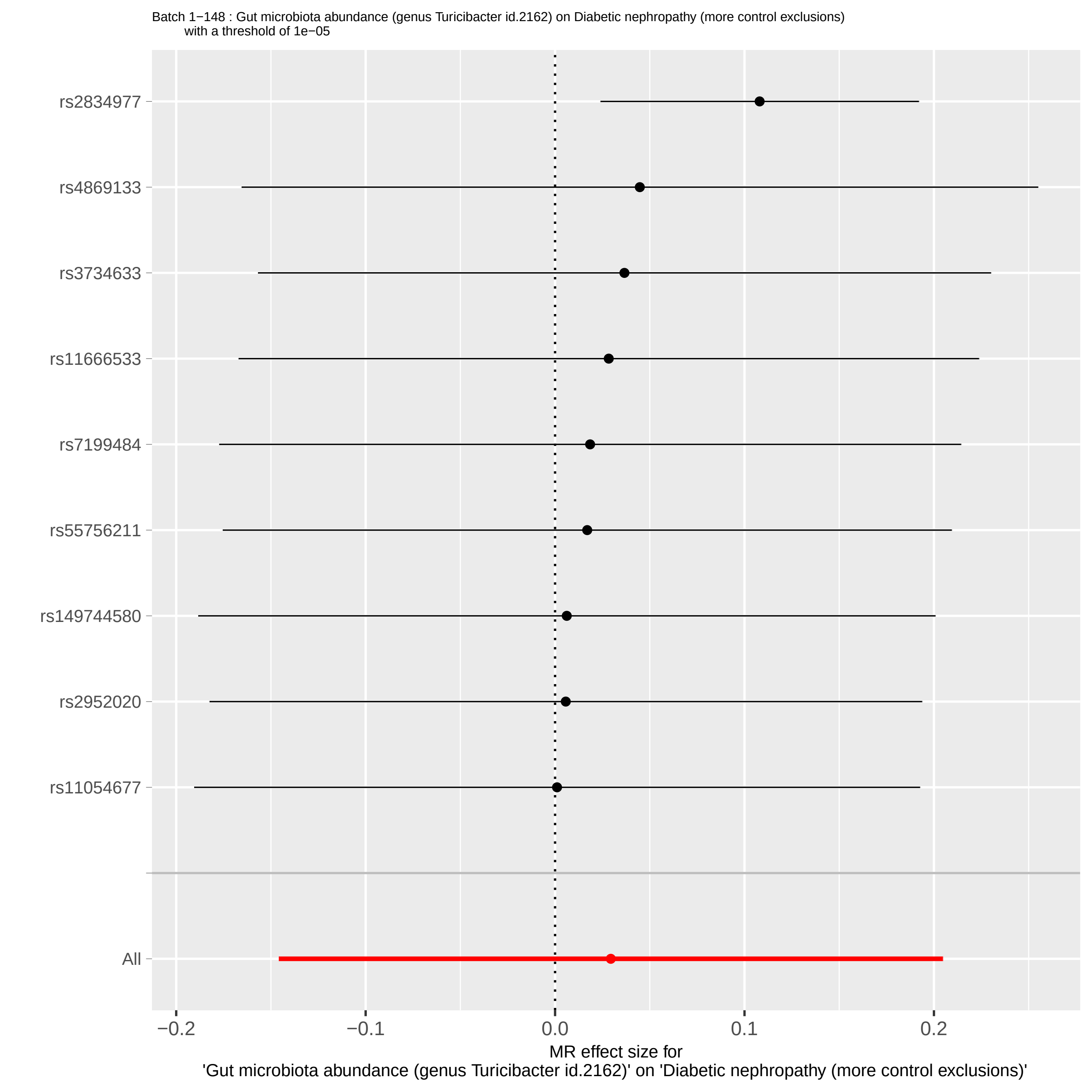
Batch 1−148 : Gut microbiota abundance (genus Turicibacter id.2162) on Diabetic nephropathy (more control exclusions)
with a threshold of 1e−05
rs2834977
rs4869133
rs3734633
rs11666533
rs7199484
rs55756211
rs149744580
rs2952020
rs11054677
All
−0.2
−0.1
0.0
0.1
0.2
MR effect size for
'Gut microbiota abundance (genus Turicibacter id.2162)' on 'Diabetic nephropathy (more control exclusions)'

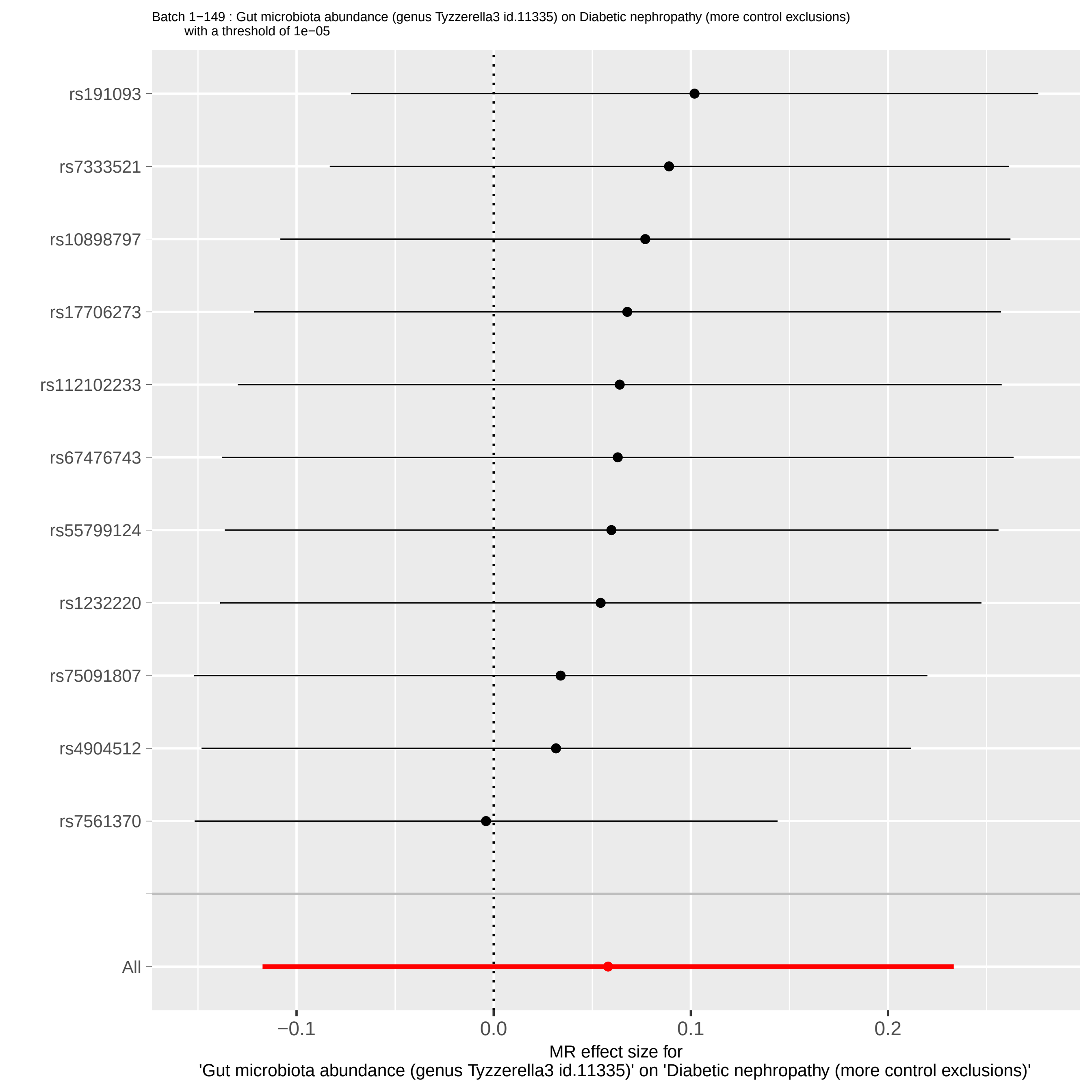

Batch 1−149 : Gut microbiota abundance (genus Tyzzerella3 id.11335) on Diabetic nephropathy (more control exclusions)
with a threshold of 1e−05
rs191093
rs7333521
rs10898797
rs17706273
rs112102233
rs67476743
rs55799124
rs1232220
rs75091807
rs4904512
rs7561370
All
−0.1
0.0
0.1
0.2
MR effect size for
'Gut microbiota abundance (genus Tyzzerella3 id.11335)' on 'Diabetic nephropathy (more control exclusions)'

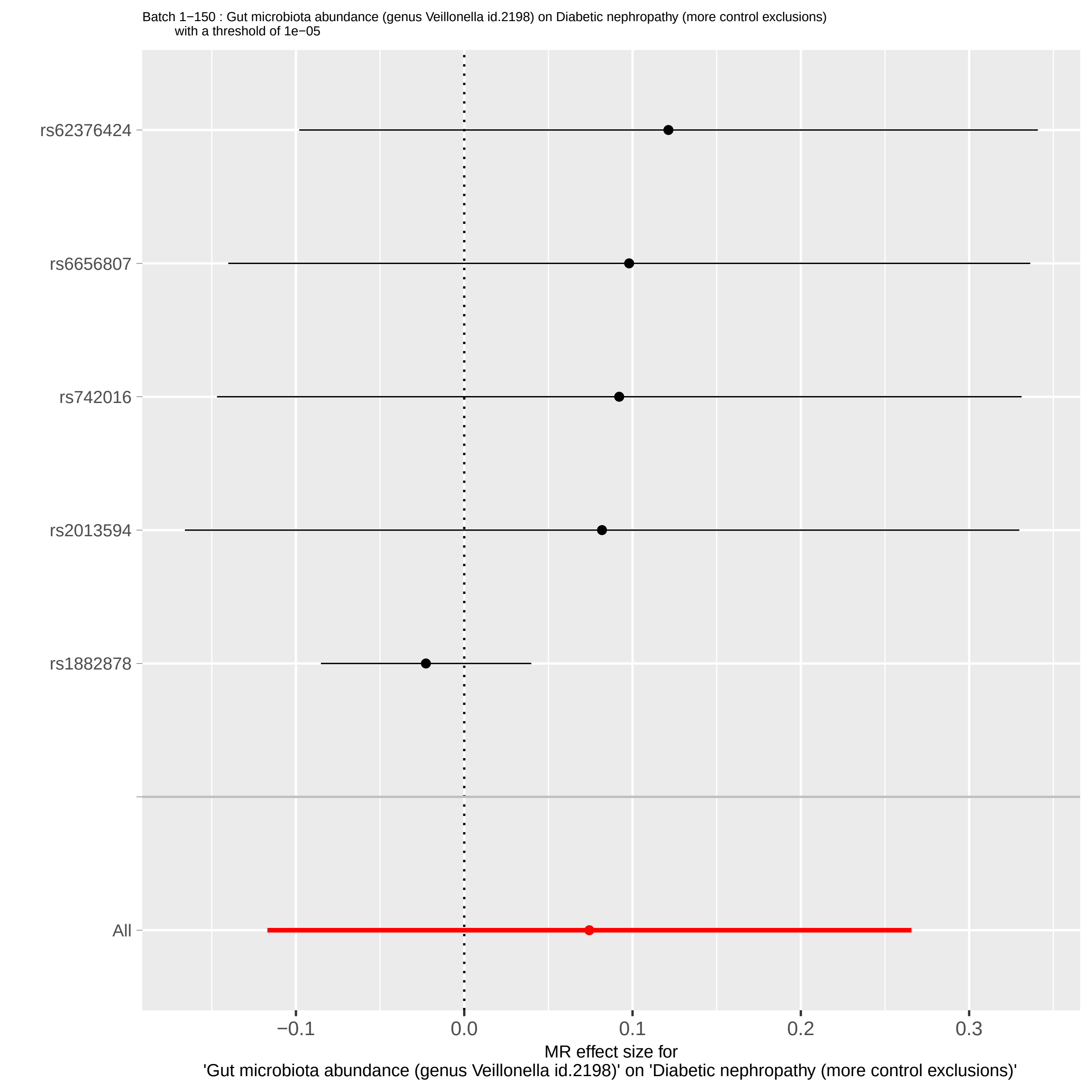

Batch 1−150 : Gut microbiota abundance (genus Veillonella id.2198) on Diabetic nephropathy (more control exclusions)
with a threshold of 1e−05
rs62376424
rs6656807
rs742016
rs2013594
rs1882878
All
−0.1
0.0
0.1
0.2
0.3
MR effect size for
'Gut microbiota abundance (genus Veillonella id.2198)' on 'Diabetic nephropathy (more control exclusions)'

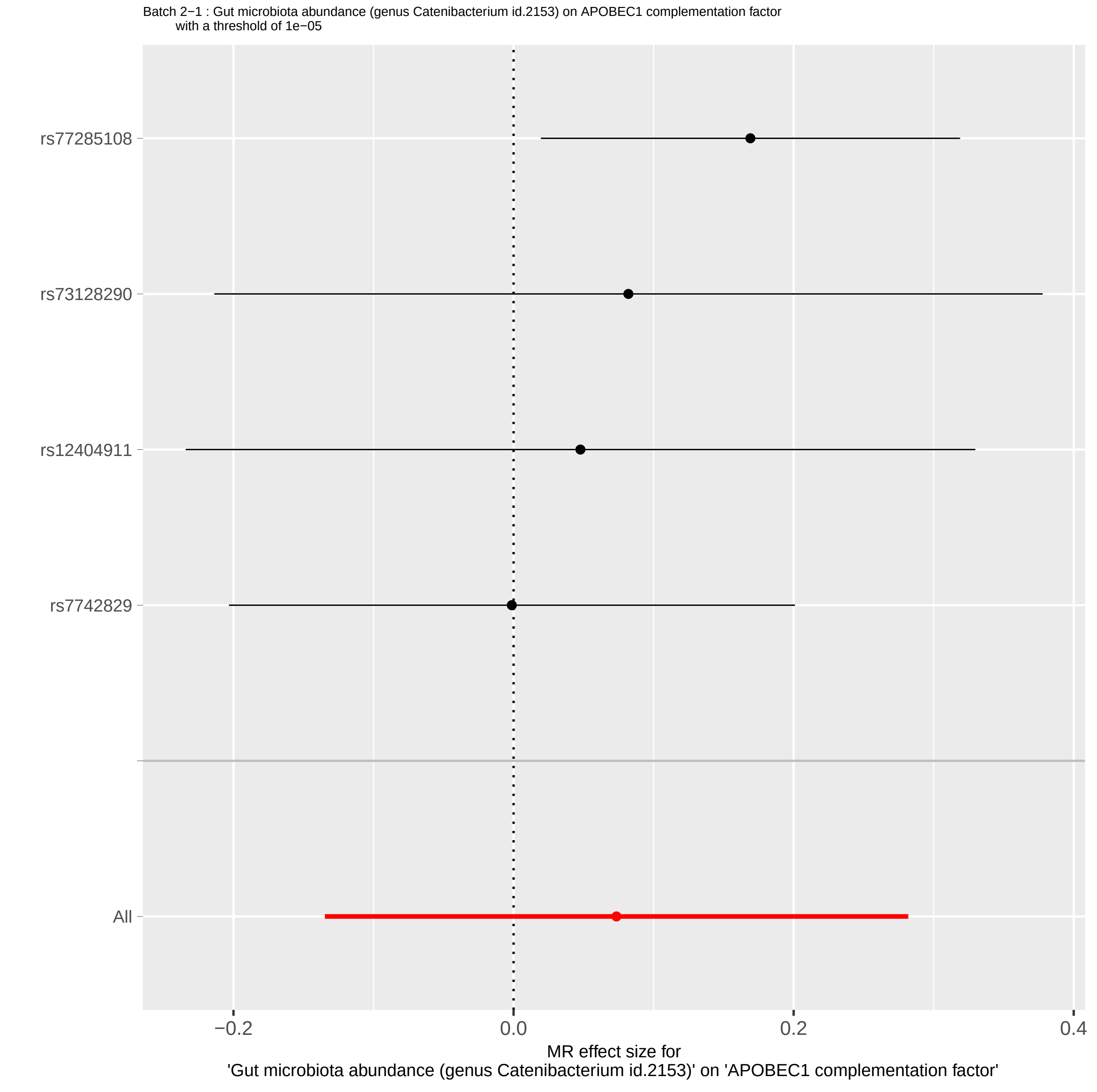

Batch 2−1 : Gut microbiota abundance (genus Catenibacterium id.2153) on APOBEC1 complementation factor
with a threshold of 1e−05
rs77285108
rs73128290
rs12404911
rs7742829
All
−0.2
0.0
0.2
0.4
MR effect size for
'Gut microbiota abundance (genus Catenibacterium id.2153)' on 'APOBEC1 complementation factor'

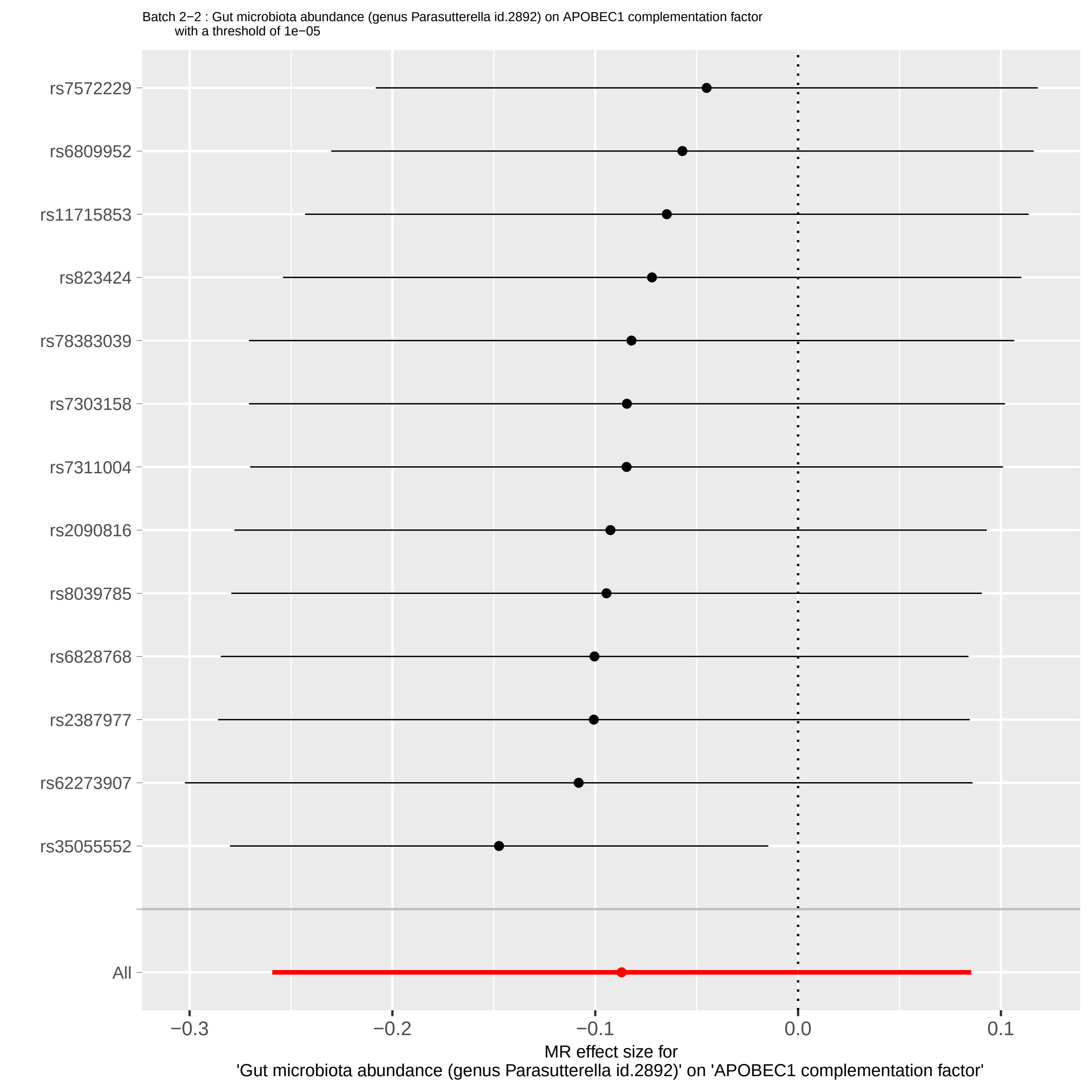

Batch 2−2 : Gut microbiota abundance (genus Parasutterella id.2892) on APOBEC1 complementation factor
with a threshold of 1e−05
rs7572229
rs6809952
rs11715853
rs823424
rs78383039
rs7303158
rs7311004
rs2090816
rs8039785
rs6828768
rs2387977
rs62273907
rs35055552
All
−0.3
−0.2
−0.1
0.0
0.1
MR effect size for
'Gut microbiota abundance (genus Parasutterella id.2892)' on 'APOBEC1 complementation factor'

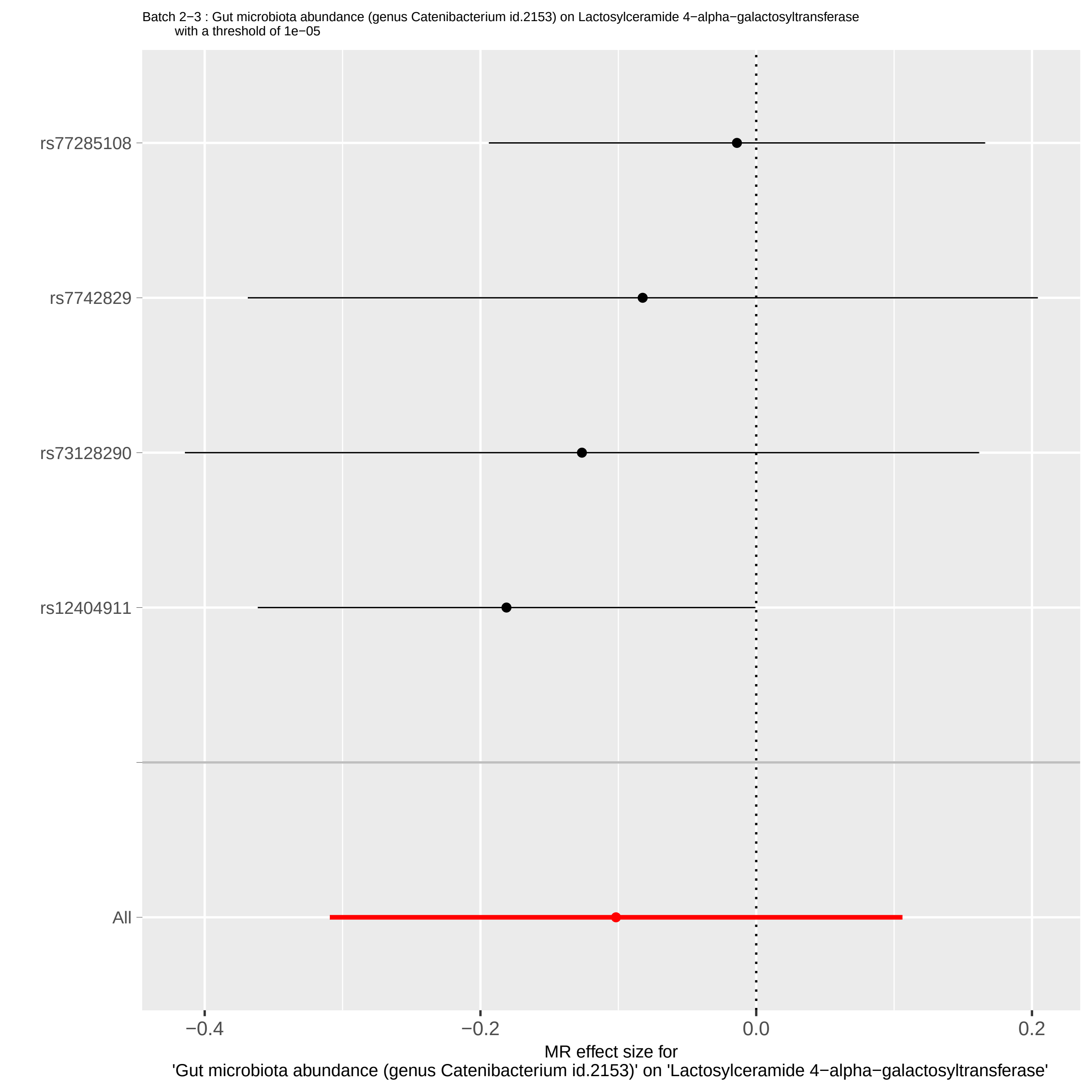

Batch 2−3 : Gut microbiota abundance (genus Catenibacterium id.2153) on Lactosylceramide 4−alpha−galactosyltransferase
with a threshold of 1e−05
rs77285108
rs7742829
rs73128290
rs12404911
All
−0.4
−0.2
0.0
0.2
MR effect size for
'Gut microbiota abundance (genus Catenibacterium id.2153)' on 'Lactosylceramide 4−alpha−galactosyltransferase'

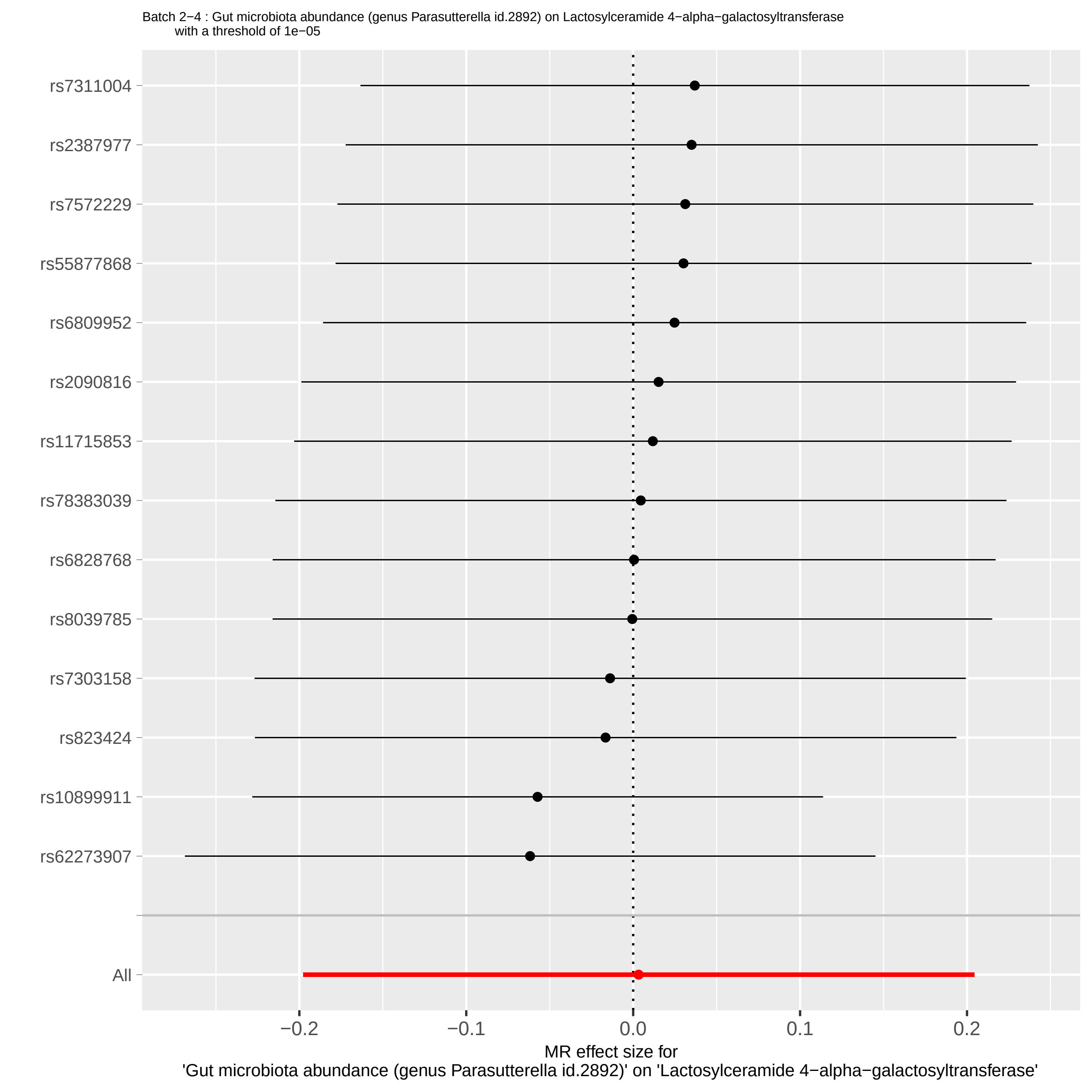

Batch 2−4 : Gut microbiota abundance (genus Parasutterella id.2892) on Lactosylceramide 4−alpha−galactosyltransferase
with a threshold of 1e−05
rs7311004
rs2387977
rs7572229
rs55877868
rs6809952
rs2090816
rs11715853
rs78383039
rs6828768
rs8039785
rs7303158
rs823424
rs10899911
rs62273907
All
−0.2
−0.1
0.0
0.1
0.2
MR effect size for
'Gut microbiota abundance (genus Parasutterella id.2892)' on 'Lactosylceramide 4−alpha−galactosyltransferase'

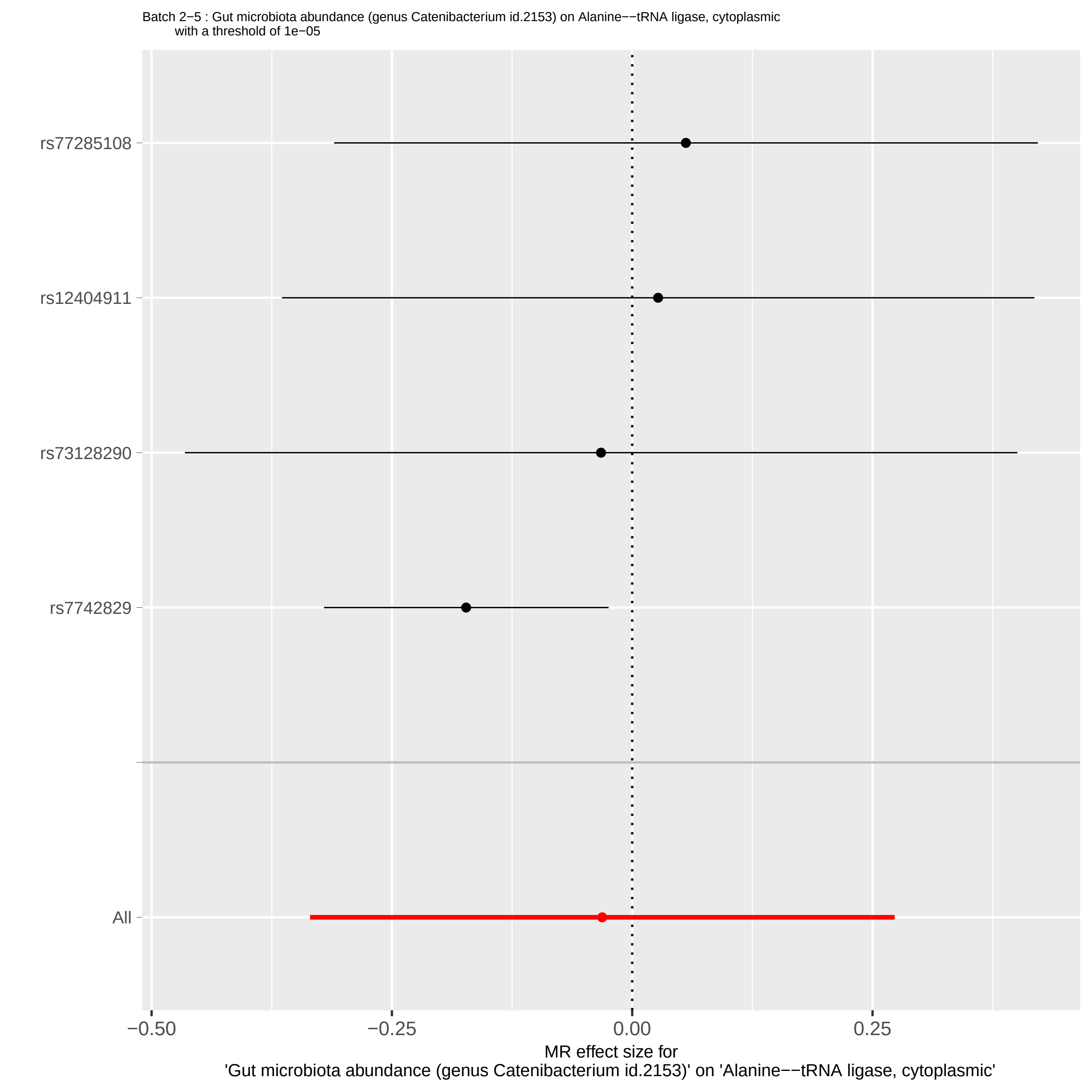

Batch 2−5 : Gut microbiota abundance (genus Catenibacterium id.2153) on Alanine−−tRNA ligase, cytoplasmic
with a threshold of 1e−05
rs77285108
rs12404911
rs73128290
rs7742829
All
−0.50
−0.25
0.00
0.25
MR effect size for
'Gut microbiota abundance (genus Catenibacterium id.2153)' on 'Alanine−−tRNA ligase, cytoplasmic'

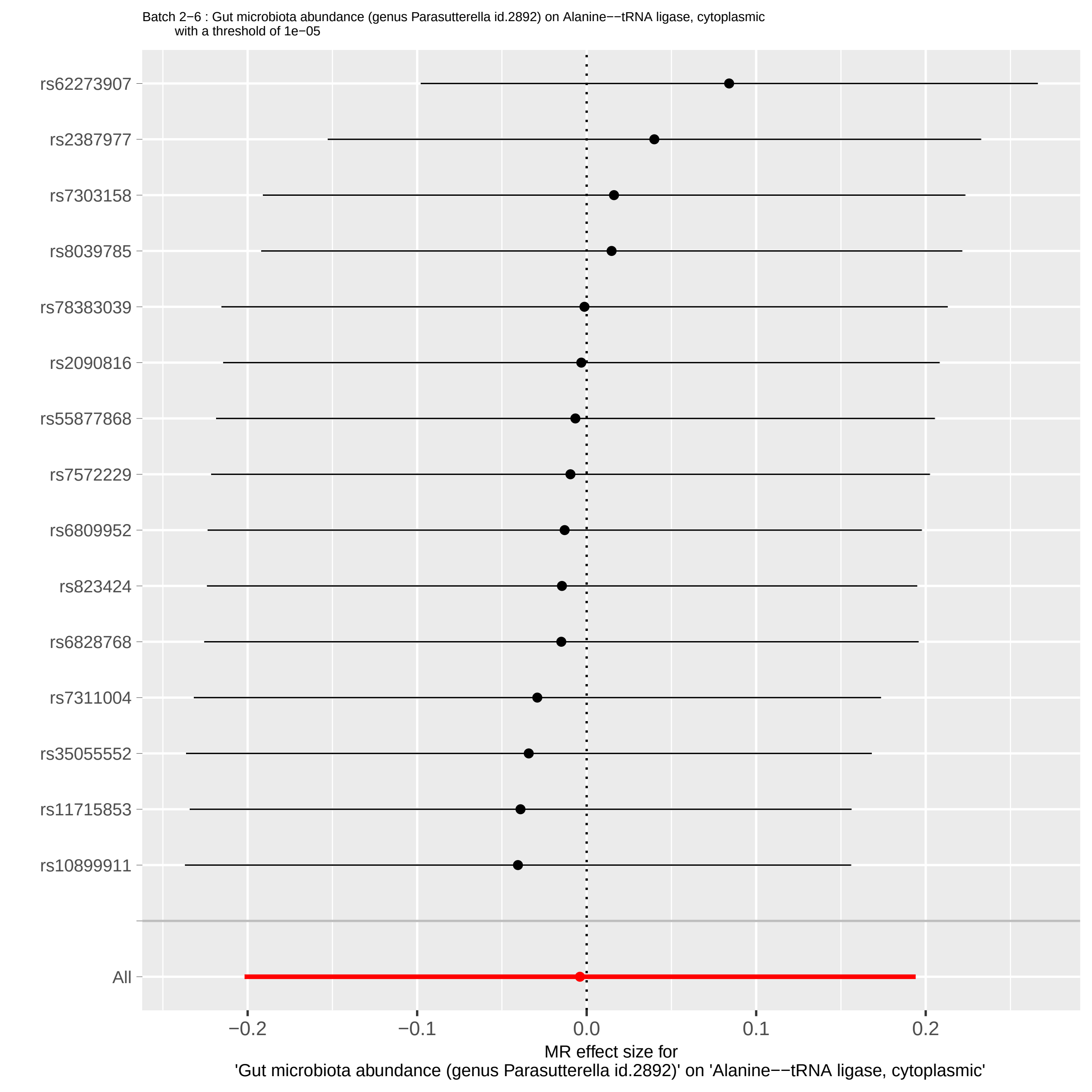
Batch 2−6 : Gut microbiota abundance (genus Parasutterella id.2892) on Alanine−−tRNA ligase, cytoplasmic
with a threshold of 1e−05
rs62273907
rs2387977
rs7303158
rs8039785
rs78383039
rs2090816
rs55877868
rs7572229
rs6809952
rs823424
rs6828768
rs7311004
rs35055552
rs11715853
rs10899911
All
−0.2
−0.1
0.0
0.1
0.2
MR effect size for
'Gut microbiota abundance (genus Parasutterella id.2892)' on 'Alanine−−tRNA ligase, cytoplasmic'

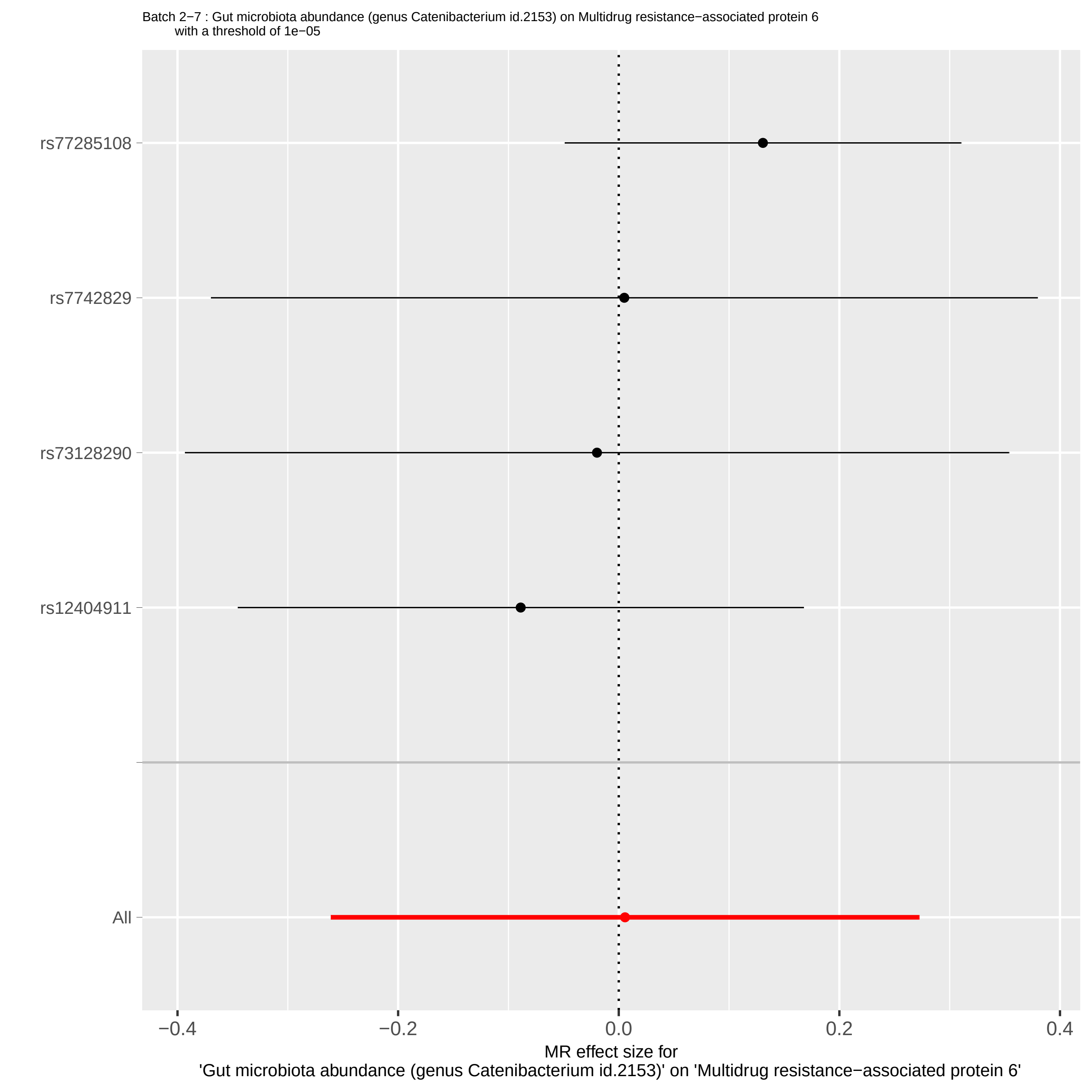
Batch 2−7 : Gut microbiota abundance (genus Catenibacterium id.2153) on Multidrug resistance−associated protein 6
with a threshold of 1e−05
rs77285108
rs7742829
rs73128290
rs12404911
All
−0.4
−0.2
0.0
0.2
0.4
MR effect size for
'Gut microbiota abundance (genus Catenibacterium id.2153)' on 'Multidrug resistance−associated protein 6'

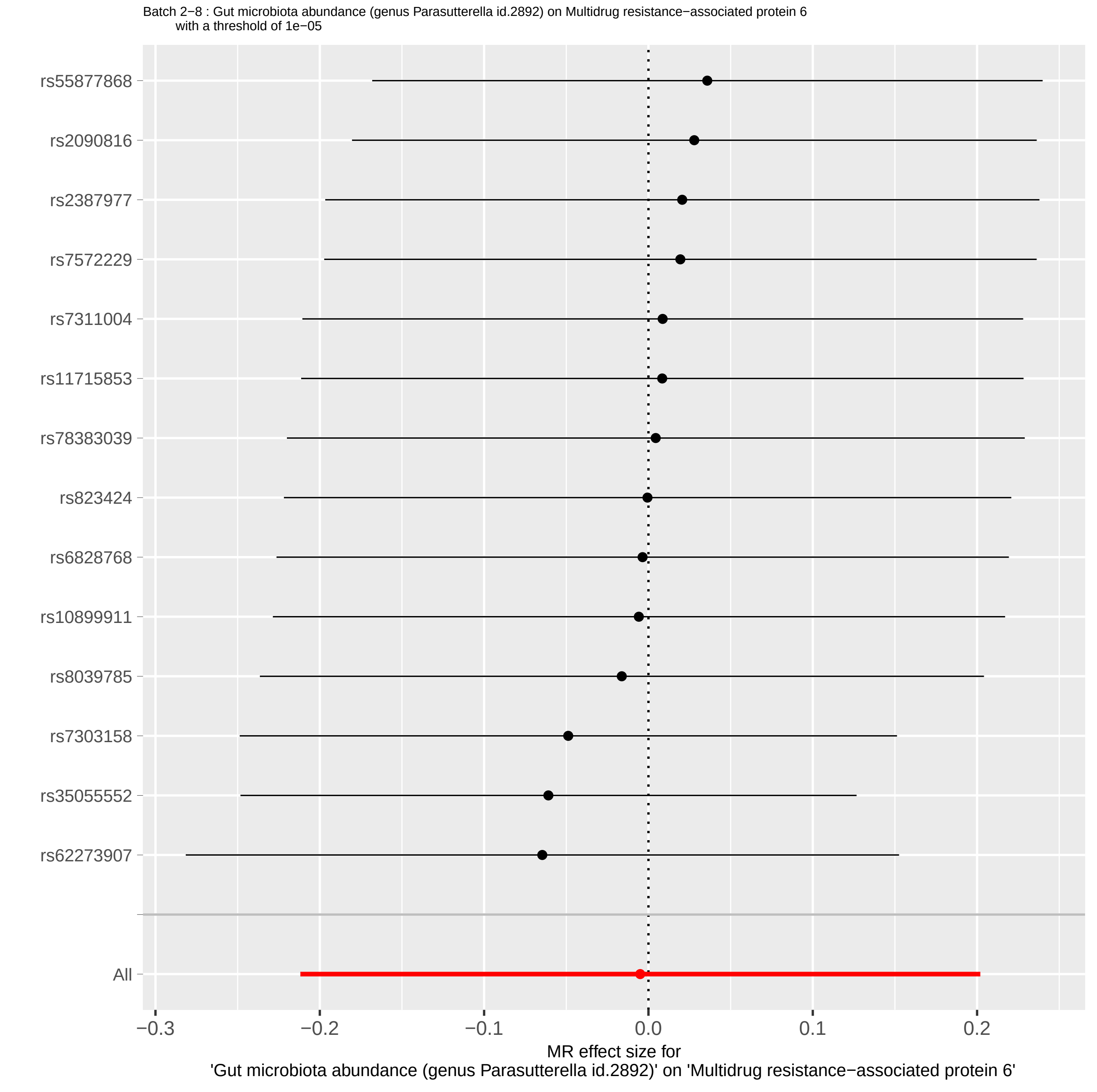

Batch 2−8 : Gut microbiota abundance (genus Parasutterella id.2892) on Multidrug resistance−associated protein 6
with a threshold of 1e−05
rs55877868
rs2090816
rs2387977
rs7572229
rs7311004
rs11715853
rs78383039
rs823424
rs6828768
rs10899911
rs8039785
rs7303158
rs35055552
rs62273907
All
−0.3
−0.2
−0.1
0.0
0.1
0.2
MR effect size for
'Gut microbiota abundance (genus Parasutterella id.2892)' on 'Multidrug resistance−associated protein 6'

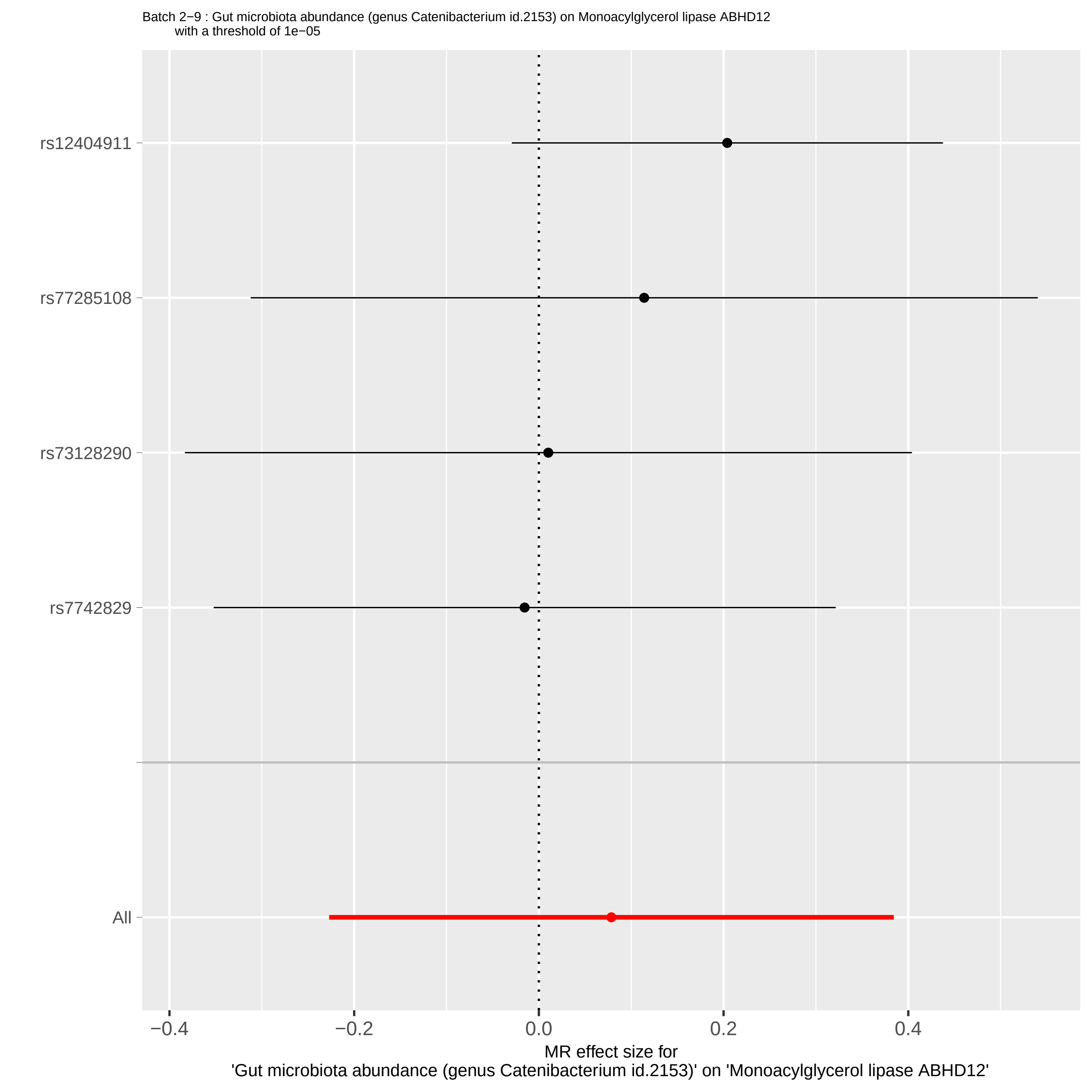

Batch 2−9 : Gut microbiota abundance (genus Catenibacterium id.2153) on Monoacylglycerol lipase ABHD12
with a threshold of 1e−05
rs12404911
rs77285108
rs73128290
rs7742829
All
−0.4
−0.2
0.0
0.2
0.4
MR effect size for
'Gut microbiota abundance (genus Catenibacterium id.2153)' on 'Monoacylglycerol lipase ABHD12'

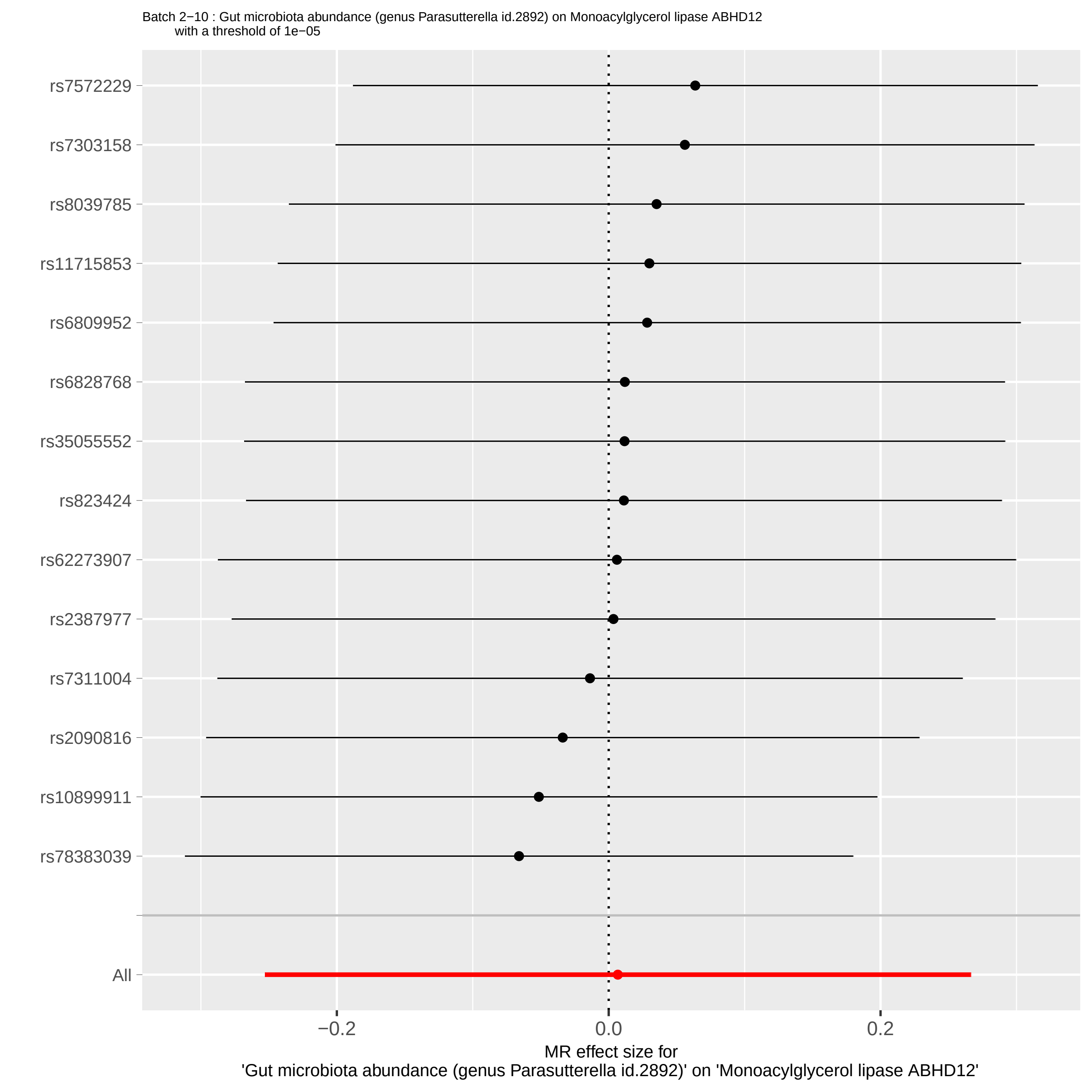

Batch 2−10 : Gut microbiota abundance (genus Parasutterella id.2892) on Monoacylglycerol lipase ABHD12
with a threshold of 1e−05
rs7572229
rs7303158
rs8039785
rs11715853
rs6809952
rs6828768
rs35055552
rs823424
rs62273907
rs2387977
rs7311004
rs2090816
rs10899911
rs78383039
All
−0.2
0.0
0.2
MR effect size for
'Gut microbiota abundance (genus Parasutterella id.2892)' on 'Monoacylglycerol lipase ABHD12'

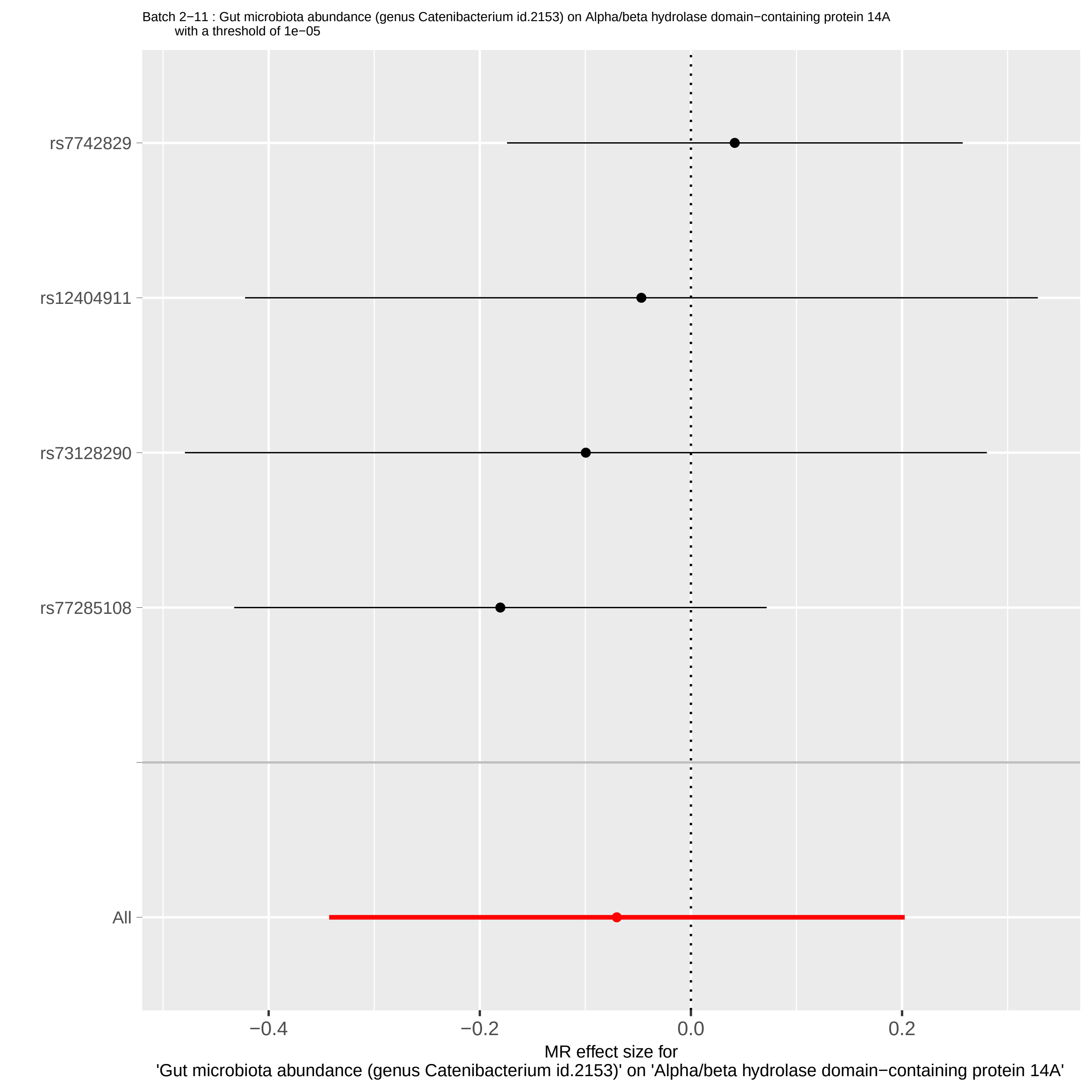

Batch 2−11 : Gut microbiota abundance (genus Catenibacterium id.2153) on Alpha/beta hydrolase domain−containing protein 14A
with a threshold of 1e−05
rs7742829
rs12404911
rs73128290
rs77285108
All
−0.4
−0.2
0.0
0.2
MR effect size for
'Gut microbiota abundance (genus Catenibacterium id.2153)' on 'Alpha/beta hydrolase domain−containing protein 14A'

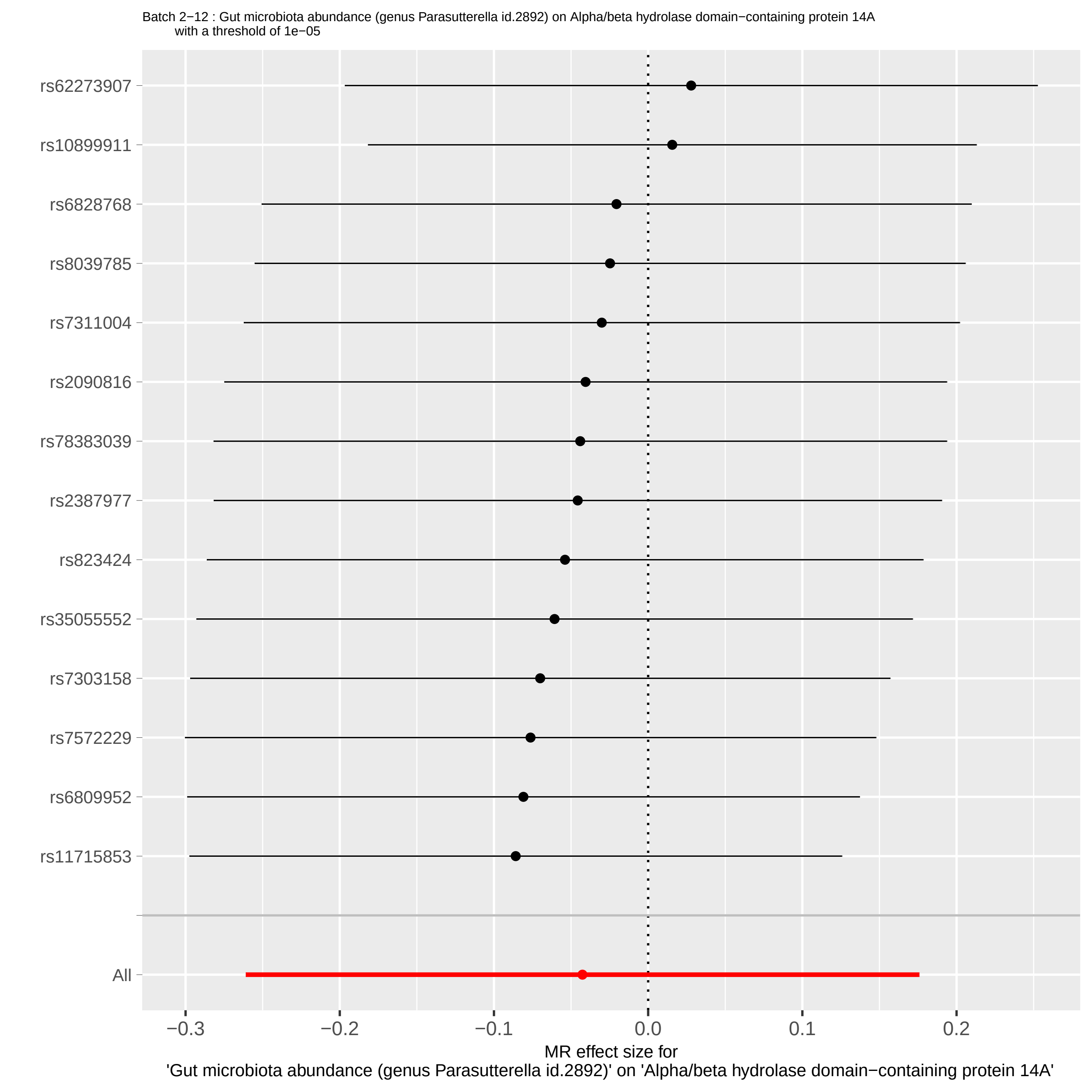

Batch 2−12 : Gut microbiota abundance (genus Parasutterella id.2892) on Alpha/beta hydrolase domain−containing protein 14A
with a threshold of 1e−05
rs62273907
rs10899911
rs6828768
rs8039785
rs7311004
rs2090816
rs78383039
rs2387977
rs823424
rs35055552
rs7303158
rs7572229
rs6809952
rs11715853
All
−0.3
−0.2
−0.1
0.0
0.1
0.2
MR effect size for
'Gut microbiota abundance (genus Parasutterella id.2892)' on 'Alpha/beta hydrolase domain−containing protein 14A'

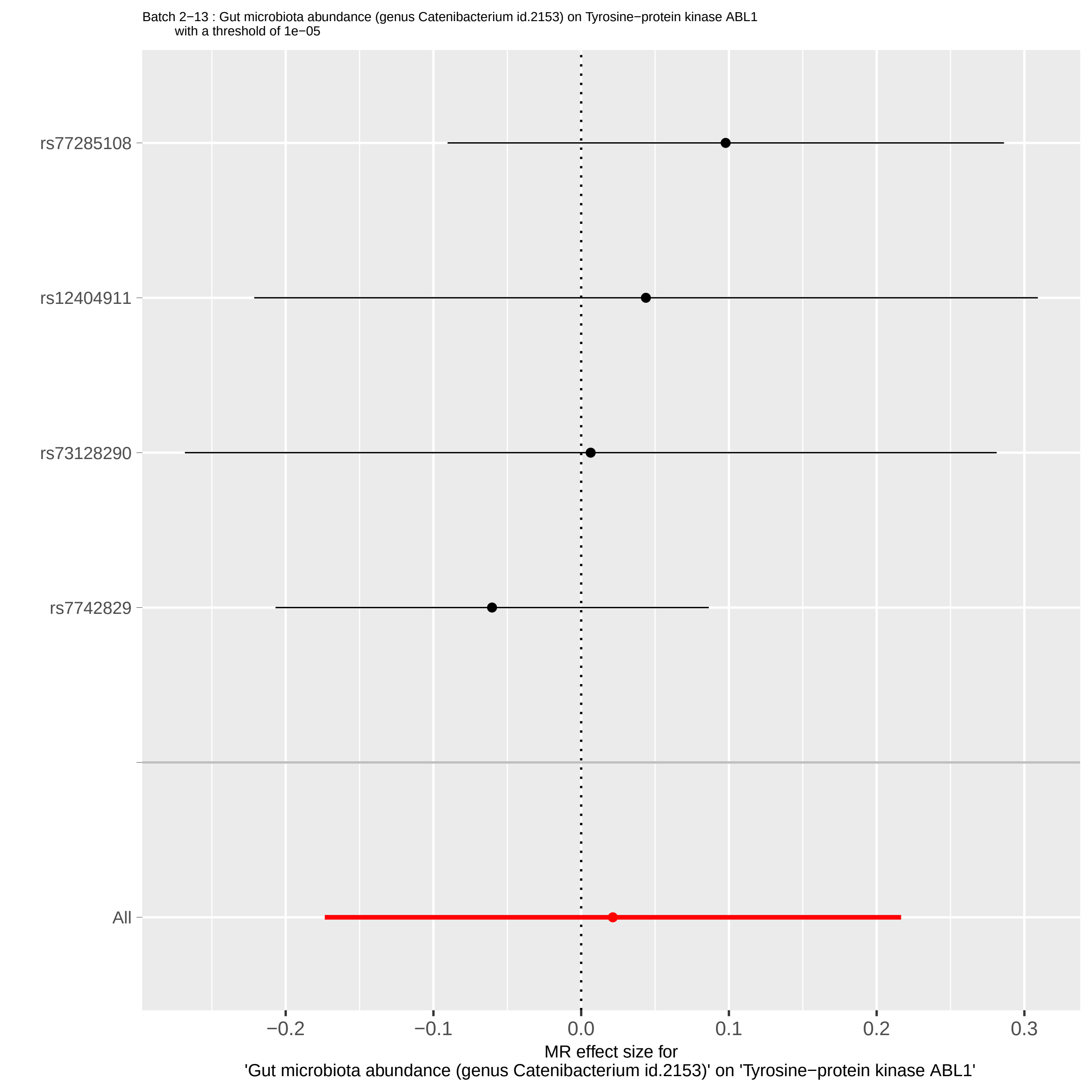
Batch 2−13 : Gut microbiota abundance (genus Catenibacterium id.2153) on Tyrosine−protein kinase ABL1
with a threshold of 1e−05
rs77285108
rs12404911
rs73128290
rs7742829
All
−0.2
−0.1
0.0
0.1
0.2
0.3
MR effect size for
'Gut microbiota abundance (genus Catenibacterium id.2153)' on 'Tyrosine−protein kinase ABL1'

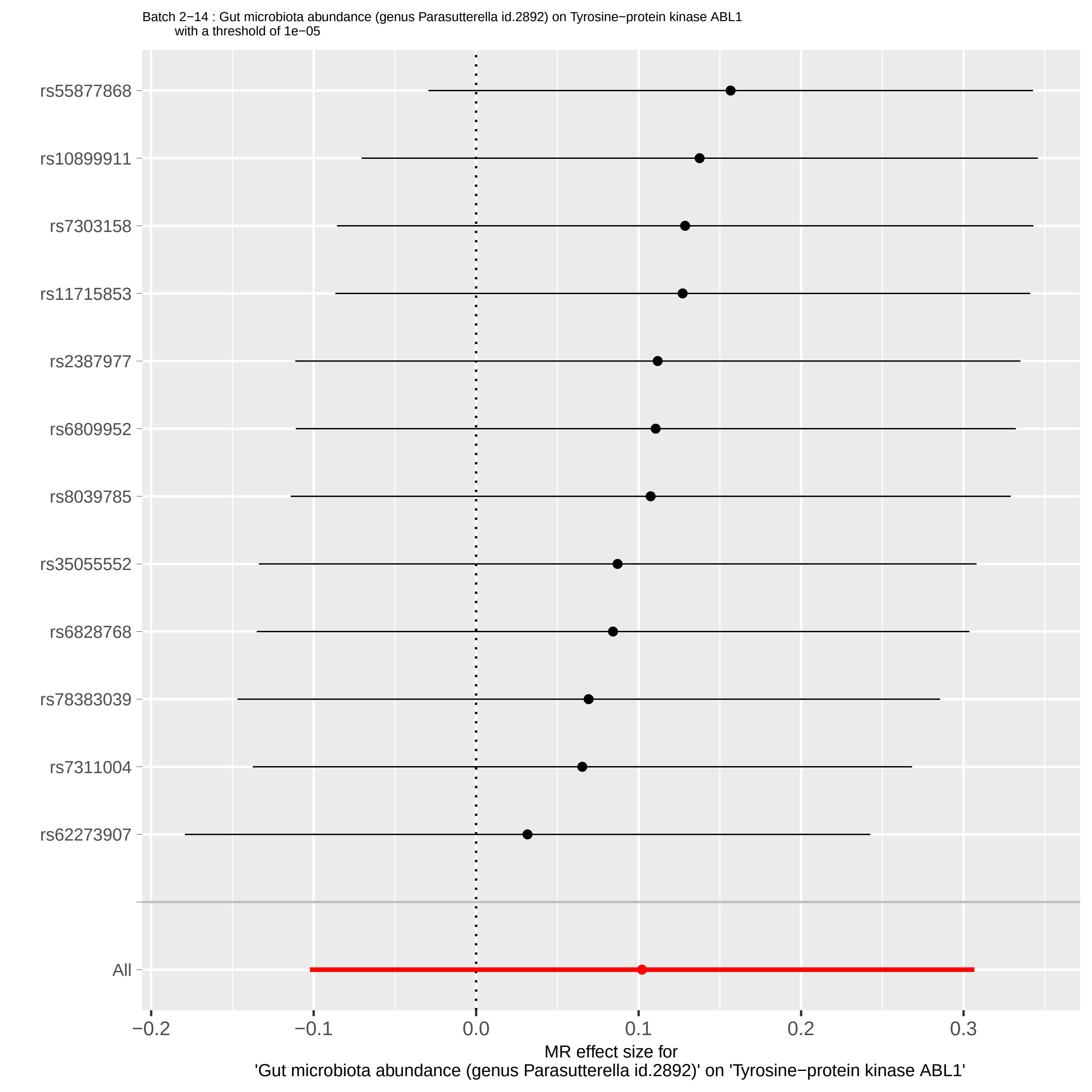
Batch 2−14 : Gut microbiota abundance (genus Parasutterella id.2892) on Tyrosine−protein kinase ABL1
with a threshold of 1e−05
rs55877868
rs10899911
rs7303158
rs11715853
rs2387977
rs6809952
rs8039785
rs35055552
rs6828768
rs78383039
rs7311004
rs62273907
All
−0.2
−0.1
0.0
0.1
0.2
0.3
MR effect size for
'Gut microbiota abundance (genus Parasutterella id.2892)' on 'Tyrosine−protein kinase ABL1'

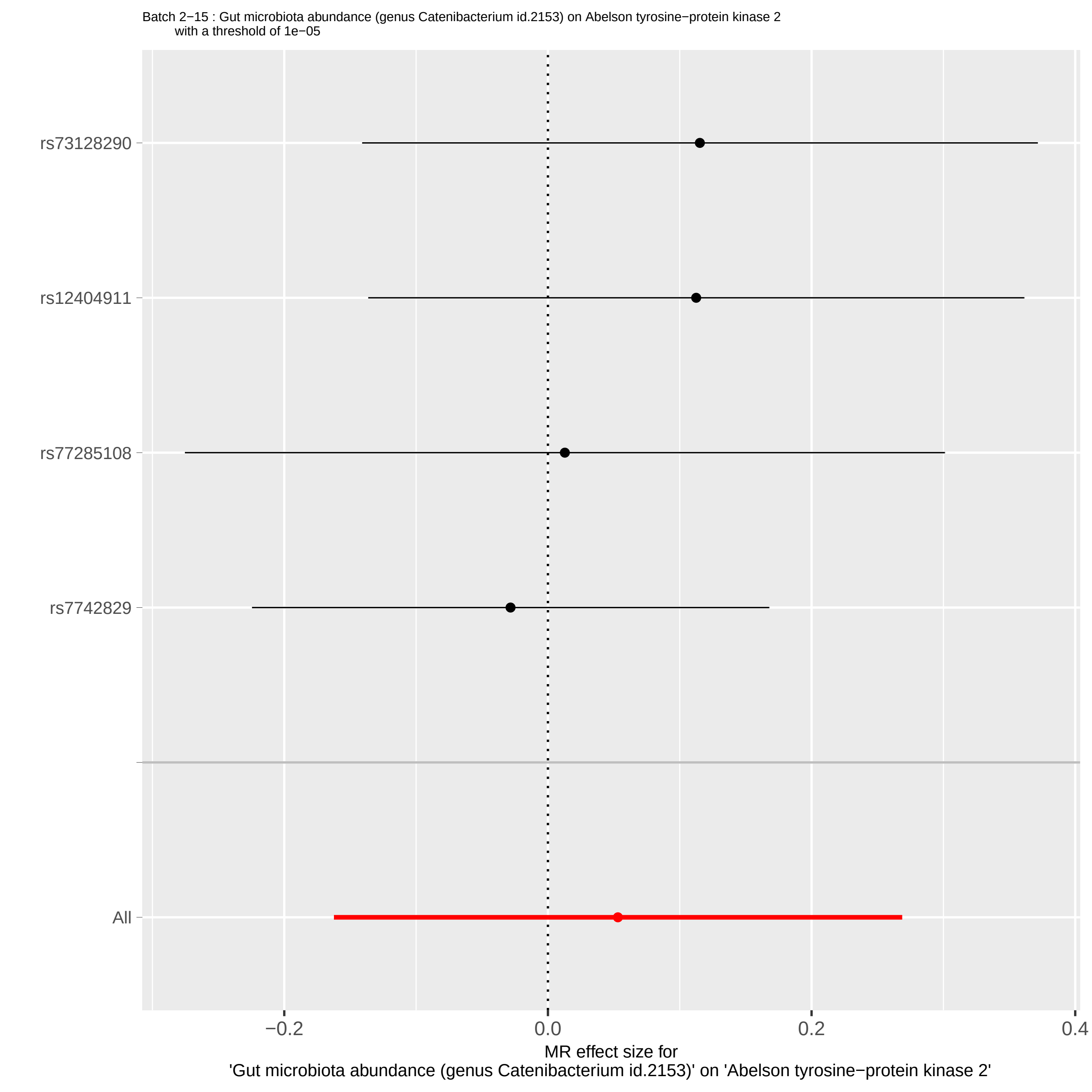
Batch 2−15 : Gut microbiota abundance (genus Catenibacterium id.2153) on Abelson tyrosine−protein kinase 2
with a threshold of 1e−05
rs73128290
rs12404911
rs77285108
rs7742829
All
−0.2
0.0
0.2
0.4
MR effect size for
'Gut microbiota abundance (genus Catenibacterium id.2153)' on 'Abelson tyrosine−protein kinase 2'

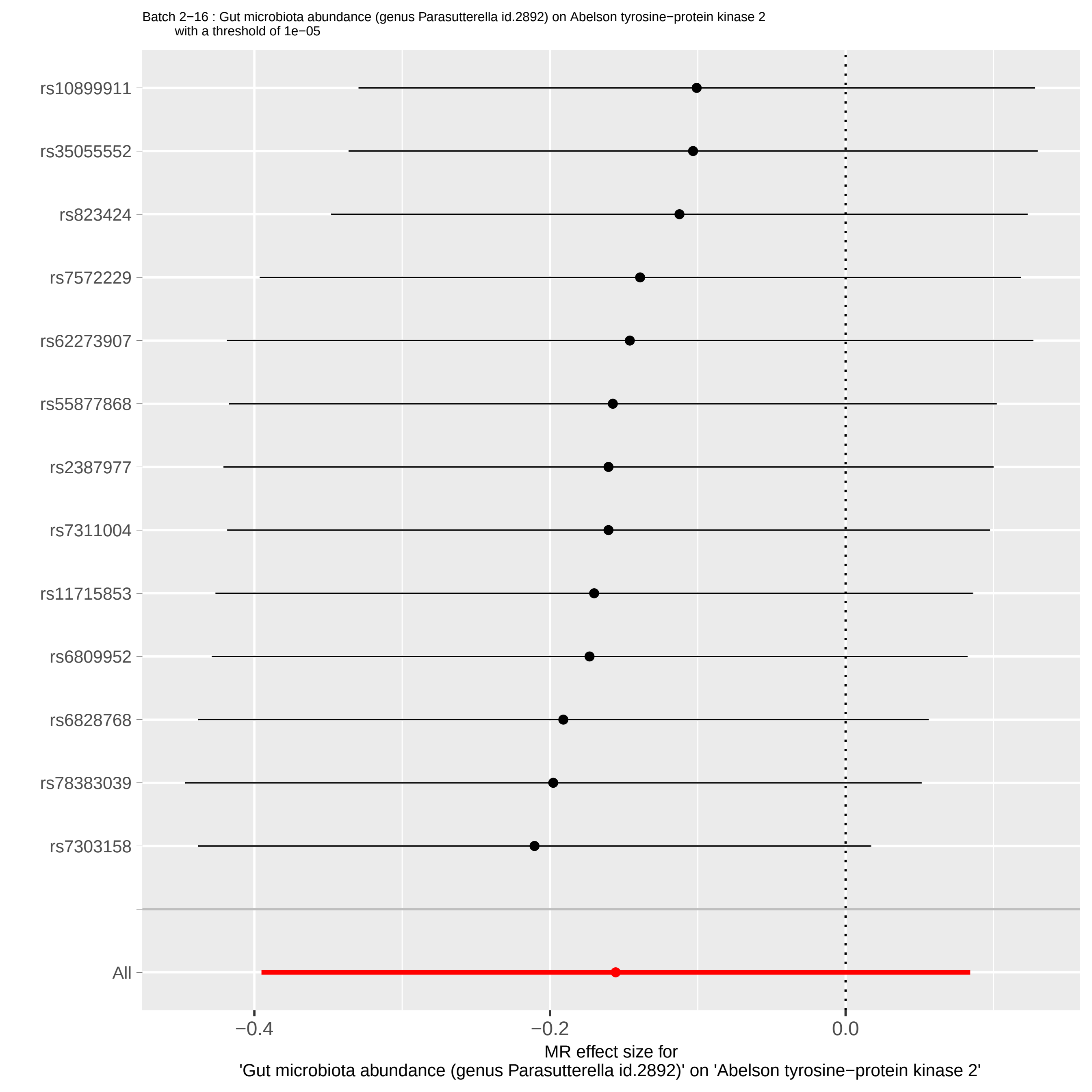

Batch 2−16 : Gut microbiota abundance (genus Parasutterella id.2892) on Abelson tyrosine−protein kinase 2
with a threshold of 1e−05
rs10899911
rs35055552
rs823424
rs7572229
rs62273907
rs55877868
rs2387977
rs7311004
rs11715853
rs6809952
rs6828768
rs78383039
rs7303158
All
−0.4
−0.2
0.0
MR effect size for
'Gut microbiota abundance (genus Parasutterella id.2892)' on 'Abelson tyrosine−protein kinase 2'

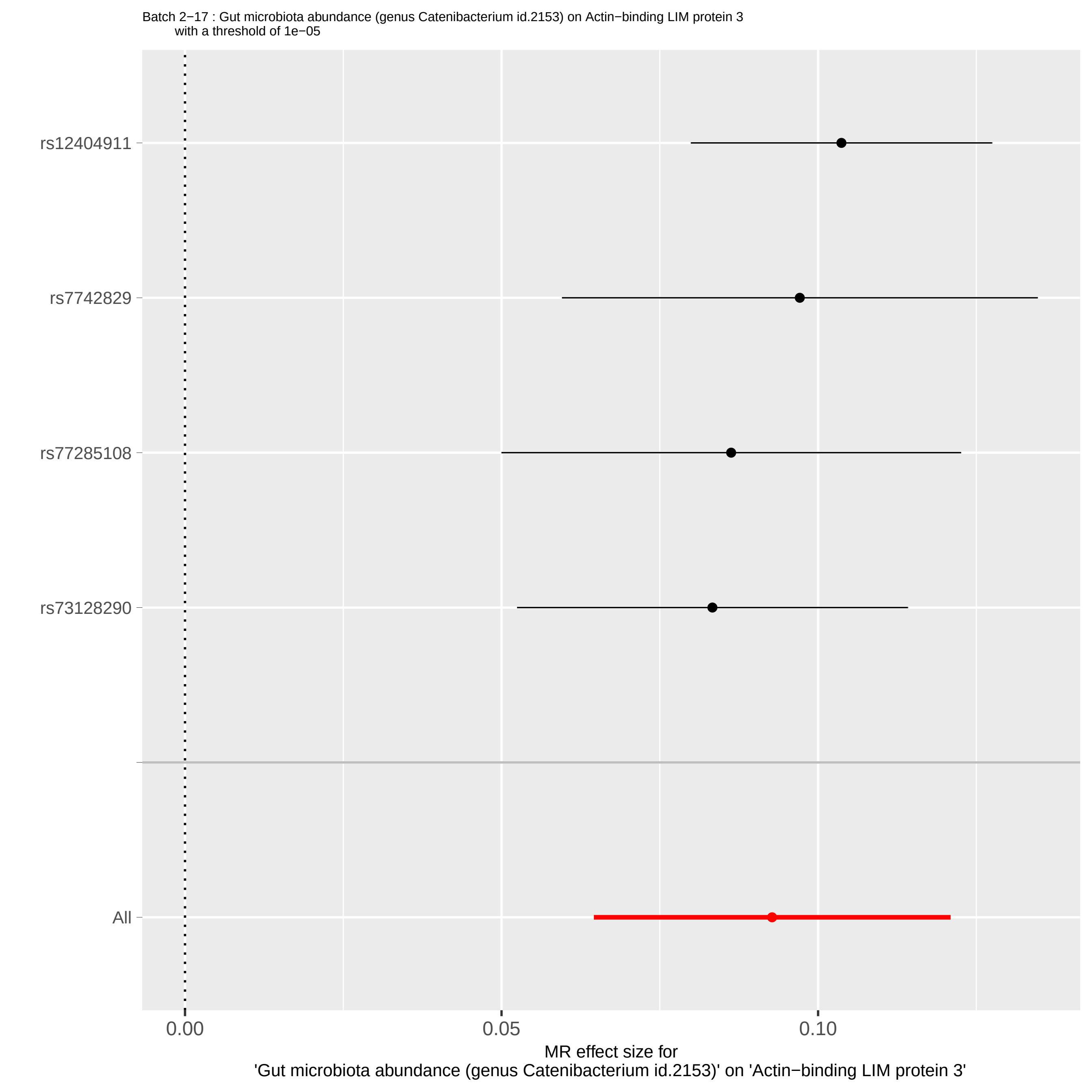
Batch 2−17 : Gut microbiota abundance (genus Catenibacterium id.2153) on Actin−binding LIM protein 3
with a threshold of 1e−05
rs12404911
rs7742829
rs77285108
rs73128290
All
0.00
0.05
0.10
MR effect size for
'Gut microbiota abundance (genus Catenibacterium id.2153)' on 'Actin−binding LIM protein 3'

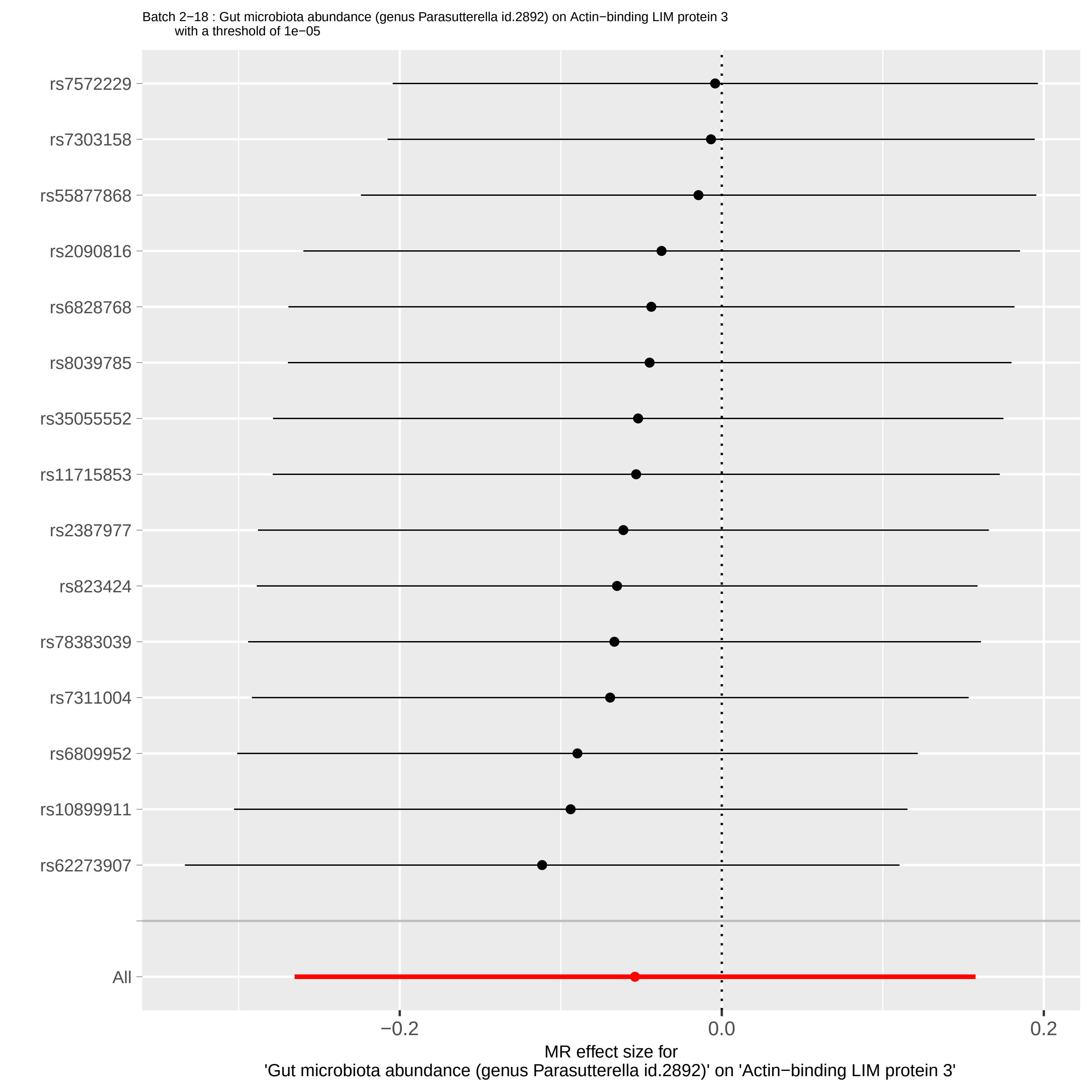
Batch 2−18 : Gut microbiota abundance (genus Parasutterella id.2892) on Actin−binding LIM protein 3
with a threshold of 1e−05
rs7572229
rs7303158
rs55877868
rs2090816
rs6828768
rs8039785
rs35055552
rs11715853
rs2387977
rs823424
rs78383039
rs7311004
rs6809952
rs10899911
rs62273907
All
−0.2
0.0
0.2
MR effect size for
'Gut microbiota abundance (genus Parasutterella id.2892)' on 'Actin−binding LIM protein 3'

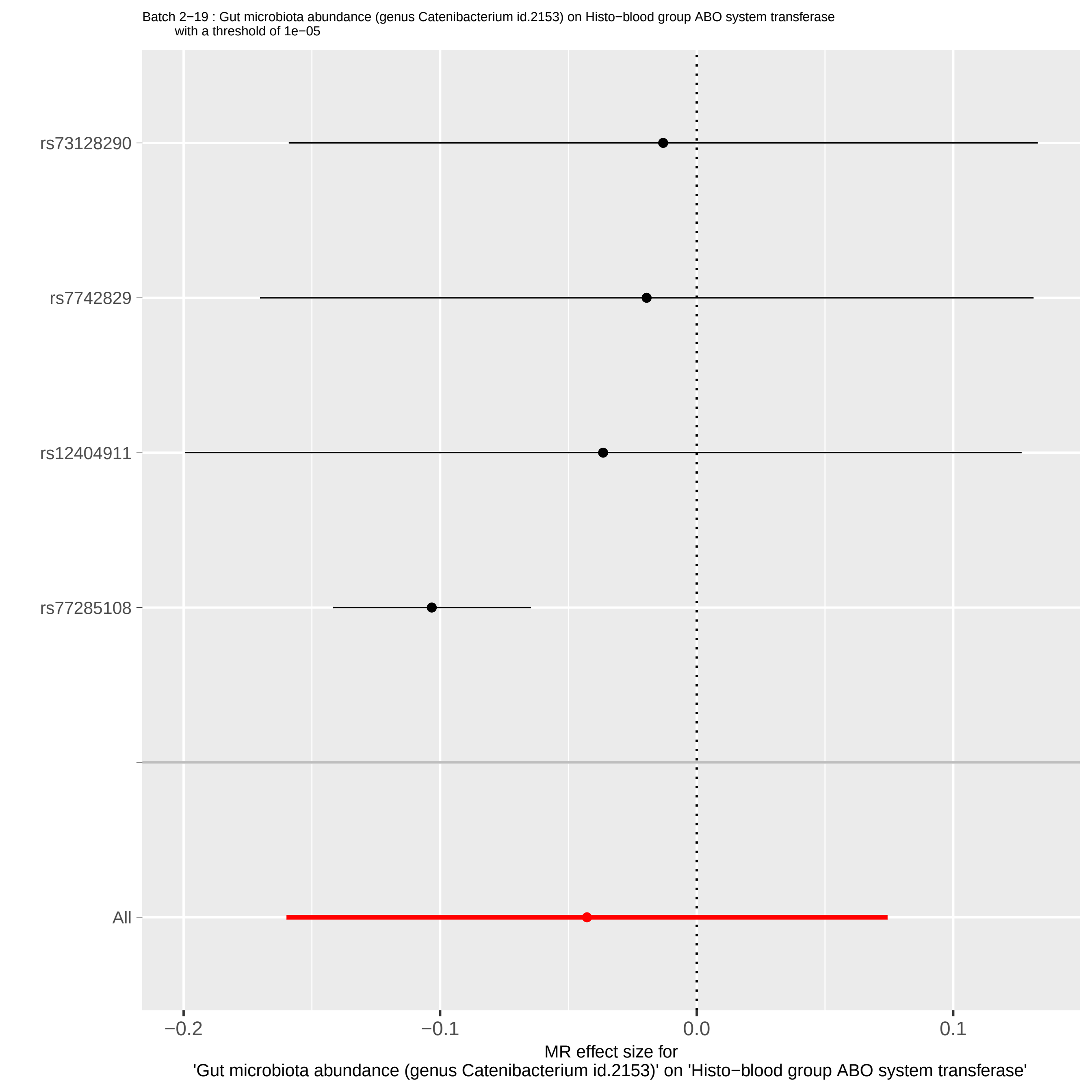
Batch 2−19 : Gut microbiota abundance (genus Catenibacterium id.2153) on Histo−blood group ABO system transferase
with a threshold of 1e−05
rs73128290
rs7742829
rs12404911
rs77285108
All
−0.2
−0.1
0.0
0.1
MR effect size for
'Gut microbiota abundance (genus Catenibacterium id.2153)' on 'Histo−blood group ABO system transferase'

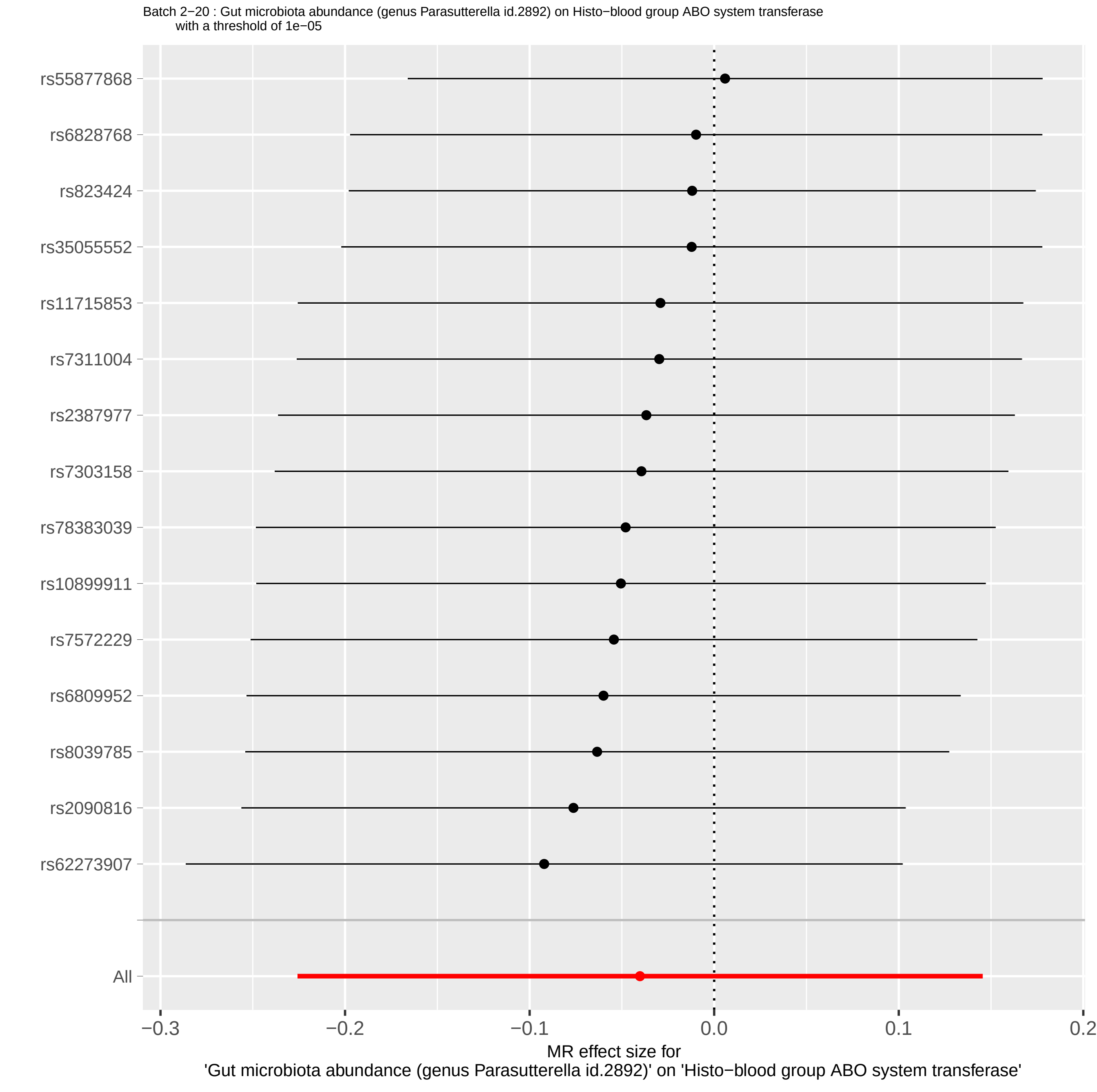
Batch 2−20 : Gut microbiota abundance (genus Parasutterella id.2892) on Histo−blood group ABO system transferase
with a threshold of 1e−05
rs55877868
rs6828768
rs823424
rs35055552
rs11715853
rs7311004
rs2387977
rs7303158
rs78383039
rs10899911
rs7572229
rs6809952
rs8039785
rs2090816
rs62273907
All
−0.3
−0.2
−0.1
0.0
0.1
0.2
MR effect size for
'Gut microbiota abundance (genus Parasutterella id.2892)' on 'Histo−blood group ABO system transferase'

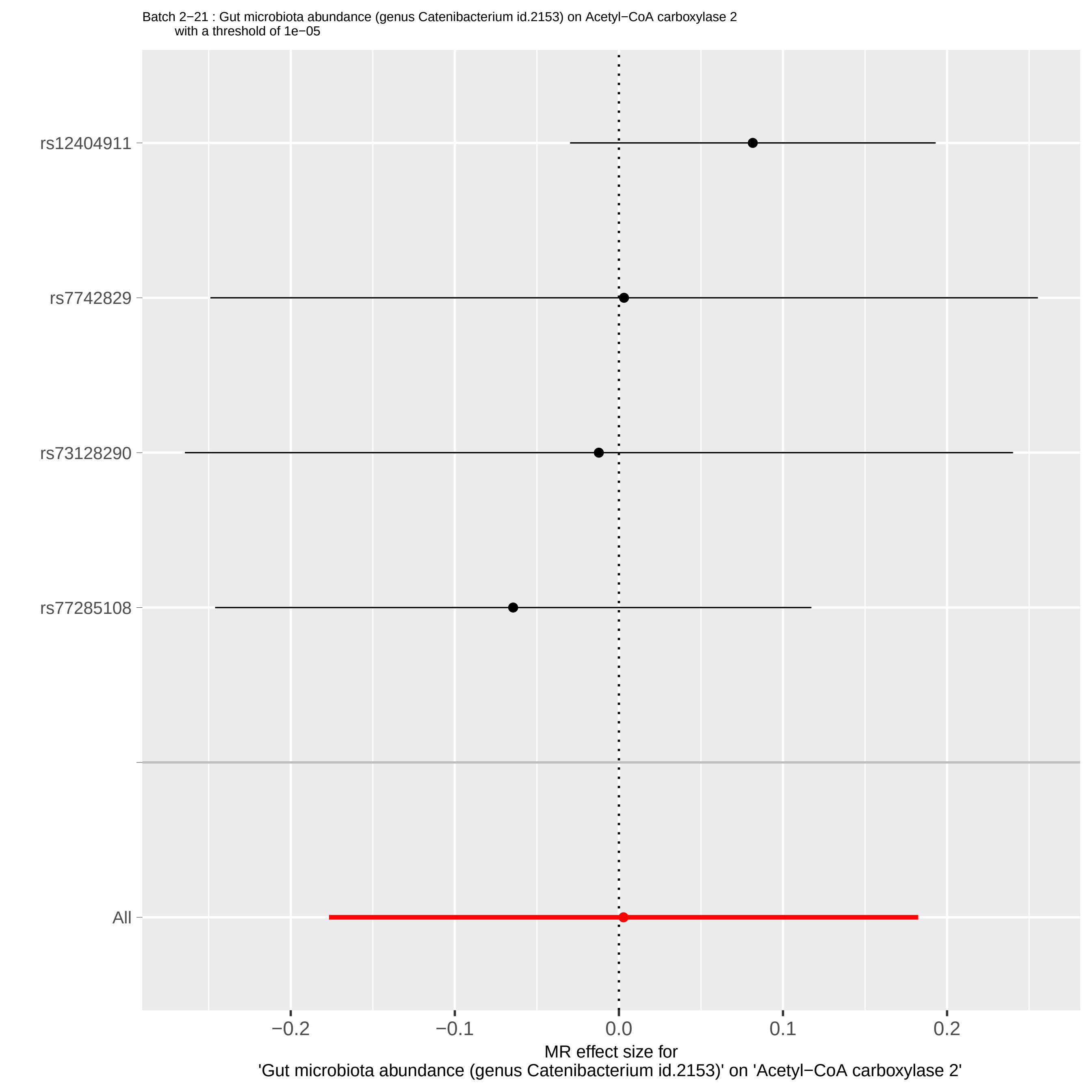

Batch 2−21 : Gut microbiota abundance (genus Catenibacterium id.2153) on Acetyl−CoA carboxylase 2
with a threshold of 1e−05
rs12404911
rs7742829
rs73128290
rs77285108
All
−0.2
−0.1
0.0
0.1
0.2
MR effect size for
'Gut microbiota abundance (genus Catenibacterium id.2153)' on 'Acetyl−CoA carboxylase 2'

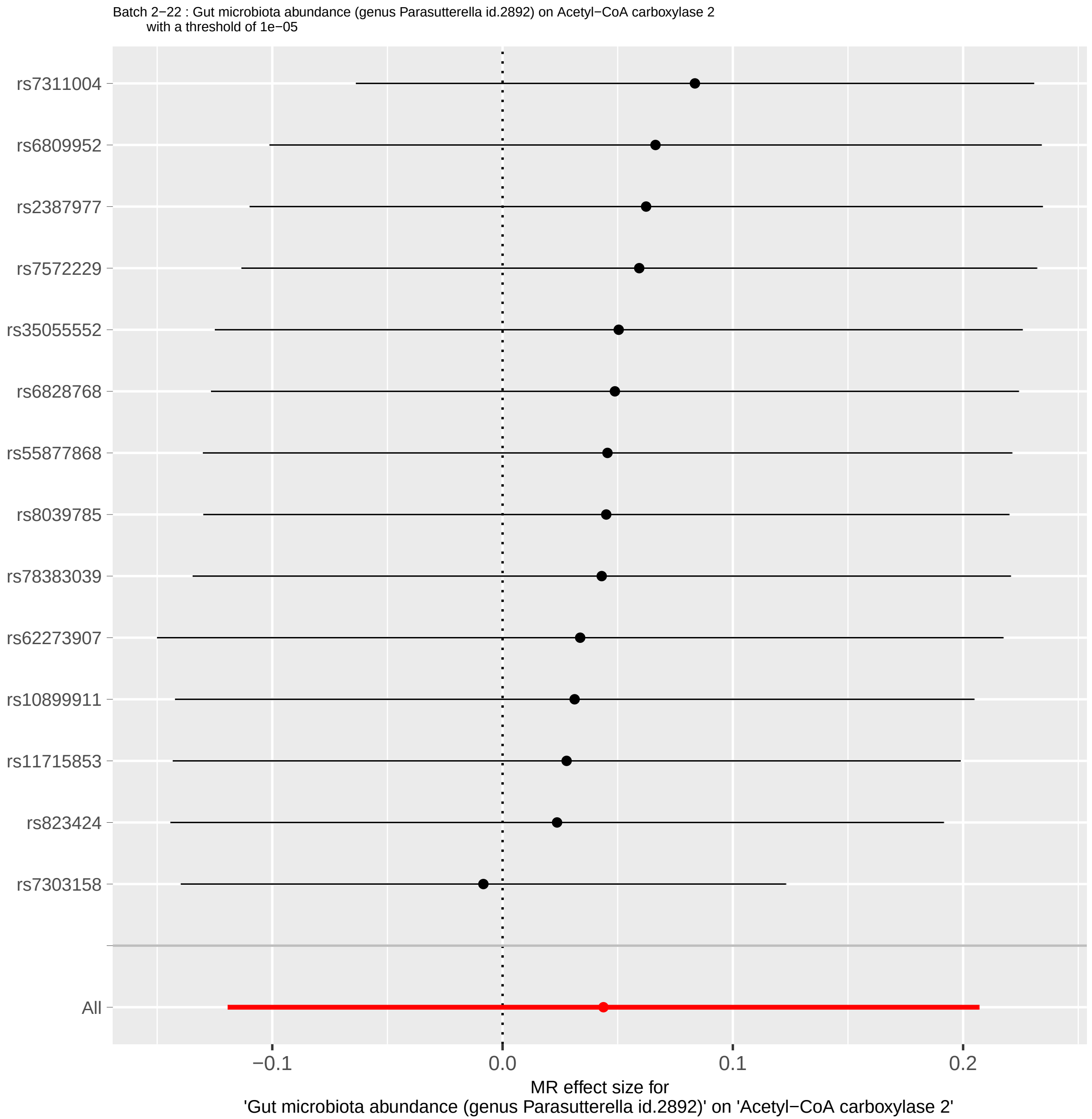

Batch 2−22 : Gut microbiota abundance (genus Parasutterella id.2892) on Acetyl−CoA carboxylase 2
with a threshold of 1e−05
rs7311004
rs6809952
rs2387977
rs7572229
rs35055552
rs6828768
rs55877868
rs8039785
rs78383039
rs62273907
rs10899911
rs11715853
rs823424
rs7303158
All
−0.1
0.0
0.1
0.2
MR effect size for
'Gut microbiota abundance (genus Parasutterella id.2892)' on 'Acetyl−CoA carboxylase 2'

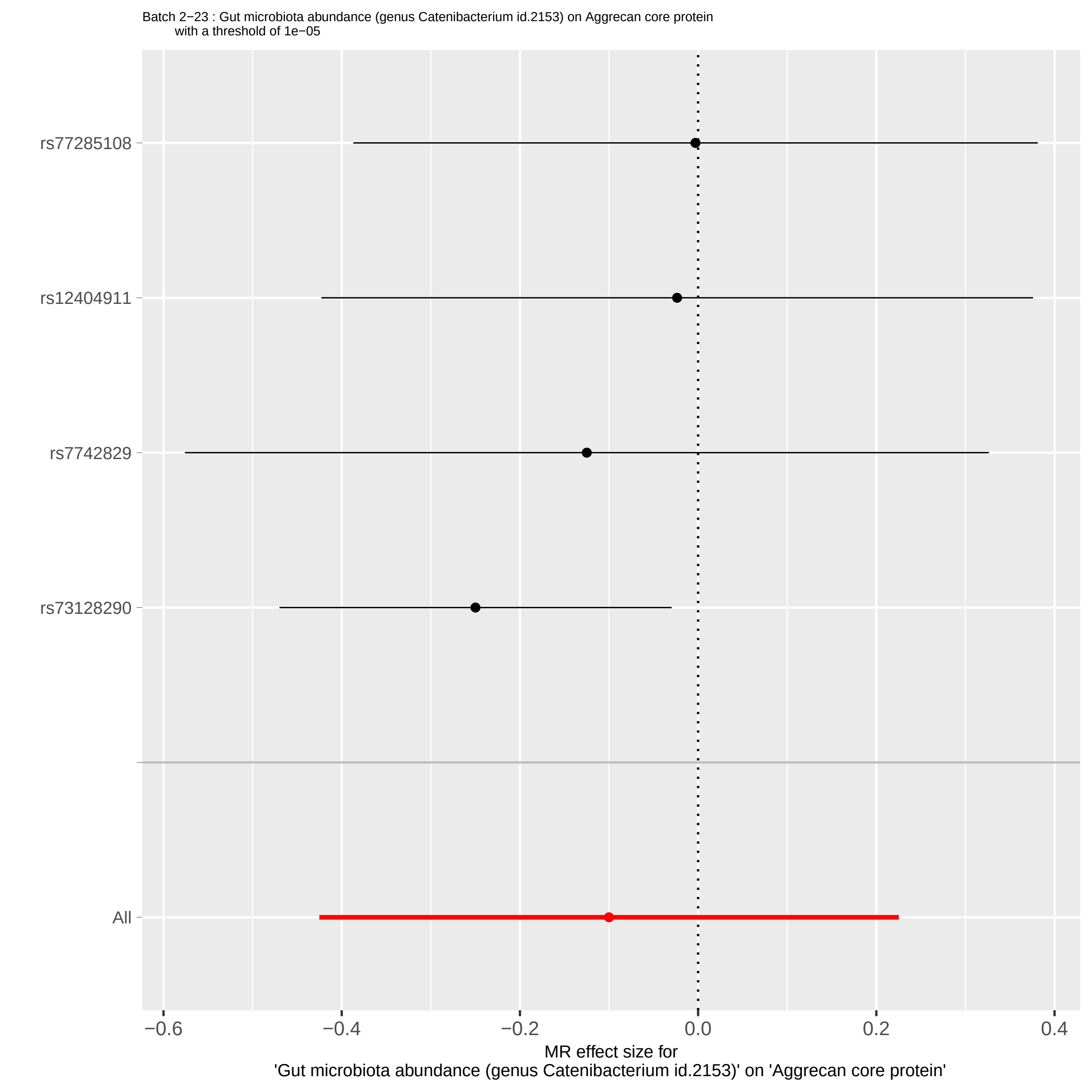
Batch 2−23 : Gut microbiota abundance (genus Catenibacterium id.2153) on Aggrecan core protein
with a threshold of 1e−05
rs77285108
rs12404911
rs7742829
rs73128290
All
−0.6
−0.4
−0.2
0.0
0.2
0.4
MR effect size for
'Gut microbiota abundance (genus Catenibacterium id.2153)' on 'Aggrecan core protein'

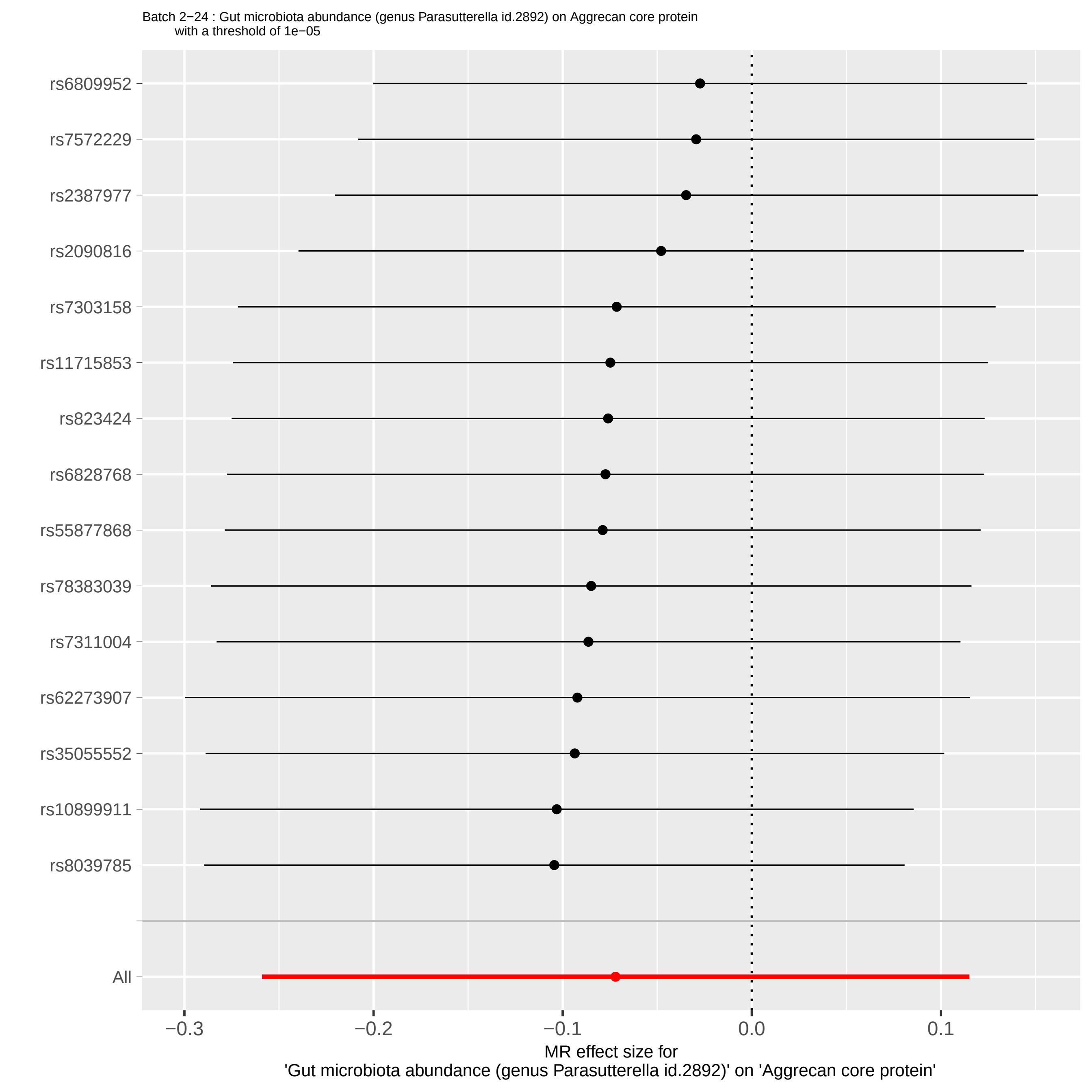

Batch 2−24 : Gut microbiota abundance (genus Parasutterella id.2892) on Aggrecan core protein
with a threshold of 1e−05
rs6809952
rs7572229
rs2387977
rs2090816
rs7303158
rs11715853
rs823424
rs6828768
rs55877868
rs78383039
rs7311004
rs62273907
rs35055552
rs10899911
rs8039785
All
−0.3
−0.2
−0.1
0.0
0.1
MR effect size for
'Gut microbiota abundance (genus Parasutterella id.2892)' on 'Aggrecan core protein'

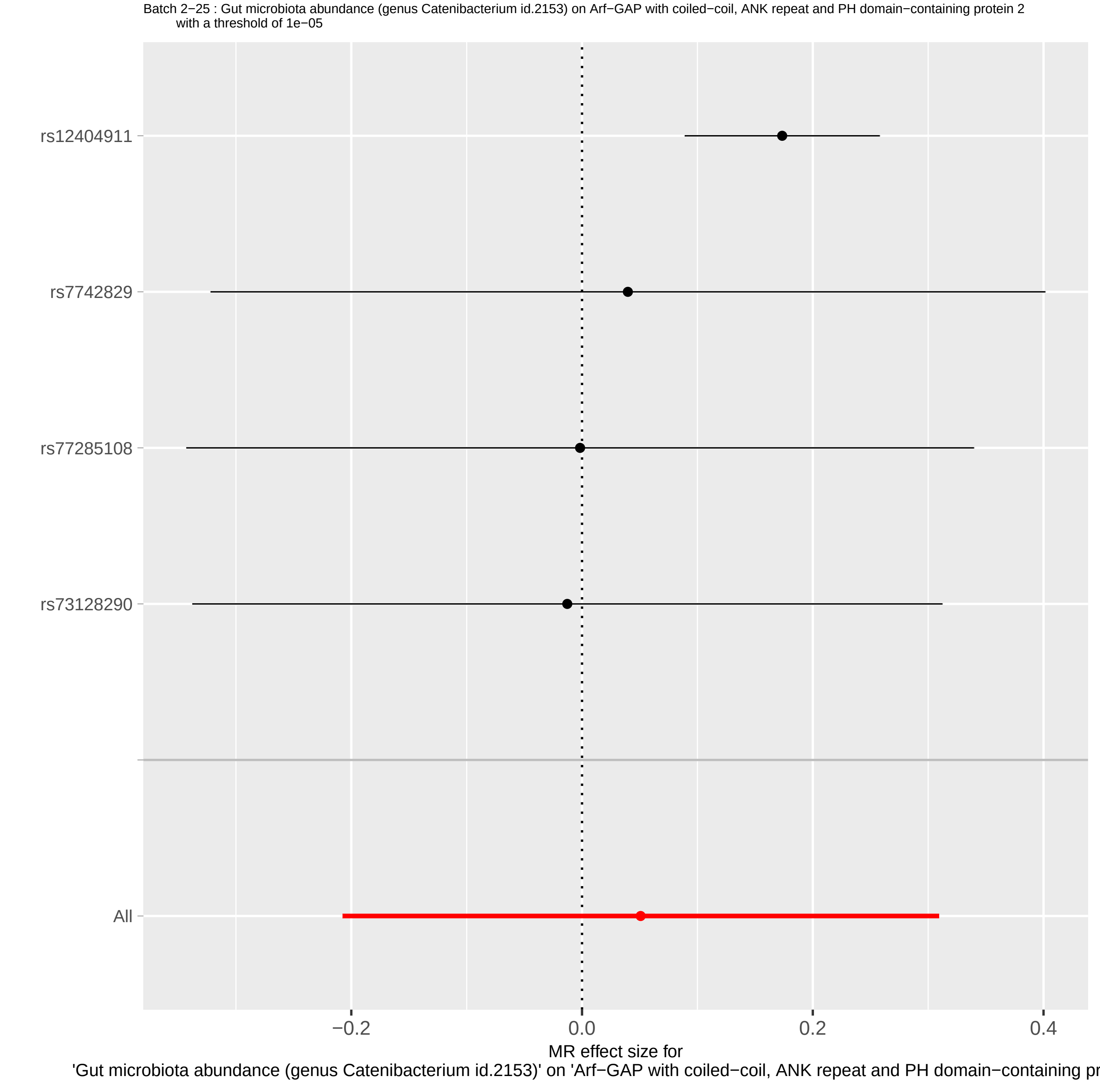

Batch 2−25 : Gut microbiota abundance (genus Catenibacterium id.2153) on Arf−GAP with coiled−coil, ANK repeat and PH domain−containing protein 2
with a threshold of 1e−05
rs12404911
rs7742829
rs77285108
rs73128290
All
−0.2
0.0
0.2
0.4
MR effect size for
'Gut microbiota abundance (genus Catenibacterium id.2153)' on 'Arf−GAP with coiled−coil, ANK repeat and PH domain−containing pr

Batch 2−26 : Gut microbiota abundance (genus Parasutterella id.2892) on Arf−GAP with coiled−coil, ANK repeat and PH domain−containing protein 2 with a threshold of 1e−05

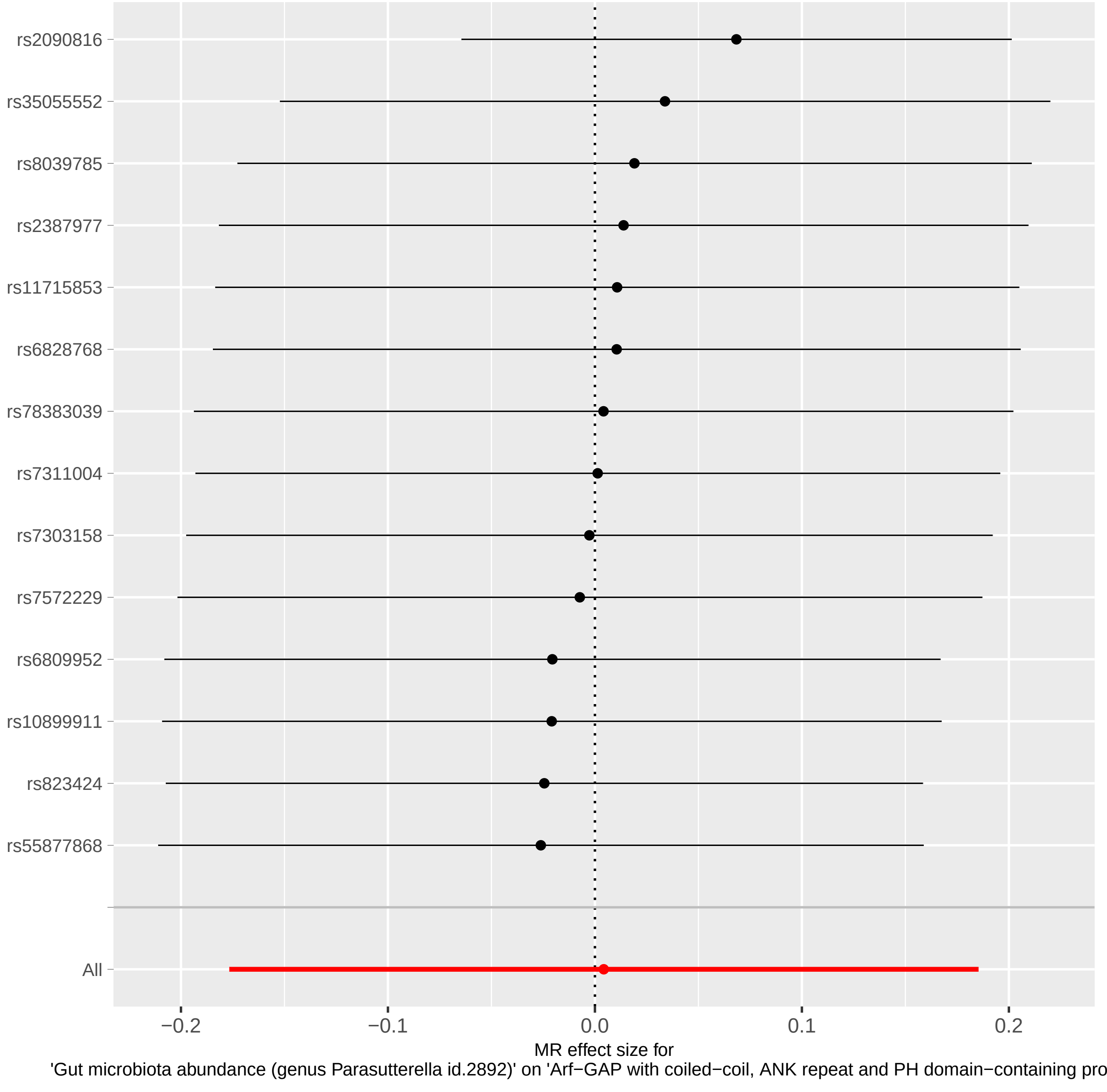

Batch 2−27 : Gut microbiota abundance (genus Catenibacterium id.2153) on Acyl−CoA−binding domain−containing protein 6
with a threshold of 1e−05

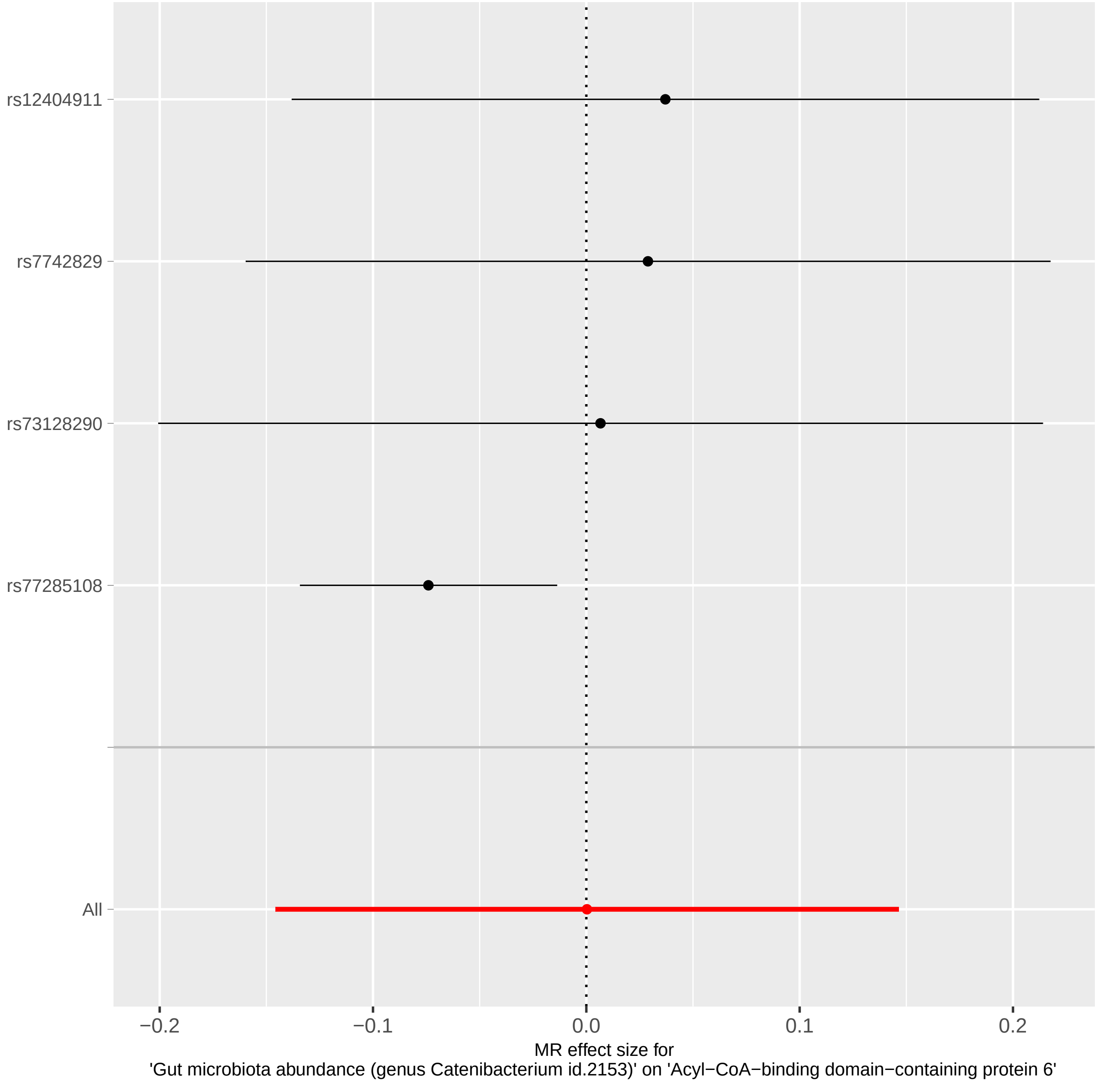

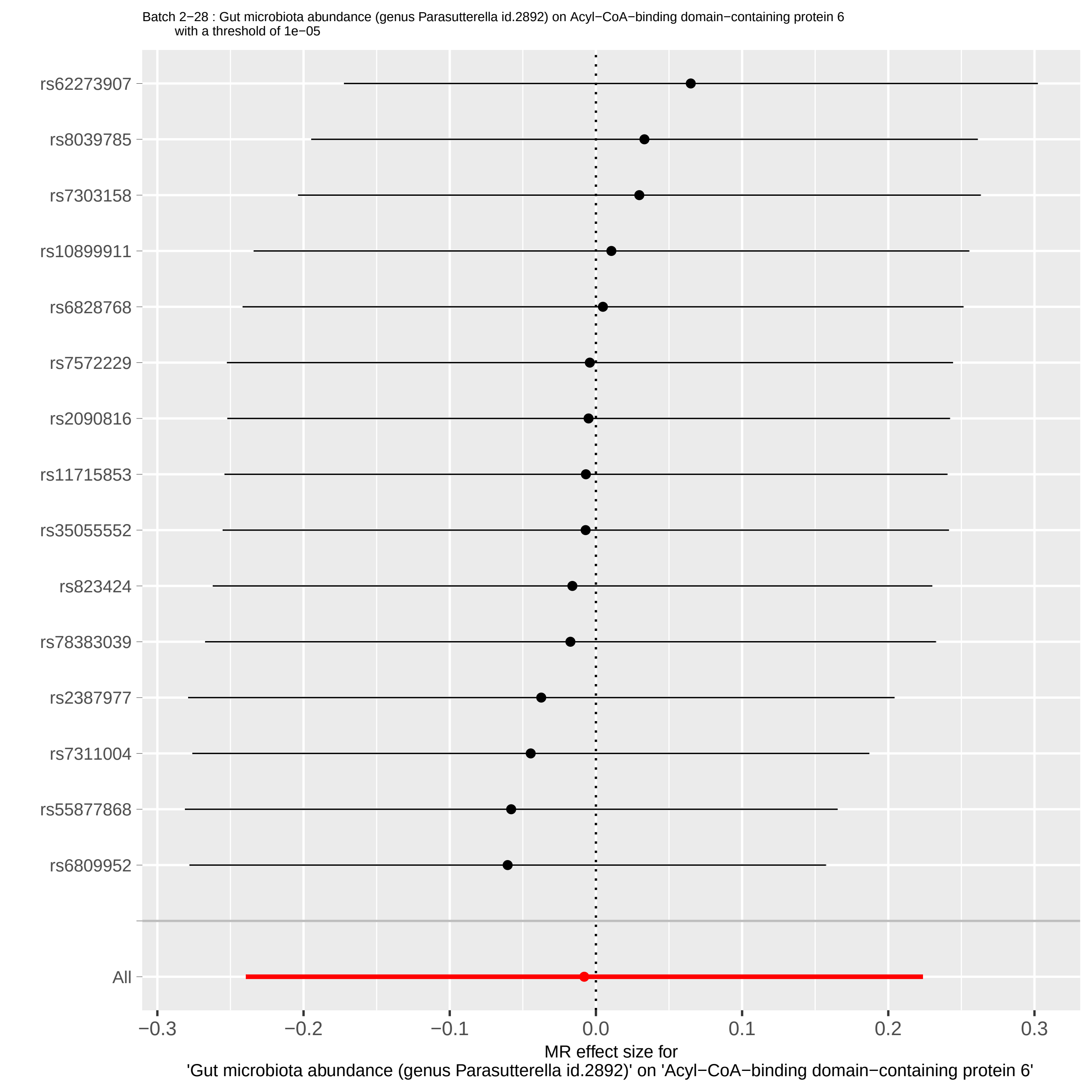

Batch 2−28 : Gut microbiota abundance (genus Parasutterella id.2892) on Acyl−CoA−binding domain−containing protein 6
with a threshold of 1e−05
rs62273907
rs8039785
rs7303158
rs10899911
rs6828768
rs7572229
rs2090816
rs11715853
rs35055552
rs823424
rs78383039
rs2387977
rs7311004
rs55877868
rs6809952
All
−0.3
−0.2
−0.1
0.0
0.1
0.2
0.3
MR effect size for
'Gut microbiota abundance (genus Parasutterella id.2892)' on 'Acyl−CoA−binding domain−containing protein 6'

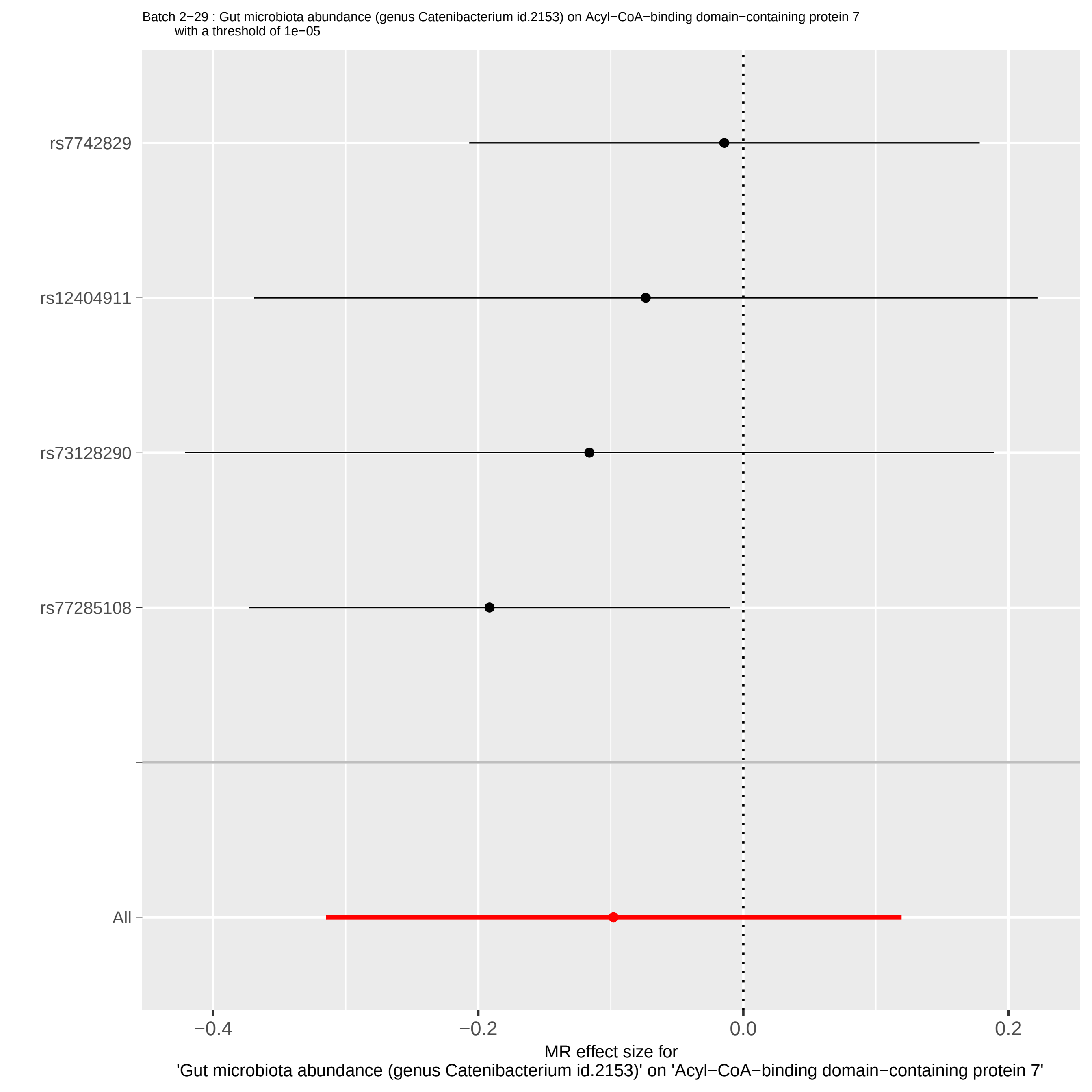

Batch 2−29 : Gut microbiota abundance (genus Catenibacterium id.2153) on Acyl−CoA−binding domain−containing protein 7
with a threshold of 1e−05
rs7742829
rs12404911
rs73128290
rs77285108
All
−0.4
−0.2
0.0
0.2
MR effect size for
'Gut microbiota abundance (genus Catenibacterium id.2153)' on 'Acyl−CoA−binding domain−containing protein 7'

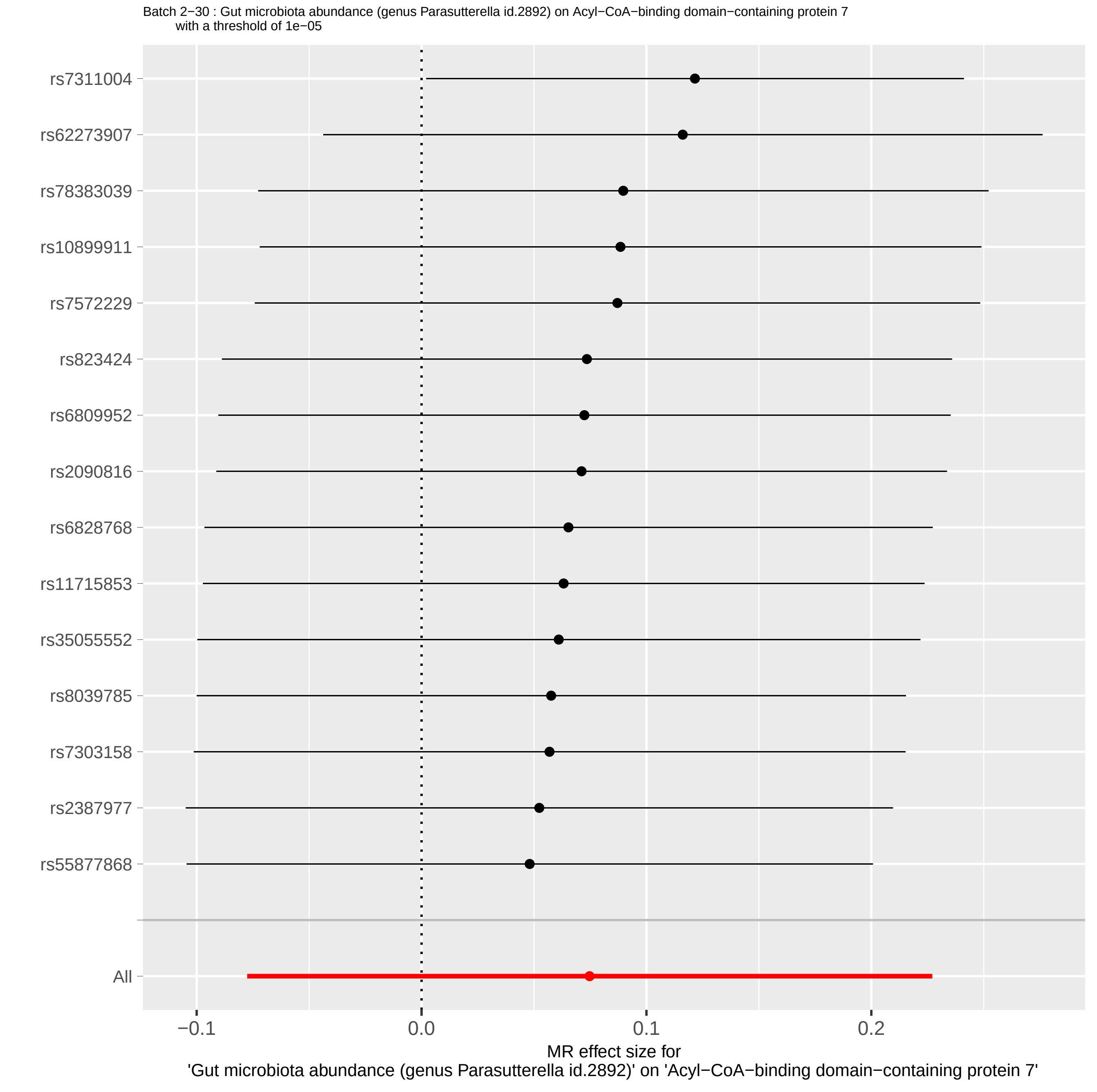

Batch 2−30 : Gut microbiota abundance (genus Parasutterella id.2892) on Acyl−CoA−binding domain−containing protein 7
with a threshold of 1e−05
rs7311004
rs62273907
rs78383039
rs10899911
rs7572229
rs823424
rs6809952
rs2090816
rs6828768
rs11715853
rs35055552
rs8039785
rs7303158
rs2387977
rs55877868
All
−0.1
0.0
0.1
0.2
MR effect size for
'Gut microbiota abundance (genus Parasutterella id.2892)' on 'Acyl−CoA−binding domain−containing protein 7'

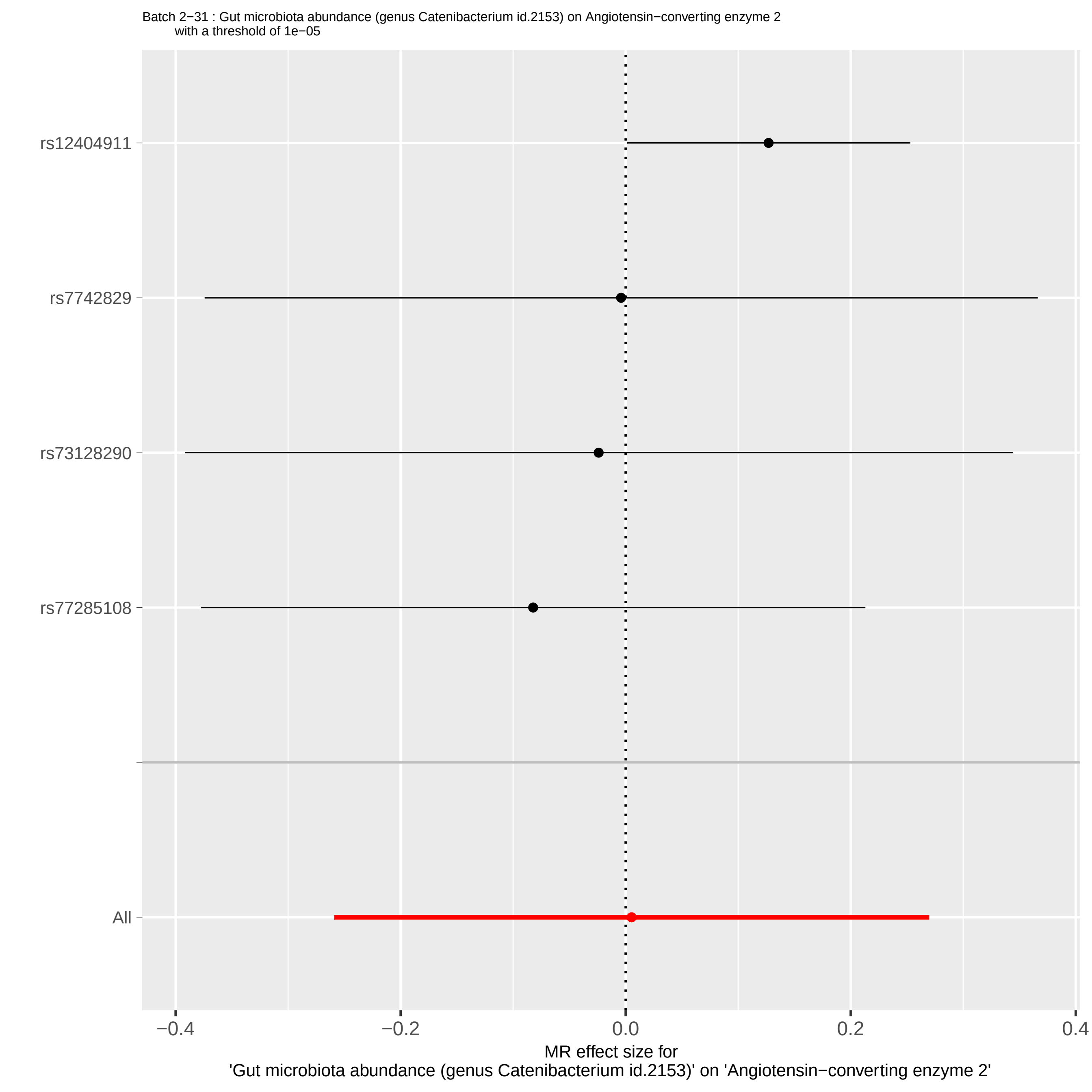
Batch 2−31 : Gut microbiota abundance (genus Catenibacterium id.2153) on Angiotensin−converting enzyme 2
with a threshold of 1e−05
rs12404911
rs7742829
rs73128290
rs77285108
All
−0.4
−0.2
0.0
0.2
0.4
MR effect size for
'Gut microbiota abundance (genus Catenibacterium id.2153)' on 'Angiotensin−converting enzyme 2'

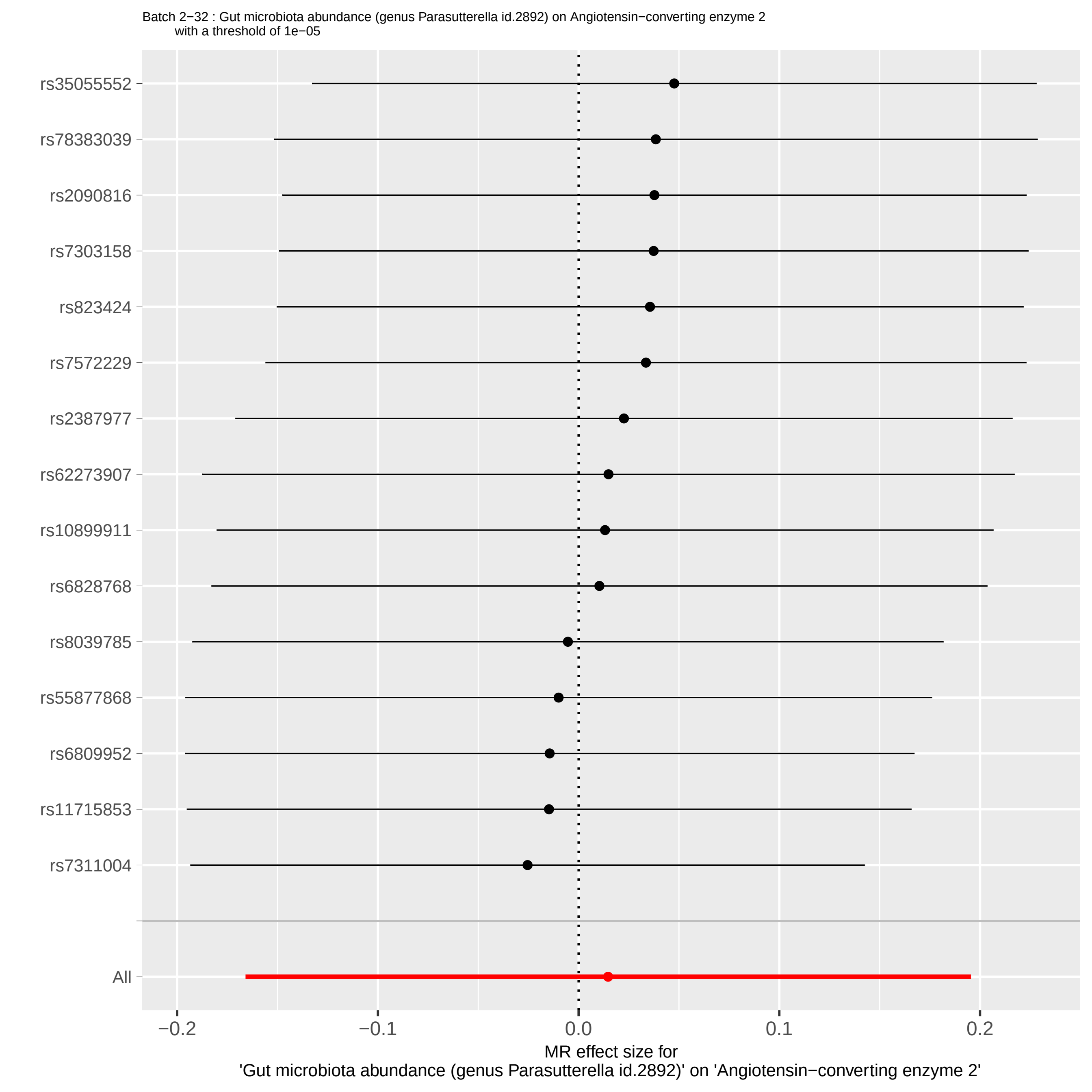
Batch 2−32 : Gut microbiota abundance (genus Parasutterella id.2892) on Angiotensin−converting enzyme 2
with a threshold of 1e−05
rs35055552
rs78383039
rs2090816
rs7303158
rs823424
rs7572229
rs2387977
rs62273907
rs10899911
rs6828768
rs8039785
rs55877868
rs6809952
rs11715853
rs7311004
All
−0.2
−0.1
0.0
0.1
0.2
MR effect size for
'Gut microbiota abundance (genus Parasutterella id.2892)' on 'Angiotensin−converting enzyme 2'

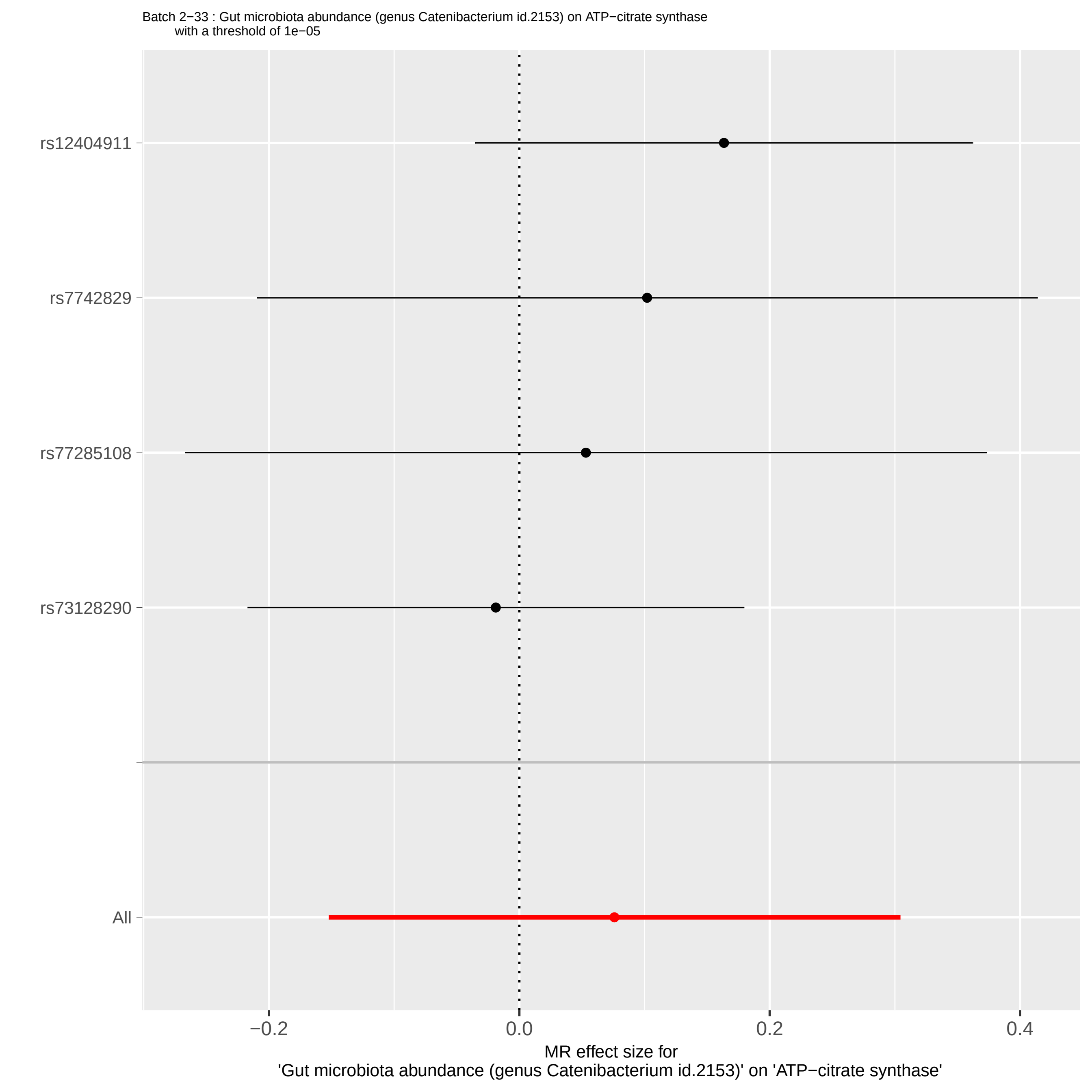
Batch 2−33 : Gut microbiota abundance (genus Catenibacterium id.2153) on ATP−citrate synthase
with a threshold of 1e−05
rs12404911
rs7742829
rs77285108
rs73128290
All
−0.2
0.0
0.2
0.4
MR effect size for
'Gut microbiota abundance (genus Catenibacterium id.2153)' on 'ATP−citrate synthase'

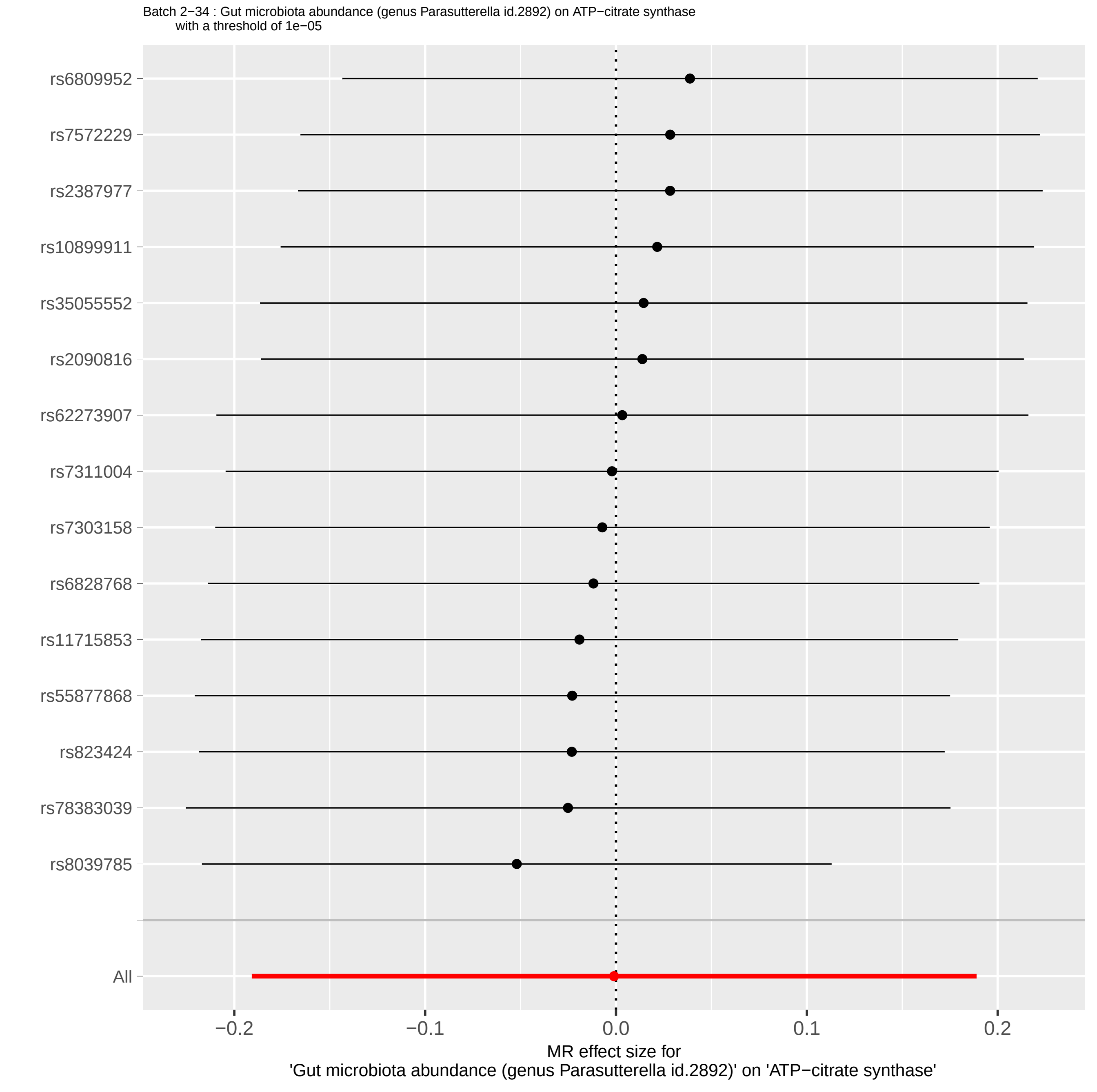

Batch 2−34 : Gut microbiota abundance (genus Parasutterella id.2892) on ATP−citrate synthase
with a threshold of 1e−05
rs6809952
rs7572229
rs2387977
rs10899911
rs35055552
rs2090816
rs62273907
rs7311004
rs7303158
rs6828768
rs11715853
rs55877868
rs823424
rs78383039
rs8039785
All
−0.2
−0.1
0.0
0.1
0.2
MR effect size for
'Gut microbiota abundance (genus Parasutterella id.2892)' on 'ATP−citrate synthase'

Batch 2−35 : Gut microbiota abundance (genus Catenibacterium id.2153) on Low molecular weight phosphotyrosine protein phosphatase with a threshold of 1e−05

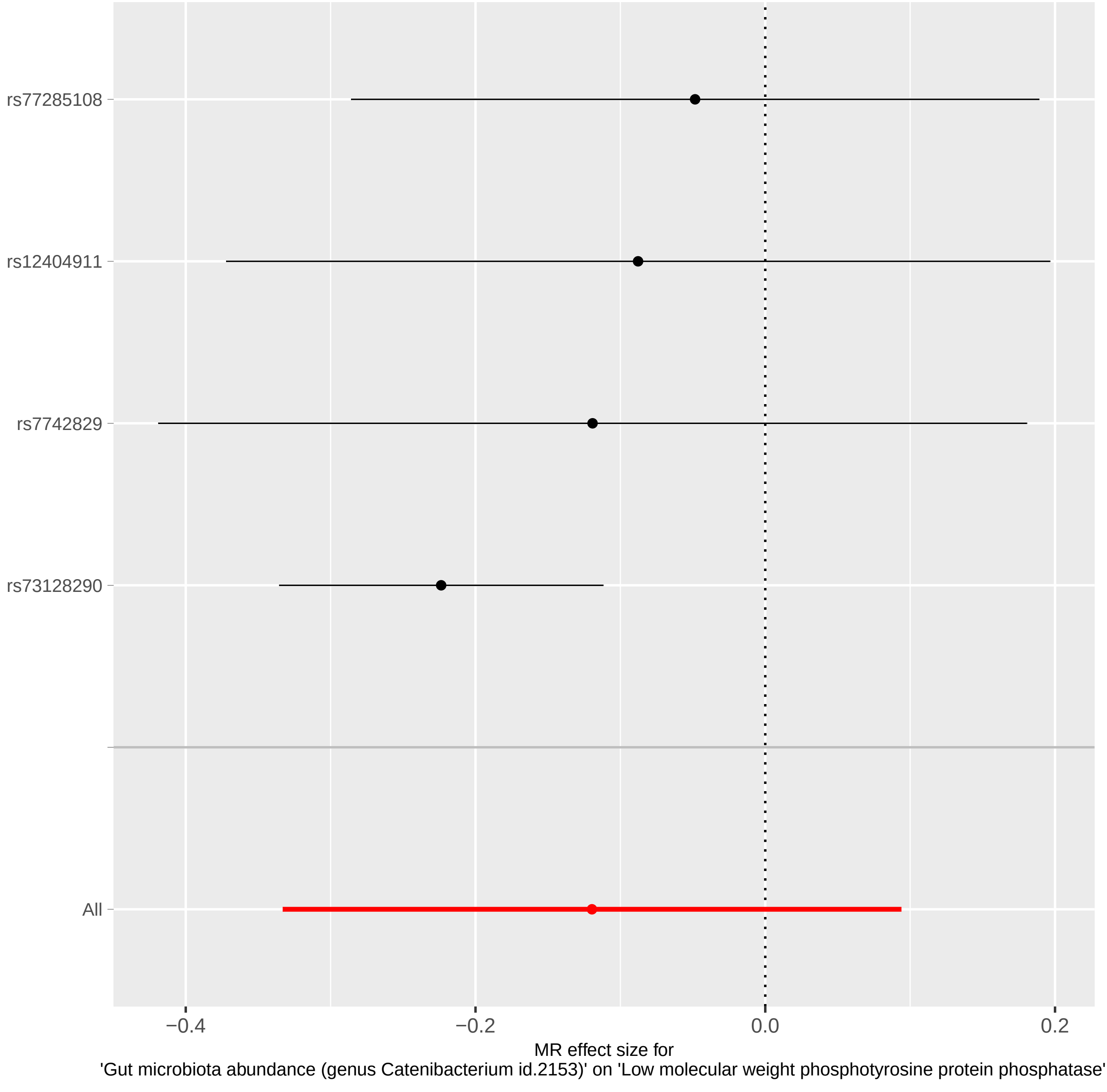

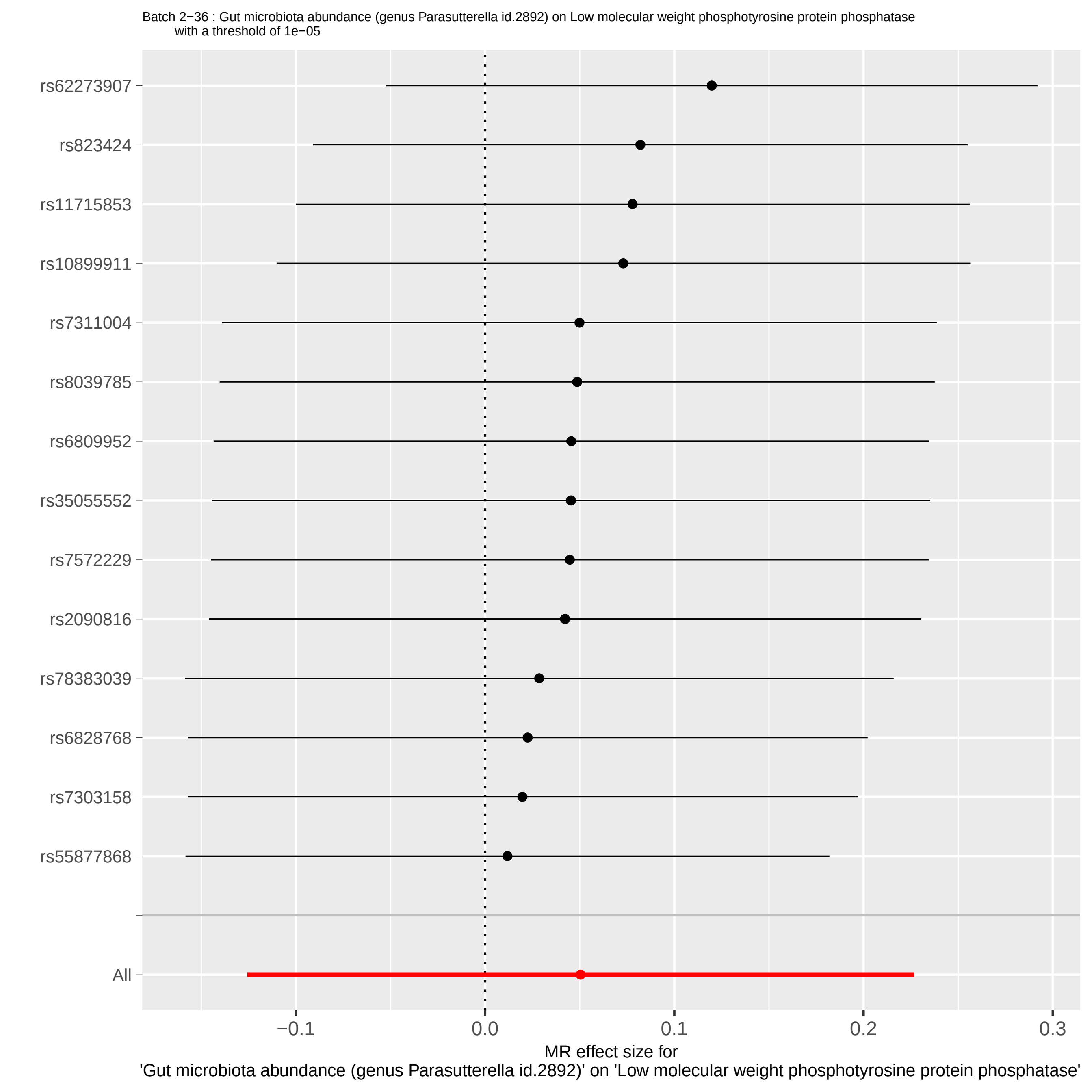
Batch 2−36 : Gut microbiota abundance (genus Parasutterella id.2892) on Low molecular weight phosphotyrosine protein phosphatase with a threshold of 1e−05
rs62273907
rs823424
rs11715853
rs10899911
rs7311004
rs8039785
rs6809952
rs35055552
rs7572229
rs2090816
rs78383039
rs6828768
rs7303158
rs55877868
All
−0.1
0.0
0.1
0.2
0.3
MR effect size for
'Gut microbiota abundance (genus Parasutterella id.2892)' on 'Low molecular weight phosphotyrosine protein phosphatase'

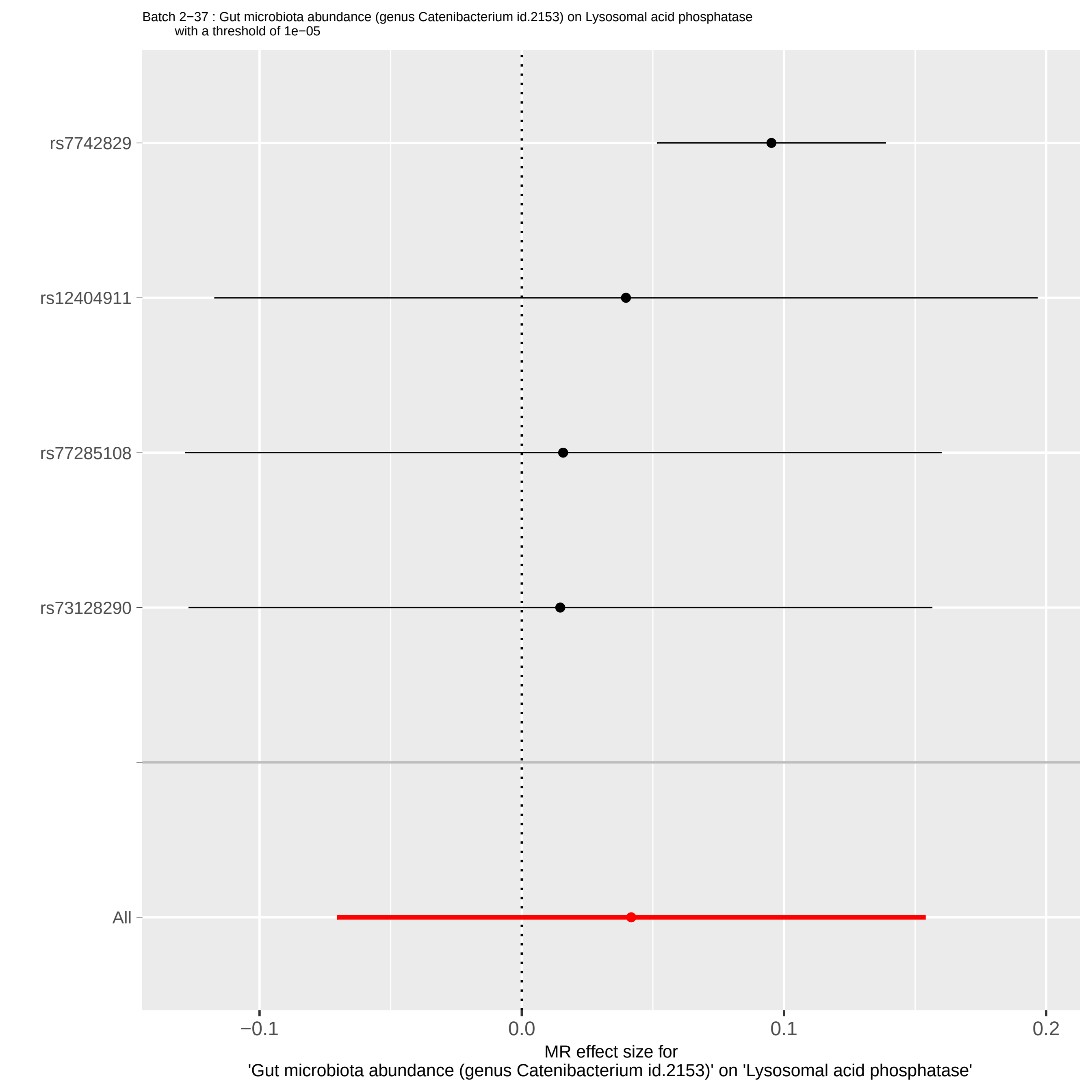
Batch 2−37 : Gut microbiota abundance (genus Catenibacterium id.2153) on Lysosomal acid phosphatase
with a threshold of 1e−05
rs7742829
rs12404911
rs77285108
rs73128290
All
−0.1
0.0
0.1
0.2
MR effect size for
'Gut microbiota abundance (genus Catenibacterium id.2153)' on 'Lysosomal acid phosphatase'

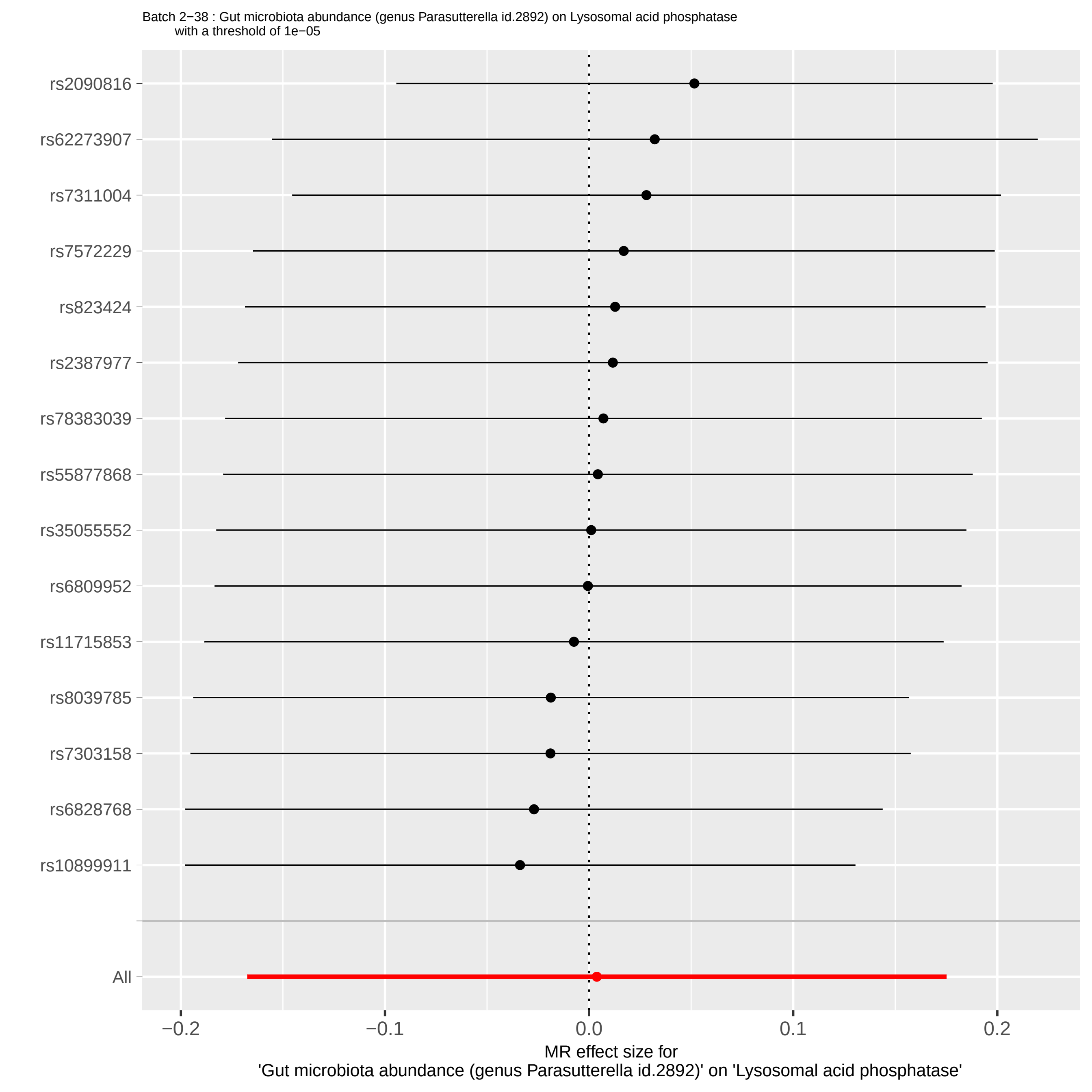

Batch 2−38 : Gut microbiota abundance (genus Parasutterella id.2892) on Lysosomal acid phosphatase
with a threshold of 1e−05
rs2090816
rs62273907
rs7311004
rs7572229
rs823424
rs2387977
rs78383039
rs55877868
rs35055552
rs6809952
rs11715853
rs8039785
rs7303158
rs6828768
rs10899911
All
−0.2
−0.1
0.0
0.1
0.2
MR effect size for
'Gut microbiota abundance (genus Parasutterella id.2892)' on 'Lysosomal acid phosphatase'

Batch 2−39 : Gut microbiota abundance (genus Catenibacterium id.2153) on Tartrate−resistant acid phosphatase type 5 with a threshold of 1e−05

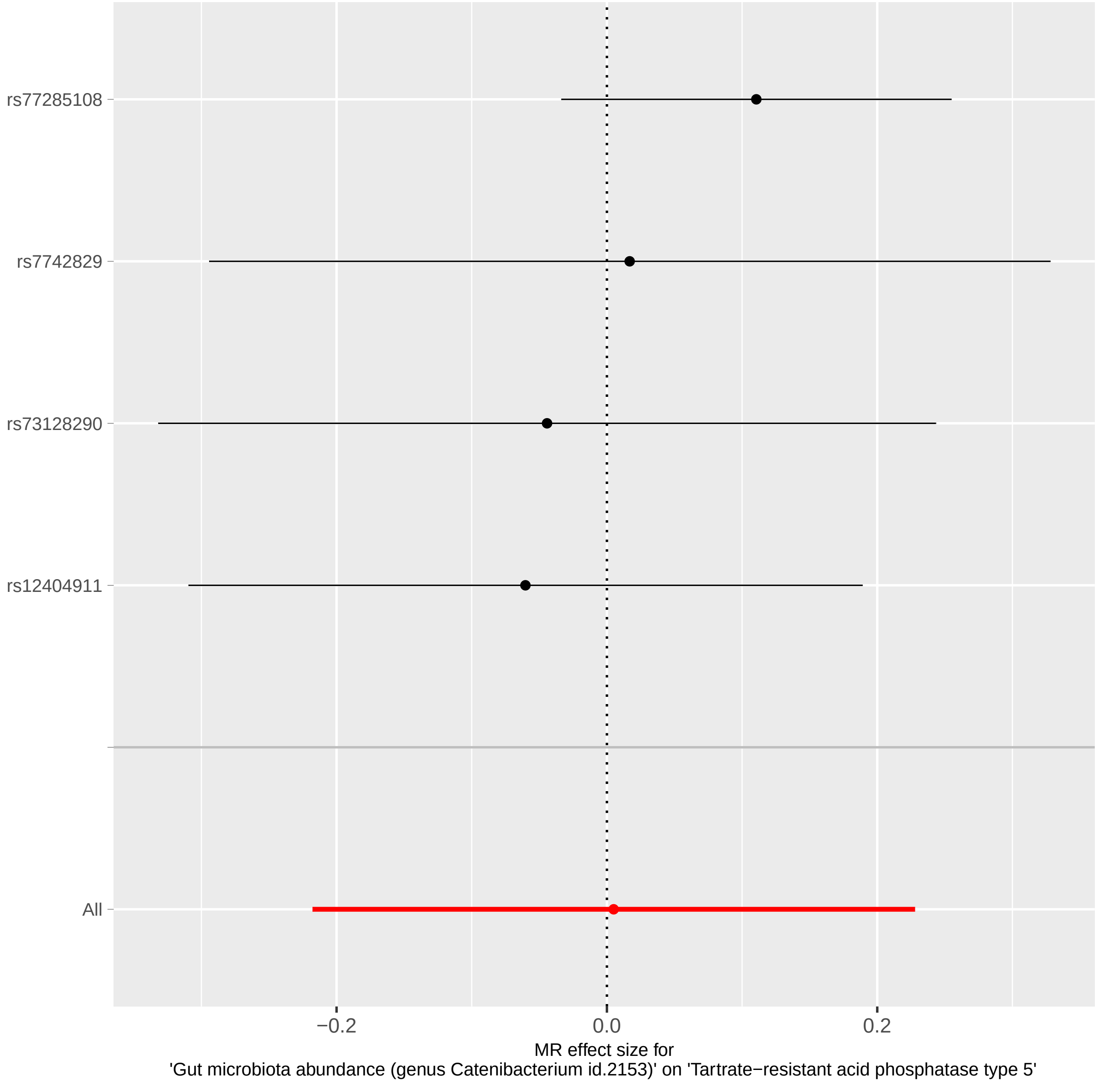

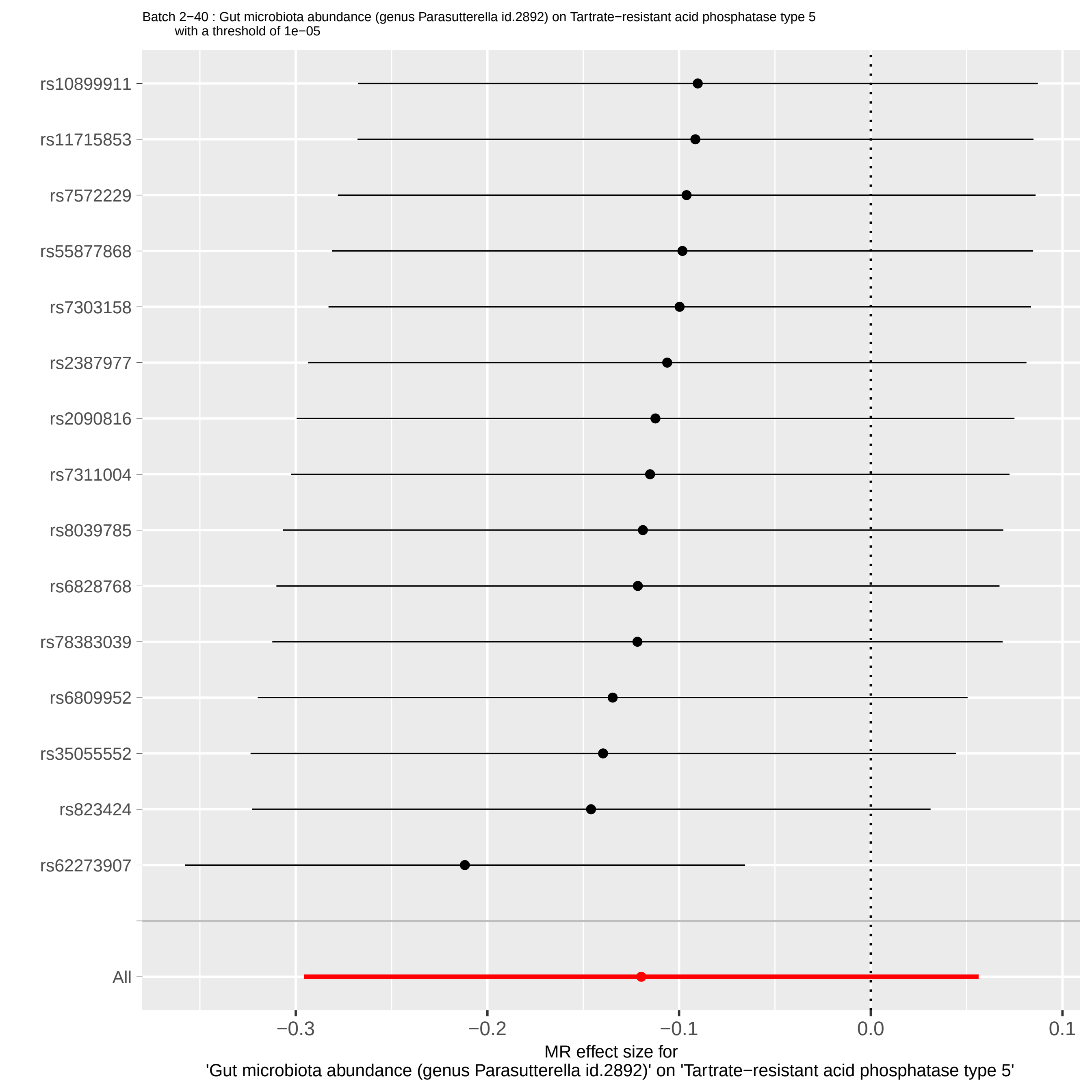

Batch 2−40 : Gut microbiota abundance (genus Parasutterella id.2892) on Tartrate−resistant acid phosphatase type 5
with a threshold of 1e−05
rs10899911
rs11715853
rs7572229
rs55877868
rs7303158
rs2387977
rs2090816
rs7311004
rs8039785
rs6828768
rs78383039
rs6809952
rs35055552
rs823424
rs62273907
All
−0.3
−0.2
−0.1
0.0
0.1
MR effect size for
'Gut microbiota abundance (genus Parasutterella id.2892)' on 'Tartrate−resistant acid phosphatase type 5'

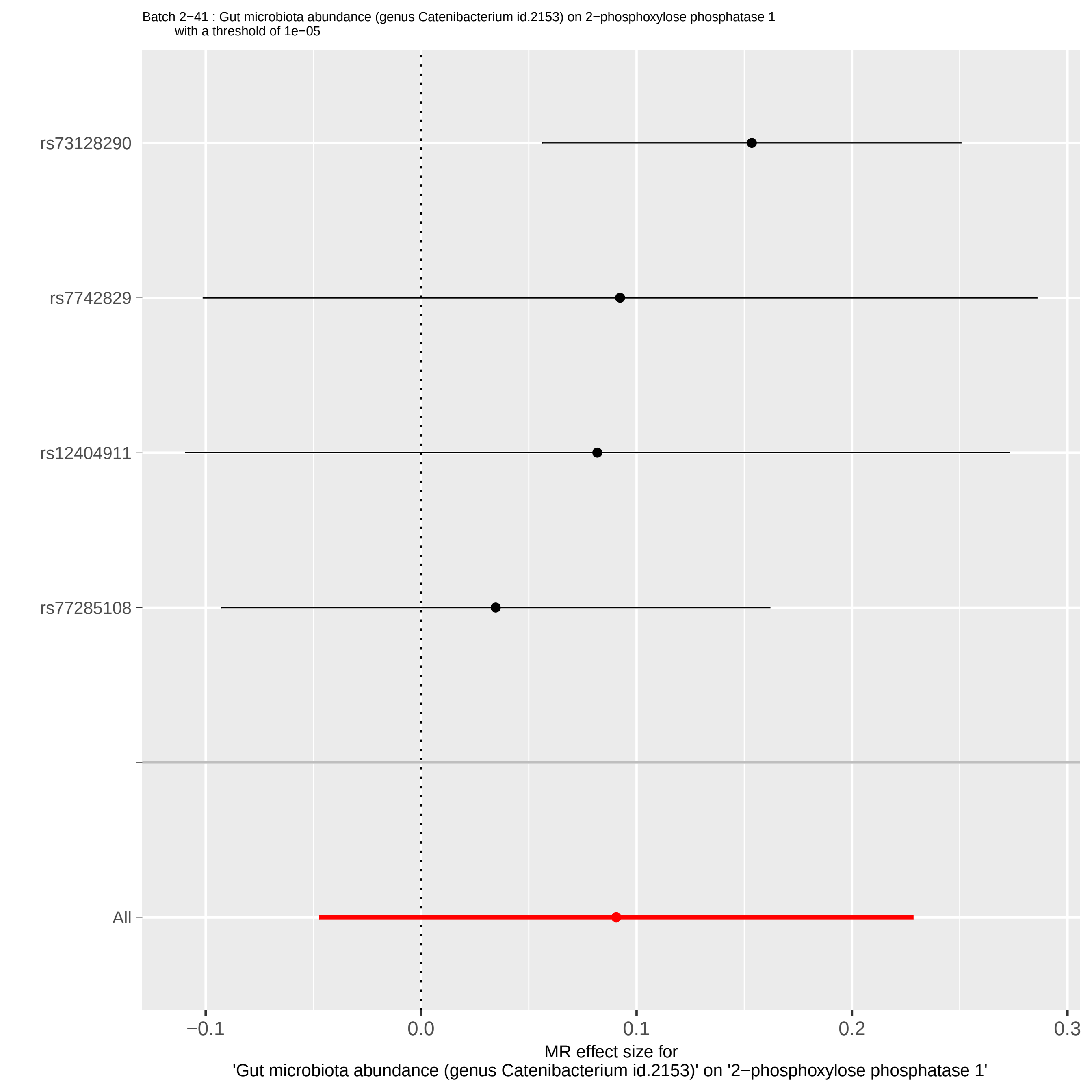
Batch 2−41 : Gut microbiota abundance (genus Catenibacterium id.2153) on 2−phosphoxylose phosphatase 1
with a threshold of 1e−05
rs73128290
rs7742829
rs12404911
rs77285108
All
−0.1
0.0
0.1
0.2
0.3
MR effect size for
'Gut microbiota abundance (genus Catenibacterium id.2153)' on '2−phosphoxylose phosphatase 1'

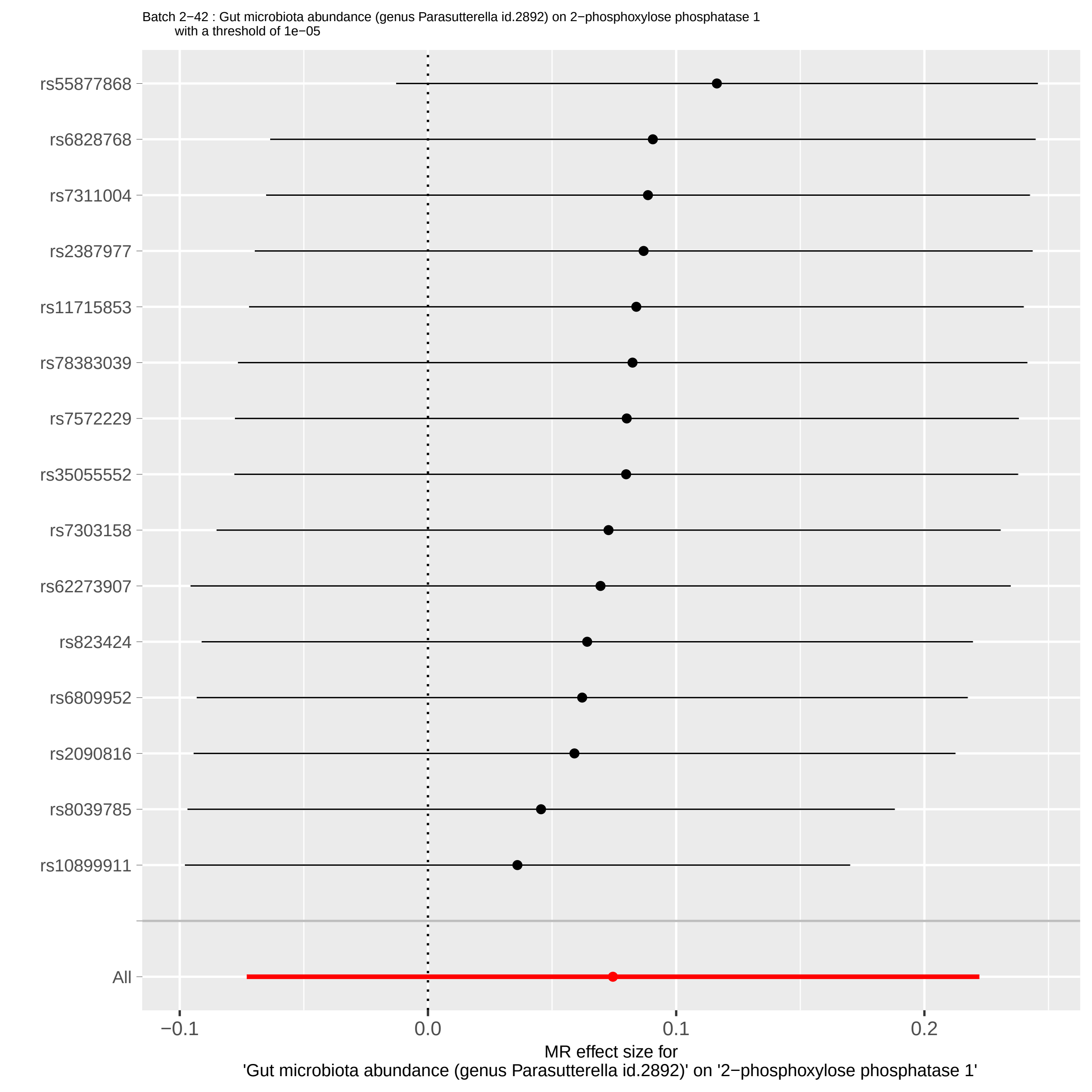
Batch 2−42 : Gut microbiota abundance (genus Parasutterella id.2892) on 2−phosphoxylose phosphatase 1
with a threshold of 1e−05
rs55877868
rs6828768
rs7311004
rs2387977
rs11715853
rs78383039
rs7572229
rs35055552
rs7303158
rs62273907
rs823424
rs6809952
rs2090816
rs8039785
rs10899911
All
−0.1
0.0
0.1
0.2
MR effect size for
'Gut microbiota abundance (genus Parasutterella id.2892)' on '2−phosphoxylose phosphatase 1'

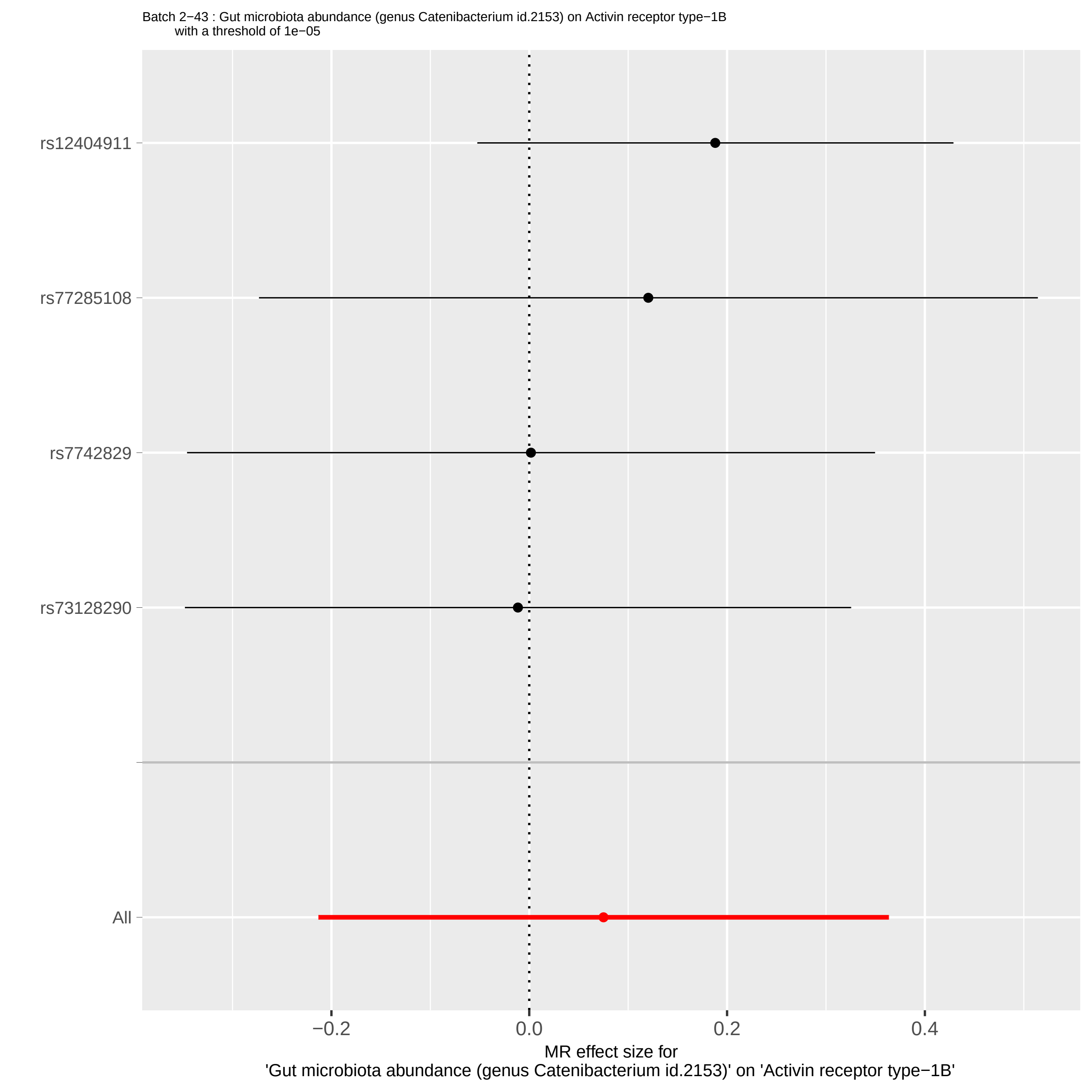

Batch 2−43 : Gut microbiota abundance (genus Catenibacterium id.2153) on Activin receptor type−1B
with a threshold of 1e−05
rs12404911
rs77285108
rs7742829
rs73128290
All
−0.2
0.0
0.2
0.4
MR effect size for
'Gut microbiota abundance (genus Catenibacterium id.2153)' on 'Activin receptor type−1B'

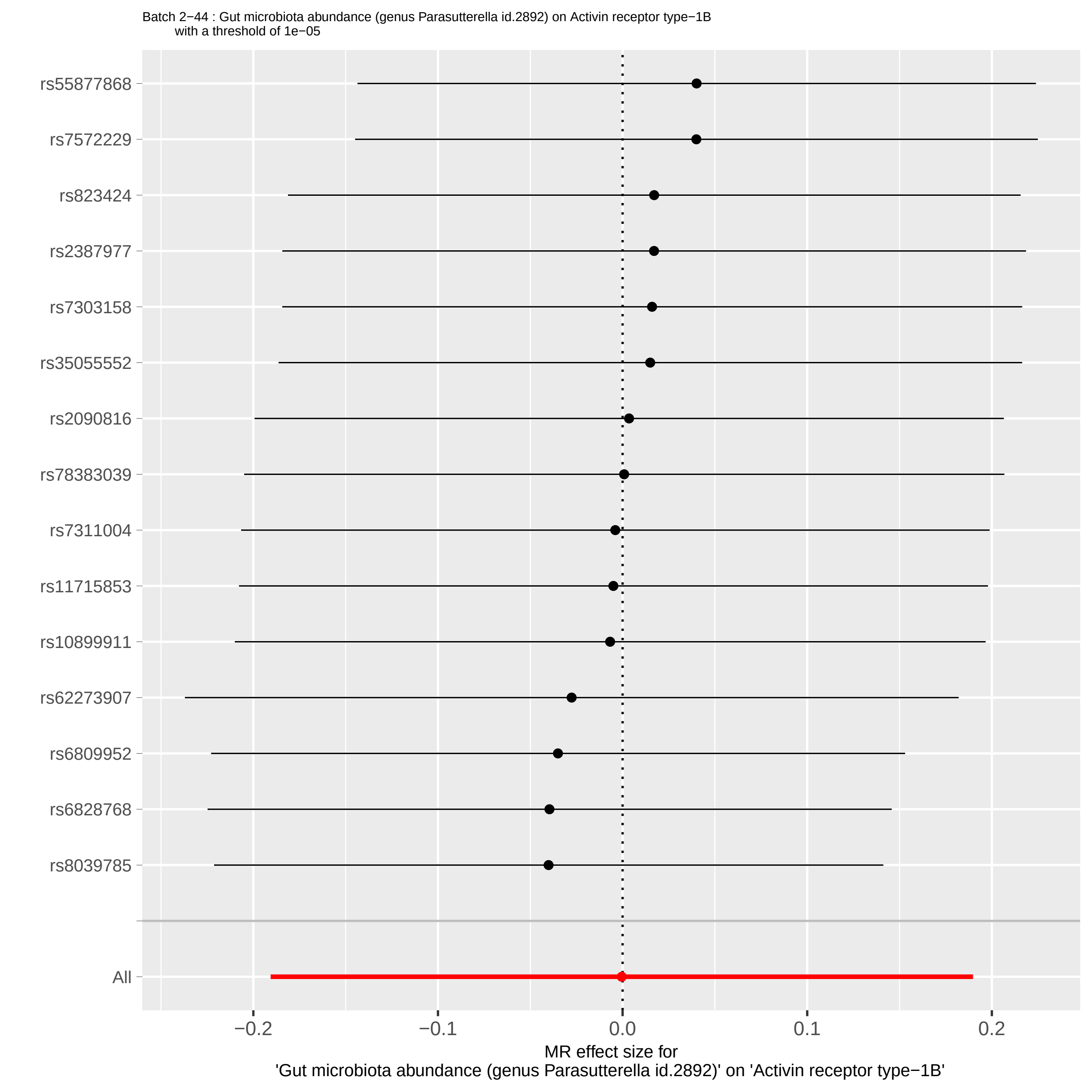

Batch 2−44 : Gut microbiota abundance (genus Parasutterella id.2892) on Activin receptor type−1B
with a threshold of 1e−05
rs55877868
rs7572229
rs823424
rs2387977
rs7303158
rs35055552
rs2090816
rs78383039
rs7311004
rs11715853
rs10899911
rs62273907
rs6809952
rs6828768
rs8039785
All
−0.2
−0.1
0.0
0.1
0.2
MR effect size for
'Gut microbiota abundance (genus Parasutterella id.2892)' on 'Activin receptor type−1B'

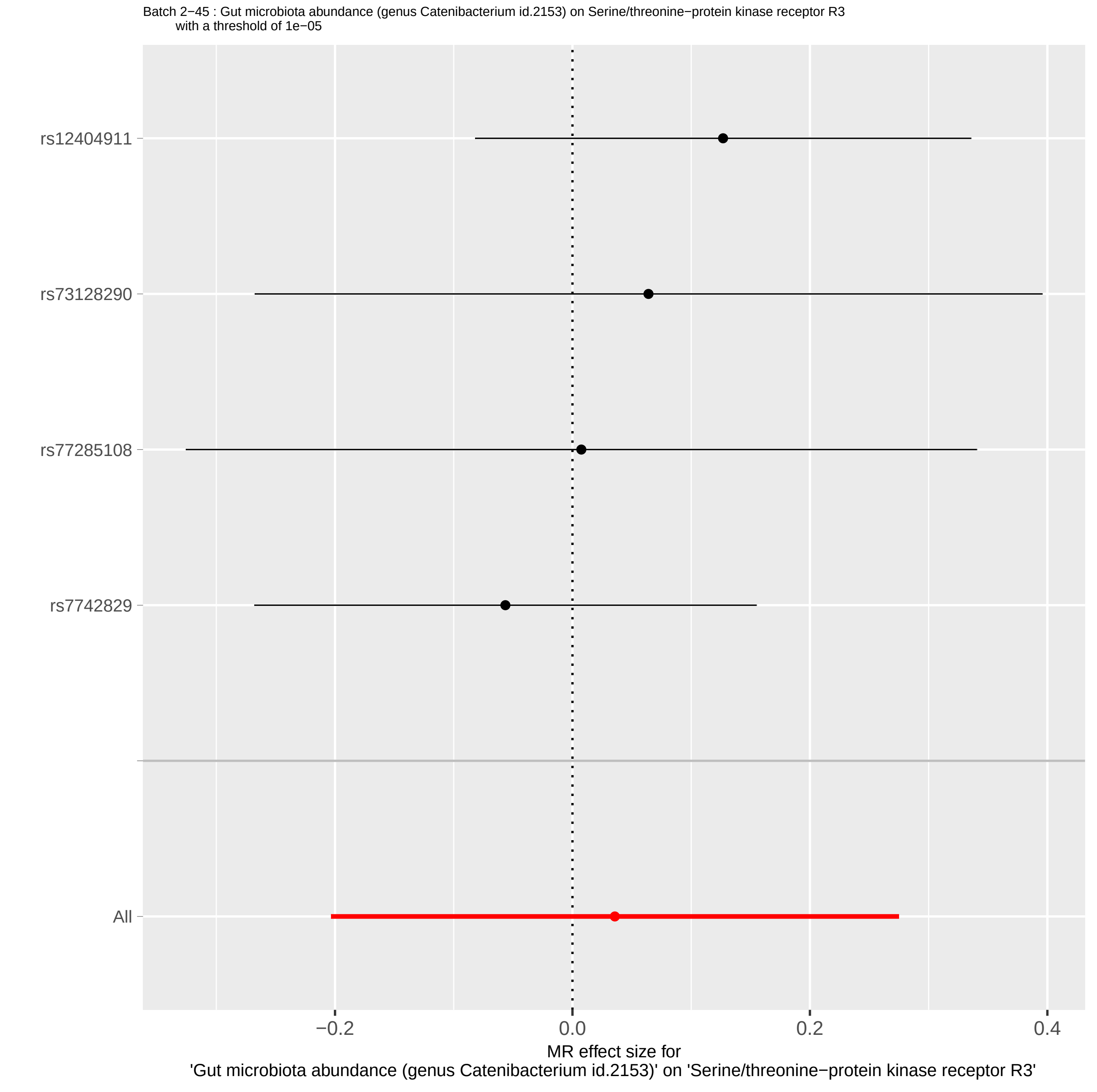
Batch 2−45 : Gut microbiota abundance (genus Catenibacterium id.2153) on Serine/threonine−protein kinase receptor R3
with a threshold of 1e−05
rs12404911
rs73128290
rs77285108
rs7742829
All
−0.2
0.0
0.2
0.4
MR effect size for
'Gut microbiota abundance (genus Catenibacterium id.2153)' on 'Serine/threonine−protein kinase receptor R3'

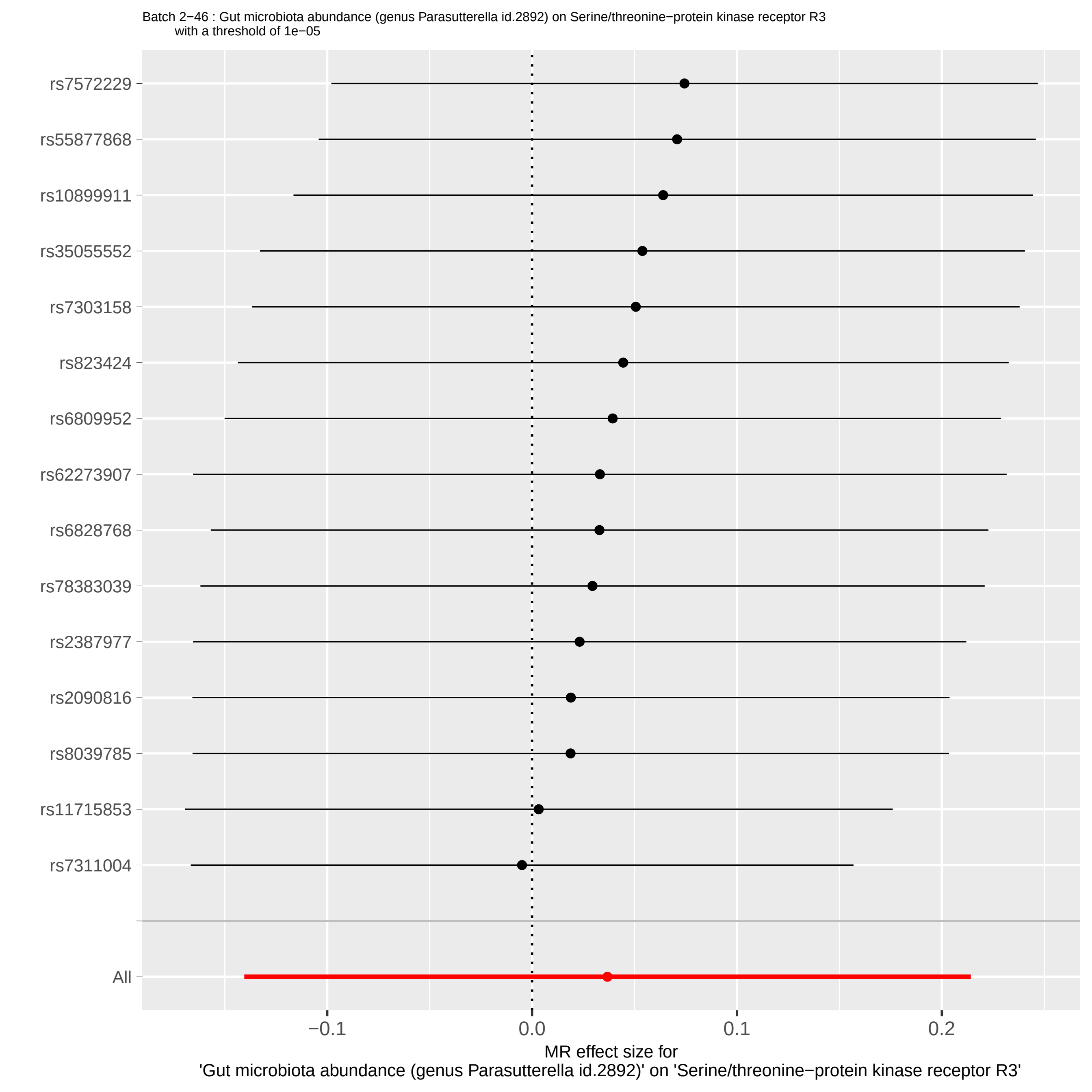

Batch 2−46 : Gut microbiota abundance (genus Parasutterella id.2892) on Serine/threonine−protein kinase receptor R3
with a threshold of 1e−05
rs7572229
rs55877868
rs10899911
rs35055552
rs7303158
rs823424
rs6809952
rs62273907
rs6828768
rs78383039
rs2387977
rs2090816
rs8039785
rs11715853
rs7311004
All
−0.1
0.0
0.1
0.2
MR effect size for
'Gut microbiota abundance (genus Parasutterella id.2892)' on 'Serine/threonine−protein kinase receptor R3'

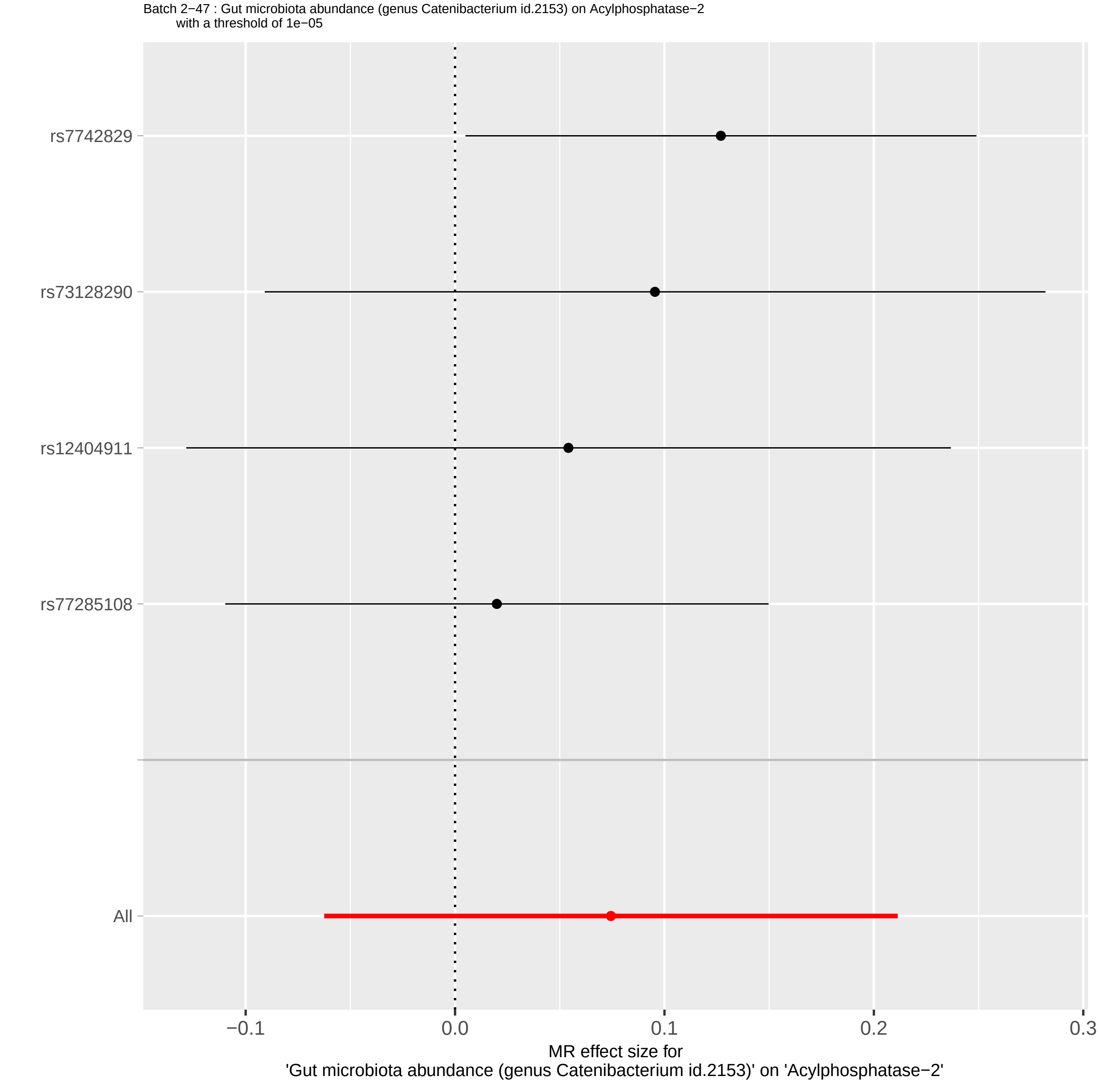

Batch 2−47 : Gut microbiota abundance (genus Catenibacterium id.2153) on Acylphosphatase−2
with a threshold of 1e−05
rs7742829
rs73128290
rs12404911
rs77285108
All
−0.1
0.0
0.1
0.2
0.3
MR effect size for
'Gut microbiota abundance (genus Catenibacterium id.2153)' on 'Acylphosphatase−2'

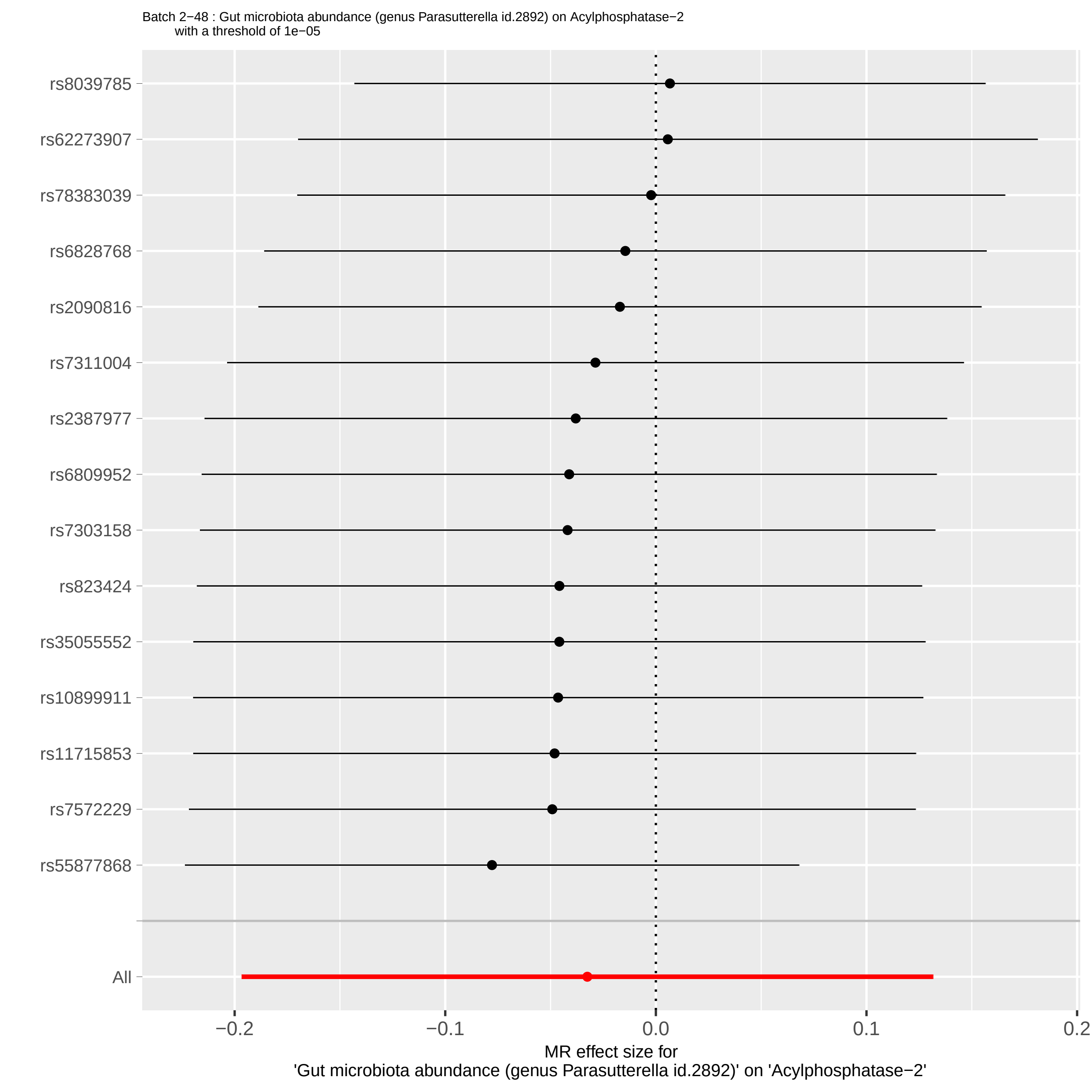

Batch 2−48 : Gut microbiota abundance (genus Parasutterella id.2892) on Acylphosphatase−2
with a threshold of 1e−05
rs8039785
rs62273907
rs78383039
rs6828768
rs2090816
rs7311004
rs2387977
rs6809952
rs7303158
rs823424
rs35055552
rs10899911
rs11715853
rs7572229
rs55877868
All
−0.2
−0.1
0.0
0.1
0.2
MR effect size for
'Gut microbiota abundance (genus Parasutterella id.2892)' on 'Acylphosphatase−2'

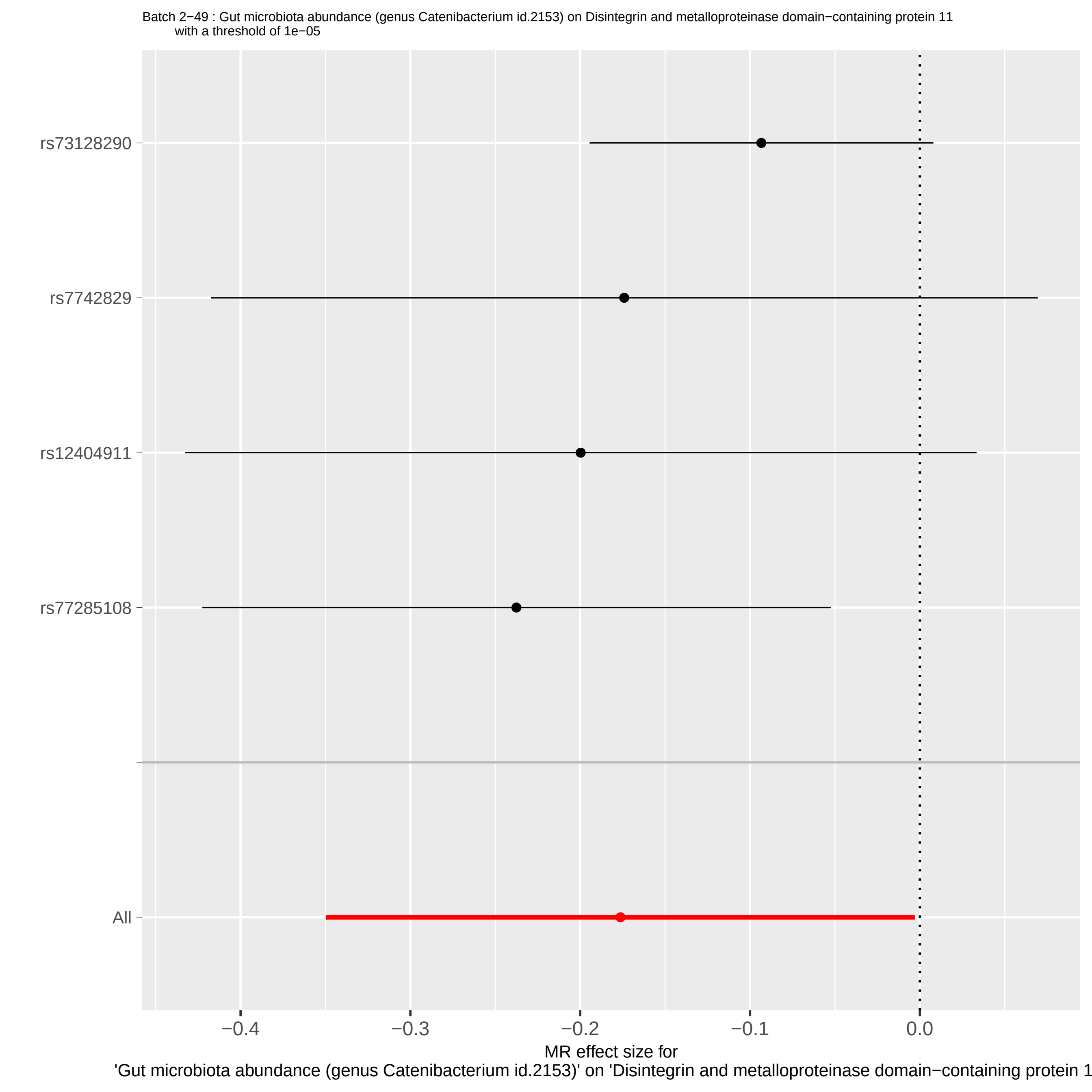

Batch 2−49 : Gut microbiota abundance (genus Catenibacterium id.2153) on Disintegrin and metalloproteinase domain−containing protein 11
with a threshold of 1e−05
rs73128290
rs7742829
rs12404911
rs77285108
All
−0.4
−0.3
−0.2
−0.1
0.0
MR effect size for
'Gut microbiota abundance (genus Catenibacterium id.2153)' on 'Disintegrin and metalloproteinase domain−containing protein 1

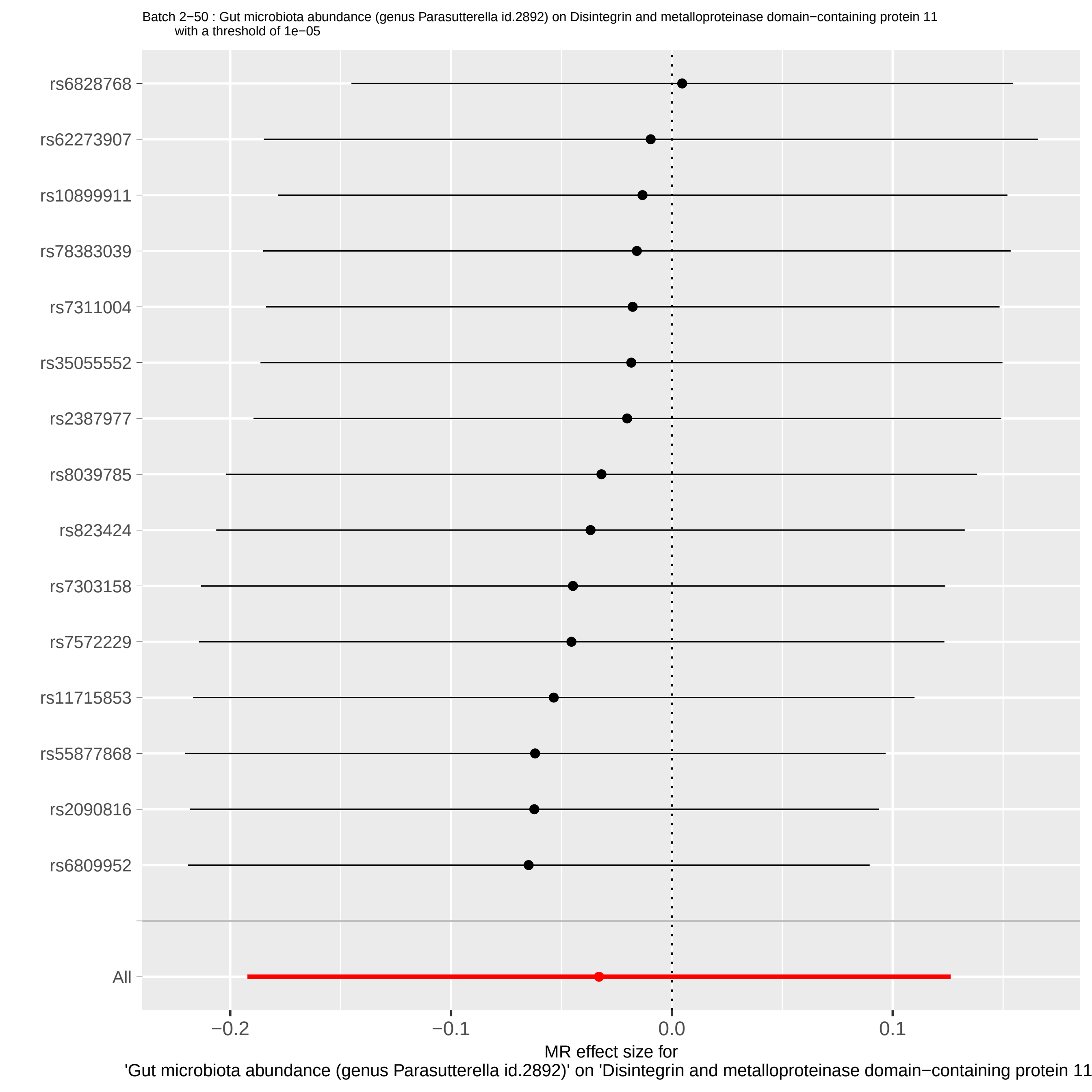

Batch 2−50 : Gut microbiota abundance (genus Parasutterella id.2892) on Disintegrin and metalloproteinase domain−containing protein 11
with a threshold of 1e−05
rs6828768
rs62273907
rs10899911
rs78383039
rs7311004
rs35055552
rs2387977
rs8039785
rs823424
rs7303158
rs7572229
rs11715853
rs55877868
rs2090816
rs6809952
All
−0.2
−0.1
0.0
0.1
MR effect size for
'Gut microbiota abundance (genus Parasutterella id.2892)' on 'Disintegrin and metalloproteinase domain−containing protein 11

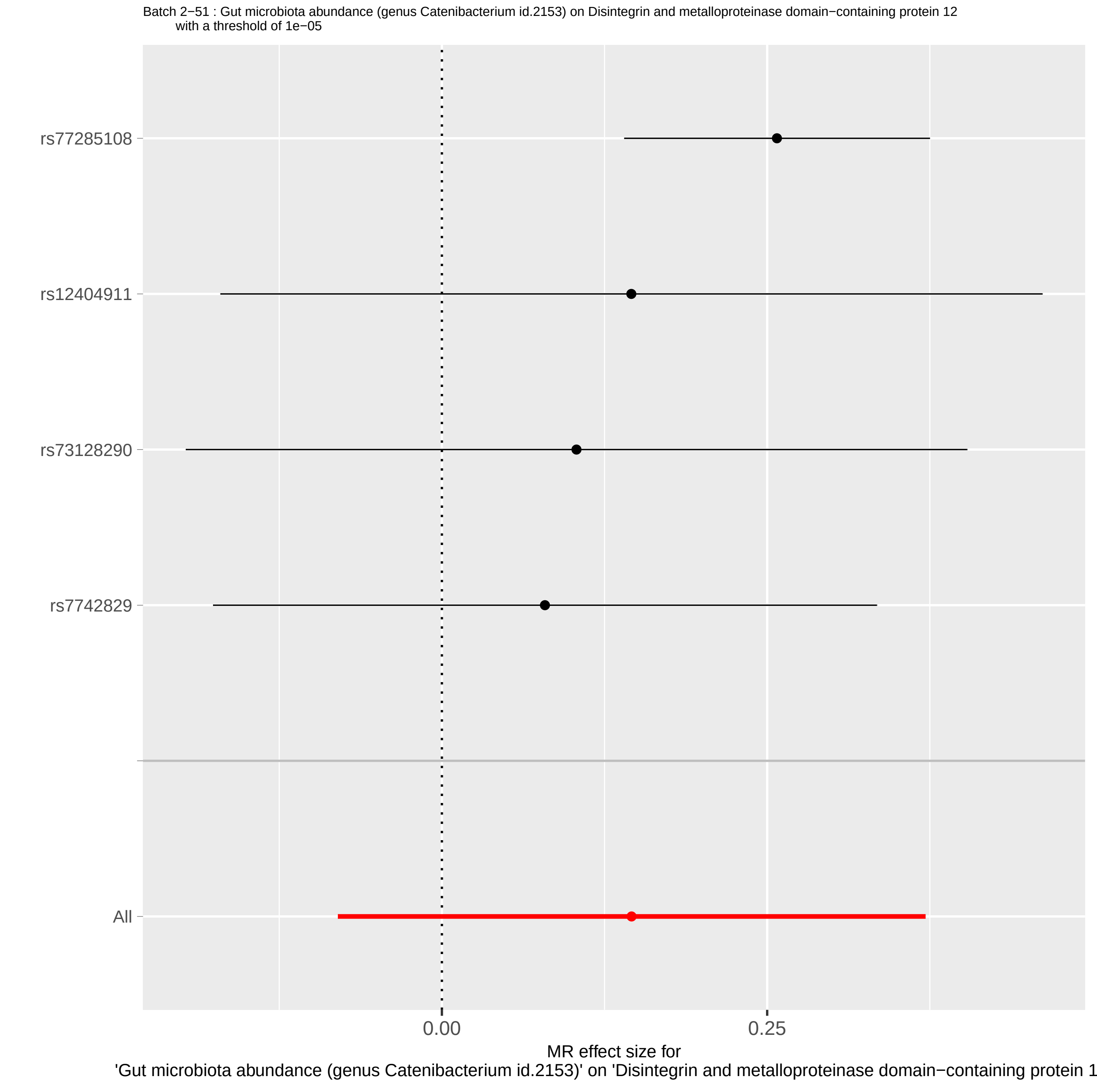

Batch 2−51 : Gut microbiota abundance (genus Catenibacterium id.2153) on Disintegrin and metalloproteinase domain−containing protein 12
with a threshold of 1e−05
rs77285108
rs12404911
rs73128290
rs7742829
All
0.00
0.25
MR effect size for
'Gut microbiota abundance (genus Catenibacterium id.2153)' on 'Disintegrin and metalloproteinase domain−containing protein 1

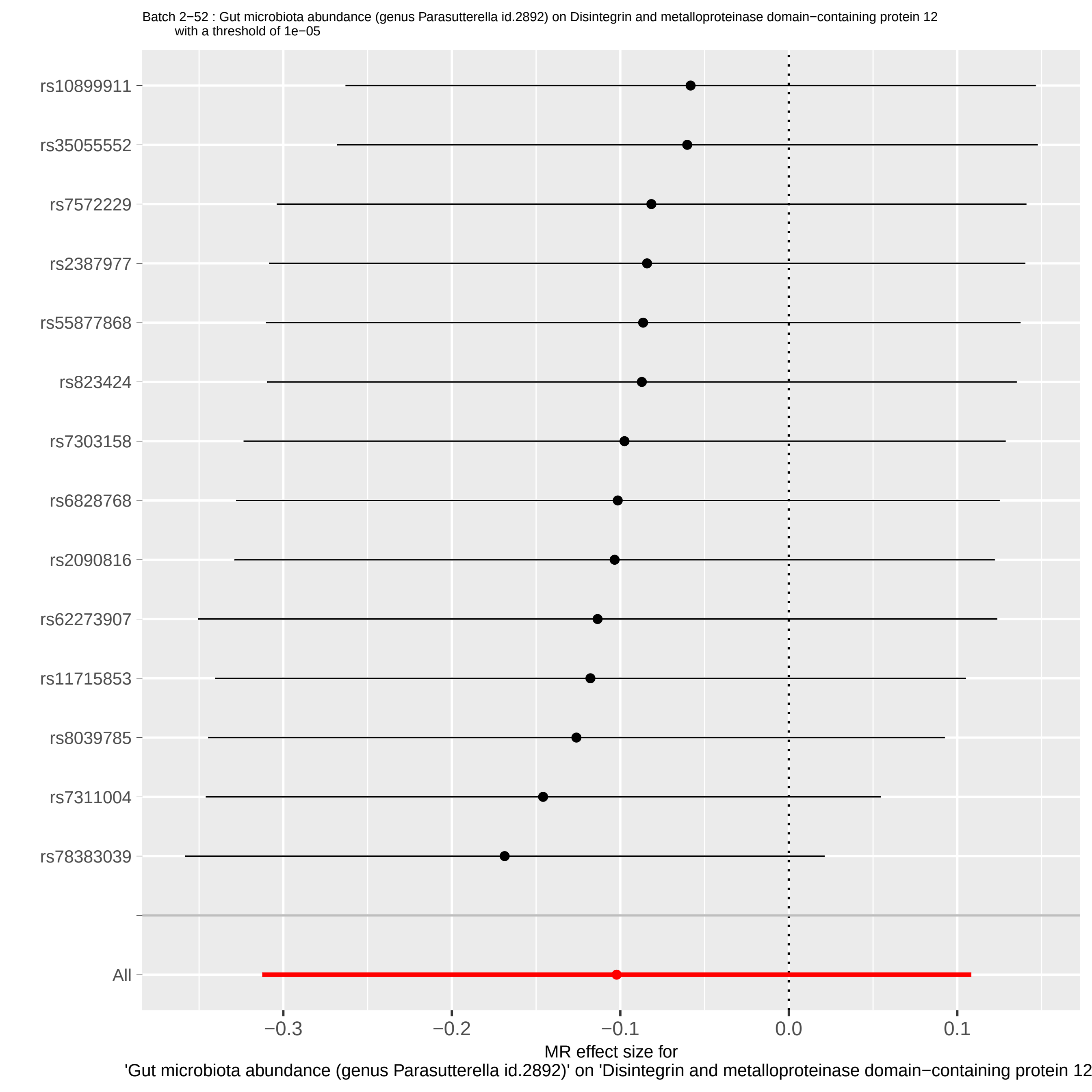
Batch 2−52 : Gut microbiota abundance (genus Parasutterella id.2892) on Disintegrin and metalloproteinase domain−containing protein 12
with a threshold of 1e−05
rs10899911
rs35055552
rs7572229
rs2387977
rs55877868
rs823424
rs7303158
rs6828768
rs2090816
rs62273907
rs11715853
rs8039785
rs7311004
rs78383039
All
−0.3
−0.2
−0.1
0.0
0.1
MR effect size for
'Gut microbiota abundance (genus Parasutterella id.2892)' on 'Disintegrin and metalloproteinase domain−containing protein 12

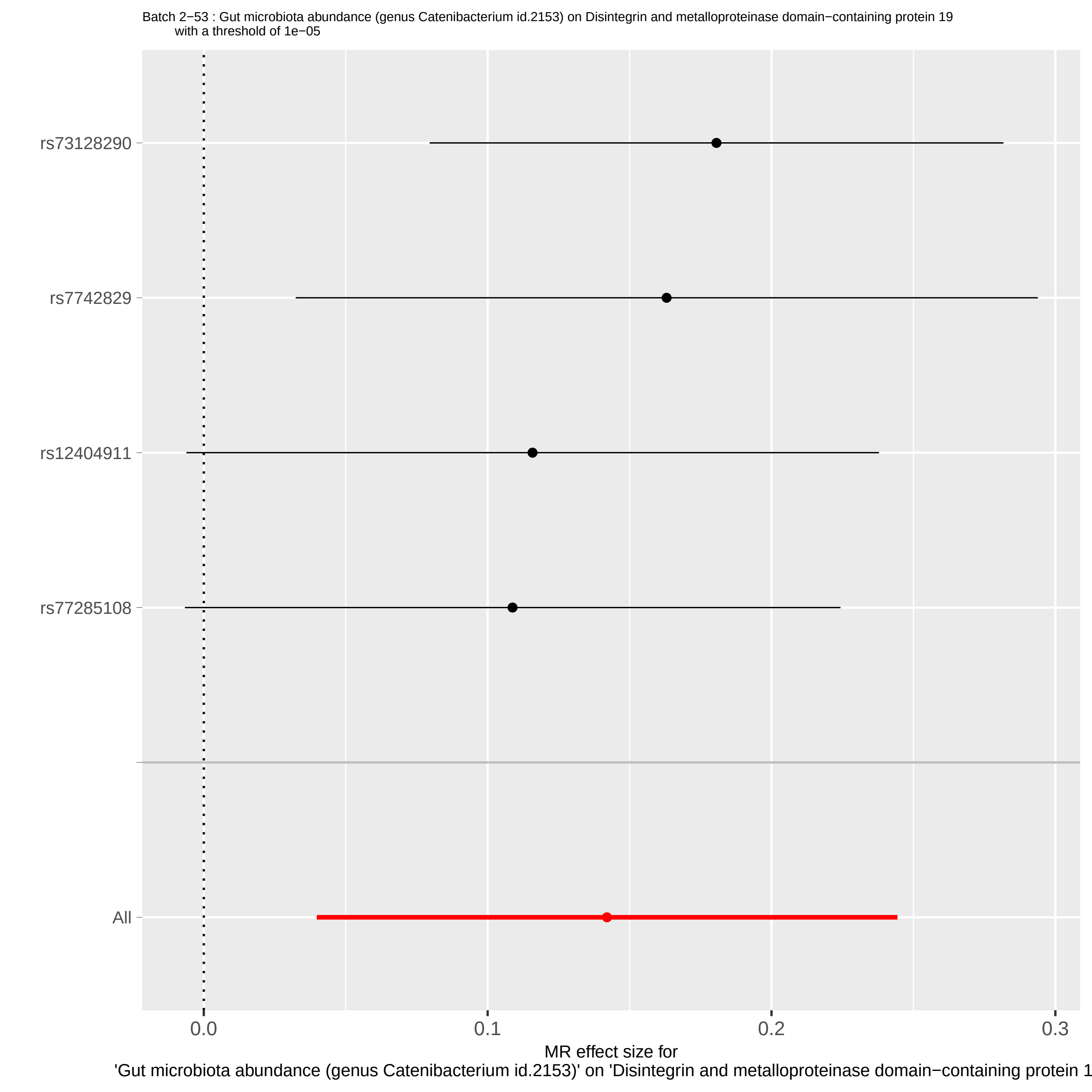
Batch 2−53 : Gut microbiota abundance (genus Catenibacterium id.2153) on Disintegrin and metalloproteinase domain−containing protein 19
with a threshold of 1e−05
rs73128290
rs7742829
rs12404911
rs77285108
All
0.0
0.1
0.2
0.3
MR effect size for
'Gut microbiota abundance (genus Catenibacterium id.2153)' on 'Disintegrin and metalloproteinase domain−containing protein 1

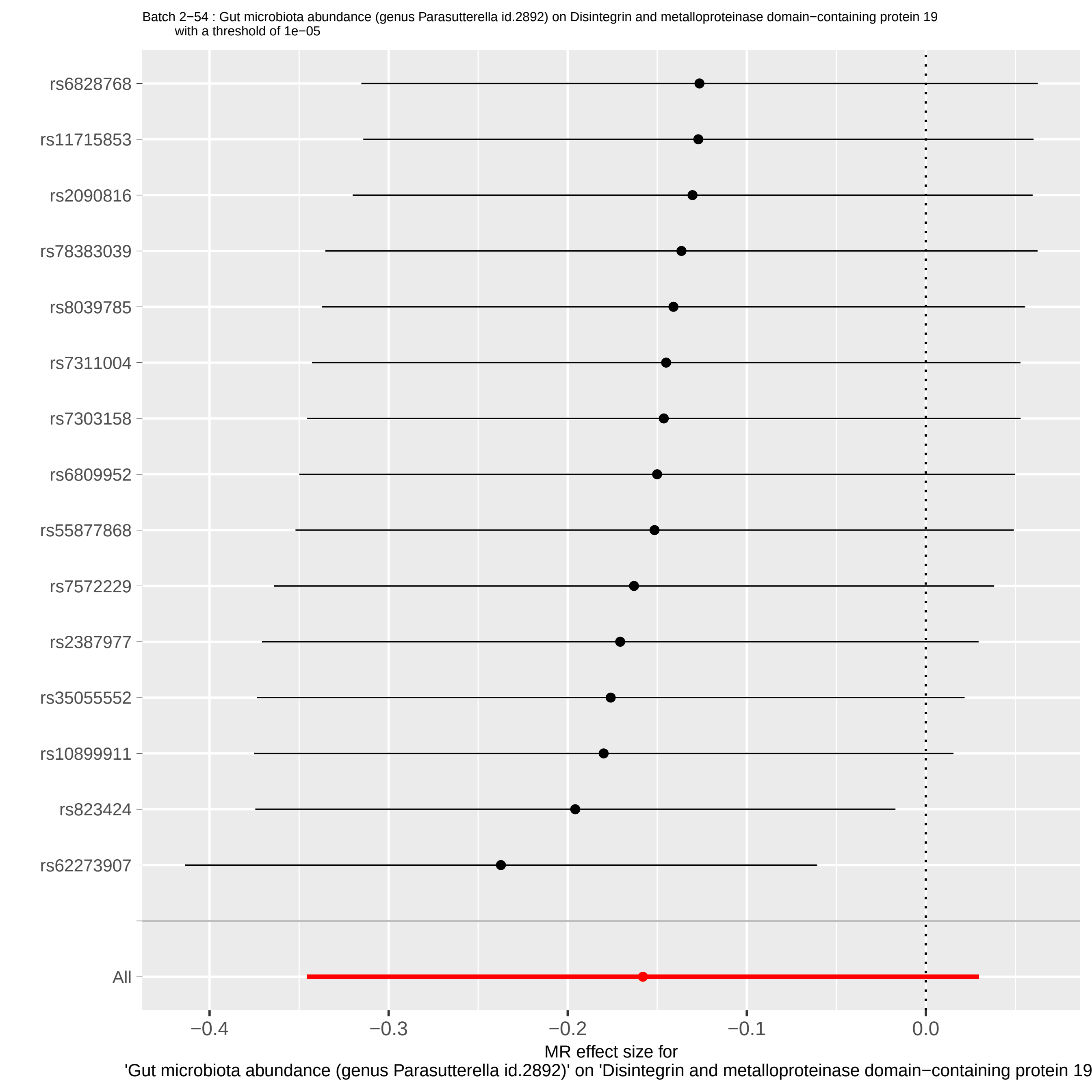

Batch 2−54 : Gut microbiota abundance (genus Parasutterella id.2892) on Disintegrin and metalloproteinase domain−containing protein 19
with a threshold of 1e−05
rs6828768
rs11715853
rs2090816
rs78383039
rs8039785
rs7311004
rs7303158
rs6809952
rs55877868
rs7572229
rs2387977
rs35055552
rs10899911
rs823424
rs62273907
All
−0.4
−0.3
−0.2
−0.1
0.0
MR effect size for
'Gut microbiota abundance (genus Parasutterella id.2892)' on 'Disintegrin and metalloproteinase domain−containing protein 19'

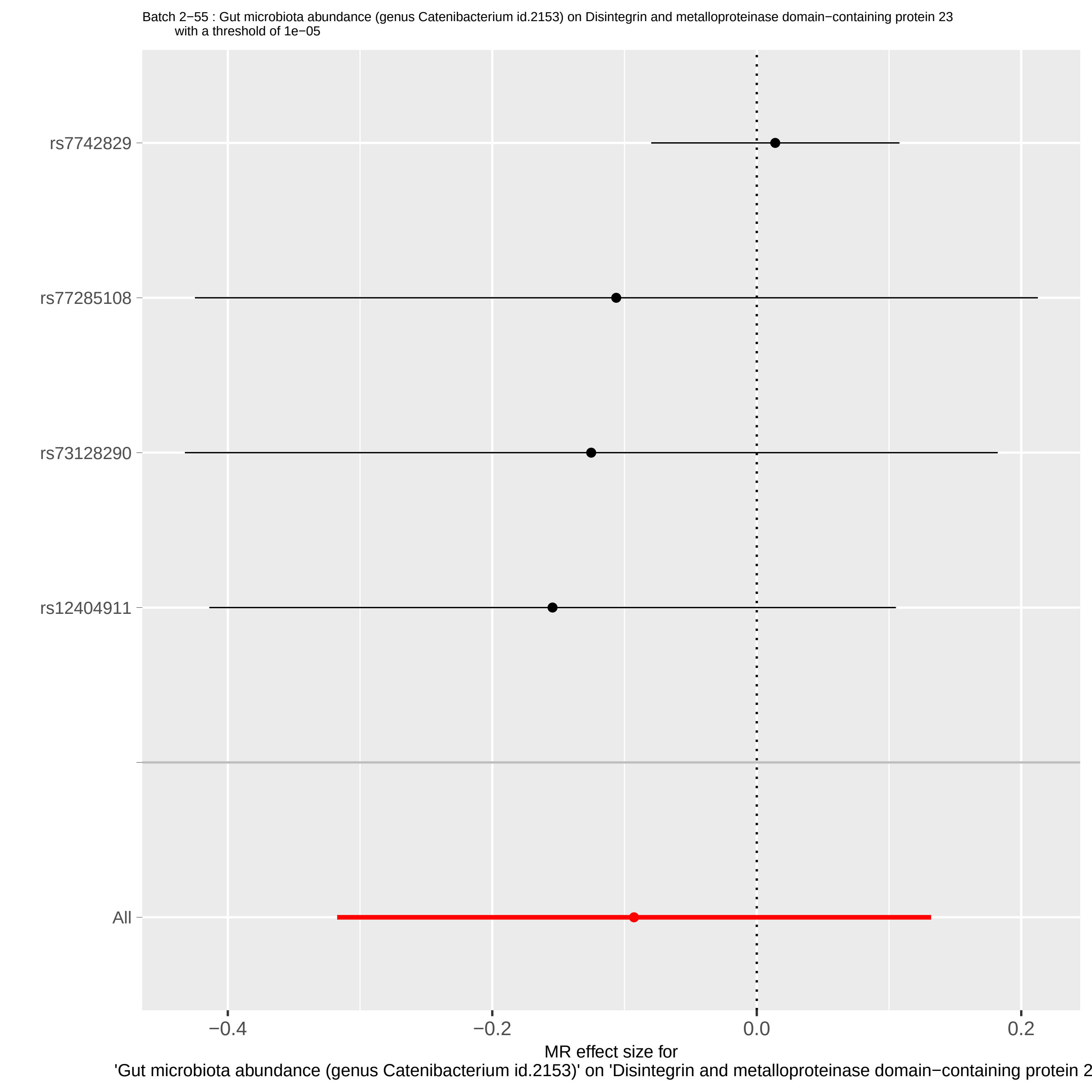

Batch 2−55 : Gut microbiota abundance (genus Catenibacterium id.2153) on Disintegrin and metalloproteinase domain−containing protein 23
with a threshold of 1e−05
rs7742829
rs77285108
rs73128290
rs12404911
All
−0.4
−0.2
0.0
0.2
MR effect size for
'Gut microbiota abundance (genus Catenibacterium id.2153)' on 'Disintegrin and metalloproteinase domain−containing protein 2

Batch 2−56 : Gut microbiota abundance (genus Parasutterella id.2892) on Disintegrin and metalloproteinase domain−containing protein 23 with a threshold of 1e−05

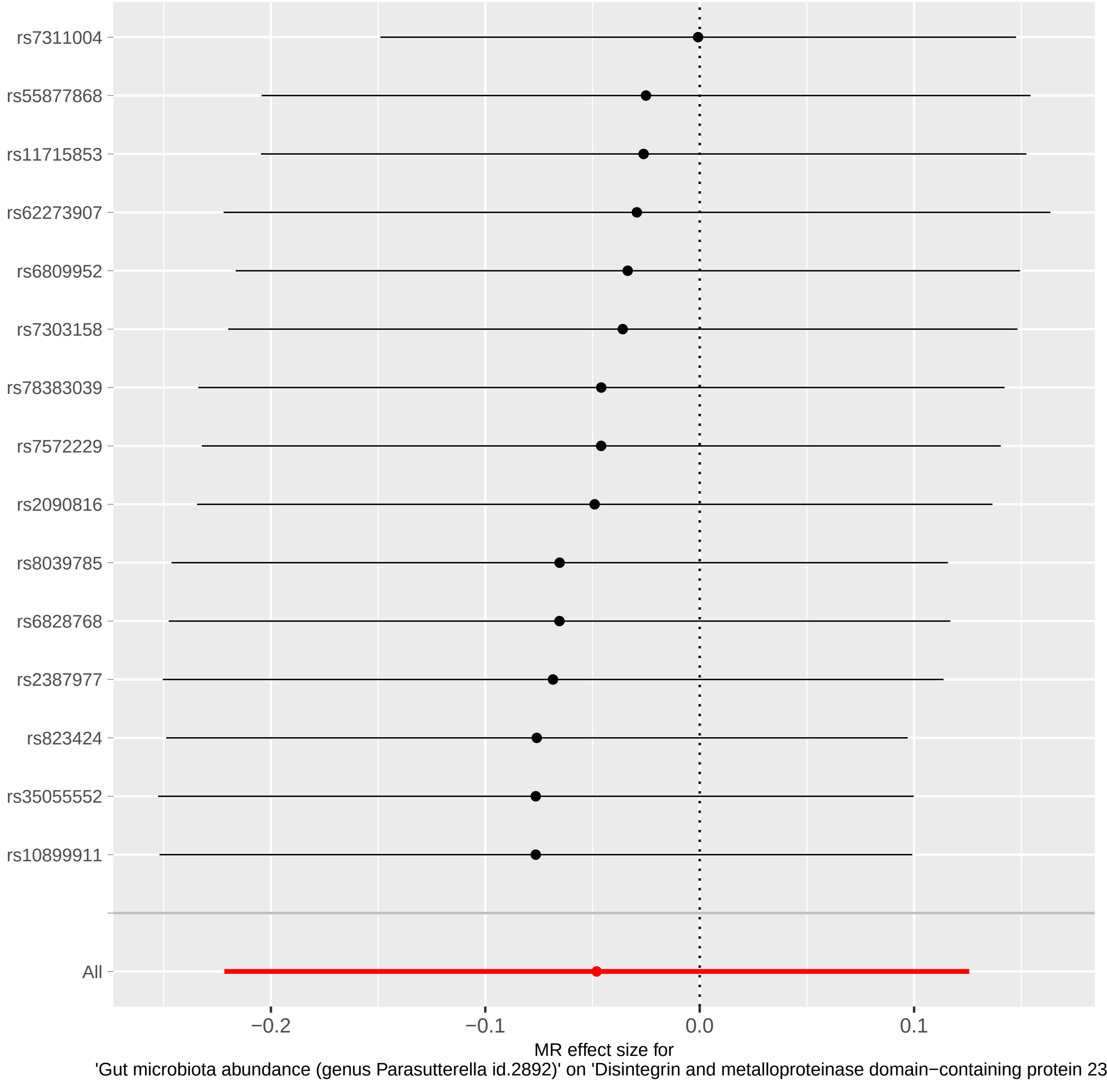

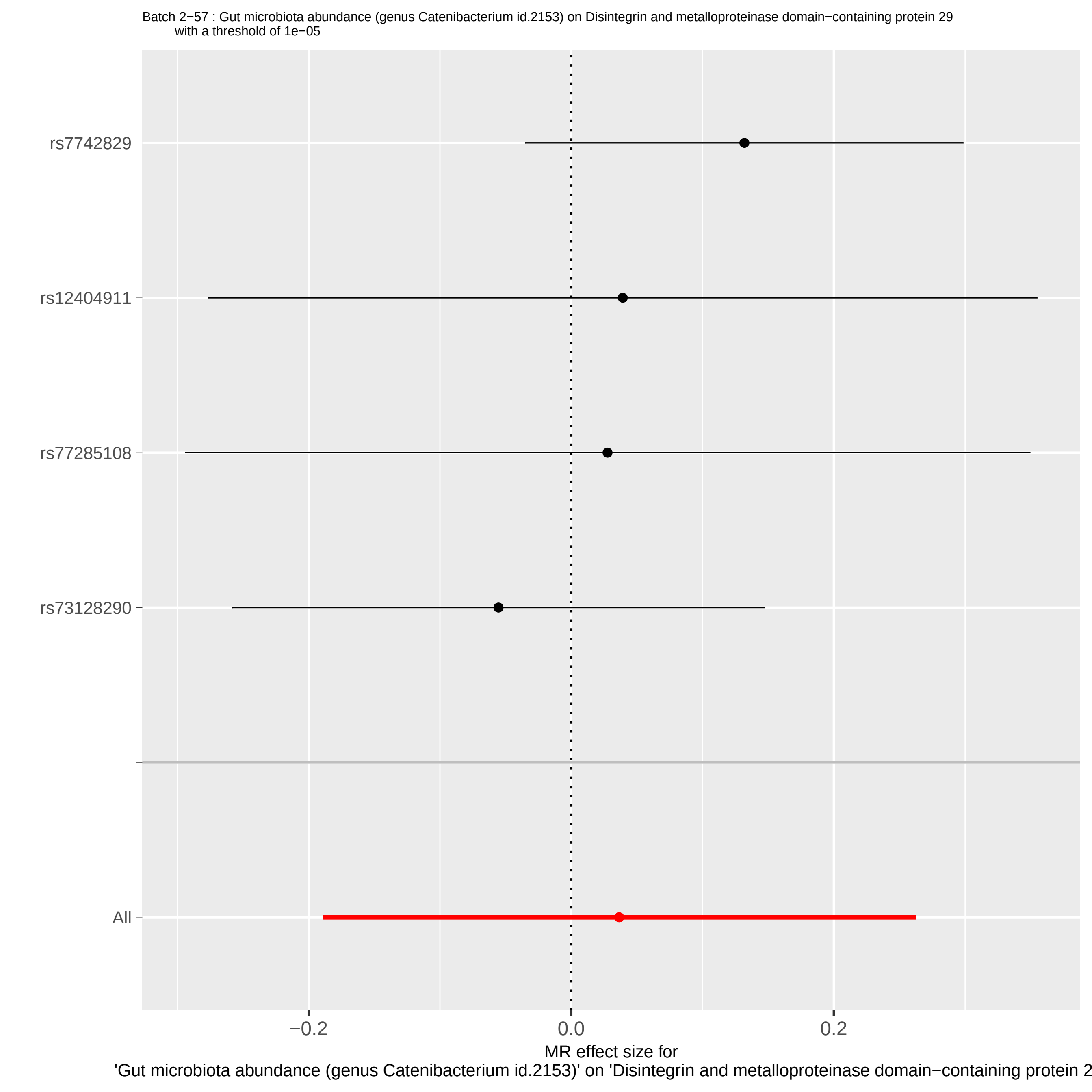
Batch 2−57 : Gut microbiota abundance (genus Catenibacterium id.2153) on Disintegrin and metalloproteinase domain−containing protein 29
with a threshold of 1e−05
rs7742829
rs12404911
rs77285108
rs73128290
All
−0.2
0.0
0.2
MR effect size for
'Gut microbiota abundance (genus Catenibacterium id.2153)' on 'Disintegrin and metalloproteinase domain−containing protein 2

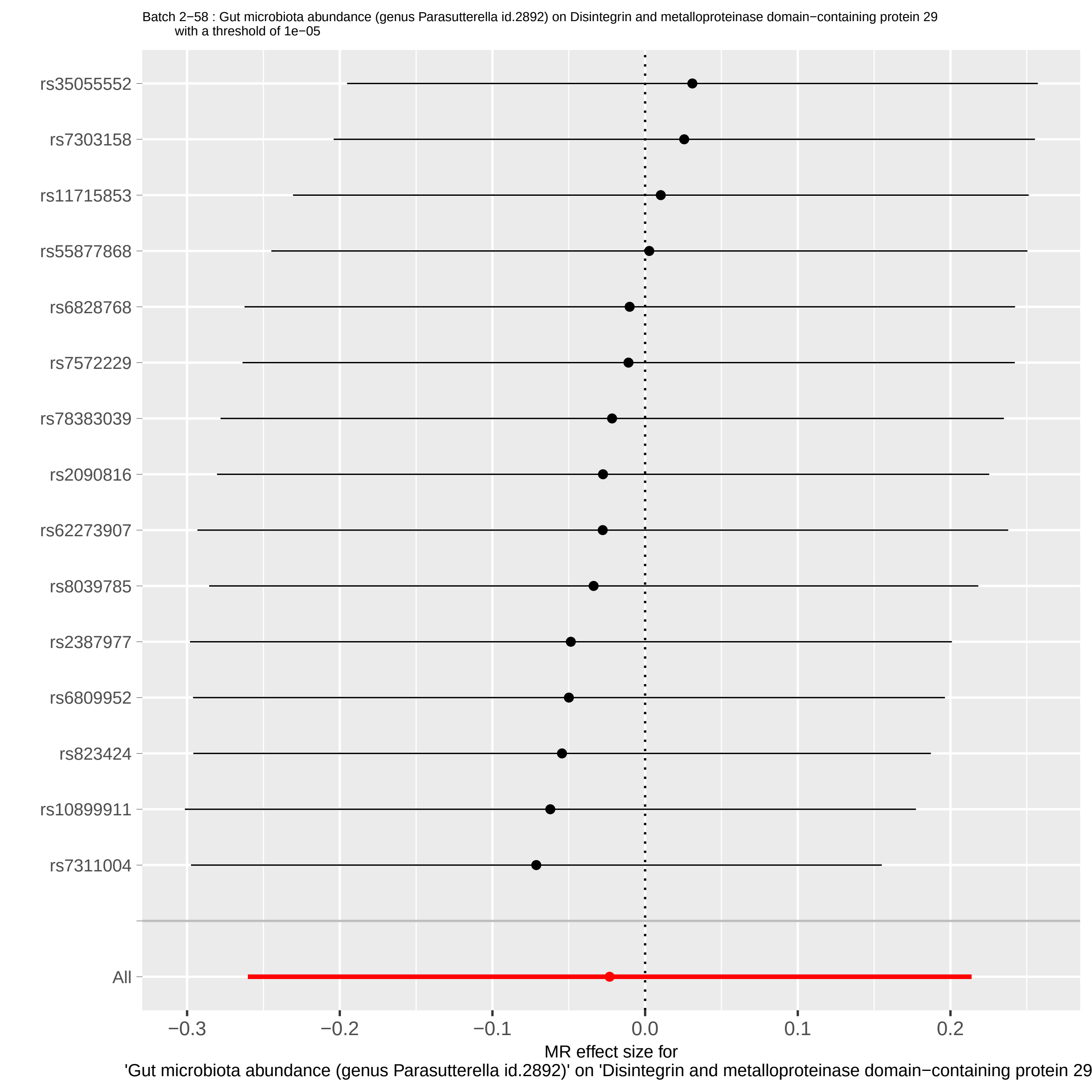

Batch 2−58 : Gut microbiota abundance (genus Parasutterella id.2892) on Disintegrin and metalloproteinase domain−containing protein 29
with a threshold of 1e−05
rs35055552
rs7303158
rs11715853
rs55877868
rs6828768
rs7572229
rs78383039
rs2090816
rs62273907
rs8039785
rs2387977
rs6809952
rs823424
rs10899911
rs7311004
All
−0.3
−0.2
−0.1
0.0
0.1
0.2
MR effect size for
'Gut microbiota abundance (genus Parasutterella id.2892)' on 'Disintegrin and metalloproteinase domain−containing protein 29'

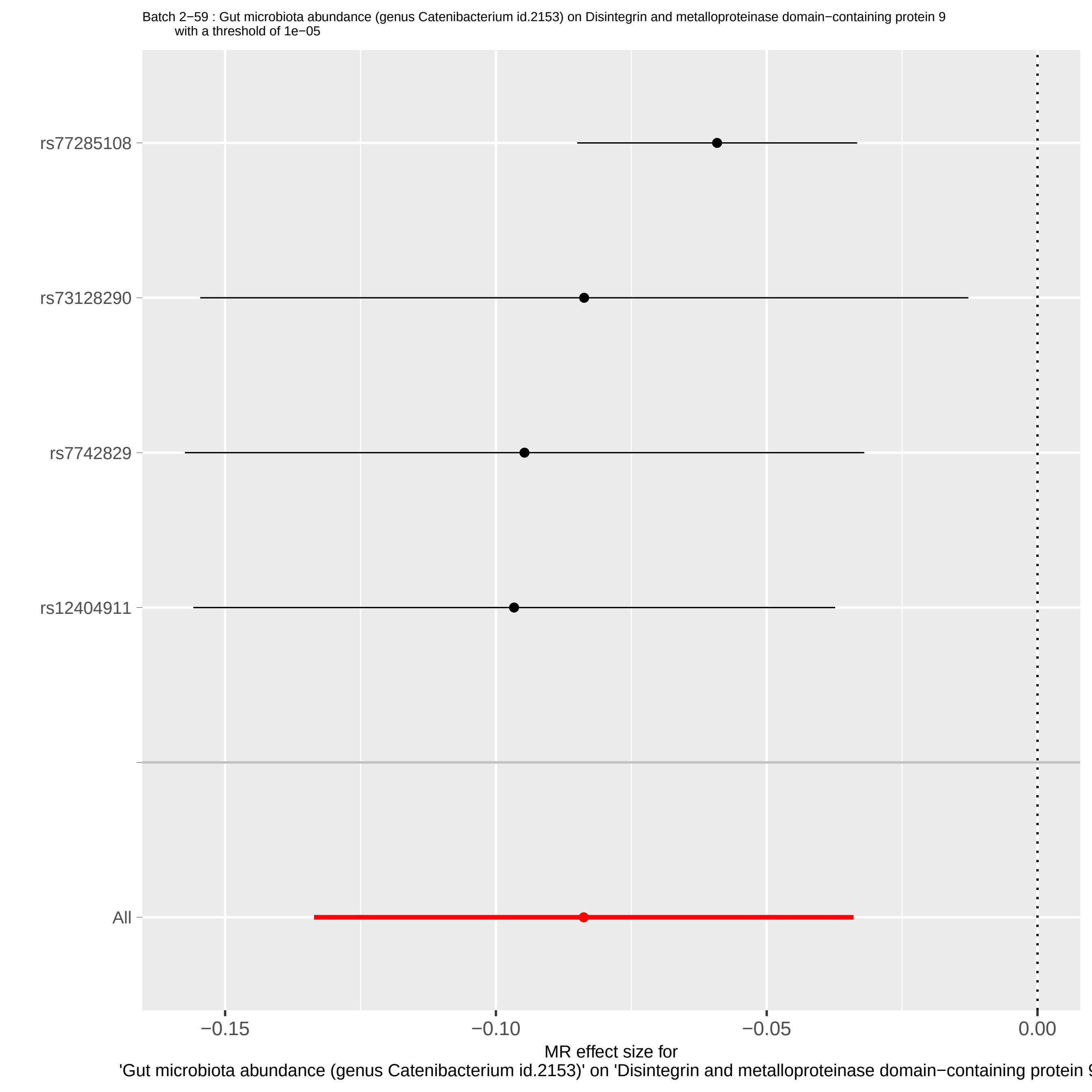

Batch 2−59 : Gut microbiota abundance (genus Catenibacterium id.2153) on Disintegrin and metalloproteinase domain−containing protein 9
with a threshold of 1e−05
rs77285108
rs73128290
rs7742829
rs12404911
All
−0.15
−0.10
−0.05
0.00
MR effect size for
'Gut microbiota abundance (genus Catenibacterium id.2153)' on 'Disintegrin and metalloproteinase domain−containing protein 9

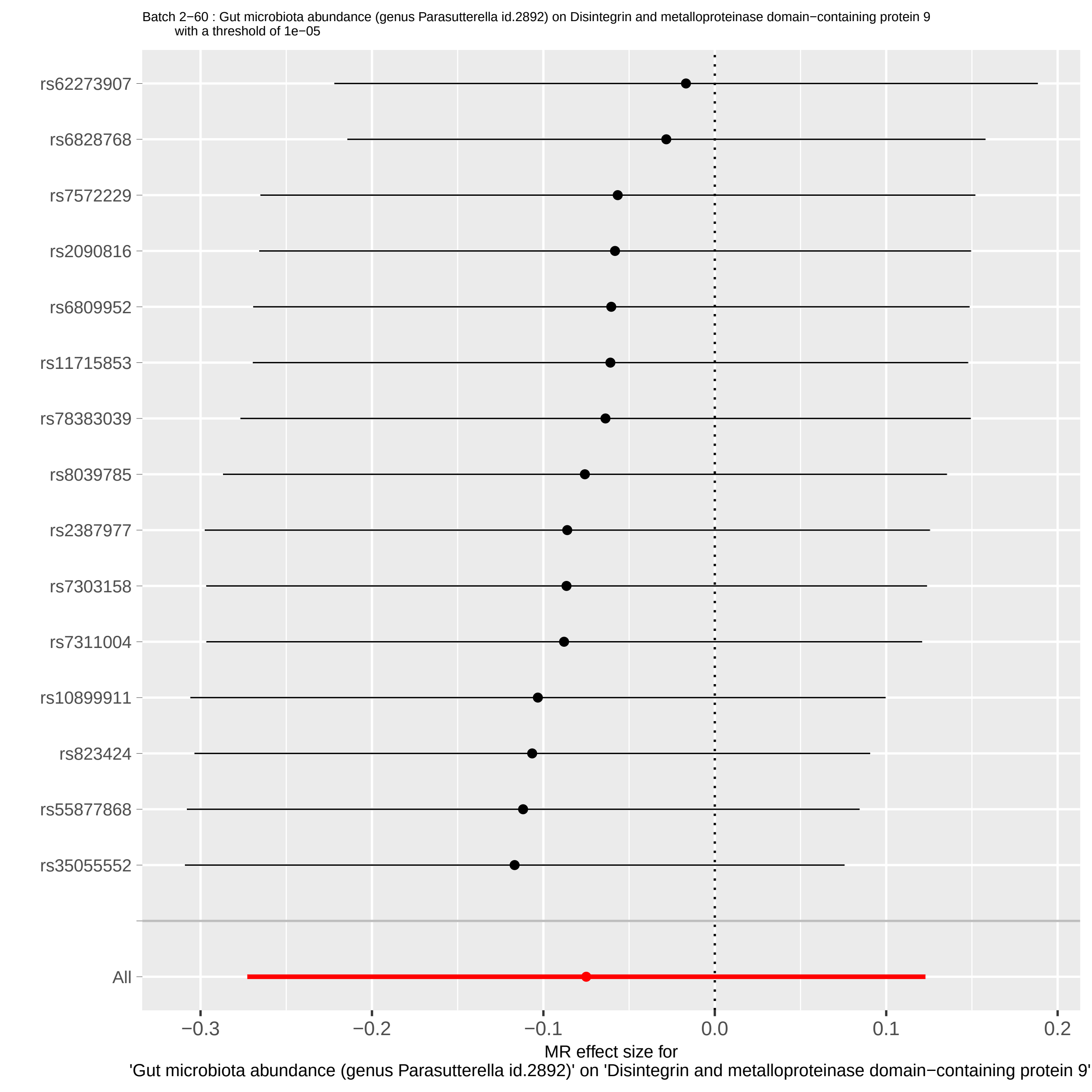
Batch 2−60 : Gut microbiota abundance (genus Parasutterella id.2892) on Disintegrin and metalloproteinase domain−containing protein 9
with a threshold of 1e−05
rs62273907
rs6828768
rs7572229
rs2090816
rs6809952
rs11715853
rs78383039
rs8039785
rs2387977
rs7303158
rs7311004
rs10899911
rs823424
rs55877868
rs35055552
All
−0.3
−0.2
−0.1
0.0
0.1
0.2
MR effect size for
'Gut microbiota abundance (genus Parasutterella id.2892)' on 'Disintegrin and metalloproteinase domain−containing protein 9'

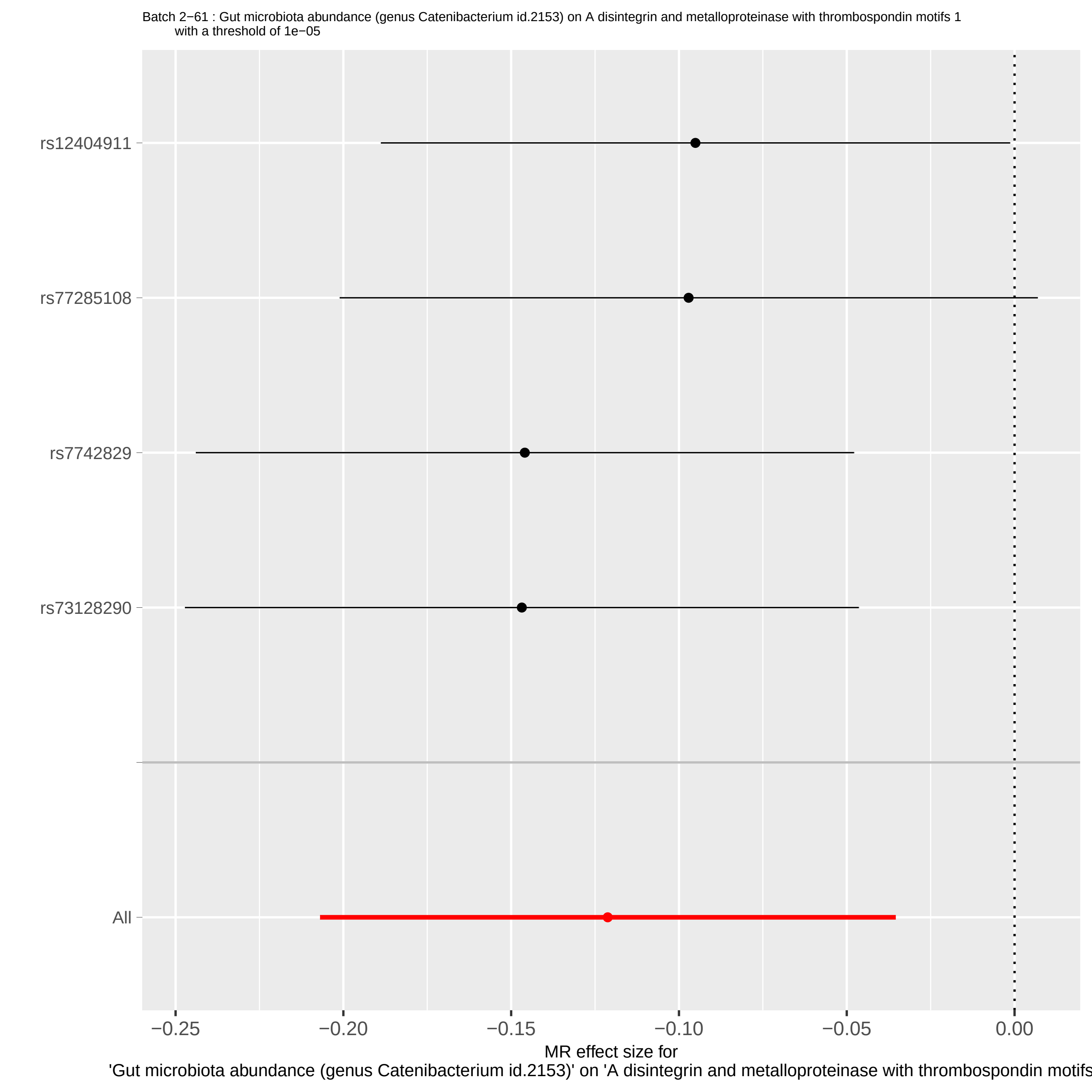

Batch 2−61 : Gut microbiota abundance (genus Catenibacterium id.2153) on A disintegrin and metalloproteinase with thrombospondin motifs 1
with a threshold of 1e−05
rs12404911
rs77285108
rs7742829
rs73128290
All
−0.25
−0.20
−0.15
−0.10
−0.05
0.00
MR effect size for
'Gut microbiota abundance (genus Catenibacterium id.2153)' on 'A disintegrin and metalloproteinase with thrombospondin motifs

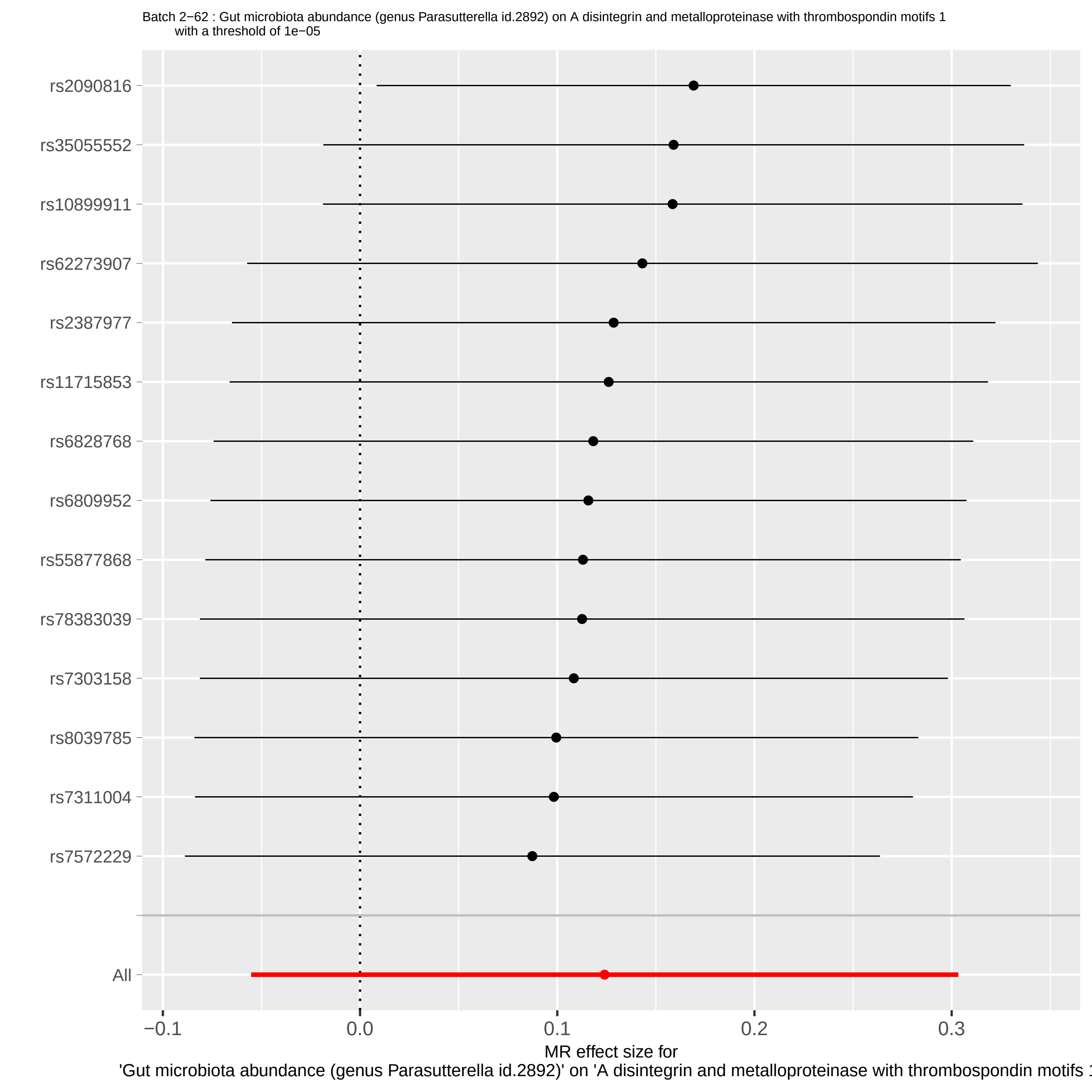
Batch 2−62 : Gut microbiota abundance (genus Parasutterella id.2892) on A disintegrin and metalloproteinase with thrombospondin motifs 1 with a threshold of 1e−05
rs2090816
rs35055552
rs10899911
rs62273907
rs2387977
rs11715853
rs6828768
rs6809952
rs55877868
rs78383039
rs7303158
rs8039785
rs7311004
rs7572229
All
−0.1
0.0
0.1
0.2
0.3
MR effect size for
'Gut microbiota abundance (genus Parasutterella id.2892)' on 'A disintegrin and metalloproteinase with thrombospondin motifs 1

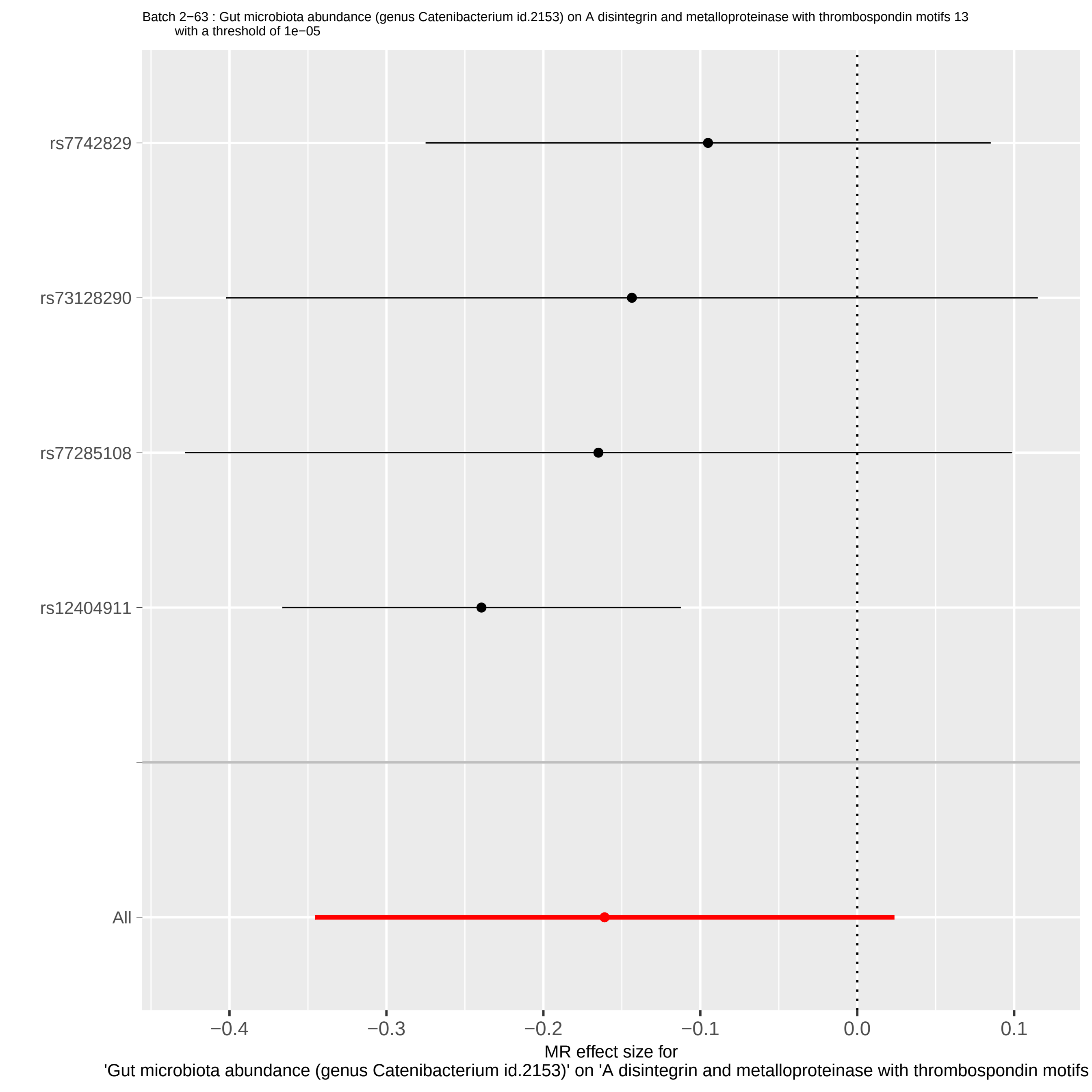
Batch 2−63 : Gut microbiota abundance (genus Catenibacterium id.2153) on A disintegrin and metalloproteinase with thrombospondin motifs 13
with a threshold of 1e−05
rs7742829
rs73128290
rs77285108
rs12404911
All
−0.4
−0.3
−0.2
−0.1
0.0
0.1
MR effect size for
'Gut microbiota abundance (genus Catenibacterium id.2153)' on 'A disintegrin and metalloproteinase with thrombospondin motifs

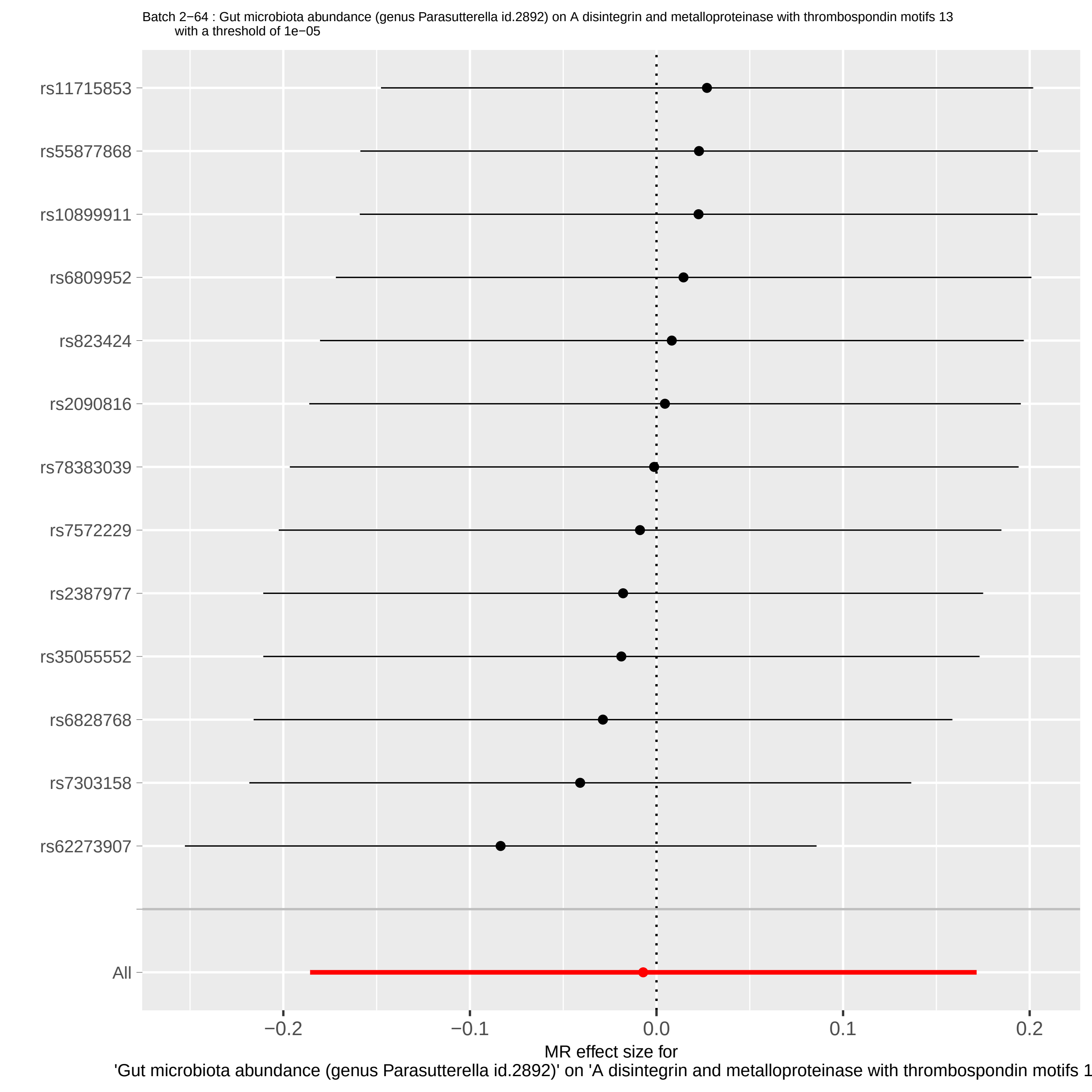
Batch 2−64 : Gut microbiota abundance (genus Parasutterella id.2892) on A disintegrin and metalloproteinase with thrombospondin motifs 13
with a threshold of 1e−05
rs11715853
rs55877868
rs10899911
rs6809952
rs823424
rs2090816
rs78383039
rs7572229
rs2387977
rs35055552
rs6828768
rs7303158
rs62273907
All
−0.2
−0.1
0.0
0.1
0.2
MR effect size for
'Gut microbiota abundance (genus Parasutterella id.2892)' on 'A disintegrin and metalloproteinase with thrombospondin motifs 1

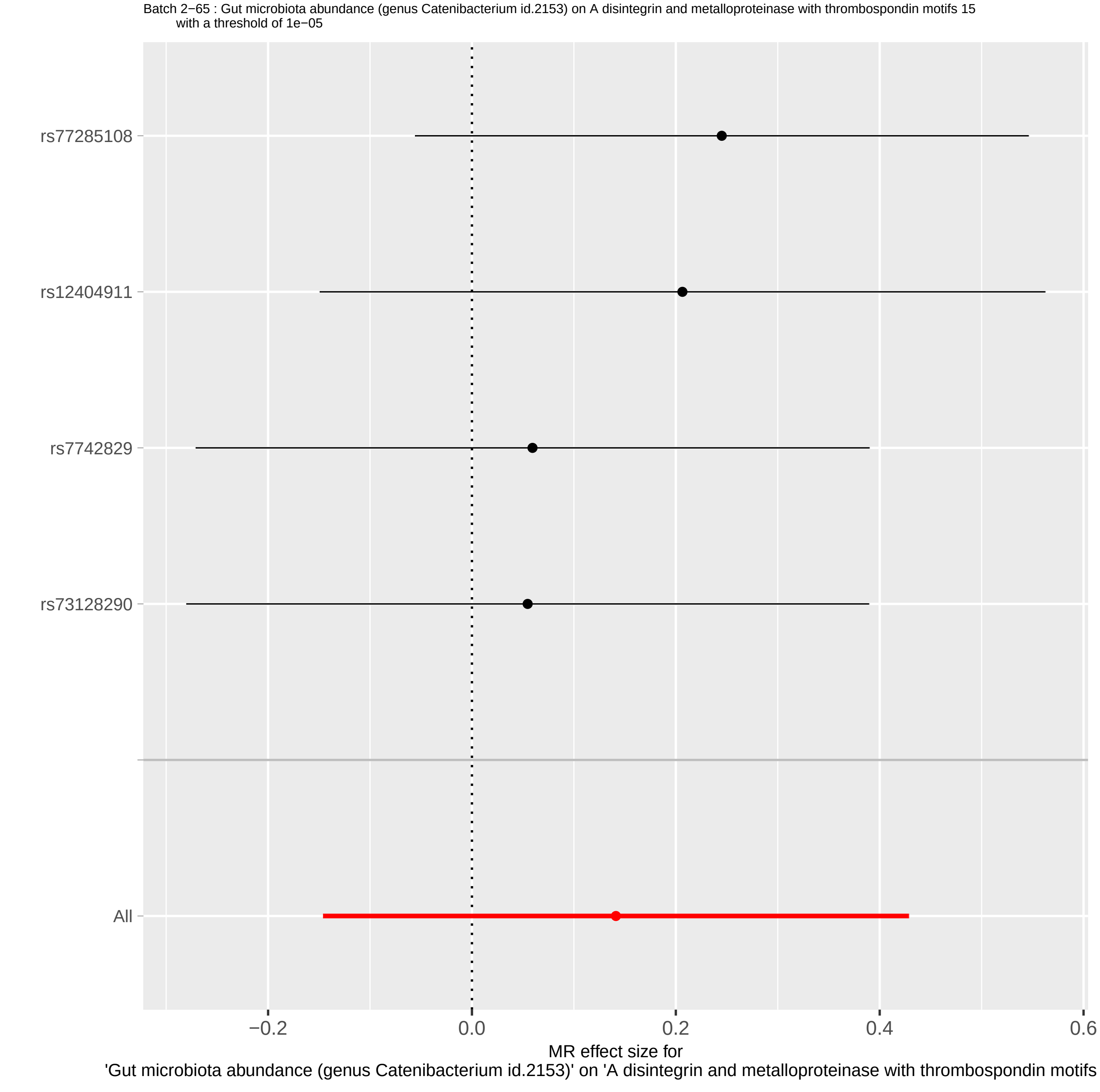

Batch 2−65 : Gut microbiota abundance (genus Catenibacterium id.2153) on A disintegrin and metalloproteinase with thrombospondin motifs 15
with a threshold of 1e−05
rs77285108
rs12404911
rs7742829
rs73128290
All
−0.2
0.0
0.2
0.4
0.6
MR effect size for
'Gut microbiota abundance (genus Catenibacterium id.2153)' on 'A disintegrin and metalloproteinase with thrombospondin motifs

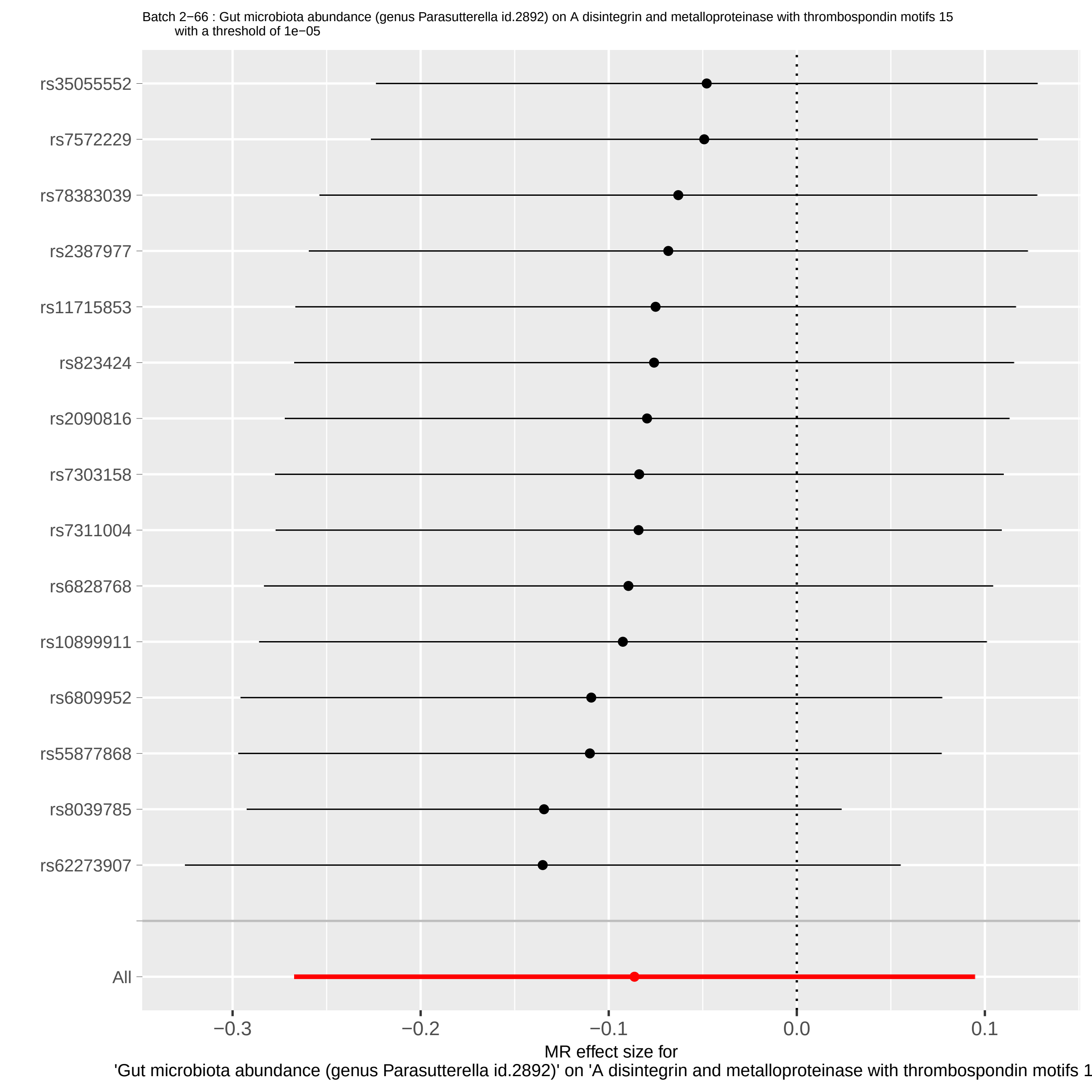

Batch 2−66 : Gut microbiota abundance (genus Parasutterella id.2892) on A disintegrin and metalloproteinase with thrombospondin motifs 15
with a threshold of 1e−05
rs35055552
rs7572229
rs78383039
rs2387977
rs11715853
rs823424
rs2090816
rs7303158
rs7311004
rs6828768
rs10899911
rs6809952
rs55877868
rs8039785
rs62273907
All
−0.3
−0.2
−0.1
0.0
0.1
MR effect size for
'Gut microbiota abundance (genus Parasutterella id.2892)' on 'A disintegrin and metalloproteinase with thrombospondin motifs 1

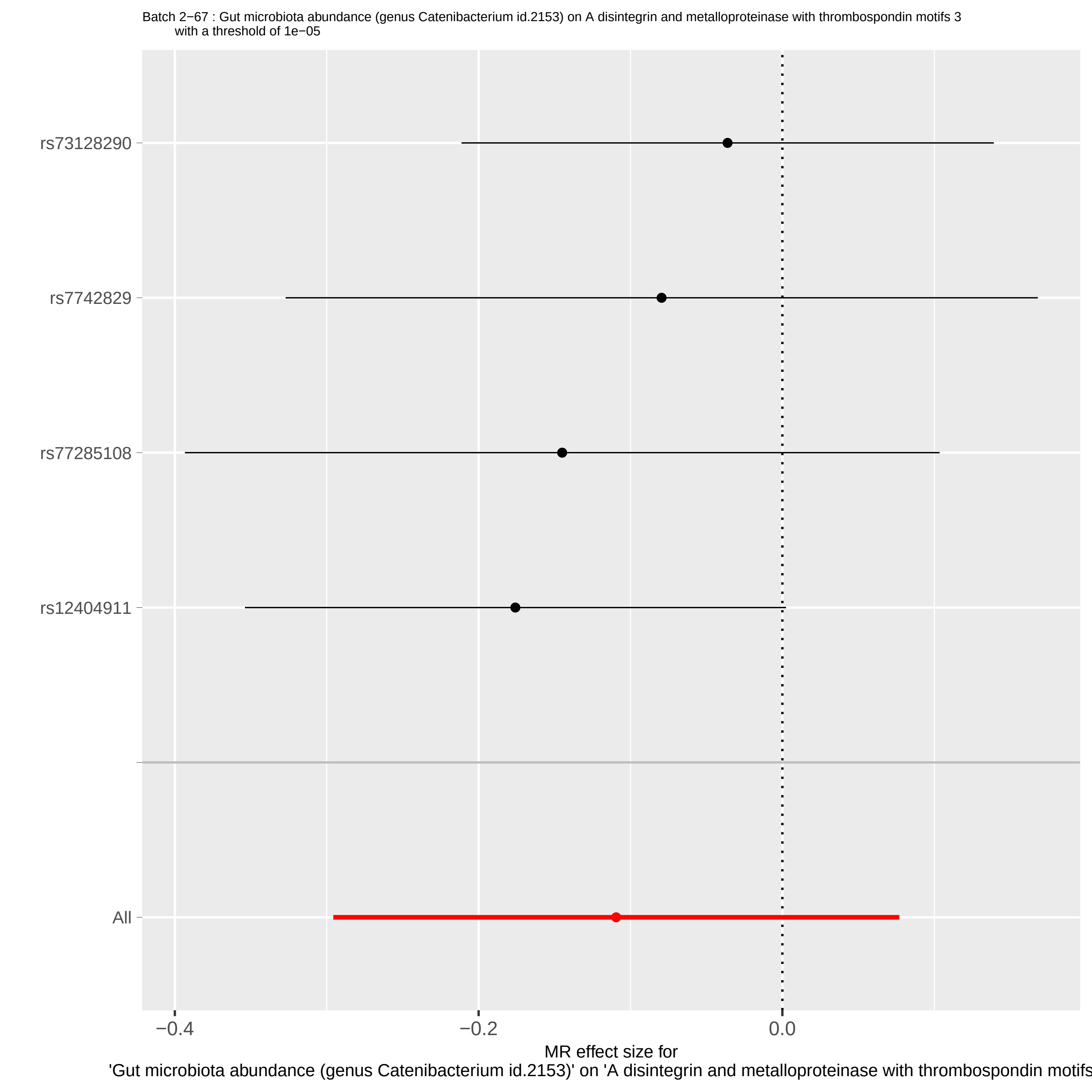

Batch 2−67 : Gut microbiota abundance (genus Catenibacterium id.2153) on A disintegrin and metalloproteinase with thrombospondin motifs 3
with a threshold of 1e−05
rs73128290
rs7742829
rs77285108
rs12404911
All
−0.4
−0.2
0.0
MR effect size for
'Gut microbiota abundance (genus Catenibacterium id.2153)' on 'A disintegrin and metalloproteinase with thrombospondin motifs

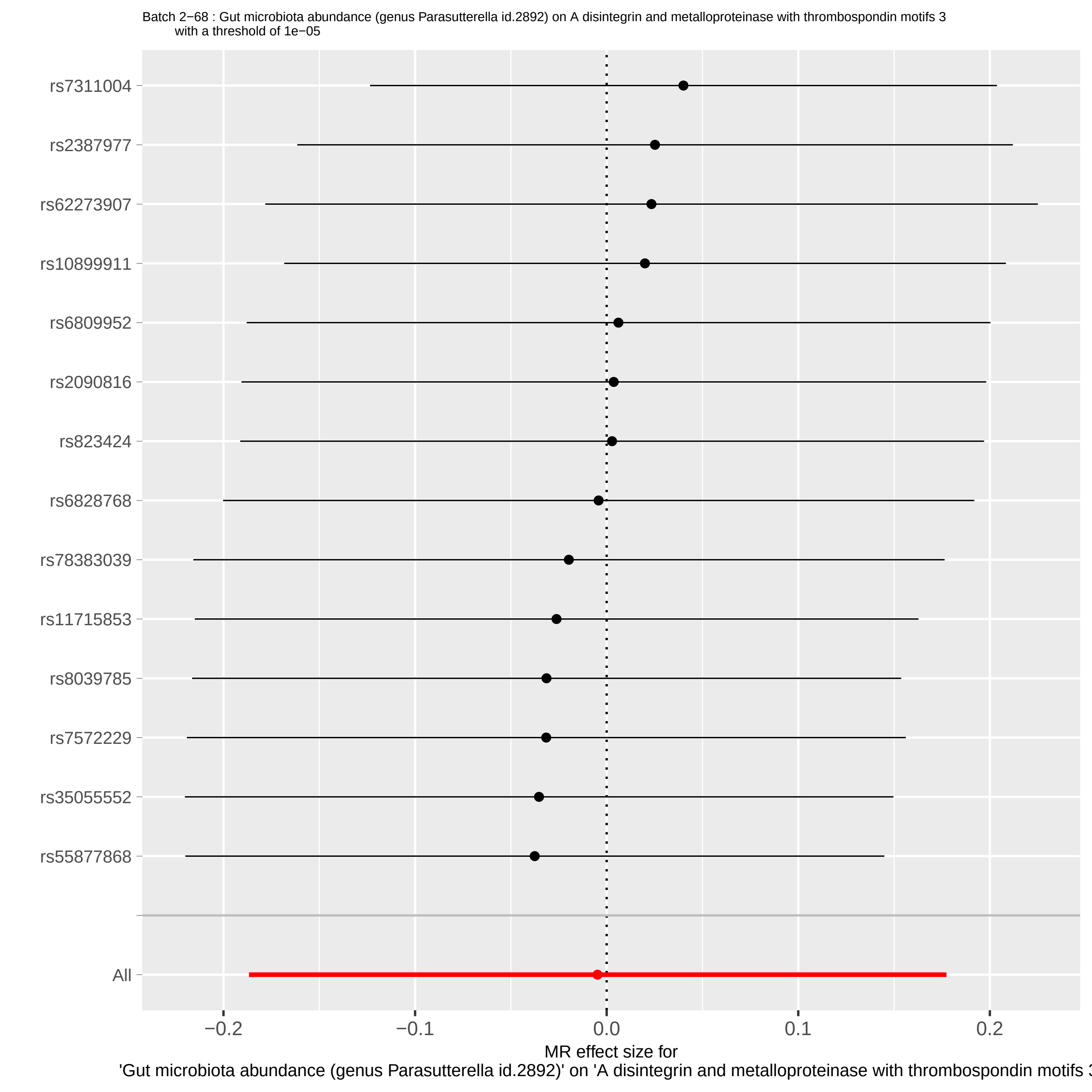

Batch 2−68 : Gut microbiota abundance (genus Parasutterella id.2892) on A disintegrin and metalloproteinase with thrombospondin motifs 3 with a threshold of 1e−05
rs7311004
rs2387977
rs62273907
rs10899911
rs6809952
rs2090816
rs823424
rs6828768
rs78383039
rs11715853
rs8039785
rs7572229
rs35055552
rs55877868
All
−0.2
−0.1
0.0
0.1
0.2
MR effect size for
'Gut microbiota abundance (genus Parasutterella id.2892)' on 'A disintegrin and metalloproteinase with thrombospondin motifs 3

Batch 2−69 : Gut microbiota abundance (genus Catenibacterium id.2153) on A disintegrin and metalloproteinase with thrombospondin motifs 4 with a threshold of 1e−05

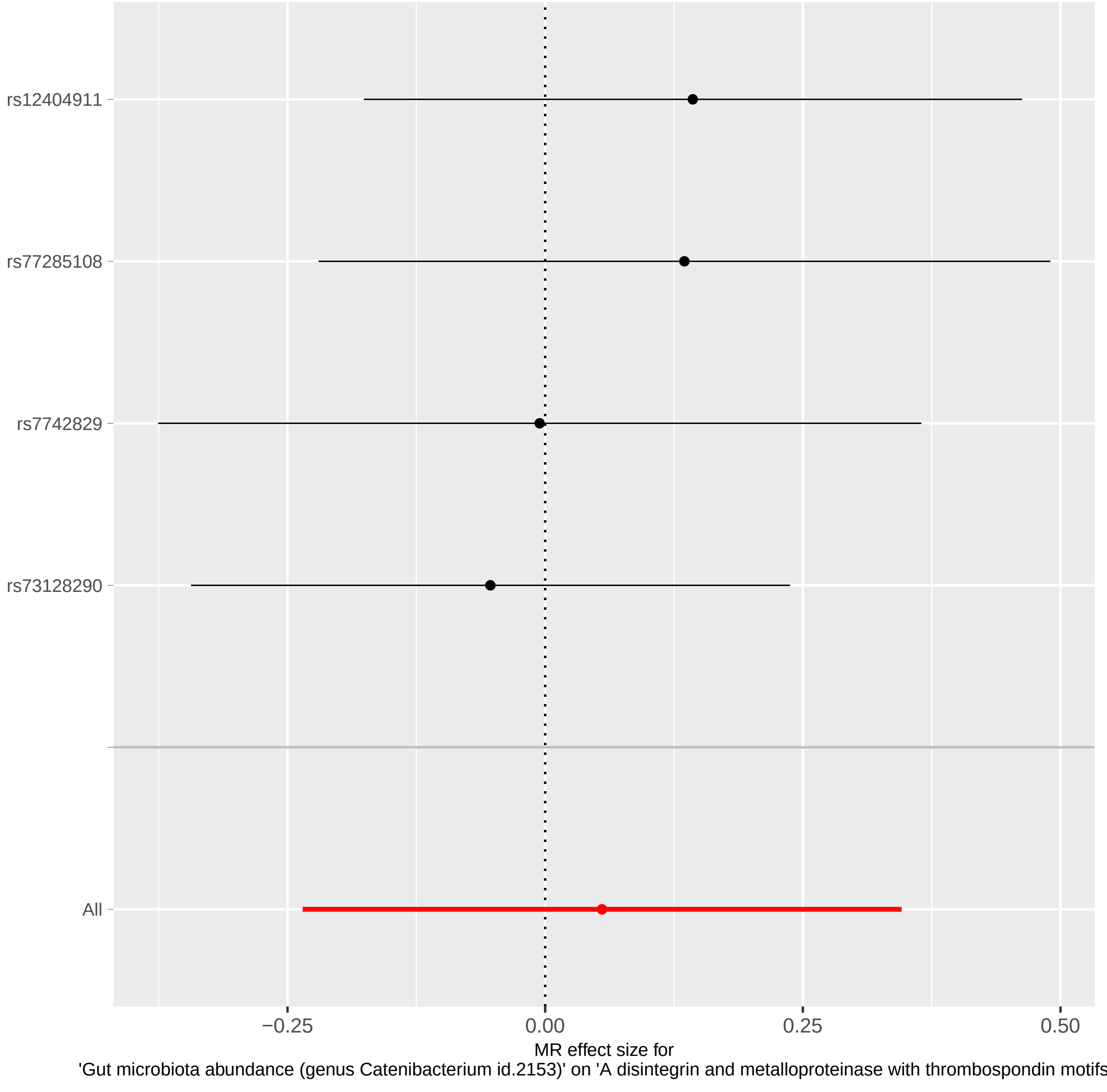

Batch 2−70 : Gut microbiota abundance (genus Parasutterella id.2892) on A disintegrin and metalloproteinase with thrombospondin motifs 4 with a threshold of 1e−05

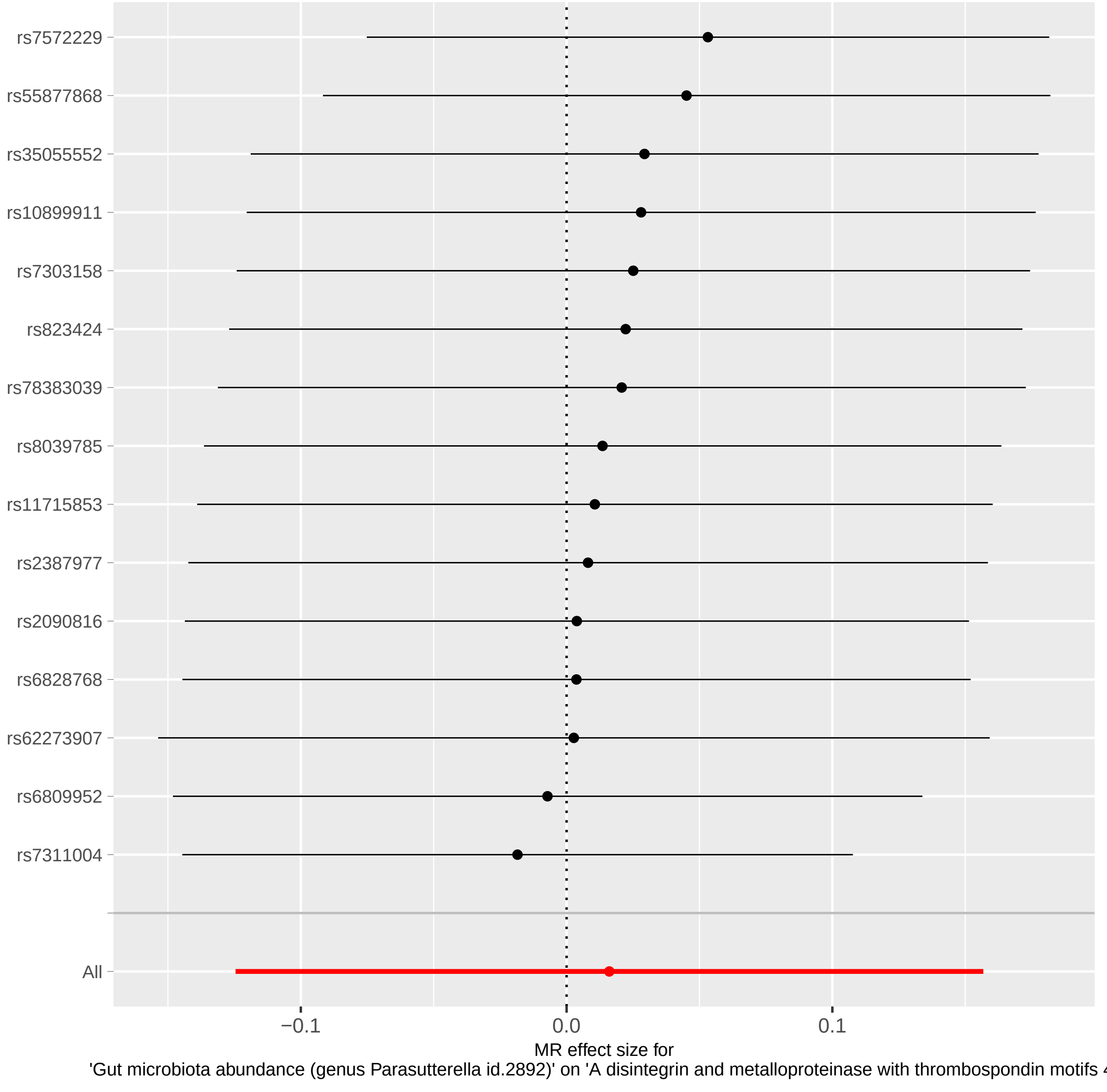

Batch 2−71 : Gut microbiota abundance (genus Catenibacterium id.2153) on A disintegrin and metalloproteinase with thrombospondin motifs 5
with a threshold of 1e−05

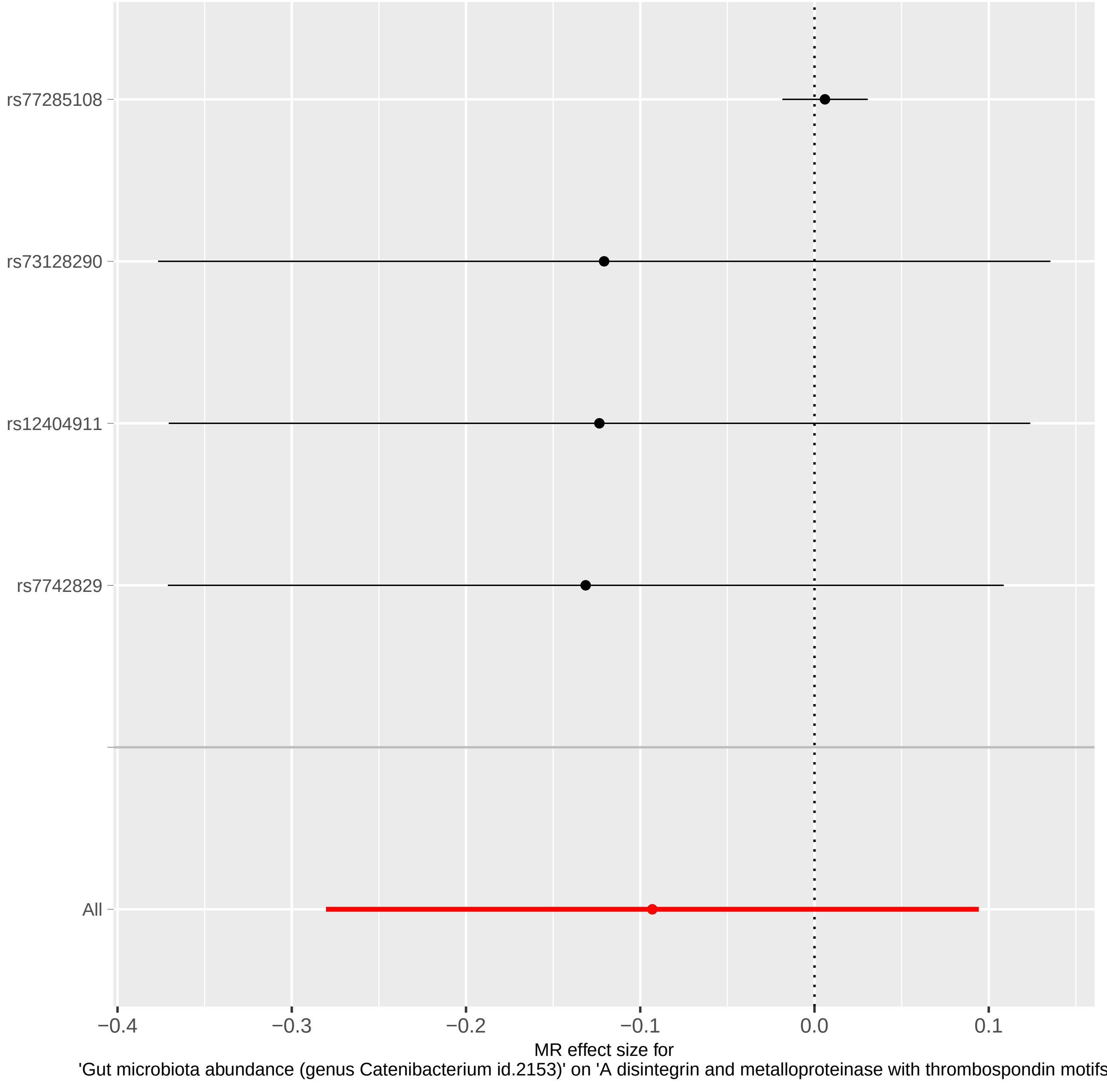

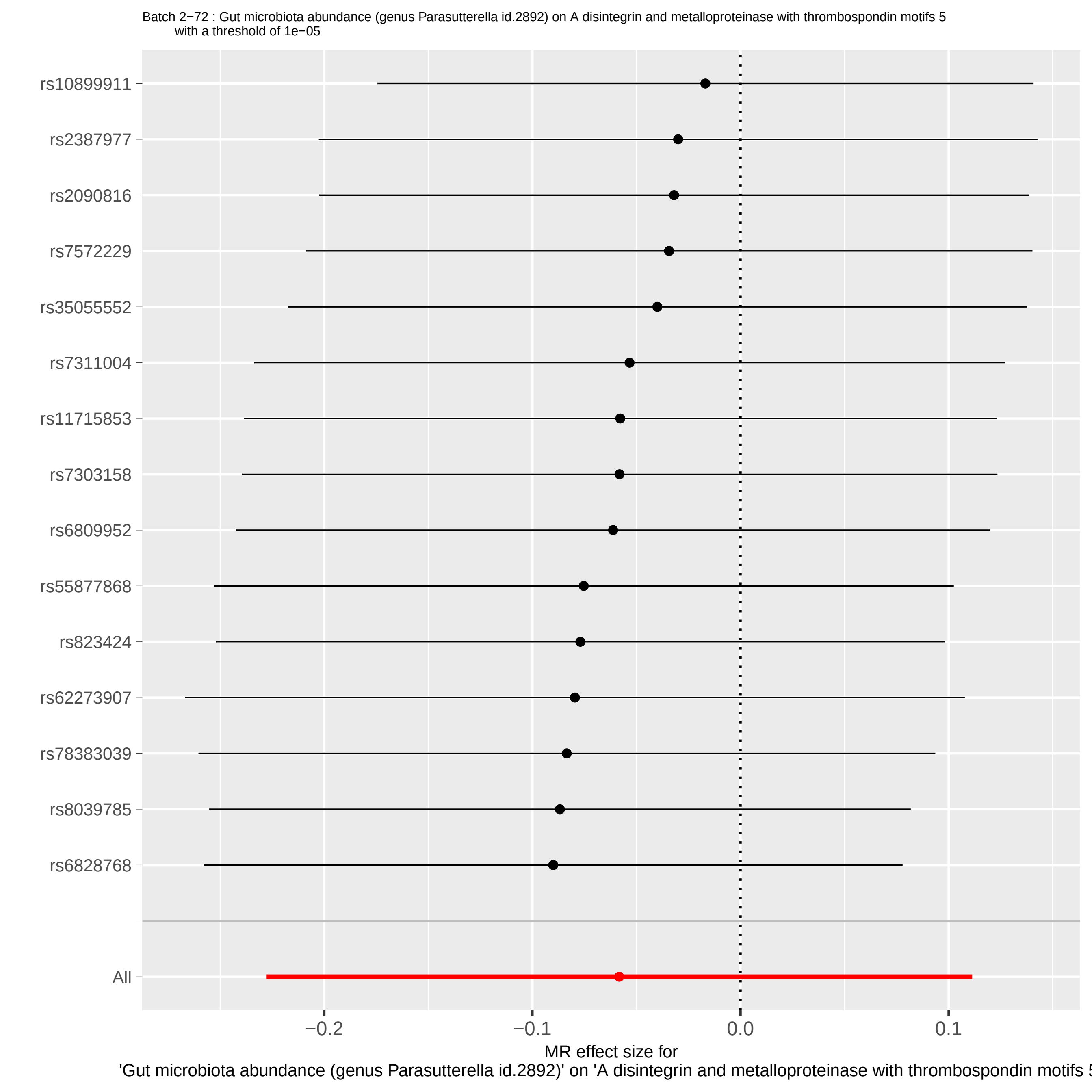

Batch 2−72 : Gut microbiota abundance (genus Parasutterella id.2892) on A disintegrin and metalloproteinase with thrombospondin motifs 5
with a threshold of 1e−05
rs10899911
rs2387977
rs2090816
rs7572229
rs35055552
rs7311004
rs11715853
rs7303158
rs6809952
rs55877868
rs823424
rs62273907
rs78383039
rs8039785
rs6828768
All
−0.2
−0.1
0.0
0.1
MR effect size for
'Gut microbiota abundance (genus Parasutterella id.2892)' on 'A disintegrin and metalloproteinase with thrombospondin motifs 5

Batch 2−73 : Gut microbiota abundance (genus Catenibacterium id.2153) on A disintegrin and metalloproteinase with thrombospondin motifs 6 with a threshold of 1e−05

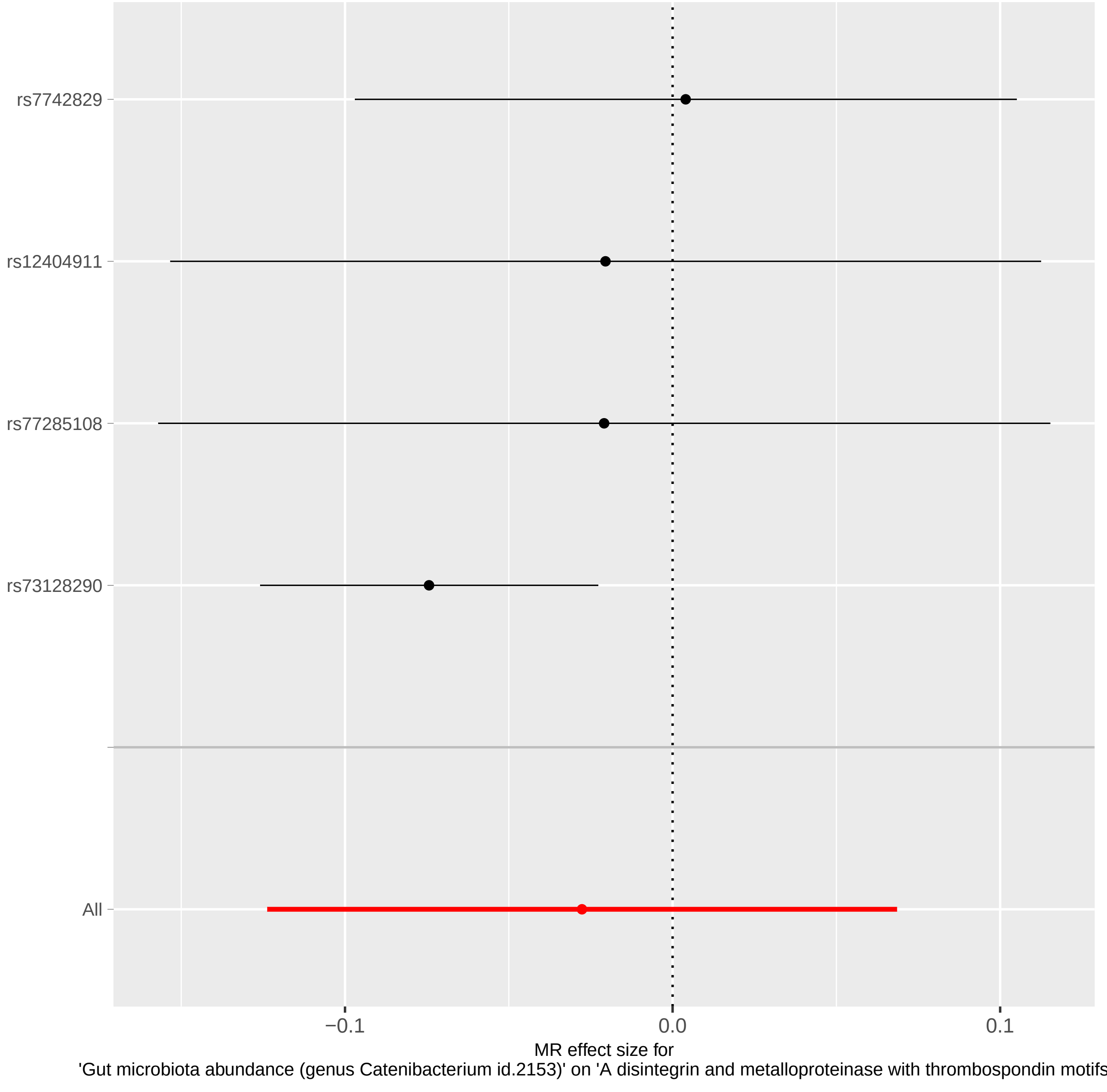

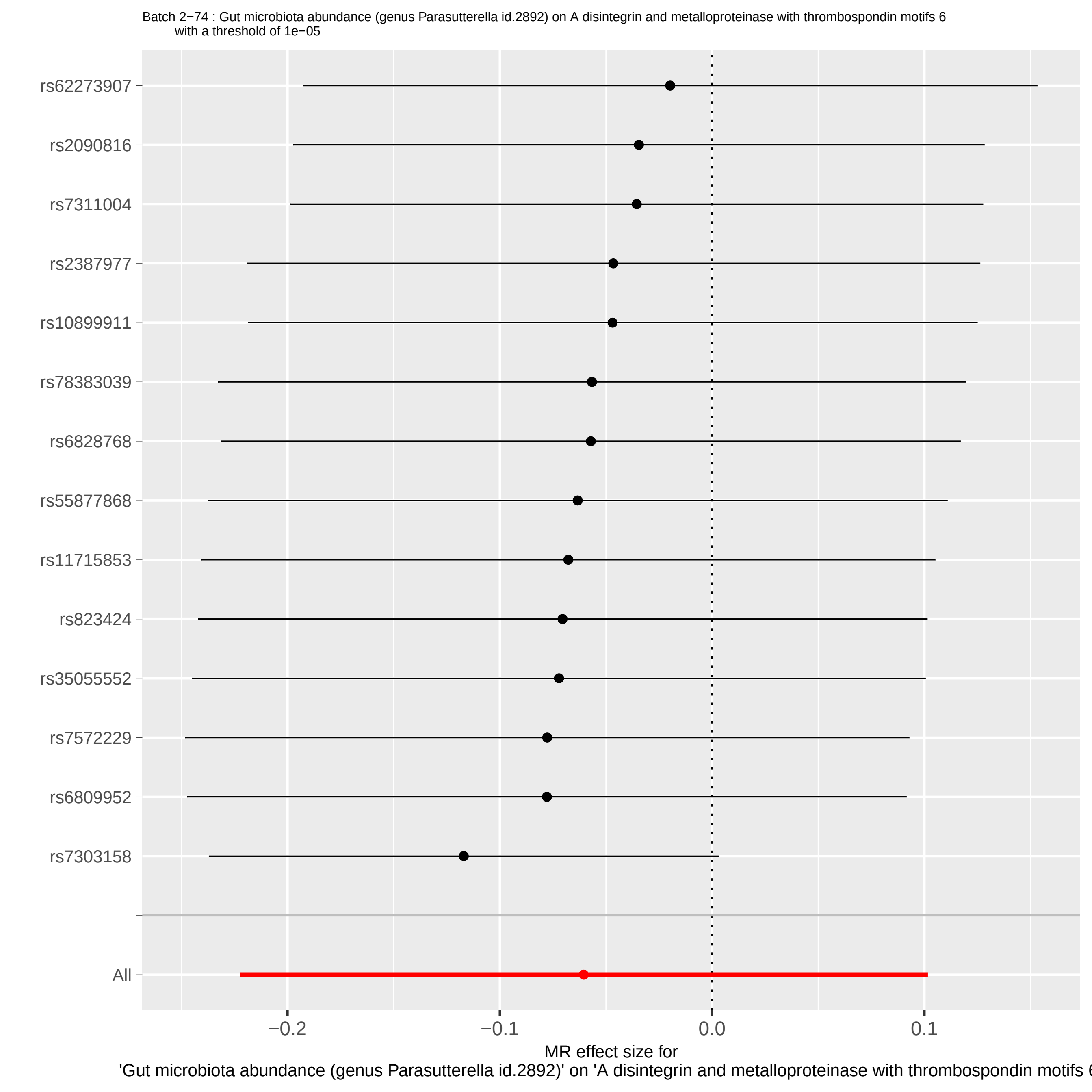

Batch 2−74 : Gut microbiota abundance (genus Parasutterella id.2892) on A disintegrin and metalloproteinase with thrombospondin motifs 6
with a threshold of 1e−05
rs62273907
rs2090816
rs7311004
rs2387977
rs10899911
rs78383039
rs6828768
rs55877868
rs11715853
rs823424
rs35055552
rs7572229
rs6809952
rs7303158
All
−0.2
−0.1
0.0
0.1
MR effect size for
'Gut microbiota abundance (genus Parasutterella id.2892)' on 'A disintegrin and metalloproteinase with thrombospondin motifs 6

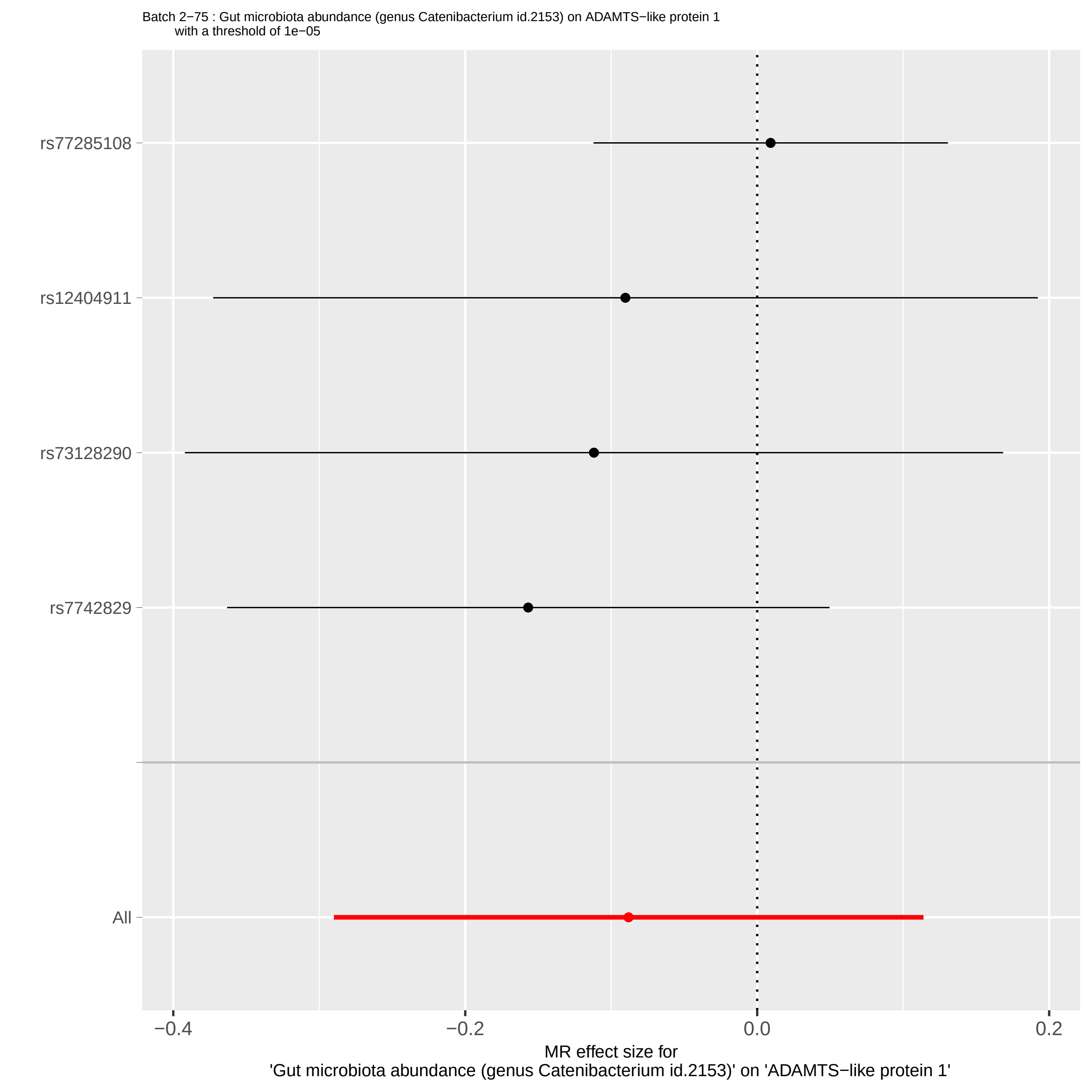
Batch 2−75 : Gut microbiota abundance (genus Catenibacterium id.2153) on ADAMTS−like protein 1
with a threshold of 1e−05
rs77285108
rs12404911
rs73128290
rs7742829
All
−0.4
−0.2
0.0
0.2
MR effect size for
'Gut microbiota abundance (genus Catenibacterium id.2153)' on 'ADAMTS−like protein 1'

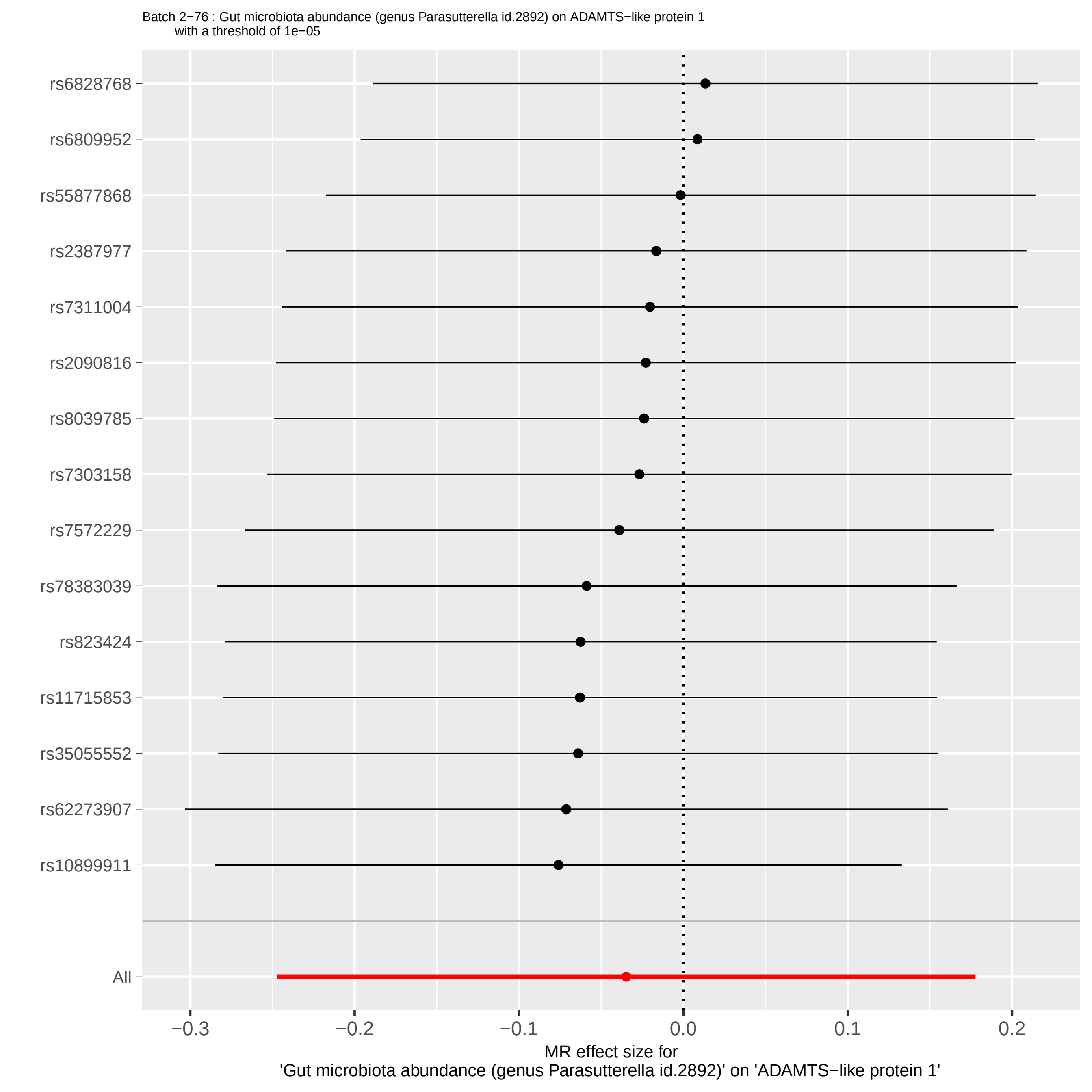

Batch 2−76 : Gut microbiota abundance (genus Parasutterella id.2892) on ADAMTS−like protein 1
with a threshold of 1e−05
rs6828768
rs6809952
rs55877868
rs2387977
rs7311004
rs2090816
rs8039785
rs7303158
rs7572229
rs78383039
rs823424
rs11715853
rs35055552
rs62273907
rs10899911
All
−0.3
−0.2
−0.1
0.0
0.1
0.2
MR effect size for
'Gut microbiota abundance (genus Parasutterella id.2892)' on 'ADAMTS−like protein 1

Batch 2−77 : Gut microbiota abundance (genus Catenibacterium id.2153) on AarF domain−containing protein kinase 4
with a threshold of 1e−05

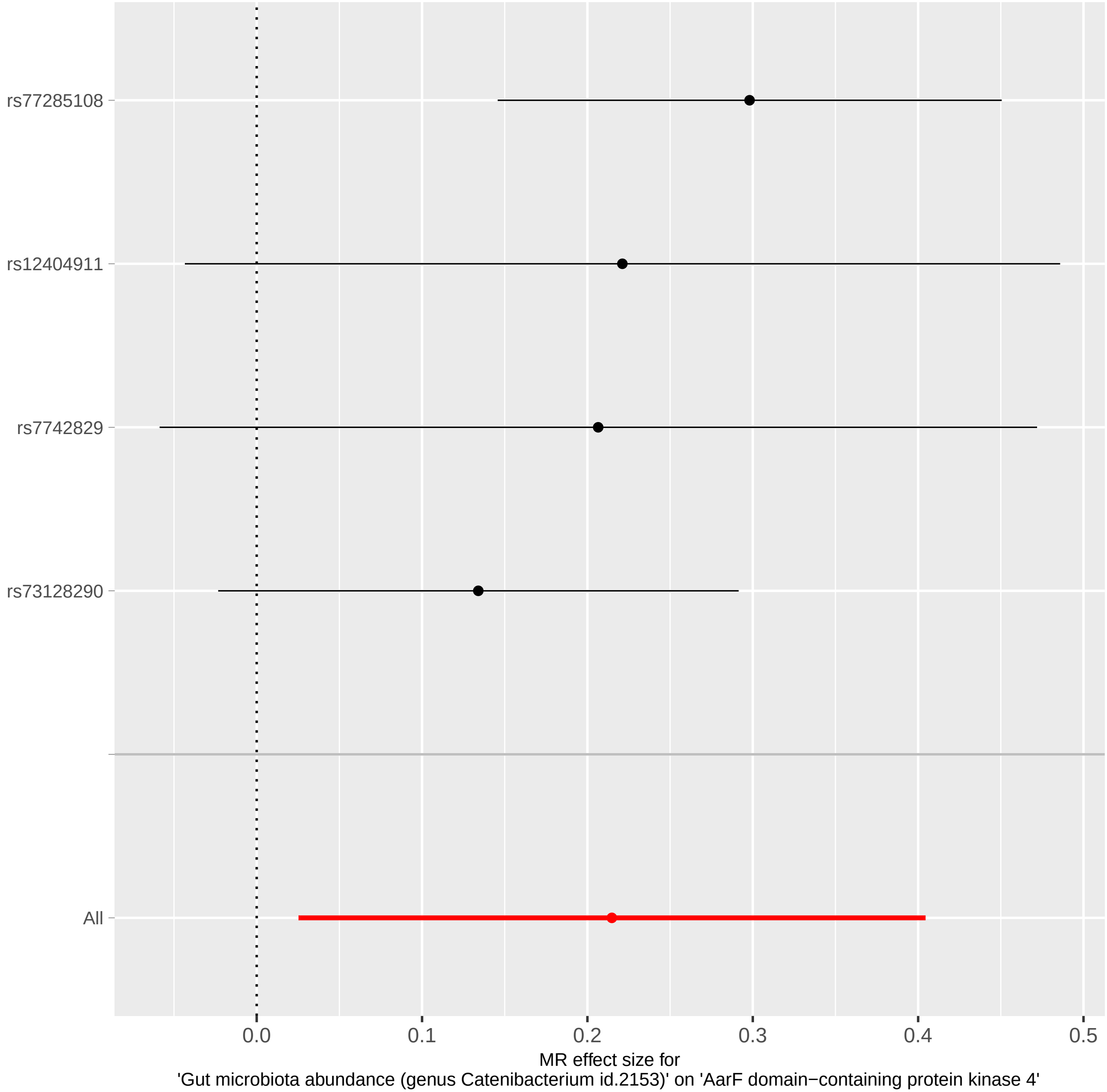

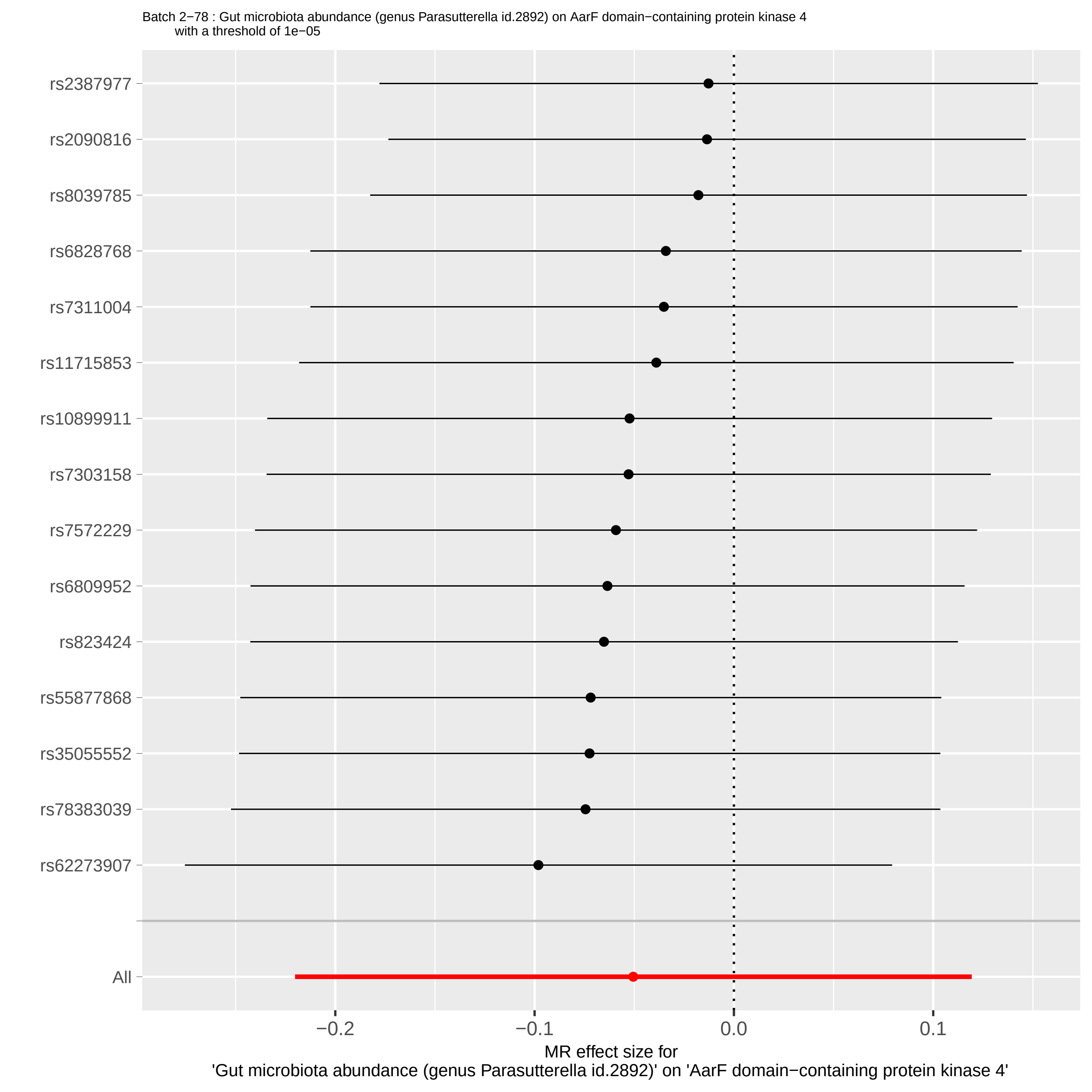

Batch 2−78 : Gut microbiota abundance (genus Parasutterella id.2892) on AarF domain−containing protein kinase 4
with a threshold of 1e−05
rs2387977
rs2090816
rs8039785
rs6828768
rs7311004
rs11715853
rs10899911
rs7303158
rs7572229
rs6809952
rs823424
rs55877868
rs35055552
rs78383039
rs62273907
All
−0.2
−0.1
0.0
0.1
MR effect size for
'Gut microbiota abundance (genus Parasutterella id.2892)' on 'AarF domain−containing protein kinase 4'

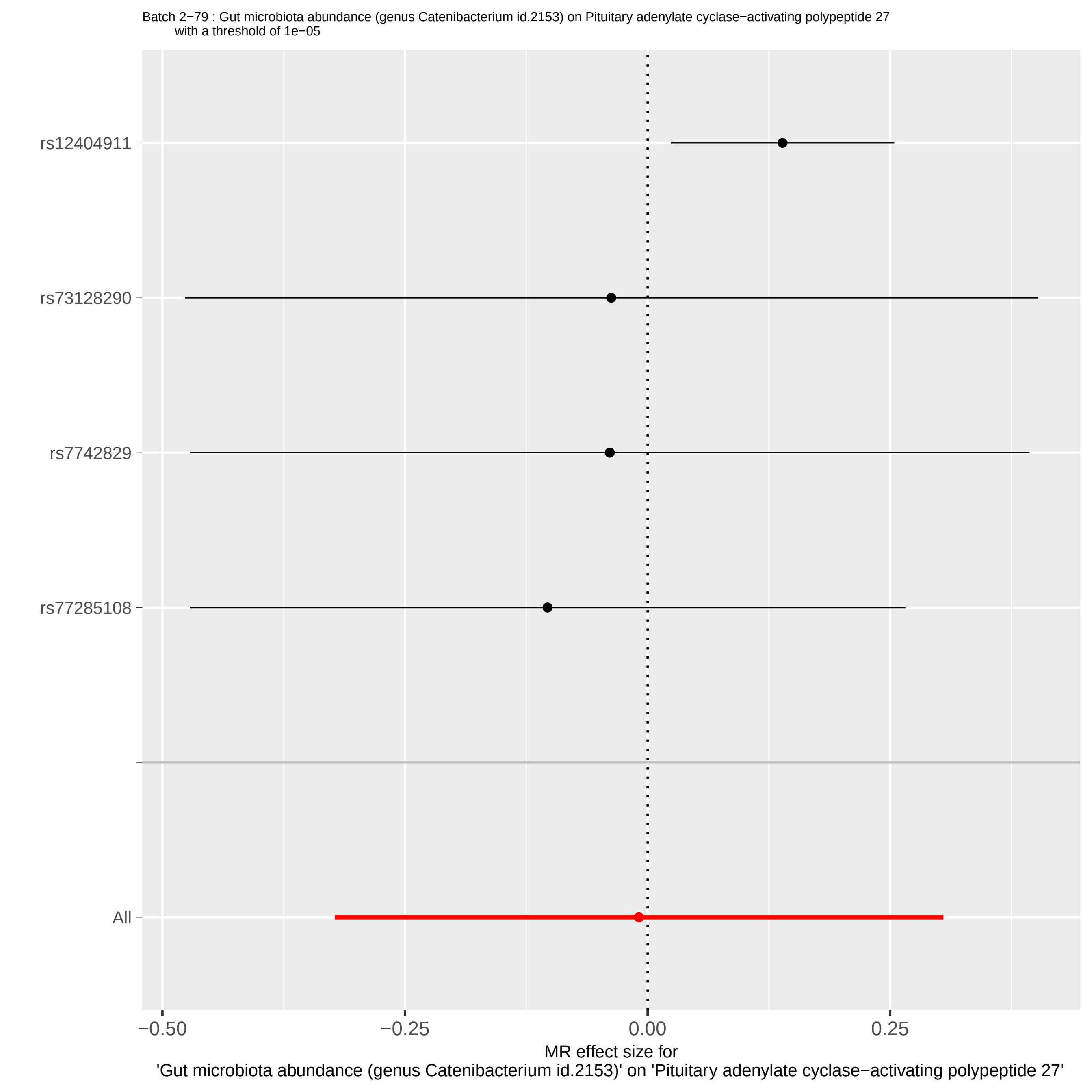

Batch 2−79 : Gut microbiota abundance (genus Catenibacterium id.2153) on Pituitary adenylate cyclase−activating polypeptide 27
with a threshold of 1e−05
rs12404911
rs73128290
rs7742829
rs77285108
All
−0.50
−0.25
0.00
0.25
MR effect size for
'Gut microbiota abundance (genus Catenibacterium id.2153)' on 'Pituitary adenylate cyclase−activating polypeptide 27'

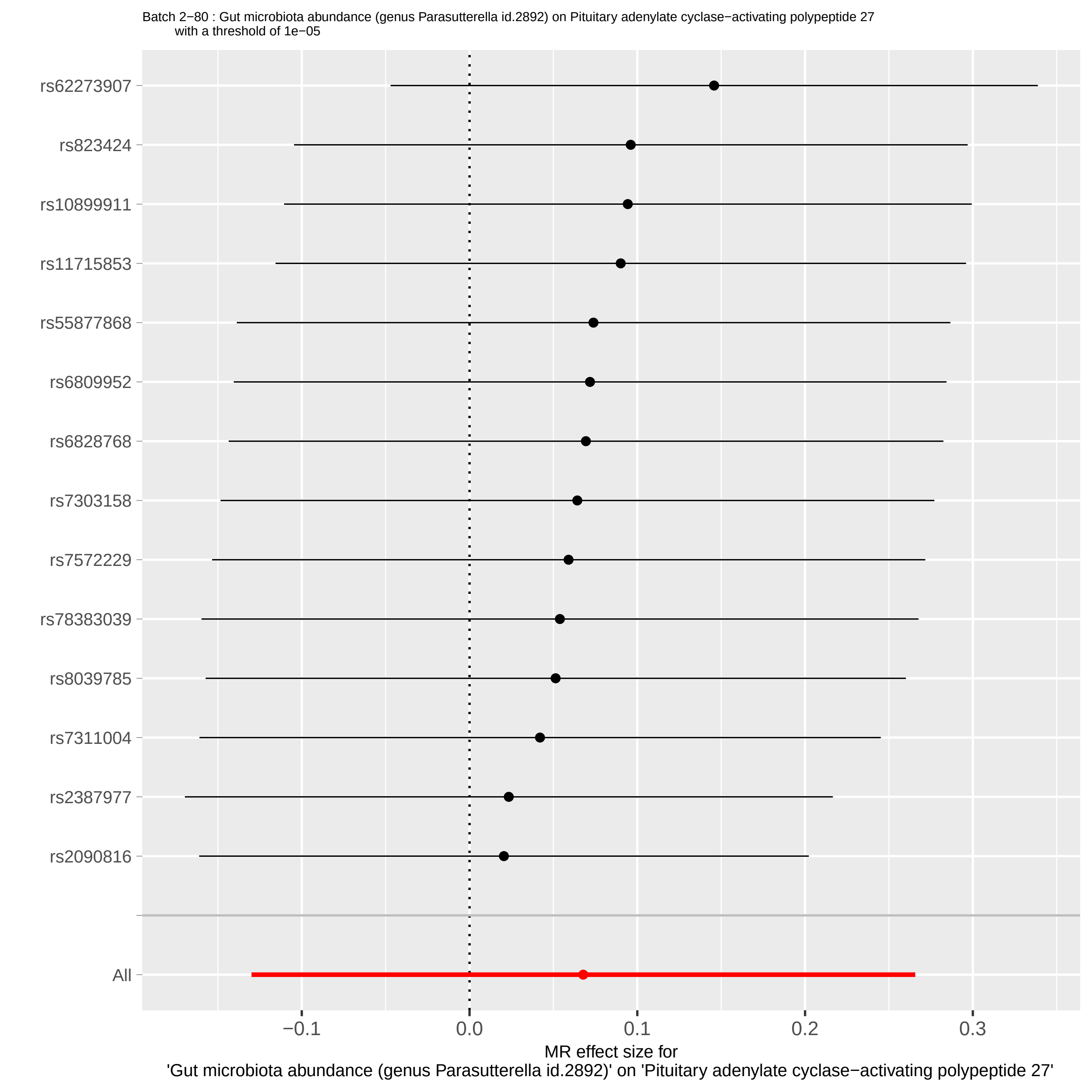

Batch 2−80 : Gut microbiota abundance (genus Parasutterella id.2892) on Pituitary adenylate cyclase−activating polypeptide 27
with a threshold of 1e−05
rs62273907
rs823424
rs10899911
rs11715853
rs55877868
rs6809952
rs6828768
rs7303158
rs7572229
rs78383039
rs8039785
rs7311004
rs2387977
rs2090816
All
−0.1
0.0
0.1
0.2
0.3
MR effect size for
'Gut microbiota abundance (genus Parasutterella id.2892)' on 'Pituitary adenylate cyclase−activating polypeptide 27'

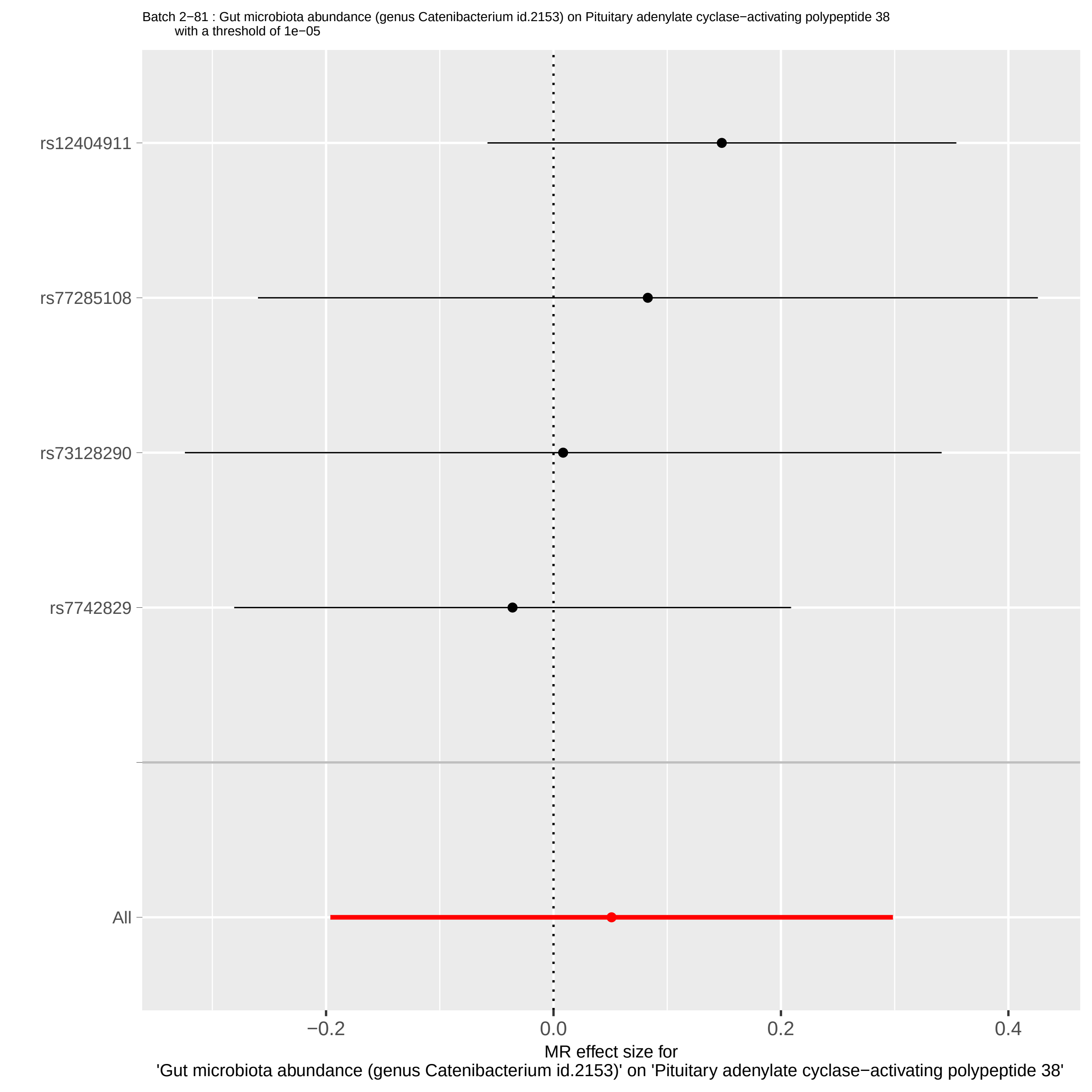
Batch 2−81 : Gut microbiota abundance (genus Catenibacterium id.2153) on Pituitary adenylate cyclase−activating polypeptide 38
with a threshold of 1e−05
rs12404911
rs77285108
rs73128290
rs7742829
All
−0.2
0.0
0.2
0.4
MR effect size for
'Gut microbiota abundance (genus Catenibacterium id.2153)' on 'Pituitary adenylate cyclase−activating polypeptide 38'

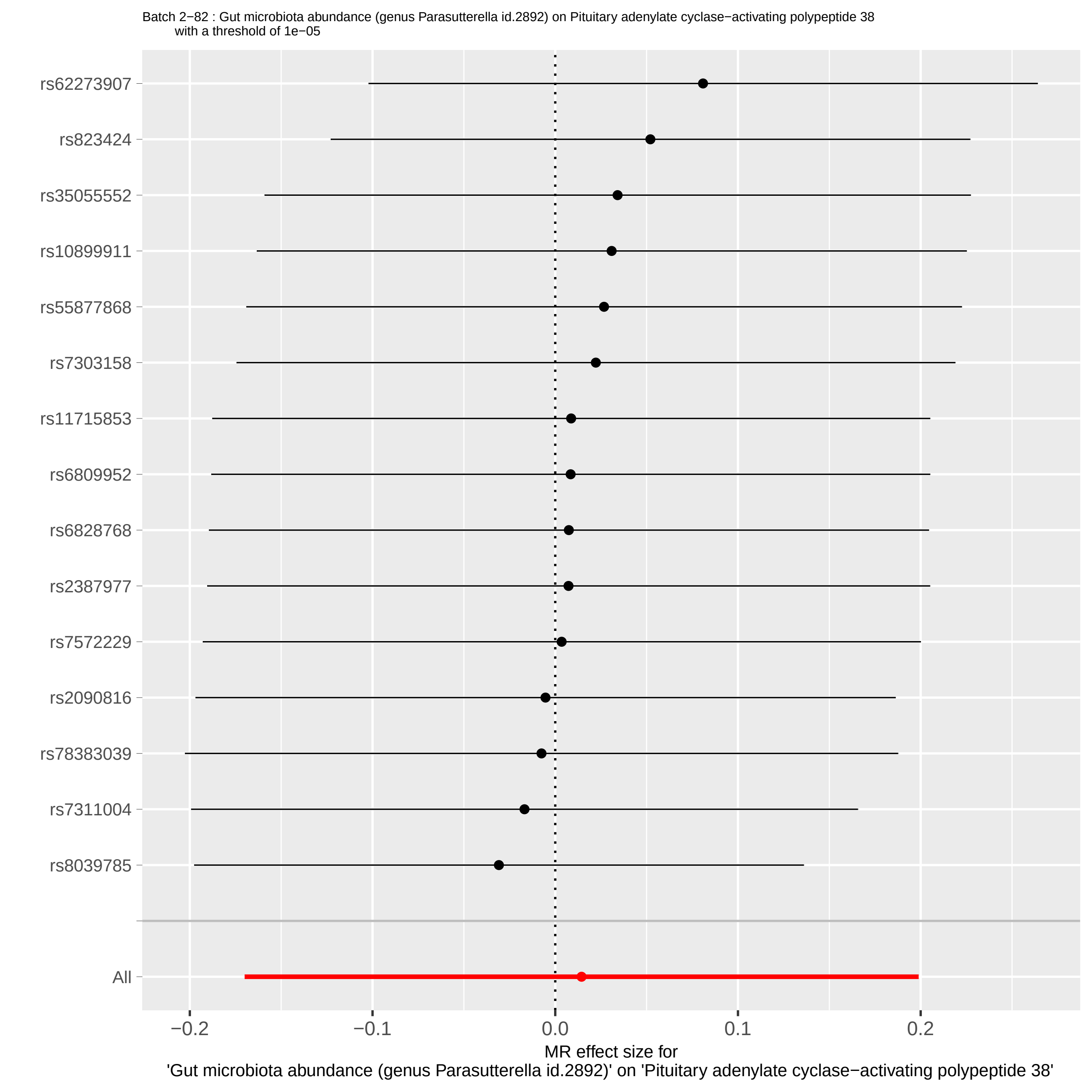

Batch 2−82 : Gut microbiota abundance (genus Parasutterella id.2892) on Pituitary adenylate cyclase−activating polypeptide 38
with a threshold of 1e−05
rs62273907
rs823424
rs35055552
rs10899911
rs55877868
rs7303158
rs11715853
rs6809952
rs6828768
rs2387977
rs7572229
rs2090816
rs78383039
rs7311004
rs8039785
All
−0.2
−0.1
0.0
0.1
0.2
MR effect size for
'Gut microbiota abundance (genus Parasutterella id.2892)' on 'Pituitary adenylate cyclase−activating polypeptide 38'

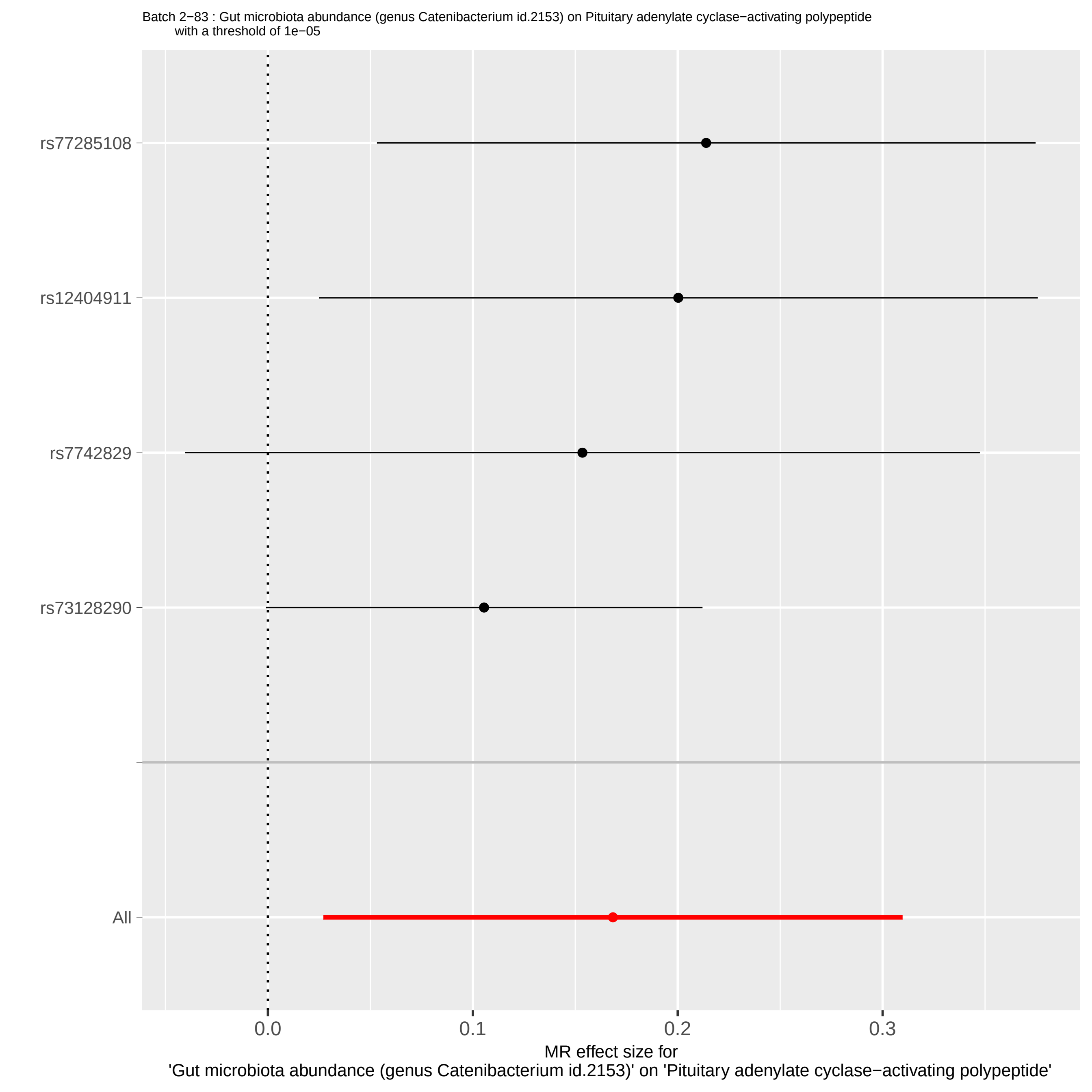

Batch 2−83 : Gut microbiota abundance (genus Catenibacterium id.2153) on Pituitary adenylate cyclase−activating polypeptide
with a threshold of 1e−05
rs77285108
rs12404911
rs7742829
rs73128290
All
0.0
0.1
0.2
0.3
MR effect size for
'Gut microbiota abundance (genus Catenibacterium id.2153)' on 'Pituitary adenylate cyclase−activating polypeptide'

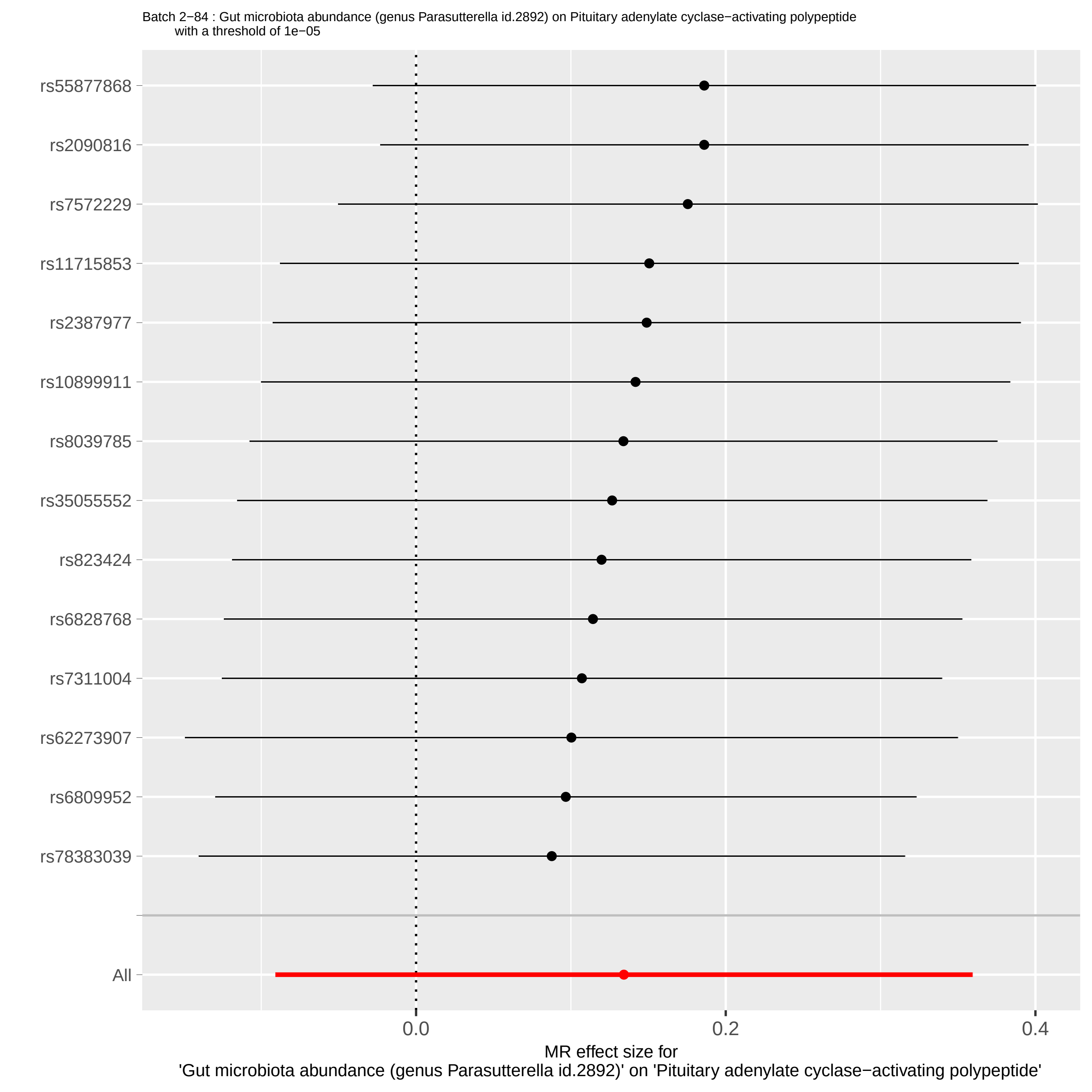
Batch 2−84 : Gut microbiota abundance (genus Parasutterella id.2892) on Pituitary adenylate cyclase−activating polypeptide
with a threshold of 1e−05
rs55877868
rs2090816
rs7572229
rs11715853
rs2387977
rs10899911
rs8039785
rs35055552
rs823424
rs6828768
rs7311004
rs62273907
rs6809952
rs78383039
All
0.0
0.2
0.4
MR effect size for
'Gut microbiota abundance (genus Parasutterella id.2892)' on 'Pituitary adenylate cyclase−activating polypeptide'

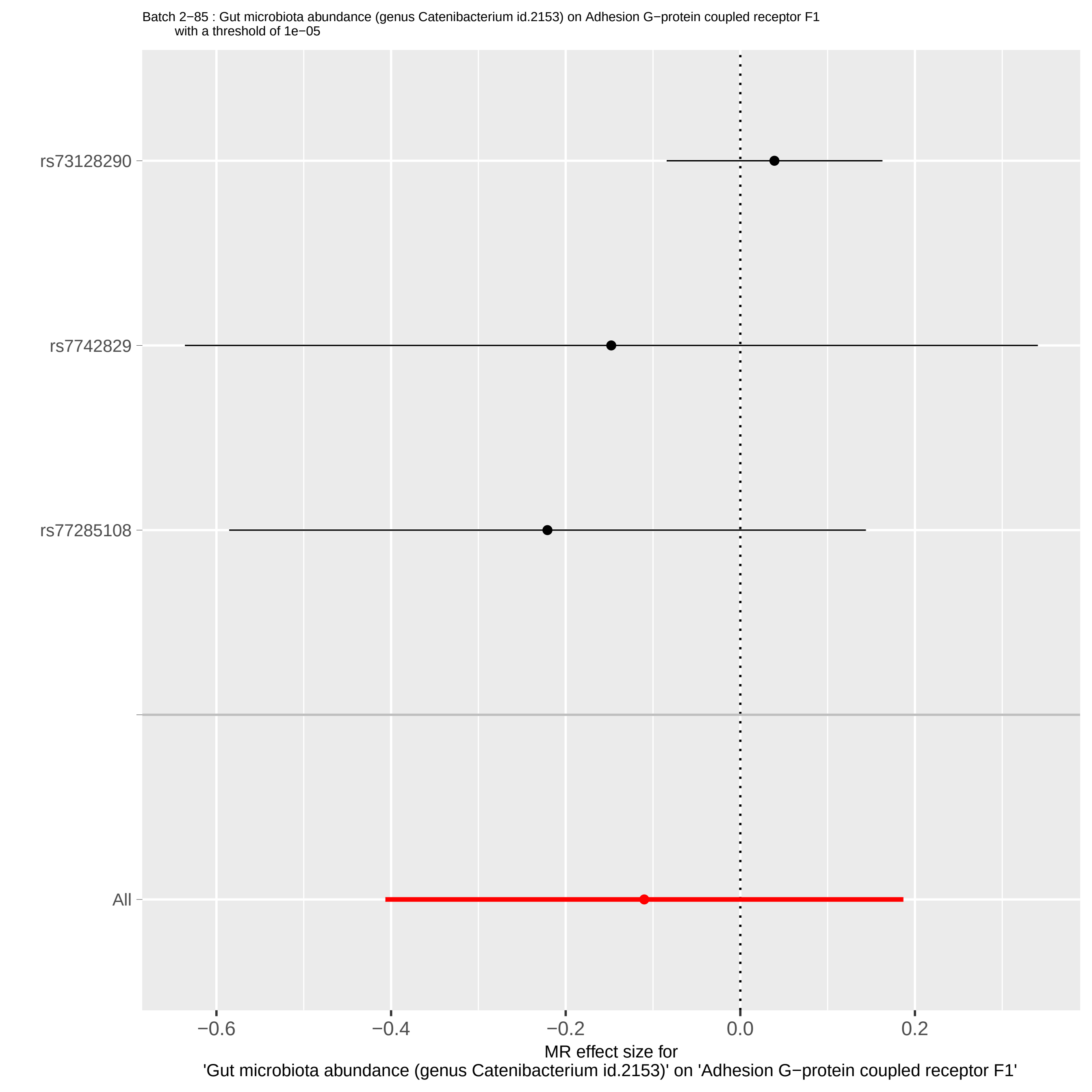
Batch 2−85 : Gut microbiota abundance (genus Catenibacterium id.2153) on Adhesion G−protein coupled receptor F1
with a threshold of 1e−05
rs73128290
rs7742829
rs77285108
All
−0.6
−0.4
−0.2
0.0
0.2
MR effect size for
'Gut microbiota abundance (genus Catenibacterium id.2153)' on 'Adhesion G−protein coupled receptor F1'

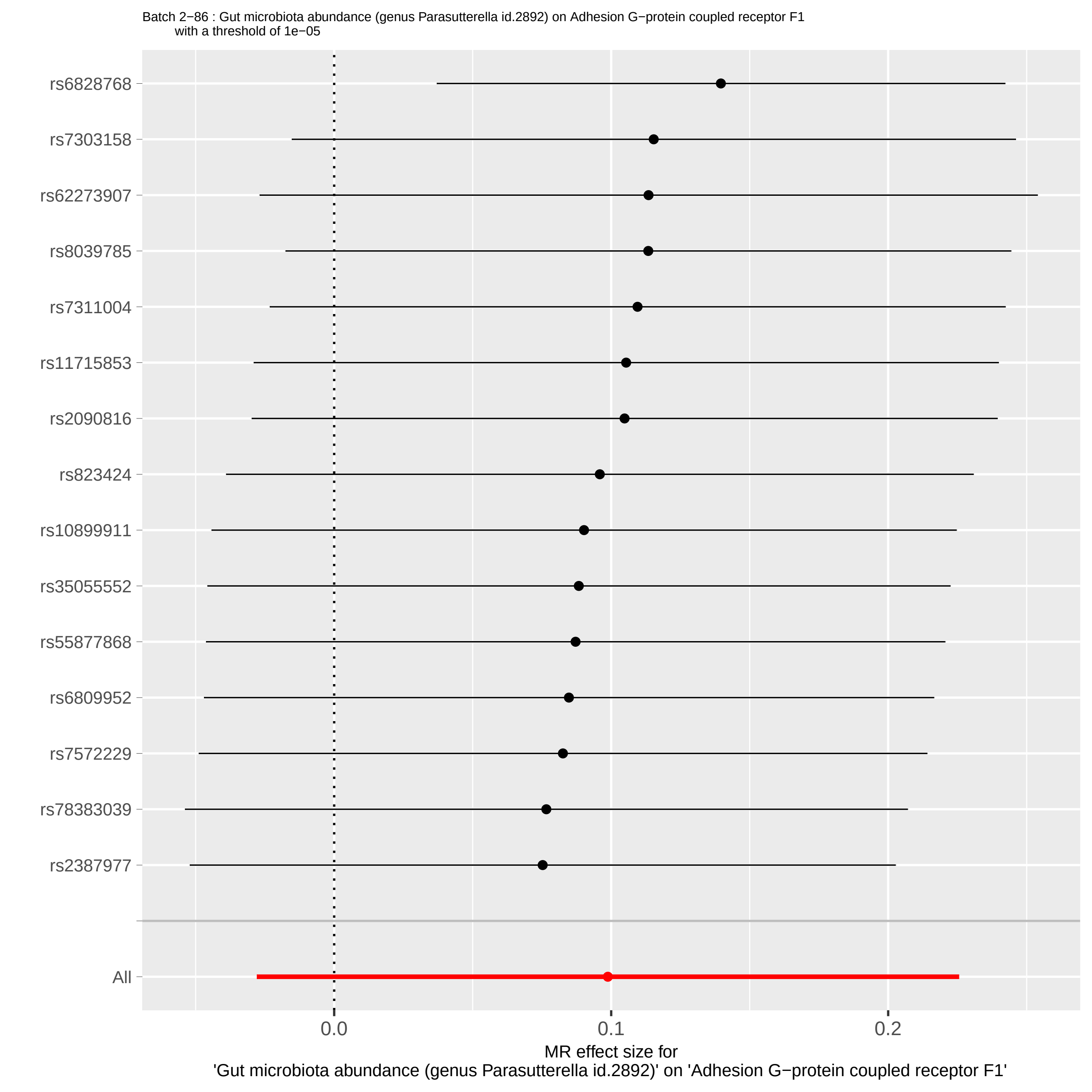

Batch 2−86 : Gut microbiota abundance (genus Parasutterella id.2892) on Adhesion G−protein coupled receptor F1
with a threshold of 1e−05
rs6828768
rs7303158
rs62273907
rs8039785
rs7311004
rs11715853
rs2090816
rs823424
rs10899911
rs35055552
rs55877868
rs6809952
rs7572229
rs78383039
rs2387977
All
0.0
0.1
0.2
MR effect size for
'Gut microbiota abundance (genus Parasutterella id.2892)' on 'Adhesion G−protein coupled receptor F1'

Batch 2−87 : Gut microbiota abundance (genus Catenibacterium id.2153) on Adhesion G protein−coupled receptor F5
with a threshold of 1e−05

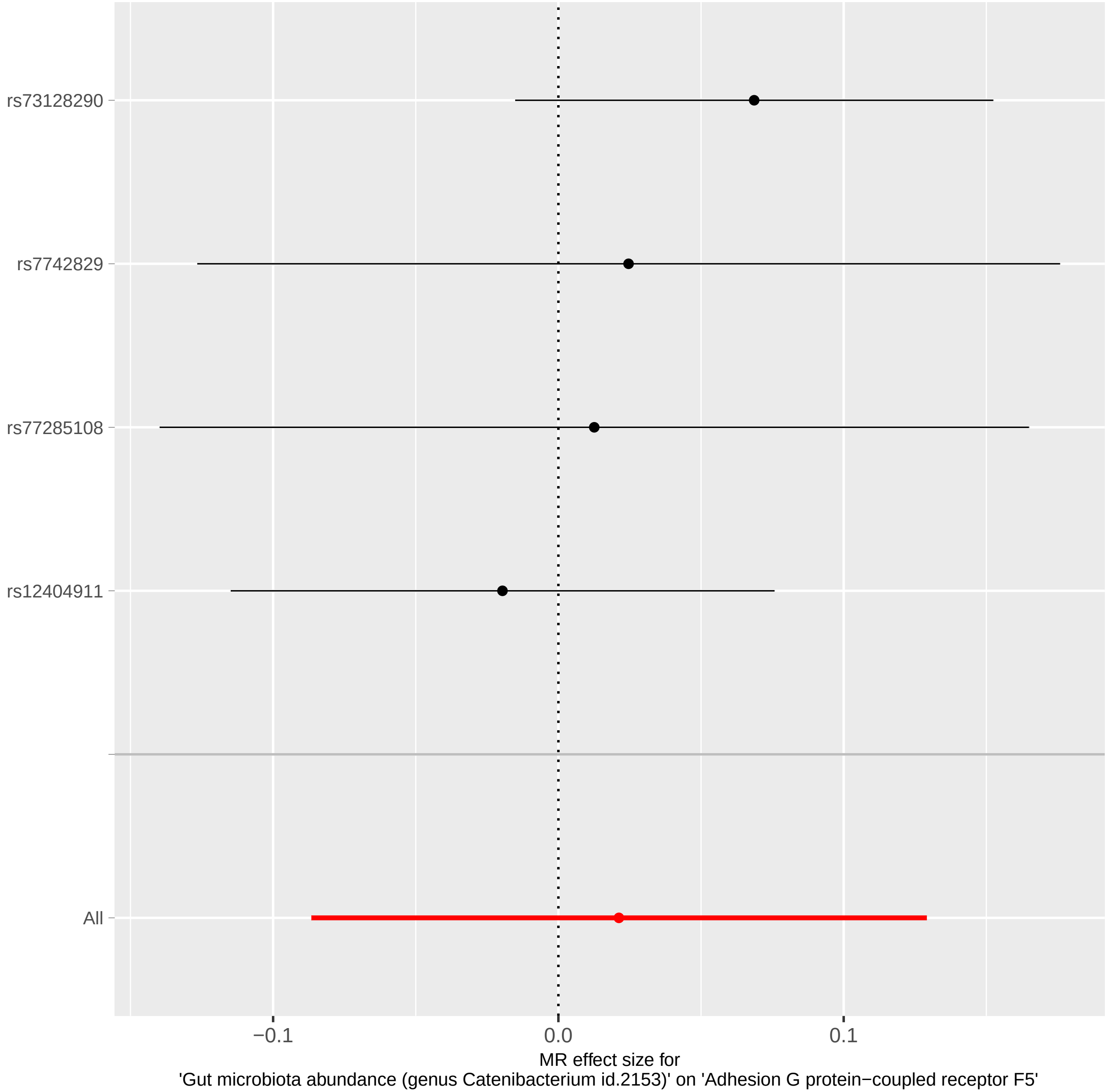

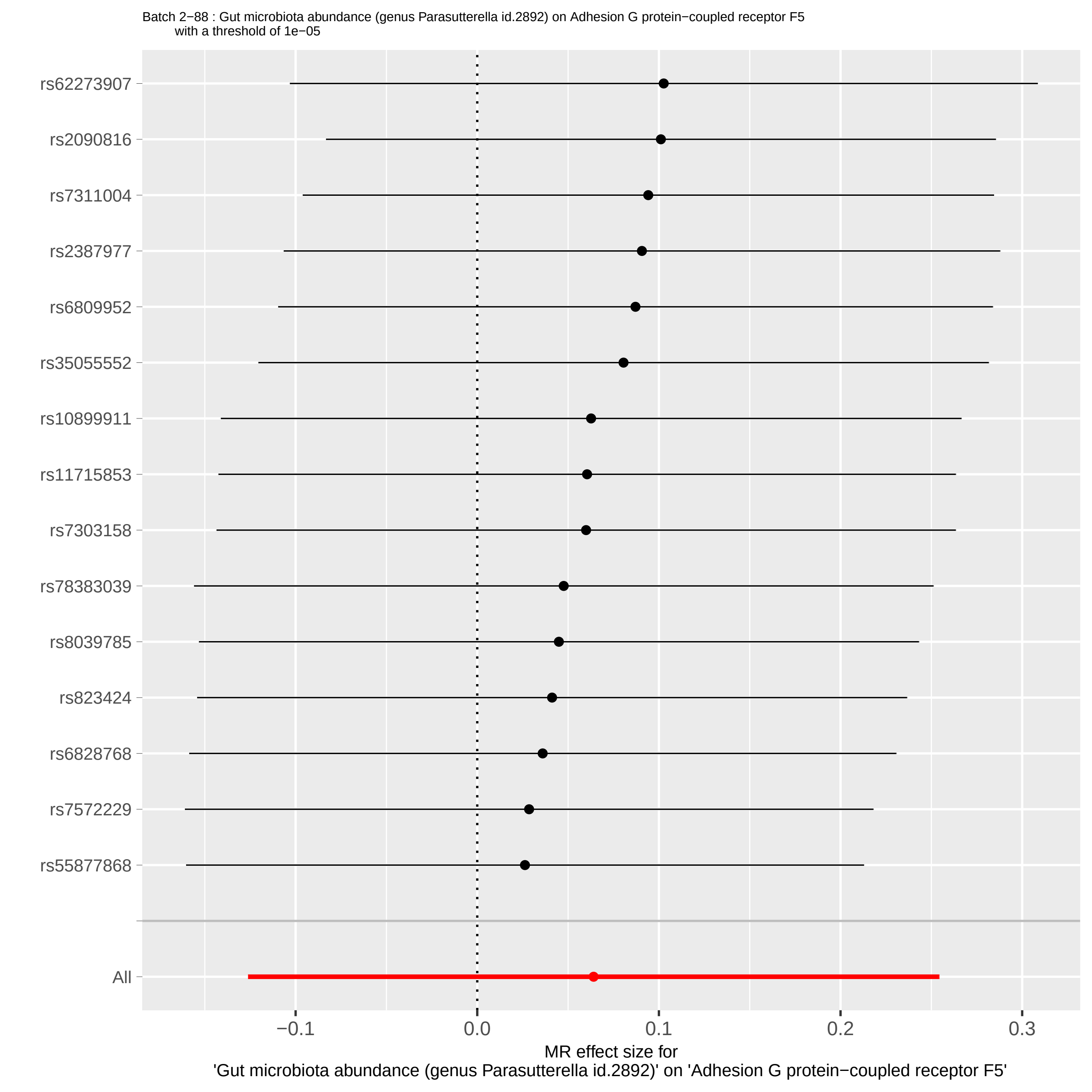

Batch 2−88 : Gut microbiota abundance (genus Parasutterella id.2892) on Adhesion G protein−coupled receptor F5
with a threshold of 1e−05
rs62273907
rs2090816
rs7311004
rs2387977
rs6809952
rs35055552
rs10899911
rs11715853
rs7303158
rs78383039
rs8039785
rs823424
rs6828768
rs7572229
rs55877868
All
−0.1
0.0
0.1
0.2
0.3
MR effect size for
'Gut microbiota abundance (genus Parasutterella id.2892)' on 'Adhesion G protein−coupled receptor F5'

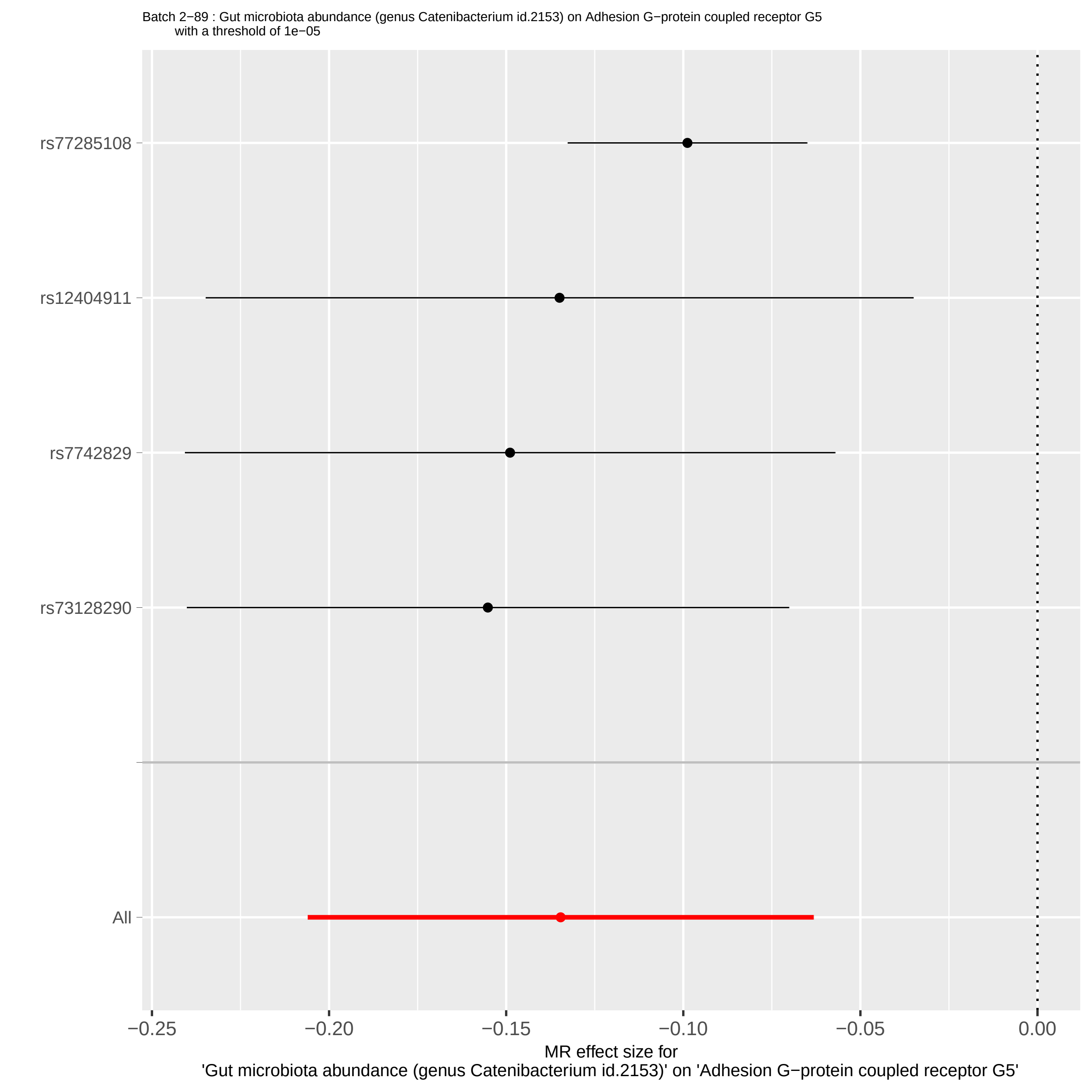

Batch 2−89 : Gut microbiota abundance (genus Catenibacterium id.2153) on Adhesion G−protein coupled receptor G5
with a threshold of 1e−05
rs77285108
rs12404911
rs7742829
rs73128290
All
−0.25
−0.20
−0.15
−0.10
−0.05
0.00
MR effect size for
'Gut microbiota abundance (genus Catenibacterium id.2153)' on 'Adhesion G−protein coupled receptor G5'

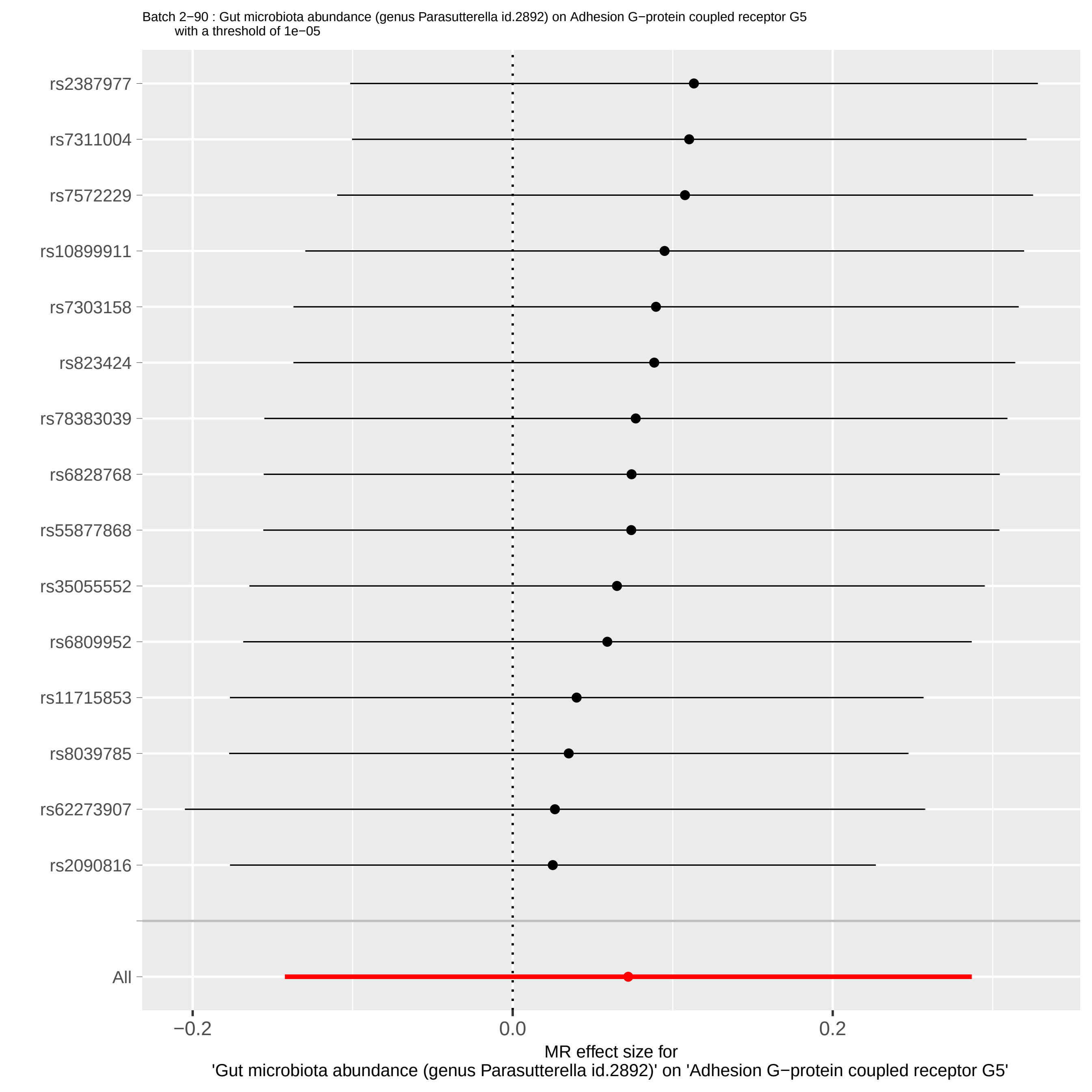

Batch 2−90 : Gut microbiota abundance (genus Parasutterella id.2892) on Adhesion G−protein coupled receptor G5
with a threshold of 1e−05
rs2387977
rs7311004
rs7572229
rs10899911
rs7303158
rs823424
rs78383039
rs6828768
rs55877868
rs35055552
rs6809952
rs11715853
rs8039785
rs62273907
rs2090816
All
−0.2
0.0
0.2
MR effect size for
'Gut microbiota abundance (genus Parasutterella id.2892)' on 'Adhesion G−protein coupled receptor G5'

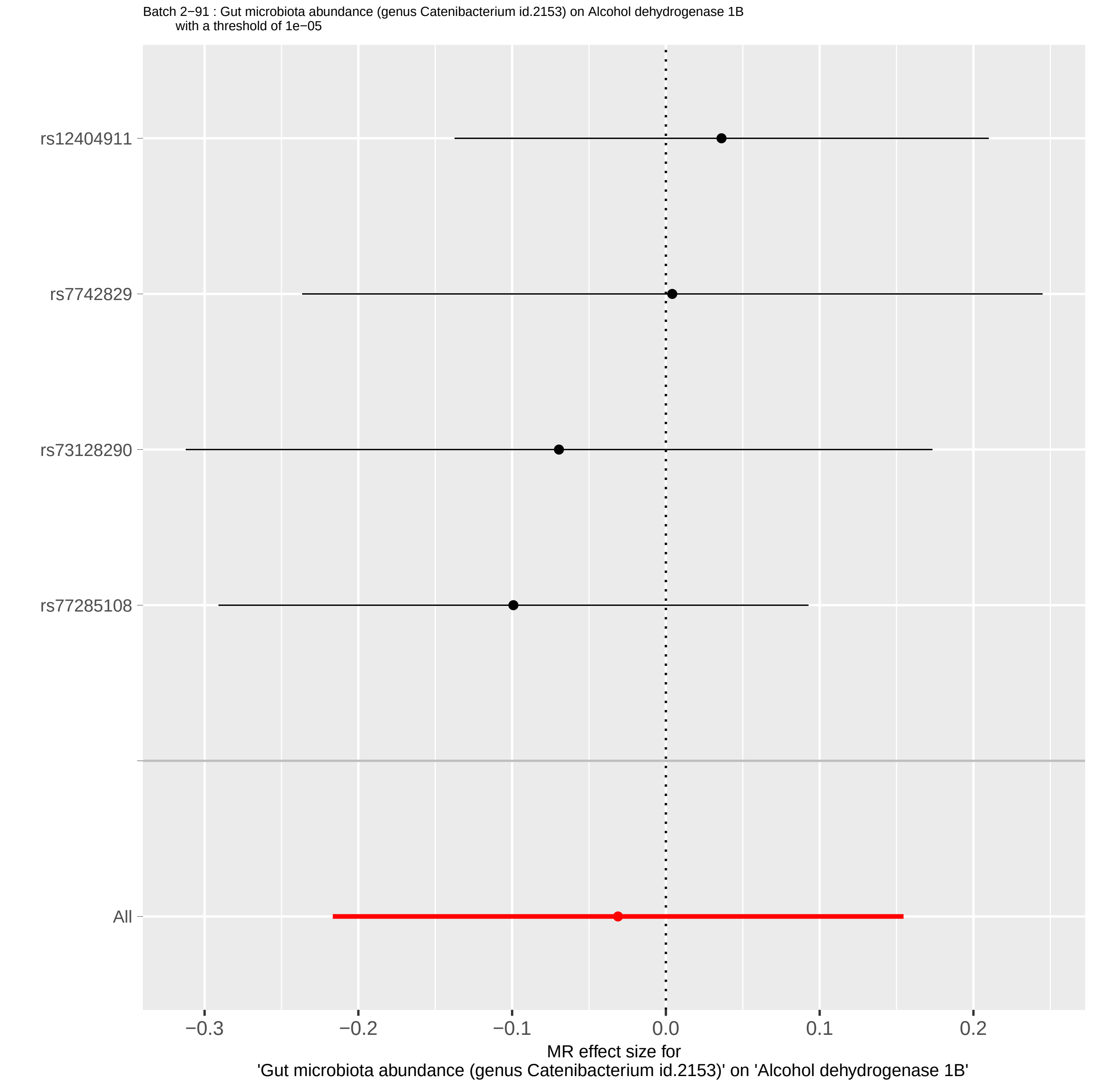
Batch 2−91 : Gut microbiota abundance (genus Catenibacterium id.2153) on Alcohol dehydrogenase 1B
with a threshold of 1e−05
rs12404911
rs7742829
rs73128290
rs77285108
All
−0.3
−0.2
−0.1
0.0
0.1
0.2
MR effect size for
'Gut microbiota abundance (genus Catenibacterium id.2153)' on 'Alcohol dehydrogenase 1B'

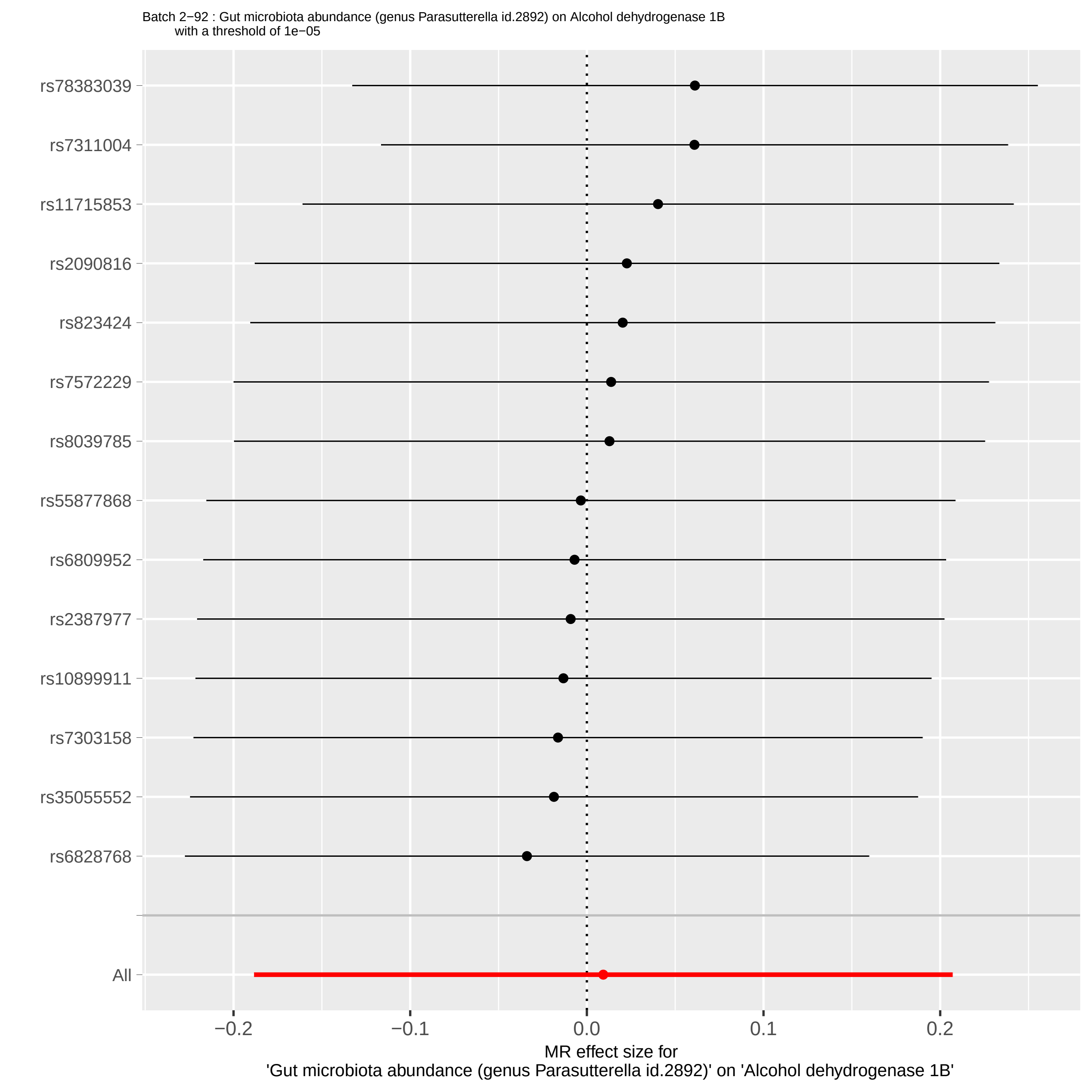
Batch 2−92 : Gut microbiota abundance (genus Parasutterella id.2892) on Alcohol dehydrogenase 1B
with a threshold of 1e−05
rs78383039
rs7311004
rs11715853
rs2090816
rs823424
rs7572229
rs8039785
rs55877868
rs6809952
rs2387977
rs10899911
rs7303158
rs35055552
rs6828768
All
−0.2
−0.1
0.0
0.1
0.2
MR effect size for
'Gut microbiota abundance (genus Parasutterella id.2892)' on 'Alcohol dehydrogenase 1B'

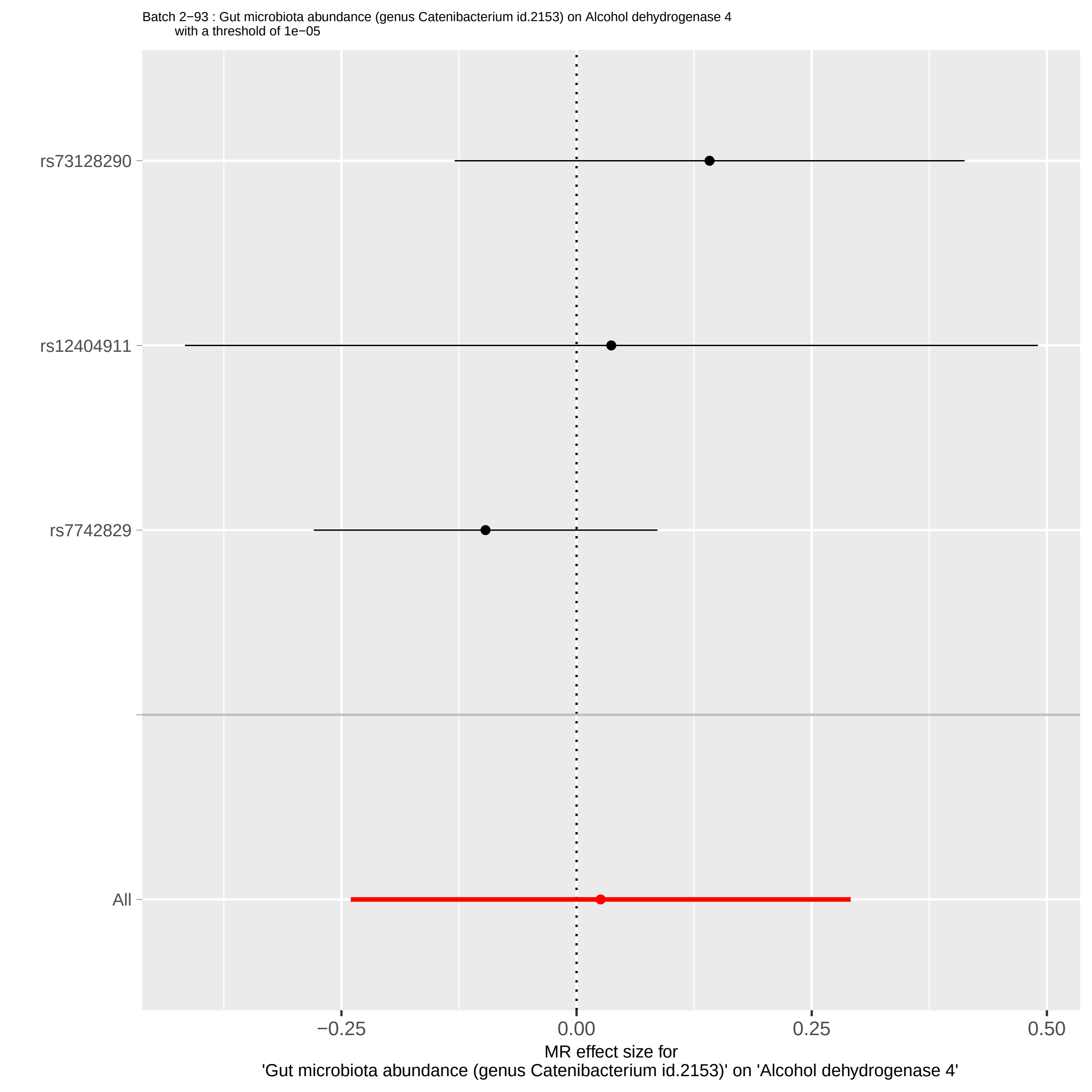

Batch 2−93 : Gut microbiota abundance (genus Catenibacterium id.2153) on Alcohol dehydrogenase 4
with a threshold of 1e−05
rs73128290
rs12404911
rs7742829
All
−0.25
0.00
0.25
0.50
MR effect size for
'Gut microbiota abundance (genus Catenibacterium id.2153)' on 'Alcohol dehydrogenase 4'

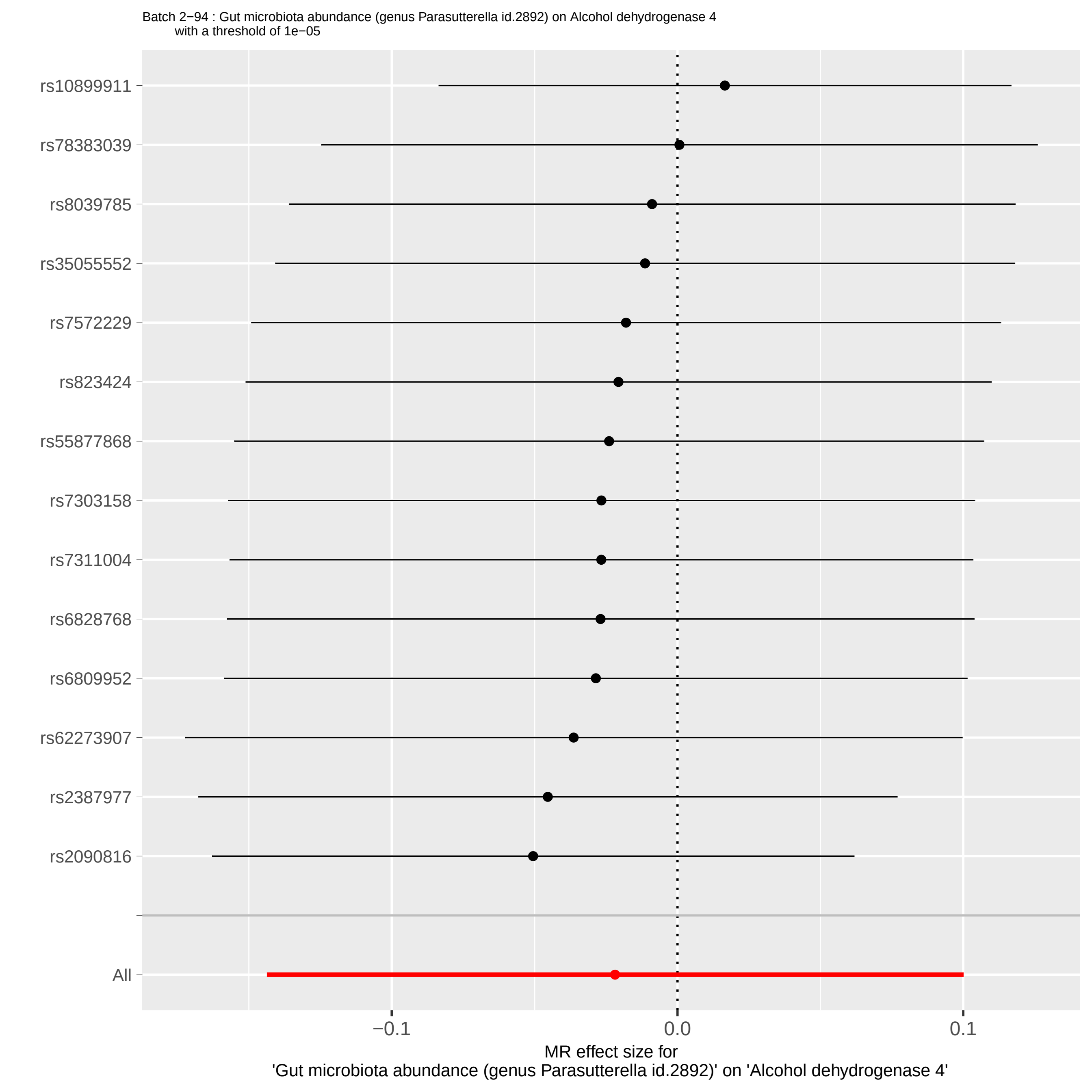
Batch 2−94 : Gut microbiota abundance (genus Parasutterella id.2892) on Alcohol dehydrogenase 4
with a threshold of 1e−05
rs10899911
rs78383039
rs8039785
rs35055552
rs7572229
rs823424
rs55877868
rs7303158
rs7311004
rs6828768
rs6809952
rs62273907
rs2387977
rs2090816
All
−0.1
0.0
0.1
MR effect size for
'Gut microbiota abundance (genus Parasutterella id.2892)' on 'Alcohol dehydrogenase 4'

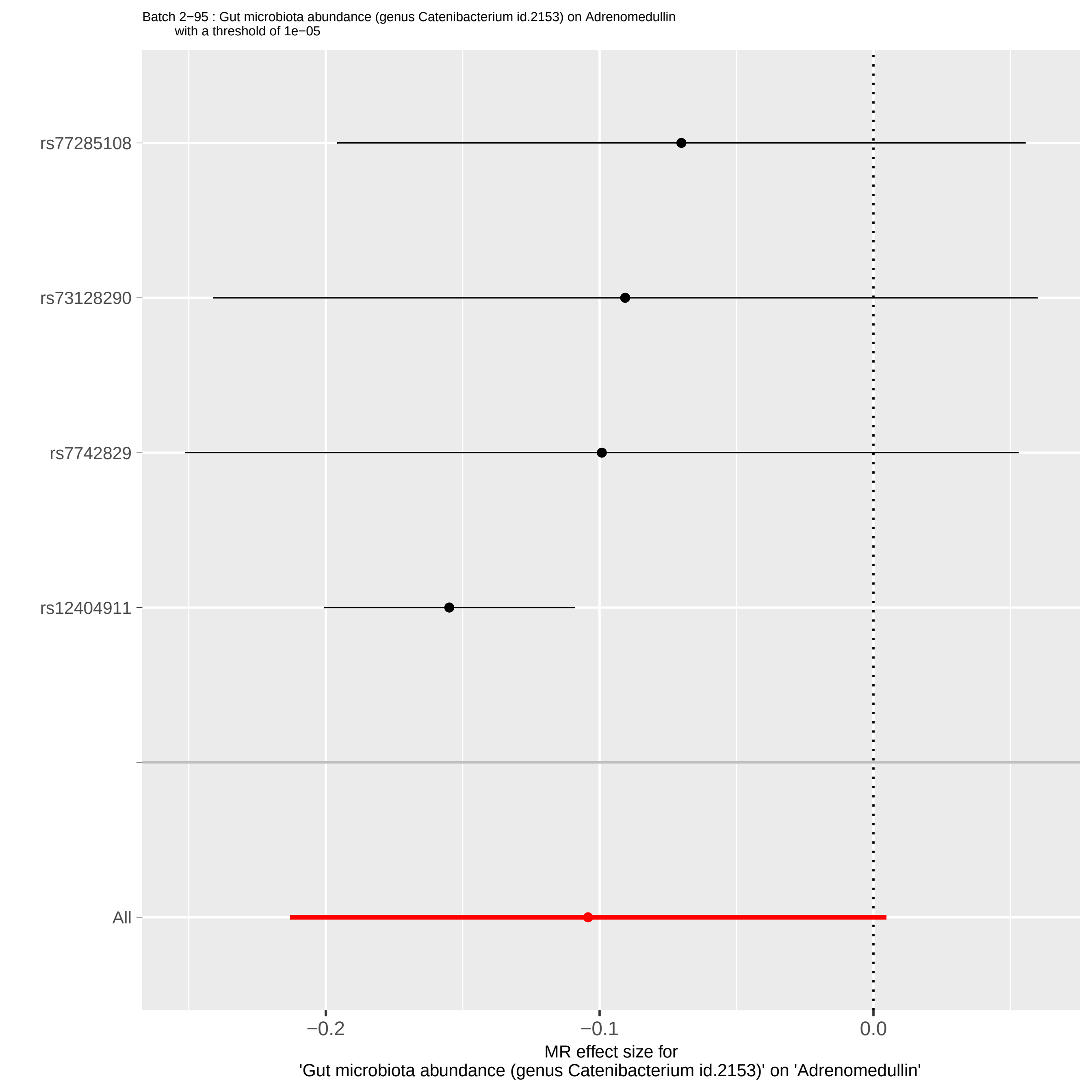
Batch 2−95 : Gut microbiota abundance (genus Catenibacterium id.2153) on Adrenomedullin
with a threshold of 1e−05
rs77285108
rs73128290
rs7742829
rs12404911
All
−0.2
−0.1
0.0
MR effect size for
'Gut microbiota abundance (genus Catenibacterium id.2153)' on 'Adrenomedullin'

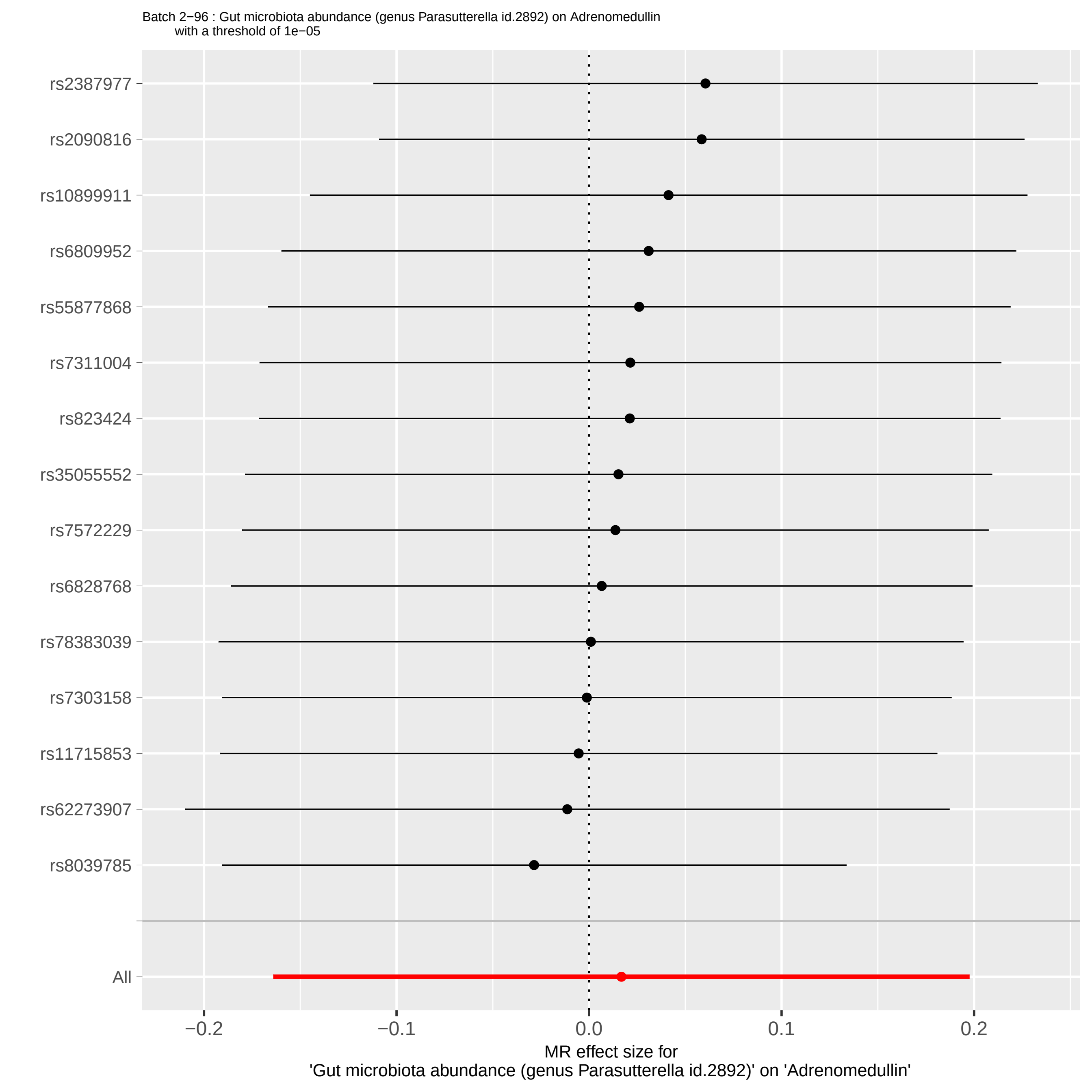
Batch 2−96 : Gut microbiota abundance (genus Parasutterella id.2892) on Adrenomedullin
with a threshold of 1e−05
rs2387977
rs2090816
rs10899911
rs6809952
rs55877868
rs7311004
rs823424
rs35055552
rs7572229
rs6828768
rs78383039
rs7303158
rs11715853
rs62273907
rs8039785
All
−0.2
−0.1
0.0
0.1
0.2
MR effect size for
'Gut microbiota abundance (genus Parasutterella id.2892)' on 'Adrenomedullin'

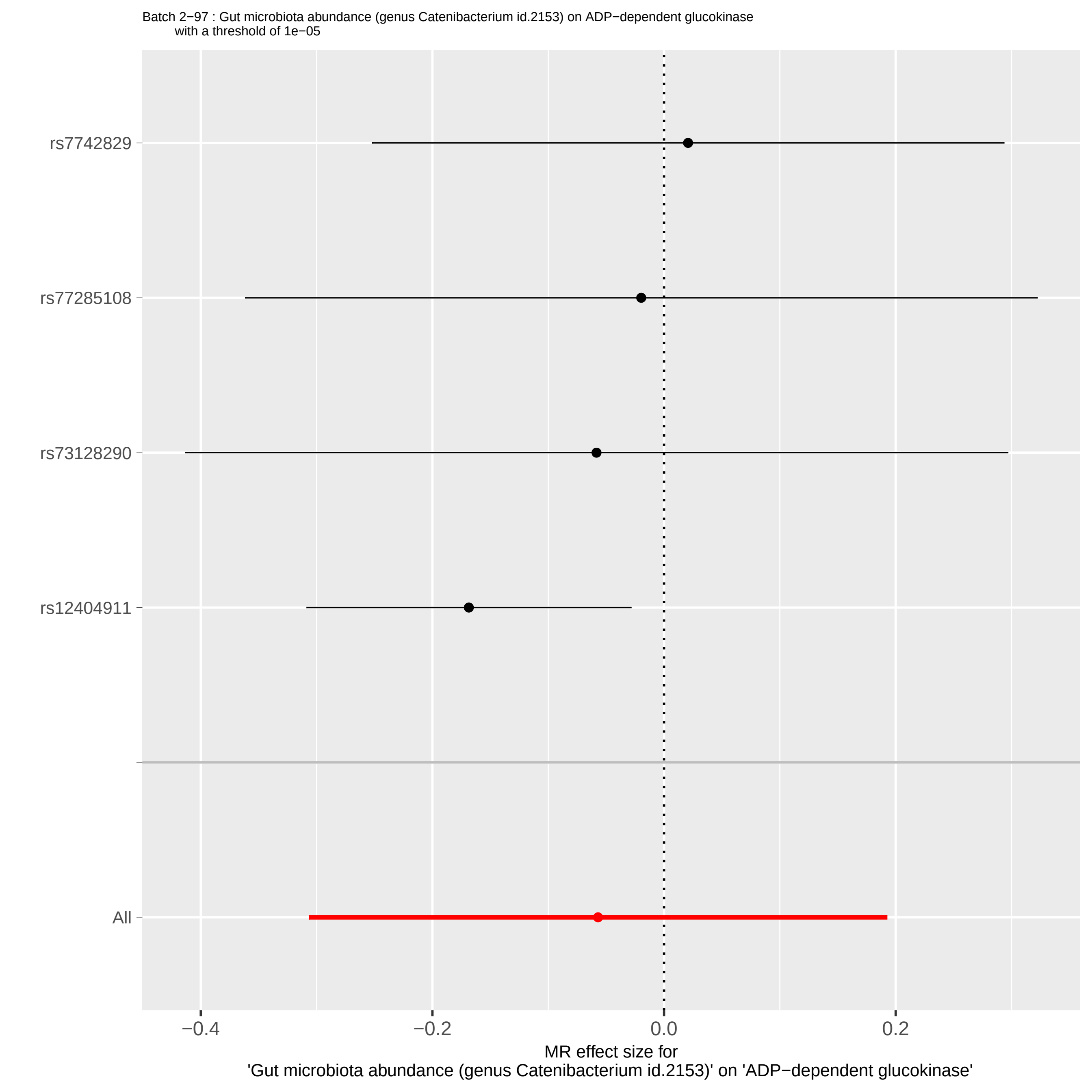
Batch 2−97 : Gut microbiota abundance (genus Catenibacterium id.2153) on ADP−dependent glucokinase
with a threshold of 1e−05
rs7742829
rs77285108
rs73128290
rs12404911
All
−0.4
−0.2
0.0
0.2
MR effect size for
'Gut microbiota abundance (genus Catenibacterium id.2153)' on 'ADP−dependent glucokinase'

Batch 2−98 : Gut microbiota abundance (genus Parasutterella id.2892) on ADP−dependent glucokinase
with a threshold of 1e−05

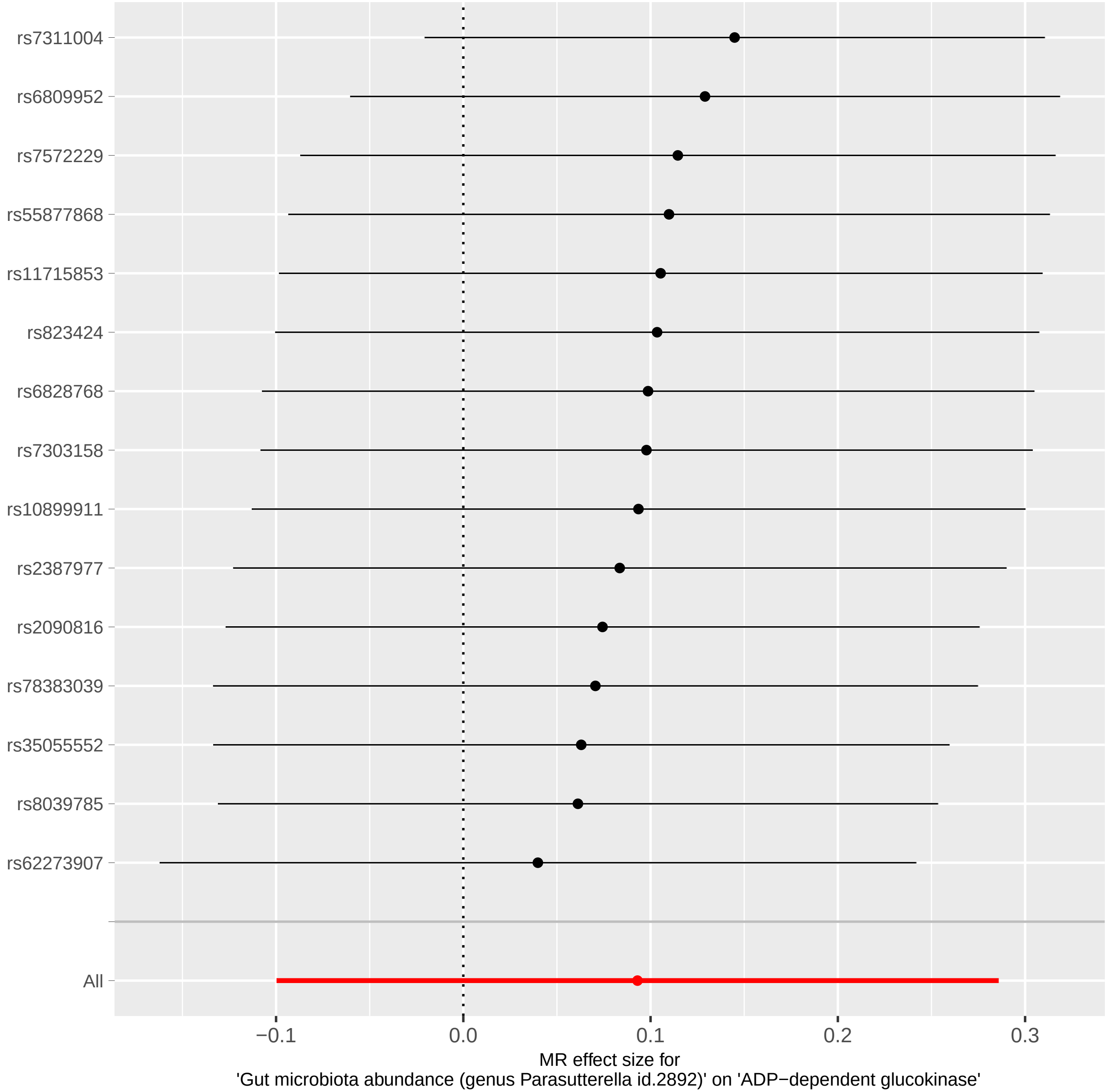

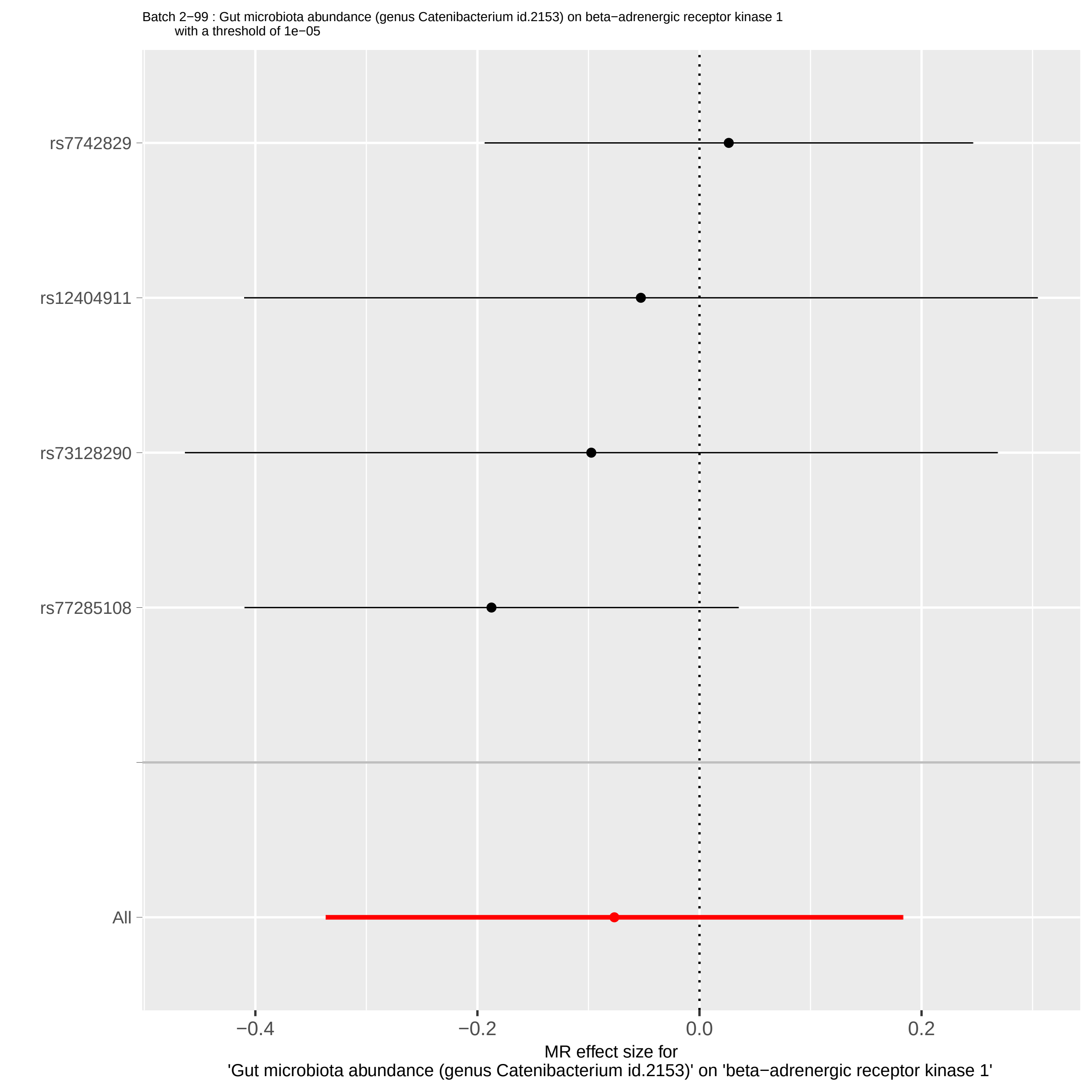

Batch 2−99 : Gut microbiota abundance (genus Catenibacterium id.2153) on beta−adrenergic receptor kinase 1
with a threshold of 1e−05
rs7742829
rs12404911
rs73128290
rs77285108
All
−0.4
−0.2
0.0
0.2
MR effect size for
'Gut microbiota abundance (genus Catenibacterium id.2153)' on 'beta−adrenergic receptor kinase 1'

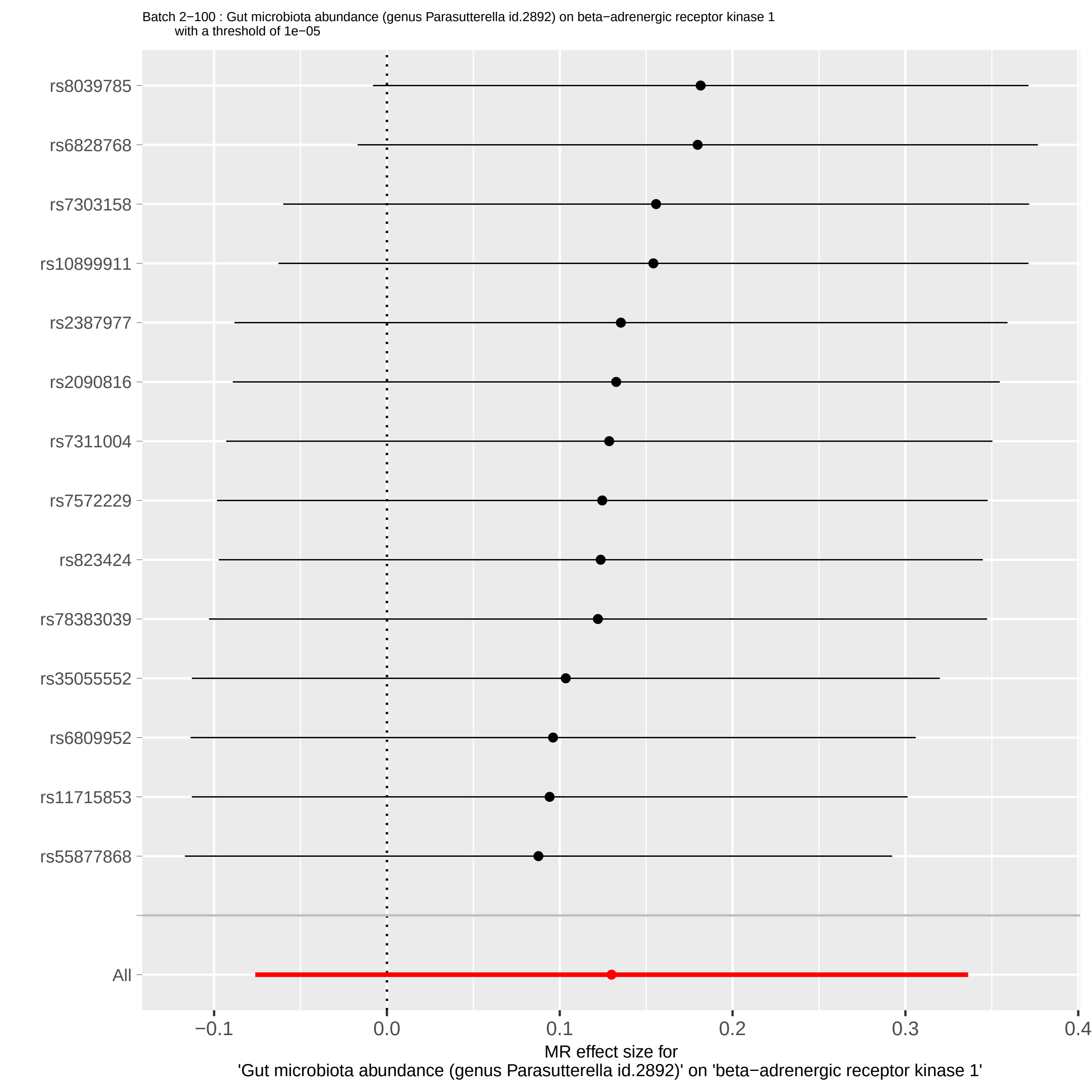

Batch 2−100 : Gut microbiota abundance (genus Parasutterella id.2892) on beta−adrenergic receptor kinase 1
with a threshold of 1e−05
rs8039785
rs6828768
rs7303158
rs10899911
rs2387977
rs2090816
rs7311004
rs7572229
rs823424
rs78383039
rs35055552
rs6809952
rs11715853
rs55877868
All
−0.1
0.0
0.1
0.2
0.3
0.4
MR effect size for
'Gut microbiota abundance (genus Parasutterella id.2892)' on 'beta−adrenergic receptor kinase 1'

Batch 2−101 : Gut microbiota abundance (genus Catenibacterium id.2153) on Adenylosuccinate synthetase isozyme 2
with a threshold of 1e−05

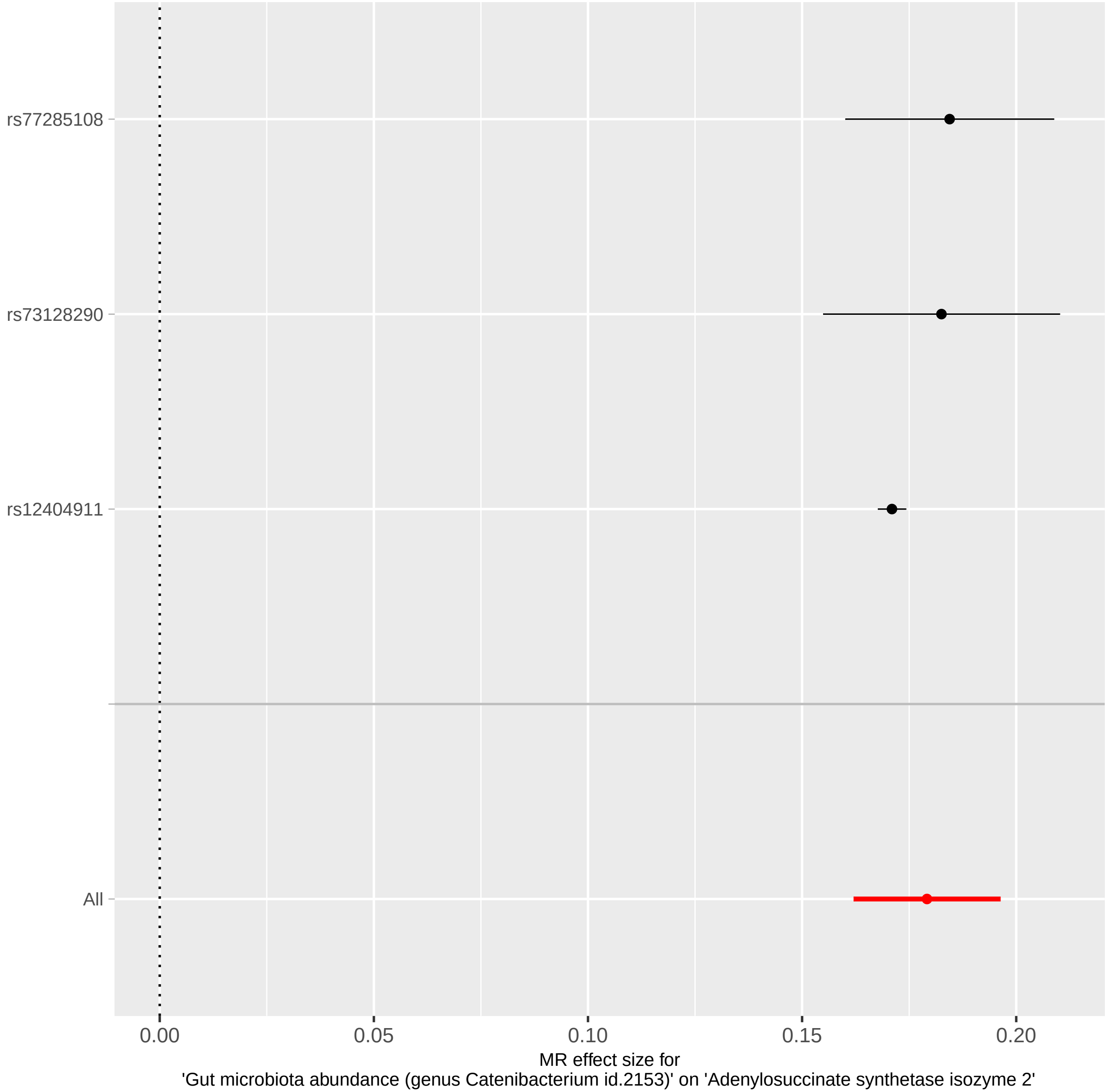

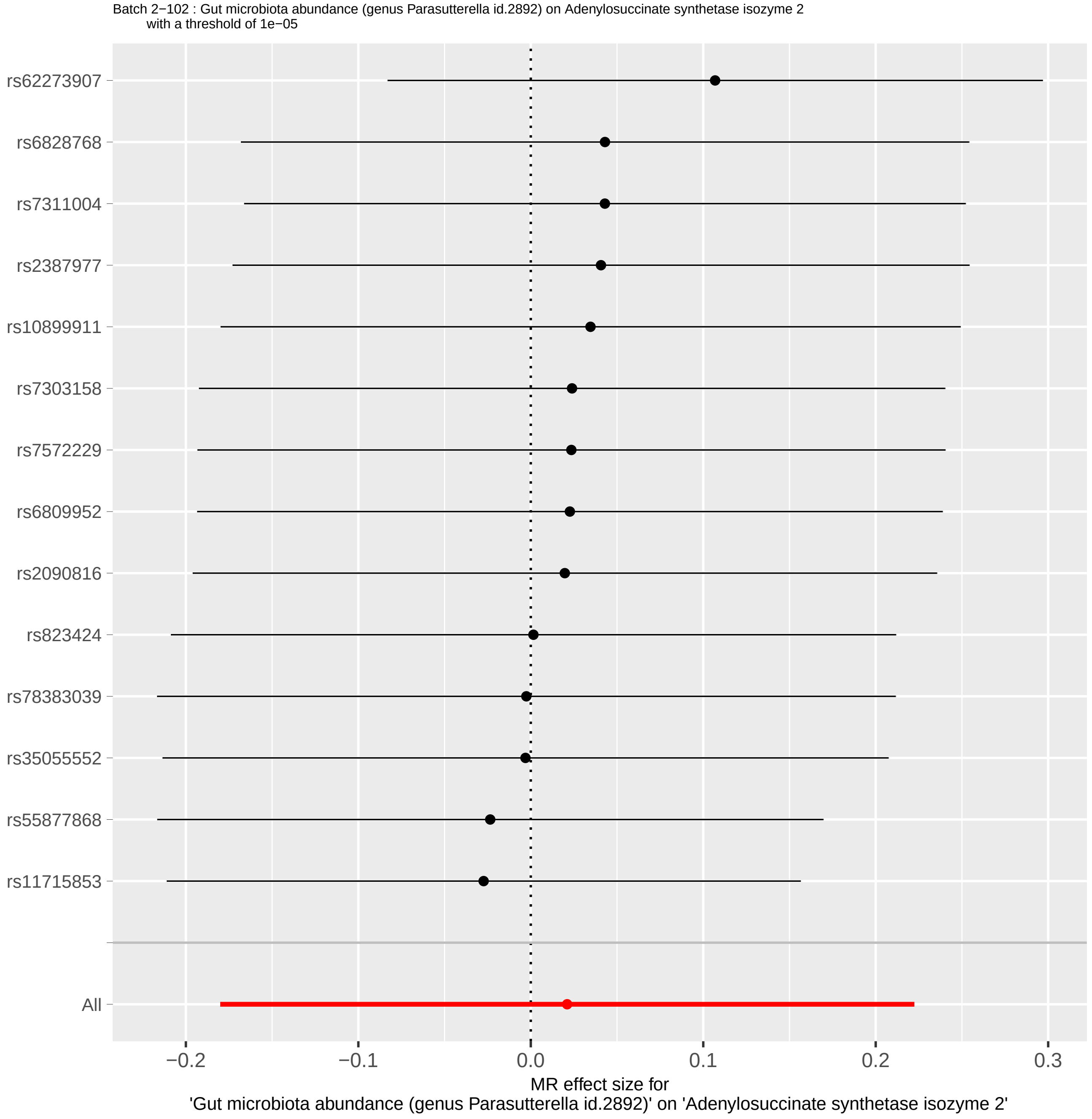
Batch 2−102 : Gut microbiota abundance (genus Parasutterella id.2892) on Adenylosuccinate synthetase isozyme 2
with a threshold of 1e−05
rs62273907
rs6828768
rs7311004
rs2387977
rs10899911
rs7303158
rs7572229
rs6809952
rs2090816
rs823424
rs78383039
rs35055552
rs55877868
rs11715853
All
−0.2
−0.1
0.0
0.1
0.2
0.3
MR effect size for
'Gut microbiota abundance (genus Parasutterella id.2892)' on 'Adenylosuccinate synthetase isozyme 2'

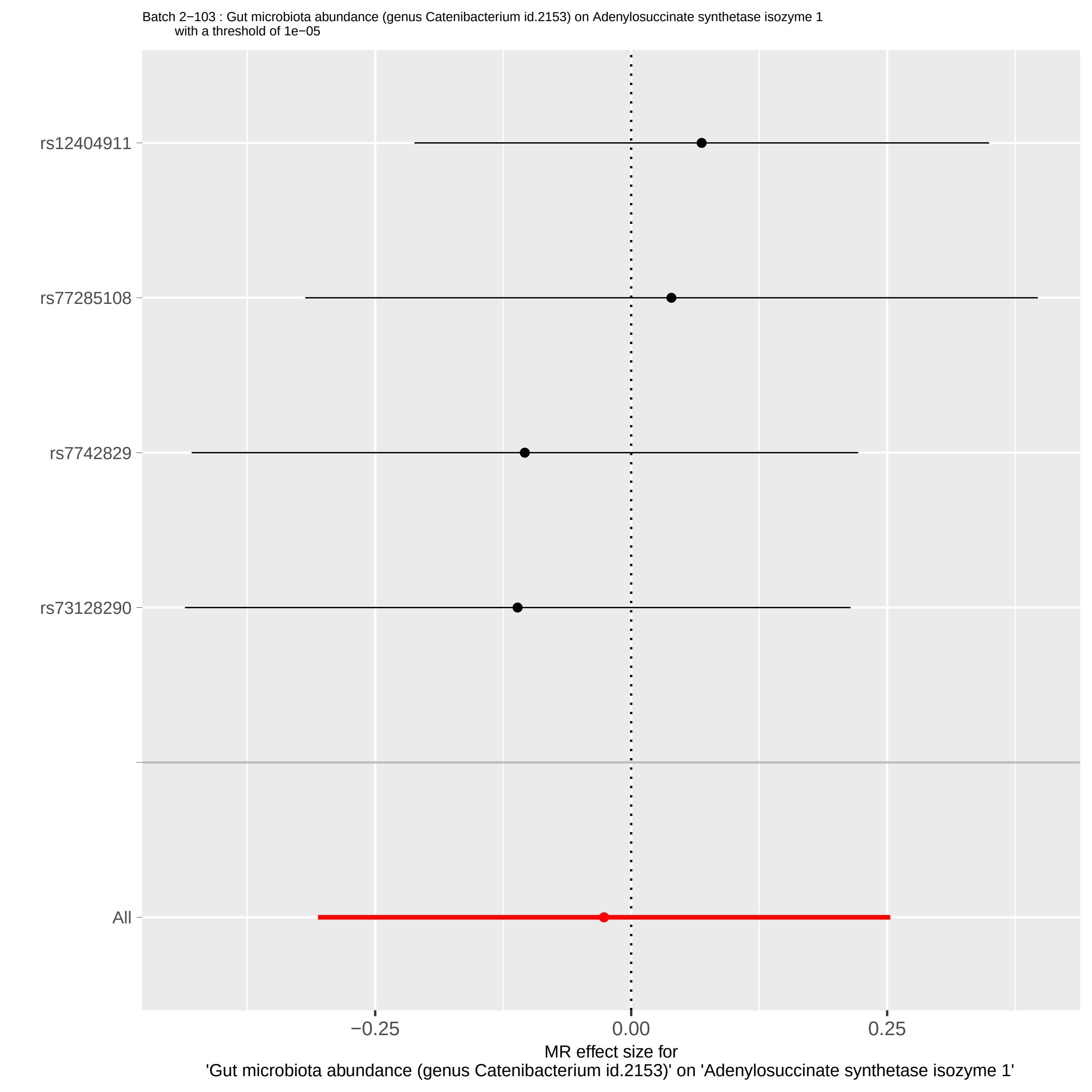
Batch 2−103 : Gut microbiota abundance (genus Catenibacterium id.2153) on Adenylosuccinate synthetase isozyme 1
with a threshold of 1e−05
rs12404911
rs77285108
rs7742829
rs73128290
All
−0.25
0.00
0.25
MR effect size for
'Gut microbiota abundance (genus Catenibacterium id.2153)' on 'Adenylosuccinate synthetase isozyme 1'

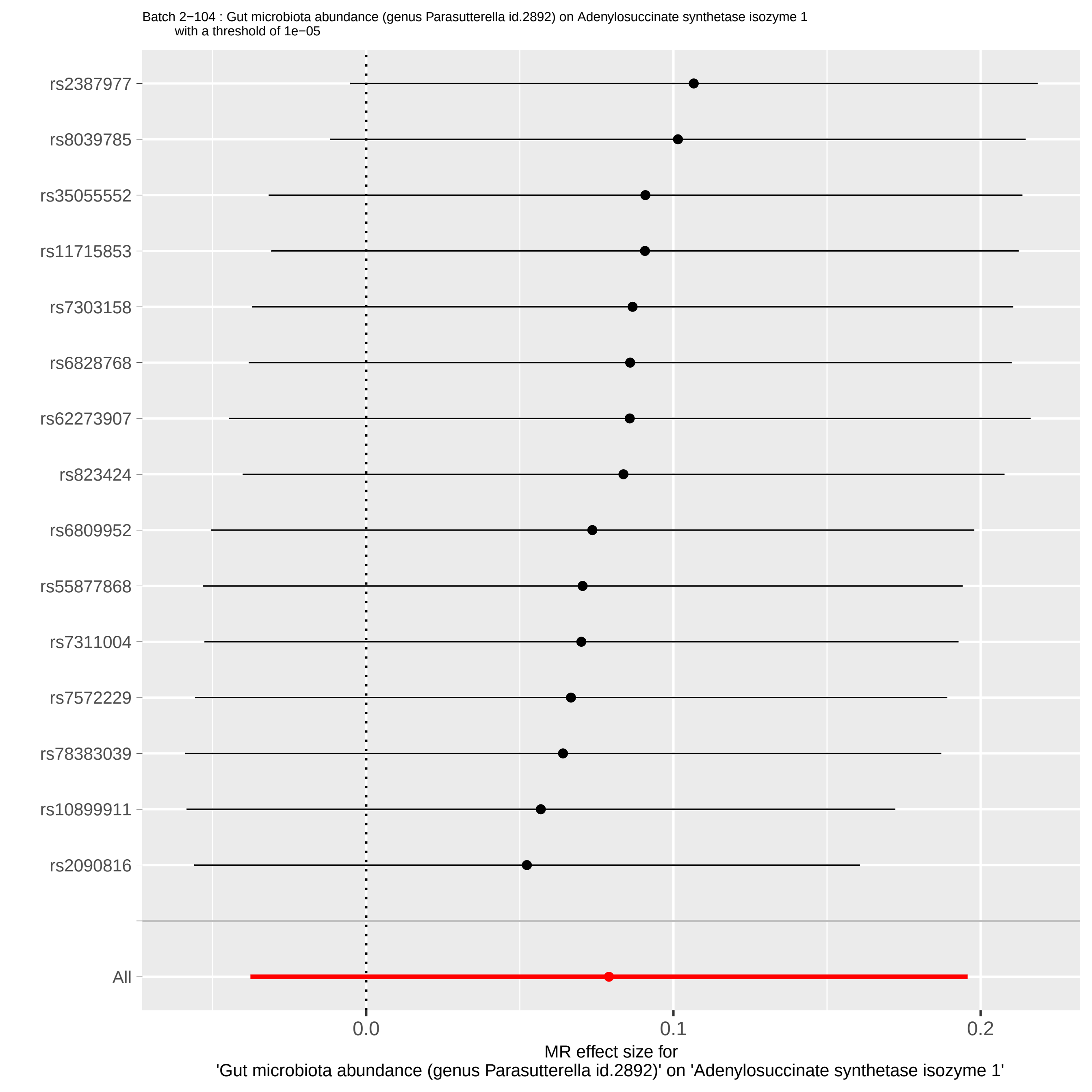

Batch 2−104 : Gut microbiota abundance (genus Parasutterella id.2892) on Adenylosuccinate synthetase isozyme 1
with a threshold of 1e−05
rs2387977
rs8039785
rs35055552
rs11715853
rs7303158
rs6828768
rs62273907
rs823424
rs6809952
rs55877868
rs7311004
rs7572229
rs78383039
rs10899911
rs2090816
All
0.0
0.1
0.2
MR effect size for
'Gut microbiota abundance (genus Parasutterella id.2892)' on 'Adenylosuccinate synthetase isozyme 1'

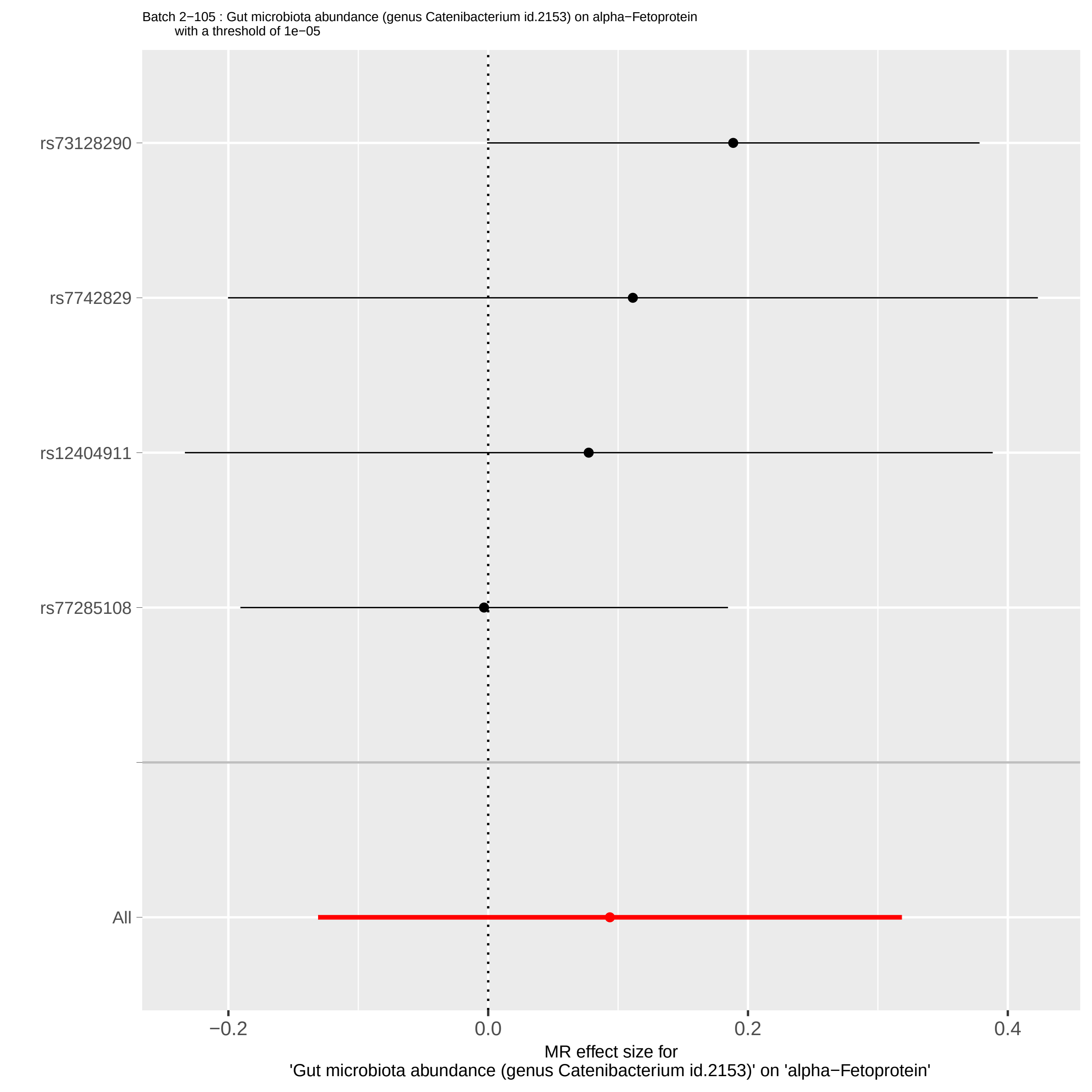
Batch 2−105 : Gut microbiota abundance (genus Catenibacterium id.2153) on alpha−Fetoprotein
with a threshold of 1e−05
rs73128290
rs7742829
rs12404911
rs77285108
All
−0.2
0.0
0.2
0.4
MR effect size for
'Gut microbiota abundance (genus Catenibacterium id.2153)' on 'alpha−Fetoprotein'

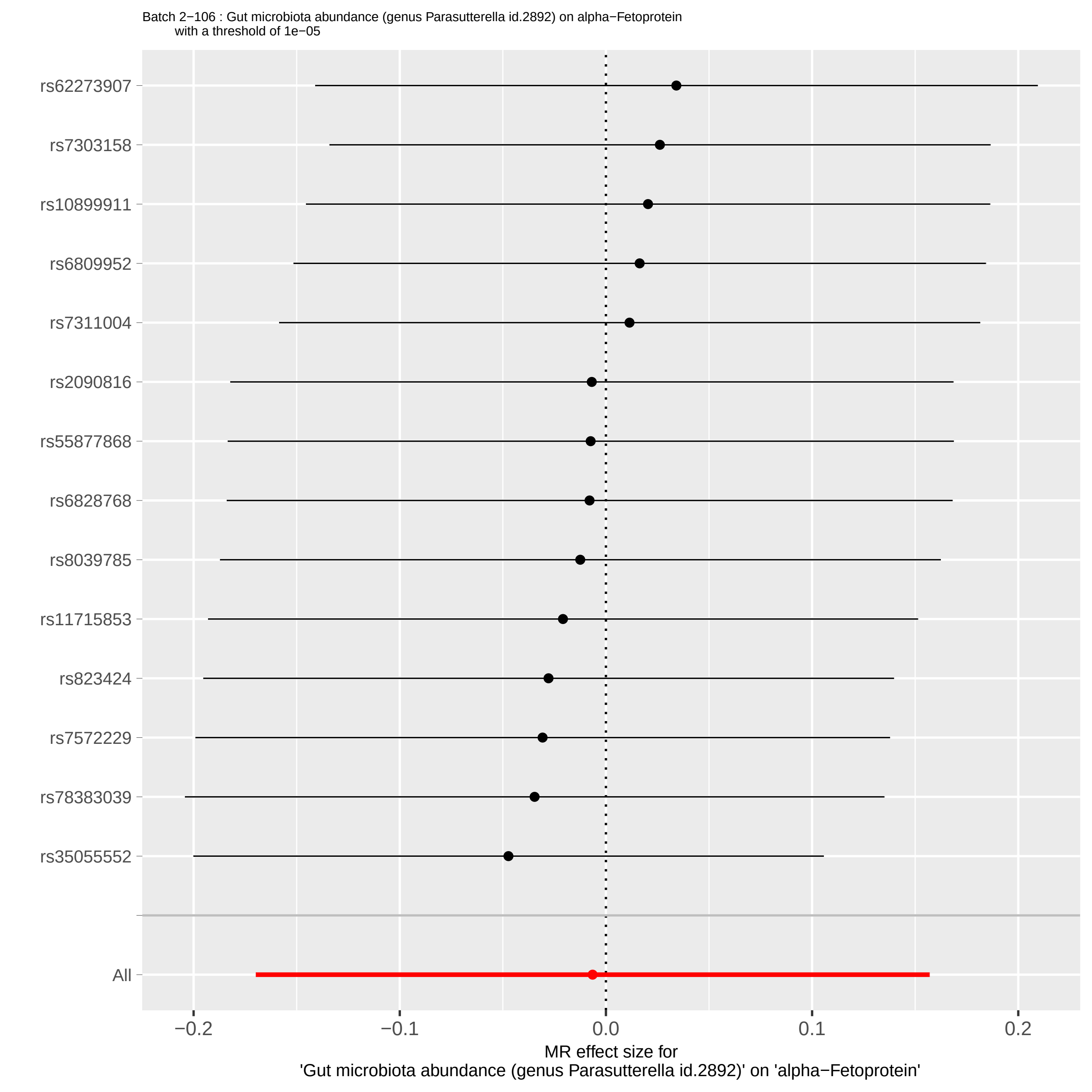
Batch 2−106 : Gut microbiota abundance (genus Parasutterella id.2892) on alpha−Fetoprotein
with a threshold of 1e−05
rs62273907
rs7303158
rs10899911
rs6809952
rs7311004
rs2090816
rs55877868
rs6828768
rs8039785
rs11715853
rs823424
rs7572229
rs78383039
rs35055552
All
−0.2
−0.1
0.0
0.1
0.2
MR effect size for
'Gut microbiota abundance (genus Parasutterella id.2892)' on 'alpha−Fetoprotein'

Batch 2−107 : Gut microbiota abundance (genus Catenibacterium id.2153) on Advanced glycosylation end product−specific receptor, soluble
with a threshold of 1e−05

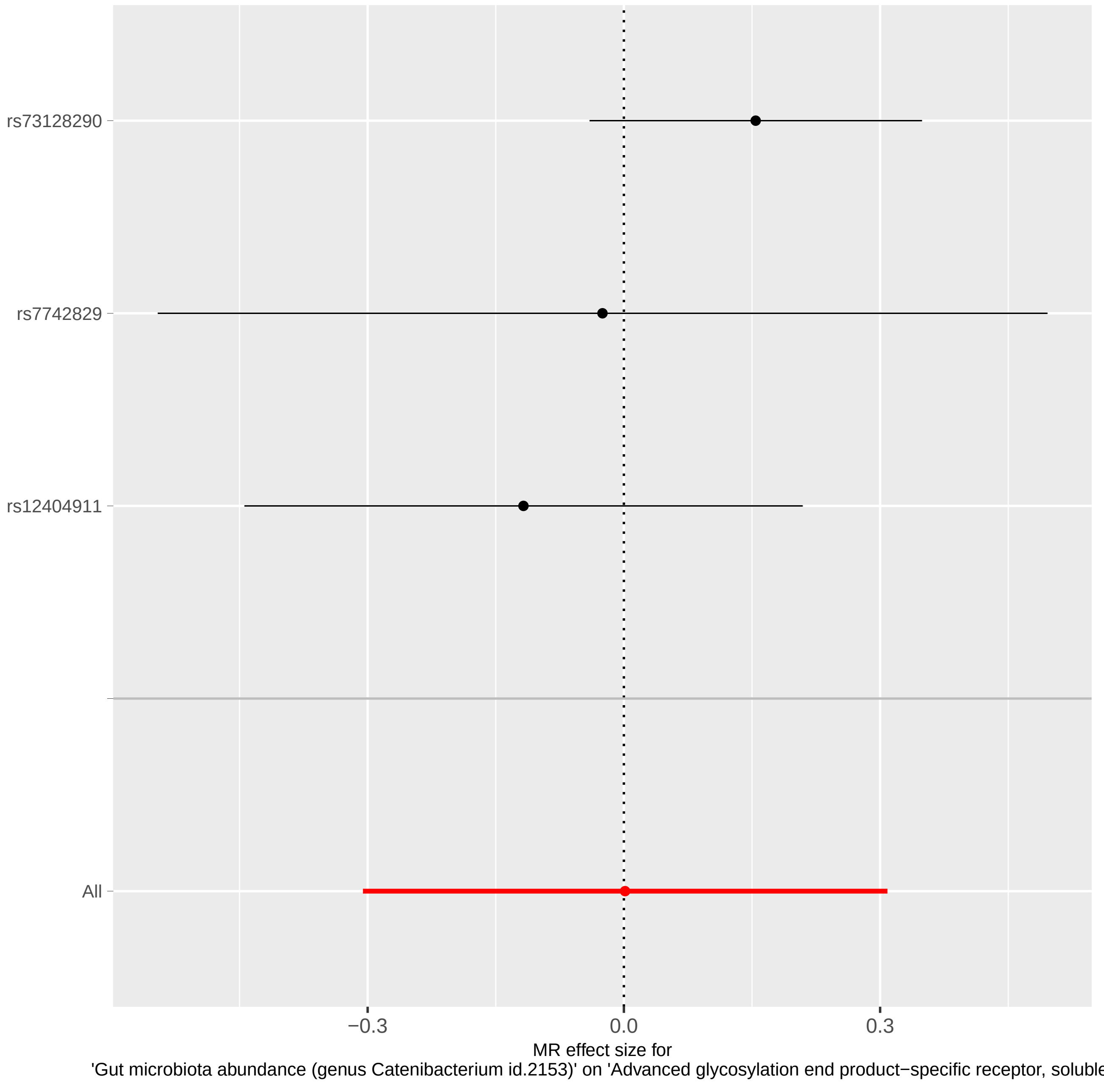

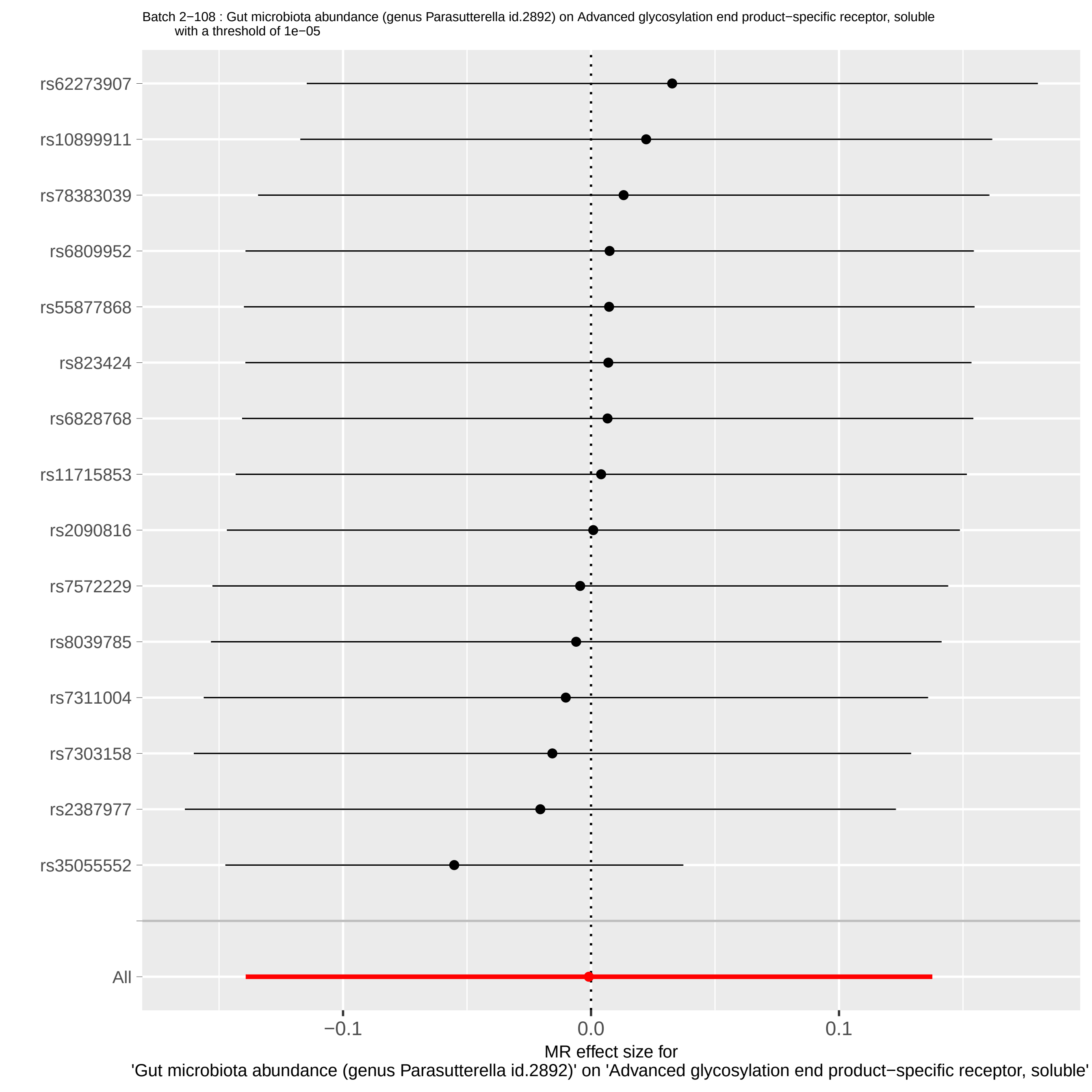

Batch 2−108 : Gut microbiota abundance (genus Parasutterella id.2892) on Advanced glycosylation end product−specific receptor, soluble with a threshold of 1e−05
rs62273907
rs10899911
rs78383039
rs6809952
rs55877868
rs823424
rs6828768
rs11715853
rs2090816
rs7572229
rs8039785
rs7311004
rs7303158
rs2387977
rs35055552
All
−0.1
0.0
0.1
MR effect size for
'Gut microbiota abundance (genus Parasutterella id.2892)' on 'Advanced glycosylation end product−specific receptor, soluble'

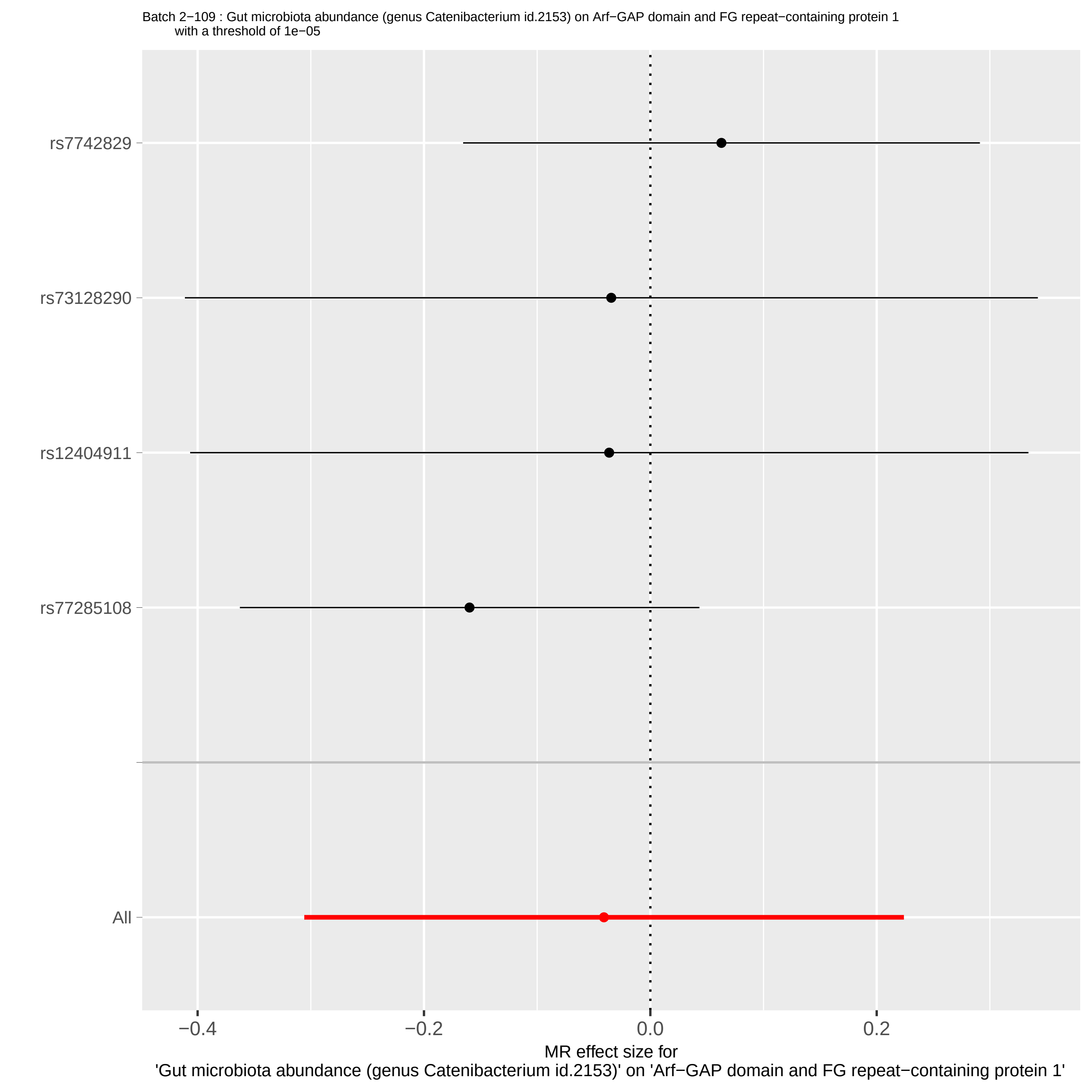
Batch 2−109 : Gut microbiota abundance (genus Catenibacterium id.2153) on Arf−GAP domain and FG repeat−containing protein 1
with a threshold of 1e−05
rs7742829
rs73128290
rs12404911
rs77285108
All
−0.4
−0.2
0.0
0.2
MR effect size for
'Gut microbiota abundance (genus Catenibacterium id.2153)' on 'Arf−GAP domain and FG repeat−containing protein 1'

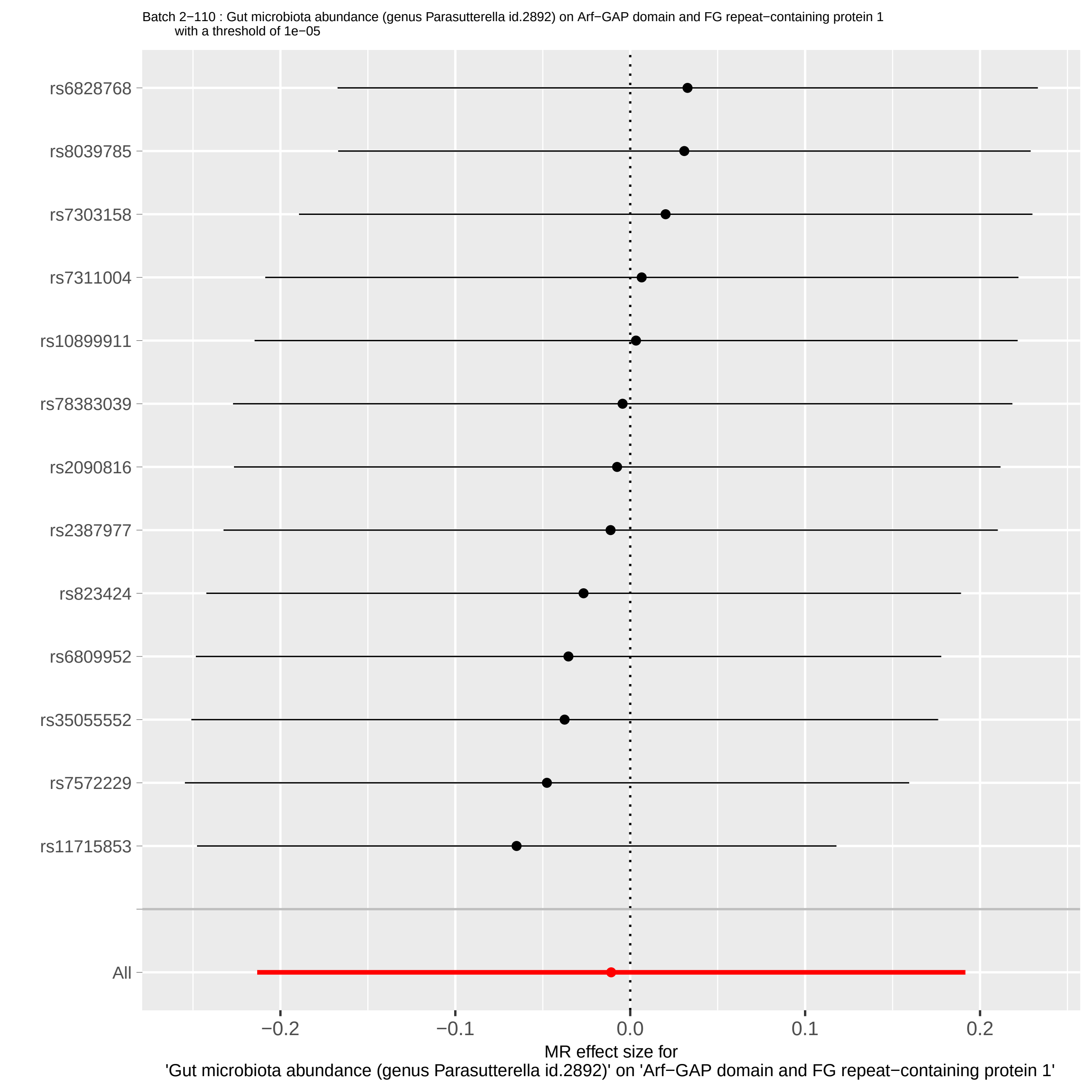

Batch 2−110 : Gut microbiota abundance (genus Parasutterella id.2892) on Arf−GAP domain and FG repeat−containing protein 1
with a threshold of 1e−05
rs6828768
rs8039785
rs7303158
rs7311004
rs10899911
rs78383039
rs2090816
rs2387977
rs823424
rs6809952
rs35055552
rs7572229
rs11715853
All
−0.2
−0.1
0.0
0.1
0.2
MR effect size for
'Gut microbiota abundance (genus Parasutterella id.2892)' on 'Arf−GAP domain and FG repeat−containing protein 1'

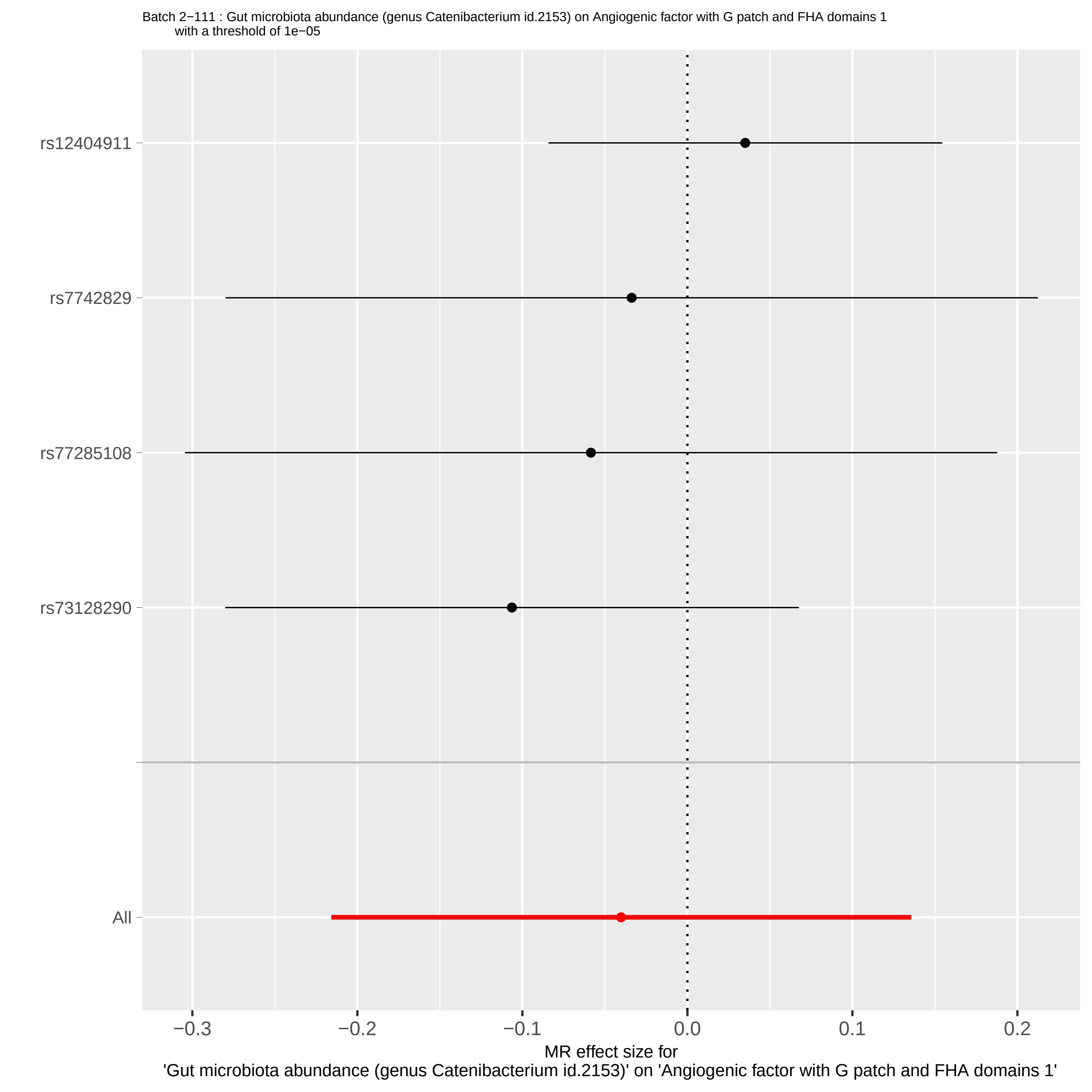

Batch 2−111 : Gut microbiota abundance (genus Catenibacterium id.2153) on Angiogenic factor with G patch and FHA domains 1
with a threshold of 1e−05
rs12404911
rs7742829
rs77285108
rs73128290
All
−0.3
−0.2
−0.1
0.0
0.1
0.2
MR effect size for
'Gut microbiota abundance (genus Catenibacterium id.2153)' on 'Angiogenic factor with G patch and FHA domains 1'

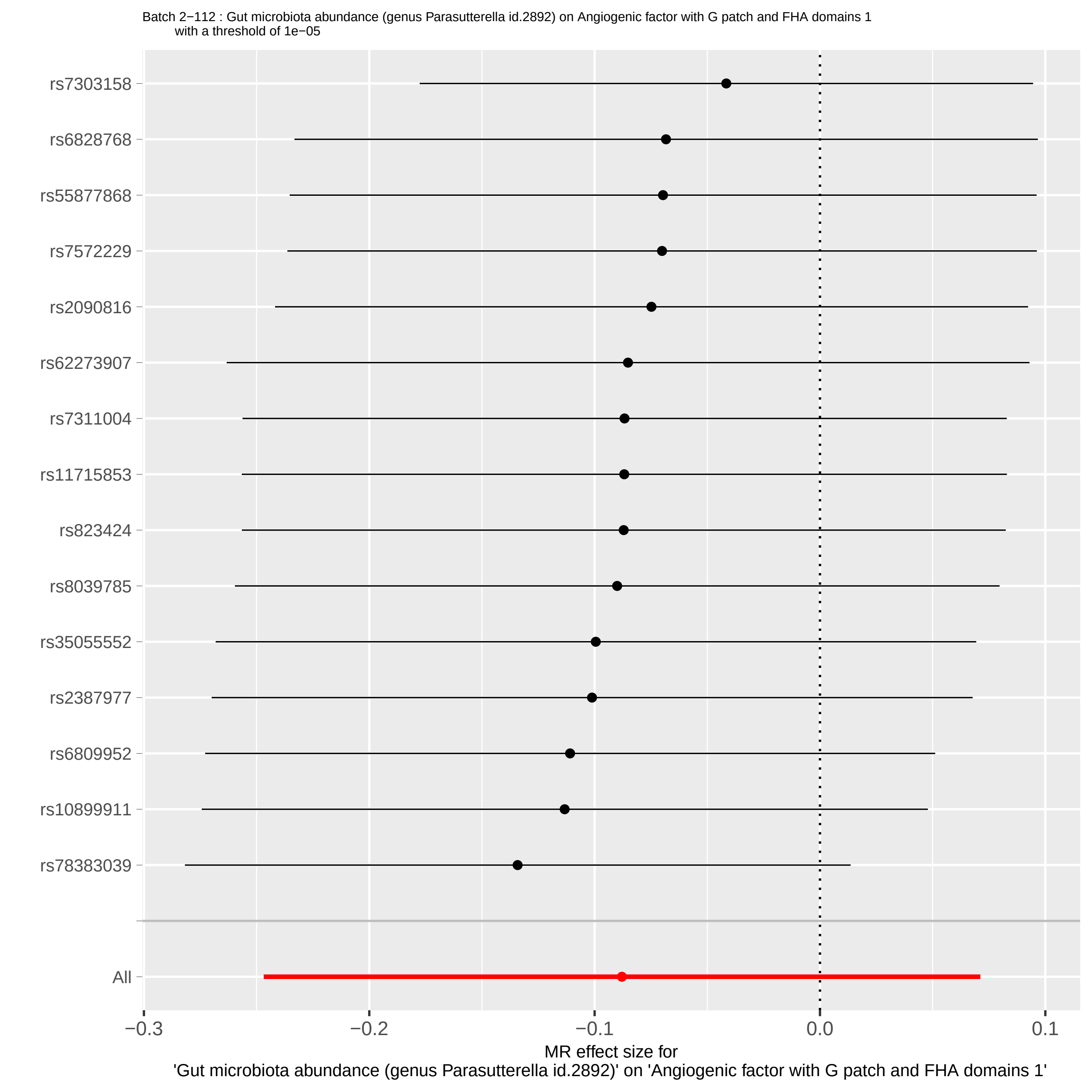

Batch 2−112 : Gut microbiota abundance (genus Parasutterella id.2892) on Angiogenic factor with G patch and FHA domains 1
with a threshold of 1e−05
rs7303158
rs6828768
rs55877868
rs7572229
rs2090816
rs62273907
rs7311004
rs11715853
rs823424
rs8039785
rs35055552
rs2387977
rs6809952
rs10899911
rs78383039
All
−0.3
−0.2
−0.1
0.0
0.1
MR effect size for
'Gut microbiota abundance (genus Parasutterella id.2892)' on 'Angiogenic factor with G patch and FHA domains 1'

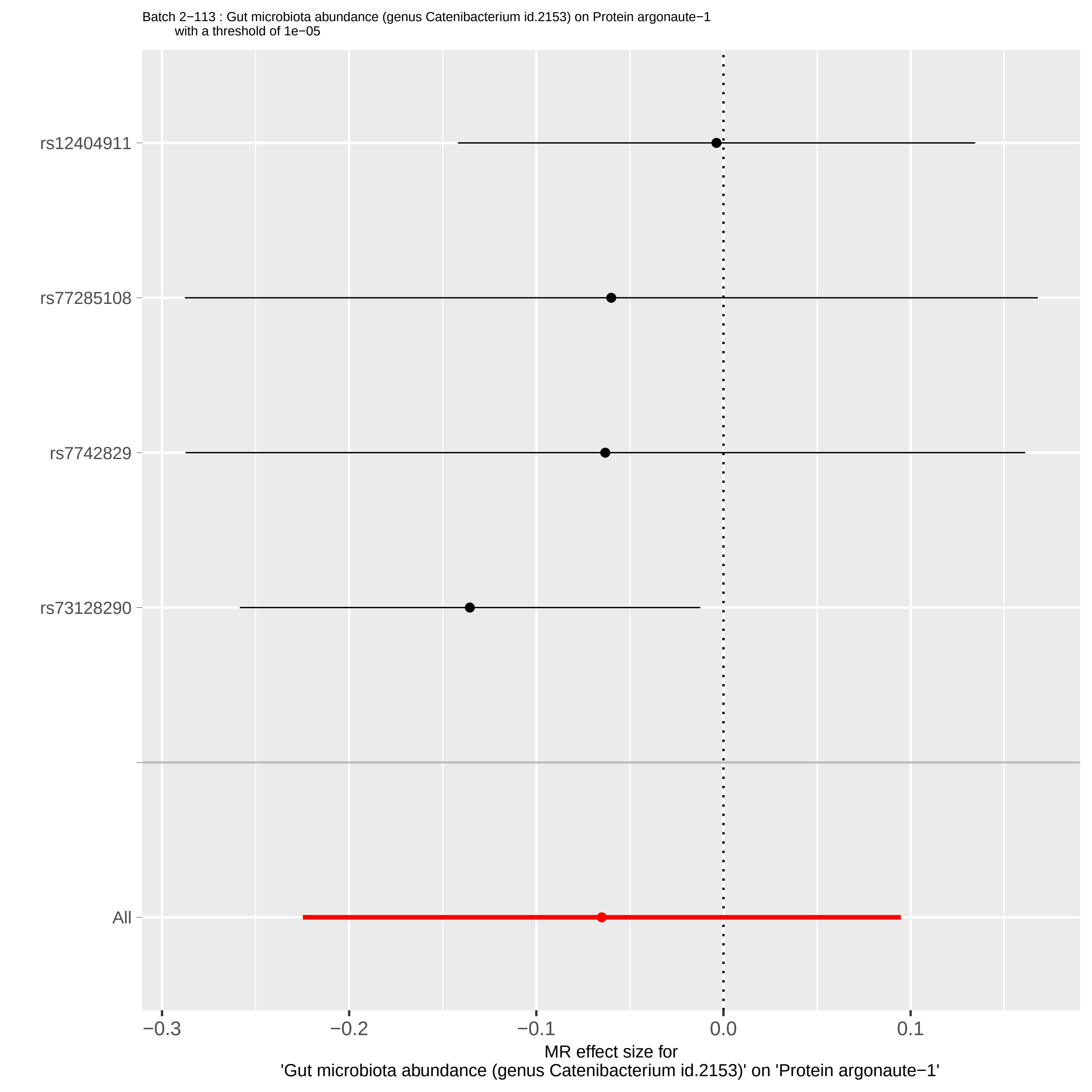
Batch 2−113 : Gut microbiota abundance (genus Catenibacterium id.2153) on Protein argonaute−1
with a threshold of 1e−05
rs12404911
rs77285108
rs7742829
rs73128290
All
−0.3
−0.2
−0.1
0.0
0.1
MR effect size for
'Gut microbiota abundance (genus Catenibacterium id.2153)' on 'Protein argonaute−1'

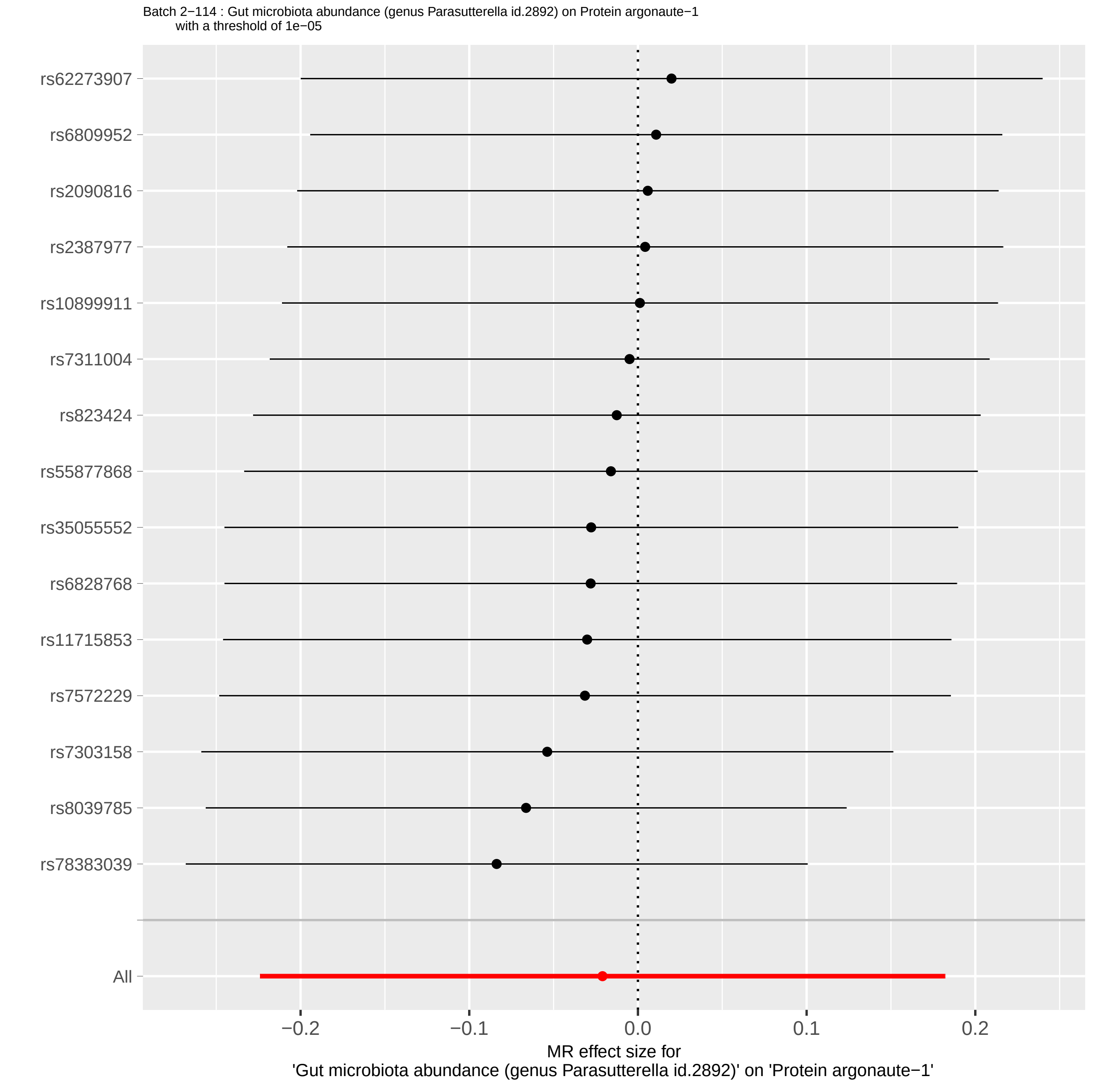
Batch 2−114 : Gut microbiota abundance (genus Parasutterella id.2892) on Protein argonaute−1
with a threshold of 1e−05
rs62273907
rs6809952
rs2090816
rs2387977
rs10899911
rs7311004
rs823424
rs55877868
rs35055552
rs6828768
rs11715853
rs7572229
rs7303158
rs8039785
rs78383039
All
−0.2
−0.1
0.0
0.1
0.2
MR effect size for
'Gut microbiota abundance (genus Parasutterella id.2892)' on 'Protein argonaute−1'

Batch 2−115 : Gut microbiota abundance (genus Catenibacterium id.2153) on Anterior gradient protein 2 homolog
with a threshold of 1e−05

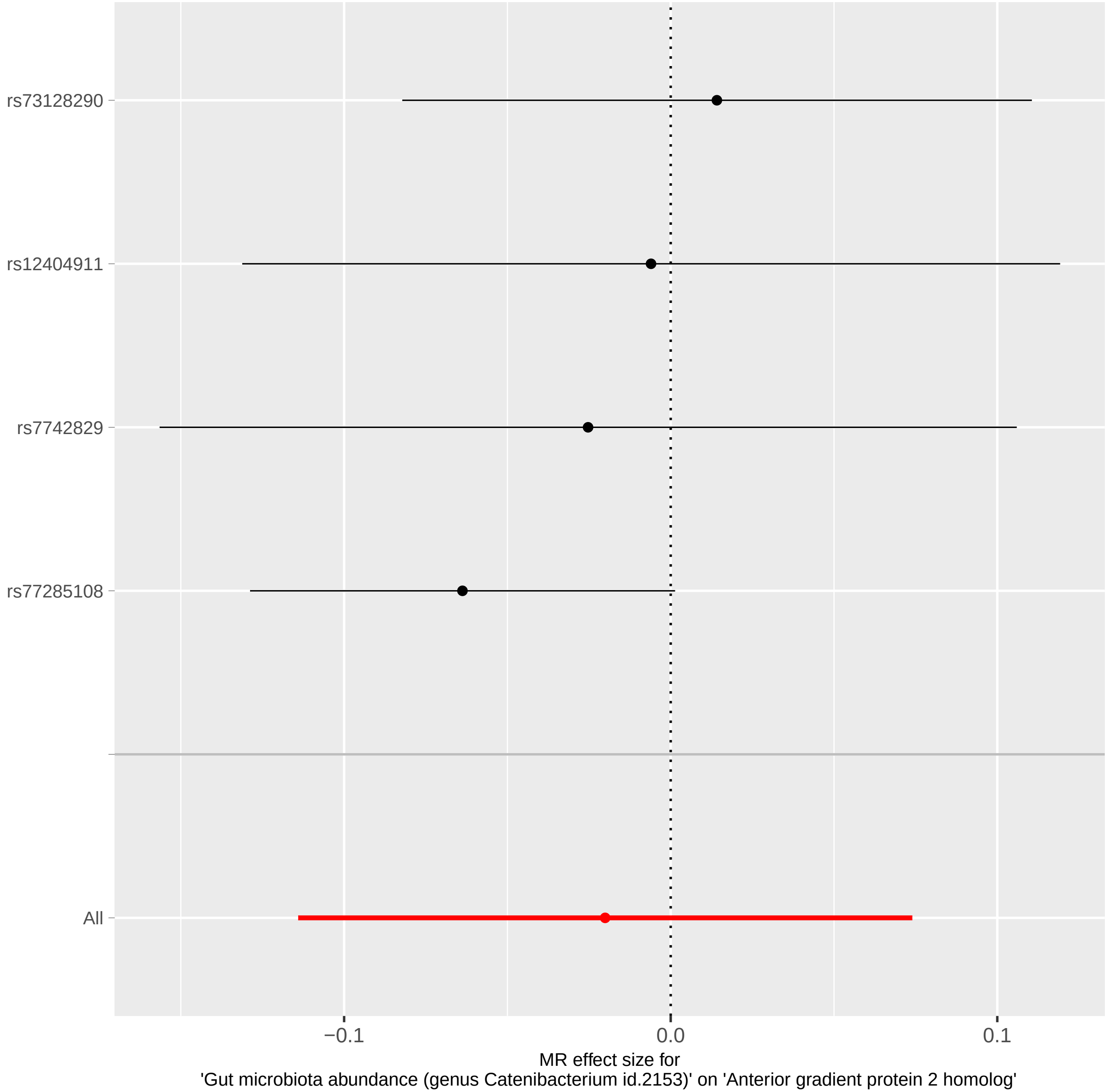

Batch 2−116 : Gut microbiota abundance (genus Parasutterella id.2892) on Anterior gradient protein 2 homolog
with a threshold of 1e−05

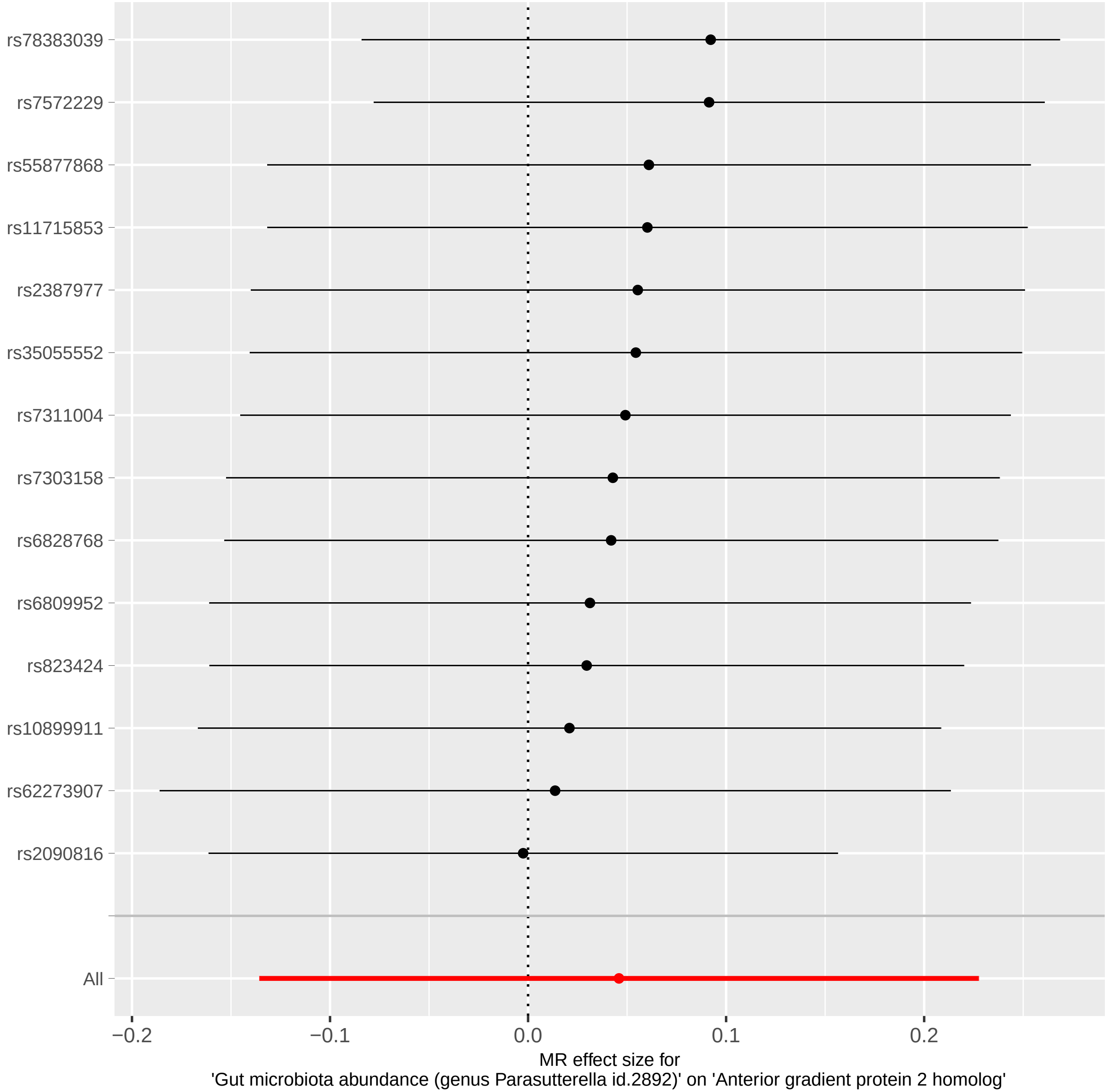

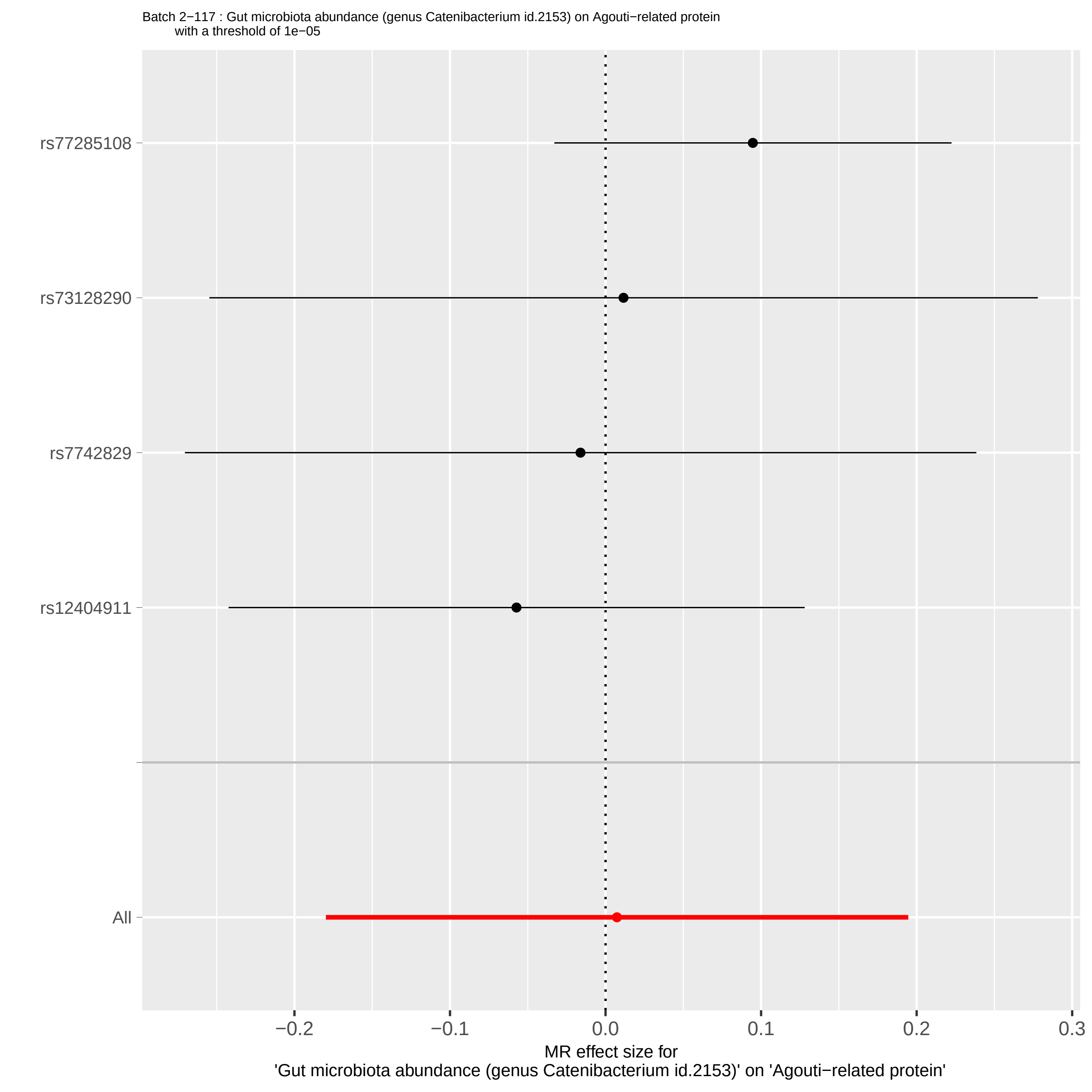

Batch 2−117 : Gut microbiota abundance (genus Catenibacterium id.2153) on Agouti−related protein
with a threshold of 1e−05
rs77285108
rs73128290
rs7742829
rs12404911
All
−0.2
−0.1
0.0
0.1
0.2
0.3
MR effect size for
'Gut microbiota abundance (genus Catenibacterium id.2153)' on 'Agouti−related protein'

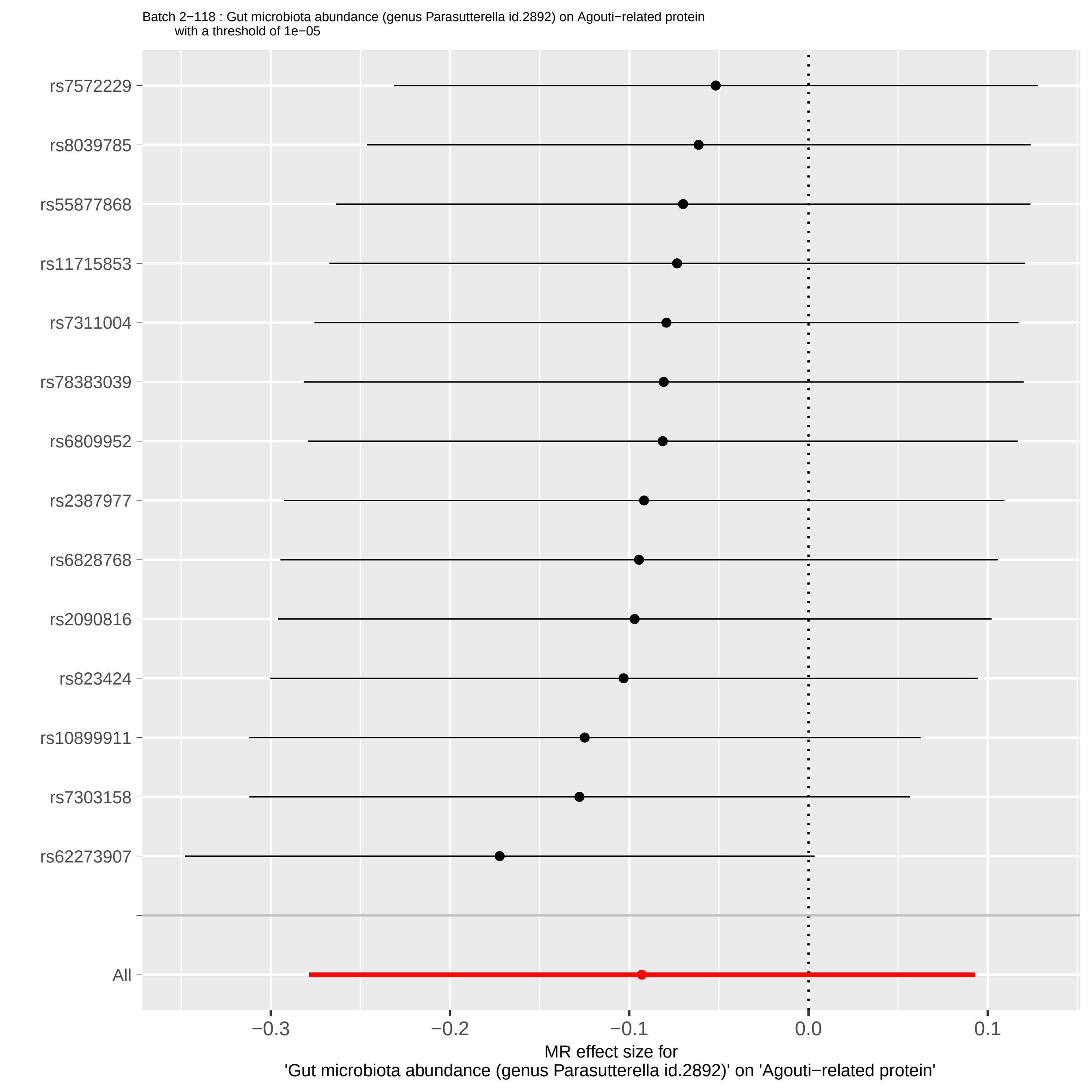

Batch 2−118 : Gut microbiota abundance (genus Parasutterella id.2892) on Agouti−related protein
with a threshold of 1e−05
rs7572229
rs8039785
rs55877868
rs11715853
rs7311004
rs78383039
rs6809952
rs2387977
rs6828768
rs2090816
rs823424
rs10899911
rs7303158
rs62273907
All
−0.3
−0.2
−0.1
0.0
0.1
MR effect size for
'Gut microbiota abundance (genus Parasutterella id.2892)' on 'Agouti−related protein'

Batch 2−119 : Gut microbiota abundance (genus Catenibacterium id.2153) on Activator of 90 kDa heat shock protein ATPase homolog 1 with a threshold of 1e−05

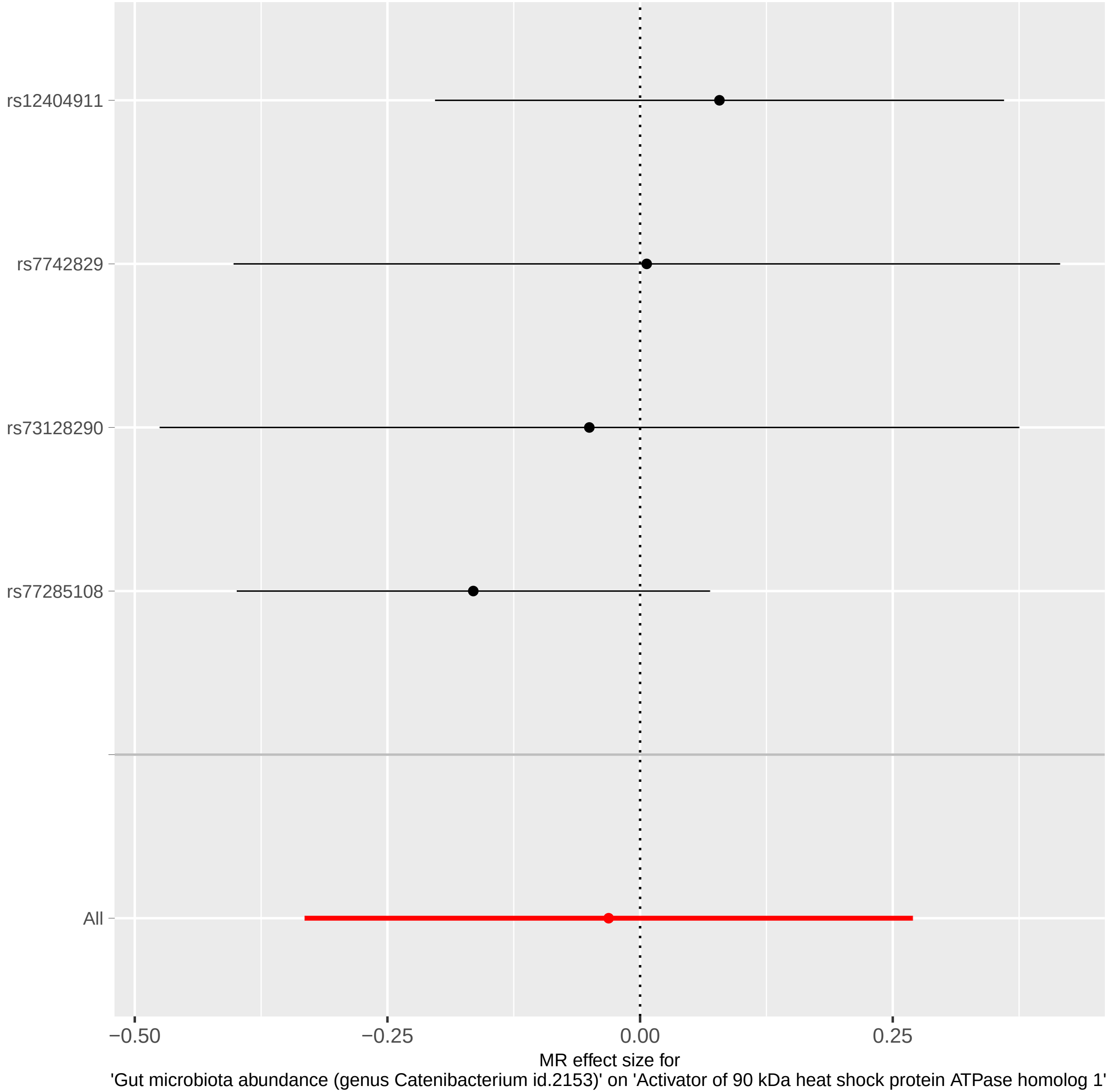

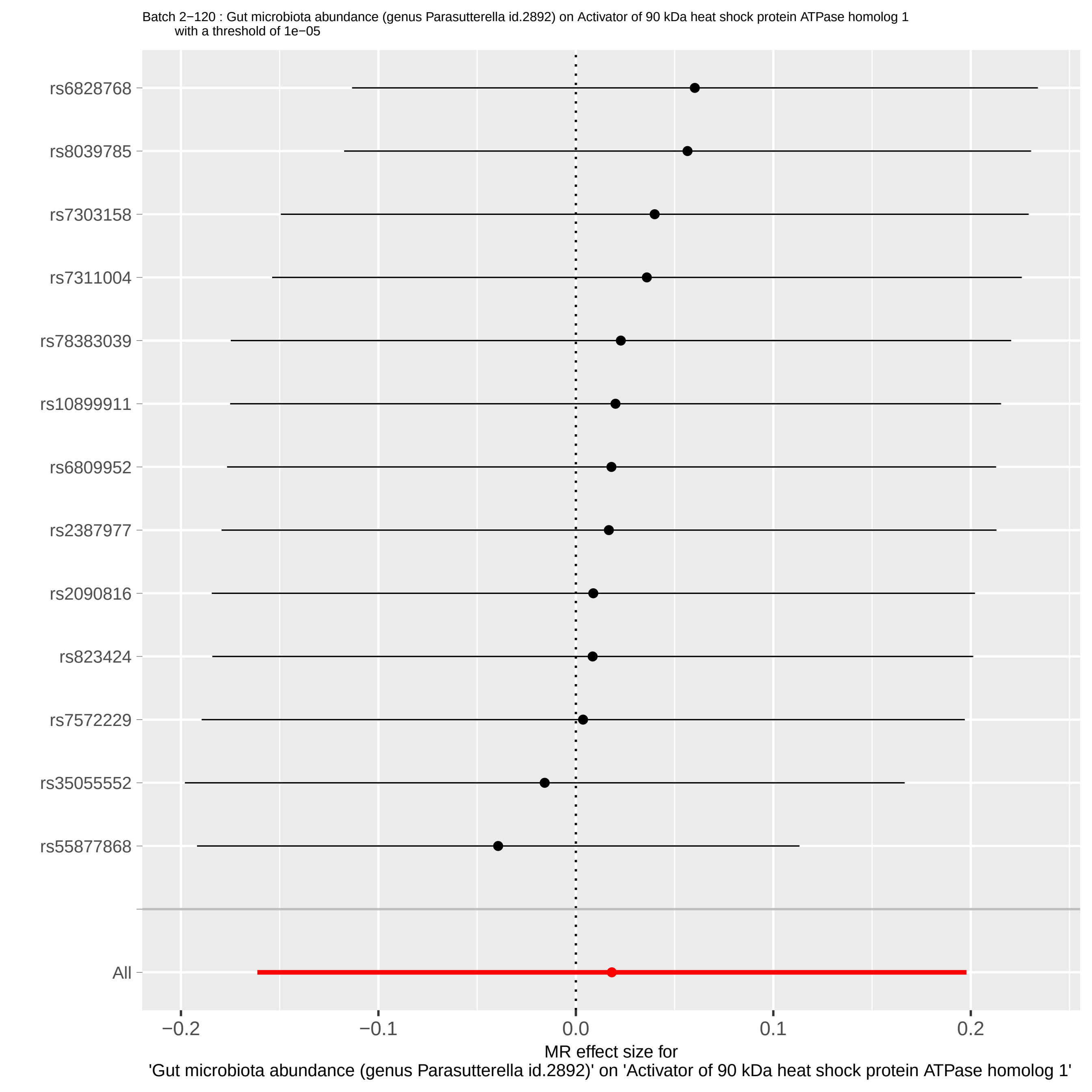

Batch 2−120 : Gut microbiota abundance (genus Parasutterella id.2892) on Activator of 90 kDa heat shock protein ATPase homolog 1
with a threshold of 1e−05
rs6828768
rs8039785
rs7303158
rs7311004
rs78383039
rs10899911
rs6809952
rs2387977
rs2090816
rs823424
rs7572229
rs35055552
rs55877868
All
−0.2
−0.1
0.0
0.1
0.2
MR effect size for
'Gut microbiota abundance (genus Parasutterella id.2892)' on 'Activator of 90 kDa heat shock protein ATPase homolog 1'

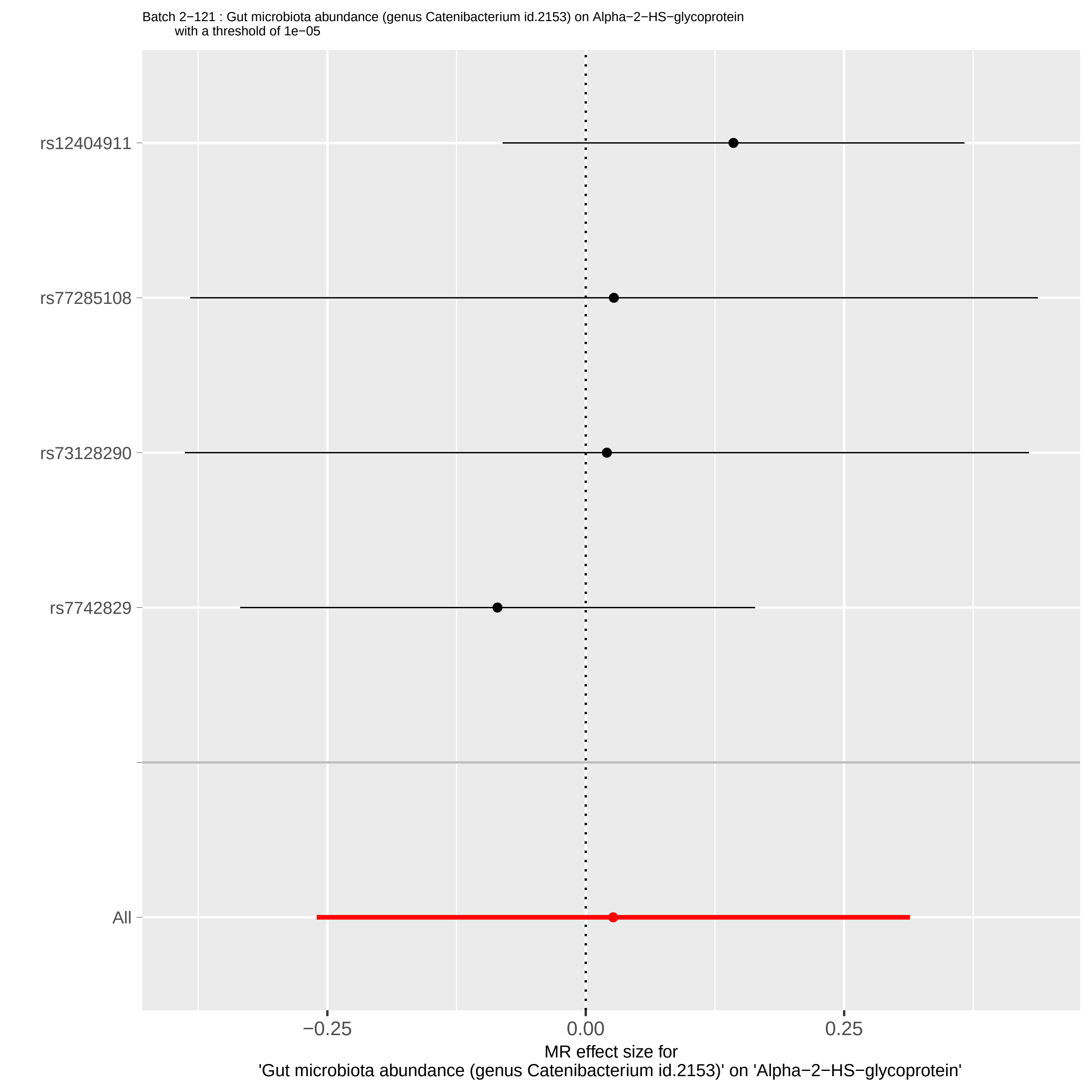
Batch 2−121 : Gut microbiota abundance (genus Catenibacterium id.2153) on Alpha−2−HS−glycoprotein
with a threshold of 1e−05
rs12404911
rs77285108
rs73128290
rs7742829
All
−0.25
0.00
0.25
MR effect size for
'Gut microbiota abundance (genus Catenibacterium id.2153)' on 'Alpha−2−HS−glycoprotein'

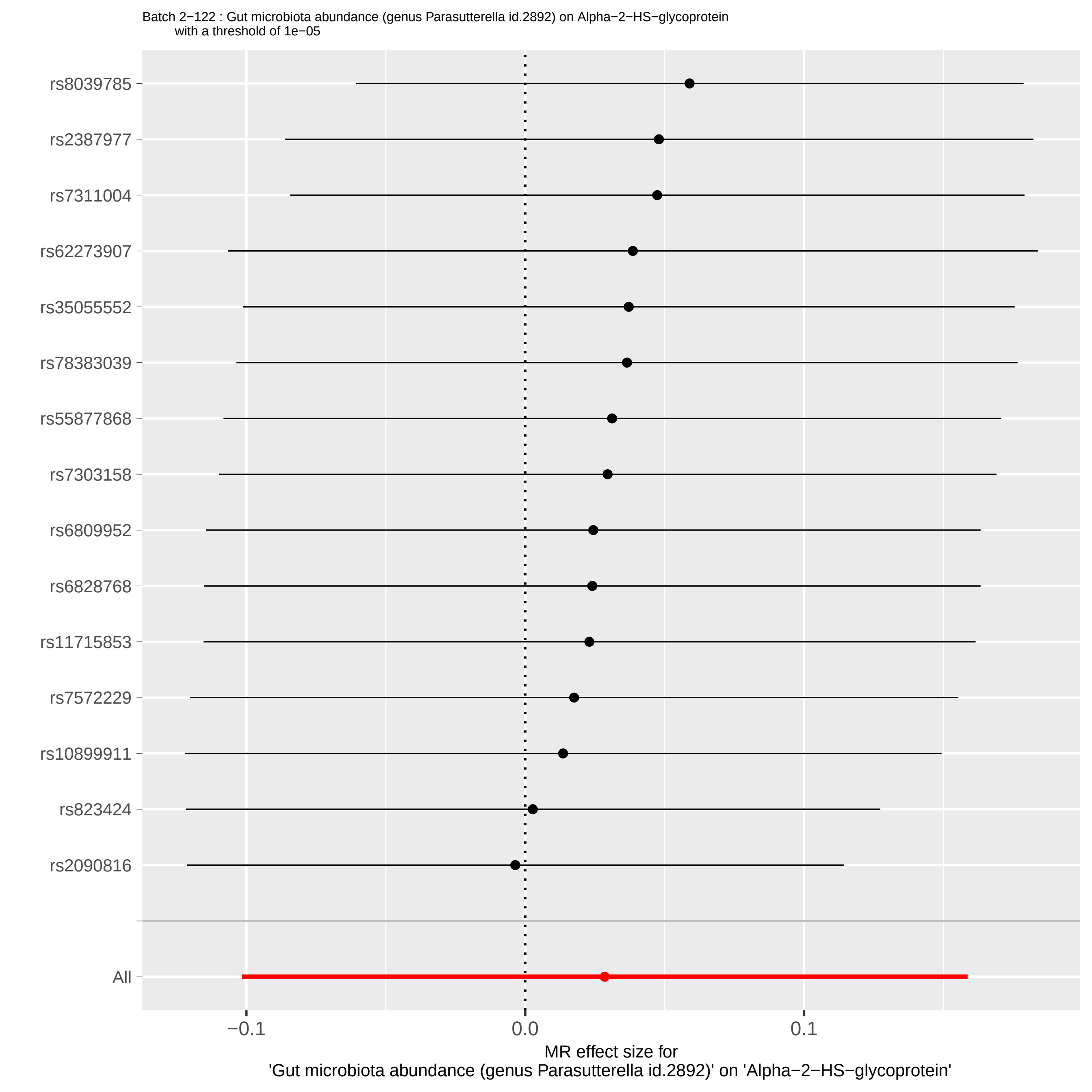

Batch 2−122 : Gut microbiota abundance (genus Parasutterella id.2892) on Alpha−2−HS−glycoprotein
with a threshold of 1e−05
rs8039785
rs2387977
rs7311004
rs62273907
rs35055552
rs78383039
rs55877868
rs7303158
rs6809952
rs6828768
rs11715853
rs7572229
rs10899911
rs823424
rs2090816
All
−0.1
0.0
0.1
MR effect size for
'Gut microbiota abundance (genus Parasutterella id.2892)' on 'Alpha−2−HS−glycoprotein'

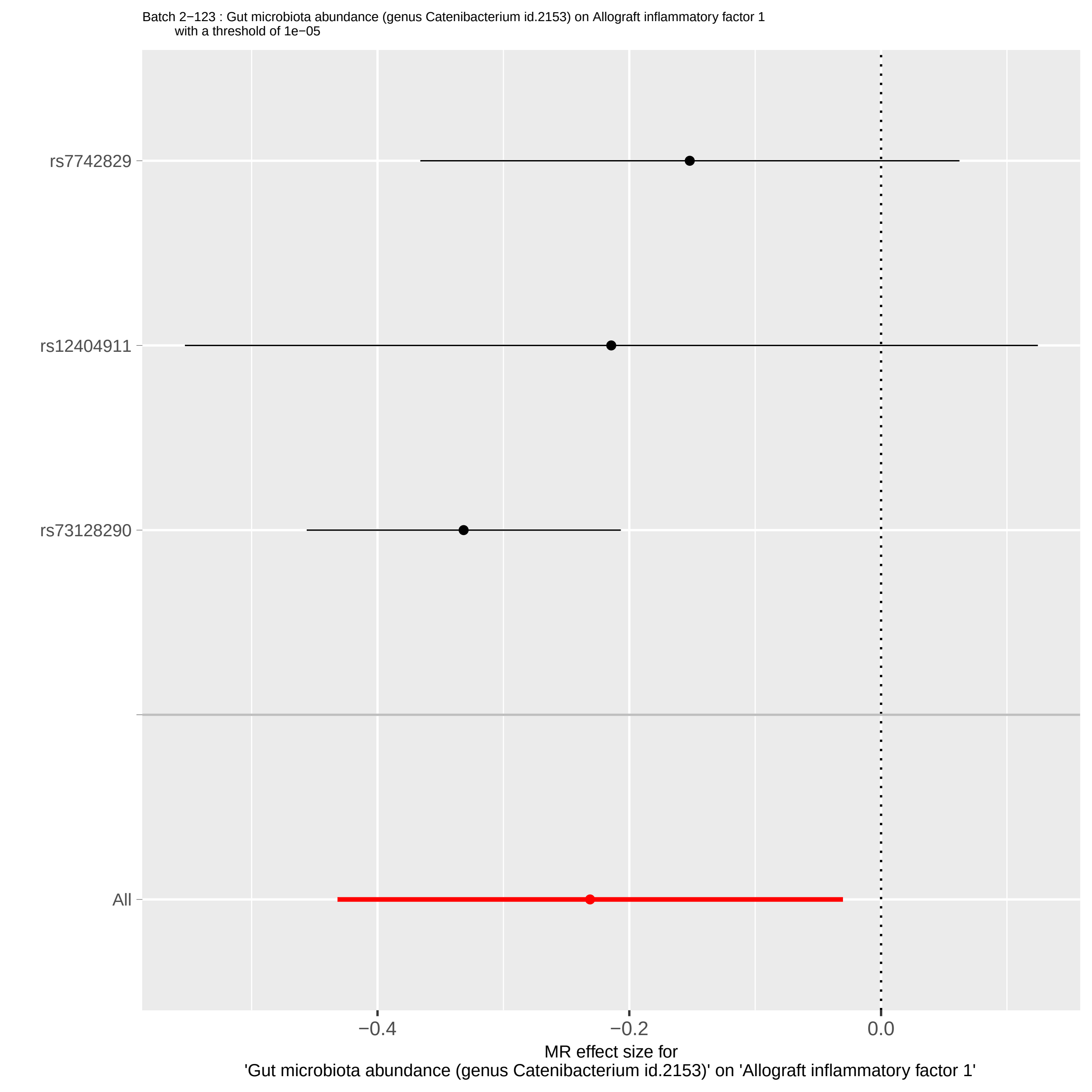

Batch 2−123 : Gut microbiota abundance (genus Catenibacterium id.2153) on Allograft inflammatory factor 1
with a threshold of 1e−05
rs7742829
rs12404911
rs73128290
All
−0.4
−0.2
0.0
MR effect size for
'Gut microbiota abundance (genus Catenibacterium id.2153)' on 'Allograft inflammatory factor 1'

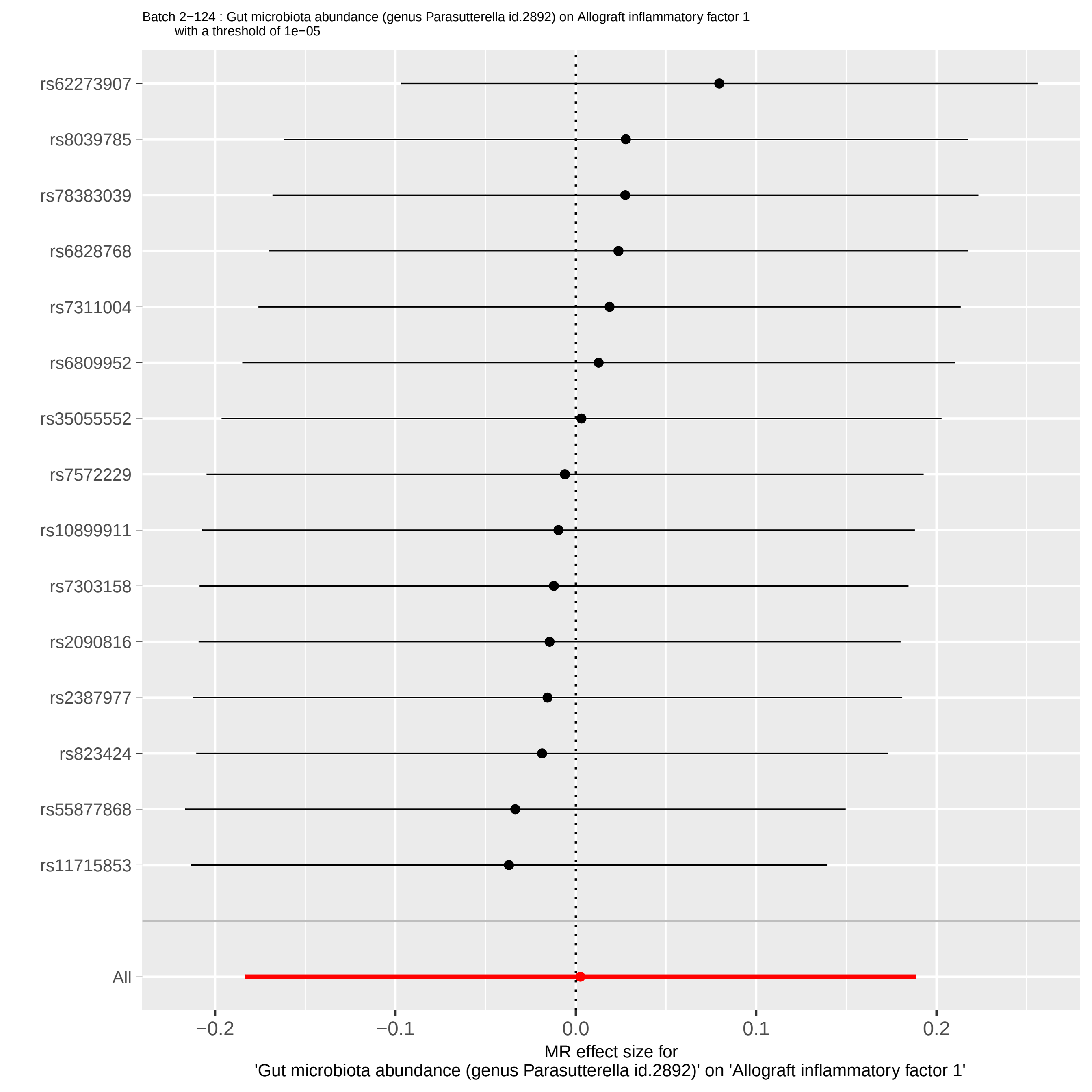

Batch 2−124 : Gut microbiota abundance (genus Parasutterella id.2892) on Allograft inflammatory factor 1
with a threshold of 1e−05
rs62273907
rs8039785
rs78383039
rs6828768
rs7311004
rs6809952
rs35055552
rs7572229
rs10899911
rs7303158
rs2090816
rs2387977
rs823424
rs55877868
rs11715853
All
−0.2
−0.1
0.0
0.1
0.2
MR effect size for
'Gut microbiota abundance (genus Parasutterella id.2892)' on 'Allograft inflammatory factor 1'

Batch 2−125 : Gut microbiota abundance (genus Catenibacterium id.2153) on Apoptosis−inducing factor 1, mitochondrial
with a threshold of 1e−05

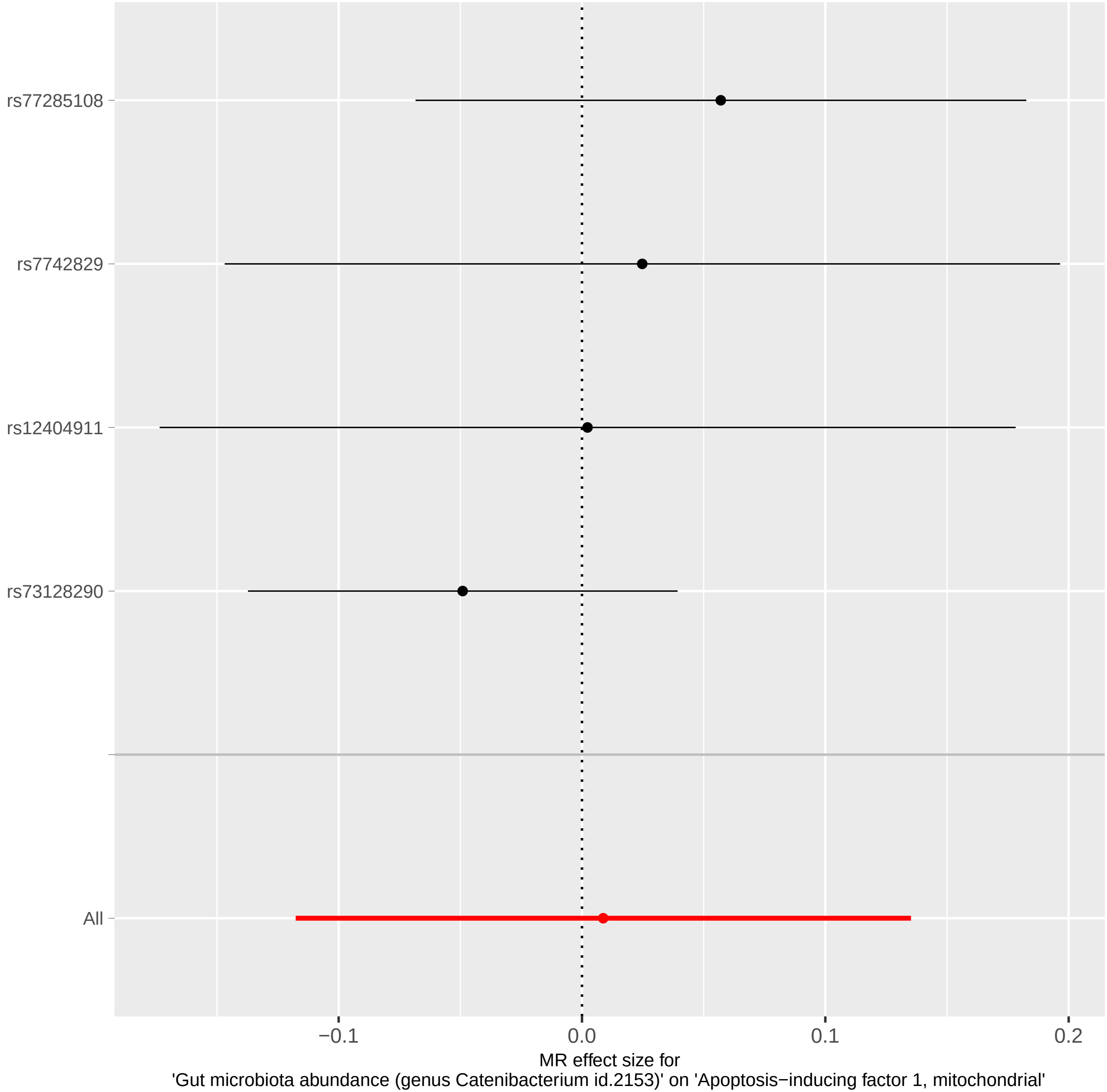

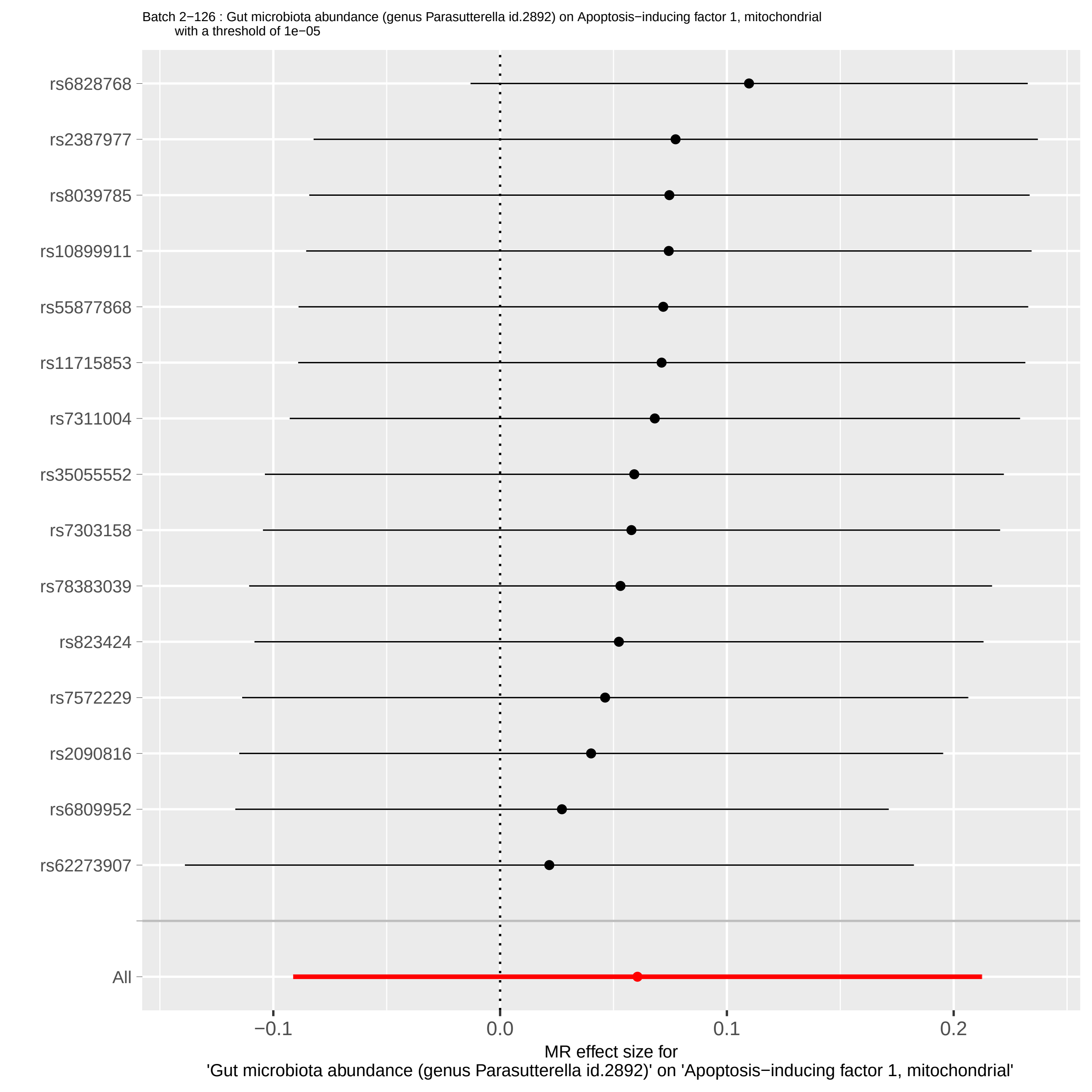
Batch 2−126 : Gut microbiota abundance (genus Parasutterella id.2892) on Apoptosis−inducing factor 1, mitochondrial
with a threshold of 1e−05
rs6828768
rs2387977
rs8039785
rs10899911
rs55877868
rs11715853
rs7311004
rs35055552
rs7303158
rs78383039
rs823424
rs7572229
rs2090816
rs6809952
rs62273907
All
−0.1
0.0
0.1
0.2
MR effect size for
'Gut microbiota abundance (genus Parasutterella id.2892)' on 'Apoptosis−inducing factor 1, mitochondrial'

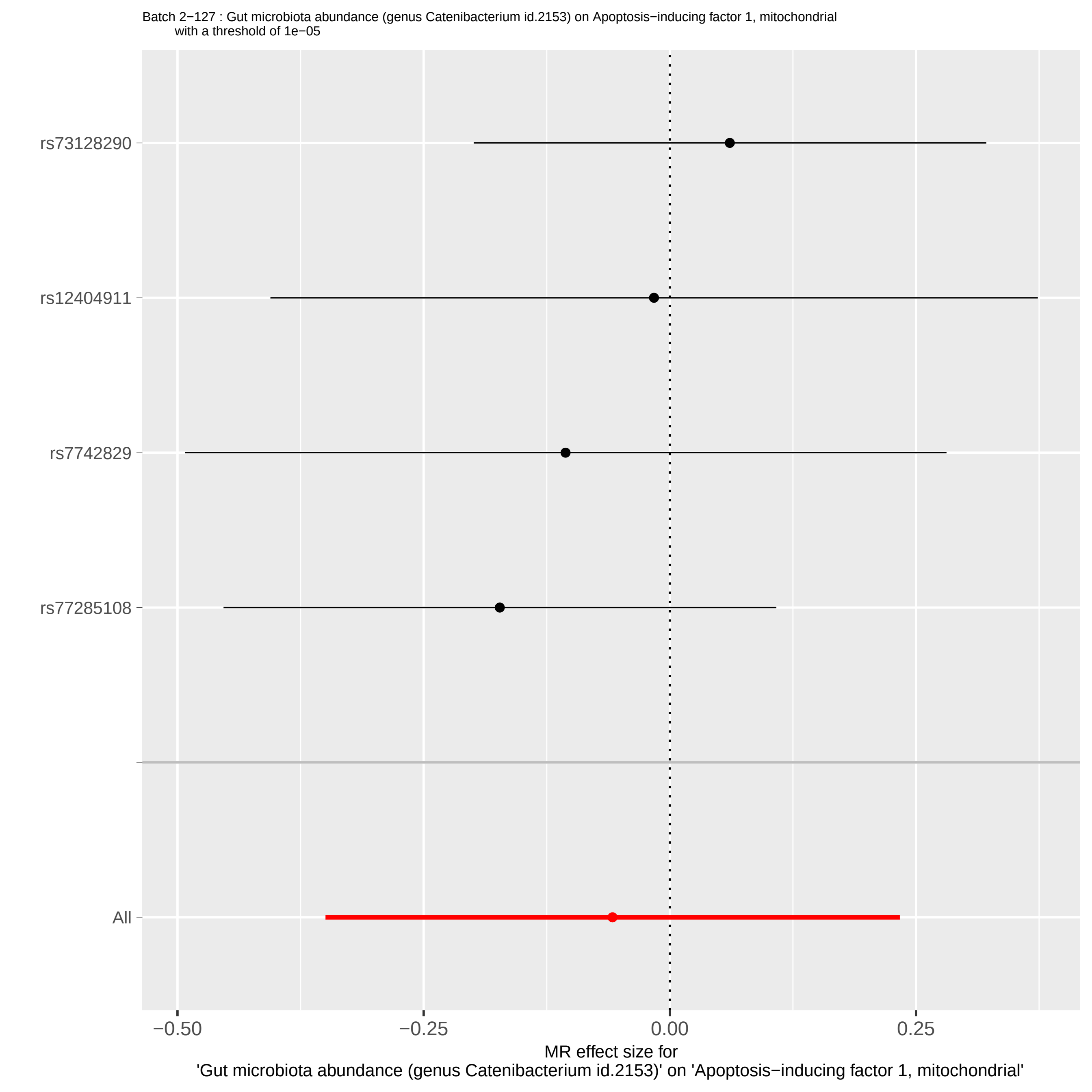

Batch 2−127 : Gut microbiota abundance (genus Catenibacterium id.2153) on Apoptosis−inducing factor 1, mitochondrial
with a threshold of 1e−05
rs73128290
rs12404911
rs7742829
rs77285108
All
−0.50
−0.25
0.00
0.25
MR effect size for
'Gut microbiota abundance (genus Catenibacterium id.2153)' on 'Apoptosis−inducing factor 1, mitochondrial'

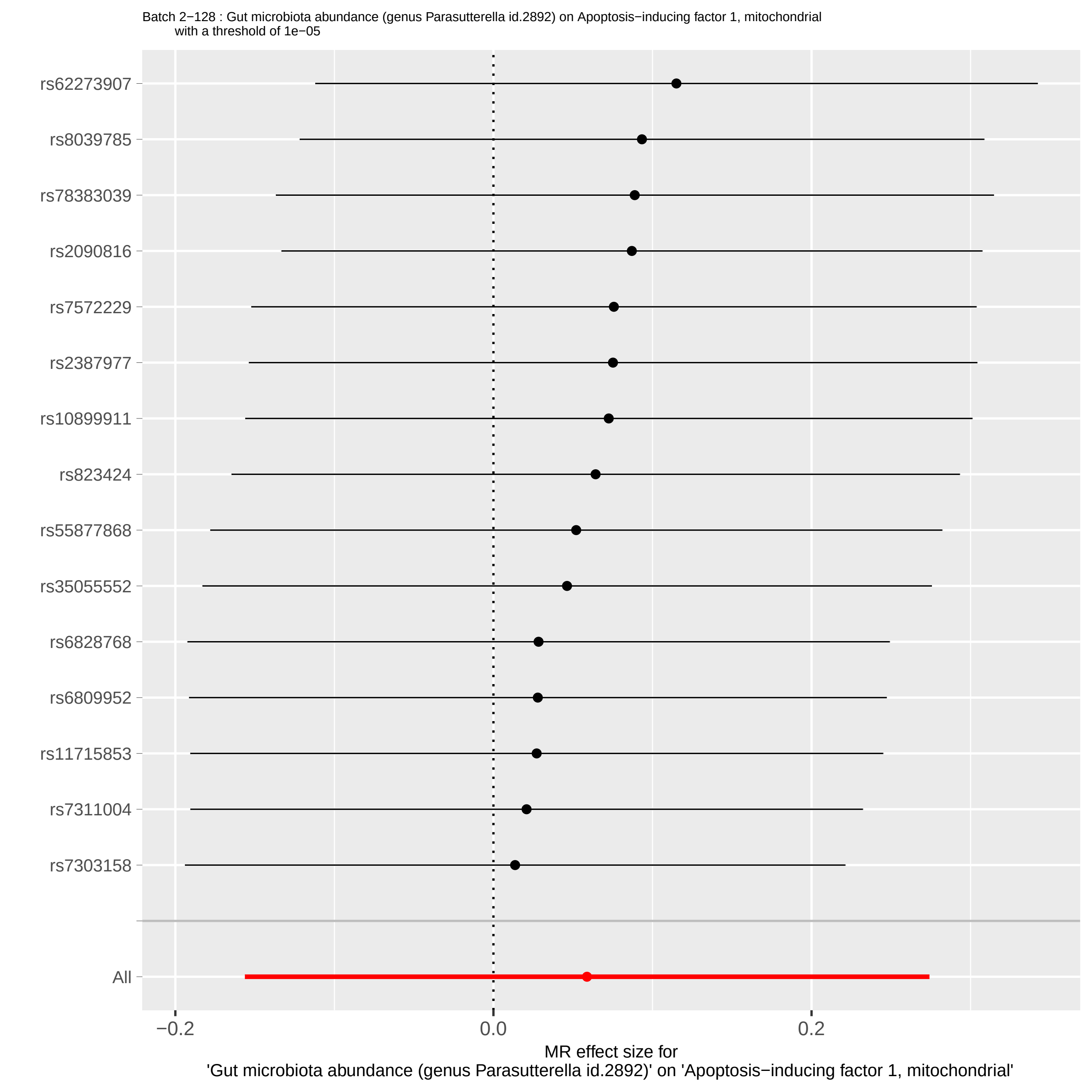

Batch 2−128 : Gut microbiota abundance (genus Parasutterella id.2892) on Apoptosis−inducing factor 1, mitochondrial
with a threshold of 1e−05
rs62273907
rs8039785
rs78383039
rs2090816
rs7572229
rs2387977
rs10899911
rs823424
rs55877868
rs35055552
rs6828768
rs6809952
rs11715853
rs7311004
rs7303158
All
−0.2
0.0
0.2
MR effect size for
'Gut microbiota abundance (genus Parasutterella id.2892)' on 'Apoptosis−inducing factor 1, mitochondrial'

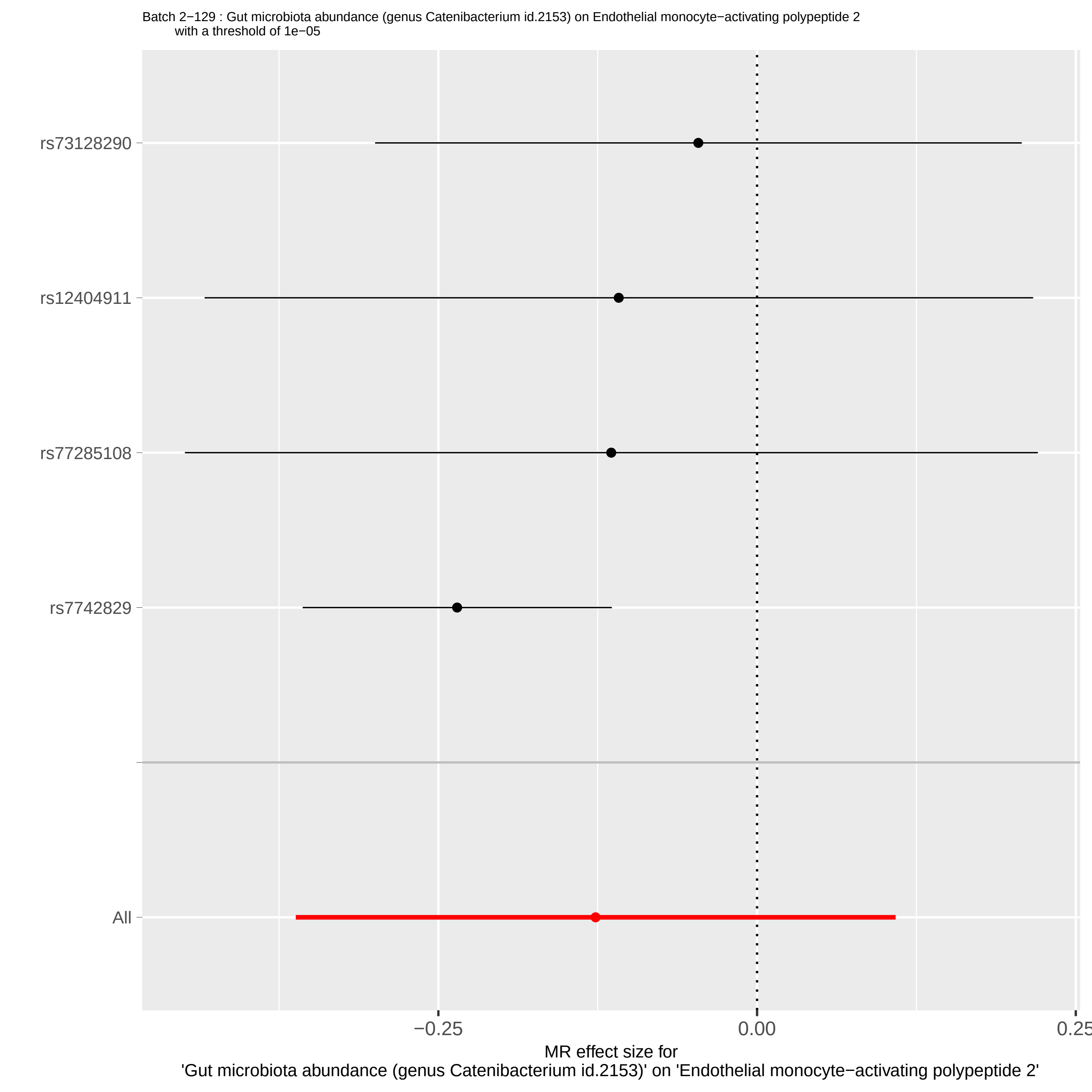

Batch 2−129 : Gut microbiota abundance (genus Catenibacterium id.2153) on Endothelial monocyte−activating polypeptide 2
with a threshold of 1e−05
rs73128290
rs12404911
rs77285108
rs7742829
All
−0.25
0.00
0.25
MR effect size for
'Gut microbiota abundance (genus Catenibacterium id.2153)' on 'Endothelial monocyte−activating polypeptide 2'

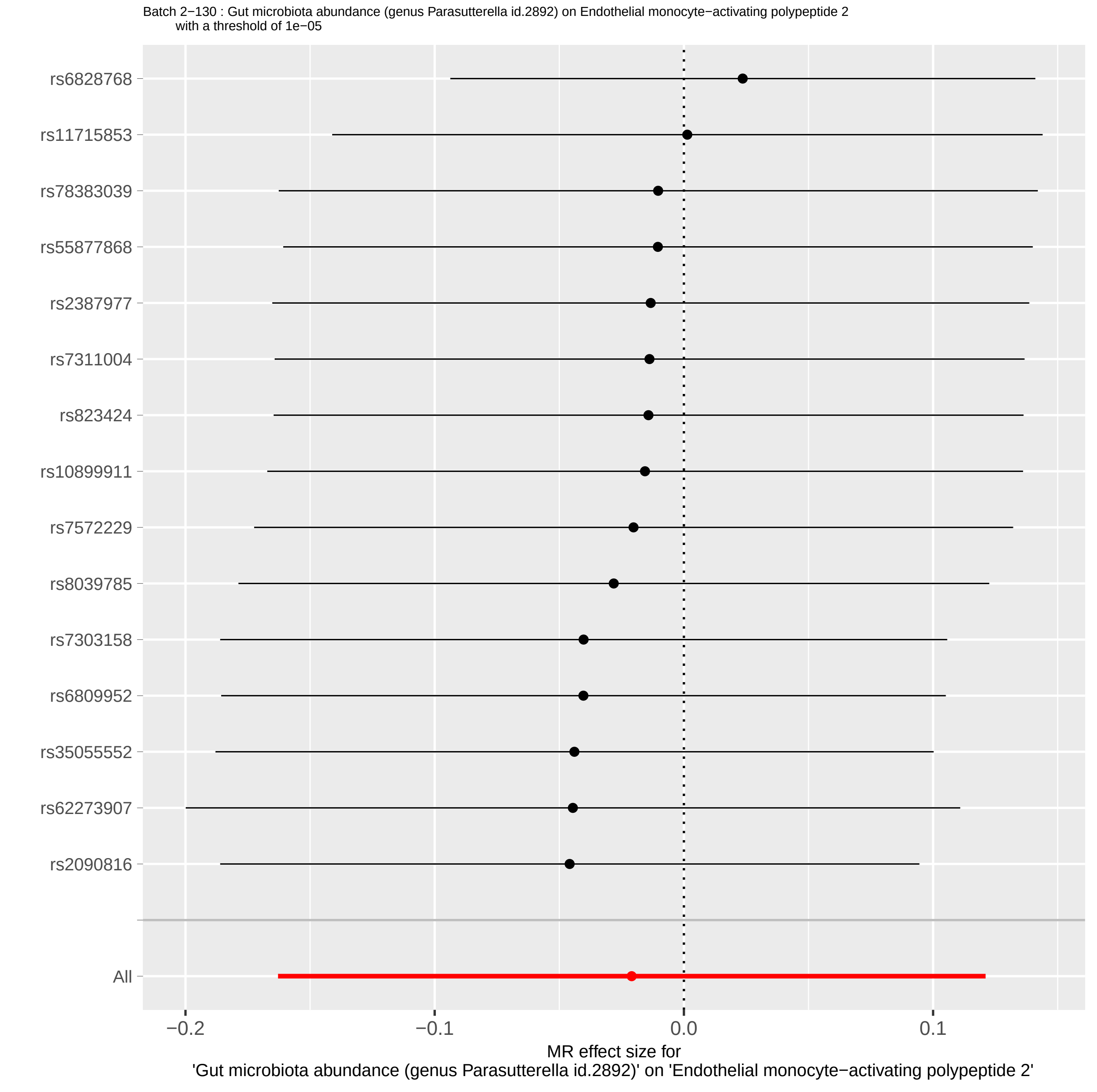

Batch 2−130 : Gut microbiota abundance (genus Parasutterella id.2892) on Endothelial monocyte−activating polypeptide 2
with a threshold of 1e−05
rs6828768
rs11715853
rs78383039
rs55877868
rs2387977
rs7311004
rs823424
rs10899911
rs7572229
rs8039785
rs7303158
rs6809952
rs35055552
rs62273907
rs2090816
All
−0.2
−0.1
0.0
0.1
MR effect size for
'Gut microbiota abundance (genus Parasutterella id.2892)' on 'Endothelial monocyte−activating polypeptide 2'

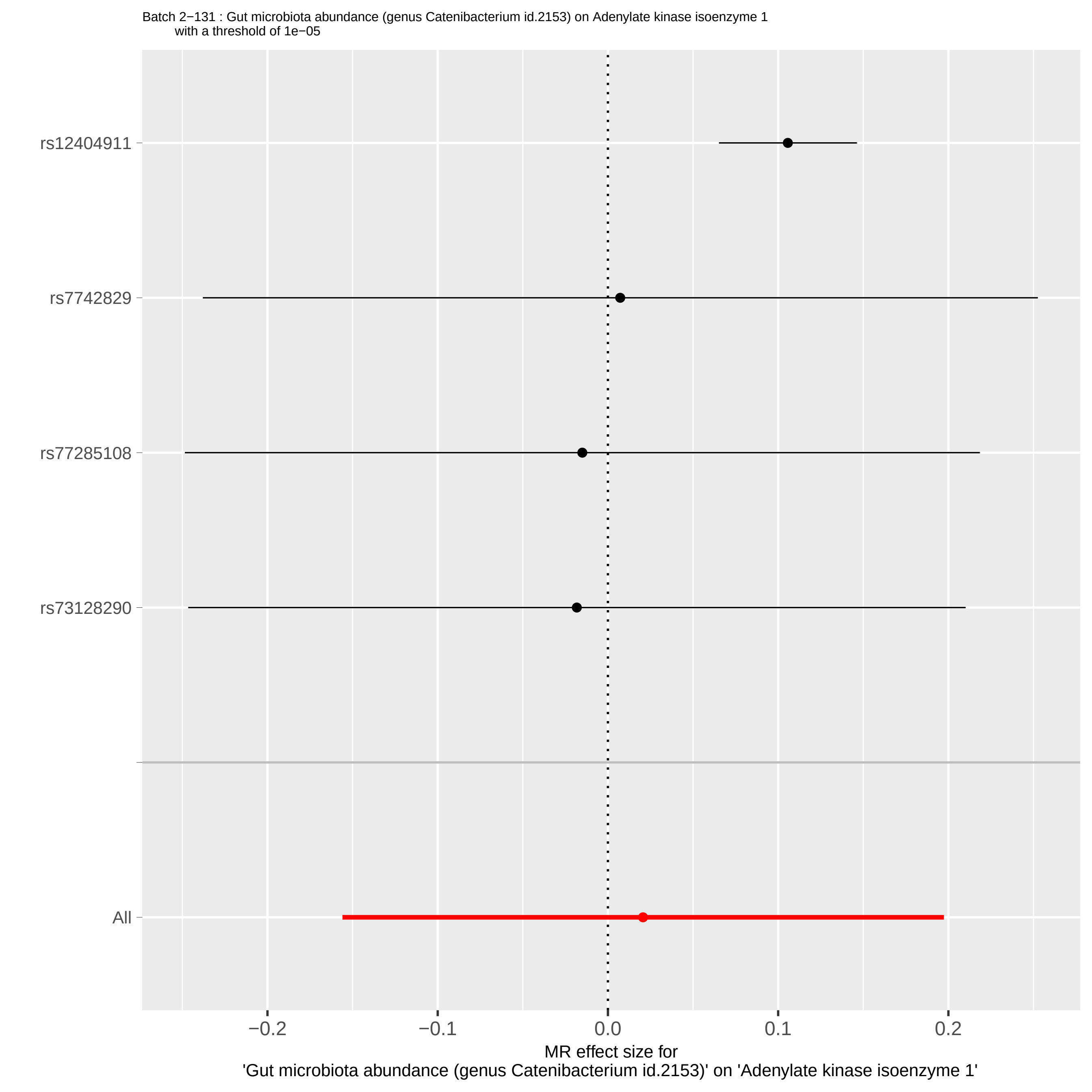

Batch 2−131 : Gut microbiota abundance (genus Catenibacterium id.2153) on Adenylate kinase isoenzyme 1
with a threshold of 1e−05
rs12404911
rs7742829
rs77285108
rs73128290
All
−0.2
−0.1
0.0
0.1
0.2
MR effect size for
'Gut microbiota abundance (genus Catenibacterium id.2153)' on 'Adenylate kinase isoenzyme 1'

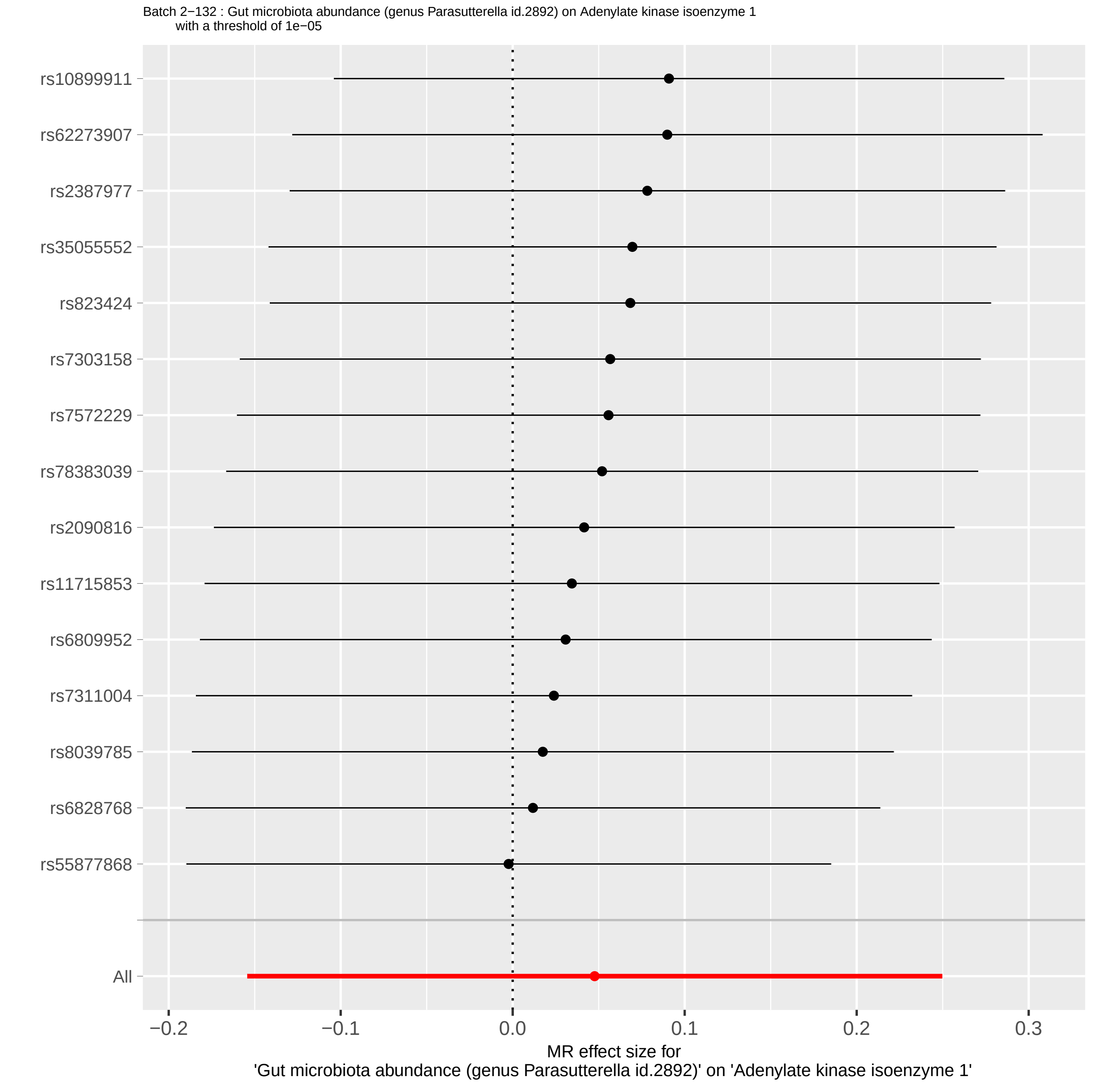

Batch 2−132 : Gut microbiota abundance (genus Parasutterella id.2892) on Adenylate kinase isoenzyme 1
with a threshold of 1e−05
rs10899911
rs62273907
rs2387977
rs35055552
rs823424
rs7303158
rs7572229
rs78383039
rs2090816
rs11715853
rs6809952
rs7311004
rs8039785
rs6828768
rs55877868
All
−0.2
−0.1
0.0
0.1
0.2
0.3
MR effect size for
'Gut microbiota abundance (genus Parasutterella id.2892)' on 'Adenylate kinase isoenzyme 1'

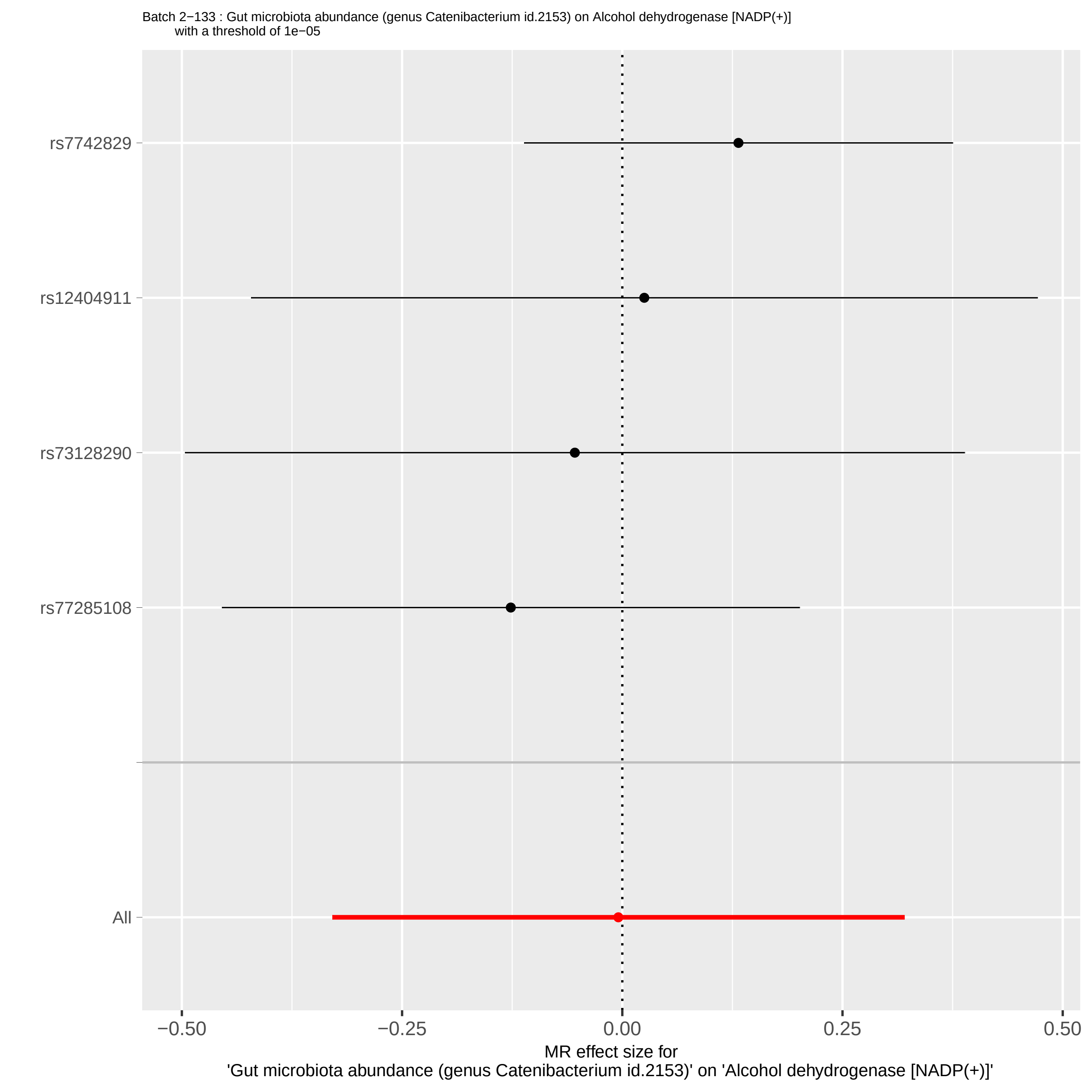
Batch 2−133 : Gut microbiota abundance (genus Catenibacterium id.2153) on Alcohol dehydrogenase [NADP(+)]
with a threshold of 1e−05
rs7742829
rs12404911
rs73128290
rs77285108
All
−0.50
−0.25
0.00
0.25
0.50
MR effect size for
'Gut microbiota abundance (genus Catenibacterium id.2153)' on 'Alcohol dehydrogenase [NADP(+)]'

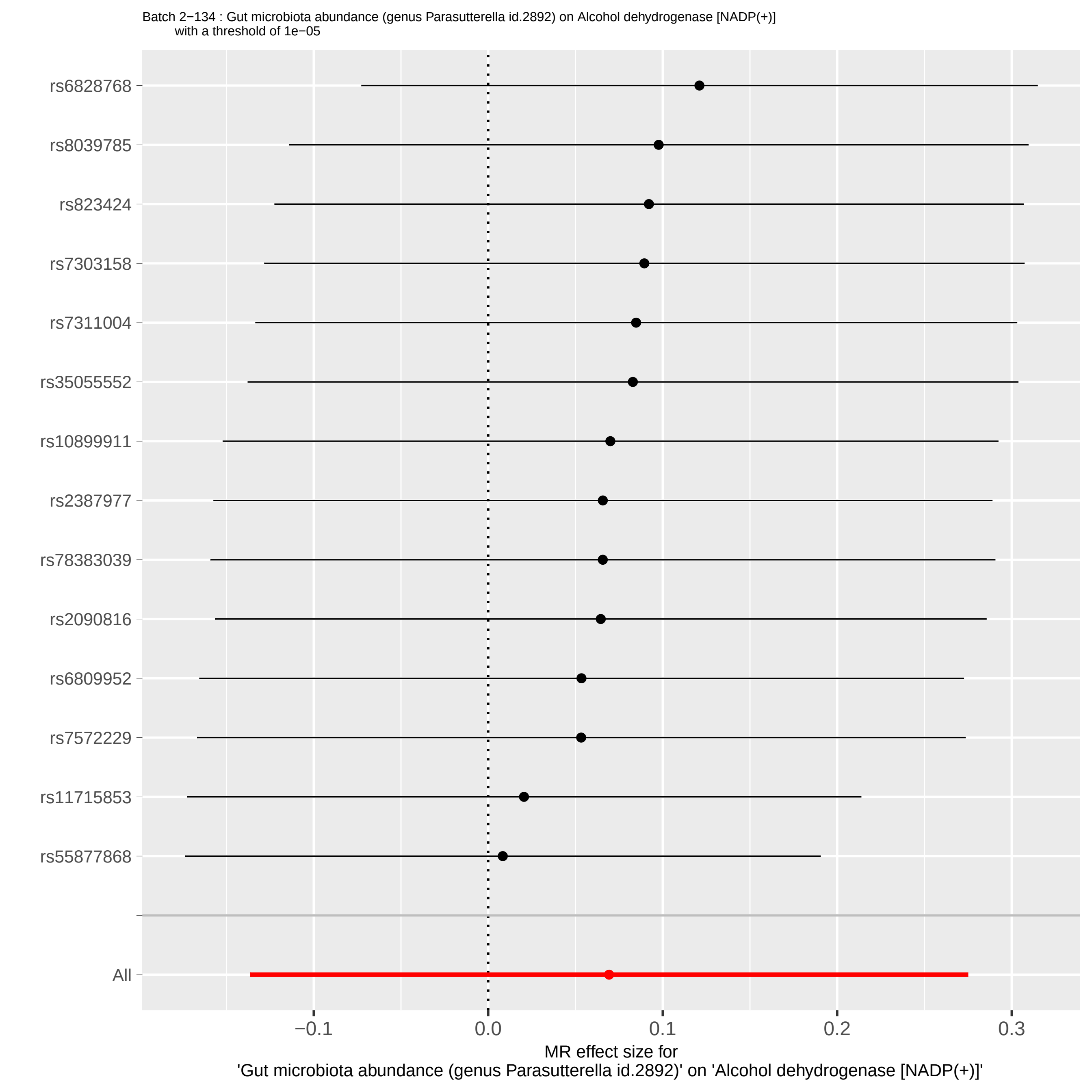

Batch 2−134 : Gut microbiota abundance (genus Parasutterella id.2892) on Alcohol dehydrogenase [NADP(+)]
with a threshold of 1e−05
rs6828768
rs8039785
rs823424
rs7303158
rs7311004
rs35055552
rs10899911
rs2387977
rs78383039
rs2090816
rs6809952
rs7572229
rs11715853
rs55877868
All
−0.1
0.0
0.1
0.2
0.3
MR effect size for
'Gut microbiota abundance (genus Parasutterella id.2892)' on 'Alcohol dehydrogenase [NADP(+)]'

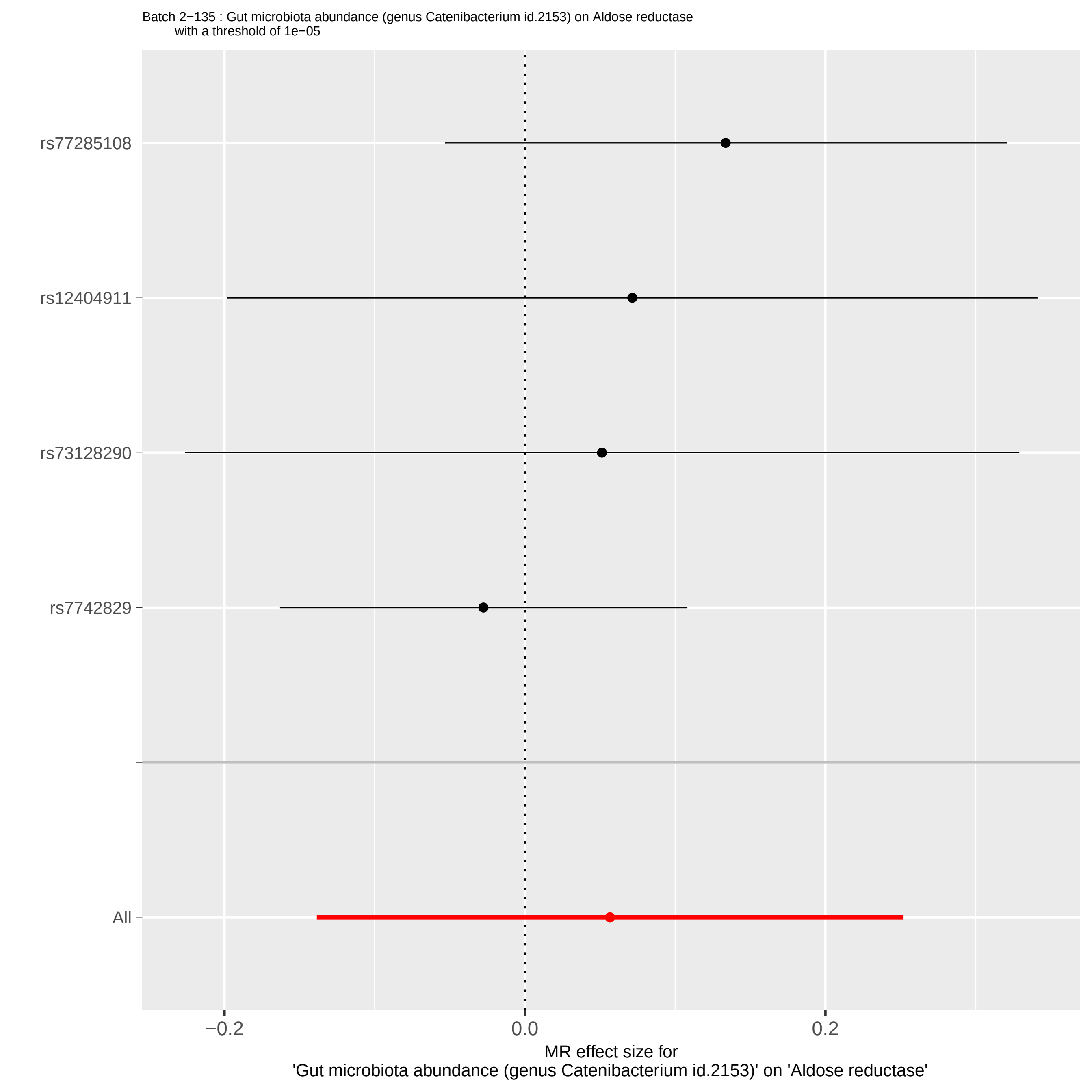

Batch 2−135 : Gut microbiota abundance (genus Catenibacterium id.2153) on Aldose reductase
with a threshold of 1e−05
rs77285108
rs12404911
rs73128290
rs7742829
All
−0.2
0.0
0.2
MR effect size for
'Gut microbiota abundance (genus Catenibacterium id.2153)' on 'Aldose reductase'

Batch 2−136 : Gut microbiota abundance (genus Parasutterella id.2892) on Aldose reductase
with a threshold of 1e−05

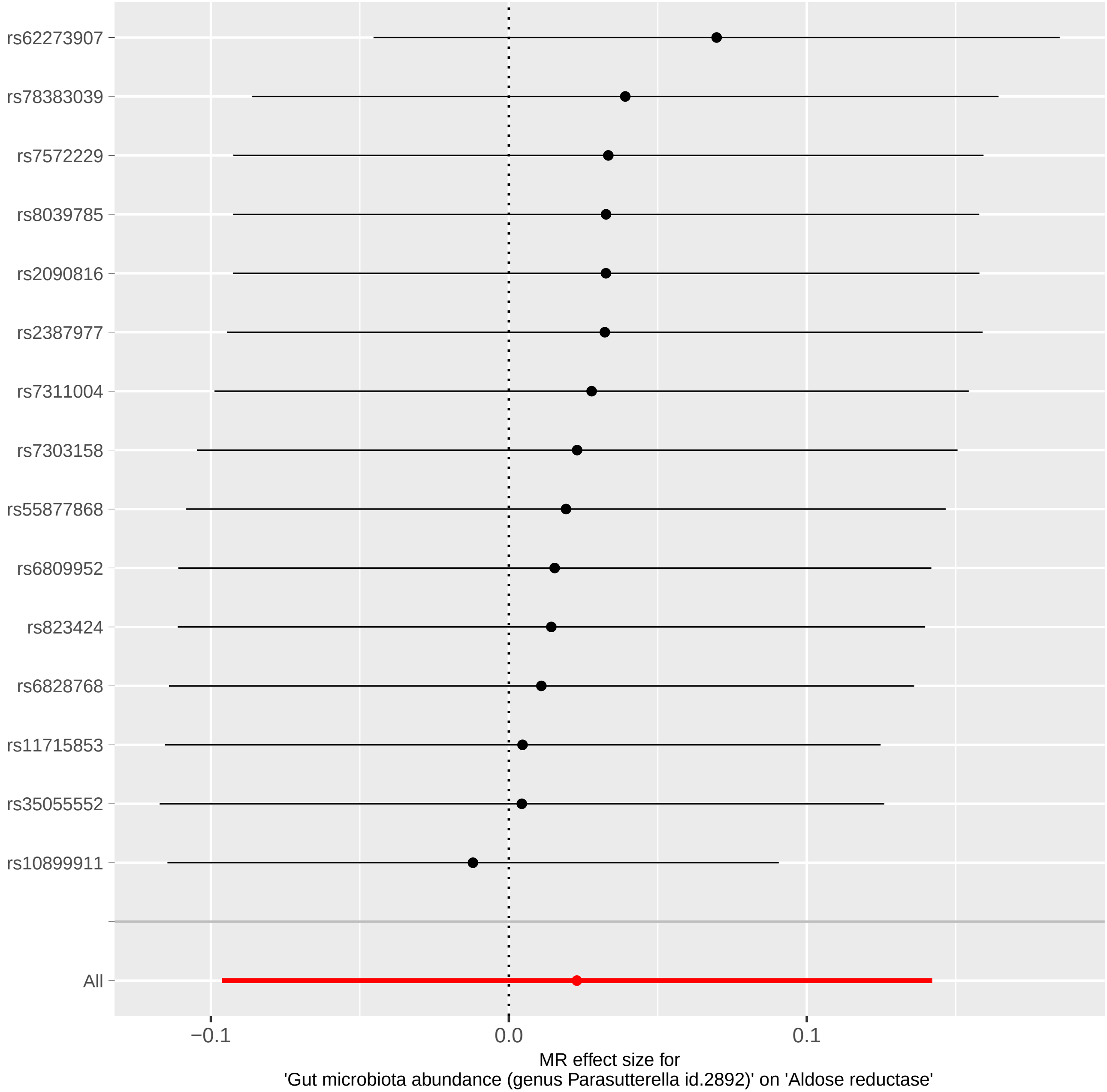

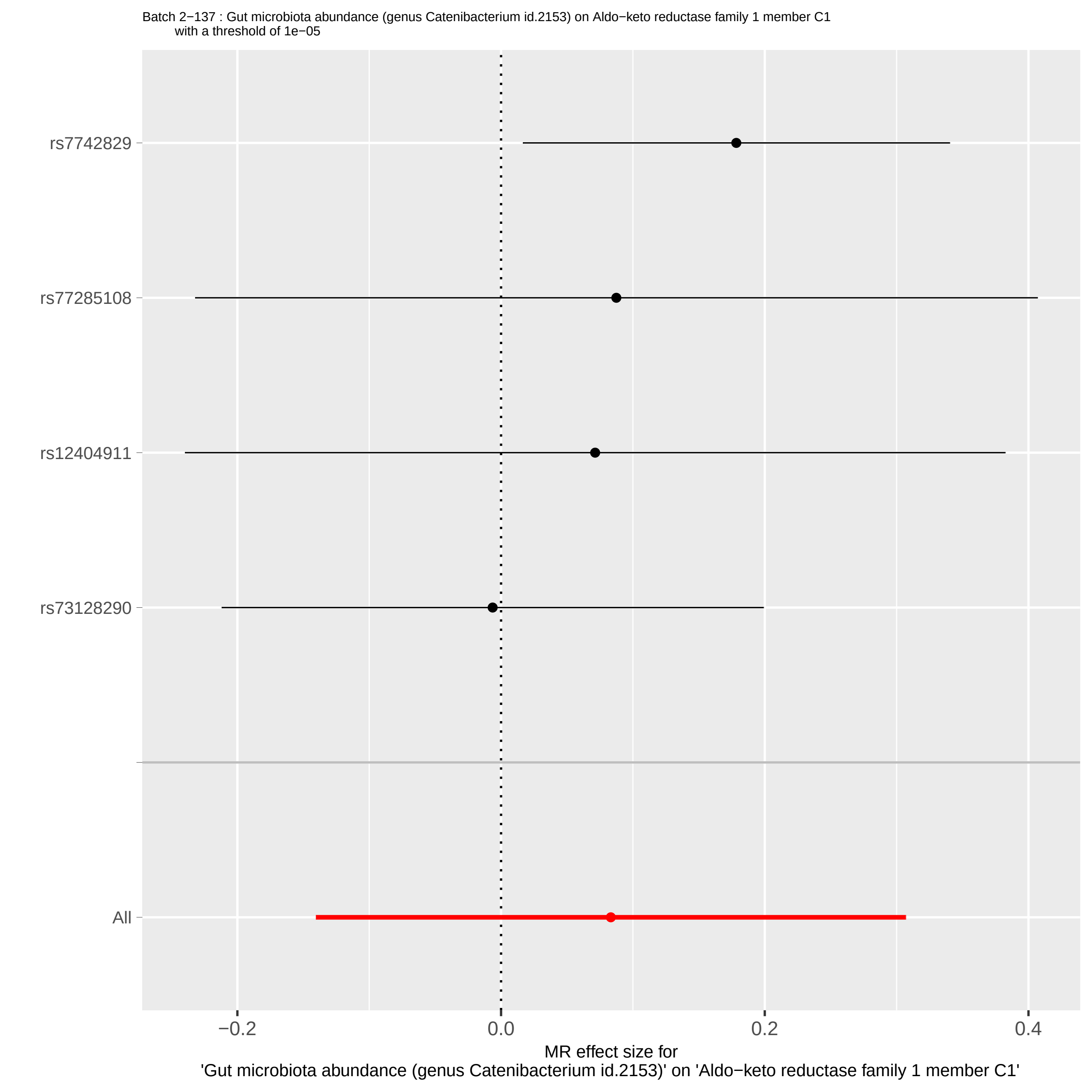

Batch 2−137 : Gut microbiota abundance (genus Catenibacterium id.2153) on Aldo−keto reductase family 1 member C1
with a threshold of 1e−05
rs7742829
rs77285108
rs12404911
rs73128290
All
−0.2
0.0
0.2
0.4
MR effect size for
'Gut microbiota abundance (genus Catenibacterium id.2153)' on 'Aldo−keto reductase family 1 member C1'

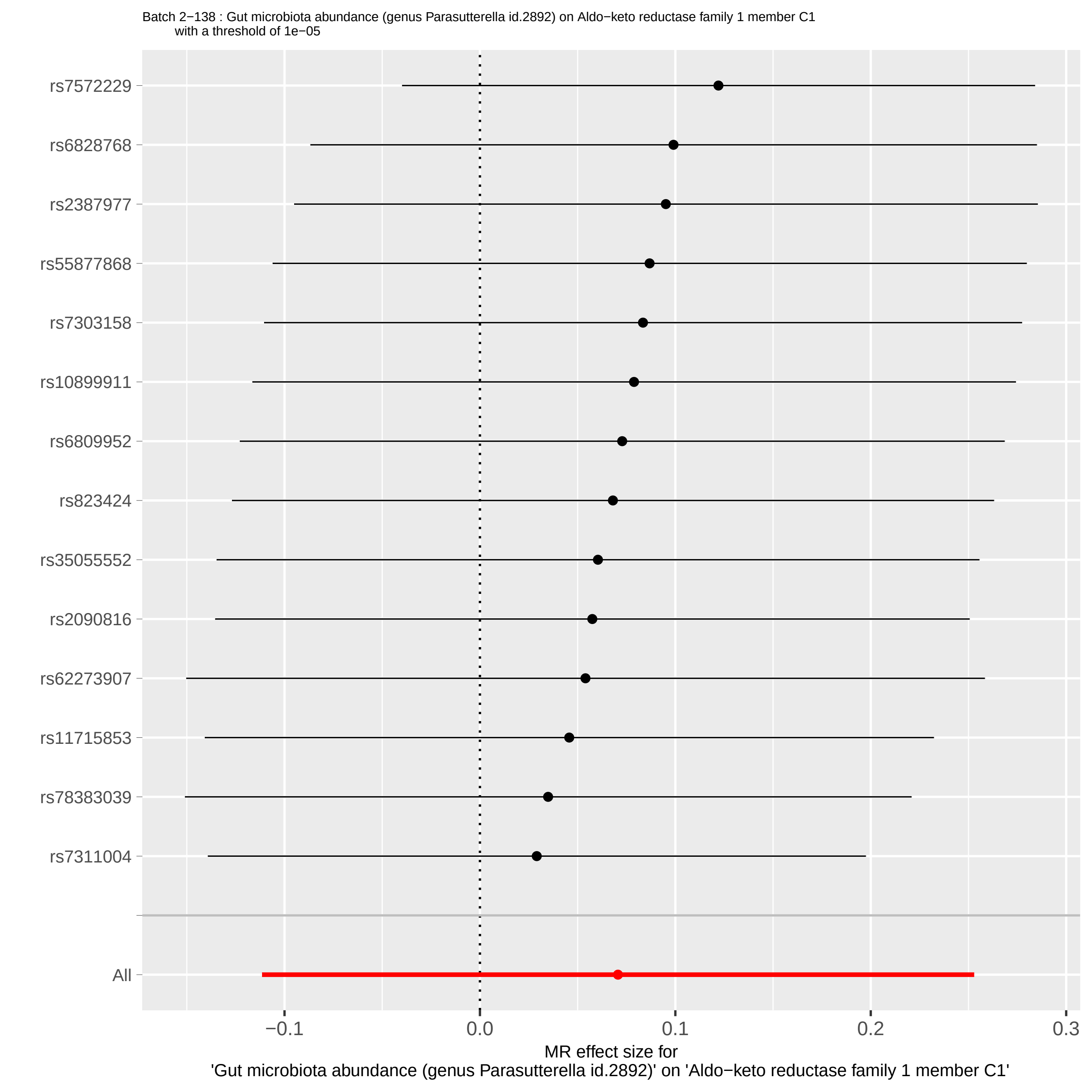

Batch 2−138 : Gut microbiota abundance (genus Parasutterella id.2892) on Aldo−keto reductase family 1 member C1
with a threshold of 1e−05
rs7572229
rs6828768
rs2387977
rs55877868
rs7303158
rs10899911
rs6809952
rs823424
rs35055552
rs2090816
rs62273907
rs11715853
rs78383039
rs7311004
All
−0.1
0.0
0.1
0.2
0.3
MR effect size for
'Gut microbiota abundance (genus Parasutterella id.2892)' on 'Aldo−keto reductase family 1 member C1'

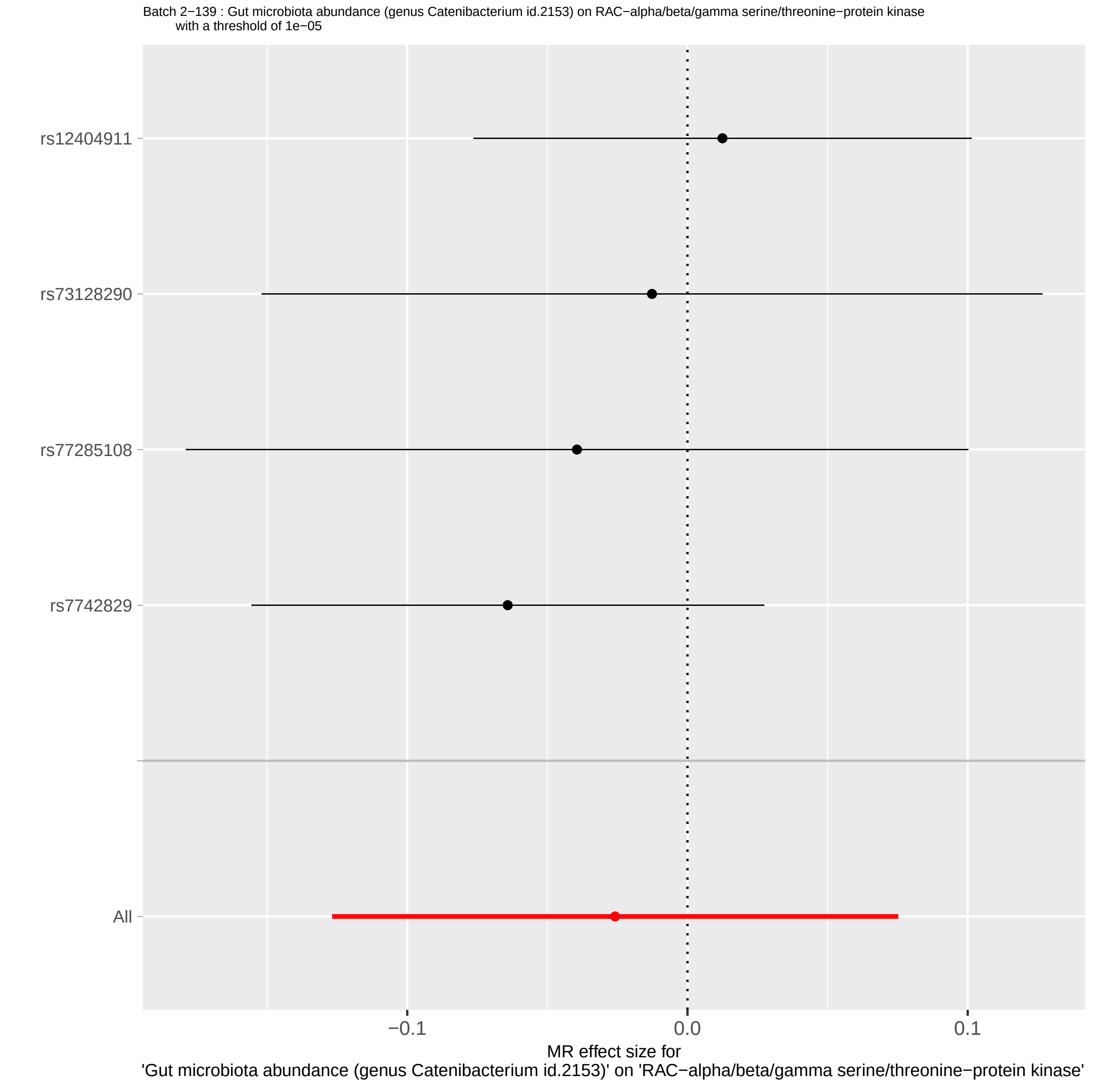

Batch 2−139 : Gut microbiota abundance (genus Catenibacterium id.2153) on RAC−alpha/beta/gamma serine/threonine−protein kinase
with a threshold of 1e−05
rs12404911
rs73128290
rs77285108
rs7742829
All
−0.1
0.0
0.1
MR effect size for
'Gut microbiota abundance (genus Catenibacterium id.2153)' on 'RAC−alpha/beta/gamma serine/threonine−protein kinase'

Batch 2−140 : Gut microbiota abundance (genus Parasutterella id.2892) on RAC−alpha/beta/gamma serine/threonine−protein kinase with a threshold of 1e−05

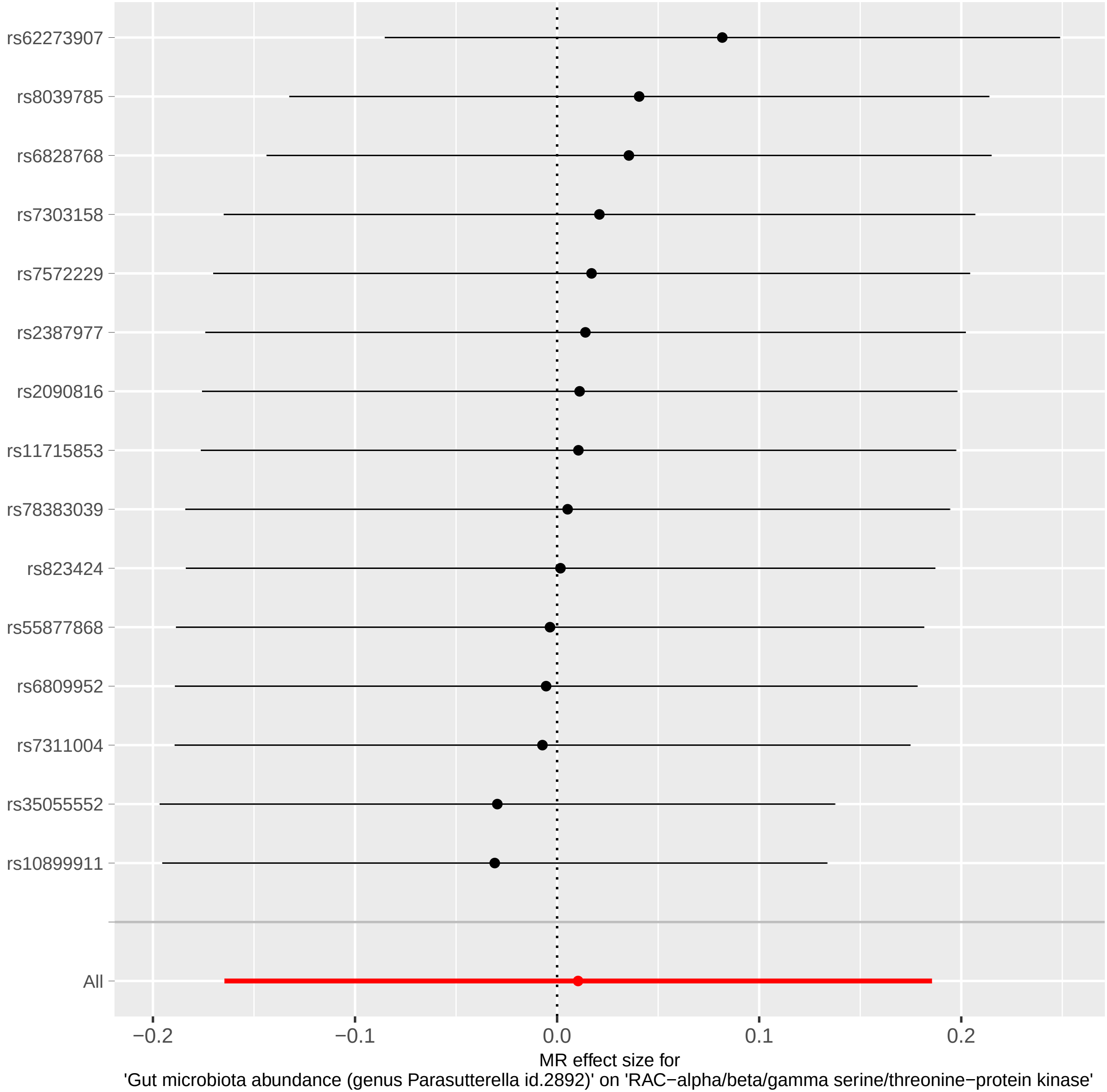

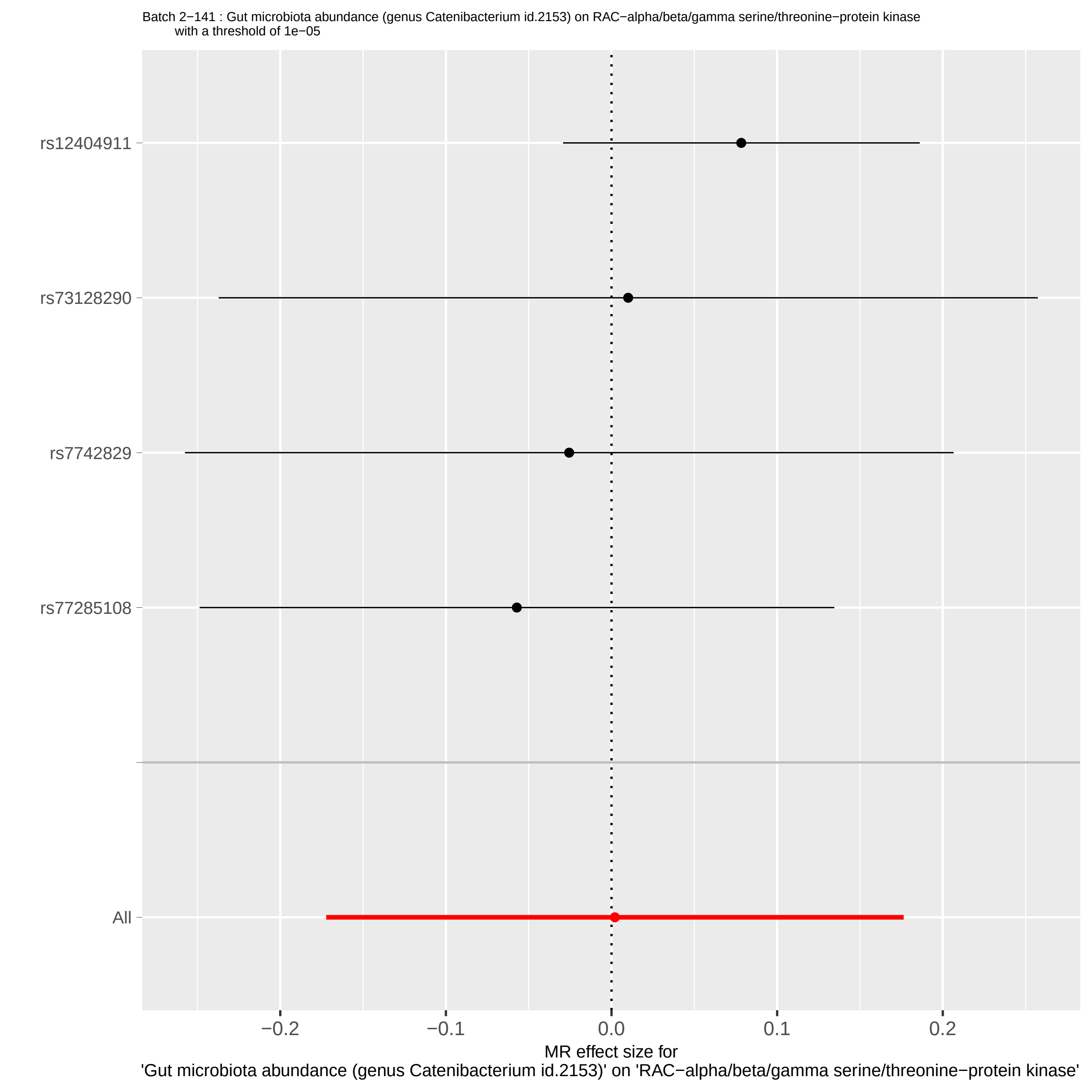

Batch 2−141 : Gut microbiota abundance (genus Catenibacterium id.2153) on RAC−alpha/beta/gamma serine/threonine−protein kinase
with a threshold of 1e−05
rs12404911
rs73128290
rs7742829
rs77285108
All
−0.2
−0.1
0.0
0.1
0.2
MR effect size for
'Gut microbiota abundance (genus Catenibacterium id.2153)' on 'RAC−alpha/beta/gamma serine/threonine−protein kinase'

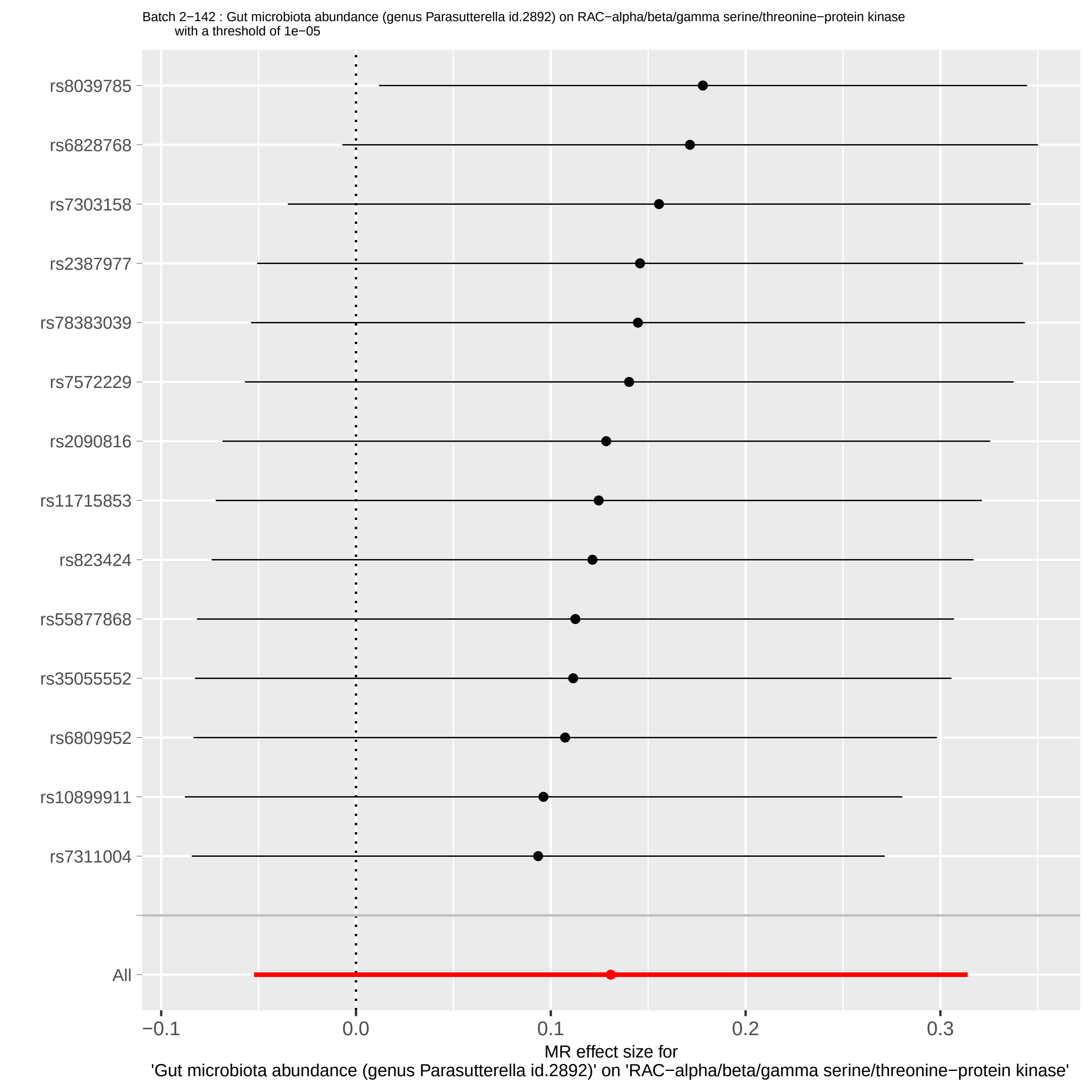
Batch 2−142 : Gut microbiota abundance (genus Parasutterella id.2892) on RAC−alpha/beta/gamma serine/threonine−protein kinase with a threshold of 1e−05
rs8039785
rs6828768
rs7303158
rs2387977
rs78383039
rs7572229
rs2090816
rs11715853
rs823424
rs55877868
rs35055552
rs6809952
rs10899911
rs7311004
All
−0.1
0.0
0.1
0.2
0.3
MR effect size for
'Gut microbiota abundance (genus Parasutterella id.2892)' on 'RAC−alpha/beta/gamma serine/threonine−protein kinase'

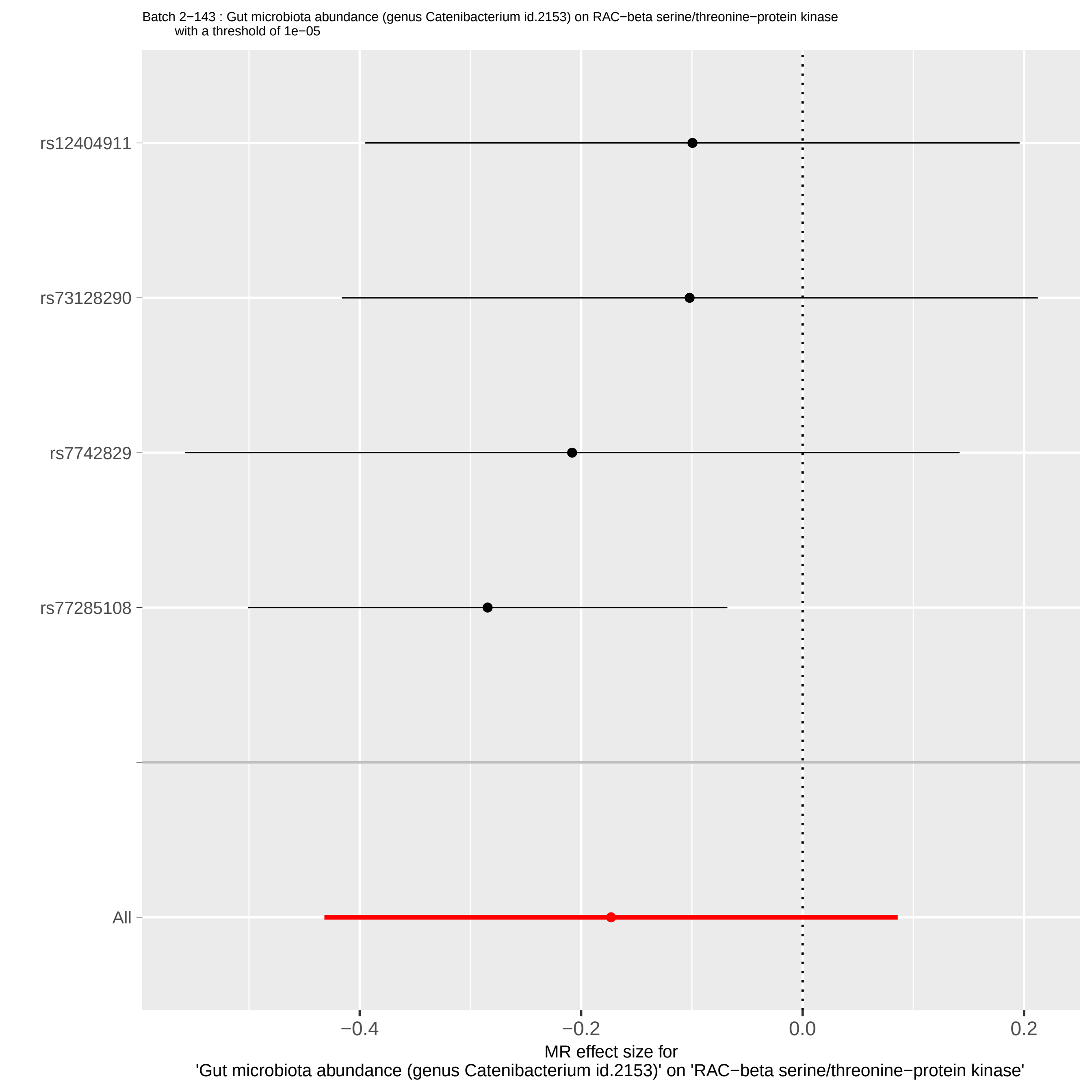

Batch 2−143 : Gut microbiota abundance (genus Catenibacterium id.2153) on RAC−beta serine/threonine−protein kinase
with a threshold of 1e−05
rs12404911
rs73128290
rs7742829
rs77285108
All
−0.4
−0.2
0.0
0.2
MR effect size for
'Gut microbiota abundance (genus Catenibacterium id.2153)' on 'RAC−beta serine/threonine−protein kinase'

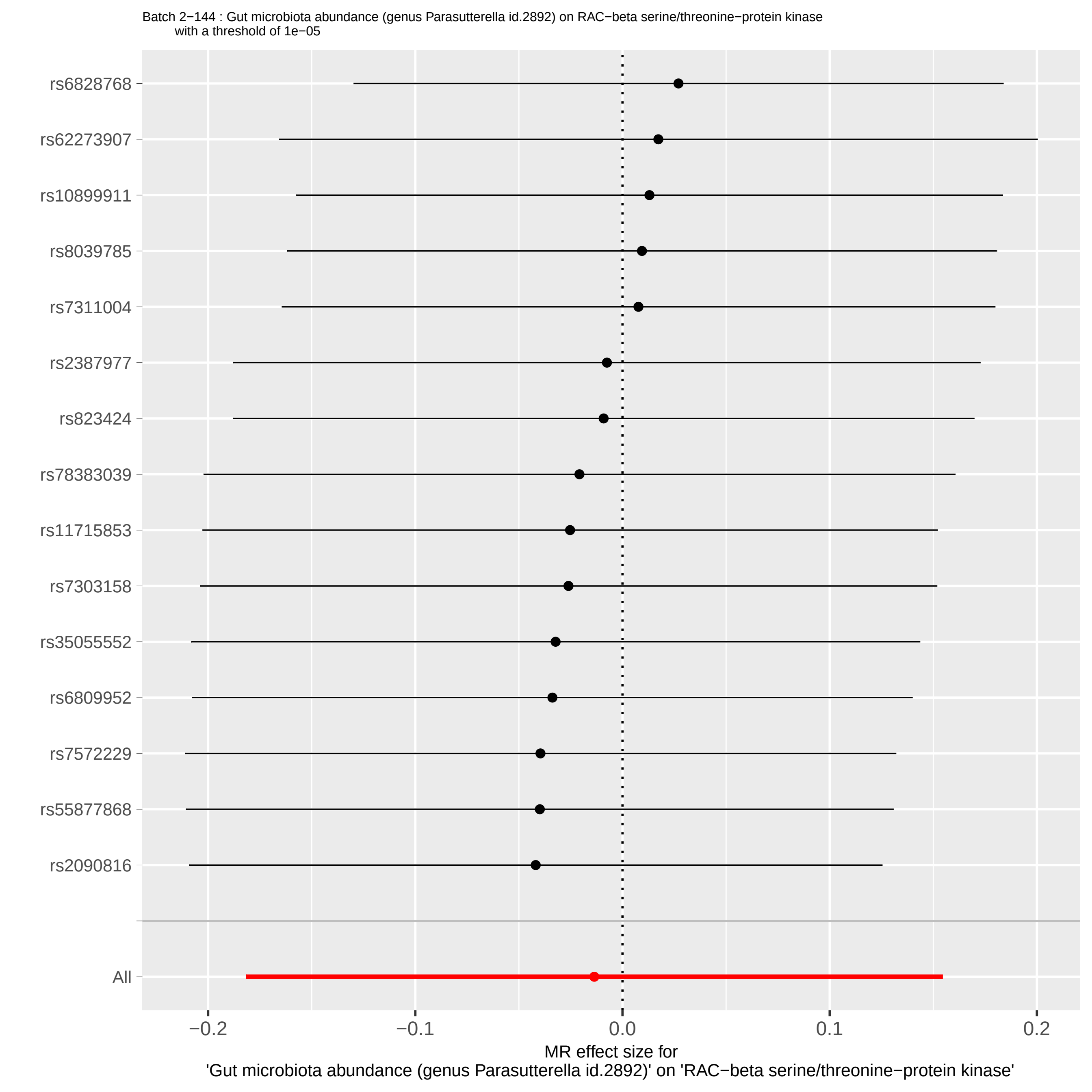
Batch 2−144 : Gut microbiota abundance (genus Parasutterella id.2892) on RAC−beta serine/threonine−protein kinase
with a threshold of 1e−05
rs6828768
rs62273907
rs10899911
rs8039785
rs7311004
rs2387977
rs823424
rs78383039
rs11715853
rs7303158
rs35055552
rs6809952
rs7572229
rs55877868
rs2090816
All
−0.2
−0.1
0.0
0.1
0.2
MR effect size for
'Gut microbiota abundance (genus Parasutterella id.2892)' on 'RAC−beta serine/threonine−protein kinase'

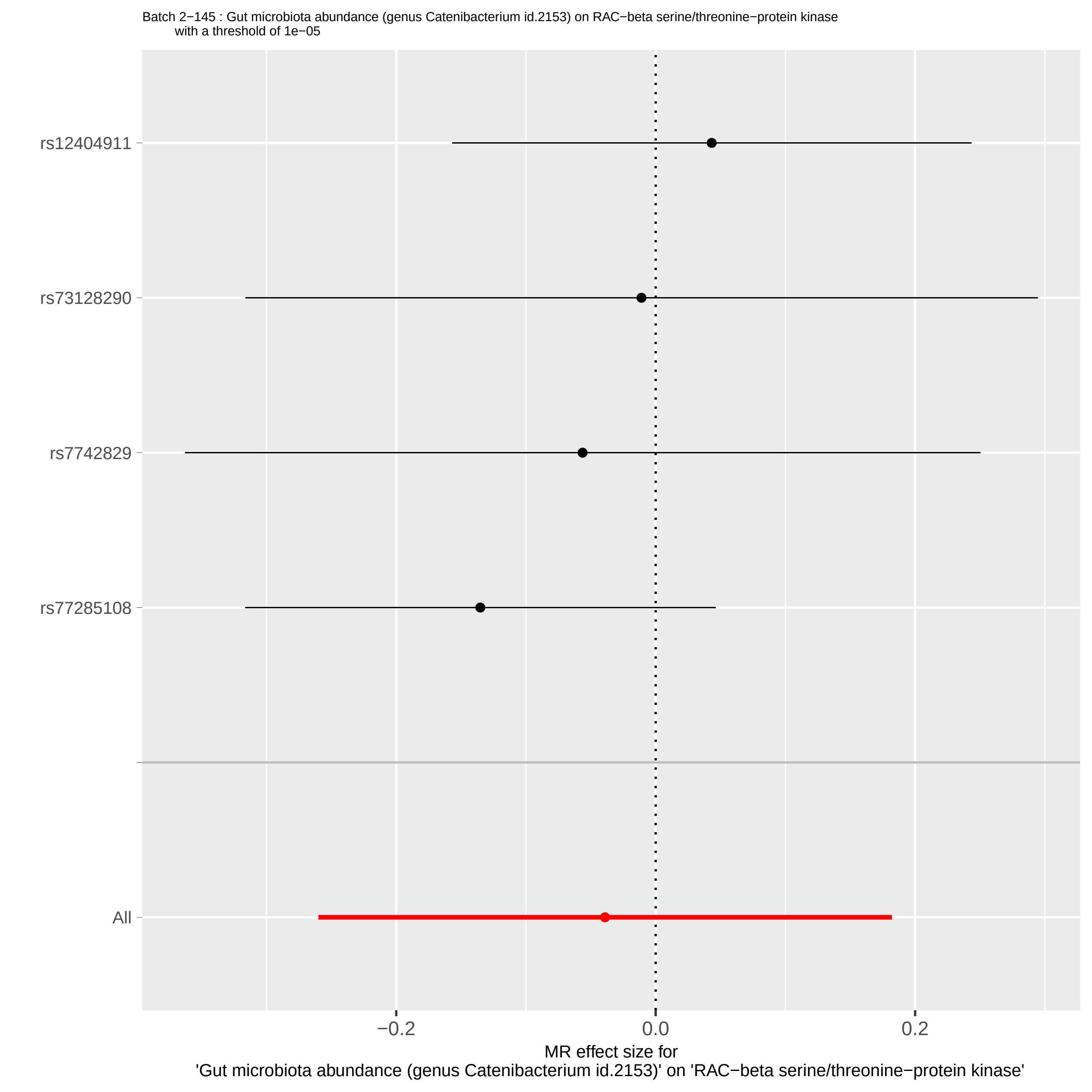

Batch 2−145 : Gut microbiota abundance (genus Catenibacterium id.2153) on RAC−beta serine/threonine−protein kinase
with a threshold of 1e−05
rs12404911
rs73128290
rs7742829
rs77285108
All
−0.2
0.0
0.2
MR effect size for
'Gut microbiota abundance (genus Catenibacterium id.2153)' on 'RAC−beta serine/threonine−protein kinase'

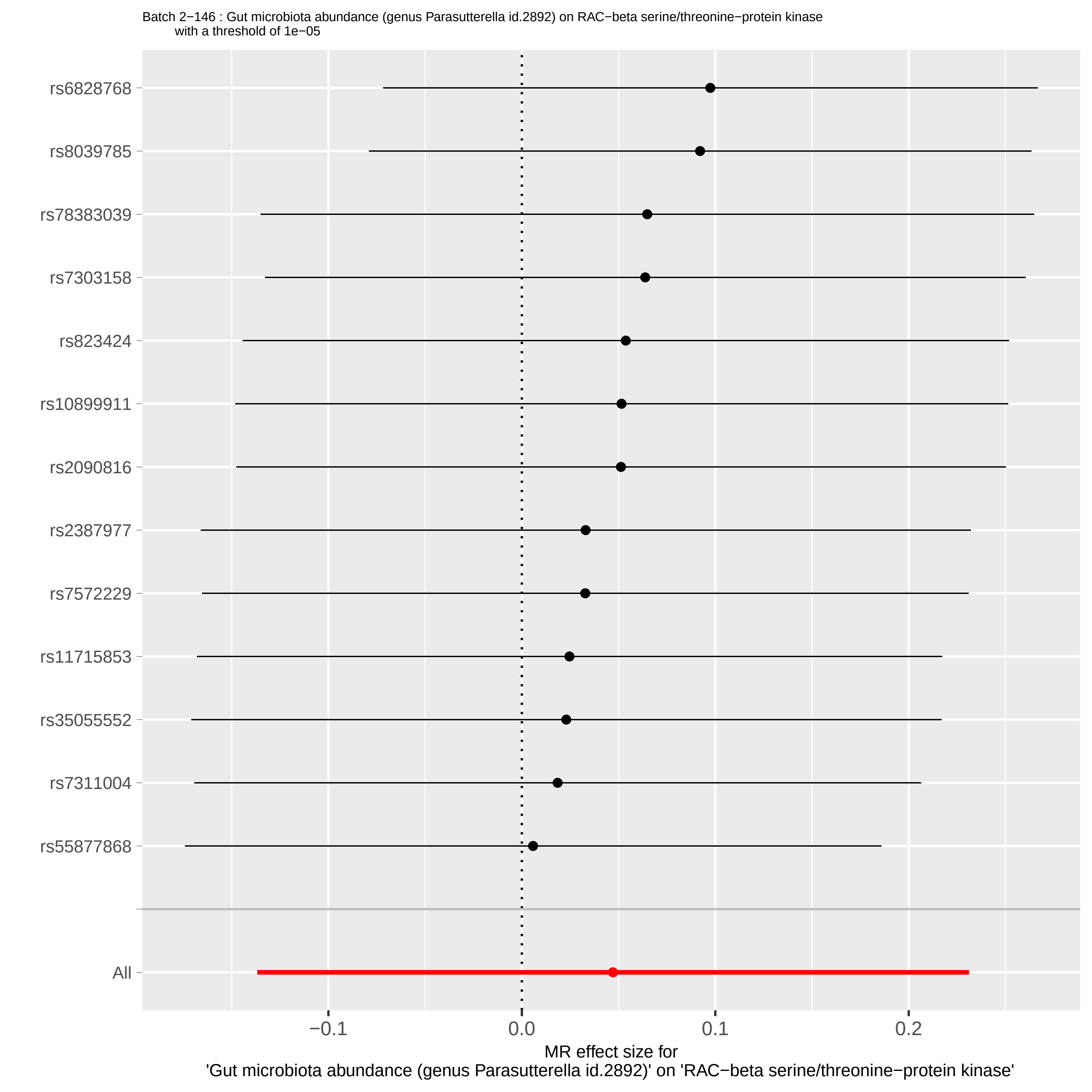
Batch 2−146 : Gut microbiota abundance (genus Parasutterella id.2892) on RAC−beta serine/threonine−protein kinase
with a threshold of 1e−05
rs6828768
rs8039785
rs78383039
rs7303158
rs823424
rs10899911
rs2090816
rs2387977
rs7572229
rs11715853
rs35055552
rs7311004
rs55877868
All
−0.1
0.0
0.1
0.2
MR effect size for
'Gut microbiota abundance (genus Parasutterella id.2892)' on 'RAC−beta serine/threonine−protein kinase'

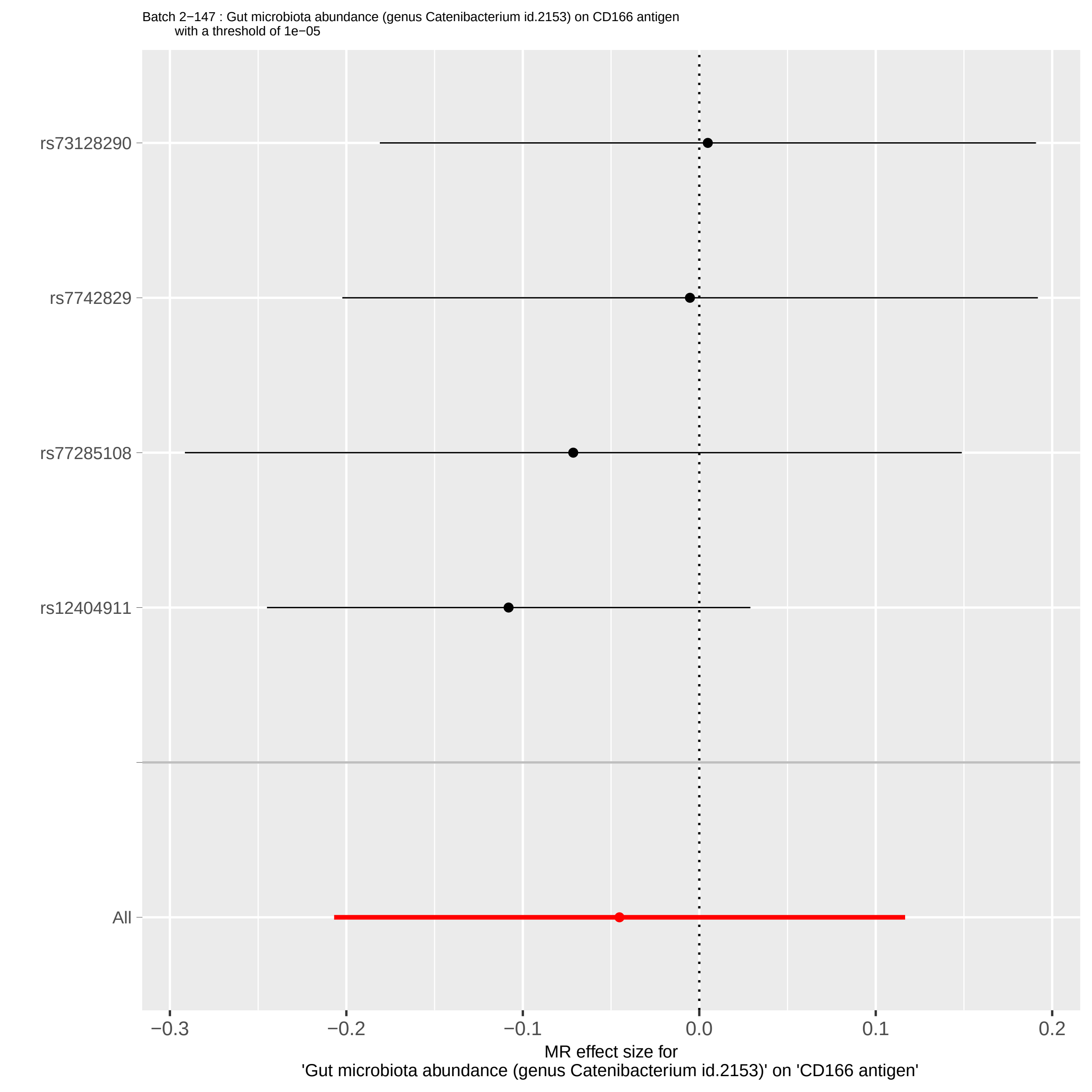
Batch 2−147 : Gut microbiota abundance (genus Catenibacterium id.2153) on CD166 antigen
with a threshold of 1e−05
rs73128290
rs7742829
rs77285108
rs12404911
All
−0.3
−0.2
−0.1
0.0
0.1
0.2
MR effect size for
'Gut microbiota abundance (genus Catenibacterium id.2153)' on 'CD166 antigen'

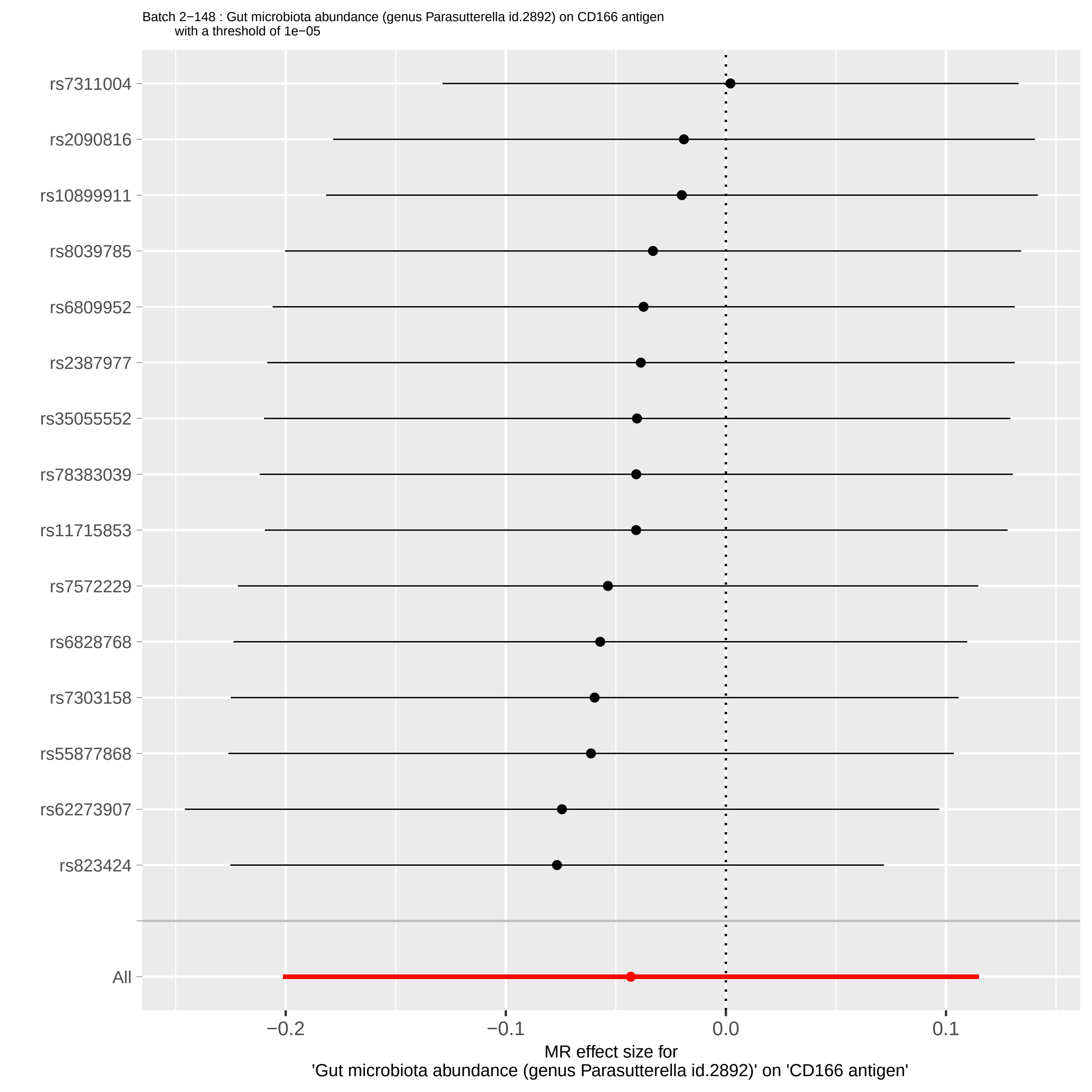
Batch 2−148 : Gut microbiota abundance (genus Parasutterella id.2892) on CD166 antigen
with a threshold of 1e−05
rs7311004
rs2090816
rs10899911
rs8039785
rs6809952
rs2387977
rs35055552
rs78383039
rs11715853
rs7572229
rs6828768
rs7303158
rs55877868
rs62273907
rs823424
All
−0.2
−0.1
0.0
0.1
MR effect size for
'Gut microbiota abundance (genus Parasutterella id.2892)' on 'CD166 antigen'

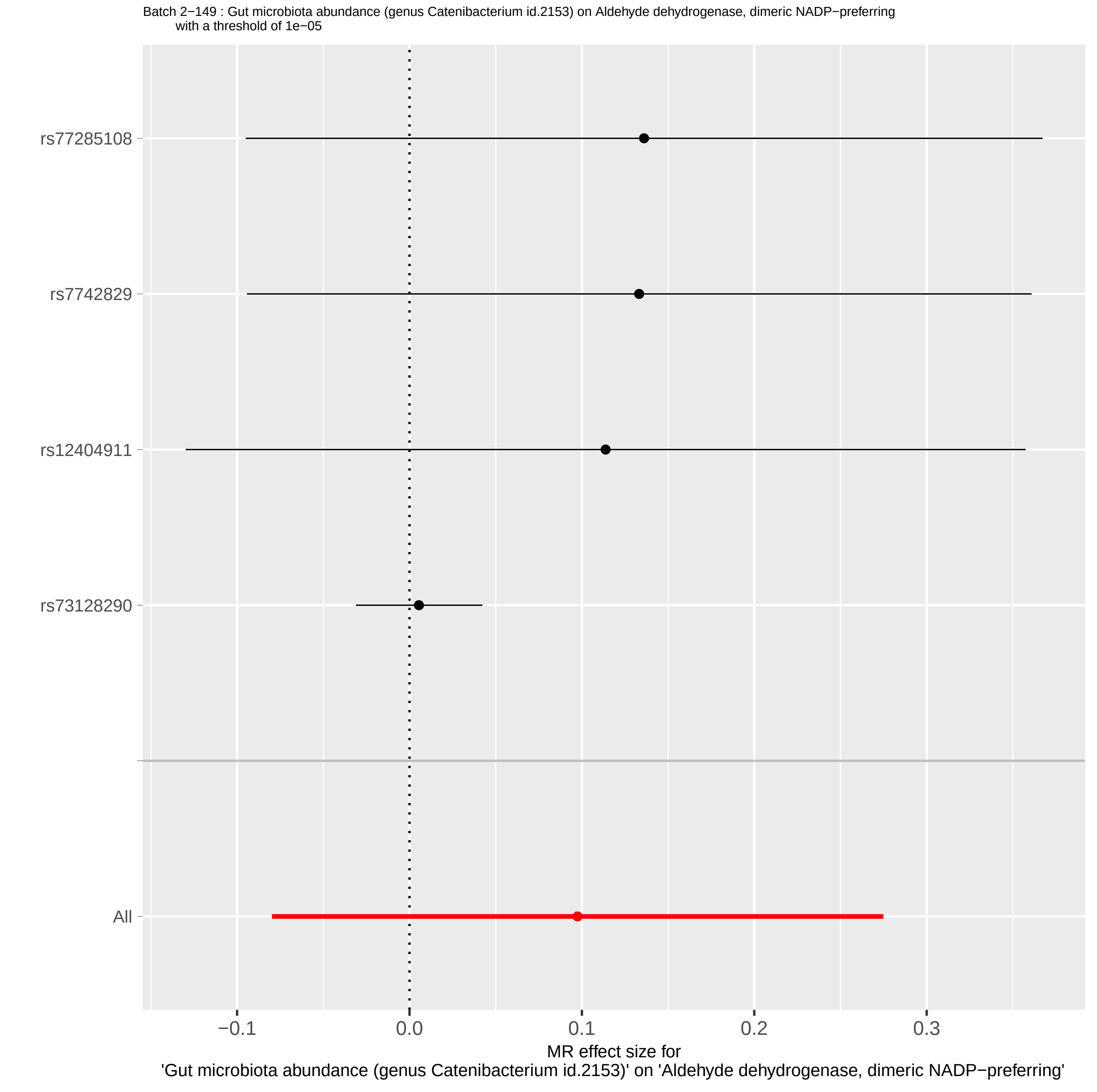

Batch 2−149 : Gut microbiota abundance (genus Catenibacterium id.2153) on Aldehyde dehydrogenase, dimeric NADP−preferring with a threshold of 1e−05
rs77285108
rs7742829
rs12404911
rs73128290
All
−0.1
0.0
0.1
0.2
0.3
MR effect size for
'Gut microbiota abundance (genus Catenibacterium id.2153)' on 'Aldehyde dehydrogenase, dimeric NADP−preferring'

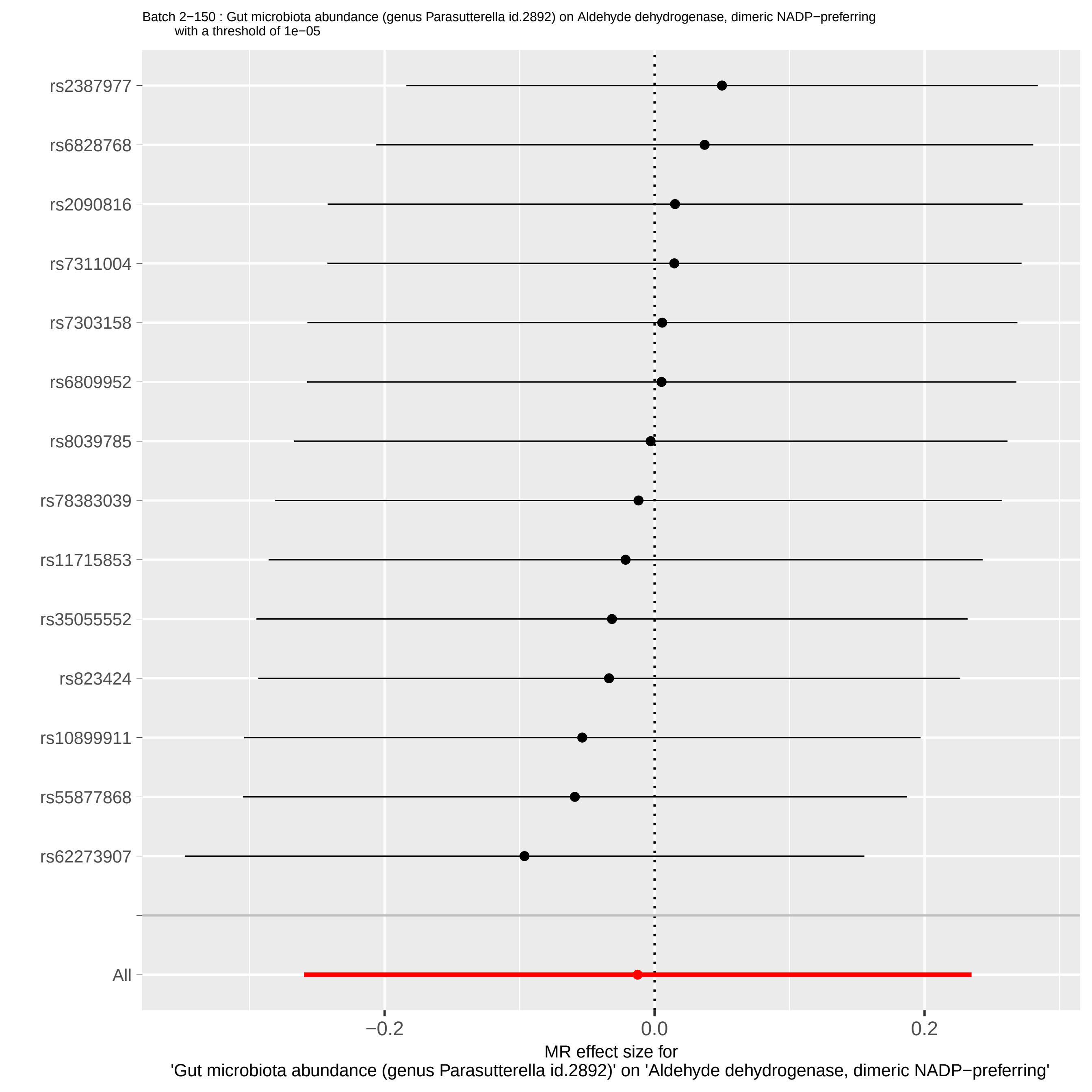

Batch 2−150 : Gut microbiota abundance (genus Parasutterella id.2892) on Aldehyde dehydrogenase, dimeric NADP−preferring
with a threshold of 1e−05
rs2387977
rs6828768
rs2090816
rs7311004
rs7303158
rs6809952
rs8039785
rs78383039
rs11715853
rs35055552
rs823424
rs10899911
rs55877868
rs62273907
All
−0.2
0.0
0.2
MR effect size for
'Gut microbiota abundance (genus Parasutterella id.2892)' on 'Aldehyde dehydrogenase, dimeric NADP−preferring'

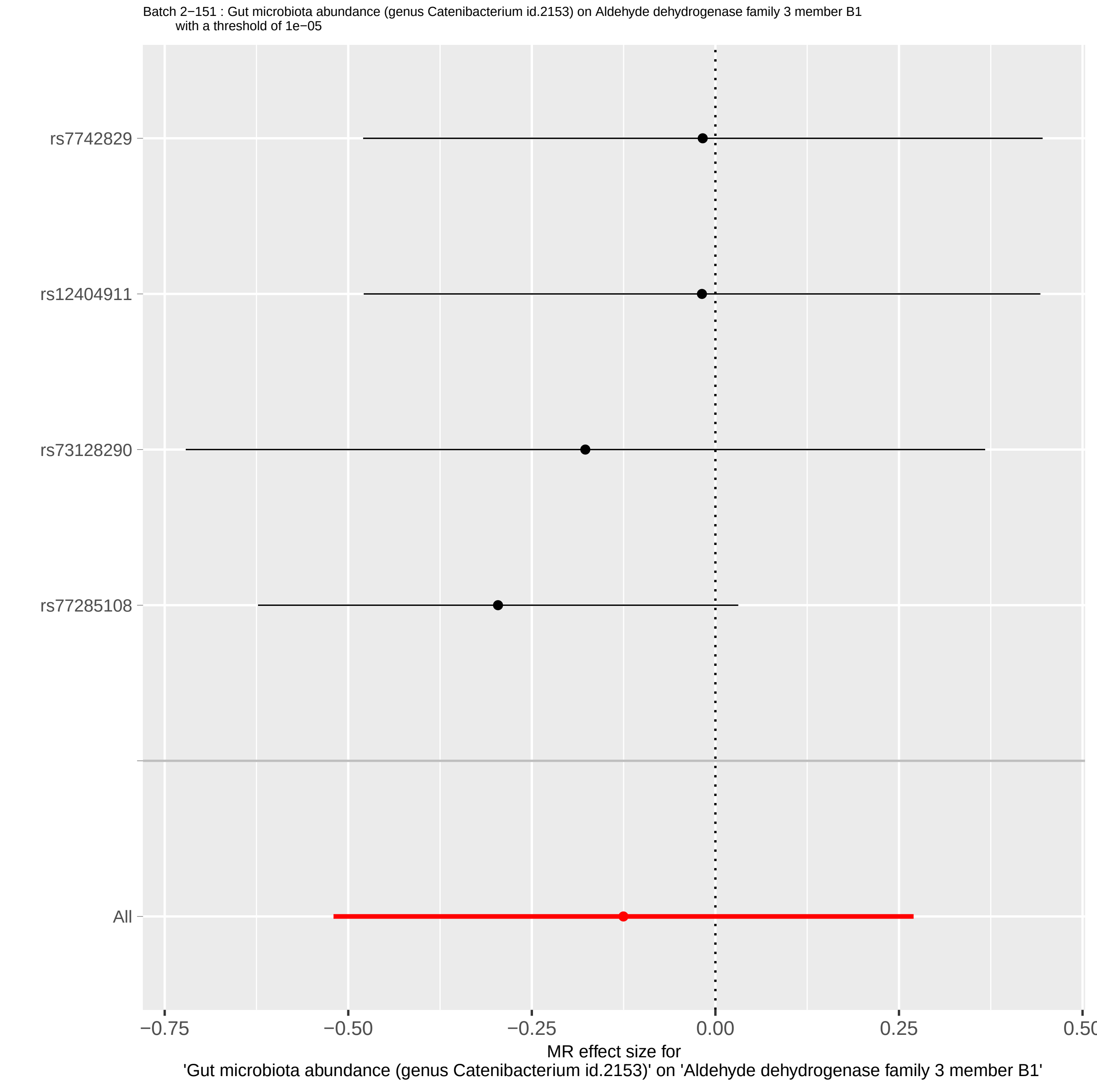
Batch 2−151 : Gut microbiota abundance (genus Catenibacterium id.2153) on Aldehyde dehydrogenase family 3 member B1
with a threshold of 1e−05
rs7742829
rs12404911
rs73128290
rs77285108
All
−0.75
−0.50
−0.25
0.00
0.25
0.50
MR effect size for
'Gut microbiota abundance (genus Catenibacterium id.2153)' on 'Aldehyde dehydrogenase family 3 member B1'

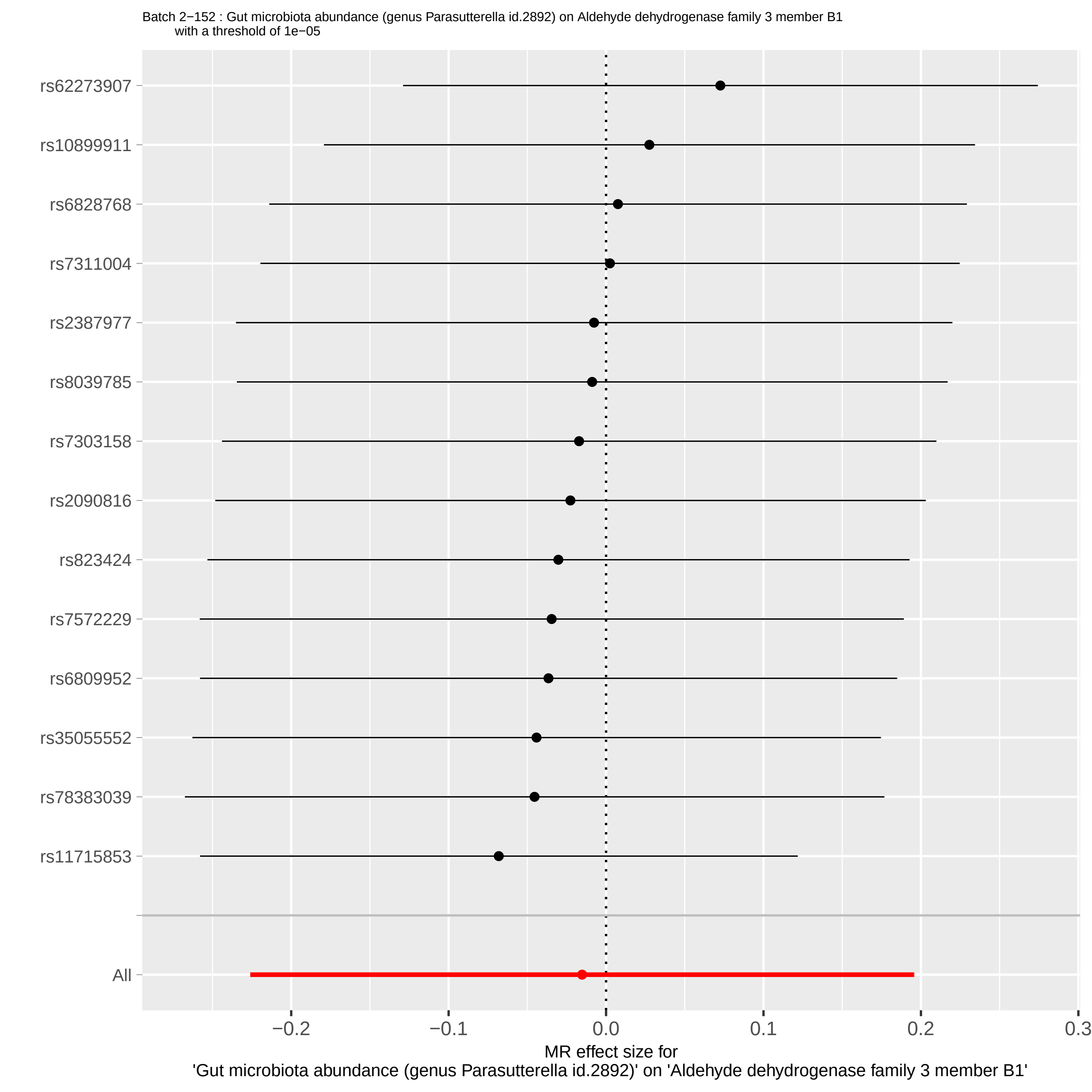

Batch 2−152 : Gut microbiota abundance (genus Parasutterella id.2892) on Aldehyde dehydrogenase family 3 member B1
with a threshold of 1e−05
rs62273907
rs10899911
rs6828768
rs7311004
rs2387977
rs8039785
rs7303158
rs2090816
rs823424
rs7572229
rs6809952
rs35055552
rs78383039
rs11715853
All
−0.2
−0.1
0.0
0.1
0.2
0.3
MR effect size for
'Gut microbiota abundance (genus Parasutterella id.2892)' on 'Aldehyde dehydrogenase family 3 member B1'

Batch 2−153 : Gut microbiota abundance (genus Catenibacterium id.2153) on Fructose−bisphosphate aldolase A
with a threshold of 1e−05

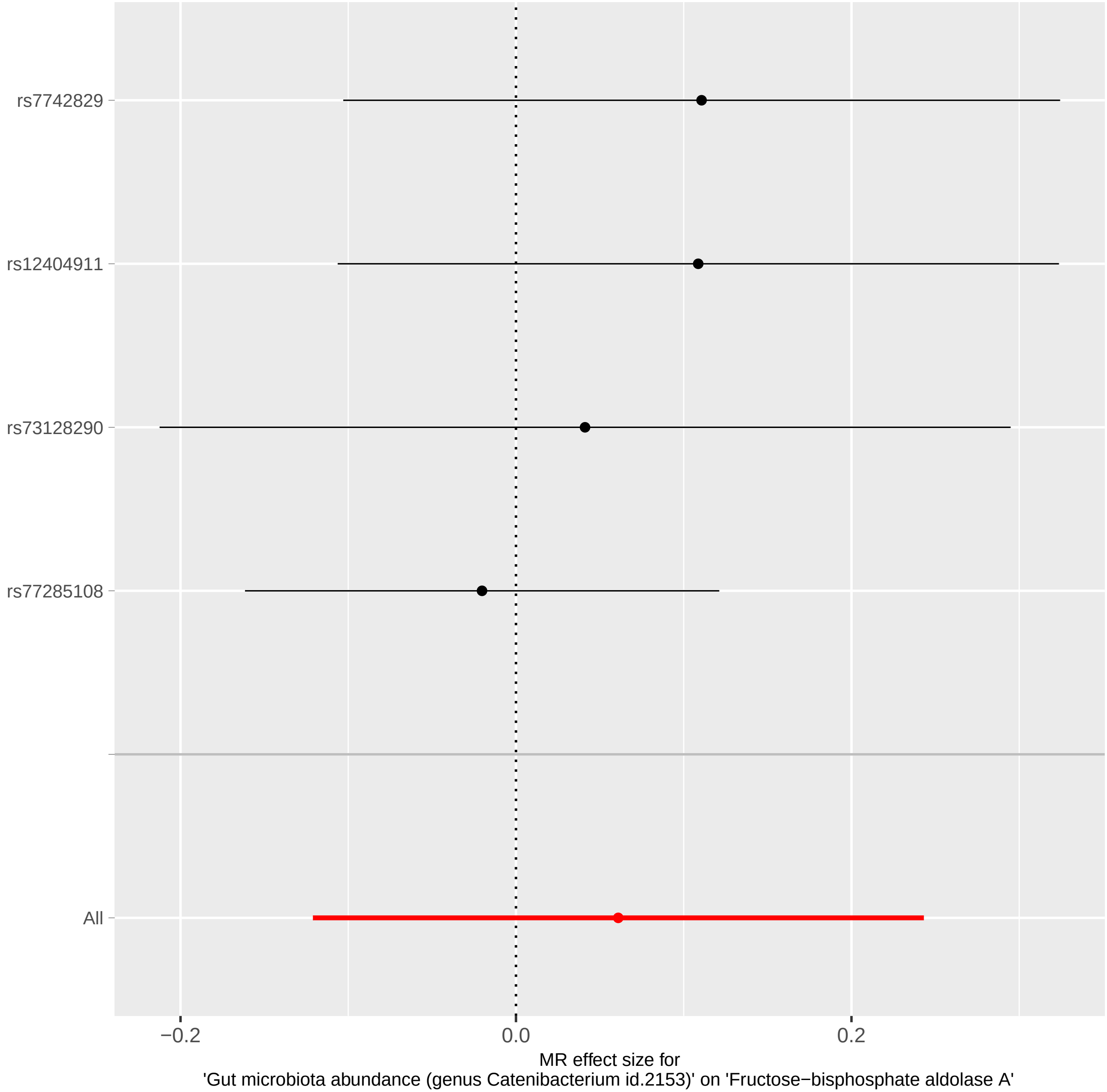

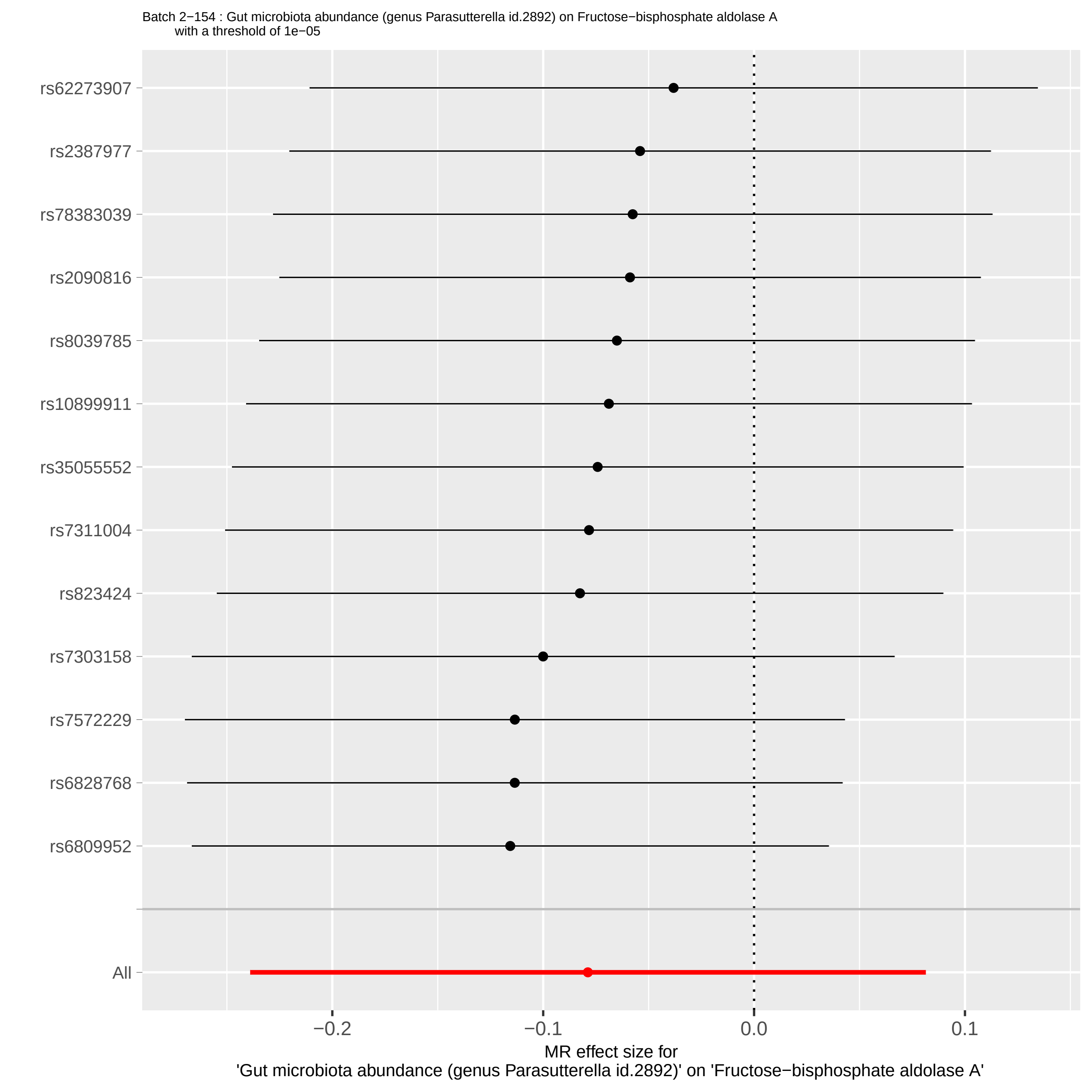

Batch 2−154 : Gut microbiota abundance (genus Parasutterella id.2892) on Fructose−bisphosphate aldolase A
with a threshold of 1e−05
rs62273907
rs2387977
rs78383039
rs2090816
rs8039785
rs10899911
rs35055552
rs7311004
rs823424
rs7303158
rs7572229
rs6828768
rs6809952
All
−0.2
−0.1
0.0
0.1
MR effect size for
'Gut microbiota abundance (genus Parasutterella id.2892)' on 'Fructose−bisphosphate aldolase A'

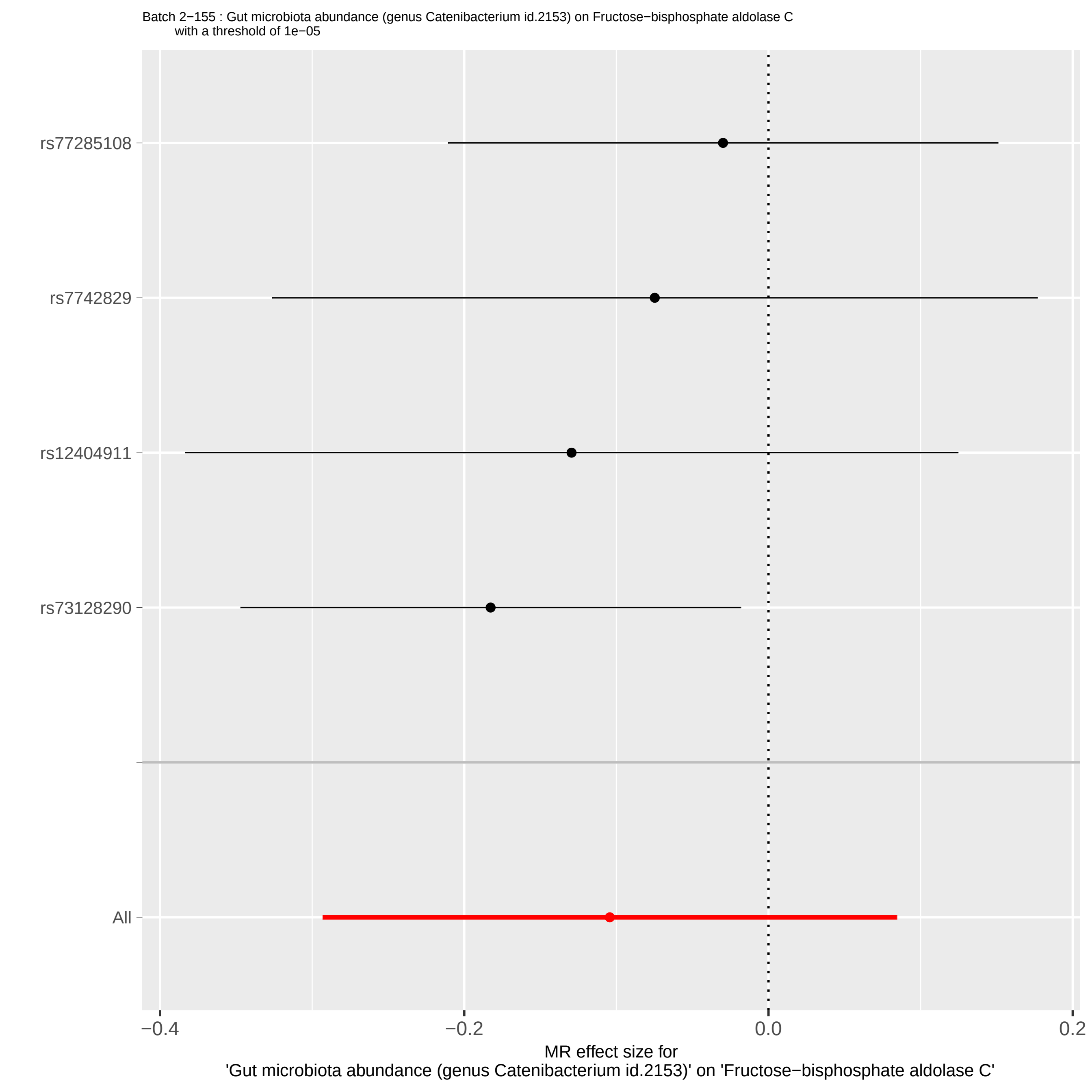

Batch 2−155 : Gut microbiota abundance (genus Catenibacterium id.2153) on Fructose−bisphosphate aldolase C
with a threshold of 1e−05
rs77285108
rs7742829
rs12404911
rs73128290
All
−0.4
−0.2
0.0
0.2
MR effect size for
'Gut microbiota abundance (genus Catenibacterium id.2153)' on 'Fructose−bisphosphate aldolase C'

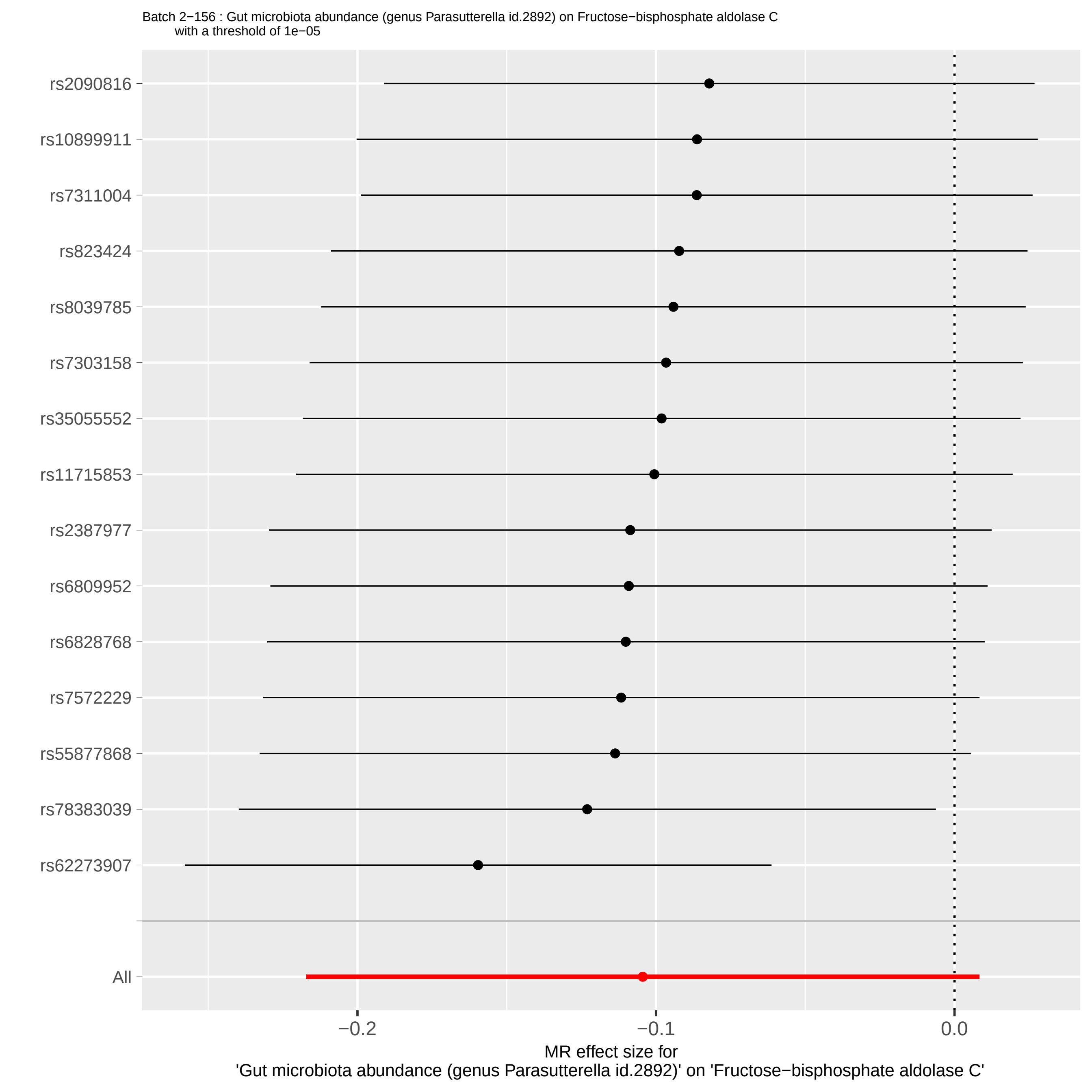

Batch 2−156 : Gut microbiota abundance (genus Parasutterella id.2892) on Fructose−bisphosphate aldolase C
with a threshold of 1e−05
rs2090816
rs10899911
rs7311004
rs823424
rs8039785
rs7303158
rs35055552
rs11715853
rs2387977
rs6809952
rs6828768
rs7572229
rs55877868
rs78383039
rs62273907
All
−0.2
−0.1
0.0
MR effect size for
'Gut microbiota abundance (genus Parasutterella id.2892)' on 'Fructose−bisphosphate aldolase C'

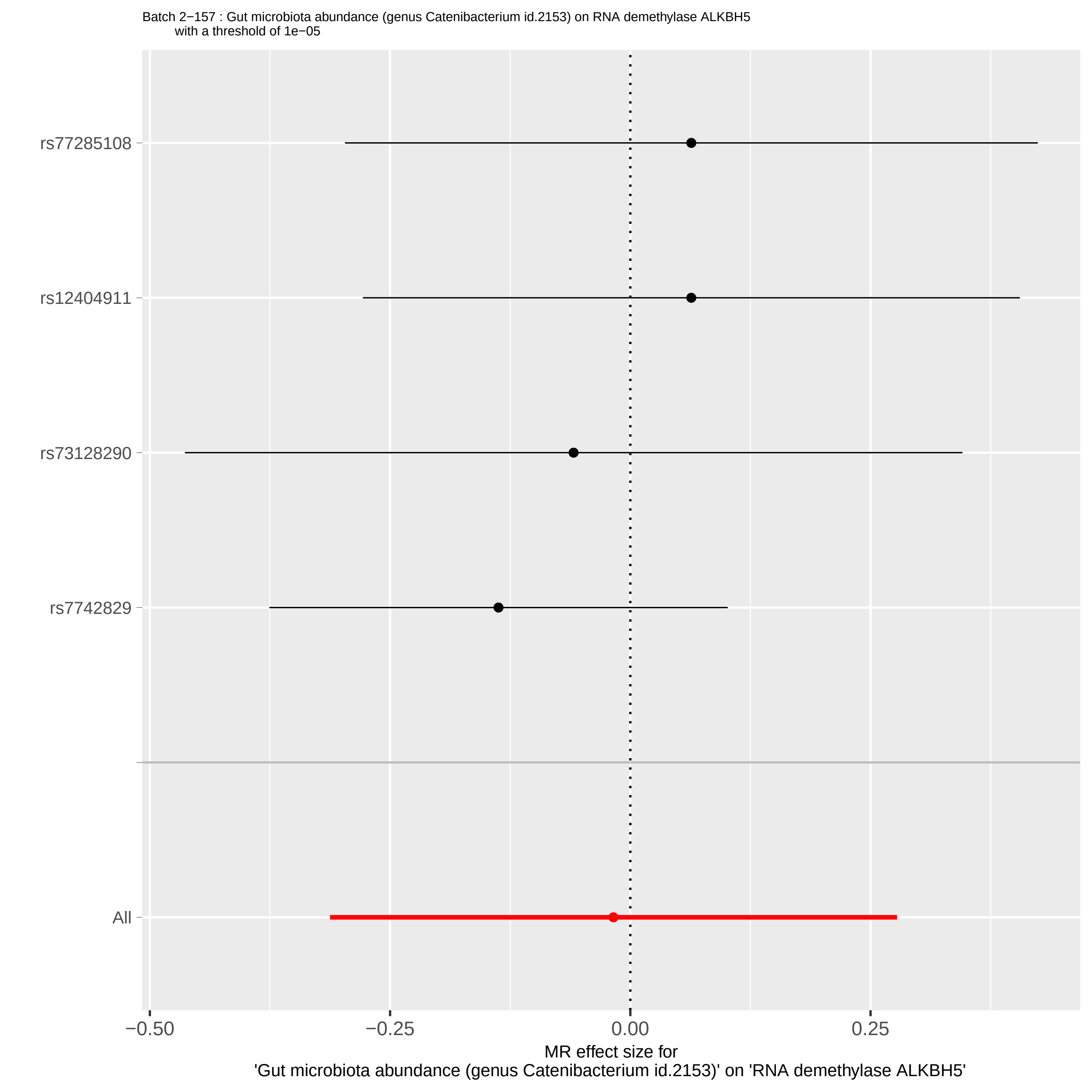
Batch 2−157 : Gut microbiota abundance (genus Catenibacterium id.2153) on RNA demethylase ALKBH5
with a threshold of 1e−05
rs77285108
rs12404911
rs73128290
rs7742829
All
−0.50
−0.25
0.00
0.25
MR effect size for
'Gut microbiota abundance (genus Catenibacterium id.2153)' on 'RNA demethylase ALKBH5'

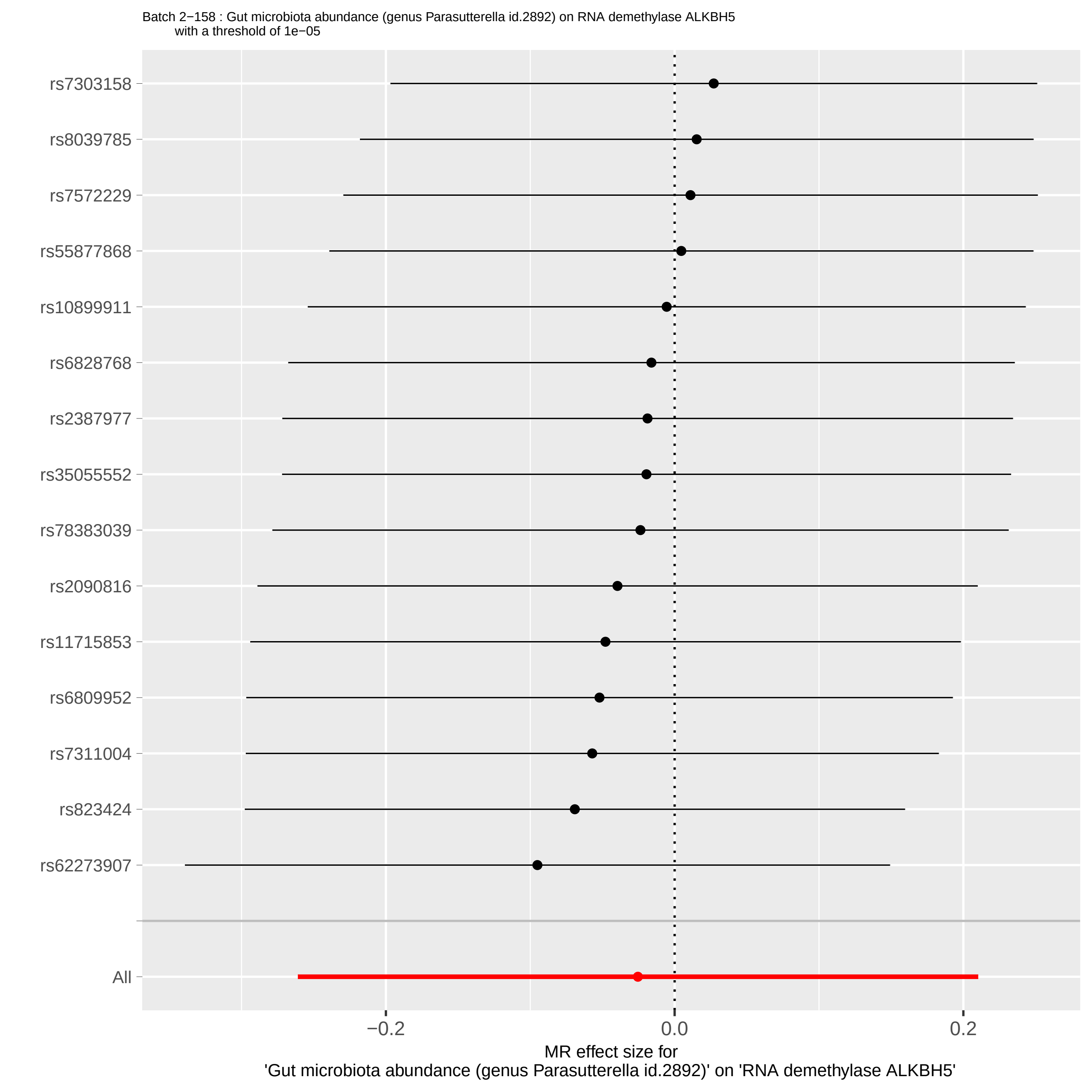
Batch 2−158 : Gut microbiota abundance (genus Parasutterella id.2892) on RNA demethylase ALKBH5
with a threshold of 1e−05
rs7303158
rs8039785
rs7572229
rs55877868
rs10899911
rs6828768
rs2387977
rs35055552
rs78383039
rs2090816
rs11715853
rs6809952
rs7311004
rs823424
rs62273907
All
−0.2
0.0
0.2
MR effect size for
'Gut microbiota abundance (genus Parasutterella id.2892)' on 'RNA demethylase ALKBH5'

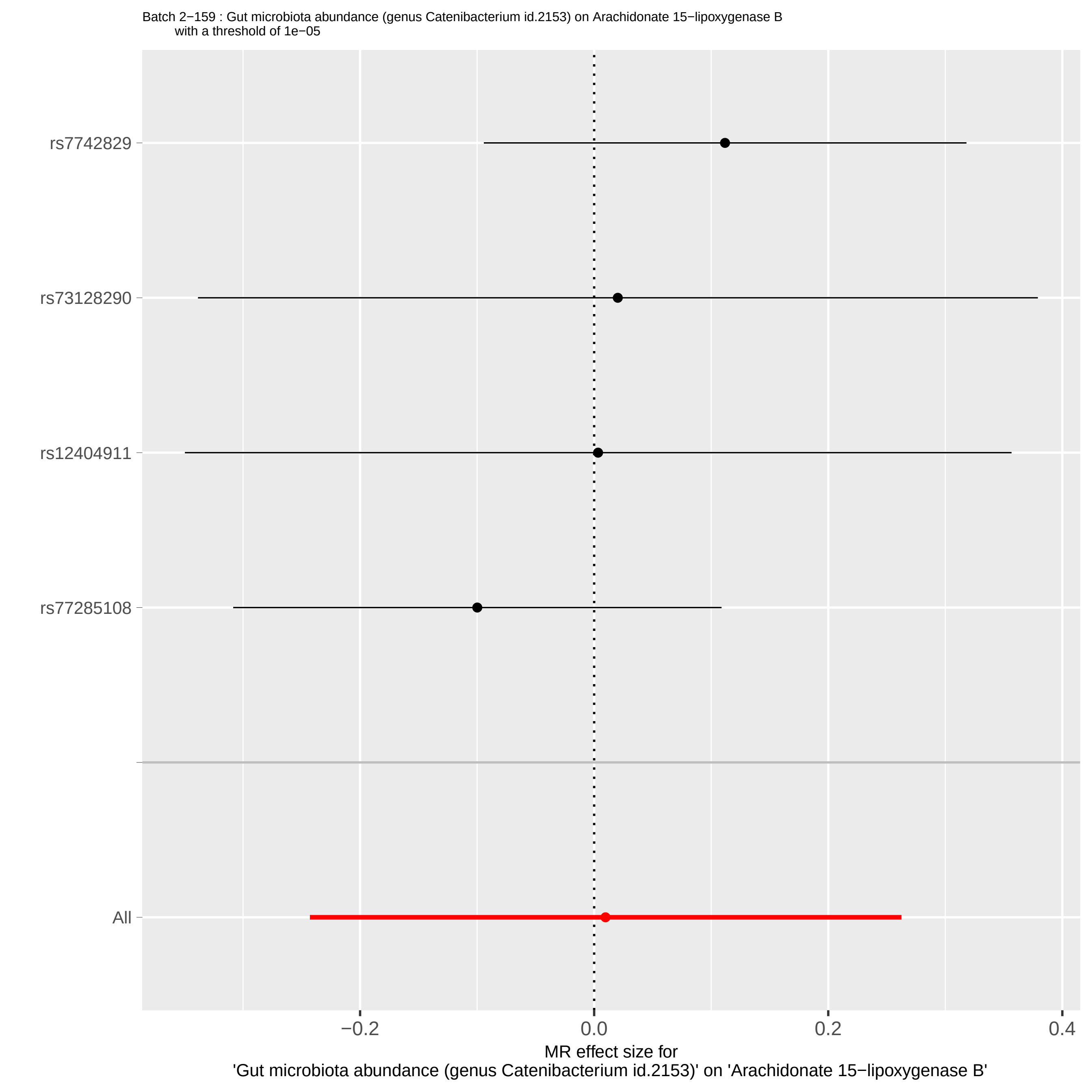

Batch 2−159 : Gut microbiota abundance (genus Catenibacterium id.2153) on Arachidonate 15−lipoxygenase B
with a threshold of 1e−05
rs7742829
rs73128290
rs12404911
rs77285108
All
−0.2
0.0
0.2
0.4
MR effect size for
'Gut microbiota abundance (genus Catenibacterium id.2153)' on 'Arachidonate 15−lipoxygenase B'

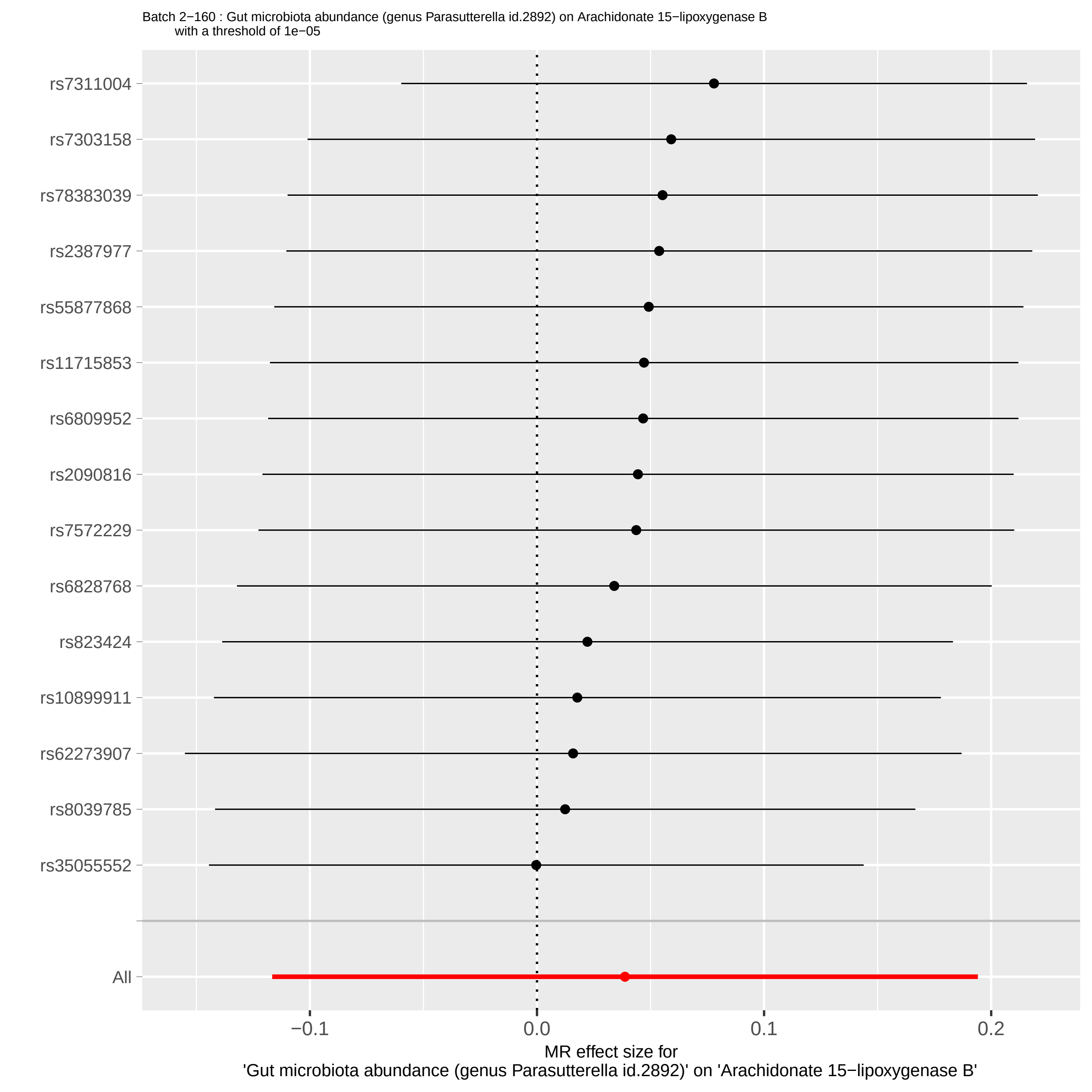

Batch 2−160 : Gut microbiota abundance (genus Parasutterella id.2892) on Arachidonate 15−lipoxygenase B
with a threshold of 1e−05
rs7311004
rs7303158
rs78383039
rs2387977
rs55877868
rs11715853
rs6809952
rs2090816
rs7572229
rs6828768
rs823424
rs10899911
rs62273907
rs8039785
rs35055552
All
−0.1
0.0
0.1
0.2
MR effect size for
'Gut microbiota abundance (genus Parasutterella id.2892)' on 'Arachidonate 15−lipoxygenase B'

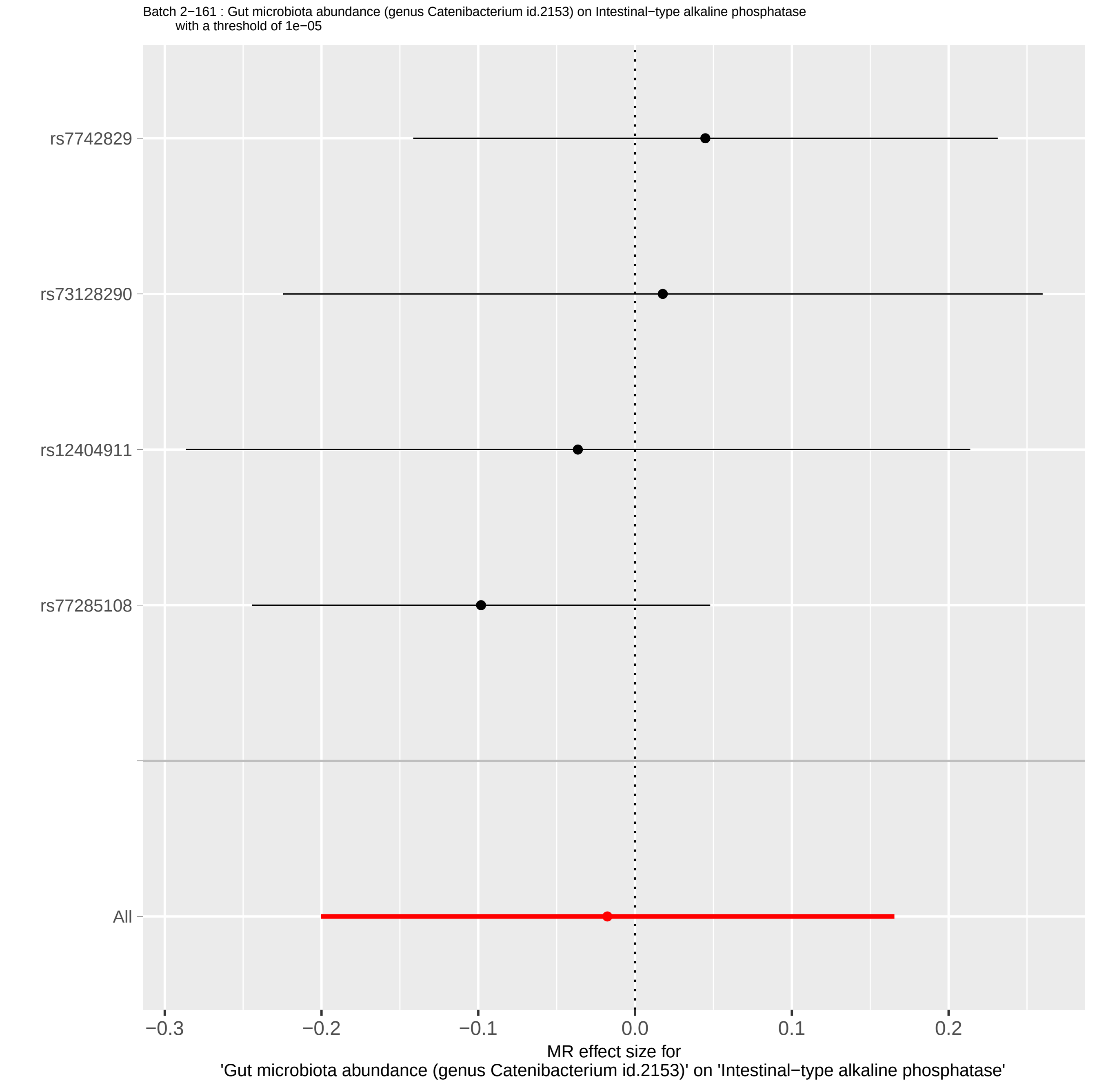

Batch 2−161 : Gut microbiota abundance (genus Catenibacterium id.2153) on Intestinal−type alkaline phosphatase
with a threshold of 1e−05
rs7742829
rs73128290
rs12404911
rs77285108
All
−0.3
−0.2
−0.1
0.0
0.1
0.2
MR effect size for
'Gut microbiota abundance (genus Catenibacterium id.2153)' on 'Intestinal−type alkaline phosphatase'

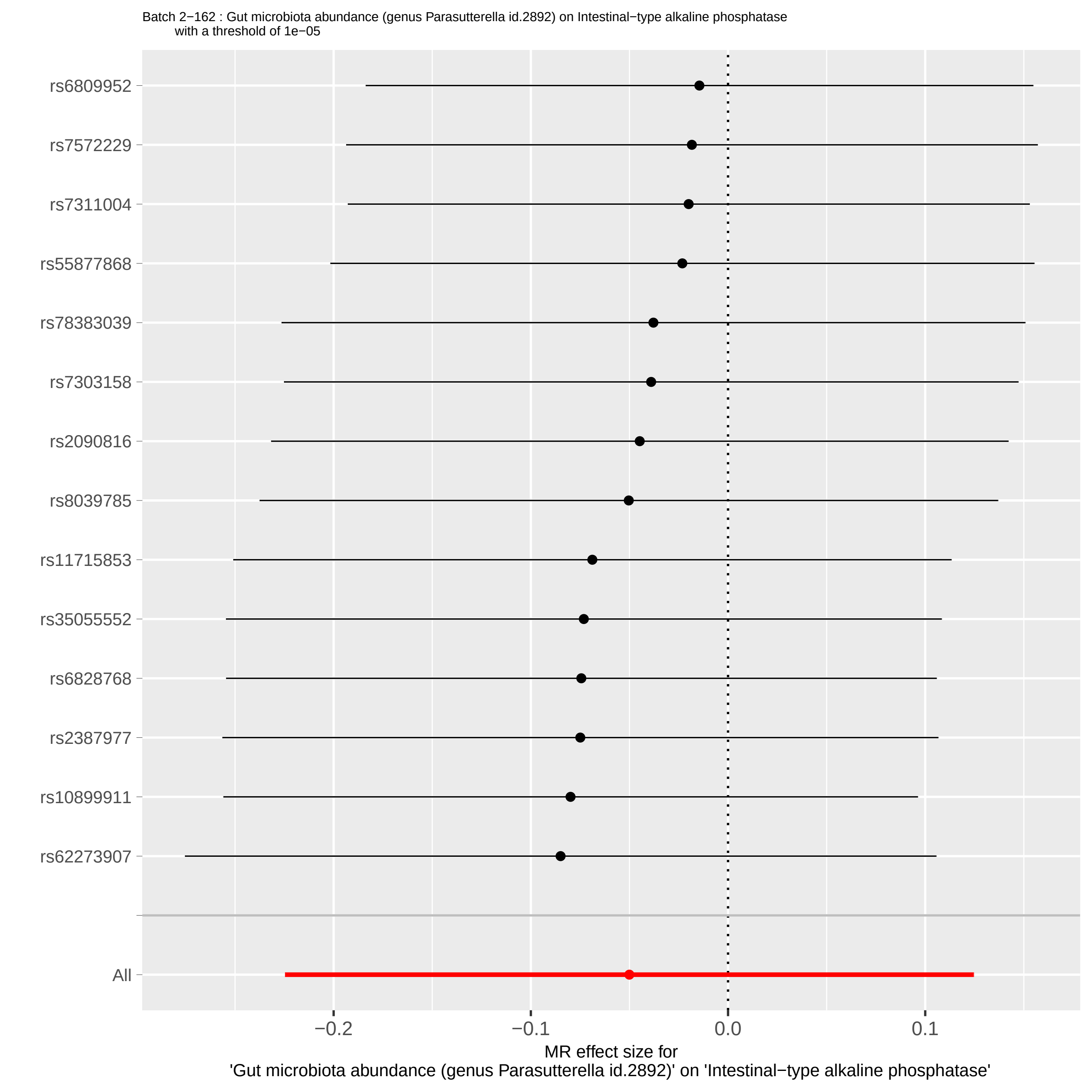
Batch 2−162 : Gut microbiota abundance (genus Parasutterella id.2892) on Intestinal−type alkaline phosphatase
with a threshold of 1e−05
rs6809952
rs7572229
rs7311004
rs55877868
rs78383039
rs7303158
rs2090816
rs8039785
rs11715853
rs35055552
rs6828768
rs2387977
rs10899911
rs62273907
All
−0.2
−0.1
0.0
0.1
MR effect size for
'Gut microbiota abundance (genus Parasutterella id.2892)' on 'Intestinal−type alkaline phosphatase'

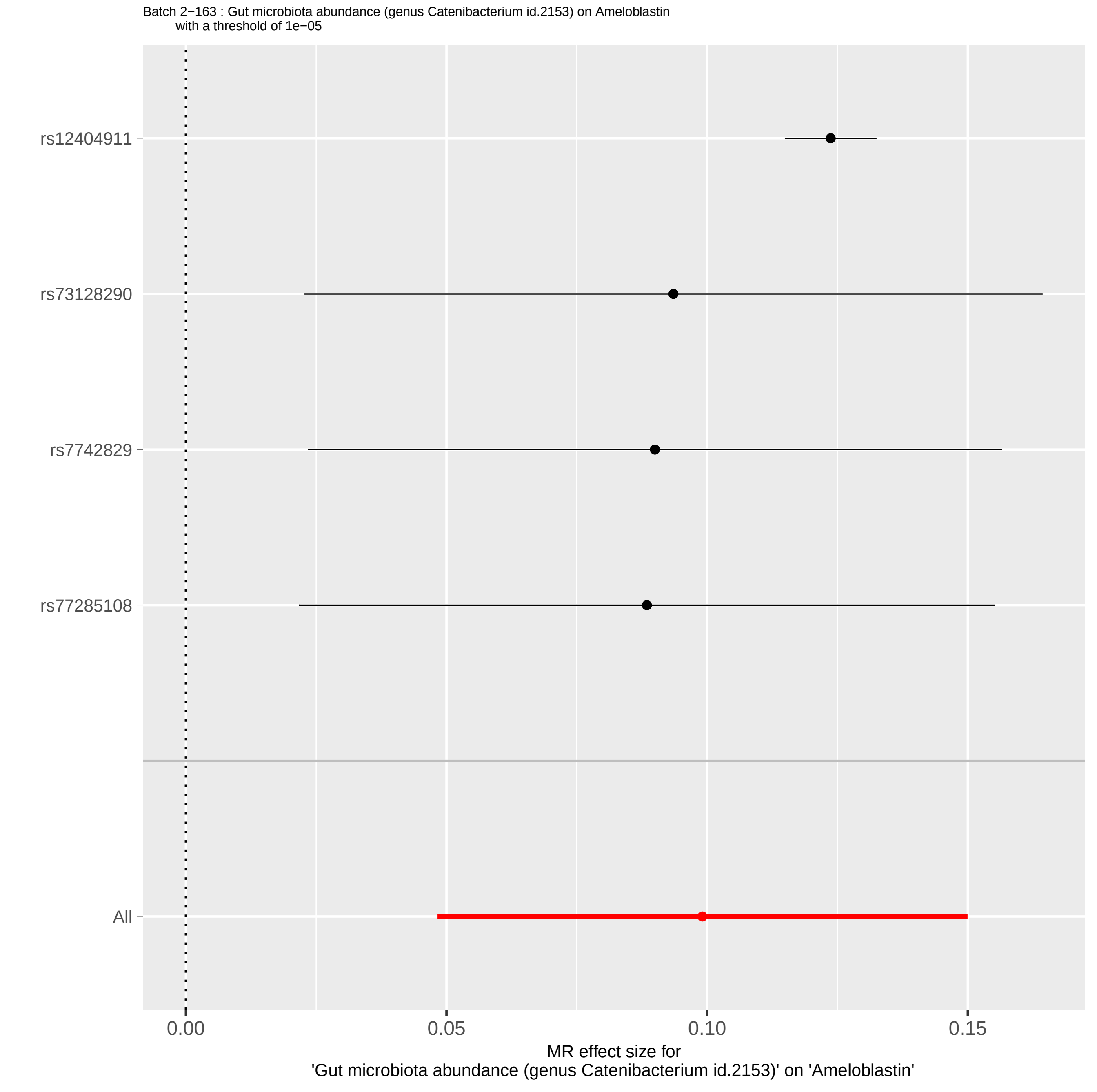
Batch 2−163 : Gut microbiota abundance (genus Catenibacterium id.2153) on Ameloblastin
with a threshold of 1e−05
rs12404911
rs73128290
rs7742829
rs77285108
All
0.00
0.05
0.10
0.15
MR effect size for
'Gut microbiota abundance (genus Catenibacterium id.2153)' on 'Ameloblastin'

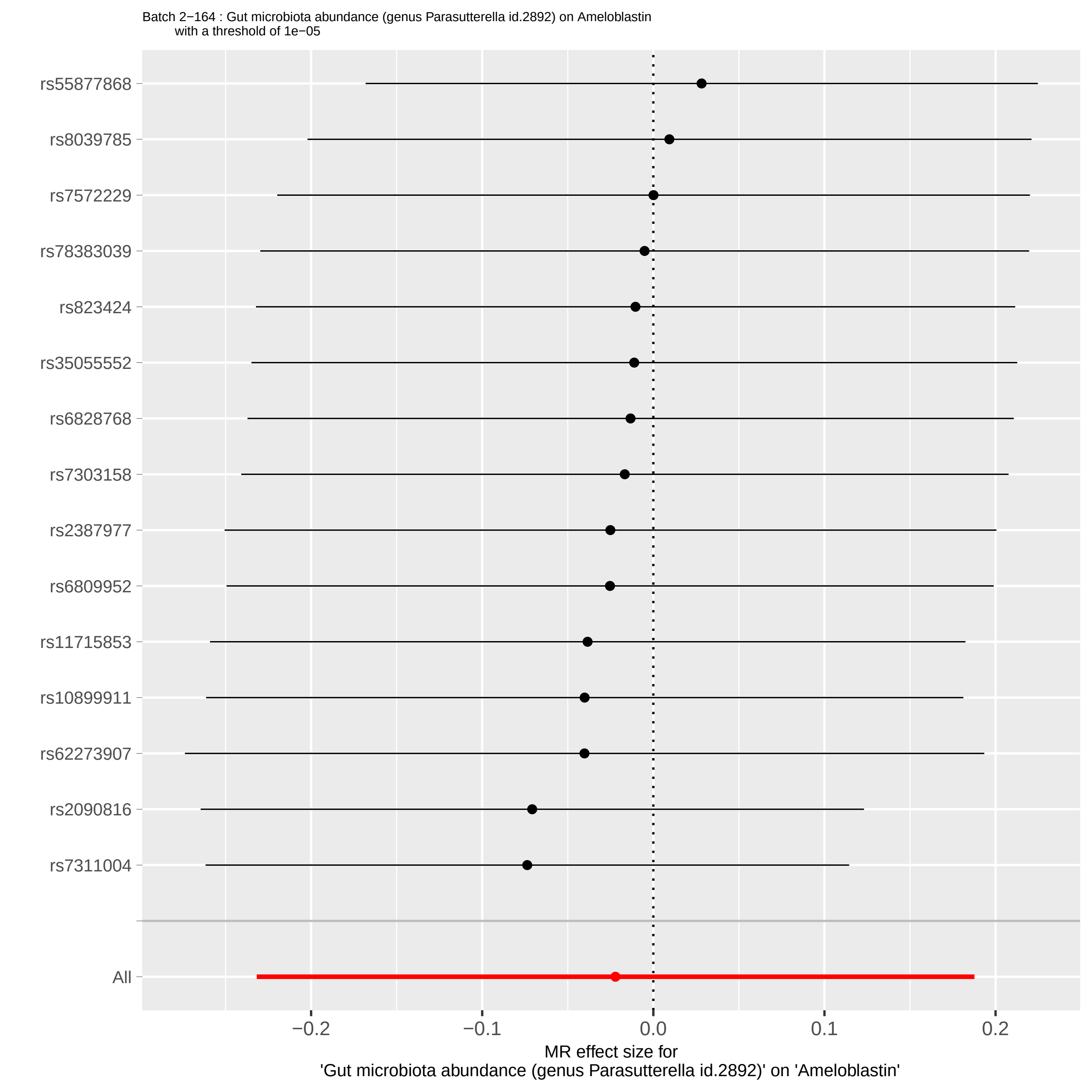
Batch 2−164 : Gut microbiota abundance (genus Parasutterella id.2892) on Ameloblastin
with a threshold of 1e−05
rs55877868
rs8039785
rs7572229
rs78383039
rs823424
rs35055552
rs6828768
rs7303158
rs2387977
rs6809952
rs11715853
rs10899911
rs62273907
rs2090816
rs7311004
All
−0.2
−0.1
0.0
0.1
0.2
MR effect size for
'Gut microbiota abundance (genus Parasutterella id.2892)' on 'Ameloblastin'

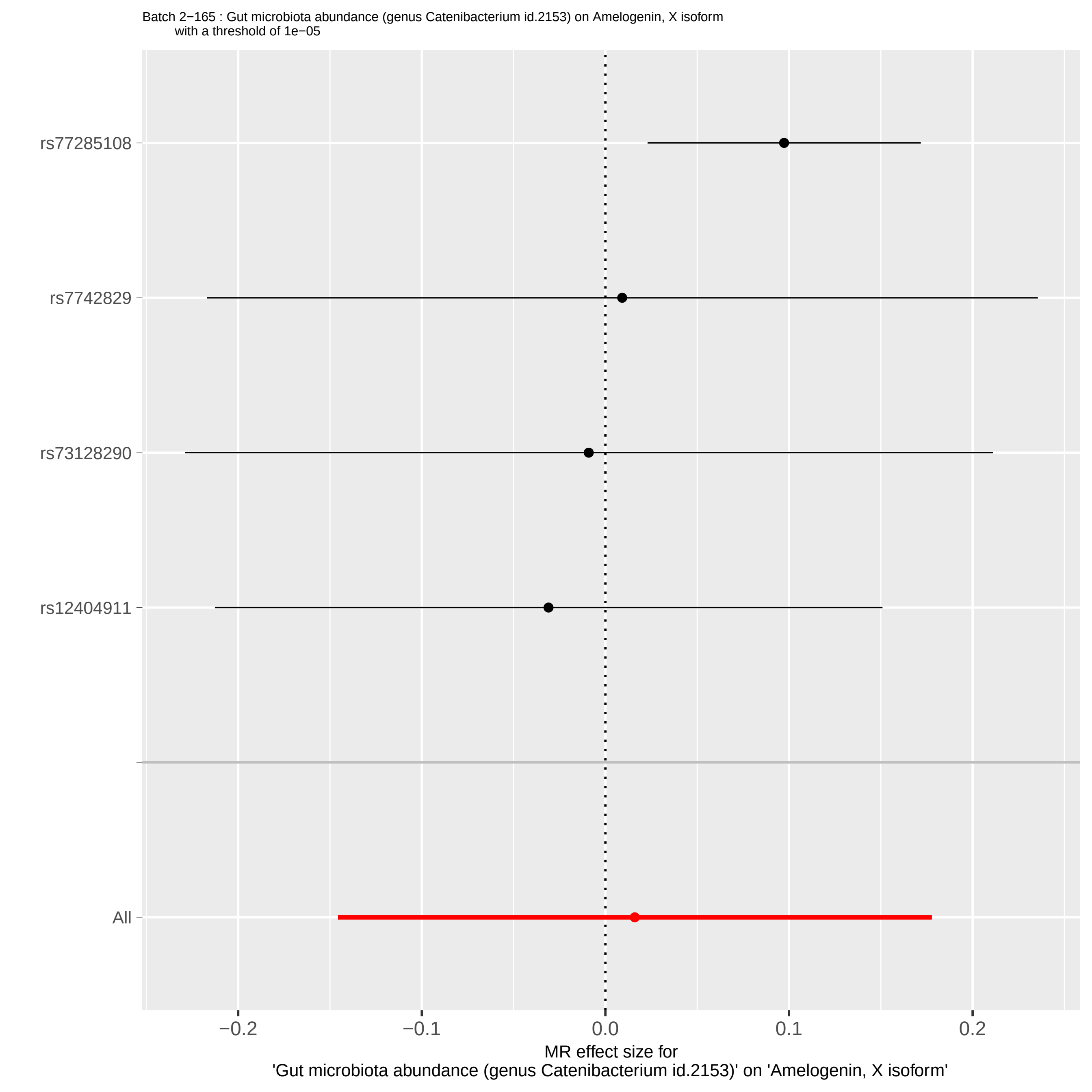

Batch 2−165 : Gut microbiota abundance (genus Catenibacterium id.2153) on Amelogenin, X isoform
with a threshold of 1e−05
rs77285108
rs7742829
rs73128290
rs12404911
All
−0.2
−0.1
0.0
0.1
0.2
MR effect size for
'Gut microbiota abundance (genus Catenibacterium id.2153)' on 'Amelogenin, X isoform'

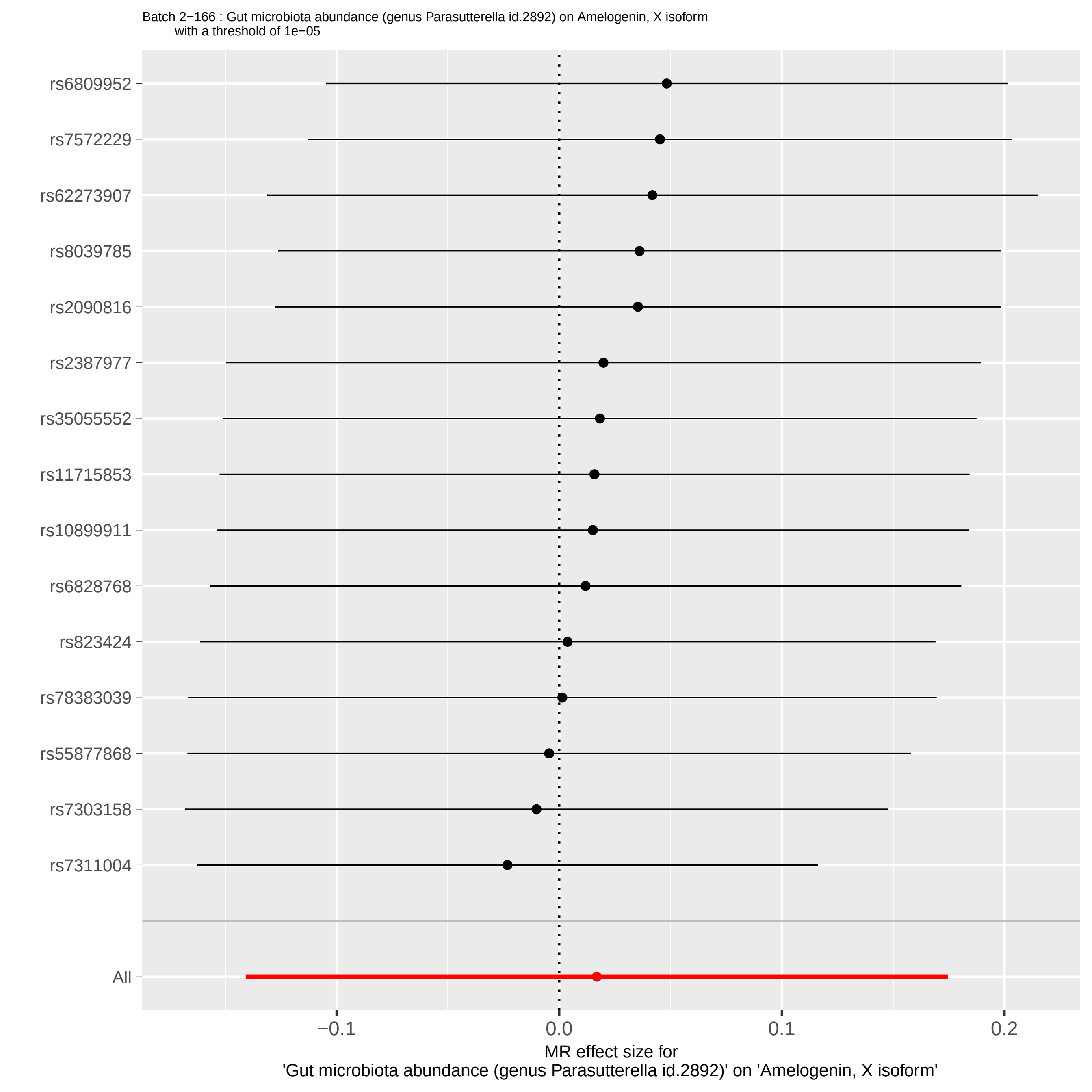

Batch 2−166 : Gut microbiota abundance (genus Parasutterella id.2892) on Amelogenin, X isoform
with a threshold of 1e−05
rs6809952
rs7572229
rs62273907
rs8039785
rs2090816
rs2387977
rs35055552
rs11715853
rs10899911
rs6828768
rs823424
rs78383039
rs55877868
rs7303158
rs7311004
All
−0.1
0.0
0.1
0.2
MR effect size for
'Gut microbiota abundance (genus Parasutterella id.2892)' on 'Amelogenin, X isoform'

Batch 2−167 : Gut microbiota abundance (genus Catenibacterium id.2153) on Muellerian−inhibiting factor
with a threshold of 1e−05

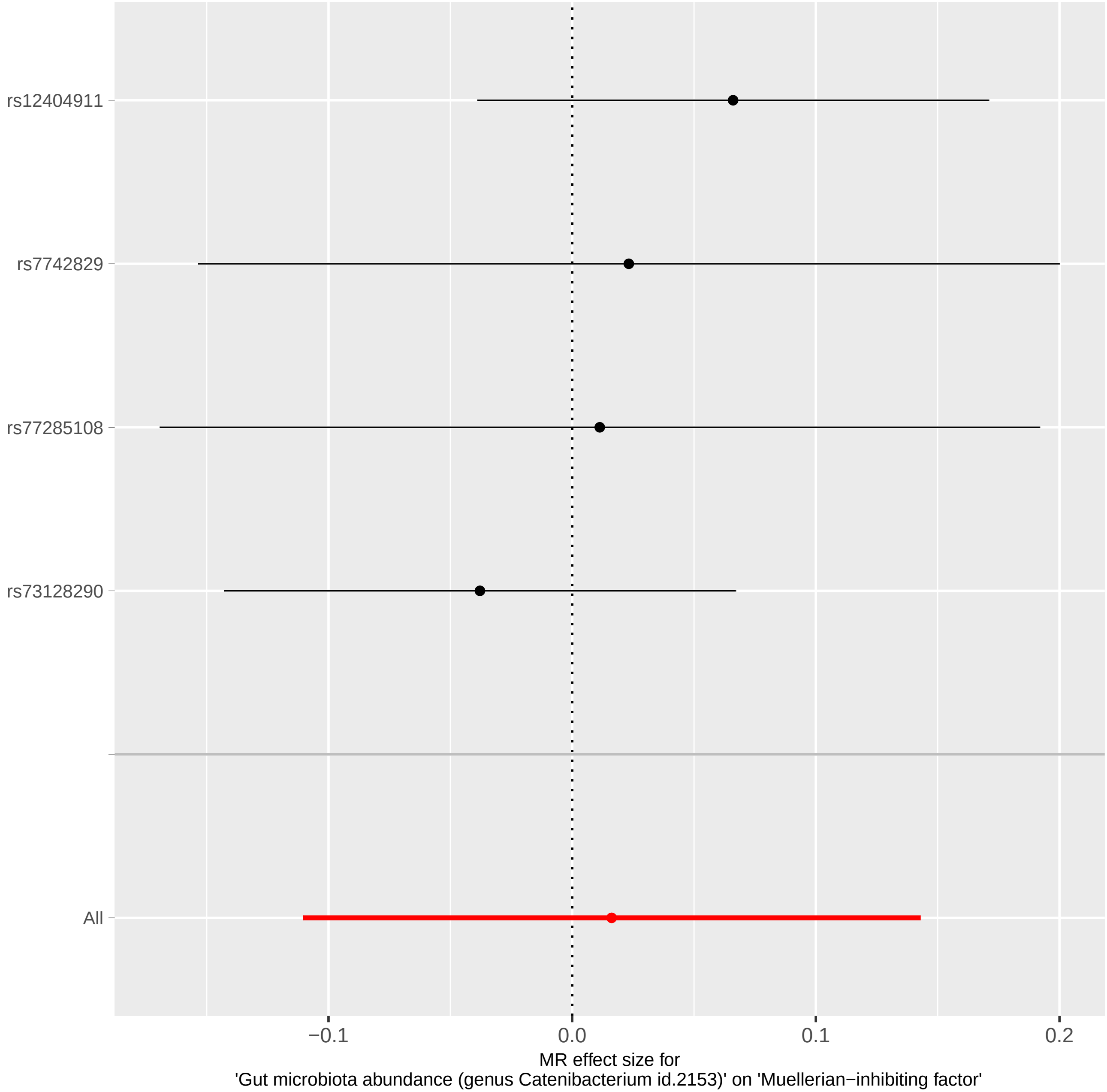

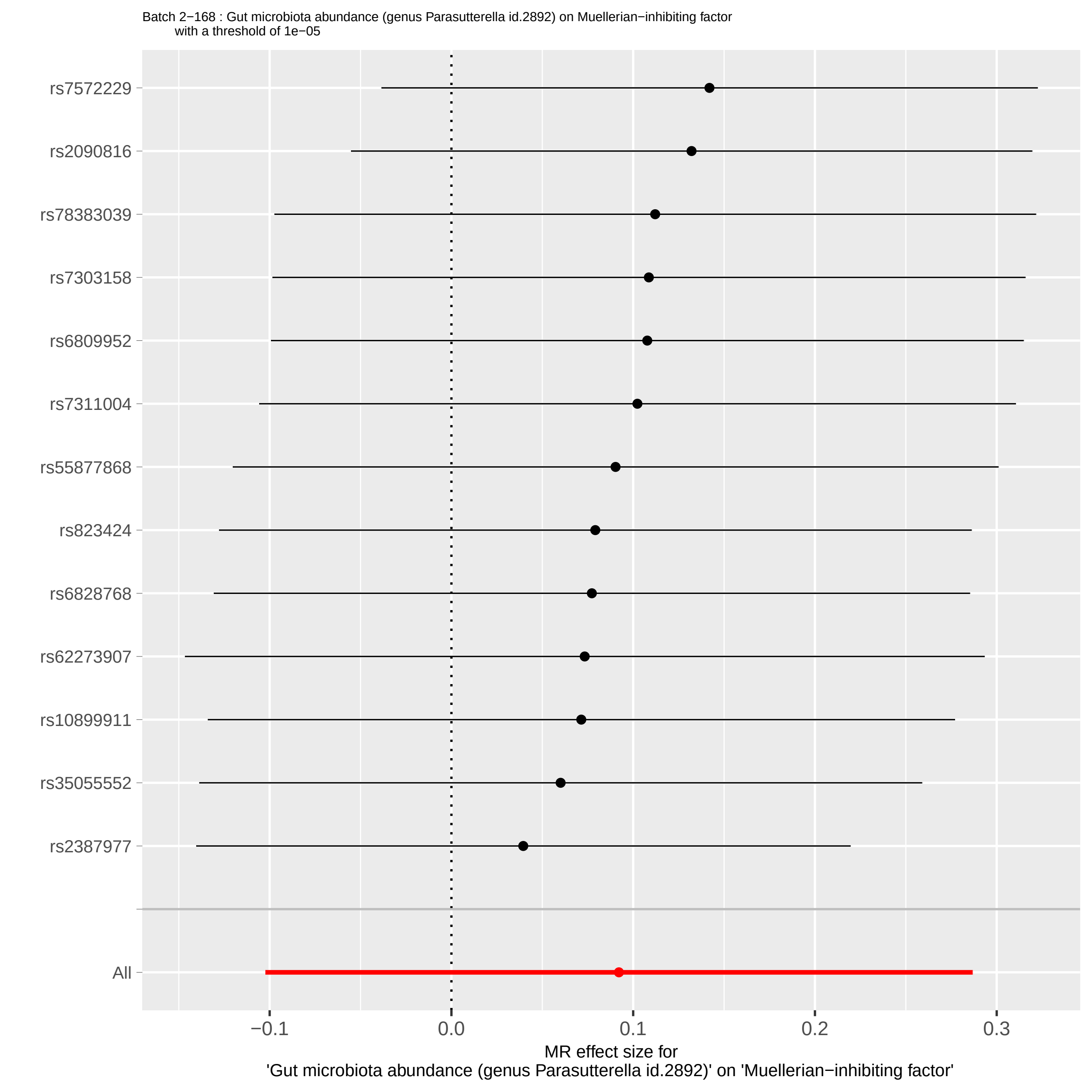

Batch 2−168 : Gut microbiota abundance (genus Parasutterella id.2892) on Muellerian−inhibiting factor
with a threshold of 1e−05
rs7572229
rs2090816
rs78383039
rs7303158
rs6809952
rs7311004
rs55877868
rs823424
rs6828768
rs62273907
rs10899911
rs35055552
rs2387977
All
−0.1
0.0
0.1
0.2
0.3
MR effect size for
'Gut microbiota abundance (genus Parasutterella id.2892)' on 'Muellerian−inhibiting factor'

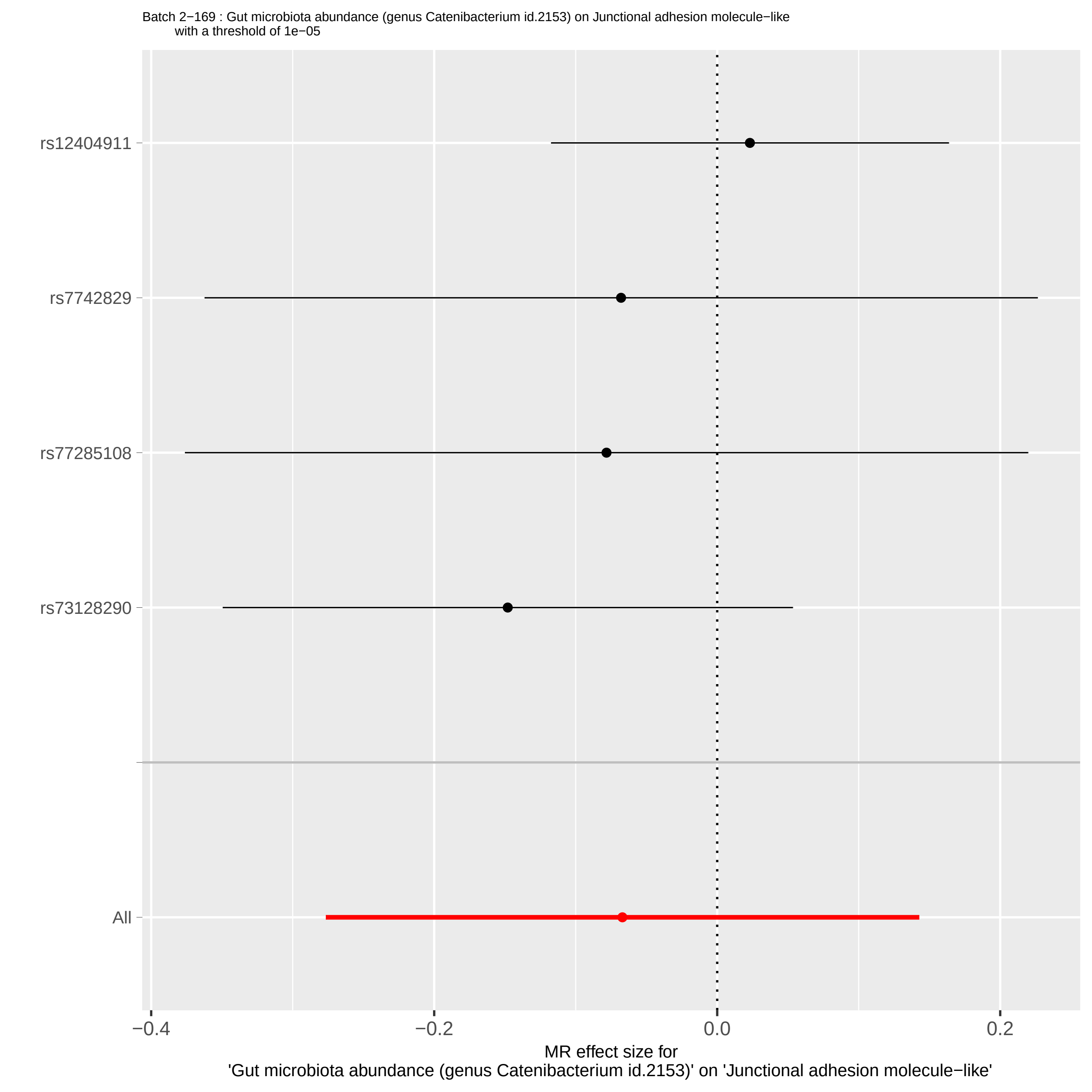
Batch 2−169 : Gut microbiota abundance (genus Catenibacterium id.2153) on Junctional adhesion molecule−like
with a threshold of 1e−05
rs12404911
rs7742829
rs77285108
rs73128290
All
−0.4
−0.2
0.0
0.2
MR effect size for
'Gut microbiota abundance (genus Catenibacterium id.2153)' on 'Junctional adhesion molecule−like'

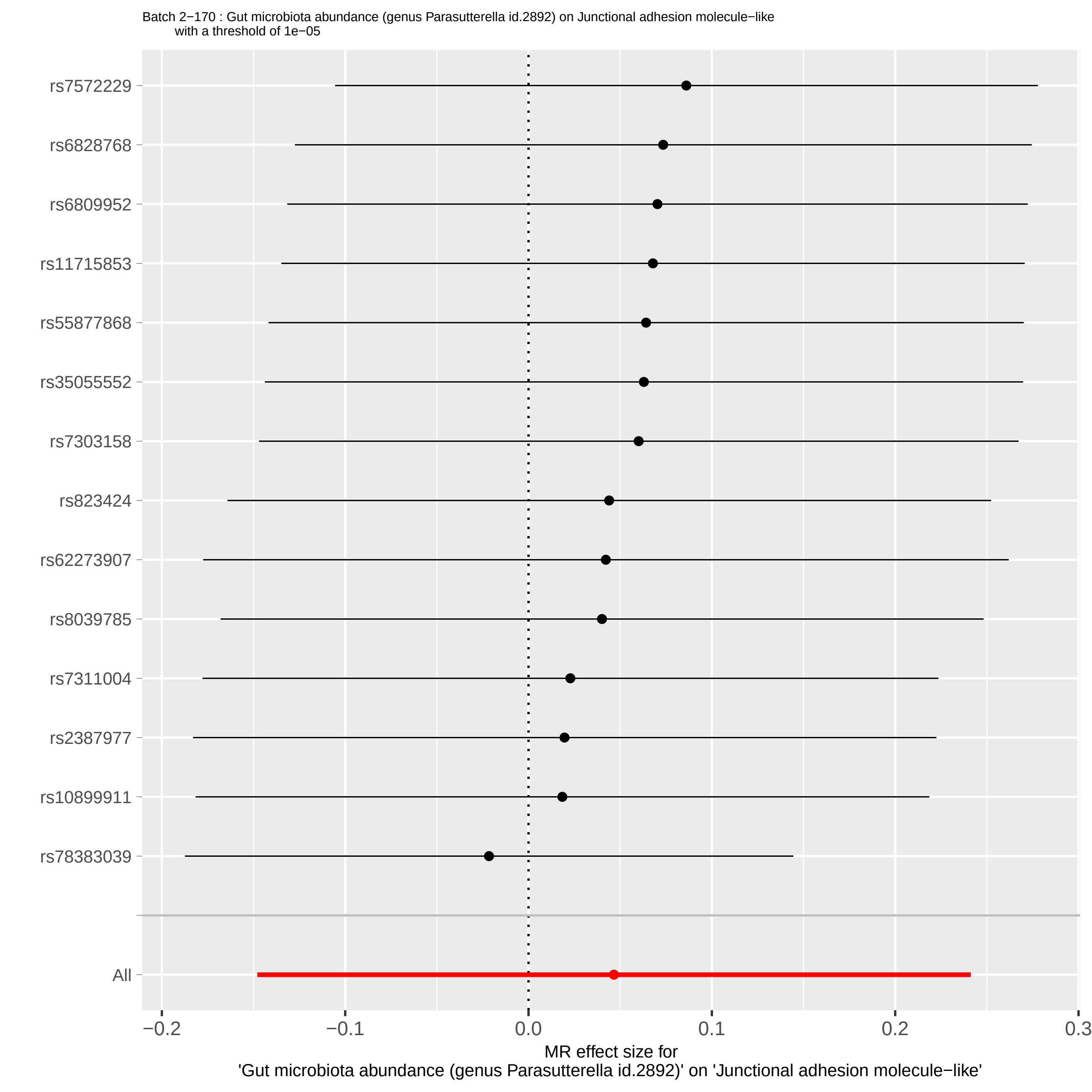

Batch 2−170 : Gut microbiota abundance (genus Parasutterella id.2892) on Junctional adhesion molecule−like
with a threshold of 1e−05
rs7572229
rs6828768
rs6809952
rs11715853
rs55877868
rs35055552
rs7303158
rs823424
rs62273907
rs8039785
rs7311004
rs2387977
rs10899911
rs78383039
All
−0.2
−0.1
0.0
0.1
0.2
0.3
MR effect size for
'Gut microbiota abundance (genus Parasutterella id.2892)' on 'Junctional adhesion molecule−like'

Batch 2−171 : Gut microbiota abundance (genus Catenibacterium id.2153) on Junctional adhesion molecule−like
with a threshold of 1e−05

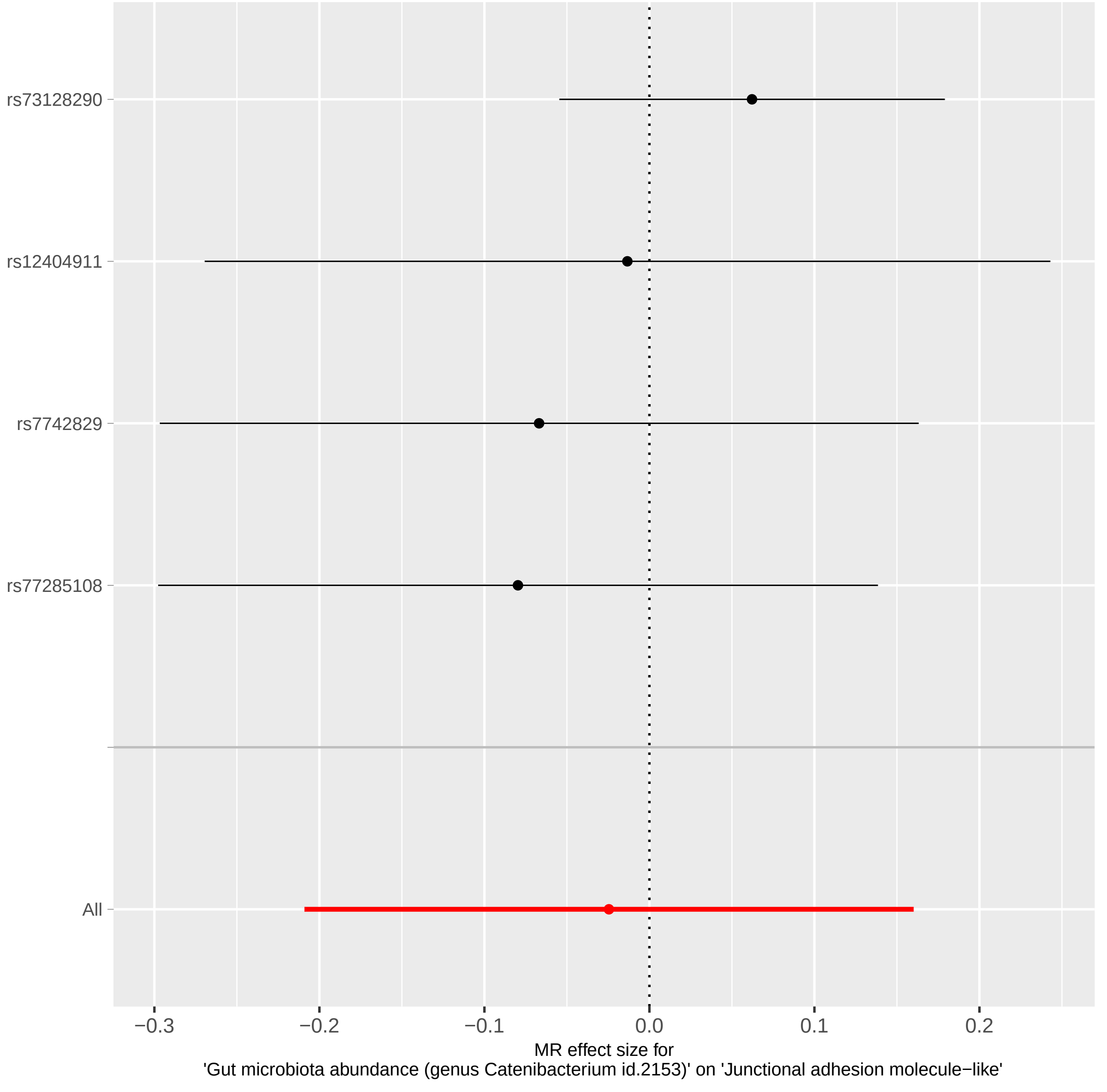

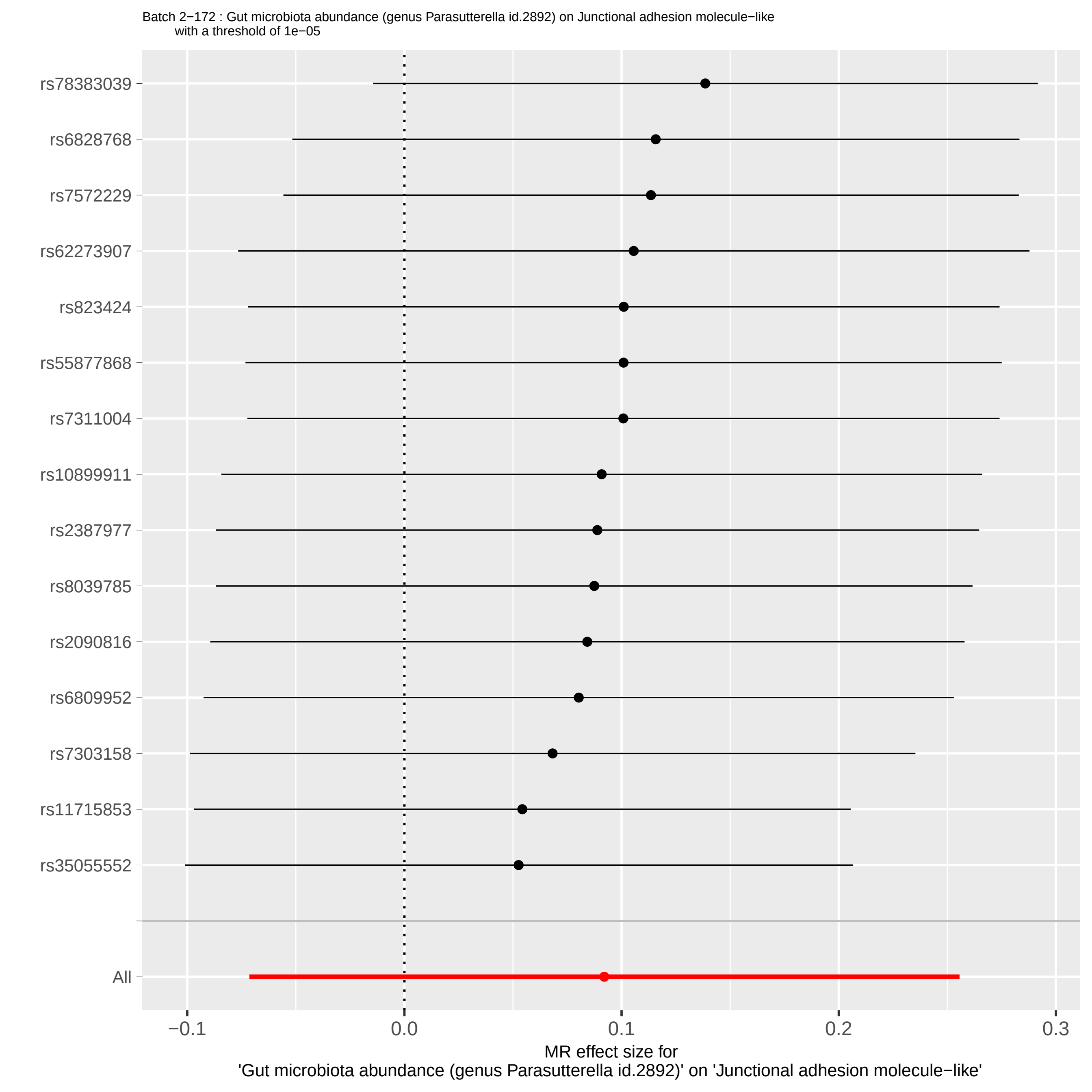
Batch 2−172 : Gut microbiota abundance (genus Parasutterella id.2892) on Junctional adhesion molecule−like
with a threshold of 1e−05
rs78383039
rs6828768
rs7572229
rs62273907
rs823424
rs55877868
rs7311004
rs10899911
rs2387977
rs8039785
rs2090816
rs6809952
rs7303158
rs11715853
rs35055552
All
−0.1
0.0
0.1
0.2
0.3
MR effect size for
'Gut microbiota abundance (genus Parasutterella id.2892)' on 'Junctional adhesion molecule−like'

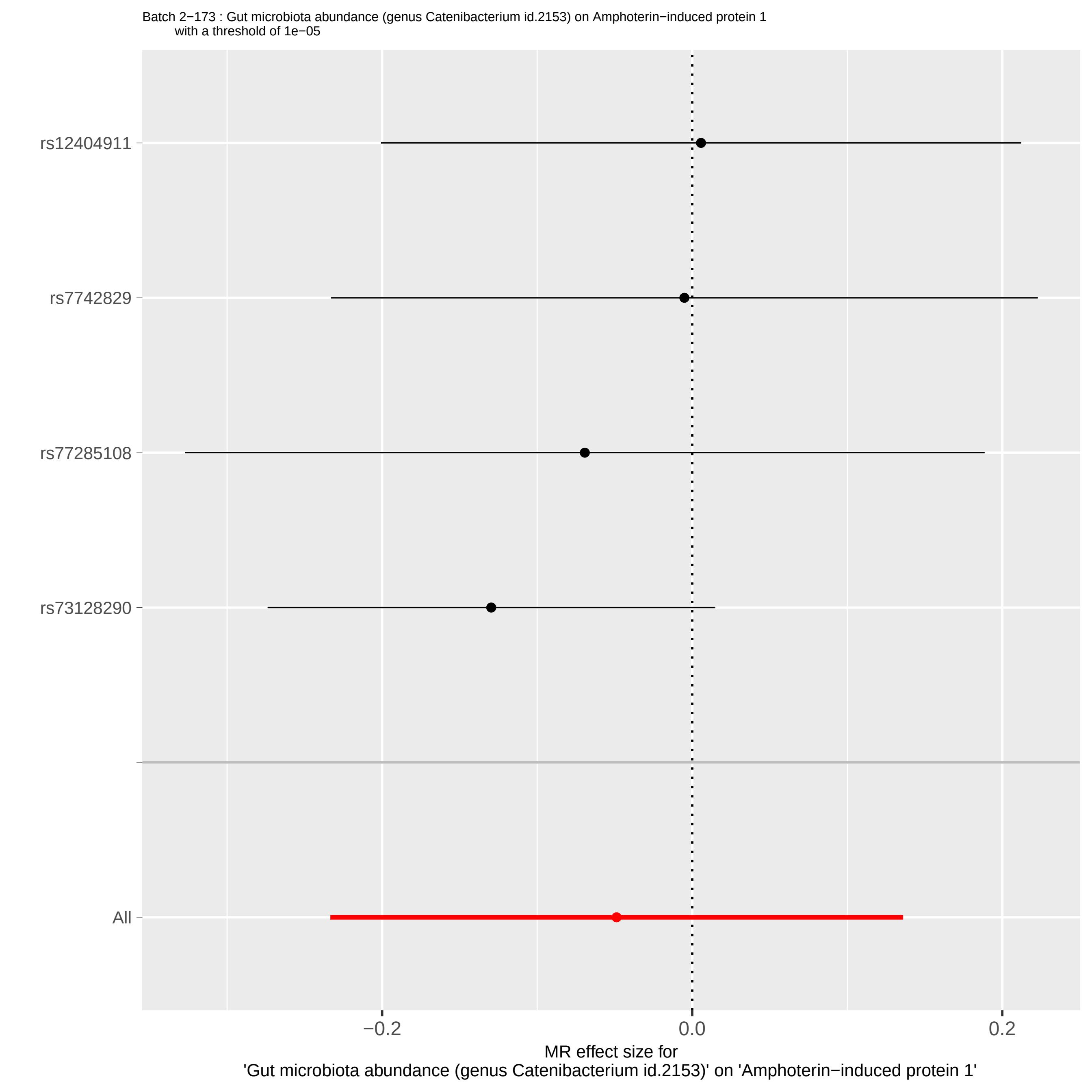

Batch 2−173 : Gut microbiota abundance (genus Catenibacterium id.2153) on Amphoterin−induced protein 1
with a threshold of 1e−05
rs12404911
rs7742829
rs77285108
rs73128290
All
−0.2
0.0
0.2
MR effect size for
'Gut microbiota abundance (genus Catenibacterium id.2153)' on 'Amphoterin−induced protein 1'

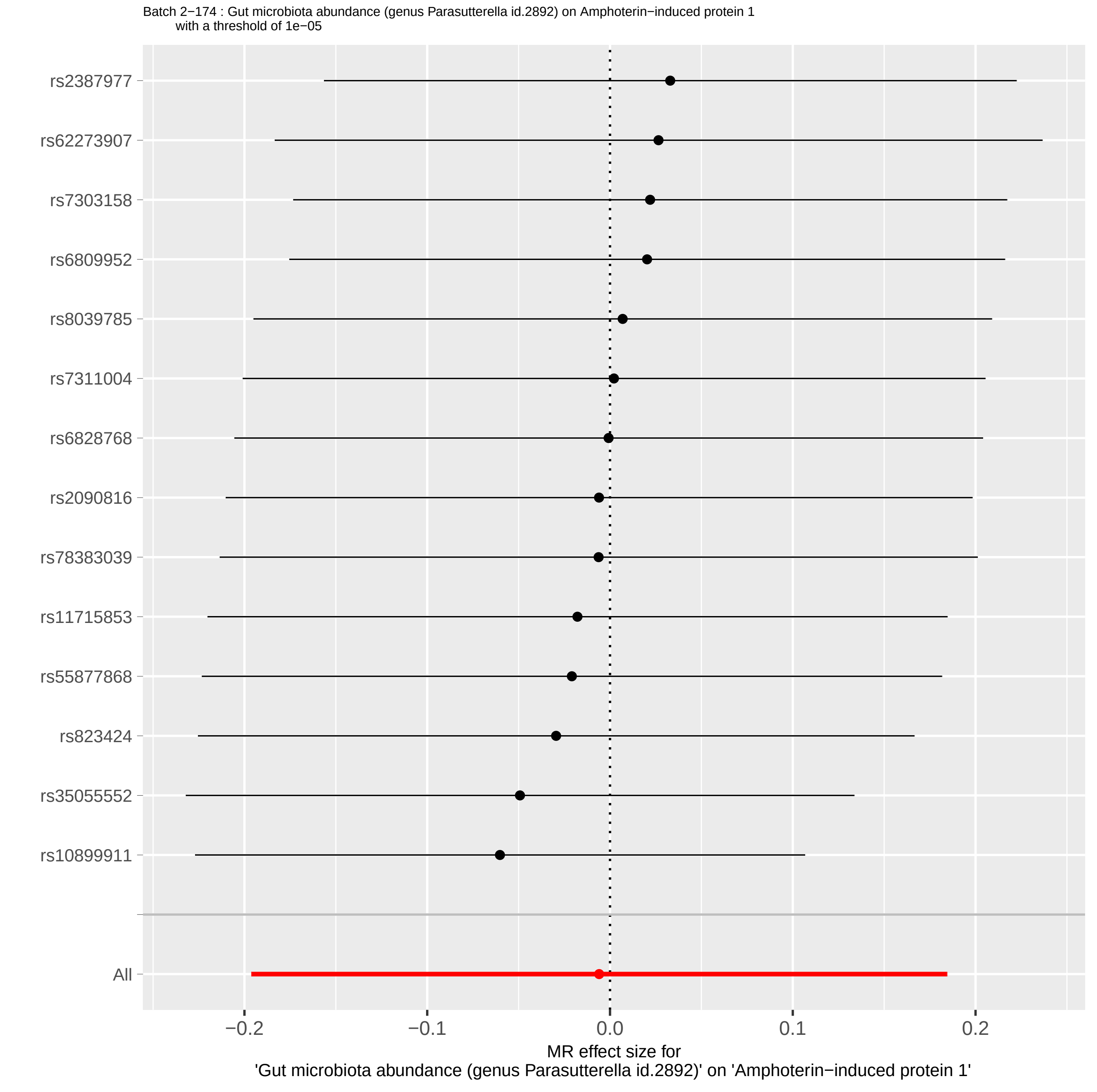

Batch 2−174 : Gut microbiota abundance (genus Parasutterella id.2892) on Amphoterin−induced protein 1
with a threshold of 1e−05
rs2387977
rs62273907
rs7303158
rs6809952
rs8039785
rs7311004
rs6828768
rs2090816
rs78383039
rs11715853
rs55877868
rs823424
rs35055552
rs10899911
All
−0.2
−0.1
0.0
0.1
0.2
MR effect size for
'Gut microbiota abundance (genus Parasutterella id.2892)' on 'Amphoterin−induced protein 1'

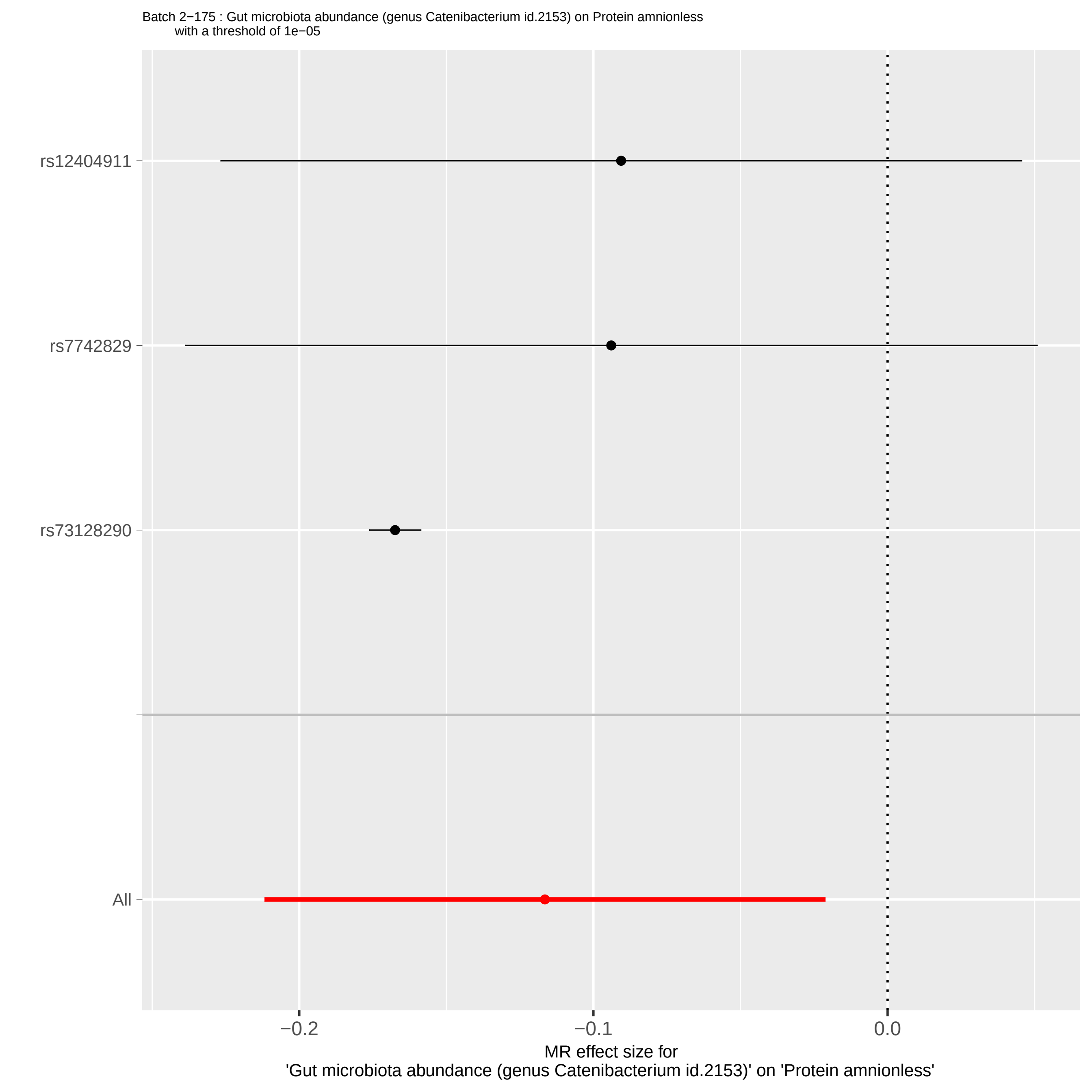

Batch 2−175 : Gut microbiota abundance (genus Catenibacterium id.2153) on Protein amnionless
with a threshold of 1e−05
rs12404911
rs7742829
rs73128290
All
−0.2
−0.1
0.0
MR effect size for
'Gut microbiota abundance (genus Catenibacterium id.2153)' on 'Protein amnionless'

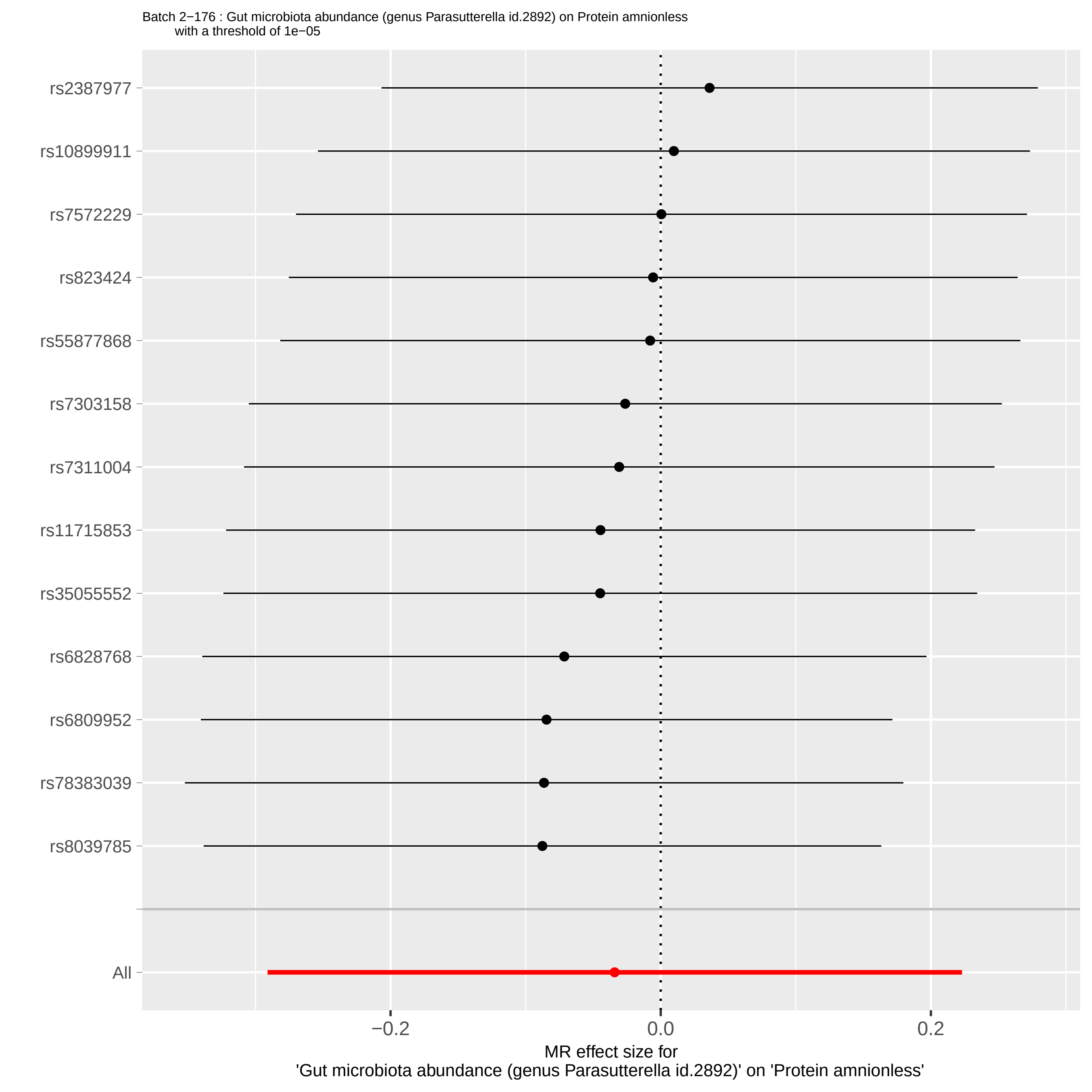
Batch 2−176 : Gut microbiota abundance (genus Parasutterella id.2892) on Protein amnionless
with a threshold of 1e−05
rs2387977
rs10899911
rs7572229
rs823424
rs55877868
rs7303158
rs7311004
rs11715853
rs35055552
rs6828768
rs6809952
rs78383039
rs8039785
All
−0.2
0.0
0.2
MR effect size for
'Gut microbiota abundance (genus Parasutterella id.2892)' on 'Protein amnionless'

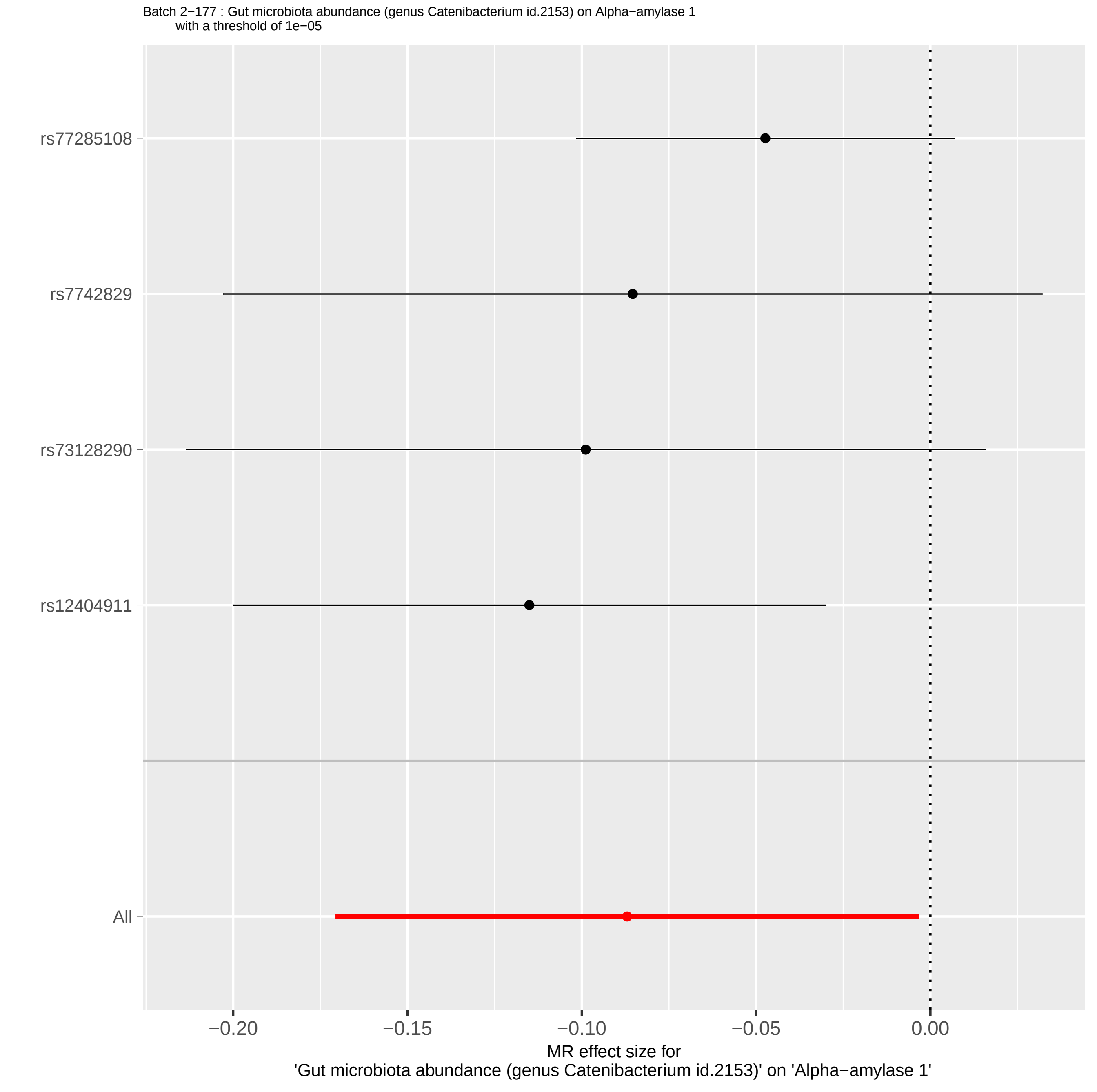
Batch 2−177 : Gut microbiota abundance (genus Catenibacterium id.2153) on Alpha−amylase 1
with a threshold of 1e−05
rs77285108
rs7742829
rs73128290
rs12404911
All
−0.20
−0.15
−0.10
−0.05
0.00
MR effect size for
'Gut microbiota abundance (genus Catenibacterium id.2153)' on 'Alpha−amylase 1'

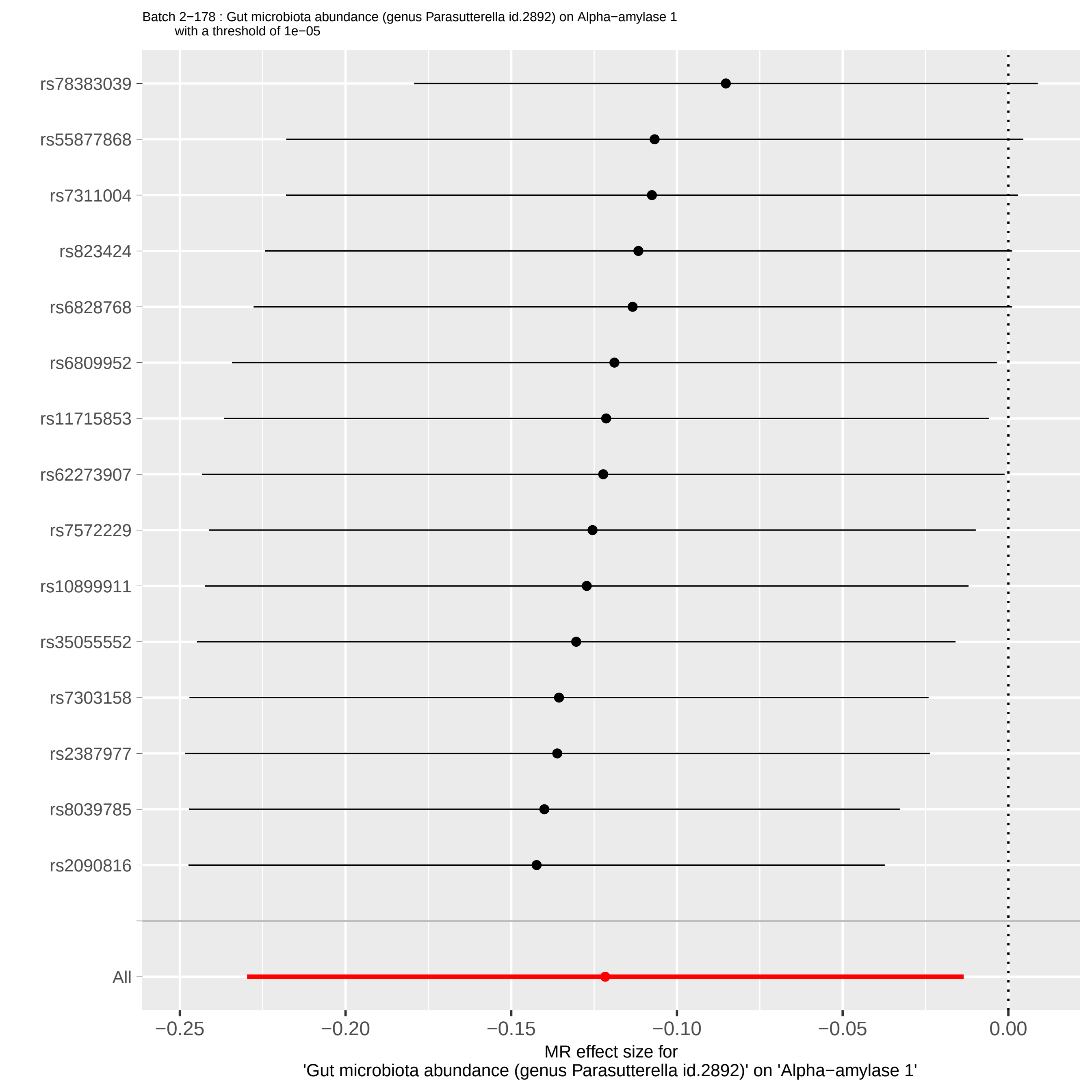

Batch 2−178 : Gut microbiota abundance (genus Parasutterella id.2892) on Alpha−amylase 1
with a threshold of 1e−05
rs78383039
rs55877868
rs7311004
rs823424
rs6828768
rs6809952
rs11715853
rs62273907
rs7572229
rs10899911
rs35055552
rs7303158
rs2387977
rs8039785
rs2090816
All
−0.25
−0.20
−0.15
−0.10
−0.05
0.00
MR effect size for
'Gut microbiota abundance (genus Parasutterella id.2892)' on 'Alpha−amylase 1'

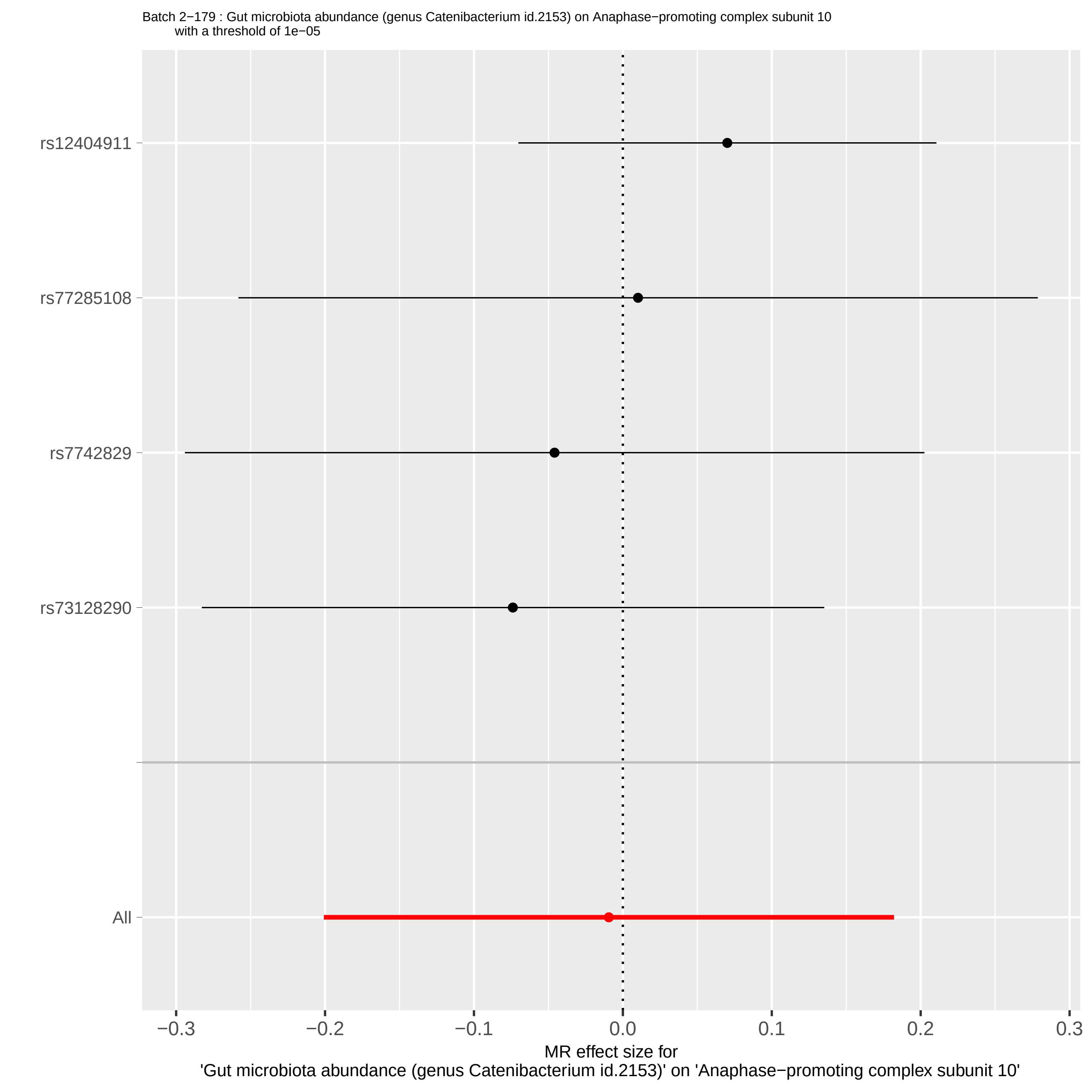

Batch 2−179 : Gut microbiota abundance (genus Catenibacterium id.2153) on Anaphase−promoting complex subunit 10
with a threshold of 1e−05
rs12404911
rs77285108
rs7742829
rs73128290
All
−0.3
−0.2
−0.1
0.0
0.1
0.2
0.3
MR effect size for
'Gut microbiota abundance (genus Catenibacterium id.2153)' on 'Anaphase−promoting complex subunit 10'

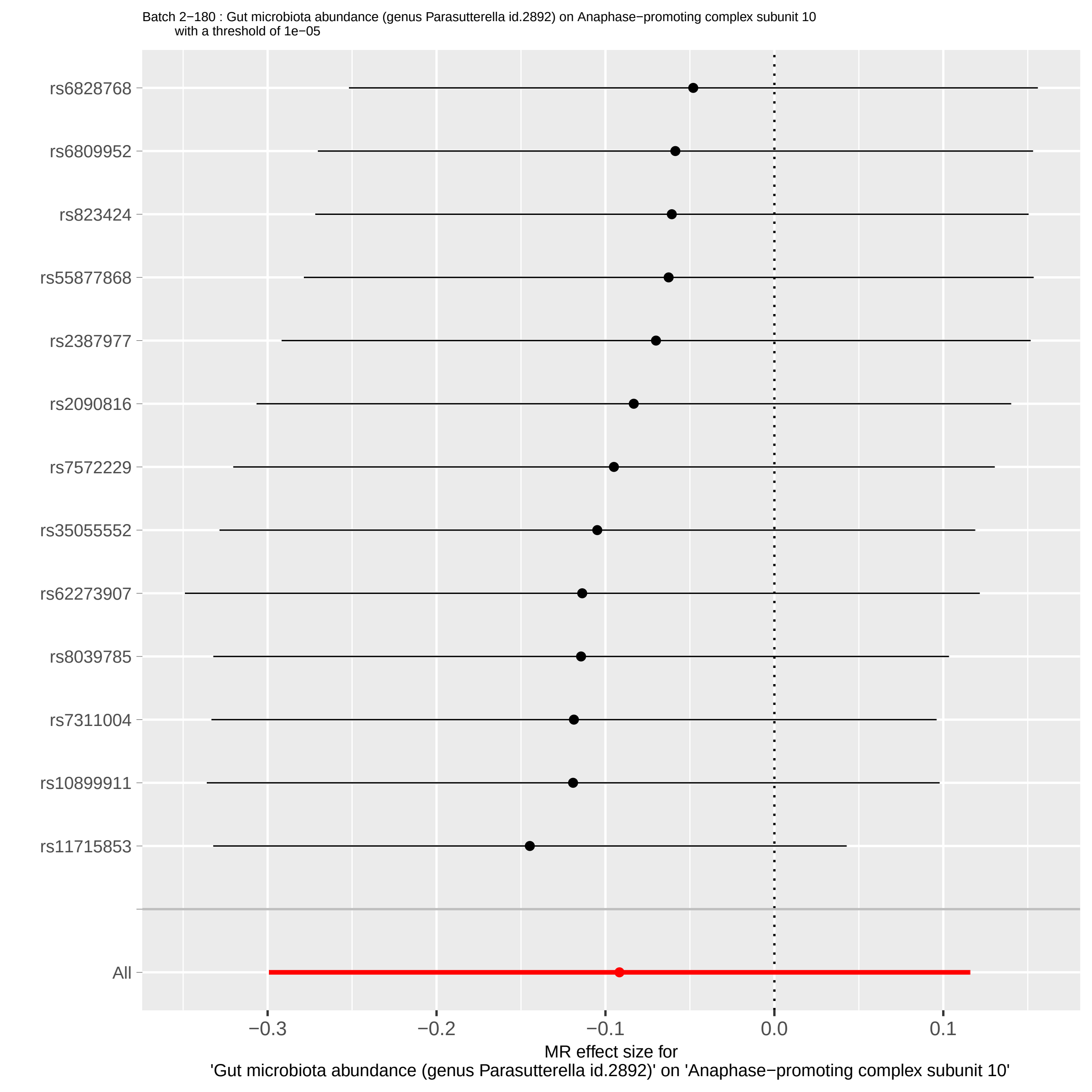
Batch 2−180 : Gut microbiota abundance (genus Parasutterella id.2892) on Anaphase−promoting complex subunit 10
with a threshold of 1e−05
rs6828768
rs6809952
rs823424
rs55877868
rs2387977
rs2090816
rs7572229
rs35055552
rs62273907
rs8039785
rs7311004
rs10899911
rs11715853
All
−0.3
−0.2
−0.1
0.0
0.1
MR effect size for
'Gut microbiota abundance (genus Parasutterella id.2892)' on 'Anaphase−promoting complex subunit 10'

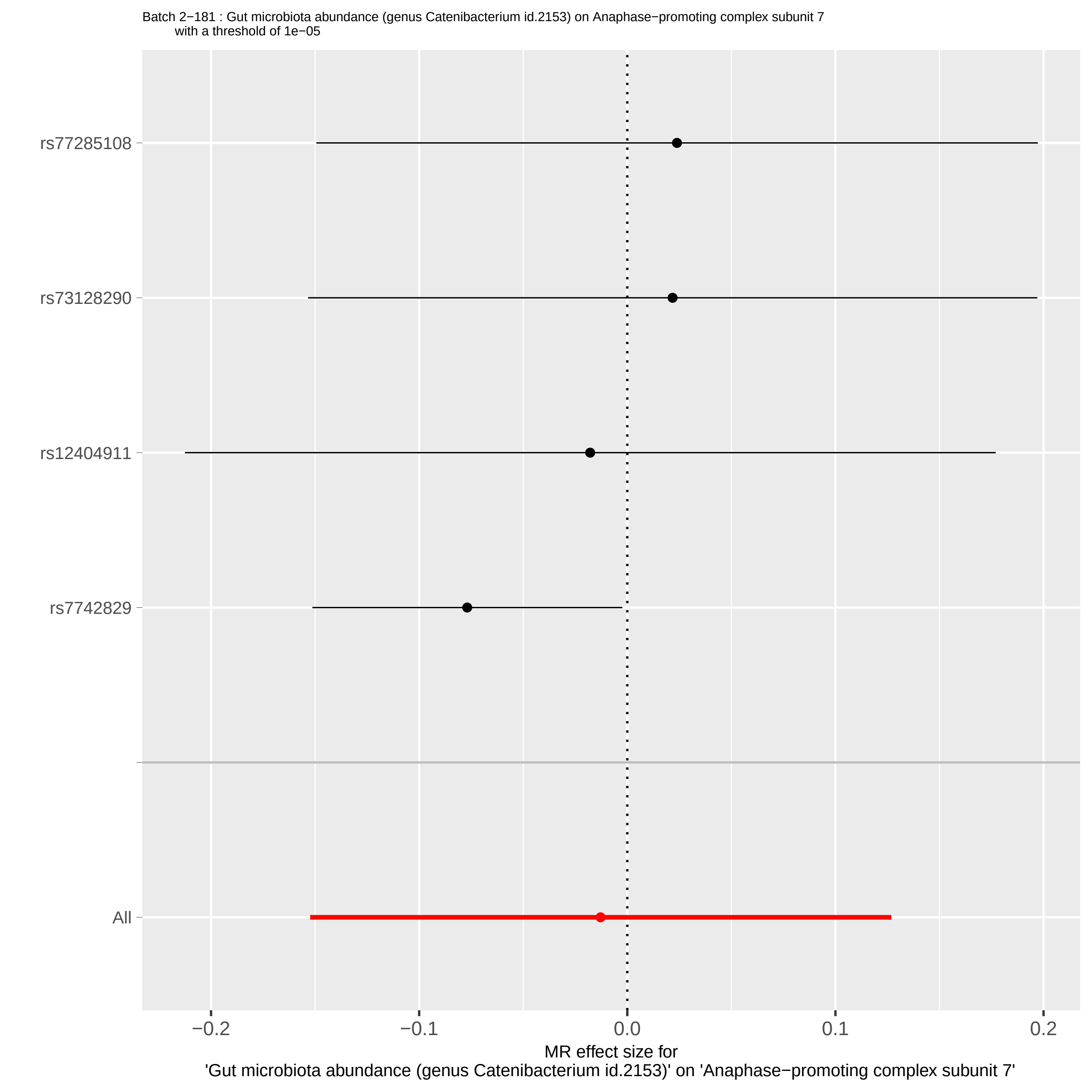
Batch 2−181 : Gut microbiota abundance (genus Catenibacterium id.2153) on Anaphase−promoting complex subunit 7
with a threshold of 1e−05
rs77285108
rs73128290
rs12404911
rs7742829
All
−0.2
−0.1
0.0
0.1
0.2
MR effect size for
'Gut microbiota abundance (genus Catenibacterium id.2153)' on 'Anaphase−promoting complex subunit 7'

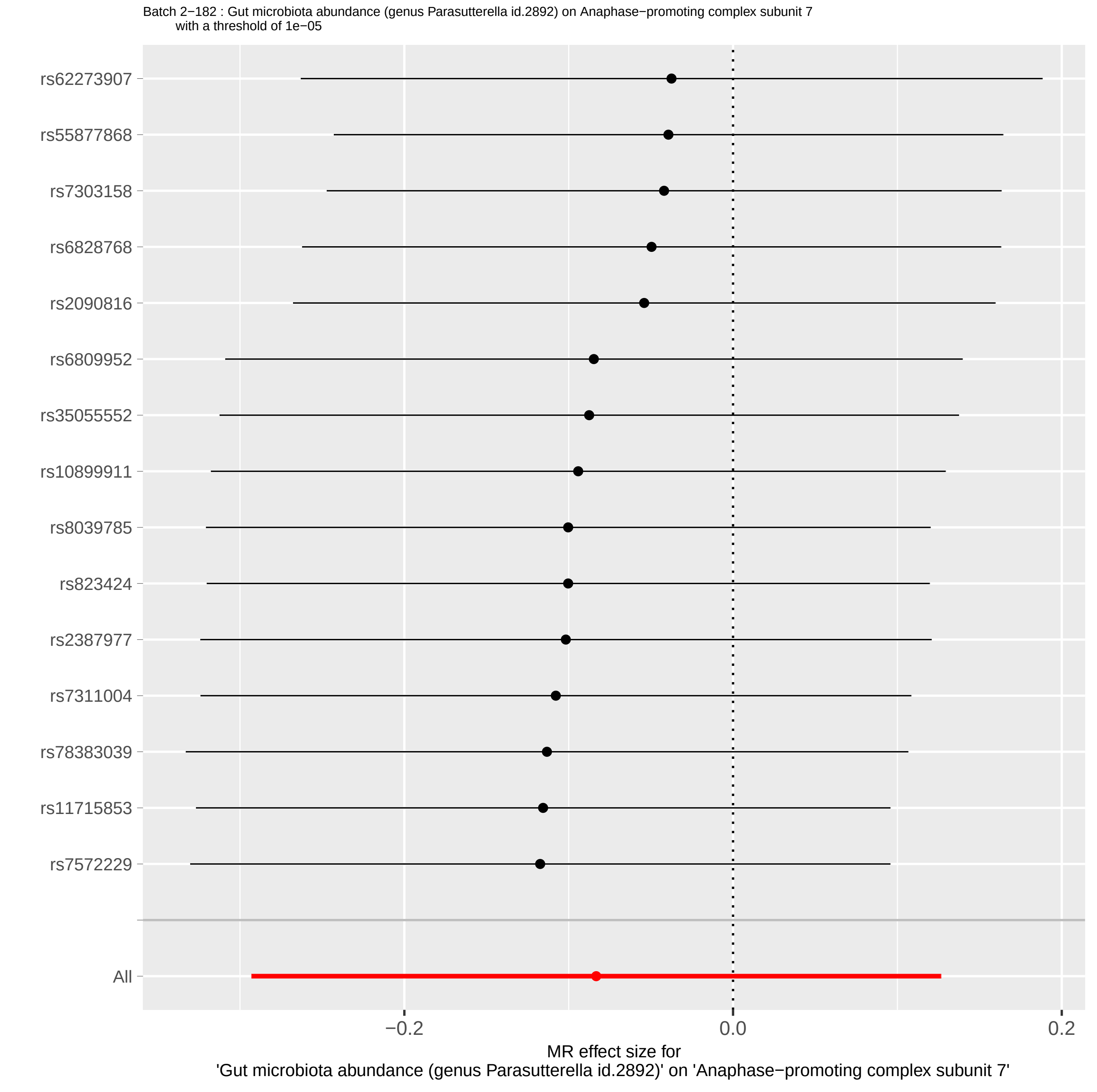

Batch 2−182 : Gut microbiota abundance (genus Parasutterella id.2892) on Anaphase−promoting complex subunit 7
with a threshold of 1e−05
rs62273907
rs55877868
rs7303158
rs6828768
rs2090816
rs6809952
rs35055552
rs10899911
rs8039785
rs823424
rs2387977
rs7311004
rs78383039
rs11715853
rs7572229
All
−0.2
0.0
0.2
MR effect size for
'Gut microbiota abundance (genus Parasutterella id.2892)' on 'Anaphase−promoting complex subunit 7'

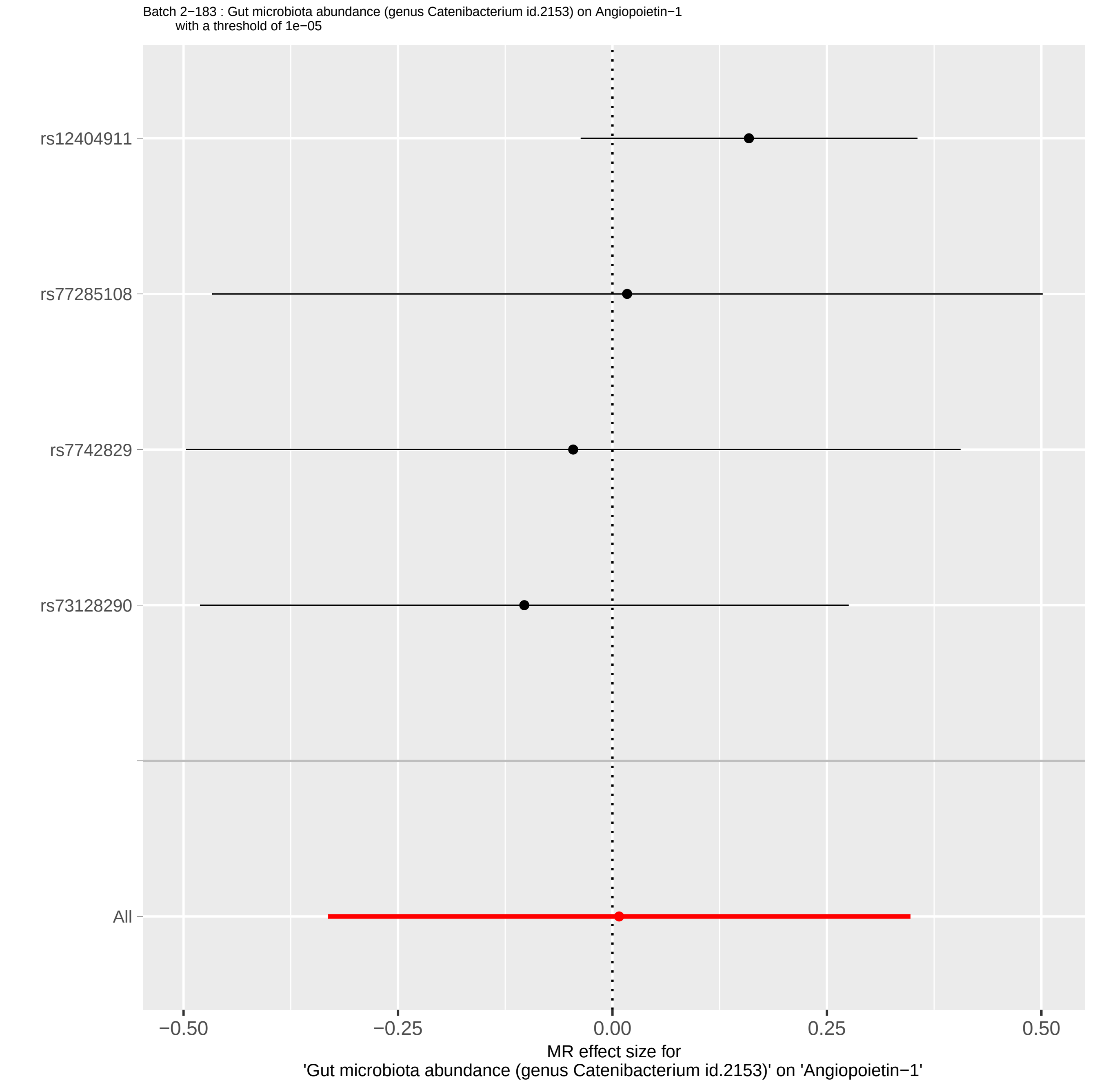

Batch 2−183 : Gut microbiota abundance (genus Catenibacterium id.2153) on Angiopoietin−1
with a threshold of 1e−05
rs12404911
rs77285108
rs7742829
rs73128290
All
−0.50
−0.25
0.00
0.25
0.50
MR effect size for
'Gut microbiota abundance (genus Catenibacterium id.2153)' on 'Angiopoietin−1'

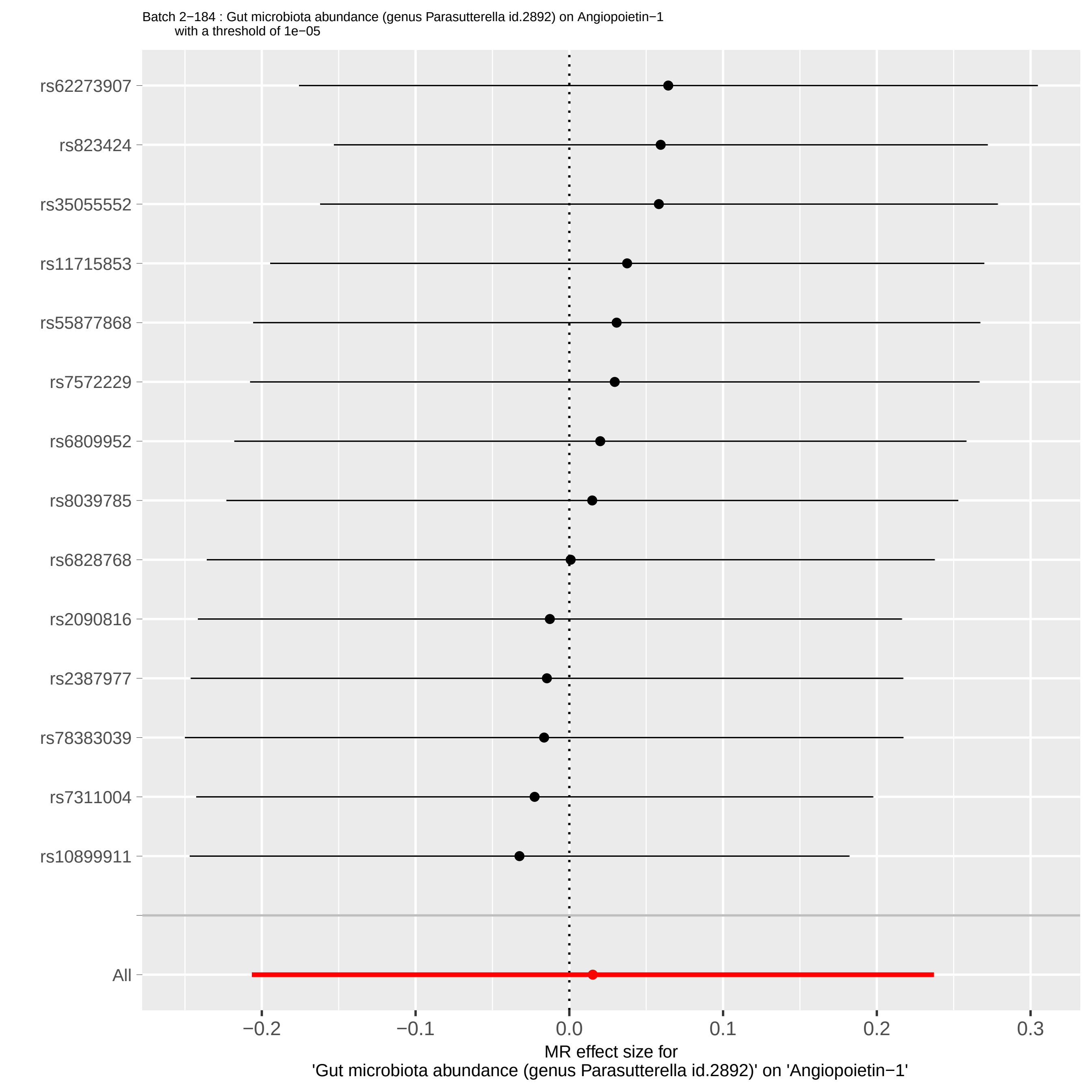

Batch 2−184 : Gut microbiota abundance (genus Parasutterella id.2892) on Angiopoietin−1
with a threshold of 1e−05
rs62273907
rs823424
rs35055552
rs11715853
rs55877868
rs7572229
rs6809952
rs8039785
rs6828768
rs2090816
rs2387977
rs78383039
rs7311004
rs10899911
All
−0.2
−0.1
0.0
0.1
0.2
0.3
MR effect size for
'Gut microbiota abundance (genus Parasutterella id.2892)' on 'Angiopoietin−1'

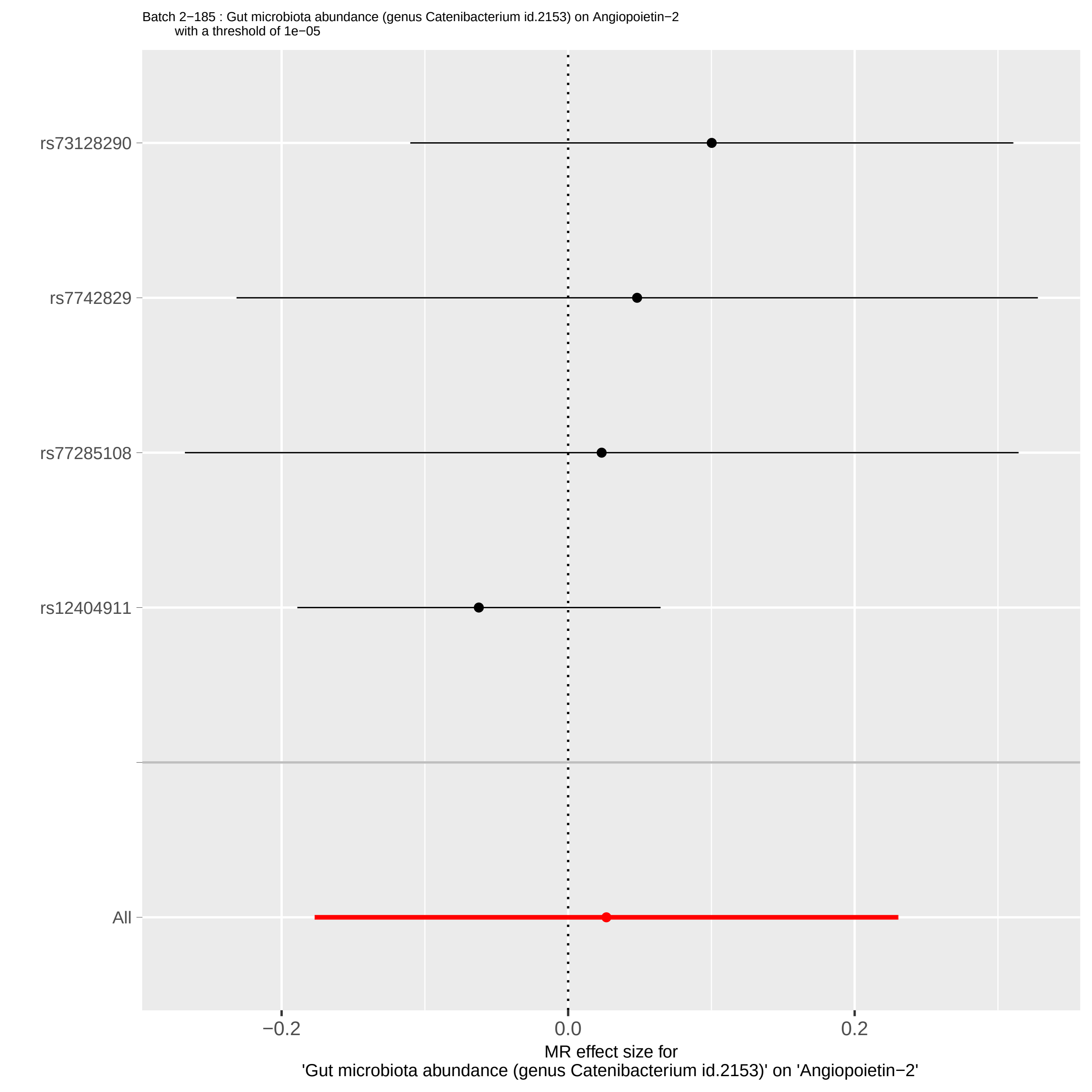

Batch 2−185 : Gut microbiota abundance (genus Catenibacterium id.2153) on Angiopoietin−2
with a threshold of 1e−05
rs73128290
rs7742829
rs77285108
rs12404911
All
−0.2
0.0
0.2
MR effect size for
'Gut microbiota abundance (genus Catenibacterium id.2153)' on 'Angiopoietin−2'

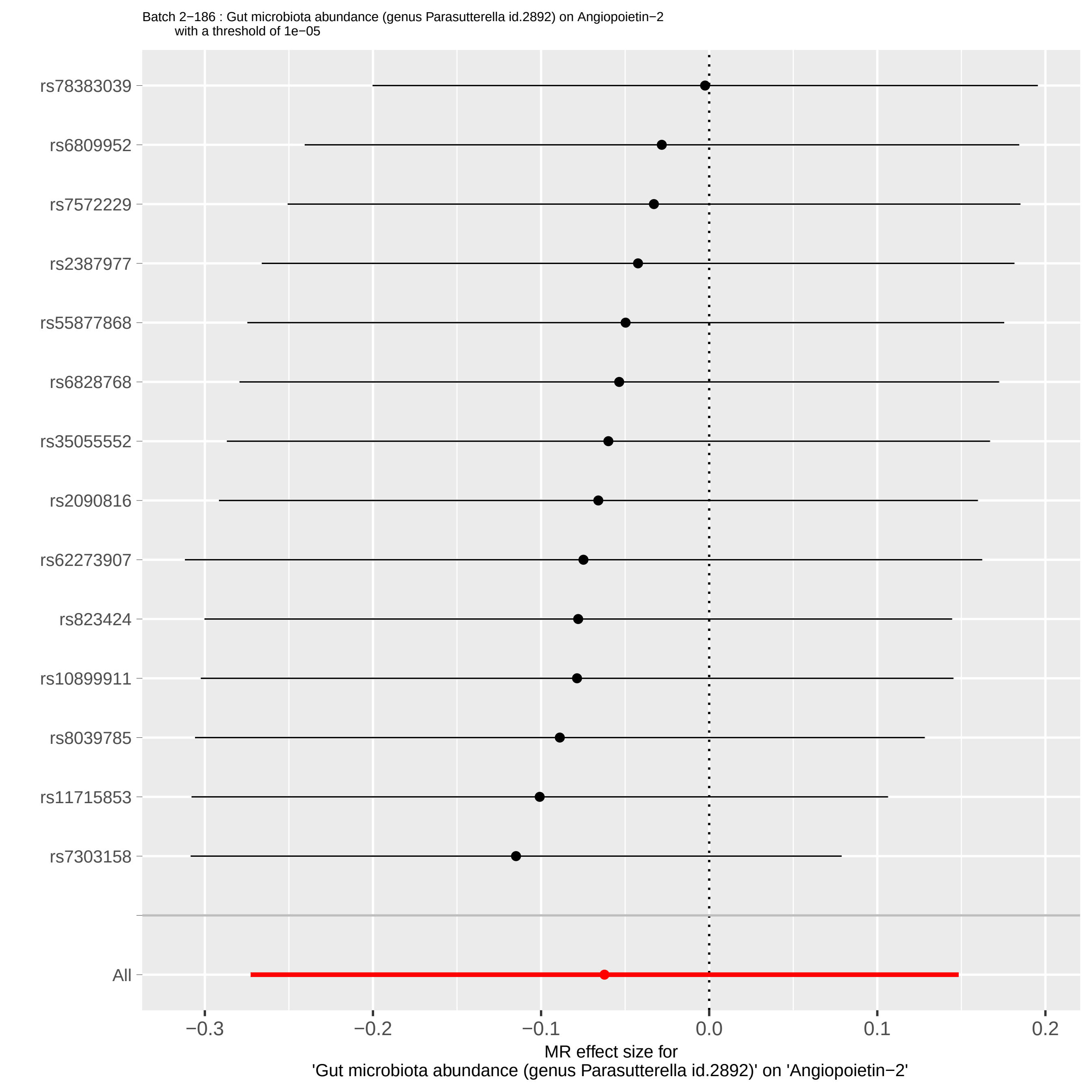

Batch 2−186 : Gut microbiota abundance (genus Parasutterella id.2892) on Angiopoietin−2
with a threshold of 1e−05
rs78383039
rs6809952
rs7572229
rs2387977
rs55877868
rs6828768
rs35055552
rs2090816
rs62273907
rs823424
rs10899911
rs8039785
rs11715853
rs7303158
All
−0.3
−0.2
−0.1
0.0
0.1
0.2
MR effect size for
'Gut microbiota abundance (genus Parasutterella id.2892)' on 'Angiopoietin−2'

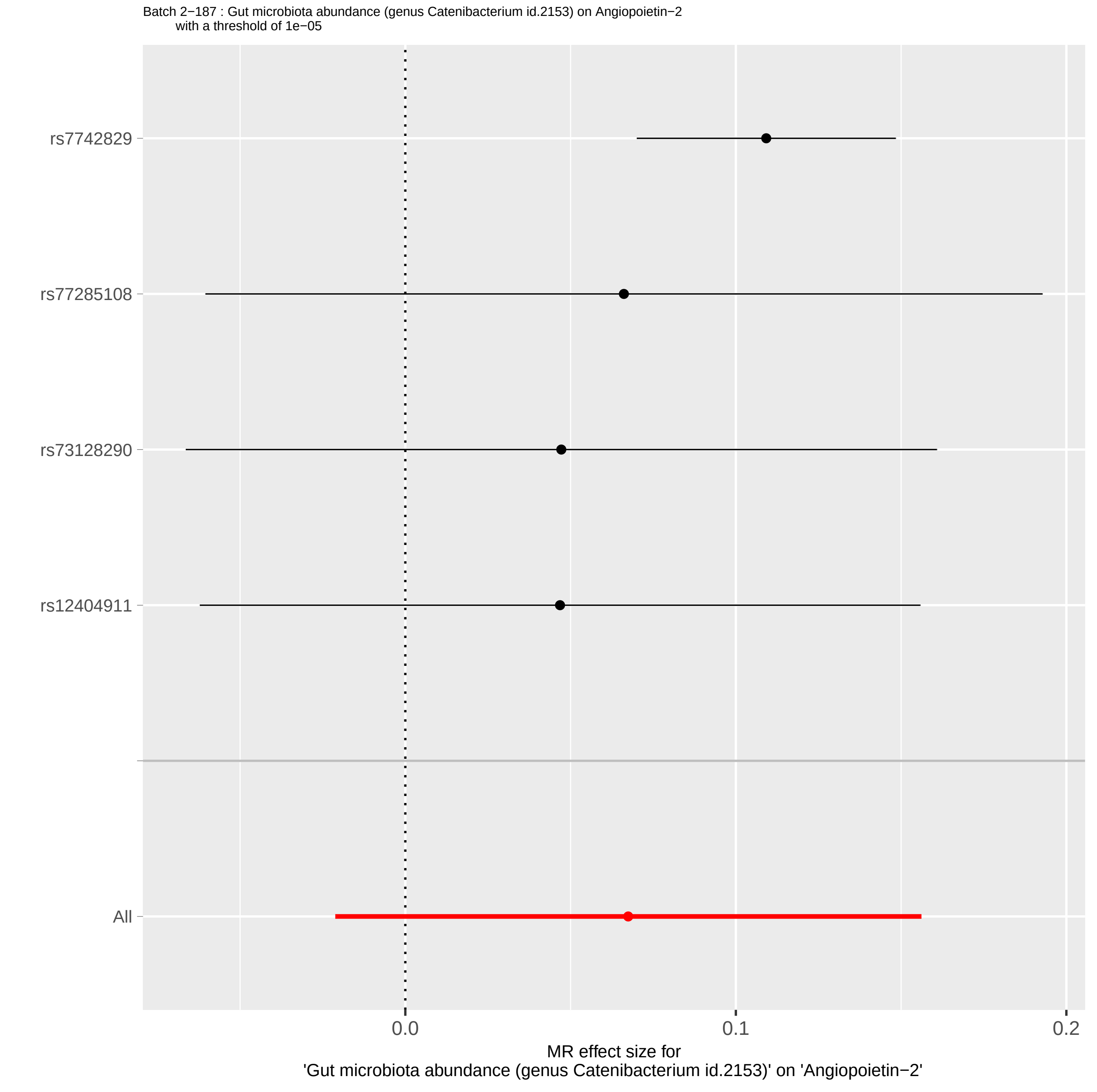

Batch 2−187 : Gut microbiota abundance (genus Catenibacterium id.2153) on Angiopoietin−2
with a threshold of 1e−05
rs7742829
rs77285108
rs73128290
rs12404911
All
0.0
0.1
0.2
MR effect size for
'Gut microbiota abundance (genus Catenibacterium id.2153)' on 'Angiopoietin−2'

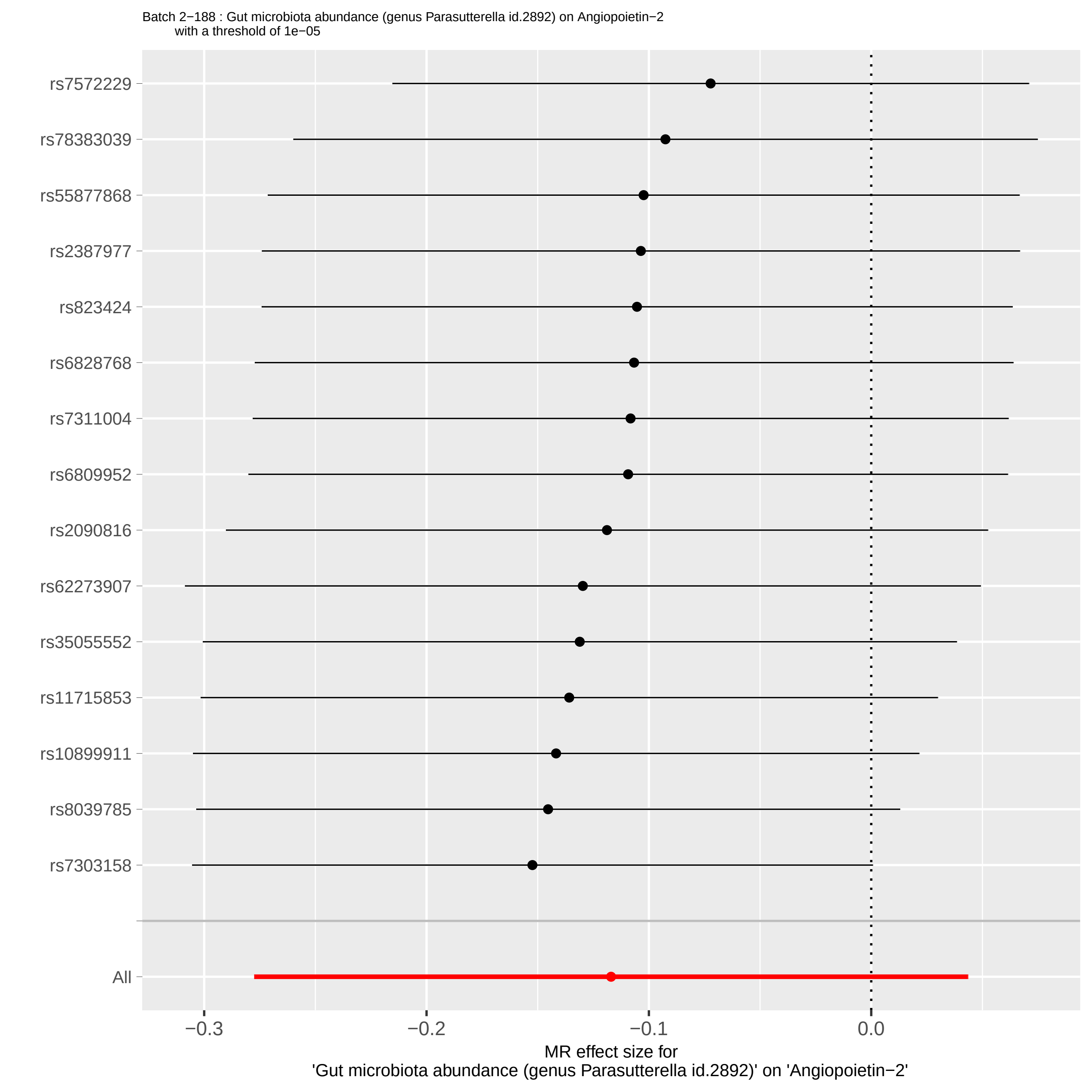

Batch 2−188 : Gut microbiota abundance (genus Parasutterella id.2892) on Angiopoietin−2
with a threshold of 1e−05
rs7572229
rs78383039
rs55877868
rs2387977
rs823424
rs6828768
rs7311004
rs6809952
rs2090816
rs62273907
rs35055552
rs11715853
rs10899911
rs8039785
rs7303158
All
−0.3
−0.2
−0.1
0.0
MR effect size for
'Gut microbiota abundance (genus Parasutterella id.2892)' on 'Angiopoietin−2'

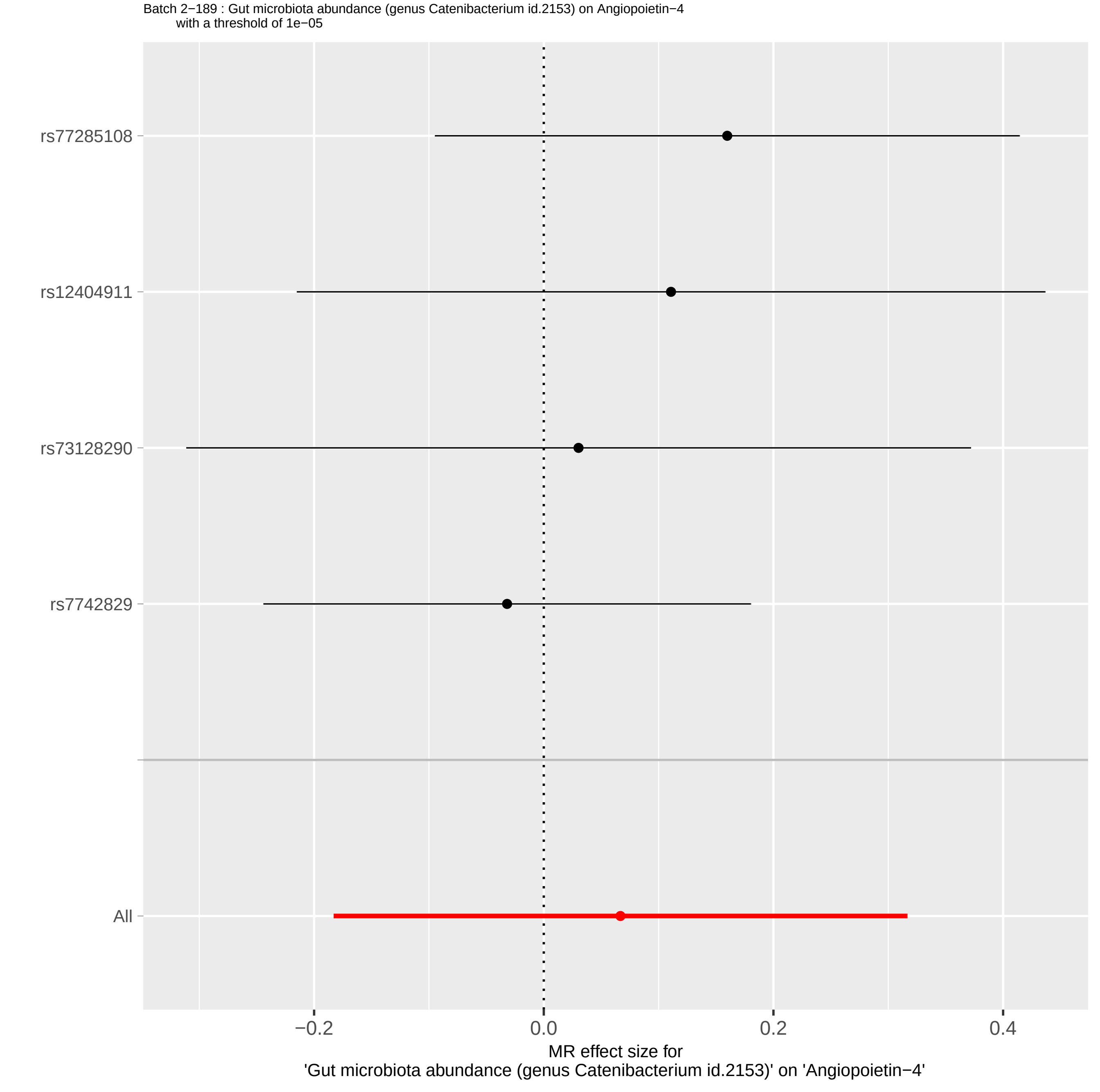

Batch 2−189 : Gut microbiota abundance (genus Catenibacterium id.2153) on Angiopoietin−4
with a threshold of 1e−05
rs77285108
rs12404911
rs73128290
rs7742829
All
−0.2
0.0
0.2
0.4
MR effect size for
'Gut microbiota abundance (genus Catenibacterium id.2153)' on 'Angiopoietin−4'

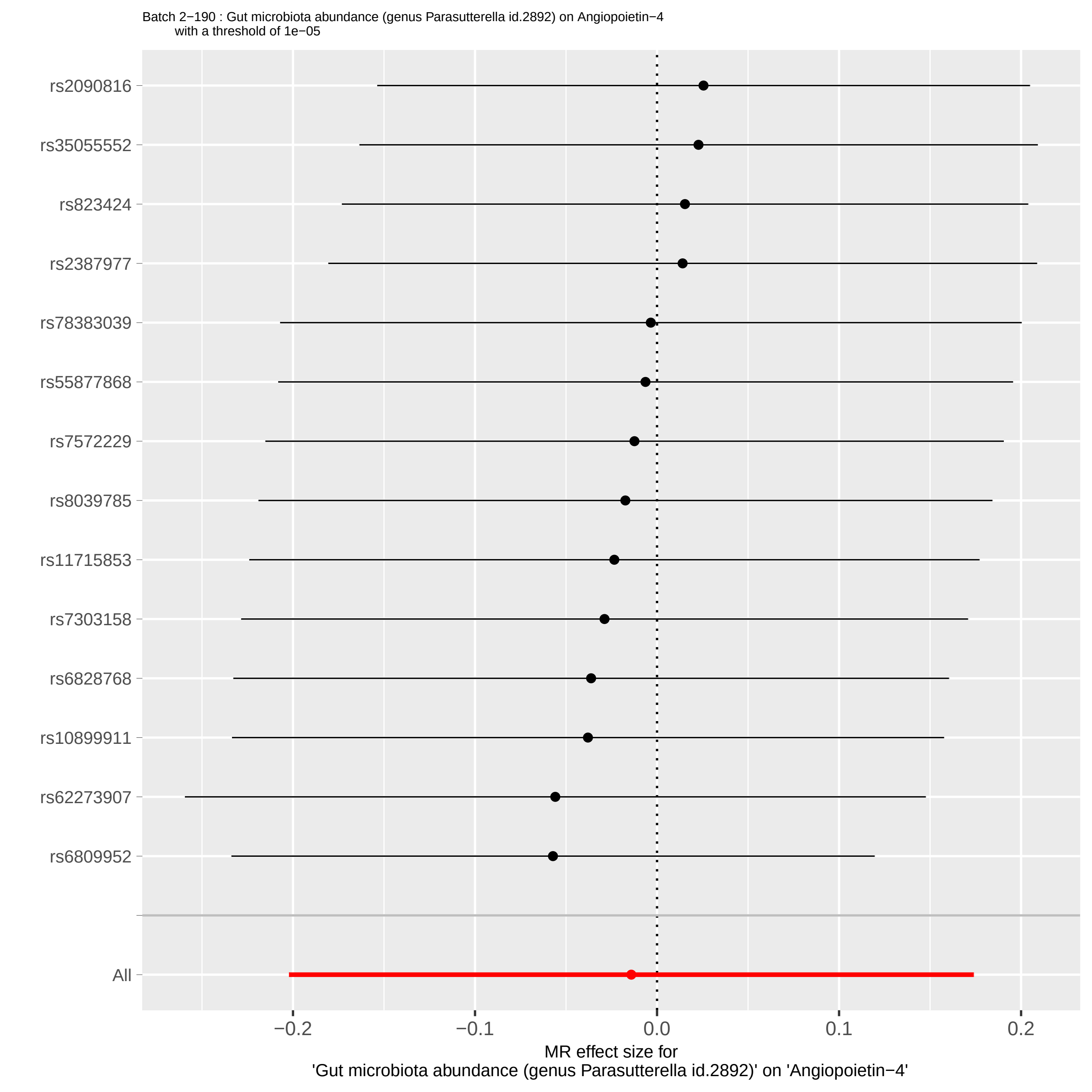

Batch 2−190 : Gut microbiota abundance (genus Parasutterella id.2892) on Angiopoietin−4
with a threshold of 1e−05
rs2090816
rs35055552
rs823424
rs2387977
rs78383039
rs55877868
rs7572229
rs8039785
rs11715853
rs7303158
rs6828768
rs10899911
rs62273907
rs6809952
All
−0.2
−0.1
0.0
0.1
0.2
MR effect size for
'Gut microbiota abundance (genus Parasutterella id.2892)' on 'Angiopoietin−4'

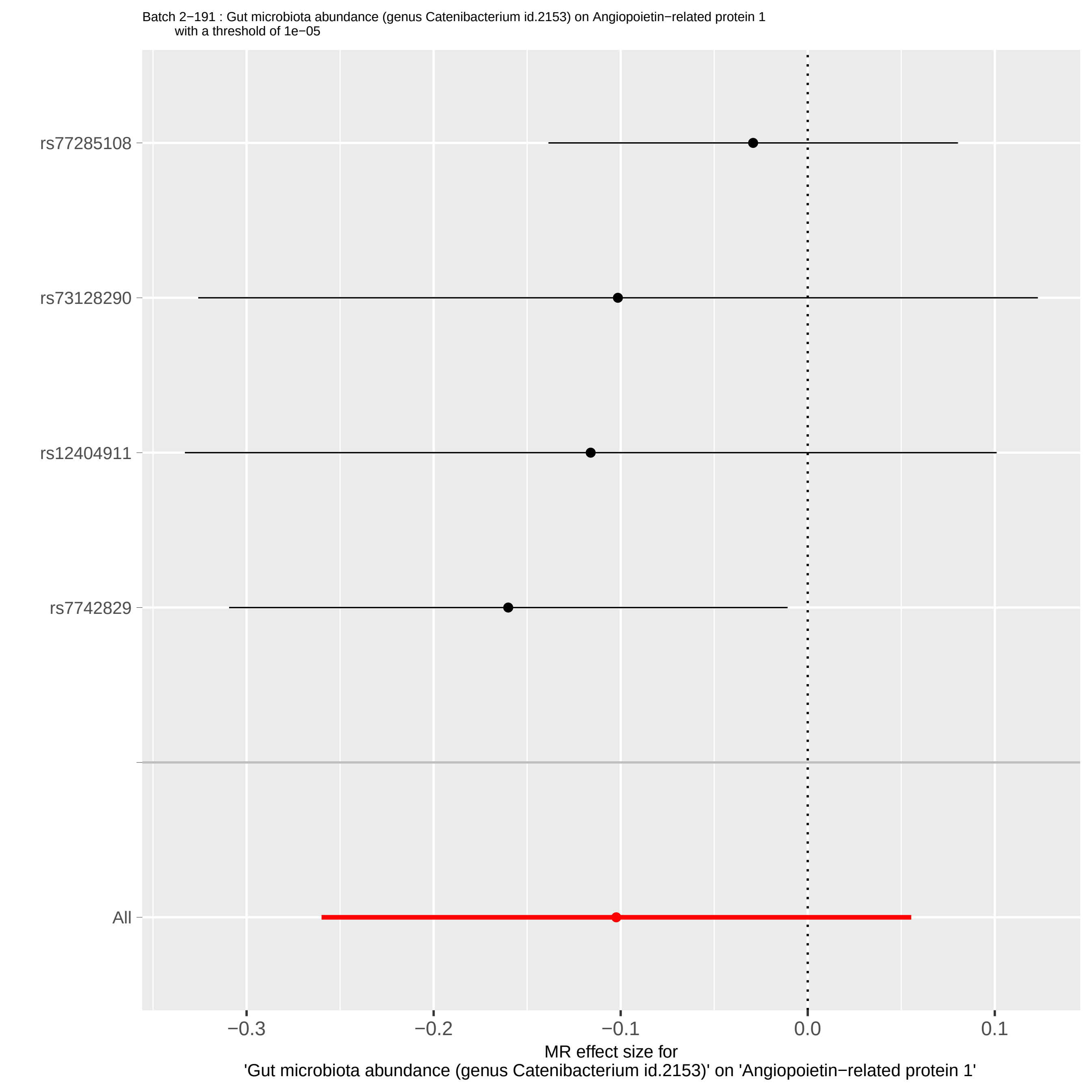
Batch 2−191 : Gut microbiota abundance (genus Catenibacterium id.2153) on Angiopoietin−related protein 1
with a threshold of 1e−05
rs77285108
rs73128290
rs12404911
rs7742829
All
−0.3
−0.2
−0.1
0.0
0.1
MR effect size for
'Gut microbiota abundance (genus Catenibacterium id.2153)' on 'Angiopoietin−related protein 1'

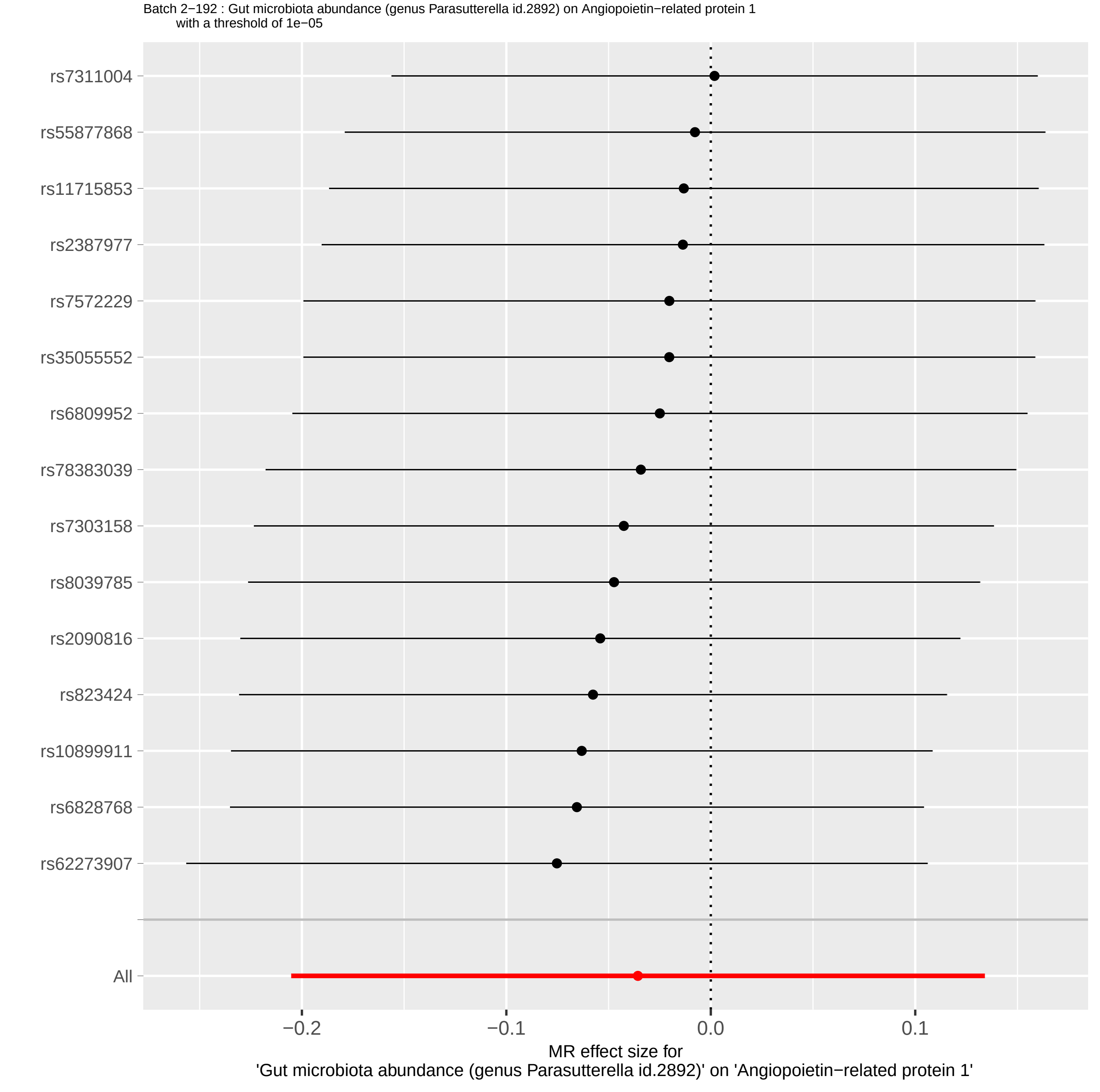

Batch 2−192 : Gut microbiota abundance (genus Parasutterella id.2892) on Angiopoietin−related protein 1
with a threshold of 1e−05
rs7311004
rs55877868
rs11715853
rs2387977
rs7572229
rs35055552
rs6809952
rs78383039
rs7303158
rs8039785
rs2090816
rs823424
rs10899911
rs6828768
rs62273907
All
−0.2
−0.1
0.0
0.1
MR effect size for
'Gut microbiota abundance (genus Parasutterella id.2892)' on 'Angiopoietin−related protein 1'

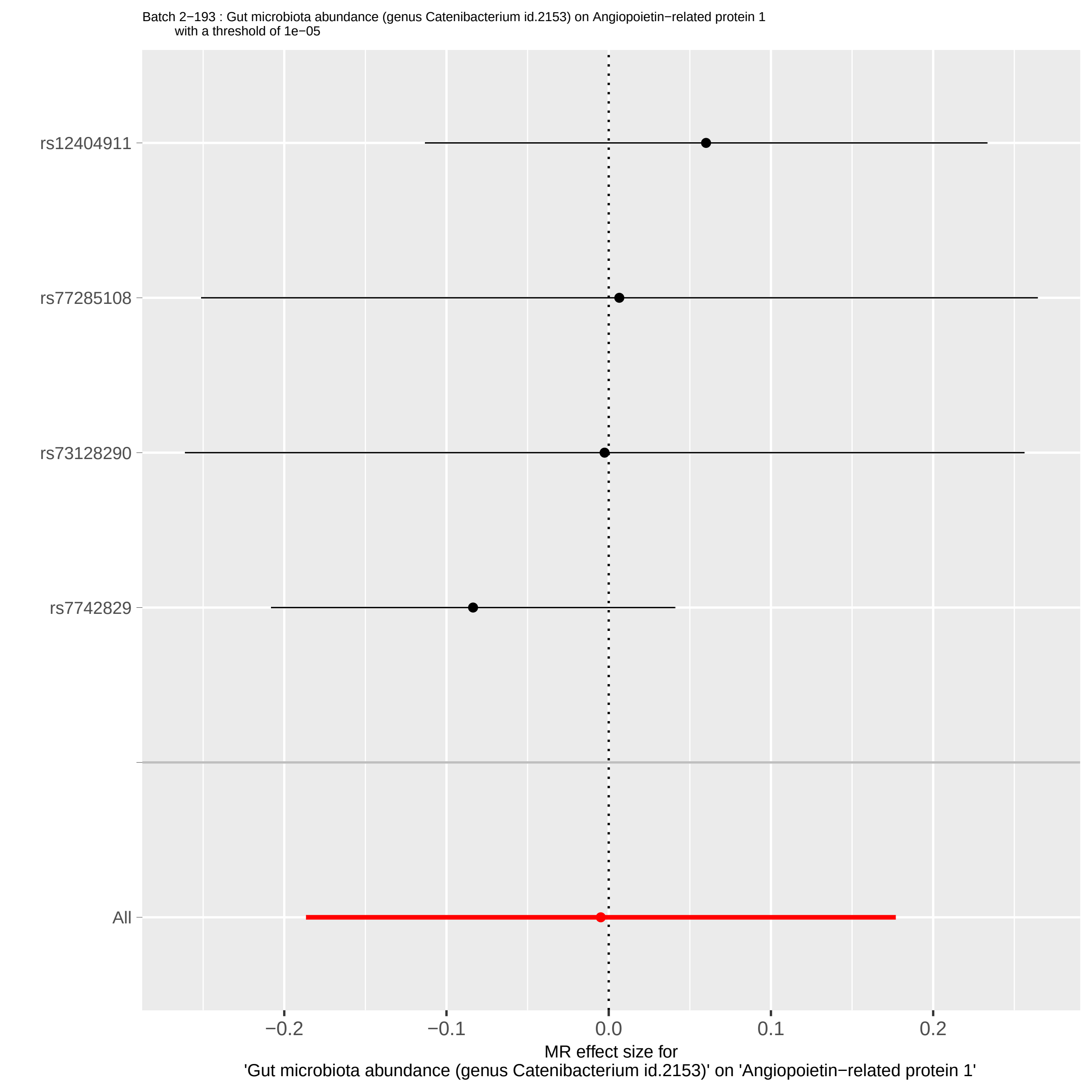

Batch 2−193 : Gut microbiota abundance (genus Catenibacterium id.2153) on Angiopoietin−related protein 1
with a threshold of 1e−05
rs12404911
rs77285108
rs73128290
rs7742829
All
−0.2
−0.1
0.0
0.1
0.2
MR effect size for
'Gut microbiota abundance (genus Catenibacterium id.2153)' on 'Angiopoietin−related protein 1'

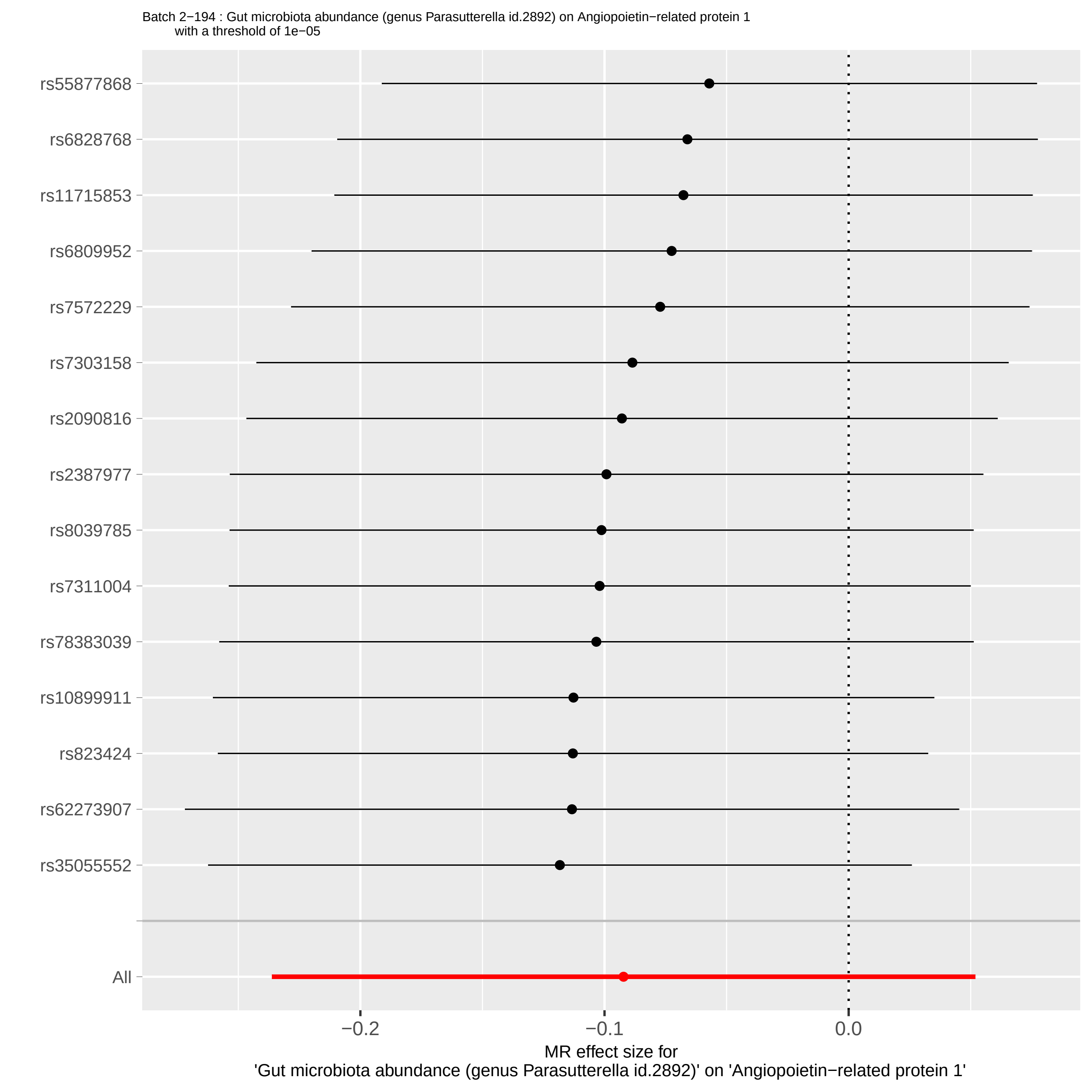
Batch 2−194 : Gut microbiota abundance (genus Parasutterella id.2892) on Angiopoietin−related protein 1
with a threshold of 1e−05
rs55877868
rs6828768
rs11715853
rs6809952
rs7572229
rs7303158
rs2090816
rs2387977
rs8039785
rs7311004
rs78383039
rs10899911
rs823424
rs62273907
rs35055552
All
−0.2
−0.1
0.0
MR effect size for
'Gut microbiota abundance (genus Parasutterella id.2892)' on 'Angiopoietin−related protein 1'

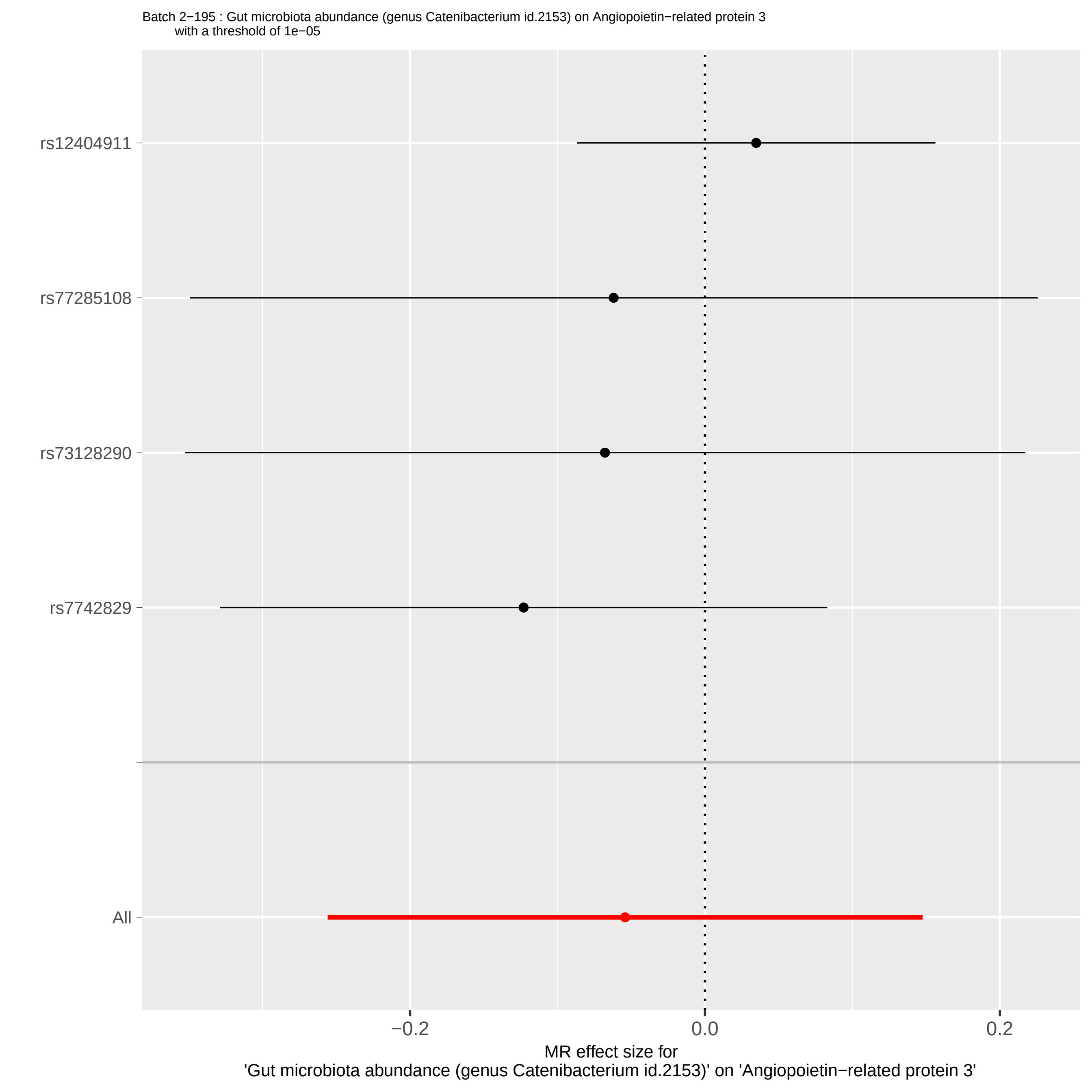
Batch 2−195 : Gut microbiota abundance (genus Catenibacterium id.2153) on Angiopoietin−related protein 3
with a threshold of 1e−05
rs12404911
rs77285108
rs73128290
rs7742829
All
−0.2
0.0
0.2
MR effect size for
'Gut microbiota abundance (genus Catenibacterium id.2153)' on 'Angiopoietin−related protein 3'

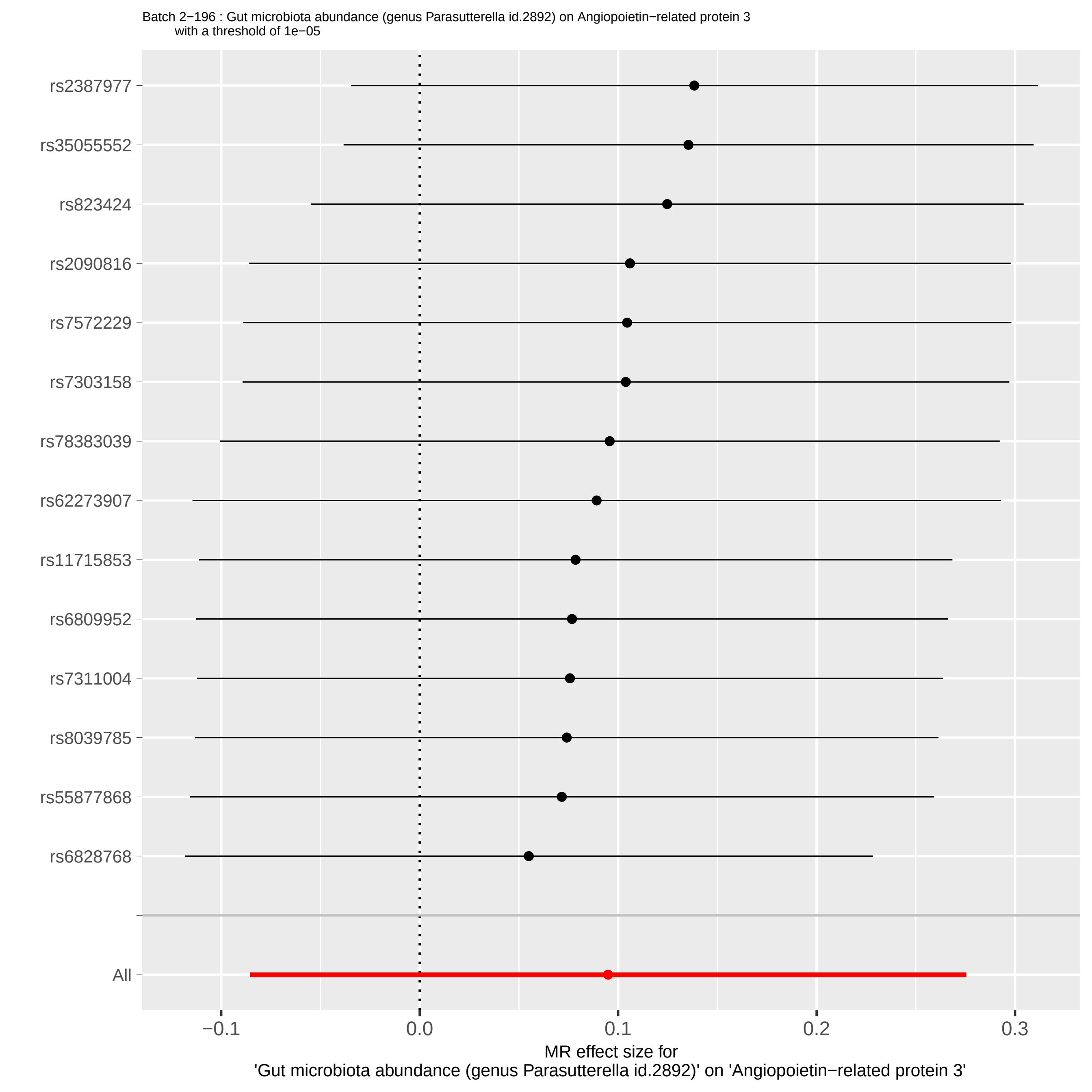

Batch 2−196 : Gut microbiota abundance (genus Parasutterella id.2892) on Angiopoietin−related protein 3
with a threshold of 1e−05
rs2387977
rs35055552
rs823424
rs2090816
rs7572229
rs7303158
rs78383039
rs62273907
rs11715853
rs6809952
rs7311004
rs8039785
rs55877868
rs6828768
All
−0.1
0.0
0.1
0.2
0.3
MR effect size for
'Gut microbiota abundance (genus Parasutterella id.2892)' on 'Angiopoietin−related protein 3'

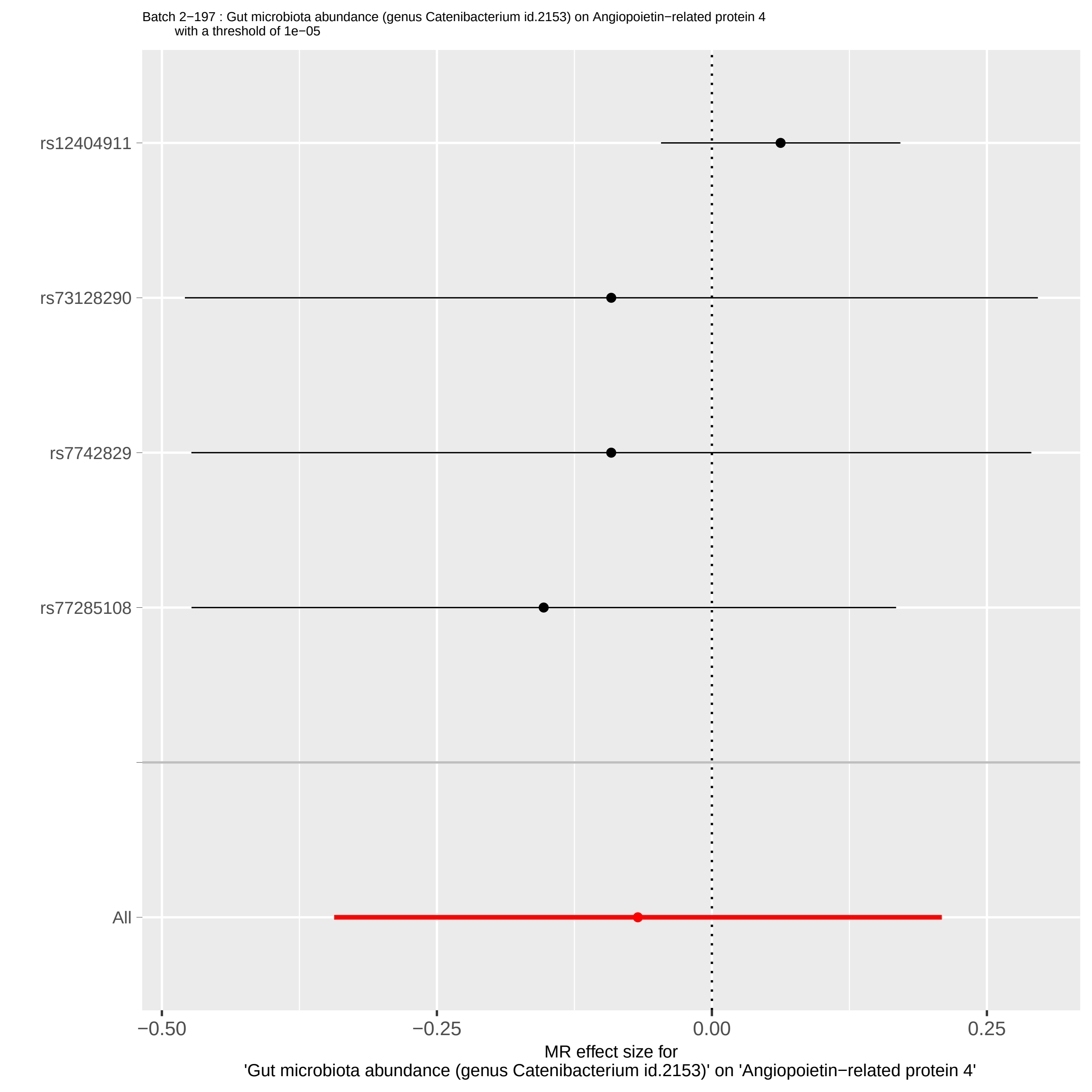

Batch 2−197 : Gut microbiota abundance (genus Catenibacterium id.2153) on Angiopoietin−related protein 4
with a threshold of 1e−05
rs12404911
rs73128290
rs7742829
rs77285108
All
−0.50
−0.25
0.00
0.25
MR effect size for
'Gut microbiota abundance (genus Catenibacterium id.2153)' on 'Angiopoietin−related protein 4'

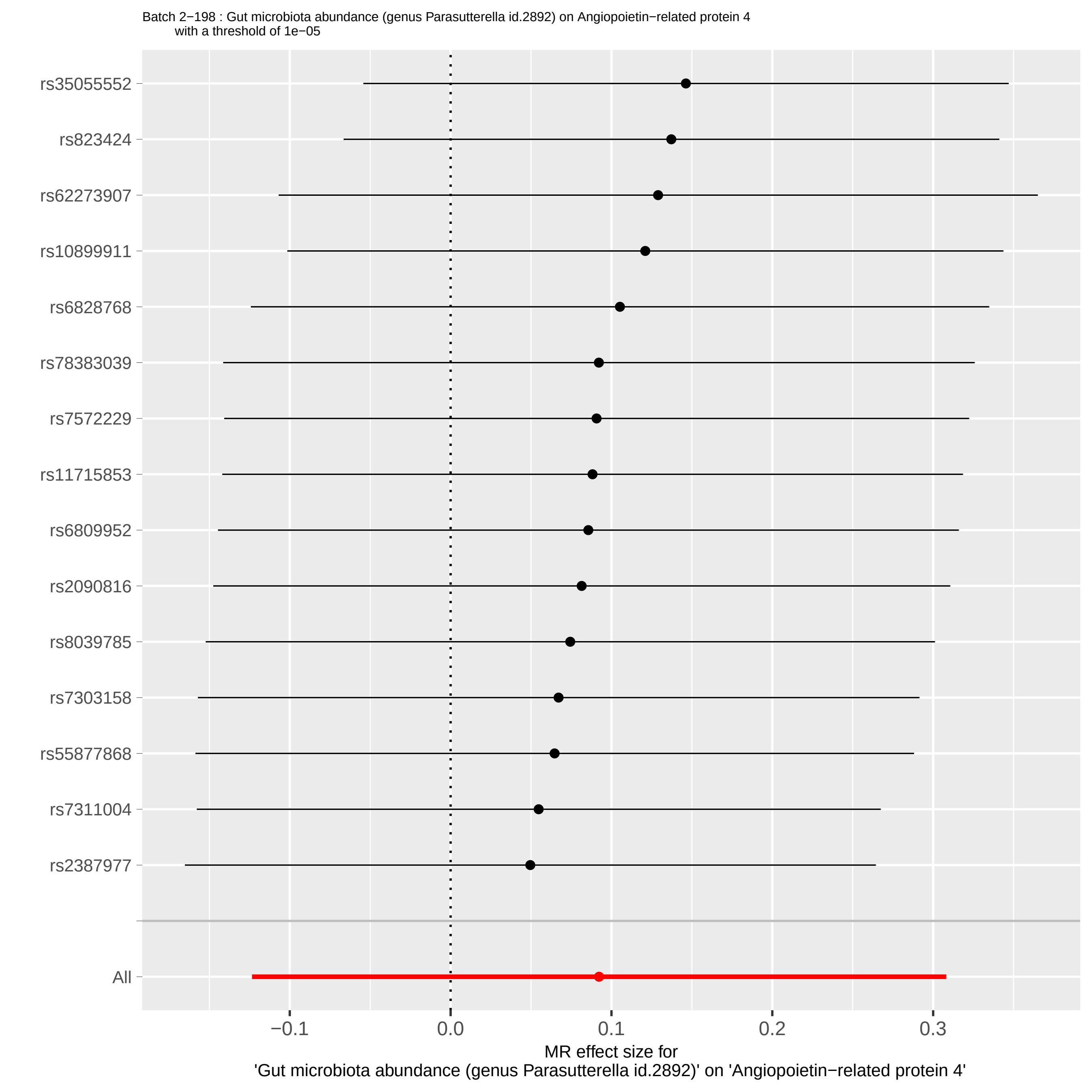

Batch 2−198 : Gut microbiota abundance (genus Parasutterella id.2892) on Angiopoietin−related protein 4
with a threshold of 1e−05
rs35055552
rs823424
rs62273907
rs10899911
rs6828768
rs78383039
rs7572229
rs11715853
rs6809952
rs2090816
rs8039785
rs7303158
rs55877868
rs7311004
rs2387977
All
−0.1
0.0
0.1
0.2
0.3
MR effect size for
'Gut microbiota abundance (genus Parasutterella id.2892)' on 'Angiopoietin−related protein 4'

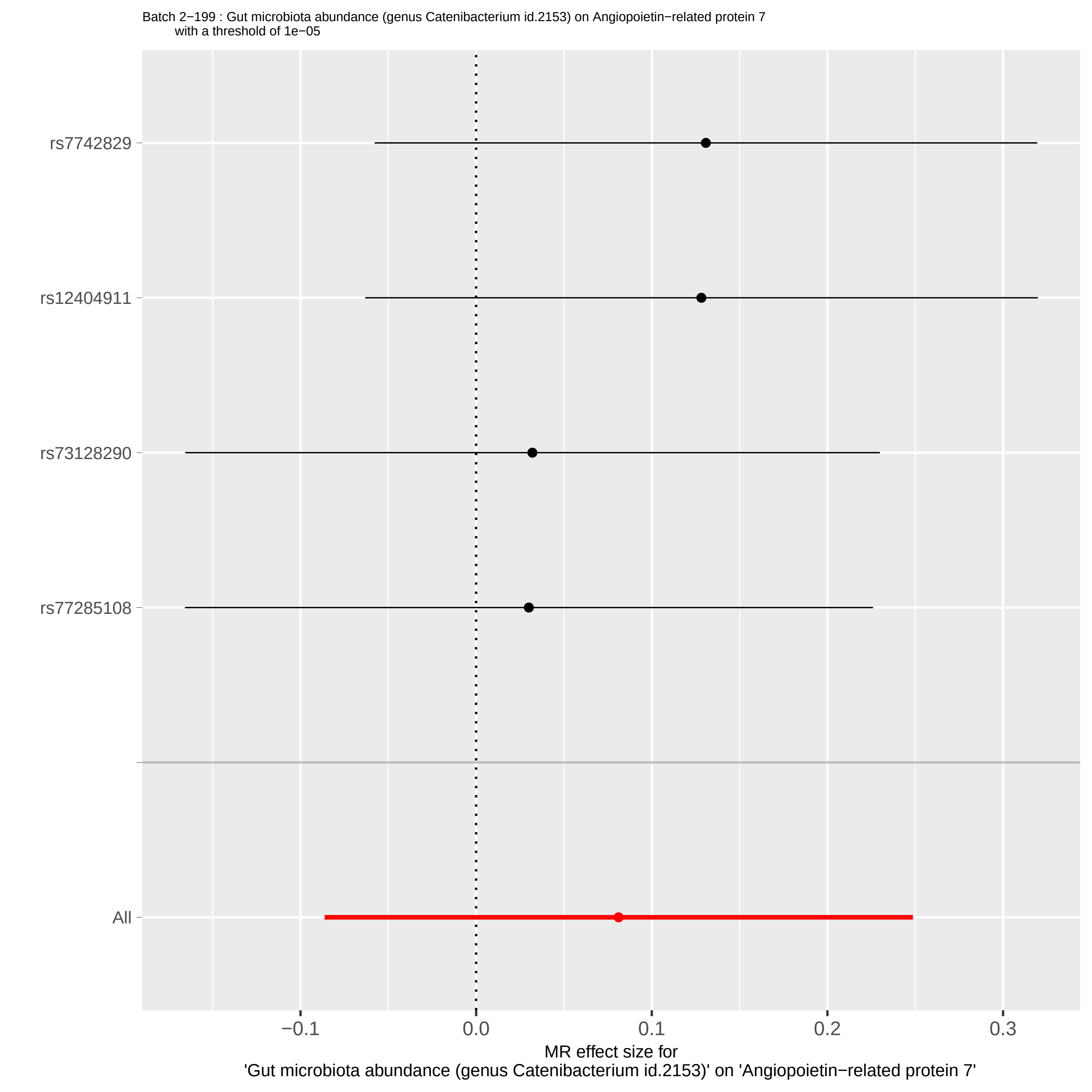
Batch 2−199 : Gut microbiota abundance (genus Catenibacterium id.2153) on Angiopoietin−related protein 7
with a threshold of 1e−05
rs7742829
rs12404911
rs73128290
rs77285108
All
−0.1
0.0
0.1
0.2
0.3
MR effect size for
'Gut microbiota abundance (genus Catenibacterium id.2153)' on 'Angiopoietin−related protein 7'

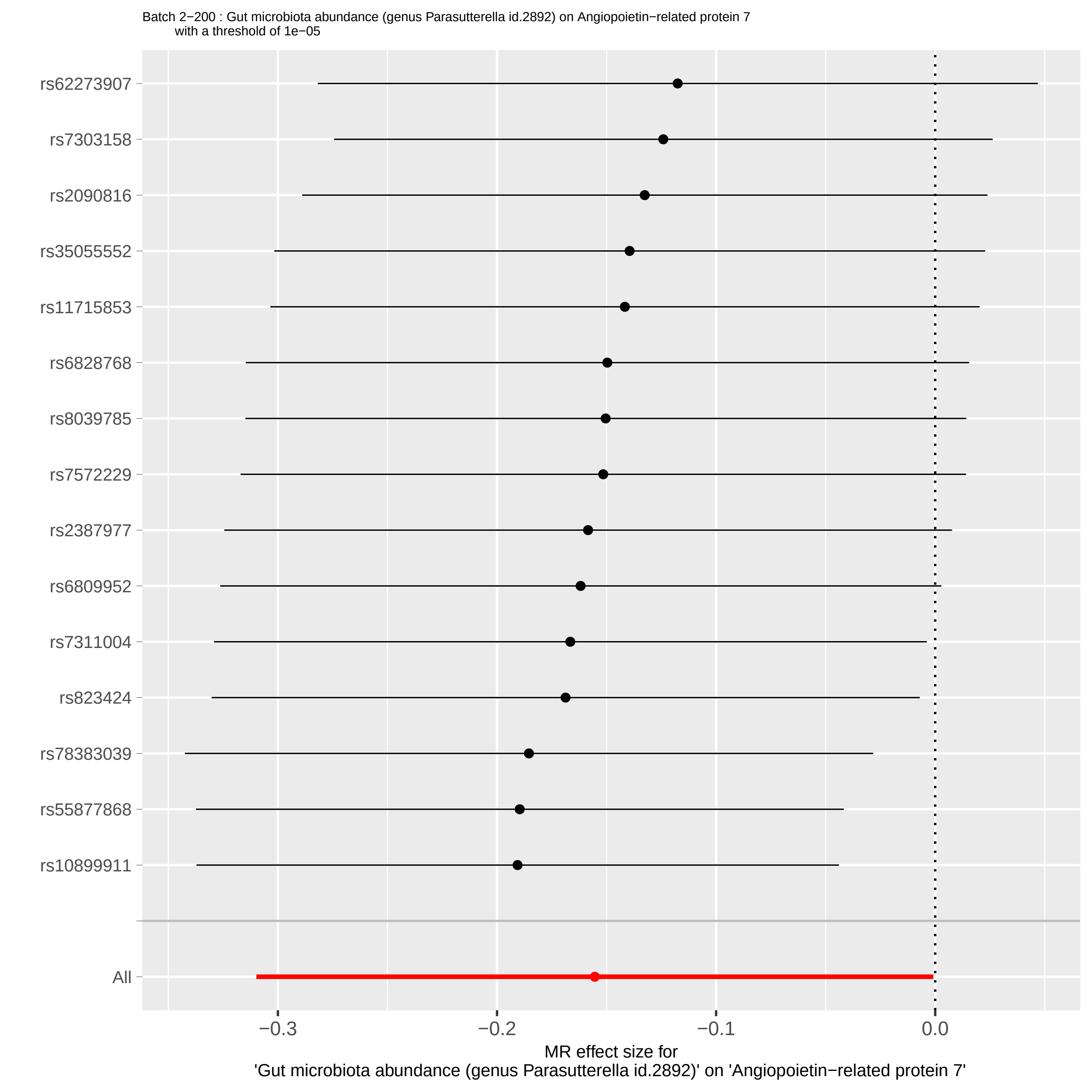

Batch 2−200 : Gut microbiota abundance (genus Parasutterella id.2892) on Angiopoietin−related protein 7
with a threshold of 1e−05
rs62273907
rs7303158
rs2090816
rs35055552
rs11715853
rs6828768
rs8039785
rs7572229
rs2387977
rs6809952
rs7311004
rs823424
rs78383039
rs55877868
rs10899911
All
−0.3
−0.2
−0.1
0.0
MR effect size for
'Gut microbiota abundance (genus Parasutterella id.2892)' on 'Angiopoietin−related protein 7'

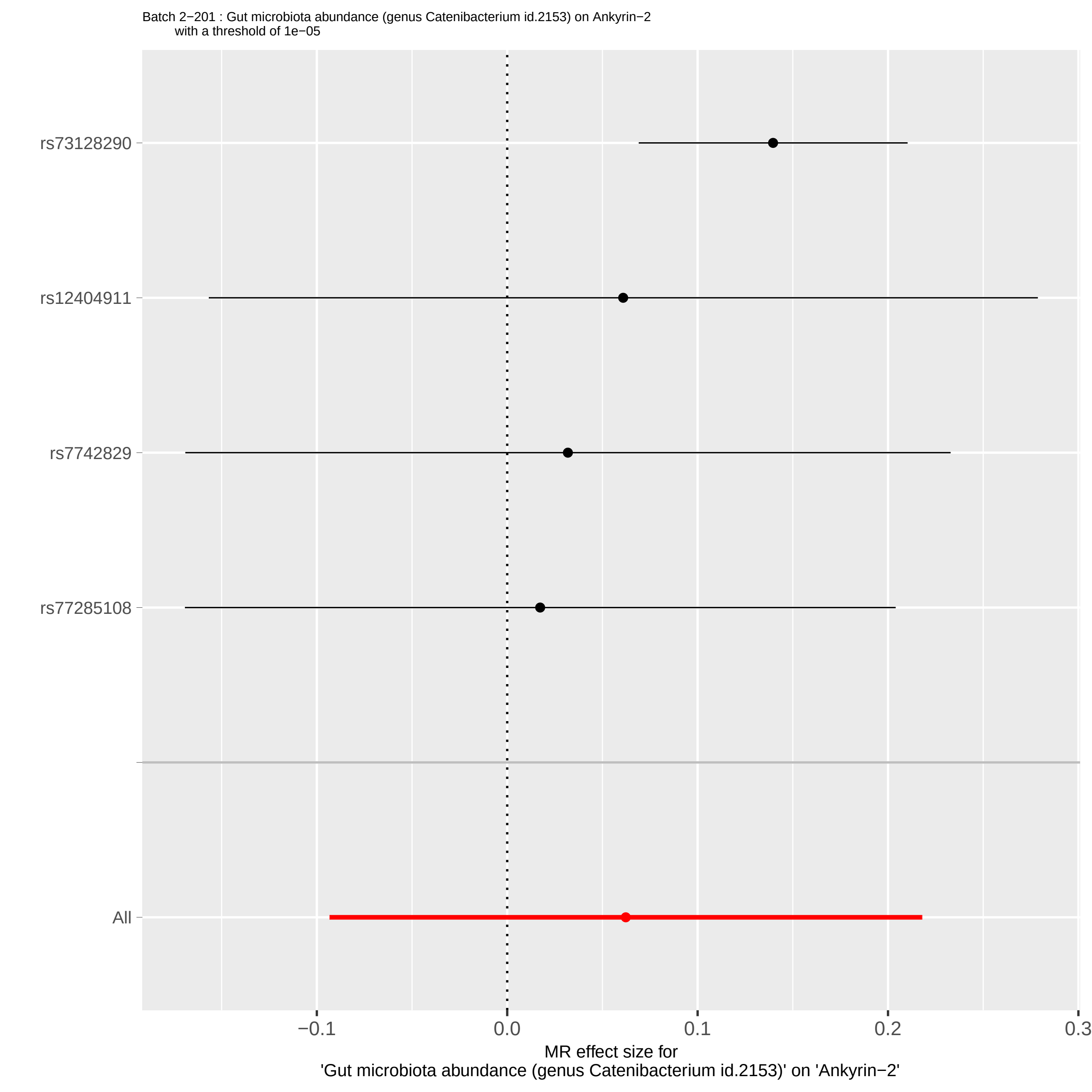
Batch 2−201 : Gut microbiota abundance (genus Catenibacterium id.2153) on Ankyrin−2
with a threshold of 1e−05
rs73128290
rs12404911
rs7742829
rs77285108
All
−0.1
0.0
0.1
0.2
0.3
MR effect size for
'Gut microbiota abundance (genus Catenibacterium id.2153)' on 'Ankyrin−2'

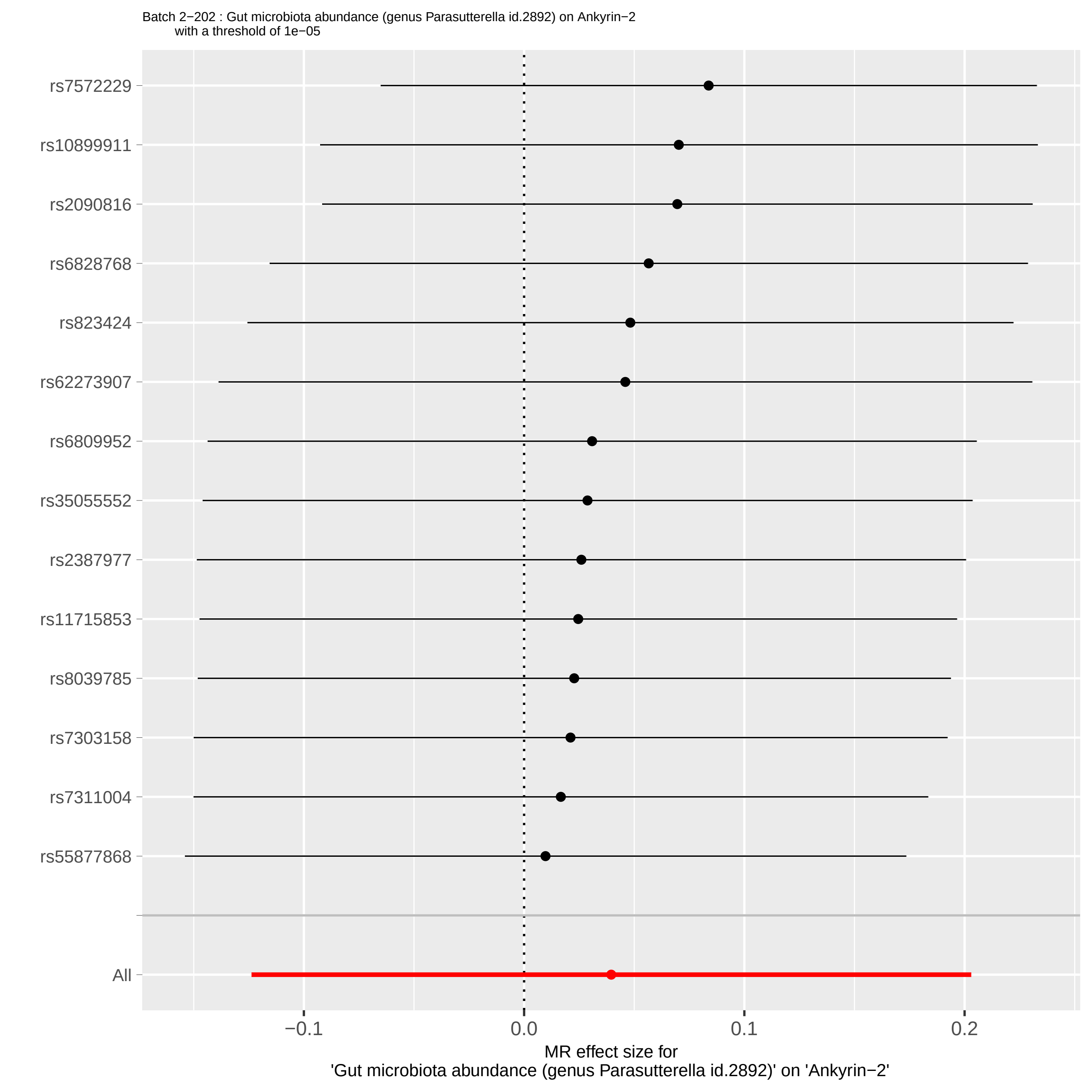

Batch 2−202 : Gut microbiota abundance (genus Parasutterella id.2892) on Ankyrin−2
with a threshold of 1e−05
rs7572229
rs10899911
rs2090816
rs6828768
rs823424
rs62273907
rs6809952
rs35055552
rs2387977
rs11715853
rs8039785
rs7303158
rs7311004
rs55877868
All
−0.1
0.0
0.1
0.2
MR effect size for
'Gut microbiota abundance (genus Parasutterella id.2892)' on 'Ankyrin−2'

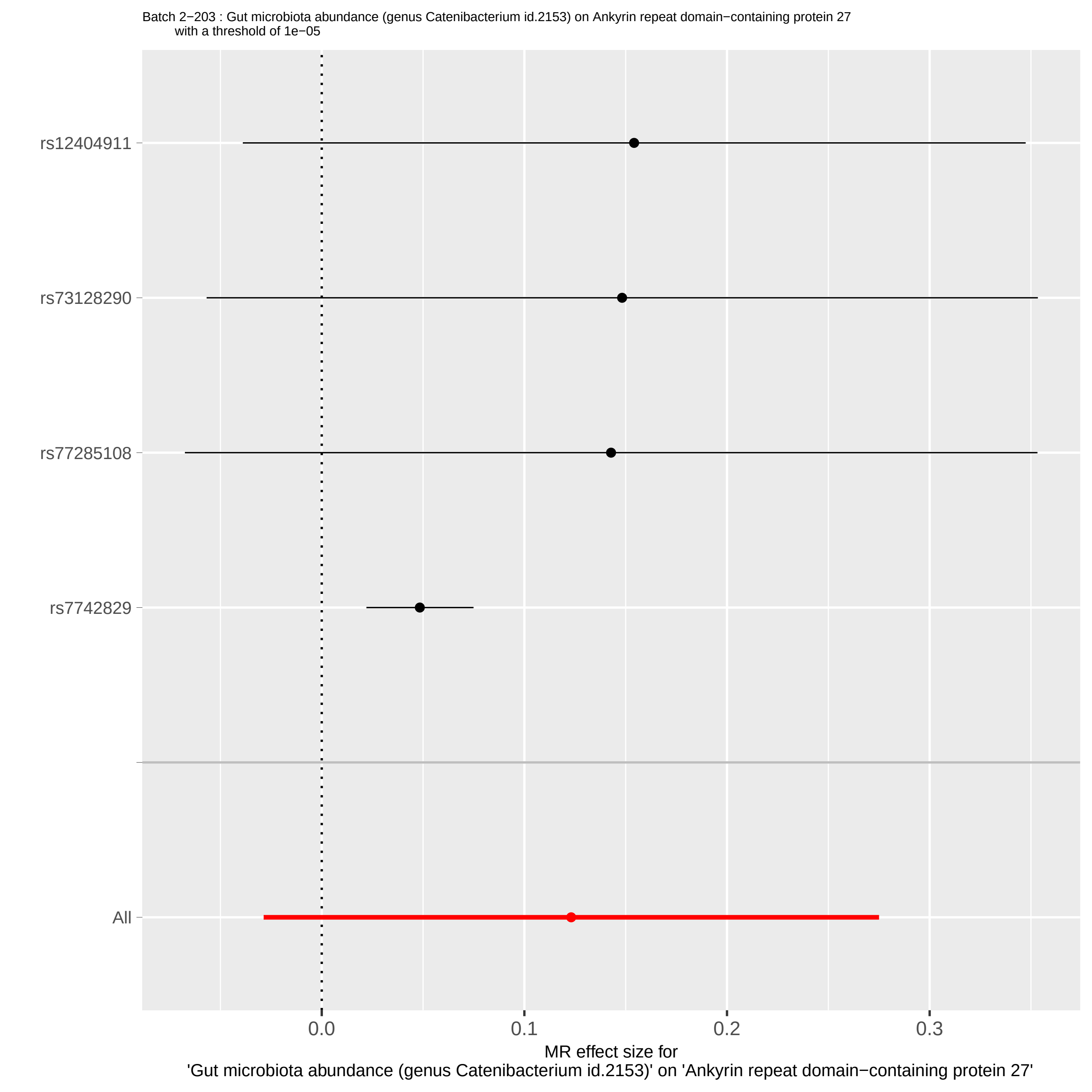
Batch 2−203 : Gut microbiota abundance (genus Catenibacterium id.2153) on Ankyrin repeat domain−containing protein 27
with a threshold of 1e−05
rs12404911
rs73128290
rs77285108
rs7742829
All
0.0
0.1
0.2
0.3
MR effect size for
'Gut microbiota abundance (genus Catenibacterium id.2153)' on 'Ankyrin repeat domain−containing protein 27'

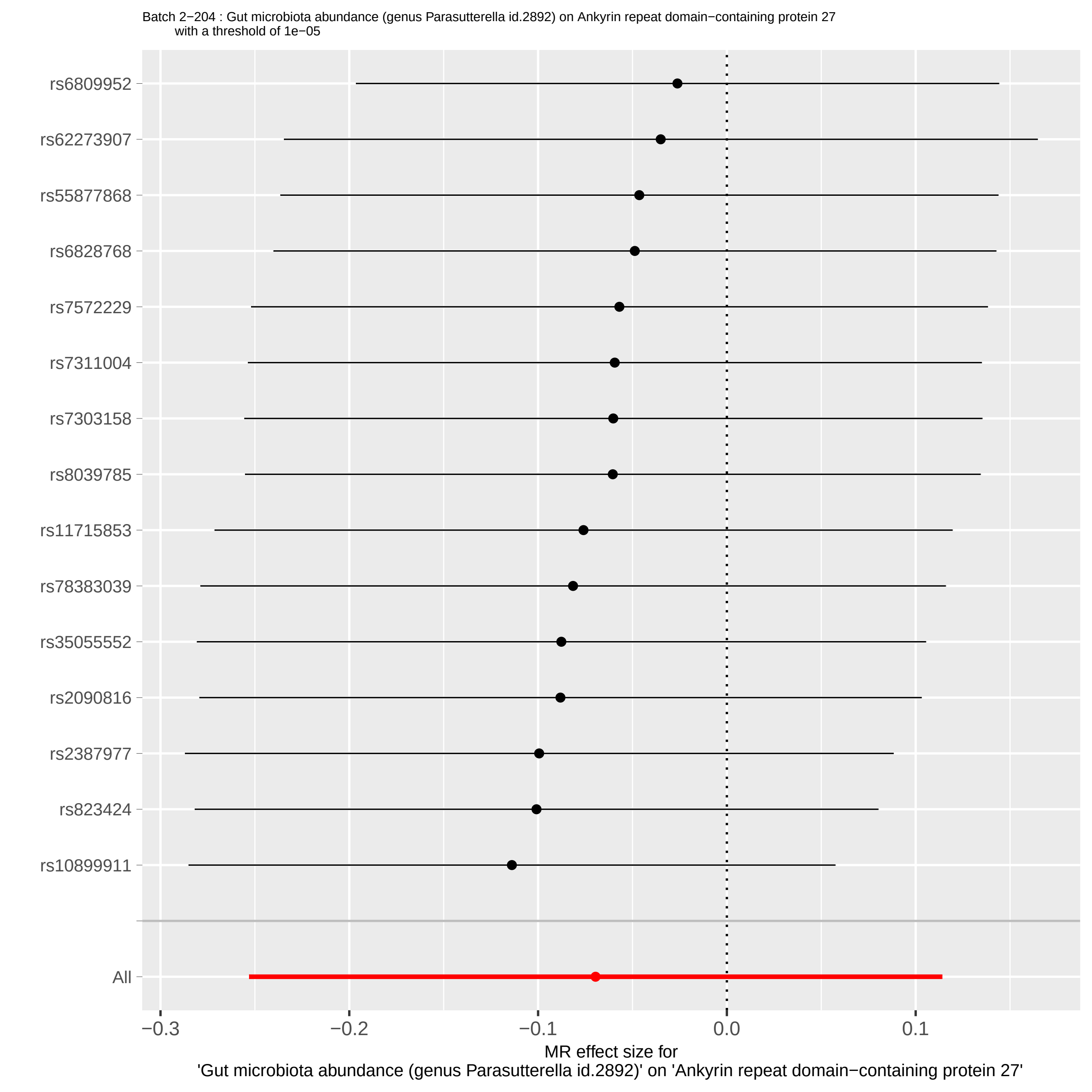
Batch 2−204 : Gut microbiota abundance (genus Parasutterella id.2892) on Ankyrin repeat domain−containing protein 27
with a threshold of 1e−05
rs6809952
rs62273907
rs55877868
rs6828768
rs7572229
rs7311004
rs7303158
rs8039785
rs11715853
rs78383039
rs35055552
rs2090816
rs2387977
rs823424
rs10899911
All
−0.3
−0.2
−0.1
0.0
0.1
MR effect size for
'Gut microbiota abundance (genus Parasutterella id.2892)' on 'Ankyrin repeat domain−containing protein 27'

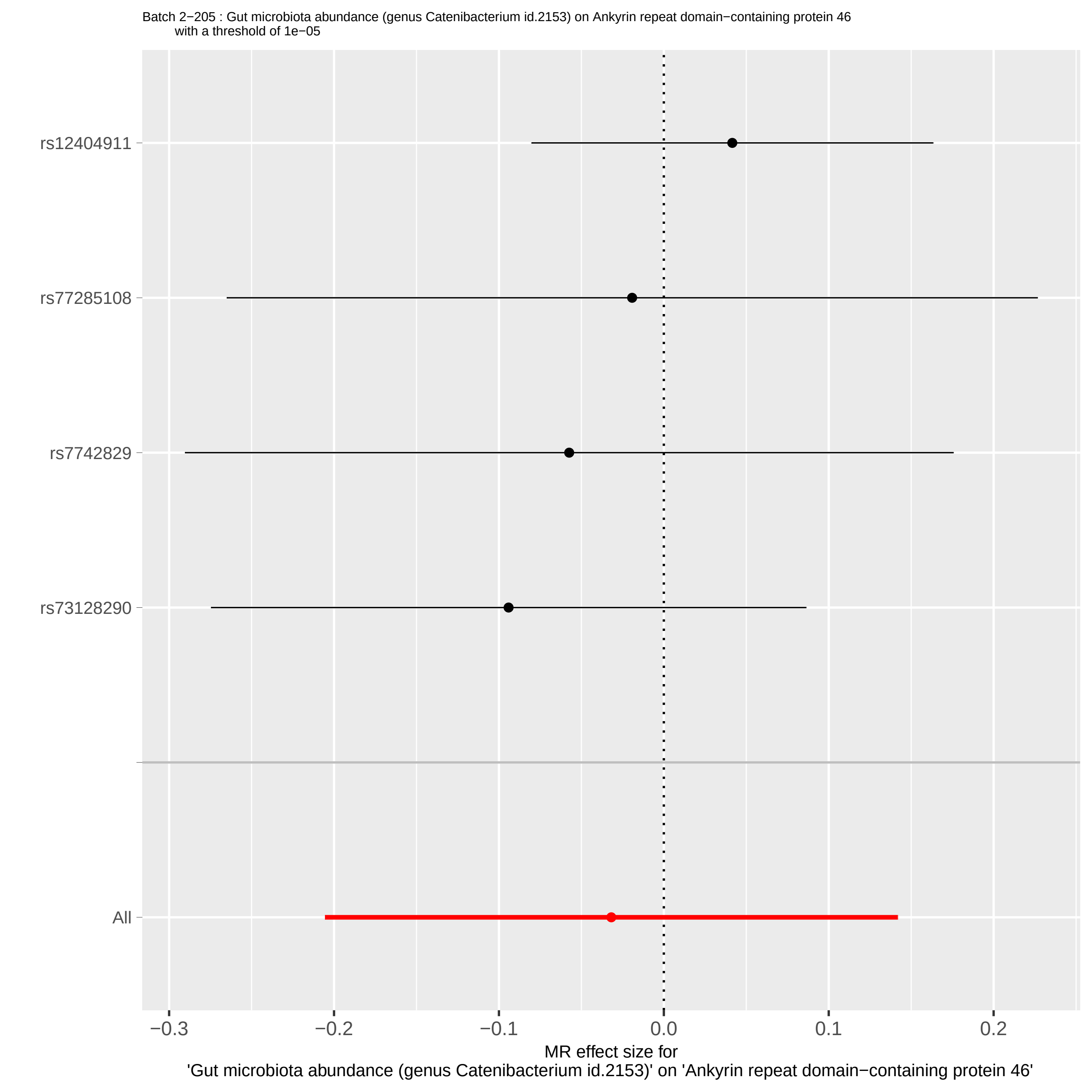
Batch 2−205 : Gut microbiota abundance (genus Catenibacterium id.2153) on Ankyrin repeat domain−containing protein 46
with a threshold of 1e−05
rs12404911
rs77285108
rs7742829
rs73128290
All
−0.3
−0.2
−0.1
0.0
0.1
0.2
MR effect size for
'Gut microbiota abundance (genus Catenibacterium id.2153)' on 'Ankyrin repeat domain−containing protein 46'

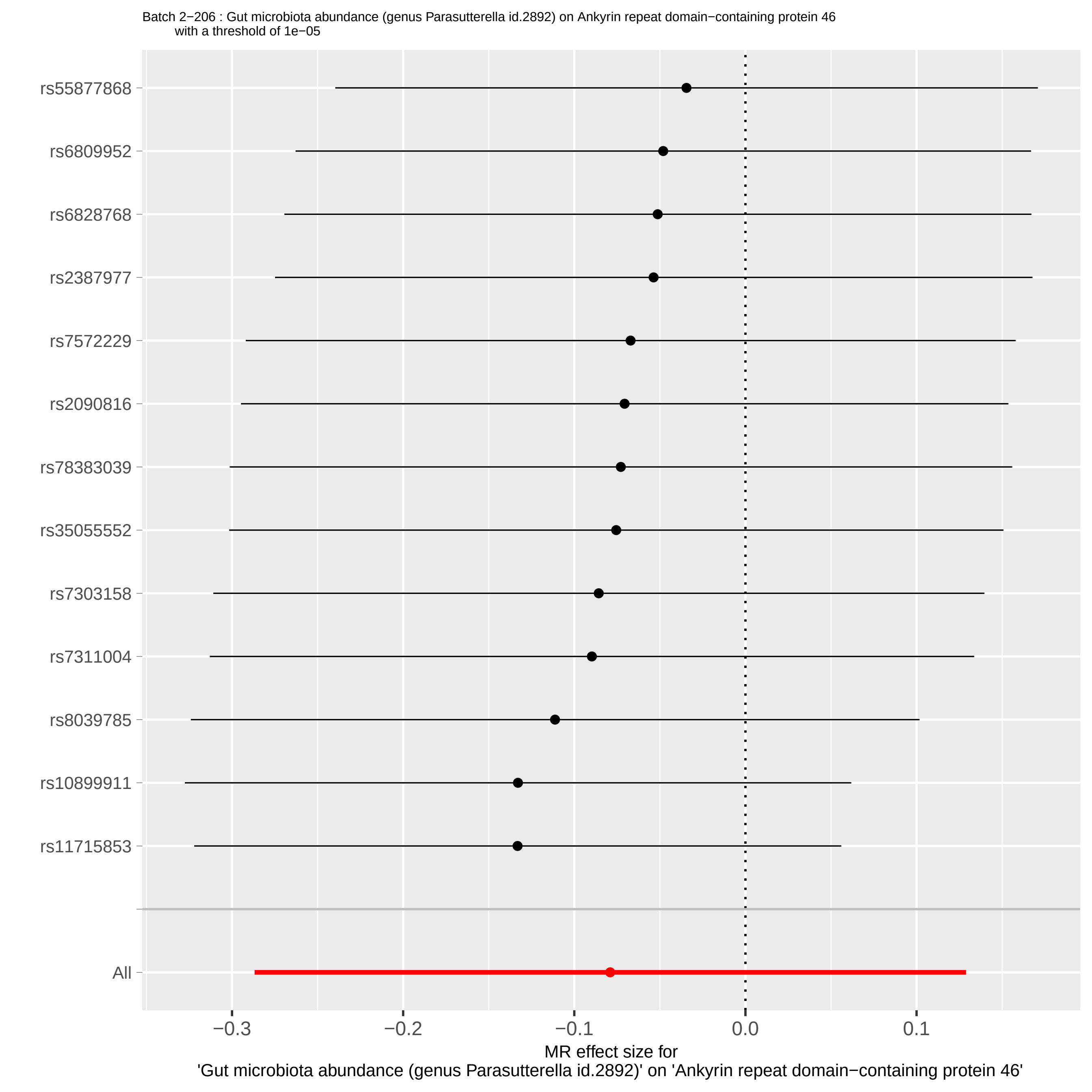
Batch 2−206 : Gut microbiota abundance (genus Parasutterella id.2892) on Ankyrin repeat domain−containing protein 46
with a threshold of 1e−05
rs55877868
rs6809952
rs6828768
rs2387977
rs7572229
rs2090816
rs78383039
rs35055552
rs7303158
rs7311004
rs8039785
rs10899911
rs11715853
All
−0.3
−0.2
−0.1
0.0
0.1
MR effect size for
'Gut microbiota abundance (genus Parasutterella id.2892)' on 'Ankyrin repeat domain−containing protein 46'

Batch 2−207 : Gut microbiota abundance (genus Catenibacterium id.2153) on Acidic leucine−rich nuclear phosphoprotein 32 family member A
with a threshold of 1e−05

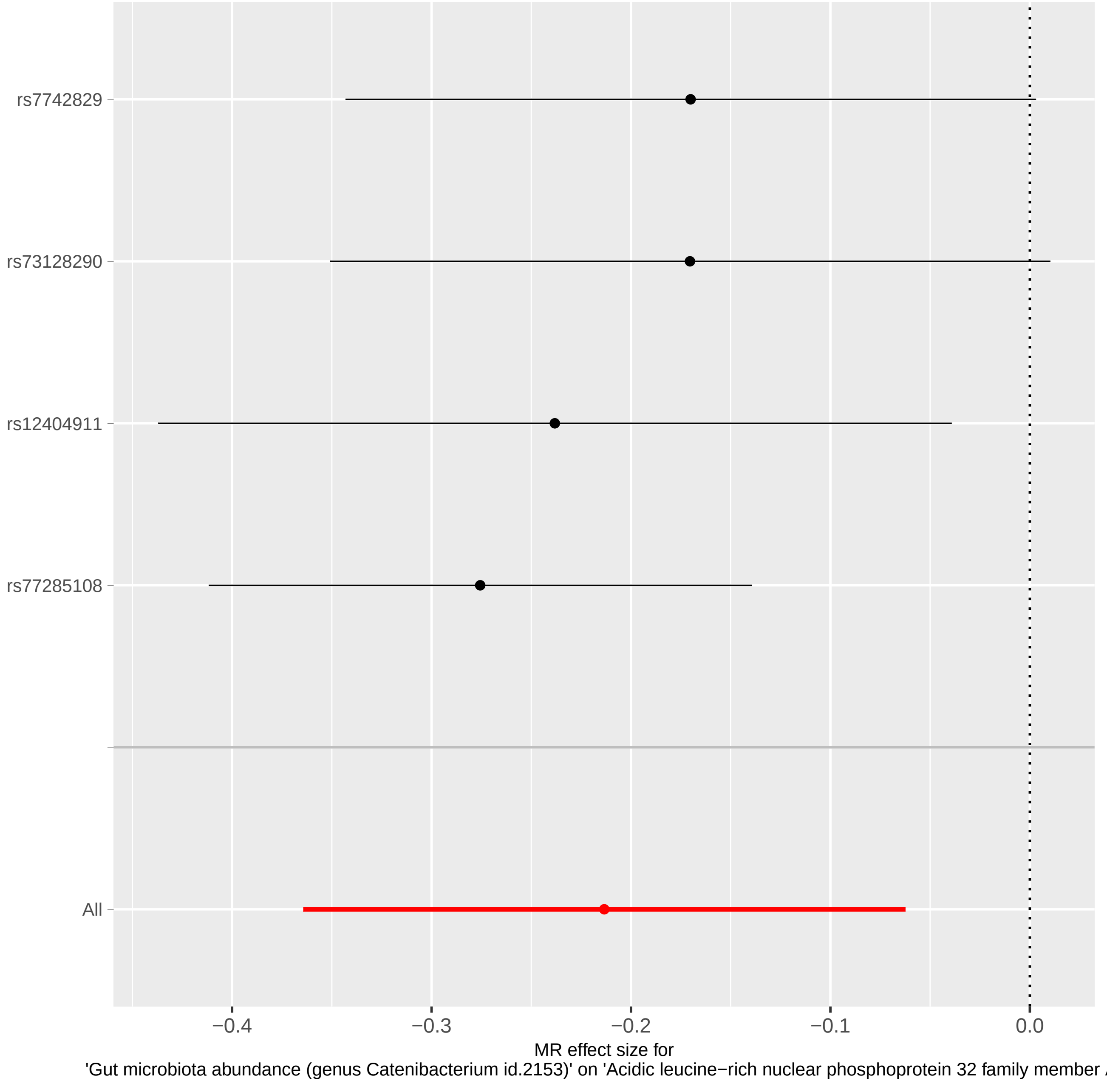

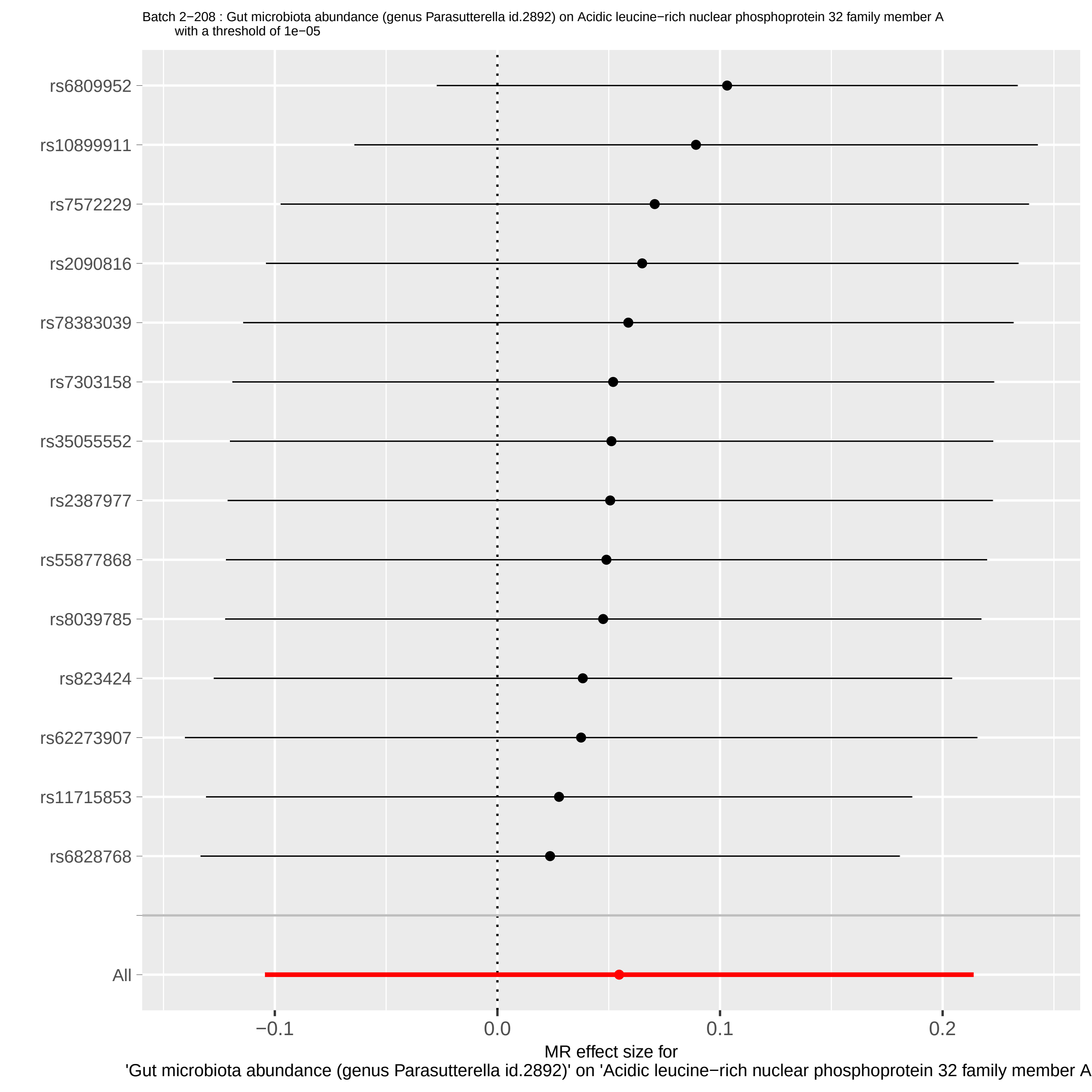

Batch 2−208 : Gut microbiota abundance (genus Parasutterella id.2892) on Acidic leucine−rich nuclear phosphoprotein 32 family member A
with a threshold of 1e−05
rs6809952
rs10899911
rs7572229
rs2090816
rs78383039
rs7303158
rs35055552
rs2387977
rs55877868
rs8039785
rs823424
rs62273907
rs11715853
rs6828768
All
−0.1
0.0
0.1
0.2
MR effect size for
'Gut microbiota abundance (genus Parasutterella id.2892)' on 'Acidic leucine−rich nuclear phosphoprotein 32 family member A

Batch 2−209 : Gut microbiota abundance (genus Catenibacterium id.2153) on Acidic leucine−rich nuclear phosphoprotein 32 family member B with a threshold of 1e−05

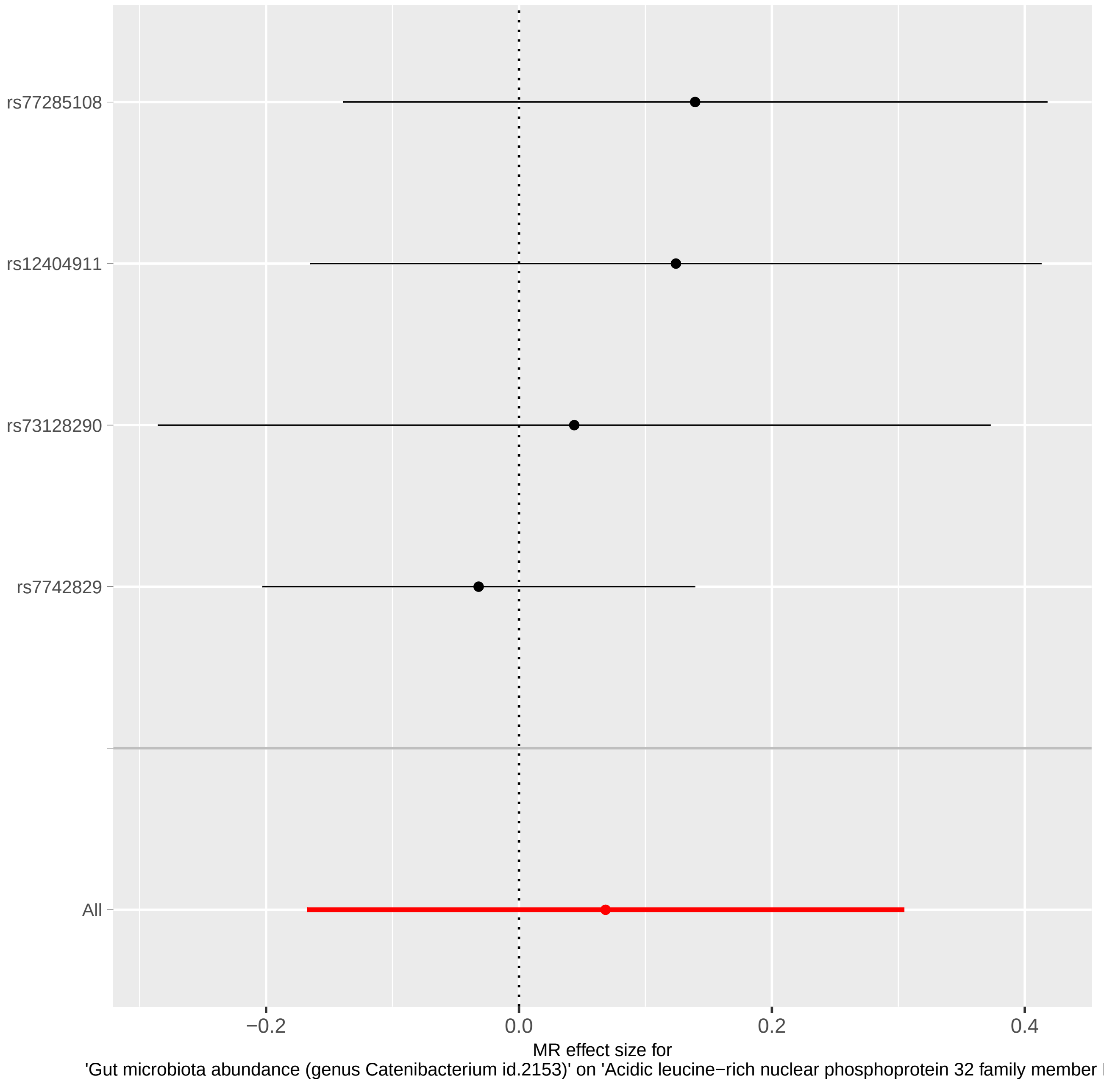

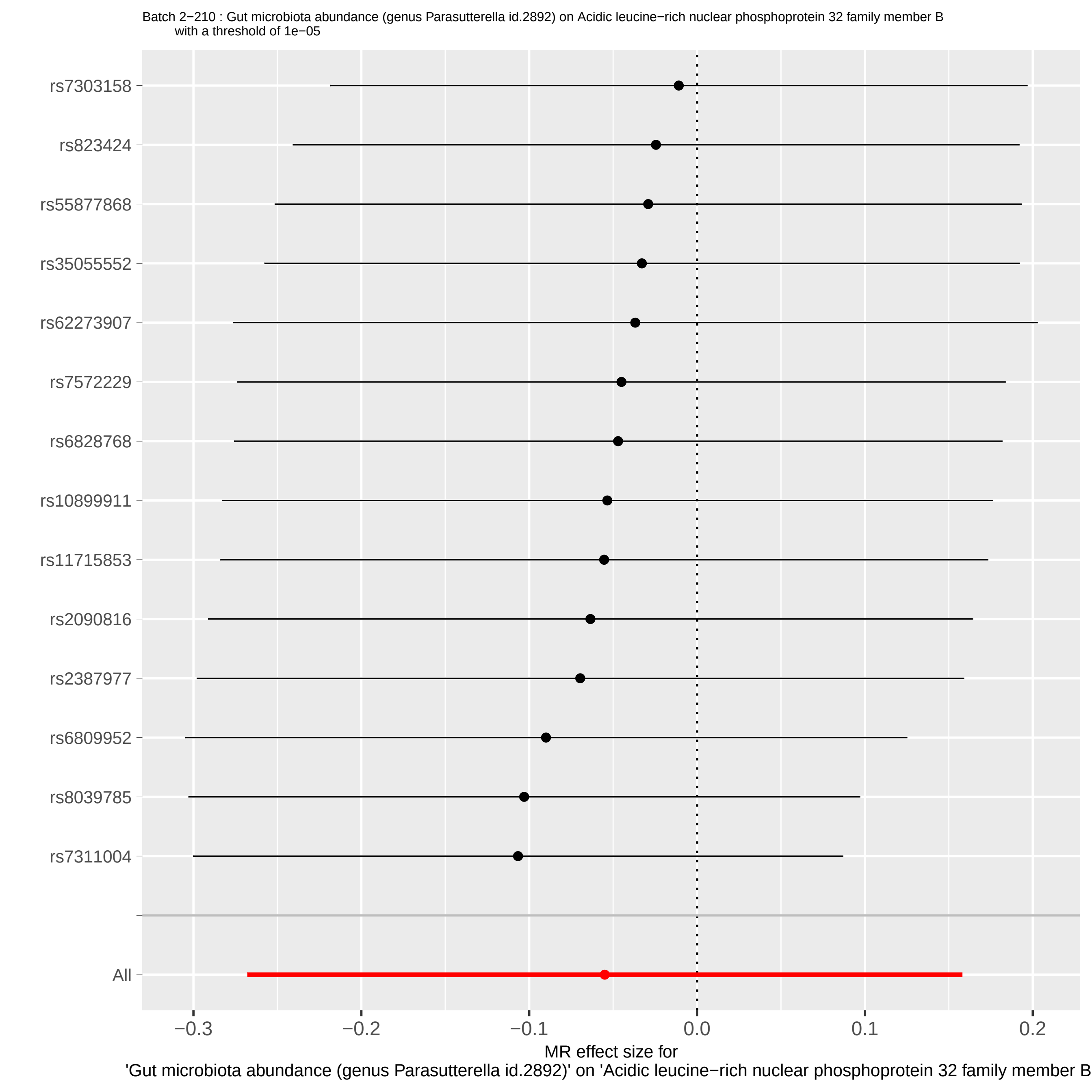

Batch 2−210 : Gut microbiota abundance (genus Parasutterella id.2892) on Acidic leucine−rich nuclear phosphoprotein 32 family member B
with a threshold of 1e−05
rs7303158
rs823424
rs55877868
rs35055552
rs62273907
rs7572229
rs6828768
rs10899911
rs11715853
rs2090816
rs2387977
rs6809952
rs8039785
rs7311004
All
−0.3
−0.2
−0.1
0.0
0.1
0.2
MR effect size for
'Gut microbiota abundance (genus Parasutterella id.2892)' on 'Acidic leucine−rich nuclear phosphoprotein 32 family member B

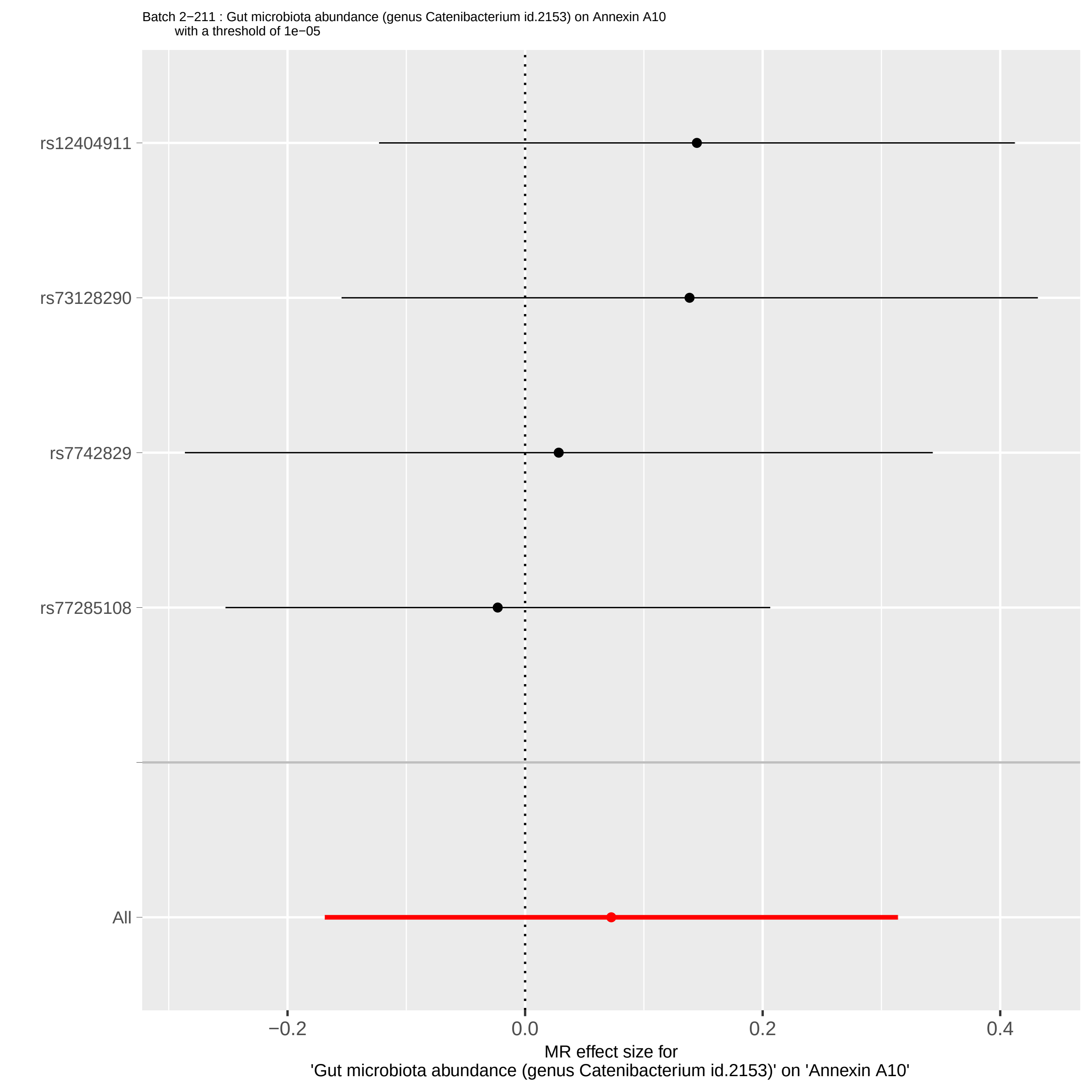
Batch 2−211 : Gut microbiota abundance (genus Catenibacterium id.2153) on Annexin A10
with a threshold of 1e−05
rs12404911
rs73128290
rs7742829
rs77285108
All
−0.2
0.0
0.2
0.4
MR effect size for
'Gut microbiota abundance (genus Catenibacterium id.2153)' on 'Annexin A10'

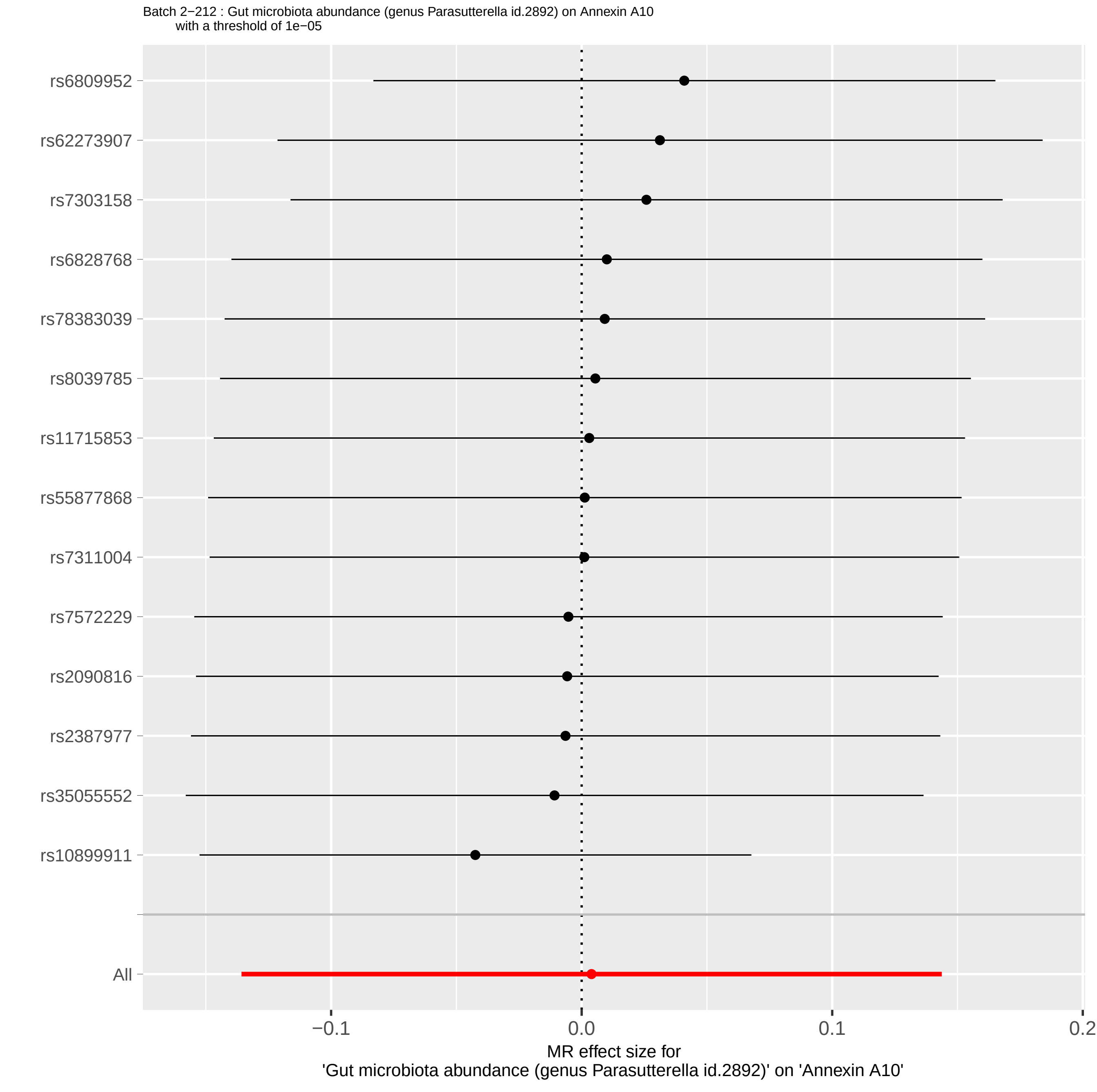
Batch 2−212 : Gut microbiota abundance (genus Parasutterella id.2892) on Annexin A10
with a threshold of 1e−05
rs6809952
rs62273907
rs7303158
rs6828768
rs78383039
rs8039785
rs11715853
rs55877868
rs7311004
rs7572229
rs2090816
rs2387977
rs35055552
rs10899911
All
−0.1
0.0
0.1
0.2
MR effect size for
'Gut microbiota abundance (genus Parasutterella id.2892)' on 'Annexin A10'

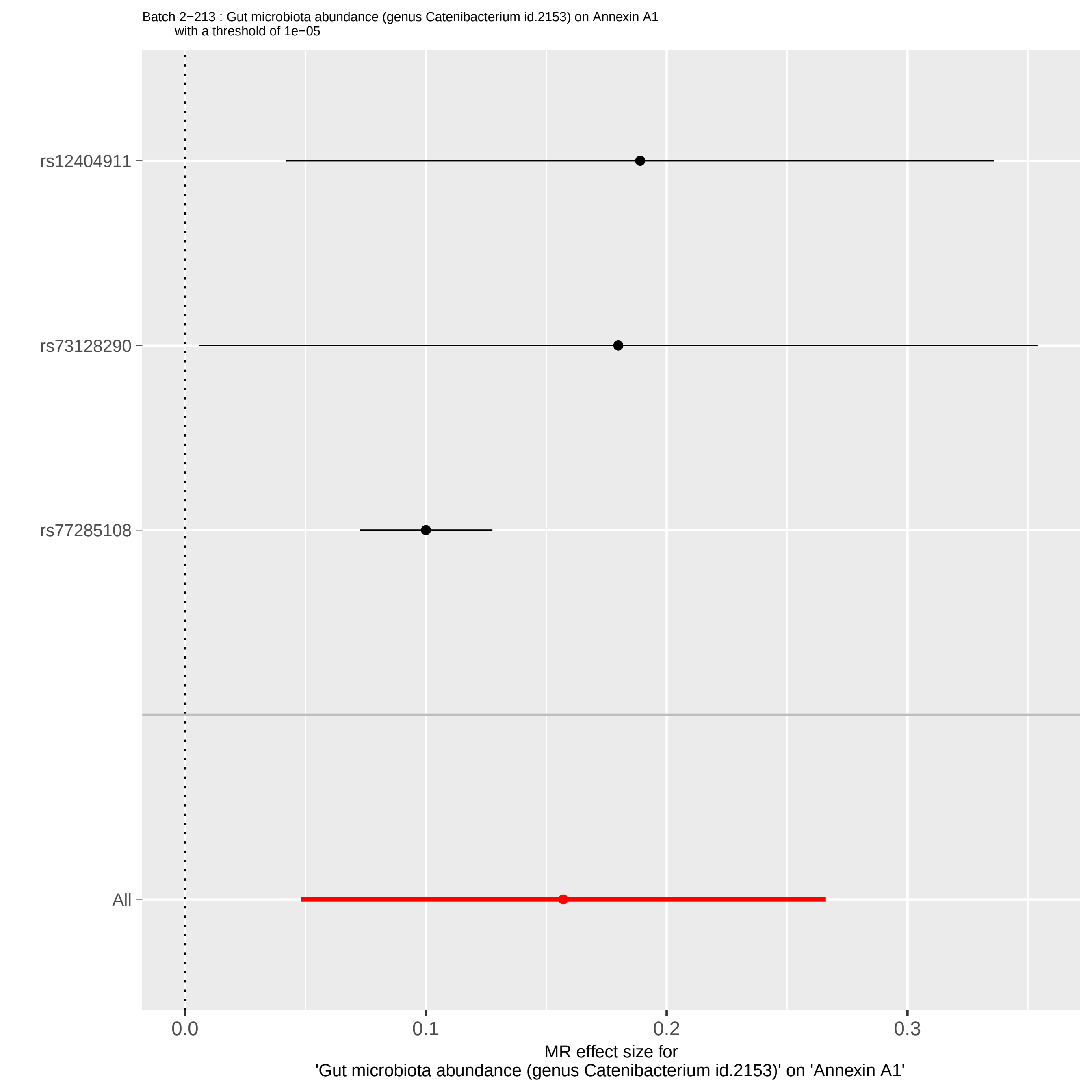

Batch 2−213 : Gut microbiota abundance (genus Catenibacterium id.2153) on Annexin A1
with a threshold of 1e−05
rs12404911
rs73128290
rs77285108
All
0.0
0.1
0.2
0.3
MR effect size for
'Gut microbiota abundance (genus Catenibacterium id.2153)' on 'Annexin A1'

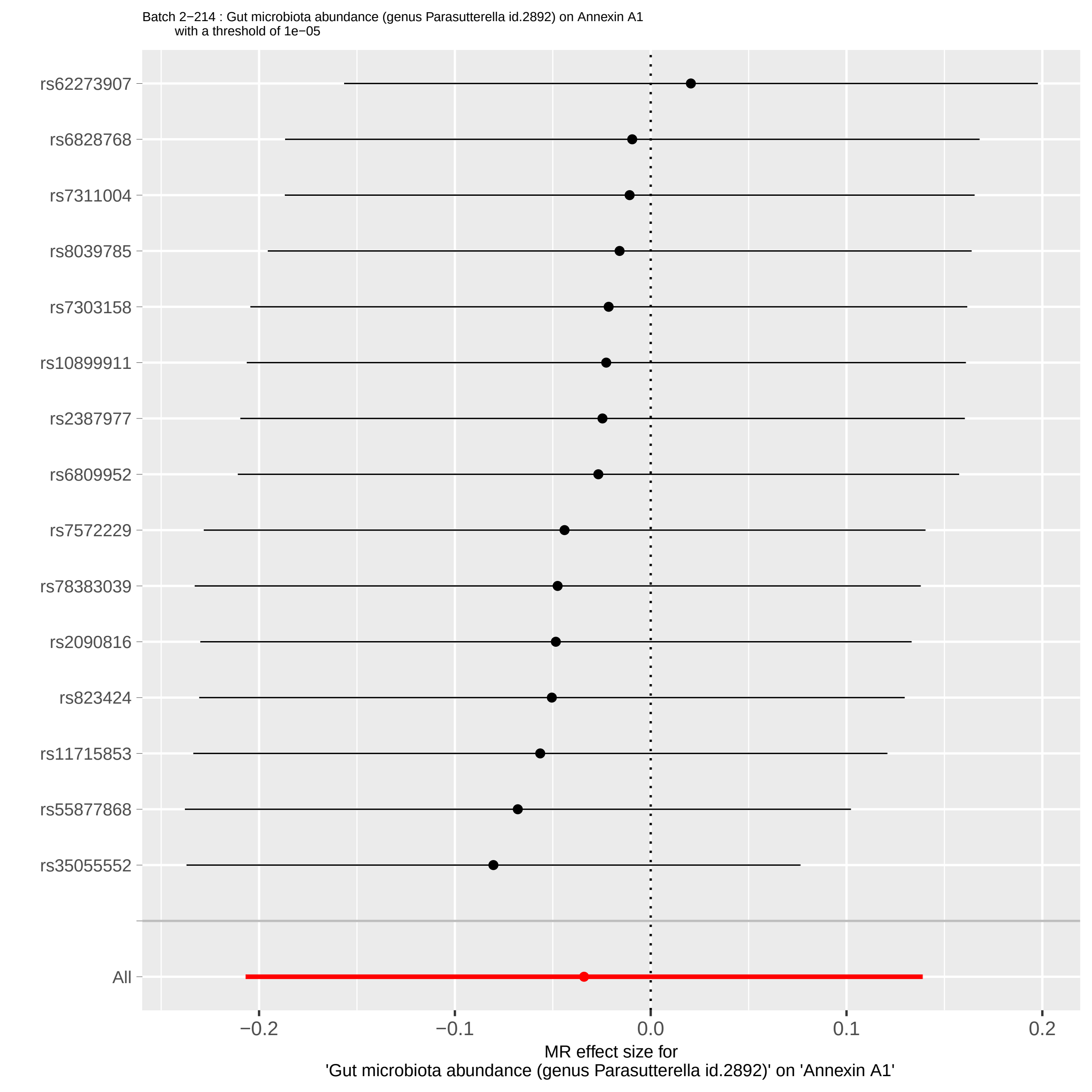
Batch 2−214 : Gut microbiota abundance (genus Parasutterella id.2892) on Annexin A1
with a threshold of 1e−05
rs62273907
rs6828768
rs7311004
rs8039785
rs7303158
rs10899911
rs2387977
rs6809952
rs7572229
rs78383039
rs2090816
rs823424
rs11715853
rs55877868
rs35055552
All
−0.2
−0.1
0.0
0.1
0.2
MR effect size for
'Gut microbiota abundance (genus Parasutterella id.2892)' on 'Annexin A1'

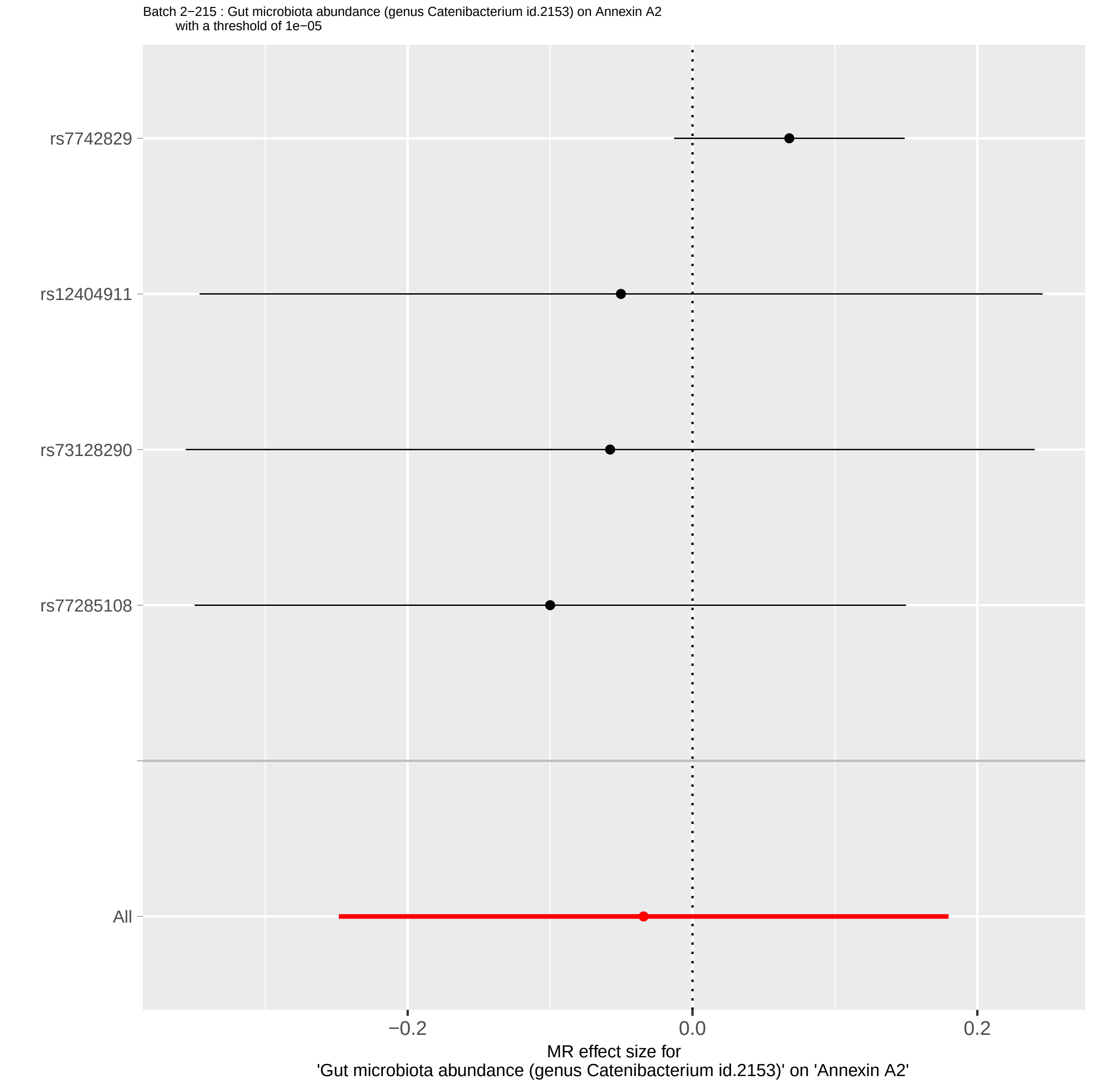

Batch 2−215 : Gut microbiota abundance (genus Catenibacterium id.2153) on Annexin A2
with a threshold of 1e−05
rs7742829
rs12404911
rs73128290
rs77285108
All
−0.2
0.0
0.2
MR effect size for
'Gut microbiota abundance (genus Catenibacterium id.2153)' on 'Annexin A2'

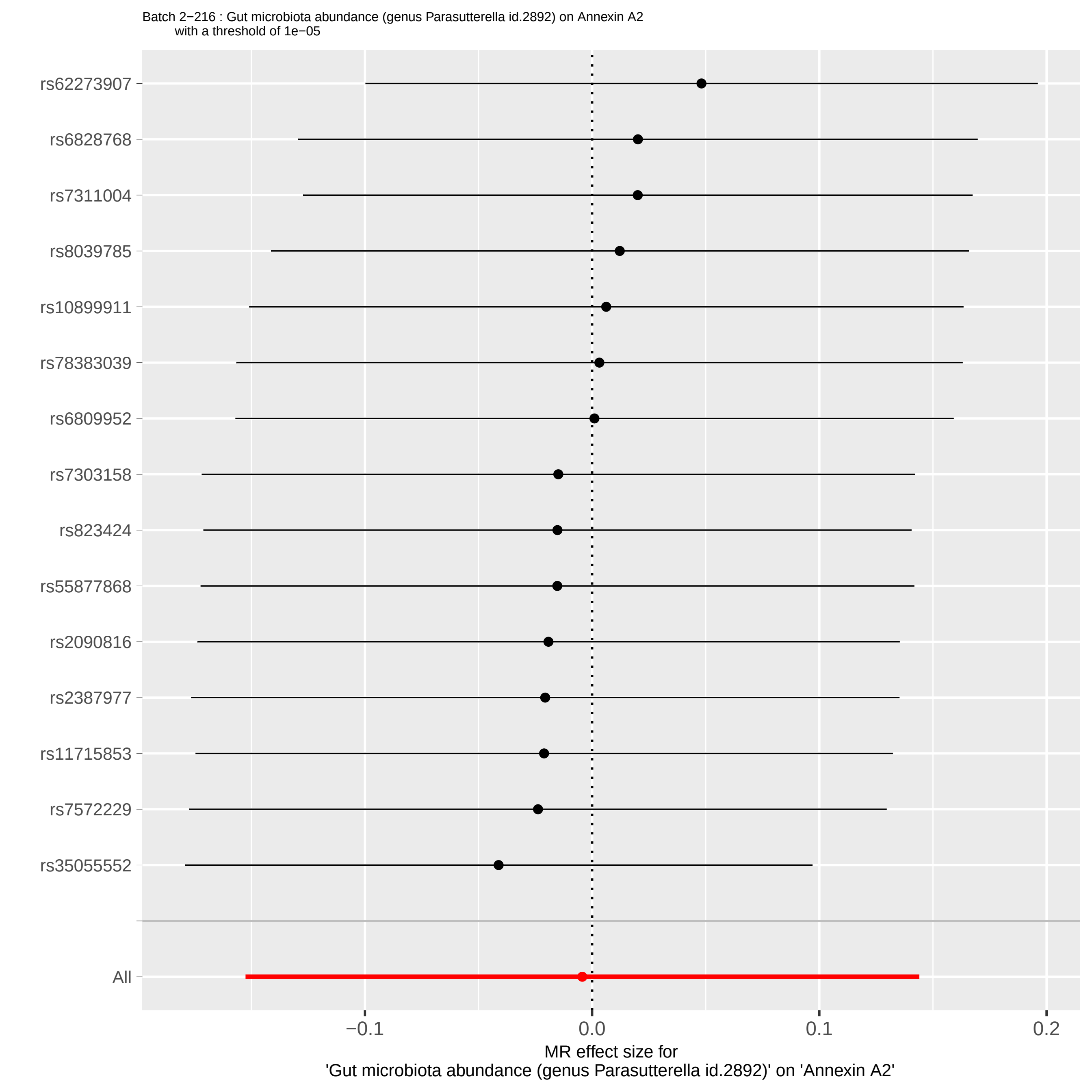

Batch 2−216 : Gut microbiota abundance (genus Parasutterella id.2892) on Annexin A2
with a threshold of 1e−05
rs62273907
rs6828768
rs7311004
rs8039785
rs10899911
rs78383039
rs6809952
rs7303158
rs823424
rs55877868
rs2090816
rs2387977
rs11715853
rs7572229
rs35055552
All
−0.1
0.0
0.1
0.2
MR effect size for
'Gut microbiota abundance (genus Parasutterella id.2892)' on 'Annexin A2'

Batch 2−217 : Gut microbiota abundance (genus Catenibacterium id.2153) on Annexin A2
with a threshold of 1e−05

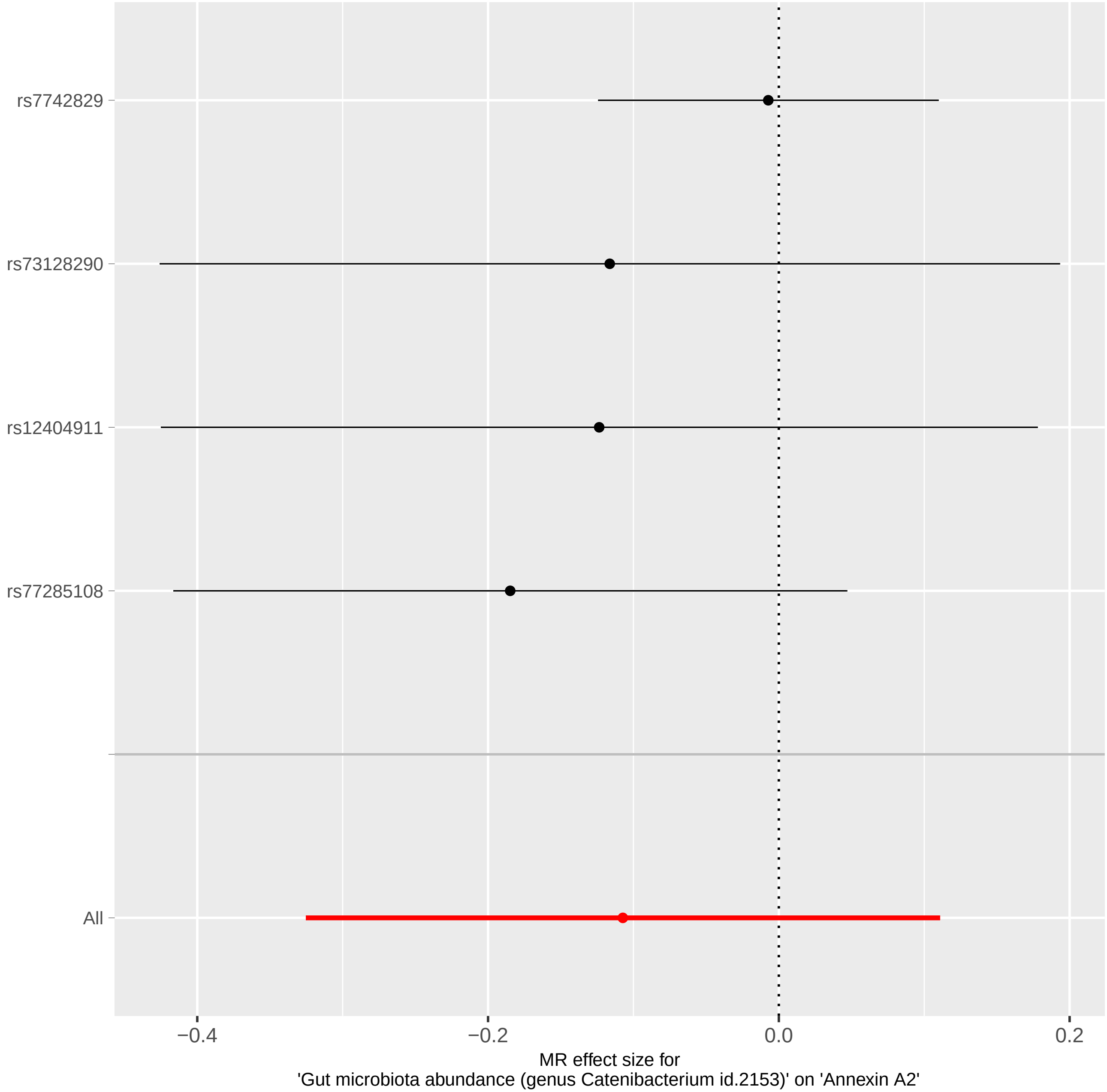

Batch 2−218 : Gut microbiota abundance (genus Parasutterella id.2892) on Annexin A2
with a threshold of 1e−05

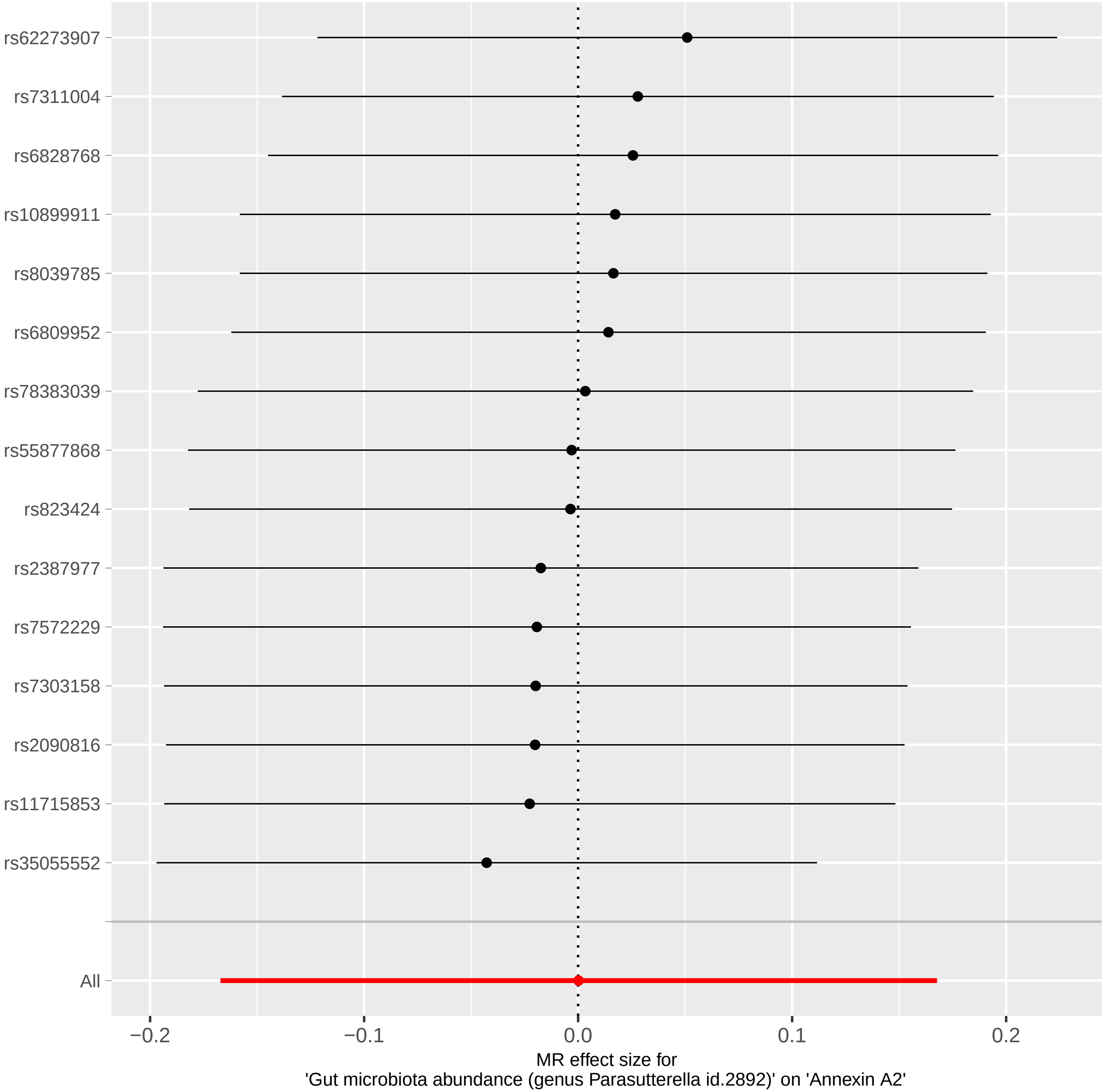

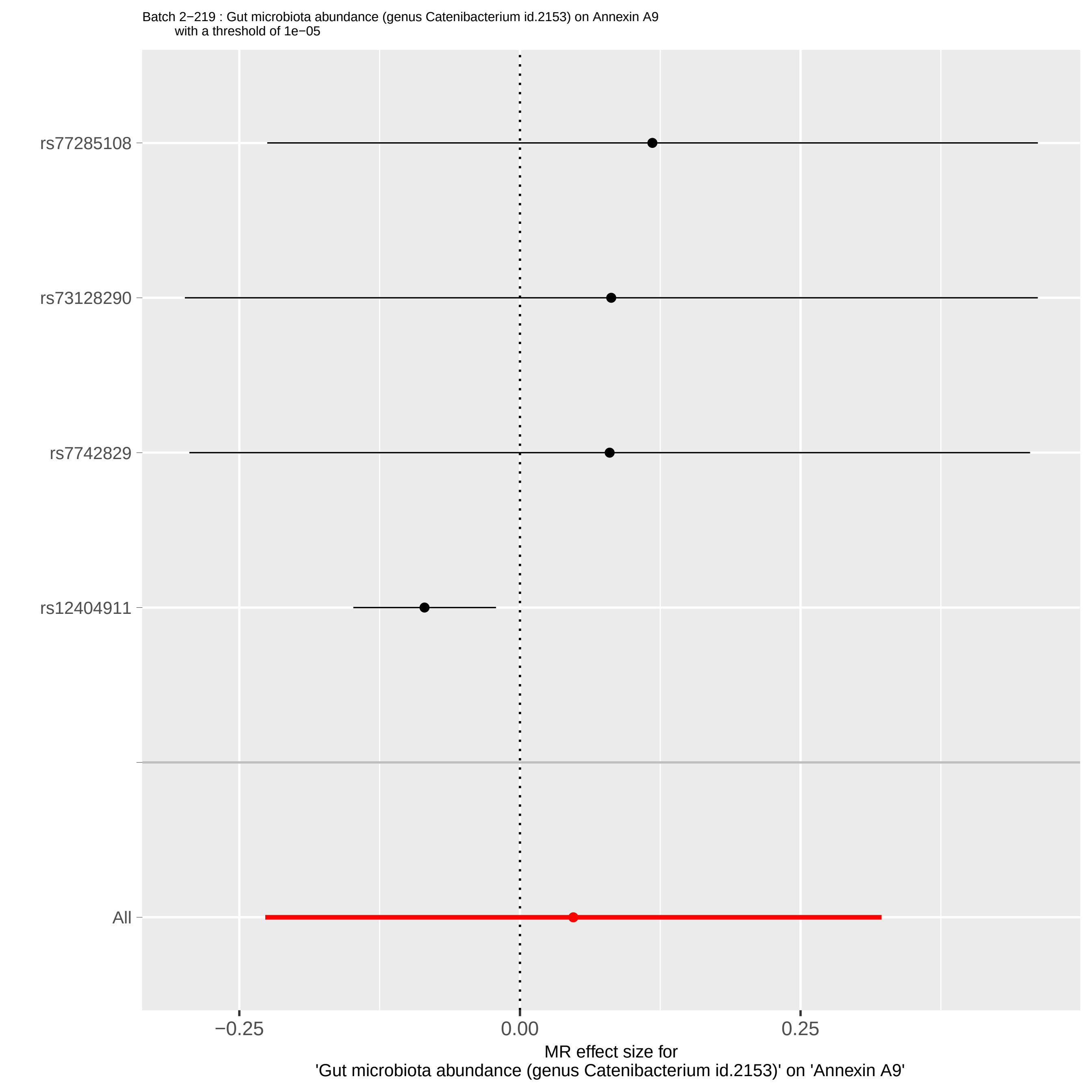
Batch 2−219 : Gut microbiota abundance (genus Catenibacterium id.2153) on Annexin A9
with a threshold of 1e−05
rs77285108
rs73128290
rs7742829
rs12404911
All
−0.25
0.00
0.25
MR effect size for
'Gut microbiota abundance (genus Catenibacterium id.2153)' on 'Annexin A9'

Batch 2−220 : Gut microbiota abundance (genus Parasutterella id.2892) on Annexin A9
with a threshold of 1e−05

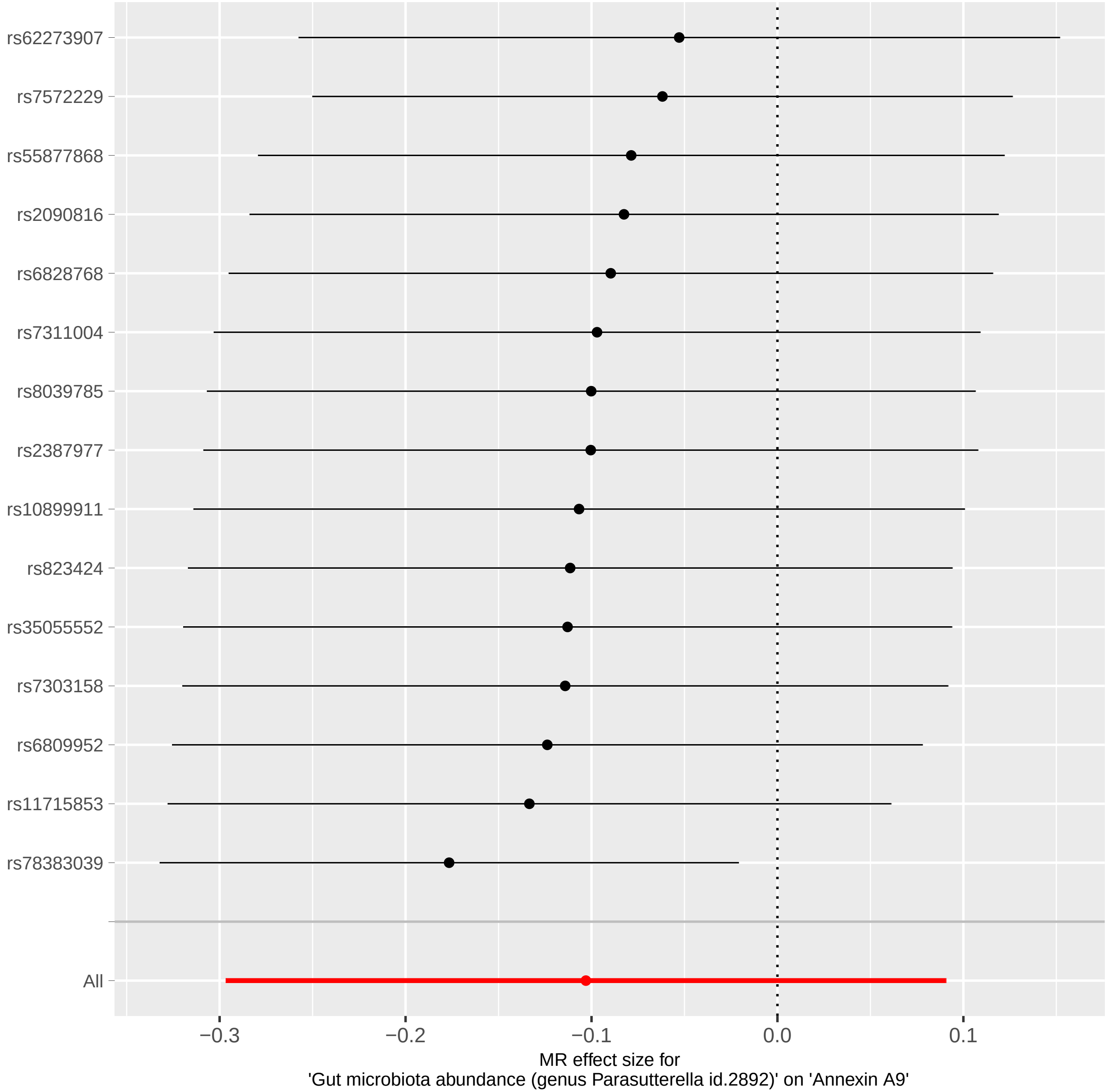

Batch 2−221 : Gut microbiota abundance (genus Catenibacterium id.2153) on Retina−specific copper amine oxidase
with a threshold of 1e−05

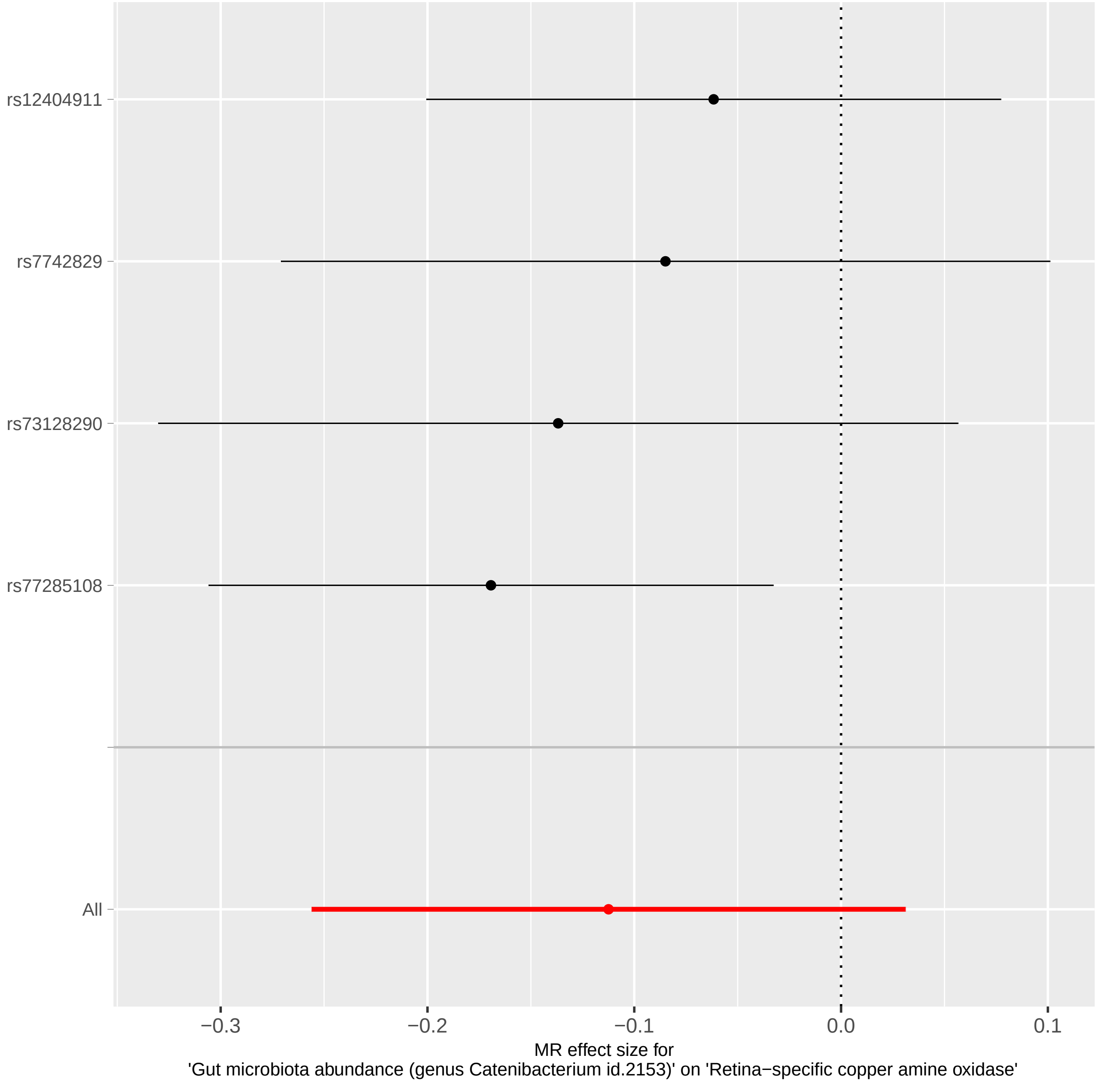

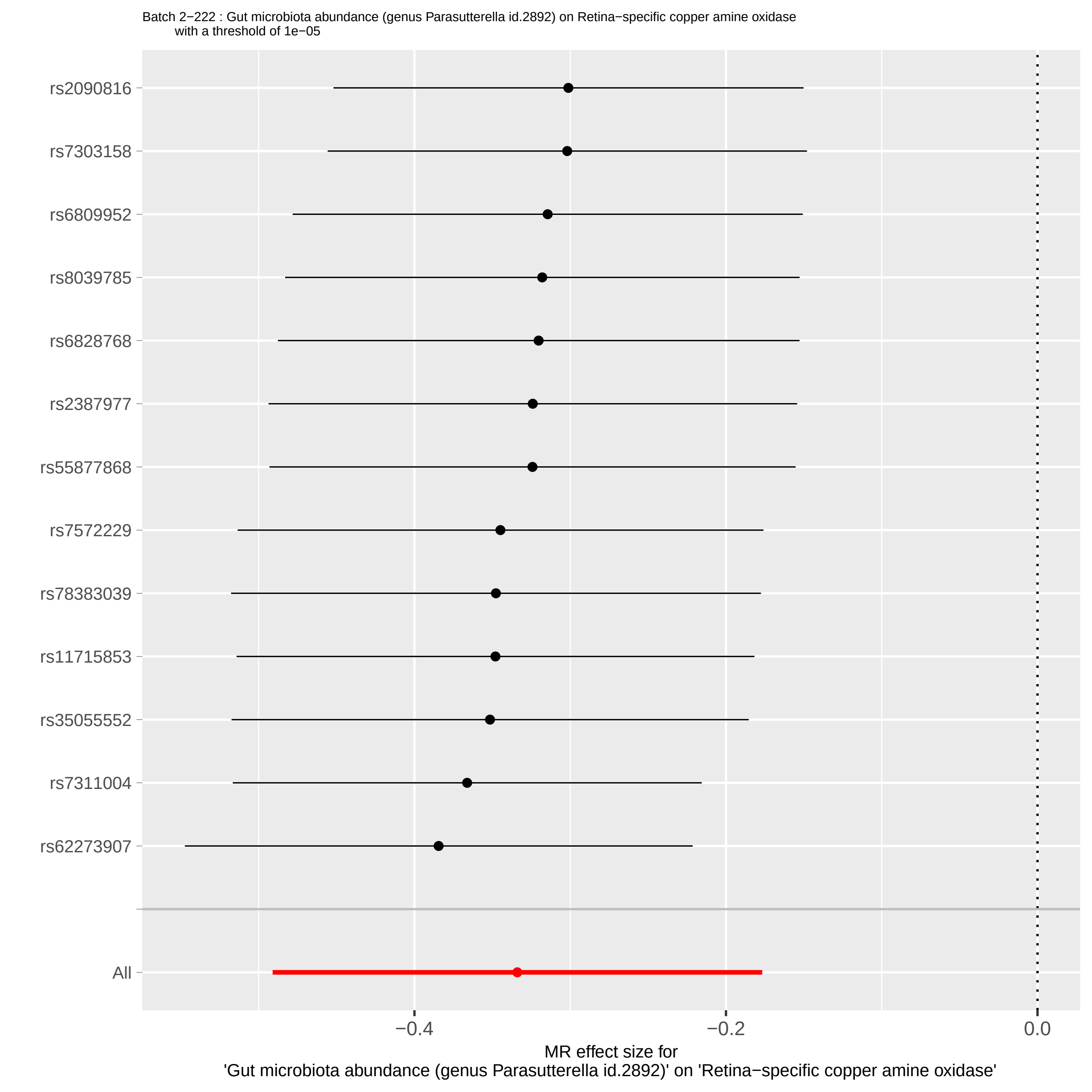
Batch 2−222 : Gut microbiota abundance (genus Parasutterella id.2892) on Retina−specific copper amine oxidase
with a threshold of 1e−05
rs2090816
rs7303158
rs6809952
rs8039785
rs6828768
rs2387977
rs55877868
rs7572229
rs78383039
rs11715853
rs35055552
rs7311004
rs62273907
All
−0.4
−0.2
0.0
MR effect size for
'Gut microbiota abundance (genus Parasutterella id.2892)' on 'Retina−specific copper amine oxidase'

Batch 2−223 : Gut microbiota abundance (genus Catenibacterium id.2153) on AP−4 complex subunit mu−1
with a threshold of 1e−05

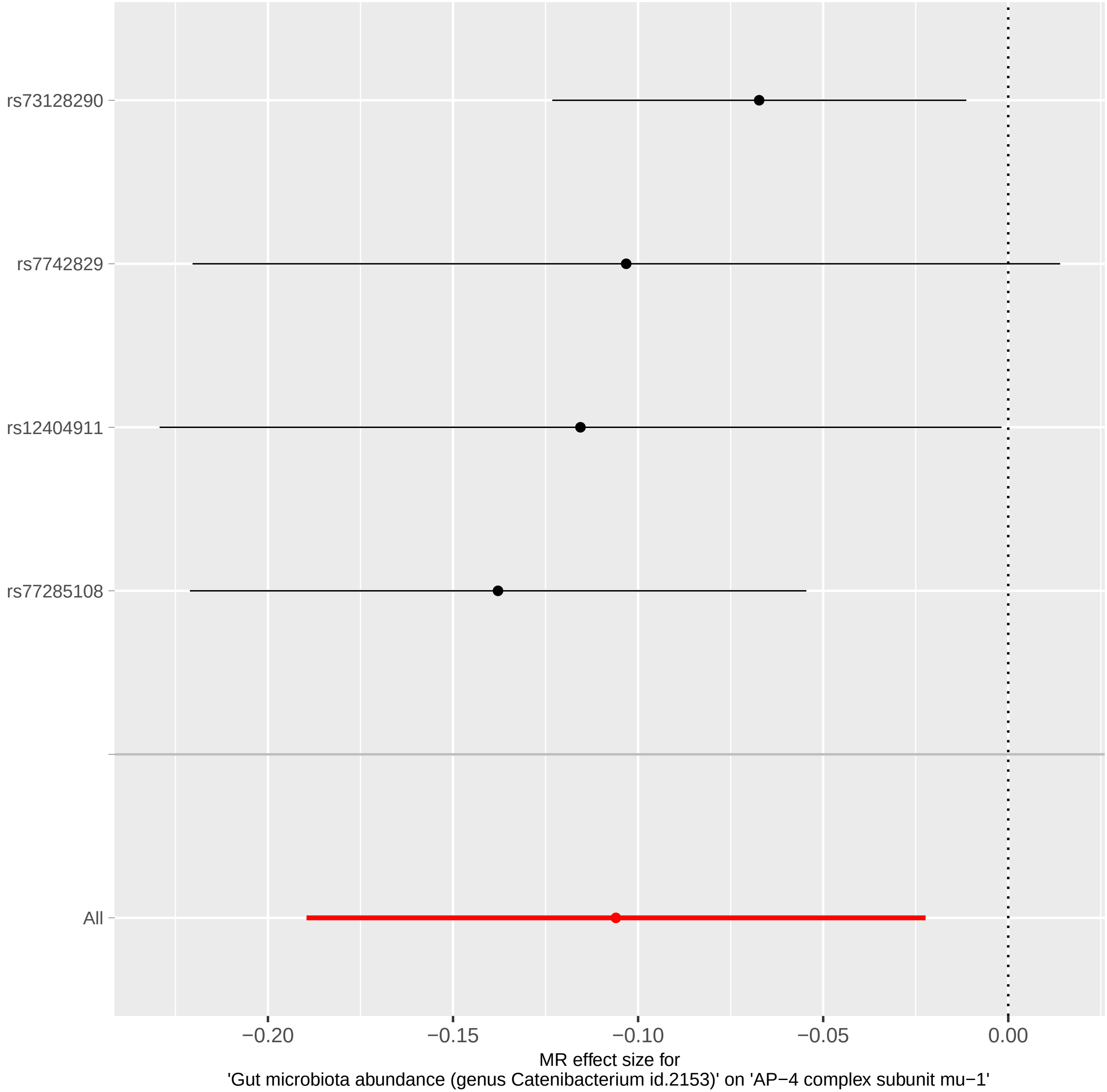

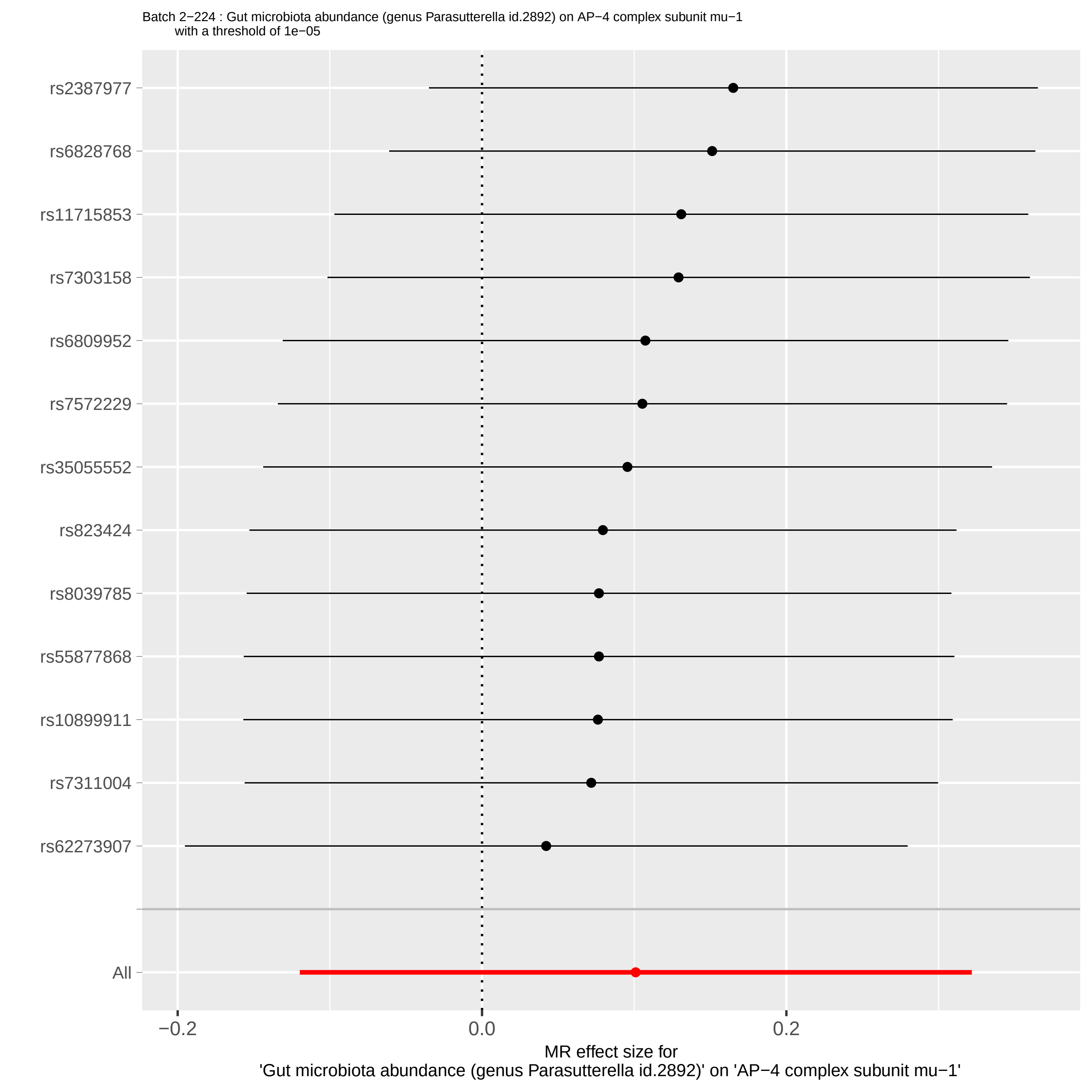
Batch 2−224 : Gut microbiota abundance (genus Parasutterella id.2892) on AP−4 complex subunit mu−1
with a threshold of 1e−05
rs2387977
rs6828768
rs11715853
rs7303158
rs6809952
rs7572229
rs35055552
rs823424
rs8039785
rs55877868
rs10899911
rs7311004
rs62273907
All
−0.2
0.0
0.2
MR effect size for
'Gut microbiota abundance (genus Parasutterella id.2892)' on 'AP−4 complex subunit mu−1'

Batch 2−225 : Gut microbiota abundance (genus Catenibacterium id.2153) on Apoptotic protease−activating factor 1
with a threshold of 1e−05

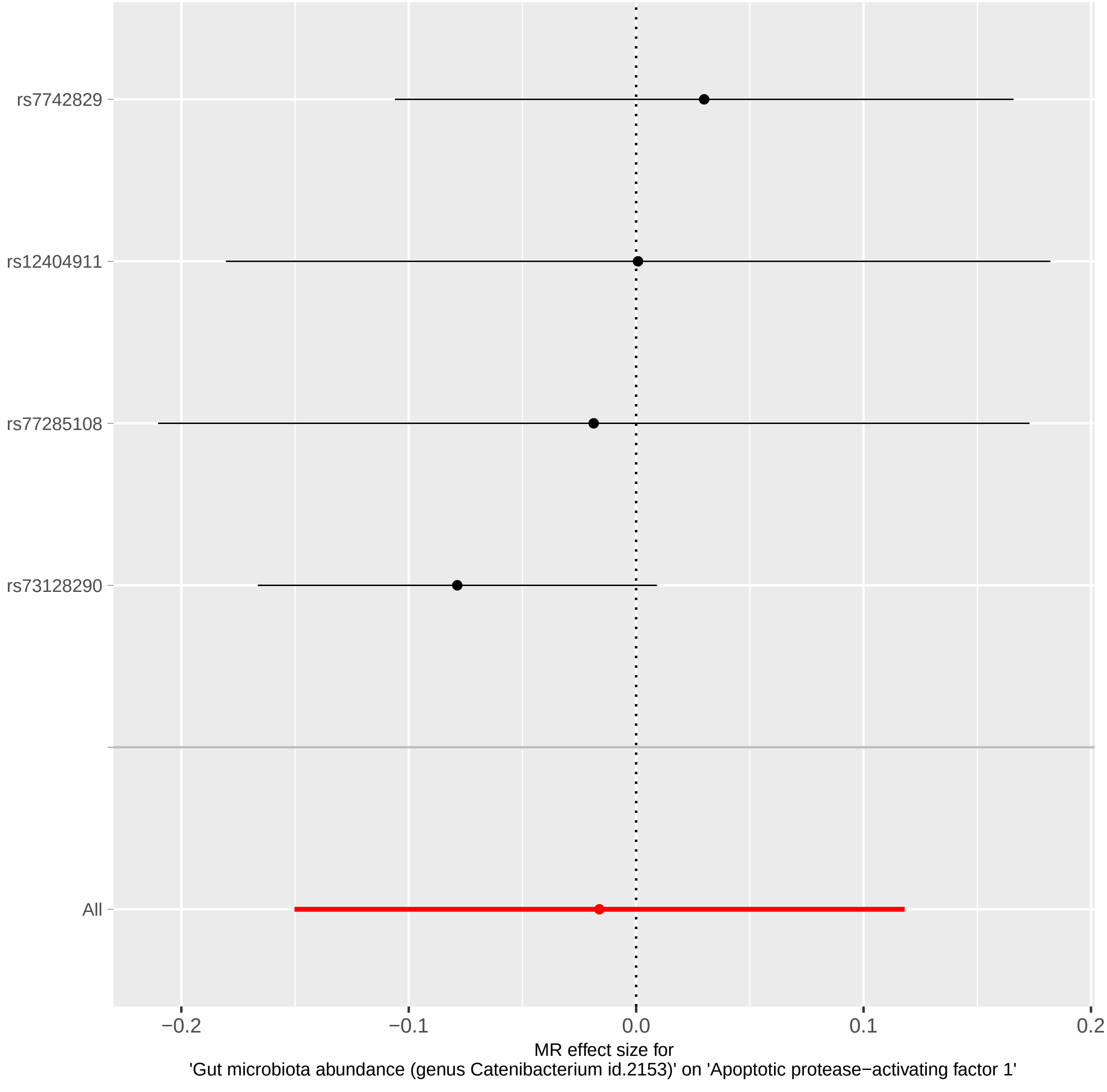

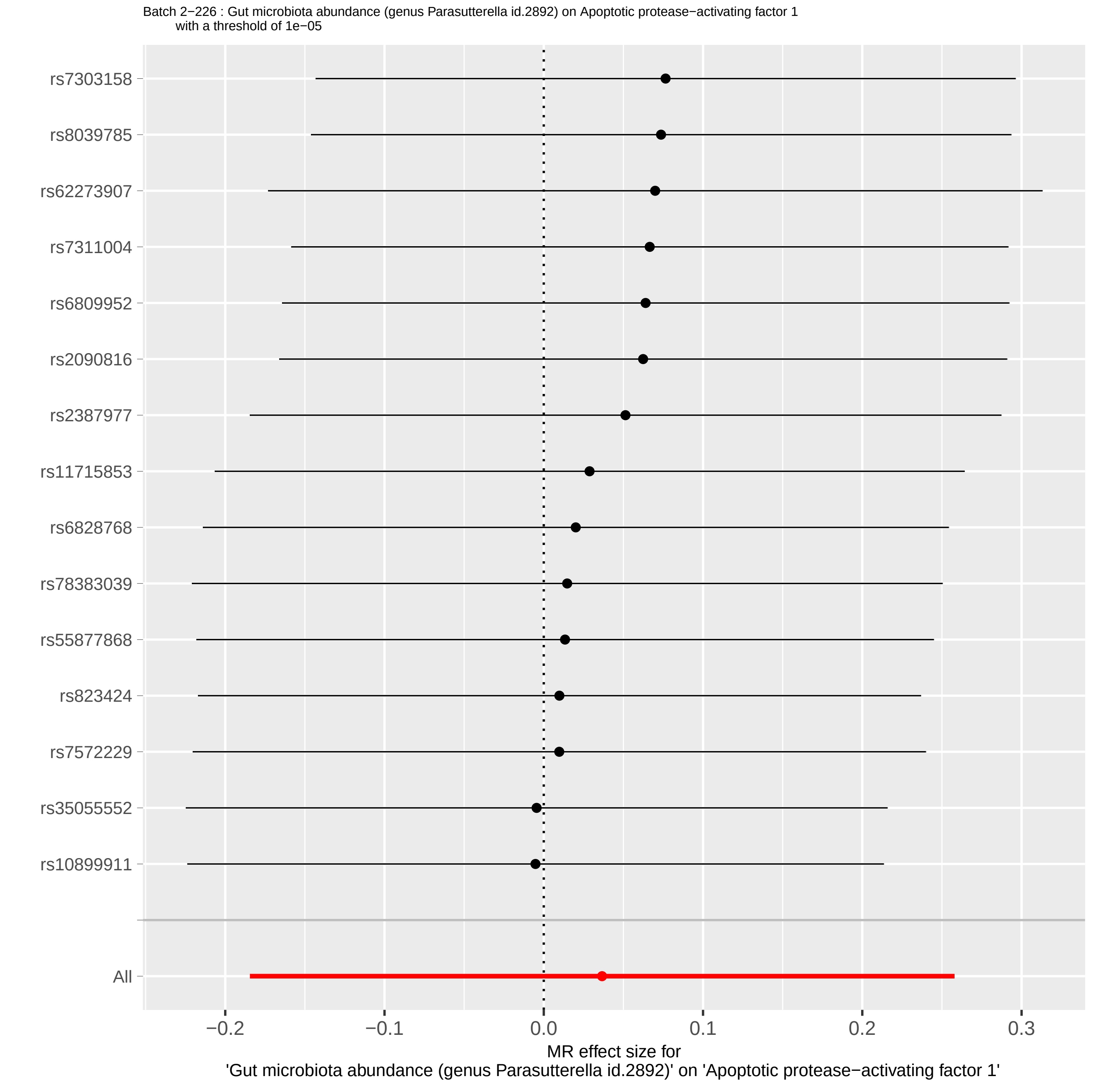
Batch 2−226 : Gut microbiota abundance (genus Parasutterella id.2892) on Apoptotic protease−activating factor 1
with a threshold of 1e−05
rs7303158
rs8039785
rs62273907
rs7311004
rs6809952
rs2090816
rs2387977
rs11715853
rs6828768
rs78383039
rs55877868
rs823424
rs7572229
rs35055552
rs10899911
All
−0.2
−0.1
0.0
0.1
0.2
0.3
MR effect size for
'Gut microbiota abundance (genus Parasutterella id.2892)' on 'Apoptotic protease−activating factor 1'

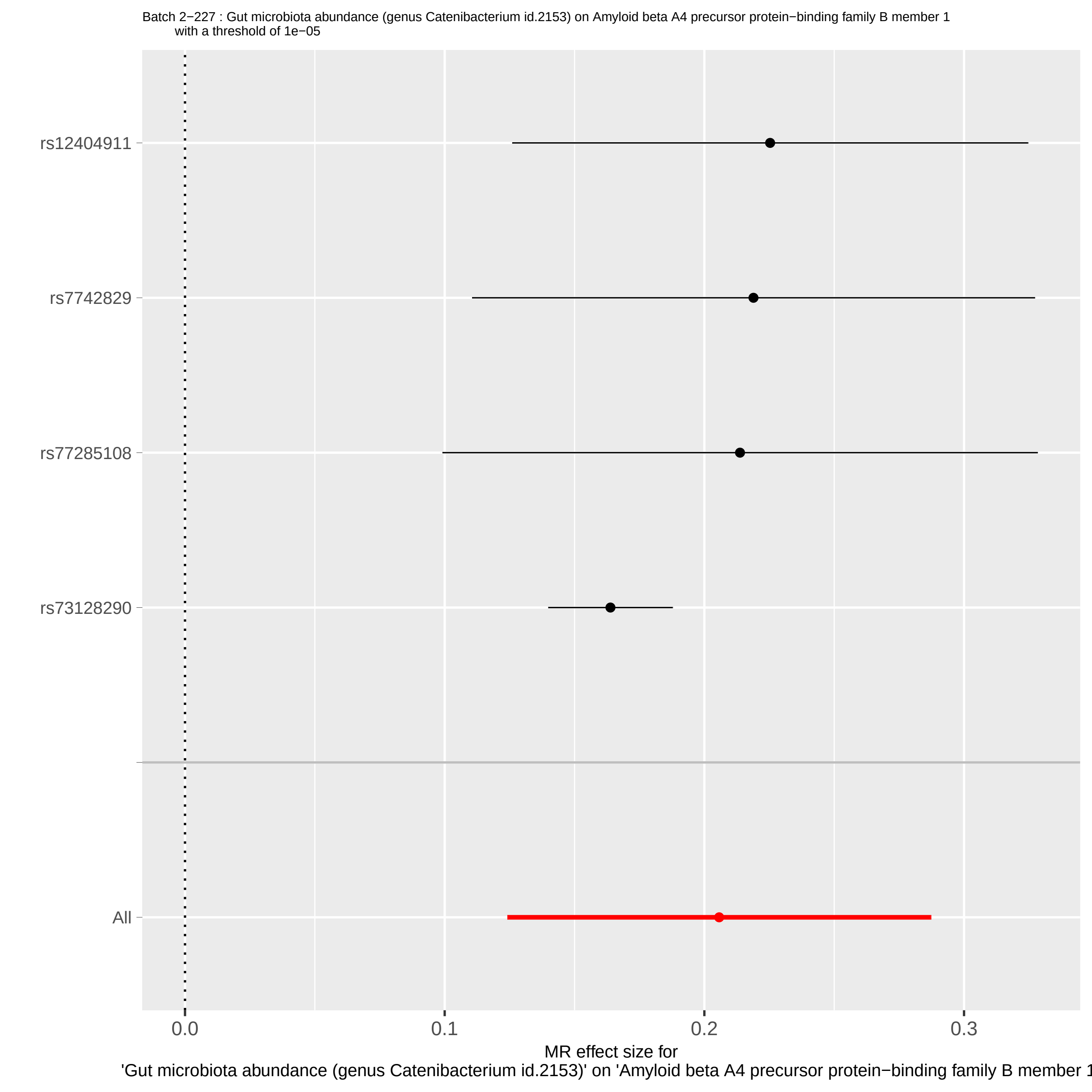
Batch 2−227 : Gut microbiota abundance (genus Catenibacterium id.2153) on Amyloid beta A4 precursor protein−binding family B member 1
with a threshold of 1e−05
rs12404911
rs7742829
rs77285108
rs73128290
All
0.0
0.1
0.2
0.3
MR effect size for
'Gut microbiota abundance (genus Catenibacterium id.2153)' on 'Amyloid beta A4 precursor protein−binding family B member 1

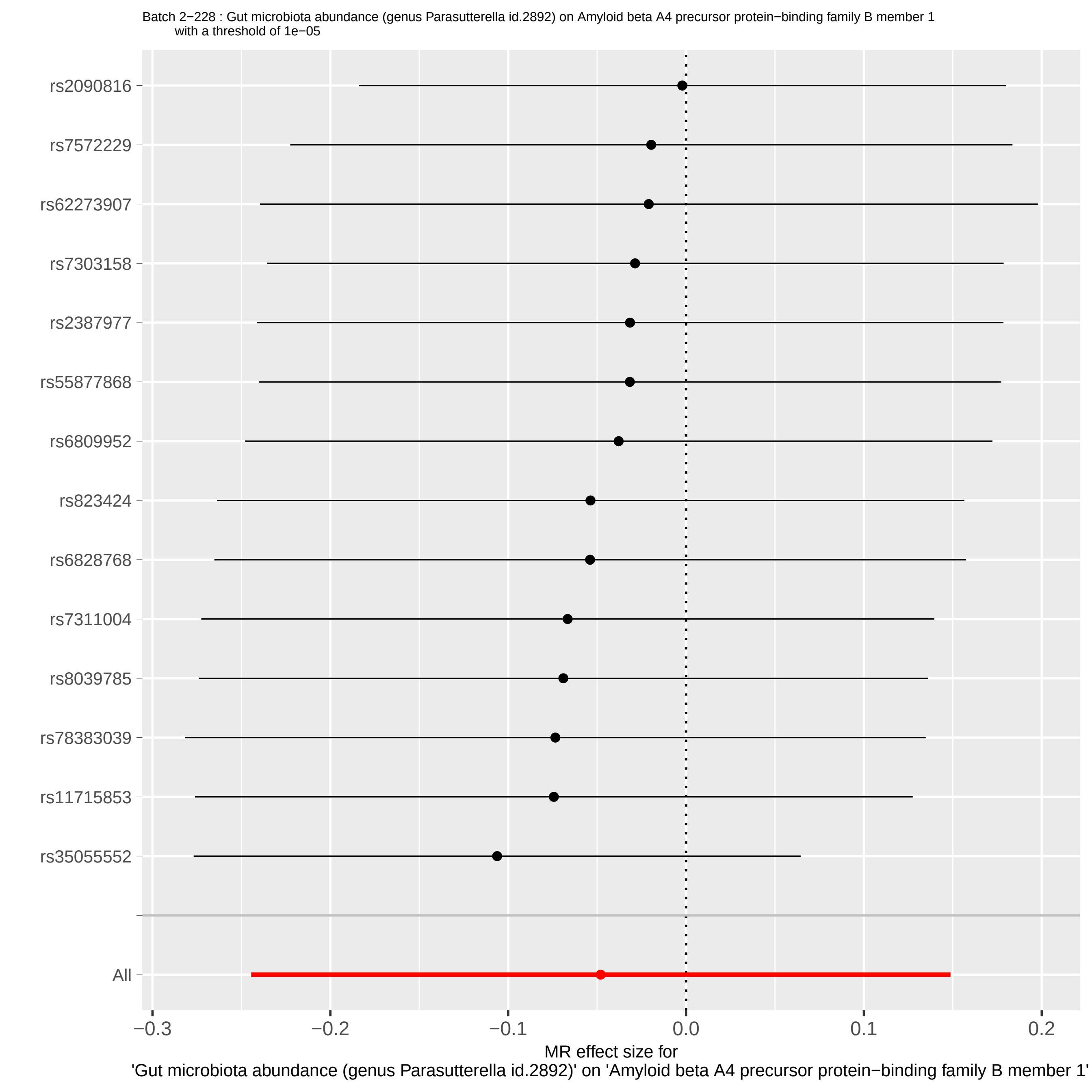
Batch 2−228 : Gut microbiota abundance (genus Parasutterella id.2892) on Amyloid beta A4 precursor protein−binding family B member 1 with a threshold of 1e−05
rs2090816
rs7572229
rs62273907
rs7303158
rs2387977
rs55877868
rs6809952
rs823424
rs6828768
rs7311004
rs8039785
rs78383039
rs11715853
rs35055552
All
−0.3
−0.2
−0.1
0.0
0.1
0.2
MR effect size for
'Gut microbiota abundance (genus Parasutterella id.2892)' on 'Amyloid beta A4 precursor protein−binding family B member 1'

Batch 2−229 : Gut microbiota abundance (genus Catenibacterium id.2153) on Amyloid beta A4 precursor protein−binding family B member 1 with a threshold of 1e−05

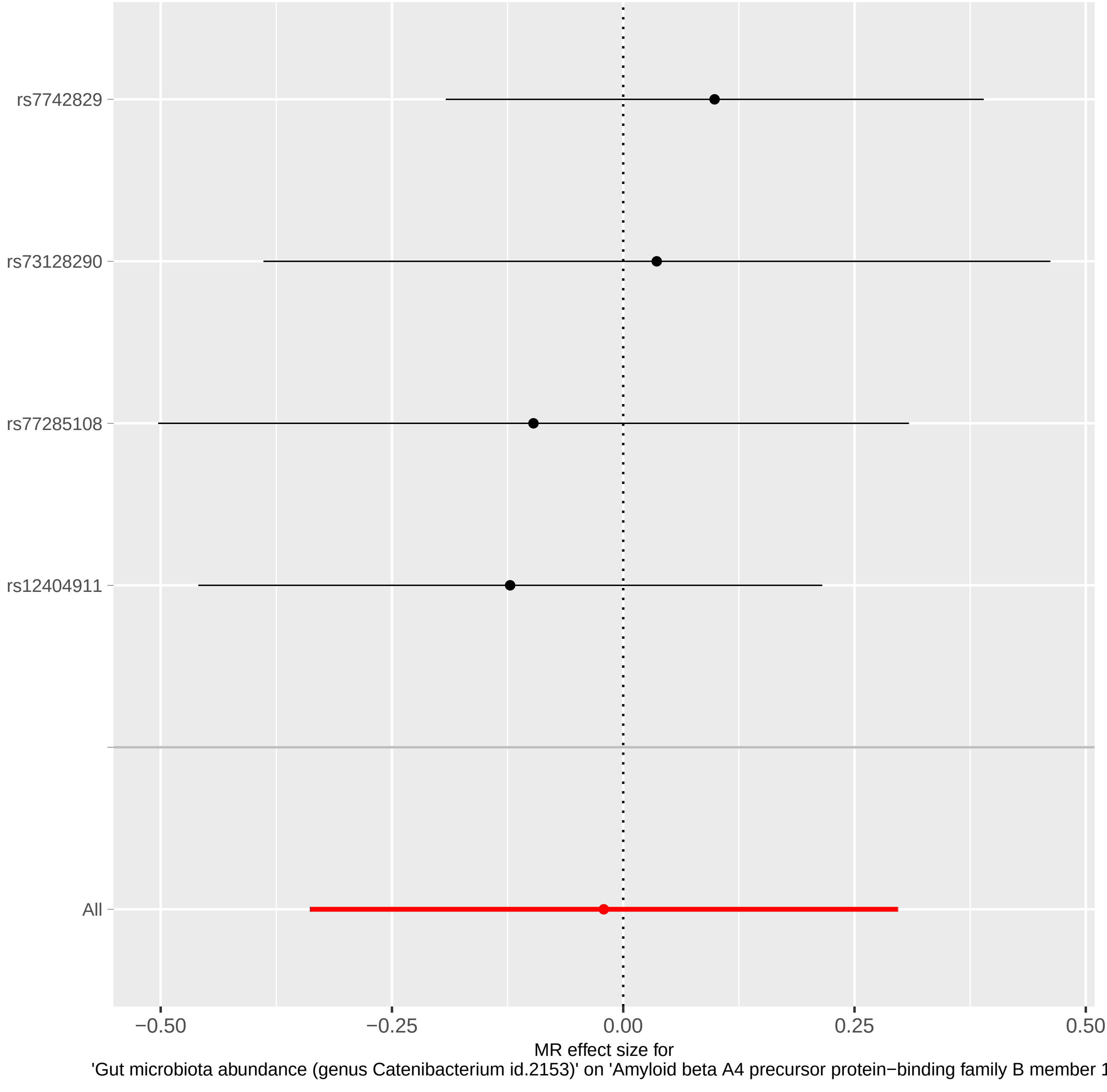

Batch 2−230 : Gut microbiota abundance (genus Parasutterella id.2892) on Amyloid beta A4 precursor protein−binding family B member 1
with a threshold of 1e−05

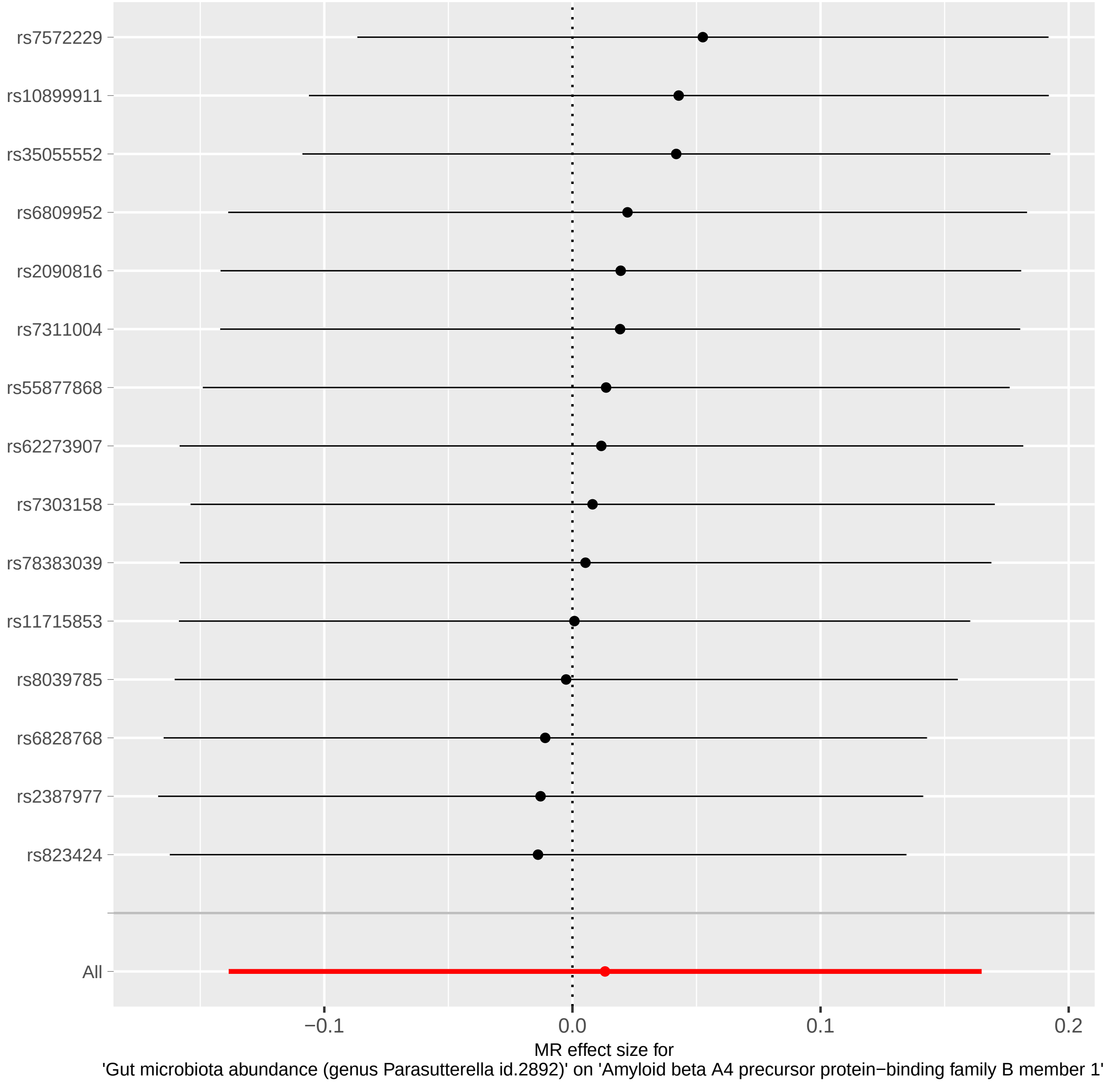

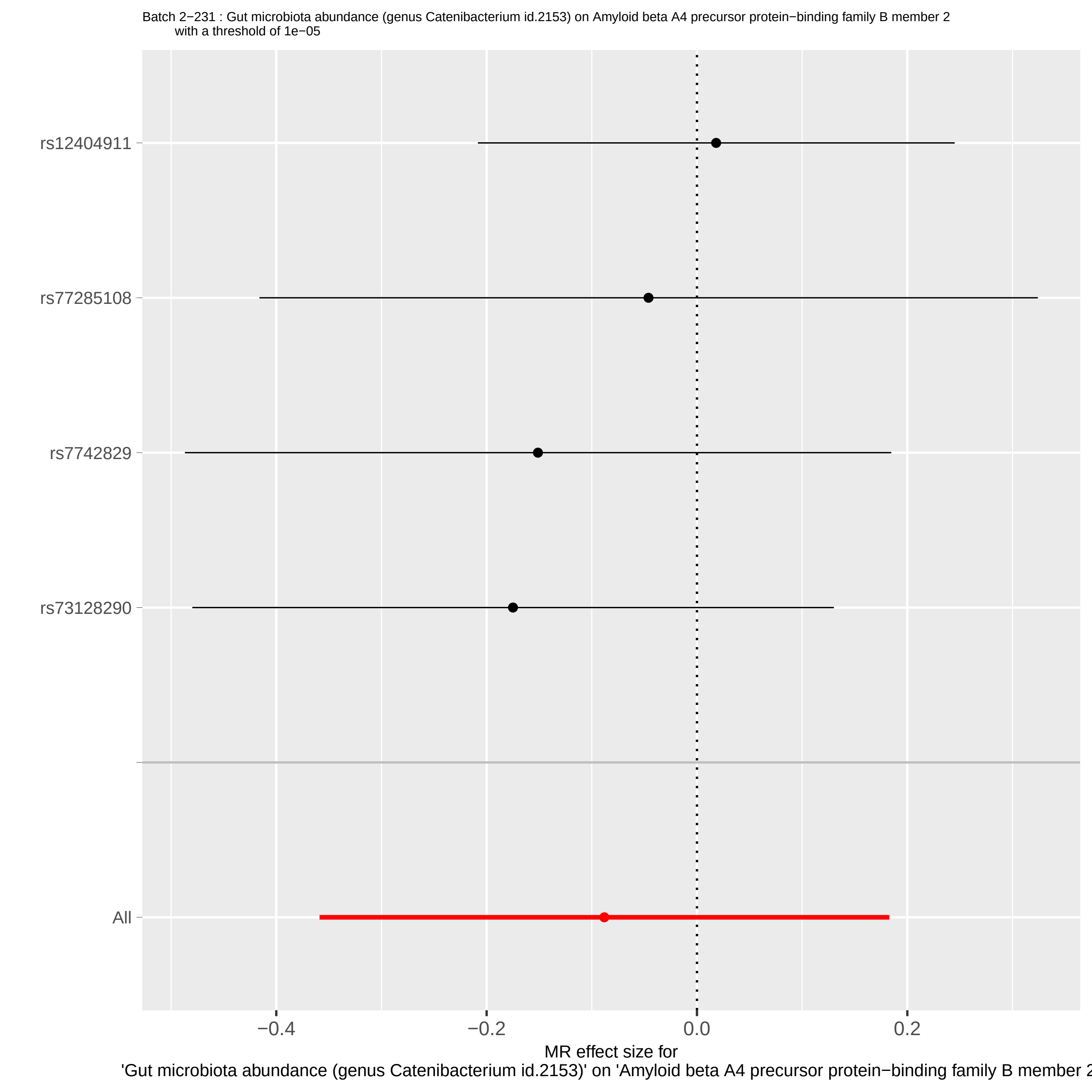

Batch 2−231 : Gut microbiota abundance (genus Catenibacterium id.2153) on Amyloid beta A4 precursor protein−binding family B member 2
with a threshold of 1e−05
rs12404911
rs77285108
rs7742829
rs73128290
All
−0.4
−0.2
0.0
0.2
MR effect size for
'Gut microbiota abundance (genus Catenibacterium id.2153)' on 'Amyloid beta A4 precursor protein−binding family B member 2

Batch 2−232 : Gut microbiota abundance (genus Parasutterella id.2892) on Amyloid beta A4 precursor protein−binding family B member 2
with a threshold of 1e−05

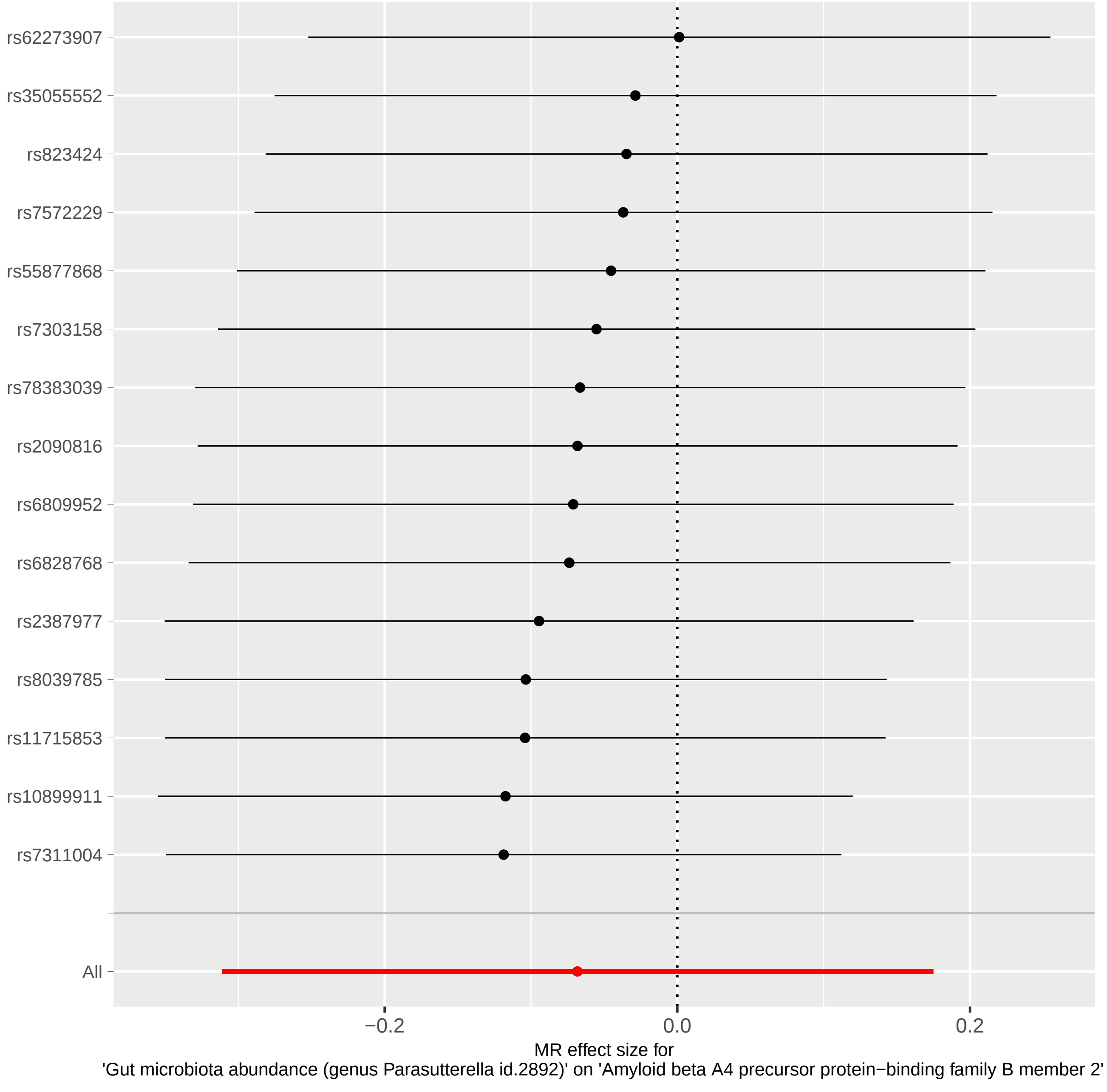

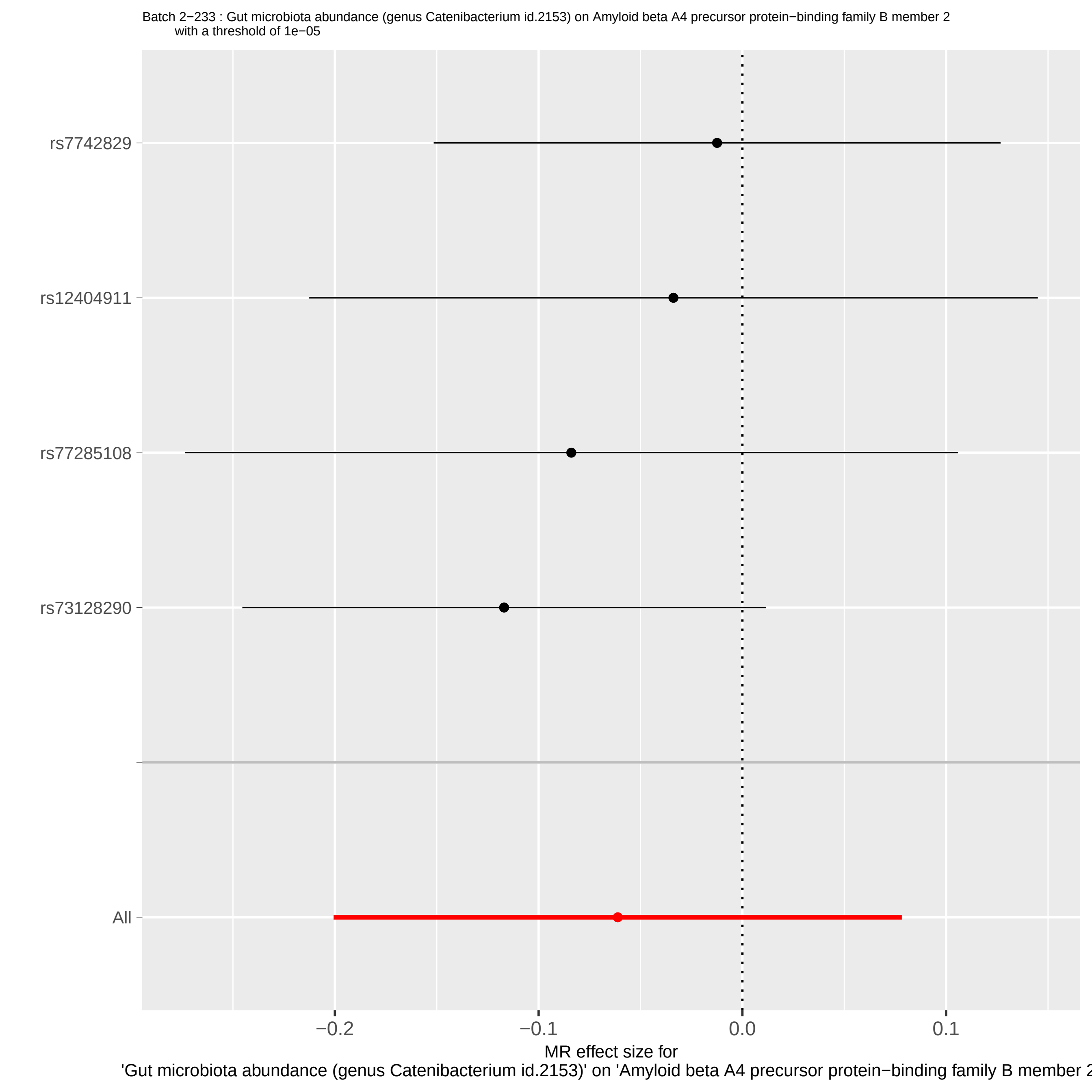
Batch 2−233 : Gut microbiota abundance (genus Catenibacterium id.2153) on Amyloid beta A4 precursor protein−binding family B member 2 with a threshold of 1e−05
rs7742829
rs12404911
rs77285108
rs73128290
All
−0.2
−0.1
0.0
0.1
MR effect size for
'Gut microbiota abundance (genus Catenibacterium id.2153)' on 'Amyloid beta A4 precursor protein−binding family B member 2

Batch 2−234 : Gut microbiota abundance (genus Parasutterella id.2892) on Amyloid beta A4 precursor protein−binding family B member 2
with a threshold of 1e−05

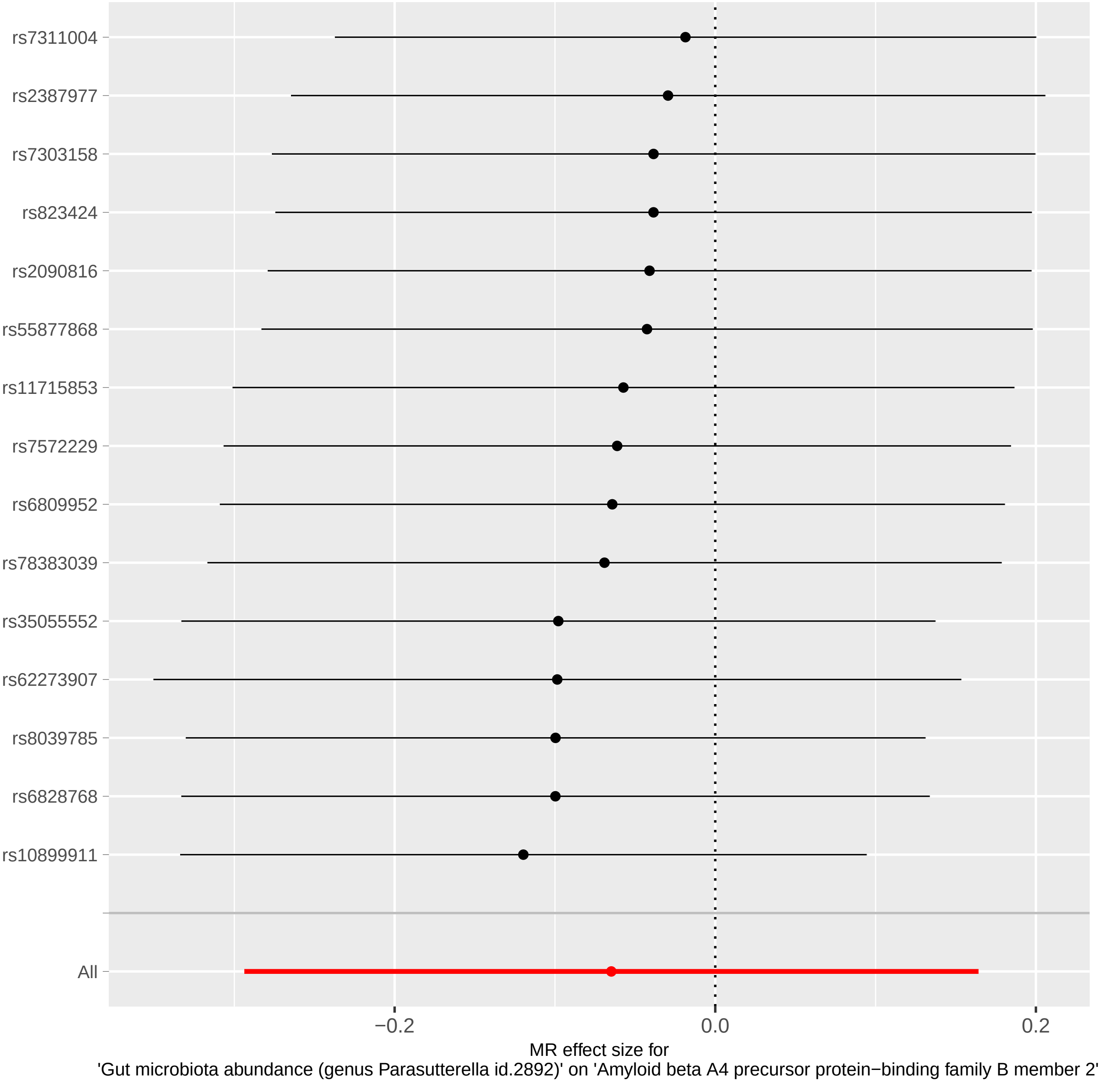

Batch 2−235 : Gut microbiota abundance (genus Catenibacterium id.2153) on Amyloid beta A4 precursor protein−binding family B member 3 with a threshold of 1e−05

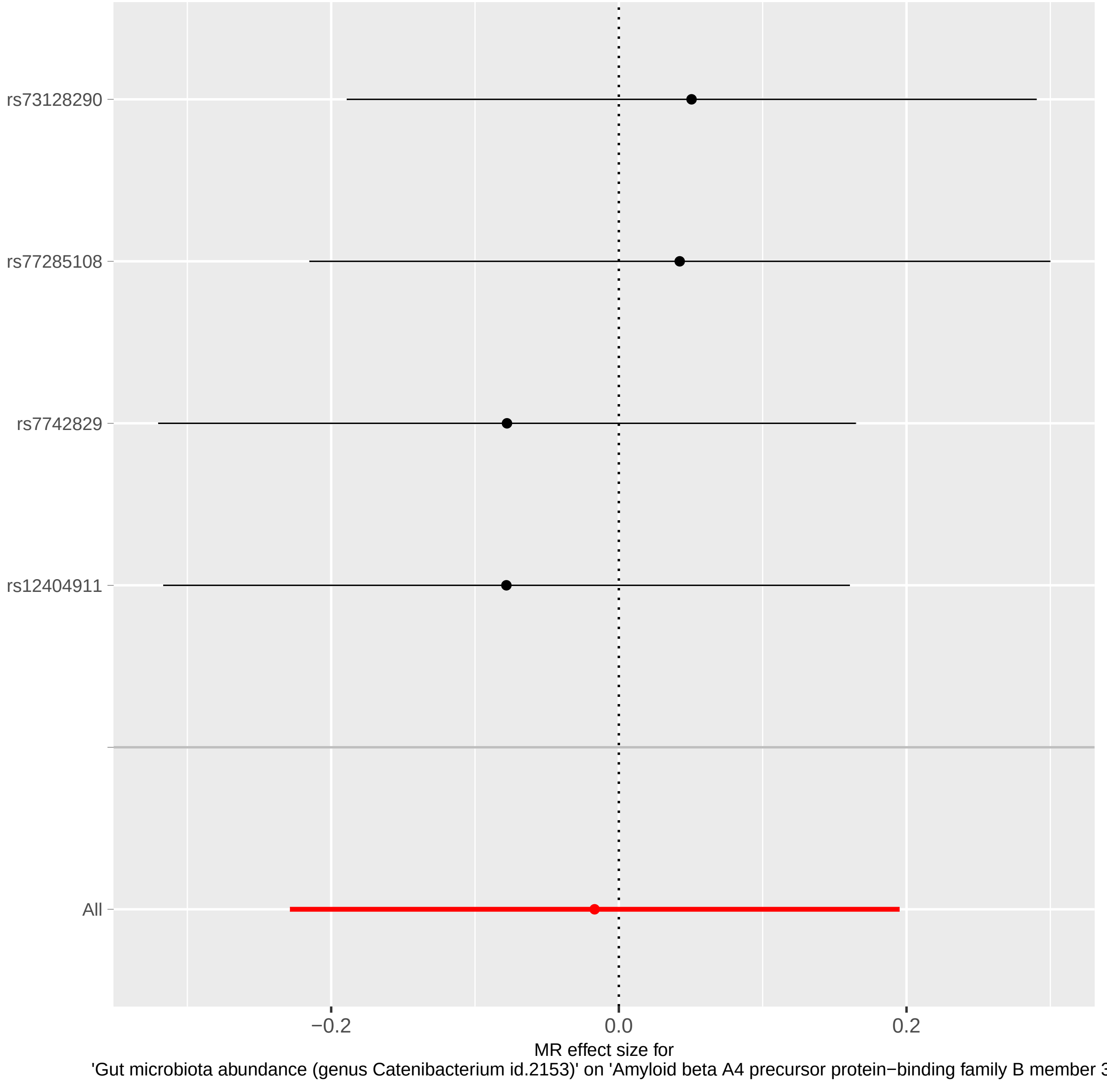

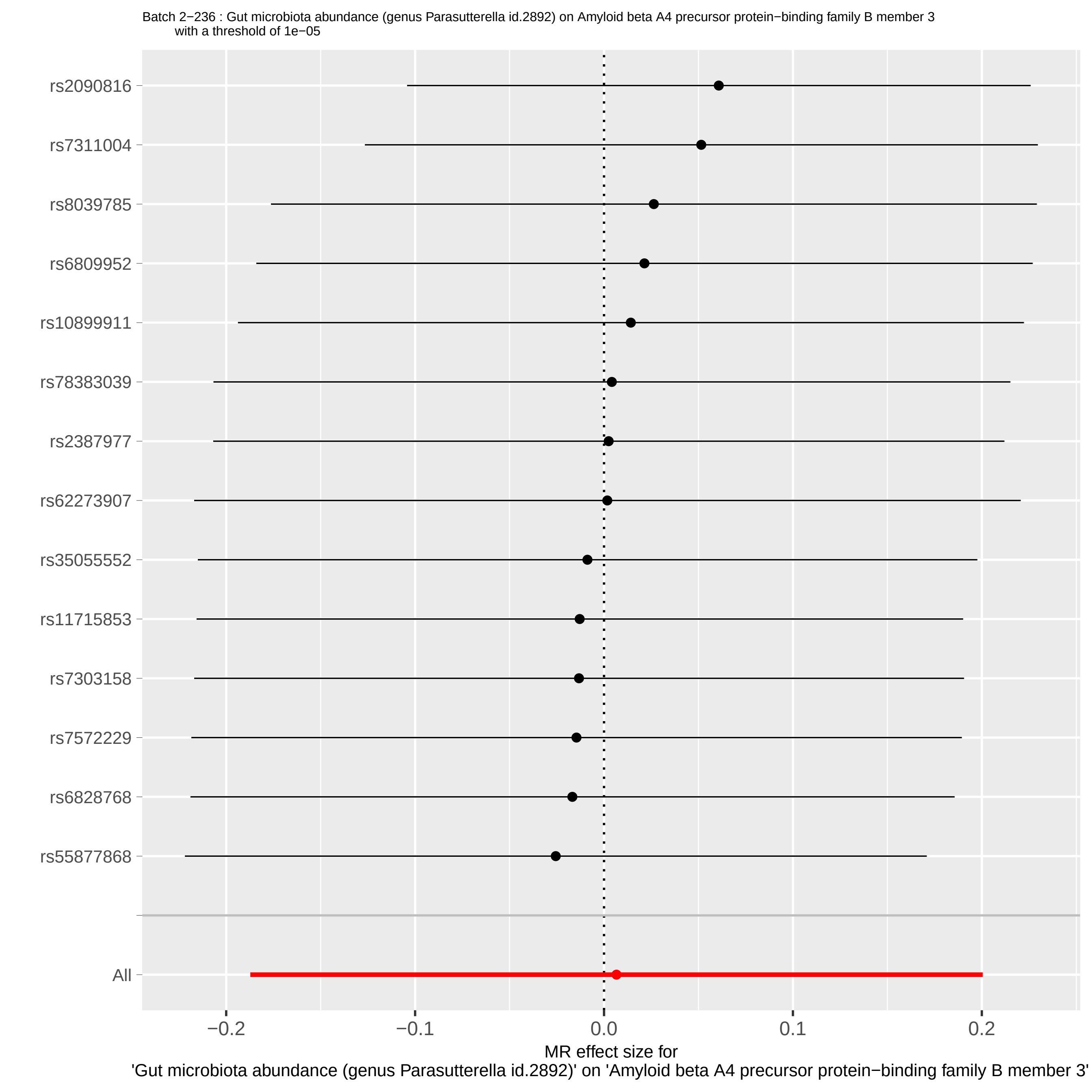

Batch 2−236 : Gut microbiota abundance (genus Parasutterella id.2892) on Amyloid beta A4 precursor protein−binding family B member 3
with a threshold of 1e−05
rs2090816
rs7311004
rs8039785
rs6809952
rs10899911
rs78383039
rs2387977
rs62273907
rs35055552
rs11715853
rs7303158
rs7572229
rs6828768
rs55877868
All
−0.2
−0.1
0.0
0.1
0.2
MR effect size for
'Gut microbiota abundance (genus Parasutterella id.2892)' on 'Amyloid beta A4 precursor protein−binding family B member 3'

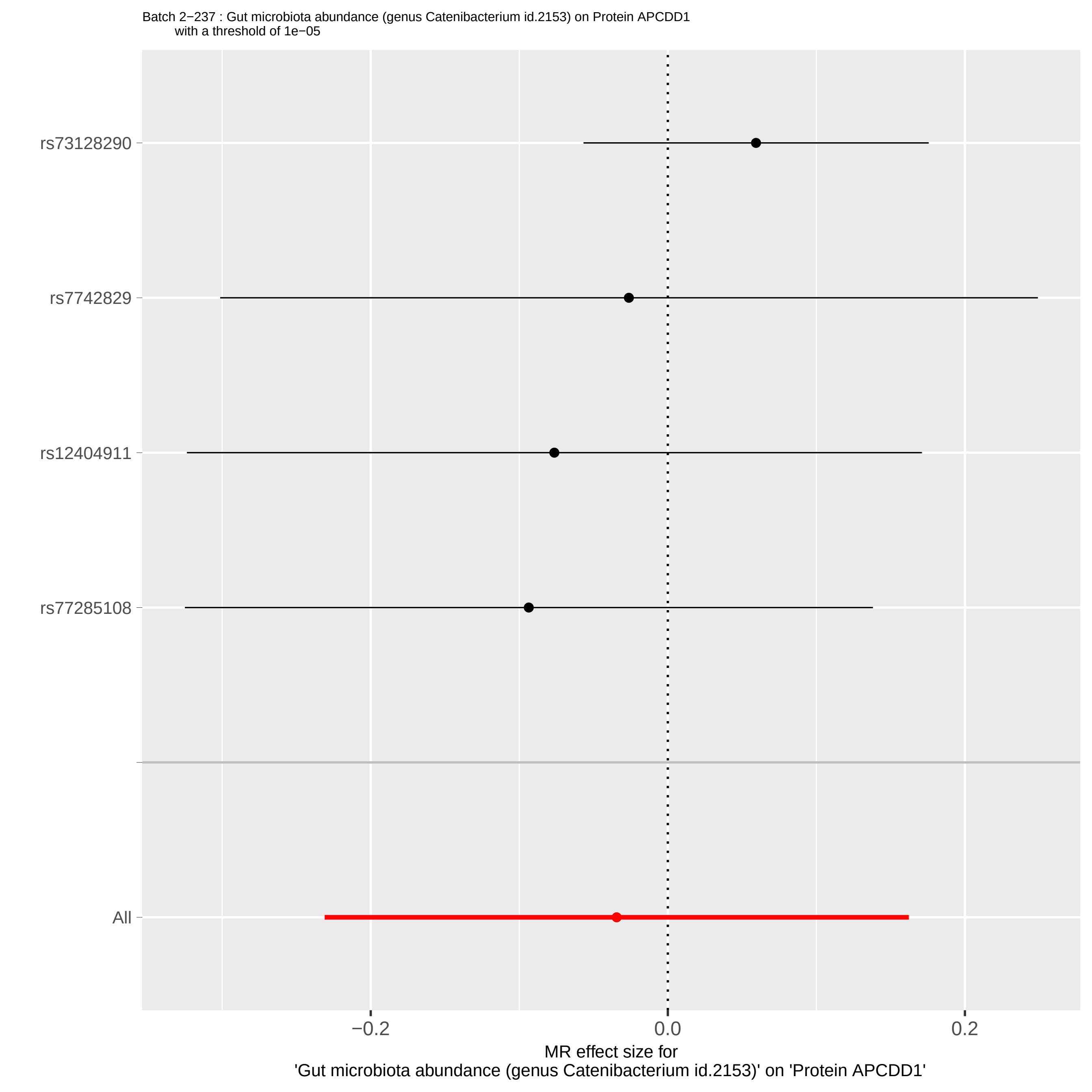

Batch 2−237 : Gut microbiota abundance (genus Catenibacterium id.2153) on Protein APCDD1
with a threshold of 1e−05
rs73128290
rs7742829
rs12404911
rs77285108
All
−0.2
0.0
0.2
MR effect size for
'Gut microbiota abundance (genus Catenibacterium id.2153)' on 'Protein APCDD1'

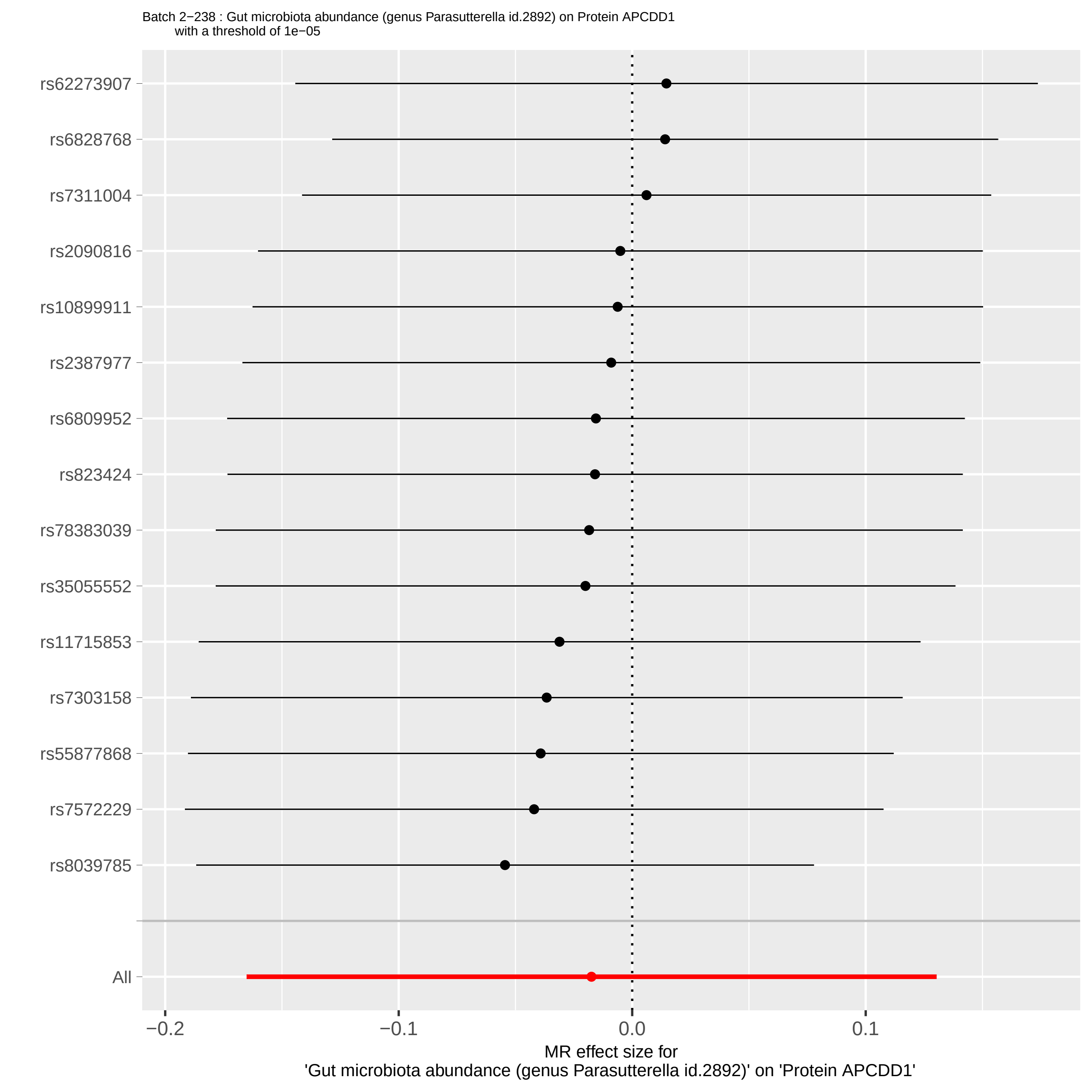

Batch 2−238 : Gut microbiota abundance (genus Parasutterella id.2892) on Protein APCDD1
with a threshold of 1e−05
rs62273907
rs6828768
rs7311004
rs2090816
rs10899911
rs2387977
rs6809952
rs823424
rs78383039
rs35055552
rs11715853
rs7303158
rs55877868
rs7572229
rs8039785
All
−0.2
−0.1
0.0
0.1
MR effect size for
'Gut microbiota abundance (genus Parasutterella id.2892)' on 'Protein APCDD1'

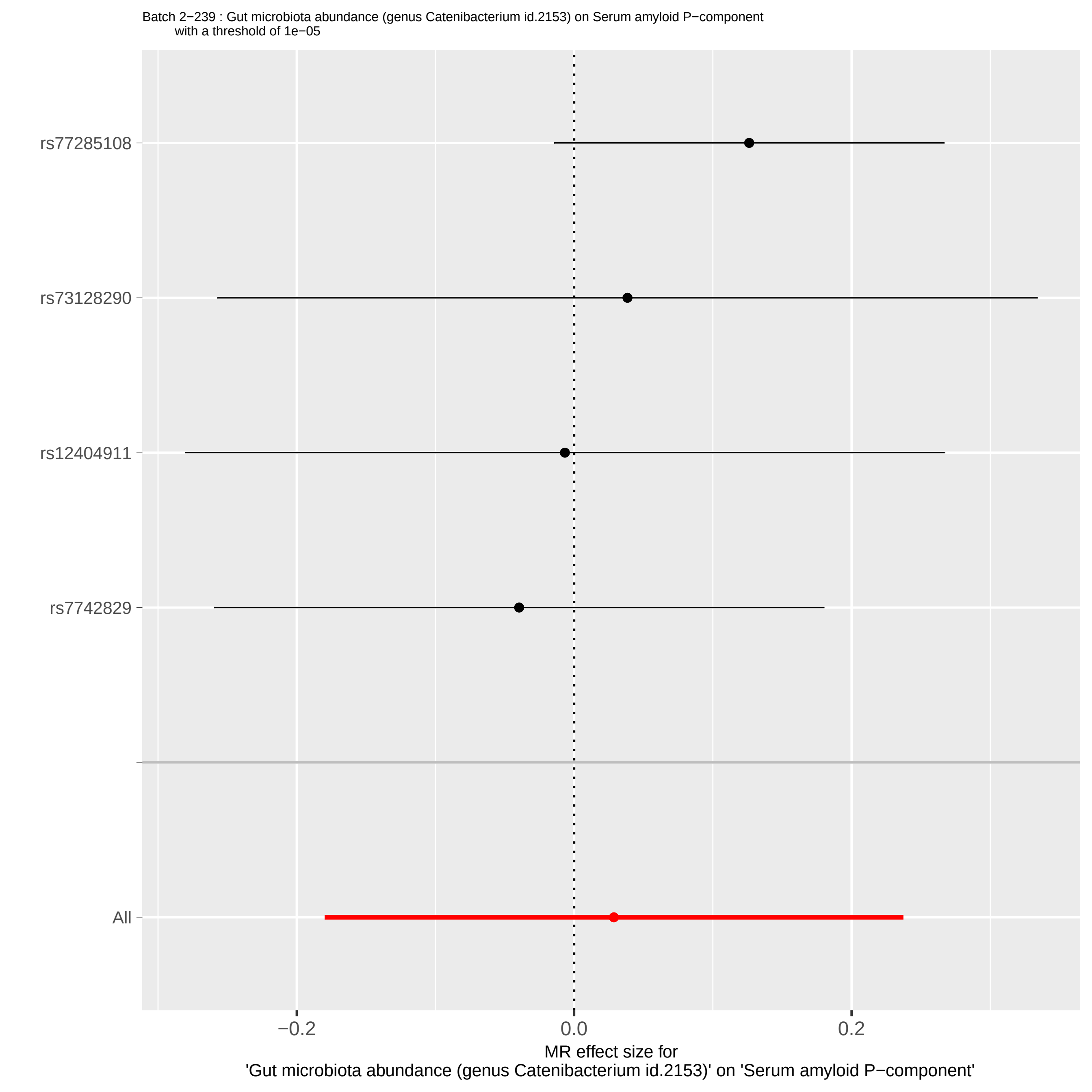

Batch 2−239 : Gut microbiota abundance (genus Catenibacterium id.2153) on Serum amyloid P−component
with a threshold of 1e−05
rs77285108
rs73128290
rs12404911
rs7742829
All
−0.2
0.0
0.2
MR effect size for
'Gut microbiota abundance (genus Catenibacterium id.2153)' on 'Serum amyloid P−component'

Batch 2−240 : Gut microbiota abundance (genus Parasutterella id.2892) on Serum amyloid P−component
with a threshold of 1e−05

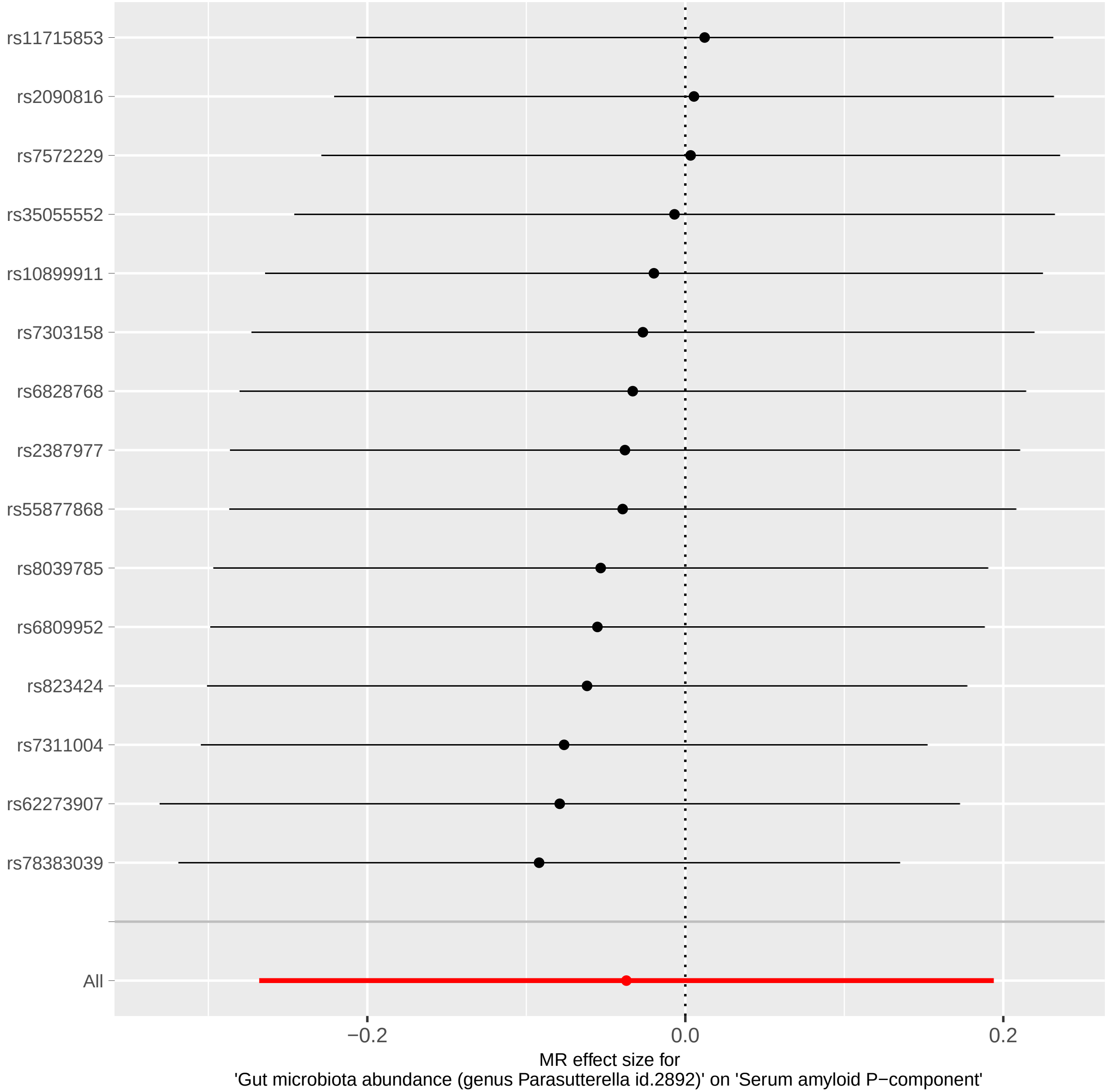

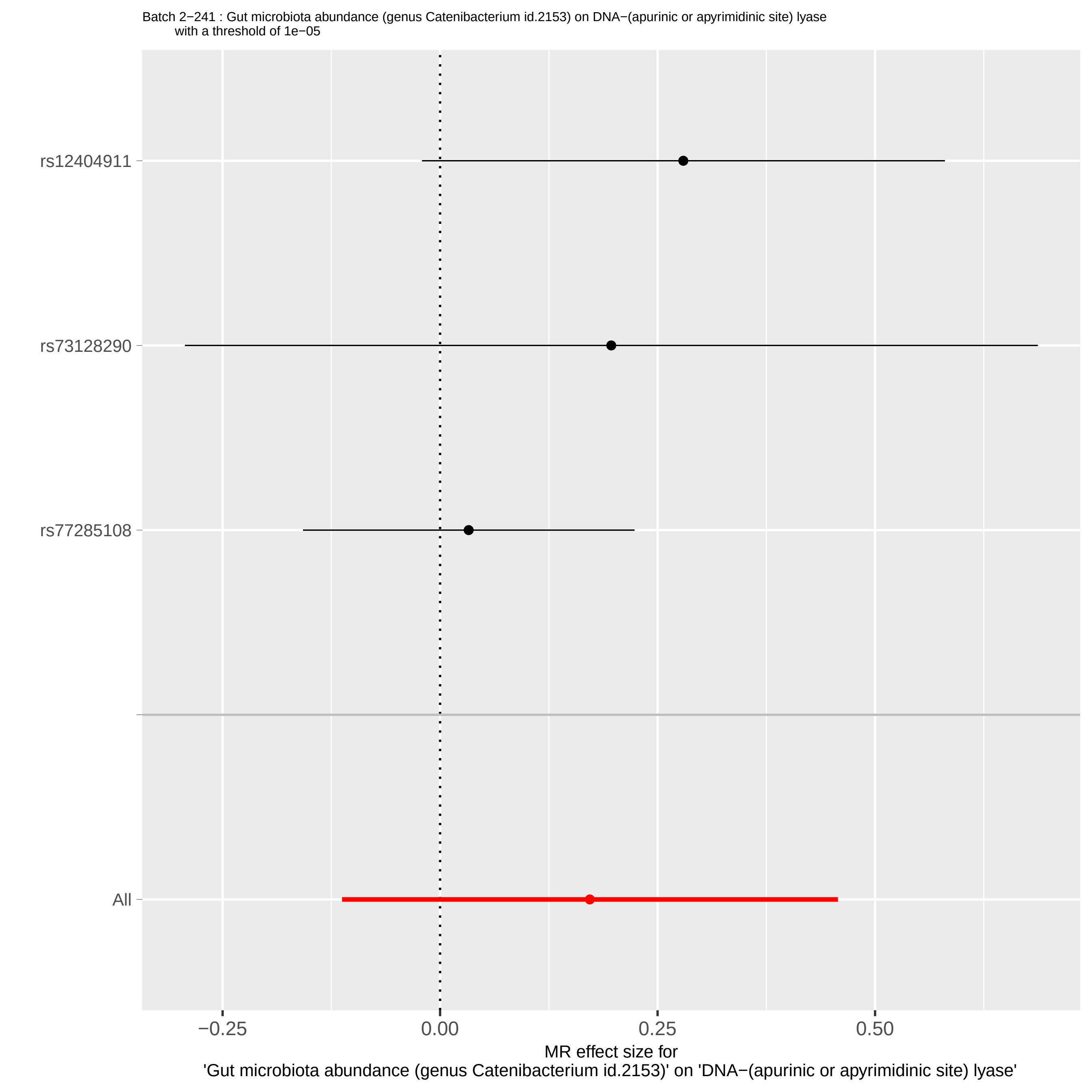
Batch 2−241 : Gut microbiota abundance (genus Catenibacterium id.2153) on DNA−(apurinic or apyrimidinic site) lyase
with a threshold of 1e−05
rs12404911
rs73128290
rs77285108
All
−0.25
0.00
0.25
0.50
MR effect size for
'Gut microbiota abundance (genus Catenibacterium id.2153)' on 'DNA−(apurinic or apyrimidinic site) lyase'

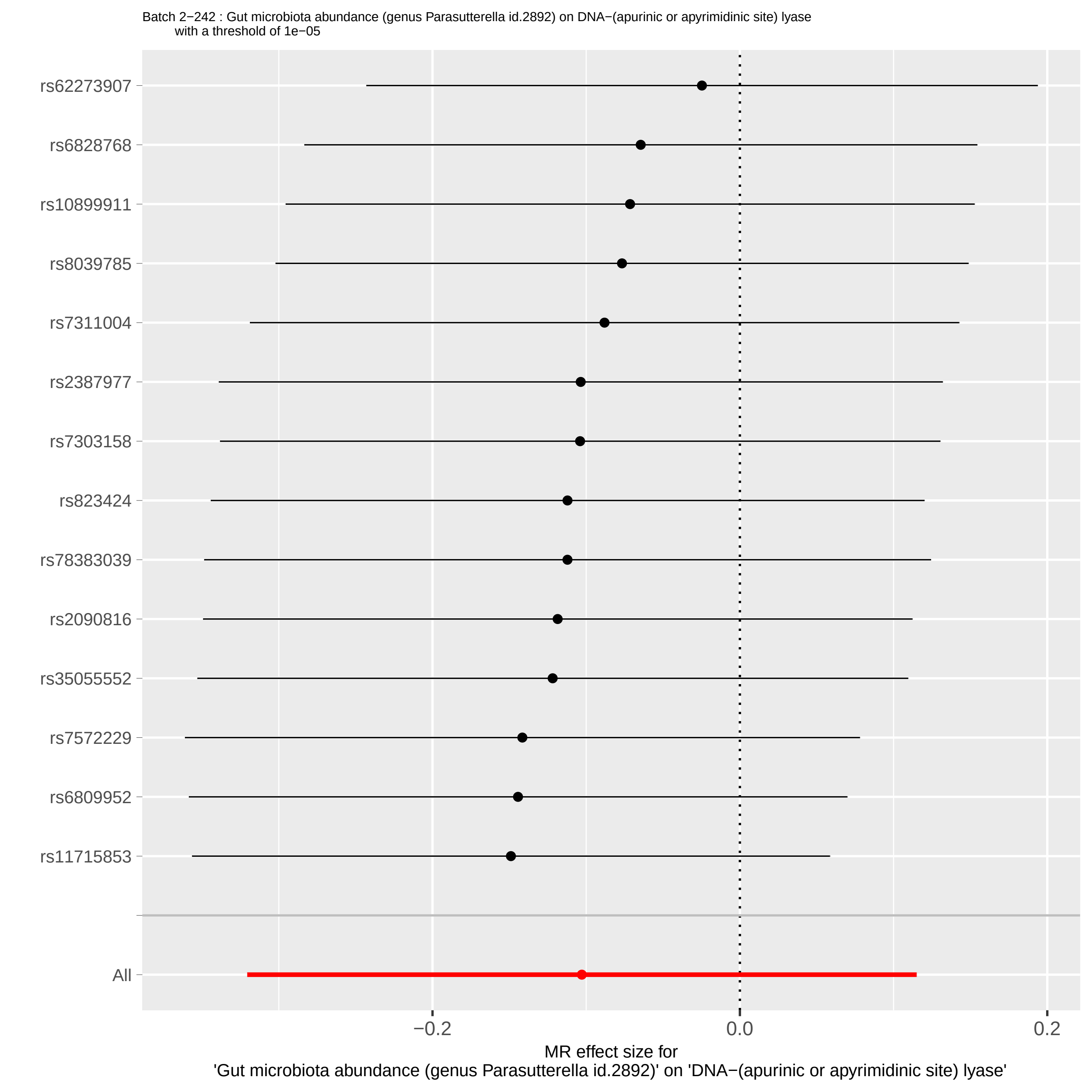

Batch 2−242 : Gut microbiota abundance (genus Parasutterella id.2892) on DNA−(apurinic or apyrimidinic site) lyase
with a threshold of 1e−05
rs62273907
rs6828768
rs10899911
rs8039785
rs7311004
rs2387977
rs7303158
rs823424
rs78383039
rs2090816
rs35055552
rs7572229
rs6809952
rs11715853
All
−0.2
0.0
0.2
MR effect size for
'Gut microbiota abundance (genus Parasutterella id.2892)' on 'DNA−(apurinic or apyrimidinic site) lyase'

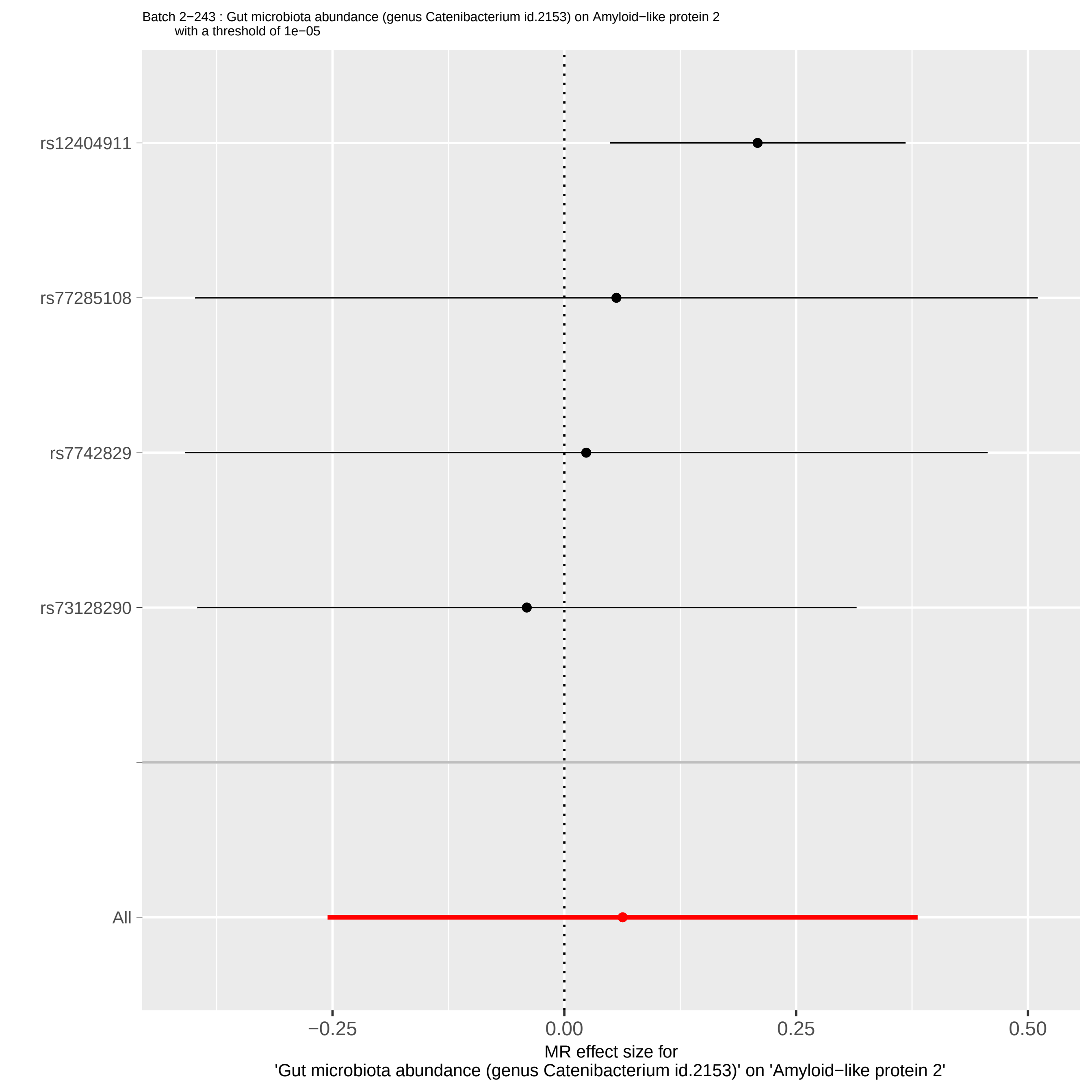

Batch 2−243 : Gut microbiota abundance (genus Catenibacterium id.2153) on Amyloid−like protein 2
with a threshold of 1e−05
rs12404911
rs77285108
rs7742829
rs73128290
All
−0.25
0.00
0.25
0.50
MR effect size for
'Gut microbiota abundance (genus Catenibacterium id.2153)' on 'Amyloid−like protein 2'

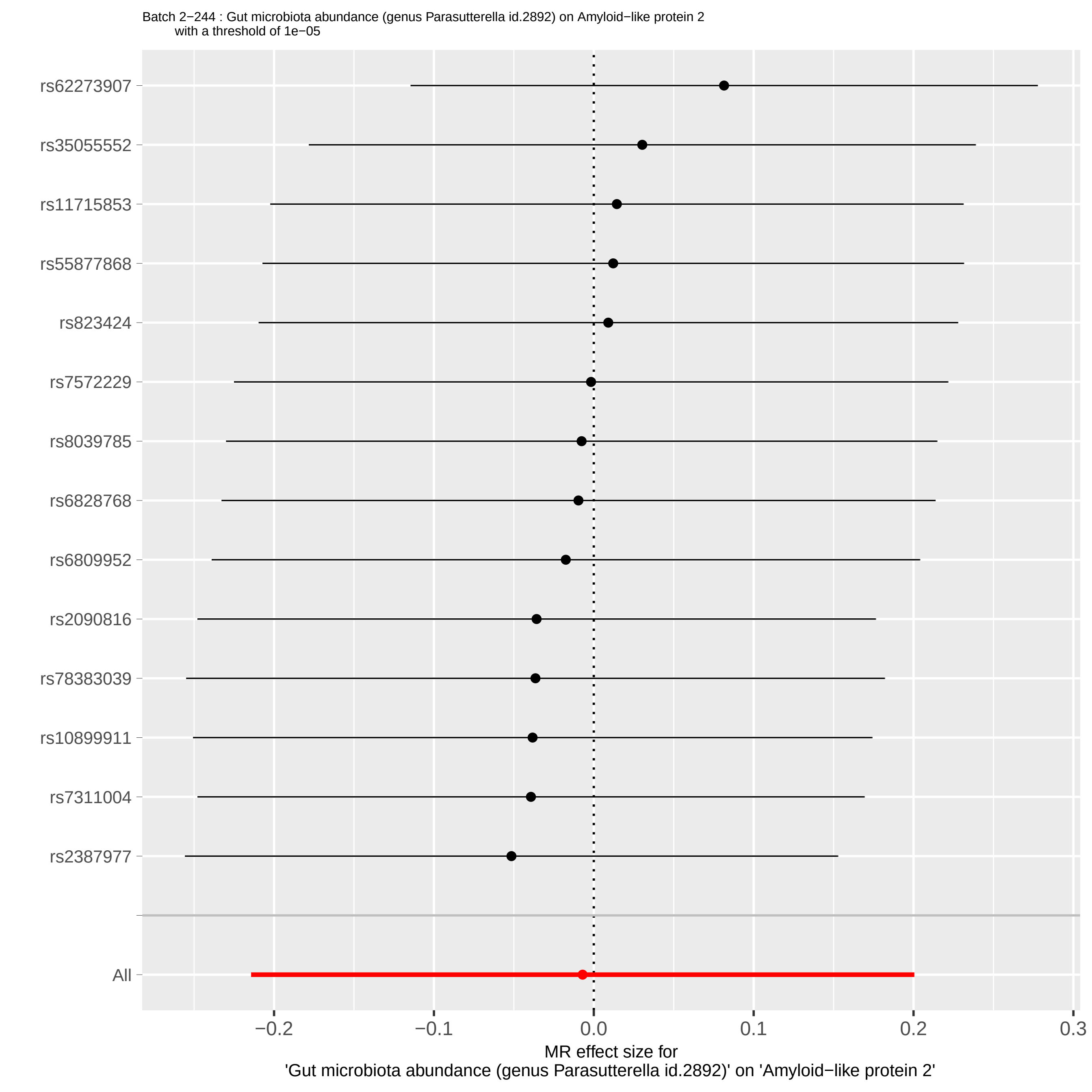

Batch 2−244 : Gut microbiota abundance (genus Parasutterella id.2892) on Amyloid−like protein 2
with a threshold of 1e−05
rs62273907
rs35055552
rs11715853
rs55877868
rs823424
rs7572229
rs8039785
rs6828768
rs6809952
rs2090816
rs78383039
rs10899911
rs7311004
rs2387977
All
−0.2
−0.1
0.0
0.1
0.2
0.3
MR effect size for
'Gut microbiota abundance (genus Parasutterella id.2892)' on 'Amyloid−like protein 2'

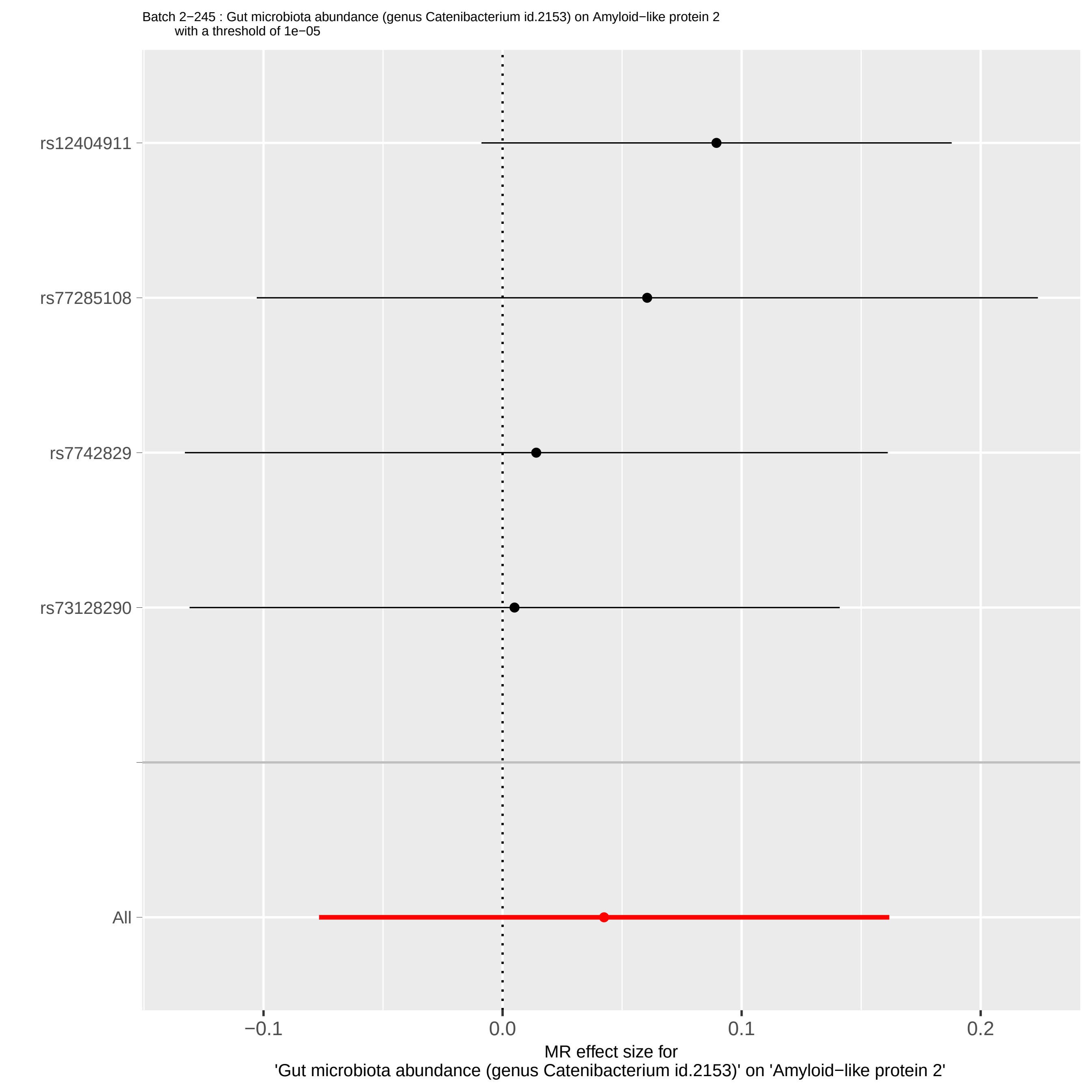

Batch 2−245 : Gut microbiota abundance (genus Catenibacterium id.2153) on Amyloid−like protein 2
with a threshold of 1e−05
rs12404911
rs77285108
rs7742829
rs73128290
All
−0.1
0.0
0.1
0.2
MR effect size for
'Gut microbiota abundance (genus Catenibacterium id.2153)' on 'Amyloid−like protein 2'

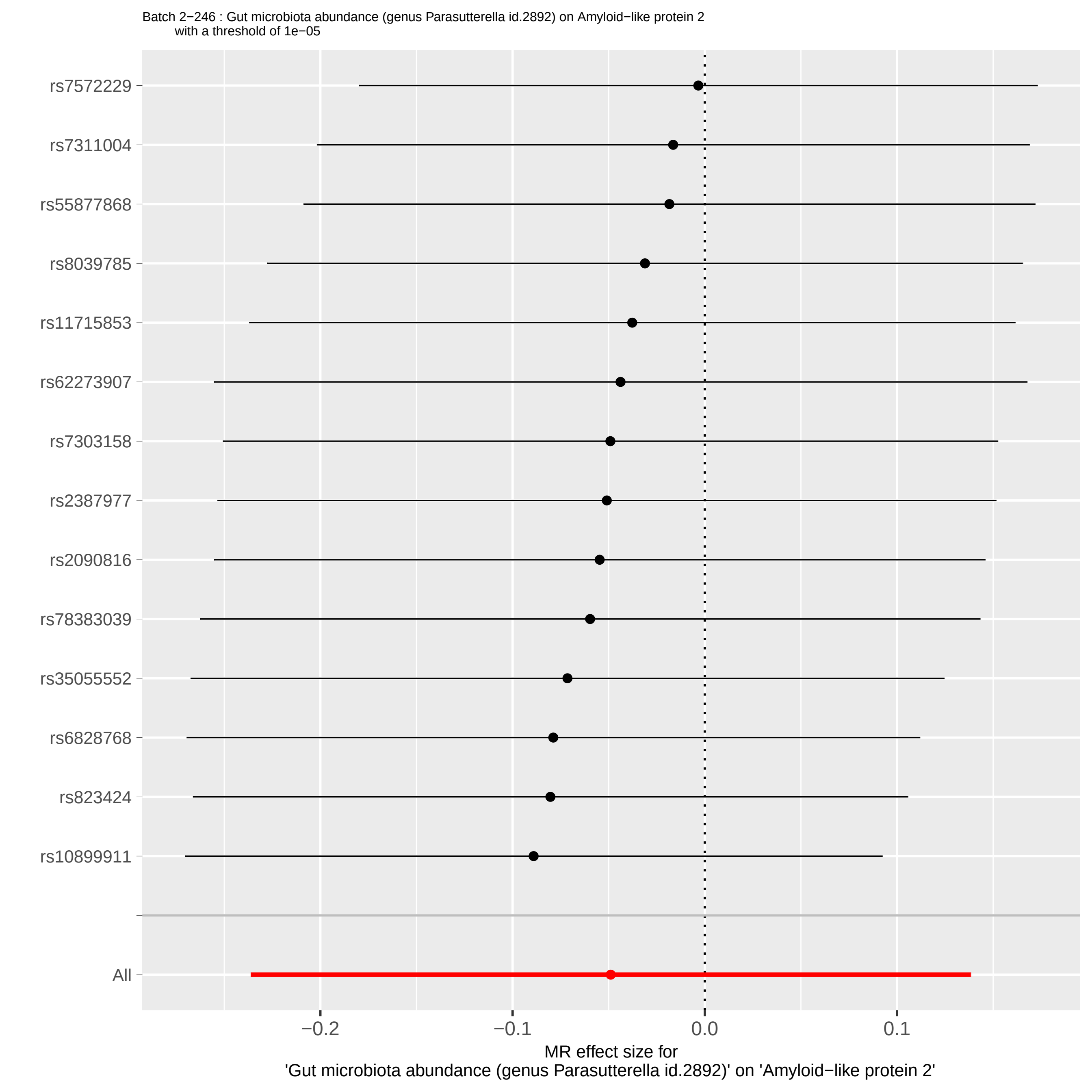

Batch 2−246 : Gut microbiota abundance (genus Parasutterella id.2892) on Amyloid−like protein 2
with a threshold of 1e−05
rs7572229
rs7311004
rs55877868
rs8039785
rs11715853
rs62273907
rs7303158
rs2387977
rs2090816
rs78383039
rs35055552
rs6828768
rs823424
rs10899911
All
−0.2
−0.1
0.0
0.1
MR effect size for
'Gut microbiota abundance (genus Parasutterella id.2892)' on 'Amyloid−like protein 2'

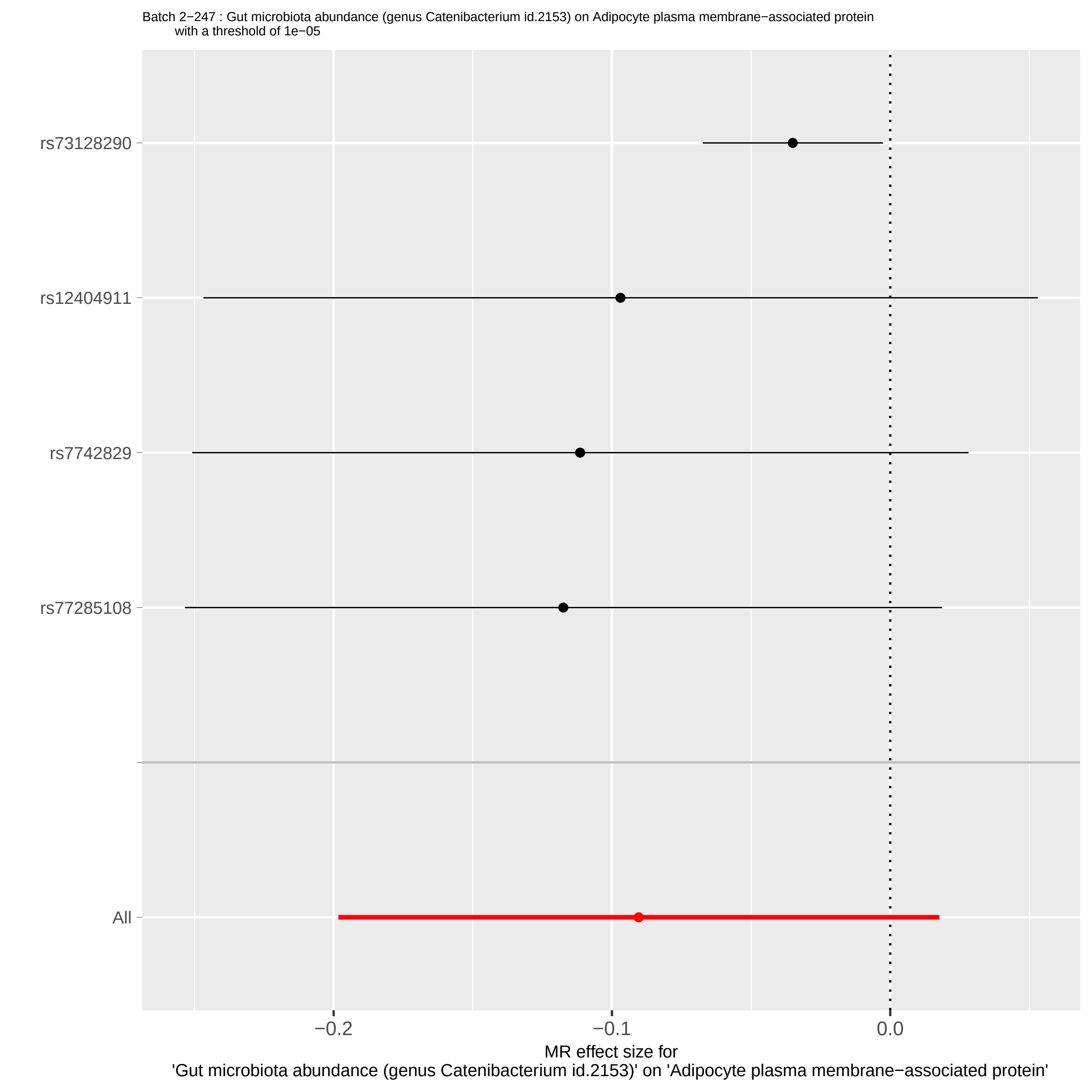

Batch 2−247 : Gut microbiota abundance (genus Catenibacterium id.2153) on Adipocyte plasma membrane−associated protein
with a threshold of 1e−05
rs73128290
rs12404911
rs7742829
rs77285108
All
−0.2
−0.1
0.0
MR effect size for
'Gut microbiota abundance (genus Catenibacterium id.2153)' on 'Adipocyte plasma membrane−associated protein'

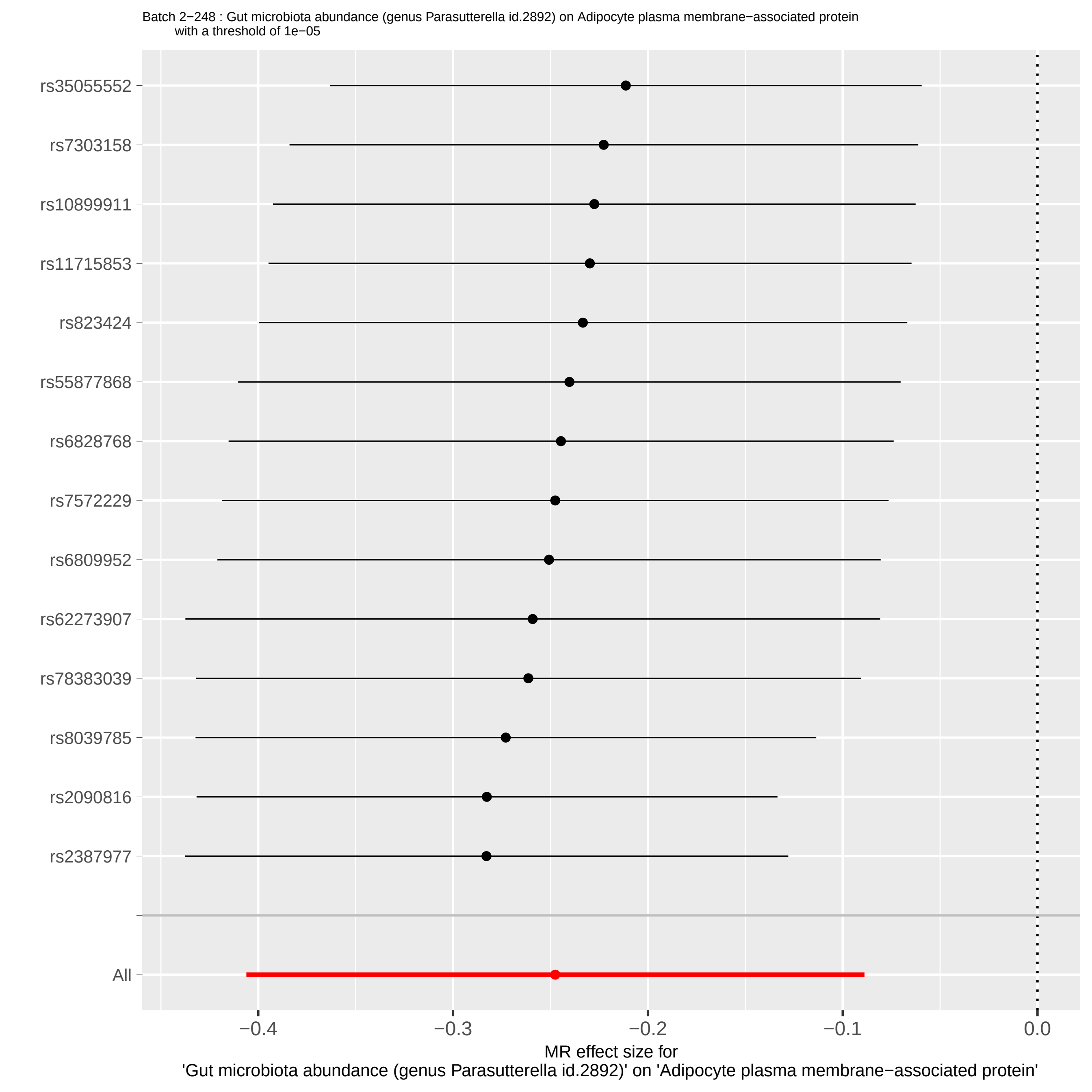

Batch 2−248 : Gut microbiota abundance (genus Parasutterella id.2892) on Adipocyte plasma membrane−associated protein
with a threshold of 1e−05
rs35055552
rs7303158
rs10899911
rs11715853
rs823424
rs55877868
rs6828768
rs7572229
rs6809952
rs62273907
rs78383039
rs8039785
rs2090816
rs2387977
All
−0.4
−0.3
−0.2
−0.1
0.0
MR effect size for
'Gut microbiota abundance (genus Parasutterella id.2892)' on 'Adipocyte plasma membrane−associated protein'

Batch 2−249 : Gut microbiota abundance (genus Catenibacterium id.2153) on Apolipoprotein A−V
with a threshold of 1e−05

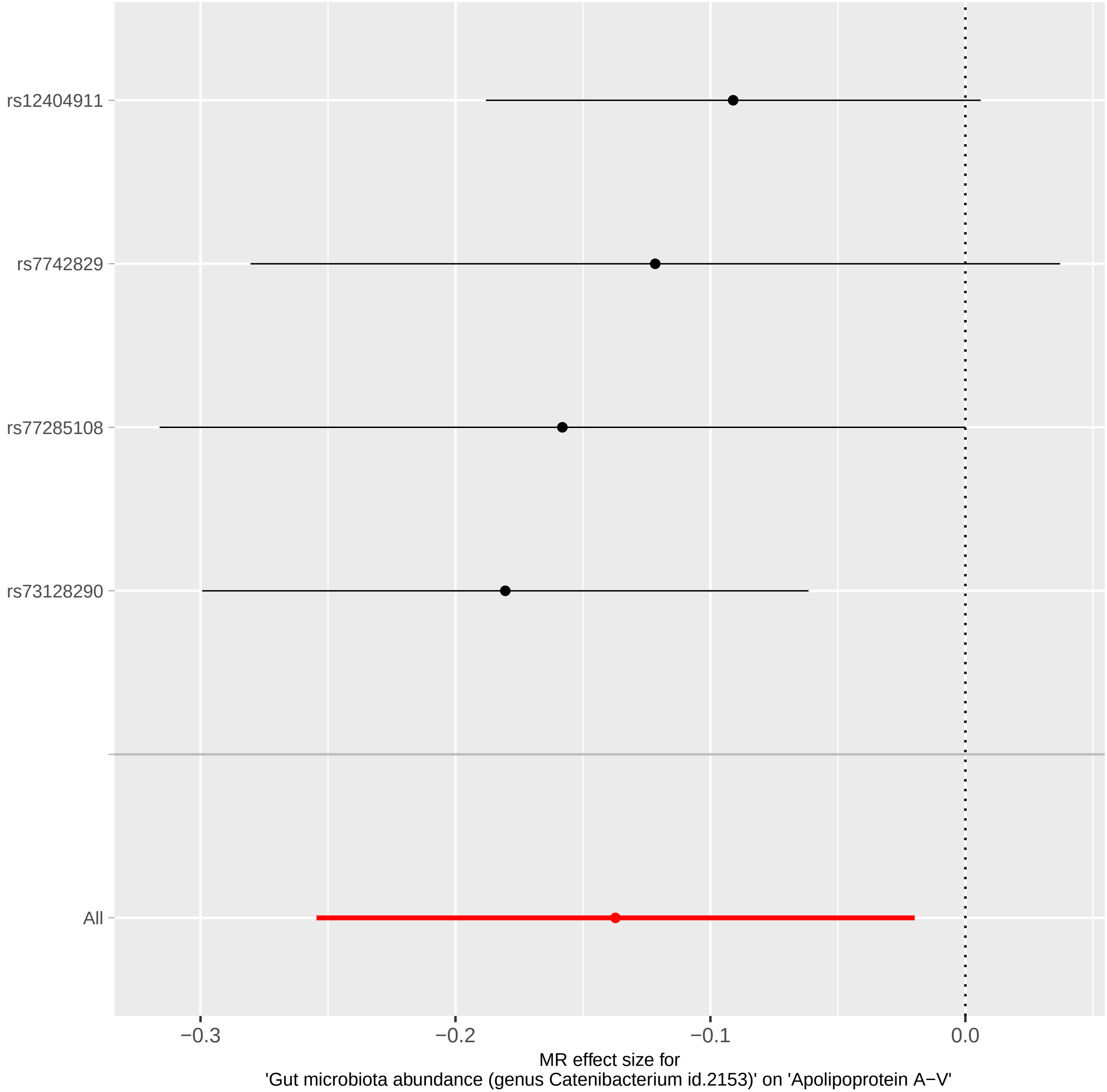

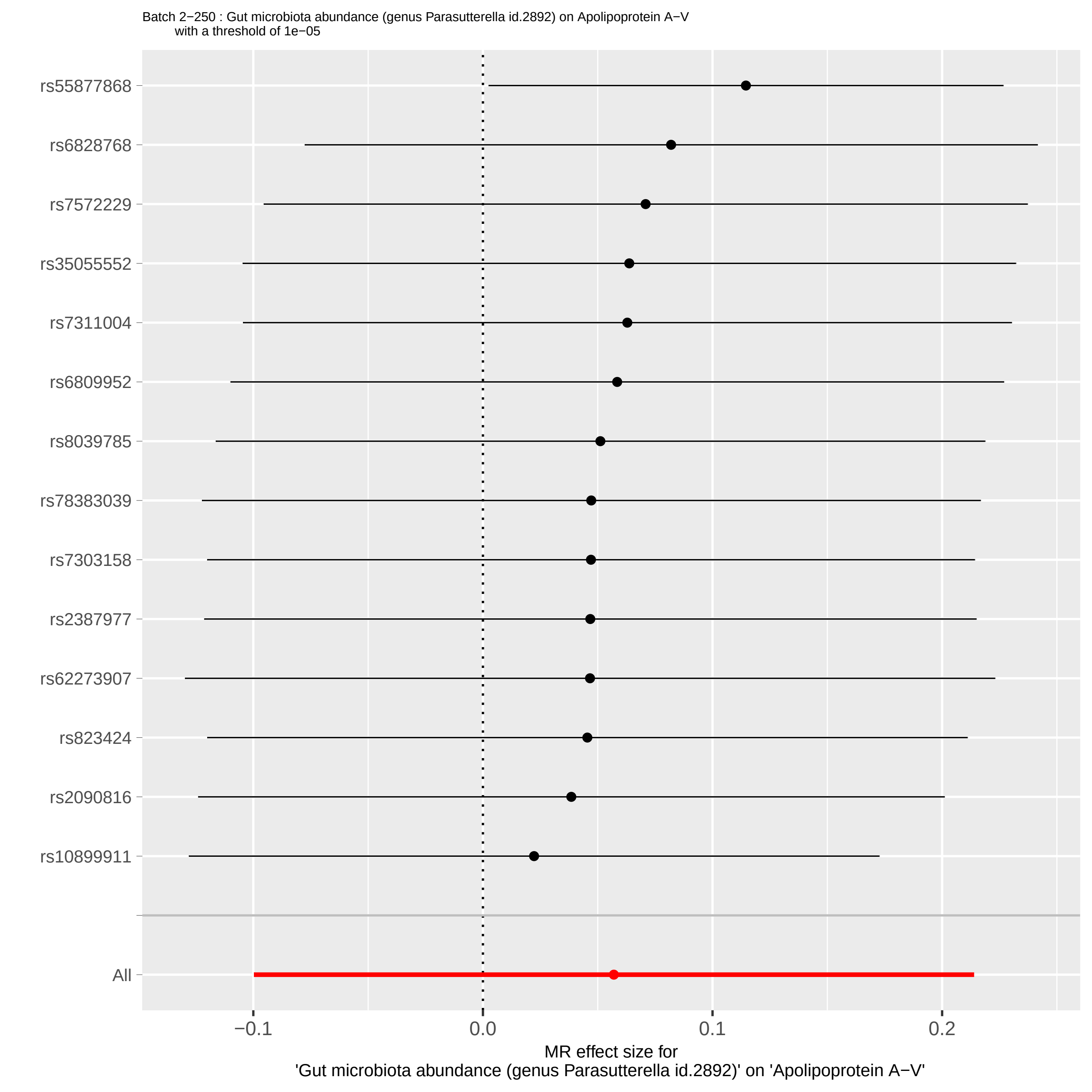
Batch 2−250 : Gut microbiota abundance (genus Parasutterella id.2892) on Apolipoprotein A−V
with a threshold of 1e−05
rs55877868
rs6828768
rs7572229
rs35055552
rs7311004
rs6809952
rs8039785
rs78383039
rs7303158
rs2387977
rs62273907
rs823424
rs2090816
rs10899911
All
−0.1
0.0
0.1
0.2
MR effect size for
'Gut microbiota abundance (genus Parasutterella id.2892)' on 'Apolipoprotein A−V'

Batch 2−251 : Gut microbiota abundance (genus Catenibacterium id.2153) on Apolipoprotein A−V
with a threshold of 1e−05

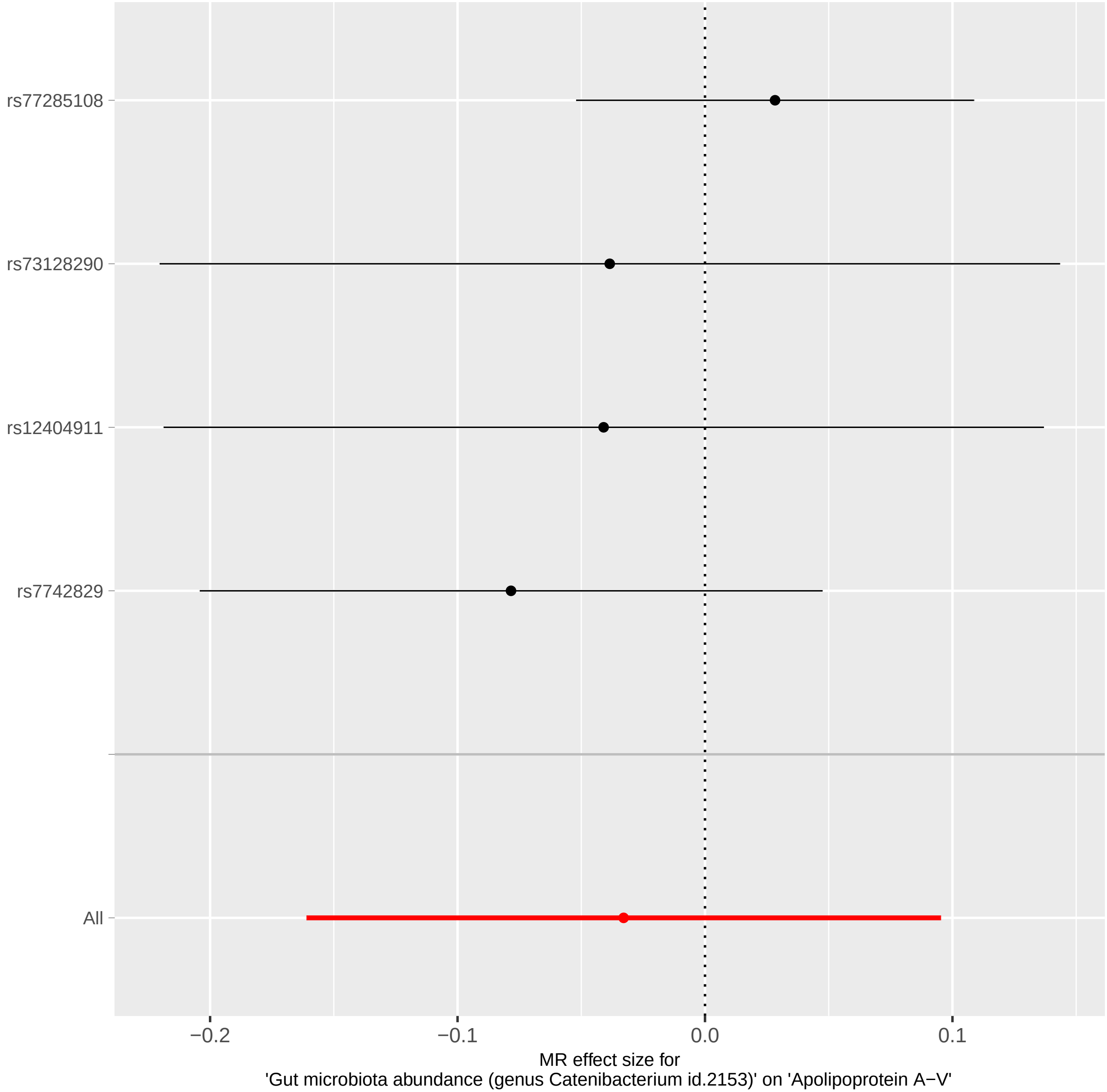

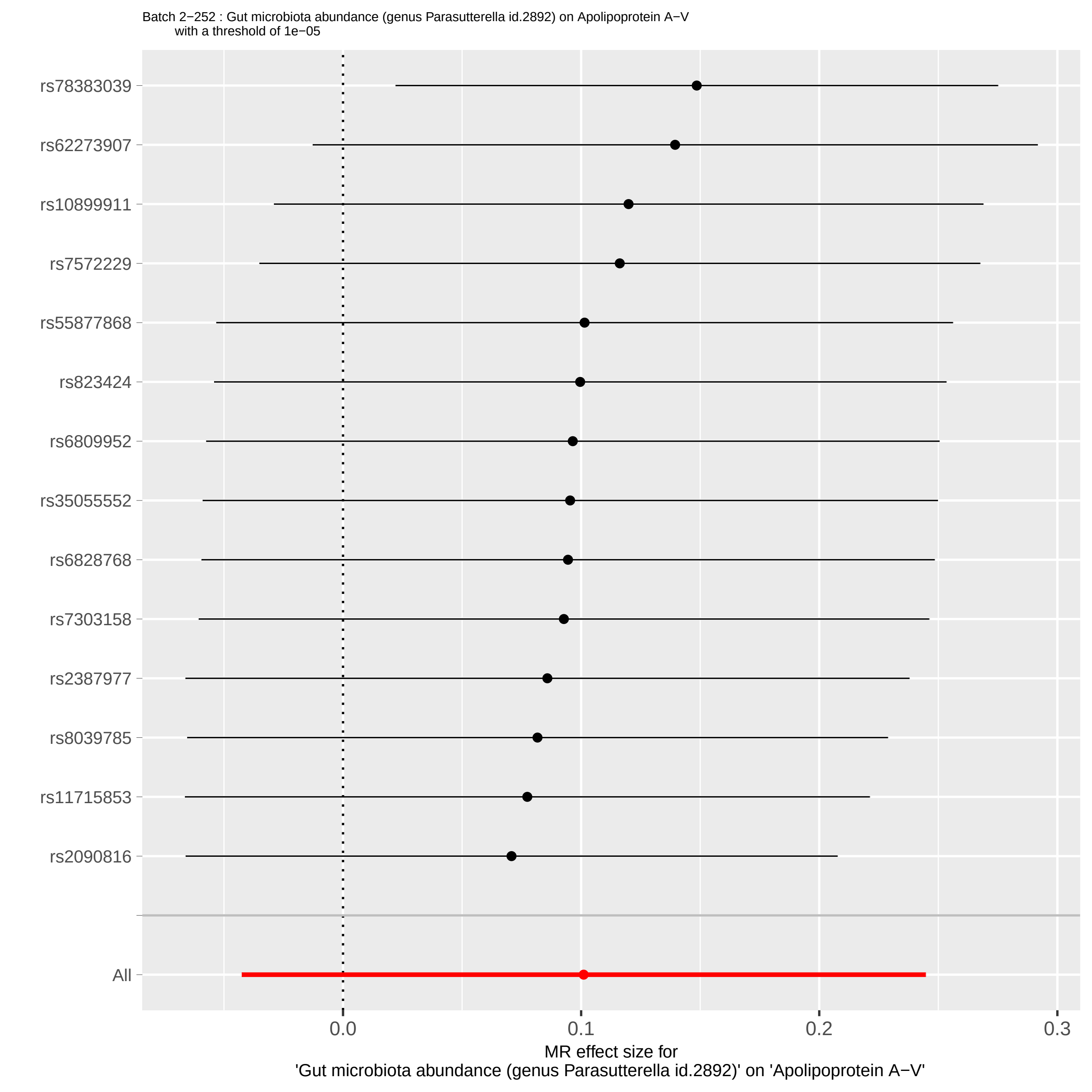

Batch 2−252 : Gut microbiota abundance (genus Parasutterella id.2892) on Apolipoprotein A−V
with a threshold of 1e−05
rs78383039
rs62273907
rs10899911
rs7572229
rs55877868
rs823424
rs6809952
rs35055552
rs6828768
rs7303158
rs2387977
rs8039785
rs11715853
rs2090816
All
0.0
0.1
0.2
0.3
MR effect size for
'Gut microbiota abundance (genus Parasutterella id.2892)' on 'Apolipoprotein A−V'

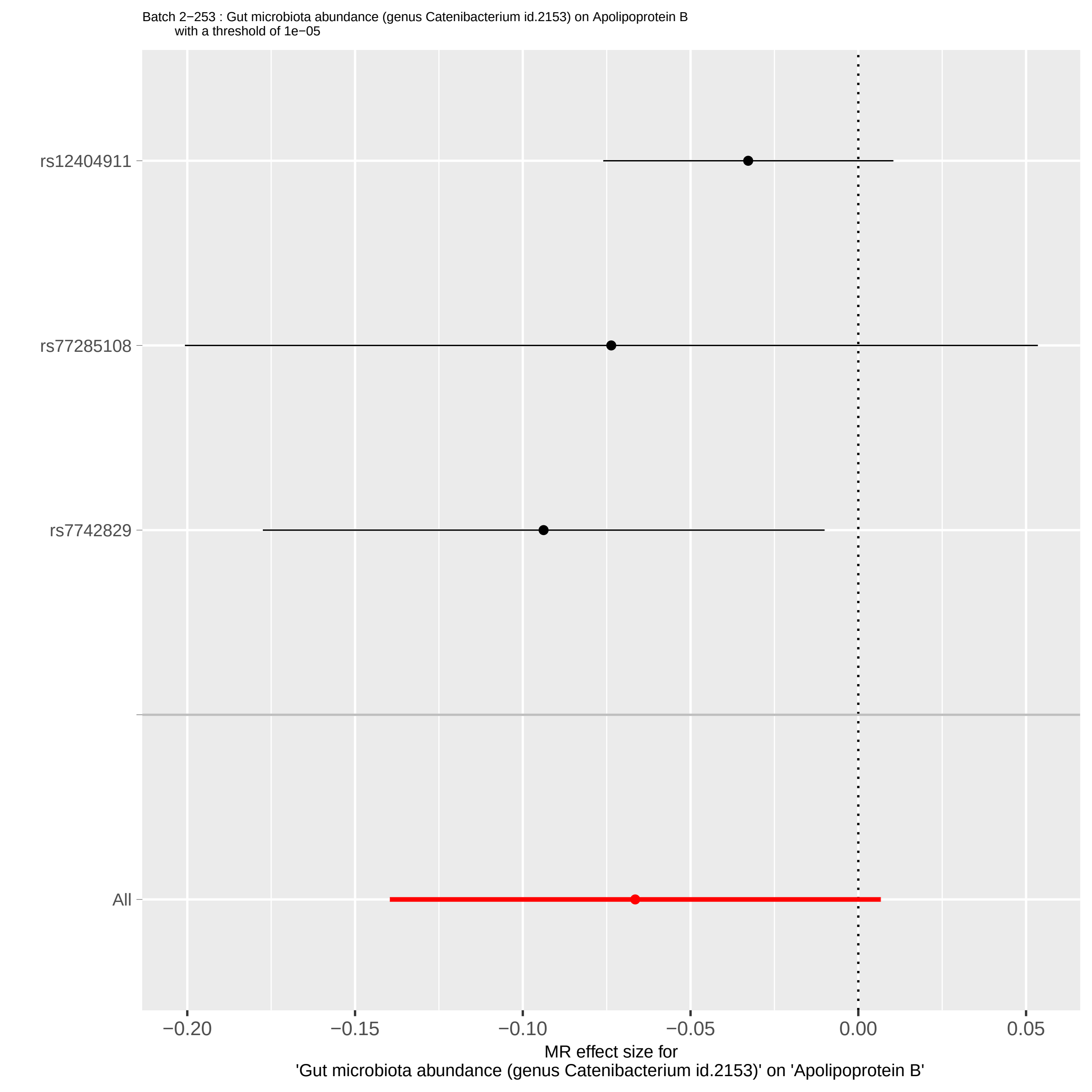

Batch 2−253 : Gut microbiota abundance (genus Catenibacterium id.2153) on Apolipoprotein B
with a threshold of 1e−05
rs12404911
rs77285108
rs7742829
All
−0.20
−0.15
−0.10
−0.05
0.00
0.05
MR effect size for
'Gut microbiota abundance (genus Catenibacterium id.2153)' on 'Apolipoprotein B'

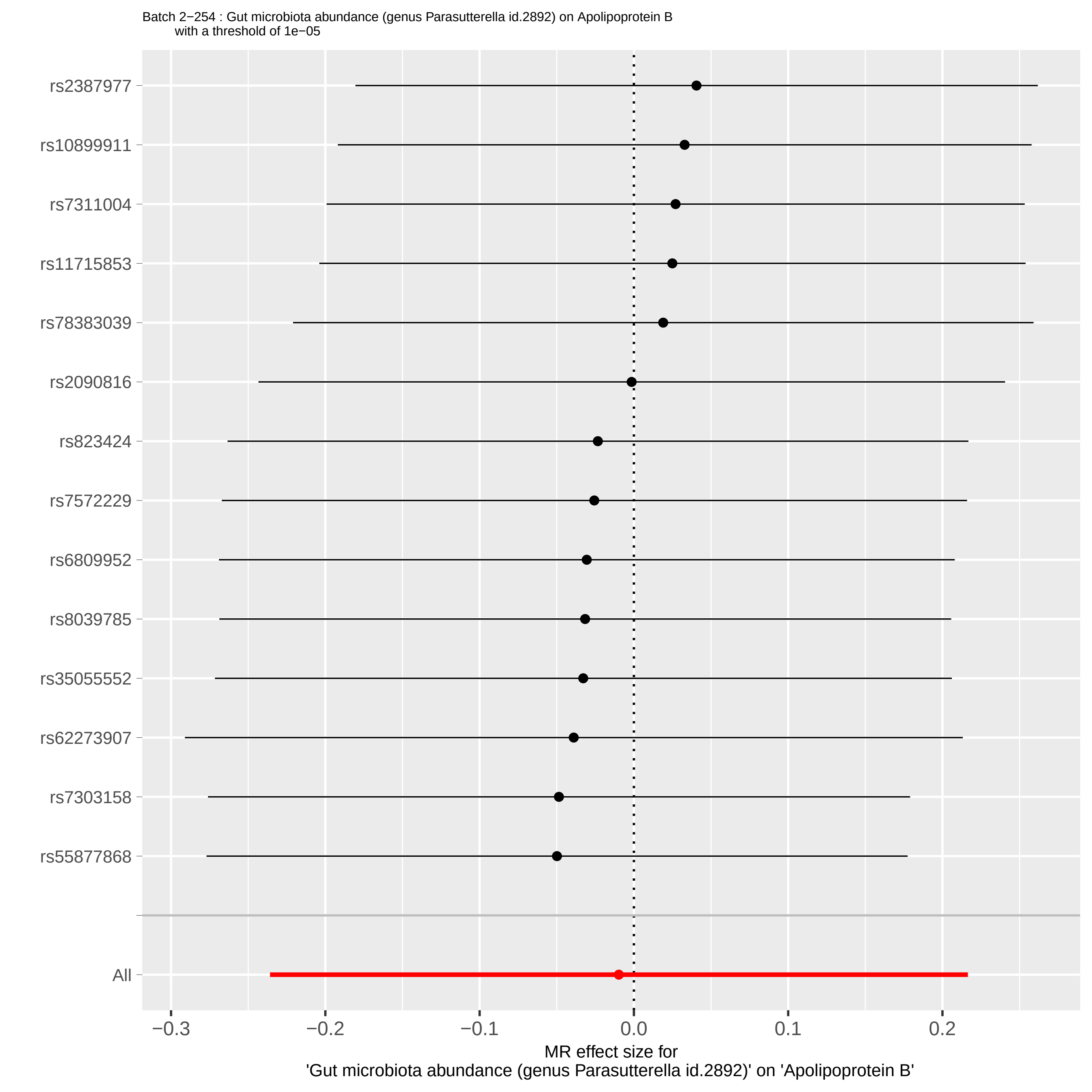

Batch 2−254 : Gut microbiota abundance (genus Parasutterella id.2892) on Apolipoprotein B
with a threshold of 1e−05
rs2387977
rs10899911
rs7311004
rs11715853
rs78383039
rs2090816
rs823424
rs7572229
rs6809952
rs8039785
rs35055552
rs62273907
rs7303158
rs55877868
All
−0.3
−0.2
−0.1
0.0
0.1
0.2
MR effect size for
'Gut microbiota abundance (genus Parasutterella id.2892)' on 'Apolipoprotein B'

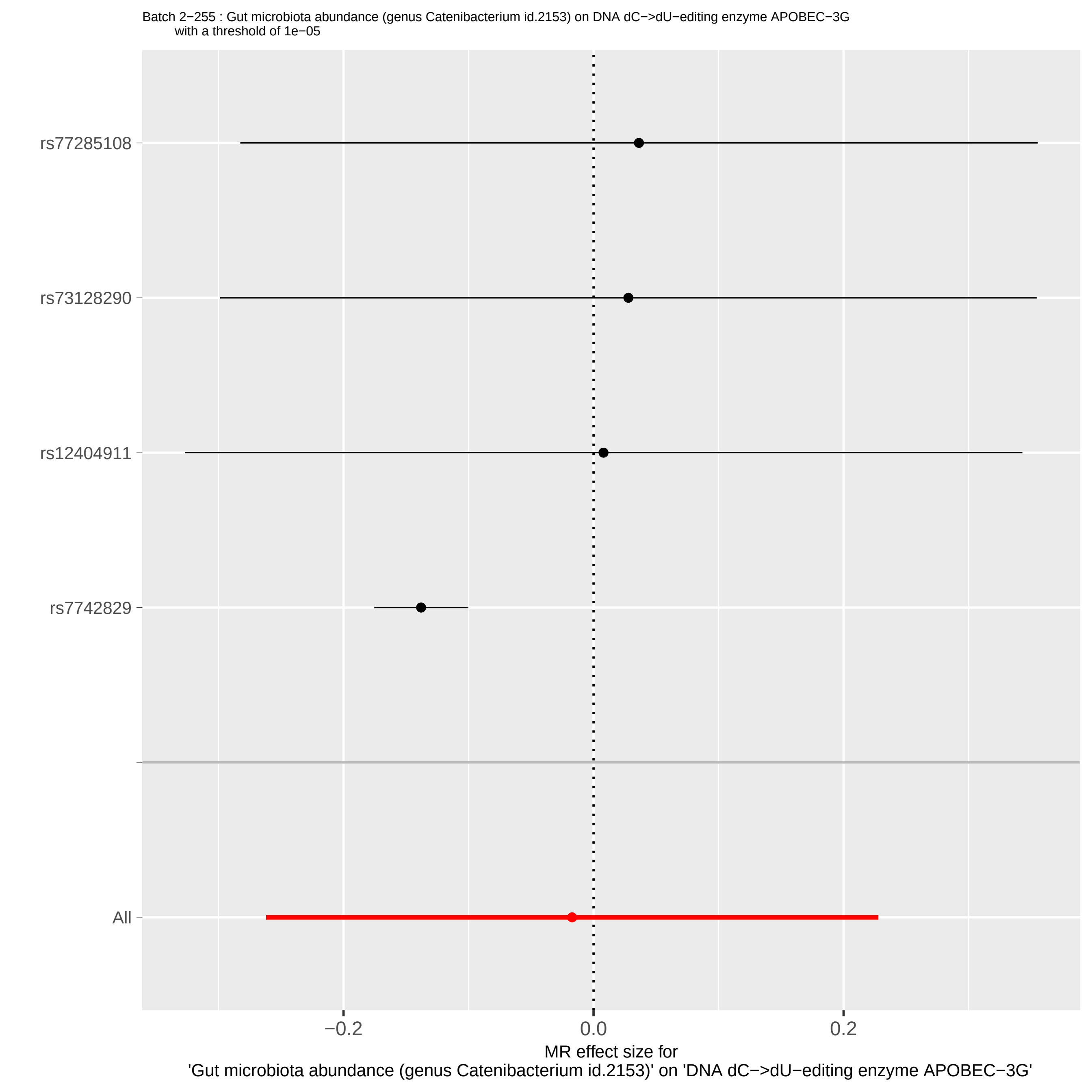
Batch 2−255 : Gut microbiota abundance (genus Catenibacterium id.2153) on DNA dC−>dU−editing enzyme APOBEC−3G
with a threshold of 1e−05
rs77285108
rs73128290
rs12404911
rs7742829
All
−0.2
0.0
0.2
MR effect size for
'Gut microbiota abundance (genus Catenibacterium id.2153)' on 'DNA dC−>dU−editing enzyme APOBEC−3G'

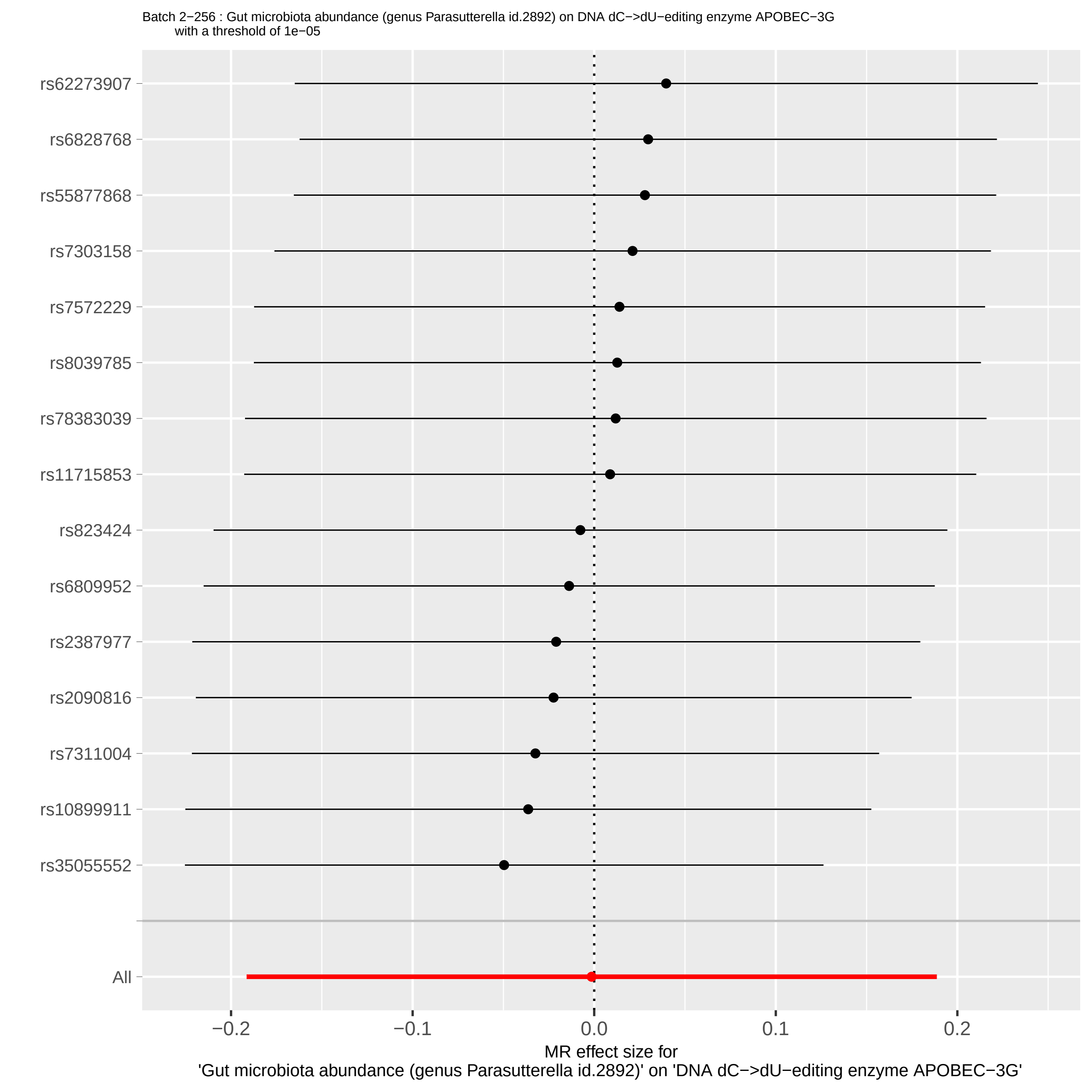

Batch 2−256 : Gut microbiota abundance (genus Parasutterella id.2892) on DNA dC−>dU−editing enzyme APOBEC−3G
with a threshold of 1e−05
rs62273907
rs6828768
rs55877868
rs7303158
rs7572229
rs8039785
rs78383039
rs11715853
rs823424
rs6809952
rs2387977
rs2090816
rs7311004
rs10899911
rs35055552
All
−0.2
−0.1
0.0
0.1
0.2
MR effect size for
'Gut microbiota abundance (genus Parasutterella id.2892)' on 'DNA dC−>dU−editing enzyme APOBEC−3G'

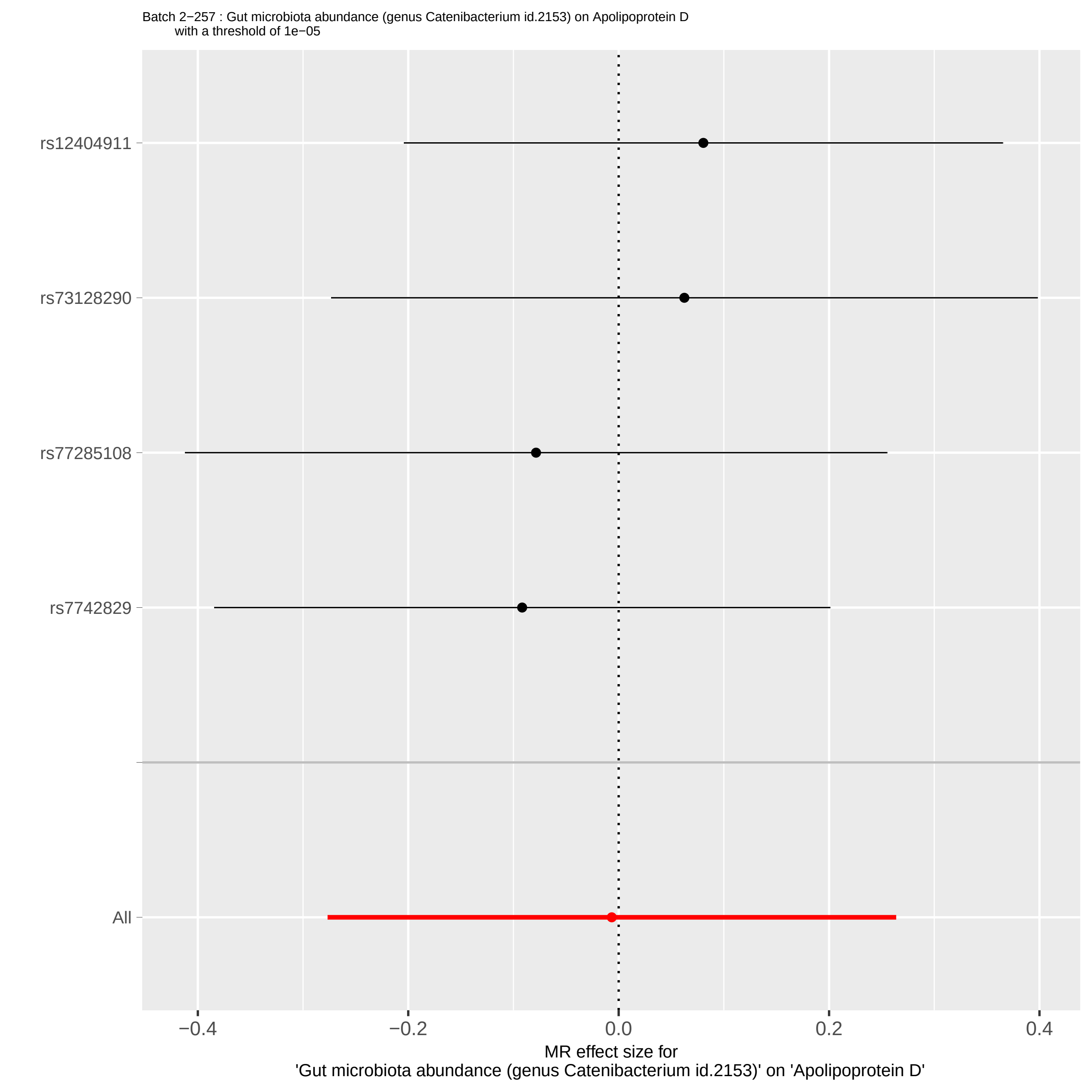

Batch 2−257 : Gut microbiota abundance (genus Catenibacterium id.2153) on Apolipoprotein D
with a threshold of 1e−05
rs12404911
rs73128290
rs77285108
rs7742829
All
−0.4
−0.2
0.0
0.2
0.4
MR effect size for
'Gut microbiota abundance (genus Catenibacterium id.2153)' on 'Apolipoprotein D'

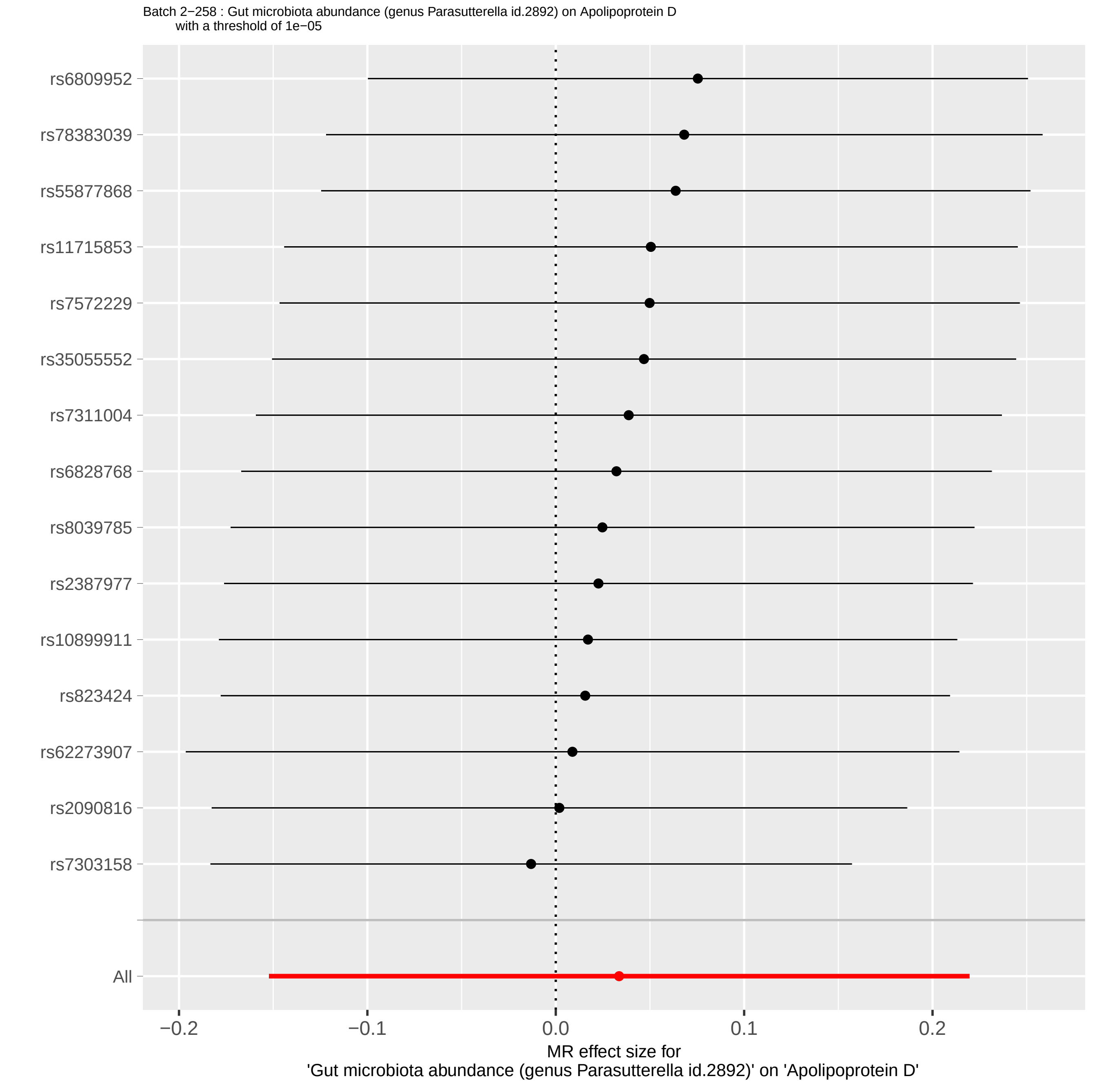

Batch 2−258 : Gut microbiota abundance (genus Parasutterella id.2892) on Apolipoprotein D
with a threshold of 1e−05
rs6809952
rs78383039
rs55877868
rs11715853
rs7572229
rs35055552
rs7311004
rs6828768
rs8039785
rs2387977
rs10899911
rs823424
rs62273907
rs2090816
rs7303158
All
−0.2
−0.1
0.0
0.1
0.2
MR effect size for
'Gut microbiota abundance (genus Parasutterella id.2892)' on 'Apolipoprotein D'

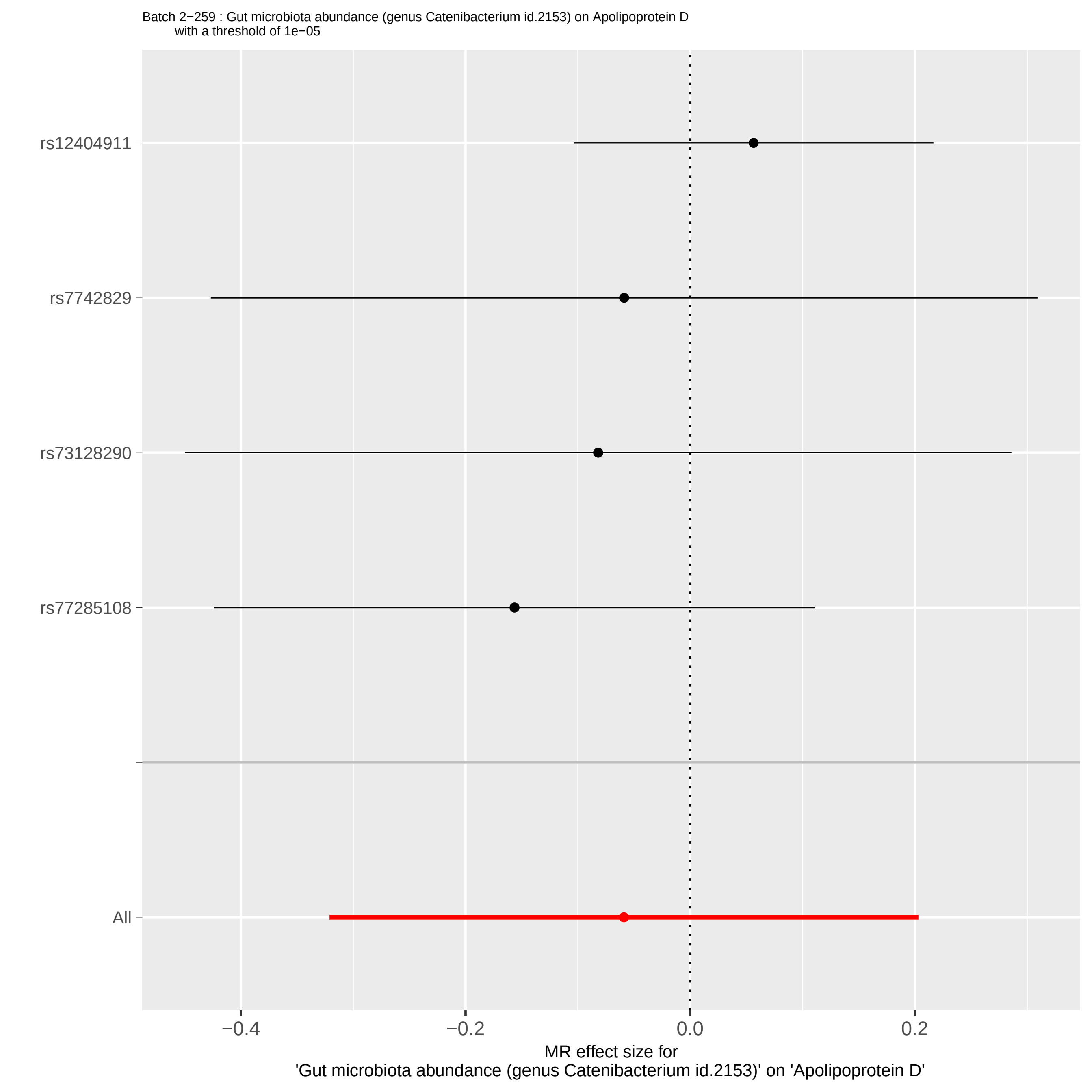
Batch 2−259 : Gut microbiota abundance (genus Catenibacterium id.2153) on Apolipoprotein D
with a threshold of 1e−05
rs12404911
rs7742829
rs73128290
rs77285108
All
−0.4
−0.2
0.0
0.2
MR effect size for
'Gut microbiota abundance (genus Catenibacterium id.2153)' on 'Apolipoprotein D'

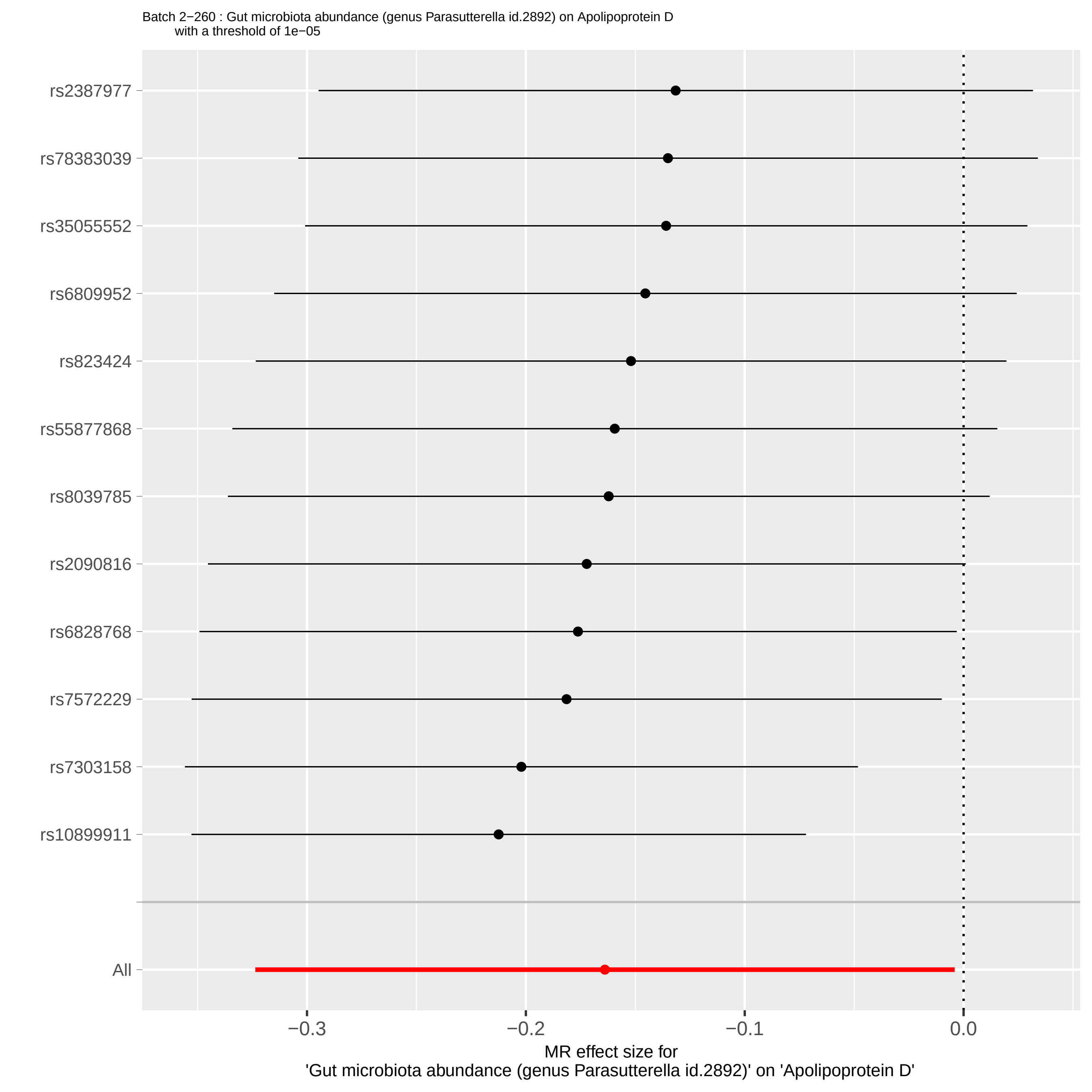

Batch 2−260 : Gut microbiota abundance (genus Parasutterella id.2892) on Apolipoprotein D
with a threshold of 1e−05
rs2387977
rs78383039
rs35055552
rs6809952
rs823424
rs55877868
rs8039785
rs2090816
rs6828768
rs7572229
rs7303158
rs10899911
All
−0.3
−0.2
−0.1
0.0
MR effect size for
'Gut microbiota abundance (genus Parasutterella id.2892)' on 'Apolipoprotein D'

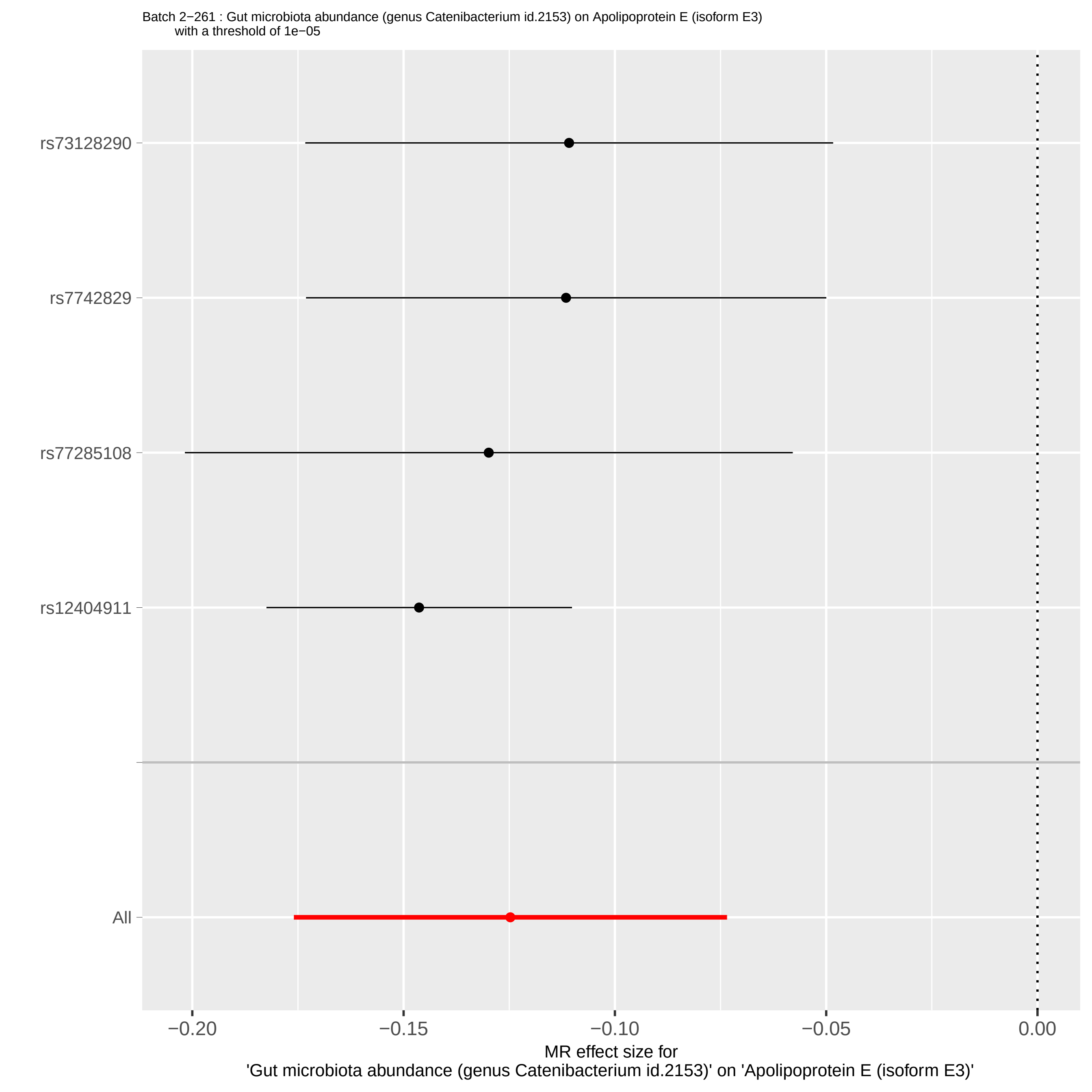
Batch 2−261 : Gut microbiota abundance (genus Catenibacterium id.2153) on Apolipoprotein E (isoform E3)
with a threshold of 1e−05
rs73128290
rs7742829
rs77285108
rs12404911
All
−0.20
−0.15
−0.10
−0.05
0.00
MR effect size for
'Gut microbiota abundance (genus Catenibacterium id.2153)' on 'Apolipoprotein E (isoform E3)'

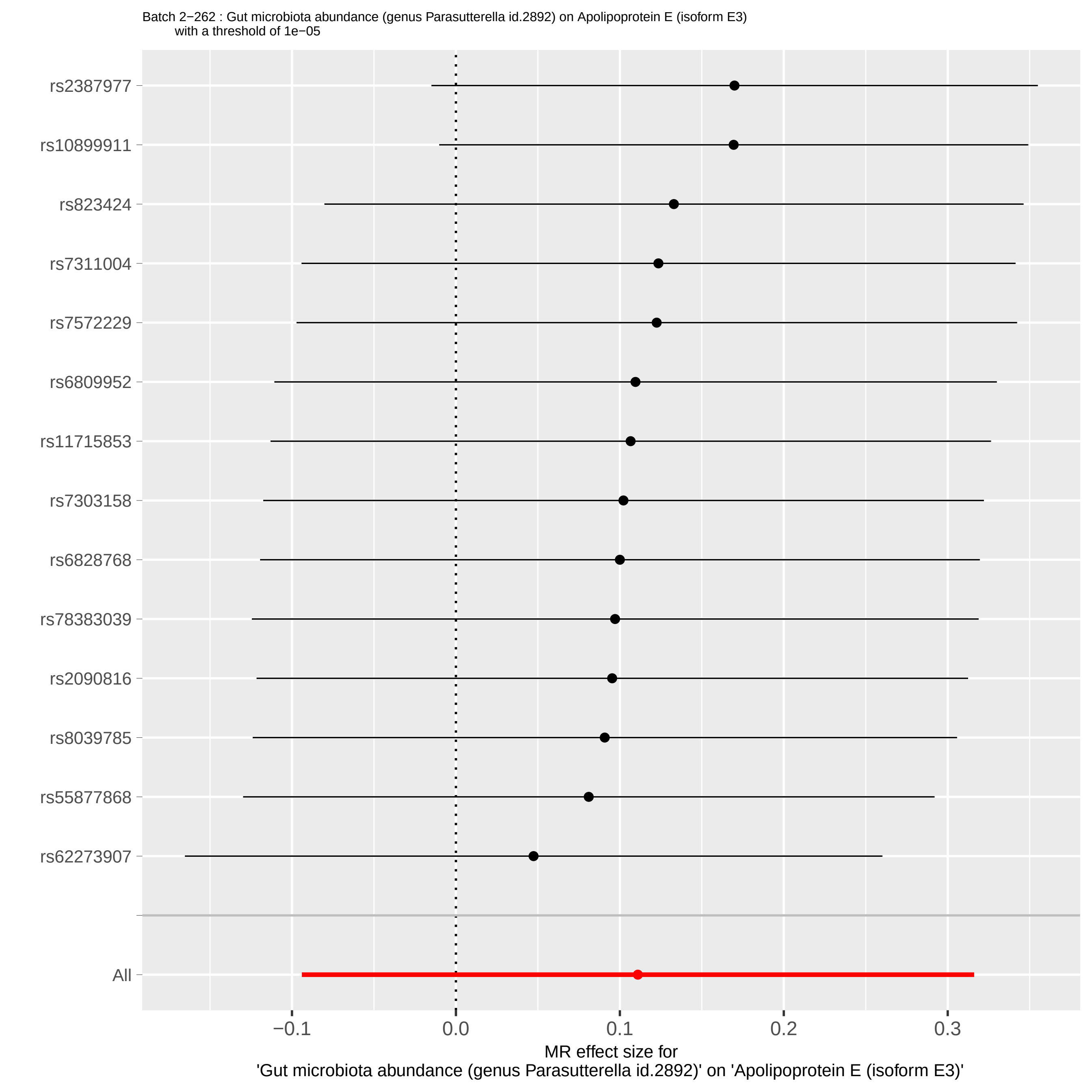
Batch 2−262 : Gut microbiota abundance (genus Parasutterella id.2892) on Apolipoprotein E (isoform E3)
with a threshold of 1e−05
rs2387977
rs10899911
rs823424
rs7311004
rs7572229
rs6809952
rs11715853
rs7303158
rs6828768
rs78383039
rs2090816
rs8039785
rs55877868
rs62273907
All
−0.1
0.0
0.1
0.2
0.3
MR effect size for
'Gut microbiota abundance (genus Parasutterella id.2892)' on 'Apolipoprotein E (isoform E3)'

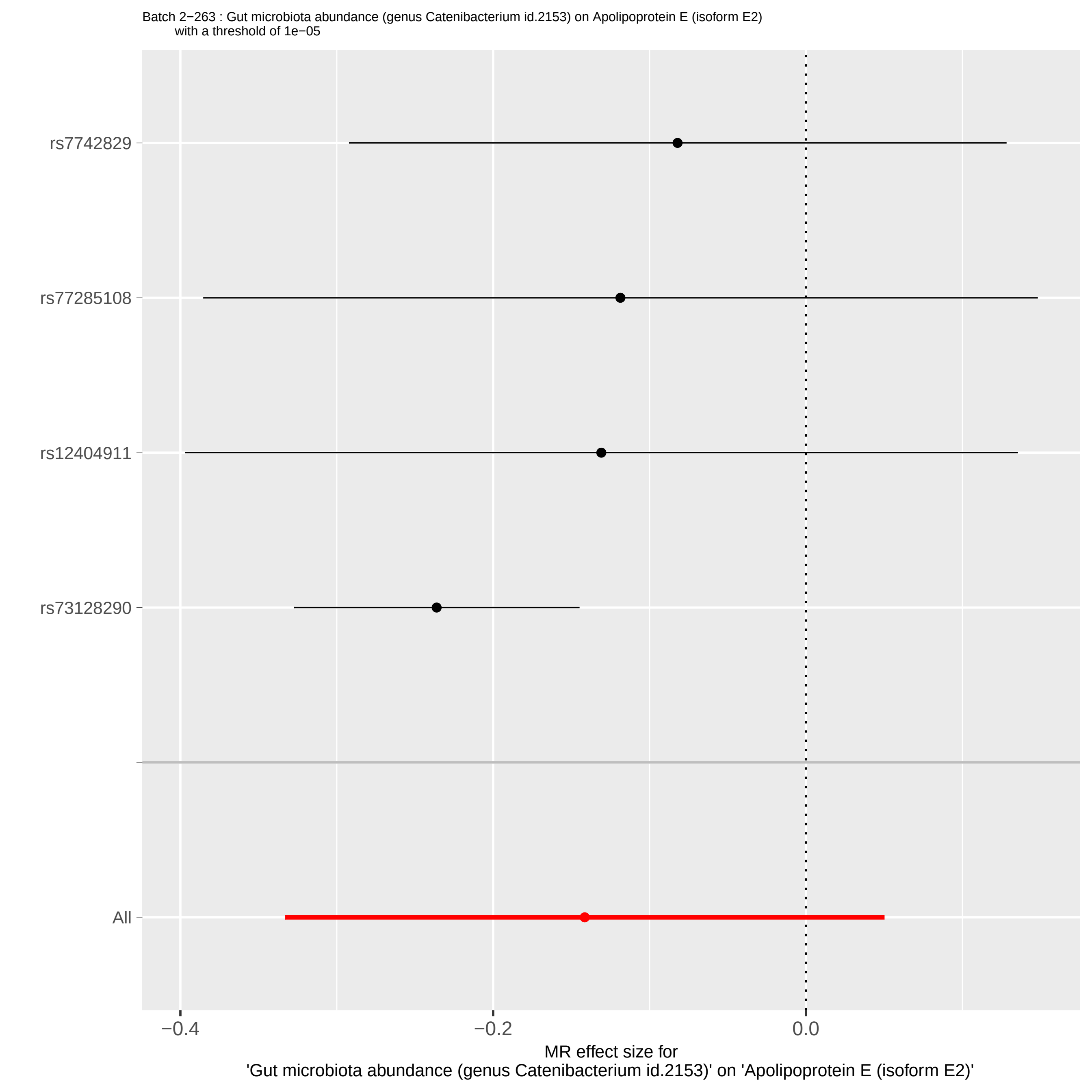
Batch 2−263 : Gut microbiota abundance (genus Catenibacterium id.2153) on Apolipoprotein E (isoform E2)
with a threshold of 1e−05
rs7742829
rs77285108
rs12404911
rs73128290
All
−0.4
−0.2
0.0
MR effect size for
'Gut microbiota abundance (genus Catenibacterium id.2153)' on 'Apolipoprotein E (isoform E2)'

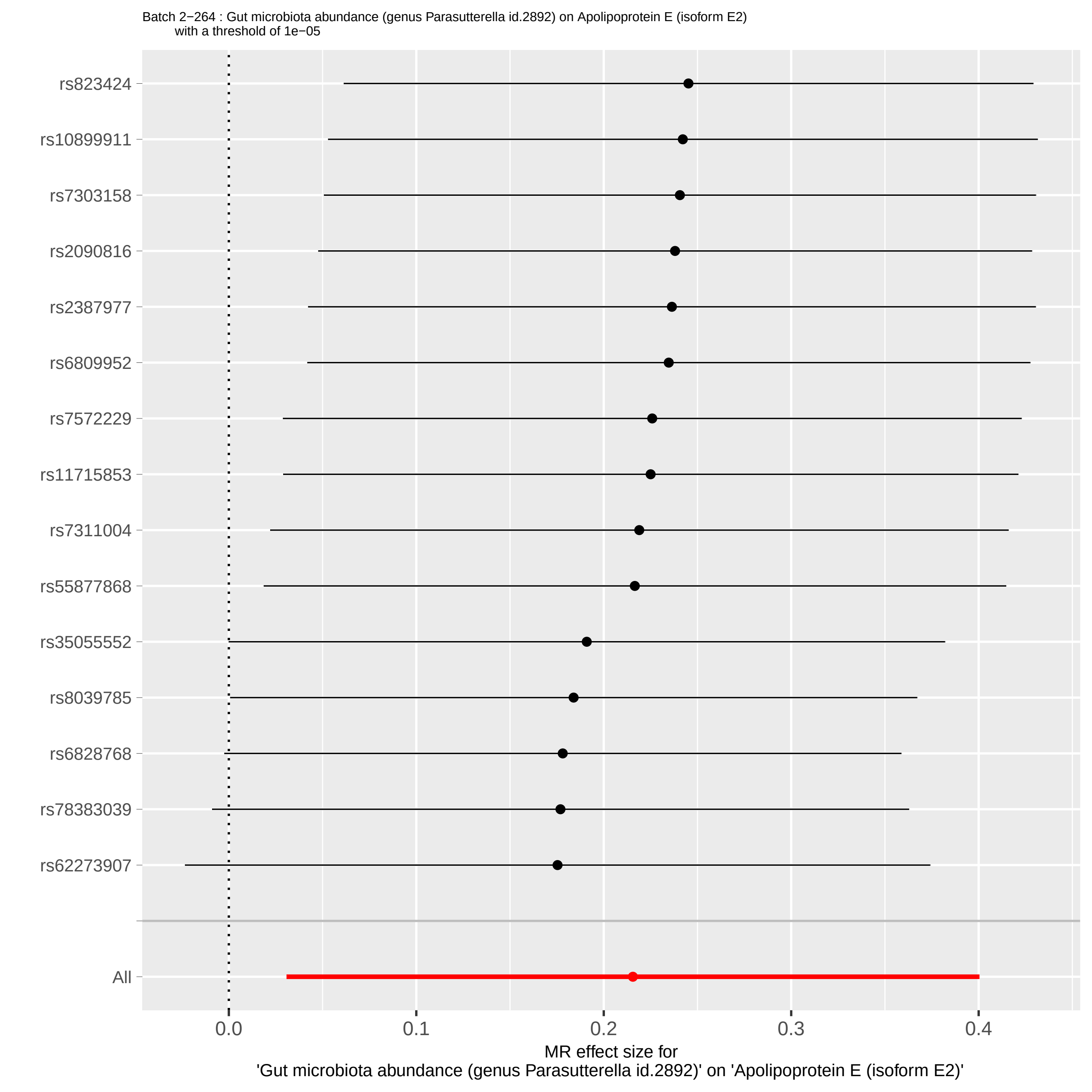
Batch 2−264 : Gut microbiota abundance (genus Parasutterella id.2892) on Apolipoprotein E (isoform E2)
with a threshold of 1e−05
rs823424
rs10899911
rs7303158
rs2090816
rs2387977
rs6809952
rs7572229
rs11715853
rs7311004
rs55877868
rs35055552
rs8039785
rs6828768
rs78383039
rs62273907
All
0.0
0.1
0.2
0.3
0.4
MR effect size for
'Gut microbiota abundance (genus Parasutterella id.2892)' on 'Apolipoprotein E (isoform E2)'

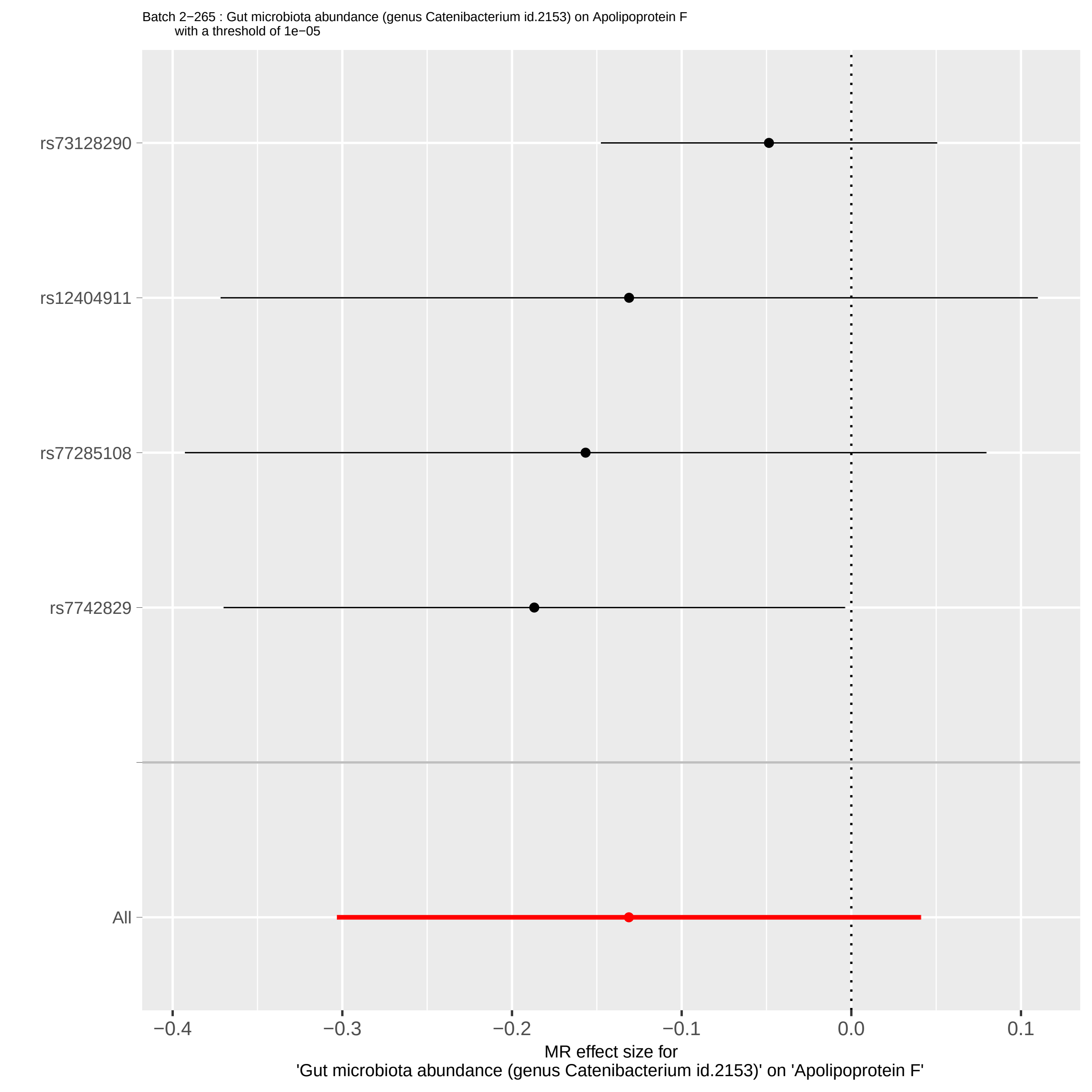
Batch 2−265 : Gut microbiota abundance (genus Catenibacterium id.2153) on Apolipoprotein F
with a threshold of 1e−05
rs73128290
rs12404911
rs77285108
rs7742829
All
−0.4
−0.3
−0.2
−0.1
0.0
0.1
MR effect size for
'Gut microbiota abundance (genus Catenibacterium id.2153)' on 'Apolipoprotein F'

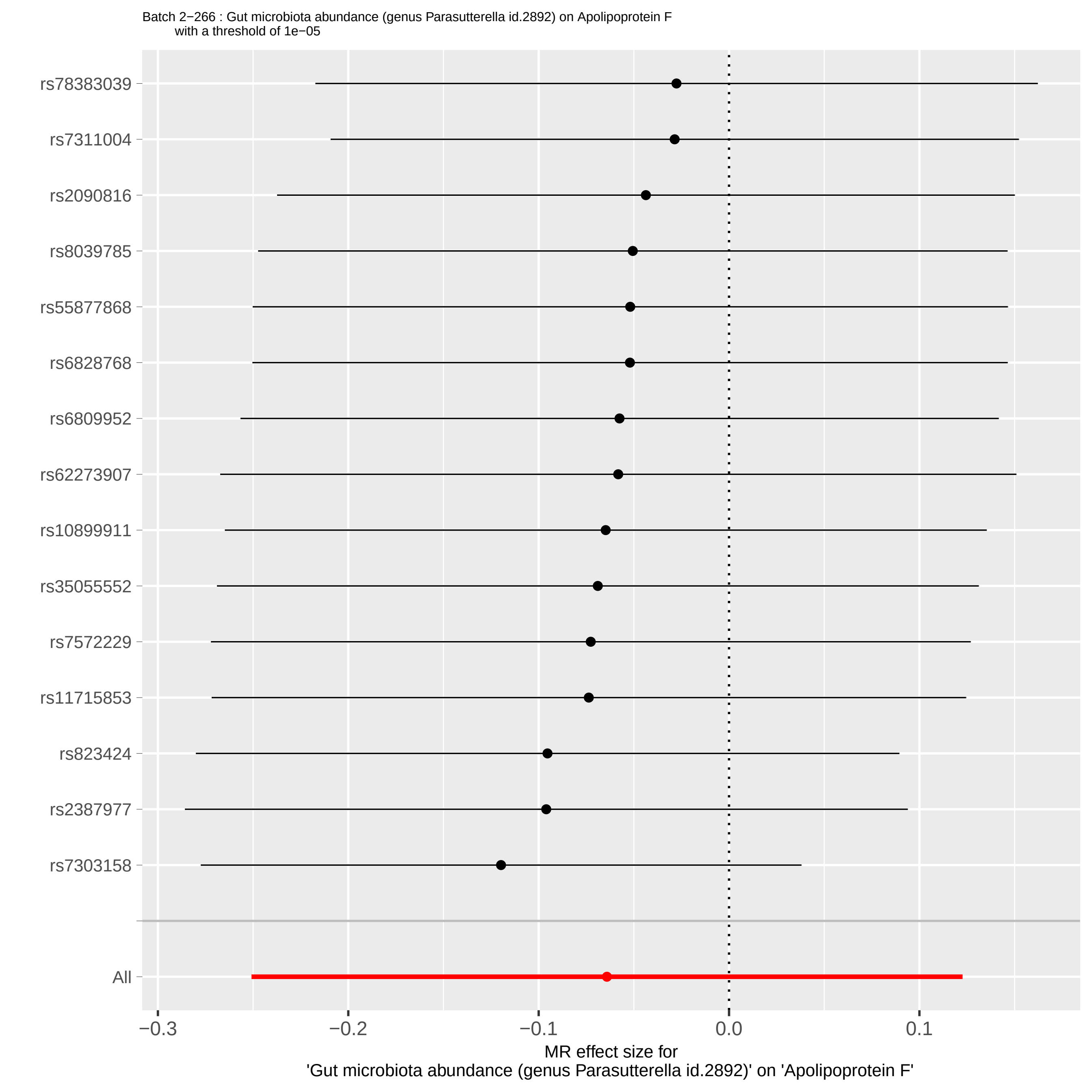
Batch 2−266 : Gut microbiota abundance (genus Parasutterella id.2892) on Apolipoprotein F
with a threshold of 1e−05
rs78383039
rs7311004
rs2090816
rs8039785
rs55877868
rs6828768
rs6809952
rs62273907
rs10899911
rs35055552
rs7572229
rs11715853
rs823424
rs2387977
rs7303158
All
−0.3
−0.2
−0.1
0.0
0.1
MR effect size for
'Gut microbiota abundance (genus Parasutterella id.2892)' on 'Apolipoprotein F'

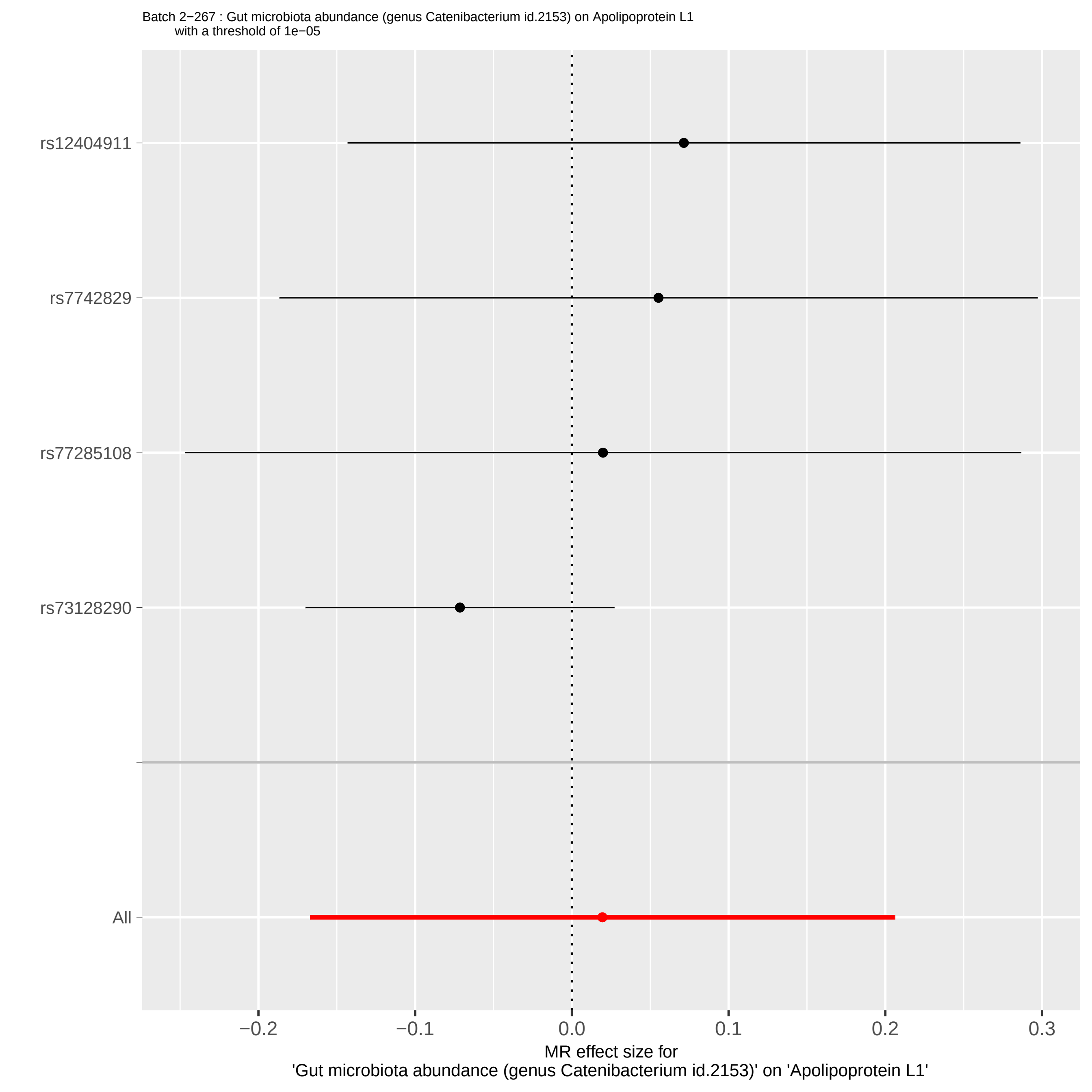
Batch 2−267 : Gut microbiota abundance (genus Catenibacterium id.2153) on Apolipoprotein L1
with a threshold of 1e−05
rs12404911
rs7742829
rs77285108
rs73128290
All
−0.2
−0.1
0.0
0.1
0.2
0.3
MR effect size for
'Gut microbiota abundance (genus Catenibacterium id.2153)' on 'Apolipoprotein L1'

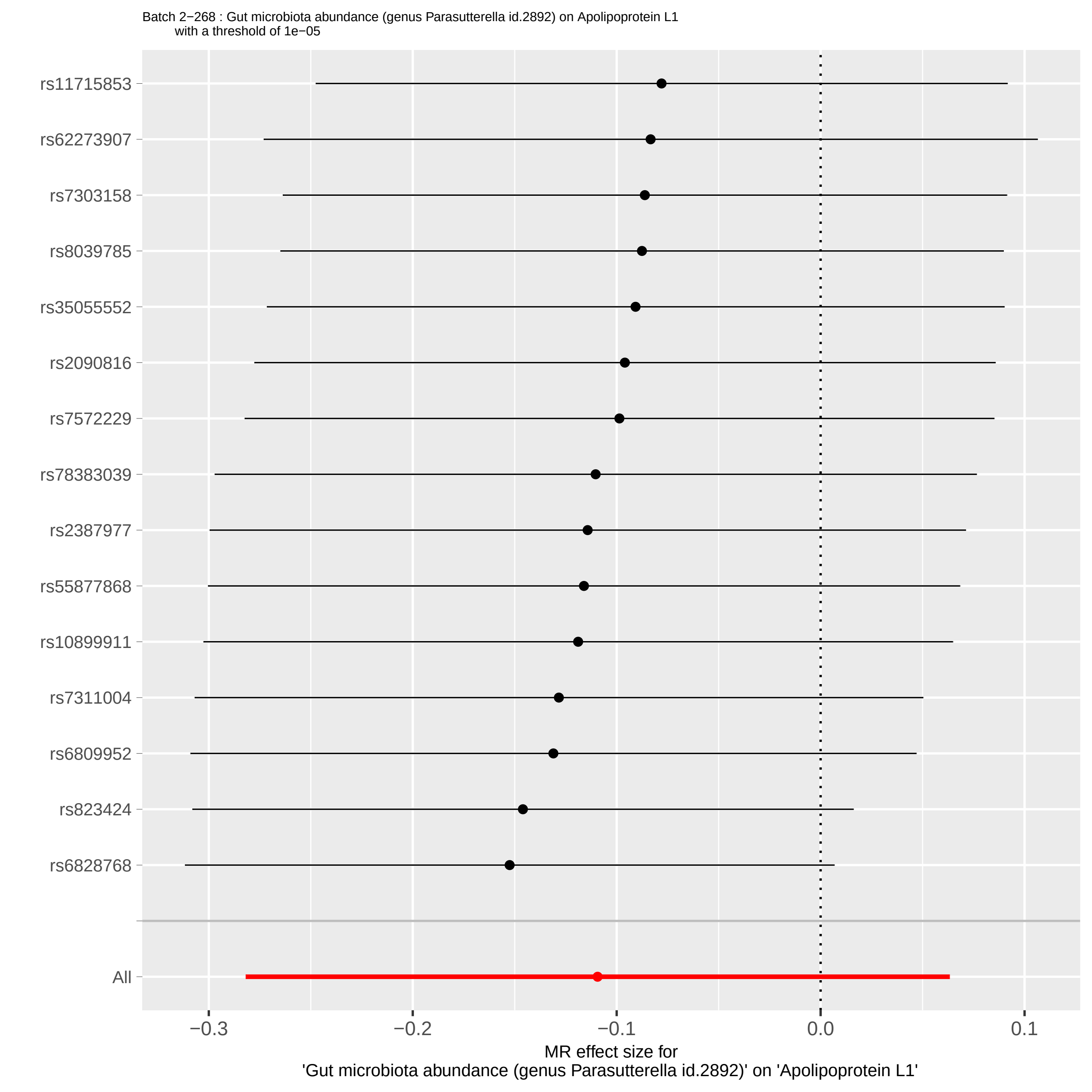

Batch 2−268 : Gut microbiota abundance (genus Parasutterella id.2892) on Apolipoprotein L1
with a threshold of 1e−05
rs11715853
rs62273907
rs7303158
rs8039785
rs35055552
rs2090816
rs7572229
rs78383039
rs2387977
rs55877868
rs10899911
rs7311004
rs6809952
rs823424
rs6828768
All
−0.3
−0.2
−0.1
0.0
0.1
MR effect size for
'Gut microbiota abundance (genus Parasutterella id.2892)' on 'Apolipoprotein L1'

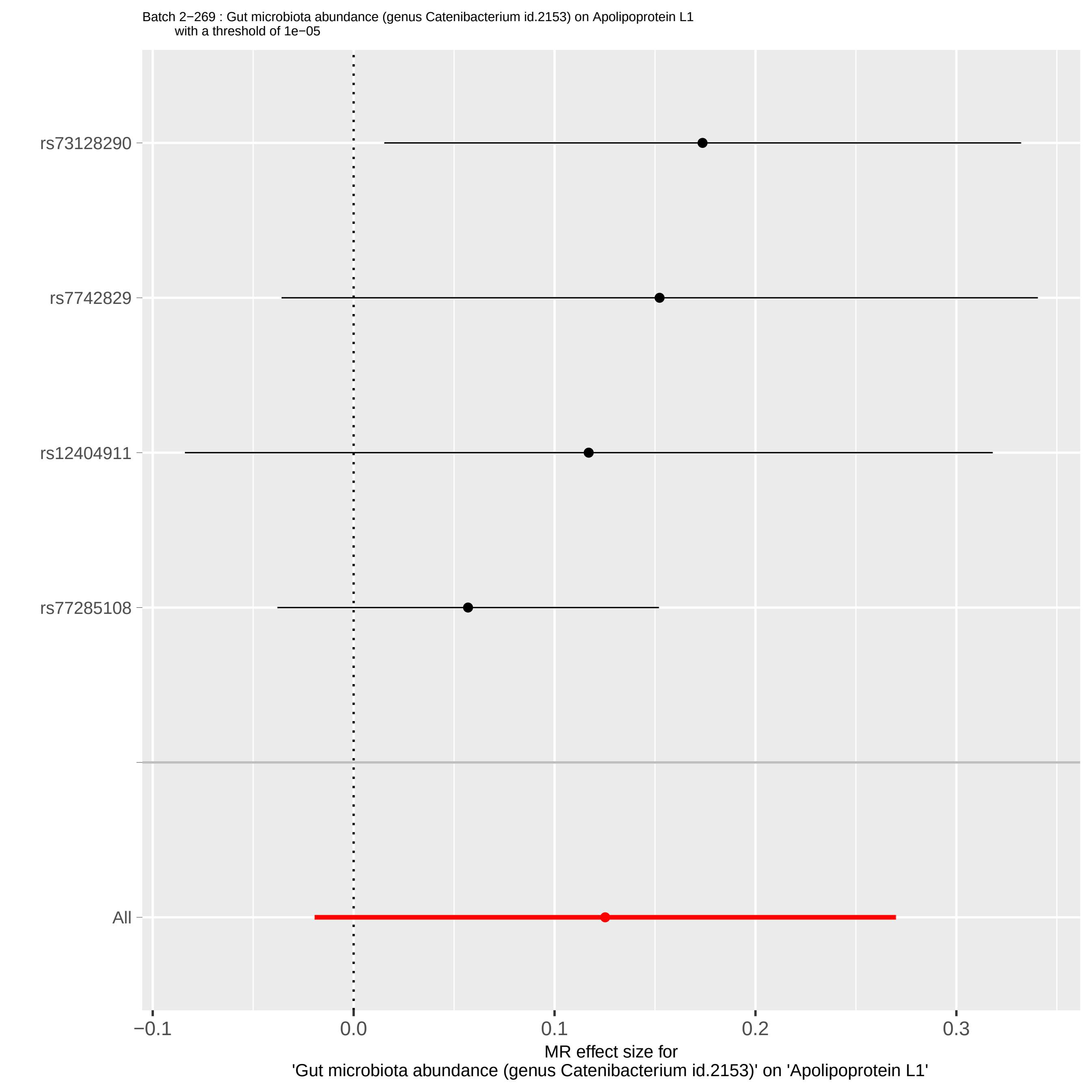
Batch 2−269 : Gut microbiota abundance (genus Catenibacterium id.2153) on Apolipoprotein L1
with a threshold of 1e−05
rs73128290
rs7742829
rs12404911
rs77285108
All
−0.1
0.0
0.1
0.2
0.3
MR effect size for
'Gut microbiota abundance (genus Catenibacterium id.2153)' on 'Apolipoprotein L1'

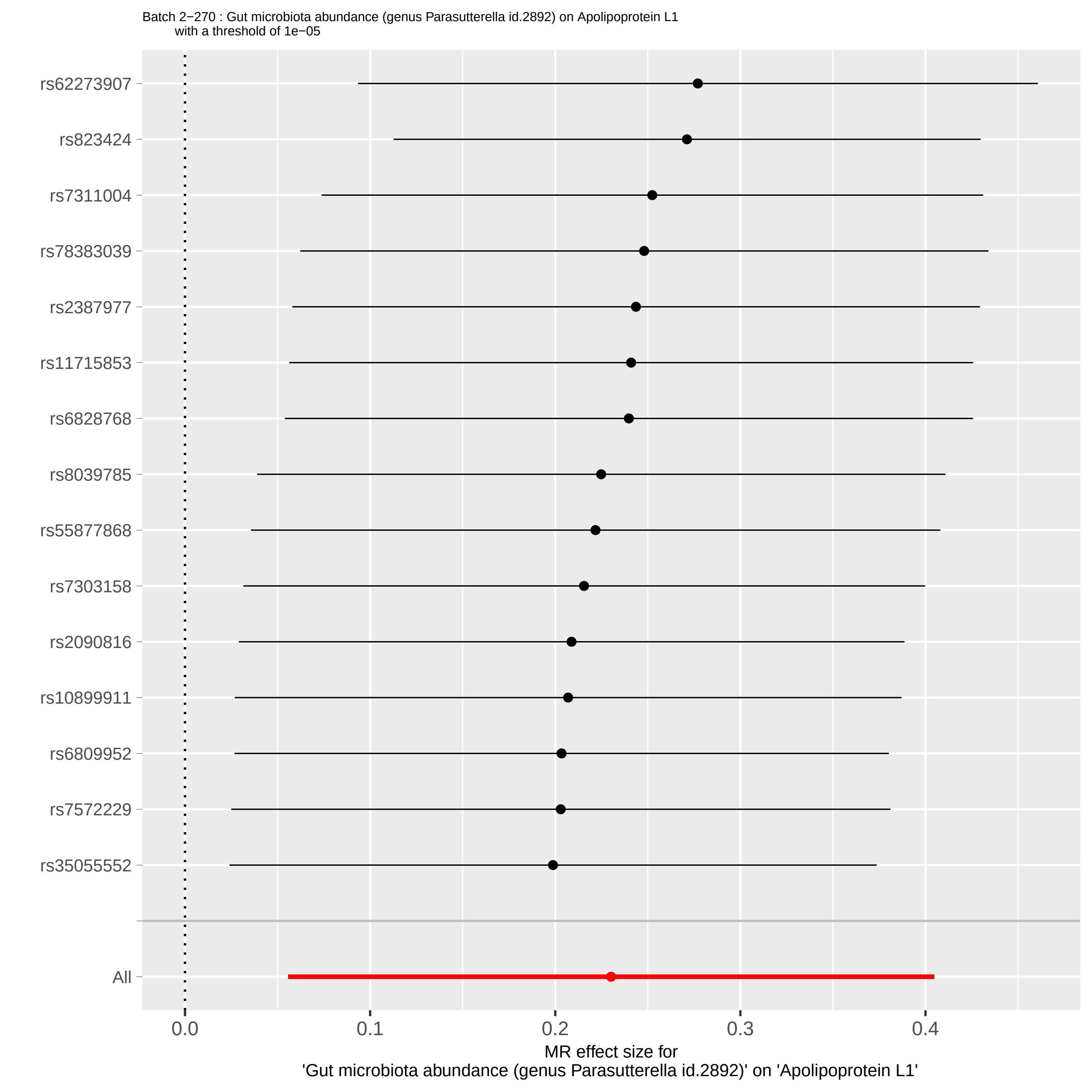

Batch 2−270 : Gut microbiota abundance (genus Parasutterella id.2892) on Apolipoprotein L1
with a threshold of 1e−05
rs62273907
rs823424
rs7311004
rs78383039
rs2387977
rs11715853
rs6828768
rs8039785
rs55877868
rs7303158
rs2090816
rs10899911
rs6809952
rs7572229
rs35055552
All
0.0
0.1
0.2
0.3
0.4
MR effect size for
'Gut microbiota abundance (genus Parasutterella id.2892)' on 'Apolipoprotein L1'

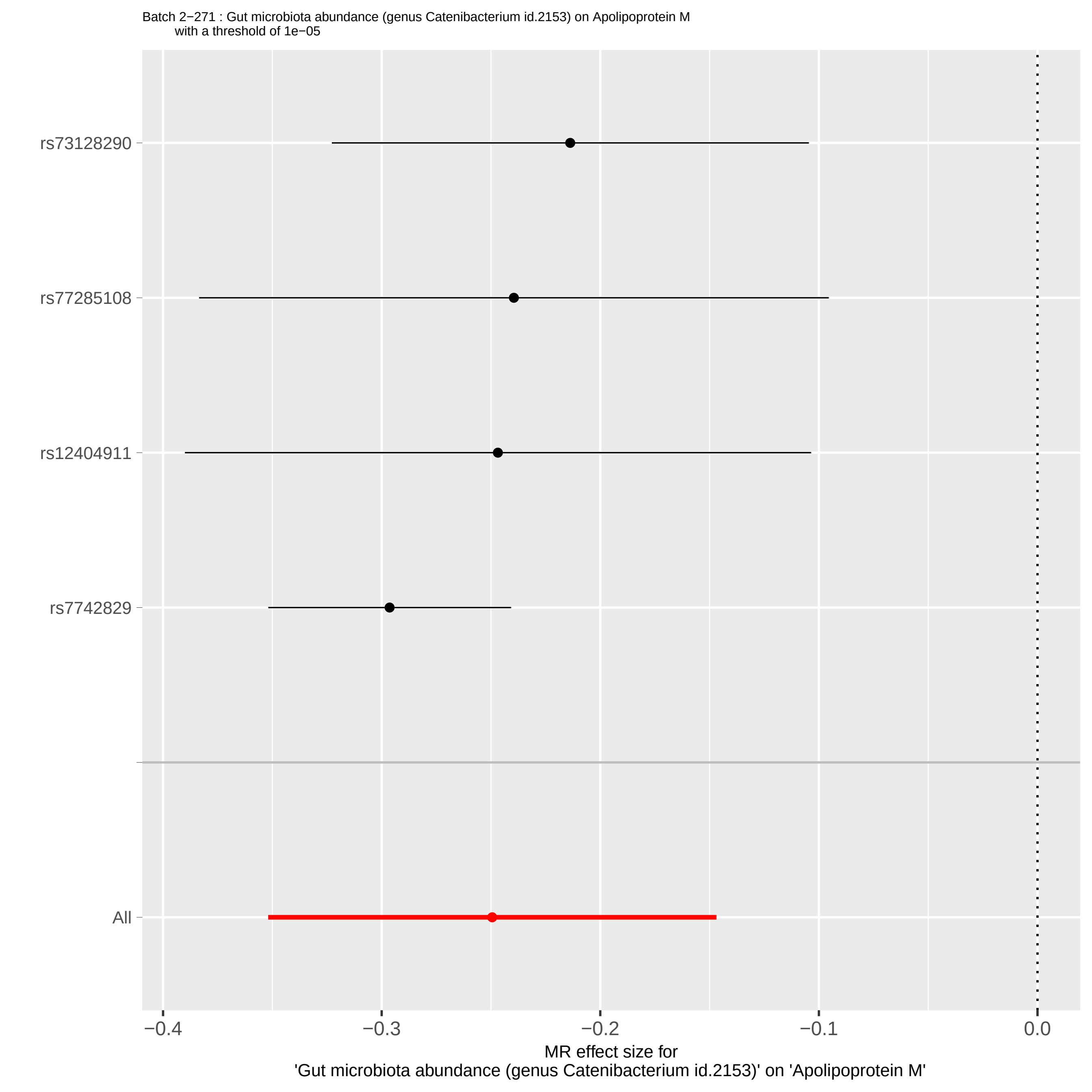

Batch 2−271 : Gut microbiota abundance (genus Catenibacterium id.2153) on Apolipoprotein M
with a threshold of 1e−05
rs73128290
rs77285108
rs12404911
rs7742829
All
−0.4
−0.3
−0.2
−0.1
0.0
MR effect size for
'Gut microbiota abundance (genus Catenibacterium id.2153)' on 'Apolipoprotein M'

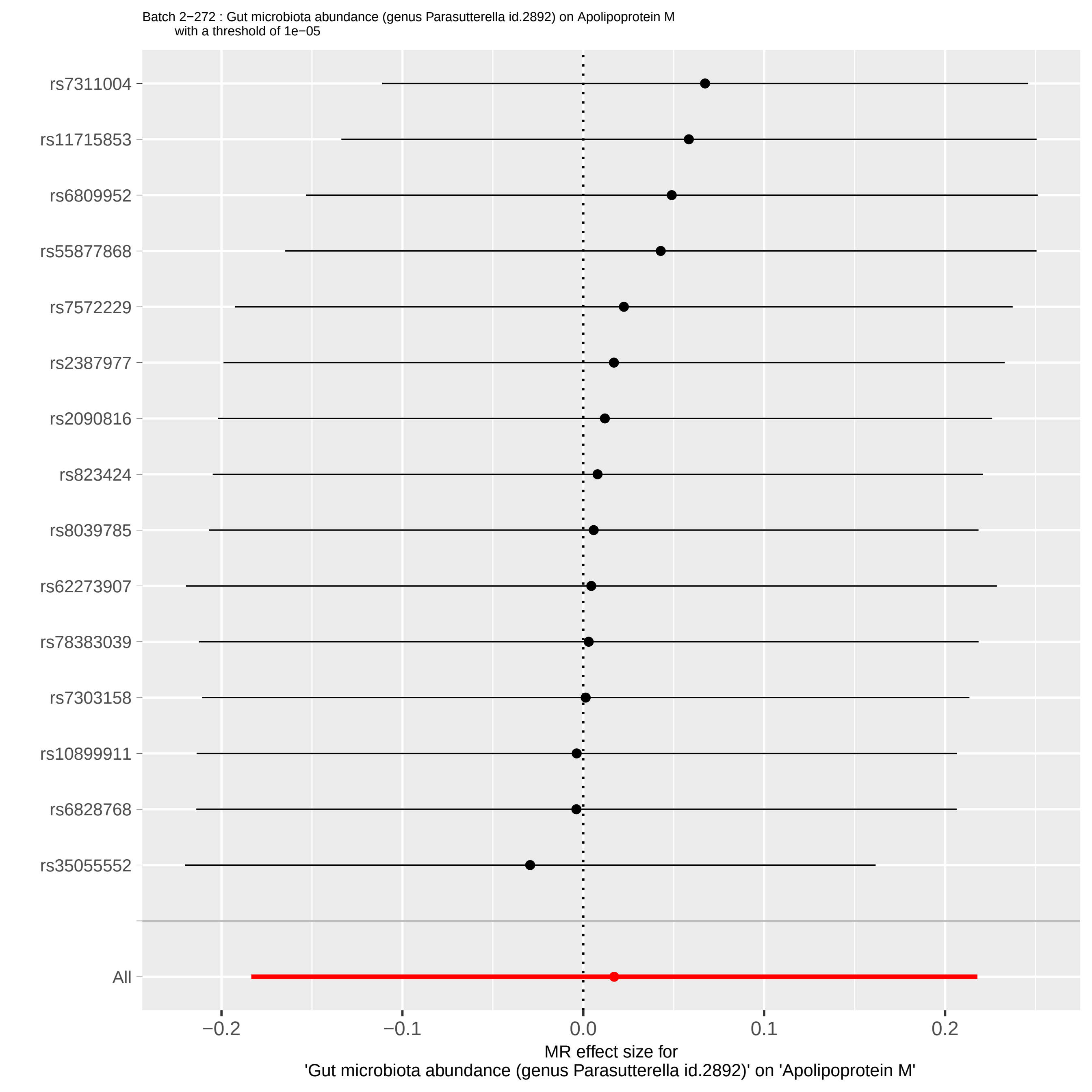

Batch 2−272 : Gut microbiota abundance (genus Parasutterella id.2892) on Apolipoprotein M
with a threshold of 1e−05
rs7311004
rs11715853
rs6809952
rs55877868
rs7572229
rs2387977
rs2090816
rs823424
rs8039785
rs62273907
rs78383039
rs7303158
rs10899911
rs6828768
rs35055552
All
−0.2
−0.1
0.0
0.1
0.2
MR effect size for
'Gut microbiota abundance (genus Parasutterella id.2892)' on 'Apolipoprotein M'

Batch 2−273 : Gut microbiota abundance (genus Catenibacterium id.2153) on Apolipoprotein M
with a threshold of 1e−05

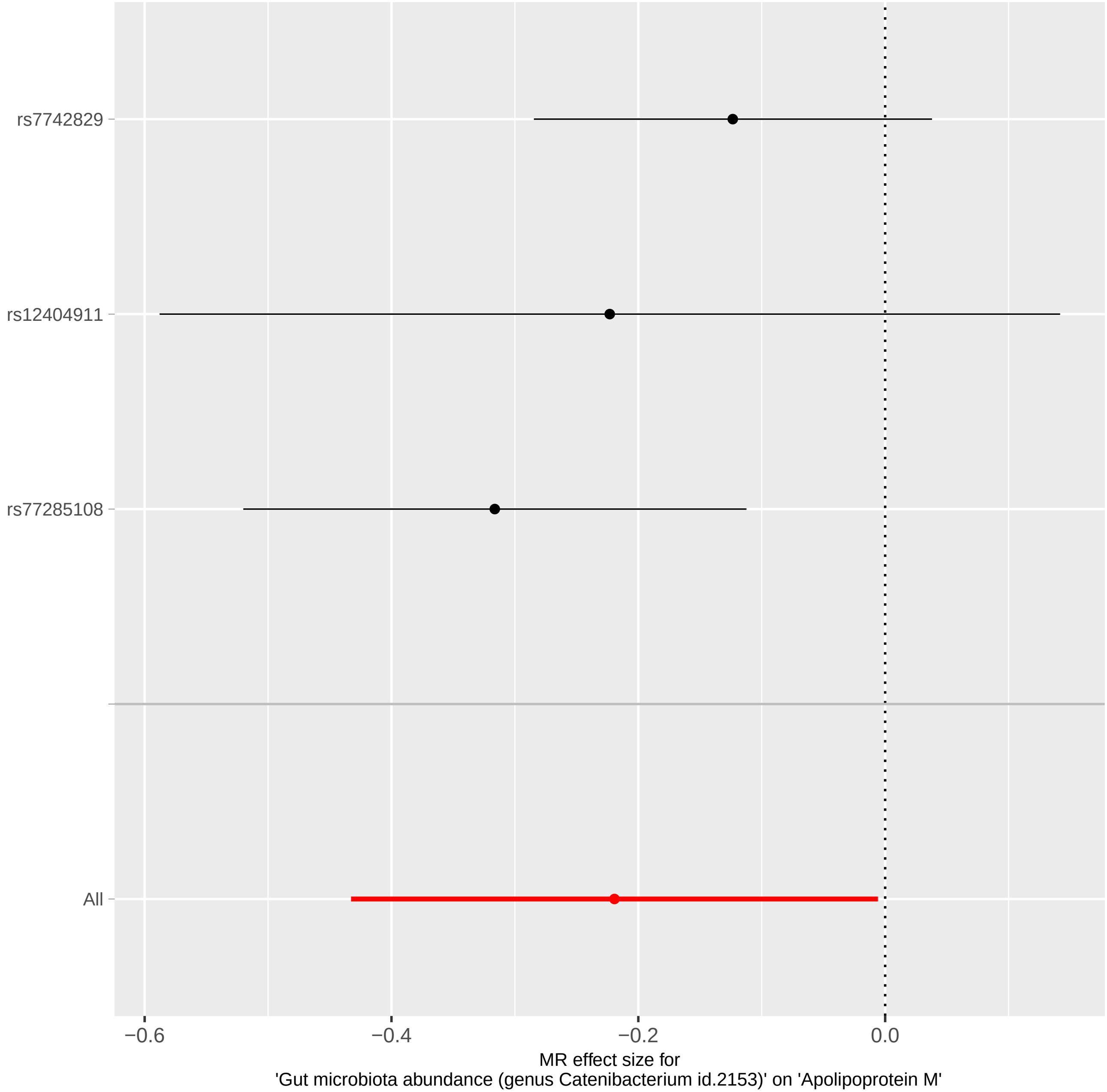

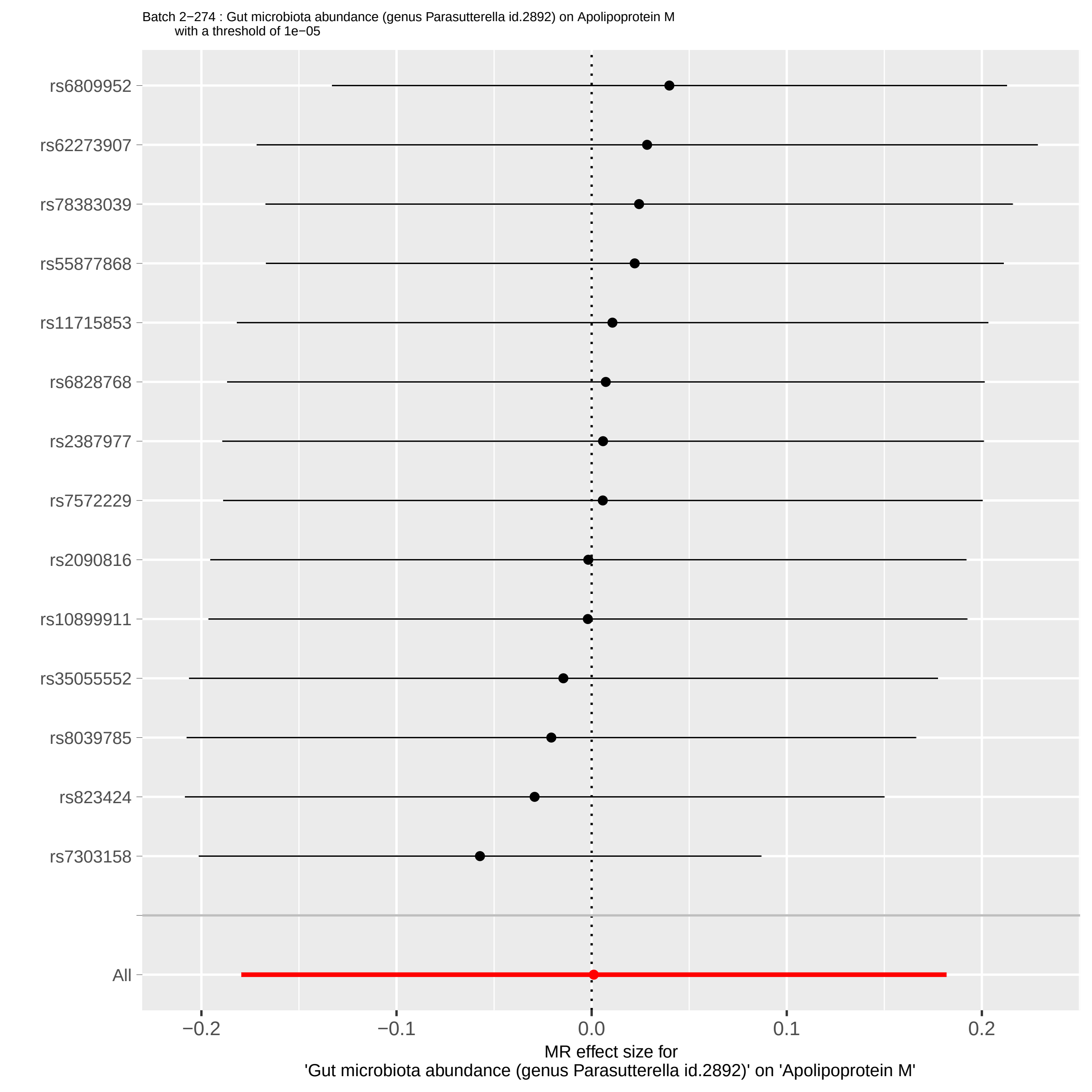

Batch 2−274 : Gut microbiota abundance (genus Parasutterella id.2892) on Apolipoprotein M
with a threshold of 1e−05
rs6809952
rs62273907
rs78383039
rs55877868
rs11715853
rs6828768
rs2387977
rs7572229
rs2090816
rs10899911
rs35055552
rs8039785
rs823424
rs7303158
All
−0.2
−0.1
0.0
0.1
0.2
MR effect size for
'Gut microbiota abundance (genus Parasutterella id.2892)' on 'Apolipoprotein M'

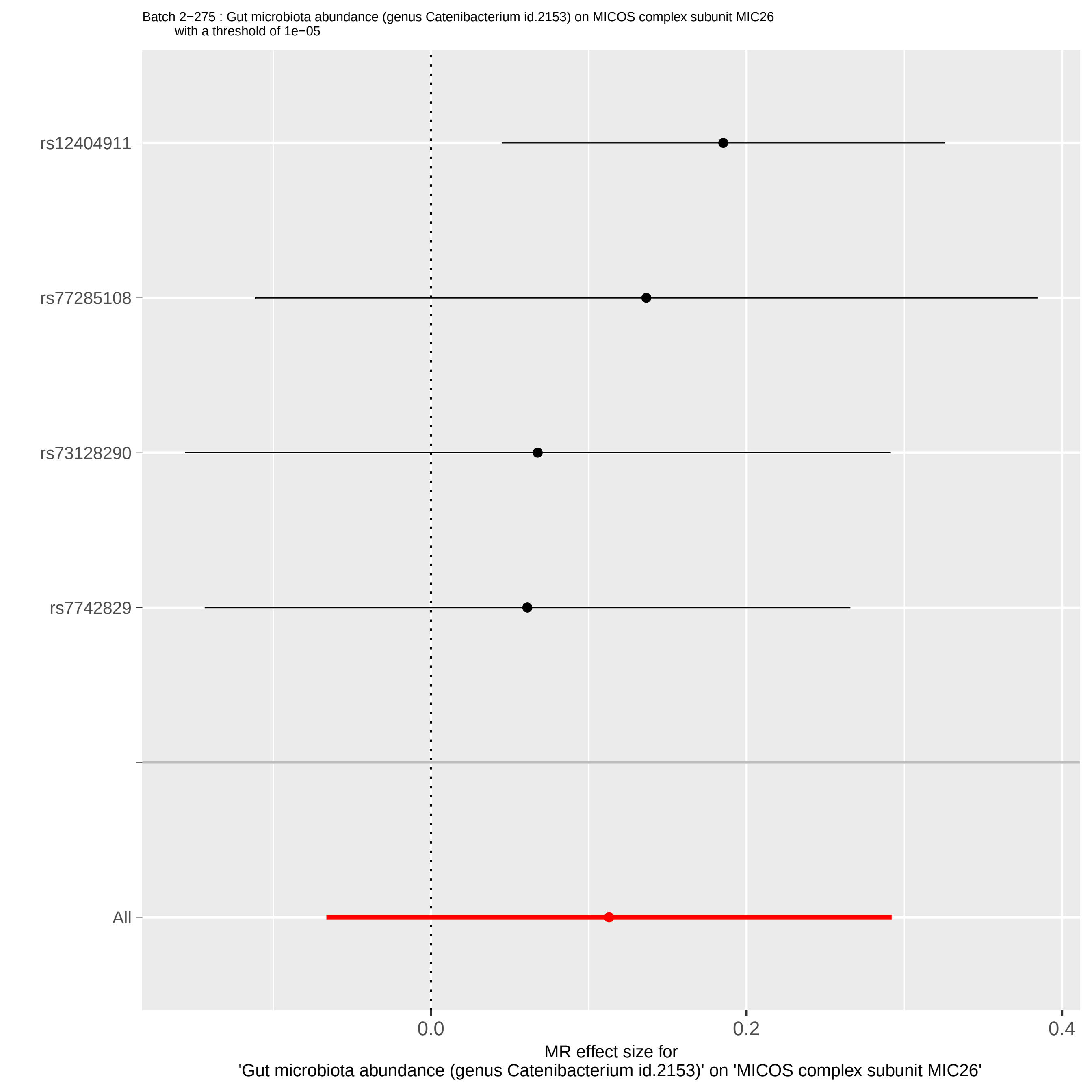
Batch 2−275 : Gut microbiota abundance (genus Catenibacterium id.2153) on MICOS complex subunit MIC26
with a threshold of 1e−05
rs12404911
rs77285108
rs73128290
rs7742829
All
0.0
0.2
0.4
MR effect size for
'Gut microbiota abundance (genus Catenibacterium id.2153)' on 'MICOS complex subunit MIC26'

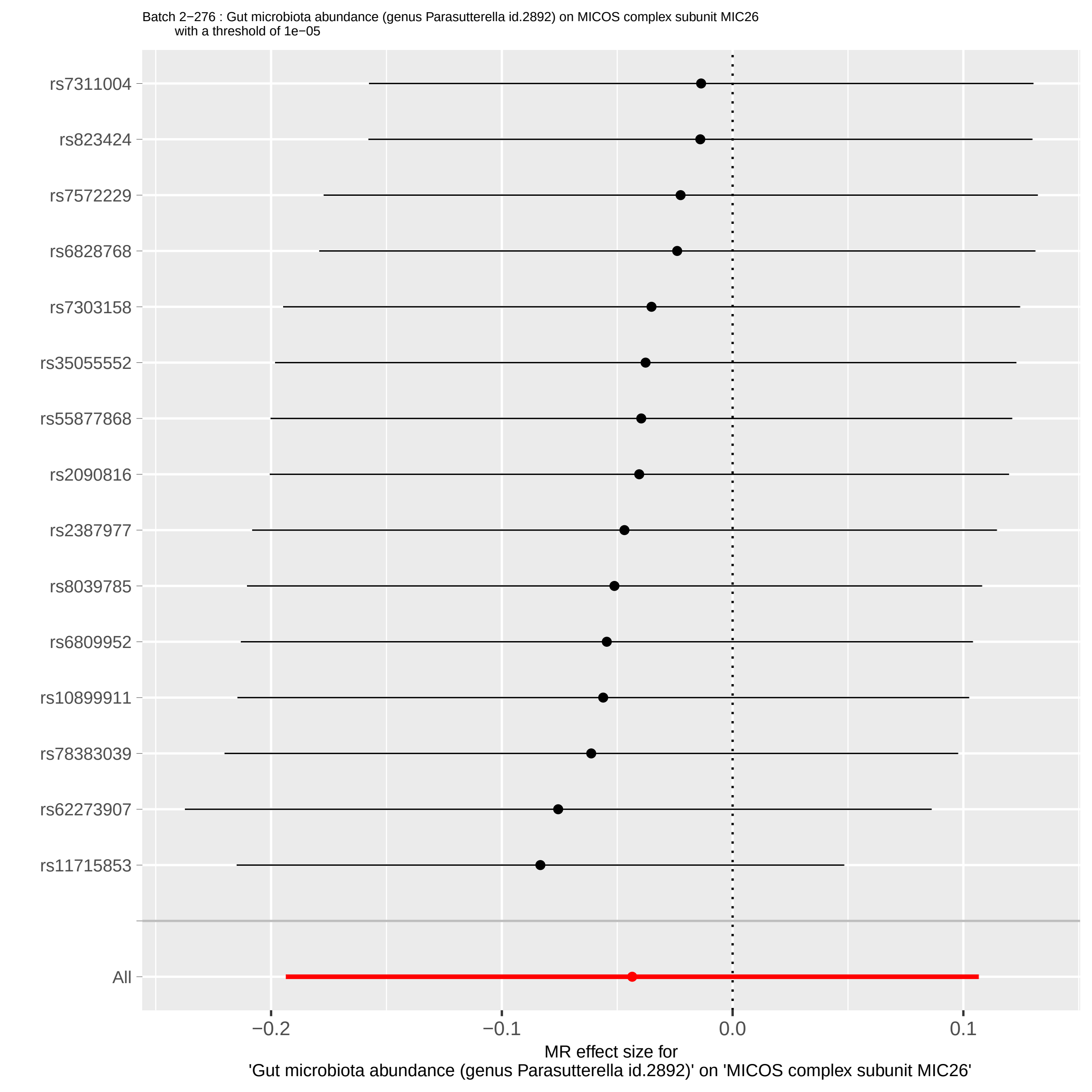

Batch 2−276 : Gut microbiota abundance (genus Parasutterella id.2892) on MICOS complex subunit MIC26
with a threshold of 1e−05
rs7311004
rs823424
rs7572229
rs6828768
rs7303158
rs35055552
rs55877868
rs2090816
rs2387977
rs8039785
rs6809952
rs10899911
rs78383039
rs62273907
rs11715853
All
−0.2
−0.1
0.0
0.1
MR effect size for
'Gut microbiota abundance (genus Parasutterella id.2892)' on 'MICOS complex subunit MIC26'

Batch 2−277 : Gut microbiota abundance (genus Catenibacterium id.2153) on Amyloid beta A4 protein
with a threshold of 1e−05

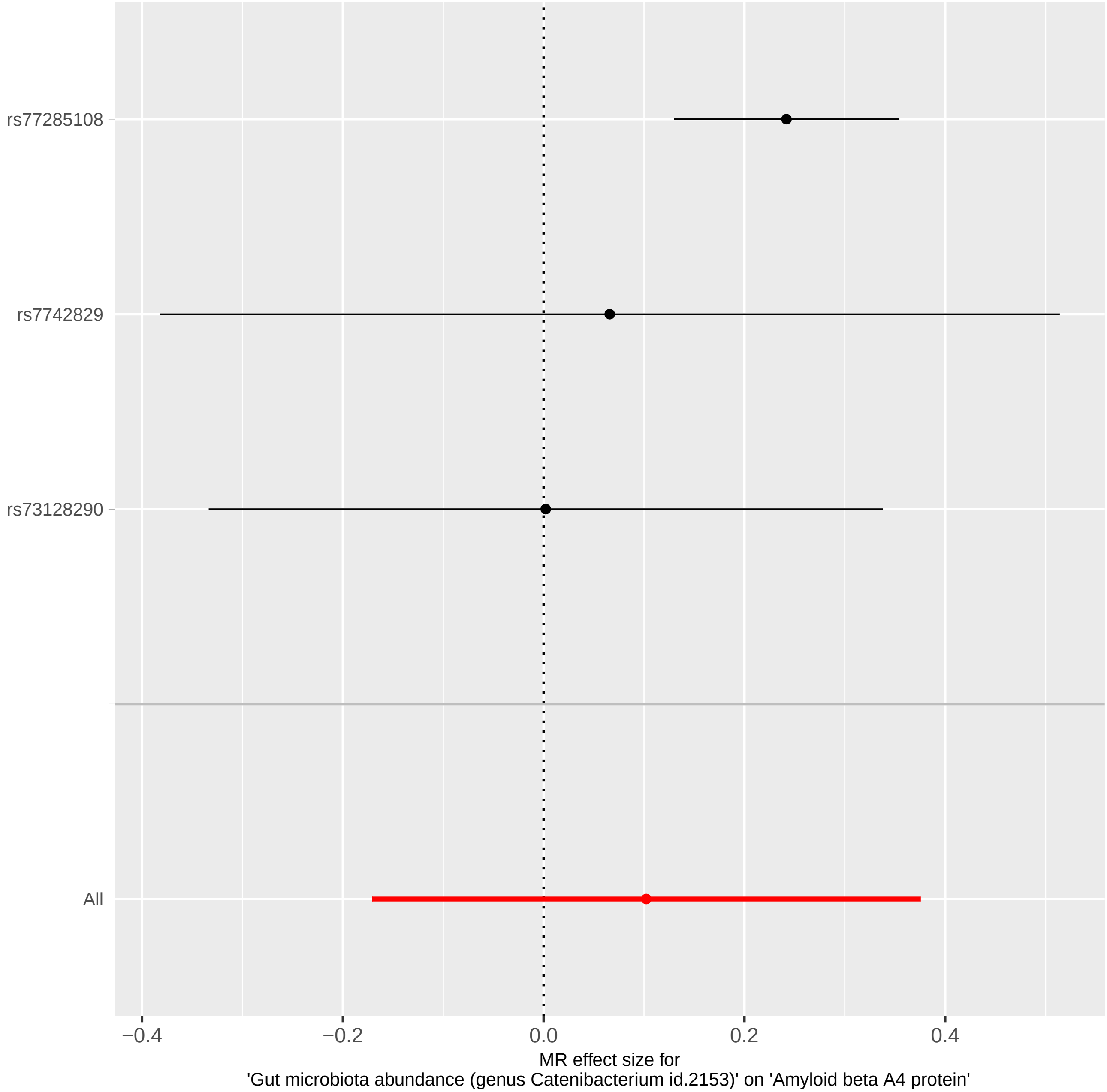

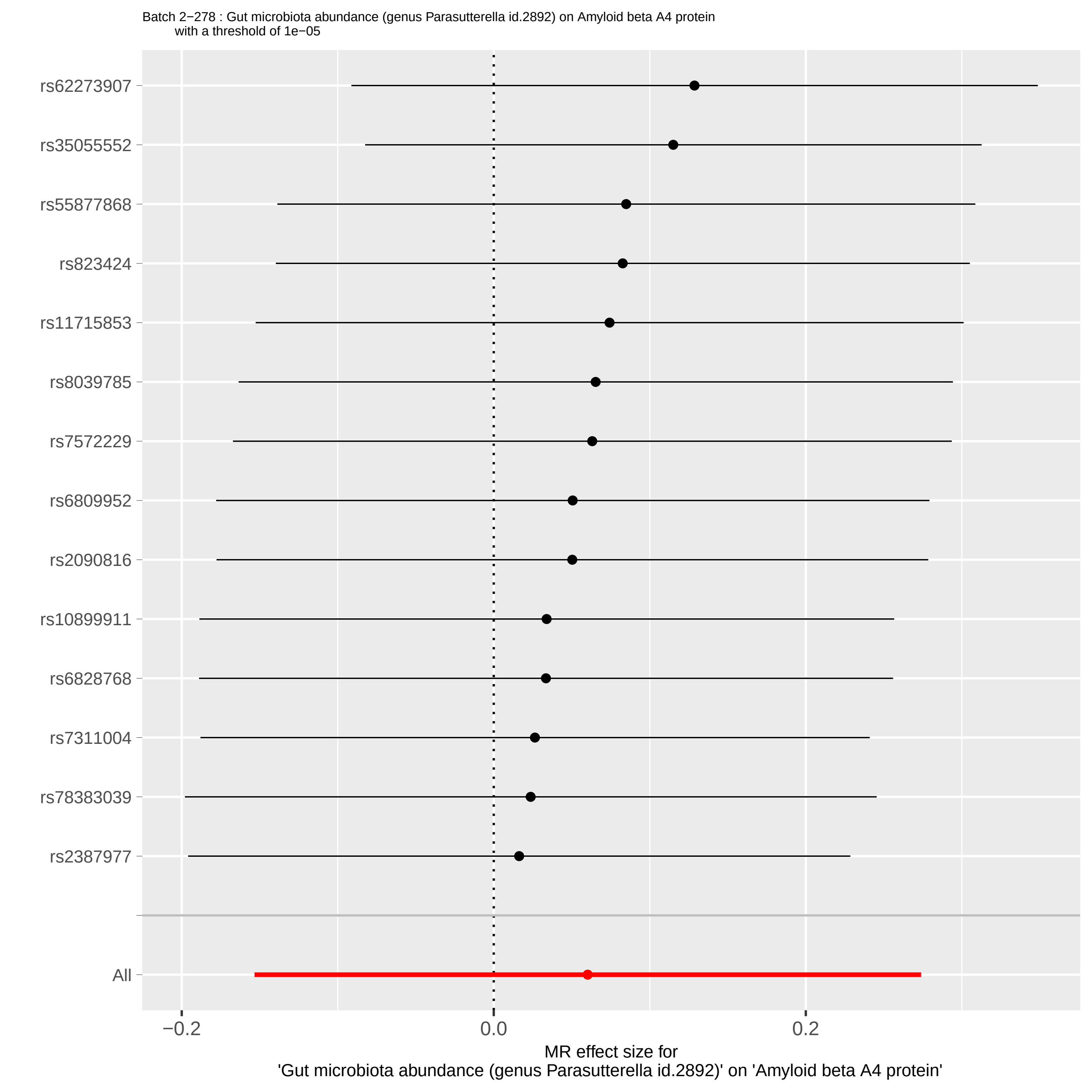
Batch 2−278 : Gut microbiota abundance (genus Parasutterella id.2892) on Amyloid beta A4 protein
with a threshold of 1e−05
rs62273907
rs35055552
rs55877868
rs823424
rs11715853
rs8039785
rs7572229
rs6809952
rs2090816
rs10899911
rs6828768
rs7311004
rs78383039
rs2387977
All
−0.2
0.0
0.2
MR effect size for
'Gut microbiota abundance (genus Parasutterella id.2892)' on 'Amyloid beta A4 protein'

Batch 2−279 : Gut microbiota abundance (genus Catenibacterium id.2153) on DCC−interacting protein 13−alpha
with a threshold of 1e−05

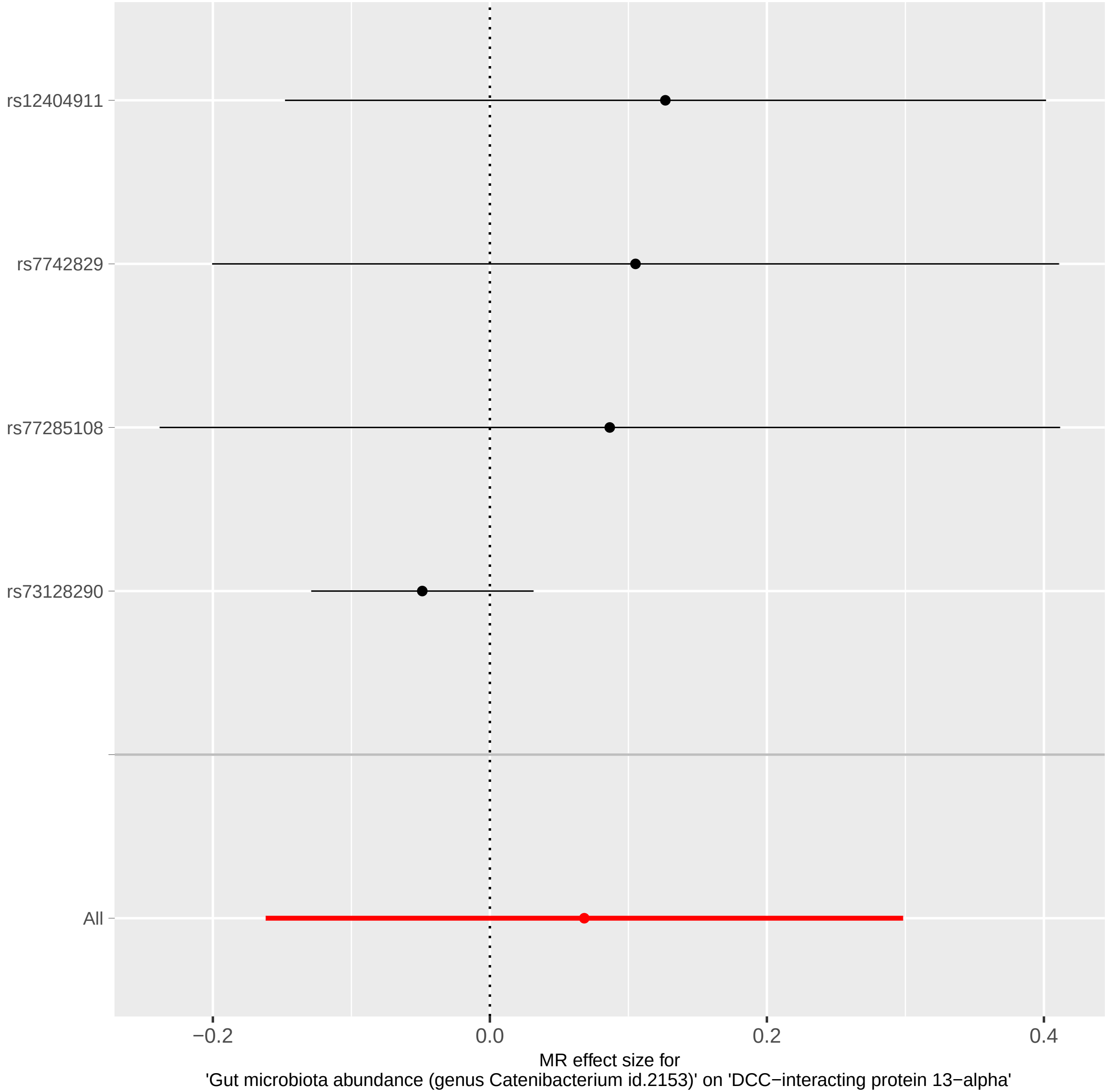

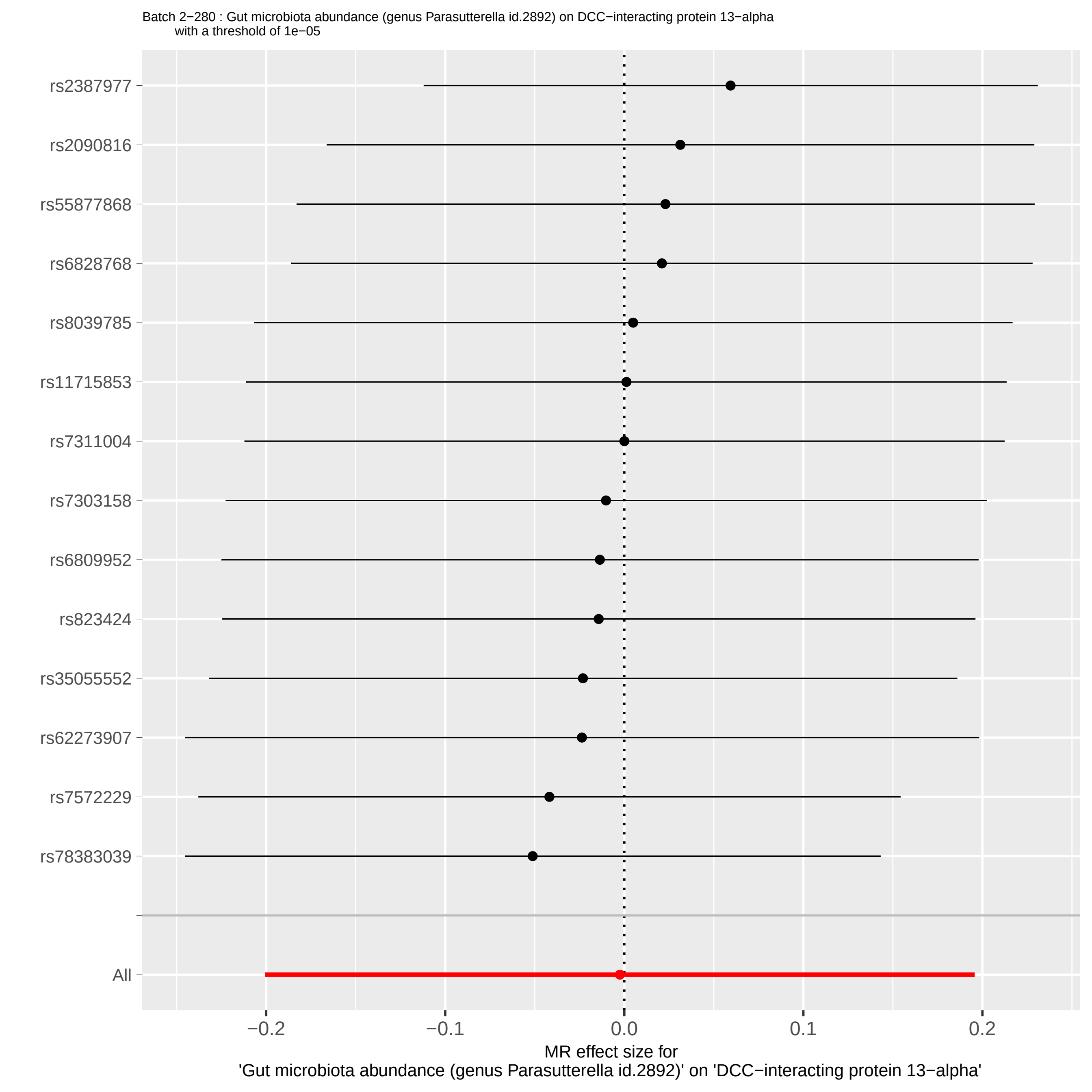

Batch 2−280 : Gut microbiota abundance (genus Parasutterella id.2892) on DCC−interacting protein 13−alpha with a threshold of 1e−05
rs2387977
rs2090816
rs55877868
rs6828768
rs8039785
rs11715853
rs7311004
rs7303158
rs6809952
rs823424
rs35055552
rs62273907
rs7572229
rs78383039
All
−0.2
−0.1
0.0
0.1
0.2
MR effect size for
'Gut microbiota abundance (genus Parasutterella id.2892)' on 'DCC−interacting protein 13−alpha'

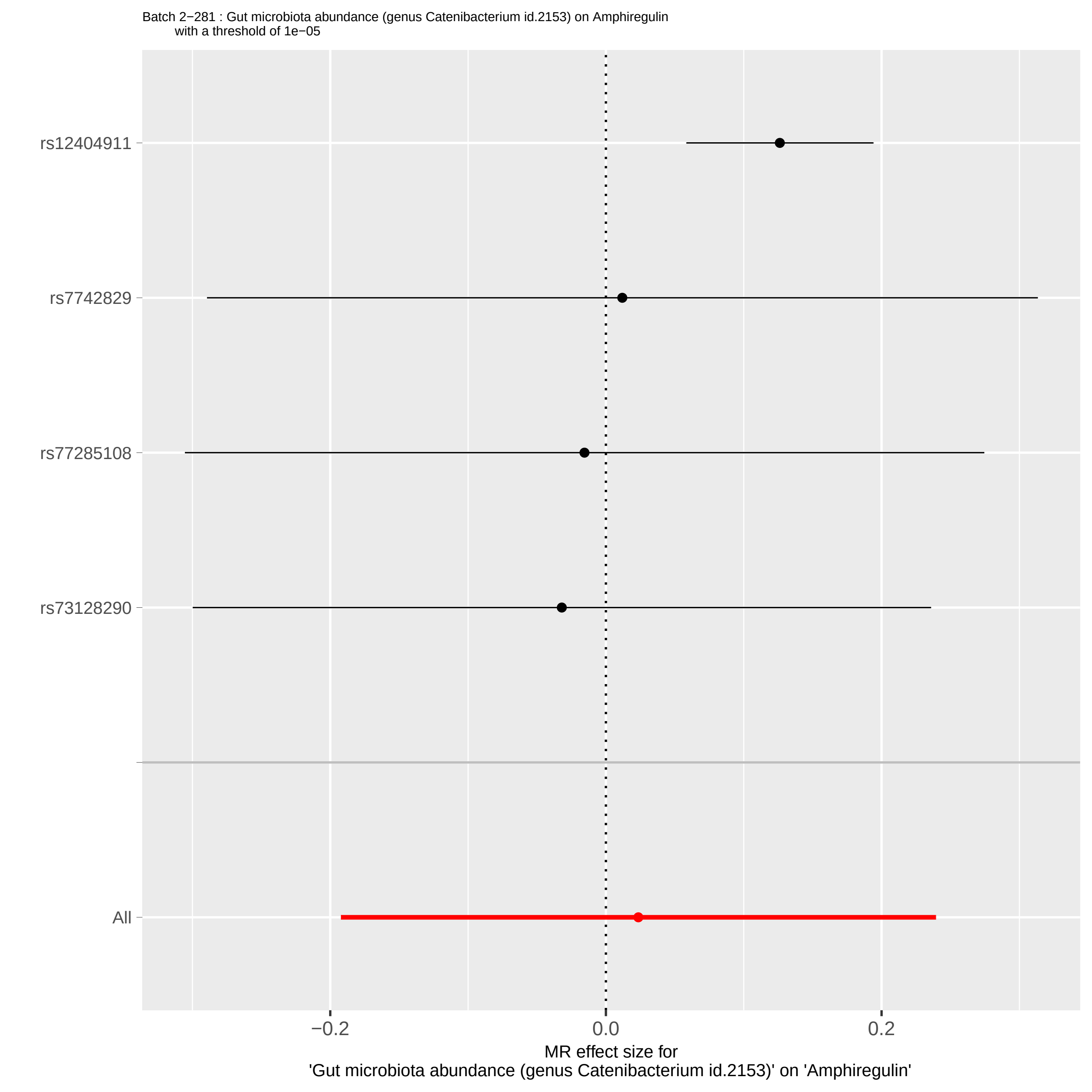

Batch 2−281 : Gut microbiota abundance (genus Catenibacterium id.2153) on Amphiregulin
with a threshold of 1e−05
rs12404911
rs7742829
rs77285108
rs73128290
All
−0.2
0.0
0.2
MR effect size for
'Gut microbiota abundance (genus Catenibacterium id.2153)' on 'Amphiregulin'

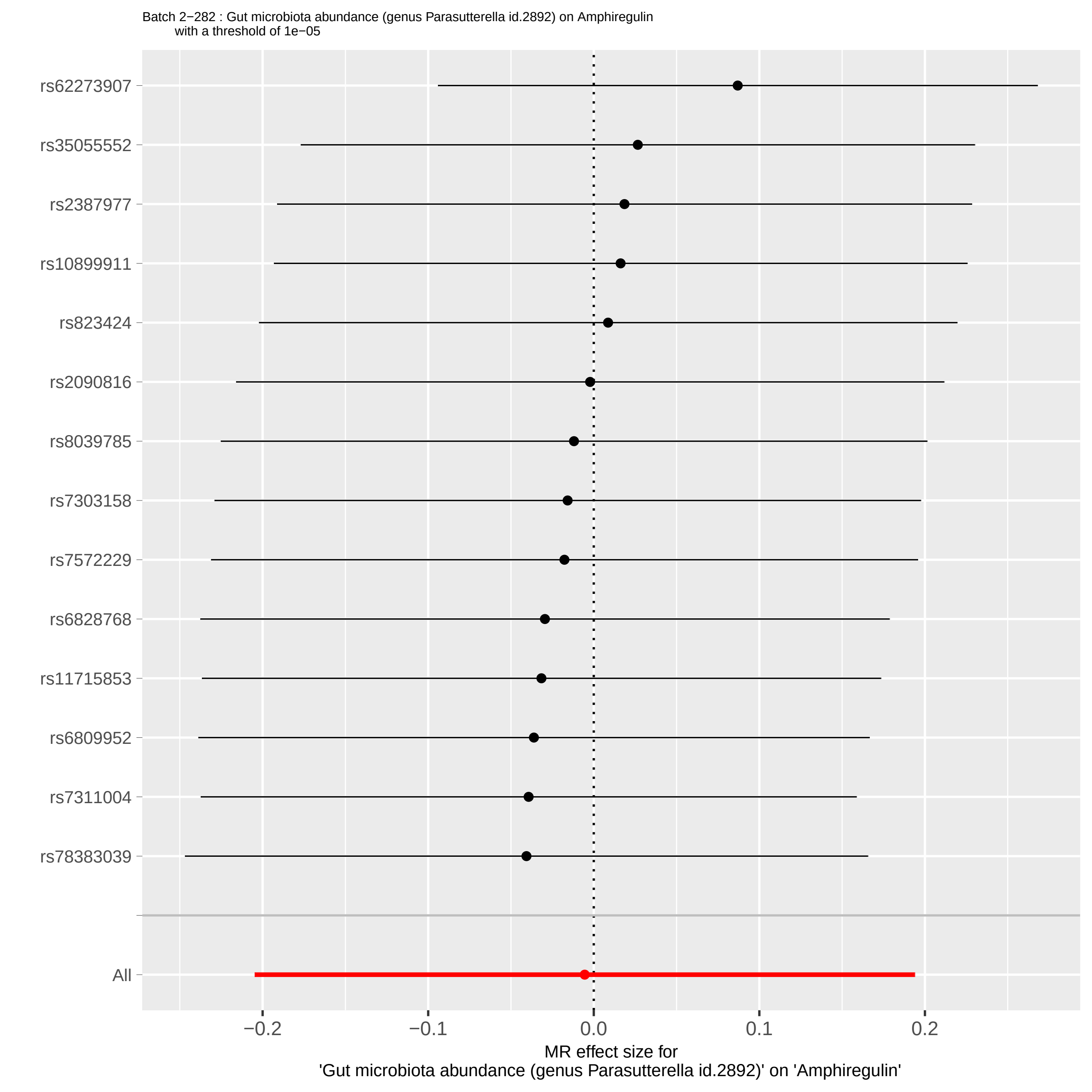

Batch 2−282 : Gut microbiota abundance (genus Parasutterella id.2892) on Amphiregulin
with a threshold of 1e−05
rs62273907
rs35055552
rs2387977
rs10899911
rs823424
rs2090816
rs8039785
rs7303158
rs7572229
rs6828768
rs11715853
rs6809952
rs7311004
rs78383039
All
−0.2
−0.1
0.0
0.1
0.2
MR effect size for
'Gut microbiota abundance (genus Parasutterella id.2892)' on 'Amphiregulin'

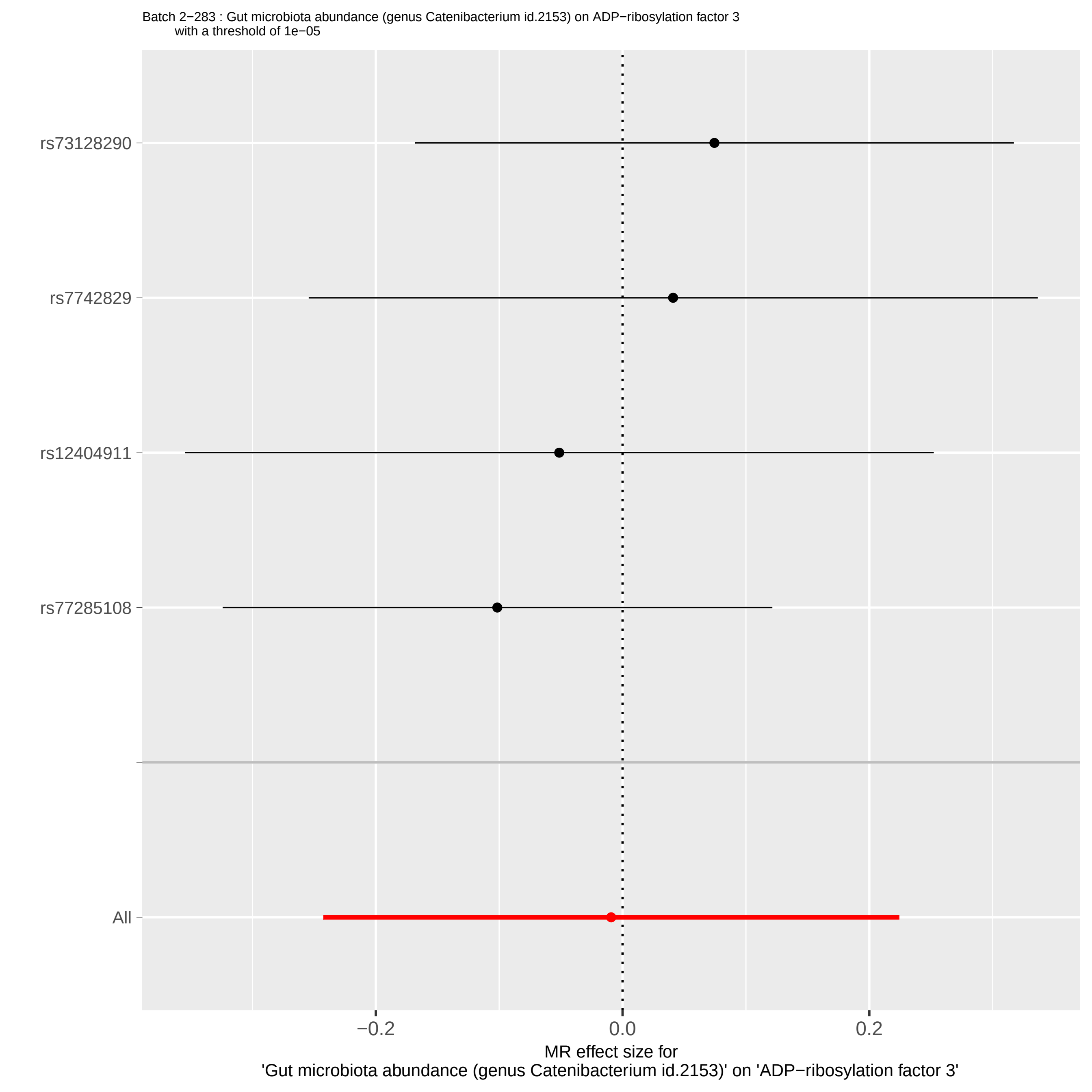

Batch 2−283 : Gut microbiota abundance (genus Catenibacterium id.2153) on ADP−ribosylation factor 3
with a threshold of 1e−05
rs73128290
rs7742829
rs12404911
rs77285108
All
−0.2
0.0
0.2
MR effect size for
'Gut microbiota abundance (genus Catenibacterium id.2153)' on 'ADP−ribosylation factor 3'

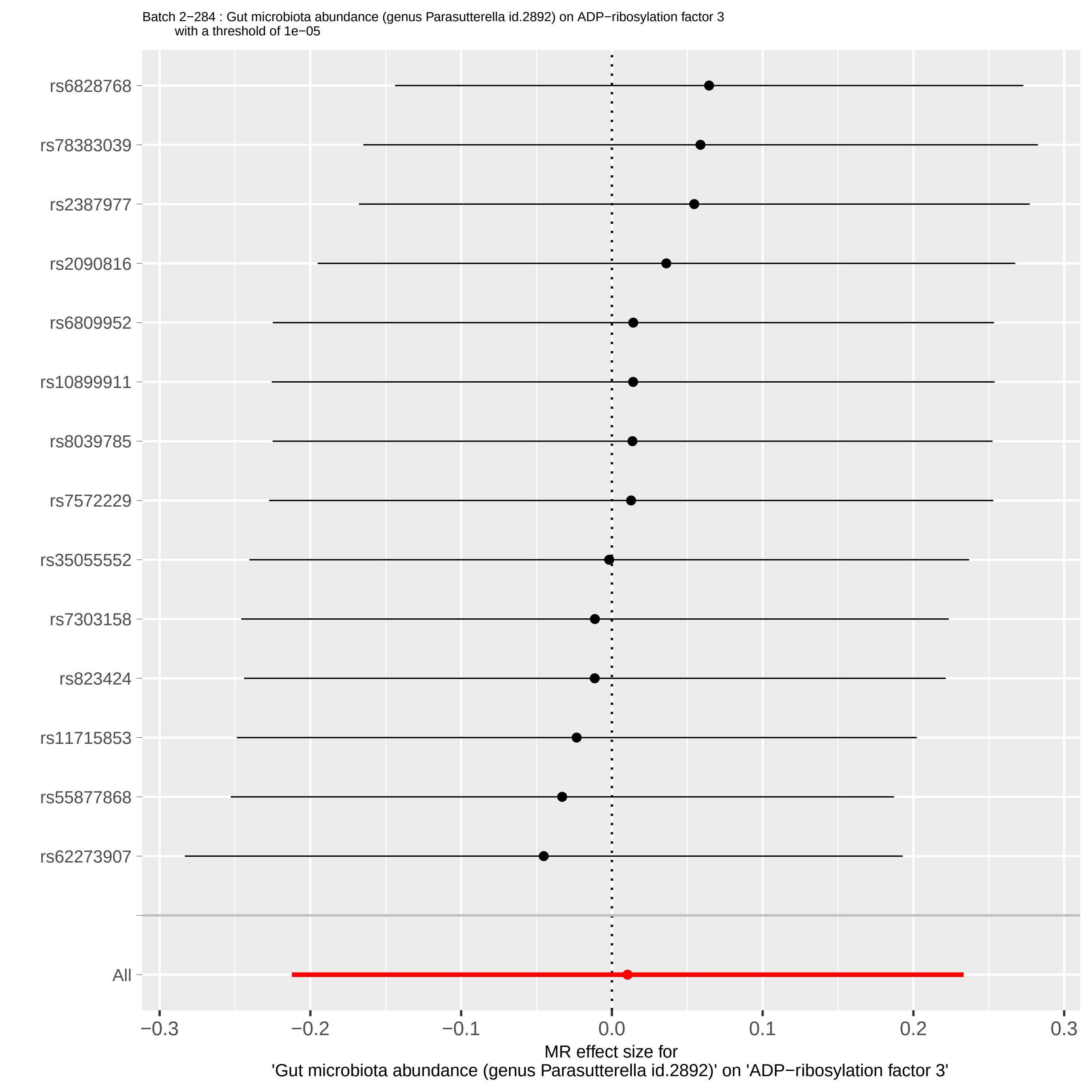
Batch 2−284 : Gut microbiota abundance (genus Parasutterella id.2892) on ADP−ribosylation factor 3
with a threshold of 1e−05
rs6828768
rs78383039
rs2387977
rs2090816
rs6809952
rs10899911
rs8039785
rs7572229
rs35055552
rs7303158
rs823424
rs11715853
rs55877868
rs62273907
All
−0.3
−0.2
−0.1
0.0
0.1
0.2
0.3
MR effect size for
'Gut microbiota abundance (genus Parasutterella id.2892)' on 'ADP−ribosylation factor 3'

Batch 2−285 : Gut microbiota abundance (genus Catenibacterium id.2153) on ADP−ribosylation factor 6
with a threshold of 1e−05

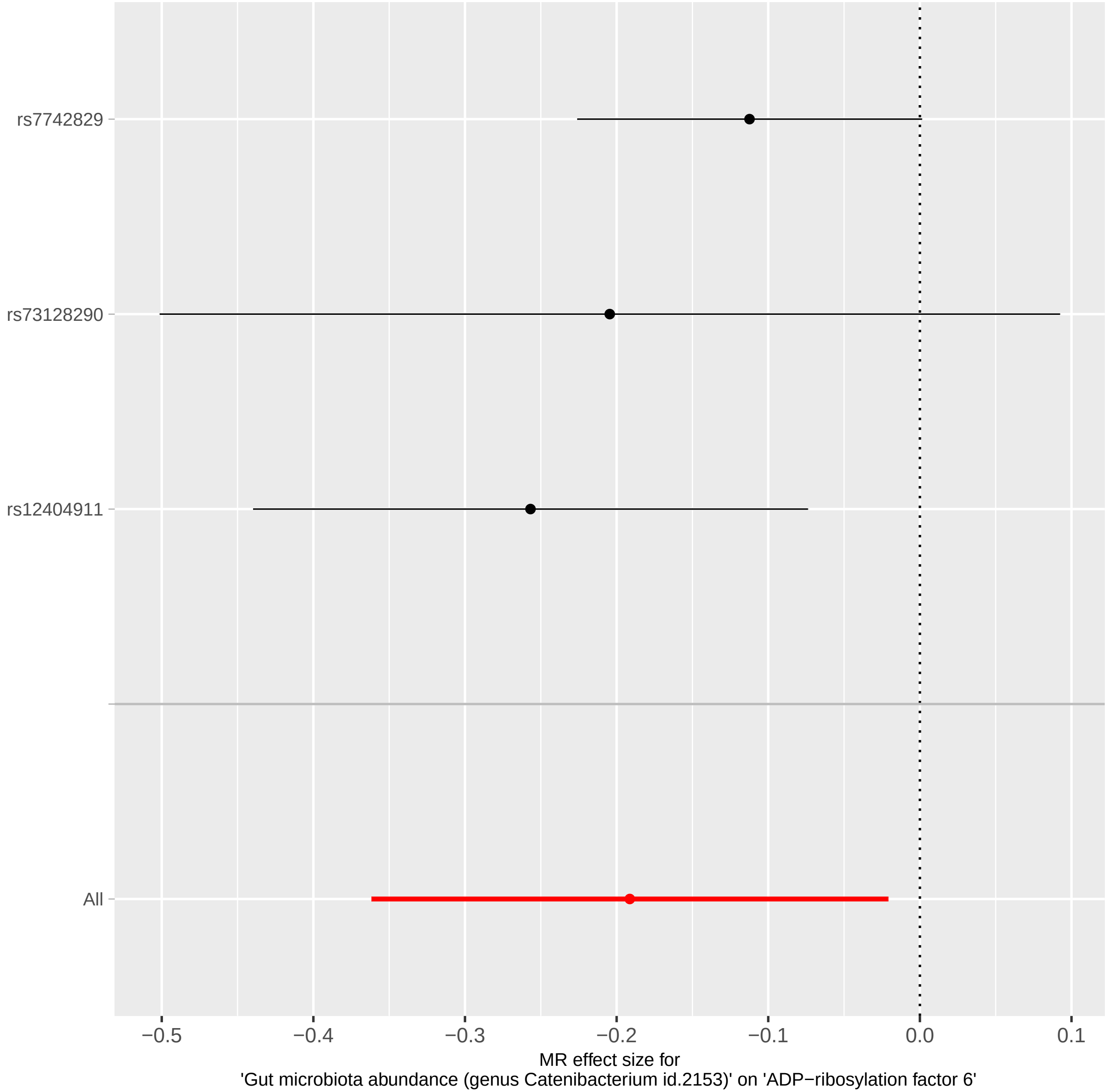

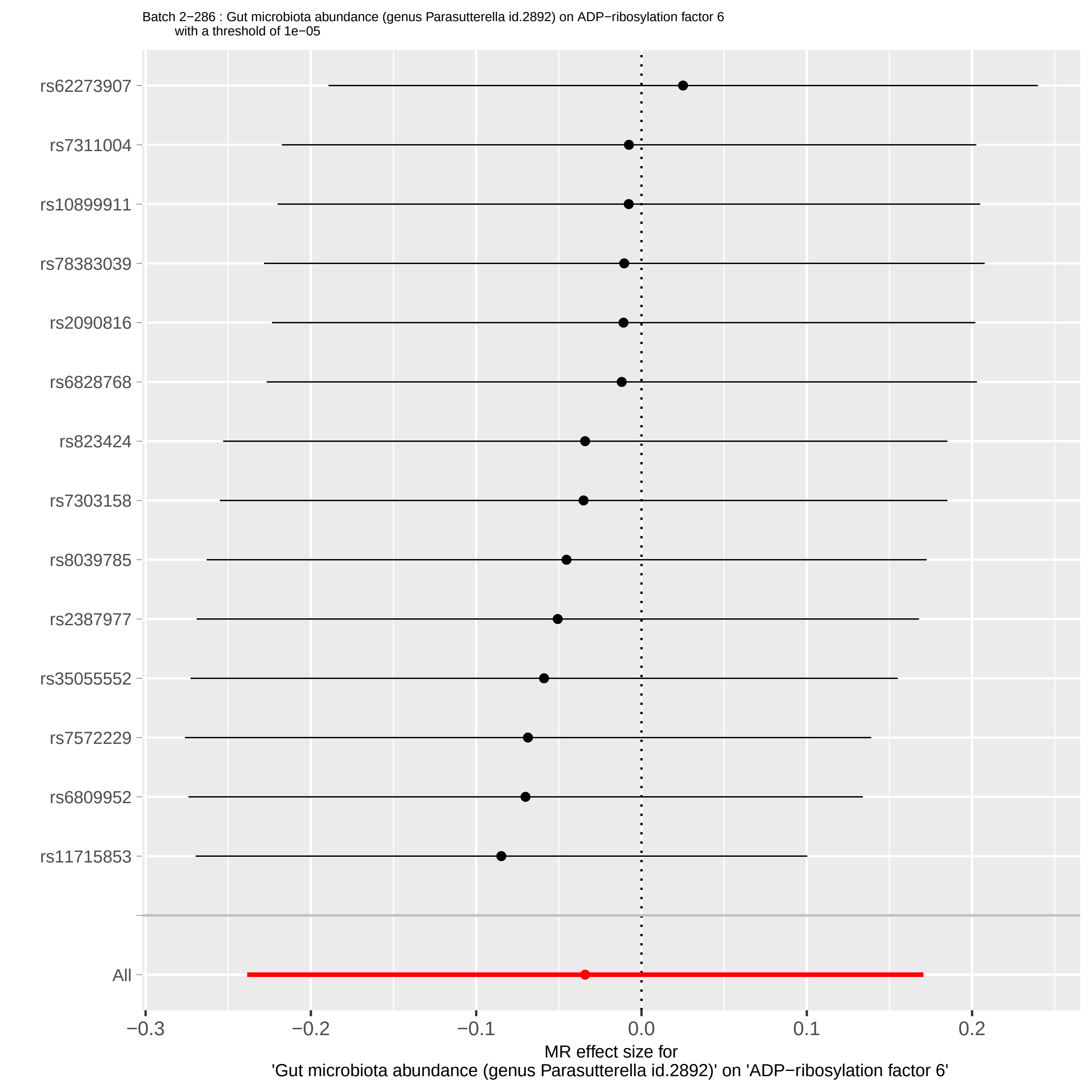
Batch 2−286 : Gut microbiota abundance (genus Parasutterella id.2892) on ADP−ribosylation factor 6
with a threshold of 1e−05
rs62273907
rs7311004
rs10899911
rs78383039
rs2090816
rs6828768
rs823424
rs7303158
rs8039785
rs2387977
rs35055552
rs7572229
rs6809952
rs11715853
All
−0.3
−0.2
−0.1
0.0
0.1
0.2
MR effect size for
'Gut microbiota abundance (genus Parasutterella id.2892)' on 'ADP−ribosylation factor 6'

Batch 2−287 : Gut microbiota abundance (genus Catenibacterium id.2153) on ADP−ribosylation factor GTPase−activating protein 2 with a threshold of 1e−05

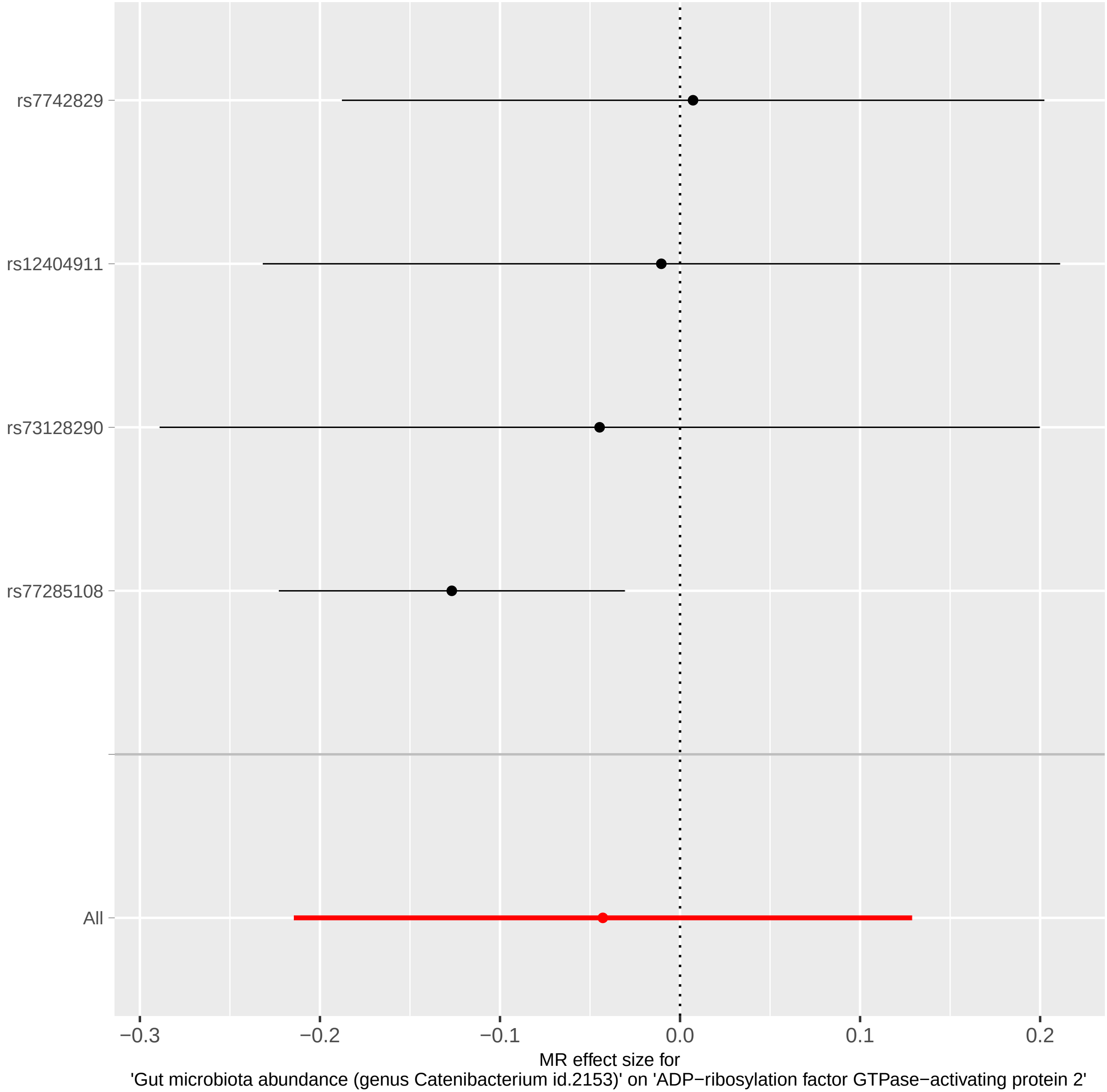

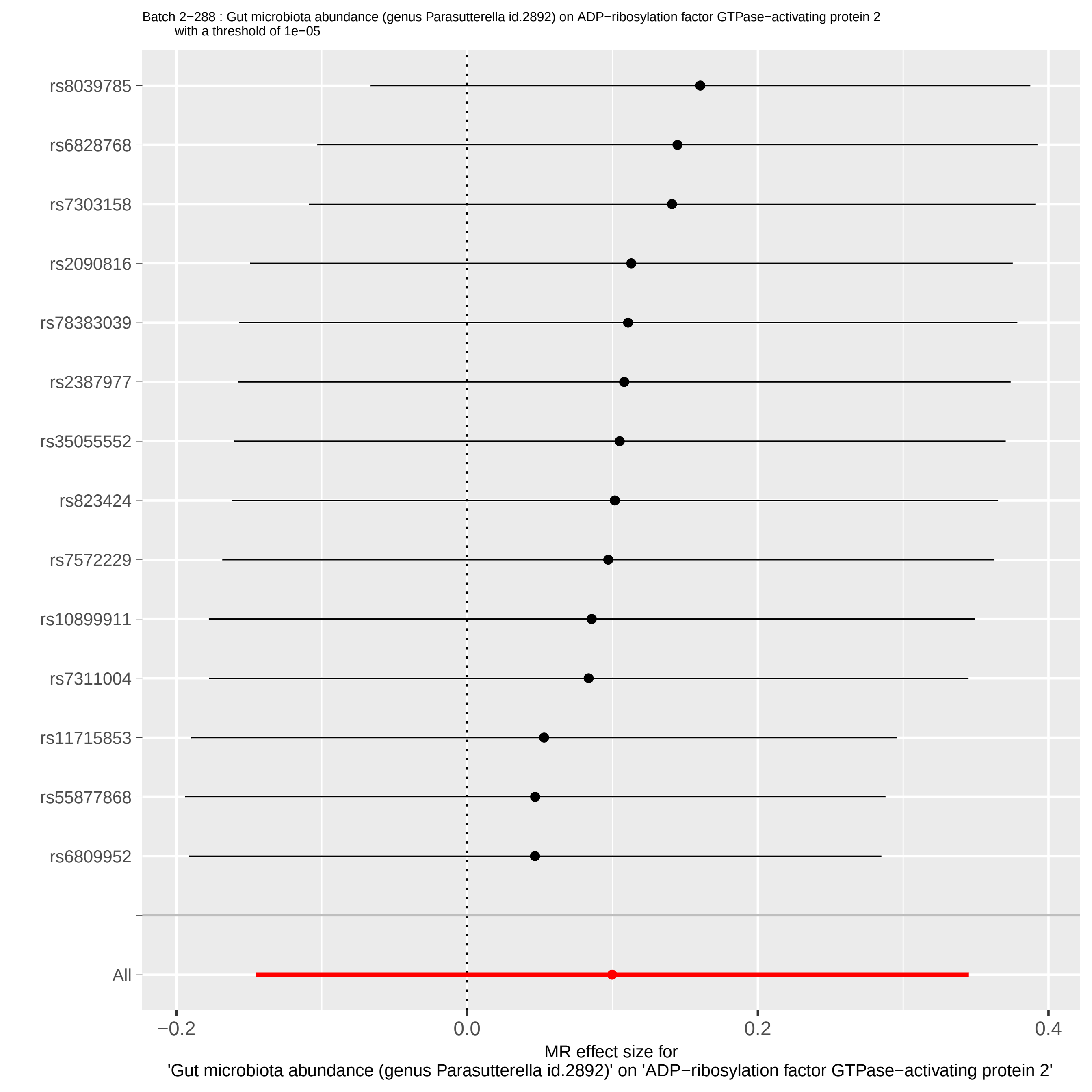

Batch 2−288 : Gut microbiota abundance (genus Parasutterella id.2892) on ADP−ribosylation factor GTPase−activating protein 2
with a threshold of 1e−05
rs8039785
rs6828768
rs7303158
rs2090816
rs78383039
rs2387977
rs35055552
rs823424
rs7572229
rs10899911
rs7311004
rs11715853
rs55877868
rs6809952
All
−0.2
0.0
0.2
0.4
MR effect size for
'Gut microbiota abundance (genus Parasutterella id.2892)' on 'ADP−ribosylation factor GTPase−activating protein 2'

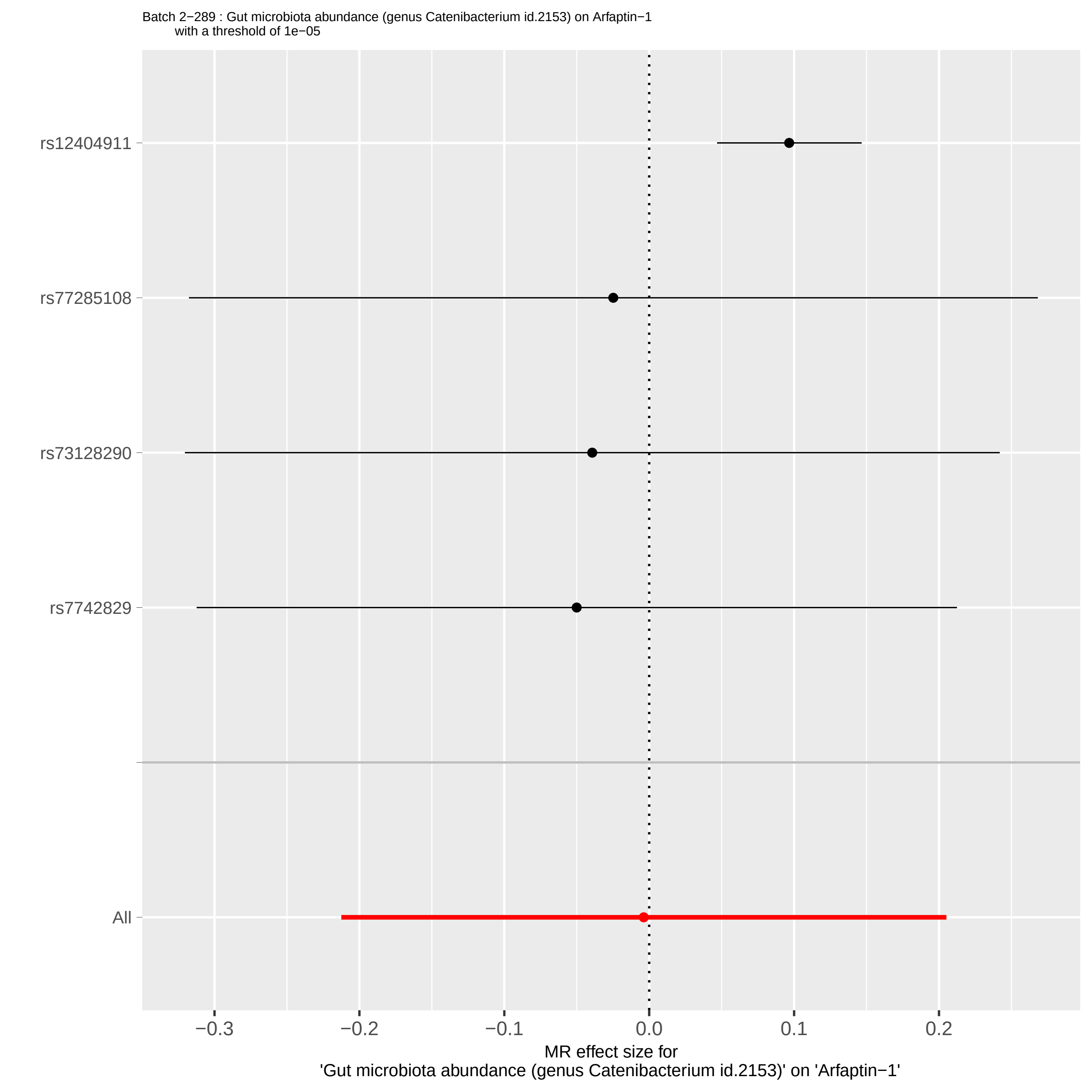

Batch 2−289 : Gut microbiota abundance (genus Catenibacterium id.2153) on Arfaptin−1
with a threshold of 1e−05
rs12404911
rs77285108
rs73128290
rs7742829
All
−0.3
−0.2
−0.1
0.0
0.1
0.2
MR effect size for
'Gut microbiota abundance (genus Catenibacterium id.2153)' on 'Arfaptin−1'

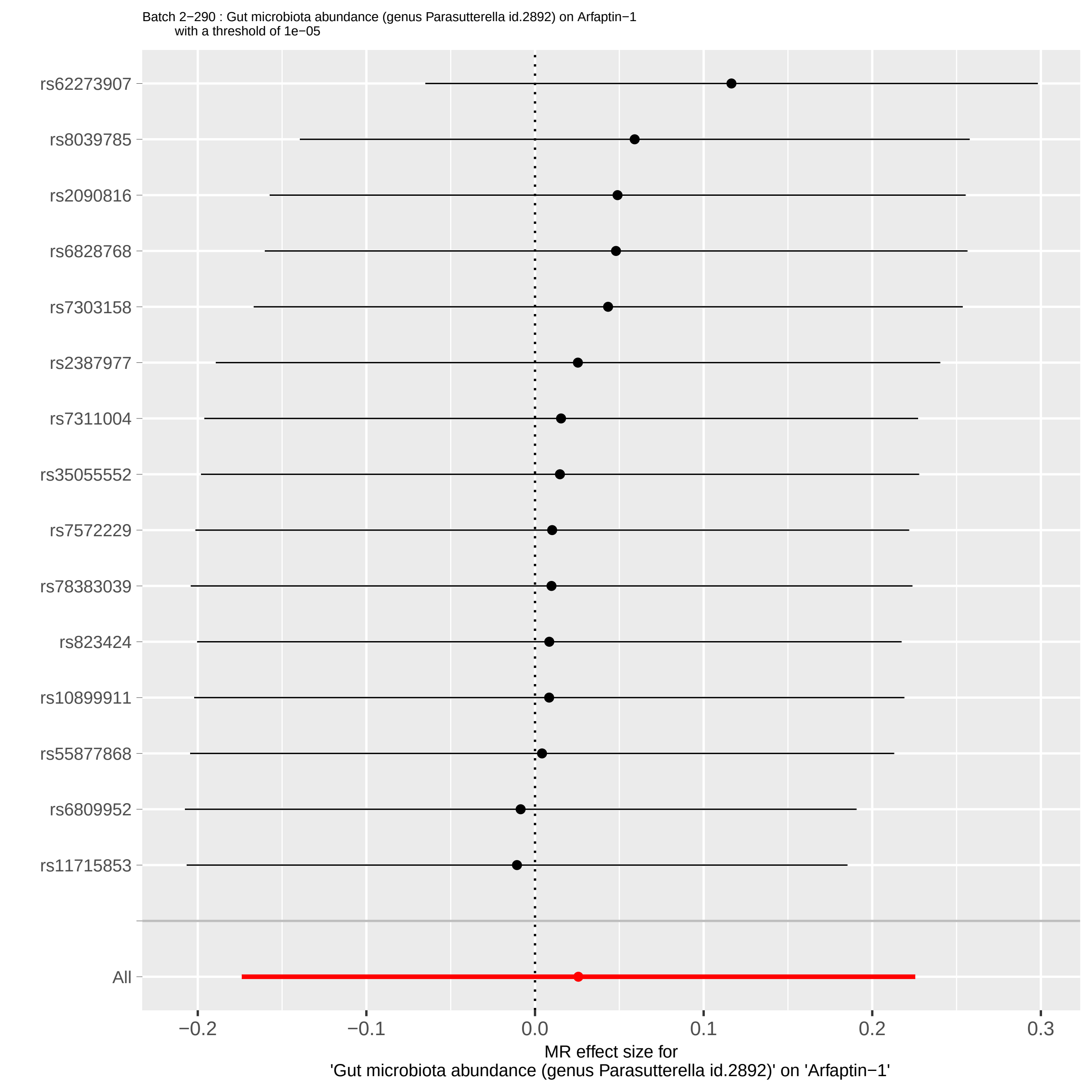
Batch 2−290 : Gut microbiota abundance (genus Parasutterella id.2892) on Arfaptin−1
with a threshold of 1e−05
rs62273907
rs8039785
rs2090816
rs6828768
rs7303158
rs2387977
rs7311004
rs35055552
rs7572229
rs78383039
rs823424
rs10899911
rs55877868
rs6809952
rs11715853
All
−0.2
−0.1
0.0
0.1
0.2
0.3
MR effect size for
'Gut microbiota abundance (genus Parasutterella id.2892)' on 'Arfaptin−1'

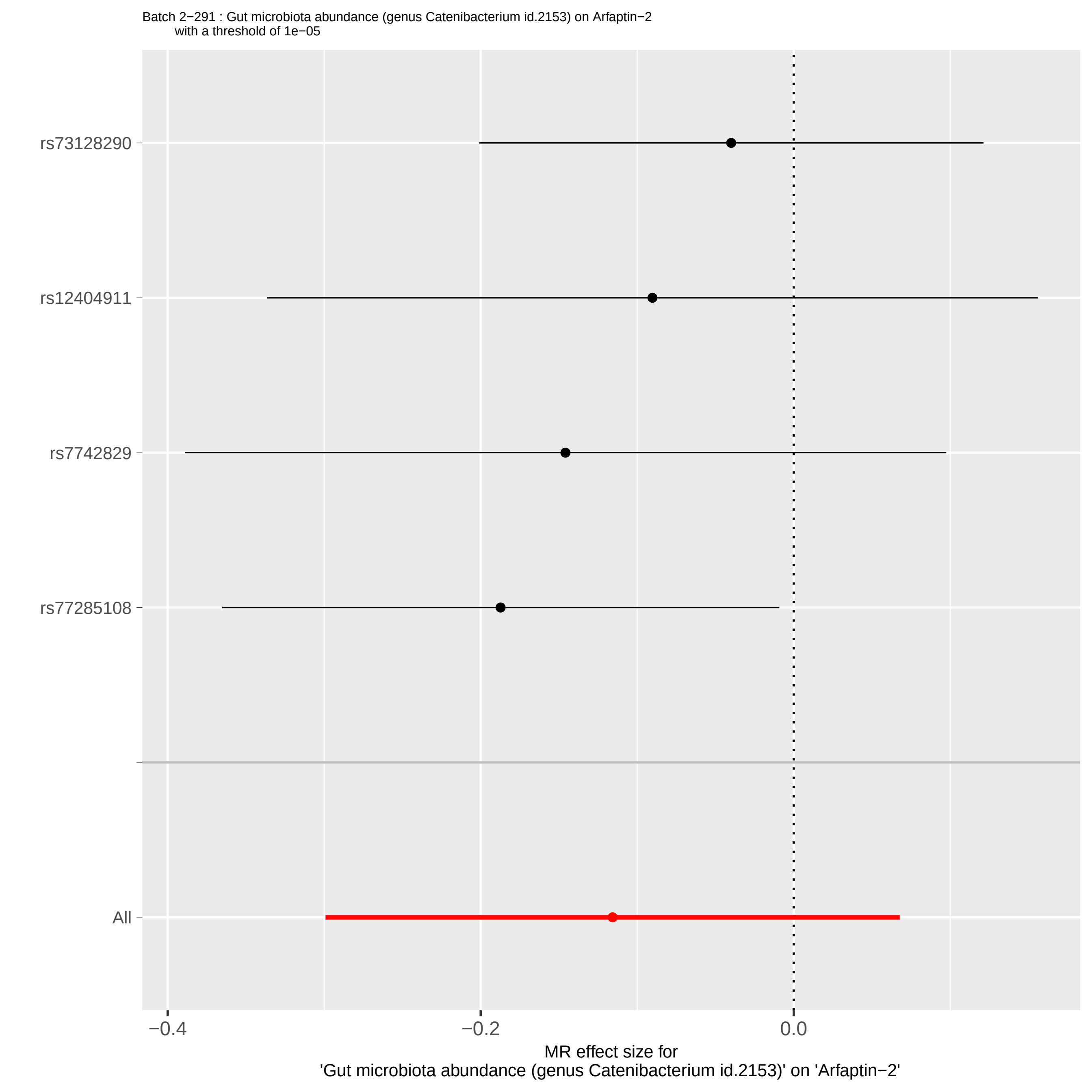
Batch 2−291 : Gut microbiota abundance (genus Catenibacterium id.2153) on Arfaptin−2
with a threshold of 1e−05
rs73128290
rs12404911
rs7742829
rs77285108
All
−0.4
−0.2
0.0
MR effect size for
'Gut microbiota abundance (genus Catenibacterium id.2153)' on 'Arfaptin−2'

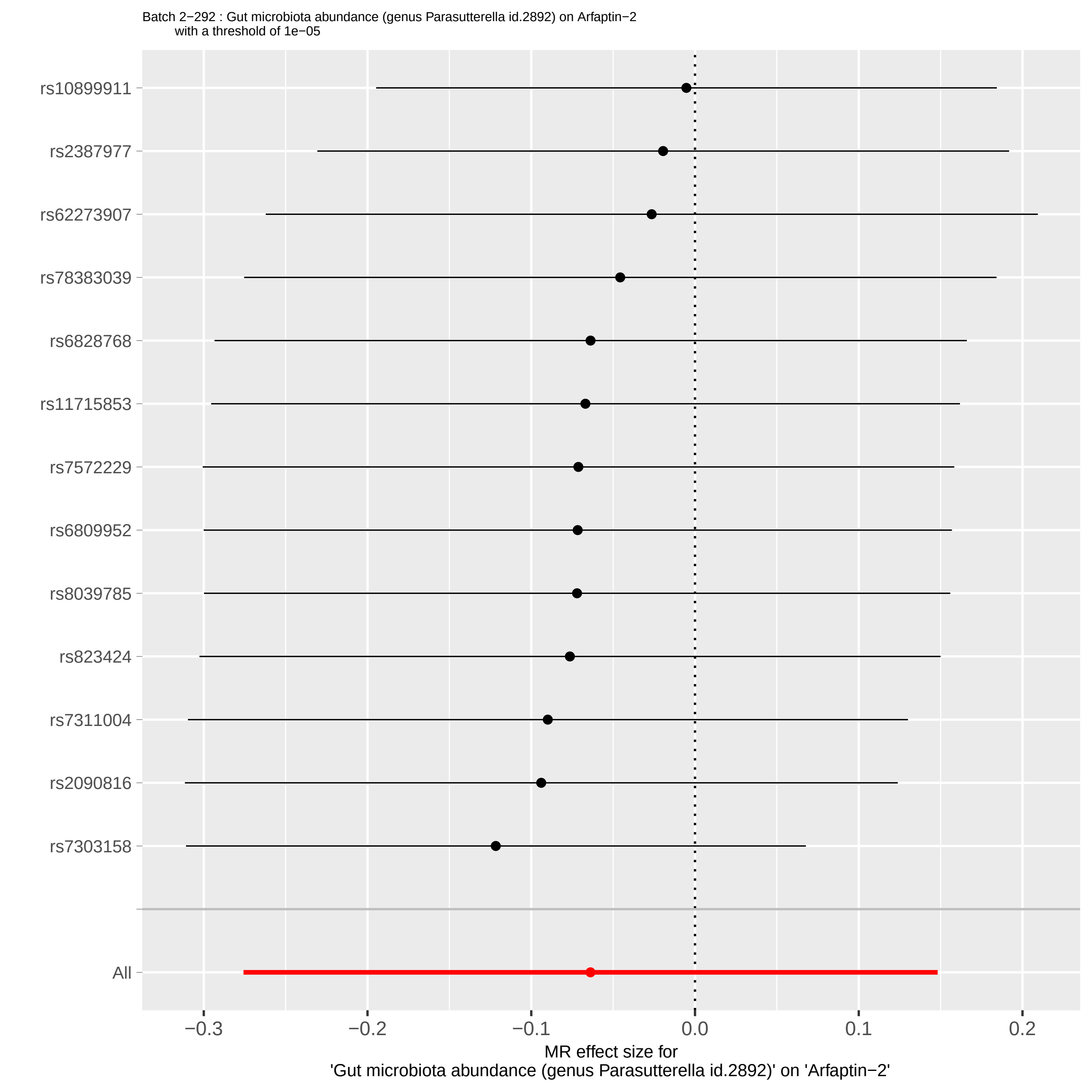
Batch 2−292 : Gut microbiota abundance (genus Parasutterella id.2892) on Arfaptin−2
with a threshold of 1e−05
rs10899911
rs2387977
rs62273907
rs78383039
rs6828768
rs11715853
rs7572229
rs6809952
rs8039785
rs823424
rs7311004
rs2090816
rs7303158
All
−0.3
−0.2
−0.1
0.0
0.1
0.2
MR effect size for
'Gut microbiota abundance (genus Parasutterella id.2892)' on 'Arfaptin−2'

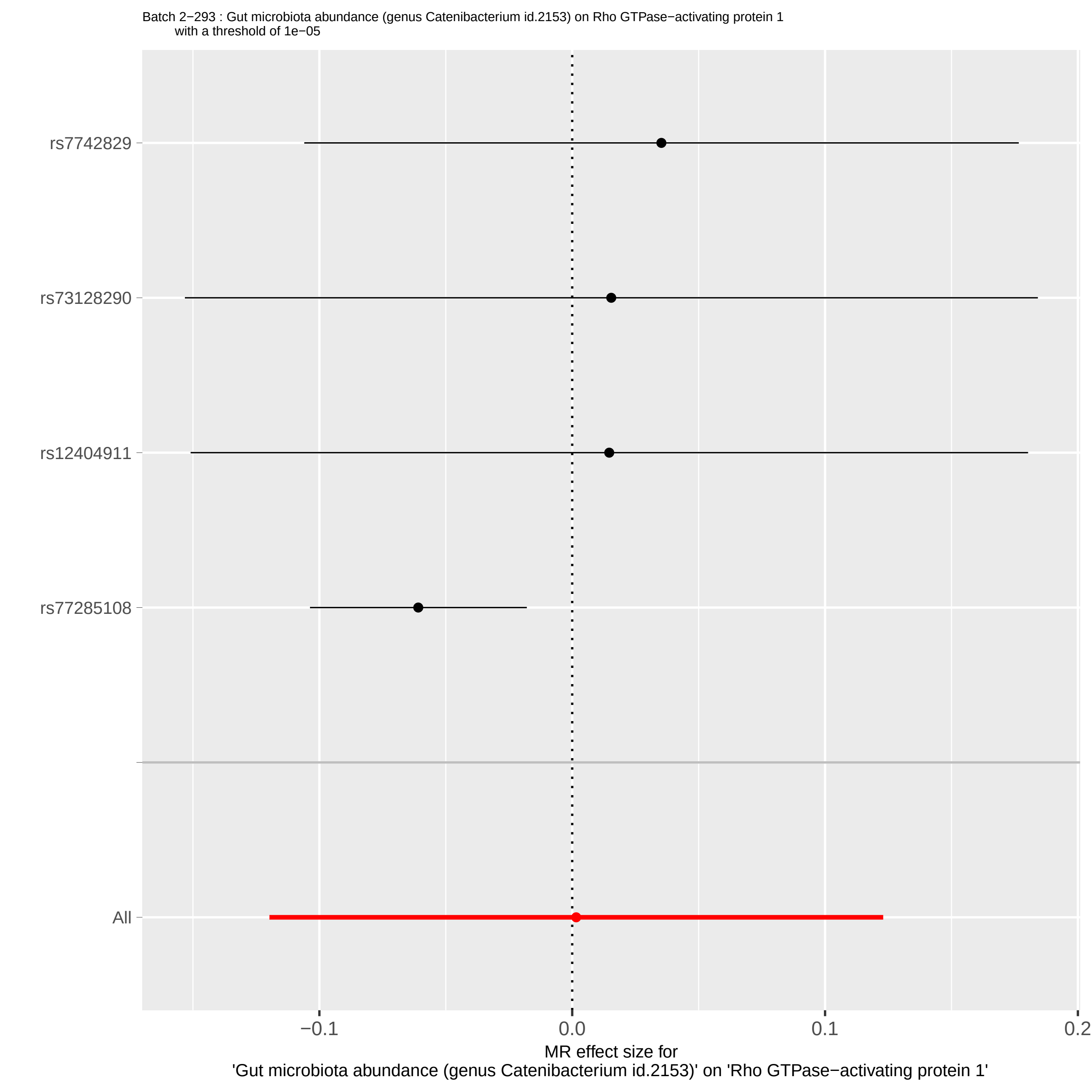

Batch 2−293 : Gut microbiota abundance (genus Catenibacterium id.2153) on Rho GTPase−activating protein 1
with a threshold of 1e−05
rs7742829
rs73128290
rs12404911
rs77285108
All
−0.1
0.0
0.1
0.2
MR effect size for
'Gut microbiota abundance (genus Catenibacterium id.2153)' on 'Rho GTPase−activating protein 1'

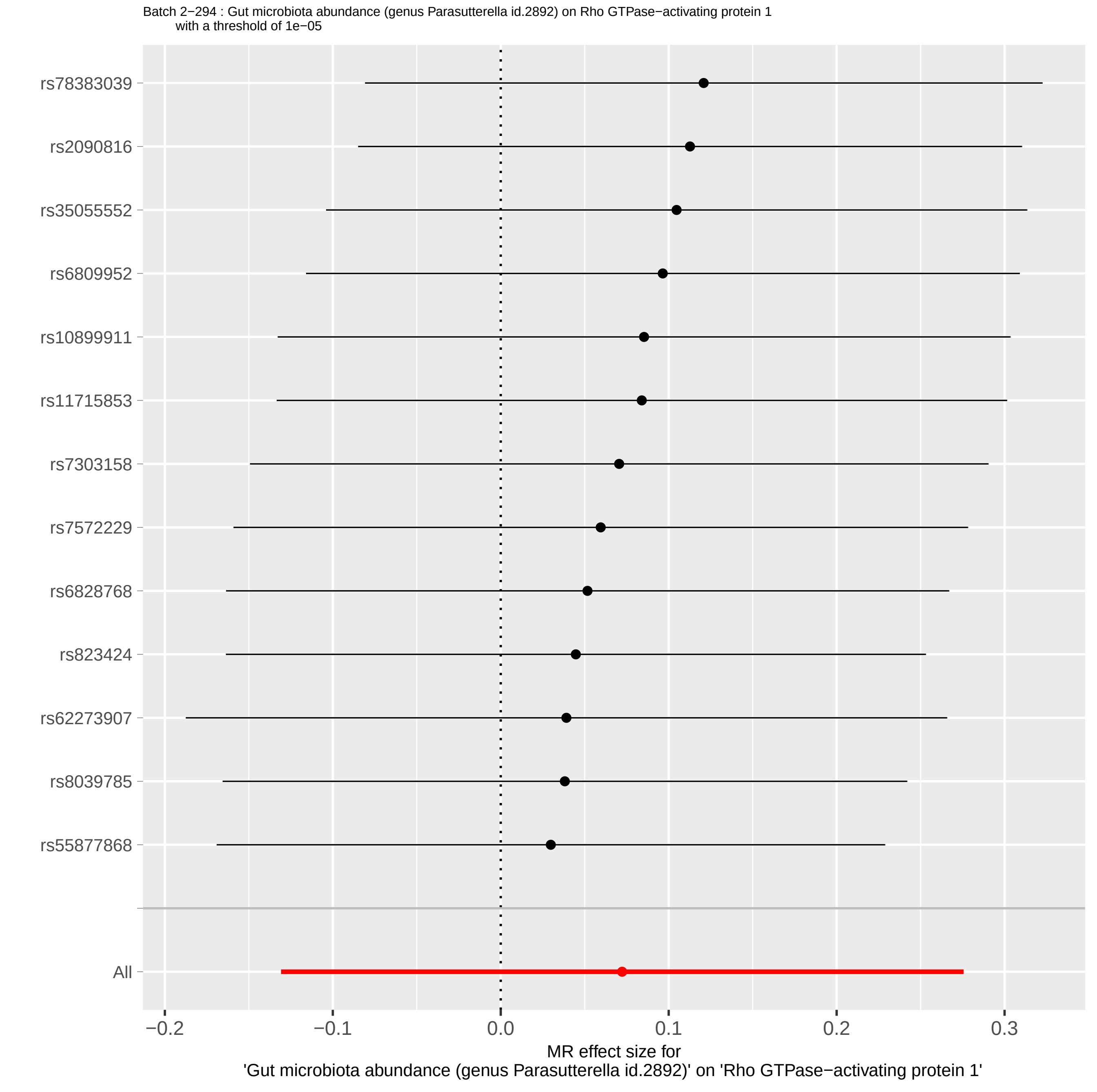

Batch 2−294 : Gut microbiota abundance (genus Parasutterella id.2892) on Rho GTPase−activating protein 1
with a threshold of 1e−05
rs78383039
rs2090816
rs35055552
rs6809952
rs10899911
rs11715853
rs7303158
rs7572229
rs6828768
rs823424
rs62273907
rs8039785
rs55877868
All
−0.2
−0.1
0.0
0.1
0.2
0.3
MR effect size for
'Gut microbiota abundance (genus Parasutterella id.2892)' on 'Rho GTPase−activating protein 1'

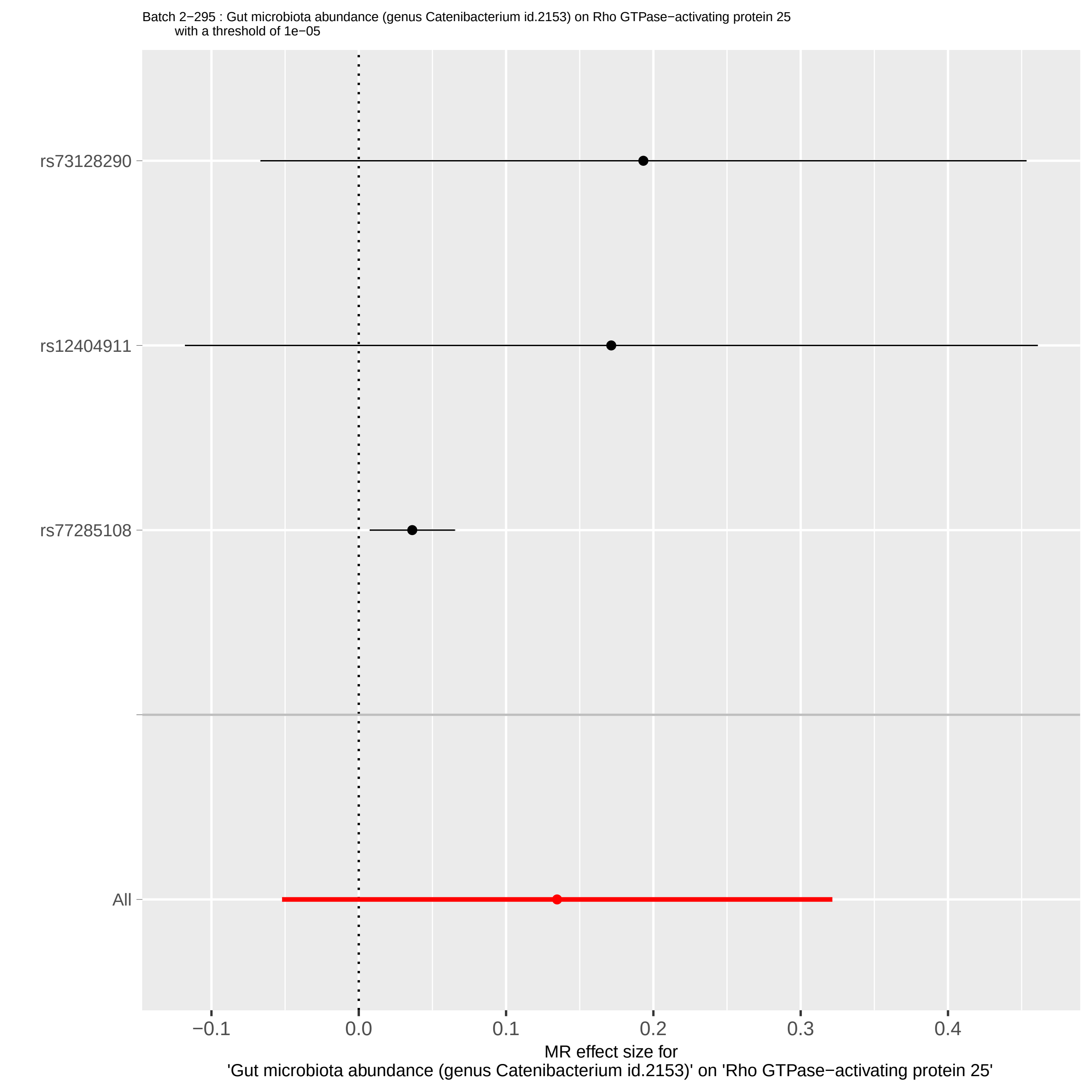
Batch 2−295 : Gut microbiota abundance (genus Catenibacterium id.2153) on Rho GTPase−activating protein 25
with a threshold of 1e−05
rs73128290
rs12404911
rs77285108
All
−0.1
0.0
0.1
0.2
0.3
0.4
MR effect size for
'Gut microbiota abundance (genus Catenibacterium id.2153)' on 'Rho GTPase−activating protein 25'

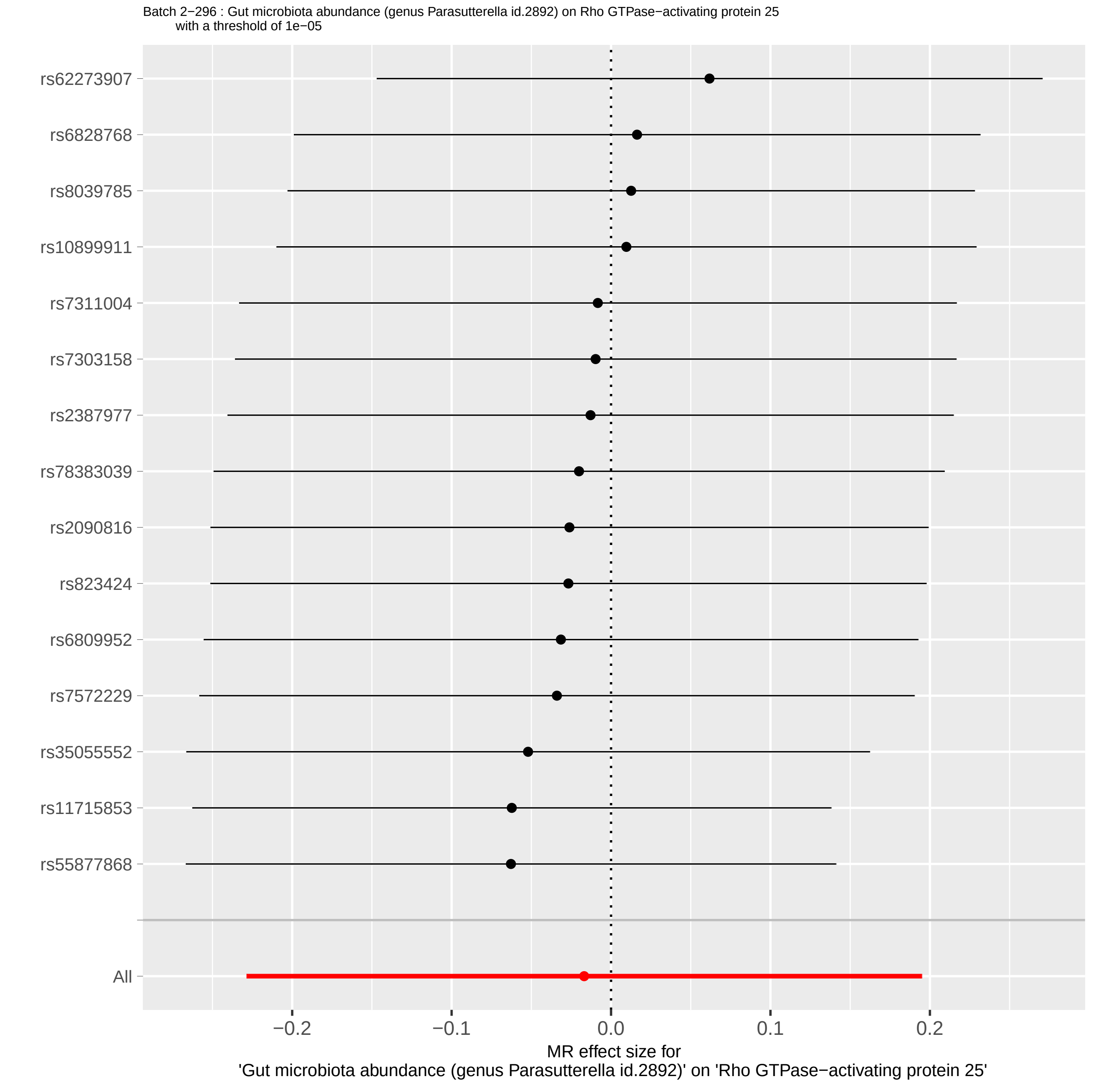

Batch 2−296 : Gut microbiota abundance (genus Parasutterella id.2892) on Rho GTPase−activating protein 25
with a threshold of 1e−05
rs62273907
rs6828768
rs8039785
rs10899911
rs7311004
rs7303158
rs2387977
rs78383039
rs2090816
rs823424
rs6809952
rs7572229
rs35055552
rs11715853
rs55877868
All
−0.2
−0.1
0.0
0.1
0.2
MR effect size for
'Gut microbiota abundance (genus Parasutterella id.2892)' on 'Rho GTPase−activating protein 25'

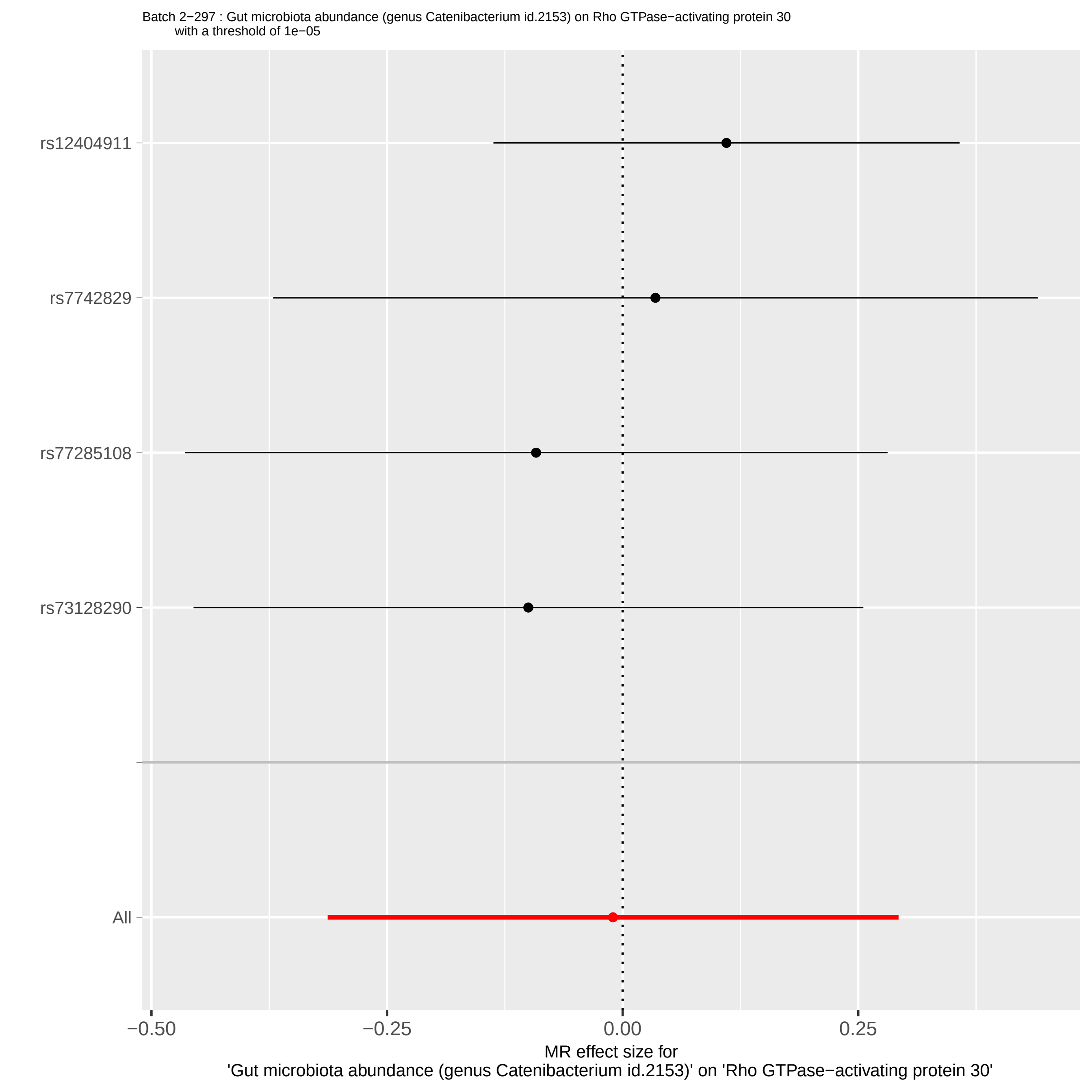

Batch 2−297 : Gut microbiota abundance (genus Catenibacterium id.2153) on Rho GTPase−activating protein 30
with a threshold of 1e−05
rs12404911
rs7742829
rs77285108
rs73128290
All
−0.50
−0.25
0.00
0.25
MR effect size for
'Gut microbiota abundance (genus Catenibacterium id.2153)' on 'Rho GTPase−activating protein 30'

Batch 2−298 : Gut microbiota abundance (genus Parasutterella id.2892) on Rho GTPase−activating protein 30
with a threshold of 1e−05

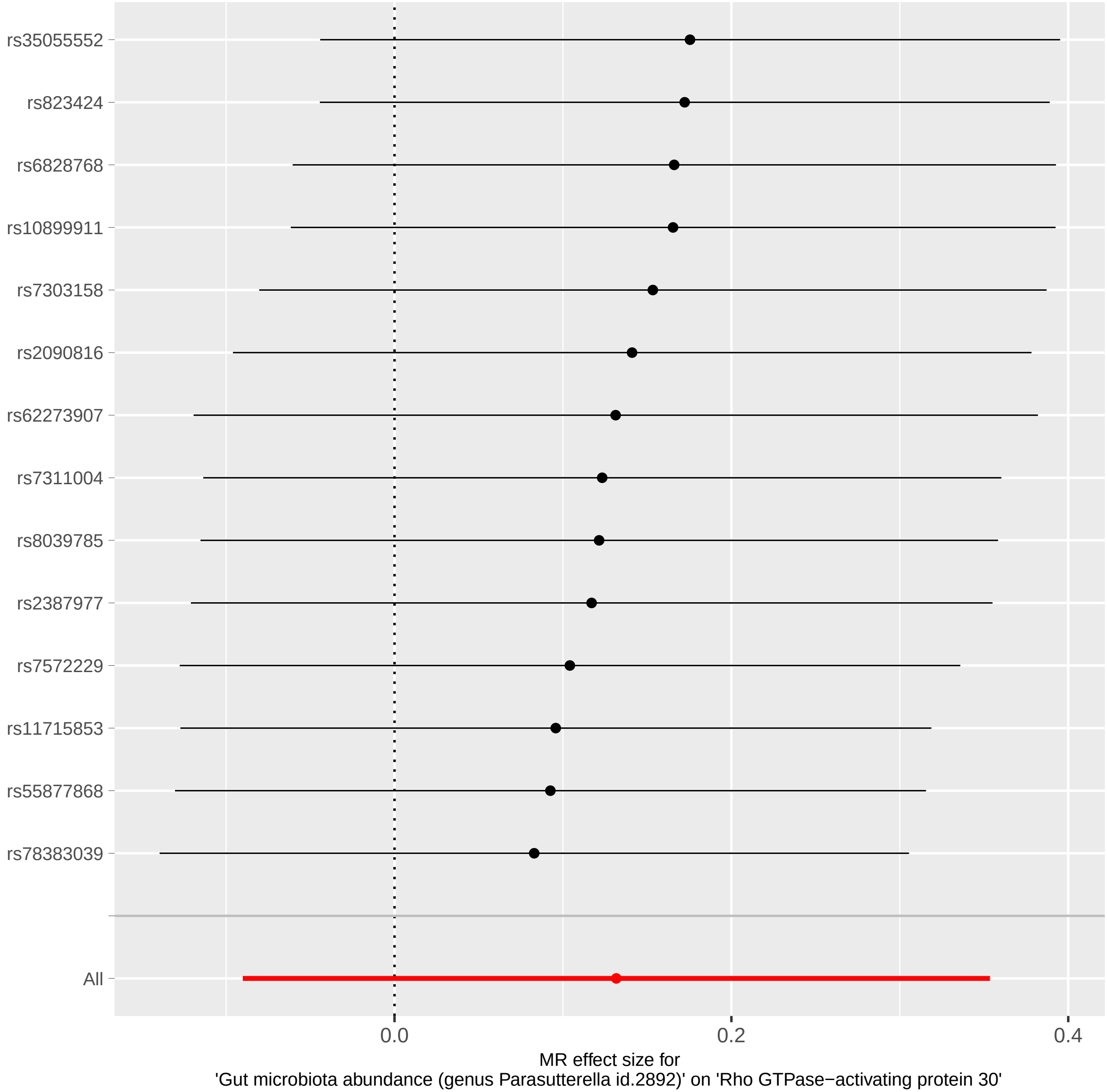

Batch 2−299 : Gut microbiota abundance (genus Catenibacterium id.2153) on Rho GTPase−activating protein 36
with a threshold of 1e−05

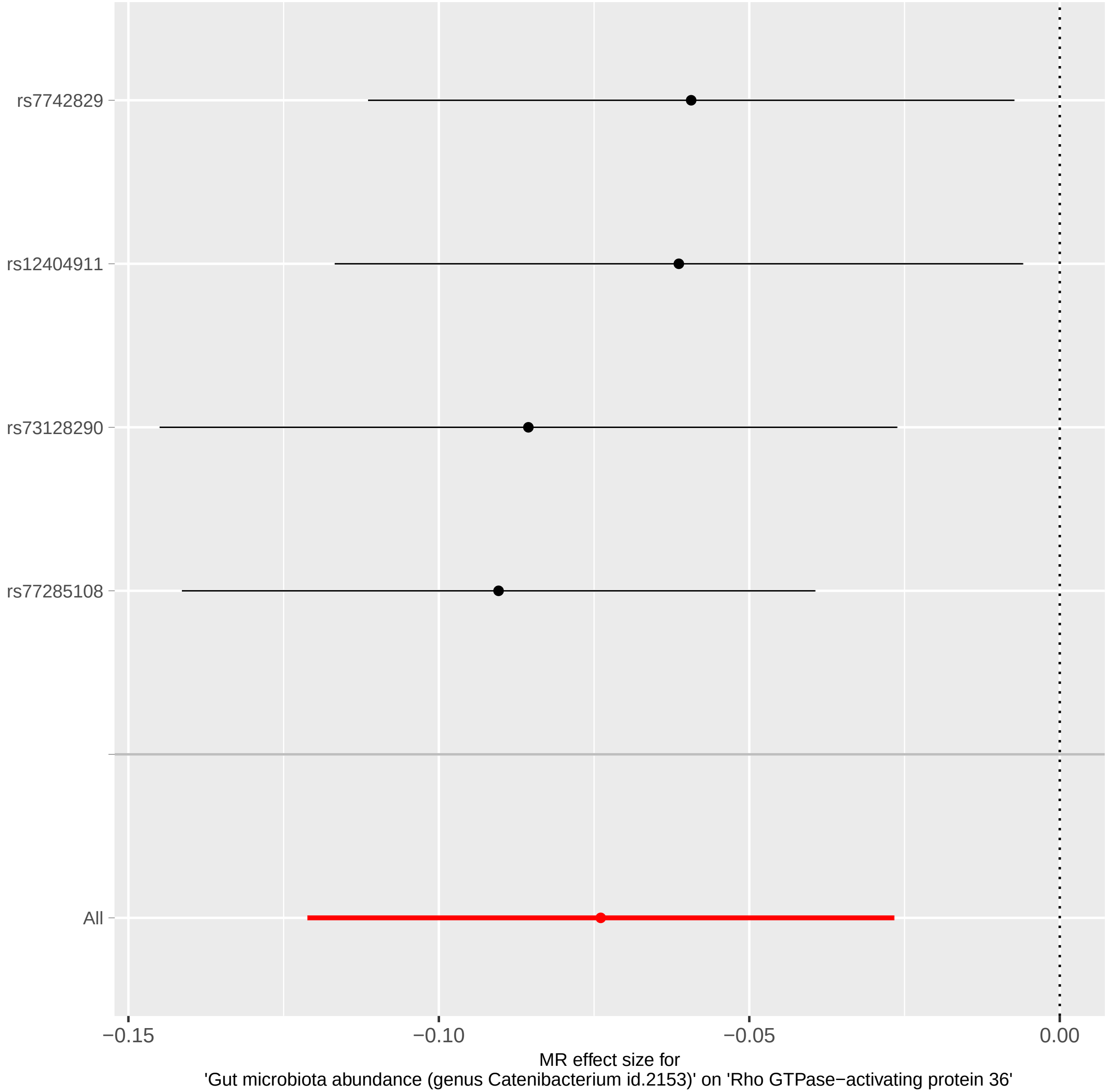

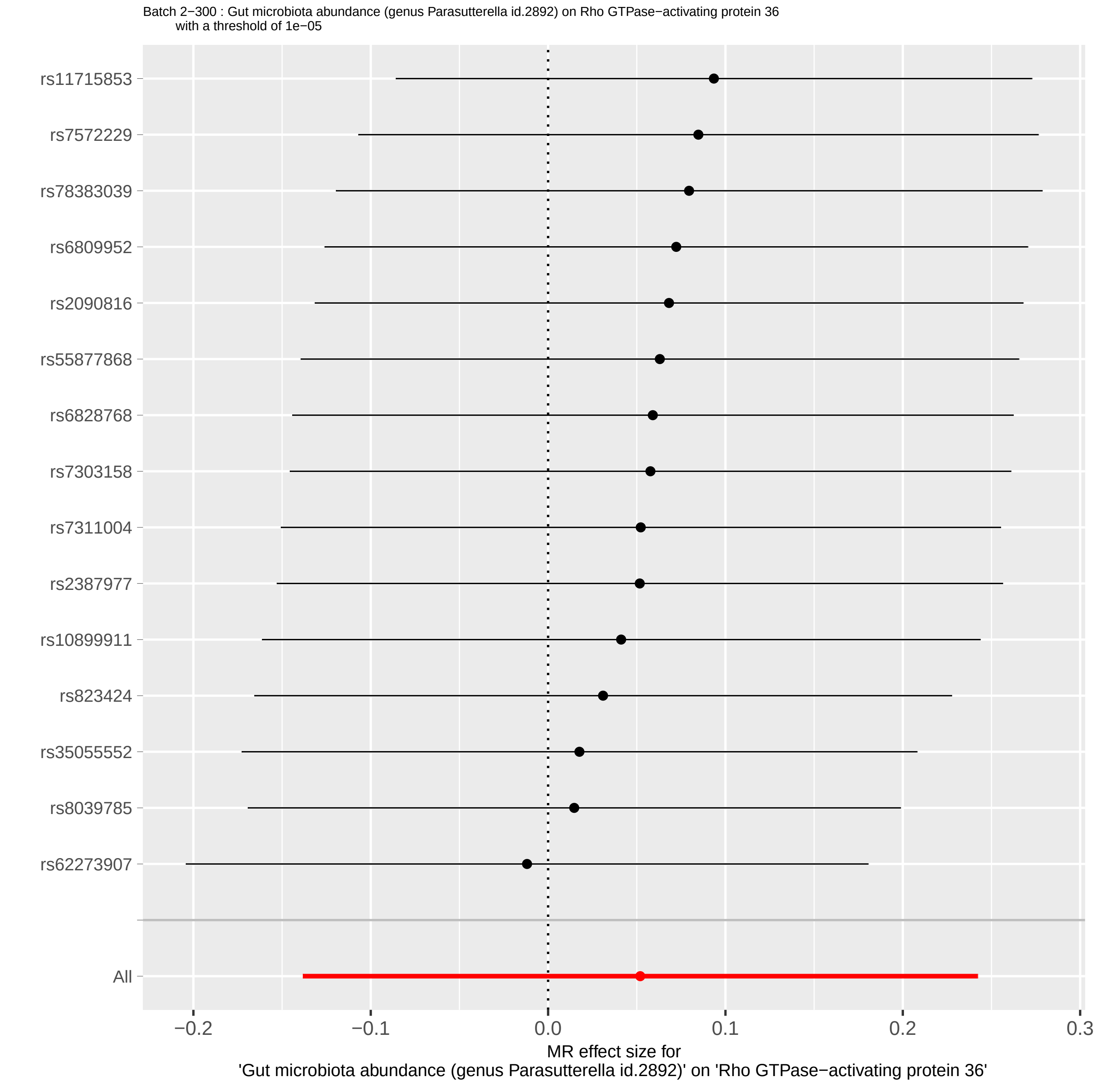

Batch 2−300 : Gut microbiota abundance (genus Parasutterella id.2892) on Rho GTPase−activating protein 36
with a threshold of 1e−05
rs11715853
rs7572229
rs78383039
rs6809952
rs2090816
rs55877868
rs6828768
rs7303158
rs7311004
rs2387977
rs10899911
rs823424
rs35055552
rs8039785
rs62273907
All
−0.2
−0.1
0.0
0.1
0.2
0.3
MR effect size for
'Gut microbiota abundance (genus Parasutterella id.2892)' on 'Rho GTPase−activating protein 36'

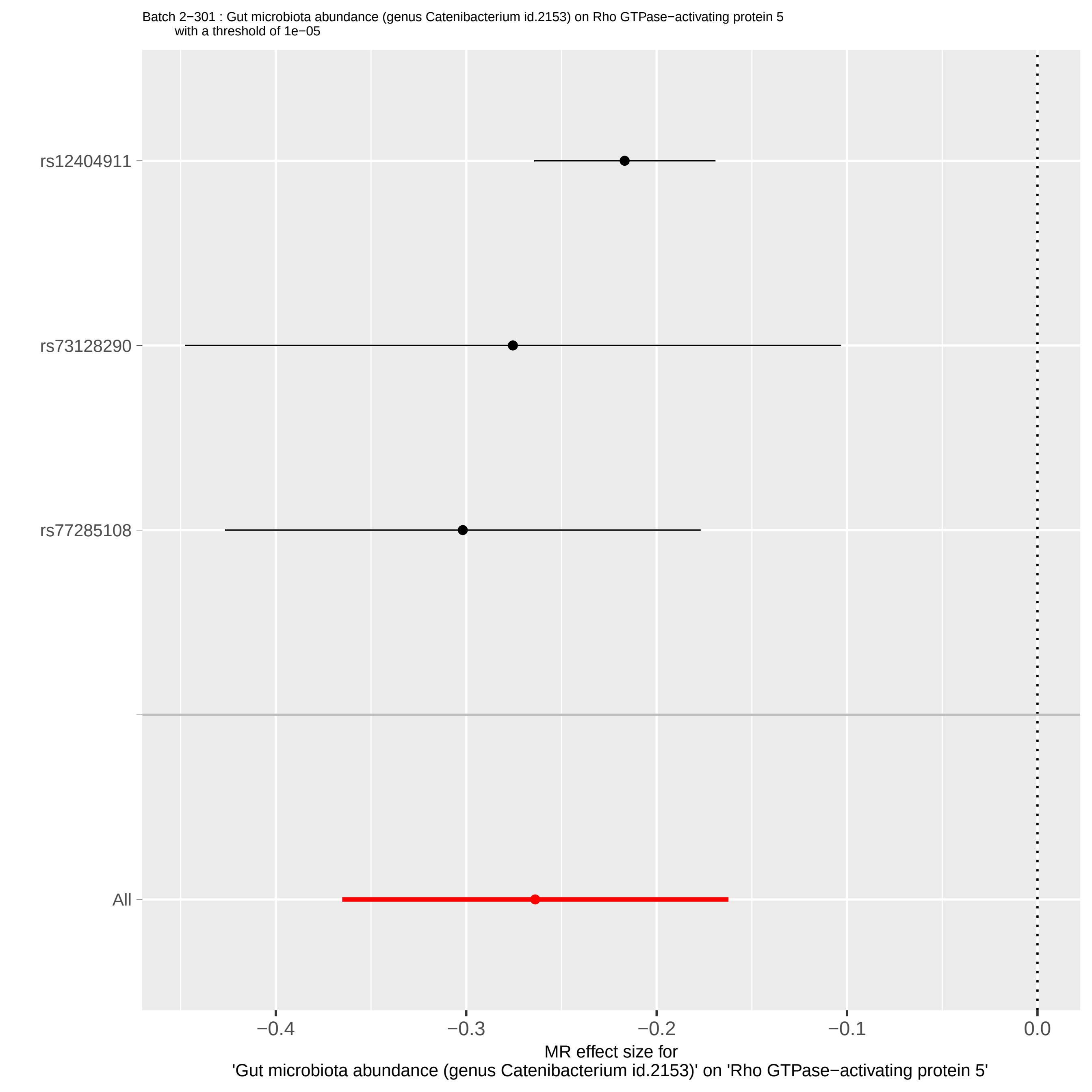

Batch 2−301 : Gut microbiota abundance (genus Catenibacterium id.2153) on Rho GTPase−activating protein 5
with a threshold of 1e−05
rs12404911
rs73128290
rs77285108
All
−0.4
−0.3
−0.2
−0.1
0.0
MR effect size for
'Gut microbiota abundance (genus Catenibacterium id.2153)' on 'Rho GTPase−activating protein 5'

Batch 2−302 : Gut microbiota abundance (genus Parasutterella id.2892) on Rho GTPase−activating protein 5
with a threshold of 1e−05

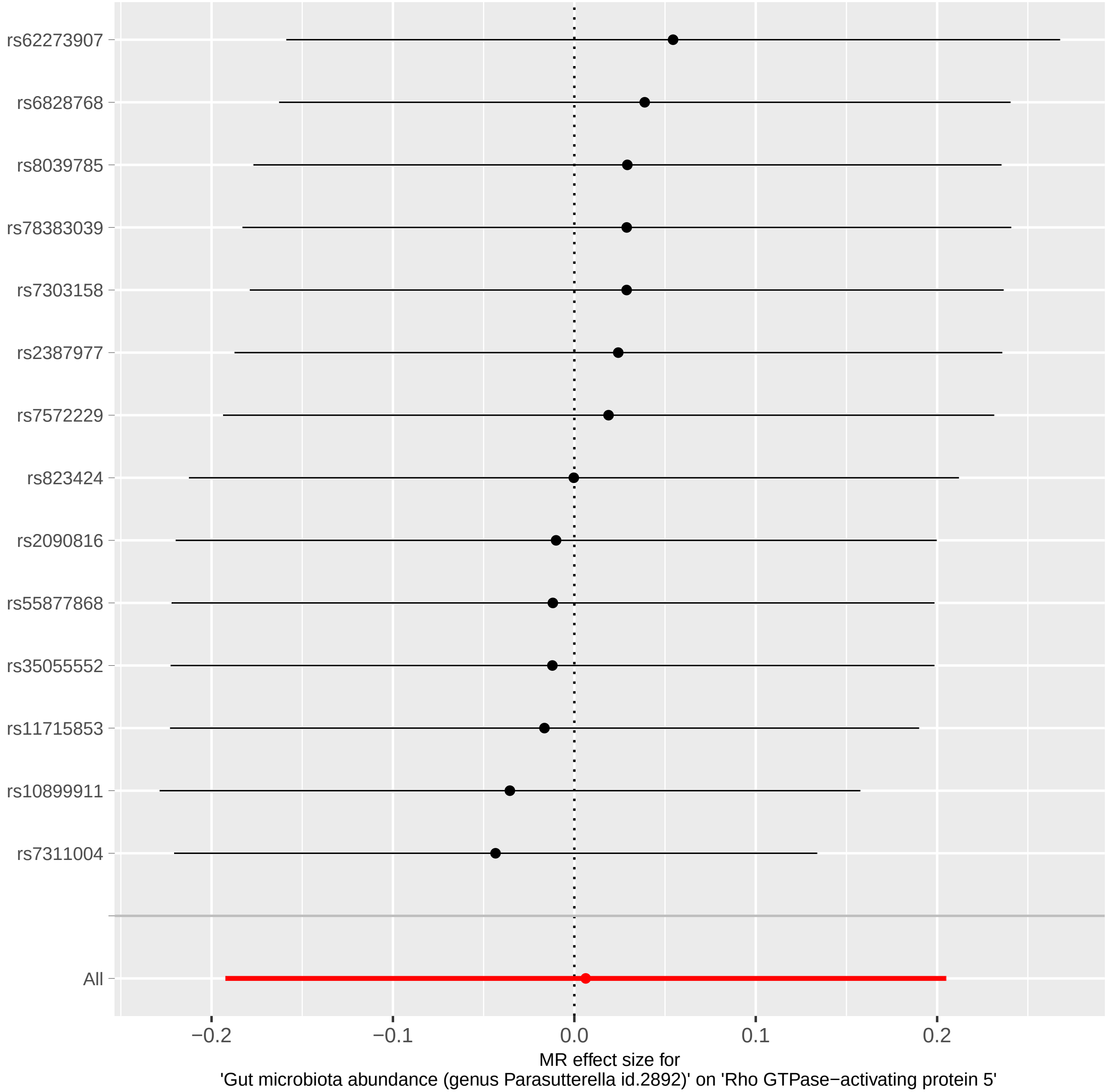

Batch 2−303 : Gut microbiota abundance (genus Catenibacterium id.2153) on Rho GDP−dissociation inhibitor 1
with a threshold of 1e−05

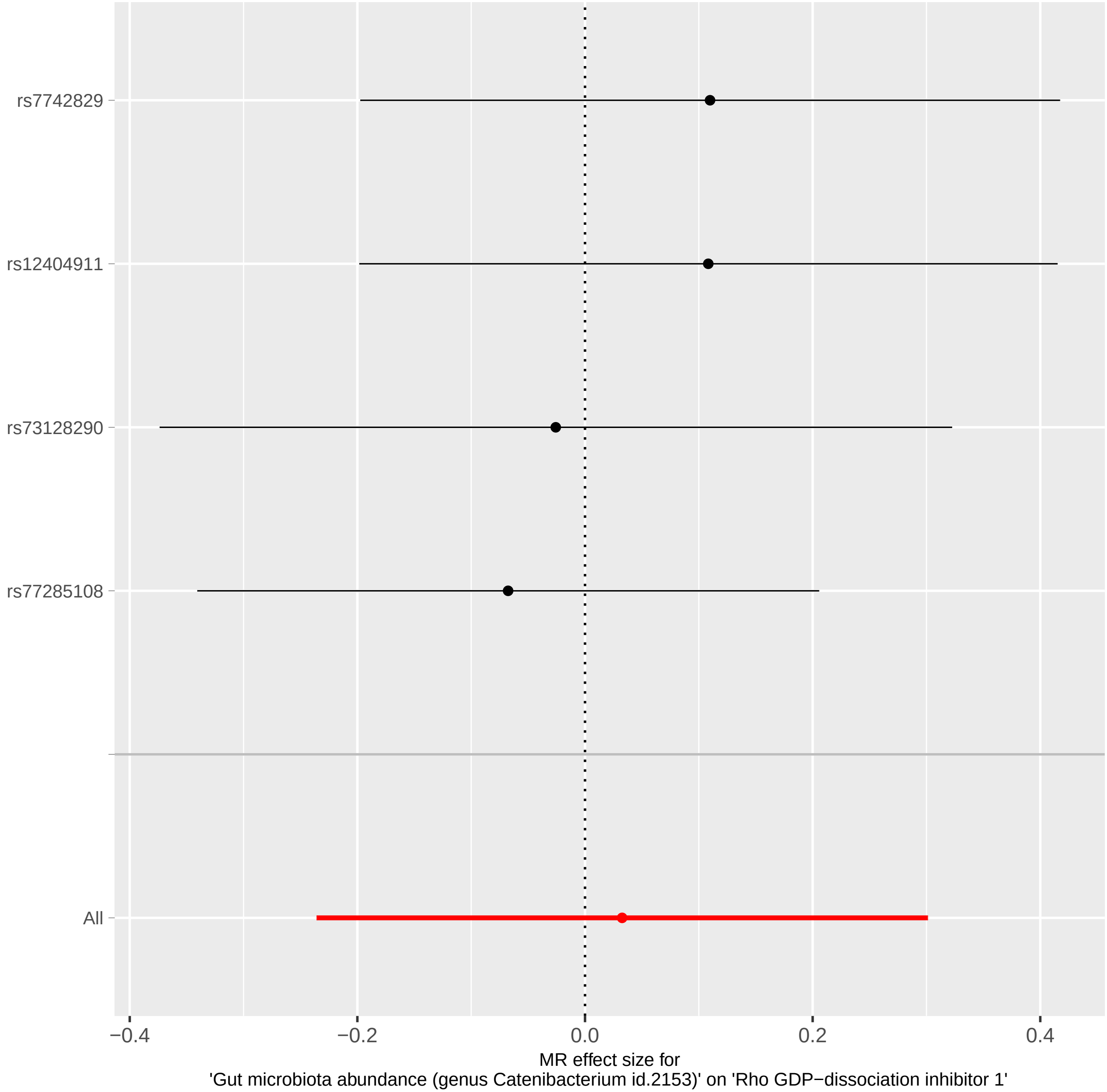

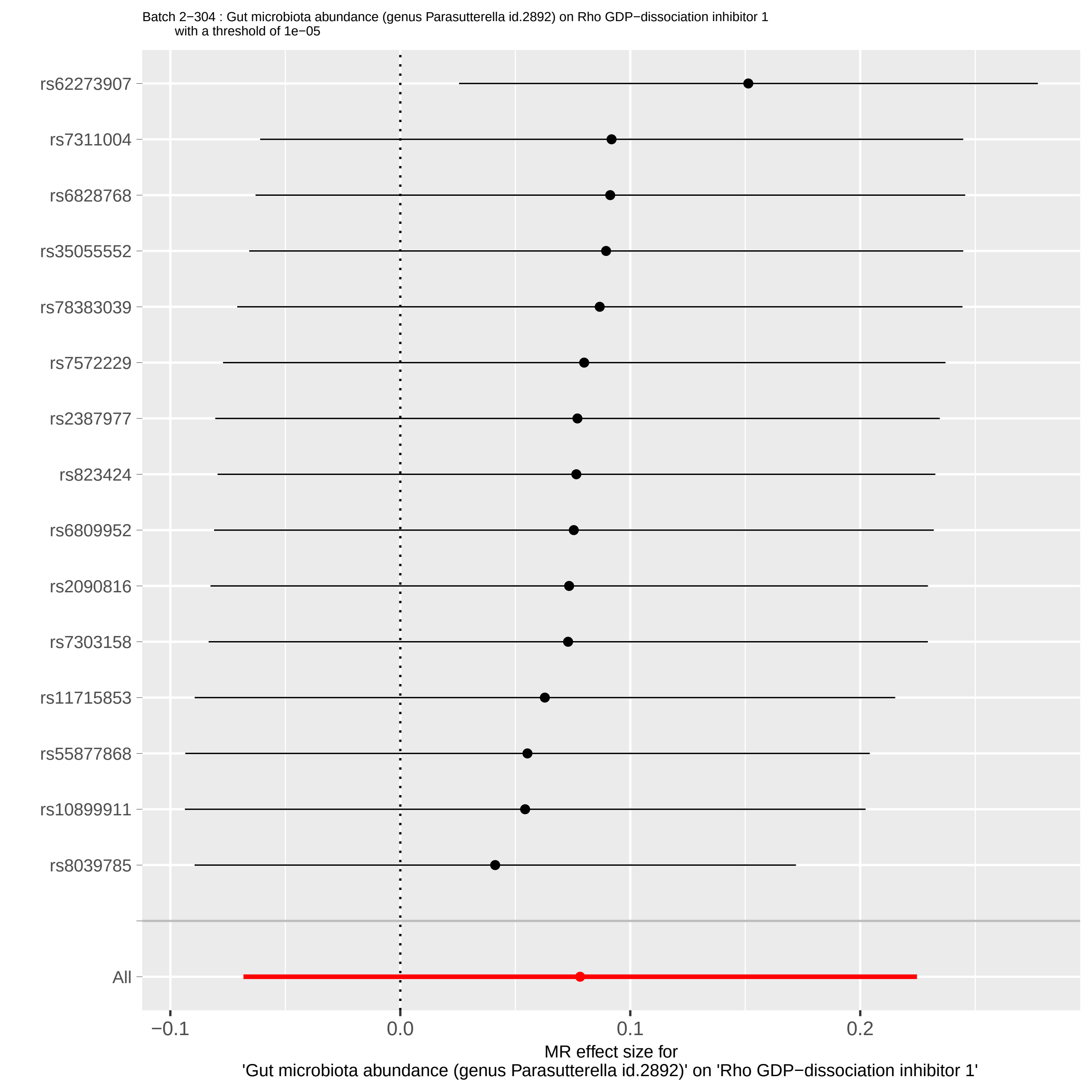
Batch 2−304 : Gut microbiota abundance (genus Parasutterella id.2892) on Rho GDP−dissociation inhibitor 1
with a threshold of 1e−05
rs62273907
rs7311004
rs6828768
rs35055552
rs78383039
rs7572229
rs2387977
rs823424
rs6809952
rs2090816
rs7303158
rs11715853
rs55877868
rs10899911
rs8039785
All
−0.1
0.0
0.1
0.2
MR effect size for
'Gut microbiota abundance (genus Parasutterella id.2892)' on 'Rho GDP−dissociation inhibitor 1'

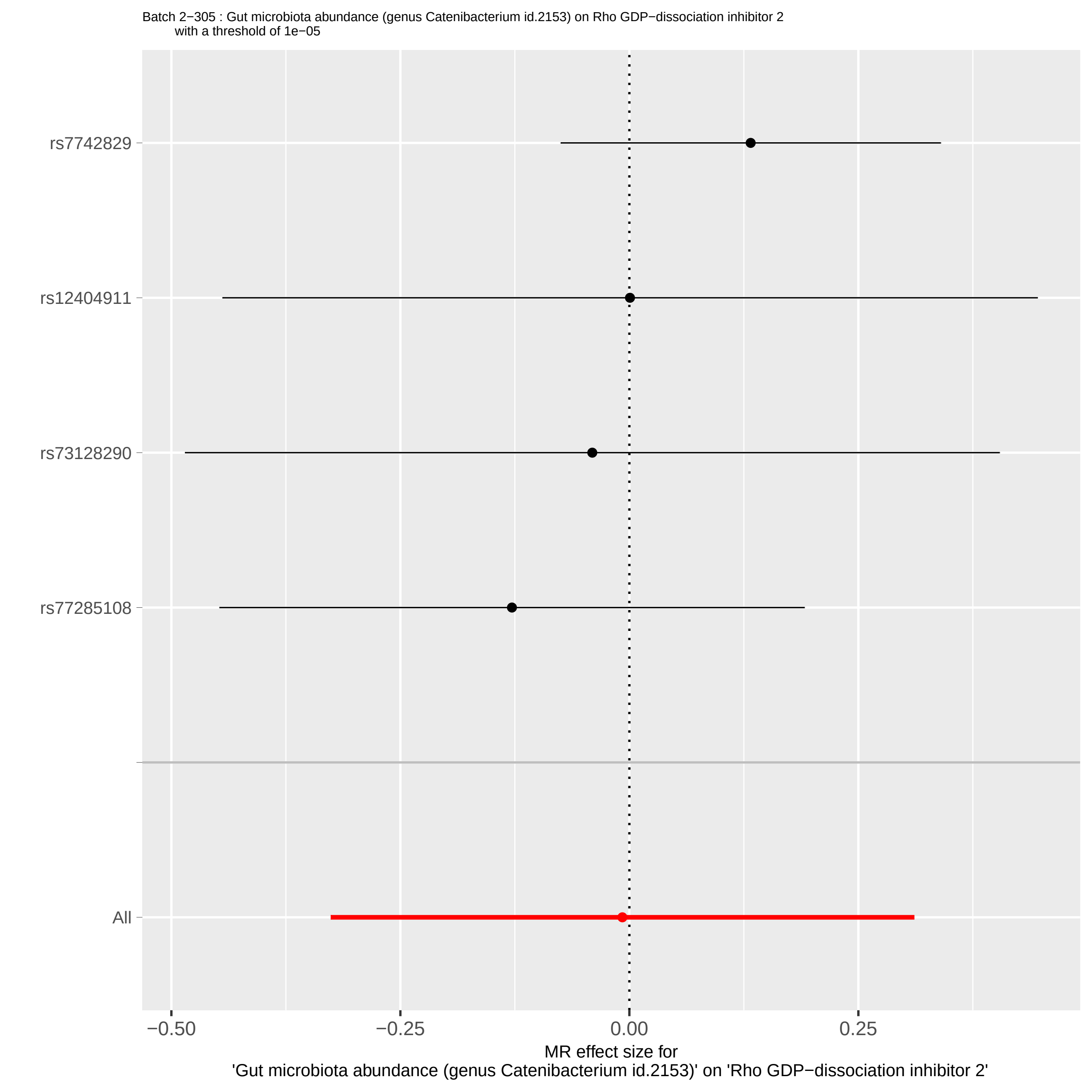

Batch 2−305 : Gut microbiota abundance (genus Catenibacterium id.2153) on Rho GDP−dissociation inhibitor 2
with a threshold of 1e−05
rs7742829
rs12404911
rs73128290
rs77285108
All
−0.50
−0.25
0.00
0.25
MR effect size for
'Gut microbiota abundance (genus Catenibacterium id.2153)' on 'Rho GDP−dissociation inhibitor 2'

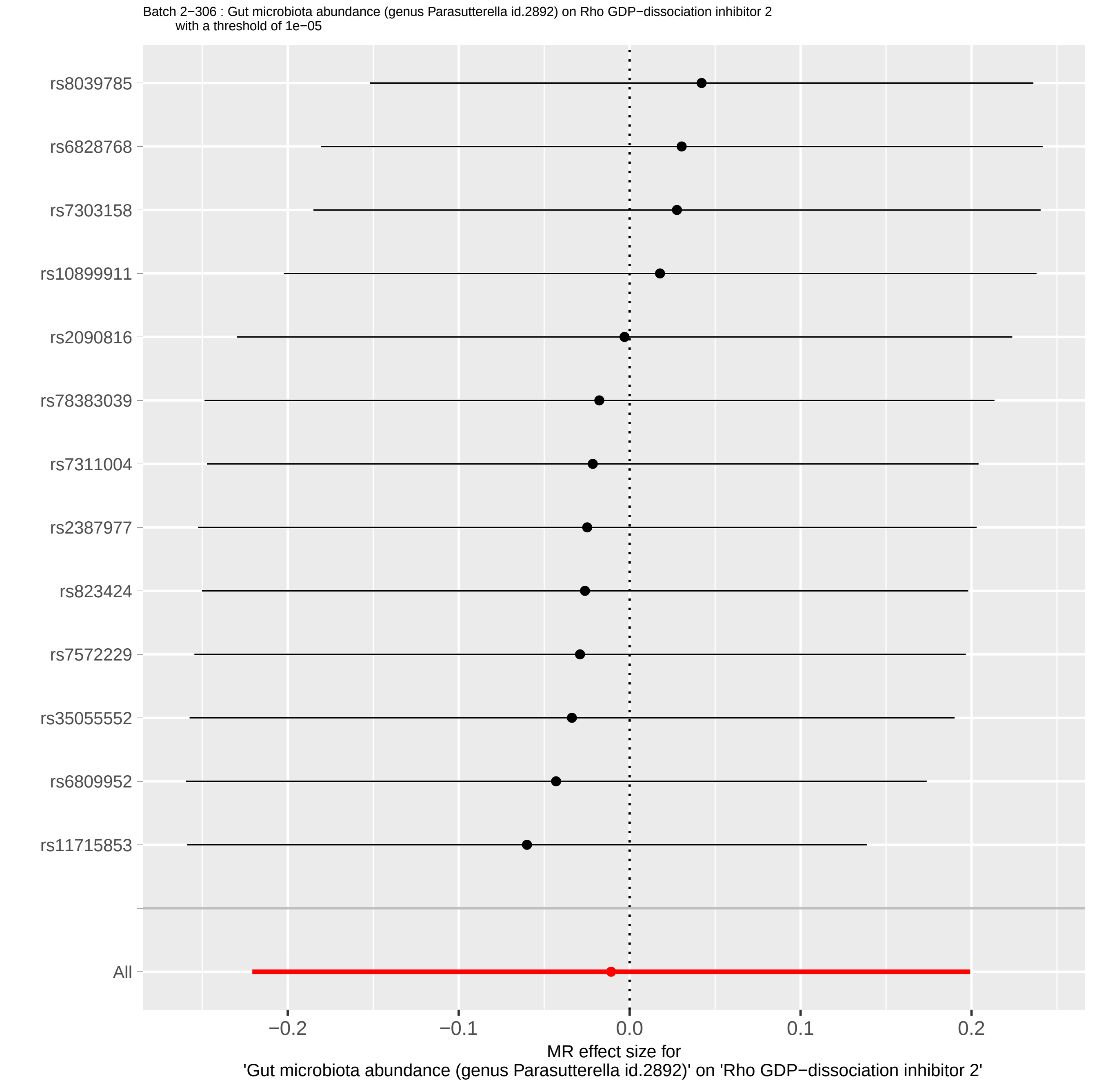

Batch 2−306 : Gut microbiota abundance (genus Parasutterella id.2892) on Rho GDP−dissociation inhibitor 2
with a threshold of 1e−05
rs8039785
rs6828768
rs7303158
rs10899911
rs2090816
rs78383039
rs7311004
rs2387977
rs823424
rs7572229
rs35055552
rs6809952
rs11715853
All
−0.2
−0.1
0.0
0.1
0.2
MR effect size for
'Gut microbiota abundance (genus Parasutterella id.2892)' on 'Rho GDP−dissociation inhibitor 2'

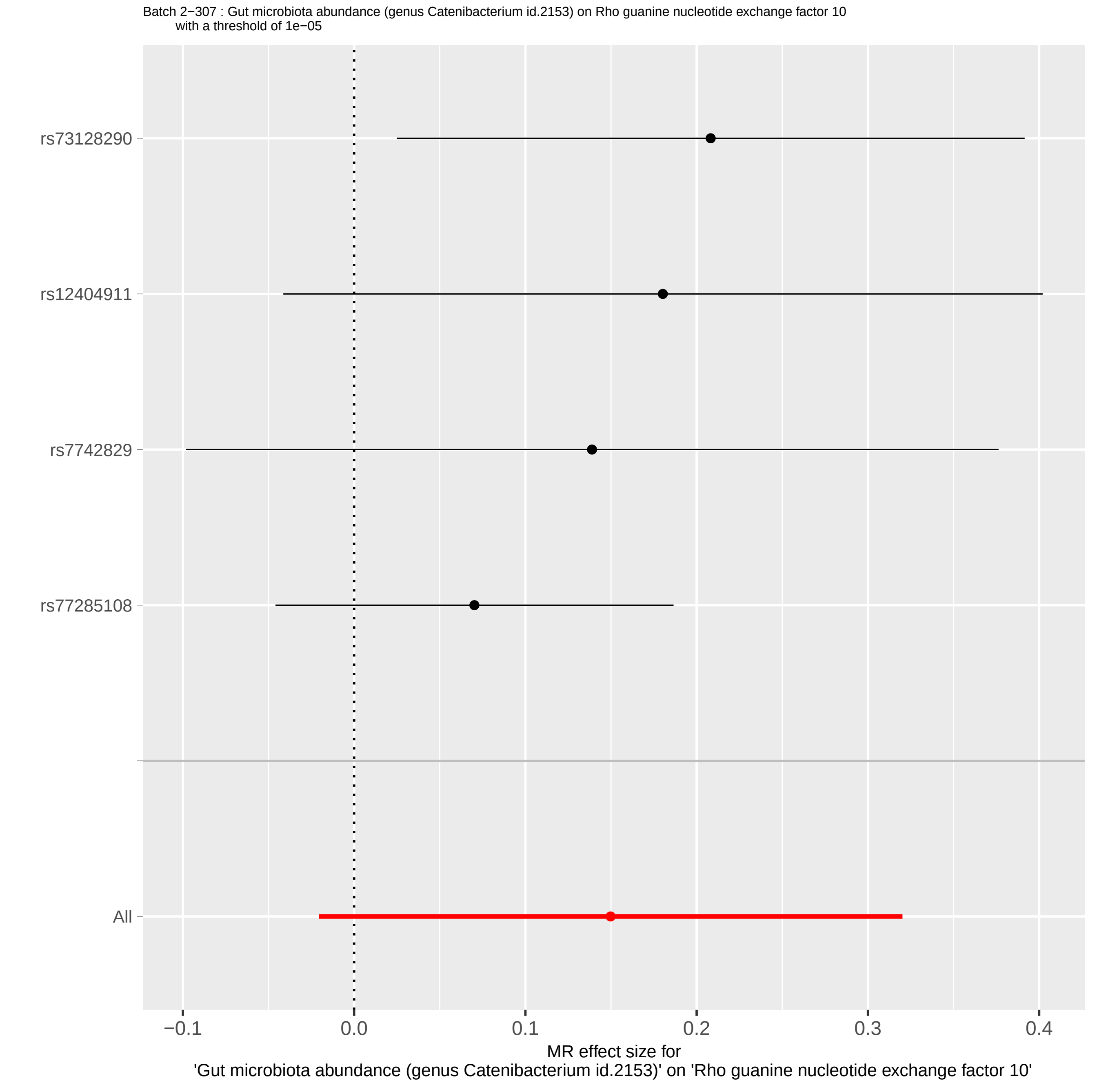
Batch 2−307 : Gut microbiota abundance (genus Catenibacterium id.2153) on Rho guanine nucleotide exchange factor 10
with a threshold of 1e−05
rs73128290
rs12404911
rs7742829
rs77285108
All
−0.1
0.0
0.1
0.2
0.3
0.4
MR effect size for
'Gut microbiota abundance (genus Catenibacterium id.2153)' on 'Rho guanine nucleotide exchange factor 10'

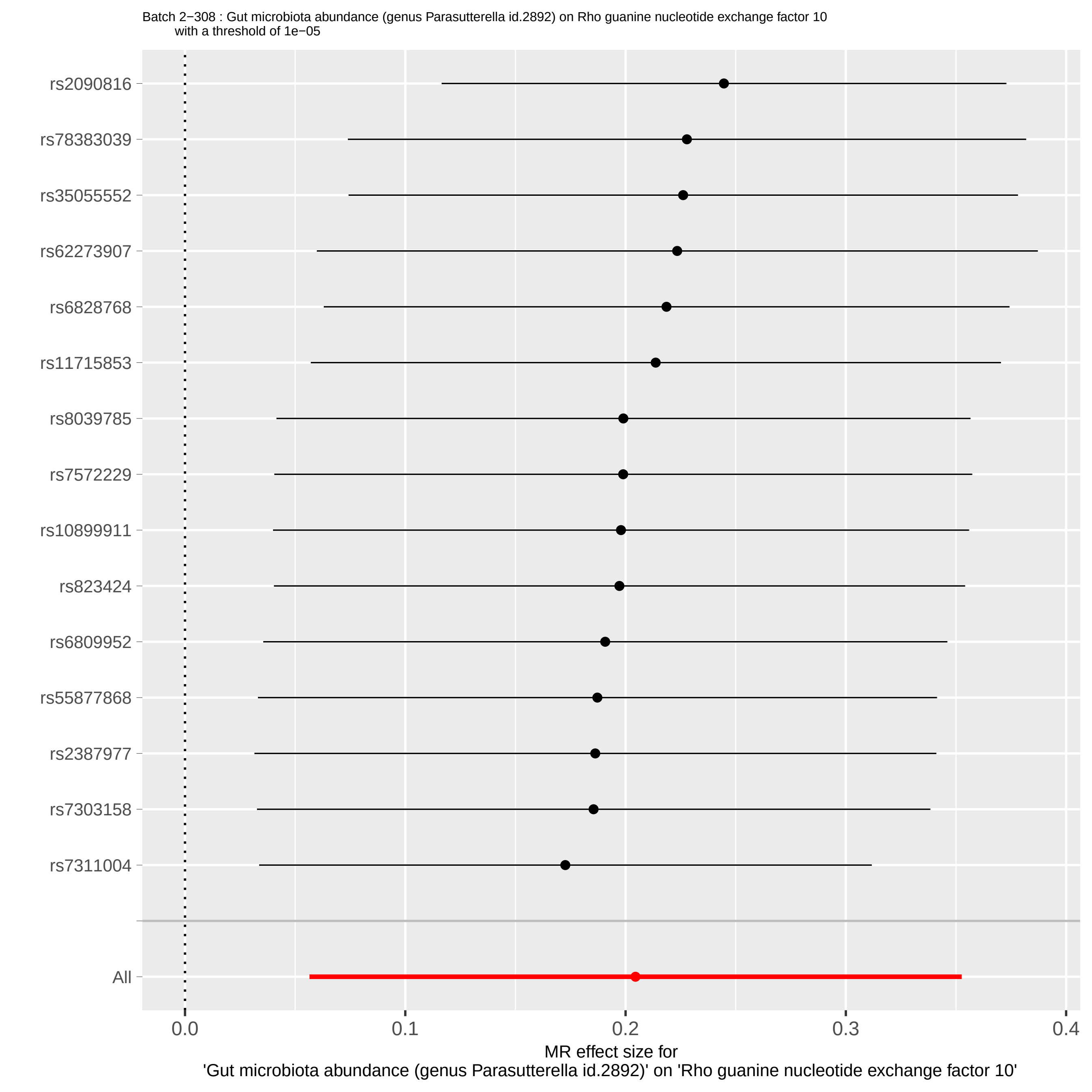

Batch 2−308 : Gut microbiota abundance (genus Parasutterella id.2892) on Rho guanine nucleotide exchange factor 10
with a threshold of 1e−05
rs2090816
rs78383039
rs35055552
rs62273907
rs6828768
rs11715853
rs8039785
rs7572229
rs10899911
rs823424
rs6809952
rs55877868
rs2387977
rs7303158
rs7311004
All
0.0
0.1
0.2
0.3
0.4
MR effect size for
'Gut microbiota abundance (genus Parasutterella id.2892)' on 'Rho guanine nucleotide exchange factor 10'

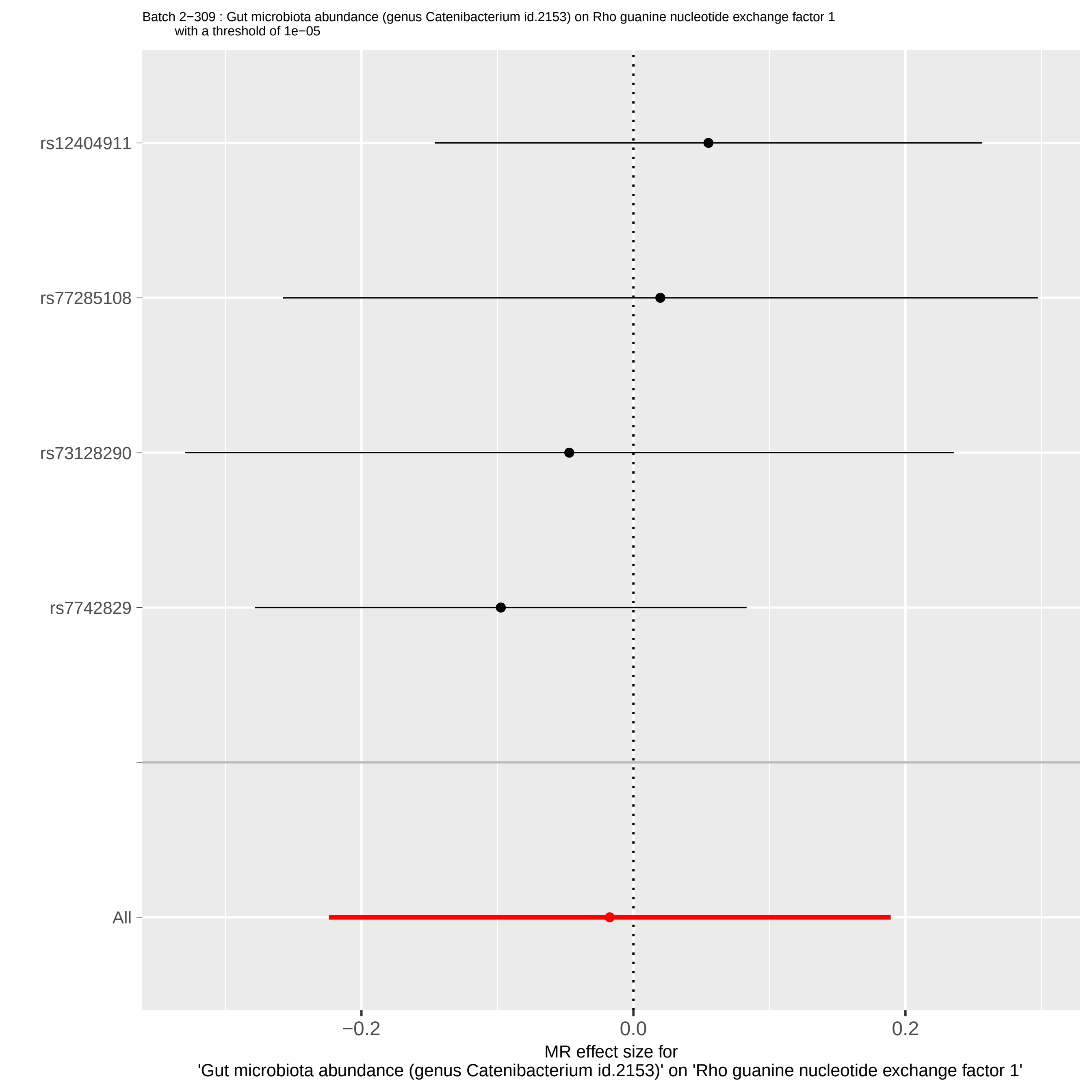

Batch 2−309 : Gut microbiota abundance (genus Catenibacterium id.2153) on Rho guanine nucleotide exchange factor 1
with a threshold of 1e−05
rs12404911
rs77285108
rs73128290
rs7742829
All
−0.2
0.0
0.2
MR effect size for
'Gut microbiota abundance (genus Catenibacterium id.2153)' on 'Rho guanine nucleotide exchange factor 1'

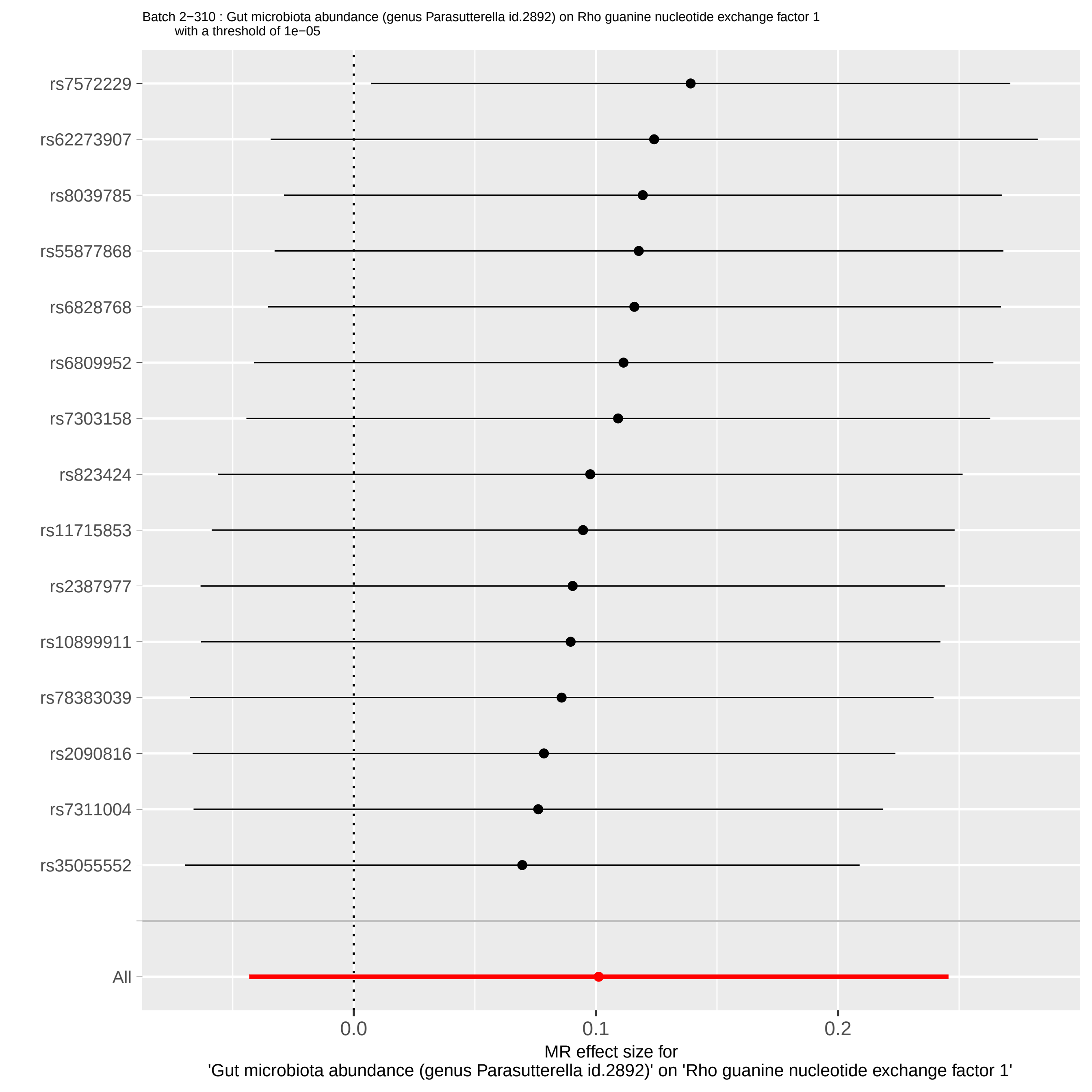

Batch 2−310 : Gut microbiota abundance (genus Parasutterella id.2892) on Rho guanine nucleotide exchange factor 1
with a threshold of 1e−05
rs7572229
rs62273907
rs8039785
rs55877868
rs6828768
rs6809952
rs7303158
rs823424
rs11715853
rs2387977
rs10899911
rs78383039
rs2090816
rs7311004
rs35055552
All
0.0
0.1
0.2
MR effect size for
'Gut microbiota abundance (genus Parasutterella id.2892)' on 'Rho guanine nucleotide exchange factor 1'

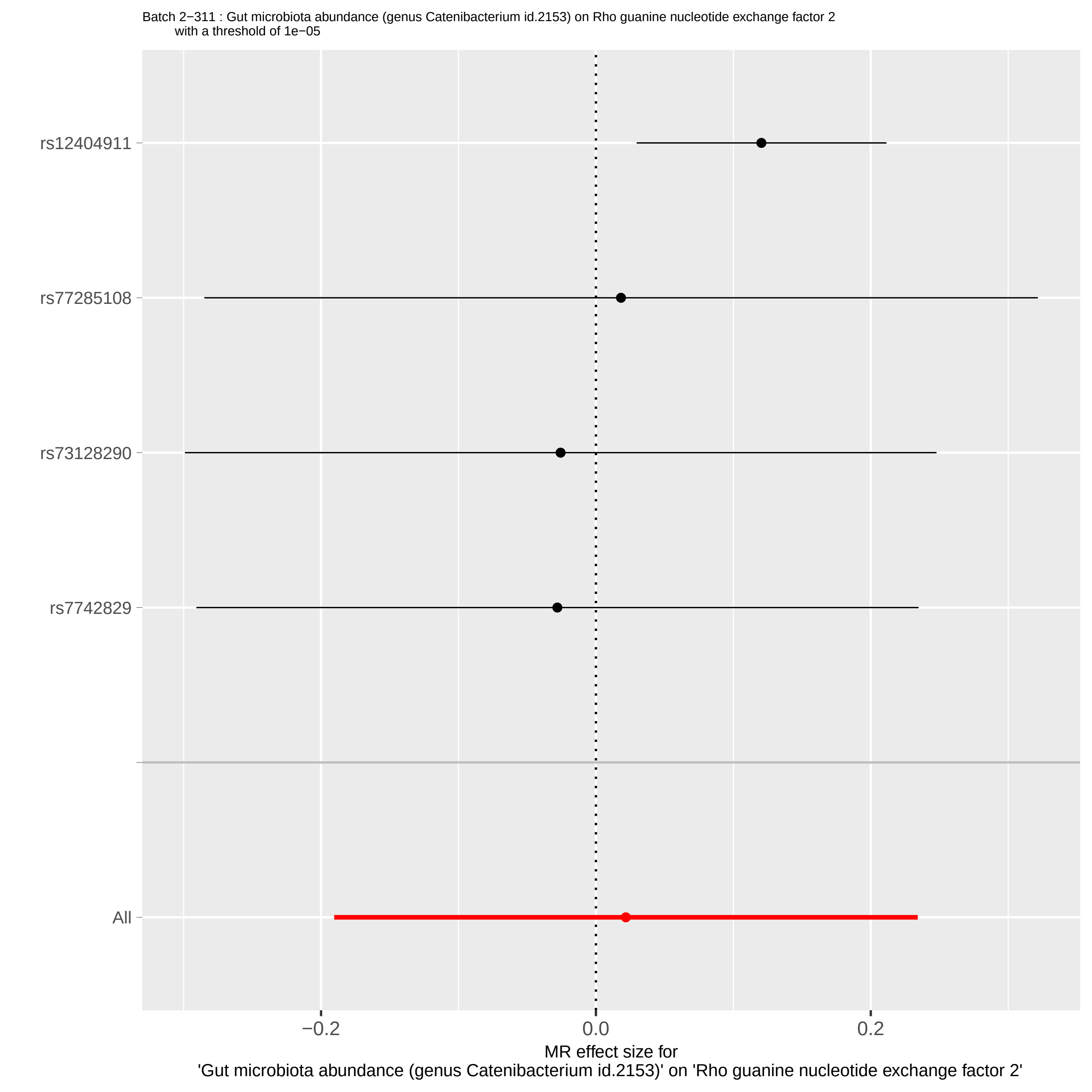

Batch 2−311 : Gut microbiota abundance (genus Catenibacterium id.2153) on Rho guanine nucleotide exchange factor 2
with a threshold of 1e−05
rs12404911
rs77285108
rs73128290
rs7742829
All
−0.2
0.0
0.2
MR effect size for
'Gut microbiota abundance (genus Catenibacterium id.2153)' on 'Rho guanine nucleotide exchange factor 2'

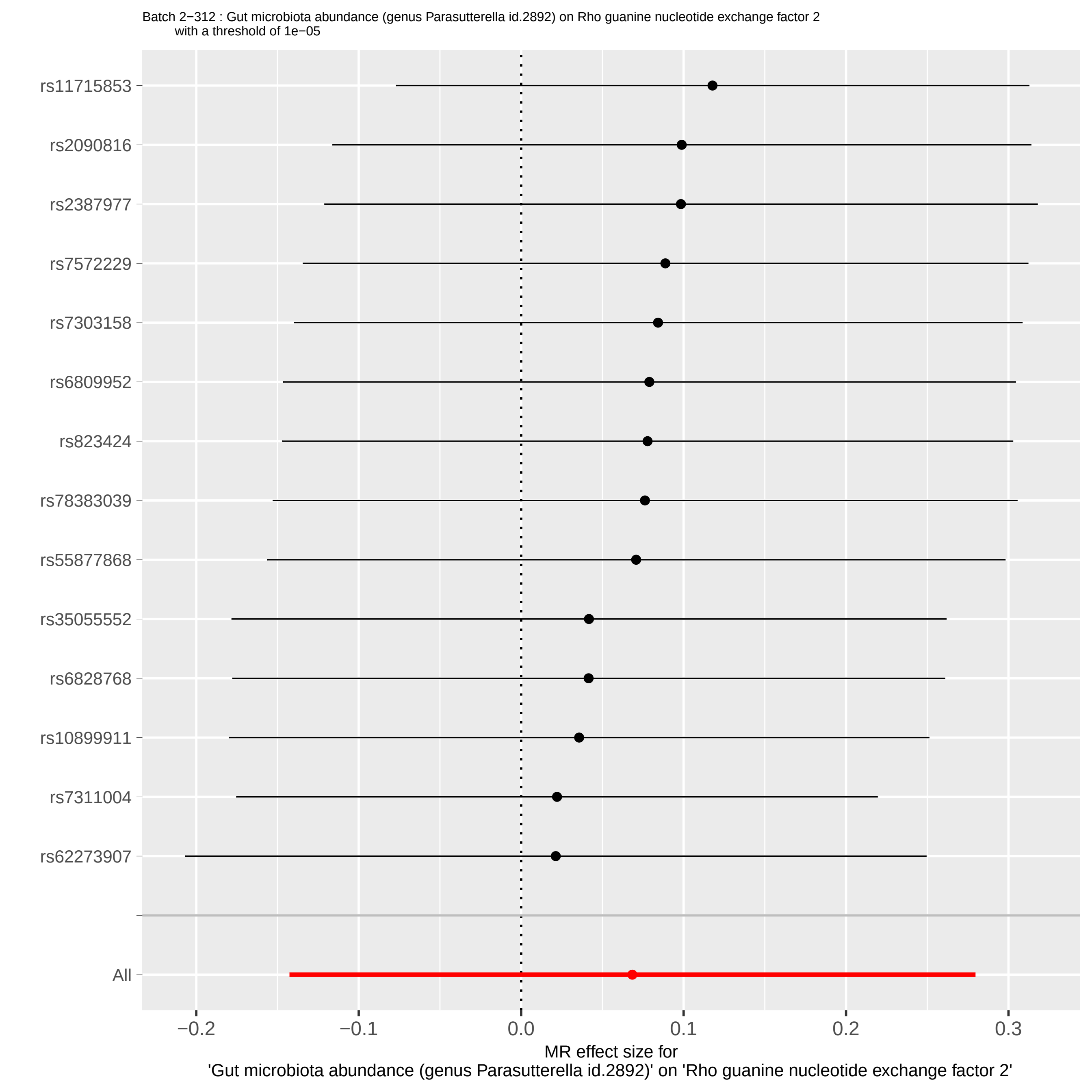

Batch 2−312 : Gut microbiota abundance (genus Parasutterella id.2892) on Rho guanine nucleotide exchange factor 2
with a threshold of 1e−05
rs11715853
rs2090816
rs2387977
rs7572229
rs7303158
rs6809952
rs823424
rs78383039
rs55877868
rs35055552
rs6828768
rs10899911
rs7311004
rs62273907
All
−0.2
−0.1
0.0
0.1
0.2
0.3
MR effect size for
'Gut microbiota abundance (genus Parasutterella id.2892)' on 'Rho guanine nucleotide exchange factor 2'

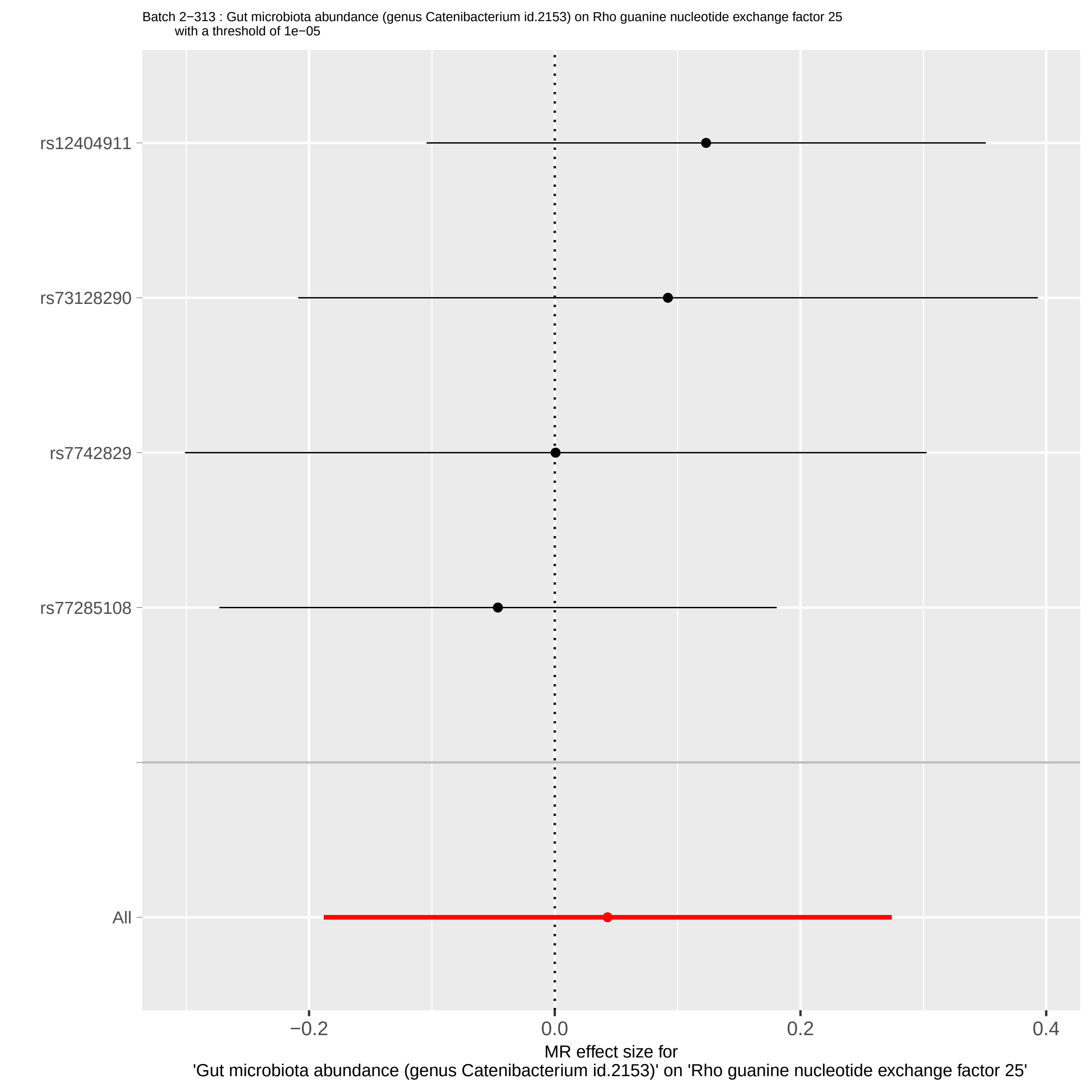

Batch 2−313 : Gut microbiota abundance (genus Catenibacterium id.2153) on Rho guanine nucleotide exchange factor 25
with a threshold of 1e−05
rs12404911
rs73128290
rs7742829
rs77285108
All
−0.2
0.0
0.2
0.4
MR effect size for
'Gut microbiota abundance (genus Catenibacterium id.2153)' on 'Rho guanine nucleotide exchange factor 25'

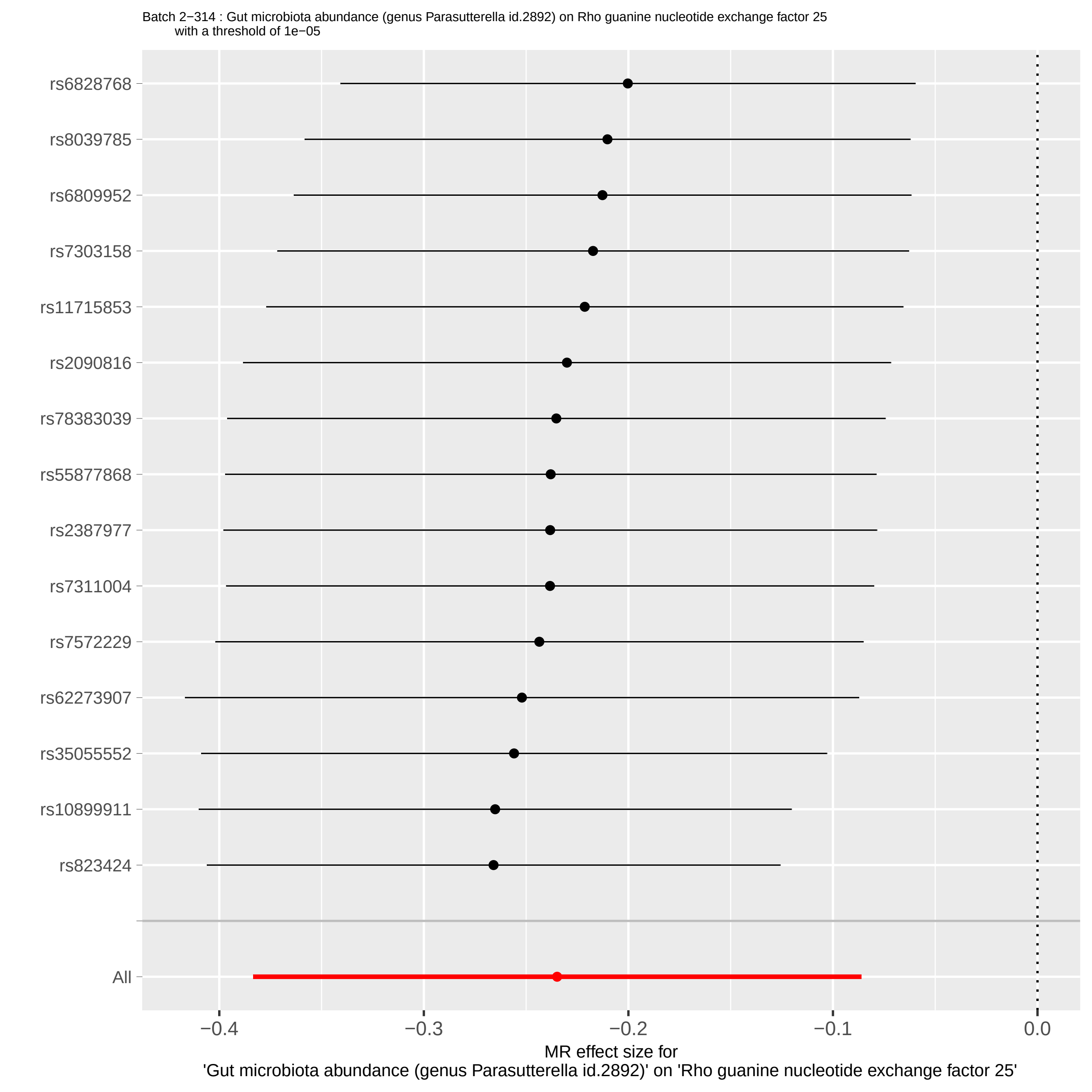

Batch 2−314 : Gut microbiota abundance (genus Parasutterella id.2892) on Rho guanine nucleotide exchange factor 25
with a threshold of 1e−05
rs6828768
rs8039785
rs6809952
rs7303158
rs11715853
rs2090816
rs78383039
rs55877868
rs2387977
rs7311004
rs7572229
rs62273907
rs35055552
rs10899911
rs823424
All
−0.4
−0.3
−0.2
−0.1
0.0
MR effect size for
'Gut microbiota abundance (genus Parasutterella id.2892)' on 'Rho guanine nucleotide exchange factor 25'

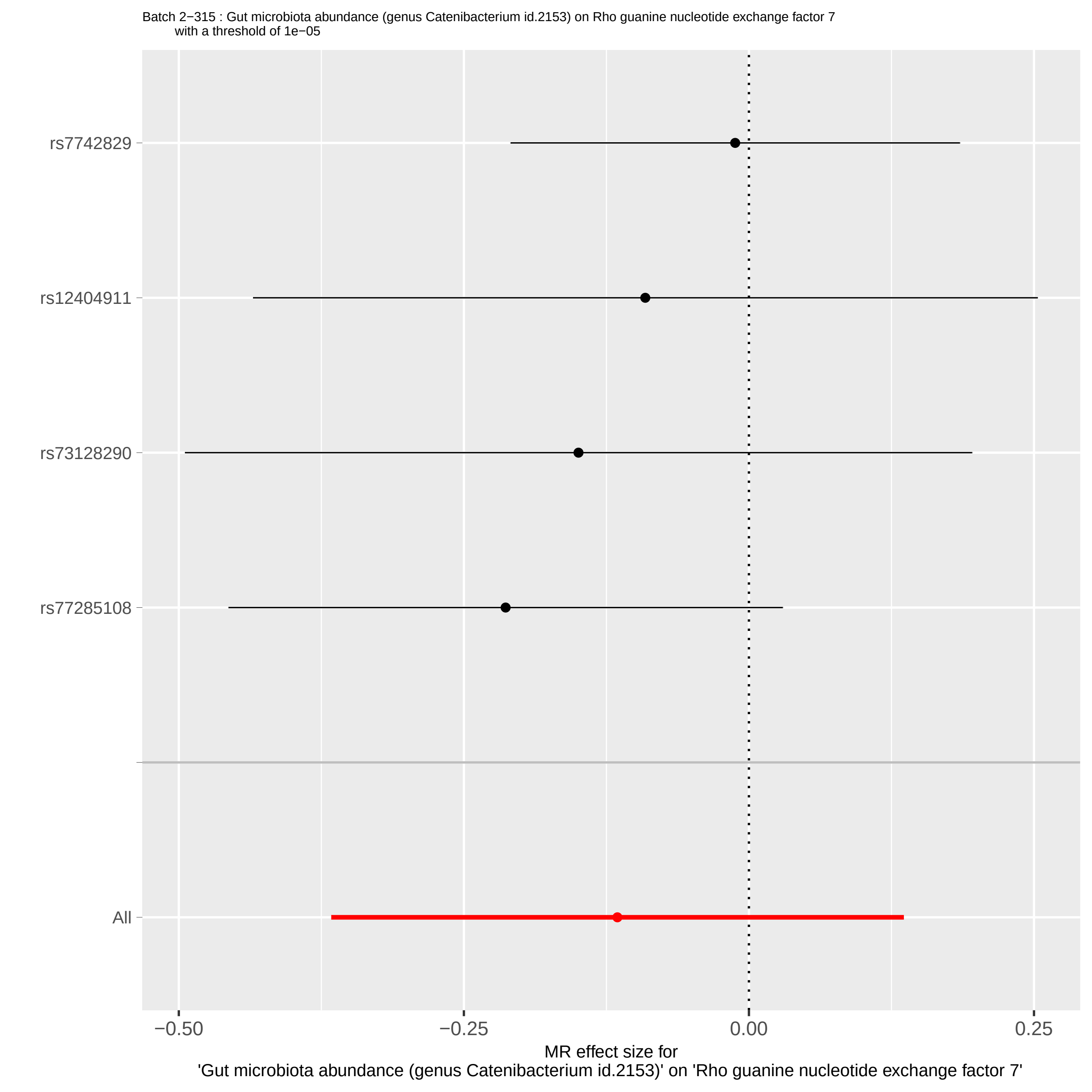
Batch 2−315 : Gut microbiota abundance (genus Catenibacterium id.2153) on Rho guanine nucleotide exchange factor 7
with a threshold of 1e−05
rs7742829
rs12404911
rs73128290
rs77285108
All
−0.50
−0.25
0.00
0.25
MR effect size for
'Gut microbiota abundance (genus Catenibacterium id.2153)' on 'Rho guanine nucleotide exchange factor 7'

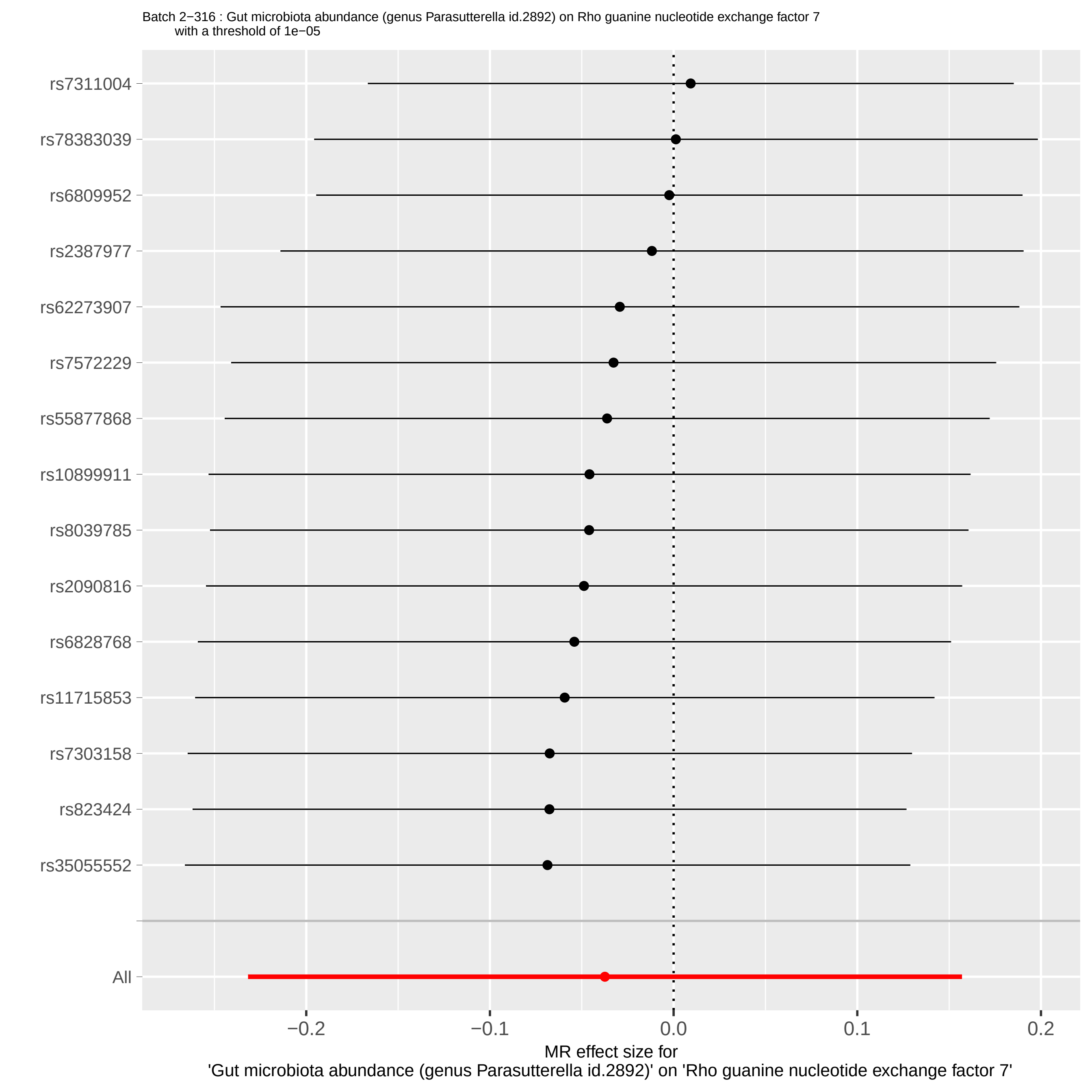

Batch 2−316 : Gut microbiota abundance (genus Parasutterella id.2892) on Rho guanine nucleotide exchange factor 7
with a threshold of 1e−05
rs7311004
rs78383039
rs6809952
rs2387977
rs62273907
rs7572229
rs55877868
rs10899911
rs8039785
rs2090816
rs6828768
rs11715853
rs7303158
rs823424
rs35055552
All
−0.2
−0.1
0.0
0.1
0.2
MR effect size for
'Gut microbiota abundance (genus Parasutterella id.2892)' on 'Rho guanine nucleotide exchange factor 7'

Batch 2−317 : Gut microbiota abundance (genus Catenibacterium id.2153) on AT−rich interactive domain−containing protein 3A
with a threshold of 1e−05

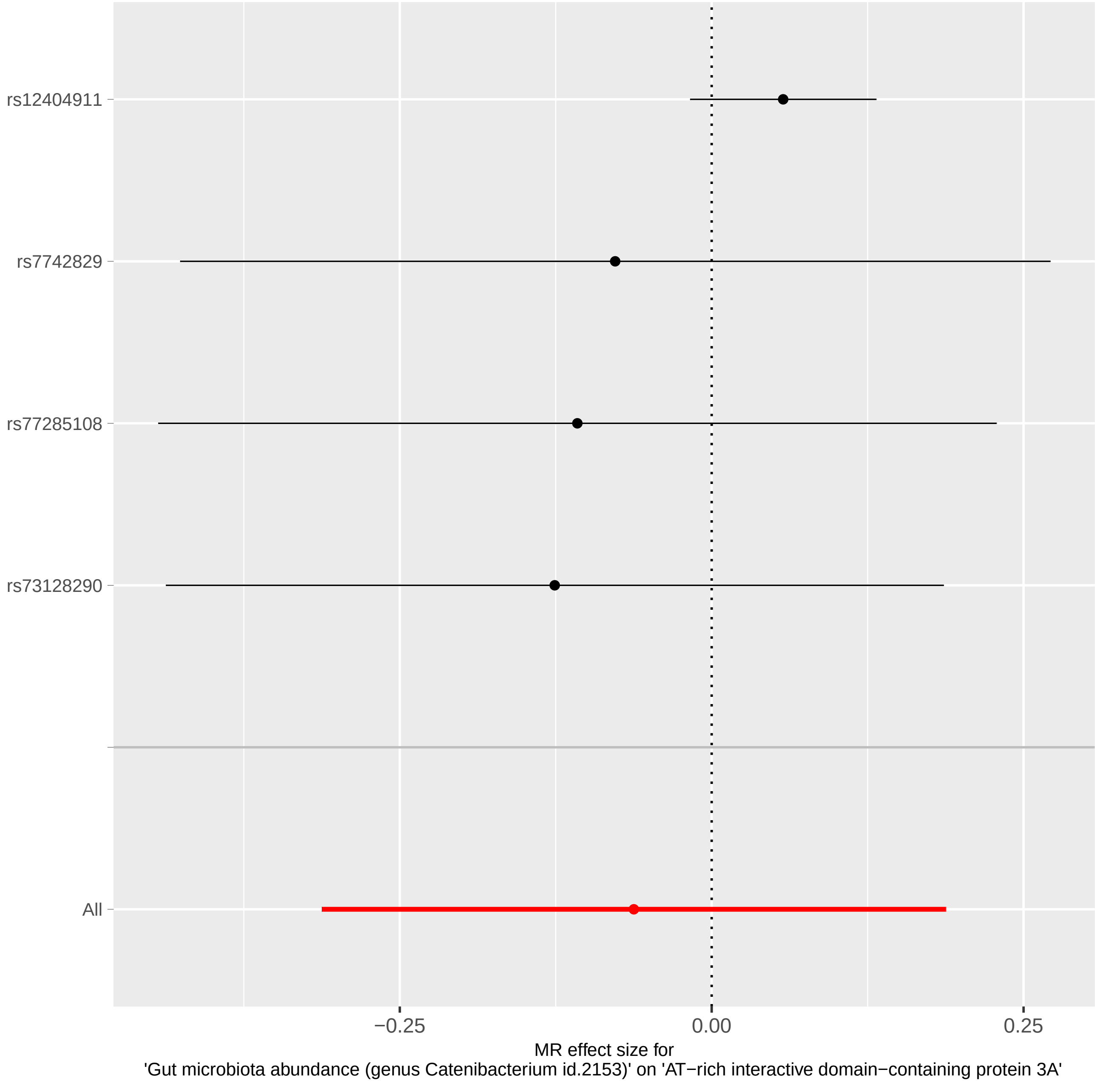

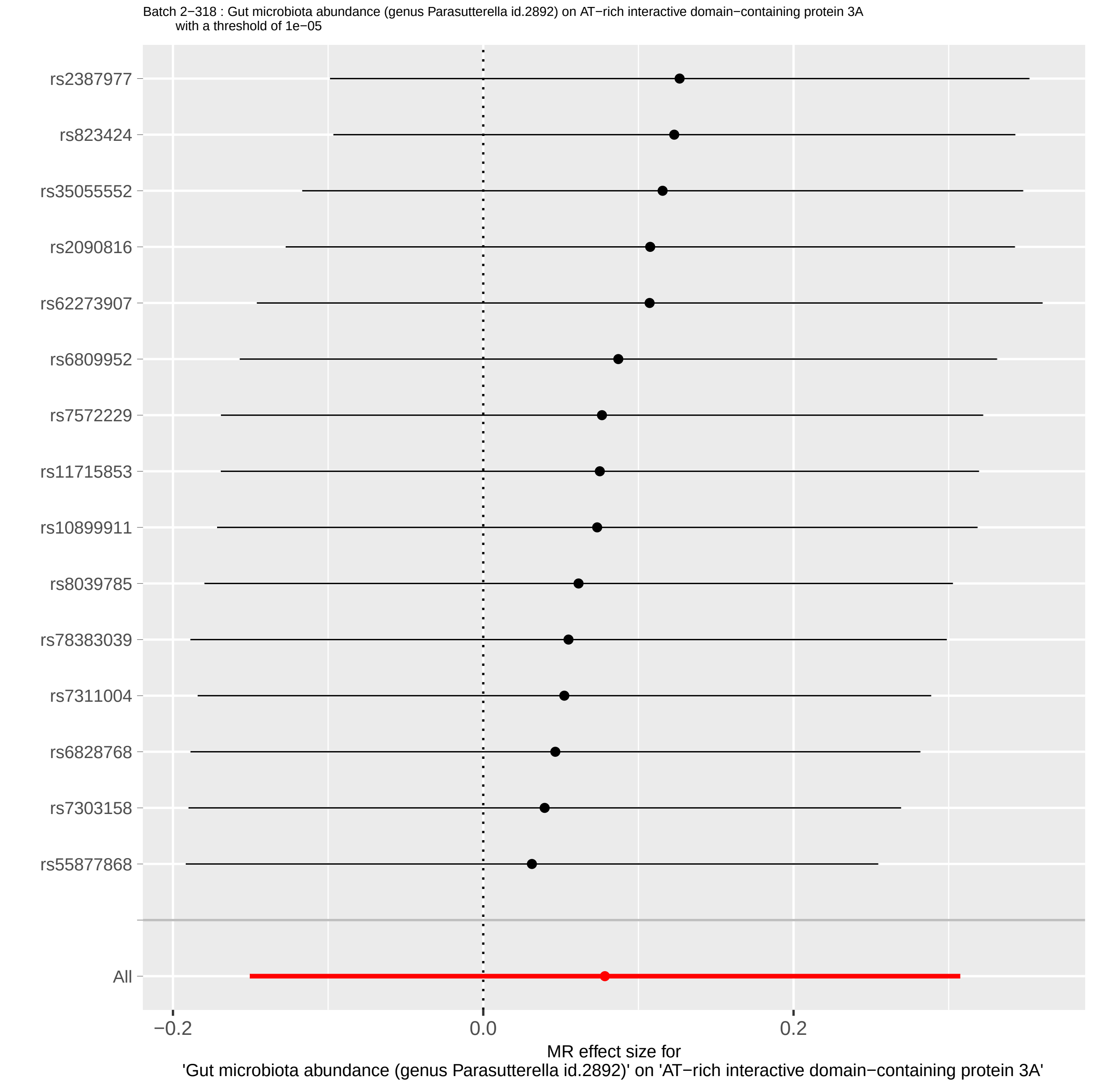

Batch 2−318 : Gut microbiota abundance (genus Parasutterella id.2892) on AT−rich interactive domain−containing protein 3A
with a threshold of 1e−05
rs2387977
rs823424
rs35055552
rs2090816
rs62273907
rs6809952
rs7572229
rs11715853
rs10899911
rs8039785
rs78383039
rs7311004
rs6828768
rs7303158
rs55877868
All
−0.2
0.0
0.2
MR effect size for
'Gut microbiota abundance (genus Parasutterella id.2892)' on 'AT−rich interactive domain−containing protein 3A'

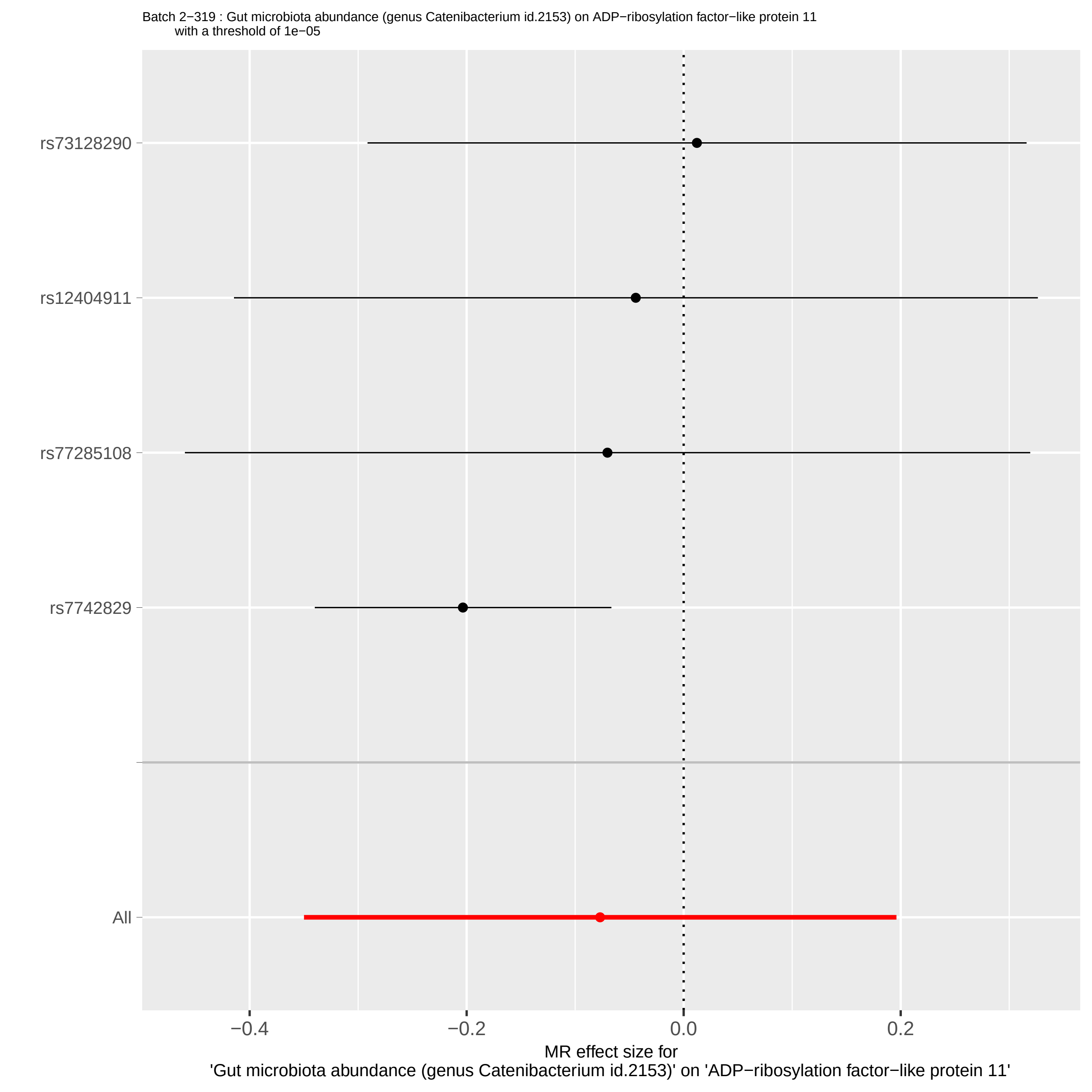

Batch 2−319 : Gut microbiota abundance (genus Catenibacterium id.2153) on ADP−ribosylation factor−like protein 11
with a threshold of 1e−05
rs73128290
rs12404911
rs77285108
rs7742829
All
−0.4
−0.2
0.0
0.2
MR effect size for
'Gut microbiota abundance (genus Catenibacterium id.2153)' on 'ADP−ribosylation factor−like protein 11'

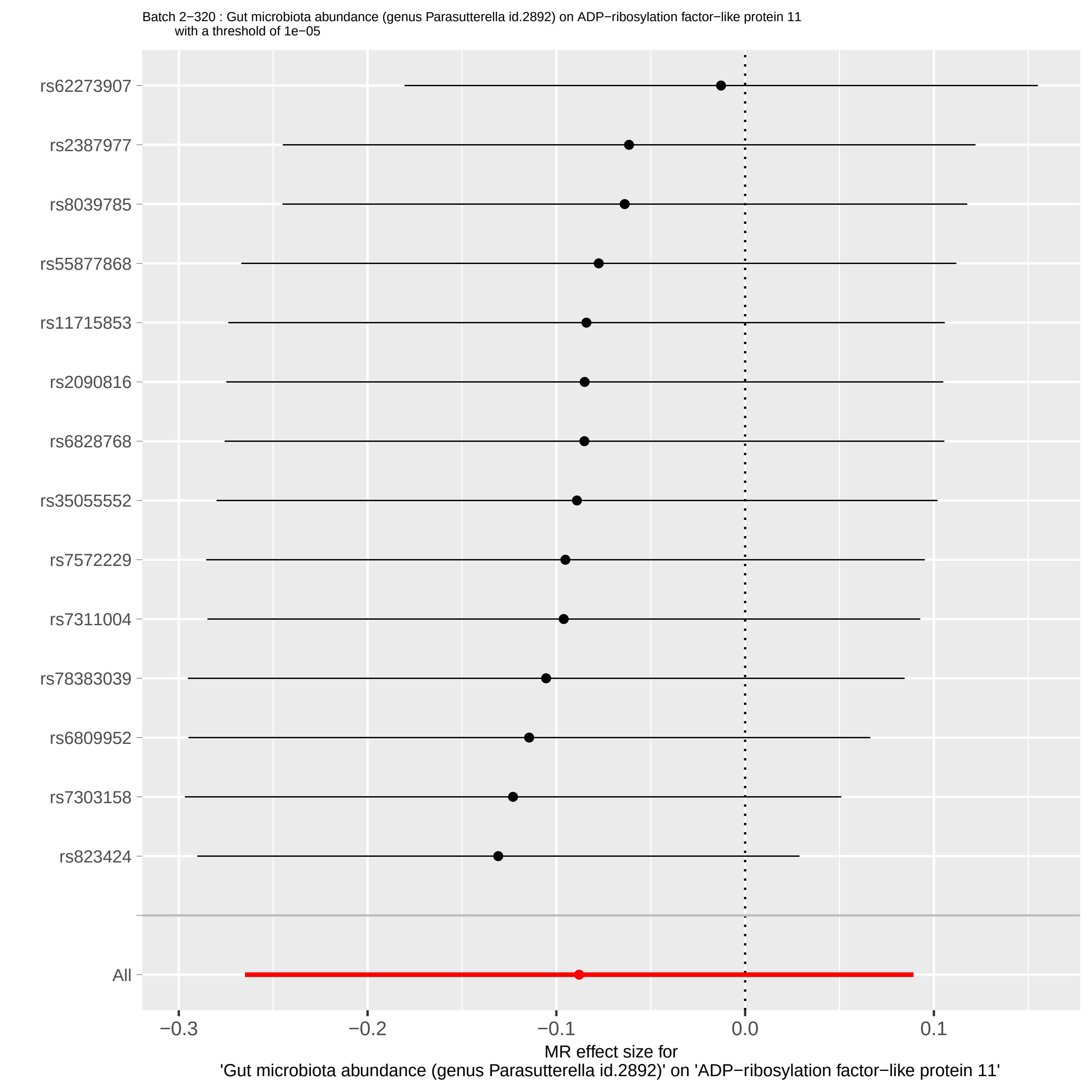

Batch 2−320 : Gut microbiota abundance (genus Parasutterella id.2892) on ADP−ribosylation factor−like protein 11
with a threshold of 1e−05
rs62273907
rs2387977
rs8039785
rs55877868
rs11715853
rs2090816
rs6828768
rs35055552
rs7572229
rs7311004
rs78383039
rs6809952
rs7303158
rs823424
All
−0.3
−0.2
−0.1
0.0
0.1
MR effect size for
'Gut microbiota abundance (genus Parasutterella id.2892)' on 'ADP−ribosylation factor−like protein 11'

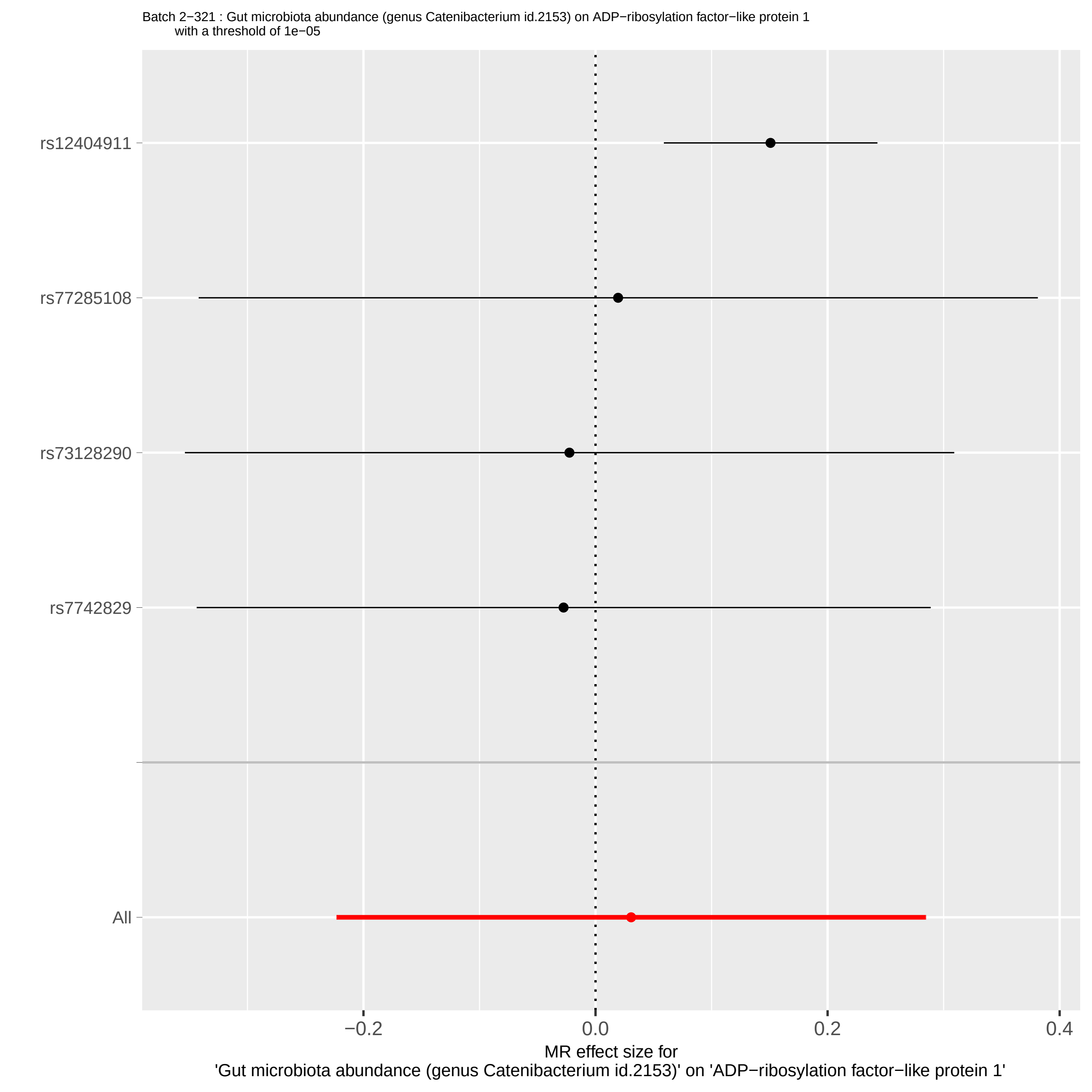

Batch 2−321 : Gut microbiota abundance (genus Catenibacterium id.2153) on ADP−ribosylation factor−like protein 1
with a threshold of 1e−05
rs12404911
rs77285108
rs73128290
rs7742829
All
−0.2
0.0
0.2
0.4
MR effect size for
'Gut microbiota abundance (genus Catenibacterium id.2153)' on 'ADP−ribosylation factor−like protein 1'

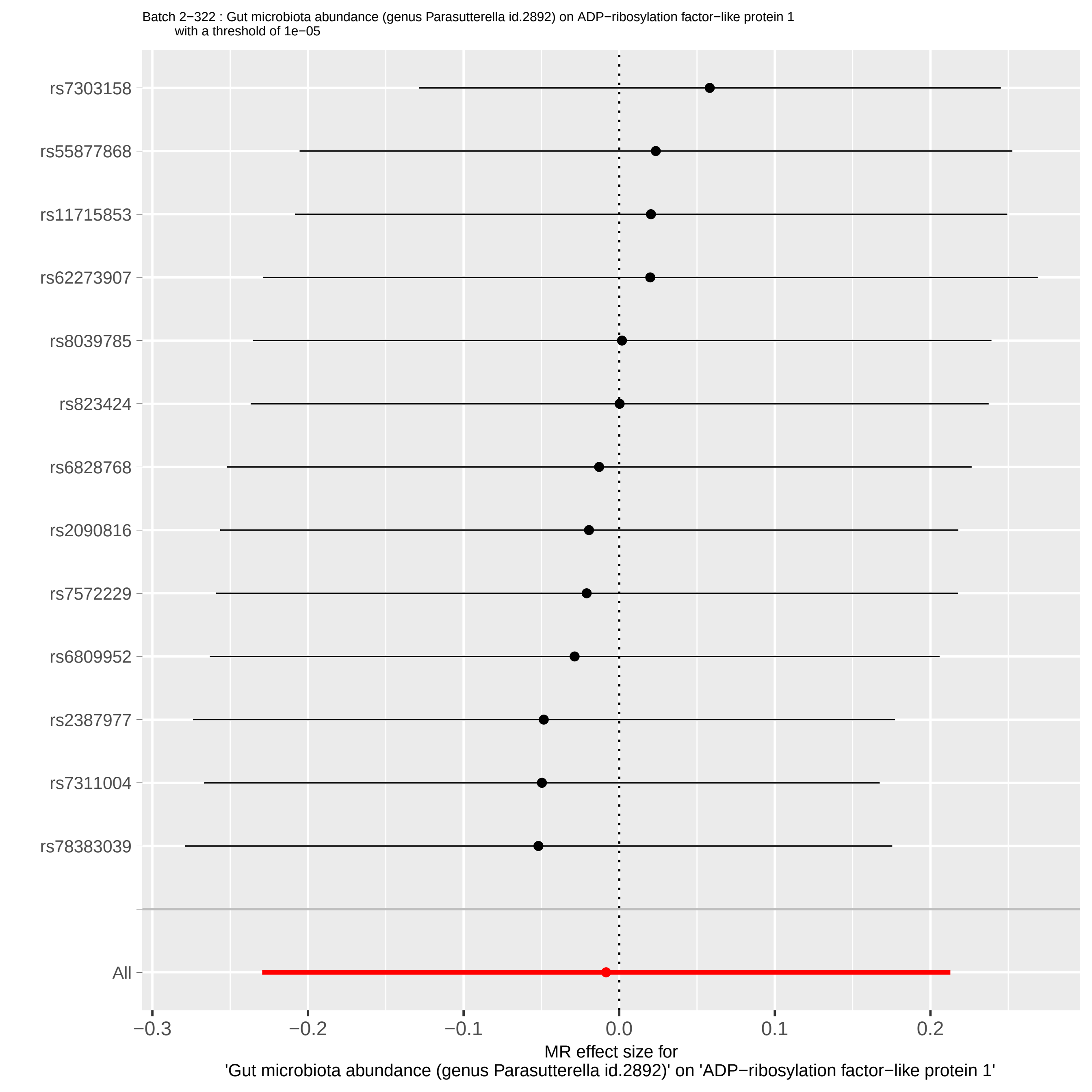

Batch 2−322 : Gut microbiota abundance (genus Parasutterella id.2892) on ADP−ribosylation factor−like protein 1
with a threshold of 1e−05
rs7303158
rs55877868
rs11715853
rs62273907
rs8039785
rs823424
rs6828768
rs2090816
rs7572229
rs6809952
rs2387977
rs7311004
rs78383039
All
−0.3
−0.2
−0.1
0.0
0.1
0.2
MR effect size for
'Gut microbiota abundance (genus Parasutterella id.2892)' on 'ADP−ribosylation factor−like protein 1'

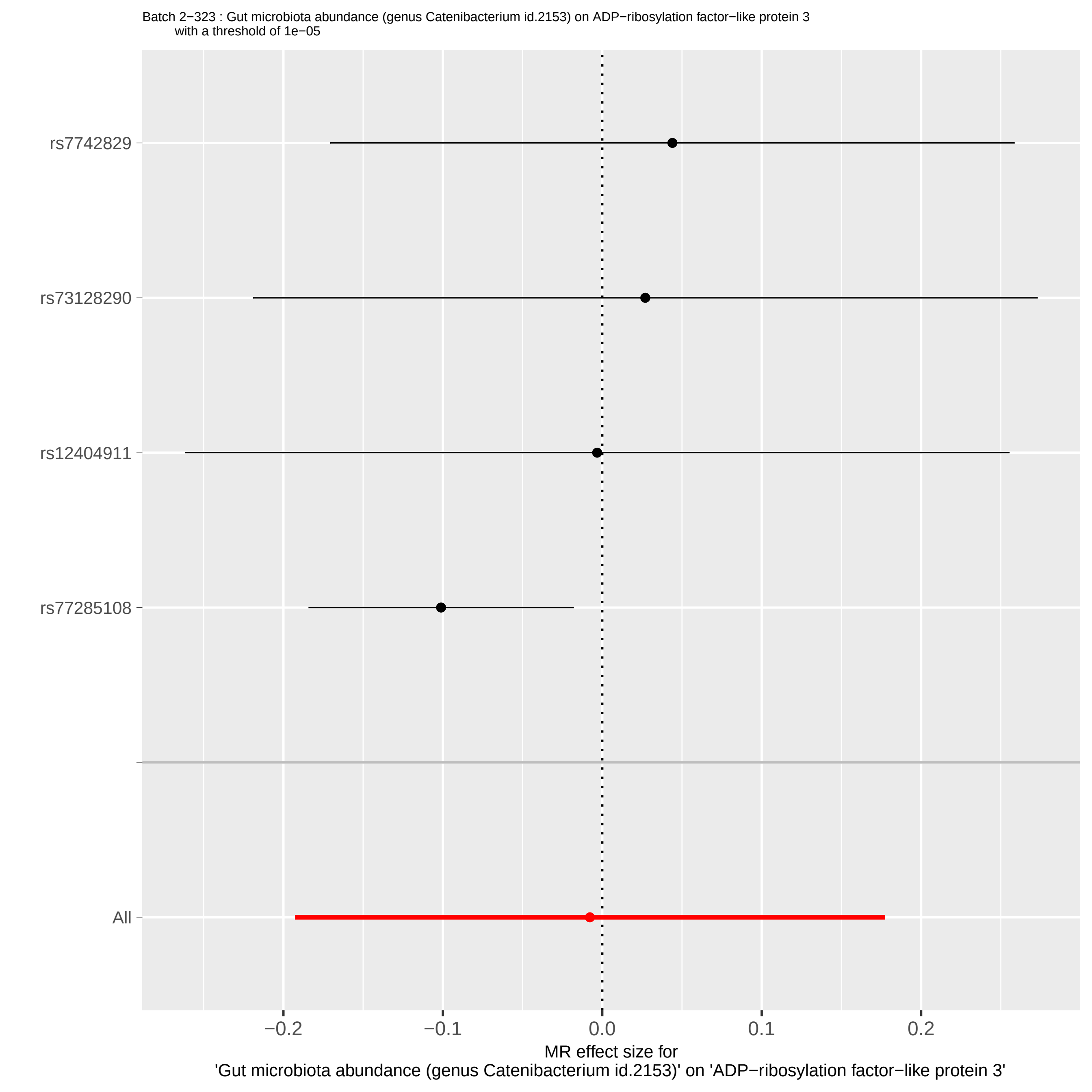

Batch 2−323 : Gut microbiota abundance (genus Catenibacterium id.2153) on ADP−ribosylation factor−like protein 3
with a threshold of 1e−05
rs7742829
rs73128290
rs12404911
rs77285108
All
−0.2
−0.1
0.0
0.1
0.2
MR effect size for
'Gut microbiota abundance (genus Catenibacterium id.2153)' on 'ADP−ribosylation factor−like protein 3'

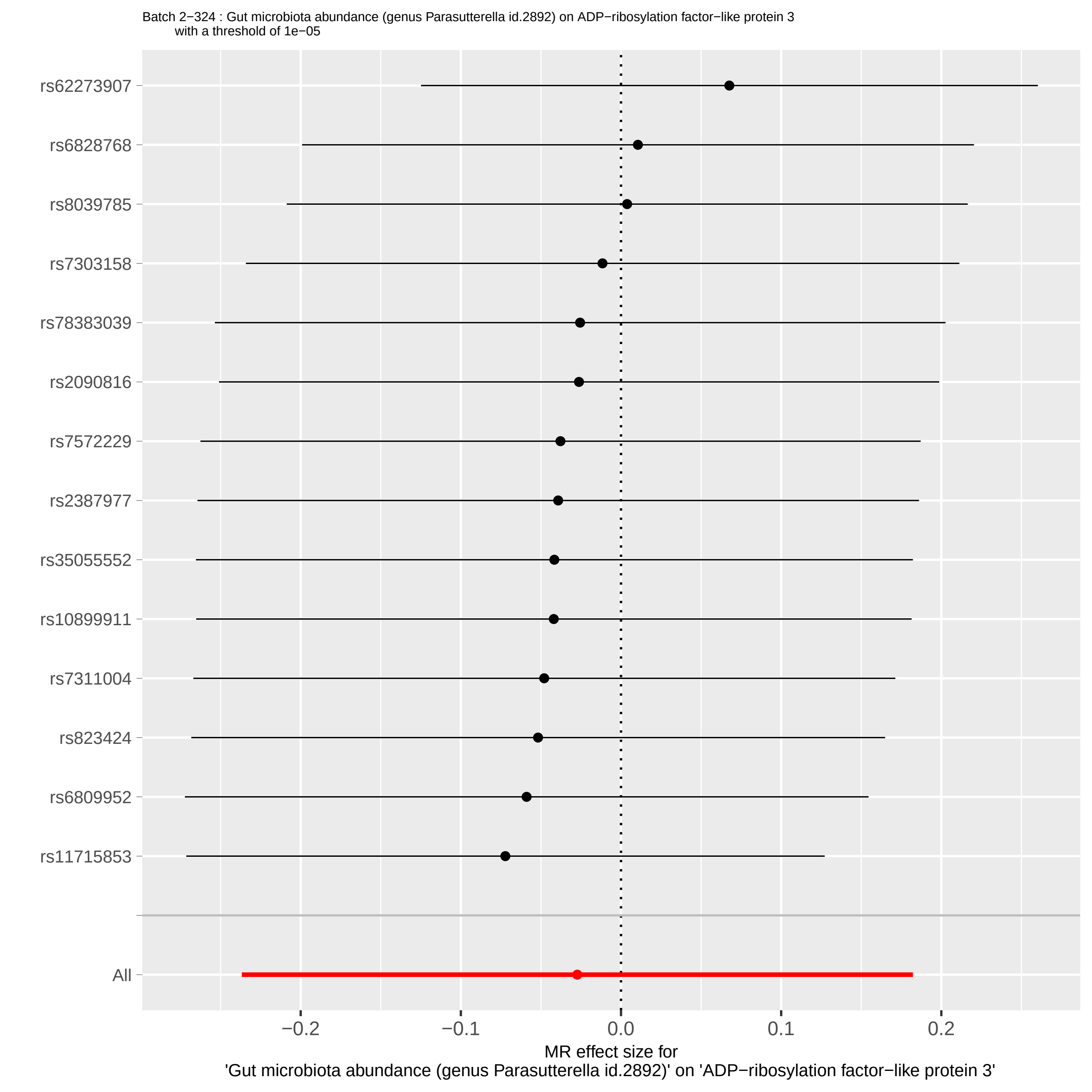
Batch 2−324 : Gut microbiota abundance (genus Parasutterella id.2892) on ADP−ribosylation factor−like protein 3
with a threshold of 1e−05
rs62273907
rs6828768
rs8039785
rs7303158
rs78383039
rs2090816
rs7572229
rs2387977
rs35055552
rs10899911
rs7311004
rs823424
rs6809952
rs11715853
All
−0.2
−0.1
0.0
0.1
0.2
MR effect size for
'Gut microbiota abundance (genus Parasutterella id.2892)' on 'ADP−ribosylation factor−like protein 3'

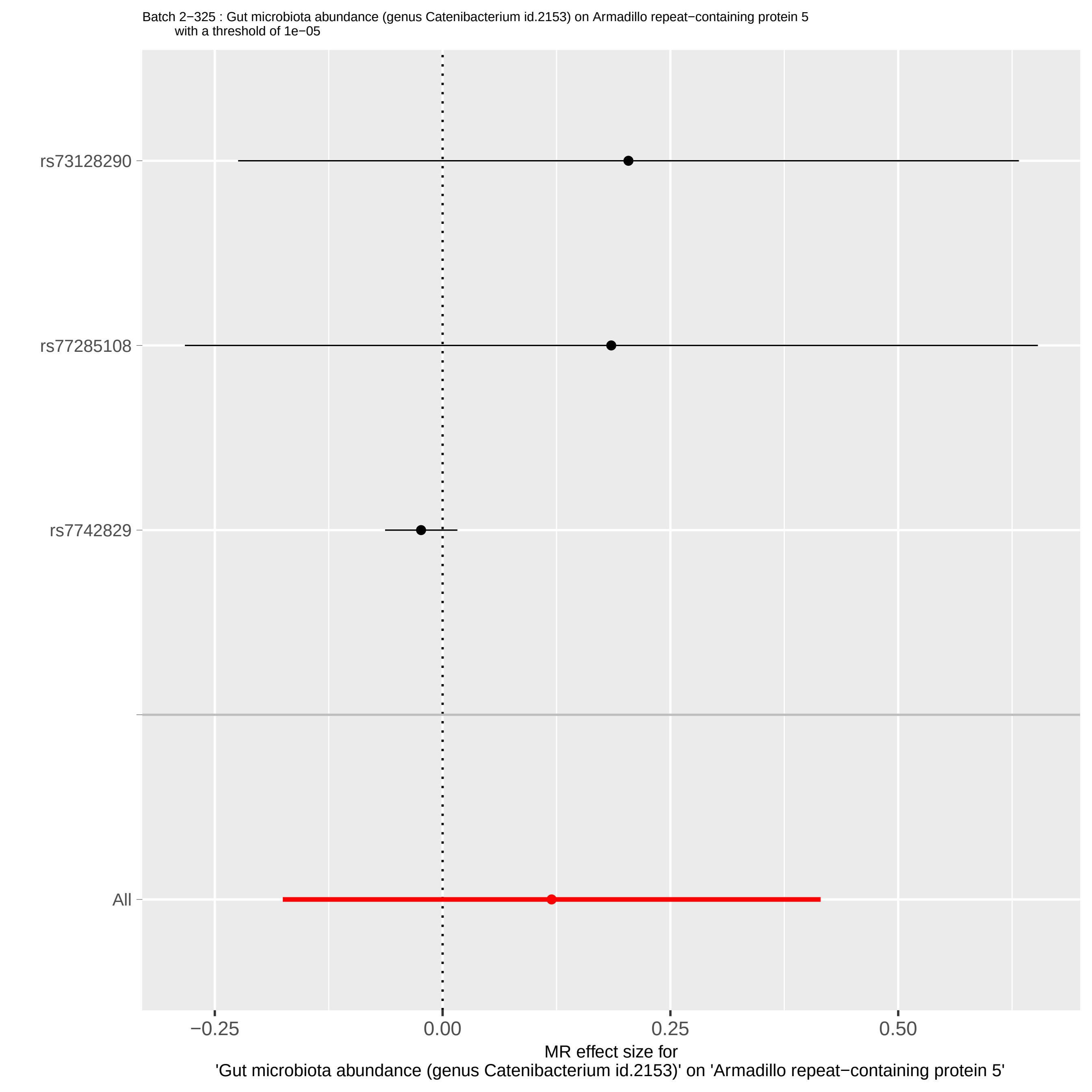
Batch 2−325 : Gut microbiota abundance (genus Catenibacterium id.2153) on Armadillo repeat−containing protein 5
with a threshold of 1e−05
rs73128290
rs77285108
rs7742829
All
−0.25
0.00
0.25
0.50
MR effect size for
'Gut microbiota abundance (genus Catenibacterium id.2153)' on 'Armadillo repeat−containing protein 5'

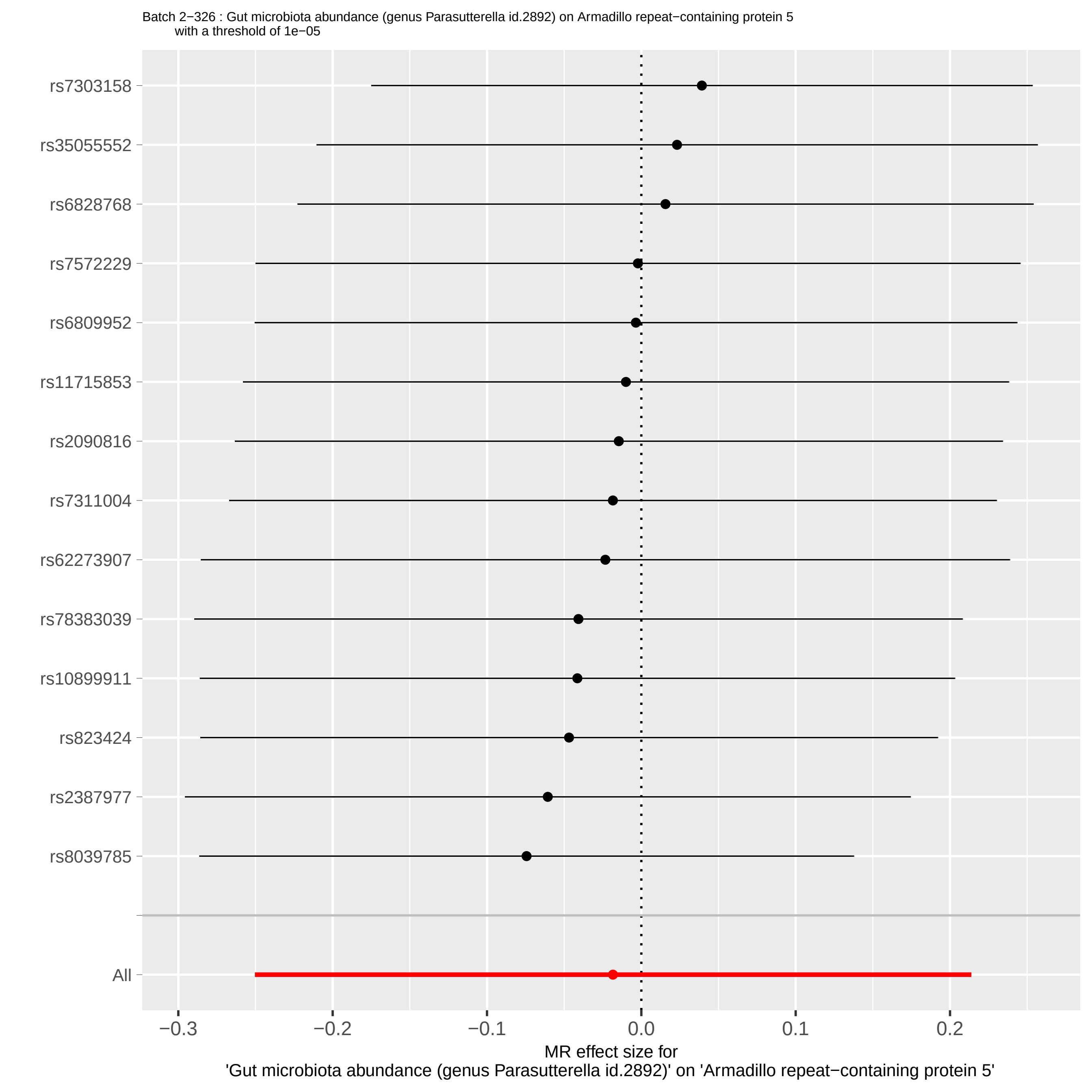

Batch 2−326 : Gut microbiota abundance (genus Parasutterella id.2892) on Armadillo repeat−containing protein 5
with a threshold of 1e−05
rs7303158
rs35055552
rs6828768
rs7572229
rs6809952
rs11715853
rs2090816
rs7311004
rs62273907
rs78383039
rs10899911
rs823424
rs2387977
rs8039785
All
−0.3
−0.2
−0.1
0.0
0.1
0.2
MR effect size for
'Gut microbiota abundance (genus Parasutterella id.2892)' on 'Armadillo repeat−containing protein 5'

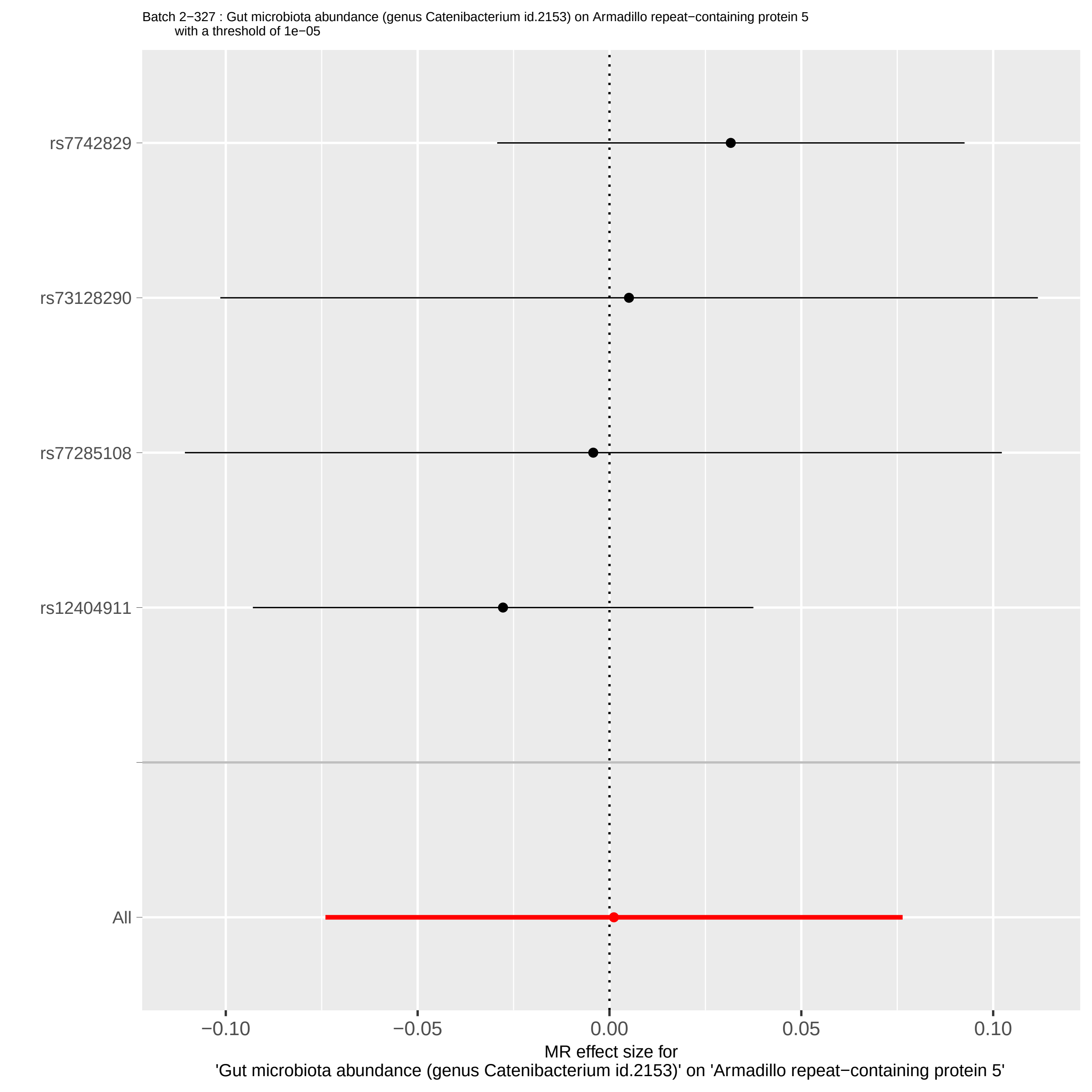

Batch 2−327 : Gut microbiota abundance (genus Catenibacterium id.2153) on Armadillo repeat−containing protein 5
with a threshold of 1e−05
rs7742829
rs73128290
rs77285108
rs12404911
All
−0.10
−0.05
0.00
0.05
0.10
MR effect size for
'Gut microbiota abundance (genus Catenibacterium id.2153)' on 'Armadillo repeat−containing protein 5'

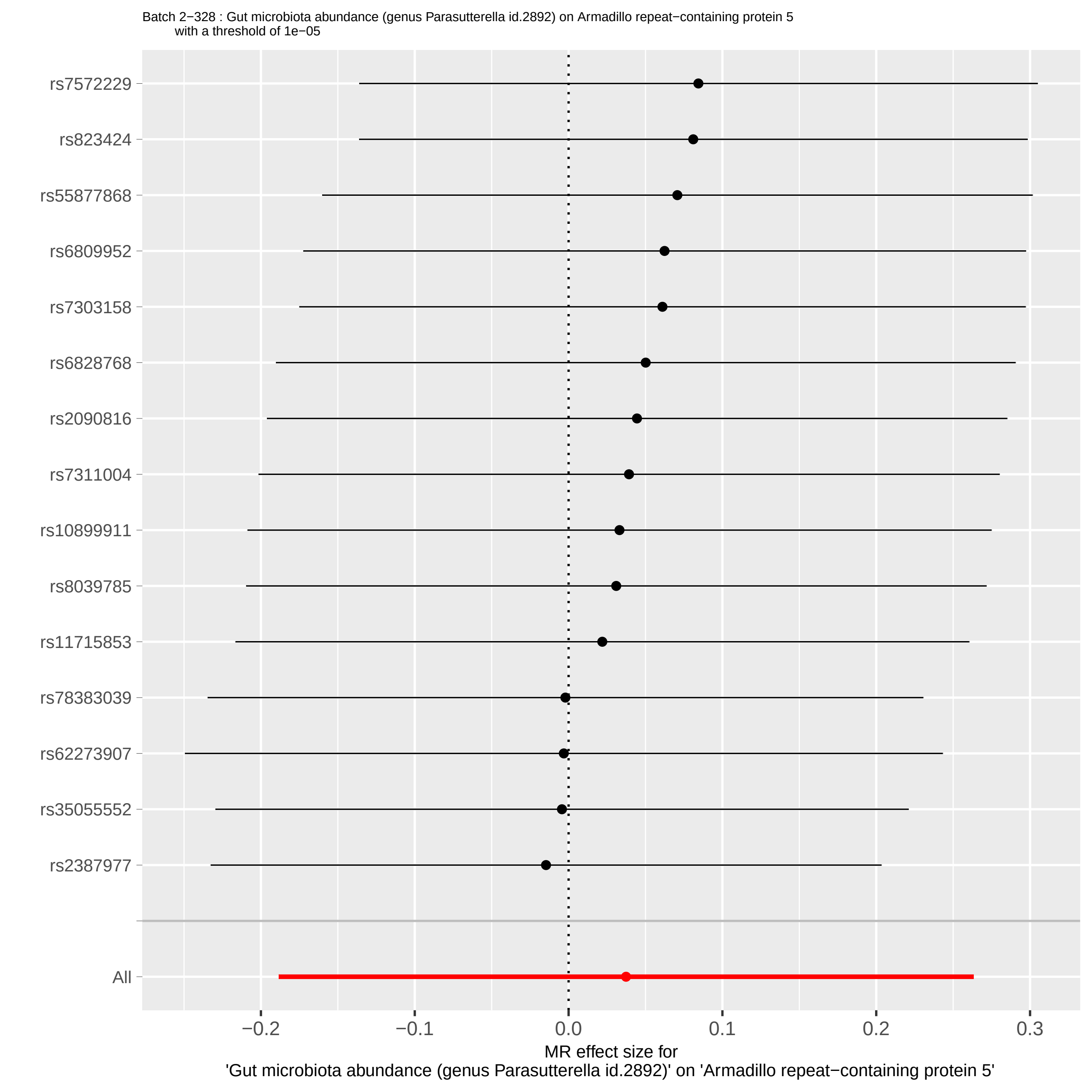

Batch 2−328 : Gut microbiota abundance (genus Parasutterella id.2892) on Armadillo repeat−containing protein 5
with a threshold of 1e−05
rs7572229
rs823424
rs55877868
rs6809952
rs7303158
rs6828768
rs2090816
rs7311004
rs10899911
rs8039785
rs11715853
rs78383039
rs62273907
rs35055552
rs2387977
All
−0.2
−0.1
0.0
0.1
0.2
0.3
MR effect size for
'Gut microbiota abundance (genus Parasutterella id.2892)' on 'Armadillo repeat−containing protein 5'

Batch 2−329 : Gut microbiota abundance (genus Catenibacterium id.2153) on Armadillo repeat−containing protein 5
with a threshold of 1e−05

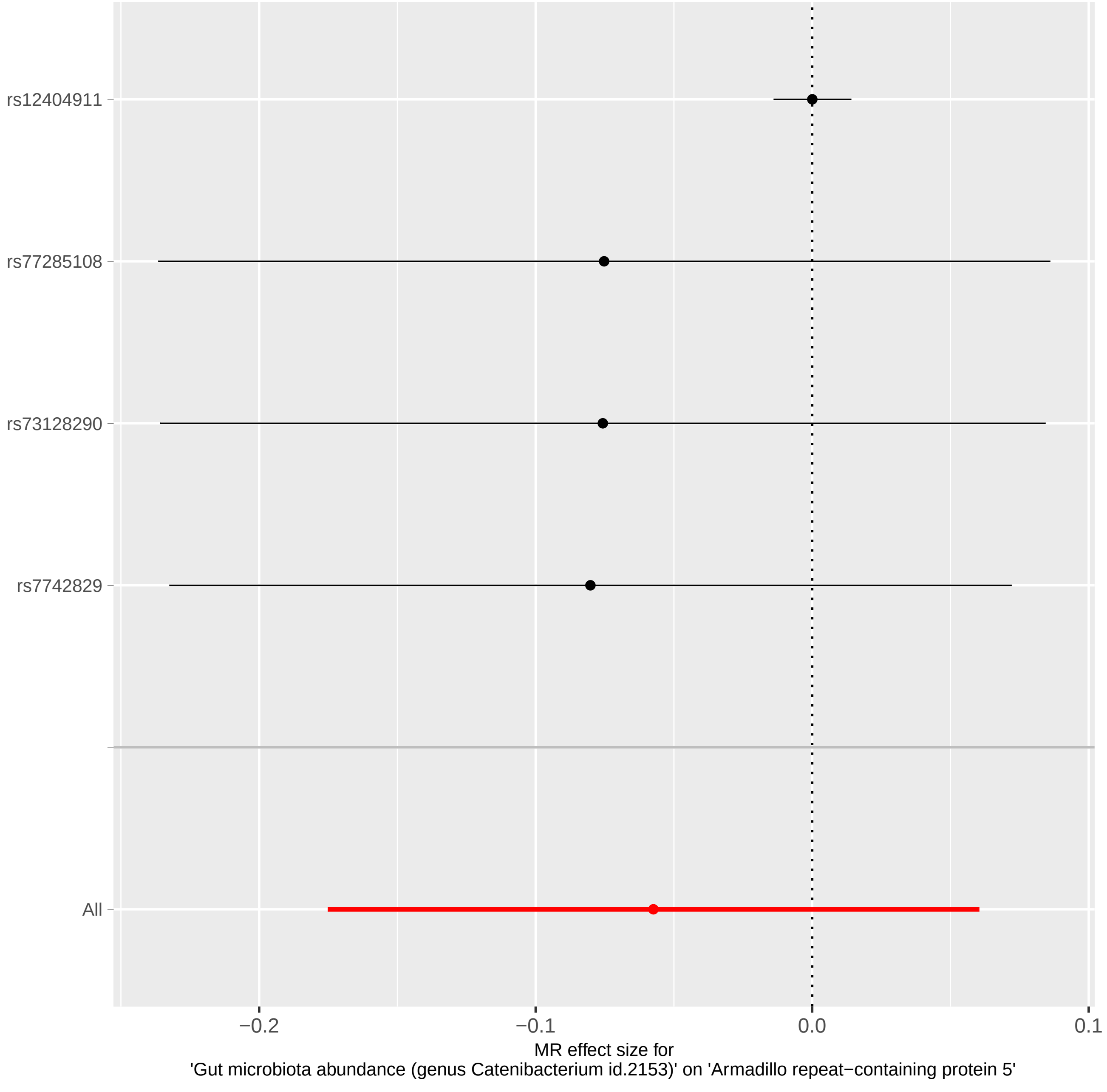

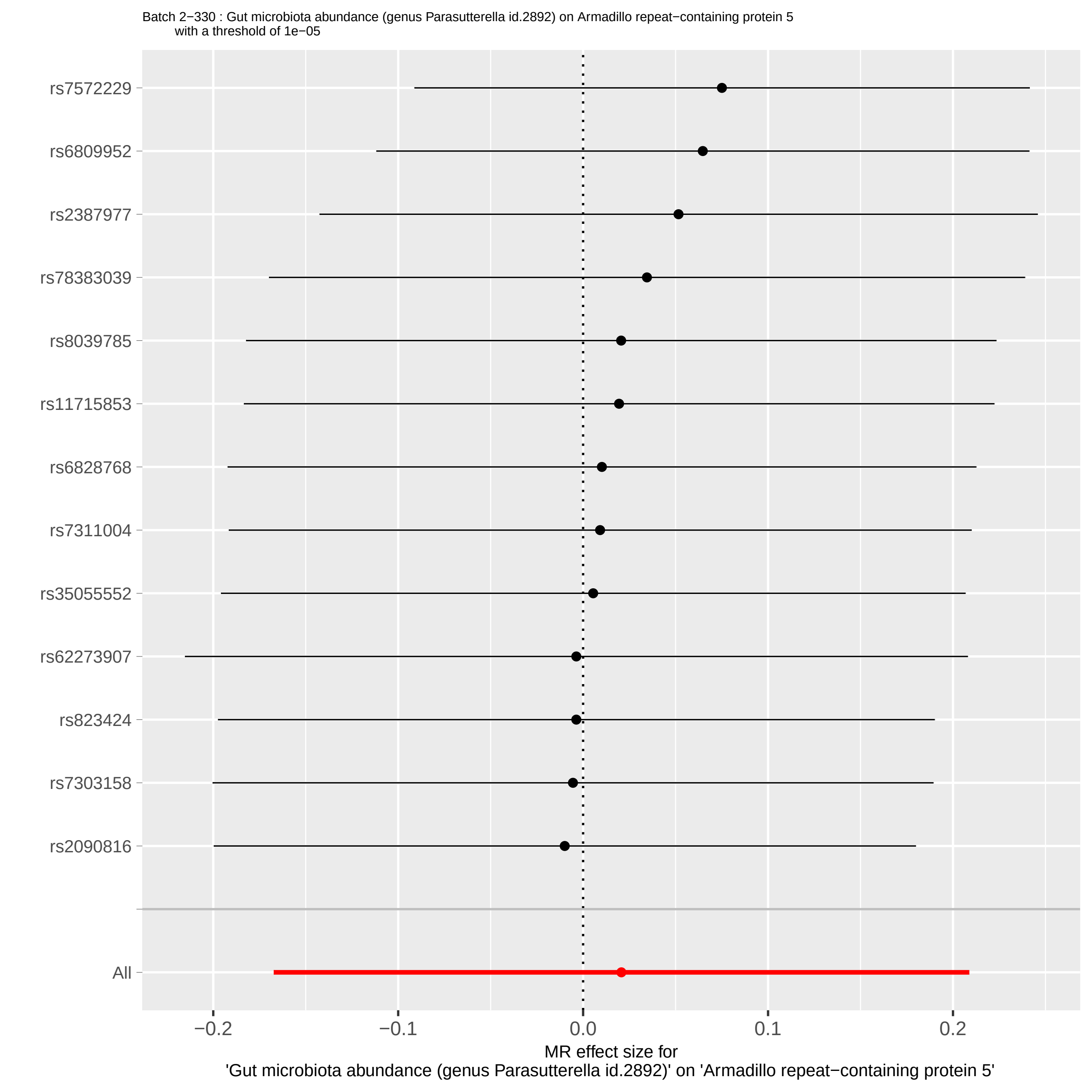

Batch 2−330 : Gut microbiota abundance (genus Parasutterella id.2892) on Armadillo repeat−containing protein 5
with a threshold of 1e−05
rs7572229
rs6809952
rs2387977
rs78383039
rs8039785
rs11715853
rs6828768
rs7311004
rs35055552
rs62273907
rs823424
rs7303158
rs2090816
All
−0.2
−0.1
0.0
0.1
0.2
MR effect size for
'Gut microbiota abundance (genus Parasutterella id.2892)' on 'Armadillo repeat−containing protein 5'

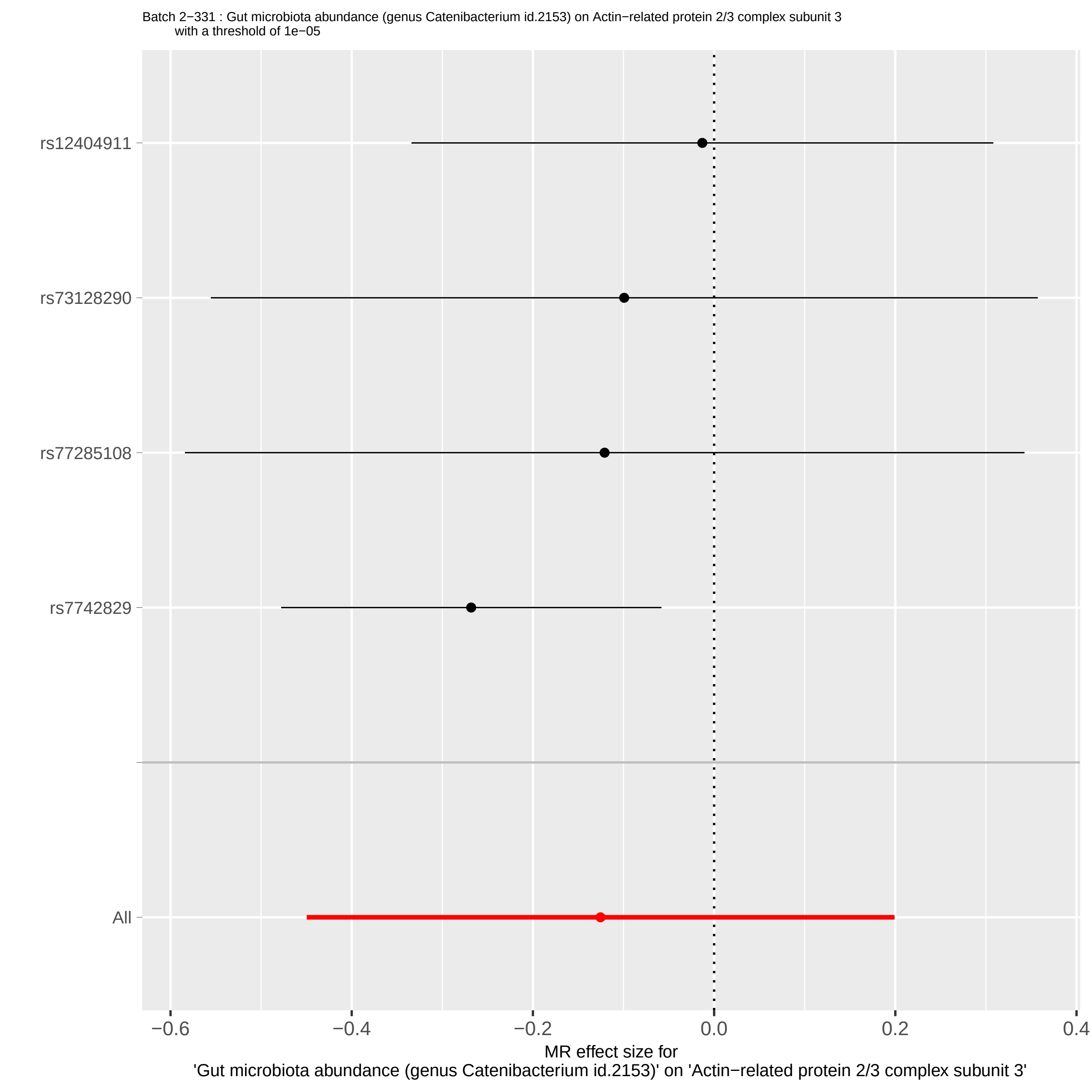
Batch 2−331 : Gut microbiota abundance (genus Catenibacterium id.2153) on Actin−related protein 2/3 complex subunit 3
with a threshold of 1e−05
rs12404911
rs73128290
rs77285108
rs7742829
All
−0.6
−0.4
−0.2
0.0
0.2
0.4
MR effect size for
'Gut microbiota abundance (genus Catenibacterium id.2153)' on 'Actin−related protein 2/3 complex subunit 3'

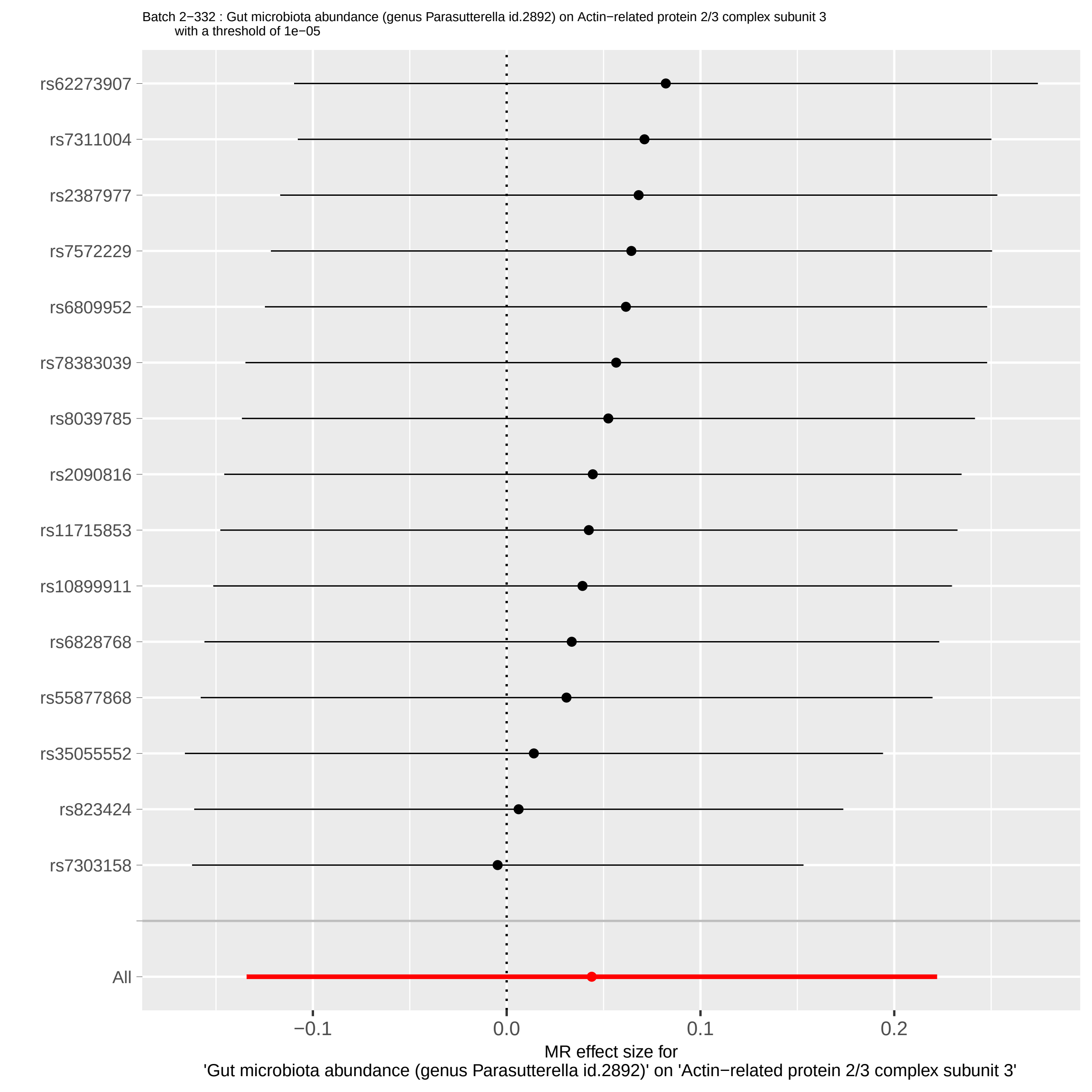

Batch 2−332 : Gut microbiota abundance (genus Parasutterella id.2892) on Actin−related protein 2/3 complex subunit 3
with a threshold of 1e−05
rs62273907
rs7311004
rs2387977
rs7572229
rs6809952
rs78383039
rs8039785
rs2090816
rs11715853
rs10899911
rs6828768
rs55877868
rs35055552
rs823424
rs7303158
All
−0.1
0.0
0.1
0.2
MR effect size for
'Gut microbiota abundance (genus Parasutterella id.2892)' on 'Actin−related protein 2/3 complex subunit 3'

Batch 2−333 : Gut microbiota abundance (genus Catenibacterium id.2153) on cAMP−regulated phosphoprotein 19
with a threshold of 1e−05

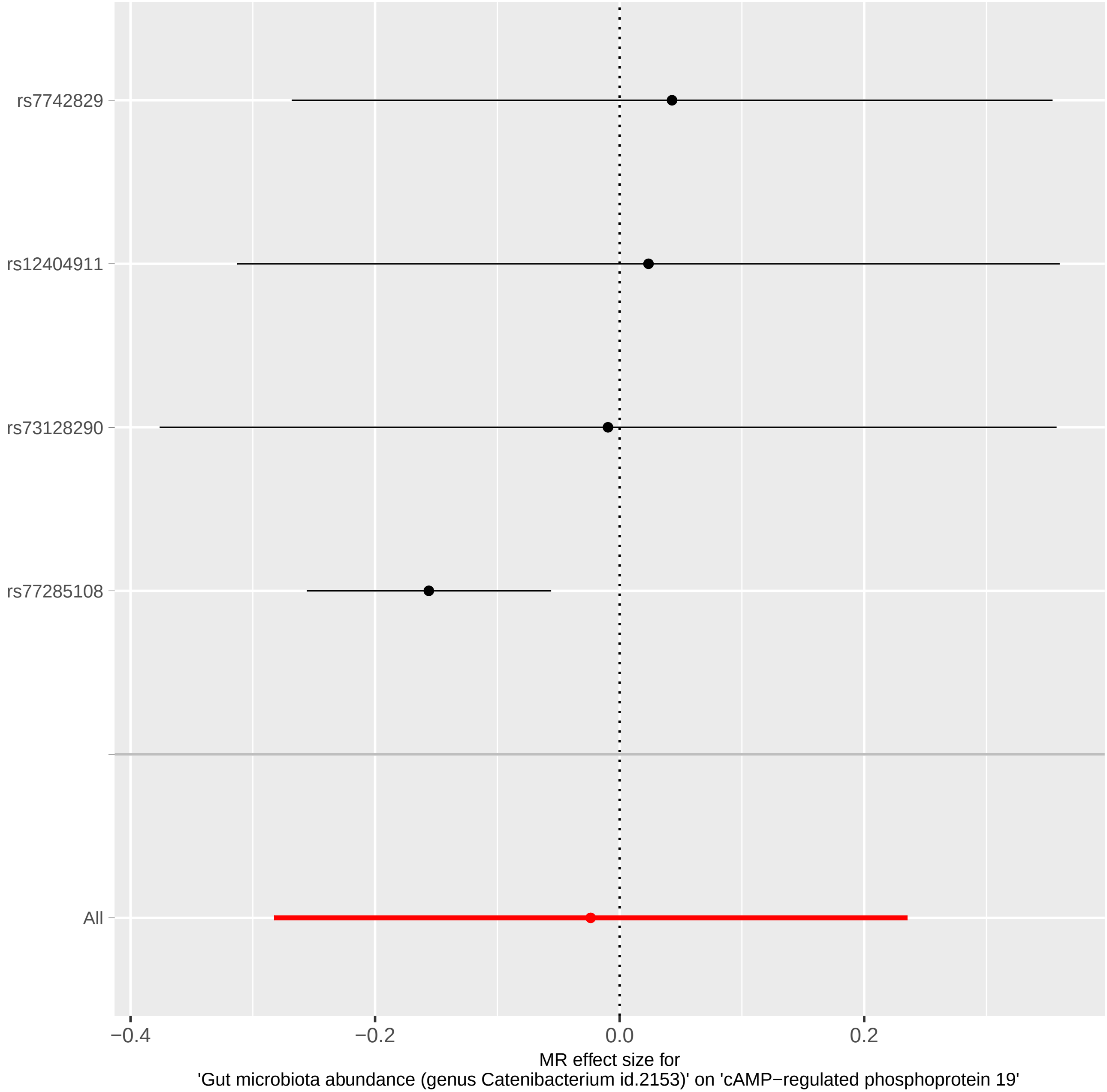

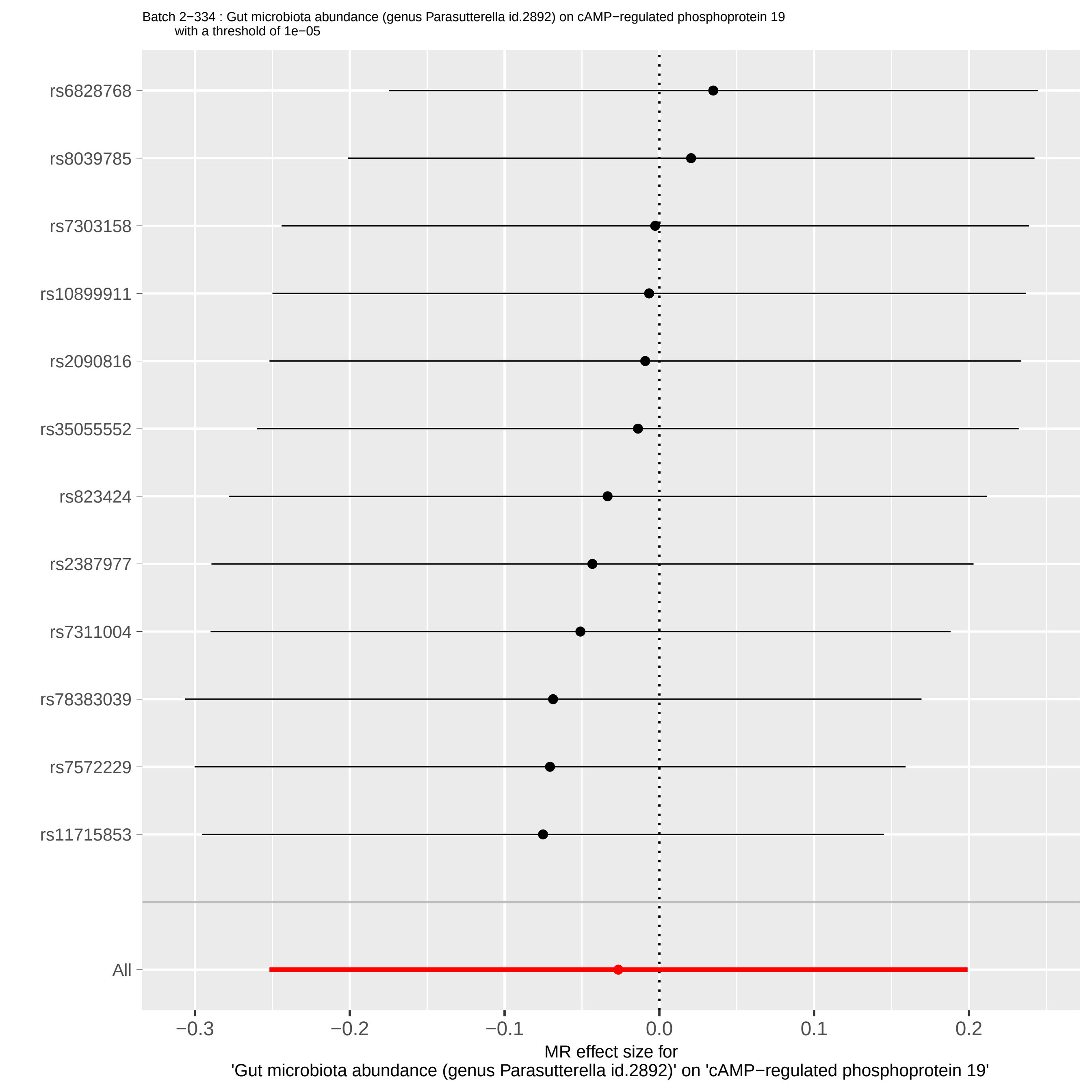

Batch 2−334 : Gut microbiota abundance (genus Parasutterella id.2892) on cAMP−regulated phosphoprotein 19
with a threshold of 1e−05
rs6828768
rs8039785
rs7303158
rs10899911
rs2090816
rs35055552
rs823424
rs2387977
rs7311004
rs78383039
rs7572229
rs11715853
All
−0.3
−0.2
−0.1
0.0
0.1
0.2
MR effect size for
'Gut microbiota abundance (genus Parasutterella id.2892)' on 'cAMP−regulated phosphoprotein 19'

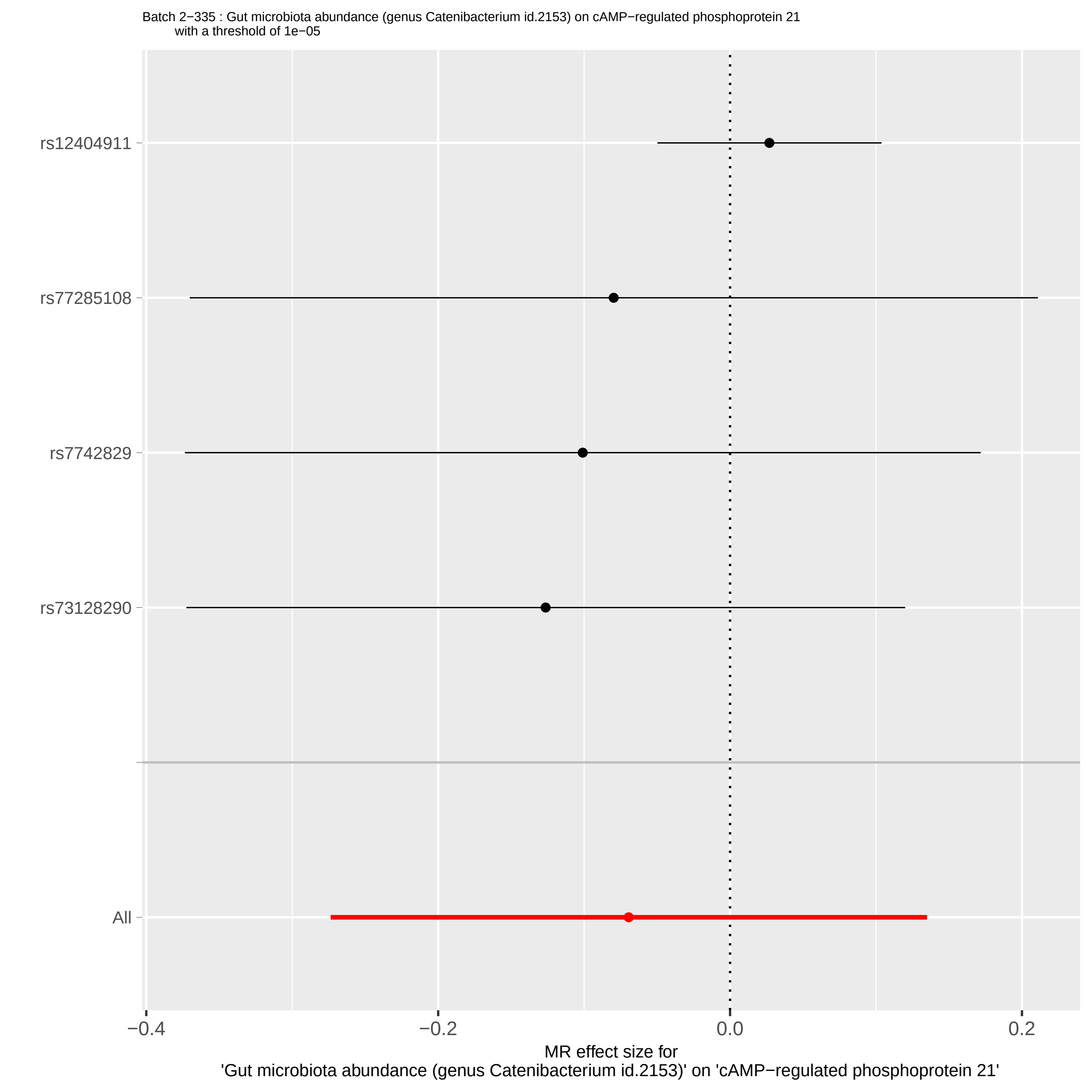
Batch 2−335 : Gut microbiota abundance (genus Catenibacterium id.2153) on cAMP−regulated phosphoprotein 21
with a threshold of 1e−05
rs12404911
rs77285108
rs7742829
rs73128290
All
−0.4
−0.2
0.0
0.2
MR effect size for
'Gut microbiota abundance (genus Catenibacterium id.2153)' on 'cAMP−regulated phosphoprotein 21'

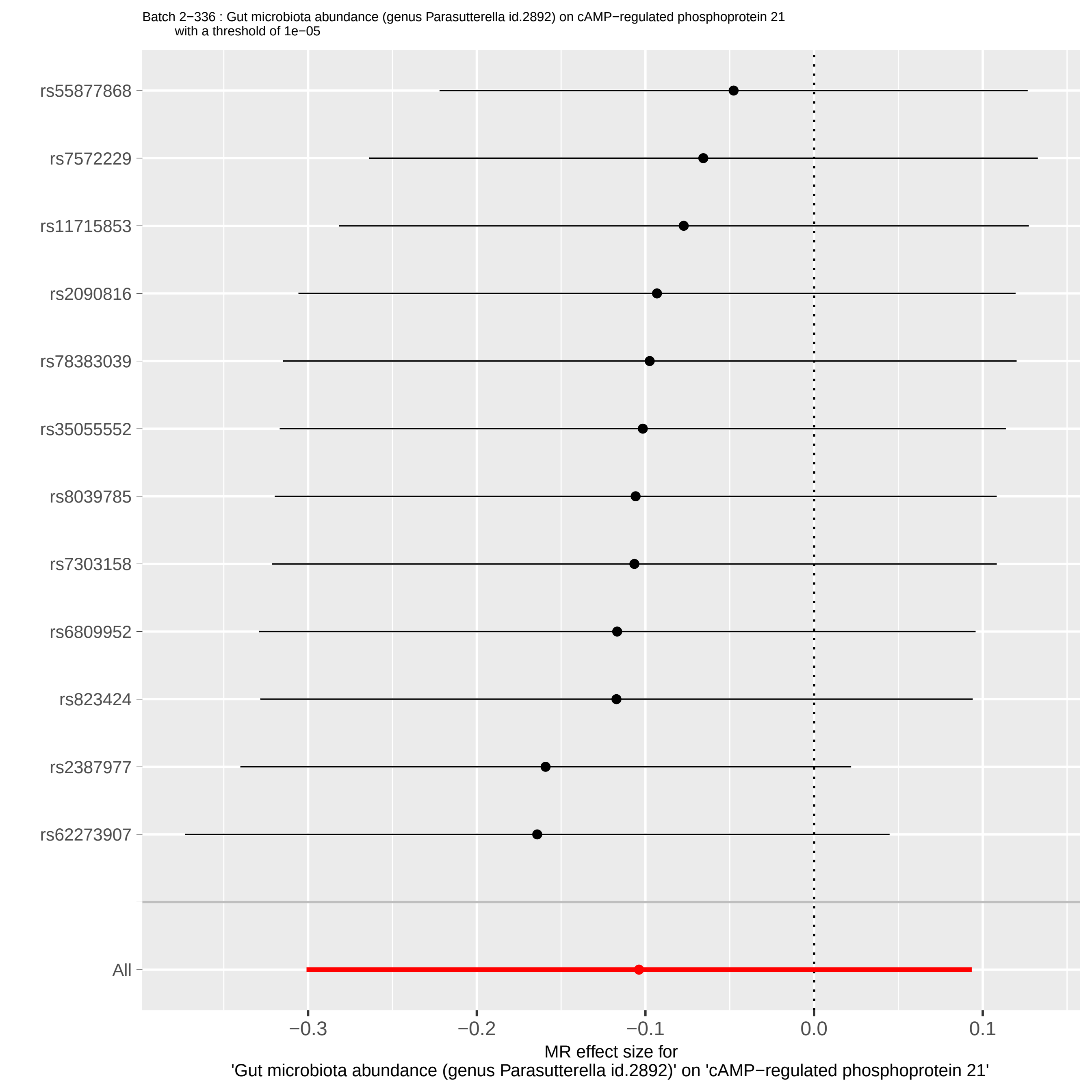
Batch 2−336 : Gut microbiota abundance (genus Parasutterella id.2892) on cAMP−regulated phosphoprotein 21
with a threshold of 1e−05
rs55877868
rs7572229
rs11715853
rs2090816
rs78383039
rs35055552
rs8039785
rs7303158
rs6809952
rs823424
rs2387977
rs62273907
All
−0.3
−0.2
−0.1
0.0
0.1
MR effect size for
'Gut microbiota abundance (genus Parasutterella id.2892)' on 'cAMP−regulated phosphoprotein 21'

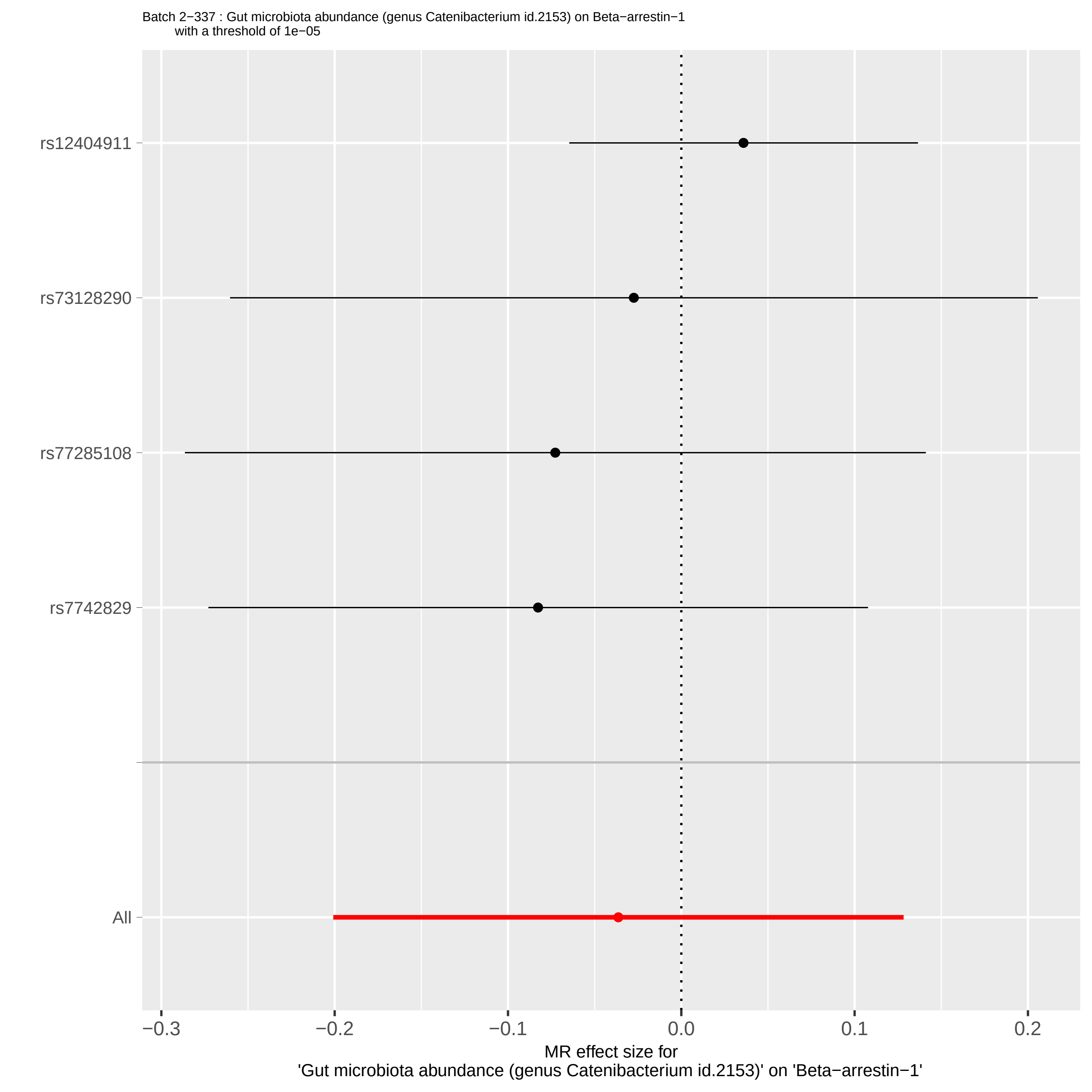

Batch 2−337 : Gut microbiota abundance (genus Catenibacterium id.2153) on Beta−arrestin−1
with a threshold of 1e−05
rs12404911
rs73128290
rs77285108
rs7742829
All
−0.3
−0.2
−0.1
0.0
0.1
0.2
MR effect size for
'Gut microbiota abundance (genus Catenibacterium id.2153)' on 'Beta−arrestin−1'

Batch 2−338 : Gut microbiota abundance (genus Parasutterella id.2892) on Beta−arrestin−1
with a threshold of 1e−05

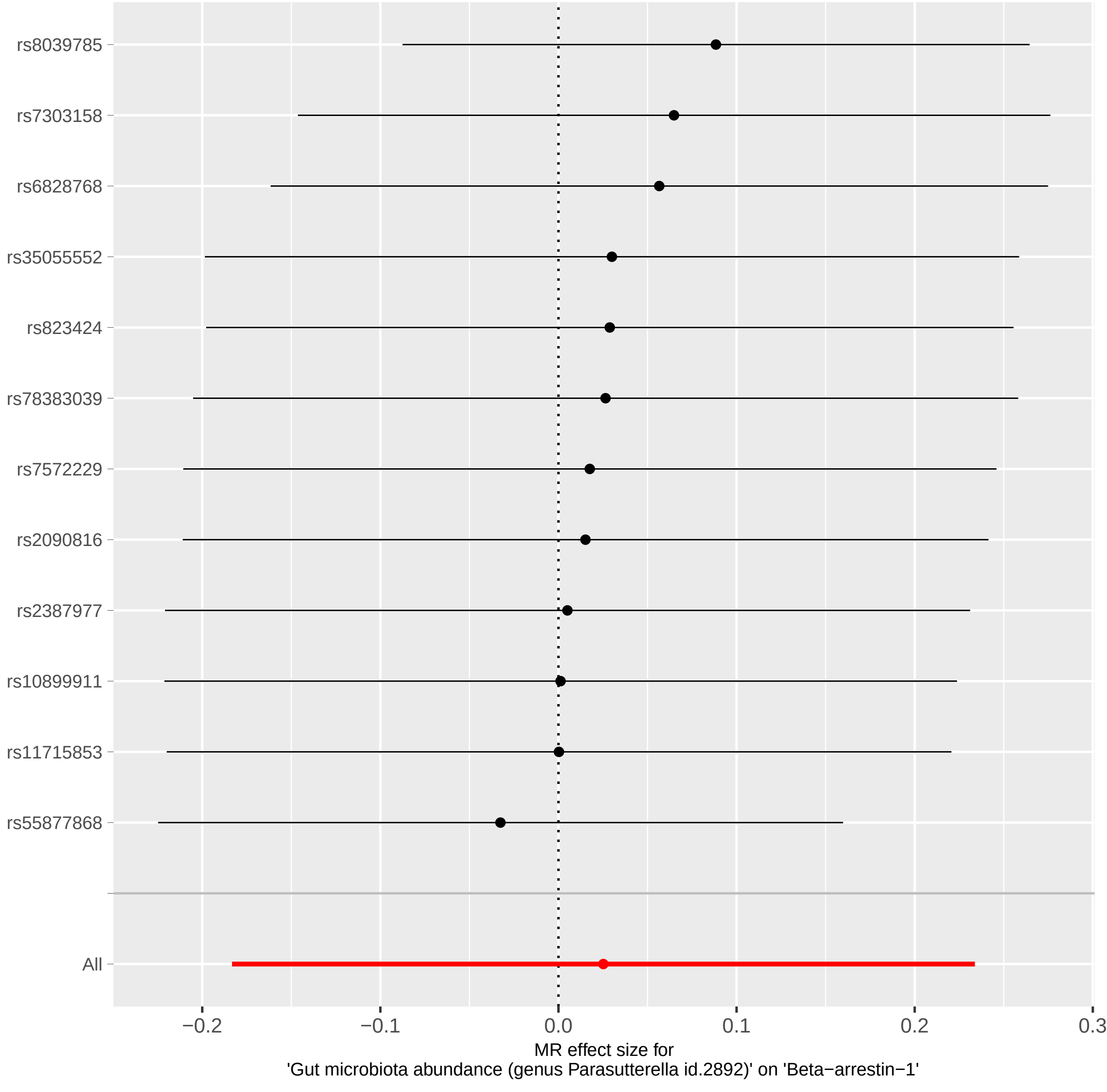

Batch 2−339 : Gut microbiota abundance (genus Catenibacterium id.2153) on Arrestin domain−containing protein 3
with a threshold of 1e−05

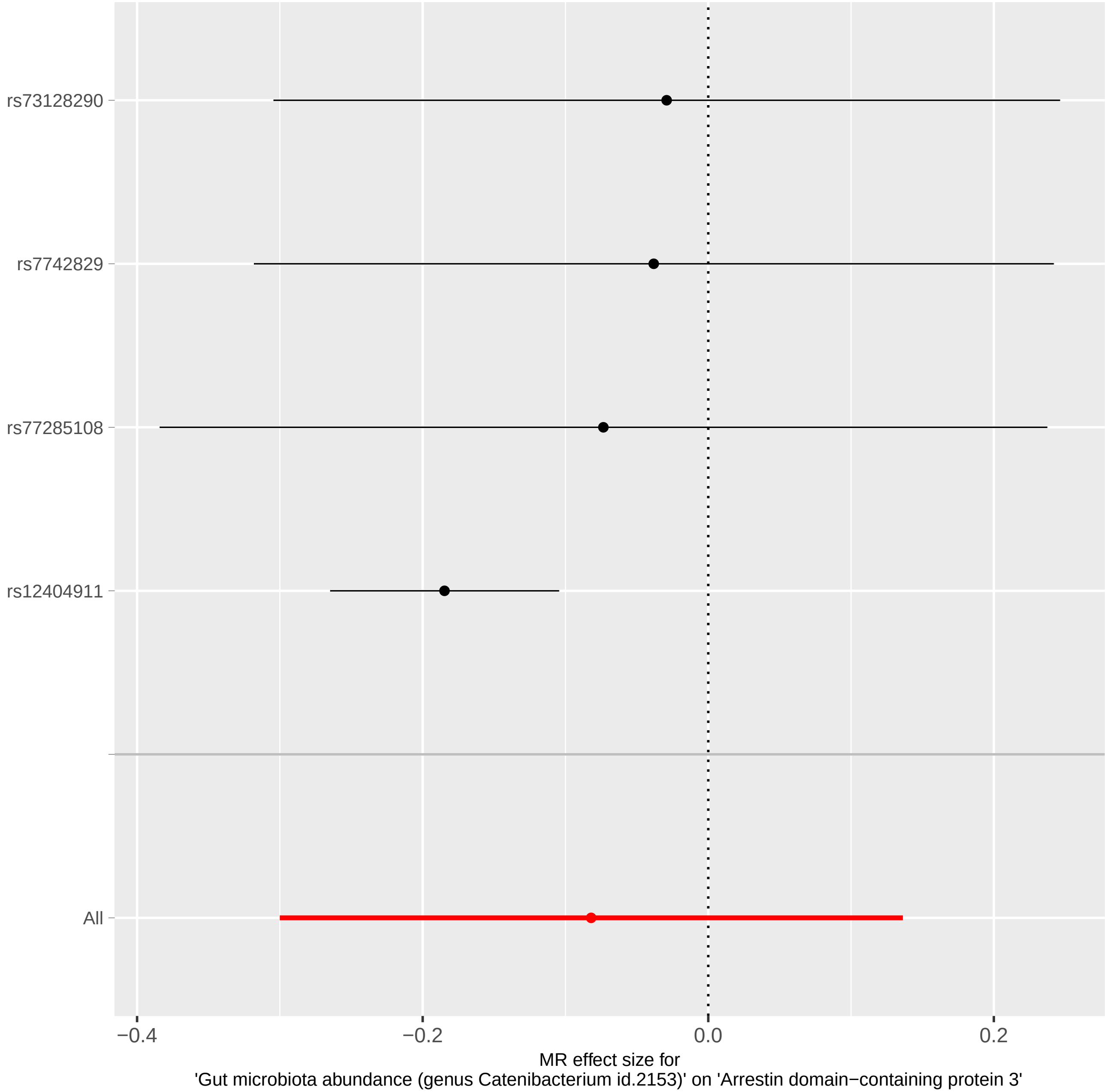

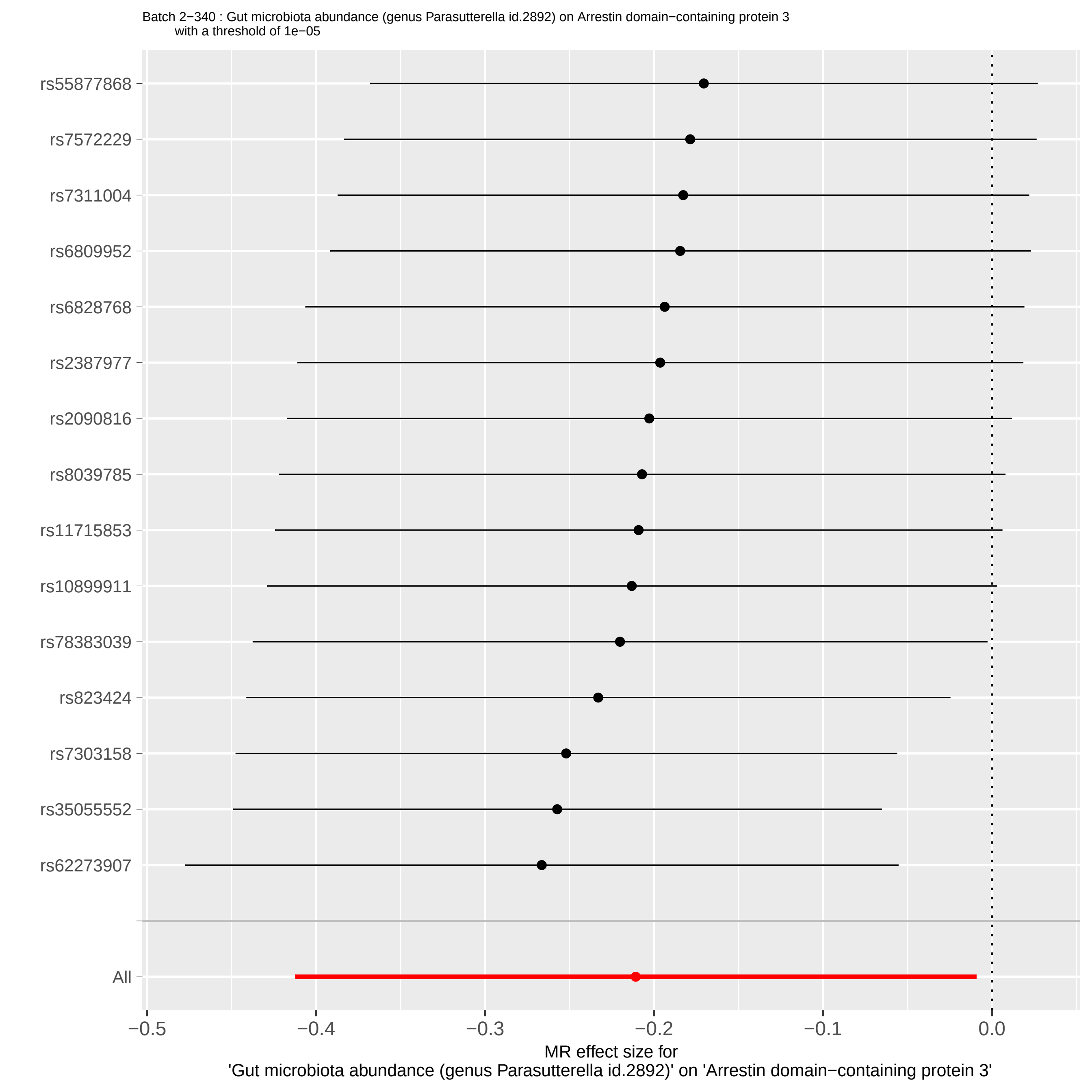

Batch 2−340 : Gut microbiota abundance (genus Parasutterella id.2892) on Arrestin domain−containing protein 3
with a threshold of 1e−05
rs55877868
rs7572229
rs7311004
rs6809952
rs6828768
rs2387977
rs2090816
rs8039785
rs11715853
rs10899911
rs78383039
rs823424
rs7303158
rs35055552
rs62273907
All
−0.5
−0.4
−0.3
−0.2
−0.1
0.0
MR effect size for
'Gut microbiota abundance (genus Parasutterella id.2892)' on 'Arrestin domain−containing protein 3'

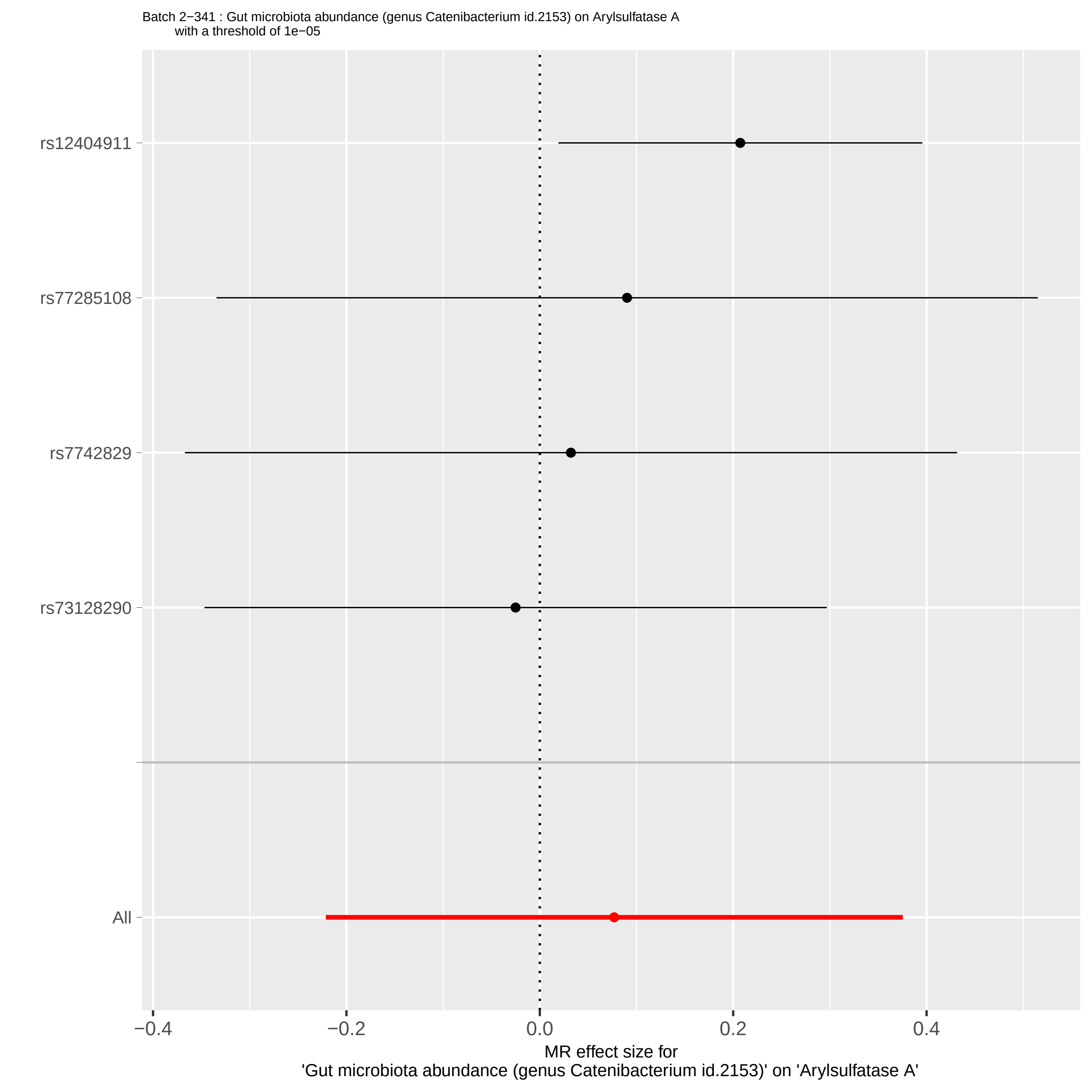
Batch 2−341 : Gut microbiota abundance (genus Catenibacterium id.2153) on Arylsulfatase A
with a threshold of 1e−05
rs12404911
rs77285108
rs7742829
rs73128290
All
−0.4
−0.2
0.0
0.2
0.4
MR effect size for
'Gut microbiota abundance (genus Catenibacterium id.2153)' on 'Arylsulfatase A'

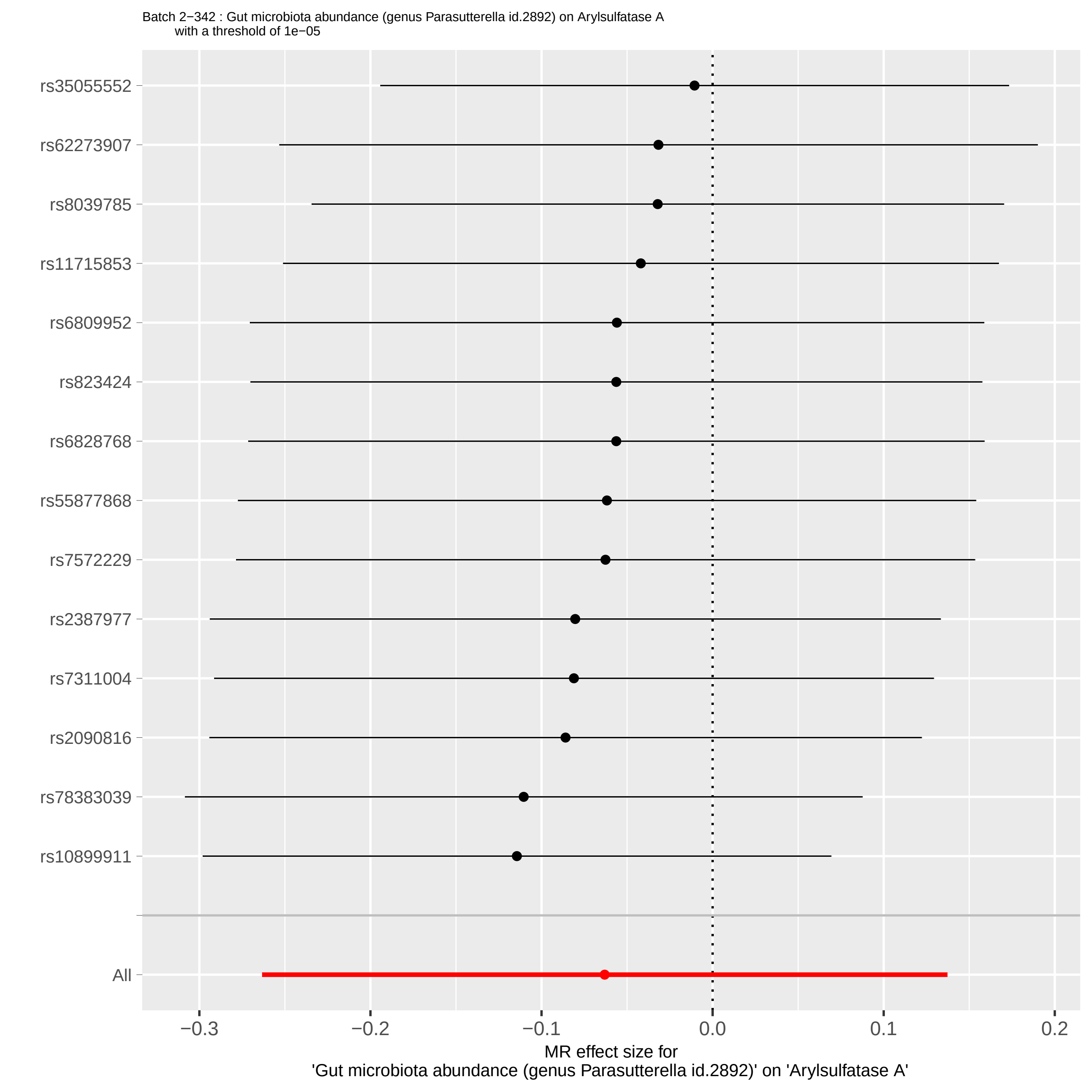

Batch 2−342 : Gut microbiota abundance (genus Parasutterella id.2892) on Arylsulfatase A
with a threshold of 1e−05
rs35055552
rs62273907
rs8039785
rs11715853
rs6809952
rs823424
rs6828768
rs55877868
rs7572229
rs2387977
rs7311004
rs2090816
rs78383039
rs10899911
All
−0.3
−0.2
−0.1
0.0
0.1
0.2
MR effect size for
'Gut microbiota abundance (genus Parasutterella id.2892)' on 'Arylsulfatase A'

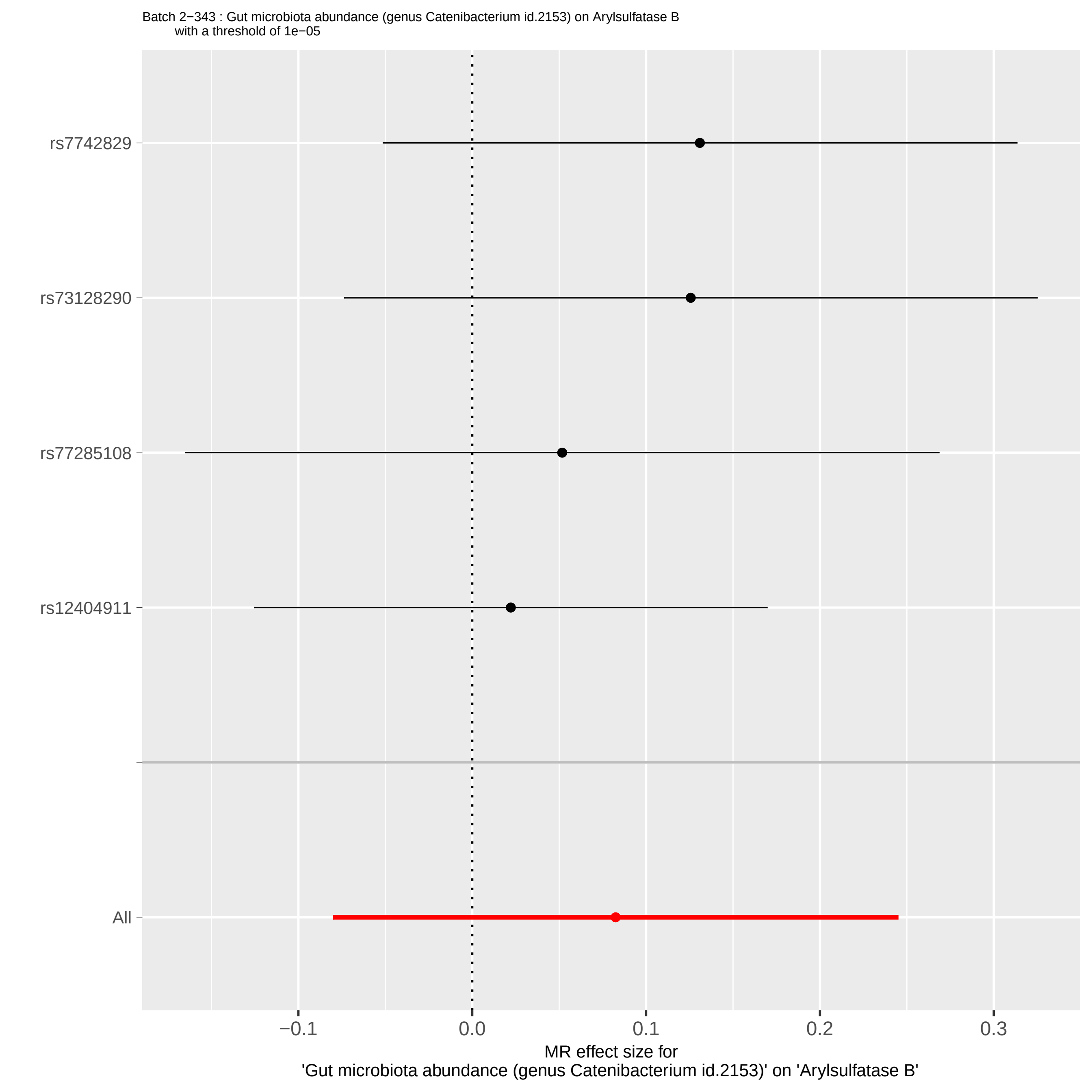
Batch 2−343 : Gut microbiota abundance (genus Catenibacterium id.2153) on Arylsulfatase B
with a threshold of 1e−05
rs7742829
rs73128290
rs77285108
rs12404911
All
−0.1
0.0
0.1
0.2
0.3
MR effect size for
'Gut microbiota abundance (genus Catenibacterium id.2153)' on 'Arylsulfatase B'

Batch 2−344 : Gut microbiota abundance (genus Parasutterella id.2892) on Arylsulfatase B
with a threshold of 1e−05

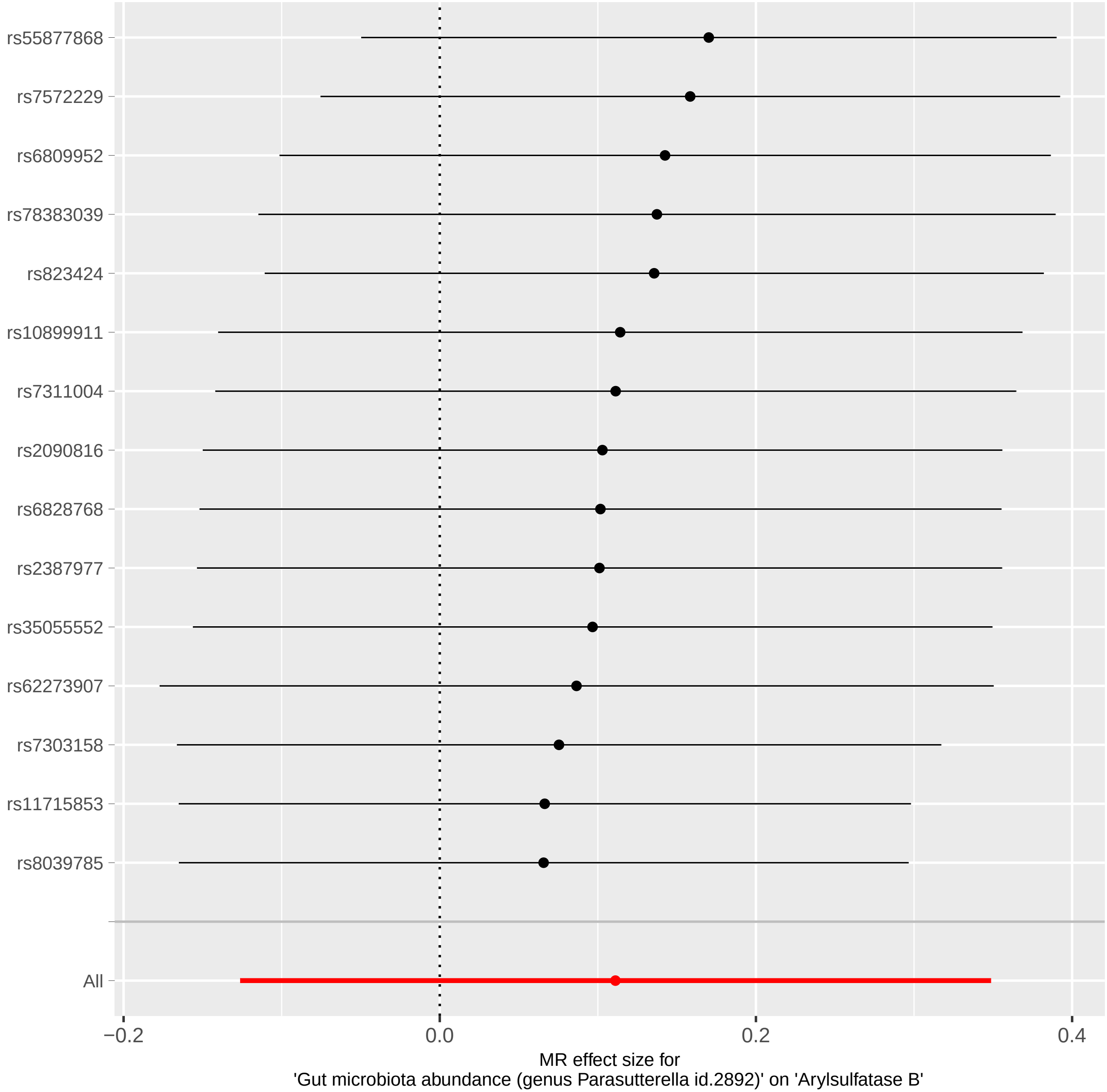

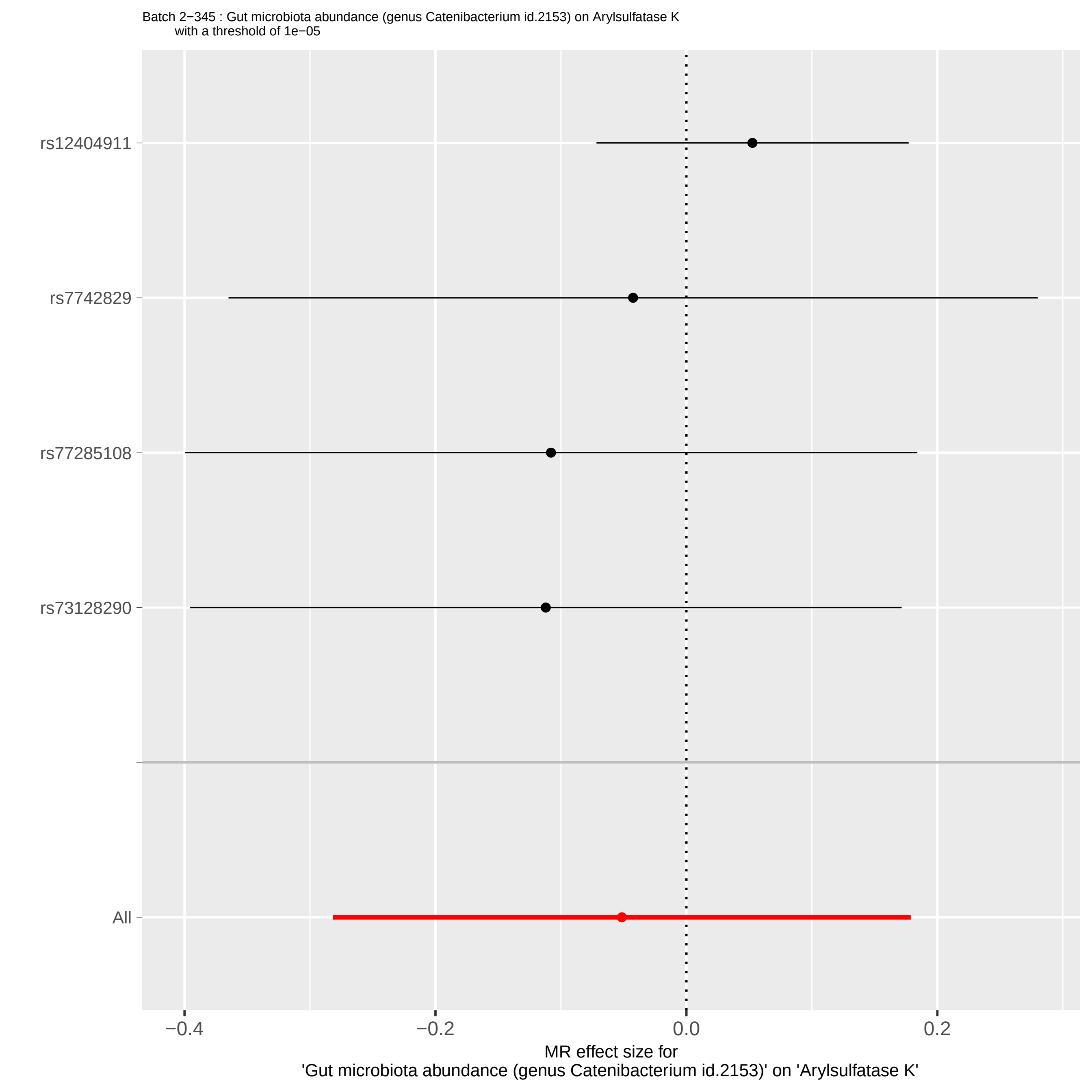

Batch 2−345 : Gut microbiota abundance (genus Catenibacterium id.2153) on Arylsulfatase K
with a threshold of 1e−05
rs12404911
rs7742829
rs77285108
rs73128290
All
−0.4
−0.2
0.0
0.2
MR effect size for
'Gut microbiota abundance (genus Catenibacterium id.2153)' on 'Arylsulfatase K'

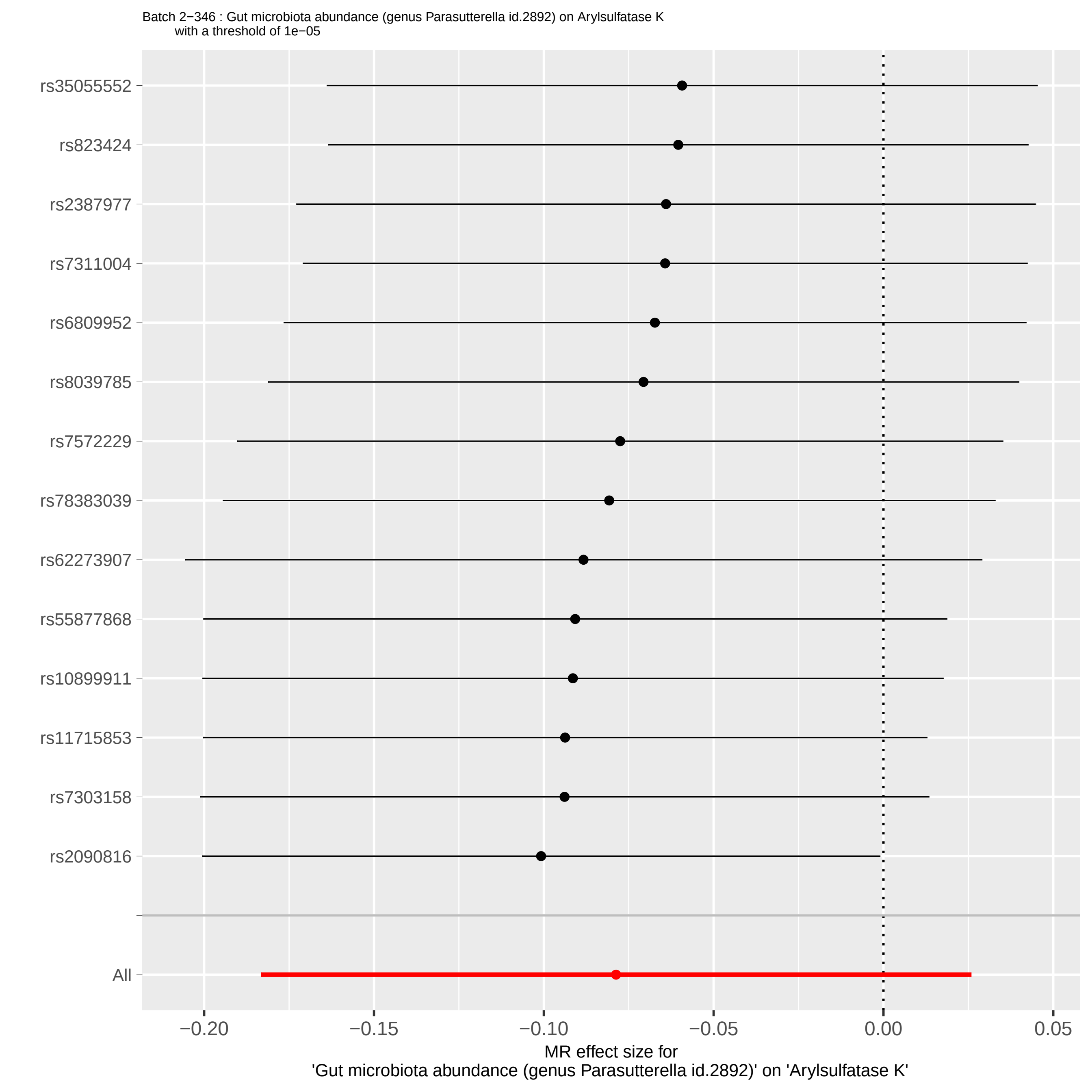

Batch 2−346 : Gut microbiota abundance (genus Parasutterella id.2892) on Arylsulfatase K
with a threshold of 1e−05
rs35055552
rs823424
rs2387977
rs7311004
rs6809952
rs8039785
rs7572229
rs78383039
rs62273907
rs55877868
rs10899911
rs11715853
rs7303158
rs2090816
All
−0.20
−0.15
−0.10
−0.05
0.00
0.05
MR effect size for
'Gut microbiota abundance (genus Parasutterella id.2892)' on 'Arylsulfatase K'

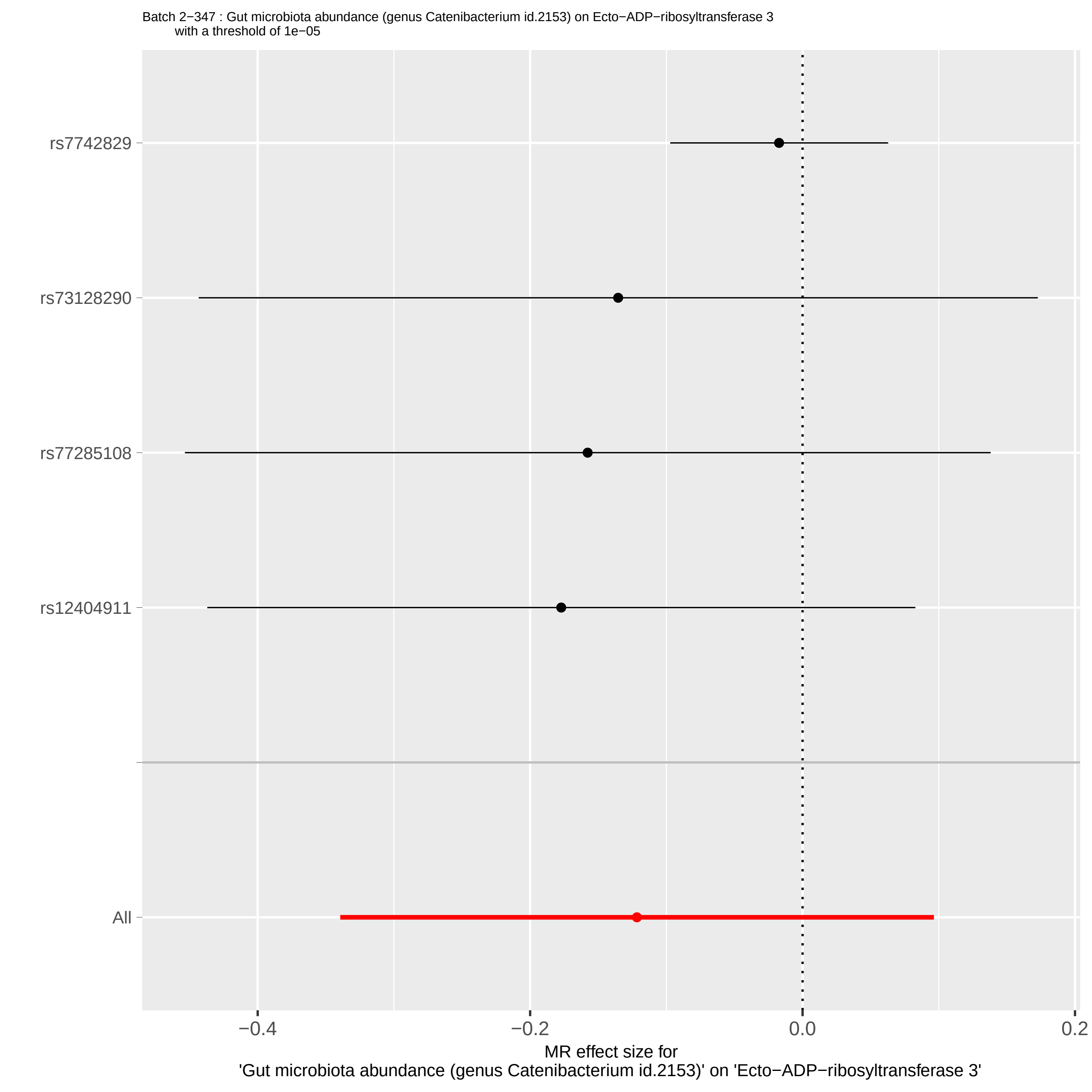
Batch 2−347 : Gut microbiota abundance (genus Catenibacterium id.2153) on Ecto−ADP−ribosyltransferase 3
with a threshold of 1e−05
rs7742829
rs73128290
rs77285108
rs12404911
All
−0.4
−0.2
0.0
0.2
MR effect size for
'Gut microbiota abundance (genus Catenibacterium id.2153)' on 'Ecto−ADP−ribosyltransferase 3'

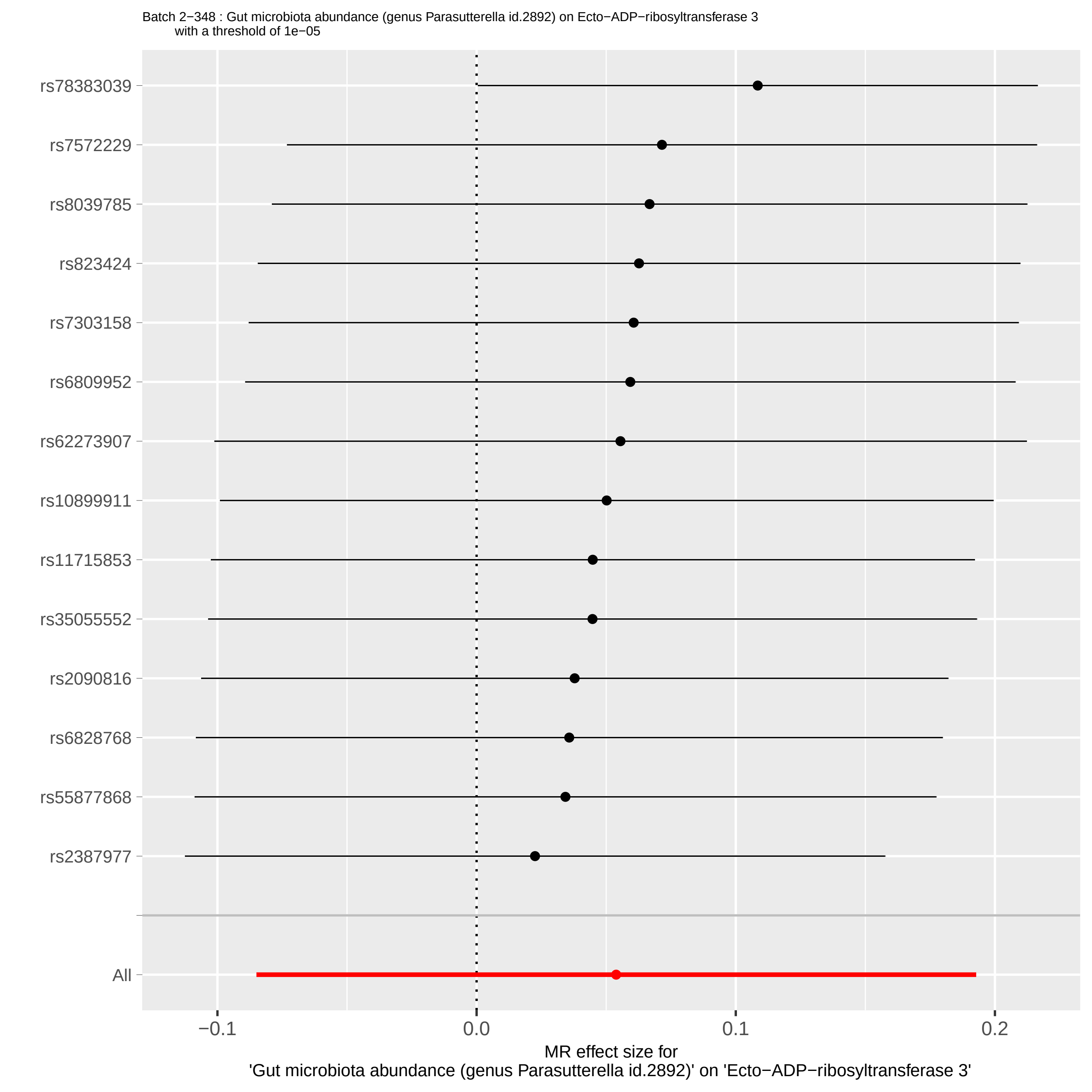

Batch 2−348 : Gut microbiota abundance (genus Parasutterella id.2892) on Ecto−ADP−ribosyltransferase 3
with a threshold of 1e−05
rs78383039
rs7572229
rs8039785
rs823424
rs7303158
rs6809952
rs62273907
rs10899911
rs11715853
rs35055552
rs2090816
rs6828768
rs55877868
rs2387977
All
−0.1
0.0
0.1
0.2
MR effect size for
'Gut microbiota abundance (genus Parasutterella id.2892)' on 'Ecto−ADP−ribosyltransferase 3'

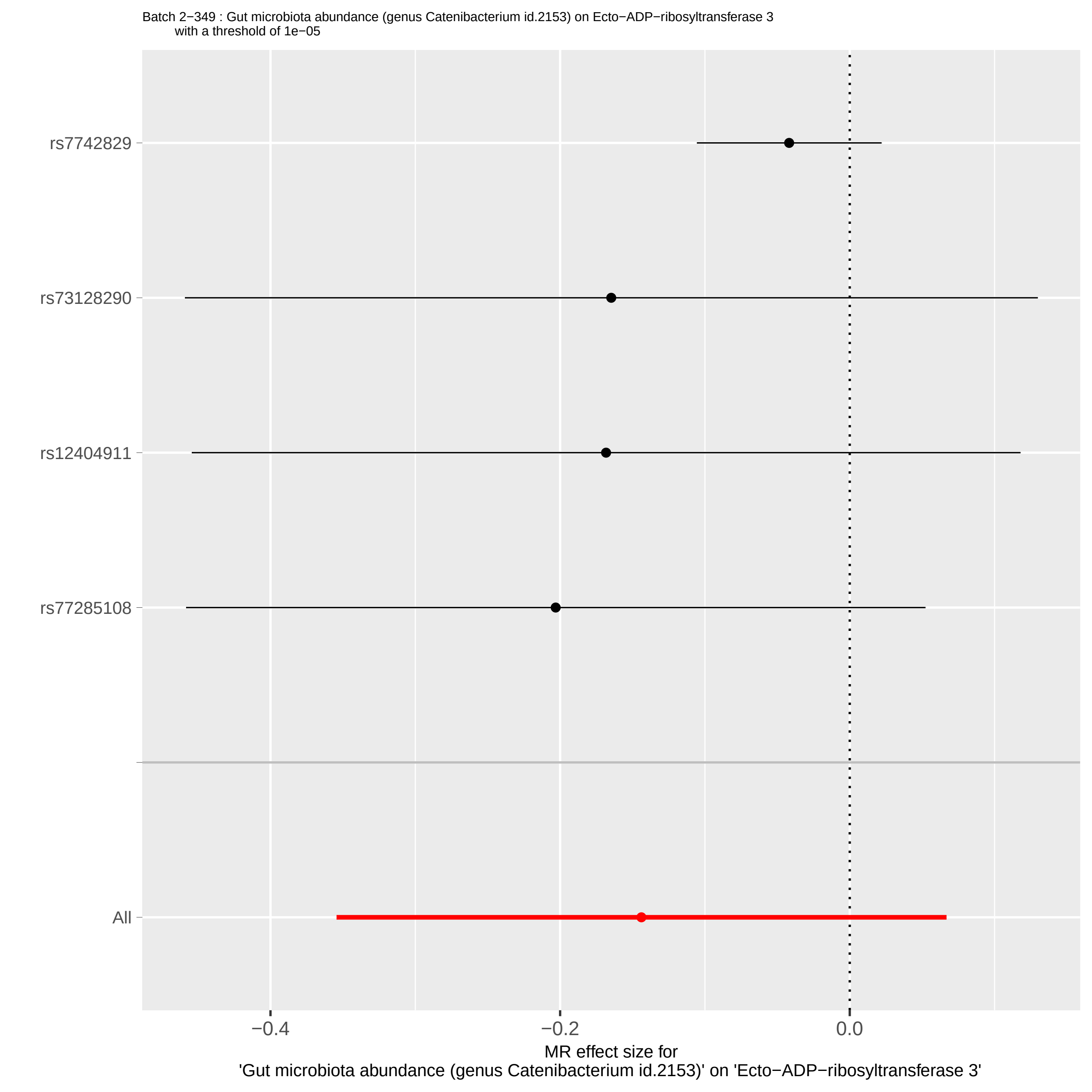

Batch 2−349 : Gut microbiota abundance (genus Catenibacterium id.2153) on Ecto−ADP−ribosyltransferase 3
with a threshold of 1e−05
rs7742829
rs73128290
rs12404911
rs77285108
All
−0.4
−0.2
0.0
MR effect size for
'Gut microbiota abundance (genus Catenibacterium id.2153)' on 'Ecto−ADP−ribosyltransferase 3'

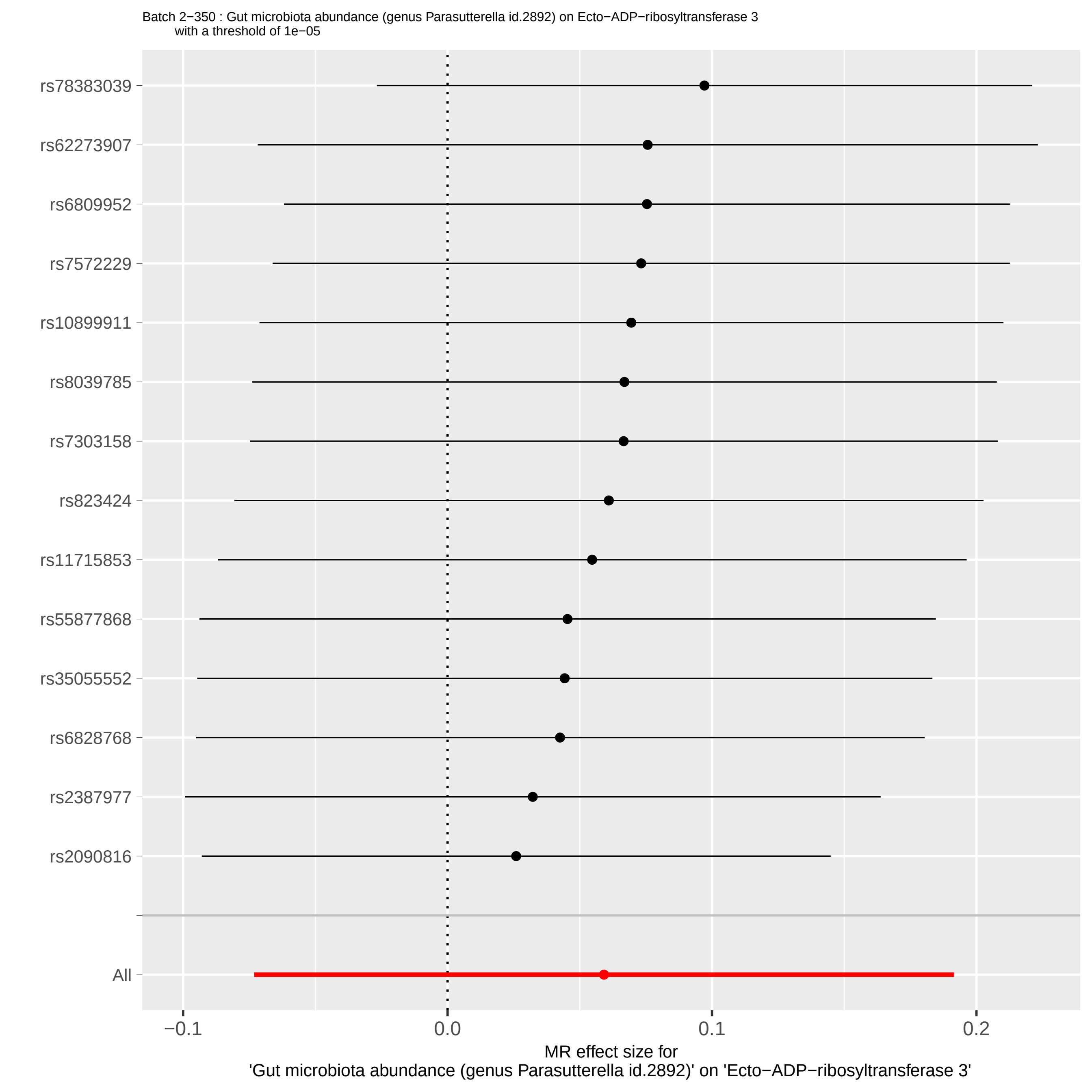
Batch 2−350 : Gut microbiota abundance (genus Parasutterella id.2892) on Ecto−ADP−ribosyltransferase 3
with a threshold of 1e−05
rs78383039
rs62273907
rs6809952
rs7572229
rs10899911
rs8039785
rs7303158
rs823424
rs11715853
rs55877868
rs35055552
rs6828768
rs2387977
rs2090816
All
−0.1
0.0
0.1
0.2
MR effect size for
'Gut microbiota abundance (genus Parasutterella id.2892)' on 'Ecto−ADP−ribosyltransferase 3'

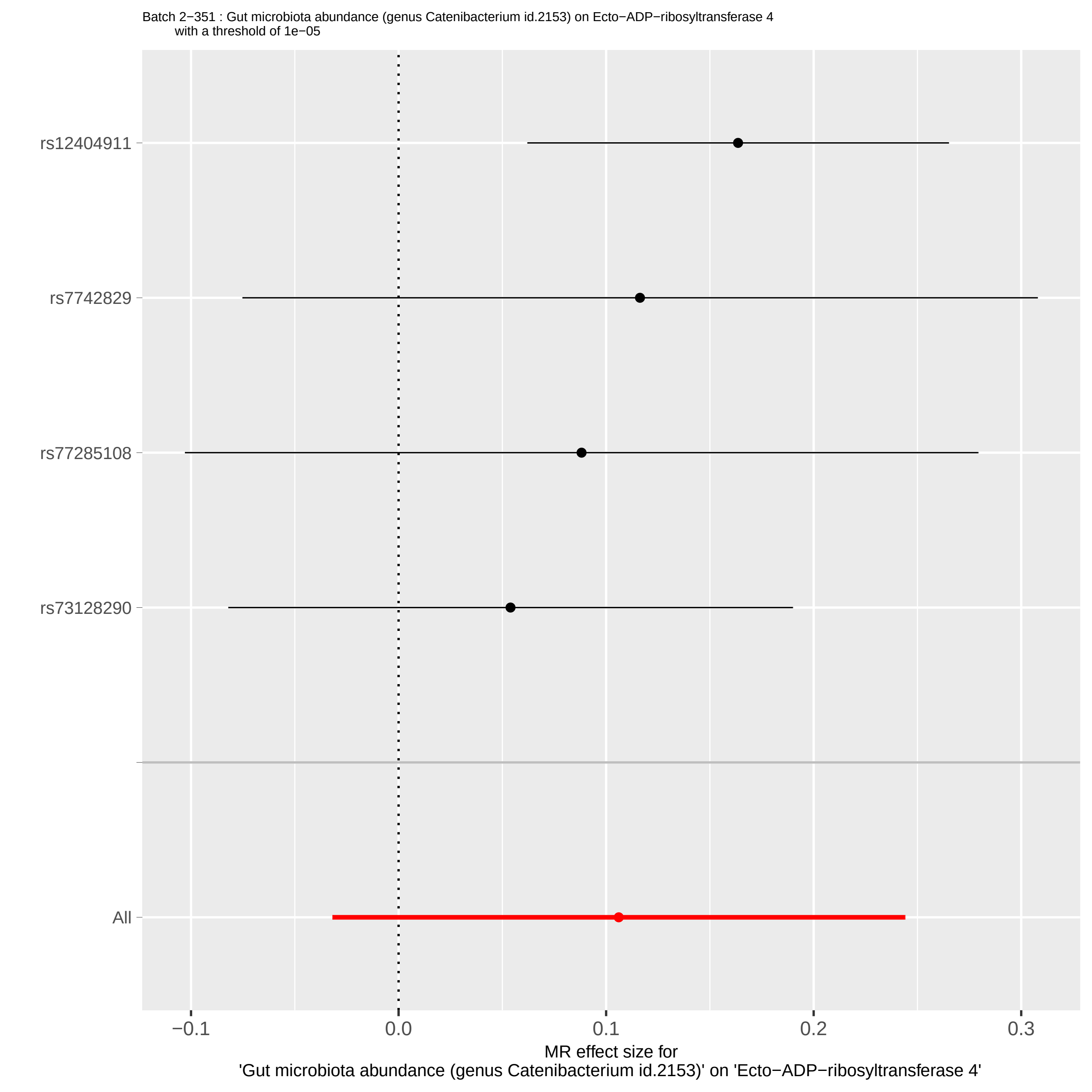

Batch 2−351 : Gut microbiota abundance (genus Catenibacterium id.2153) on Ecto−ADP−ribosyltransferase 4
with a threshold of 1e−05
rs12404911
rs7742829
rs77285108
rs73128290
All
−0.1
0.0
0.1
0.2
0.3
MR effect size for
'Gut microbiota abundance (genus Catenibacterium id.2153)' on 'Ecto−ADP−ribosyltransferase 4'

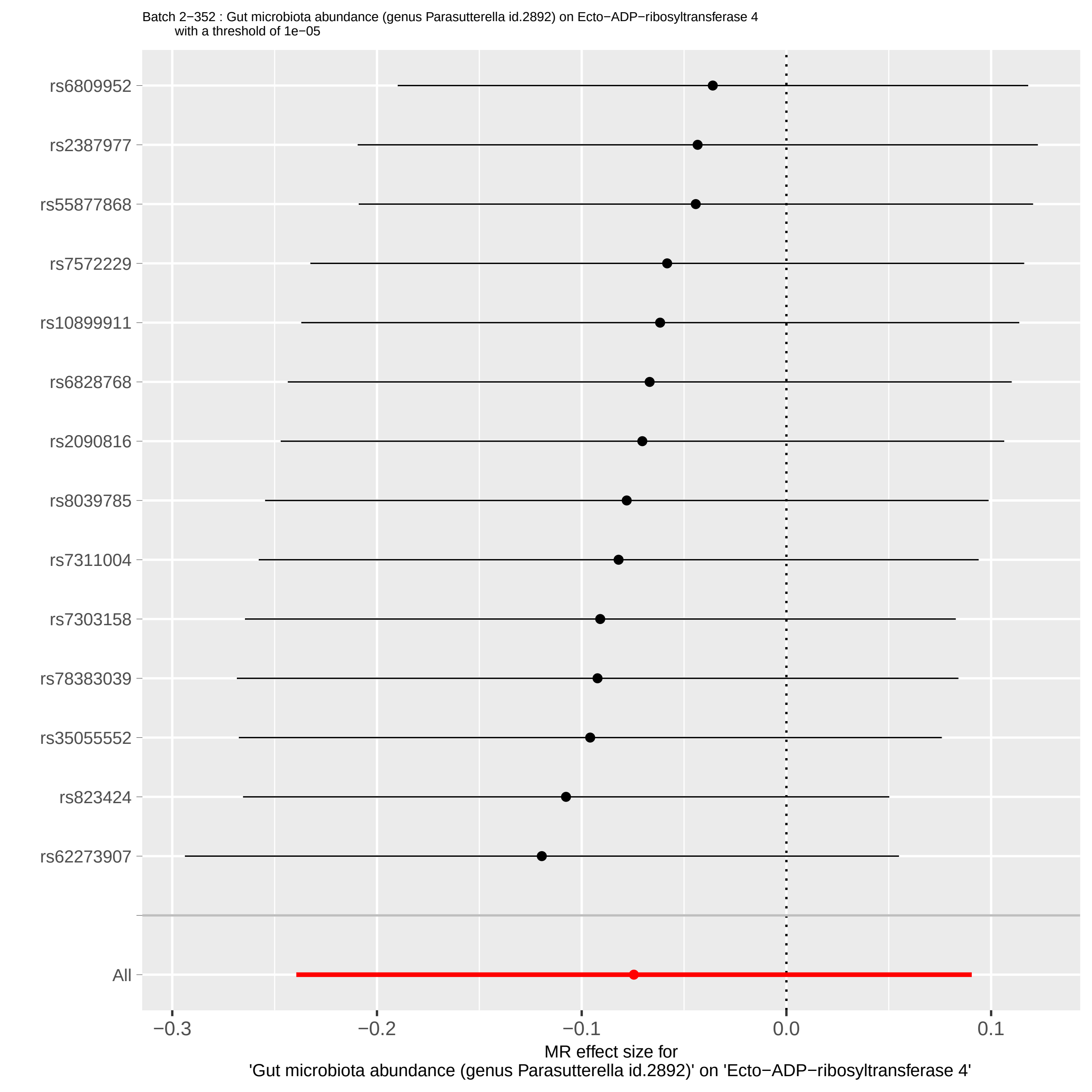

Batch 2−352 : Gut microbiota abundance (genus Parasutterella id.2892) on Ecto−ADP−ribosyltransferase 4
with a threshold of 1e−05
rs6809952
rs2387977
rs55877868
rs7572229
rs10899911
rs6828768
rs2090816
rs8039785
rs7311004
rs7303158
rs78383039
rs35055552
rs823424
rs62273907
All
−0.3
−0.2
−0.1
0.0
0.1
MR effect size for
'Gut microbiota abundance (genus Parasutterella id.2892)' on 'Ecto−ADP−ribosyltransferase 4'

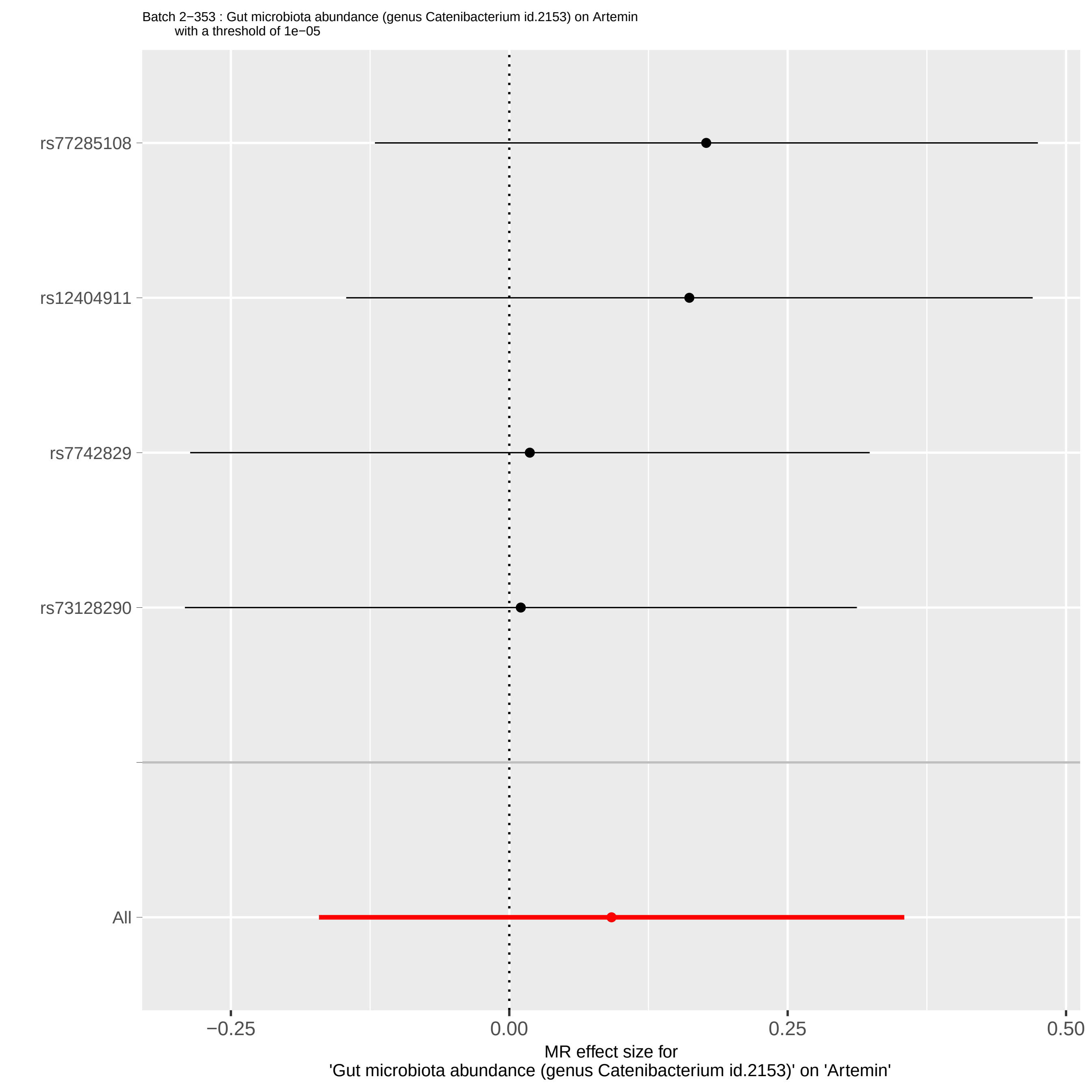
Batch 2−353 : Gut microbiota abundance (genus Catenibacterium id.2153) on Artemin
with a threshold of 1e−05
rs77285108
rs12404911
rs7742829
rs73128290
All
−0.25
0.00
0.25
0.50
MR effect size for
'Gut microbiota abundance (genus Catenibacterium id.2153)' on 'Artemin'

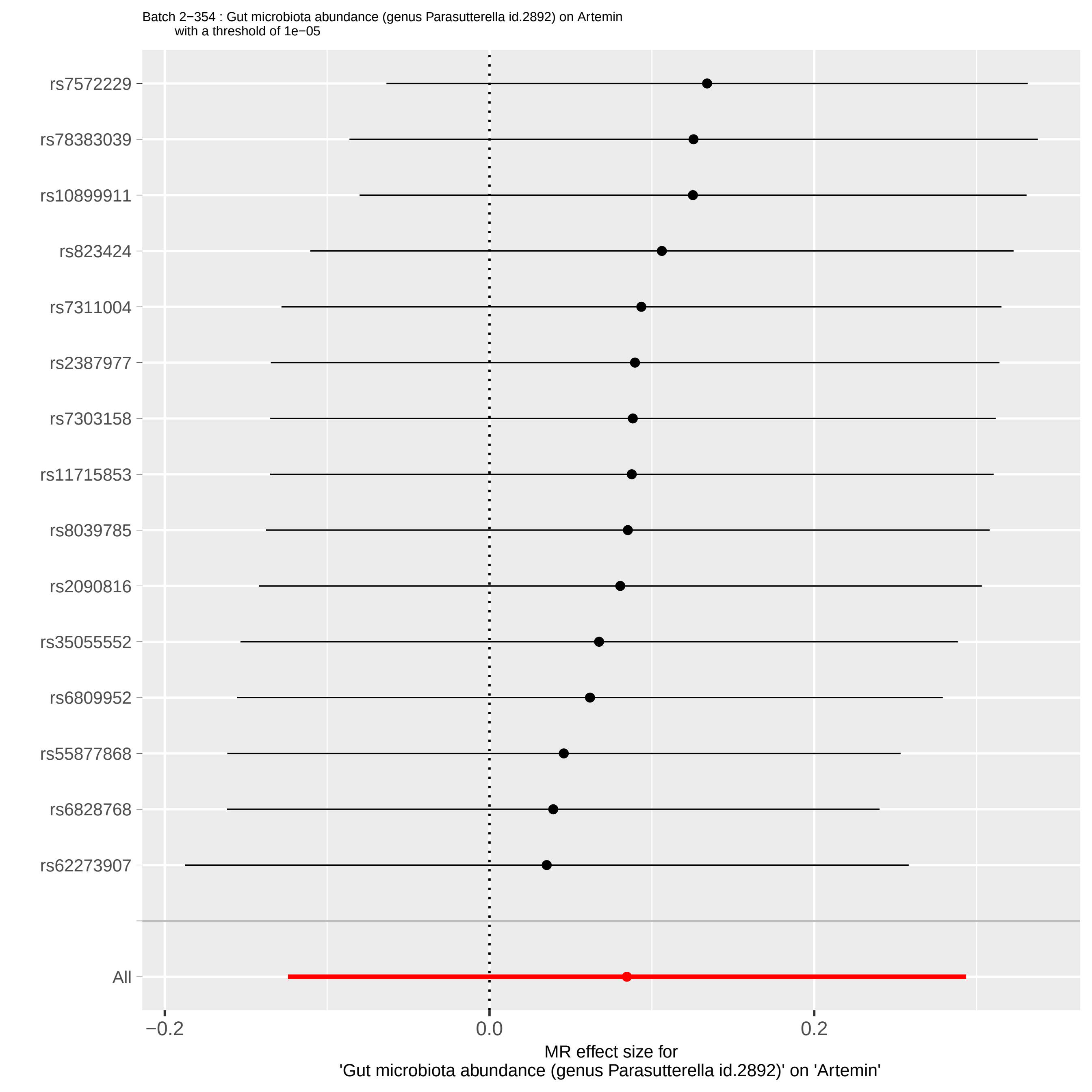

Batch 2−354 : Gut microbiota abundance (genus Parasutterella id.2892) on Artemin
with a threshold of 1e−05
rs7572229
rs78383039
rs10899911
rs823424
rs7311004
rs2387977
rs7303158
rs11715853
rs8039785
rs2090816
rs35055552
rs6809952
rs55877868
rs6828768
rs62273907
All
−0.2
0.0
0.2
MR effect size for
'Gut microbiota abundance (genus Parasutterella id.2892)' on 'Artemin'

Batch 2−355 : Gut microbiota abundance (genus Catenibacterium id.2153) on Acid ceramidase
with a threshold of 1e−05

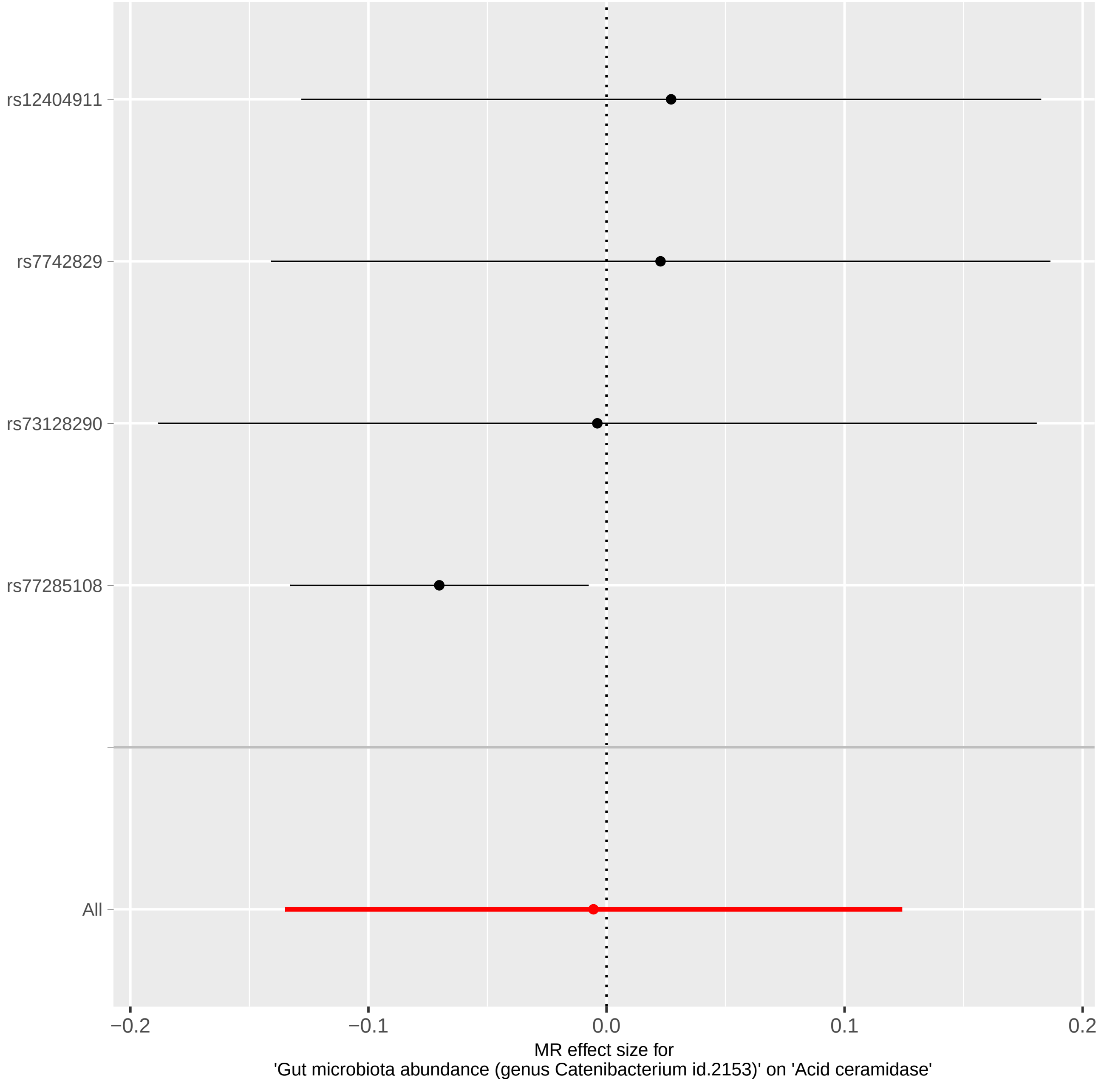

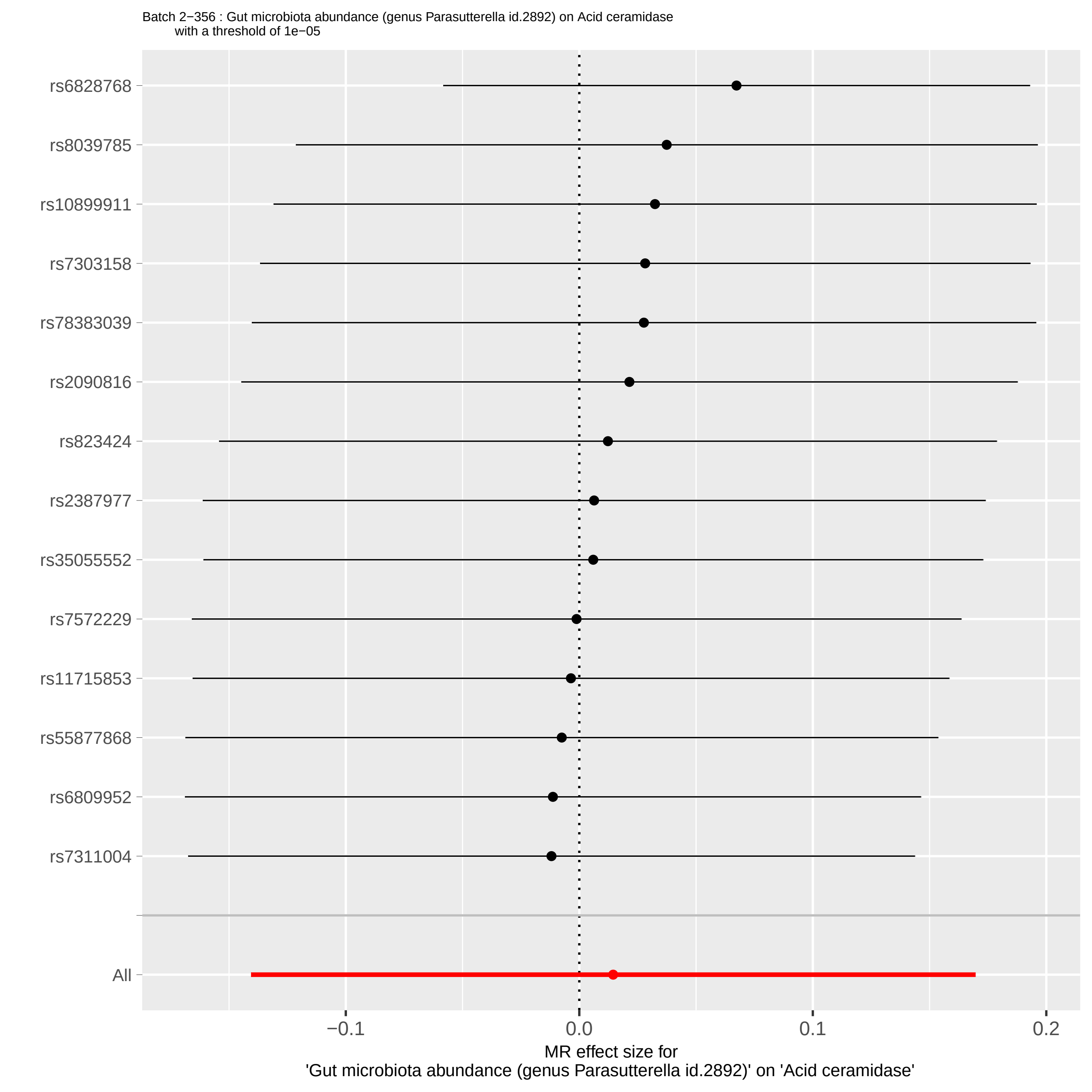

Batch 2−356 : Gut microbiota abundance (genus Parasutterella id.2892) on Acid ceramidase
with a threshold of 1e−05
rs6828768
rs8039785
rs10899911
rs7303158
rs78383039
rs2090816
rs823424
rs2387977
rs35055552
rs7572229
rs11715853
rs55877868
rs6809952
rs7311004
All
−0.1
0.0
0.1
0.2
MR effect size for
'Gut microbiota abundance (genus Parasutterella id.2892)' on 'Acid ceramidase'

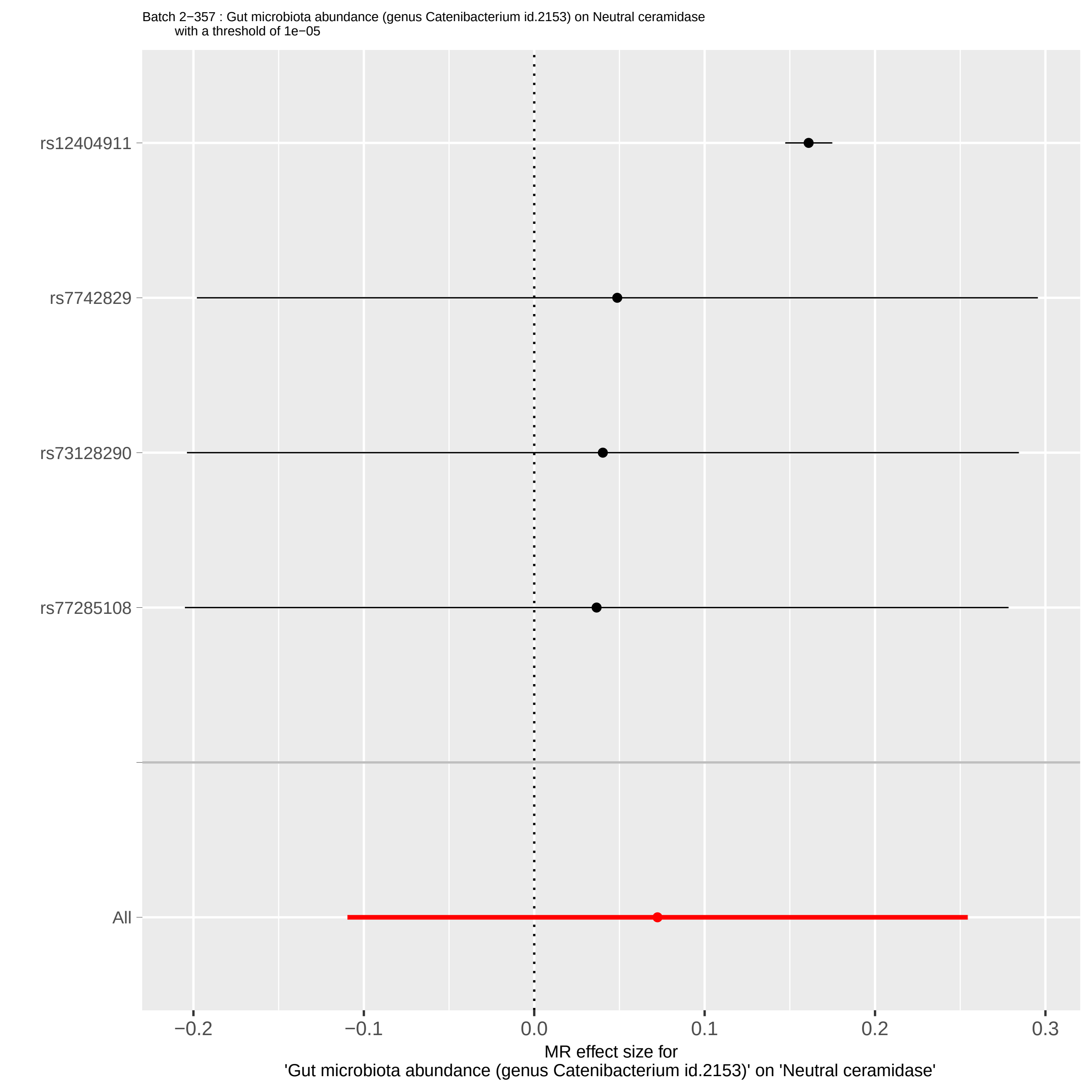

Batch 2−357 : Gut microbiota abundance (genus Catenibacterium id.2153) on Neutral ceramidase
with a threshold of 1e−05
rs12404911
rs7742829
rs73128290
rs77285108
All
−0.2
−0.1
0.0
0.1
0.2
0.3
MR effect size for
'Gut microbiota abundance (genus Catenibacterium id.2153)' on 'Neutral ceramidase'

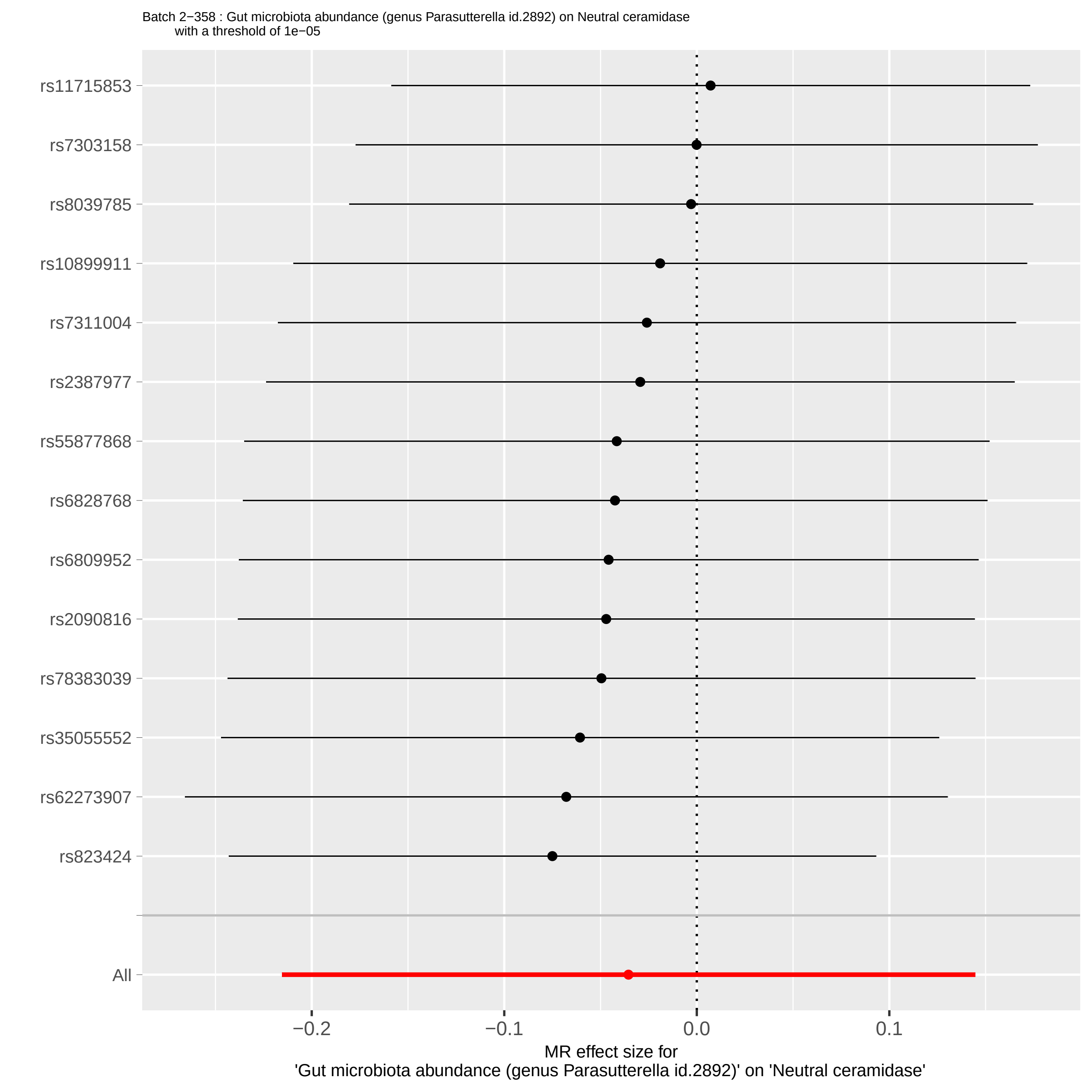
Batch 2−358 : Gut microbiota abundance (genus Parasutterella id.2892) on Neutral ceramidase
with a threshold of 1e−05
rs11715853
rs7303158
rs8039785
rs10899911
rs7311004
rs2387977
rs55877868
rs6828768
rs6809952
rs2090816
rs78383039
rs35055552
rs62273907
rs823424
All
−0.2
−0.1
0.0
0.1
MR effect size for
'Gut microbiota abundance (genus Parasutterella id.2892)' on 'Neutral ceramidase'

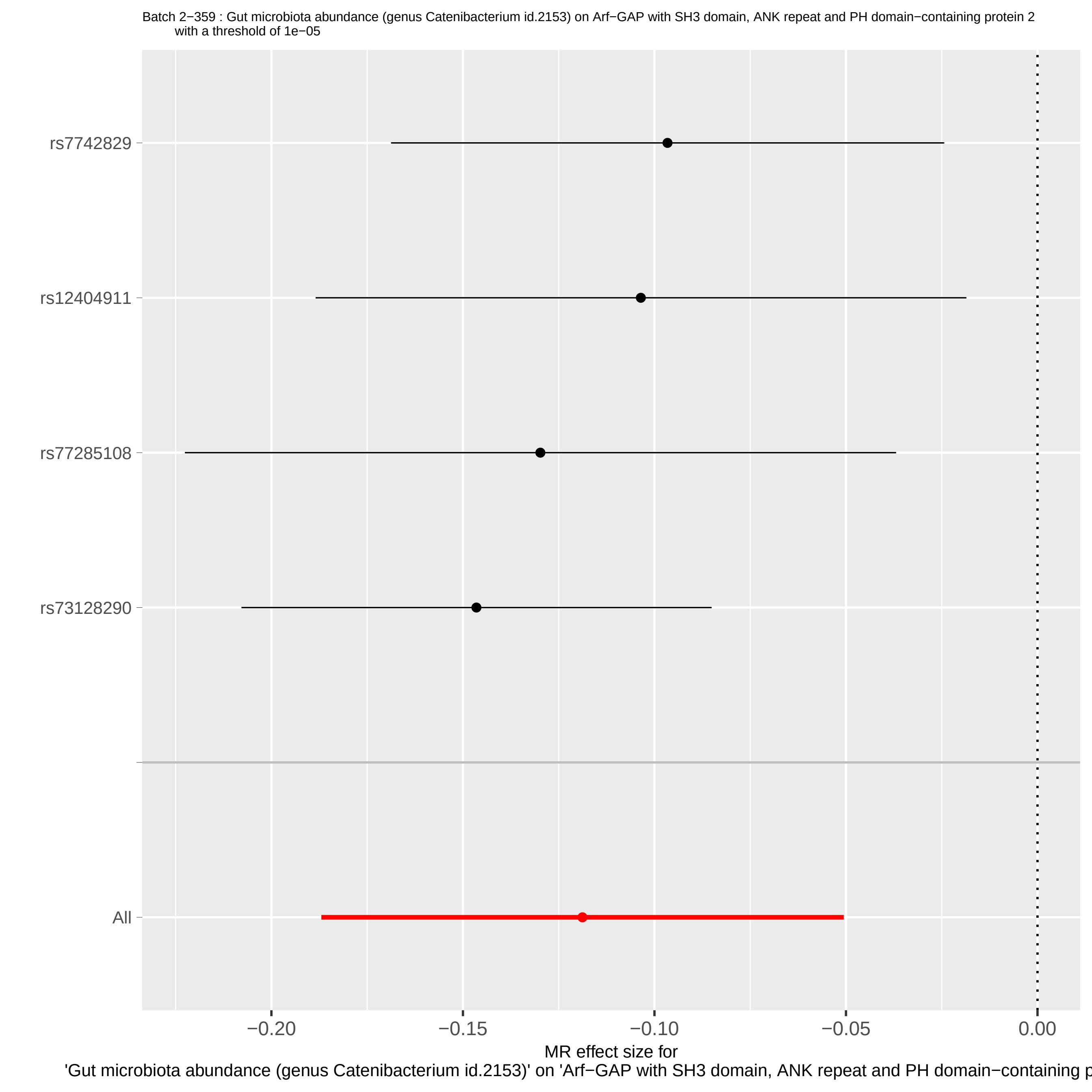
Batch 2−359 : Gut microbiota abundance (genus Catenibacterium id.2153) on Arf−GAP with SH3 domain, ANK repeat and PH domain−containing protein 2
with a threshold of 1e−05
rs7742829
rs12404911
rs77285108
rs73128290
All
−0.20
−0.15
−0.10
−0.05
0.00
MR effect size for
'Gut microbiota abundance (genus Catenibacterium id.2153)' on 'Arf−GAP with SH3 domain, ANK repeat and PH domain−containing p

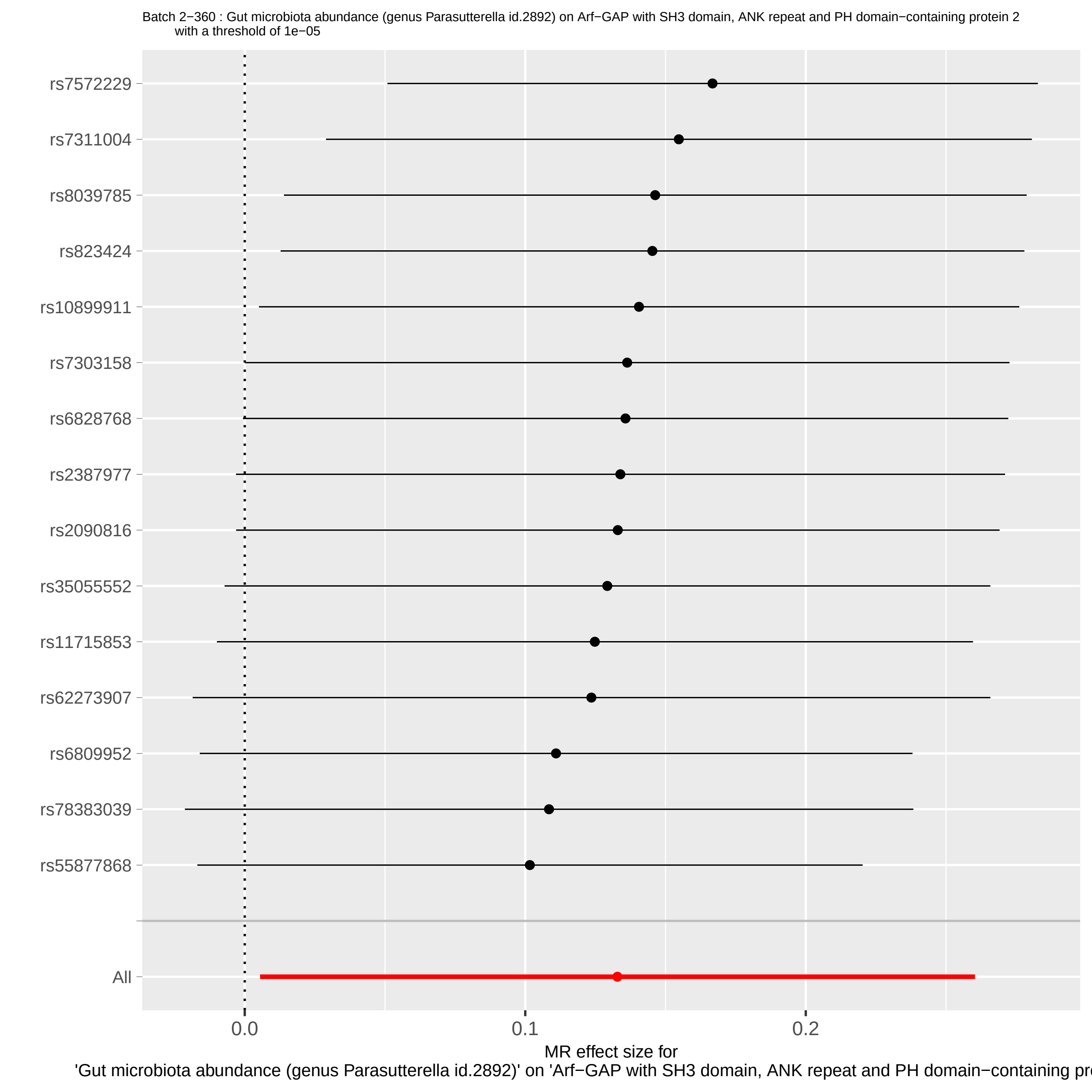

Batch 2−360 : Gut microbiota abundance (genus Parasutterella id.2892) on Arf−GAP with SH3 domain, ANK repeat and PH domain−containing protein 2
with a threshold of 1e−05
rs7572229
rs7311004
rs8039785
rs823424
rs10899911
rs7303158
rs6828768
rs2387977
rs2090816
rs35055552
rs11715853
rs62273907
rs6809952
rs78383039
rs55877868
All
0.0
0.1
0.2
MR effect size for
'Gut microbiota abundance (genus Parasutterella id.2892)' on 'Arf−GAP with SH3 domain, ANK repeat and PH domain−containing pr

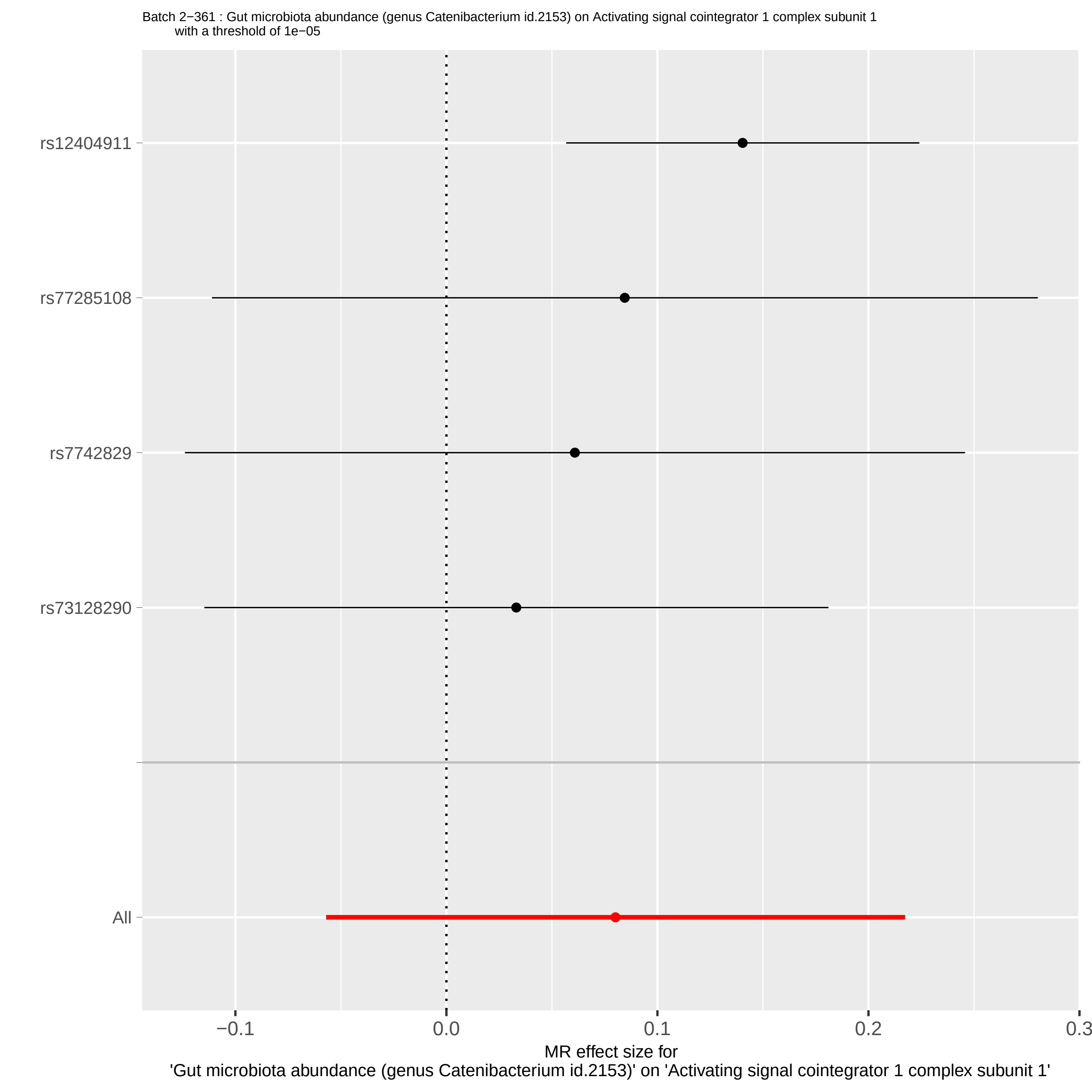

Batch 2−361 : Gut microbiota abundance (genus Catenibacterium id.2153) on Activating signal cointegrator 1 complex subunit 1
with a threshold of 1e−05
rs12404911
rs77285108
rs7742829
rs73128290
All
−0.1
0.0
0.1
0.2
0.3
MR effect size for
'Gut microbiota abundance (genus Catenibacterium id.2153)' on 'Activating signal cointegrator 1 complex subunit 1'

Batch 2−362 : Gut microbiota abundance (genus Parasutterella id.2892) on Activating signal cointegrator 1 complex subunit 1
with a threshold of 1e−05

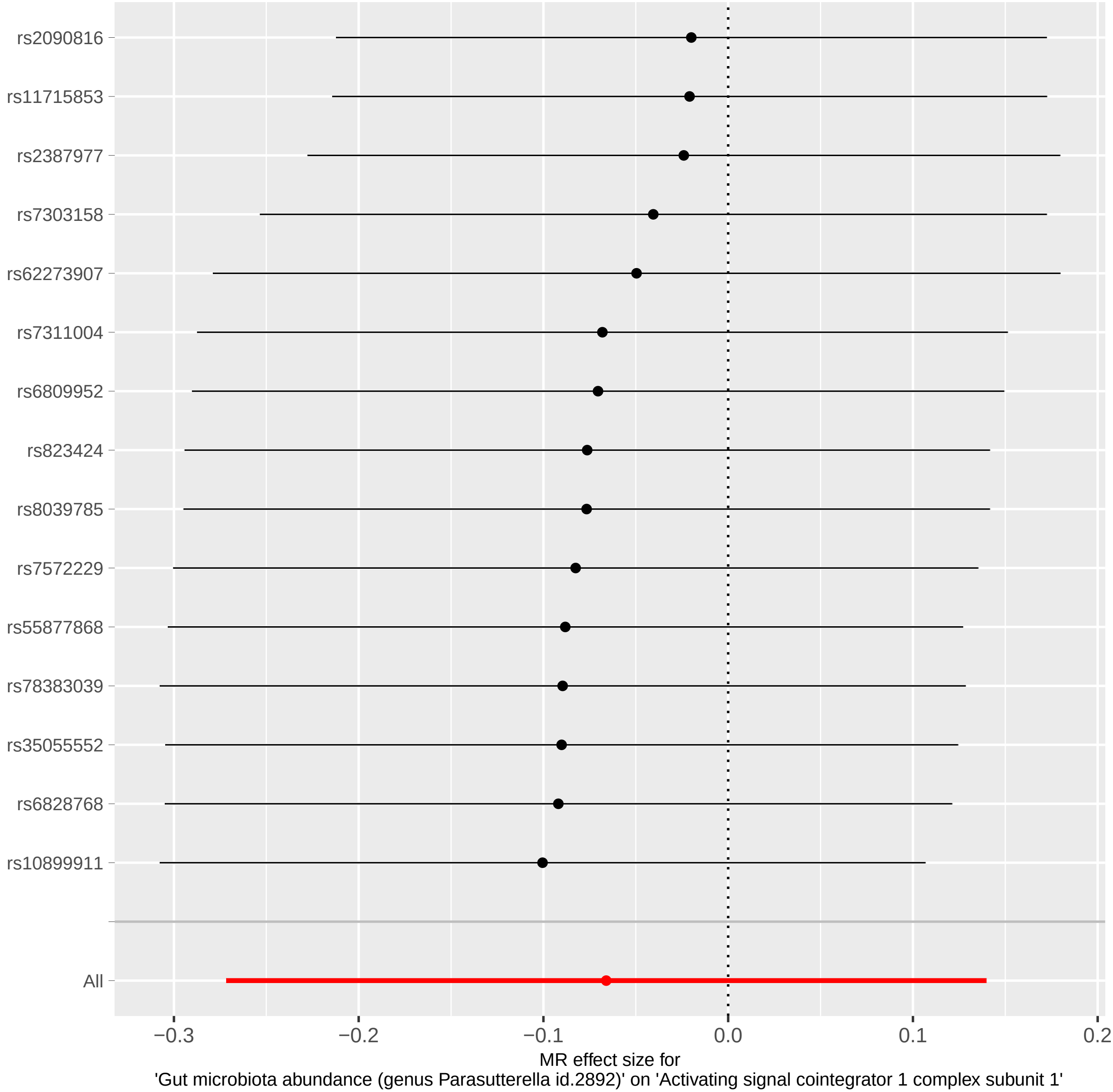

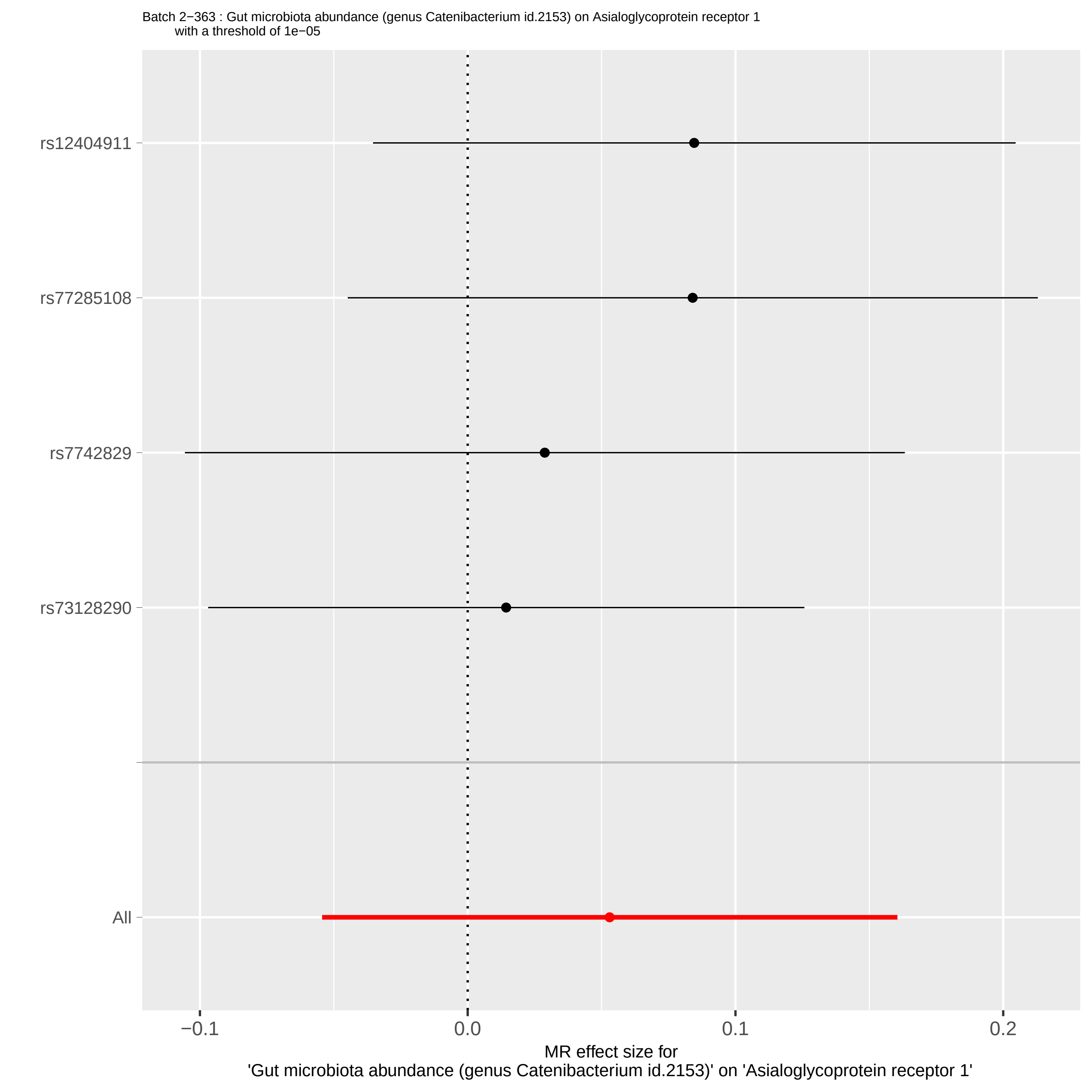

Batch 2−363 : Gut microbiota abundance (genus Catenibacterium id.2153) on Asialoglycoprotein receptor 1
with a threshold of 1e−05
rs12404911
rs77285108
rs7742829
rs73128290
All
−0.1
0.0
0.1
0.2
MR effect size for
'Gut microbiota abundance (genus Catenibacterium id.2153)' on 'Asialoglycoprotein receptor 1'

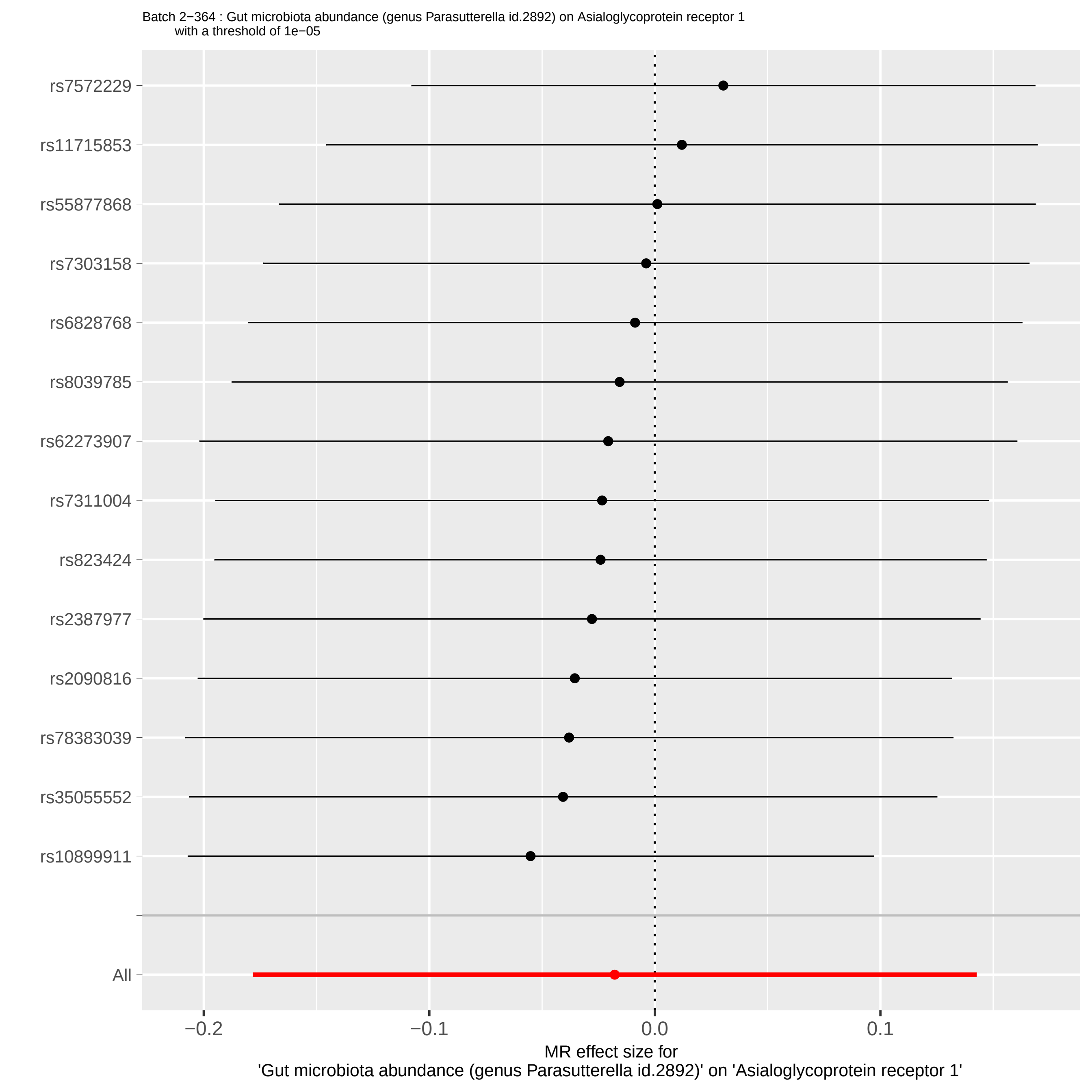
Batch 2−364 : Gut microbiota abundance (genus Parasutterella id.2892) on Asialoglycoprotein receptor 1
with a threshold of 1e−05
rs7572229
rs11715853
rs55877868
rs7303158
rs6828768
rs8039785
rs62273907
rs7311004
rs823424
rs2387977
rs2090816
rs78383039
rs35055552
rs10899911
All
−0.2
−0.1
0.0
0.1
MR effect size for
'Gut microbiota abundance (genus Parasutterella id.2892)' on 'Asialoglycoprotein receptor 1'

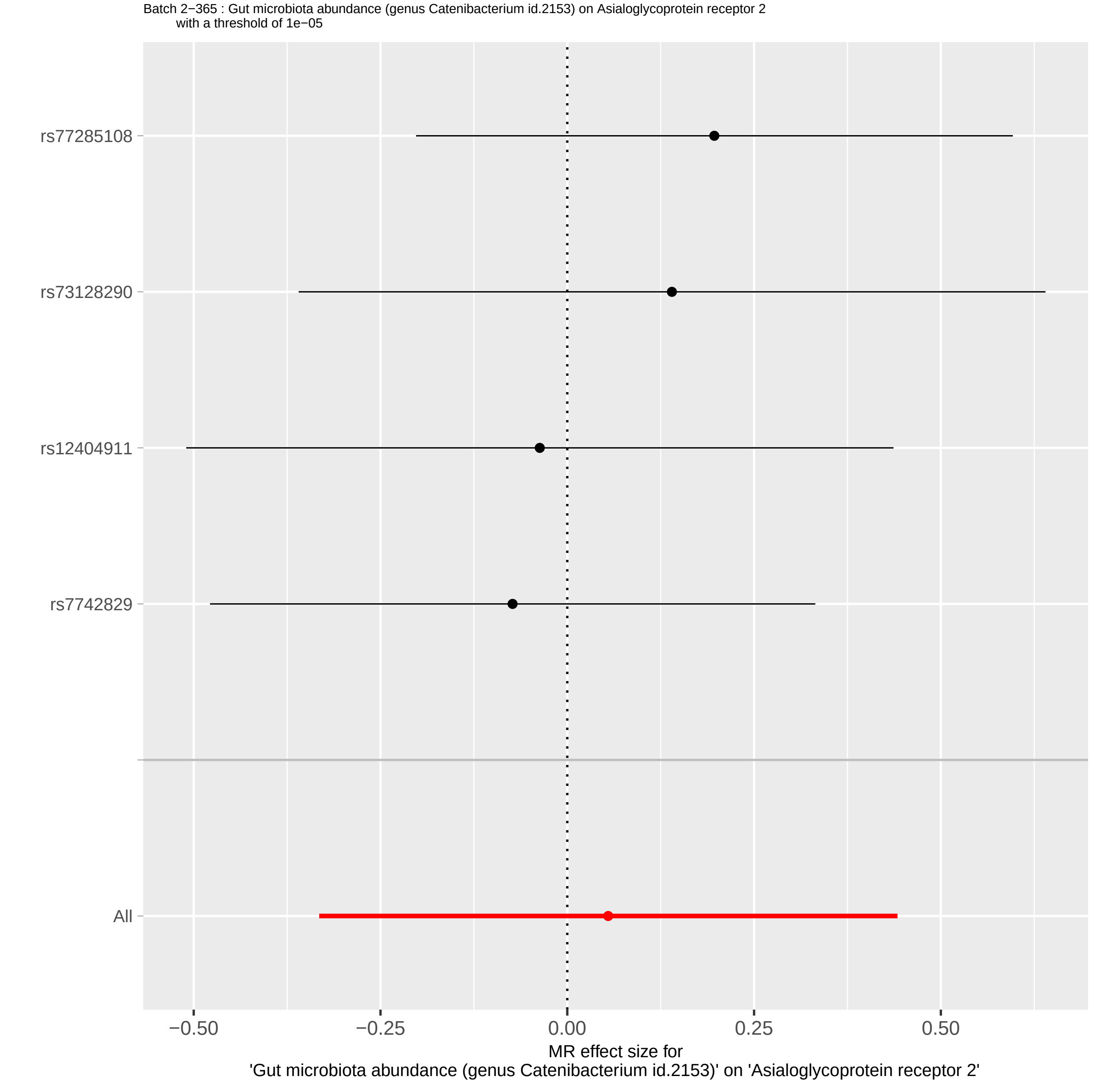

Batch 2−365 : Gut microbiota abundance (genus Catenibacterium id.2153) on Asialoglycoprotein receptor 2
with a threshold of 1e−05
rs77285108
rs73128290
rs12404911
rs7742829
All
−0.50
−0.25
0.00
0.25
0.50
MR effect size for
'Gut microbiota abundance (genus Catenibacterium id.2153)' on 'Asialoglycoprotein receptor 2'

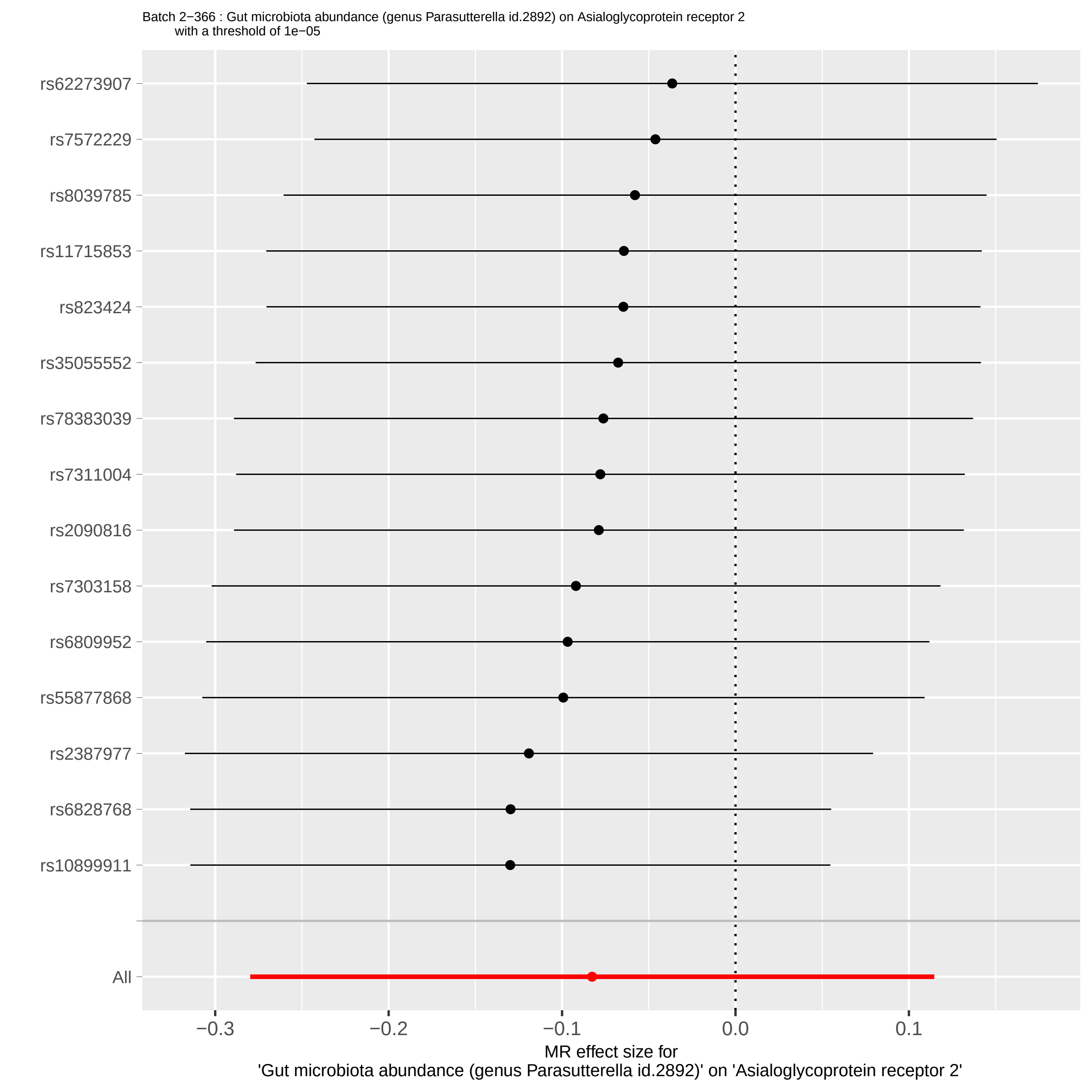
Batch 2−366 : Gut microbiota abundance (genus Parasutterella id.2892) on Asialoglycoprotein receptor 2
with a threshold of 1e−05
rs62273907
rs7572229
rs8039785
rs11715853
rs823424
rs35055552
rs78383039
rs7311004
rs2090816
rs7303158
rs6809952
rs55877868
rs2387977
rs6828768
rs10899911
All
−0.3
−0.2
−0.1
0.0
0.1
MR effect size for
'Gut microbiota abundance (genus Parasutterella id.2892)' on 'Asialoglycoprotein receptor 2'

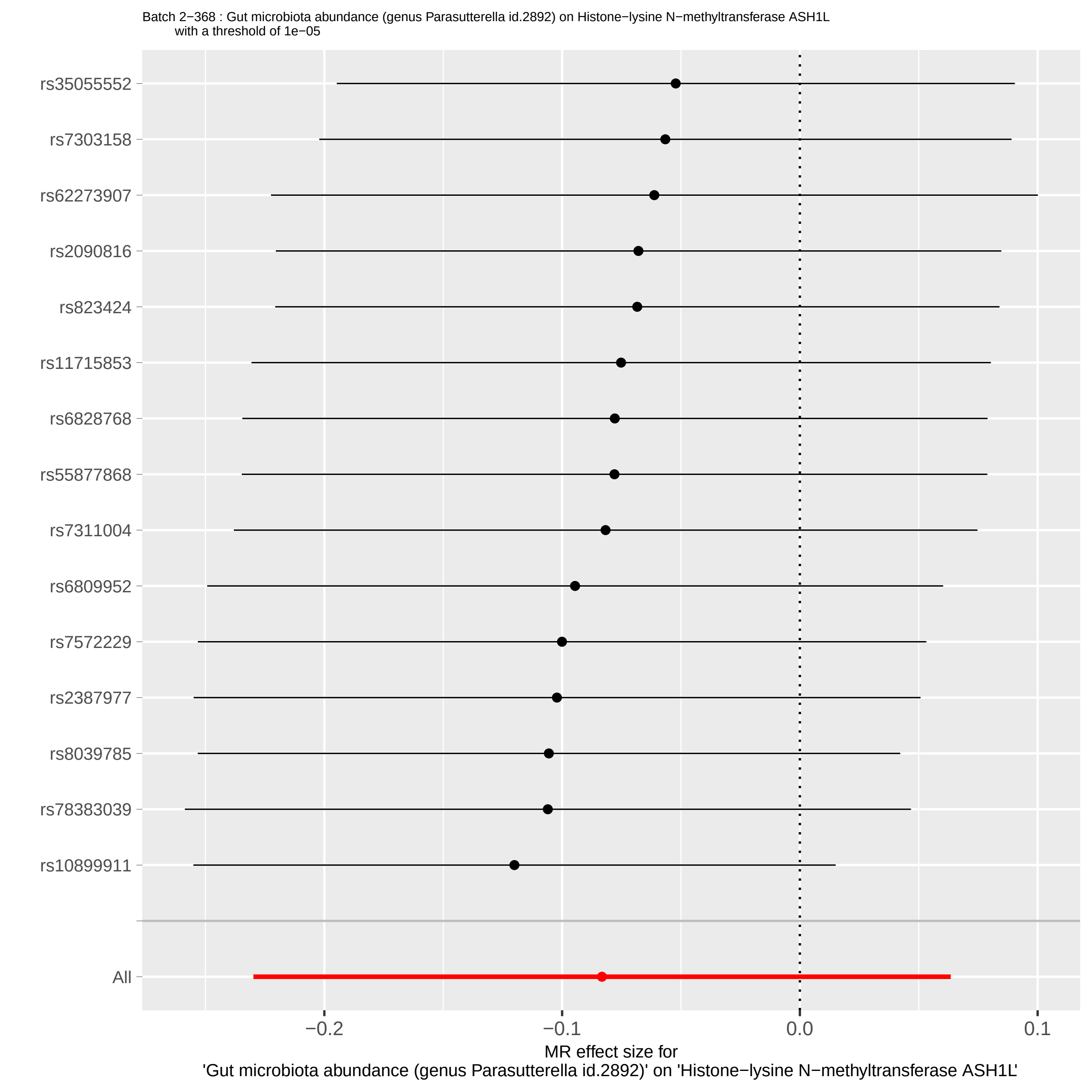
Batch 2−368 : Gut microbiota abundance (genus Parasutterella id.2892) on Histone−lysine N−methyltransferase ASH1L
with a threshold of 1e−05
rs35055552
rs7303158
rs62273907
rs2090816
rs823424
rs11715853
rs6828768
rs55877868
rs7311004
rs6809952
rs7572229
rs2387977
rs8039785
rs78383039
rs10899911
All
−0.2
−0.1
0.0
0.1
MR effect size for
'Gut microbiota abundance (genus Parasutterella id.2892)' on 'Histone−lysine N−methyltransferase ASH1L'

Batch 2−369 : Gut microbiota abundance (genus Catenibacterium id.2153) on Set1/Ash2 histone methyltransferase complex subunit ASH2
with a threshold of 1e−05

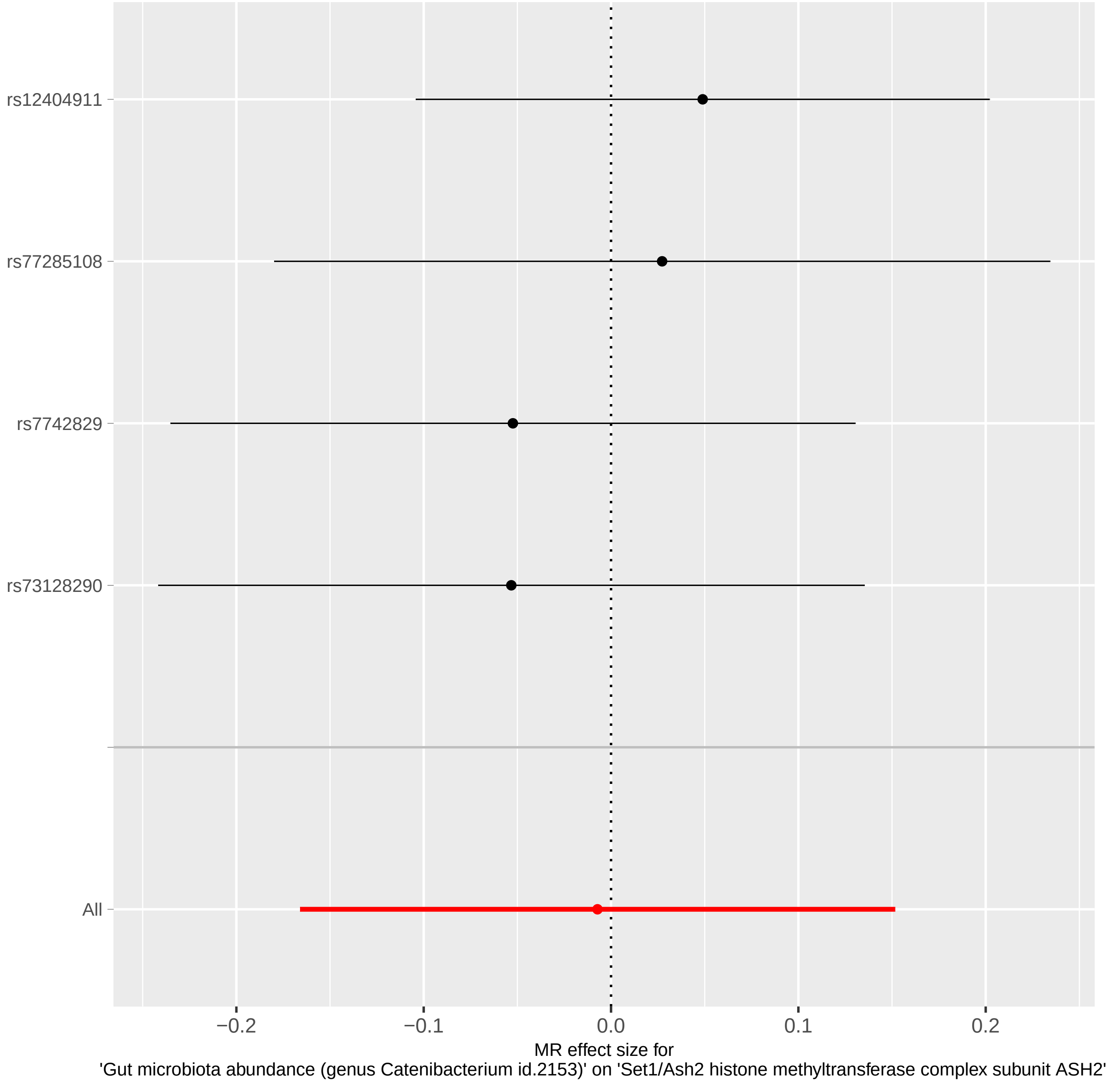

Batch 2−370 : Gut microbiota abundance (genus Parasutterella id.2892) on Set1/Ash2 histone methyltransferase complex subunit ASH2 with a threshold of 1e−05

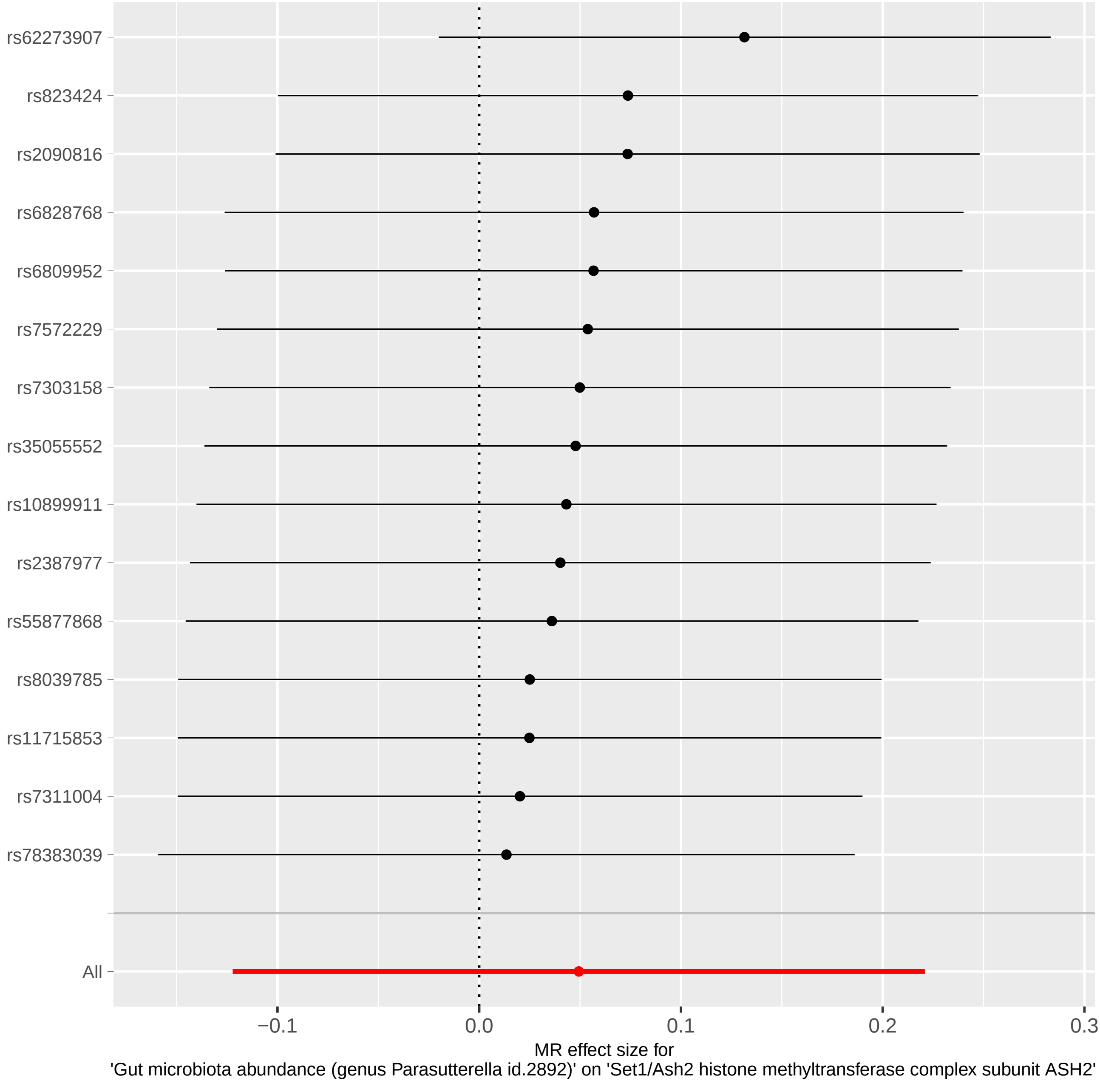

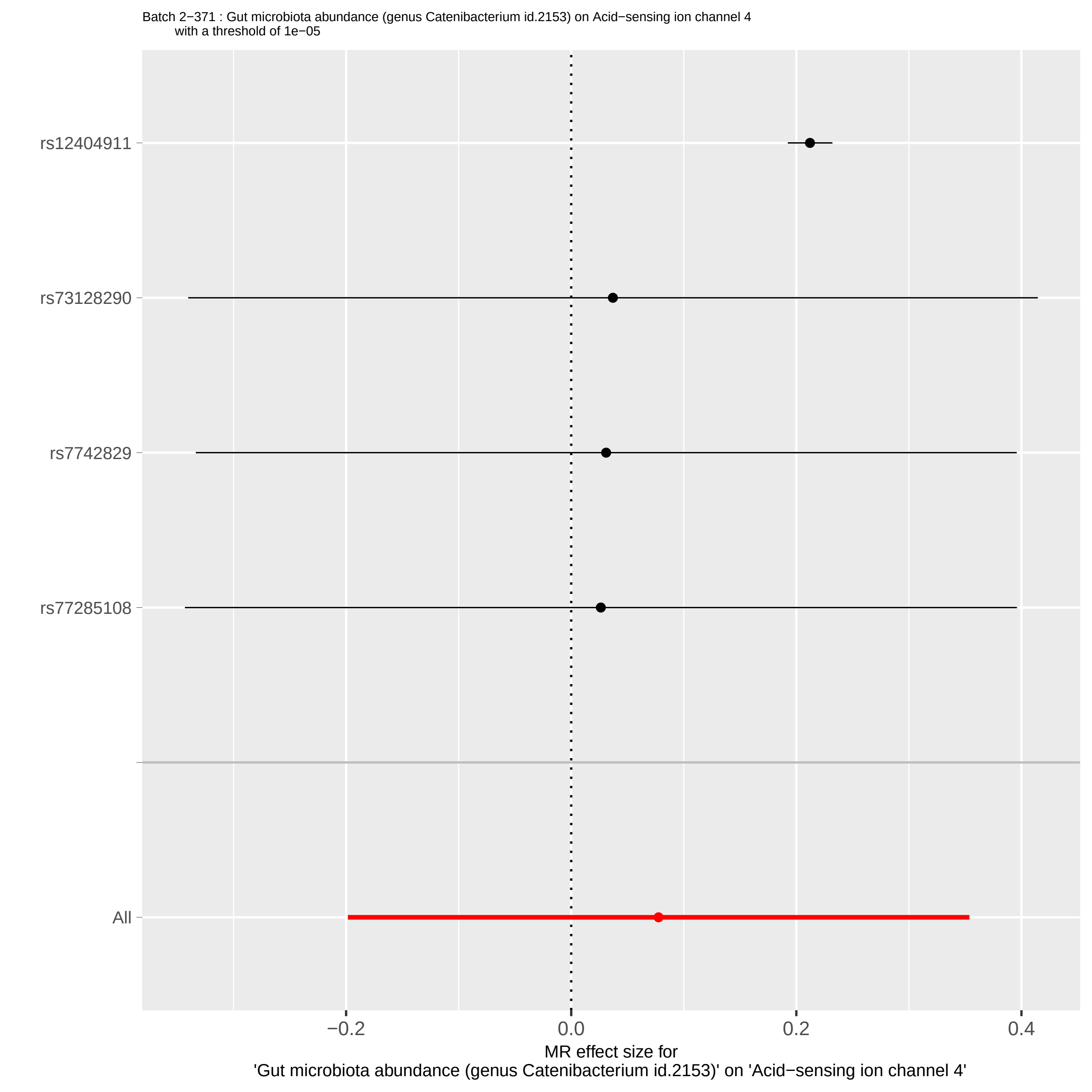
Batch 2−371 : Gut microbiota abundance (genus Catenibacterium id.2153) on Acid−sensing ion channel 4
with a threshold of 1e−05
rs12404911
rs73128290
rs7742829
rs77285108
All
−0.2
0.0
0.2
0.4
MR effect size for
'Gut microbiota abundance (genus Catenibacterium id.2153)' on 'Acid−sensing ion channel 4'

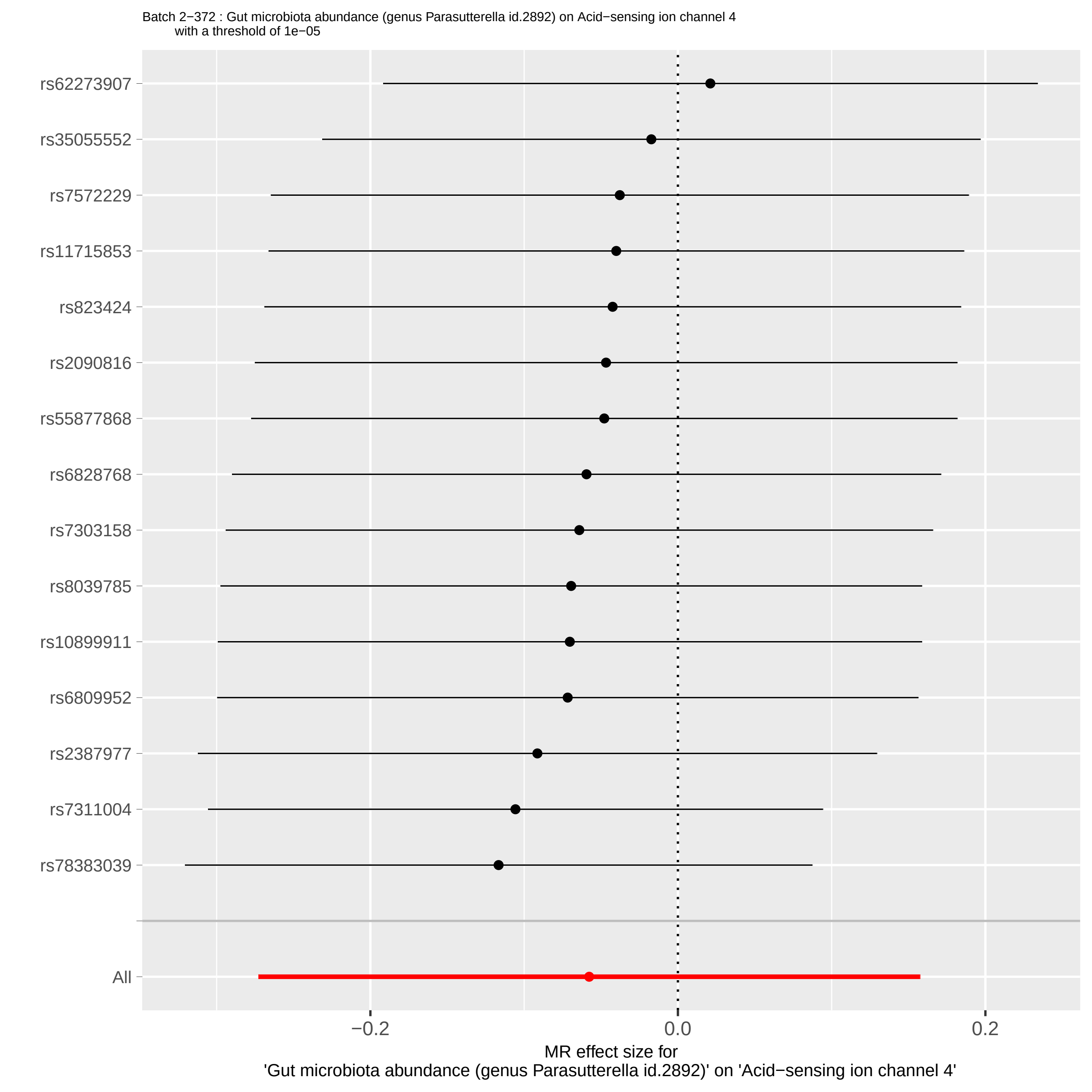
Batch 2−372 : Gut microbiota abundance (genus Parasutterella id.2892) on Acid−sensing ion channel 4
with a threshold of 1e−05
rs62273907
rs35055552
rs7572229
rs11715853
rs823424
rs2090816
rs55877868
rs6828768
rs7303158
rs8039785
rs10899911
rs6809952
rs2387977
rs7311004
rs78383039
All
−0.2
0.0
0.2
MR effect size for
'Gut microbiota abundance (genus Parasutterella id.2892)' on 'Acid−sensing ion channel 4'

Batch 2−373 : Gut microbiota abundance (genus Catenibacterium id.2153) on Agouti−signaling protein
with a threshold of 1e−05

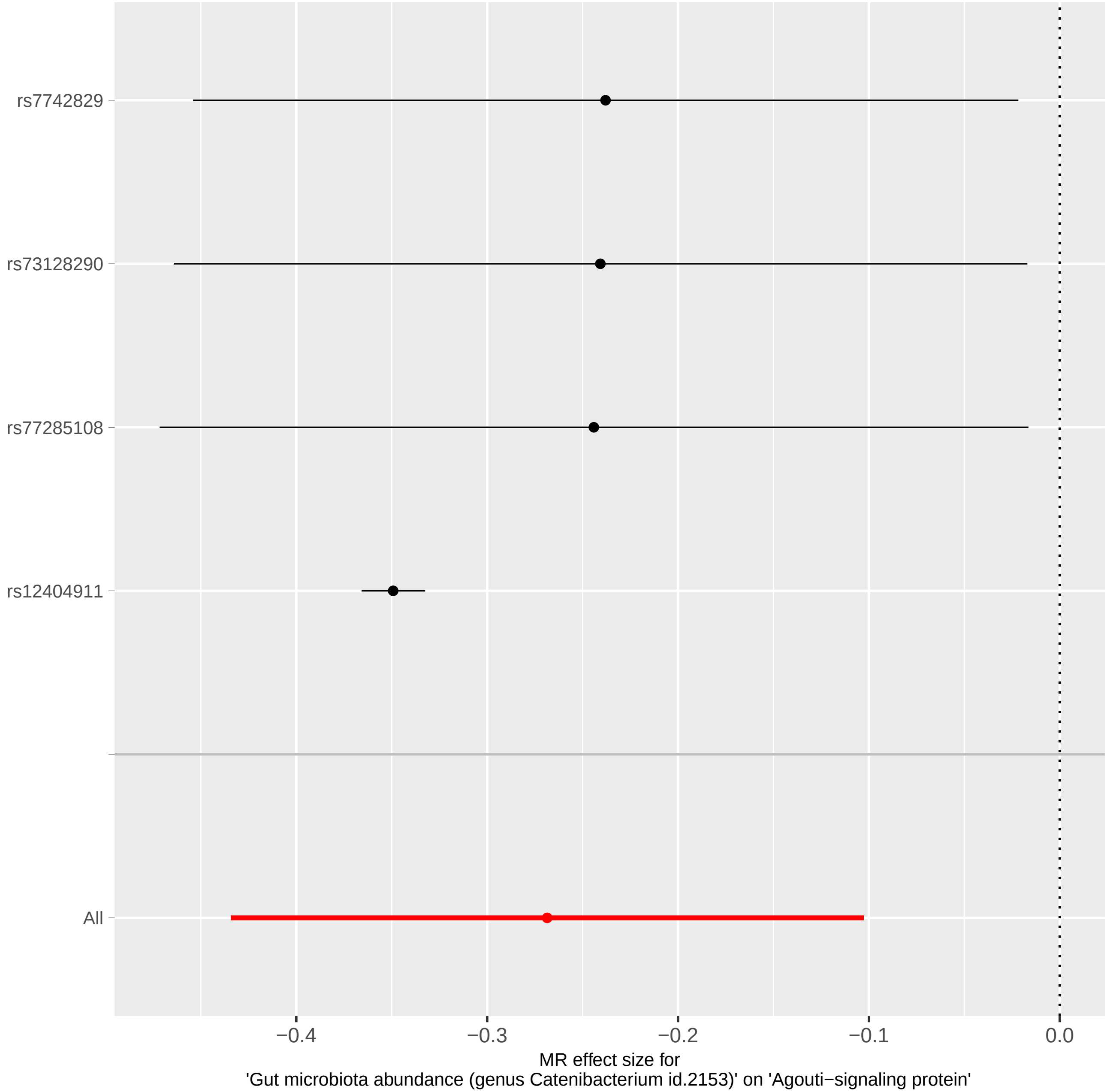

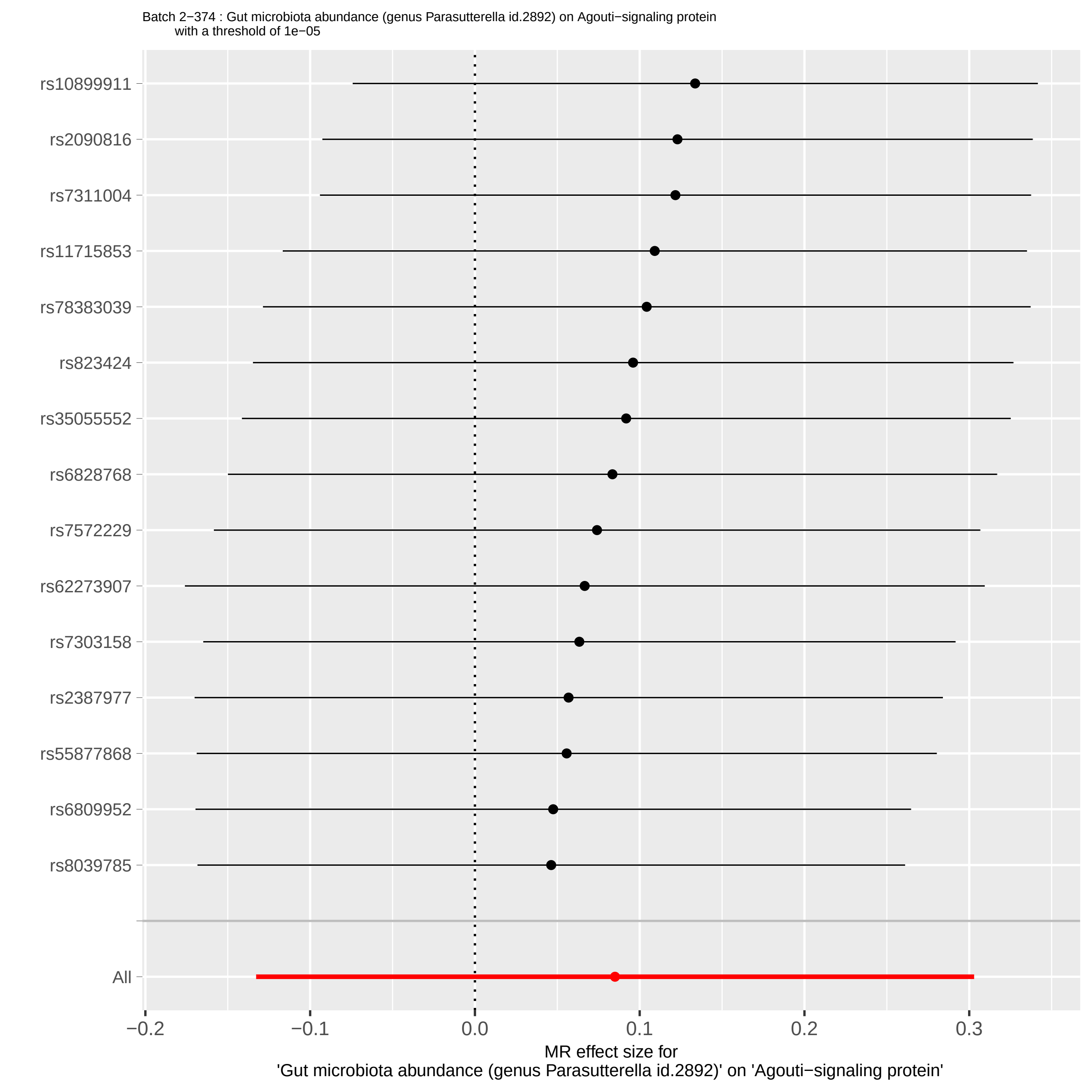
Batch 2−374 : Gut microbiota abundance (genus Parasutterella id.2892) on Agouti−signaling protein
with a threshold of 1e−05
rs10899911
rs2090816
rs7311004
rs11715853
rs78383039
rs823424
rs35055552
rs6828768
rs7572229
rs62273907
rs7303158
rs2387977
rs55877868
rs6809952
rs8039785
All
−0.2
−0.1
0.0
0.1
0.2
0.3
MR effect size for
'Gut microbiota abundance (genus Parasutterella id.2892)' on 'Agouti−signaling protein'

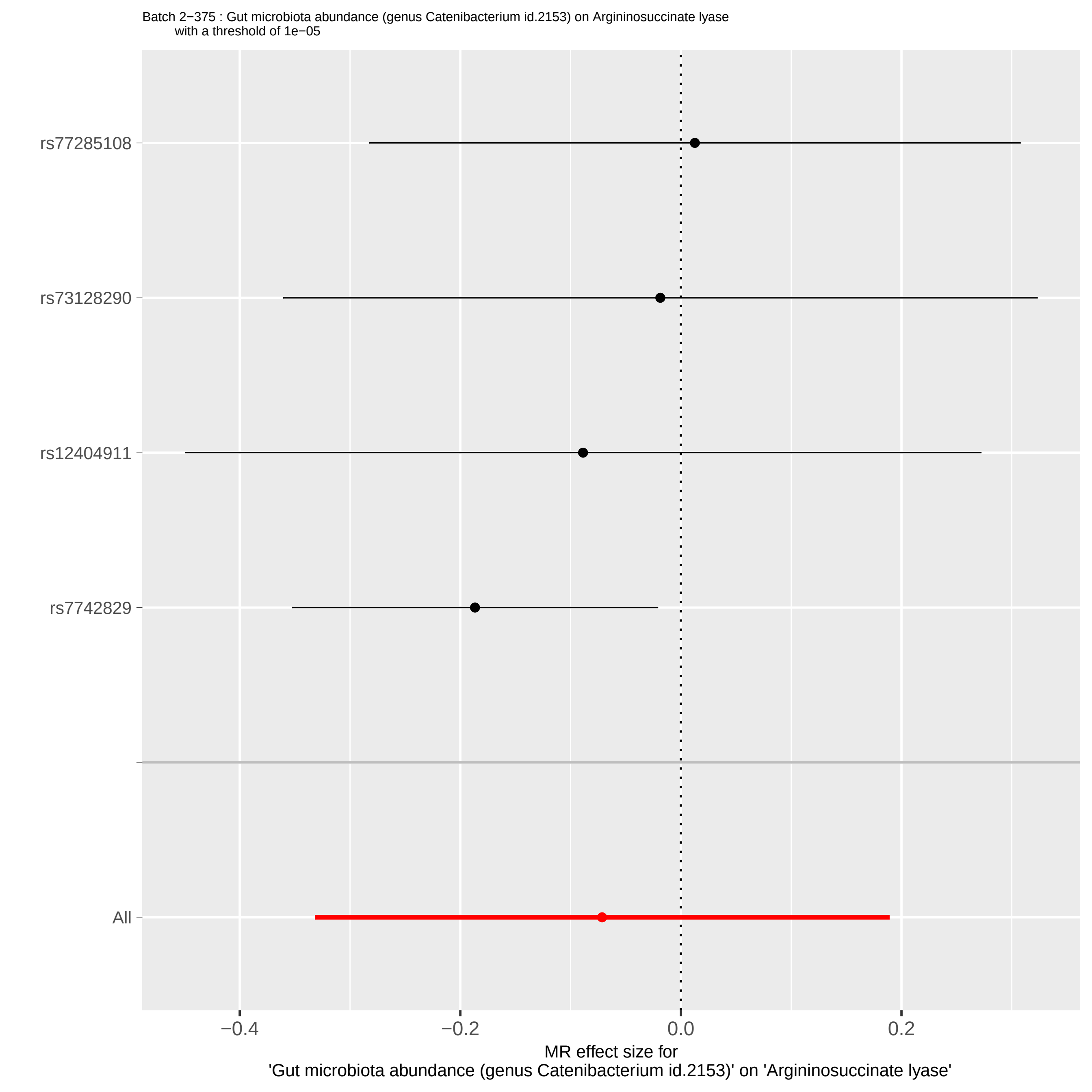
Batch 2−375 : Gut microbiota abundance (genus Catenibacterium id.2153) on Argininosuccinate lyase
with a threshold of 1e−05
rs77285108
rs73128290
rs12404911
rs7742829
All
−0.4
−0.2
0.0
0.2
MR effect size for
'Gut microbiota abundance (genus Catenibacterium id.2153)' on 'Argininosuccinate lyase'

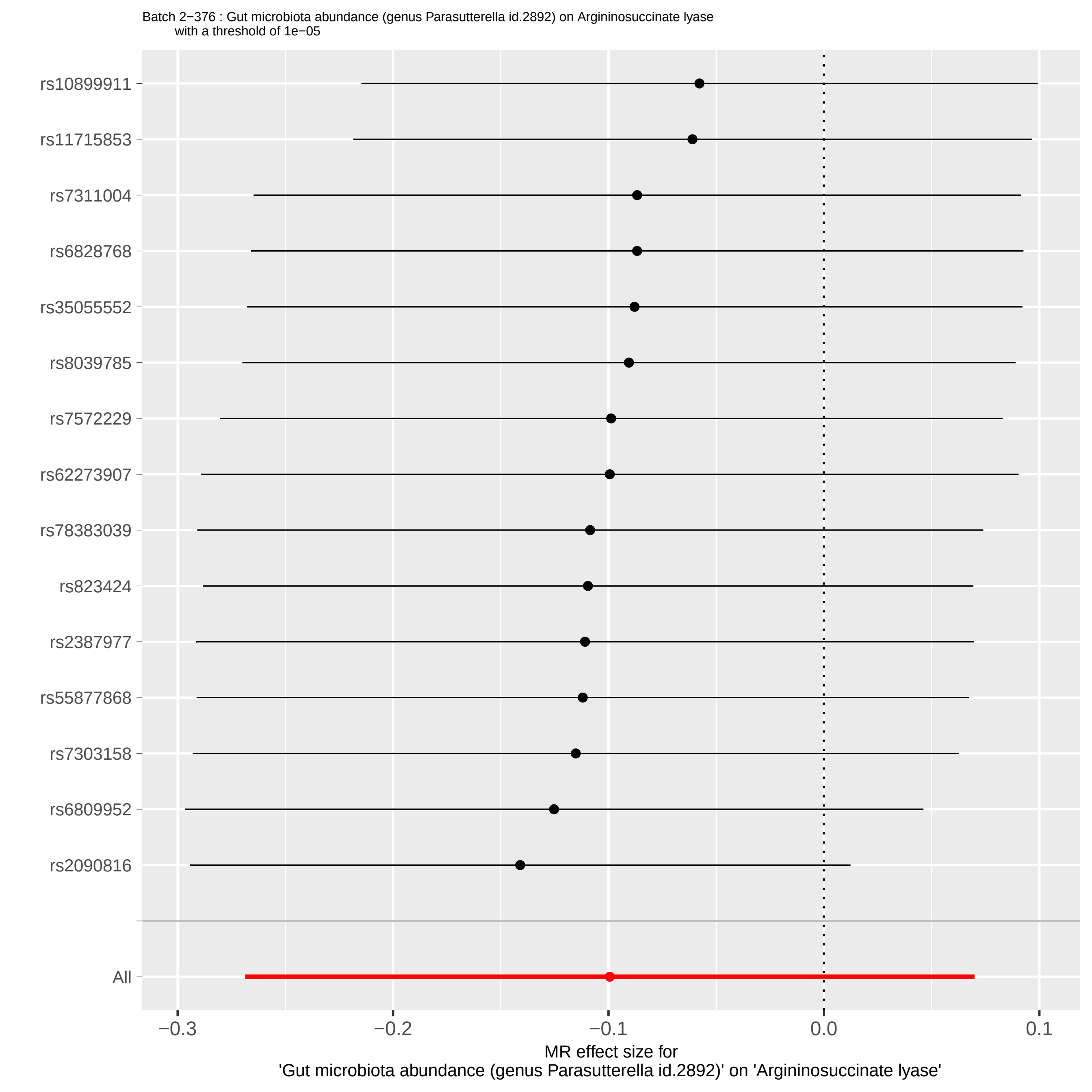
Batch 2−376 : Gut microbiota abundance (genus Parasutterella id.2892) on Argininosuccinate lyase
with a threshold of 1e−05
rs10899911
rs11715853
rs7311004
rs6828768
rs35055552
rs8039785
rs7572229
rs62273907
rs78383039
rs823424
rs2387977
rs55877868
rs7303158
rs6809952
rs2090816
All
−0.3
−0.2
−0.1
0.0
0.1
MR effect size for
'Gut microbiota abundance (genus Parasutterella id.2892)' on 'Argininosuccinate lyase'

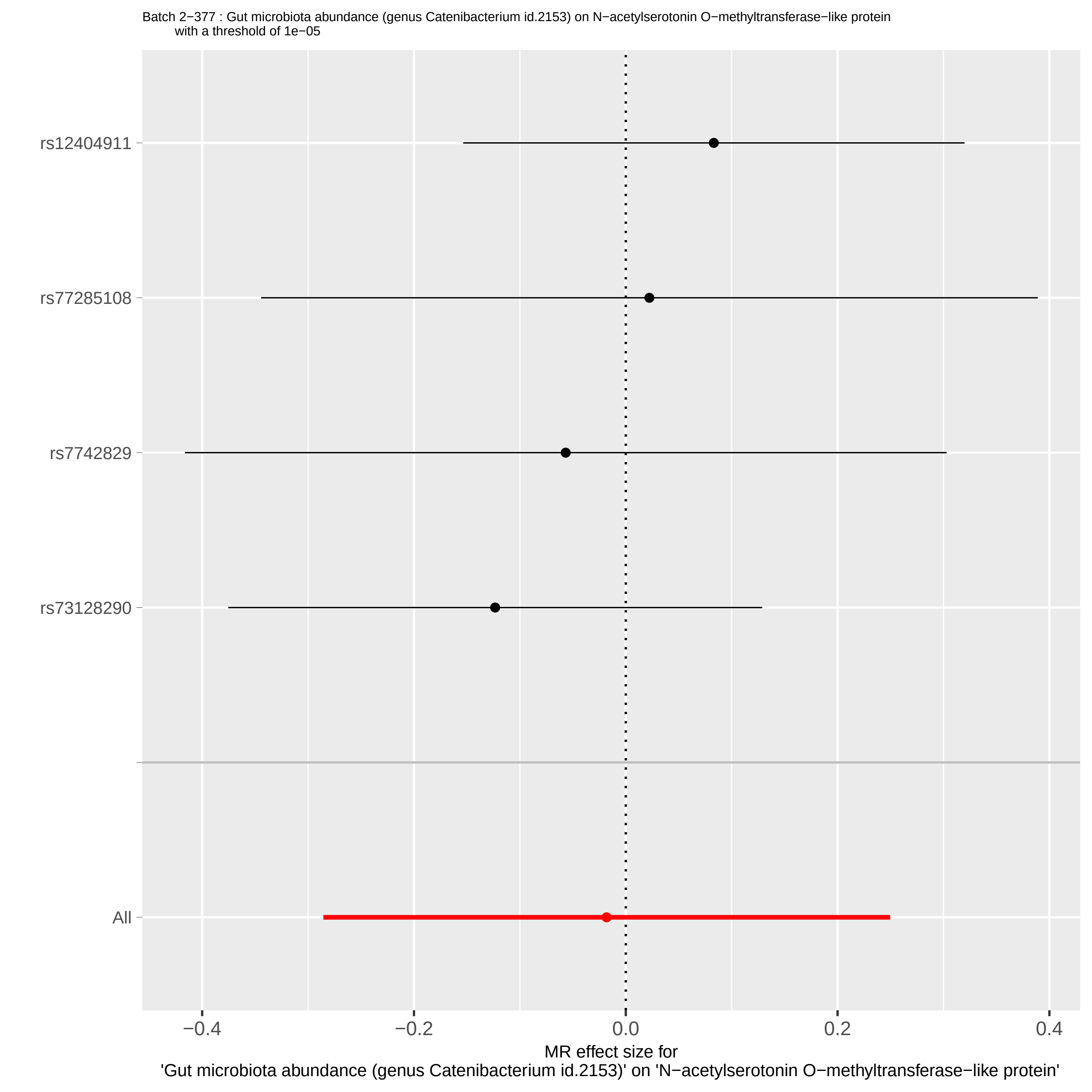

Batch 2−377 : Gut microbiota abundance (genus Catenibacterium id.2153) on N−acetylserotonin O−methyltransferase−like protein
with a threshold of 1e−05
rs12404911
rs77285108
rs7742829
rs73128290
All
−0.4
−0.2
0.0
0.2
0.4
MR effect size for
'Gut microbiota abundance (genus Catenibacterium id.2153)' on 'N−acetylserotonin O−methyltransferase−like protein'

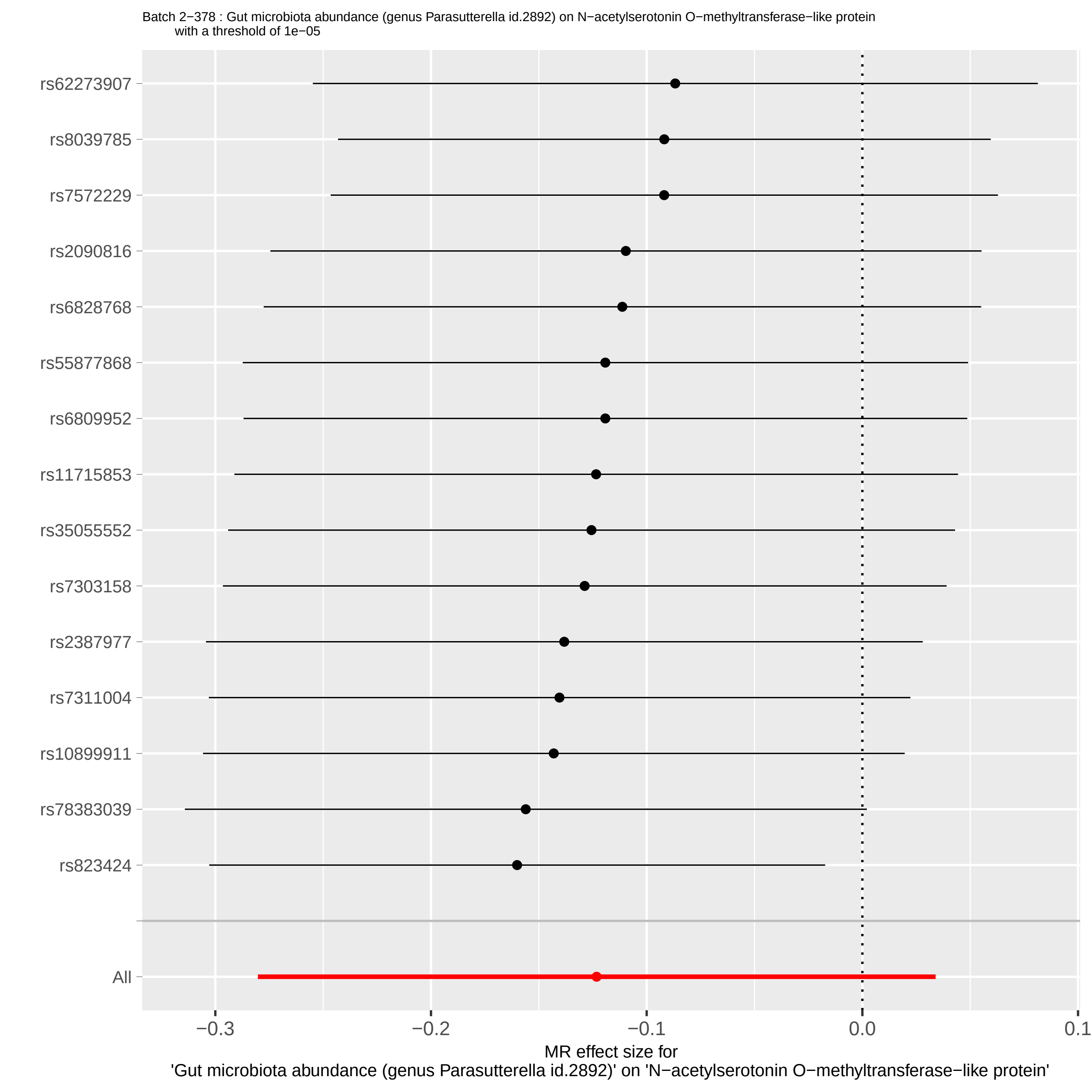
Batch 2−378 : Gut microbiota abundance (genus Parasutterella id.2892) on N−acetylserotonin O−methyltransferase−like protein
with a threshold of 1e−05
rs62273907
rs8039785
rs7572229
rs2090816
rs6828768
rs55877868
rs6809952
rs11715853
rs35055552
rs7303158
rs2387977
rs7311004
rs10899911
rs78383039
rs823424
All
−0.3
−0.2
−0.1
0.0
0.1
MR effect size for
'Gut microbiota abundance (genus Parasutterella id.2892)' on 'N−acetylserotonin O−methyltransferase−like protein'

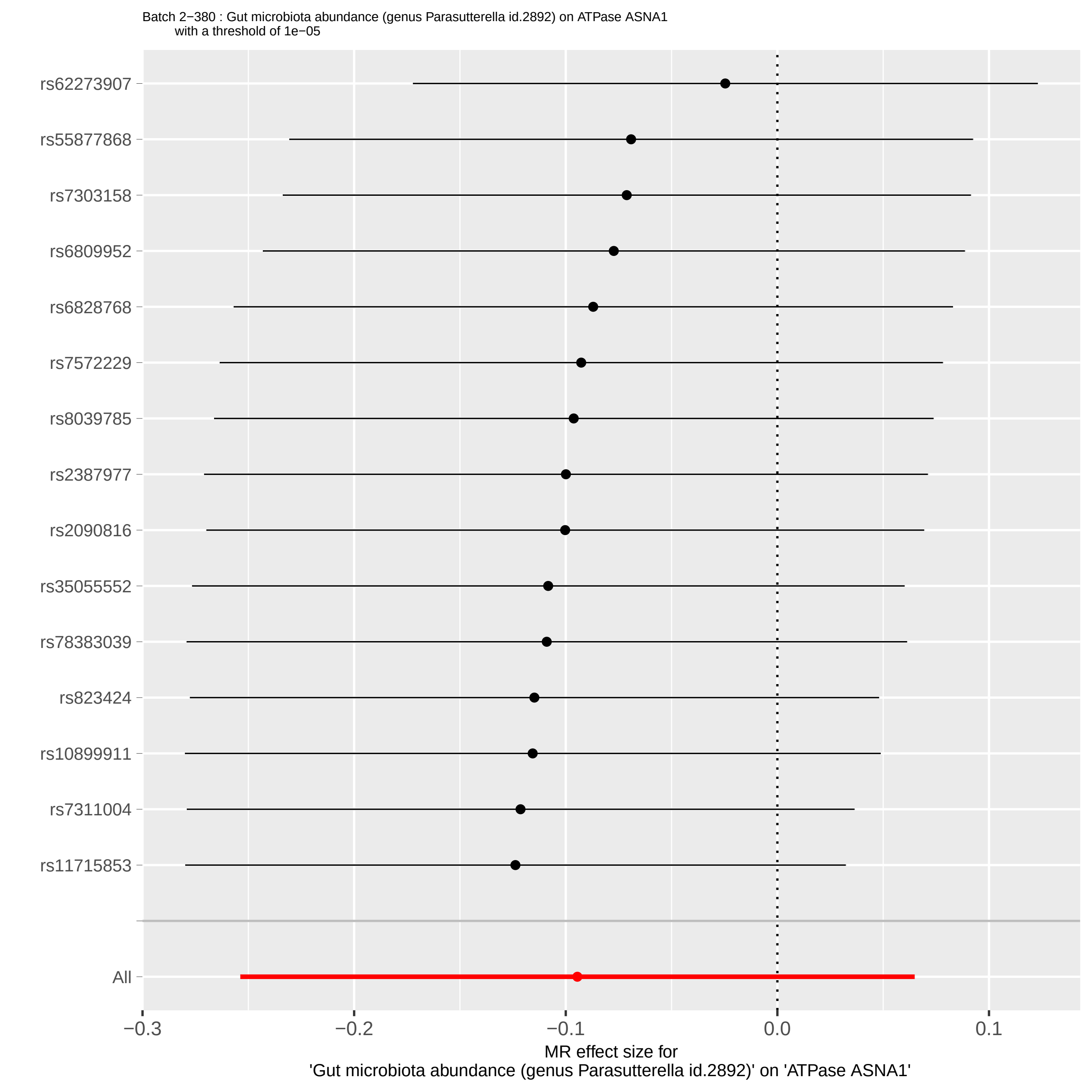

Batch 2−380 : Gut microbiota abundance (genus Parasutterella id.2892) on ATPase ASNA1
with a threshold of 1e−05
rs62273907
rs55877868
rs7303158
rs6809952
rs6828768
rs7572229
rs8039785
rs2387977
rs2090816
rs35055552
rs78383039
rs823424
rs10899911
rs7311004
rs11715853
All
−0.3
−0.2
−0.1
0.0
0.1
MR effect size for
'Gut microbiota abundance (genus Parasutterella id.2892)' on 'ATPase ASNA1'

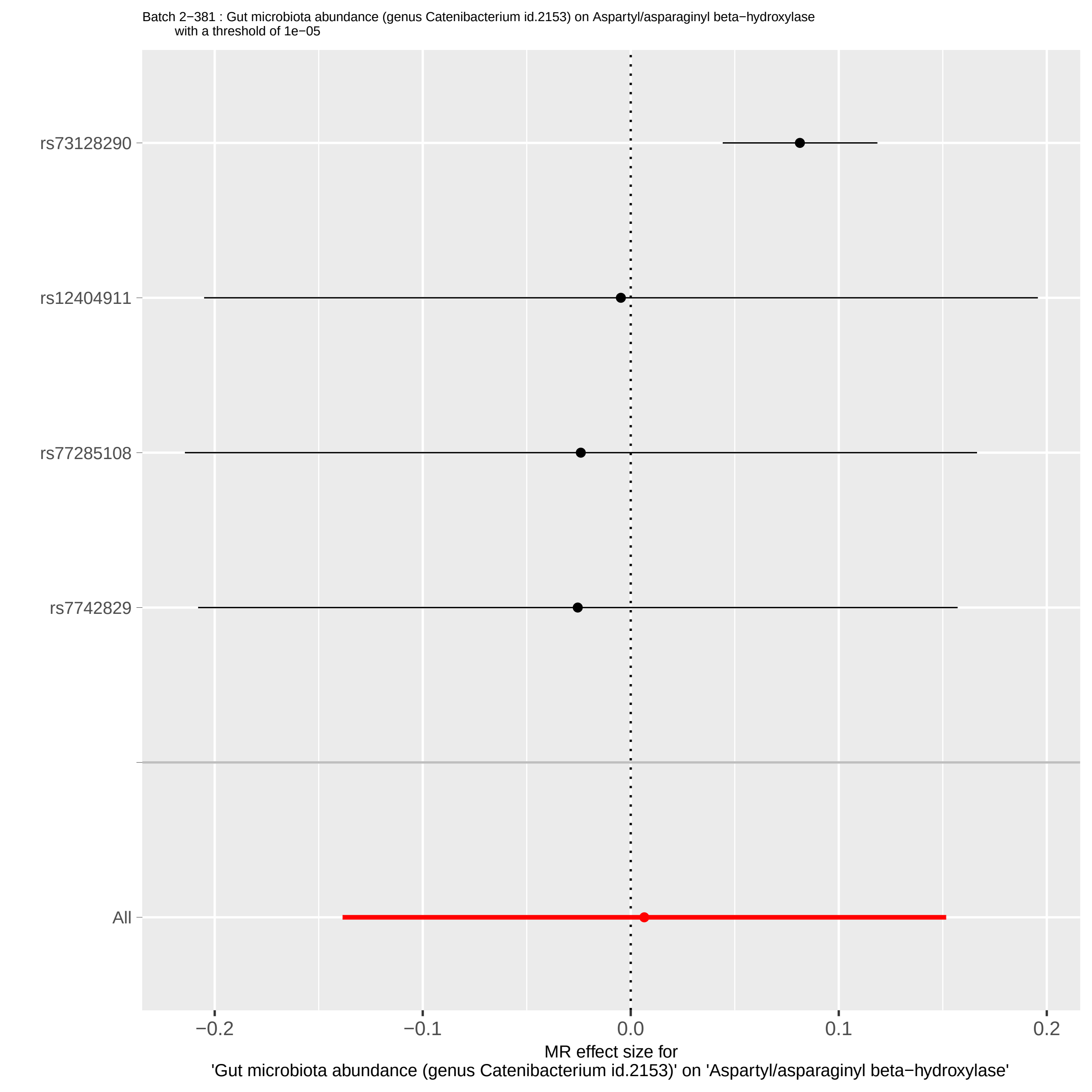
Batch 2−381 : Gut microbiota abundance (genus Catenibacterium id.2153) on Aspartyl/asparaginyl beta−hydroxylase
with a threshold of 1e−05
rs73128290
rs12404911
rs77285108
rs7742829
All
−0.2
−0.1
0.0
0.1
0.2
MR effect size for
'Gut microbiota abundance (genus Catenibacterium id.2153)' on 'Aspartyl/asparaginyl beta−hydroxylase'

Batch 2−382 : Gut microbiota abundance (genus Parasutterella id.2892) on Aspartyl/asparaginyl beta−hydroxylase with a threshold of 1e−05

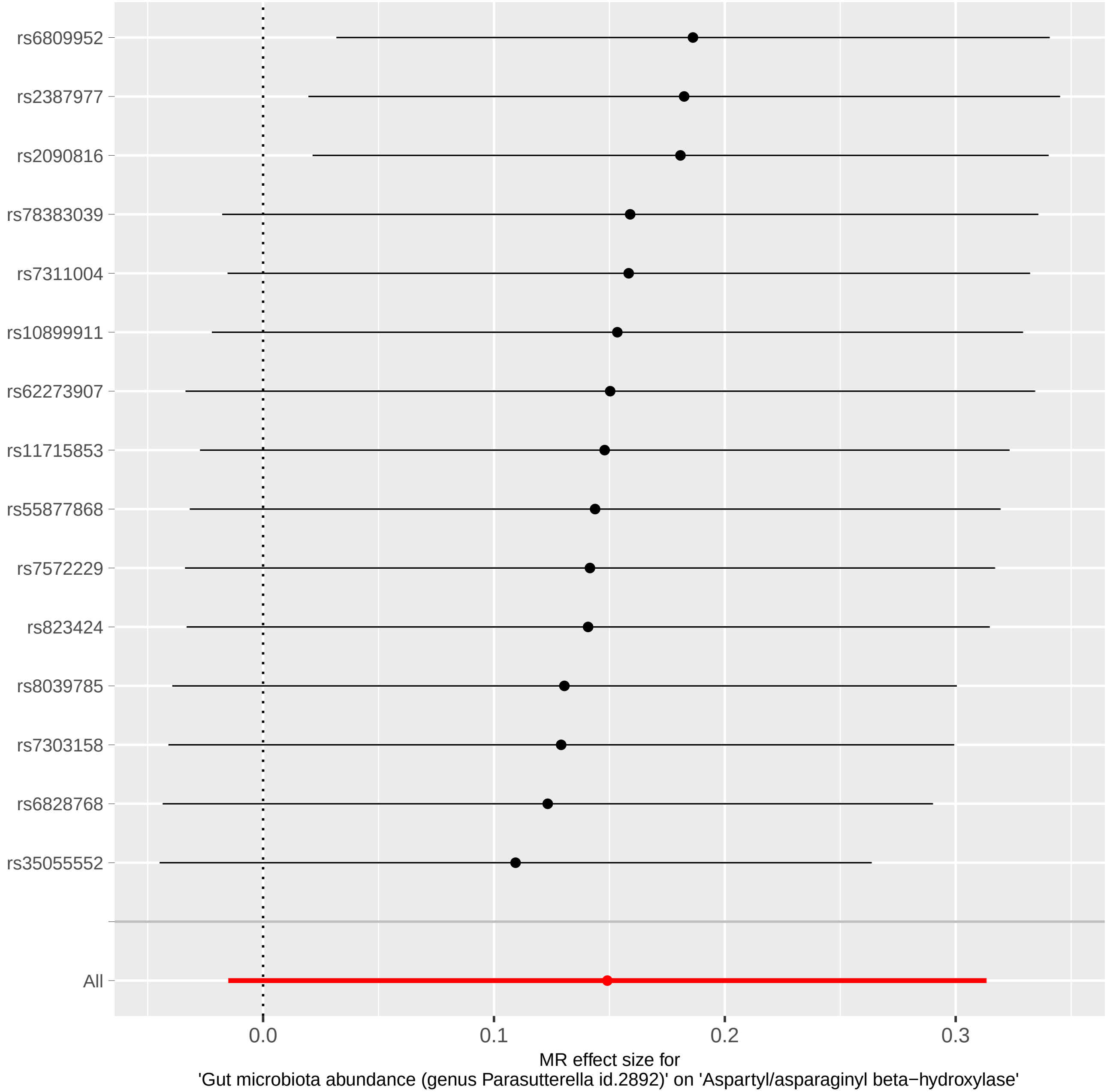

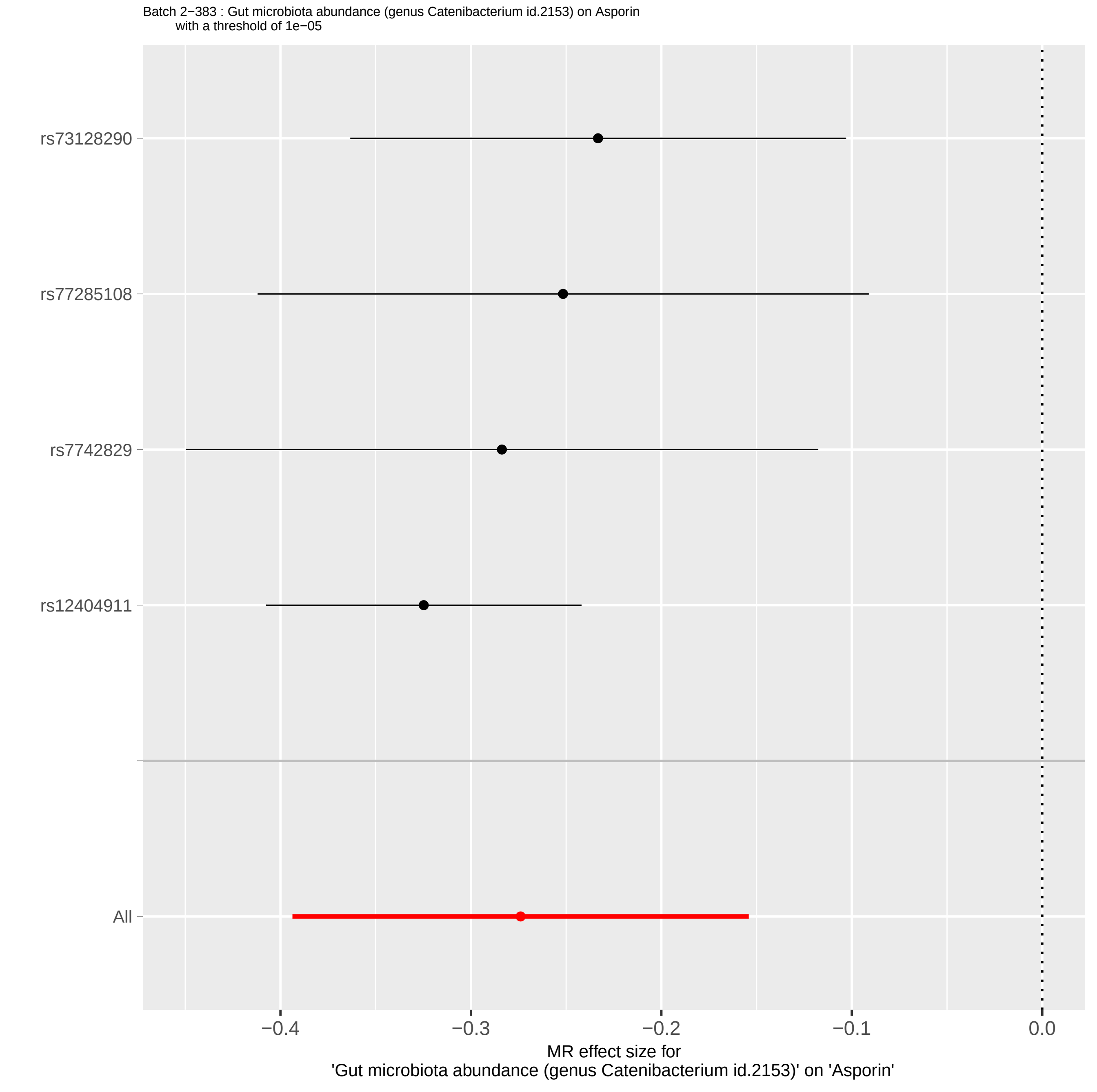
Batch 2−383 : Gut microbiota abundance (genus Catenibacterium id.2153) on Asporin
with a threshold of 1e−05
rs73128290
rs77285108
rs7742829
rs12404911
All
−0.4
−0.3
−0.2
−0.1
0.0
MR effect size for
'Gut microbiota abundance (genus Catenibacterium id.2153)' on 'Asporin'

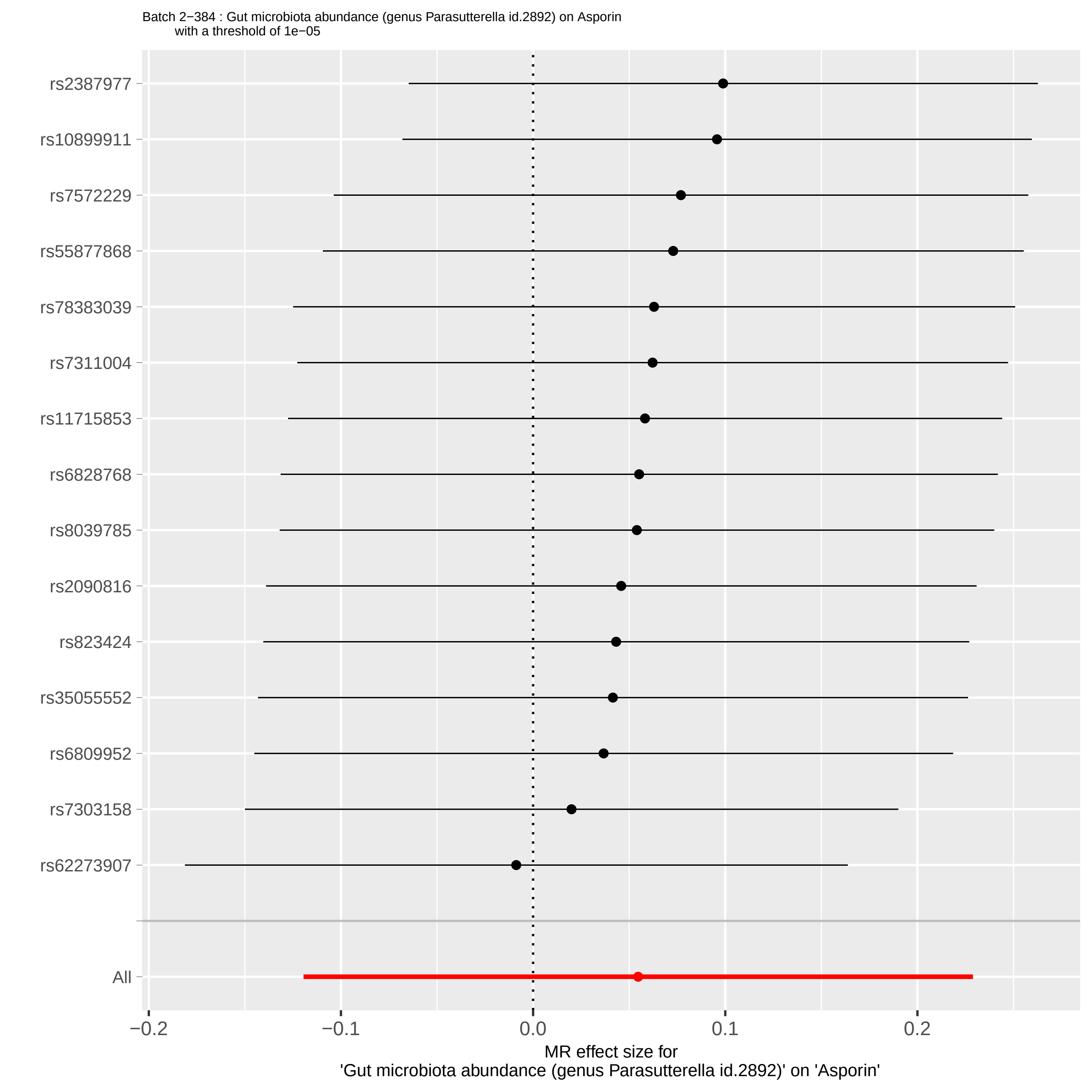

Batch 2−384 : Gut microbiota abundance (genus Parasutterella id.2892) on Asporin
with a threshold of 1e−05
rs2387977
rs10899911
rs7572229
rs55877868
rs78383039
rs7311004
rs11715853
rs6828768
rs8039785
rs2090816
rs823424
rs35055552
rs6809952
rs7303158
rs62273907
All
−0.2
−0.1
0.0
0.1
0.2
MR effect size for
'Gut microbiota abundance (genus Parasutterella id.2892)' on 'Asporin'

Batch 2−385 : Gut microbiota abundance (genus Catenibacterium id.2153) on Retroviral−like aspartic protease 1
with a threshold of 1e−05

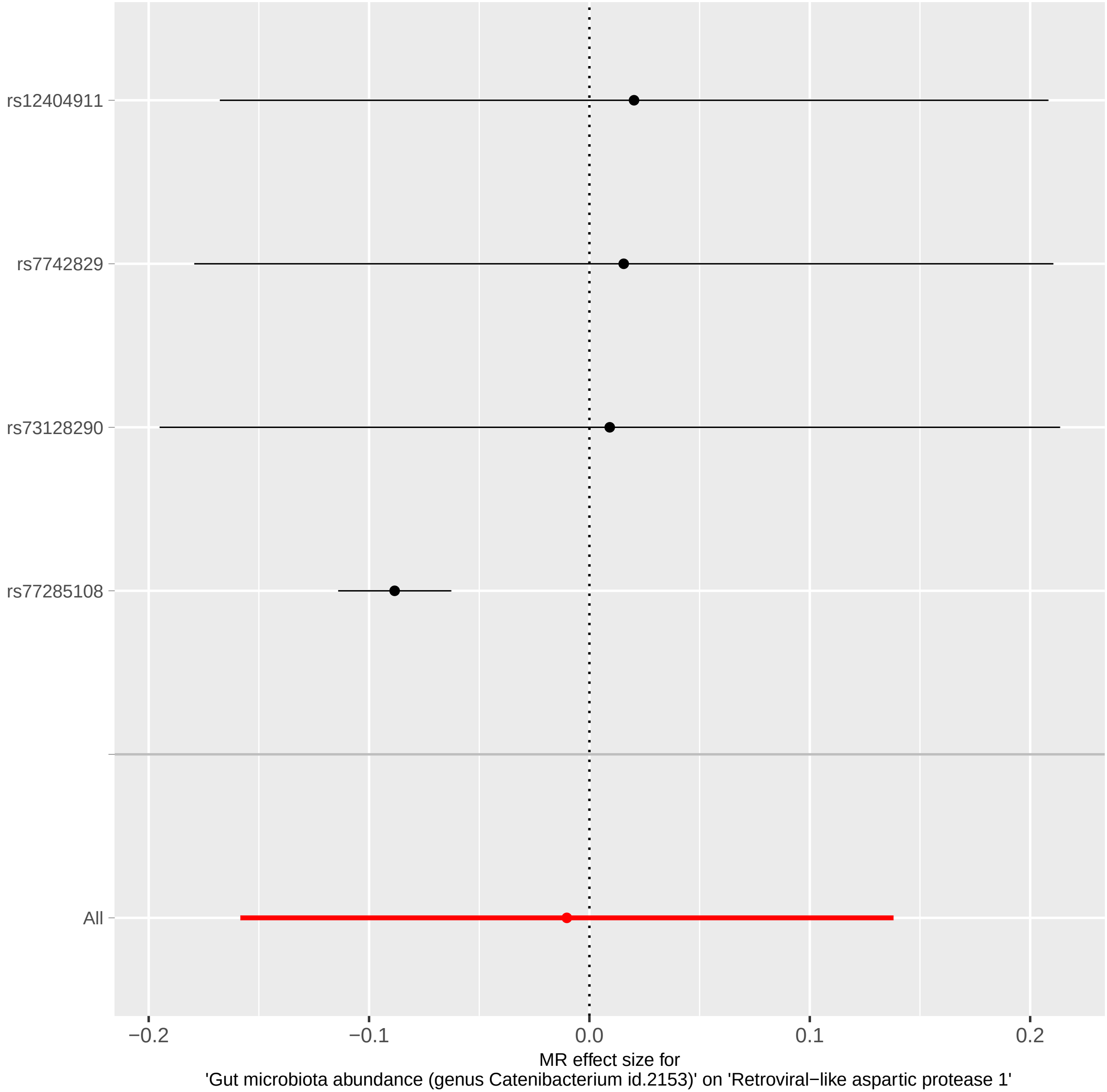

Batch 2−386 : Gut microbiota abundance (genus Parasutterella id.2892) on Retroviral−like aspartic protease 1
with a threshold of 1e−05

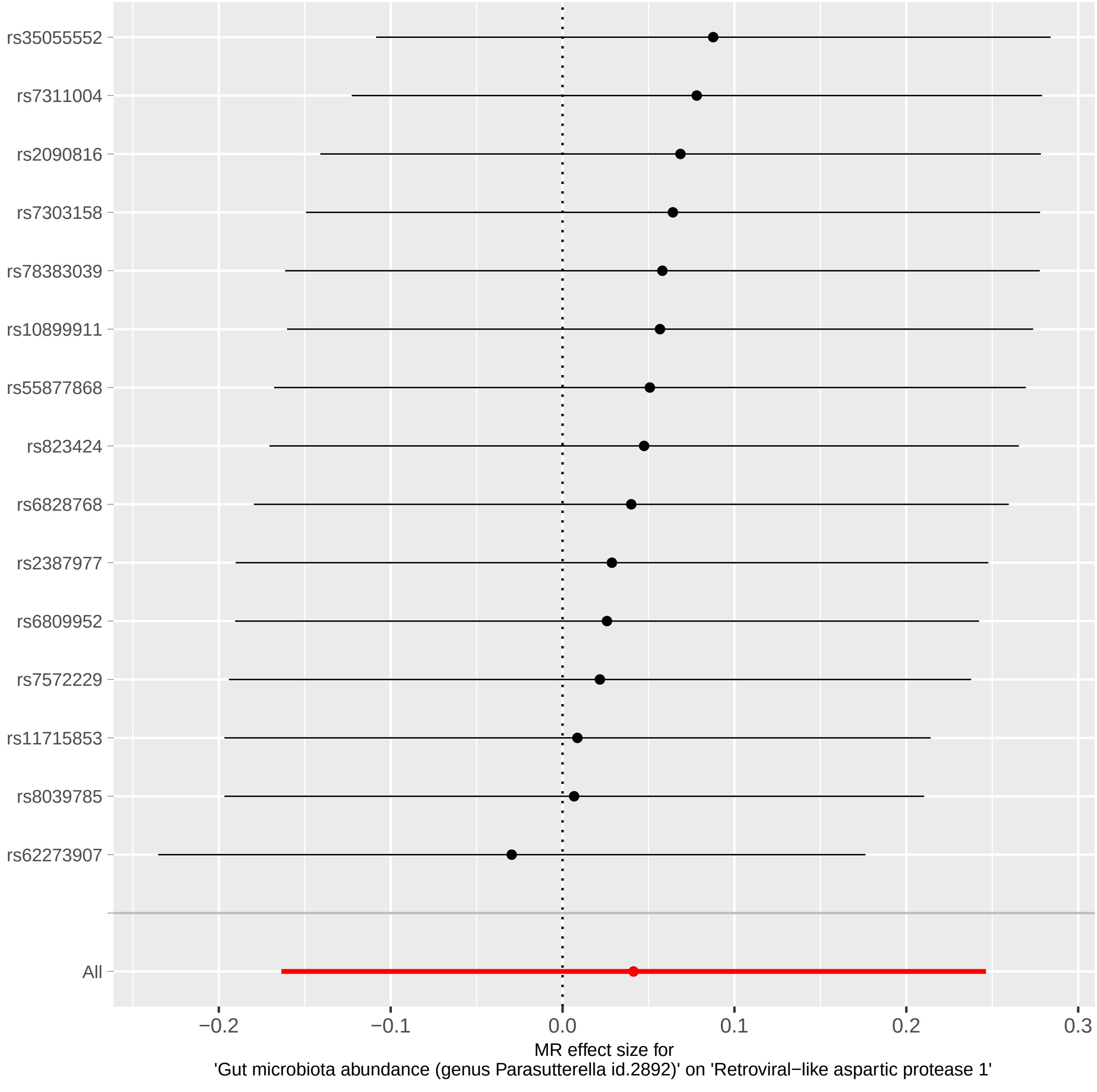

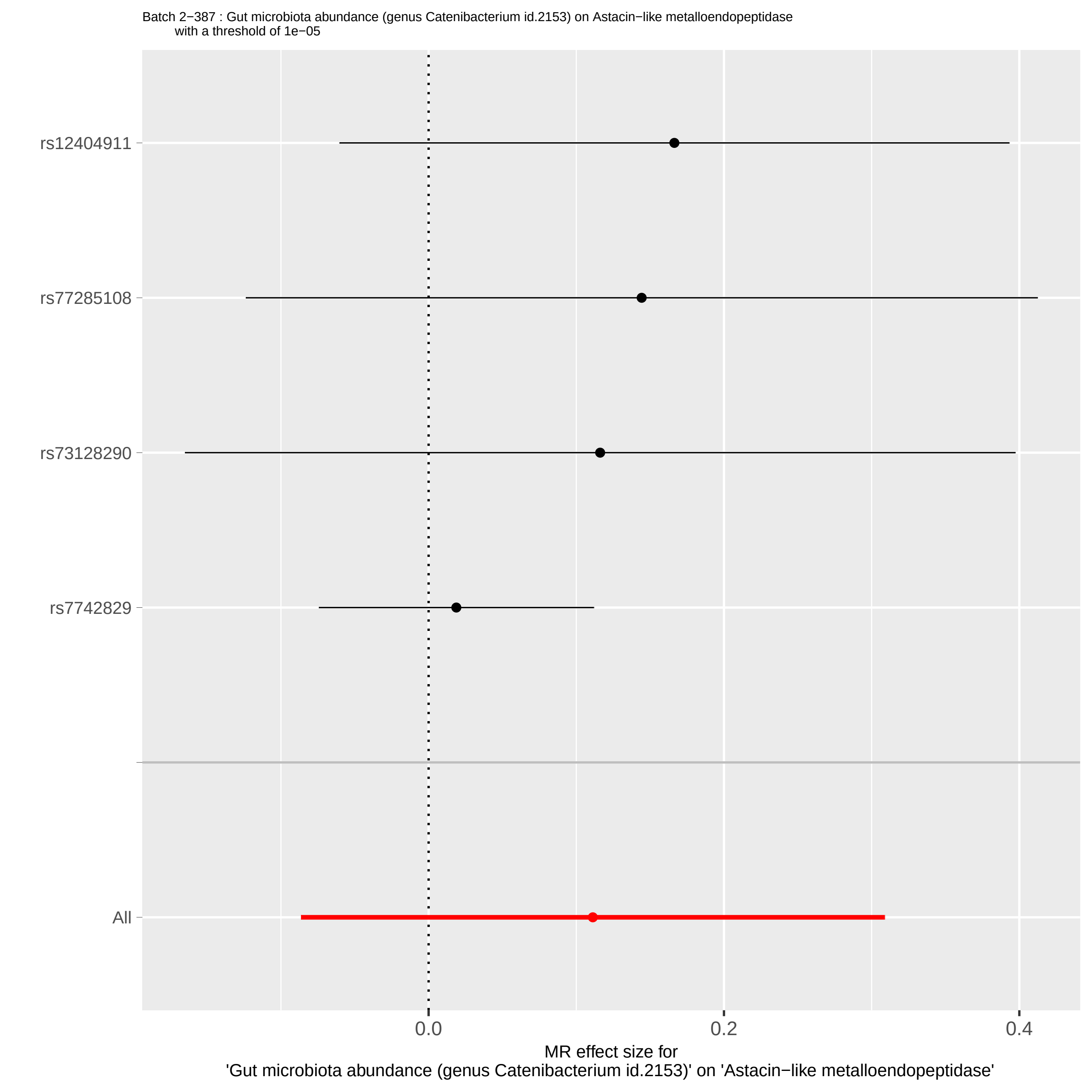

Batch 2−387 : Gut microbiota abundance (genus Catenibacterium id.2153) on Astacin−like metalloendopeptidase
with a threshold of 1e−05
rs12404911
rs77285108
rs73128290
rs7742829
All
0.0
0.2
0.4
MR effect size for
'Gut microbiota abundance (genus Catenibacterium id.2153)' on 'Astacin−like metalloendopeptidase'

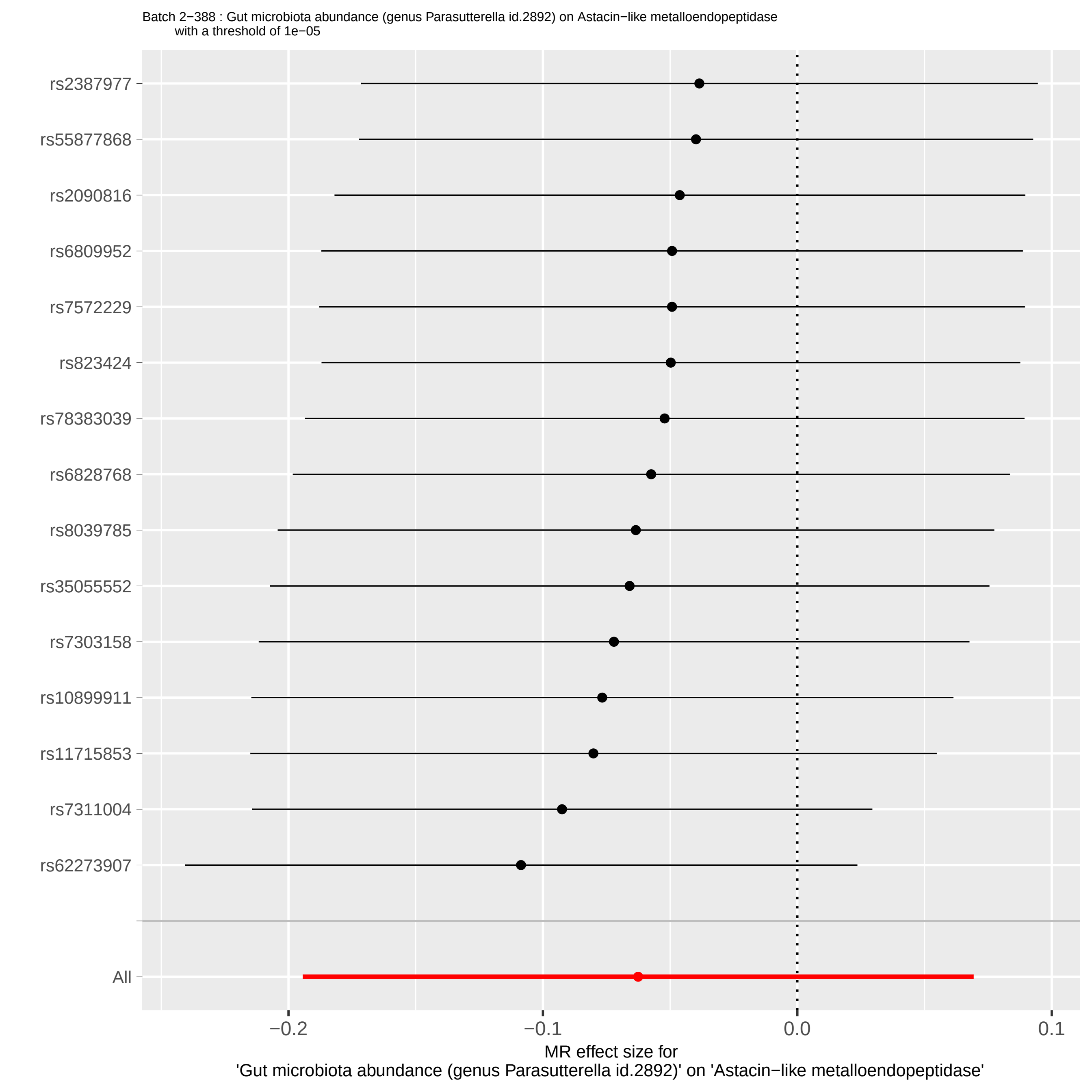

Batch 2−388 : Gut microbiota abundance (genus Parasutterella id.2892) on Astacin−like metalloendopeptidase
with a threshold of 1e−05
rs2387977
rs55877868
rs2090816
rs6809952
rs7572229
rs823424
rs78383039
rs6828768
rs8039785
rs35055552
rs7303158
rs10899911
rs11715853
rs7311004
rs62273907
All
−0.2
−0.1
0.0
0.1
MR effect size for
'Gut microbiota abundance (genus Parasutterella id.2892)' on 'Astacin−like metalloendopeptidase'

Batch 2−389 : Gut microbiota abundance (genus Catenibacterium id.2153) on ATPase family AAA domain−containing protein 1 with a threshold of 1e−05

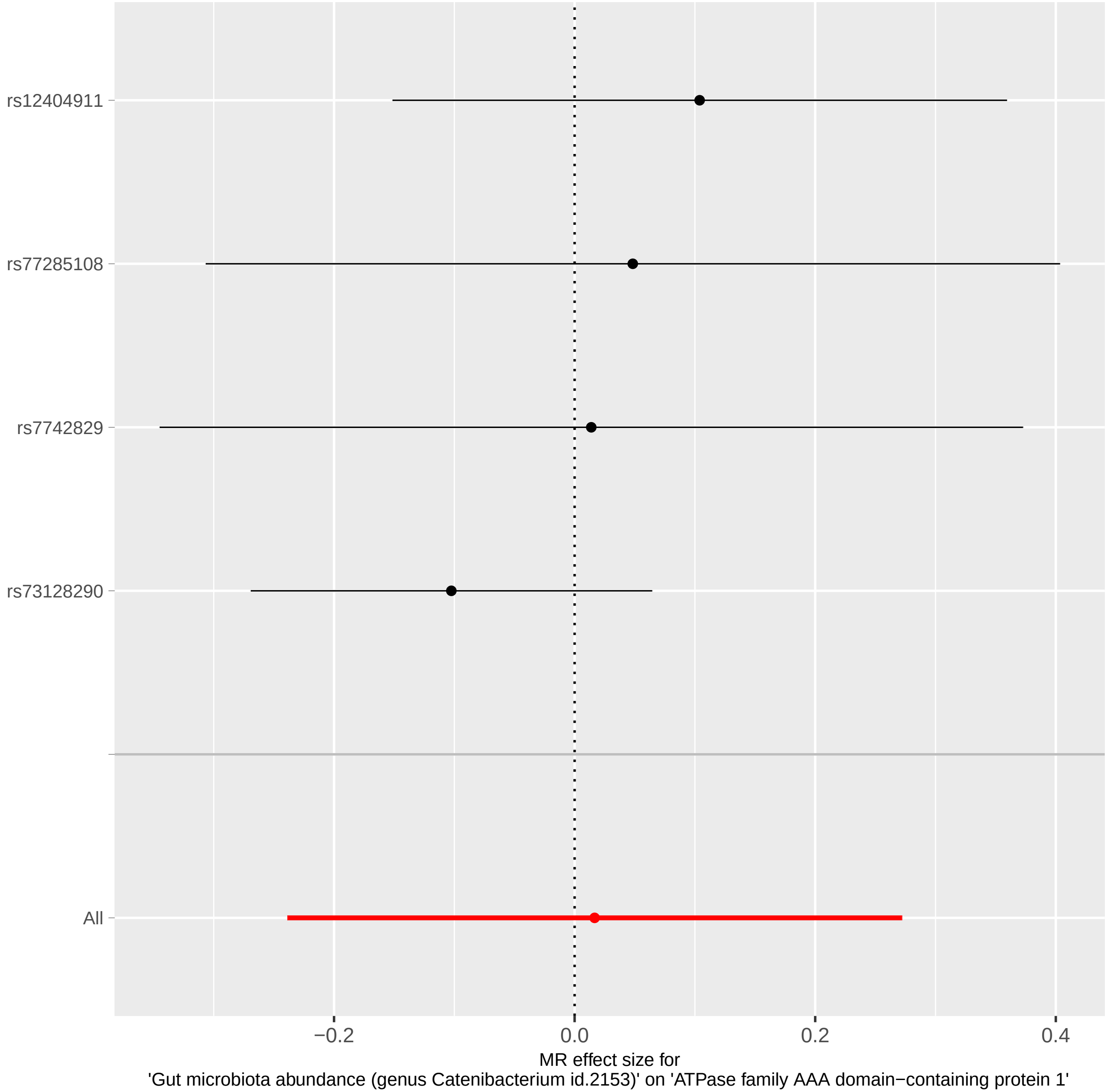

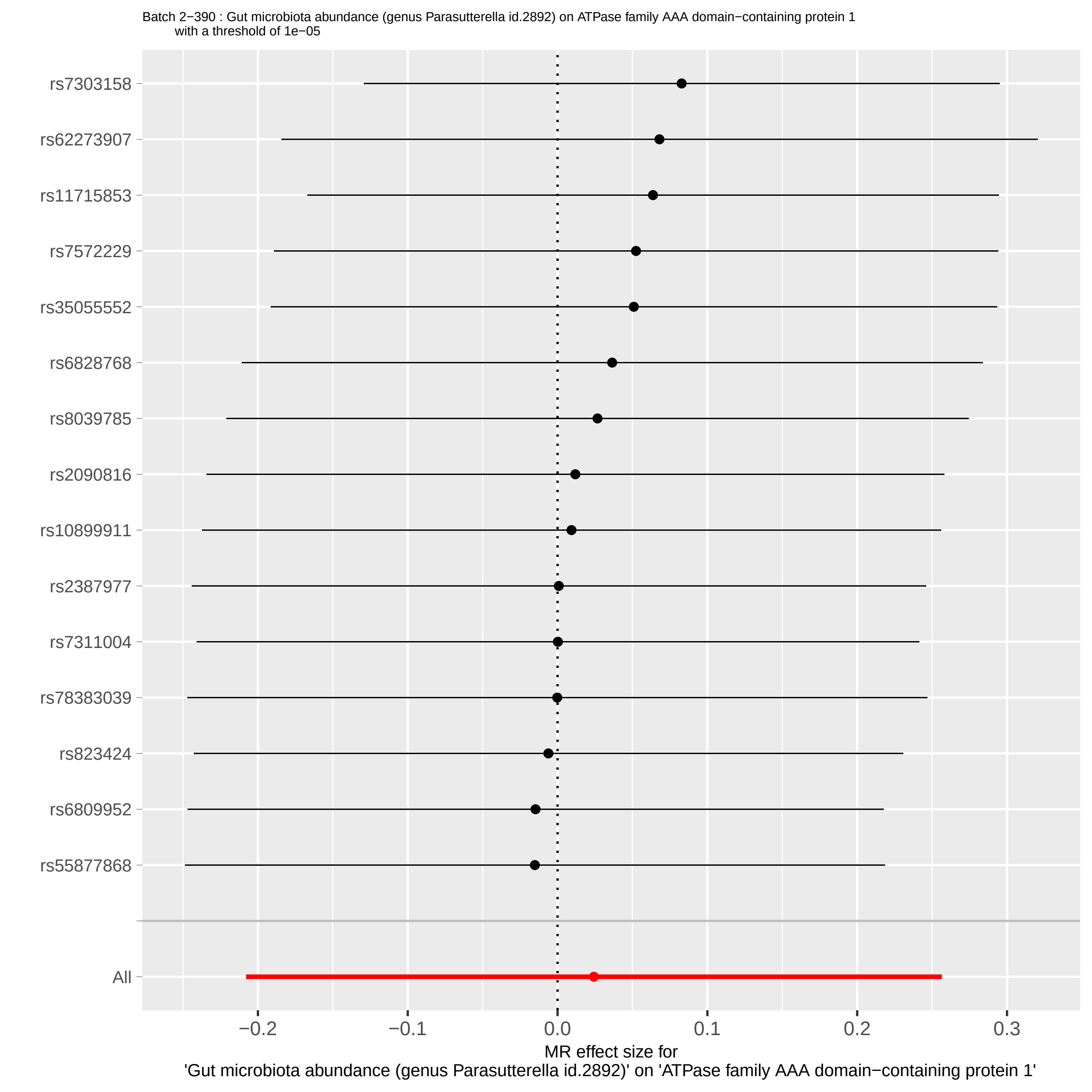

Batch 2−390 : Gut microbiota abundance (genus Parasutterella id.2892) on ATPase family AAA domain−containing protein 1
with a threshold of 1e−05
rs7303158
rs62273907
rs11715853
rs7572229
rs35055552
rs6828768
rs8039785
rs2090816
rs10899911
rs2387977
rs7311004
rs78383039
rs823424
rs6809952
rs55877868
All
−0.2
−0.1
0.0
0.1
0.2
0.3
MR effect size for
'Gut microbiota abundance (genus Parasutterella id.2892)' on 'ATPase family AAA domain−containing protein 1'

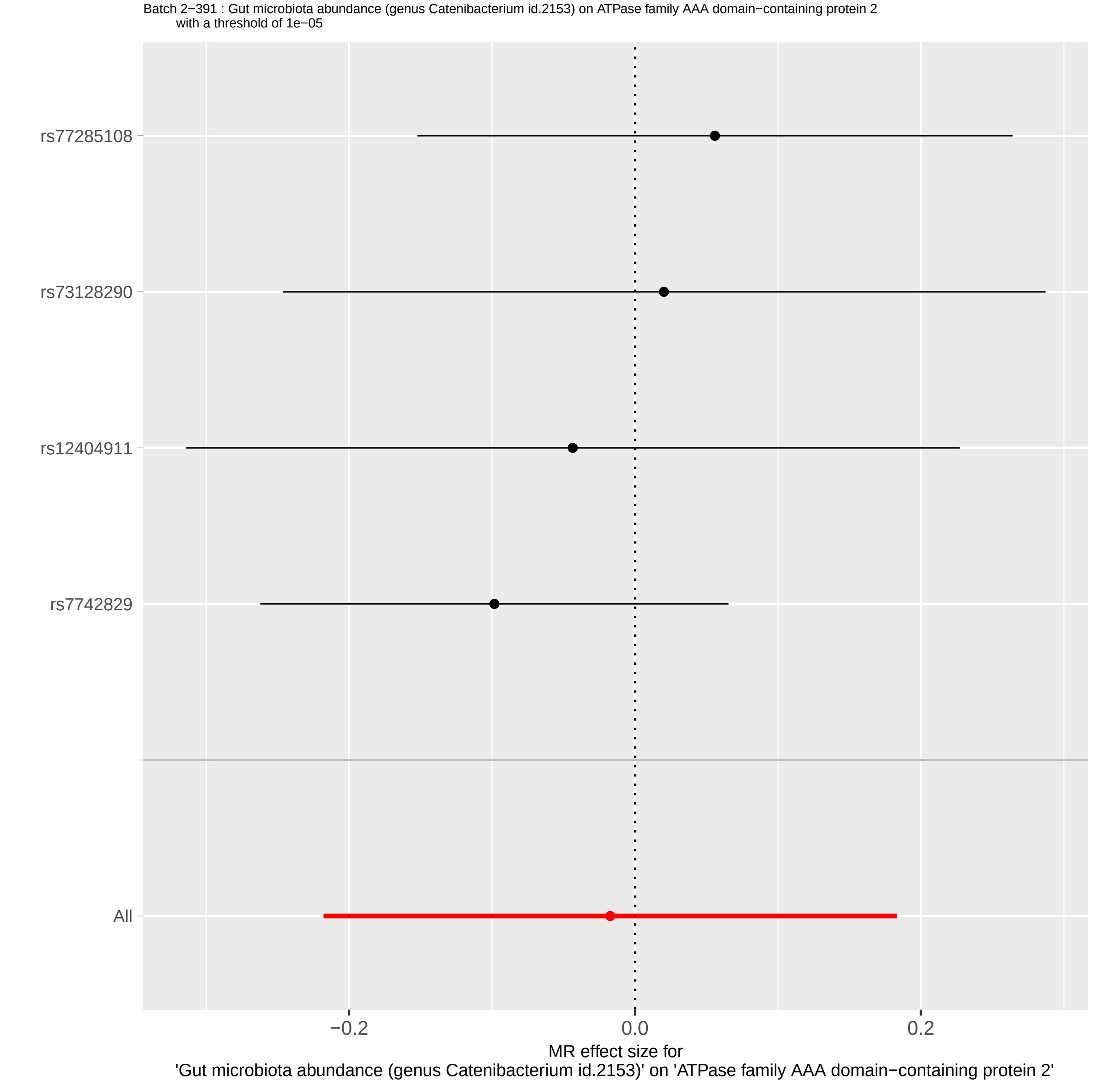

Batch 2−391 : Gut microbiota abundance (genus Catenibacterium id.2153) on ATPase family AAA domain−containing protein 2
with a threshold of 1e−05
rs77285108
rs73128290
rs12404911
rs7742829
All
−0.2
0.0
0.2
MR effect size for
'Gut microbiota abundance (genus Catenibacterium id.2153)' on 'ATPase family AAA domain−containing protein 2'

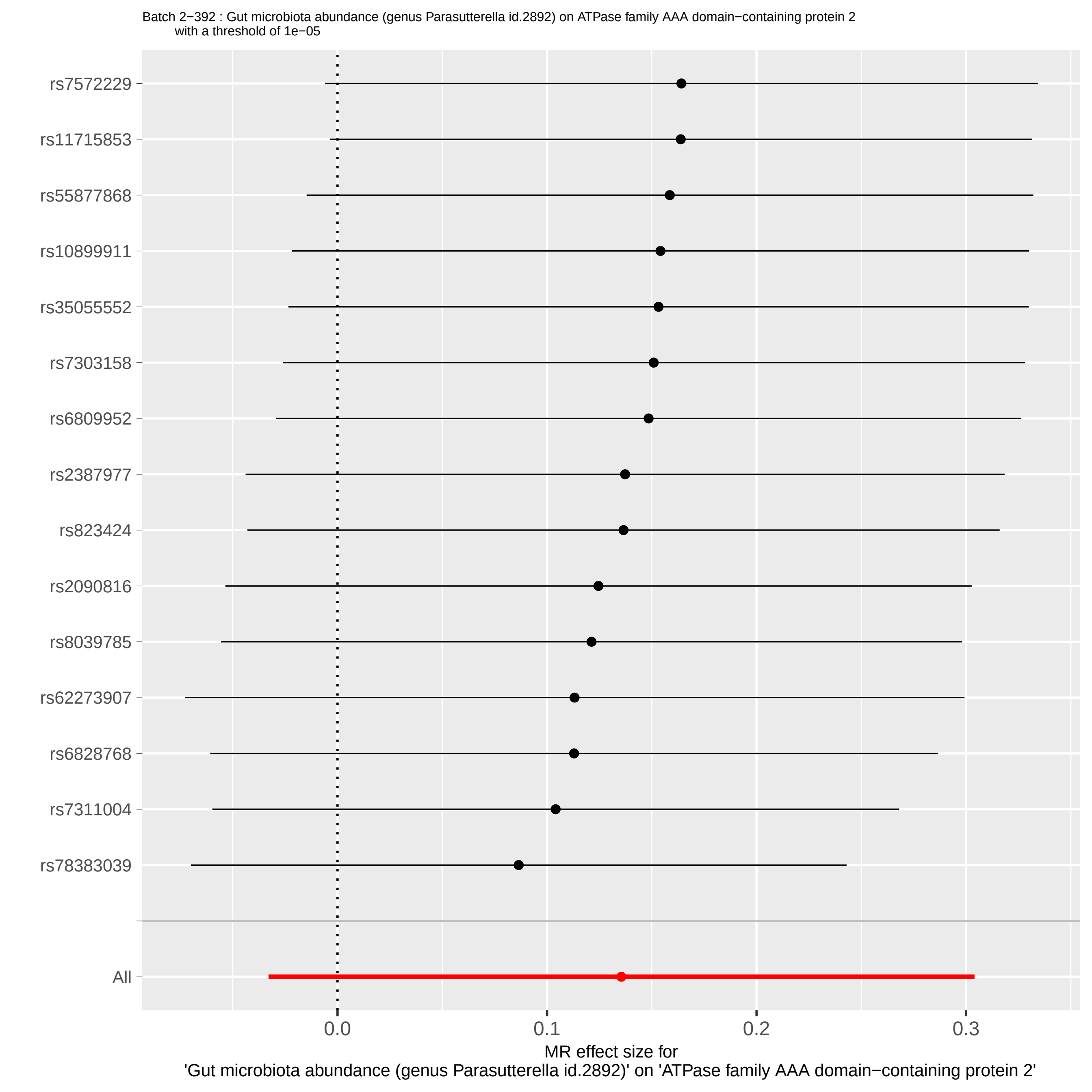

Batch 2−392 : Gut microbiota abundance (genus Parasutterella id.2892) on ATPase family AAA domain−containing protein 2
with a threshold of 1e−05
rs7572229
rs11715853
rs55877868
rs10899911
rs35055552
rs7303158
rs6809952
rs2387977
rs823424
rs2090816
rs8039785
rs62273907
rs6828768
rs7311004
rs78383039
All
0.0
0.1
0.2
0.3
MR effect size for
'Gut microbiota abundance (genus Parasutterella id.2892)' on 'ATPase family AAA domain−containing protein 2'

Batch 2−393 : Gut microbiota abundance (genus Catenibacterium id.2153) on Cyclic AMP−dependent transcription factor ATF−6 alpha
with a threshold of 1e−05

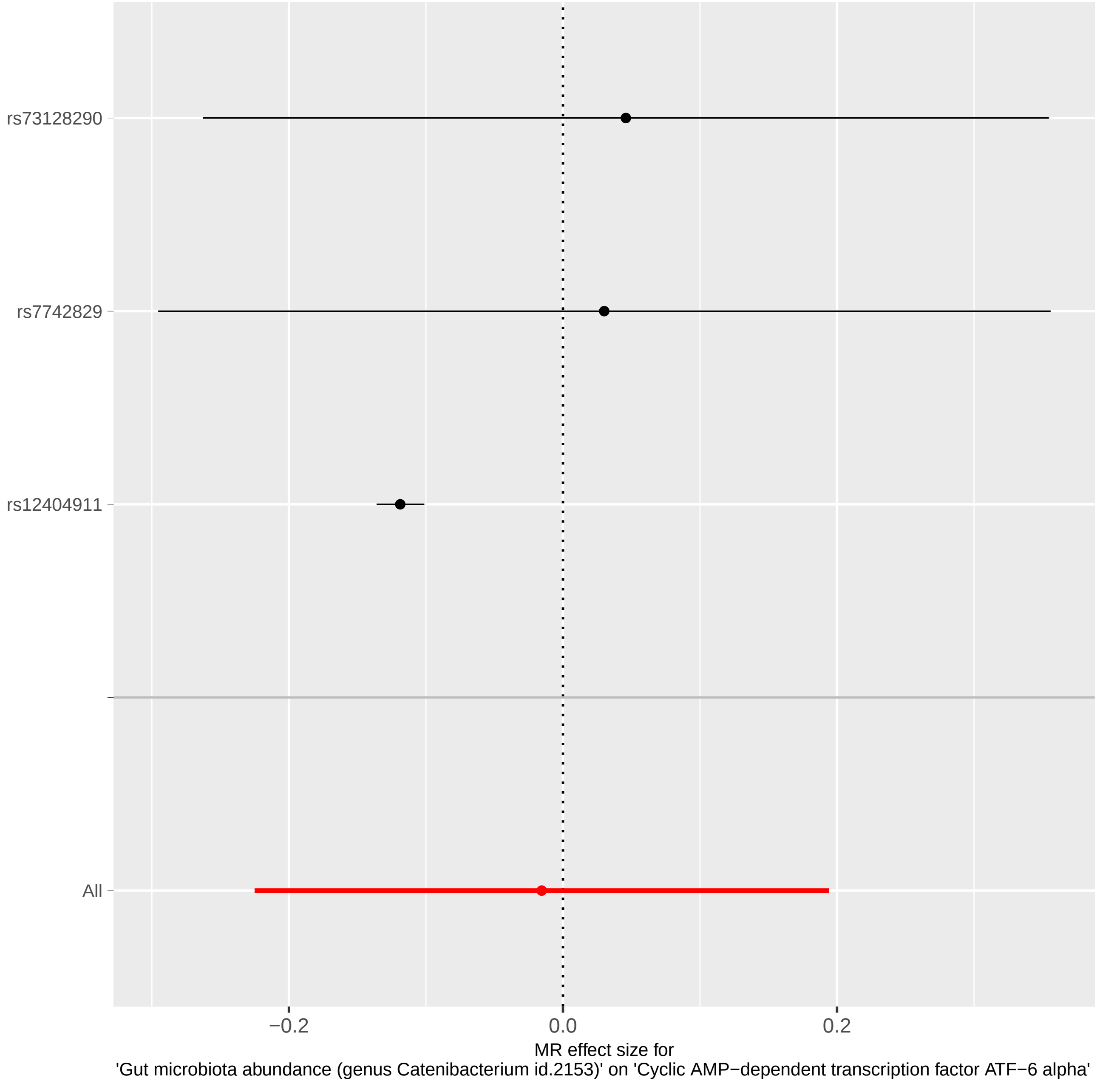

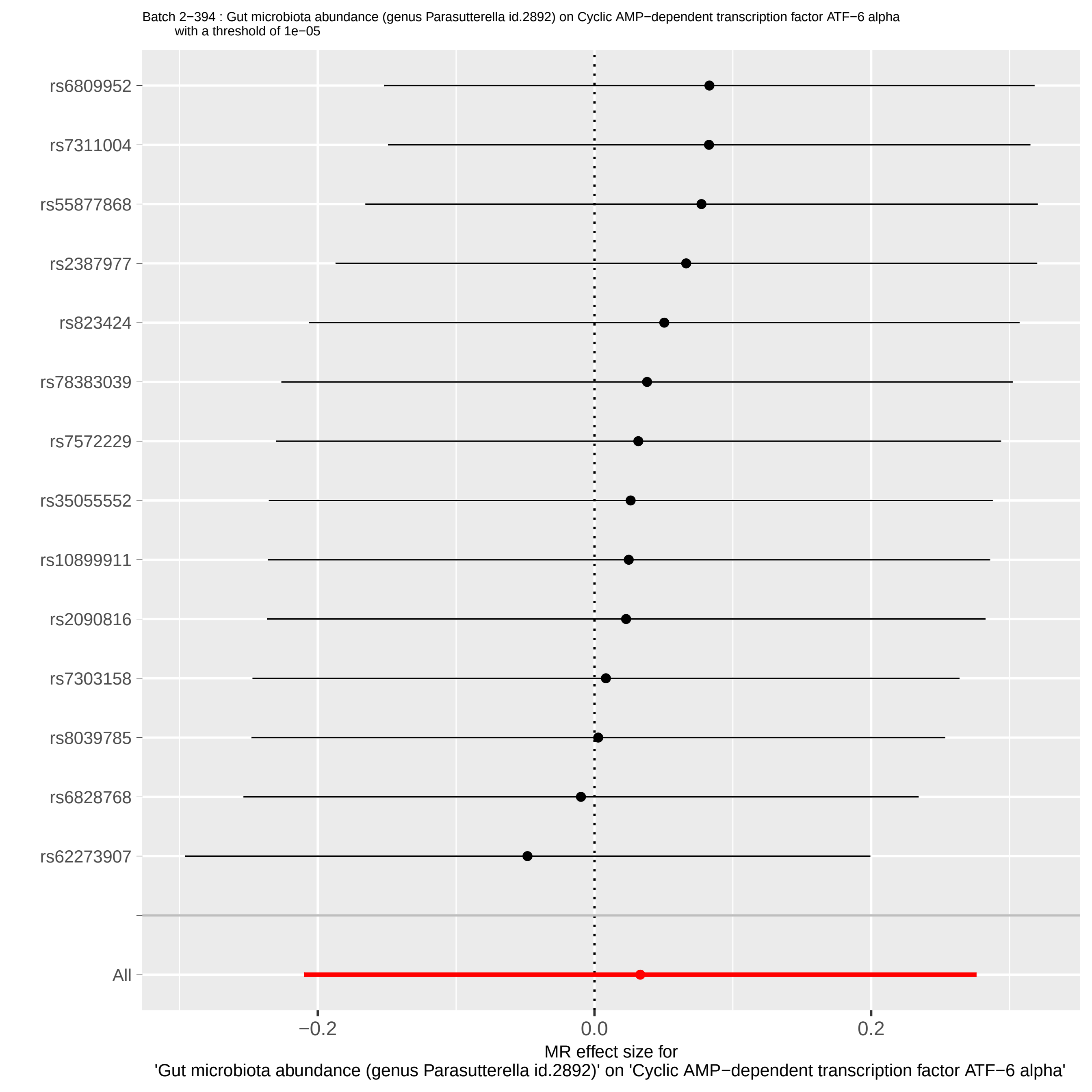

Batch 2−394 : Gut microbiota abundance (genus Parasutterella id.2892) on Cyclic AMP−dependent transcription factor ATF−6 alpha
with a threshold of 1e−05
rs6809952
rs7311004
rs55877868
rs2387977
rs823424
rs78383039
rs7572229
rs35055552
rs10899911
rs2090816
rs7303158
rs8039785
rs6828768
rs62273907
All
−0.2
0.0
0.2
MR effect size for
'Gut microbiota abundance (genus Parasutterella id.2892)' on 'Cyclic AMP−dependent transcription factor ATF−6 alpha'

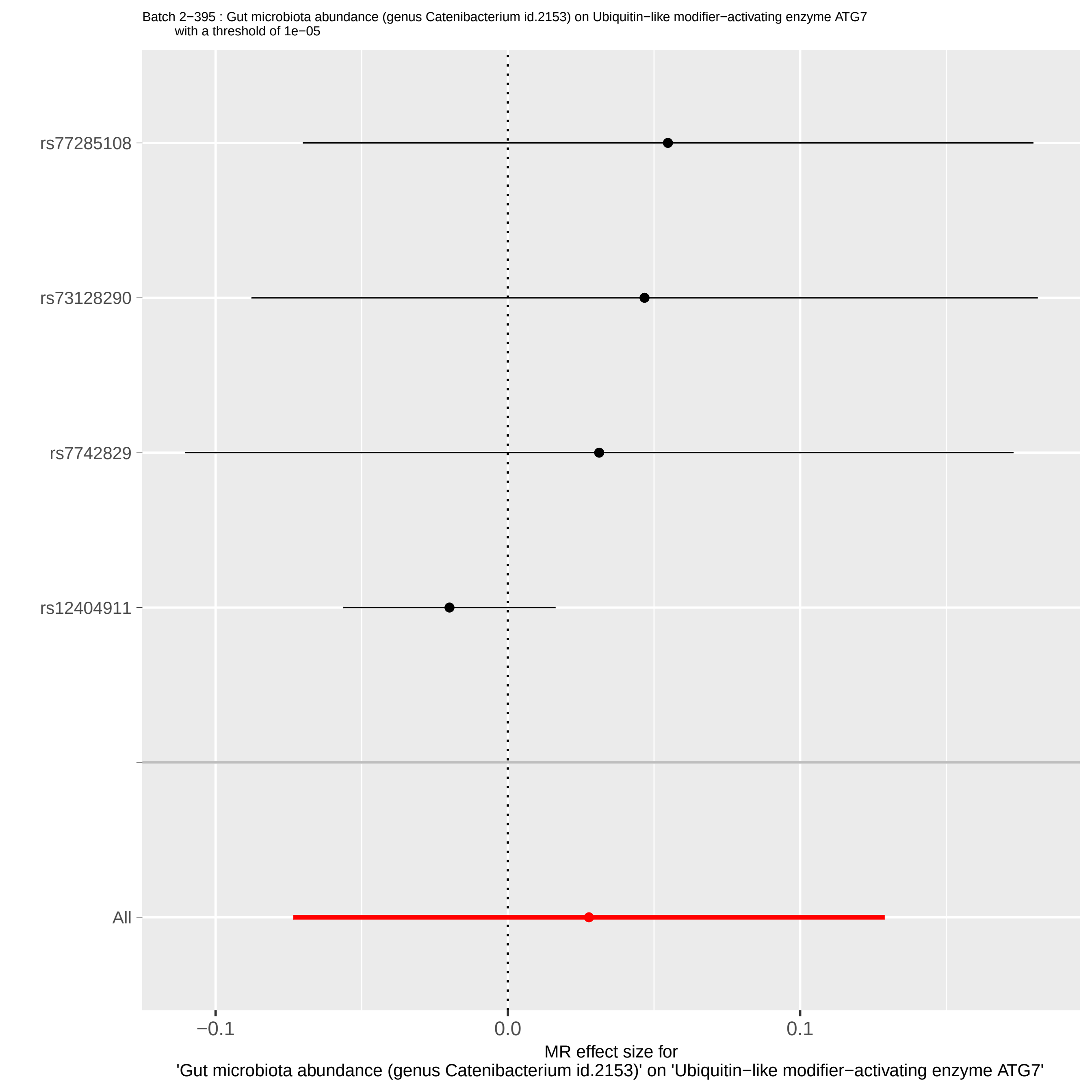
Batch 2−395 : Gut microbiota abundance (genus Catenibacterium id.2153) on Ubiquitin−like modifier−activating enzyme ATG7
with a threshold of 1e−05
rs77285108
rs73128290
rs7742829
rs12404911
All
−0.1
0.0
0.1
MR effect size for
'Gut microbiota abundance (genus Catenibacterium id.2153)' on 'Ubiquitin−like modifier−activating enzyme ATG7'

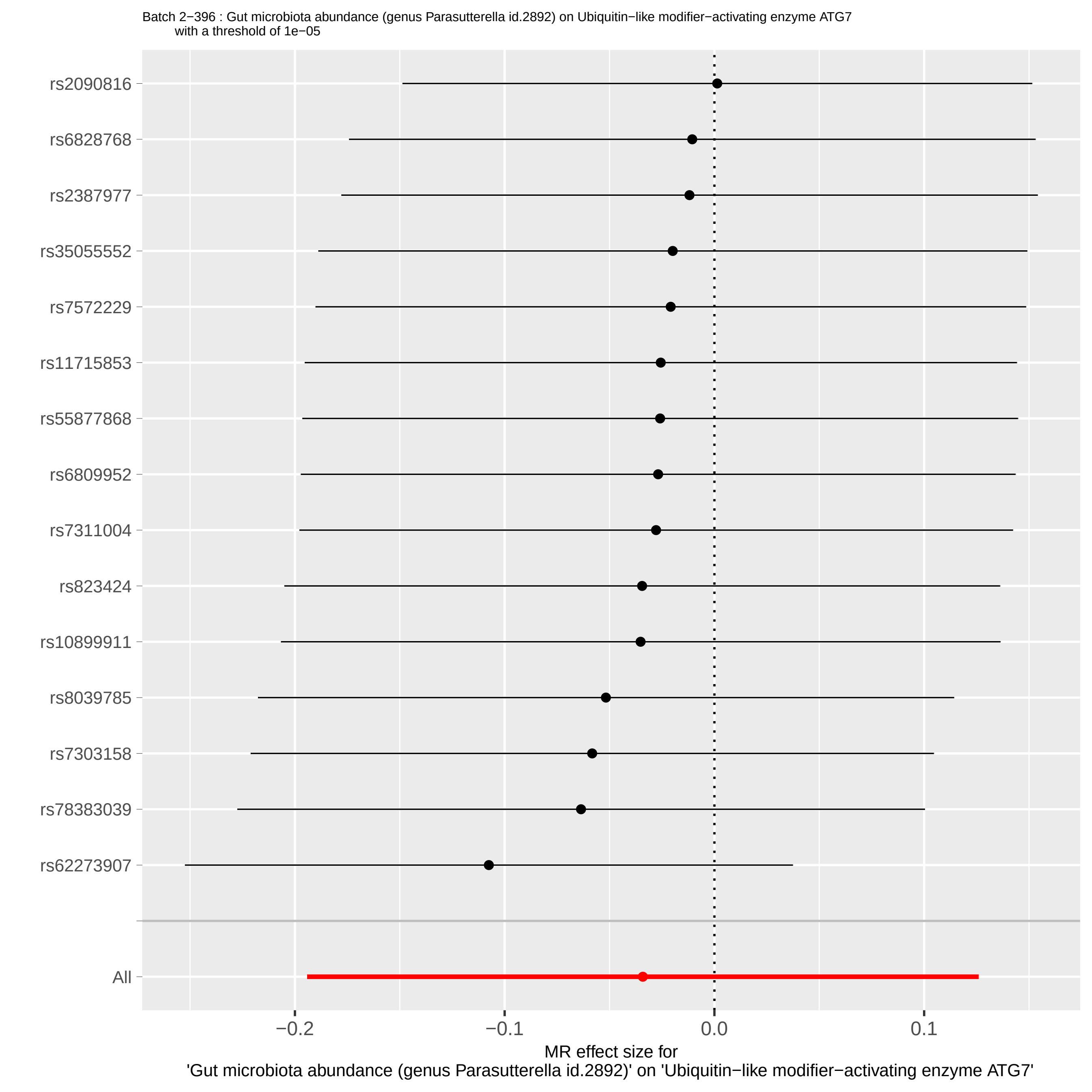
Batch 2−396 : Gut microbiota abundance (genus Parasutterella id.2892) on Ubiquitin−like modifier−activating enzyme ATG7
with a threshold of 1e−05
rs2090816
rs6828768
rs2387977
rs35055552
rs7572229
rs11715853
rs55877868
rs6809952
rs7311004
rs823424
rs10899911
rs8039785
rs7303158
rs78383039
rs62273907
All
−0.2
−0.1
0.0
0.1
MR effect size for
'Gut microbiota abundance (genus Parasutterella id.2892)' on 'Ubiquitin−like modifier−activating enzyme ATG7'

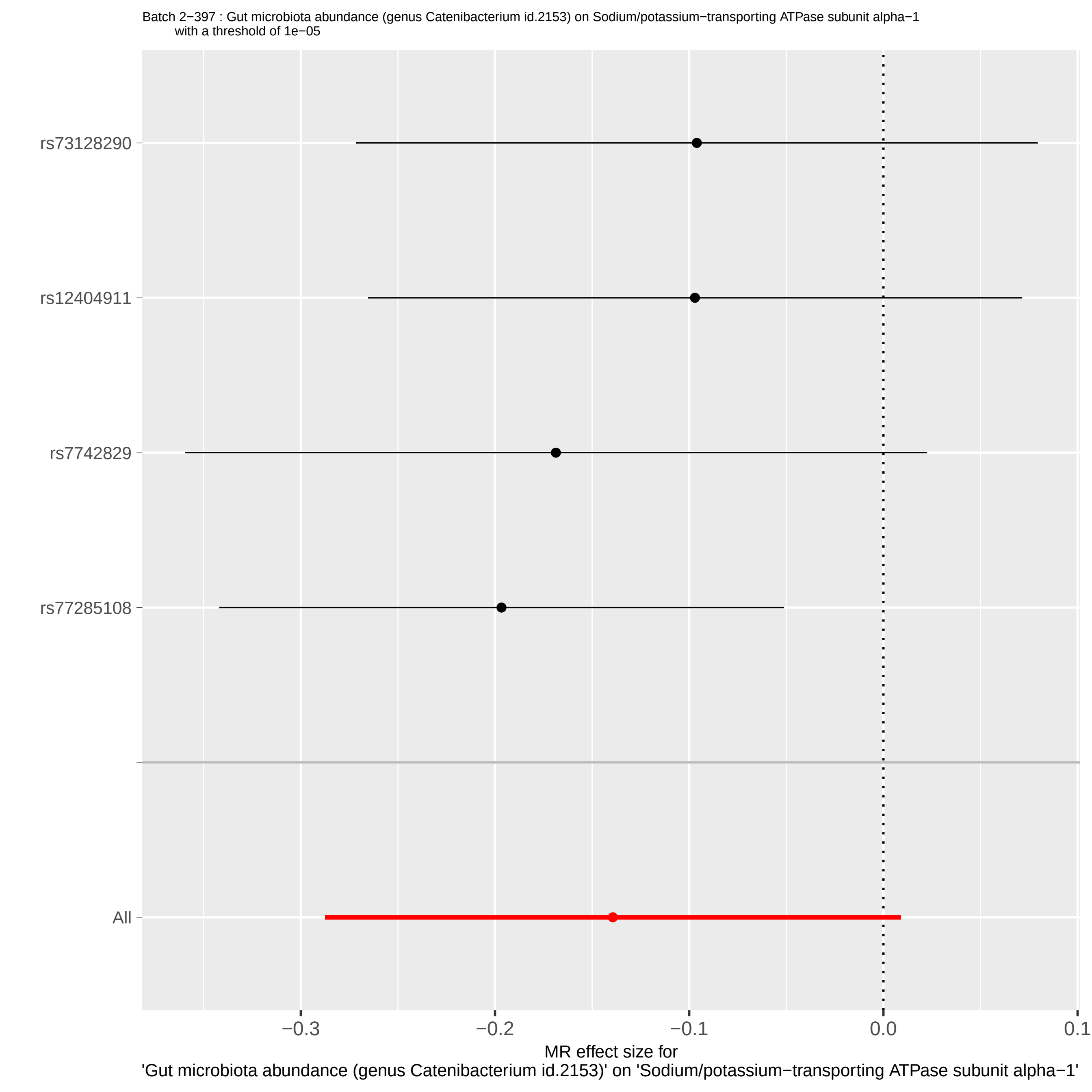

Batch 2−397 : Gut microbiota abundance (genus Catenibacterium id.2153) on Sodium/potassium−transporting ATPase subunit alpha−1
with a threshold of 1e−05
rs73128290
rs12404911
rs7742829
rs77285108
All
−0.3
−0.2
−0.1
0.0
0.1
MR effect size for
'Gut microbiota abundance (genus Catenibacterium id.2153)' on 'Sodium/potassium−transporting ATPase subunit alpha−1'

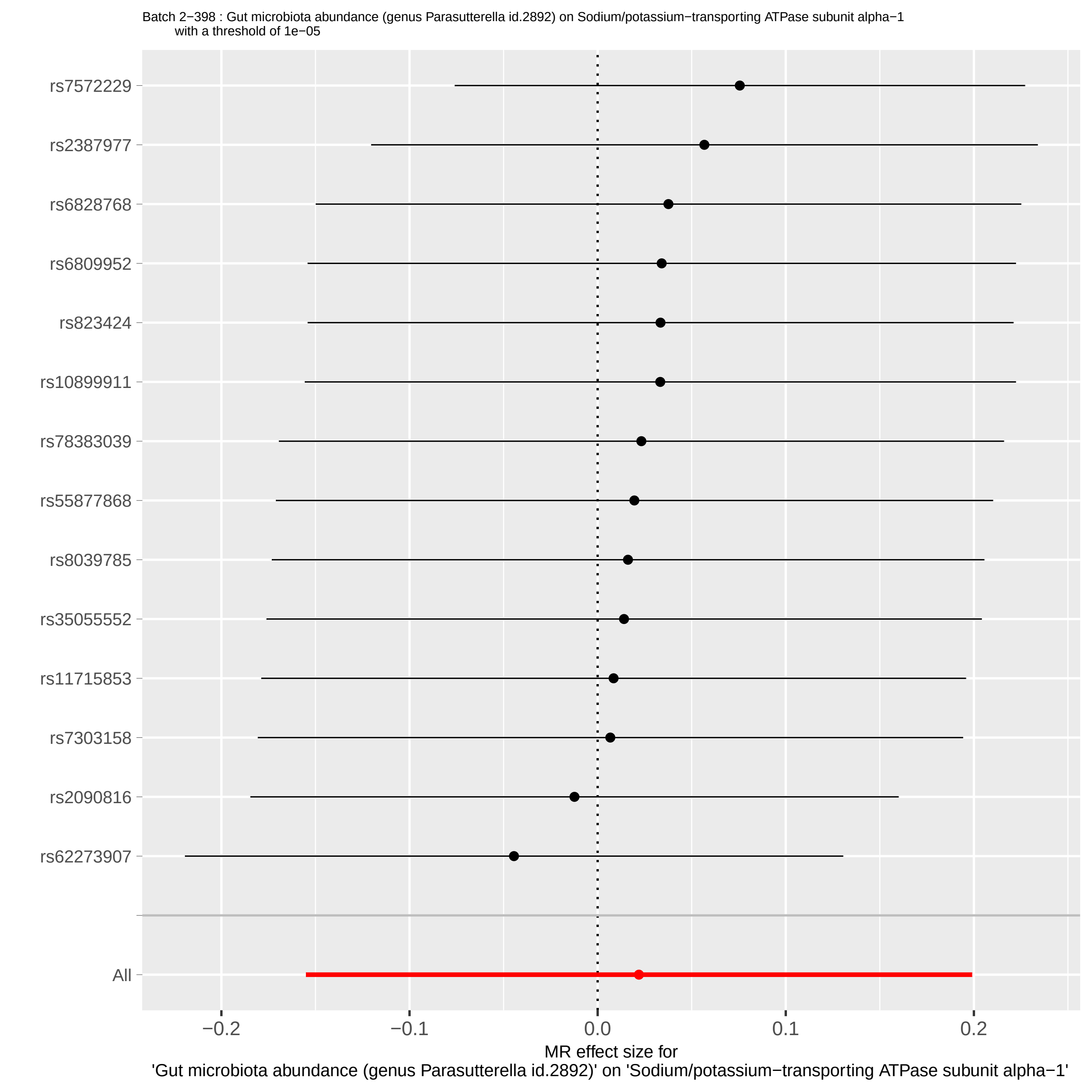
Batch 2−398 : Gut microbiota abundance (genus Parasutterella id.2892) on Sodium/potassium−transporting ATPase subunit alpha−1
with a threshold of 1e−05
rs7572229
rs2387977
rs6828768
rs6809952
rs823424
rs10899911
rs78383039
rs55877868
rs8039785
rs35055552
rs11715853
rs7303158
rs2090816
rs62273907
All
−0.2
−0.1
0.0
0.1
0.2
MR effect size for
'Gut microbiota abundance (genus Parasutterella id.2892)' on 'Sodium/potassium−transporting ATPase subunit alpha−1'

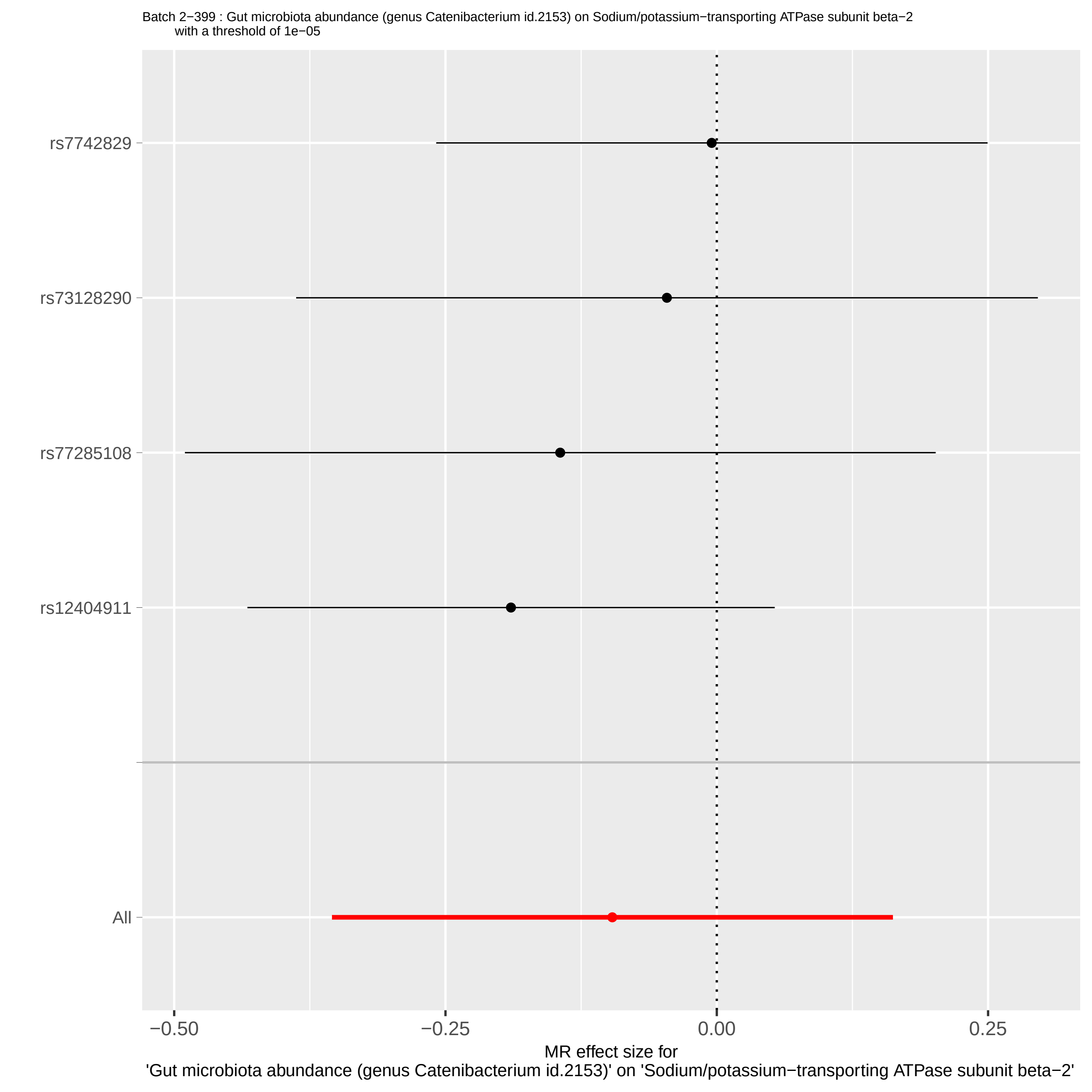
Batch 2−399 : Gut microbiota abundance (genus Catenibacterium id.2153) on Sodium/potassium−transporting ATPase subunit beta−2
with a threshold of 1e−05
rs7742829
rs73128290
rs77285108
rs12404911
All
−0.50
−0.25
0.00
0.25
MR effect size for
'Gut microbiota abundance (genus Catenibacterium id.2153)' on 'Sodium/potassium−transporting ATPase subunit beta−2'

Batch 2−400 : Gut microbiota abundance (genus Parasutterella id.2892) on Sodium/potassium−transporting ATPase subunit beta−2
with a threshold of 1e−05

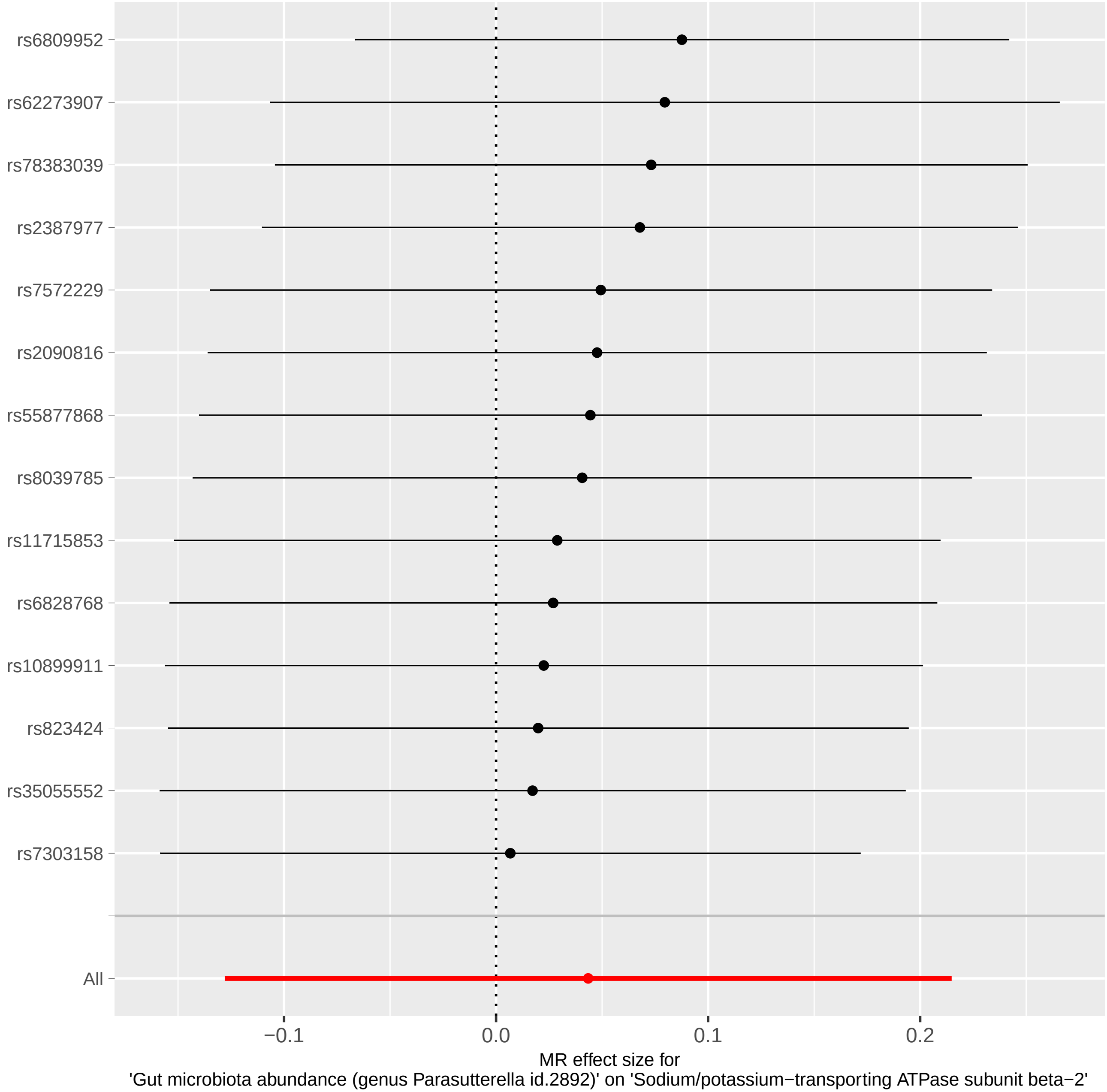

Batch 2−401 : Gut microbiota abundance (genus Catenibacterium id.2153) on Sarcoplasmic/endoplasmic reticulum calcium ATPase 3
with a threshold of 1e−05

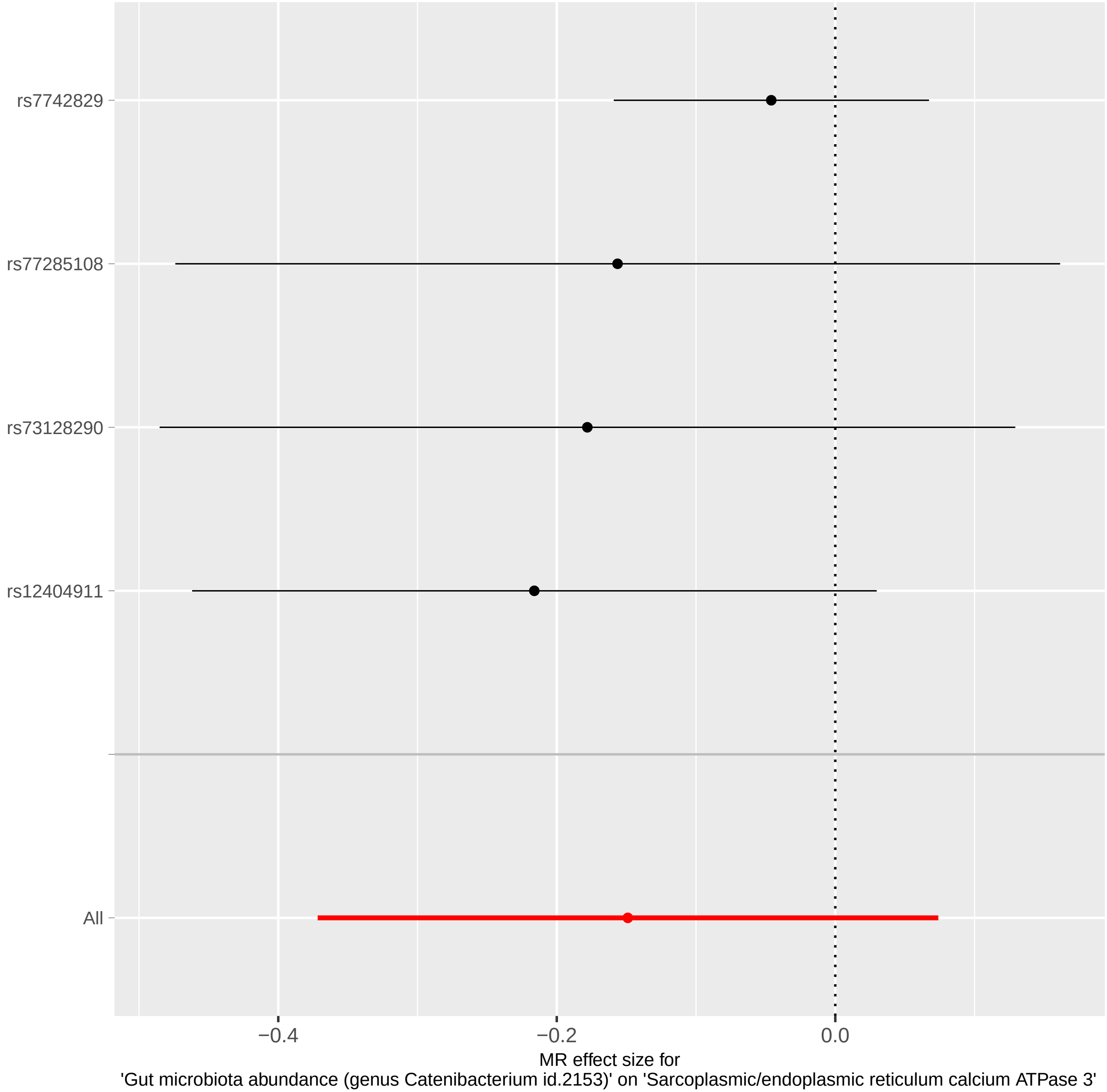

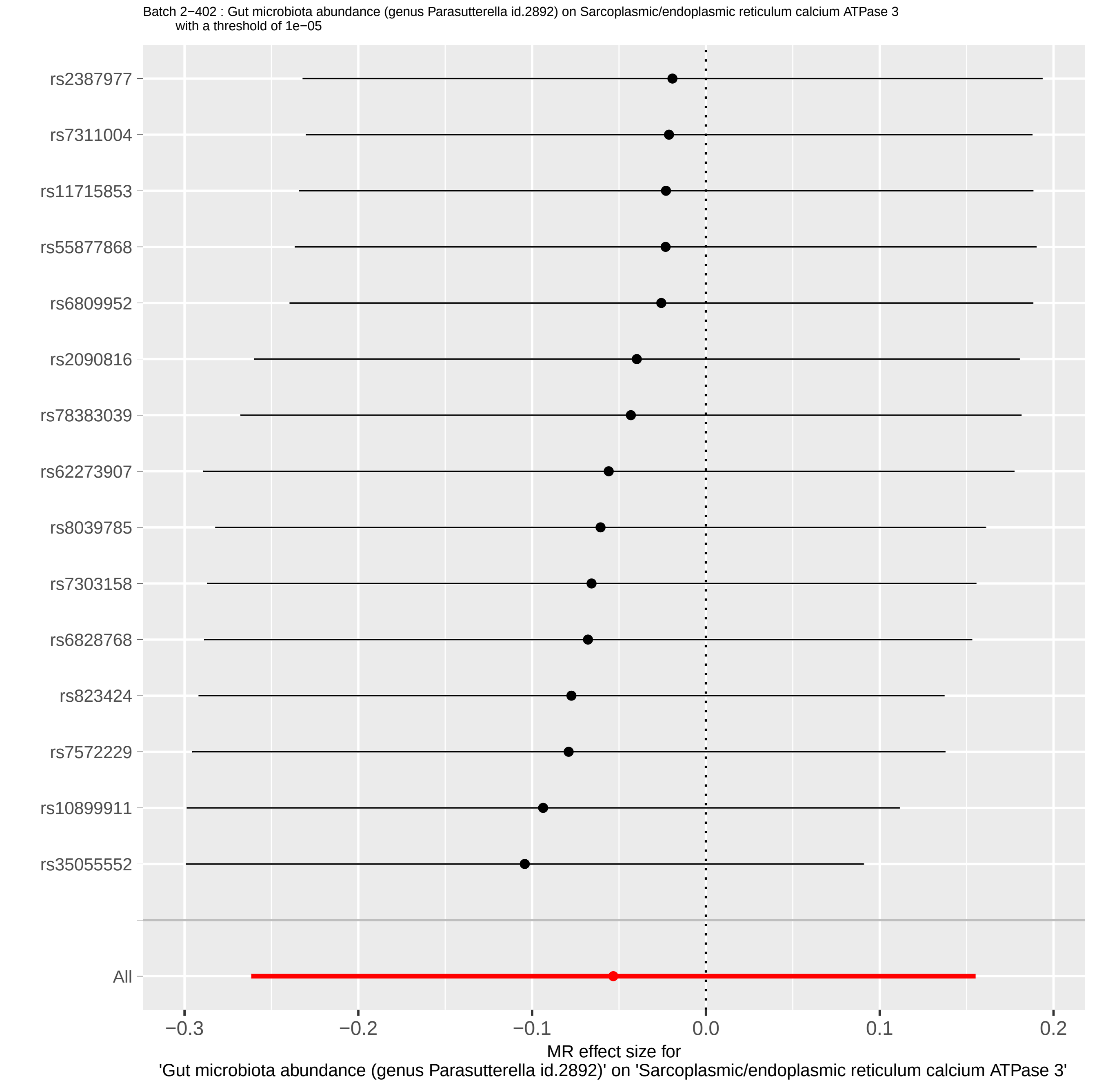
Batch 2−402 : Gut microbiota abundance (genus Parasutterella id.2892) on Sarcoplasmic/endoplasmic reticulum calcium ATPase 3
with a threshold of 1e−05
rs2387977
rs7311004
rs11715853
rs55877868
rs6809952
rs2090816
rs78383039
rs62273907
rs8039785
rs7303158
rs6828768
rs823424
rs7572229
rs10899911
rs35055552
All
−0.3
−0.2
−0.1
0.0
0.1
0.2
MR effect size for
'Gut microbiota abundance (genus Parasutterella id.2892)' on 'Sarcoplasmic/endoplasmic reticulum calcium ATPase 3'

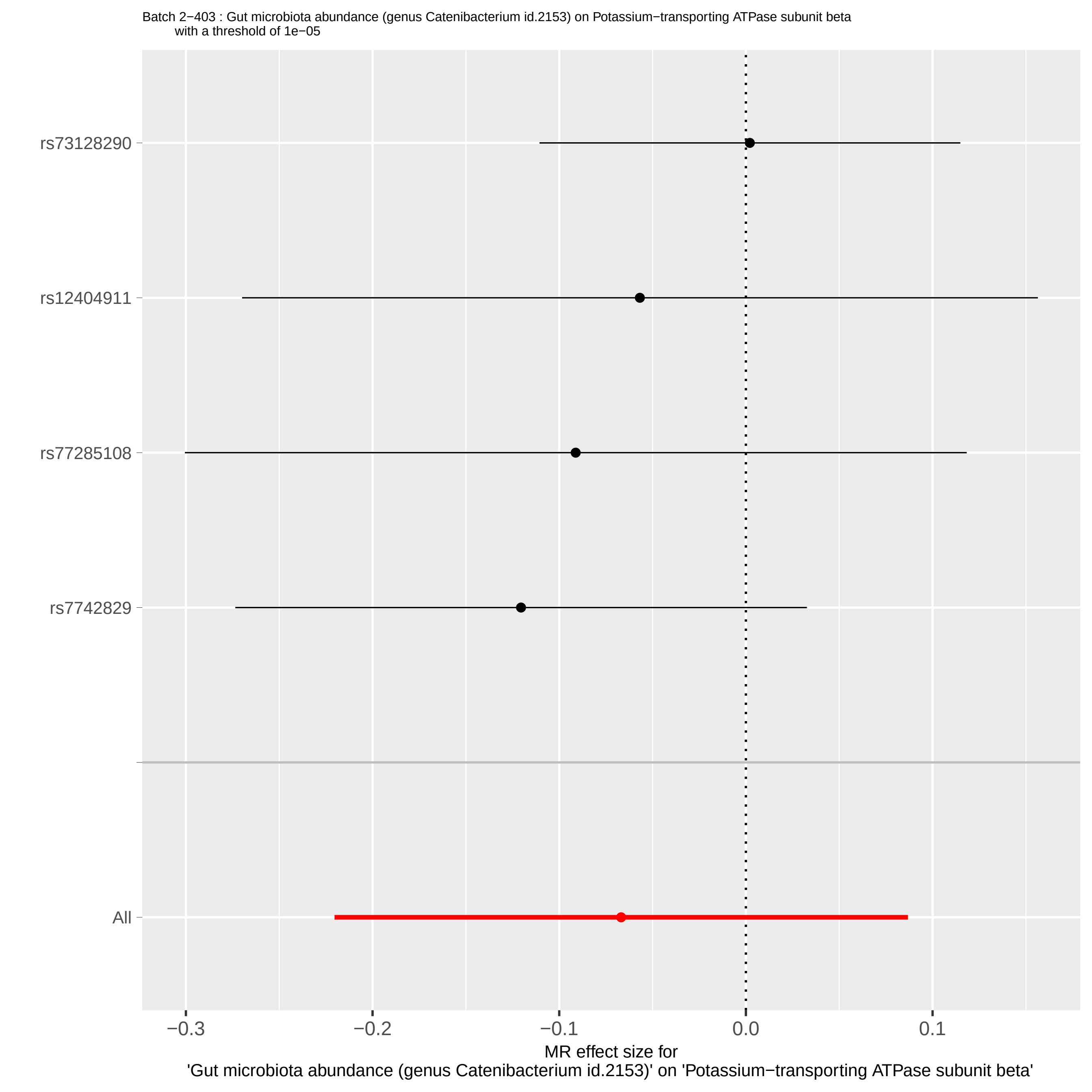

Batch 2−403 : Gut microbiota abundance (genus Catenibacterium id.2153) on Potassium−transporting ATPase subunit beta
with a threshold of 1e−05
rs73128290
rs12404911
rs77285108
rs7742829
All
−0.3
−0.2
−0.1
0.0
0.1
MR effect size for
'Gut microbiota abundance (genus Catenibacterium id.2153)' on 'Potassium−transporting ATPase subunit beta'

Batch 2−404 : Gut microbiota abundance (genus Parasutterella id.2892) on Potassium−transporting ATPase subunit beta
with a threshold of 1e−05

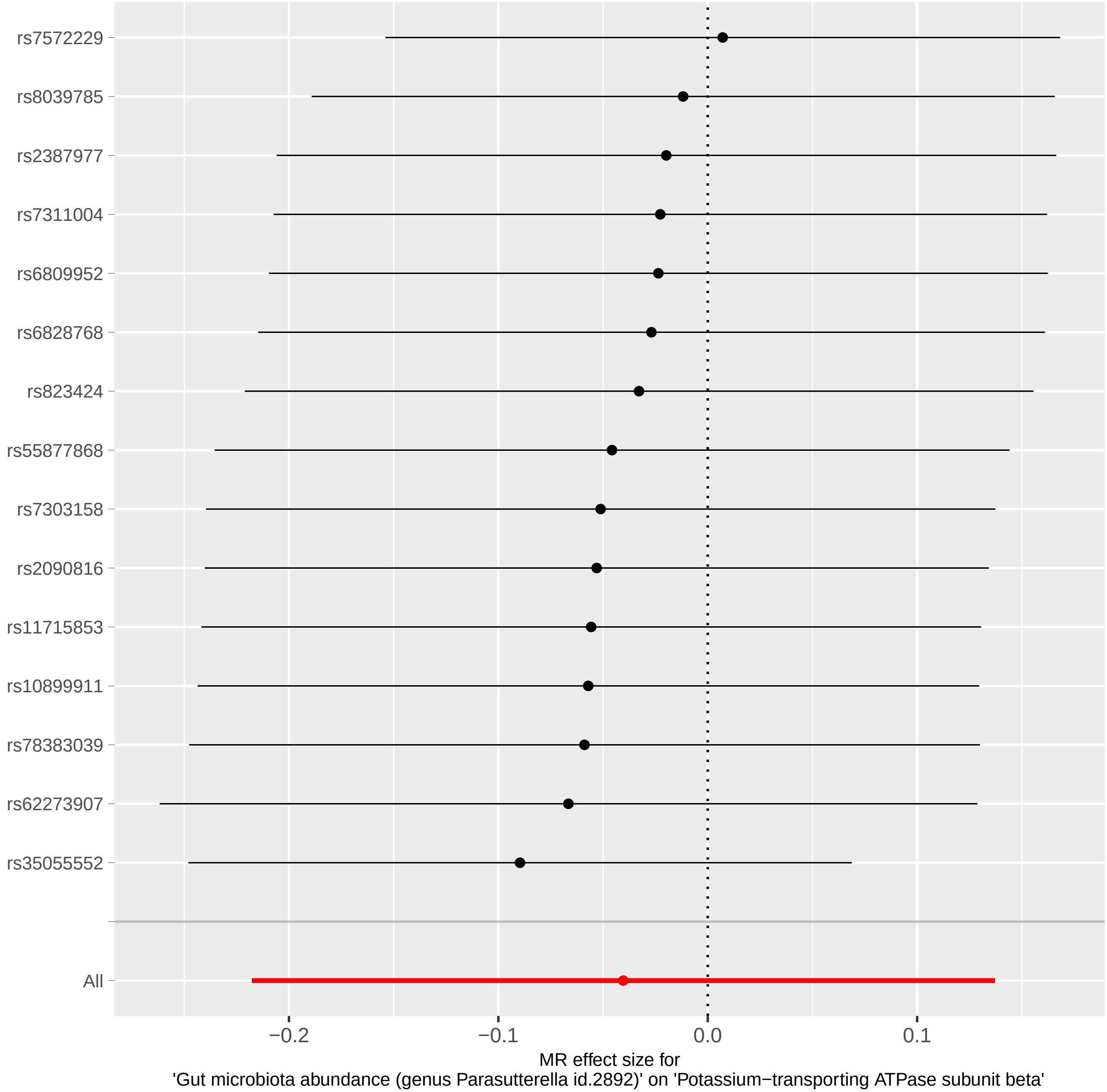

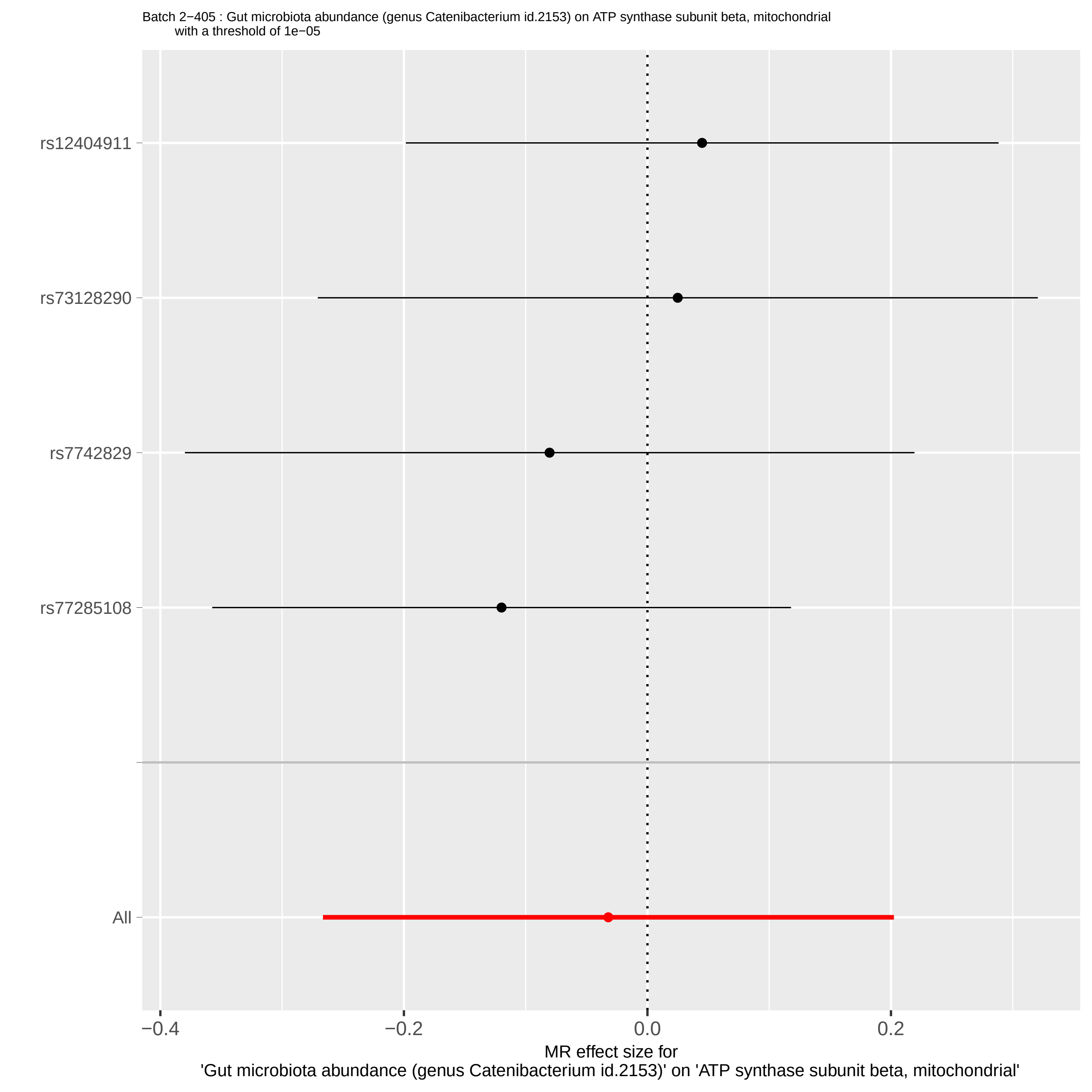

Batch 2−405 : Gut microbiota abundance (genus Catenibacterium id.2153) on ATP synthase subunit beta, mitochondrial
with a threshold of 1e−05
rs12404911
rs73128290
rs7742829
rs77285108
All
−0.4
−0.2
0.0
0.2
MR effect size for
'Gut microbiota abundance (genus Catenibacterium id.2153)' on 'ATP synthase subunit beta, mitochondrial'

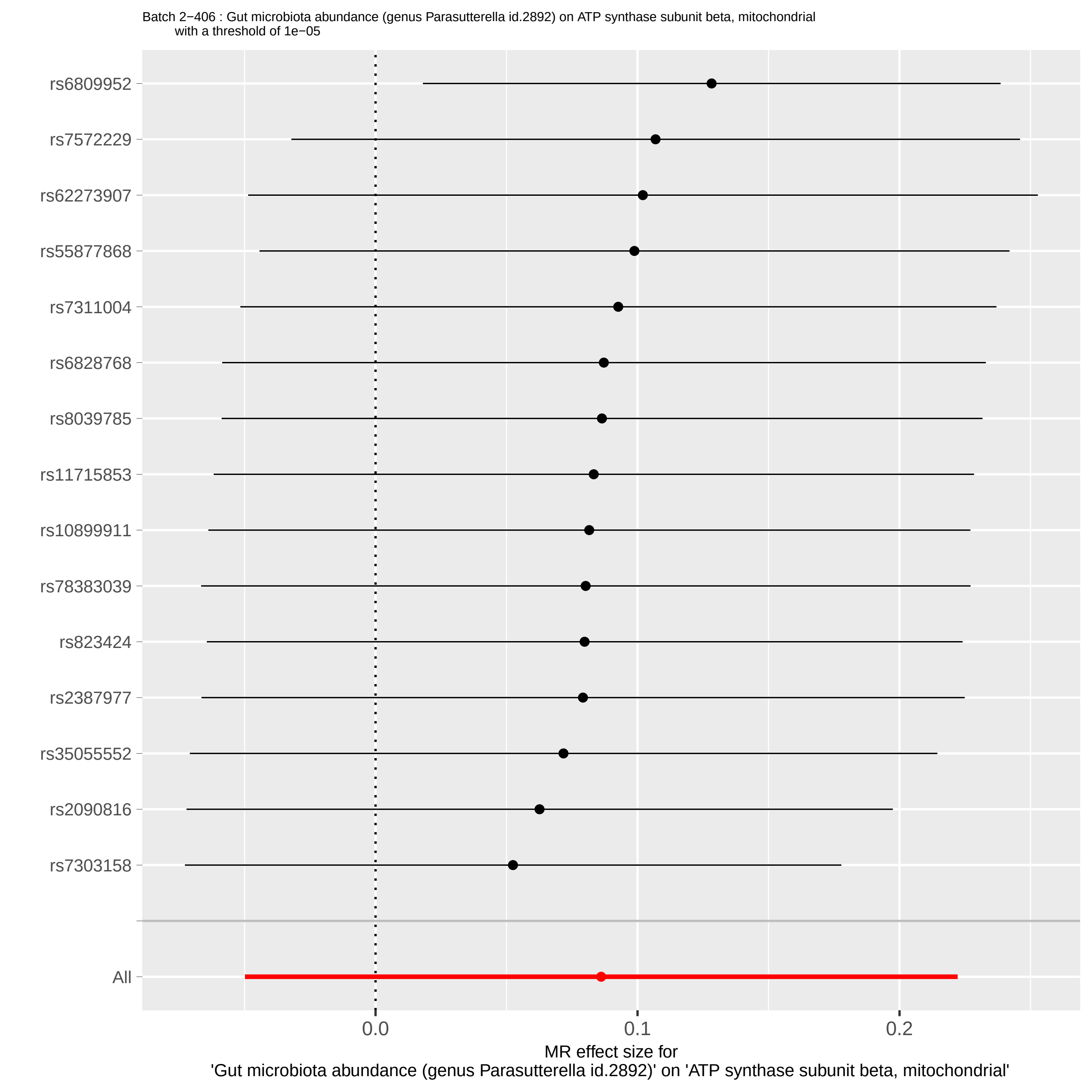
Batch 2−406 : Gut microbiota abundance (genus Parasutterella id.2892) on ATP synthase subunit beta, mitochondrial
with a threshold of 1e−05
rs6809952
rs7572229
rs62273907
rs55877868
rs7311004
rs6828768
rs8039785
rs11715853
rs10899911
rs78383039
rs823424
rs2387977
rs35055552
rs2090816
rs7303158
All
0.0
0.1
0.2
MR effect size for
'Gut microbiota abundance (genus Parasutterella id.2892)' on 'ATP synthase subunit beta, mitochondrial'

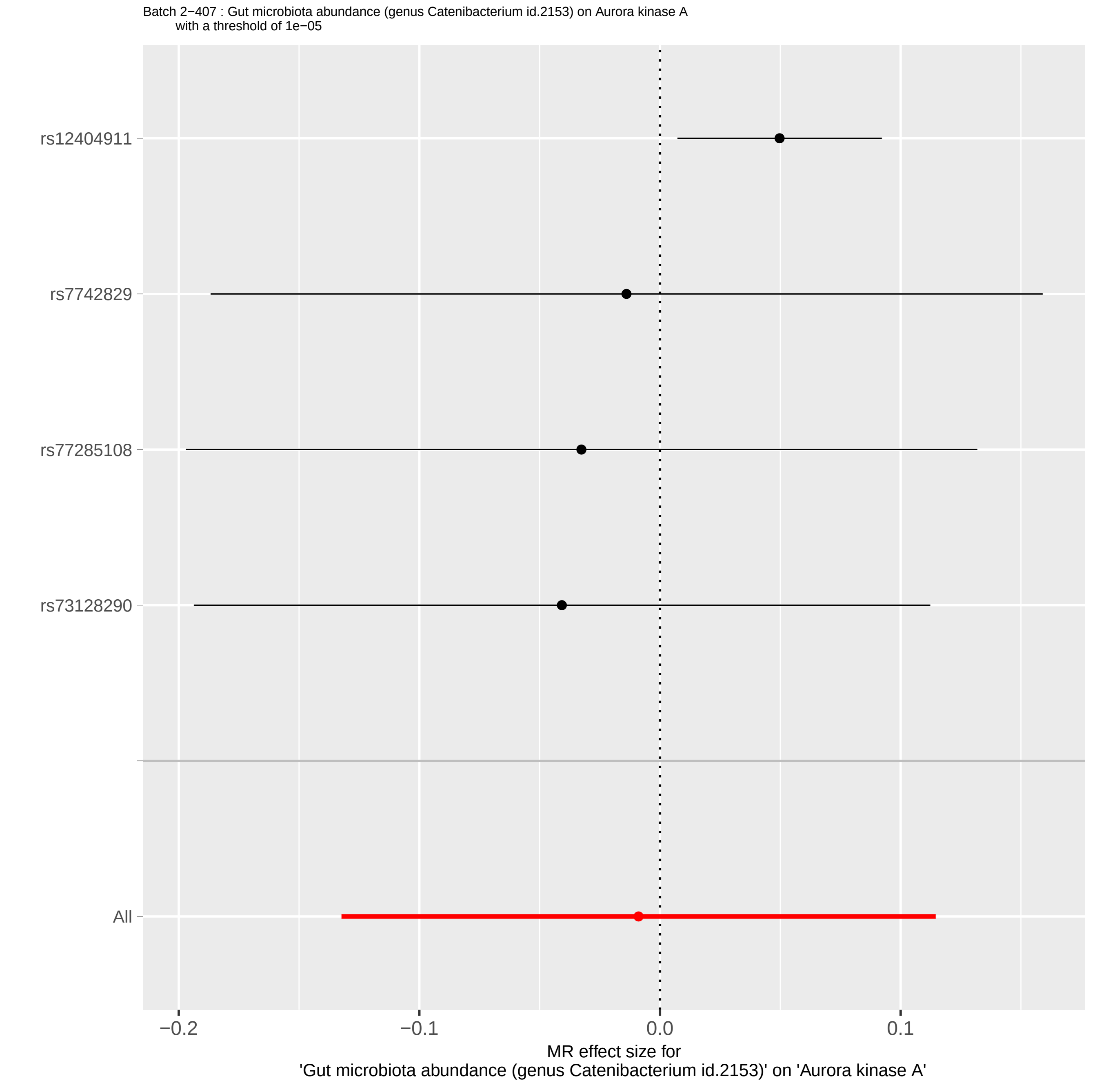

Batch 2−407 : Gut microbiota abundance (genus Catenibacterium id.2153) on Aurora kinase A
with a threshold of 1e−05
rs12404911
rs7742829
rs77285108
rs73128290
All
−0.2
−0.1
0.0
0.1
MR effect size for
'Gut microbiota abundance (genus Catenibacterium id.2153)' on 'Aurora kinase A'

Batch 2−408 : Gut microbiota abundance (genus Parasutterella id.2892) on Aurora kinase A
with a threshold of 1e−05

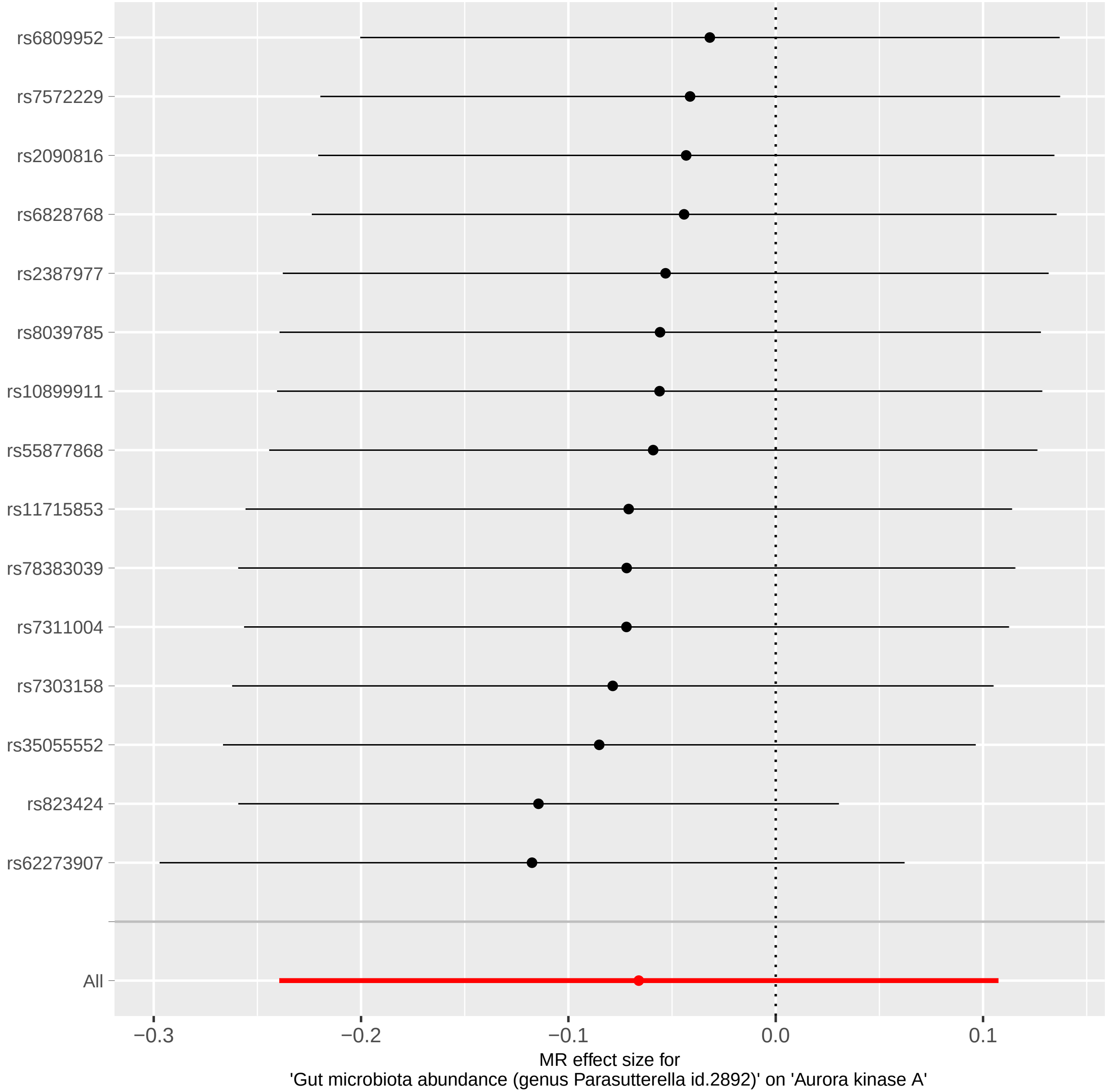

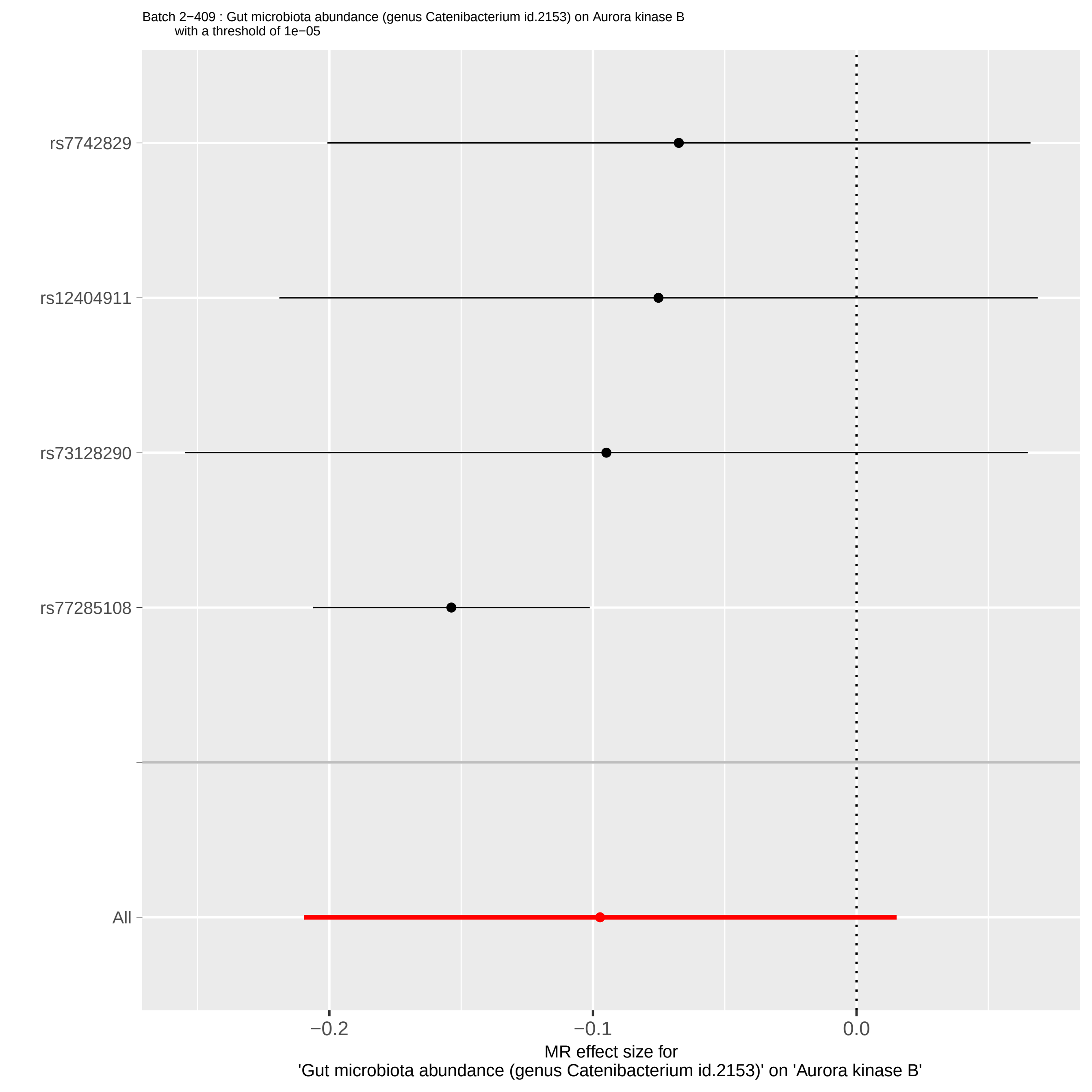

Batch 2−409 : Gut microbiota abundance (genus Catenibacterium id.2153) on Aurora kinase B
with a threshold of 1e−05
rs7742829
rs12404911
rs73128290
rs77285108
All
−0.2
−0.1
0.0
MR effect size for
'Gut microbiota abundance (genus Catenibacterium id.2153)' on 'Aurora kinase B'

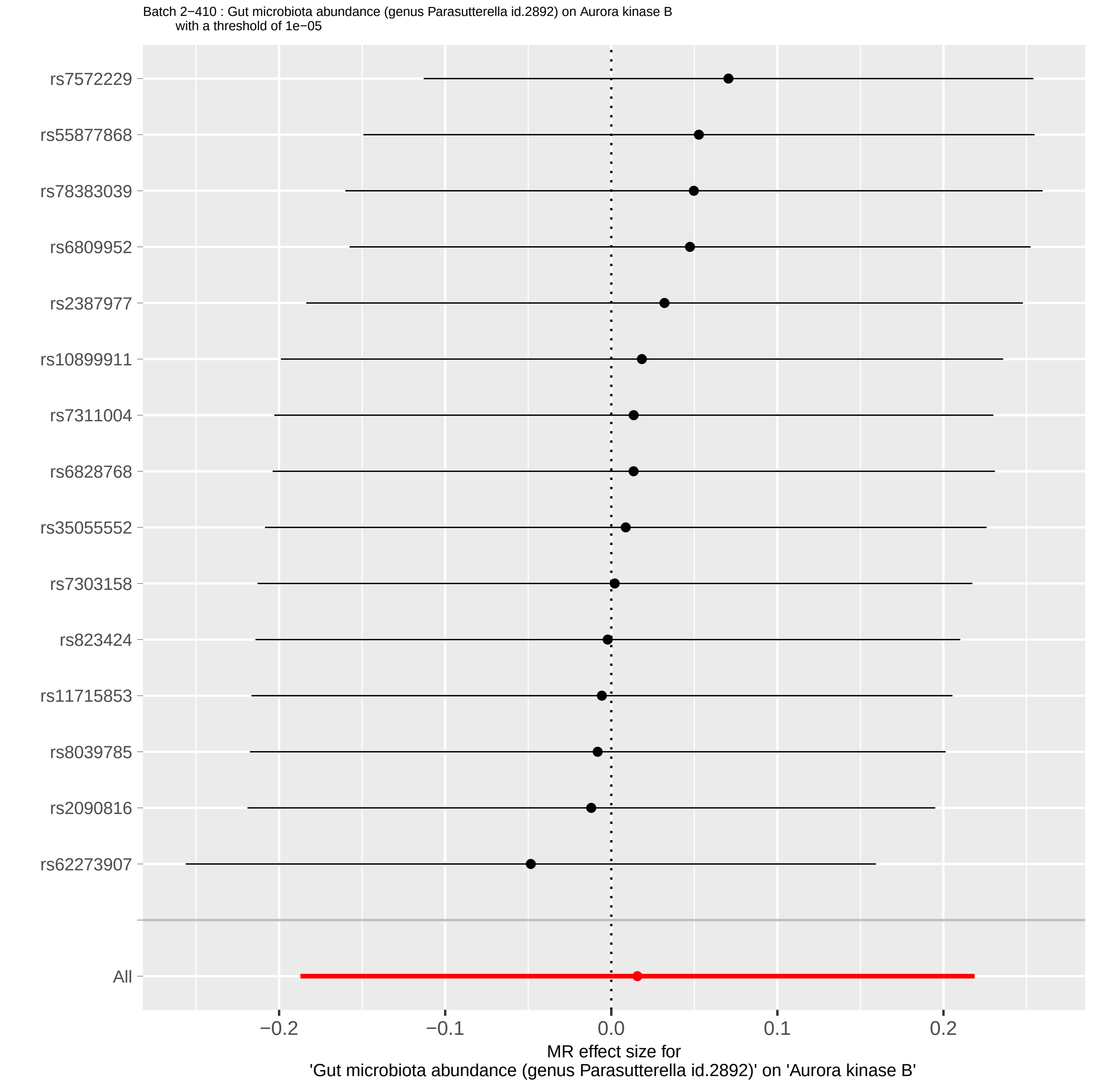

Batch 2−410 : Gut microbiota abundance (genus Parasutterella id.2892) on Aurora kinase B
with a threshold of 1e−05
rs7572229
rs55877868
rs78383039
rs6809952
rs2387977
rs10899911
rs7311004
rs6828768
rs35055552
rs7303158
rs823424
rs11715853
rs8039785
rs2090816
rs62273907
All
−0.2
−0.1
0.0
0.1
0.2
MR effect size for
'Gut microbiota abundance (genus Parasutterella id.2892)' on 'Aurora kinase B'

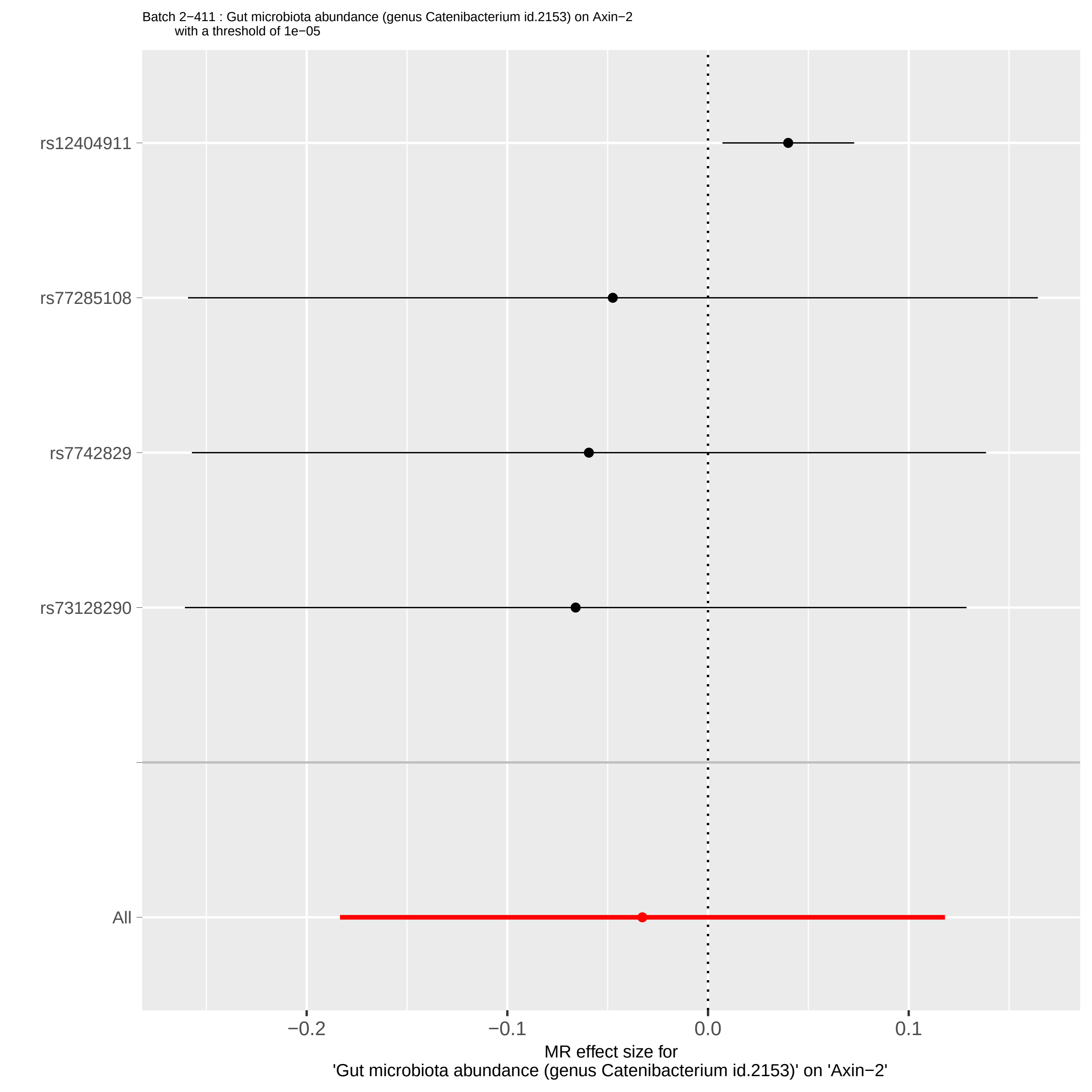

Batch 2−411 : Gut microbiota abundance (genus Catenibacterium id.2153) on Axin−2
with a threshold of 1e−05
rs12404911
rs77285108
rs7742829
rs73128290
All
−0.2
−0.1
0.0
0.1
MR effect size for
'Gut microbiota abundance (genus Catenibacterium id.2153)' on 'Axin−2'

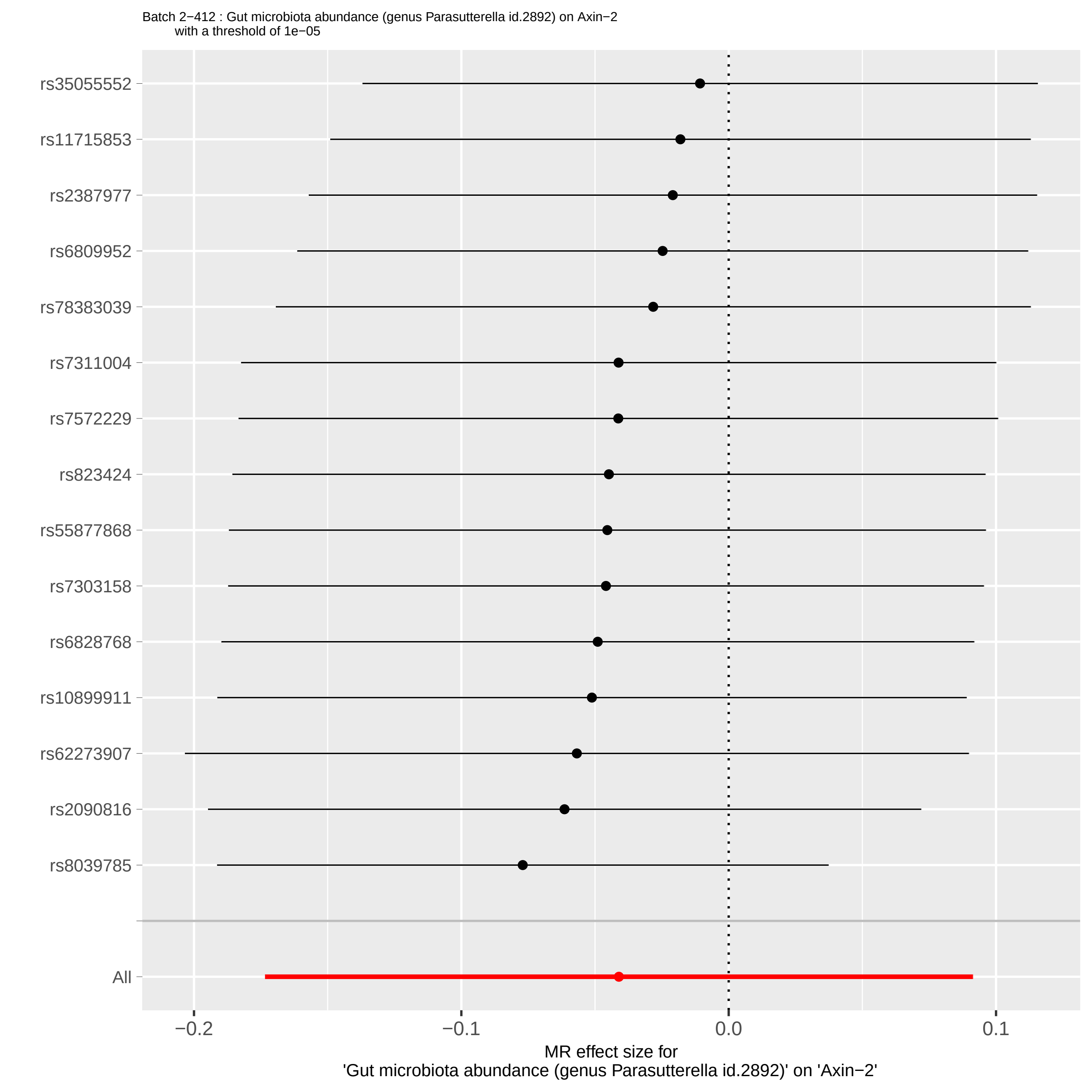
Batch 2−412 : Gut microbiota abundance (genus Parasutterella id.2892) on Axin−2
with a threshold of 1e−05
rs35055552
rs11715853
rs2387977
rs6809952
rs78383039
rs7311004
rs7572229
rs823424
rs55877868
rs7303158
rs6828768
rs10899911
rs62273907
rs2090816
rs8039785
All
−0.2
−0.1
0.0
0.1
MR effect size for
'Gut microbiota abundance (genus Parasutterella id.2892)' on 'Axin−2'

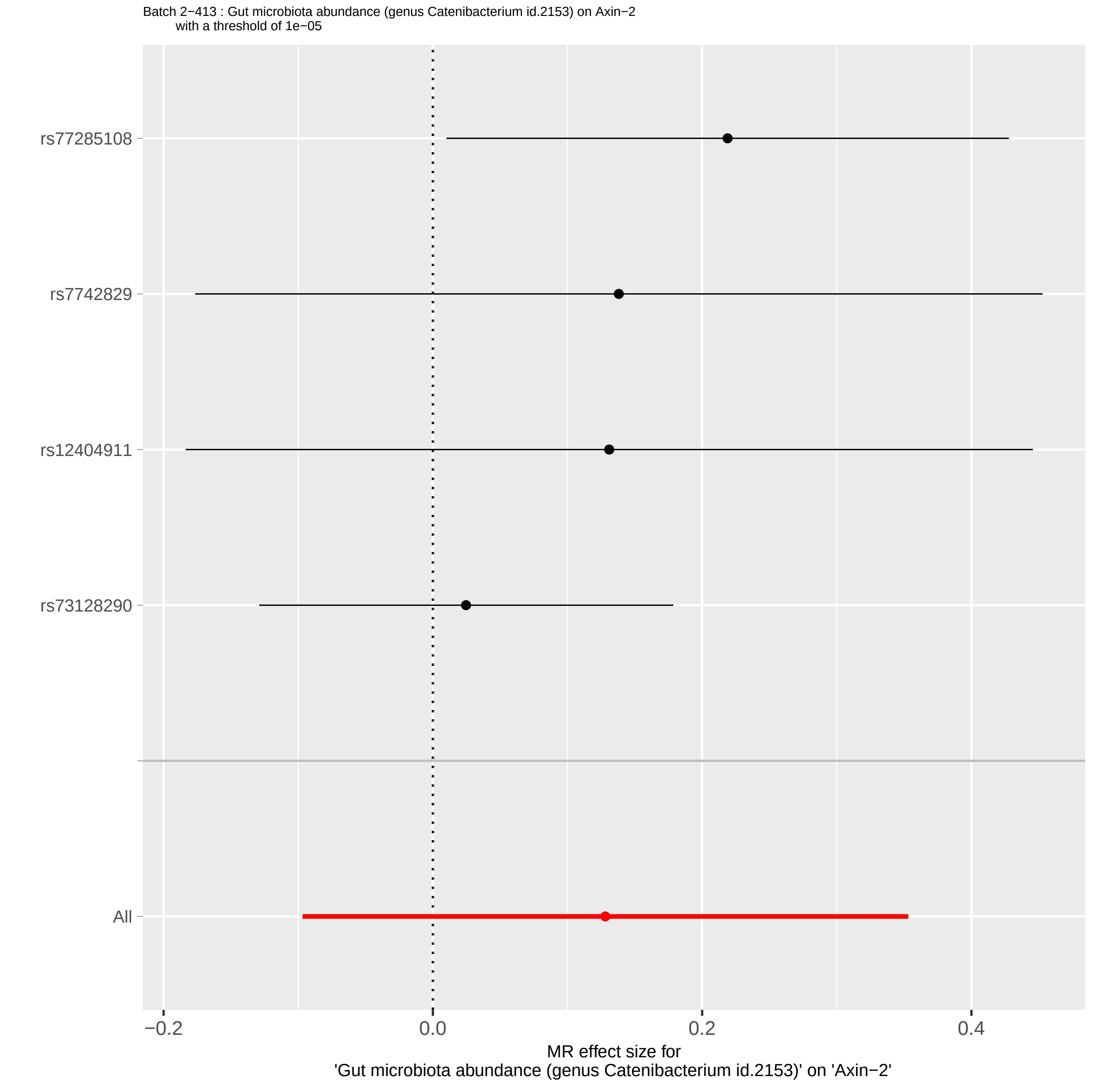
Batch 2−413 : Gut microbiota abundance (genus Catenibacterium id.2153) on Axin−2
with a threshold of 1e−05
rs77285108
rs7742829
rs12404911
rs73128290
All
−0.2
0.0
0.2
0.4
MR effect size for
'Gut microbiota abundance (genus Catenibacterium id.2153)' on 'Axin−2'

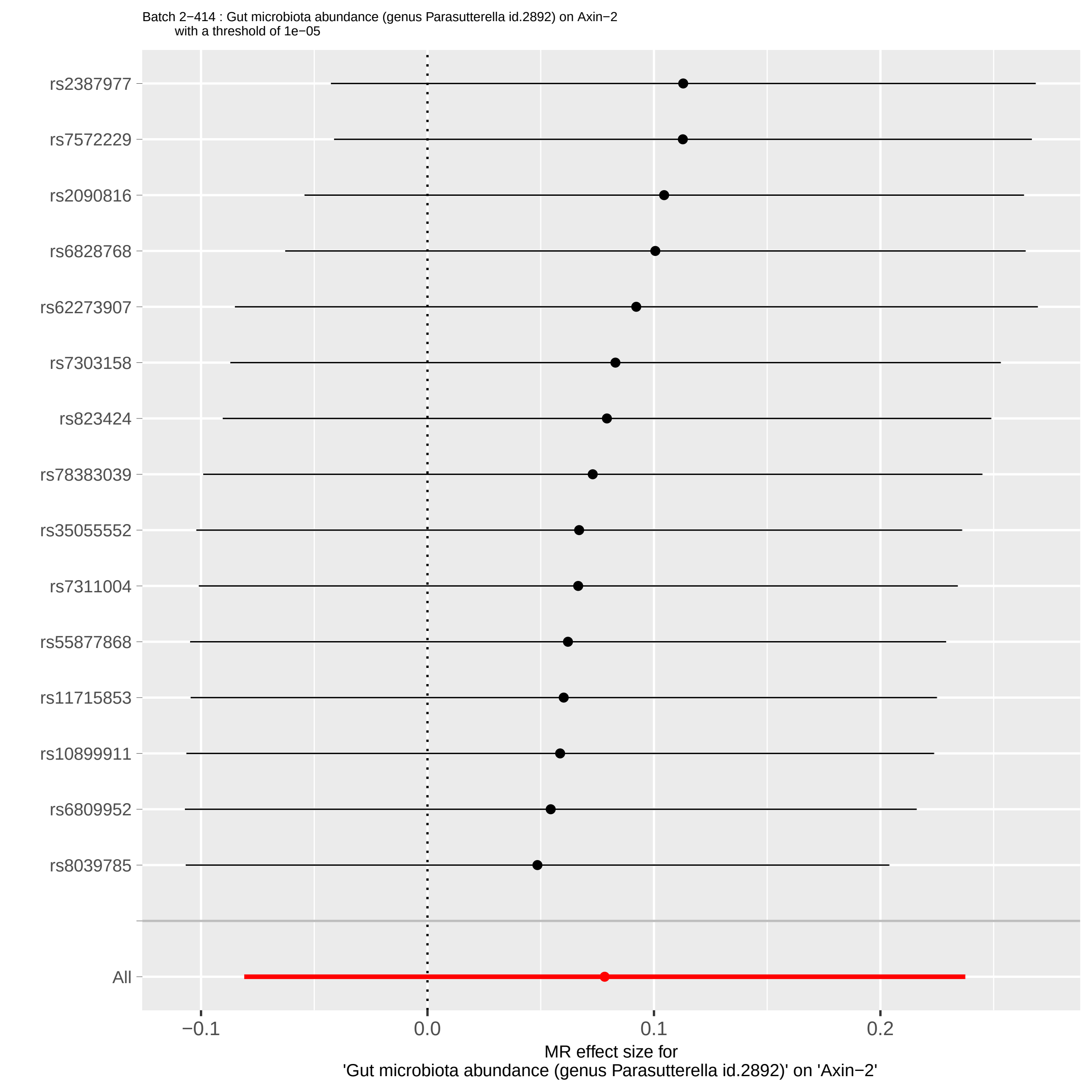
Batch 2−414 : Gut microbiota abundance (genus Parasutterella id.2892) on Axin−2
with a threshold of 1e−05
rs2387977
rs7572229
rs2090816
rs6828768
rs62273907
rs7303158
rs823424
rs78383039
rs35055552
rs7311004
rs55877868
rs11715853
rs10899911
rs6809952
rs8039785
All
−0.1
0.0
0.1
0.2
MR effect size for
'Gut microbiota abundance (genus Parasutterella id.2892)' on 'Axin−2'

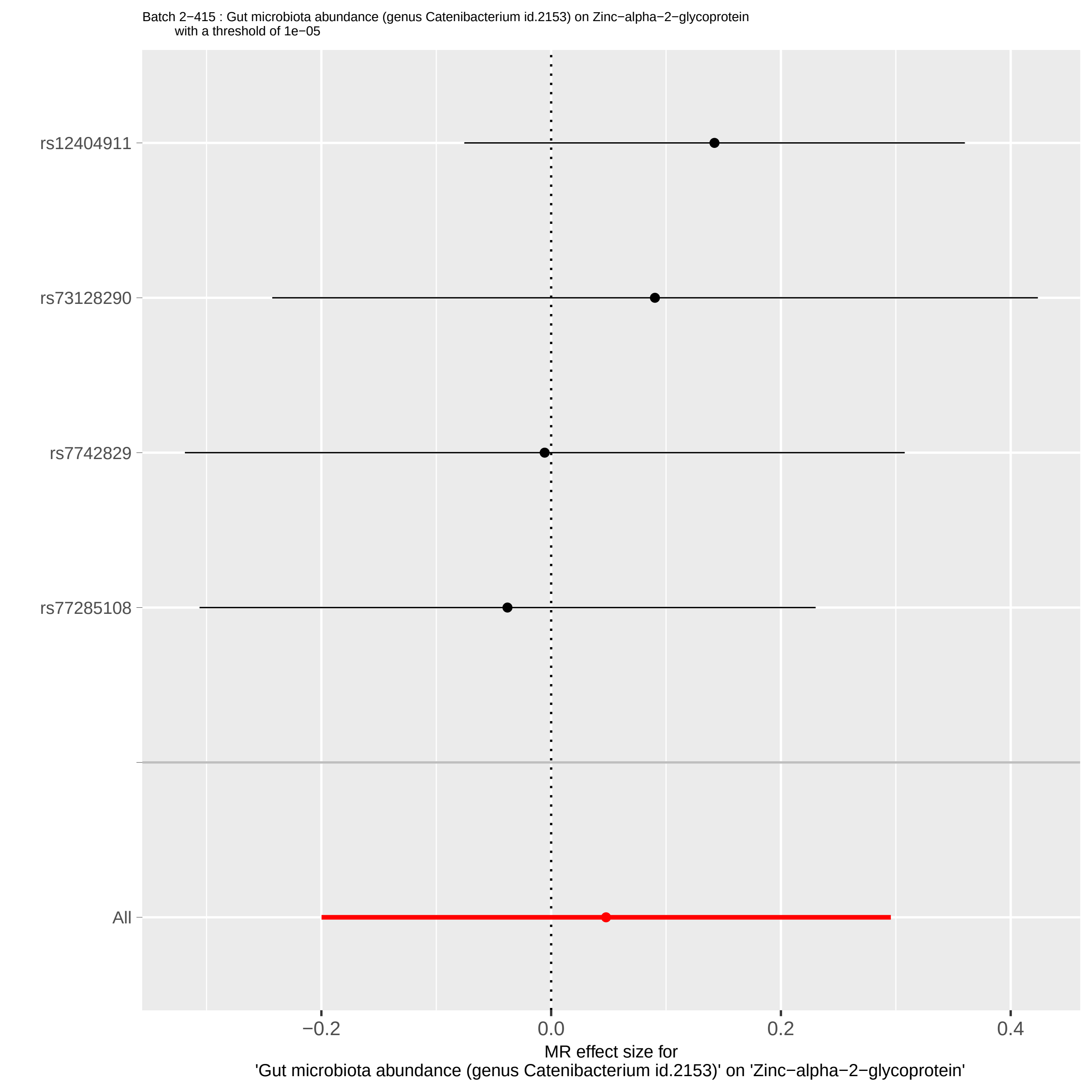
Batch 2−415 : Gut microbiota abundance (genus Catenibacterium id.2153) on Zinc−alpha−2−glycoprotein
with a threshold of 1e−05
rs12404911
rs73128290
rs7742829
rs77285108
All
−0.2
0.0
0.2
0.4
MR effect size for
'Gut microbiota abundance (genus Catenibacterium id.2153)' on 'Zinc−alpha−2−glycoprotein'

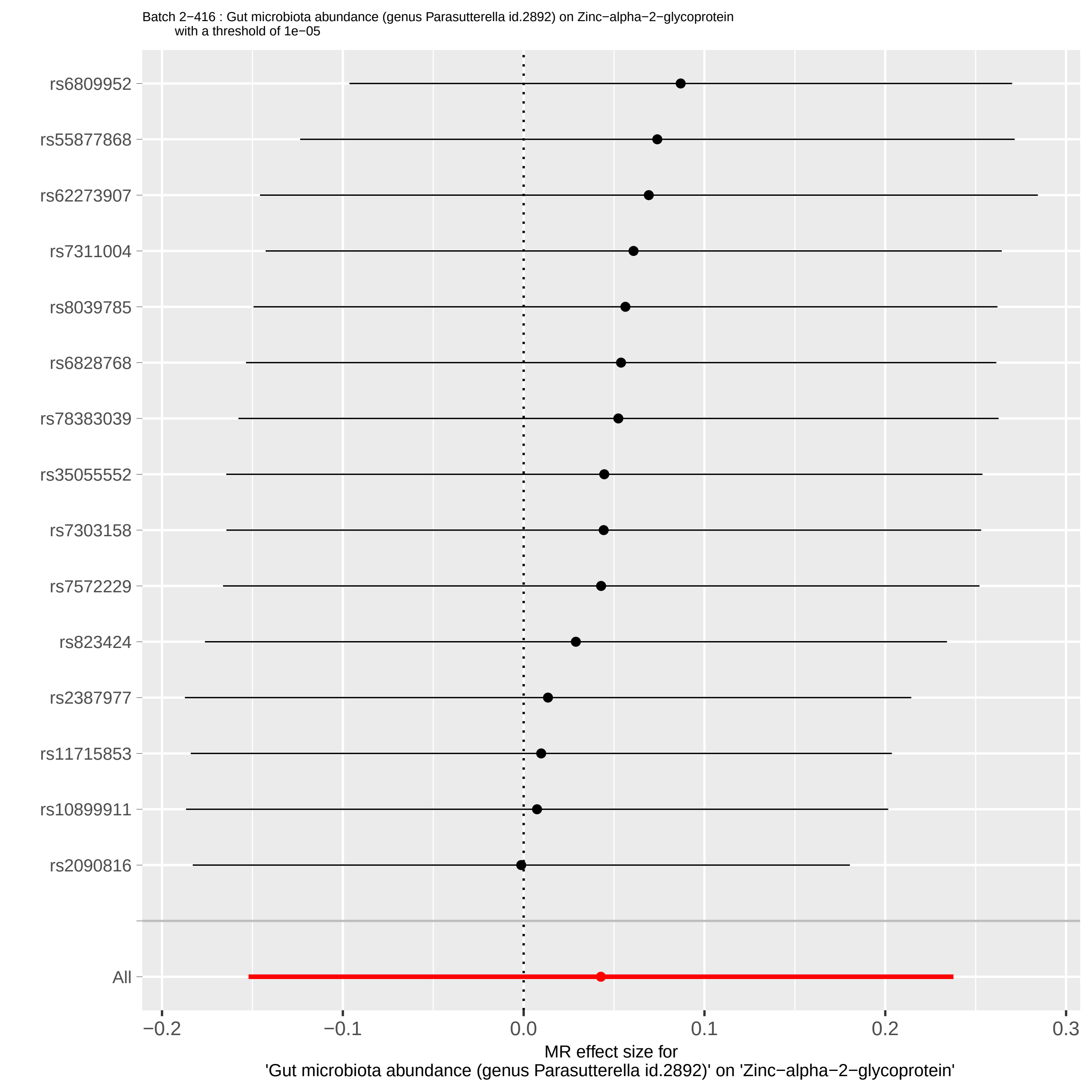
Batch 2−416 : Gut microbiota abundance (genus Parasutterella id.2892) on Zinc−alpha−2−glycoprotein
with a threshold of 1e−05
rs6809952
rs55877868
rs62273907
rs7311004
rs8039785
rs6828768
rs78383039
rs35055552
rs7303158
rs7572229
rs823424
rs2387977
rs11715853
rs10899911
rs2090816
All
−0.2
−0.1
0.0
0.1
0.2
0.3
MR effect size for
'Gut microbiota abundance (genus Parasutterella id.2892)' on 'Zinc−alpha−2−glycoprotein'

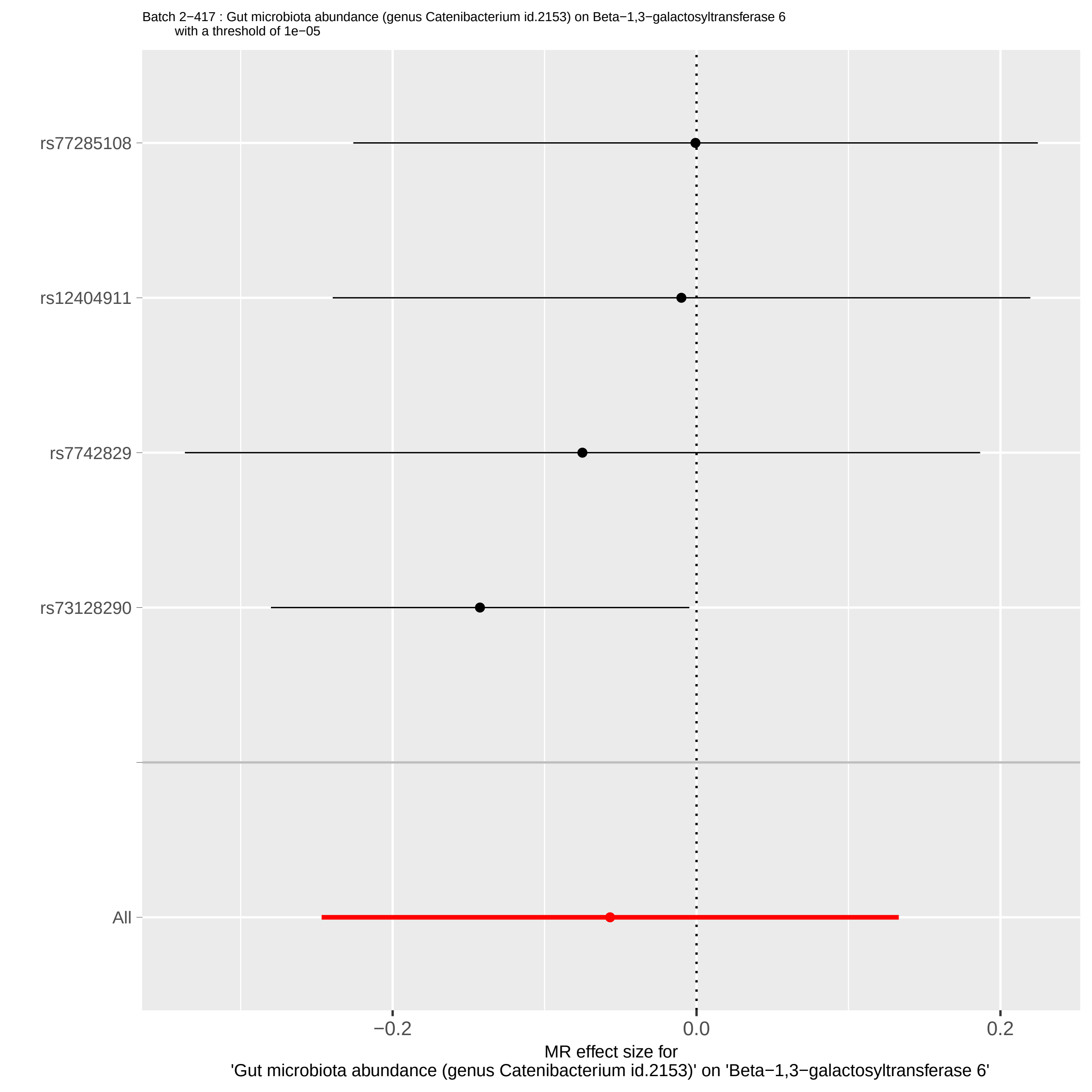
Batch 2−417 : Gut microbiota abundance (genus Catenibacterium id.2153) on Beta−1,3−galactosyltransferase 6
with a threshold of 1e−05
rs77285108
rs12404911
rs7742829
rs73128290
All
−0.2
0.0
0.2
MR effect size for
'Gut microbiota abundance (genus Catenibacterium id.2153)' on 'Beta−1,3−galactosyltransferase 6'

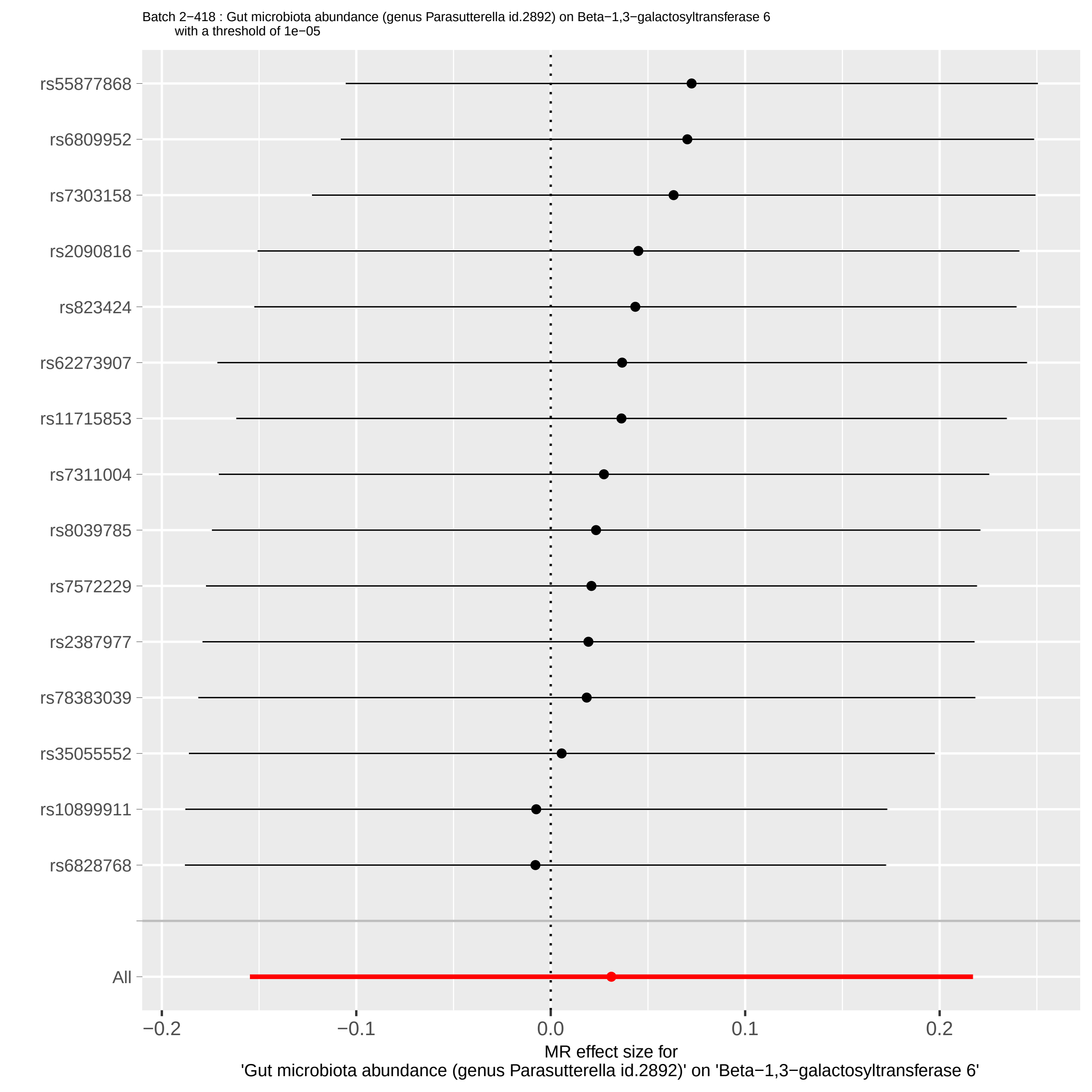
Batch 2−418 : Gut microbiota abundance (genus Parasutterella id.2892) on Beta−1,3−galactosyltransferase 6
with a threshold of 1e−05
rs55877868
rs6809952
rs7303158
rs2090816
rs823424
rs62273907
rs11715853
rs7311004
rs8039785
rs7572229
rs2387977
rs78383039
rs35055552
rs10899911
rs6828768
All
−0.2
−0.1
0.0
0.1
0.2
MR effect size for
'Gut microbiota abundance (genus Parasutterella id.2892)' on 'Beta−1,3−galactosyltransferase 6'

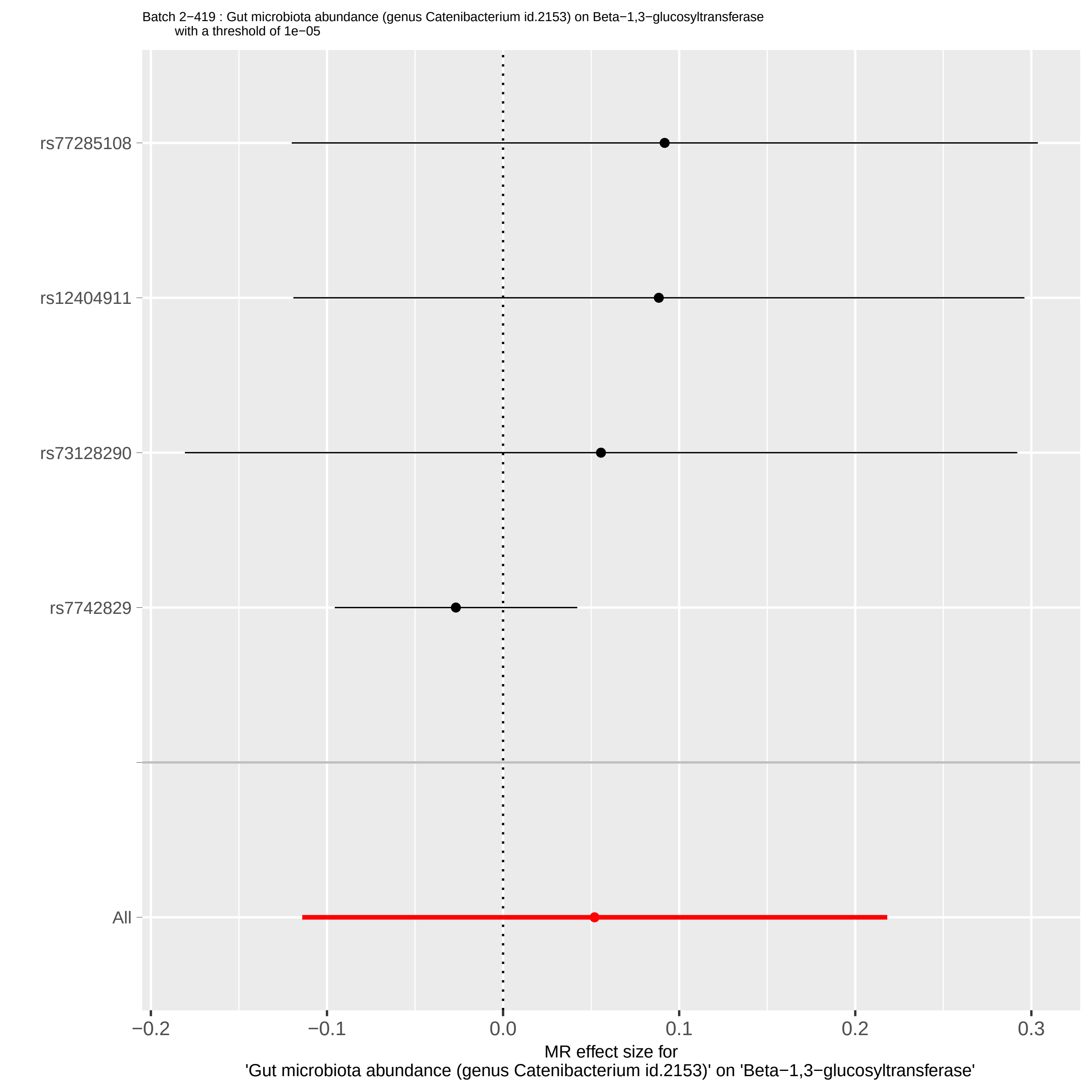
Batch 2−419 : Gut microbiota abundance (genus Catenibacterium id.2153) on Beta−1,3−glucosyltransferase
with a threshold of 1e−05
rs77285108
rs12404911
rs73128290
rs7742829
All
−0.2
−0.1
0.0
0.1
0.2
0.3
MR effect size for
'Gut microbiota abundance (genus Catenibacterium id.2153)' on 'Beta−1,3−glucosyltransferase'

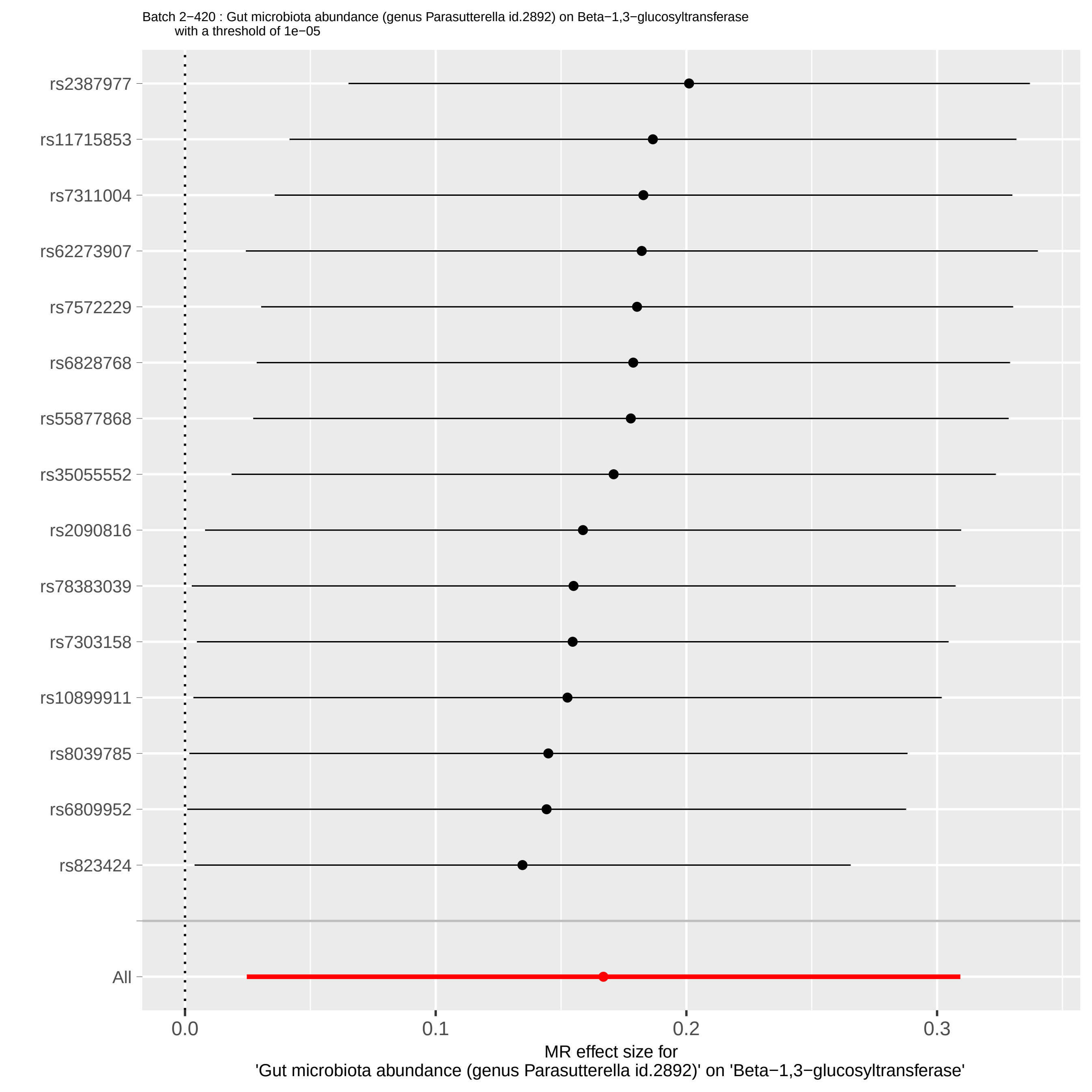

Batch 2−420 : Gut microbiota abundance (genus Parasutterella id.2892) on Beta−1,3−glucosyltransferase
with a threshold of 1e−05
rs2387977
rs11715853
rs7311004
rs62273907
rs7572229
rs6828768
rs55877868
rs35055552
rs2090816
rs78383039
rs7303158
rs10899911
rs8039785
rs6809952
rs823424
All
0.0
0.1
0.2
0.3
MR effect size for
'Gut microbiota abundance (genus Parasutterella id.2892)' on 'Beta−1,3−glucosyltransferase'

Batch 2−421 : Gut microbiota abundance (genus Catenibacterium id.2153) on Galactosylgalactosylxylosylprotein 3−beta−glucuronosyltransferase 3
with a threshold of 1e−05

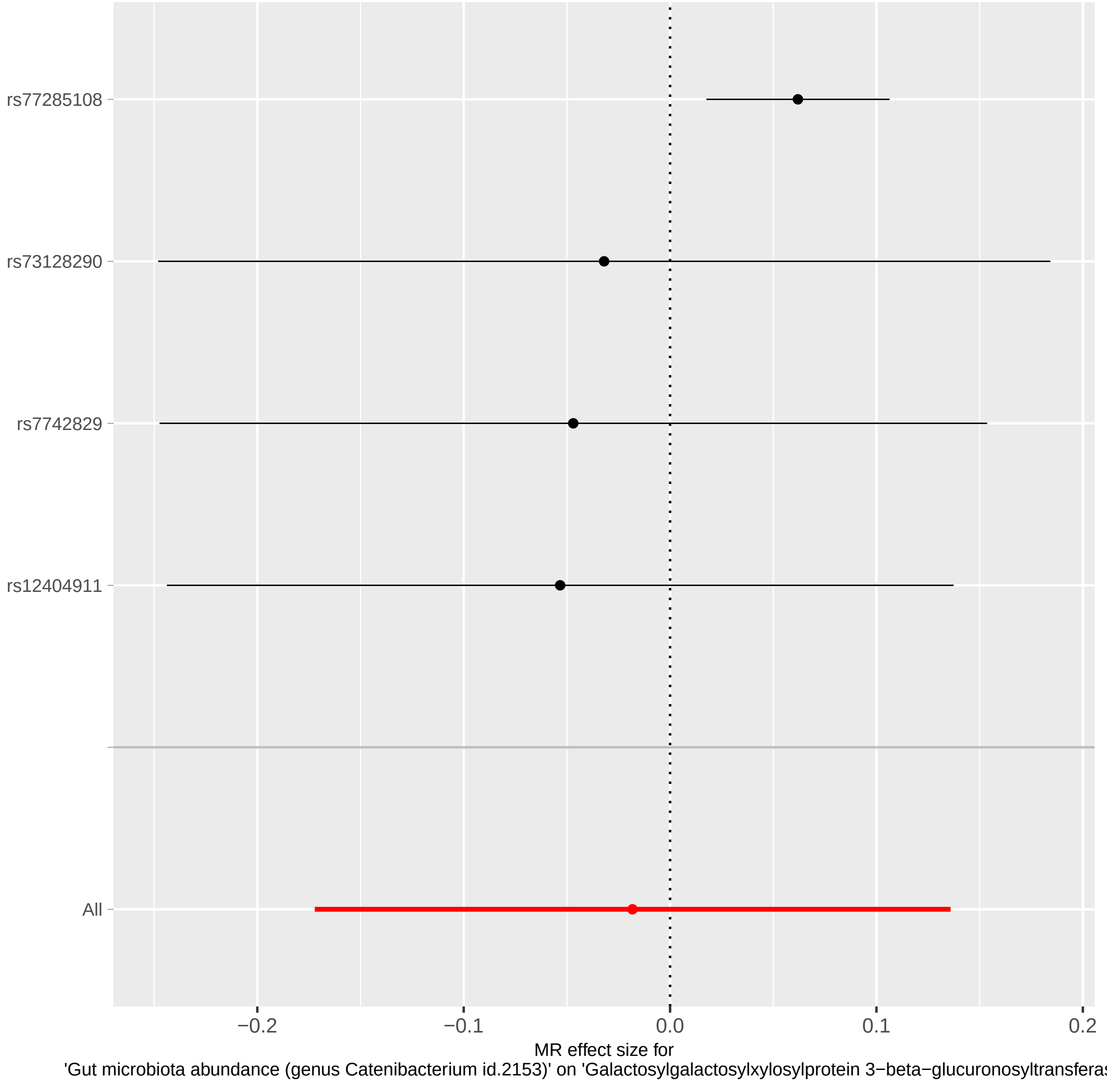

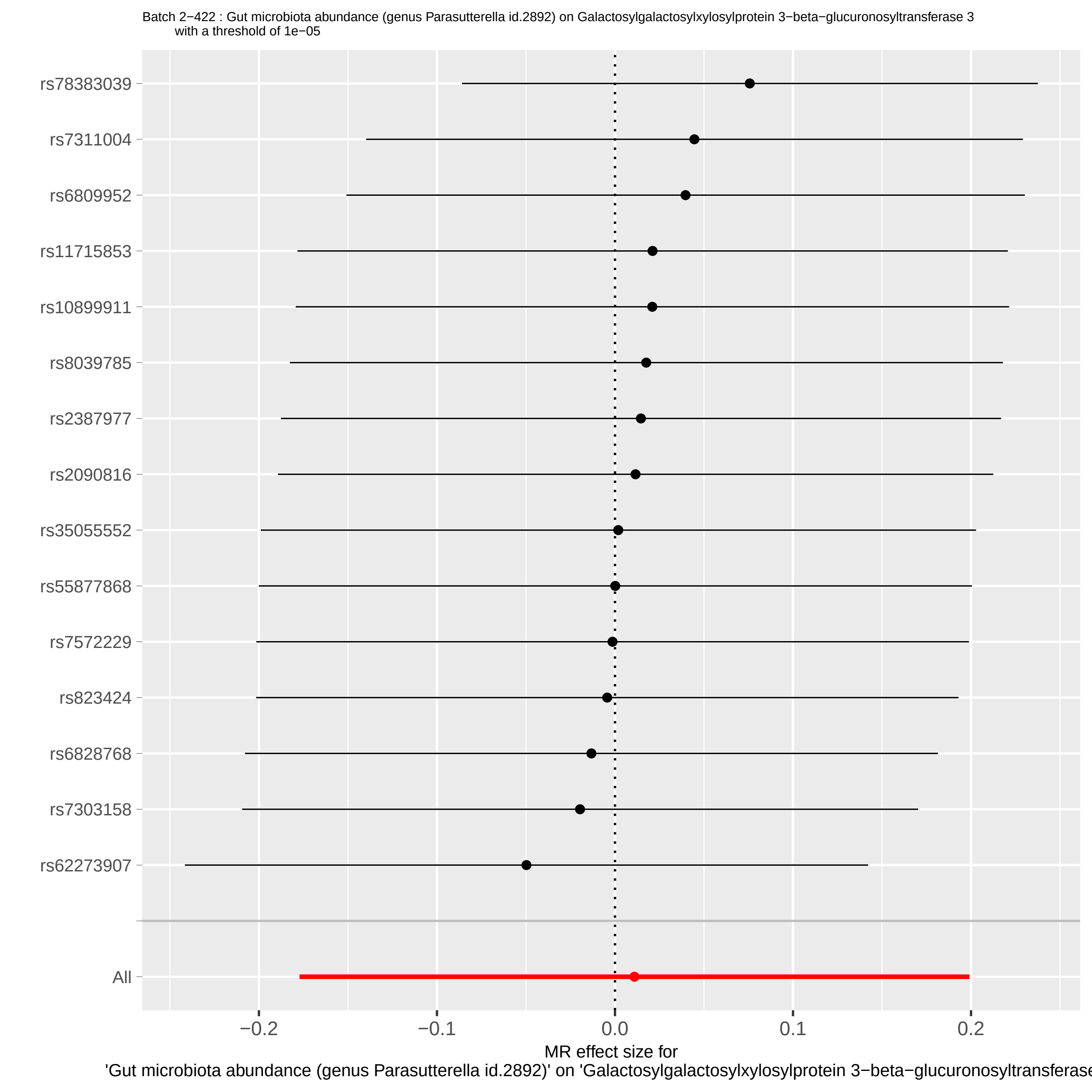
Batch 2−422 : Gut microbiota abundance (genus Parasutterella id.2892) on Galactosylgalactosylxylosylprotein 3−beta−glucuronosyltransferase 3
with a threshold of 1e−05
rs78383039
rs7311004
rs6809952
rs11715853
rs10899911
rs8039785
rs2387977
rs2090816
rs35055552
rs55877868
rs7572229
rs823424
rs6828768
rs7303158
rs62273907
All
−0.2
−0.1
0.0
0.1
0.2
MR effect size for
'Gut microbiota abundance (genus Parasutterella id.2892)' on 'Galactosylgalactosylxylosylprotein 3−beta−glucuronosyltransferase

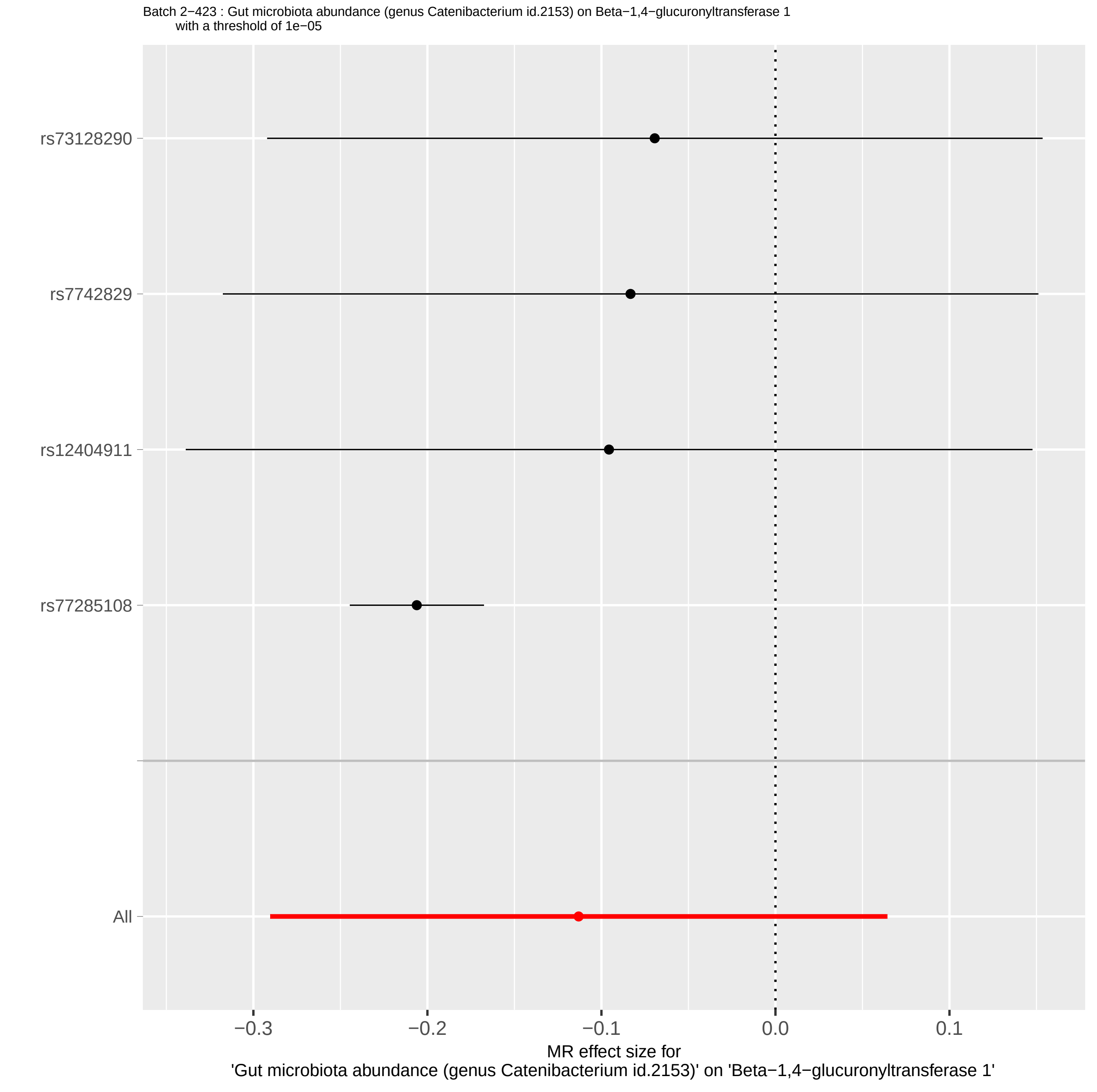

Batch 2−423 : Gut microbiota abundance (genus Catenibacterium id.2153) on Beta−1,4−glucuronyltransferase 1
with a threshold of 1e−05
rs73128290
rs7742829
rs12404911
rs77285108
All
−0.3
−0.2
−0.1
0.0
0.1
MR effect size for
'Gut microbiota abundance (genus Catenibacterium id.2153)' on 'Beta−1,4−glucuronyltransferase 1'

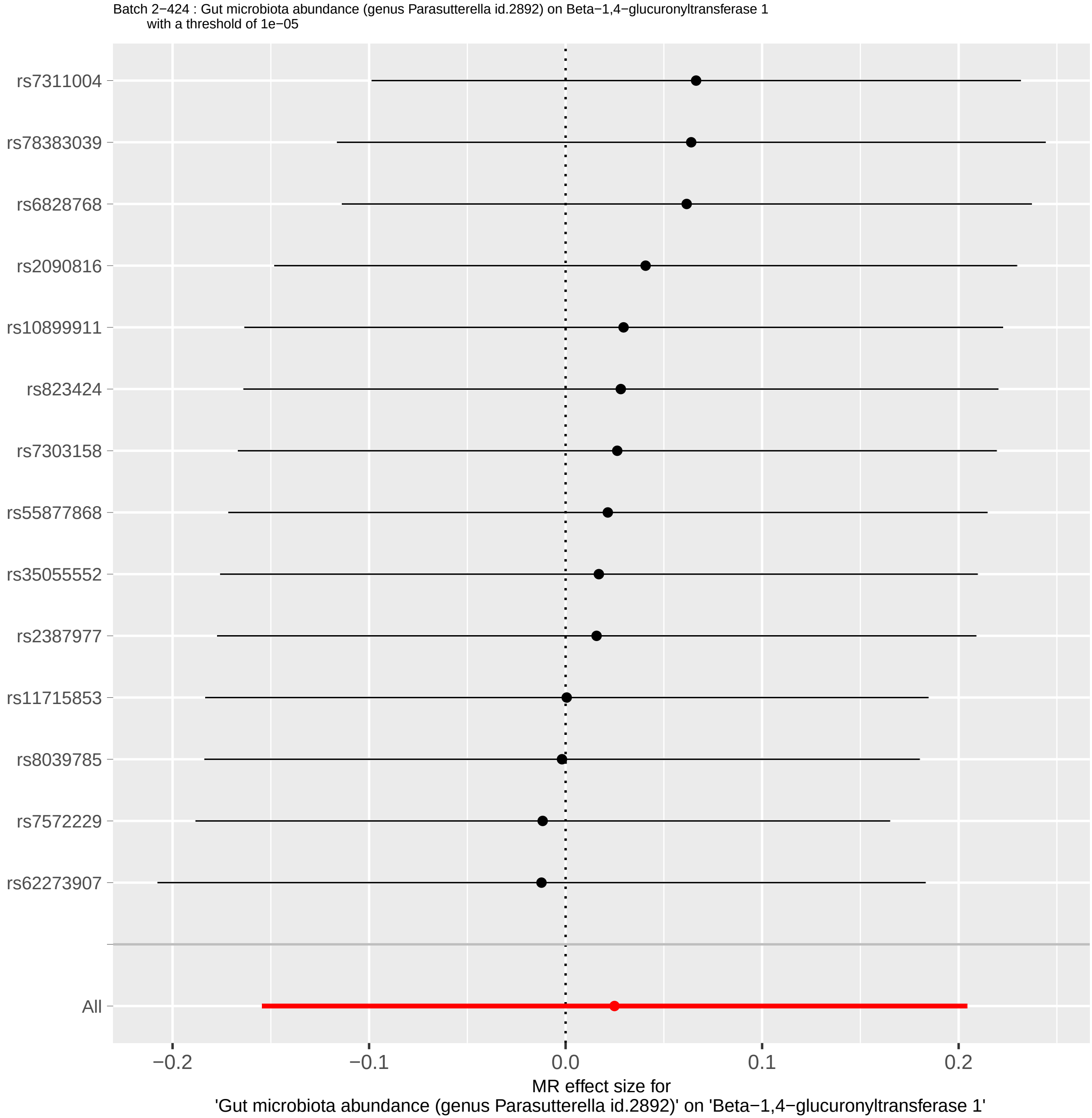
Batch 2−424 : Gut microbiota abundance (genus Parasutterella id.2892) on Beta−1,4−glucuronyltransferase 1
with a threshold of 1e−05
rs7311004
rs78383039
rs6828768
rs2090816
rs10899911
rs823424
rs7303158
rs55877868
rs35055552
rs2387977
rs11715853
rs8039785
rs7572229
rs62273907
All
−0.2
−0.1
0.0
0.1
0.2
MR effect size for
'Gut microbiota abundance (genus Parasutterella id.2892)' on 'Beta−1,4−glucuronyltransferase 1'

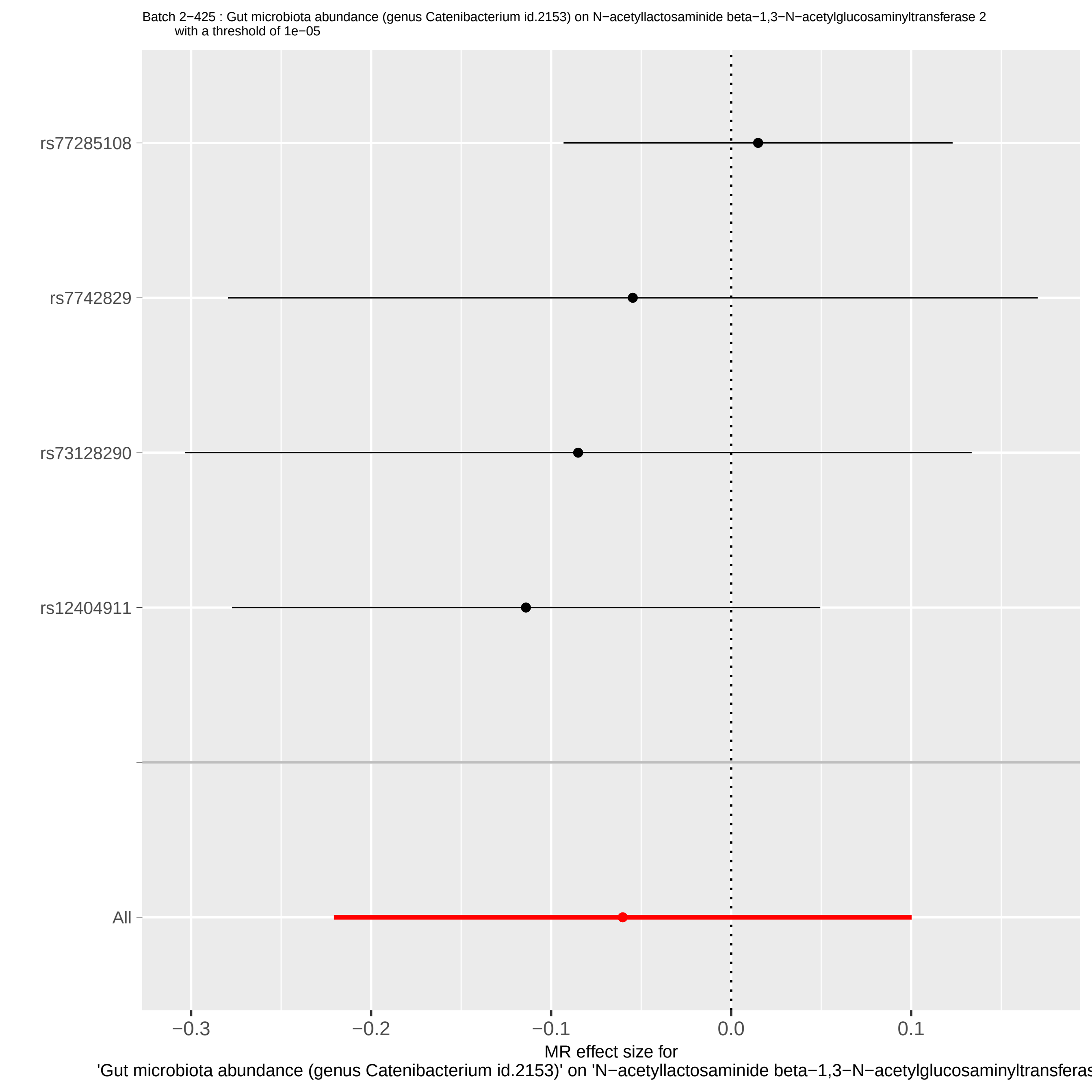

Batch 2−425 : Gut microbiota abundance (genus Catenibacterium id.2153) on N−acetyllactosaminide beta−1,3−N−acetylglucosaminyltransferase 2
with a threshold of 1e−05
rs77285108
rs7742829
rs73128290
rs12404911
All
−0.3
−0.2
−0.1
0.0
0.1
MR effect size for
'Gut microbiota abundance (genus Catenibacterium id.2153)' on 'N−acetyllactosaminide beta−1,3−N−acetylglucosaminyltransferas

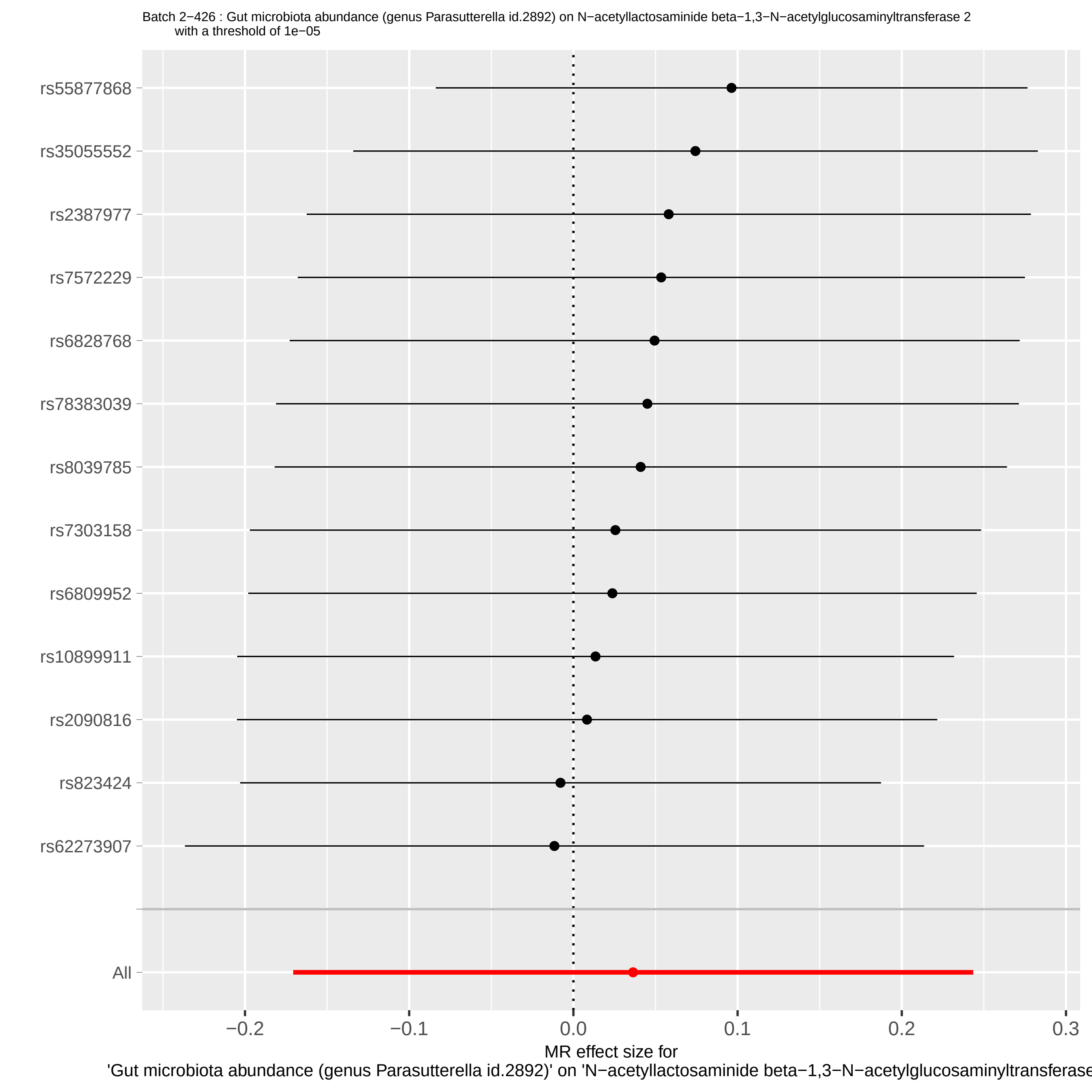

Batch 2−426 : Gut microbiota abundance (genus Parasutterella id.2892) on N−acetyllactosaminide beta−1,3−N−acetylglucosaminyltransferase 2
with a threshold of 1e−05
rs55877868
rs35055552
rs2387977
rs7572229
rs6828768
rs78383039
rs8039785
rs7303158
rs6809952
rs10899911
rs2090816
rs823424
rs62273907
All
−0.2
−0.1
0.0
0.1
0.2
0.3
MR effect size for
'Gut microbiota abundance (genus Parasutterella id.2892)' on 'N−acetyllactosaminide beta−1,3−N−acetylglucosaminyltransferase

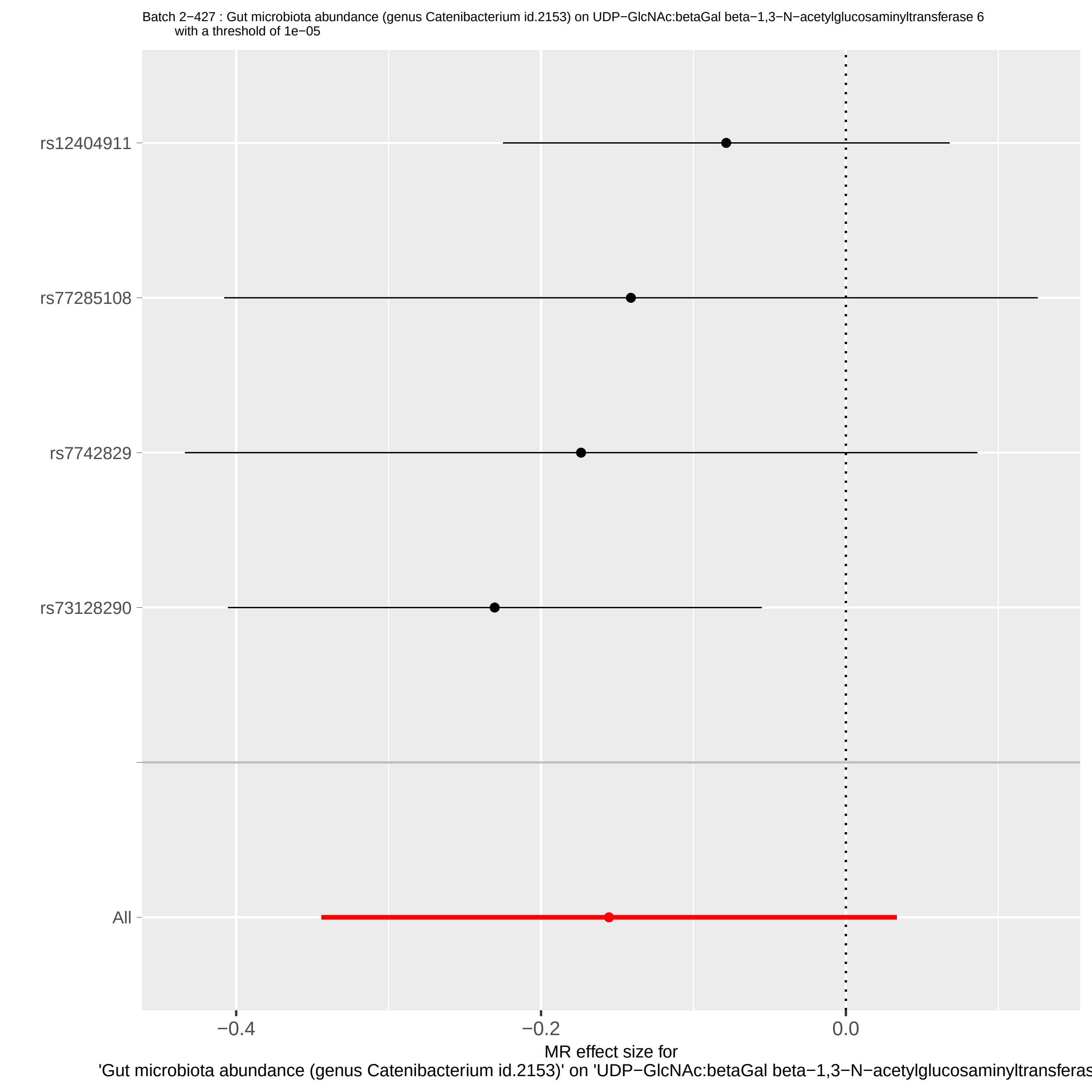
Batch 2−427 : Gut microbiota abundance (genus Catenibacterium id.2153) on UDP−GlcNAc:betaGal beta−1,3−N−acetylglucosaminyltransferase 6
with a threshold of 1e−05
rs12404911
rs77285108
rs7742829
rs73128290
All
−0.4
−0.2
0.0
MR effect size for
'Gut microbiota abundance (genus Catenibacterium id.2153)' on 'UDP−GlcNAc:betaGal beta−1,3−N−acetylglucosaminyltransferas

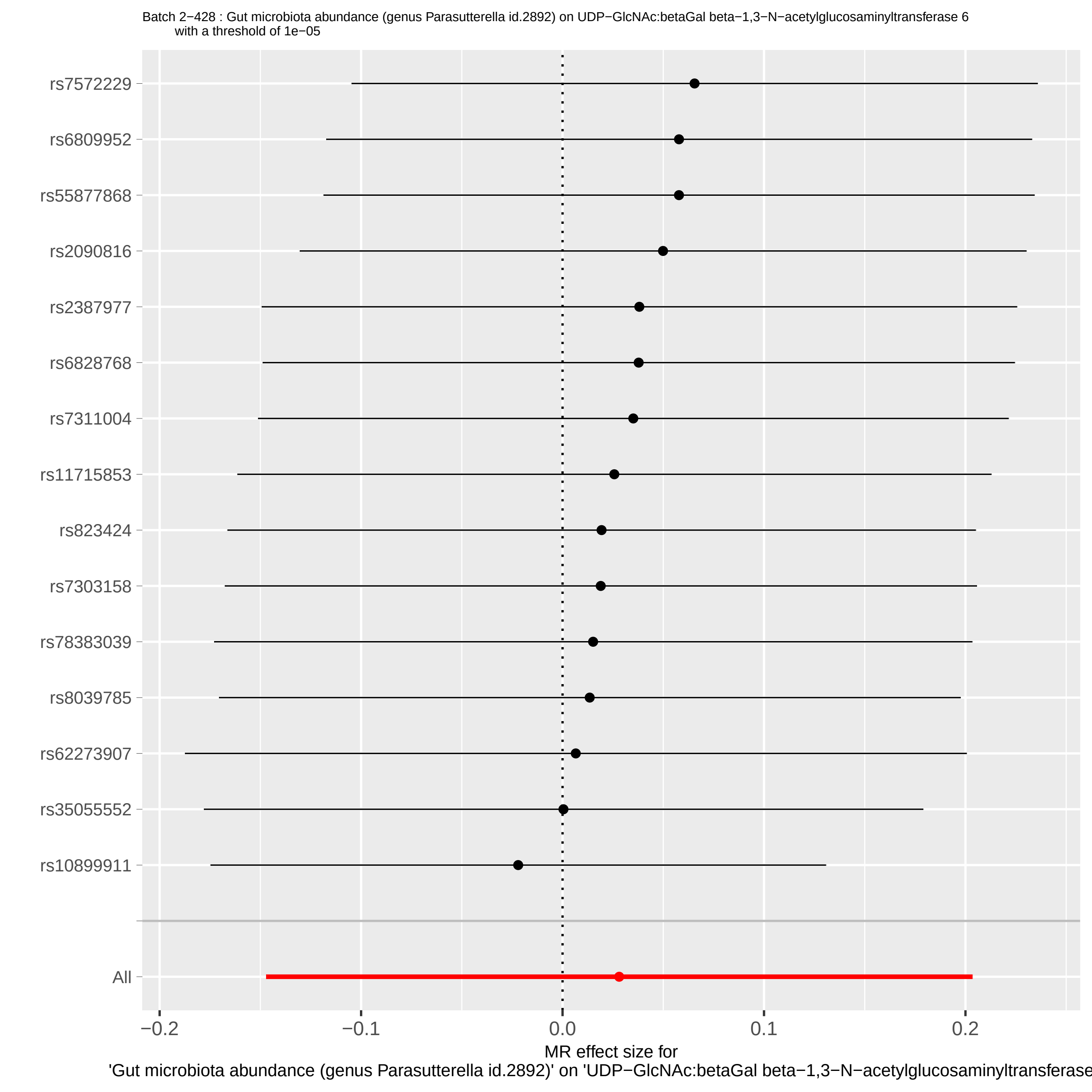
Batch 2−428 : Gut microbiota abundance (genus Parasutterella id.2892) on UDP−GlcNAc:betaGal beta−1,3−N−acetylglucosaminyltransferase 6
with a threshold of 1e−05
rs7572229
rs6809952
rs55877868
rs2090816
rs2387977
rs6828768
rs7311004
rs11715853
rs823424
rs7303158
rs78383039
rs8039785
rs62273907
rs35055552
rs10899911
All
−0.2
−0.1
0.0
0.1
0.2
MR effect size for
'Gut microbiota abundance (genus Parasutterella id.2892)' on 'UDP−GlcNAc:betaGal beta−1,3−N−acetylglucosaminyltransferase

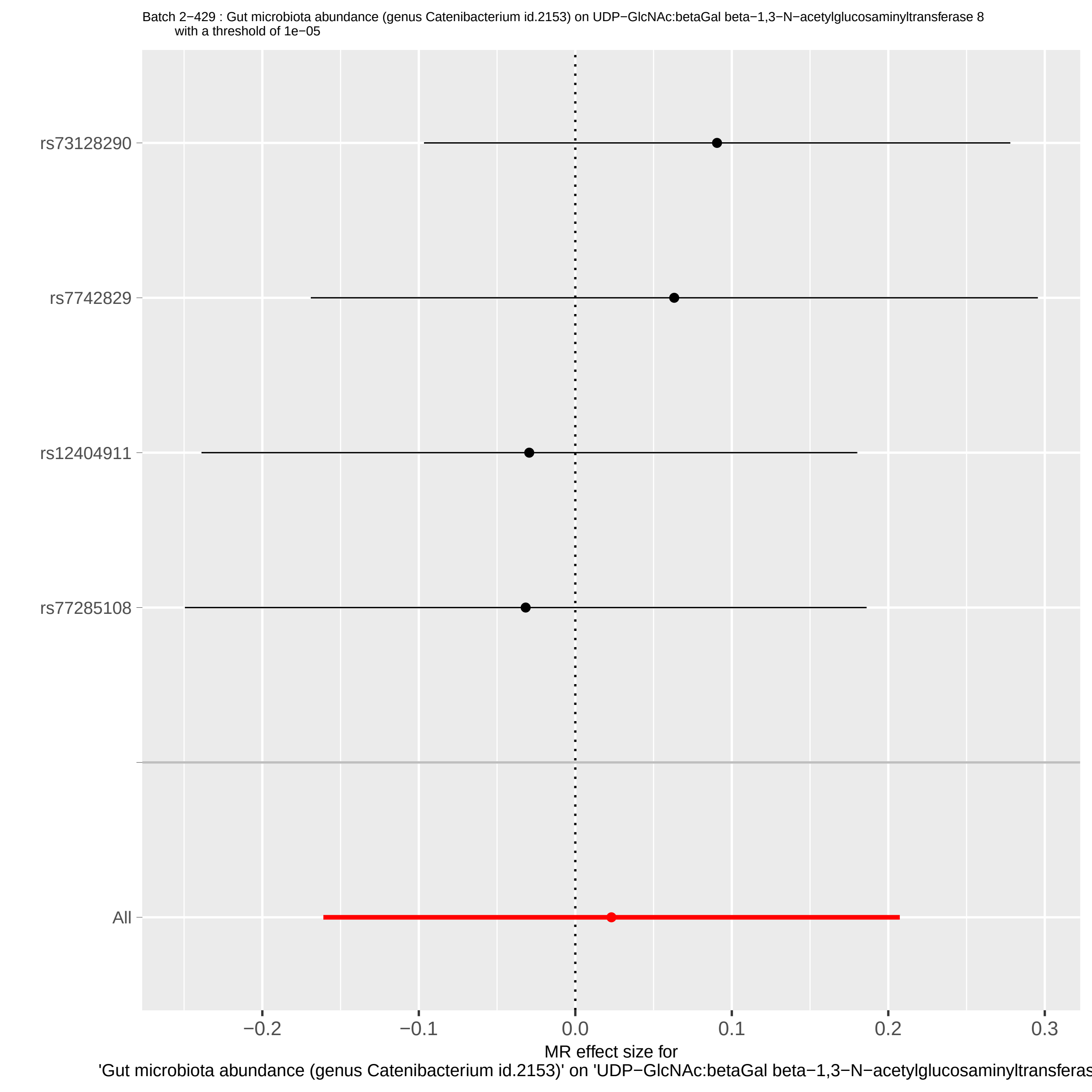

Batch 2−429 : Gut microbiota abundance (genus Catenibacterium id.2153) on UDP−GlcNAc:betaGal beta−1,3−N−acetylglucosaminyltransferase 8
with a threshold of 1e−05
rs73128290
rs7742829
rs12404911
rs77285108
All
−0.2
−0.1
0.0
0.1
0.2
0.3
MR effect size for
'Gut microbiota abundance (genus Catenibacterium id.2153)' on 'UDP−GlcNAc:betaGal beta−1,3−N−acetylglucosaminyltransferas

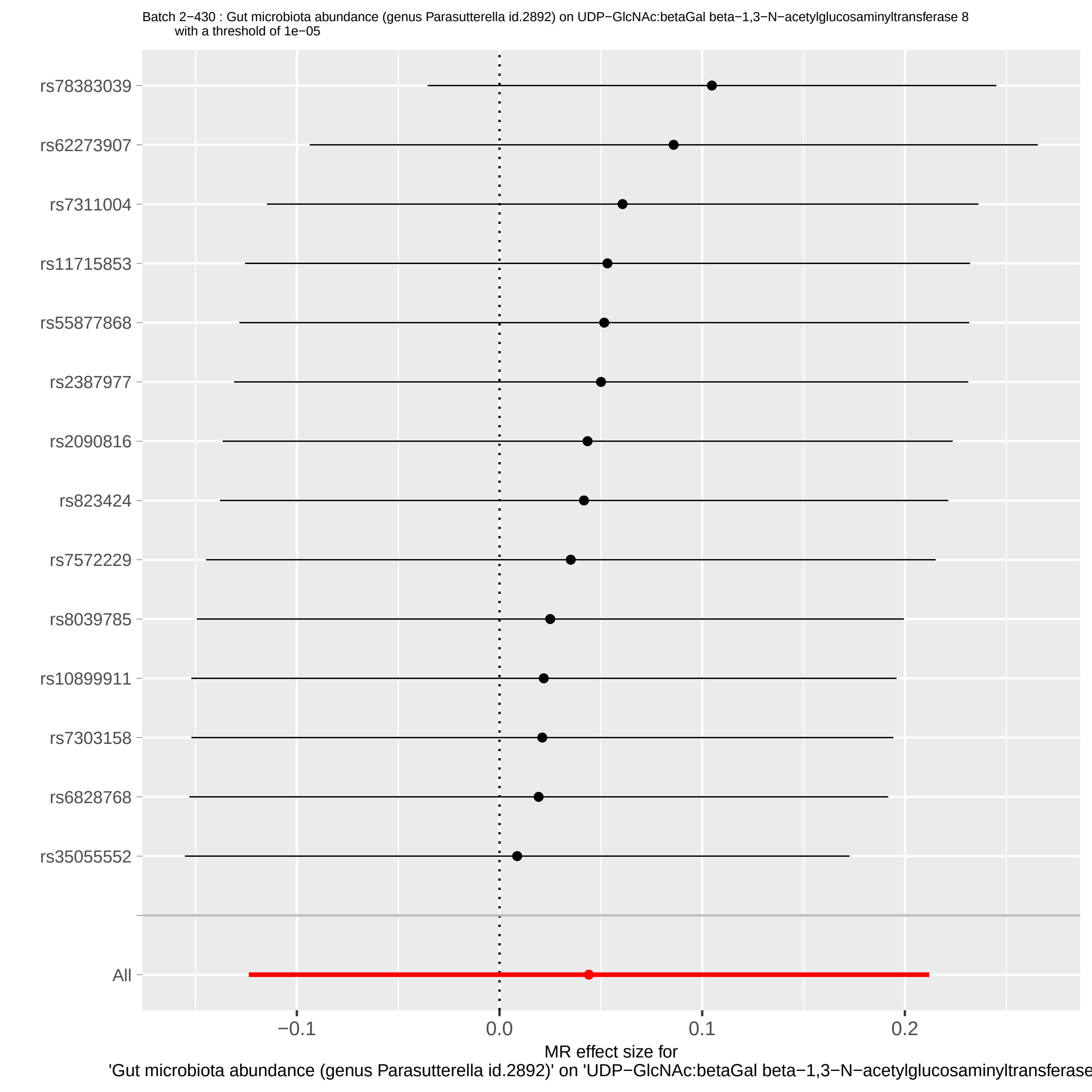

Batch 2−430 : Gut microbiota abundance (genus Parasutterella id.2892) on UDP−GlcNAc:betaGal beta−1,3−N−acetylglucosaminyltransferase 8
with a threshold of 1e−05
rs78383039
rs62273907
rs7311004
rs11715853
rs55877868
rs2387977
rs2090816
rs823424
rs7572229
rs8039785
rs10899911
rs7303158
rs6828768
rs35055552
All
−0.1
0.0
0.1
0.2
MR effect size for
'Gut microbiota abundance (genus Parasutterella id.2892)' on 'UDP−GlcNAc:betaGal beta−1,3−N−acetylglucosaminyltransferase

Batch 2−431 : Gut microbiota abundance (genus Catenibacterium id.2153) on Beta−1,4−galactosyltransferase 1
with a threshold of 1e−05

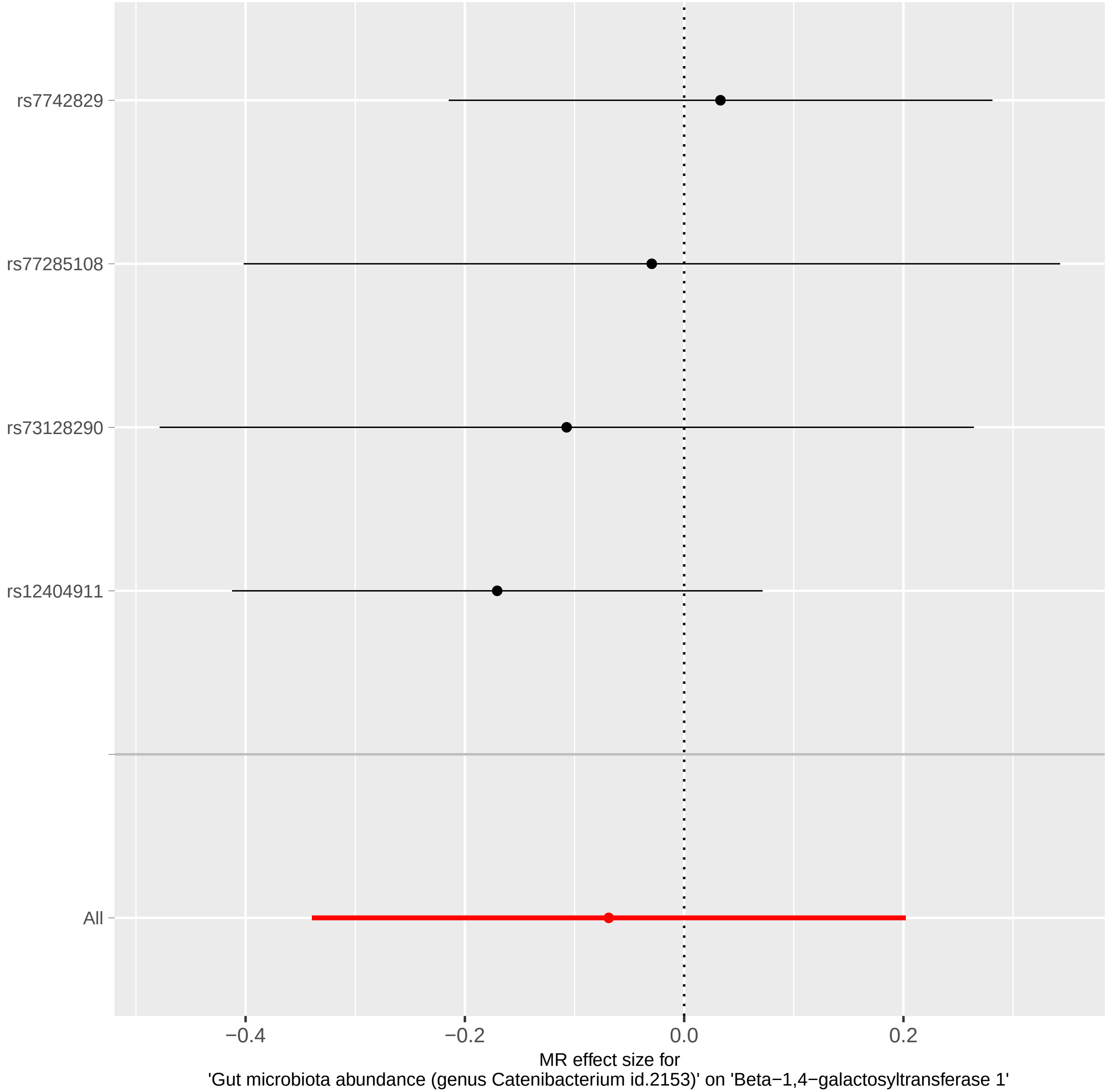

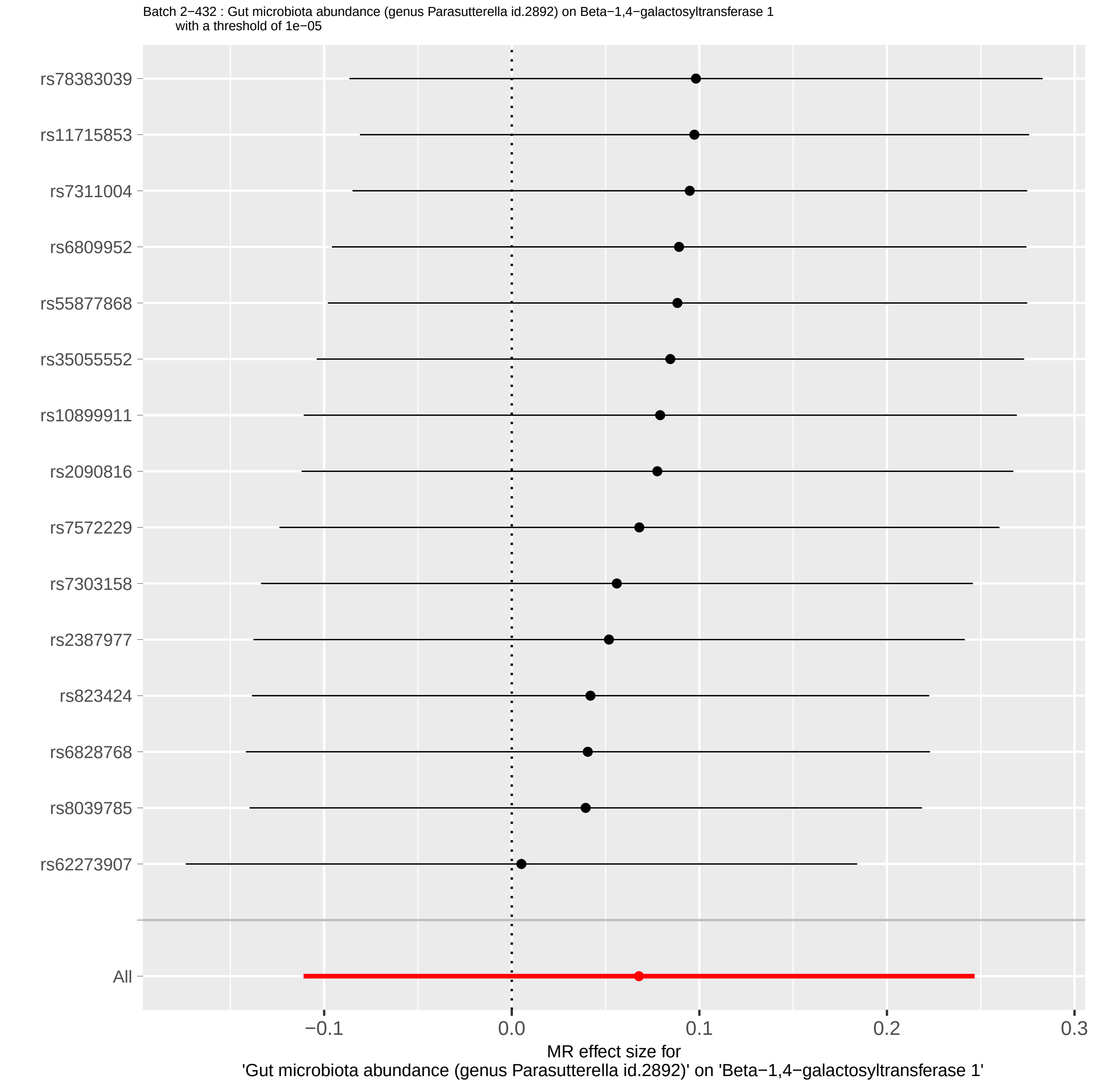
Batch 2−432 : Gut microbiota abundance (genus Parasutterella id.2892) on Beta−1,4−galactosyltransferase 1
with a threshold of 1e−05
rs78383039
rs11715853
rs7311004
rs6809952
rs55877868
rs35055552
rs10899911
rs2090816
rs7572229
rs7303158
rs2387977
rs823424
rs6828768
rs8039785
rs62273907
All
−0.1
0.0
0.1
0.2
0.3
MR effect size for
'Gut microbiota abundance (genus Parasutterella id.2892)' on 'Beta−1,4−galactosyltransferase 1'

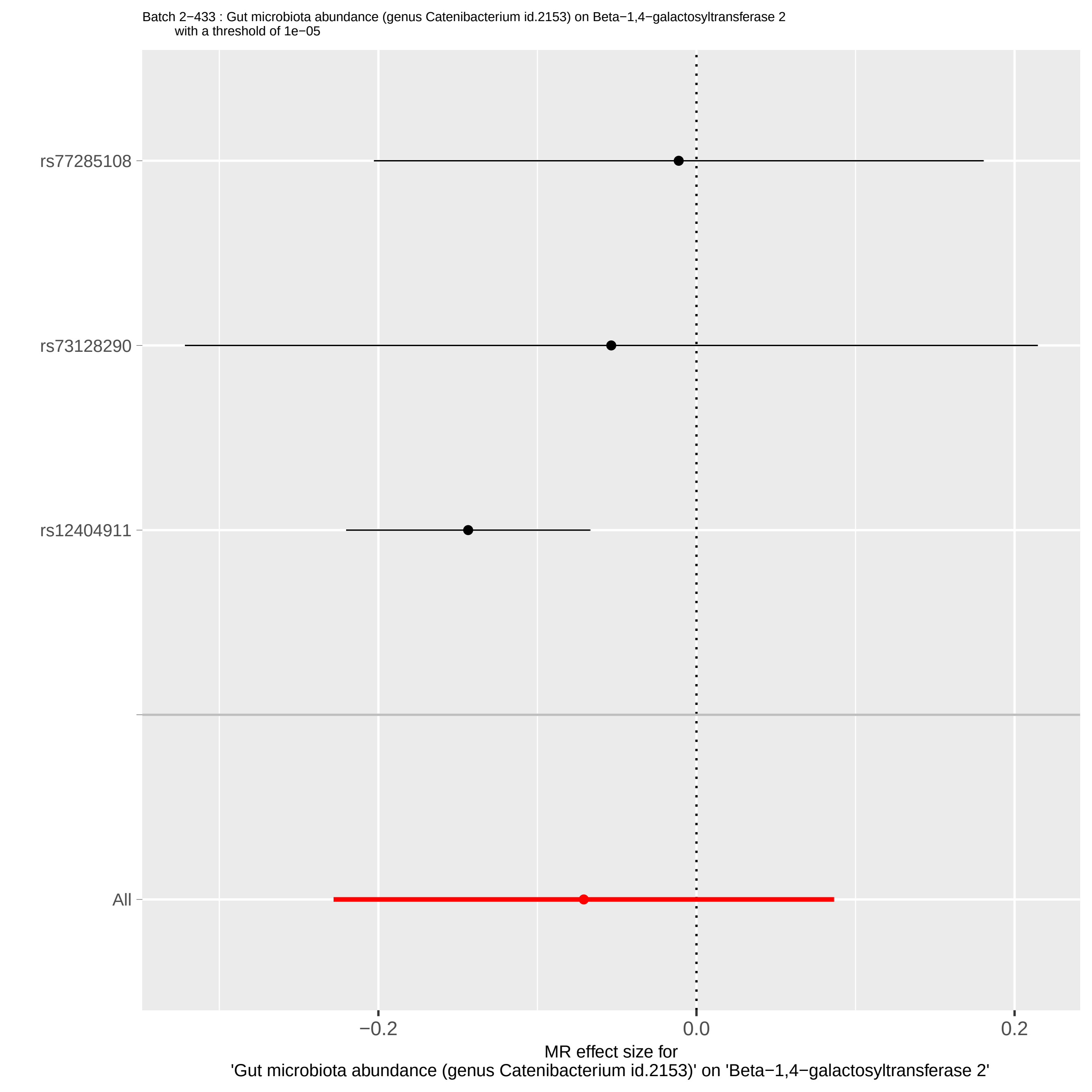

Batch 2−433 : Gut microbiota abundance (genus Catenibacterium id.2153) on Beta−1,4−galactosyltransferase 2
with a threshold of 1e−05
rs77285108
rs73128290
rs12404911
All
−0.2
0.0
0.2
MR effect size for
'Gut microbiota abundance (genus Catenibacterium id.2153)' on 'Beta−1,4−galactosyltransferase 2'

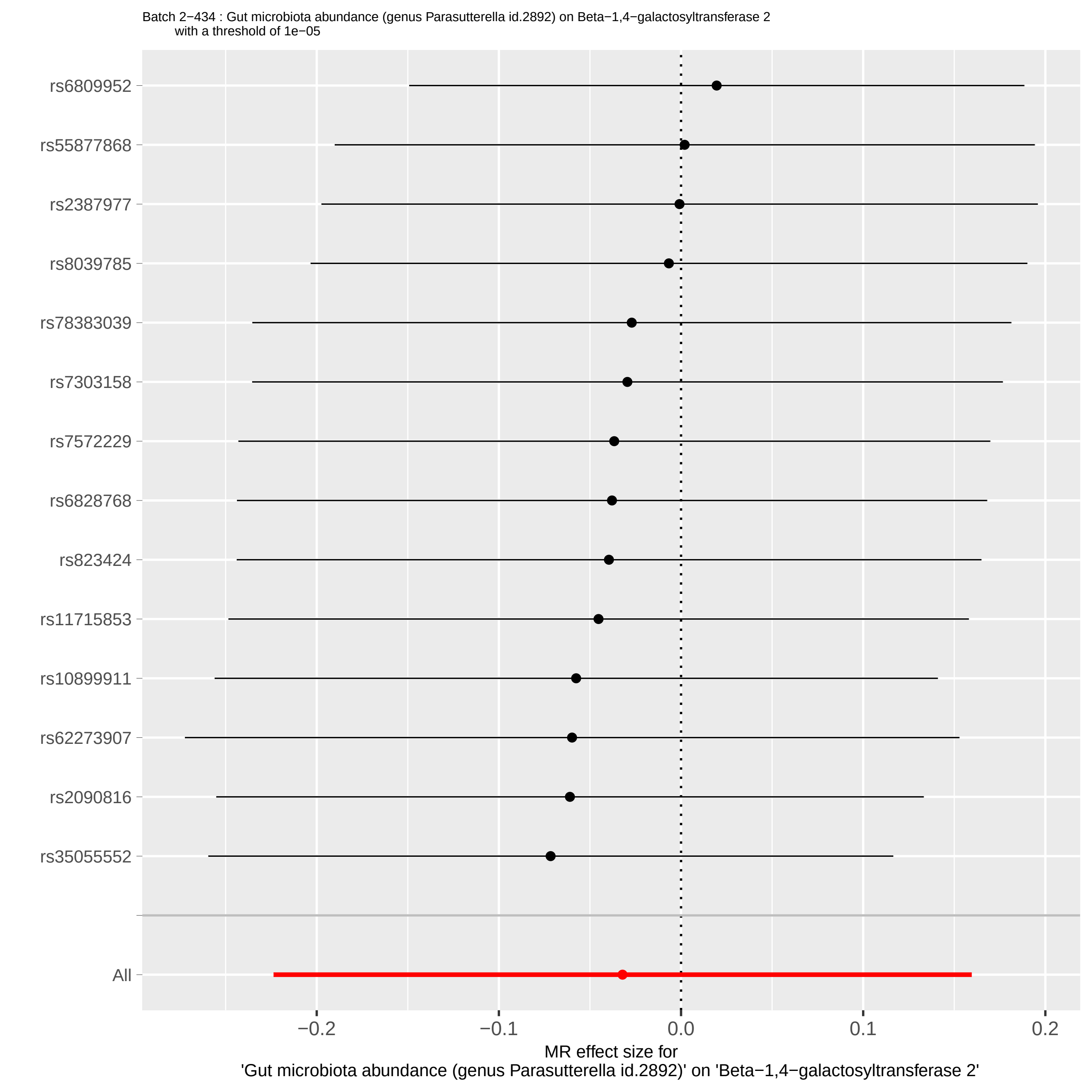

Batch 2−434 : Gut microbiota abundance (genus Parasutterella id.2892) on Beta−1,4−galactosyltransferase 2
with a threshold of 1e−05
rs6809952
rs55877868
rs2387977
rs8039785
rs78383039
rs7303158
rs7572229
rs6828768
rs823424
rs11715853
rs10899911
rs62273907
rs2090816
rs35055552
All
−0.2
−0.1
0.0
0.1
0.2
MR effect size for
'Gut microbiota abundance (genus Parasutterella id.2892)' on 'Beta−1,4−galactosyltransferase 2'

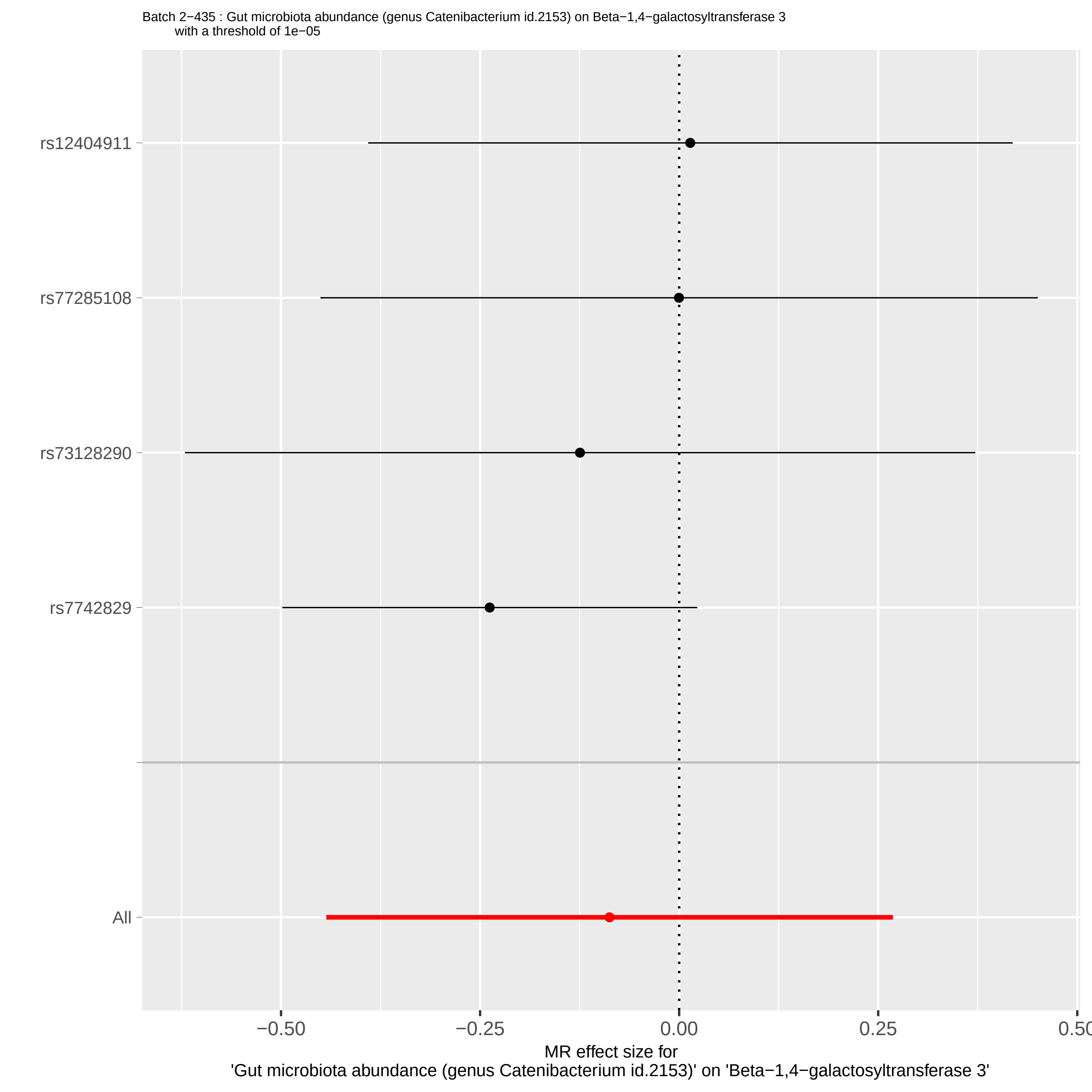

Batch 2−435 : Gut microbiota abundance (genus Catenibacterium id.2153) on Beta−1,4−galactosyltransferase 3
with a threshold of 1e−05
rs12404911
rs77285108
rs73128290
rs7742829
All
−0.50
−0.25
0.00
0.25
0.50
MR effect size for
'Gut microbiota abundance (genus Catenibacterium id.2153)' on 'Beta−1,4−galactosyltransferase 3'

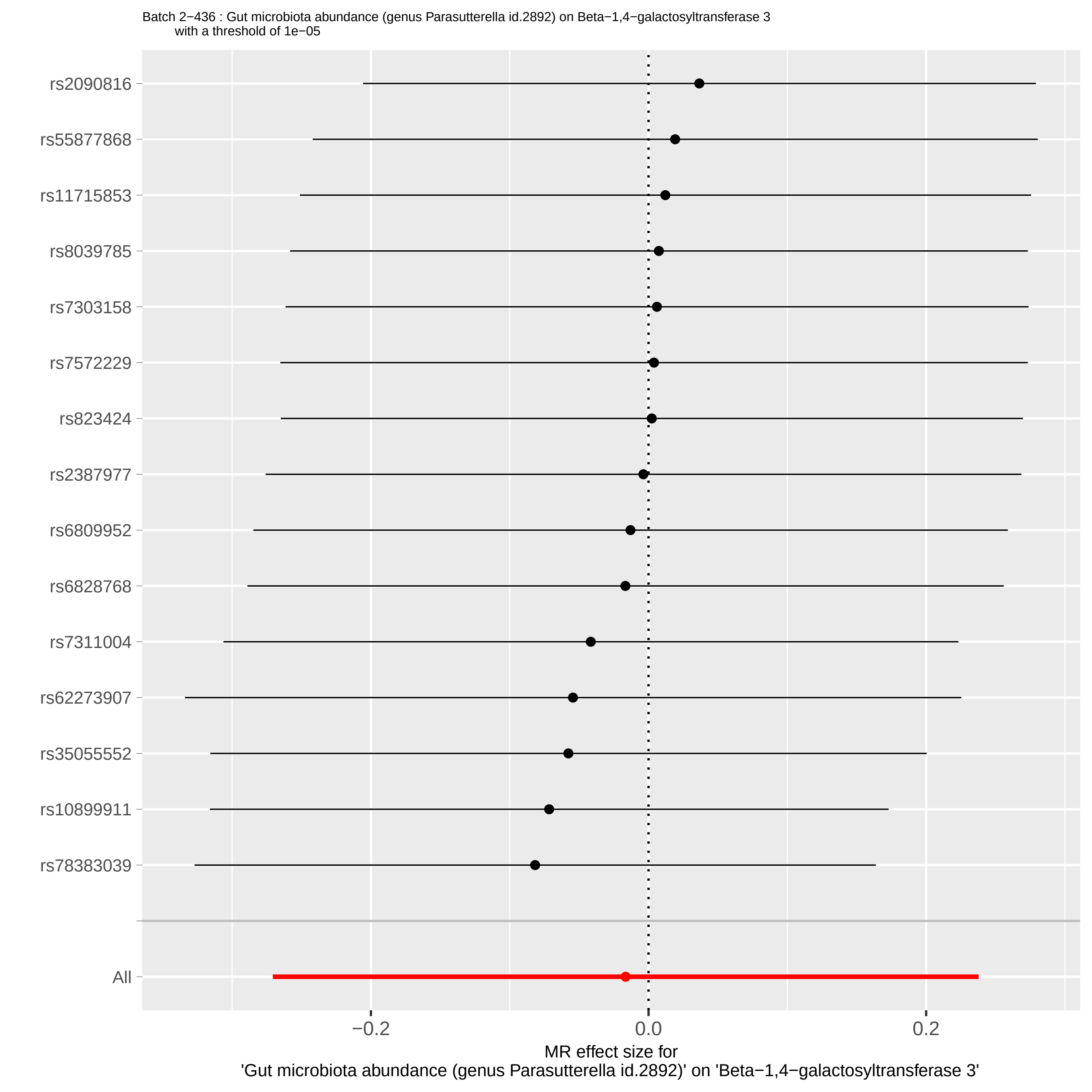

Batch 2−436 : Gut microbiota abundance (genus Parasutterella id.2892) on Beta−1,4−galactosyltransferase 3
with a threshold of 1e−05
rs2090816
rs55877868
rs11715853
rs8039785
rs7303158
rs7572229
rs823424
rs2387977
rs6809952
rs6828768
rs7311004
rs62273907
rs35055552
rs10899911
rs78383039
All
−0.2
0.0
0.2
MR effect size for
'Gut microbiota abundance (genus Parasutterella id.2892)' on 'Beta−1,4−galactosyltransferase 3'

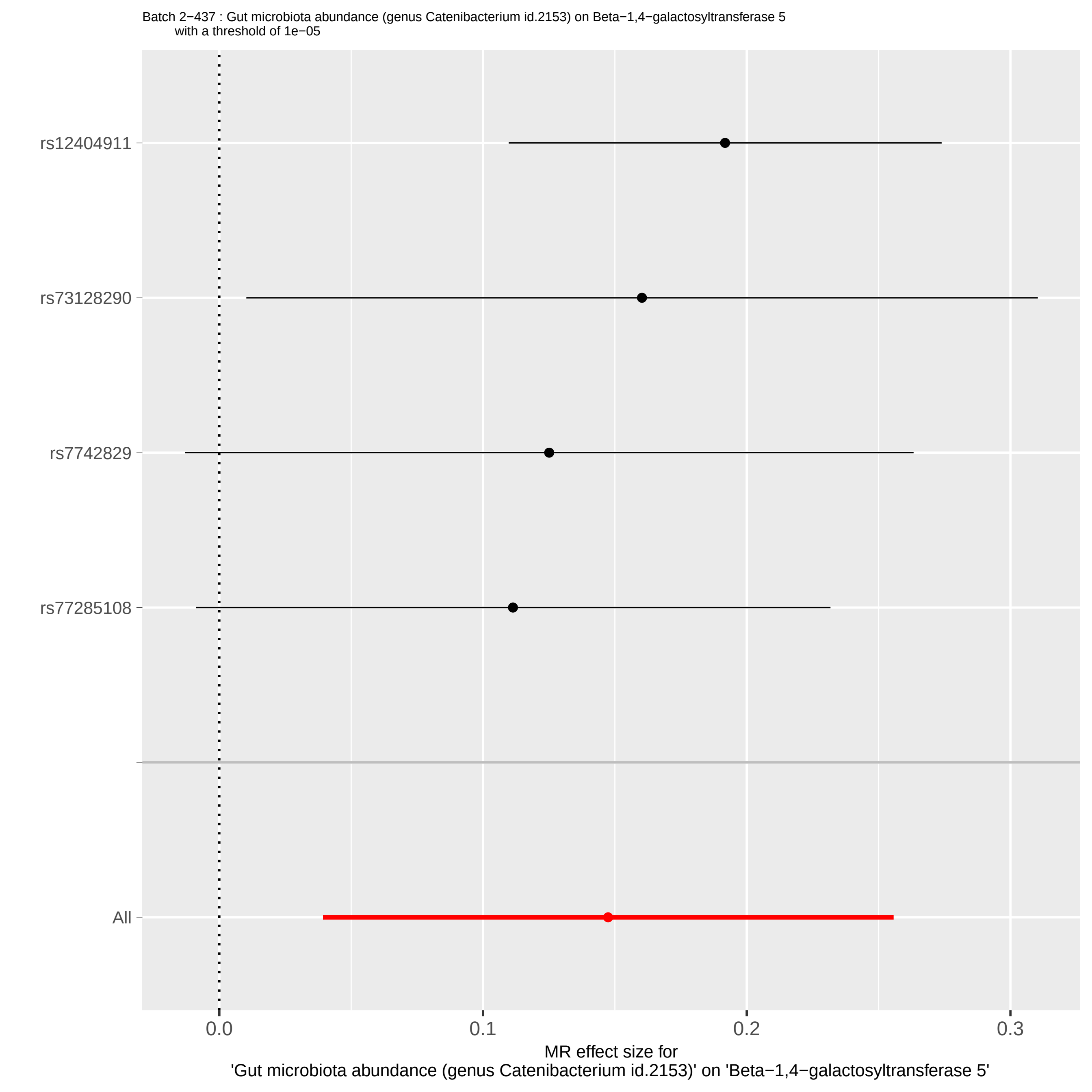
Batch 2−437 : Gut microbiota abundance (genus Catenibacterium id.2153) on Beta−1,4−galactosyltransferase 5
with a threshold of 1e−05
rs12404911
rs73128290
rs7742829
rs77285108
All
0.0
0.1
0.2
0.3
MR effect size for
'Gut microbiota abundance (genus Catenibacterium id.2153)' on 'Beta−1,4−galactosyltransferase 5'

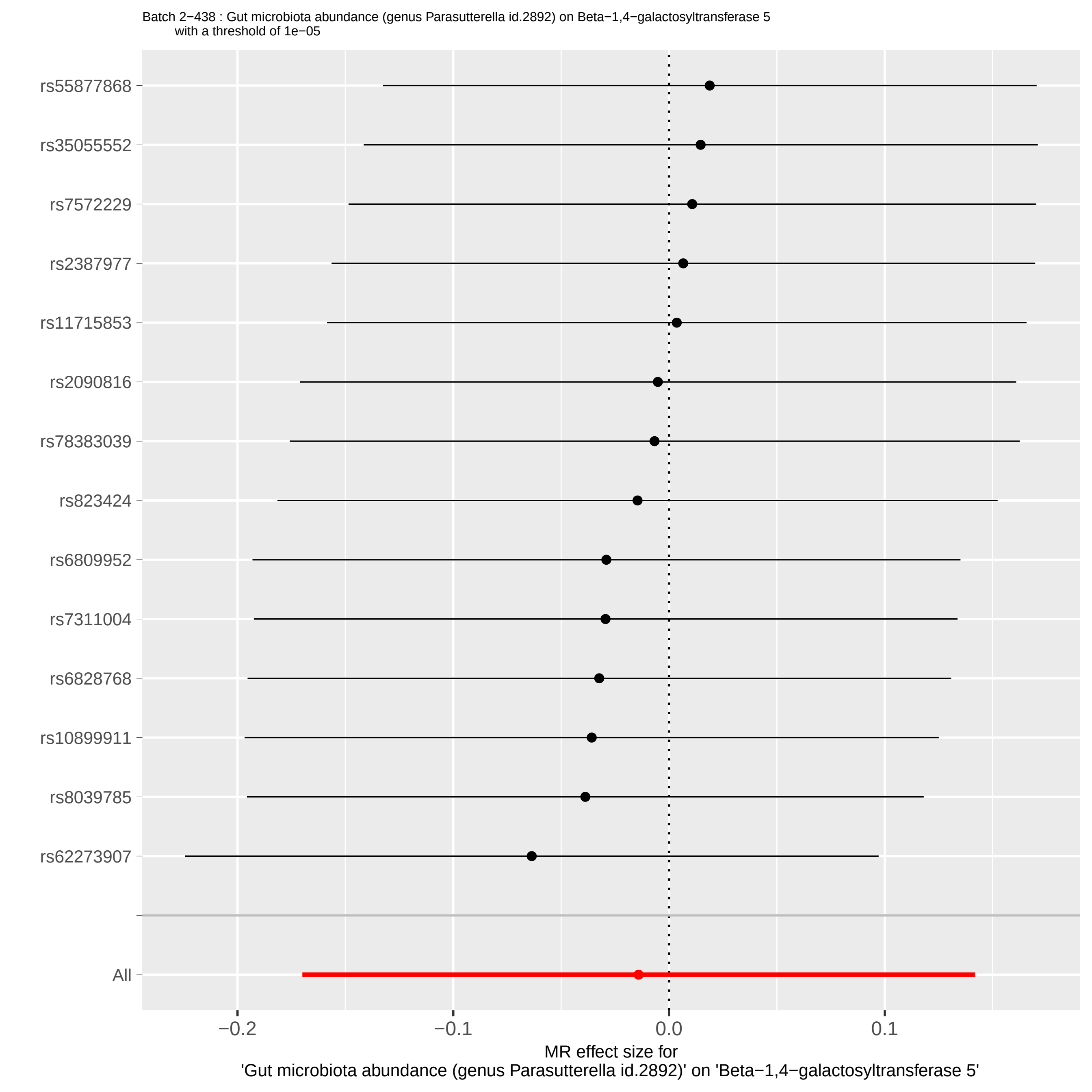

Batch 2−438 : Gut microbiota abundance (genus Parasutterella id.2892) on Beta−1,4−galactosyltransferase 5
with a threshold of 1e−05
rs55877868
rs35055552
rs7572229
rs2387977
rs11715853
rs2090816
rs78383039
rs823424
rs6809952
rs7311004
rs6828768
rs10899911
rs8039785
rs62273907
All
−0.2
−0.1
0.0
0.1
MR effect size for
'Gut microbiota abundance (genus Parasutterella id.2892)' on 'Beta−1,4−galactosyltransferase 5'

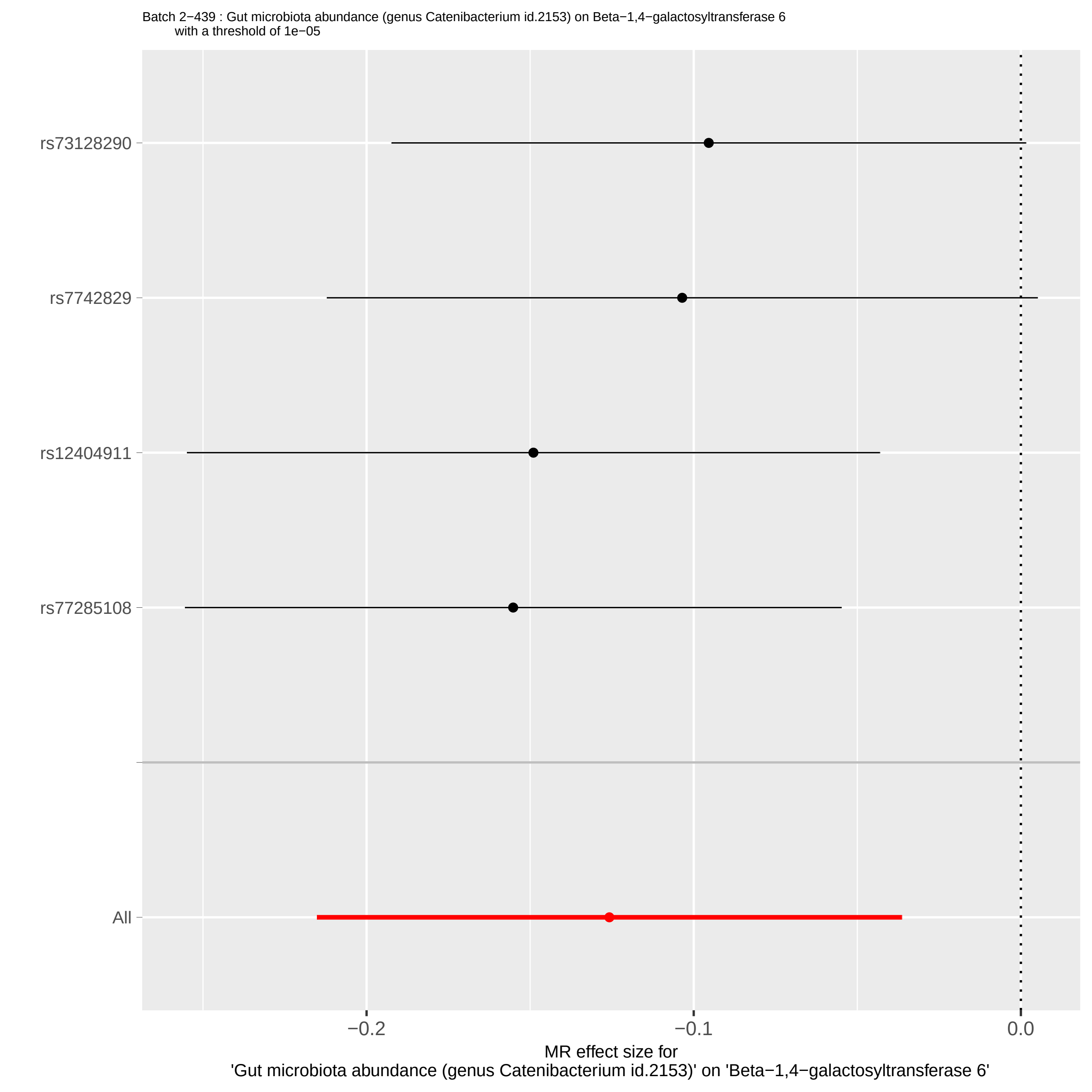

Batch 2−439 : Gut microbiota abundance (genus Catenibacterium id.2153) on Beta−1,4−galactosyltransferase 6
with a threshold of 1e−05
rs73128290
rs7742829
rs12404911
rs77285108
All
−0.2
−0.1
0.0
MR effect size for
'Gut microbiota abundance (genus Catenibacterium id.2153)' on 'Beta−1,4−galactosyltransferase 6'

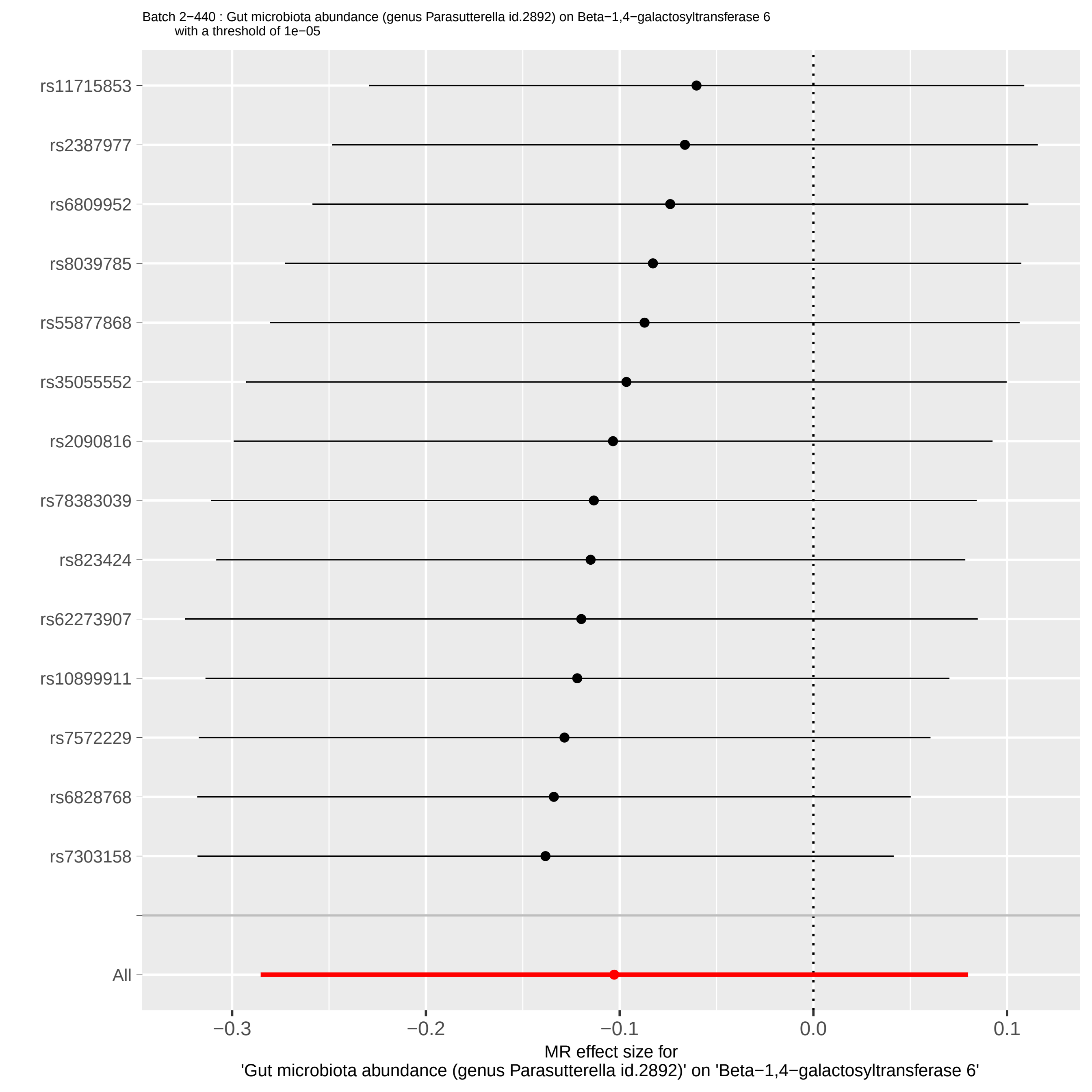

Batch 2−440 : Gut microbiota abundance (genus Parasutterella id.2892) on Beta−1,4−galactosyltransferase 6
with a threshold of 1e−05
rs11715853
rs2387977
rs6809952
rs8039785
rs55877868
rs35055552
rs2090816
rs78383039
rs823424
rs62273907
rs10899911
rs7572229
rs6828768
rs7303158
All
−0.3
−0.2
−0.1
0.0
0.1
MR effect size for
'Gut microbiota abundance (genus Parasutterella id.2892)' on 'Beta−1,4−galactosyltransferase 6'

Batch 2−441 : Gut microbiota abundance (genus Catenibacterium id.2153) on Beta−1,4−galactosyltransferase 7
with a threshold of 1e−05

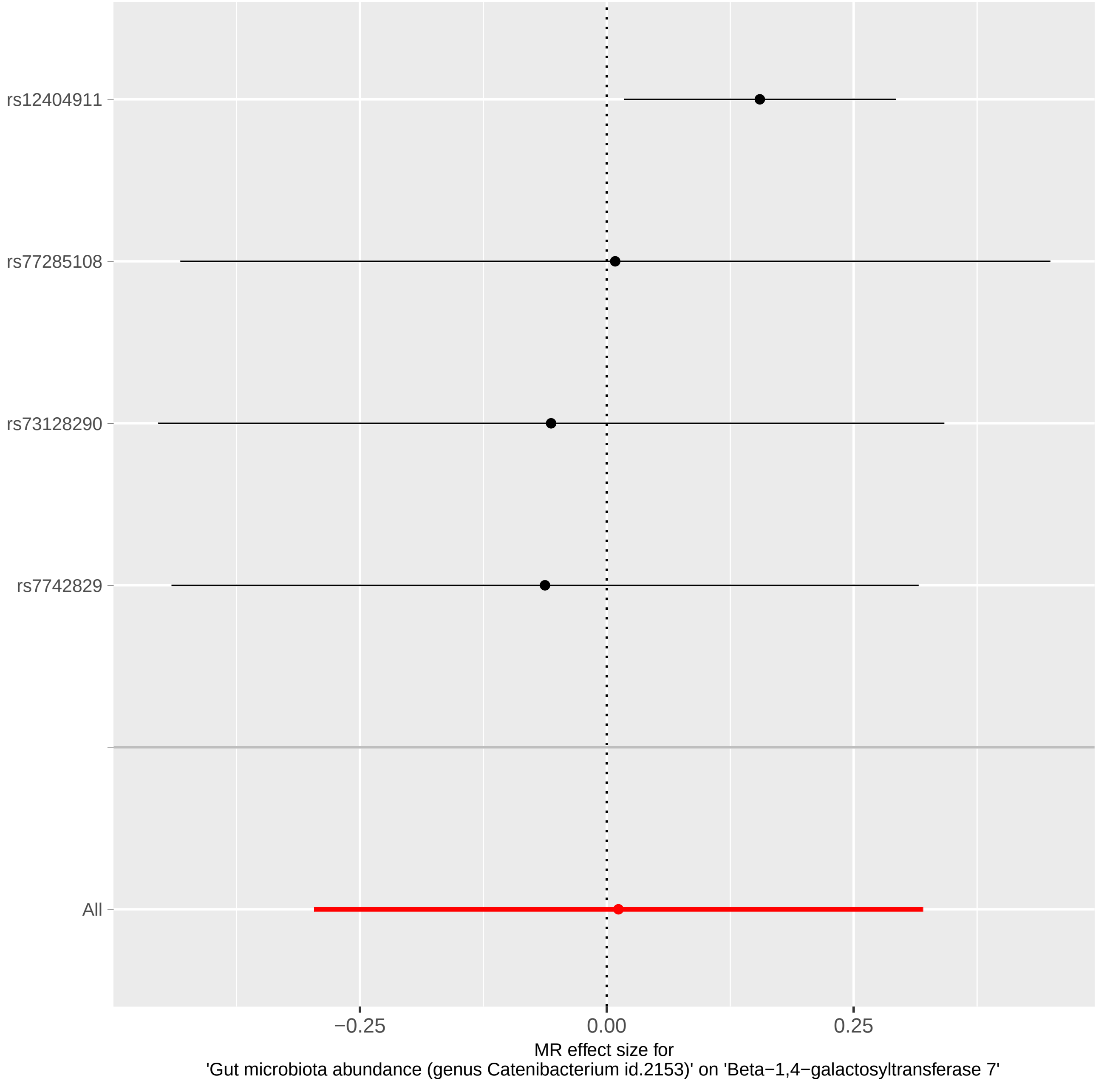

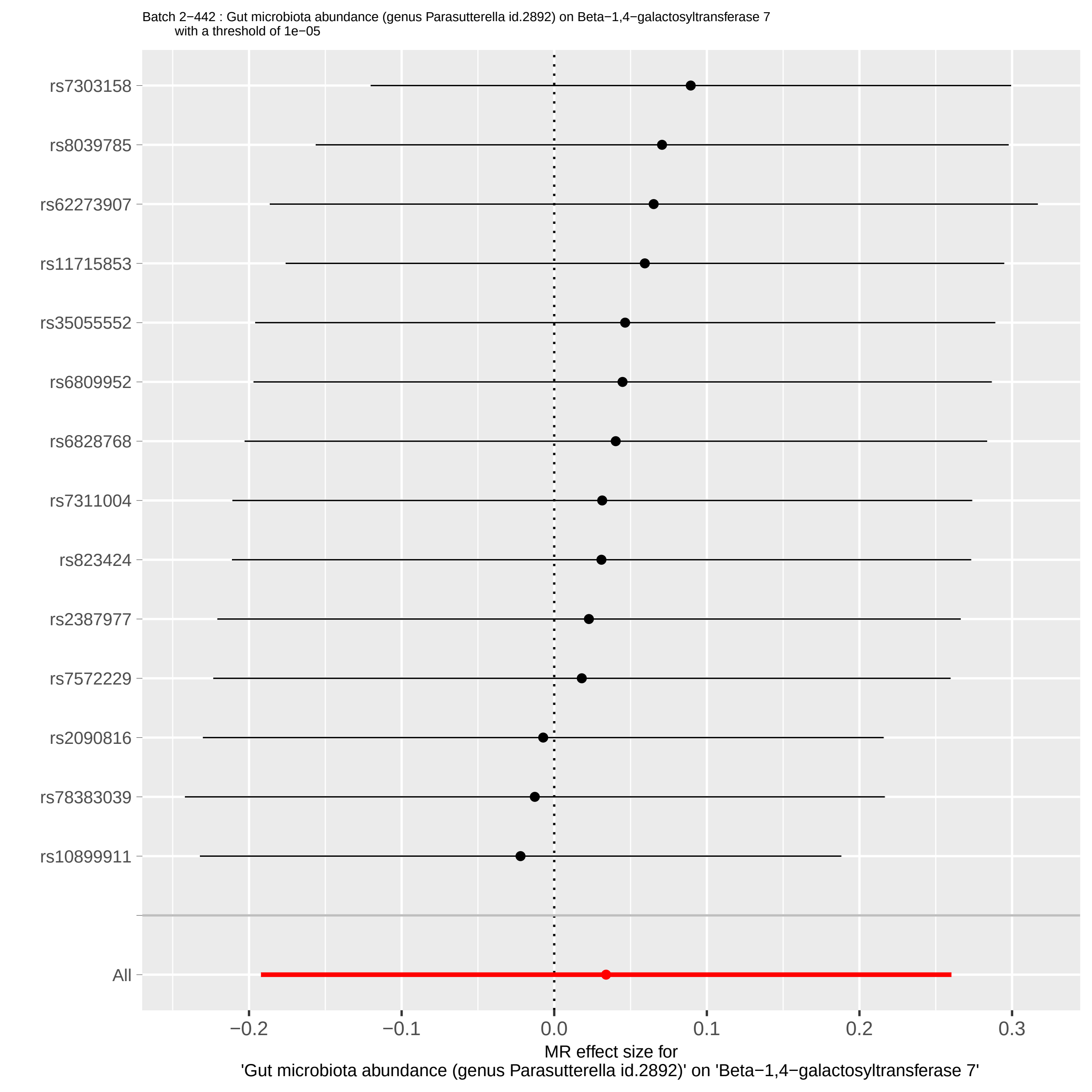
Batch 2−442 : Gut microbiota abundance (genus Parasutterella id.2892) on Beta−1,4−galactosyltransferase 7
with a threshold of 1e−05
rs7303158
rs8039785
rs62273907
rs11715853
rs35055552
rs6809952
rs6828768
rs7311004
rs823424
rs2387977
rs7572229
rs2090816
rs78383039
rs10899911
All
−0.2
−0.1
0.0
0.1
0.2
0.3
MR effect size for
'Gut microbiota abundance (genus Parasutterella id.2892)' on 'Beta−1,4−galactosyltransferase 7'

Batch 2−443 : Gut microbiota abundance (genus Catenibacterium id.2153) on Transcription regulator protein BACH1
with a threshold of 1e−05

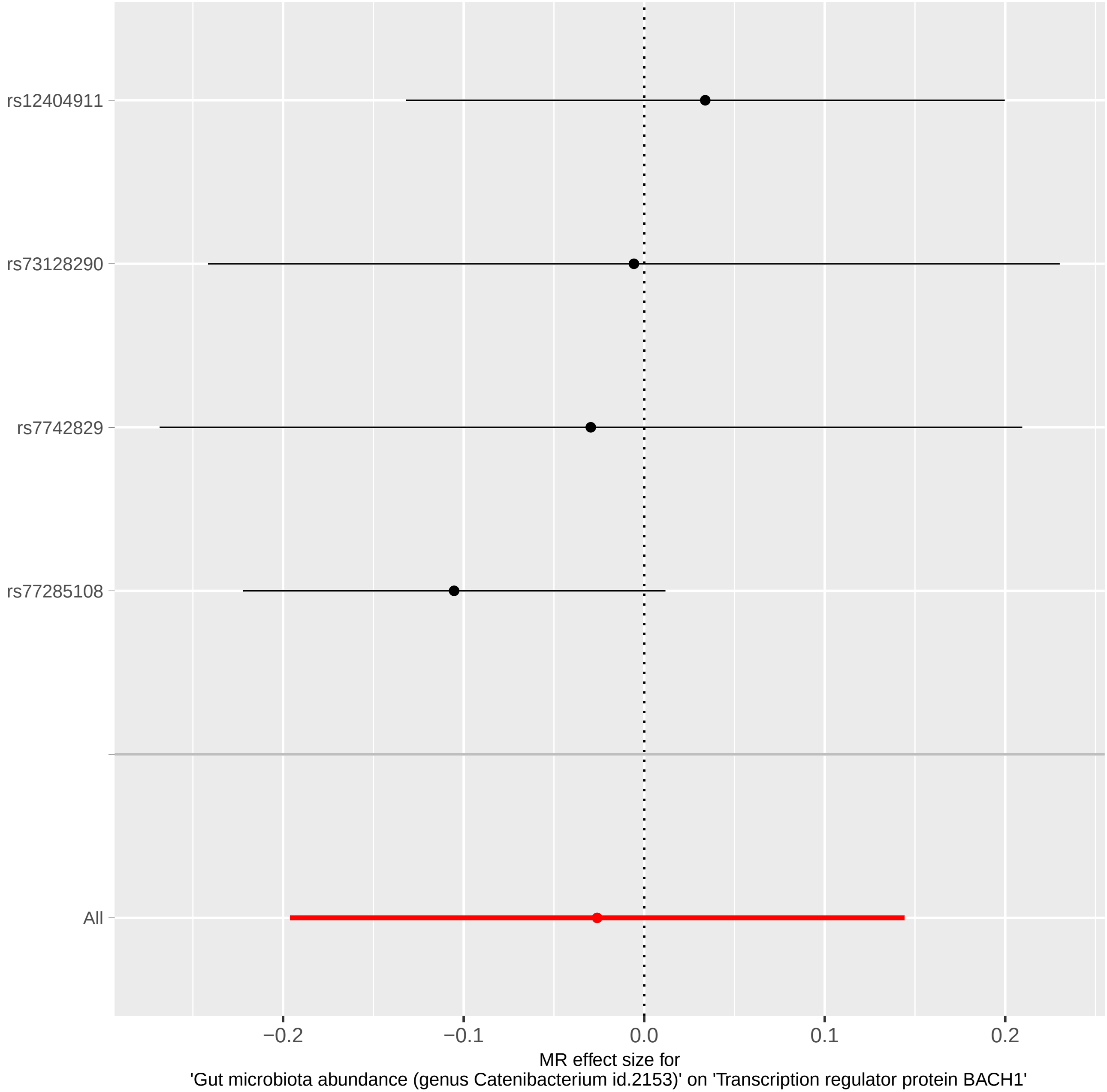

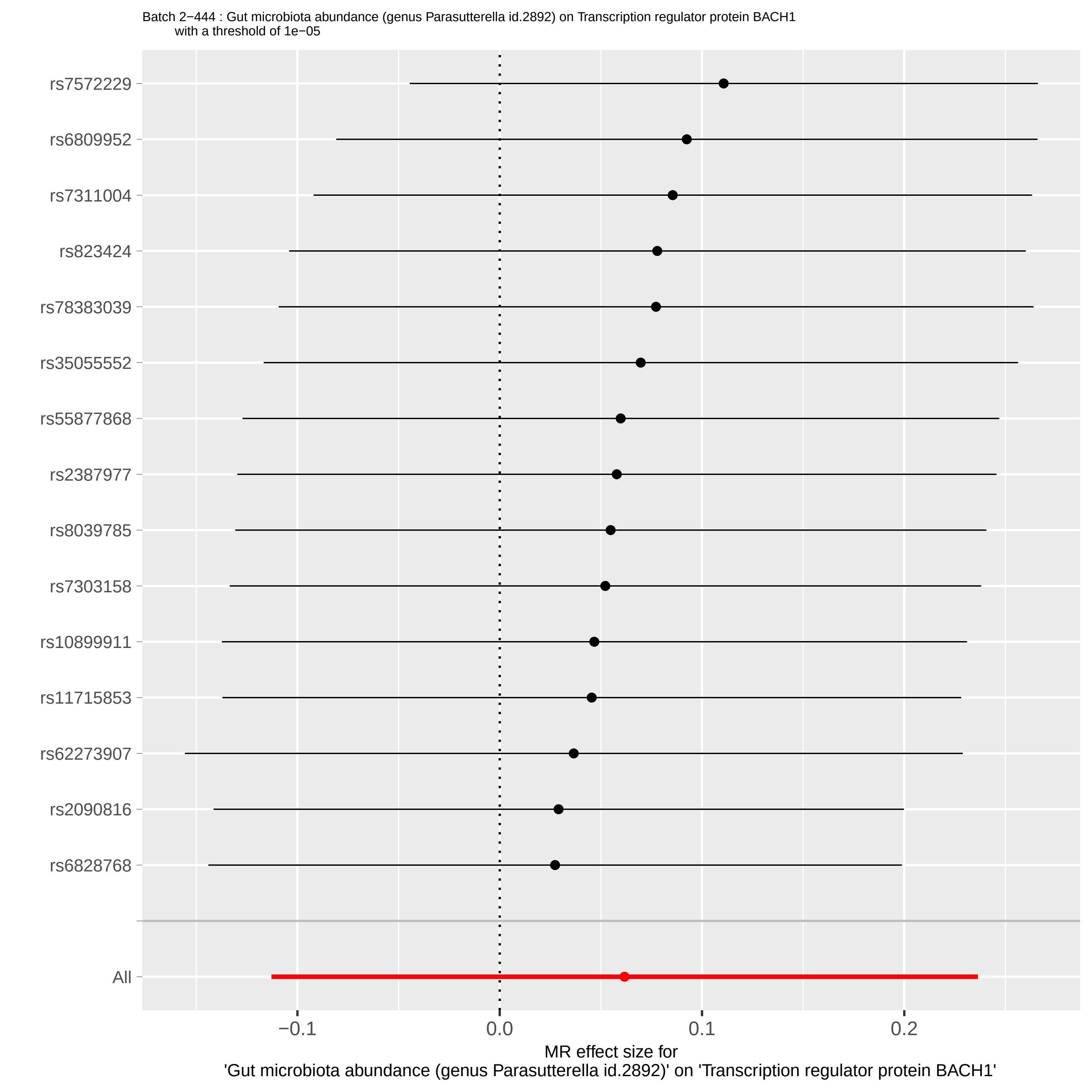

Batch 2−444 : Gut microbiota abundance (genus Parasutterella id.2892) on Transcription regulator protein BACH1
with a threshold of 1e−05
rs7572229
rs6809952
rs7311004
rs823424
rs78383039
rs35055552
rs55877868
rs2387977
rs8039785
rs7303158
rs10899911
rs11715853
rs62273907
rs2090816
rs6828768
All
−0.1
0.0
0.1
0.2
MR effect size for
'Gut microbiota abundance (genus Parasutterella id.2892)' on 'Transcription regulator protein BACH1'

Batch 2−445 : Gut microbiota abundance (genus Catenibacterium id.2153) on Transcription regulator protein BACH2
with a threshold of 1e−05

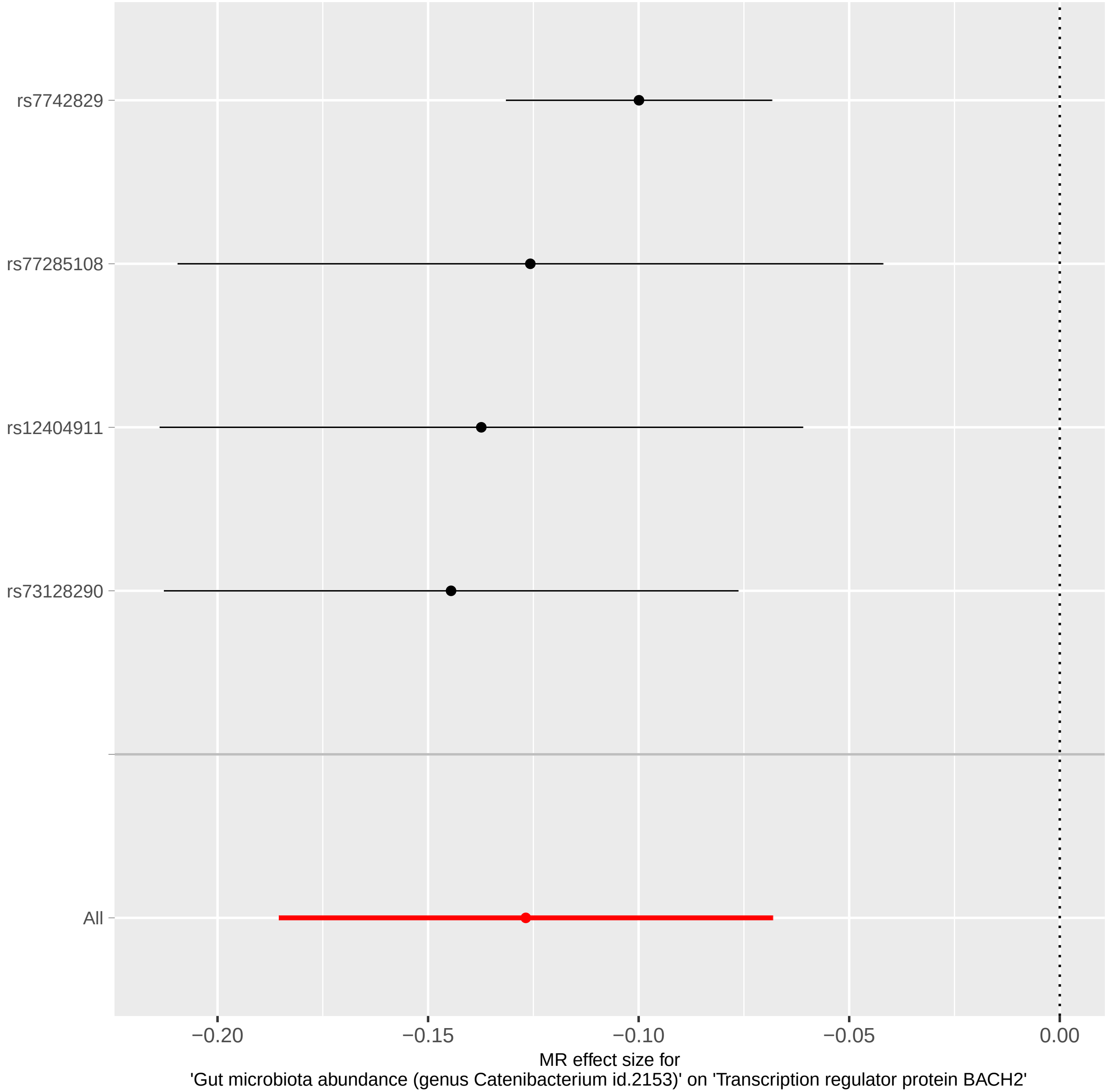

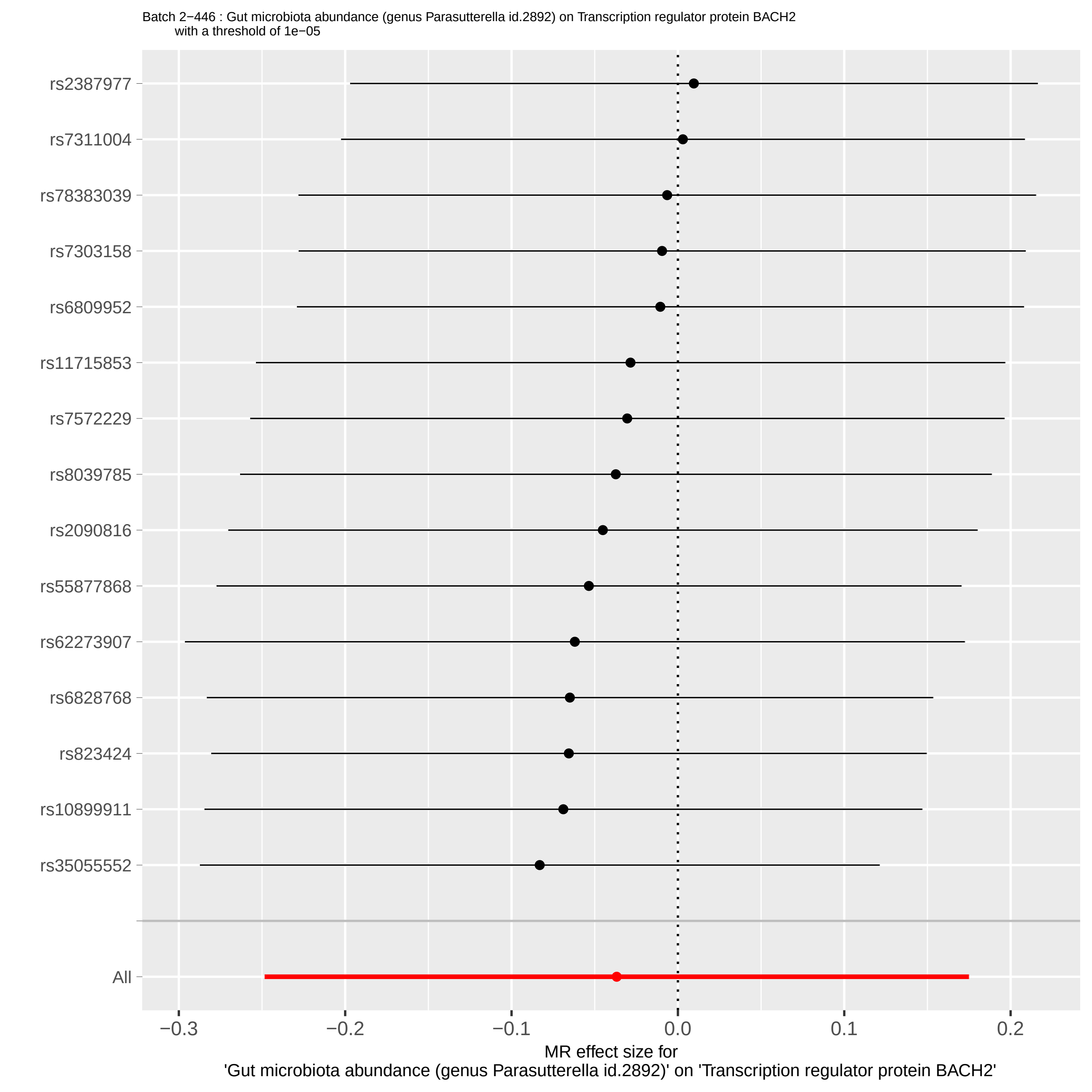
Batch 2−446 : Gut microbiota abundance (genus Parasutterella id.2892) on Transcription regulator protein BACH2
with a threshold of 1e−05
rs2387977
rs7311004
rs78383039
rs7303158
rs6809952
rs11715853
rs7572229
rs8039785
rs2090816
rs55877868
rs62273907
rs6828768
rs823424
rs10899911
rs35055552
All
−0.3
−0.2
−0.1
0.0
0.1
0.2
MR effect size for
'Gut microbiota abundance (genus Parasutterella id.2892)' on 'Transcription regulator protein BACH2'

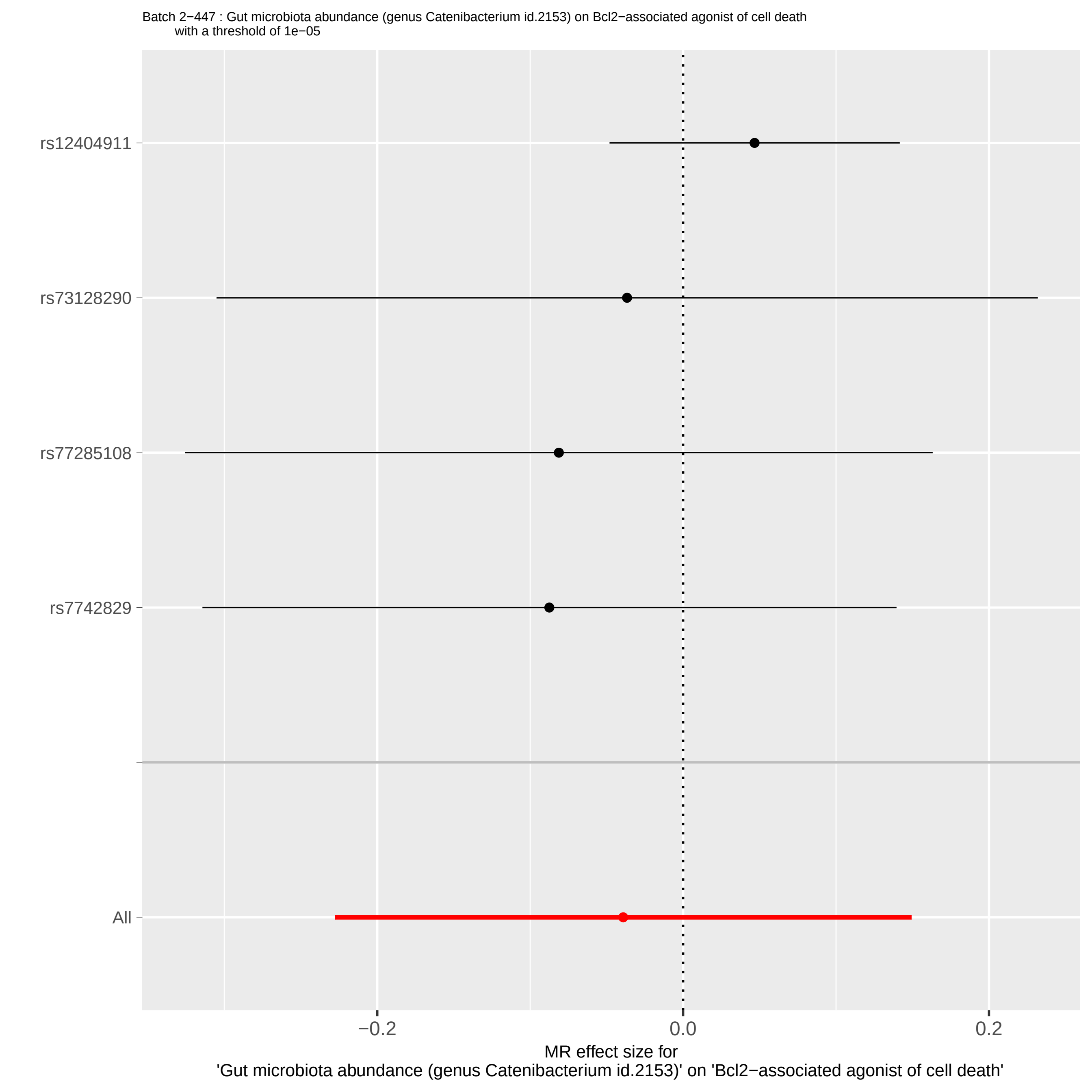
Batch 2−447 : Gut microbiota abundance (genus Catenibacterium id.2153) on Bcl2−associated agonist of cell death
with a threshold of 1e−05
rs12404911
rs73128290
rs77285108
rs7742829
All
−0.2
0.0
0.2
MR effect size for
'Gut microbiota abundance (genus Catenibacterium id.2153)' on 'Bcl2−associated agonist of cell death'

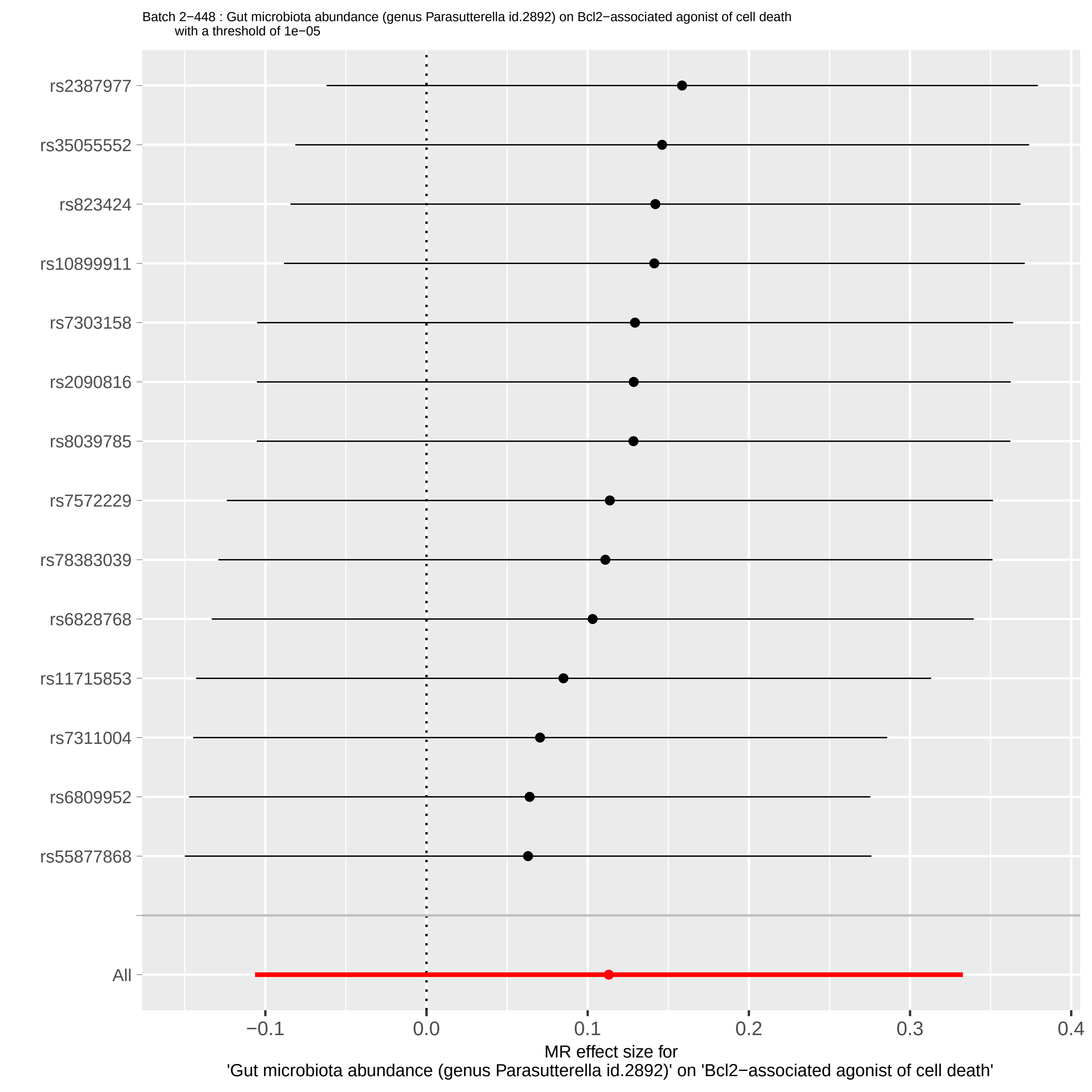
Batch 2−448 : Gut microbiota abundance (genus Parasutterella id.2892) on Bcl2−associated agonist of cell death
with a threshold of 1e−05
rs2387977
rs35055552
rs823424
rs10899911
rs7303158
rs2090816
rs8039785
rs7572229
rs78383039
rs6828768
rs11715853
rs7311004
rs6809952
rs55877868
All
−0.1
0.0
0.1
0.2
0.3
0.4
MR effect size for
'Gut microbiota abundance (genus Parasutterella id.2892)' on 'Bcl2−associated agonist of cell death'

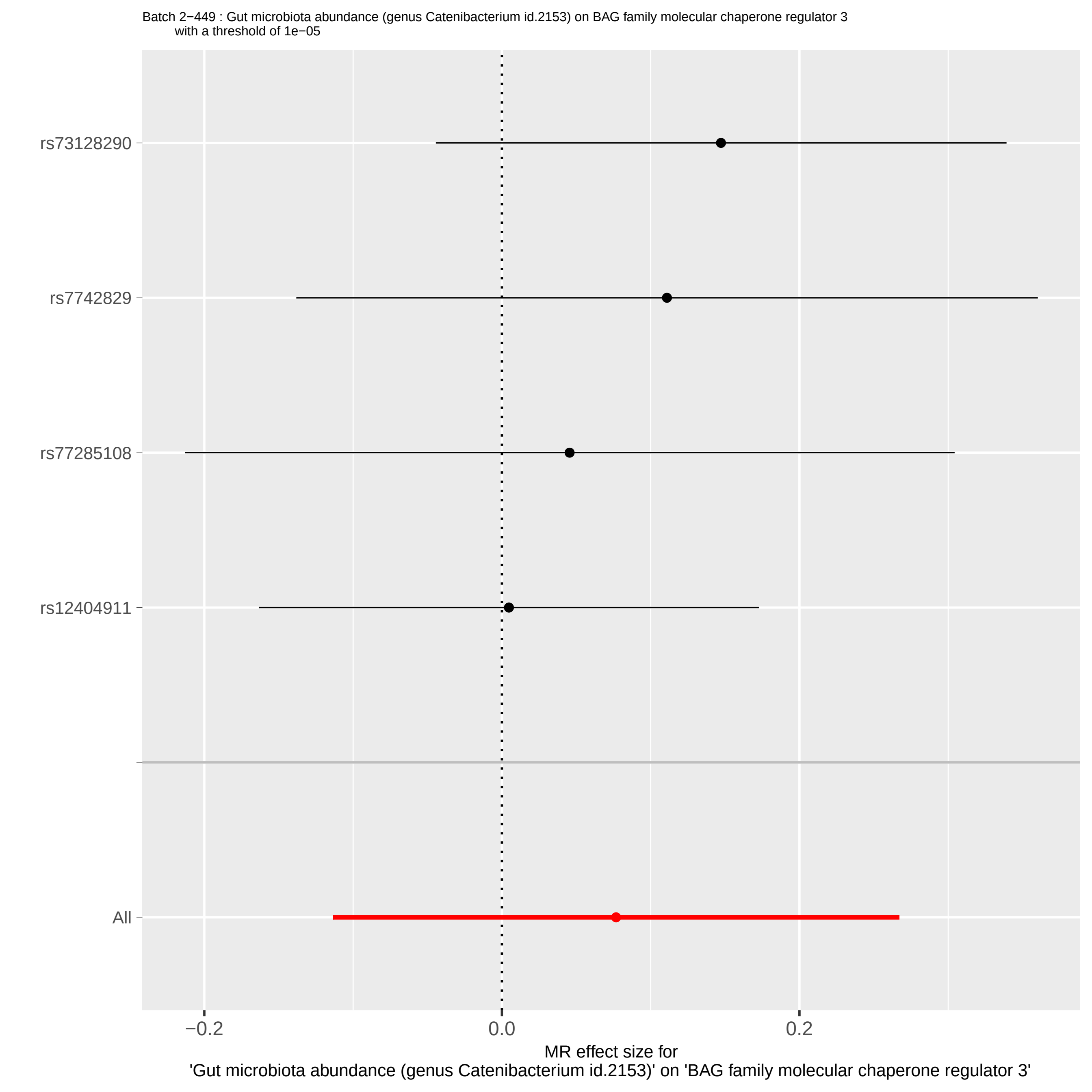
Batch 2−449 : Gut microbiota abundance (genus Catenibacterium id.2153) on BAG family molecular chaperone regulator 3
with a threshold of 1e−05
rs73128290
rs7742829
rs77285108
rs12404911
All
−0.2
0.0
0.2
MR effect size for
'Gut microbiota abundance (genus Catenibacterium id.2153)' on 'BAG family molecular chaperone regulator 3'

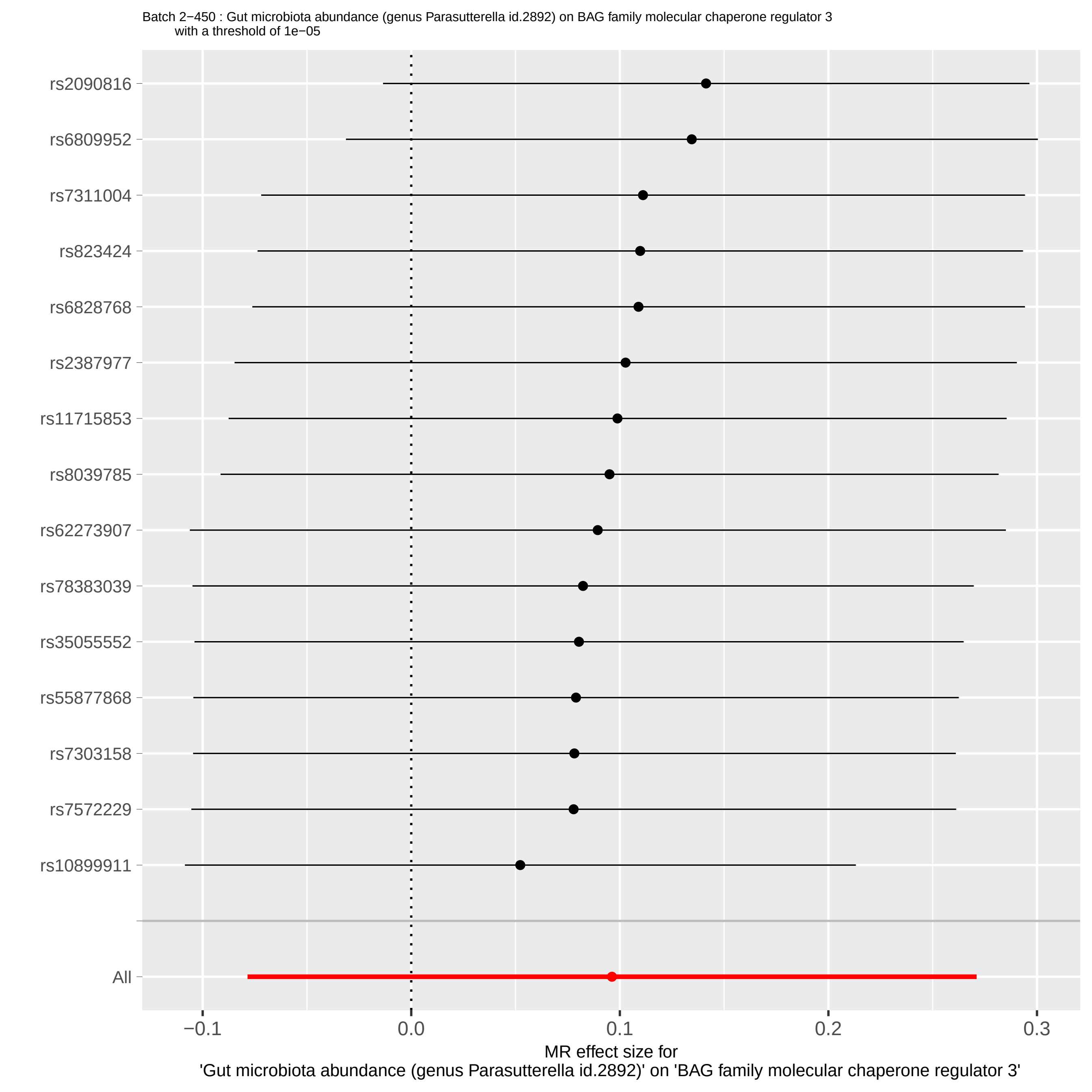
Batch 2−450 : Gut microbiota abundance (genus Parasutterella id.2892) on BAG family molecular chaperone regulator 3
with a threshold of 1e−05
rs2090816
rs6809952
rs7311004
rs823424
rs6828768
rs2387977
rs11715853
rs8039785
rs62273907
rs78383039
rs35055552
rs55877868
rs7303158
rs7572229
rs10899911
All
−0.1
0.0
0.1
0.2
0.3
MR effect size for
'Gut microbiota abundance (genus Parasutterella id.2892)' on 'BAG family molecular chaperone regulator 3'

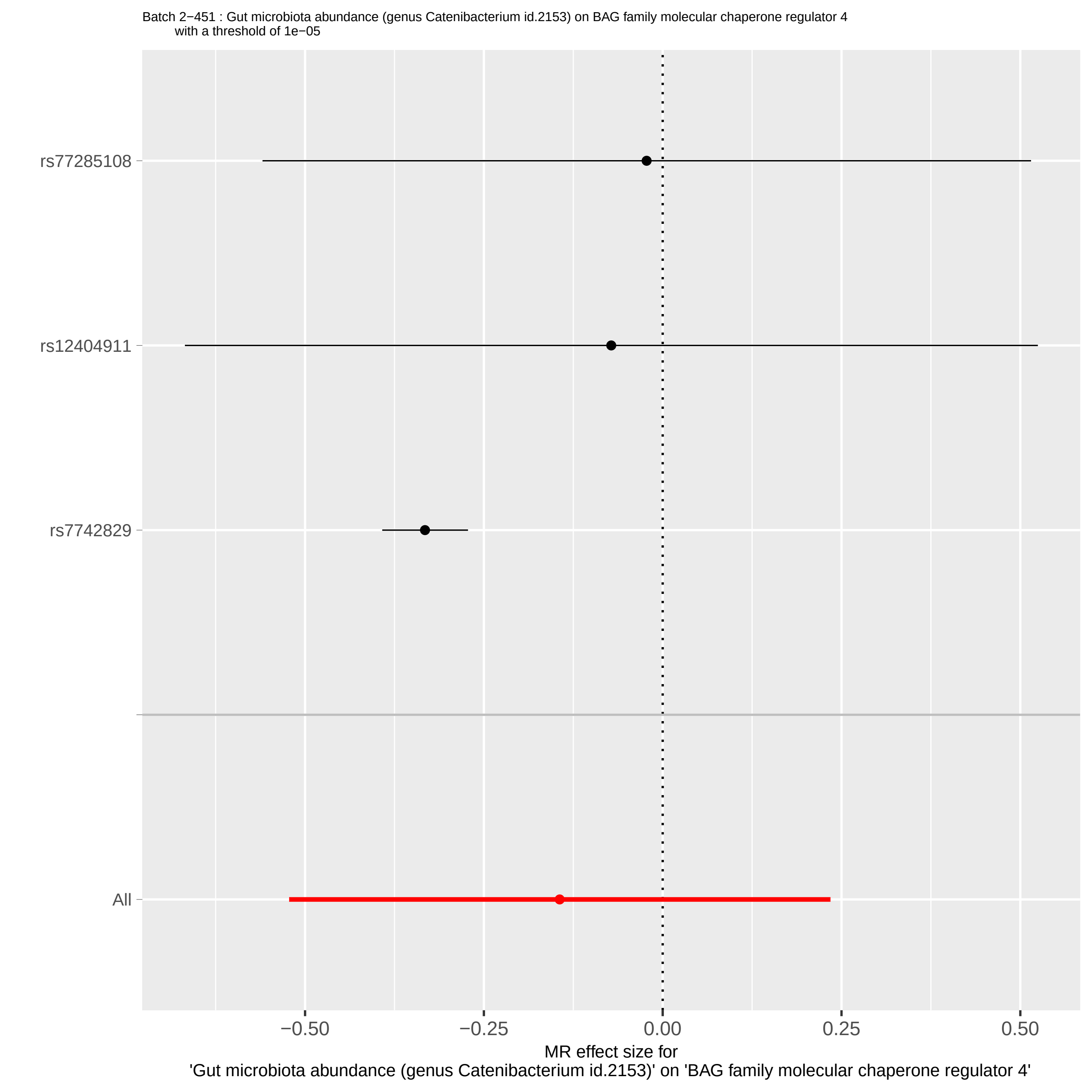
Batch 2−451 : Gut microbiota abundance (genus Catenibacterium id.2153) on BAG family molecular chaperone regulator 4
with a threshold of 1e−05
rs77285108
rs12404911
rs7742829
All
−0.50
−0.25
0.00
0.25
0.50
MR effect size for
'Gut microbiota abundance (genus Catenibacterium id.2153)' on 'BAG family molecular chaperone regulator 4'

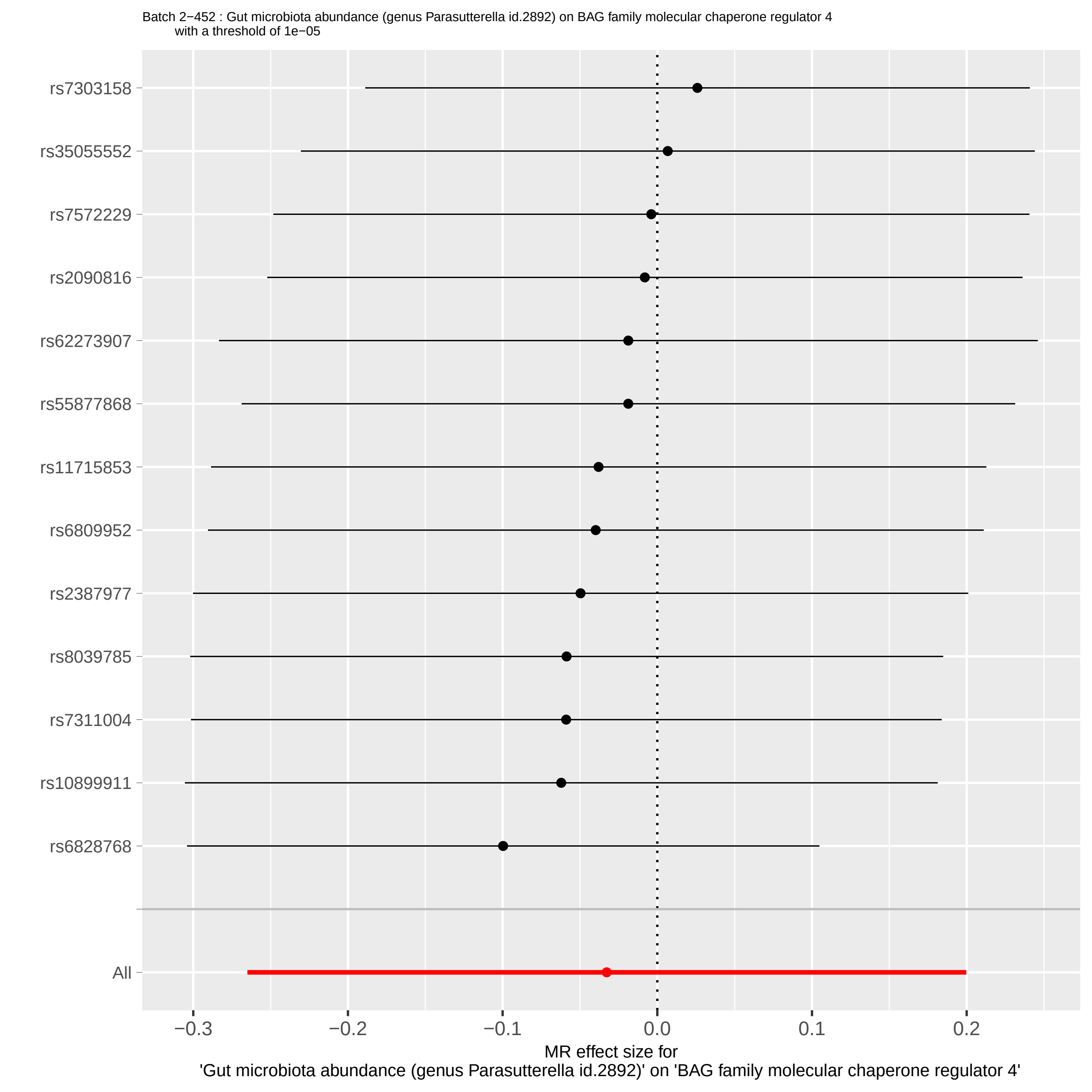

Batch 2−452 : Gut microbiota abundance (genus Parasutterella id.2892) on BAG family molecular chaperone regulator 4
with a threshold of 1e−05
rs7303158
rs35055552
rs7572229
rs2090816
rs62273907
rs55877868
rs11715853
rs6809952
rs2387977
rs8039785
rs7311004
rs10899911
rs6828768
All
−0.3
−0.2
−0.1
0.0
0.1
0.2
MR effect size for
'Gut microbiota abundance (genus Parasutterella id.2892)' on 'BAG family molecular chaperone regulator 4'

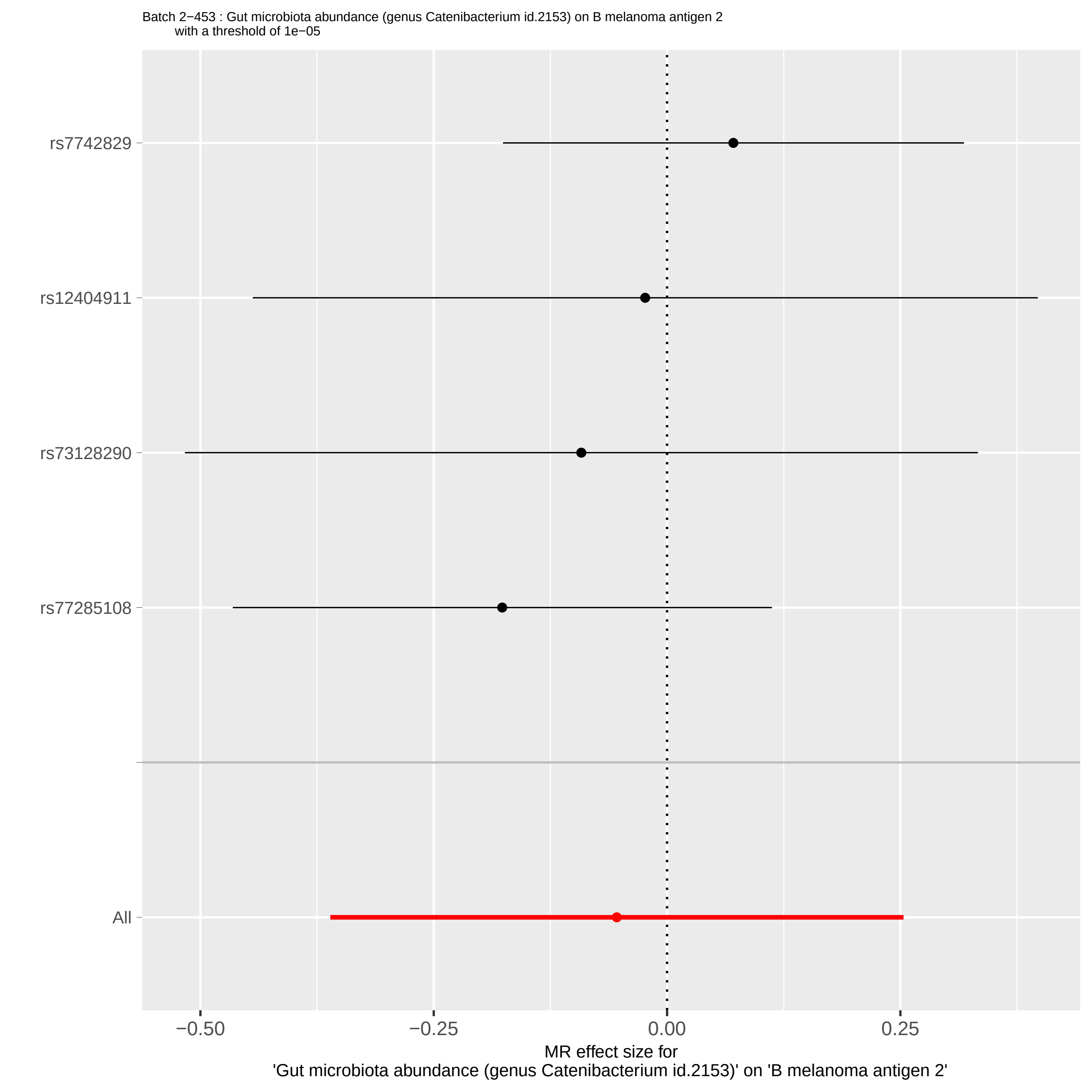

Batch 2−453 : Gut microbiota abundance (genus Catenibacterium id.2153) on B melanoma antigen 2
with a threshold of 1e−05
rs7742829
rs12404911
rs73128290
rs77285108
All
−0.50
−0.25
0.00
0.25
MR effect size for
'Gut microbiota abundance (genus Catenibacterium id.2153)' on 'B melanoma antigen 2'

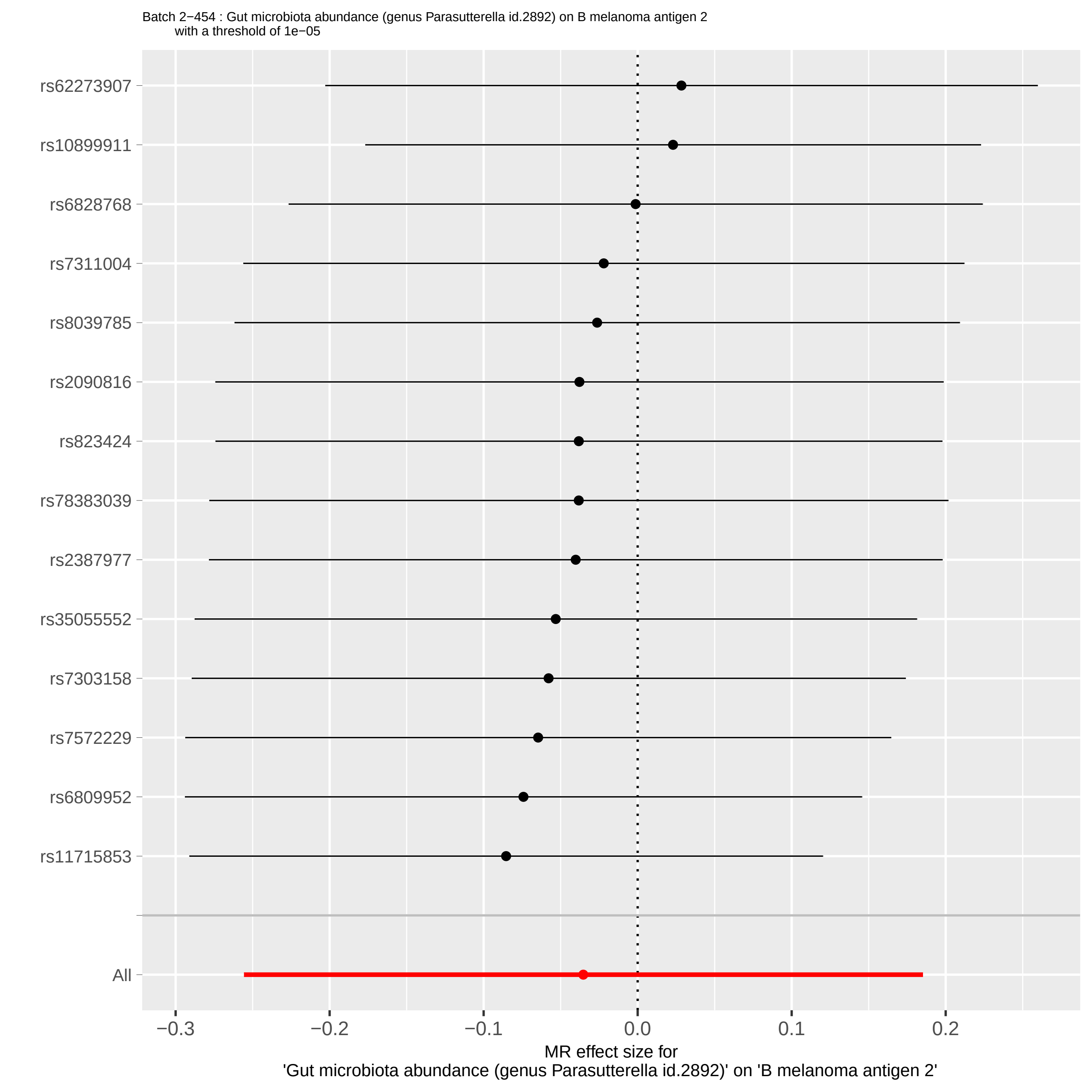

Batch 2−454 : Gut microbiota abundance (genus Parasutterella id.2892) on B melanoma antigen 2
with a threshold of 1e−05
rs62273907
rs10899911
rs6828768
rs7311004
rs8039785
rs2090816
rs823424
rs78383039
rs2387977
rs35055552
rs7303158
rs7572229
rs6809952
rs11715853
All
−0.3
−0.2
−0.1
0.0
0.1
0.2
MR effect size for
'Gut microbiota abundance (genus Parasutterella id.2892)' on 'B melanoma antigen 2'

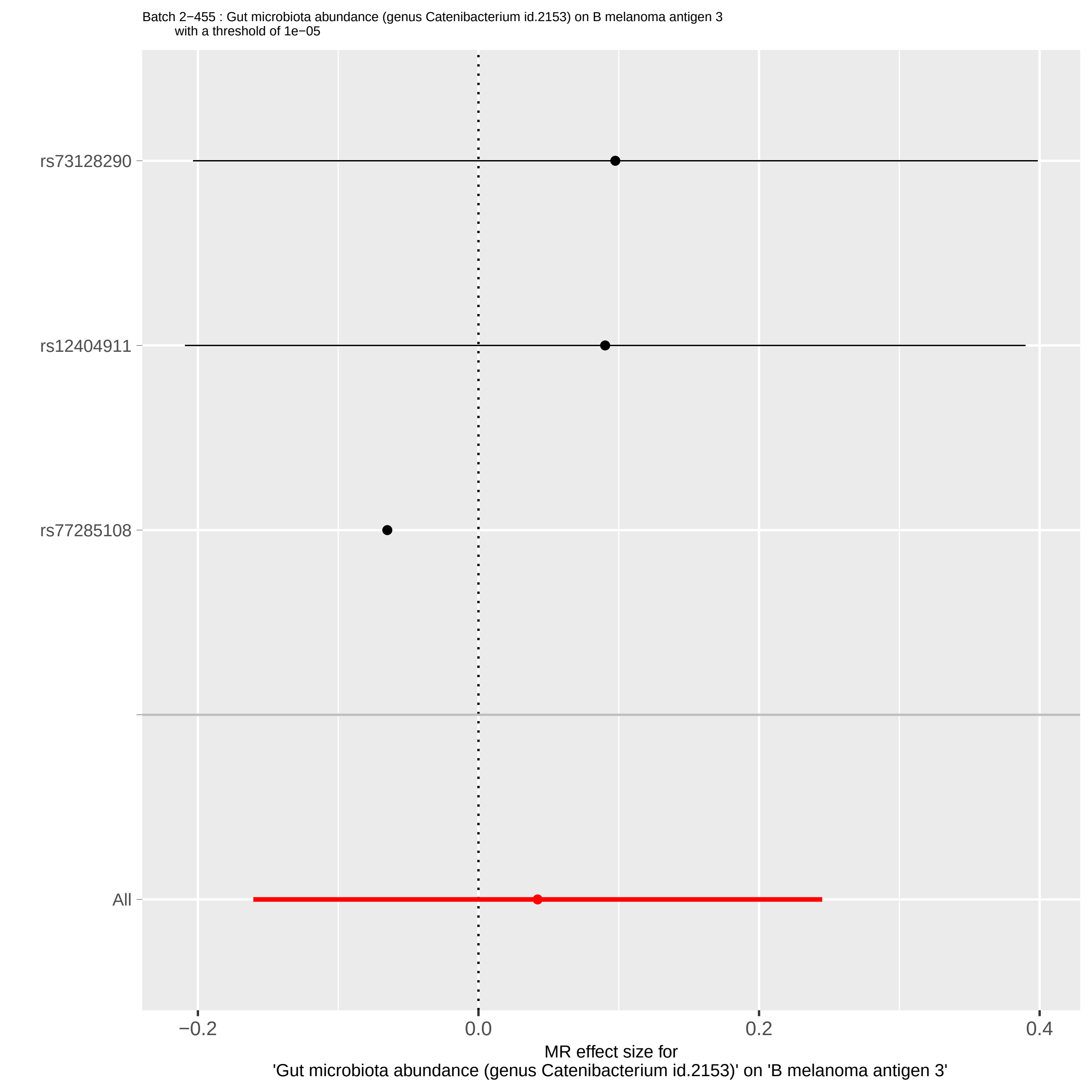
Batch 2−455 : Gut microbiota abundance (genus Catenibacterium id.2153) on B melanoma antigen 3
with a threshold of 1e−05
rs73128290
rs12404911
rs77285108
All
−0.2
0.0
0.2
0.4
MR effect size for
'Gut microbiota abundance (genus Catenibacterium id.2153)' on 'B melanoma antigen 3'

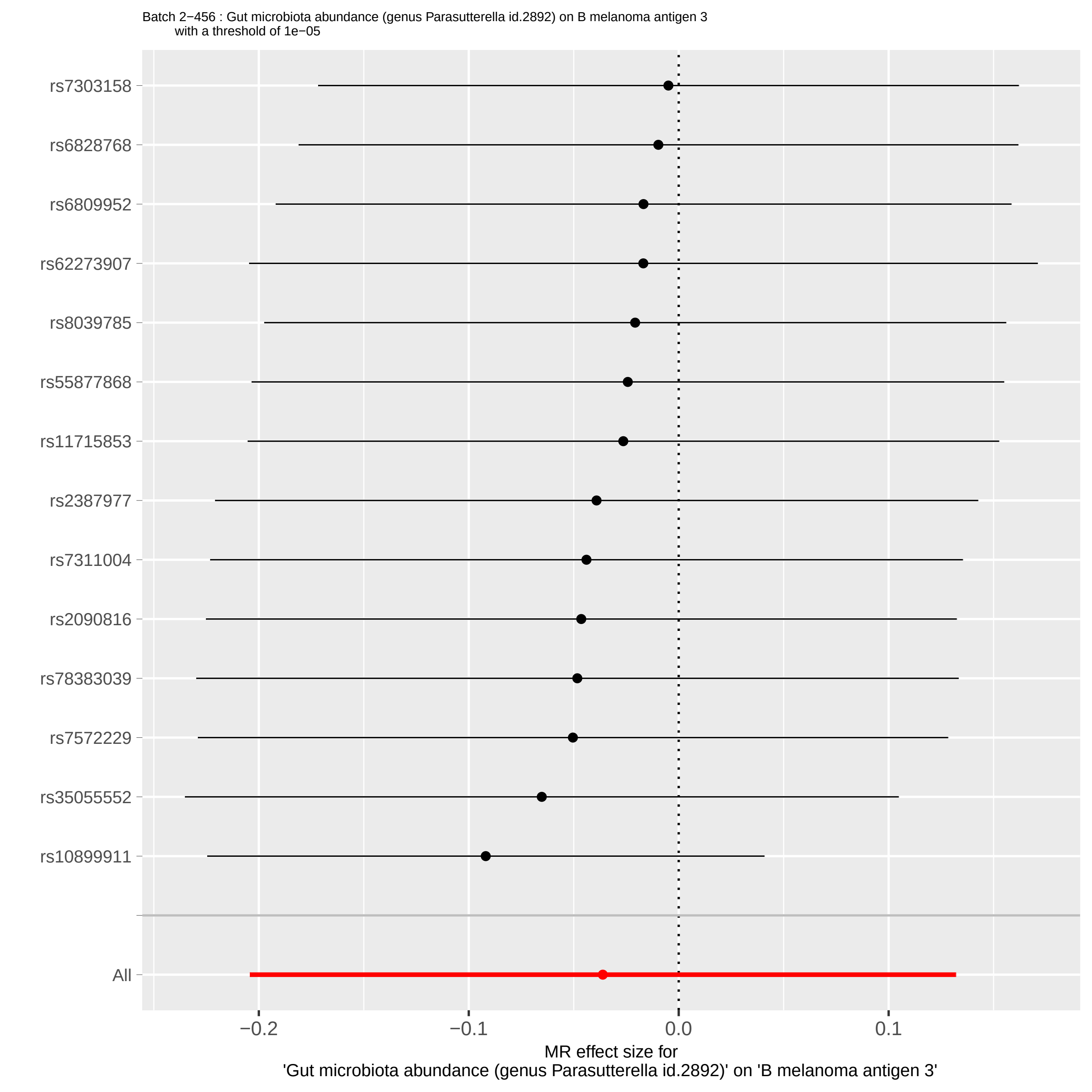
Batch 2−456 : Gut microbiota abundance (genus Parasutterella id.2892) on B melanoma antigen 3
with a threshold of 1e−05
rs7303158
rs6828768
rs6809952
rs62273907
rs8039785
rs55877868
rs11715853
rs2387977
rs7311004
rs2090816
rs78383039
rs7572229
rs35055552
rs10899911
All
−0.2
−0.1
0.0
0.1
MR effect size for
'Gut microbiota abundance (genus Parasutterella id.2892)' on 'B melanoma antigen 3'

Batch 2−457 : Gut microbiota abundance (genus Catenibacterium id.2153) on BRCA1−associated RING domain protein 1
with a threshold of 1e−05

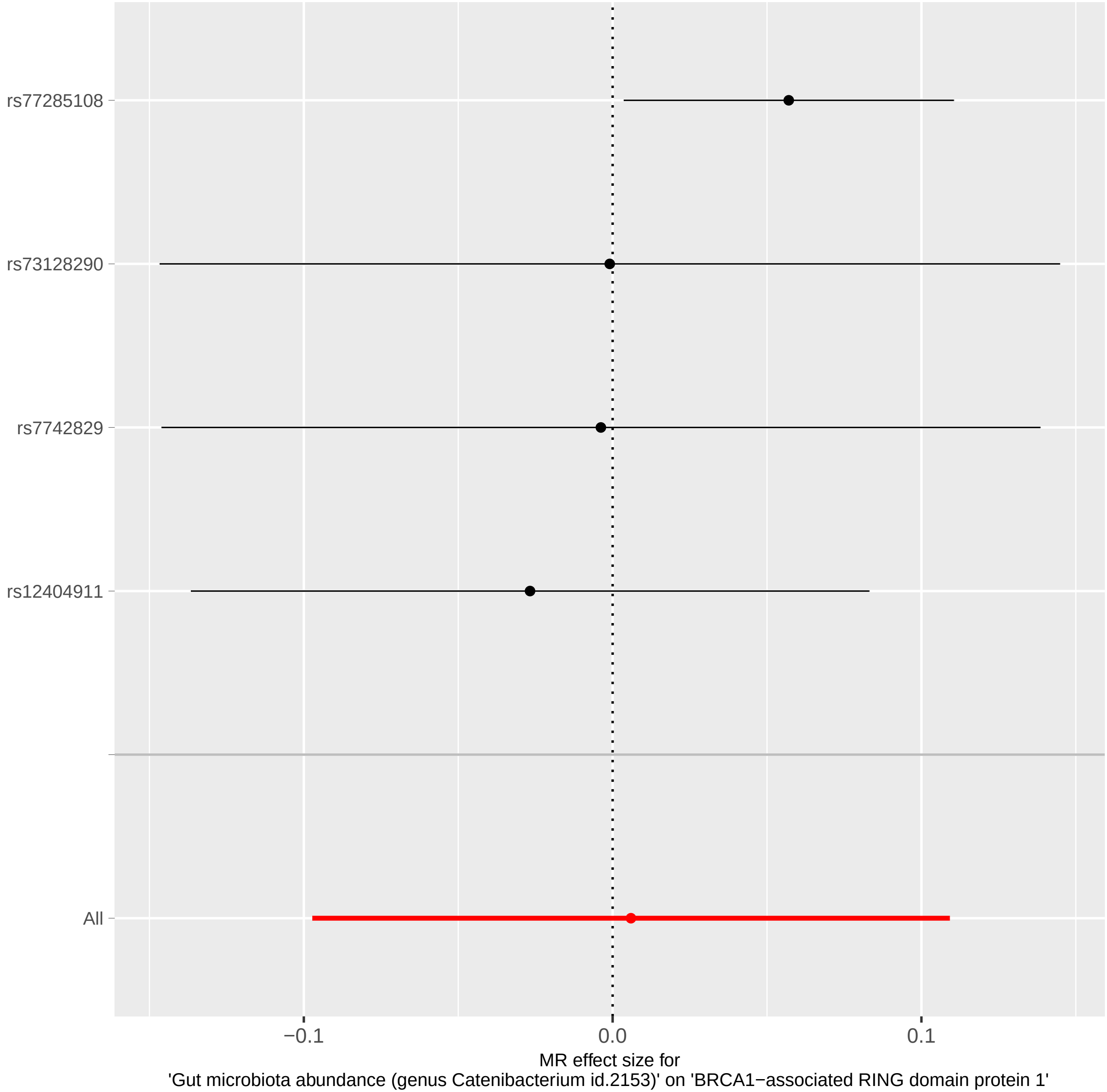

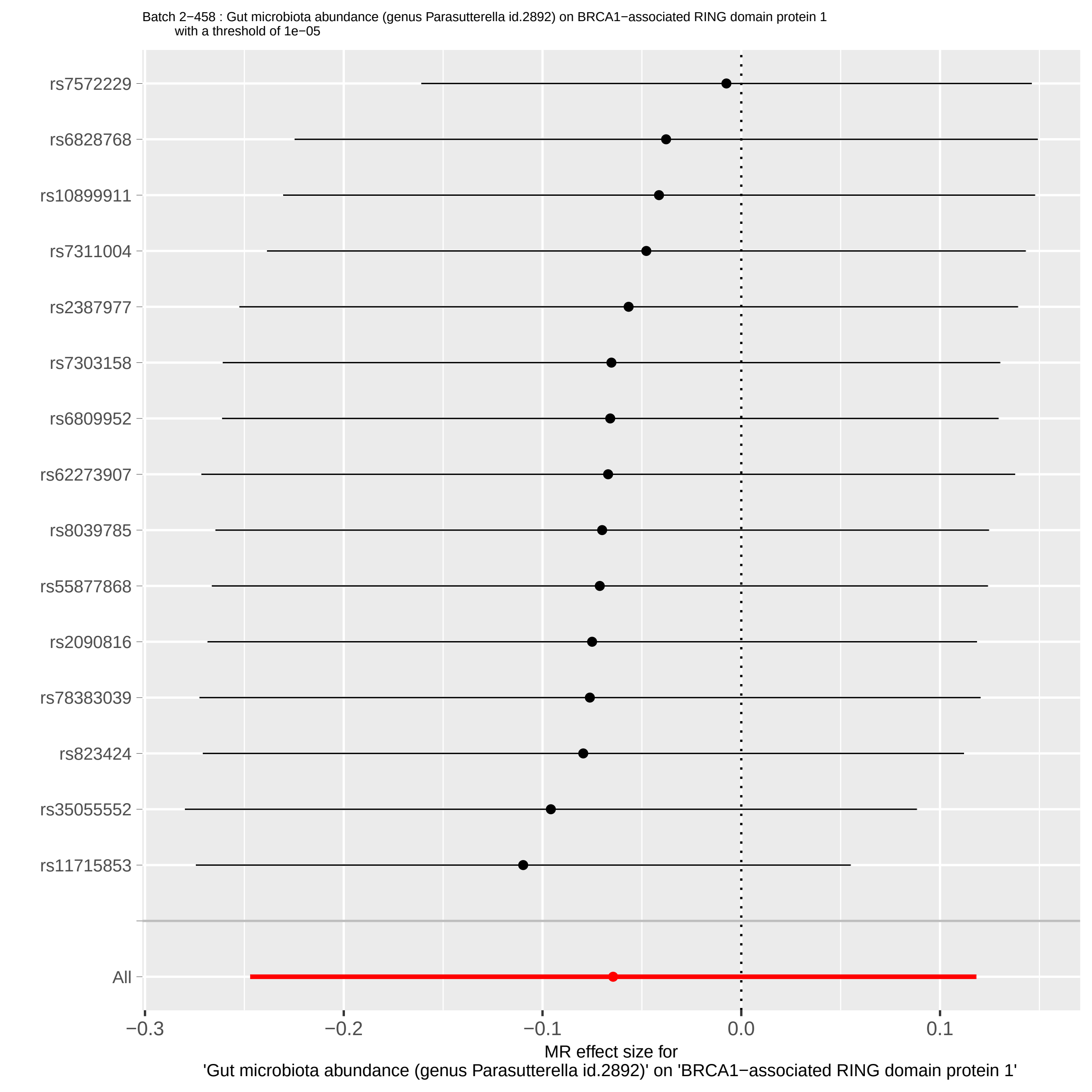

Batch 2−458 : Gut microbiota abundance (genus Parasutterella id.2892) on BRCA1−associated RING domain protein 1
with a threshold of 1e−05
rs7572229
rs6828768
rs10899911
rs7311004
rs2387977
rs7303158
rs6809952
rs62273907
rs8039785
rs55877868
rs2090816
rs78383039
rs823424
rs35055552
rs11715853
All
−0.3
−0.2
−0.1
0.0
0.1
MR effect size for
'Gut microbiota abundance (genus Parasutterella id.2892)' on 'BRCA1−associated RING domain protein 1'

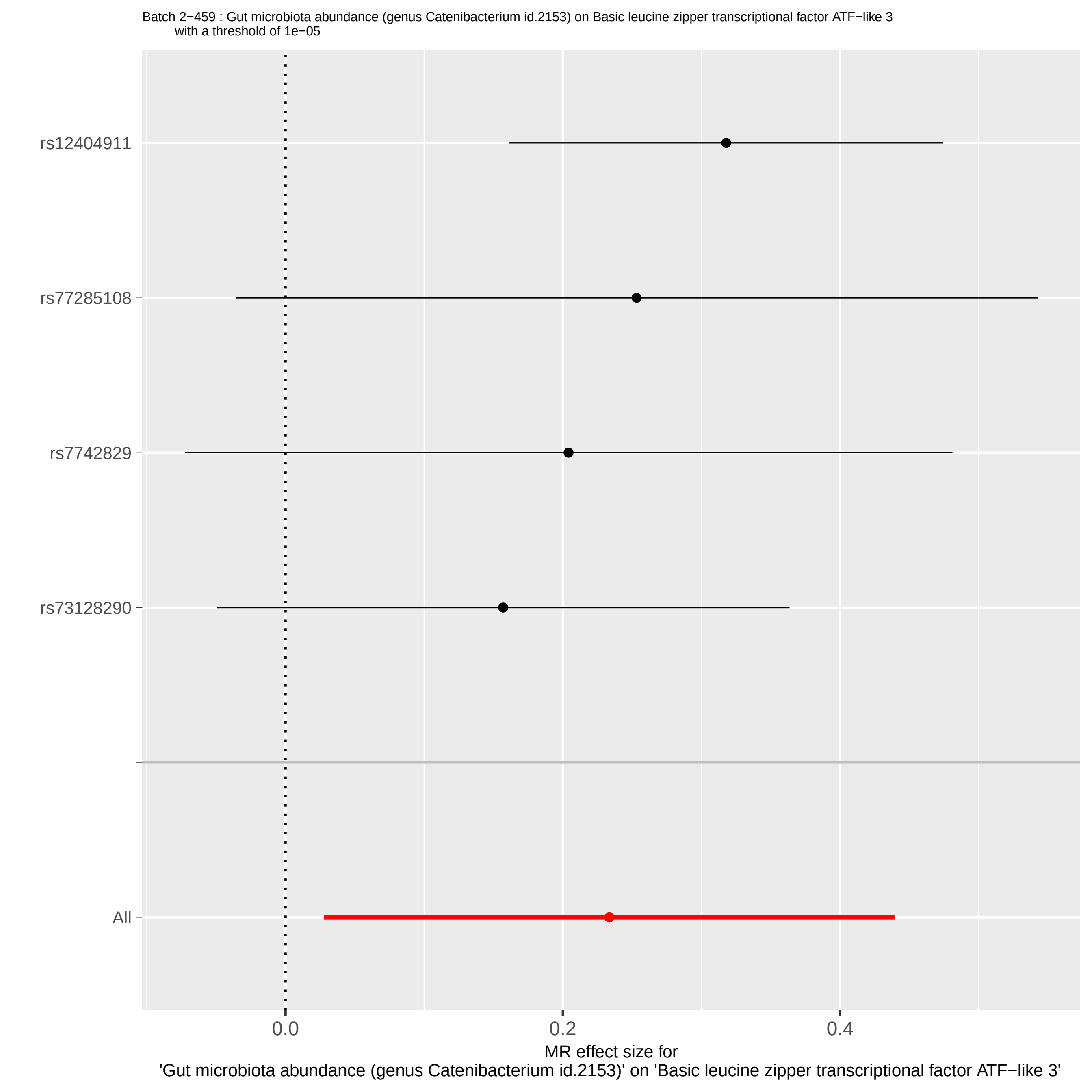
Batch 2−459 : Gut microbiota abundance (genus Catenibacterium id.2153) on Basic leucine zipper transcriptional factor ATF−like 3
with a threshold of 1e−05
rs12404911
rs77285108
rs7742829
rs73128290
All
0.0
0.2
0.4
MR effect size for
'Gut microbiota abundance (genus Catenibacterium id.2153)' on 'Basic leucine zipper transcriptional factor ATF−like 3'

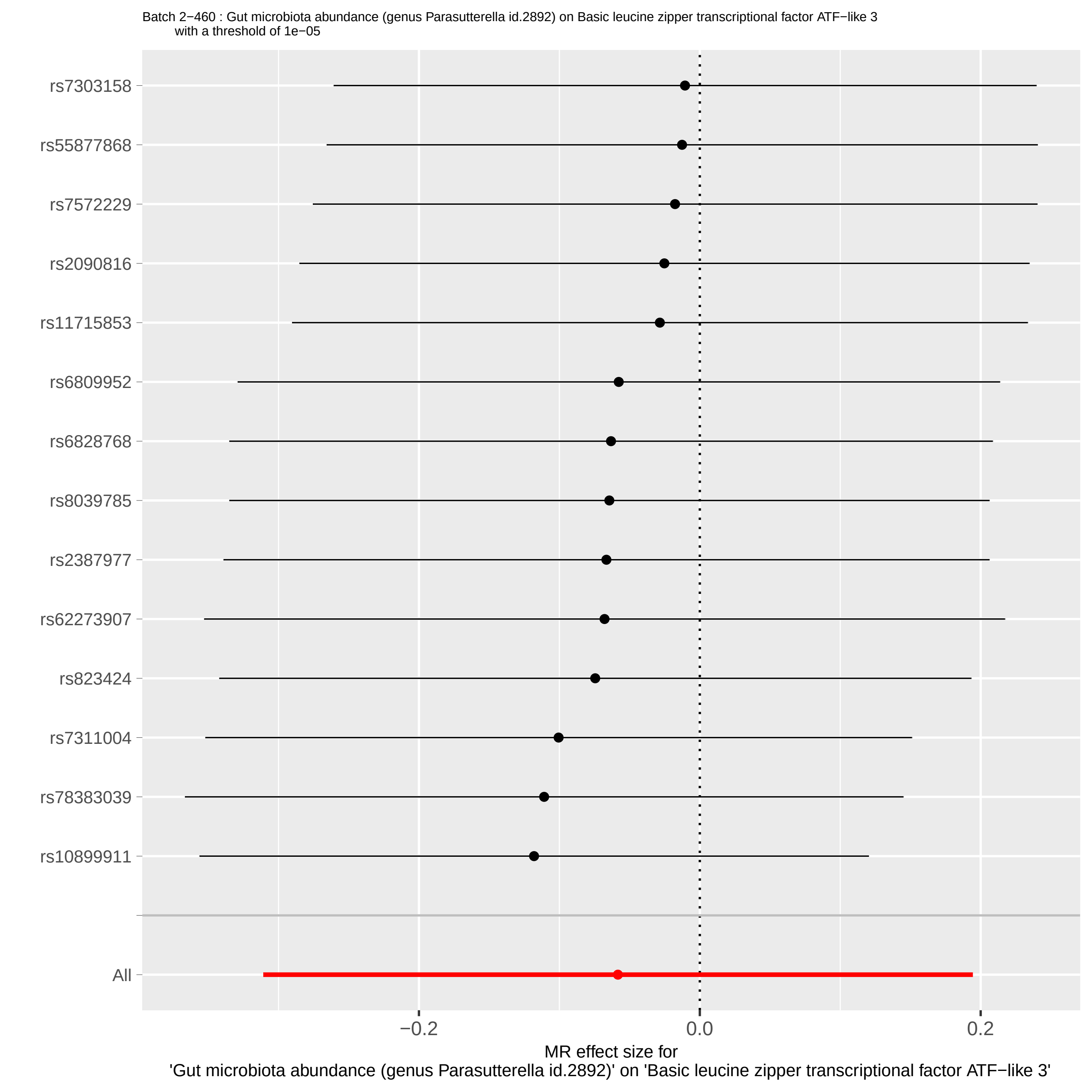

Batch 2−460 : Gut microbiota abundance (genus Parasutterella id.2892) on Basic leucine zipper transcriptional factor ATF−like 3
with a threshold of 1e−05
rs7303158
rs55877868
rs7572229
rs2090816
rs11715853
rs6809952
rs6828768
rs8039785
rs2387977
rs62273907
rs823424
rs7311004
rs78383039
rs10899911
All
−0.2
0.0
0.2
MR effect size for
'Gut microbiota abundance (genus Parasutterella id.2892)' on 'Basic leucine zipper transcriptional factor ATF−like 3'

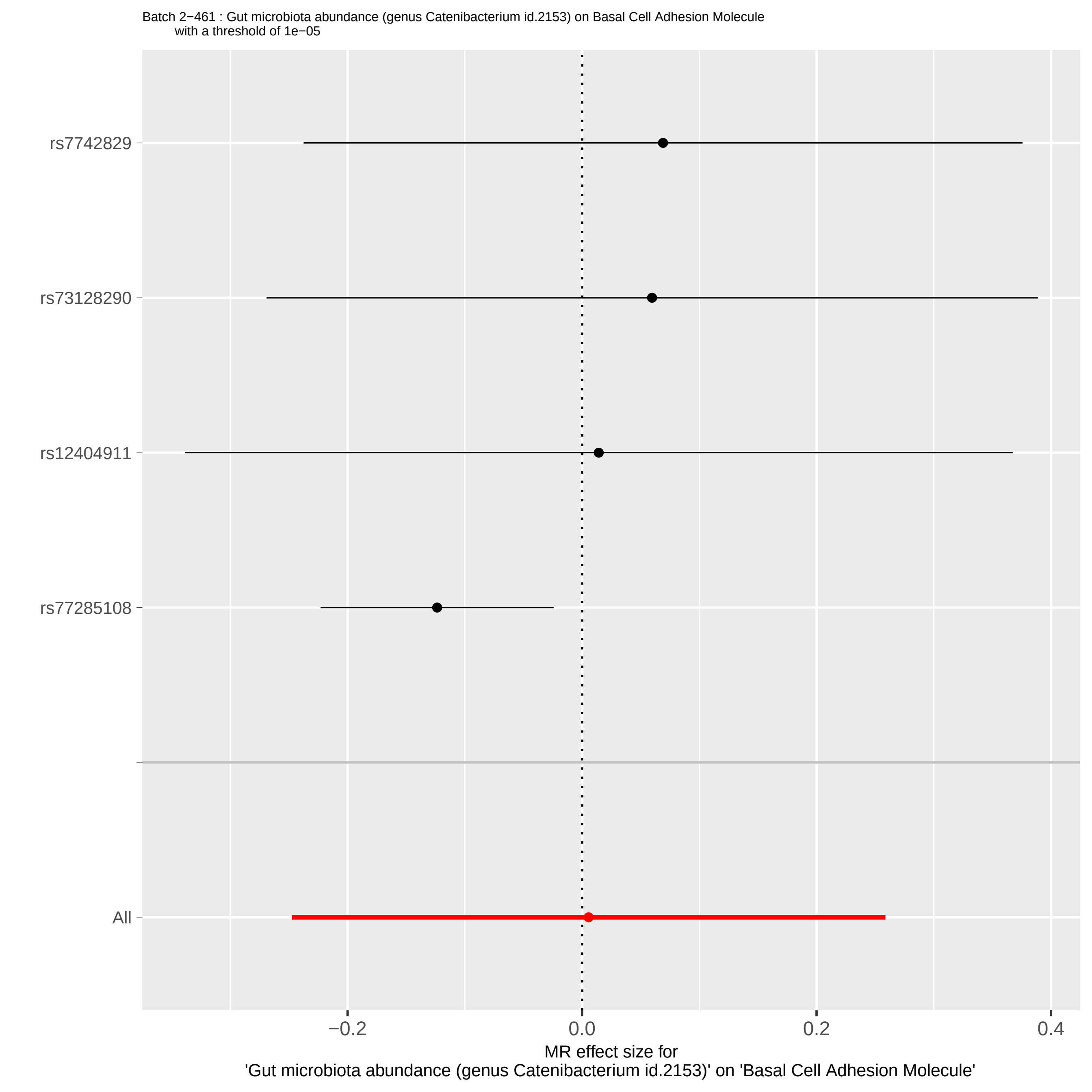

Batch 2−461 : Gut microbiota abundance (genus Catenibacterium id.2153) on Basal Cell Adhesion Molecule
with a threshold of 1e−05
rs7742829
rs73128290
rs12404911
rs77285108
All
−0.2
0.0
0.2
0.4
MR effect size for
'Gut microbiota abundance (genus Catenibacterium id.2153)' on 'Basal Cell Adhesion Molecule'

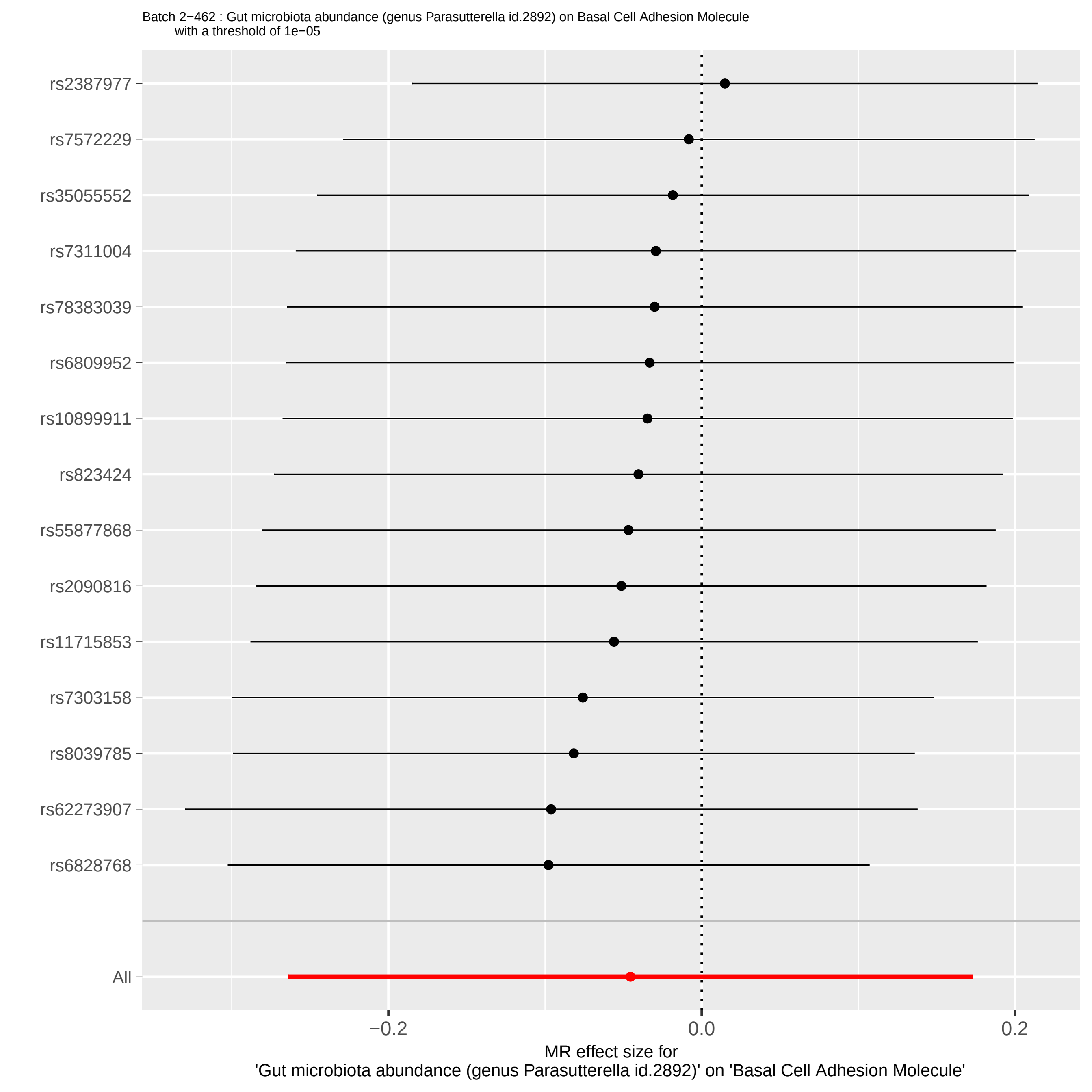
Batch 2−462 : Gut microbiota abundance (genus Parasutterella id.2892) on Basal Cell Adhesion Molecule
with a threshold of 1e−05
rs2387977
rs7572229
rs35055552
rs7311004
rs78383039
rs6809952
rs10899911
rs823424
rs55877868
rs2090816
rs11715853
rs7303158
rs8039785
rs62273907
rs6828768
All
−0.2
0.0
0.2
MR effect size for
'Gut microbiota abundance (genus Parasutterella id.2892)' on 'Basal Cell Adhesion Molecule'

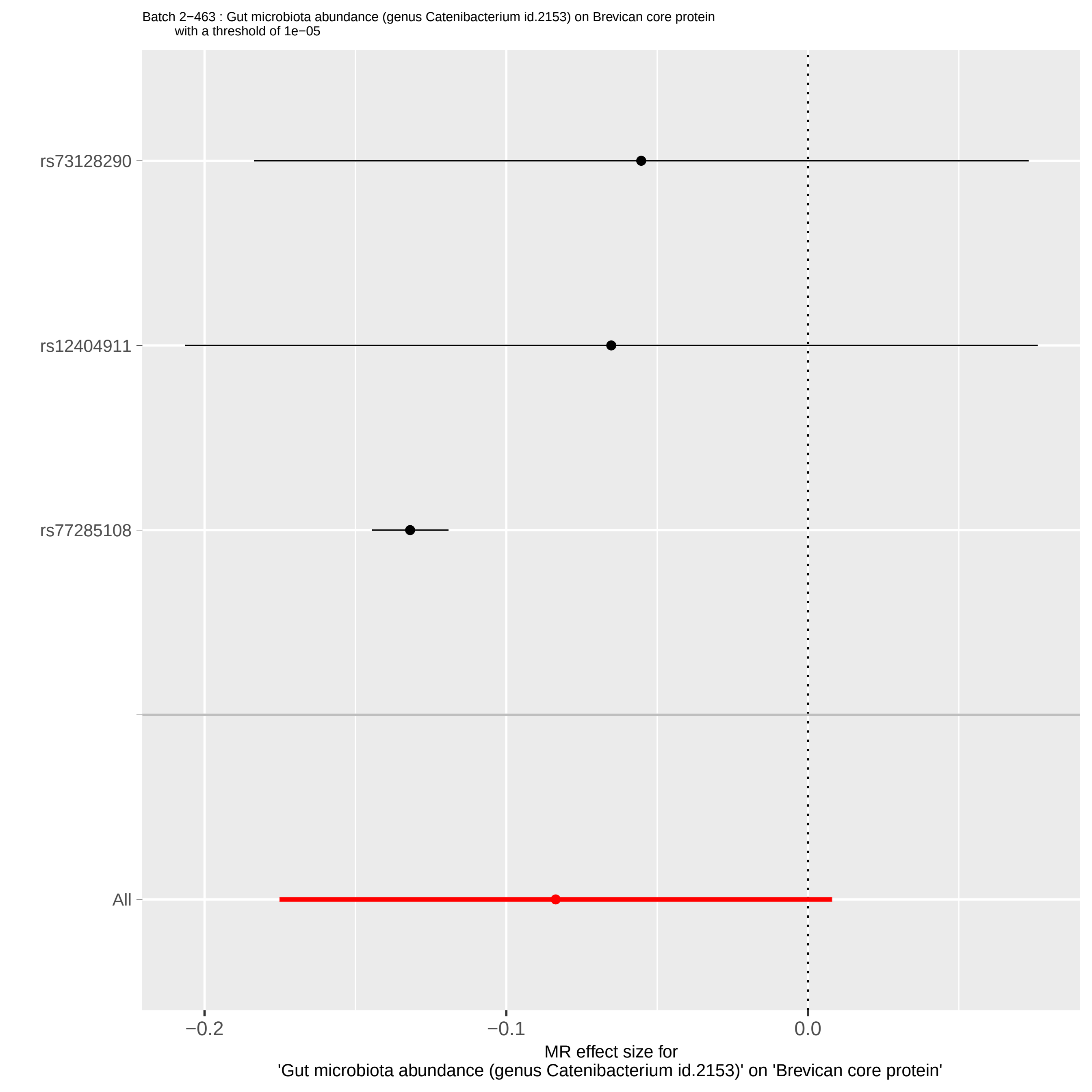
Batch 2−463 : Gut microbiota abundance (genus Catenibacterium id.2153) on Brevican core protein
with a threshold of 1e−05
rs73128290
rs12404911
rs77285108
All
−0.2
−0.1
0.0
MR effect size for
'Gut microbiota abundance (genus Catenibacterium id.2153)' on 'Brevican core protein'

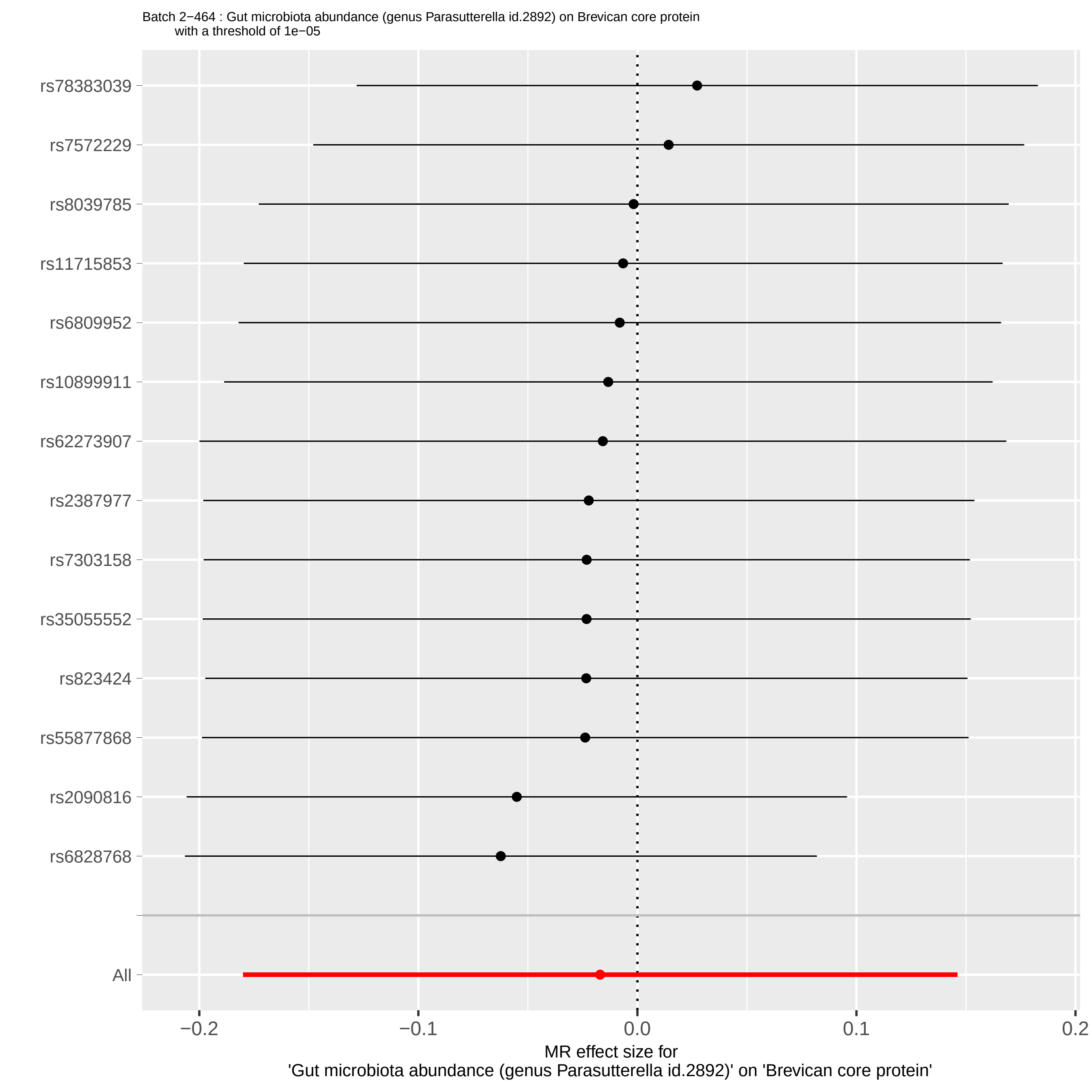

Batch 2−464 : Gut microbiota abundance (genus Parasutterella id.2892) on Brevican core protein
with a threshold of 1e−05
rs78383039
rs7572229
rs8039785
rs11715853
rs6809952
rs10899911
rs62273907
rs2387977
rs7303158
rs35055552
rs823424
rs55877868
rs2090816
rs6828768
All
−0.2
−0.1
0.0
0.1
0.2
MR effect size for
'Gut microbiota abundance (genus Parasutterella id.2892)' on 'Brevican core protein'

Batch 2−465 : Gut microbiota abundance (genus Catenibacterium id.2153) on B−cell receptor−associated protein 29
with a threshold of 1e−05

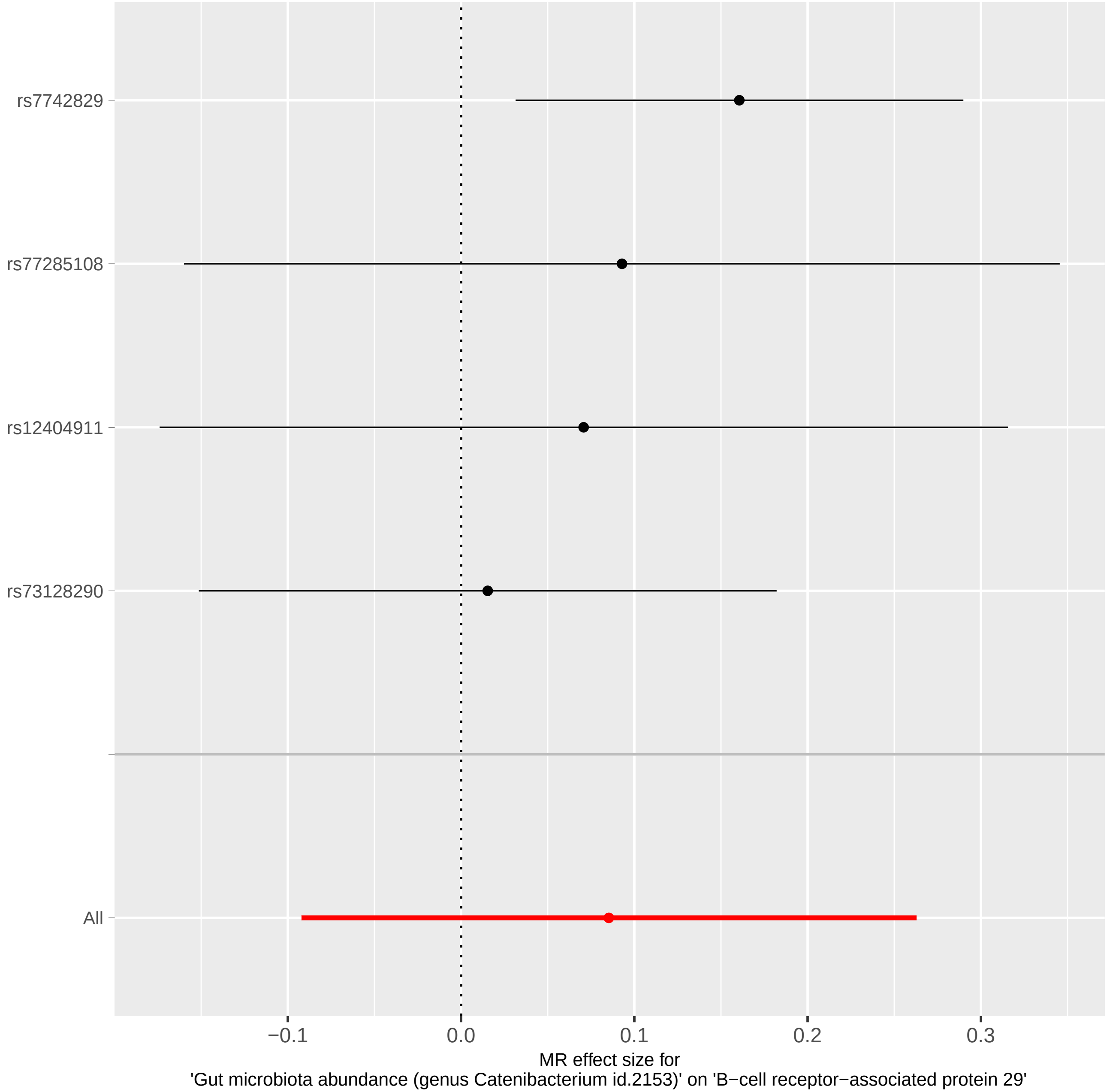

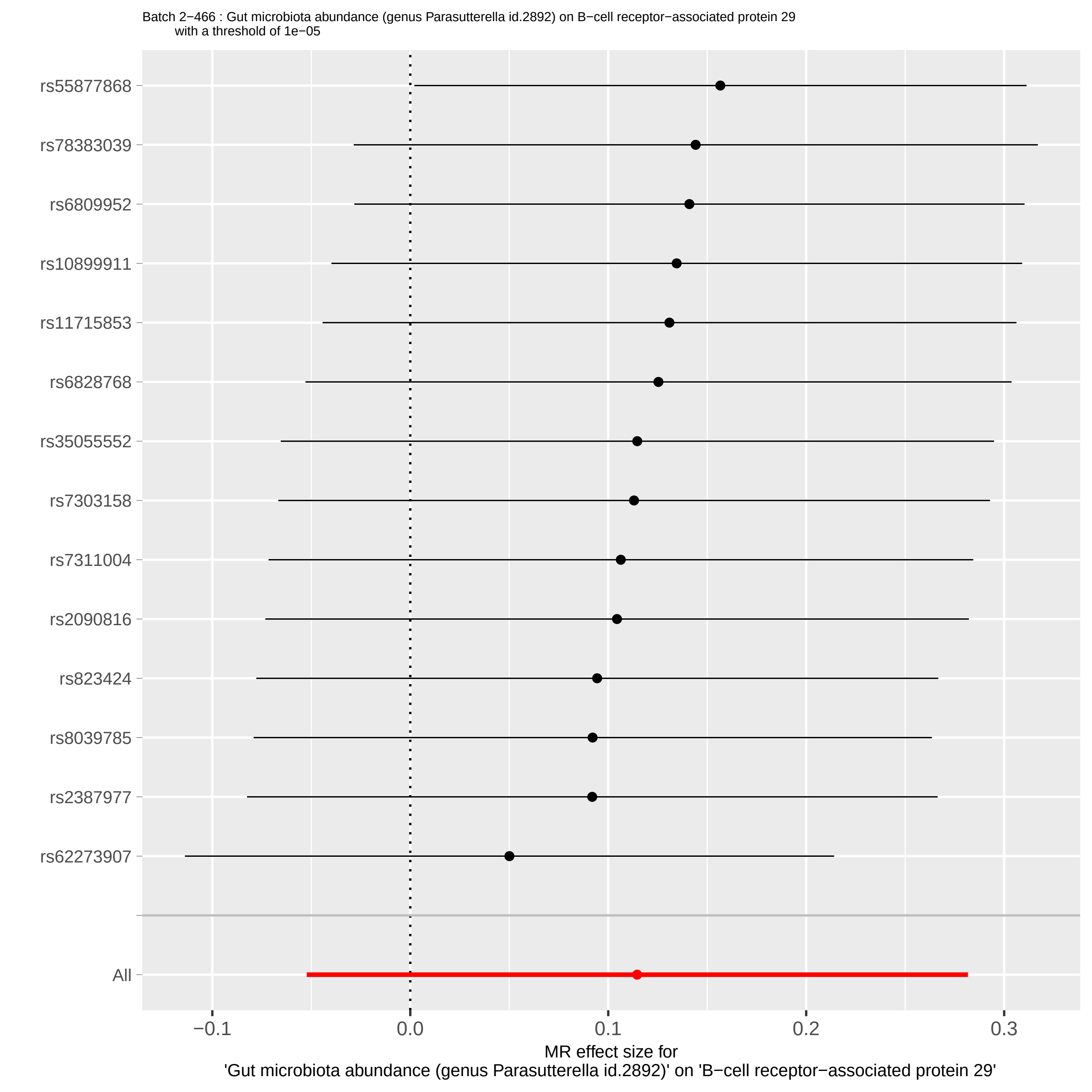
Batch 2−466 : Gut microbiota abundance (genus Parasutterella id.2892) on B−cell receptor−associated protein 29
with a threshold of 1e−05
rs55877868
rs78383039
rs6809952
rs10899911
rs11715853
rs6828768
rs35055552
rs7303158
rs7311004
rs2090816
rs823424
rs8039785
rs2387977
rs62273907
All
−0.1
0.0
0.1
0.2
0.3
MR effect size for
'Gut microbiota abundance (genus Parasutterella id.2892)' on 'B−cell receptor−associated protein 29'

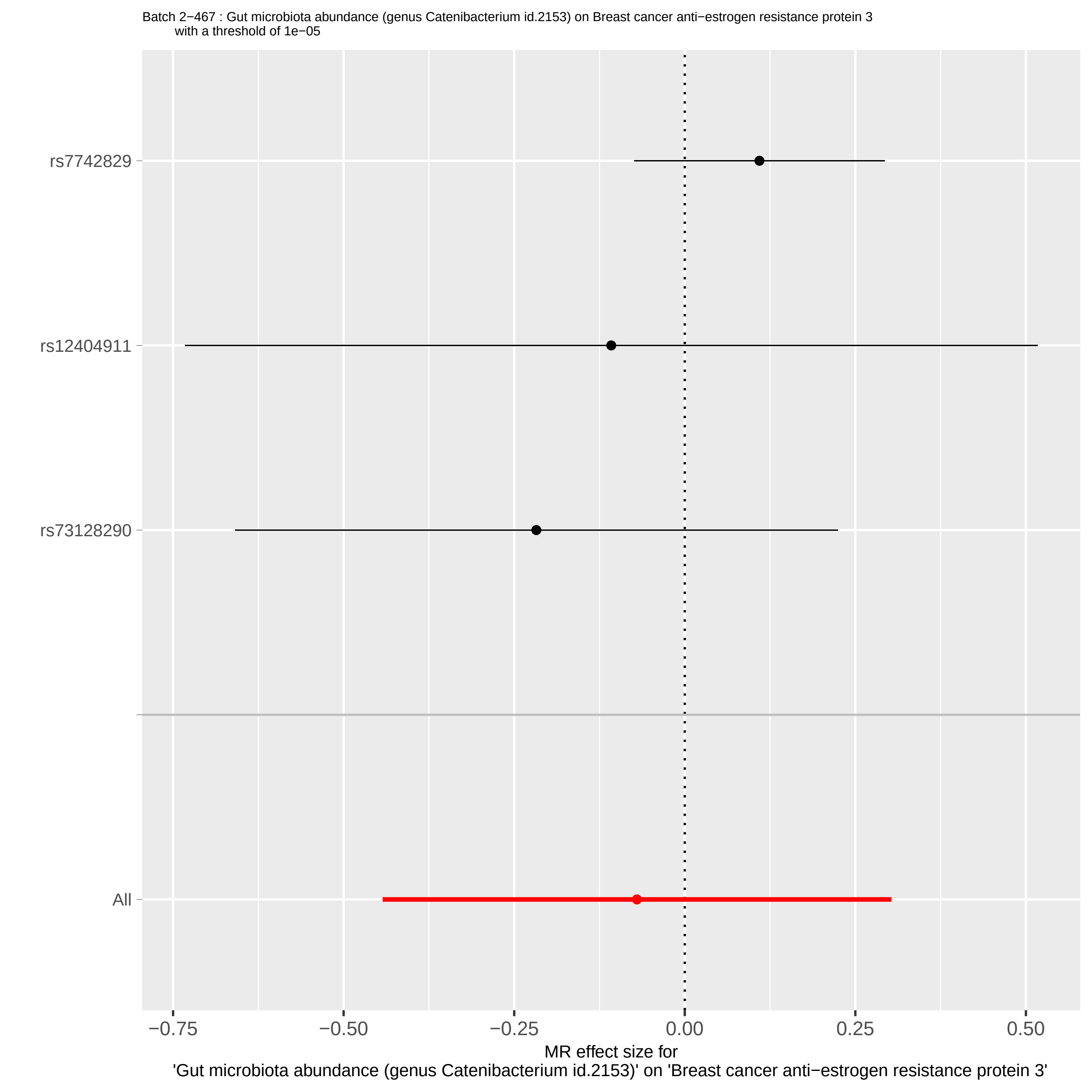

Batch 2−467 : Gut microbiota abundance (genus Catenibacterium id.2153) on Breast cancer anti−estrogen resistance protein 3
with a threshold of 1e−05
rs7742829
rs12404911
rs73128290
All
−0.75
−0.50
−0.25
0.00
0.25
0.50
MR effect size for
'Gut microbiota abundance (genus Catenibacterium id.2153)' on 'Breast cancer anti−estrogen resistance protein 3'

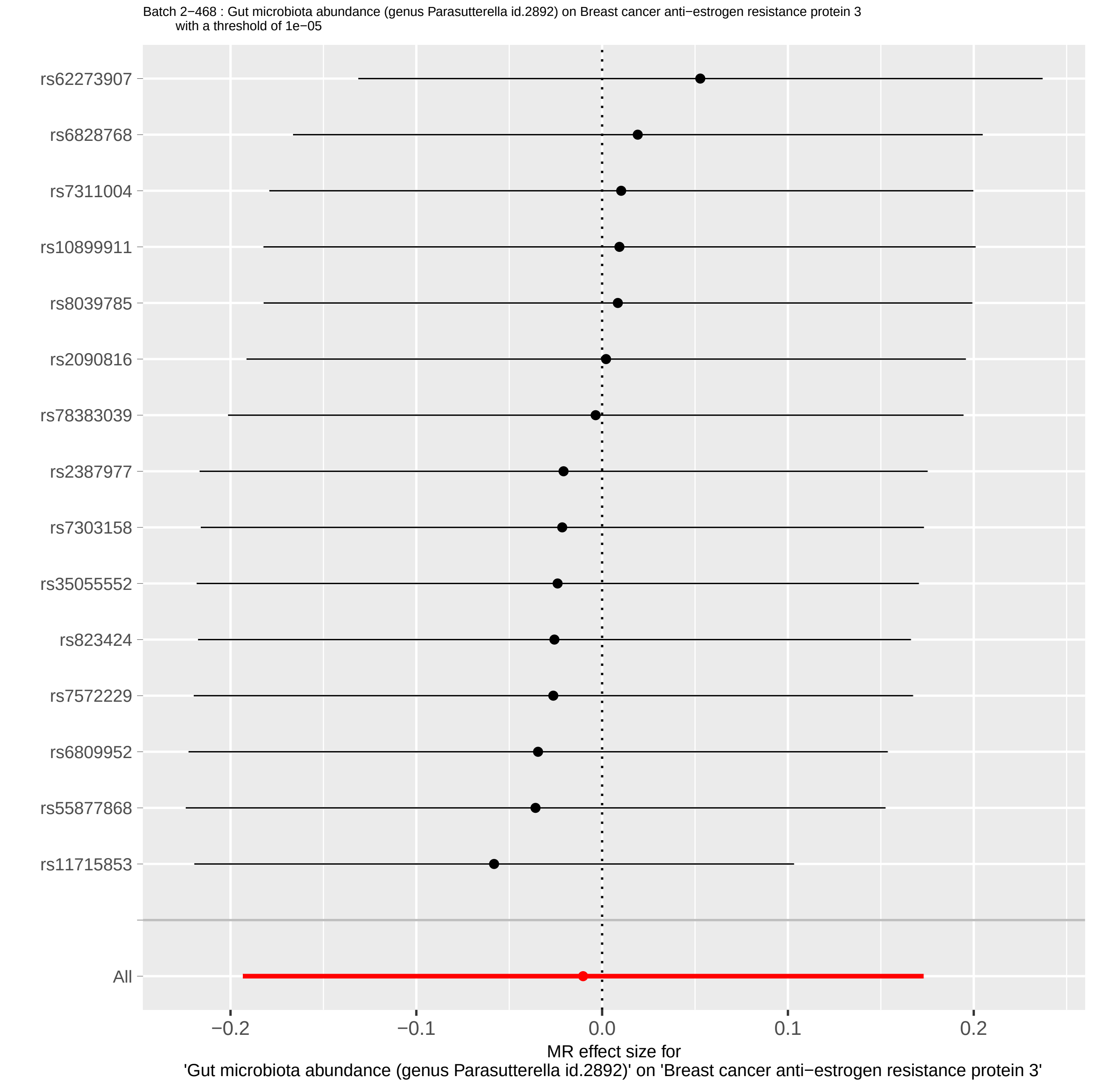
Batch 2−468 : Gut microbiota abundance (genus Parasutterella id.2892) on Breast cancer anti−estrogen resistance protein 3
with a threshold of 1e−05
rs62273907
rs6828768
rs7311004
rs10899911
rs8039785
rs2090816
rs78383039
rs2387977
rs7303158
rs35055552
rs823424
rs7572229
rs6809952
rs55877868
rs11715853
All
−0.2
−0.1
0.0
0.1
0.2
MR effect size for
'Gut microbiota abundance (genus Parasutterella id.2892)' on 'Breast cancer anti−estrogen resistance protein 3'

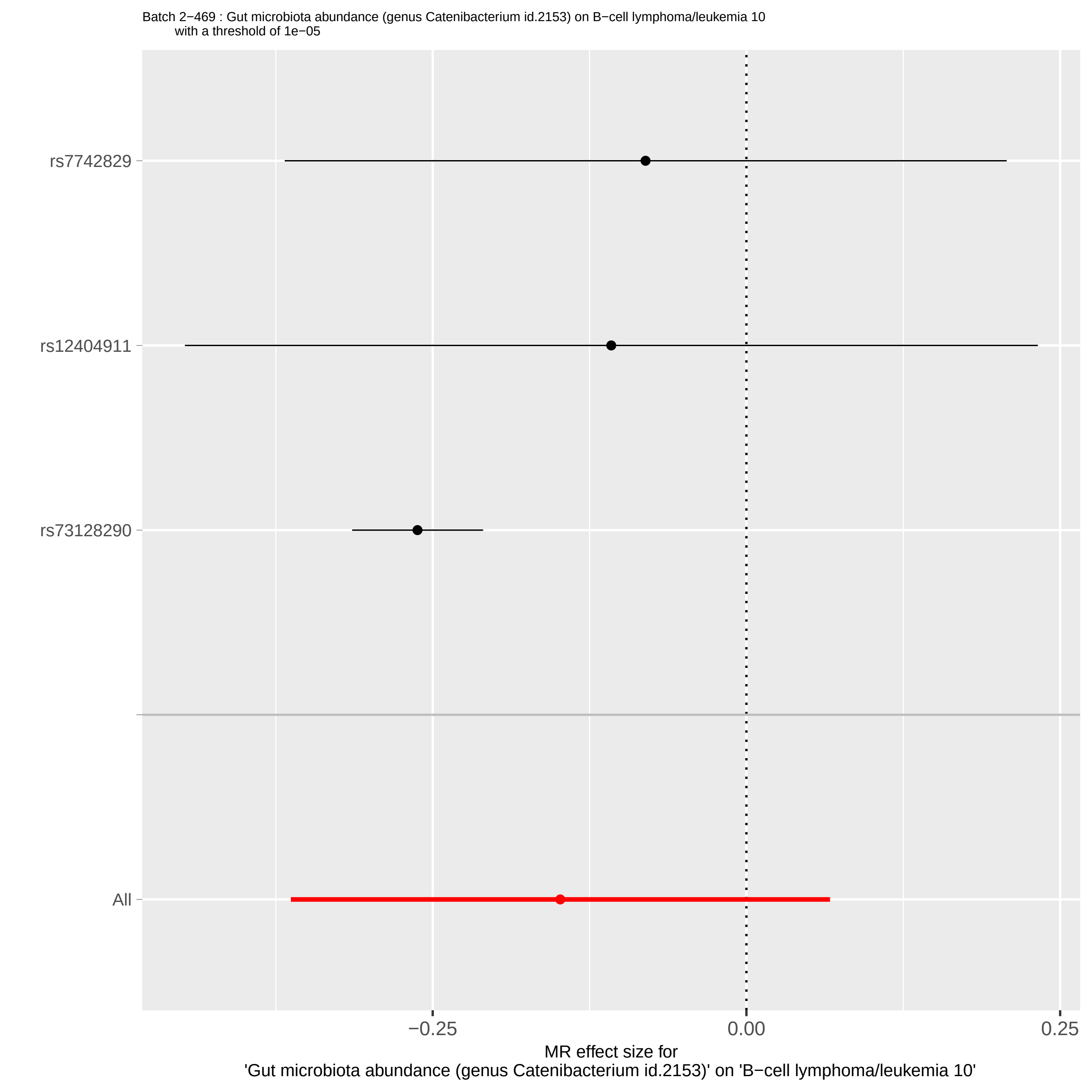
Batch 2−469 : Gut microbiota abundance (genus Catenibacterium id.2153) on B−cell lymphoma/leukemia 10
with a threshold of 1e−05
rs7742829
rs12404911
rs73128290
All
−0.25
0.00
0.25
MR effect size for
'Gut microbiota abundance (genus Catenibacterium id.2153)' on 'B−cell lymphoma/leukemia 10'

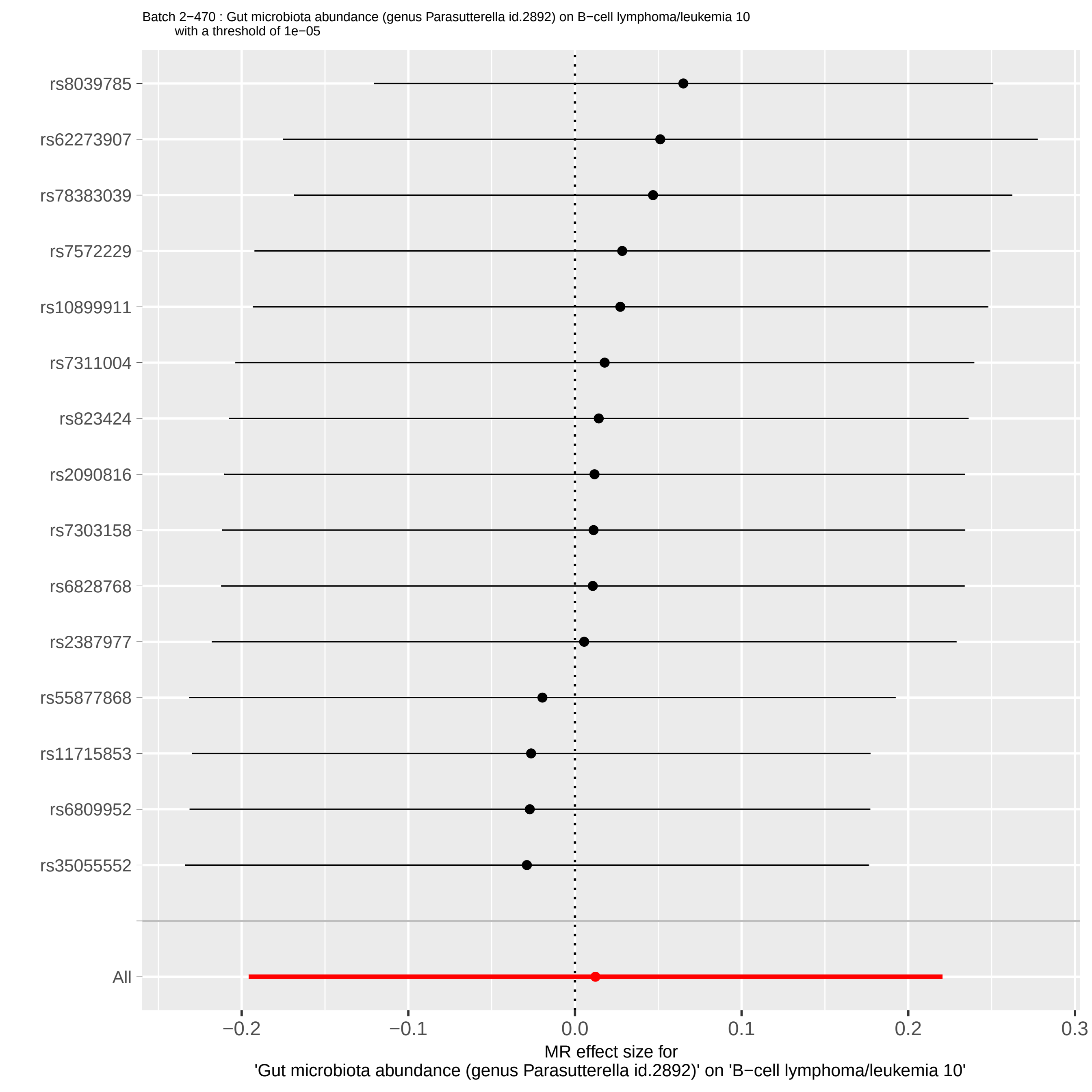
Batch 2−470 : Gut microbiota abundance (genus Parasutterella id.2892) on B−cell lymphoma/leukemia 10
with a threshold of 1e−05
rs8039785
rs62273907
rs78383039
rs7572229
rs10899911
rs7311004
rs823424
rs2090816
rs7303158
rs6828768
rs2387977
rs55877868
rs11715853
rs6809952
rs35055552
All
−0.2
−0.1
0.0
0.1
0.2
0.3
MR effect size for
'Gut microbiota abundance (genus Parasutterella id.2892)' on 'B−cell lymphoma/leukemia 10'

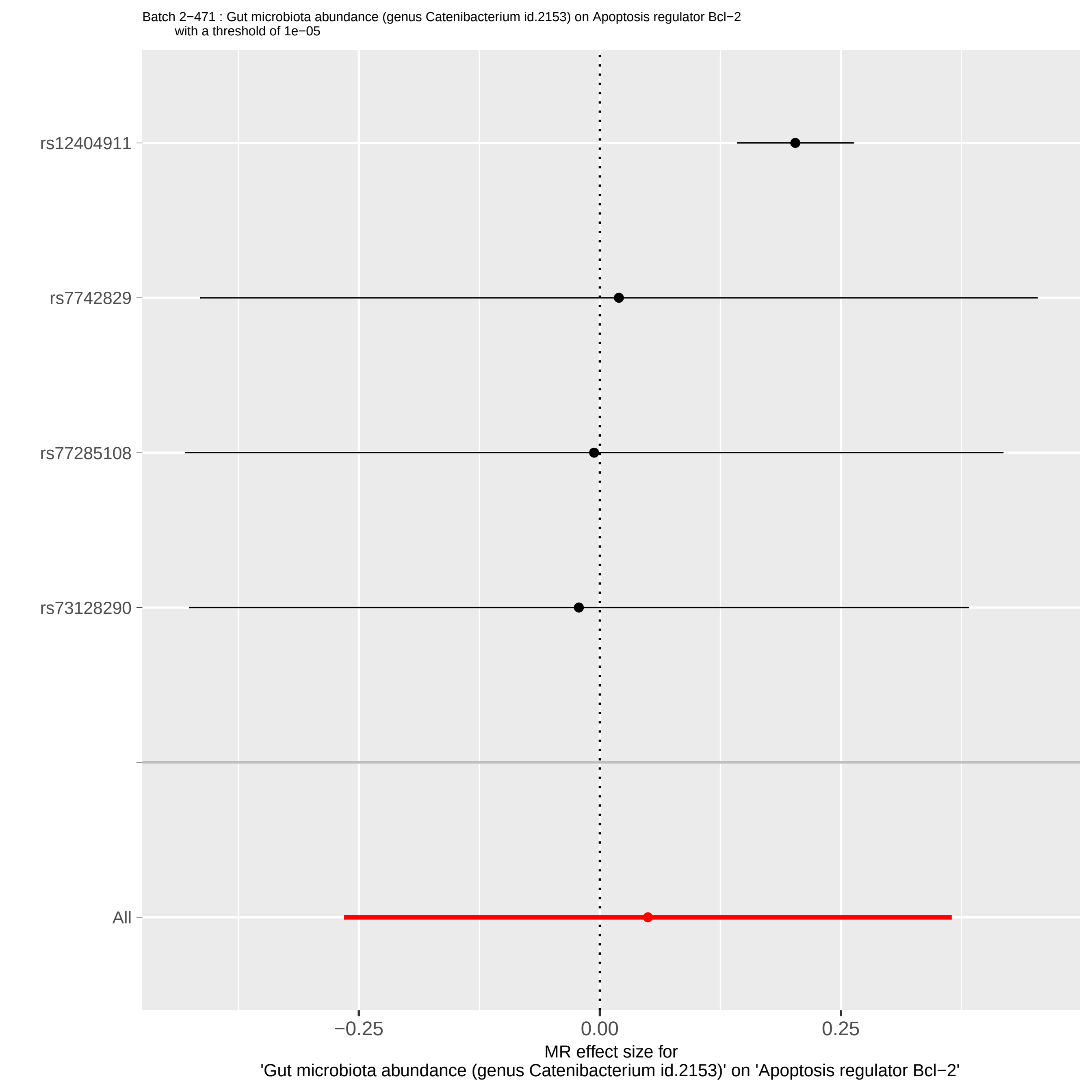

Batch 2−471 : Gut microbiota abundance (genus Catenibacterium id.2153) on Apoptosis regulator Bcl−2
with a threshold of 1e−05
rs12404911
rs7742829
rs77285108
rs73128290
All
−0.25
0.00
0.25
MR effect size for
'Gut microbiota abundance (genus Catenibacterium id.2153)' on 'Apoptosis regulator Bcl−2'

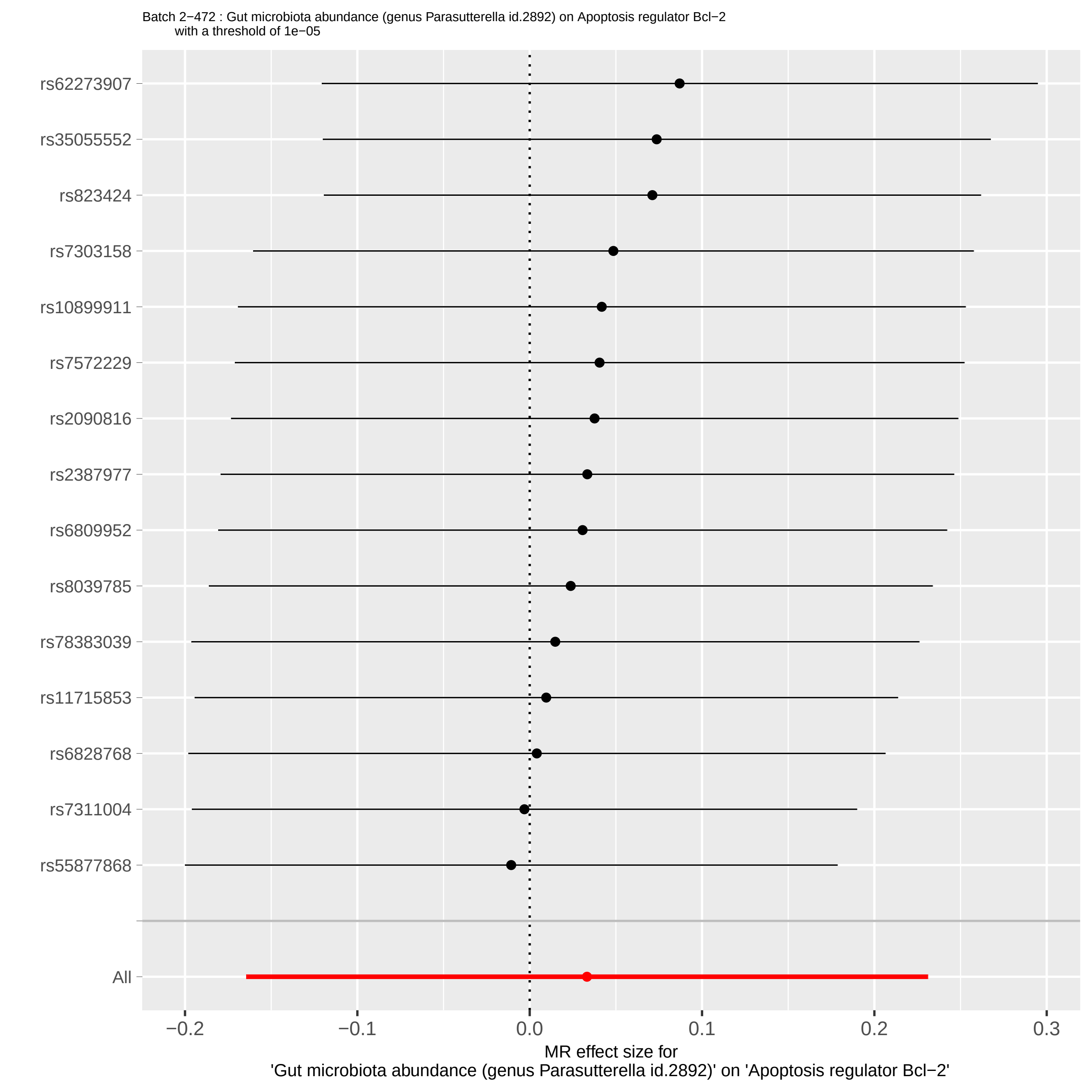

Batch 2−472 : Gut microbiota abundance (genus Parasutterella id.2892) on Apoptosis regulator Bcl−2
with a threshold of 1e−05
rs62273907
rs35055552
rs823424
rs7303158
rs10899911
rs7572229
rs2090816
rs2387977
rs6809952
rs8039785
rs78383039
rs11715853
rs6828768
rs7311004
rs55877868
All
−0.2
−0.1
0.0
0.1
0.2
0.3
MR effect size for
'Gut microbiota abundance (genus Parasutterella id.2892)' on 'Apoptosis regulator Bcl−2'

Batch 2−473 : Gut microbiota abundance (genus Catenibacterium id.2153) on Bcl−2−related protein A1
with a threshold of 1e−05

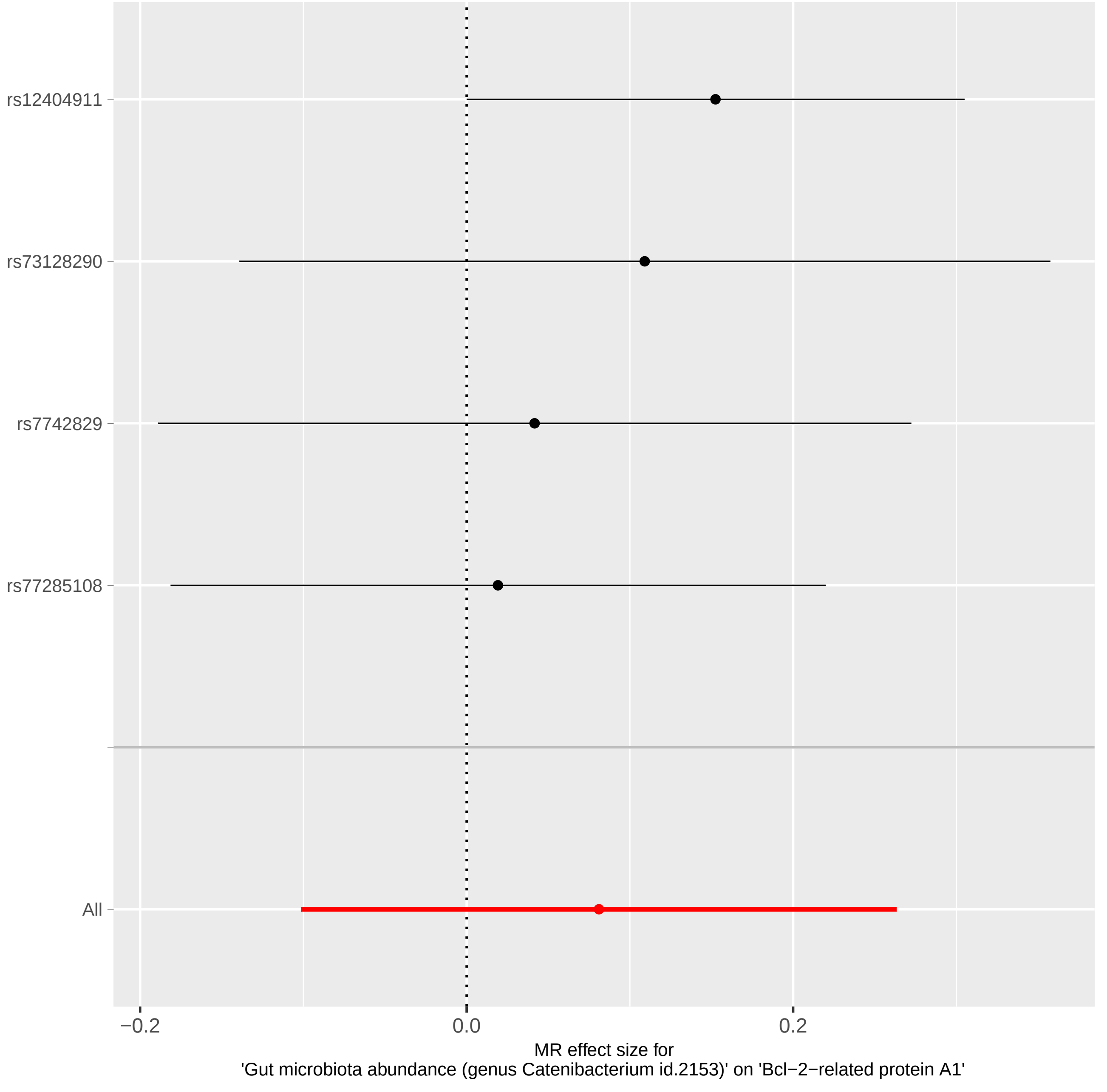

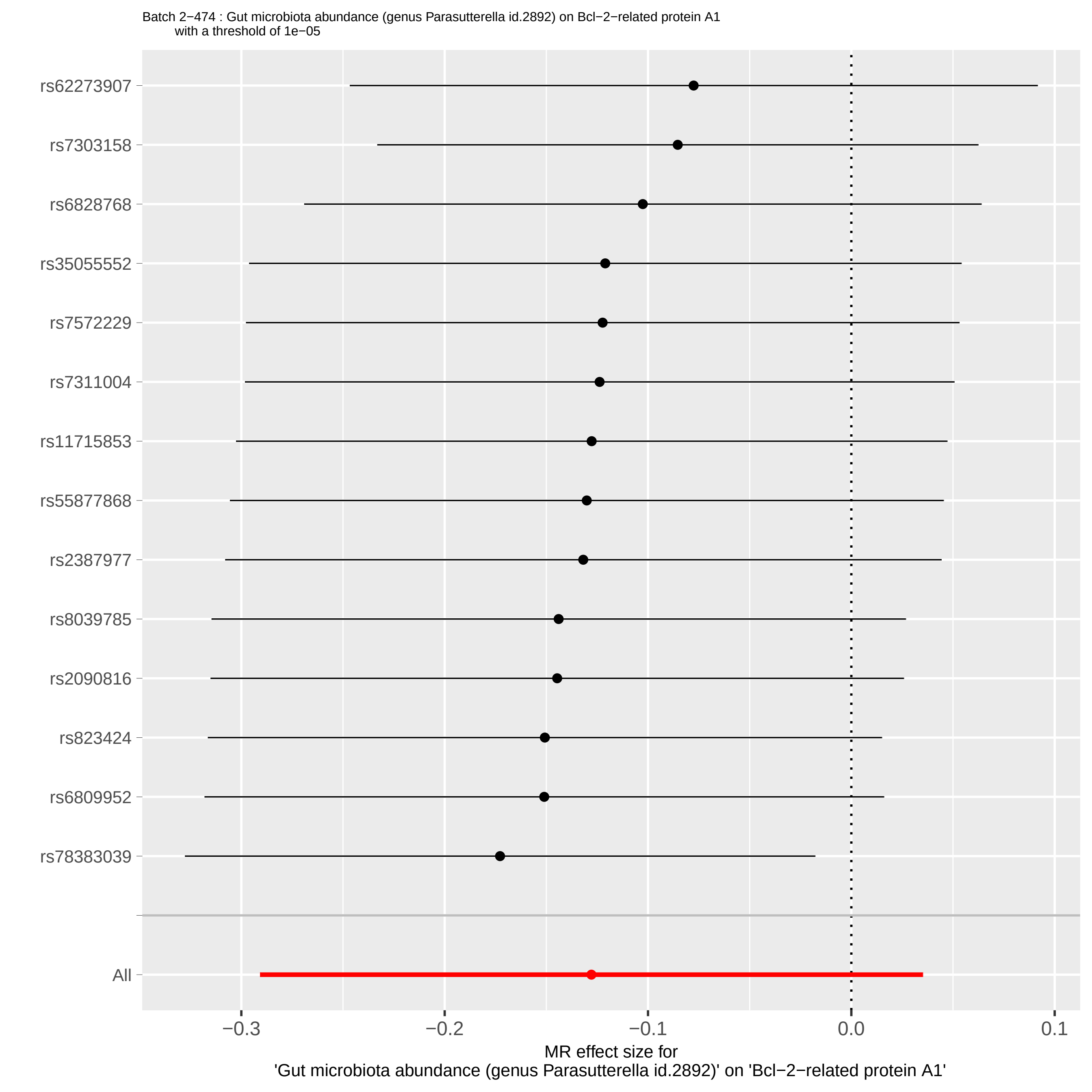
Batch 2−474 : Gut microbiota abundance (genus Parasutterella id.2892) on Bcl−2−related protein A1
with a threshold of 1e−05
rs62273907
rs7303158
rs6828768
rs35055552
rs7572229
rs7311004
rs11715853
rs55877868
rs2387977
rs8039785
rs2090816
rs823424
rs6809952
rs78383039
All
−0.3
−0.2
−0.1
0.0
0.1
MR effect size for
'Gut microbiota abundance (genus Parasutterella id.2892)' on 'Bcl−2−related protein A1'

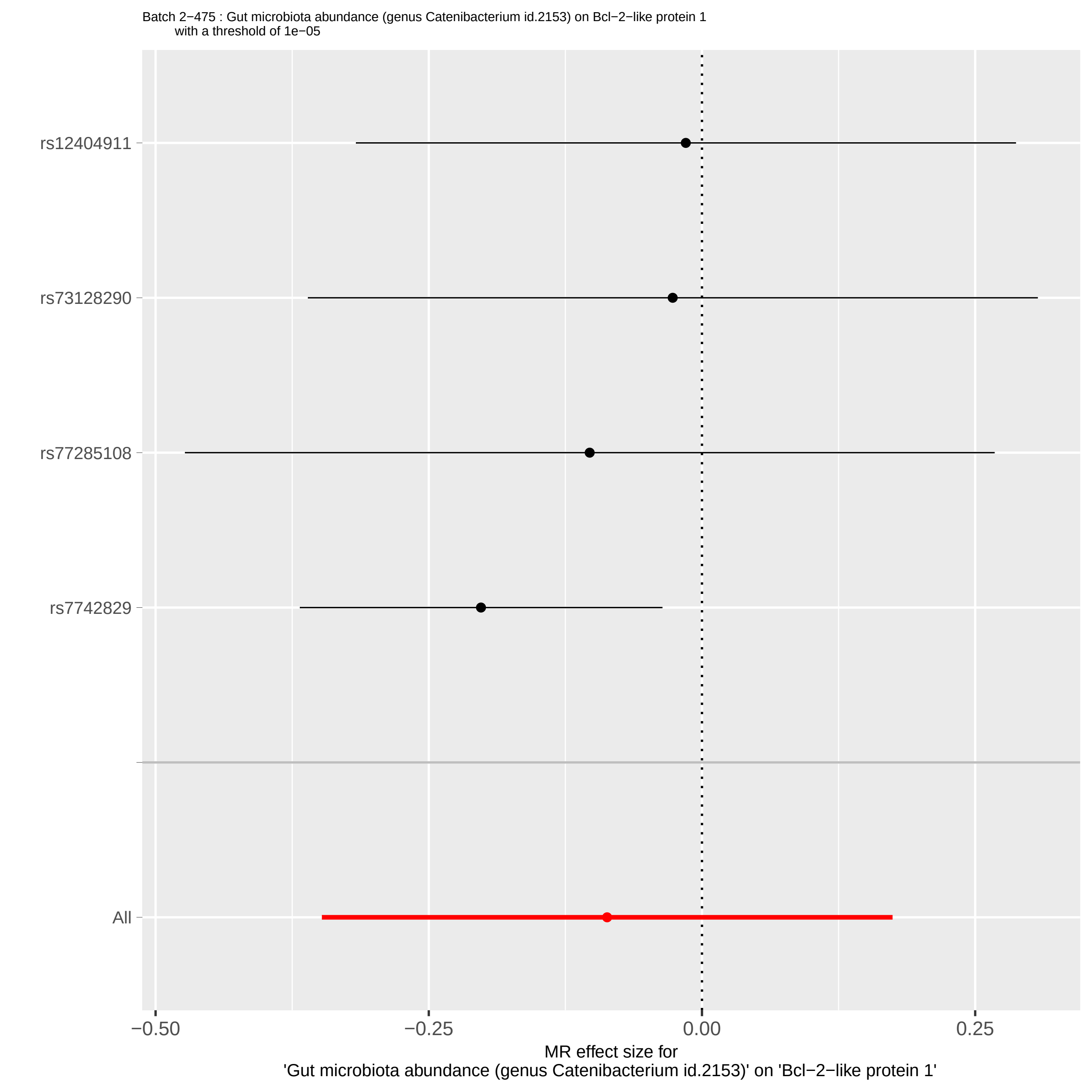
Batch 2−475 : Gut microbiota abundance (genus Catenibacterium id.2153) on Bcl−2−like protein 1
with a threshold of 1e−05
rs12404911
rs73128290
rs77285108
rs7742829
All
−0.50
−0.25
0.00
0.25
MR effect size for
'Gut microbiota abundance (genus Catenibacterium id.2153)' on 'Bcl−2−like protein 1'

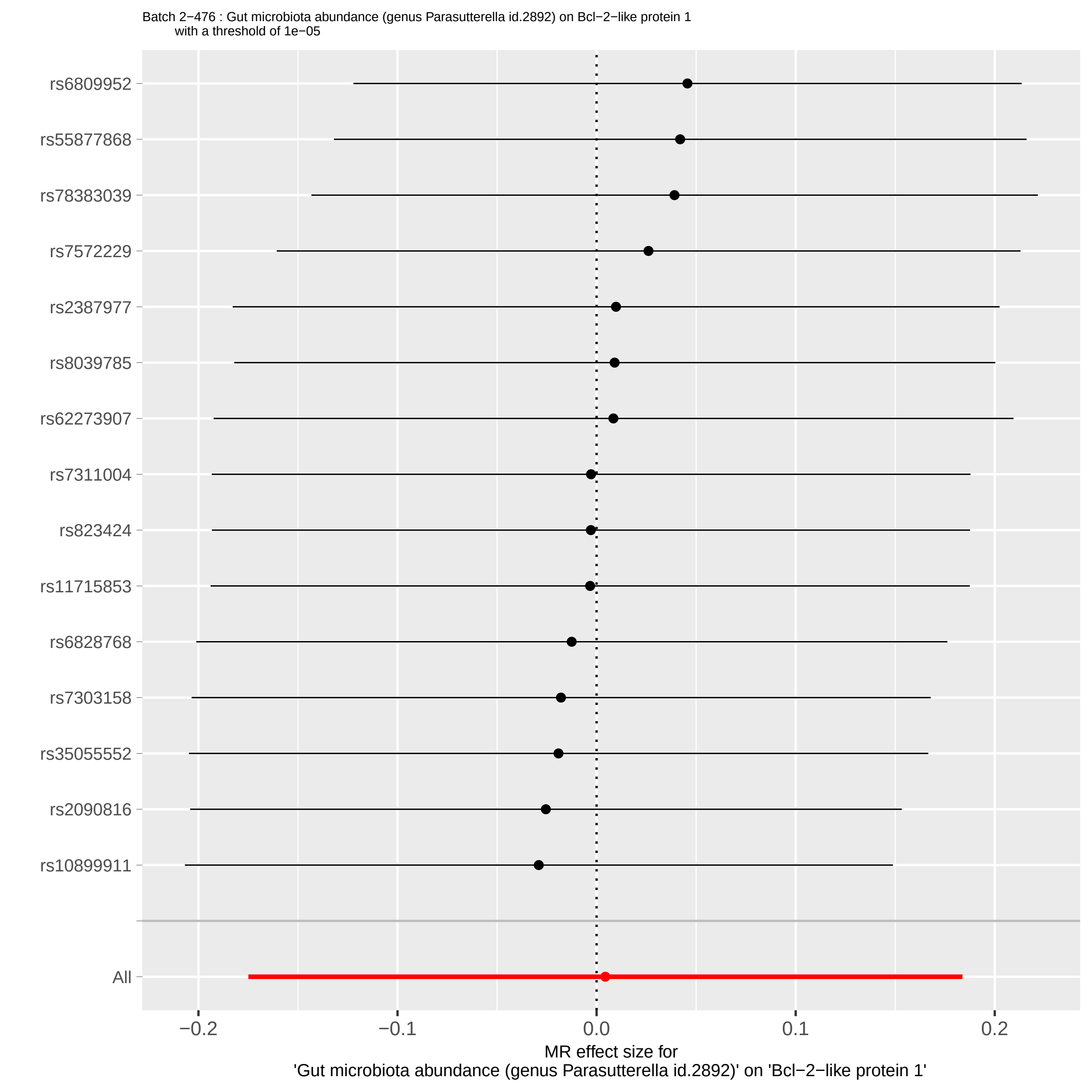

Batch 2−476 : Gut microbiota abundance (genus Parasutterella id.2892) on Bcl−2−like protein 1
with a threshold of 1e−05
rs6809952
rs55877868
rs78383039
rs7572229
rs2387977
rs8039785
rs62273907
rs7311004
rs823424
rs11715853
rs6828768
rs7303158
rs35055552
rs2090816
rs10899911
All
−0.2
−0.1
0.0
0.1
0.2
MR effect size for
'Gut microbiota abundance (genus Parasutterella id.2892)' on 'Bcl−2−like protein 1'

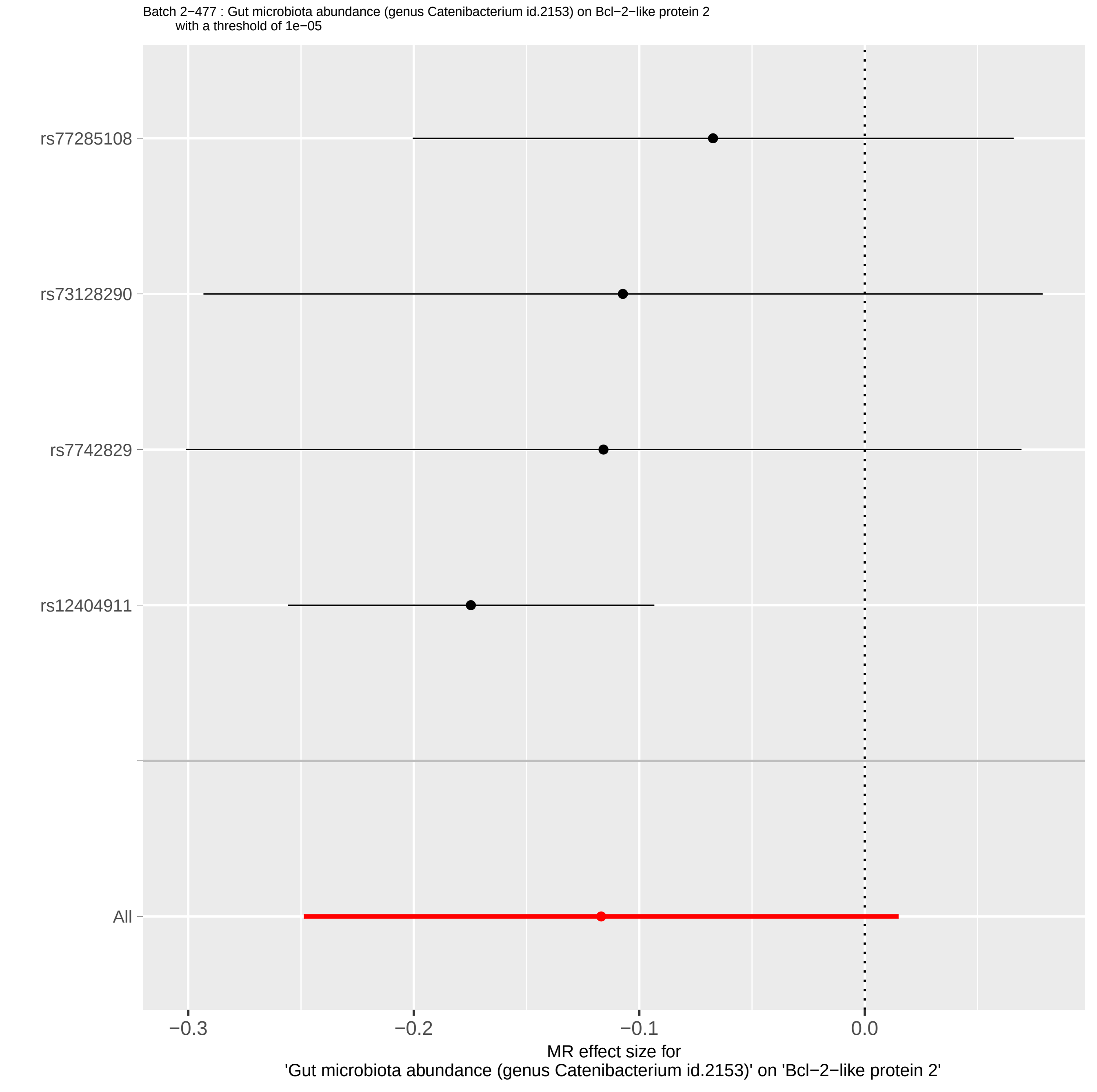
Batch 2−477 : Gut microbiota abundance (genus Catenibacterium id.2153) on Bcl−2−like protein 2
with a threshold of 1e−05
rs77285108
rs73128290
rs7742829
rs12404911
All
−0.3
−0.2
−0.1
0.0
MR effect size for
'Gut microbiota abundance (genus Catenibacterium id.2153)' on 'Bcl−2−like protein 2'

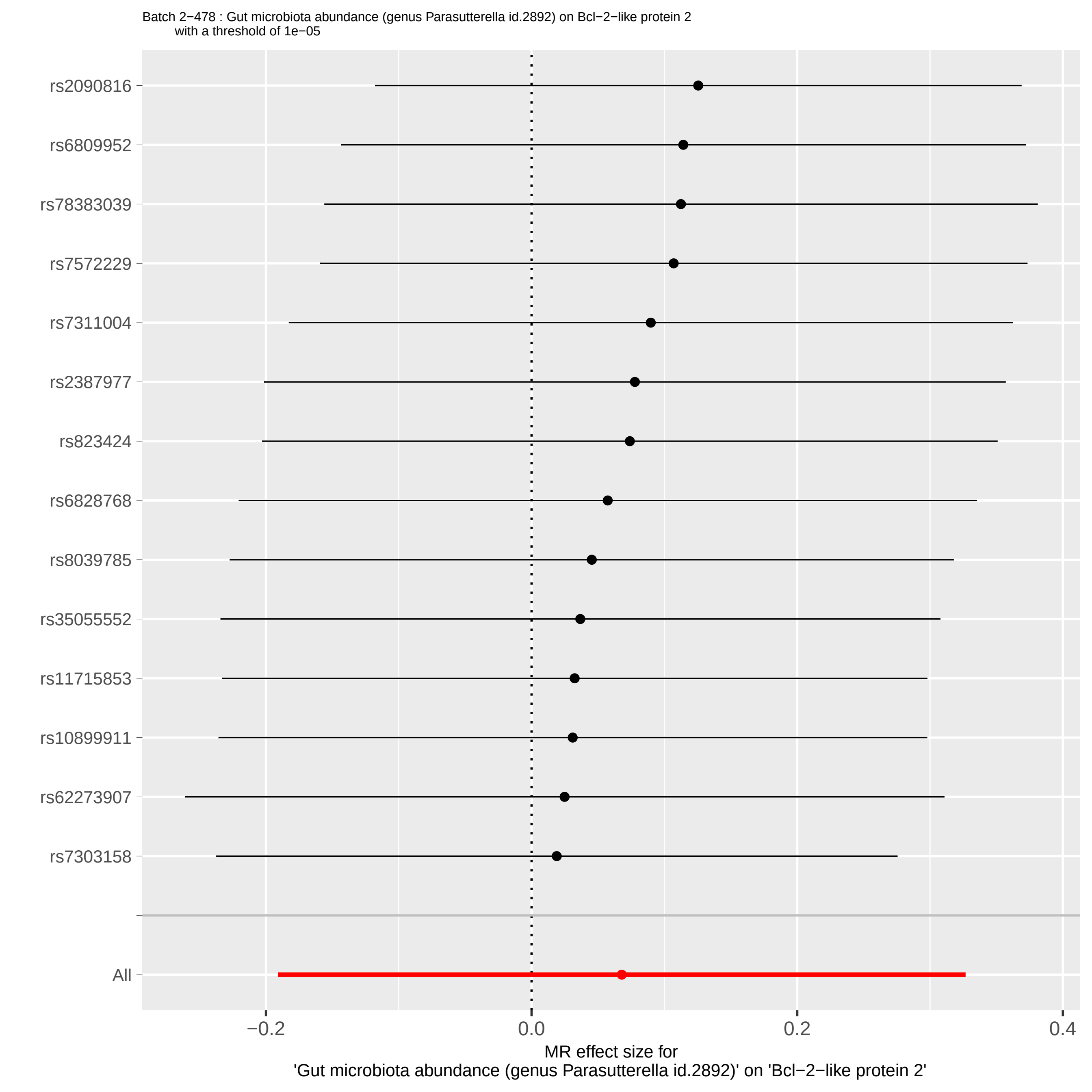

Batch 2−478 : Gut microbiota abundance (genus Parasutterella id.2892) on Bcl−2−like protein 2
with a threshold of 1e−05
rs2090816
rs6809952
rs78383039
rs7572229
rs7311004
rs2387977
rs823424
rs6828768
rs8039785
rs35055552
rs11715853
rs10899911
rs62273907
rs7303158
All
−0.2
0.0
0.2
0.4
MR effect size for
'Gut microbiota abundance (genus Parasutterella id.2892)' on 'Bcl−2−like protein 2'

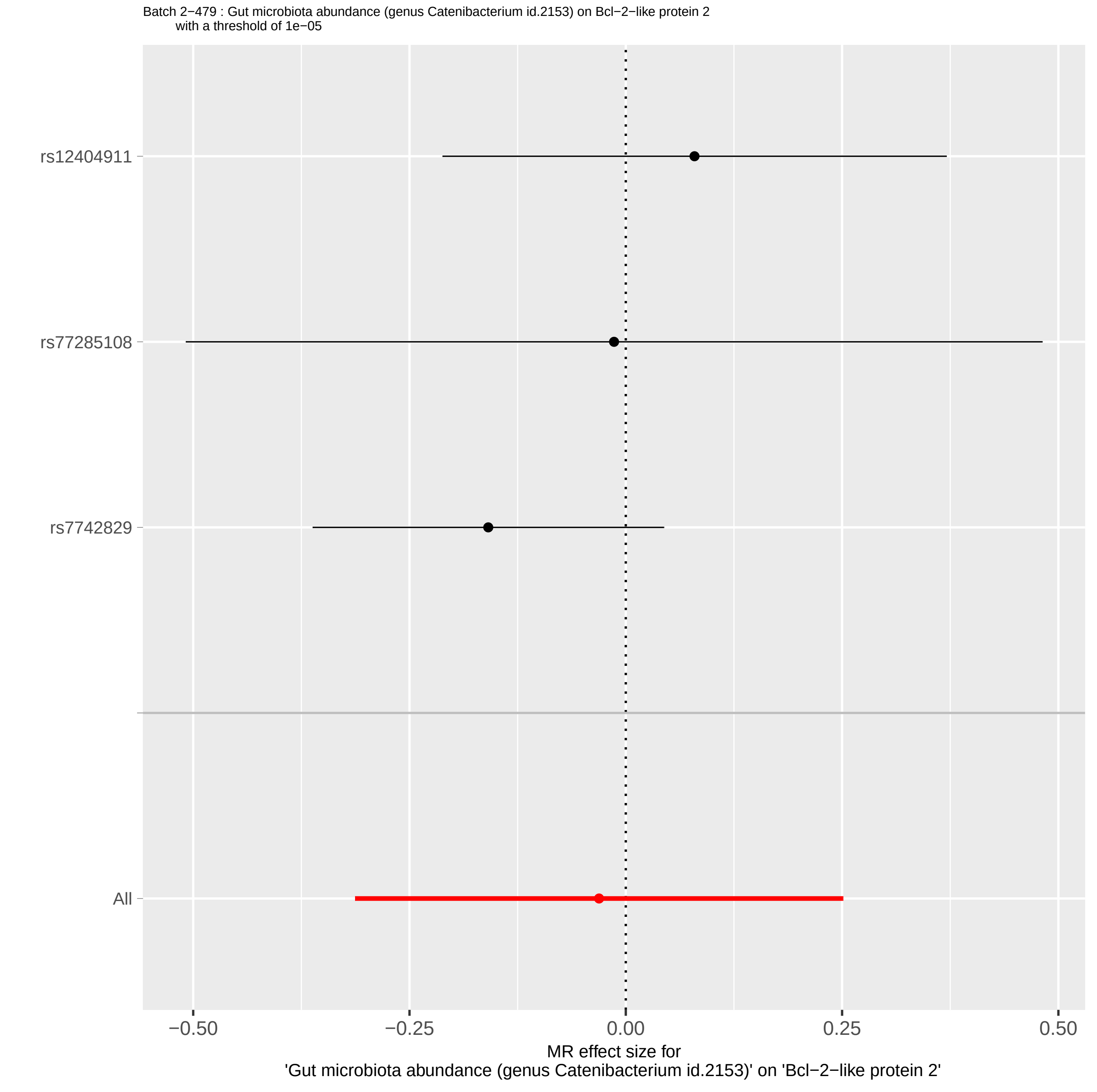

Batch 2−479 : Gut microbiota abundance (genus Catenibacterium id.2153) on Bcl−2−like protein 2
with a threshold of 1e−05
rs12404911
rs77285108
rs7742829
All
−0.50
−0.25
0.00
0.25
0.50
MR effect size for
'Gut microbiota abundance (genus Catenibacterium id.2153)' on 'Bcl−2−like protein 2'

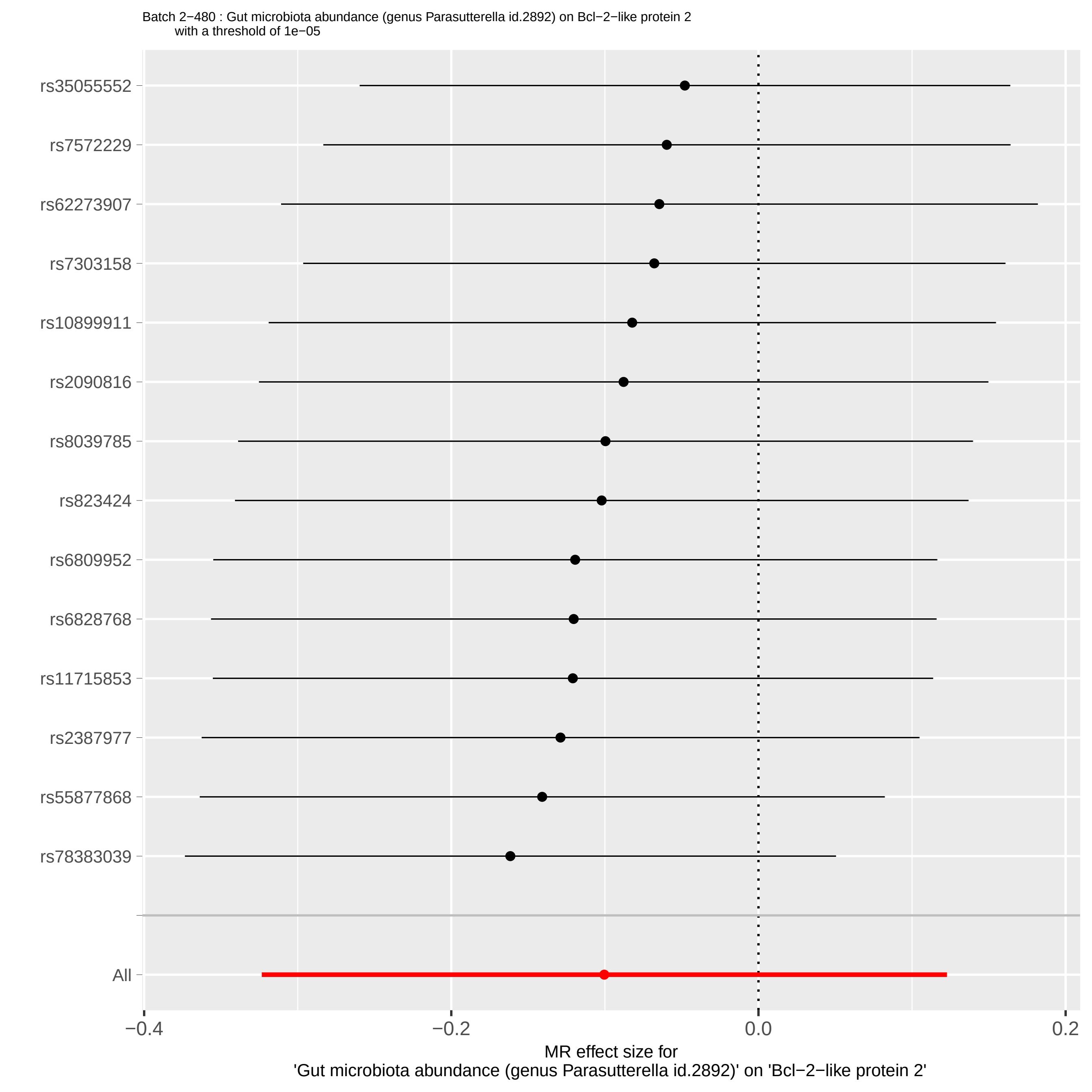

Batch 2−480 : Gut microbiota abundance (genus Parasutterella id.2892) on Bcl−2−like protein 2
with a threshold of 1e−05
rs35055552
rs7572229
rs62273907
rs7303158
rs10899911
rs2090816
rs8039785
rs823424
rs6809952
rs6828768
rs11715853
rs2387977
rs55877868
rs78383039
All
−0.4
−0.2
0.0
0.2
MR effect size for
'Gut microbiota abundance (genus Parasutterella id.2892)' on 'Bcl−2−like protein 2'

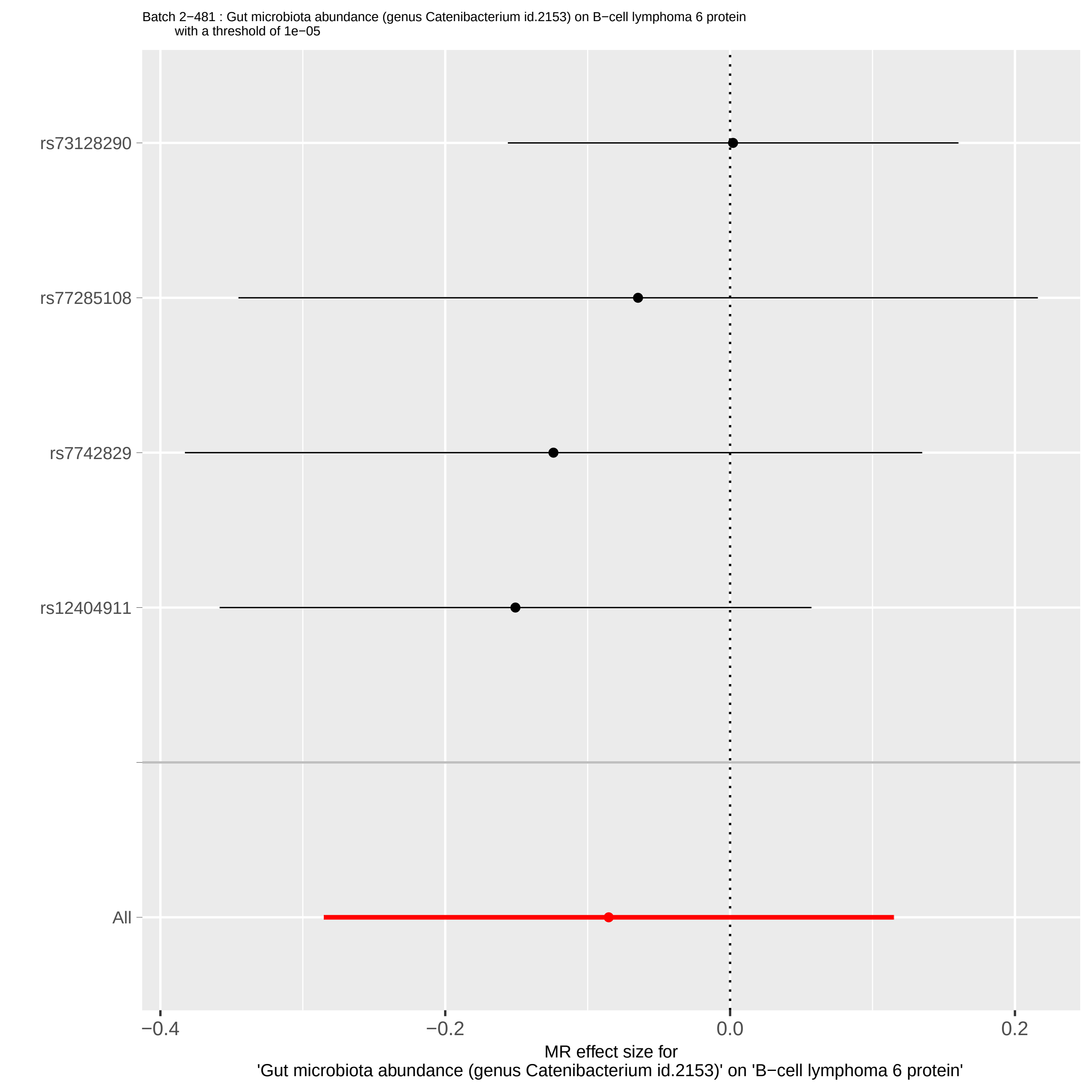

Batch 2−481 : Gut microbiota abundance (genus Catenibacterium id.2153) on B−cell lymphoma 6 protein
with a threshold of 1e−05
rs73128290
rs77285108
rs7742829
rs12404911
All
−0.4
−0.2
0.0
0.2
MR effect size for
'Gut microbiota abundance (genus Catenibacterium id.2153)' on 'B−cell lymphoma 6 protein'

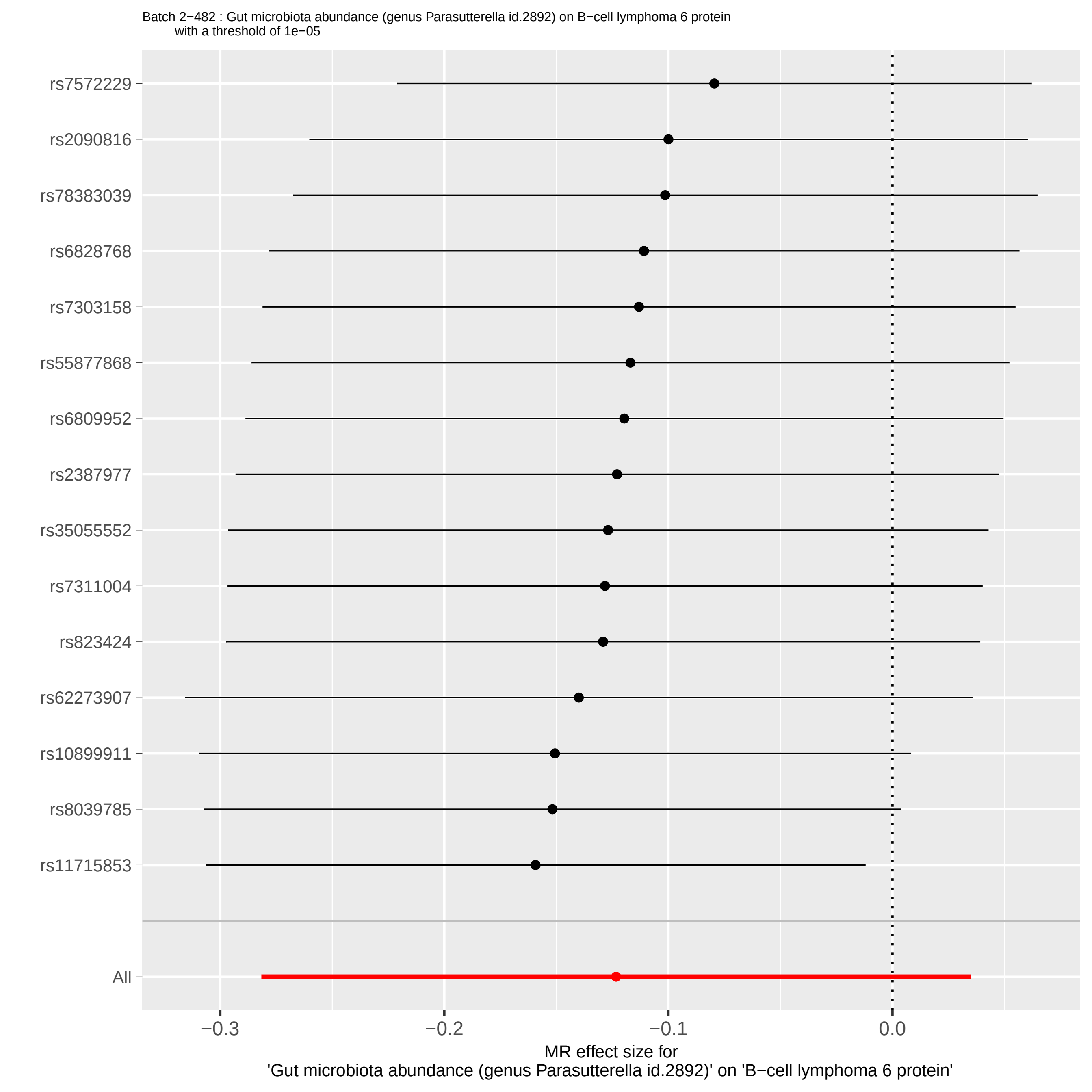

Batch 2−482 : Gut microbiota abundance (genus Parasutterella id.2892) on B−cell lymphoma 6 protein
with a threshold of 1e−05
rs7572229
rs2090816
rs78383039
rs6828768
rs7303158
rs55877868
rs6809952
rs2387977
rs35055552
rs7311004
rs823424
rs62273907
rs10899911
rs8039785
rs11715853
All
−0.3
−0.2
−0.1
0.0
MR effect size for
'Gut microbiota abundance (genus Parasutterella id.2892)' on 'B−cell lymphoma 6 protein'

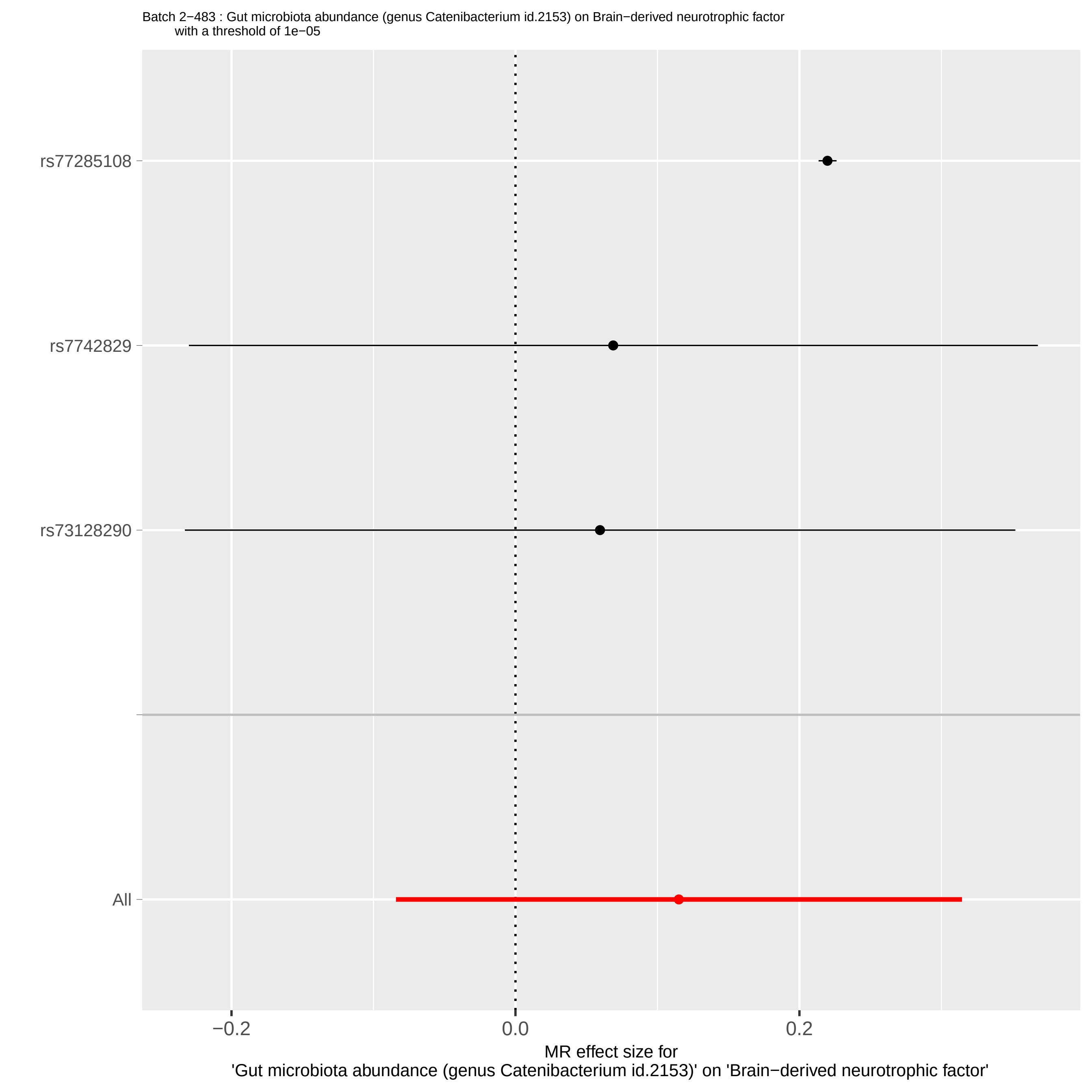
Batch 2−483 : Gut microbiota abundance (genus Catenibacterium id.2153) on Brain−derived neurotrophic factor
with a threshold of 1e−05
rs77285108
rs7742829
rs73128290
All
−0.2
0.0
0.2
MR effect size for
'Gut microbiota abundance (genus Catenibacterium id.2153)' on 'Brain−derived neurotrophic factor'

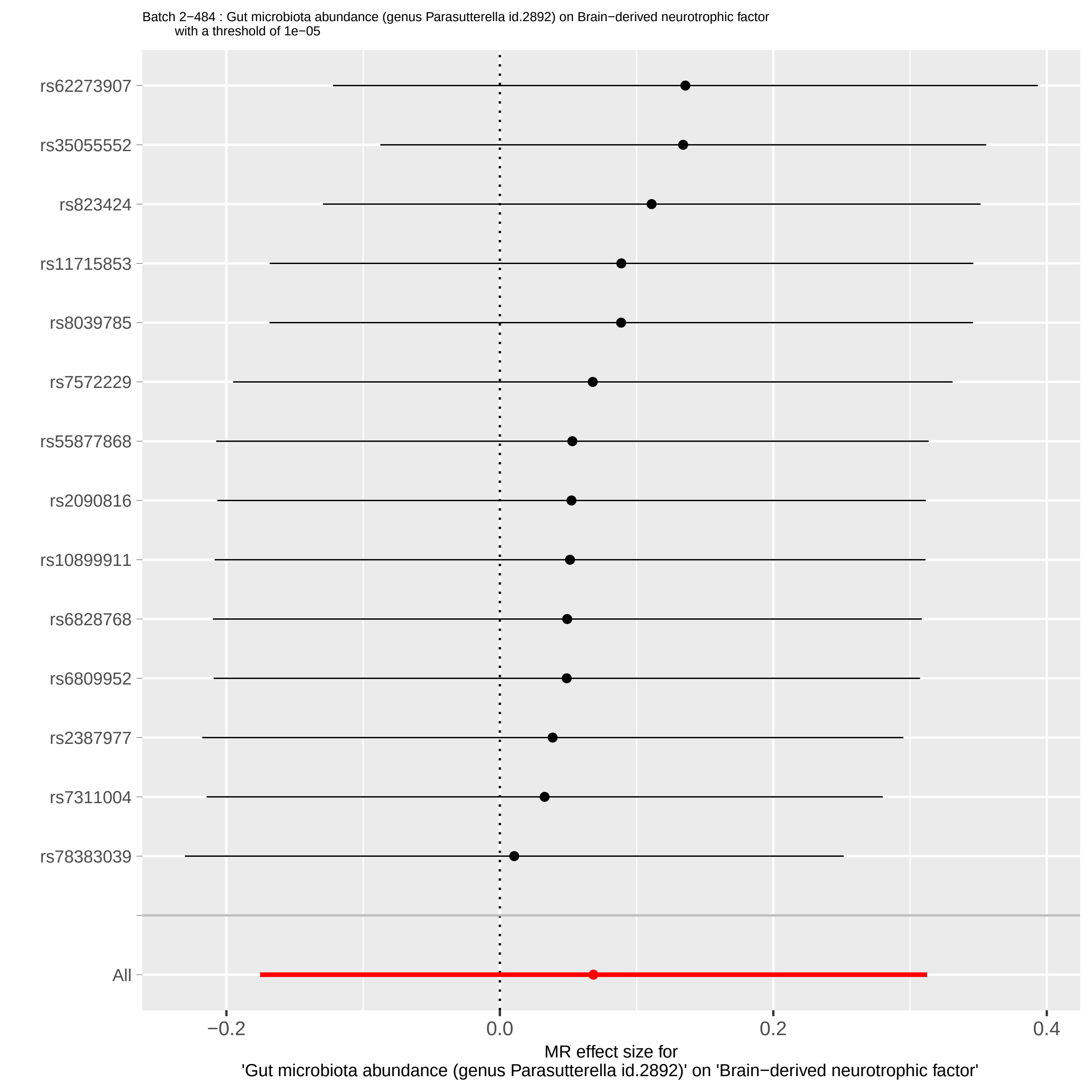
Batch 2−484 : Gut microbiota abundance (genus Parasutterella id.2892) on Brain−derived neurotrophic factor
with a threshold of 1e−05
rs62273907
rs35055552
rs823424
rs11715853
rs8039785
rs7572229
rs55877868
rs2090816
rs10899911
rs6828768
rs6809952
rs2387977
rs7311004
rs78383039
All
−0.2
0.0
0.2
0.4
MR effect size for
'Gut microbiota abundance (genus Parasutterella id.2892)' on 'Brain−derived neurotrophic factor'

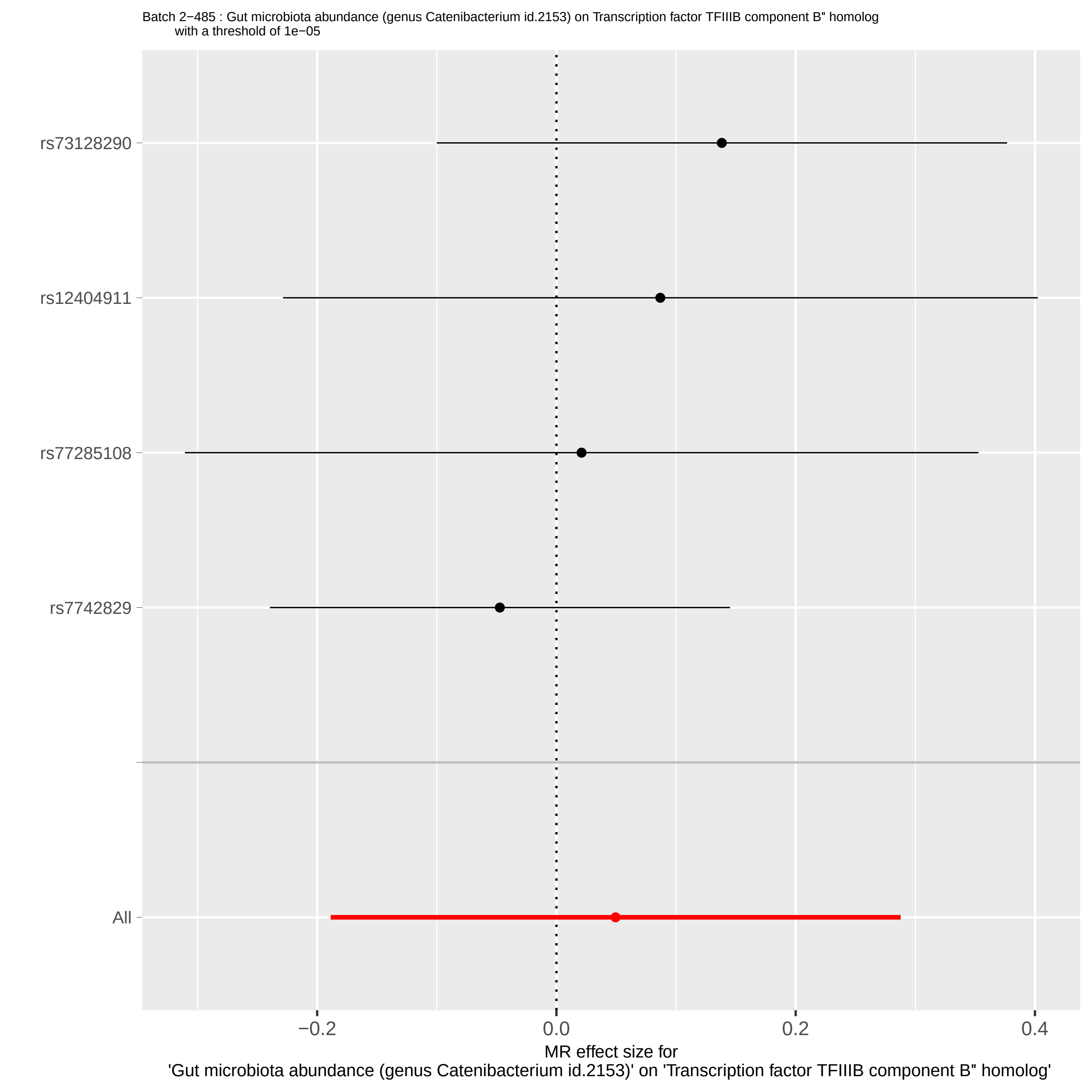

Batch 2−485 : Gut microbiota abundance (genus Catenibacterium id.2153) on Transcription factor TFIIIB component B" homolog
with a threshold of 1e−05
rs73128290
rs12404911
rs77285108
rs7742829
All
−0.2
0.0
0.2
0.4
MR effect size for
'Gut microbiota abundance (genus Catenibacterium id.2153)' on 'Transcription factor TFIIIB component B" homolog'

Batch 2−486 : Gut microbiota abundance (genus Parasutterella id.2892) on Transcription factor TFIIIB component B" homolog
with a threshold of 1e−05

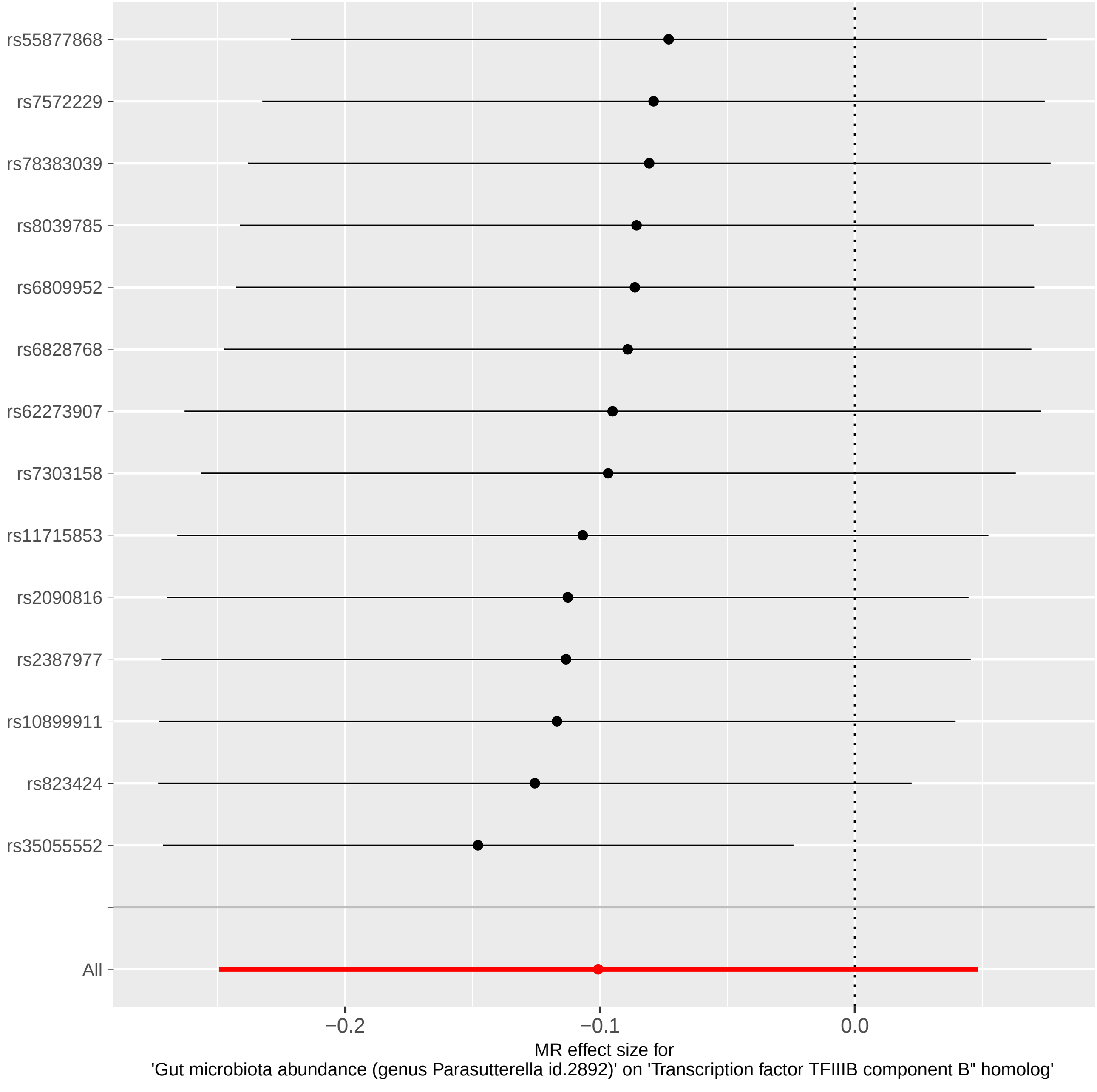

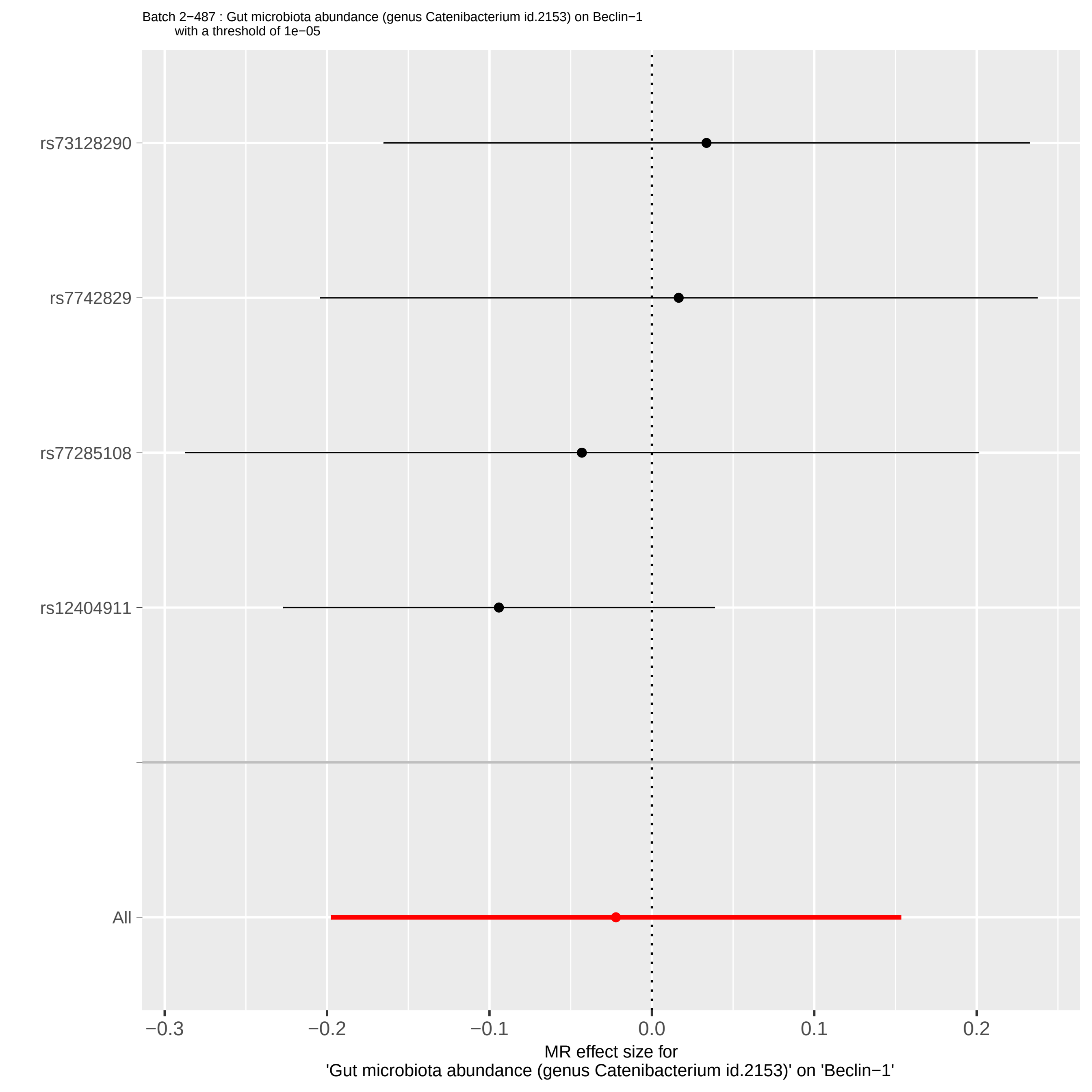
Batch 2−487 : Gut microbiota abundance (genus Catenibacterium id.2153) on Beclin−1
with a threshold of 1e−05
rs73128290
rs7742829
rs77285108
rs12404911
All
−0.3
−0.2
−0.1
0.0
0.1
0.2
MR effect size for
'Gut microbiota abundance (genus Catenibacterium id.2153)' on 'Beclin−1'

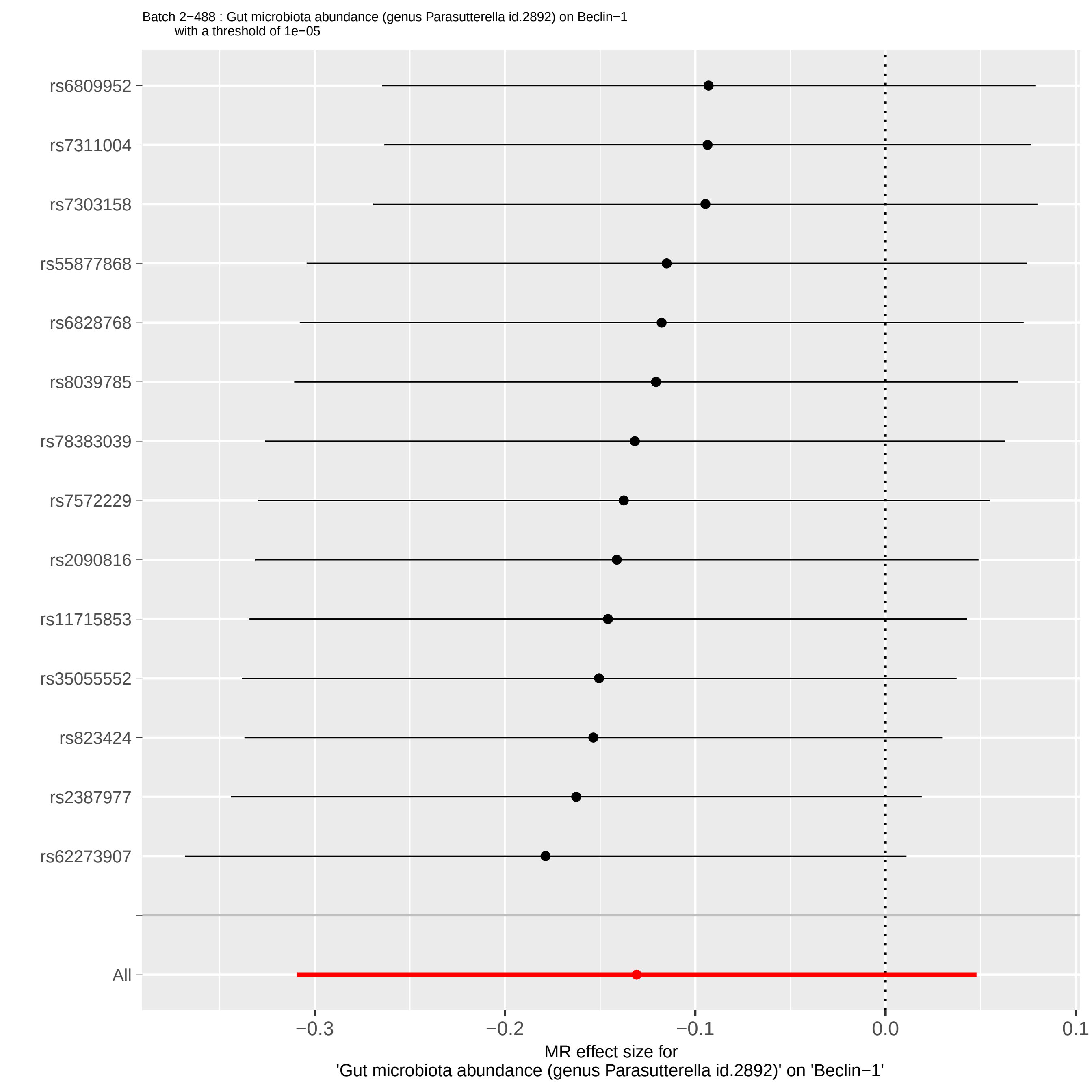

Batch 2−488 : Gut microbiota abundance (genus Parasutterella id.2892) on Beclin−1
with a threshold of 1e−05
rs6809952
rs7311004
rs7303158
rs55877868
rs6828768
rs8039785
rs78383039
rs7572229
rs2090816
rs11715853
rs35055552
rs823424
rs2387977
rs62273907
All
−0.3
−0.2
−0.1
0.0
0.1
MR effect size for
'Gut microbiota abundance (genus Parasutterella id.2892)' on 'Beclin−1'

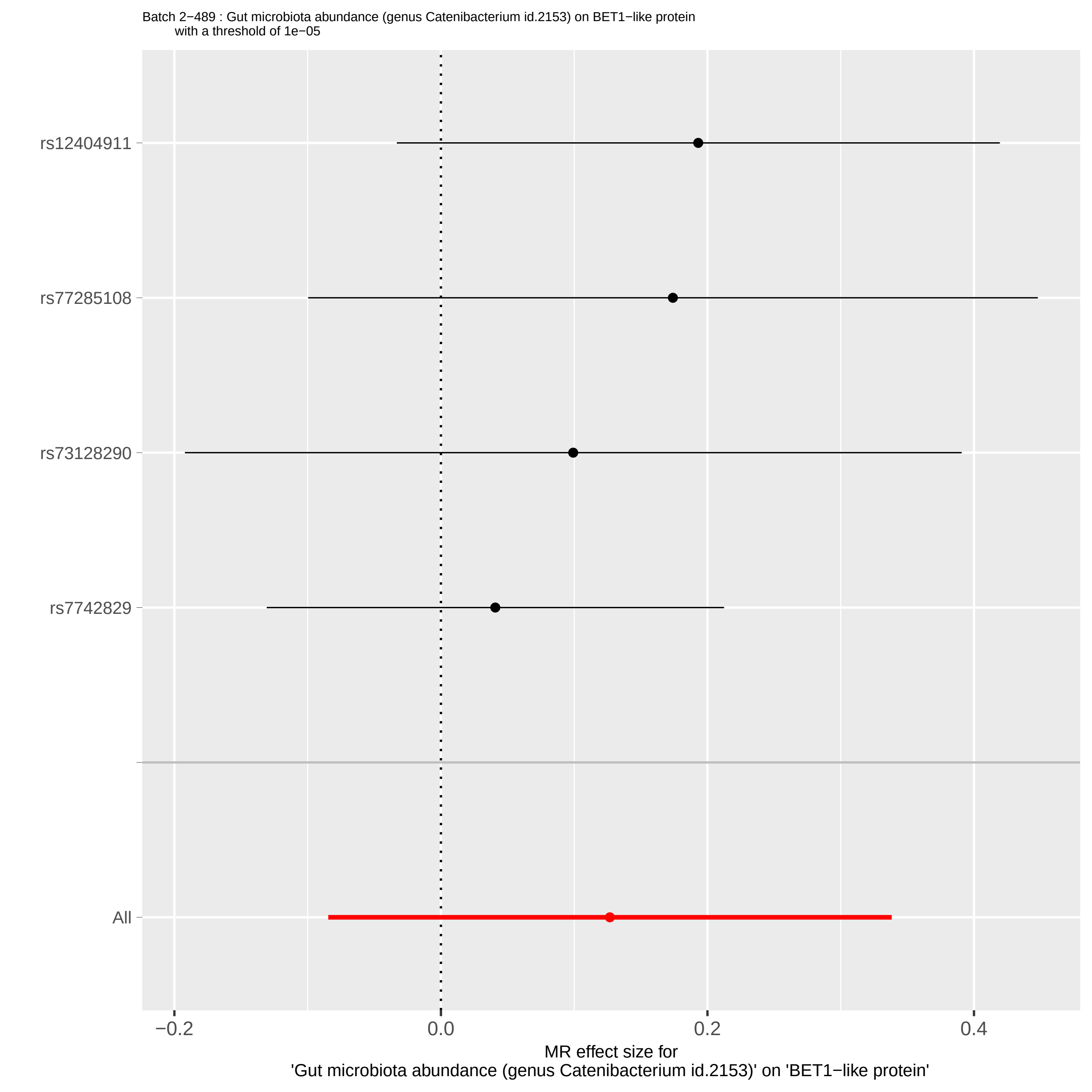
Batch 2−489 : Gut microbiota abundance (genus Catenibacterium id.2153) on BET1−like protein
with a threshold of 1e−05
rs12404911
rs77285108
rs73128290
rs7742829
All
−0.2
0.0
0.2
0.4
MR effect size for
'Gut microbiota abundance (genus Catenibacterium id.2153)' on 'BET1−like protein'

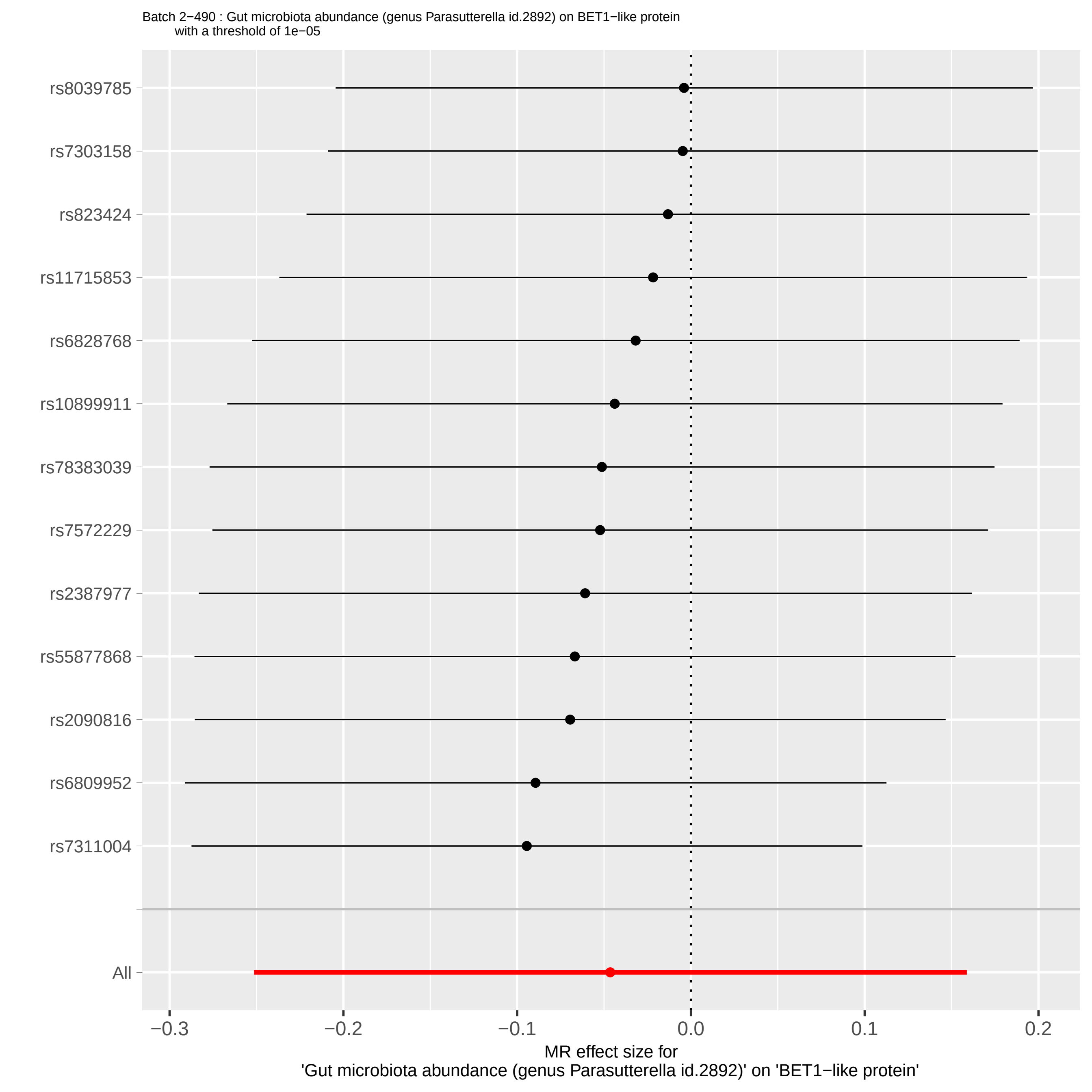

Batch 2−490 : Gut microbiota abundance (genus Parasutterella id.2892) on BET1−like protein
with a threshold of 1e−05
rs8039785
rs7303158
rs823424
rs11715853
rs6828768
rs10899911
rs78383039
rs7572229
rs2387977
rs55877868
rs2090816
rs6809952
rs7311004
All
−0.3
−0.2
−0.1
0.0
0.1
0.2
MR effect size for
'Gut microbiota abundance (genus Parasutterella id.2892)' on 'BET1−like protein'

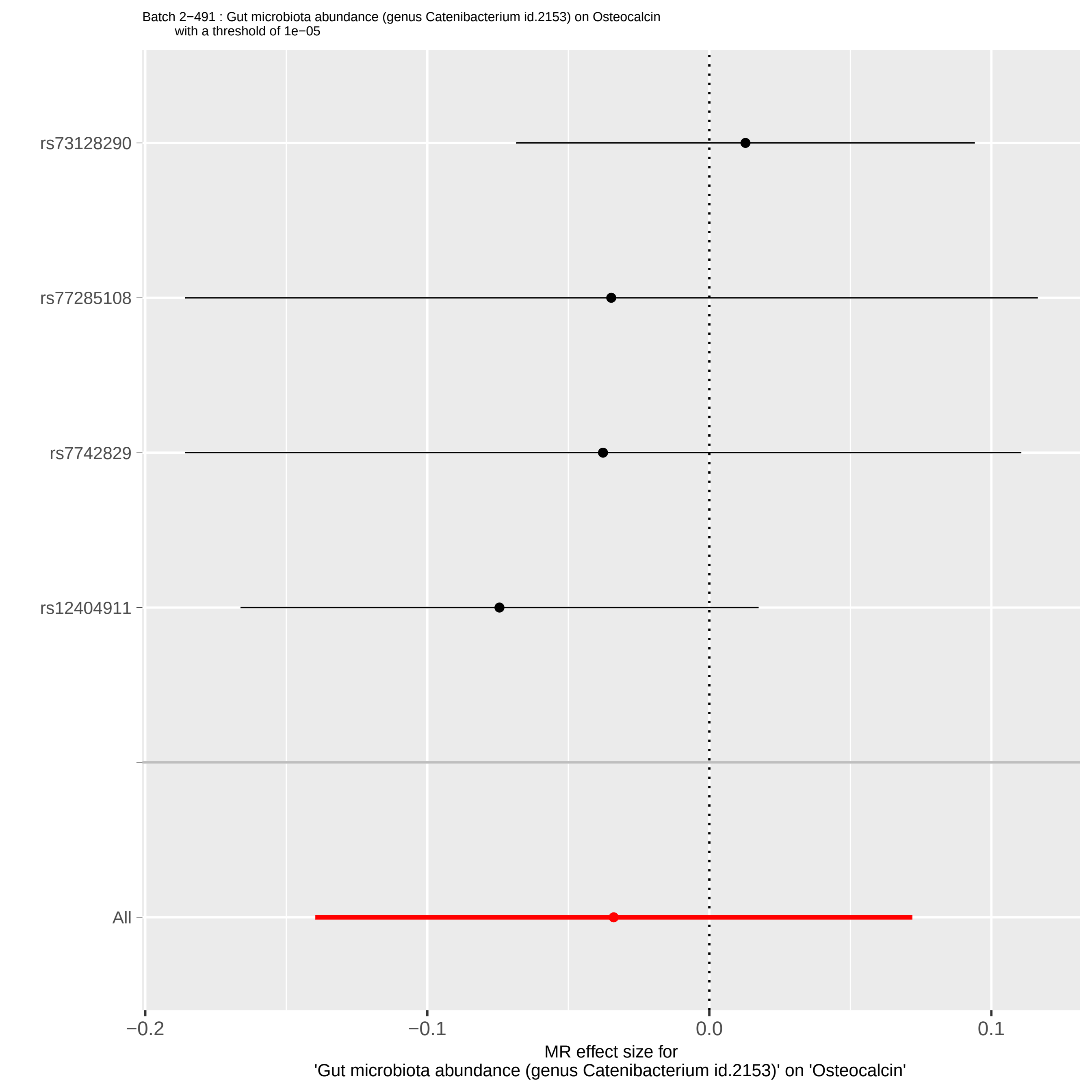

Batch 2−491 : Gut microbiota abundance (genus Catenibacterium id.2153) on Osteocalcin
with a threshold of 1e−05
rs73128290
rs77285108
rs7742829
rs12404911
All
−0.2
−0.1
0.0
0.1
MR effect size for
'Gut microbiota abundance (genus Catenibacterium id.2153)' on 'Osteocalcin'

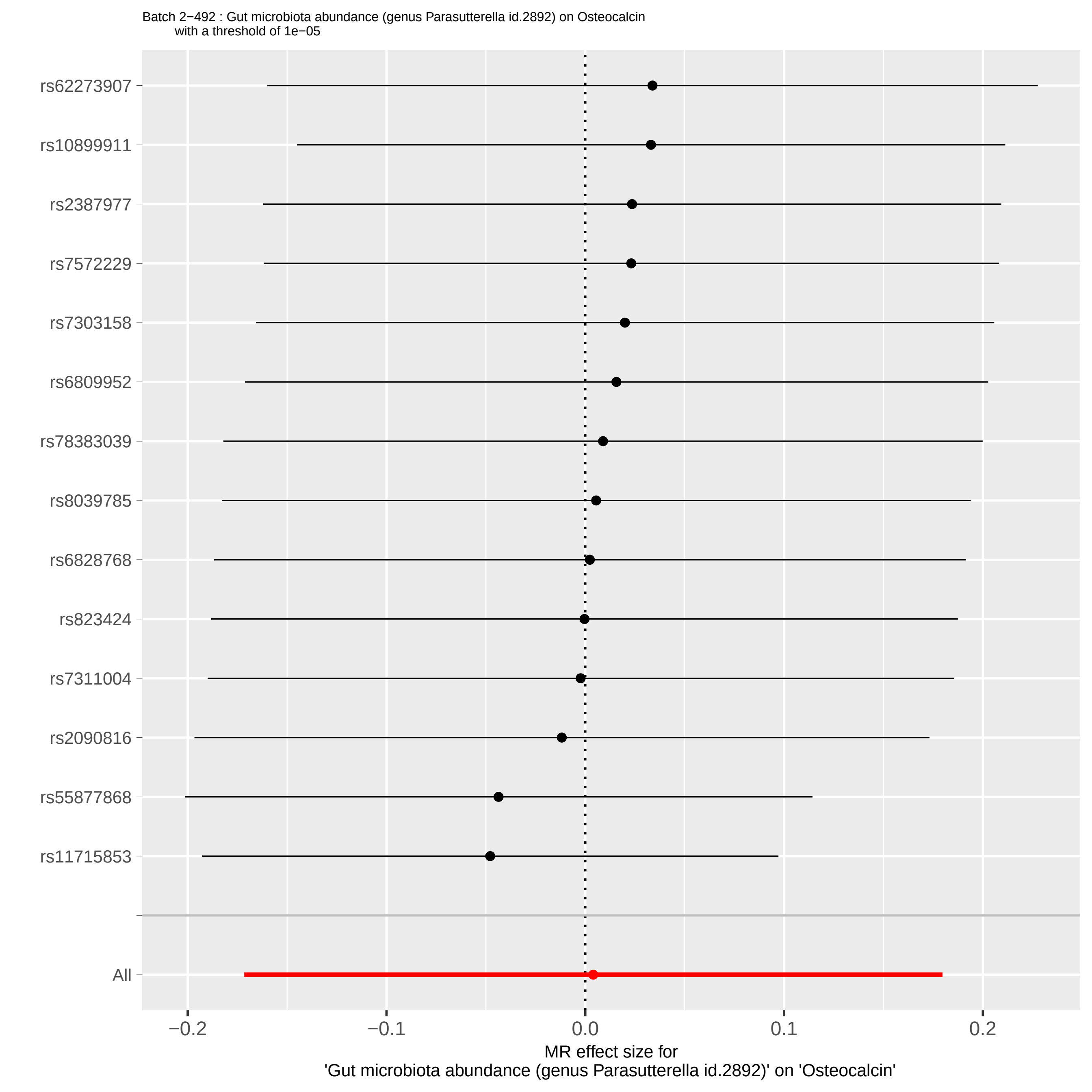

Batch 2−492 : Gut microbiota abundance (genus Parasutterella id.2892) on Osteocalcin
with a threshold of 1e−05
rs62273907
rs10899911
rs2387977
rs7572229
rs7303158
rs6809952
rs78383039
rs8039785
rs6828768
rs823424
rs7311004
rs2090816
rs55877868
rs11715853
All
−0.2
−0.1
0.0
0.1
0.2
MR effect size for
'Gut microbiota abundance (genus Parasutterella id.2892)' on 'Osteocalcin'

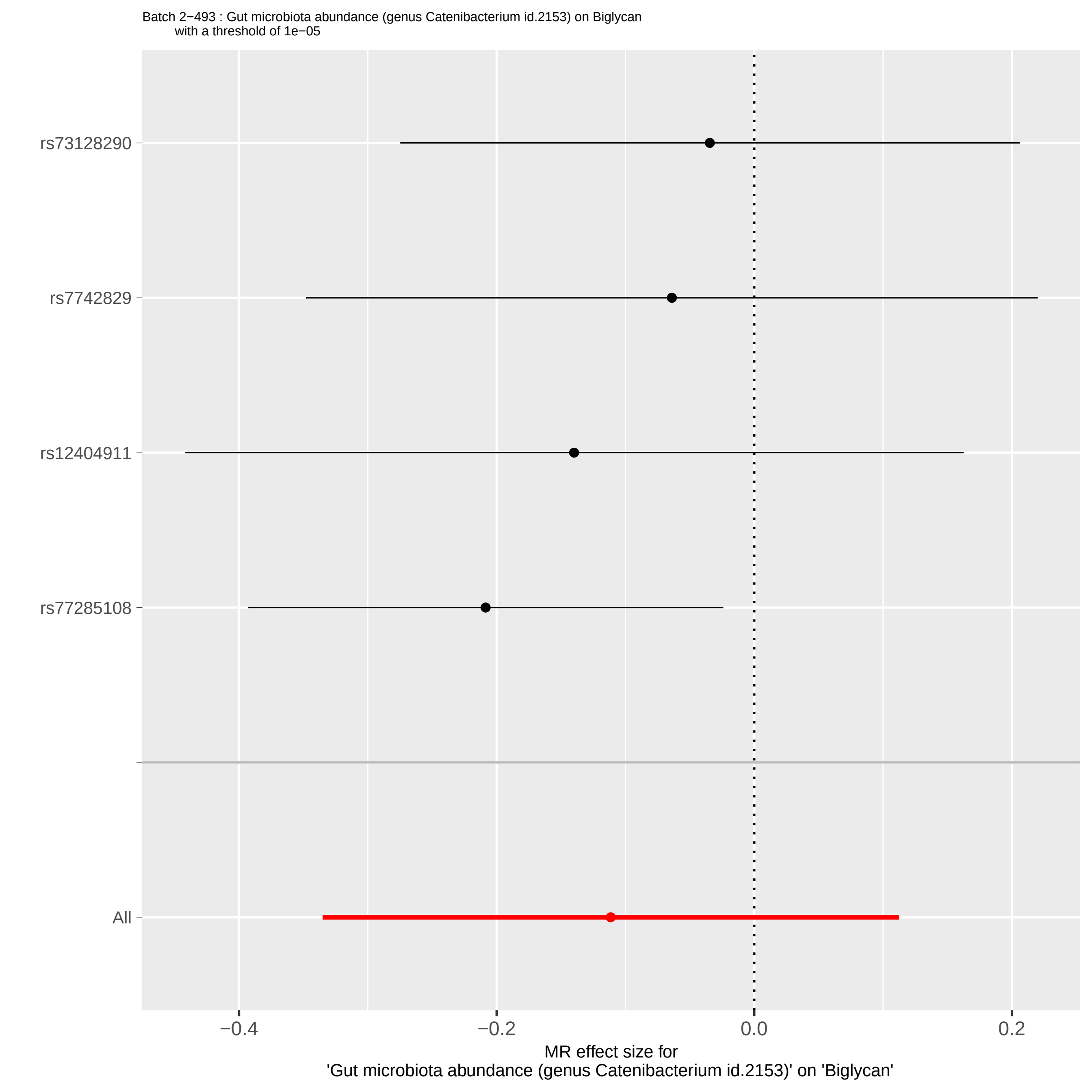

Batch 2−493 : Gut microbiota abundance (genus Catenibacterium id.2153) on Biglycan
with a threshold of 1e−05
rs73128290
rs7742829
rs12404911
rs77285108
All
−0.4
−0.2
0.0
0.2
MR effect size for
'Gut microbiota abundance (genus Catenibacterium id.2153)' on 'Biglycan'

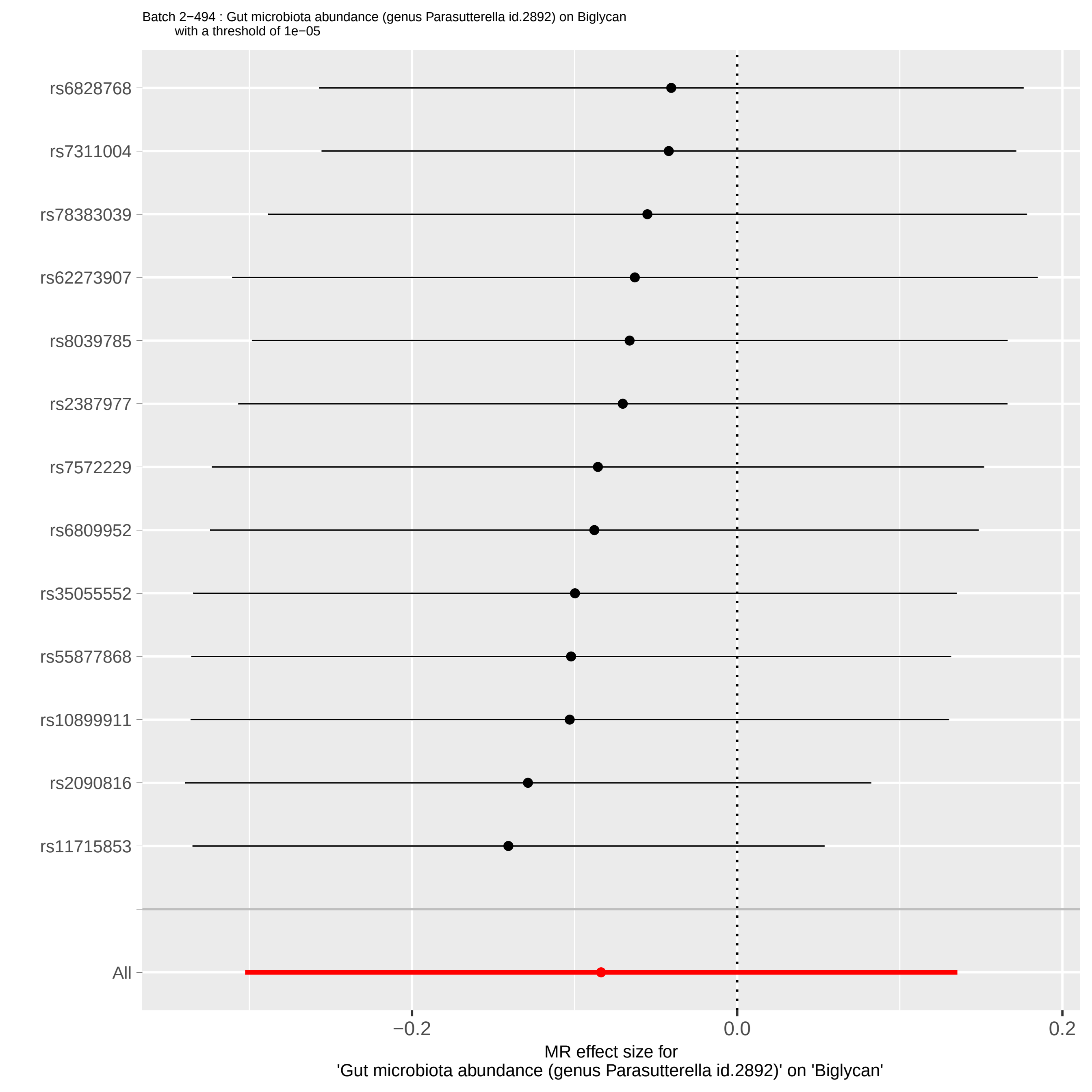

Batch 2−494 : Gut microbiota abundance (genus Parasutterella id.2892) on Biglycan
with a threshold of 1e−05
rs6828768
rs7311004
rs78383039
rs62273907
rs8039785
rs2387977
rs7572229
rs6809952
rs35055552
rs55877868
rs10899911
rs2090816
rs11715853
All
−0.2
0.0
0.2
MR effect size for
'Gut microbiota abundance (genus Parasutterella id.2892)' on 'Biglycan'

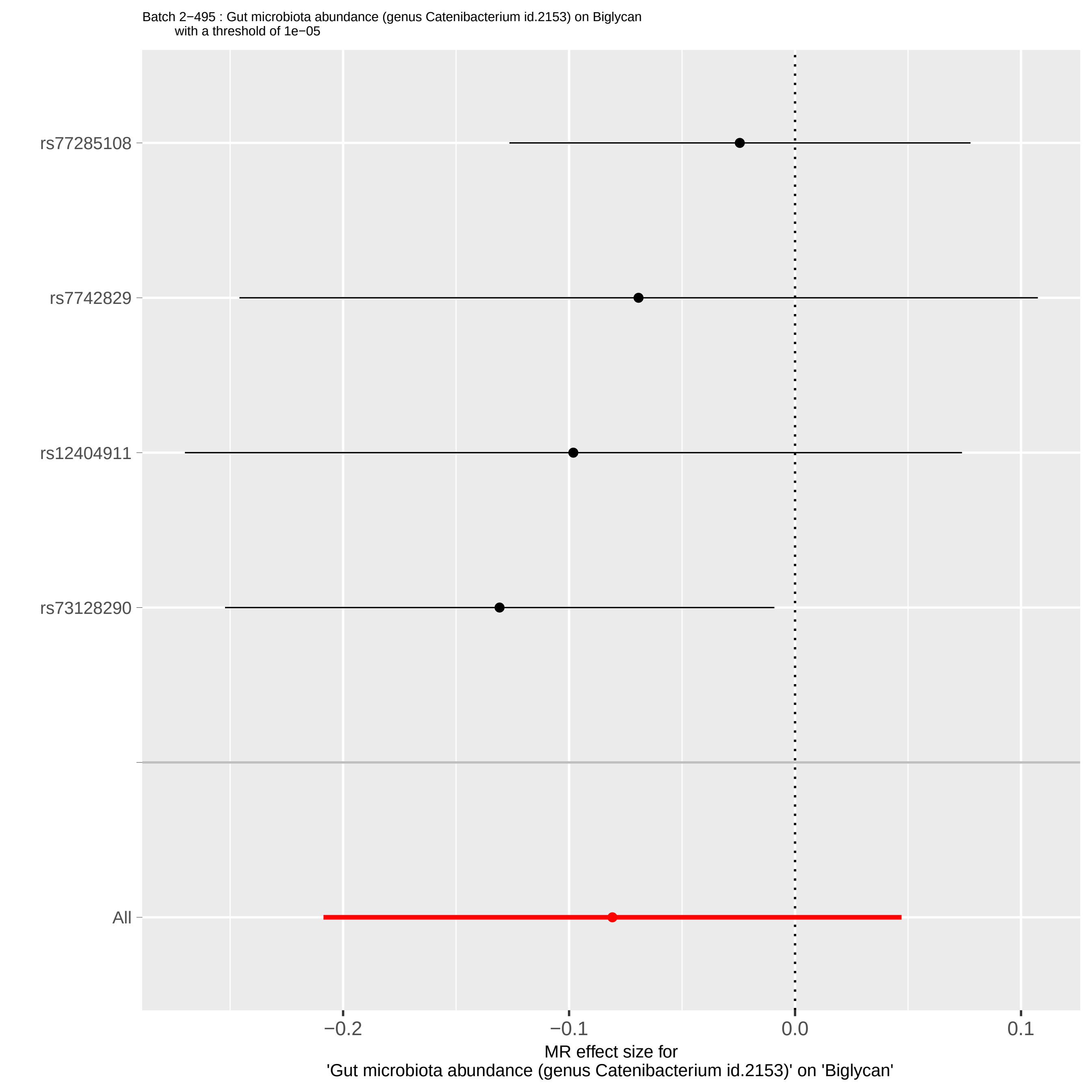

Batch 2−495 : Gut microbiota abundance (genus Catenibacterium id.2153) on Biglycan
with a threshold of 1e−05
rs77285108
rs7742829
rs12404911
rs73128290
All
−0.2
−0.1
0.0
0.1
MR effect size for
'Gut microbiota abundance (genus Catenibacterium id.2153)' on 'Biglycan'

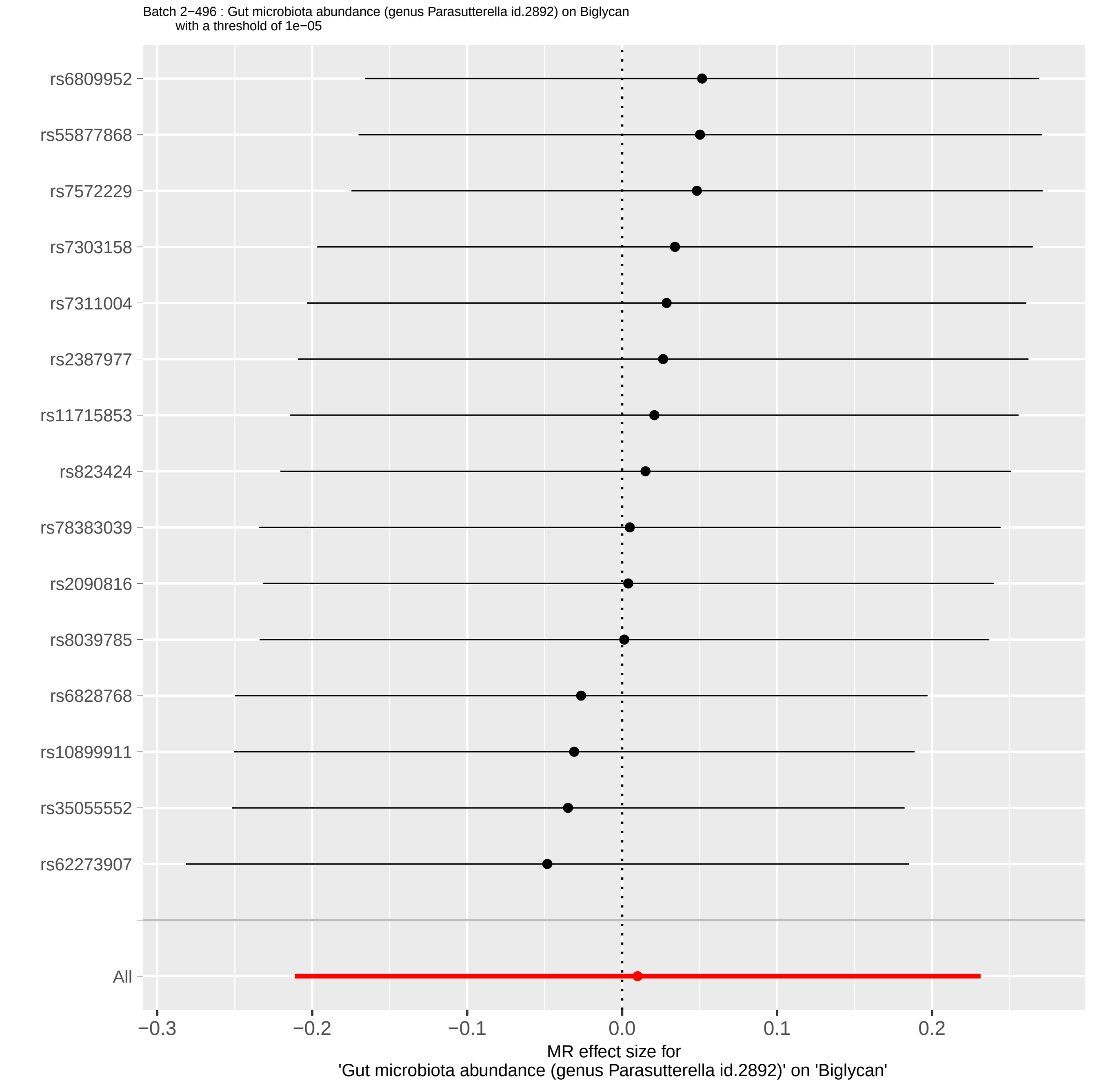
Batch 2−496 : Gut microbiota abundance (genus Parasutterella id.2892) on Biglycan
with a threshold of 1e−05
rs6809952
rs55877868
rs7572229
rs7303158
rs7311004
rs2387977
rs11715853
rs823424
rs78383039
rs2090816
rs8039785
rs6828768
rs10899911
rs35055552
rs62273907
All
−0.3
−0.2
−0.1
0.0
0.1
0.2
MR effect size for
'Gut microbiota abundance (genus Parasutterella id.2892)' on 'Biglycan'

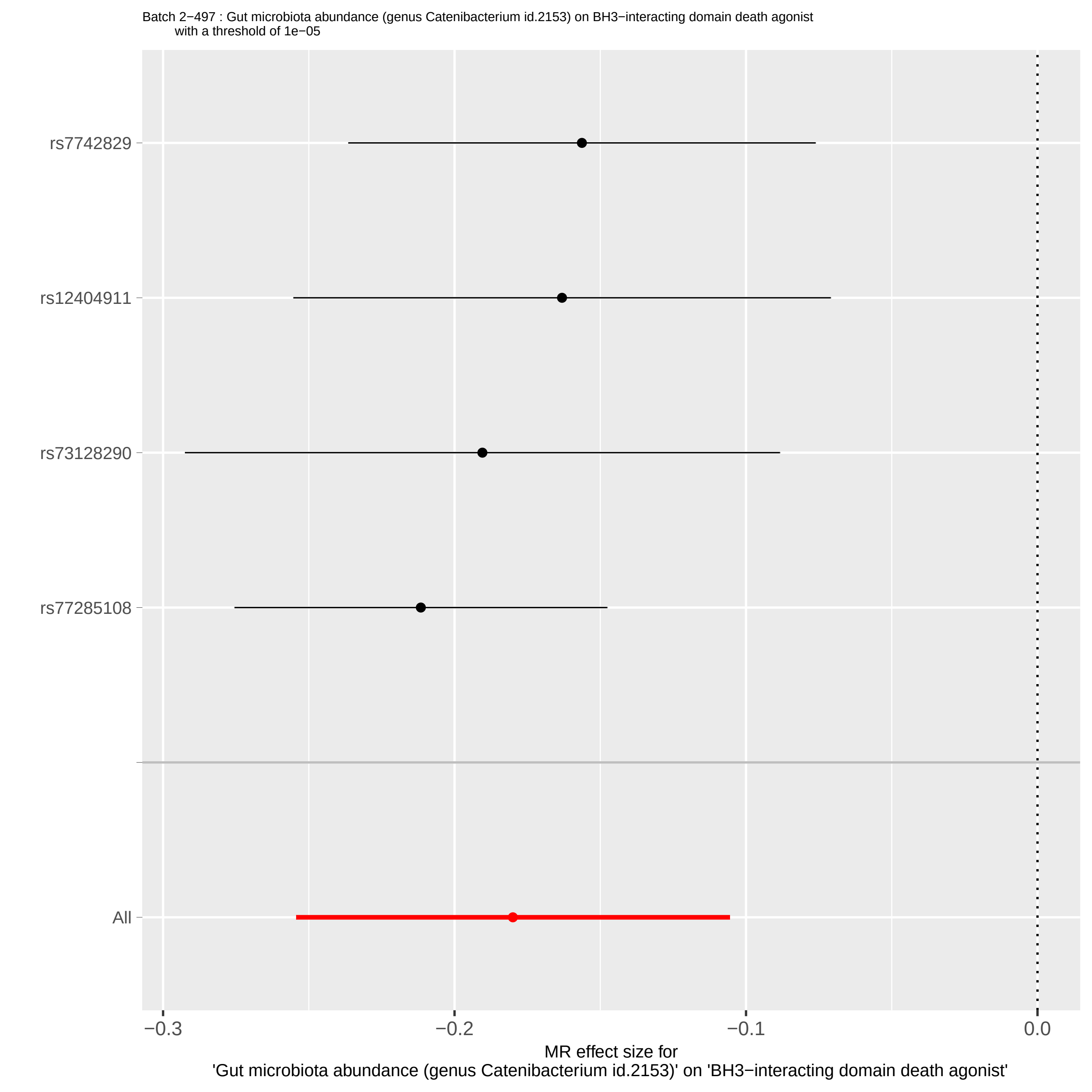

Batch 2−497 : Gut microbiota abundance (genus Catenibacterium id.2153) on BH3−interacting domain death agonist
with a threshold of 1e−05
rs7742829
rs12404911
rs73128290
rs77285108
All
−0.3
−0.2
−0.1
0.0
MR effect size for
'Gut microbiota abundance (genus Catenibacterium id.2153)' on 'BH3−interacting domain death agonist'

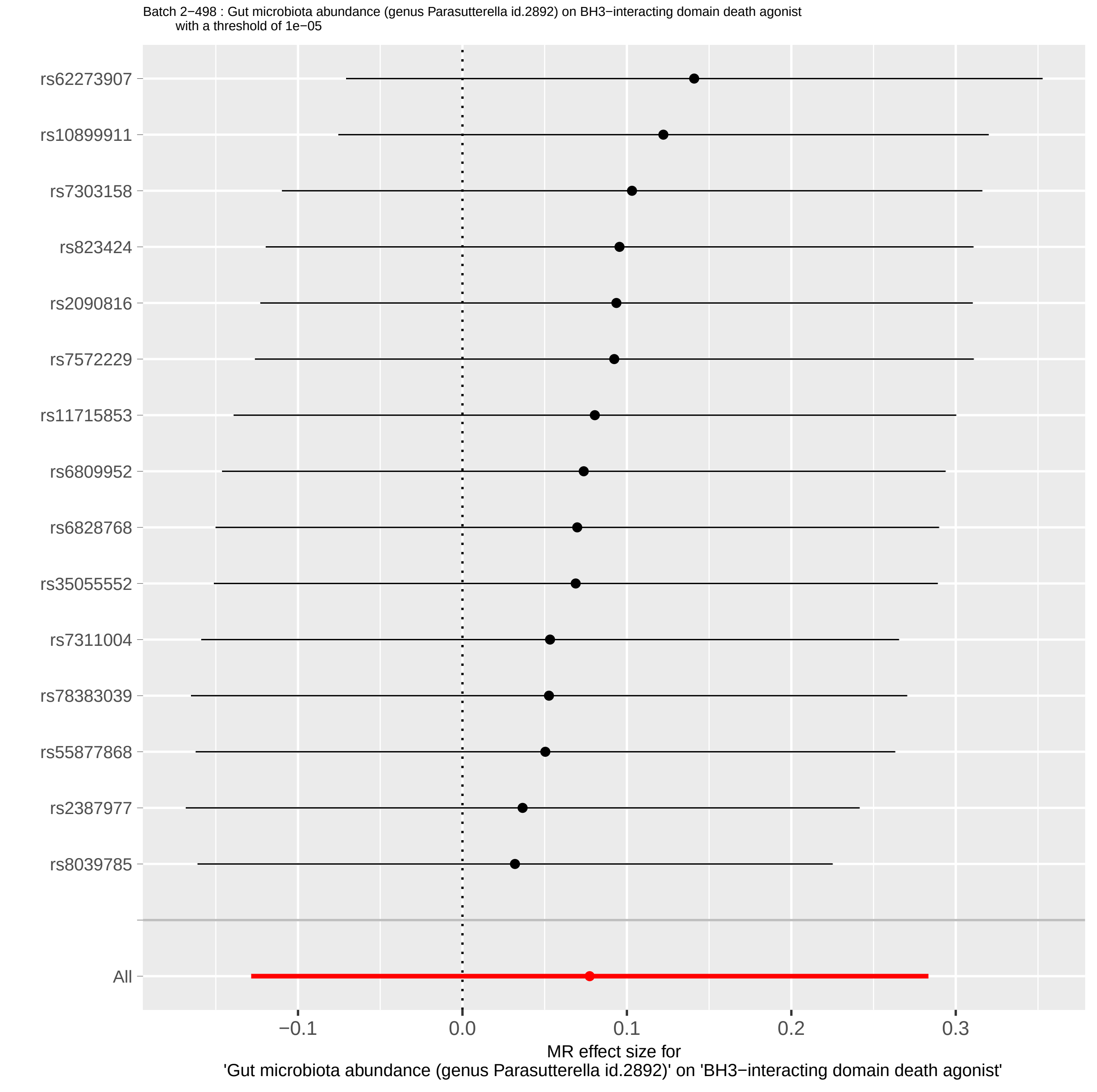

Batch 2−498 : Gut microbiota abundance (genus Parasutterella id.2892) on BH3−interacting domain death agonist
with a threshold of 1e−05
rs62273907
rs10899911
rs7303158
rs823424
rs2090816
rs7572229
rs11715853
rs6809952
rs6828768
rs35055552
rs7311004
rs78383039
rs55877868
rs2387977
rs8039785
All
−0.1
0.0
0.1
0.2
0.3
MR effect size for
'Gut microbiota abundance (genus Parasutterella id.2892)' on 'BH3−interacting domain death agonist'

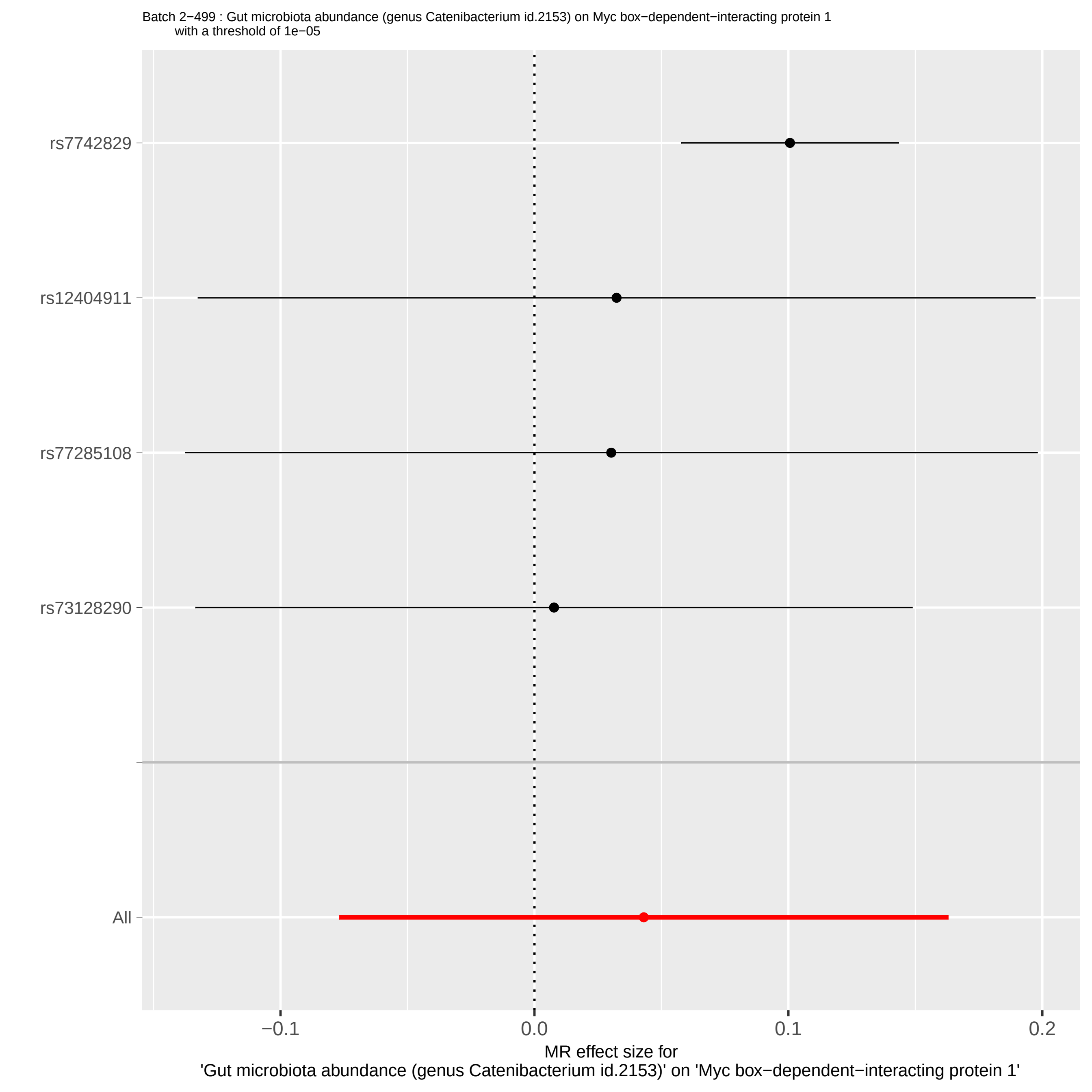
Batch 2−499 : Gut microbiota abundance (genus Catenibacterium id.2153) on Myc box−dependent−interacting protein 1
with a threshold of 1e−05
rs7742829
rs12404911
rs77285108
rs73128290
All
−0.1
0.0
0.1
0.2
MR effect size for
'Gut microbiota abundance (genus Catenibacterium id.2153)' on 'Myc box−dependent−interacting protein 1'

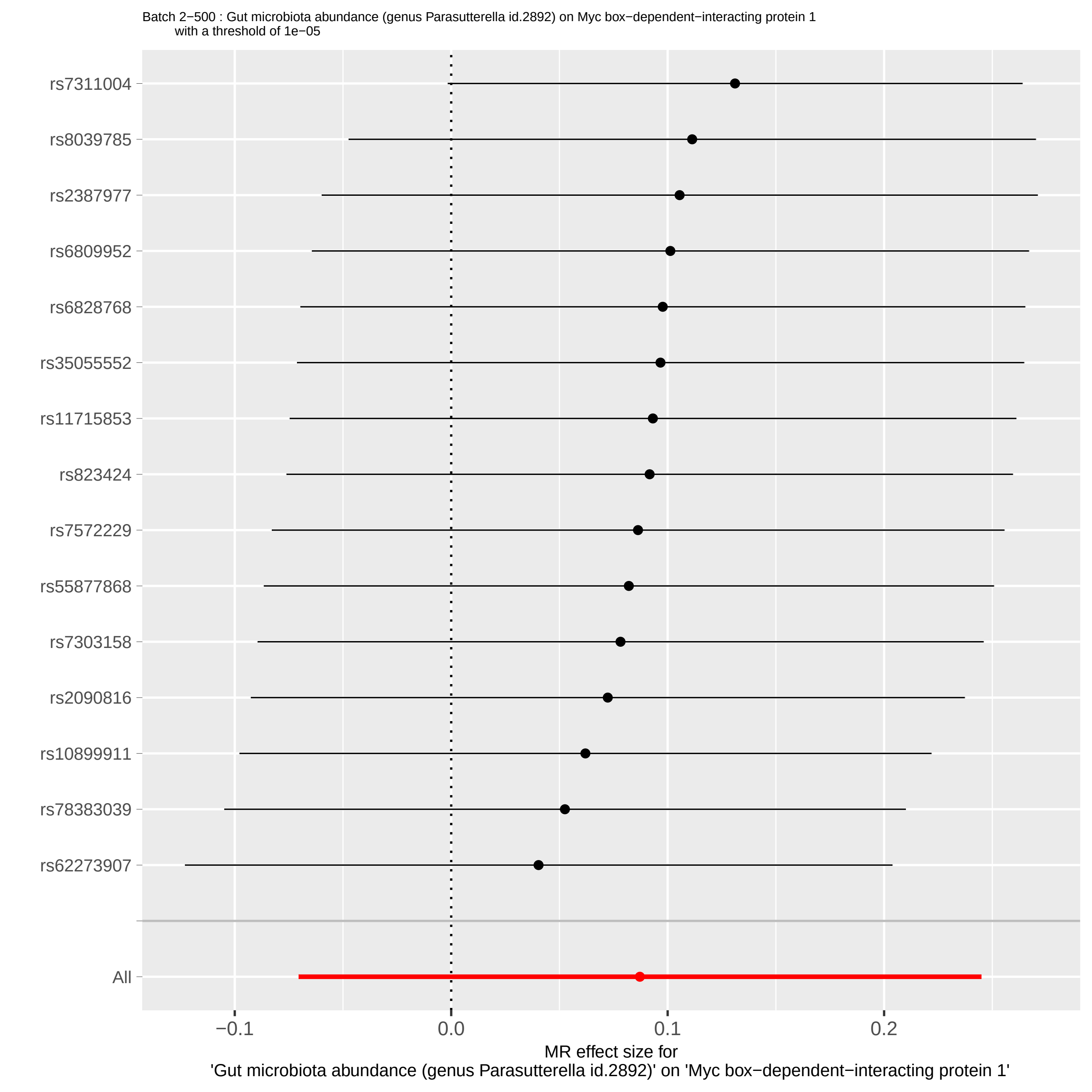

Batch 2−500 : Gut microbiota abundance (genus Parasutterella id.2892) on Myc box−dependent−interacting protein 1
with a threshold of 1e−05
rs7311004
rs8039785
rs2387977
rs6809952
rs6828768
rs35055552
rs11715853
rs823424
rs7572229
rs55877868
rs7303158
rs2090816
rs10899911
rs78383039
rs62273907
All
−0.1
0.0
0.1
0.2
MR effect size for
'Gut microbiota abundance (genus Parasutterella id.2892)' on 'Myc box−dependent−interacting protein 1'

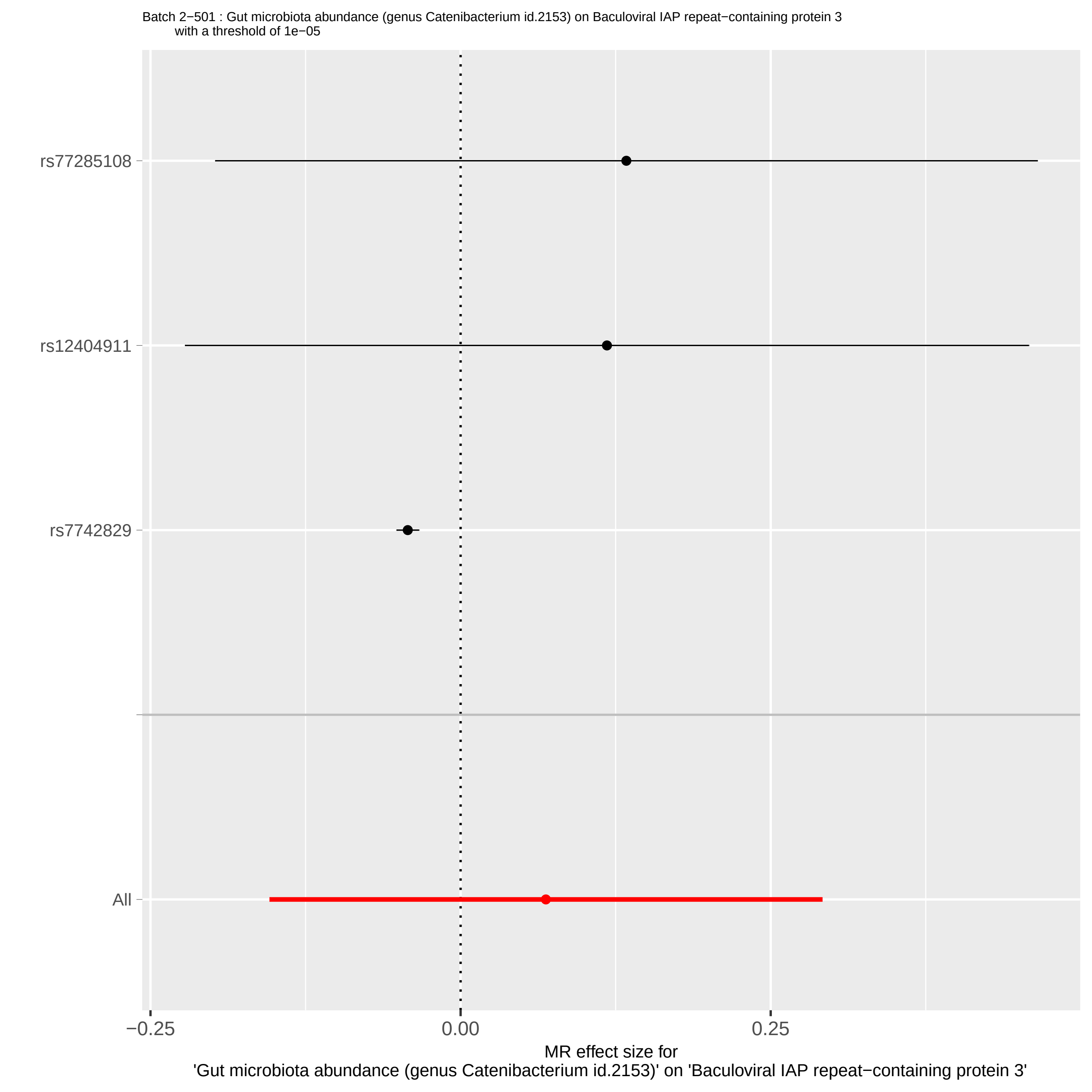
Batch 2−501 : Gut microbiota abundance (genus Catenibacterium id.2153) on Baculoviral IAP repeat−containing protein 3
with a threshold of 1e−05
rs77285108
rs12404911
rs7742829
All
−0.25
0.00
0.25
MR effect size for
'Gut microbiota abundance (genus Catenibacterium id.2153)' on 'Baculoviral IAP repeat−containing protein 3'

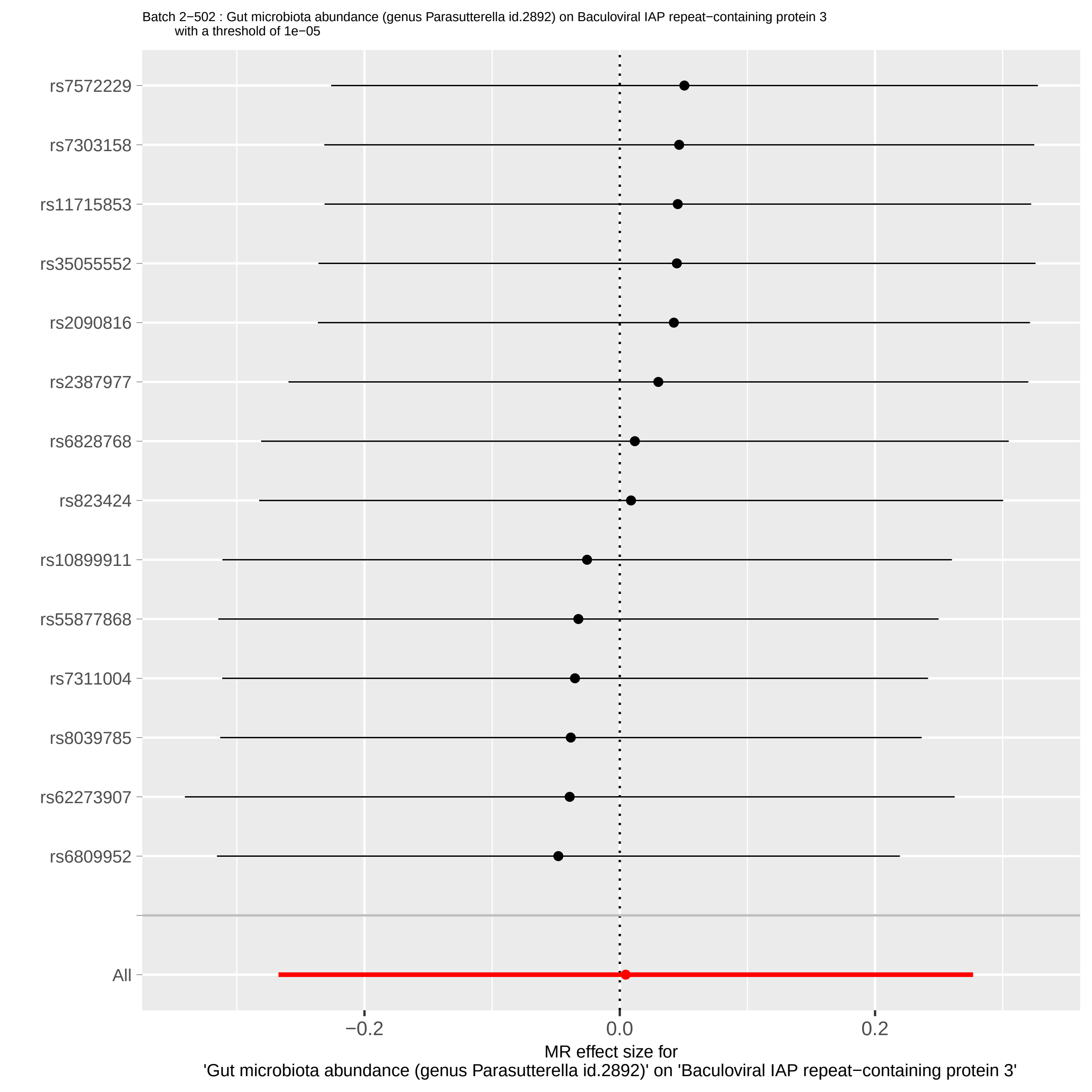
Batch 2−502 : Gut microbiota abundance (genus Parasutterella id.2892) on Baculoviral IAP repeat−containing protein 3
with a threshold of 1e−05
rs7572229
rs7303158
rs11715853
rs35055552
rs2090816
rs2387977
rs6828768
rs823424
rs10899911
rs55877868
rs7311004
rs8039785
rs62273907
rs6809952
All
−0.2
0.0
0.2
MR effect size for
'Gut microbiota abundance (genus Parasutterella id.2892)' on 'Baculoviral IAP repeat−containing protein 3'

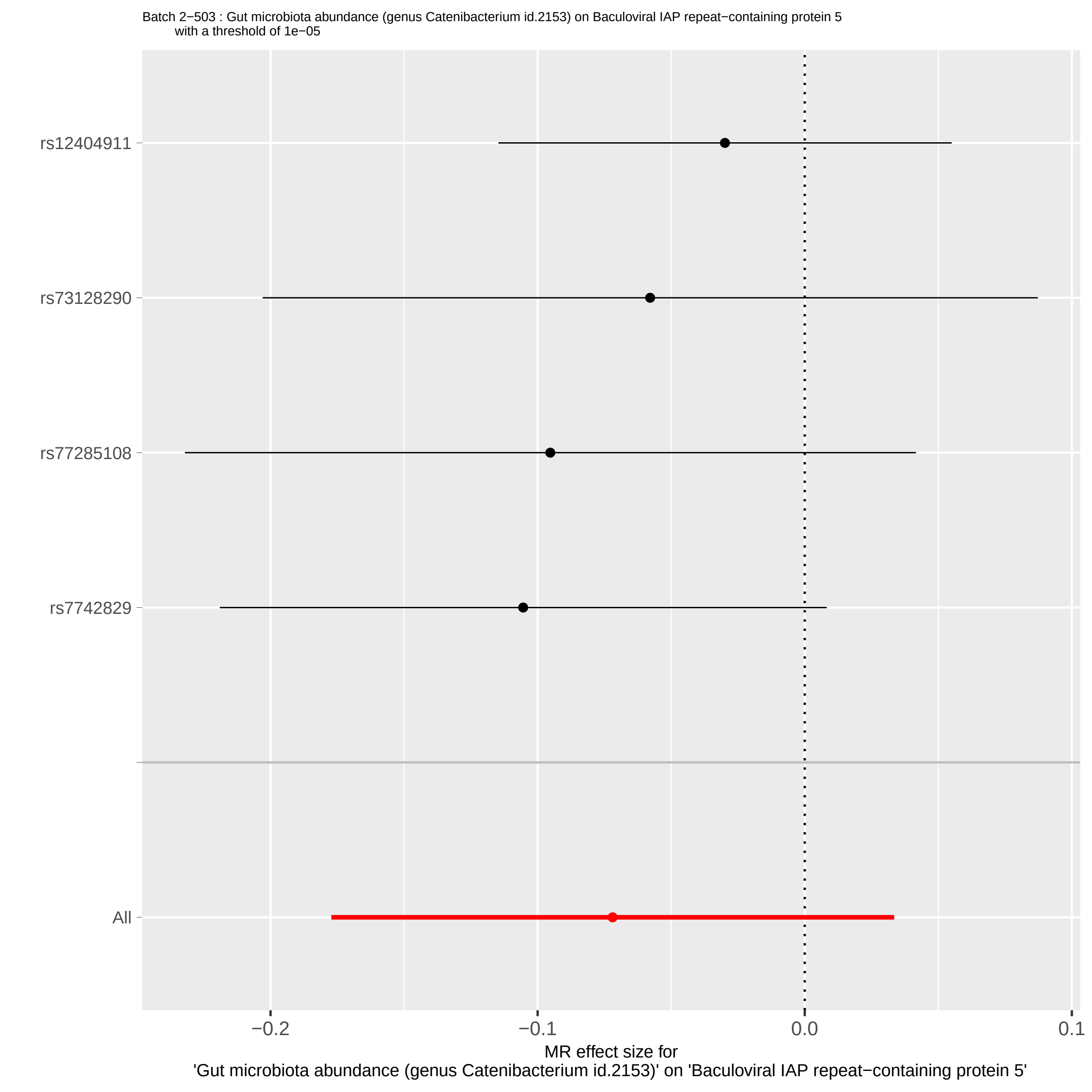

Batch 2−503 : Gut microbiota abundance (genus Catenibacterium id.2153) on Baculoviral IAP repeat−containing protein 5
with a threshold of 1e−05
rs12404911
rs73128290
rs77285108
rs7742829
All
−0.2
−0.1
0.0
0.1
MR effect size for
'Gut microbiota abundance (genus Catenibacterium id.2153)' on 'Baculoviral IAP repeat−containing protein 5'

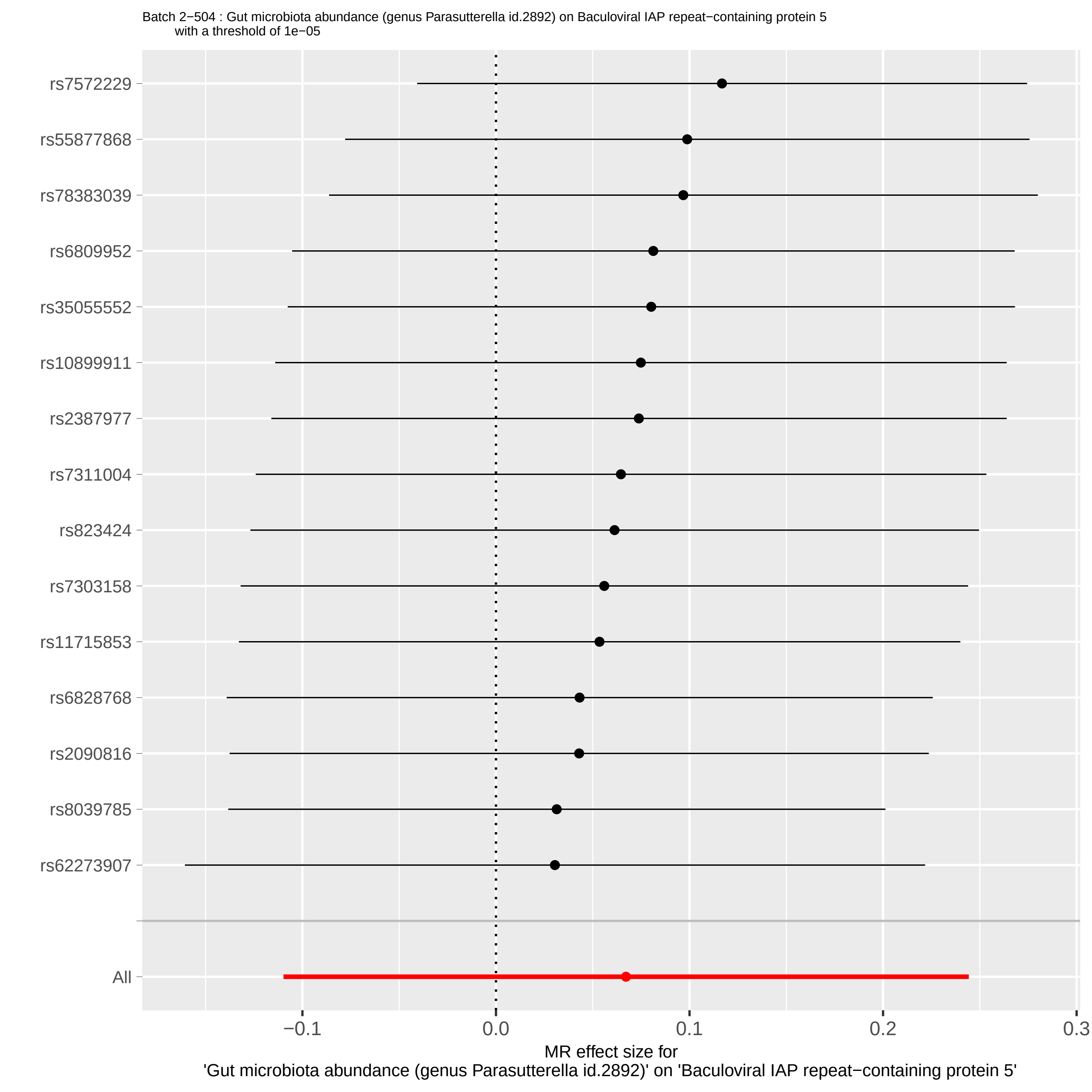

Batch 2−504 : Gut microbiota abundance (genus Parasutterella id.2892) on Baculoviral IAP repeat−containing protein 5
with a threshold of 1e−05
rs7572229
rs55877868
rs78383039
rs6809952
rs35055552
rs10899911
rs2387977
rs7311004
rs823424
rs7303158
rs11715853
rs6828768
rs2090816
rs8039785
rs62273907
All
−0.1
0.0
0.1
0.2
0.3
MR effect size for
'Gut microbiota abundance (genus Parasutterella id.2892)' on 'Baculoviral IAP repeat−containing protein 5'

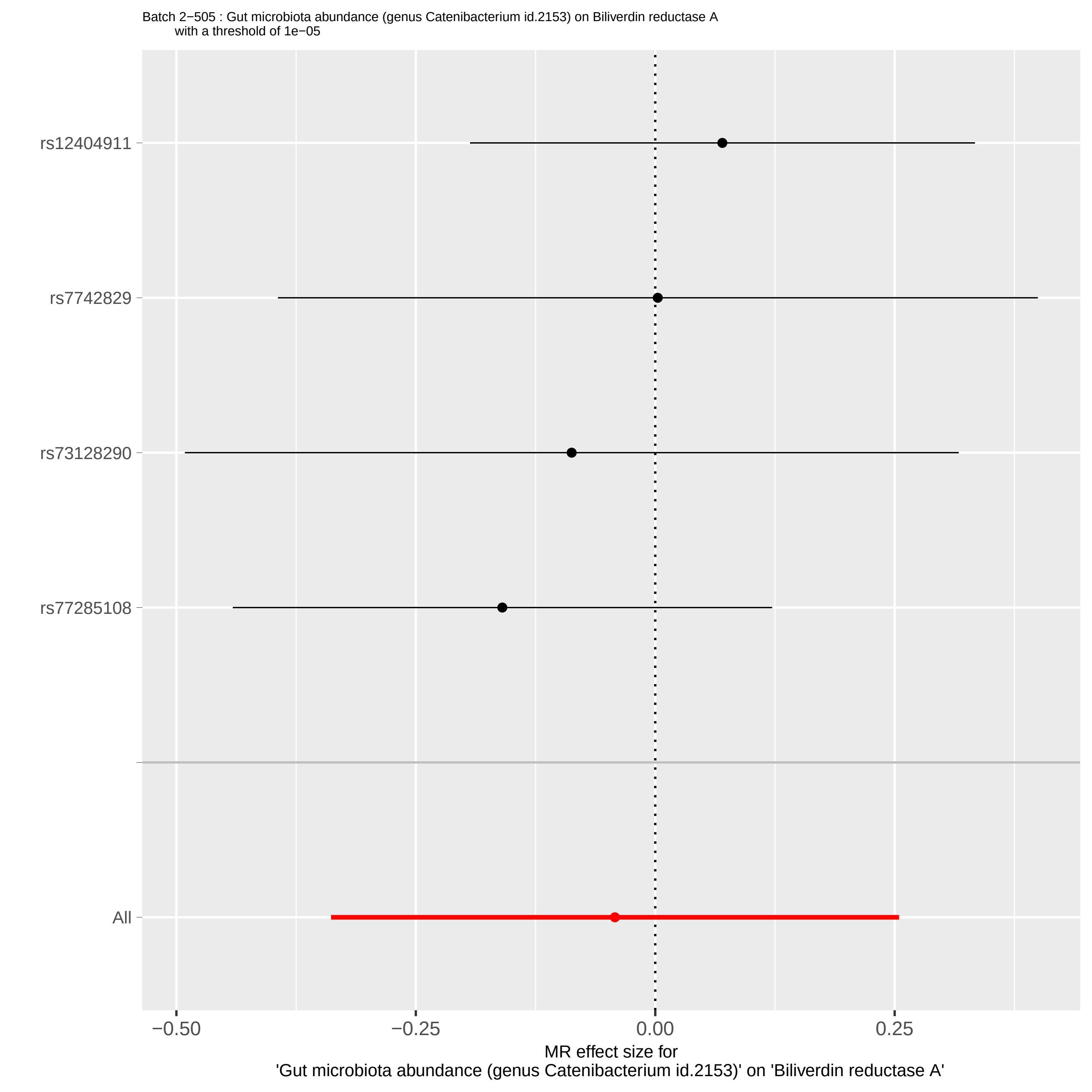
Batch 2−505 : Gut microbiota abundance (genus Catenibacterium id.2153) on Biliverdin reductase A
with a threshold of 1e−05
rs12404911
rs7742829
rs73128290
rs77285108
All
−0.50
−0.25
0.00
0.25
MR effect size for
'Gut microbiota abundance (genus Catenibacterium id.2153)' on 'Biliverdin reductase A'

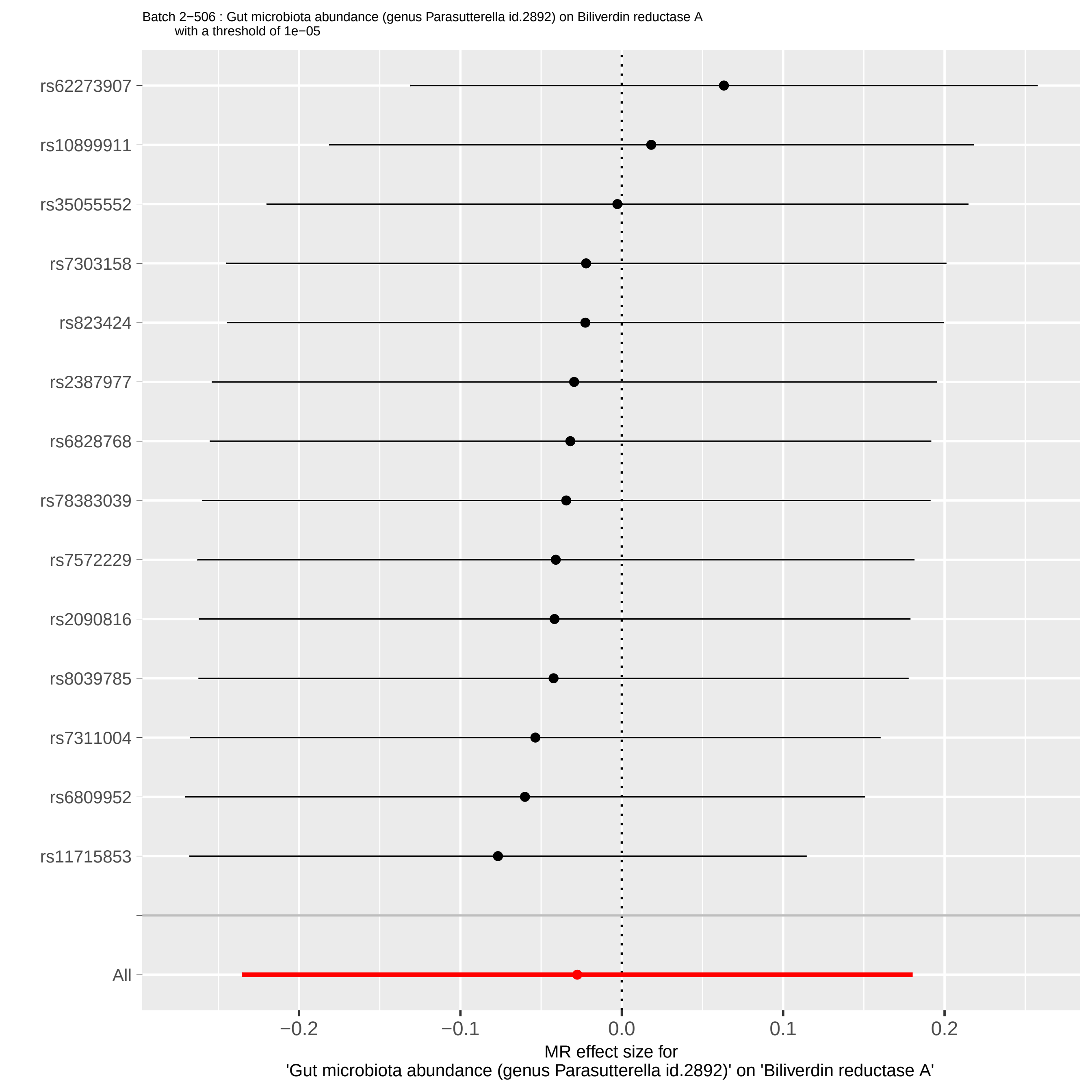
Batch 2−506 : Gut microbiota abundance (genus Parasutterella id.2892) on Biliverdin reductase A
with a threshold of 1e−05
rs62273907
rs10899911
rs35055552
rs7303158
rs823424
rs2387977
rs6828768
rs78383039
rs7572229
rs2090816
rs8039785
rs7311004
rs6809952
rs11715853
All
−0.2
−0.1
0.0
0.1
0.2
MR effect size for
'Gut microbiota abundance (genus Parasutterella id.2892)' on 'Biliverdin reductase A'

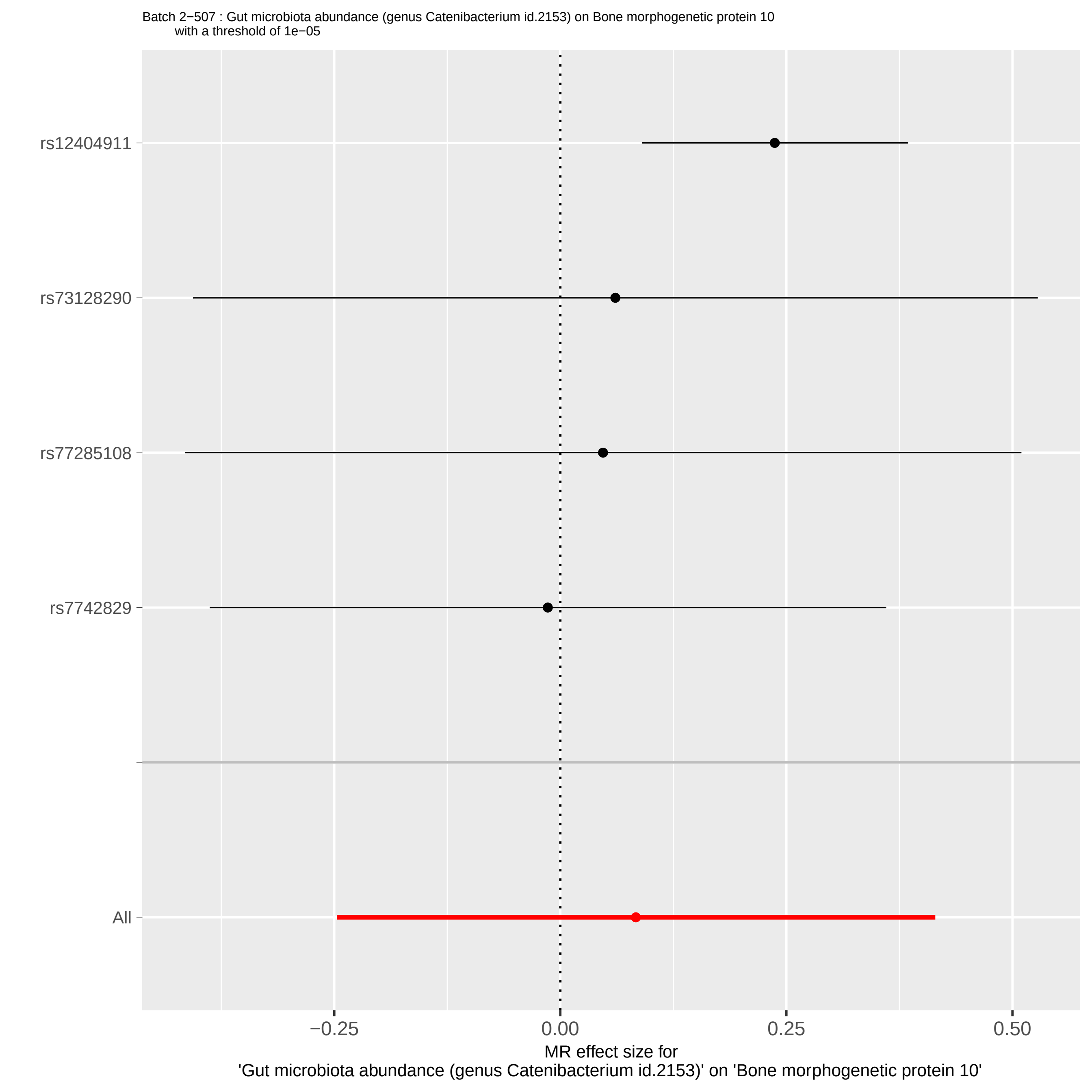

Batch 2−507 : Gut microbiota abundance (genus Catenibacterium id.2153) on Bone morphogenetic protein 10
with a threshold of 1e−05
rs12404911
rs73128290
rs77285108
rs7742829
All
−0.25
0.00
0.25
0.50
MR effect size for
'Gut microbiota abundance (genus Catenibacterium id.2153)' on 'Bone morphogenetic protein 10'

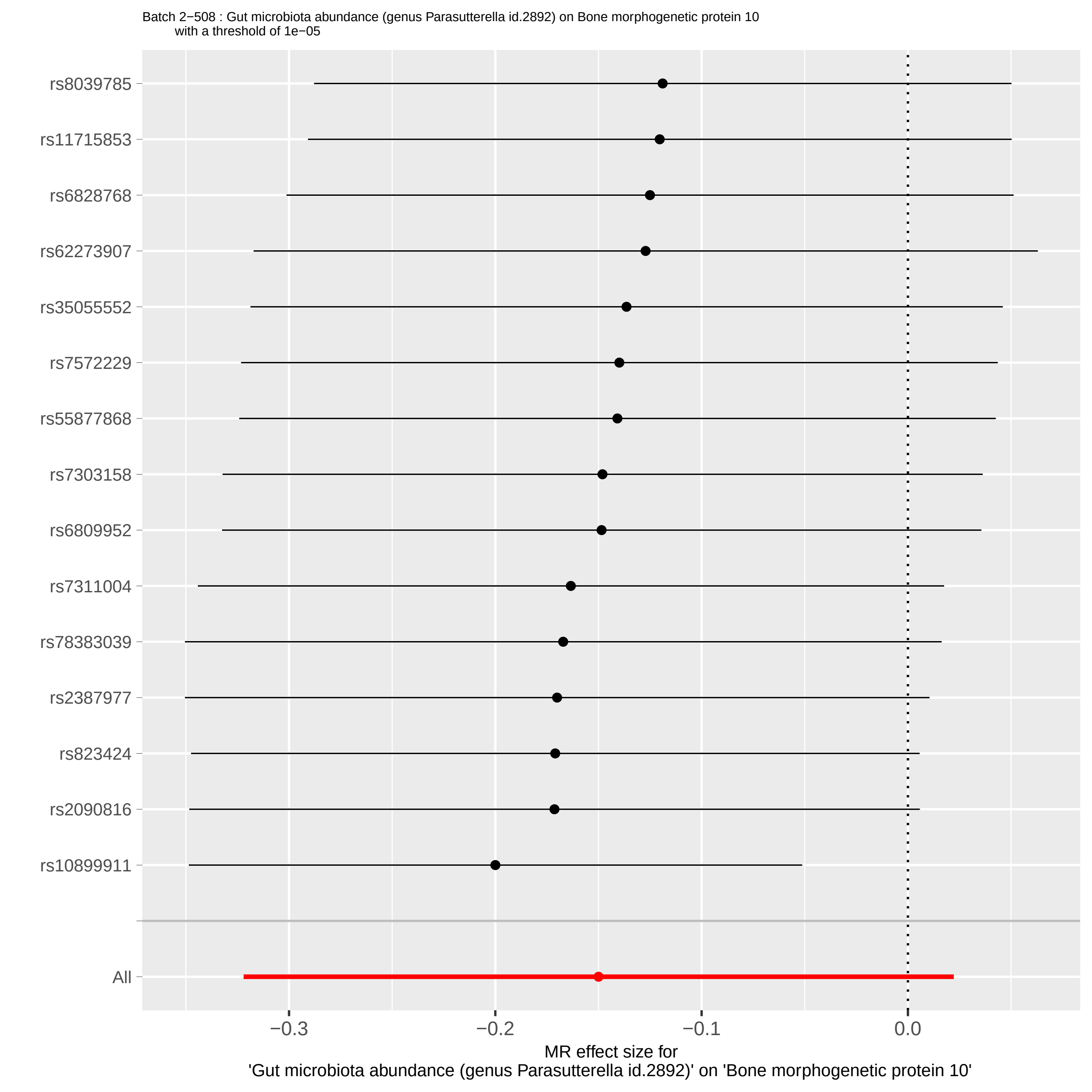

Batch 2−508 : Gut microbiota abundance (genus Parasutterella id.2892) on Bone morphogenetic protein 10
with a threshold of 1e−05
rs8039785
rs11715853
rs6828768
rs62273907
rs35055552
rs7572229
rs55877868
rs7303158
rs6809952
rs7311004
rs78383039
rs2387977
rs823424
rs2090816
rs10899911
All
−0.3
−0.2
−0.1
0.0
MR effect size for
'Gut microbiota abundance (genus Parasutterella id.2892)' on 'Bone morphogenetic protein 10'

Batch 2−509 : Gut microbiota abundance (genus Catenibacterium id.2153) on Bone morphogenetic protein 6
with a threshold of 1e−05

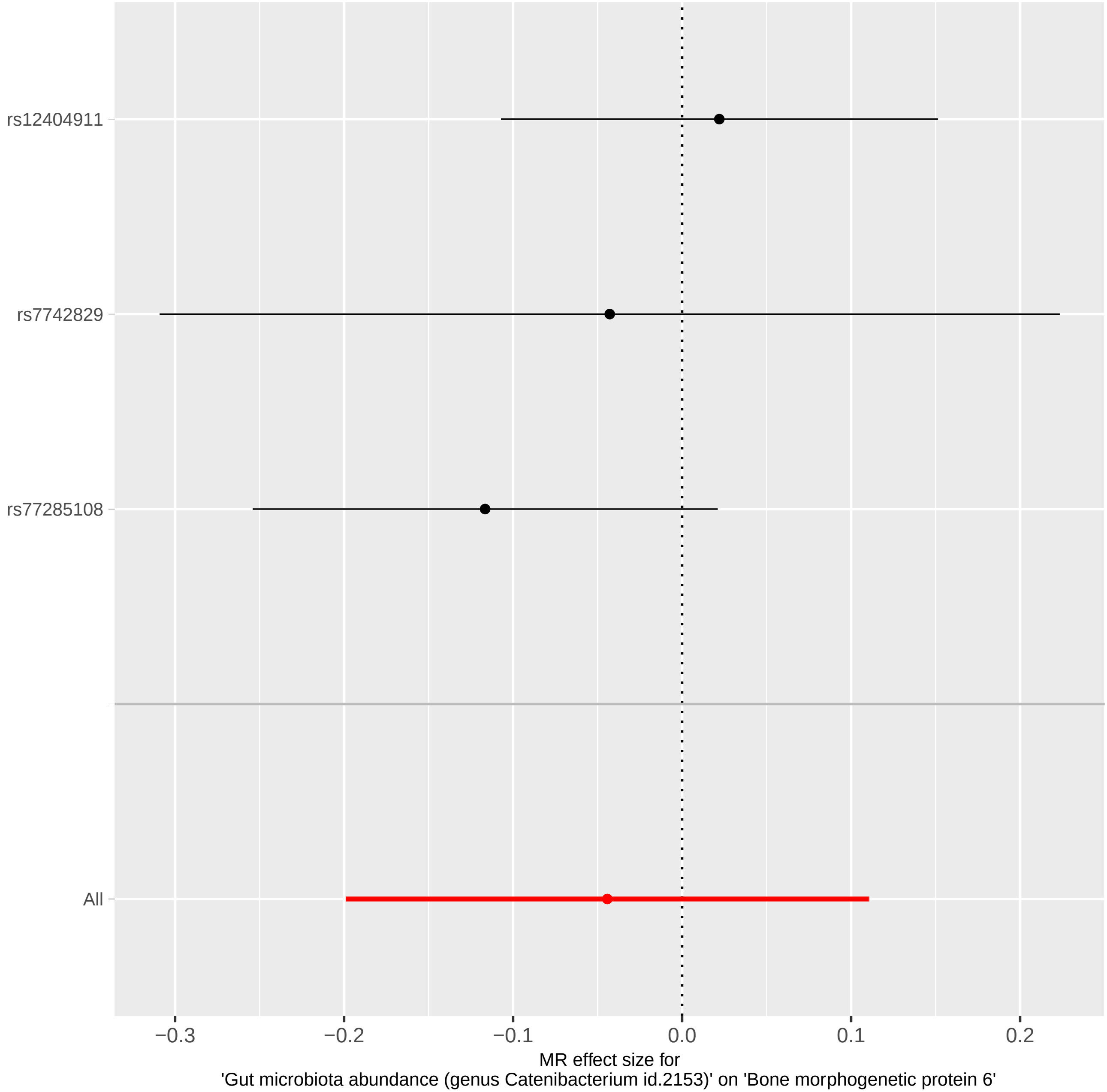

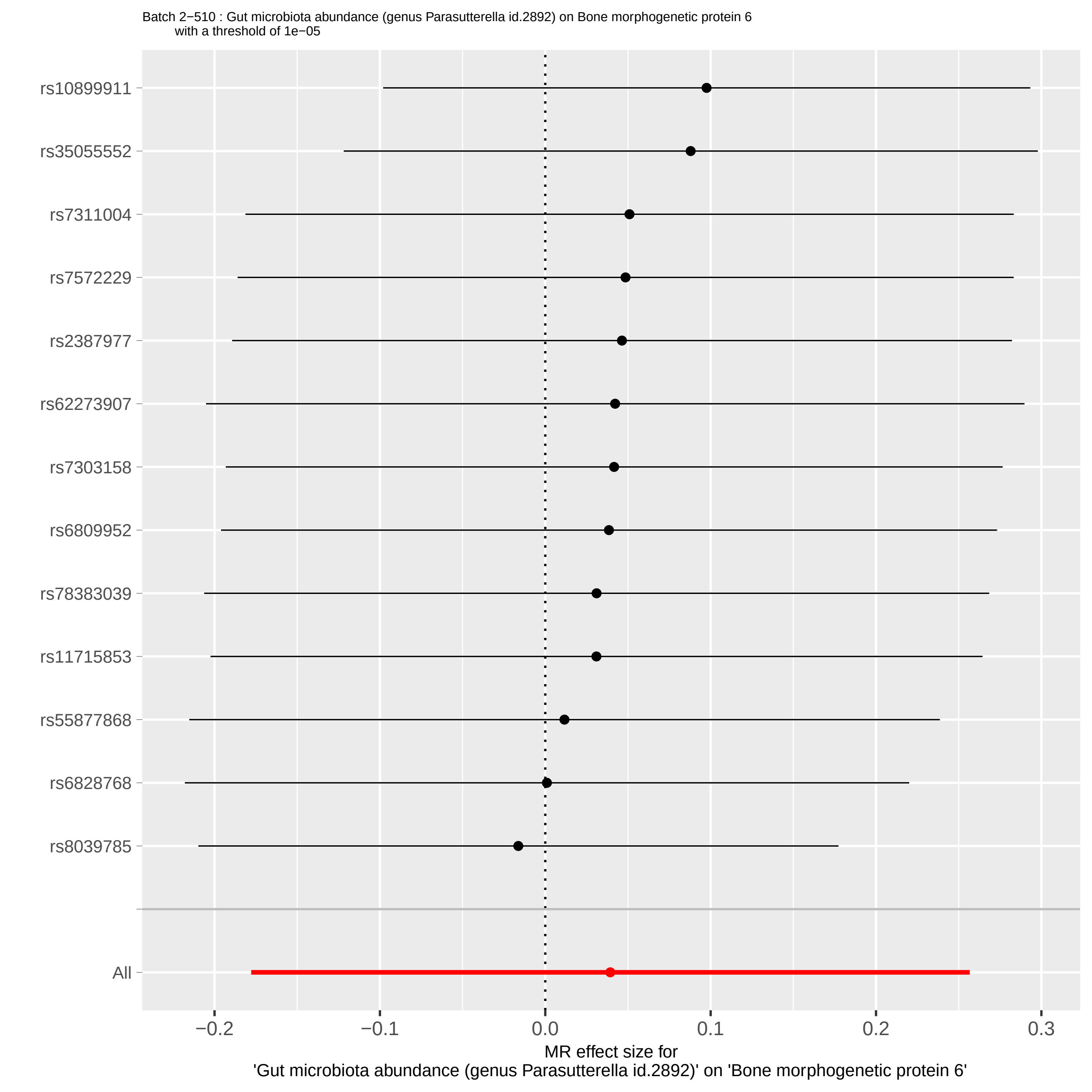
Batch 2−510 : Gut microbiota abundance (genus Parasutterella id.2892) on Bone morphogenetic protein 6
with a threshold of 1e−05
rs10899911
rs35055552
rs7311004
rs7572229
rs2387977
rs62273907
rs7303158
rs6809952
rs78383039
rs11715853
rs55877868
rs6828768
rs8039785
All
−0.2
−0.1
0.0
0.1
0.2
0.3
MR effect size for
'Gut microbiota abundance (genus Parasutterella id.2892)' on 'Bone morphogenetic protein 6'

Batch 2−512 : Gut microbiota abundance (genus Parasutterella id.2892) on Bone morphogenetic protein 6
with a threshold of 1e−05

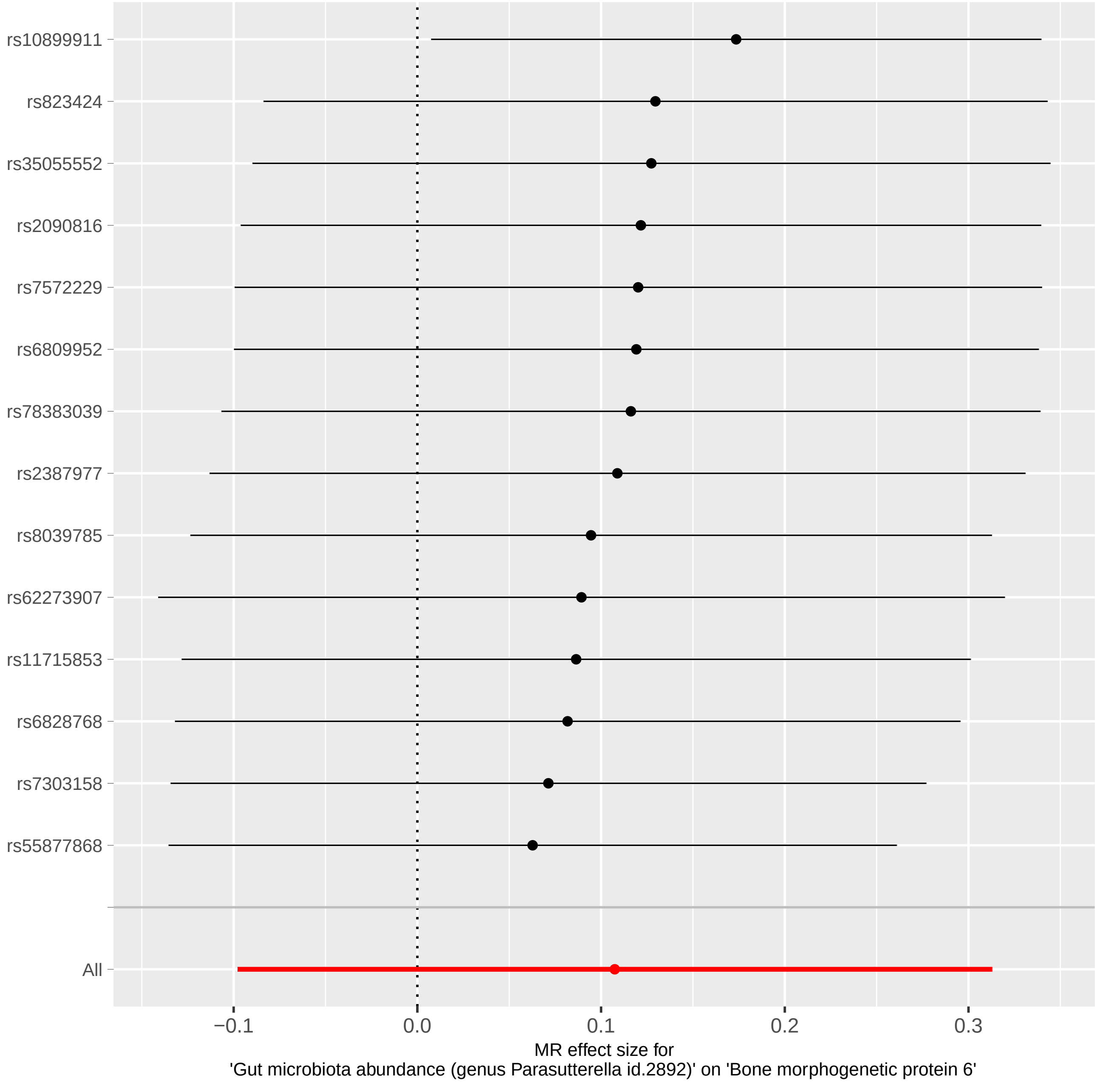

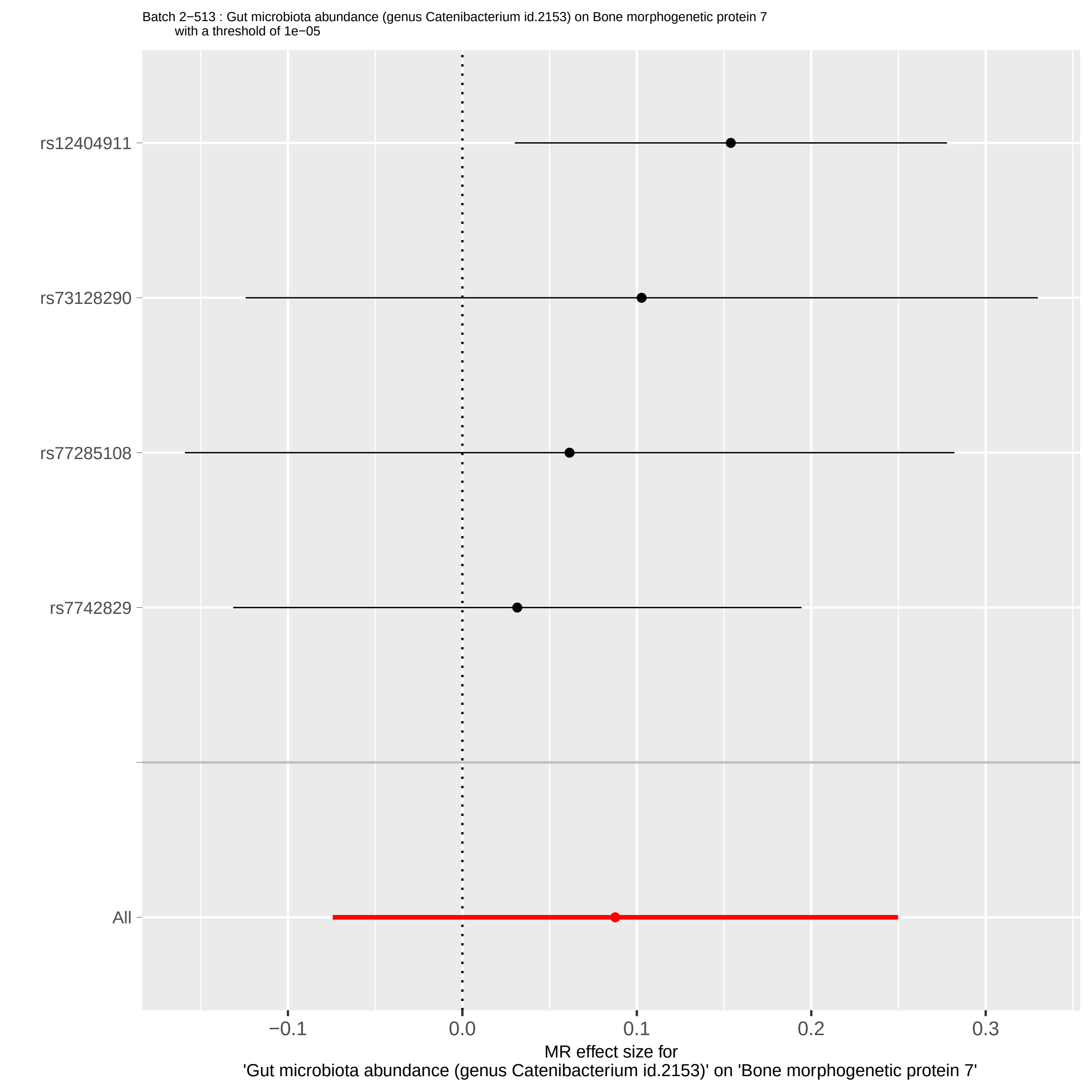
Batch 2−513 : Gut microbiota abundance (genus Catenibacterium id.2153) on Bone morphogenetic protein 7
with a threshold of 1e−05
rs12404911
rs73128290
rs77285108
rs7742829
All
−0.1
0.0
0.1
0.2
0.3
MR effect size for
'Gut microbiota abundance (genus Catenibacterium id.2153)' on 'Bone morphogenetic protein 7'

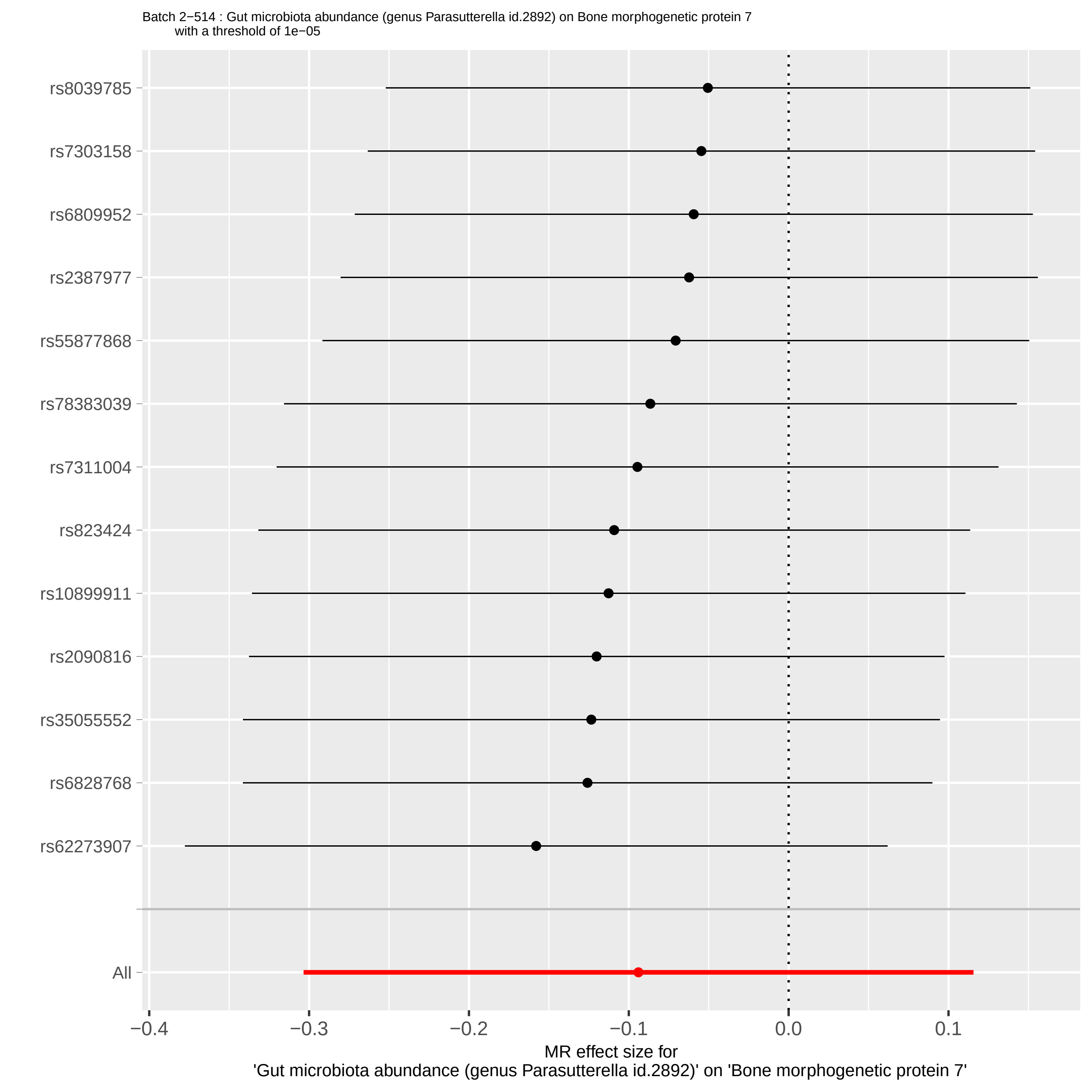
Batch 2−514 : Gut microbiota abundance (genus Parasutterella id.2892) on Bone morphogenetic protein 7
with a threshold of 1e−05
rs8039785
rs7303158
rs6809952
rs2387977
rs55877868
rs78383039
rs7311004
rs823424
rs10899911
rs2090816
rs35055552
rs6828768
rs62273907
All
−0.4
−0.3
−0.2
−0.1
0.0
0.1
MR effect size for
'Gut microbiota abundance (genus Parasutterella id.2892)' on 'Bone morphogenetic protein 7'

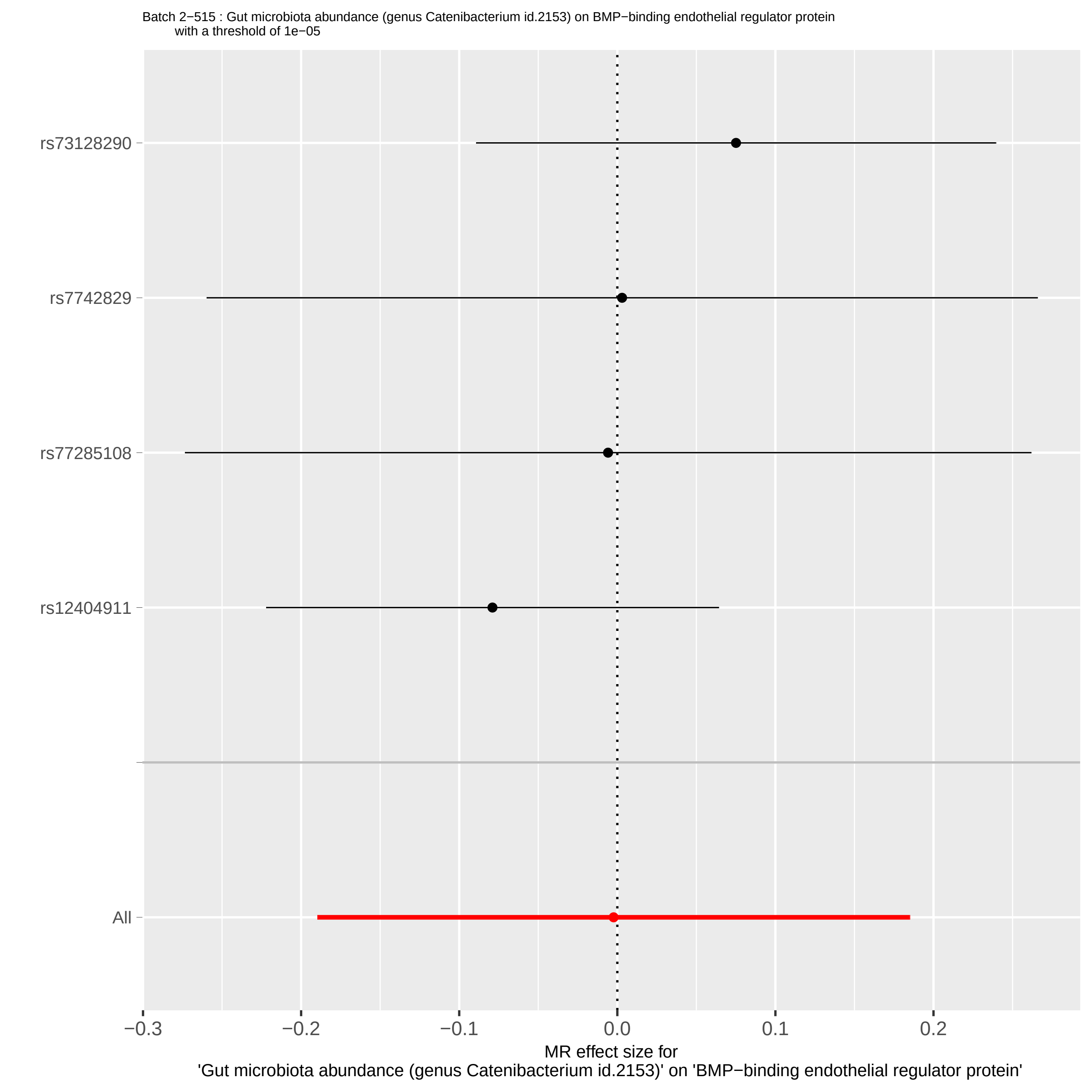
Batch 2−515 : Gut microbiota abundance (genus Catenibacterium id.2153) on BMP−binding endothelial regulator protein
with a threshold of 1e−05
rs73128290
rs7742829
rs77285108
rs12404911
All
−0.3
−0.2
−0.1
0.0
0.1
0.2
MR effect size for
'Gut microbiota abundance (genus Catenibacterium id.2153)' on 'BMP−binding endothelial regulator protein'

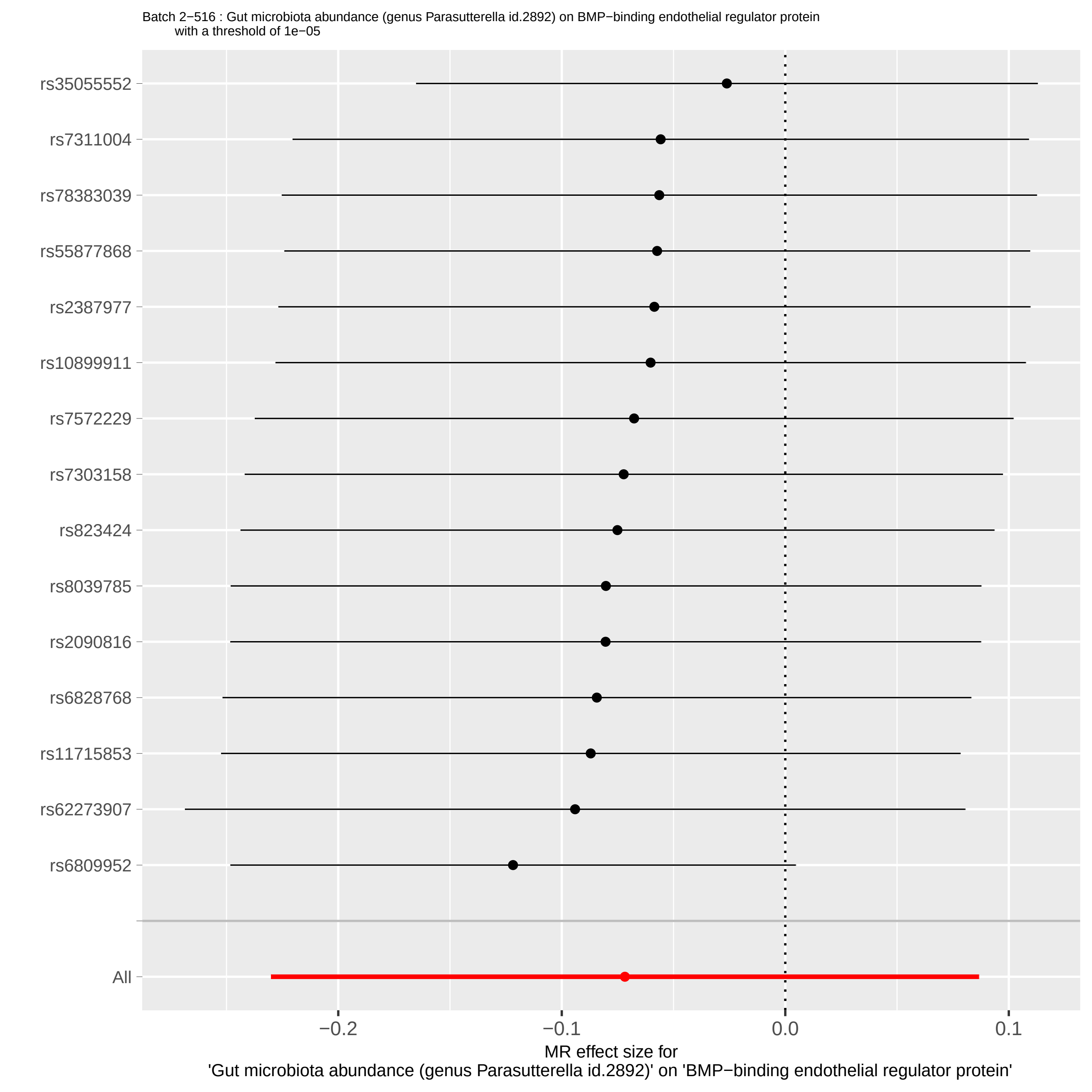
Batch 2−516 : Gut microbiota abundance (genus Parasutterella id.2892) on BMP−binding endothelial regulator protein
with a threshold of 1e−05
rs35055552
rs7311004
rs78383039
rs55877868
rs2387977
rs10899911
rs7572229
rs7303158
rs823424
rs8039785
rs2090816
rs6828768
rs11715853
rs62273907
rs6809952
All
−0.2
−0.1
0.0
0.1
MR effect size for
'Gut microbiota abundance (genus Parasutterella id.2892)' on 'BMP−binding endothelial regulator protein'

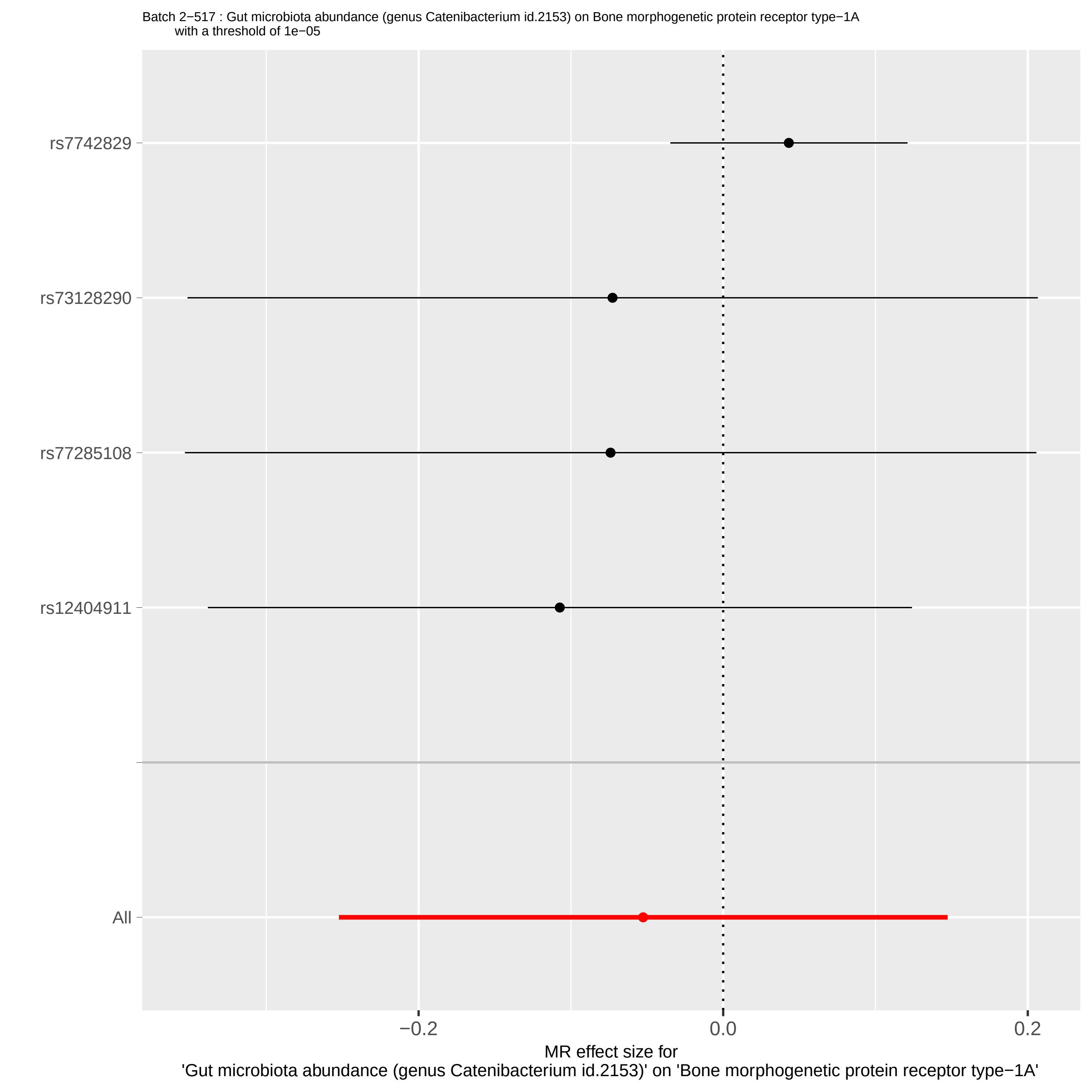
Batch 2−517 : Gut microbiota abundance (genus Catenibacterium id.2153) on Bone morphogenetic protein receptor type−1A
with a threshold of 1e−05
rs7742829
rs73128290
rs77285108
rs12404911
All
−0.2
0.0
0.2
MR effect size for
'Gut microbiota abundance (genus Catenibacterium id.2153)' on 'Bone morphogenetic protein receptor type−1A'

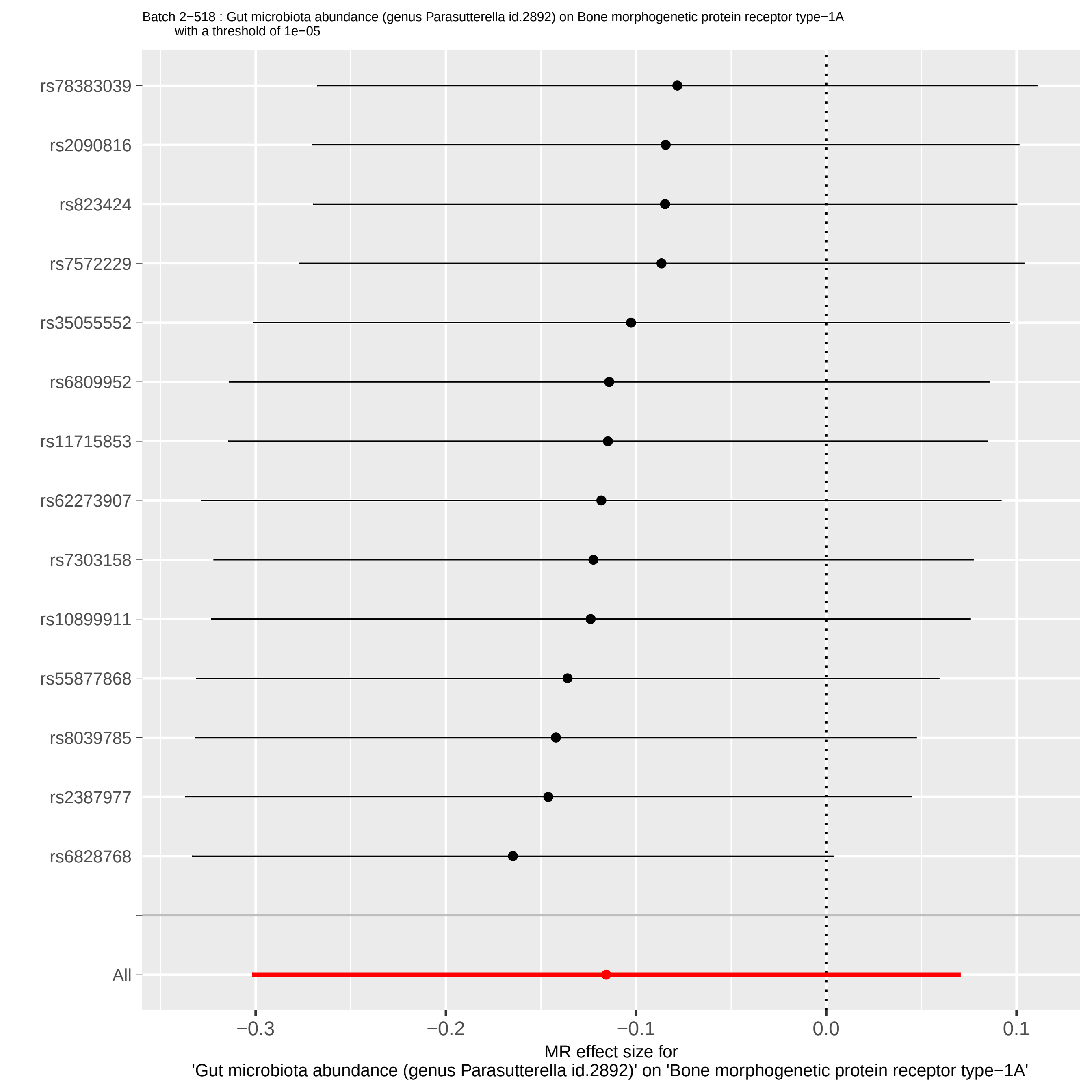
Batch 2−518 : Gut microbiota abundance (genus Parasutterella id.2892) on Bone morphogenetic protein receptor type−1A
with a threshold of 1e−05
rs78383039
rs2090816
rs823424
rs7572229
rs35055552
rs6809952
rs11715853
rs62273907
rs7303158
rs10899911
rs55877868
rs8039785
rs2387977
rs6828768
All
−0.3
−0.2
−0.1
0.0
0.1
MR effect size for
'Gut microbiota abundance (genus Parasutterella id.2892)' on 'Bone morphogenetic protein receptor type−1A'

Batch 2−519 : Gut microbiota abundance (genus Catenibacterium id.2153) on Cytoplasmic tyrosine−protein kinase BMX
with a threshold of 1e−05

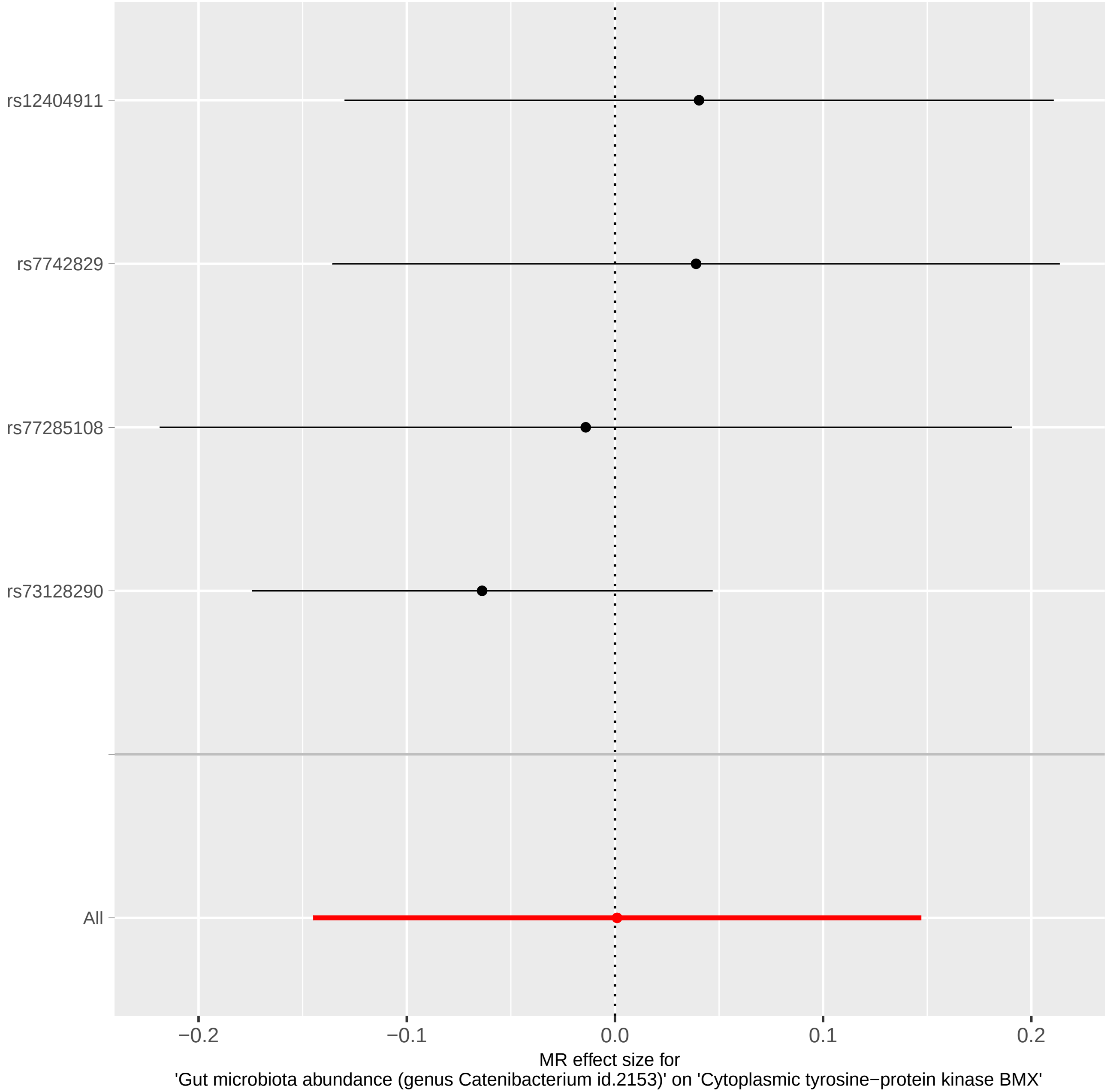

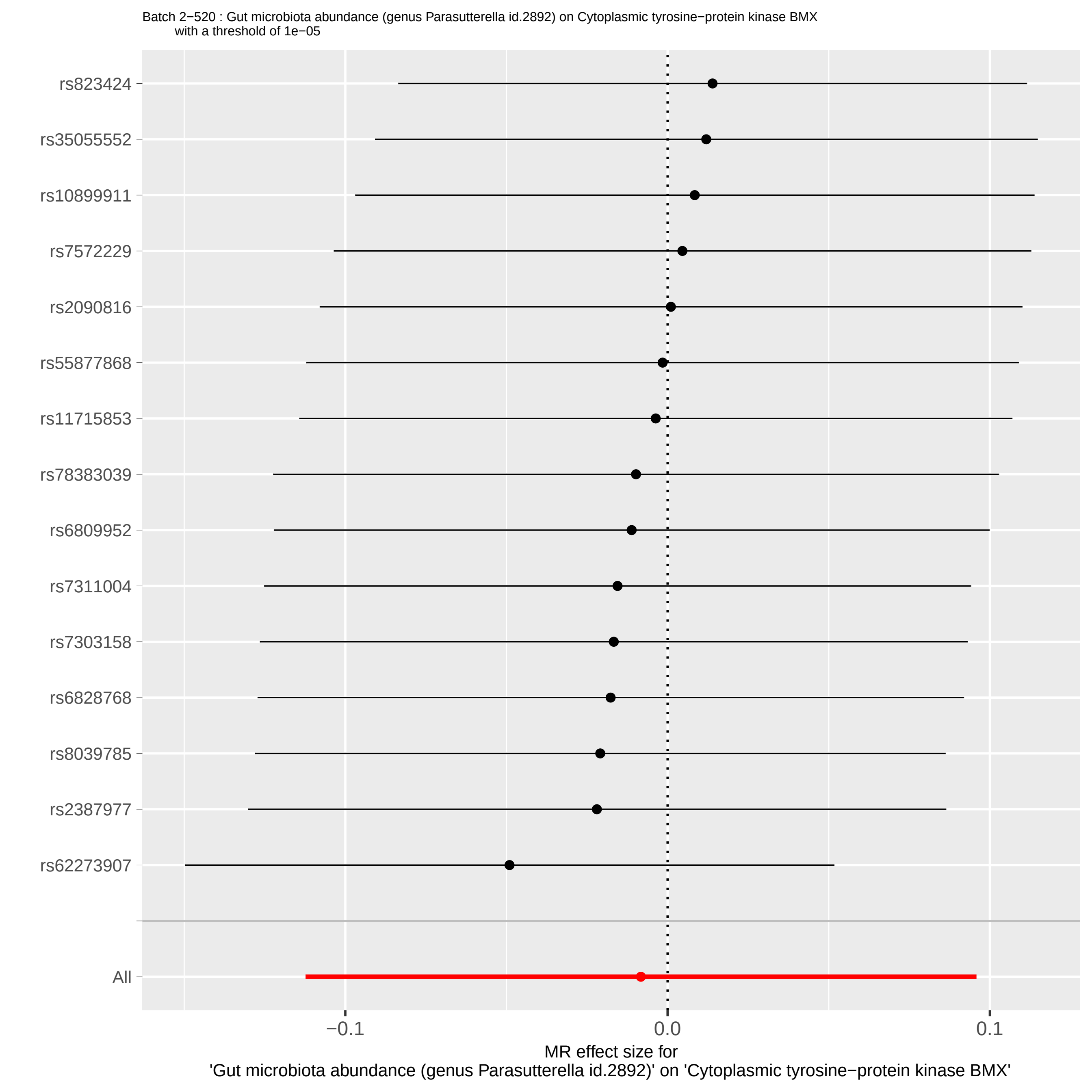

Batch 2−520 : Gut microbiota abundance (genus Parasutterella id.2892) on Cytoplasmic tyrosine−protein kinase BMX
with a threshold of 1e−05
rs823424
rs35055552
rs10899911
rs7572229
rs2090816
rs55877868
rs11715853
rs78383039
rs6809952
rs7311004
rs7303158
rs6828768
rs8039785
rs2387977
rs62273907
All
−0.1
0.0
0.1
MR effect size for
'Gut microbiota abundance (genus Parasutterella id.2892)' on 'Cytoplasmic tyrosine−protein kinase BMX'

Batch 2−521 : Gut microbiota abundance (genus Catenibacterium id.2153) on BCL2/adenovirus E1B 19 kDa protein−interacting protein 3
with a threshold of 1e−05

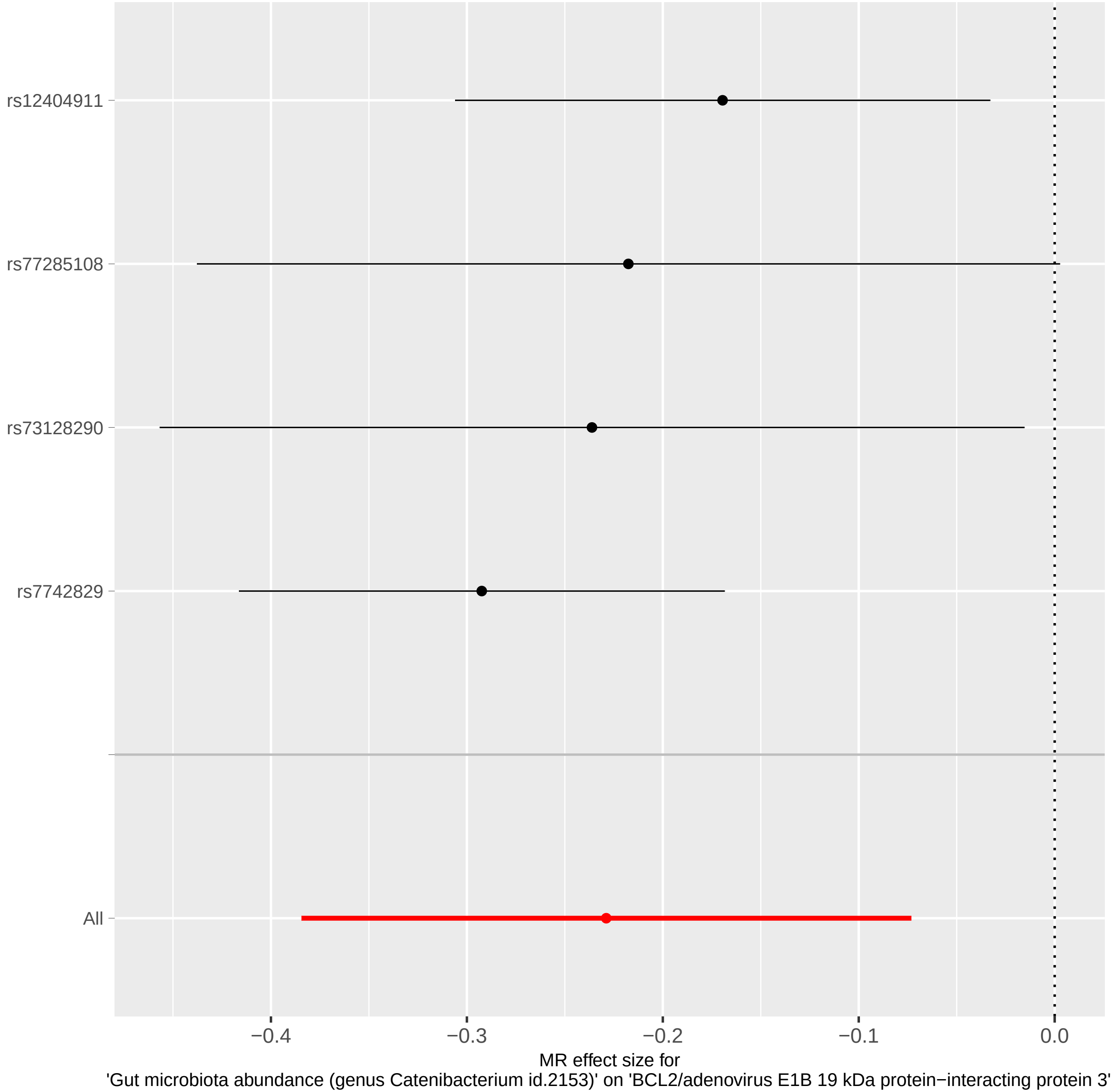

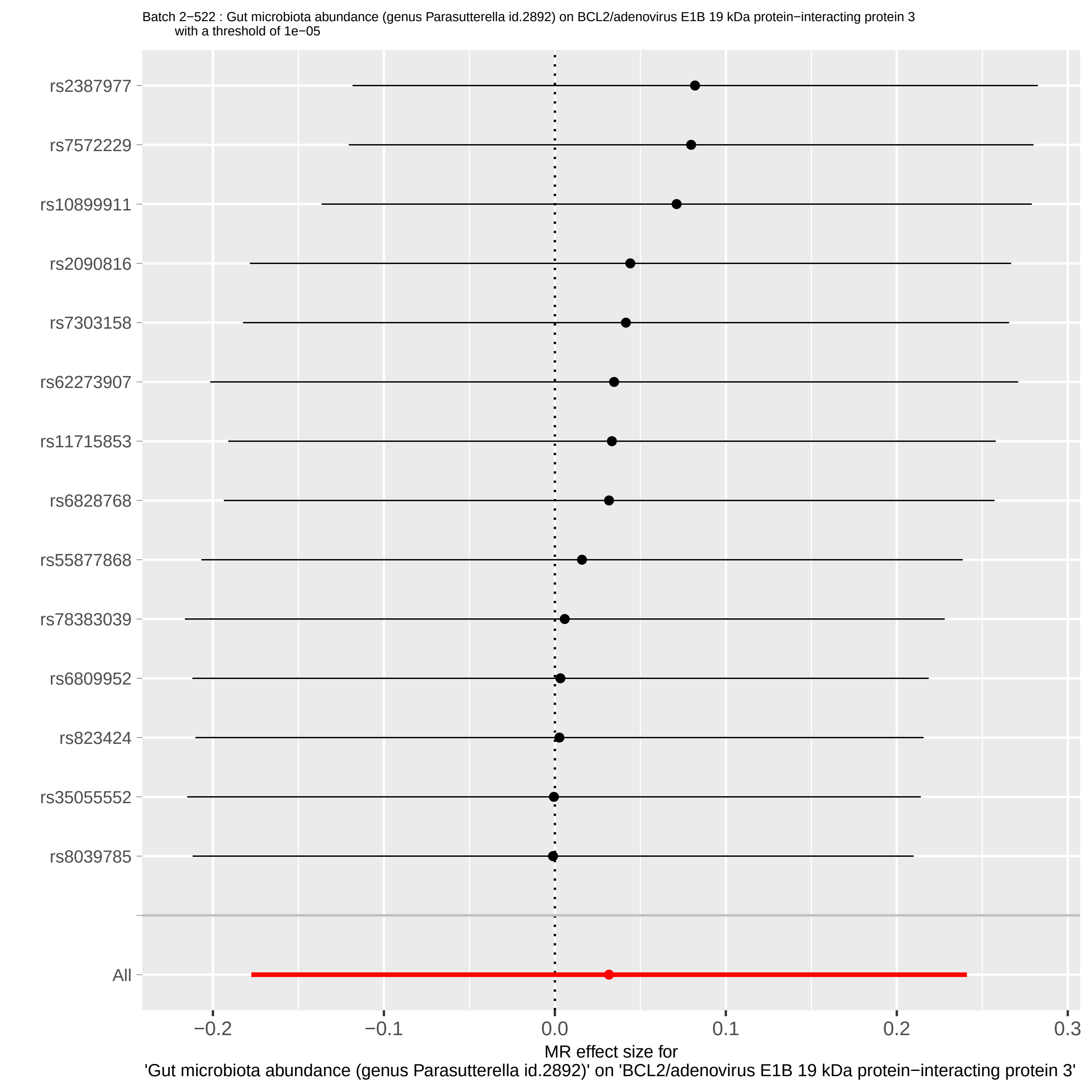

Batch 2−522 : Gut microbiota abundance (genus Parasutterella id.2892) on BCL2/adenovirus E1B 19 kDa protein−interacting protein 3
with a threshold of 1e−05
rs2387977
rs7572229
rs10899911
rs2090816
rs7303158
rs62273907
rs11715853
rs6828768
rs55877868
rs78383039
rs6809952
rs823424
rs35055552
rs8039785
All
−0.2
−0.1
0.0
0.1
0.2
0.3
MR effect size for
'Gut microbiota abundance (genus Parasutterella id.2892)' on 'BCL2/adenovirus E1B 19 kDa protein−interacting protein 3'

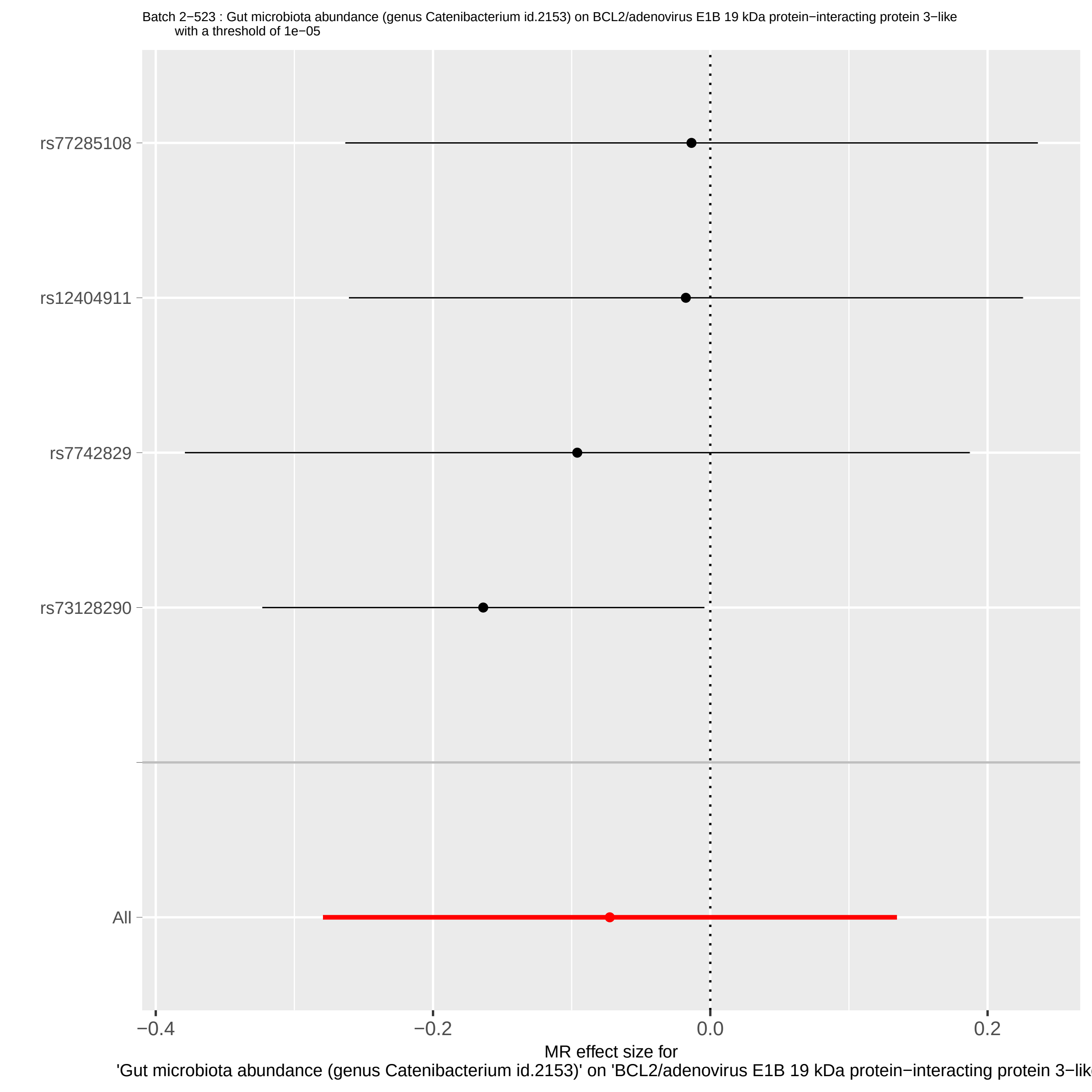
Batch 2−523 : Gut microbiota abundance (genus Catenibacterium id.2153) on BCL2/adenovirus E1B 19 kDa protein−interacting protein 3−like
with a threshold of 1e−05
rs77285108
rs12404911
rs7742829
rs73128290
All
−0.4
−0.2
0.0
0.2
MR effect size for
'Gut microbiota abundance (genus Catenibacterium id.2153)' on 'BCL2/adenovirus E1B 19 kDa protein−interacting protein 3−lik

Batch 2−524 : Gut microbiota abundance (genus Parasutterella id.2892) on BCL2/adenovirus E1B 19 kDa protein−interacting protein 3−like
with a threshold of 1e−05

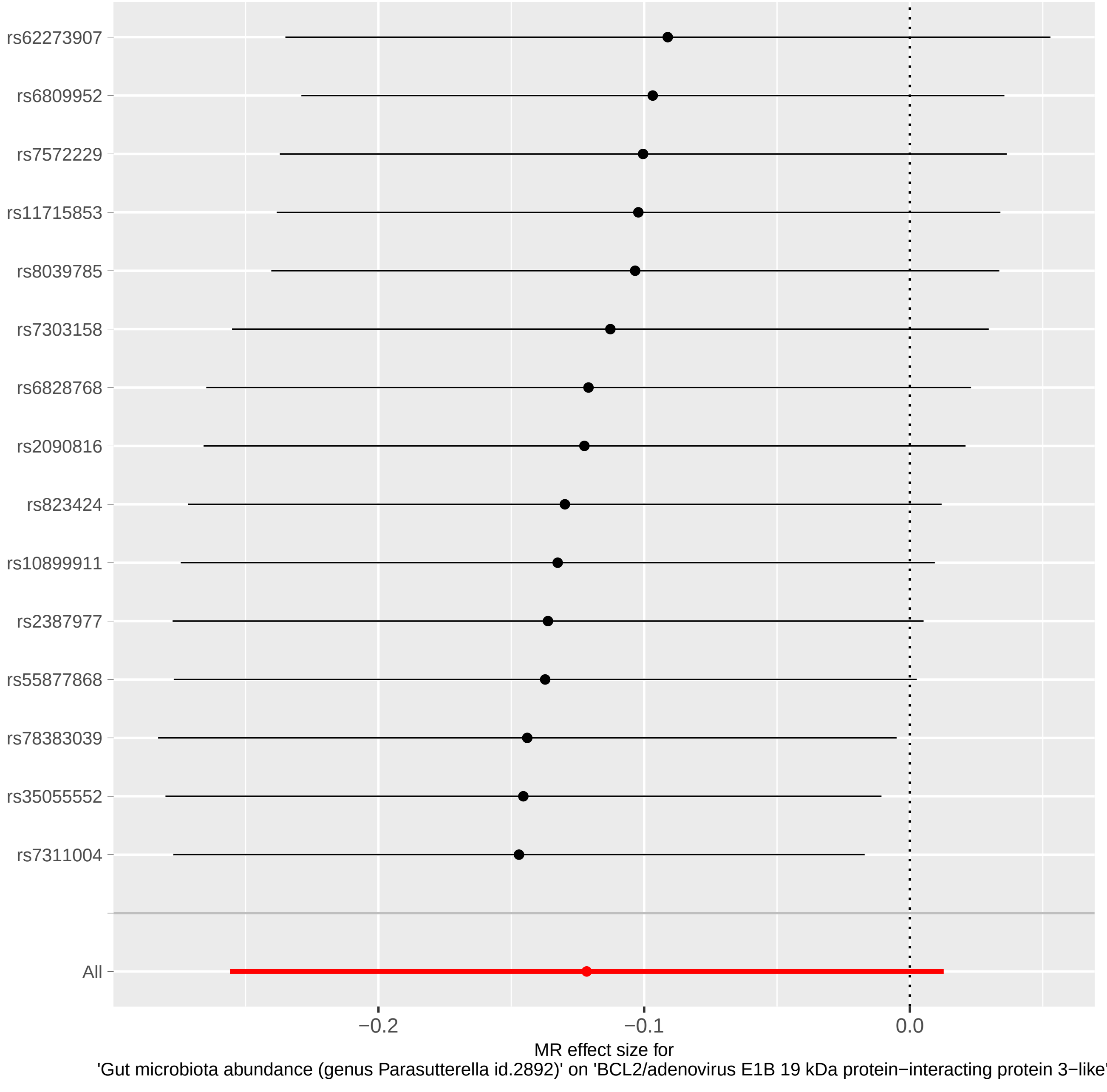

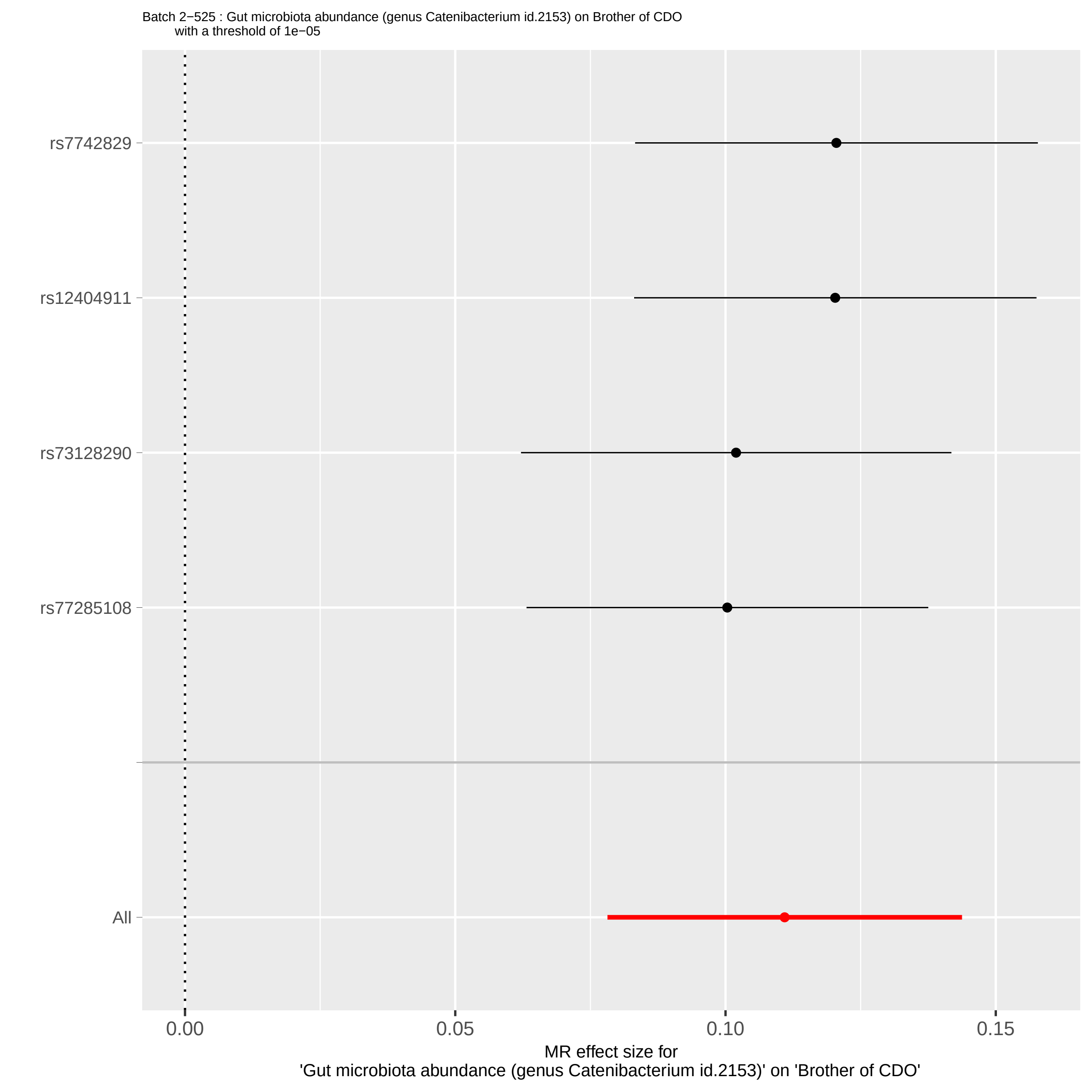
Batch 2−525 : Gut microbiota abundance (genus Catenibacterium id.2153) on Brother of CDO
with a threshold of 1e−05
rs7742829
rs12404911
rs73128290
rs77285108
All
0.00
0.05
0.10
0.15
MR effect size for
'Gut microbiota abundance (genus Catenibacterium id.2153)' on 'Brother of CDO'

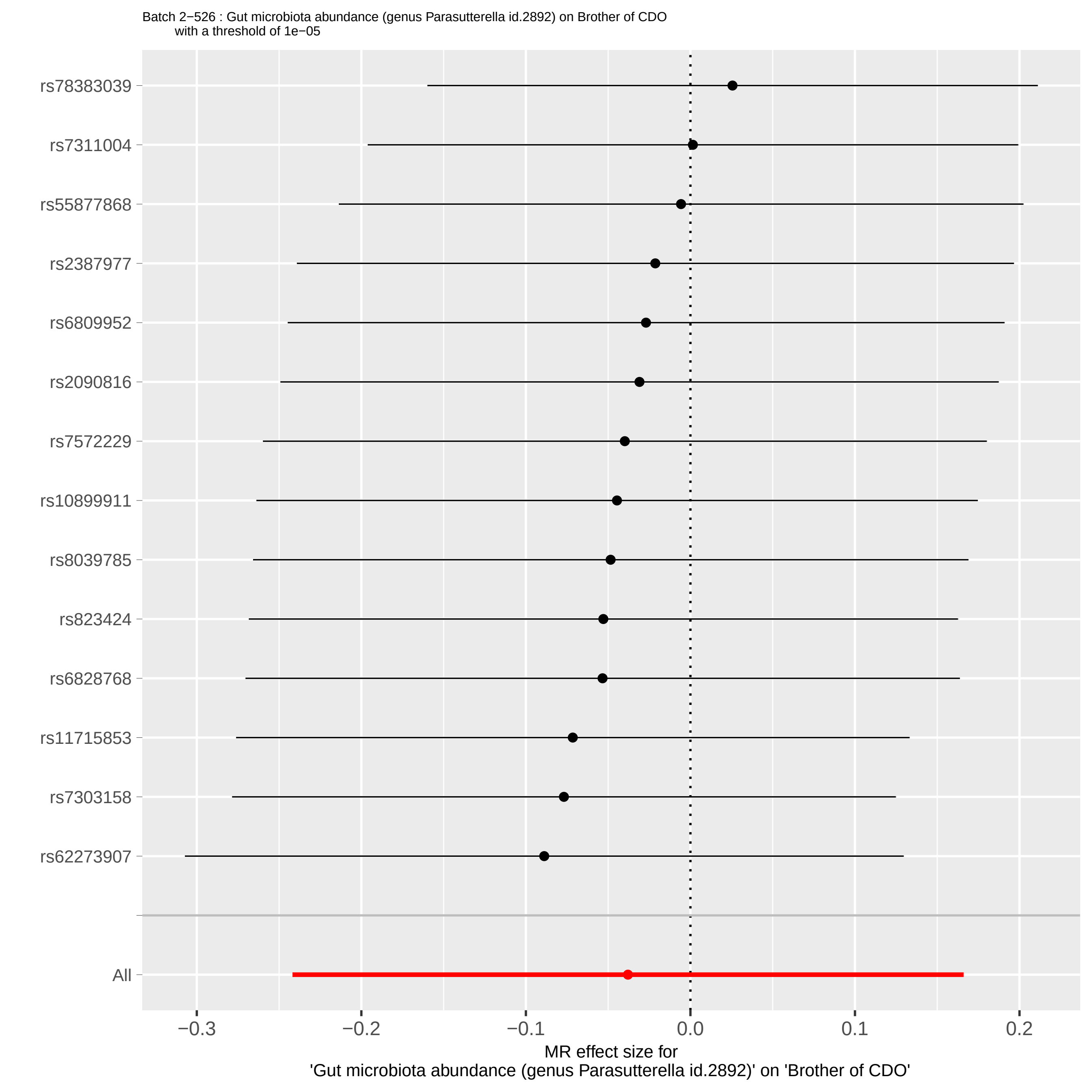

Batch 2−526 : Gut microbiota abundance (genus Parasutterella id.2892) on Brother of CDO
with a threshold of 1e−05
rs78383039
rs7311004
rs55877868
rs2387977
rs6809952
rs2090816
rs7572229
rs10899911
rs8039785
rs823424
rs6828768
rs11715853
rs7303158
rs62273907
All
−0.3
−0.2
−0.1
0.0
0.1
0.2
MR effect size for
'Gut microbiota abundance (genus Parasutterella id.2892)' on 'Brother of CDO'

Batch 2−527 : Gut microbiota abundance (genus Catenibacterium id.2153) on BolA−like protein 2
with a threshold of 1e−05

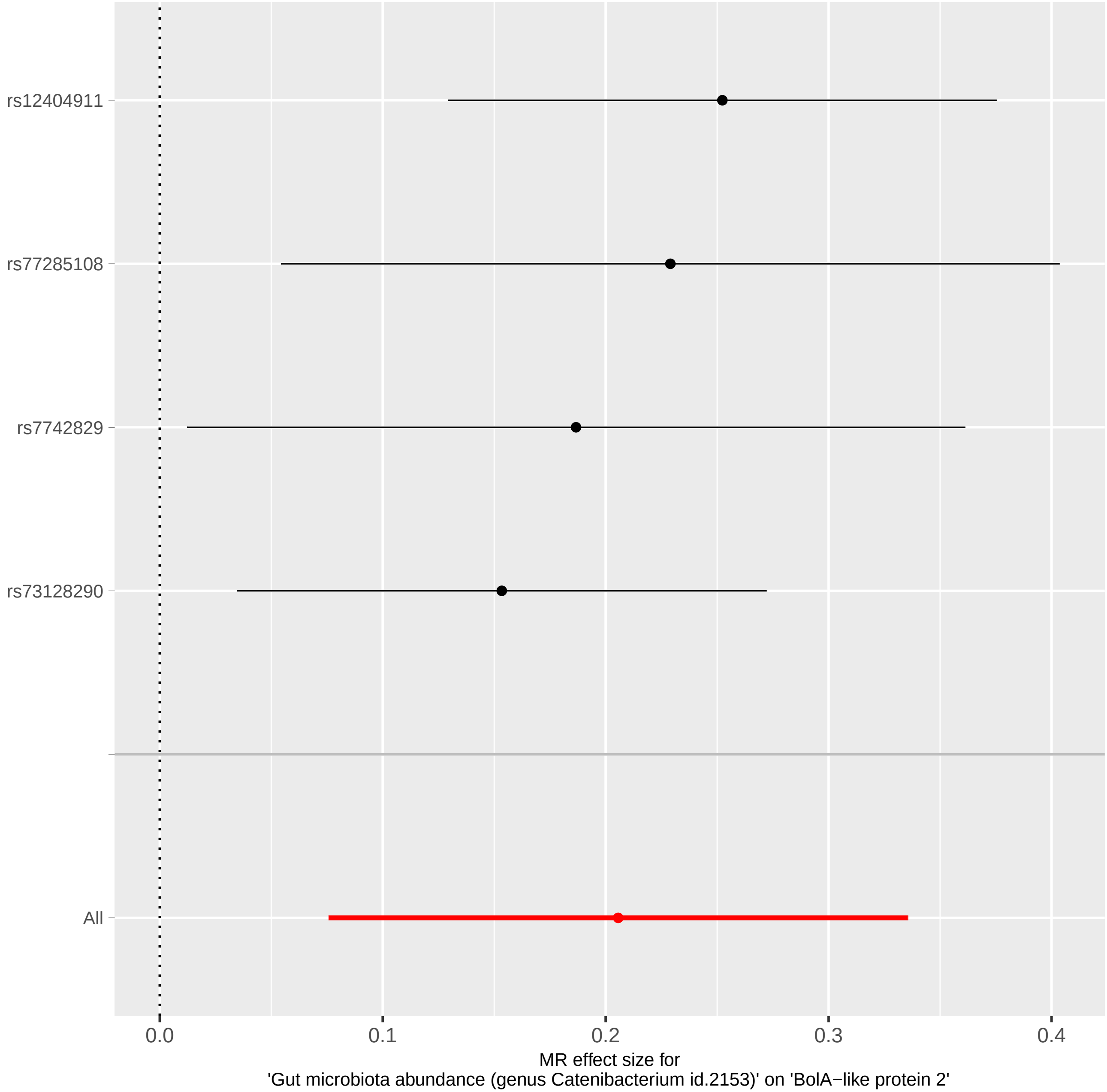

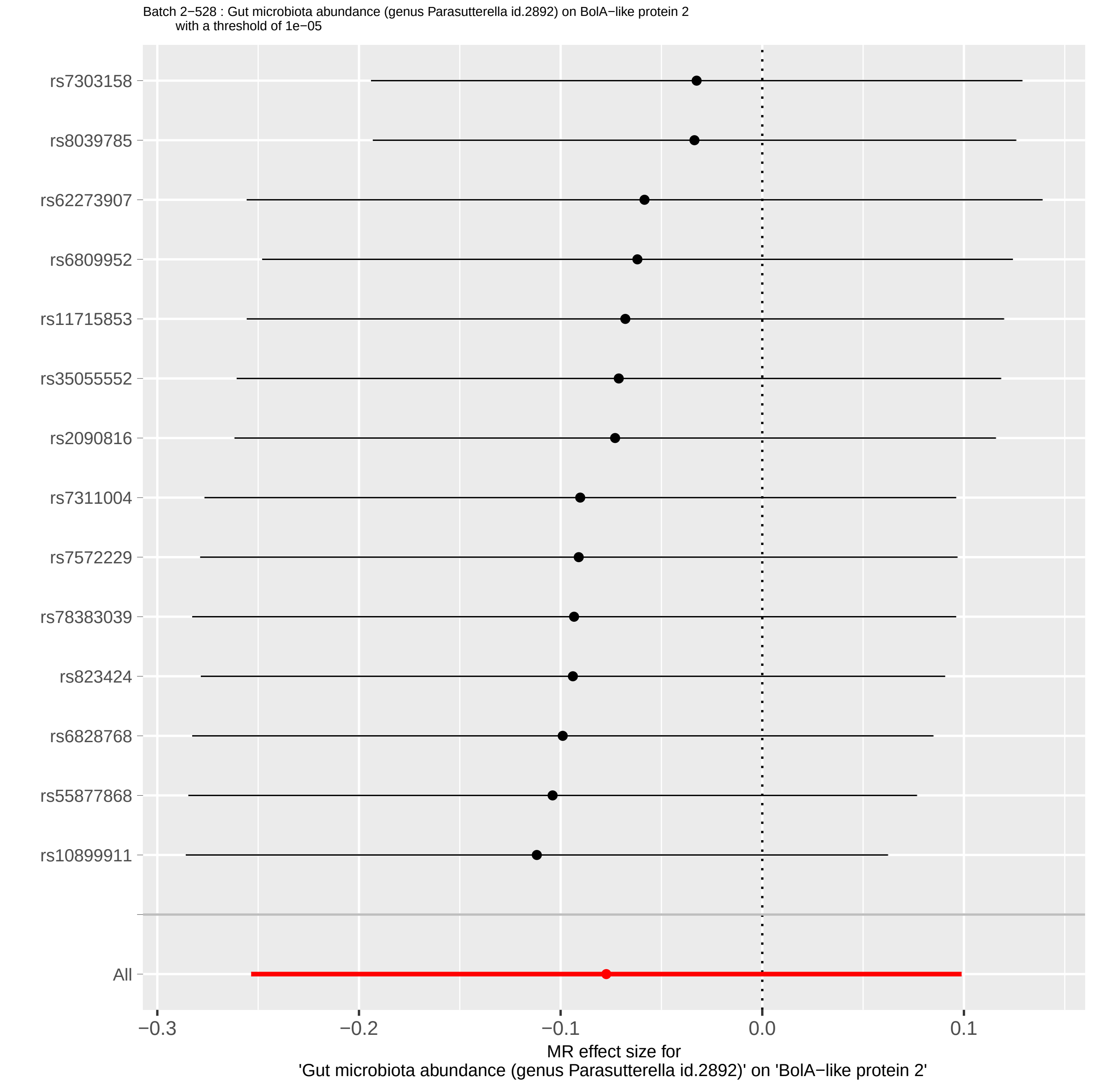
Batch 2−528 : Gut microbiota abundance (genus Parasutterella id.2892) on BolA−like protein 2
with a threshold of 1e−05
rs7303158
rs8039785
rs62273907
rs6809952
rs11715853
rs35055552
rs2090816
rs7311004
rs7572229
rs78383039
rs823424
rs6828768
rs55877868
rs10899911
All
−0.3
−0.2
−0.1
0.0
0.1
MR effect size for
'Gut microbiota abundance (genus Parasutterella id.2892)' on 'BolA−like protein 2'

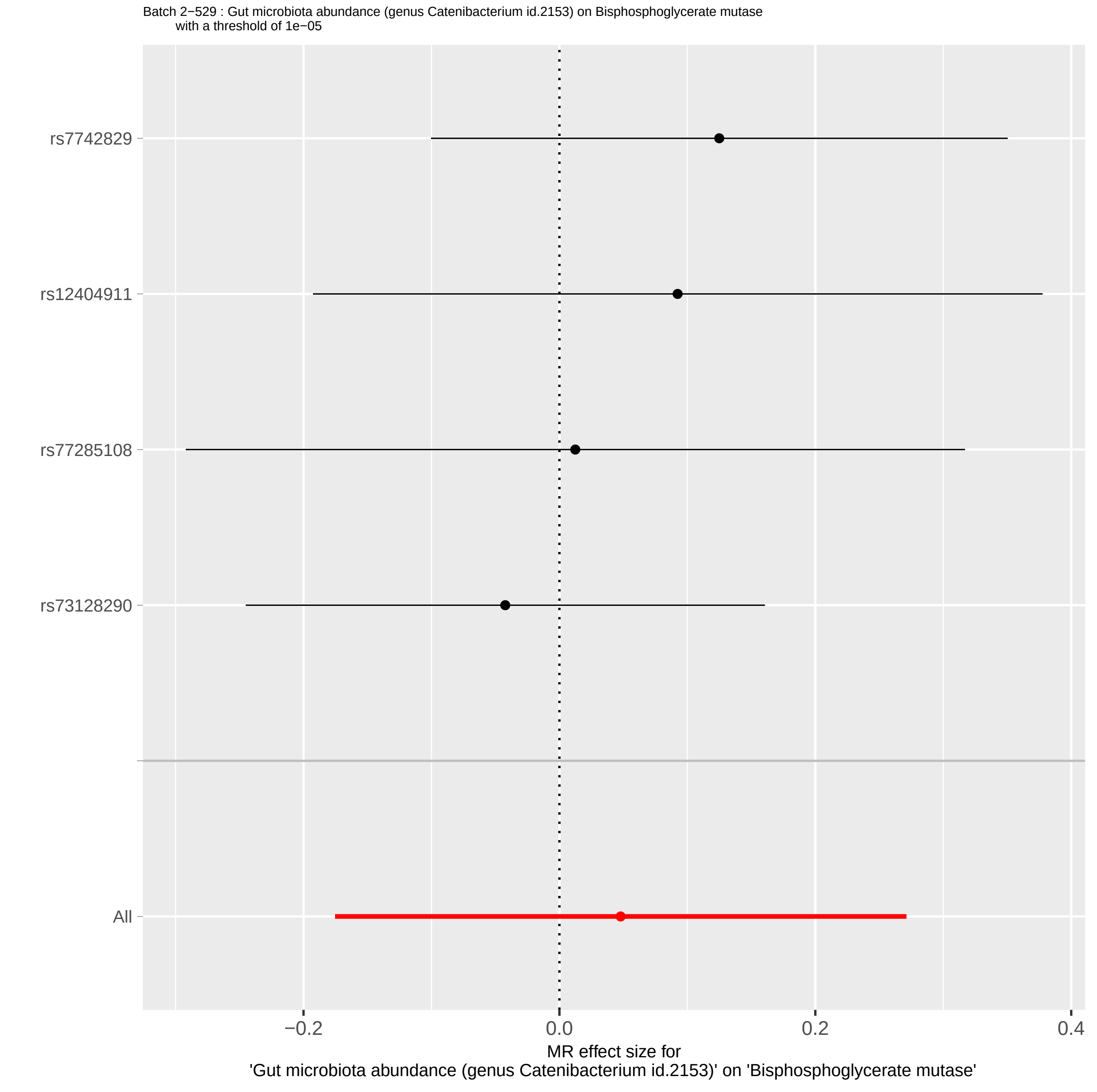

Batch 2−529 : Gut microbiota abundance (genus Catenibacterium id.2153) on Bisphosphoglycerate mutase
with a threshold of 1e−05
rs7742829
rs12404911
rs77285108
rs73128290
All
−0.2
0.0
0.2
0.4
MR effect size for
'Gut microbiota abundance (genus Catenibacterium id.2153)' on 'Bisphosphoglycerate mutase'

Batch 2−530 : Gut microbiota abundance (genus Parasutterella id.2892) on Bisphosphoglycerate mutase
with a threshold of 1e−05

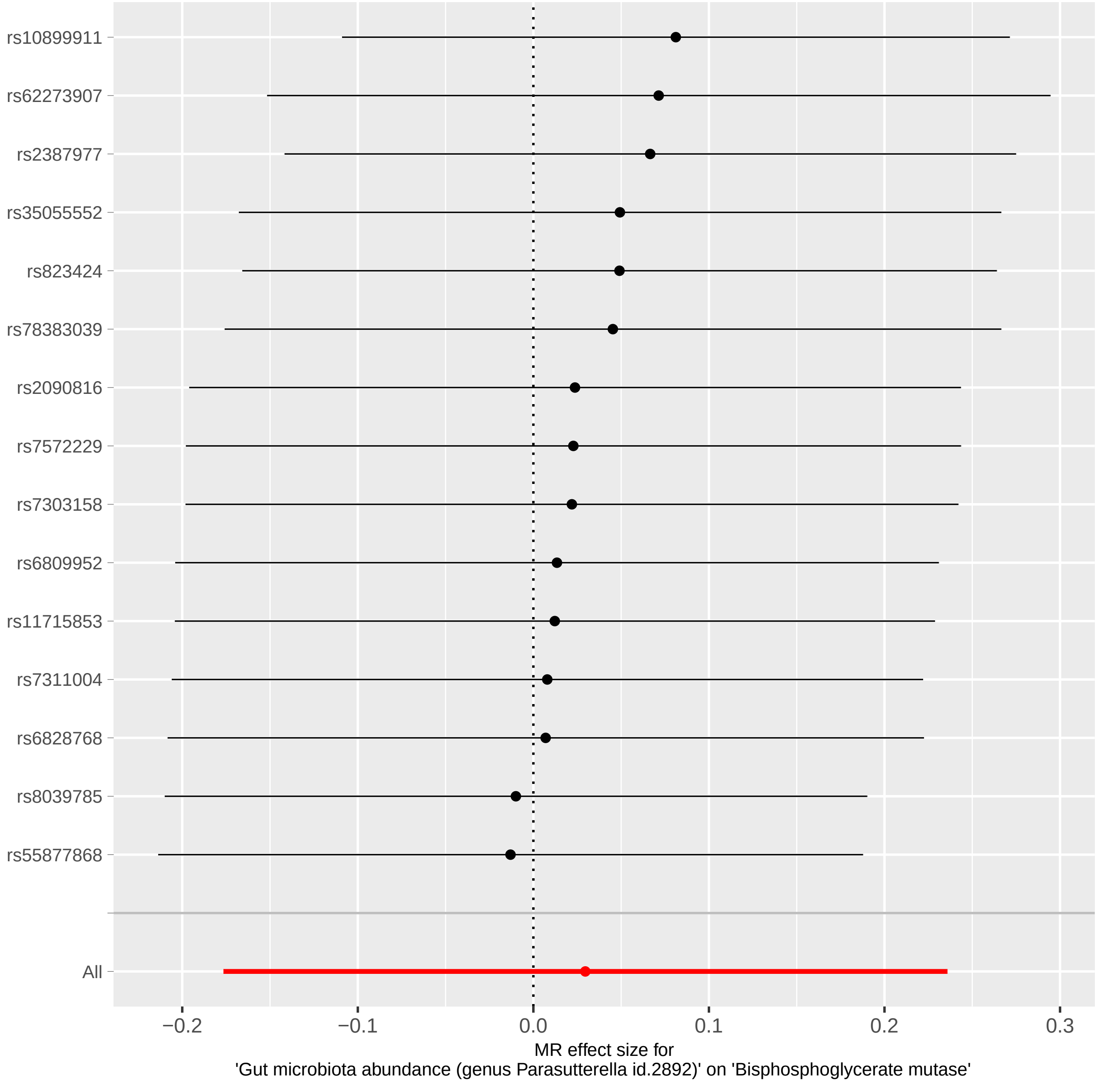

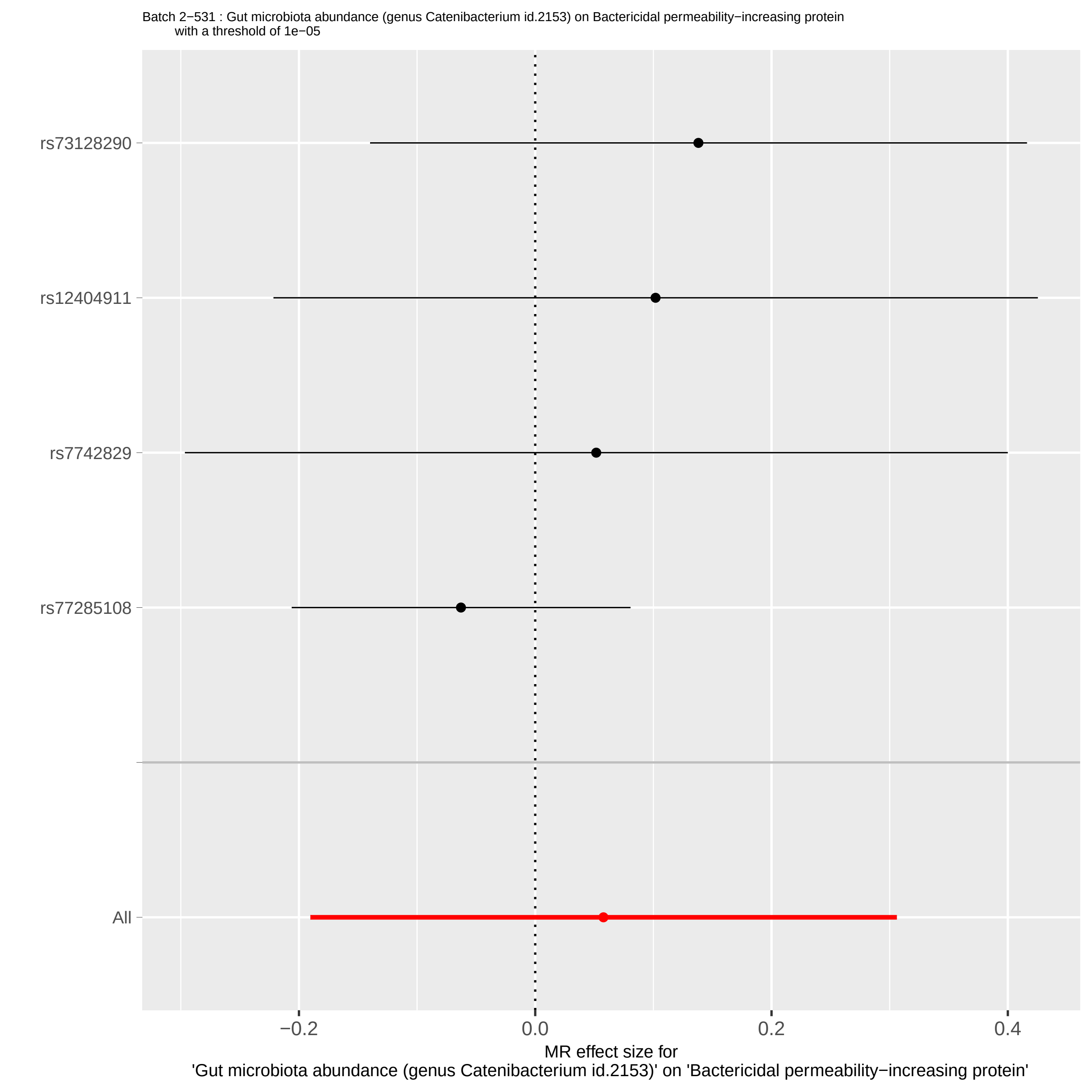
Batch 2−531 : Gut microbiota abundance (genus Catenibacterium id.2153) on Bactericidal permeability−increasing protein
with a threshold of 1e−05
rs73128290
rs12404911
rs7742829
rs77285108
All
−0.2
0.0
0.2
0.4
MR effect size for
'Gut microbiota abundance (genus Catenibacterium id.2153)' on 'Bactericidal permeability−increasing protein'

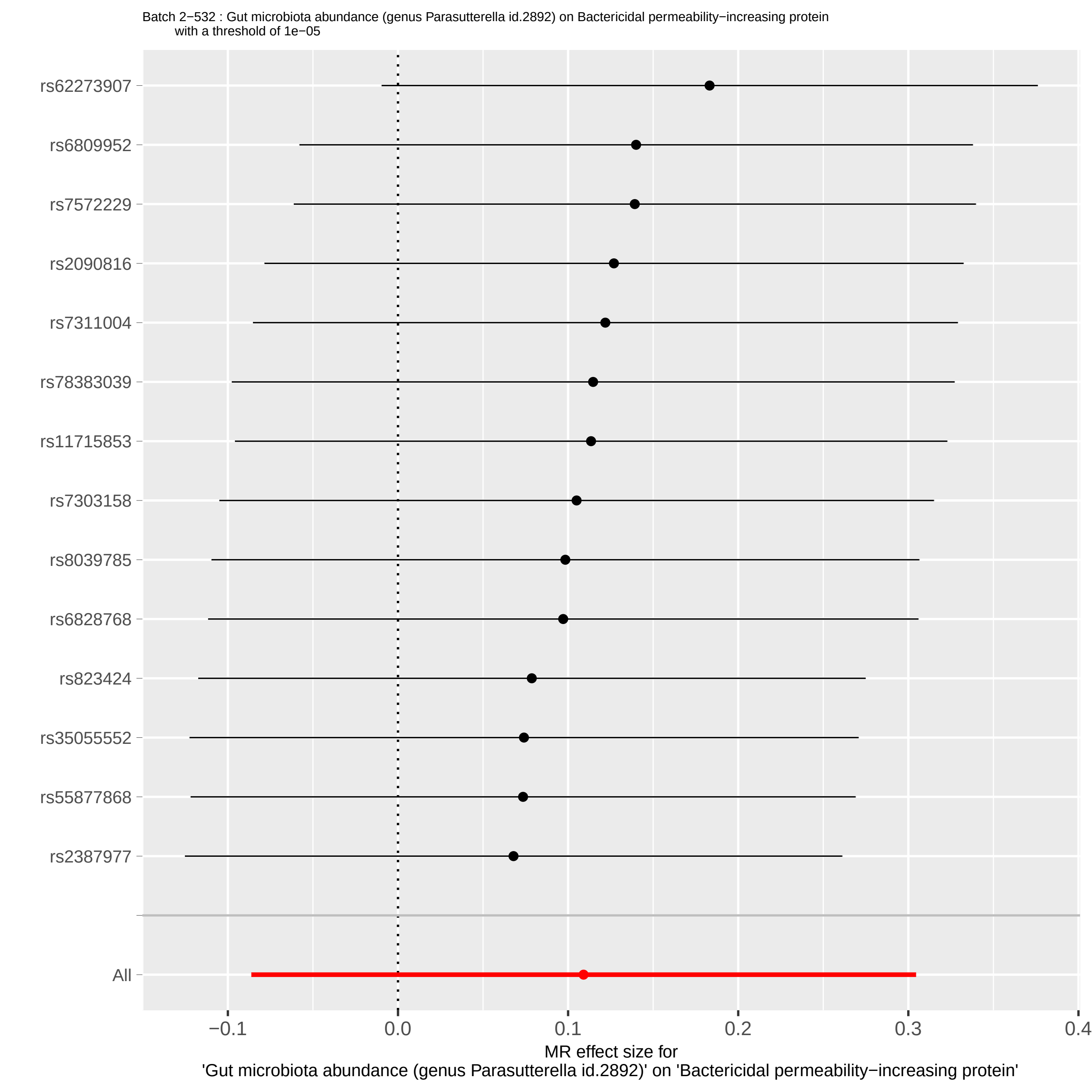
Batch 2−532 : Gut microbiota abundance (genus Parasutterella id.2892) on Bactericidal permeability−increasing protein
with a threshold of 1e−05
rs62273907
rs6809952
rs7572229
rs2090816
rs7311004
rs78383039
rs11715853
rs7303158
rs8039785
rs6828768
rs823424
rs35055552
rs55877868
rs2387977
All
−0.1
0.0
0.1
0.2
0.3
0.4
MR effect size for
'Gut microbiota abundance (genus Parasutterella id.2892)' on 'Bactericidal permeability−increasing protein'

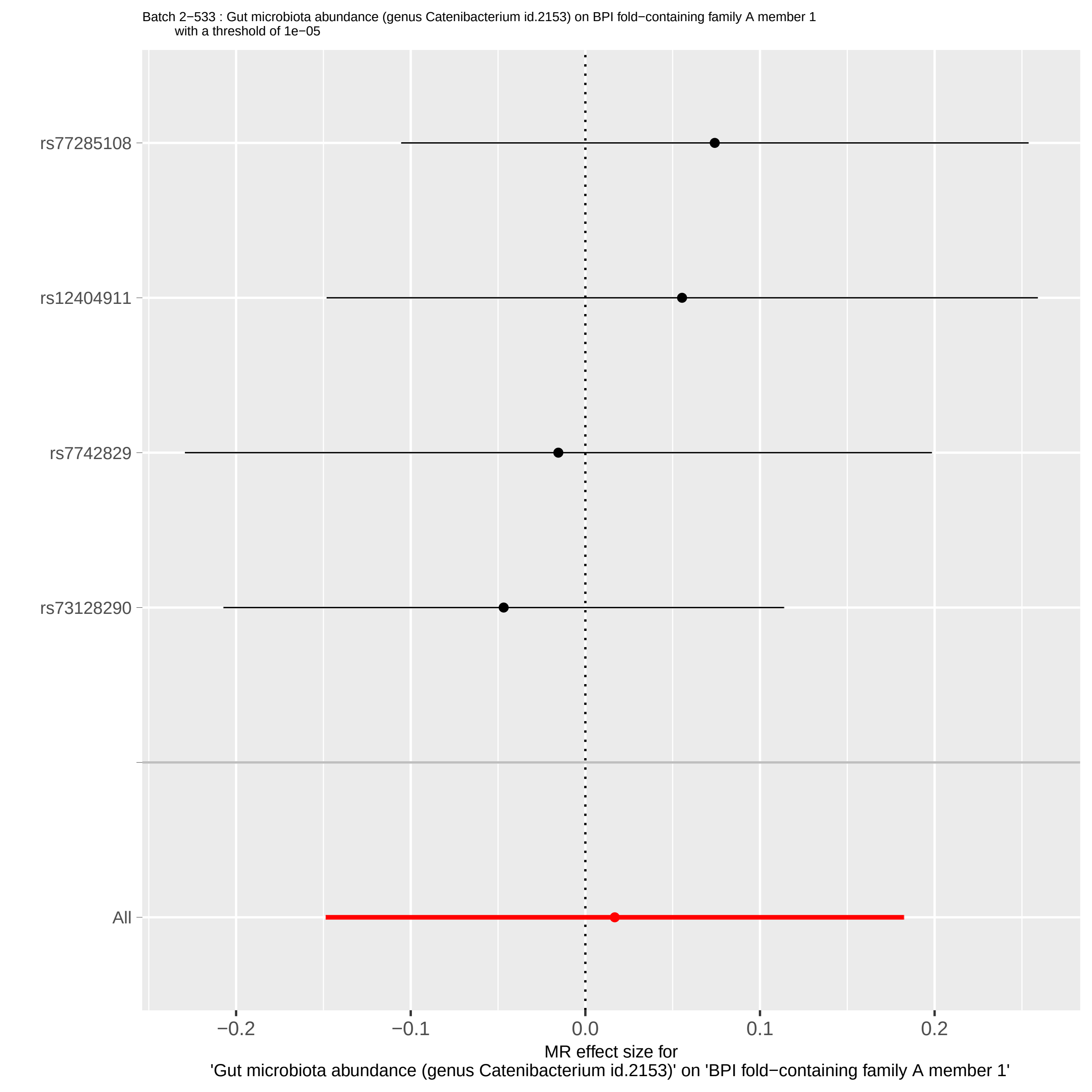
Batch 2−533 : Gut microbiota abundance (genus Catenibacterium id.2153) on BPI fold−containing family A member 1
with a threshold of 1e−05
rs77285108
rs12404911
rs7742829
rs73128290
All
−0.2
−0.1
0.0
0.1
0.2
MR effect size for
'Gut microbiota abundance (genus Catenibacterium id.2153)' on 'BPI fold−containing family A member 1'

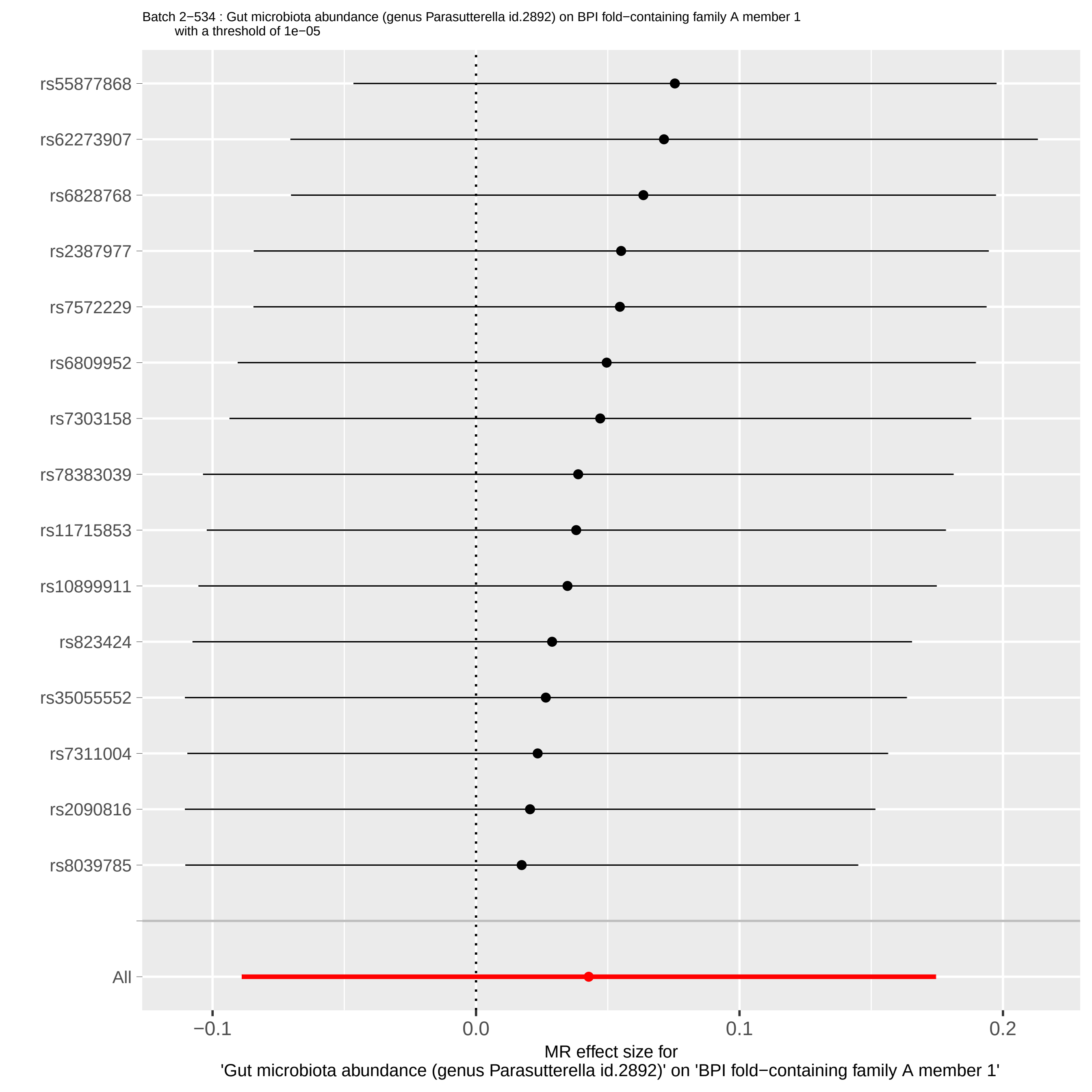
Batch 2−534 : Gut microbiota abundance (genus Parasutterella id.2892) on BPI fold−containing family A member 1
with a threshold of 1e−05
rs55877868
rs62273907
rs6828768
rs2387977
rs7572229
rs6809952
rs7303158
rs78383039
rs11715853
rs10899911
rs823424
rs35055552
rs7311004
rs2090816
rs8039785
All
−0.1
0.0
0.1
0.2
MR effect size for
'Gut microbiota abundance (genus Parasutterella id.2892)' on 'BPI fold−containing family A member 1'

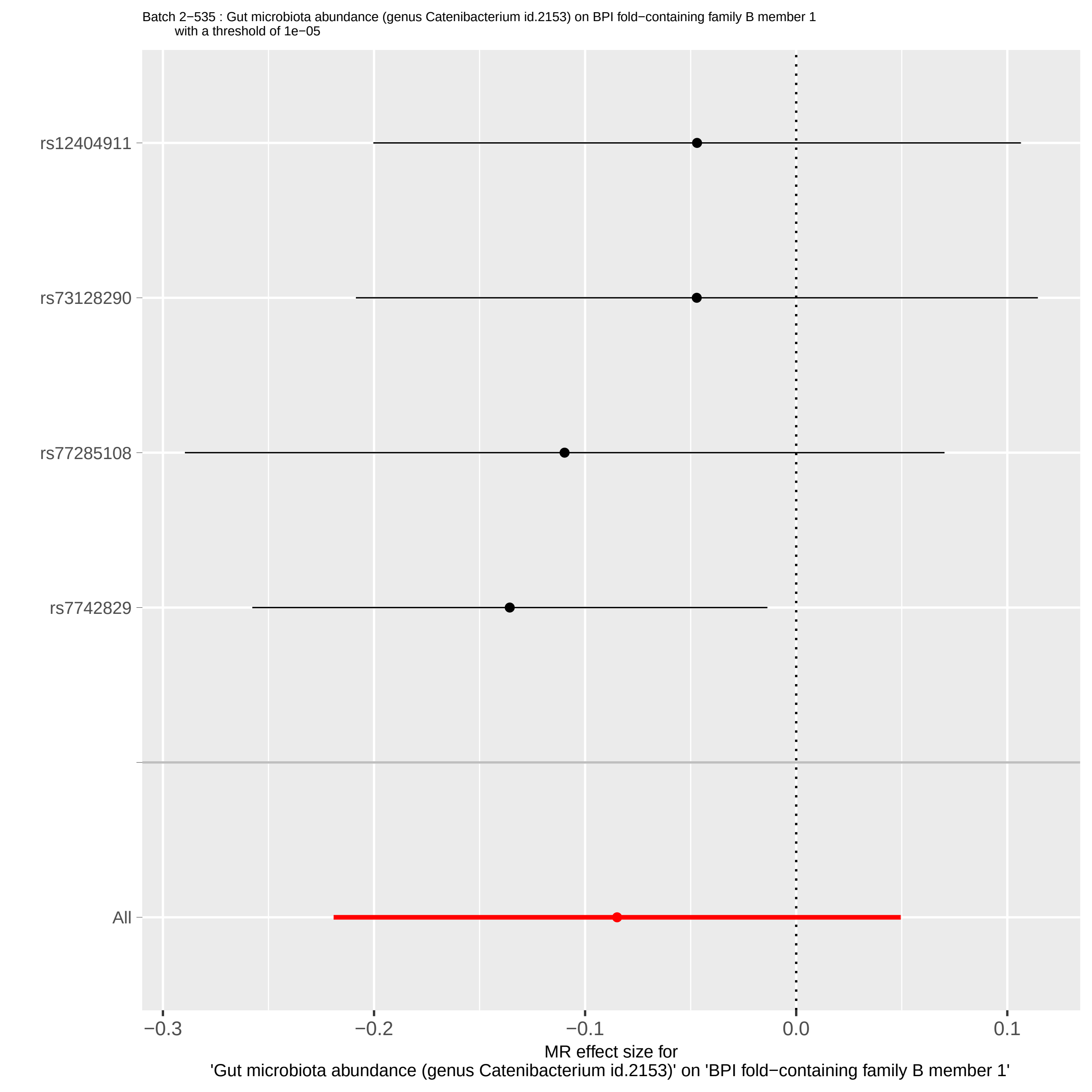

Batch 2−535 : Gut microbiota abundance (genus Catenibacterium id.2153) on BPI fold−containing family B member 1
with a threshold of 1e−05
rs12404911
rs73128290
rs77285108
rs7742829
All
−0.3
−0.2
−0.1
0.0
0.1
MR effect size for
'Gut microbiota abundance (genus Catenibacterium id.2153)' on 'BPI fold−containing family B member 1'

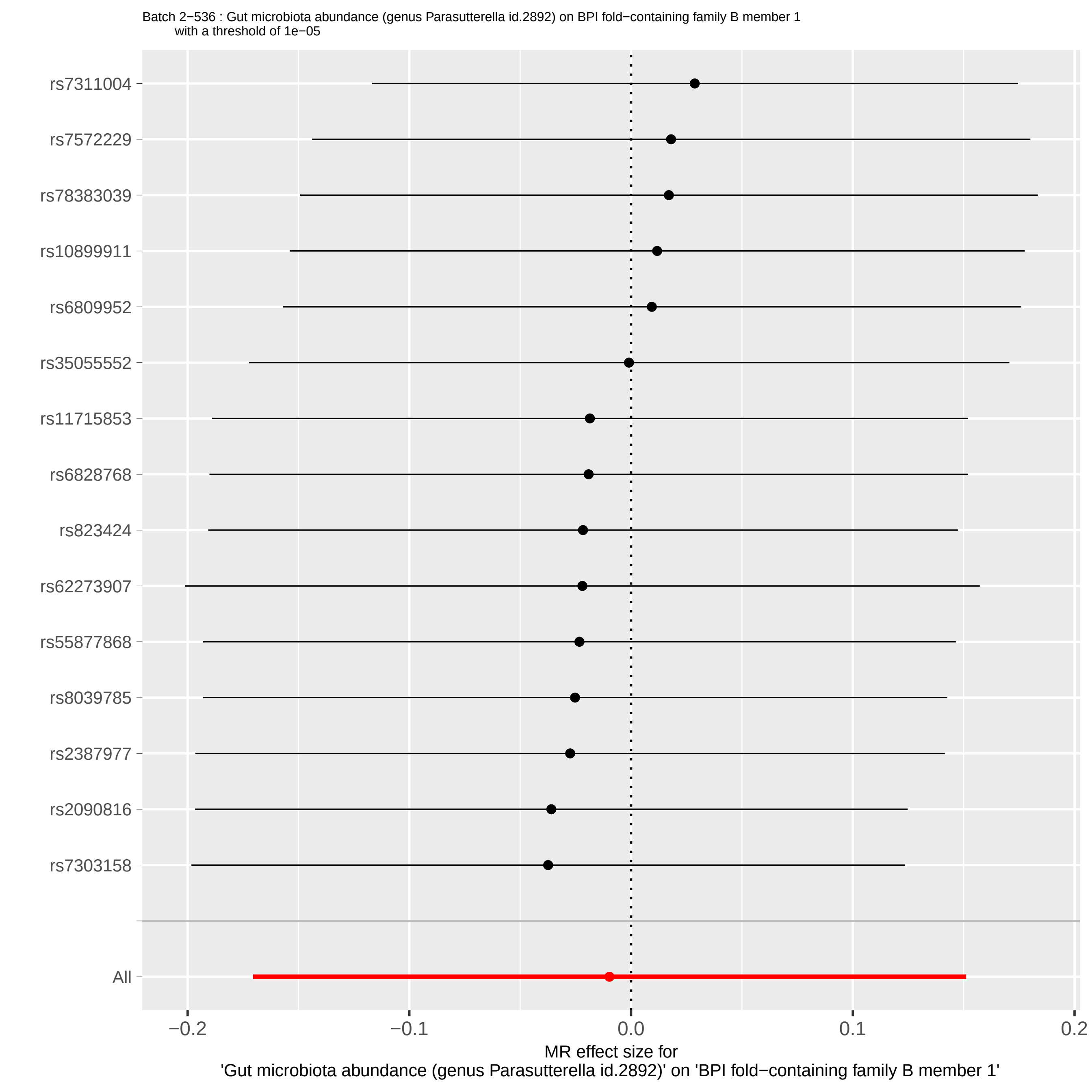

Batch 2−536 : Gut microbiota abundance (genus Parasutterella id.2892) on BPI fold−containing family B member 1
with a threshold of 1e−05
rs7311004
rs7572229
rs78383039
rs10899911
rs6809952
rs35055552
rs11715853
rs6828768
rs823424
rs62273907
rs55877868
rs8039785
rs2387977
rs2090816
rs7303158
All
−0.2
−0.1
0.0
0.1
0.2
MR effect size for
'Gut microbiota abundance (genus Parasutterella id.2892)' on 'BPI fold−containing family B member 1'

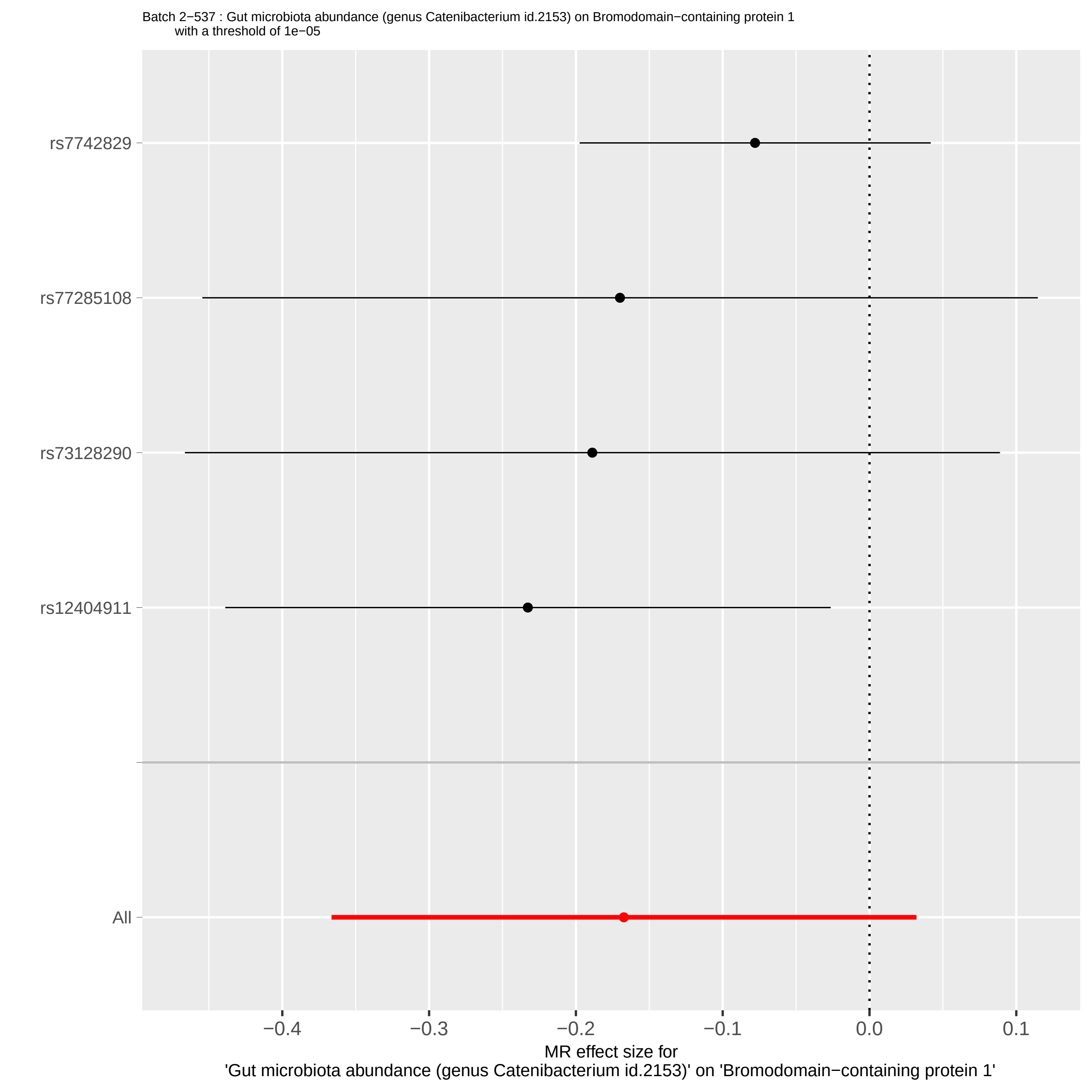
Batch 2−537 : Gut microbiota abundance (genus Catenibacterium id.2153) on Bromodomain−containing protein 1
with a threshold of 1e−05
rs7742829
rs77285108
rs73128290
rs12404911
All
−0.4
−0.3
−0.2
−0.1
0.0
0.1
MR effect size for
'Gut microbiota abundance (genus Catenibacterium id.2153)' on 'Bromodomain−containing protein 1'

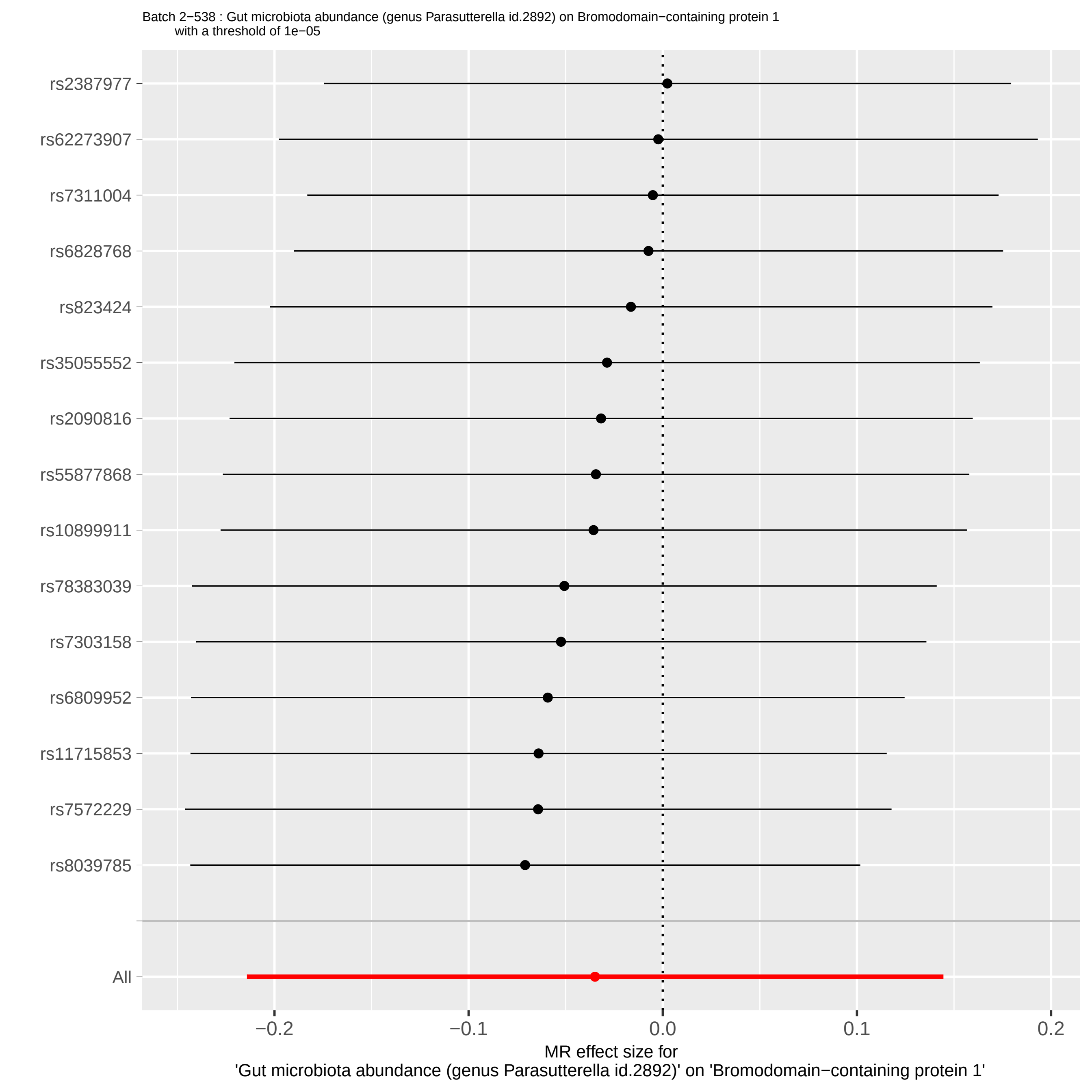

Batch 2−538 : Gut microbiota abundance (genus Parasutterella id.2892) on Bromodomain−containing protein 1
with a threshold of 1e−05
rs2387977
rs62273907
rs7311004
rs6828768
rs823424
rs35055552
rs2090816
rs55877868
rs10899911
rs78383039
rs7303158
rs6809952
rs11715853
rs7572229
rs8039785
All
−0.2
−0.1
0.0
0.1
0.2
MR effect size for
'Gut microbiota abundance (genus Parasutterella id.2892)' on 'Bromodomain−containing protein 1'

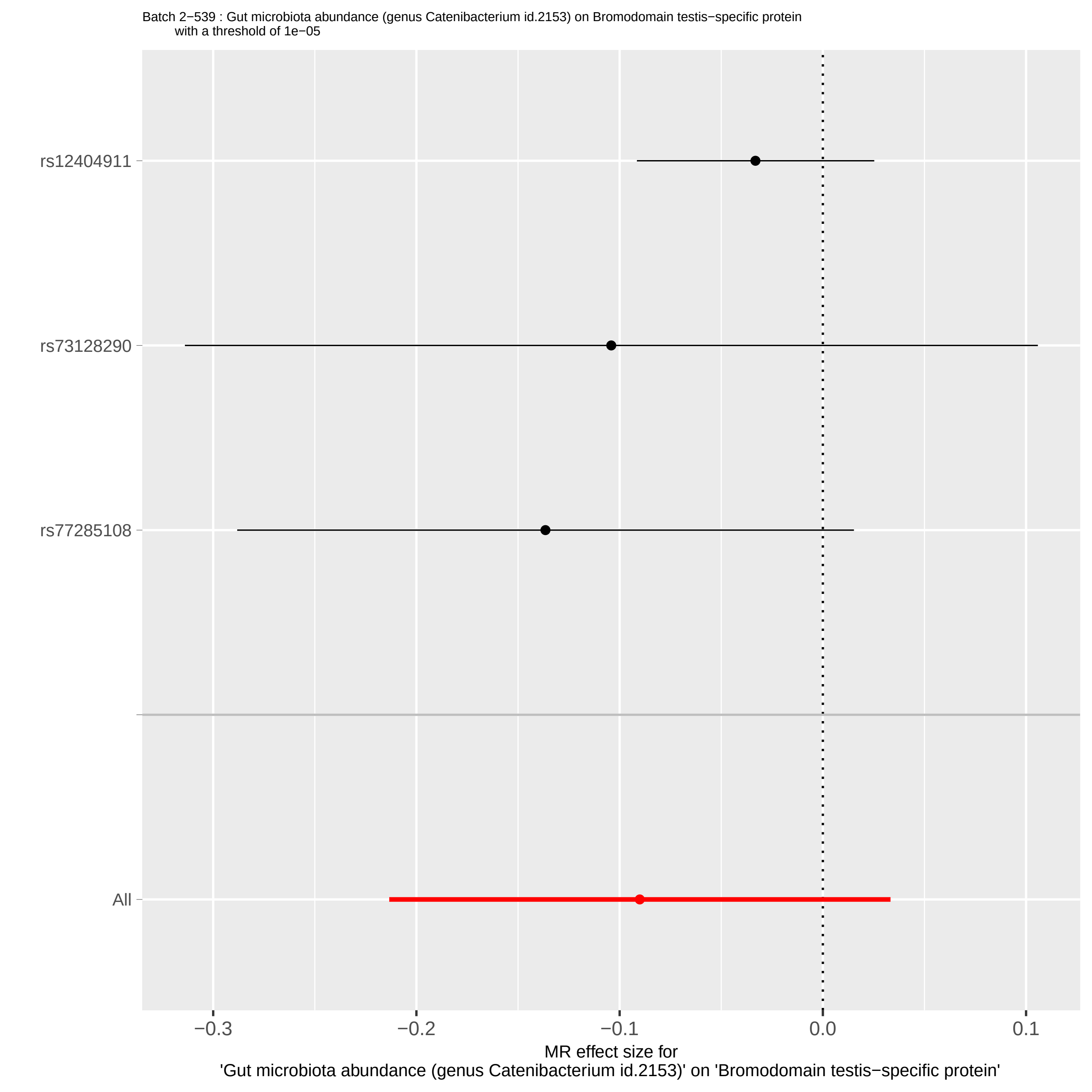
Batch 2−539 : Gut microbiota abundance (genus Catenibacterium id.2153) on Bromodomain testis−specific protein
with a threshold of 1e−05
rs12404911
rs73128290
rs77285108
All
−0.3
−0.2
−0.1
0.0
0.1
MR effect size for
'Gut microbiota abundance (genus Catenibacterium id.2153)' on 'Bromodomain testis−specific protein'

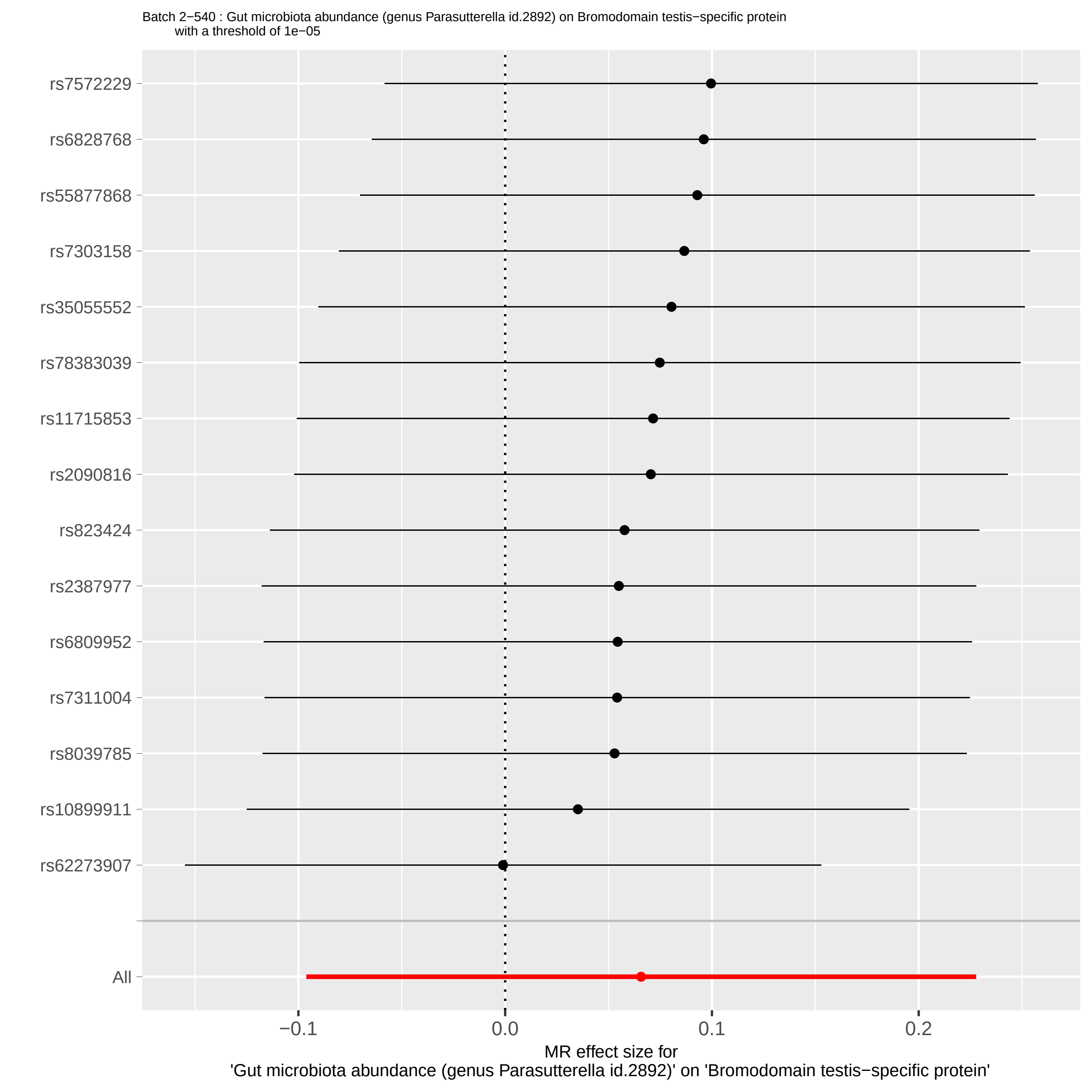

Batch 2−540 : Gut microbiota abundance (genus Parasutterella id.2892) on Bromodomain testis−specific protein
with a threshold of 1e−05
rs7572229
rs6828768
rs55877868
rs7303158
rs35055552
rs78383039
rs11715853
rs2090816
rs823424
rs2387977
rs6809952
rs7311004
rs8039785
rs10899911
rs62273907
All
−0.1
0.0
0.1
0.2
MR effect size for
'Gut microbiota abundance (genus Parasutterella id.2892)' on 'Bromodomain testis−specific protein'

Batch 2−541 : Gut microbiota abundance (genus Catenibacterium id.2153) on Transcription factor IIIB 90 kDa subunit
with a threshold of 1e−05

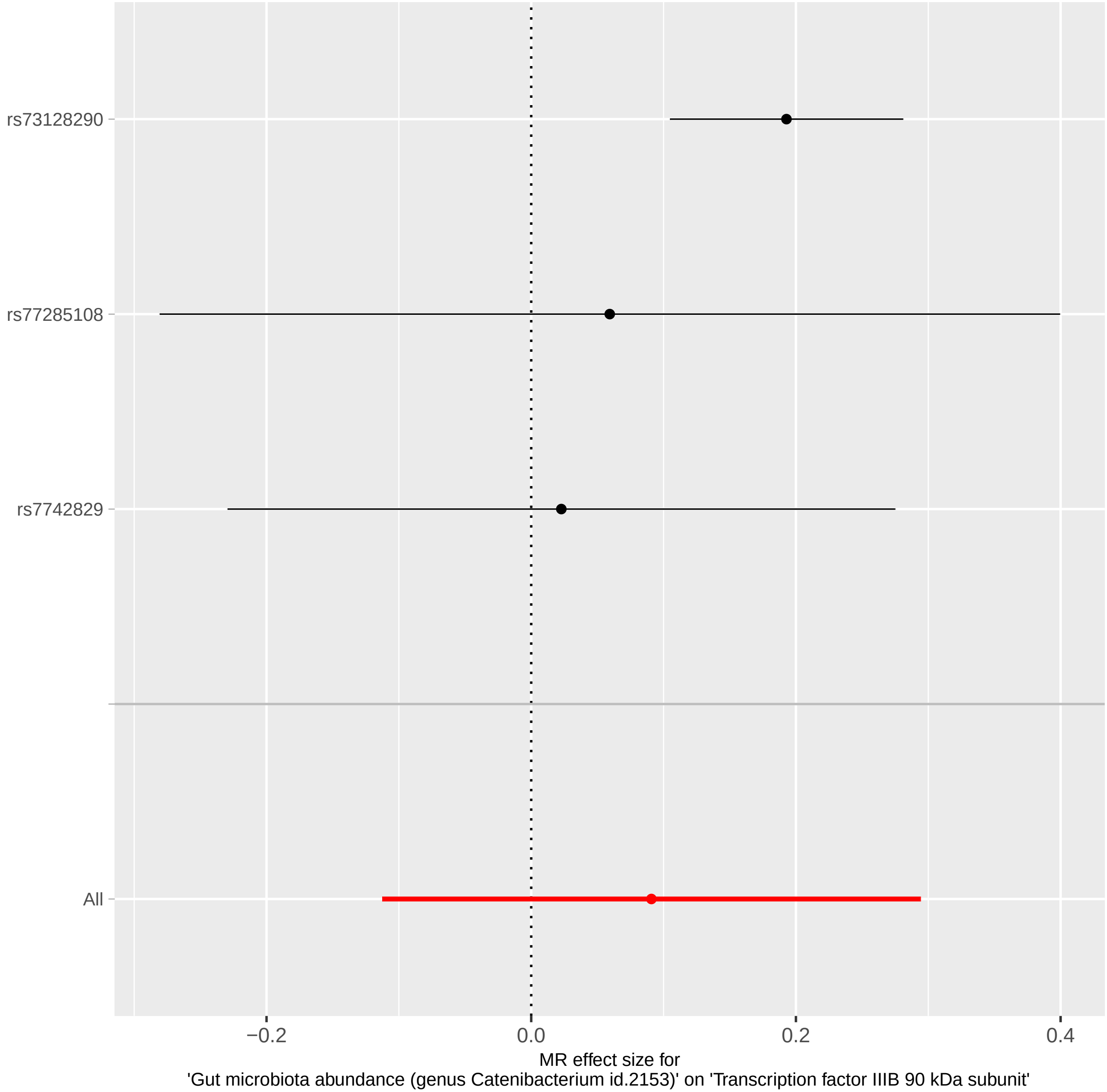

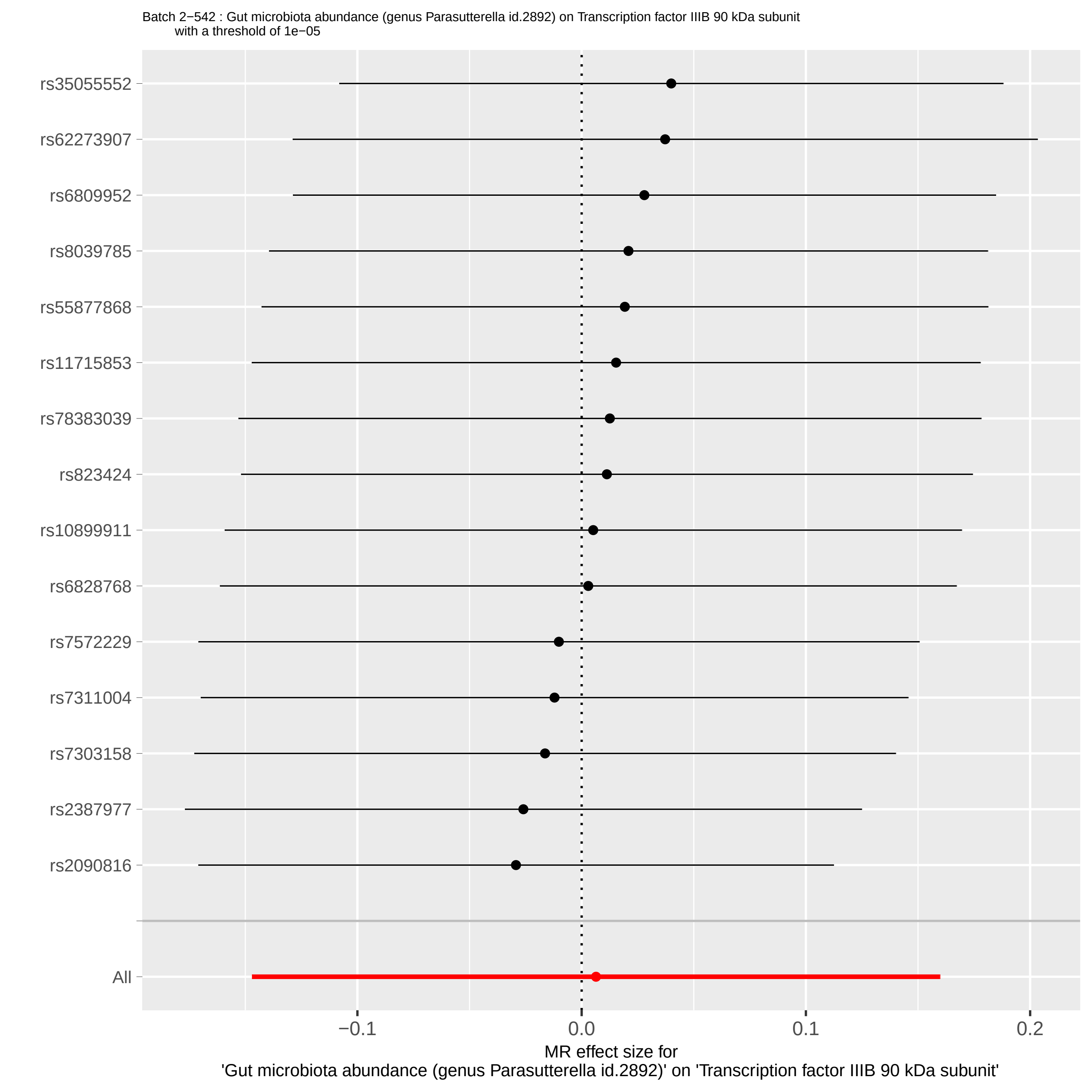
Batch 2−542 : Gut microbiota abundance (genus Parasutterella id.2892) on Transcription factor IIIB 90 kDa subunit
with a threshold of 1e−05
rs35055552
rs62273907
rs6809952
rs8039785
rs55877868
rs11715853
rs78383039
rs823424
rs10899911
rs6828768
rs7572229
rs7311004
rs7303158
rs2387977
rs2090816
All
−0.1
0.0
0.1
0.2
MR effect size for
'Gut microbiota abundance (genus Parasutterella id.2892)' on 'Transcription factor IIIB 90 kDa subunit'

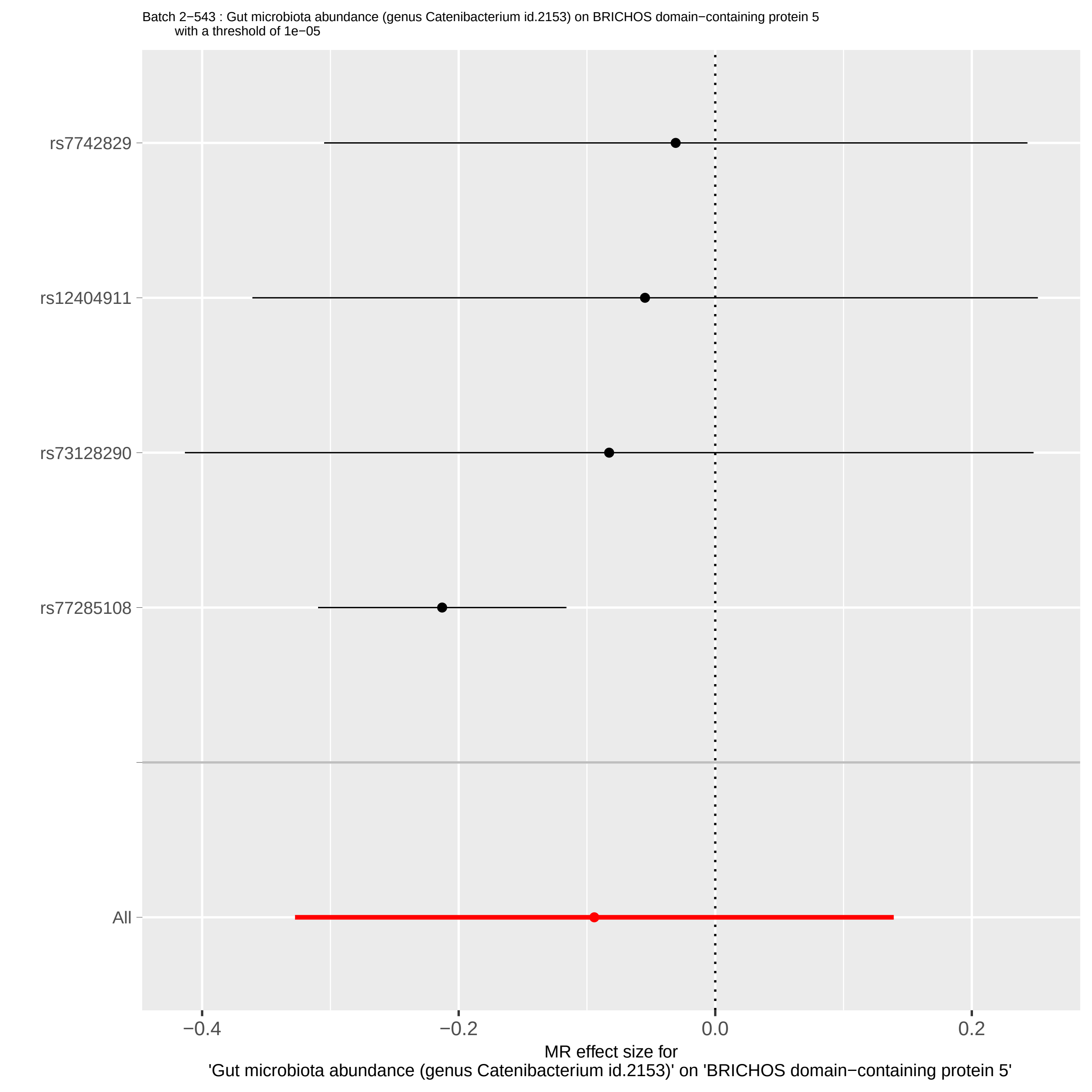
Batch 2−543 : Gut microbiota abundance (genus Catenibacterium id.2153) on BRICHOS domain−containing protein 5
with a threshold of 1e−05
rs7742829
rs12404911
rs73128290
rs77285108
All
−0.4
−0.2
0.0
0.2
MR effect size for
'Gut microbiota abundance (genus Catenibacterium id.2153)' on 'BRICHOS domain−containing protein 5'

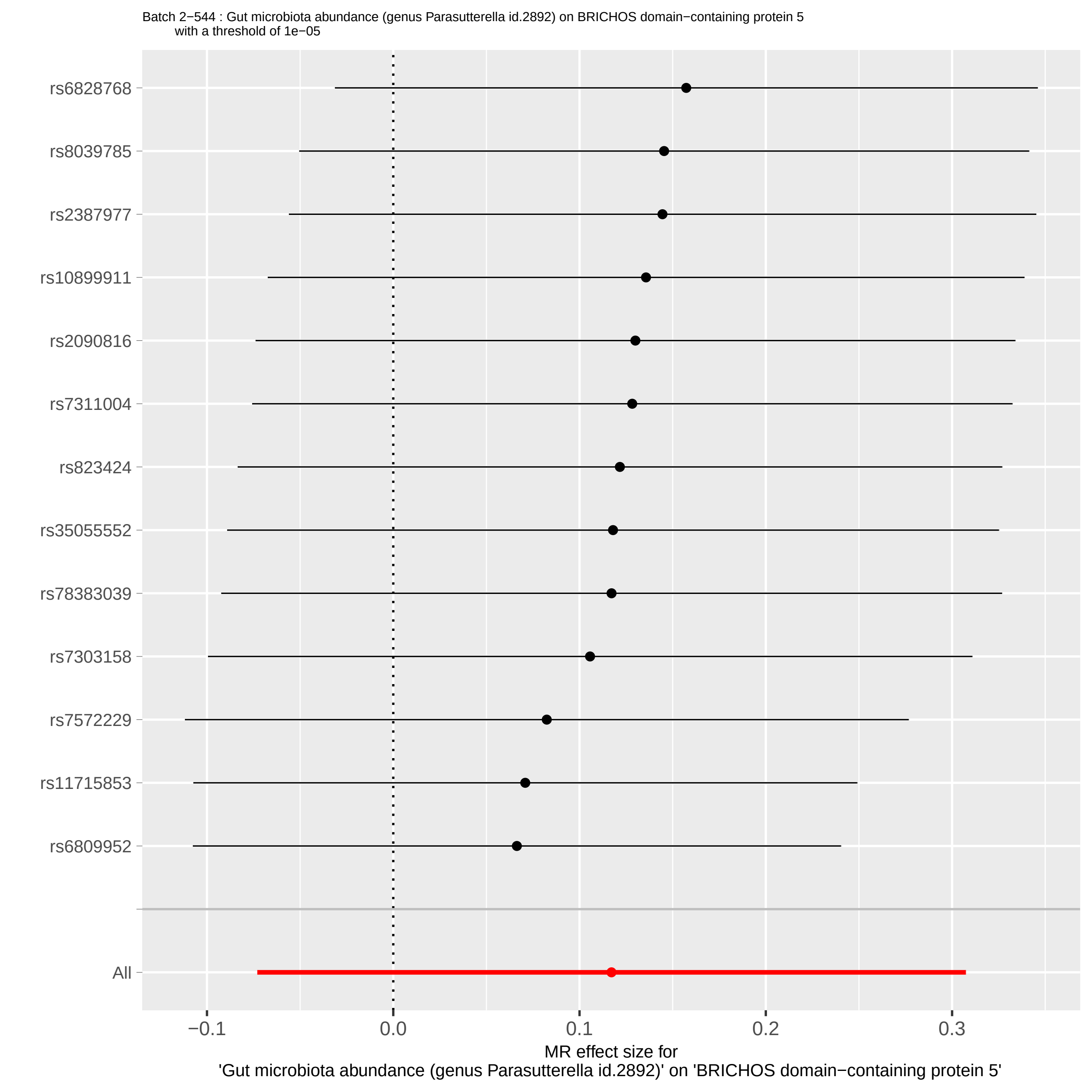

Batch 2−544 : Gut microbiota abundance (genus Parasutterella id.2892) on BRICHOS domain−containing protein 5
with a threshold of 1e−05
rs6828768
rs8039785
rs2387977
rs10899911
rs2090816
rs7311004
rs823424
rs35055552
rs78383039
rs7303158
rs7572229
rs11715853
rs6809952
All
−0.1
0.0
0.1
0.2
0.3
MR effect size for
'Gut microbiota abundance (genus Parasutterella id.2892)' on 'BRICHOS domain−containing protein 5'

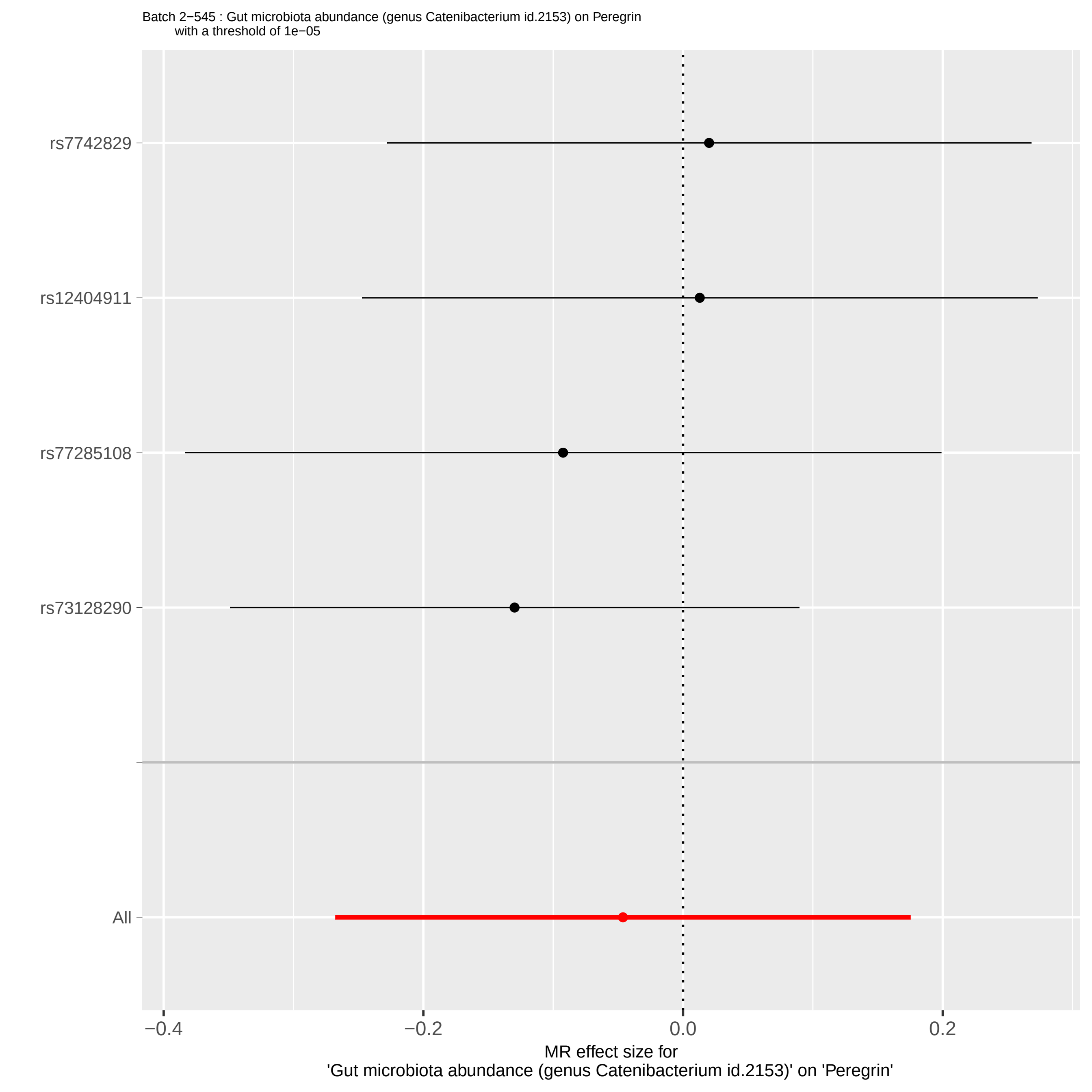
Batch 2−545 : Gut microbiota abundance (genus Catenibacterium id.2153) on Peregrin
with a threshold of 1e−05
rs7742829
rs12404911
rs77285108
rs73128290
All
−0.4
−0.2
0.0
0.2
MR effect size for
'Gut microbiota abundance (genus Catenibacterium id.2153)' on 'Peregrin'

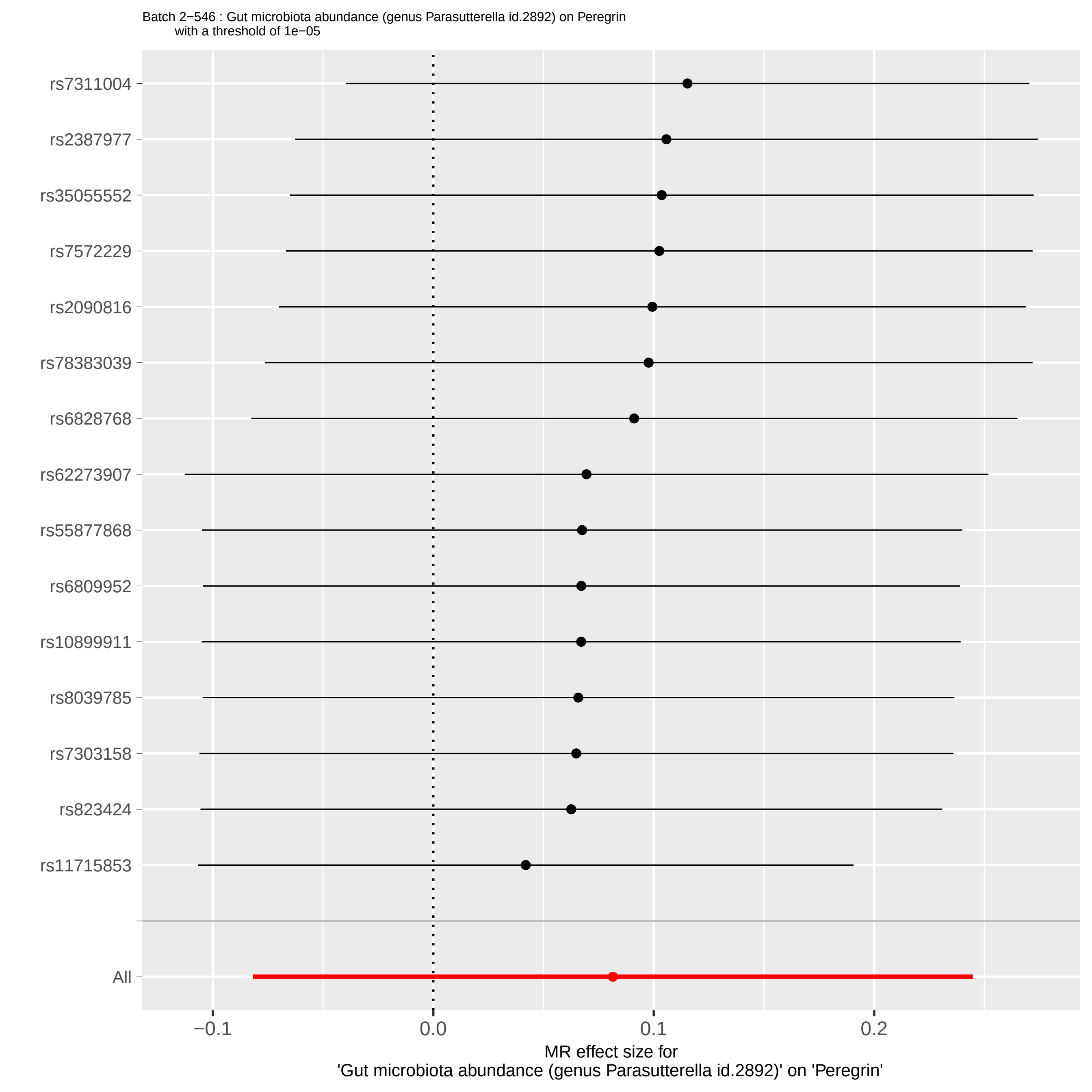

Batch 2−546 : Gut microbiota abundance (genus Parasutterella id.2892) on Peregrin
with a threshold of 1e−05
rs7311004
rs2387977
rs35055552
rs7572229
rs2090816
rs78383039
rs6828768
rs62273907
rs55877868
rs6809952
rs10899911
rs8039785
rs7303158
rs823424
rs11715853
All
−0.1
0.0
0.1
0.2
MR effect size for
'Gut microbiota abundance (genus Parasutterella id.2892)' on 'Peregrin'

Batch 2−547 : Gut microbiota abundance (genus Catenibacterium id.2153) on Serine/threonine−protein kinase BRSK2
with a threshold of 1e−05

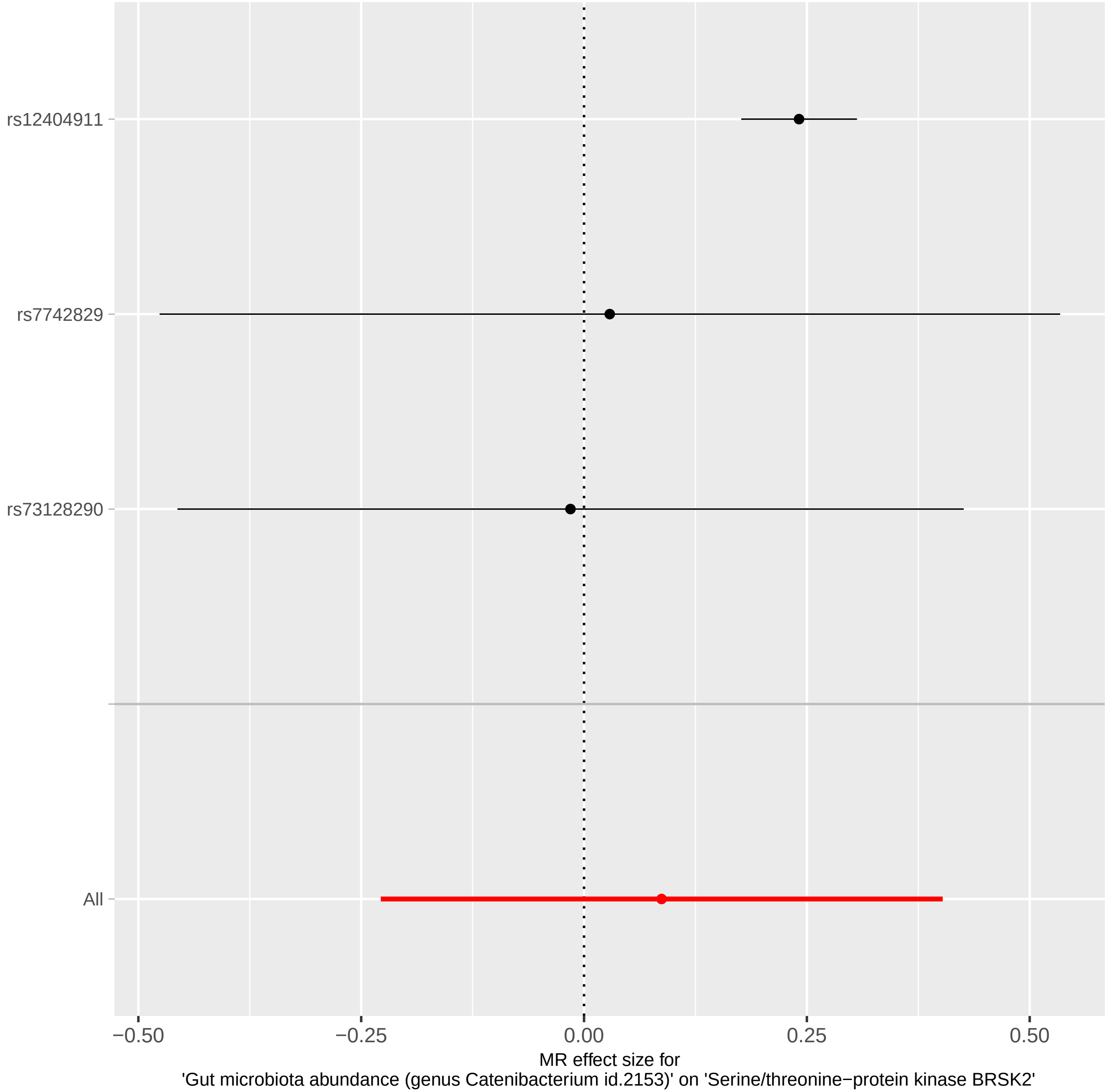

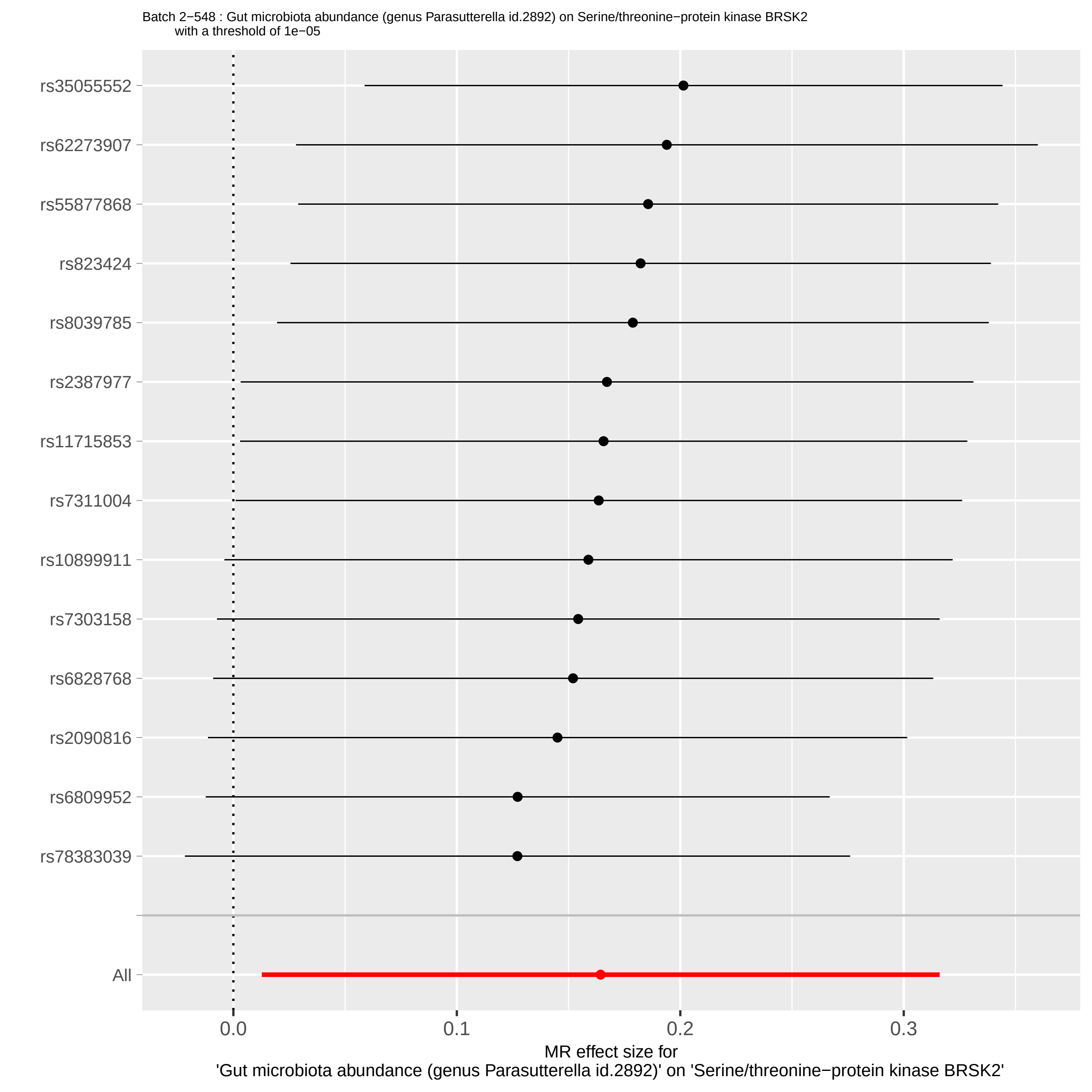
Batch 2−548 : Gut microbiota abundance (genus Parasutterella id.2892) on Serine/threonine−protein kinase BRSK2
with a threshold of 1e−05
rs35055552
rs62273907
rs55877868
rs823424
rs8039785
rs2387977
rs11715853
rs7311004
rs10899911
rs7303158
rs6828768
rs2090816
rs6809952
rs78383039
All
0.0
0.1
0.2
0.3
MR effect size for
'Gut microbiota abundance (genus Parasutterella id.2892)' on 'Serine/threonine−protein kinase BRSK2'

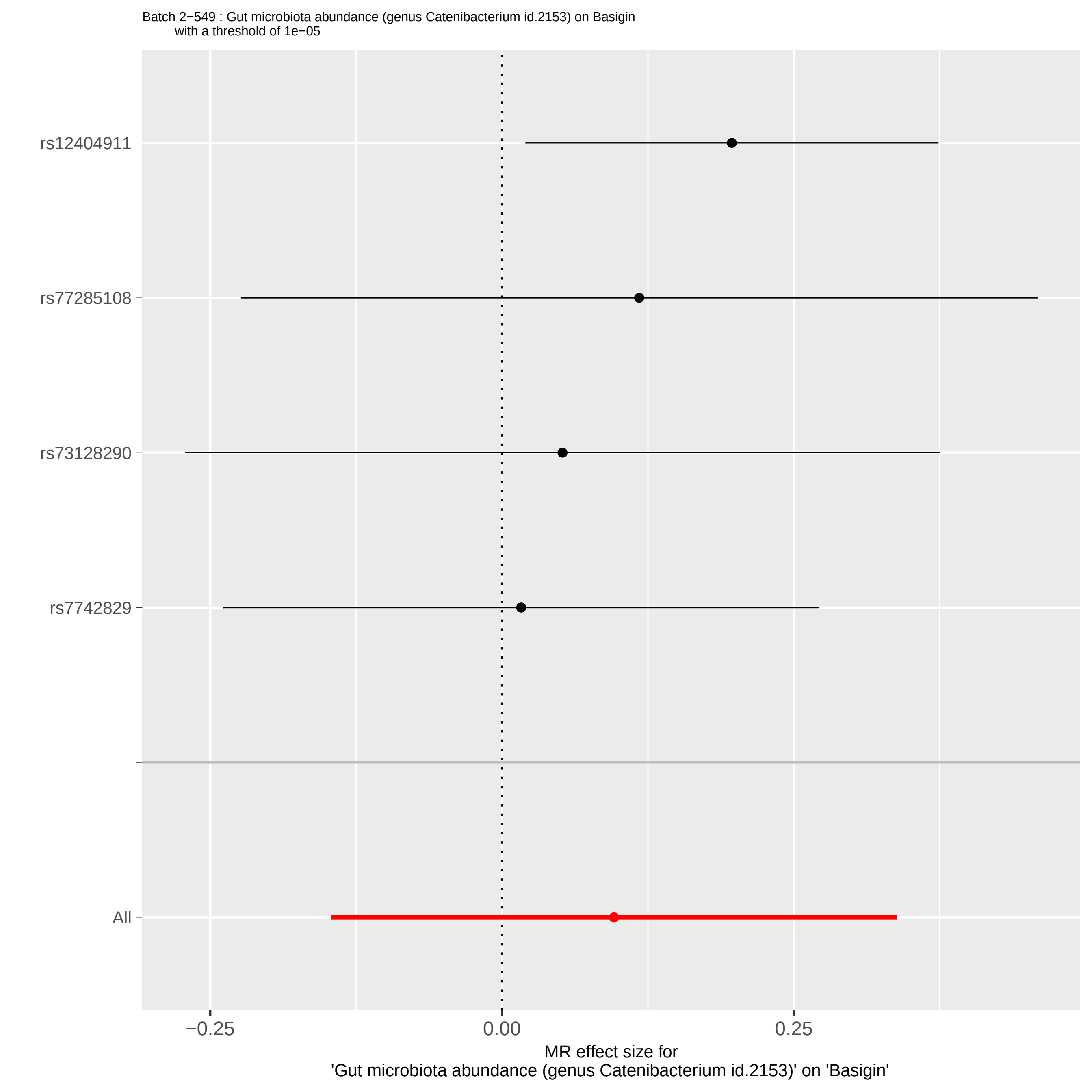

Batch 2−549 : Gut microbiota abundance (genus Catenibacterium id.2153) on Basigin
with a threshold of 1e−05
rs12404911
rs77285108
rs73128290
rs7742829
All
−0.25
0.00
0.25
MR effect size for
'Gut microbiota abundance (genus Catenibacterium id.2153)' on 'Basigin'

Batch 2−550 : Gut microbiota abundance (genus Parasutterella id.2892) on Basigin
with a threshold of 1e−05

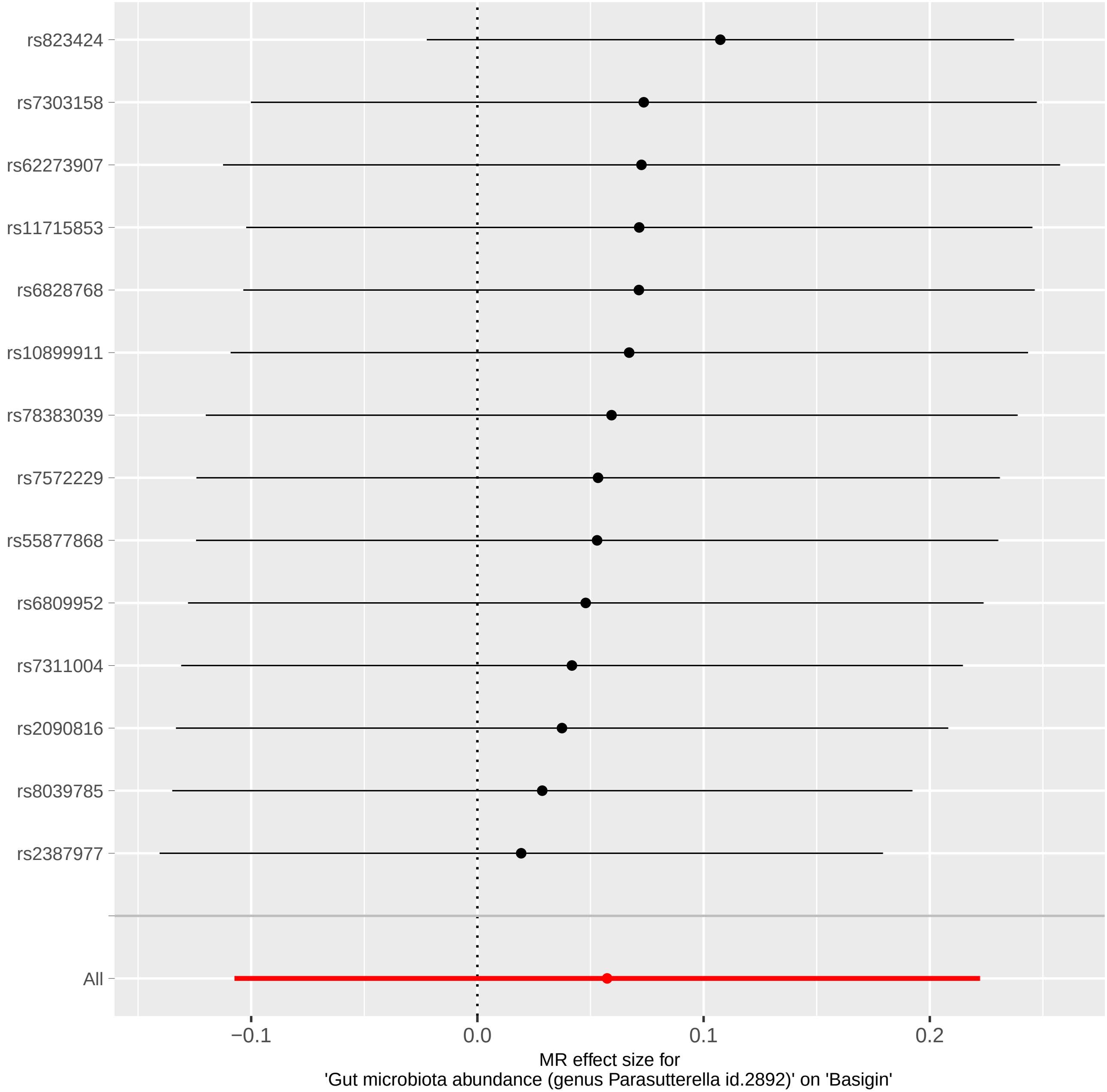

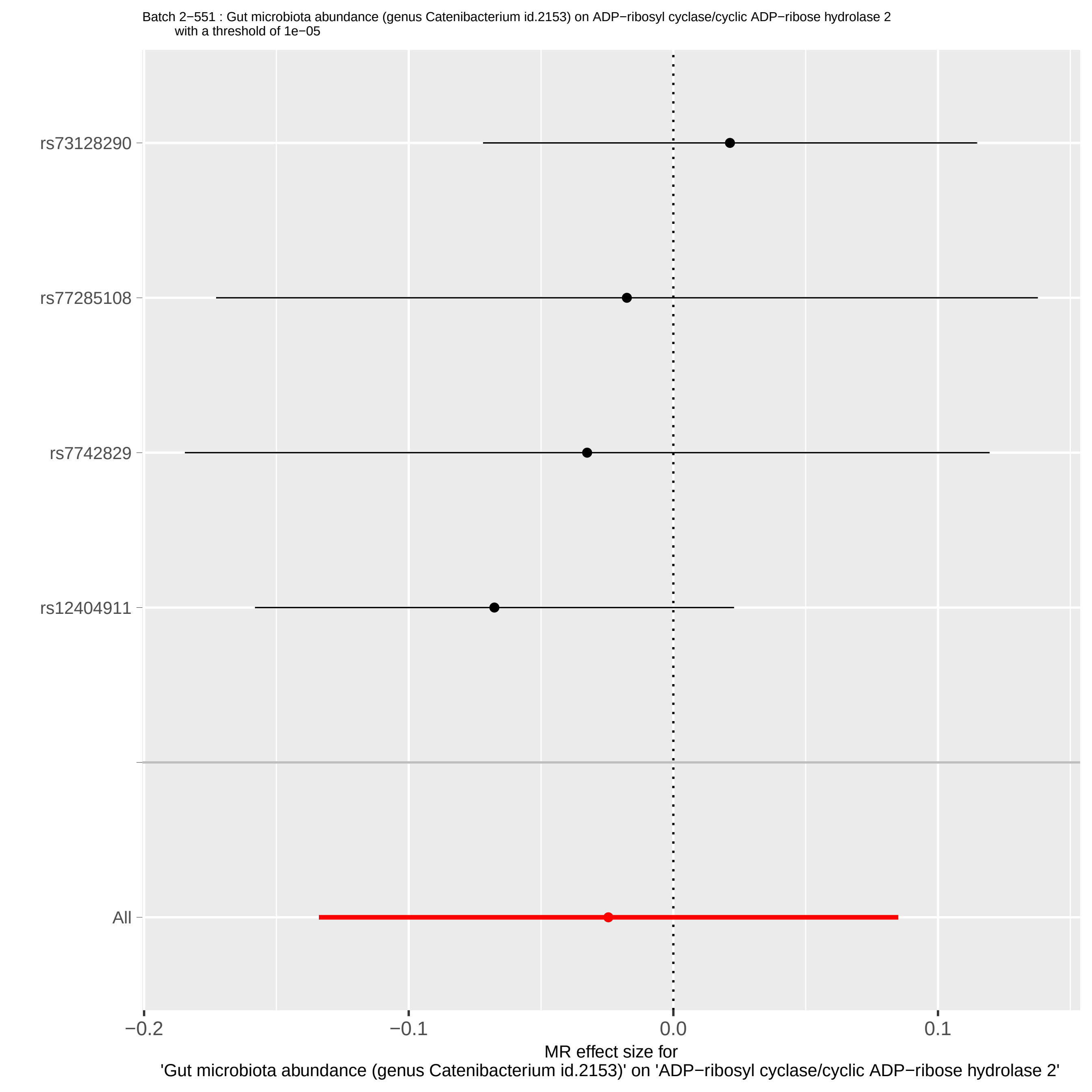

Batch 2−551 : Gut microbiota abundance (genus Catenibacterium id.2153) on ADP−ribosyl cyclase/cyclic ADP−ribose hydrolase 2
with a threshold of 1e−05
rs73128290
rs77285108
rs7742829
rs12404911
All
−0.2
−0.1
0.0
0.1
MR effect size for
'Gut microbiota abundance (genus Catenibacterium id.2153)' on 'ADP−ribosyl cyclase/cyclic ADP−ribose hydrolase 2'

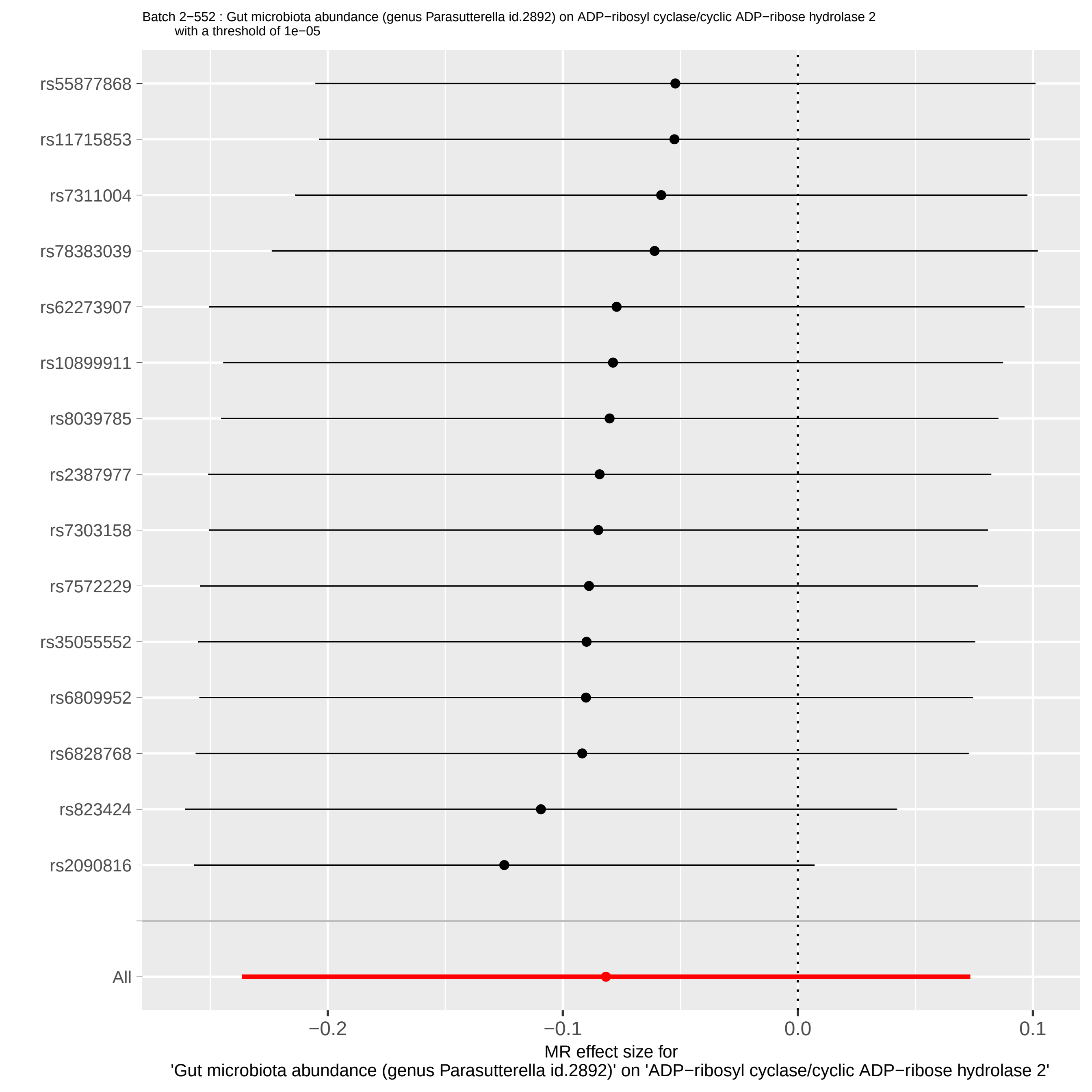
Batch 2−552 : Gut microbiota abundance (genus Parasutterella id.2892) on ADP−ribosyl cyclase/cyclic ADP−ribose hydrolase 2
with a threshold of 1e−05
rs55877868
rs11715853
rs7311004
rs78383039
rs62273907
rs10899911
rs8039785
rs2387977
rs7303158
rs7572229
rs35055552
rs6809952
rs6828768
rs823424
rs2090816
All
−0.2
−0.1
0.0
0.1
MR effect size for
'Gut microbiota abundance (genus Parasutterella id.2892)' on 'ADP−ribosyl cyclase/cyclic ADP−ribose hydrolase 2'

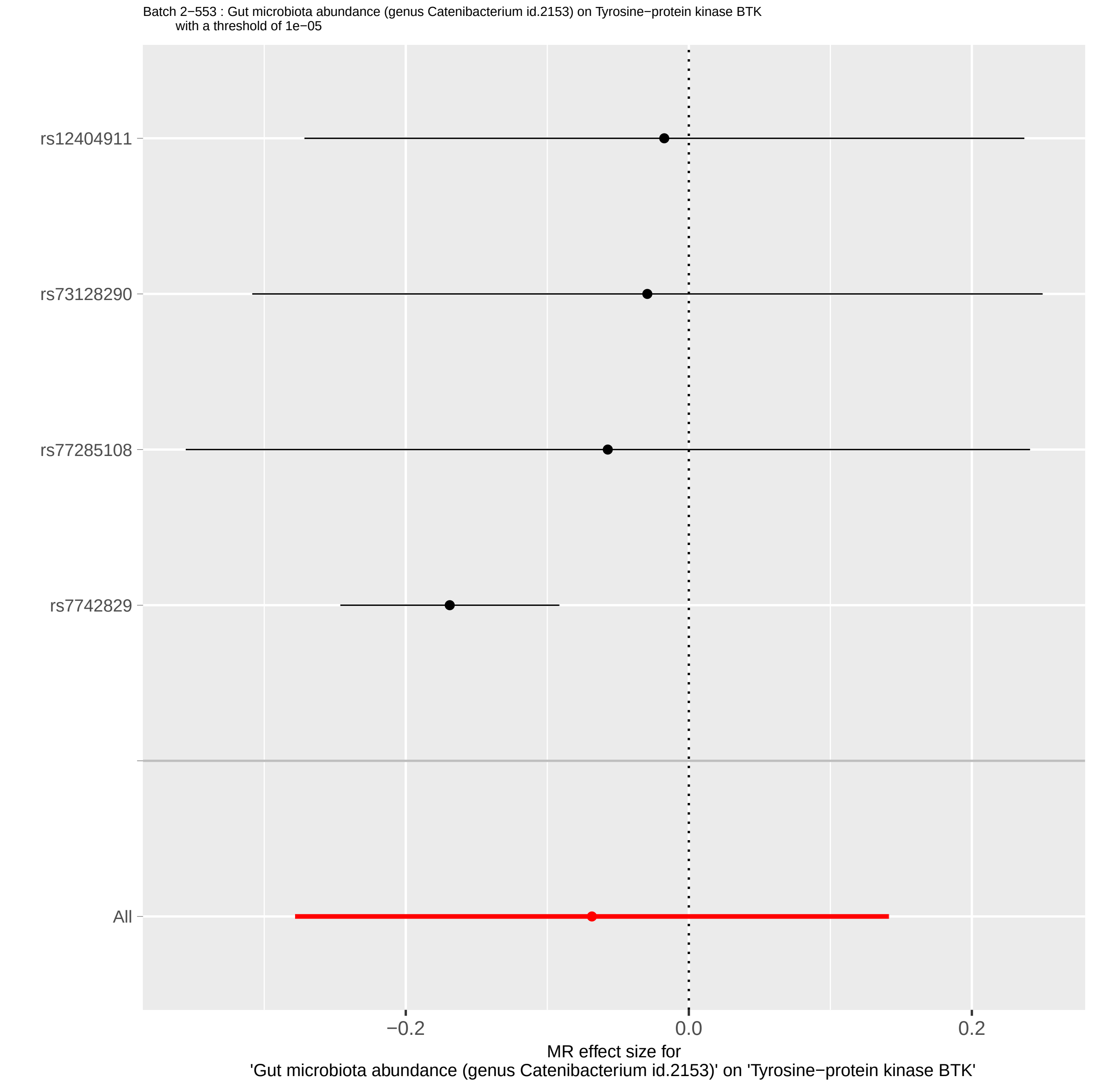
Batch 2−553 : Gut microbiota abundance (genus Catenibacterium id.2153) on Tyrosine−protein kinase BTK
with a threshold of 1e−05
rs12404911
rs73128290
rs77285108
rs7742829
All
−0.2
0.0
0.2
MR effect size for
'Gut microbiota abundance (genus Catenibacterium id.2153)' on 'Tyrosine−protein kinase BTK'

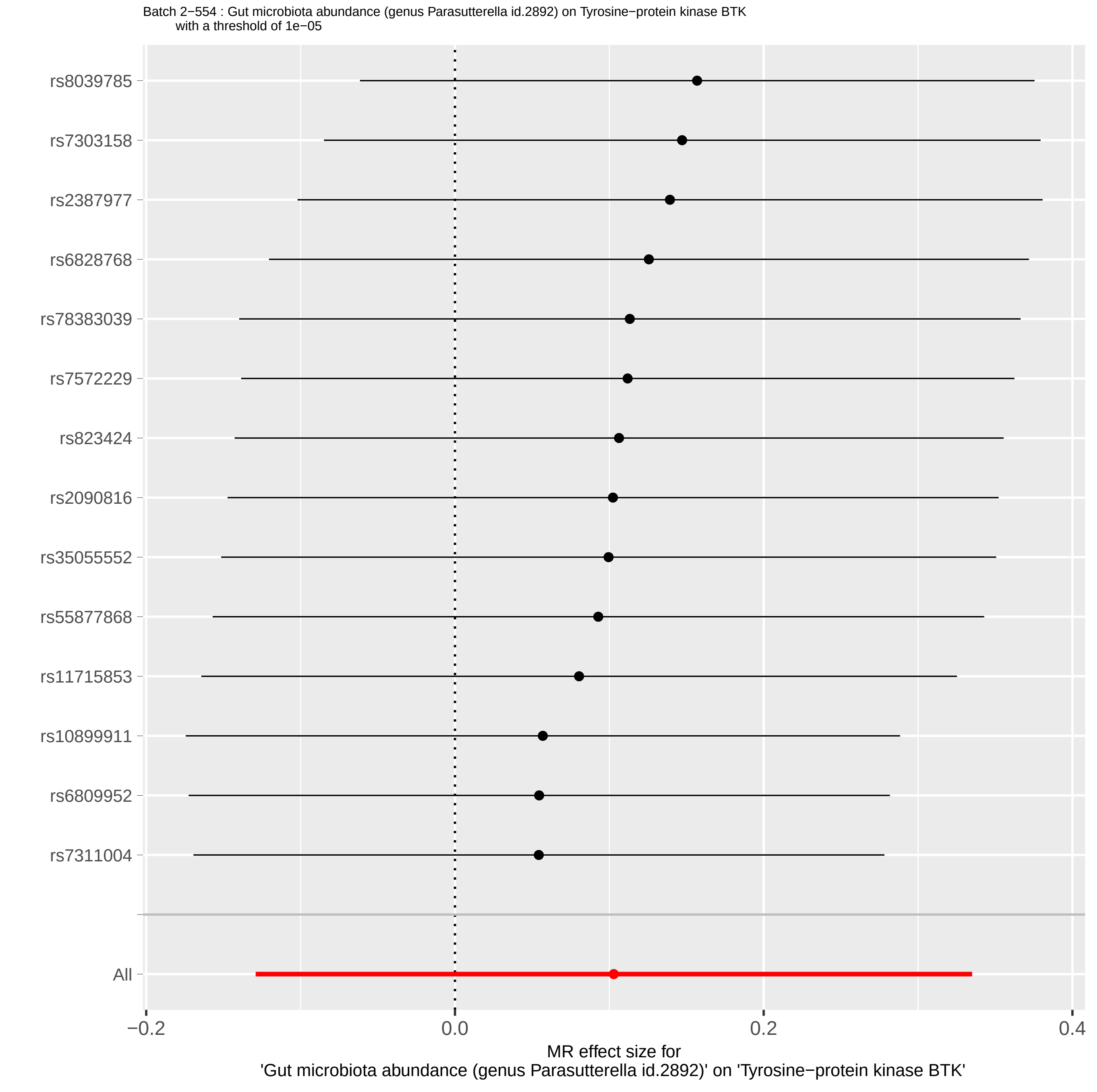

Batch 2−554 : Gut microbiota abundance (genus Parasutterella id.2892) on Tyrosine−protein kinase BTK
with a threshold of 1e−05
rs8039785
rs7303158
rs2387977
rs6828768
rs78383039
rs7572229
rs823424
rs2090816
rs35055552
rs55877868
rs11715853
rs10899911
rs6809952
rs7311004
All
−0.2
0.0
0.2
0.4
MR effect size for
'Gut microbiota abundance (genus Parasutterella id.2892)' on 'Tyrosine−protein kinase BTK'

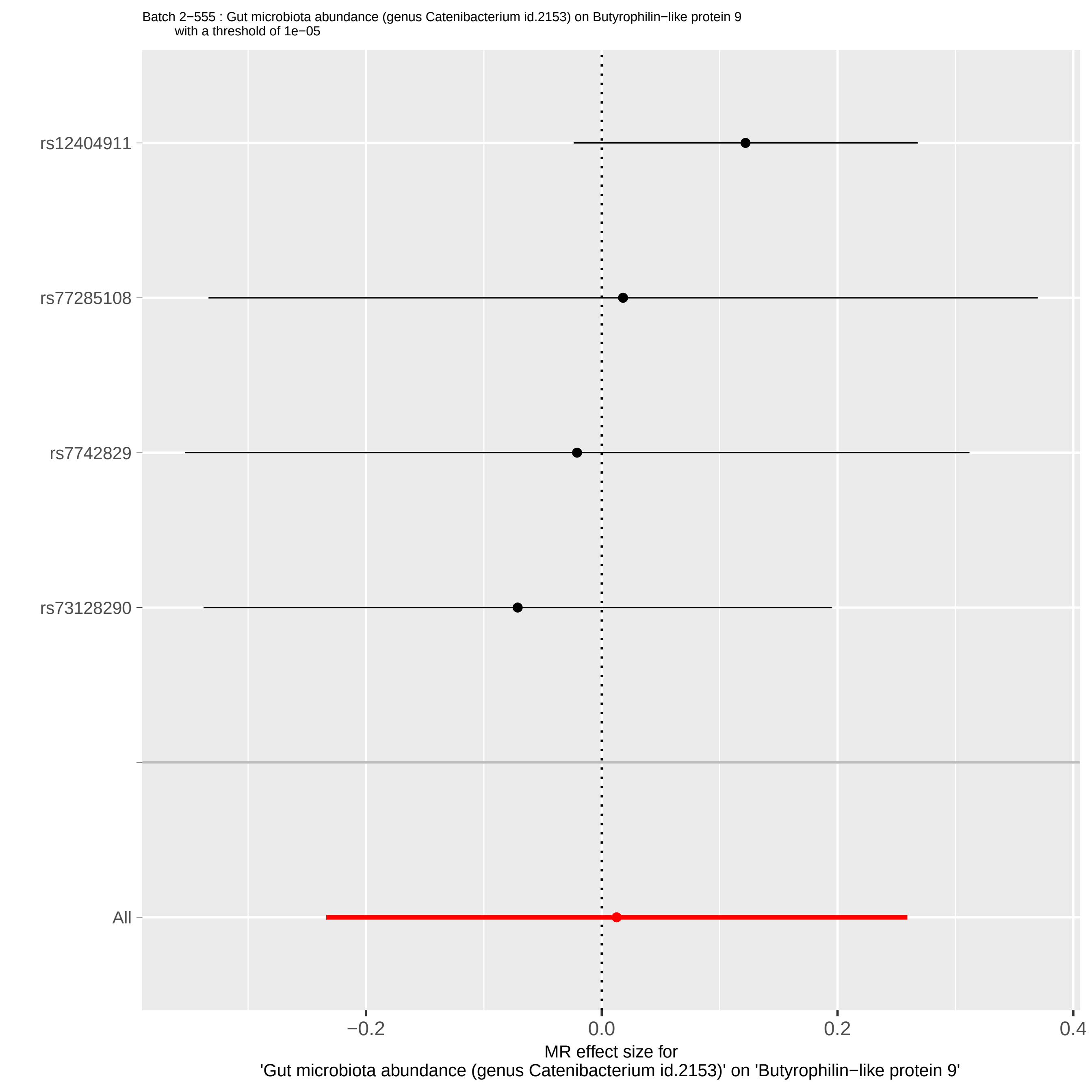

Batch 2−555 : Gut microbiota abundance (genus Catenibacterium id.2153) on Butyrophilin−like protein 9
with a threshold of 1e−05
rs12404911
rs77285108
rs7742829
rs73128290
All
−0.2
0.0
0.2
0.4
MR effect size for
'Gut microbiota abundance (genus Catenibacterium id.2153)' on 'Butyrophilin−like protein 9'

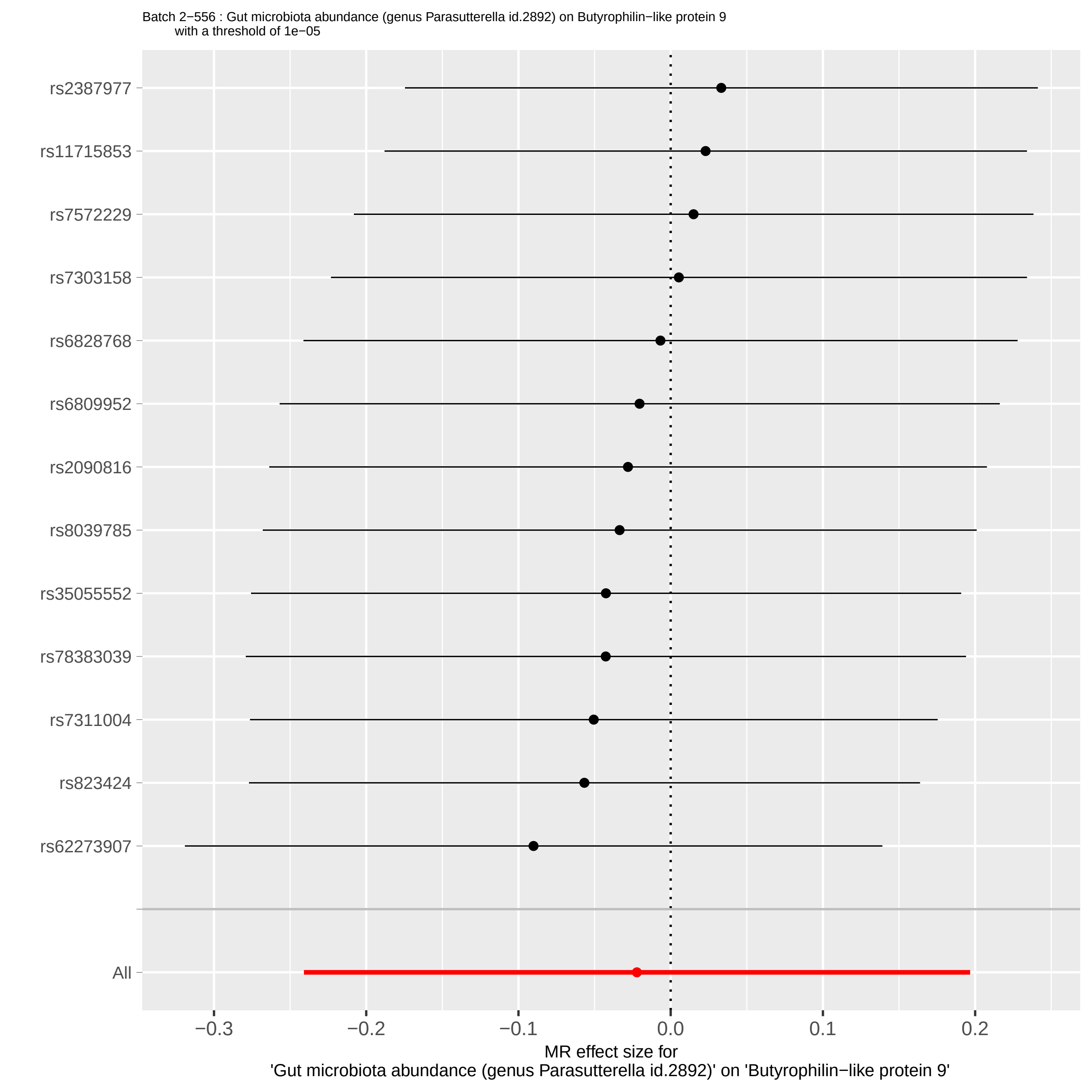

Batch 2−556 : Gut microbiota abundance (genus Parasutterella id.2892) on Butyrophilin−like protein 9
with a threshold of 1e−05
rs2387977
rs11715853
rs7572229
rs7303158
rs6828768
rs6809952
rs2090816
rs8039785
rs35055552
rs78383039
rs7311004
rs823424
rs62273907
All
−0.3
−0.2
−0.1
0.0
0.1
0.2
MR effect size for
'Gut microbiota abundance (genus Parasutterella id.2892)' on 'Butyrophilin−like protein 9'

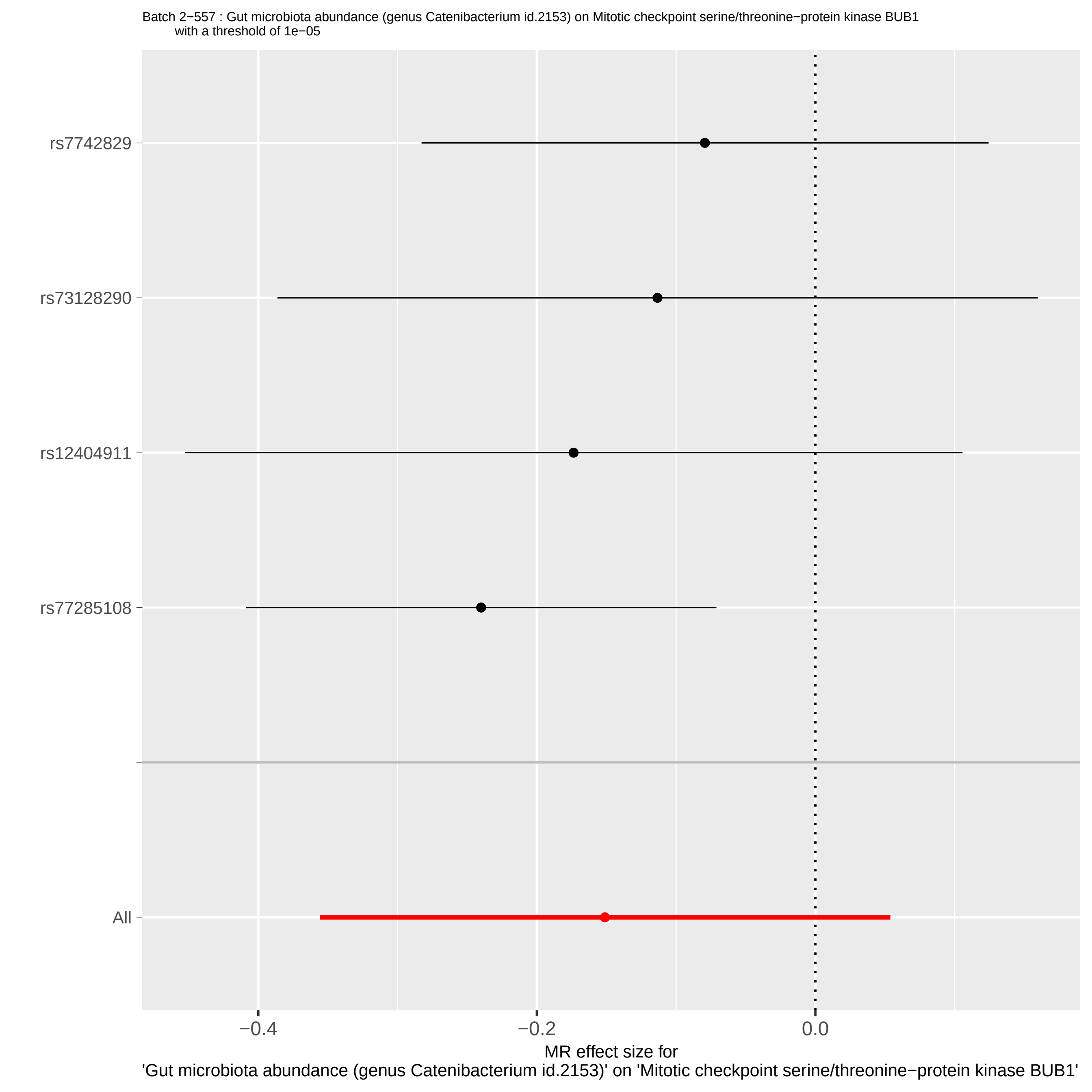
Batch 2−557 : Gut microbiota abundance (genus Catenibacterium id.2153) on Mitotic checkpoint serine/threonine−protein kinase BUB1
with a threshold of 1e−05
rs7742829
rs73128290
rs12404911
rs77285108
All
−0.4
−0.2
0.0
MR effect size for
'Gut microbiota abundance (genus Catenibacterium id.2153)' on 'Mitotic checkpoint serine/threonine−protein kinase BUB1'

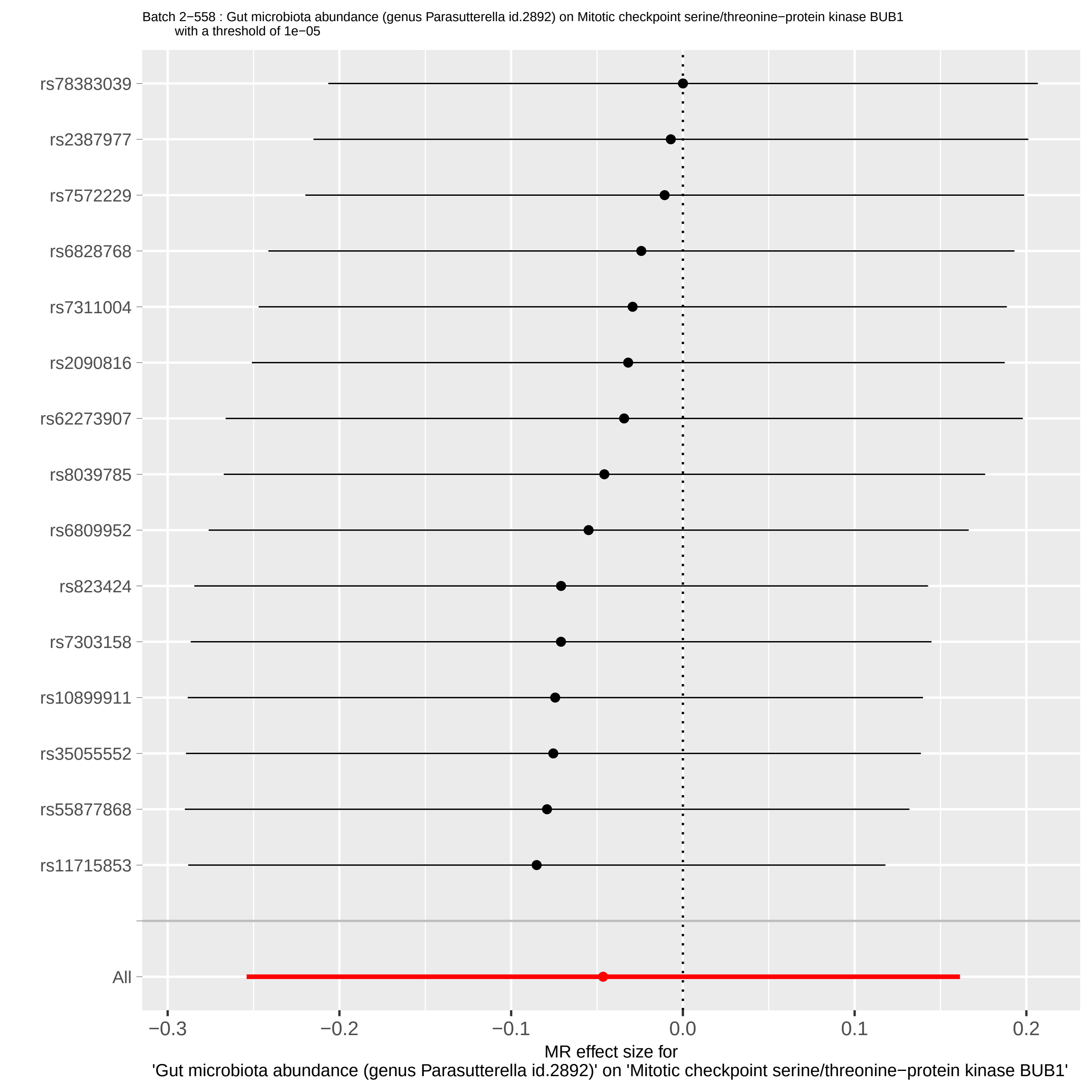

Batch 2−558 : Gut microbiota abundance (genus Parasutterella id.2892) on Mitotic checkpoint serine/threonine−protein kinase BUB1
with a threshold of 1e−05
rs78383039
rs2387977
rs7572229
rs6828768
rs7311004
rs2090816
rs62273907
rs8039785
rs6809952
rs823424
rs7303158
rs10899911
rs35055552
rs55877868
rs11715853
All
−0.3
−0.2
−0.1
0.0
0.1
0.2
MR effect size for
'Gut microbiota abundance (genus Parasutterella id.2892)' on 'Mitotic checkpoint serine/threonine−protein kinase BUB1'

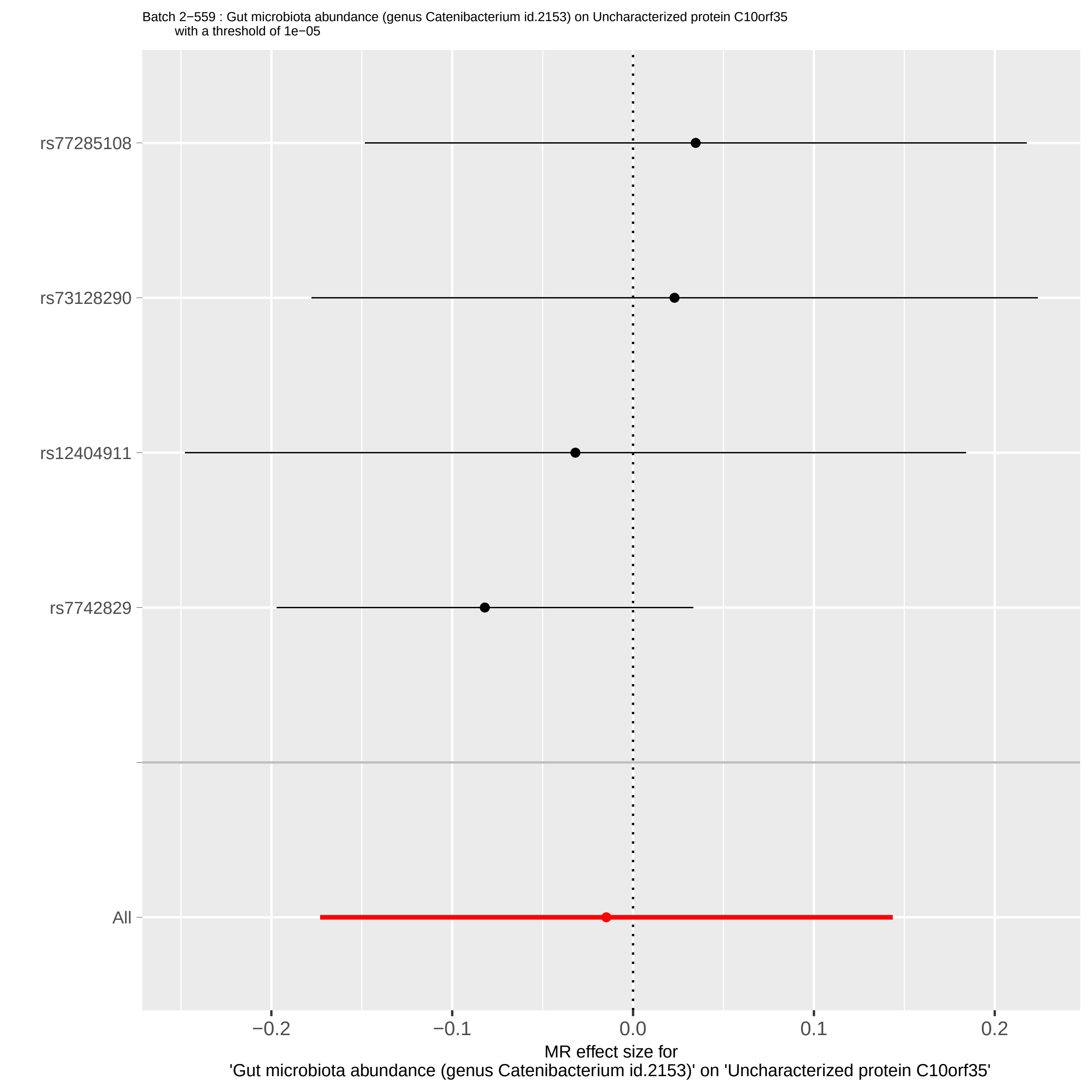

Batch 2−559 : Gut microbiota abundance (genus Catenibacterium id.2153) on Uncharacterized protein C10orf35
with a threshold of 1e−05
rs77285108
rs73128290
rs12404911
rs7742829
All
−0.2
−0.1
0.0
0.1
0.2
MR effect size for
'Gut microbiota abundance (genus Catenibacterium id.2153)' on 'Uncharacterized protein C10orf35'

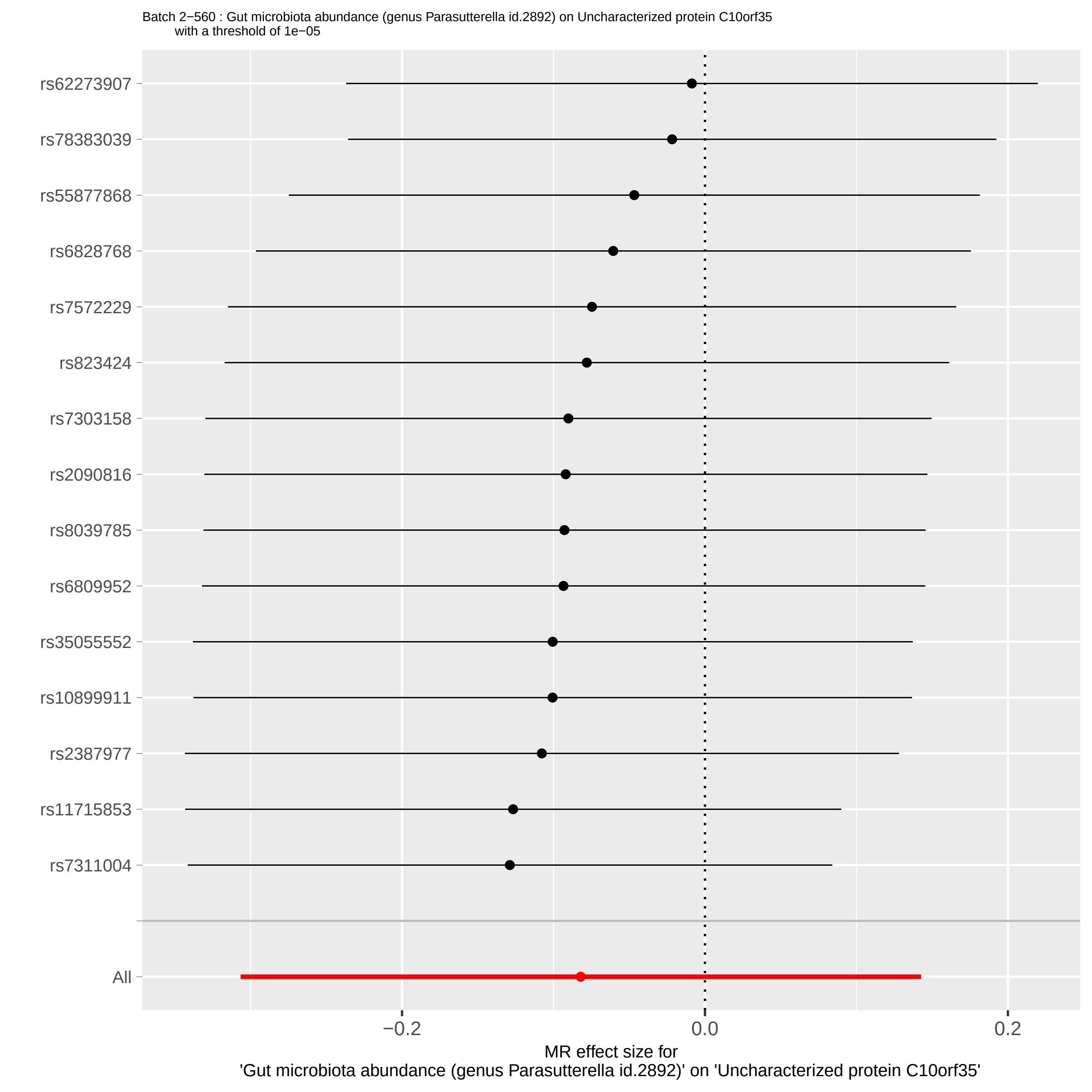
Batch 2−560 : Gut microbiota abundance (genus Parasutterella id.2892) on Uncharacterized protein C10orf35
with a threshold of 1e−05
rs62273907
rs78383039
rs55877868
rs6828768
rs7572229
rs823424
rs7303158
rs2090816
rs8039785
rs6809952
rs35055552
rs10899911
rs2387977
rs11715853
rs7311004
All
−0.2
0.0
0.2
MR effect size for
'Gut microbiota abundance (genus Parasutterella id.2892)' on 'Uncharacterized protein C10orf35'

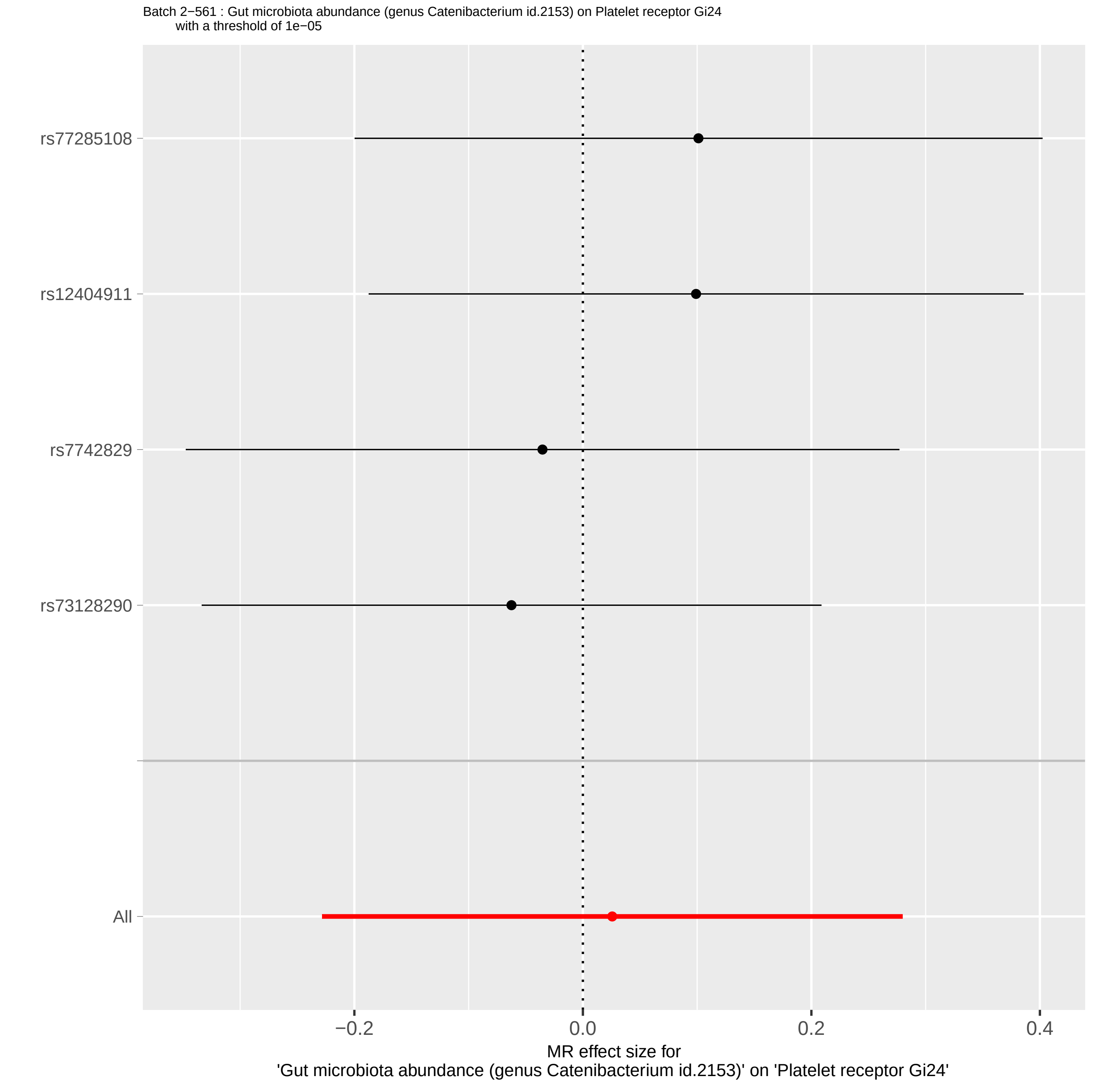
Batch 2−561 : Gut microbiota abundance (genus Catenibacterium id.2153) on Platelet receptor Gi24
with a threshold of 1e−05
rs77285108
rs12404911
rs7742829
rs73128290
All
−0.2
0.0
0.2
0.4
MR effect size for
'Gut microbiota abundance (genus Catenibacterium id.2153)' on 'Platelet receptor Gi24'

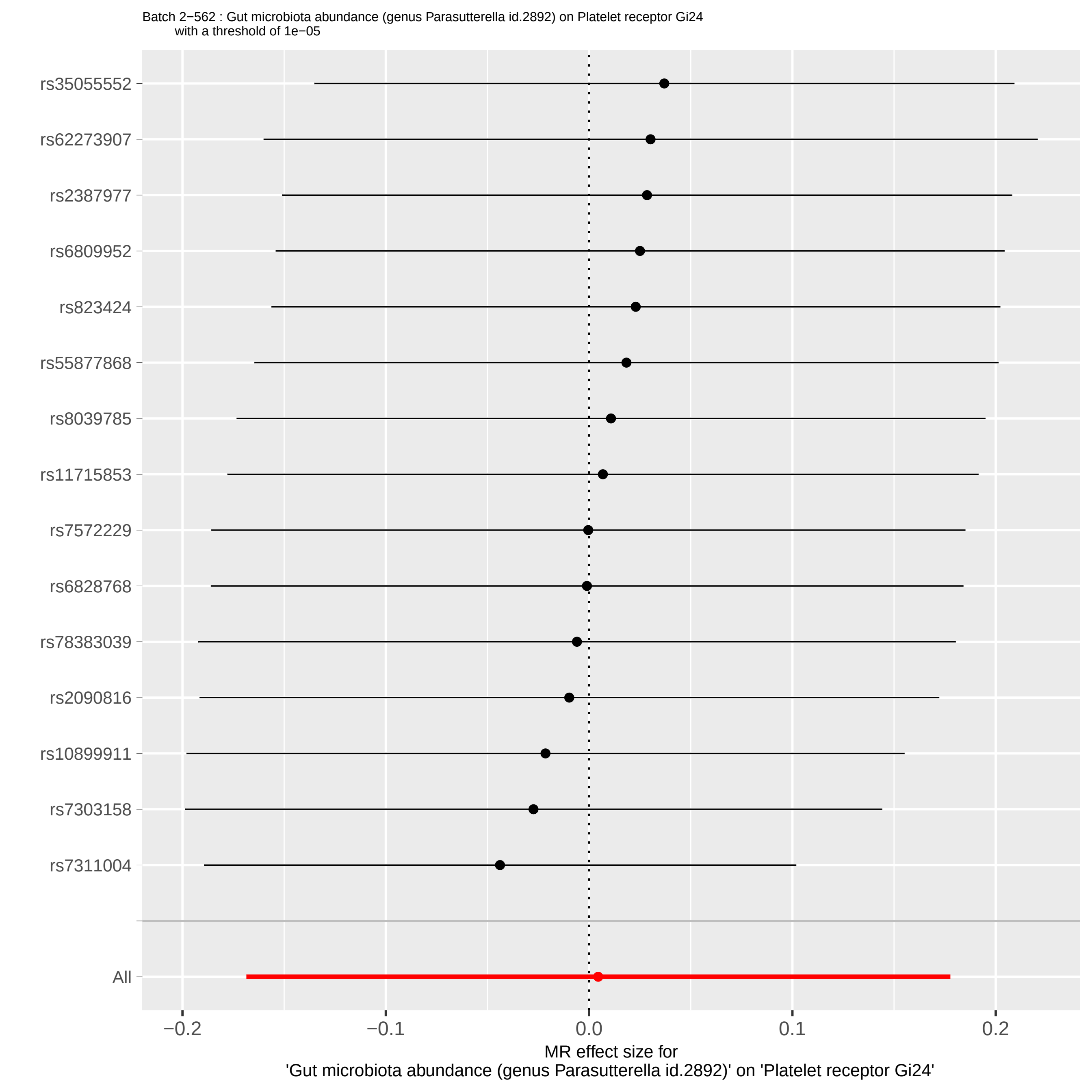

Batch 2−562 : Gut microbiota abundance (genus Parasutterella id.2892) on Platelet receptor Gi24
with a threshold of 1e−05
rs35055552
rs62273907
rs2387977
rs6809952
rs823424
rs55877868
rs8039785
rs11715853
rs7572229
rs6828768
rs78383039
rs2090816
rs10899911
rs7303158
rs7311004
All
−0.2
−0.1
0.0
0.1
0.2
MR effect size for
'Gut microbiota abundance (genus Parasutterella id.2892)' on 'Platelet receptor Gi24'

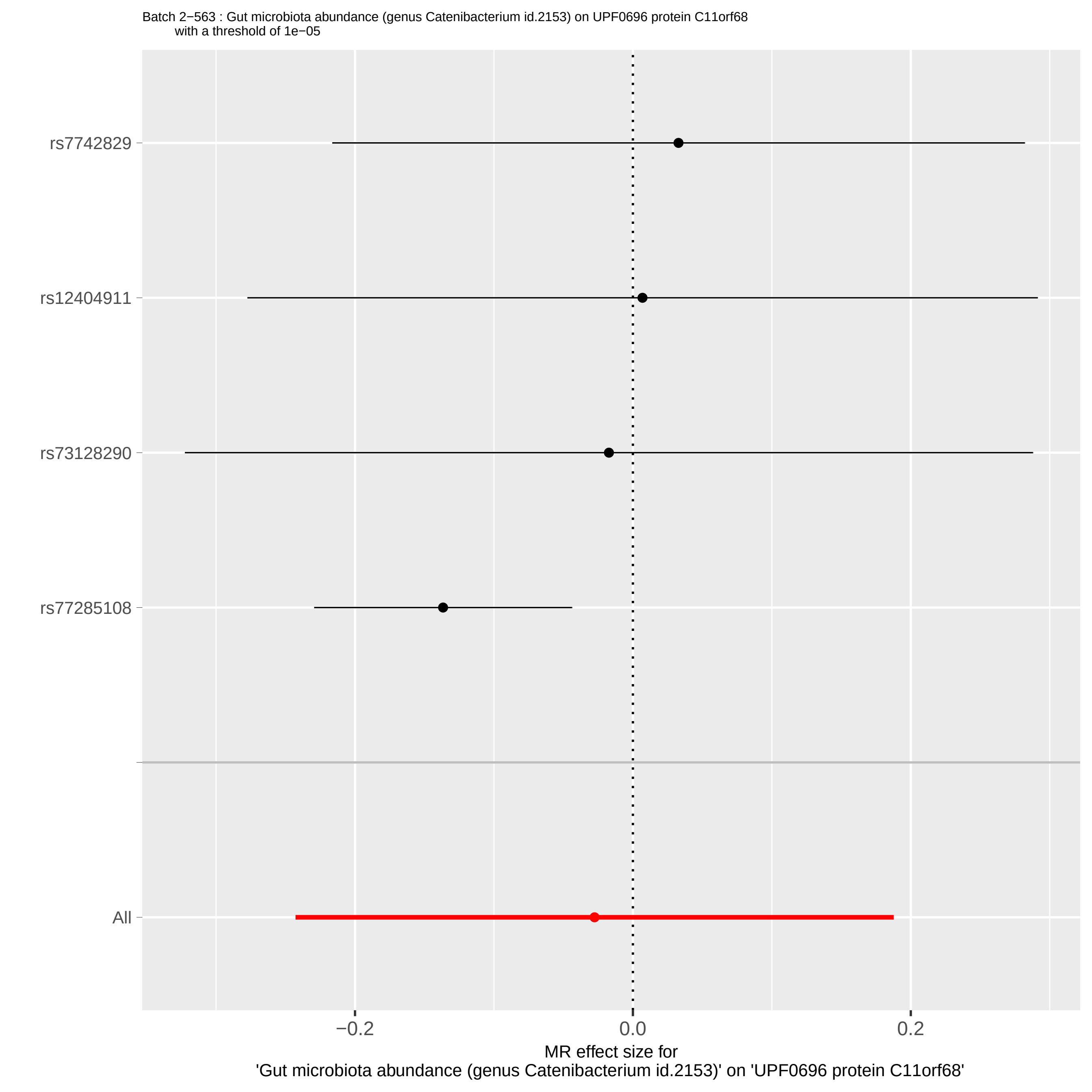

Batch 2−563 : Gut microbiota abundance (genus Catenibacterium id.2153) on UPF0696 protein C11orf68
with a threshold of 1e−05
rs7742829
rs12404911
rs73128290
rs77285108
All
−0.2
0.0
0.2
MR effect size for
'Gut microbiota abundance (genus Catenibacterium id.2153)' on 'UPF0696 protein C11orf68'

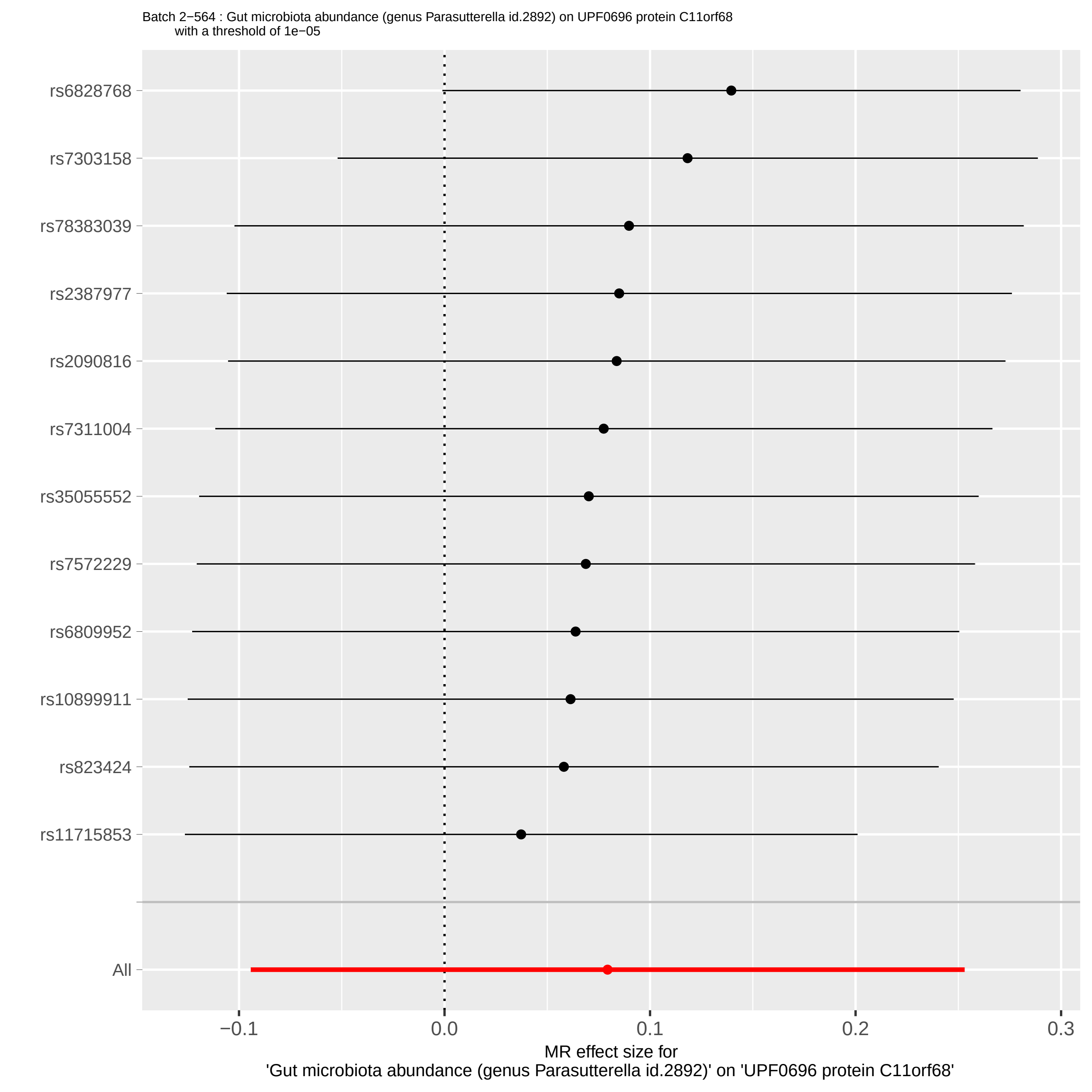
Batch 2−564 : Gut microbiota abundance (genus Parasutterella id.2892) on UPF0696 protein C11orf68
with a threshold of 1e−05
rs6828768
rs7303158
rs78383039
rs2387977
rs2090816
rs7311004
rs35055552
rs7572229
rs6809952
rs10899911
rs823424
rs11715853
All
−0.1
0.0
0.1
0.2
0.3
MR effect size for
'Gut microbiota abundance (genus Parasutterella id.2892)' on 'UPF0696 protein C11orf68'

Batch 2−565 : Gut microbiota abundance (genus Catenibacterium id.2153) on UPF0454 protein C12orf49
with a threshold of 1e−05

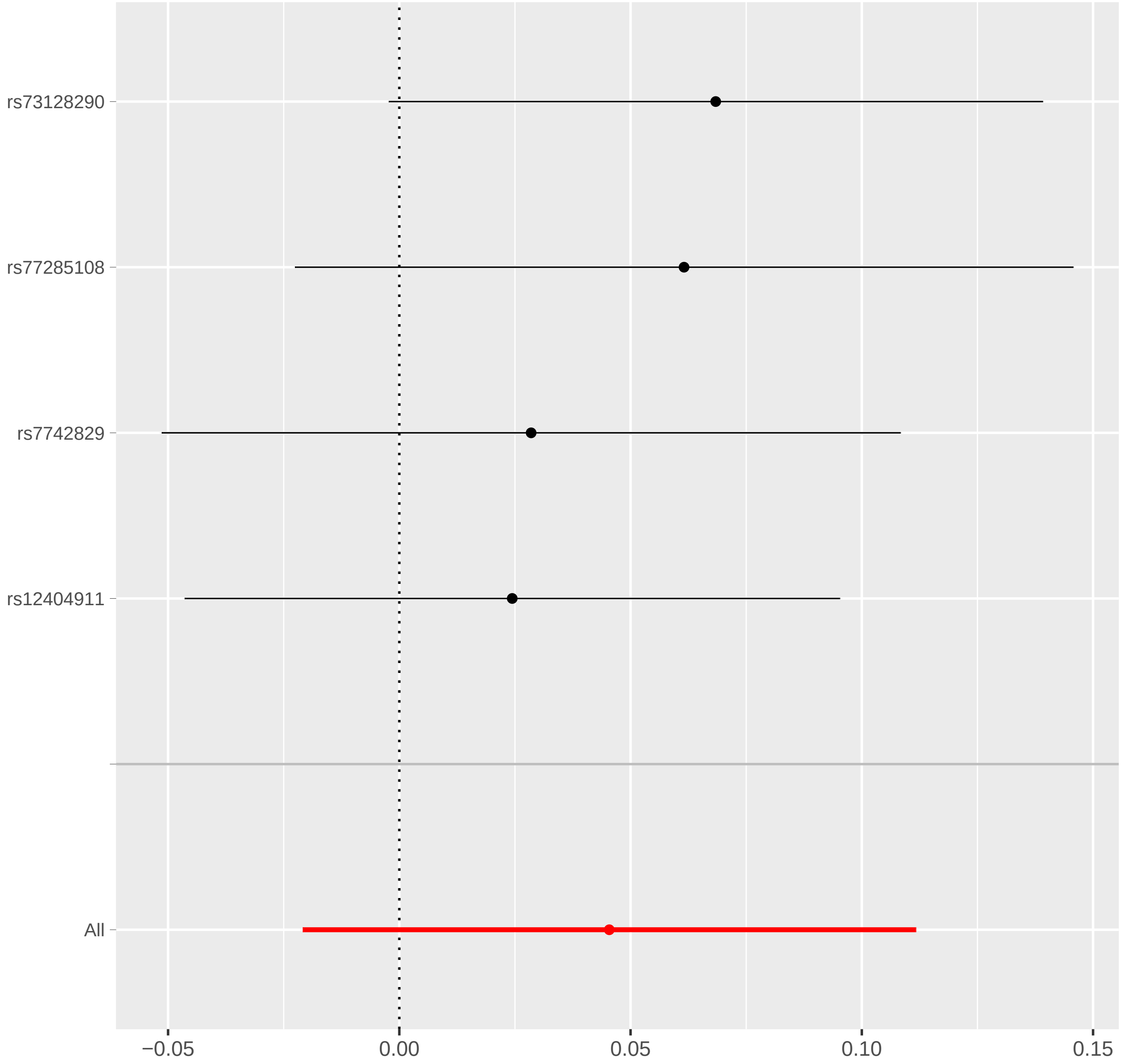

MR effect size for
'Gut microbiota abundance (genus Catenibacterium id.2153)' on 'UPF0454 protein C12orf49'

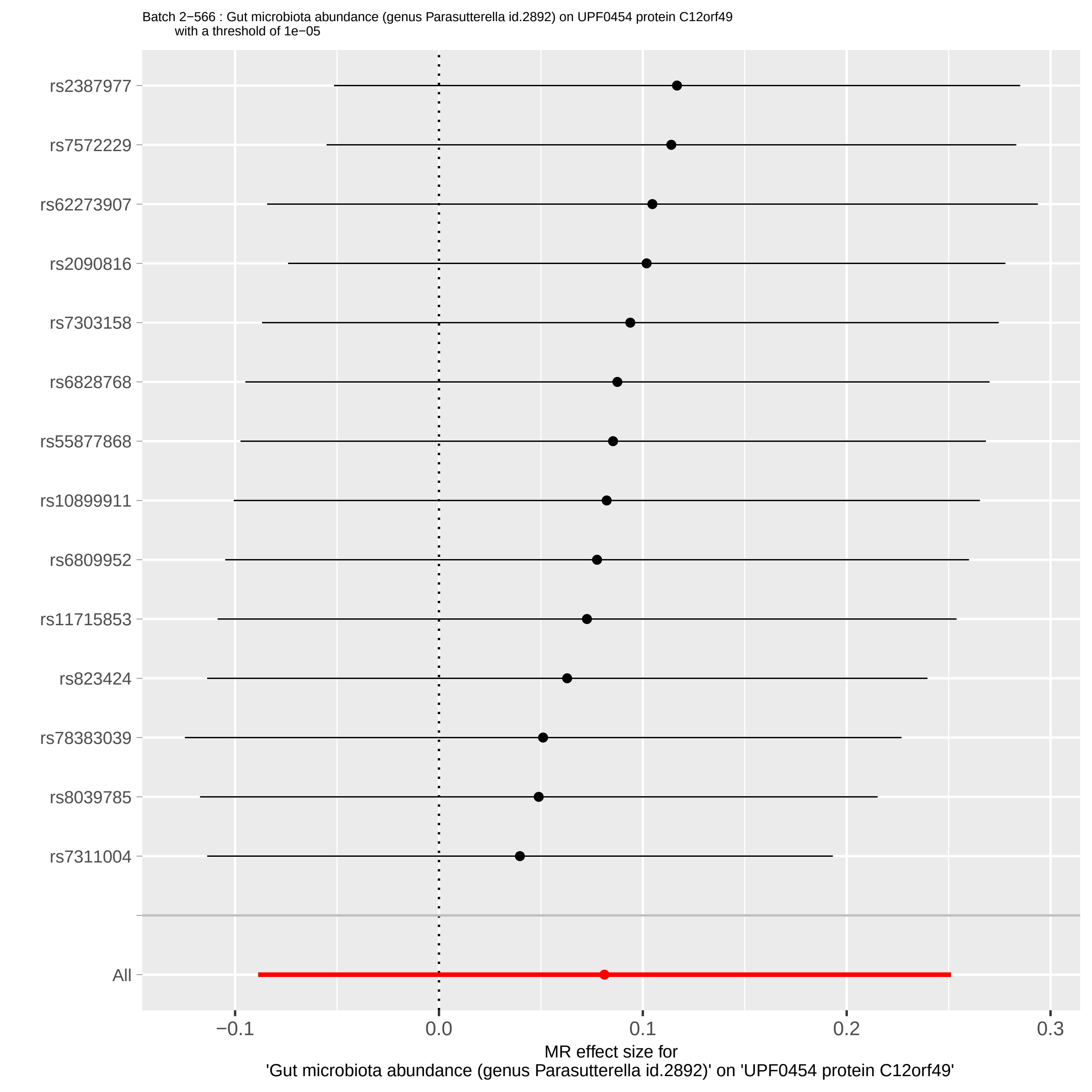

Batch 2−566 : Gut microbiota abundance (genus Parasutterella id.2892) on UPF0454 protein C12orf49
with a threshold of 1e−05
rs2387977
rs7572229
rs62273907
rs2090816
rs7303158
rs6828768
rs55877868
rs10899911
rs6809952
rs11715853
rs823424
rs78383039
rs8039785
rs7311004
All
−0.1
0.0
0.1
0.2
0.3
MR effect size for
'Gut microbiota abundance (genus Parasutterella id.2892)' on 'UPF0454 protein C12orf49'

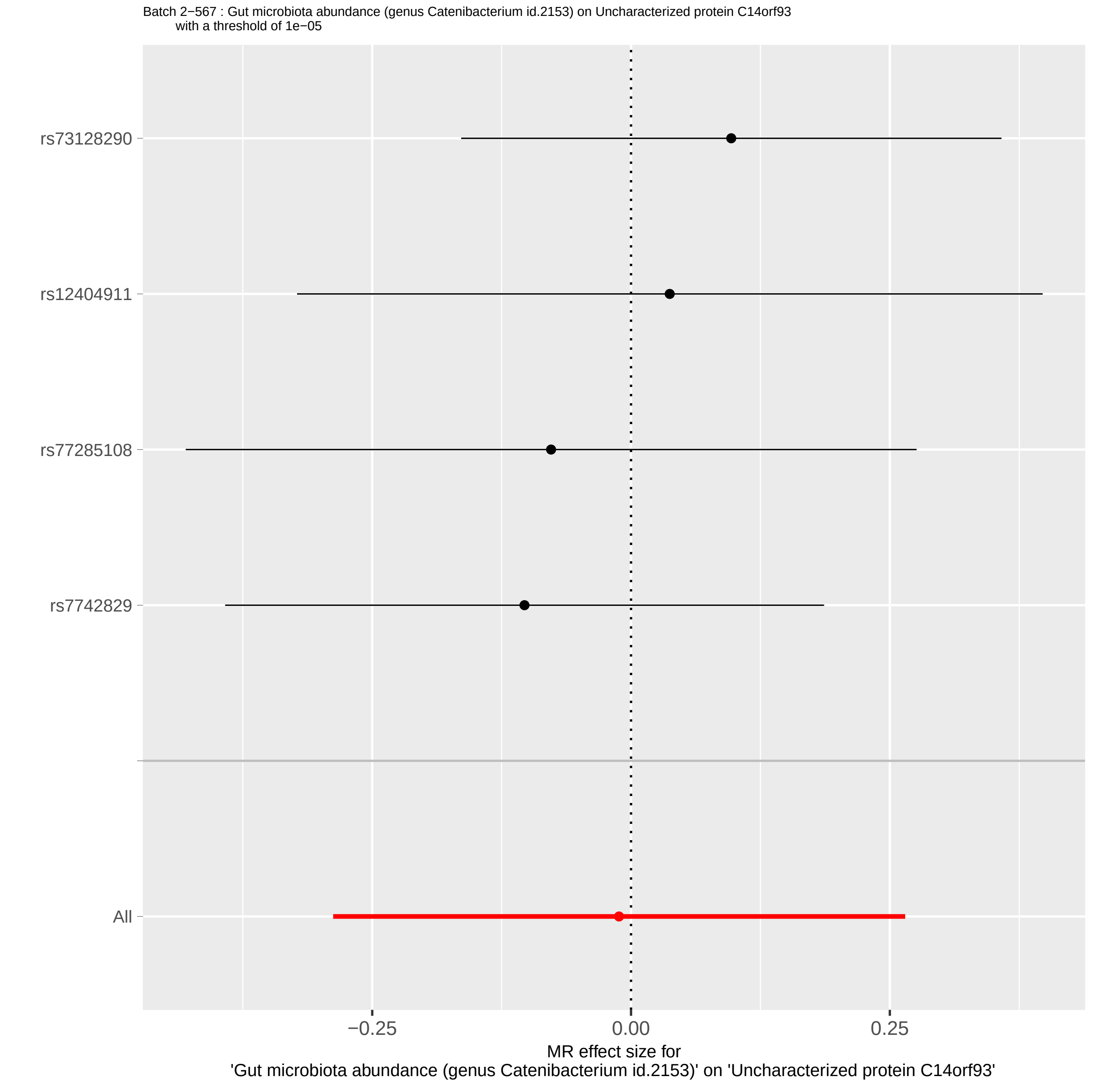

Batch 2−567 : Gut microbiota abundance (genus Catenibacterium id.2153) on Uncharacterized protein C14orf93
with a threshold of 1e−05
rs73128290
rs12404911
rs77285108
rs7742829
All
−0.25
0.00
0.25
MR effect size for
'Gut microbiota abundance (genus Catenibacterium id.2153)' on 'Uncharacterized protein C14orf93'

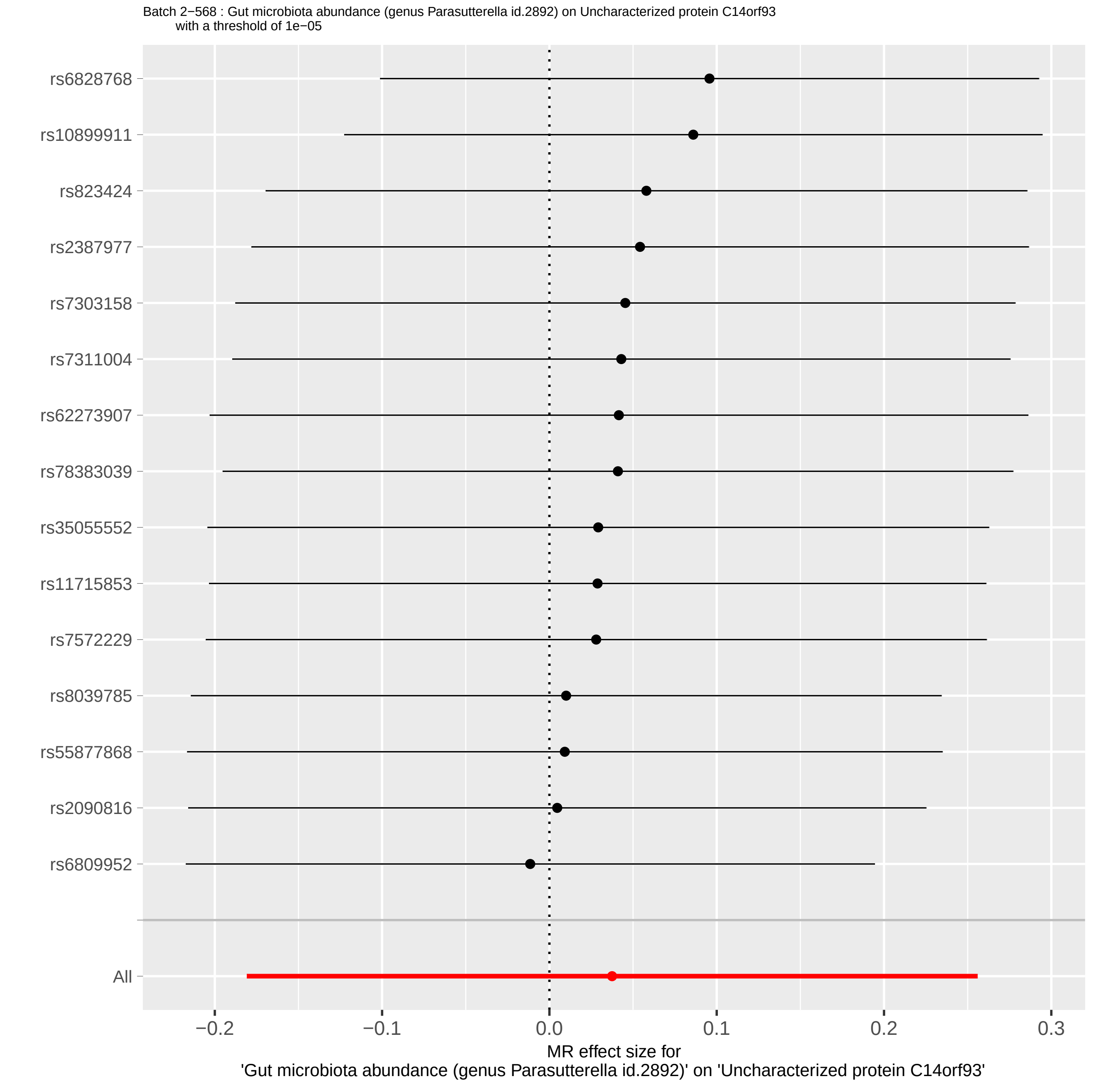

Batch 2−568 : Gut microbiota abundance (genus Parasutterella id.2892) on Uncharacterized protein C14orf93
with a threshold of 1e−05
rs6828768
rs10899911
rs823424
rs2387977
rs7303158
rs7311004
rs62273907
rs78383039
rs35055552
rs11715853
rs7572229
rs8039785
rs55877868
rs2090816
rs6809952
All
−0.2
−0.1
0.0
0.1
0.2
0.3
MR effect size for
'Gut microbiota abundance (genus Parasutterella id.2892)' on 'Uncharacterized protein C14orf93'

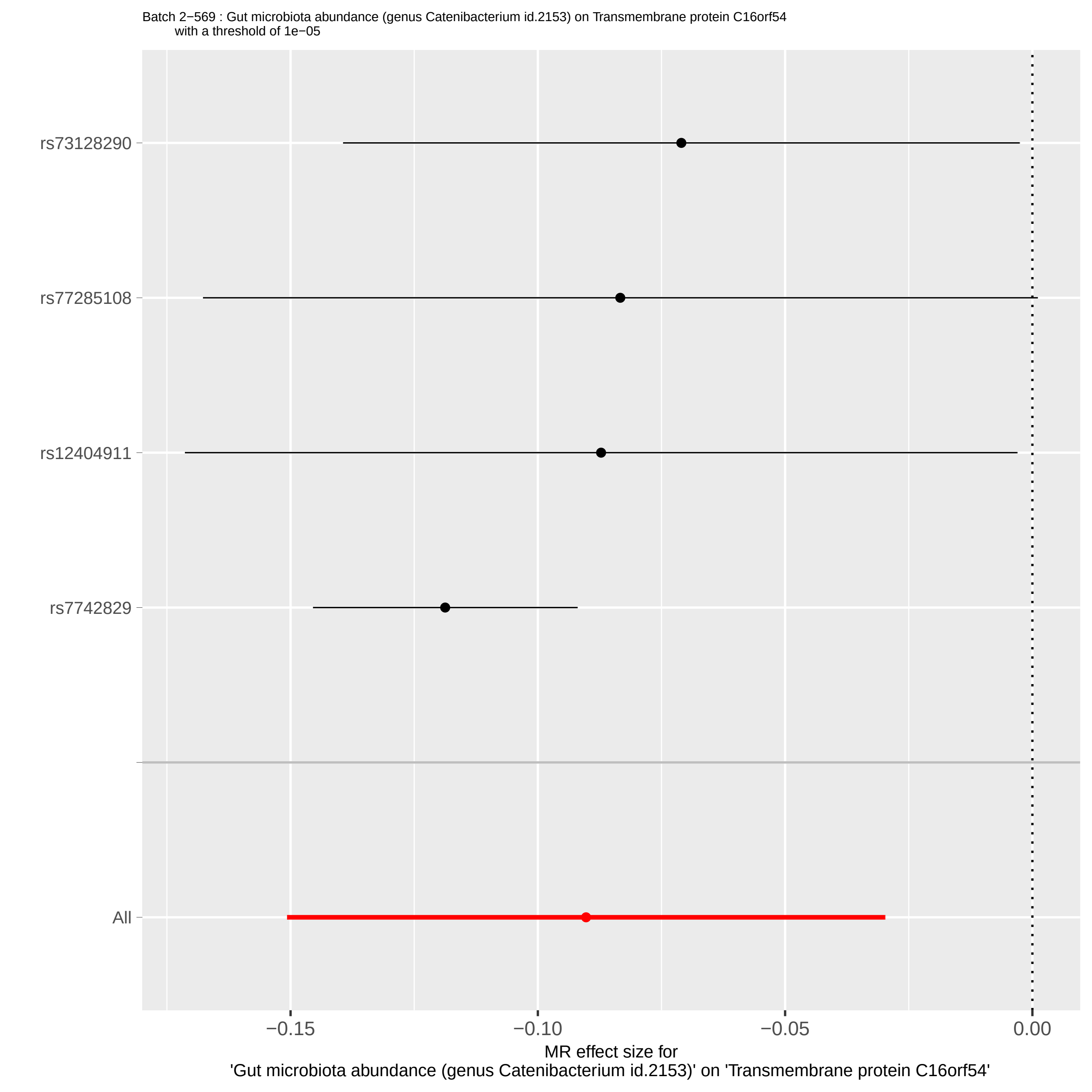

Batch 2−569 : Gut microbiota abundance (genus Catenibacterium id.2153) on Transmembrane protein C16orf54
with a threshold of 1e−05
rs73128290
rs77285108
rs12404911
rs7742829
All
−0.15
−0.10
−0.05
0.00
MR effect size for
'Gut microbiota abundance (genus Catenibacterium id.2153)' on 'Transmembrane protein C16orf54'

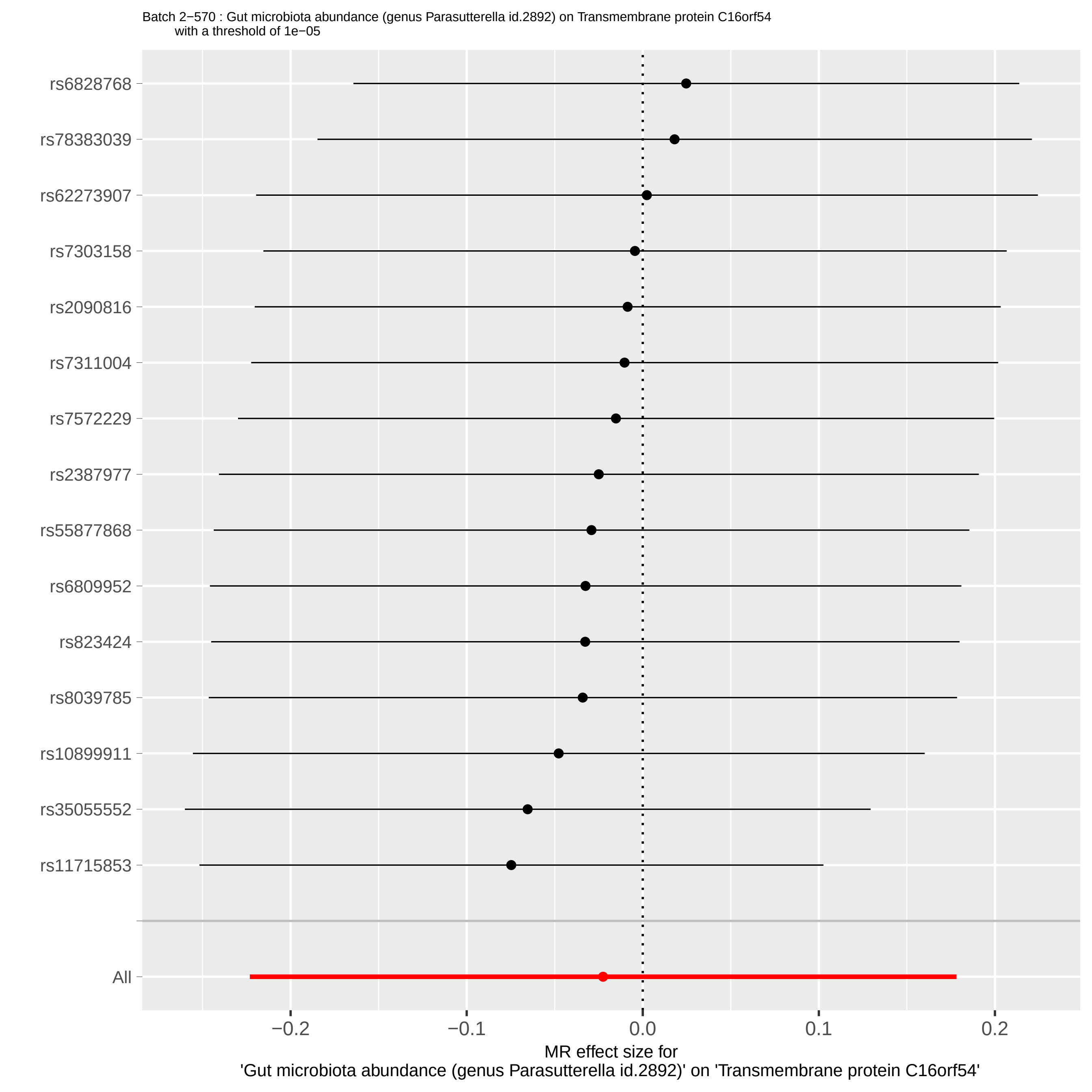

Batch 2−570 : Gut microbiota abundance (genus Parasutterella id.2892) on Transmembrane protein C16orf54
with a threshold of 1e−05
rs6828768
rs78383039
rs62273907
rs7303158
rs2090816
rs7311004
rs7572229
rs2387977
rs55877868
rs6809952
rs823424
rs8039785
rs10899911
rs35055552
rs11715853
All
−0.2
−0.1
0.0
0.1
0.2
MR effect size for
'Gut microbiota abundance (genus Parasutterella id.2892)' on 'Transmembrane protein C16orf54'

Batch 2−571 : Gut microbiota abundance (genus Catenibacterium id.2153) on Uncharacterized protein C17orf67
with a threshold of 1e−05

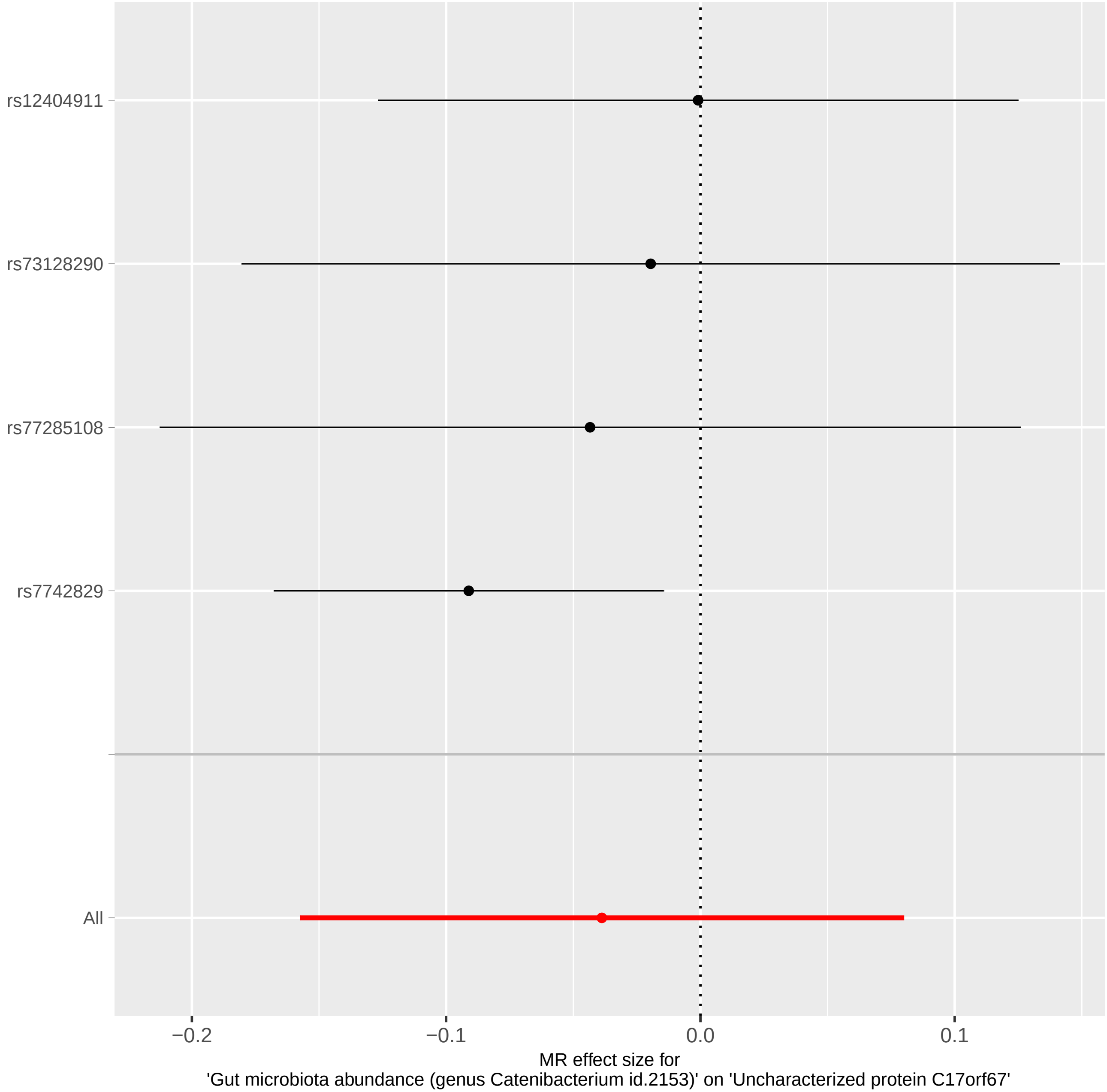

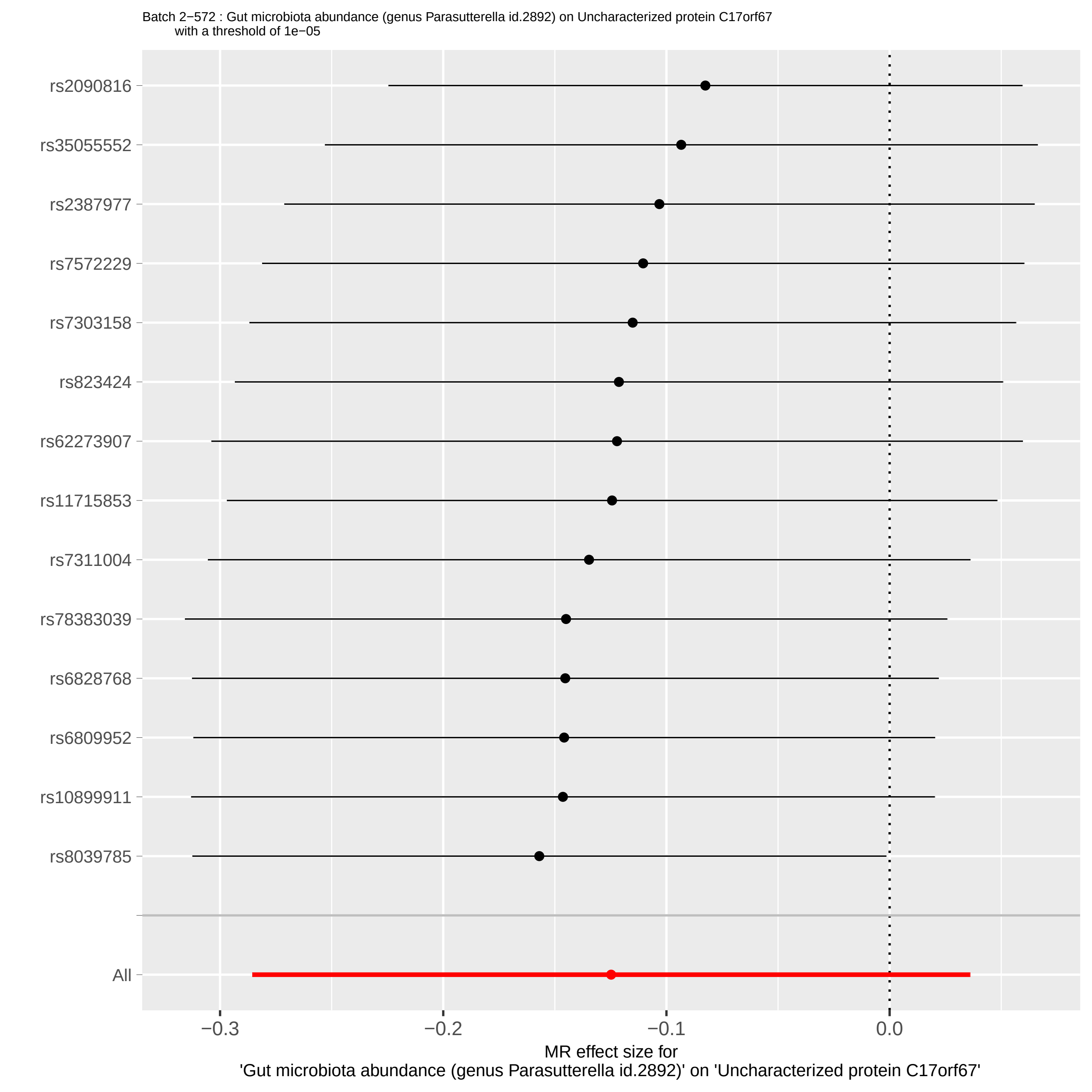

Batch 2−572 : Gut microbiota abundance (genus Parasutterella id.2892) on Uncharacterized protein C17orf67
with a threshold of 1e−05
rs2090816
rs35055552
rs2387977
rs7572229
rs7303158
rs823424
rs62273907
rs11715853
rs7311004
rs78383039
rs6828768
rs6809952
rs10899911
rs8039785
All
−0.3
−0.2
−0.1
0.0
MR effect size for
'Gut microbiota abundance (genus Parasutterella id.2892)' on 'Uncharacterized protein C17orf67'

Batch 2−573 : Gut microbiota abundance (genus Catenibacterium id.2153) on Uncharacterized protein C17orf78
with a threshold of 1e−05

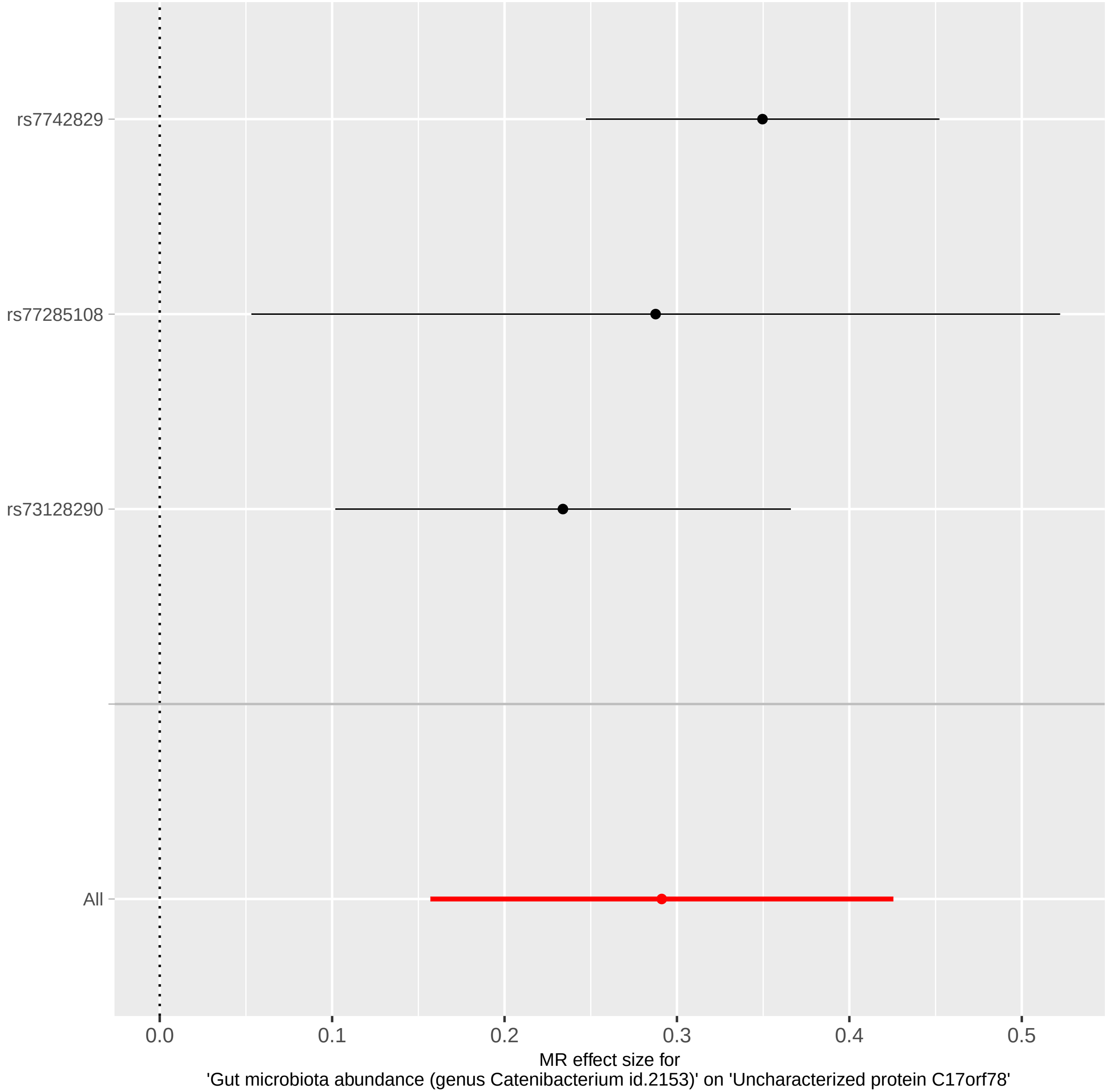

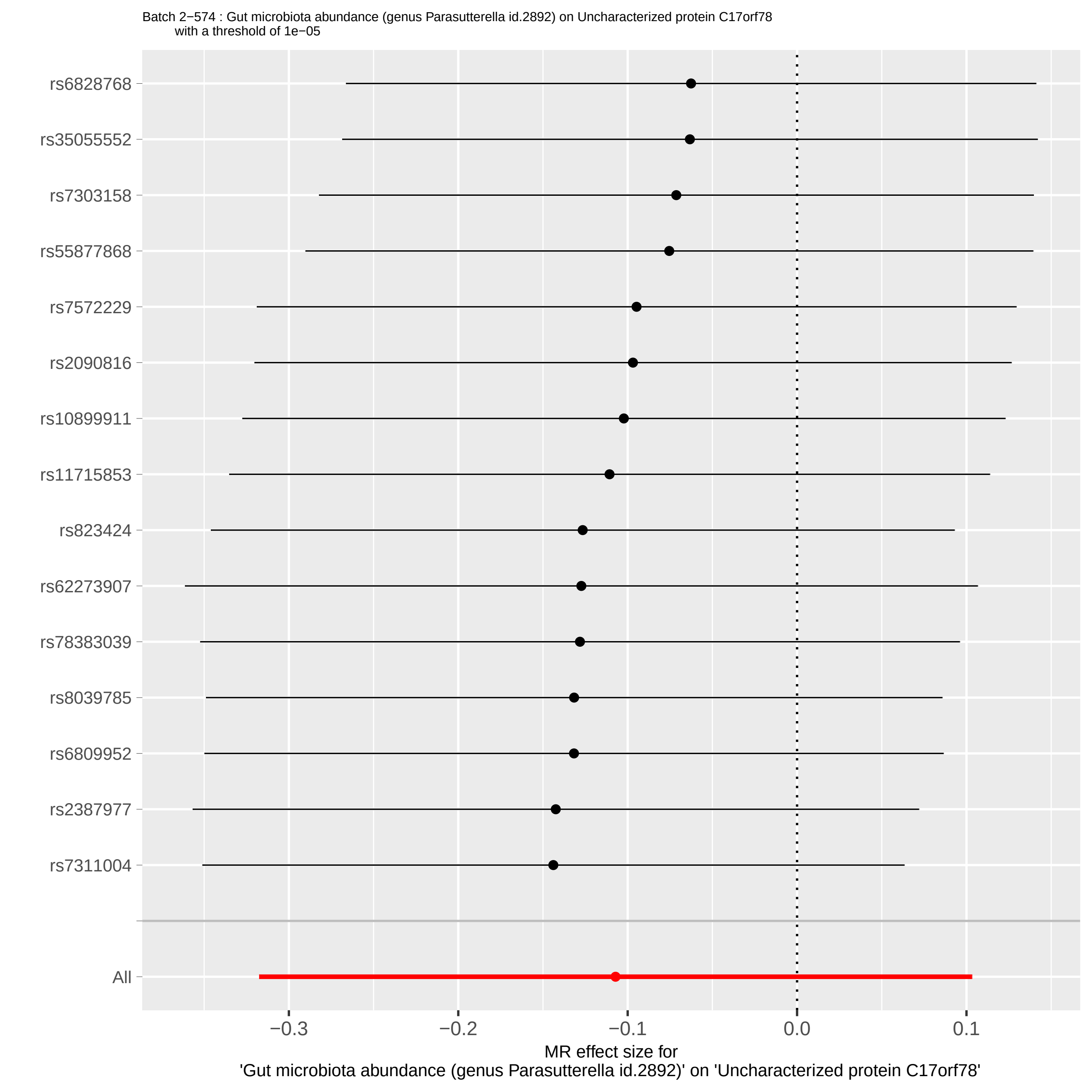

Batch 2−574 : Gut microbiota abundance (genus Parasutterella id.2892) on Uncharacterized protein C17orf78
with a threshold of 1e−05
rs6828768
rs35055552
rs7303158
rs55877868
rs7572229
rs2090816
rs10899911
rs11715853
rs823424
rs62273907
rs78383039
rs8039785
rs6809952
rs2387977
rs7311004
All
−0.3
−0.2
−0.1
0.0
0.1
MR effect size for
'Gut microbiota abundance (genus Parasutterella id.2892)' on 'Uncharacterized protein C17orf78'

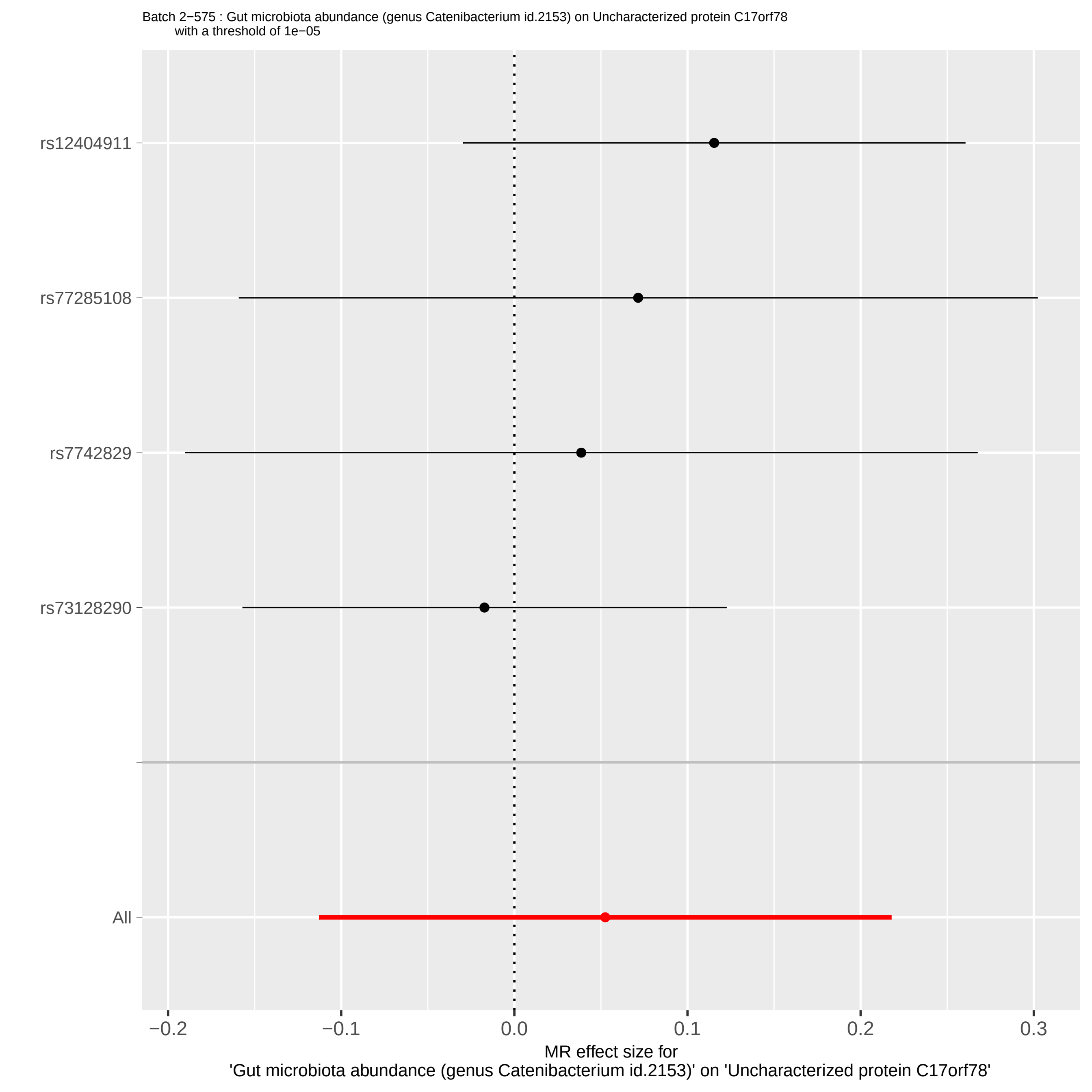
Batch 2−575 : Gut microbiota abundance (genus Catenibacterium id.2153) on Uncharacterized protein C17orf78
with a threshold of 1e−05
rs12404911
rs77285108
rs7742829
rs73128290
All
−0.2
−0.1
0.0
0.1
0.2
0.3
MR effect size for
'Gut microbiota abundance (genus Catenibacterium id.2153)' on 'Uncharacterized protein C17orf78'

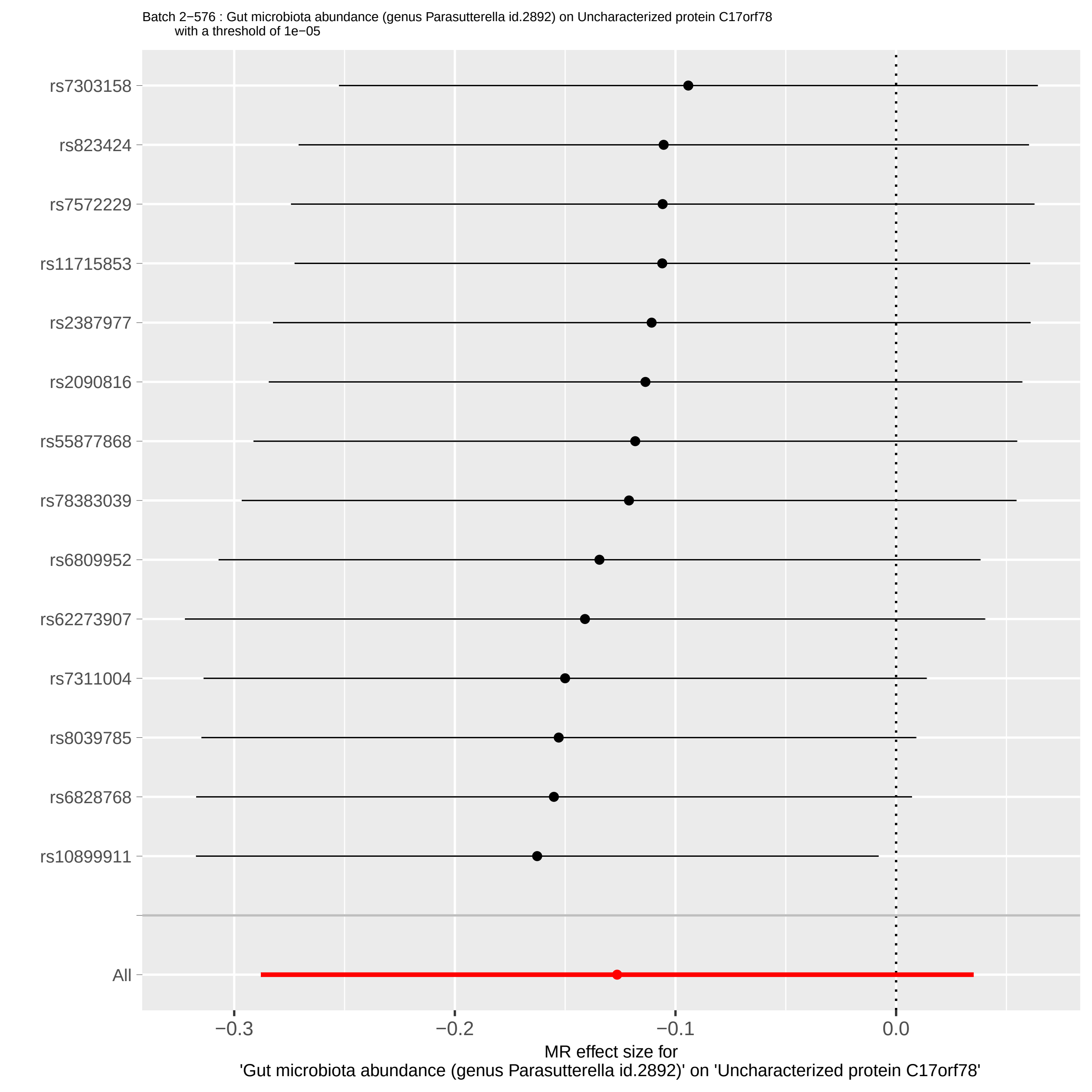

Batch 2−576 : Gut microbiota abundance (genus Parasutterella id.2892) on Uncharacterized protein C17orf78
with a threshold of 1e−05
rs7303158
rs823424
rs7572229
rs11715853
rs2387977
rs2090816
rs55877868
rs78383039
rs6809952
rs62273907
rs7311004
rs8039785
rs6828768
rs10899911
All
−0.3
−0.2
−0.1
0.0
MR effect size for
'Gut microbiota abundance (genus Parasutterella id.2892)' on 'Uncharacterized protein C17orf78'

Batch 2−577 : Gut microbiota abundance (genus Catenibacterium id.2153) on Uncharacterized protein C17orf89
with a threshold of 1e−05

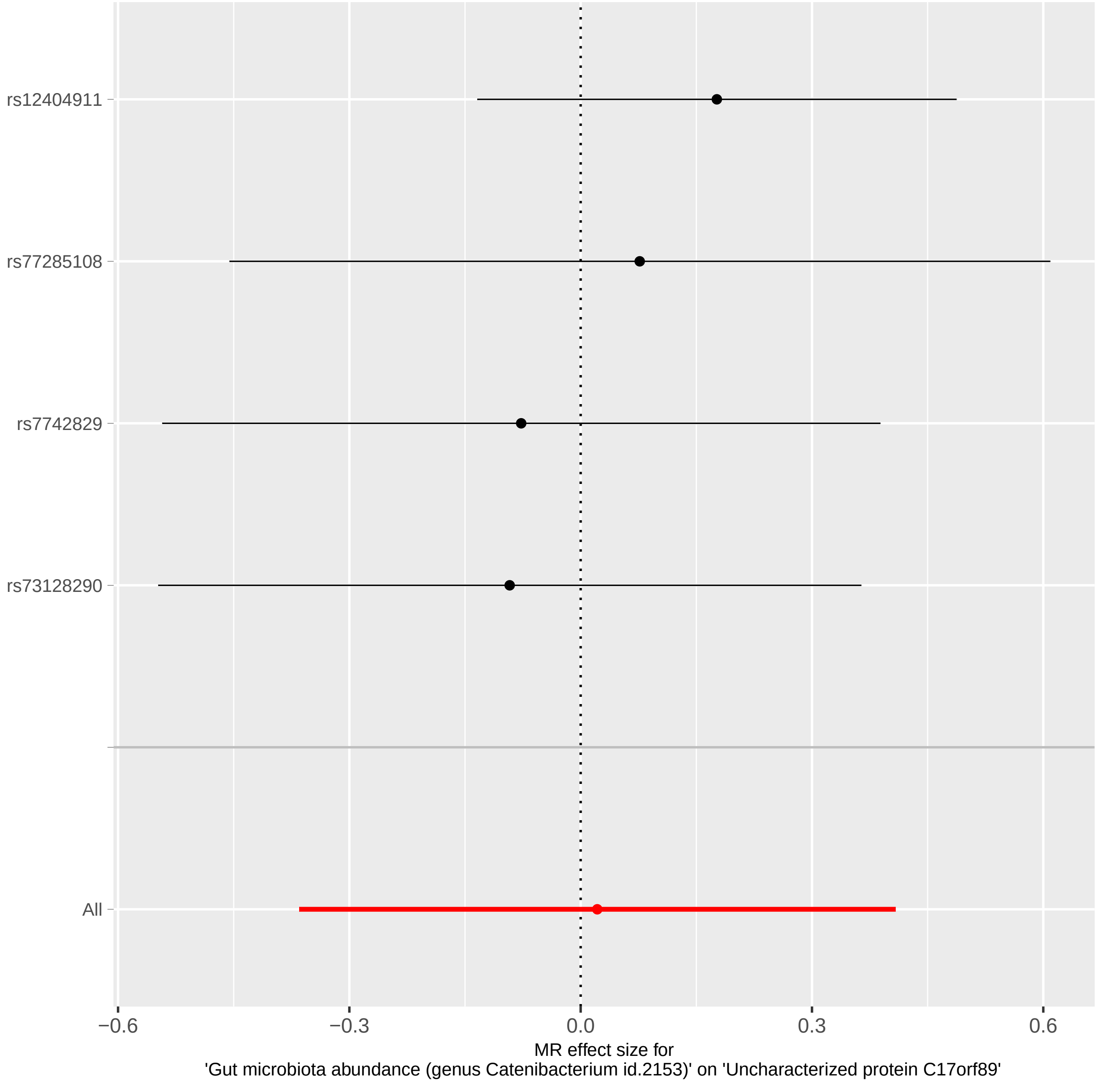

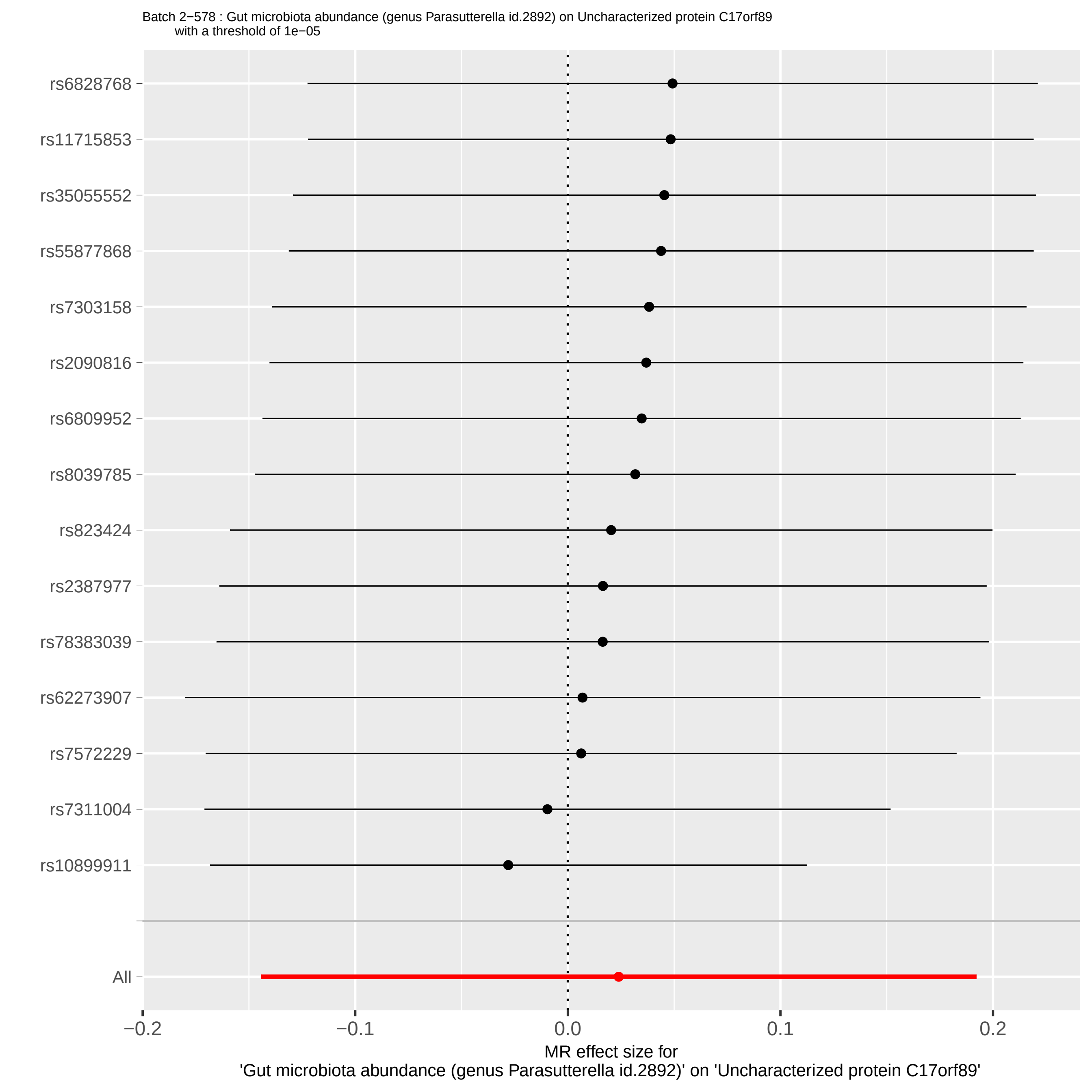
Batch 2−578 : Gut microbiota abundance (genus Parasutterella id.2892) on Uncharacterized protein C17orf89
with a threshold of 1e−05
rs6828768
rs11715853
rs35055552
rs55877868
rs7303158
rs2090816
rs6809952
rs8039785
rs823424
rs2387977
rs78383039
rs62273907
rs7572229
rs7311004
rs10899911
All
−0.2
−0.1
0.0
0.1
0.2
MR effect size for
'Gut microbiota abundance (genus Parasutterella id.2892)' on 'Uncharacterized protein C17orf89'

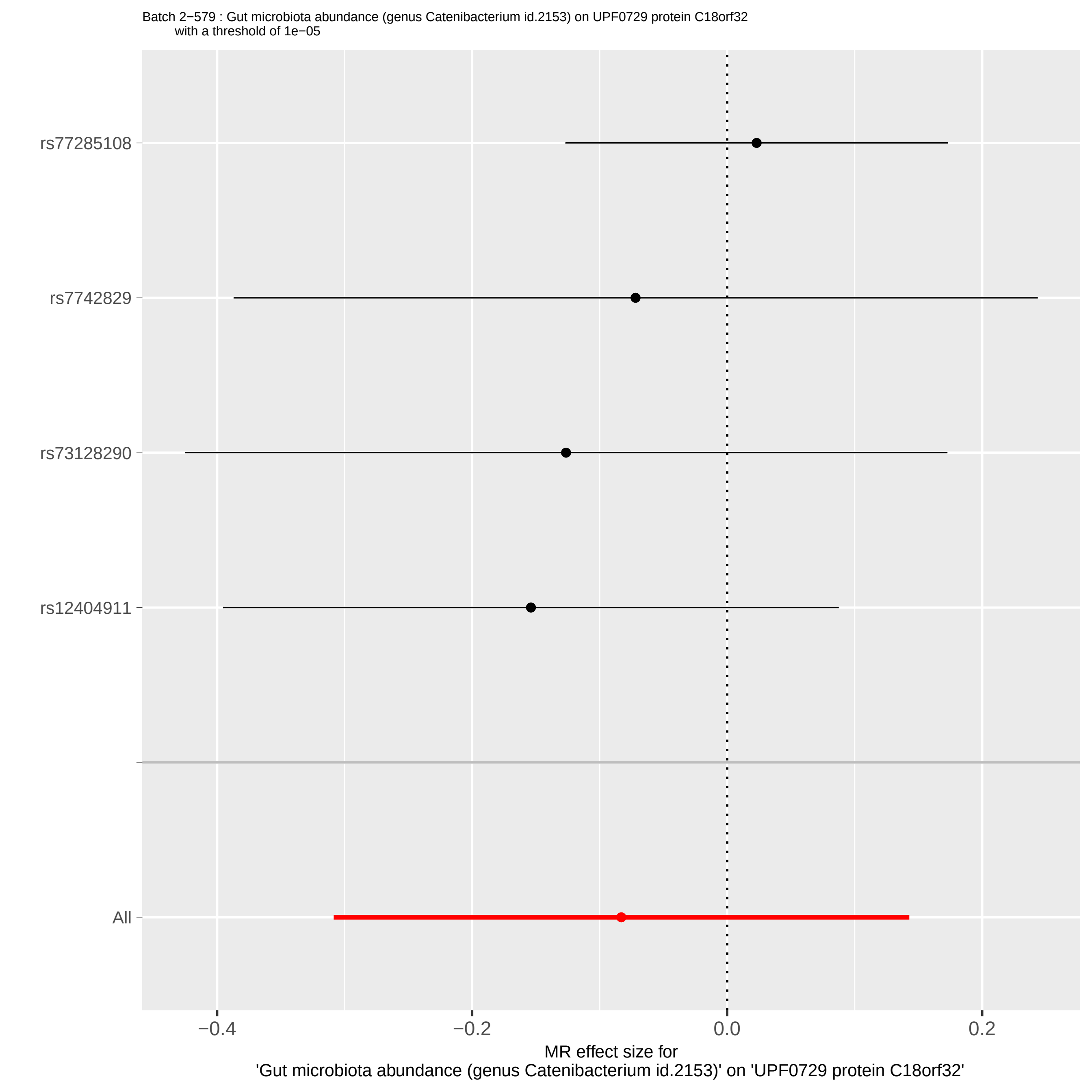
Batch 2−579 : Gut microbiota abundance (genus Catenibacterium id.2153) on UPF0729 protein C18orf32
with a threshold of 1e−05
rs77285108
rs7742829
rs73128290
rs12404911
All
−0.4
−0.2
0.0
0.2
MR effect size for
'Gut microbiota abundance (genus Catenibacterium id.2153)' on 'UPF0729 protein C18orf32'

Batch 2−580 : Gut microbiota abundance (genus Parasutterella id.2892) on UPF0729 protein C18orf32
with a threshold of 1e−05

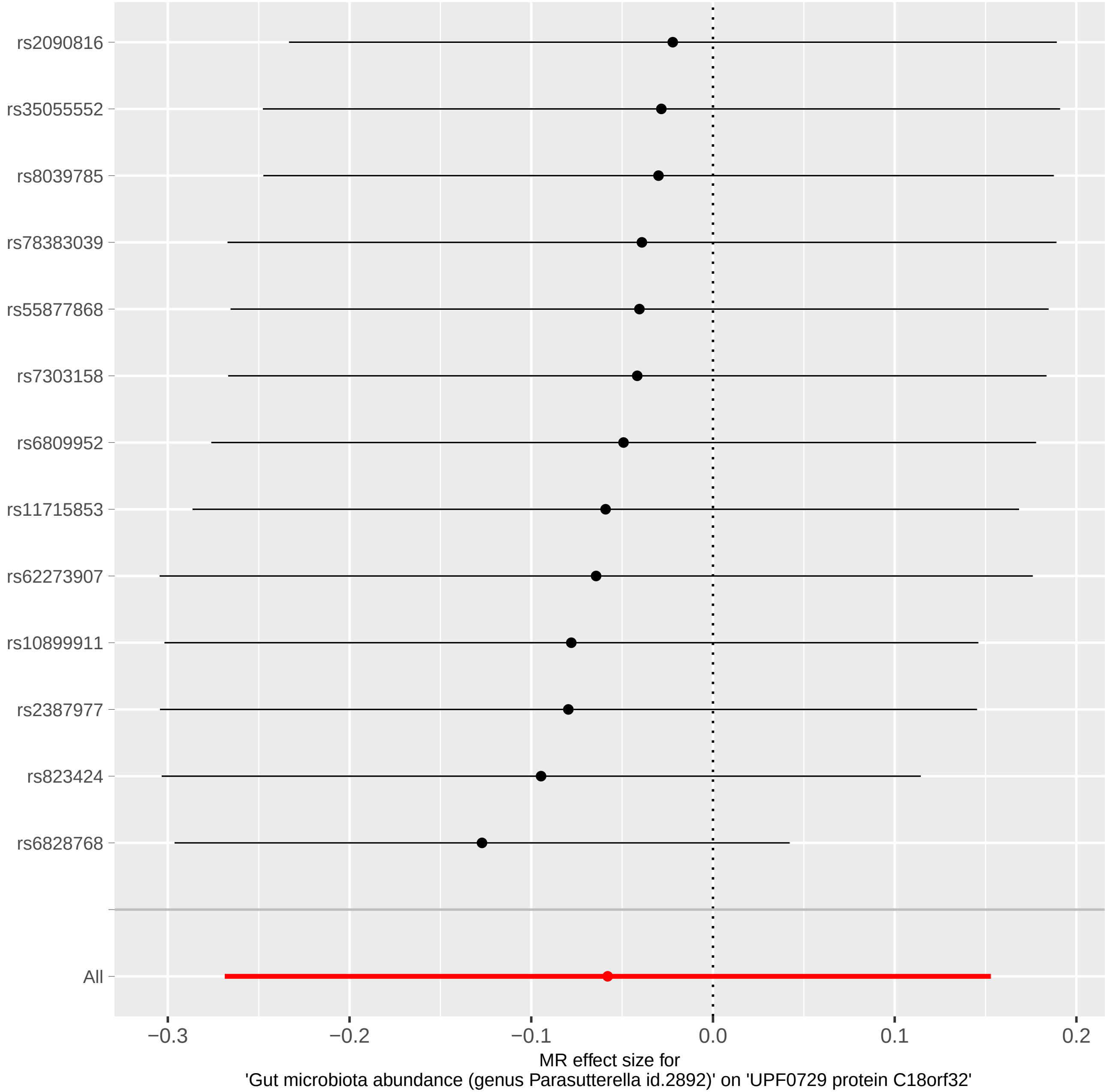

Batch 2−581 : Gut microbiota abundance (genus Catenibacterium id.2153) on Uncharacterized protein C19orf18
with a threshold of 1e−05

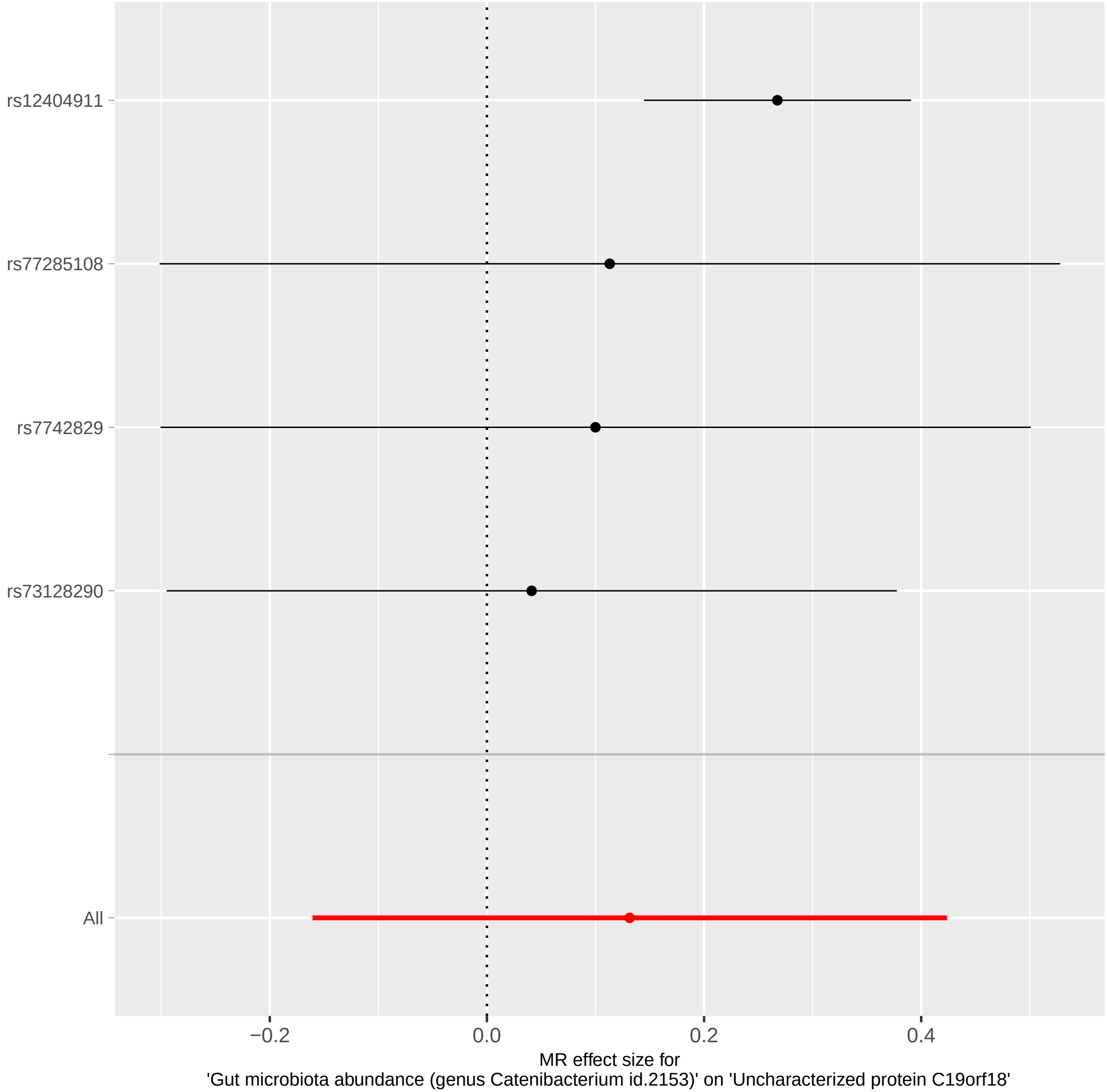

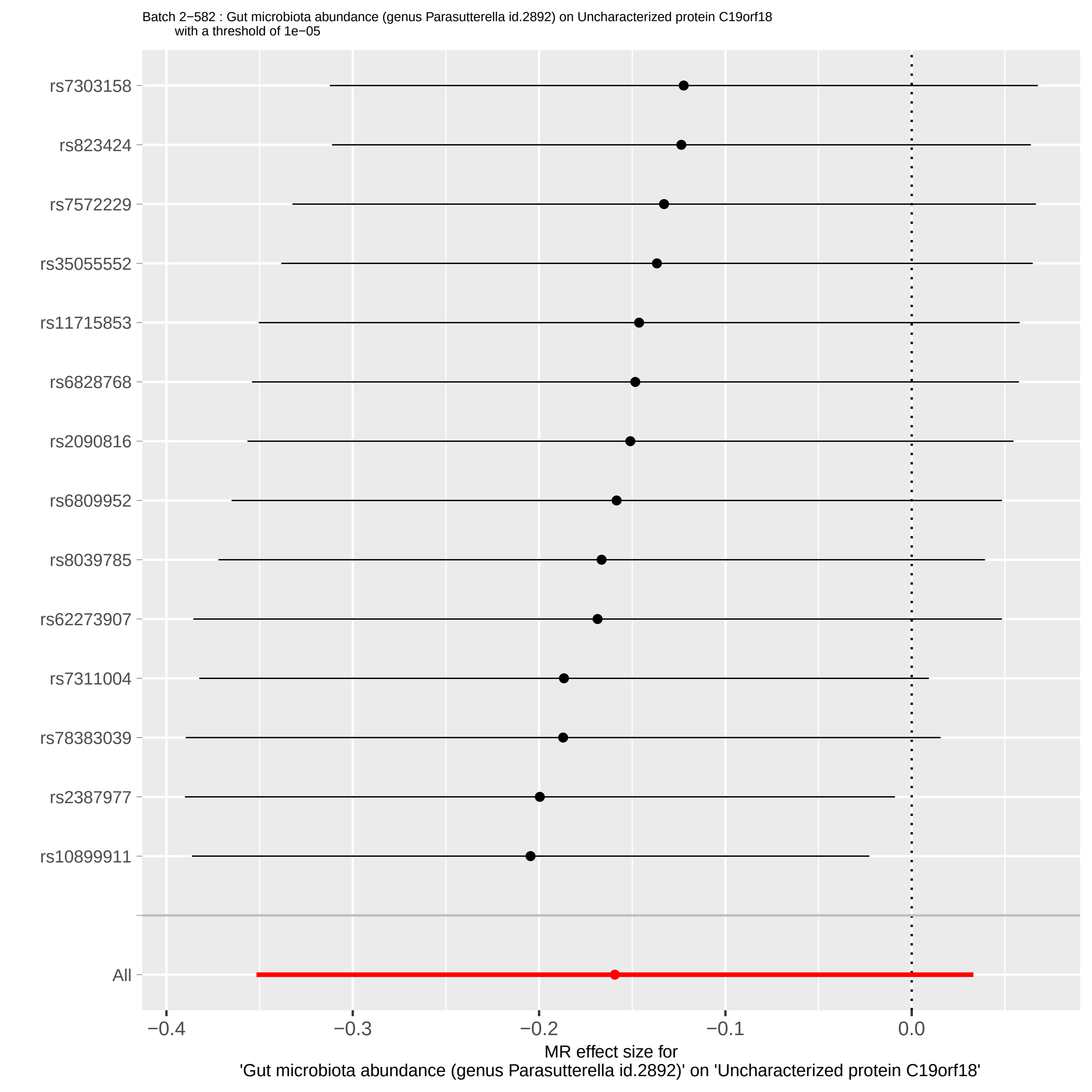
Batch 2−582 : Gut microbiota abundance (genus Parasutterella id.2892) on Uncharacterized protein C19orf18
with a threshold of 1e−05
rs7303158
rs823424
rs7572229
rs35055552
rs11715853
rs6828768
rs2090816
rs6809952
rs8039785
rs62273907
rs7311004
rs78383039
rs2387977
rs10899911
All
−0.4
−0.3
−0.2
−0.1
0.0
MR effect size for
'Gut microbiota abundance (genus Parasutterella id.2892)' on 'Uncharacterized protein C19orf18'

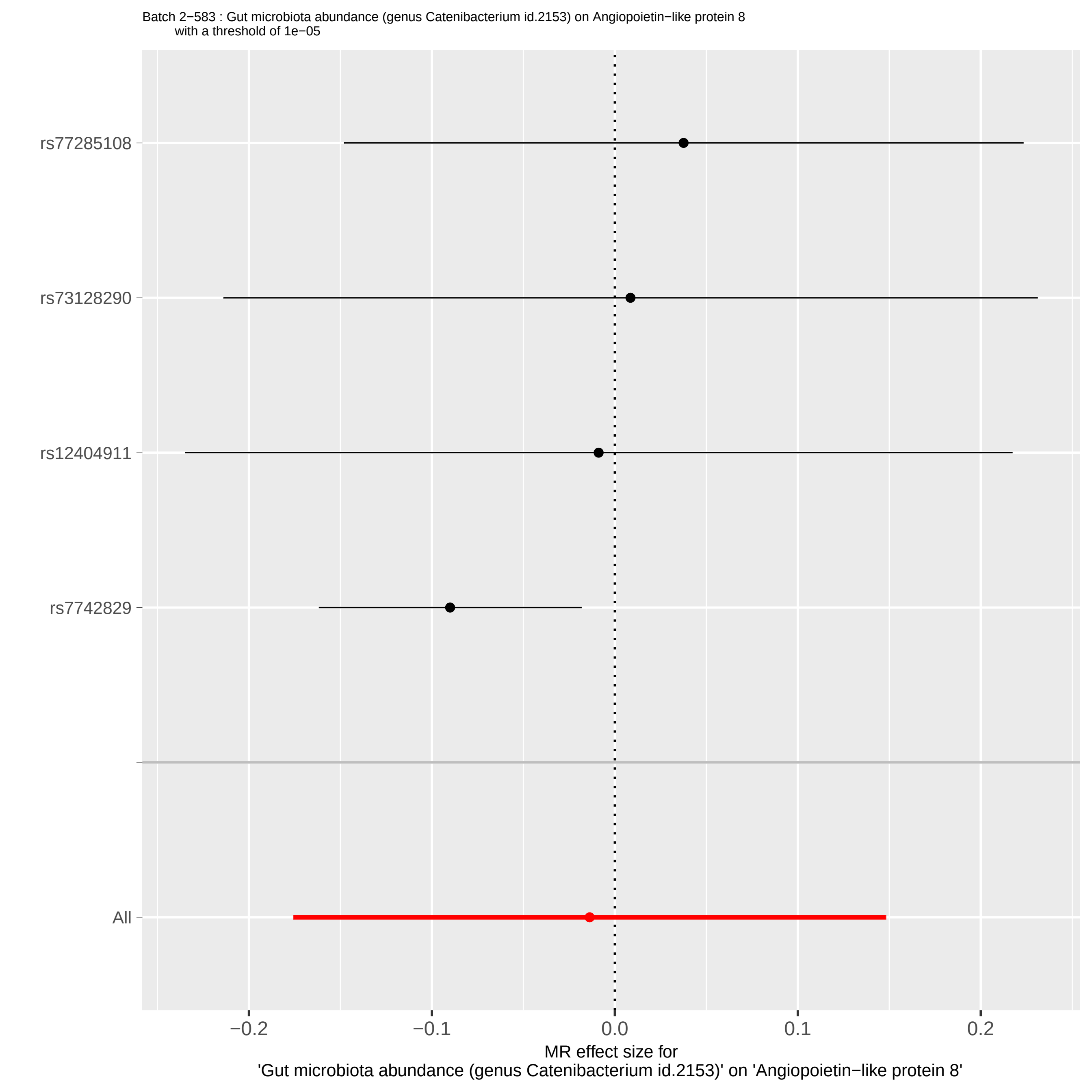

Batch 2−583 : Gut microbiota abundance (genus Catenibacterium id.2153) on Angiopoietin−like protein 8
with a threshold of 1e−05
rs77285108
rs73128290
rs12404911
rs7742829
All
−0.2
−0.1
0.0
0.1
0.2
MR effect size for
'Gut microbiota abundance (genus Catenibacterium id.2153)' on 'Angiopoietin−like protein 8'

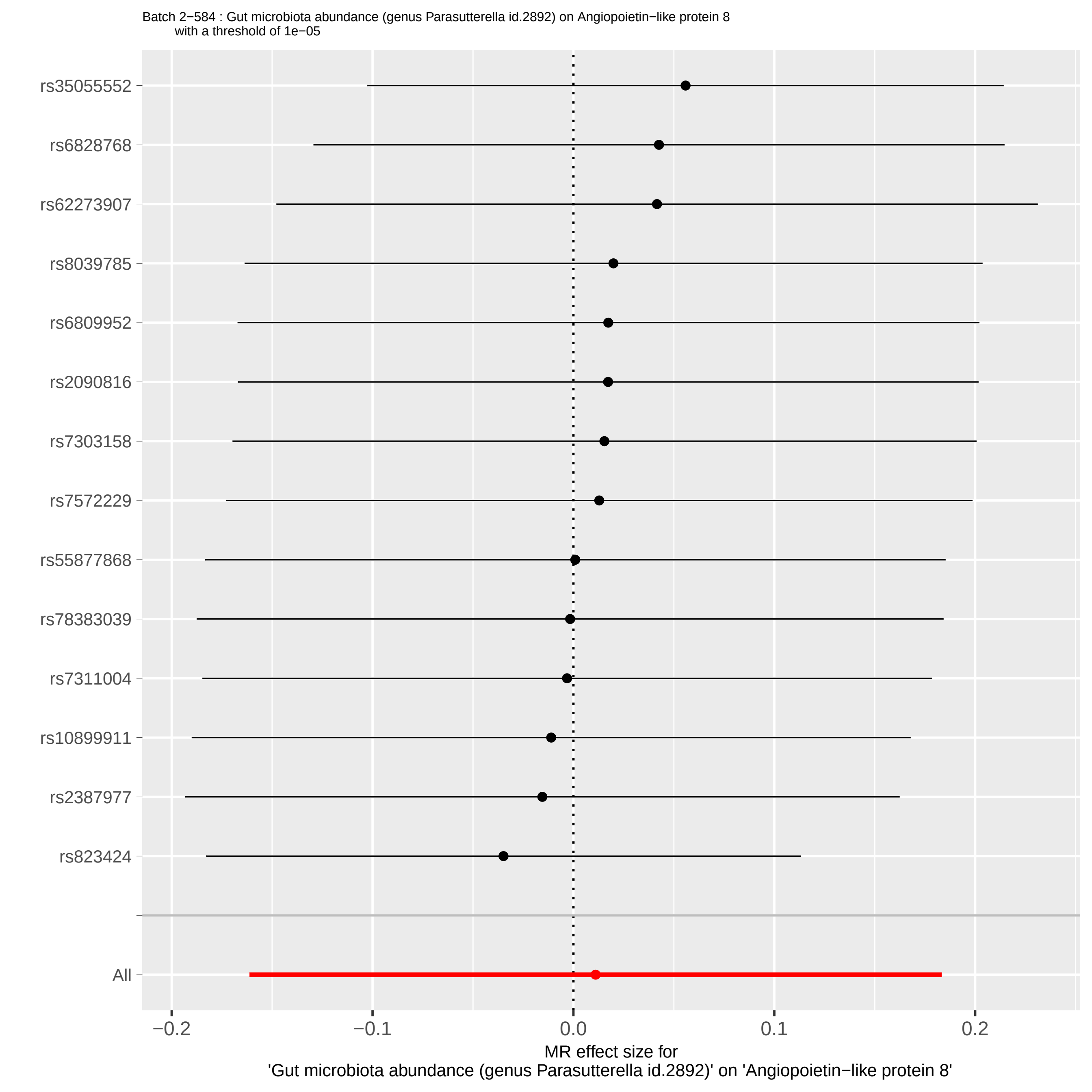

Batch 2−584 : Gut microbiota abundance (genus Parasutterella id.2892) on Angiopoietin−like protein 8
with a threshold of 1e−05
rs35055552
rs6828768
rs62273907
rs8039785
rs6809952
rs2090816
rs7303158
rs7572229
rs55877868
rs78383039
rs7311004
rs10899911
rs2387977
rs823424
All
−0.2
−0.1
0.0
0.1
0.2
MR effect size for
'Gut microbiota abundance (genus Parasutterella id.2892)' on 'Angiopoietin−like protein 8'

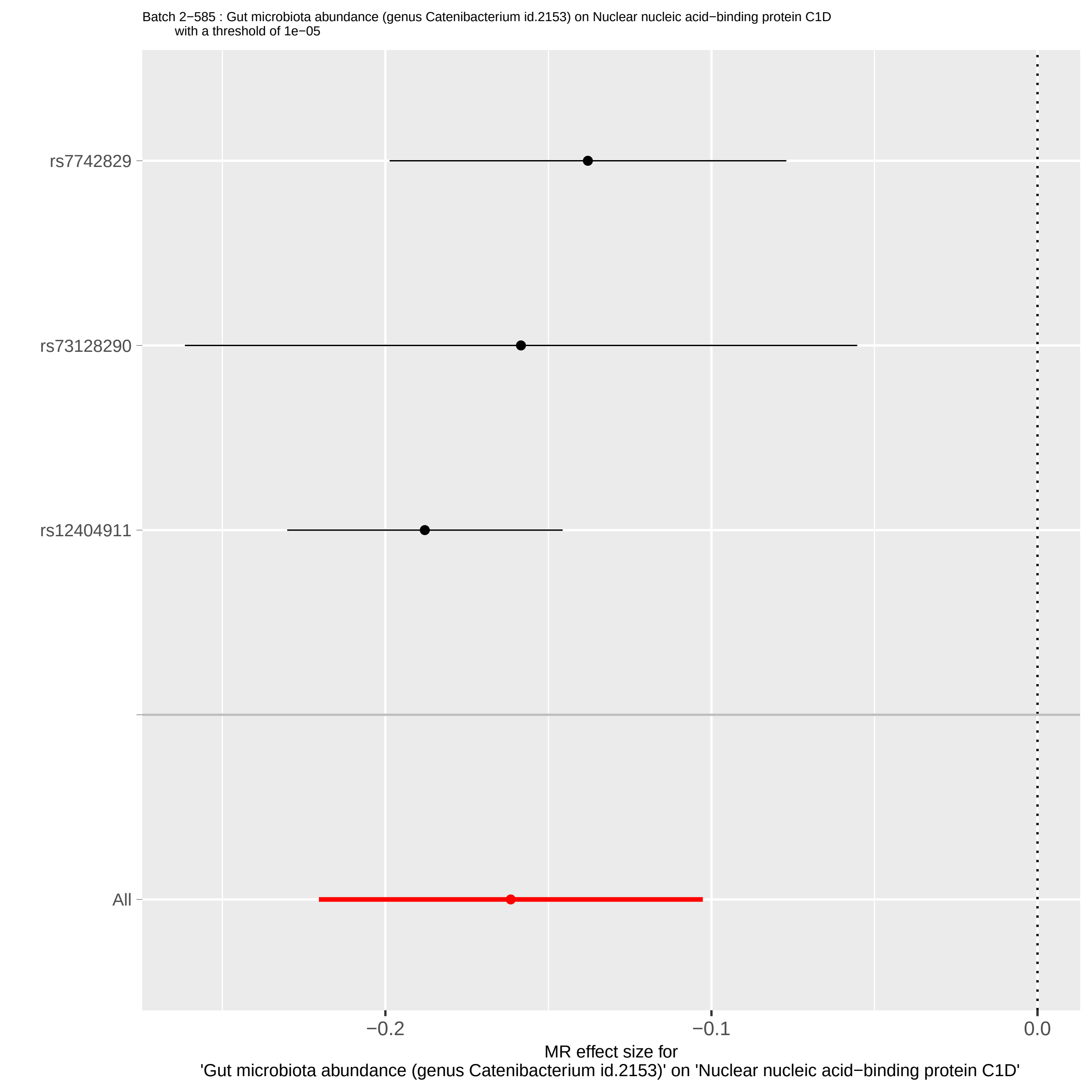

Batch 2−585 : Gut microbiota abundance (genus Catenibacterium id.2153) on Nuclear nucleic acid−binding protein C1D
with a threshold of 1e−05
rs7742829
rs73128290
rs12404911
All
−0.2
−0.1
0.0
MR effect size for
'Gut microbiota abundance (genus Catenibacterium id.2153)' on 'Nuclear nucleic acid−binding protein C1D'

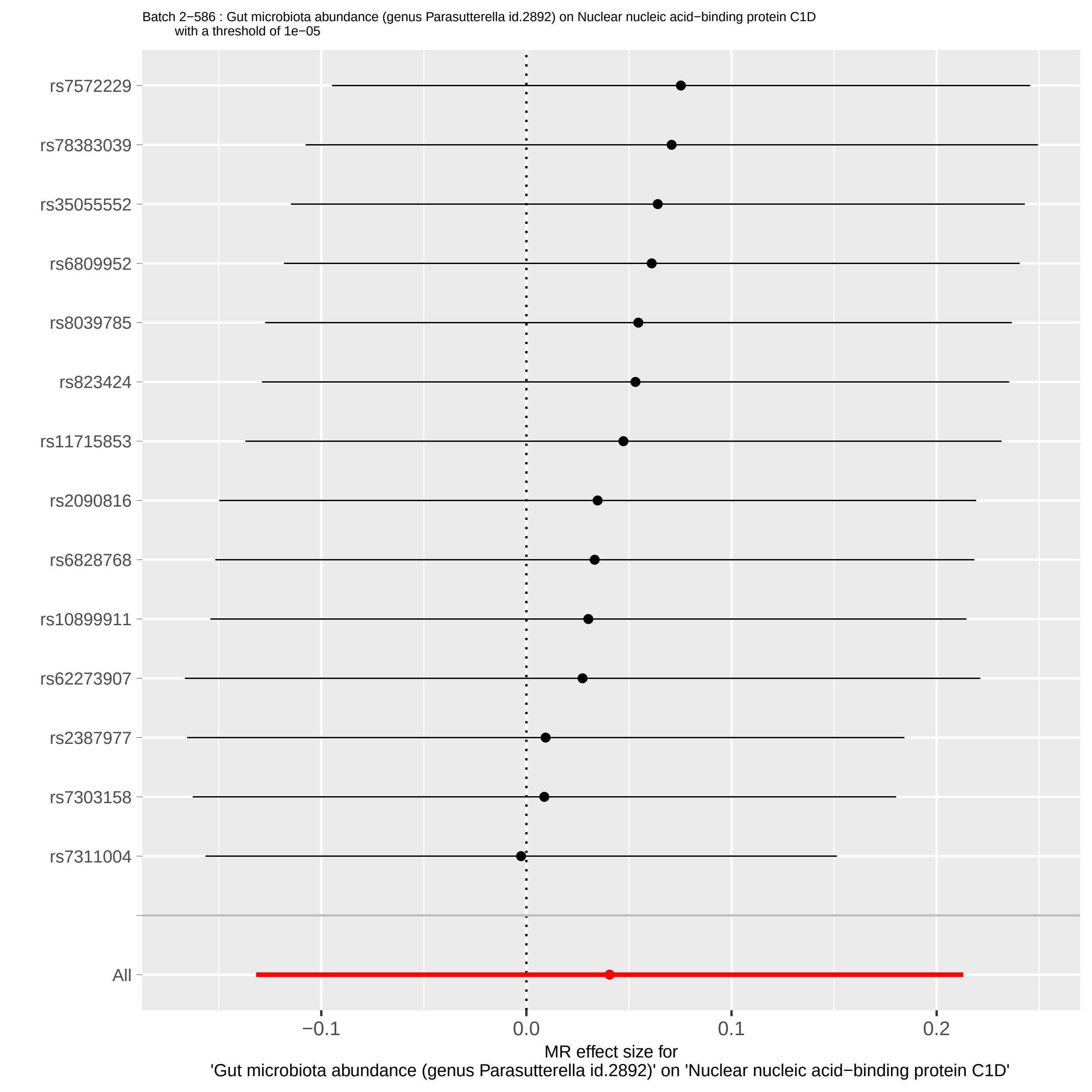
Batch 2−586 : Gut microbiota abundance (genus Parasutterella id.2892) on Nuclear nucleic acid−binding protein C1D
with a threshold of 1e−05
rs7572229
rs78383039
rs35055552
rs6809952
rs8039785
rs823424
rs11715853
rs2090816
rs6828768
rs10899911
rs62273907
rs2387977
rs7303158
rs7311004
All
−0.1
0.0
0.1
0.2
MR effect size for
'Gut microbiota abundance (genus Parasutterella id.2892)' on 'Nuclear nucleic acid−binding protein C1D'

Batch 2−587 : Gut microbiota abundance (genus Catenibacterium id.2153) on C1GALT1−specific chaperone 1
with a threshold of 1e−05

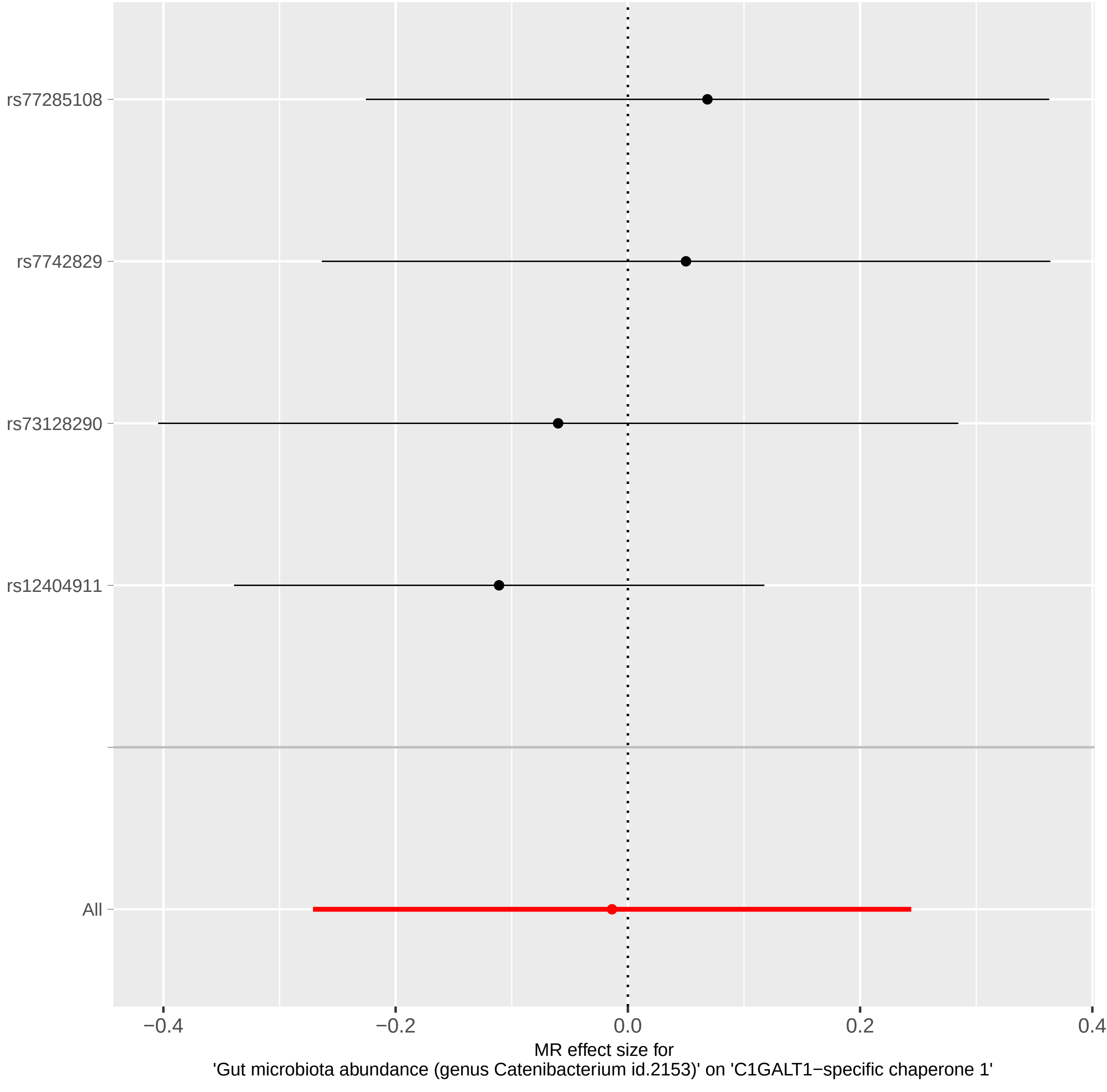

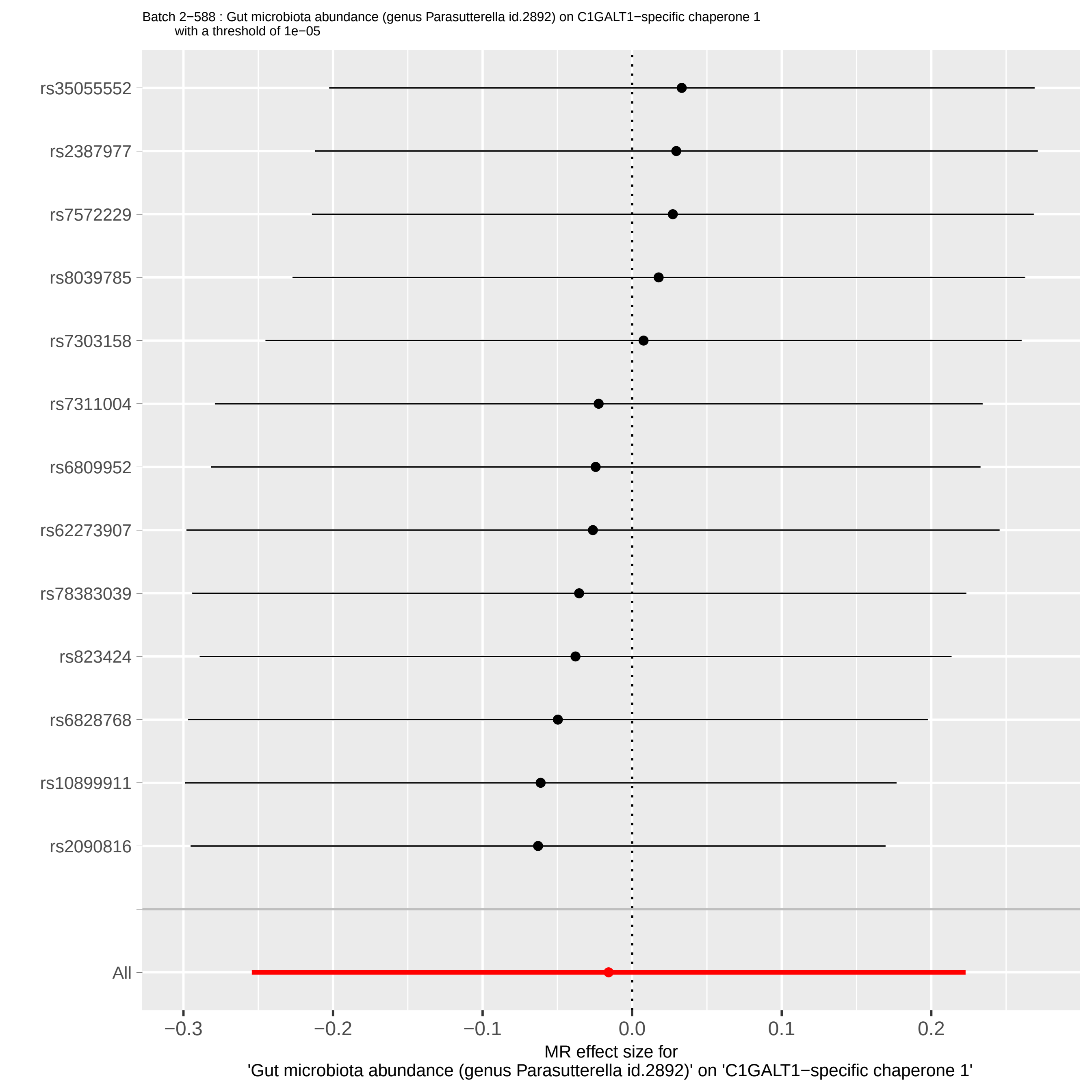
Batch 2−588 : Gut microbiota abundance (genus Parasutterella id.2892) on C1GALT1−specific chaperone 1
with a threshold of 1e−05
rs35055552
rs2387977
rs7572229
rs8039785
rs7303158
rs7311004
rs6809952
rs62273907
rs78383039
rs823424
rs6828768
rs10899911
rs2090816
All
−0.3
−0.2
−0.1
0.0
0.1
0.2
MR effect size for
'Gut microbiota abundance (genus Parasutterella id.2892)' on 'C1GALT1−specific chaperone 1'

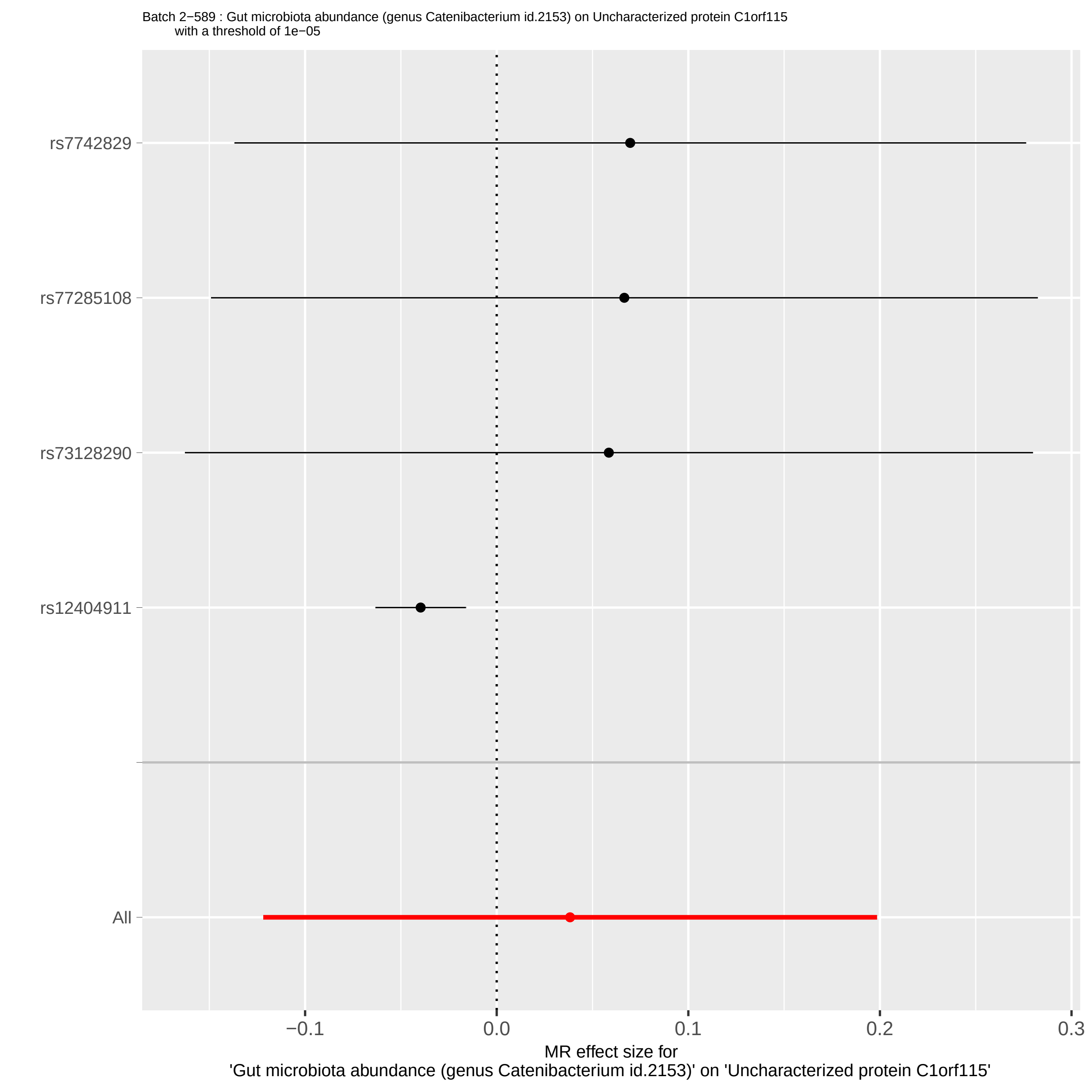

Batch 2−589 : Gut microbiota abundance (genus Catenibacterium id.2153) on Uncharacterized protein C1orf115
with a threshold of 1e−05
rs7742829
rs77285108
rs73128290
rs12404911
All
−0.1
0.0
0.1
0.2
0.3
MR effect size for
'Gut microbiota abundance (genus Catenibacterium id.2153)' on 'Uncharacterized protein C1orf115'

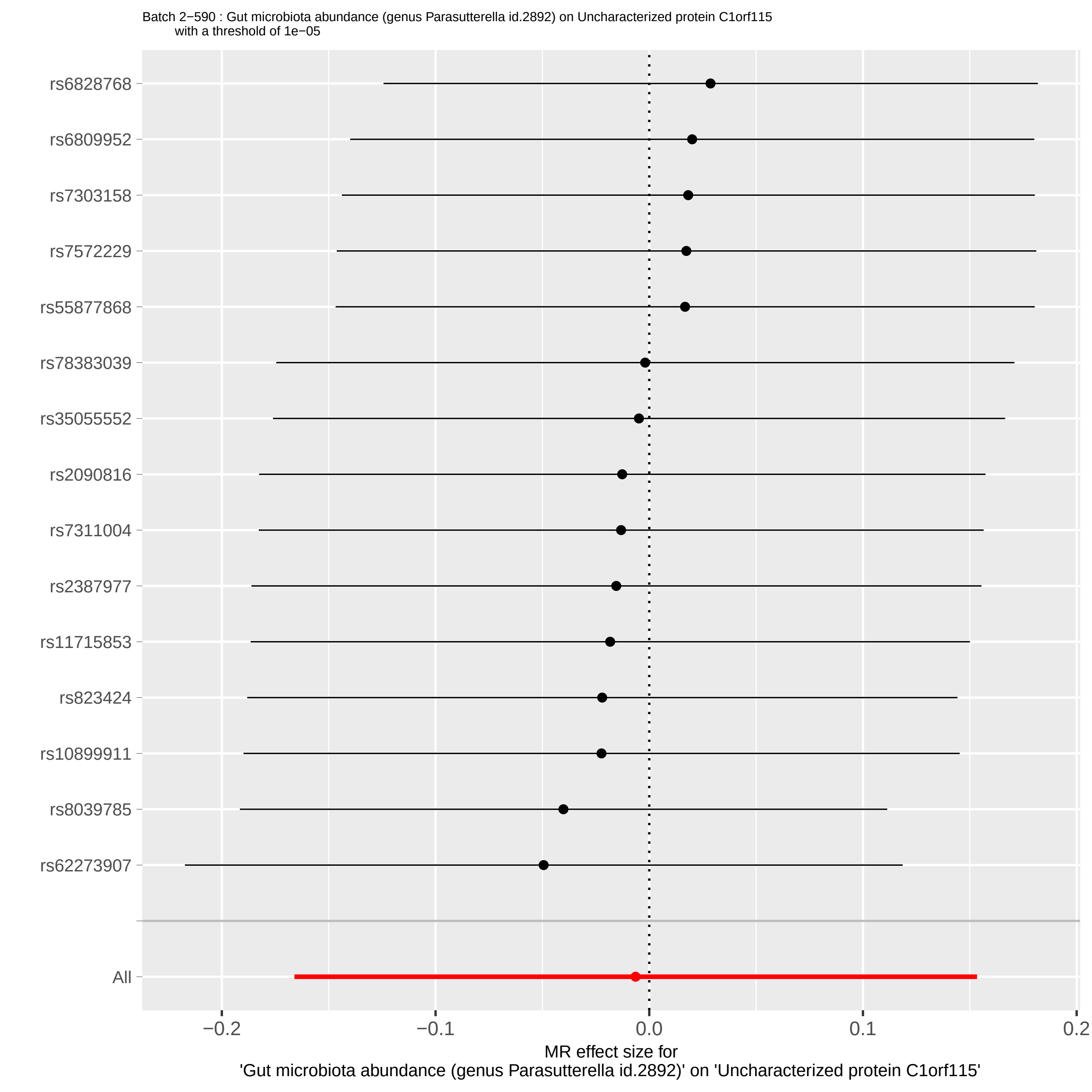
Batch 2−590 : Gut microbiota abundance (genus Parasutterella id.2892) on Uncharacterized protein C1orf115
with a threshold of 1e−05
rs6828768
rs6809952
rs7303158
rs7572229
rs55877868
rs78383039
rs35055552
rs2090816
rs7311004
rs2387977
rs11715853
rs823424
rs10899911
rs8039785
rs62273907
All
−0.2
−0.1
0.0
0.1
0.2
MR effect size for
'Gut microbiota abundance (genus Parasutterella id.2892)' on 'Uncharacterized protein C1orf115'

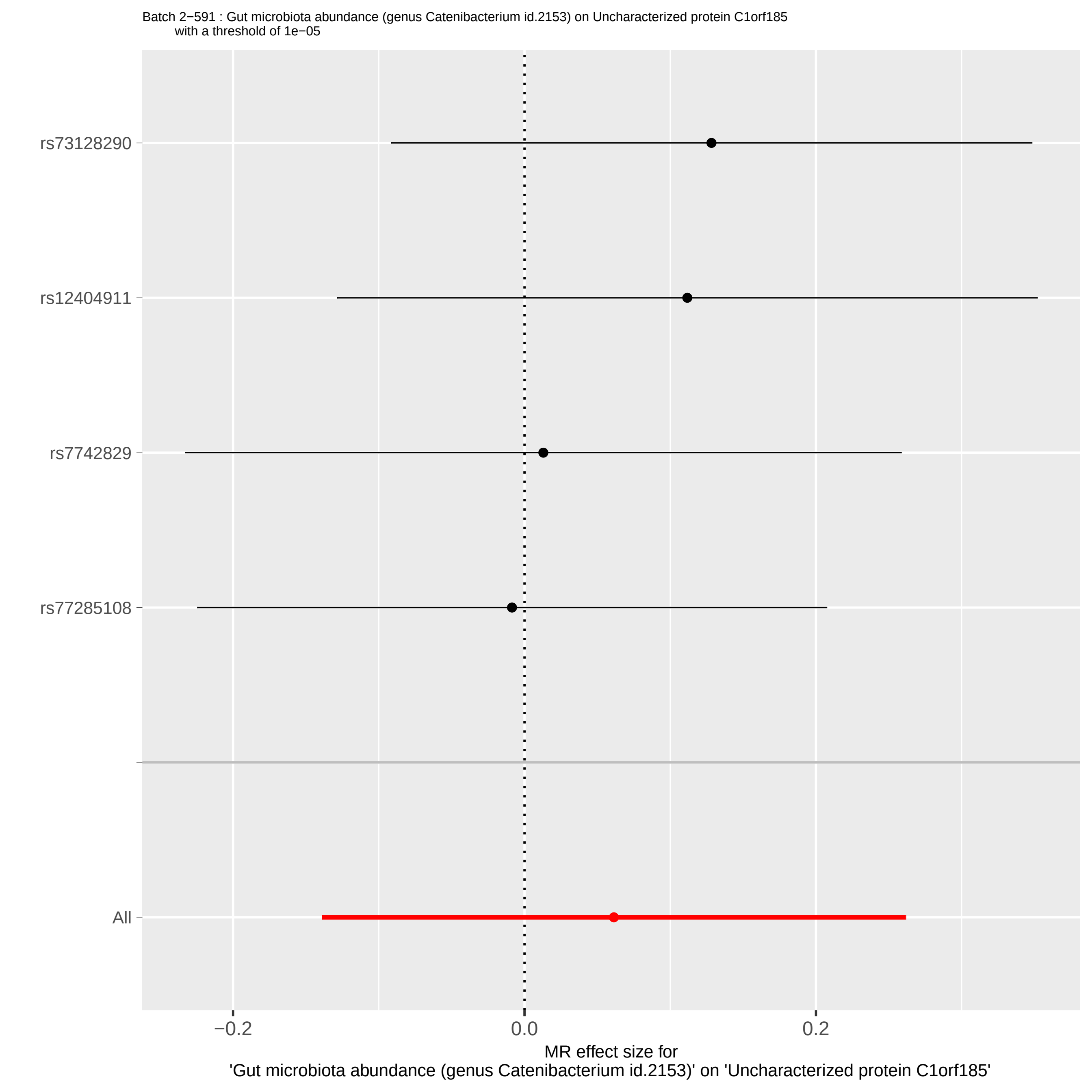

Batch 2−591 : Gut microbiota abundance (genus Catenibacterium id.2153) on Uncharacterized protein C1orf185
with a threshold of 1e−05
rs73128290
rs12404911
rs7742829
rs77285108
All
−0.2
0.0
0.2
MR effect size for
'Gut microbiota abundance (genus Catenibacterium id.2153)' on 'Uncharacterized protein C1orf185'

Batch 2−592 : Gut microbiota abundance (genus Parasutterella id.2892) on Uncharacterized protein C1orf185 with a threshold of 1e−05

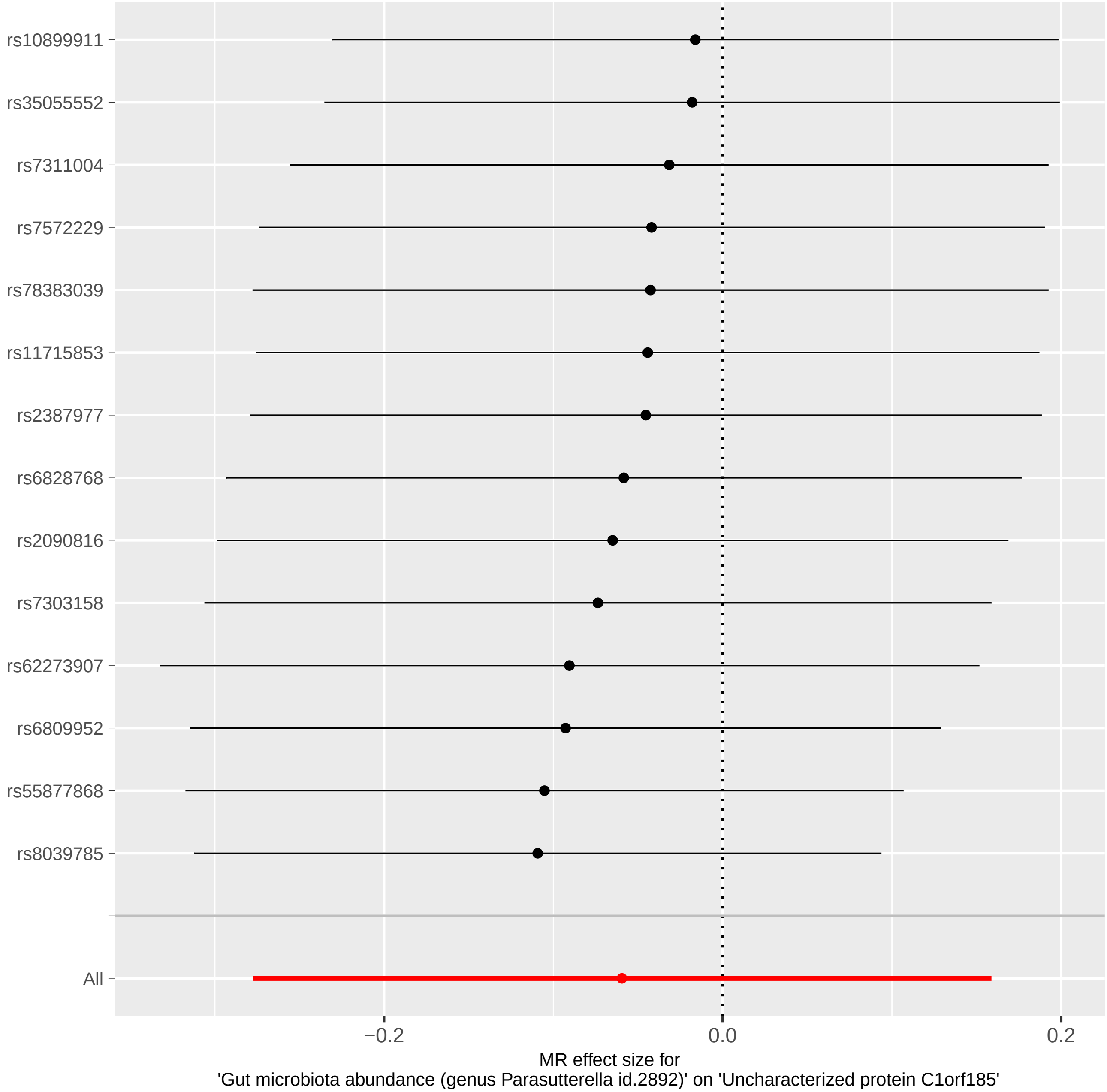

Batch 2−593 : Gut microbiota abundance (genus Catenibacterium id.2153) on Uncharacterized protein C1orf198
with a threshold of 1e−05

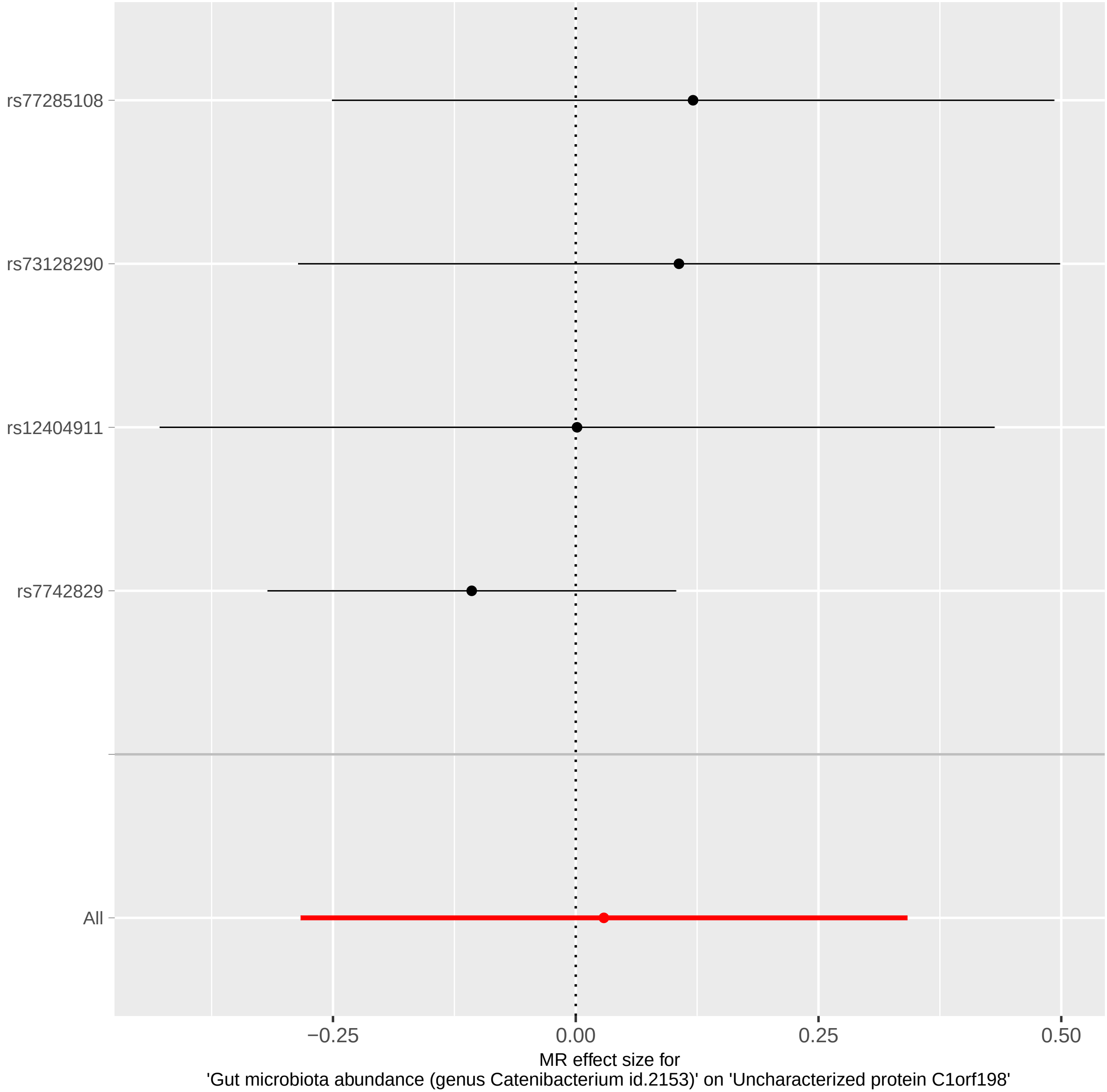

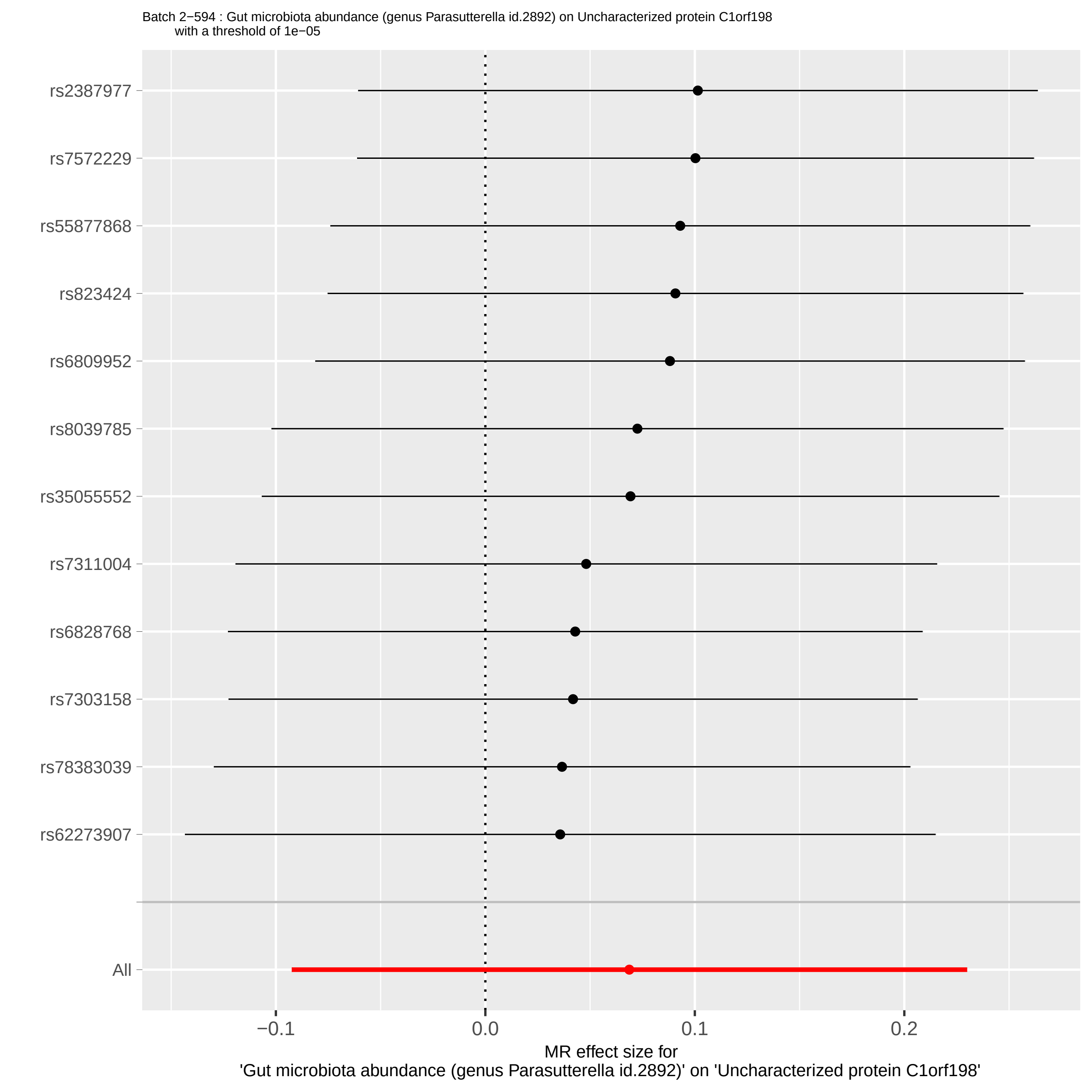
Batch 2−594 : Gut microbiota abundance (genus Parasutterella id.2892) on Uncharacterized protein C1orf198
with a threshold of 1e−05
rs2387977
rs7572229
rs55877868
rs823424
rs6809952
rs8039785
rs35055552
rs7311004
rs6828768
rs7303158
rs78383039
rs62273907
All
−0.1
0.0
0.1
0.2
MR effect size for
'Gut microbiota abundance (genus Parasutterella id.2892)' on 'Uncharacterized protein C1orf198'

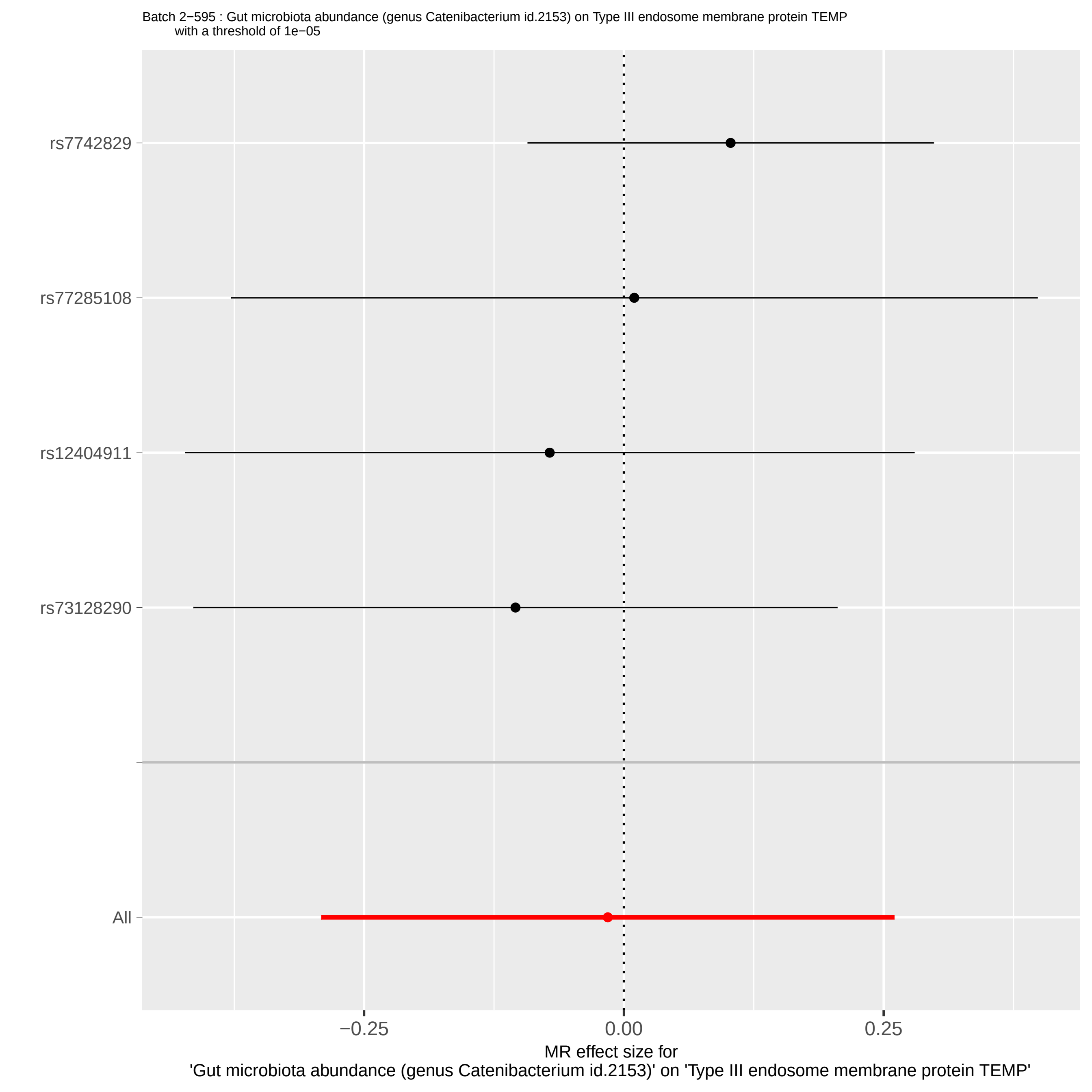

Batch 2−595 : Gut microbiota abundance (genus Catenibacterium id.2153) on Type III endosome membrane protein TEMP
with a threshold of 1e−05
rs7742829
rs77285108
rs12404911
rs73128290
All
−0.25
0.00
0.25
MR effect size for
'Gut microbiota abundance (genus Catenibacterium id.2153)' on 'Type III endosome membrane protein TEMP'

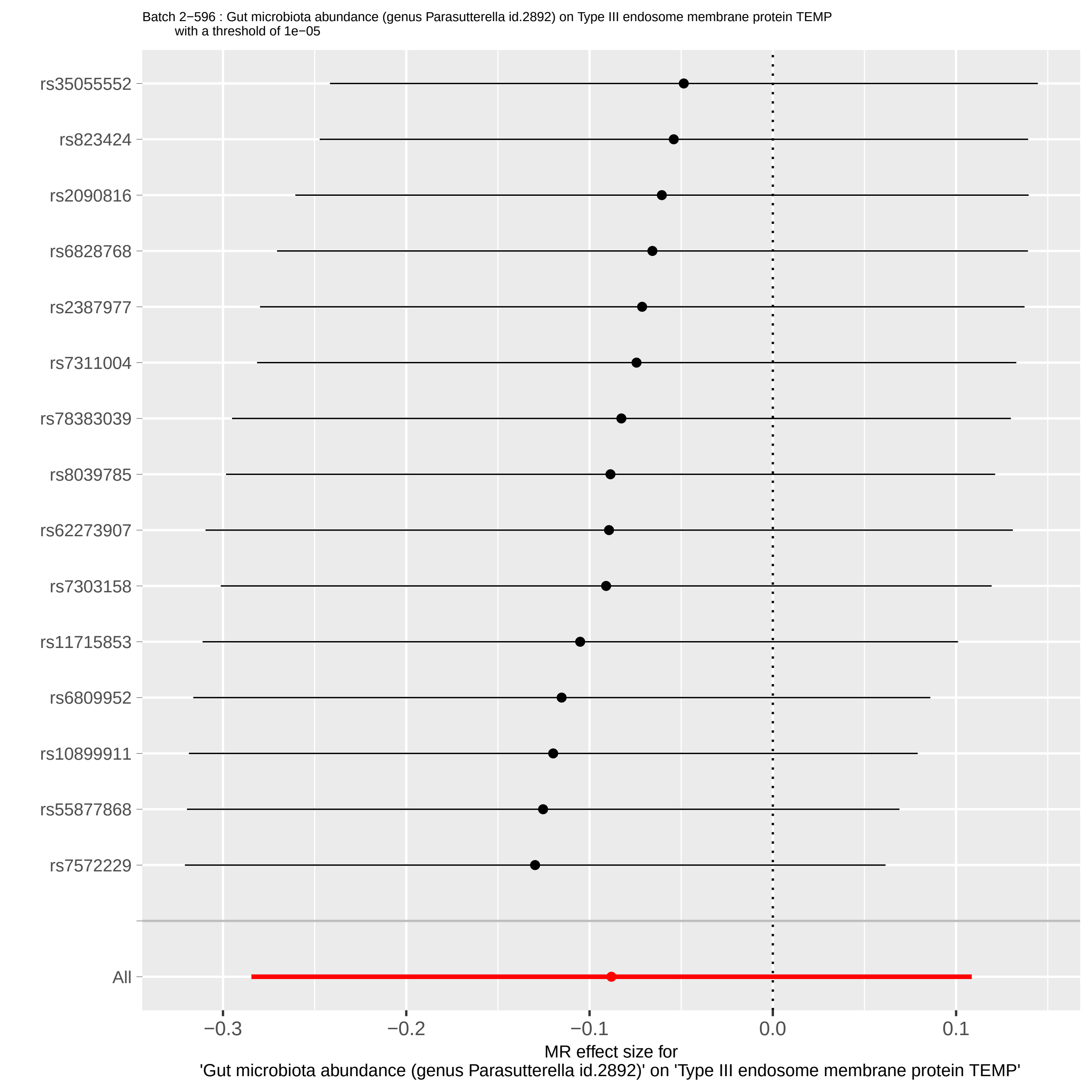

Batch 2−596 : Gut microbiota abundance (genus Parasutterella id.2892) on Type III endosome membrane protein TEMP
with a threshold of 1e−05
rs35055552
rs823424
rs2090816
rs6828768
rs2387977
rs7311004
rs78383039
rs8039785
rs62273907
rs7303158
rs11715853
rs6809952
rs10899911
rs55877868
rs7572229
All
−0.3
−0.2
−0.1
0.0
0.1
MR effect size for
'Gut microbiota abundance (genus Parasutterella id.2892)' on 'Type III endosome membrane protein TEMP'

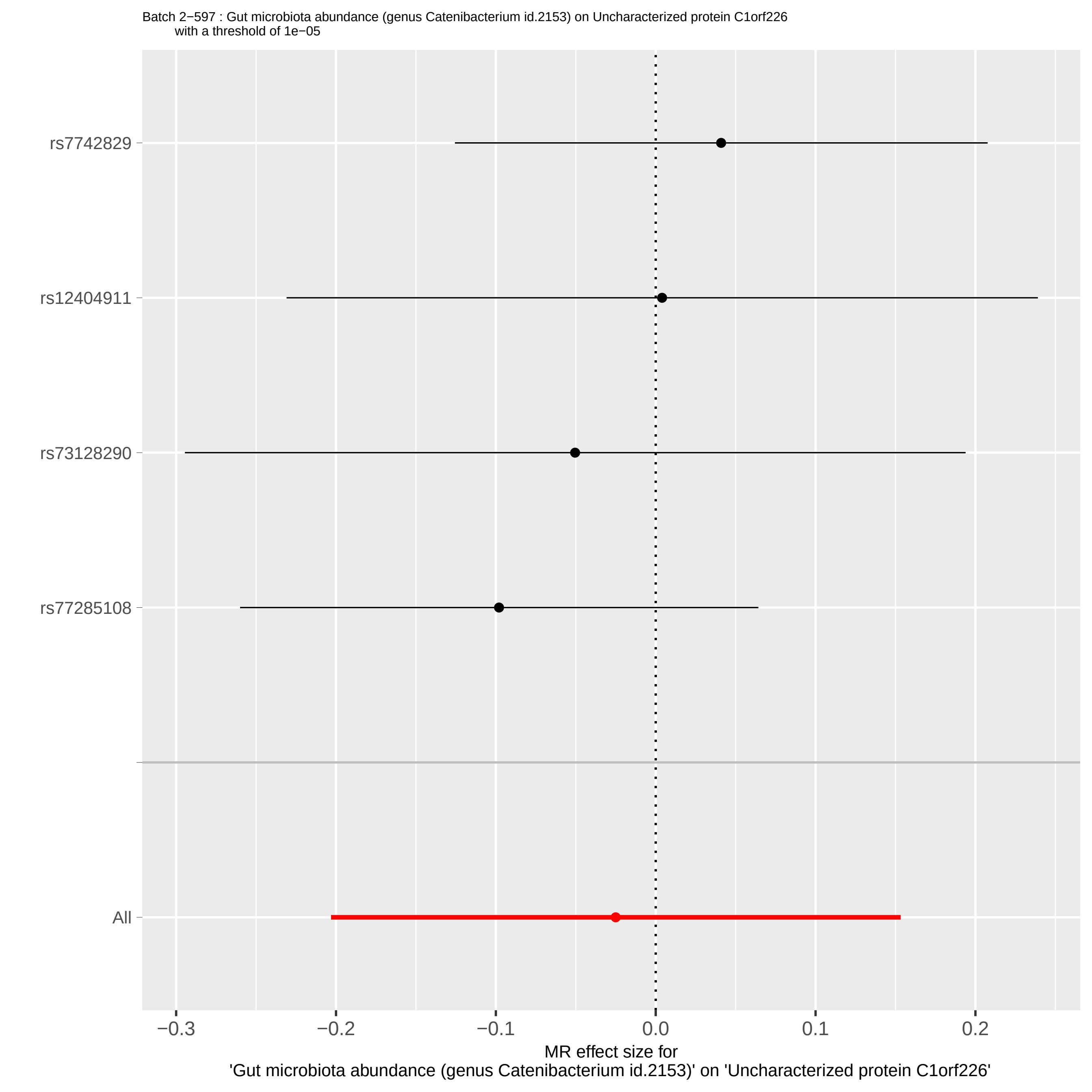

Batch 2−597 : Gut microbiota abundance (genus Catenibacterium id.2153) on Uncharacterized protein C1orf226
with a threshold of 1e−05
rs7742829
rs12404911
rs73128290
rs77285108
All
−0.3
−0.2
−0.1
0.0
0.1
0.2
MR effect size for
'Gut microbiota abundance (genus Catenibacterium id.2153)' on 'Uncharacterized protein C1orf226'

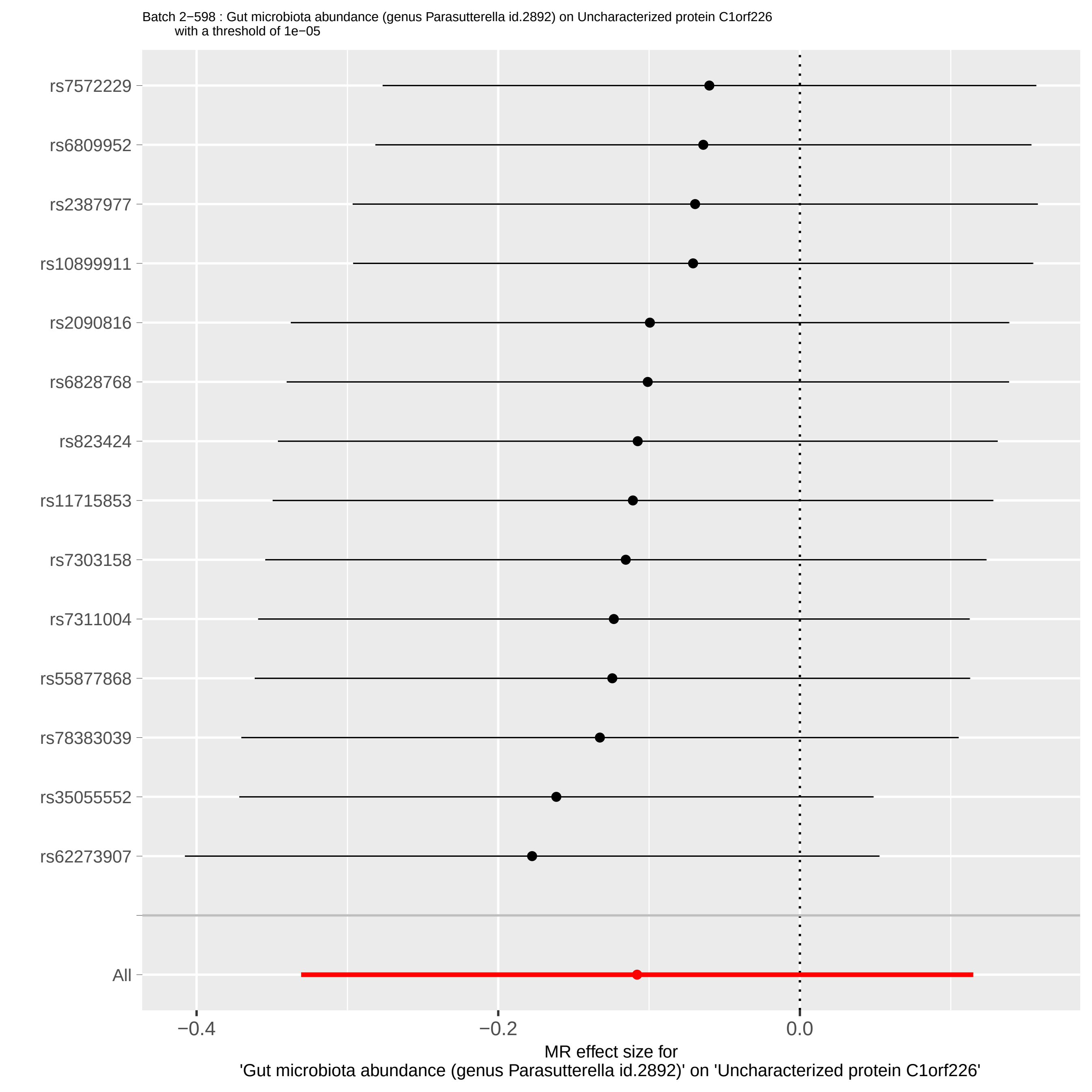

Batch 2−598 : Gut microbiota abundance (genus Parasutterella id.2892) on Uncharacterized protein C1orf226
with a threshold of 1e−05
rs7572229
rs6809952
rs2387977
rs10899911
rs2090816
rs6828768
rs823424
rs11715853
rs7303158
rs7311004
rs55877868
rs78383039
rs35055552
rs62273907
All
−0.4
−0.2
0.0
MR effect size for
'Gut microbiota abundance (genus Parasutterella id.2892)' on 'Uncharacterized protein C1orf226'

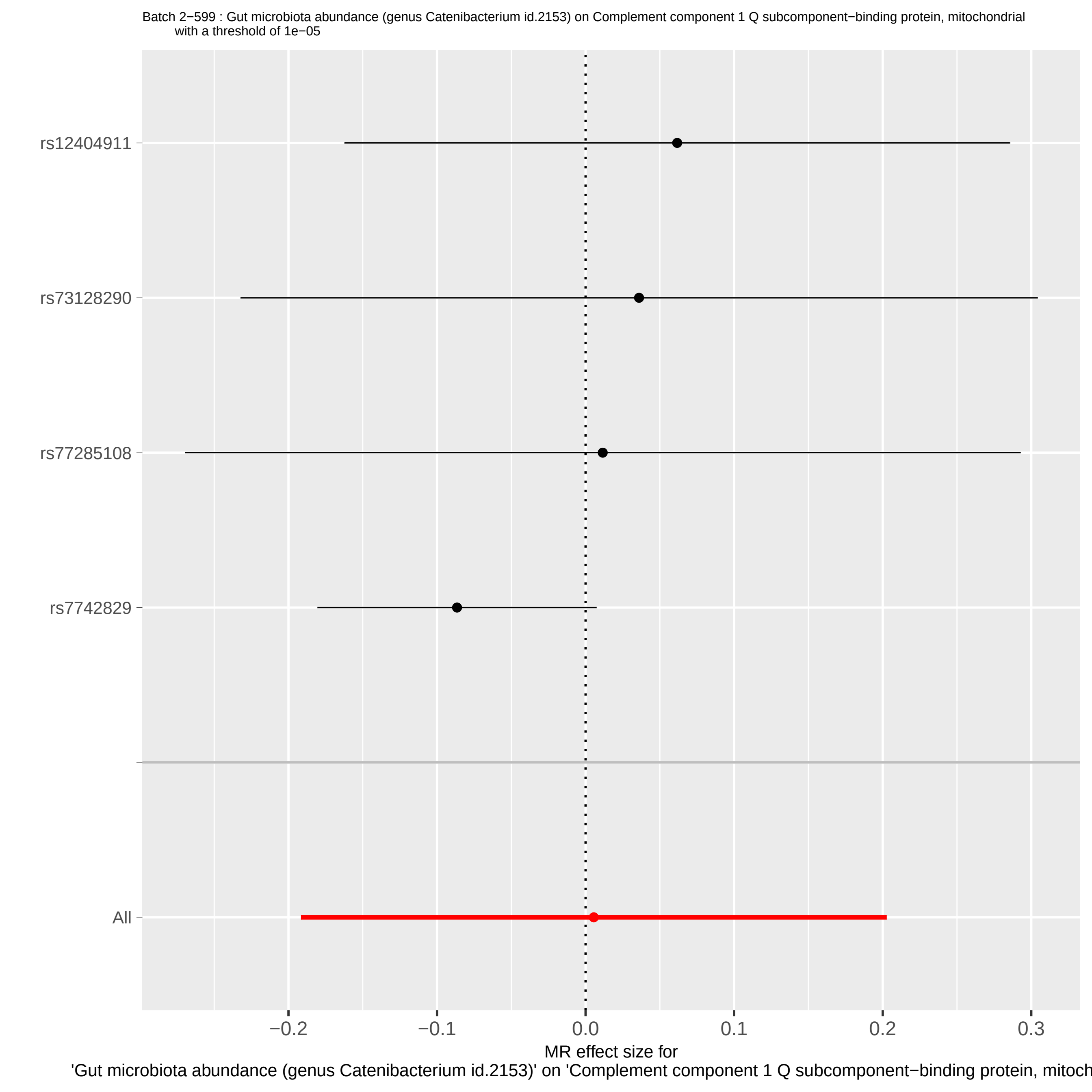
Batch 2−599 : Gut microbiota abundance (genus Catenibacterium id.2153) on Complement component 1 Q subcomponent−binding protein, mitochondrial with a threshold of 1e−05
rs12404911
rs73128290
rs77285108
rs7742829
All
−0.2
−0.1
0.0
0.1
0.2
0.3
MR effect size for
'Gut microbiota abundance (genus Catenibacterium id.2153)' on 'Complement component 1 Q subcomponent−binding protein, mitoch

Batch 2−600 : Gut microbiota abundance (genus Parasutterella id.2892) on Complement component 1 Q subcomponent−binding protein, mitochondrial with a threshold of 1e−05

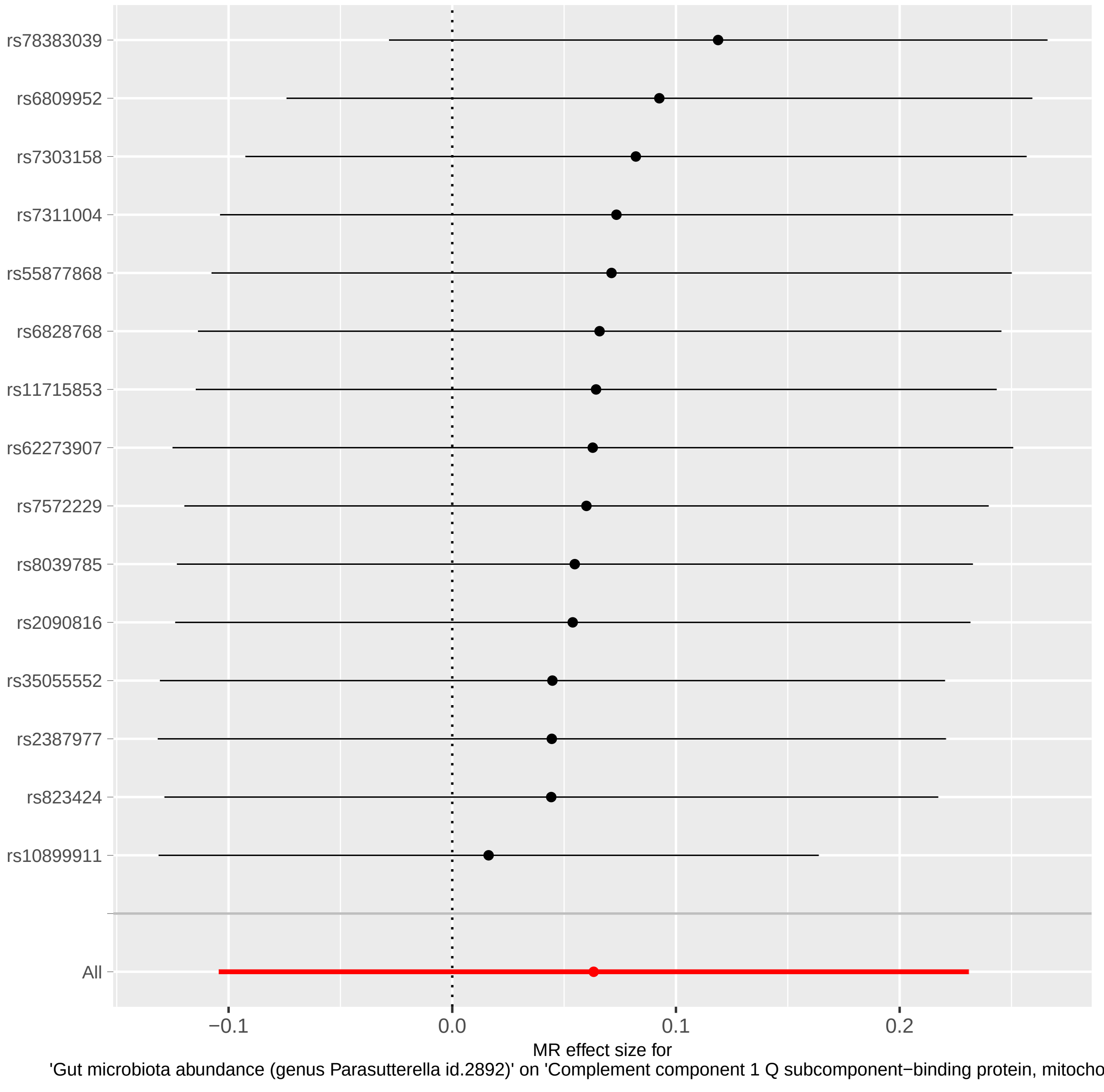

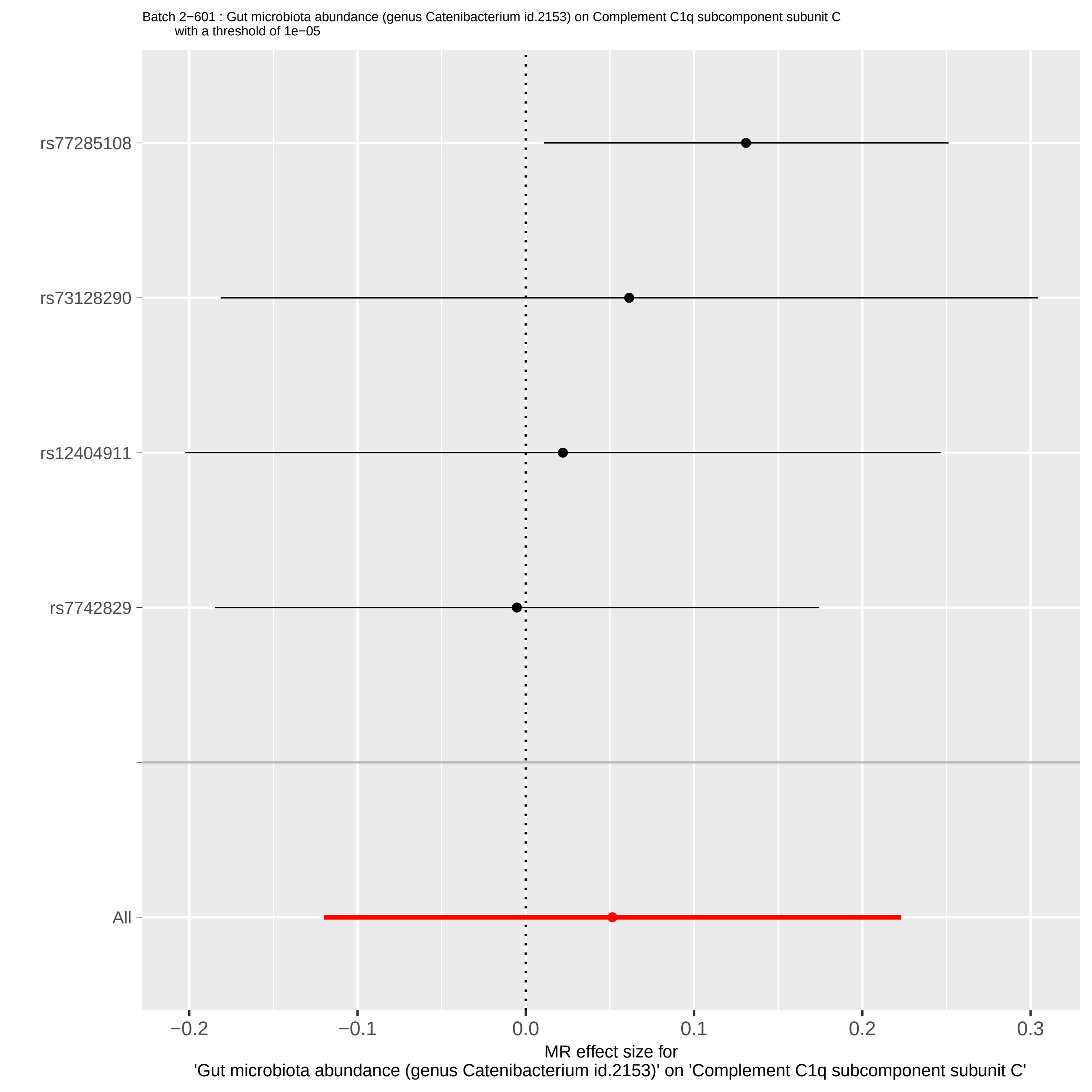
Batch 2−601 : Gut microbiota abundance (genus Catenibacterium id.2153) on Complement C1q subcomponent subunit C
with a threshold of 1e−05
rs77285108
rs73128290
rs12404911
rs7742829
All
−0.2
−0.1
0.0
0.1
0.2
0.3
MR effect size for
'Gut microbiota abundance (genus Catenibacterium id.2153)' on 'Complement C1q subcomponent subunit C'

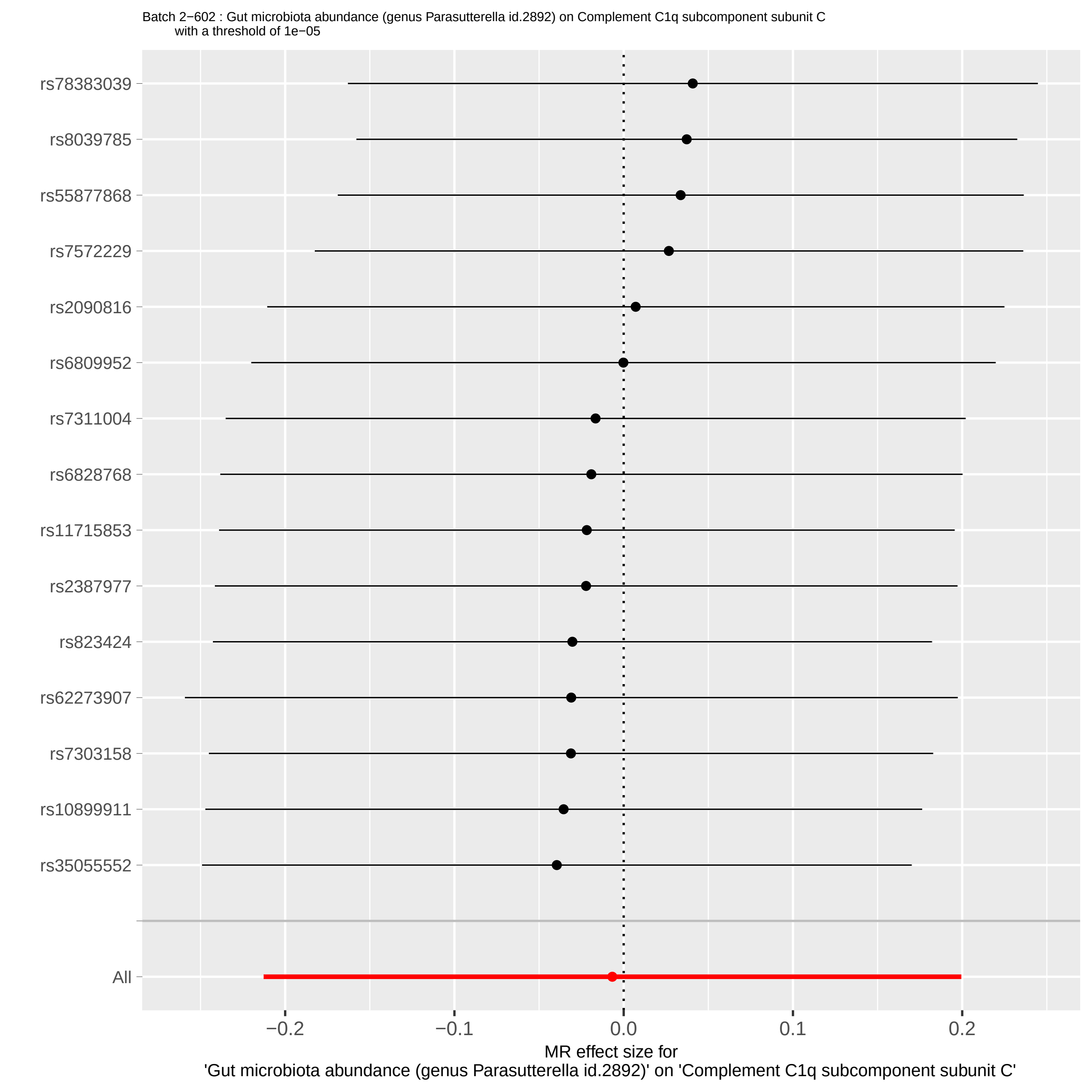

Batch 2−602 : Gut microbiota abundance (genus Parasutterella id.2892) on Complement C1q subcomponent subunit C
with a threshold of 1e−05
rs78383039
rs8039785
rs55877868
rs7572229
rs2090816
rs6809952
rs7311004
rs6828768
rs11715853
rs2387977
rs823424
rs62273907
rs7303158
rs10899911
rs35055552
All
−0.2
−0.1
0.0
0.1
0.2
MR effect size for
'Gut microbiota abundance (genus Parasutterella id.2892)' on 'Complement C1q subcomponent subunit C'

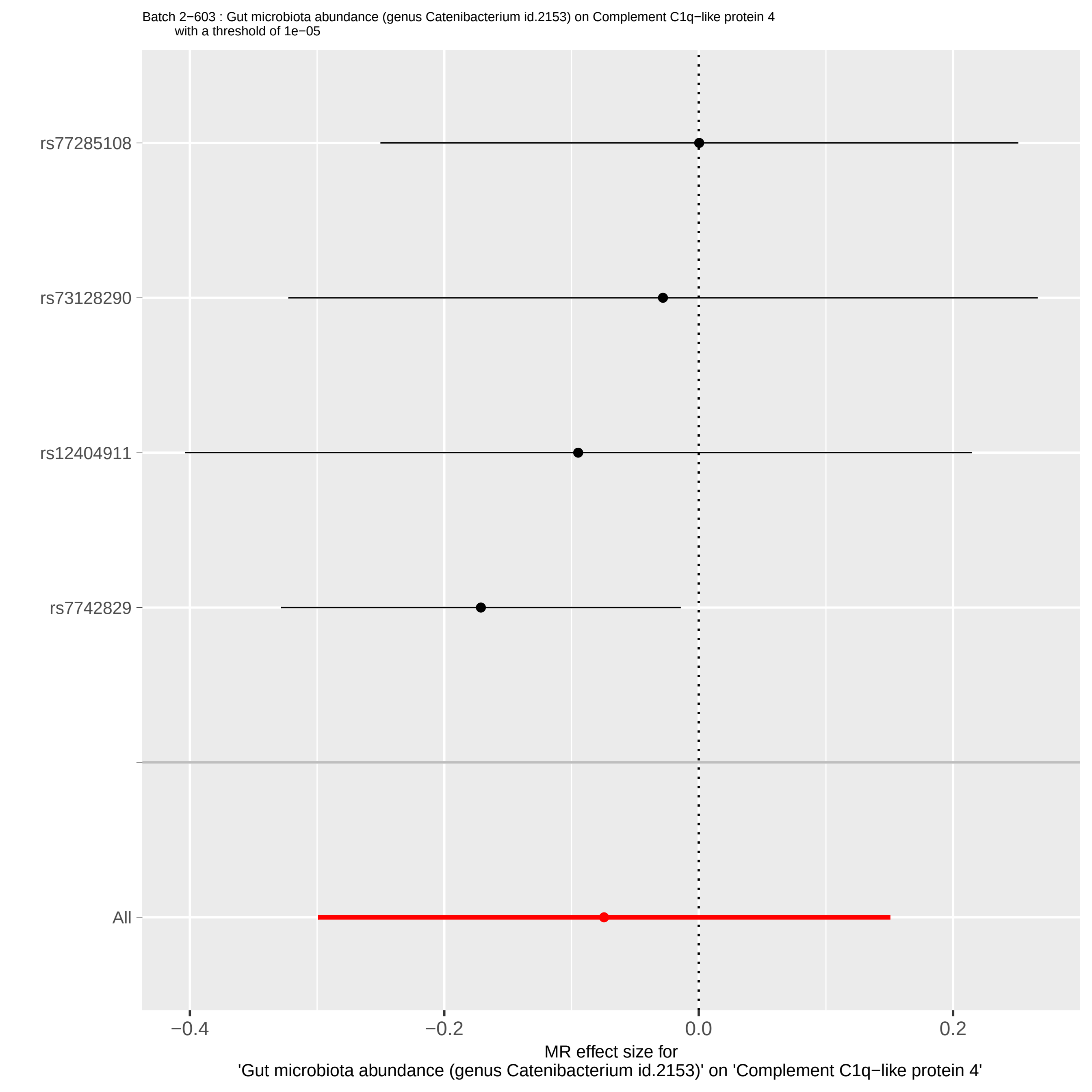
Batch 2−603 : Gut microbiota abundance (genus Catenibacterium id.2153) on Complement C1q−like protein 4
with a threshold of 1e−05
rs77285108
rs73128290
rs12404911
rs7742829
All
−0.4
−0.2
0.0
0.2
MR effect size for
'Gut microbiota abundance (genus Catenibacterium id.2153)' on 'Complement C1q−like protein 4'

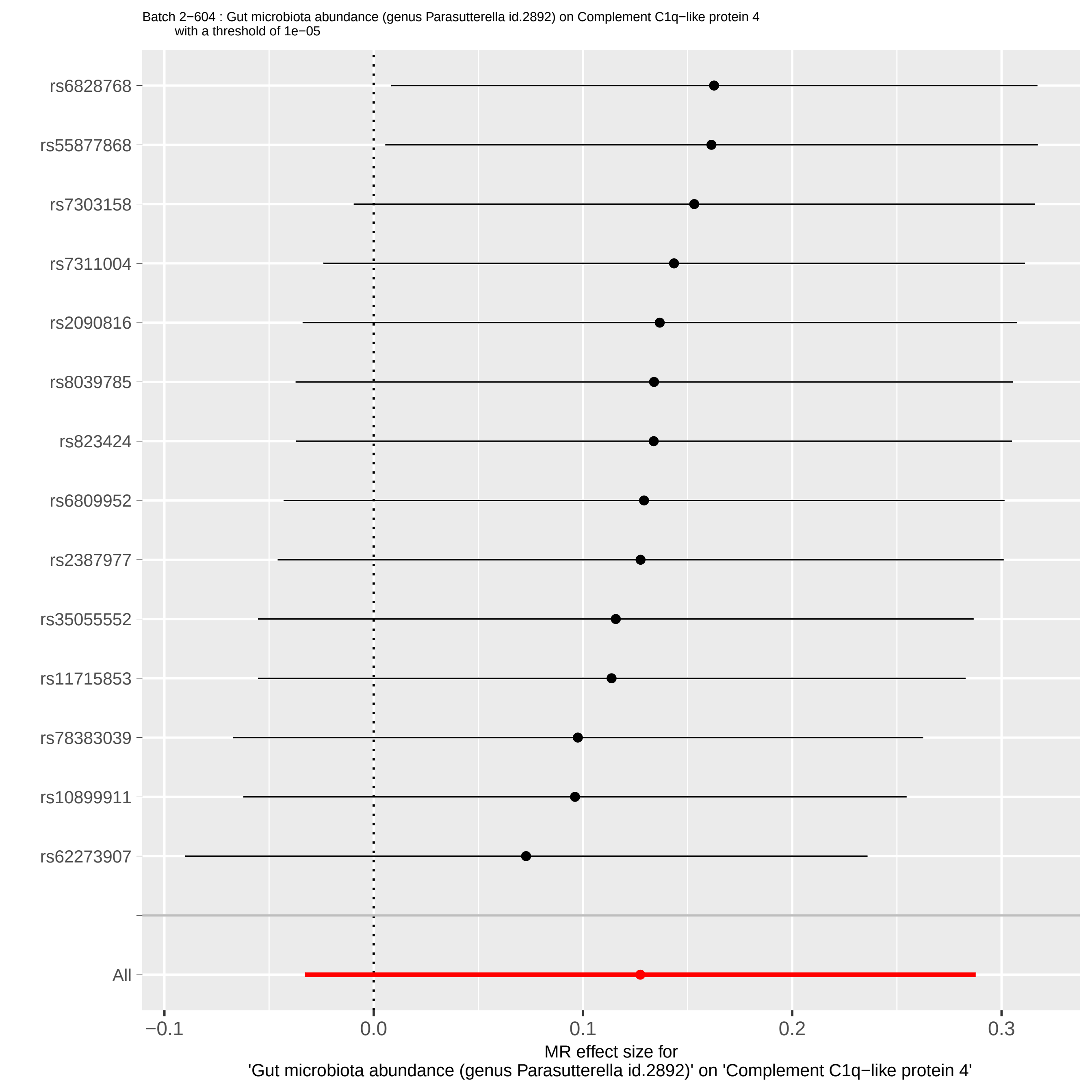
Batch 2−604 : Gut microbiota abundance (genus Parasutterella id.2892) on Complement C1q−like protein 4
with a threshold of 1e−05
rs6828768
rs55877868
rs7303158
rs7311004
rs2090816
rs8039785
rs823424
rs6809952
rs2387977
rs35055552
rs11715853
rs78383039
rs10899911
rs62273907
All
−0.1
0.0
0.1
0.2
0.3
MR effect size for
'Gut microbiota abundance (genus Parasutterella id.2892)' on 'Complement C1q−like protein 4'

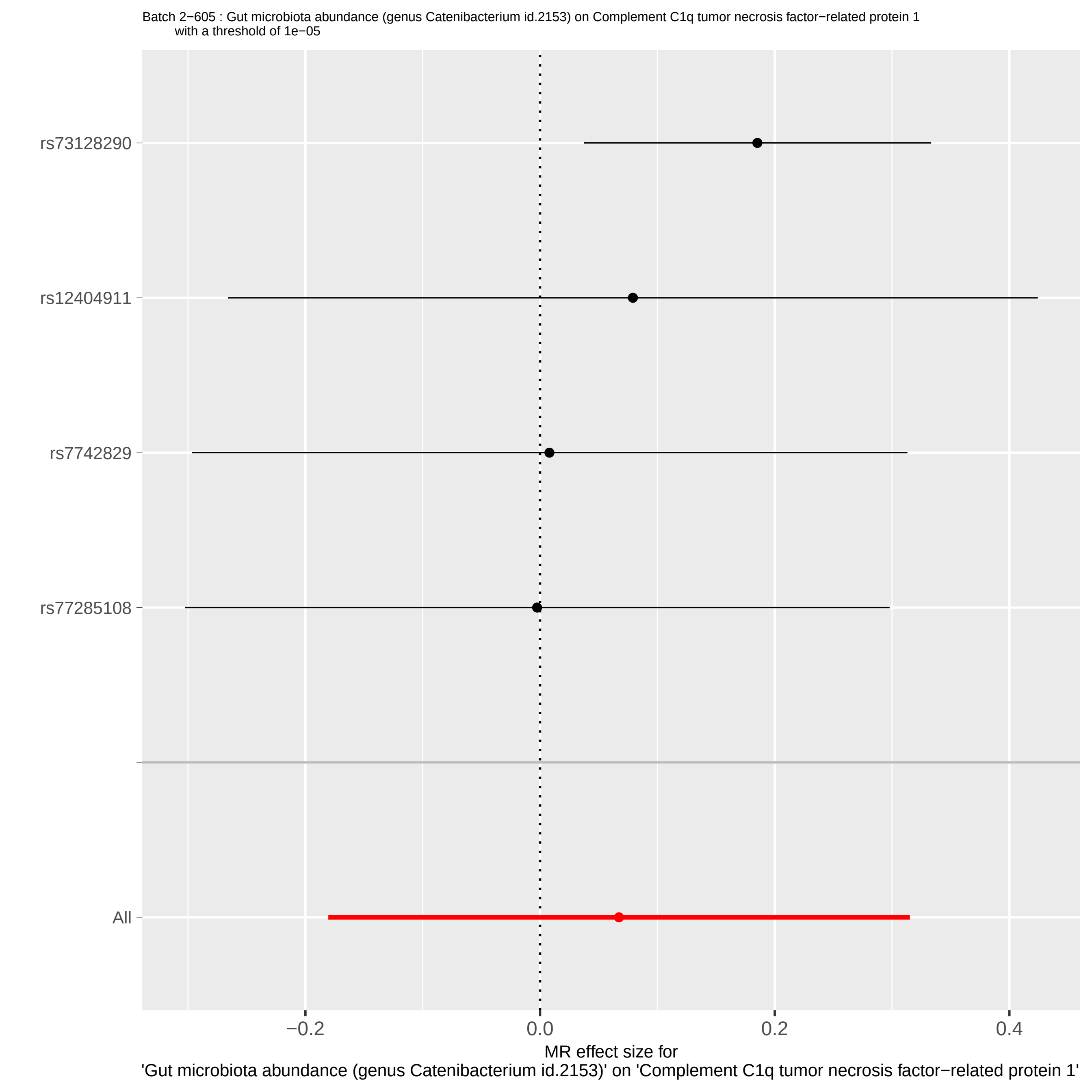
Batch 2−605 : Gut microbiota abundance (genus Catenibacterium id.2153) on Complement C1q tumor necrosis factor−related protein 1 with a threshold of 1e−05
rs73128290
rs12404911
rs7742829
rs77285108
All
−0.2
0.0
0.2
0.4
MR effect size for
'Gut microbiota abundance (genus Catenibacterium id.2153)' on 'Complement C1q tumor necrosis factor−related protein 1'

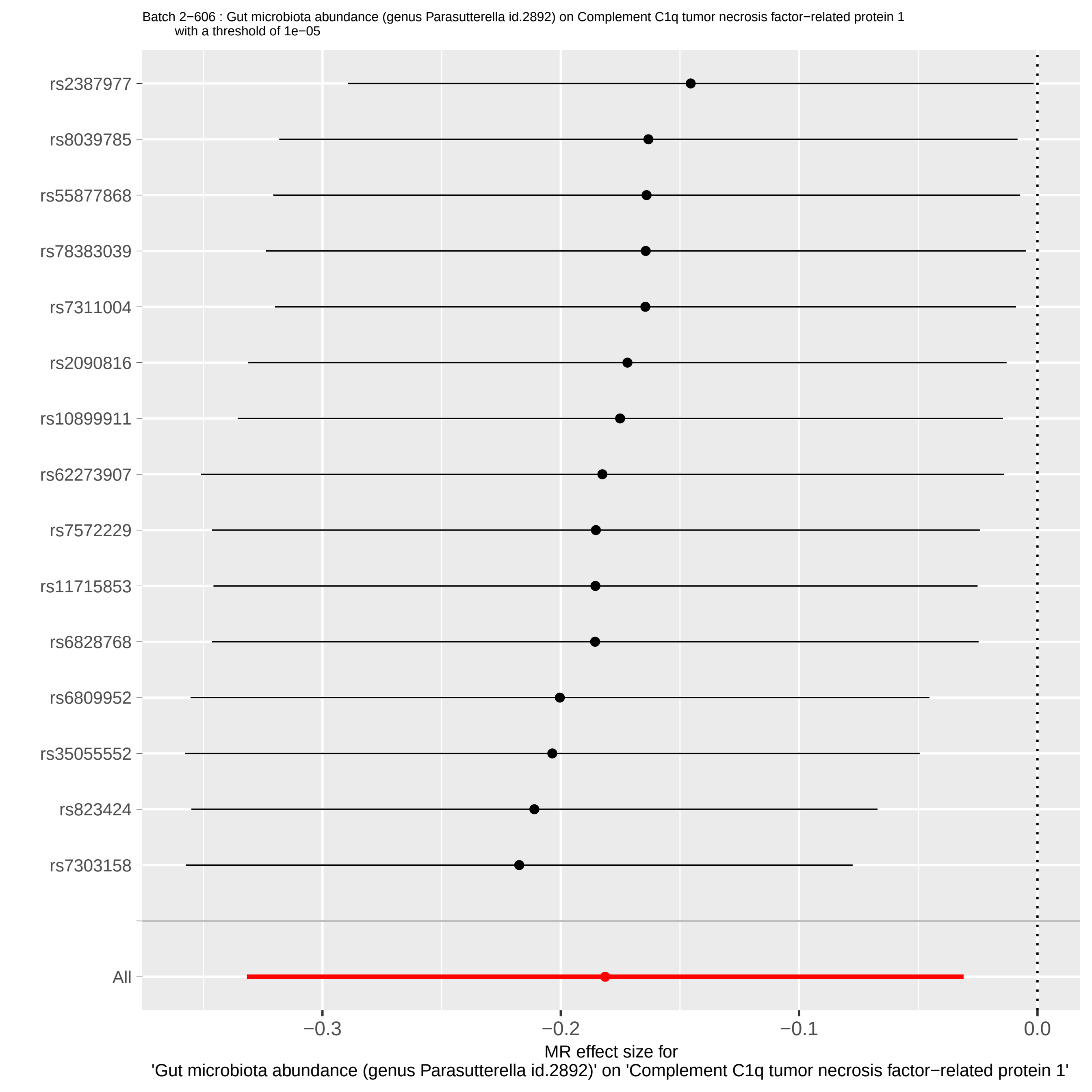
Batch 2−606 : Gut microbiota abundance (genus Parasutterella id.2892) on Complement C1q tumor necrosis factor−related protein 1
with a threshold of 1e−05
rs2387977
rs8039785
rs55877868
rs78383039
rs7311004
rs2090816
rs10899911
rs62273907
rs7572229
rs11715853
rs6828768
rs6809952
rs35055552
rs823424
rs7303158
All
−0.3
−0.2
−0.1
0.0
MR effect size for
'Gut microbiota abundance (genus Parasutterella id.2892)' on 'Complement C1q tumor necrosis factor−related protein 1'

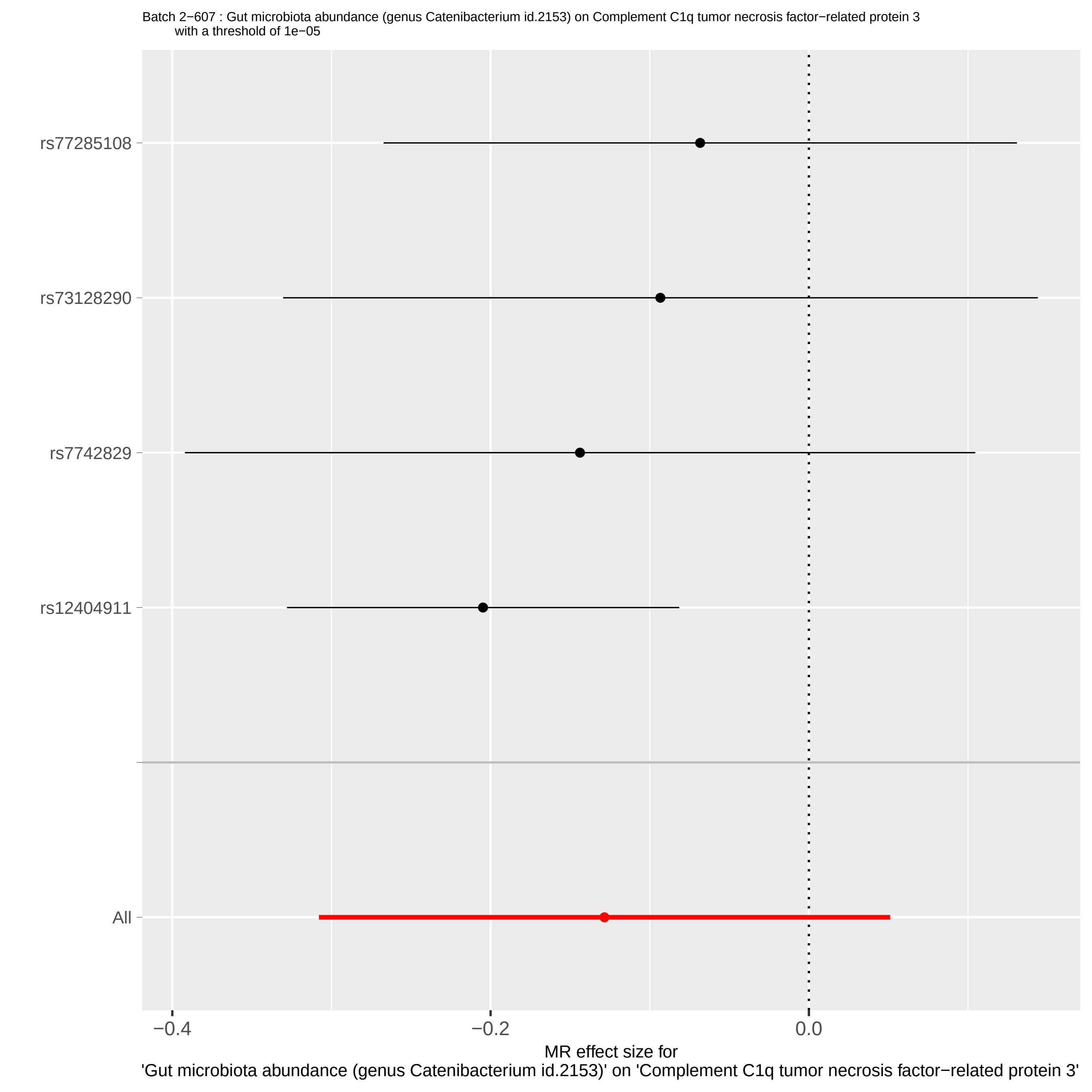

Batch 2−607 : Gut microbiota abundance (genus Catenibacterium id.2153) on Complement C1q tumor necrosis factor−related protein 3
with a threshold of 1e−05
rs77285108
rs73128290
rs7742829
rs12404911
All
−0.4
−0.2
0.0
MR effect size for
'Gut microbiota abundance (genus Catenibacterium id.2153)' on 'Complement C1q tumor necrosis factor−related protein 3'

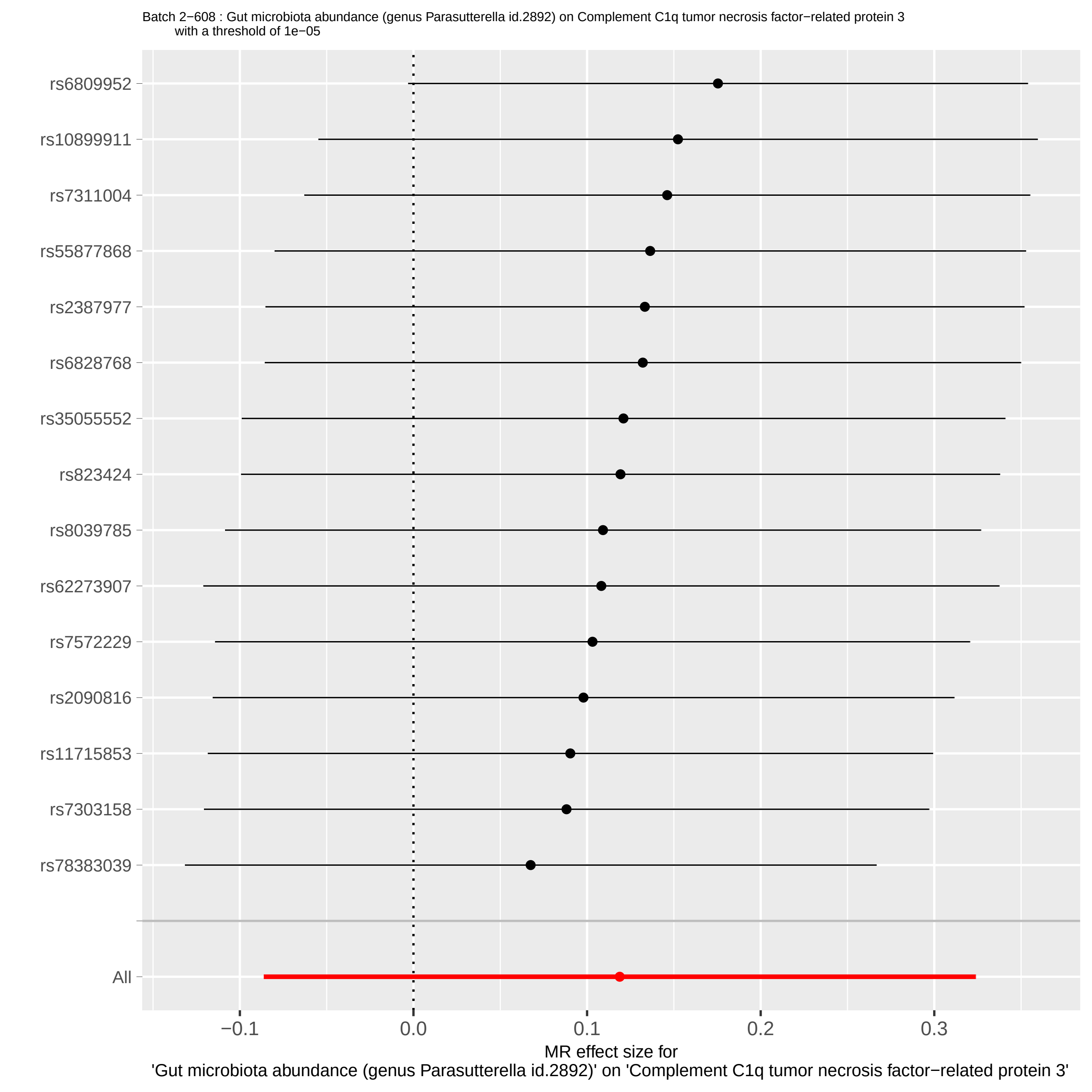

Batch 2−608 : Gut microbiota abundance (genus Parasutterella id.2892) on Complement C1q tumor necrosis factor−related protein 3
with a threshold of 1e−05
rs6809952
rs10899911
rs7311004
rs55877868
rs2387977
rs6828768
rs35055552
rs823424
rs8039785
rs62273907
rs7572229
rs2090816
rs11715853
rs7303158
rs78383039
All
−0.1
0.0
0.1
0.2
0.3
MR effect size for
'Gut microbiota abundance (genus Parasutterella id.2892)' on 'Complement C1q tumor necrosis factor−related protein 3'

Batch 2−609 : Gut microbiota abundance (genus Catenibacterium id.2153) on Complement C1q tumor necrosis factor−related protein 5
with a threshold of 1e−05

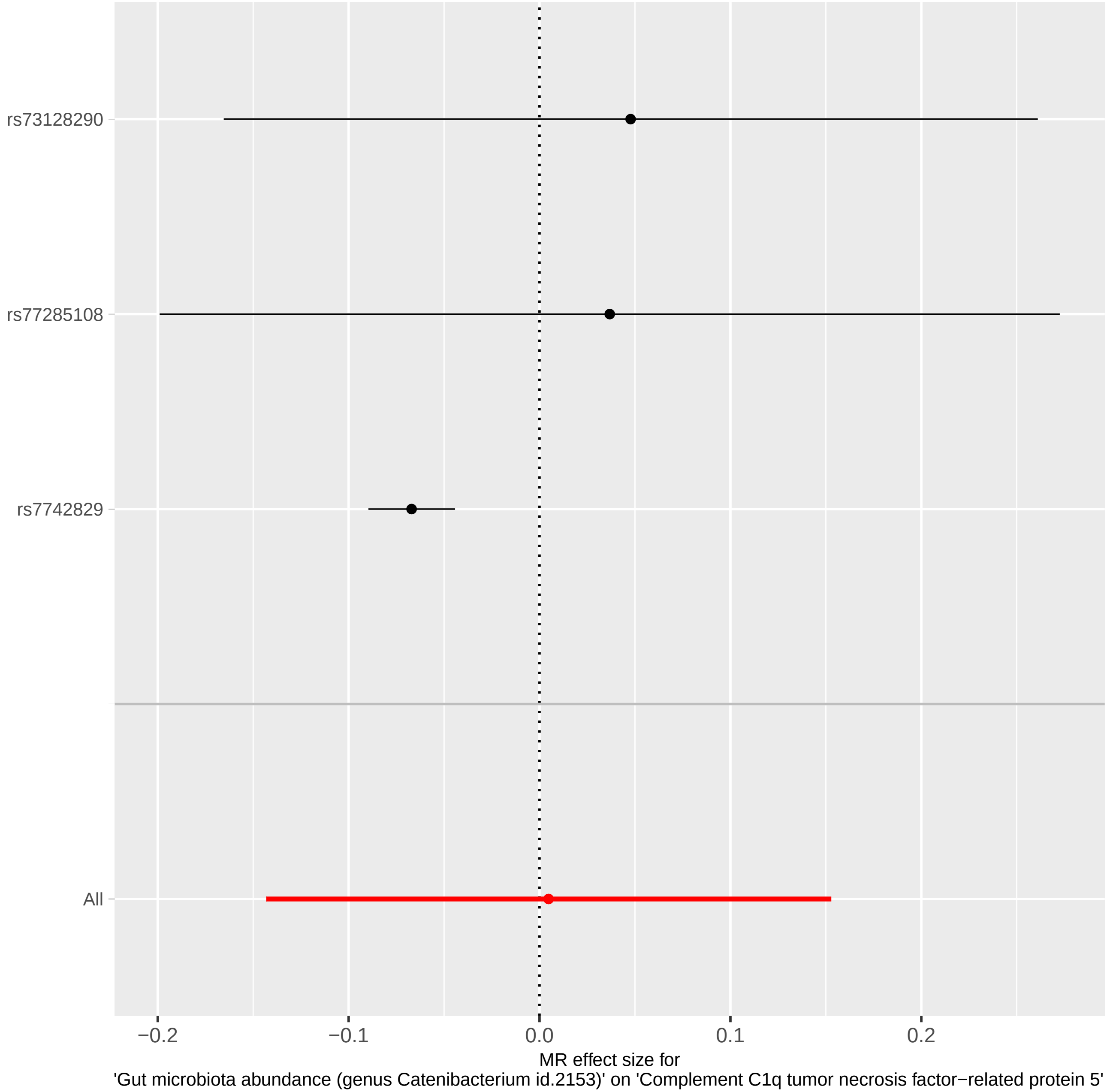

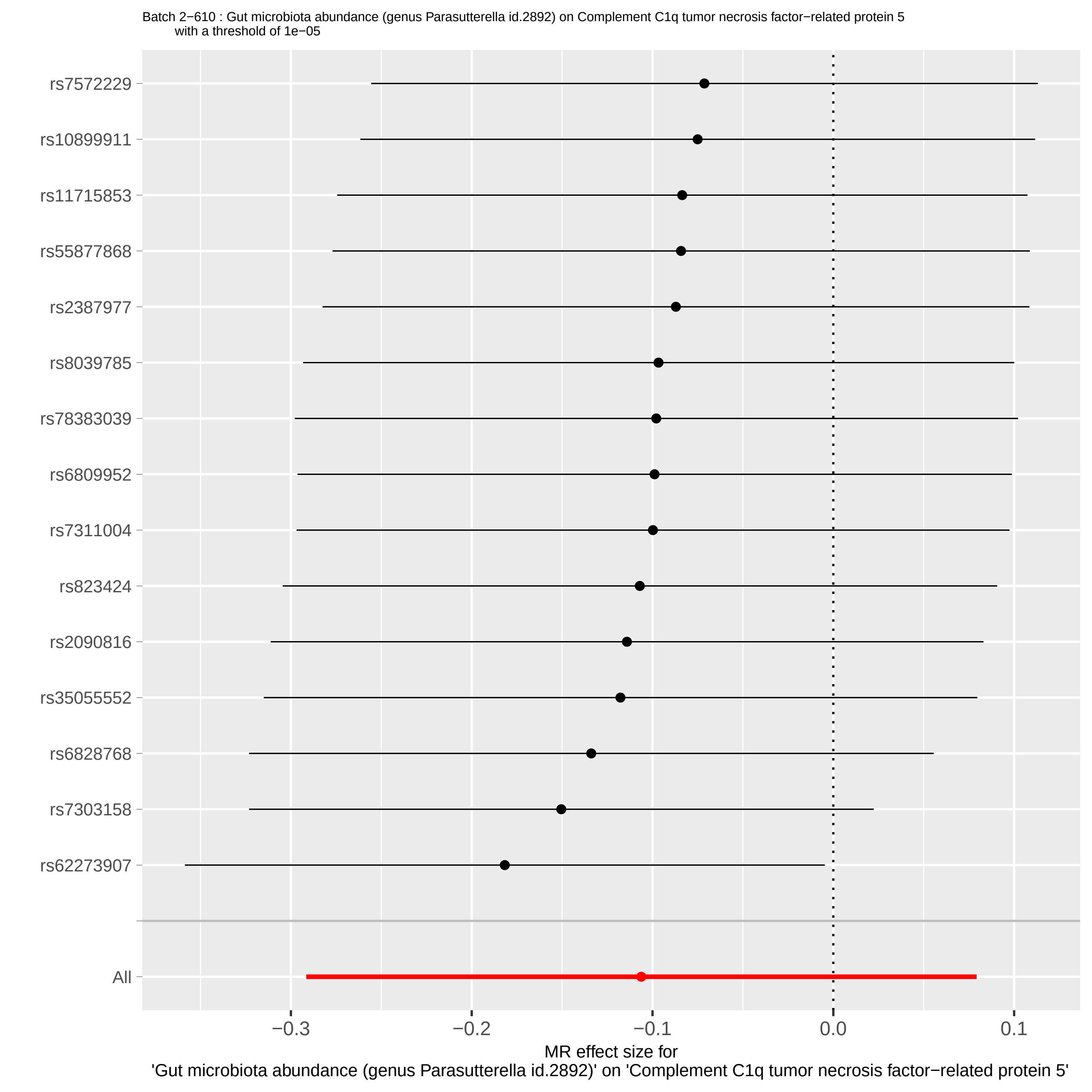

Batch 2−610 : Gut microbiota abundance (genus Parasutterella id.2892) on Complement C1q tumor necrosis factor−related protein 5
with a threshold of 1e−05
rs7572229
rs10899911
rs11715853
rs55877868
rs2387977
rs8039785
rs78383039
rs6809952
rs7311004
rs823424
rs2090816
rs35055552
rs6828768
rs7303158
rs62273907
All
−0.3
−0.2
−0.1
0.0
0.1
MR effect size for
'Gut microbiota abundance (genus Parasutterella id.2892)' on 'Complement C1q tumor necrosis factor−related protein 5'

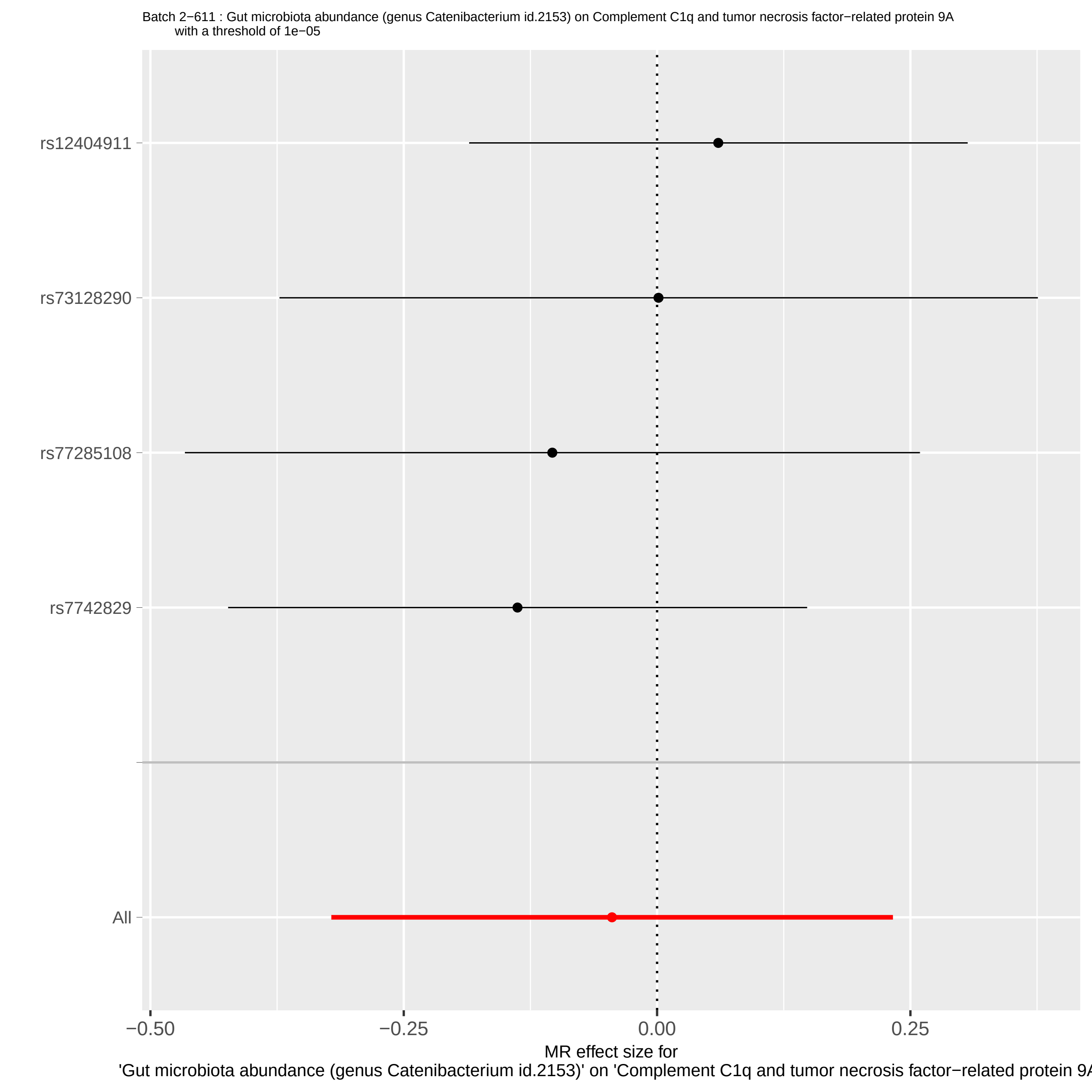
Batch 2−611 : Gut microbiota abundance (genus Catenibacterium id.2153) on Complement C1q and tumor necrosis factor−related protein 9A
with a threshold of 1e−05
rs12404911
rs73128290
rs77285108
rs7742829
All
−0.50
−0.25
0.00
0.25
MR effect size for
'Gut microbiota abundance (genus Catenibacterium id.2153)' on 'Complement C1q and tumor necrosis factor−related protein 9A

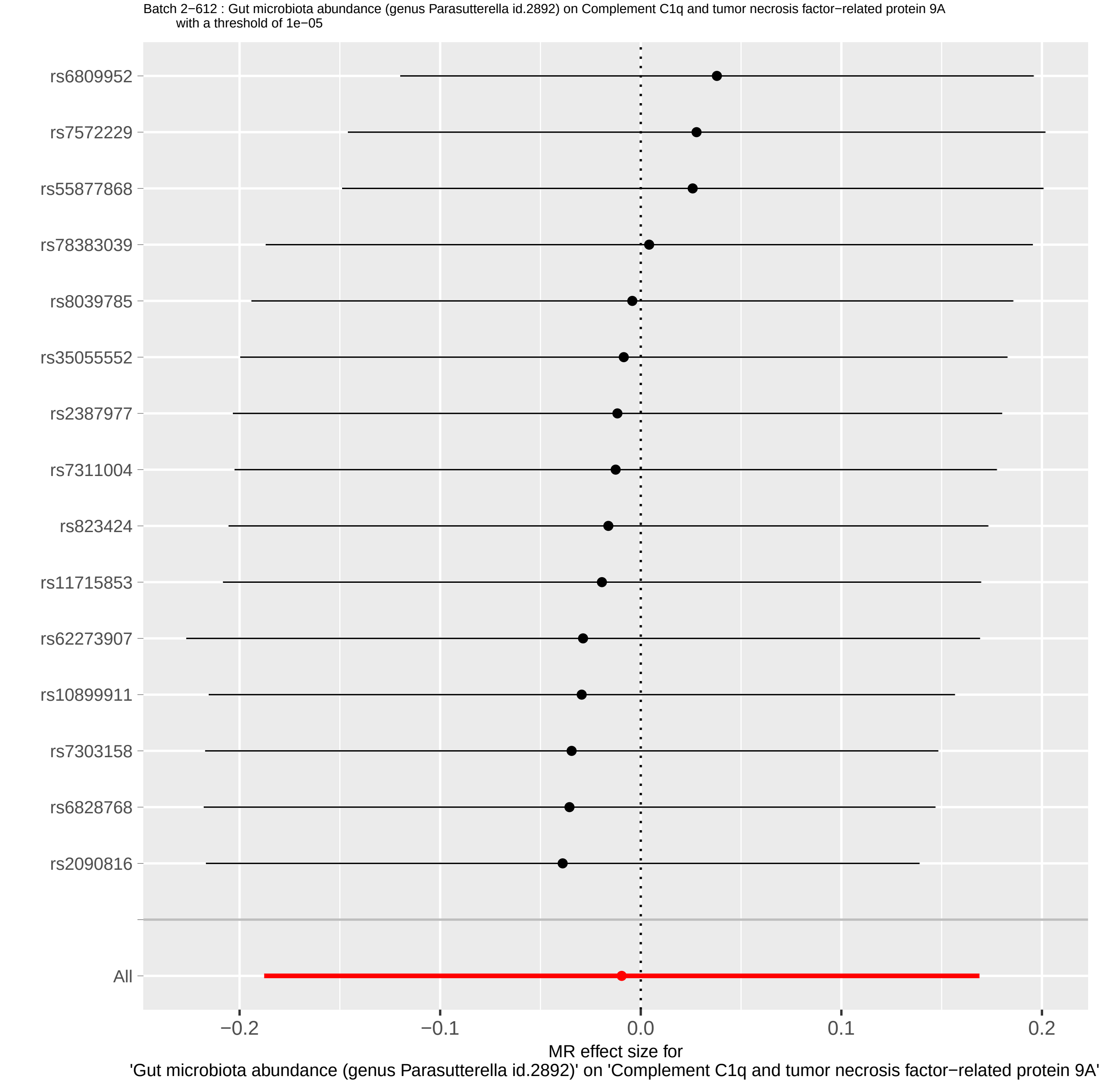
Batch 2−612 : Gut microbiota abundance (genus Parasutterella id.2892) on Complement C1q and tumor necrosis factor−related protein 9A
with a threshold of 1e−05
rs6809952
rs7572229
rs55877868
rs78383039
rs8039785
rs35055552
rs2387977
rs7311004
rs823424
rs11715853
rs62273907
rs10899911
rs7303158
rs6828768
rs2090816
All
−0.2
−0.1
0.0
0.1
0.2
MR effect size for
'Gut microbiota abundance (genus Parasutterella id.2892)' on 'Complement C1q and tumor necrosis factor−related protein 9A'

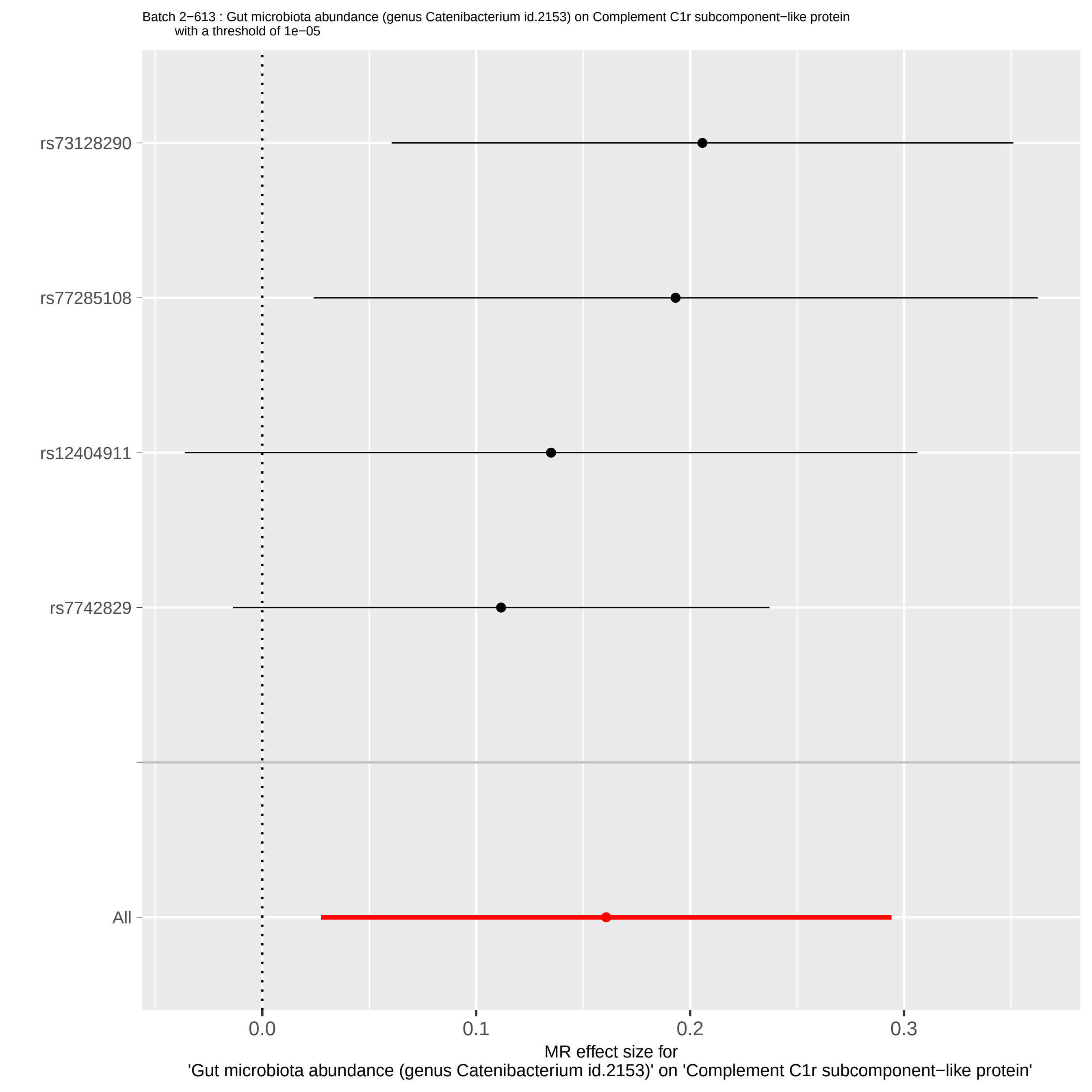

Batch 2−613 : Gut microbiota abundance (genus Catenibacterium id.2153) on Complement C1r subcomponent−like protein
with a threshold of 1e−05
rs73128290
rs77285108
rs12404911
rs7742829
All
0.0
0.1
0.2
0.3
MR effect size for
'Gut microbiota abundance (genus Catenibacterium id.2153)' on 'Complement C1r subcomponent−like protein'

Batch 2−614 : Gut microbiota abundance (genus Parasutterella id.2892) on Complement C1r subcomponent−like protein
with a threshold of 1e−05

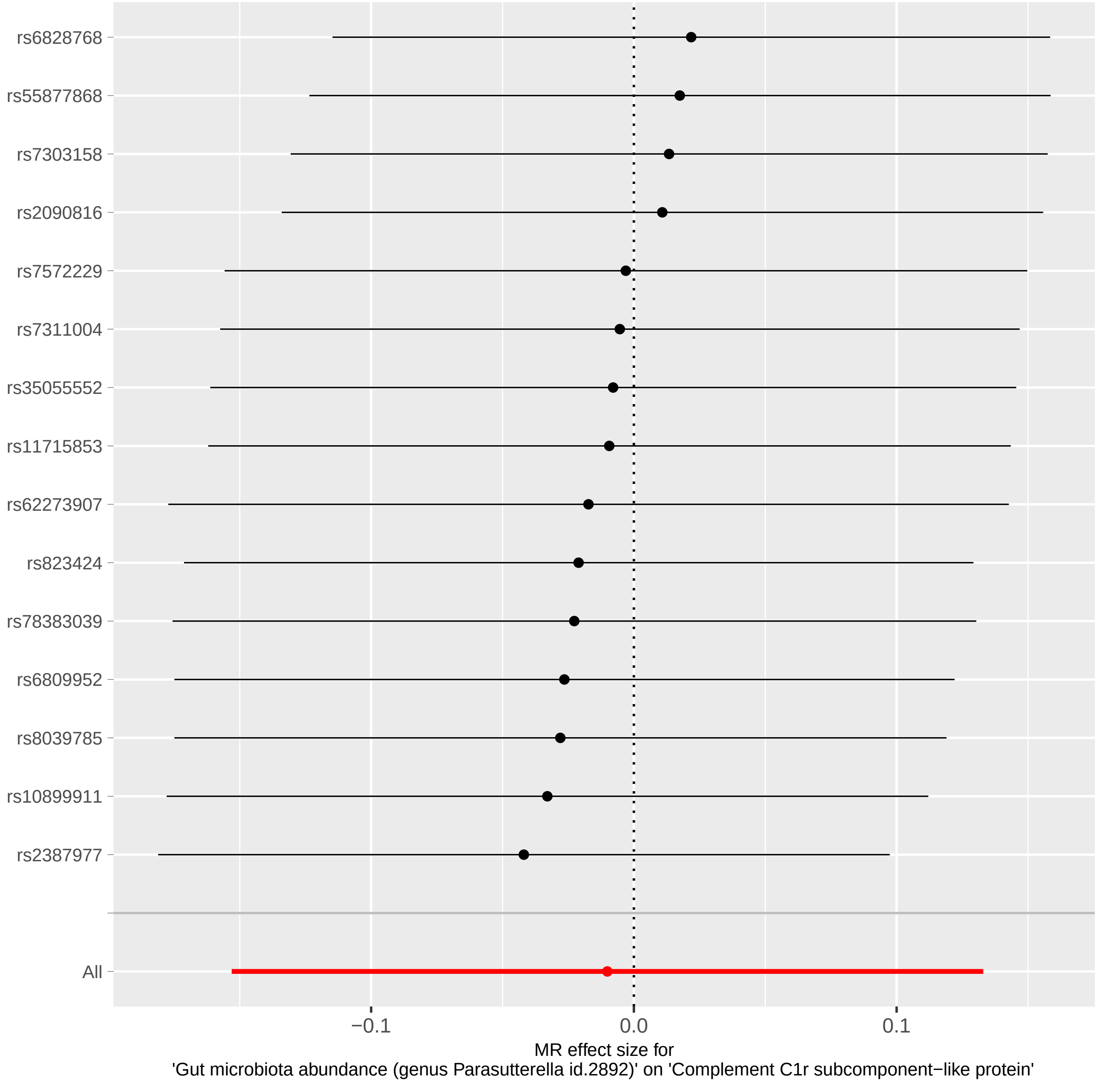

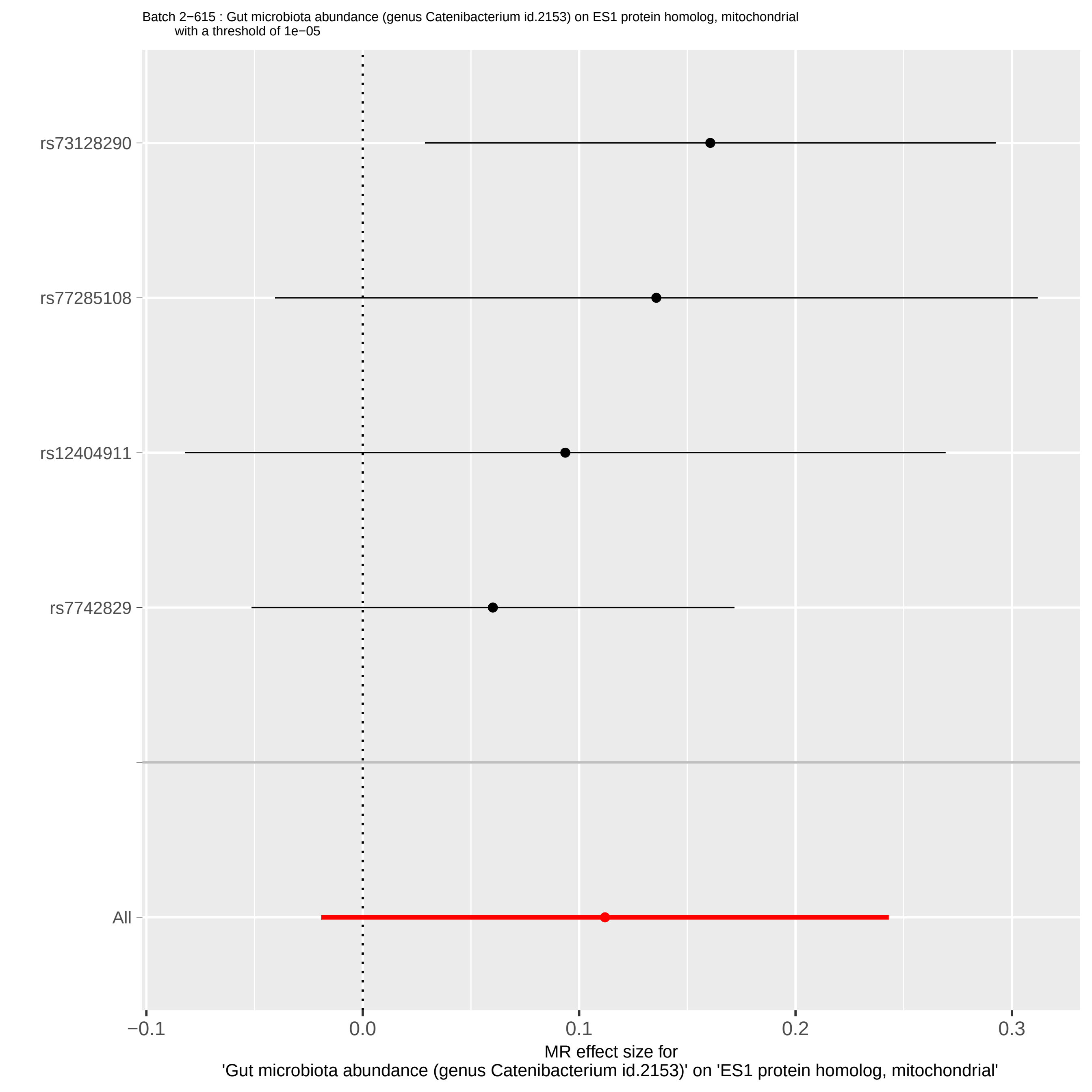

Batch 2−615 : Gut microbiota abundance (genus Catenibacterium id.2153) on ES1 protein homolog, mitochondrial
with a threshold of 1e−05
rs73128290
rs77285108
rs12404911
rs7742829
All
−0.1
0.0
0.1
0.2
0.3
MR effect size for
'Gut microbiota abundance (genus Catenibacterium id.2153)' on 'ES1 protein homolog, mitochondrial'

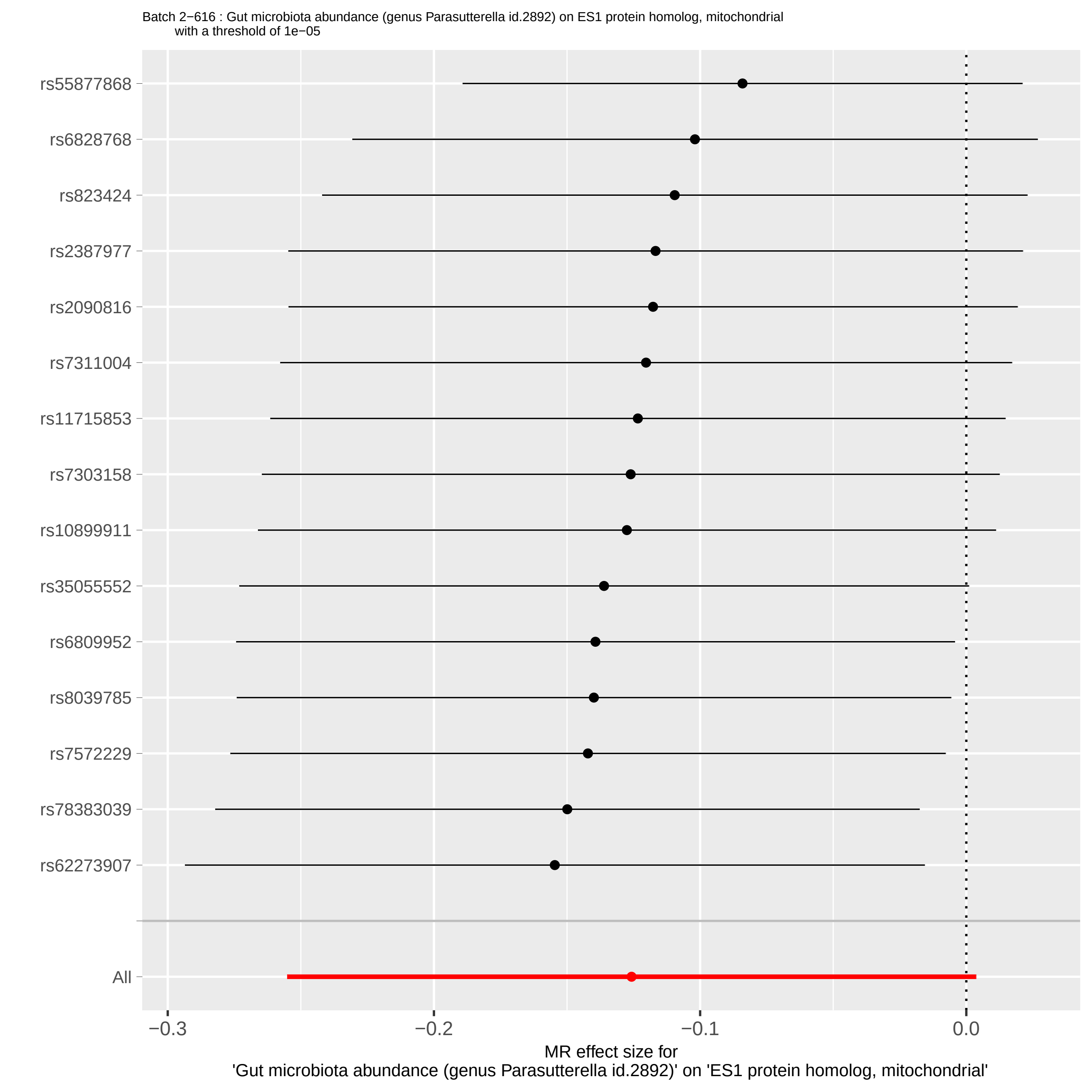
Batch 2−616 : Gut microbiota abundance (genus Parasutterella id.2892) on ES1 protein homolog, mitochondrial
with a threshold of 1e−05
rs55877868
rs6828768
rs823424
rs2387977
rs2090816
rs7311004
rs11715853
rs7303158
rs10899911
rs35055552
rs6809952
rs8039785
rs7572229
rs78383039
rs62273907
All
−0.3
−0.2
−0.1
0.0
MR effect size for
'Gut microbiota abundance (genus Parasutterella id.2892)' on 'ES1 protein homolog, mitochondrial'

Batch 2−617 : Gut microbiota abundance (genus Catenibacterium id.2153) on Uncharacterized protein C22orf15
with a threshold of 1e−05

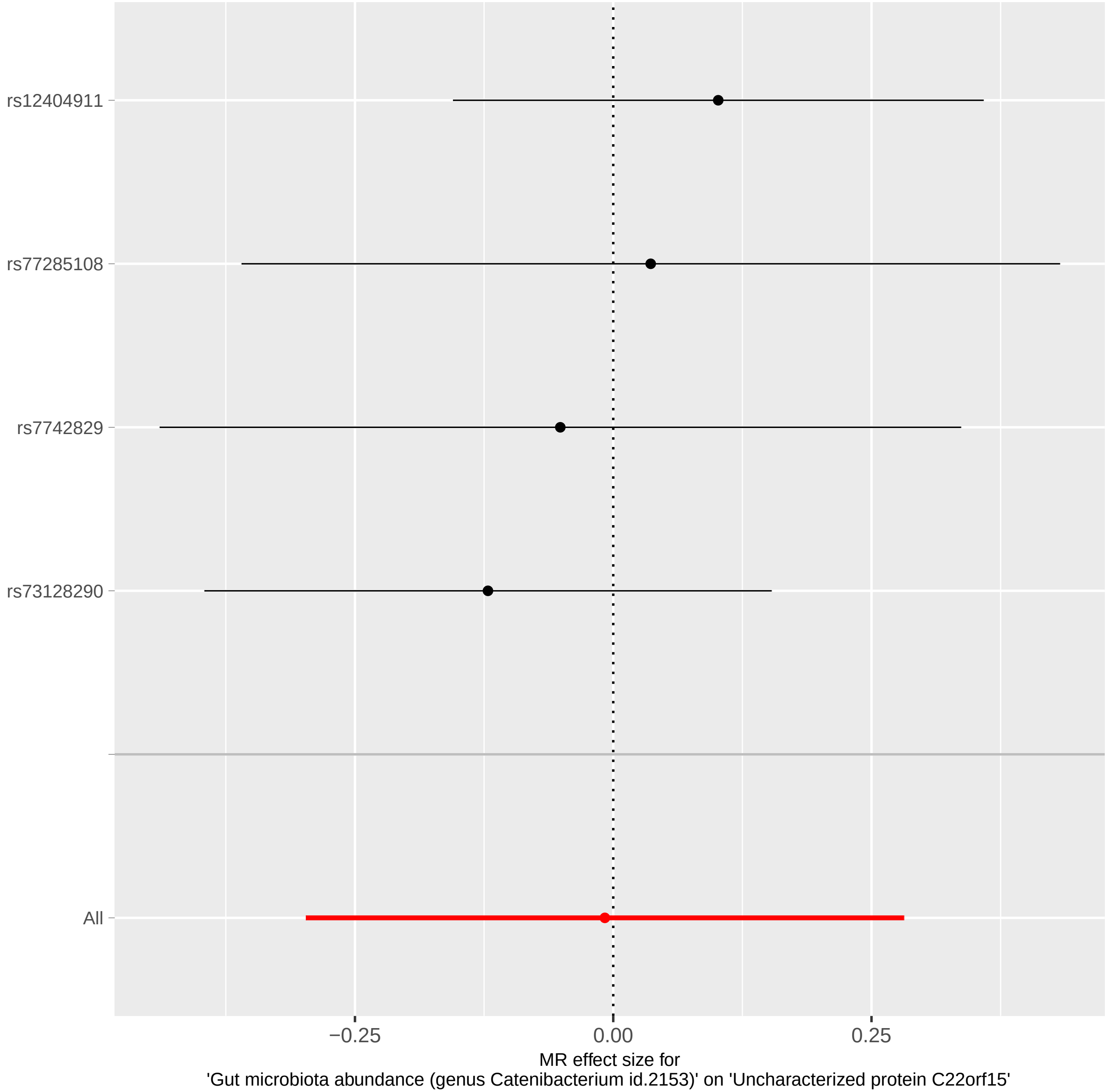

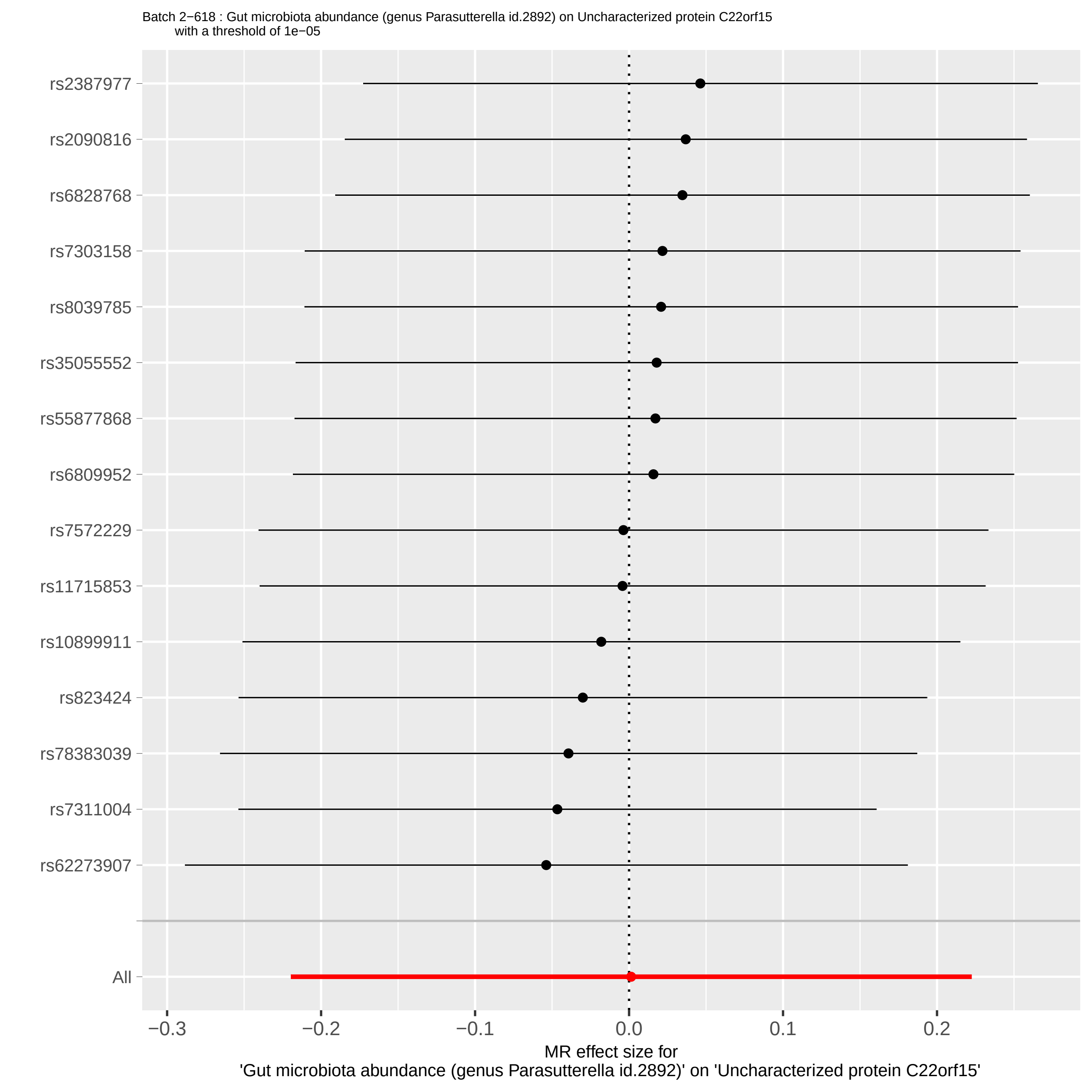
Batch 2−618 : Gut microbiota abundance (genus Parasutterella id.2892) on Uncharacterized protein C22orf15
with a threshold of 1e−05
rs2387977
rs2090816
rs6828768
rs7303158
rs8039785
rs35055552
rs55877868
rs6809952
rs7572229
rs11715853
rs10899911
rs823424
rs78383039
rs7311004
rs62273907
All
−0.3
−0.2
−0.1
0.0
0.1
0.2
MR effect size for
'Gut microbiota abundance (genus Parasutterella id.2892)' on 'Uncharacterized protein C22orf15'

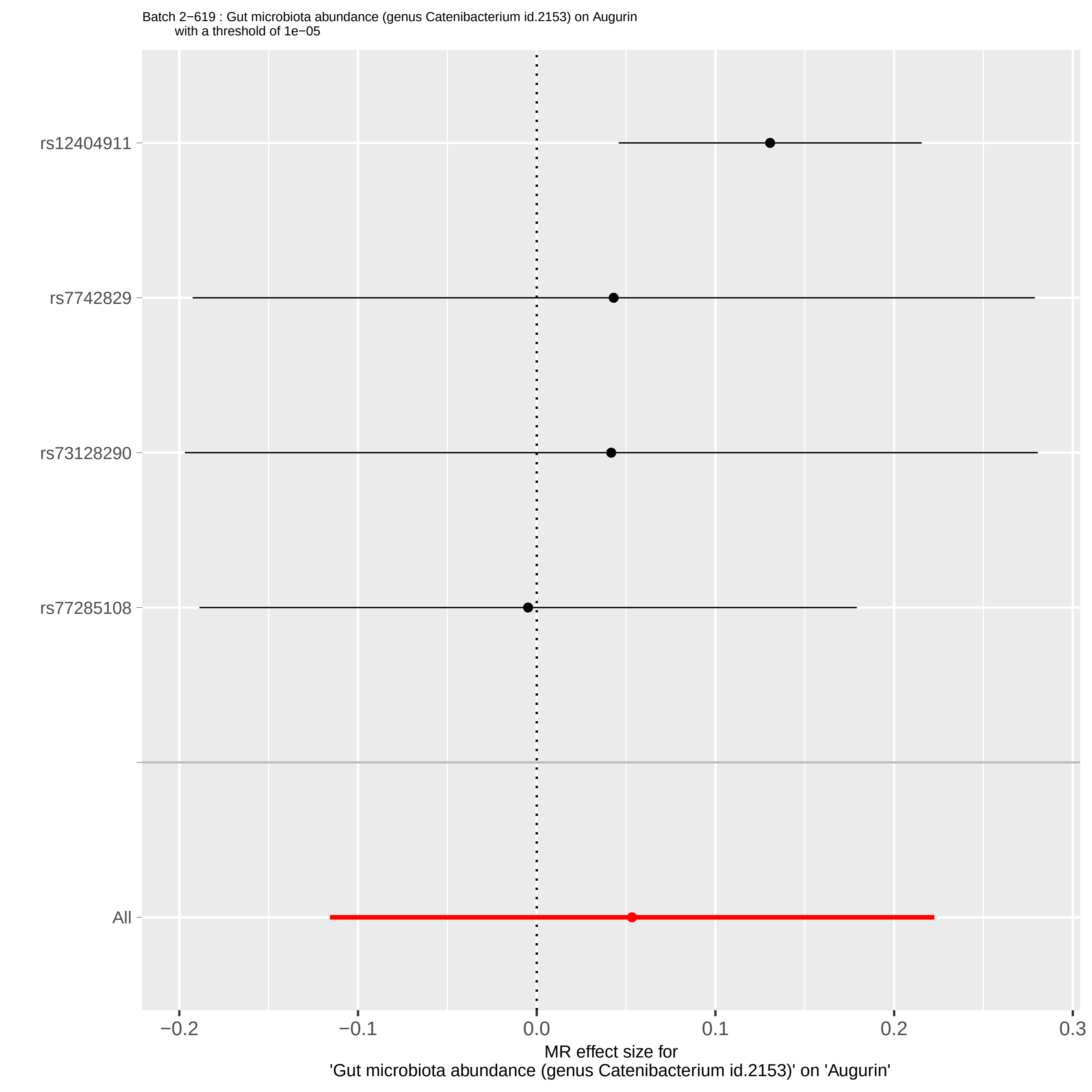

Batch 2−619 : Gut microbiota abundance (genus Catenibacterium id.2153) on Augurin
with a threshold of 1e−05
rs12404911
rs7742829
rs73128290
rs77285108
All
−0.2
−0.1
0.0
0.1
0.2
0.3
MR effect size for
'Gut microbiota abundance (genus Catenibacterium id.2153)' on 'Augurin'

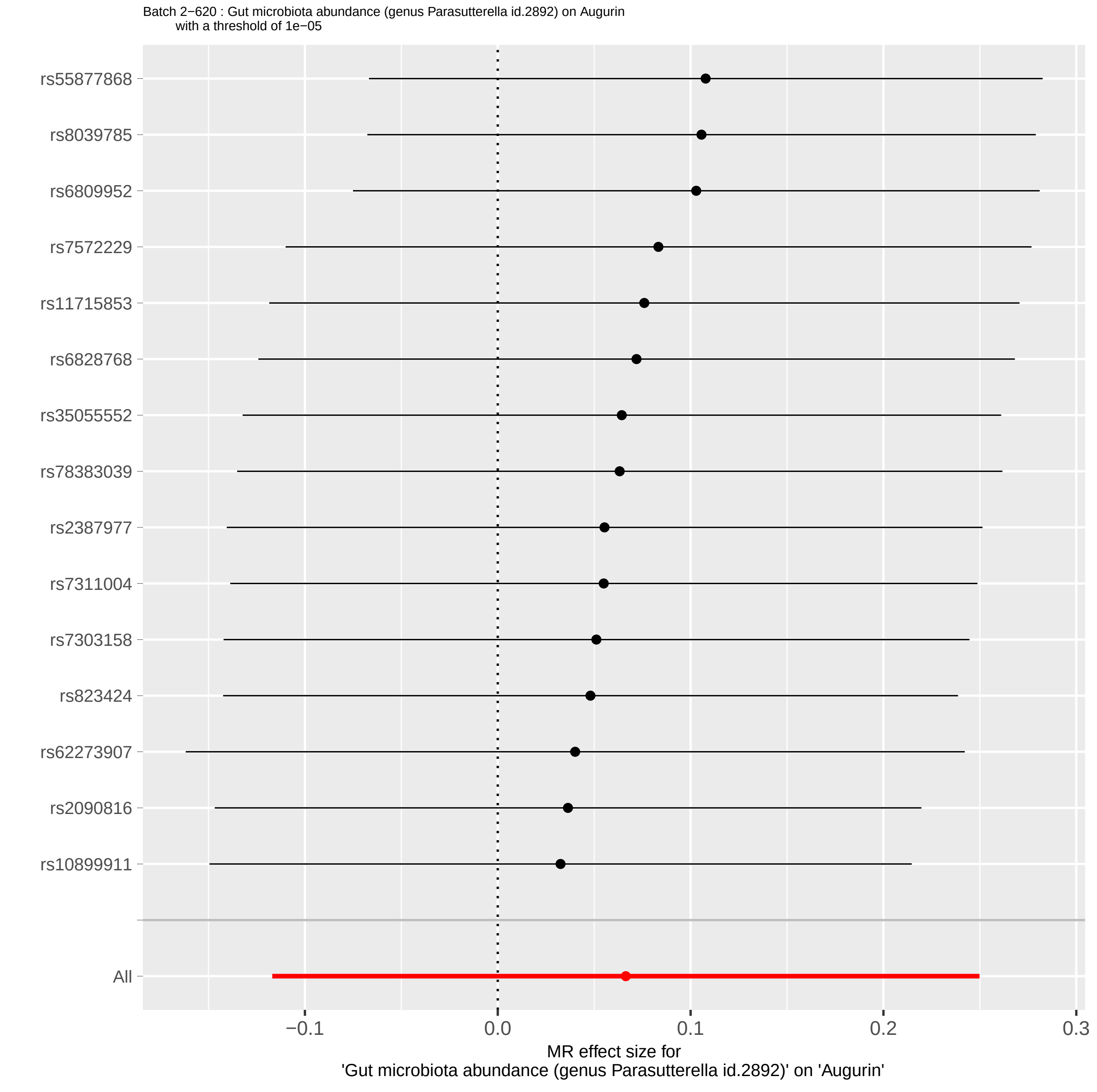
Batch 2−620 : Gut microbiota abundance (genus Parasutterella id.2892) on Augurin
with a threshold of 1e−05
rs55877868
rs8039785
rs6809952
rs7572229
rs11715853
rs6828768
rs35055552
rs78383039
rs2387977
rs7311004
rs7303158
rs823424
rs62273907
rs2090816
rs10899911
All
−0.1
0.0
0.1
0.2
0.3
MR effect size for
'Gut microbiota abundance (genus Parasutterella id.2892)' on 'Augurin'

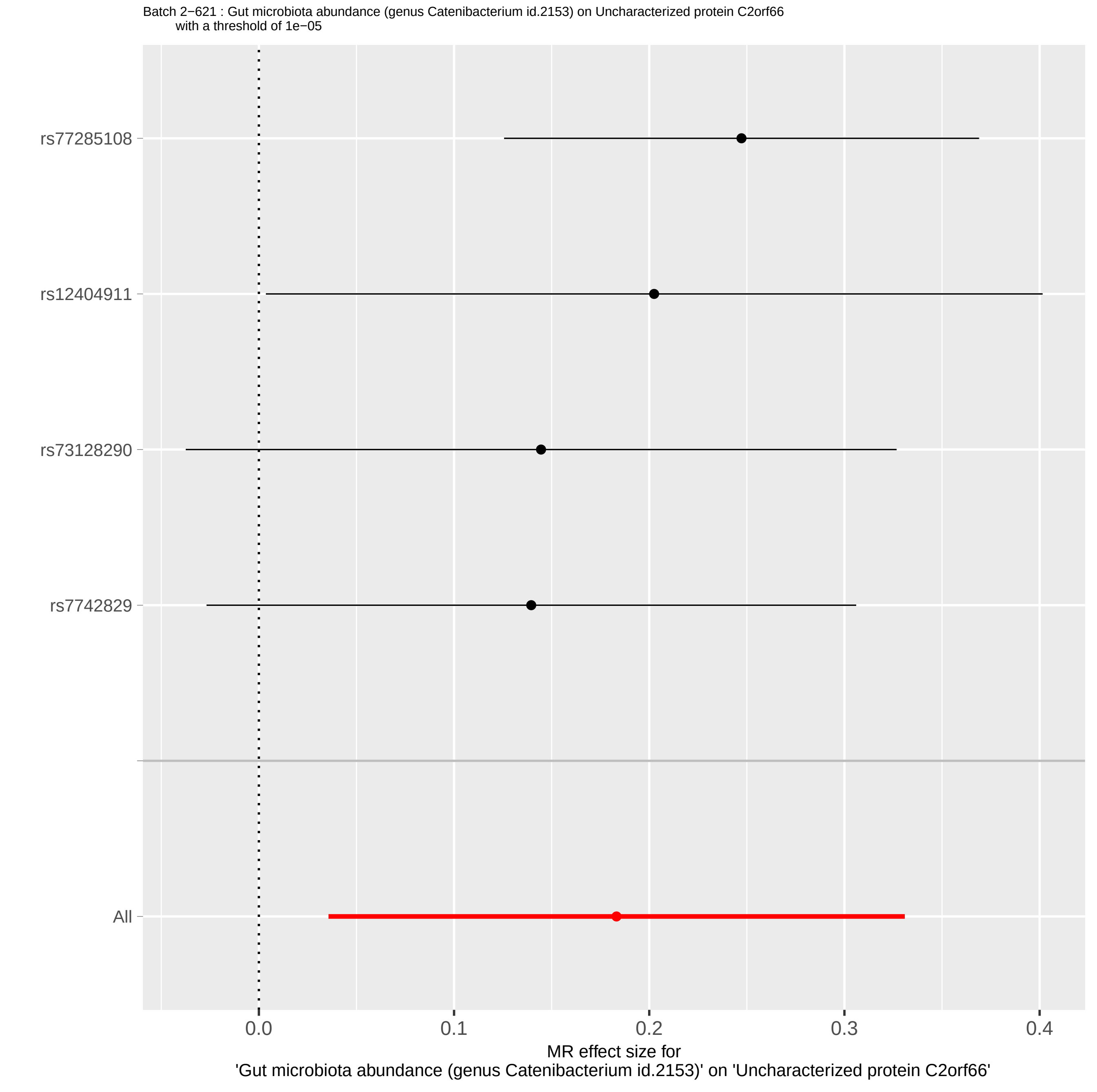

Batch 2−621 : Gut microbiota abundance (genus Catenibacterium id.2153) on Uncharacterized protein C2orf66
with a threshold of 1e−05
rs77285108
rs12404911
rs73128290
rs7742829
All
0.0
0.1
0.2
0.3
0.4
MR effect size for
'Gut microbiota abundance (genus Catenibacterium id.2153)' on 'Uncharacterized protein C2orf66'

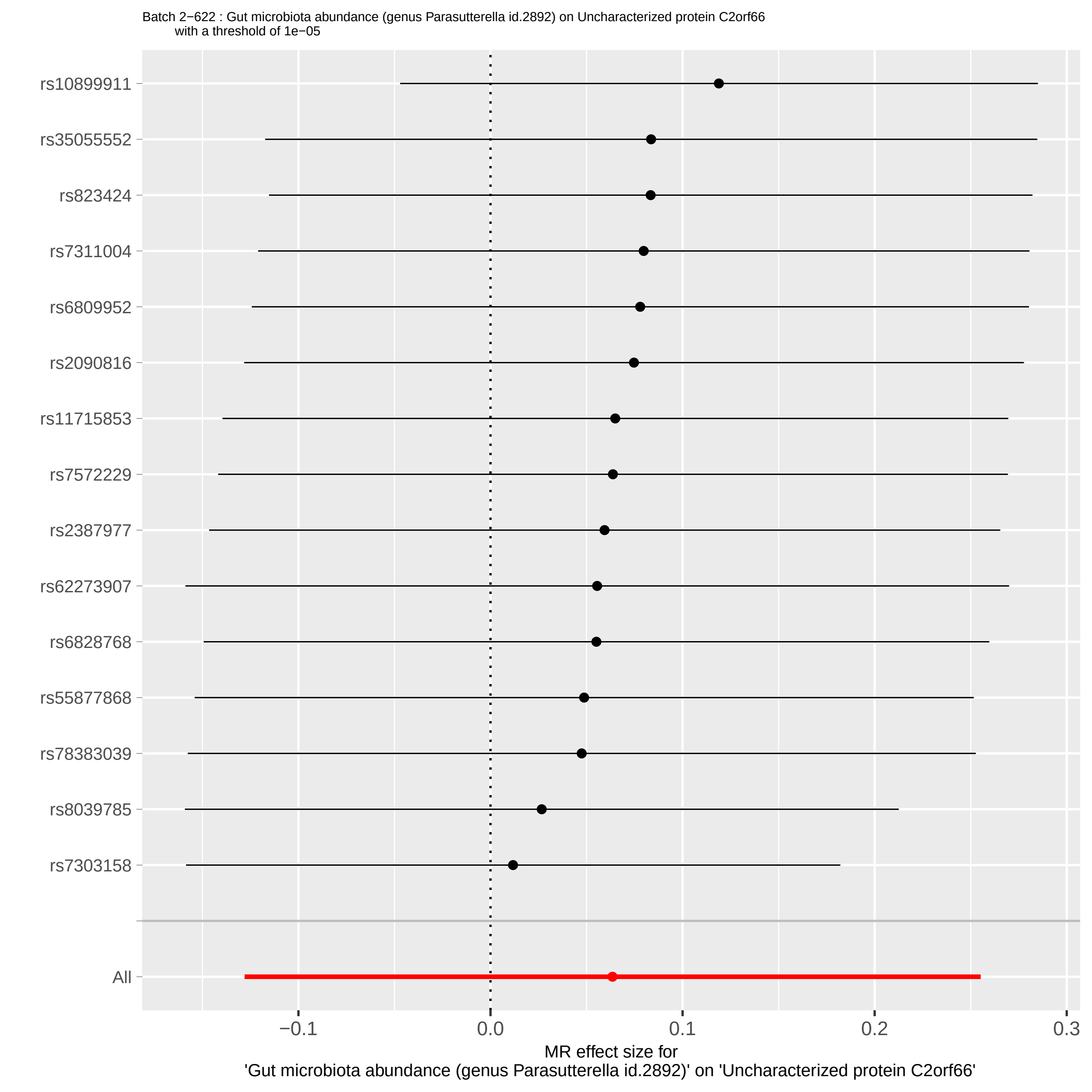
Batch 2−622 : Gut microbiota abundance (genus Parasutterella id.2892) on Uncharacterized protein C2orf66
with a threshold of 1e−05
rs10899911
rs35055552
rs823424
rs7311004
rs6809952
rs2090816
rs11715853
rs7572229
rs2387977
rs62273907
rs6828768
rs55877868
rs78383039
rs8039785
rs7303158
All
−0.1
0.0
0.1
0.2
0.3
MR effect size for
'Gut microbiota abundance (genus Parasutterella id.2892)' on 'Uncharacterized protein C2orf66'

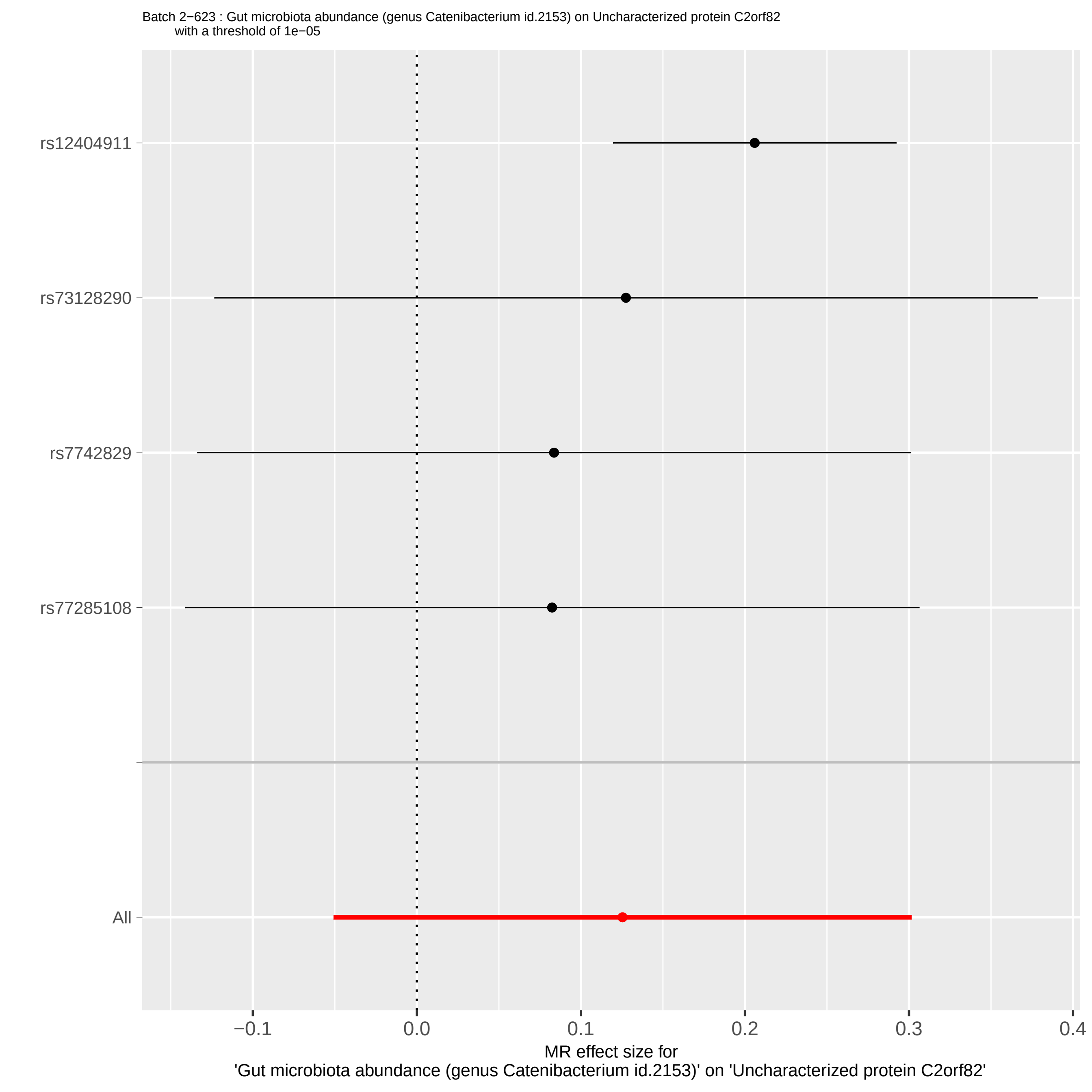

Batch 2−623 : Gut microbiota abundance (genus Catenibacterium id.2153) on Uncharacterized protein C2orf82
with a threshold of 1e−05
rs12404911
rs73128290
rs7742829
rs77285108
All
−0.1
0.0
0.1
0.2
0.3
0.4
MR effect size for
'Gut microbiota abundance (genus Catenibacterium id.2153)' on 'Uncharacterized protein C2orf82'

Batch 2−624 : Gut microbiota abundance (genus Parasutterella id.2892) on Uncharacterized protein C2orf82
with a threshold of 1e−05

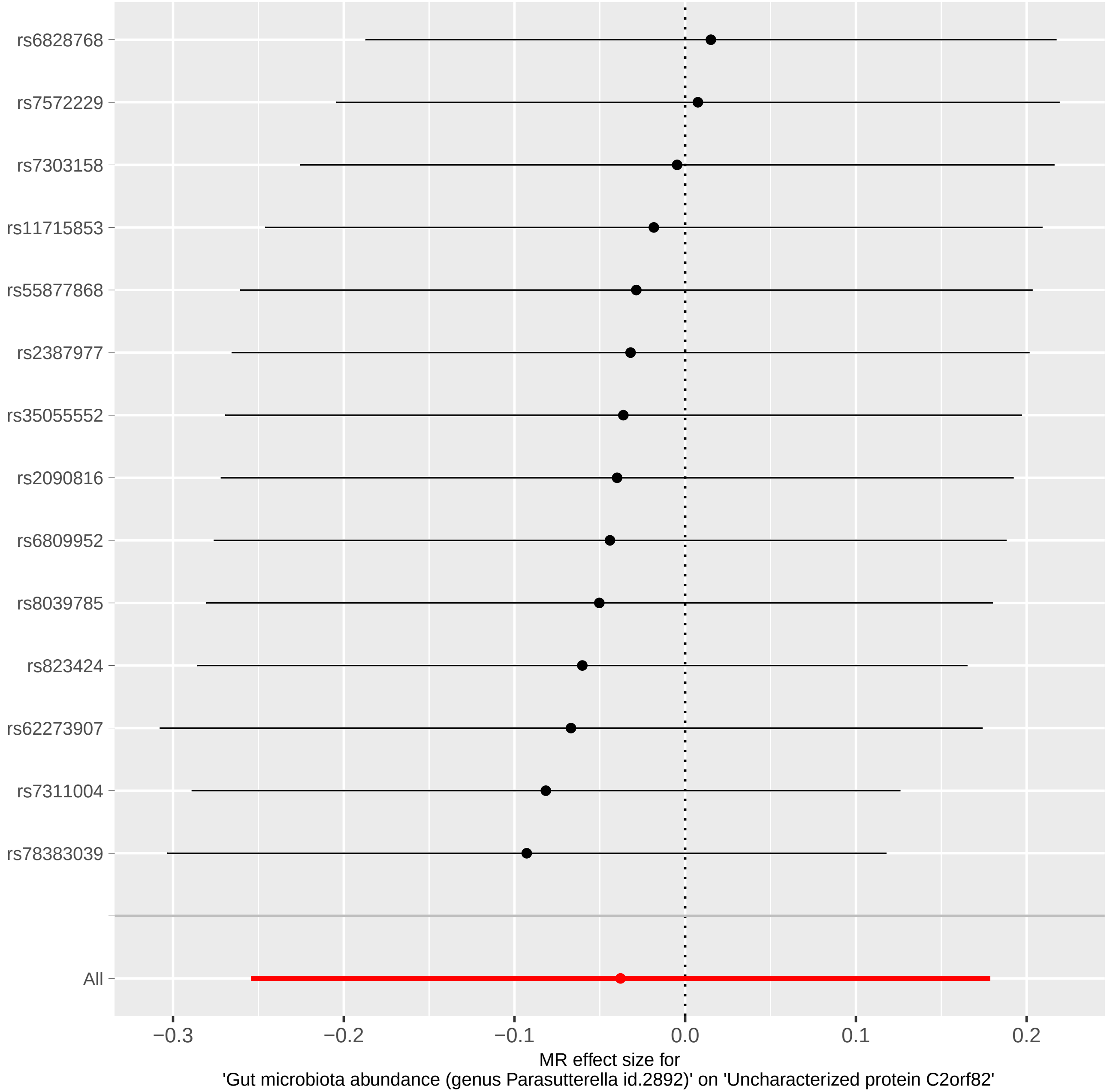

Batch 2−625 : Gut microbiota abundance (genus Catenibacterium id.2153) on C3a anaphylatoxin des Arginine
with a threshold of 1e−05

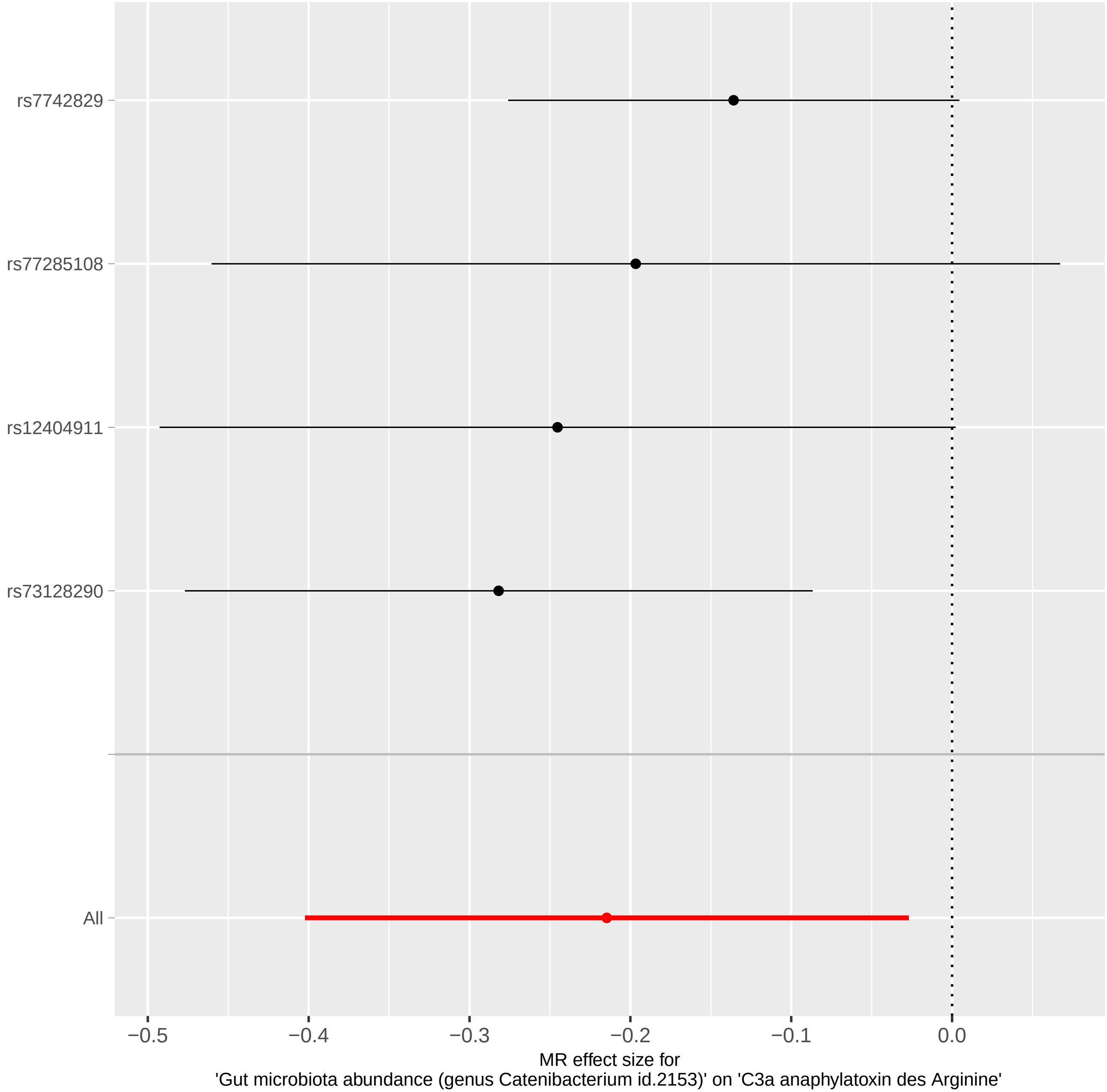

Batch 2−626 : Gut microbiota abundance (genus Parasutterella id.2892) on C3a anaphylatoxin des Arginine
with a threshold of 1e−05

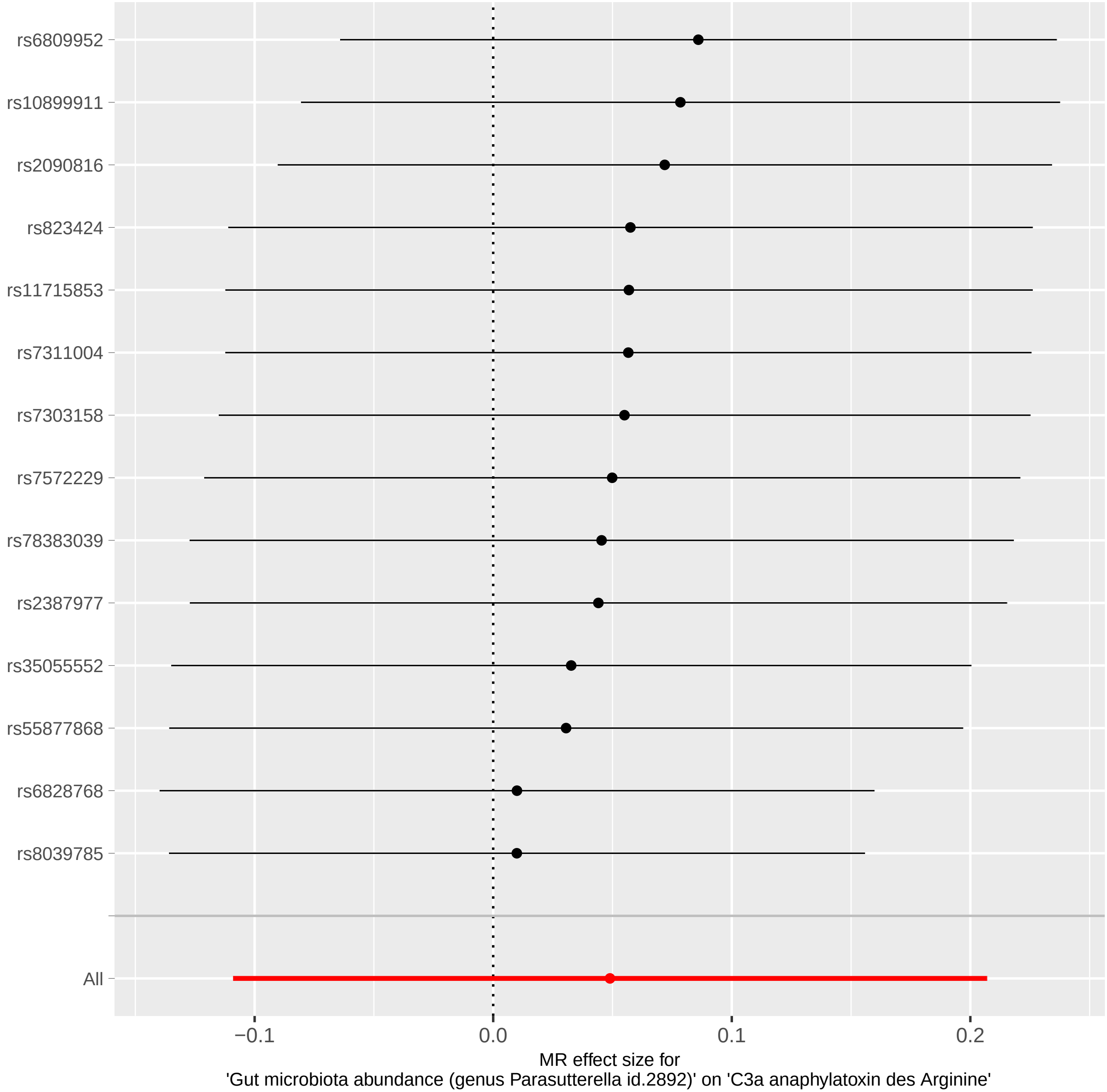

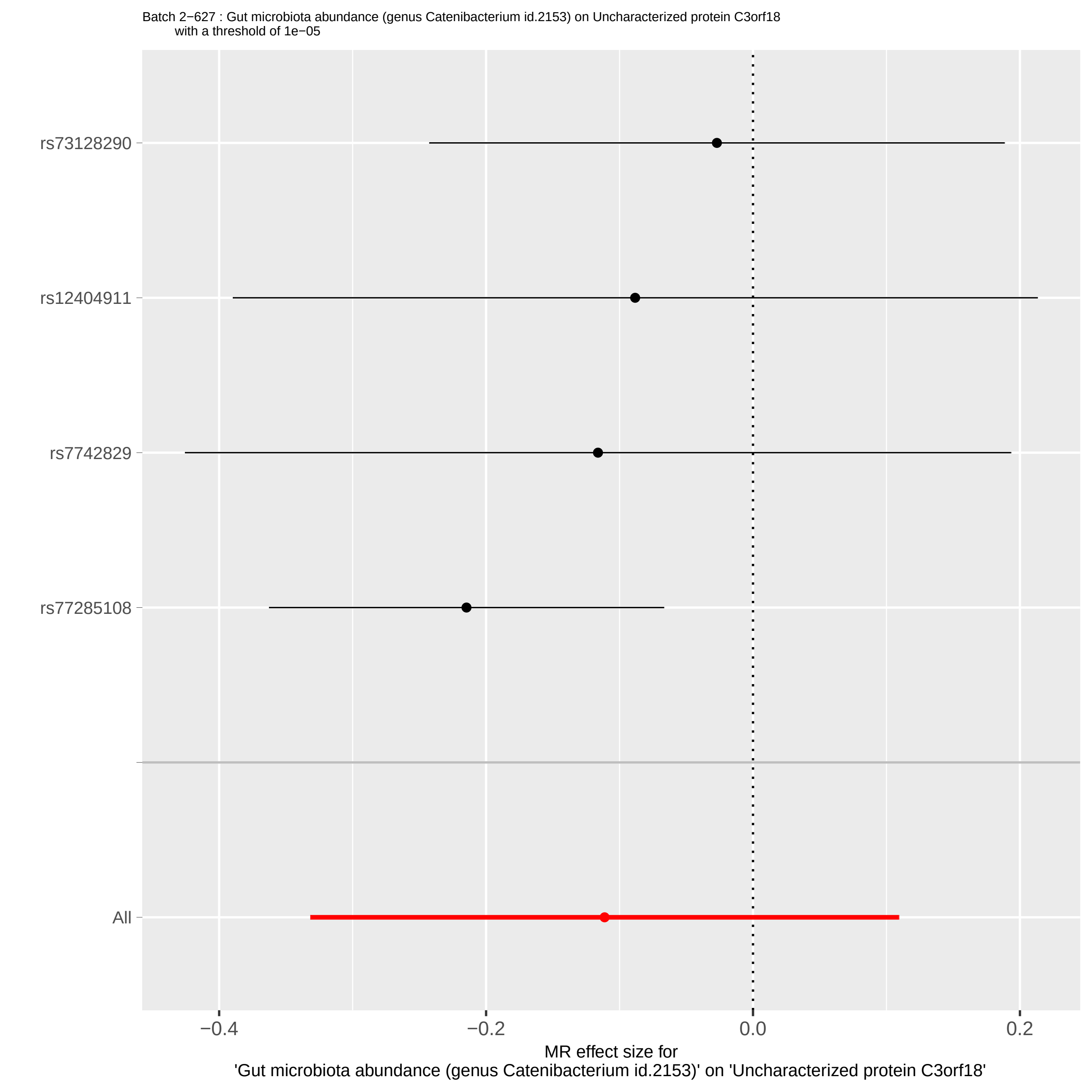

Batch 2−627 : Gut microbiota abundance (genus Catenibacterium id.2153) on Uncharacterized protein C3orf18
with a threshold of 1e−05
rs73128290
rs12404911
rs7742829
rs77285108
All
−0.4
−0.2
0.0
0.2
MR effect size for
'Gut microbiota abundance (genus Catenibacterium id.2153)' on 'Uncharacterized protein C3orf18'

Batch 2−628 : Gut microbiota abundance (genus Parasutterella id.2892) on Uncharacterized protein C3orf18
with a threshold of 1e−05

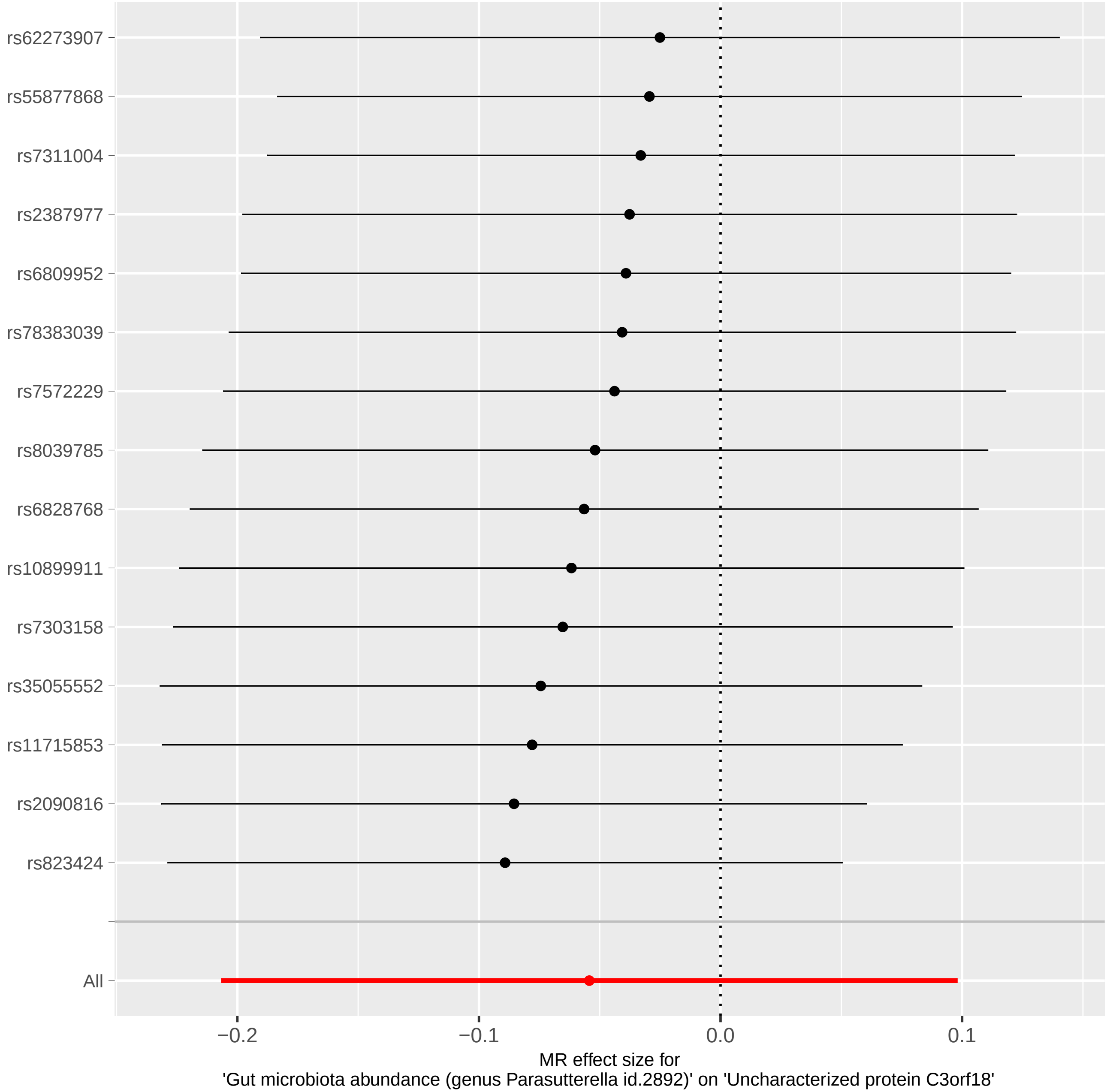

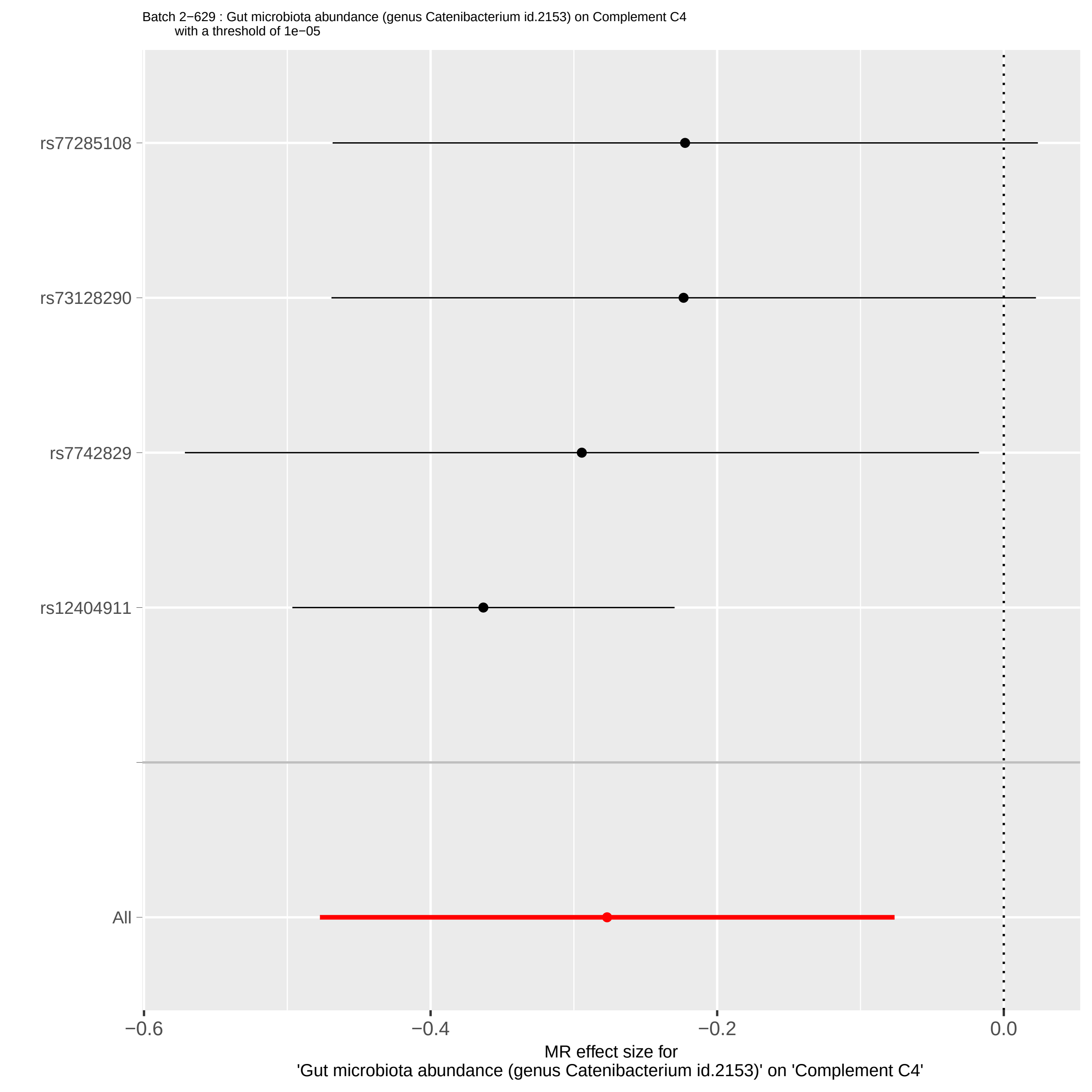
Batch 2−629 : Gut microbiota abundance (genus Catenibacterium id.2153) on Complement C4
with a threshold of 1e−05
rs77285108
rs73128290
rs7742829
rs12404911
All
−0.6
−0.4
−0.2
0.0
MR effect size for
'Gut microbiota abundance (genus Catenibacterium id.2153)' on 'Complement C4'

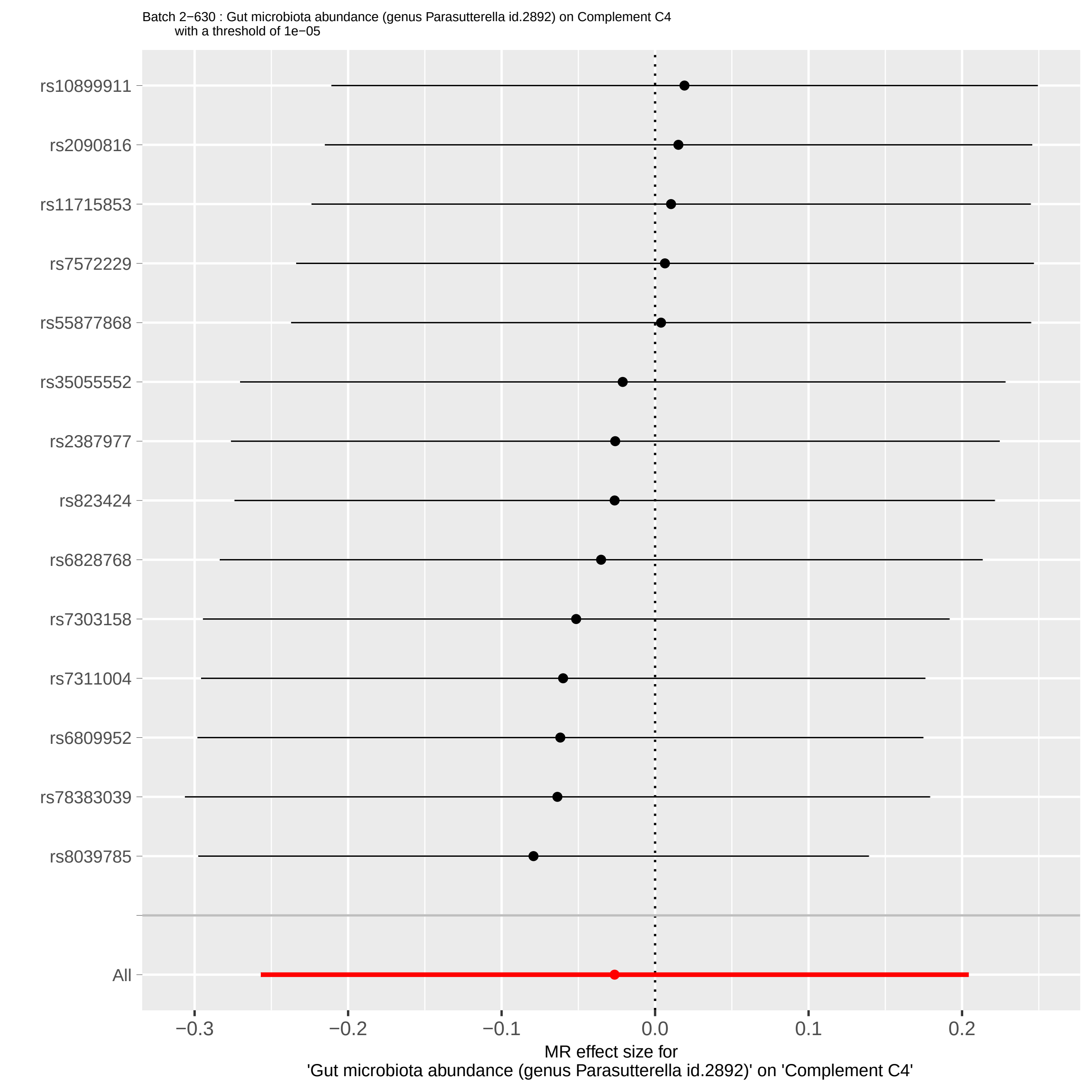

Batch 2−630 : Gut microbiota abundance (genus Parasutterella id.2892) on Complement C4
with a threshold of 1e−05
rs10899911
rs2090816
rs11715853
rs7572229
rs55877868
rs35055552
rs2387977
rs823424
rs6828768
rs7303158
rs7311004
rs6809952
rs78383039
rs8039785
All
−0.3
−0.2
−0.1
0.0
0.1
0.2
MR effect size for
'Gut microbiota abundance (genus Parasutterella id.2892)' on 'Complement C4'

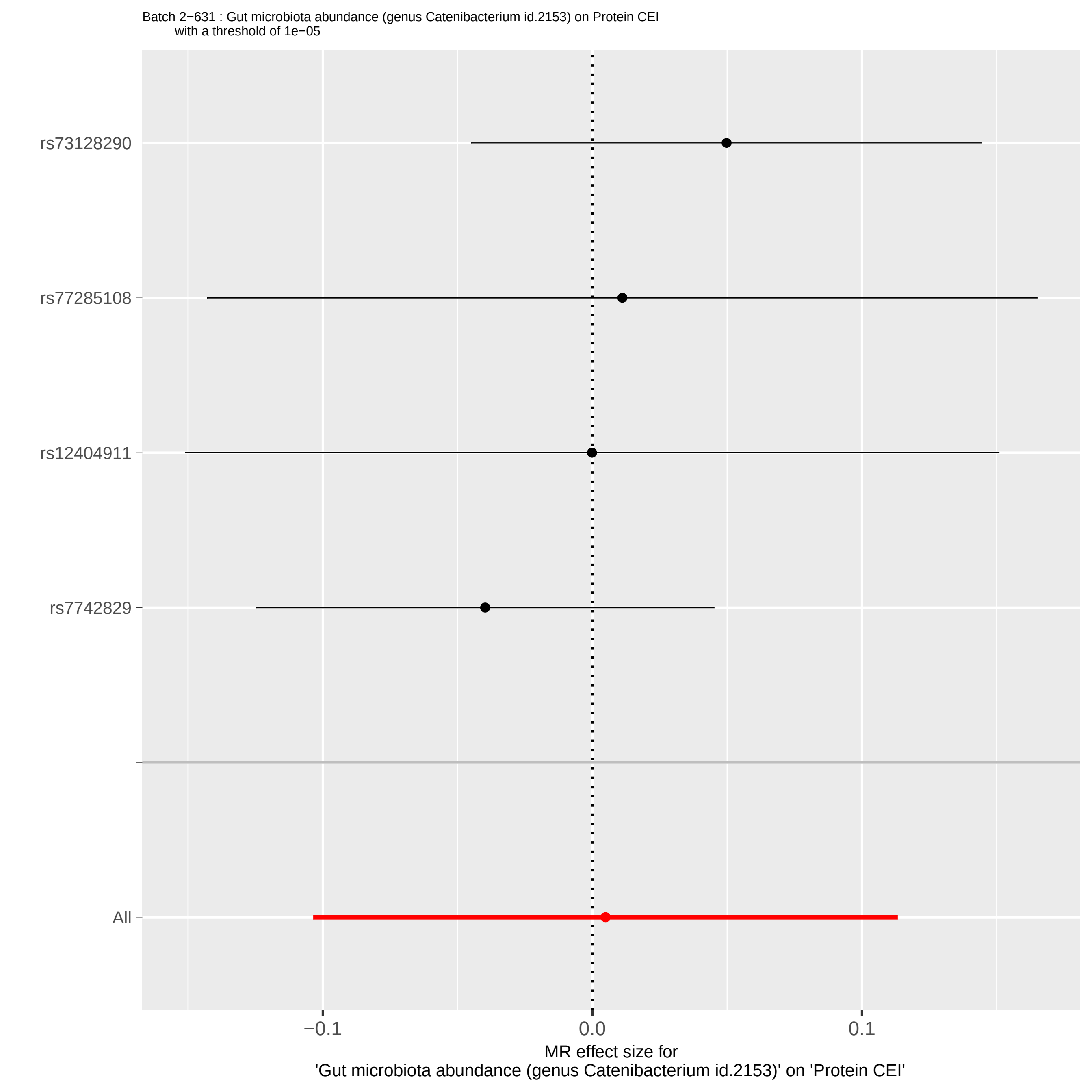

Batch 2−631 : Gut microbiota abundance (genus Catenibacterium id.2153) on Protein CEI
with a threshold of 1e−05
rs73128290
rs77285108
rs12404911
rs7742829
All
−0.1
0.0
0.1
MR effect size for
'Gut microbiota abundance (genus Catenibacterium id.2153)' on 'Protein CEI'

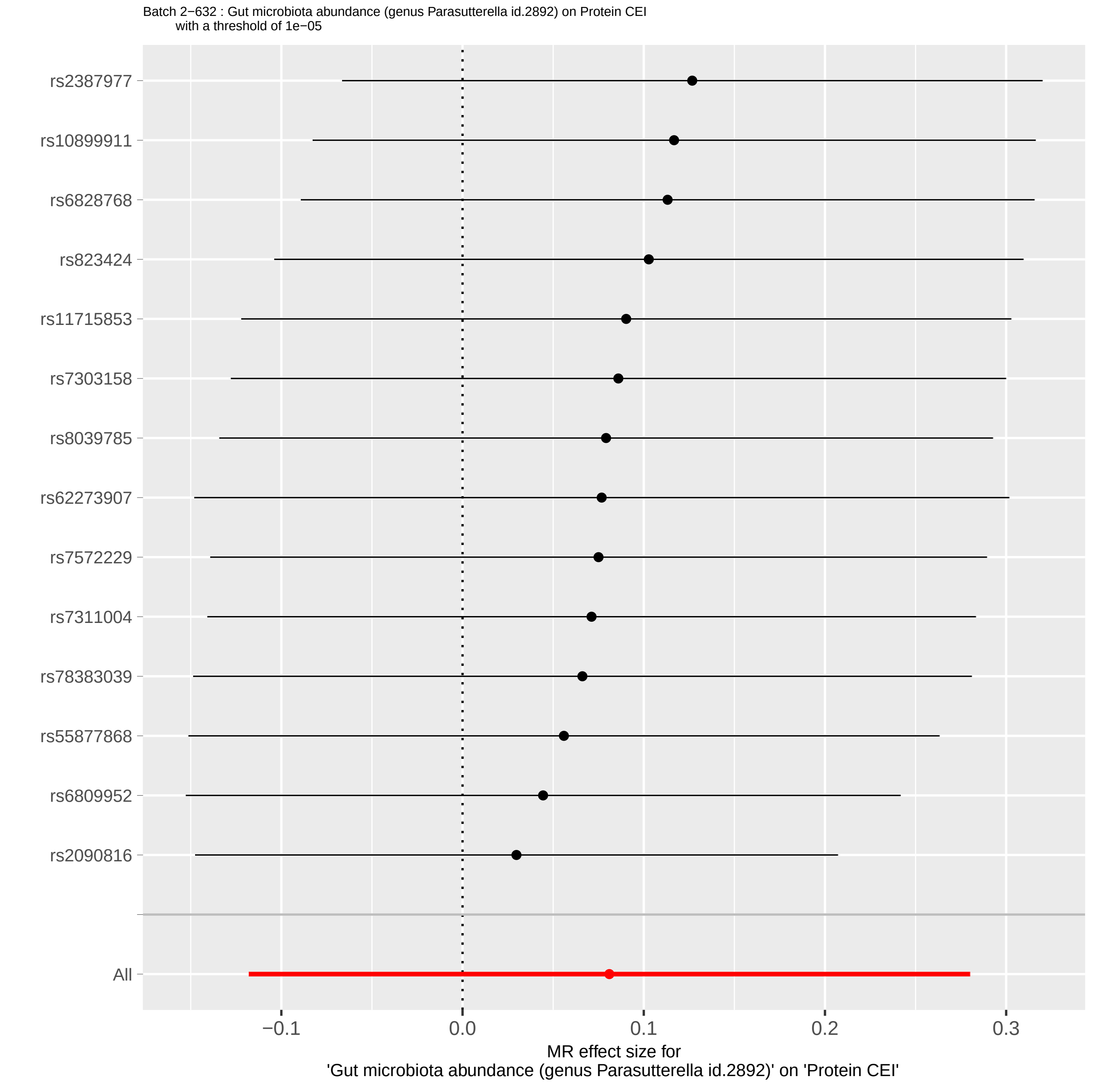

Batch 2−632 : Gut microbiota abundance (genus Parasutterella id.2892) on Protein CEI
with a threshold of 1e−05
rs2387977
rs10899911
rs6828768
rs823424
rs11715853
rs7303158
rs8039785
rs62273907
rs7572229
rs7311004
rs78383039
rs55877868
rs6809952
rs2090816
All
−0.1
0.0
0.1
0.2
0.3
MR effect size for
'Gut microbiota abundance (genus Parasutterella id.2892)' on 'Protein CEI'

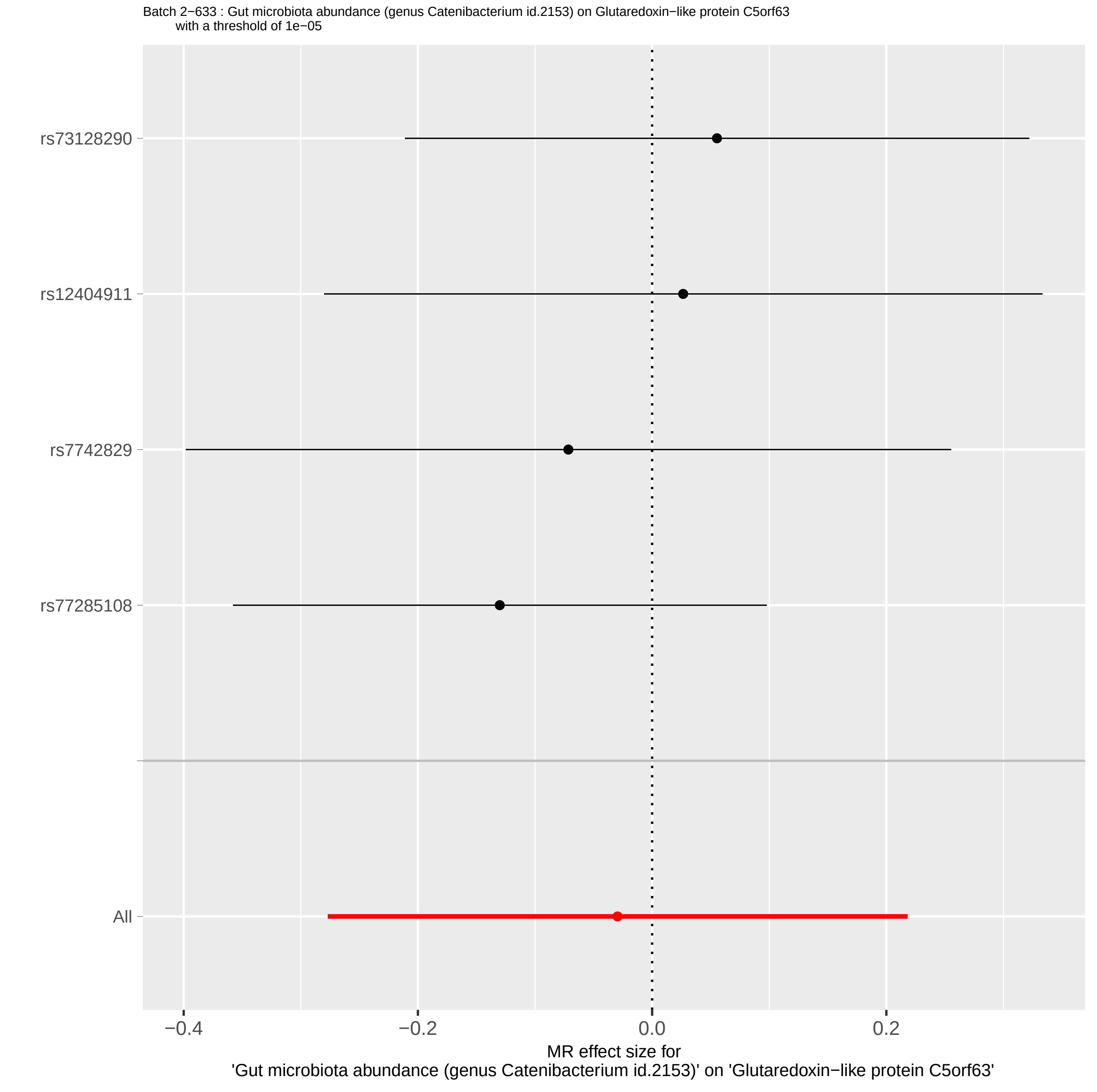

Batch 2−633 : Gut microbiota abundance (genus Catenibacterium id.2153) on Glutaredoxin−like protein C5orf63
with a threshold of 1e−05
rs73128290
rs12404911
rs7742829
rs77285108
All
−0.4
−0.2
0.0
0.2
MR effect size for
'Gut microbiota abundance (genus Catenibacterium id.2153)' on 'Glutaredoxin−like protein C5orf63'

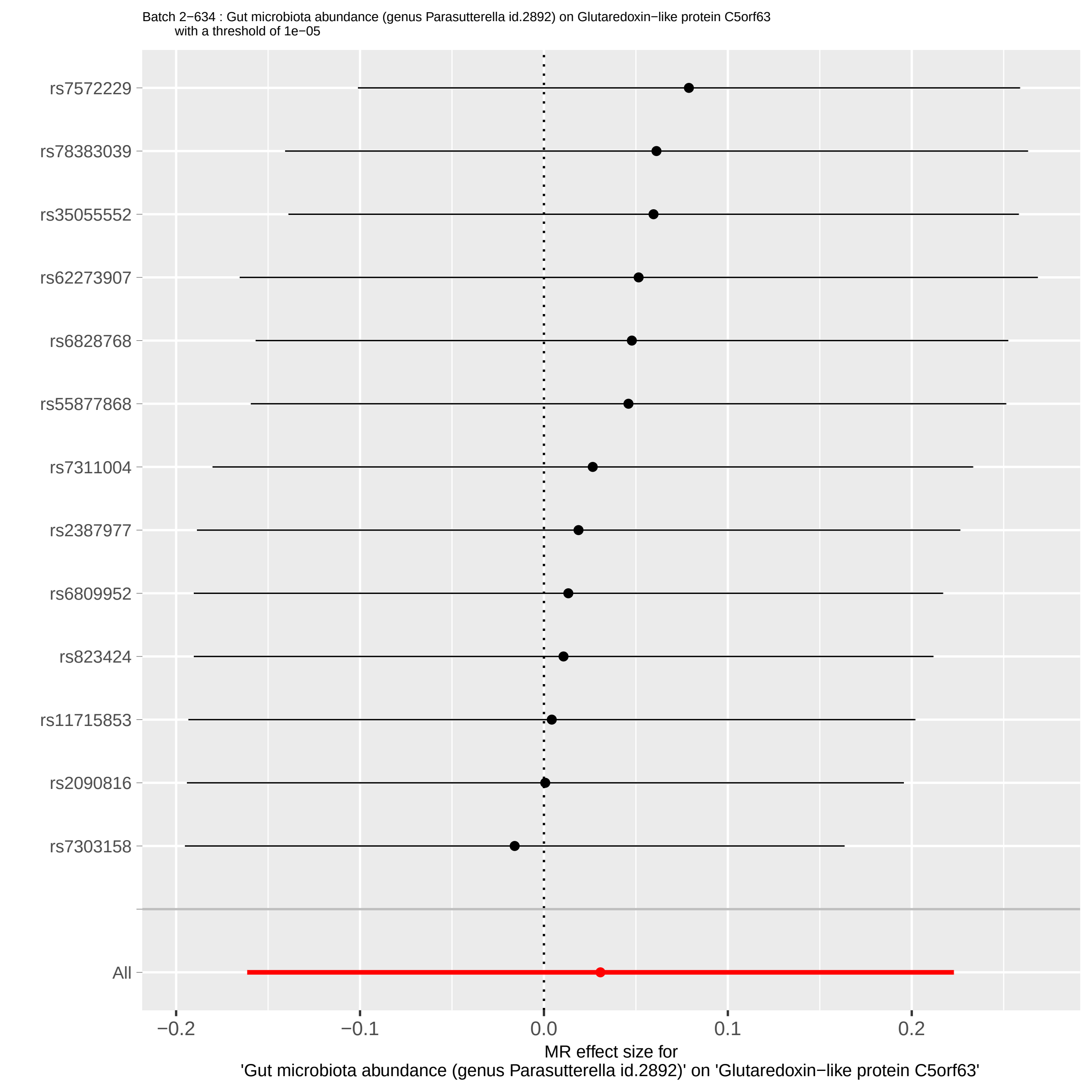

Batch 2−634 : Gut microbiota abundance (genus Parasutterella id.2892) on Glutaredoxin−like protein C5orf63
with a threshold of 1e−05
rs7572229
rs78383039
rs35055552
rs62273907
rs6828768
rs55877868
rs7311004
rs2387977
rs6809952
rs823424
rs11715853
rs2090816
rs7303158
All
−0.2
−0.1
0.0
0.1
0.2
MR effect size for
'Gut microbiota abundance (genus Parasutterella id.2892)' on 'Glutaredoxin−like protein C5orf63'

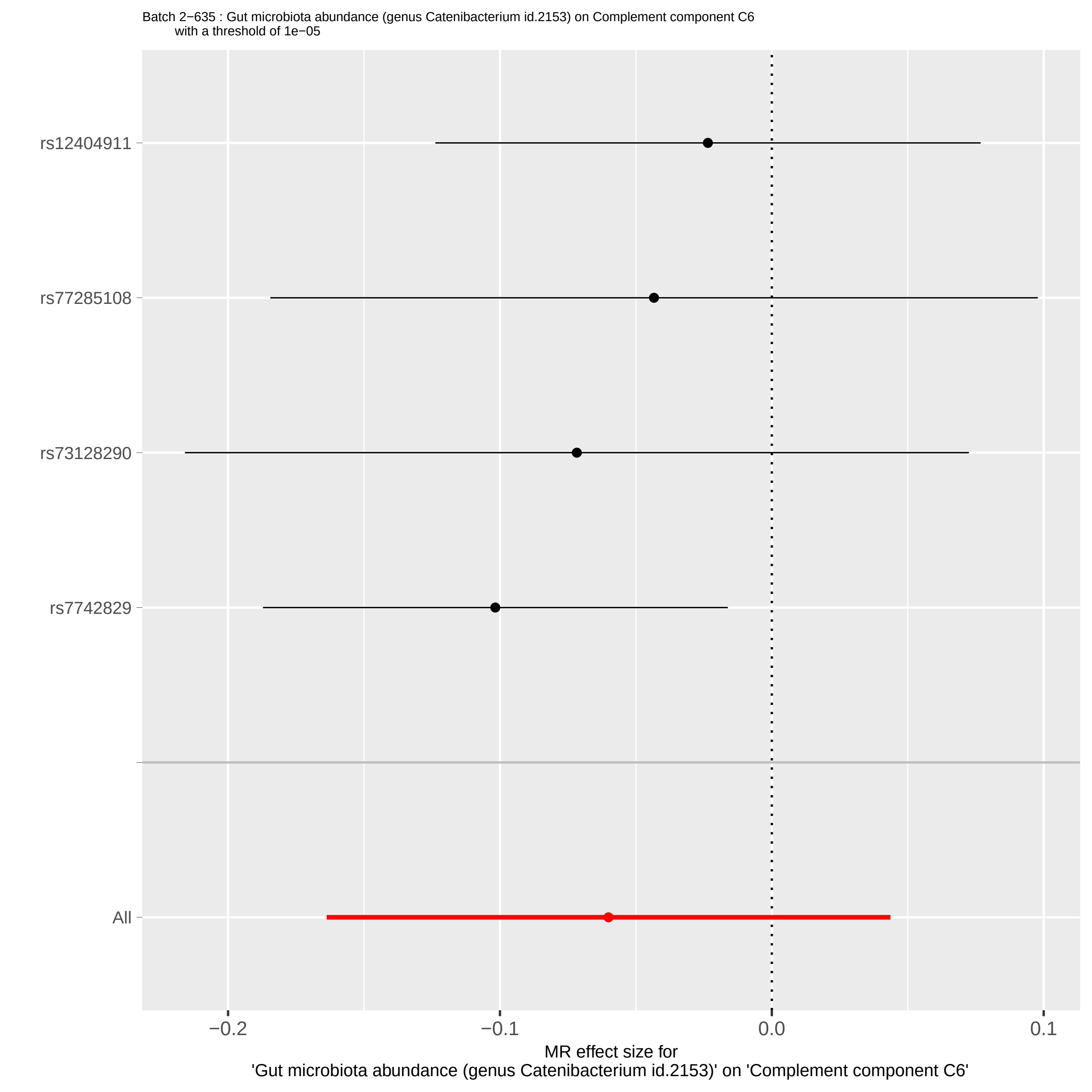

Batch 2−635 : Gut microbiota abundance (genus Catenibacterium id.2153) on Complement component C6
with a threshold of 1e−05
rs12404911
rs77285108
rs73128290
rs7742829
All
−0.2
−0.1
0.0
0.1
MR effect size for
'Gut microbiota abundance (genus Catenibacterium id.2153)' on 'Complement component C6'

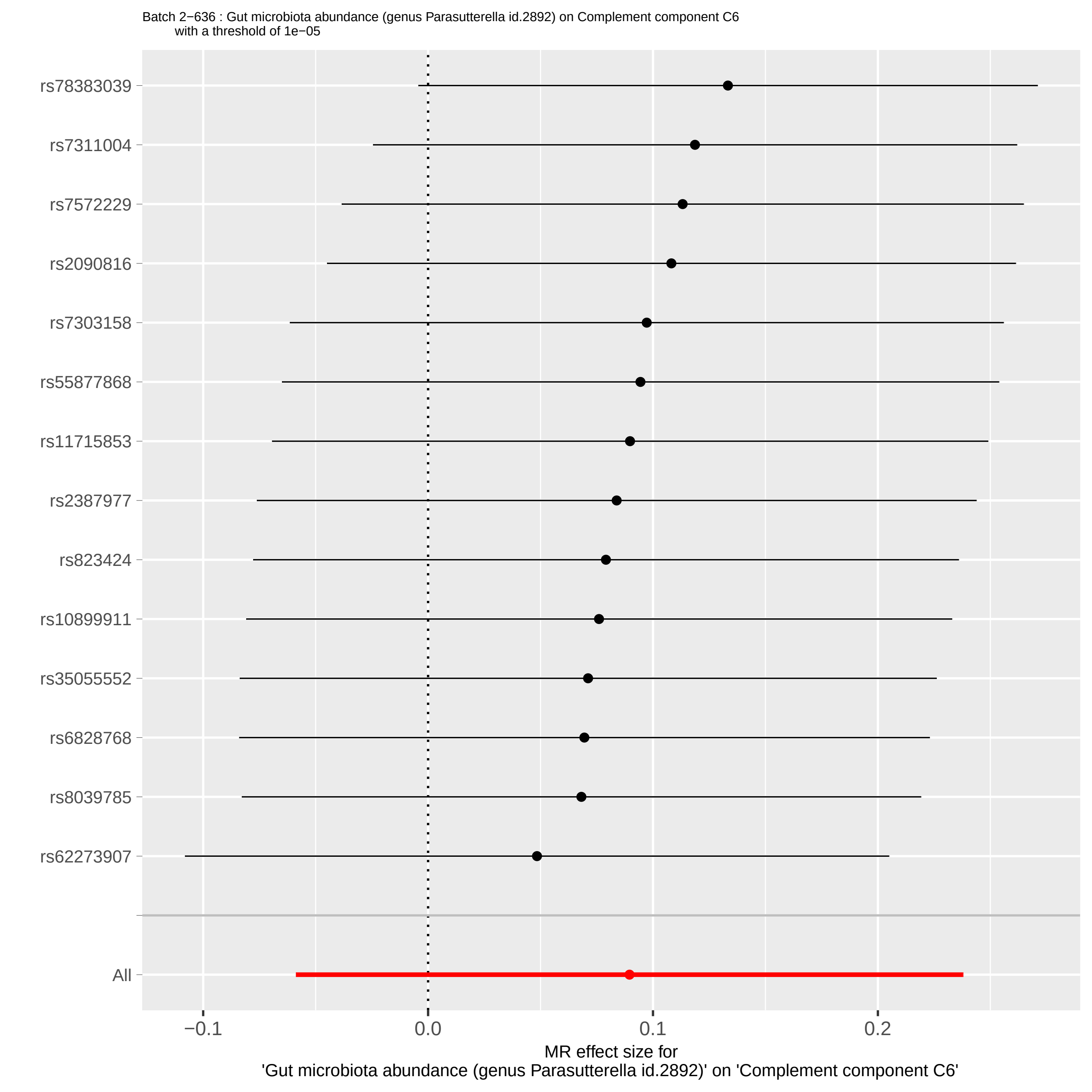
Batch 2−636 : Gut microbiota abundance (genus Parasutterella id.2892) on Complement component C6
with a threshold of 1e−05
rs78383039
rs7311004
rs7572229
rs2090816
rs7303158
rs55877868
rs11715853
rs2387977
rs823424
rs10899911
rs35055552
rs6828768
rs8039785
rs62273907
All
−0.1
0.0
0.1
0.2
MR effect size for
'Gut microbiota abundance (genus Parasutterella id.2892)' on 'Complement component C6'

Batch 2−637 : Gut microbiota abundance (genus Catenibacterium id.2153) on Uncharacterized protein C6orf226
with a threshold of 1e−05

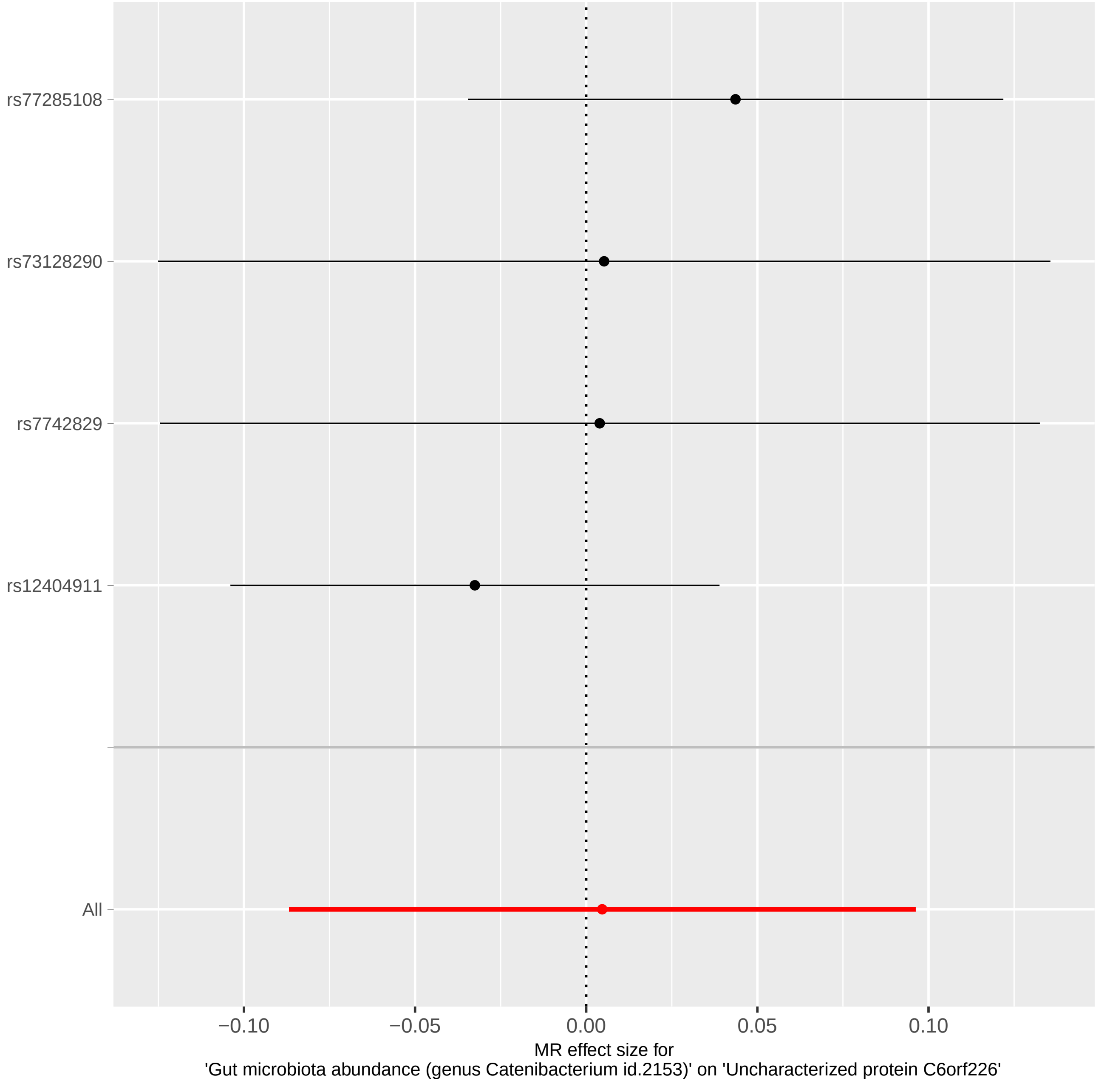

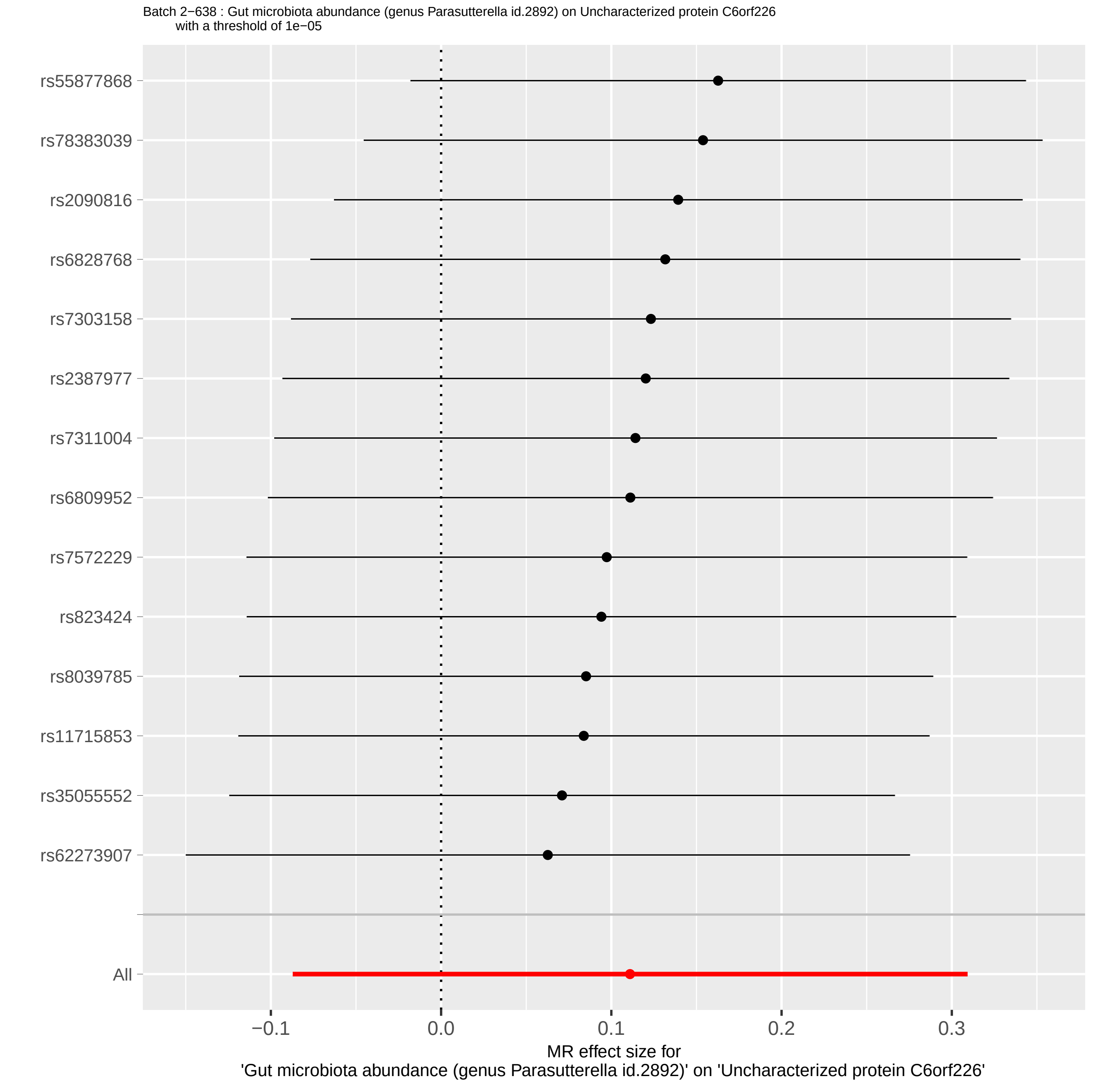

Batch 2−638 : Gut microbiota abundance (genus Parasutterella id.2892) on Uncharacterized protein C6orf226
with a threshold of 1e−05
rs55877868
rs78383039
rs2090816
rs6828768
rs7303158
rs2387977
rs7311004
rs6809952
rs7572229
rs823424
rs8039785
rs11715853
rs35055552
rs62273907
All
−0.1
0.0
0.1
0.2
0.3
MR effect size for
'Gut microbiota abundance (genus Parasutterella id.2892)' on 'Uncharacterized protein C6orf226'

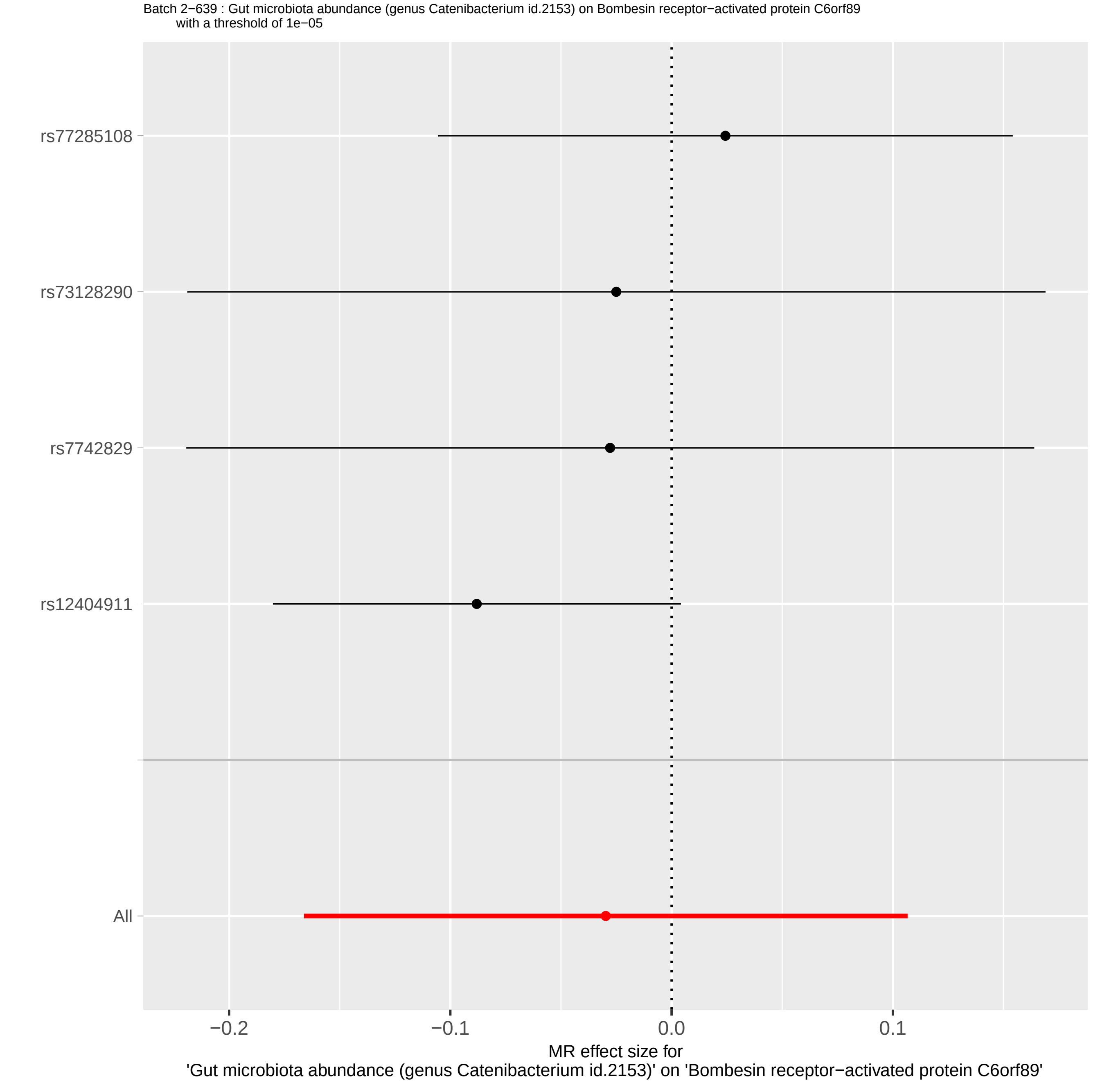

Batch 2−639 : Gut microbiota abundance (genus Catenibacterium id.2153) on Bombesin receptor−activated protein C6orf89
with a threshold of 1e−05
rs77285108
rs73128290
rs7742829
rs12404911
All
−0.2
−0.1
0.0
0.1
MR effect size for
'Gut microbiota abundance (genus Catenibacterium id.2153)' on 'Bombesin receptor−activated protein C6orf89'

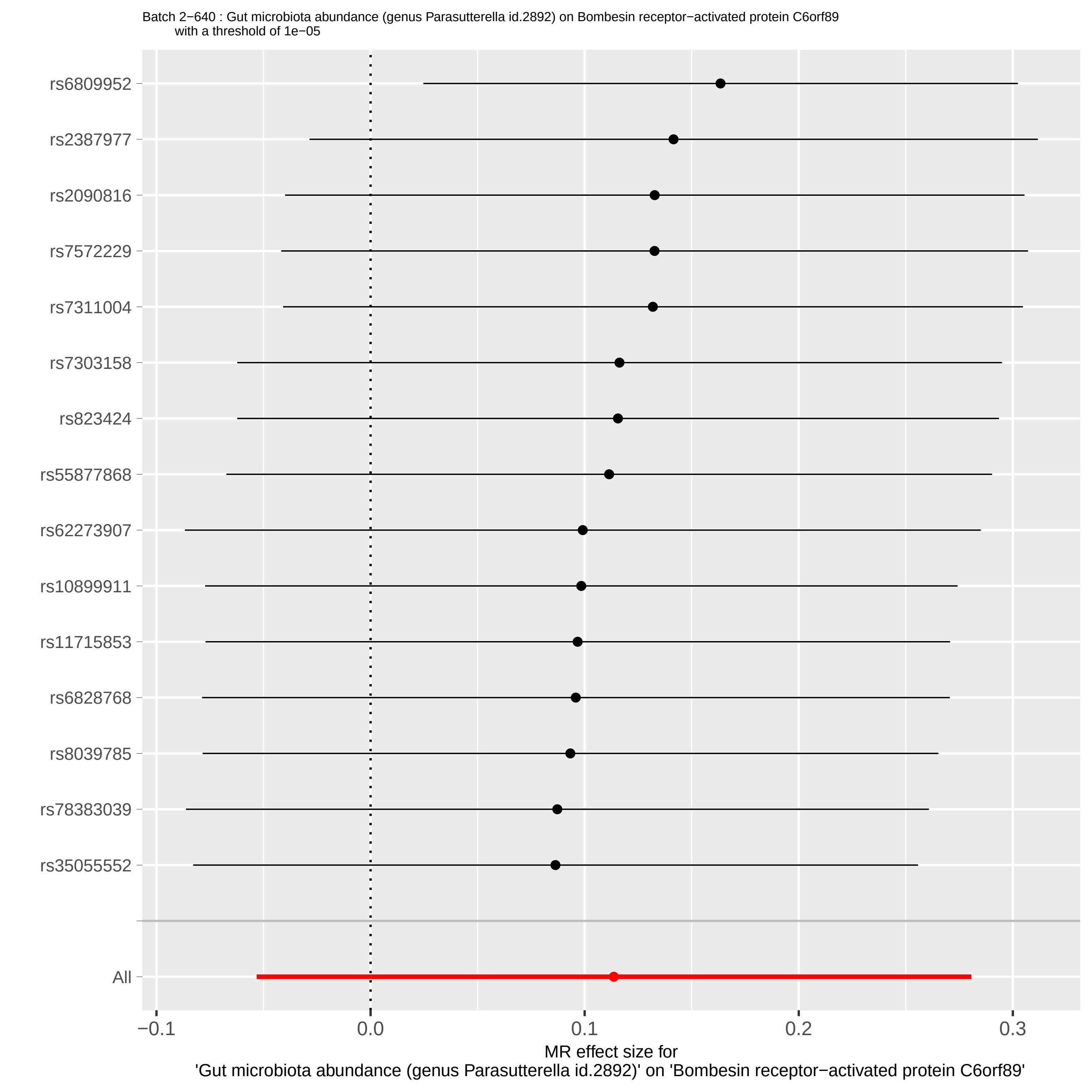

Batch 2−640 : Gut microbiota abundance (genus Parasutterella id.2892) on Bombesin receptor−activated protein C6orf89
with a threshold of 1e−05
rs6809952
rs2387977
rs2090816
rs7572229
rs7311004
rs7303158
rs823424
rs55877868
rs62273907
rs10899911
rs11715853
rs6828768
rs8039785
rs78383039
rs35055552
All
−0.1
0.0
0.1
0.2
0.3
MR effect size for
'Gut microbiota abundance (genus Parasutterella id.2892)' on 'Bombesin receptor−activated protein C6orf89'

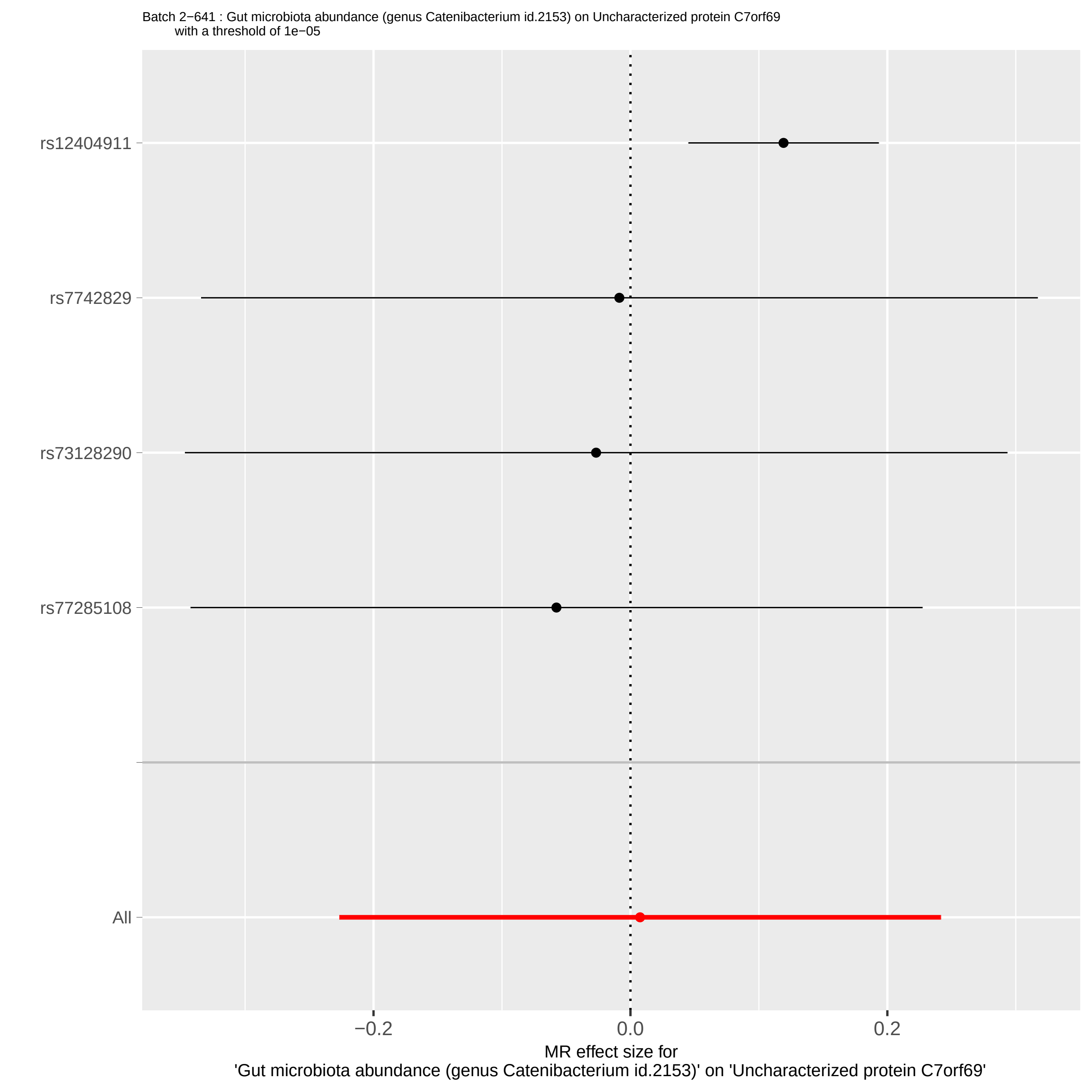
Batch 2−641 : Gut microbiota abundance (genus Catenibacterium id.2153) on Uncharacterized protein C7orf69
with a threshold of 1e−05
rs12404911
rs7742829
rs73128290
rs77285108
All
−0.2
0.0
0.2
MR effect size for
'Gut microbiota abundance (genus Catenibacterium id.2153)' on 'Uncharacterized protein C7orf69'

Batch 2−642 : Gut microbiota abundance (genus Parasutterella id.2892) on Uncharacterized protein C7orf69
with a threshold of 1e−05

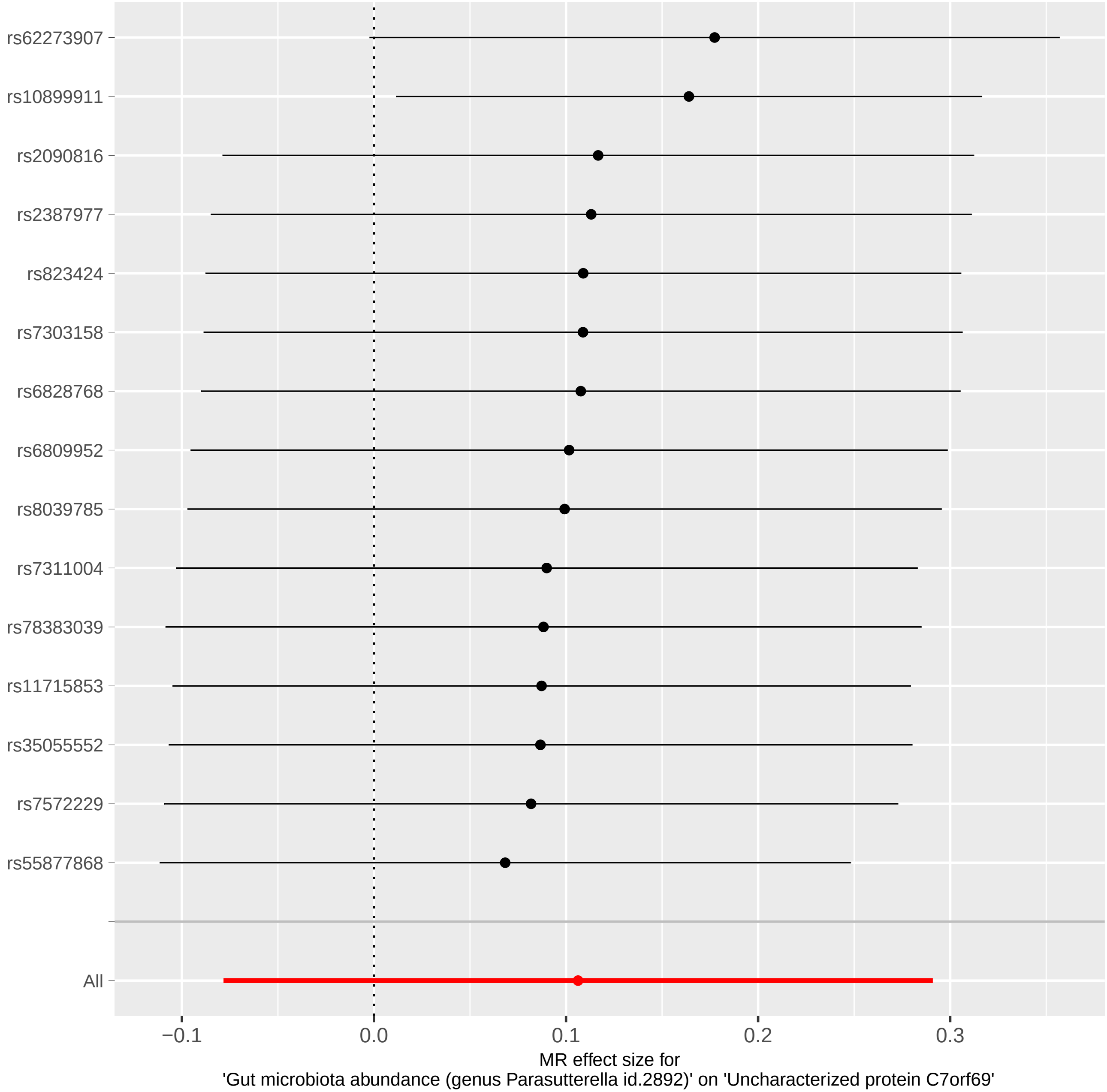

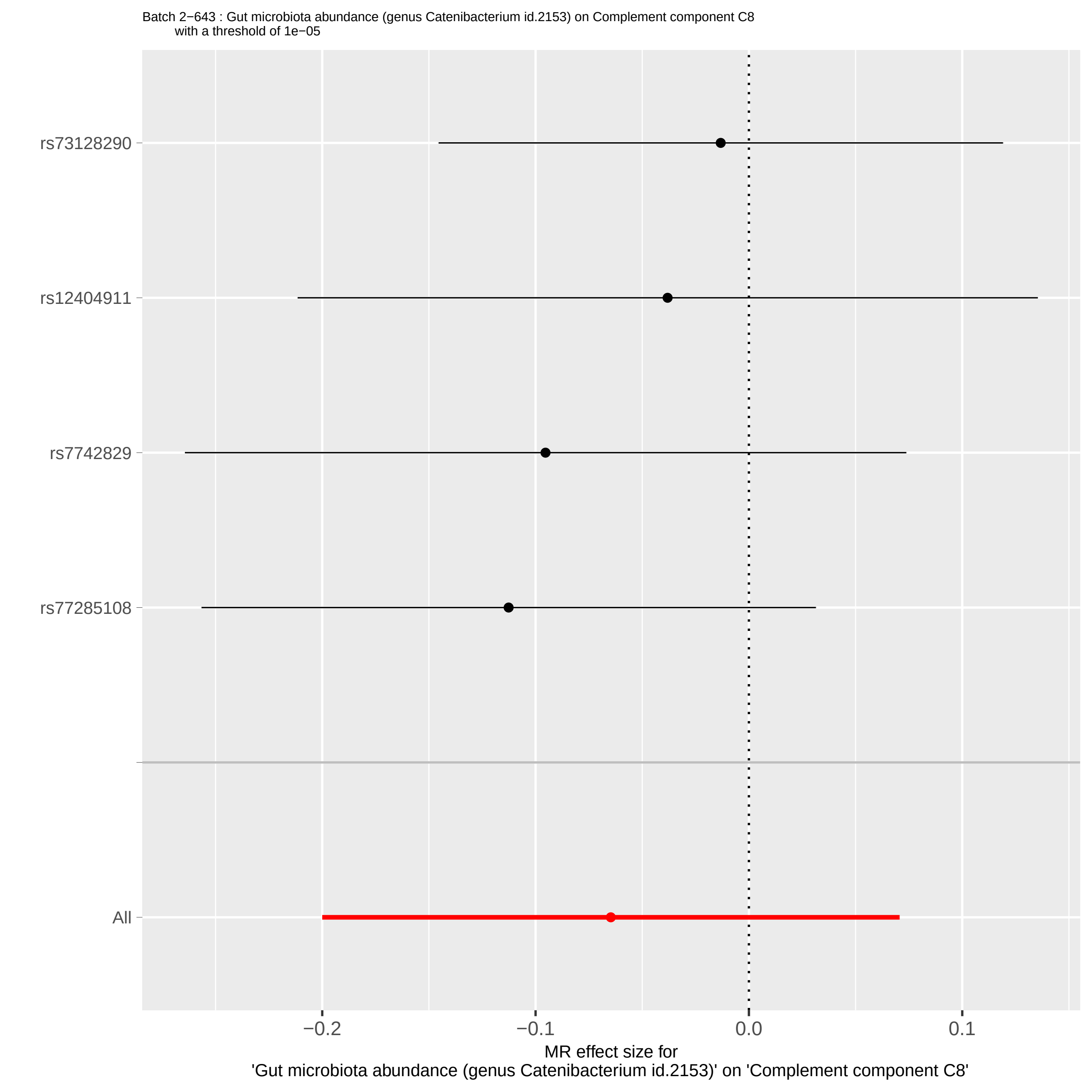

Batch 2−643 : Gut microbiota abundance (genus Catenibacterium id.2153) on Complement component C8
with a threshold of 1e−05
rs73128290
rs12404911
rs7742829
rs77285108
All
−0.2
−0.1
0.0
0.1
MR effect size for
'Gut microbiota abundance (genus Catenibacterium id.2153)' on 'Complement component C8'

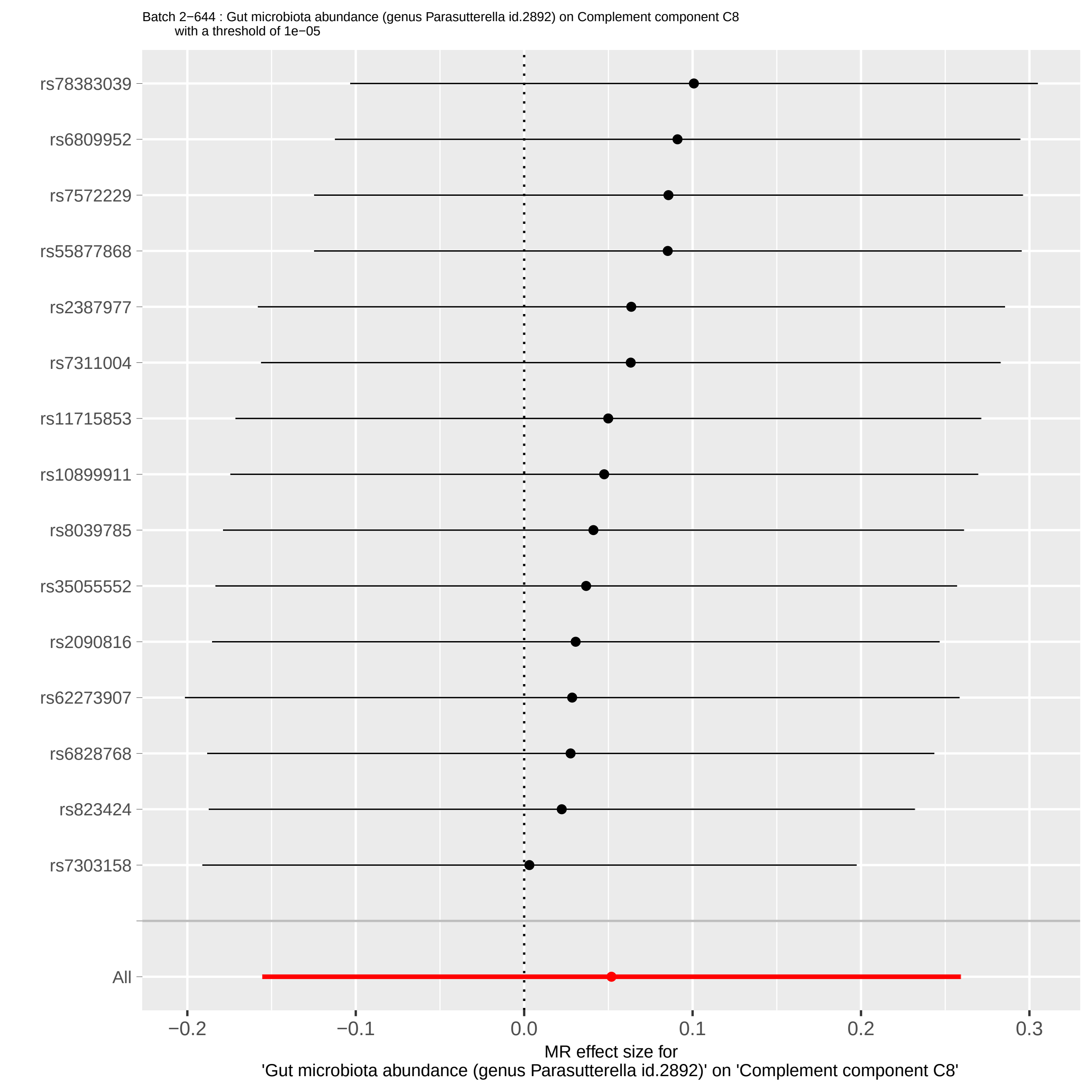

Batch 2−644 : Gut microbiota abundance (genus Parasutterella id.2892) on Complement component C8
with a threshold of 1e−05
rs78383039
rs6809952
rs7572229
rs55877868
rs2387977
rs7311004
rs11715853
rs10899911
rs8039785
rs35055552
rs2090816
rs62273907
rs6828768
rs823424
rs7303158
All
−0.2
−0.1
0.0
0.1
0.2
0.3
MR effect size for
'Gut microbiota abundance (genus Parasutterella id.2892)' on 'Complement component C8'

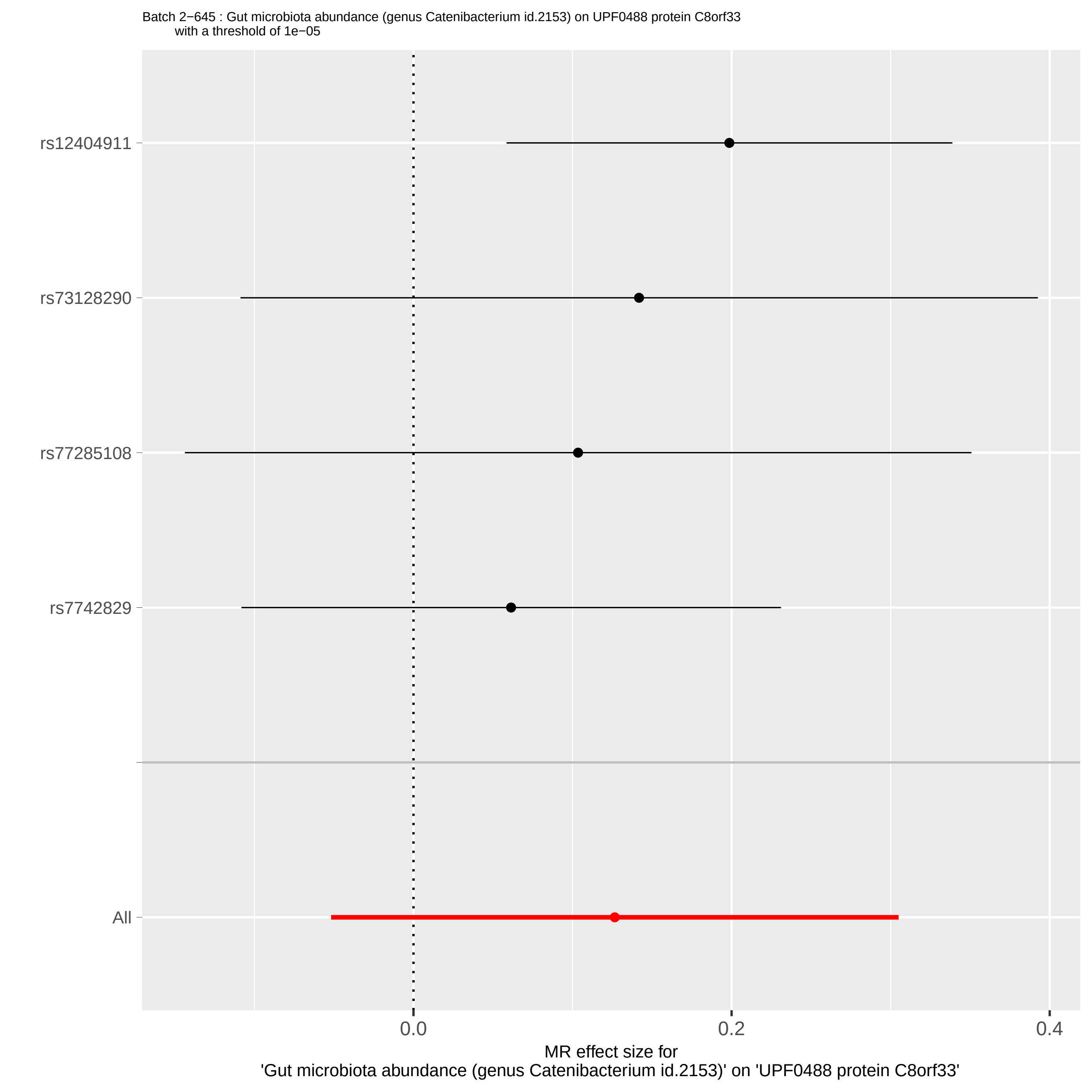
Batch 2−645 : Gut microbiota abundance (genus Catenibacterium id.2153) on UPF0488 protein C8orf33
with a threshold of 1e−05
rs12404911
rs73128290
rs77285108
rs7742829
All
0.0
0.2
0.4
MR effect size for
'Gut microbiota abundance (genus Catenibacterium id.2153)' on 'UPF0488 protein C8orf33'

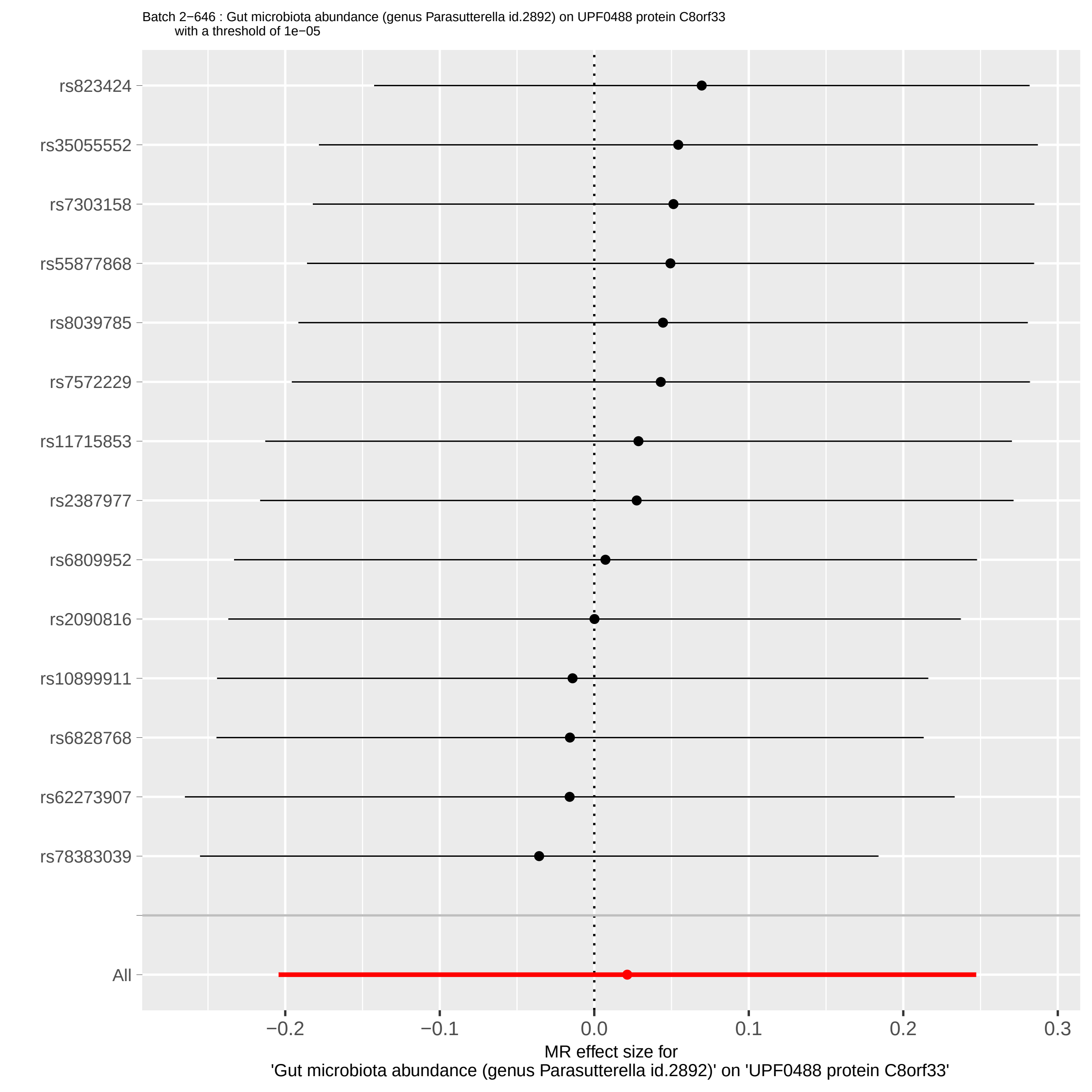

Batch 2−646 : Gut microbiota abundance (genus Parasutterella id.2892) on UPF0488 protein C8orf33
with a threshold of 1e−05
rs823424
rs35055552
rs7303158
rs55877868
rs8039785
rs7572229
rs11715853
rs2387977
rs6809952
rs2090816
rs10899911
rs6828768
rs62273907
rs78383039
All
−0.2
−0.1
0.0
0.1
0.2
0.3
MR effect size for
'Gut microbiota abundance (genus Parasutterella id.2892)' on 'UPF0488 protein C8orf33'

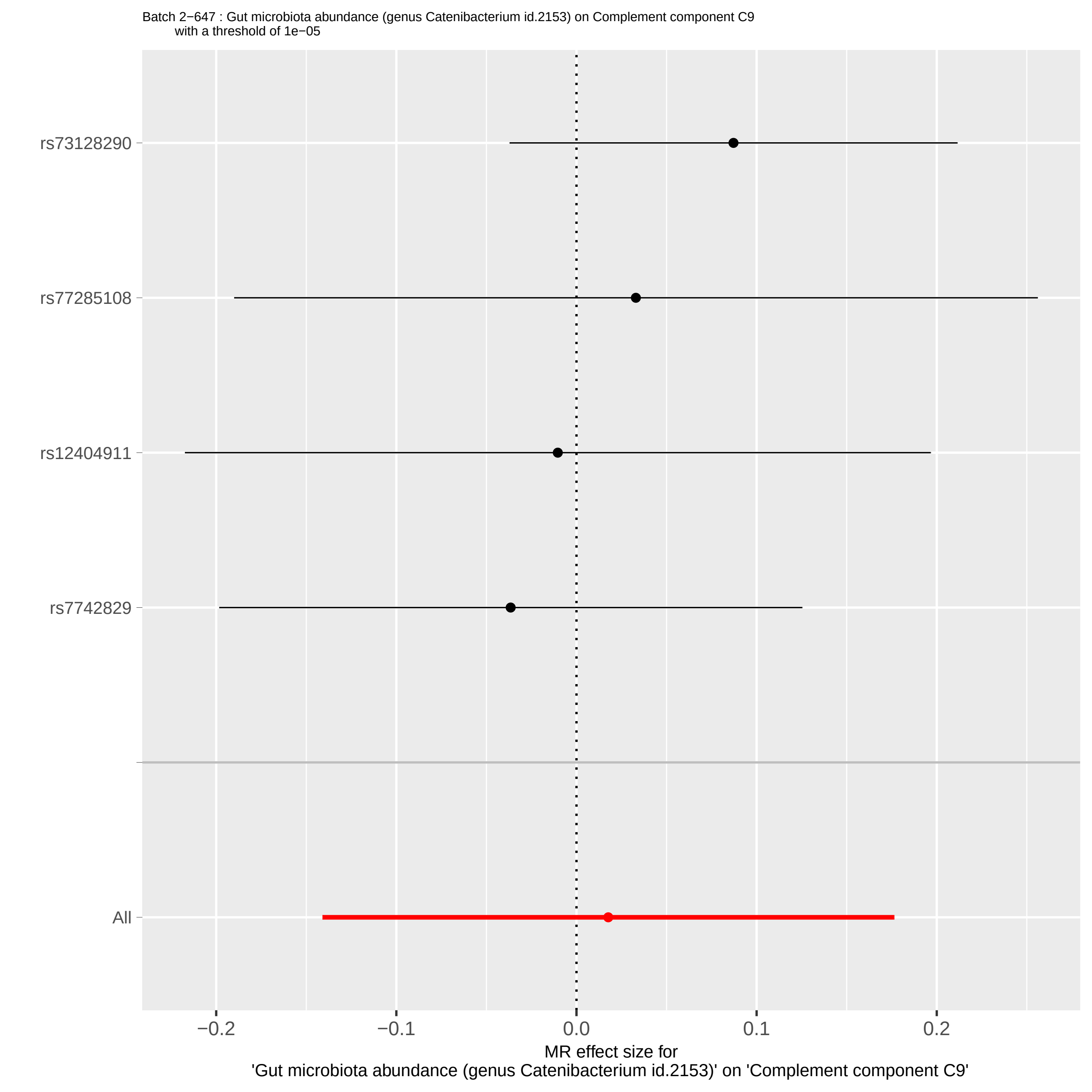
Batch 2−647 : Gut microbiota abundance (genus Catenibacterium id.2153) on Complement component C9
with a threshold of 1e−05
rs73128290
rs77285108
rs12404911
rs7742829
All
−0.2
−0.1
0.0
0.1
0.2
MR effect size for
'Gut microbiota abundance (genus Catenibacterium id.2153)' on 'Complement component C9'

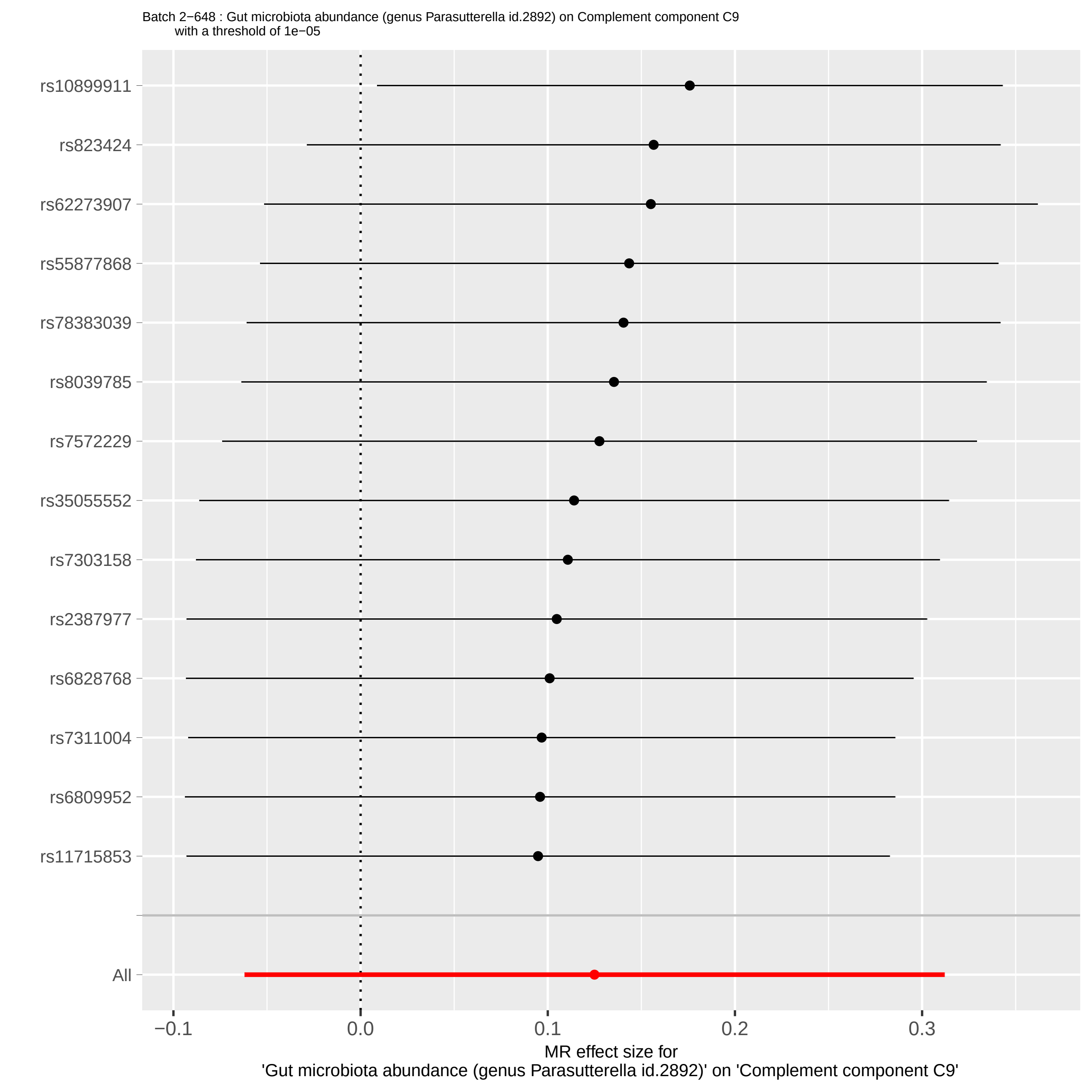

Batch 2−648 : Gut microbiota abundance (genus Parasutterella id.2892) on Complement component C9
with a threshold of 1e−05
rs10899911
rs823424
rs62273907
rs55877868
rs78383039
rs8039785
rs7572229
rs35055552
rs7303158
rs2387977
rs6828768
rs7311004
rs6809952
rs11715853
All
−0.1
0.0
0.1
0.2
0.3
MR effect size for
'Gut microbiota abundance (genus Parasutterella id.2892)' on 'Complement component C9'

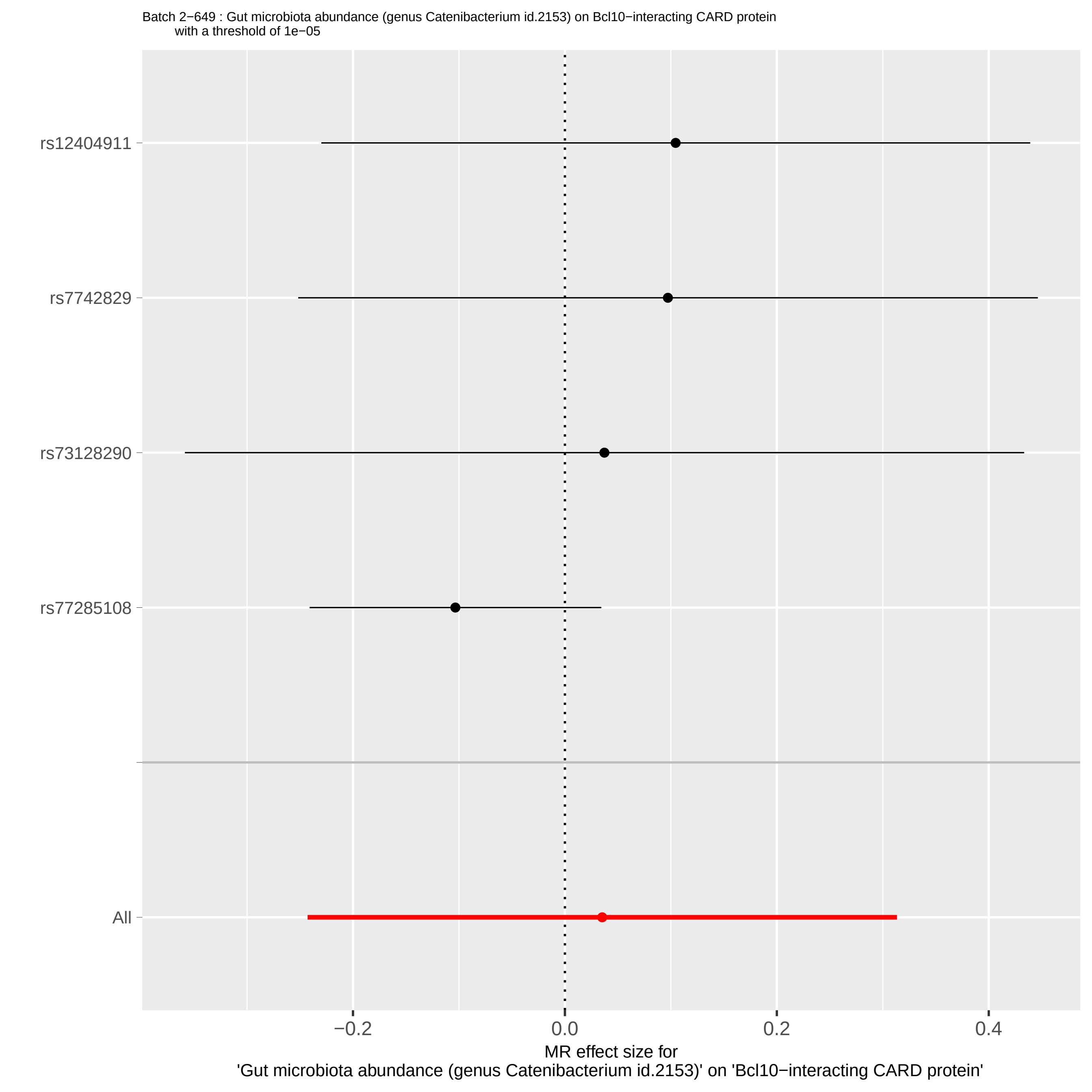
Batch 2−649 : Gut microbiota abundance (genus Catenibacterium id.2153) on Bcl10−interacting CARD protein
with a threshold of 1e−05
rs12404911
rs7742829
rs73128290
rs77285108
All
−0.2
0.0
0.2
0.4
MR effect size for
'Gut microbiota abundance (genus Catenibacterium id.2153)' on 'Bcl10−interacting CARD protein'

Batch 2−650 : Gut microbiota abundance (genus Parasutterella id.2892) on Bcl10−interacting CARD protein
with a threshold of 1e−05

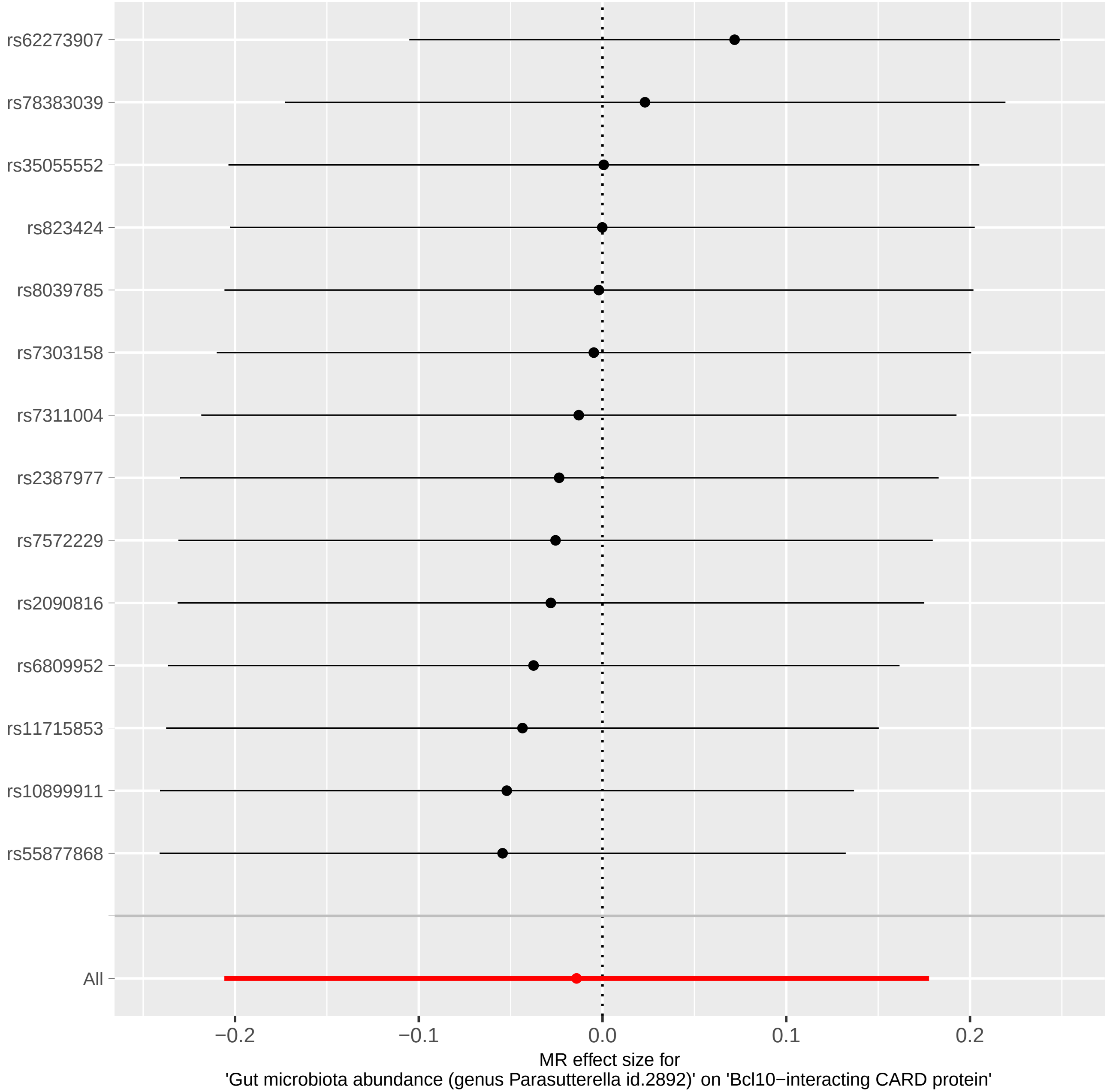

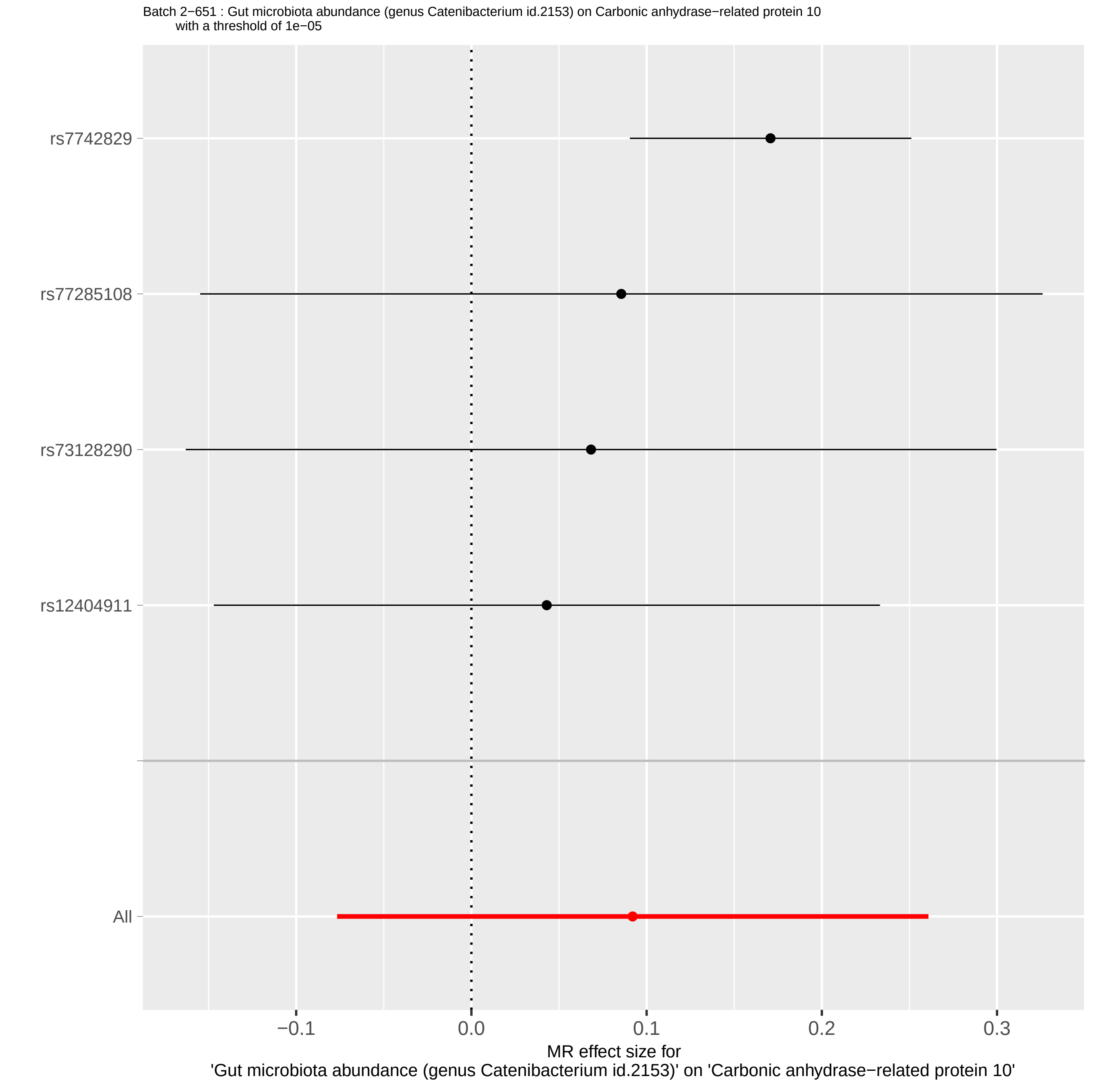
Batch 2−651 : Gut microbiota abundance (genus Catenibacterium id.2153) on Carbonic anhydrase−related protein 10
with a threshold of 1e−05
rs7742829
rs77285108
rs73128290
rs12404911
All
−0.1
0.0
0.1
0.2
0.3
MR effect size for
'Gut microbiota abundance (genus Catenibacterium id.2153)' on 'Carbonic anhydrase−related protein 10'

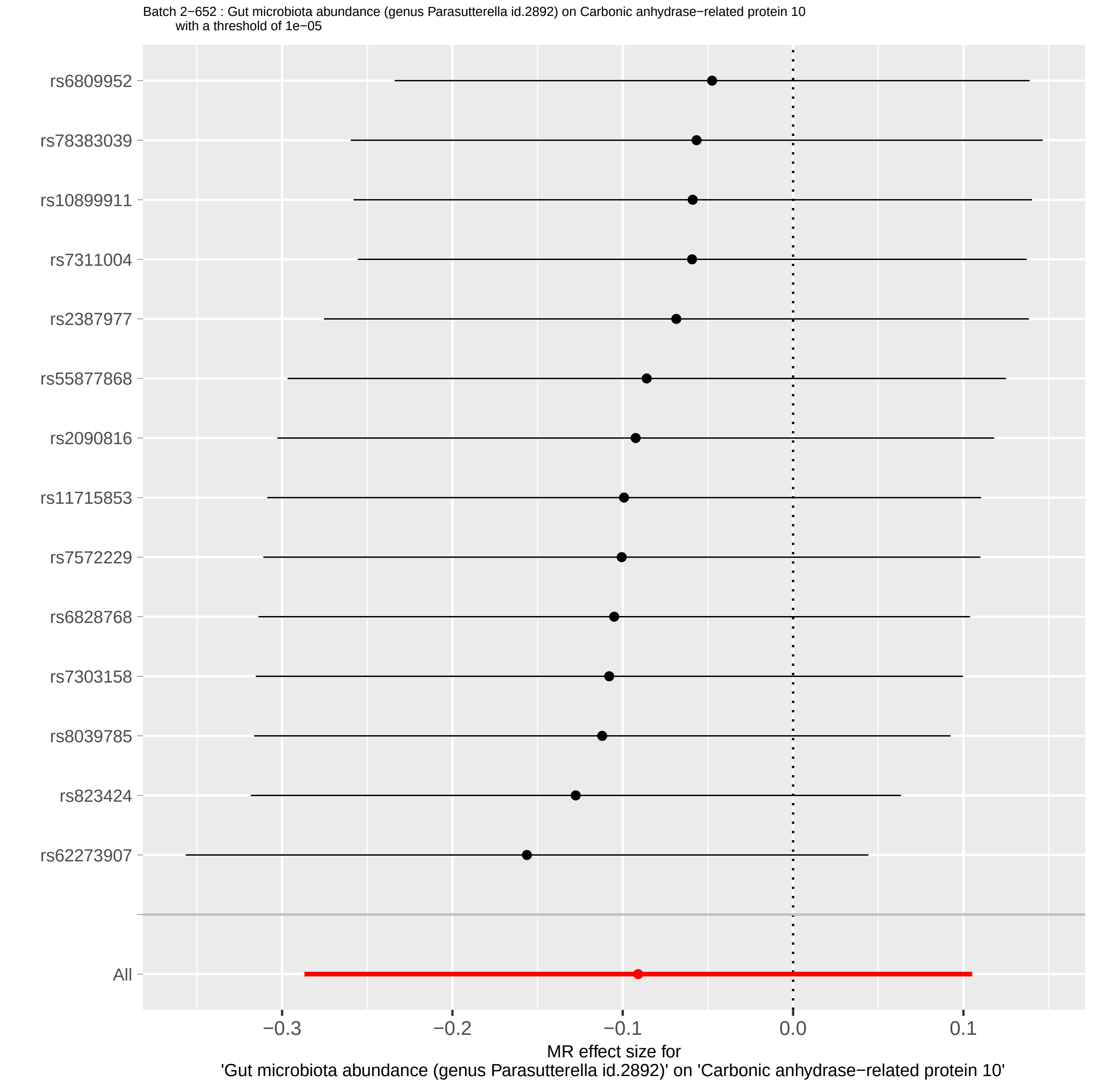

Batch 2−652 : Gut microbiota abundance (genus Parasutterella id.2892) on Carbonic anhydrase−related protein 10
with a threshold of 1e−05
rs6809952
rs78383039
rs10899911
rs7311004
rs2387977
rs55877868
rs2090816
rs11715853
rs7572229
rs6828768
rs7303158
rs8039785
rs823424
rs62273907
All
−0.3
−0.2
−0.1
0.0
0.1
MR effect size for
'Gut microbiota abundance (genus Parasutterella id.2892)' on 'Carbonic anhydrase−related protein 10'

Batch 2−653 : Gut microbiota abundance (genus Catenibacterium id.2153) on Carbonic anhydrase−related protein 10
with a threshold of 1e−05

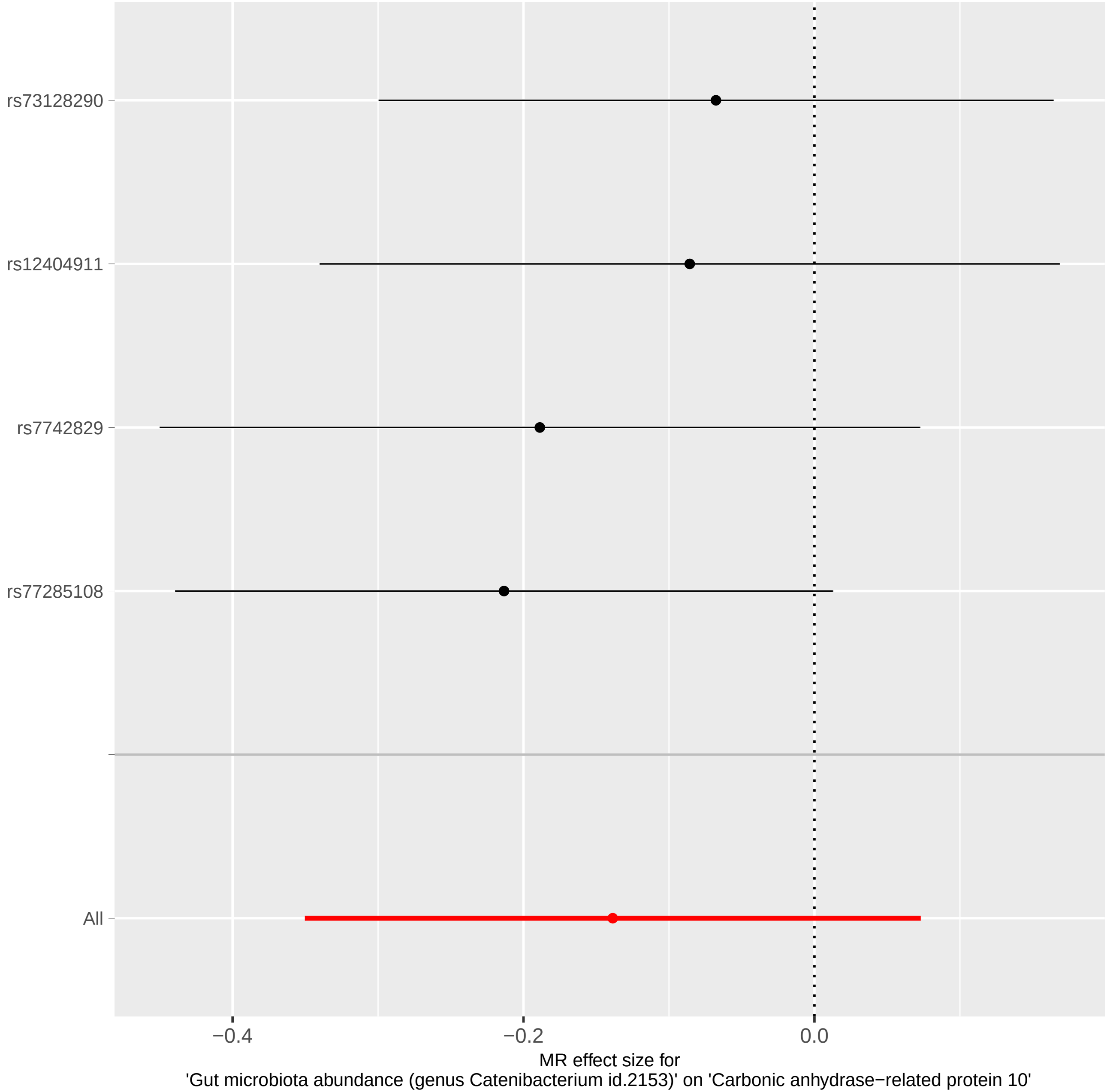

Batch 2−654 : Gut microbiota abundance (genus Parasutterella id.2892) on Carbonic anhydrase−related protein 10
with a threshold of 1e−05

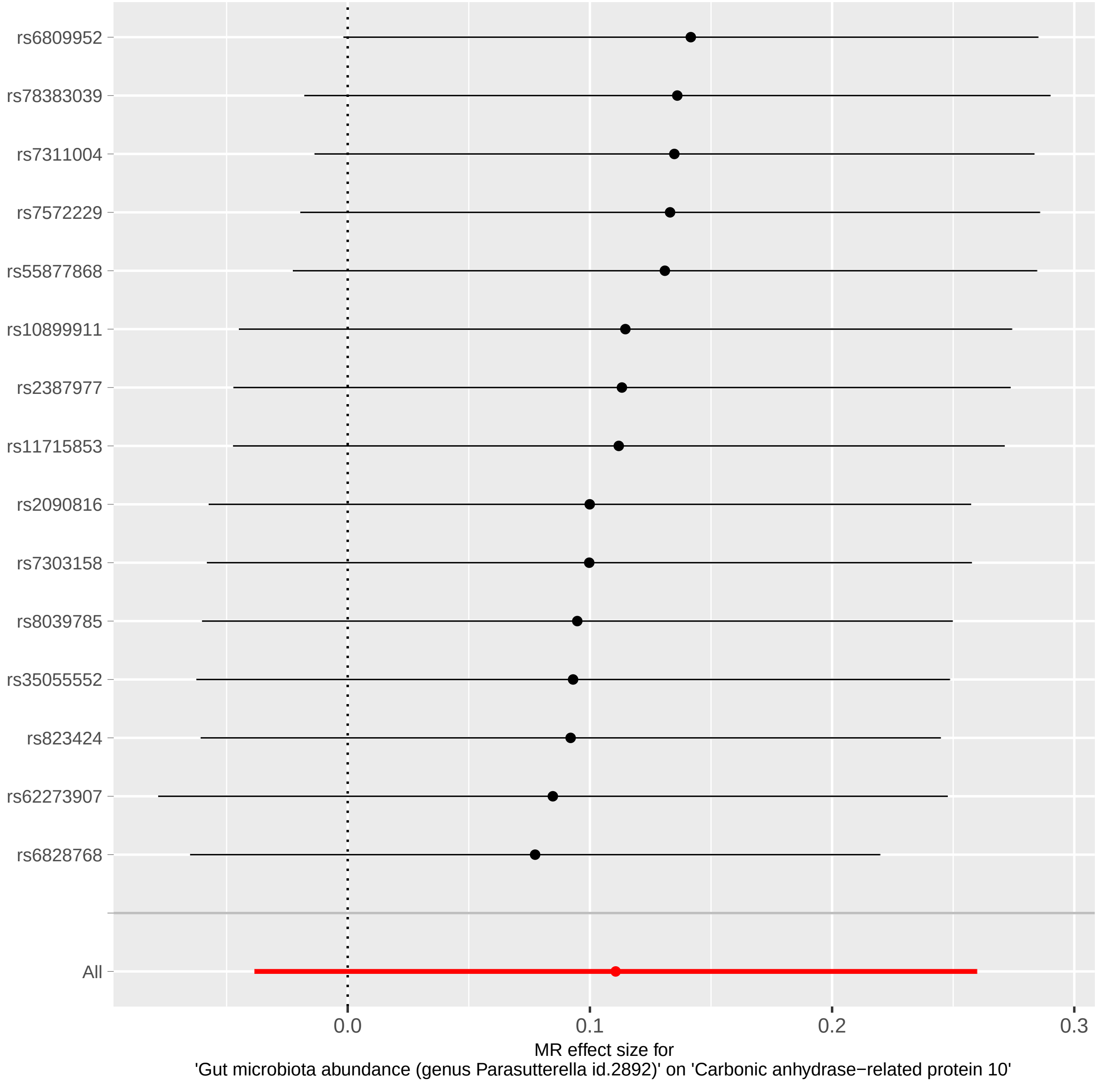

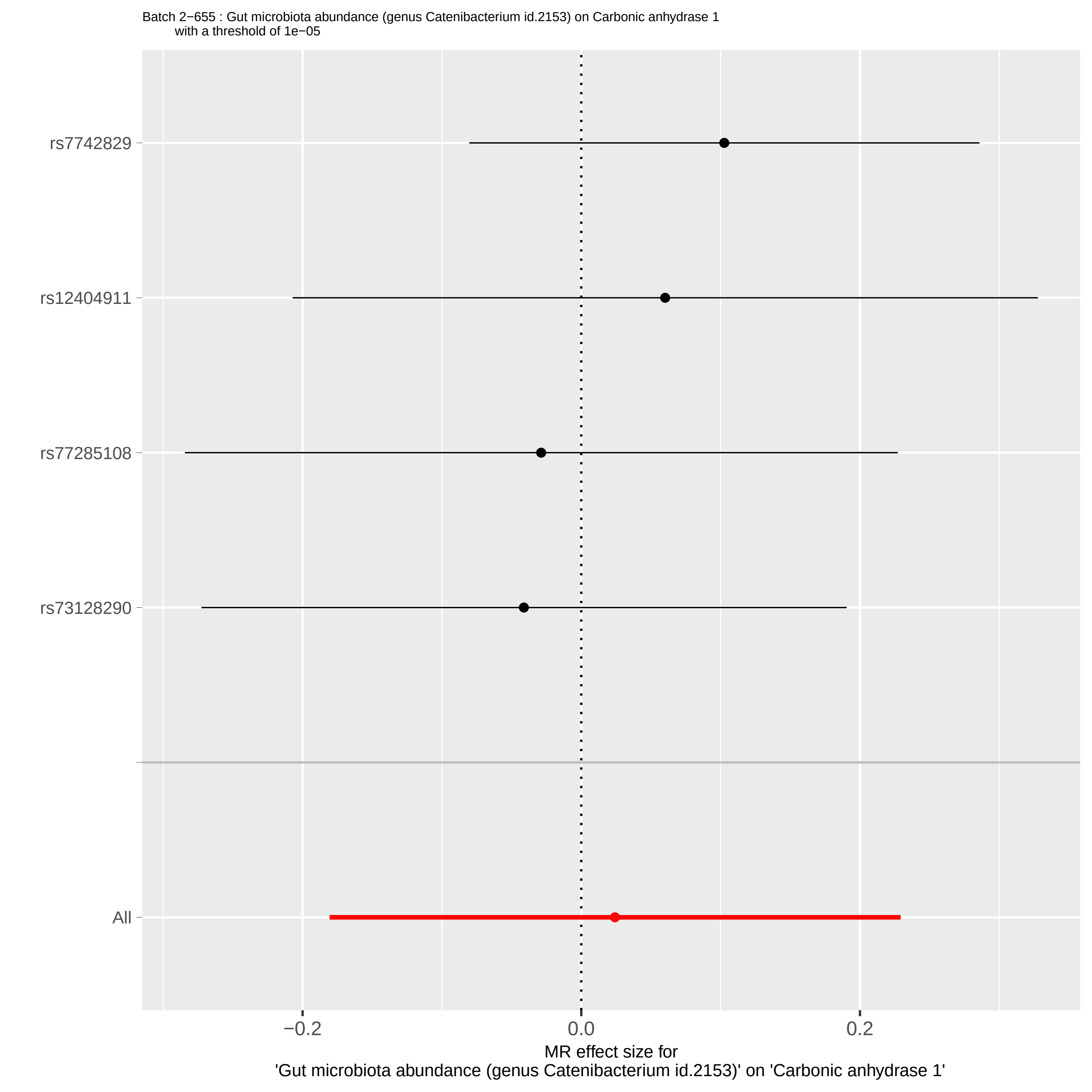
Batch 2−655 : Gut microbiota abundance (genus Catenibacterium id.2153) on Carbonic anhydrase 1
with a threshold of 1e−05
rs7742829
rs12404911
rs77285108
rs73128290
All
−0.2
0.0
0.2
MR effect size for
'Gut microbiota abundance (genus Catenibacterium id.2153)' on 'Carbonic anhydrase 1'

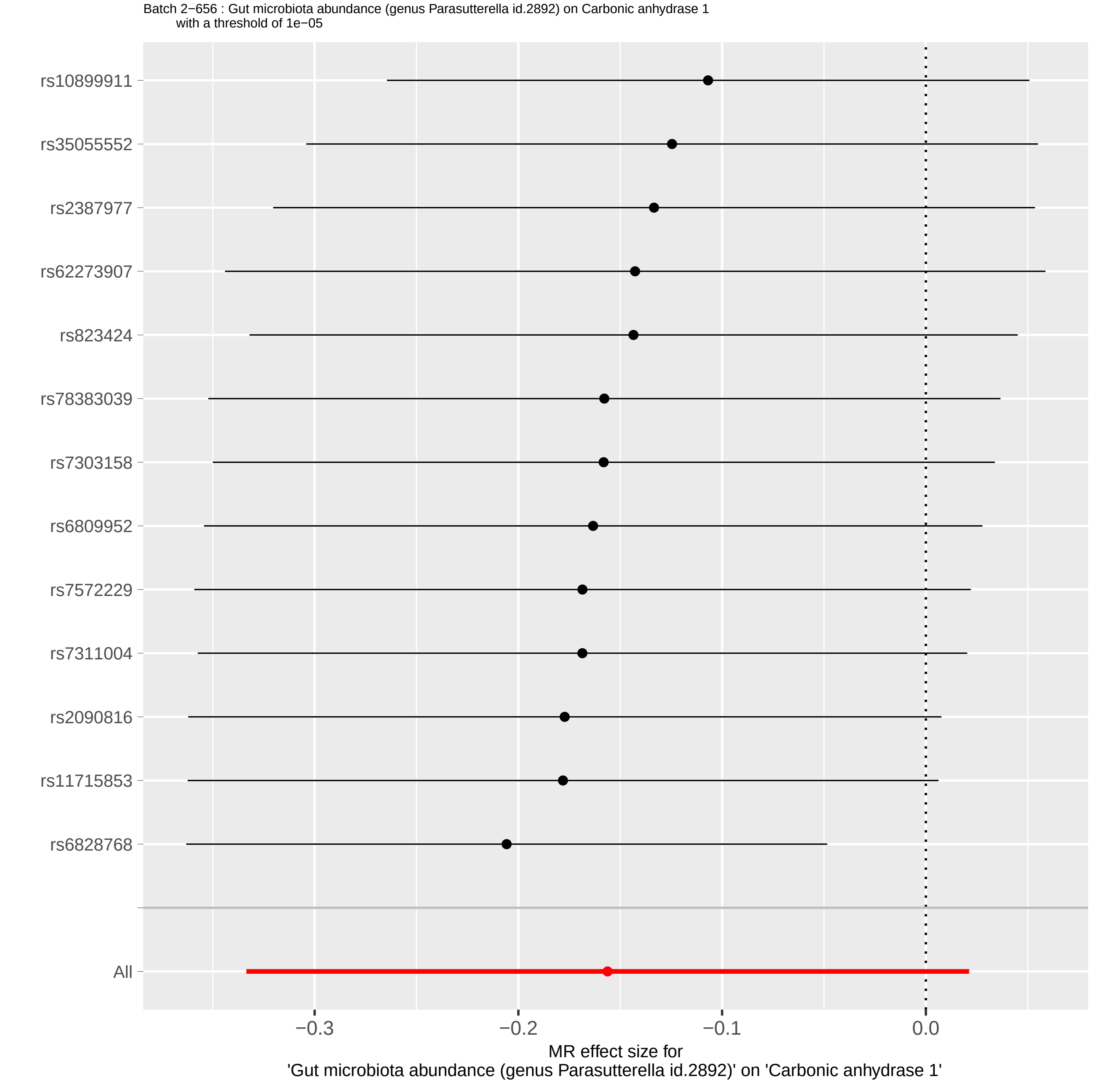
Batch 2−656 : Gut microbiota abundance (genus Parasutterella id.2892) on Carbonic anhydrase 1
with a threshold of 1e−05
rs10899911
rs35055552
rs2387977
rs62273907
rs823424
rs78383039
rs7303158
rs6809952
rs7572229
rs7311004
rs2090816
rs11715853
rs6828768
All
−0.3
−0.2
−0.1
0.0
MR effect size for
'Gut microbiota abundance (genus Parasutterella id.2892)' on 'Carbonic anhydrase 1'

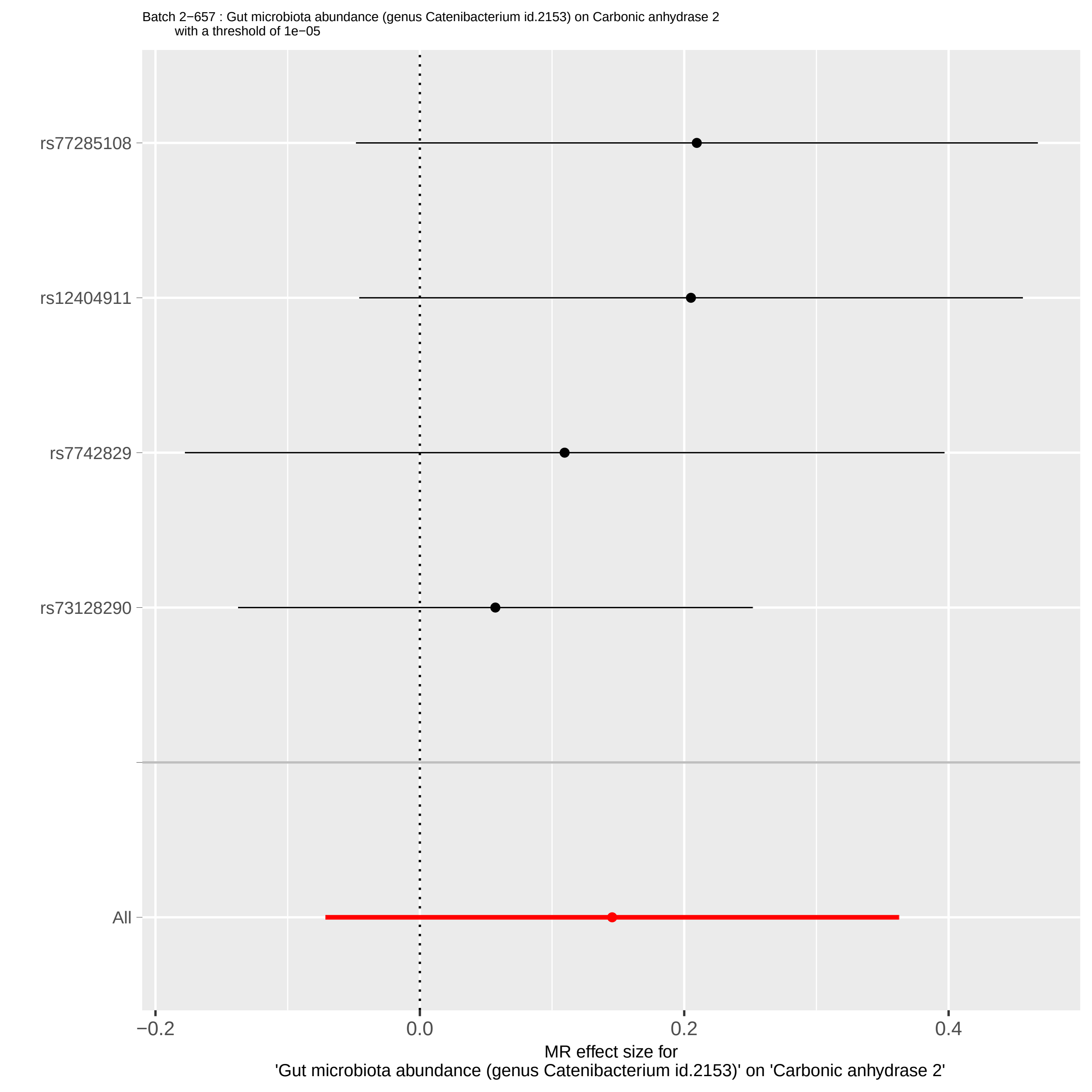

Batch 2−657 : Gut microbiota abundance (genus Catenibacterium id.2153) on Carbonic anhydrase 2
with a threshold of 1e−05
rs77285108
rs12404911
rs7742829
rs73128290
All
−0.2
0.0
0.2
0.4
MR effect size for
'Gut microbiota abundance (genus Catenibacterium id.2153)' on 'Carbonic anhydrase 2'

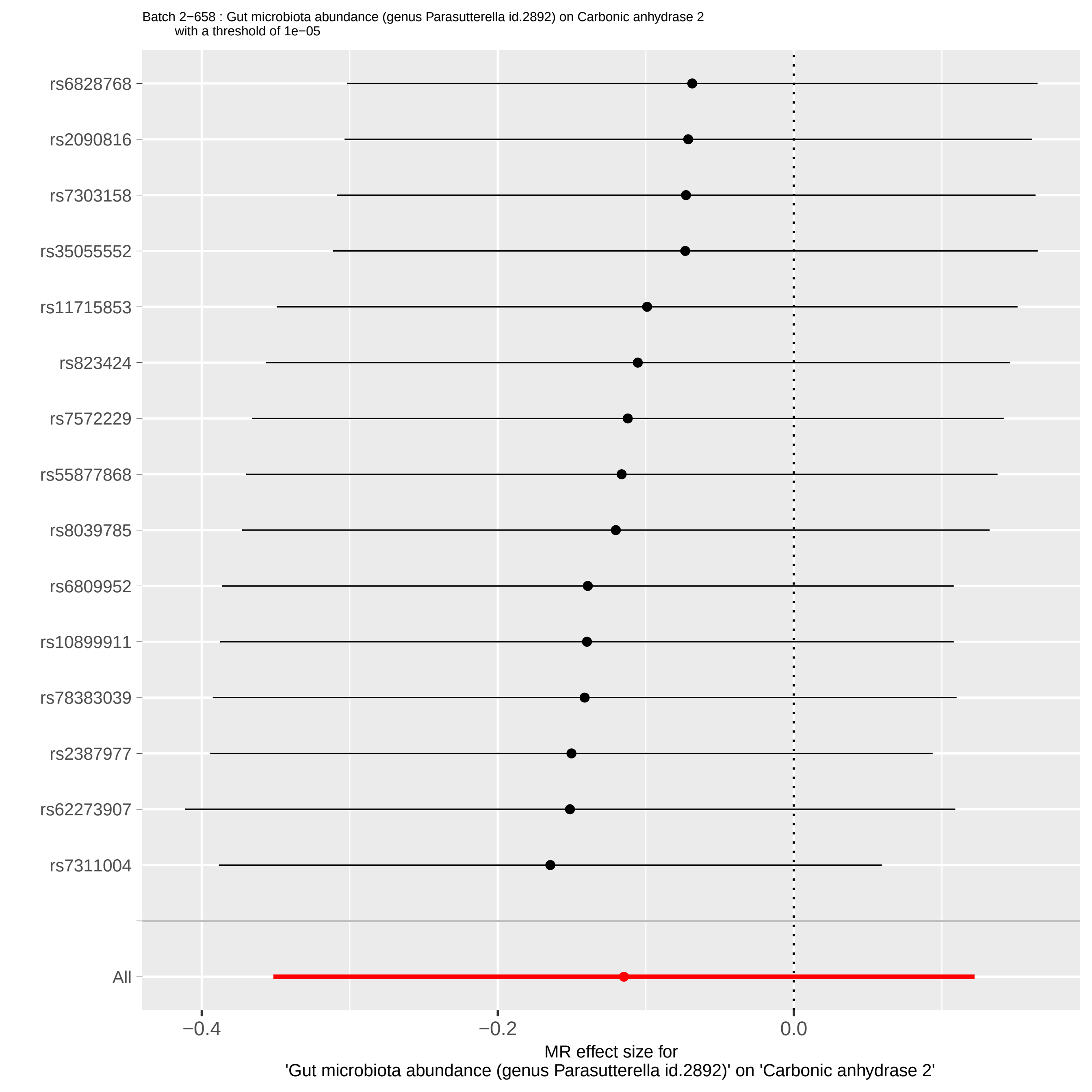
Batch 2−658 : Gut microbiota abundance (genus Parasutterella id.2892) on Carbonic anhydrase 2
with a threshold of 1e−05
rs6828768
rs2090816
rs7303158
rs35055552
rs11715853
rs823424
rs7572229
rs55877868
rs8039785
rs6809952
rs10899911
rs78383039
rs2387977
rs62273907
rs7311004
All
−0.4
−0.2
0.0
MR effect size for
'Gut microbiota abundance (genus Parasutterella id.2892)' on 'Carbonic anhydrase 2'

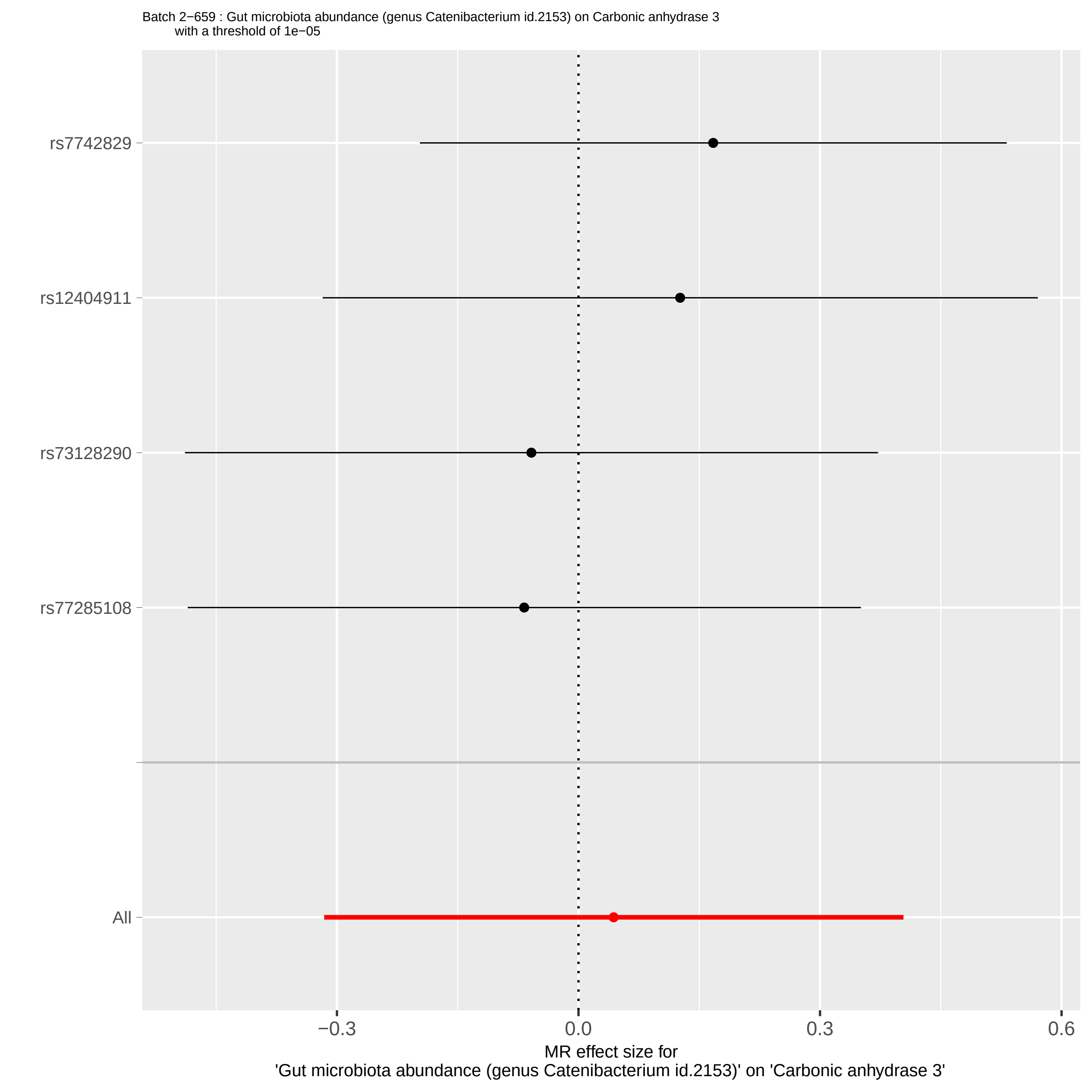
Batch 2−659 : Gut microbiota abundance (genus Catenibacterium id.2153) on Carbonic anhydrase 3
with a threshold of 1e−05
rs7742829
rs12404911
rs73128290
rs77285108
All
−0.3
0.0
0.3
0.6
MR effect size for
'Gut microbiota abundance (genus Catenibacterium id.2153)' on 'Carbonic anhydrase 3'

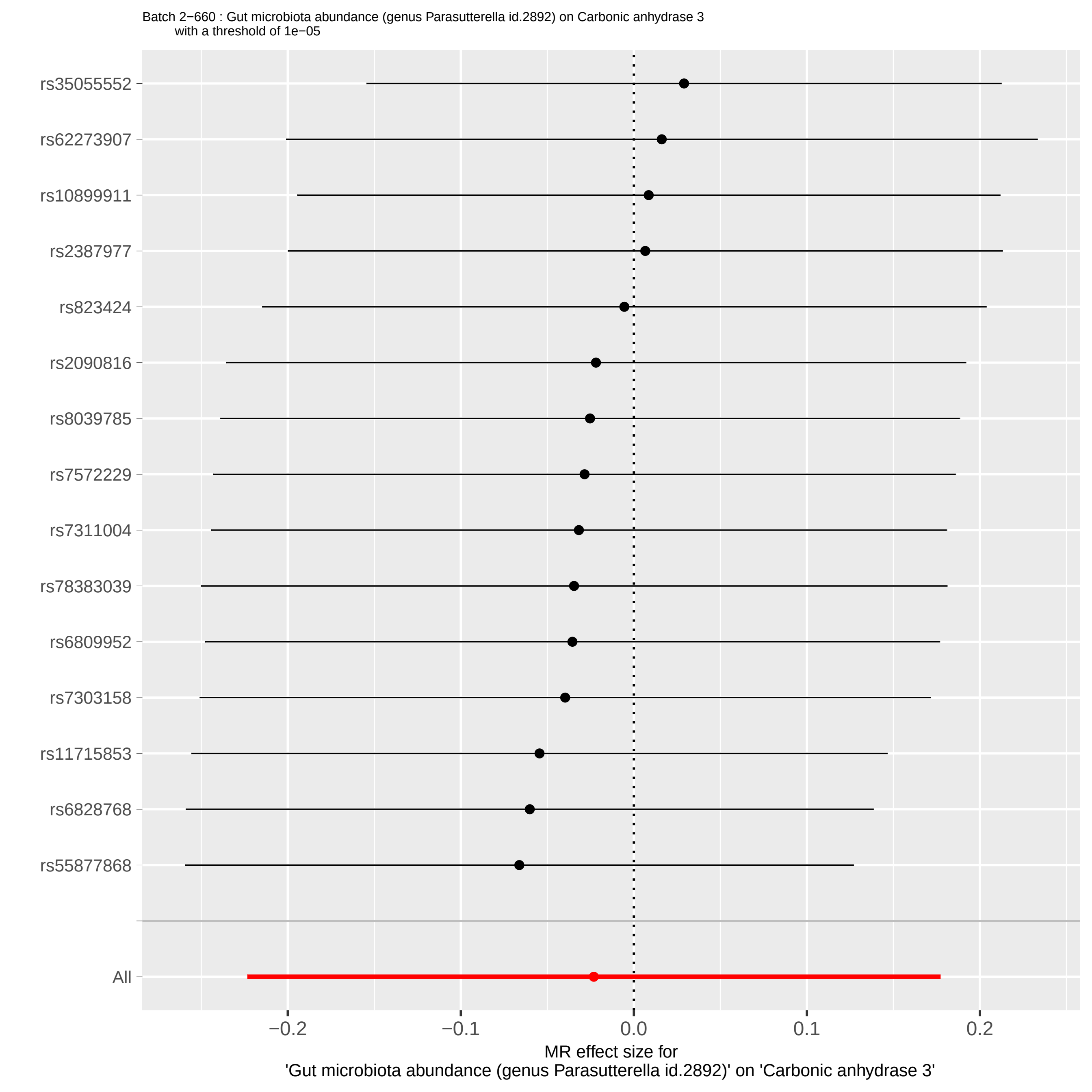

Batch 2−660 : Gut microbiota abundance (genus Parasutterella id.2892) on Carbonic anhydrase 3
with a threshold of 1e−05
rs35055552
rs62273907
rs10899911
rs2387977
rs823424
rs2090816
rs8039785
rs7572229
rs7311004
rs78383039
rs6809952
rs7303158
rs11715853
rs6828768
rs55877868
All
−0.2
−0.1
0.0
0.1
0.2
MR effect size for
'Gut microbiota abundance (genus Parasutterella id.2892)' on 'Carbonic anhydrase 3'

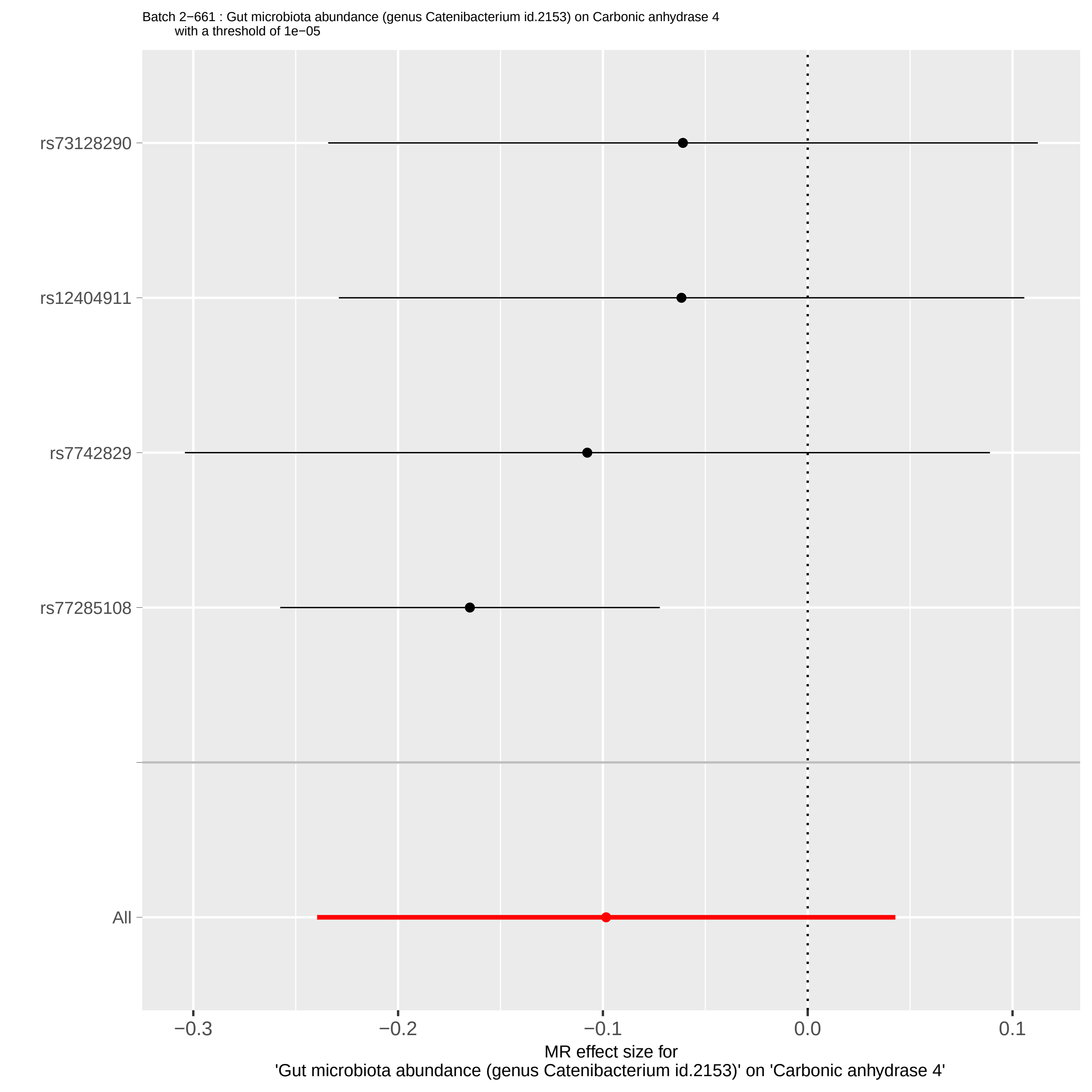

Batch 2−661 : Gut microbiota abundance (genus Catenibacterium id.2153) on Carbonic anhydrase 4
with a threshold of 1e−05
rs73128290
rs12404911
rs7742829
rs77285108
All
−0.3
−0.2
−0.1
0.0
0.1
MR effect size for
'Gut microbiota abundance (genus Catenibacterium id.2153)' on 'Carbonic anhydrase 4'

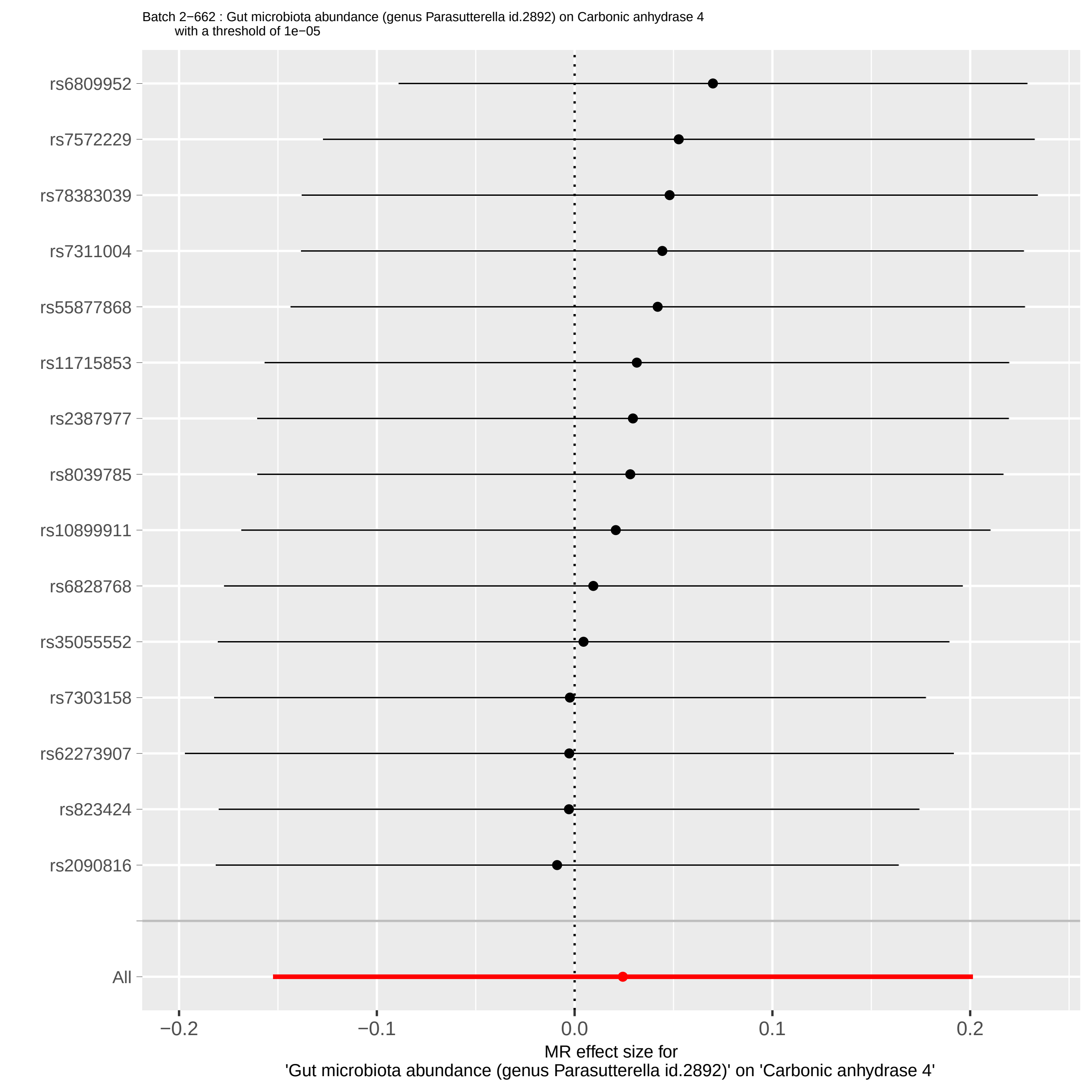

Batch 2−662 : Gut microbiota abundance (genus Parasutterella id.2892) on Carbonic anhydrase 4
with a threshold of 1e−05
rs6809952
rs7572229
rs78383039
rs7311004
rs55877868
rs11715853
rs2387977
rs8039785
rs10899911
rs6828768
rs35055552
rs7303158
rs62273907
rs823424
rs2090816
All
−0.2
−0.1
0.0
0.1
0.2
MR effect size for
'Gut microbiota abundance (genus Parasutterella id.2892)' on 'Carbonic anhydrase 4'

Batch 2−663 : Gut microbiota abundance (genus Catenibacterium id.2153) on Carbonic anhydrase 5A, mitochondrial
with a threshold of 1e−05

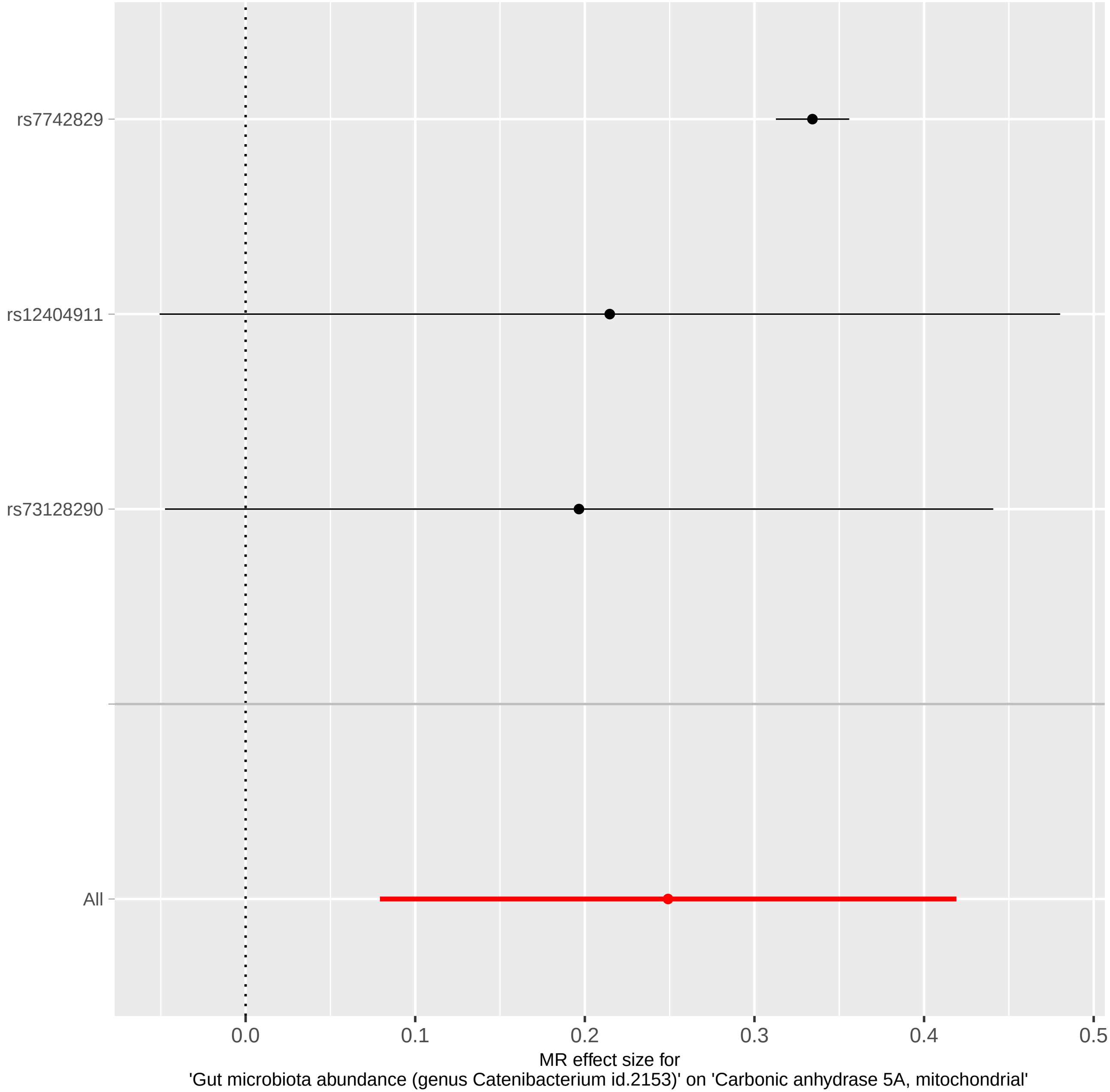

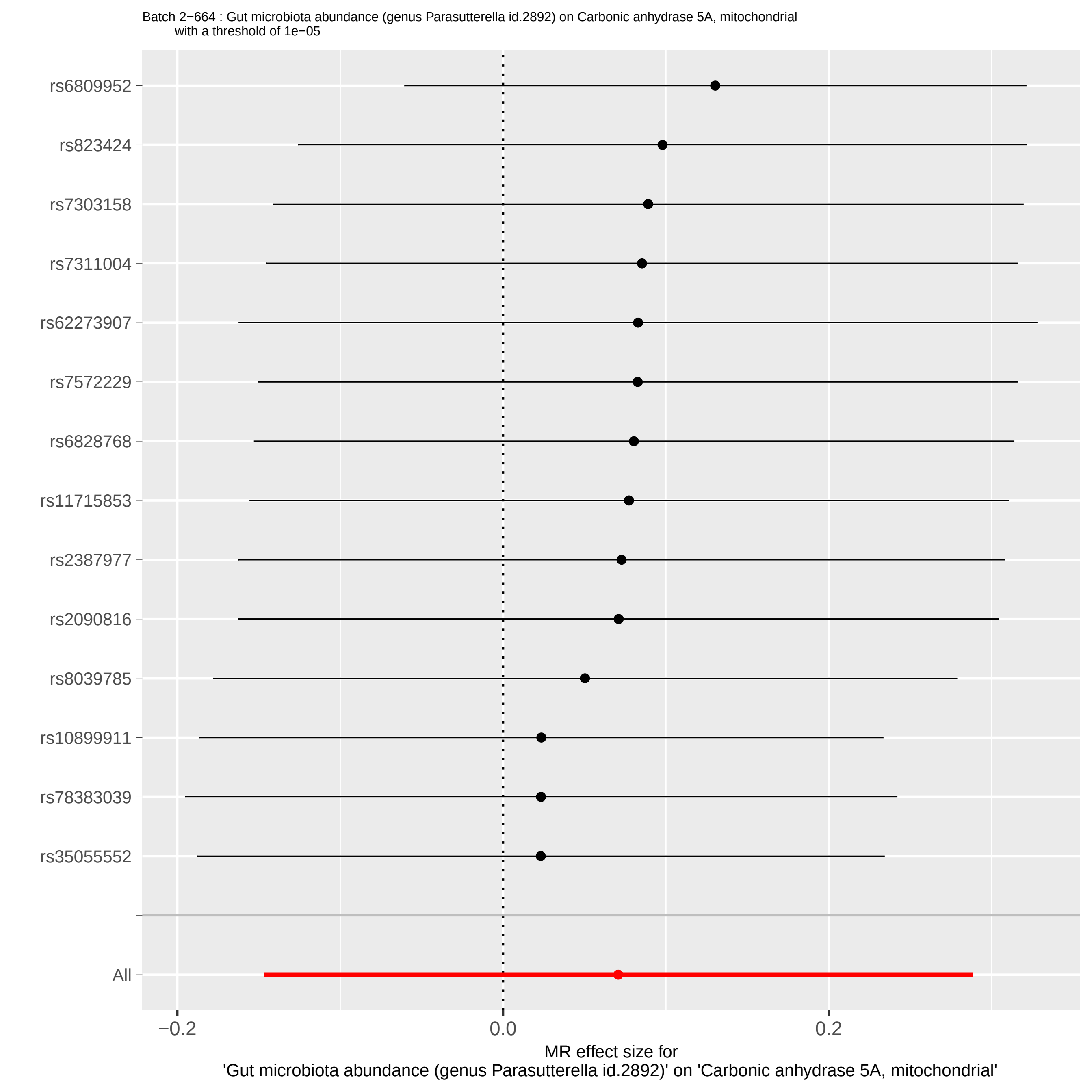
Batch 2−664 : Gut microbiota abundance (genus Parasutterella id.2892) on Carbonic anhydrase 5A, mitochondrial
with a threshold of 1e−05
rs6809952
rs823424
rs7303158
rs7311004
rs62273907
rs7572229
rs6828768
rs11715853
rs2387977
rs2090816
rs8039785
rs10899911
rs78383039
rs35055552
All
−0.2
0.0
0.2
MR effect size for
'Gut microbiota abundance (genus Parasutterella id.2892)' on 'Carbonic anhydrase 5A, mitochondrial'

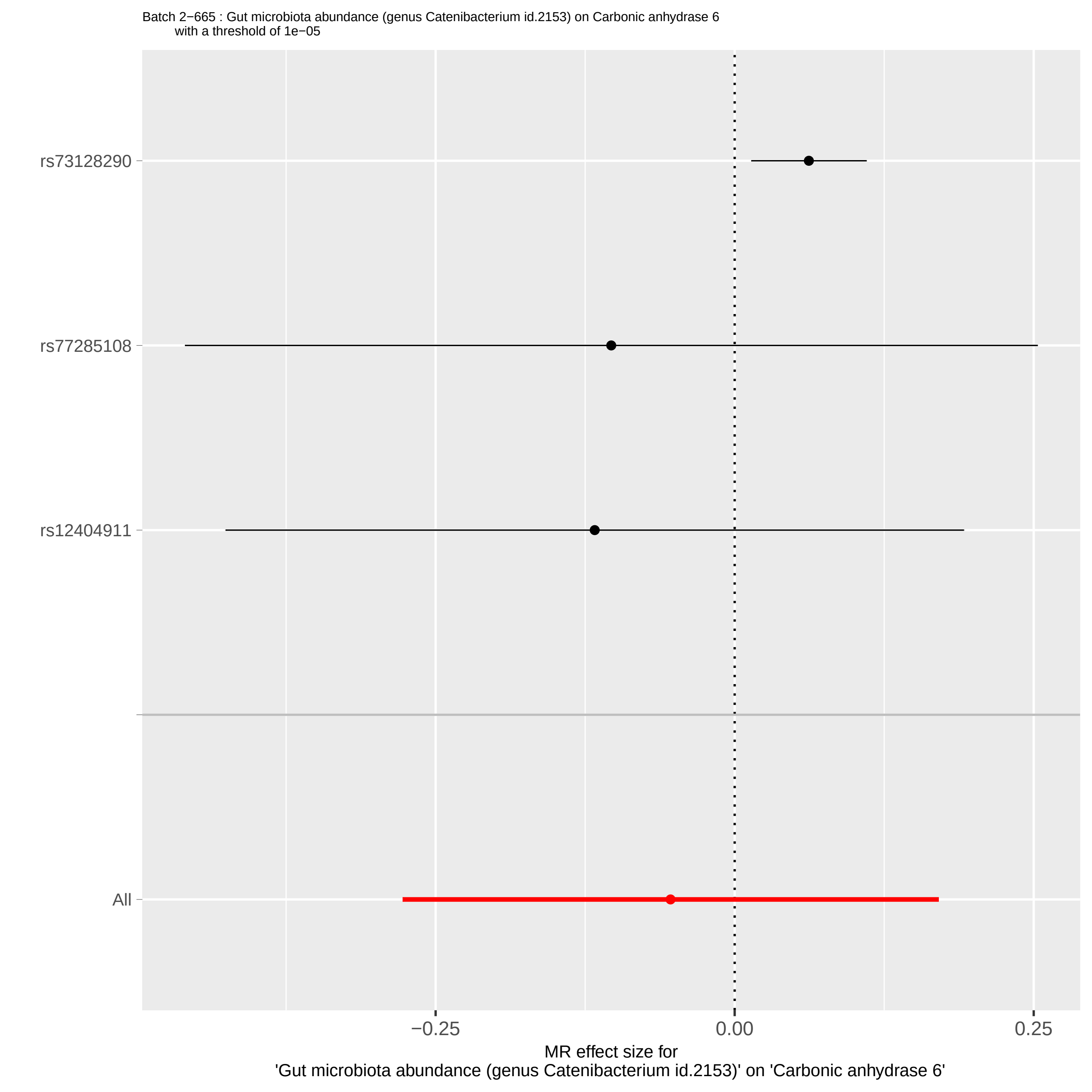
Batch 2−665 : Gut microbiota abundance (genus Catenibacterium id.2153) on Carbonic anhydrase 6
with a threshold of 1e−05
rs73128290
rs77285108
rs12404911
All
−0.25
0.00
0.25
MR effect size for
'Gut microbiota abundance (genus Catenibacterium id.2153)' on 'Carbonic anhydrase 6'

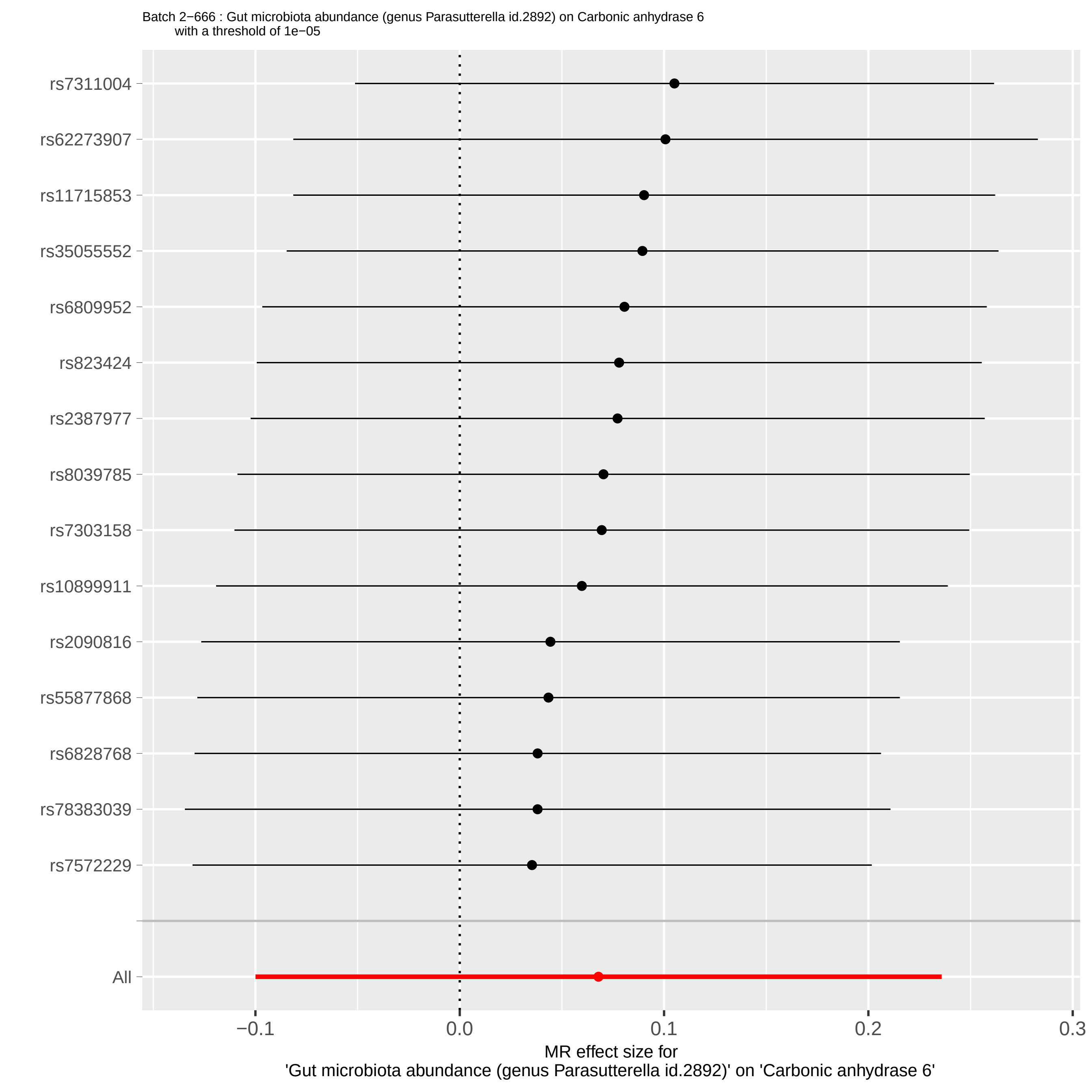
Batch 2−666 : Gut microbiota abundance (genus Parasutterella id.2892) on Carbonic anhydrase 6
with a threshold of 1e−05
rs7311004
rs62273907
rs11715853
rs35055552
rs6809952
rs823424
rs2387977
rs8039785
rs7303158
rs10899911
rs2090816
rs55877868
rs6828768
rs78383039
rs7572229
All
−0.1
0.0
0.1
0.2
0.3
MR effect size for
'Gut microbiota abundance (genus Parasutterella id.2892)' on 'Carbonic anhydrase 6'

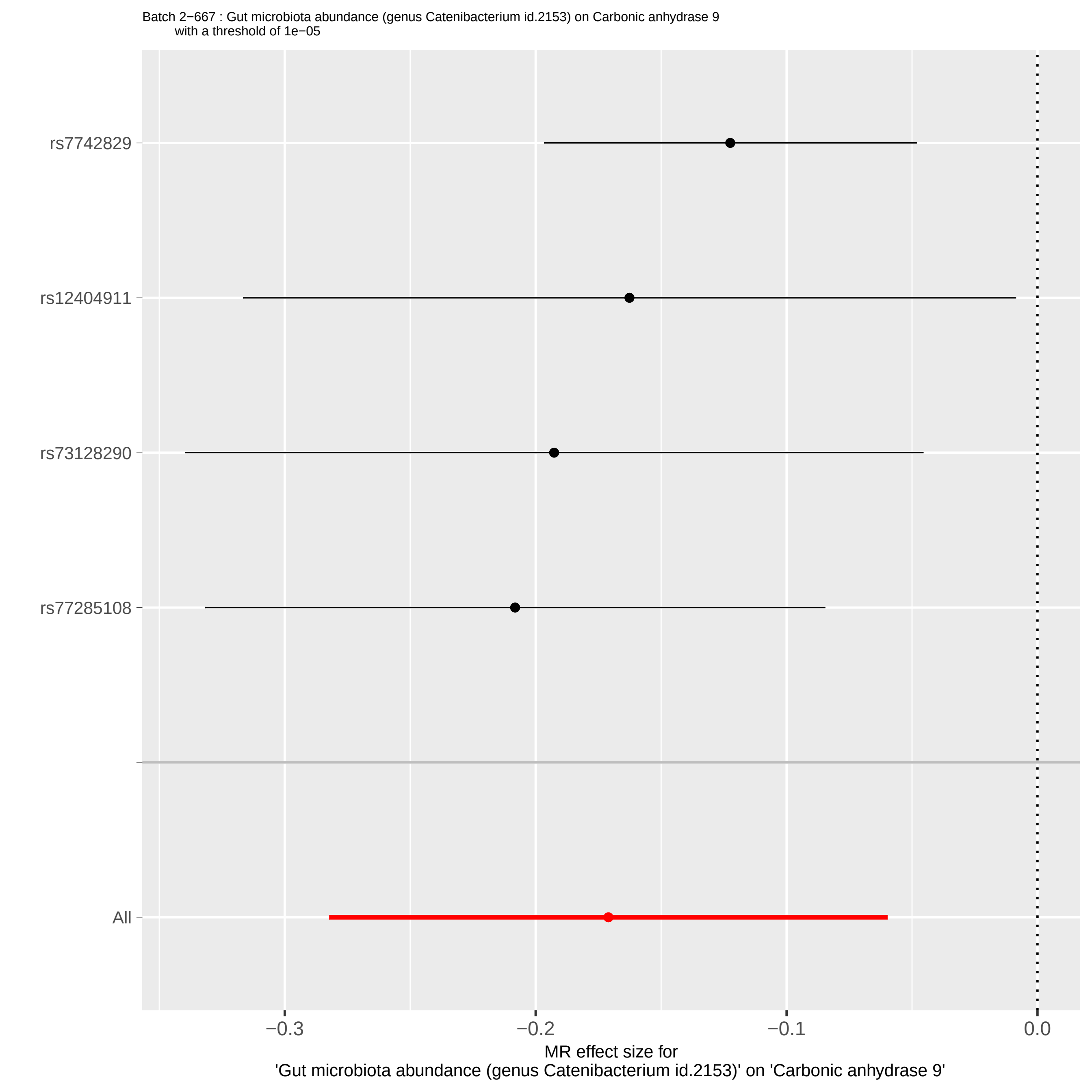
Batch 2−667 : Gut microbiota abundance (genus Catenibacterium id.2153) on Carbonic anhydrase 9
with a threshold of 1e−05
rs7742829
rs12404911
rs73128290
rs77285108
All
−0.3
−0.2
−0.1
0.0
MR effect size for
'Gut microbiota abundance (genus Catenibacterium id.2153)' on 'Carbonic anhydrase 9'

Batch 2−668 : Gut microbiota abundance (genus Parasutterella id.2892) on Carbonic anhydrase 9
with a threshold of 1e−05

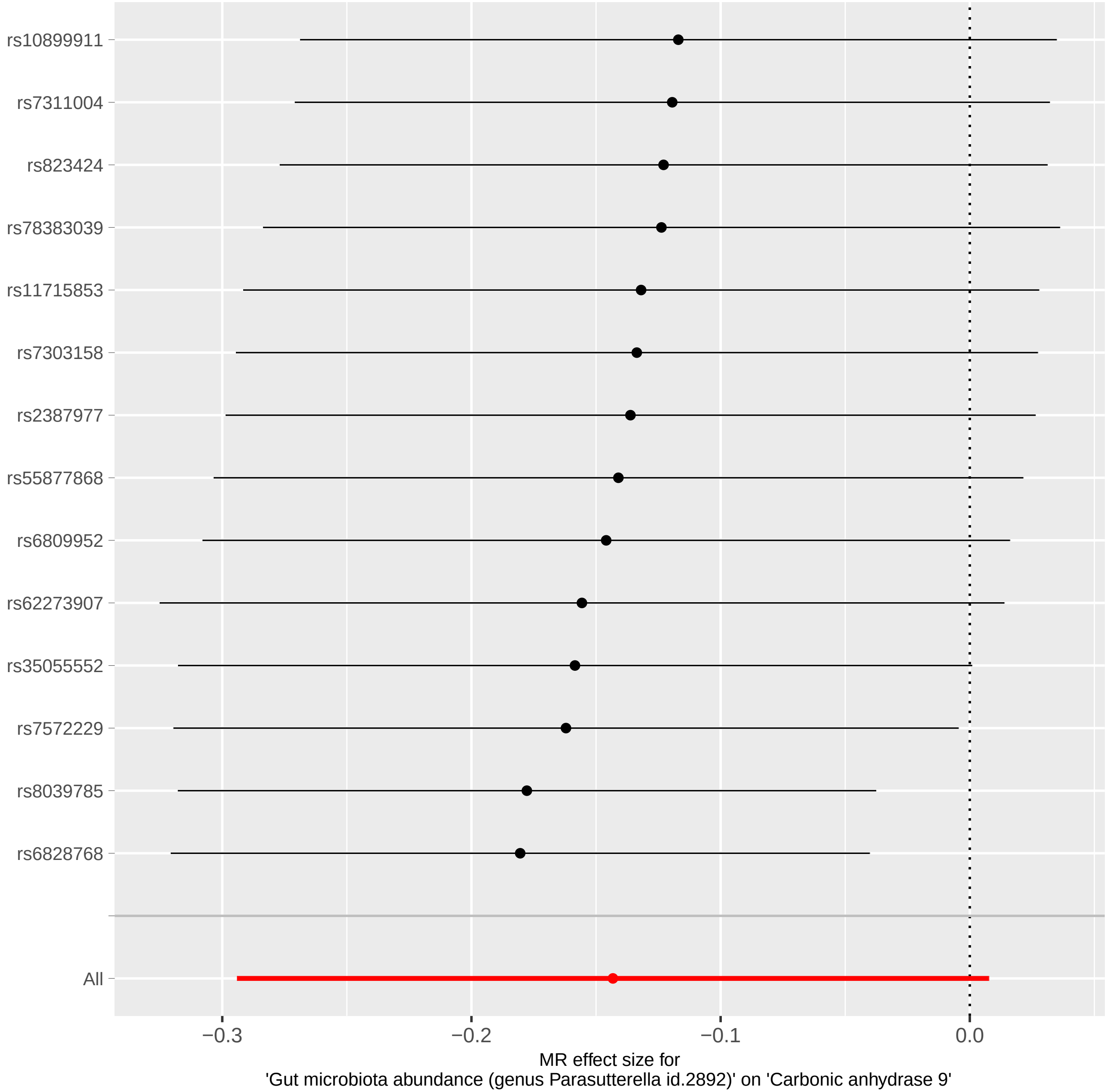

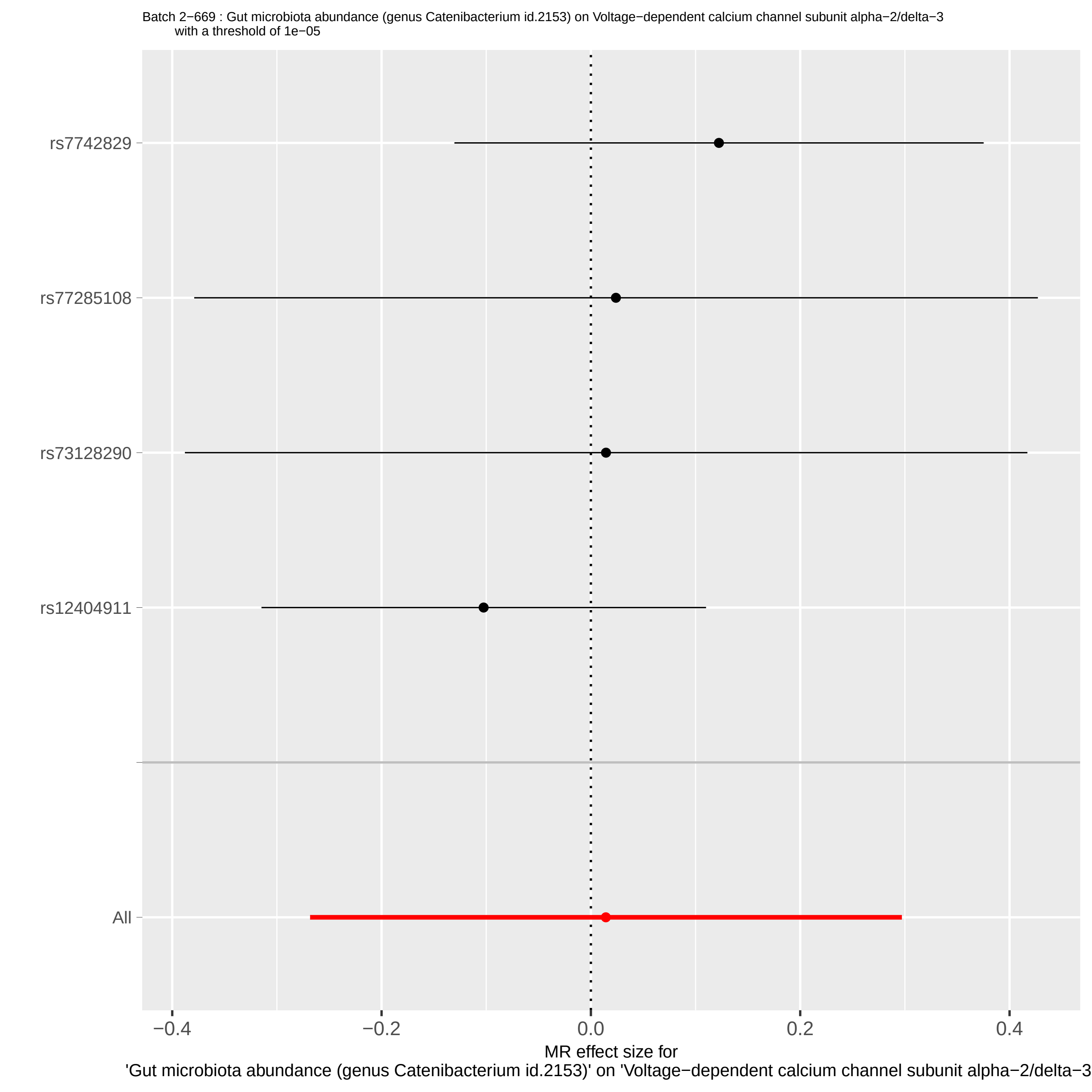

Batch 2−669 : Gut microbiota abundance (genus Catenibacterium id.2153) on Voltage−dependent calcium channel subunit alpha−2/delta−3
with a threshold of 1e−05
rs7742829
rs77285108
rs73128290
rs12404911
All
−0.4
−0.2
0.0
0.2
0.4
MR effect size for
'Gut microbiota abundance (genus Catenibacterium id.2153)' on 'Voltage−dependent calcium channel subunit alpha−2/delta−3

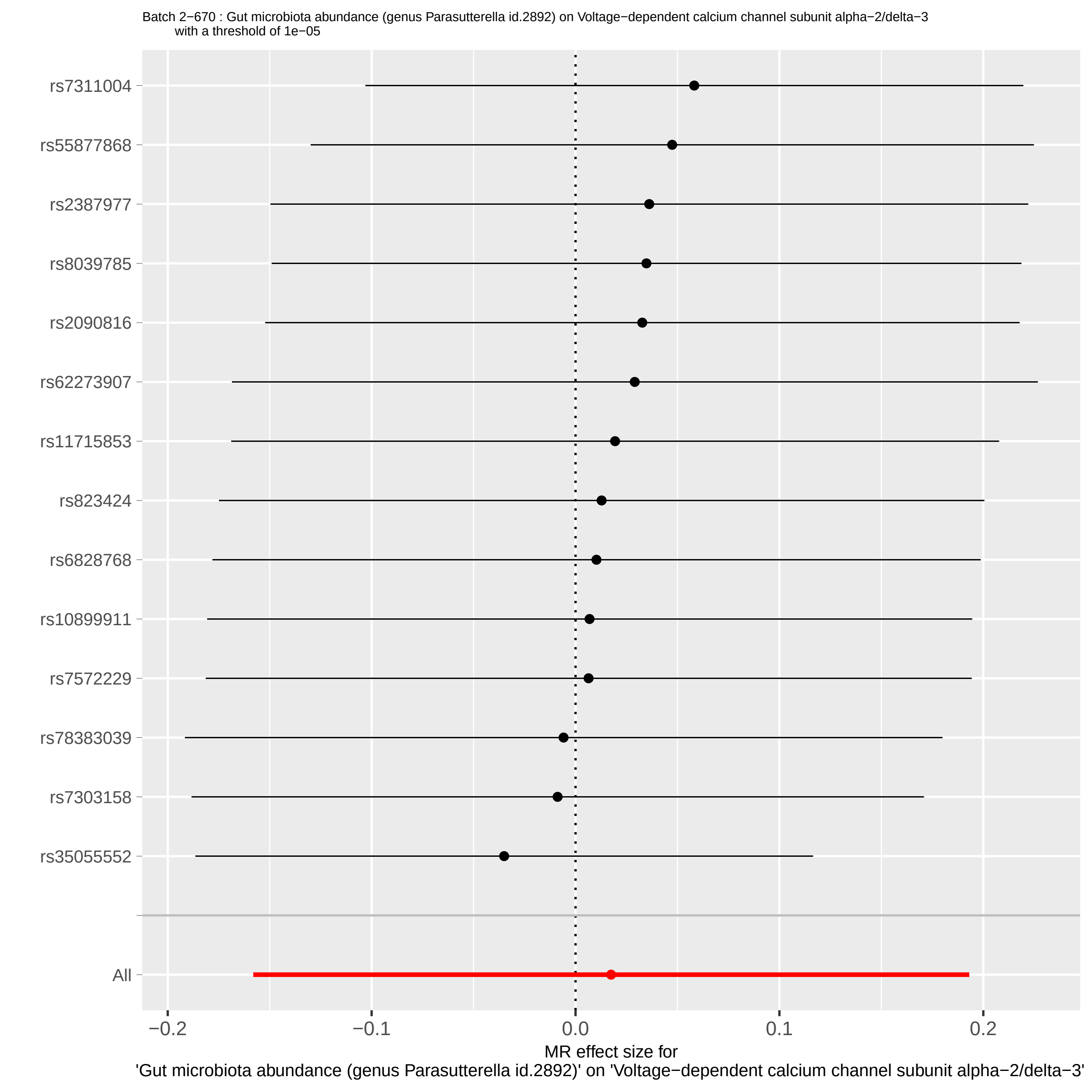

Batch 2−670 : Gut microbiota abundance (genus Parasutterella id.2892) on Voltage−dependent calcium channel subunit alpha−2/delta−3
with a threshold of 1e−05
rs7311004
rs55877868
rs2387977
rs8039785
rs2090816
rs62273907
rs11715853
rs823424
rs6828768
rs10899911
rs7572229
rs78383039
rs7303158
rs35055552
All
−0.2
−0.1
0.0
0.1
0.2
MR effect size for
'Gut microbiota abundance (genus Parasutterella id.2892)' on 'Voltage−dependent calcium channel subunit alpha−2/delta−3'

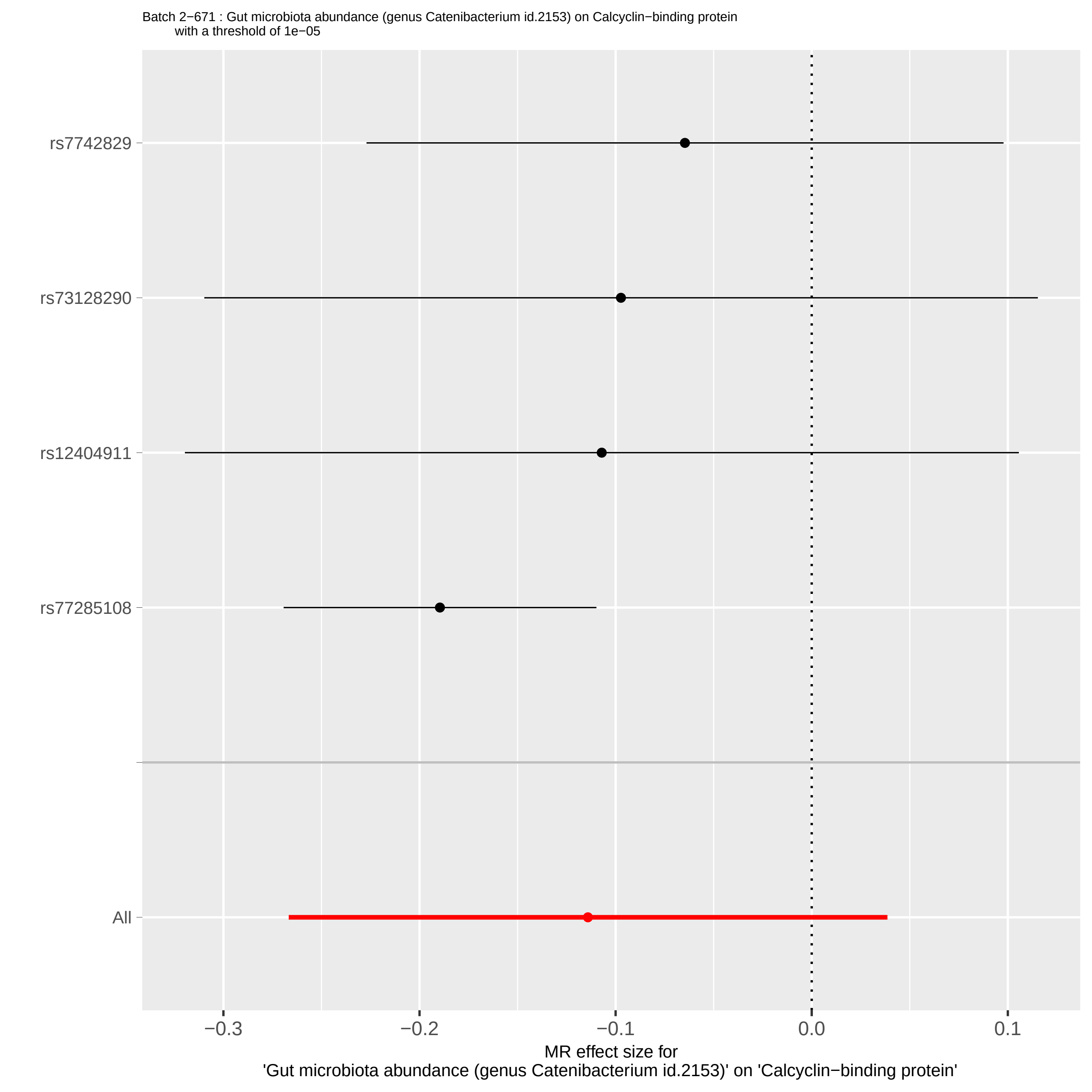
Batch 2−671 : Gut microbiota abundance (genus Catenibacterium id.2153) on Calcyclin−binding protein
with a threshold of 1e−05
rs7742829
rs73128290
rs12404911
rs77285108
All
−0.3
−0.2
−0.1
0.0
0.1
MR effect size for
'Gut microbiota abundance (genus Catenibacterium id.2153)' on 'Calcyclin−binding protein'

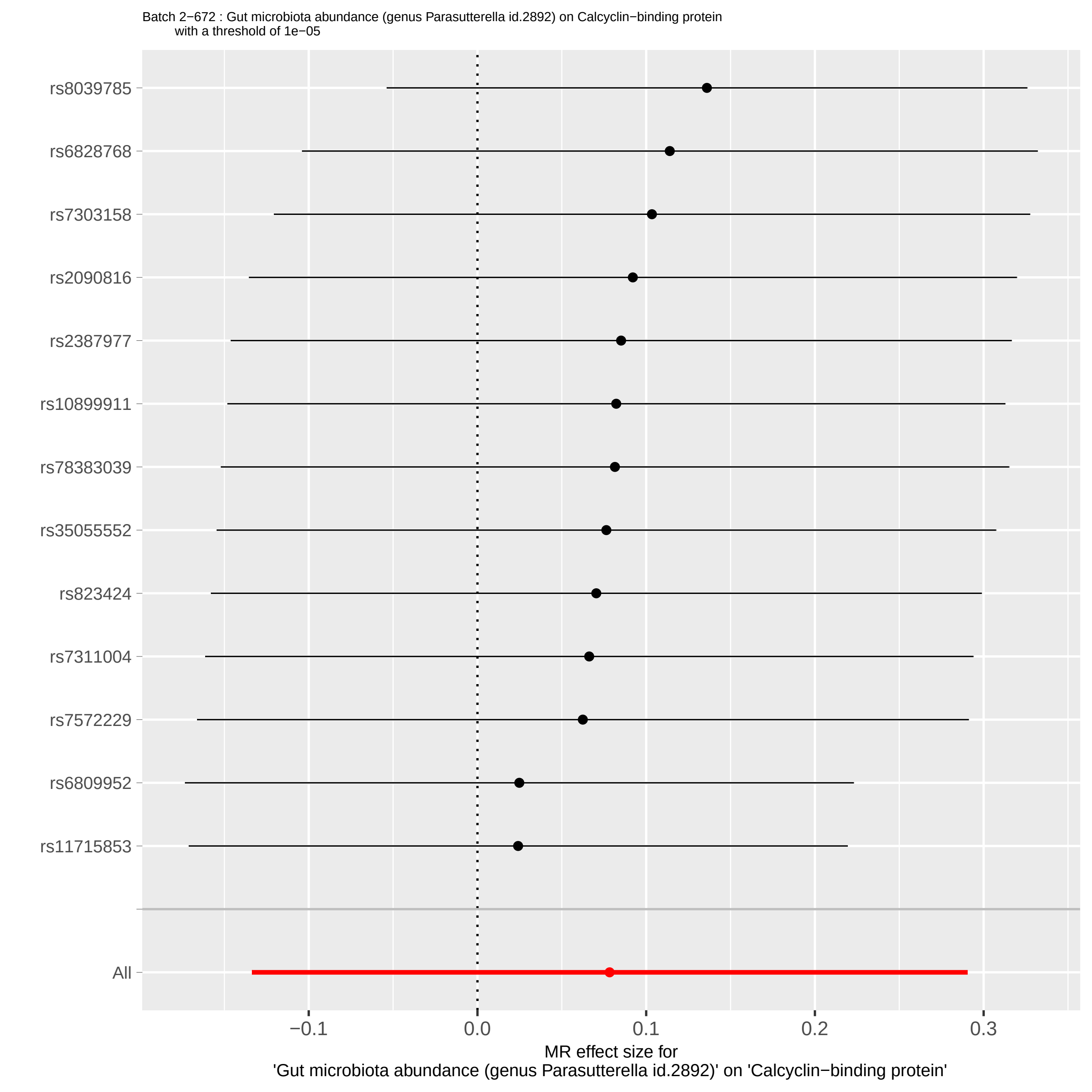

Batch 2−672 : Gut microbiota abundance (genus Parasutterella id.2892) on Calcyclin−binding protein
with a threshold of 1e−05
rs8039785
rs6828768
rs7303158
rs2090816
rs2387977
rs10899911
rs78383039
rs35055552
rs823424
rs7311004
rs7572229
rs6809952
rs11715853
All
−0.1
0.0
0.1
0.2
0.3
MR effect size for
'Gut microbiota abundance (genus Parasutterella id.2892)' on 'Calcyclin−binding protein'

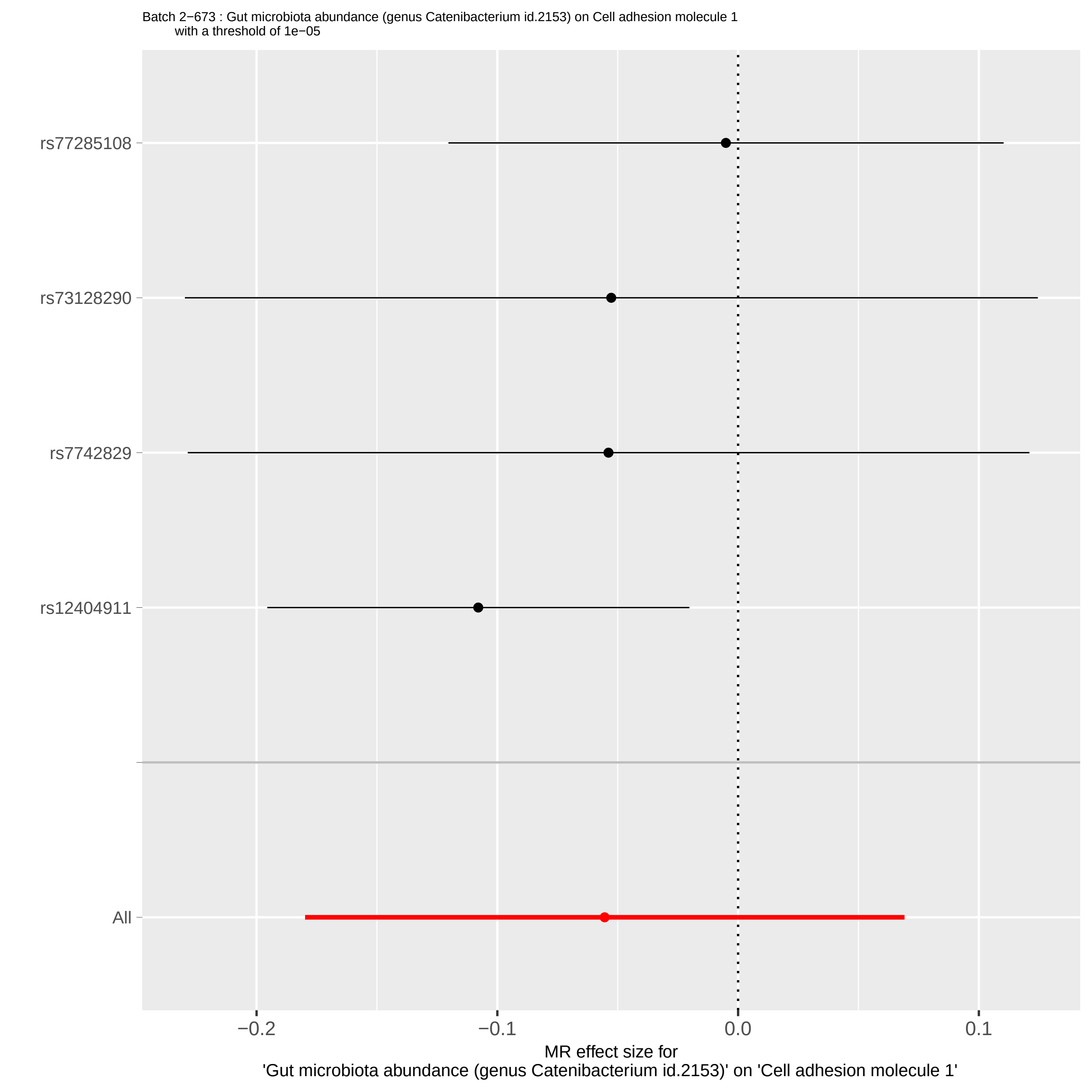

Batch 2−673 : Gut microbiota abundance (genus Catenibacterium id.2153) on Cell adhesion molecule 1
with a threshold of 1e−05
rs77285108
rs73128290
rs7742829
rs12404911
All
−0.2
−0.1
0.0
0.1
MR effect size for
'Gut microbiota abundance (genus Catenibacterium id.2153)' on 'Cell adhesion molecule 1'

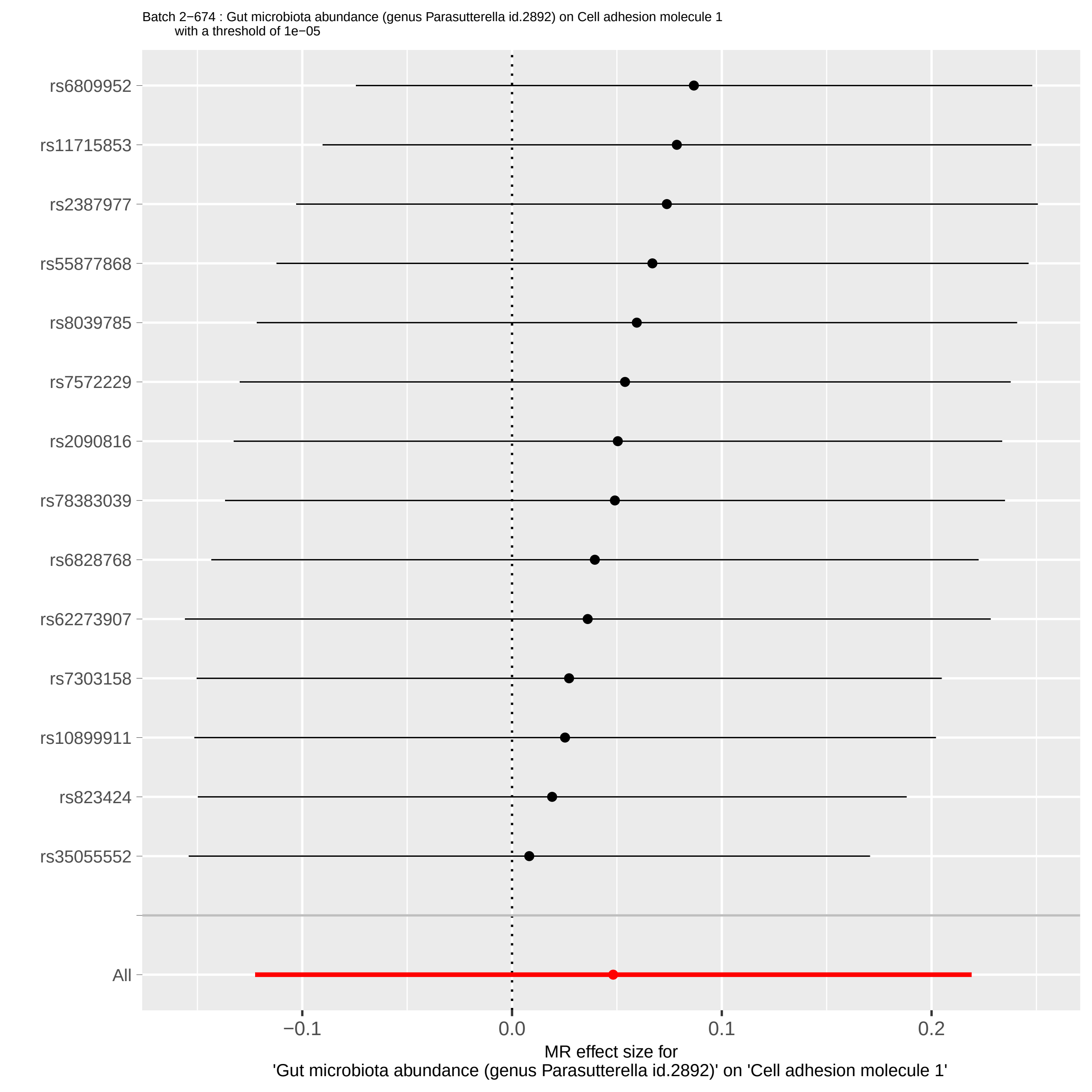

Batch 2−674 : Gut microbiota abundance (genus Parasutterella id.2892) on Cell adhesion molecule 1
with a threshold of 1e−05
rs6809952
rs11715853
rs2387977
rs55877868
rs8039785
rs7572229
rs2090816
rs78383039
rs6828768
rs62273907
rs7303158
rs10899911
rs823424
rs35055552
All
−0.1
0.0
0.1
0.2
MR effect size for
'Gut microbiota abundance (genus Parasutterella id.2892)' on 'Cell adhesion molecule 1'

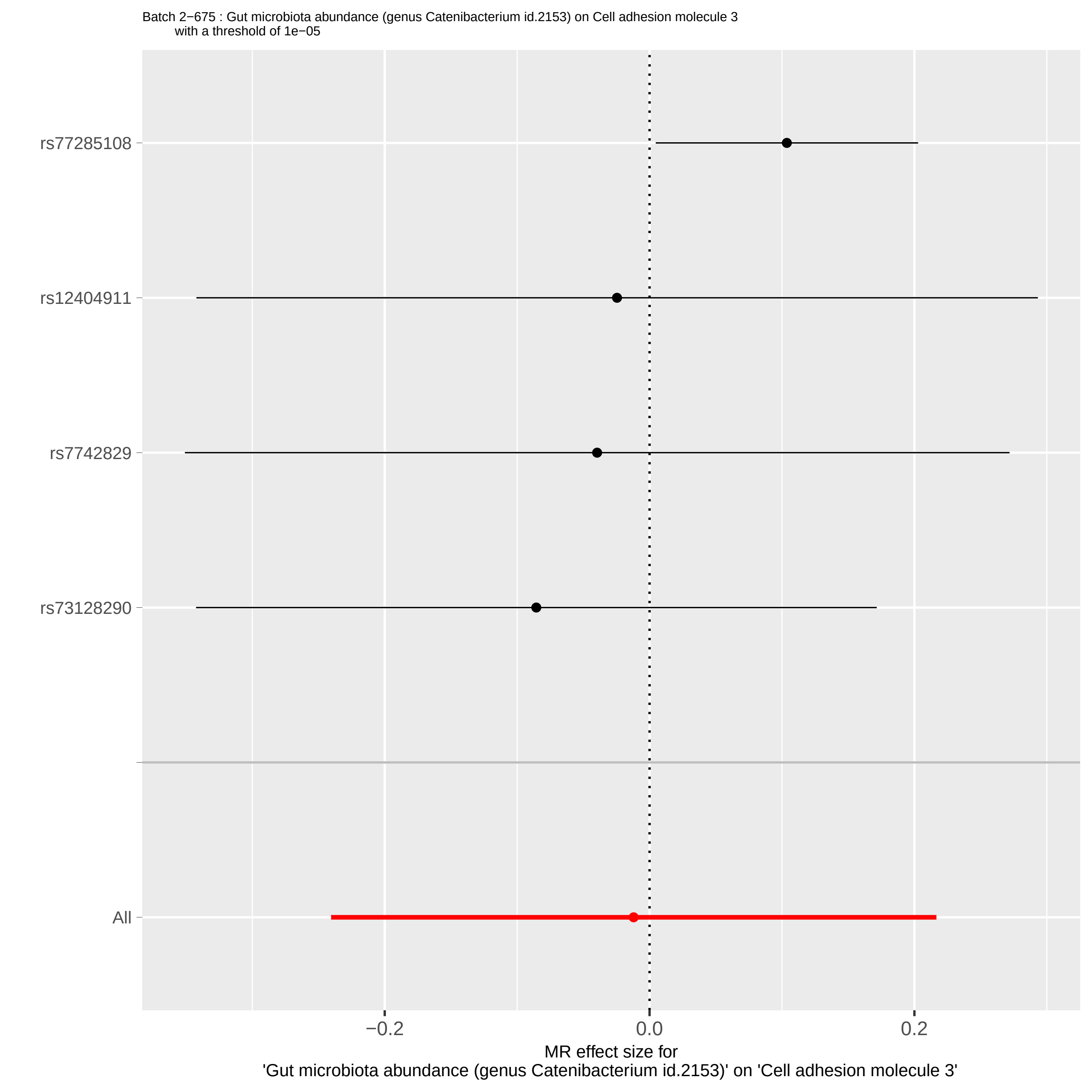
Batch 2−675 : Gut microbiota abundance (genus Catenibacterium id.2153) on Cell adhesion molecule 3
with a threshold of 1e−05
rs77285108
rs12404911
rs7742829
rs73128290
All
−0.2
0.0
0.2
MR effect size for
'Gut microbiota abundance (genus Catenibacterium id.2153)' on 'Cell adhesion molecule 3'

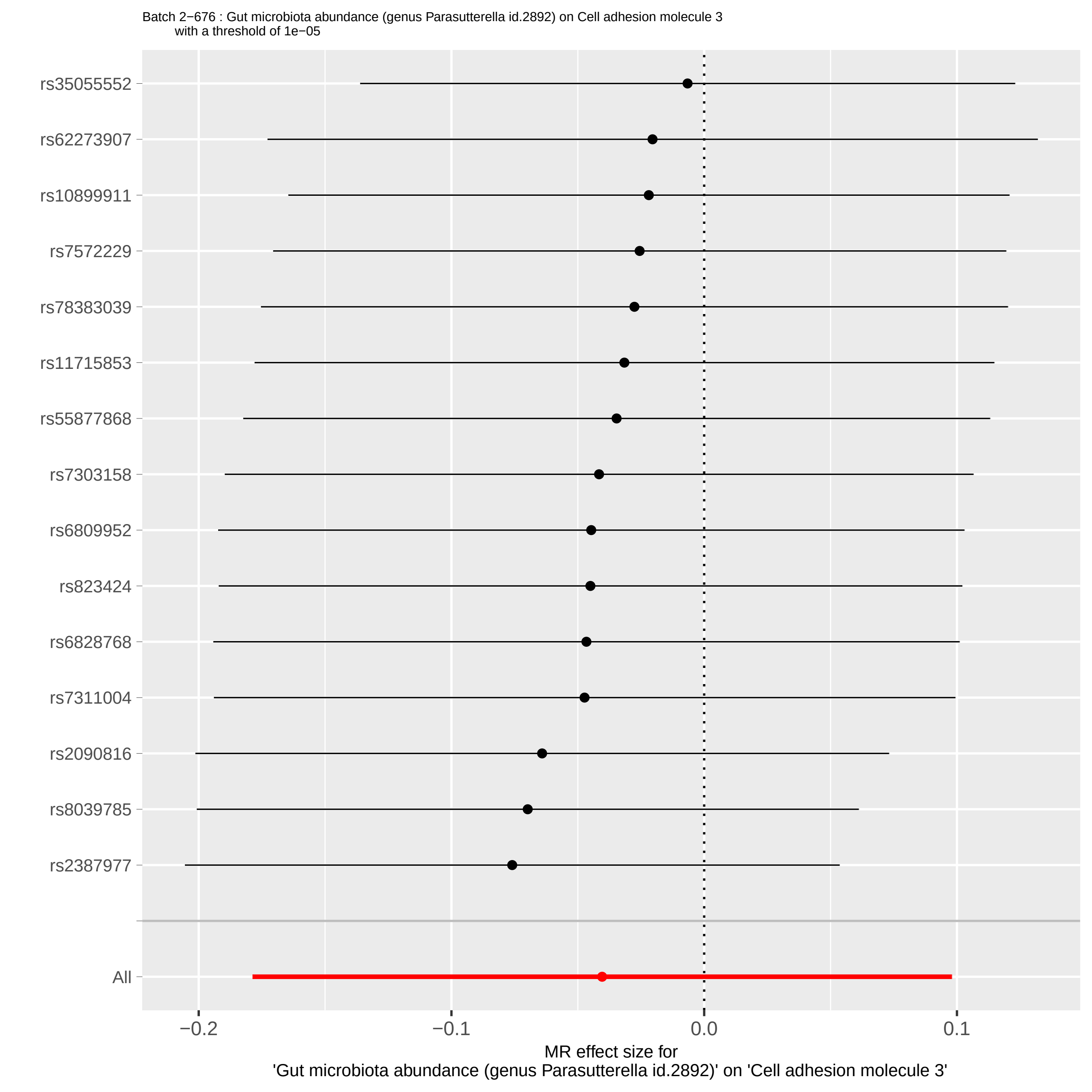

Batch 2−676 : Gut microbiota abundance (genus Parasutterella id.2892) on Cell adhesion molecule 3
with a threshold of 1e−05
rs35055552
rs62273907
rs10899911
rs7572229
rs78383039
rs11715853
rs55877868
rs7303158
rs6809952
rs823424
rs6828768
rs7311004
rs2090816
rs8039785
rs2387977
All
−0.2
−0.1
0.0
0.1
MR effect size for
'Gut microbiota abundance (genus Parasutterella id.2892)' on 'Cell adhesion molecule 3'

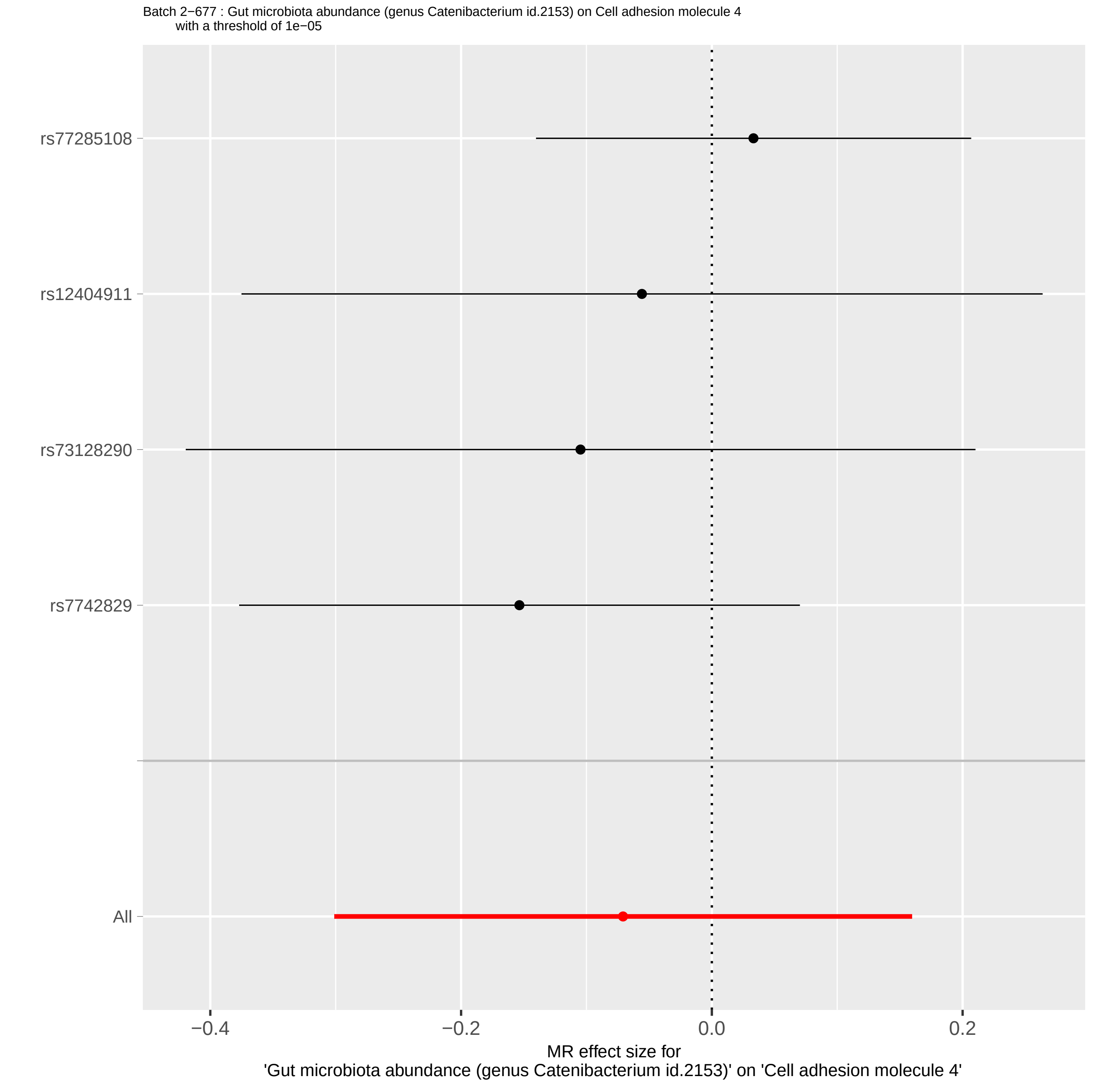
Batch 2−677 : Gut microbiota abundance (genus Catenibacterium id.2153) on Cell adhesion molecule 4
with a threshold of 1e−05
rs77285108
rs12404911
rs73128290
rs7742829
All
−0.4
−0.2
0.0
0.2
MR effect size for
'Gut microbiota abundance (genus Catenibacterium id.2153)' on 'Cell adhesion molecule 4'

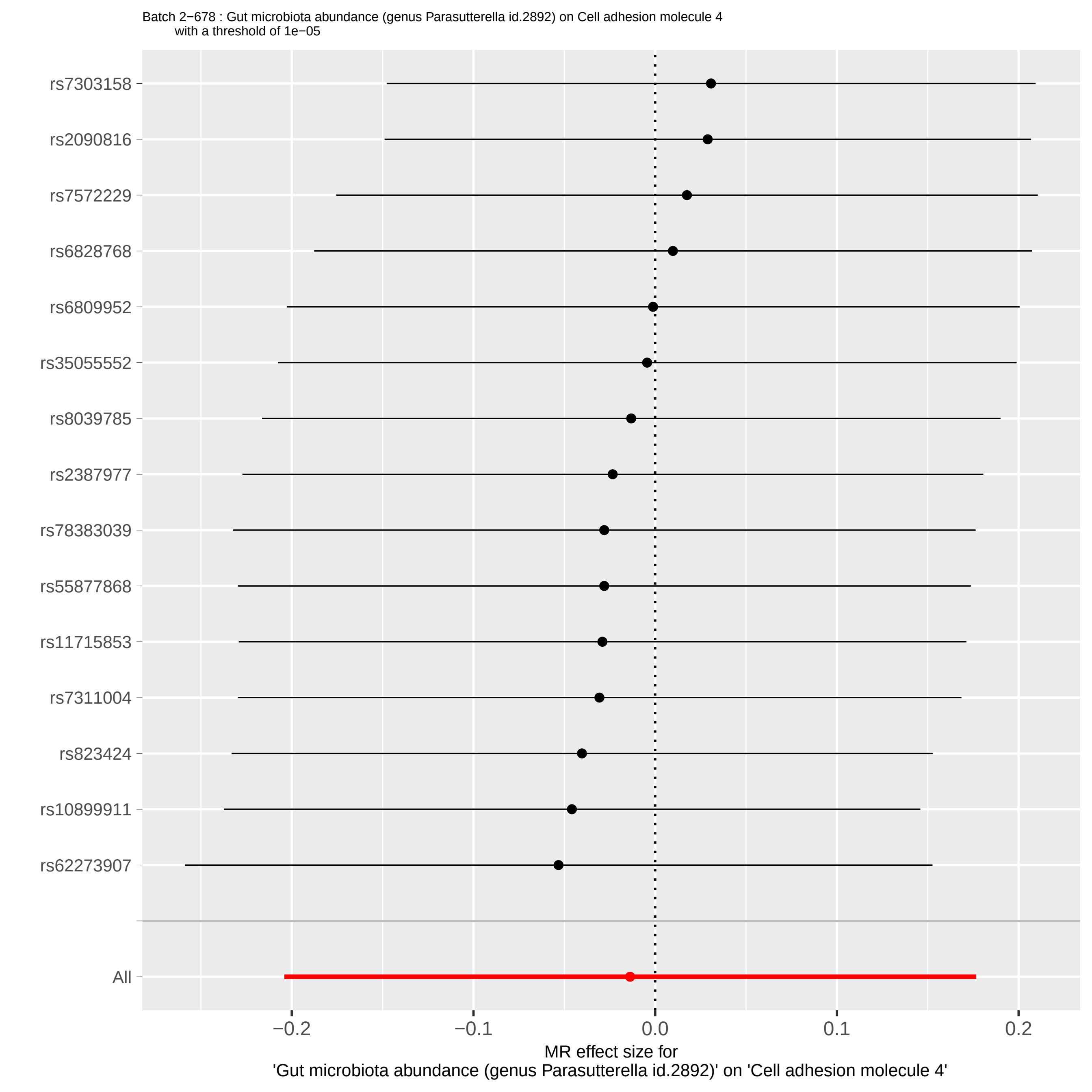
Batch 2−678 : Gut microbiota abundance (genus Parasutterella id.2892) on Cell adhesion molecule 4
with a threshold of 1e−05
rs7303158
rs2090816
rs7572229
rs6828768
rs6809952
rs35055552
rs8039785
rs2387977
rs78383039
rs55877868
rs11715853
rs7311004
rs823424
rs10899911
rs62273907
All
−0.2
−0.1
0.0
0.1
0.2
MR effect size for
'Gut microbiota abundance (genus Parasutterella id.2892)' on 'Cell adhesion molecule 4'

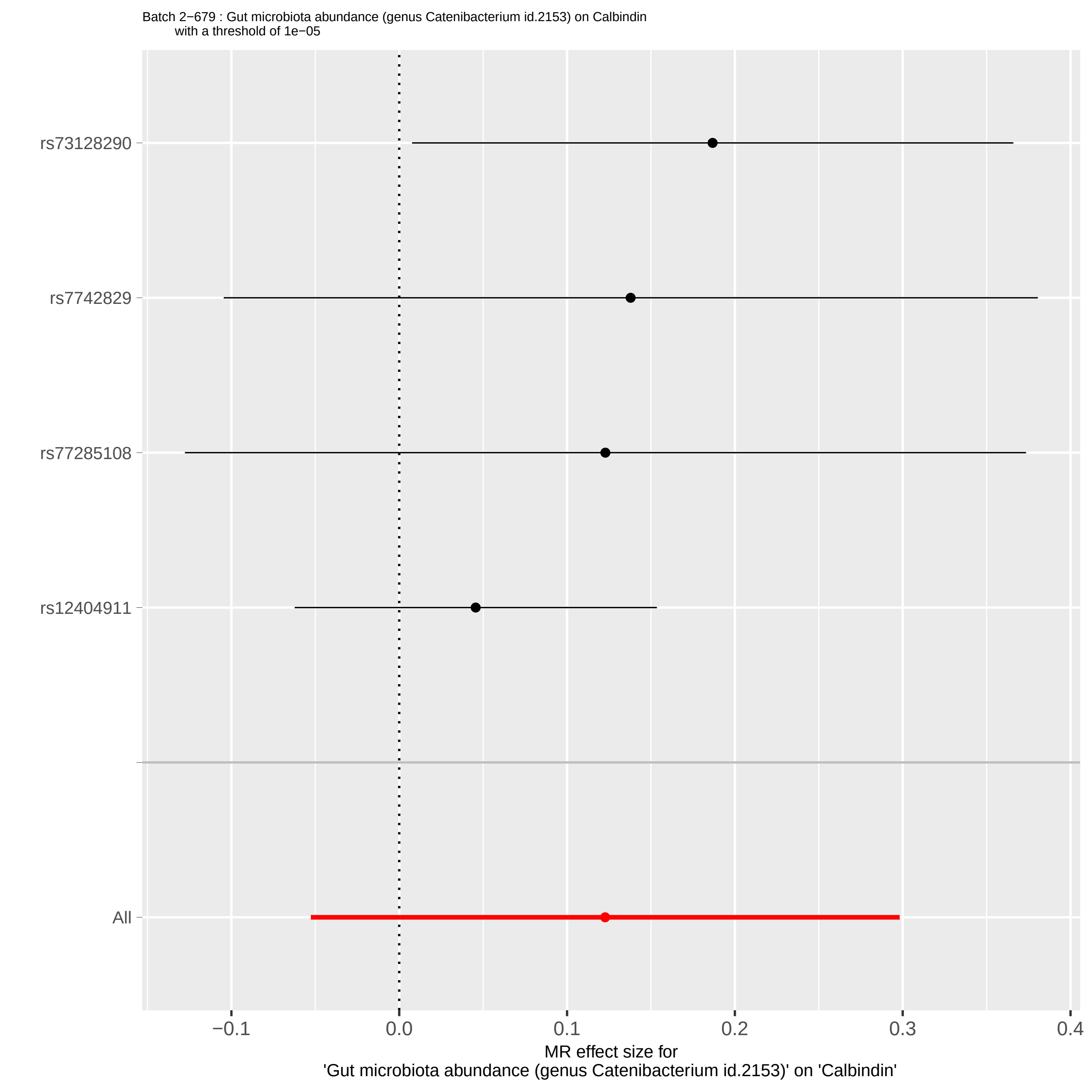

Batch 2−679 : Gut microbiota abundance (genus Catenibacterium id.2153) on Calbindin
with a threshold of 1e−05
rs73128290
rs7742829
rs77285108
rs12404911
All
−0.1
0.0
0.1
0.2
0.3
0.4
MR effect size for
'Gut microbiota abundance (genus Catenibacterium id.2153)' on 'Calbindin'

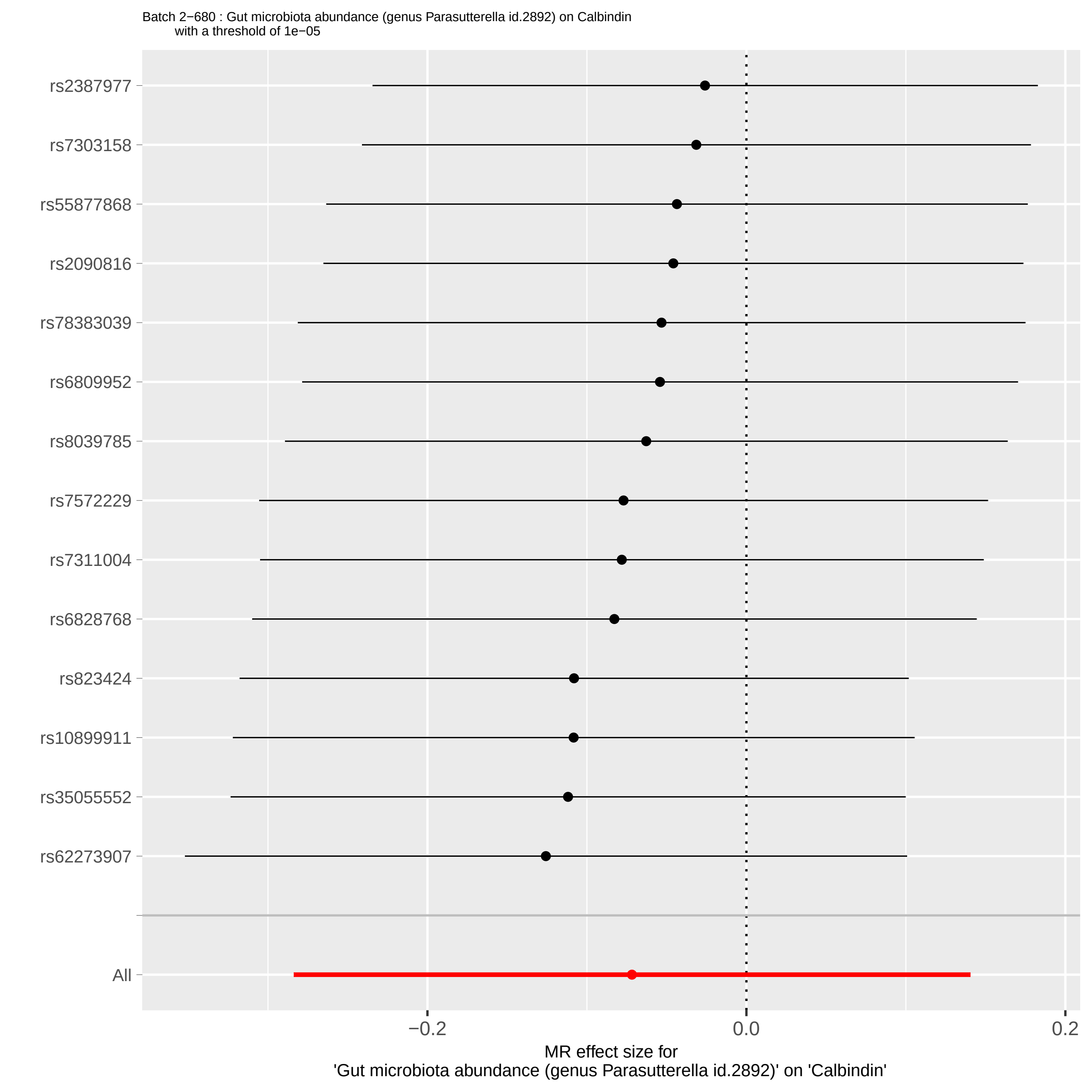
Batch 2−680 : Gut microbiota abundance (genus Parasutterella id.2892) on Calbindin
with a threshold of 1e−05
rs2387977
rs7303158
rs55877868
rs2090816
rs78383039
rs6809952
rs8039785
rs7572229
rs7311004
rs6828768
rs823424
rs10899911
rs35055552
rs62273907
All
−0.2
0.0
0.2
MR effect size for
'Gut microbiota abundance (genus Parasutterella id.2892)' on 'Calbindin'

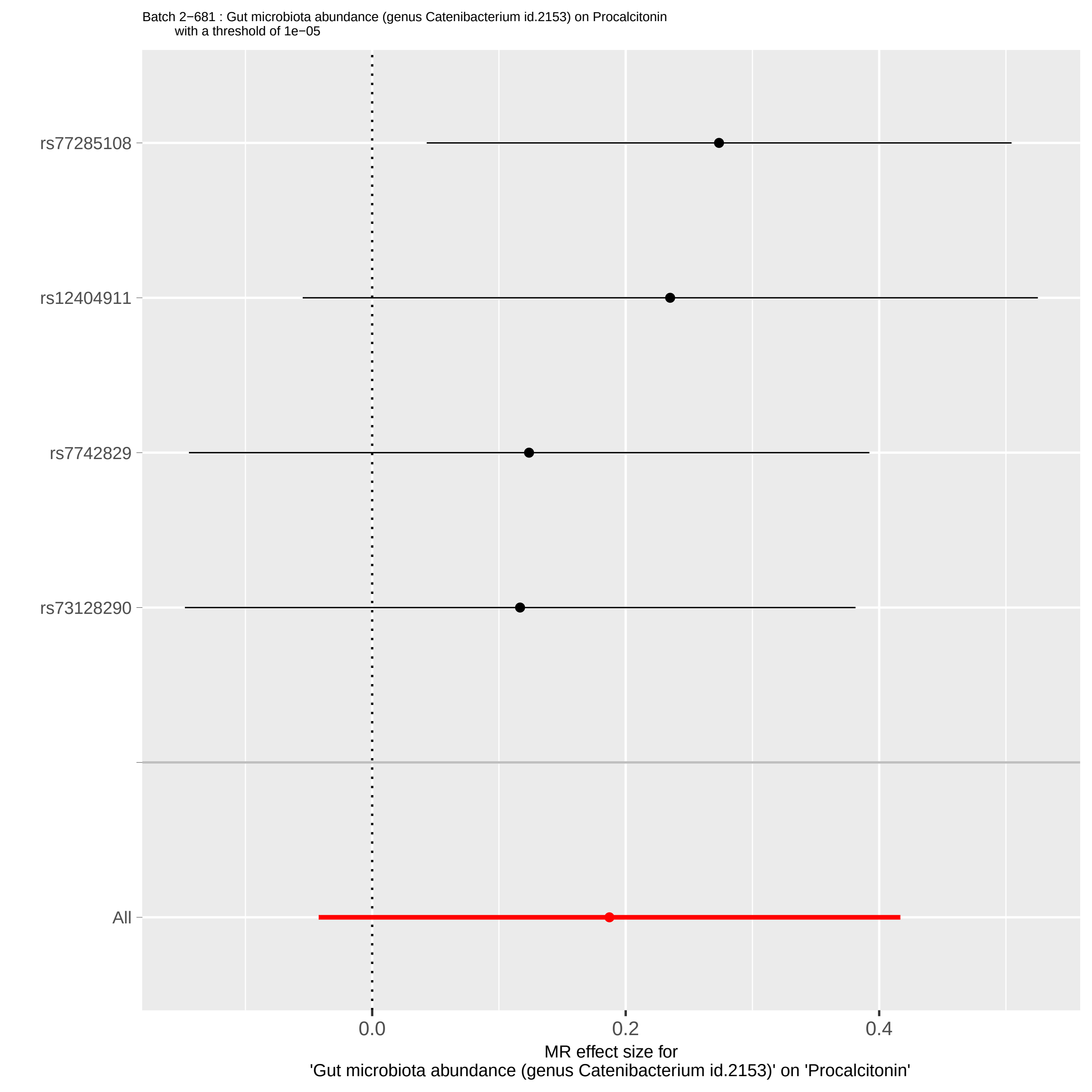
Batch 2−681 : Gut microbiota abundance (genus Catenibacterium id.2153) on Procalcitonin
with a threshold of 1e−05
rs77285108
rs12404911
rs7742829
rs73128290
All
0.0
0.2
0.4
MR effect size for
'Gut microbiota abundance (genus Catenibacterium id.2153)' on 'Procalcitonin'

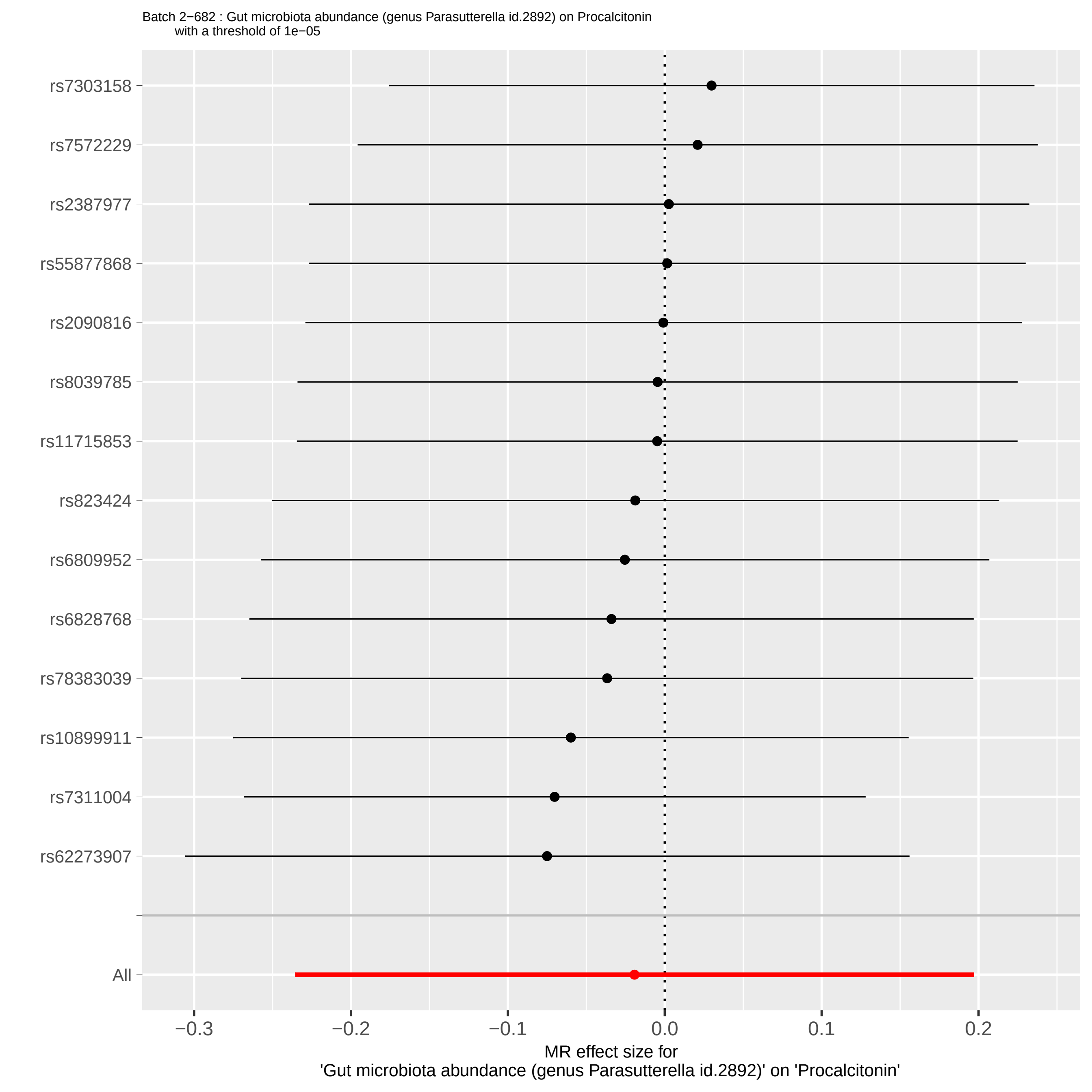
Batch 2−682 : Gut microbiota abundance (genus Parasutterella id.2892) on Procalcitonin
with a threshold of 1e−05
rs7303158
rs7572229
rs2387977
rs55877868
rs2090816
rs8039785
rs11715853
rs823424
rs6809952
rs6828768
rs78383039
rs10899911
rs7311004
rs62273907
All
−0.3
−0.2
−0.1
0.0
0.1
0.2
MR effect size for
'Gut microbiota abundance (genus Parasutterella id.2892)' on 'Procalcitonin'

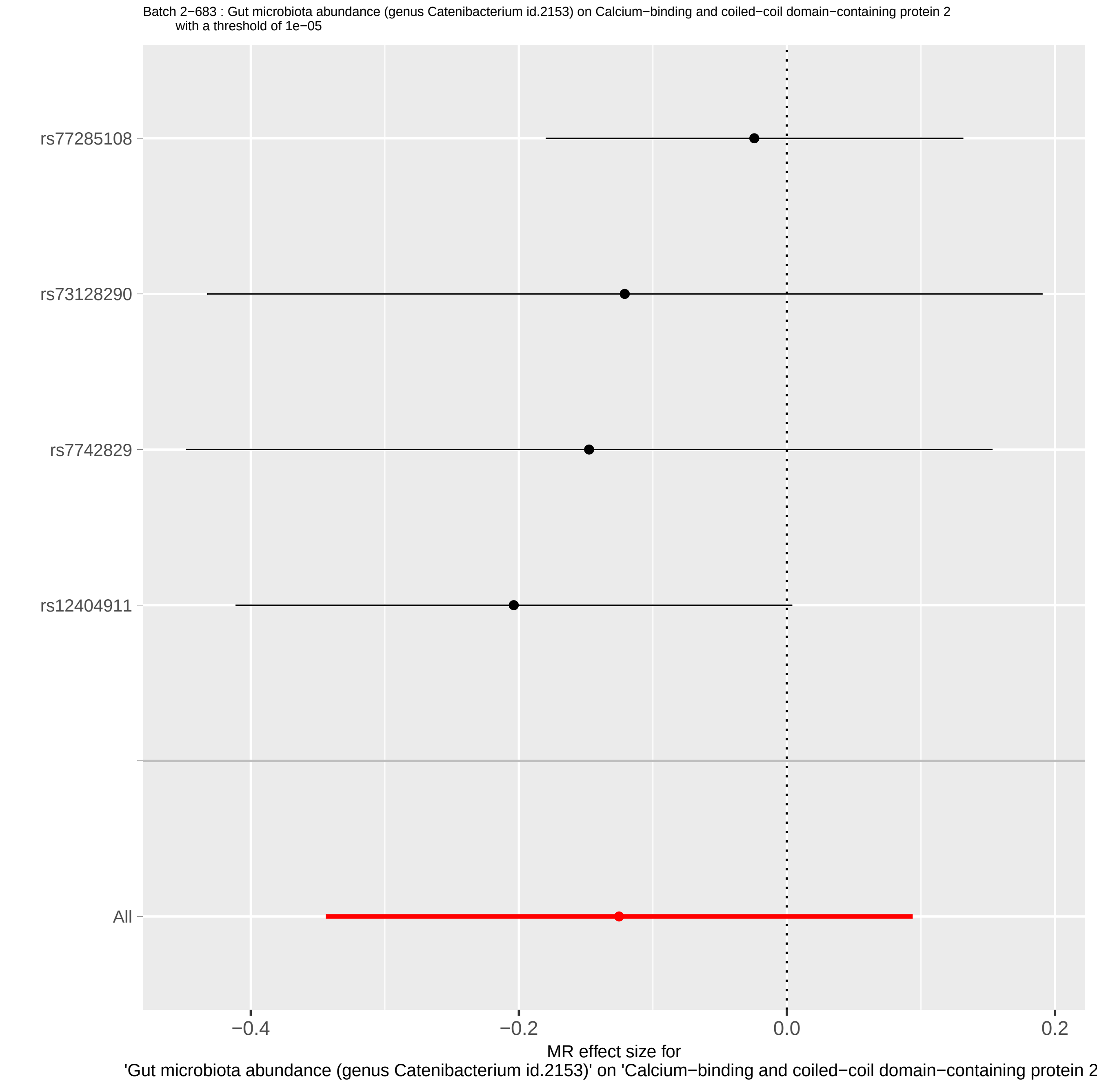

Batch 2−683 : Gut microbiota abundance (genus Catenibacterium id.2153) on Calcium−binding and coiled−coil domain−containing protein 2
with a threshold of 1e−05
rs77285108
rs73128290
rs7742829
rs12404911
All
−0.4
−0.2
0.0
0.2
MR effect size for
'Gut microbiota abundance (genus Catenibacterium id.2153)' on 'Calcium−binding and coiled−coil domain−containing protein 2

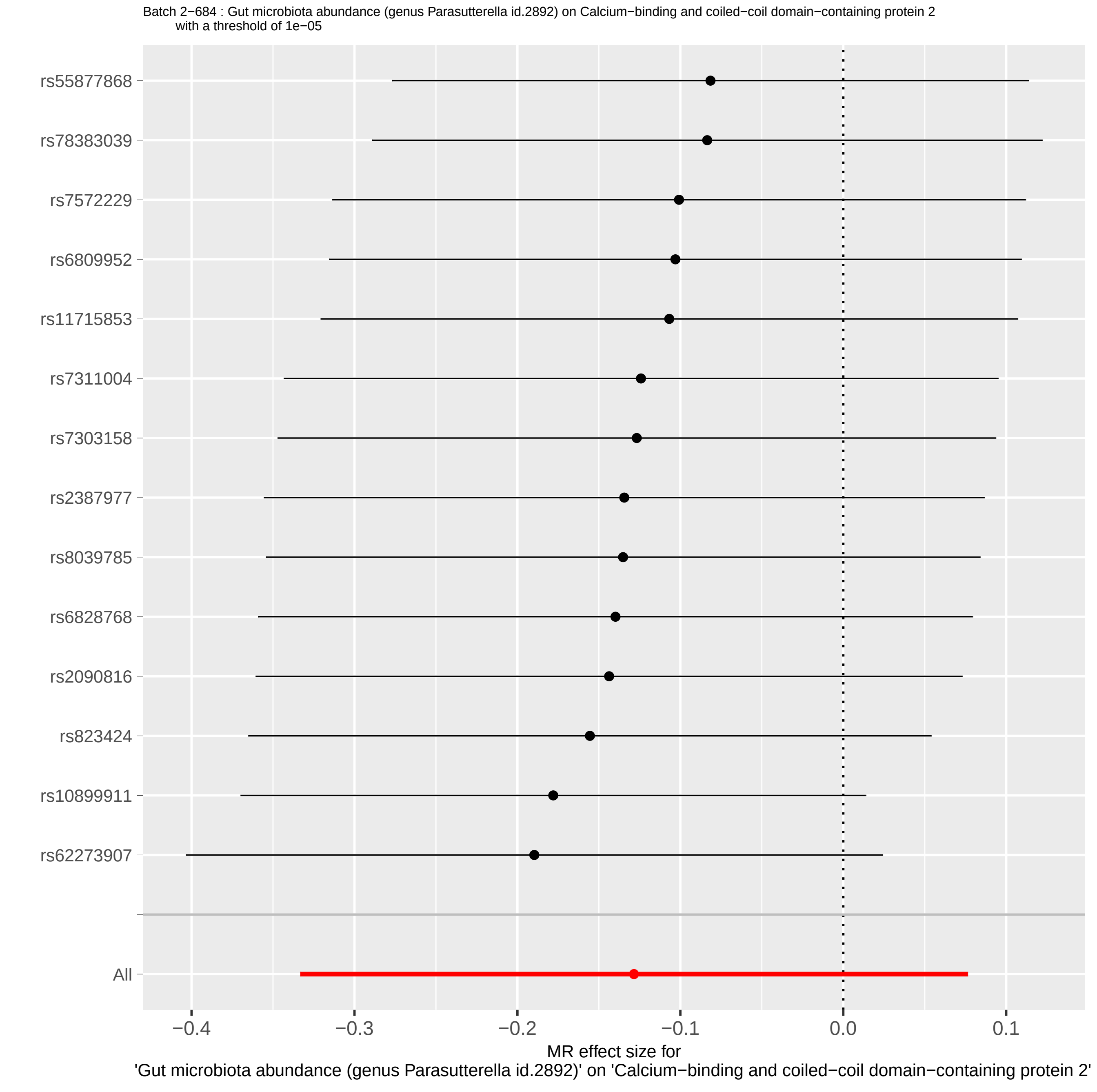
Batch 2−684 : Gut microbiota abundance (genus Parasutterella id.2892) on Calcium−binding and coiled−coil domain−containing protein 2
with a threshold of 1e−05
rs55877868
rs78383039
rs7572229
rs6809952
rs11715853
rs7311004
rs7303158
rs2387977
rs8039785
rs6828768
rs2090816
rs823424
rs10899911
rs62273907
All
−0.4
−0.3
−0.2
−0.1
0.0
0.1
MR effect size for
'Gut microbiota abundance (genus Parasutterella id.2892)' on 'Calcium−binding and coiled−coil domain−containing protein 2'

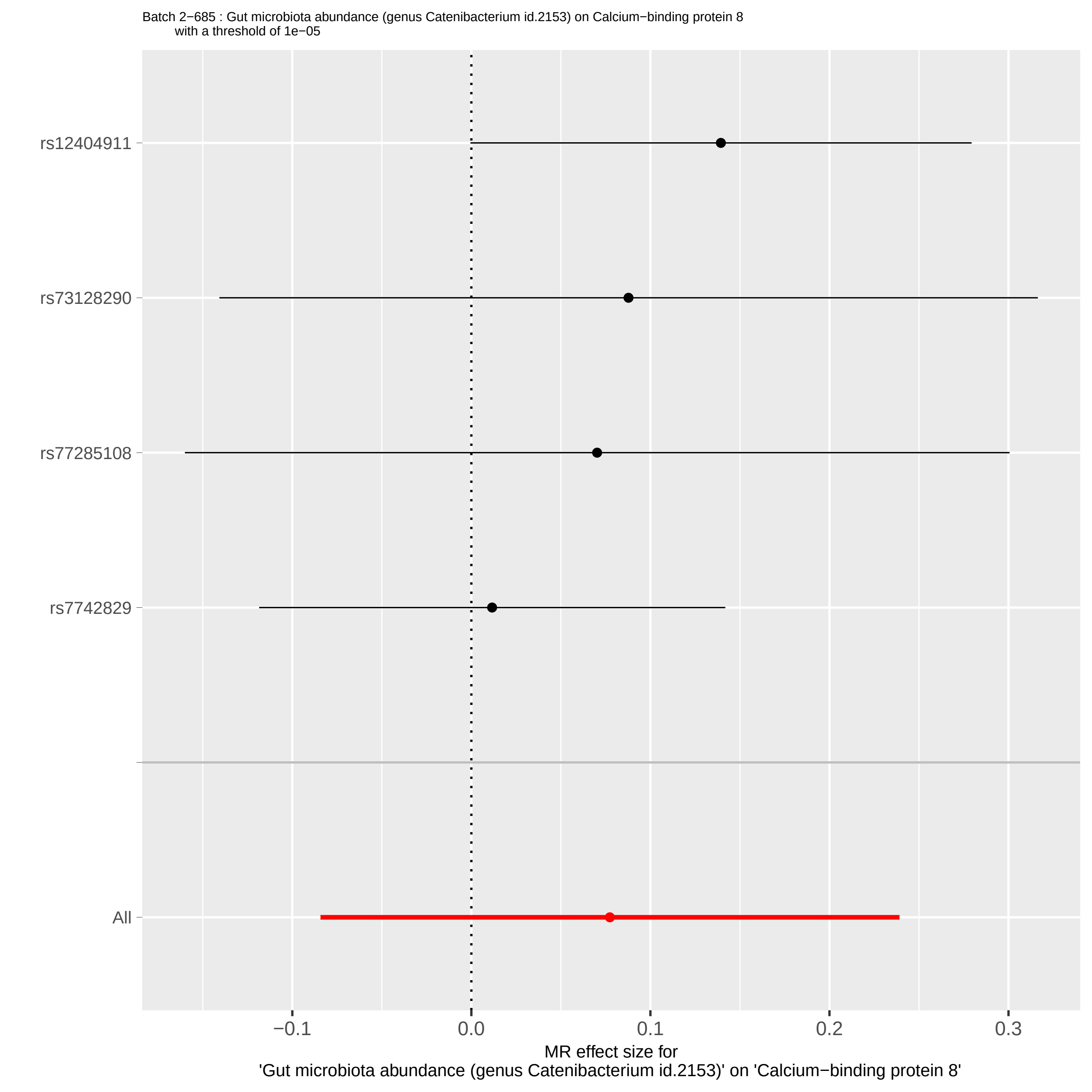

Batch 2−685 : Gut microbiota abundance (genus Catenibacterium id.2153) on Calcium−binding protein 8
with a threshold of 1e−05
rs12404911
rs73128290
rs77285108
rs7742829
All
−0.1
0.0
0.1
0.2
0.3
MR effect size for
'Gut microbiota abundance (genus Catenibacterium id.2153)' on 'Calcium−binding protein 8'

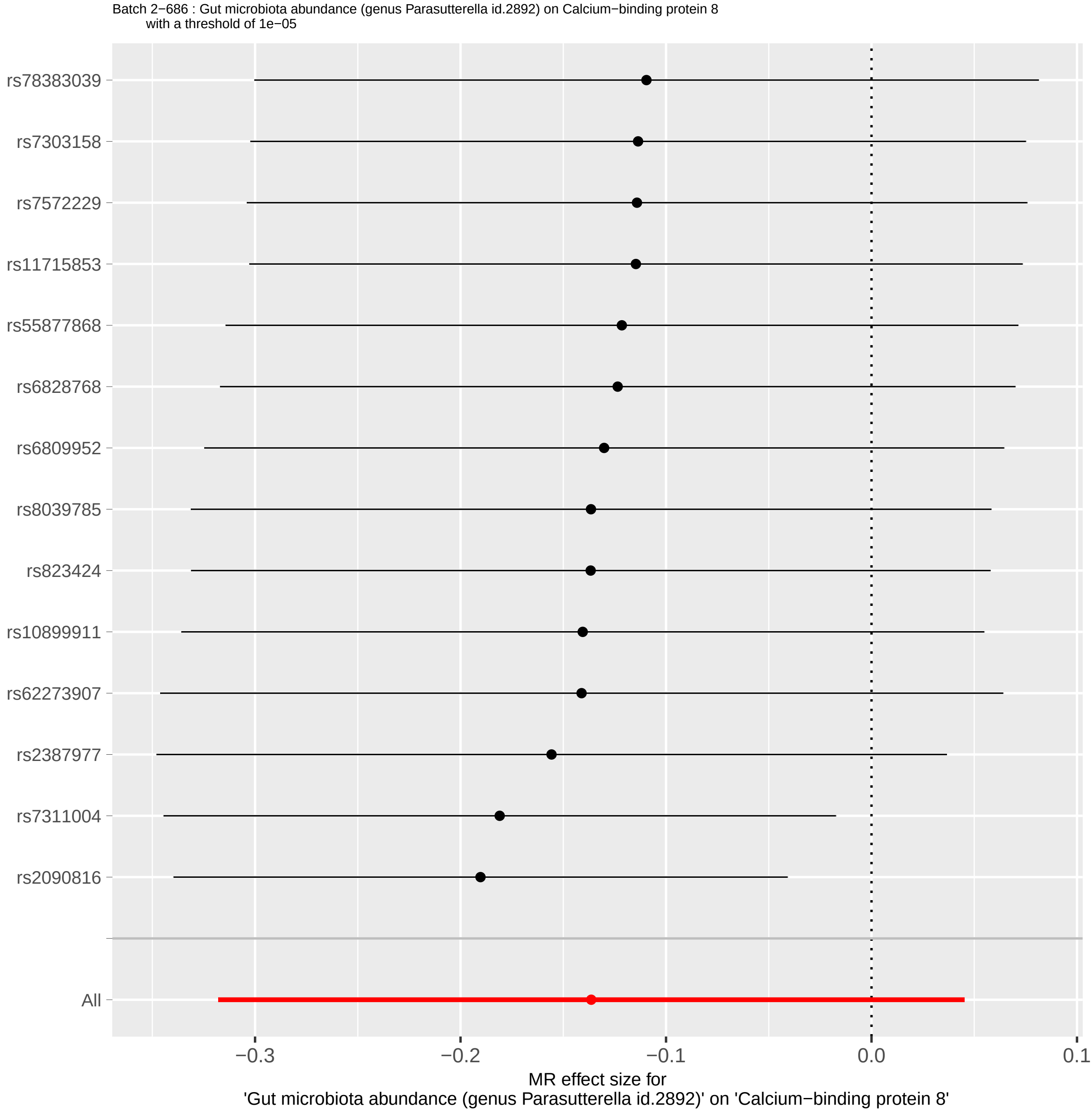
Batch 2−686 : Gut microbiota abundance (genus Parasutterella id.2892) on Calcium−binding protein 8
with a threshold of 1e−05
rs78383039
rs7303158
rs7572229
rs11715853
rs55877868
rs6828768
rs6809952
rs8039785
rs823424
rs10899911
rs62273907
rs2387977
rs7311004
rs2090816
All
−0.3
−0.2
−0.1
0.0
0.1
MR effect size for
'Gut microbiota abundance (genus Parasutterella id.2892)' on 'Calcium−binding protein 8'

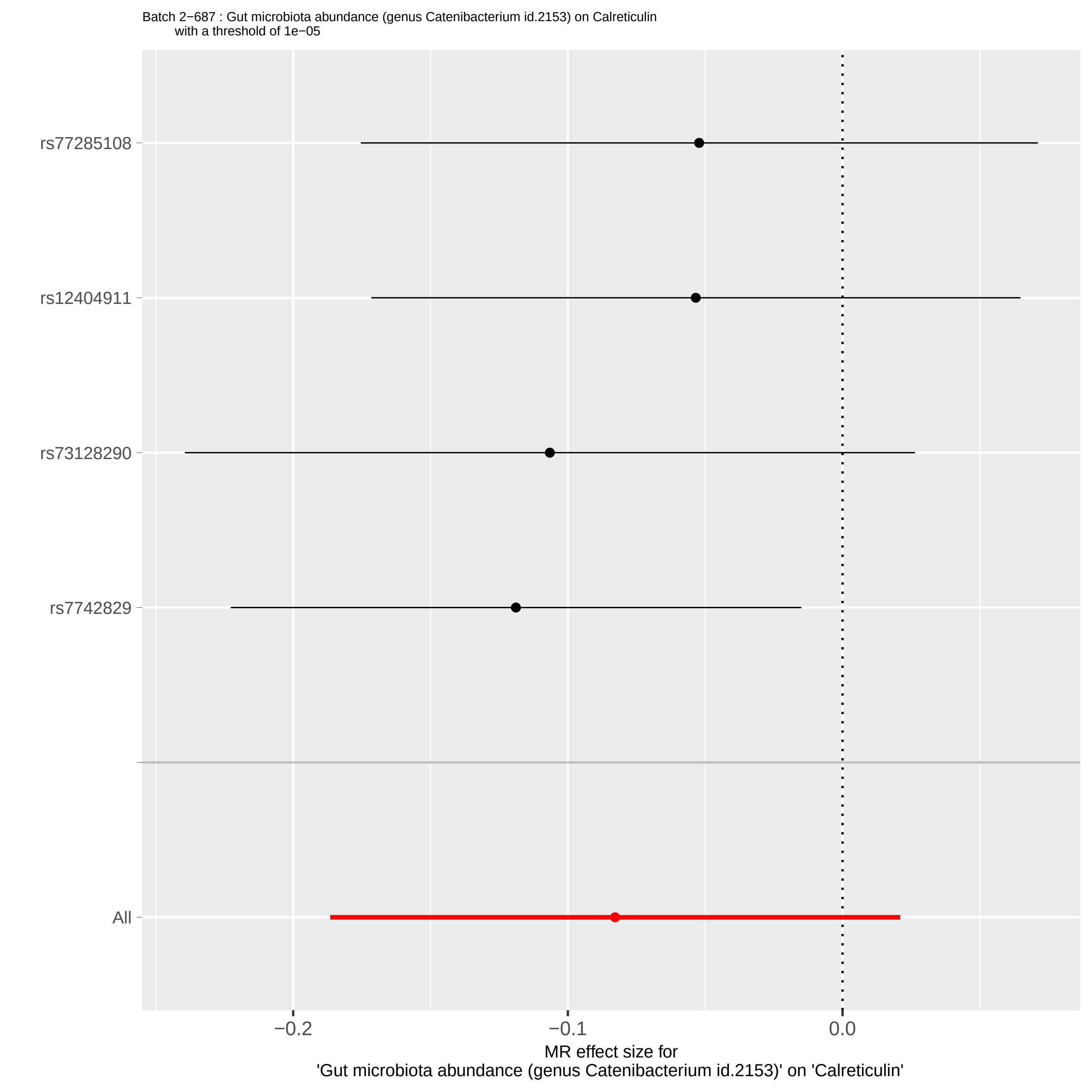
Batch 2−687 : Gut microbiota abundance (genus Catenibacterium id.2153) on Calreticulin
with a threshold of 1e−05
rs77285108
rs12404911
rs73128290
rs7742829
All
−0.2
−0.1
0.0
MR effect size for
'Gut microbiota abundance (genus Catenibacterium id.2153)' on 'Calreticulin'

Batch 2−688 : Gut microbiota abundance (genus Parasutterella id.2892) on Calreticulin
with a threshold of 1e−05

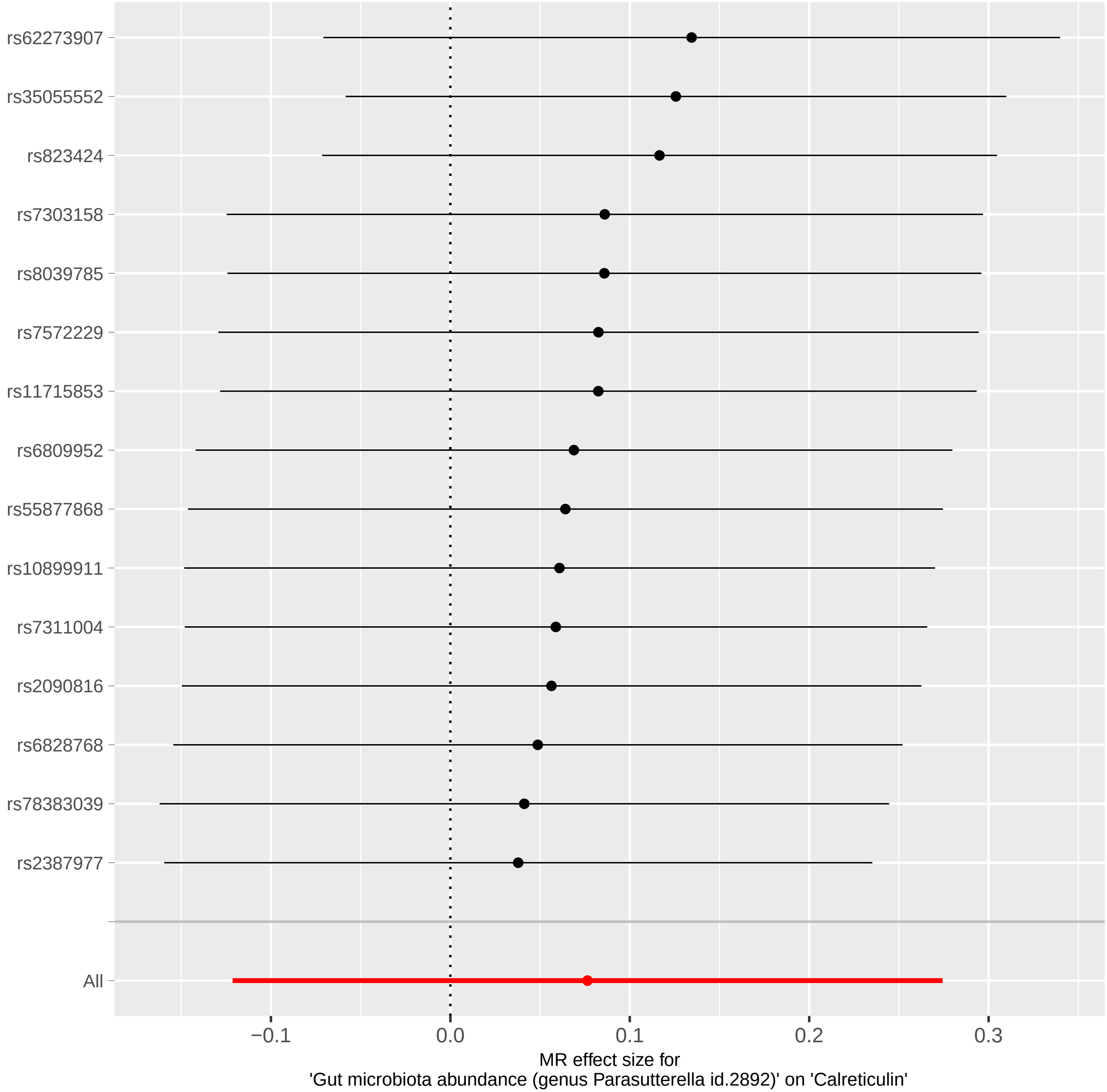

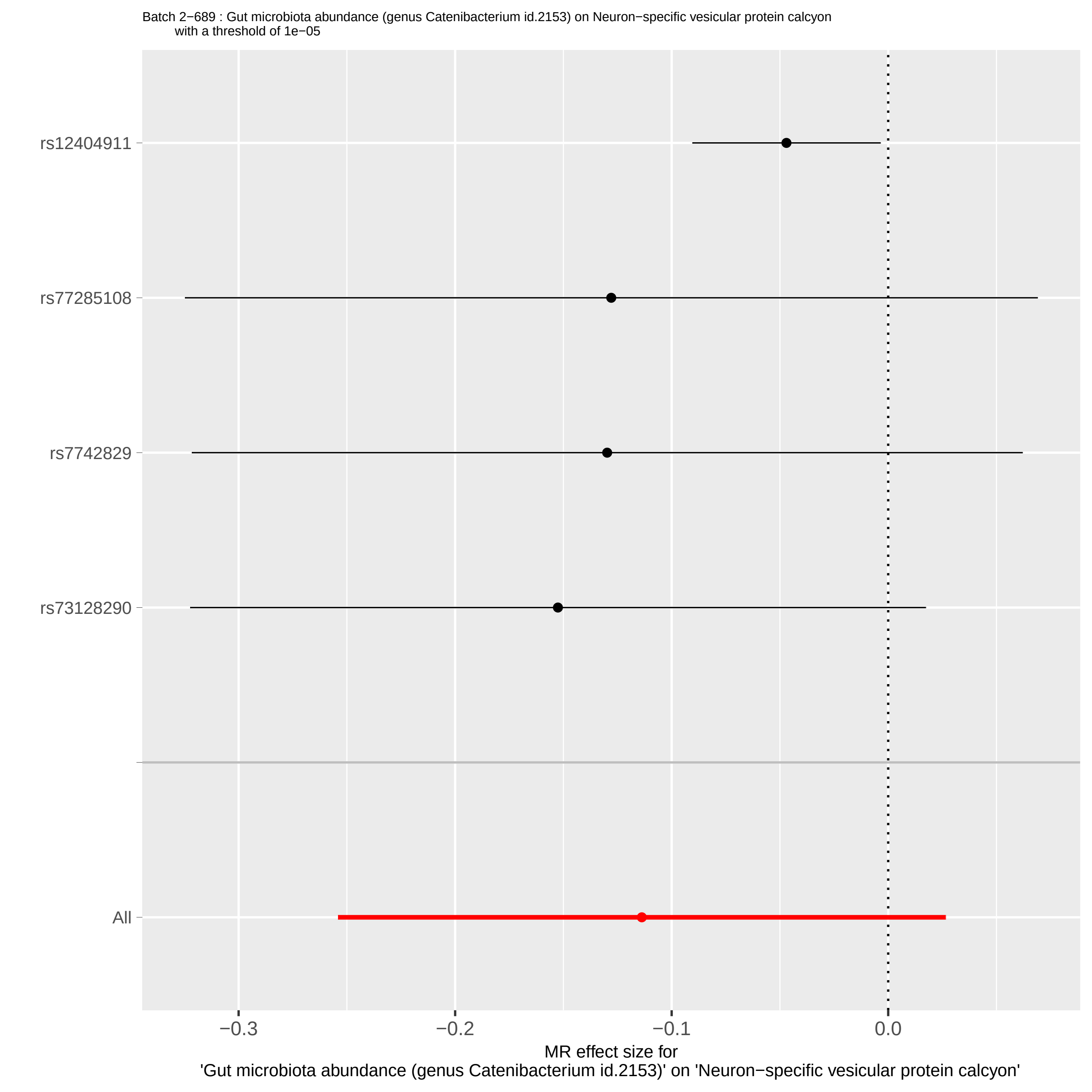

Batch 2−689 : Gut microbiota abundance (genus Catenibacterium id.2153) on Neuron−specific vesicular protein calcyon
with a threshold of 1e−05
rs12404911
rs77285108
rs7742829
rs73128290
All
−0.3
−0.2
−0.1
0.0
MR effect size for
'Gut microbiota abundance (genus Catenibacterium id.2153)' on 'Neuron−specific vesicular protein calcyon'

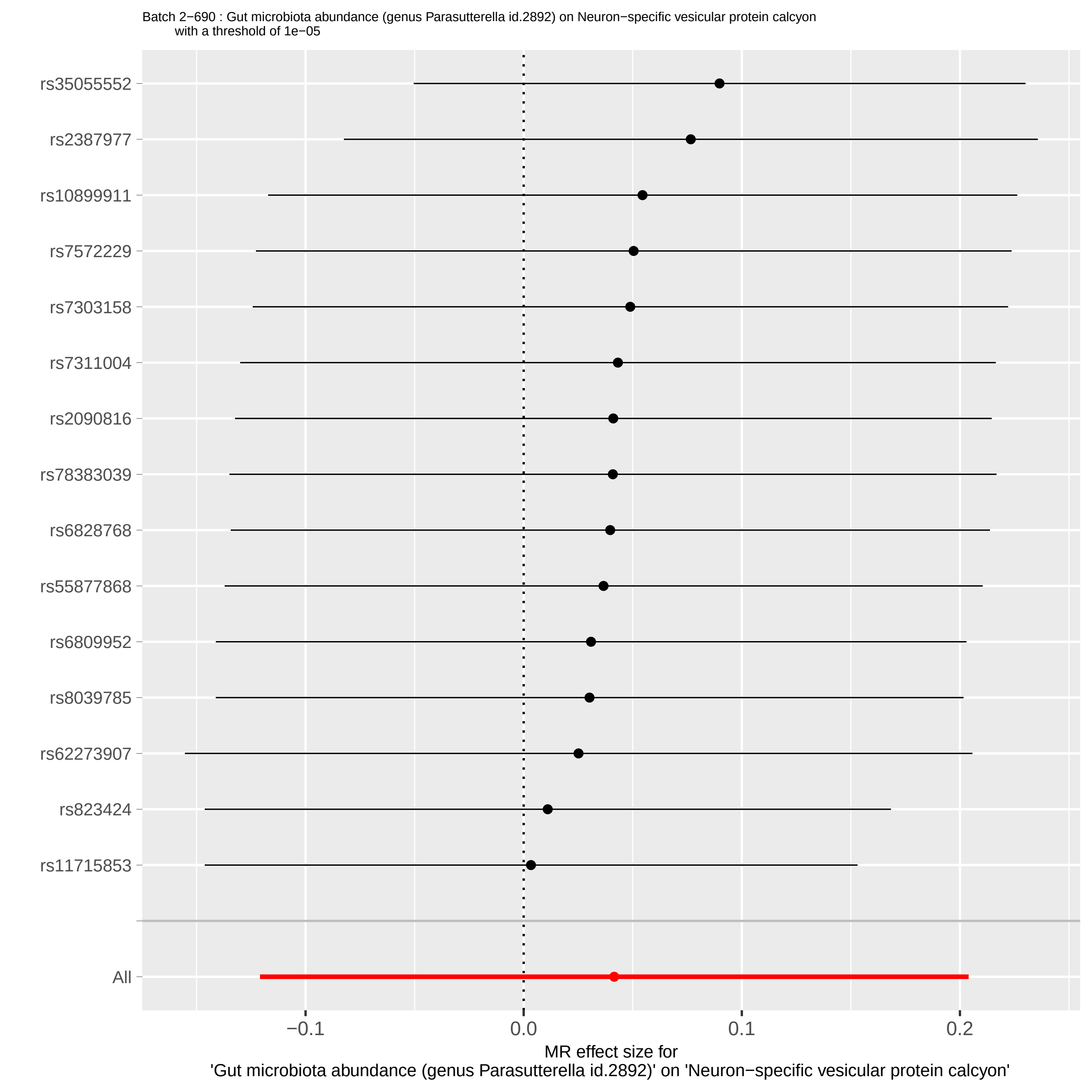

Batch 2−690 : Gut microbiota abundance (genus Parasutterella id.2892) on Neuron−specific vesicular protein calcyon
with a threshold of 1e−05
rs35055552
rs2387977
rs10899911
rs7572229
rs7303158
rs7311004
rs2090816
rs78383039
rs6828768
rs55877868
rs6809952
rs8039785
rs62273907
rs823424
rs11715853
All
−0.1
0.0
0.1
0.2
MR effect size for
'Gut microbiota abundance (genus Parasutterella id.2892)' on 'Neuron−specific vesicular protein calcyon'

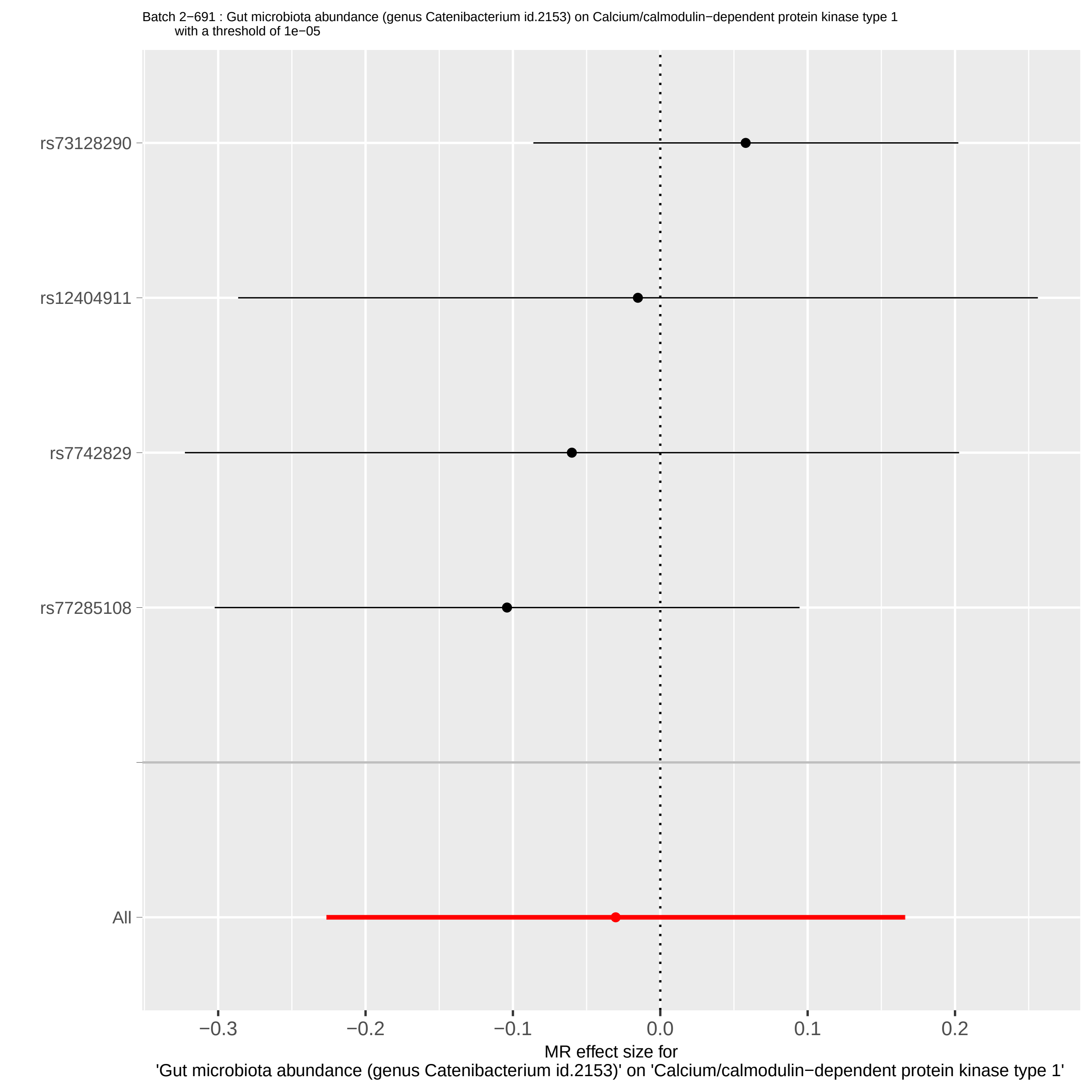

Batch 2−691 : Gut microbiota abundance (genus Catenibacterium id.2153) on Calcium/calmodulin−dependent protein kinase type 1
with a threshold of 1e−05
rs73128290
rs12404911
rs7742829
rs77285108
All
−0.3
−0.2
−0.1
0.0
0.1
0.2
MR effect size for
'Gut microbiota abundance (genus Catenibacterium id.2153)' on 'Calcium/calmodulin−dependent protein kinase type 1'

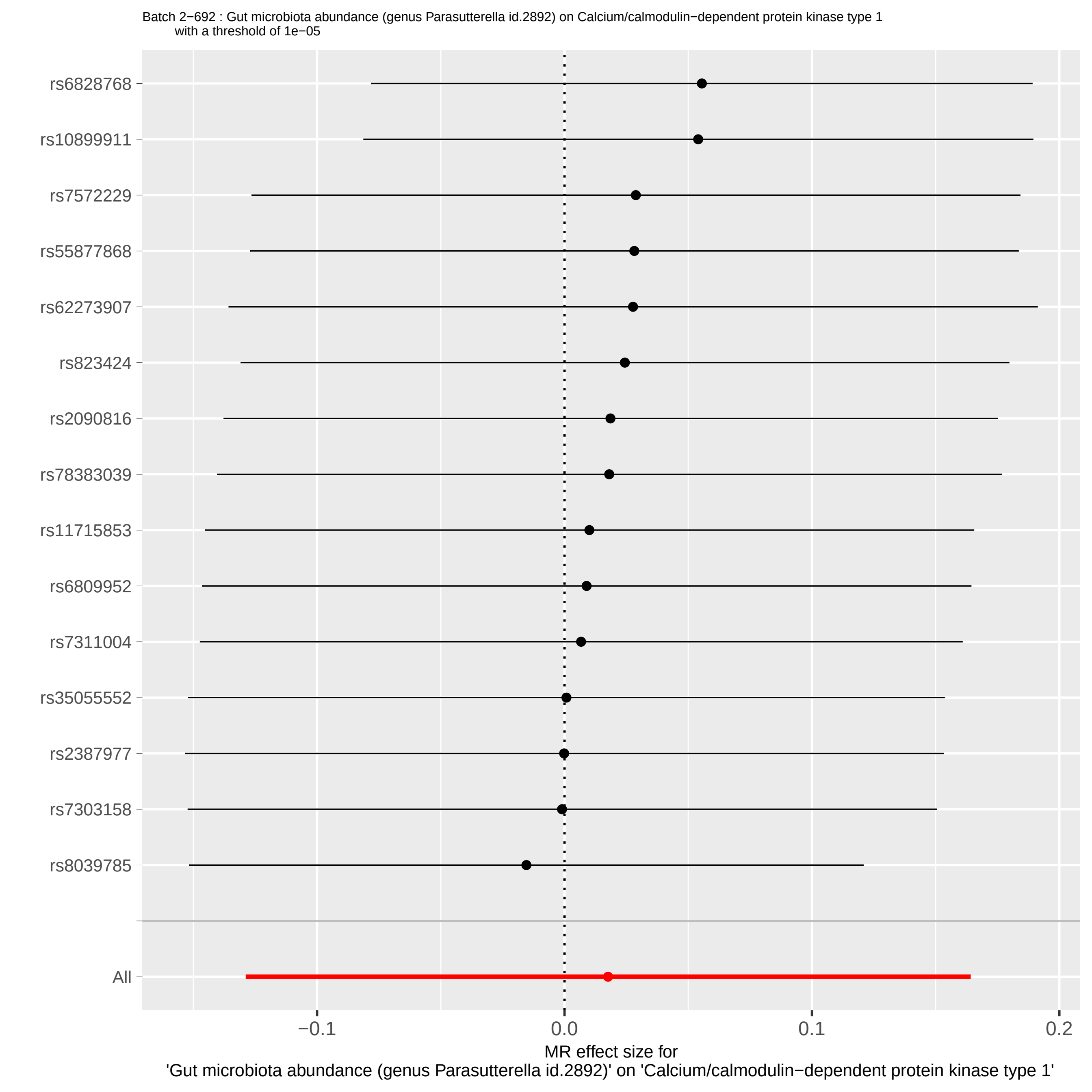

Batch 2−692 : Gut microbiota abundance (genus Parasutterella id.2892) on Calcium/calmodulin−dependent protein kinase type 1
with a threshold of 1e−05
rs6828768
rs10899911
rs7572229
rs55877868
rs62273907
rs823424
rs2090816
rs78383039
rs11715853
rs6809952
rs7311004
rs35055552
rs2387977
rs7303158
rs8039785
All
−0.1
0.0
0.1
0.2
MR effect size for
'Gut microbiota abundance (genus Parasutterella id.2892)' on 'Calcium/calmodulin−dependent protein kinase type 1'

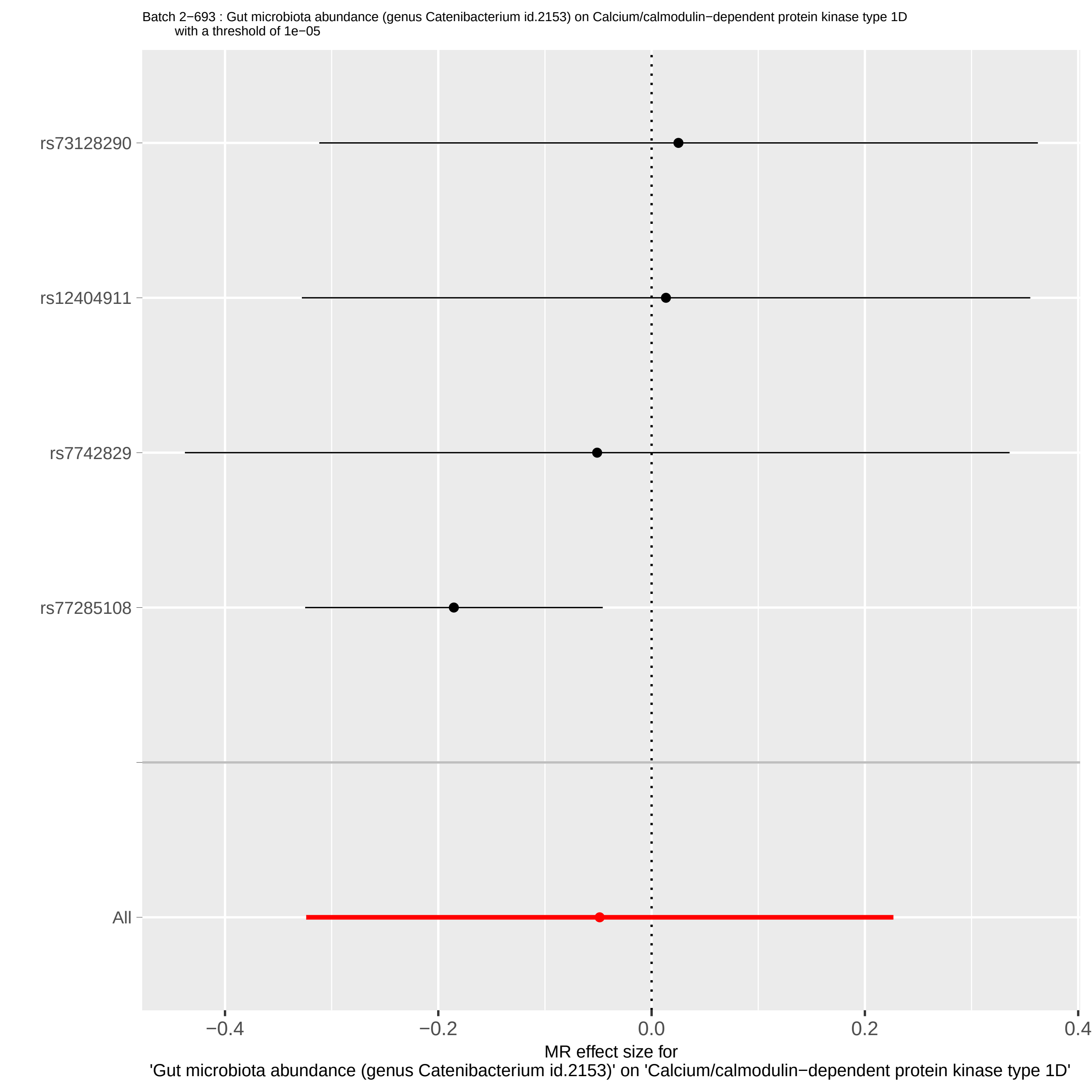

Batch 2−693 : Gut microbiota abundance (genus Catenibacterium id.2153) on Calcium/calmodulin−dependent protein kinase type 1D
with a threshold of 1e−05
rs73128290
rs12404911
rs7742829
rs77285108
All
−0.4
−0.2
0.0
0.2
0.4
MR effect size for
'Gut microbiota abundance (genus Catenibacterium id.2153)' on 'Calcium/calmodulin−dependent protein kinase type 1D'

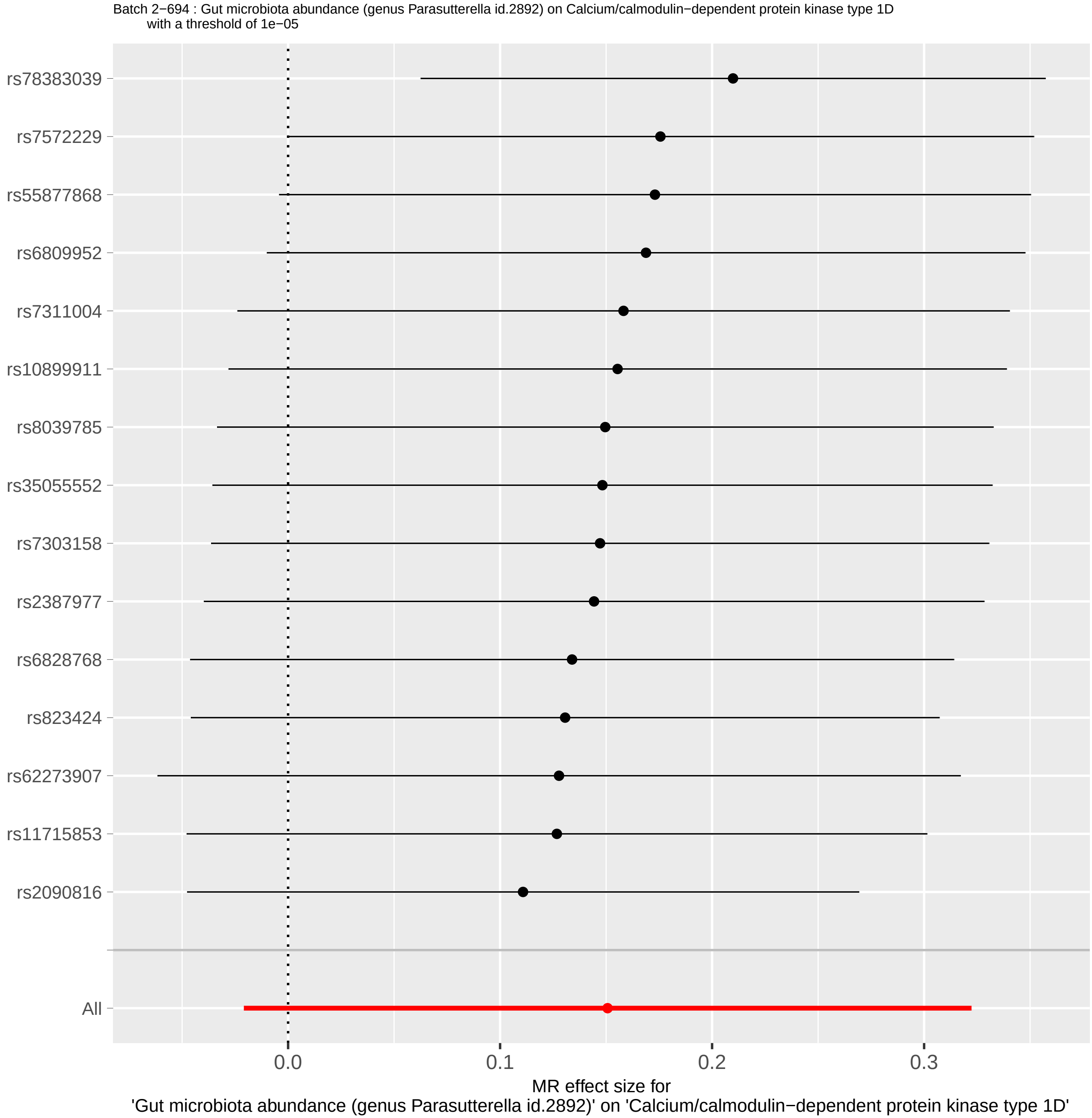

Batch 2−694 : Gut microbiota abundance (genus Parasutterella id.2892) on Calcium/calmodulin−dependent protein kinase type 1D
with a threshold of 1e−05
rs78383039
rs7572229
rs55877868
rs6809952
rs7311004
rs10899911
rs8039785
rs35055552
rs7303158
rs2387977
rs6828768
rs823424
rs62273907
rs11715853
rs2090816
All
0.0
0.1
0.2
0.3
MR effect size for
'Gut microbiota abundance (genus Parasutterella id.2892)' on 'Calcium/calmodulin−dependent protein kinase type 1D'

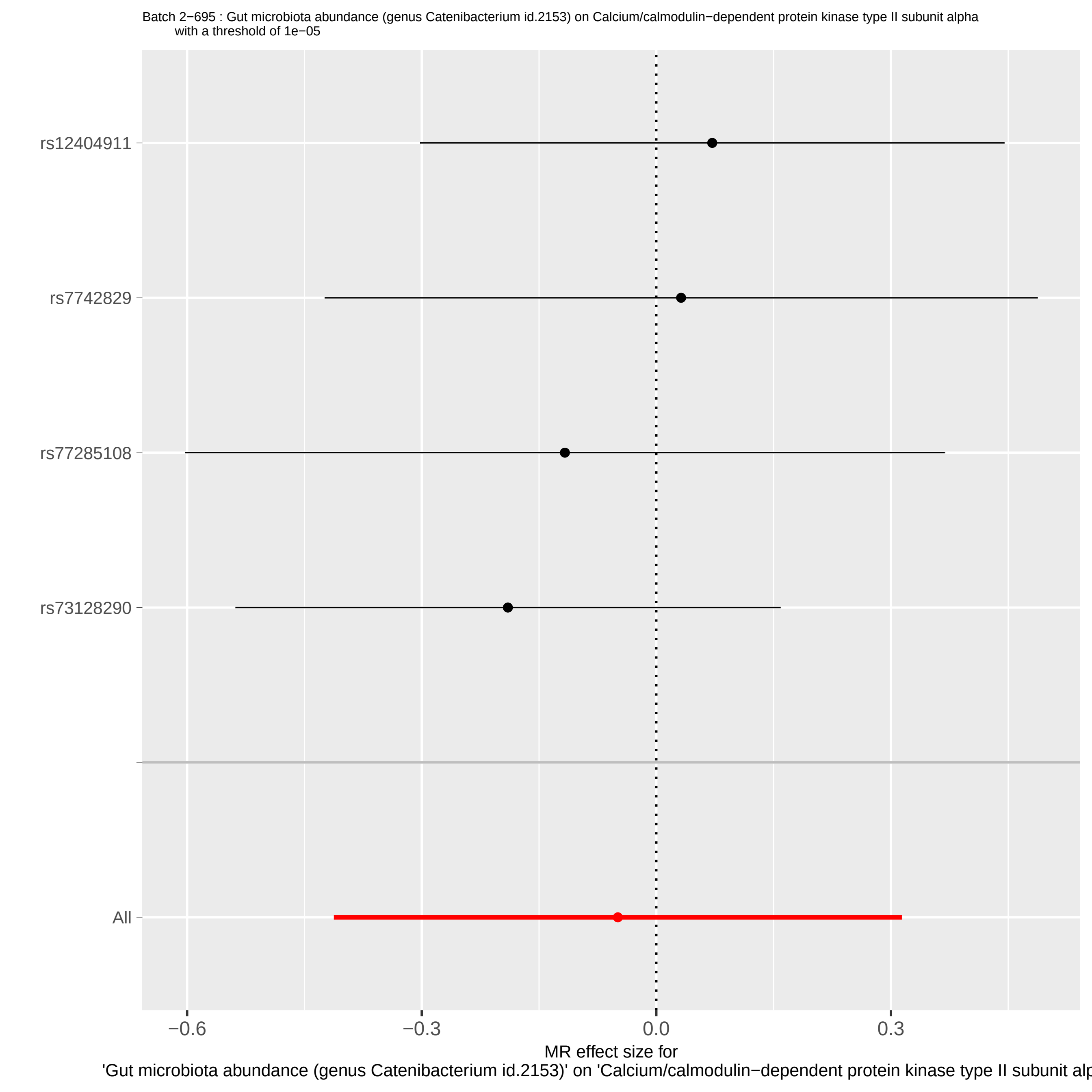
Batch 2−695 : Gut microbiota abundance (genus Catenibacterium id.2153) on Calcium/calmodulin−dependent protein kinase type II subunit alpha
with a threshold of 1e−05
rs12404911
rs7742829
rs77285108
rs73128290
All
−0.6
−0.3
0.0
0.3
MR effect size for
'Gut microbiota abundance (genus Catenibacterium id.2153)' on 'Calcium/calmodulin−dependent protein kinase type II subunit alp

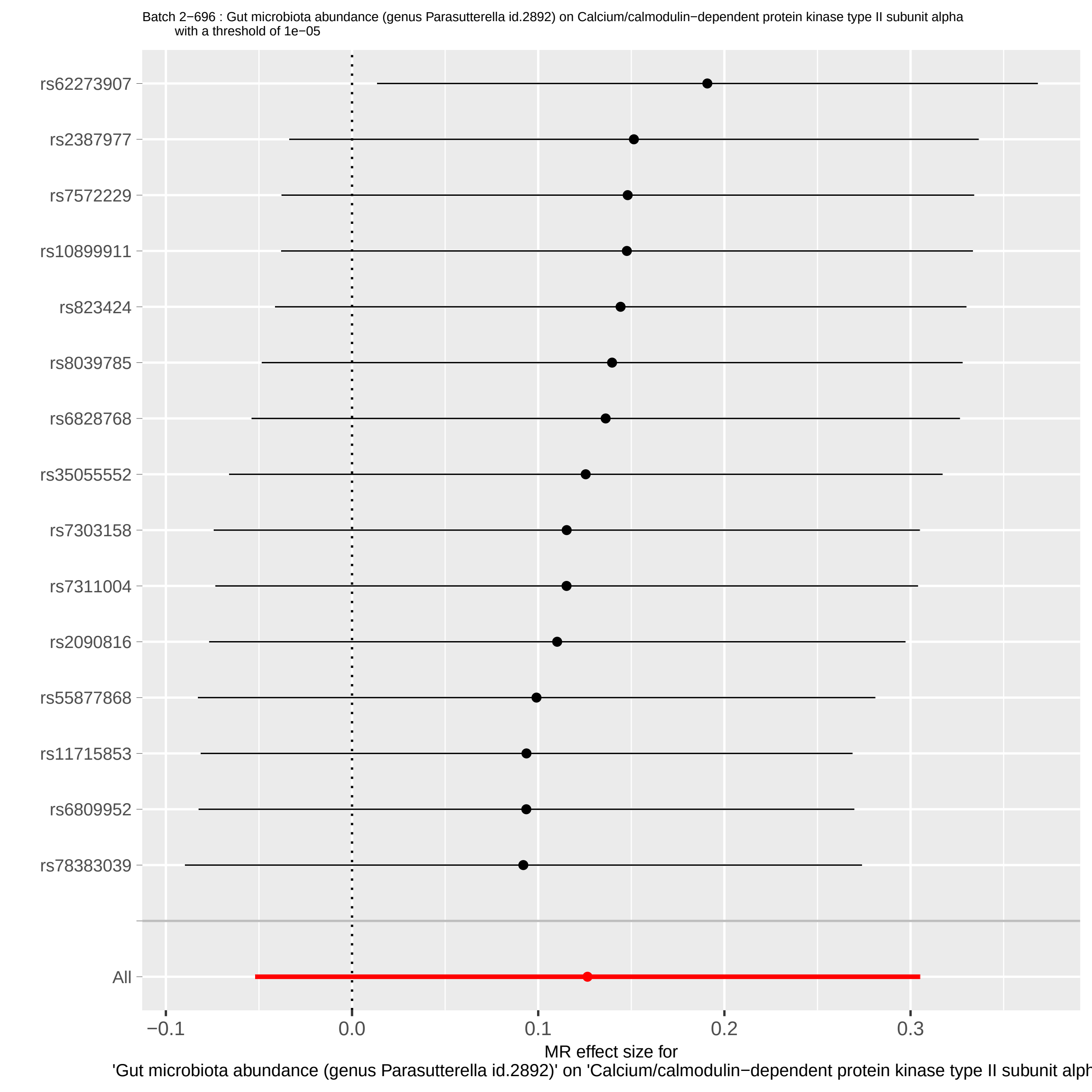

Batch 2−696 : Gut microbiota abundance (genus Parasutterella id.2892) on Calcium/calmodulin−dependent protein kinase type II subunit alpha with a threshold of 1e−05
rs62273907
rs2387977
rs7572229
rs10899911
rs823424
rs8039785
rs6828768
rs35055552
rs7303158
rs7311004
rs2090816
rs55877868
rs11715853
rs6809952
rs78383039
All
−0.1
0.0
0.1
0.2
0.3
MR effect size for
'Gut microbiota abundance (genus Parasutterella id.2892)' on 'Calcium/calmodulin−dependent protein kinase type II subunit alph

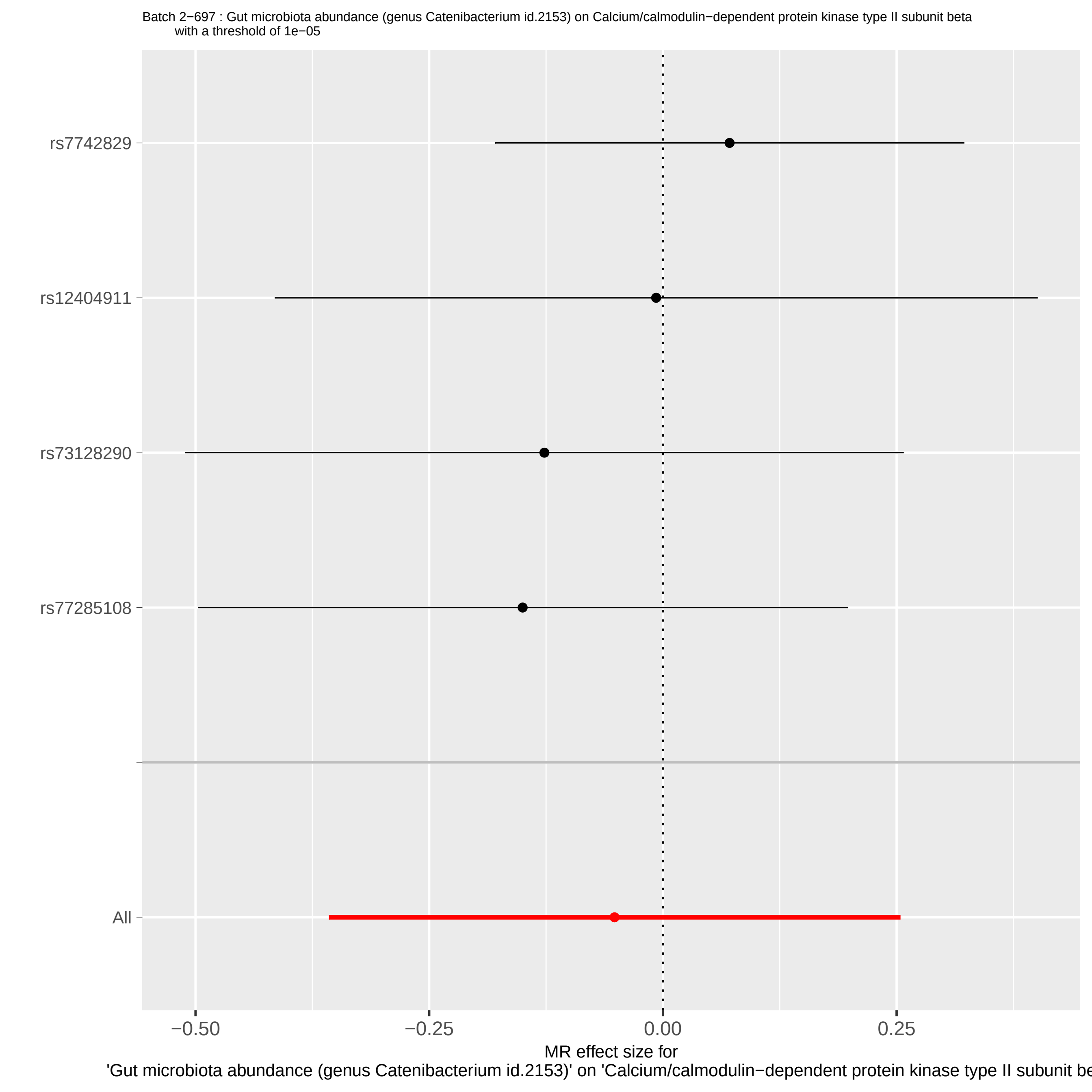
Batch 2−697 : Gut microbiota abundance (genus Catenibacterium id.2153) on Calcium/calmodulin−dependent protein kinase type II subunit beta
with a threshold of 1e−05
rs7742829
rs12404911
rs73128290
rs77285108
All
−0.50
−0.25
0.00
0.25
MR effect size for
'Gut microbiota abundance (genus Catenibacterium id.2153)' on 'Calcium/calmodulin−dependent protein kinase type II subunit be

Batch 2−698 : Gut microbiota abundance (genus Parasutterella id.2892) on Calcium/calmodulin−dependent protein kinase type II subunit beta with a threshold of 1e−05

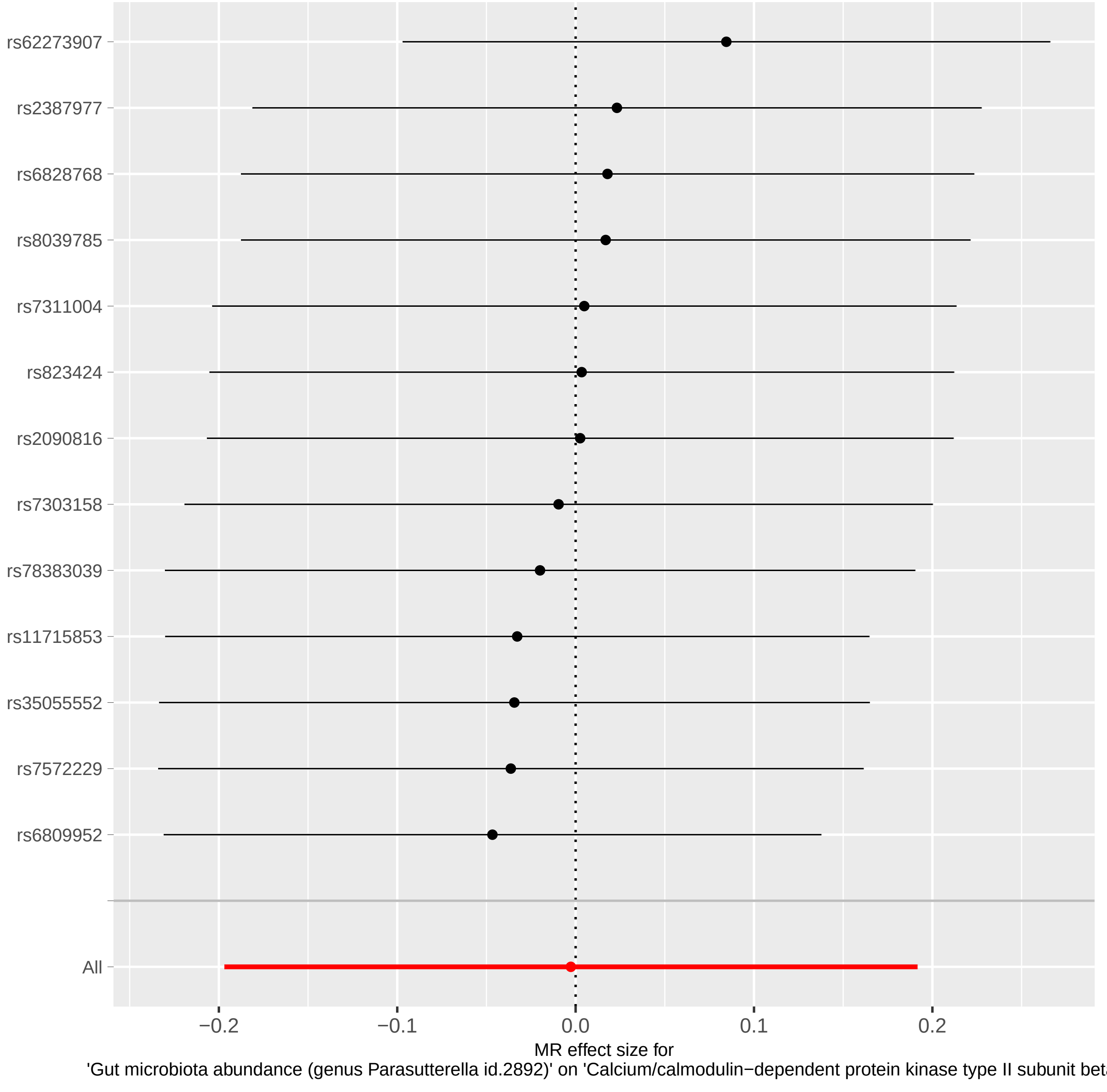

Batch 2−699 : Gut microbiota abundance (genus Catenibacterium id.2153) on Calcium/calmodulin−dependent protein kinase kinase 1
with a threshold of 1e−05

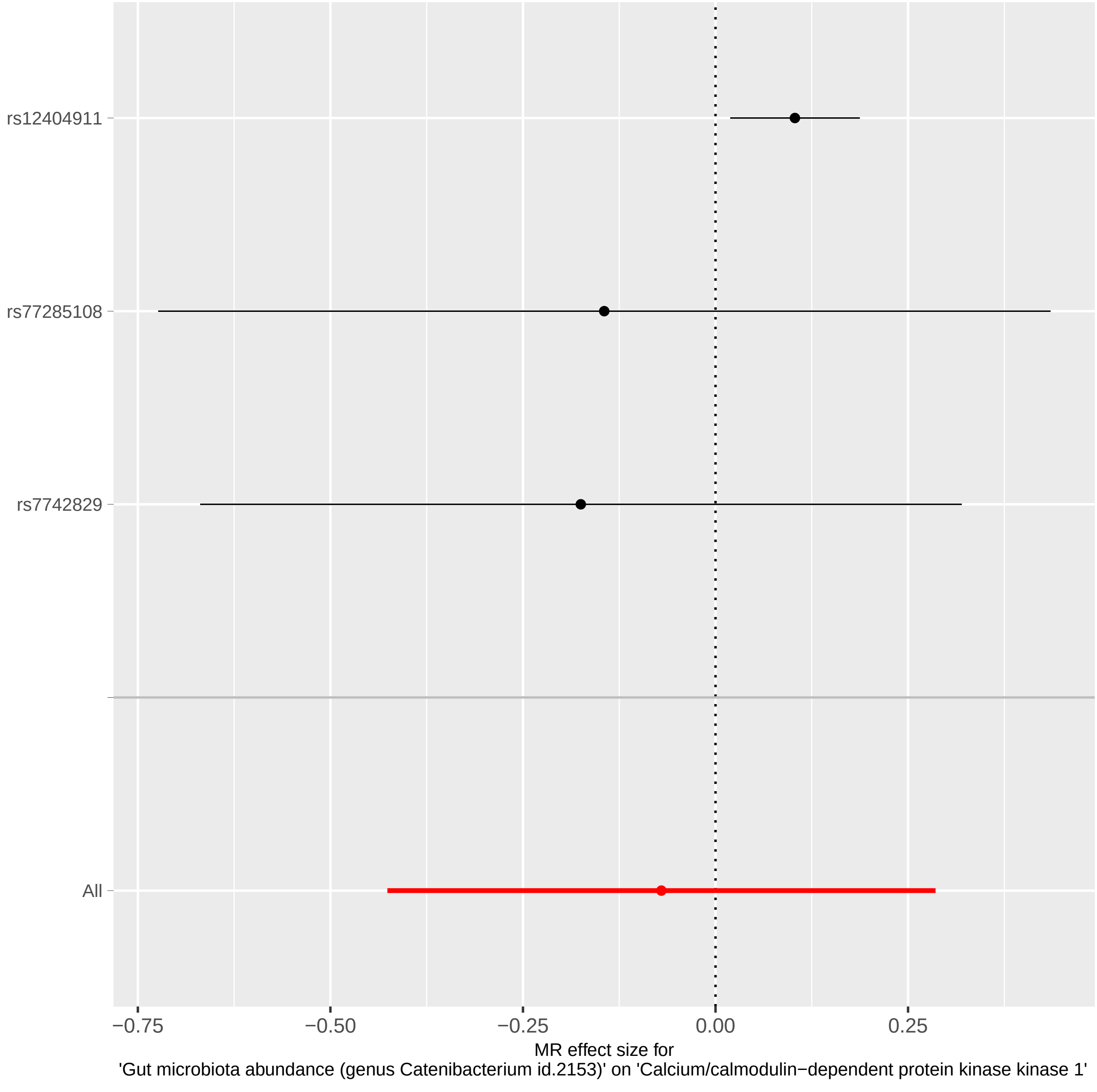

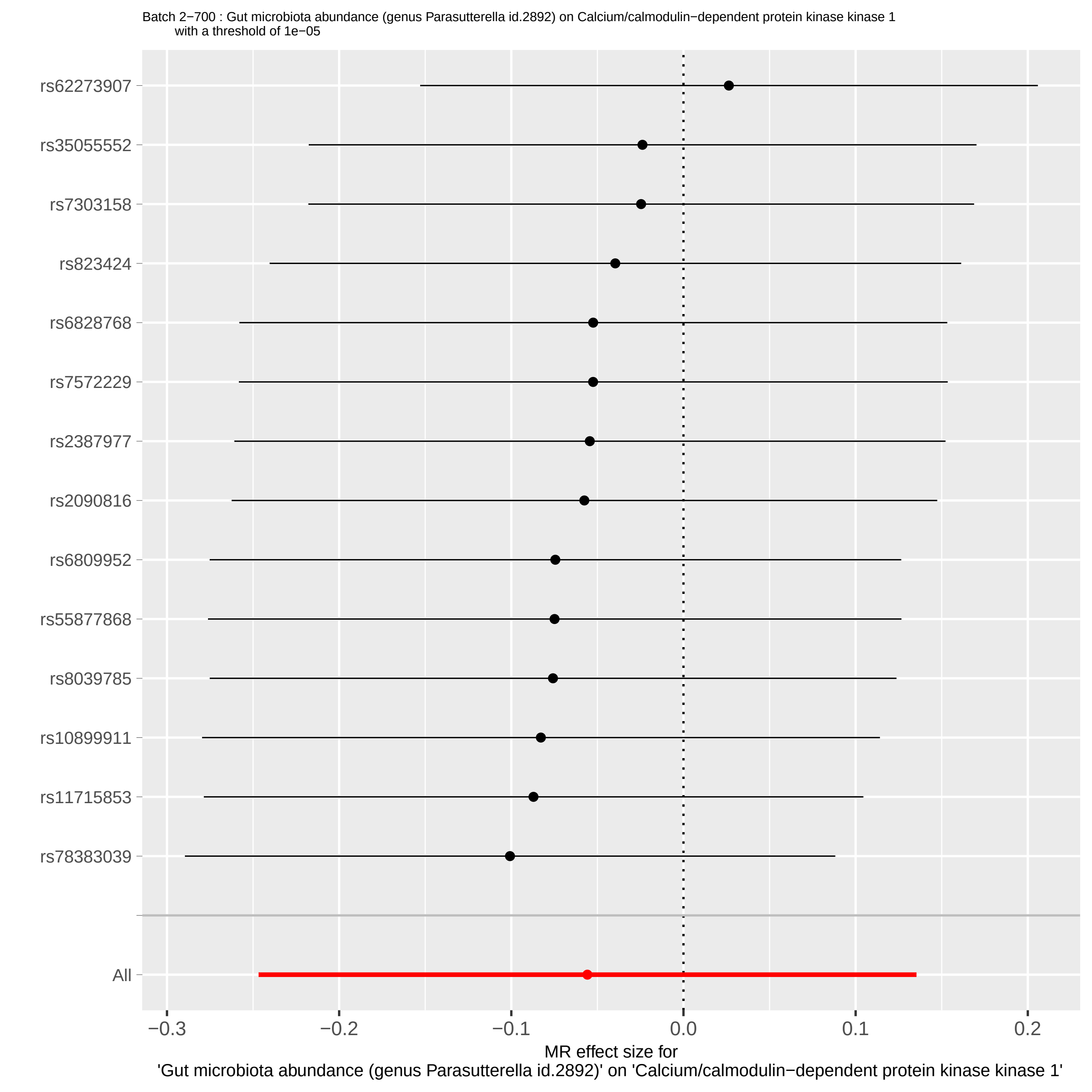
Batch 2−700 : Gut microbiota abundance (genus Parasutterella id.2892) on Calcium/calmodulin−dependent protein kinase kinase 1
with a threshold of 1e−05
rs62273907
rs35055552
rs7303158
rs823424
rs6828768
rs7572229
rs2387977
rs2090816
rs6809952
rs55877868
rs8039785
rs10899911
rs11715853
rs78383039
All
−0.3
−0.2
−0.1
0.0
0.1
0.2
MR effect size for
'Gut microbiota abundance (genus Parasutterella id.2892)' on 'Calcium/calmodulin−dependent protein kinase kinase 1'

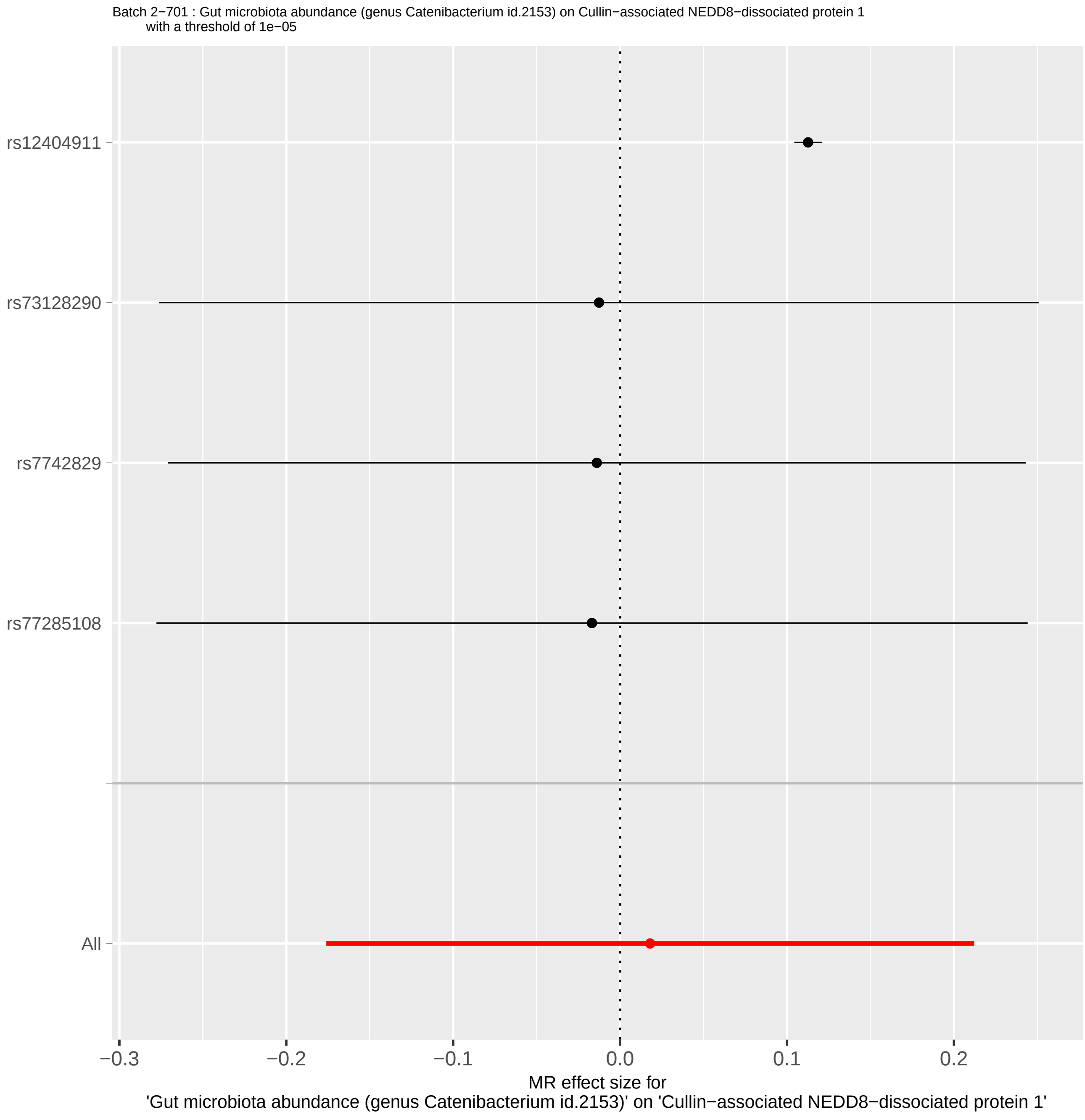
Batch 2−701 : Gut microbiota abundance (genus Catenibacterium id.2153) on Cullin−associated NEDD8−dissociated protein 1
with a threshold of 1e−05
rs12404911
rs73128290
rs7742829
rs77285108
All
−0.3
−0.2
−0.1
0.0
0.1
0.2
MR effect size for
'Gut microbiota abundance (genus Catenibacterium id.2153)' on 'Cullin−associated NEDD8−dissociated protein 1'

Batch 2−702 : Gut microbiota abundance (genus Parasutterella id.2892) on Cullin−associated NEDD8−dissociated protein 1
with a threshold of 1e−05

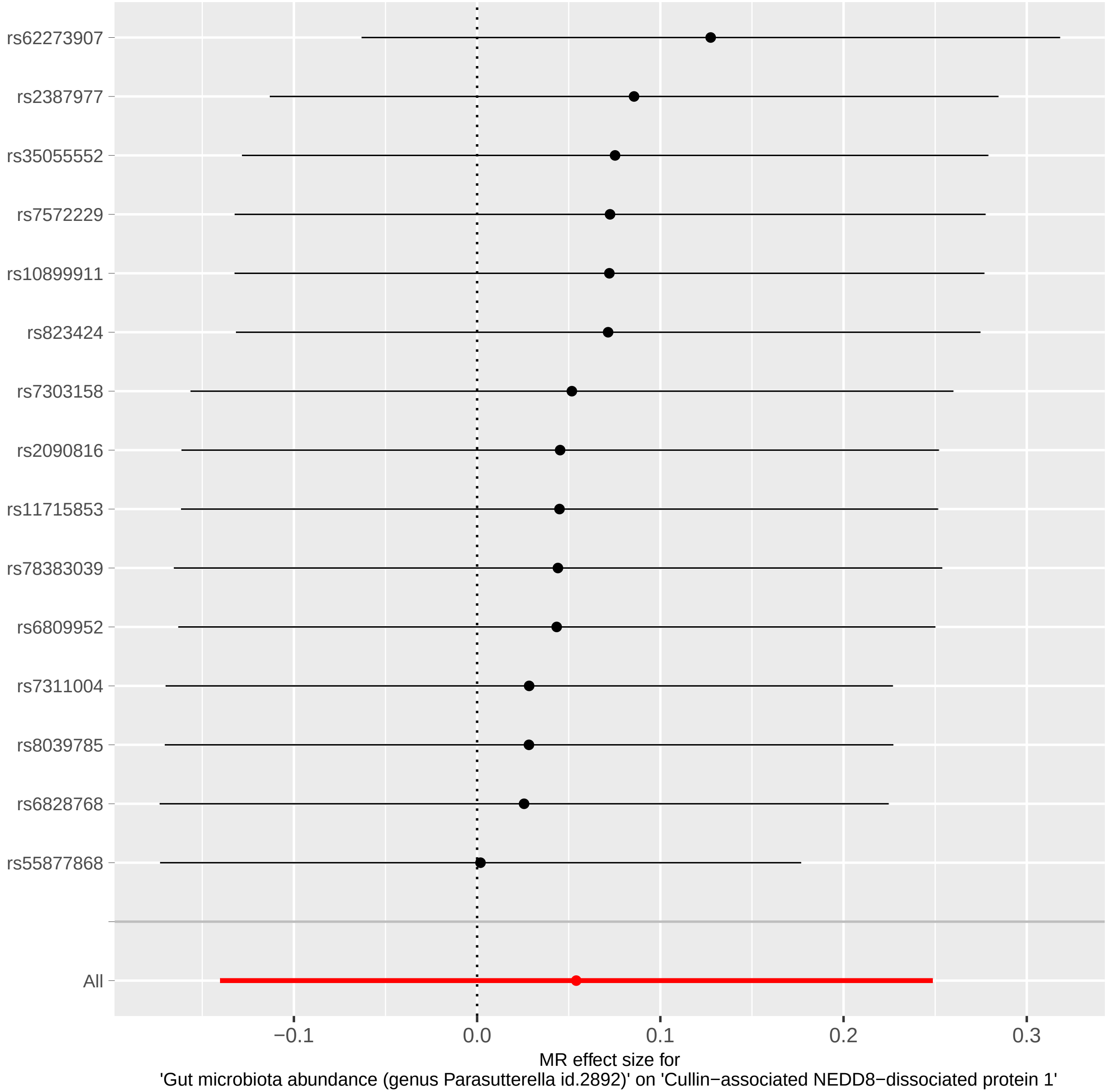

Batch 2−703 : Gut microbiota abundance (genus Catenibacterium id.2153) on Soluble calcium−activated nucleotidase 1
with a threshold of 1e−05

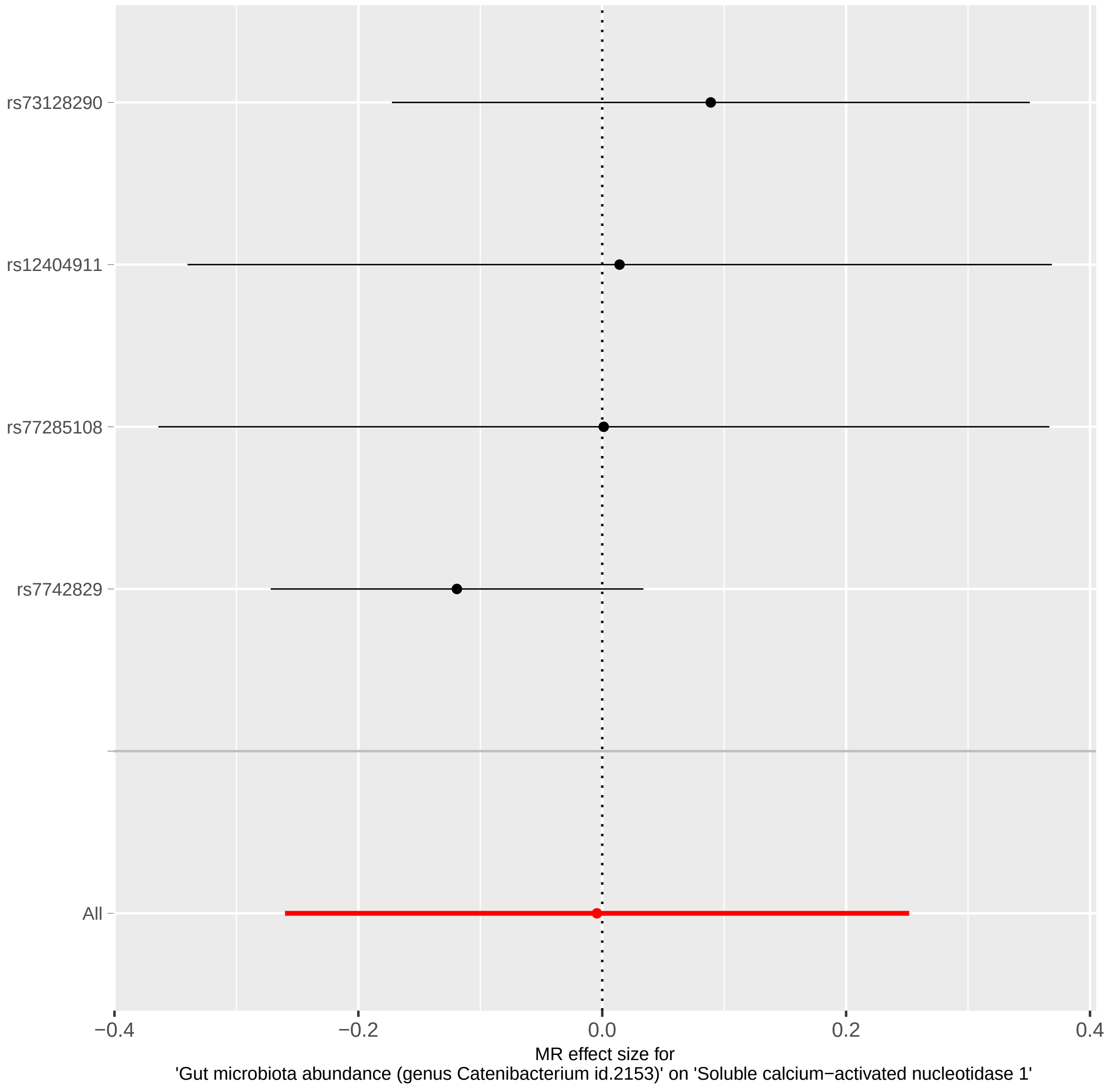

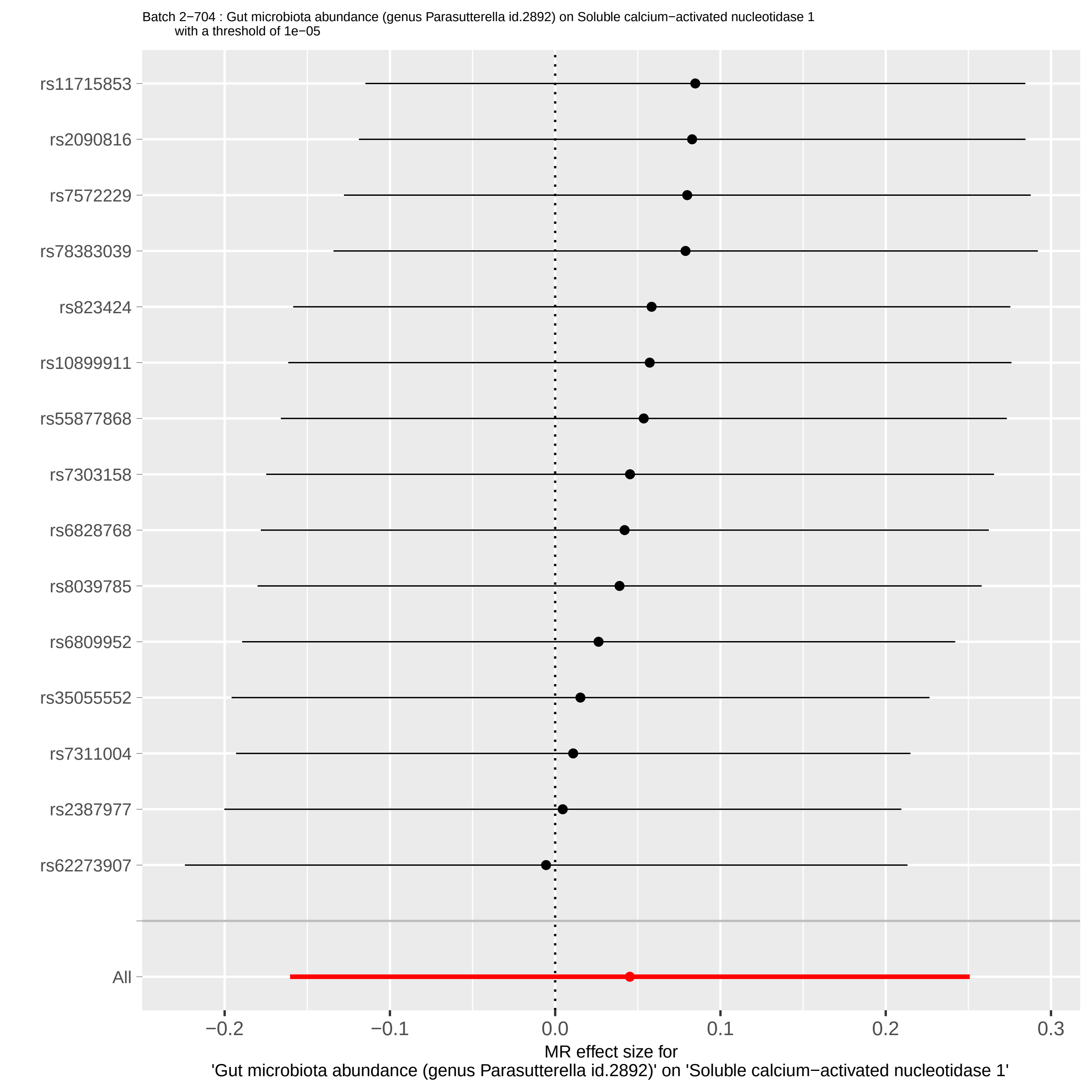

Batch 2−704 : Gut microbiota abundance (genus Parasutterella id.2892) on Soluble calcium−activated nucleotidase 1
with a threshold of 1e−05
rs11715853
rs2090816
rs7572229
rs78383039
rs823424
rs10899911
rs55877868
rs7303158
rs6828768
rs8039785
rs6809952
rs35055552
rs7311004
rs2387977
rs62273907
All
−0.2
−0.1
0.0
0.1
0.2
0.3
MR effect size for
'Gut microbiota abundance (genus Parasutterella id.2892)' on 'Soluble calcium−activated nucleotidase 1'

Batch 2−705 : Gut microbiota abundance (genus Catenibacterium id.2153) on Calnexin
with a threshold of 1e−05

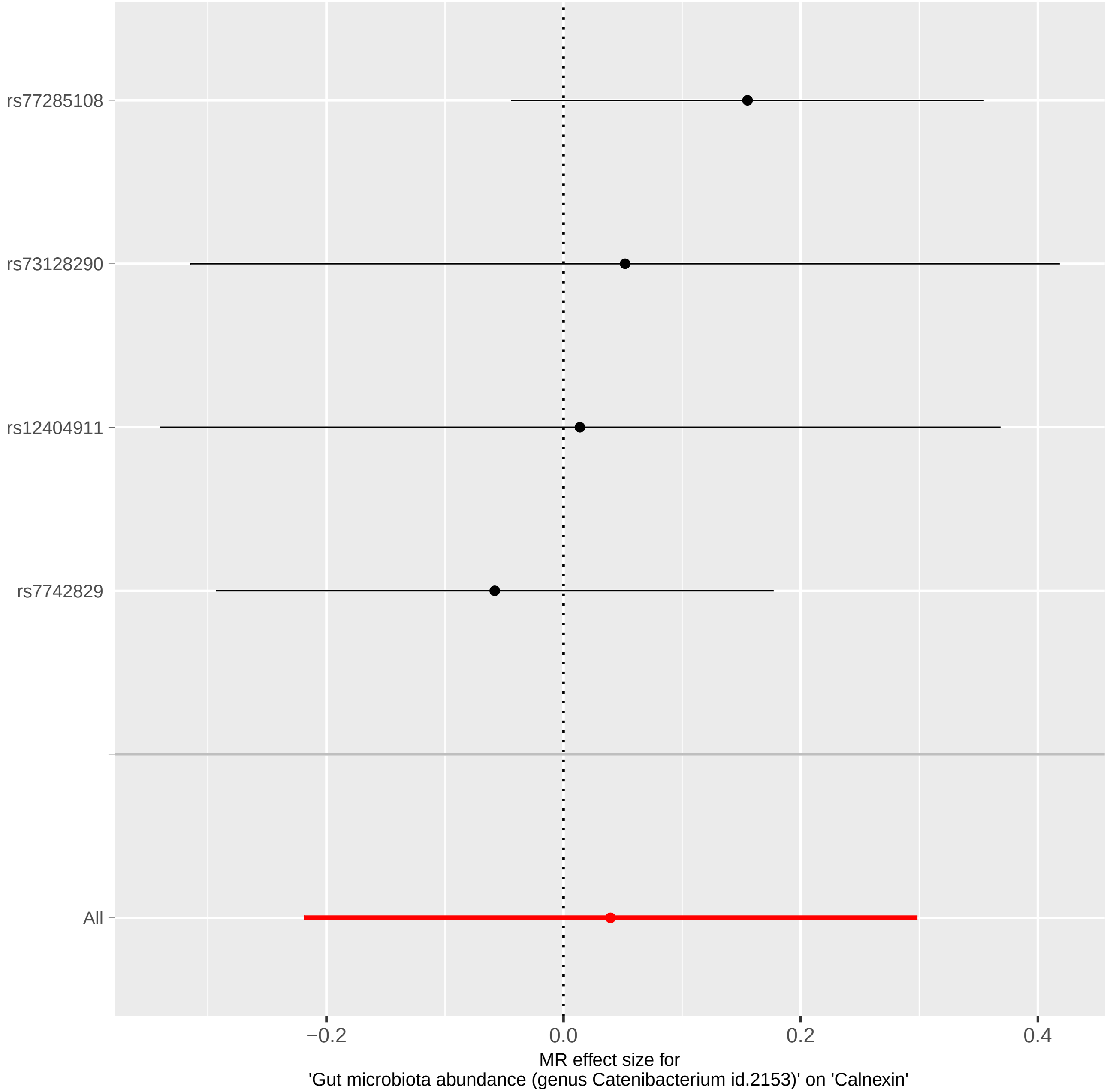

Batch 2−706 : Gut microbiota abundance (genus Parasutterella id.2892) on Calnexin
with a threshold of 1e−05

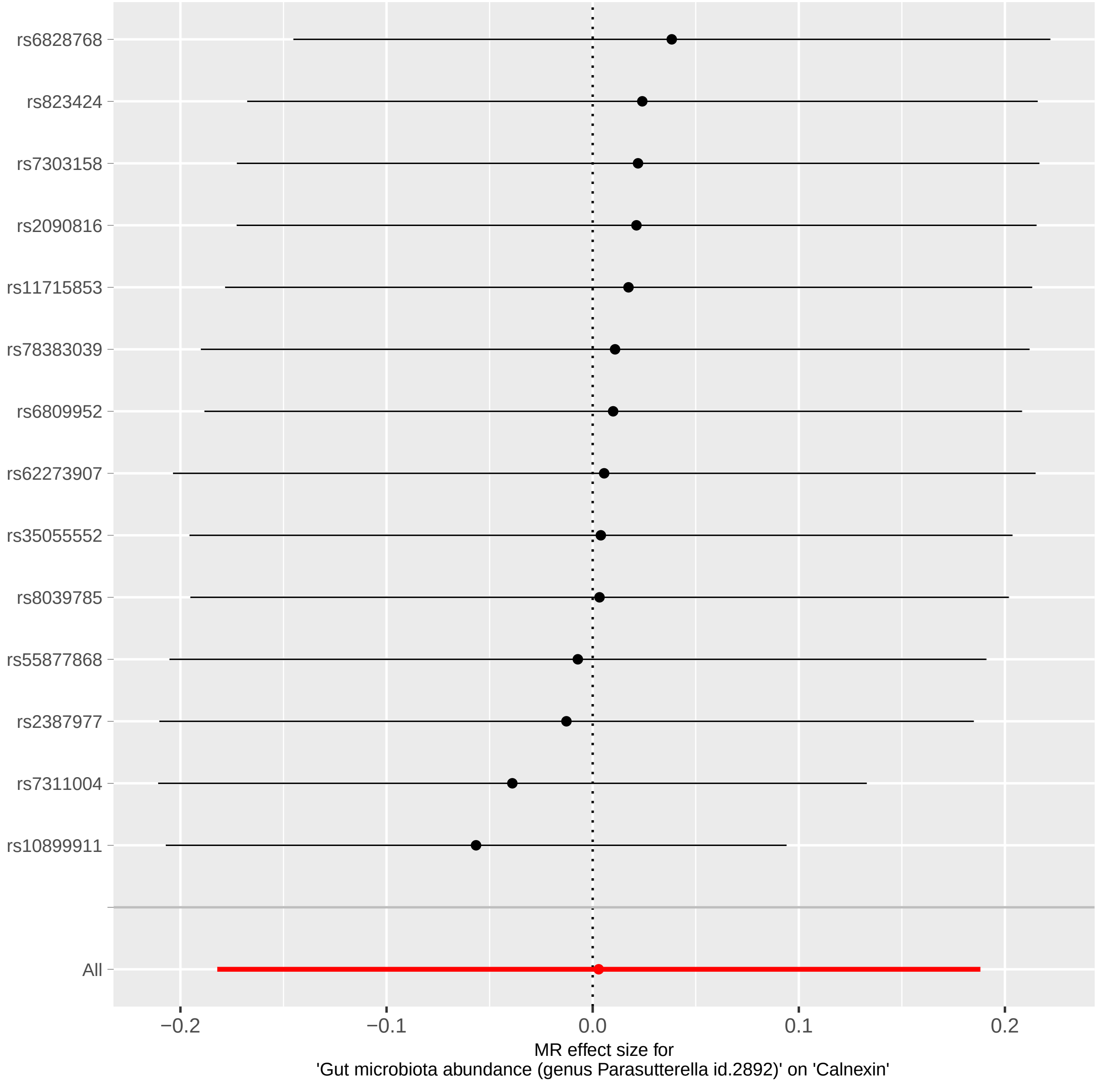

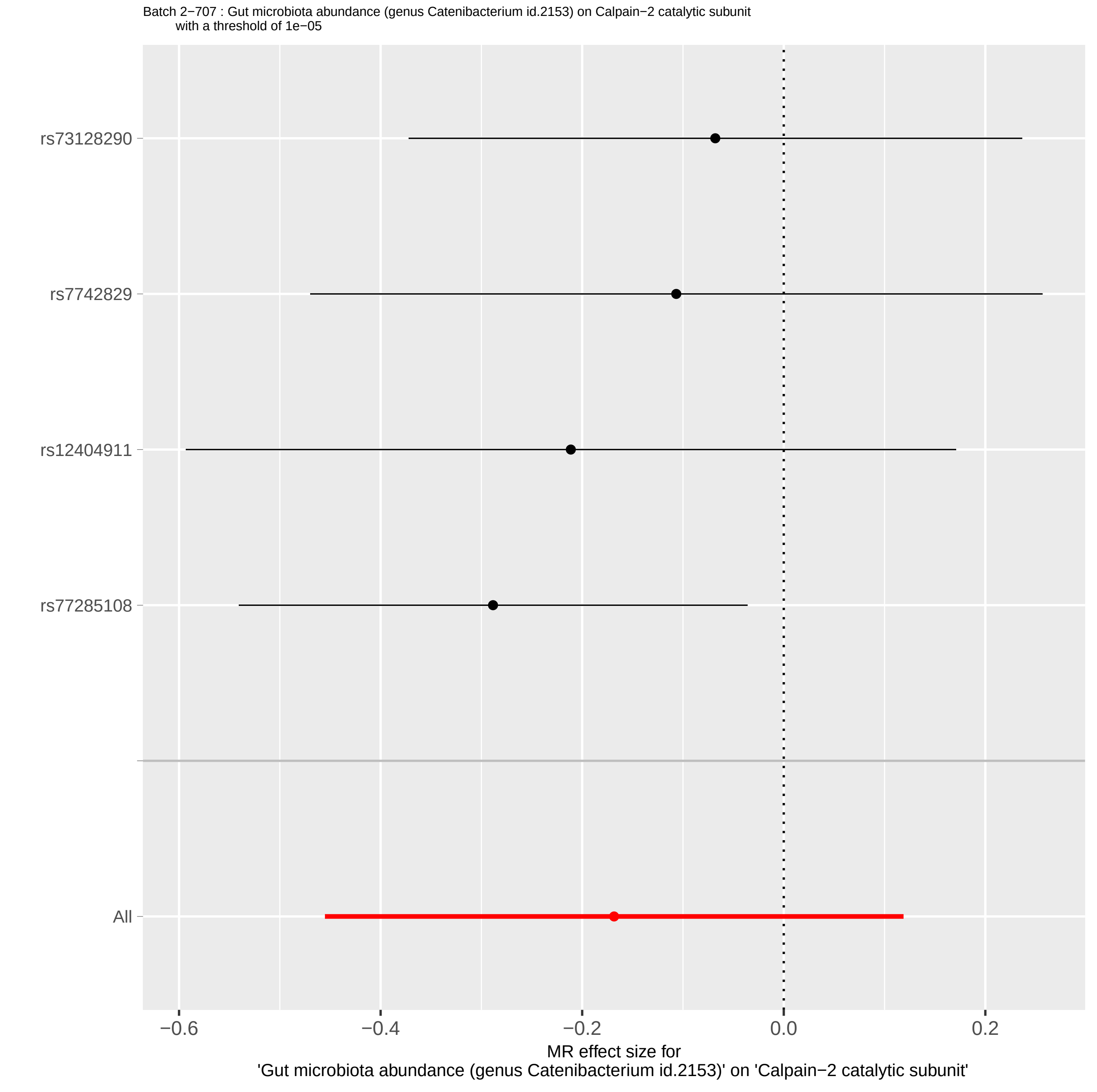
Batch 2−707 : Gut microbiota abundance (genus Catenibacterium id.2153) on Calpain−2 catalytic subunit
with a threshold of 1e−05
rs73128290
rs7742829
rs12404911
rs77285108
All
−0.6
−0.4
−0.2
0.0
0.2
MR effect size for
'Gut microbiota abundance (genus Catenibacterium id.2153)' on 'Calpain−2 catalytic subunit'

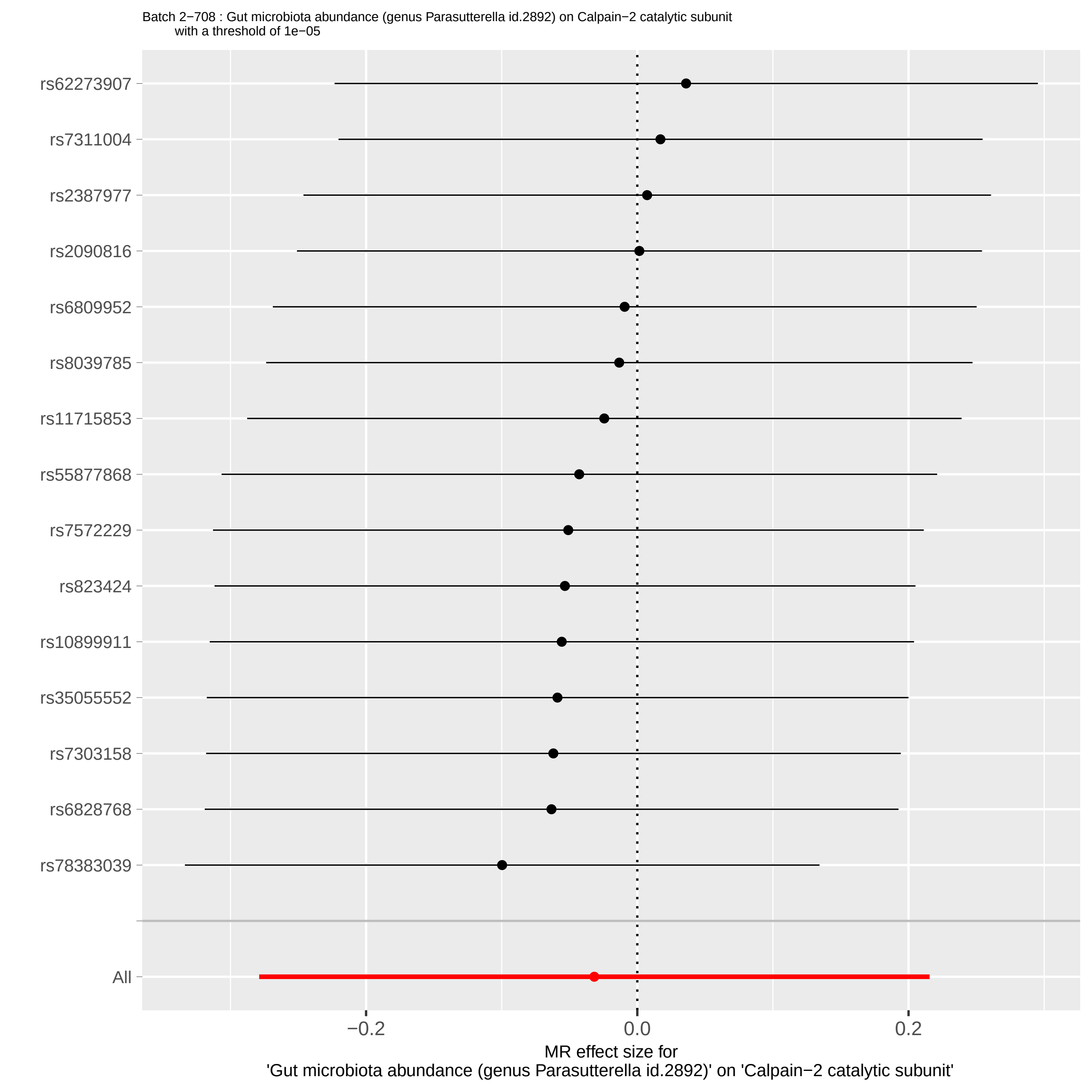

Batch 2−708 : Gut microbiota abundance (genus Parasutterella id.2892) on Calpain−2 catalytic subunit
with a threshold of 1e−05
rs62273907
rs7311004
rs2387977
rs2090816
rs6809952
rs8039785
rs11715853
rs55877868
rs7572229
rs823424
rs10899911
rs35055552
rs7303158
rs6828768
rs78383039
All
−0.2
0.0
0.2
MR effect size for
'Gut microbiota abundance (genus Parasutterella id.2892)' on 'Calpain−2 catalytic subunit'

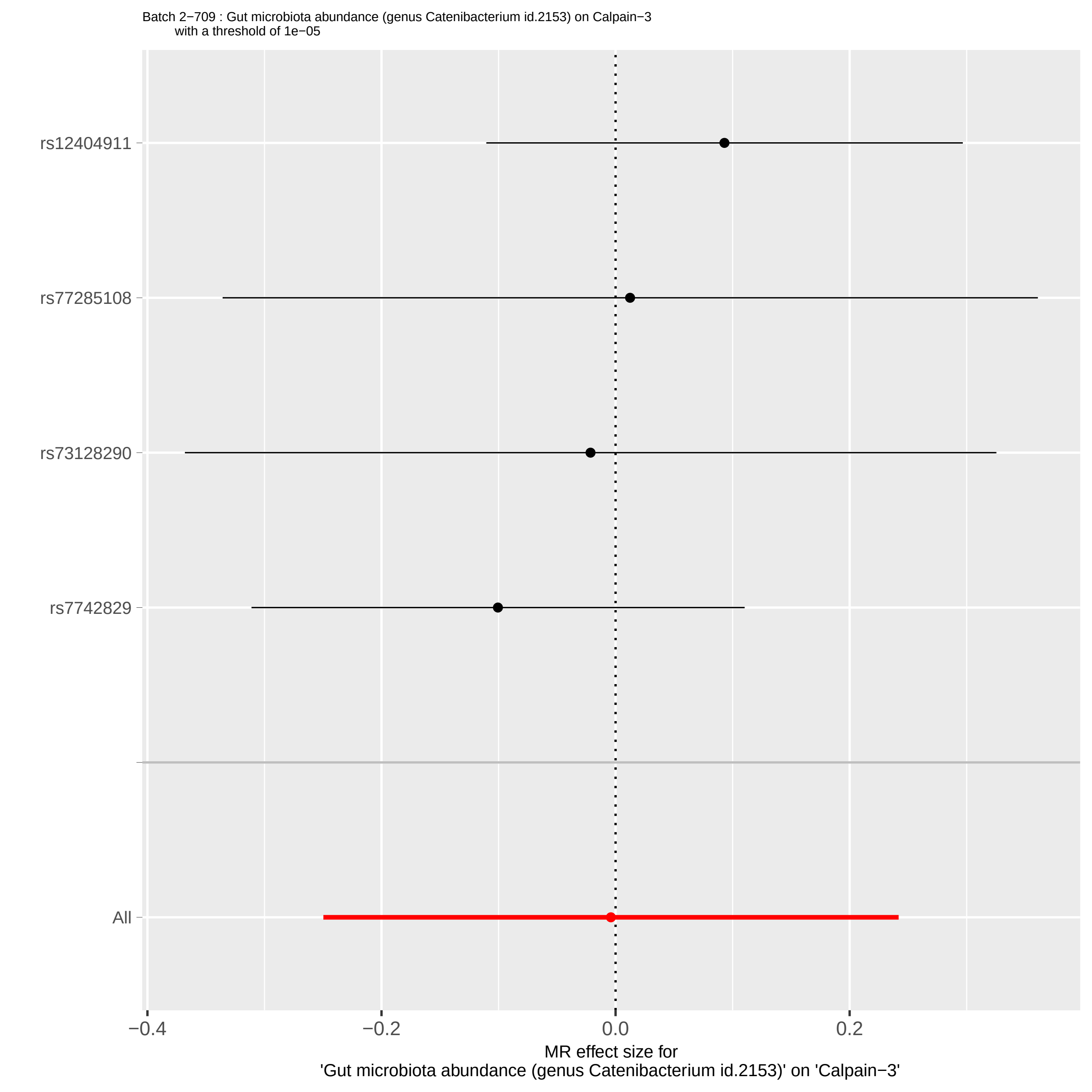

Batch 2−709 : Gut microbiota abundance (genus Catenibacterium id.2153) on Calpain−3
with a threshold of 1e−05
rs12404911
rs77285108
rs73128290
rs7742829
All
−0.4
−0.2
0.0
0.2
MR effect size for
'Gut microbiota abundance (genus Catenibacterium id.2153)' on 'Calpain−3'

Batch 2−710 : Gut microbiota abundance (genus Parasutterella id.2892) on Calpain−3
with a threshold of 1e−05

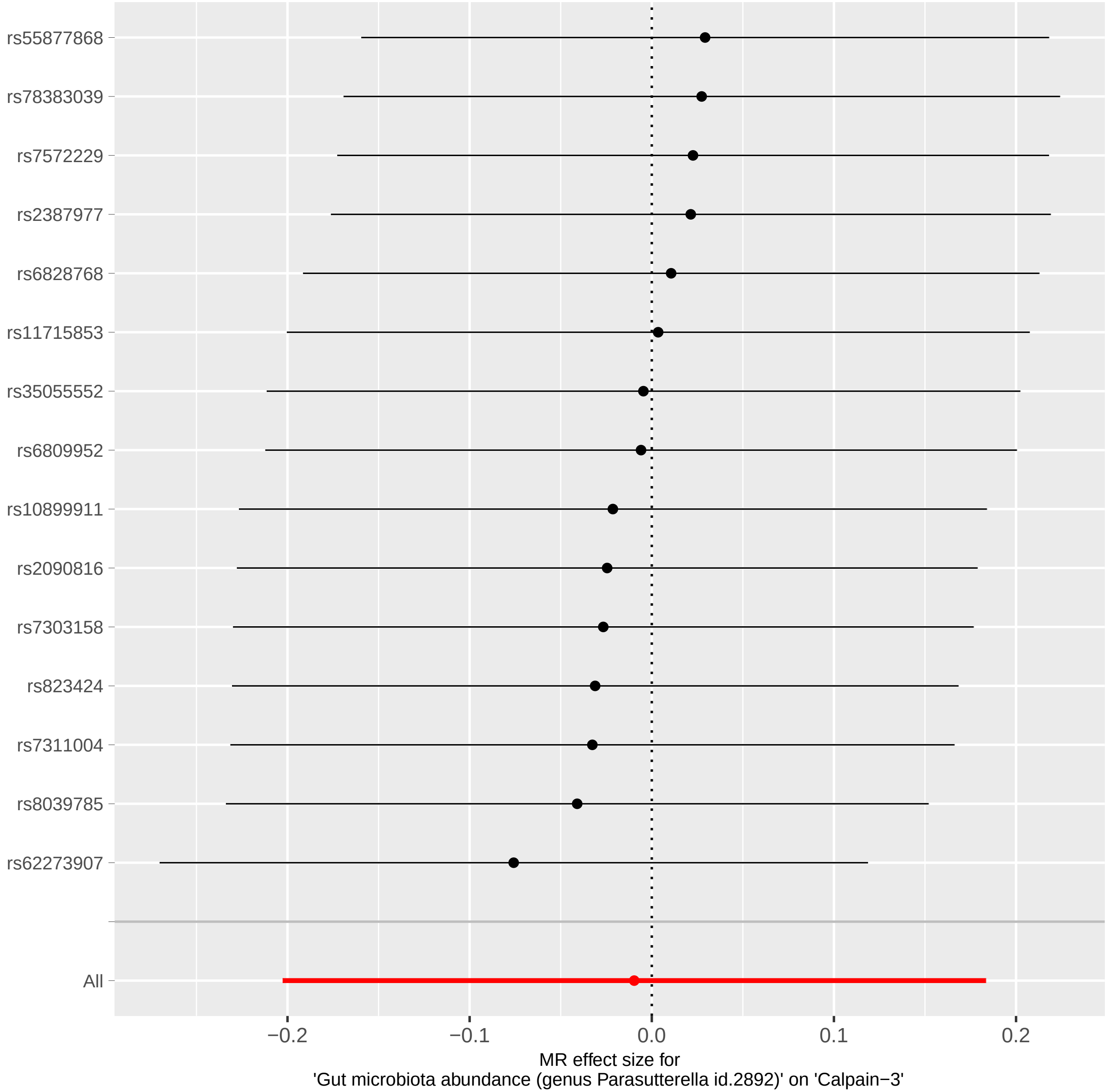

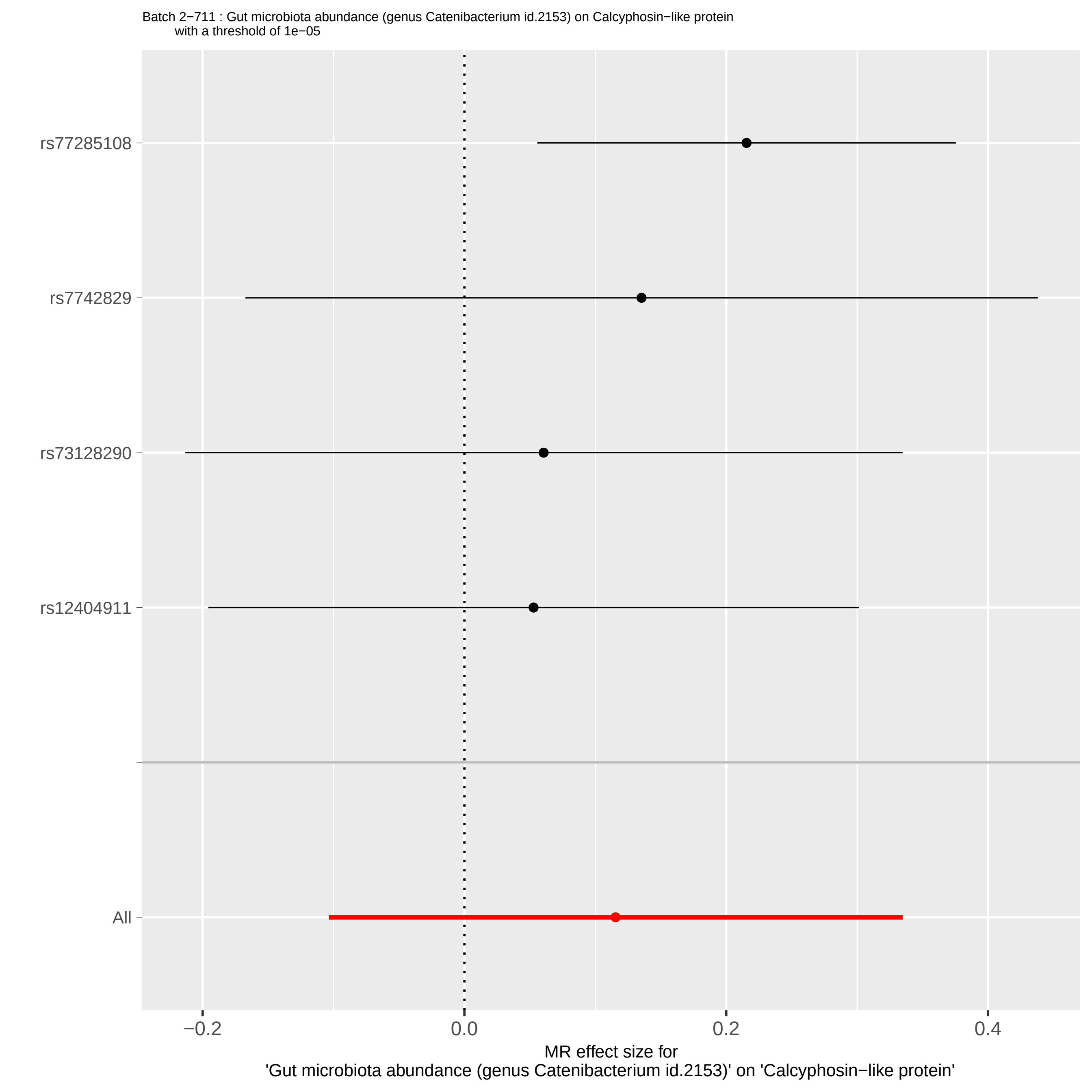

Batch 2−711 : Gut microbiota abundance (genus Catenibacterium id.2153) on Calcyphosin−like protein
with a threshold of 1e−05
rs77285108
rs7742829
rs73128290
rs12404911
All
−0.2
0.0
0.2
0.4
MR effect size for
'Gut microbiota abundance (genus Catenibacterium id.2153)' on 'Calcyphosin−like protein'

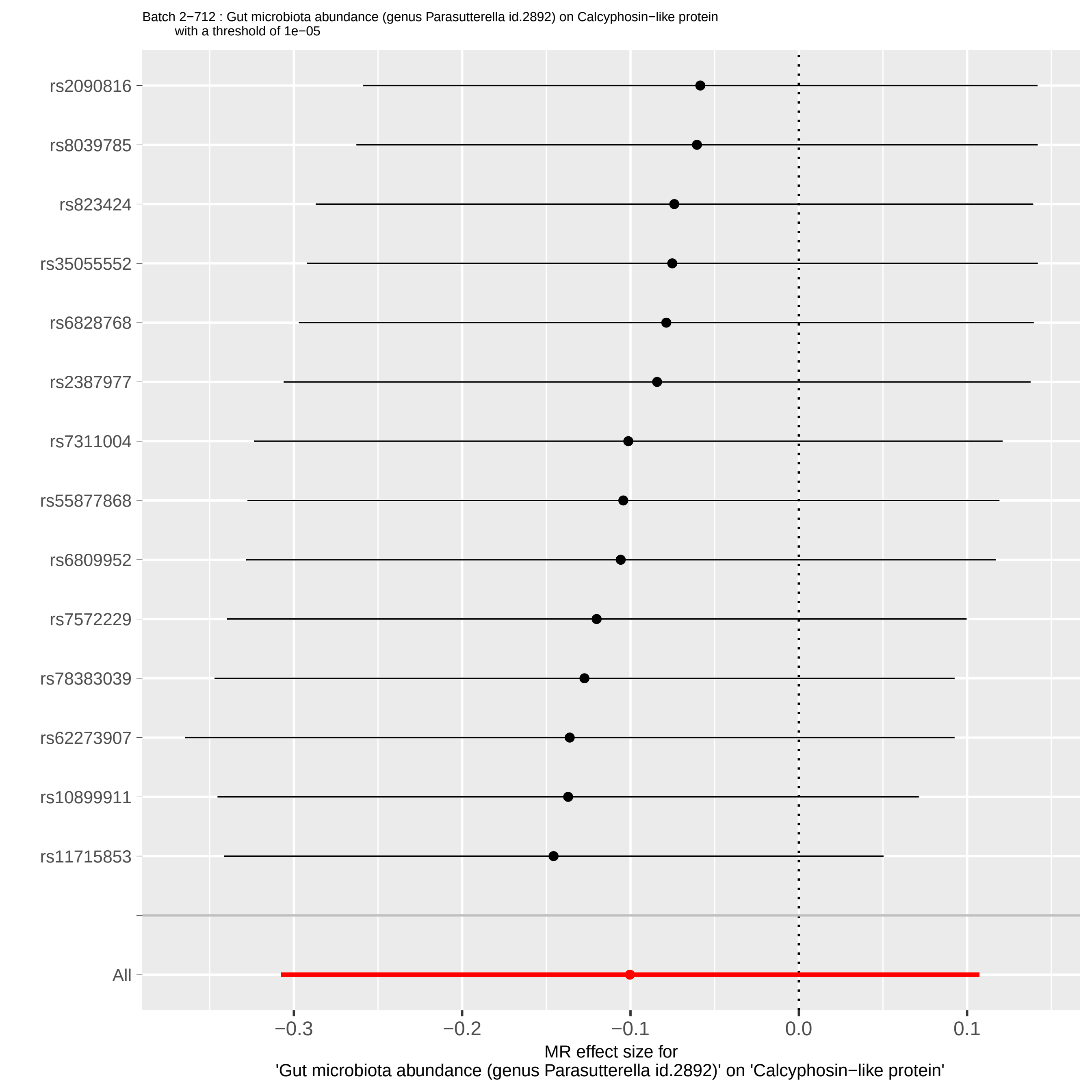

Batch 2−712 : Gut microbiota abundance (genus Parasutterella id.2892) on Calcyphosin−like protein
with a threshold of 1e−05
rs2090816
rs8039785
rs823424
rs35055552
rs6828768
rs2387977
rs7311004
rs55877868
rs6809952
rs7572229
rs78383039
rs62273907
rs10899911
rs11715853
All
−0.3
−0.2
−0.1
0.0
0.1
MR effect size for
'Gut microbiota abundance (genus Parasutterella id.2892)' on 'Calcyphosin−like protein'

Batch 2−713 : Gut microbiota abundance (genus Catenibacterium id.2153) on Calcium−regulated heat stable protein 1
with a threshold of 1e−05

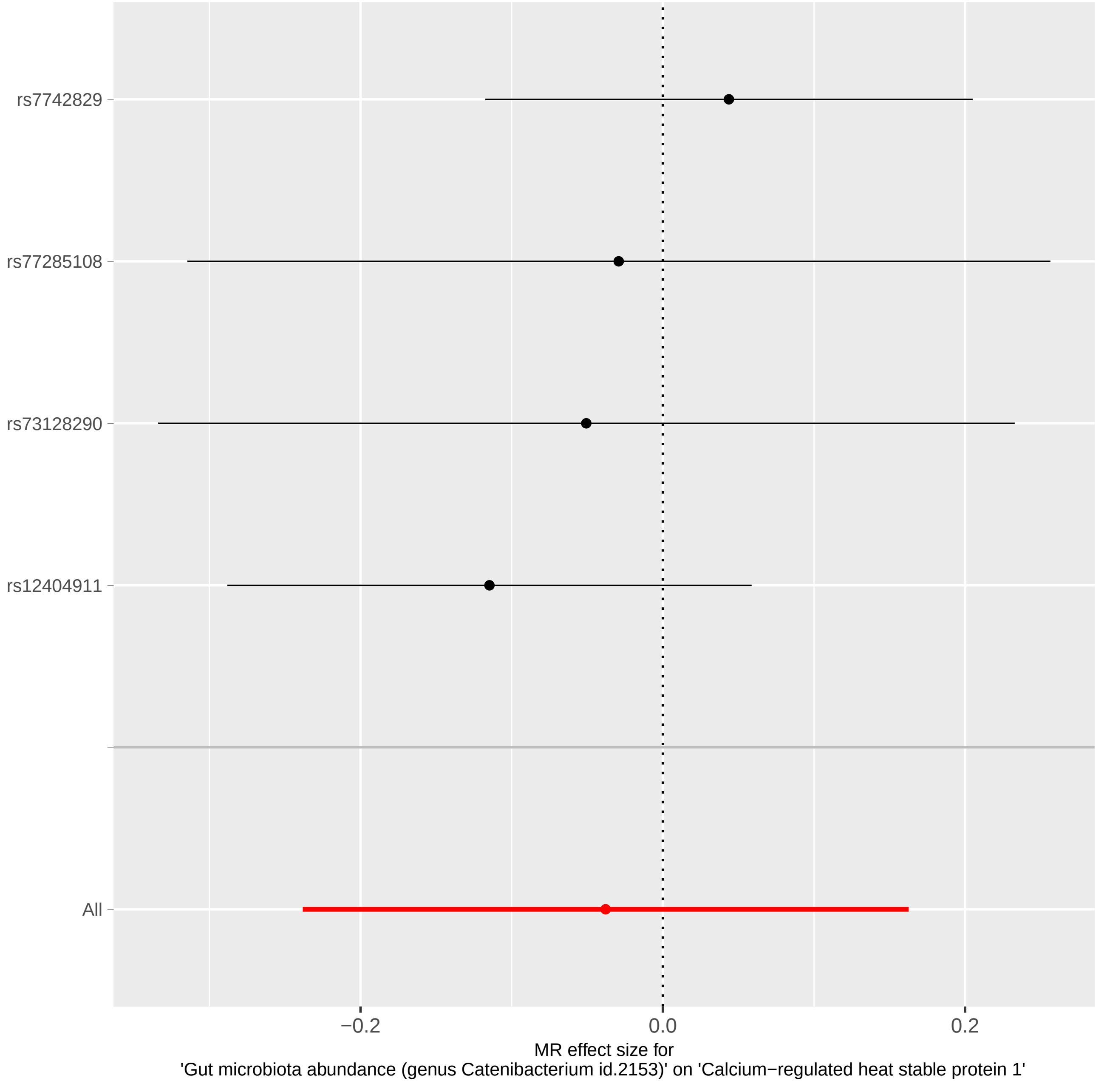

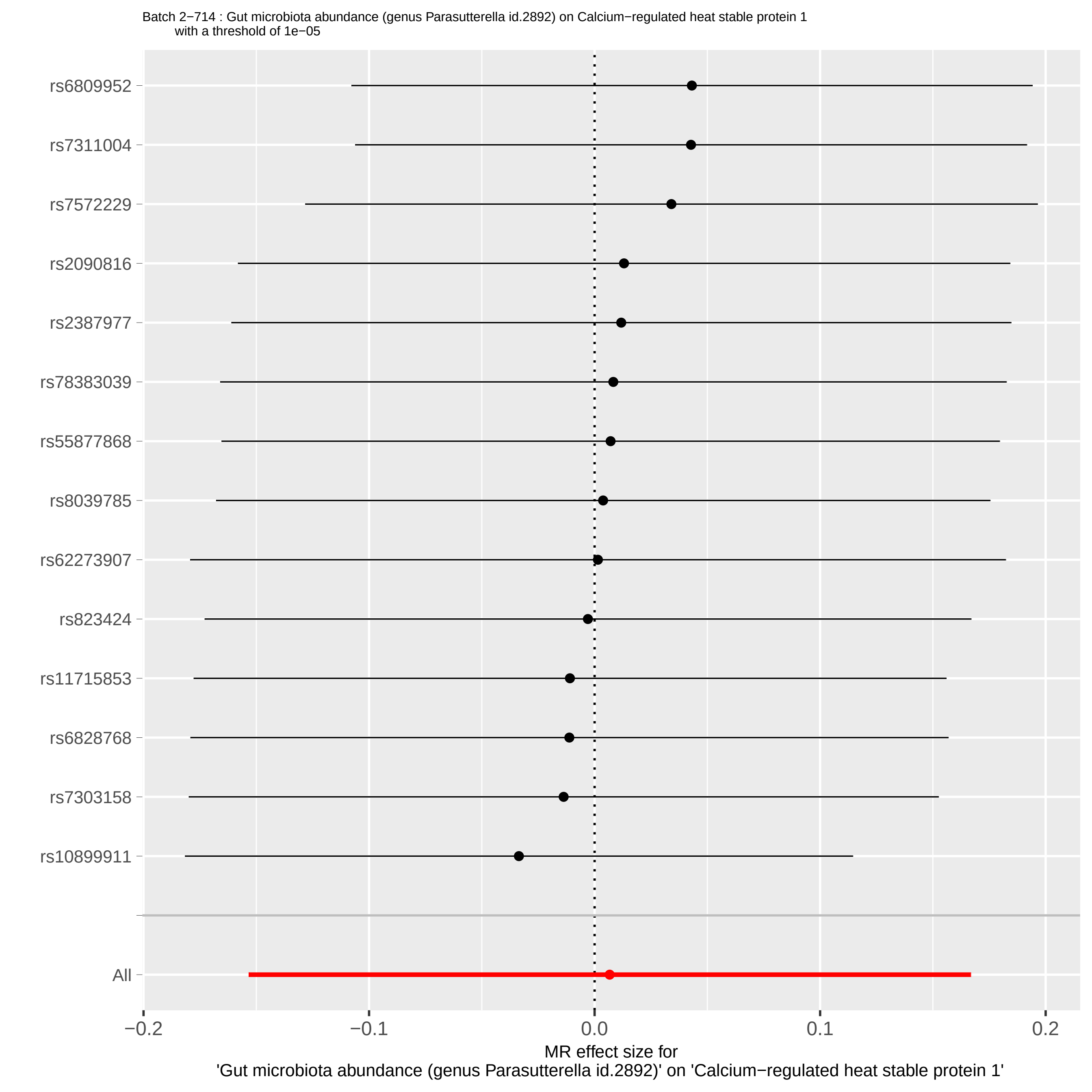

Batch 2−714 : Gut microbiota abundance (genus Parasutterella id.2892) on Calcium−regulated heat stable protein 1
with a threshold of 1e−05
rs6809952
rs7311004
rs7572229
rs2090816
rs2387977
rs78383039
rs55877868
rs8039785
rs62273907
rs823424
rs11715853
rs6828768
rs7303158
rs10899911
All
−0.2
−0.1
0.0
0.1
0.2
MR effect size for
'Gut microbiota abundance (genus Parasutterella id.2892)' on 'Calcium−regulated heat stable protein 1'

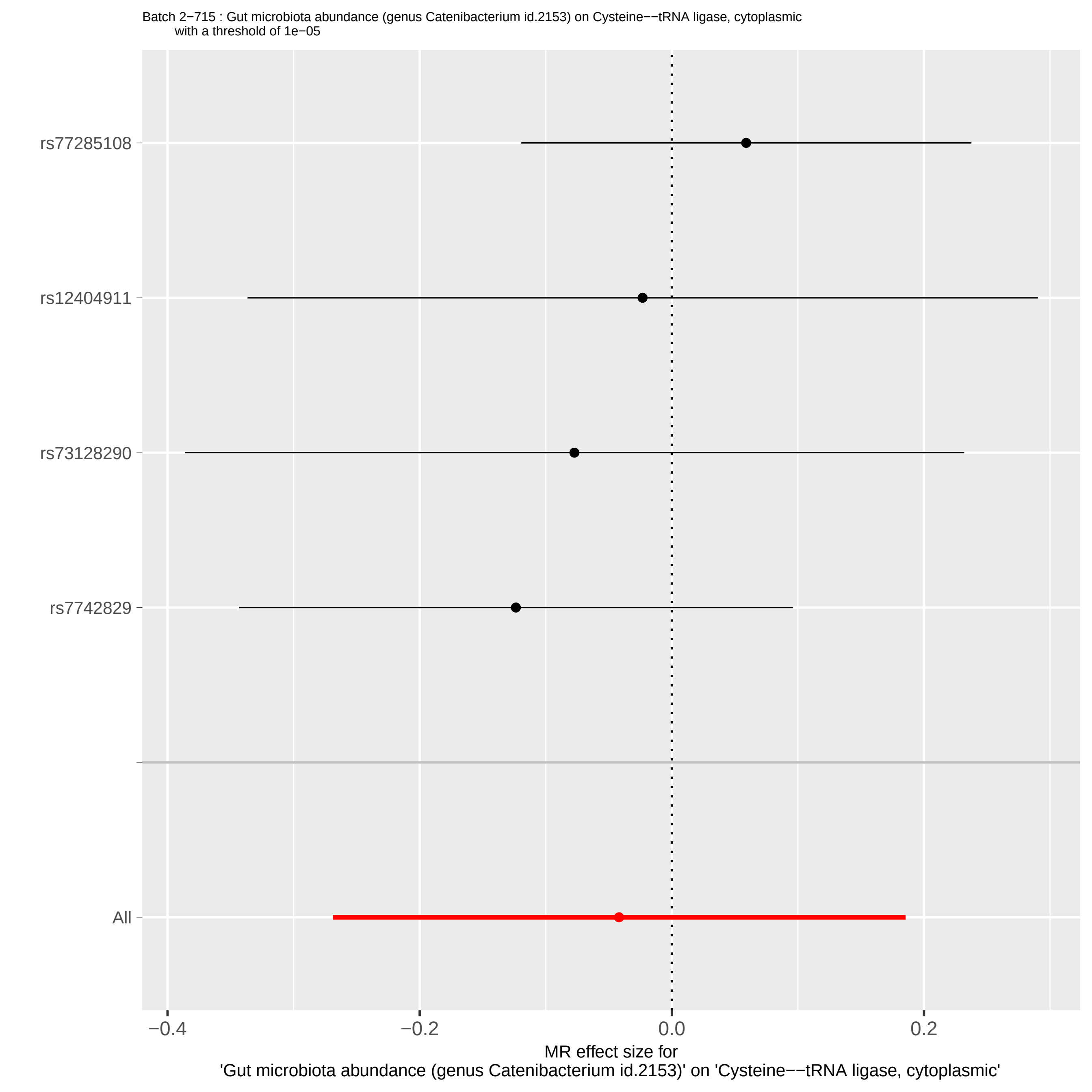
Batch 2−715 : Gut microbiota abundance (genus Catenibacterium id.2153) on Cysteine−−tRNA ligase, cytoplasmic
with a threshold of 1e−05
rs77285108
rs12404911
rs73128290
rs7742829
All
−0.4
−0.2
0.0
0.2
MR effect size for
'Gut microbiota abundance (genus Catenibacterium id.2153)' on 'Cysteine−−tRNA ligase, cytoplasmic'

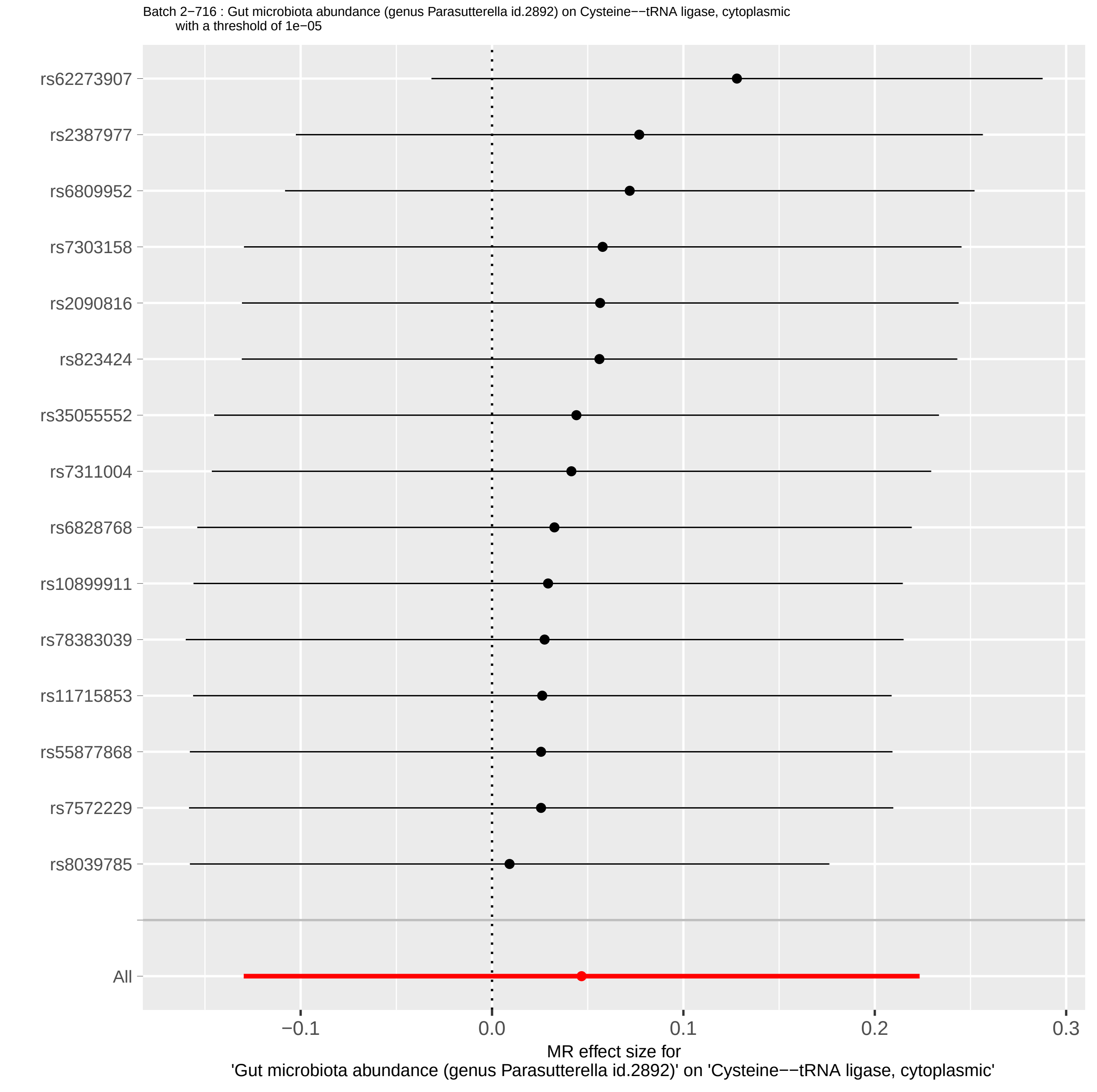
Batch 2−716 : Gut microbiota abundance (genus Parasutterella id.2892) on Cysteine−−tRNA ligase, cytoplasmic
with a threshold of 1e−05
rs62273907
rs2387977
rs6809952
rs7303158
rs2090816
rs823424
rs35055552
rs7311004
rs6828768
rs10899911
rs78383039
rs11715853
rs55877868
rs7572229
rs8039785
All
−0.1
0.0
0.1
0.2
0.3
MR effect size for
'Gut microbiota abundance (genus Parasutterella id.2892)' on 'Cysteine−−tRNA ligase, cytoplasmic'

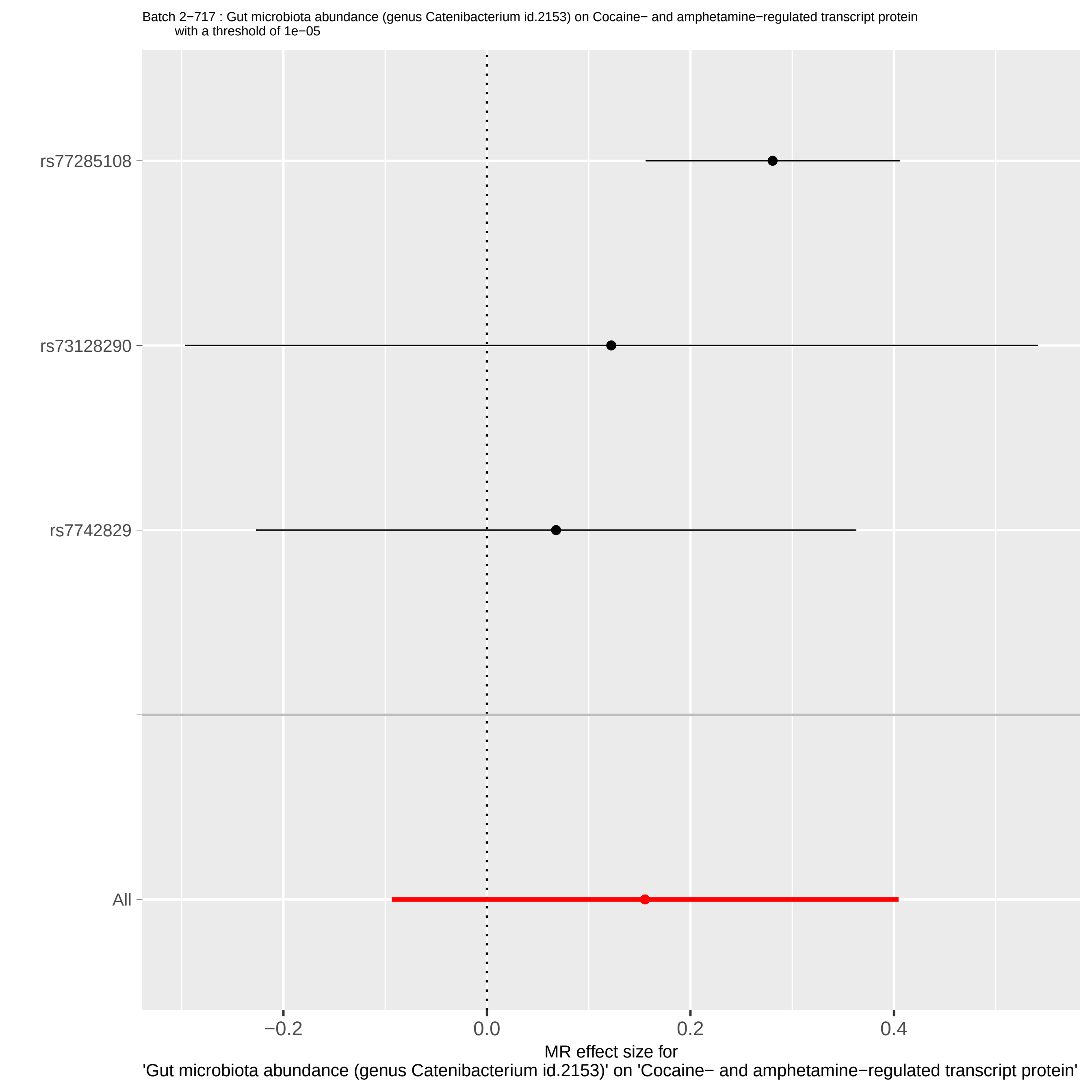
Batch 2−717 : Gut microbiota abundance (genus Catenibacterium id.2153) on Cocaine− and amphetamine−regulated transcript protein
with a threshold of 1e−05
rs77285108
rs73128290
rs7742829
All
−0.2
0.0
0.2
0.4
MR effect size for
'Gut microbiota abundance (genus Catenibacterium id.2153)' on 'Cocaine− and amphetamine−regulated transcript protein'

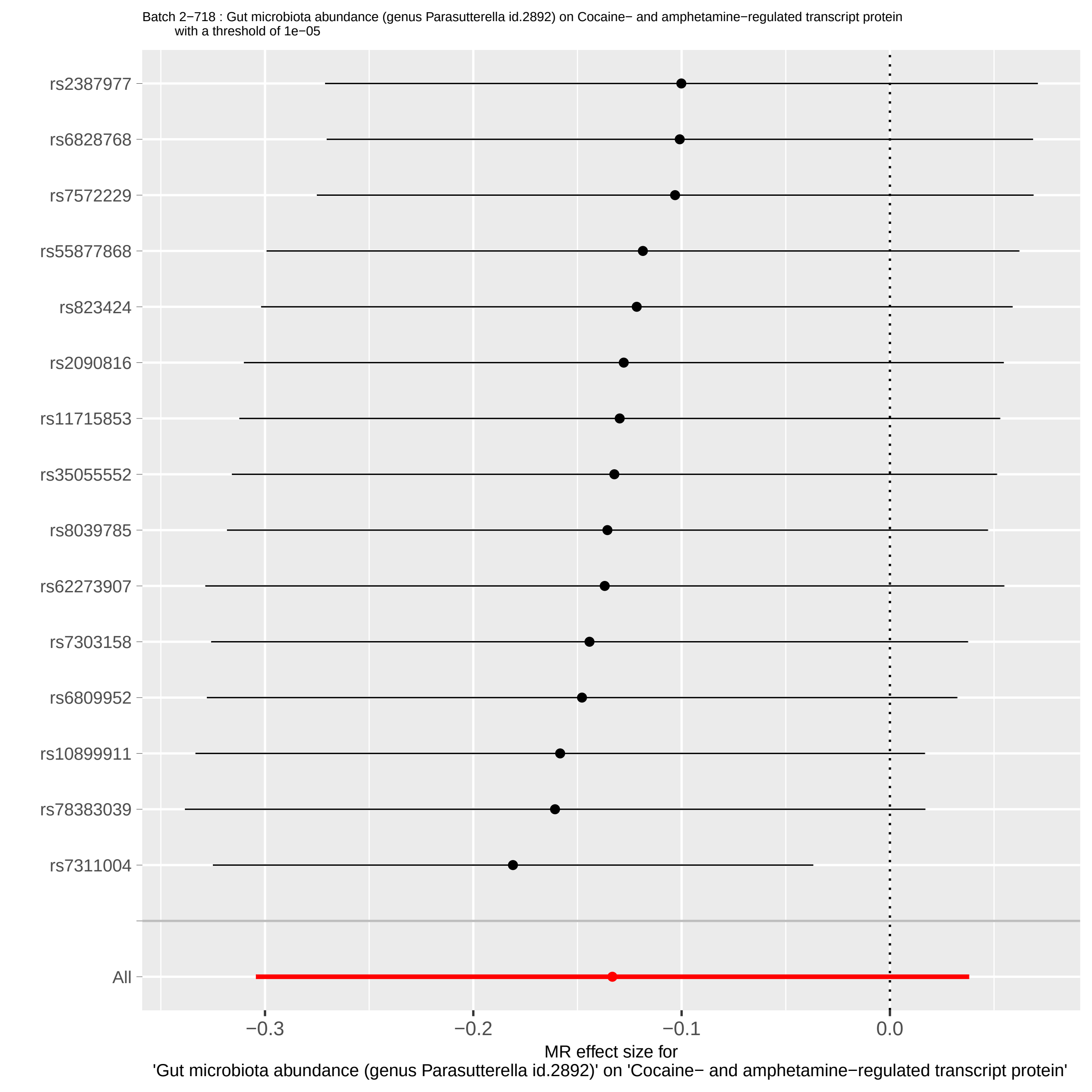

Batch 2−718 : Gut microbiota abundance (genus Parasutterella id.2892) on Cocaine− and amphetamine−regulated transcript protein with a threshold of 1e−05
rs2387977
rs6828768
rs7572229
rs55877868
rs823424
rs2090816
rs11715853
rs35055552
rs8039785
rs62273907
rs7303158
rs6809952
rs10899911
rs78383039
rs7311004
All
−0.3
−0.2
−0.1
0.0
MR effect size for
'Gut microbiota abundance (genus Parasutterella id.2892)' on 'Cocaine− and amphetamine−regulated transcript protein'

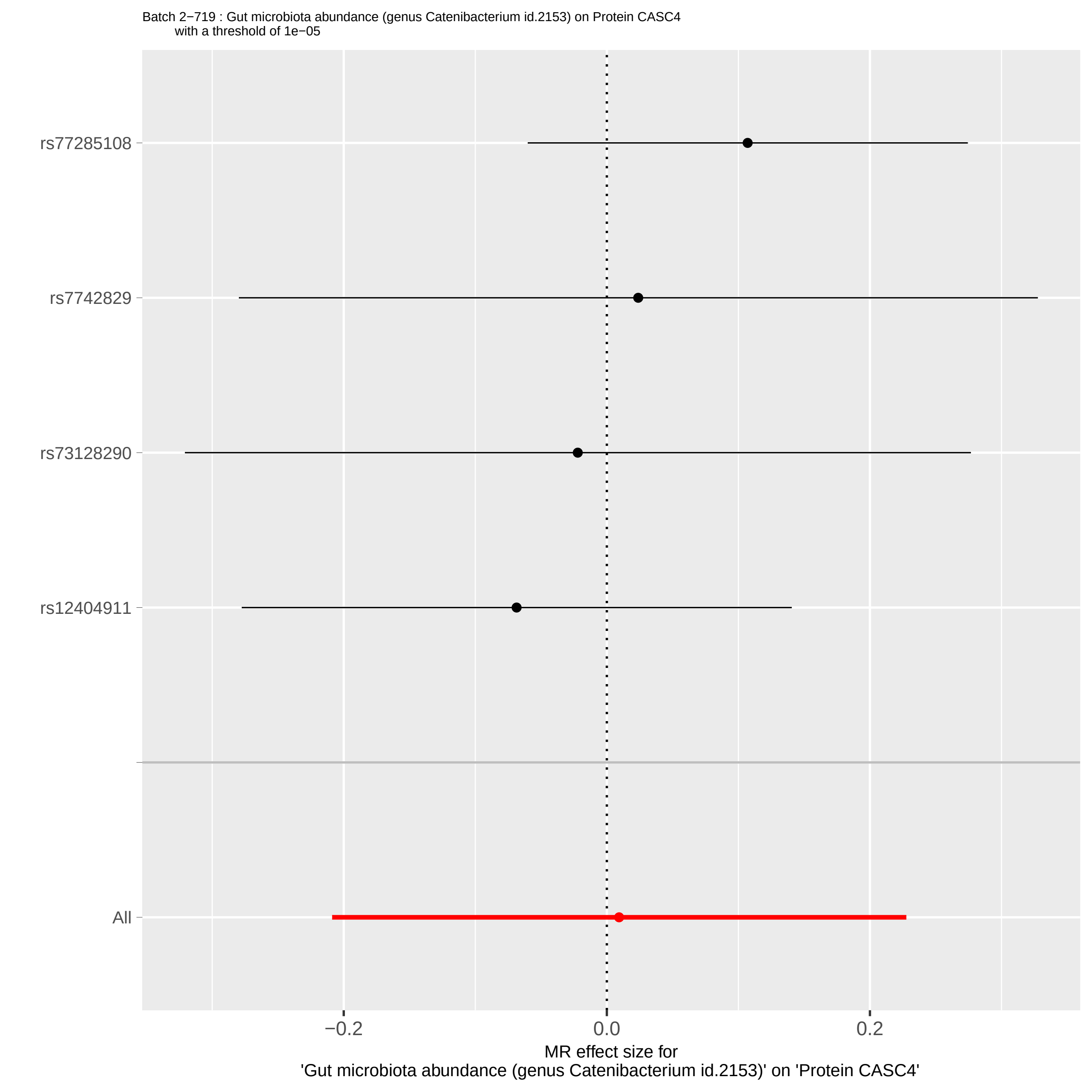
Batch 2−719 : Gut microbiota abundance (genus Catenibacterium id.2153) on Protein CASC4
with a threshold of 1e−05
rs77285108
rs7742829
rs73128290
rs12404911
All
−0.2
0.0
0.2
MR effect size for
'Gut microbiota abundance (genus Catenibacterium id.2153)' on 'Protein CASC4'

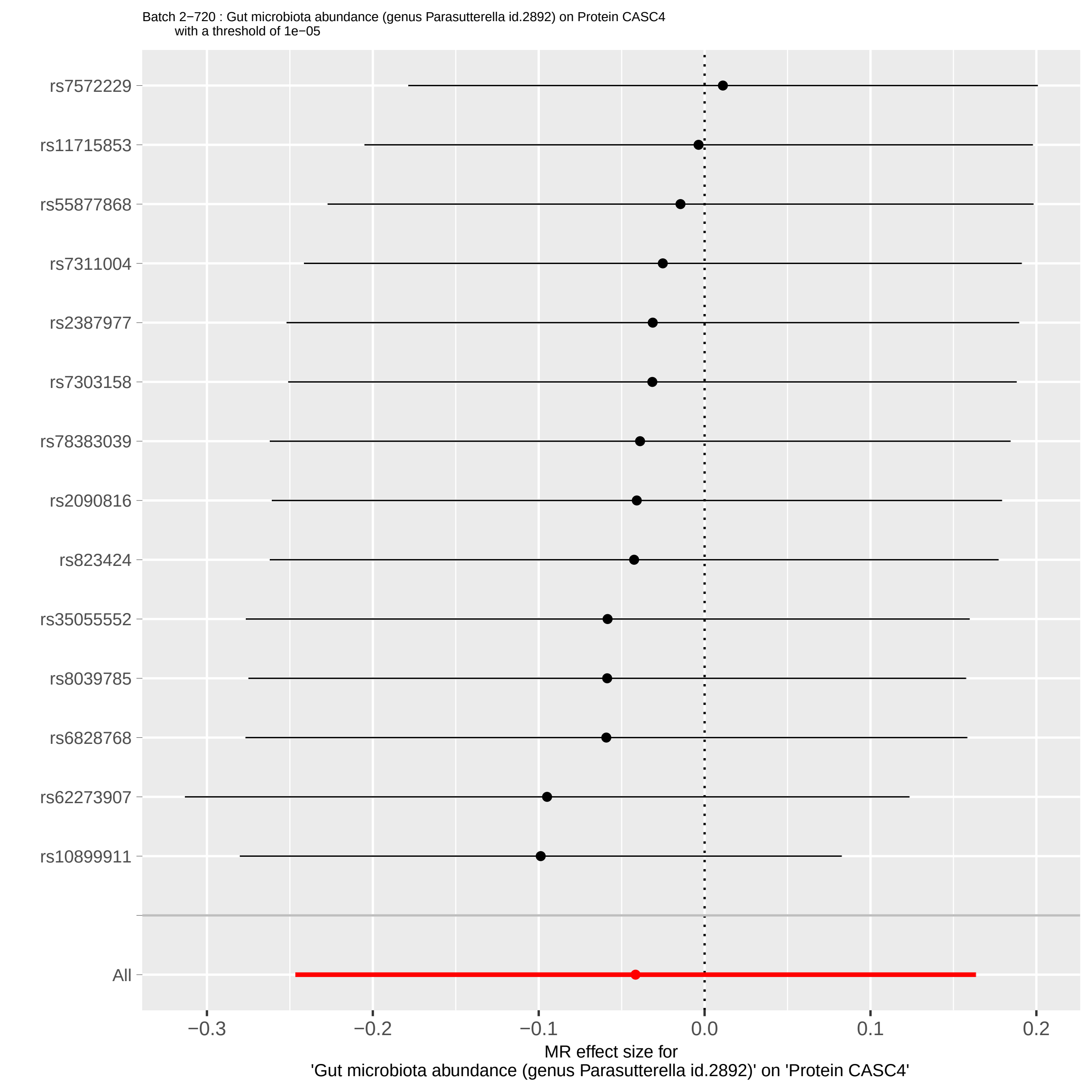
Batch 2−720 : Gut microbiota abundance (genus Parasutterella id.2892) on Protein CASC4
with a threshold of 1e−05
rs7572229
rs11715853
rs55877868
rs7311004
rs2387977
rs7303158
rs78383039
rs2090816
rs823424
rs35055552
rs8039785
rs6828768
rs62273907
rs10899911
All
−0.3
−0.2
−0.1
0.0
0.1
0.2
MR effect size for
'Gut microbiota abundance (genus Parasutterella id.2892)' on 'Protein CASC4'

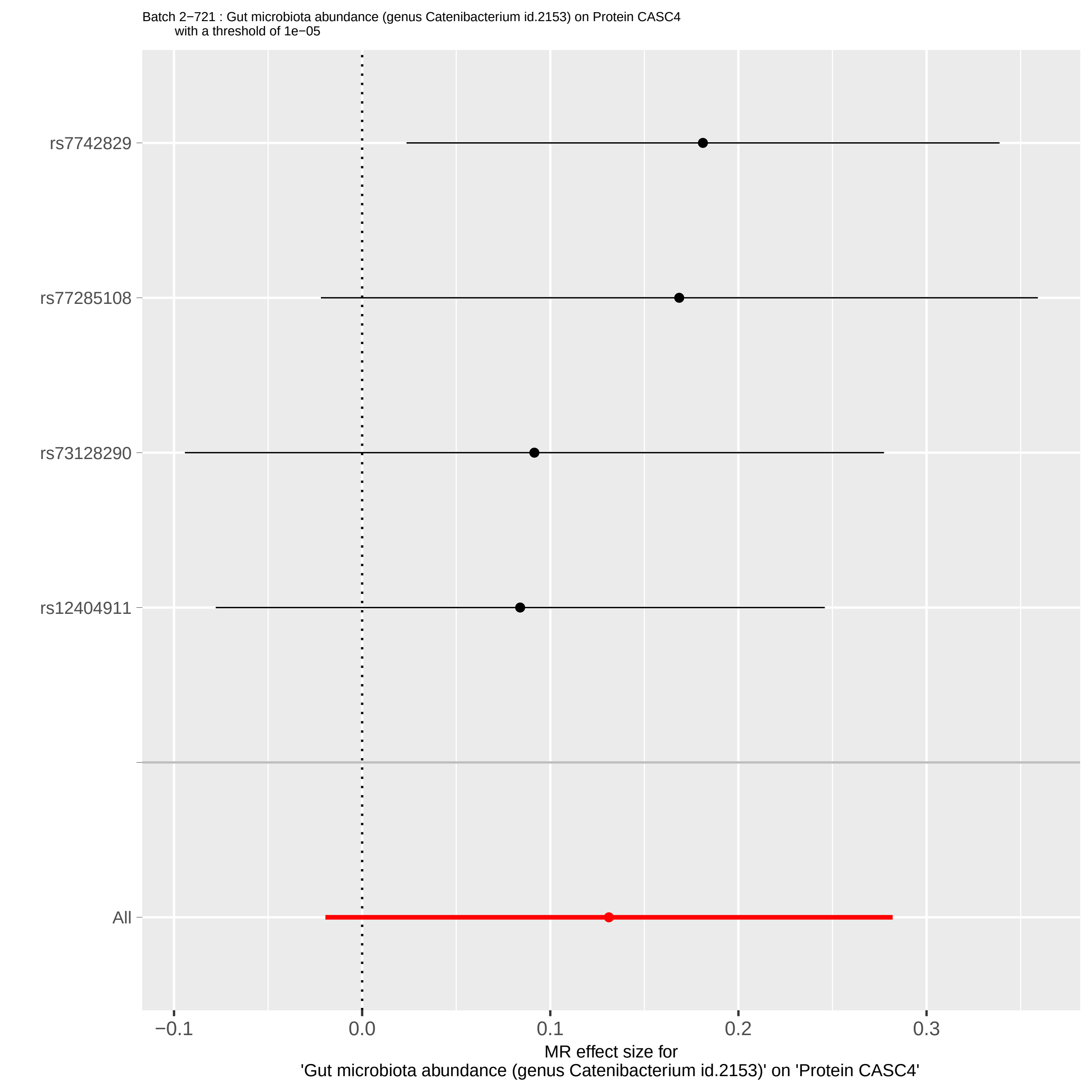

Batch 2−721 : Gut microbiota abundance (genus Catenibacterium id.2153) on Protein CASC4
with a threshold of 1e−05
rs7742829
rs77285108
rs73128290
rs12404911
All
−0.1
0.0
0.1
0.2
0.3
MR effect size for
'Gut microbiota abundance (genus Catenibacterium id.2153)' on 'Protein CASC4'

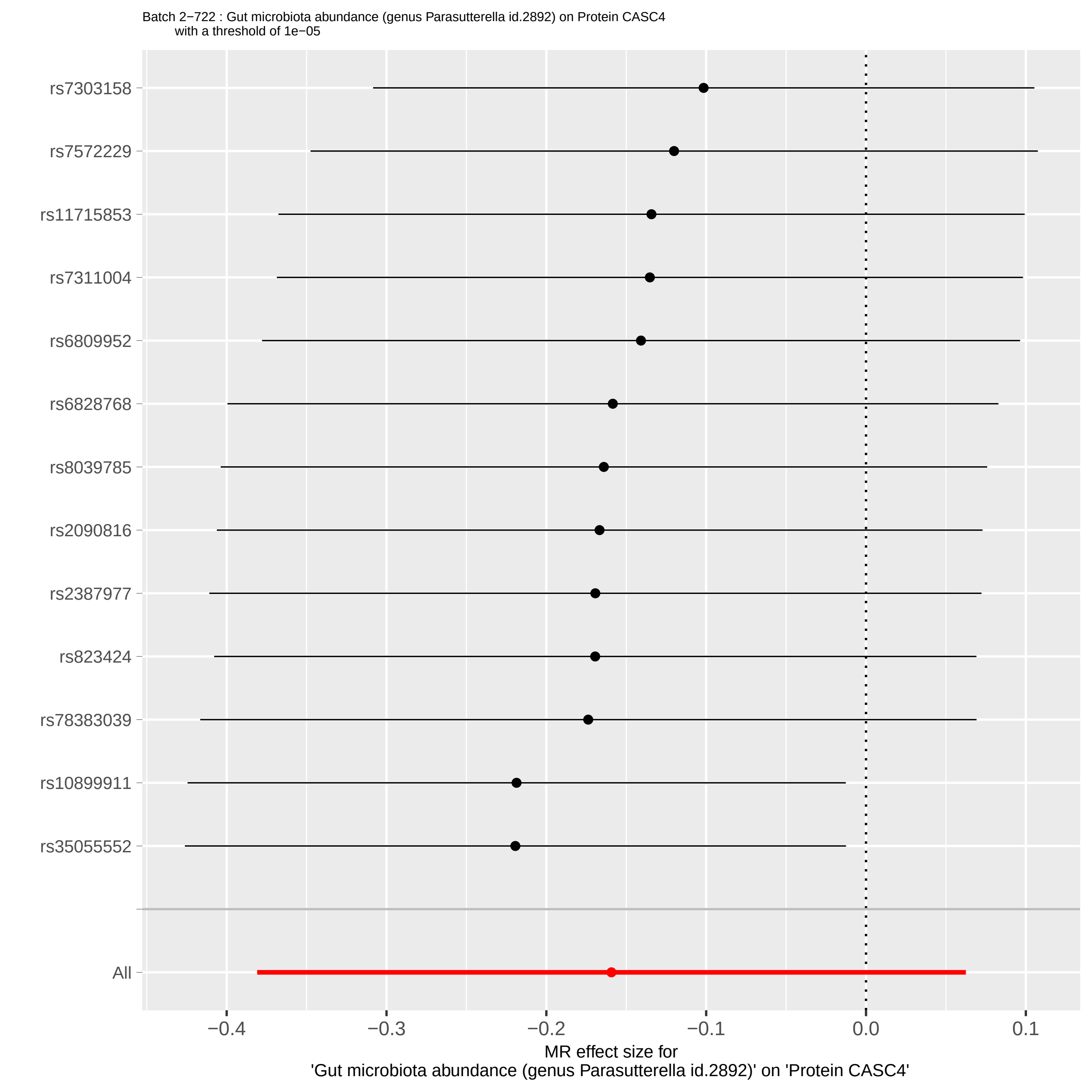

Batch 2−722 : Gut microbiota abundance (genus Parasutterella id.2892) on Protein CASC4
with a threshold of 1e−05
rs7303158
rs7572229
rs11715853
rs7311004
rs6809952
rs6828768
rs8039785
rs2090816
rs2387977
rs823424
rs78383039
rs10899911
rs35055552
All
−0.4
−0.3
−0.2
−0.1
0.0
0.1
MR effect size for
'Gut microbiota abundance (genus Parasutterella id.2892)' on 'Protein CASC4'

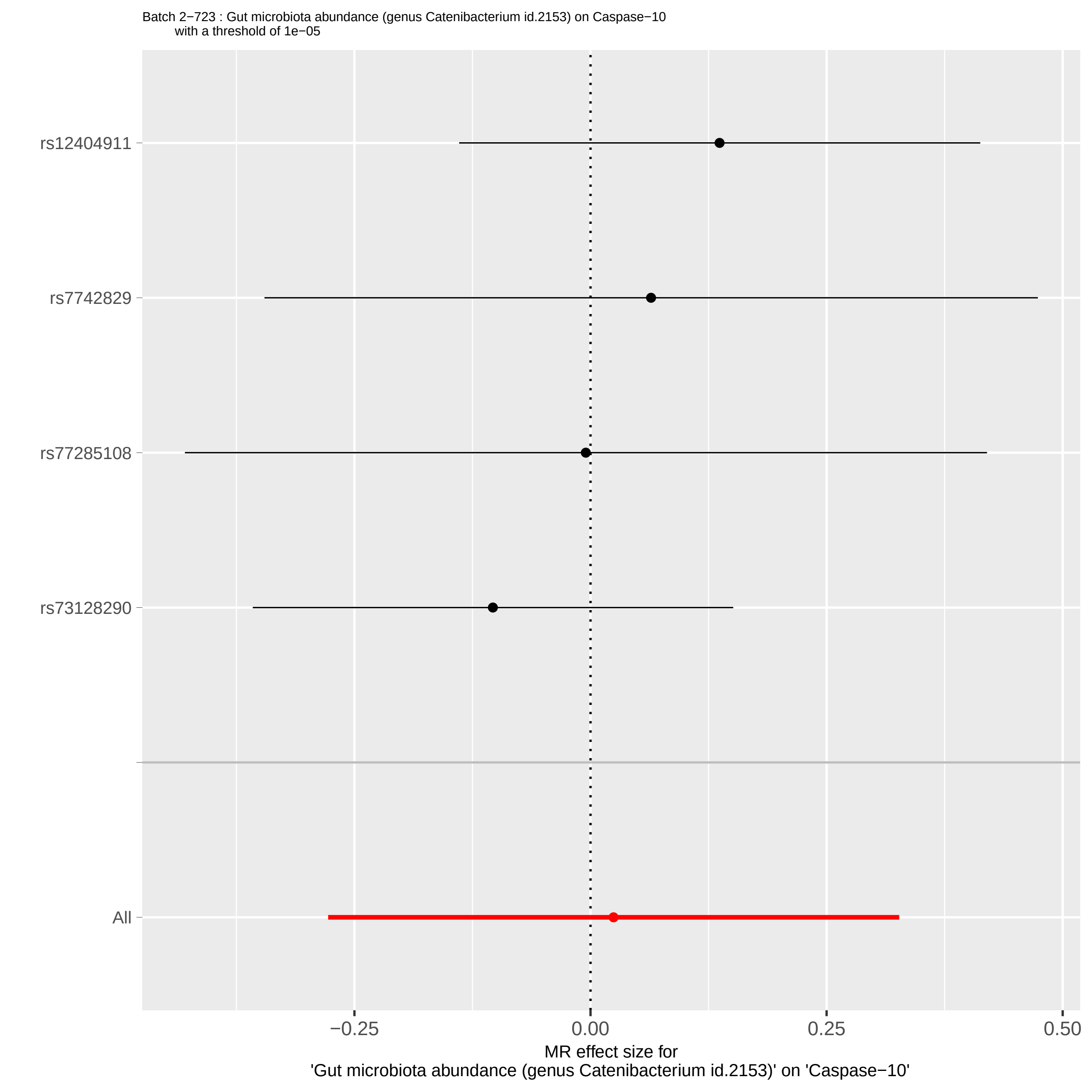

Batch 2−723 : Gut microbiota abundance (genus Catenibacterium id.2153) on Caspase−10
with a threshold of 1e−05
rs12404911
rs7742829
rs77285108
rs73128290
All
−0.25
0.00
0.25
0.50
MR effect size for
'Gut microbiota abundance (genus Catenibacterium id.2153)' on 'Caspase−10'

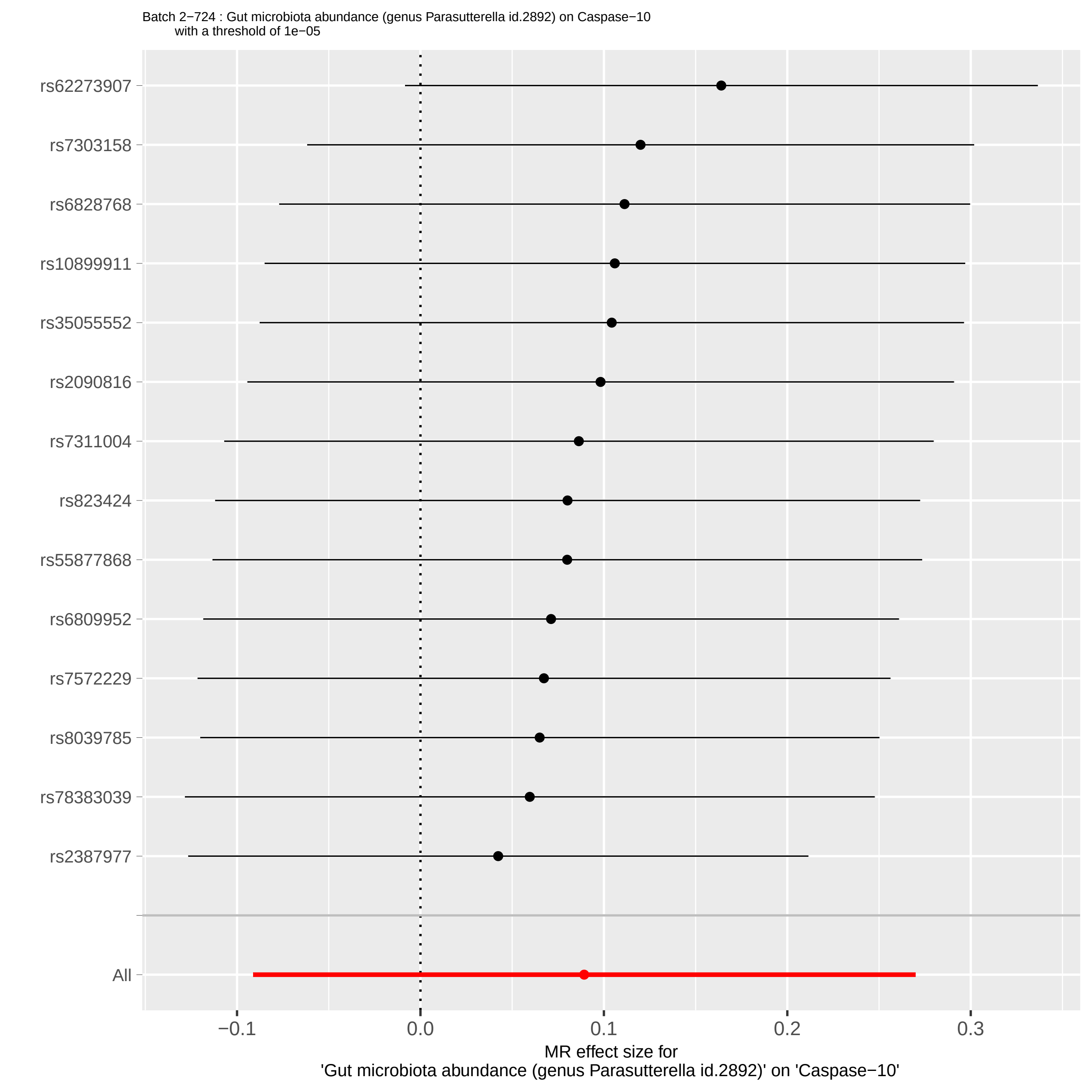

Batch 2−724 : Gut microbiota abundance (genus Parasutterella id.2892) on Caspase−10
with a threshold of 1e−05
rs62273907
rs7303158
rs6828768
rs10899911
rs35055552
rs2090816
rs7311004
rs823424
rs55877868
rs6809952
rs7572229
rs8039785
rs78383039
rs2387977
All
−0.1
0.0
0.1
0.2
0.3
MR effect size for
'Gut microbiota abundance (genus Parasutterella id.2892)' on 'Caspase−10'

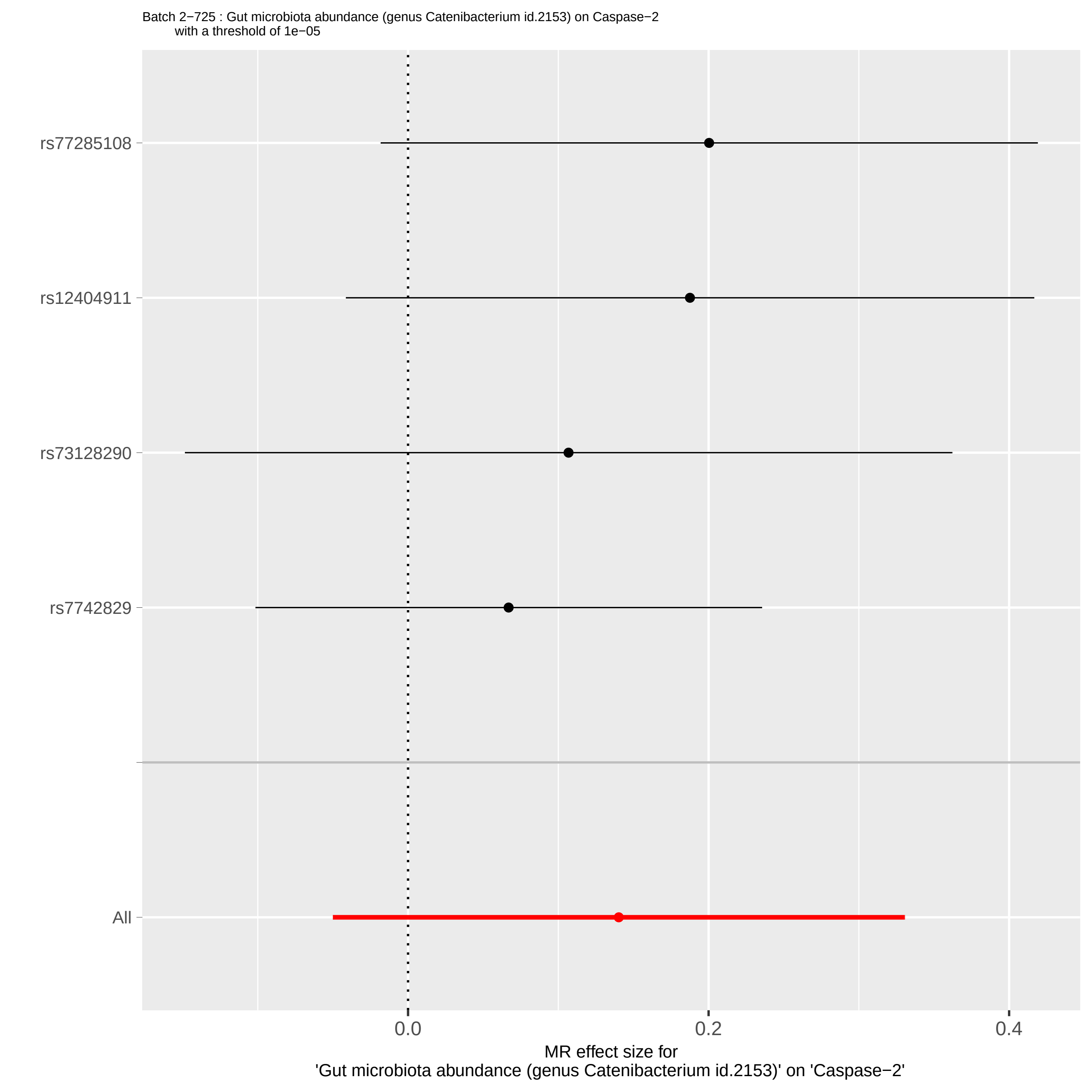
Batch 2−725 : Gut microbiota abundance (genus Catenibacterium id.2153) on Caspase−2
with a threshold of 1e−05
rs77285108
rs12404911
rs73128290
rs7742829
All
0.0
0.2
0.4
MR effect size for
'Gut microbiota abundance (genus Catenibacterium id.2153)' on 'Caspase−2'

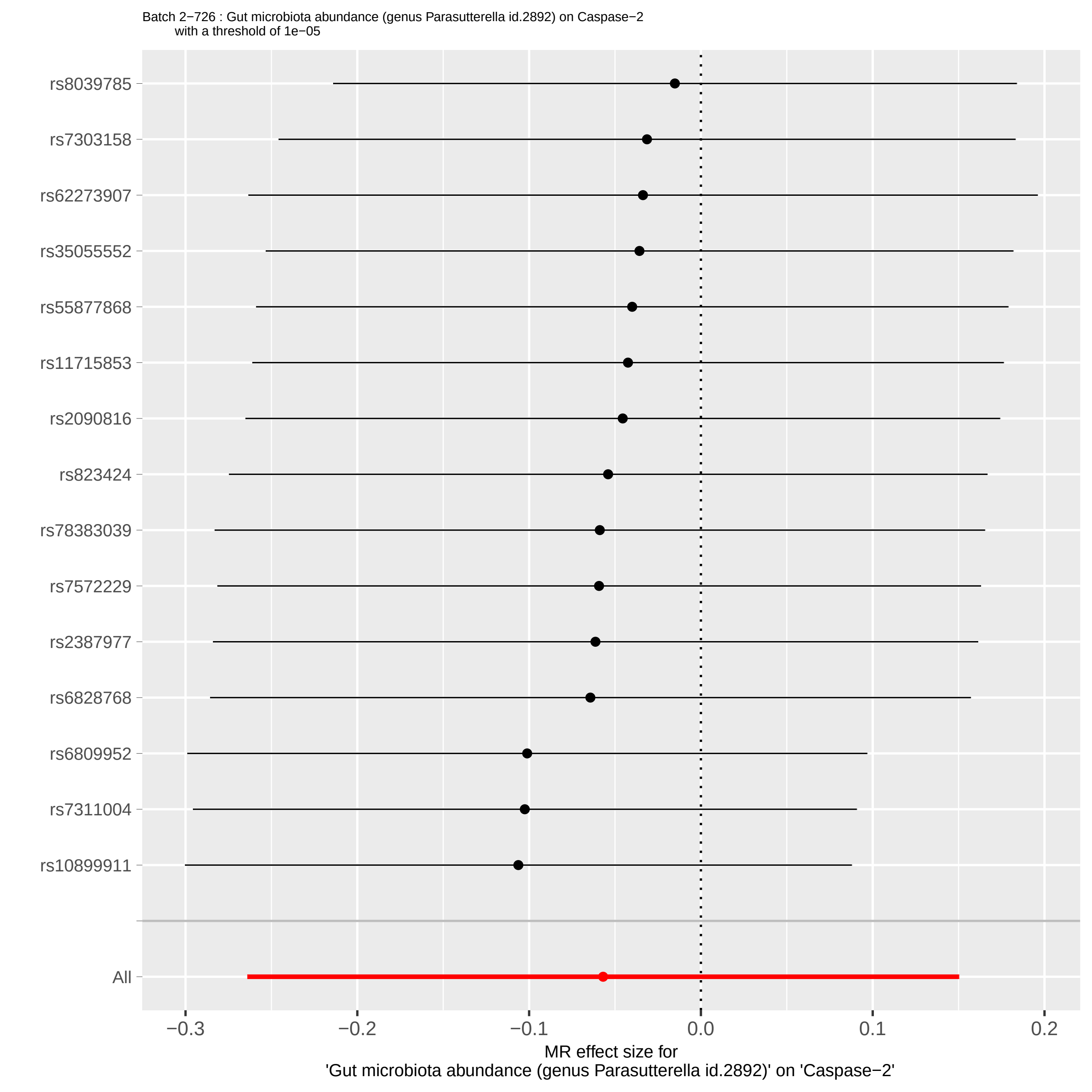

Batch 2−726 : Gut microbiota abundance (genus Parasutterella id.2892) on Caspase−2
with a threshold of 1e−05
rs8039785
rs7303158
rs62273907
rs35055552
rs55877868
rs11715853
rs2090816
rs823424
rs78383039
rs7572229
rs2387977
rs6828768
rs6809952
rs7311004
rs10899911
All
−0.3
−0.2
−0.1
0.0
0.1
0.2
MR effect size for
'Gut microbiota abundance (genus Parasutterella id.2892)' on 'Caspase−2'

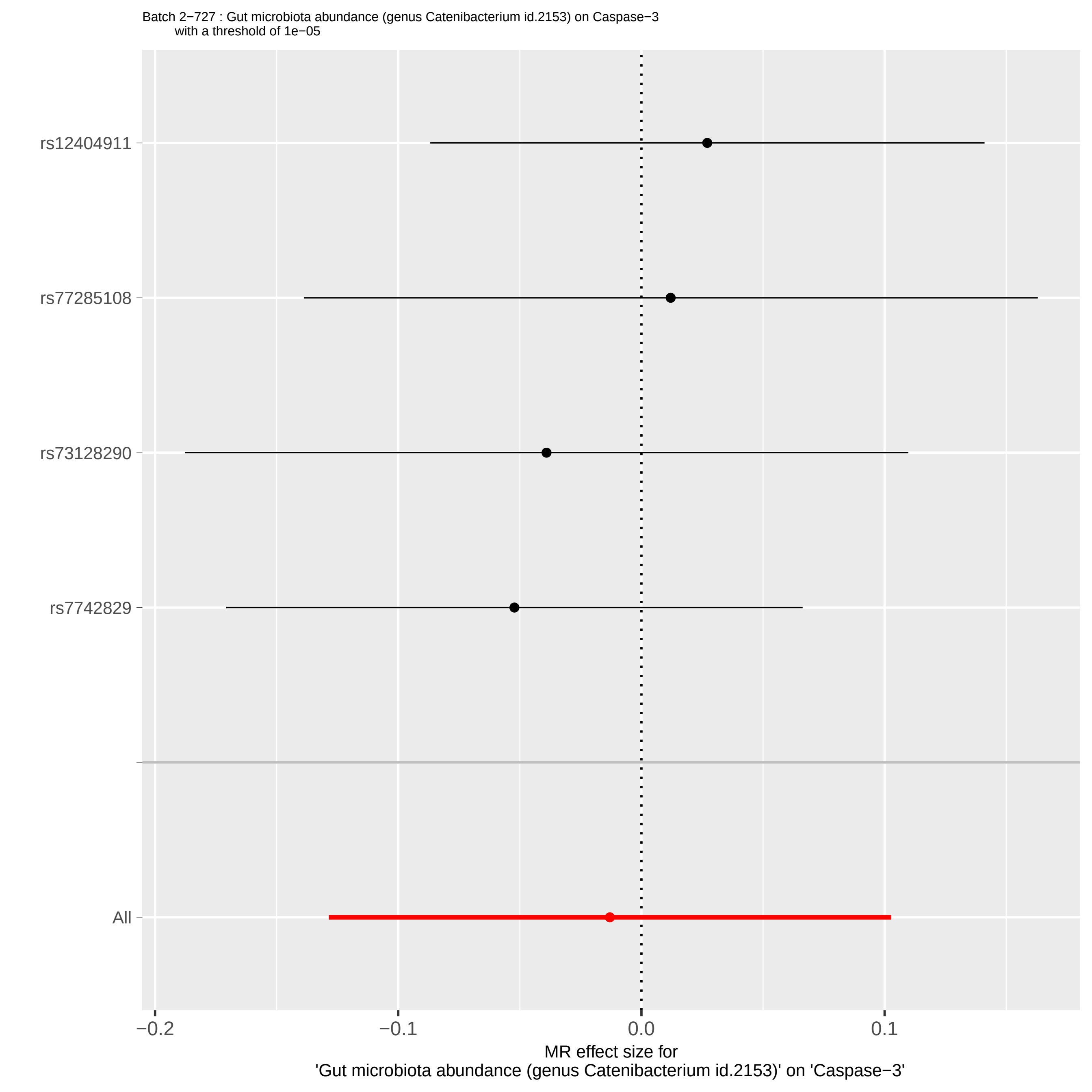

Batch 2−727 : Gut microbiota abundance (genus Catenibacterium id.2153) on Caspase−3
with a threshold of 1e−05
rs12404911
rs77285108
rs73128290
rs7742829
All
−0.2
−0.1
0.0
0.1
MR effect size for
'Gut microbiota abundance (genus Catenibacterium id.2153)' on 'Caspase−3'

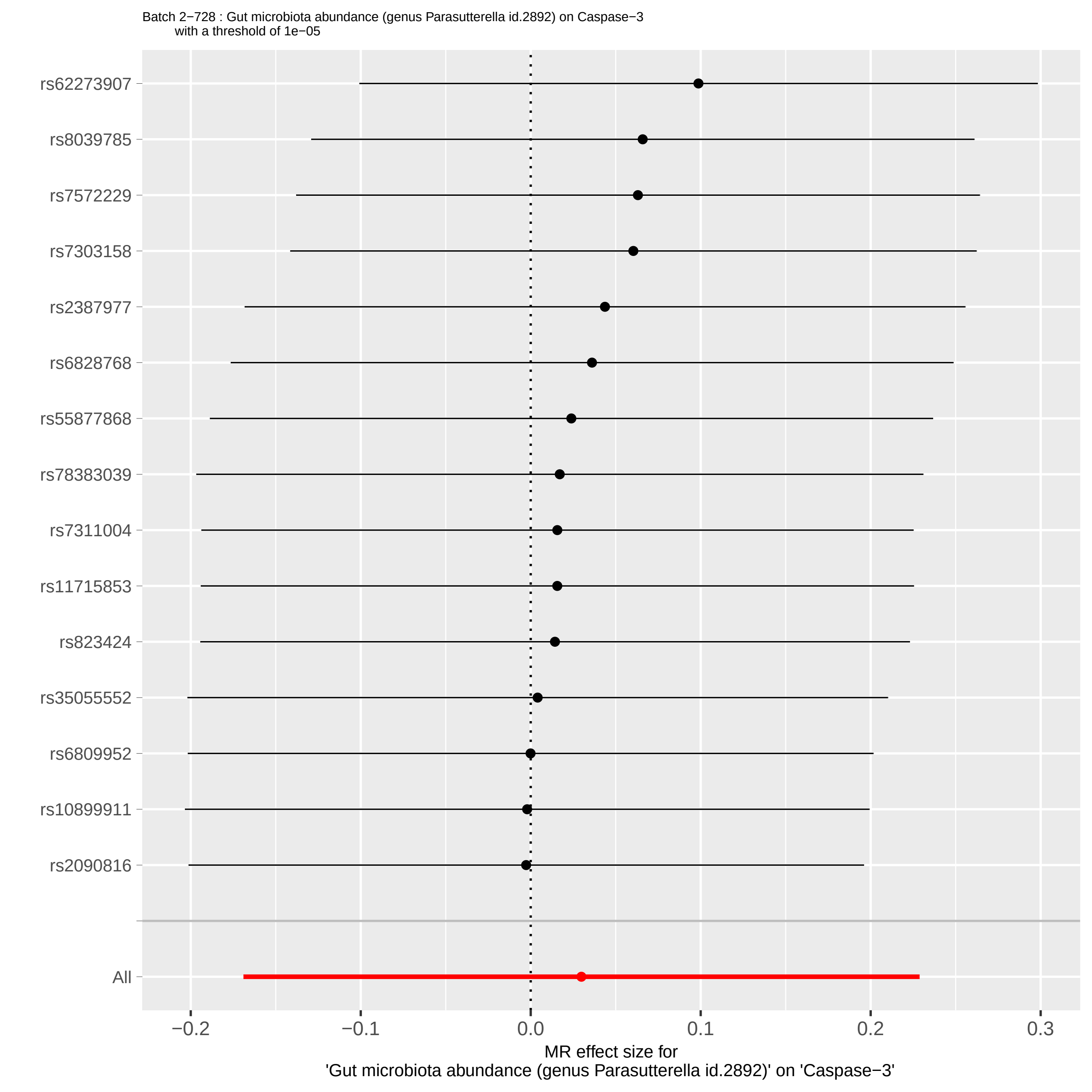

Batch 2−728 : Gut microbiota abundance (genus Parasutterella id.2892) on Caspase−3
with a threshold of 1e−05
rs62273907
rs8039785
rs7572229
rs7303158
rs2387977
rs6828768
rs55877868
rs78383039
rs7311004
rs11715853
rs823424
rs35055552
rs6809952
rs10899911
rs2090816
All
−0.2
−0.1
0.0
0.1
0.2
0.3
MR effect size for
'Gut microbiota abundance (genus Parasutterella id.2892)' on 'Caspase−3'

Batch 2−729 : Gut microbiota abundance (genus Catenibacterium id.2153) on Cas scaffolding protein family member 4
with a threshold of 1e−05

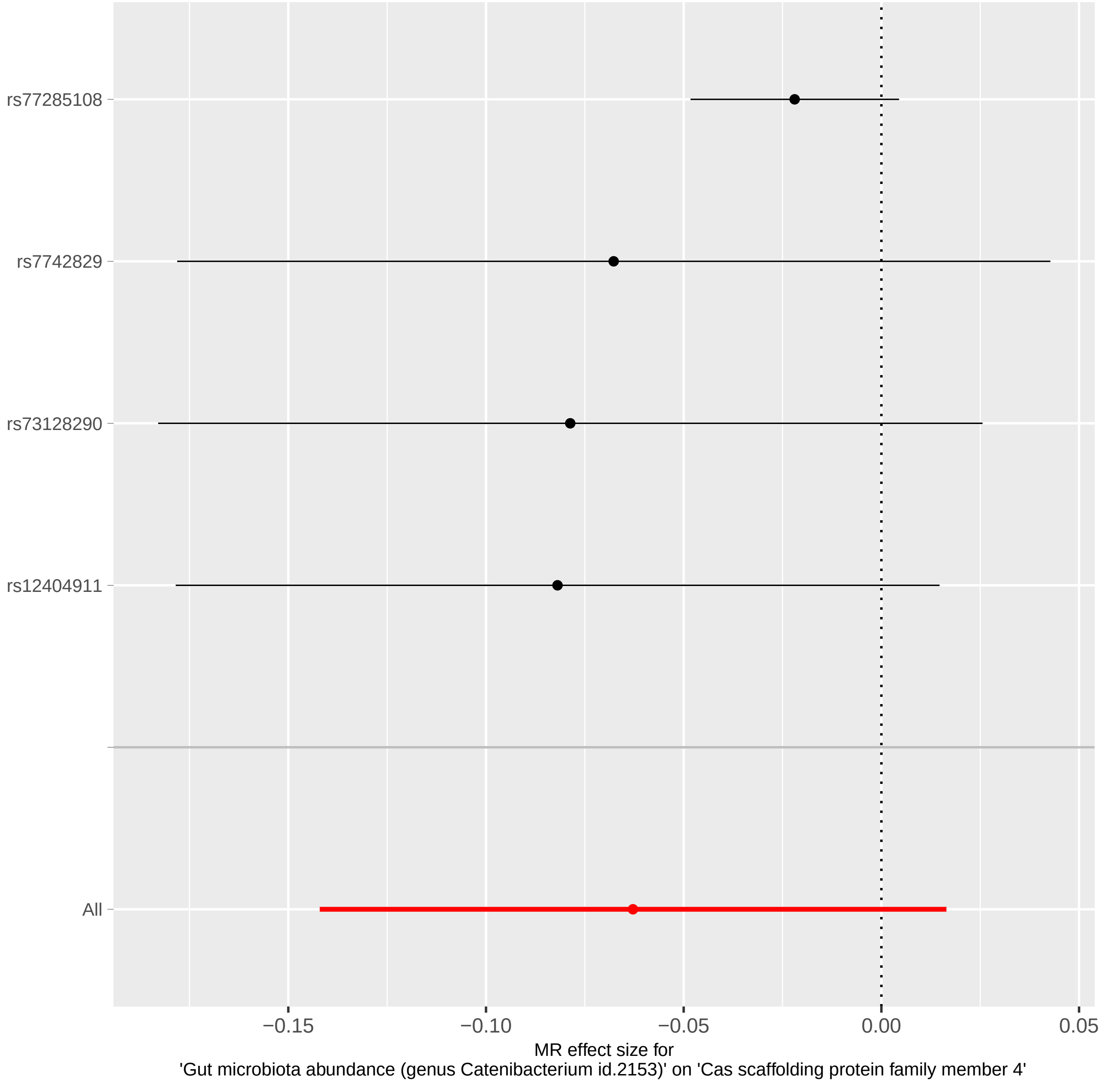

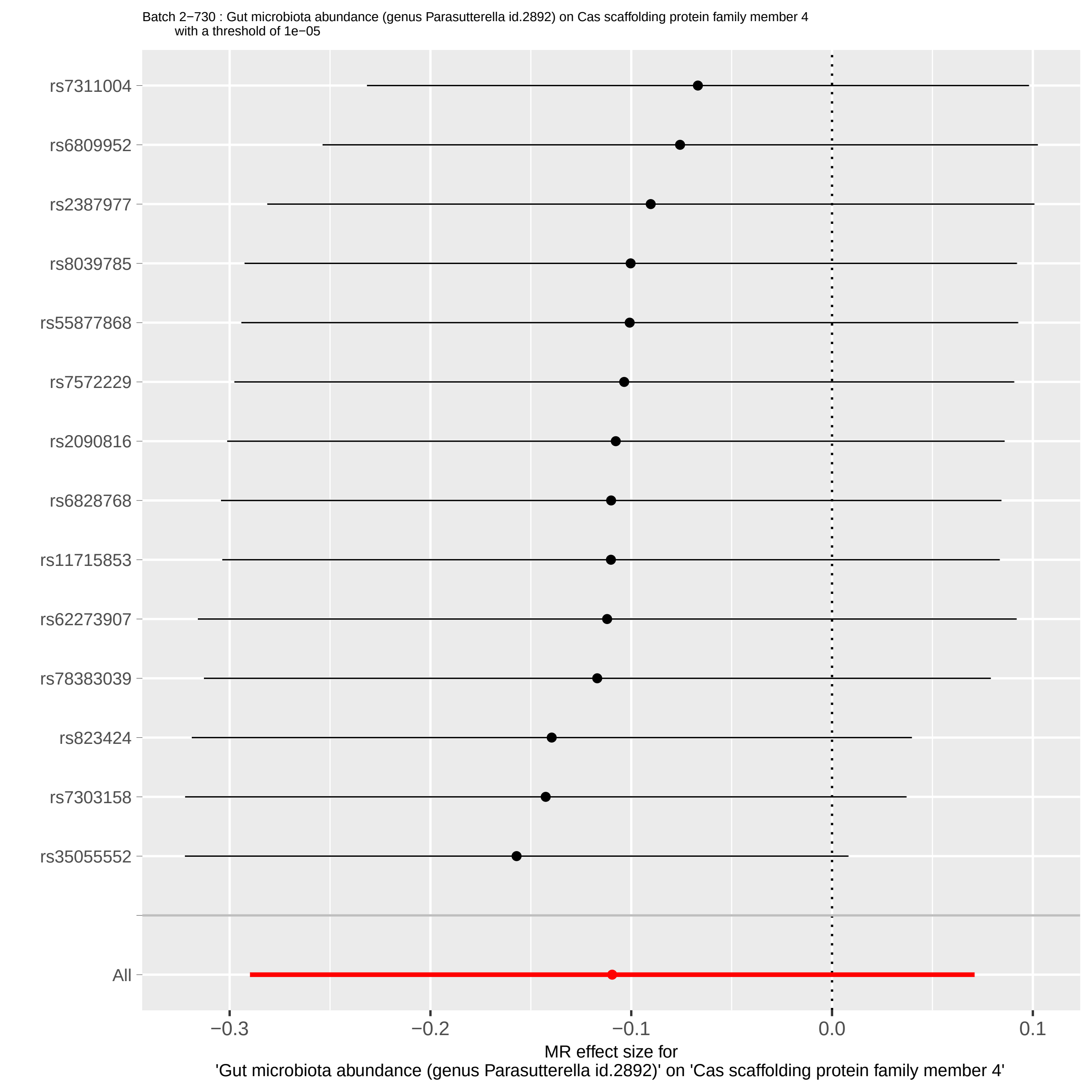
Batch 2−730 : Gut microbiota abundance (genus Parasutterella id.2892) on Cas scaffolding protein family member 4
with a threshold of 1e−05
rs7311004
rs6809952
rs2387977
rs8039785
rs55877868
rs7572229
rs2090816
rs6828768
rs11715853
rs62273907
rs78383039
rs823424
rs7303158
rs35055552
All
−0.3
−0.2
−0.1
0.0
0.1
MR effect size for
'Gut microbiota abundance (genus Parasutterella id.2892)' on 'Cas scaffolding protein family member 4'

Batch 2−731 : Gut microbiota abundance (genus Catenibacterium id.2153) on Calpastatin
with a threshold of 1e−05

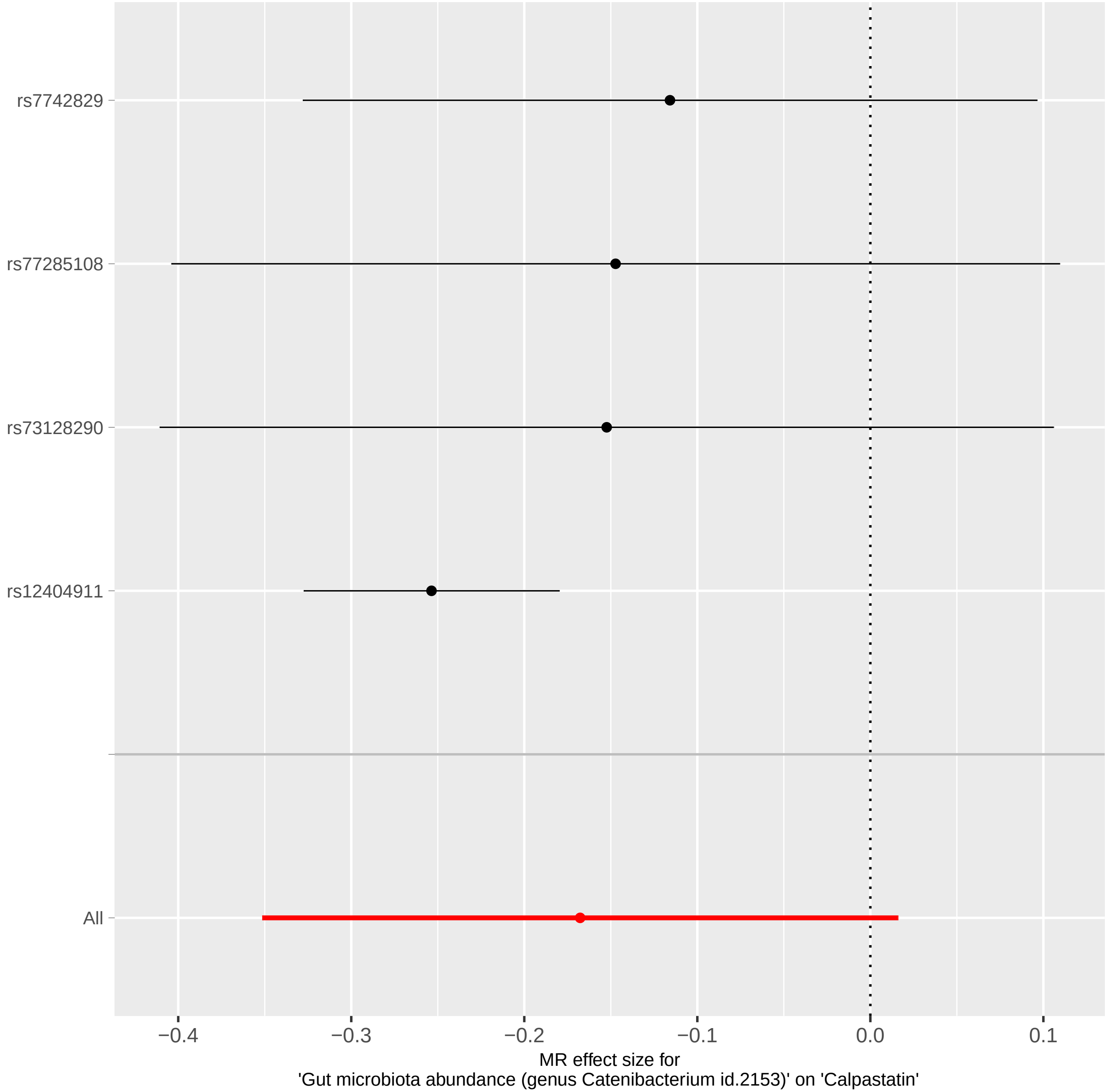

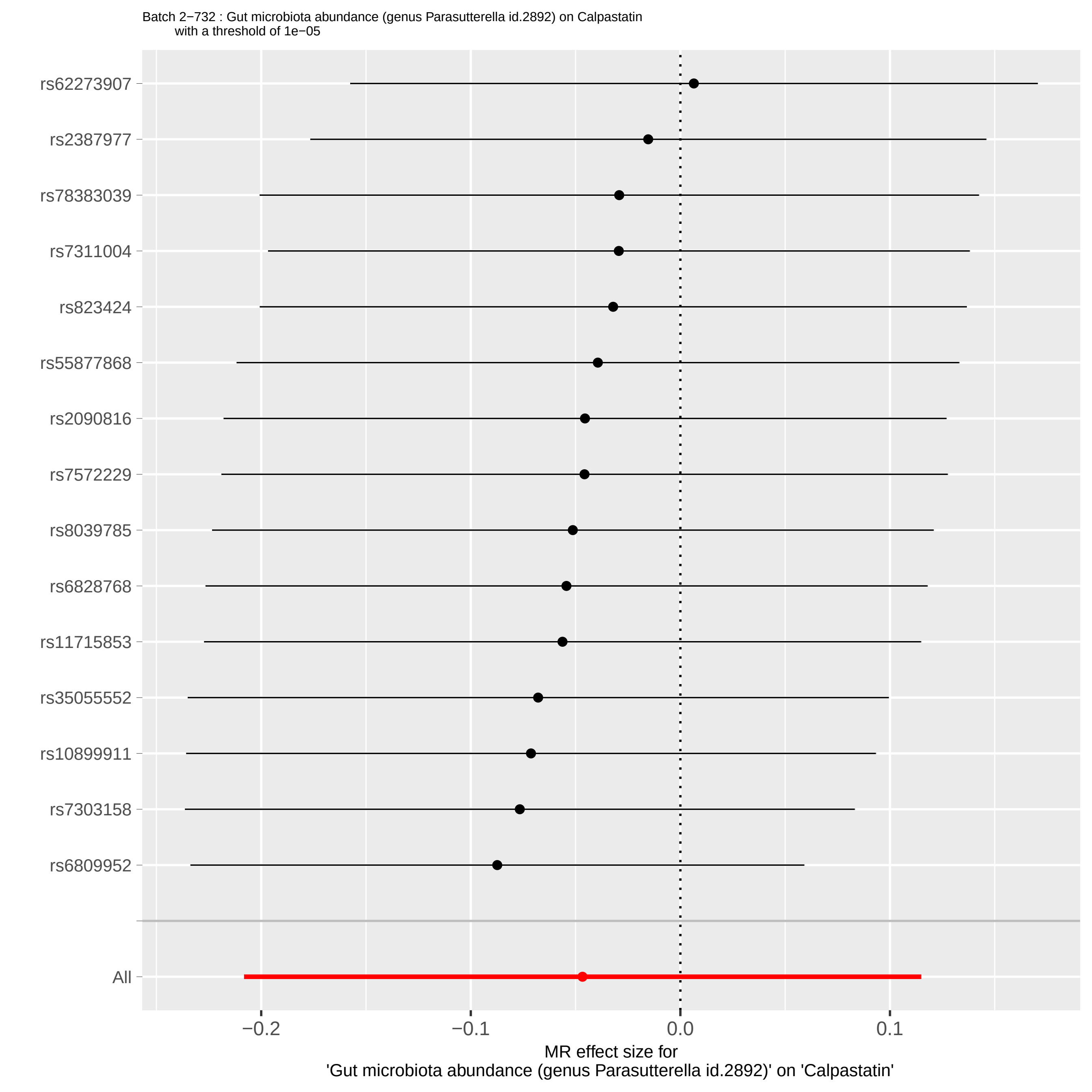

Batch 2−732 : Gut microbiota abundance (genus Parasutterella id.2892) on Calpastatin
with a threshold of 1e−05
rs62273907
rs2387977
rs78383039
rs7311004
rs823424
rs55877868
rs2090816
rs7572229
rs8039785
rs6828768
rs11715853
rs35055552
rs10899911
rs7303158
rs6809952
All
−0.2
−0.1
0.0
0.1
MR effect size for
'Gut microbiota abundance (genus Parasutterella id.2892)' on 'Calpastatin'

Batch 2−733 : Gut microbiota abundance (genus Catenibacterium id.2153) on Catalase
with a threshold of 1e−05

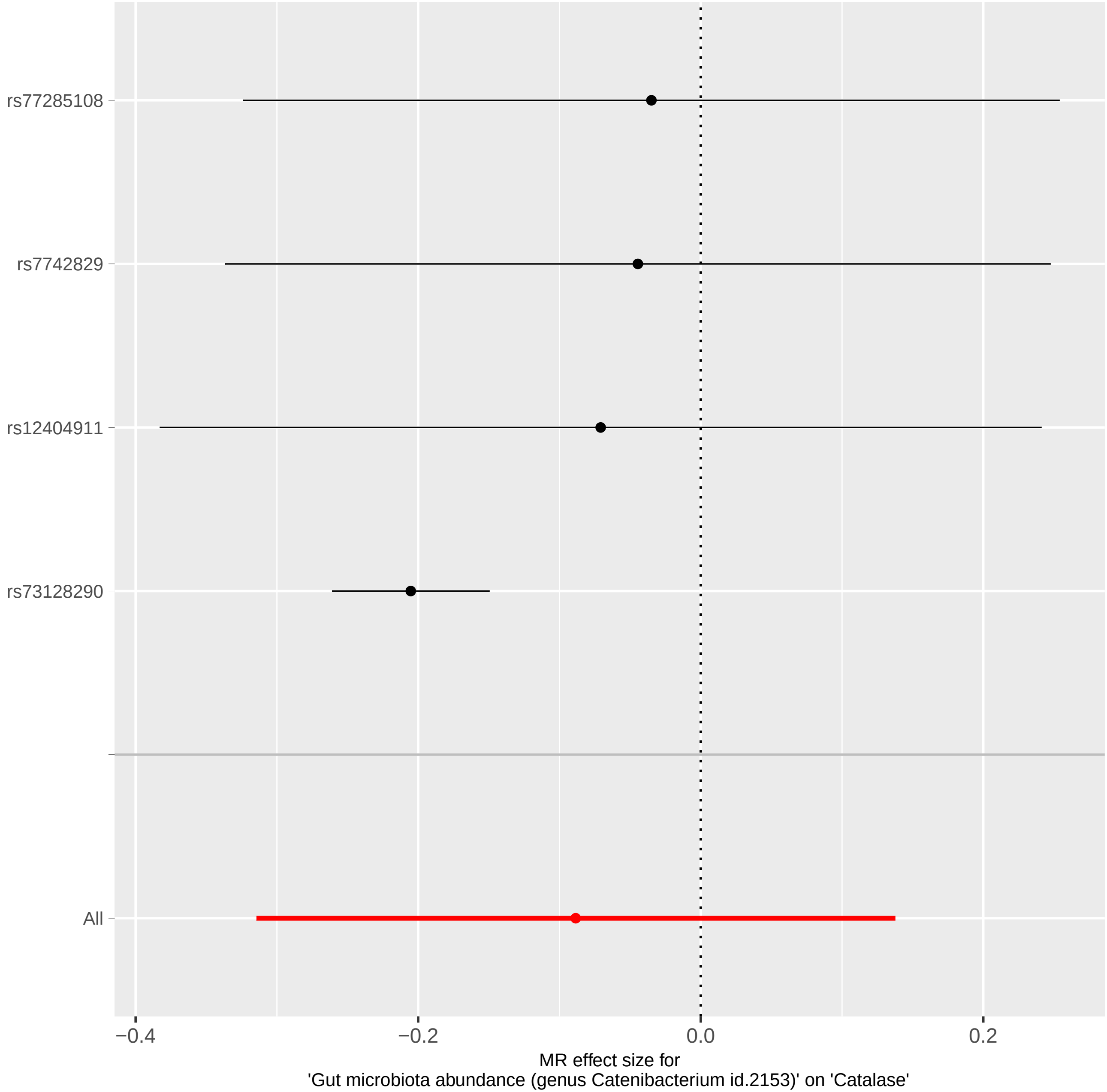

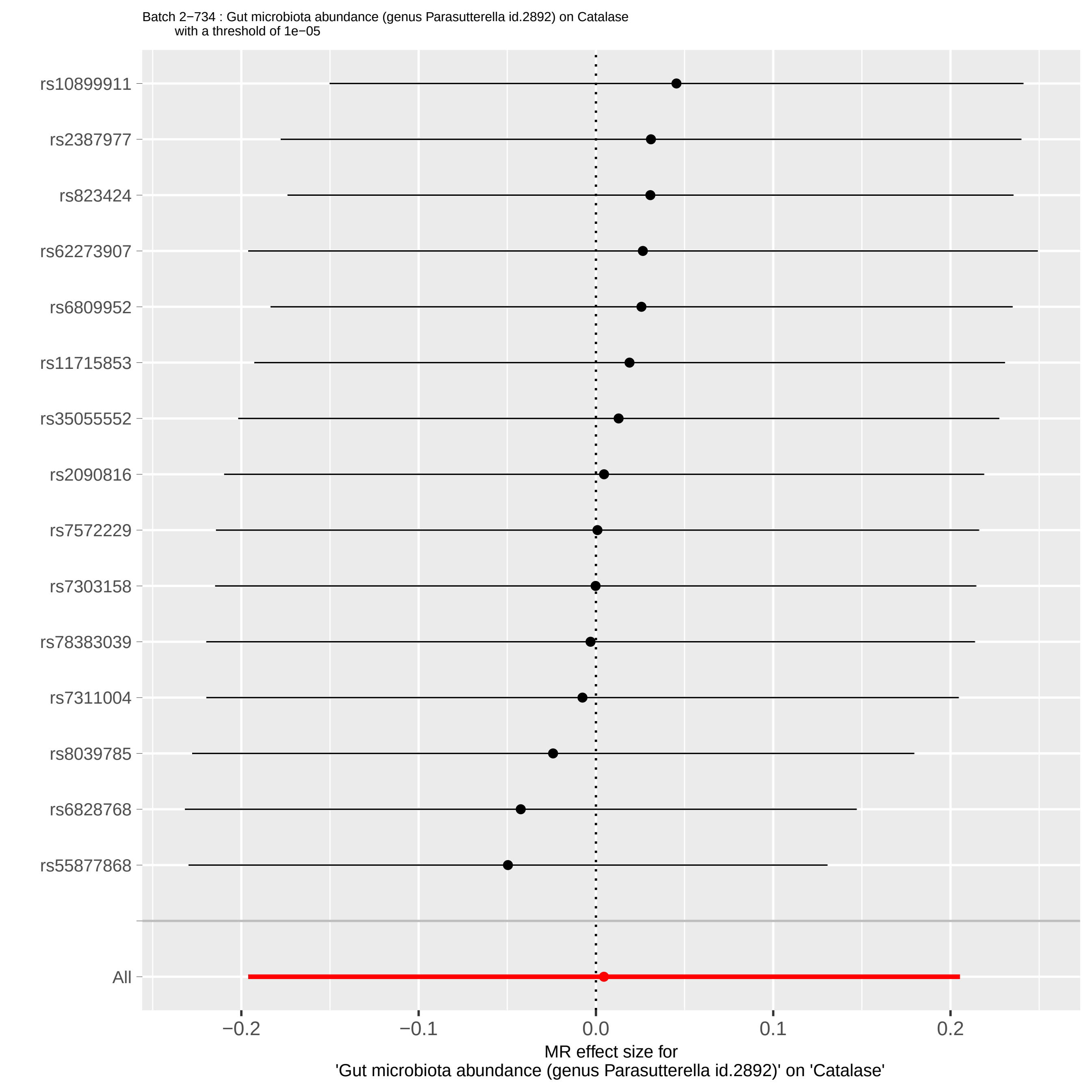

Batch 2−734 : Gut microbiota abundance (genus Parasutterella id.2892) on Catalase
with a threshold of 1e−05
rs10899911
rs2387977
rs823424
rs62273907
rs6809952
rs11715853
rs35055552
rs2090816
rs7572229
rs7303158
rs78383039
rs7311004
rs8039785
rs6828768
rs55877868
All
−0.2
−0.1
0.0
0.1
0.2
MR effect size for
'Gut microbiota abundance (genus Parasutterella id.2892)' on 'Catalase'

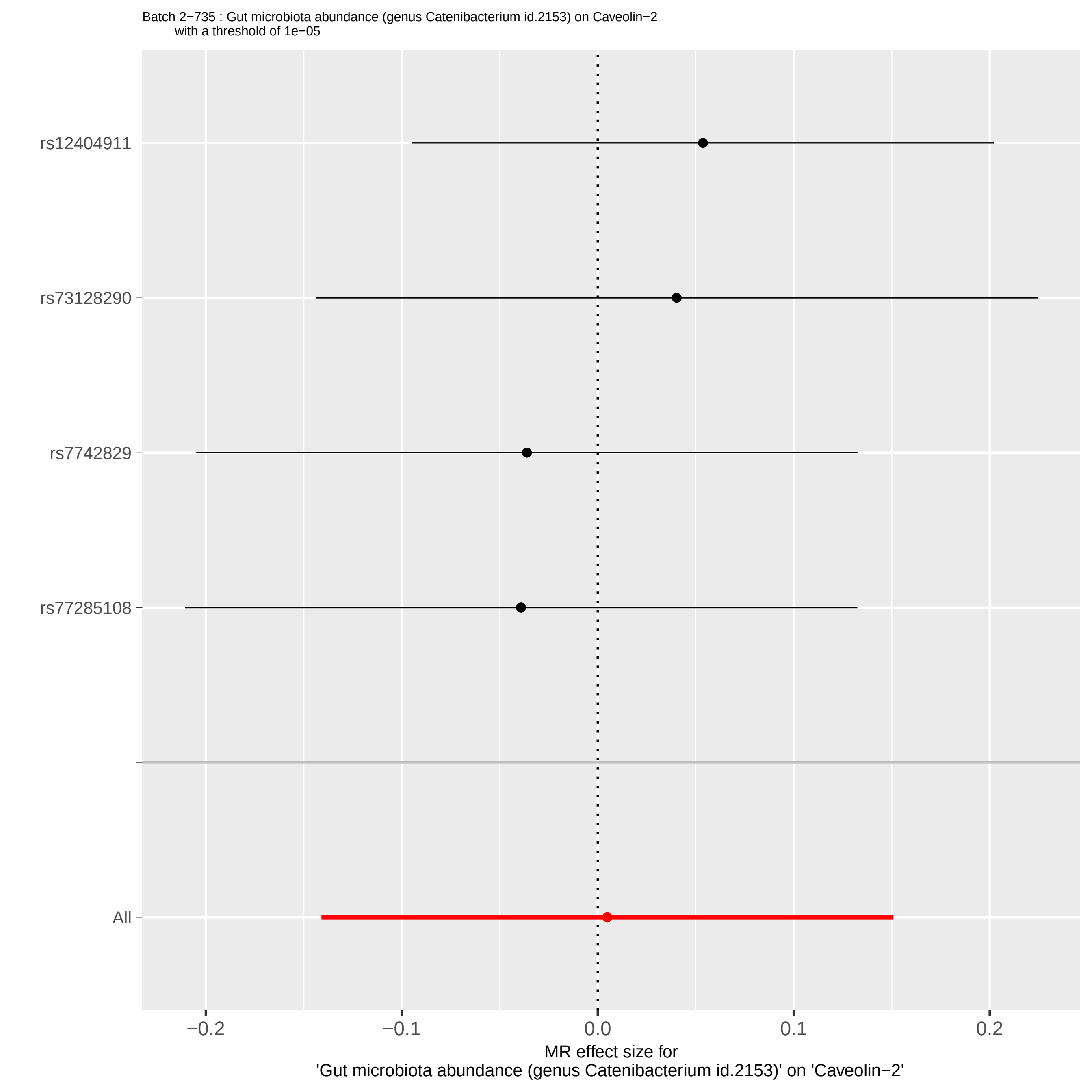
Batch 2−735 : Gut microbiota abundance (genus Catenibacterium id.2153) on Caveolin−2
with a threshold of 1e−05
rs12404911
rs73128290
rs7742829
rs77285108
All
−0.2
−0.1
0.0
0.1
0.2
MR effect size for
'Gut microbiota abundance (genus Catenibacterium id.2153)' on 'Caveolin−2'

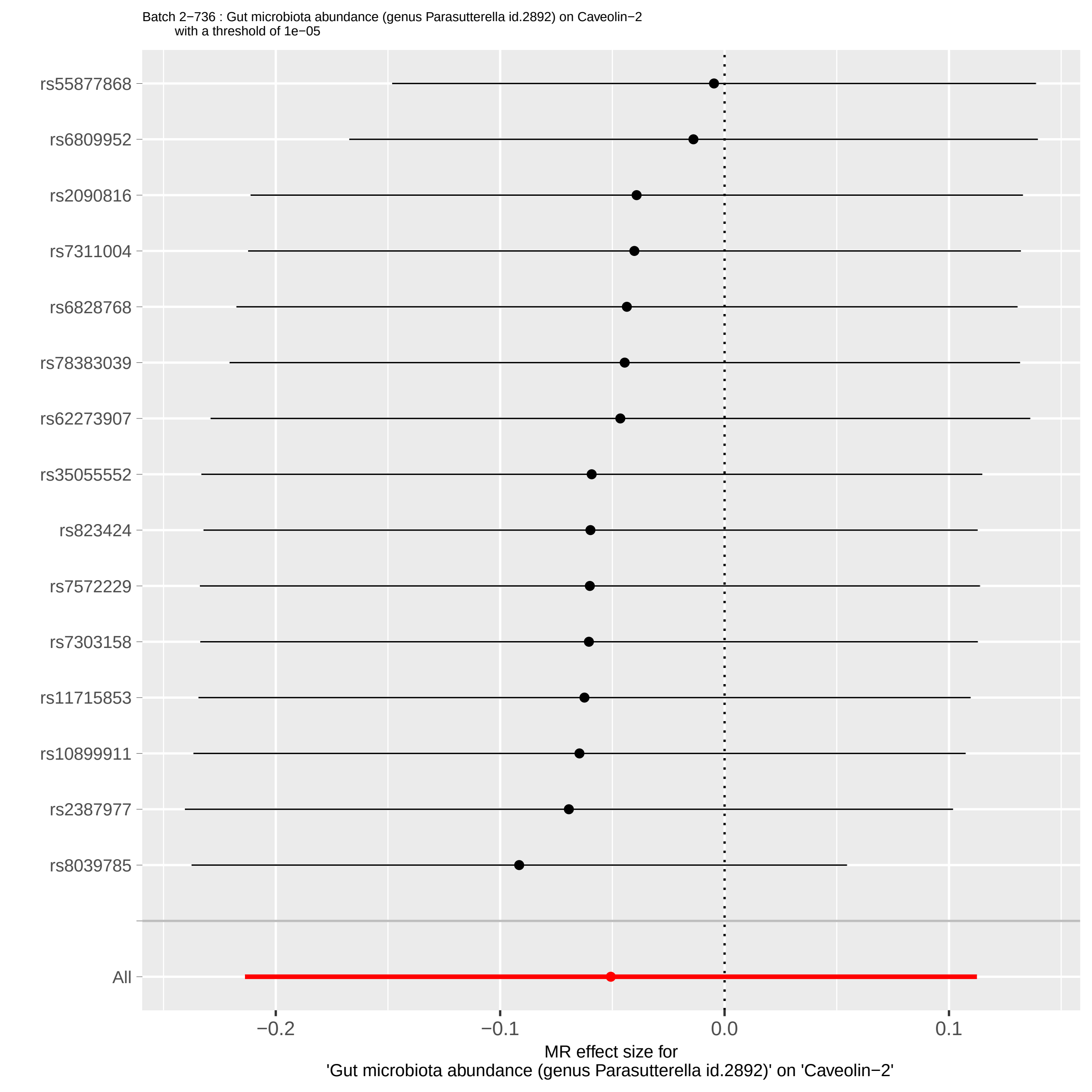
Batch 2−736 : Gut microbiota abundance (genus Parasutterella id.2892) on Caveolin−2
with a threshold of 1e−05
rs55877868
rs6809952
rs2090816
rs7311004
rs6828768
rs78383039
rs62273907
rs35055552
rs823424
rs7572229
rs7303158
rs11715853
rs10899911
rs2387977
rs8039785
All
−0.2
−0.1
0.0
0.1
MR effect size for
'Gut microbiota abundance (genus Parasutterella id.2892)' on 'Caveolin−2'

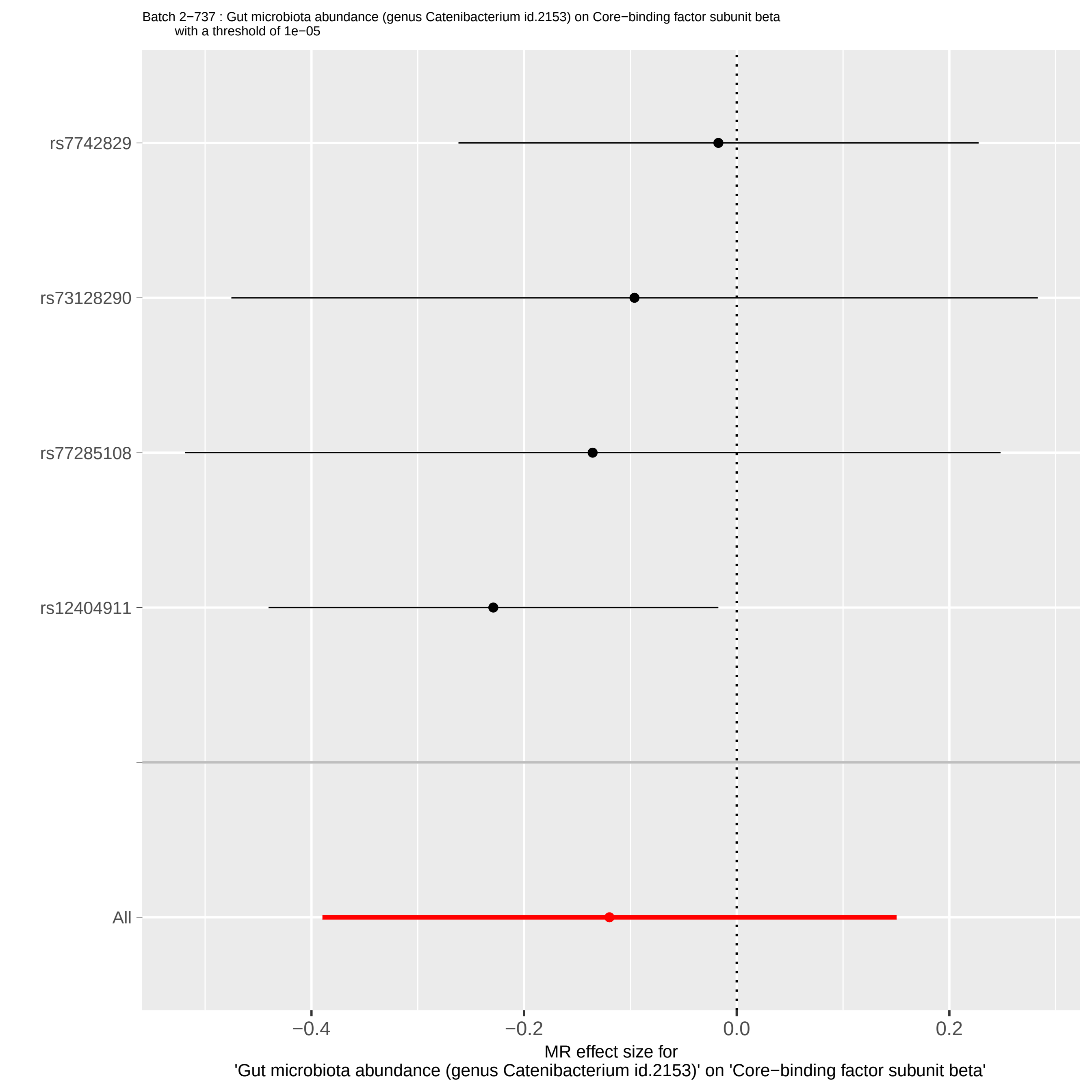
Batch 2−737 : Gut microbiota abundance (genus Catenibacterium id.2153) on Core−binding factor subunit beta
with a threshold of 1e−05
rs7742829
rs73128290
rs77285108
rs12404911
All
−0.4
−0.2
0.0
0.2
MR effect size for
'Gut microbiota abundance (genus Catenibacterium id.2153)' on 'Core−binding factor subunit beta'

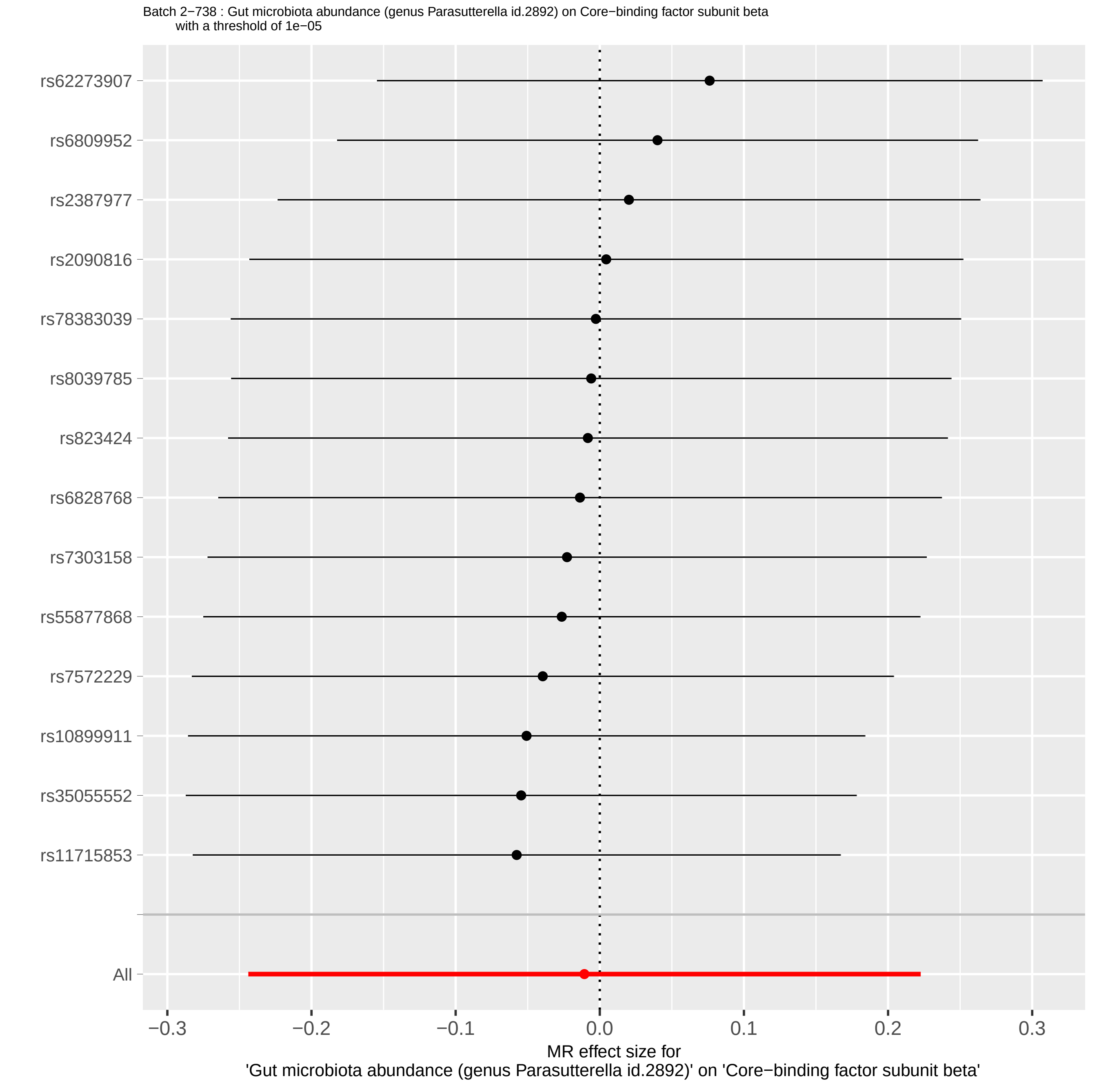
Batch 2−738 : Gut microbiota abundance (genus Parasutterella id.2892) on Core−binding factor subunit beta
with a threshold of 1e−05
rs62273907
rs6809952
rs2387977
rs2090816
rs78383039
rs8039785
rs823424
rs6828768
rs7303158
rs55877868
rs7572229
rs10899911
rs35055552
rs11715853
All
−0.3
−0.2
−0.1
0.0
0.1
0.2
0.3
MR effect size for
'Gut microbiota abundance (genus Parasutterella id.2892)' on 'Core−binding factor subunit beta'

Batch 2−739 : Gut microbiota abundance (genus Catenibacterium id.2153) on E3 ubiquitin−protein ligase CBL
with a threshold of 1e−05

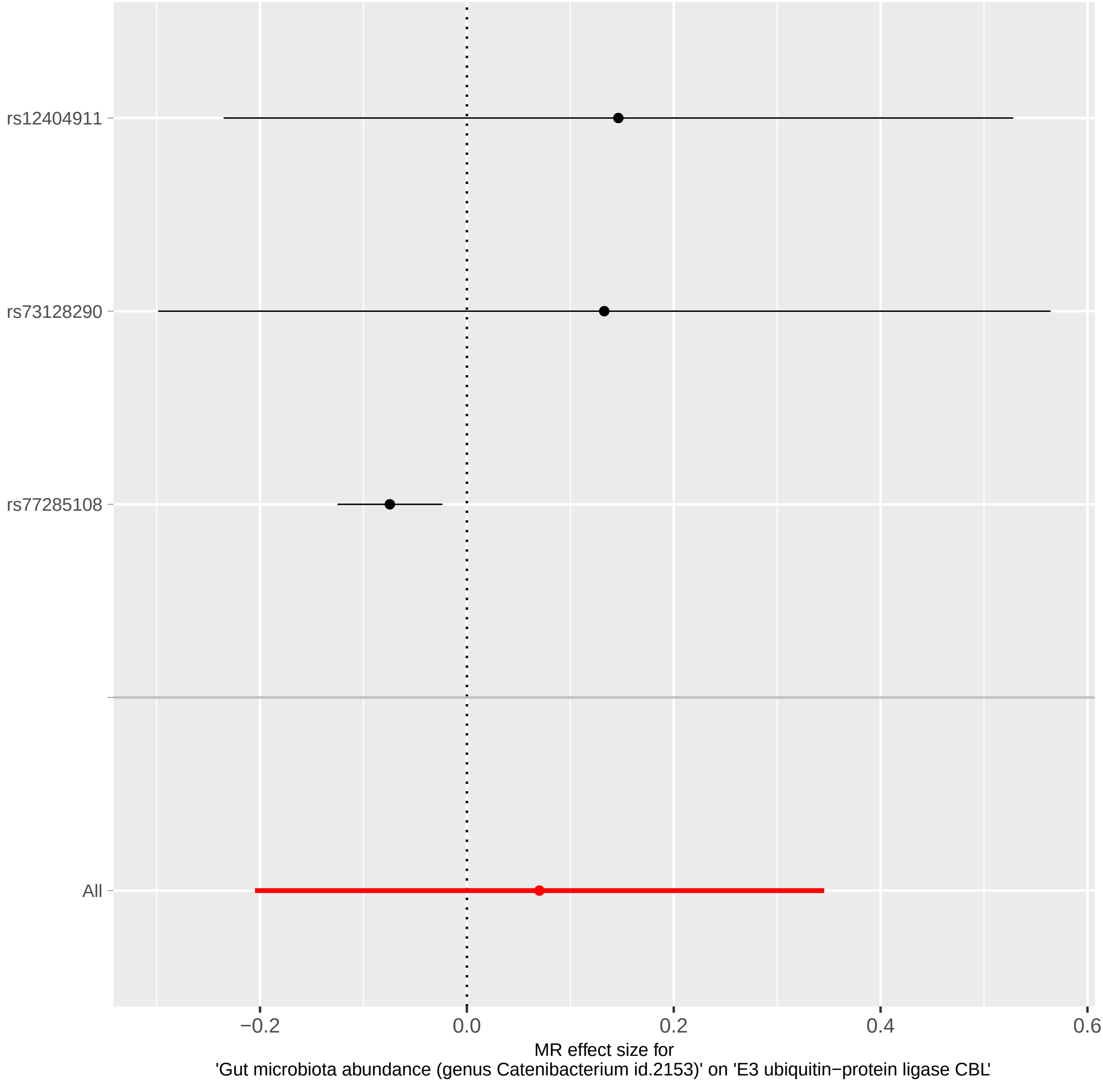

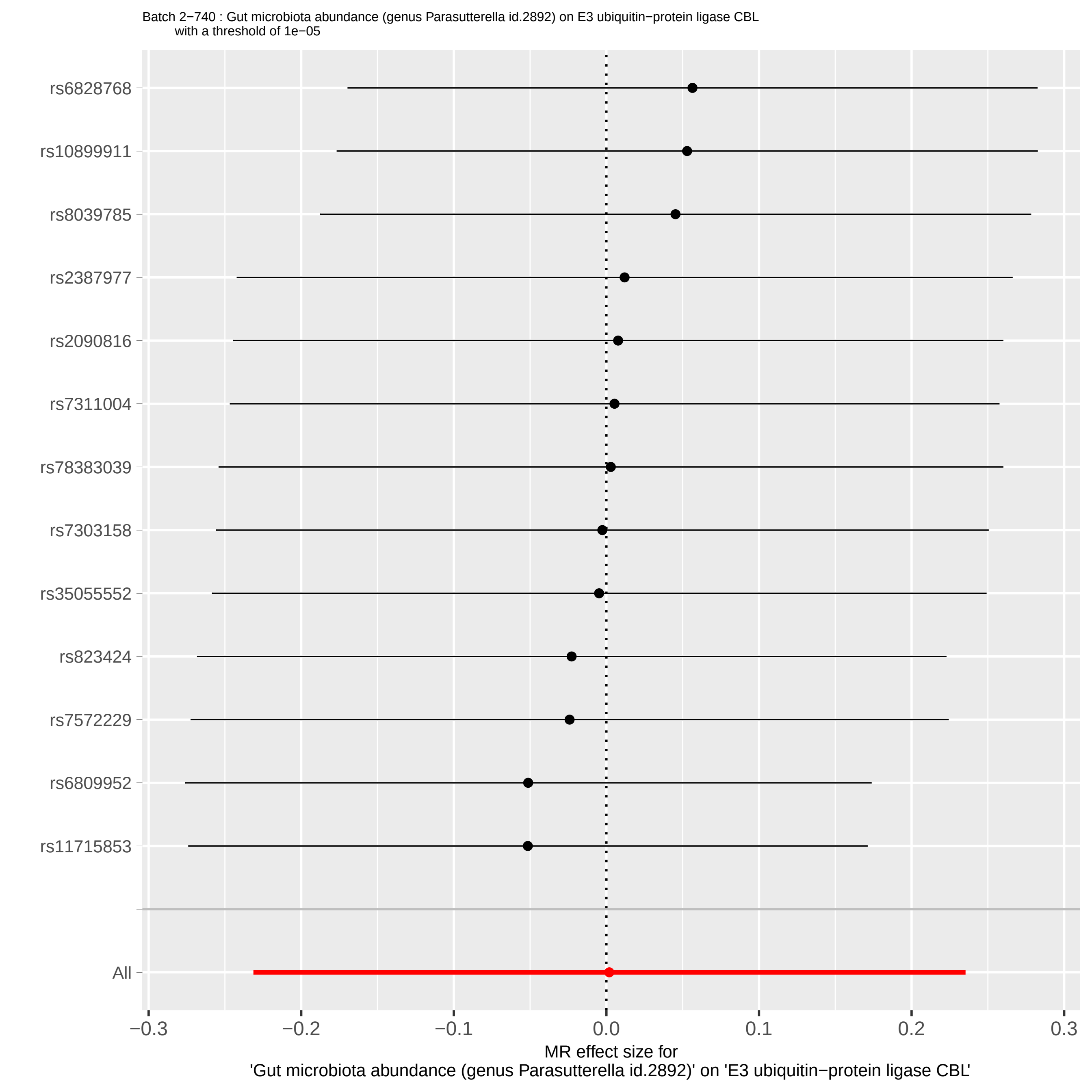

Batch 2−740 : Gut microbiota abundance (genus Parasutterella id.2892) on E3 ubiquitin−protein ligase CBL
with a threshold of 1e−05
rs6828768
rs10899911
rs8039785
rs2387977
rs2090816
rs7311004
rs78383039
rs7303158
rs35055552
rs823424
rs7572229
rs6809952
rs11715853
All
−0.3
−0.2
−0.1
0.0
0.1
0.2
0.3
MR effect size for
'Gut microbiota abundance (genus Parasutterella id.2892)' on 'E3 ubiquitin−protein ligase CBL'

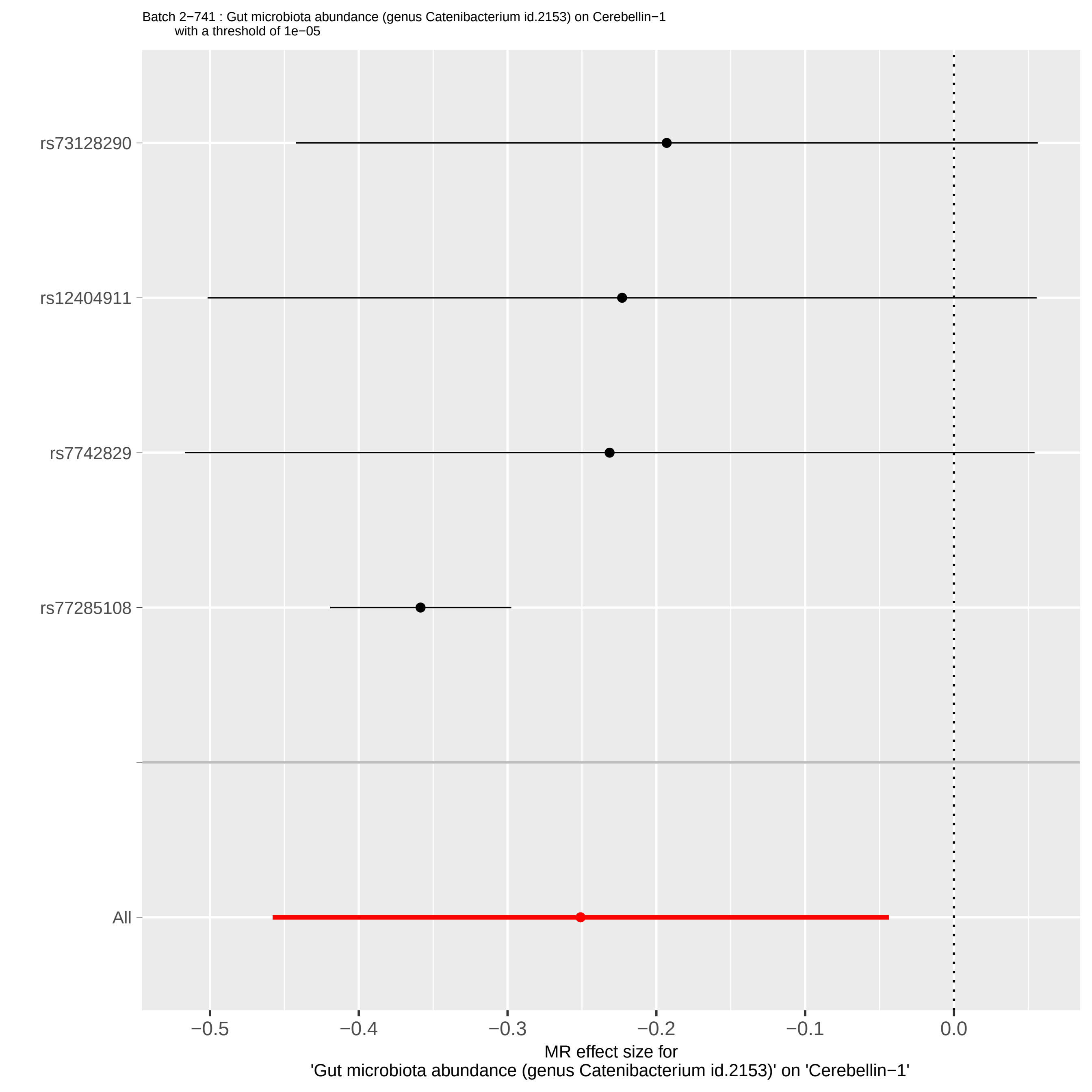
Batch 2−741 : Gut microbiota abundance (genus Catenibacterium id.2153) on Cerebellin−1
with a threshold of 1e−05
rs73128290
rs12404911
rs7742829
rs77285108
All
−0.5
−0.4
−0.3
−0.2
−0.1
0.0
MR effect size for
'Gut microbiota abundance (genus Catenibacterium id.2153)' on 'Cerebellin−1'

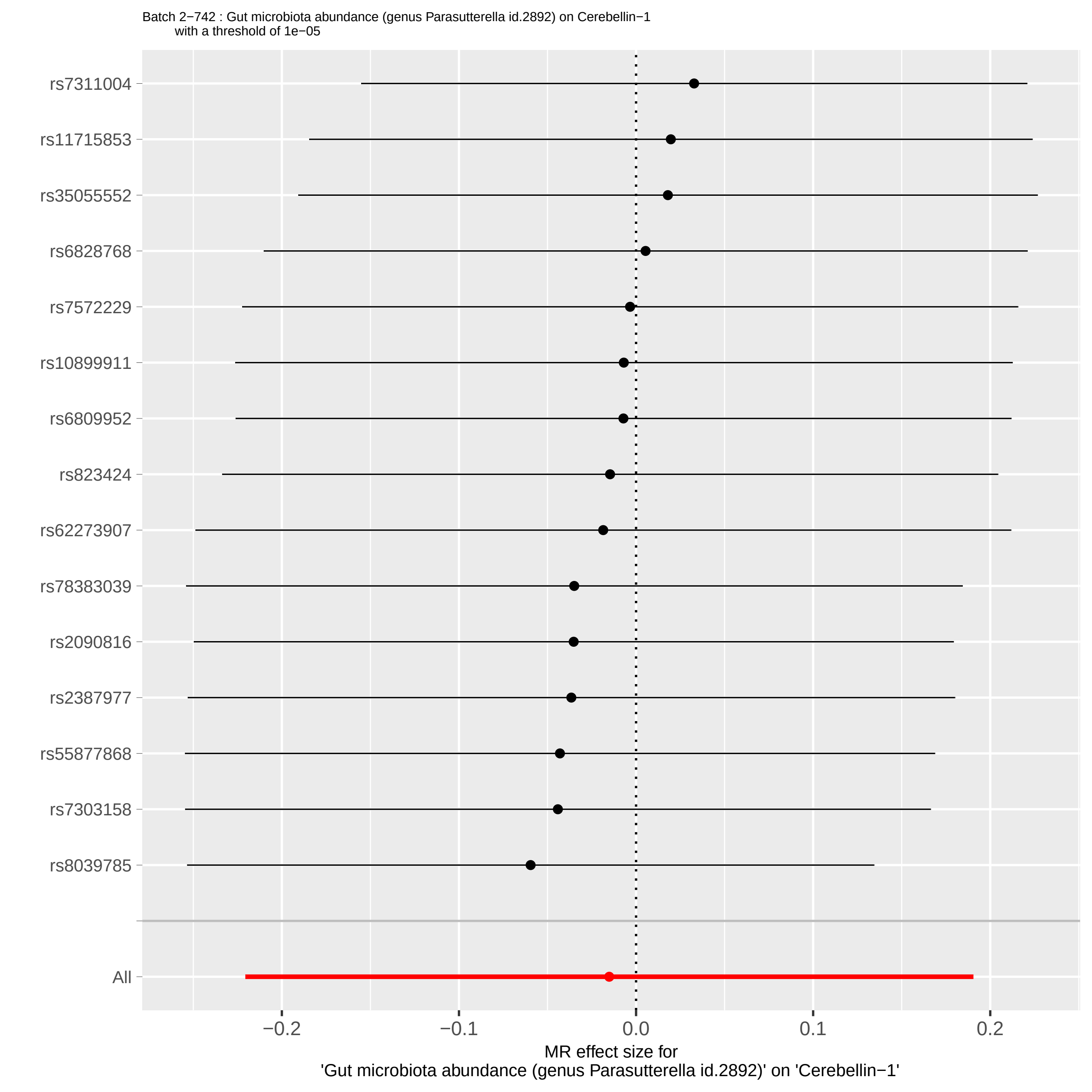
Batch 2−742 : Gut microbiota abundance (genus Parasutterella id.2892) on Cerebellin−1
with a threshold of 1e−05
rs7311004
rs11715853
rs35055552
rs6828768
rs7572229
rs10899911
rs6809952
rs823424
rs62273907
rs78383039
rs2090816
rs2387977
rs55877868
rs7303158
rs8039785
All
−0.2
−0.1
0.0
0.1
0.2
MR effect size for
'Gut microbiota abundance (genus Parasutterella id.2892)' on 'Cerebellin−1'

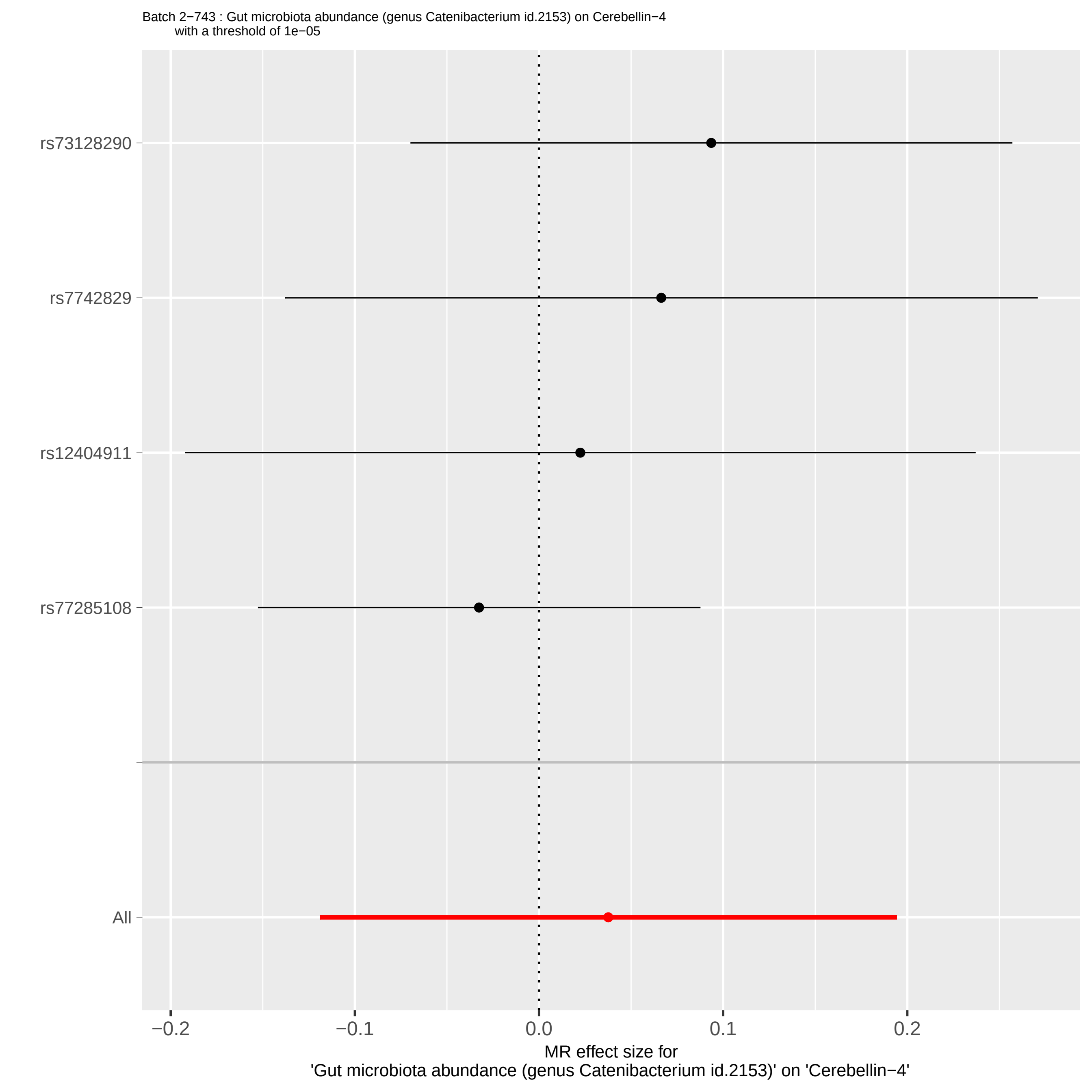
Batch 2−743 : Gut microbiota abundance (genus Catenibacterium id.2153) on Cerebellin−4
with a threshold of 1e−05
rs73128290
rs7742829
rs12404911
rs77285108
All
−0.2
−0.1
0.0
0.1
0.2
MR effect size for
'Gut microbiota abundance (genus Catenibacterium id.2153)' on 'Cerebellin−4'

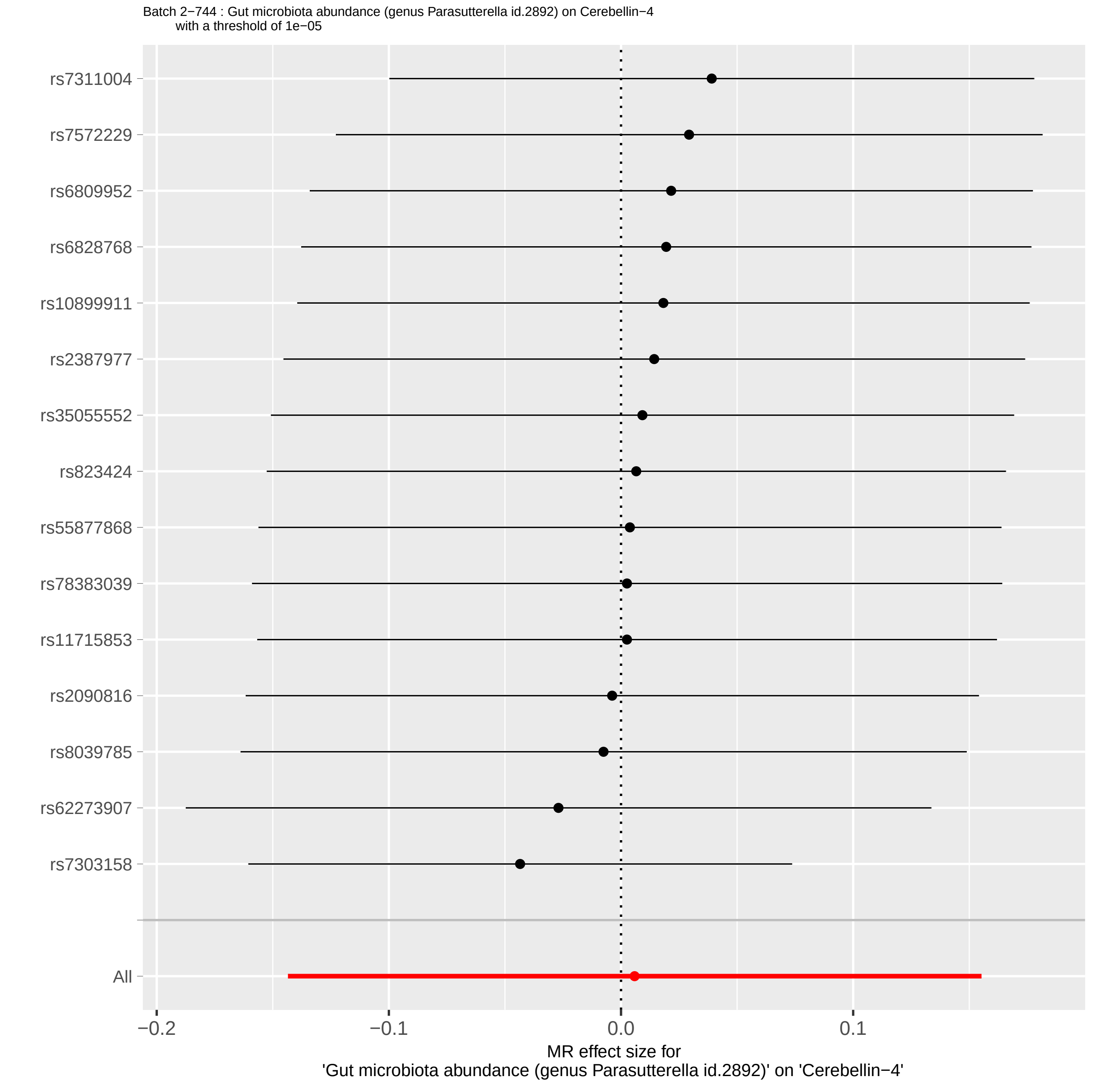
Batch 2−744 : Gut microbiota abundance (genus Parasutterella id.2892) on Cerebellin−4
with a threshold of 1e−05
rs7311004
rs7572229
rs6809952
rs6828768
rs10899911
rs2387977
rs35055552
rs823424
rs55877868
rs78383039
rs11715853
rs2090816
rs8039785
rs62273907
rs7303158
All
−0.2
−0.1
0.0
0.1
MR effect size for
'Gut microbiota abundance (genus Parasutterella id.2892)' on 'Cerebellin−4'

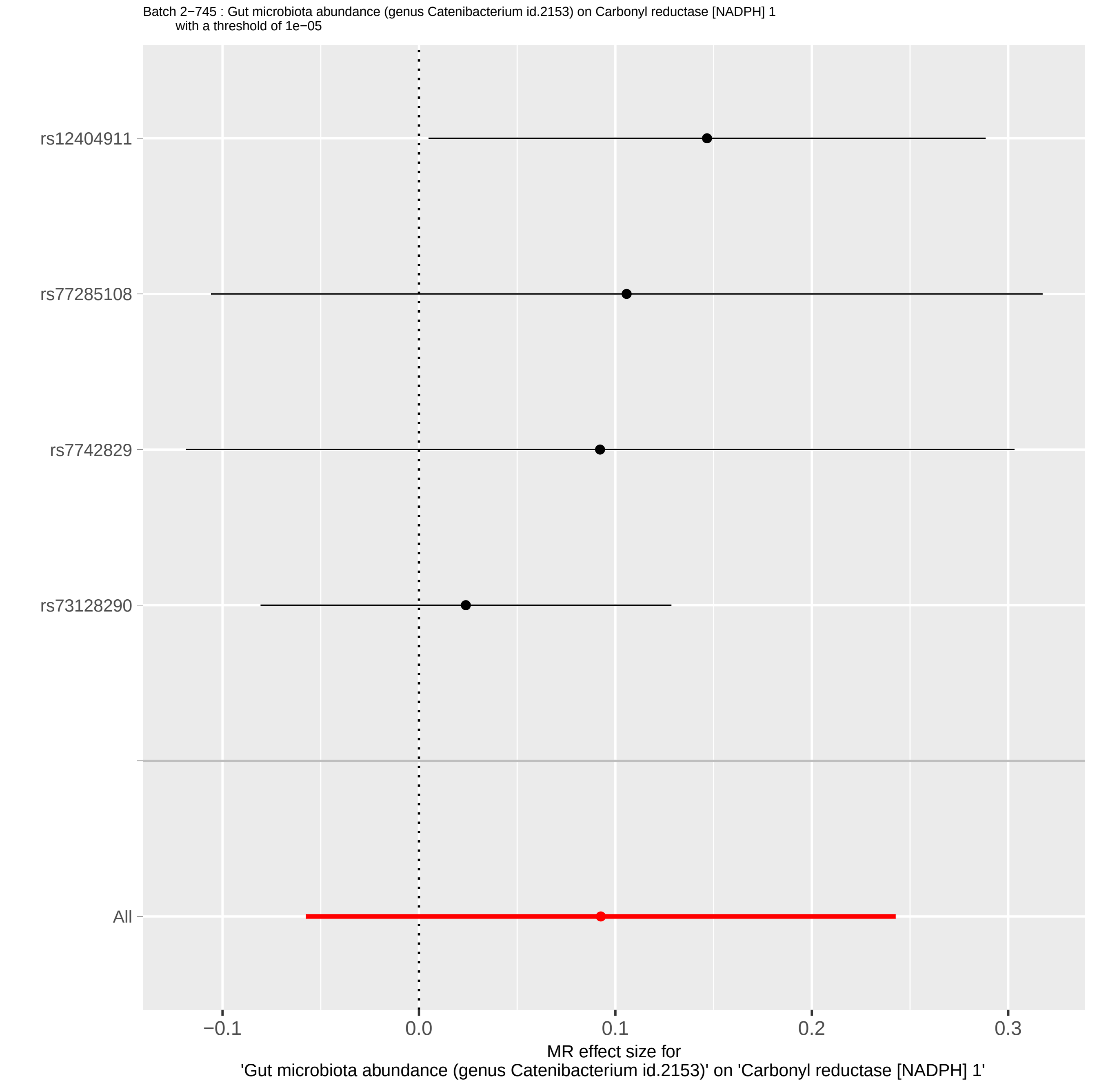

Batch 2−745 : Gut microbiota abundance (genus Catenibacterium id.2153) on Carbonyl reductase [NADPH] 1
with a threshold of 1e−05
rs12404911
rs77285108
rs7742829
rs73128290
All
−0.1
0.0
0.1
0.2
0.3
MR effect size for
'Gut microbiota abundance (genus Catenibacterium id.2153)' on 'Carbonyl reductase [NADPH] 1'

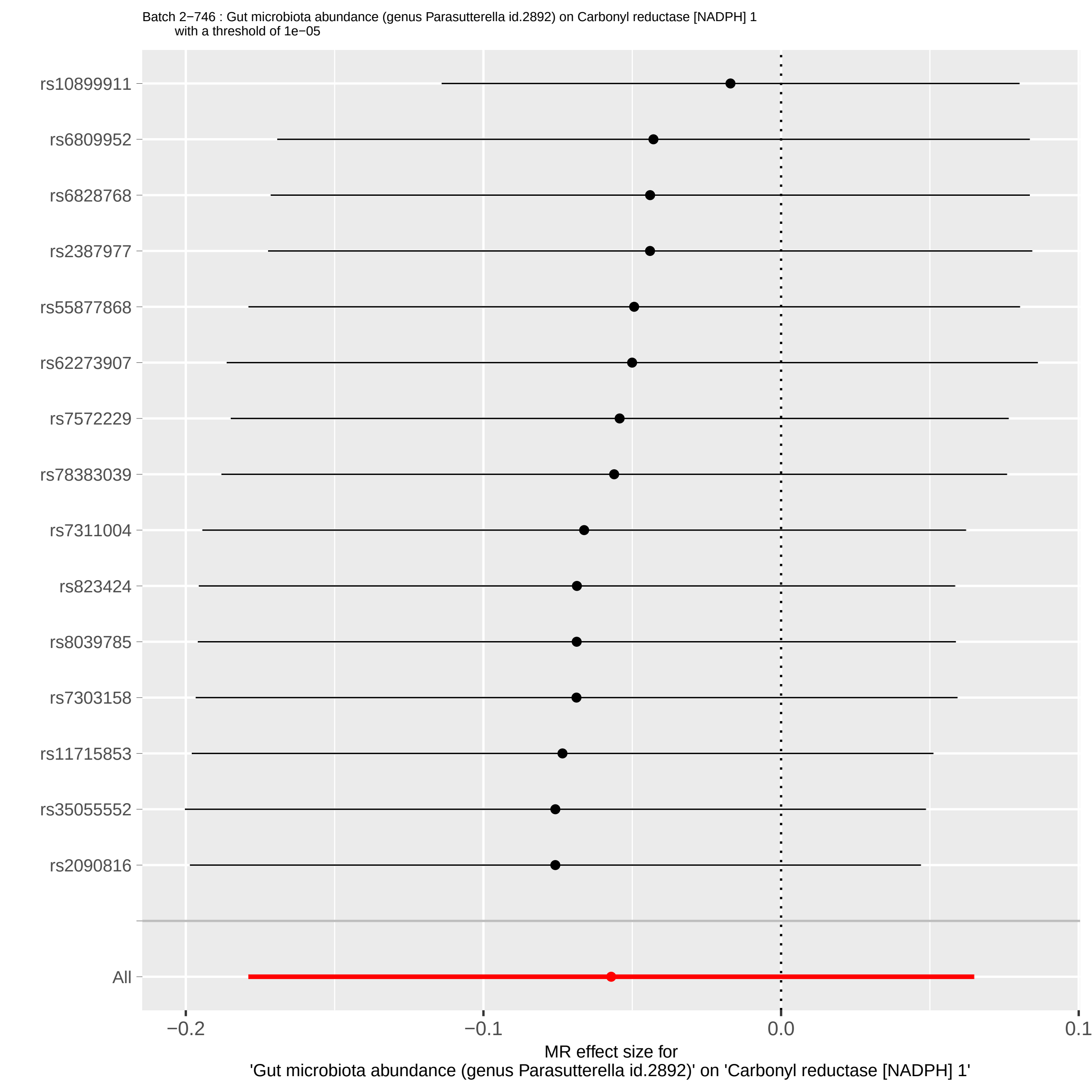
Batch 2−746 : Gut microbiota abundance (genus Parasutterella id.2892) on Carbonyl reductase [NADPH] 1
with a threshold of 1e−05
rs10899911
rs6809952
rs6828768
rs2387977
rs55877868
rs62273907
rs7572229
rs78383039
rs7311004
rs823424
rs8039785
rs7303158
rs11715853
rs35055552
rs2090816
All
−0.2
−0.1
0.0
0.1
MR effect size for
'Gut microbiota abundance (genus Parasutterella id.2892)' on 'Carbonyl reductase [NADPH] 1'

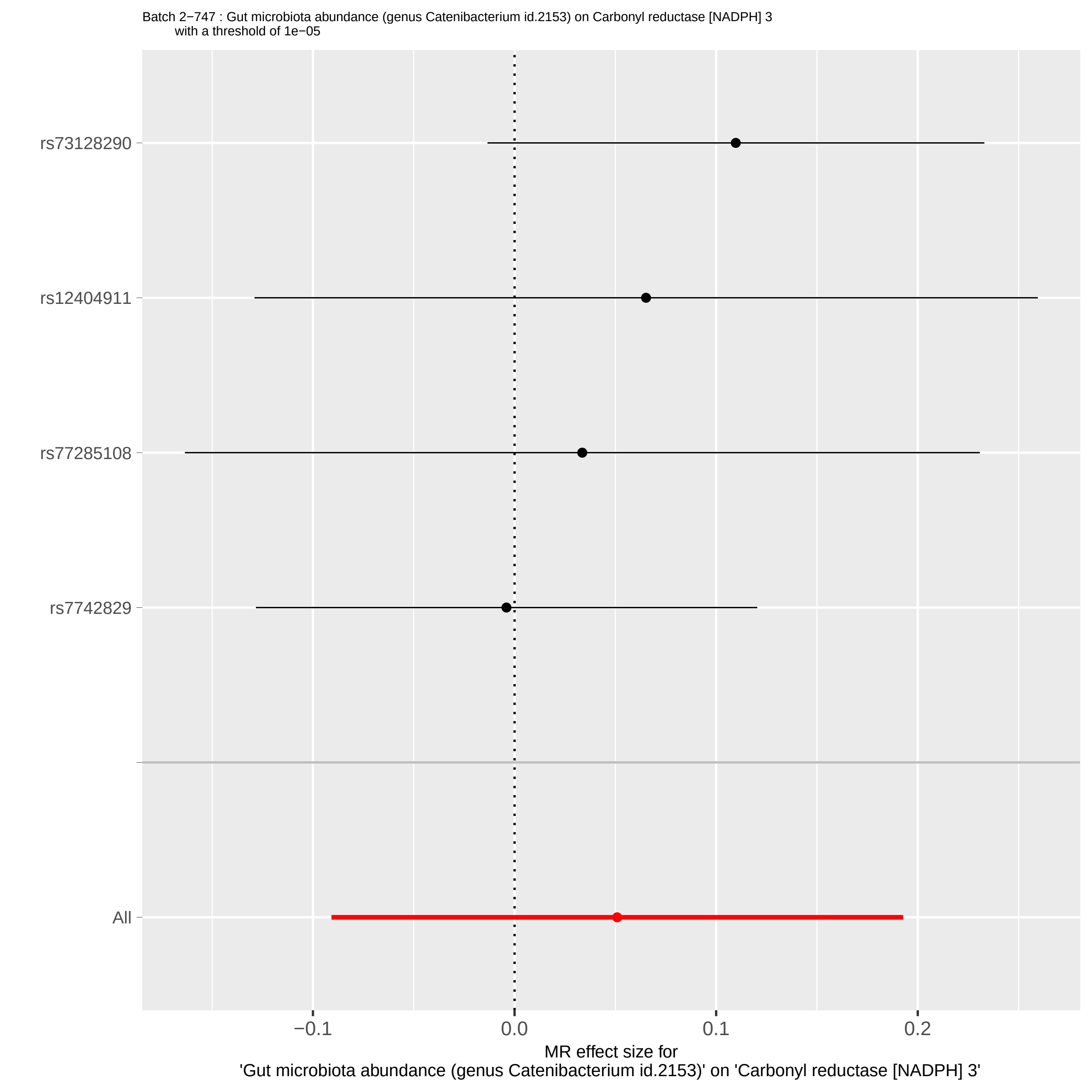

Batch 2−747 : Gut microbiota abundance (genus Catenibacterium id.2153) on Carbonyl reductase [NADPH] 3
with a threshold of 1e−05
rs73128290
rs12404911
rs77285108
rs7742829
All
−0.1
0.0
0.1
0.2
MR effect size for
'Gut microbiota abundance (genus Catenibacterium id.2153)' on 'Carbonyl reductase [NADPH] 3'

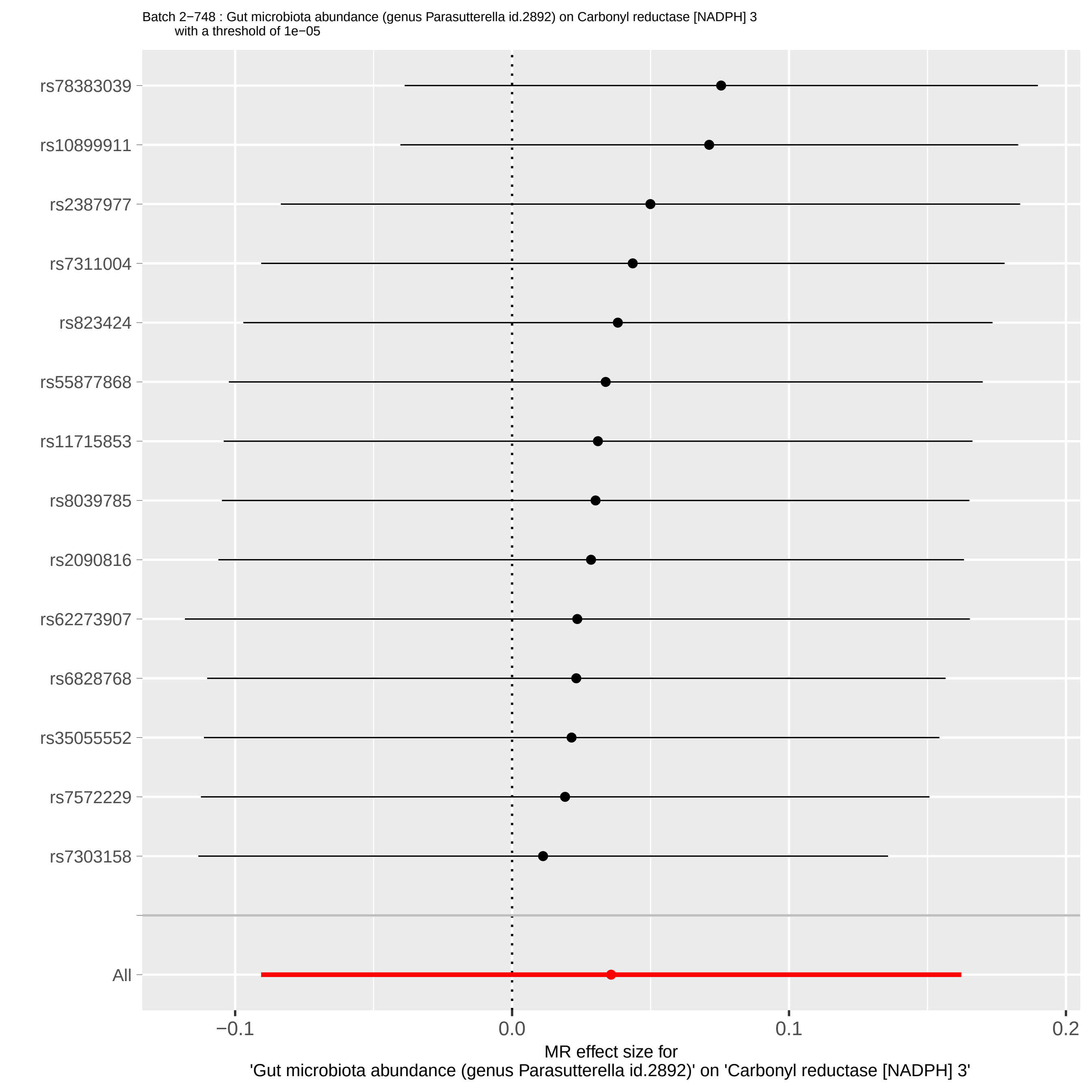

Batch 2−748 : Gut microbiota abundance (genus Parasutterella id.2892) on Carbonyl reductase [NADPH] 3
with a threshold of 1e−05
rs78383039
rs10899911
rs2387977
rs7311004
rs823424
rs55877868
rs11715853
rs8039785
rs2090816
rs62273907
rs6828768
rs35055552
rs7572229
rs7303158
All
−0.1
0.0
0.1
0.2
MR effect size for
'Gut microbiota abundance (genus Parasutterella id.2892)' on 'Carbonyl reductase [NADPH] 3'

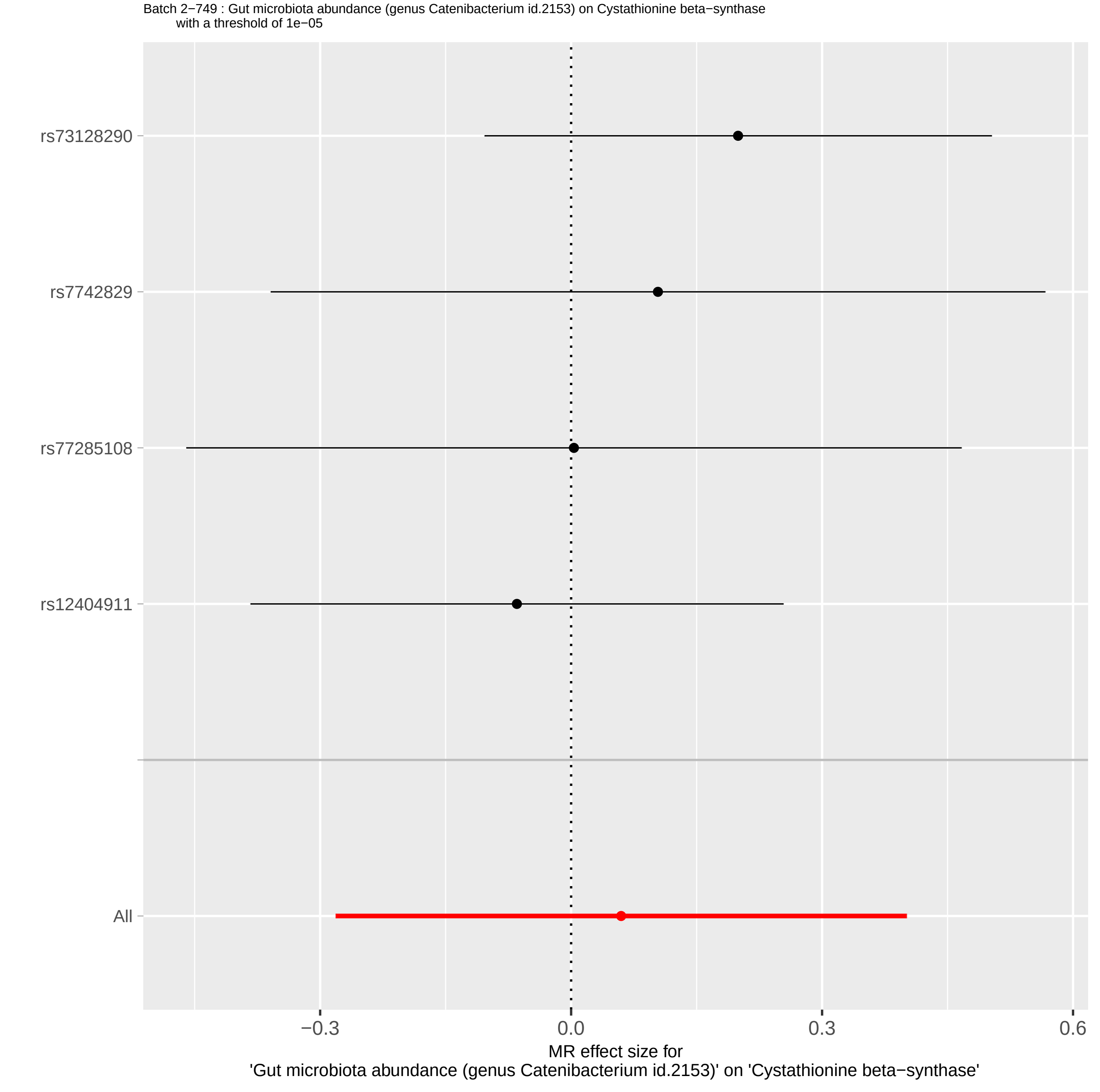

Batch 2−749 : Gut microbiota abundance (genus Catenibacterium id.2153) on Cystathionine beta−synthase
with a threshold of 1e−05
rs73128290
rs7742829
rs77285108
rs12404911
All
−0.3
0.0
0.3
0.6
MR effect size for
'Gut microbiota abundance (genus Catenibacterium id.2153)' on 'Cystathionine beta−synthase'

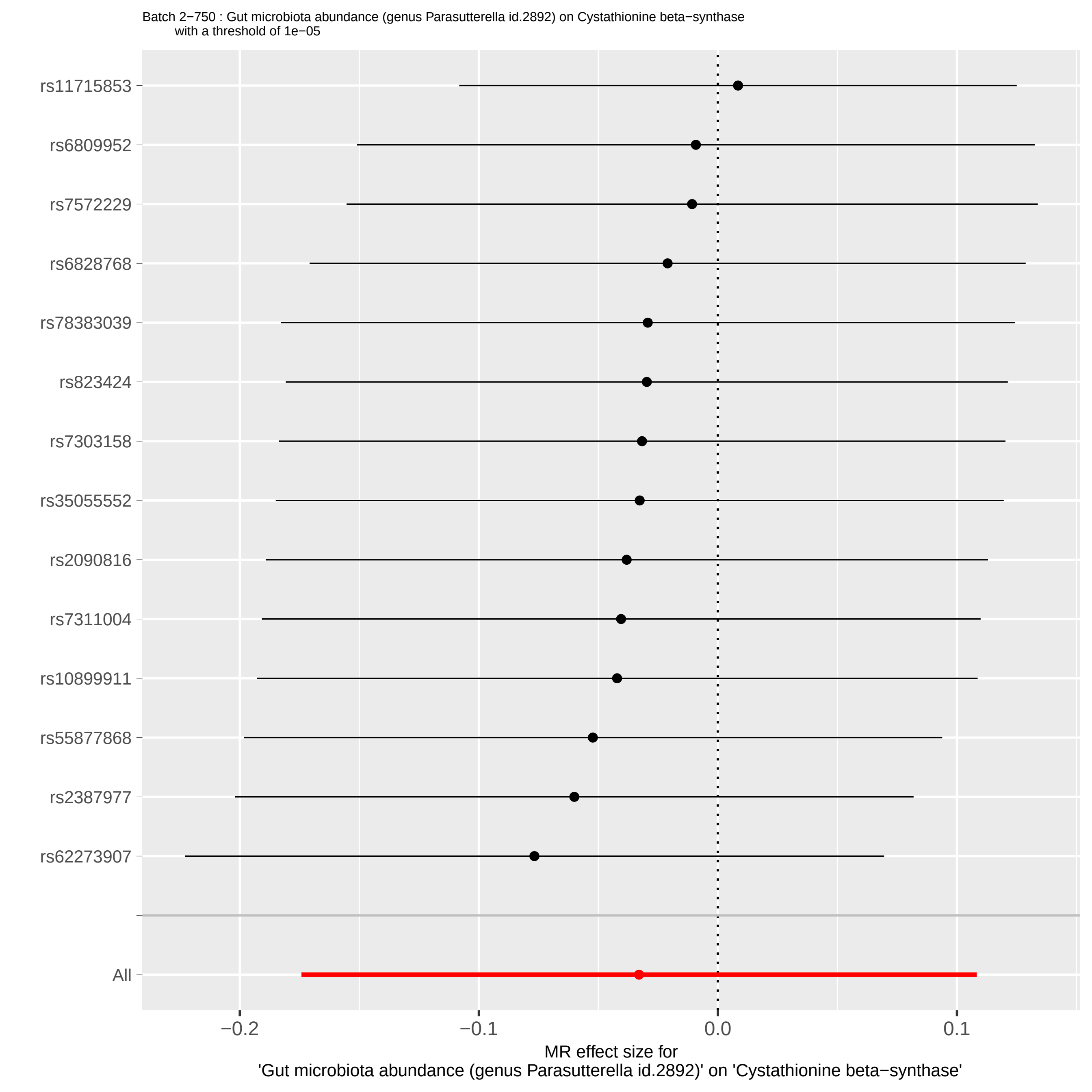

Batch 2−750 : Gut microbiota abundance (genus Parasutterella id.2892) on Cystathionine beta−synthase
with a threshold of 1e−05
rs11715853
rs6809952
rs7572229
rs6828768
rs78383039
rs823424
rs7303158
rs35055552
rs2090816
rs7311004
rs10899911
rs55877868
rs2387977
rs62273907
All
−0.2
−0.1
0.0
0.1
MR effect size for
'Gut microbiota abundance (genus Parasutterella id.2892)' on 'Cystathionine beta−synthase'

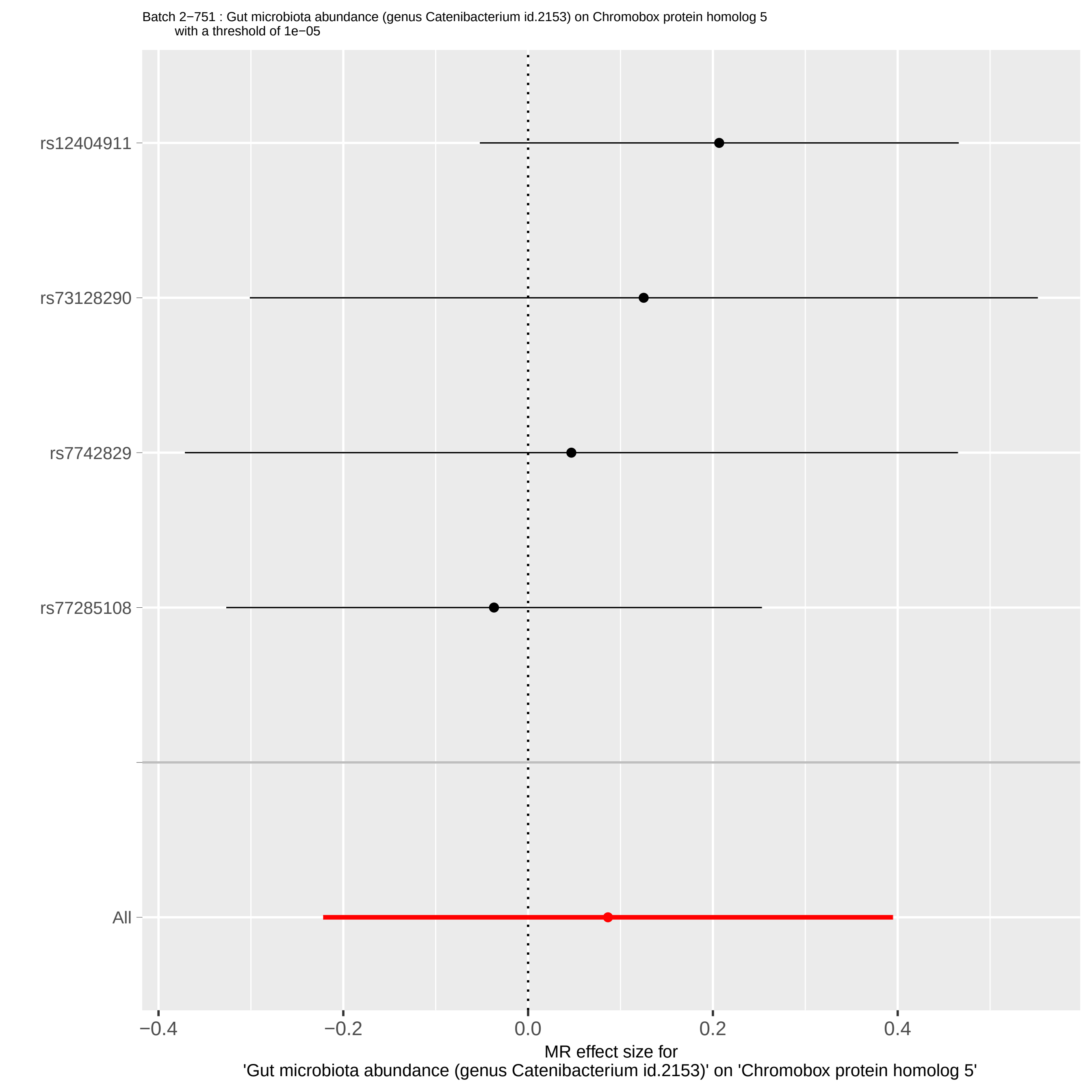

Batch 2−751 : Gut microbiota abundance (genus Catenibacterium id.2153) on Chromobox protein homolog 5
with a threshold of 1e−05
rs12404911
rs73128290
rs7742829
rs77285108
All
−0.4
−0.2
0.0
0.2
0.4
MR effect size for
'Gut microbiota abundance (genus Catenibacterium id.2153)' on 'Chromobox protein homolog 5'

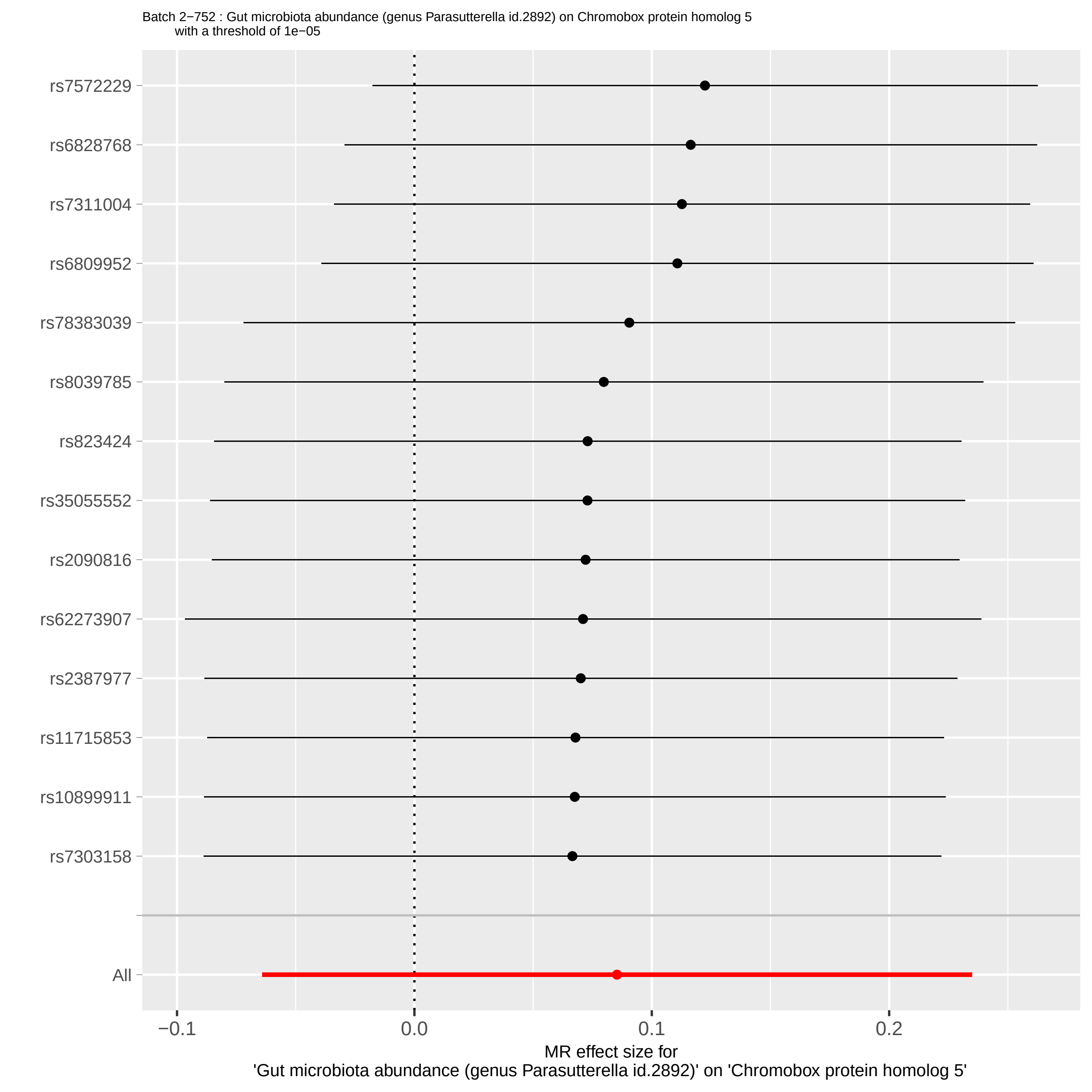

Batch 2−752 : Gut microbiota abundance (genus Parasutterella id.2892) on Chromobox protein homolog 5
with a threshold of 1e−05
rs7572229
rs6828768
rs7311004
rs6809952
rs78383039
rs8039785
rs823424
rs35055552
rs2090816
rs62273907
rs2387977
rs11715853
rs10899911
rs7303158
All
−0.1
0.0
0.1
0.2
MR effect size for
'Gut microbiota abundance (genus Parasutterella id.2892)' on 'Chromobox protein homolog 5'

Batch 2−753 : Gut microbiota abundance (genus Catenibacterium id.2153) on Chromobox protein homolog 7
with a threshold of 1e−05

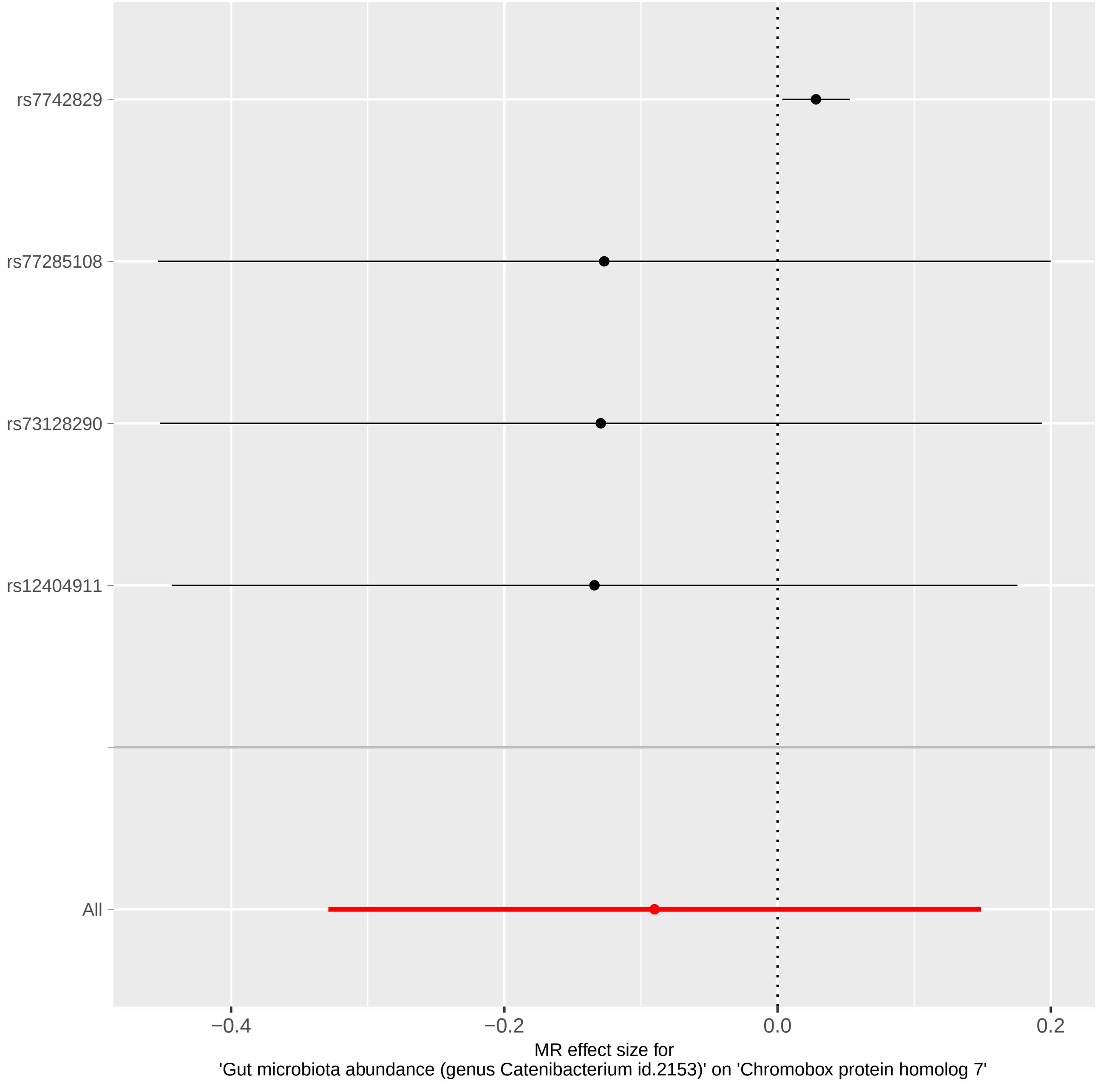

Batch 2−754 : Gut microbiota abundance (genus Parasutterella id.2892) on Chromobox protein homolog 7
with a threshold of 1e−05

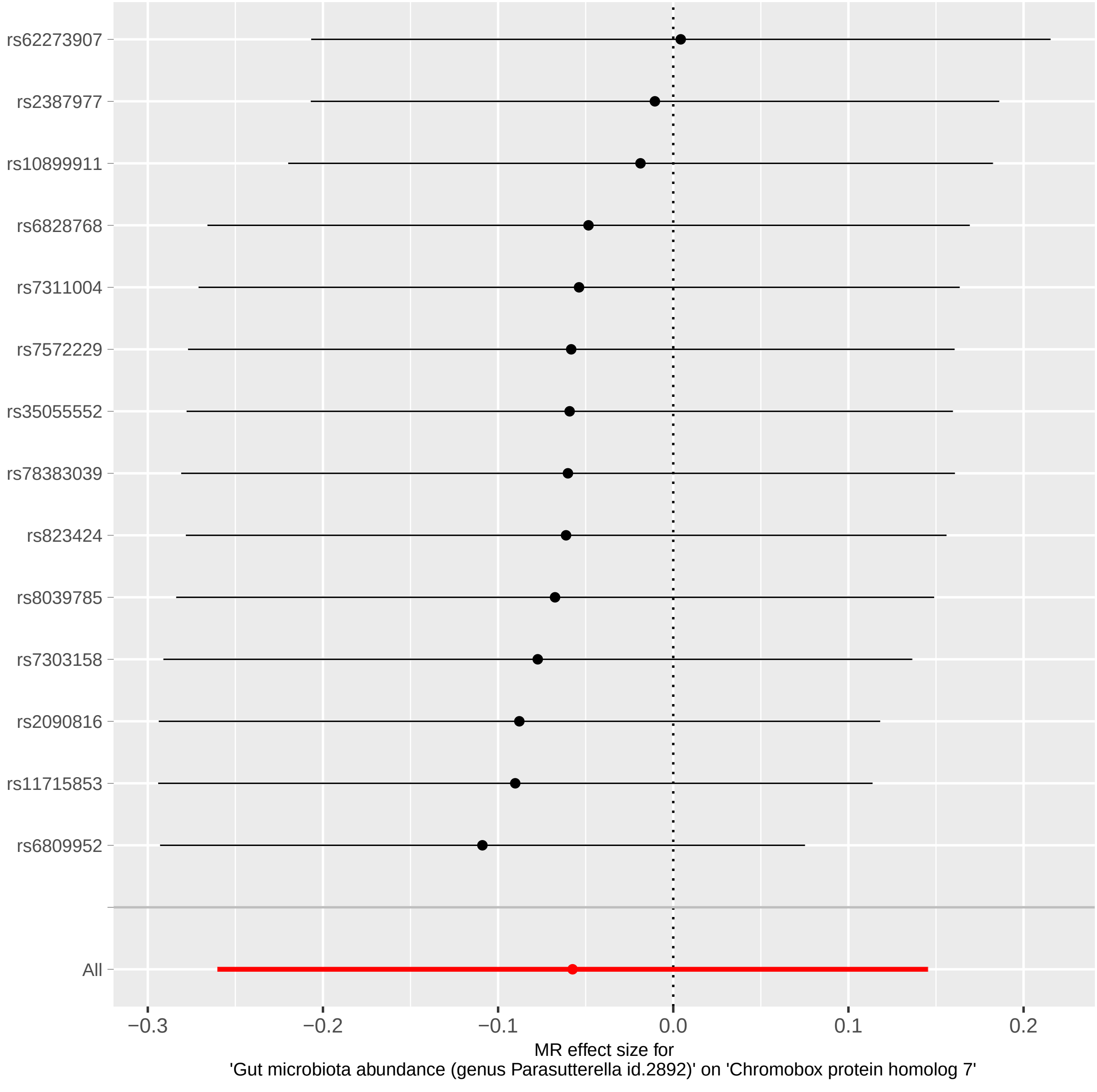

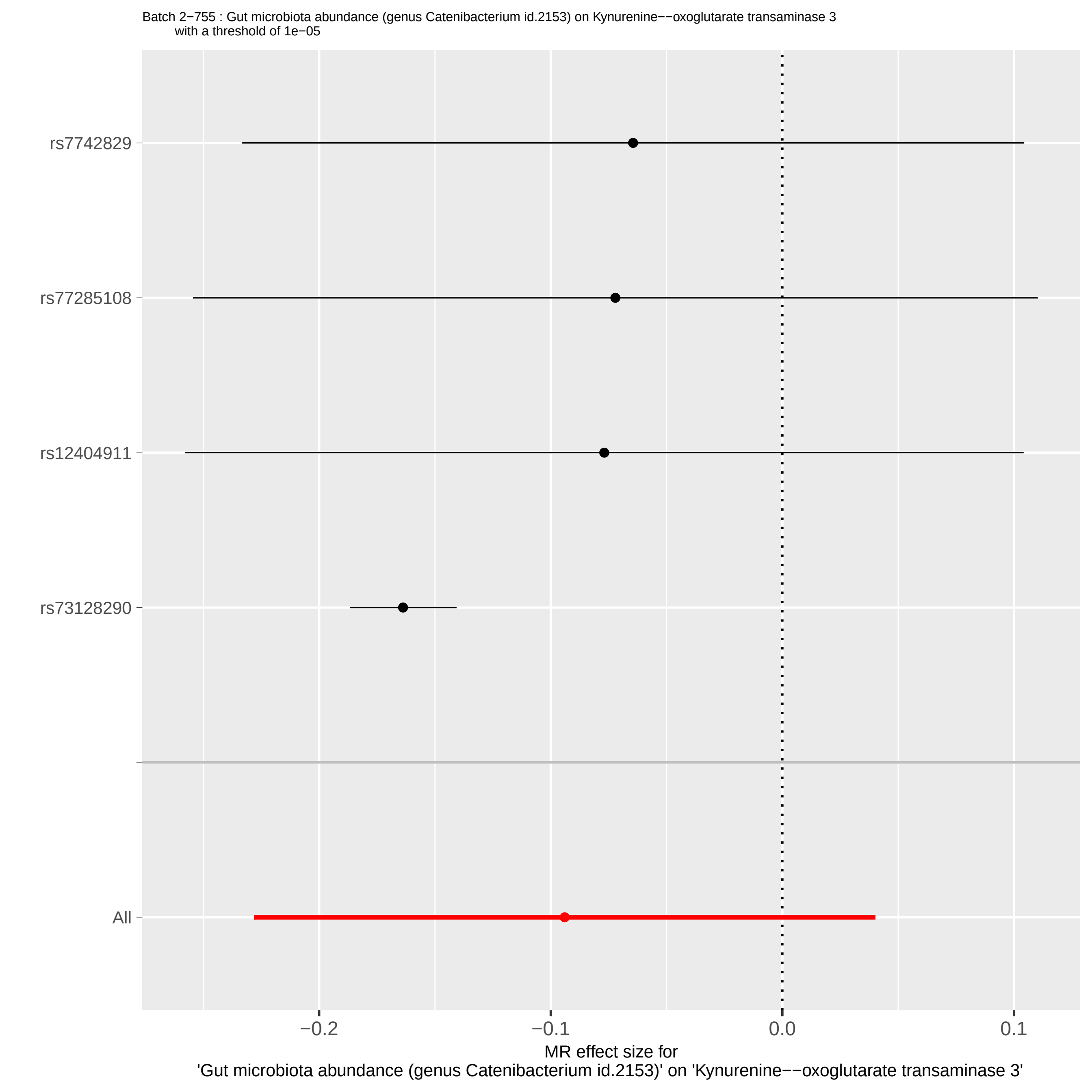
Batch 2−755 : Gut microbiota abundance (genus Catenibacterium id.2153) on Kynurenine−−oxoglutarate transaminase 3
with a threshold of 1e−05
rs7742829
rs77285108
rs12404911
rs73128290
All
−0.2
−0.1
0.0
0.1
MR effect size for
'Gut microbiota abundance (genus Catenibacterium id.2153)' on 'Kynurenine−−oxoglutarate transaminase 3'

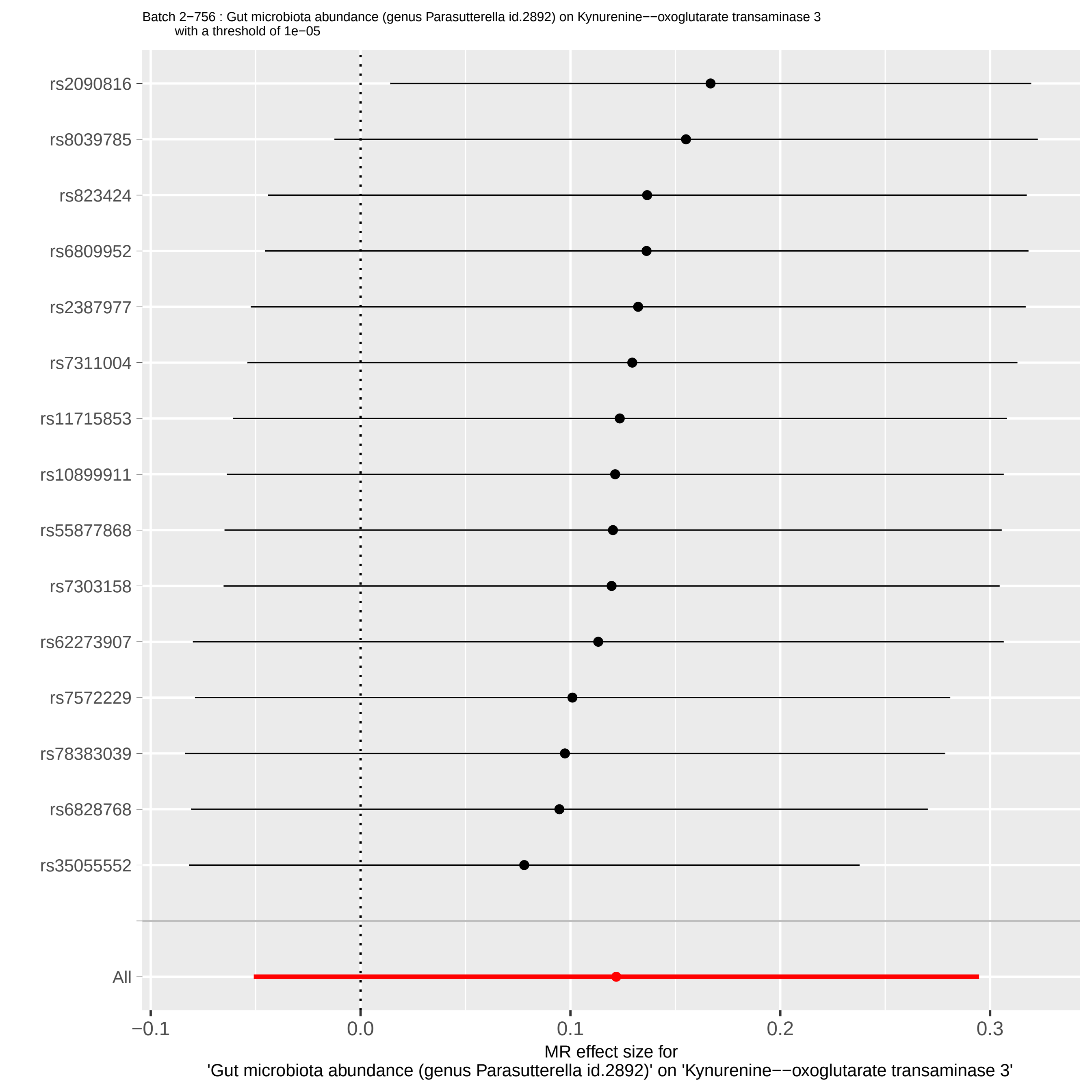

Batch 2−756 : Gut microbiota abundance (genus Parasutterella id.2892) on Kynurenine−−oxoglutarate transaminase 3
with a threshold of 1e−05
rs2090816
rs8039785
rs823424
rs6809952
rs2387977
rs7311004
rs11715853
rs10899911
rs55877868
rs7303158
rs62273907
rs7572229
rs78383039
rs6828768
rs35055552
All
−0.1
0.0
0.1
0.2
0.3
MR effect size for
'Gut microbiota abundance (genus Parasutterella id.2892)' on 'Kynurenine−−oxoglutarate transaminase 3'

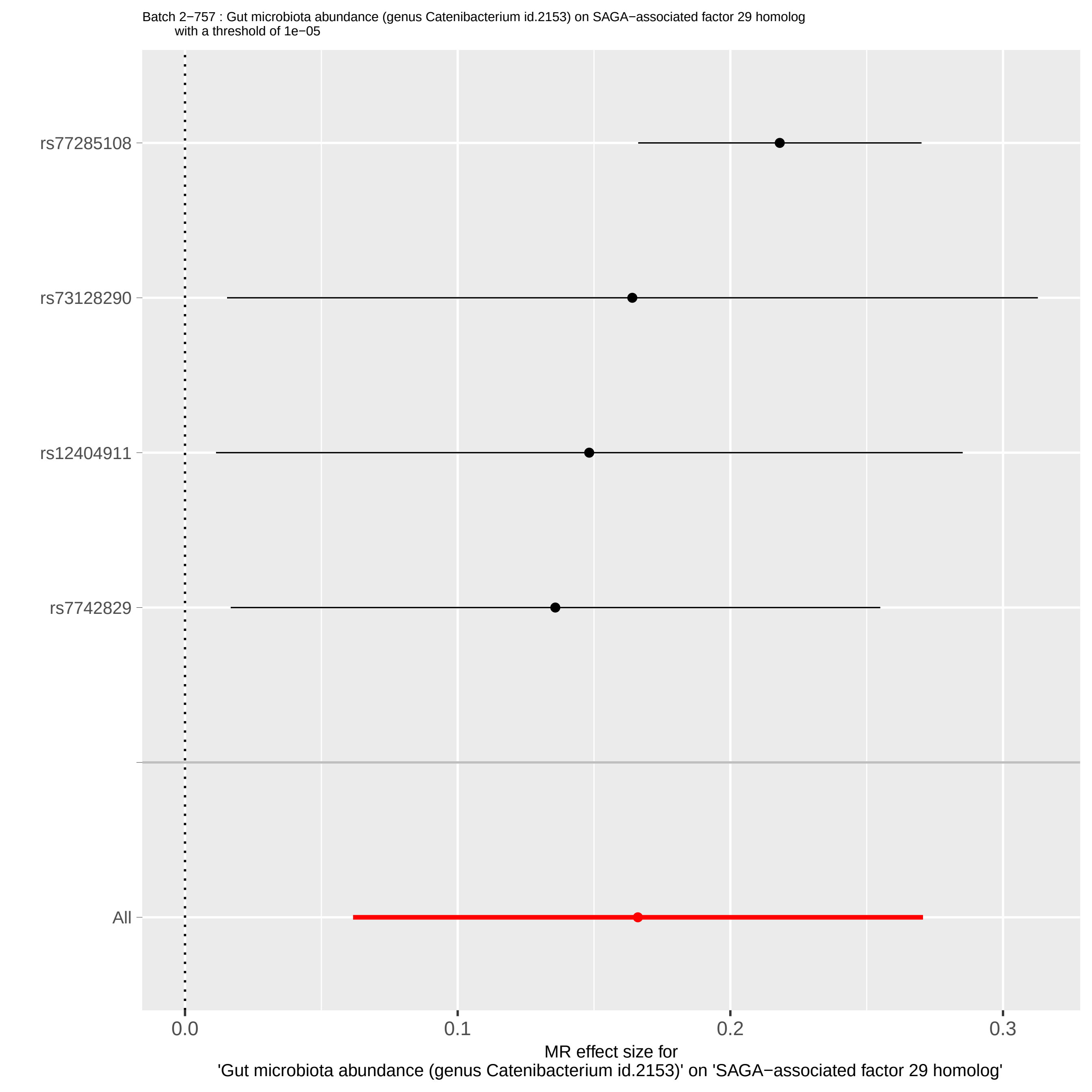
Batch 2−757 : Gut microbiota abundance (genus Catenibacterium id.2153) on SAGA−associated factor 29 homolog
with a threshold of 1e−05
rs77285108
rs73128290
rs12404911
rs7742829
All
0.0
0.1
0.2
0.3
MR effect size for
'Gut microbiota abundance (genus Catenibacterium id.2153)' on 'SAGA−associated factor 29 homolog'

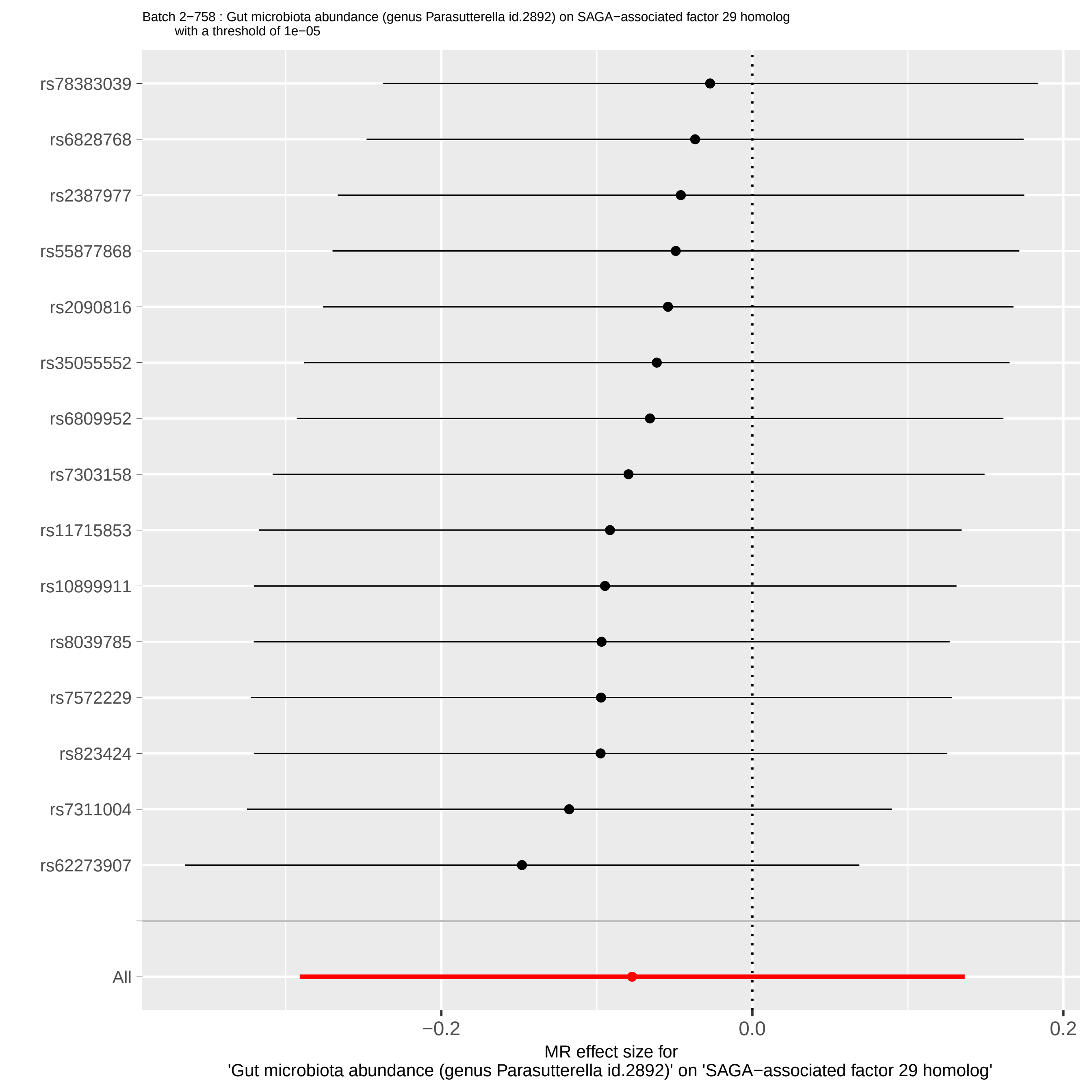
Batch 2−758 : Gut microbiota abundance (genus Parasutterella id.2892) on SAGA−associated factor 29 homolog
with a threshold of 1e−05
rs78383039
rs6828768
rs2387977
rs55877868
rs2090816
rs35055552
rs6809952
rs7303158
rs11715853
rs10899911
rs8039785
rs7572229
rs823424
rs7311004
rs62273907
All
−0.2
0.0
0.2
MR effect size for
'Gut microbiota abundance (genus Parasutterella id.2892)' on 'SAGA−associated factor 29 homolog'

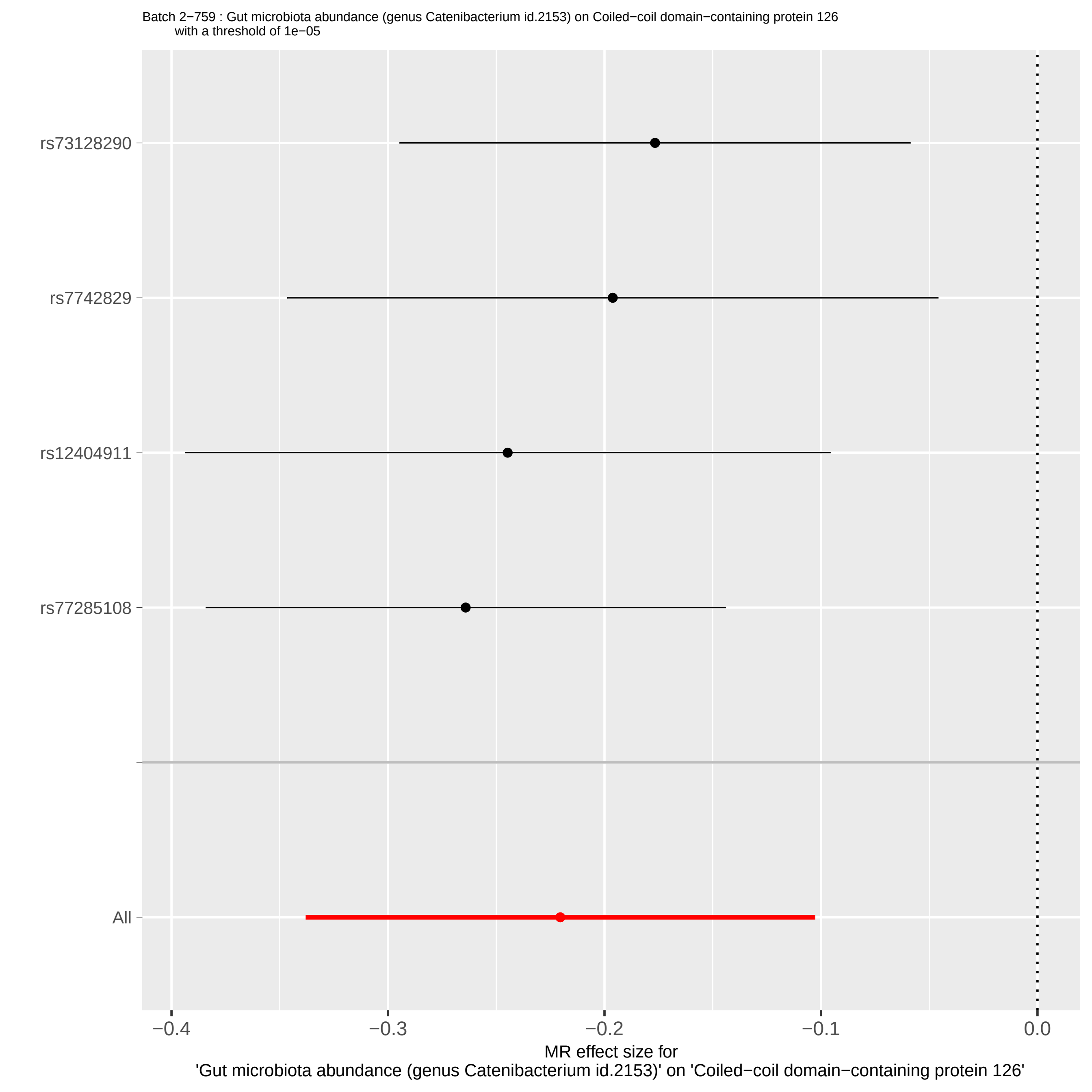

Batch 2−759 : Gut microbiota abundance (genus Catenibacterium id.2153) on Coiled−coil domain−containing protein 126
with a threshold of 1e−05
rs73128290
rs7742829
rs12404911
rs77285108
All
−0.4
−0.3
−0.2
−0.1
0.0
MR effect size for
'Gut microbiota abundance (genus Catenibacterium id.2153)' on 'Coiled−coil domain−containing protein 126'

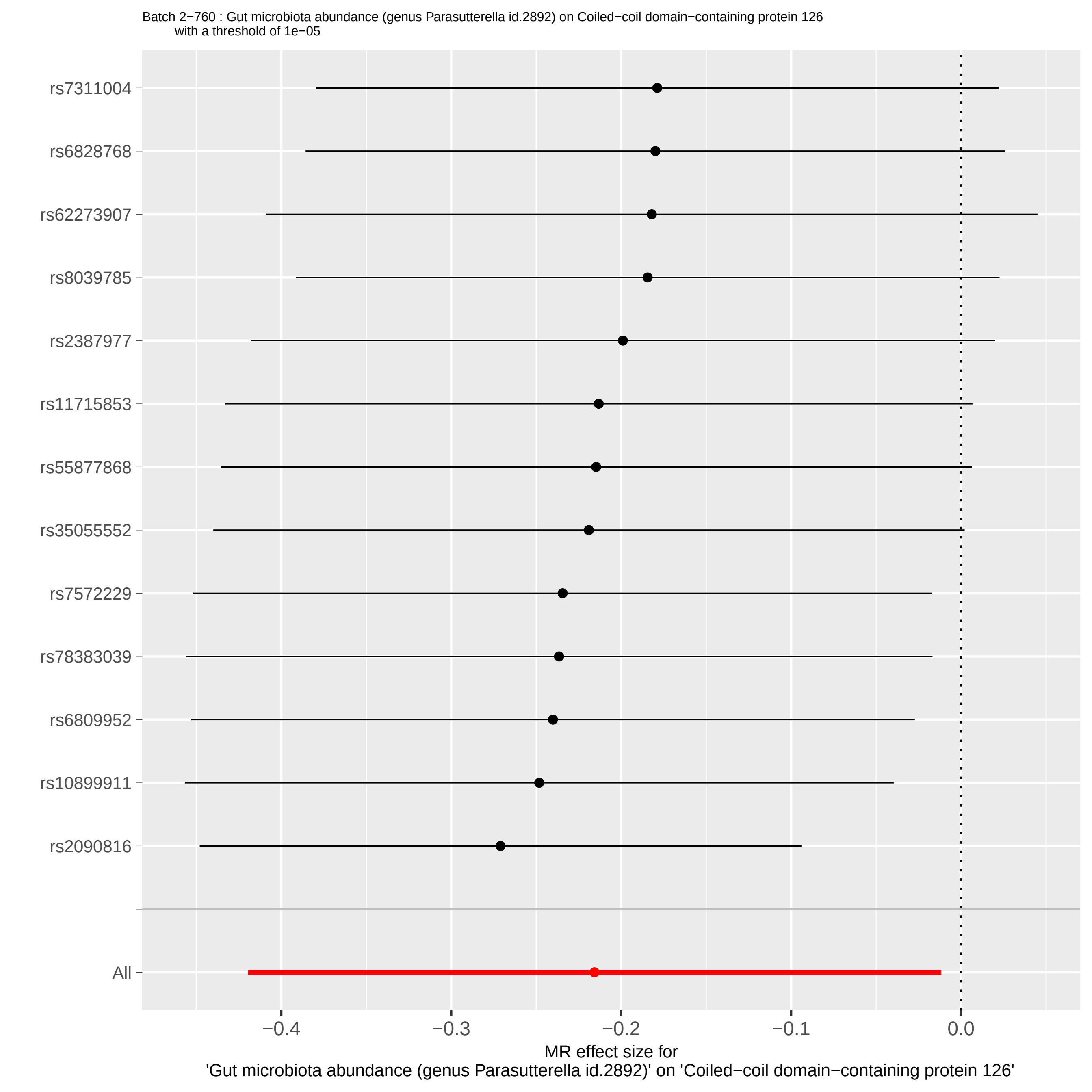
Batch 2−760 : Gut microbiota abundance (genus Parasutterella id.2892) on Coiled−coil domain−containing protein 126
with a threshold of 1e−05
rs7311004
rs6828768
rs62273907
rs8039785
rs2387977
rs11715853
rs55877868
rs35055552
rs7572229
rs78383039
rs6809952
rs10899911
rs2090816
All
−0.4
−0.3
−0.2
−0.1
0.0
MR effect size for
'Gut microbiota abundance (genus Parasutterella id.2892)' on 'Coiled−coil domain−containing protein 126'

Batch 2−761 : Gut microbiota abundance (genus Catenibacterium id.2153) on Coiled−coil domain−containing protein 134
with a threshold of 1e−05

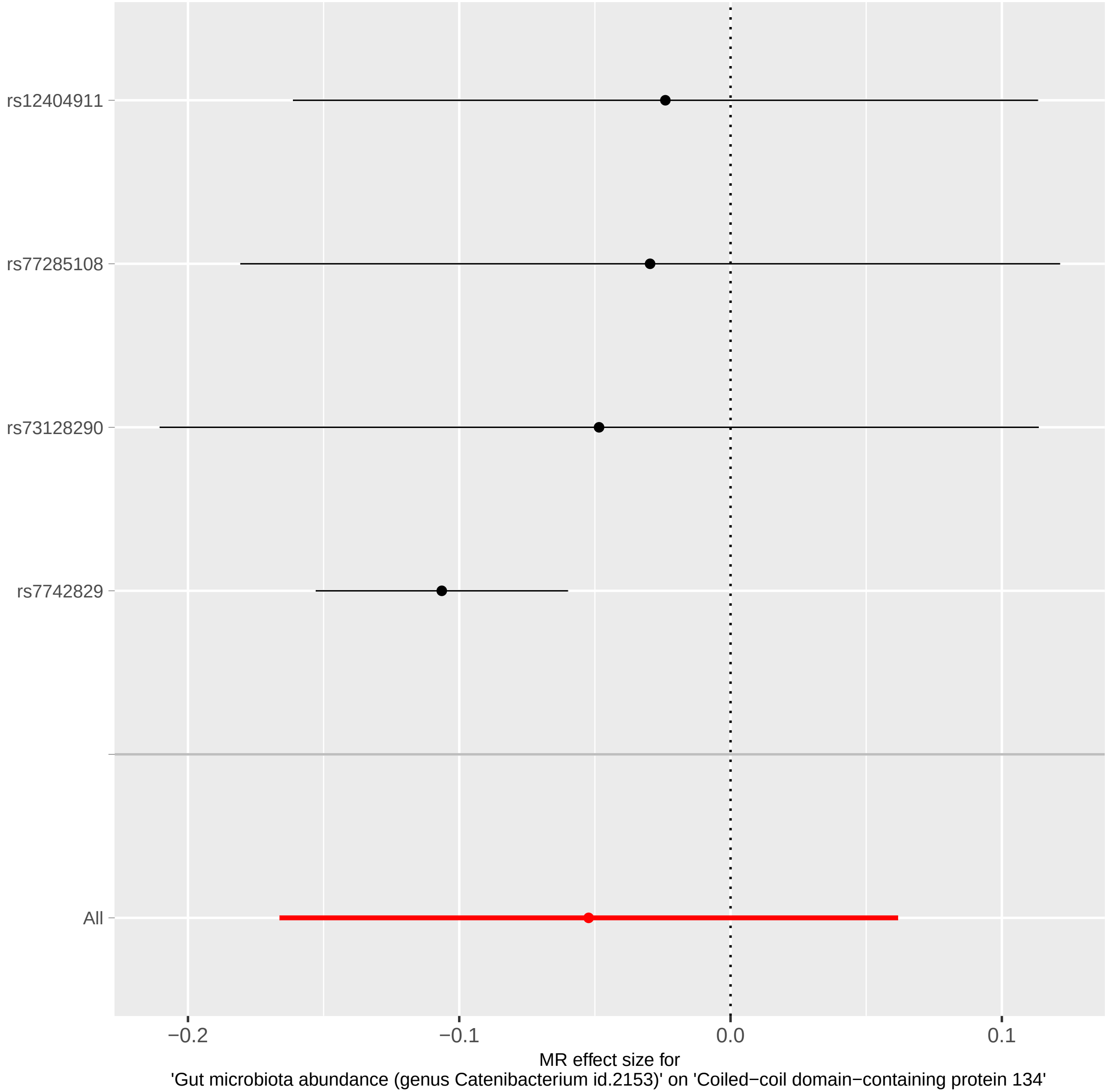

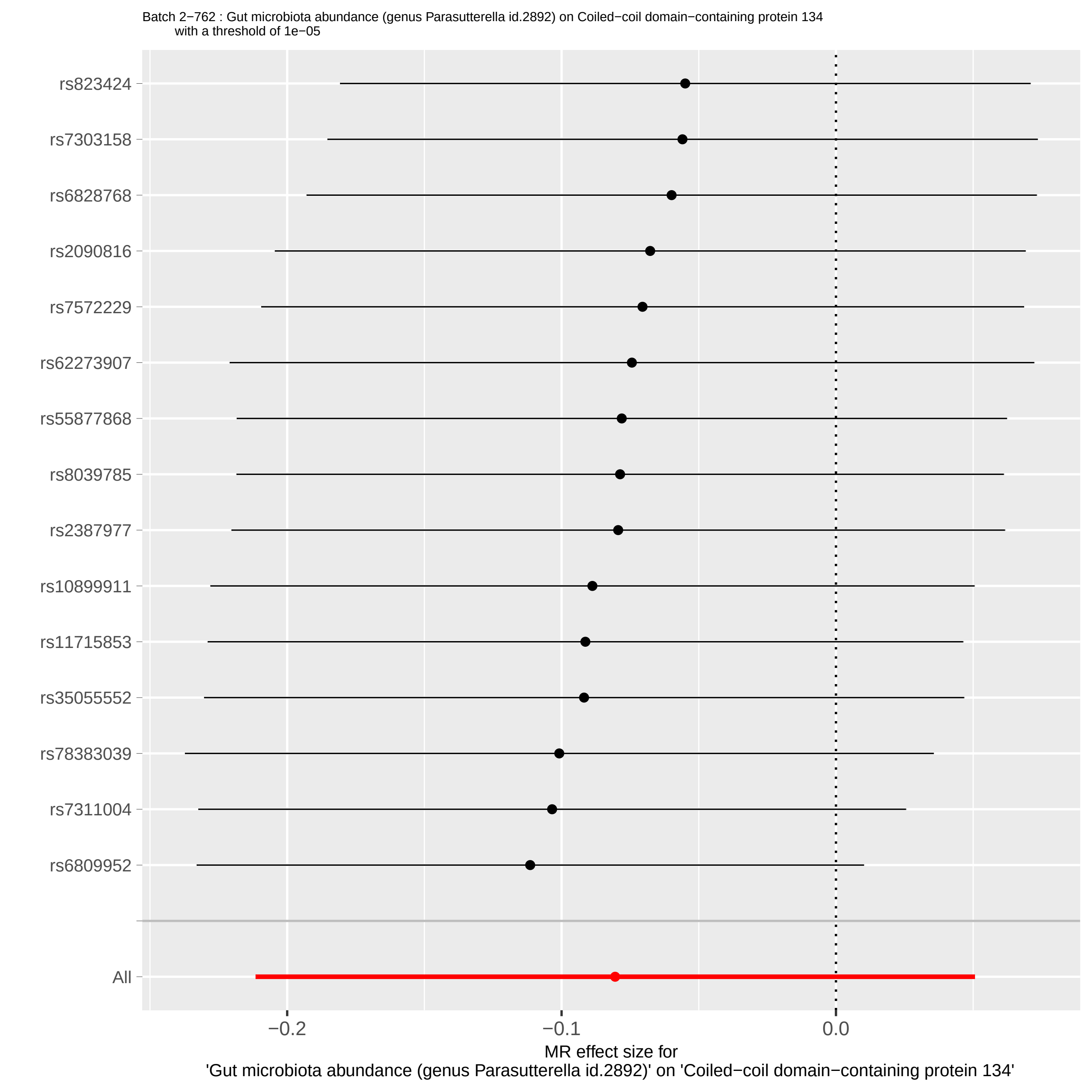
Batch 2−762 : Gut microbiota abundance (genus Parasutterella id.2892) on Coiled−coil domain−containing protein 134
with a threshold of 1e−05
rs823424
rs7303158
rs6828768
rs2090816
rs7572229
rs62273907
rs55877868
rs8039785
rs2387977
rs10899911
rs11715853
rs35055552
rs78383039
rs7311004
rs6809952
All
−0.2
−0.1
0.0
MR effect size for
'Gut microbiota abundance (genus Parasutterella id.2892)' on 'Coiled−coil domain−containing protein 134'

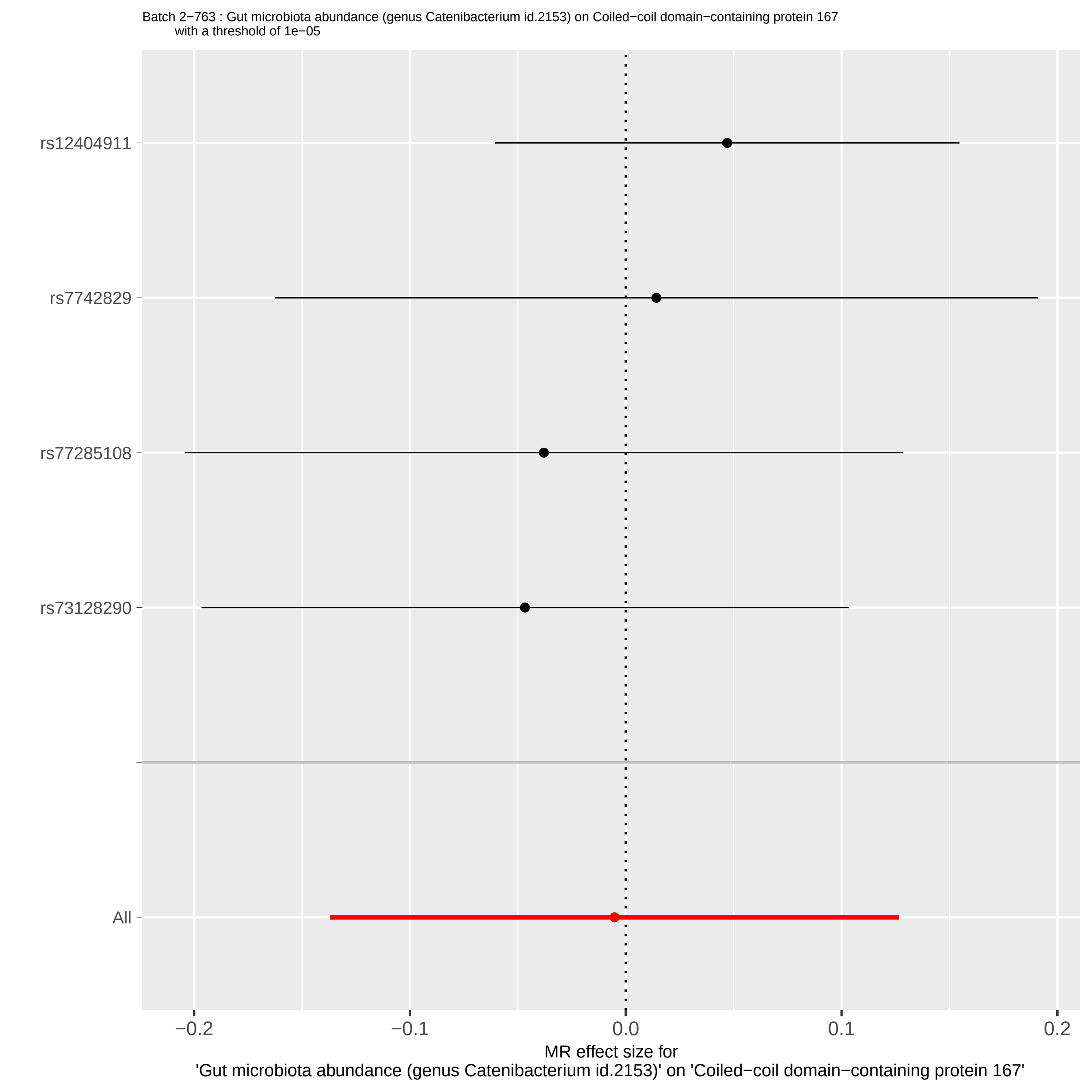

Batch 2−763 : Gut microbiota abundance (genus Catenibacterium id.2153) on Coiled−coil domain−containing protein 167
with a threshold of 1e−05
rs12404911
rs7742829
rs77285108
rs73128290
All
−0.2
−0.1
0.0
0.1
0.2
MR effect size for
'Gut microbiota abundance (genus Catenibacterium id.2153)' on 'Coiled−coil domain−containing protein 167'

Batch 2−764 : Gut microbiota abundance (genus Parasutterella id.2892) on Coiled−coil domain−containing protein 167
with a threshold of 1e−05

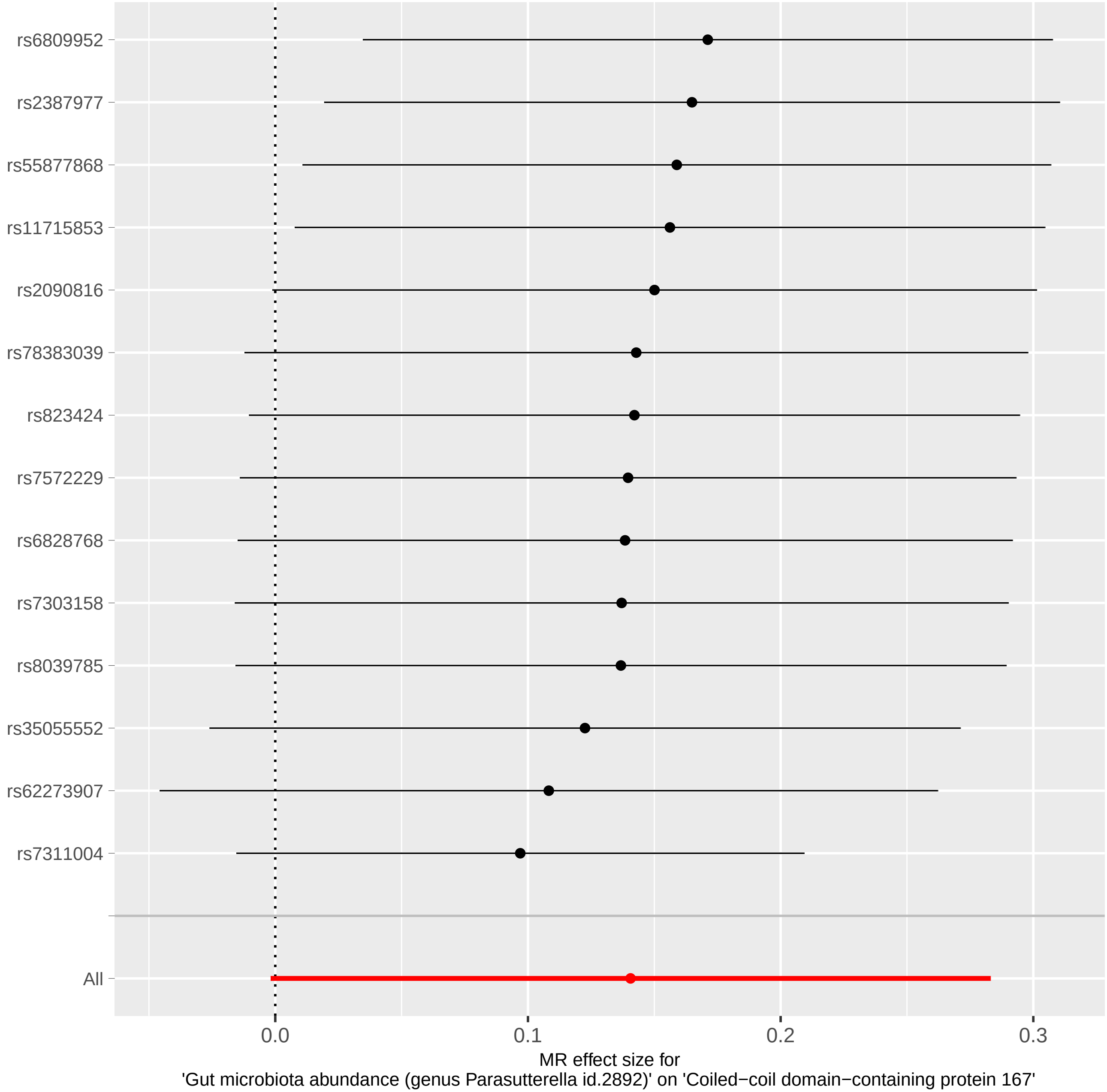

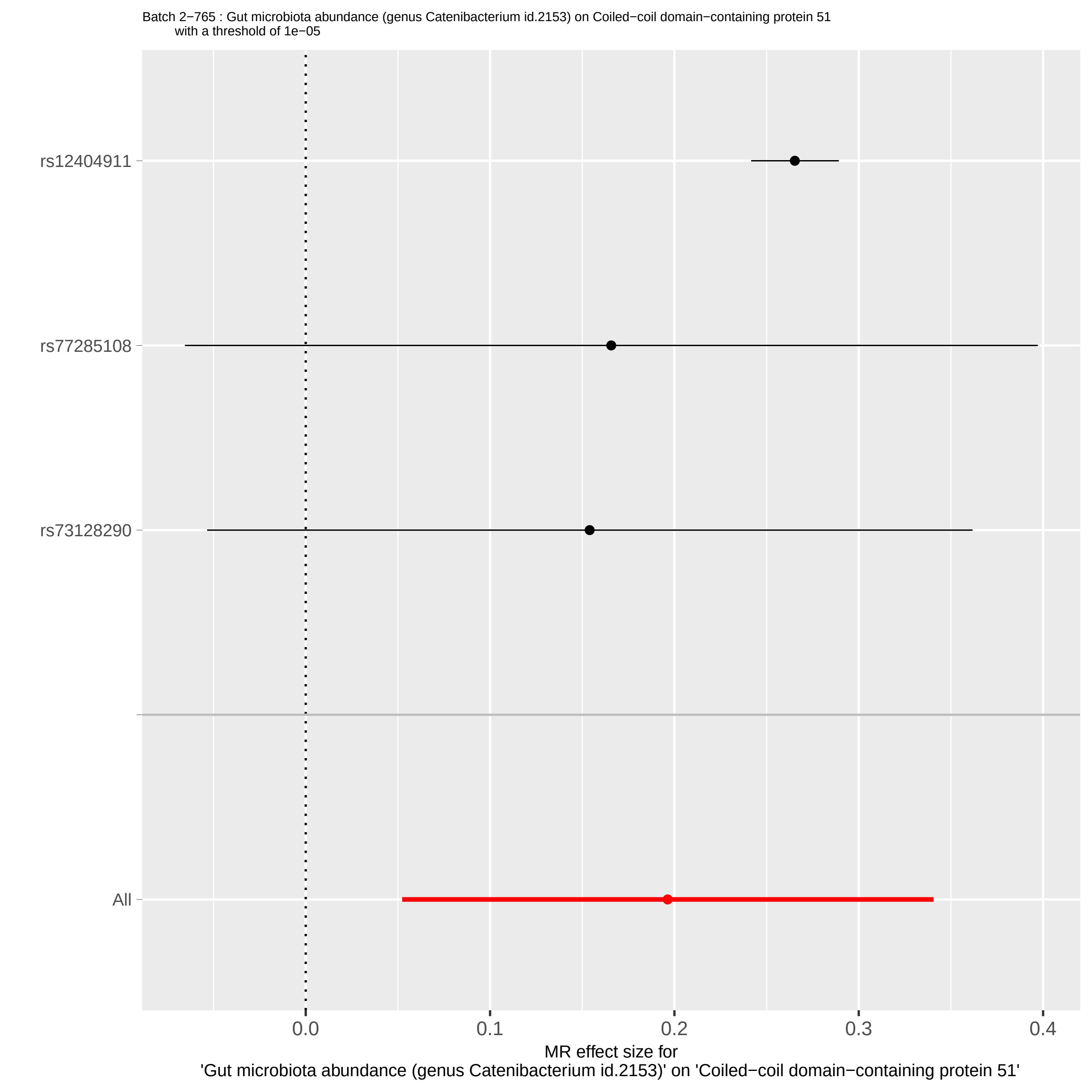

Batch 2−765 : Gut microbiota abundance (genus Catenibacterium id.2153) on Coiled−coil domain−containing protein 51
with a threshold of 1e−05
rs12404911
rs77285108
rs73128290
All
0.0
0.1
0.2
0.3
0.4
MR effect size for
'Gut microbiota abundance (genus Catenibacterium id.2153)' on 'Coiled−coil domain−containing protein 51'

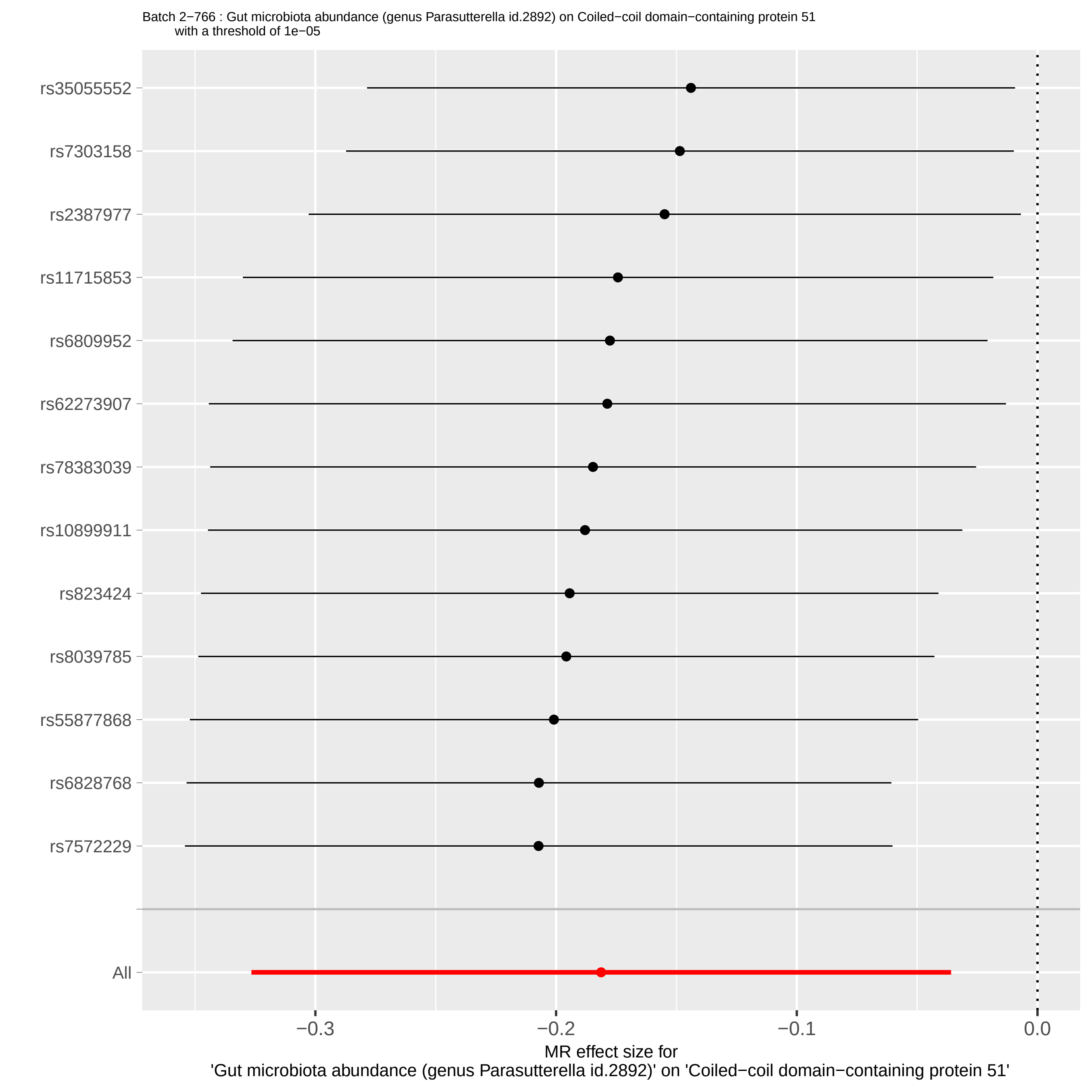
Batch 2−766 : Gut microbiota abundance (genus Parasutterella id.2892) on Coiled−coil domain−containing protein 51
with a threshold of 1e−05
rs35055552
rs7303158
rs2387977
rs11715853
rs6809952
rs62273907
rs78383039
rs10899911
rs823424
rs8039785
rs55877868
rs6828768
rs7572229
All
−0.3
−0.2
−0.1
0.0
MR effect size for
'Gut microbiota abundance (genus Parasutterella id.2892)' on 'Coiled−coil domain−containing protein 51'

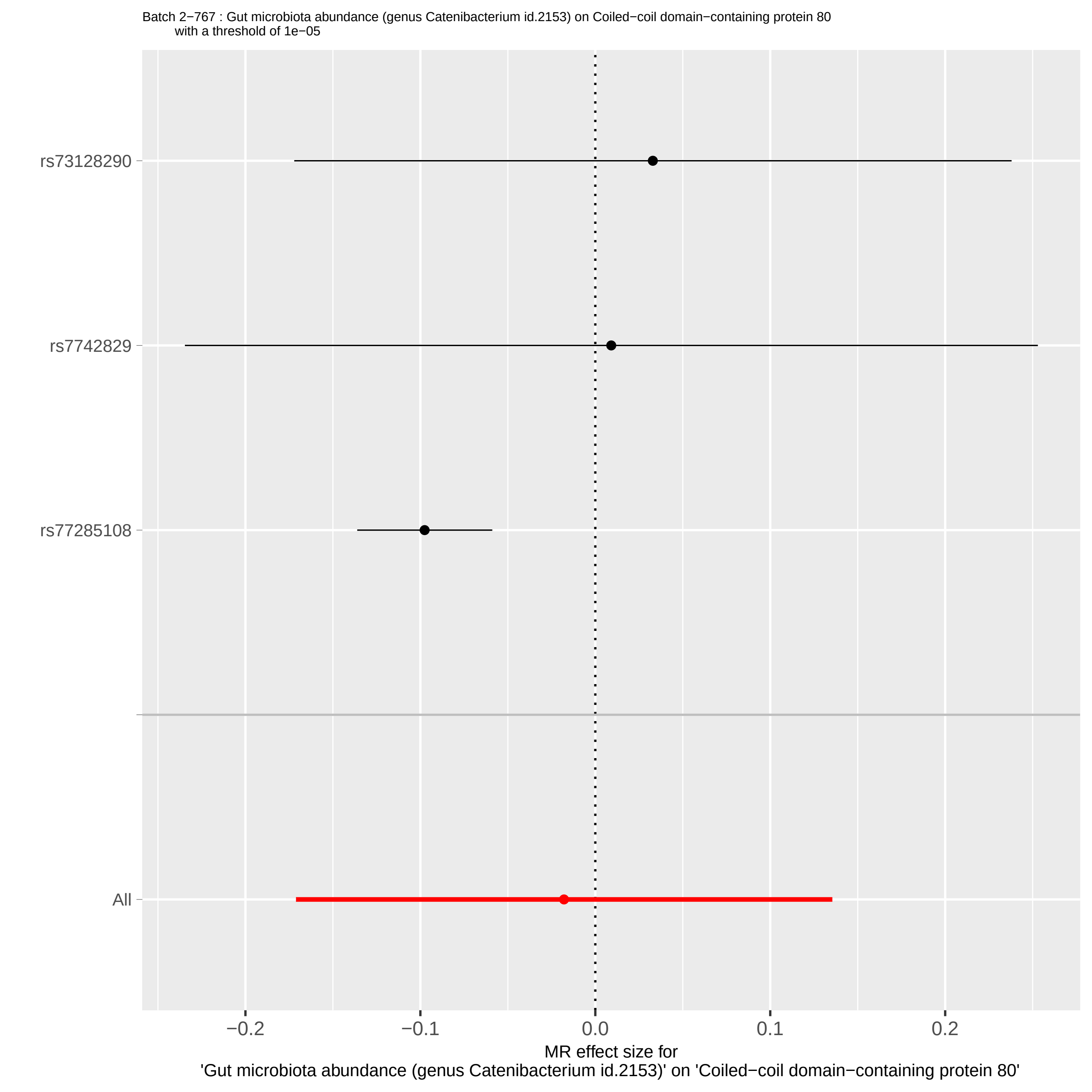

Batch 2−767 : Gut microbiota abundance (genus Catenibacterium id.2153) on Coiled−coil domain−containing protein 80
with a threshold of 1e−05
rs73128290
rs7742829
rs77285108
All
−0.2
−0.1
0.0
0.1
0.2
MR effect size for
'Gut microbiota abundance (genus Catenibacterium id.2153)' on 'Coiled−coil domain−containing protein 80'

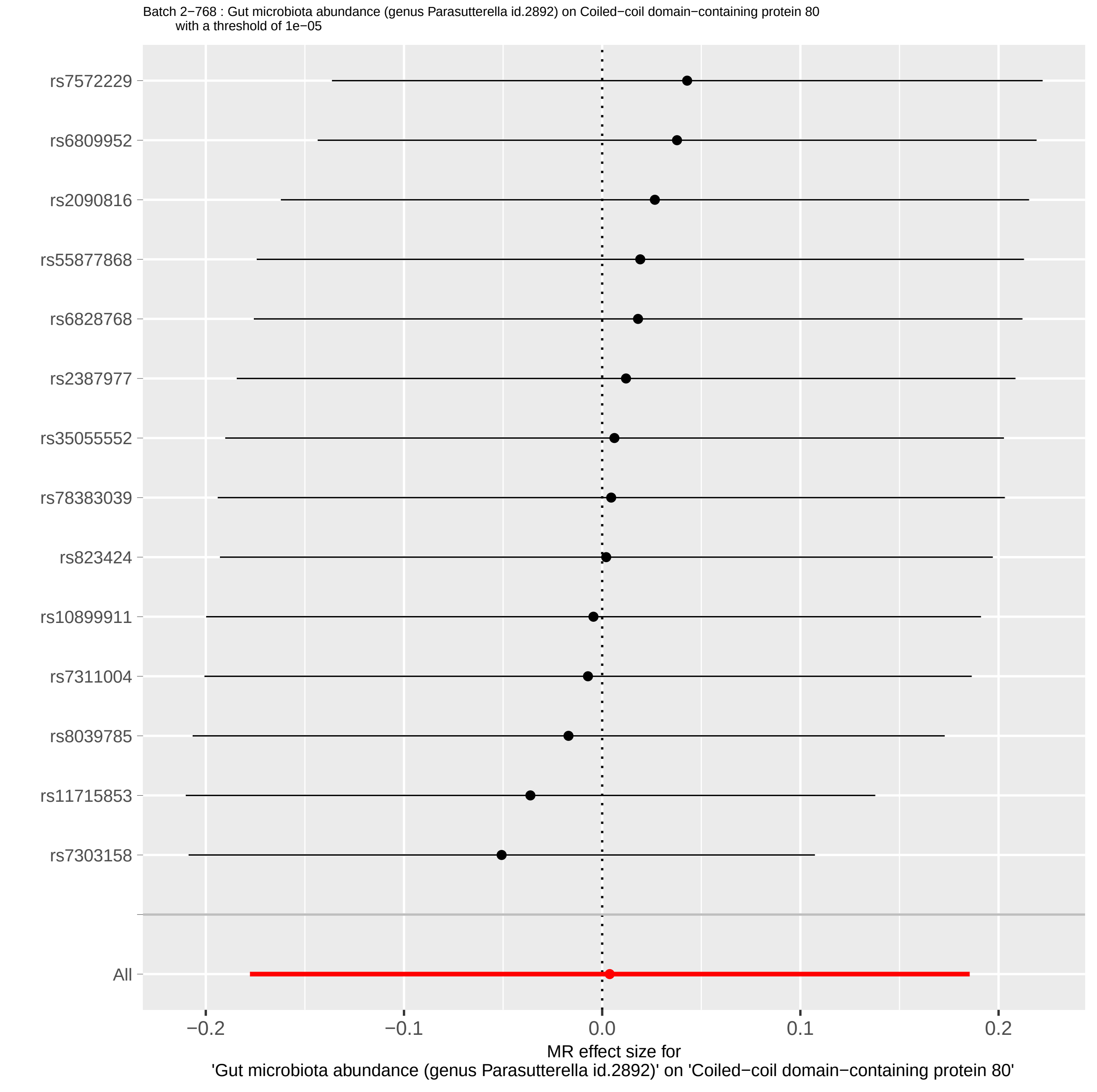

Batch 2−768 : Gut microbiota abundance (genus Parasutterella id.2892) on Coiled−coil domain−containing protein 80
with a threshold of 1e−05
rs7572229
rs6809952
rs2090816
rs55877868
rs6828768
rs2387977
rs35055552
rs78383039
rs823424
rs10899911
rs7311004
rs8039785
rs11715853
rs7303158
All
−0.2
−0.1
0.0
0.1
0.2
MR effect size for
'Gut microbiota abundance (genus Parasutterella id.2892)' on 'Coiled−coil domain−containing protein 80'

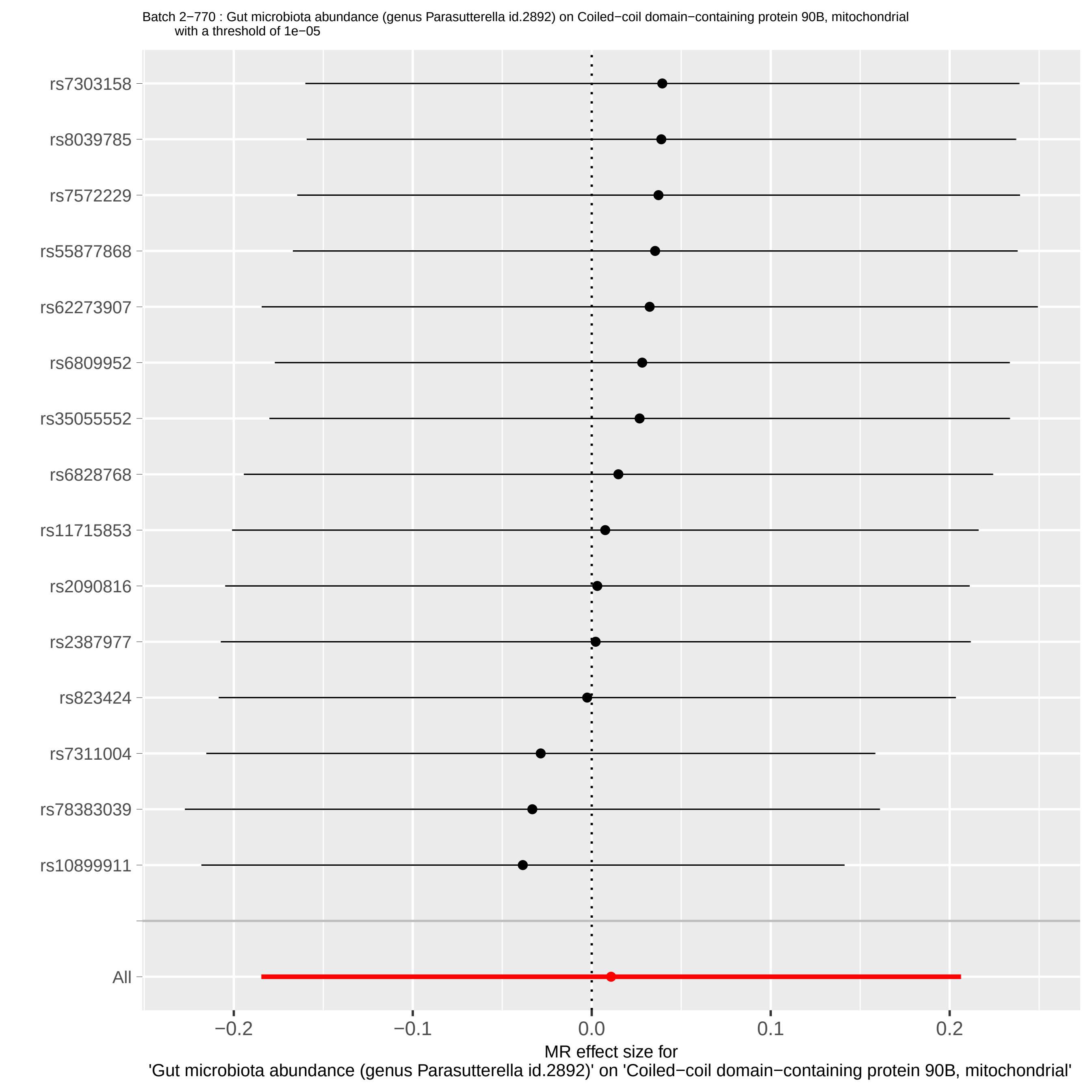

Batch 2−770 : Gut microbiota abundance (genus Parasutterella id.2892) on Coiled−coil domain−containing protein 90B, mitochondrial
with a threshold of 1e−05
rs7303158
rs8039785
rs7572229
rs55877868
rs62273907
rs6809952
rs35055552
rs6828768
rs11715853
rs2090816
rs2387977
rs823424
rs7311004
rs78383039
rs10899911
All
−0.2
−0.1
0.0
0.1
0.2
MR effect size for
'Gut microbiota abundance (genus Parasutterella id.2892)' on 'Coiled−coil domain−containing protein 90B, mitochondrial'

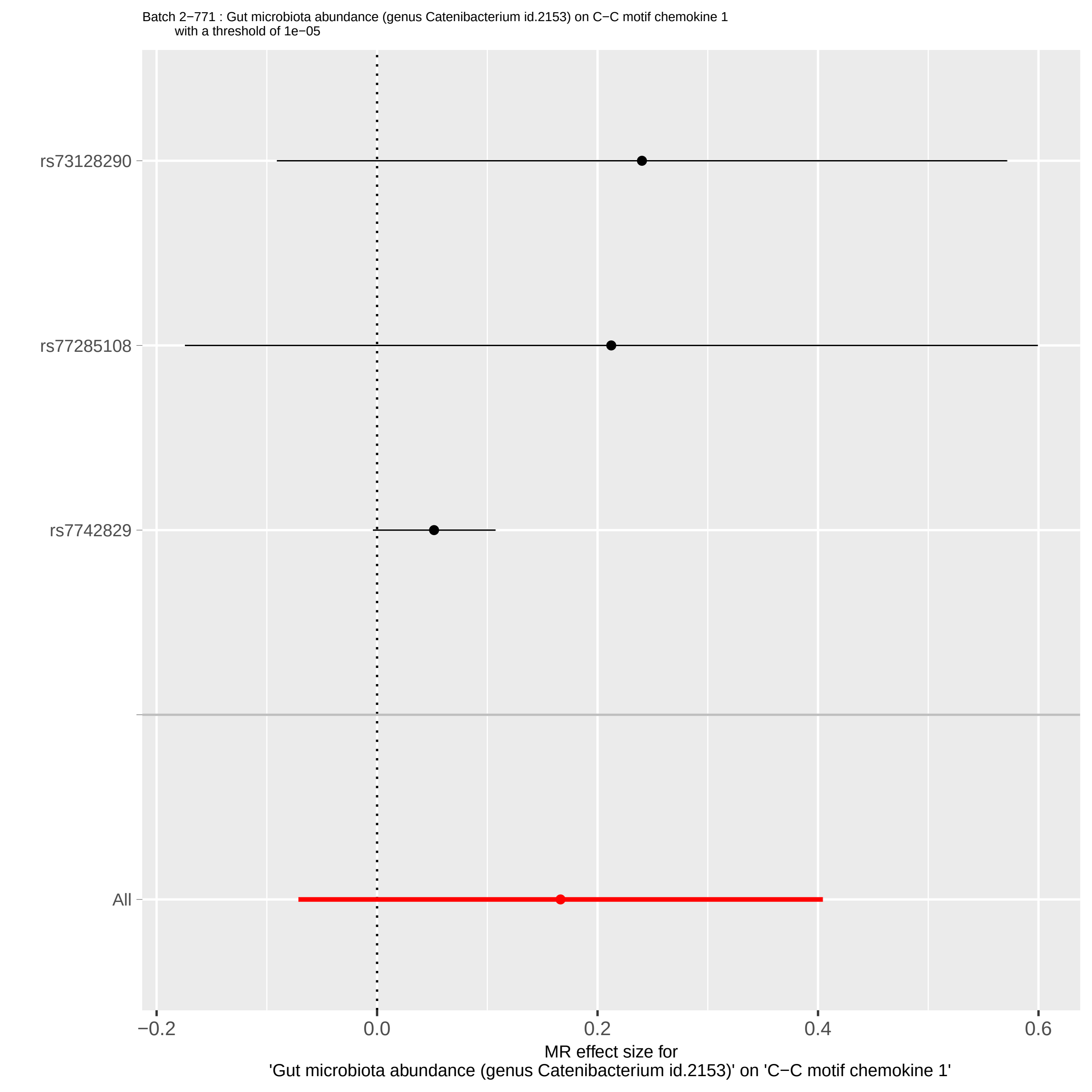
Batch 2−771 : Gut microbiota abundance (genus Catenibacterium id.2153) on C−C motif chemokine 1
with a threshold of 1e−05
rs73128290
rs77285108
rs7742829
All
−0.2
0.0
0.2
0.4
0.6
MR effect size for
'Gut microbiota abundance (genus Catenibacterium id.2153)' on 'C−C motif chemokine 1'

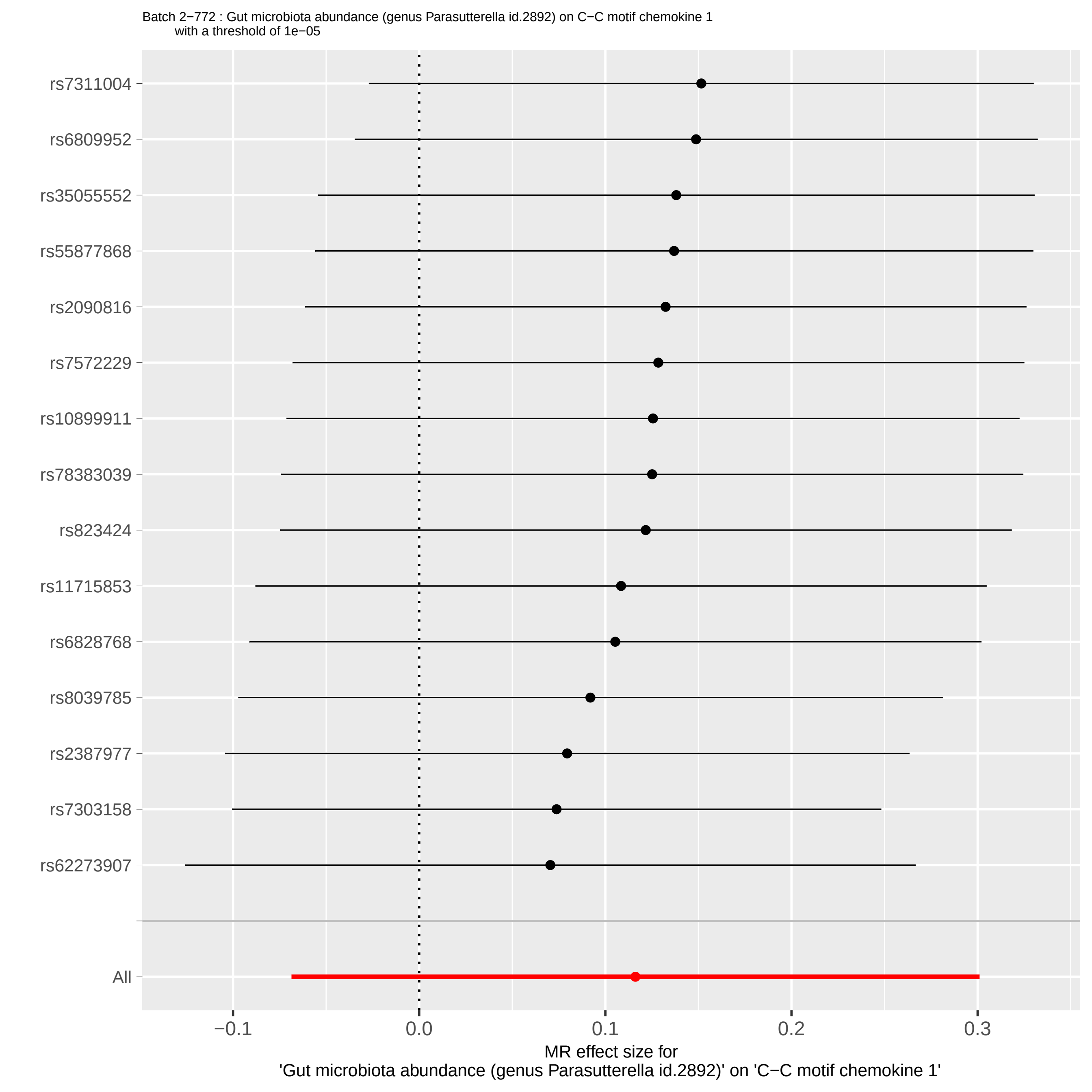

Batch 2−772 : Gut microbiota abundance (genus Parasutterella id.2892) on C−C motif chemokine 1
with a threshold of 1e−05
rs7311004
rs6809952
rs35055552
rs55877868
rs2090816
rs7572229
rs10899911
rs78383039
rs823424
rs11715853
rs6828768
rs8039785
rs2387977
rs7303158
rs62273907
All
−0.1
0.0
0.1
0.2
0.3
MR effect size for
'Gut microbiota abundance (genus Parasutterella id.2892)' on 'C−C motif chemokine 1'

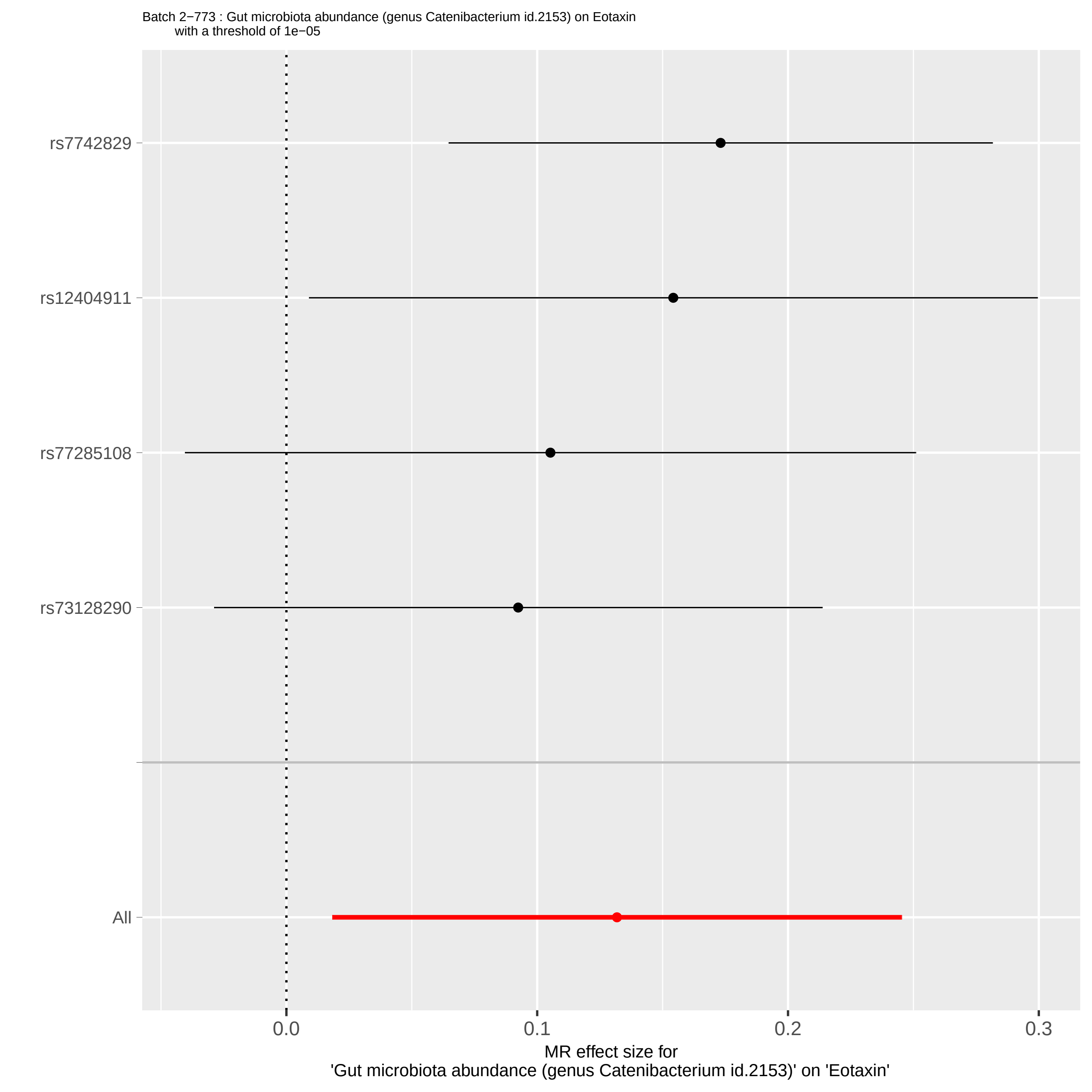

Batch 2−773 : Gut microbiota abundance (genus Catenibacterium id.2153) on Eotaxin
with a threshold of 1e−05
rs7742829
rs12404911
rs77285108
rs73128290
All
0.0
0.1
0.2
0.3
MR effect size for
'Gut microbiota abundance (genus Catenibacterium id.2153)' on 'Eotaxin'

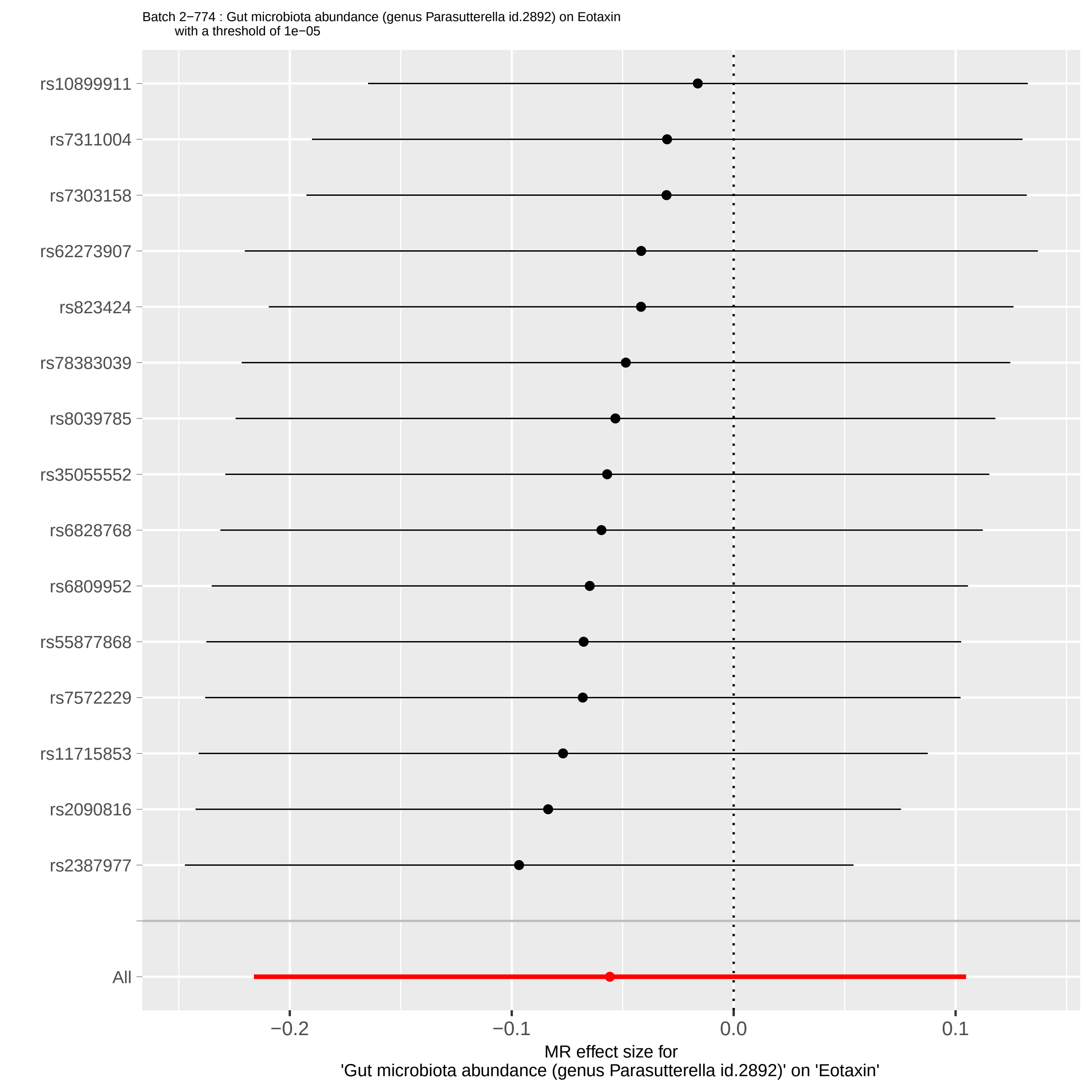

Batch 2−774 : Gut microbiota abundance (genus Parasutterella id.2892) on Eotaxin
with a threshold of 1e−05
rs10899911
rs7311004
rs7303158
rs62273907
rs823424
rs78383039
rs8039785
rs35055552
rs6828768
rs6809952
rs55877868
rs7572229
rs11715853
rs2090816
rs2387977
All
−0.2
−0.1
0.0
0.1
MR effect size for
'Gut microbiota abundance (genus Parasutterella id.2892)' on 'Eotaxin'

Batch 2−775 : Gut microbiota abundance (genus Catenibacterium id.2153) on C−C motif chemokine 1
with a threshold of 1e−05

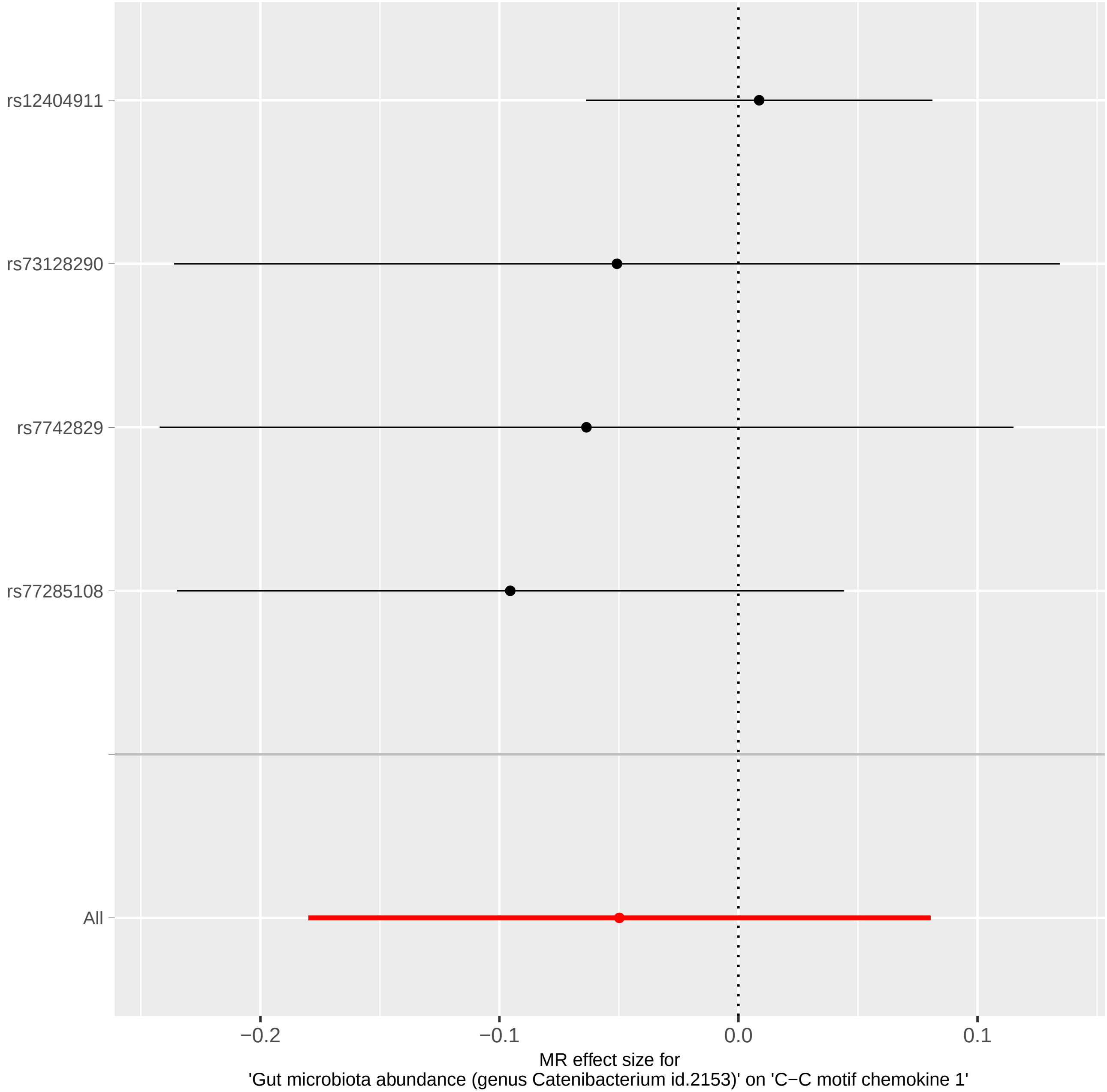

Batch 2−776 : Gut microbiota abundance (genus Parasutterella id.2892) on C−C motif chemokine 1
with a threshold of 1e−05

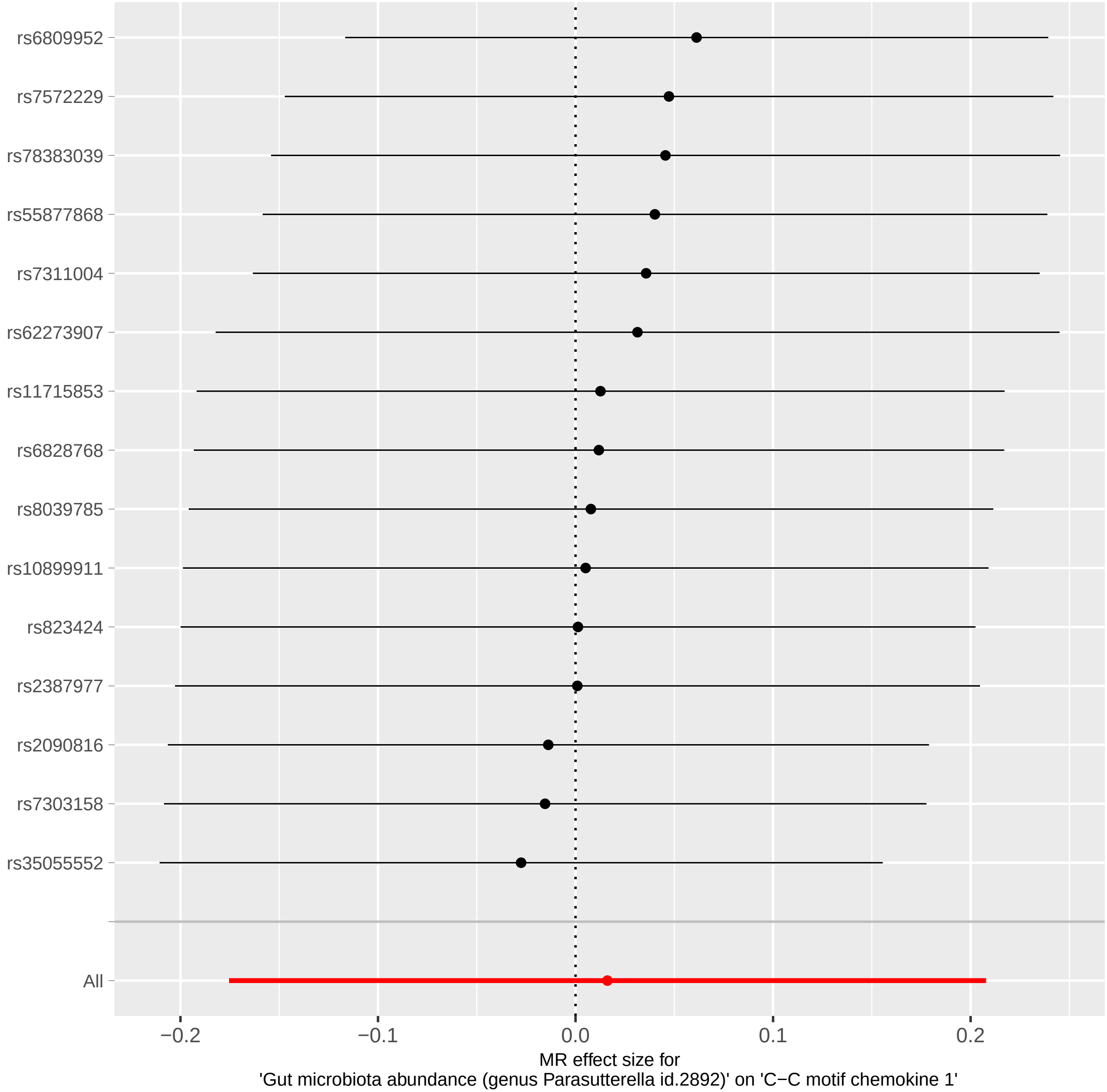

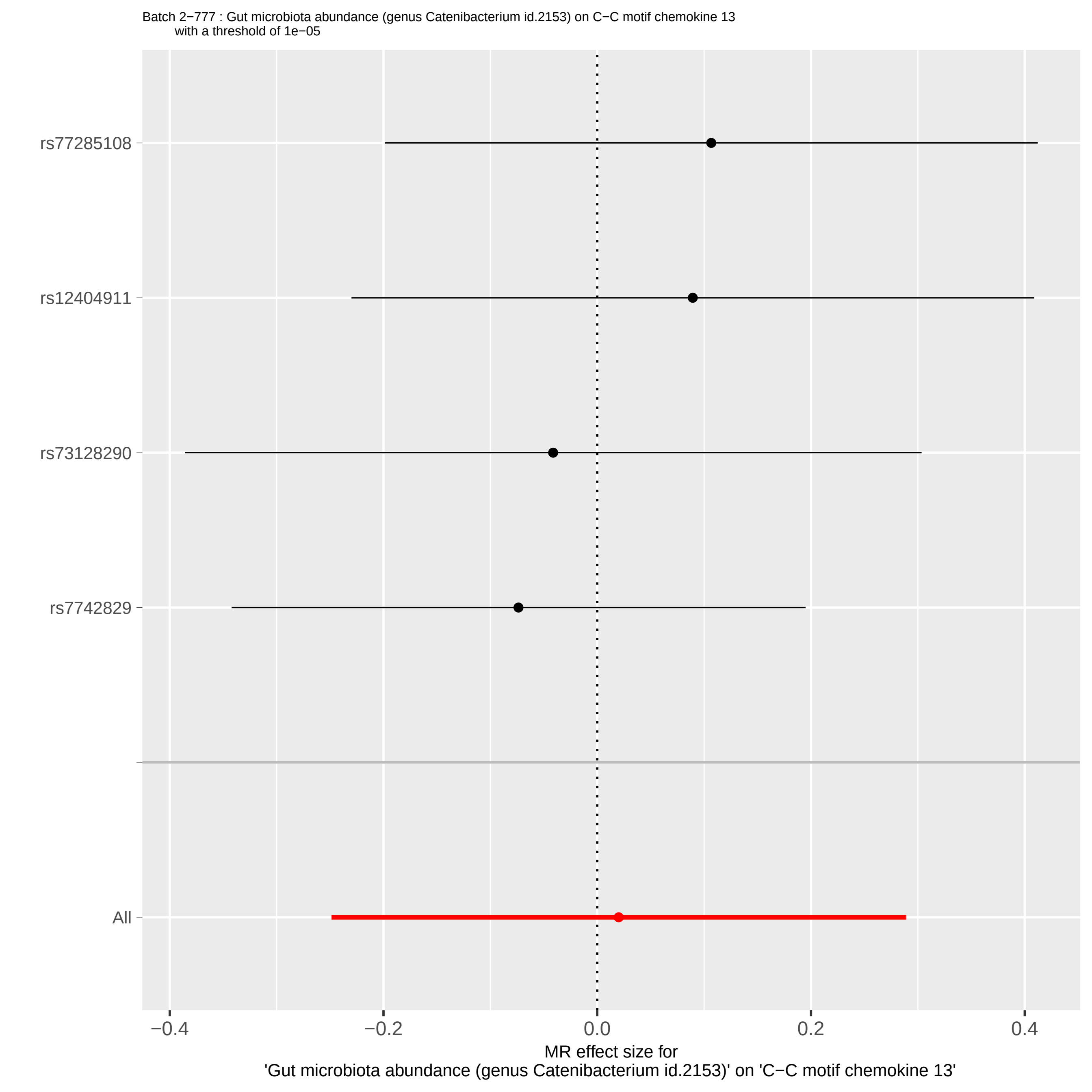
Batch 2−777 : Gut microbiota abundance (genus Catenibacterium id.2153) on C−C motif chemokine 13
with a threshold of 1e−05
rs77285108
rs12404911
rs73128290
rs7742829
All
−0.4
−0.2
0.0
0.2
0.4
MR effect size for
'Gut microbiota abundance (genus Catenibacterium id.2153)' on 'C−C motif chemokine 13'

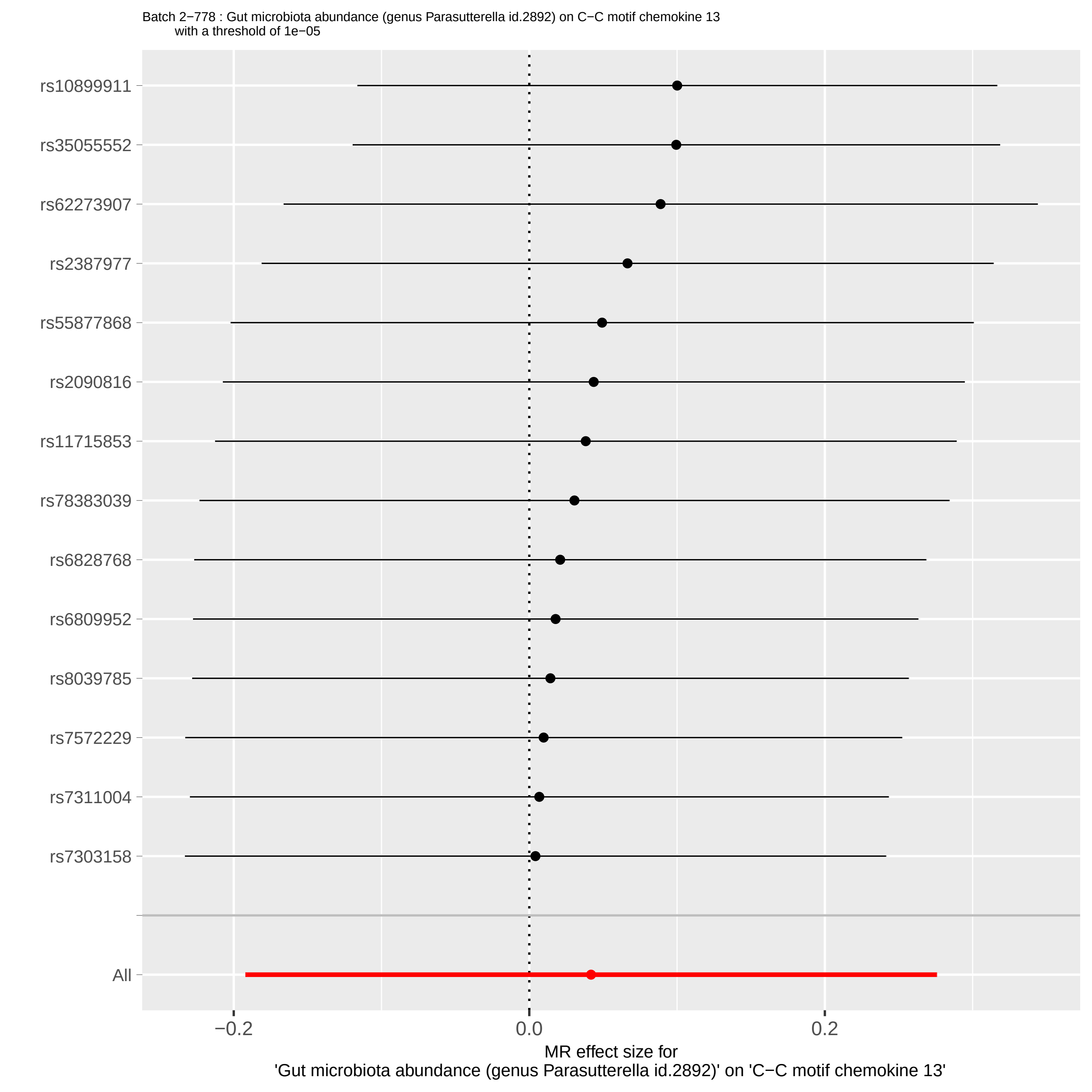

Batch 2−778 : Gut microbiota abundance (genus Parasutterella id.2892) on C−C motif chemokine 13
with a threshold of 1e−05
rs10899911
rs35055552
rs62273907
rs2387977
rs55877868
rs2090816
rs11715853
rs78383039
rs6828768
rs6809952
rs8039785
rs7572229
rs7311004
rs7303158
All
−0.2
0.0
0.2
MR effect size for
'Gut microbiota abundance (genus Parasutterella id.2892)' on 'C−C motif chemokine 13'

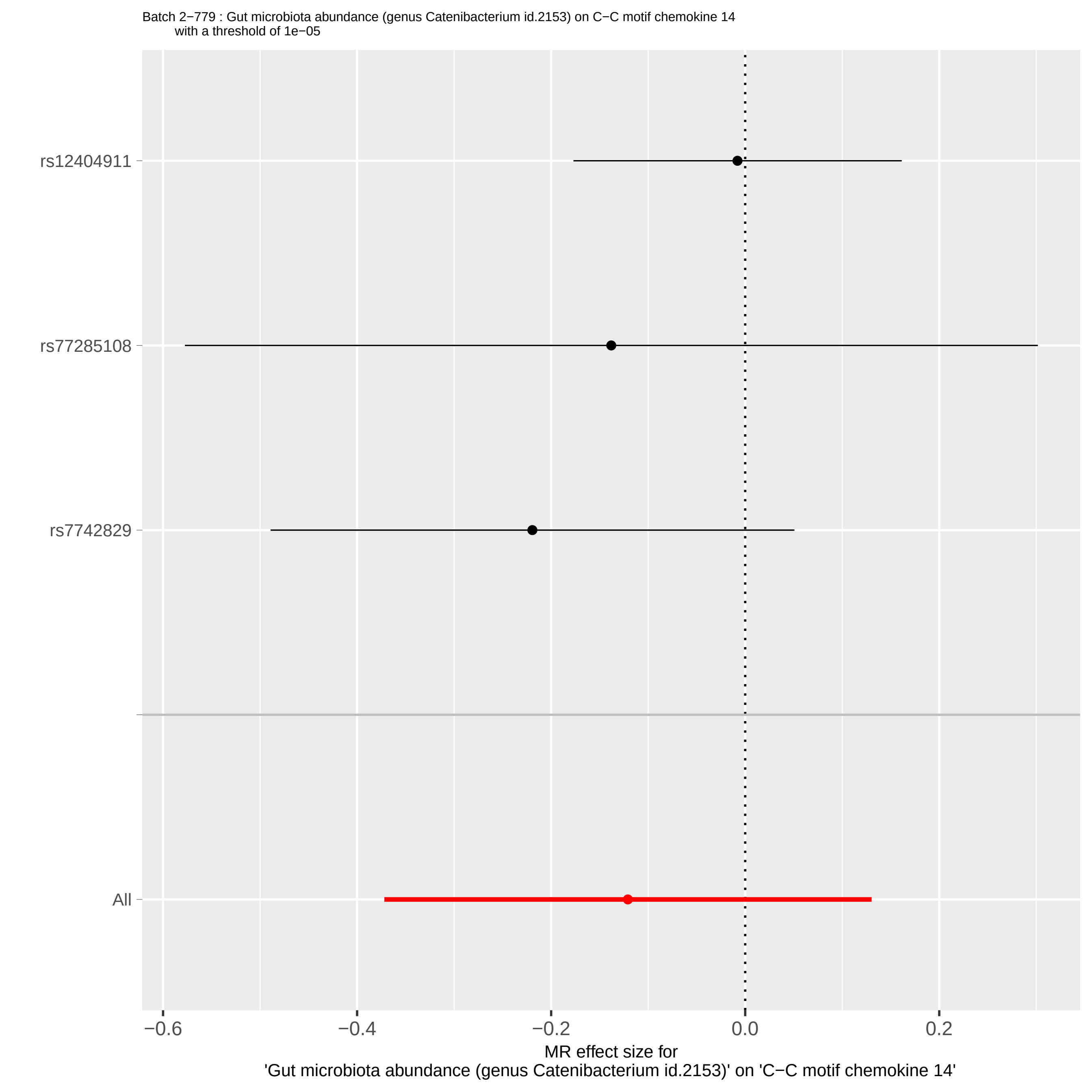

Batch 2−779 : Gut microbiota abundance (genus Catenibacterium id.2153) on C−C motif chemokine 14
with a threshold of 1e−05
rs12404911
rs77285108
rs7742829
All
−0.6
−0.4
−0.2
0.0
0.2
MR effect size for
'Gut microbiota abundance (genus Catenibacterium id.2153)' on 'C−C motif chemokine 14'

Batch 2−780 : Gut microbiota abundance (genus Parasutterella id.2892) on C−C motif chemokine 14 with a threshold of 1e−05

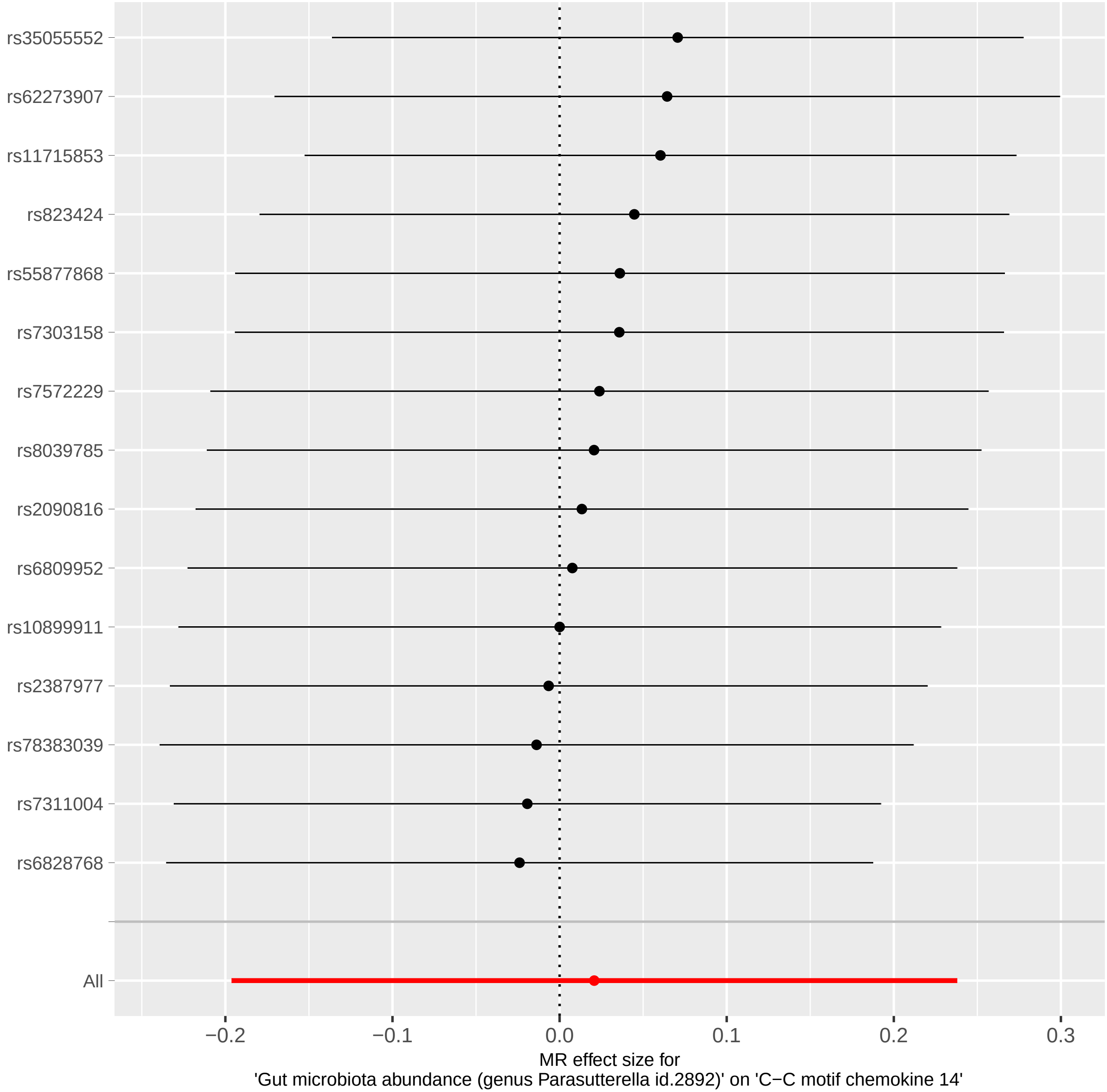

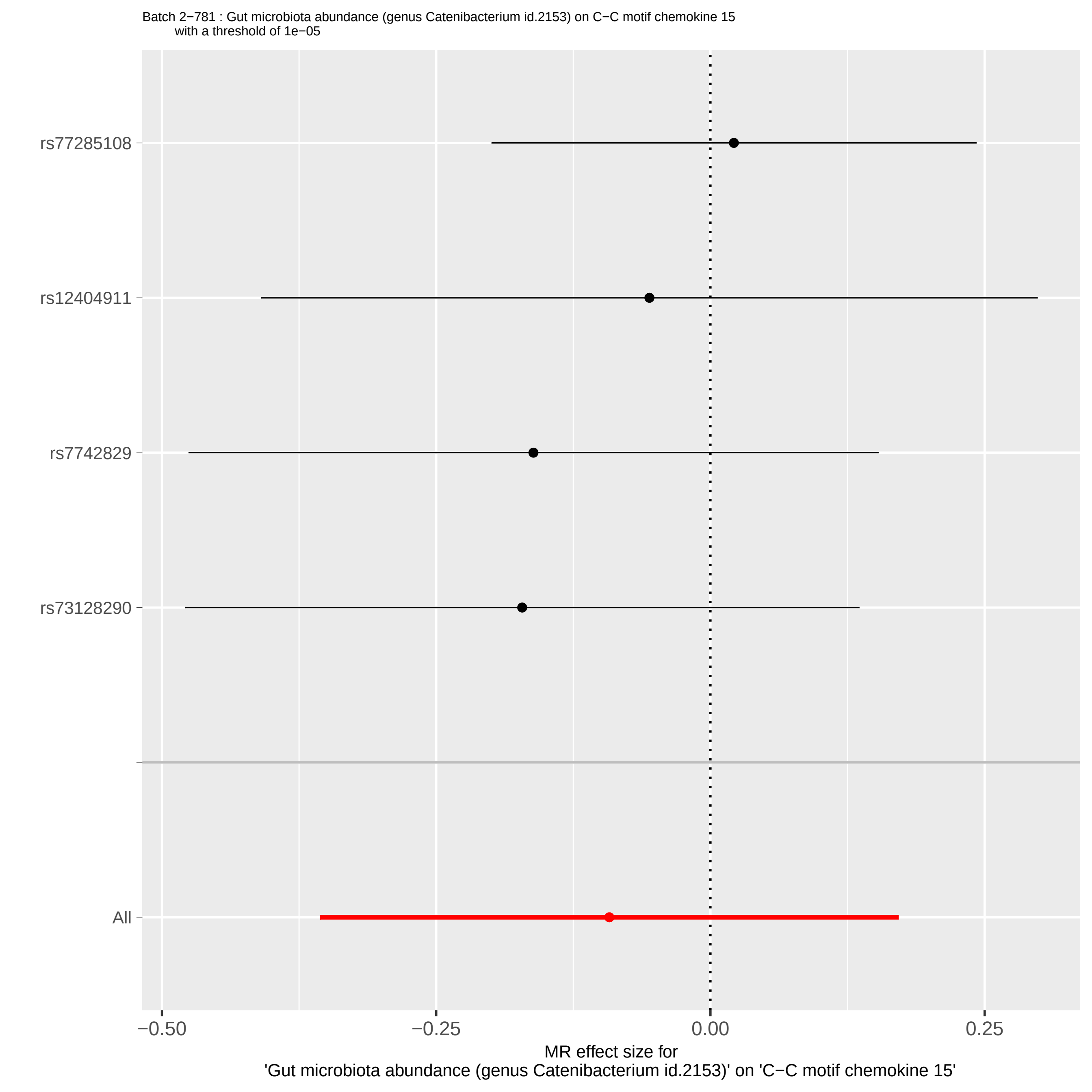
Batch 2−781 : Gut microbiota abundance (genus Catenibacterium id.2153) on C−C motif chemokine 15
with a threshold of 1e−05
rs77285108
rs12404911
rs7742829
rs73128290
All
−0.50
−0.25
0.00
0.25
MR effect size for
'Gut microbiota abundance (genus Catenibacterium id.2153)' on 'C−C motif chemokine 15'

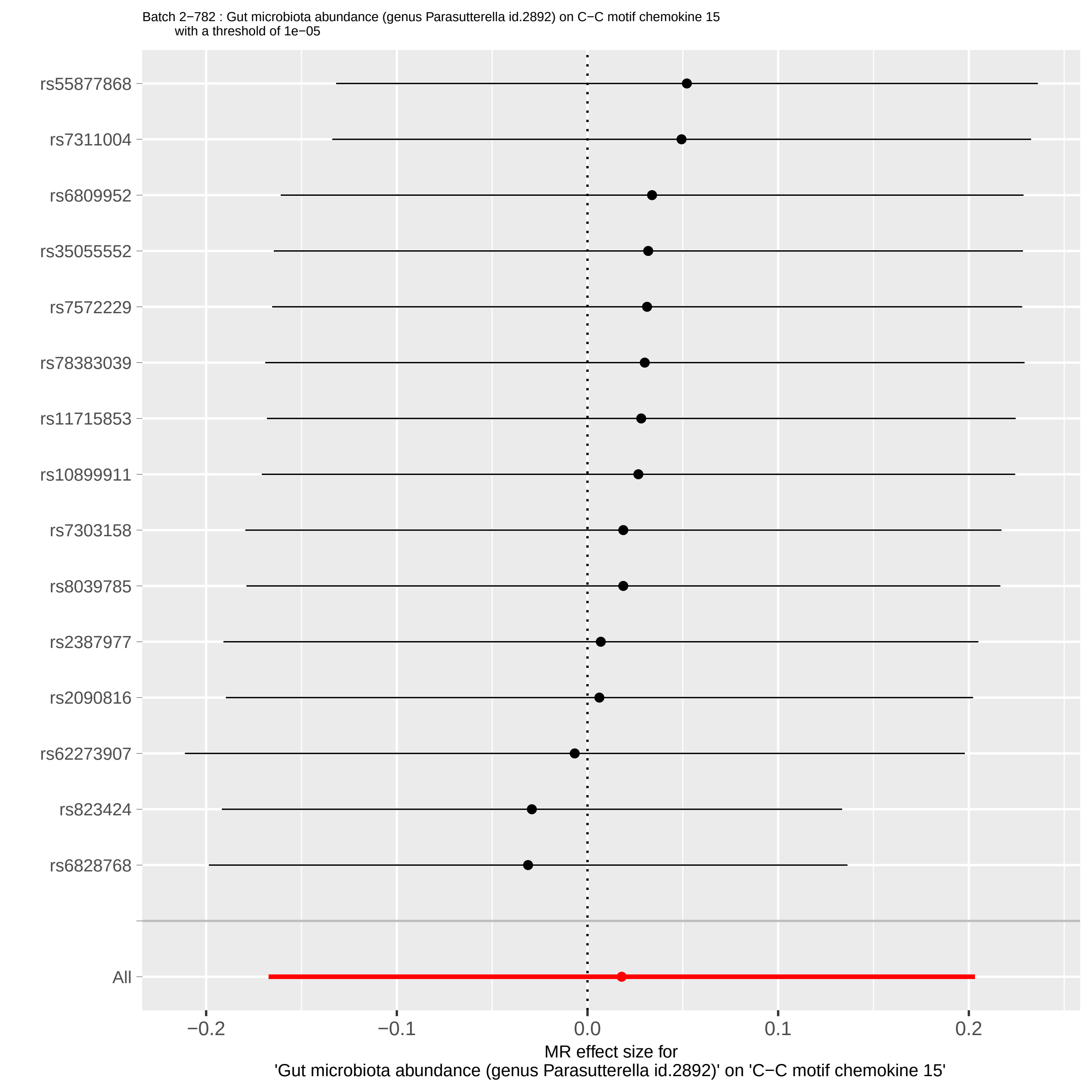
Batch 2−782 : Gut microbiota abundance (genus Parasutterella id.2892) on C−C motif chemokine 15
with a threshold of 1e−05
rs55877868
rs7311004
rs6809952
rs35055552
rs7572229
rs78383039
rs11715853
rs10899911
rs7303158
rs8039785
rs2387977
rs2090816
rs62273907
rs823424
rs6828768
All
−0.2
−0.1
0.0
0.1
0.2
MR effect size for
'Gut microbiota abundance (genus Parasutterella id.2892)' on 'C−C motif chemokine 15'

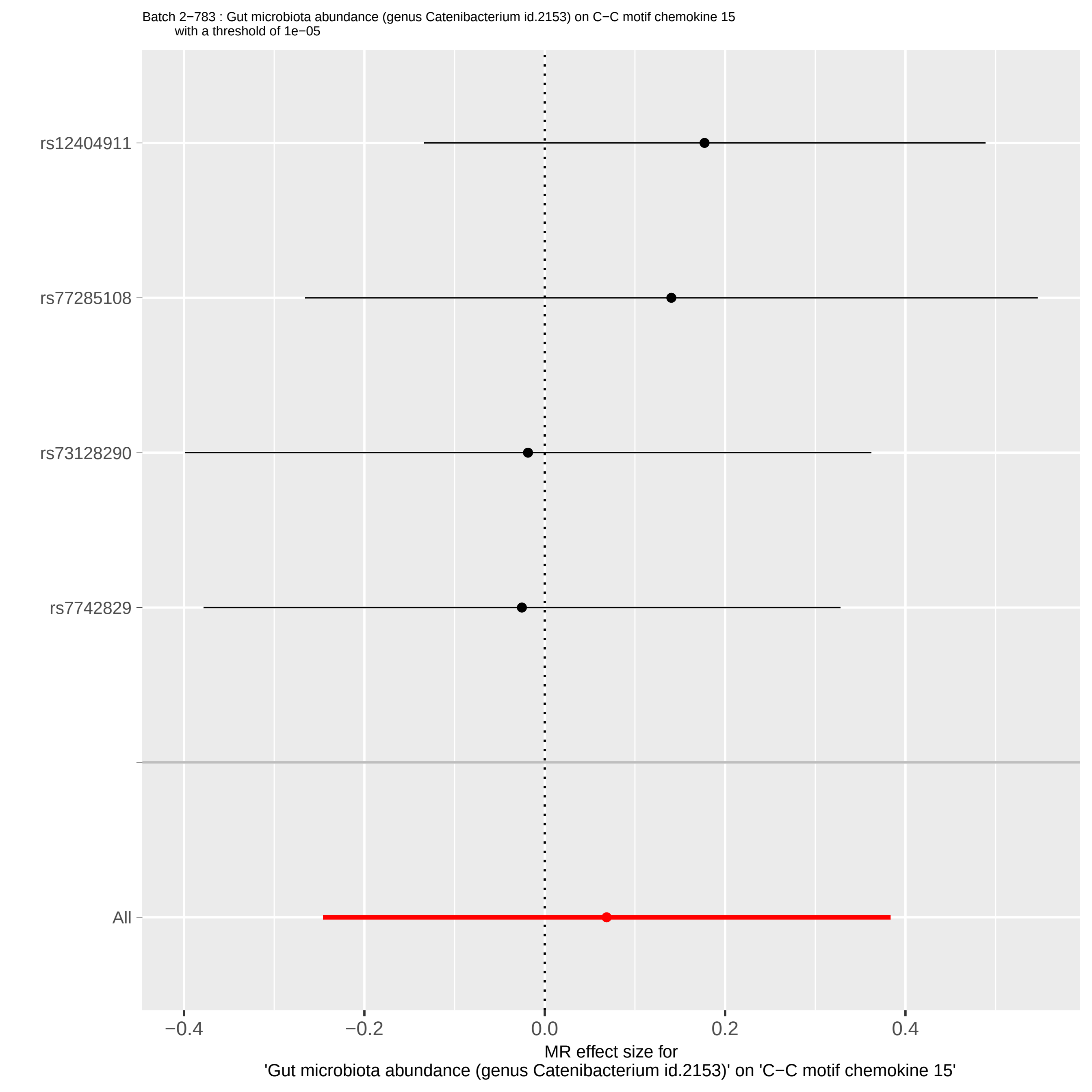

Batch 2−783 : Gut microbiota abundance (genus Catenibacterium id.2153) on C−C motif chemokine 15
with a threshold of 1e−05
rs12404911
rs77285108
rs73128290
rs7742829
All
−0.4
−0.2
0.0
0.2
0.4
MR effect size for
'Gut microbiota abundance (genus Catenibacterium id.2153)' on 'C−C motif chemokine 15'

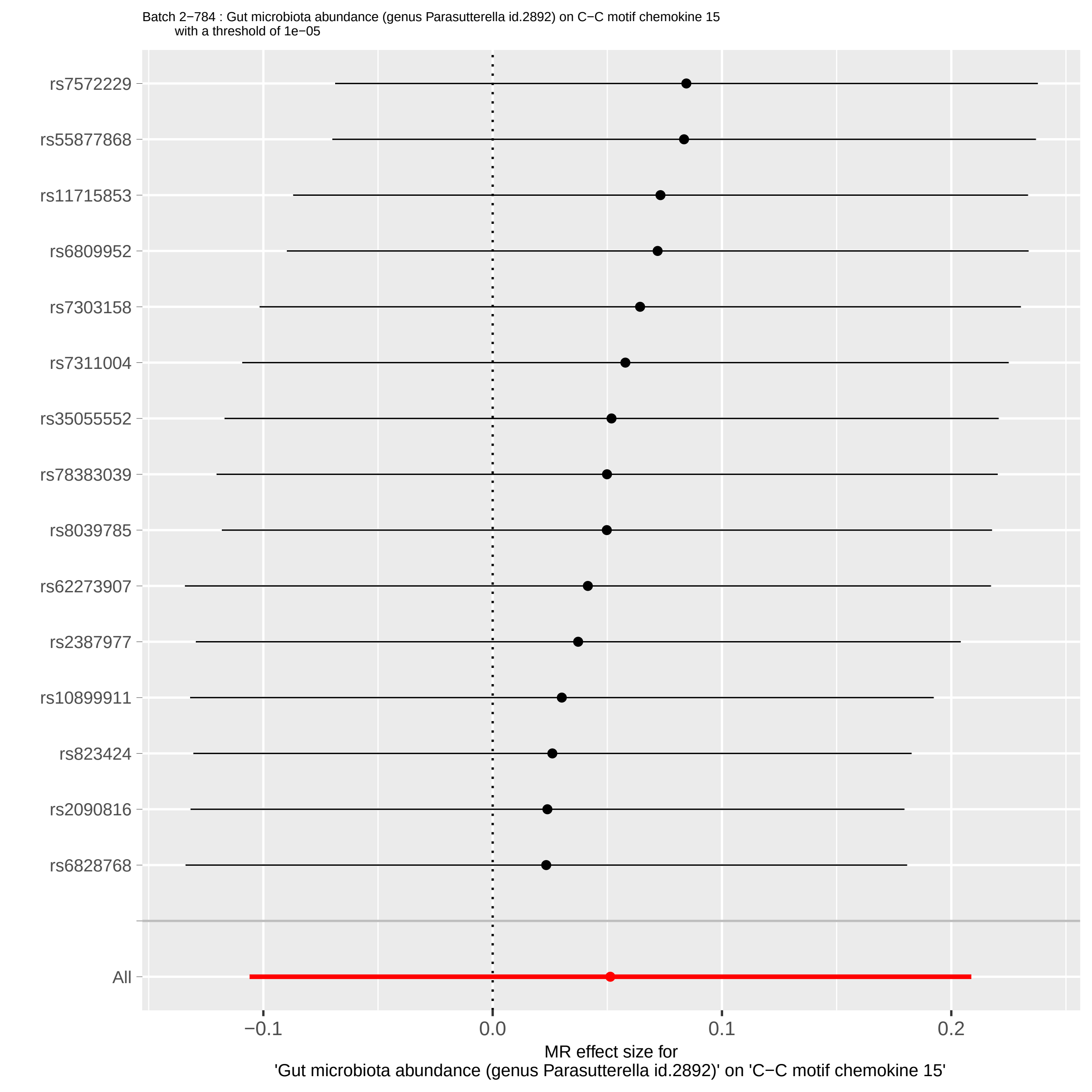

Batch 2−784 : Gut microbiota abundance (genus Parasutterella id.2892) on C−C motif chemokine 15
with a threshold of 1e−05
rs7572229
rs55877868
rs11715853
rs6809952
rs7303158
rs7311004
rs35055552
rs78383039
rs8039785
rs62273907
rs2387977
rs10899911
rs823424
rs2090816
rs6828768
All
−0.1
0.0
0.1
0.2
MR effect size for
'Gut microbiota abundance (genus Parasutterella id.2892)' on 'C−C motif chemokine 15'

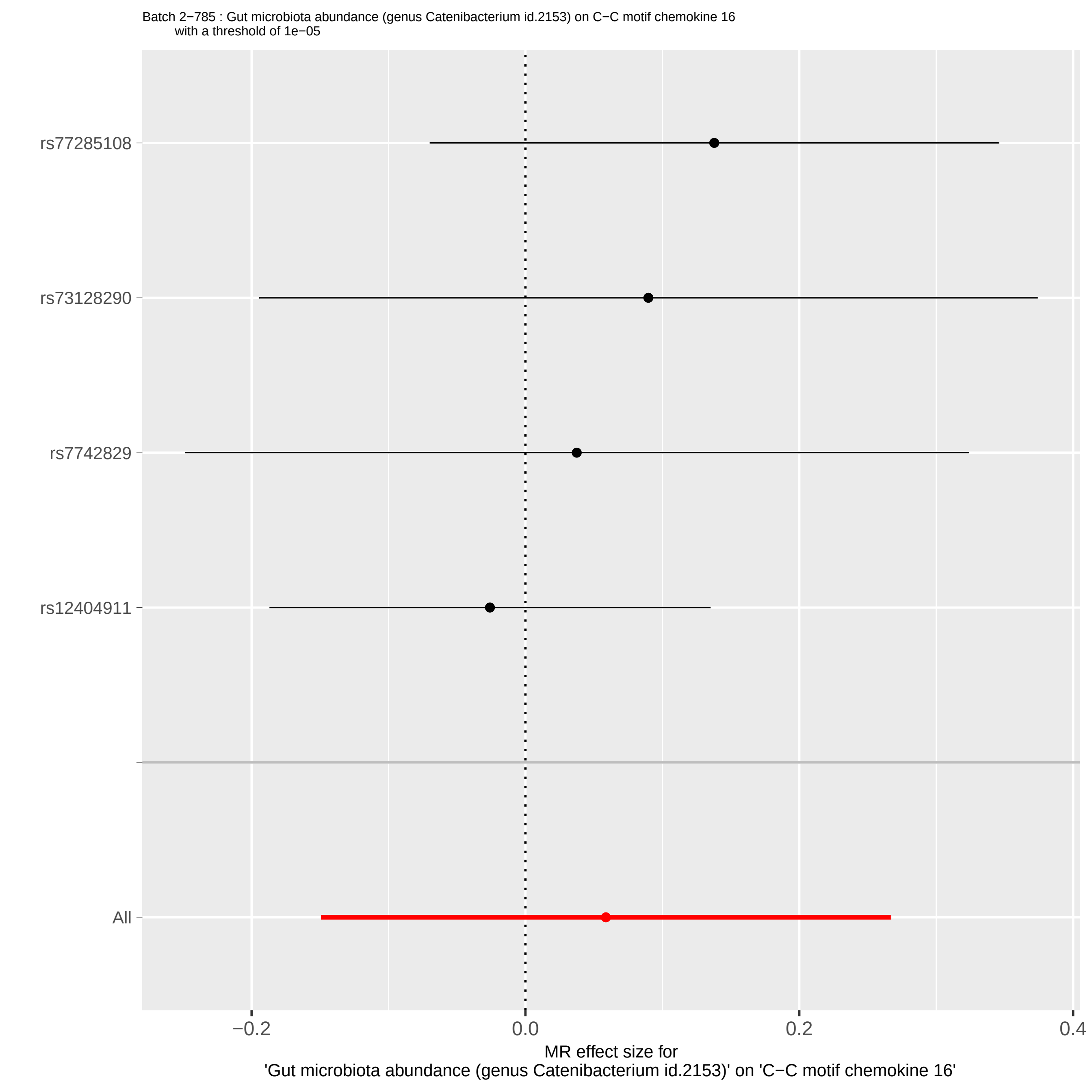
Batch 2−785 : Gut microbiota abundance (genus Catenibacterium id.2153) on C−C motif chemokine 16
with a threshold of 1e−05
rs77285108
rs73128290
rs7742829
rs12404911
All
−0.2
0.0
0.2
0.4
MR effect size for
'Gut microbiota abundance (genus Catenibacterium id.2153)' on 'C−C motif chemokine 16'

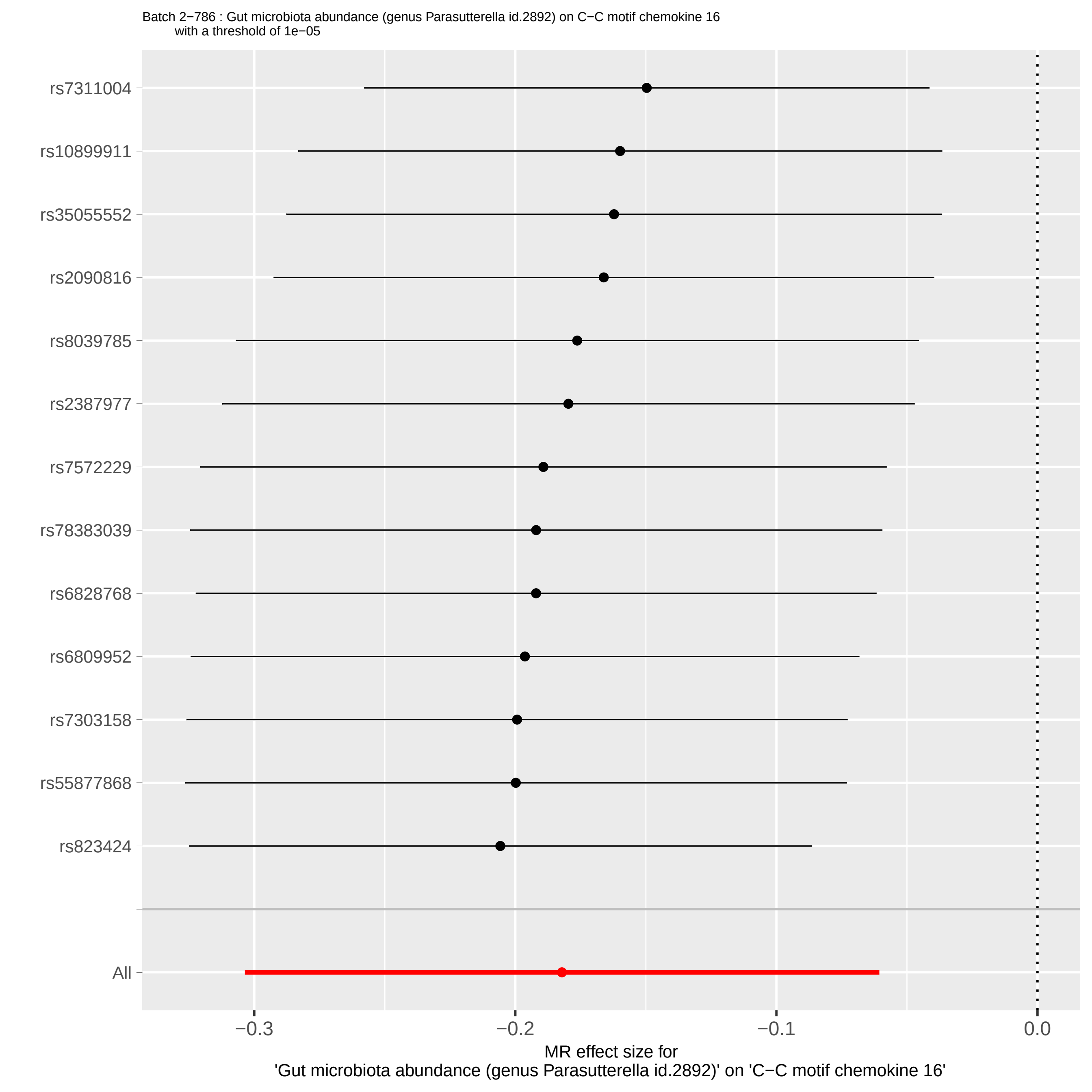
Batch 2−786 : Gut microbiota abundance (genus Parasutterella id.2892) on C−C motif chemokine 16
with a threshold of 1e−05
rs7311004
rs10899911
rs35055552
rs2090816
rs8039785
rs2387977
rs7572229
rs78383039
rs6828768
rs6809952
rs7303158
rs55877868
rs823424
All
−0.3
−0.2
−0.1
0.0
MR effect size for
'Gut microbiota abundance (genus Parasutterella id.2892)' on 'C−C motif chemokine 16'

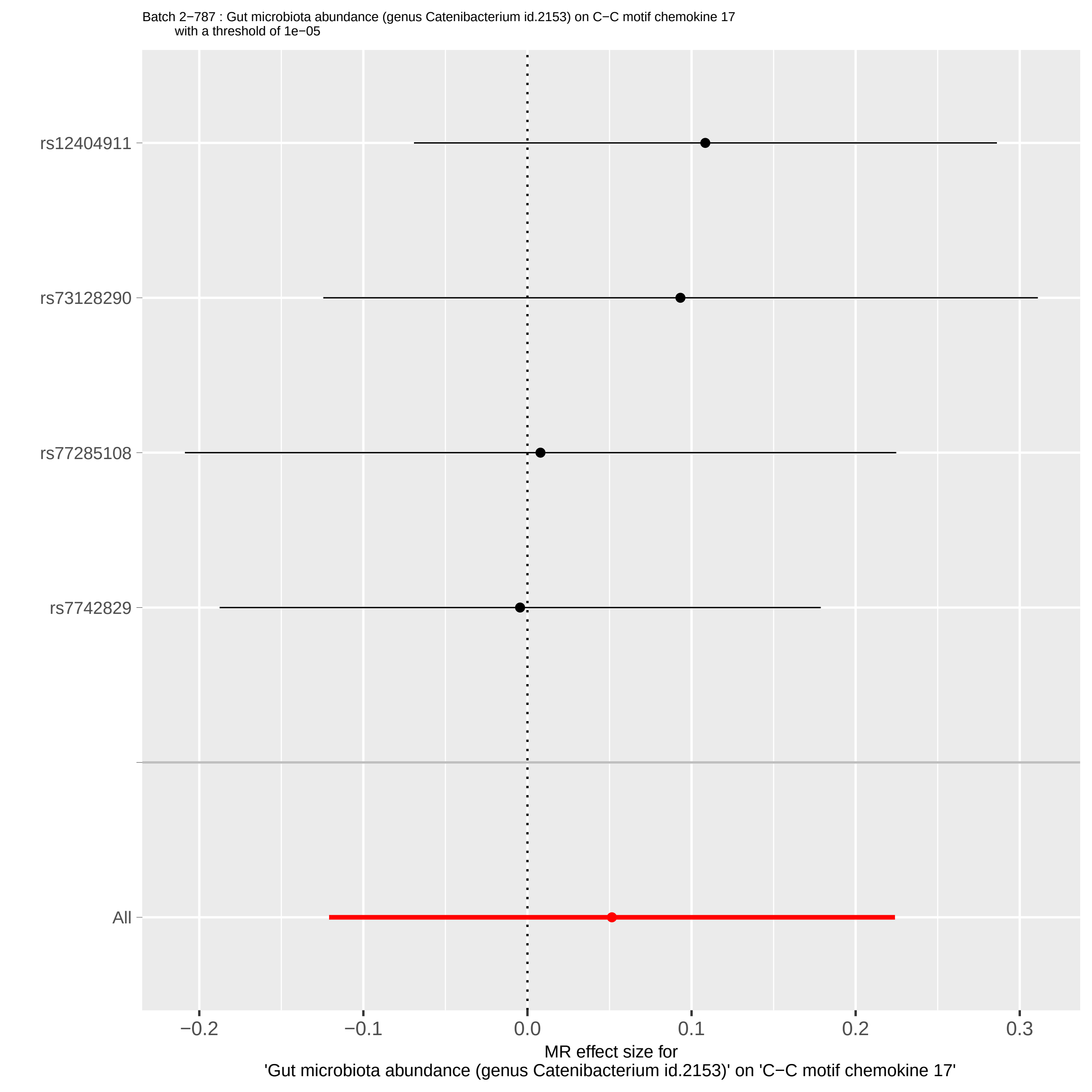
Batch 2−787 : Gut microbiota abundance (genus Catenibacterium id.2153) on C−C motif chemokine 17
with a threshold of 1e−05
rs12404911
rs73128290
rs77285108
rs7742829
All
−0.2
−0.1
0.0
0.1
0.2
0.3
MR effect size for
'Gut microbiota abundance (genus Catenibacterium id.2153)' on 'C−C motif chemokine 17'

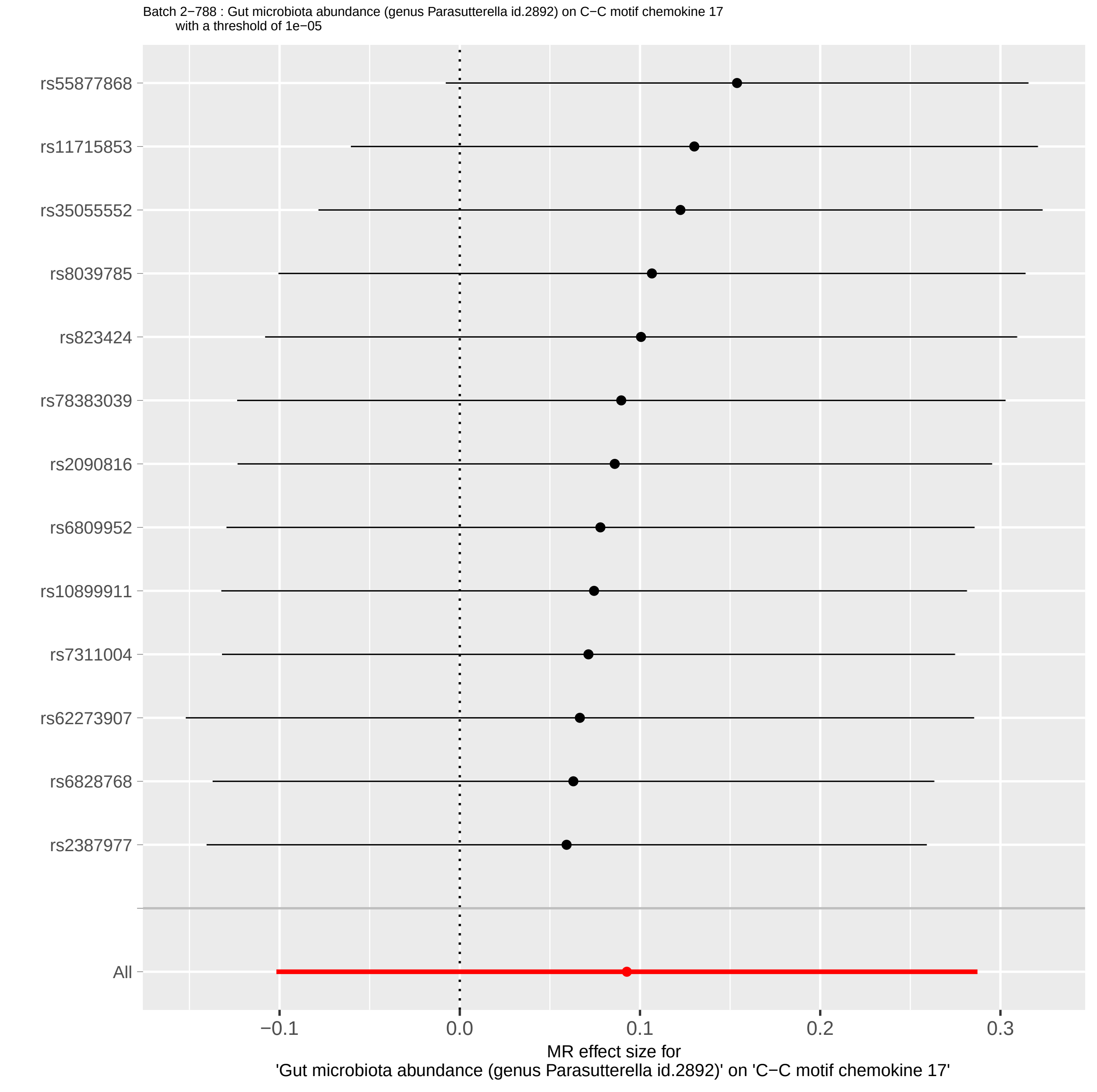
Batch 2−788 : Gut microbiota abundance (genus Parasutterella id.2892) on C−C motif chemokine 17
with a threshold of 1e−05
rs55877868
rs11715853
rs35055552
rs8039785
rs823424
rs78383039
rs2090816
rs6809952
rs10899911
rs7311004
rs62273907
rs6828768
rs2387977
All
−0.1
0.0
0.1
0.2
0.3
MR effect size for
'Gut microbiota abundance (genus Parasutterella id.2892)' on 'C−C motif chemokine 17'

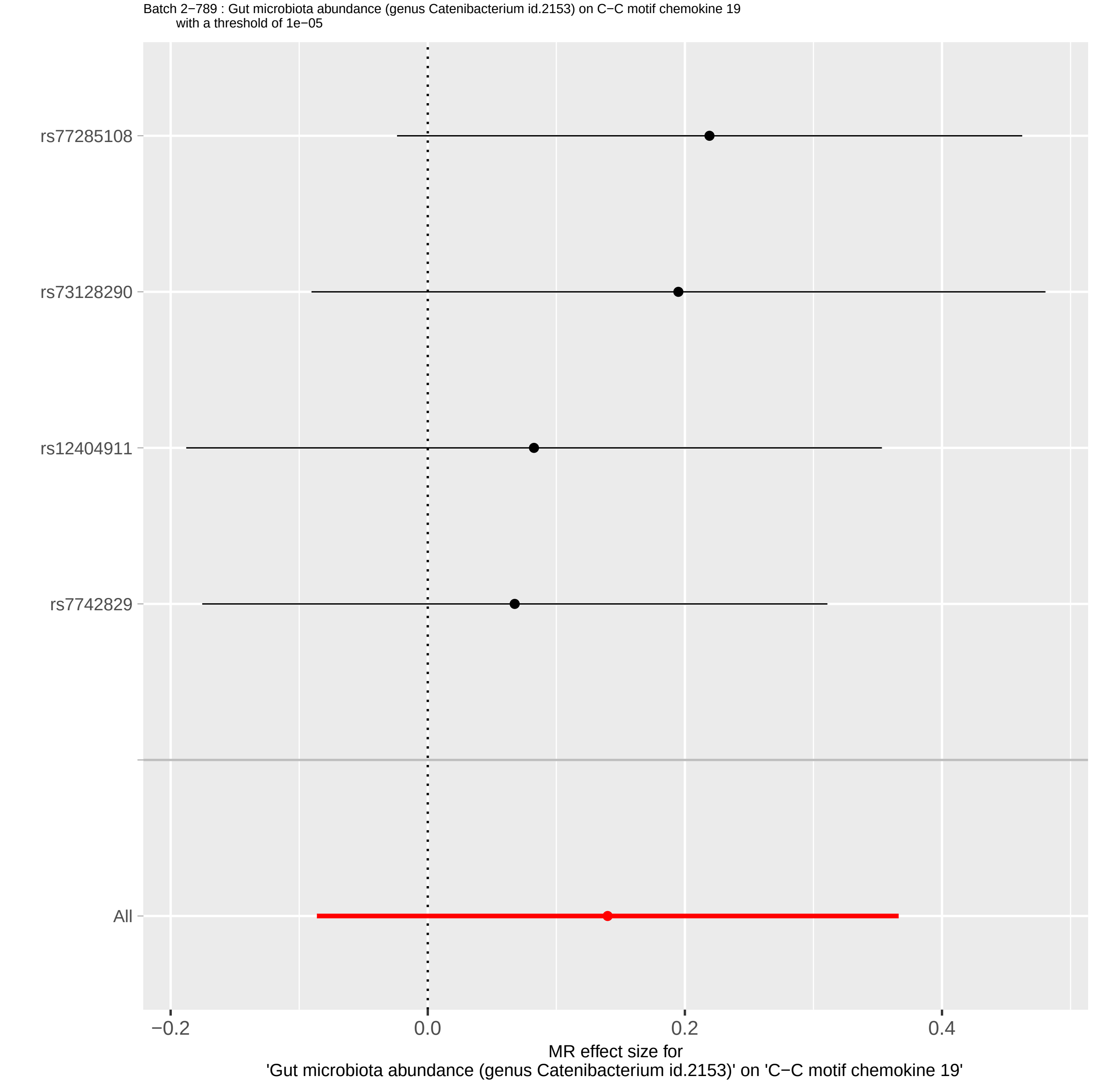

Batch 2−789 : Gut microbiota abundance (genus Catenibacterium id.2153) on C−C motif chemokine 19
with a threshold of 1e−05
rs77285108
rs73128290
rs12404911
rs7742829
All
−0.2
0.0
0.2
0.4
MR effect size for
'Gut microbiota abundance (genus Catenibacterium id.2153)' on 'C−C motif chemokine 19'

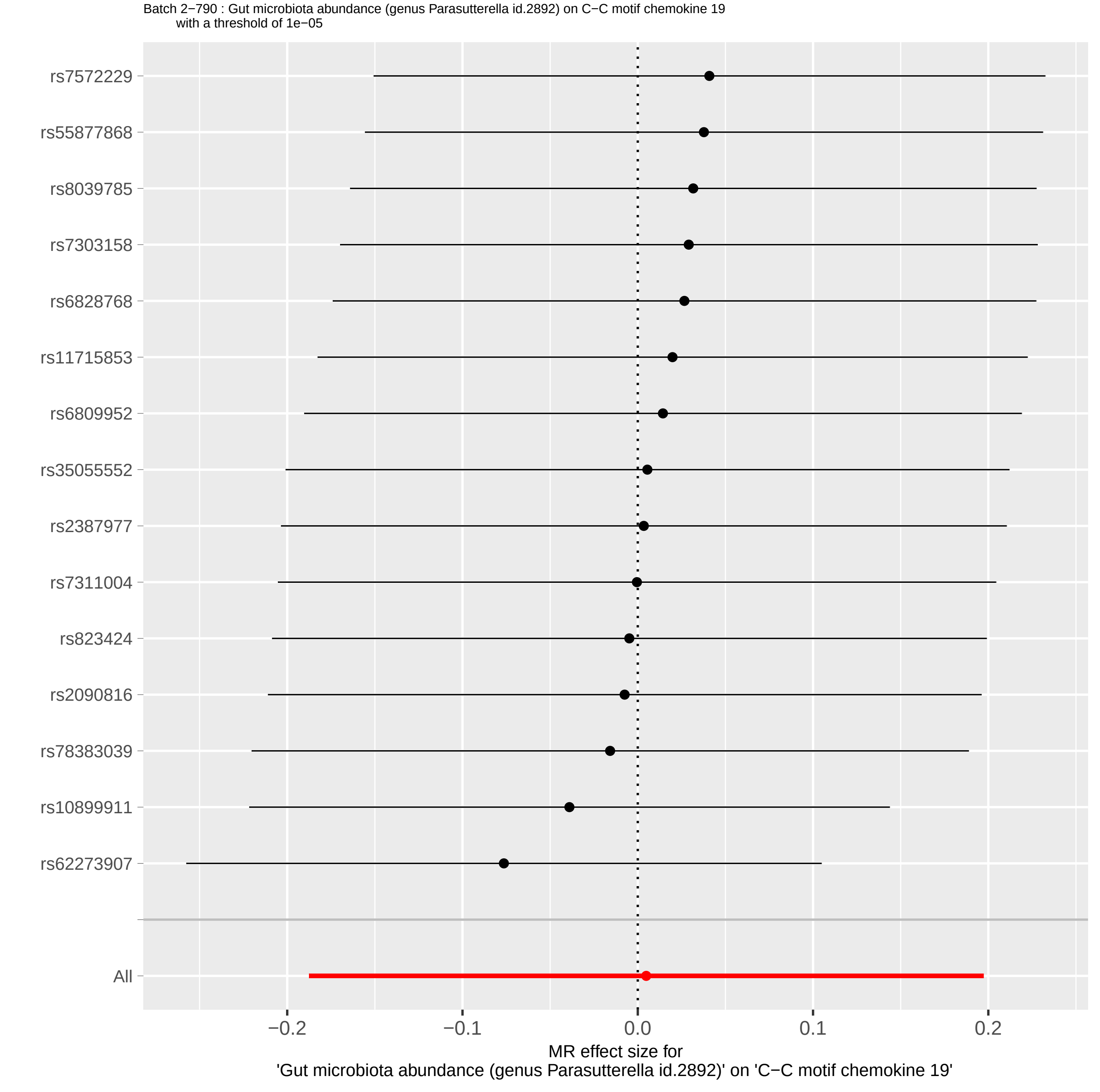

Batch 2−790 : Gut microbiota abundance (genus Parasutterella id.2892) on C−C motif chemokine 19
with a threshold of 1e−05
rs7572229
rs55877868
rs8039785
rs7303158
rs6828768
rs11715853
rs6809952
rs35055552
rs2387977
rs7311004
rs823424
rs2090816
rs78383039
rs10899911
rs62273907
All
−0.2
−0.1
0.0
0.1
0.2
MR effect size for
'Gut microbiota abundance (genus Parasutterella id.2892)' on 'C−C motif chemokine 19'

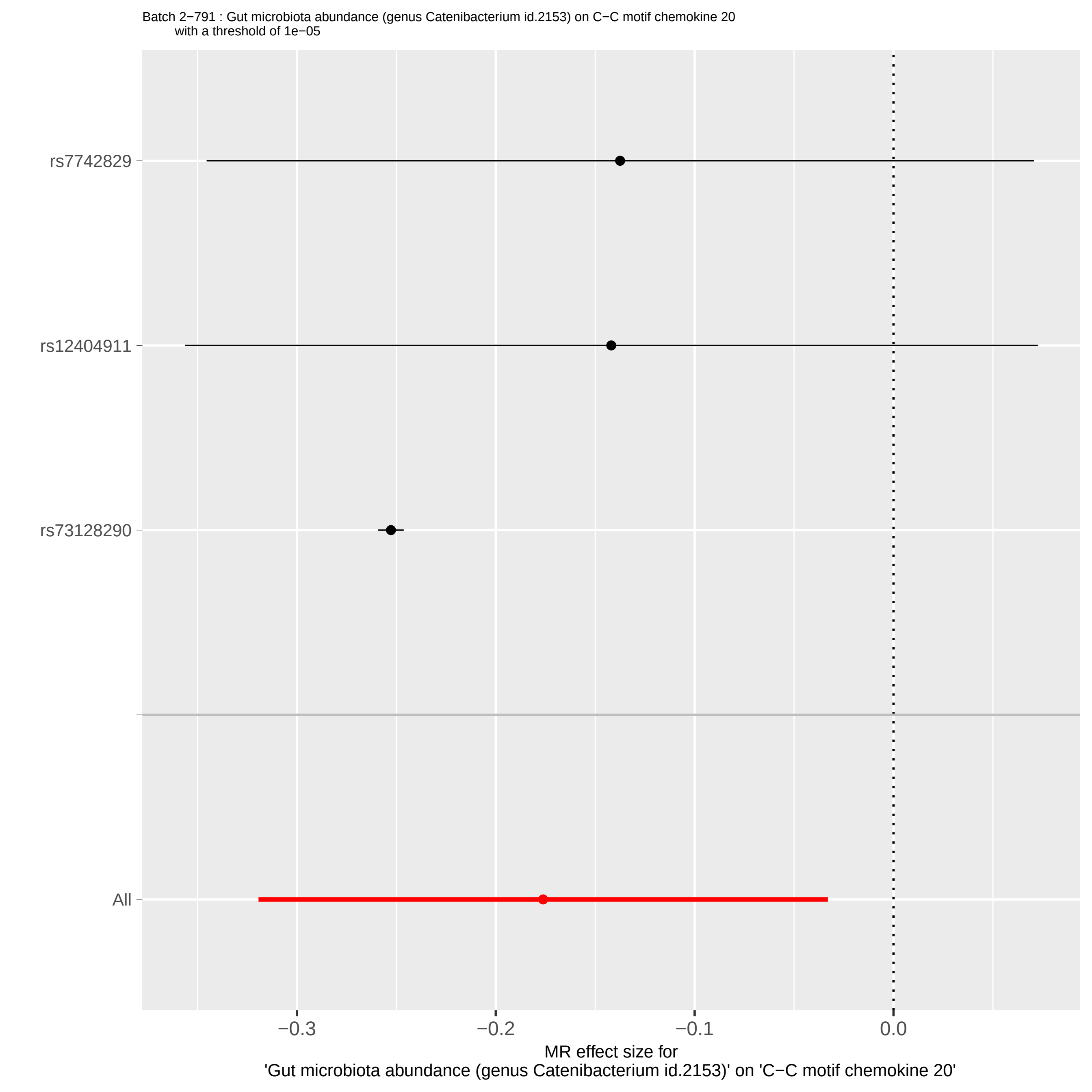
Batch 2−791 : Gut microbiota abundance (genus Catenibacterium id.2153) on C−C motif chemokine 20
with a threshold of 1e−05
rs7742829
rs12404911
rs73128290
All
−0.3
−0.2
−0.1
0.0
MR effect size for
'Gut microbiota abundance (genus Catenibacterium id.2153)' on 'C−C motif chemokine 20'

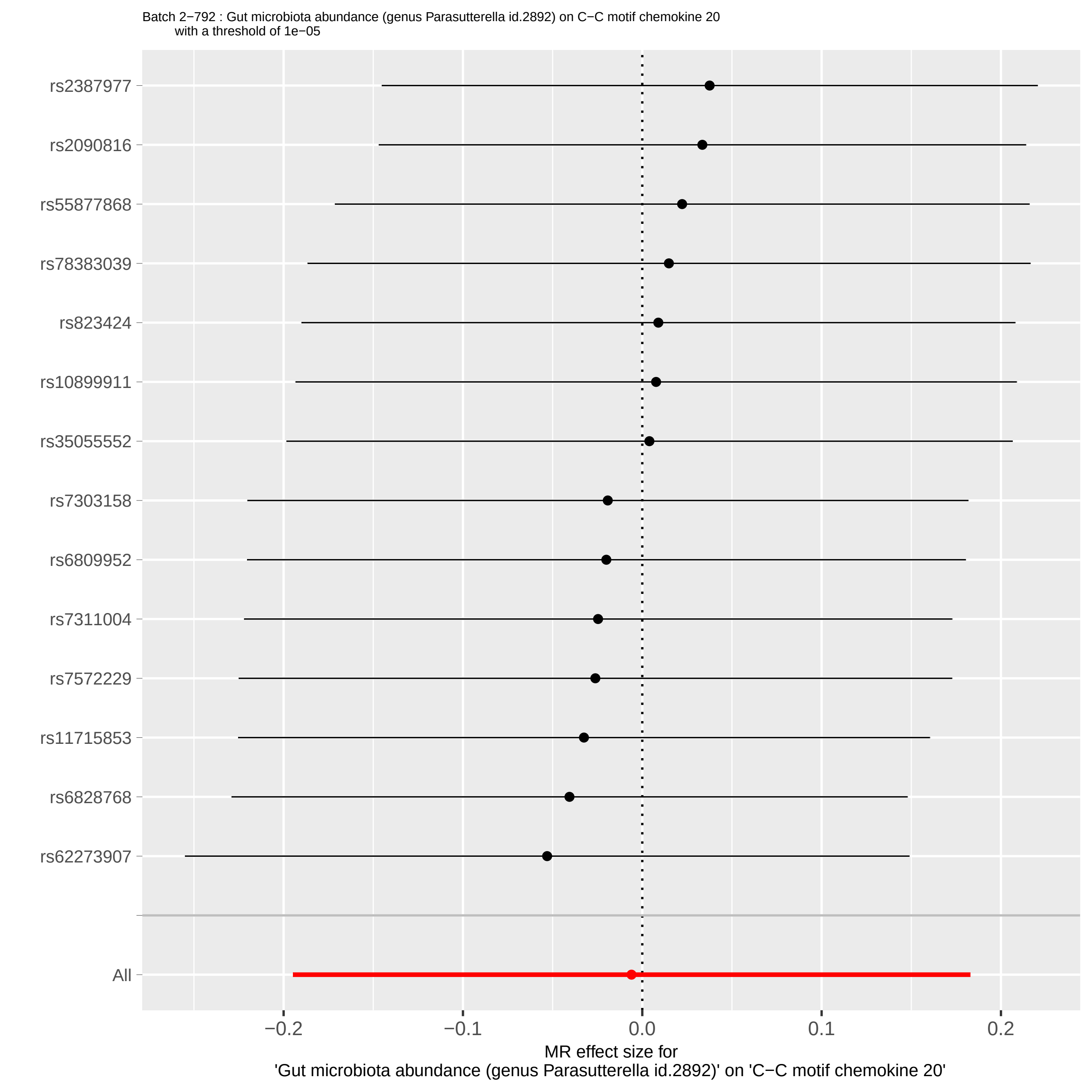

Batch 2−792 : Gut microbiota abundance (genus Parasutterella id.2892) on C−C motif chemokine 20
with a threshold of 1e−05
rs2387977
rs2090816
rs55877868
rs78383039
rs823424
rs10899911
rs35055552
rs7303158
rs6809952
rs7311004
rs7572229
rs11715853
rs6828768
rs62273907
All
−0.2
−0.1
0.0
0.1
0.2
MR effect size for
'Gut microbiota abundance (genus Parasutterella id.2892)' on 'C−C motif chemokine 20'

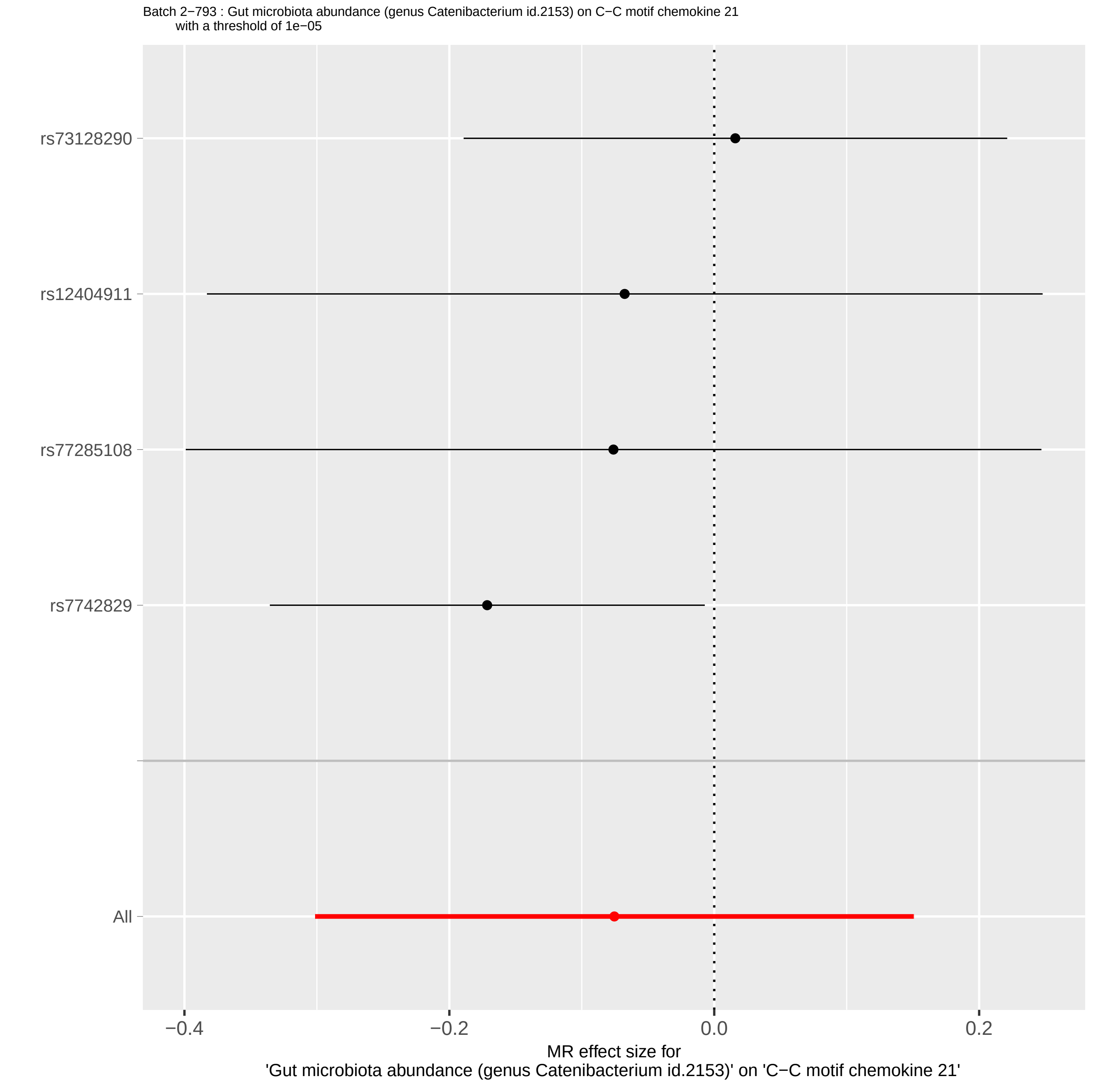
Batch 2−793 : Gut microbiota abundance (genus Catenibacterium id.2153) on C−C motif chemokine 21
with a threshold of 1e−05
rs73128290
rs12404911
rs77285108
rs7742829
All
−0.4
−0.2
0.0
0.2
MR effect size for
'Gut microbiota abundance (genus Catenibacterium id.2153)' on 'C−C motif chemokine 21'

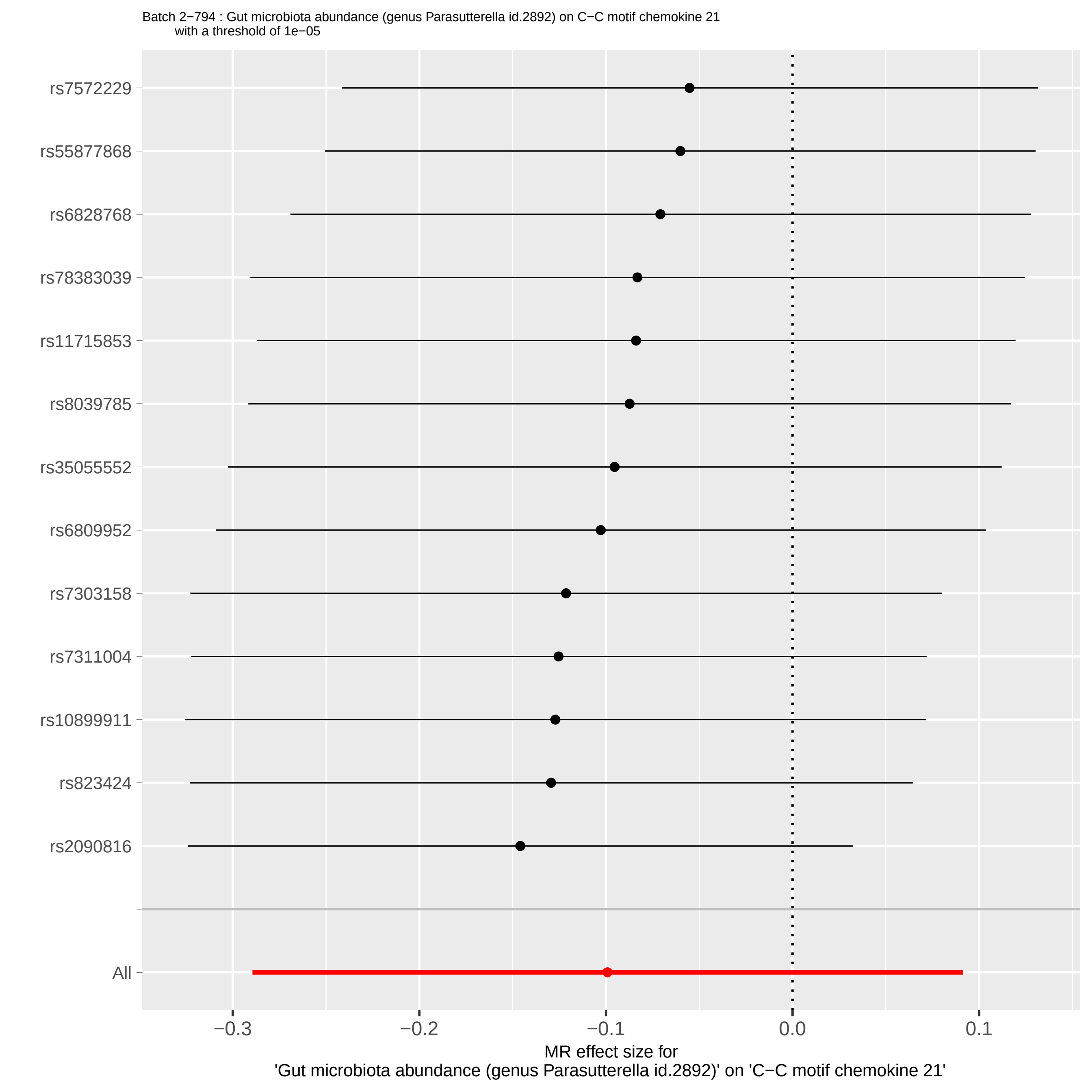

Batch 2−794 : Gut microbiota abundance (genus Parasutterella id.2892) on C−C motif chemokine 21
with a threshold of 1e−05
rs7572229
rs55877868
rs6828768
rs78383039
rs11715853
rs8039785
rs35055552
rs6809952
rs7303158
rs7311004
rs10899911
rs823424
rs2090816
All
−0.3
−0.2
−0.1
0.0
0.1
MR effect size for
'Gut microbiota abundance (genus Parasutterella id.2892)' on 'C−C motif chemokine 21'

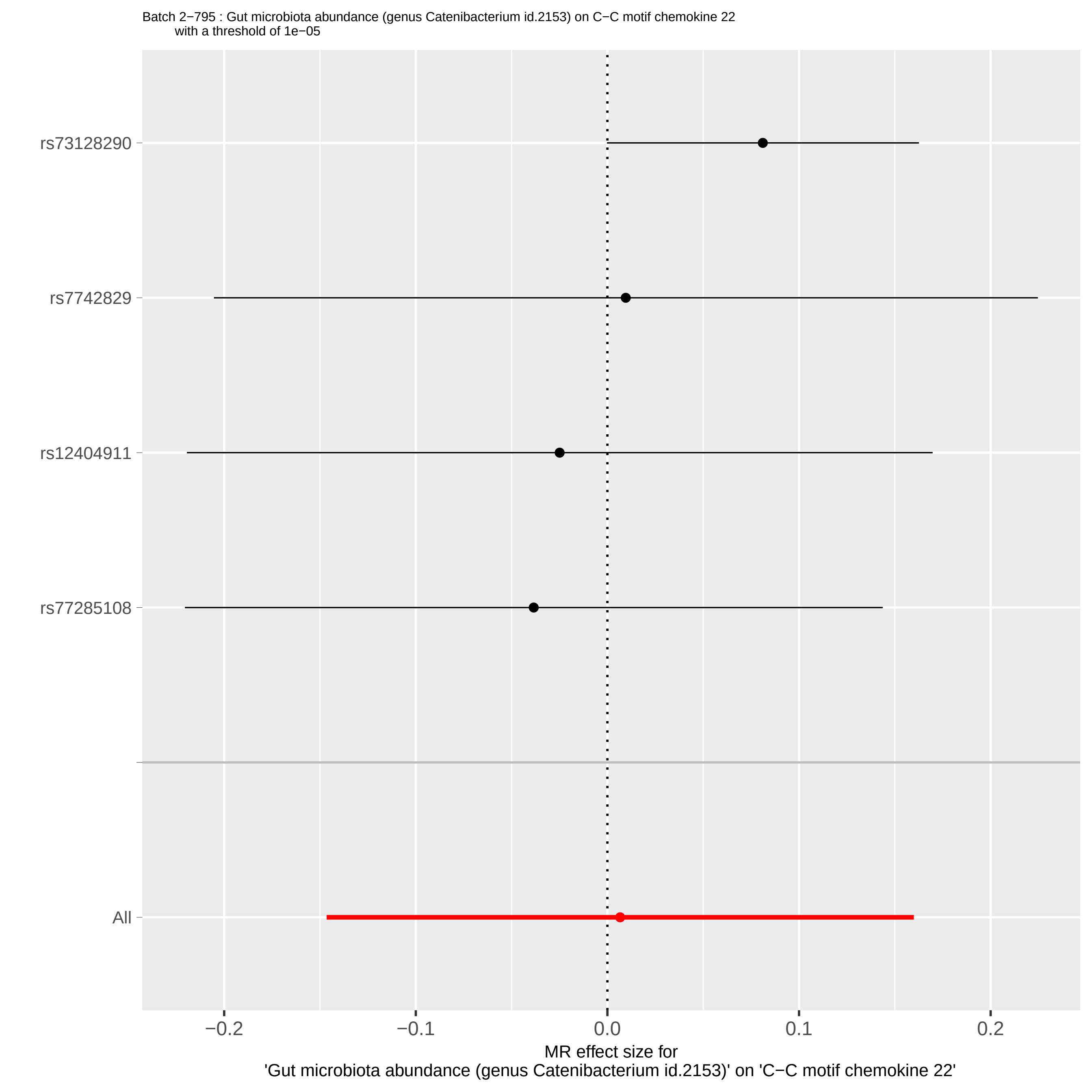

Batch 2−795 : Gut microbiota abundance (genus Catenibacterium id.2153) on C−C motif chemokine 22
with a threshold of 1e−05
rs73128290
rs7742829
rs12404911
rs77285108
All
−0.2
−0.1
0.0
0.1
0.2
MR effect size for
'Gut microbiota abundance (genus Catenibacterium id.2153)' on 'C−C motif chemokine 22'

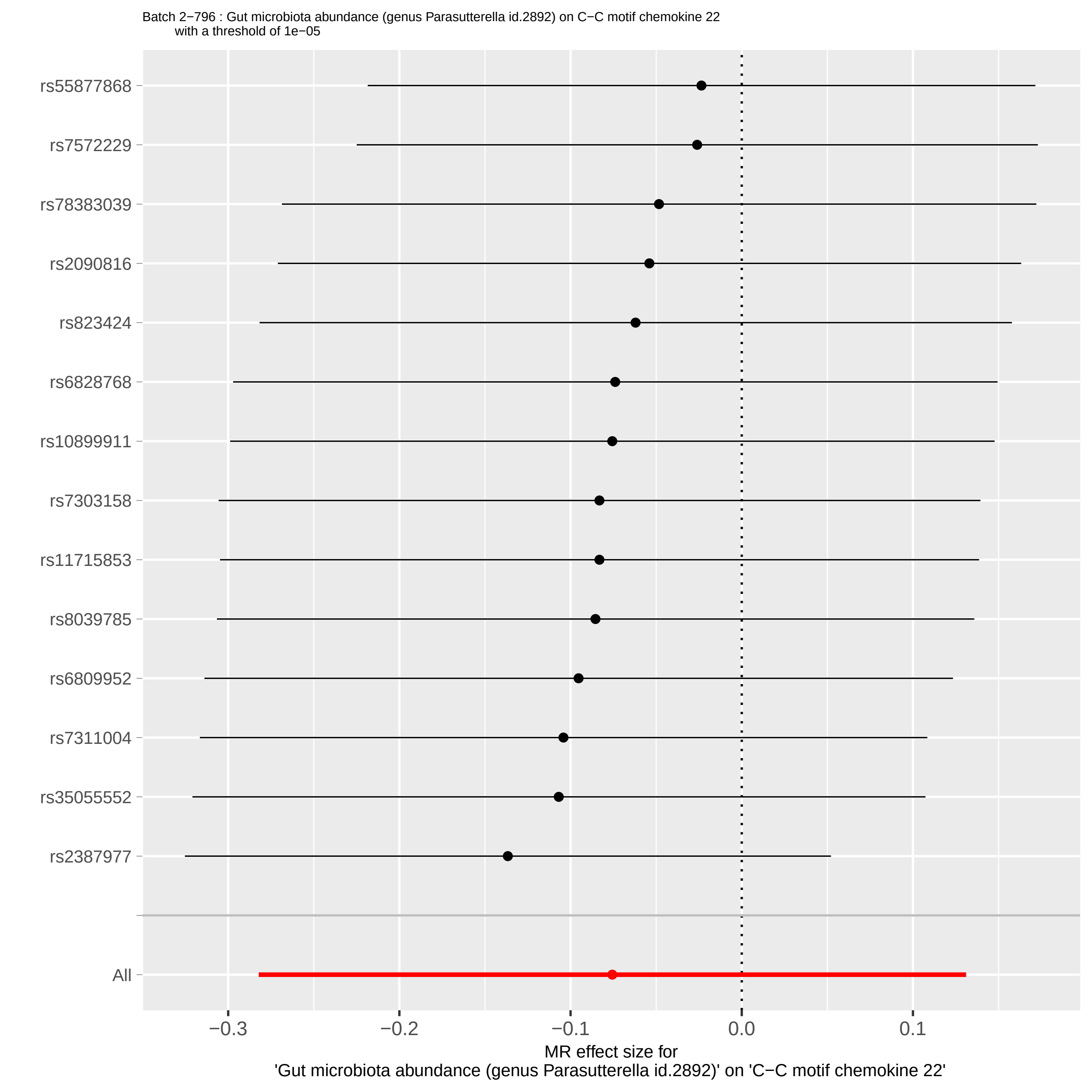
Batch 2−796 : Gut microbiota abundance (genus Parasutterella id.2892) on C−C motif chemokine 22
with a threshold of 1e−05
rs55877868
rs7572229
rs78383039
rs2090816
rs823424
rs6828768
rs10899911
rs7303158
rs11715853
rs8039785
rs6809952
rs7311004
rs35055552
rs2387977
All
−0.3
−0.2
−0.1
0.0
0.1
MR effect size for
'Gut microbiota abundance (genus Parasutterella id.2892)' on 'C−C motif chemokine 22'

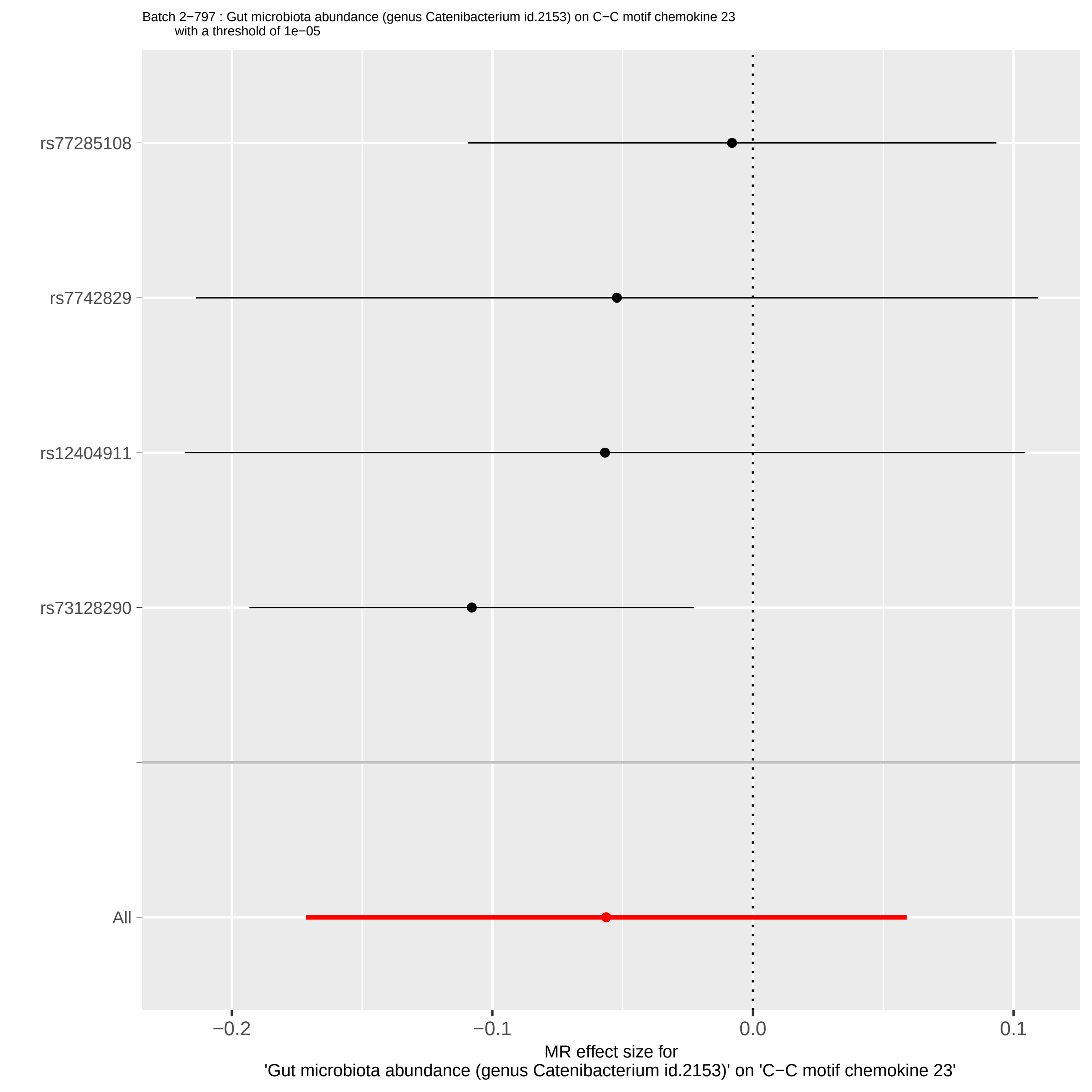
Batch 2−797 : Gut microbiota abundance (genus Catenibacterium id.2153) on C−C motif chemokine 23
with a threshold of 1e−05
rs77285108
rs7742829
rs12404911
rs73128290
All
−0.2
−0.1
0.0
0.1
MR effect size for
'Gut microbiota abundance (genus Catenibacterium id.2153)' on 'C−C motif chemokine 23'

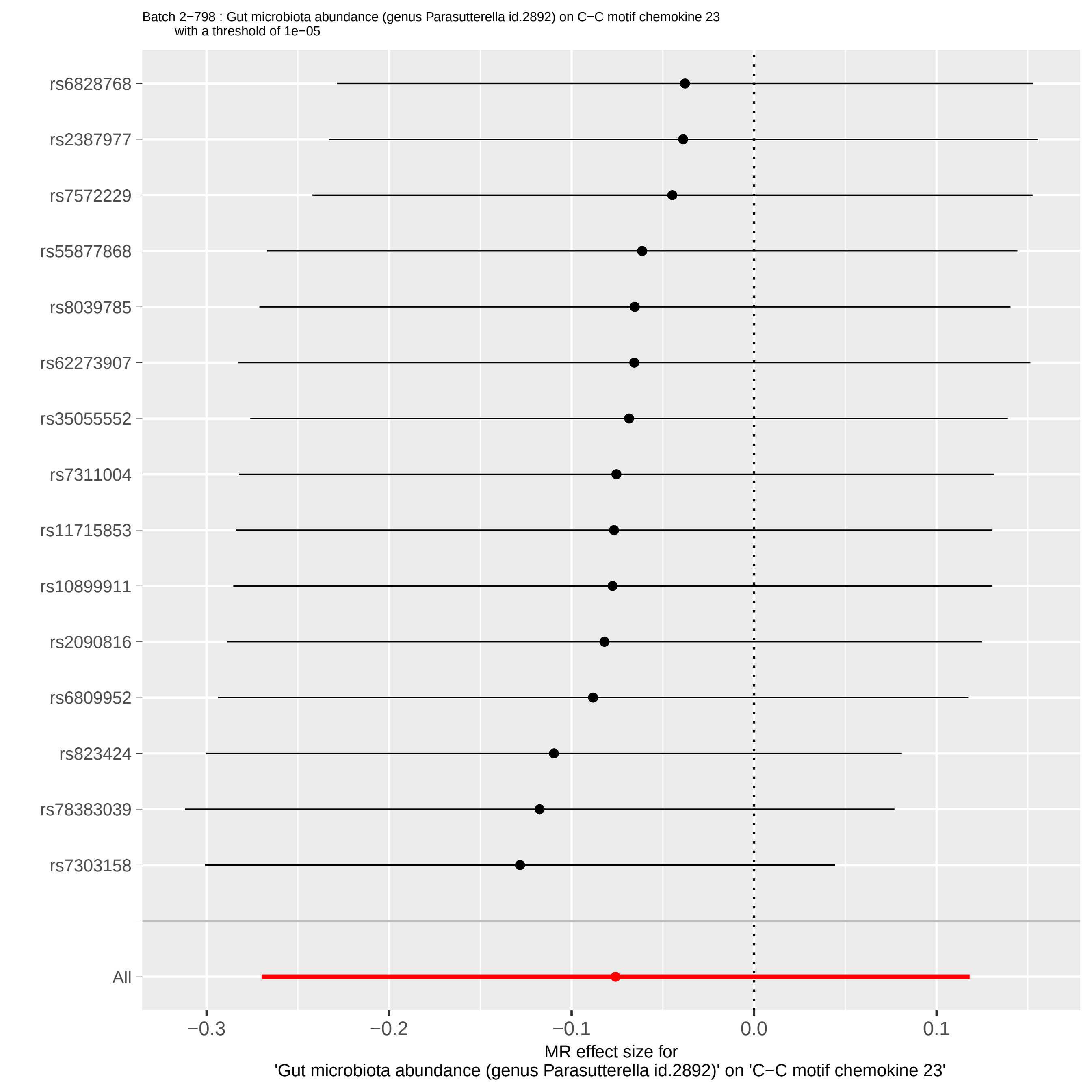

Batch 2−798 : Gut microbiota abundance (genus Parasutterella id.2892) on C−C motif chemokine 23
with a threshold of 1e−05
rs6828768
rs2387977
rs7572229
rs55877868
rs8039785
rs62273907
rs35055552
rs7311004
rs11715853
rs10899911
rs2090816
rs6809952
rs823424
rs78383039
rs7303158
All
−0.3
−0.2
−0.1
0.0
0.1
MR effect size for
'Gut microbiota abundance (genus Parasutterella id.2892)' on 'C−C motif chemokine 23'

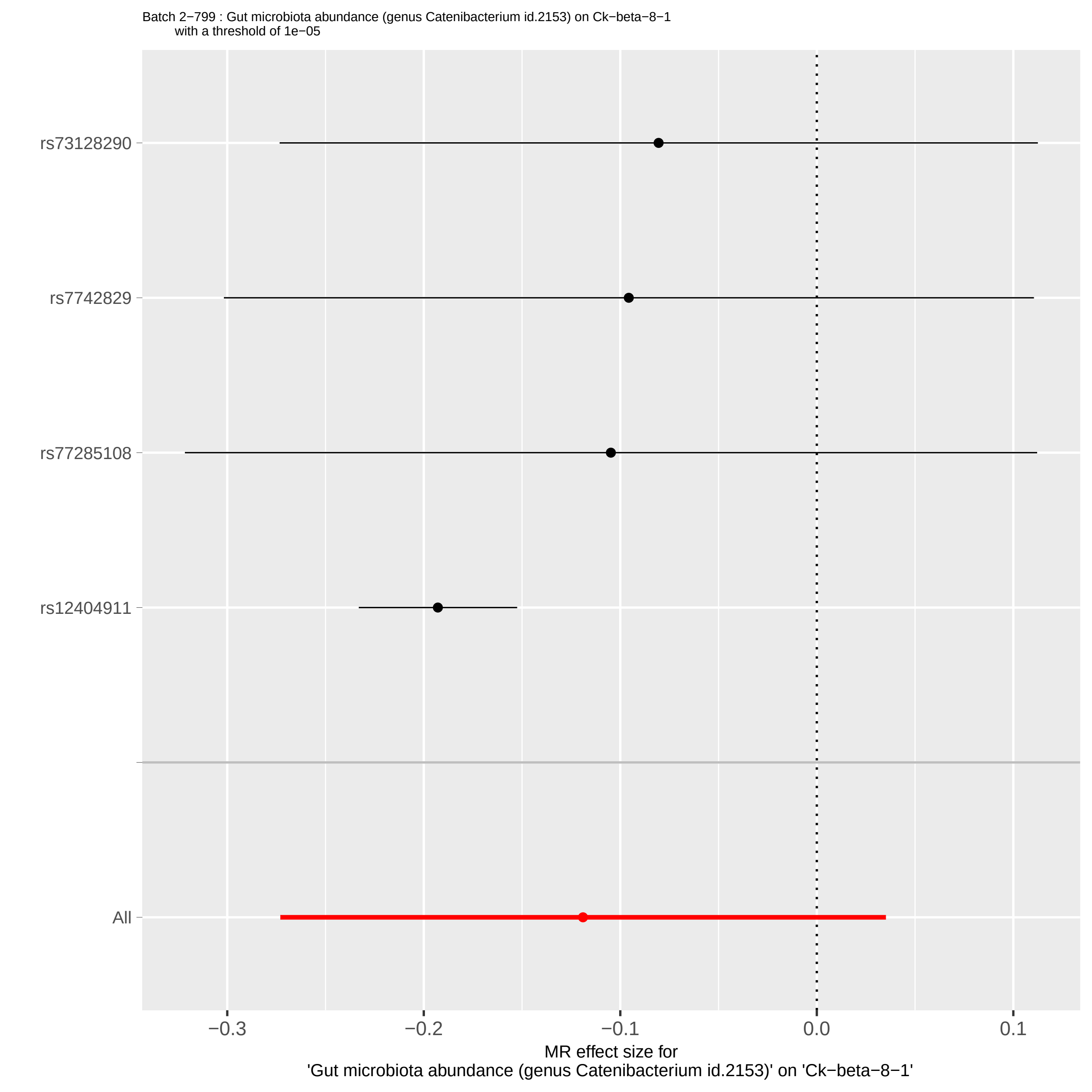

Batch 2−799 : Gut microbiota abundance (genus Catenibacterium id.2153) on Ck−beta−8−1
with a threshold of 1e−05
rs73128290
rs7742829
rs77285108
rs12404911
All
−0.3
−0.2
−0.1
0.0
0.1
MR effect size for
'Gut microbiota abundance (genus Catenibacterium id.2153)' on 'Ck−beta−8−1'

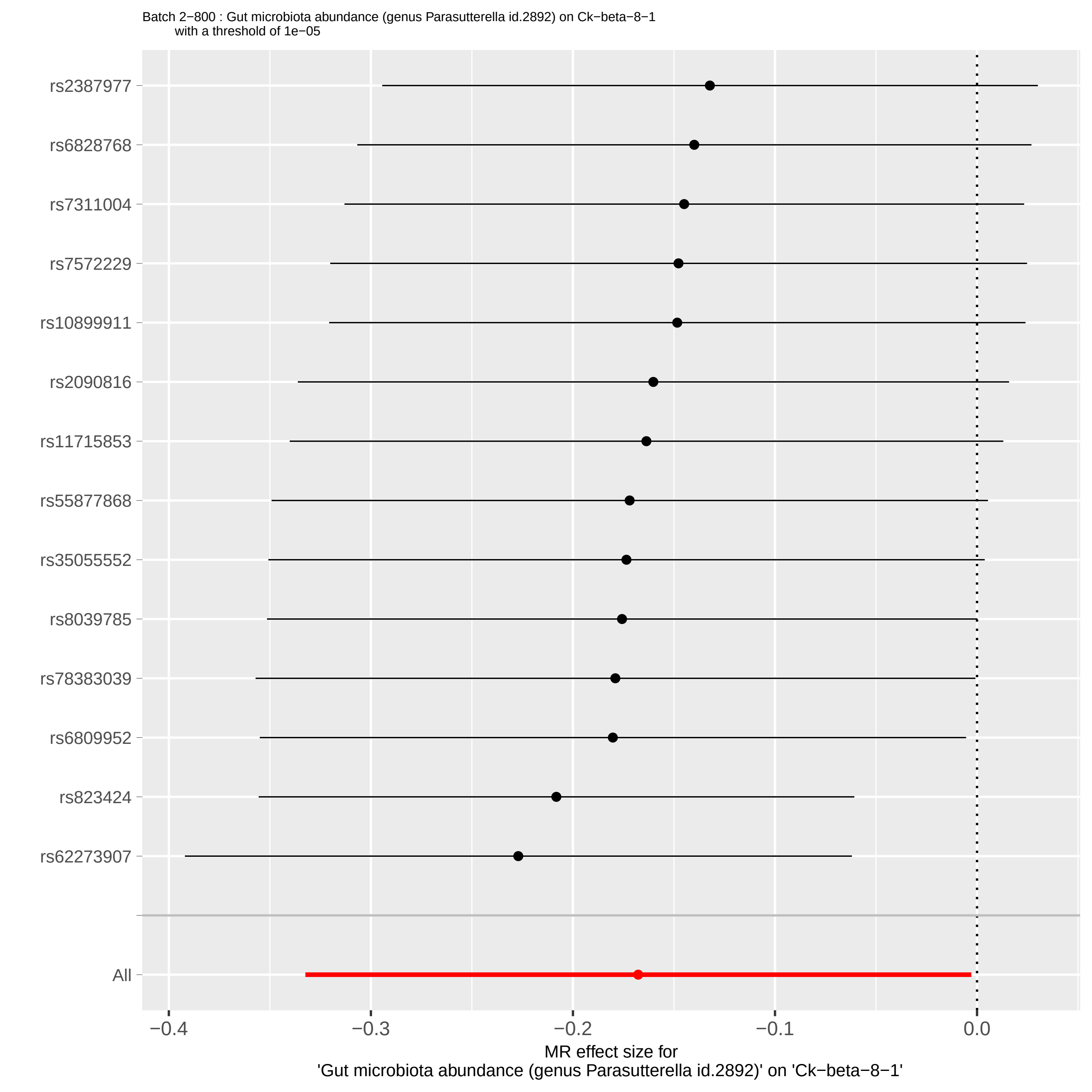

Batch 2−800 : Gut microbiota abundance (genus Parasutterella id.2892) on Ck−beta−8−1
with a threshold of 1e−05
rs2387977
rs6828768
rs7311004
rs7572229
rs10899911
rs2090816
rs11715853
rs55877868
rs35055552
rs8039785
rs78383039
rs6809952
rs823424
rs62273907
All
−0.4
−0.3
−0.2
−0.1
0.0
MR effect size for
'Gut microbiota abundance (genus Parasutterella id.2892)' on 'Ck−beta−8−1'

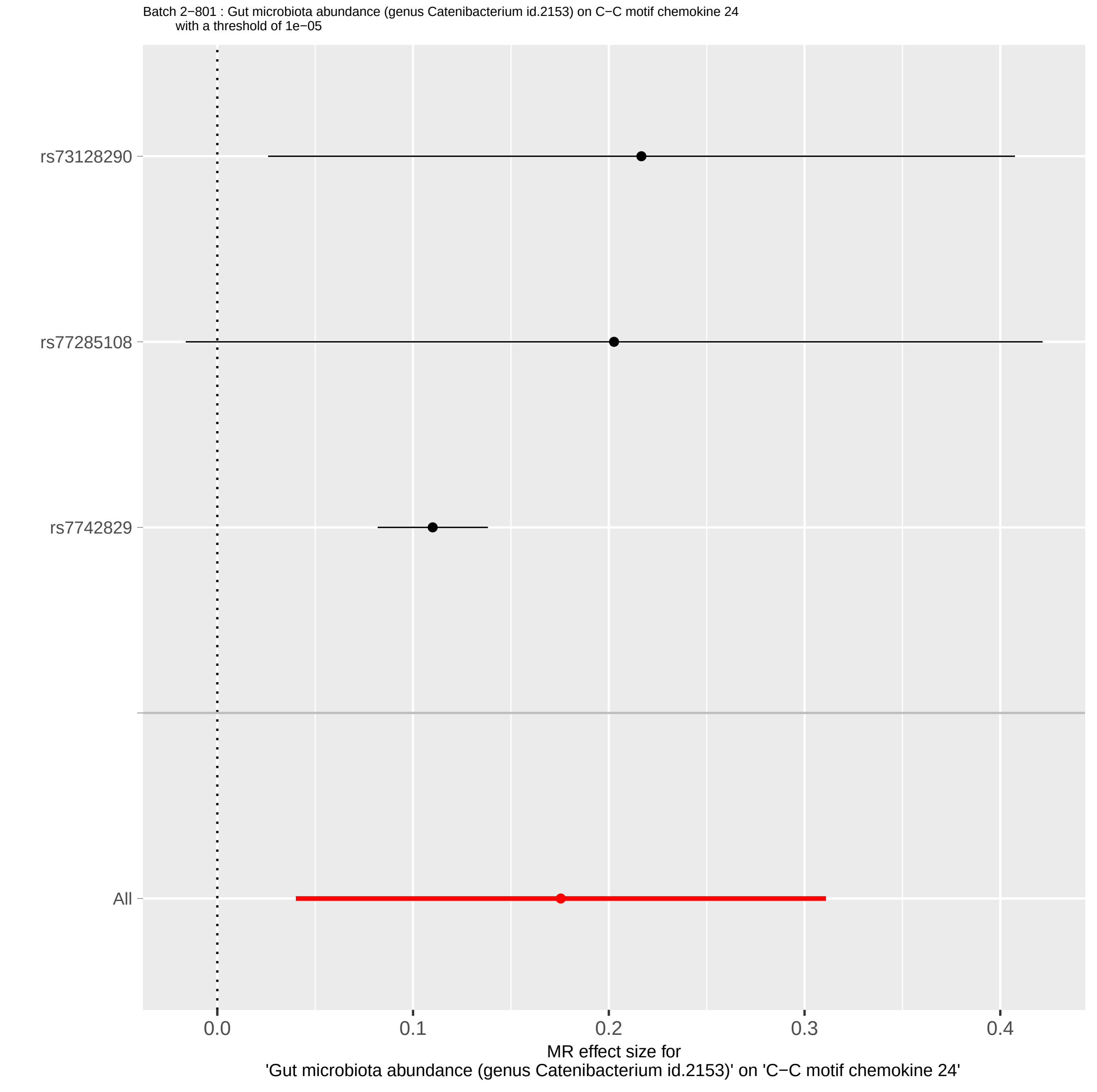
Batch 2−801 : Gut microbiota abundance (genus Catenibacterium id.2153) on C−C motif chemokine 24
with a threshold of 1e−05
rs73128290
rs77285108
rs7742829
All
0.0
0.1
0.2
0.3
0.4
MR effect size for
'Gut microbiota abundance (genus Catenibacterium id.2153)' on 'C−C motif chemokine 24'

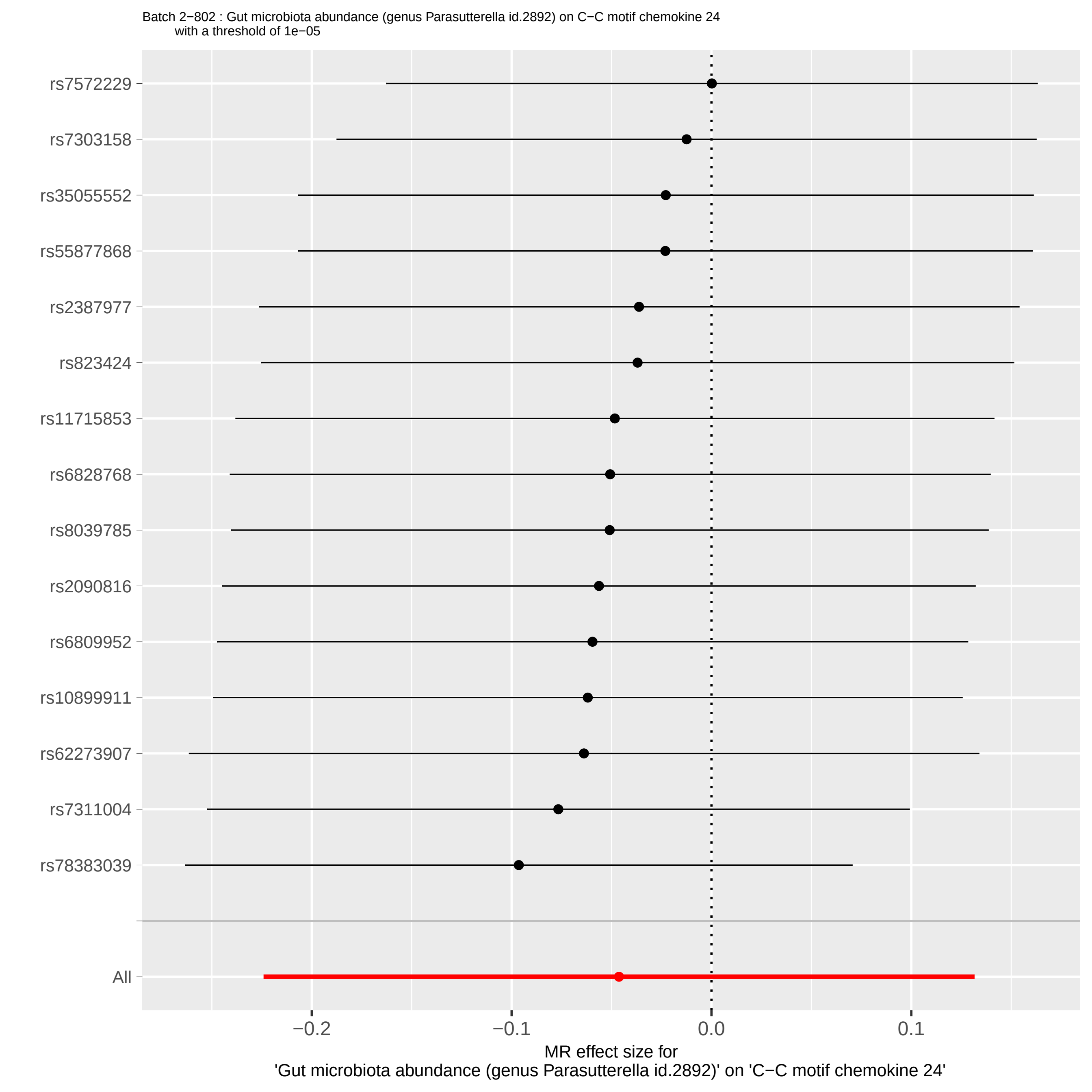
Batch 2−802 : Gut microbiota abundance (genus Parasutterella id.2892) on C−C motif chemokine 24
with a threshold of 1e−05
rs7572229
rs7303158
rs35055552
rs55877868
rs2387977
rs823424
rs11715853
rs6828768
rs8039785
rs2090816
rs6809952
rs10899911
rs62273907
rs7311004
rs78383039
All
−0.2
−0.1
0.0
0.1
MR effect size for
'Gut microbiota abundance (genus Parasutterella id.2892)' on 'C−C motif chemokine 24'

Batch 2−803 : Gut microbiota abundance (genus Catenibacterium id.2153) on C−C motif chemokine 25
with a threshold of 1e−05

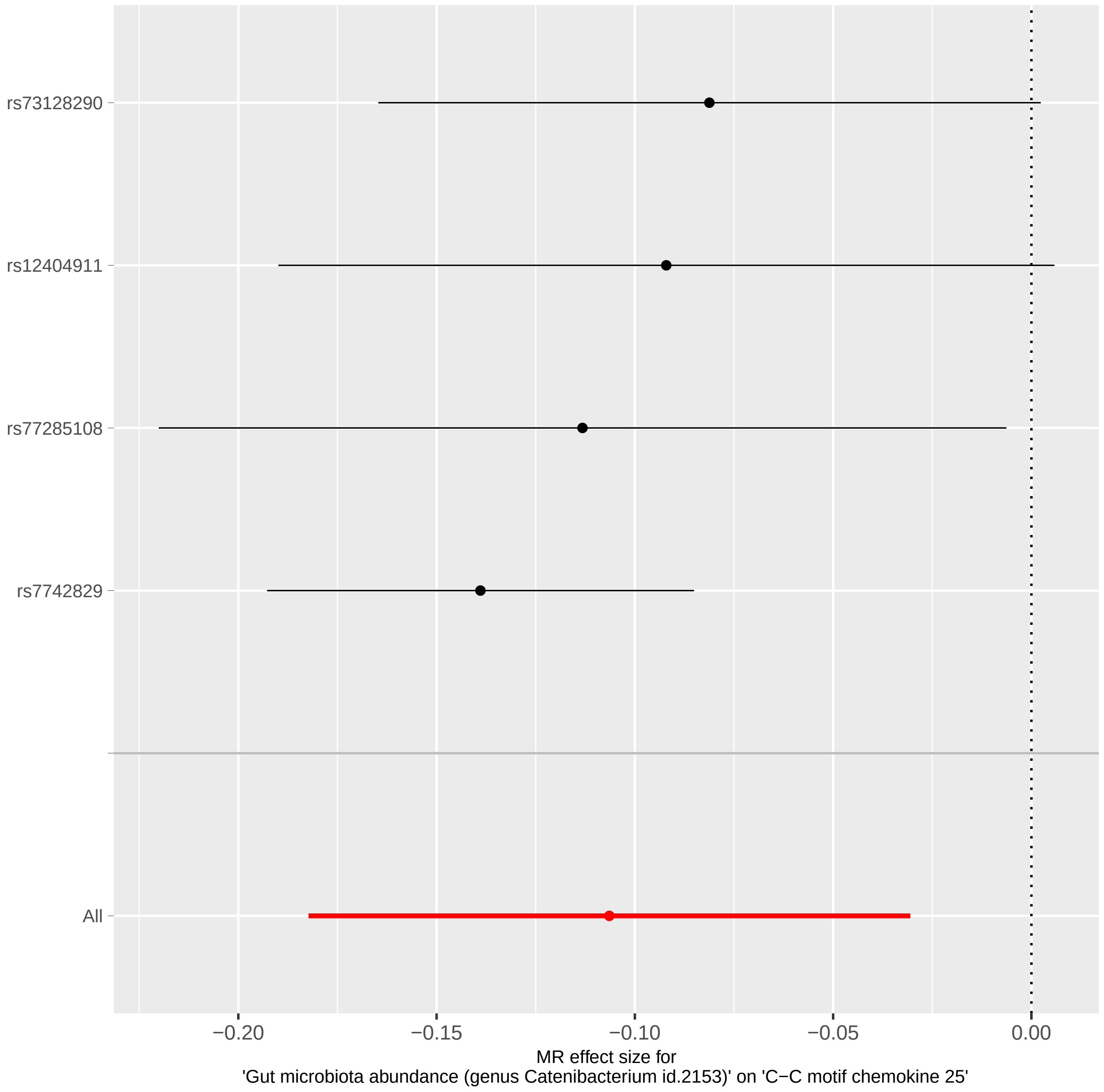

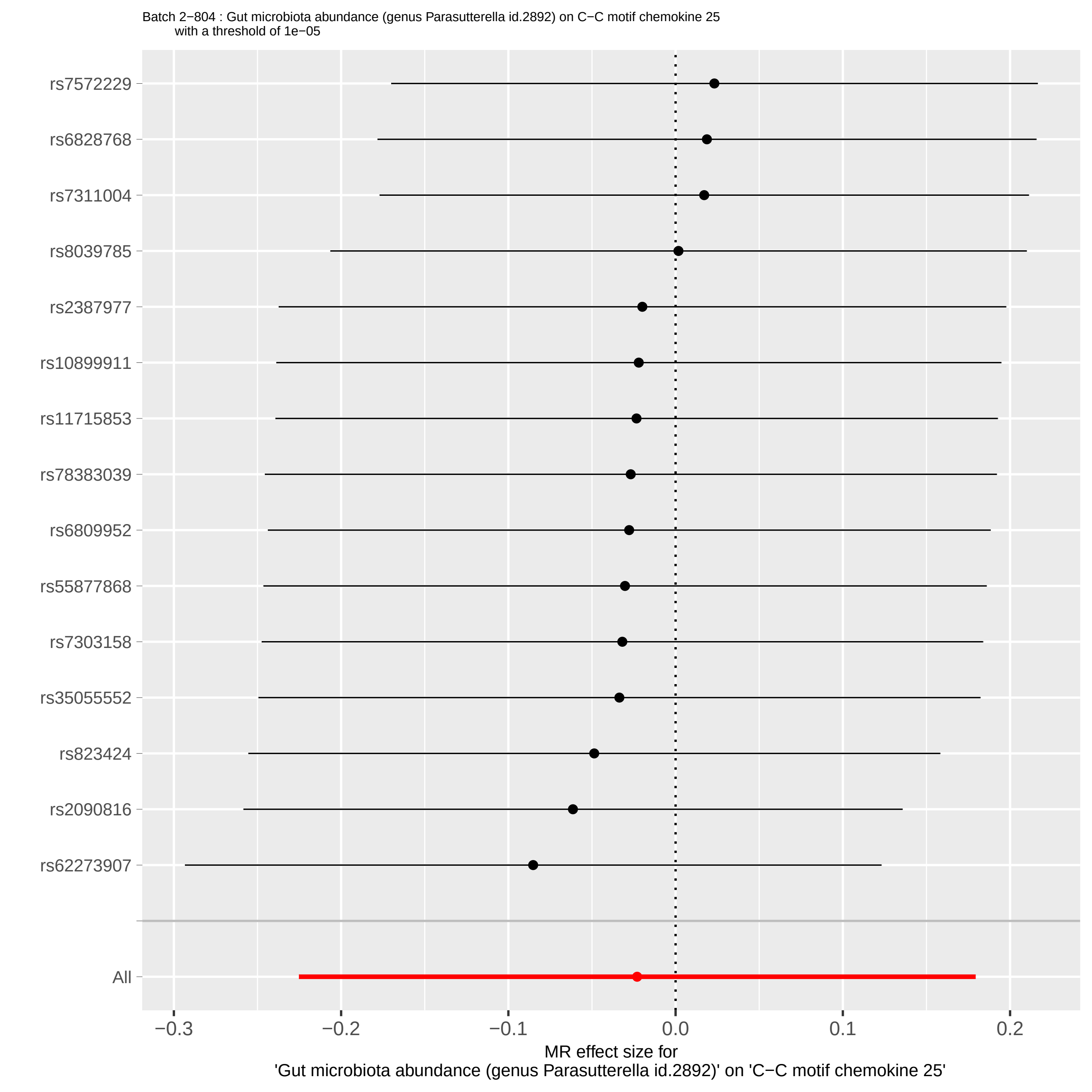

Batch 2−804 : Gut microbiota abundance (genus Parasutterella id.2892) on C−C motif chemokine 25
with a threshold of 1e−05
rs7572229
rs6828768
rs7311004
rs8039785
rs2387977
rs10899911
rs11715853
rs78383039
rs6809952
rs55877868
rs7303158
rs35055552
rs823424
rs2090816
rs62273907
All
−0.3
−0.2
−0.1
0.0
0.1
0.2
MR effect size for
'Gut microbiota abundance (genus Parasutterella id.2892)' on 'C−C motif chemokine 25'

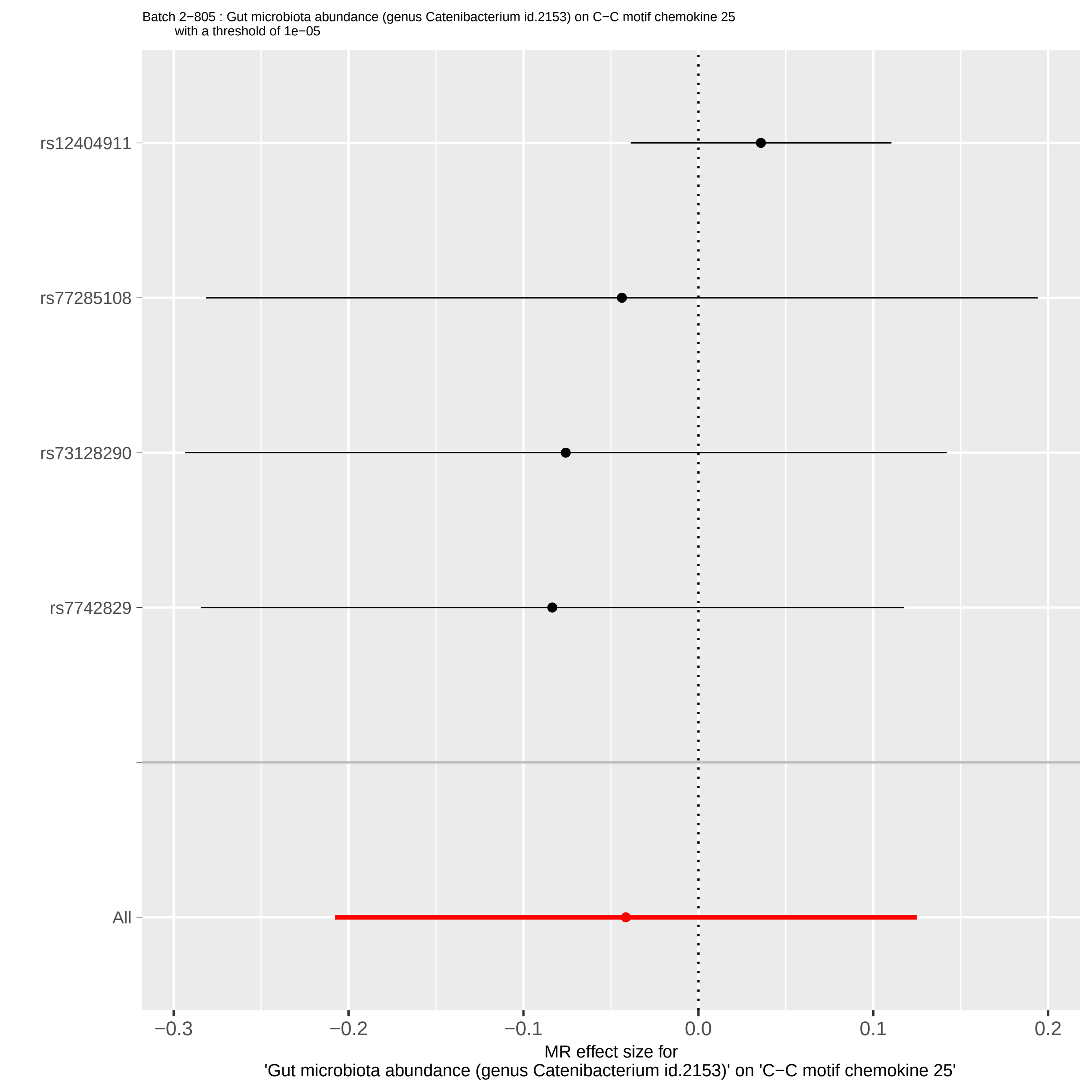

Batch 2−805 : Gut microbiota abundance (genus Catenibacterium id.2153) on C−C motif chemokine 25
with a threshold of 1e−05
rs12404911
rs77285108
rs73128290
rs7742829
All
−0.3
−0.2
−0.1
0.0
0.1
0.2
MR effect size for
'Gut microbiota abundance (genus Catenibacterium id.2153)' on 'C−C motif chemokine 25'

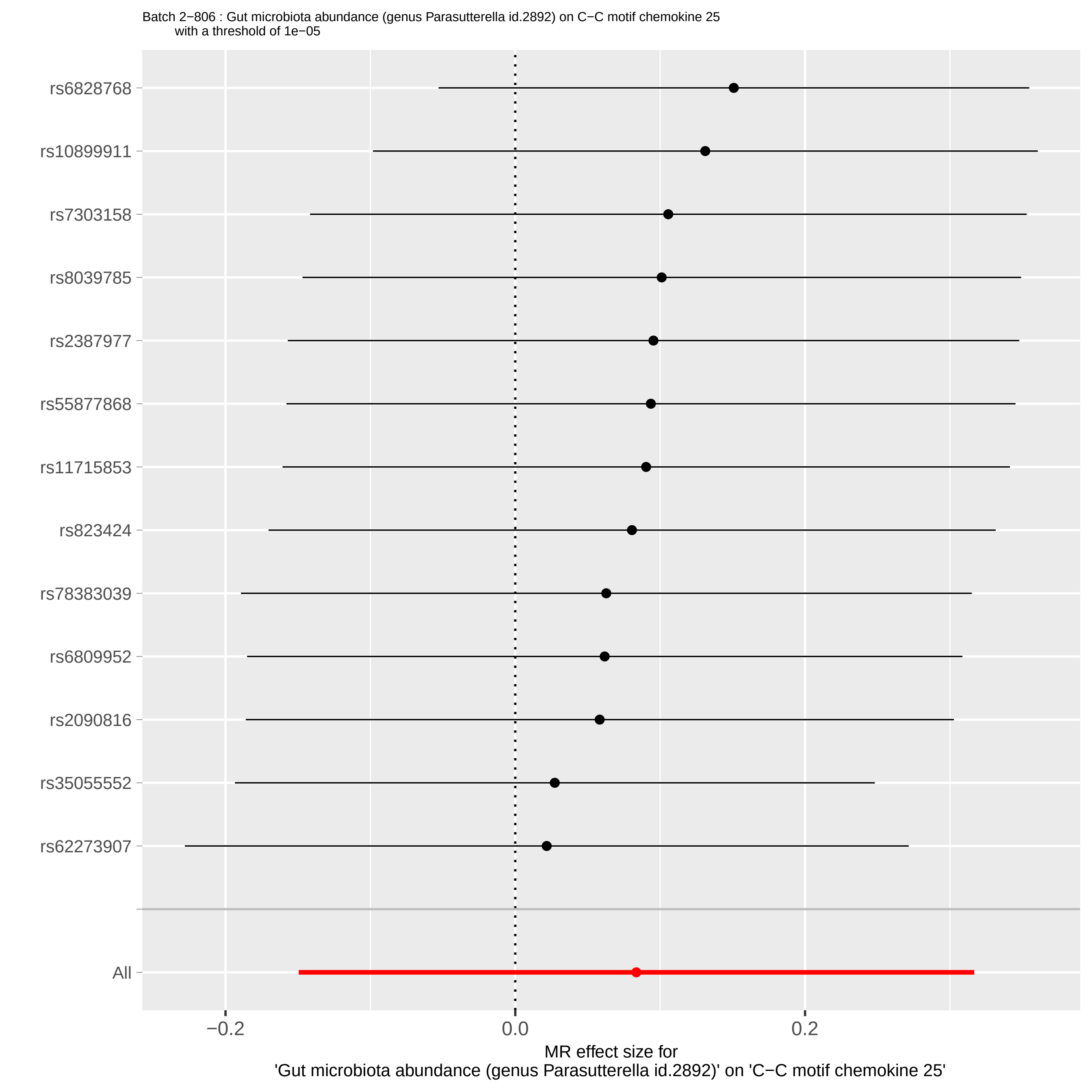
Batch 2−806 : Gut microbiota abundance (genus Parasutterella id.2892) on C−C motif chemokine 25
with a threshold of 1e−05
rs6828768
rs10899911
rs7303158
rs8039785
rs2387977
rs55877868
rs11715853
rs823424
rs78383039
rs6809952
rs2090816
rs35055552
rs62273907
All
−0.2
0.0
0.2
MR effect size for
'Gut microbiota abundance (genus Parasutterella id.2892)' on 'C−C motif chemokine 25'

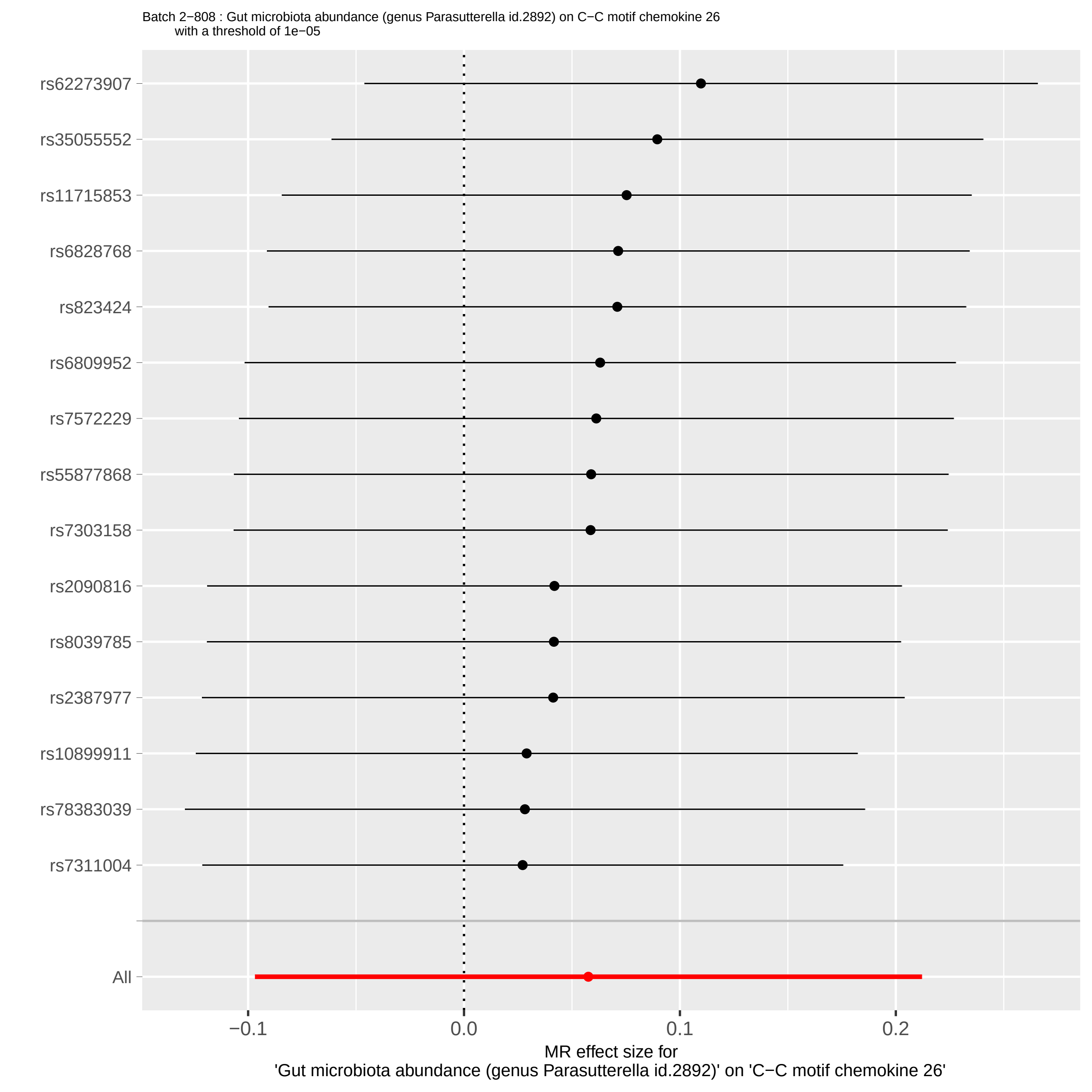

Batch 2−808 : Gut microbiota abundance (genus Parasutterella id.2892) on C−C motif chemokine 26
with a threshold of 1e−05
rs62273907
rs35055552
rs11715853
rs6828768
rs823424
rs6809952
rs7572229
rs55877868
rs7303158
rs2090816
rs8039785
rs2387977
rs10899911
rs78383039
rs7311004
All
−0.1
0.0
0.1
0.2
MR effect size for
'Gut microbiota abundance (genus Parasutterella id.2892)' on 'C−C motif chemokine 26'

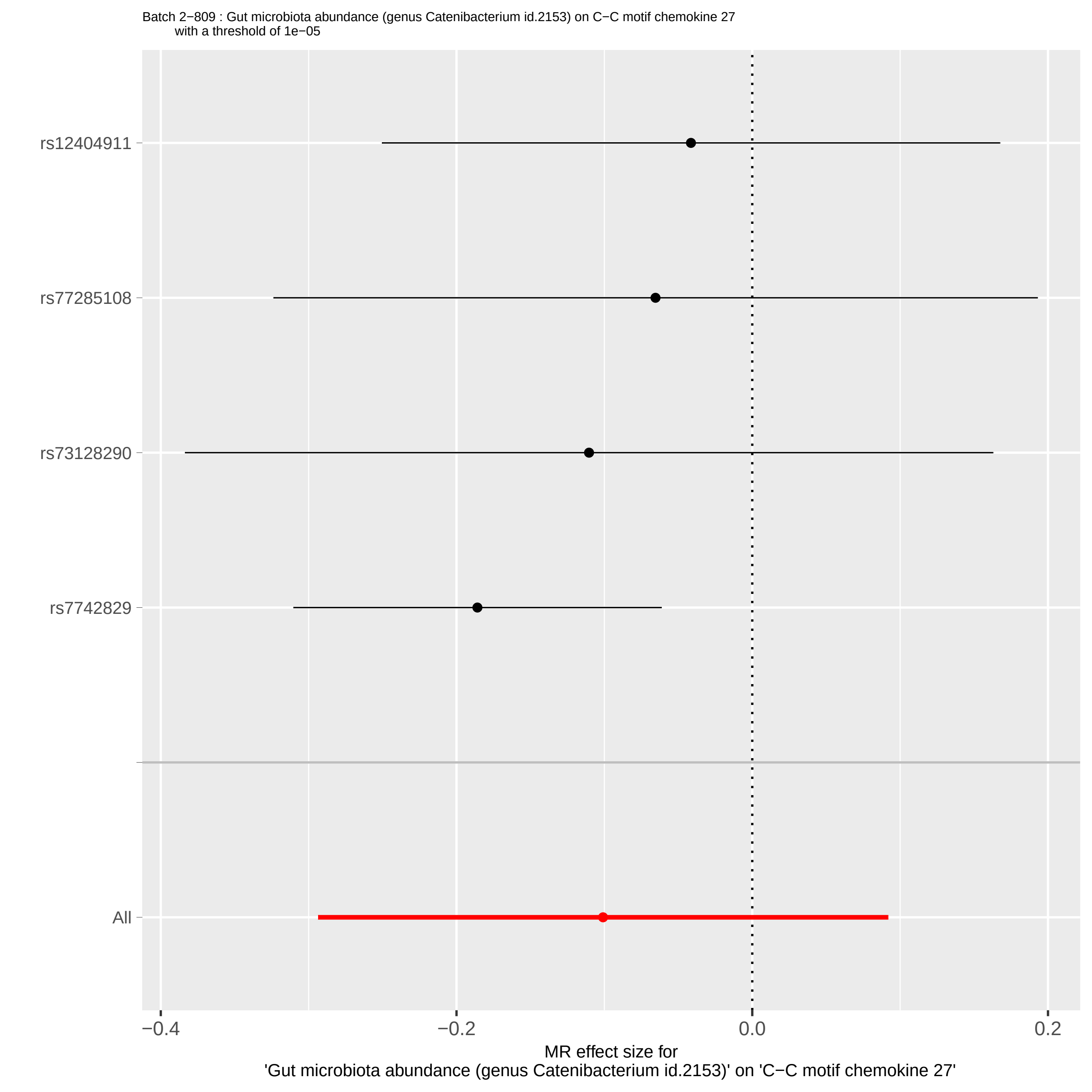
Batch 2−809 : Gut microbiota abundance (genus Catenibacterium id.2153) on C−C motif chemokine 27
with a threshold of 1e−05
rs12404911
rs77285108
rs73128290
rs7742829
All
−0.4
−0.2
0.0
0.2
MR effect size for
'Gut microbiota abundance (genus Catenibacterium id.2153)' on 'C−C motif chemokine 27'

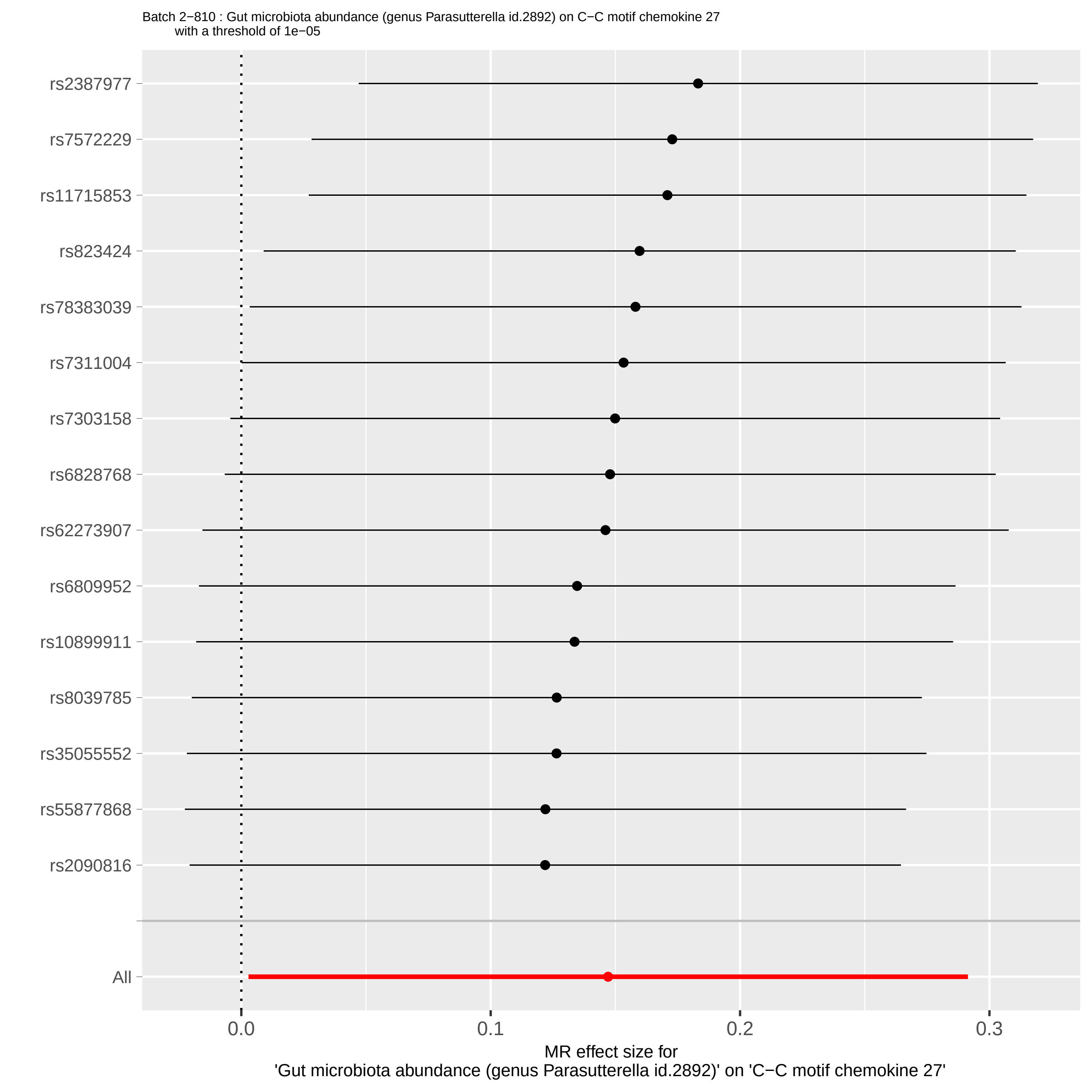
Batch 2−810 : Gut microbiota abundance (genus Parasutterella id.2892) on C−C motif chemokine 27
with a threshold of 1e−05
rs2387977
rs7572229
rs11715853
rs823424
rs78383039
rs7311004
rs7303158
rs6828768
rs62273907
rs6809952
rs10899911
rs8039785
rs35055552
rs55877868
rs2090816
All
0.0
0.1
0.2
0.3
MR effect size for
'Gut microbiota abundance (genus Parasutterella id.2892)' on 'C−C motif chemokine 27'

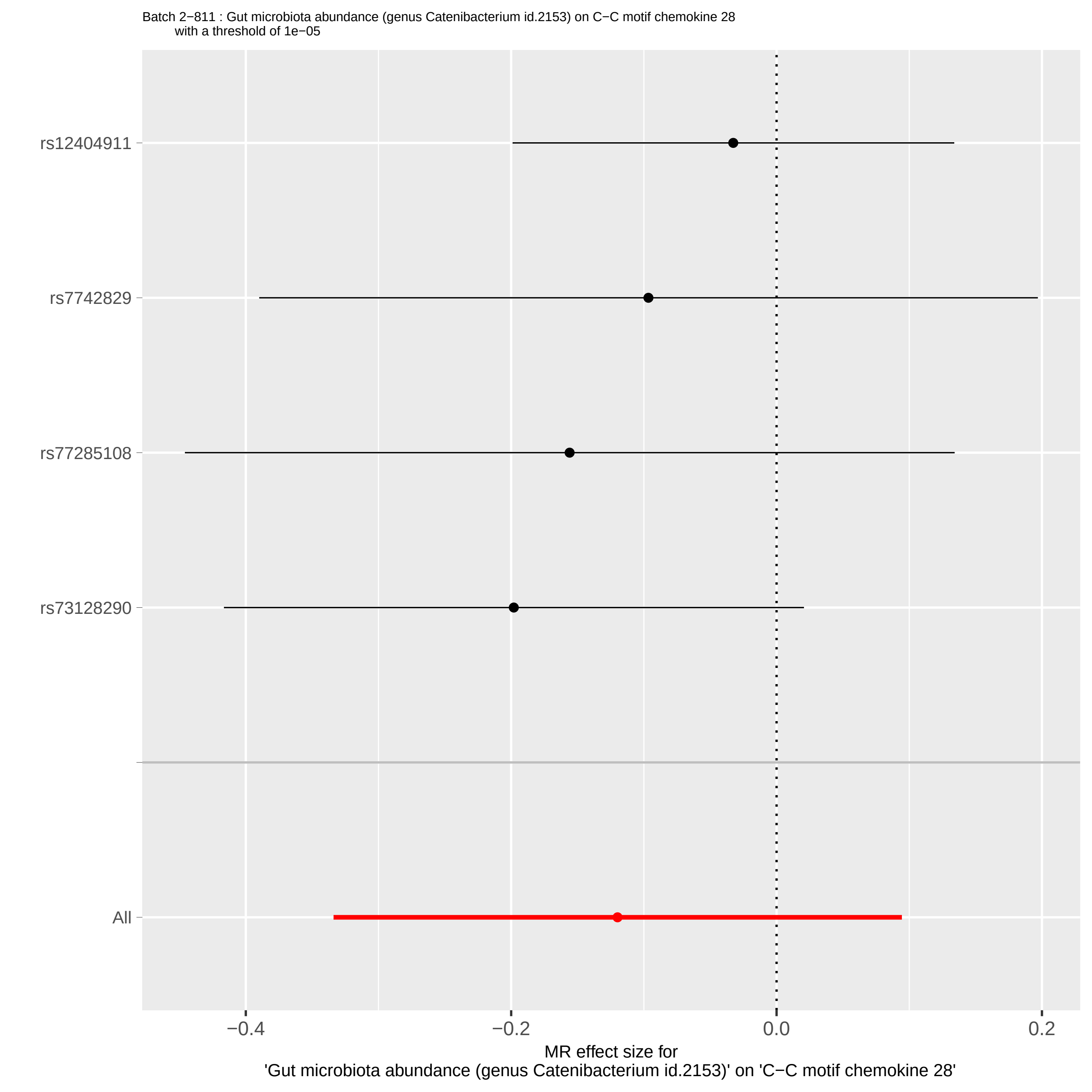

Batch 2−811 : Gut microbiota abundance (genus Catenibacterium id.2153) on C−C motif chemokine 28
with a threshold of 1e−05
rs12404911
rs7742829
rs77285108
rs73128290
All
−0.4
−0.2
0.0
0.2
MR effect size for
'Gut microbiota abundance (genus Catenibacterium id.2153)' on 'C−C motif chemokine 28'

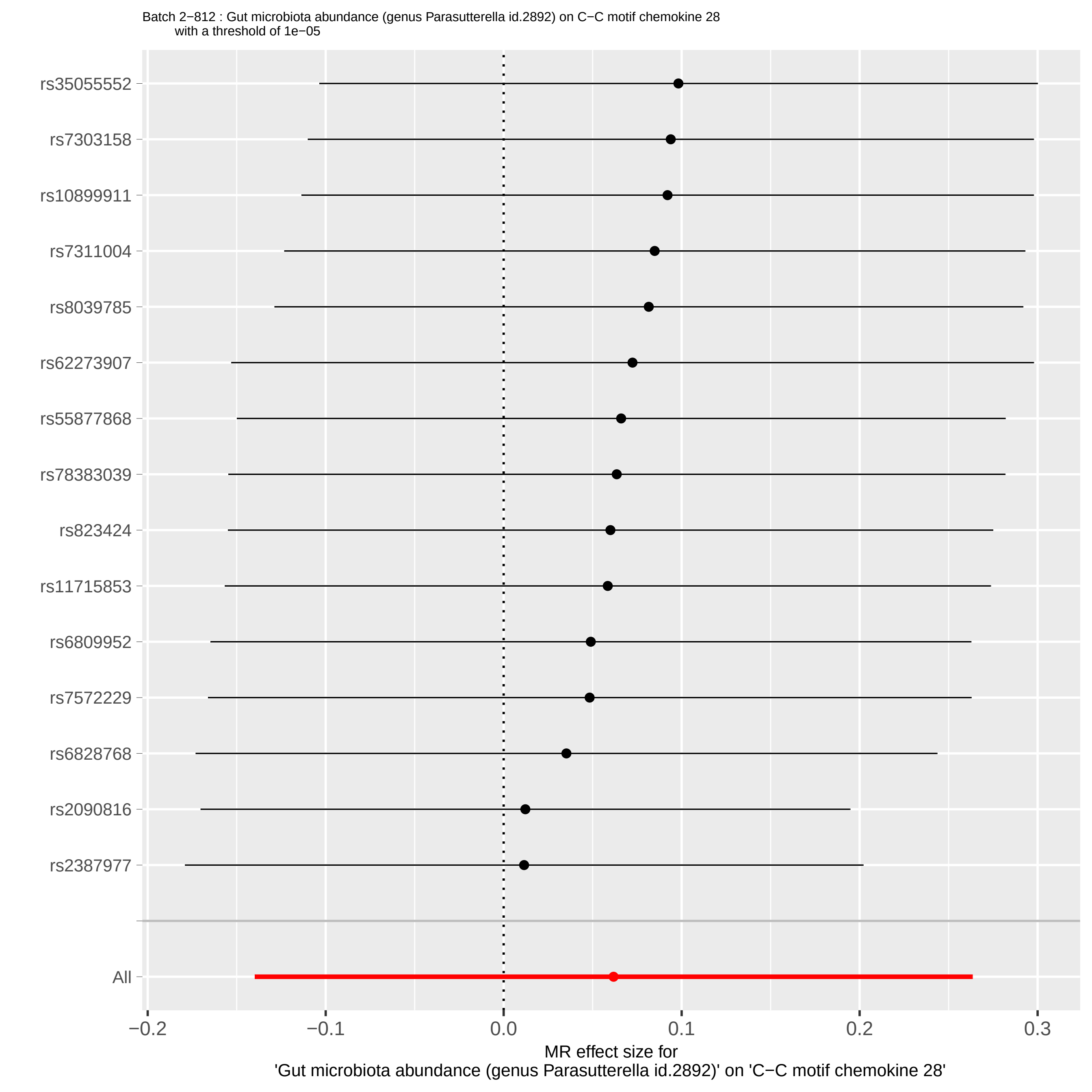
Batch 2−812 : Gut microbiota abundance (genus Parasutterella id.2892) on C−C motif chemokine 28
with a threshold of 1e−05
rs35055552
rs7303158
rs10899911
rs7311004
rs8039785
rs62273907
rs55877868
rs78383039
rs823424
rs11715853
rs6809952
rs7572229
rs6828768
rs2090816
rs2387977
All
−0.2
−0.1
0.0
0.1
0.2
0.3
MR effect size for
'Gut microbiota abundance (genus Parasutterella id.2892)' on 'C−C motif chemokine 28'

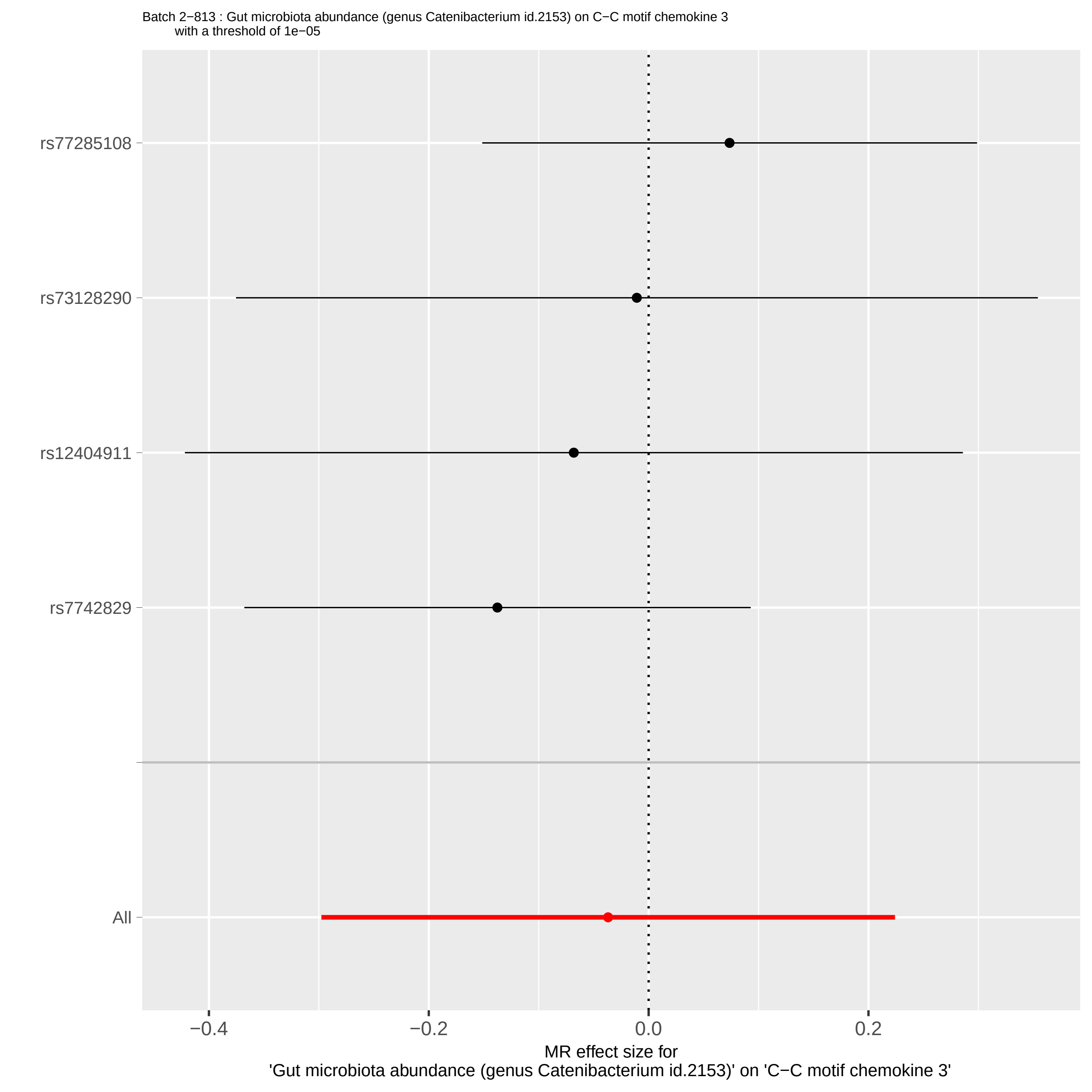
Batch 2−813 : Gut microbiota abundance (genus Catenibacterium id.2153) on C−C motif chemokine 3
with a threshold of 1e−05
rs77285108
rs73128290
rs12404911
rs7742829
All
−0.4
−0.2
0.0
0.2
MR effect size for
'Gut microbiota abundance (genus Catenibacterium id.2153)' on 'C−C motif chemokine 3'

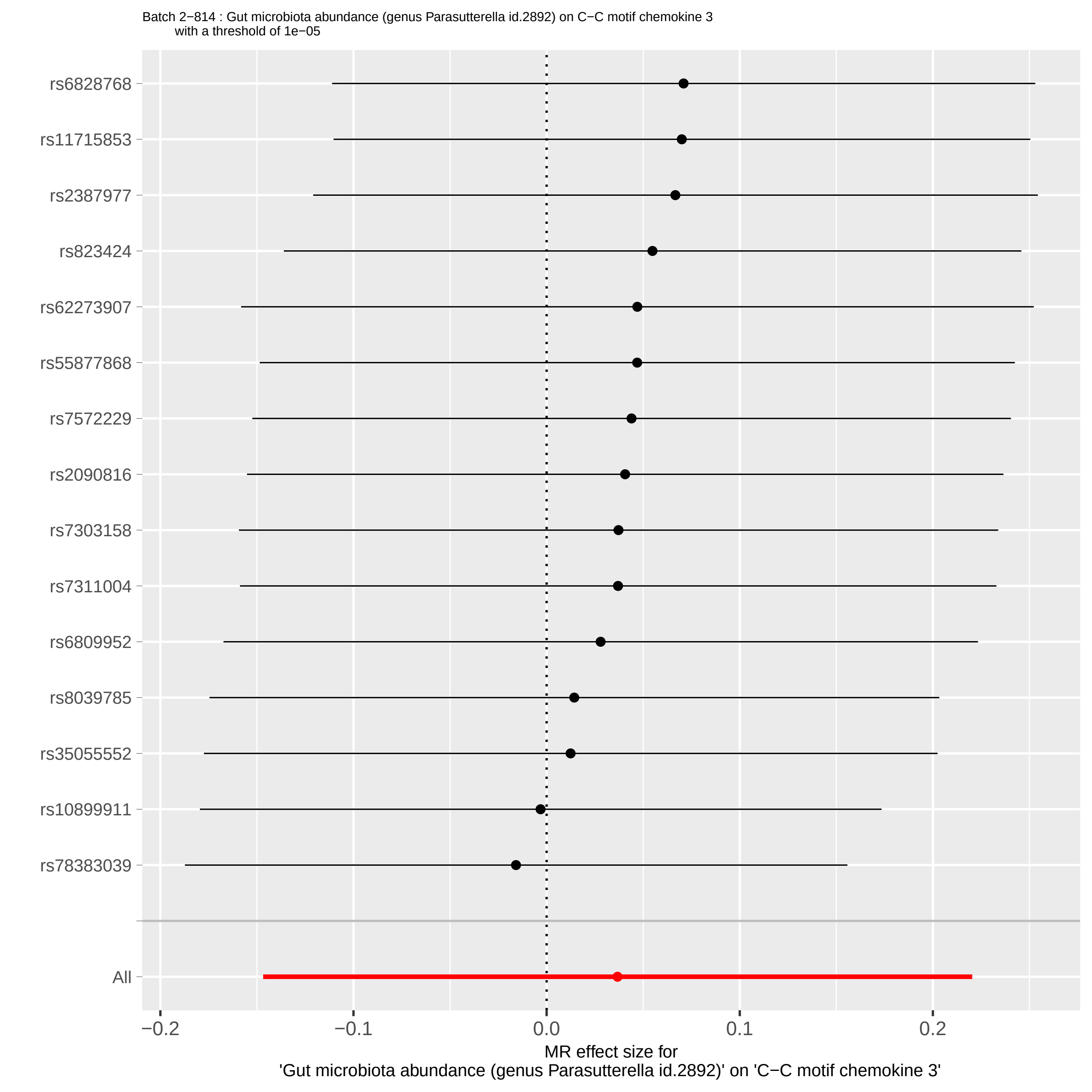
Batch 2−814 : Gut microbiota abundance (genus Parasutterella id.2892) on C−C motif chemokine 3
with a threshold of 1e−05
rs6828768
rs11715853
rs2387977
rs823424
rs62273907
rs55877868
rs7572229
rs2090816
rs7303158
rs7311004
rs6809952
rs8039785
rs35055552
rs10899911
rs78383039
All
−0.2
−0.1
0.0
0.1
0.2
MR effect size for
'Gut microbiota abundance (genus Parasutterella id.2892)' on 'C−C motif chemokine 3'

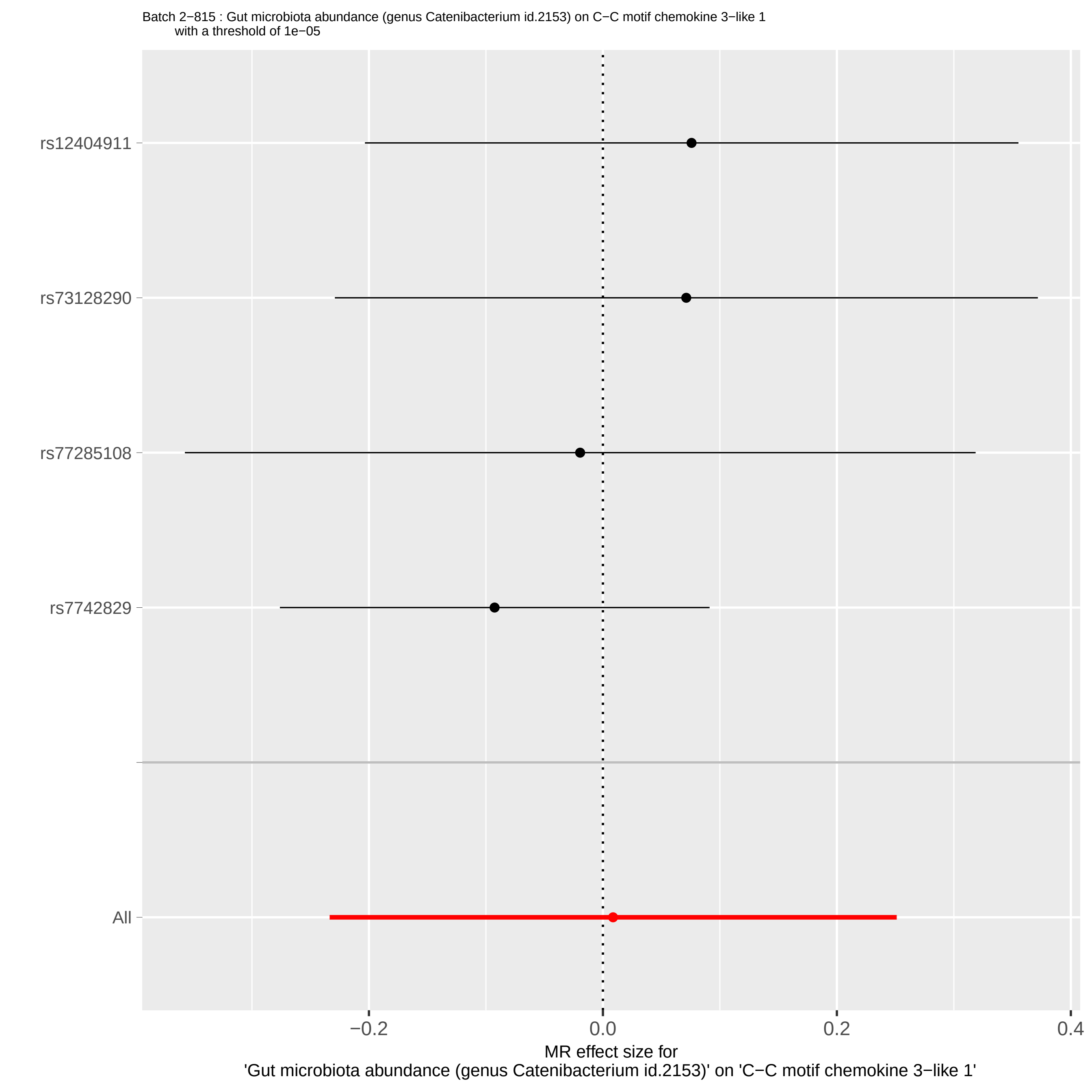
Batch 2−815 : Gut microbiota abundance (genus Catenibacterium id.2153) on C−C motif chemokine 3−like 1
with a threshold of 1e−05
rs12404911
rs73128290
rs77285108
rs7742829
All
−0.2
0.0
0.2
0.4
MR effect size for
'Gut microbiota abundance (genus Catenibacterium id.2153)' on 'C−C motif chemokine 3−like 1'

Batch 2−816 : Gut microbiota abundance (genus Parasutterella id.2892) on C−C motif chemokine 3−like 1
with a threshold of 1e−05

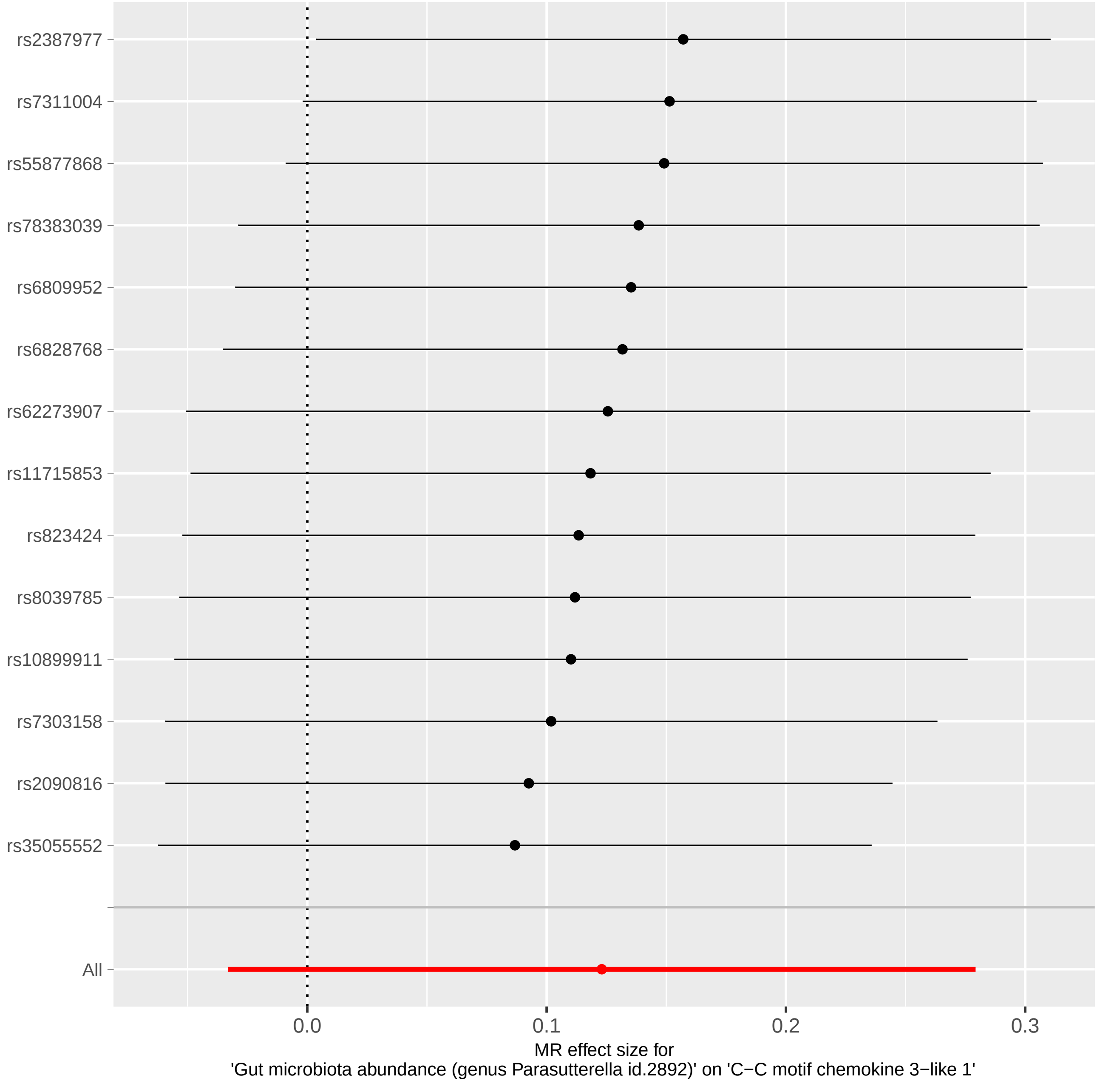

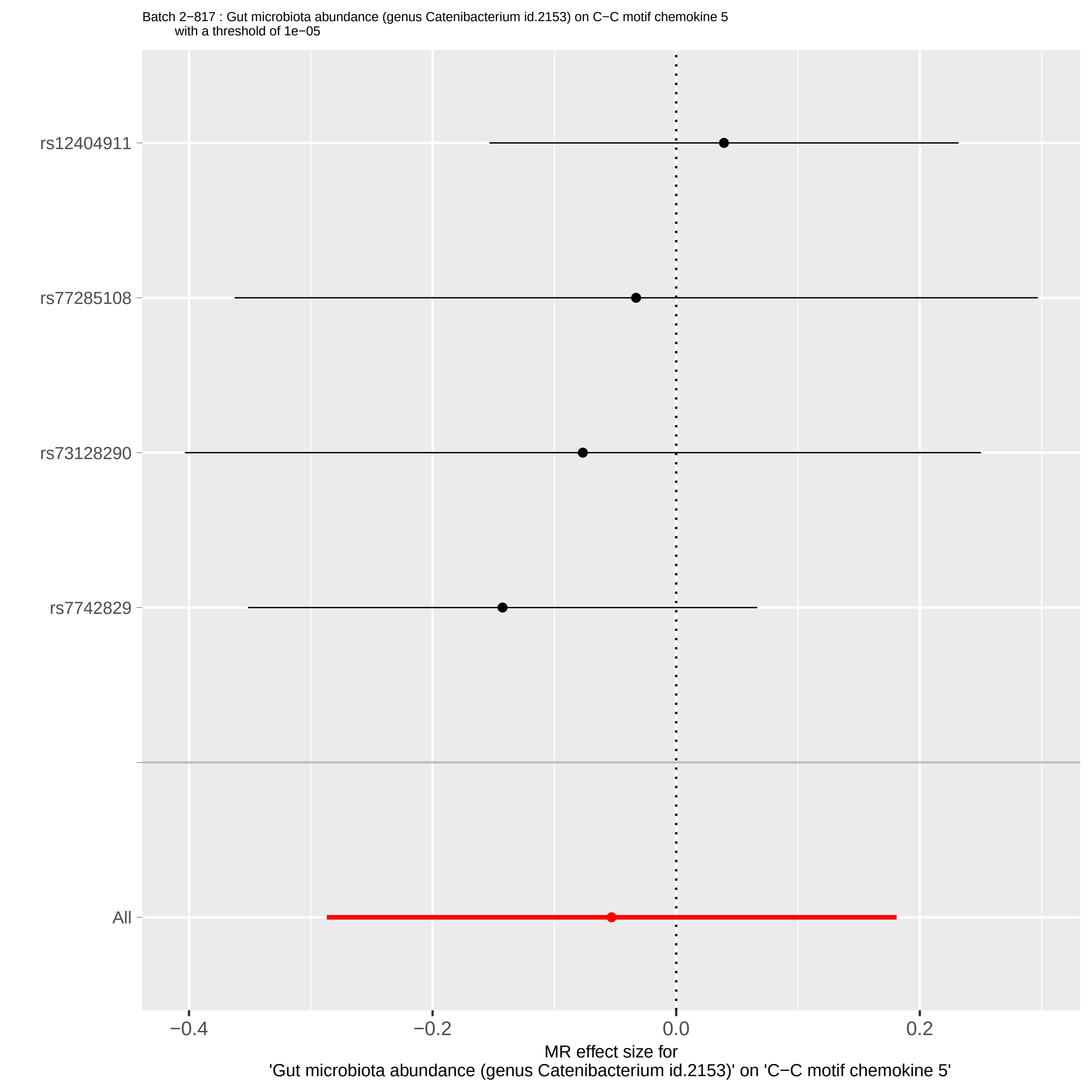

Batch 2−817 : Gut microbiota abundance (genus Catenibacterium id.2153) on C−C motif chemokine 5
with a threshold of 1e−05
rs12404911
rs77285108
rs73128290
rs7742829
All
−0.4
−0.2
0.0
0.2
MR effect size for
'Gut microbiota abundance (genus Catenibacterium id.2153)' on 'C−C motif chemokine 5'

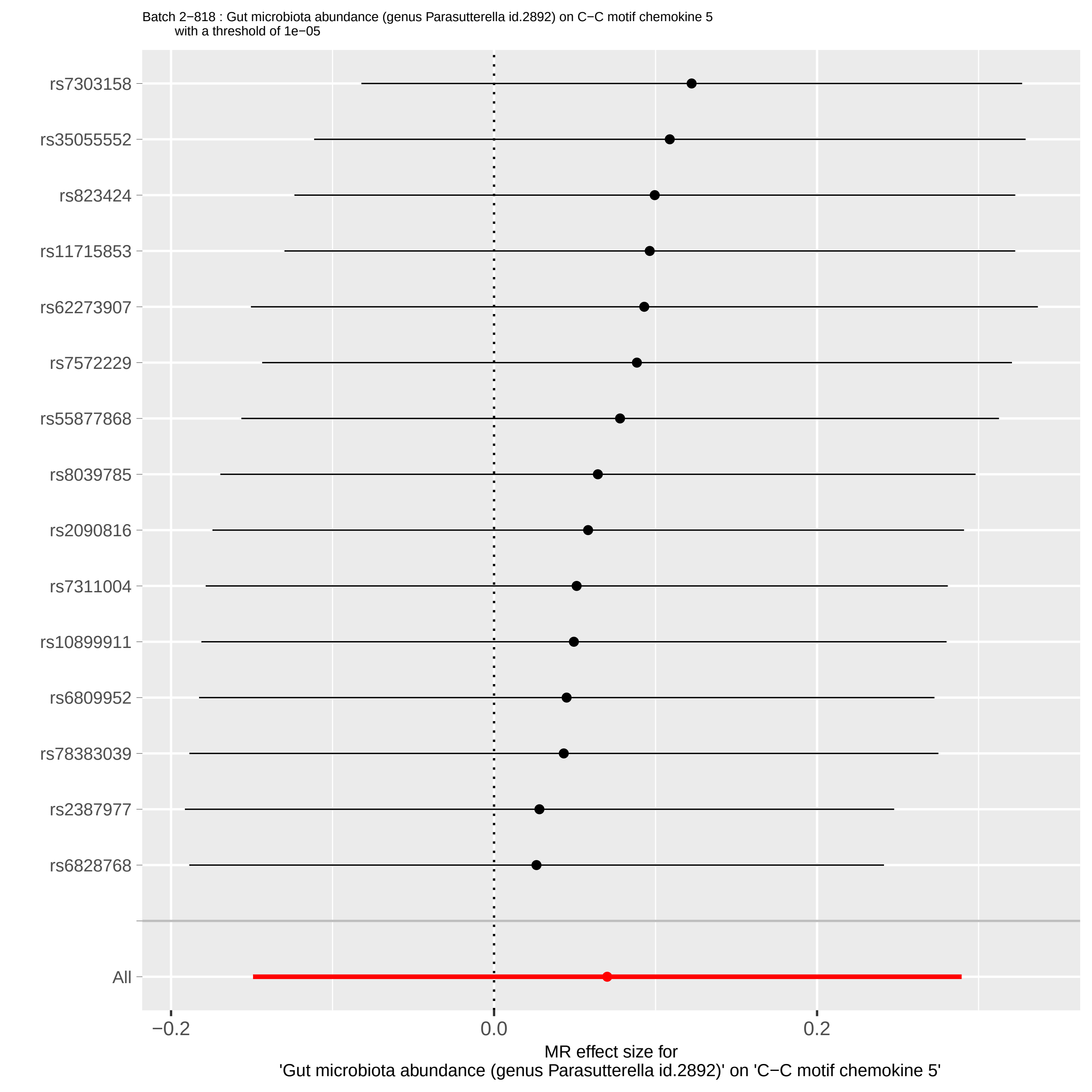

Batch 2−818 : Gut microbiota abundance (genus Parasutterella id.2892) on C−C motif chemokine 5
with a threshold of 1e−05
rs7303158
rs35055552
rs823424
rs11715853
rs62273907
rs7572229
rs55877868
rs8039785
rs2090816
rs7311004
rs10899911
rs6809952
rs78383039
rs2387977
rs6828768
All
−0.2
0.0
0.2
MR effect size for
'Gut microbiota abundance (genus Parasutterella id.2892)' on 'C−C motif chemokine 5'

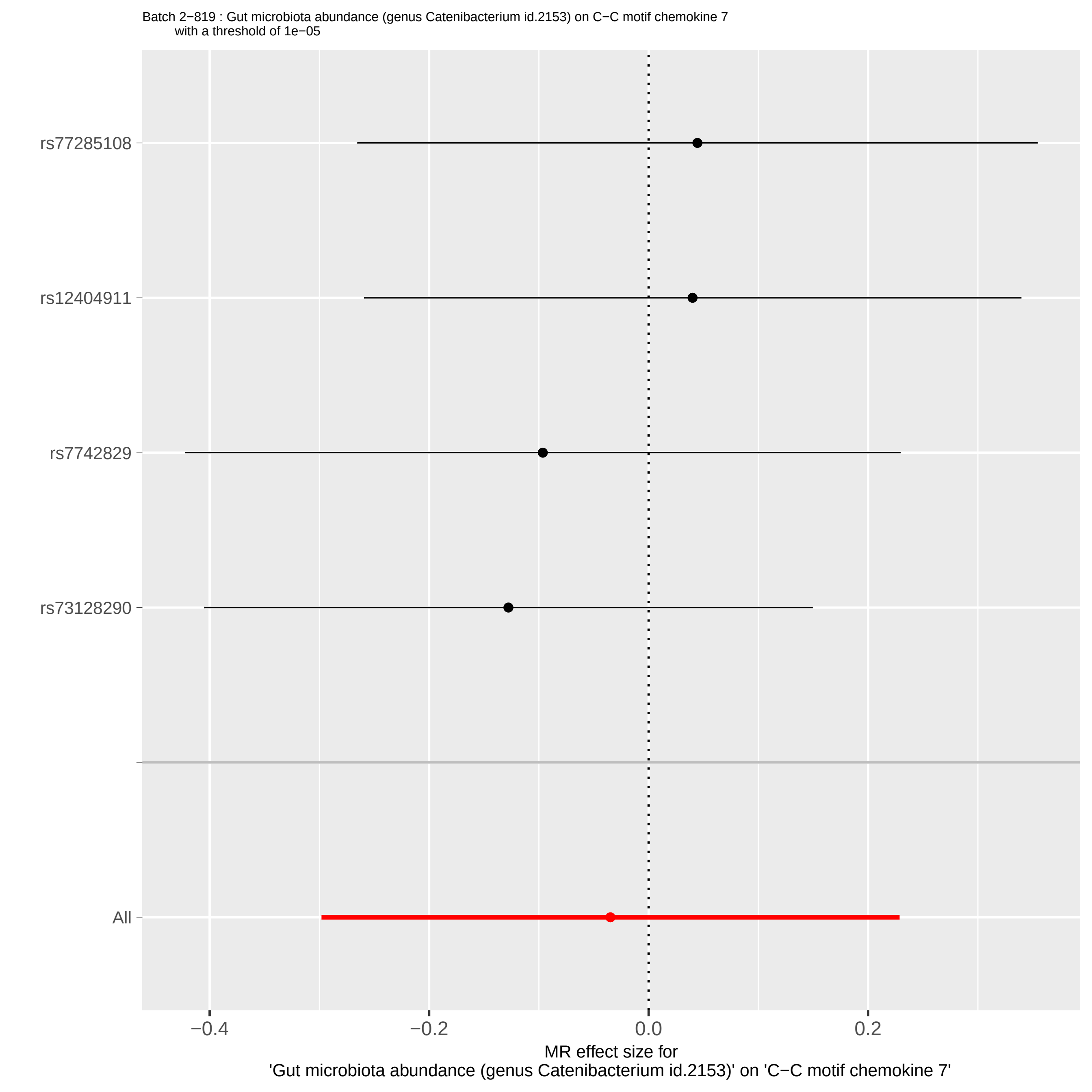

Batch 2−819 : Gut microbiota abundance (genus Catenibacterium id.2153) on C−C motif chemokine 7
with a threshold of 1e−05
rs77285108
rs12404911
rs7742829
rs73128290
All
−0.4
−0.2
0.0
0.2
MR effect size for
'Gut microbiota abundance (genus Catenibacterium id.2153)' on 'C−C motif chemokine 7'

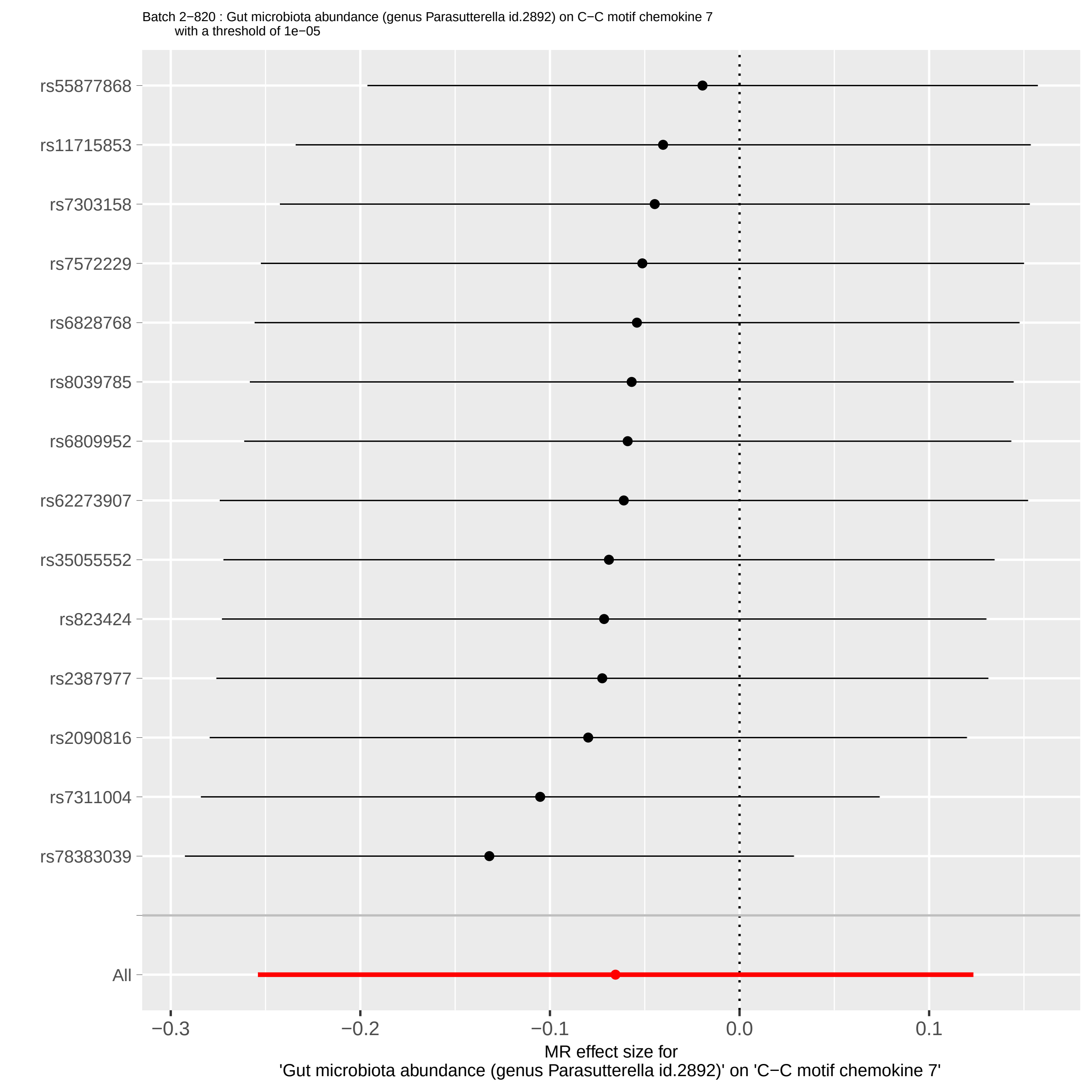
Batch 2−820 : Gut microbiota abundance (genus Parasutterella id.2892) on C−C motif chemokine 7
with a threshold of 1e−05
rs55877868
rs11715853
rs7303158
rs7572229
rs6828768
rs8039785
rs6809952
rs62273907
rs35055552
rs823424
rs2387977
rs2090816
rs7311004
rs78383039
All
−0.3
−0.2
−0.1
0.0
0.1
MR effect size for
'Gut microbiota abundance (genus Parasutterella id.2892)' on 'C−C motif chemokine 7'

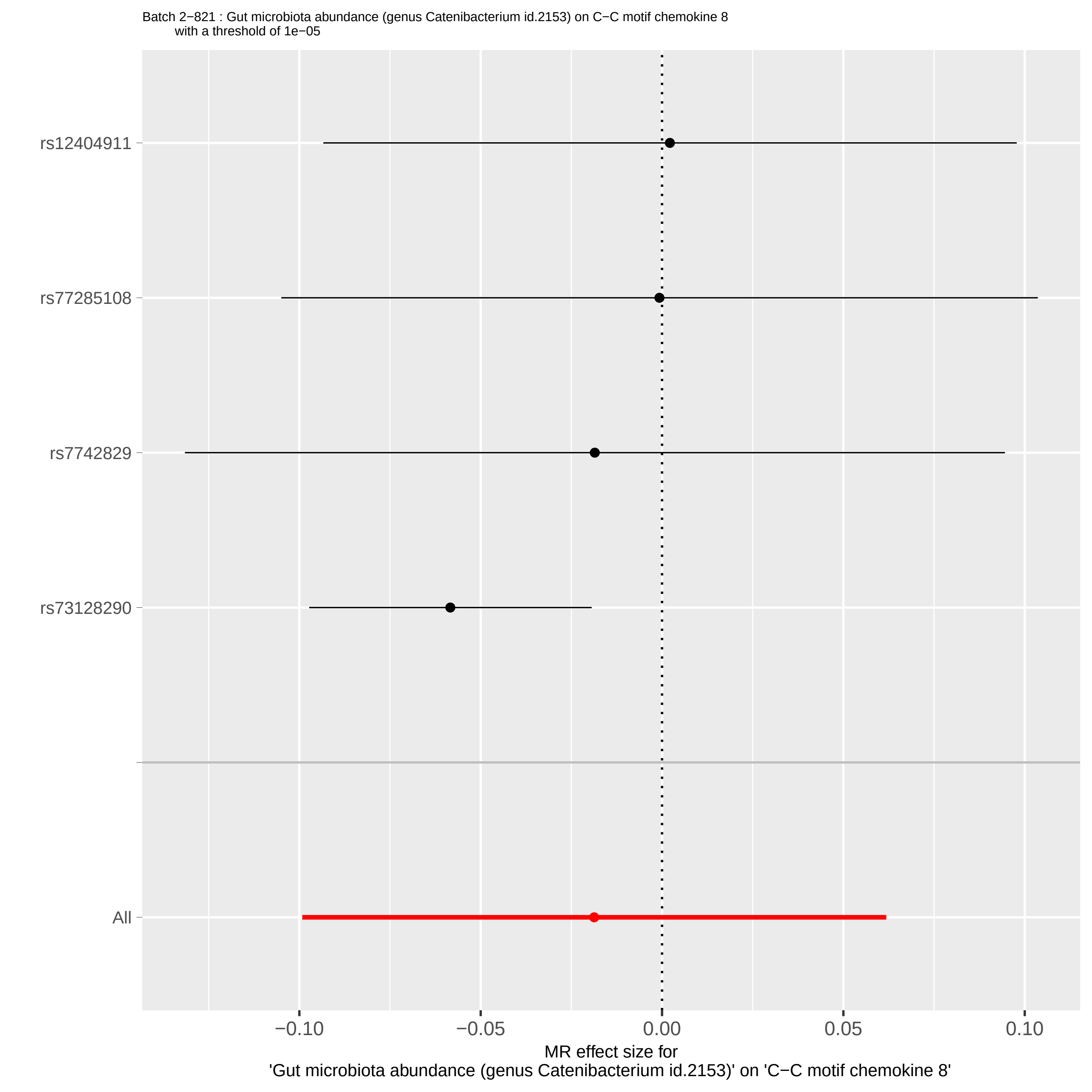

Batch 2−821 : Gut microbiota abundance (genus Catenibacterium id.2153) on C−C motif chemokine 8
with a threshold of 1e−05
rs12404911
rs77285108
rs7742829
rs73128290
All
−0.10
−0.05
0.00
0.05
0.10
MR effect size for
'Gut microbiota abundance (genus Catenibacterium id.2153)' on 'C−C motif chemokine 8'

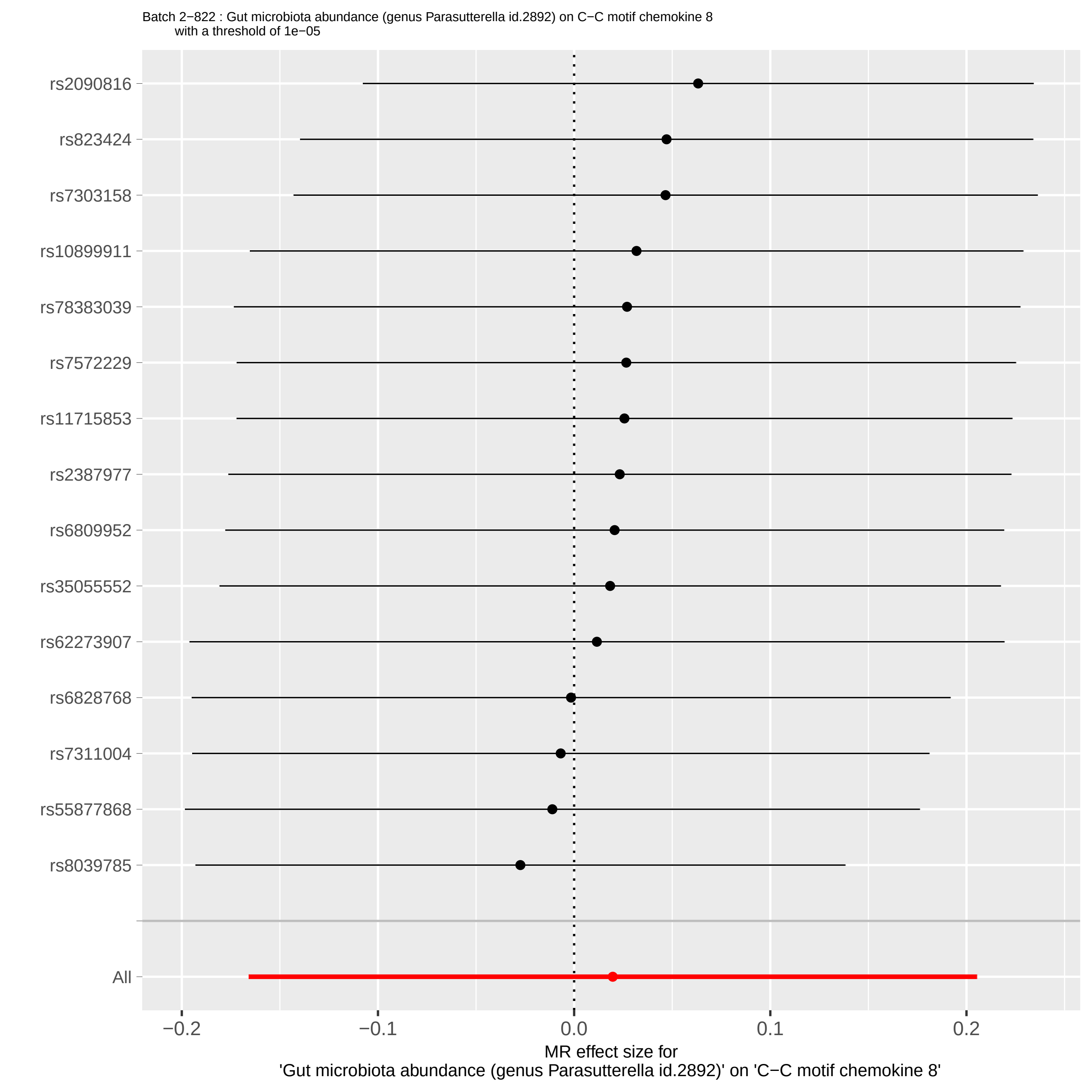

Batch 2−822 : Gut microbiota abundance (genus Parasutterella id.2892) on C−C motif chemokine 8
with a threshold of 1e−05
rs2090816
rs823424
rs7303158
rs10899911
rs78383039
rs7572229
rs11715853
rs2387977
rs6809952
rs35055552
rs62273907
rs6828768
rs7311004
rs55877868
rs8039785
All
−0.2
−0.1
0.0
0.1
0.2
MR effect size for
'Gut microbiota abundance (genus Parasutterella id.2892)' on 'C−C motif chemokine 8'

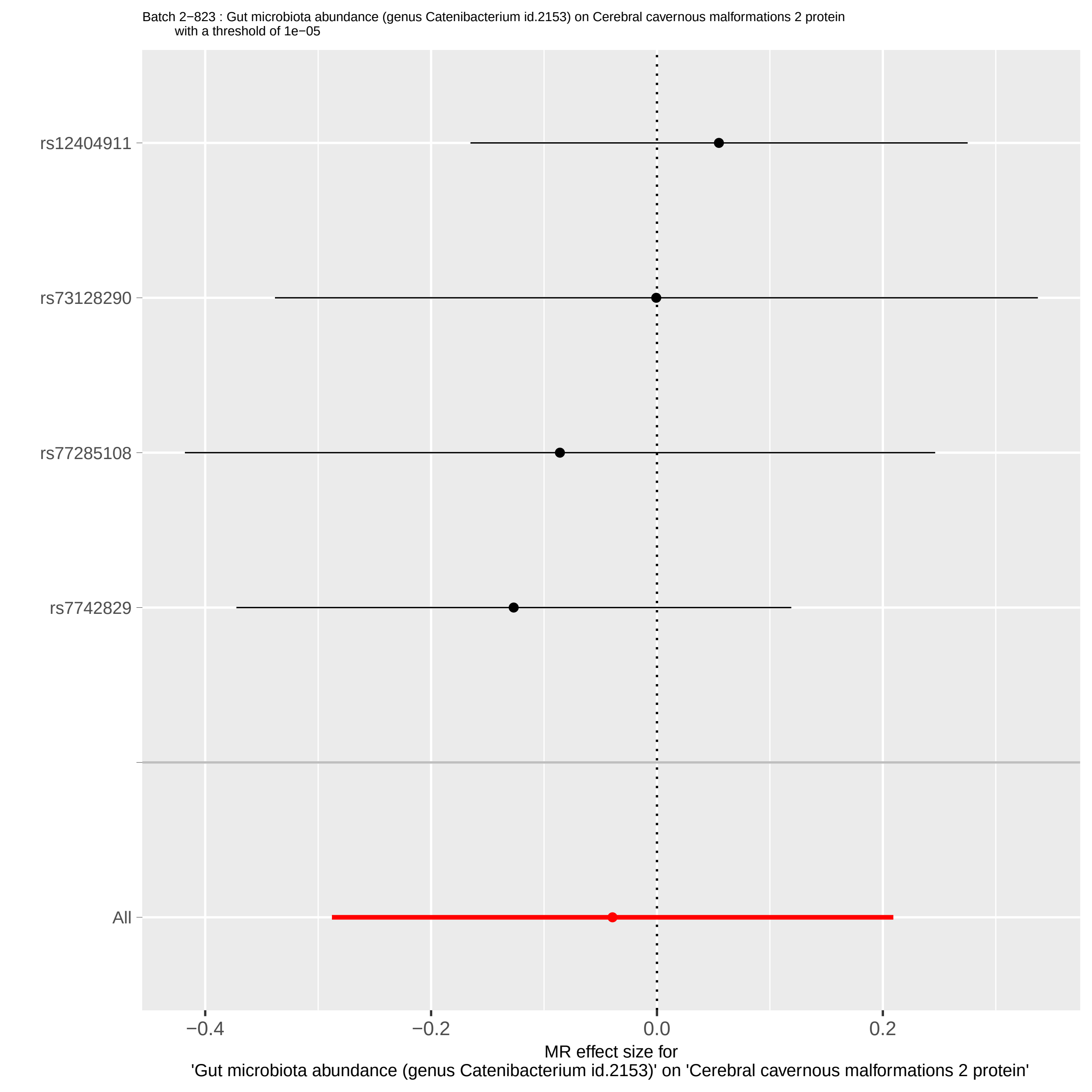

Batch 2−823 : Gut microbiota abundance (genus Catenibacterium id.2153) on Cerebral cavernous malformations 2 protein
with a threshold of 1e−05
rs12404911
rs73128290
rs77285108
rs7742829
All
−0.4
−0.2
0.0
0.2
MR effect size for
'Gut microbiota abundance (genus Catenibacterium id.2153)' on 'Cerebral cavernous malformations 2 protein'

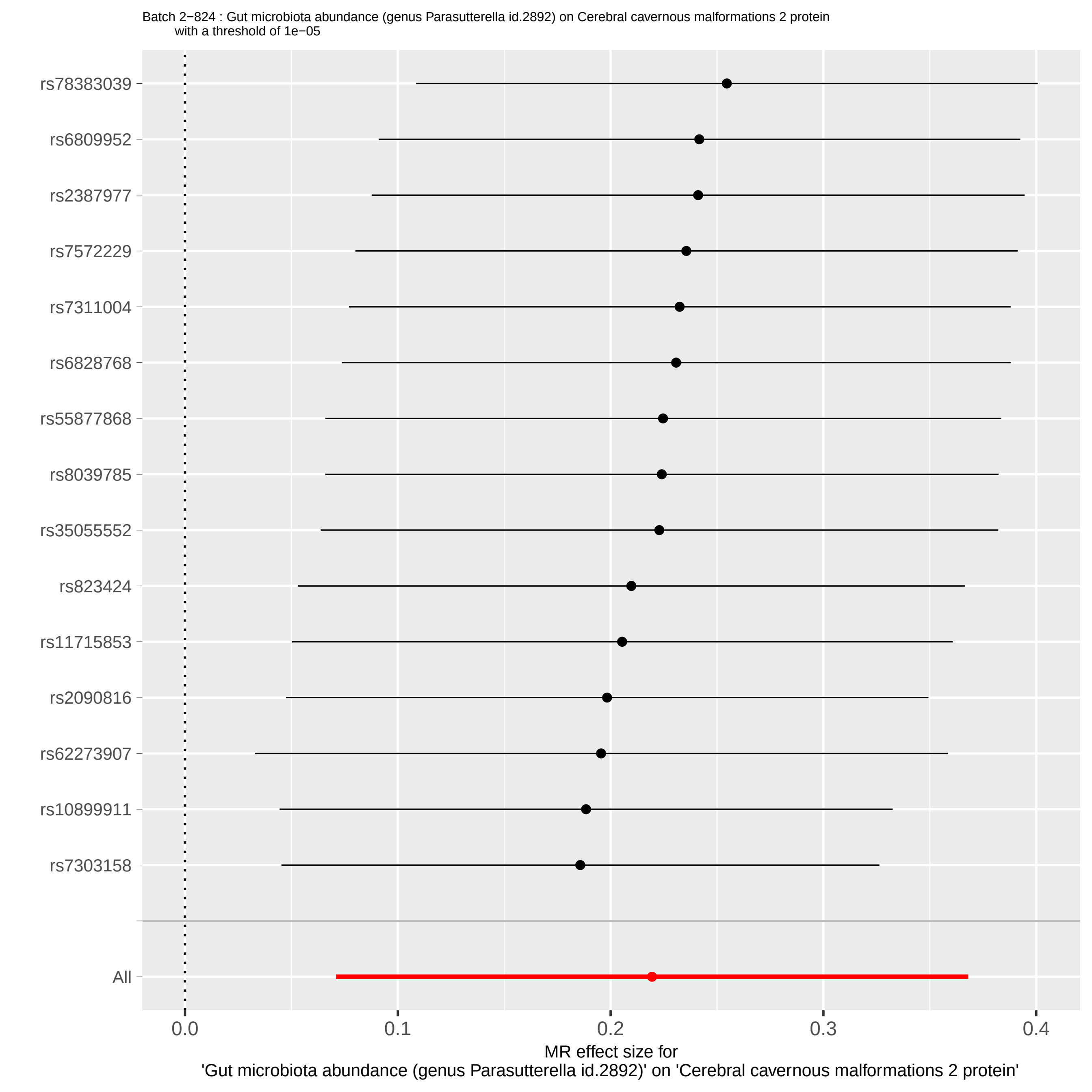
Batch 2−824 : Gut microbiota abundance (genus Parasutterella id.2892) on Cerebral cavernous malformations 2 protein
with a threshold of 1e−05
rs78383039
rs6809952
rs2387977
rs7572229
rs7311004
rs6828768
rs55877868
rs8039785
rs35055552
rs823424
rs11715853
rs2090816
rs62273907
rs10899911
rs7303158
All
0.0
0.1
0.2
0.3
0.4
MR effect size for
'Gut microbiota abundance (genus Parasutterella id.2892)' on 'Cerebral cavernous malformations 2 protein'

Batch 2−825 : Gut microbiota abundance (genus Catenibacterium id.2153) on G2/mitotic−specific cyclin−B1
with a threshold of 1e−05

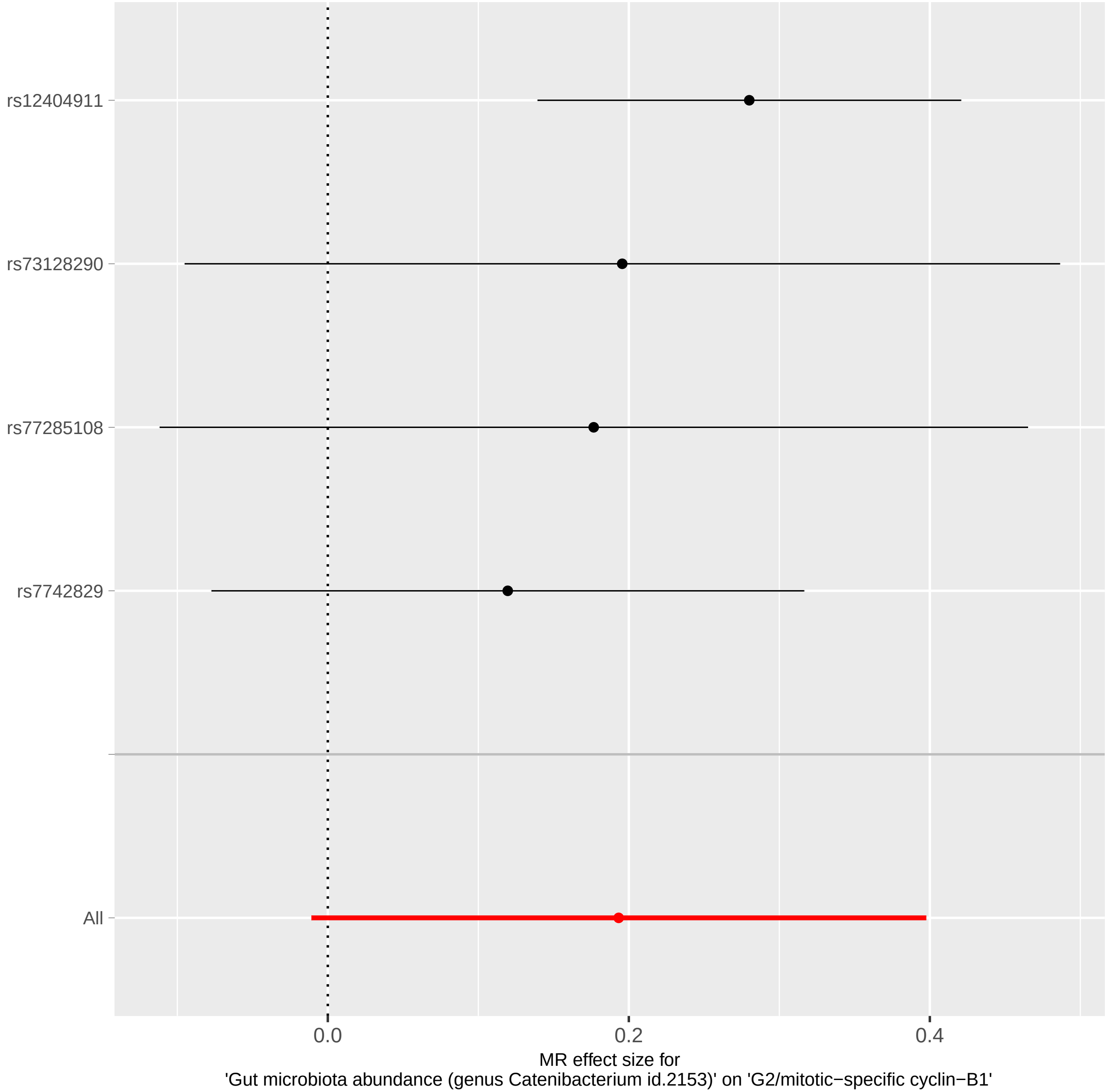

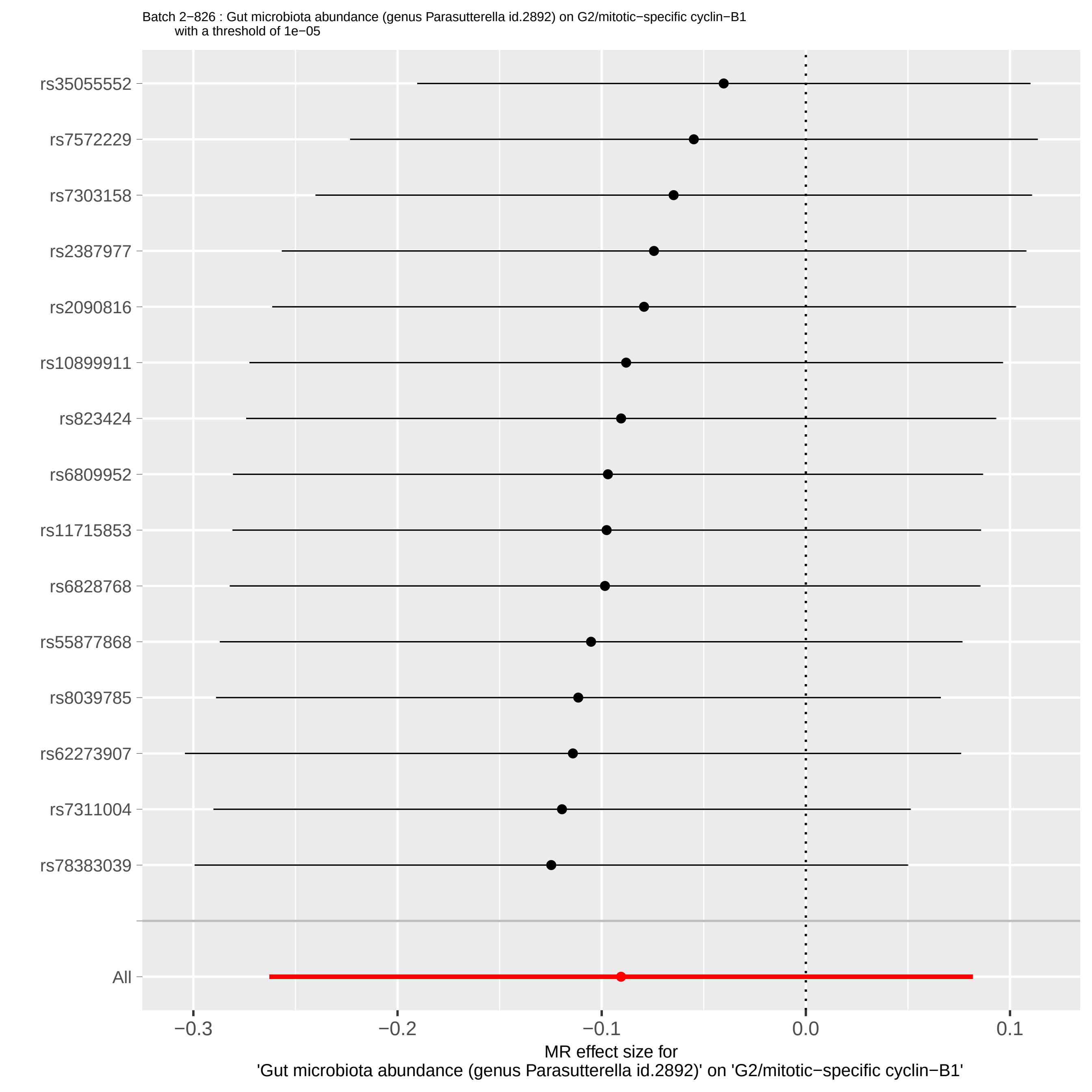

Batch 2−826 : Gut microbiota abundance (genus Parasutterella id.2892) on G2/mitotic−specific cyclin−B1
with a threshold of 1e−05
rs35055552
rs7572229
rs7303158
rs2387977
rs2090816
rs10899911
rs823424
rs6809952
rs11715853
rs6828768
rs55877868
rs8039785
rs62273907
rs7311004
rs78383039
All
−0.3
−0.2
−0.1
0.0
0.1
MR effect size for
'Gut microbiota abundance (genus Parasutterella id.2892)' on 'G2/mitotic−specific cyclin−B1'

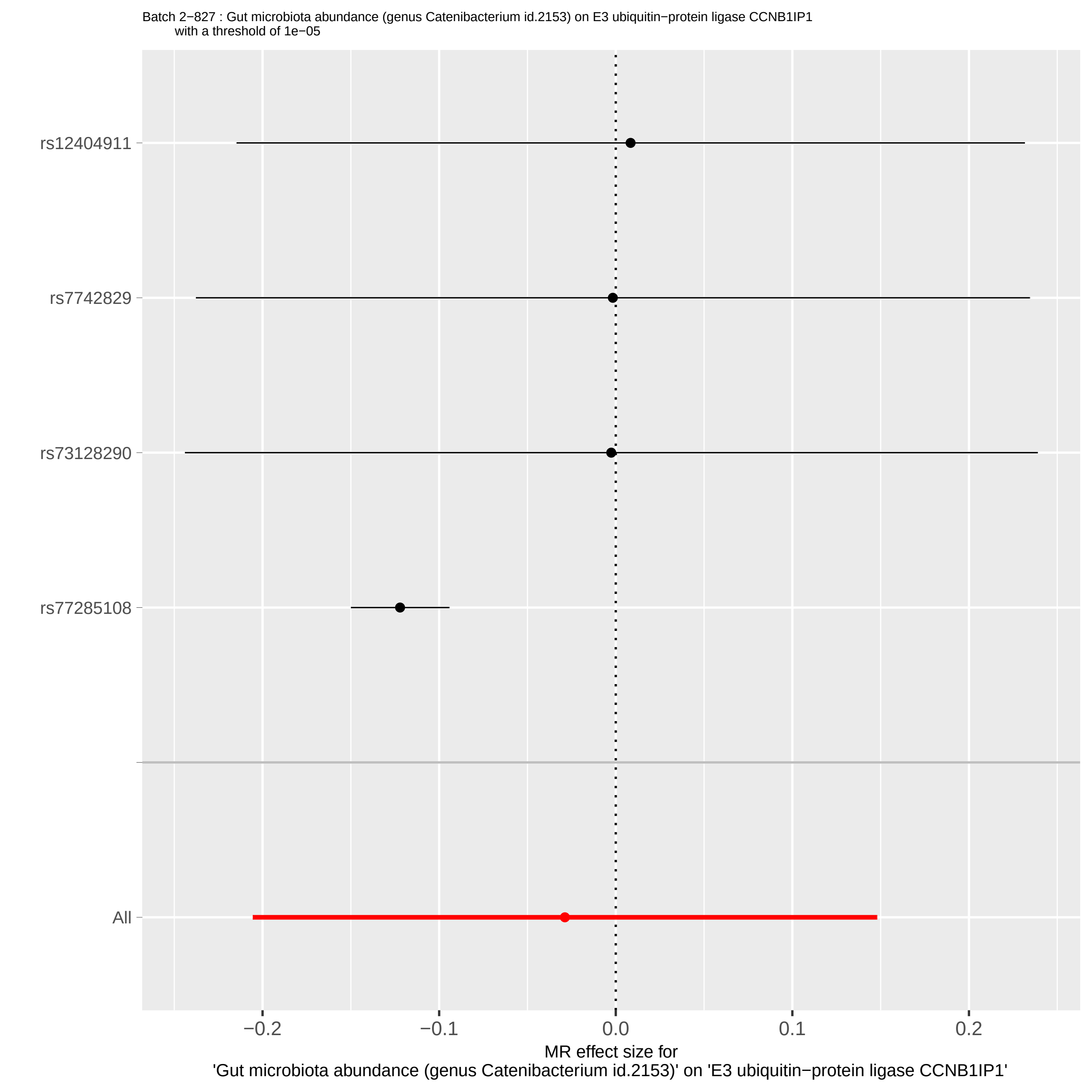
Batch 2−827 : Gut microbiota abundance (genus Catenibacterium id.2153) on E3 ubiquitin−protein ligase CCNB1IP1
with a threshold of 1e−05
rs12404911
rs7742829
rs73128290
rs77285108
All
−0.2
−0.1
0.0
0.1
0.2
MR effect size for
'Gut microbiota abundance (genus Catenibacterium id.2153)' on 'E3 ubiquitin−protein ligase CCNB1IP1'

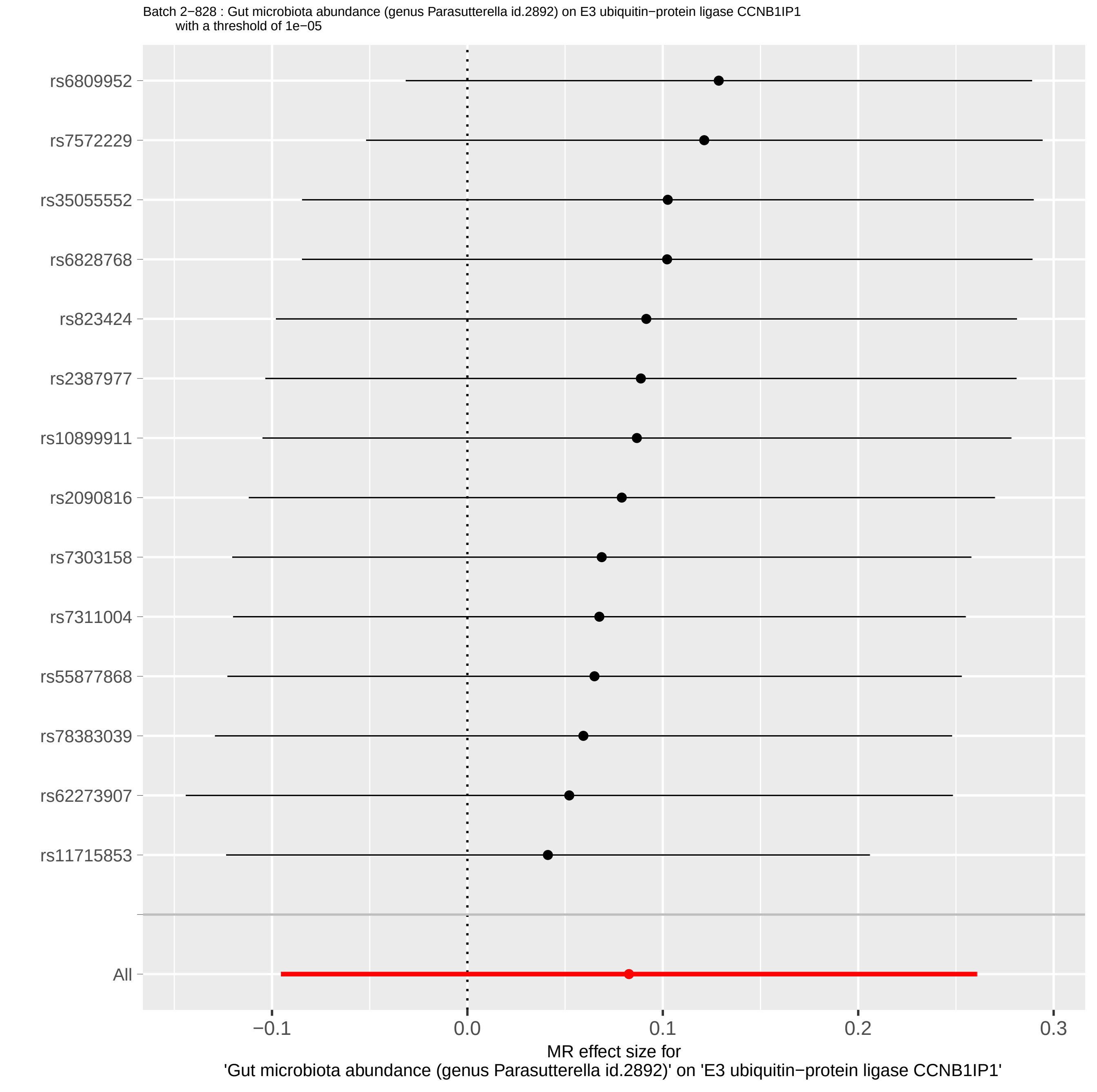

Batch 2−828 : Gut microbiota abundance (genus Parasutterella id.2892) on E3 ubiquitin−protein ligase CCNB1IP1
with a threshold of 1e−05
rs6809952
rs7572229
rs35055552
rs6828768
rs823424
rs2387977
rs10899911
rs2090816
rs7303158
rs7311004
rs55877868
rs78383039
rs62273907
rs11715853
All
−0.1
0.0
0.1
0.2
0.3
MR effect size for
'Gut microbiota abundance (genus Parasutterella id.2892)' on 'E3 ubiquitin−protein ligase CCNB1IP1'

Batch 2−829 : Gut microbiota abundance (genus Catenibacterium id.2153) on Cyclin−C
with a threshold of 1e−05

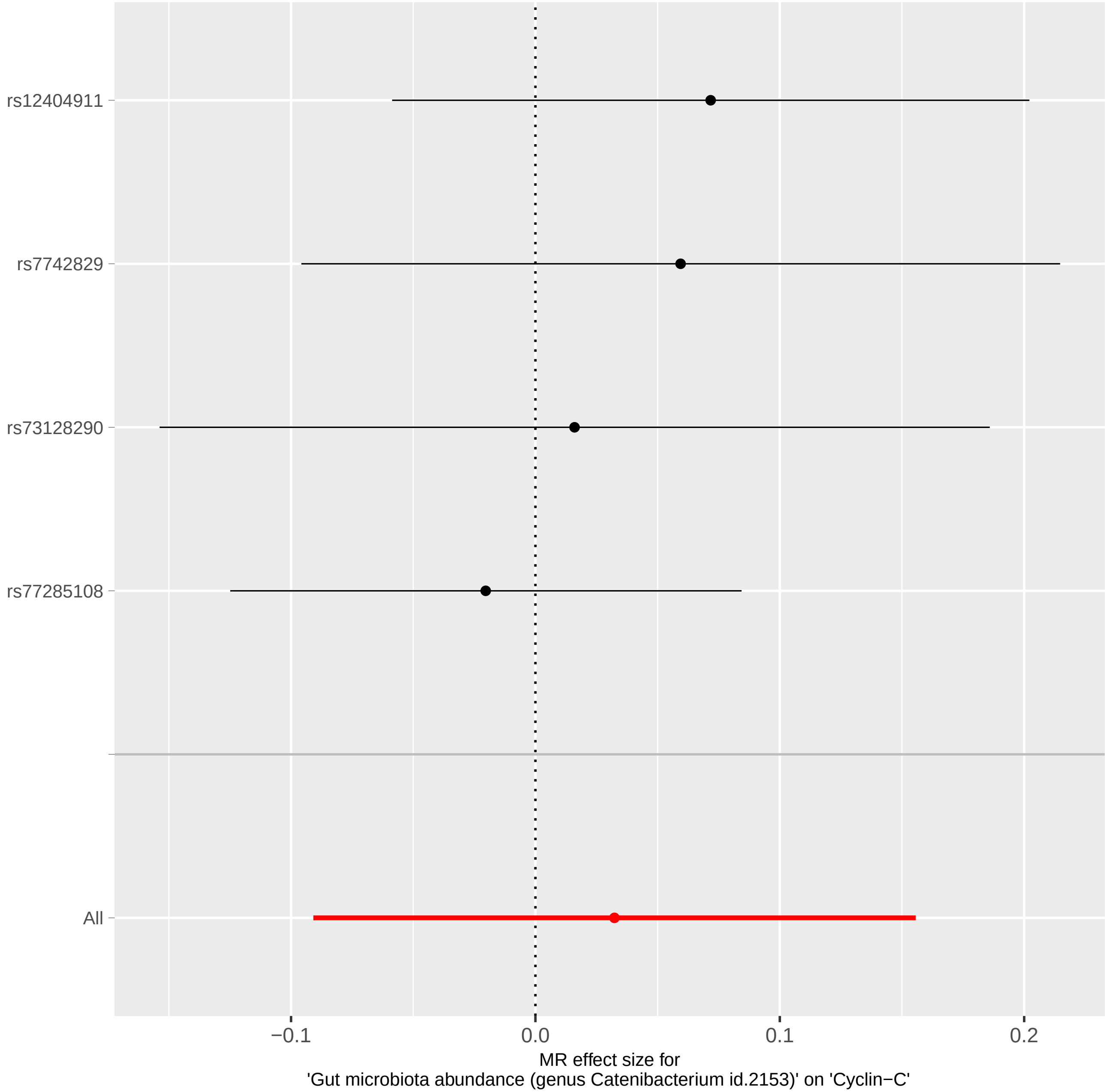

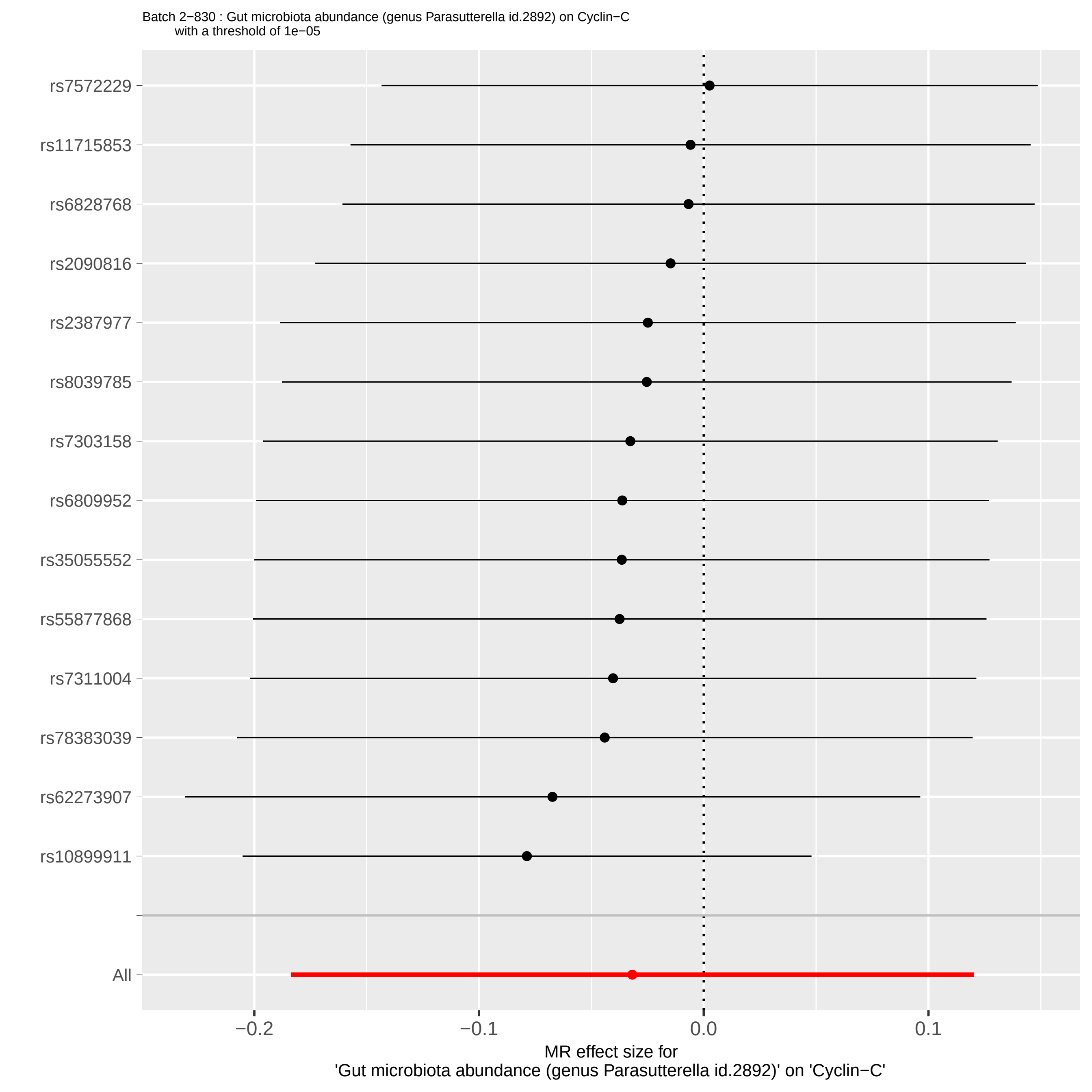

Batch 2−830 : Gut microbiota abundance (genus Parasutterella id.2892) on Cyclin−C
with a threshold of 1e−05
rs7572229
rs11715853
rs6828768
rs2090816
rs2387977
rs8039785
rs7303158
rs6809952
rs35055552
rs55877868
rs7311004
rs78383039
rs62273907
rs10899911
All
−0.2
−0.1
0.0
0.1
MR effect size for
'Gut microbiota abundance (genus Parasutterella id.2892)' on 'Cyclin−C'

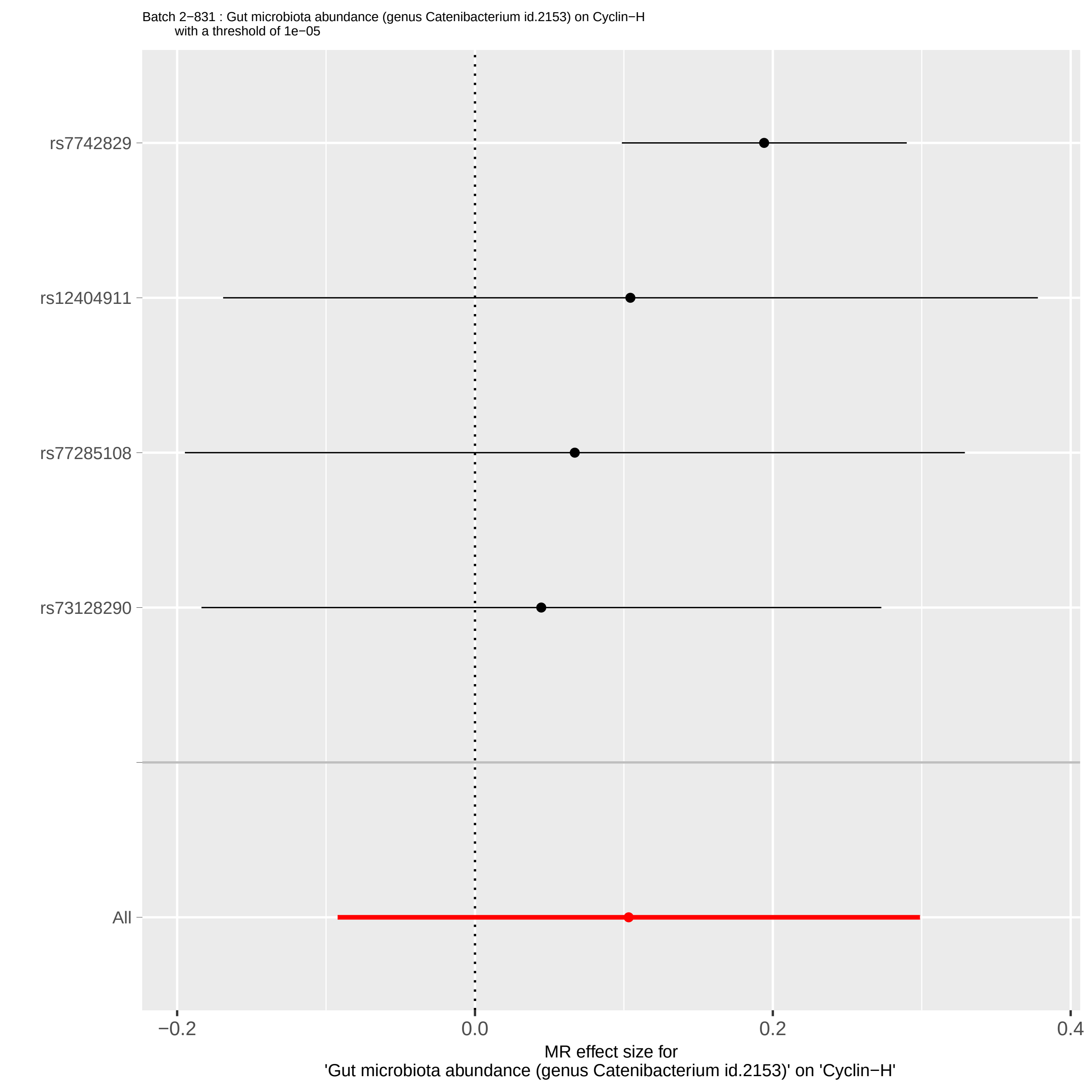
Batch 2−831 : Gut microbiota abundance (genus Catenibacterium id.2153) on Cyclin−H
with a threshold of 1e−05
rs7742829
rs12404911
rs77285108
rs73128290
All
−0.2
0.0
0.2
0.4
MR effect size for
'Gut microbiota abundance (genus Catenibacterium id.2153)' on 'Cyclin−H'

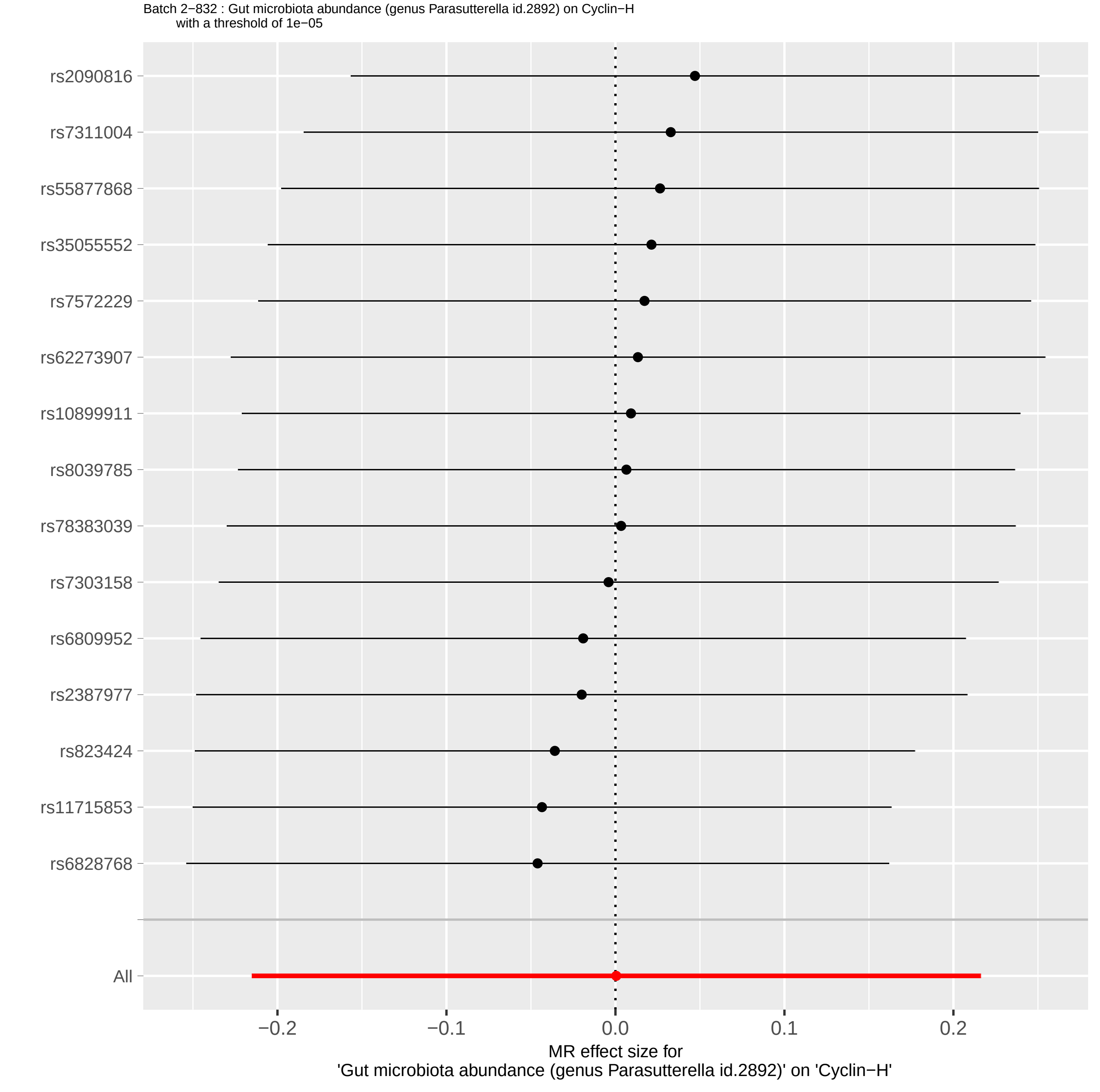

Batch 2−832 : Gut microbiota abundance (genus Parasutterella id.2892) on Cyclin−H
with a threshold of 1e−05
rs2090816
rs7311004
rs55877868
rs35055552
rs7572229
rs62273907
rs10899911
rs8039785
rs78383039
rs7303158
rs6809952
rs2387977
rs823424
rs11715853
rs6828768
All
−0.2
−0.1
0.0
0.1
0.2
MR effect size for
'Gut microbiota abundance (genus Parasutterella id.2892)' on 'Cyclin−H'

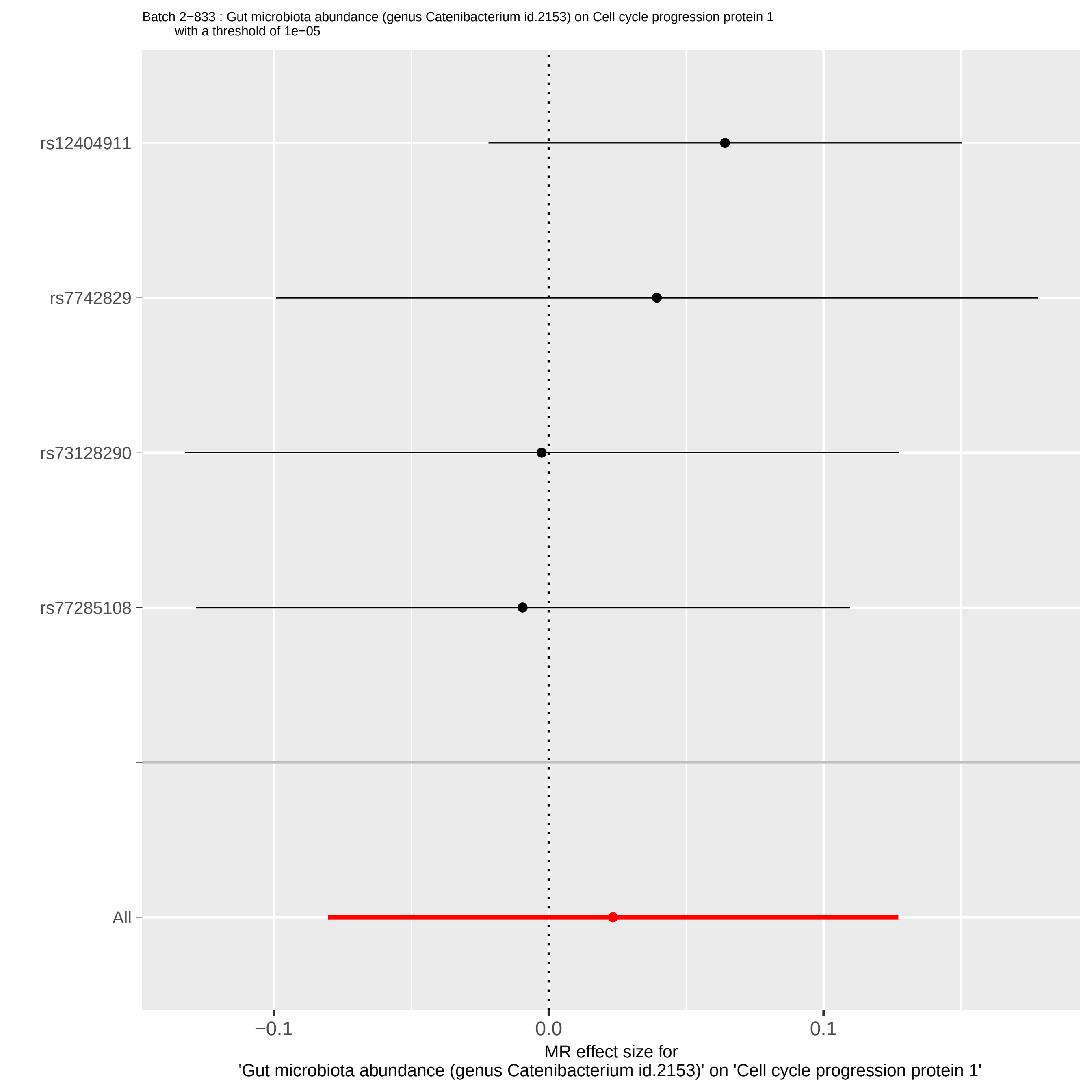

Batch 2−833 : Gut microbiota abundance (genus Catenibacterium id.2153) on Cell cycle progression protein 1
with a threshold of 1e−05
rs12404911
rs7742829
rs73128290
rs77285108
All
−0.1
0.0
0.1
MR effect size for
'Gut microbiota abundance (genus Catenibacterium id.2153)' on 'Cell cycle progression protein 1'

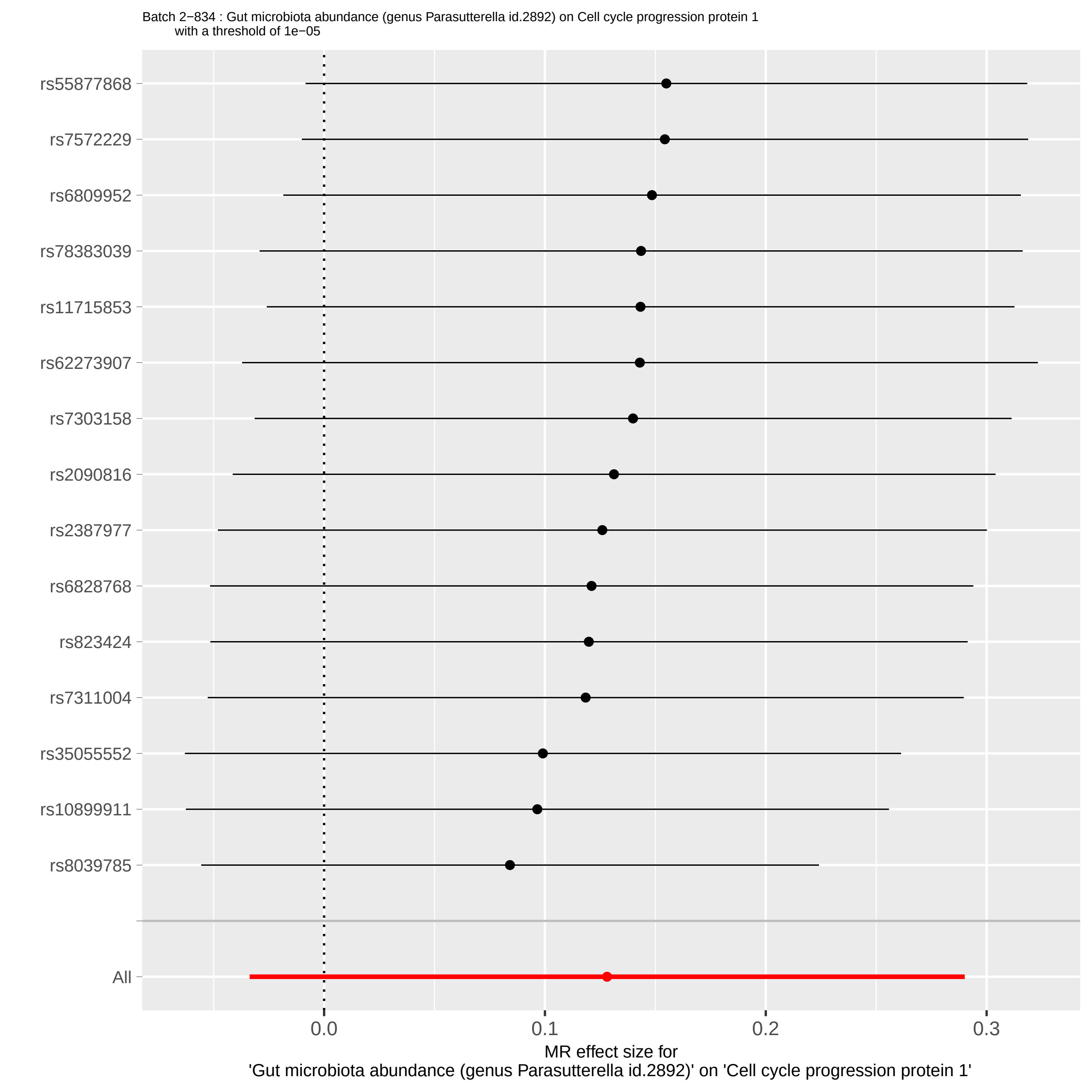
Batch 2−834 : Gut microbiota abundance (genus Parasutterella id.2892) on Cell cycle progression protein 1
with a threshold of 1e−05
rs55877868
rs7572229
rs6809952
rs78383039
rs11715853
rs62273907
rs7303158
rs2090816
rs2387977
rs6828768
rs823424
rs7311004
rs35055552
rs10899911
rs8039785
All
0.0
0.1
0.2
0.3
MR effect size for
'Gut microbiota abundance (genus Parasutterella id.2892)' on 'Cell cycle progression protein 1'

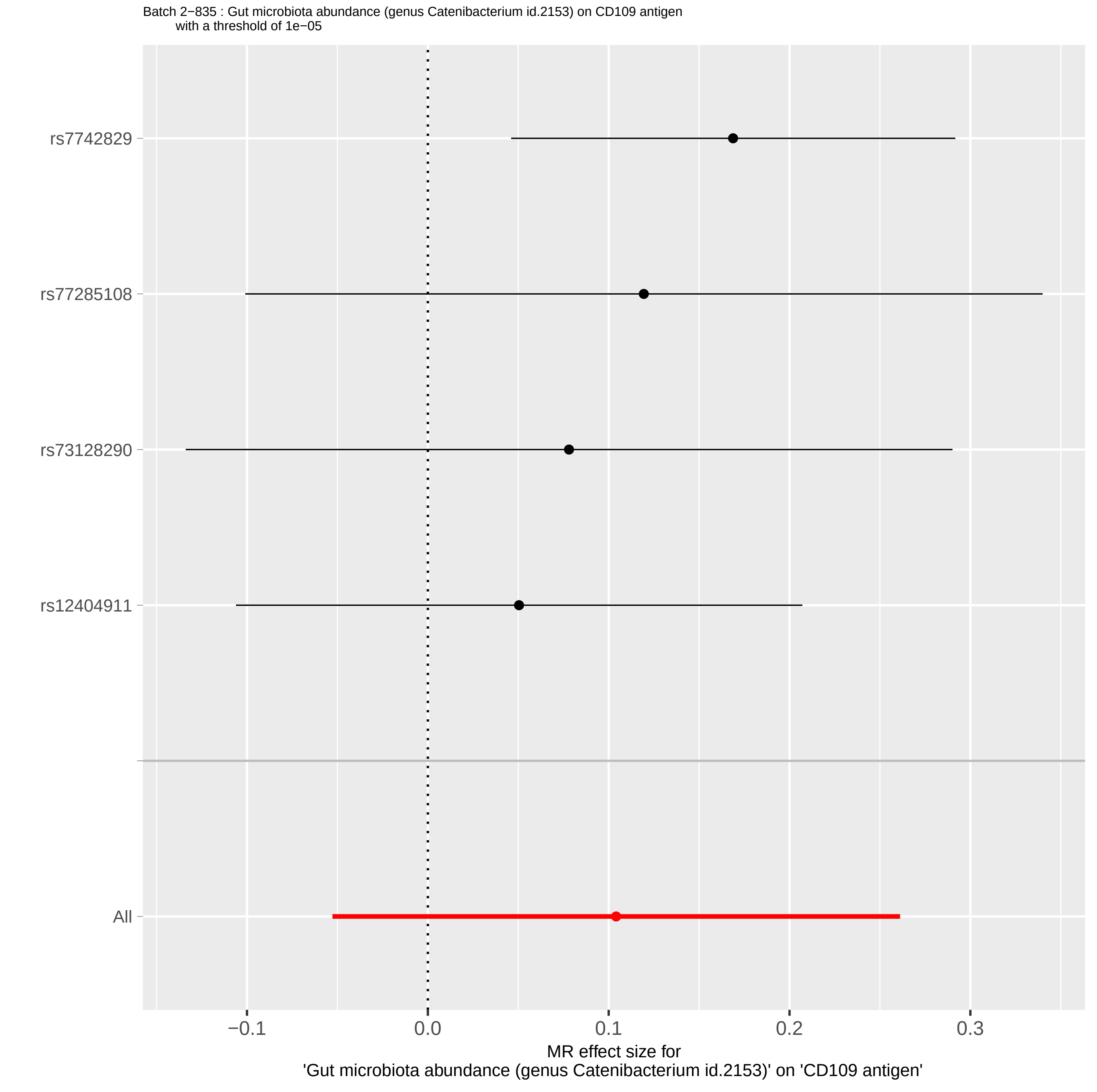
Batch 2−835 : Gut microbiota abundance (genus Catenibacterium id.2153) on CD109 antigen
with a threshold of 1e−05
rs7742829
rs77285108
rs73128290
rs12404911
All
−0.1
0.0
0.1
0.2
0.3
MR effect size for
'Gut microbiota abundance (genus Catenibacterium id.2153)' on 'CD109 antigen'

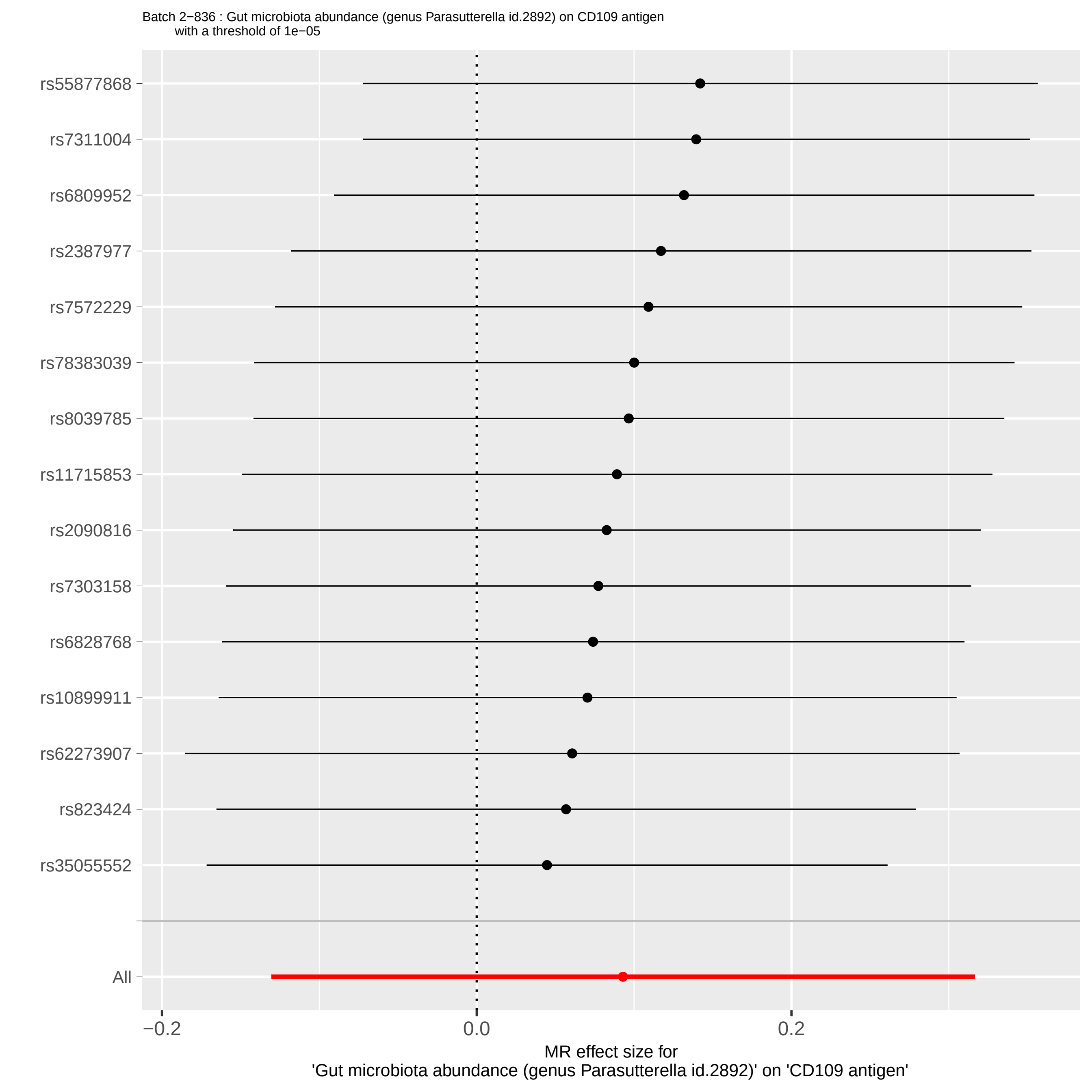
Batch 2−836 : Gut microbiota abundance (genus Parasutterella id.2892) on CD109 antigen
with a threshold of 1e−05
rs55877868
rs7311004
rs6809952
rs2387977
rs7572229
rs78383039
rs8039785
rs11715853
rs2090816
rs7303158
rs6828768
rs10899911
rs62273907
rs823424
rs35055552
All
−0.2
0.0
0.2
MR effect size for
'Gut microbiota abundance (genus Parasutterella id.2892)' on 'CD109 antigen'

Batch 2−837 : Gut microbiota abundance (genus Catenibacterium id.2153) on Scavenger receptor cysteine−rich type 1 protein M130
with a threshold of 1e−05

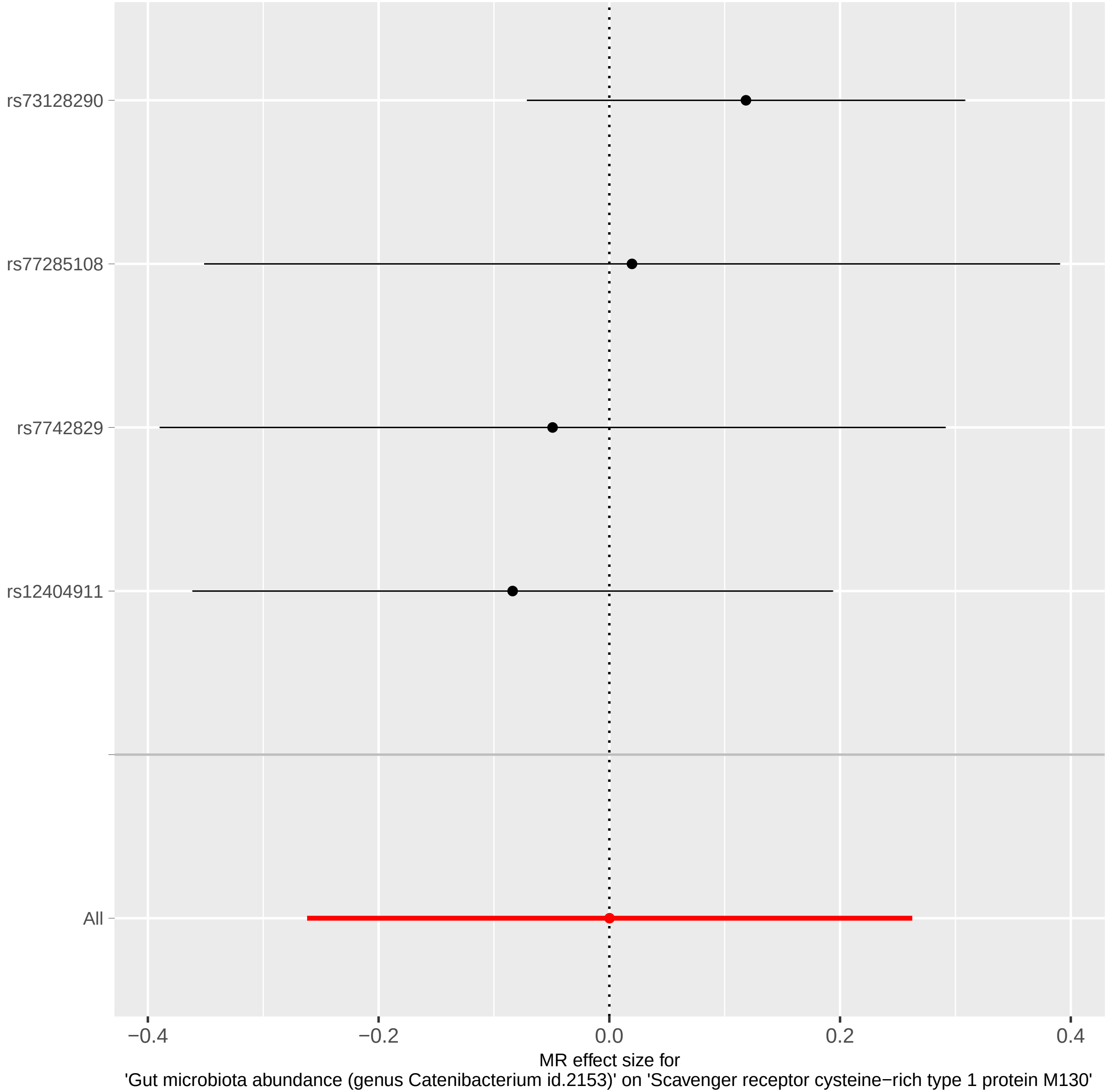

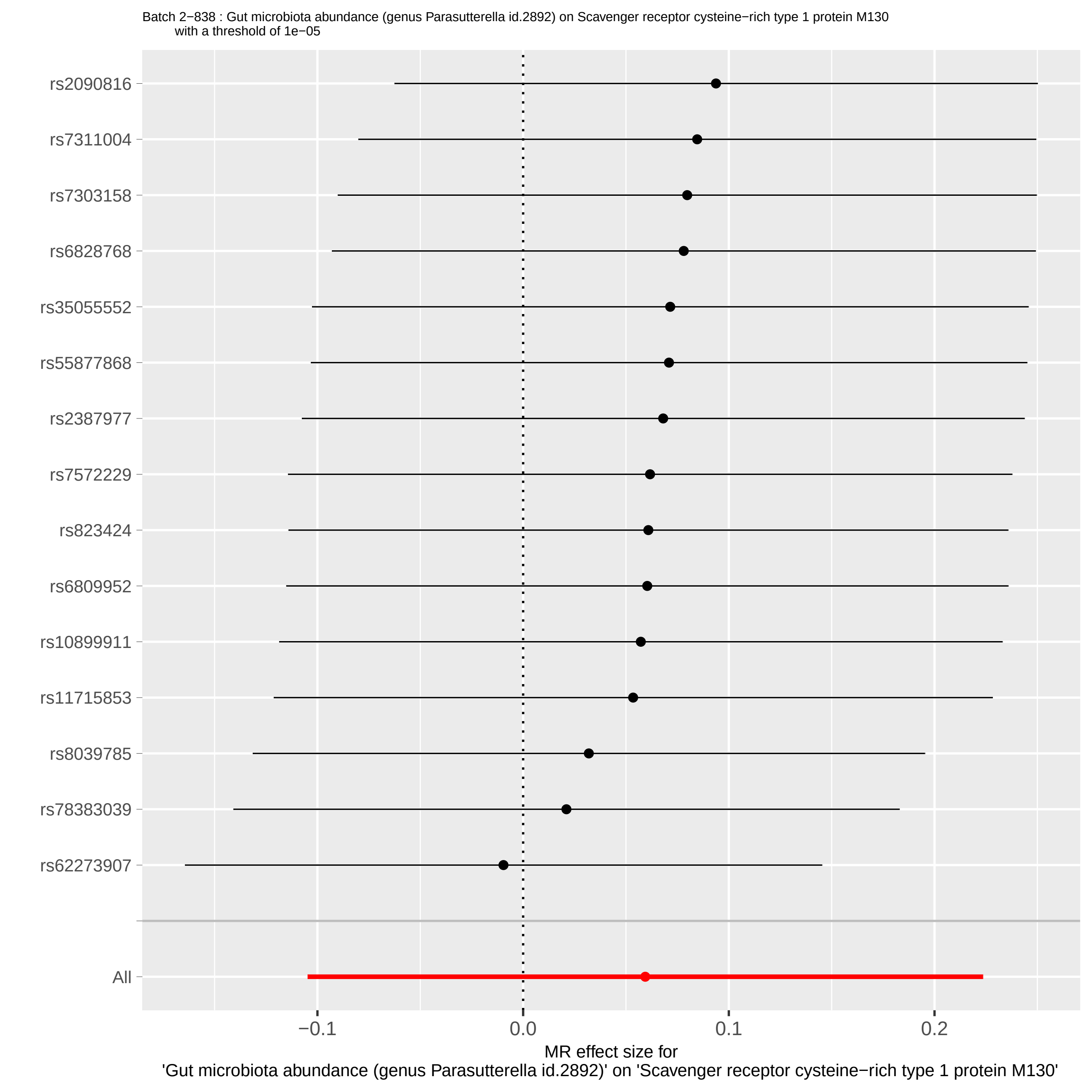

Batch 2−838 : Gut microbiota abundance (genus Parasutterella id.2892) on Scavenger receptor cysteine−rich type 1 protein M130
with a threshold of 1e−05
rs2090816
rs7311004
rs7303158
rs6828768
rs35055552
rs55877868
rs2387977
rs7572229
rs823424
rs6809952
rs10899911
rs11715853
rs8039785
rs78383039
rs62273907
All
−0.1
0.0
0.1
0.2
MR effect size for
'Gut microbiota abundance (genus Parasutterella id.2892)' on 'Scavenger receptor cysteine−rich type 1 protein M130'

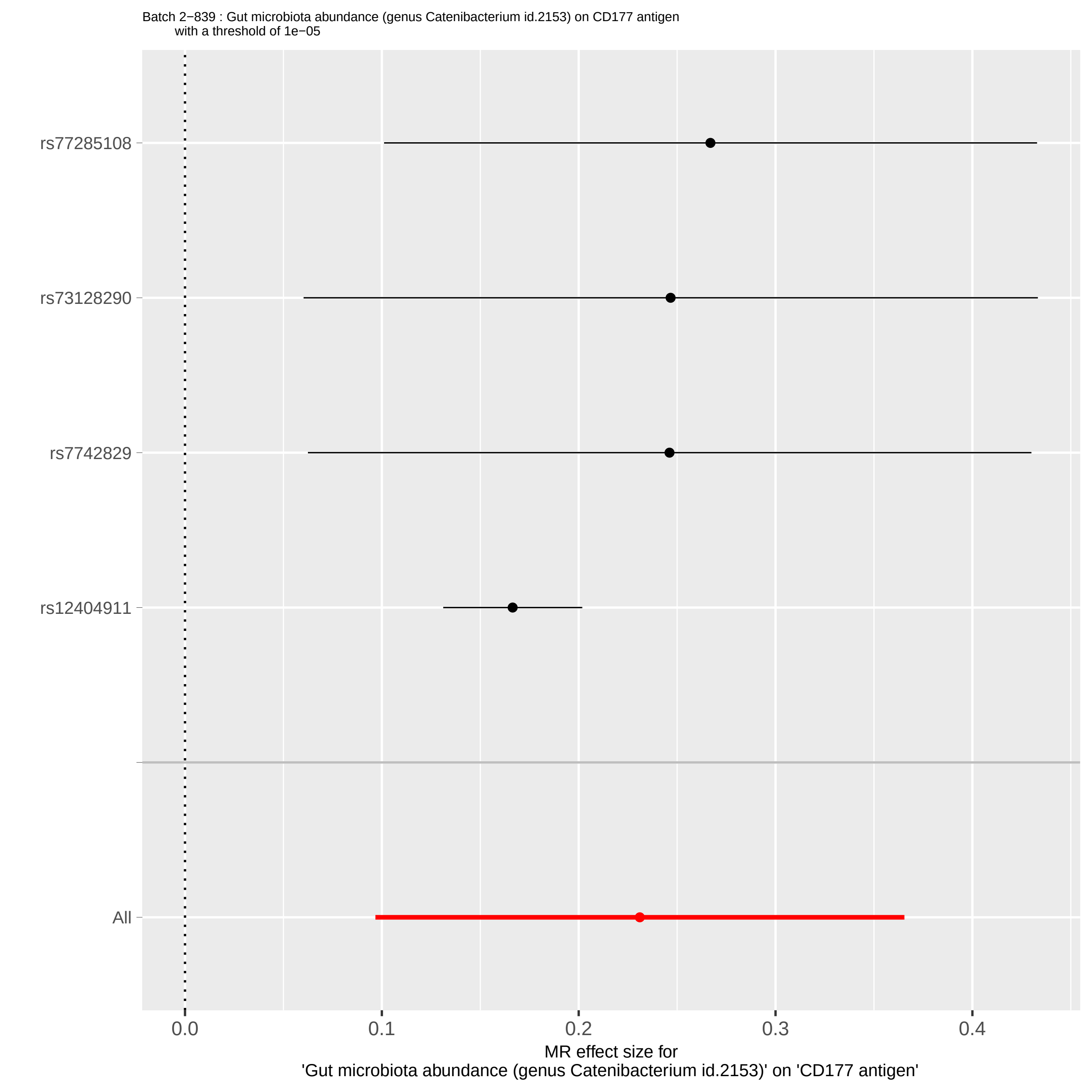
Batch 2−839 : Gut microbiota abundance (genus Catenibacterium id.2153) on CD177 antigen
with a threshold of 1e−05
rs77285108
rs73128290
rs7742829
rs12404911
All
0.0
0.1
0.2
0.3
0.4
MR effect size for
'Gut microbiota abundance (genus Catenibacterium id.2153)' on 'CD177 antigen'

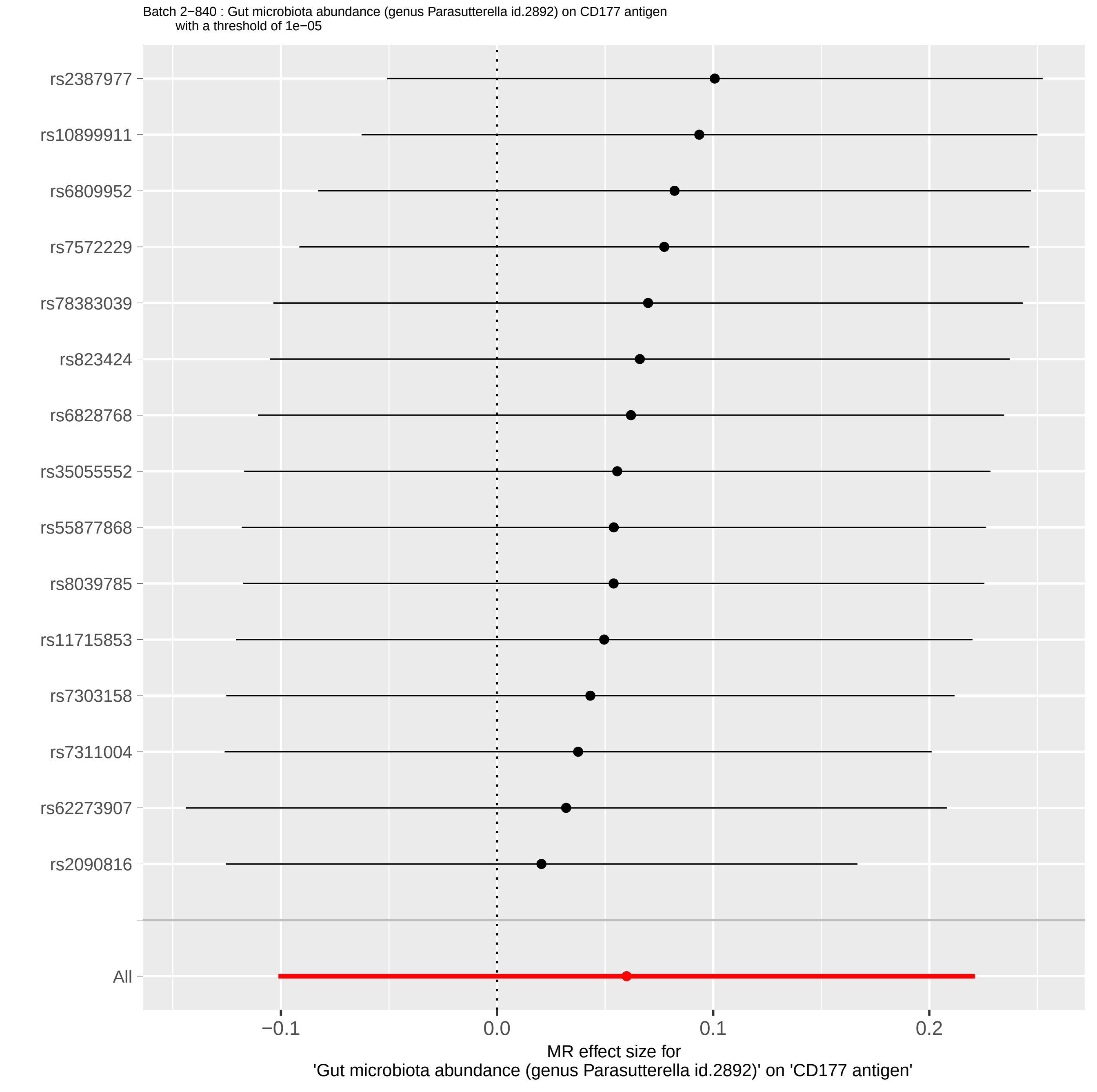

Batch 2−840 : Gut microbiota abundance (genus Parasutterella id.2892) on CD177 antigen
with a threshold of 1e−05
rs2387977
rs10899911
rs6809952
rs7572229
rs78383039
rs823424
rs6828768
rs35055552
rs55877868
rs8039785
rs11715853
rs7303158
rs7311004
rs62273907
rs2090816
All
−0.1
0.0
0.1
0.2
MR effect size for
'Gut microbiota abundance (genus Parasutterella id.2892)' on 'CD177 antigen'

Batch 2−841 : Gut microbiota abundance (genus Catenibacterium id.2153) on Antigen−presenting glycoprotein CD1d
with a threshold of 1e−05

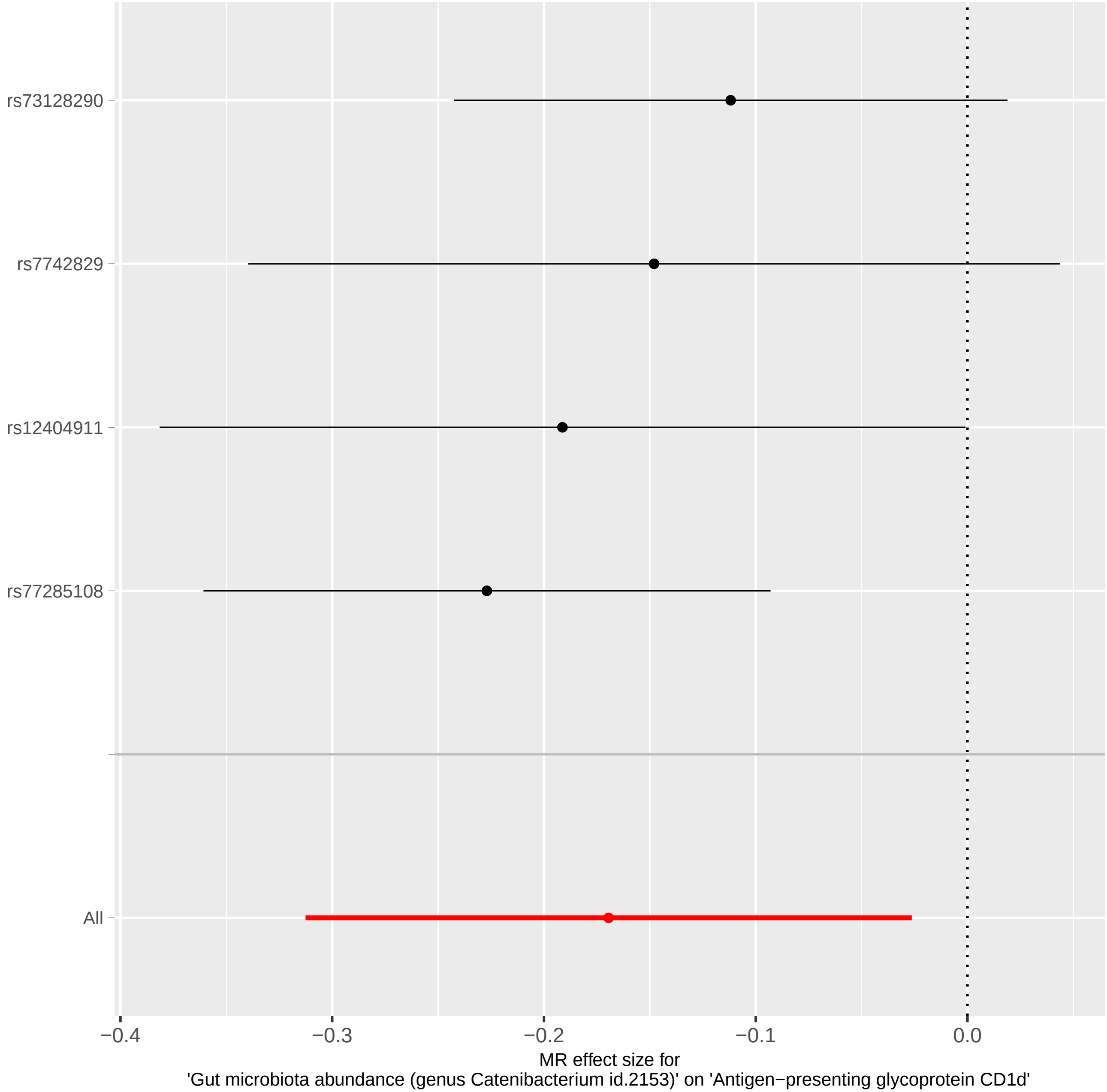

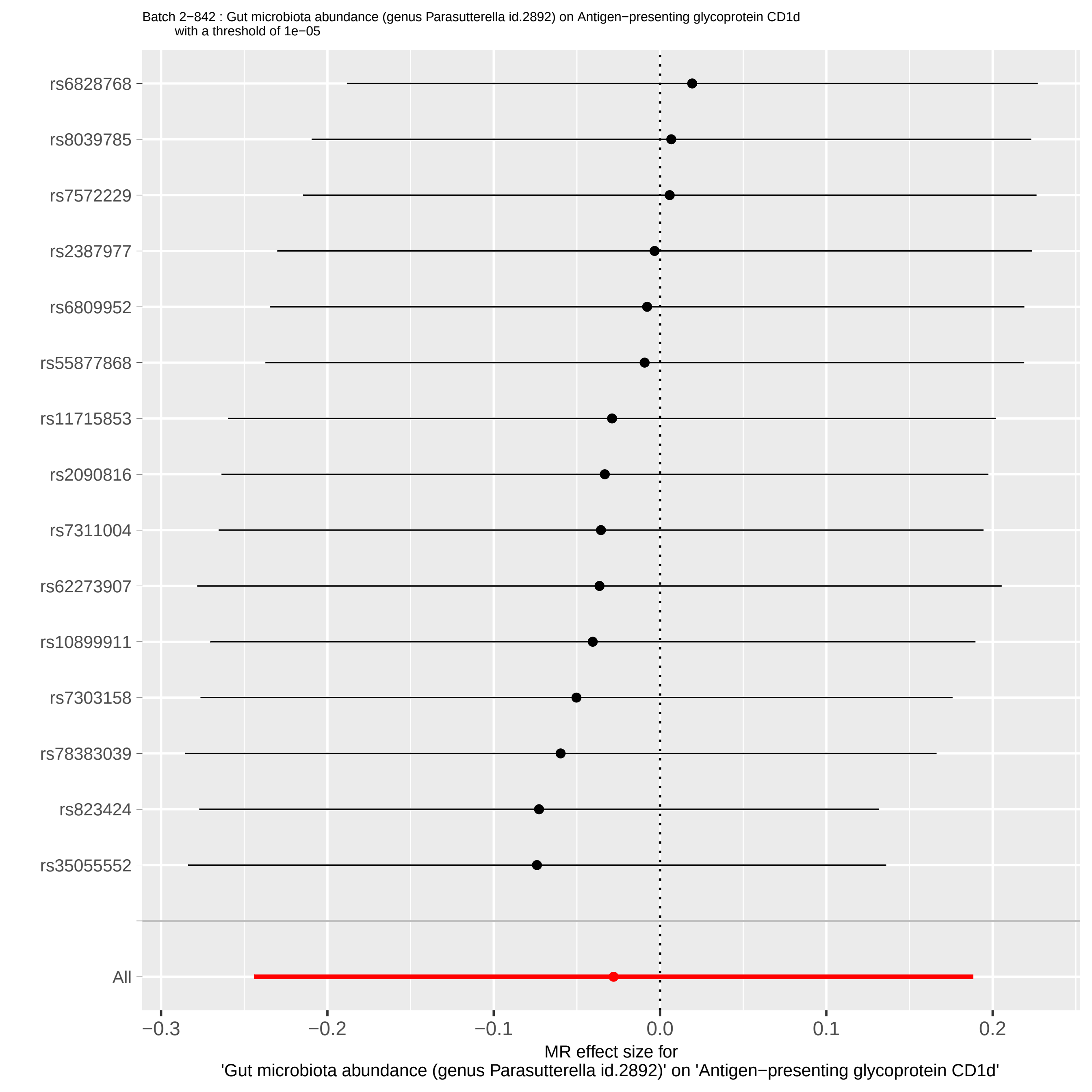

Batch 2−842 : Gut microbiota abundance (genus Parasutterella id.2892) on Antigen−presenting glycoprotein CD1d with a threshold of 1e−05
rs6828768
rs8039785
rs7572229
rs2387977
rs6809952
rs55877868
rs11715853
rs2090816
rs7311004
rs62273907
rs10899911
rs7303158
rs78383039
rs823424
rs35055552
All
−0.3
−0.2
−0.1
0.0
0.1
0.2
MR effect size for
'Gut microbiota abundance (genus Parasutterella id.2892)' on 'Antigen−presenting glycoprotein CD1d'

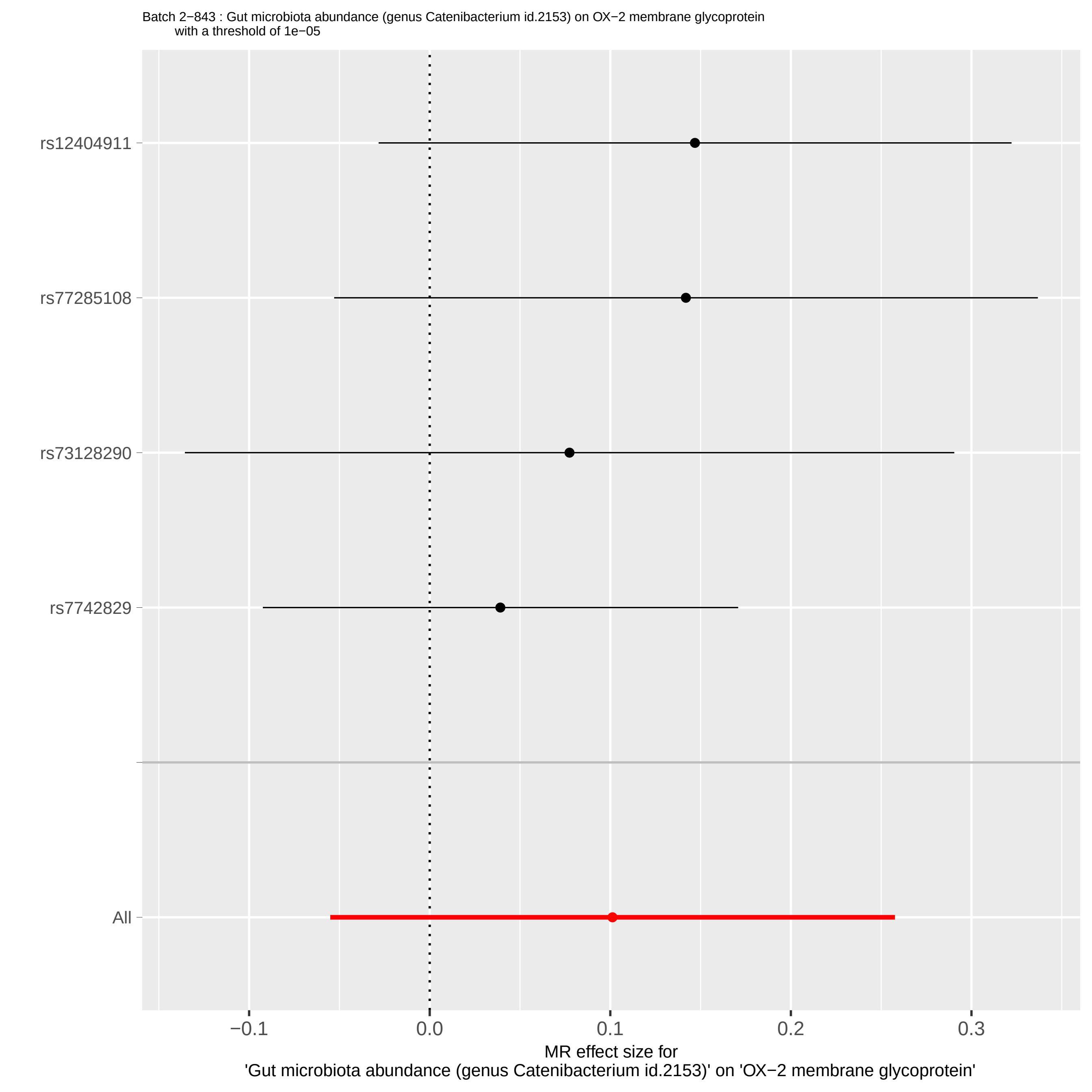

Batch 2−843 : Gut microbiota abundance (genus Catenibacterium id.2153) on OX−2 membrane glycoprotein
with a threshold of 1e−05
rs12404911
rs77285108
rs73128290
rs7742829
All
−0.1
0.0
0.1
0.2
0.3
MR effect size for
'Gut microbiota abundance (genus Catenibacterium id.2153)' on 'OX−2 membrane glycoprotein'

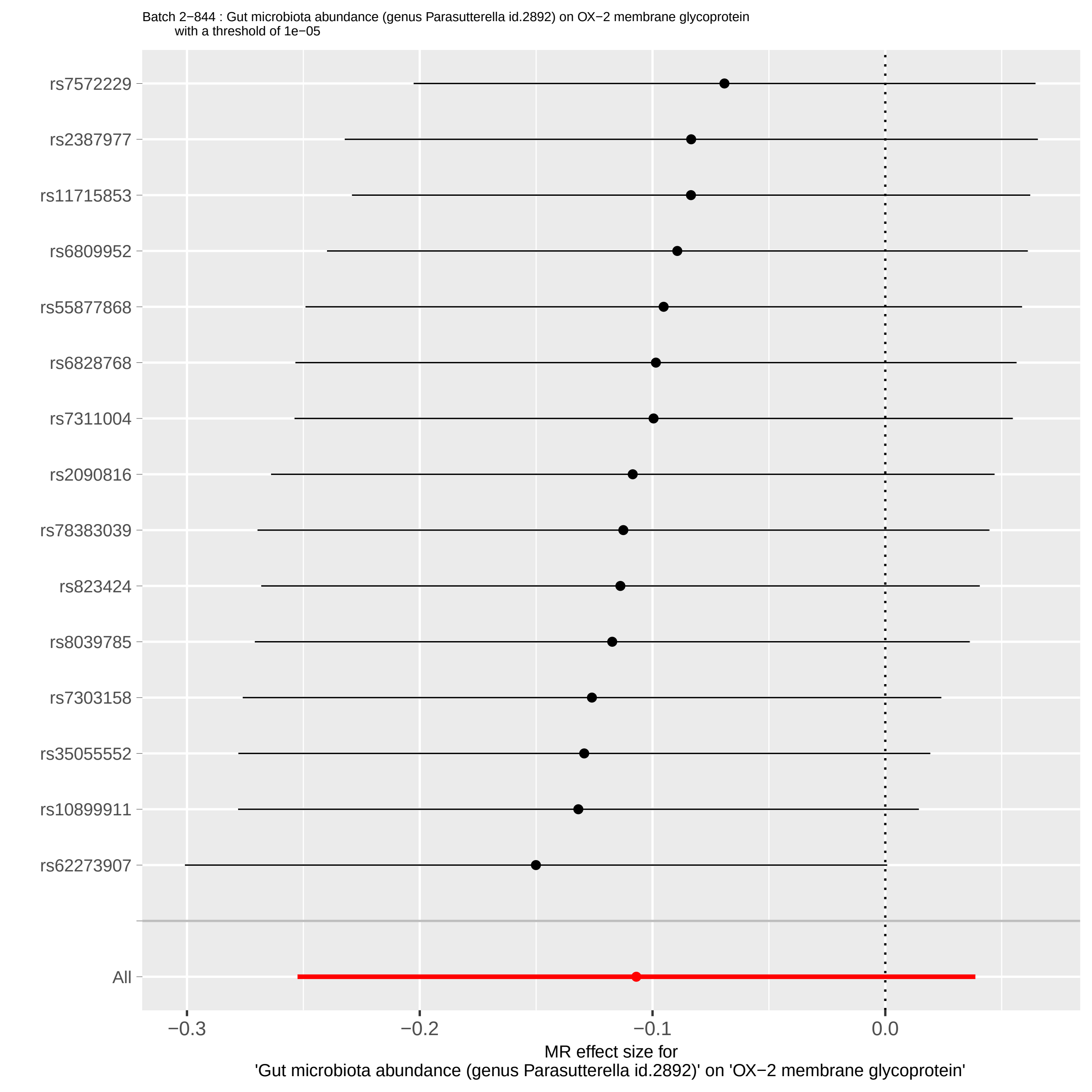

Batch 2−844 : Gut microbiota abundance (genus Parasutterella id.2892) on OX−2 membrane glycoprotein
with a threshold of 1e−05
rs7572229
rs2387977
rs11715853
rs6809952
rs55877868
rs6828768
rs7311004
rs2090816
rs78383039
rs823424
rs8039785
rs7303158
rs35055552
rs10899911
rs62273907
All
−0.3
−0.2
−0.1
0.0
MR effect size for
'Gut microbiota abundance (genus Parasutterella id.2892)' on 'OX−2 membrane glycoprotein'

Batch 2−845 : Gut microbiota abundance (genus Catenibacterium id.2153) on Cell surface glycoprotein CD200 receptor 1
with a threshold of 1e−05

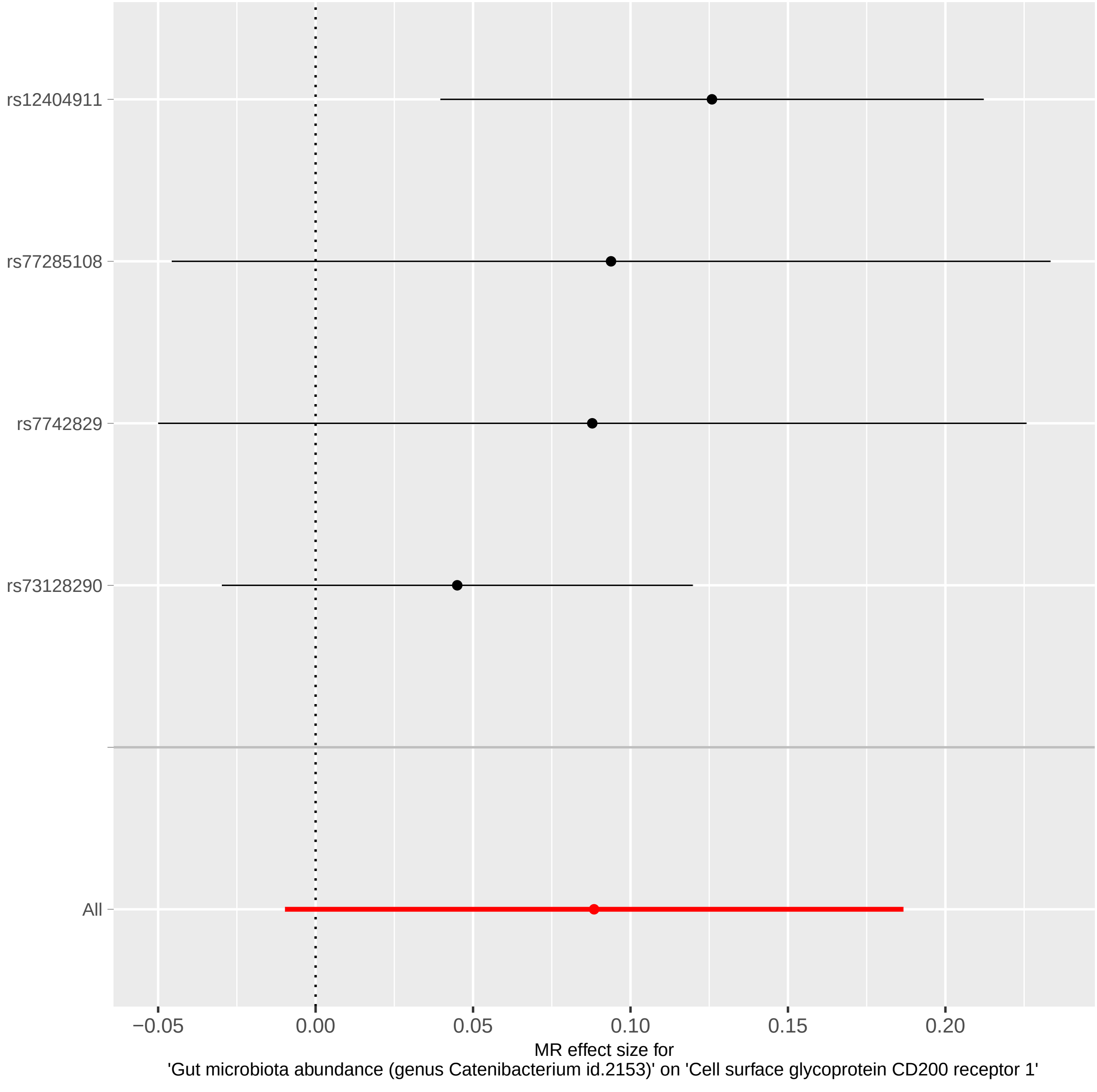

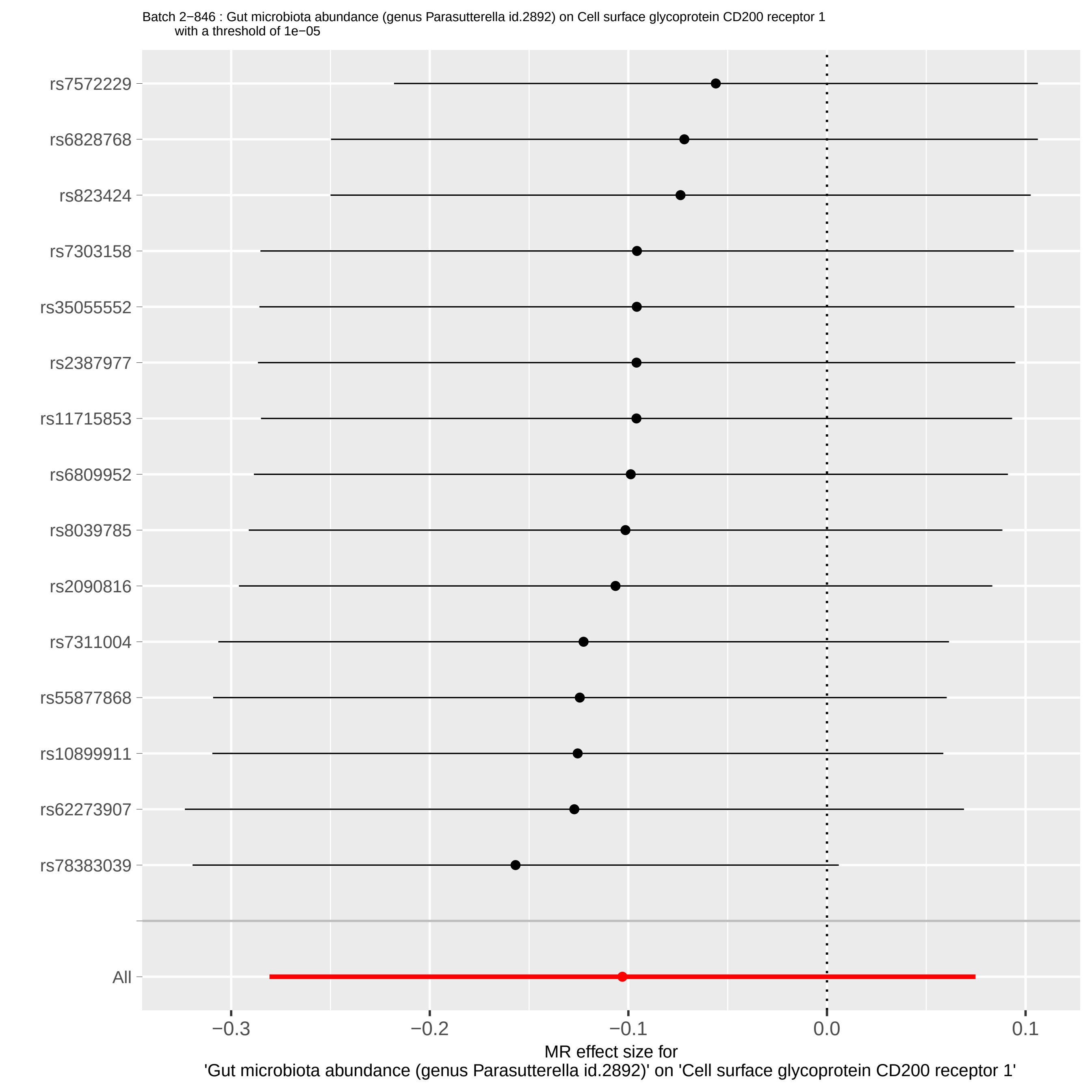

Batch 2−846 : Gut microbiota abundance (genus Parasutterella id.2892) on Cell surface glycoprotein CD200 receptor 1
with a threshold of 1e−05
rs7572229
rs6828768
rs823424
rs7303158
rs35055552
rs2387977
rs11715853
rs6809952
rs8039785
rs2090816
rs7311004
rs55877868
rs10899911
rs62273907
rs78383039
All
−0.3
−0.2
−0.1
0.0
0.1
MR effect size for
'Gut microbiota abundance (genus Parasutterella id.2892)' on 'Cell surface glycoprotein CD200 receptor 1'

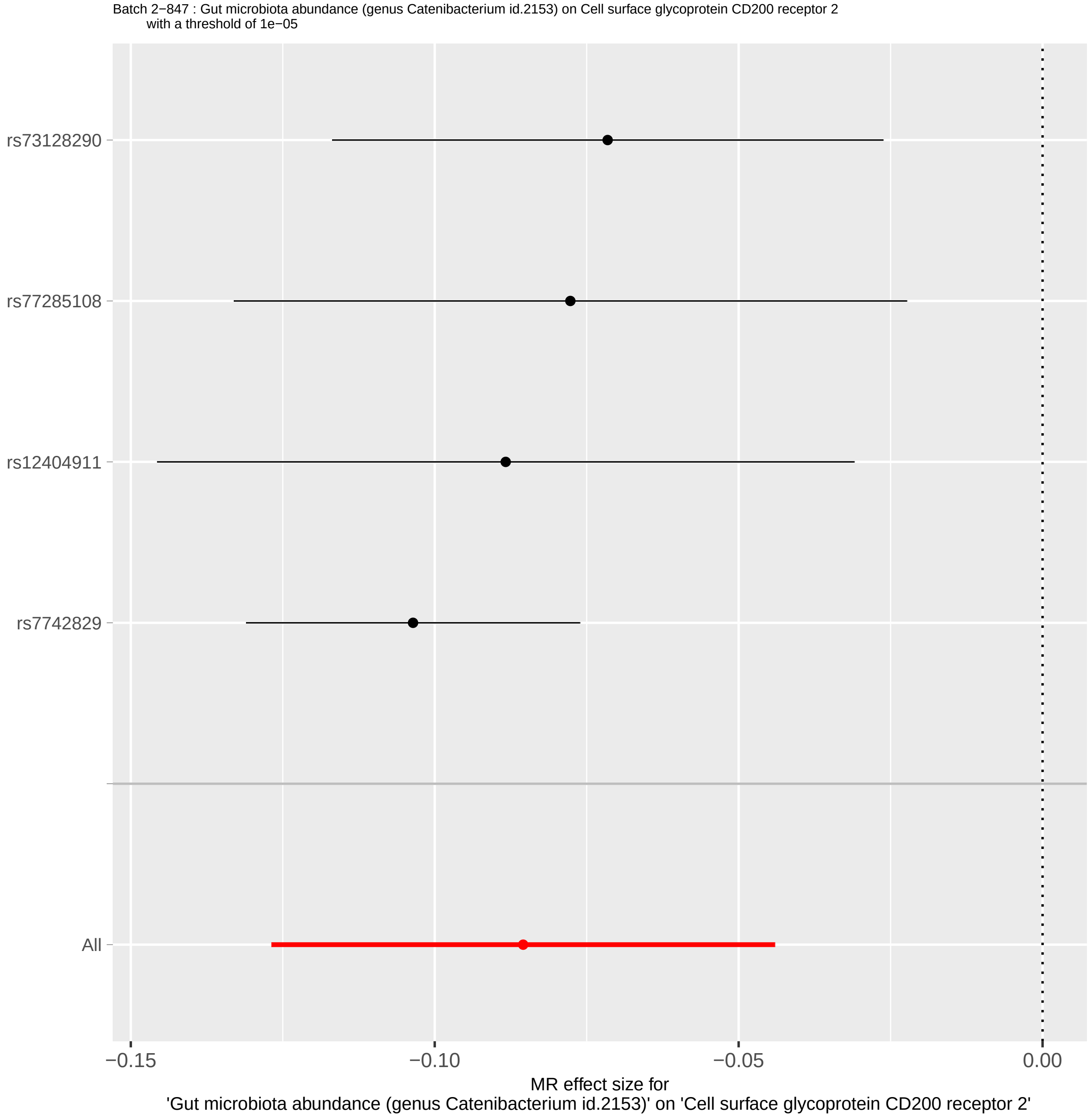
Batch 2−847 : Gut microbiota abundance (genus Catenibacterium id.2153) on Cell surface glycoprotein CD200 receptor 2
with a threshold of 1e−05
rs73128290
rs77285108
rs12404911
rs7742829
All
−0.15
−0.10
−0.05
0.00
MR effect size for
'Gut microbiota abundance (genus Catenibacterium id.2153)' on 'Cell surface glycoprotein CD200 receptor 2'

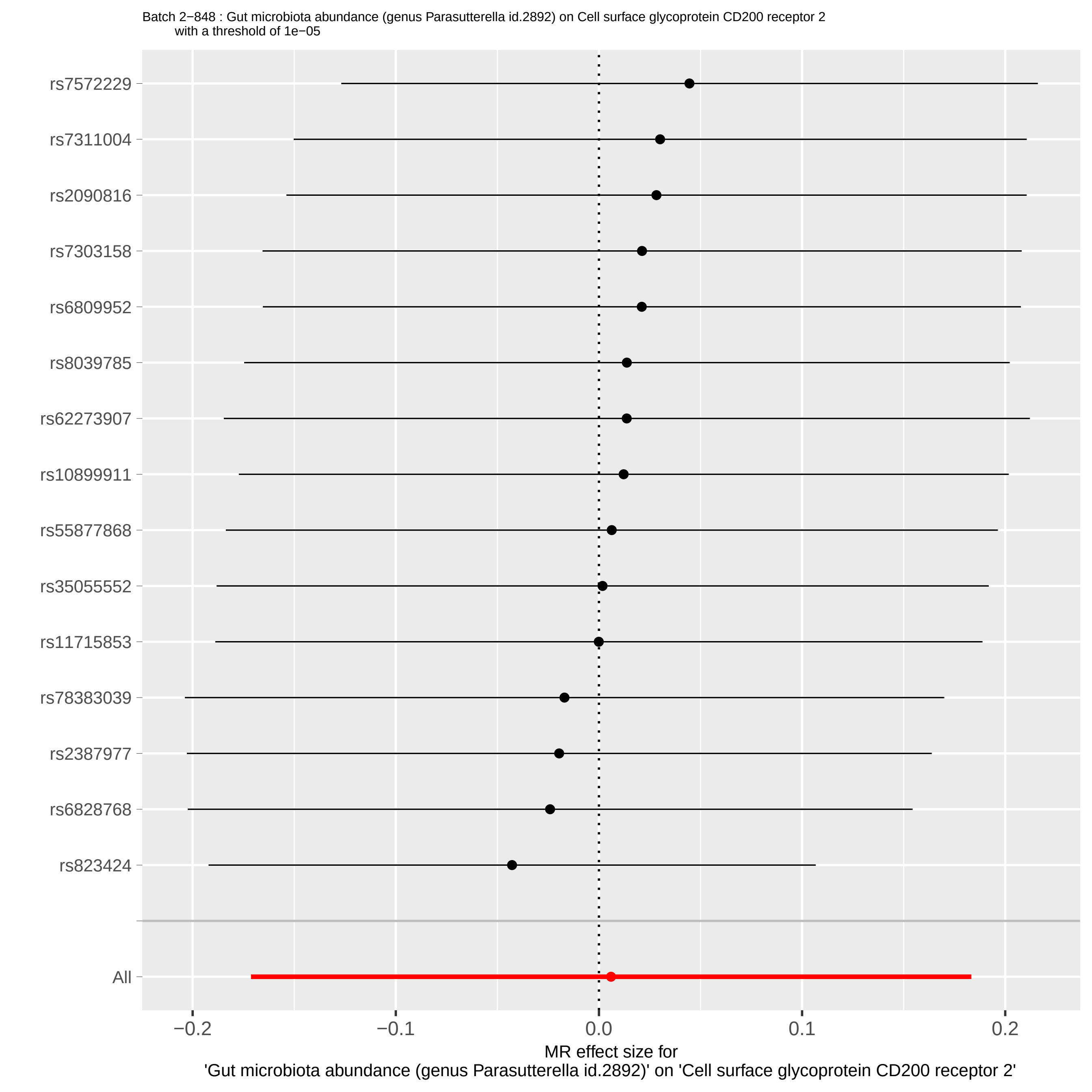

Batch 2−848 : Gut microbiota abundance (genus Parasutterella id.2892) on Cell surface glycoprotein CD200 receptor 2
with a threshold of 1e−05
rs7572229
rs7311004
rs2090816
rs7303158
rs6809952
rs8039785
rs62273907
rs10899911
rs55877868
rs35055552
rs11715853
rs78383039
rs2387977
rs6828768
rs823424
All
−0.2
−0.1
0.0
0.1
0.2
MR effect size for
'Gut microbiota abundance (genus Parasutterella id.2892)' on 'Cell surface glycoprotein CD200 receptor 2'

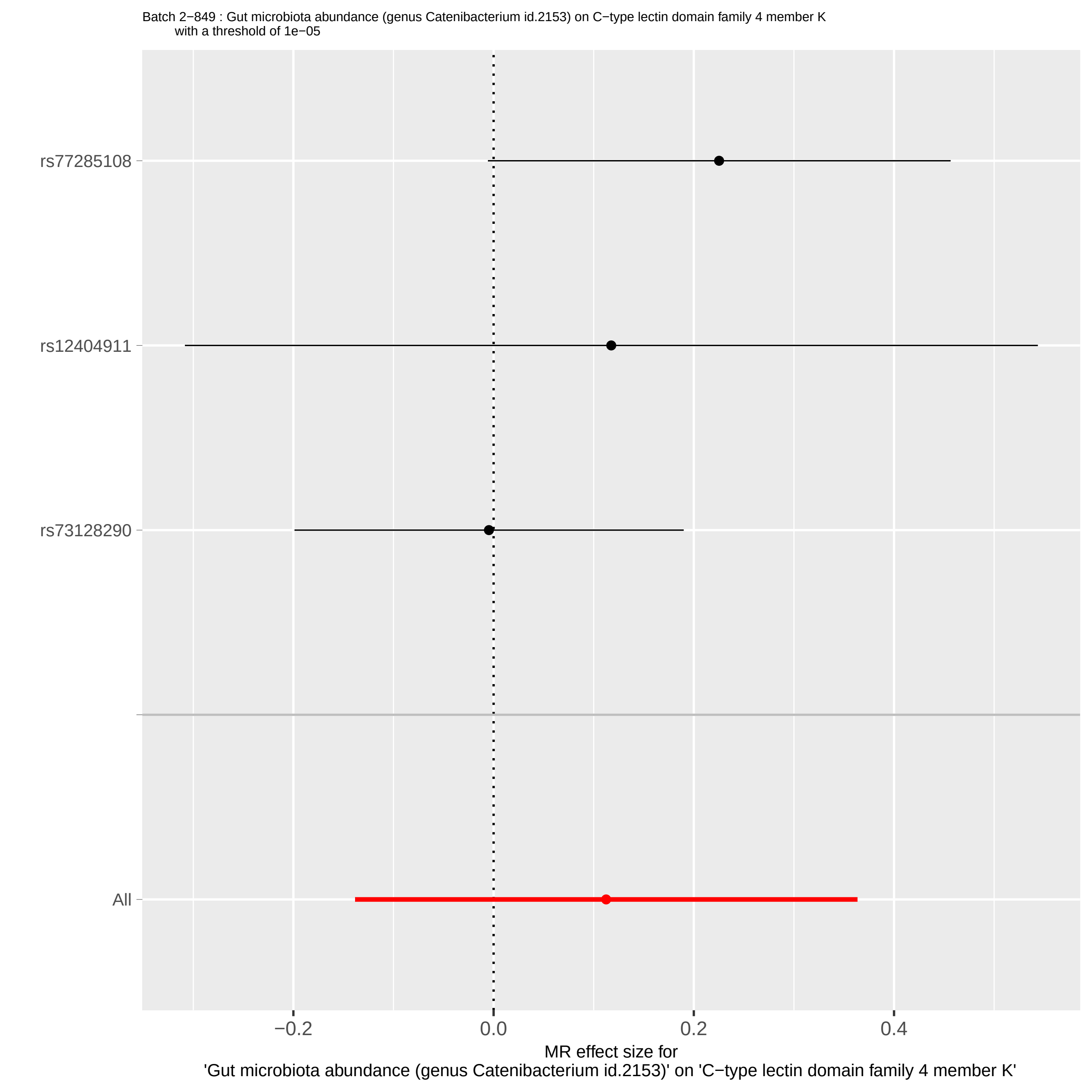
Batch 2−849 : Gut microbiota abundance (genus Catenibacterium id.2153) on C−type lectin domain family 4 member K
with a threshold of 1e−05
rs77285108
rs12404911
rs73128290
All
−0.2
0.0
0.2
0.4
MR effect size for
'Gut microbiota abundance (genus Catenibacterium id.2153)' on 'C−type lectin domain family 4 member K'

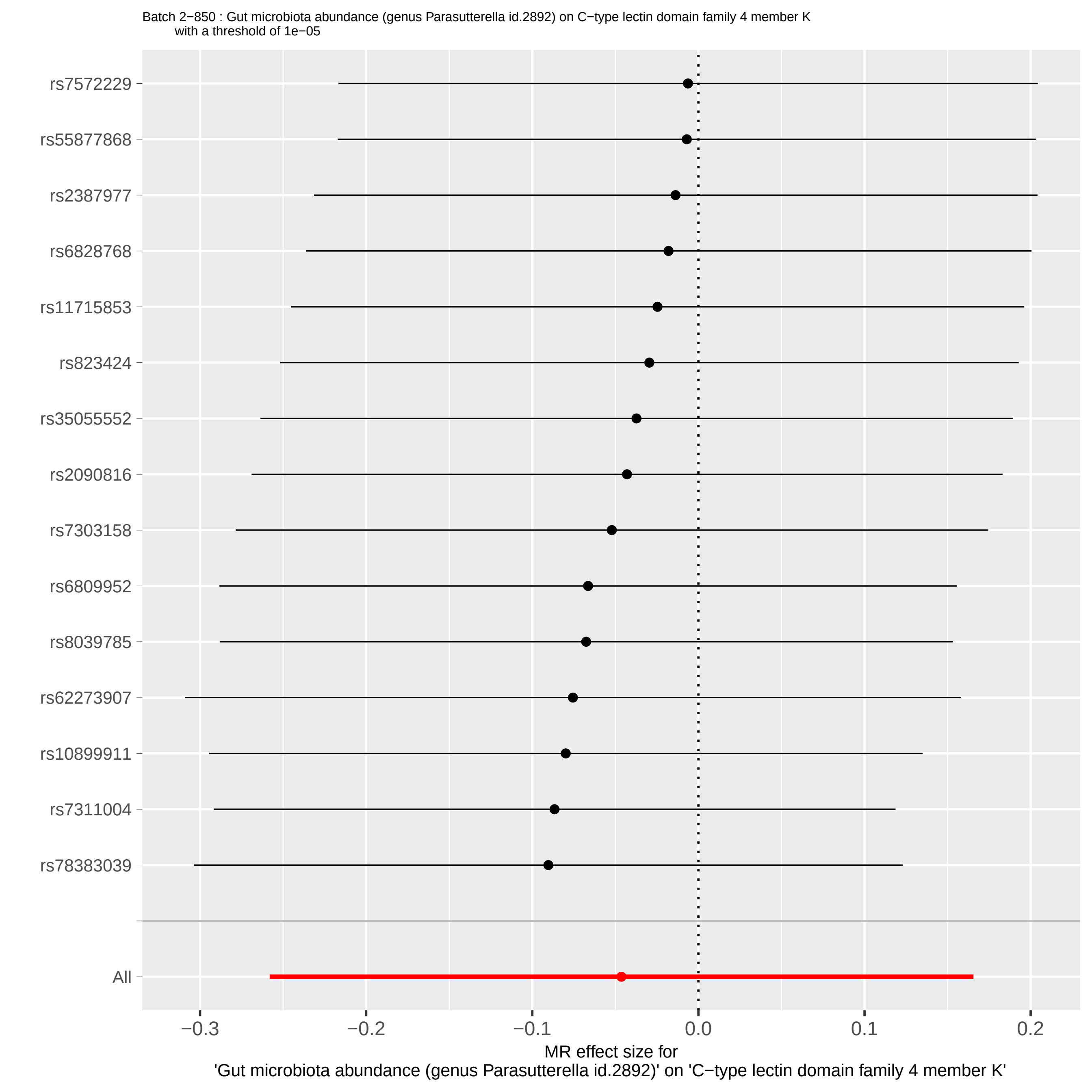

Batch 2−850 : Gut microbiota abundance (genus Parasutterella id.2892) on C−type lectin domain family 4 member K
with a threshold of 1e−05
rs7572229
rs55877868
rs2387977
rs6828768
rs11715853
rs823424
rs35055552
rs2090816
rs7303158
rs6809952
rs8039785
rs62273907
rs10899911
rs7311004
rs78383039
All
−0.3
−0.2
−0.1
0.0
0.1
0.2
MR effect size for
'Gut microbiota abundance (genus Parasutterella id.2892)' on 'C−type lectin domain family 4 member K'

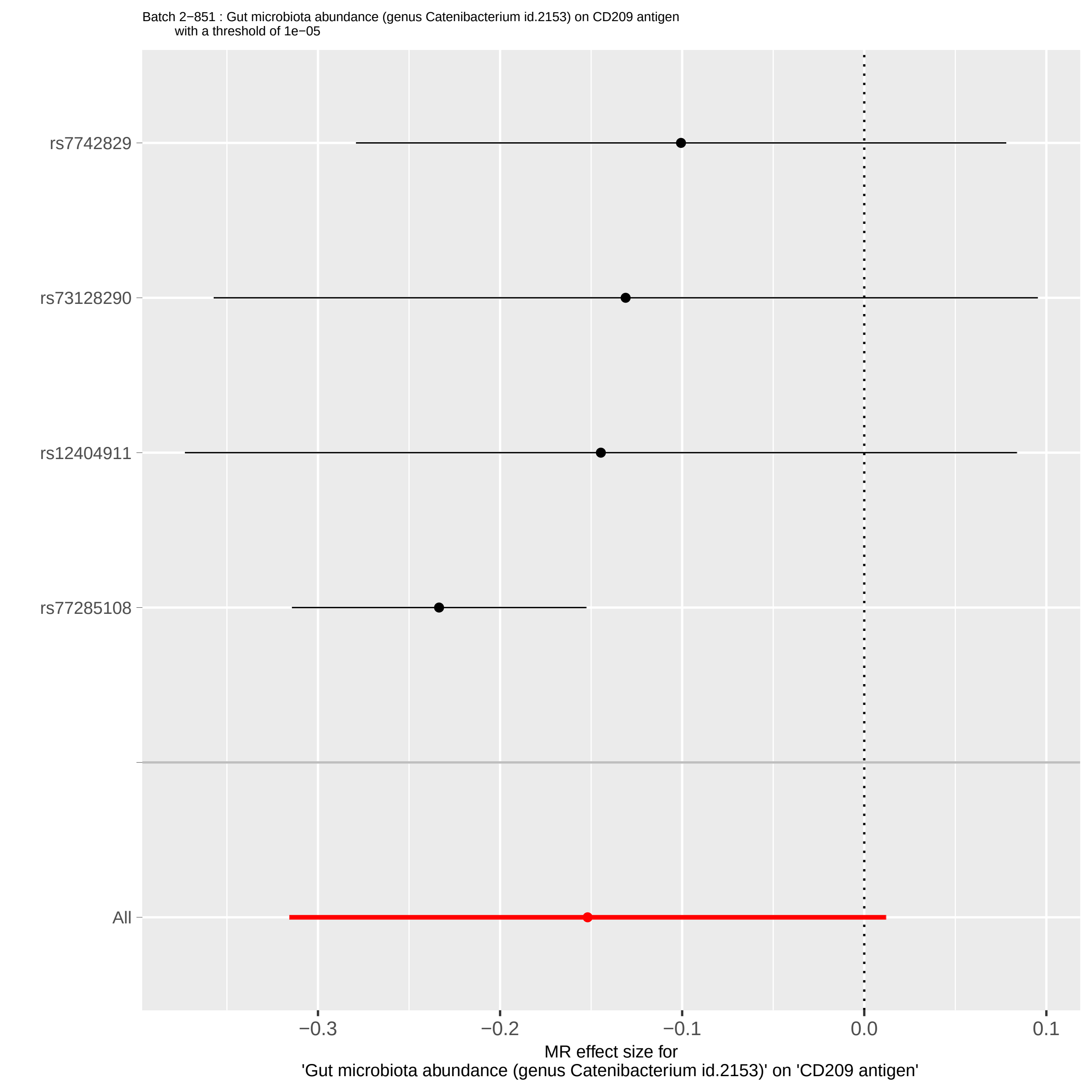

Batch 2−851 : Gut microbiota abundance (genus Catenibacterium id.2153) on CD209 antigen
with a threshold of 1e−05
rs7742829
rs73128290
rs12404911
rs77285108
All
−0.3
−0.2
−0.1
0.0
0.1
MR effect size for
'Gut microbiota abundance (genus Catenibacterium id.2153)' on 'CD209 antigen'

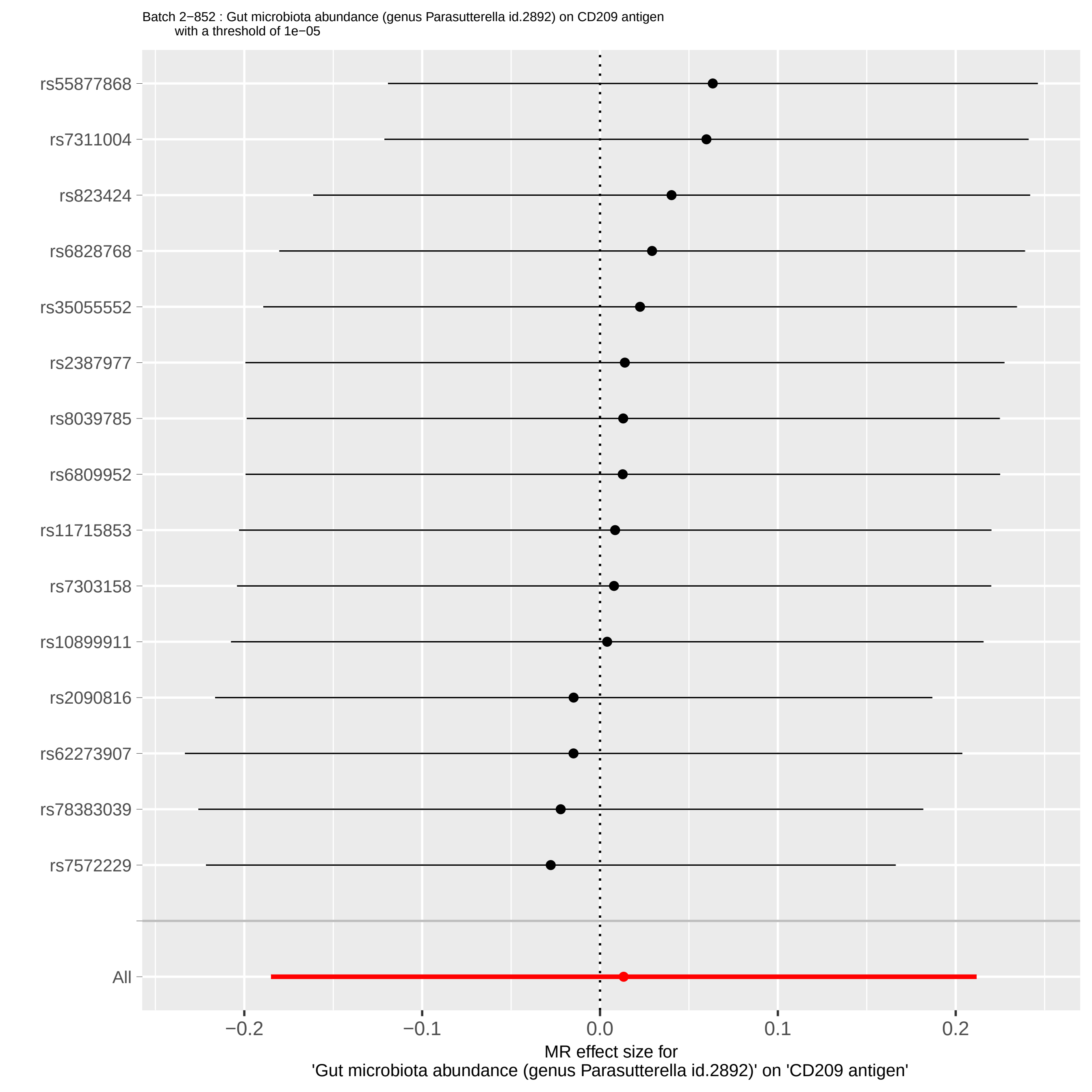

Batch 2−852 : Gut microbiota abundance (genus Parasutterella id.2892) on CD209 antigen
with a threshold of 1e−05
rs55877868
rs7311004
rs823424
rs6828768
rs35055552
rs2387977
rs8039785
rs6809952
rs11715853
rs7303158
rs10899911
rs2090816
rs62273907
rs78383039
rs7572229
All
−0.2
−0.1
0.0
0.1
0.2
MR effect size for
'Gut microbiota abundance (genus Parasutterella id.2892)' on 'CD209 antigen'

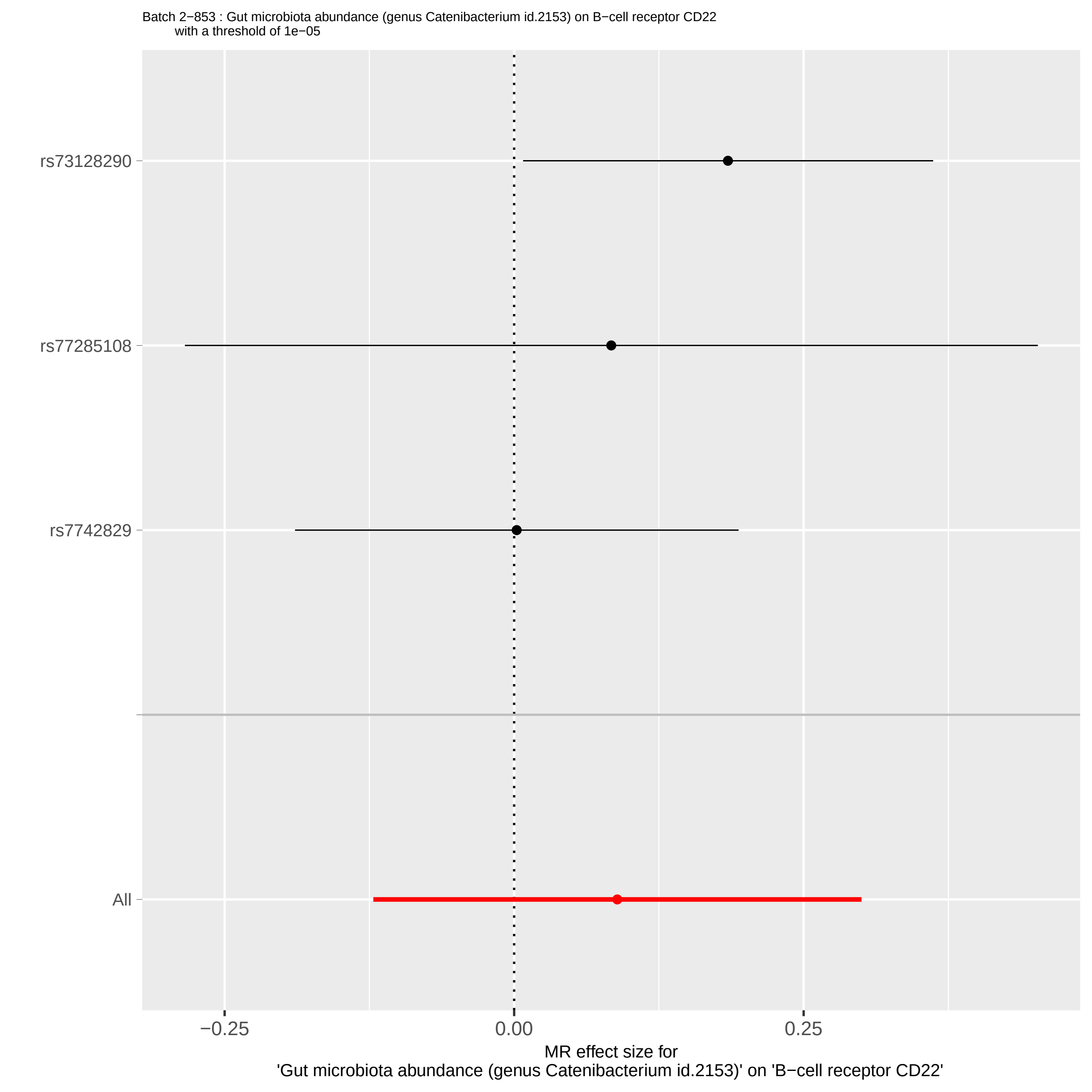

Batch 2−853 : Gut microbiota abundance (genus Catenibacterium id.2153) on B−cell receptor CD22
with a threshold of 1e−05
rs73128290
rs77285108
rs7742829
All
−0.25
0.00
0.25
MR effect size for
'Gut microbiota abundance (genus Catenibacterium id.2153)' on 'B−cell receptor CD22'

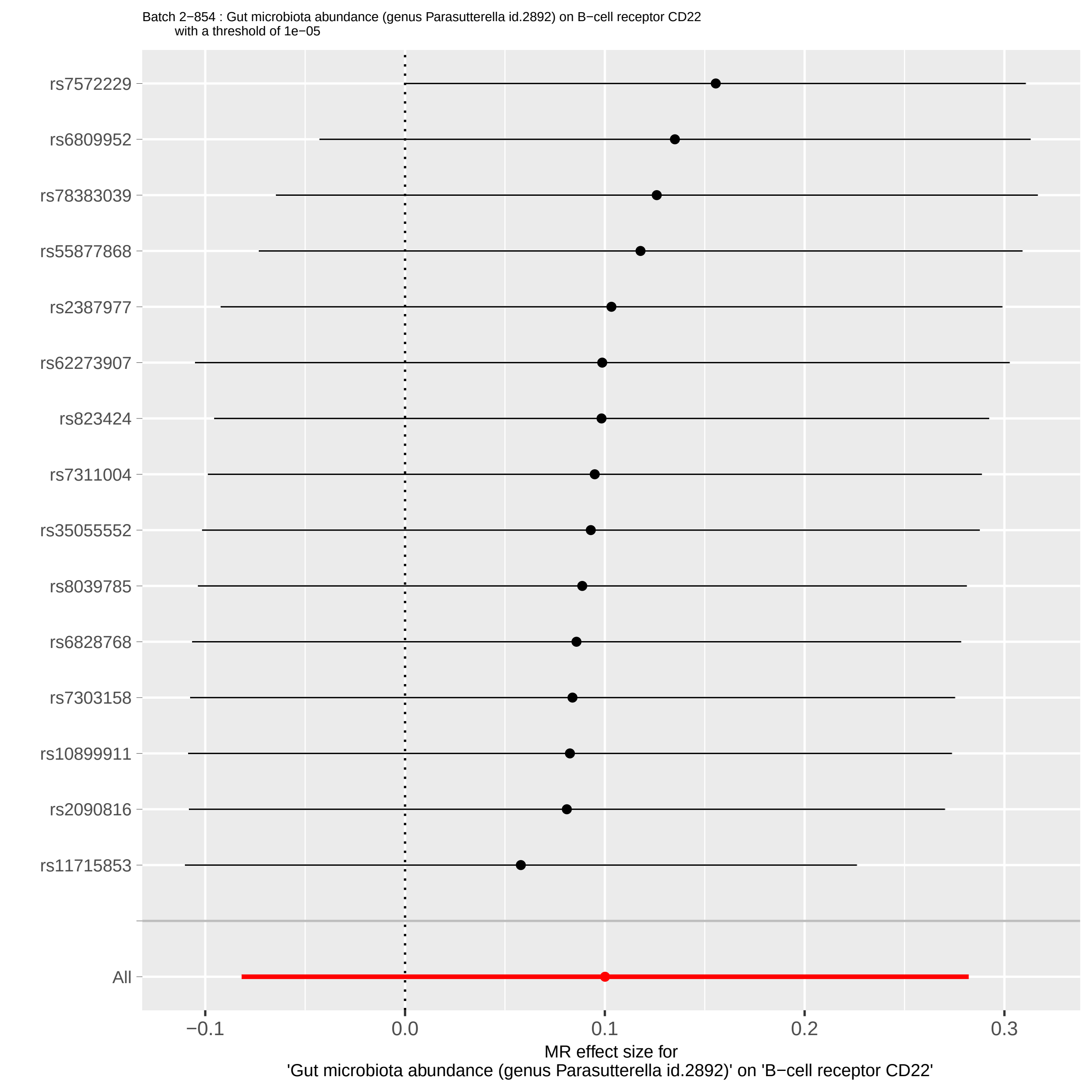
Batch 2−854 : Gut microbiota abundance (genus Parasutterella id.2892) on B−cell receptor CD22
with a threshold of 1e−05
rs7572229
rs6809952
rs78383039
rs55877868
rs2387977
rs62273907
rs823424
rs7311004
rs35055552
rs8039785
rs6828768
rs7303158
rs10899911
rs2090816
rs11715853
All
−0.1
0.0
0.1
0.2
0.3
MR effect size for
'Gut microbiota abundance (genus Parasutterella id.2892)' on 'B−cell receptor CD22'

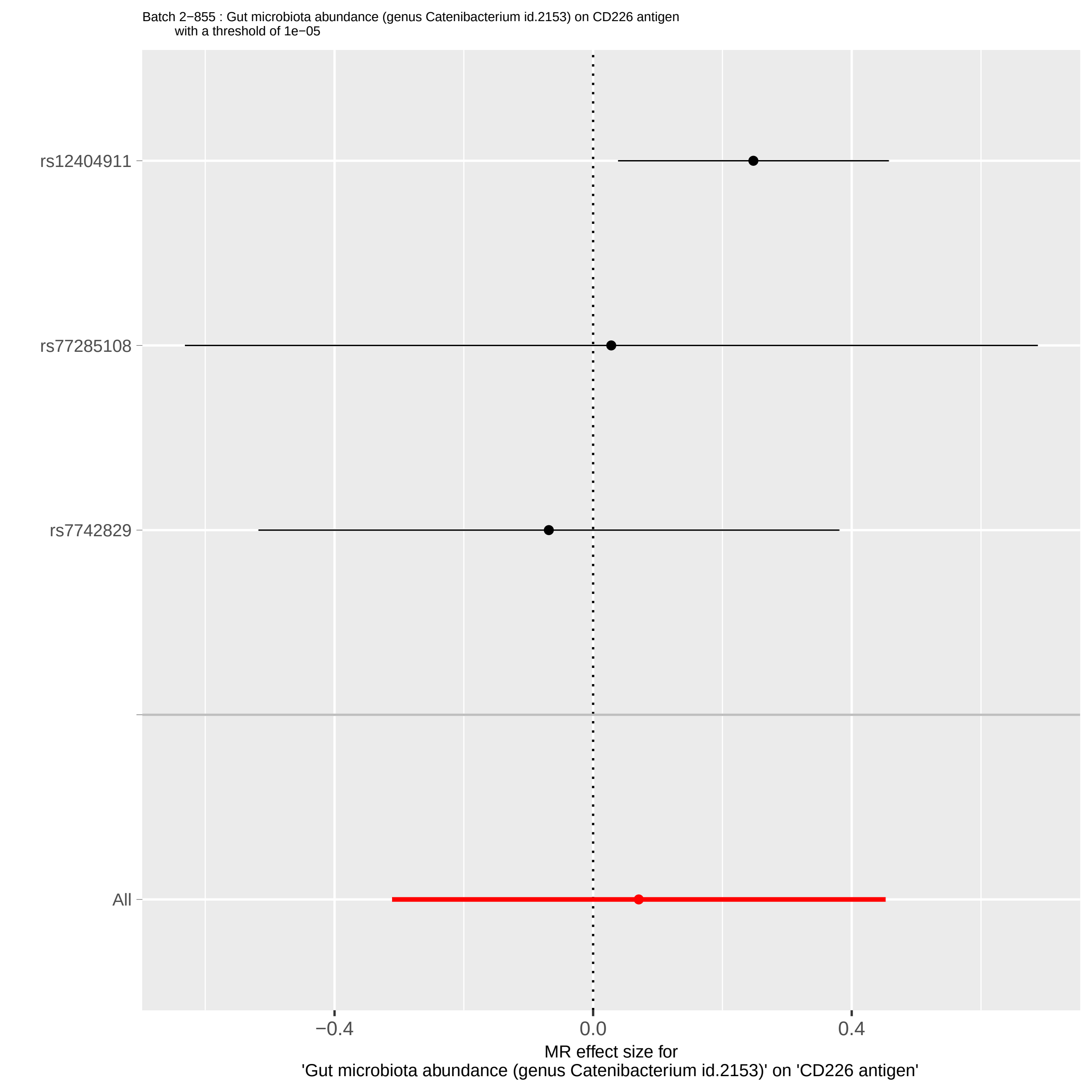

Batch 2−855 : Gut microbiota abundance (genus Catenibacterium id.2153) on CD226 antigen
with a threshold of 1e−05
rs12404911
rs77285108
rs7742829
All
−0.4
0.0
0.4
MR effect size for
'Gut microbiota abundance (genus Catenibacterium id.2153)' on 'CD226 antigen'

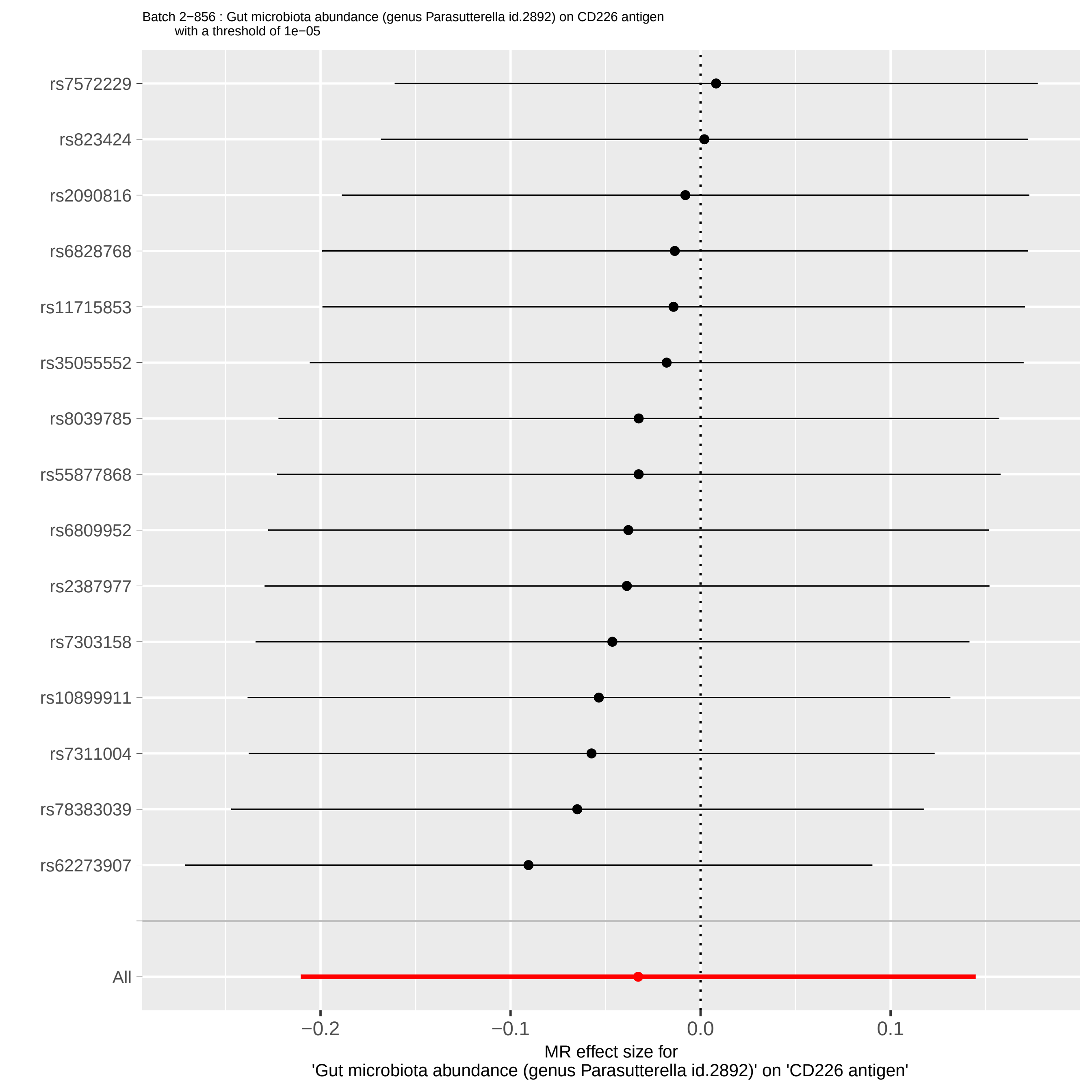
Batch 2−856 : Gut microbiota abundance (genus Parasutterella id.2892) on CD226 antigen
with a threshold of 1e−05
rs7572229
rs823424
rs2090816
rs6828768
rs11715853
rs35055552
rs8039785
rs55877868
rs6809952
rs2387977
rs7303158
rs10899911
rs7311004
rs78383039
rs62273907
All
−0.2
−0.1
0.0
0.1
MR effect size for
'Gut microbiota abundance (genus Parasutterella id.2892)' on 'CD226 antigen'

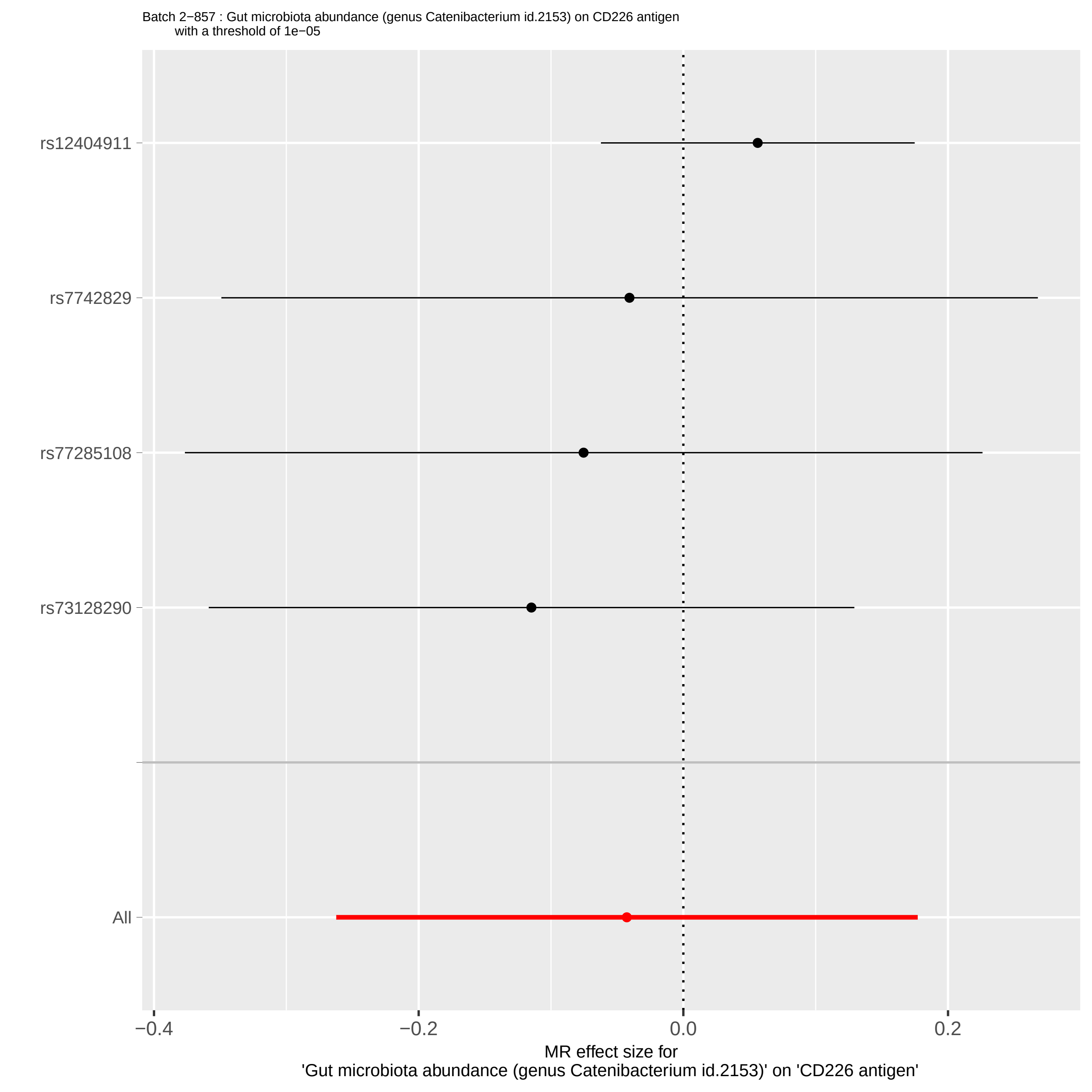

Batch 2−857 : Gut microbiota abundance (genus Catenibacterium id.2153) on CD226 antigen
with a threshold of 1e−05
rs12404911
rs7742829
rs77285108
rs73128290
All
−0.4
−0.2
0.0
0.2
MR effect size for
'Gut microbiota abundance (genus Catenibacterium id.2153)' on 'CD226 antigen'

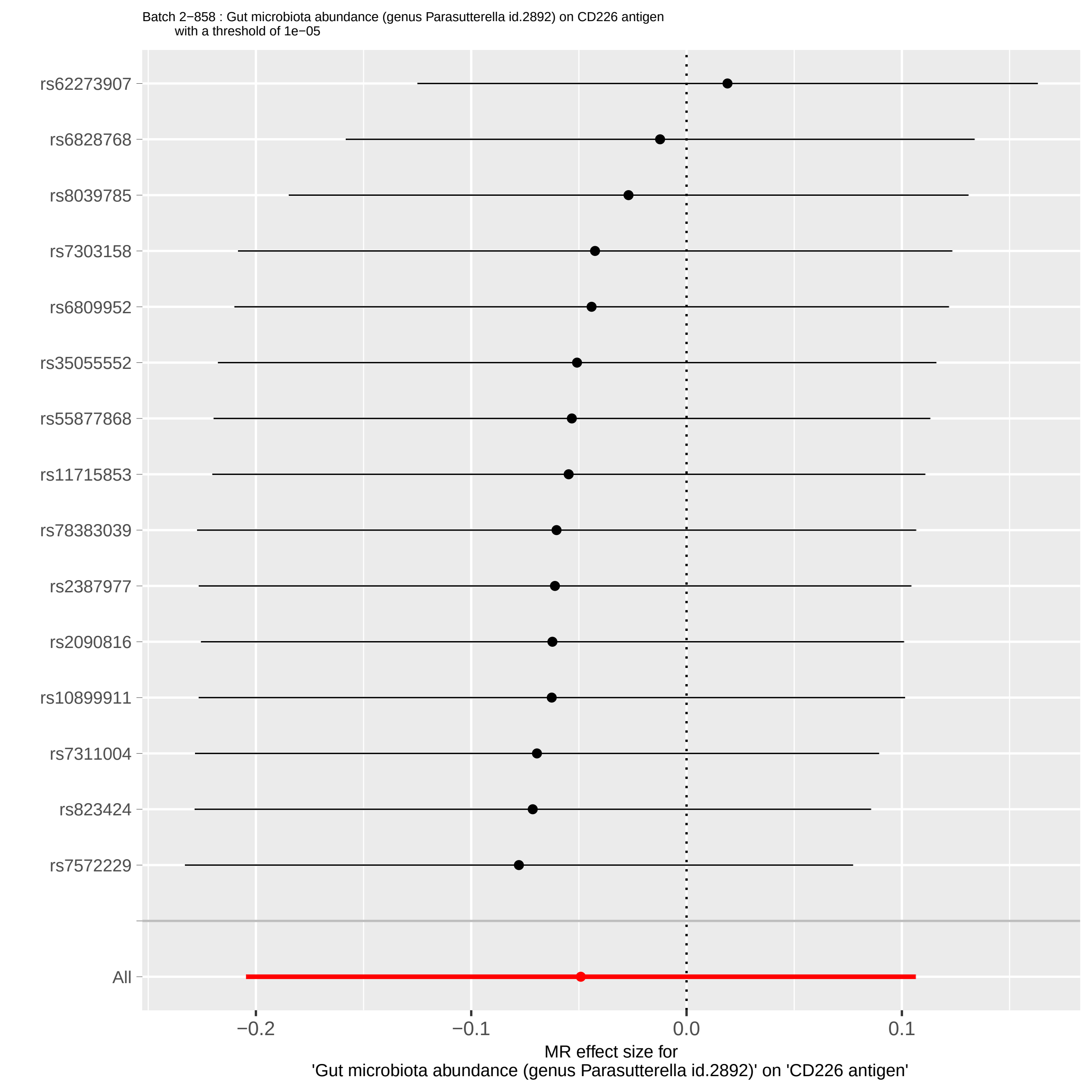

Batch 2−858 : Gut microbiota abundance (genus Parasutterella id.2892) on CD226 antigen
with a threshold of 1e−05
rs62273907
rs6828768
rs8039785
rs7303158
rs6809952
rs35055552
rs55877868
rs11715853
rs78383039
rs2387977
rs2090816
rs10899911
rs7311004
rs823424
rs7572229
All
−0.2
−0.1
0.0
0.1
MR effect size for
'Gut microbiota abundance (genus Parasutterella id.2892)' on 'CD226 antigen'

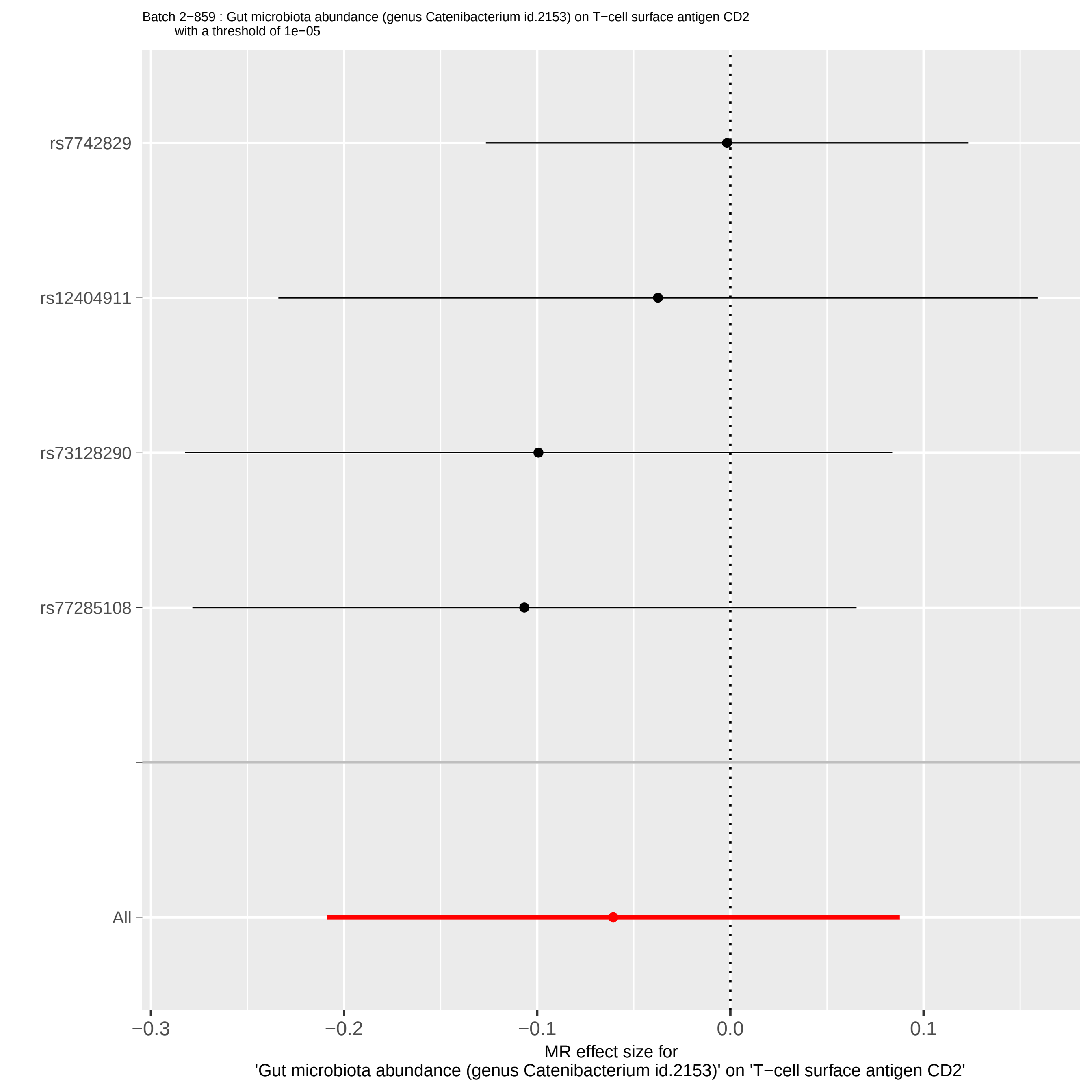

Batch 2−859 : Gut microbiota abundance (genus Catenibacterium id.2153) on T−cell surface antigen CD2
with a threshold of 1e−05
rs7742829
rs12404911
rs73128290
rs77285108
All
−0.3
−0.2
−0.1
0.0
0.1
MR effect size for
'Gut microbiota abundance (genus Catenibacterium id.2153)' on 'T−cell surface antigen CD2'

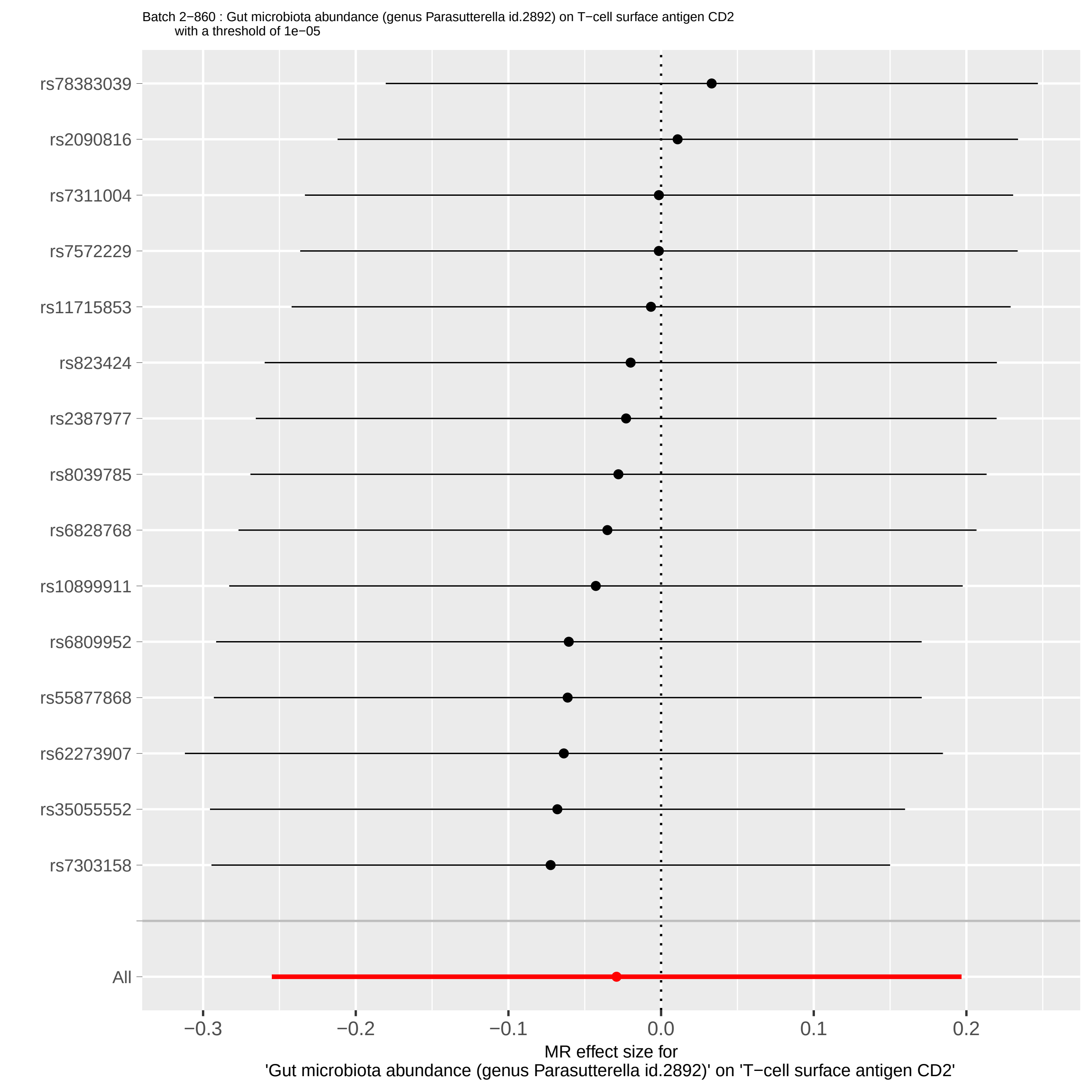

Batch 2−860 : Gut microbiota abundance (genus Parasutterella id.2892) on T−cell surface antigen CD2
with a threshold of 1e−05
rs78383039
rs2090816
rs7311004
rs7572229
rs11715853
rs823424
rs2387977
rs8039785
rs6828768
rs10899911
rs6809952
rs55877868
rs62273907
rs35055552
rs7303158
All
−0.3
−0.2
−0.1
0.0
0.1
0.2
MR effect size for
'Gut microbiota abundance (genus Parasutterella id.2892)' on 'T−cell surface antigen CD2'

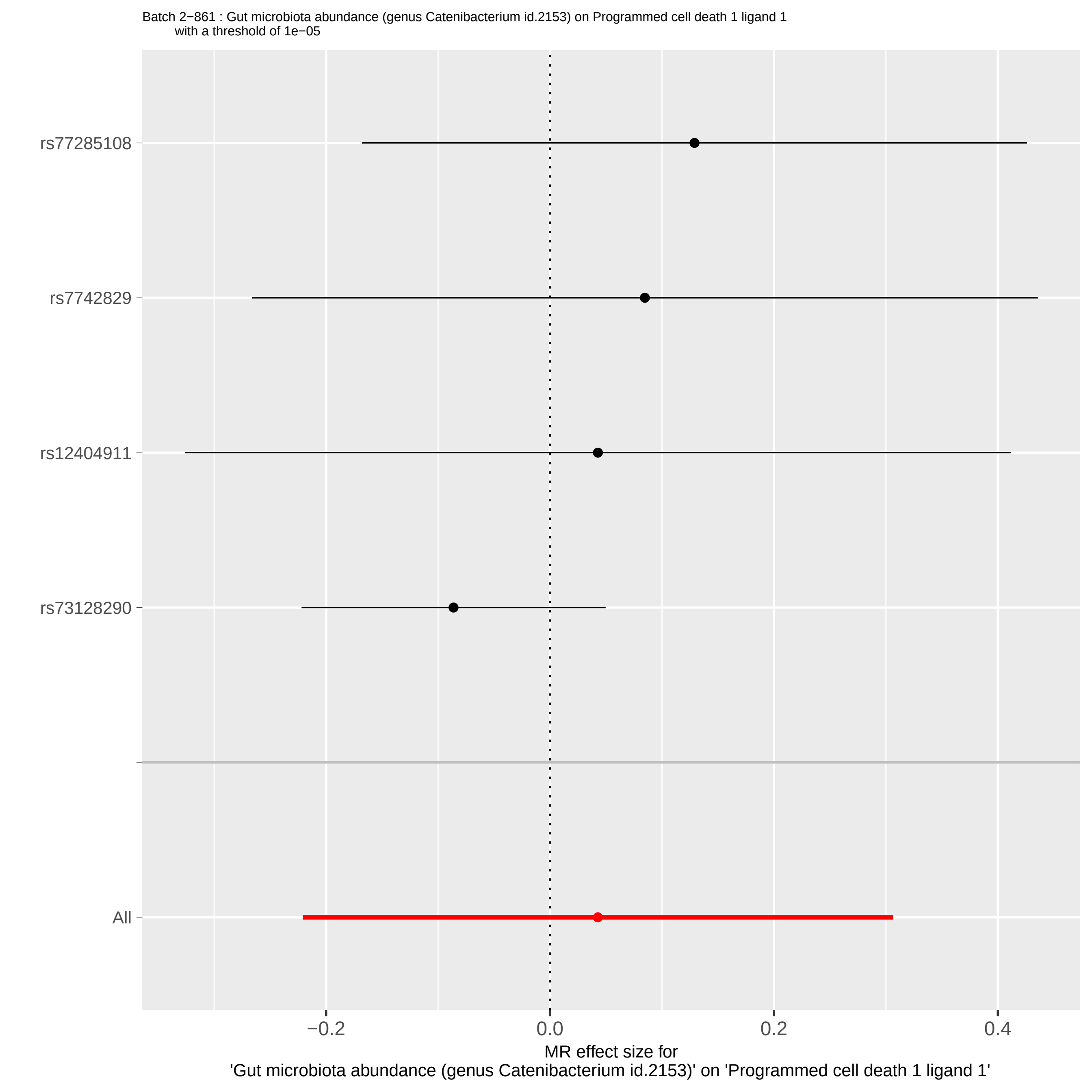
Batch 2−861 : Gut microbiota abundance (genus Catenibacterium id.2153) on Programmed cell death 1 ligand 1
with a threshold of 1e−05
rs77285108
rs7742829
rs12404911
rs73128290
All
−0.2
0.0
0.2
0.4
MR effect size for
'Gut microbiota abundance (genus Catenibacterium id.2153)' on 'Programmed cell death 1 ligand 1'

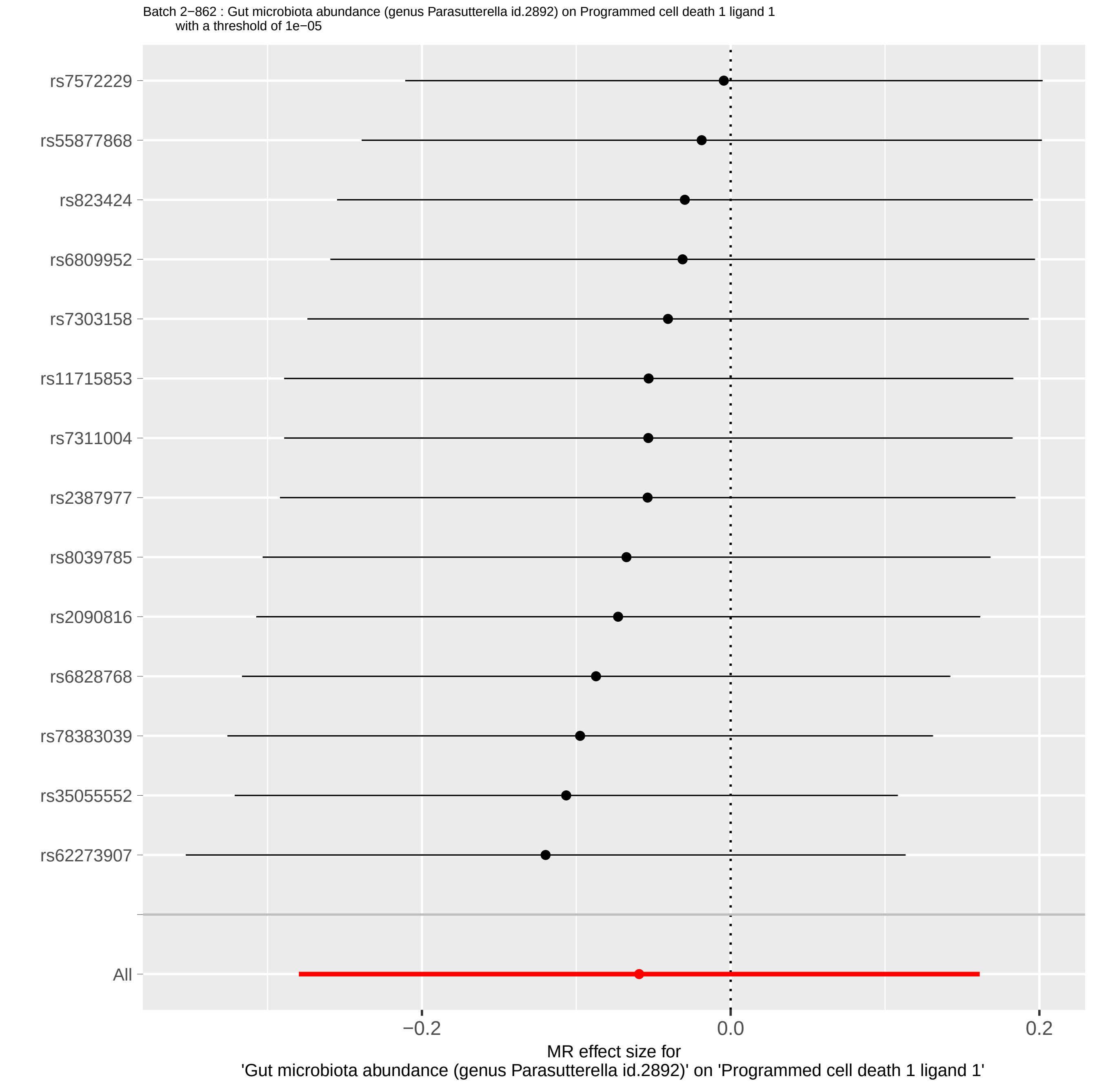
Batch 2−862 : Gut microbiota abundance (genus Parasutterella id.2892) on Programmed cell death 1 ligand 1
with a threshold of 1e−05
rs7572229
rs55877868
rs823424
rs6809952
rs7303158
rs11715853
rs7311004
rs2387977
rs8039785
rs2090816
rs6828768
rs78383039
rs35055552
rs62273907
All
−0.2
0.0
0.2
MR effect size for
'Gut microbiota abundance (genus Parasutterella id.2892)' on 'Programmed cell death 1 ligand 1'

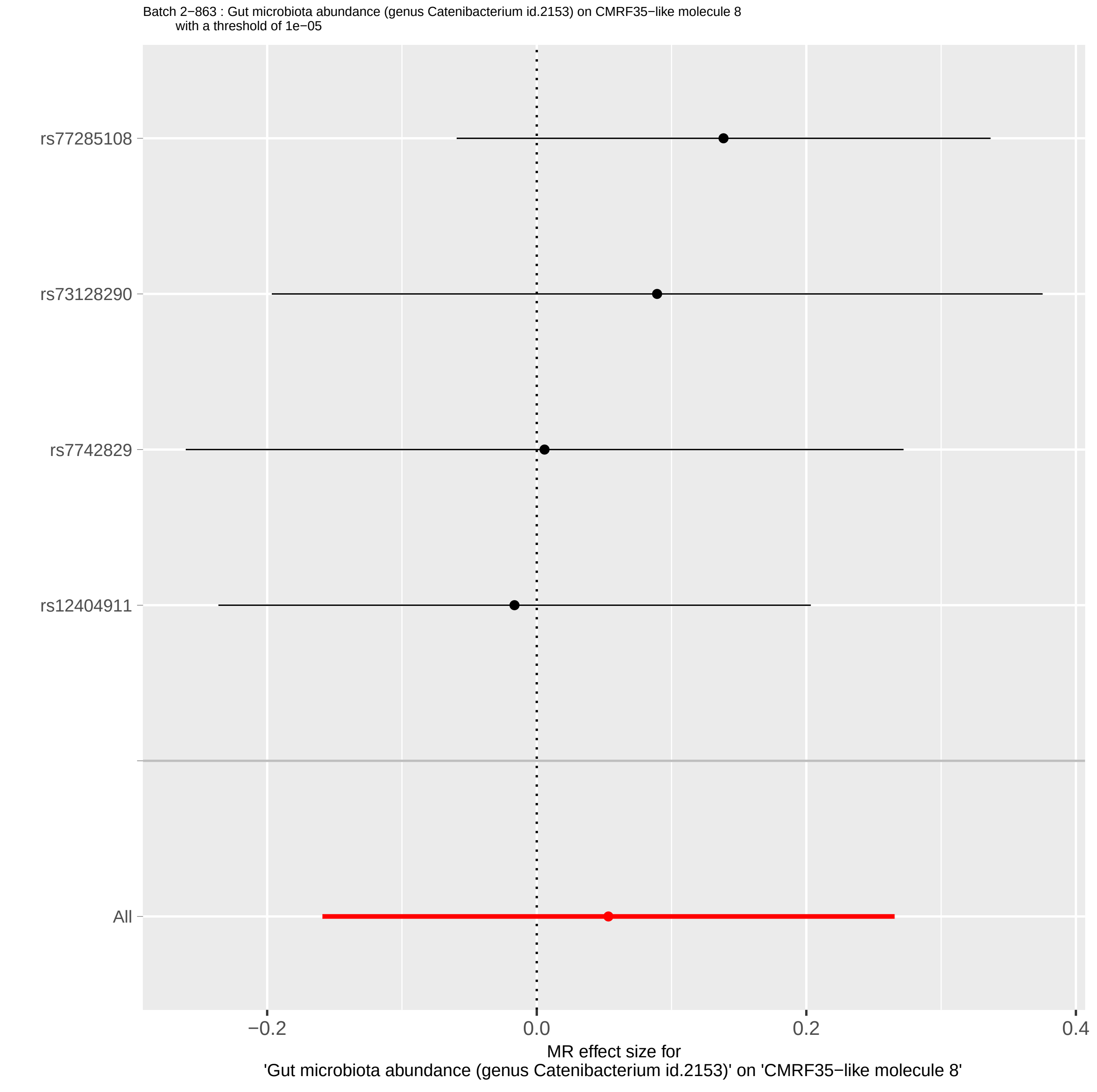
Batch 2−863 : Gut microbiota abundance (genus Catenibacterium id.2153) on CMRF35−like molecule 8
with a threshold of 1e−05
rs77285108
rs73128290
rs7742829
rs12404911
All
−0.2
0.0
0.2
0.4
MR effect size for
'Gut microbiota abundance (genus Catenibacterium id.2153)' on 'CMRF35−like molecule 8'

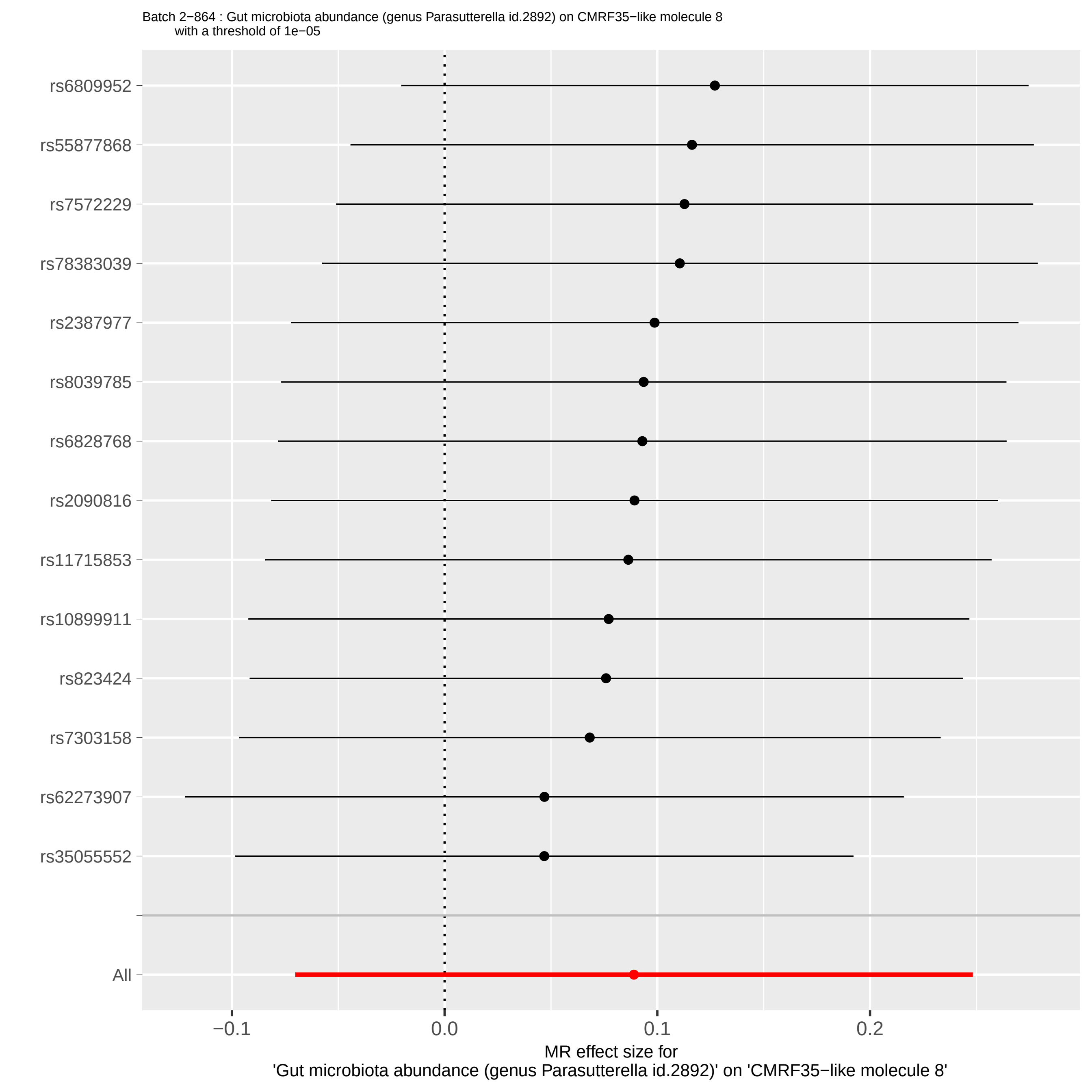

Batch 2−864 : Gut microbiota abundance (genus Parasutterella id.2892) on CMRF35−like molecule 8
with a threshold of 1e−05
rs6809952
rs55877868
rs7572229
rs78383039
rs2387977
rs8039785
rs6828768
rs2090816
rs11715853
rs10899911
rs823424
rs7303158
rs62273907
rs35055552
All
−0.1
0.0
0.1
0.2
MR effect size for
'Gut microbiota abundance (genus Parasutterella id.2892)' on 'CMRF35−like molecule 8'

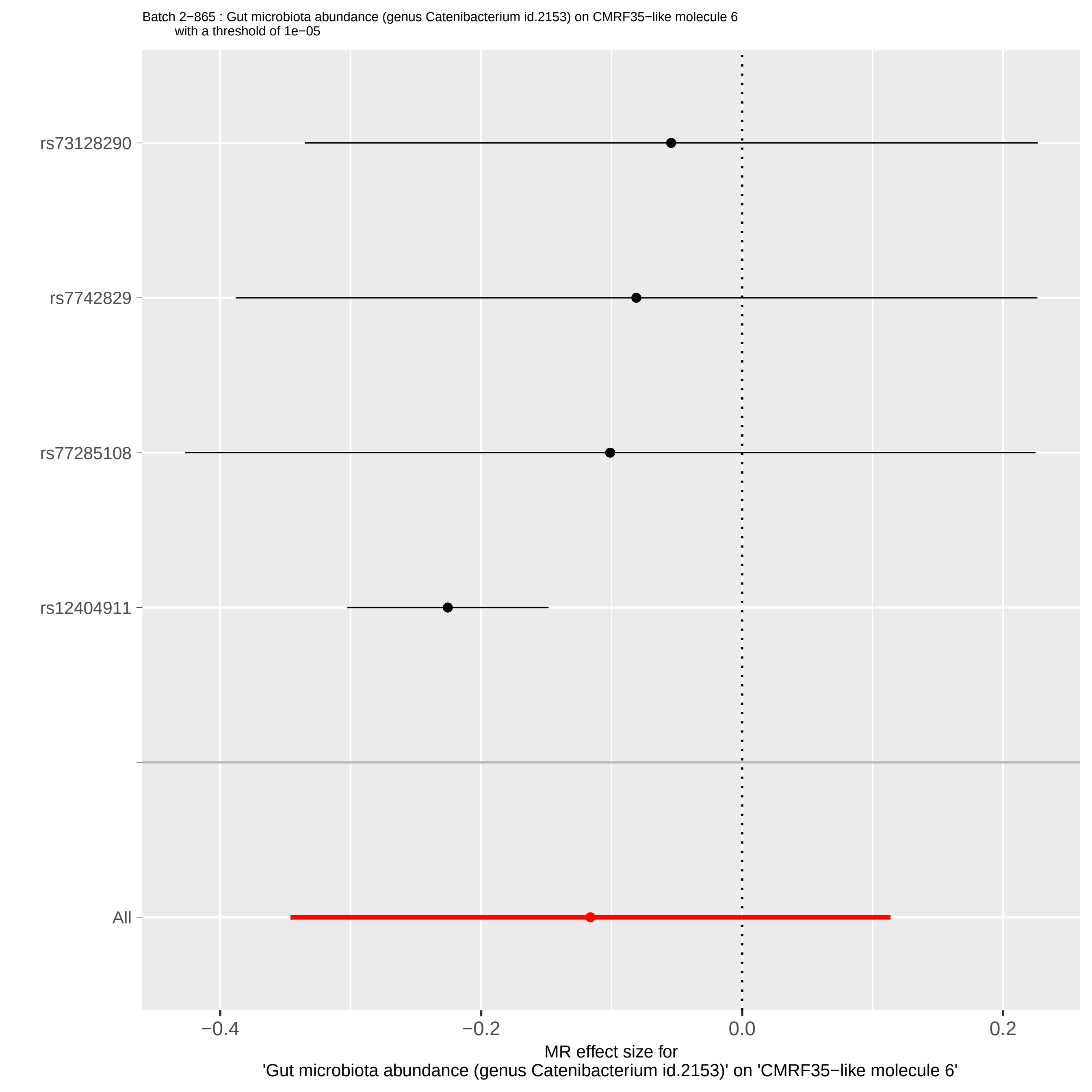
Batch 2−865 : Gut microbiota abundance (genus Catenibacterium id.2153) on CMRF35−like molecule 6
with a threshold of 1e−05
rs73128290
rs7742829
rs77285108
rs12404911
All
−0.4
−0.2
0.0
0.2
MR effect size for
'Gut microbiota abundance (genus Catenibacterium id.2153)' on 'CMRF35−like molecule 6'

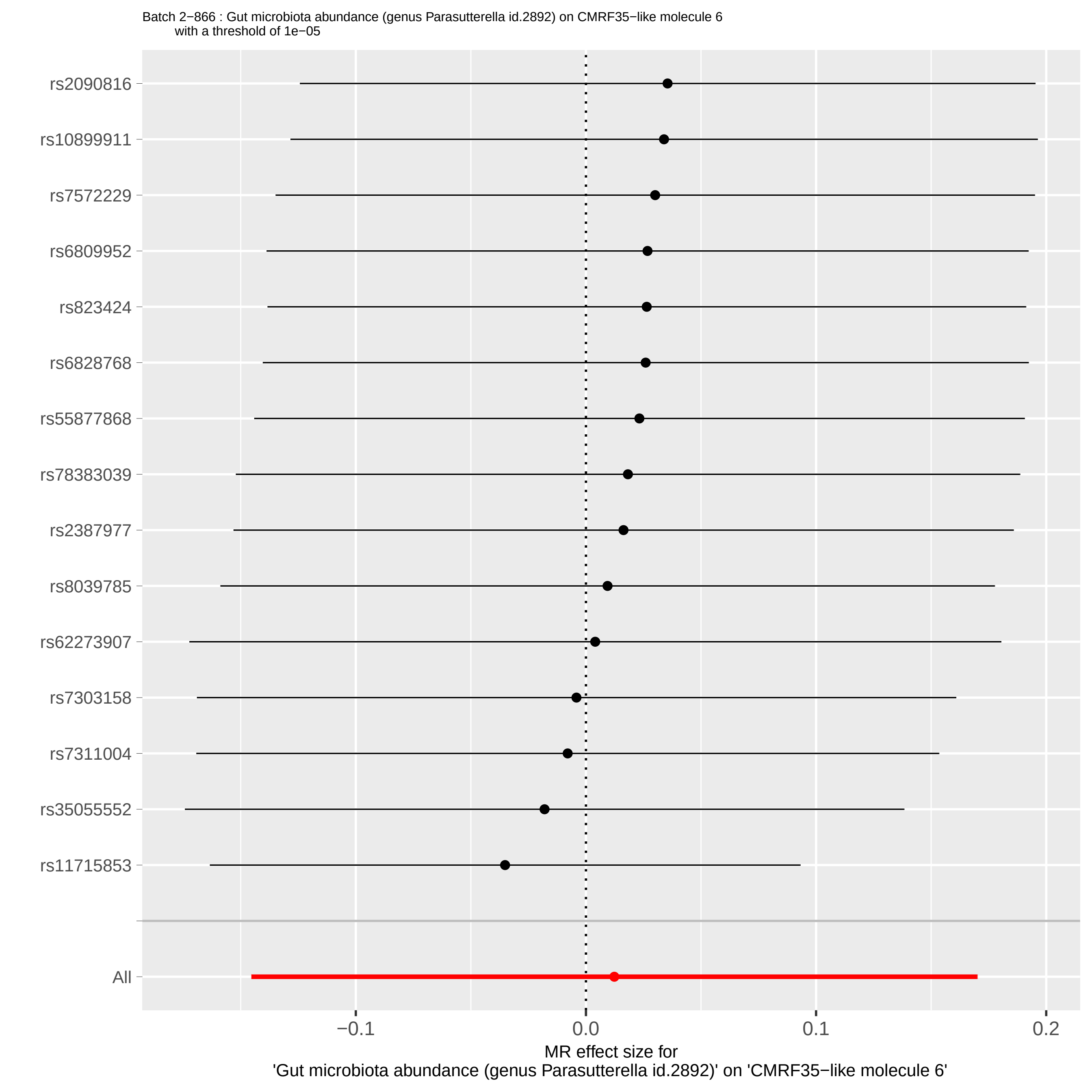

Batch 2−866 : Gut microbiota abundance (genus Parasutterella id.2892) on CMRF35−like molecule 6
with a threshold of 1e−05
rs2090816
rs10899911
rs7572229
rs6809952
rs823424
rs6828768
rs55877868
rs78383039
rs2387977
rs8039785
rs62273907
rs7303158
rs7311004
rs35055552
rs11715853
All
−0.1
0.0
0.1
0.2
MR effect size for
'Gut microbiota abundance (genus Parasutterella id.2892)' on 'CMRF35−like molecule 6'

Batch 2−867 : Gut microbiota abundance (genus Catenibacterium id.2153) on CMRF35−like molecule 6
with a threshold of 1e−05

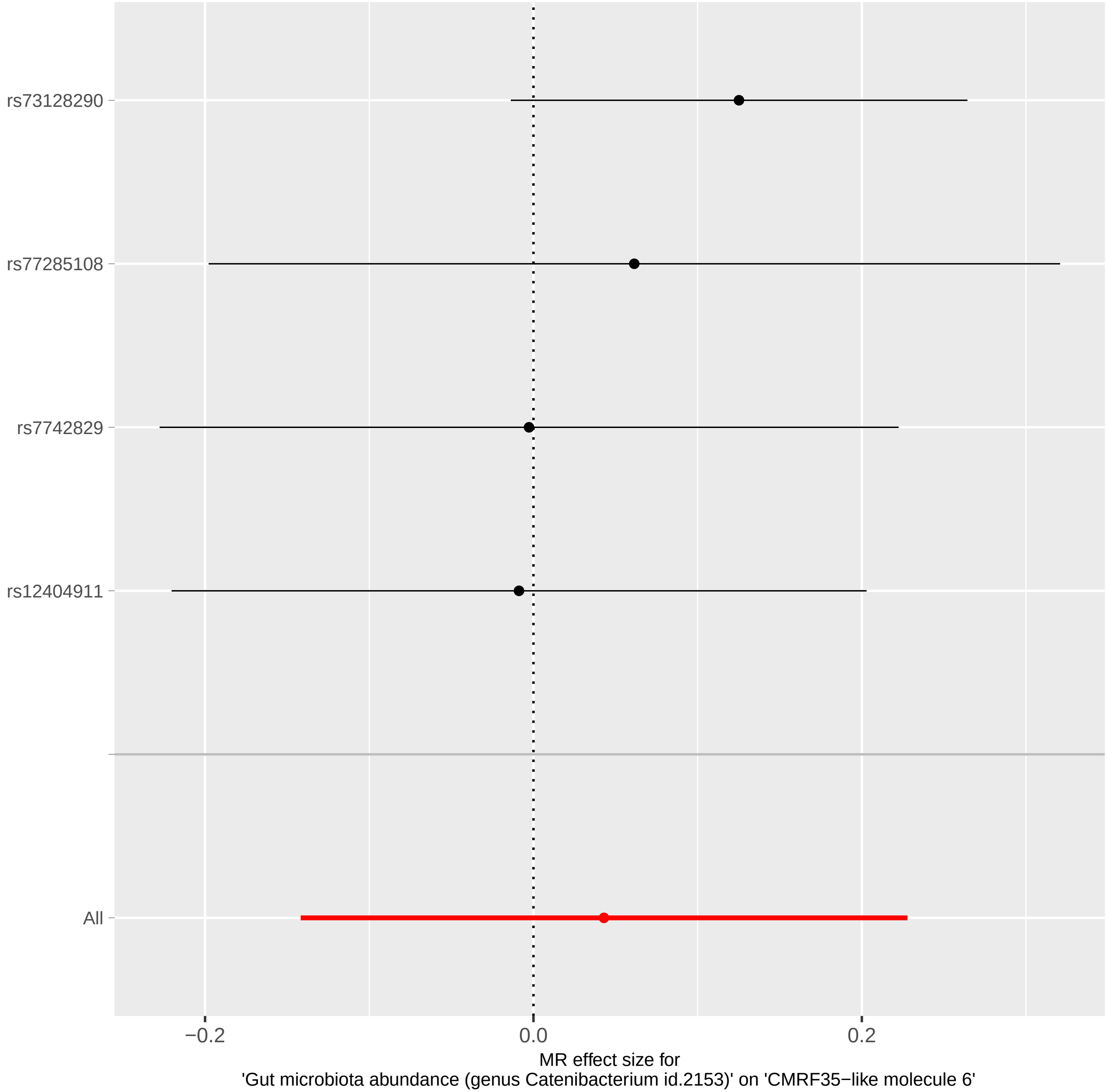

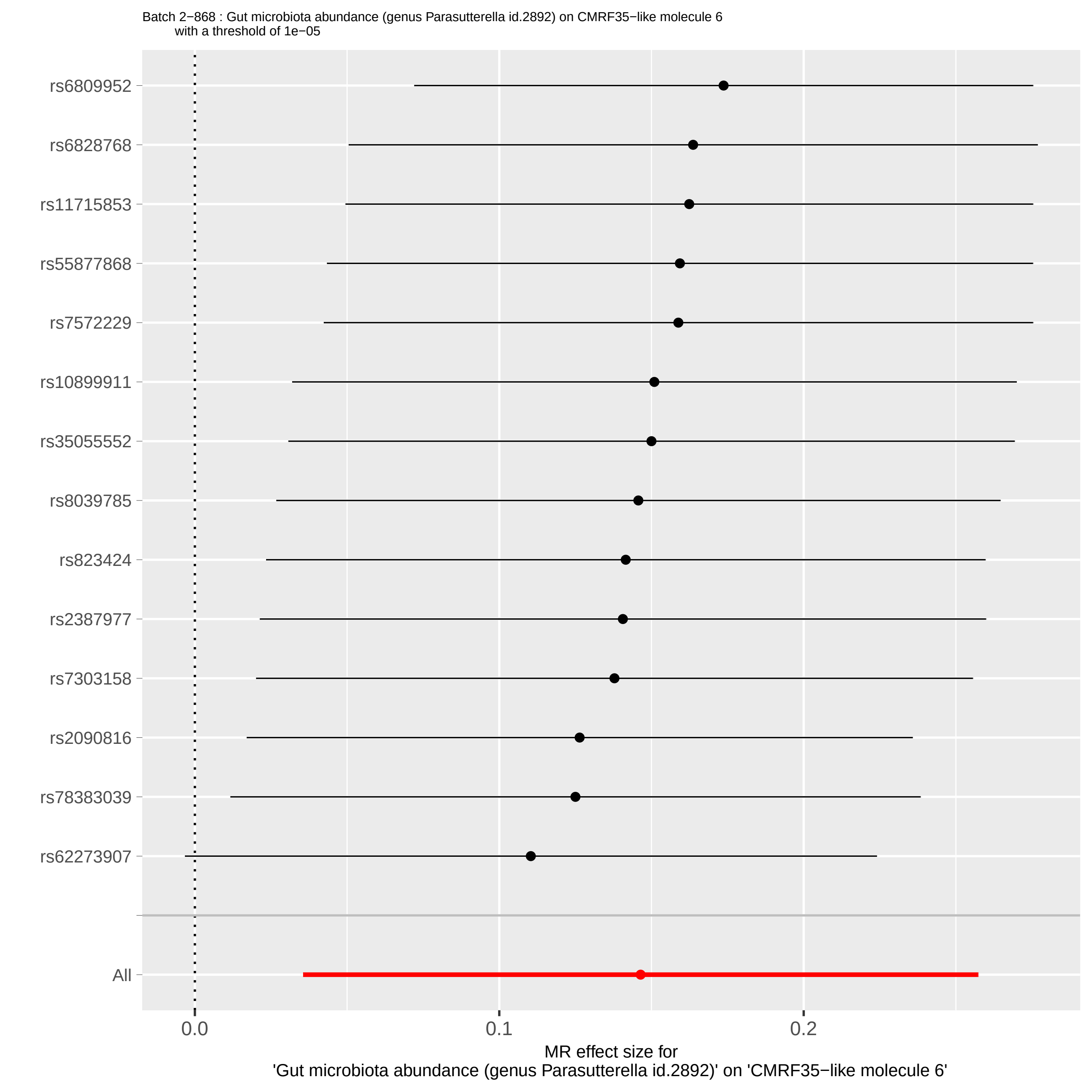
Batch 2−868 : Gut microbiota abundance (genus Parasutterella id.2892) on CMRF35−like molecule 6
with a threshold of 1e−05
rs6809952
rs6828768
rs11715853
rs55877868
rs7572229
rs10899911
rs35055552
rs8039785
rs823424
rs2387977
rs7303158
rs2090816
rs78383039
rs62273907
All
0.0
0.1
0.2
MR effect size for
'Gut microbiota abundance (genus Parasutterella id.2892)' on 'CMRF35−like molecule 6'

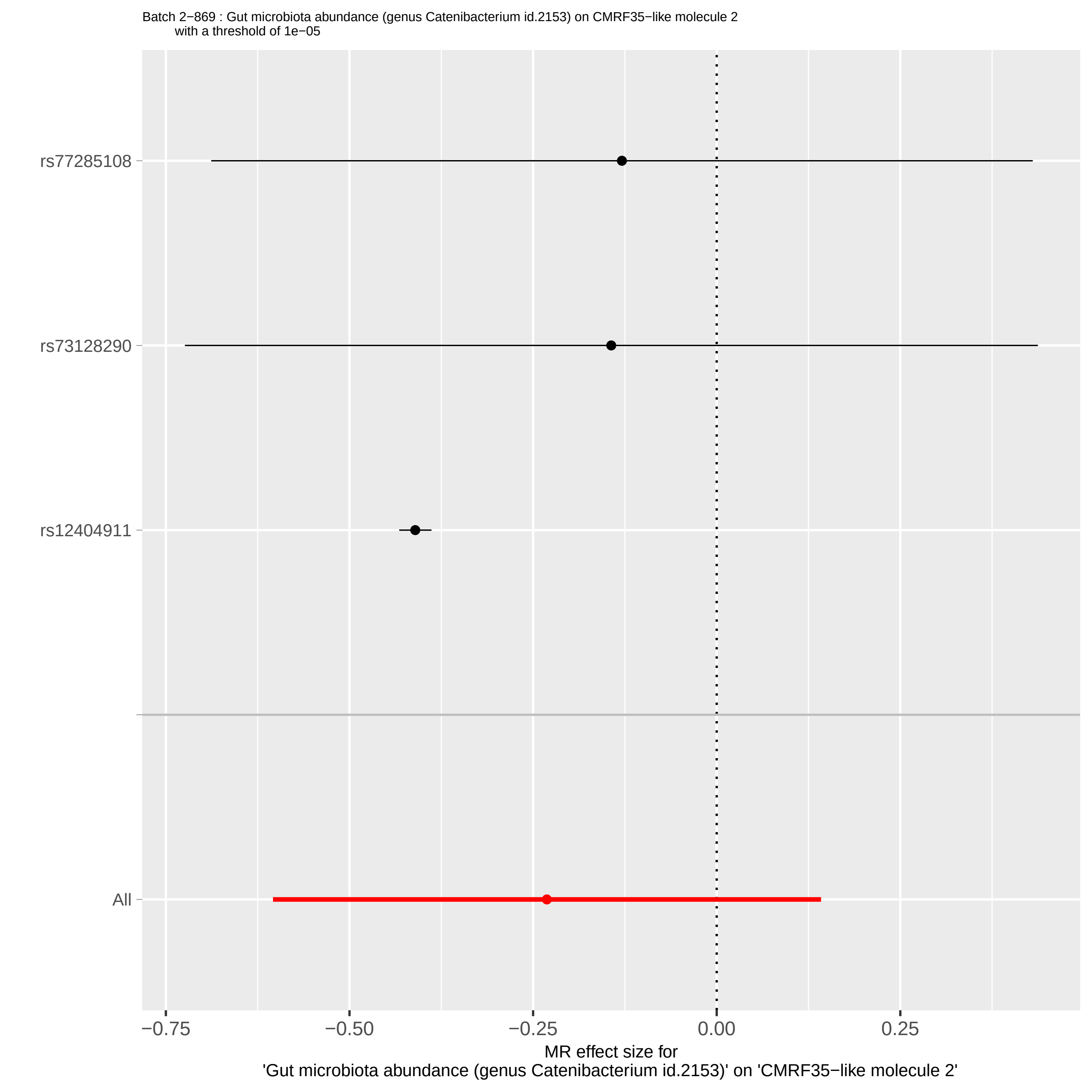
Batch 2−869 : Gut microbiota abundance (genus Catenibacterium id.2153) on CMRF35−like molecule 2
with a threshold of 1e−05
rs77285108
rs73128290
rs12404911
All
−0.75
−0.50
−0.25
0.00
0.25
MR effect size for
'Gut microbiota abundance (genus Catenibacterium id.2153)' on 'CMRF35−like molecule 2'

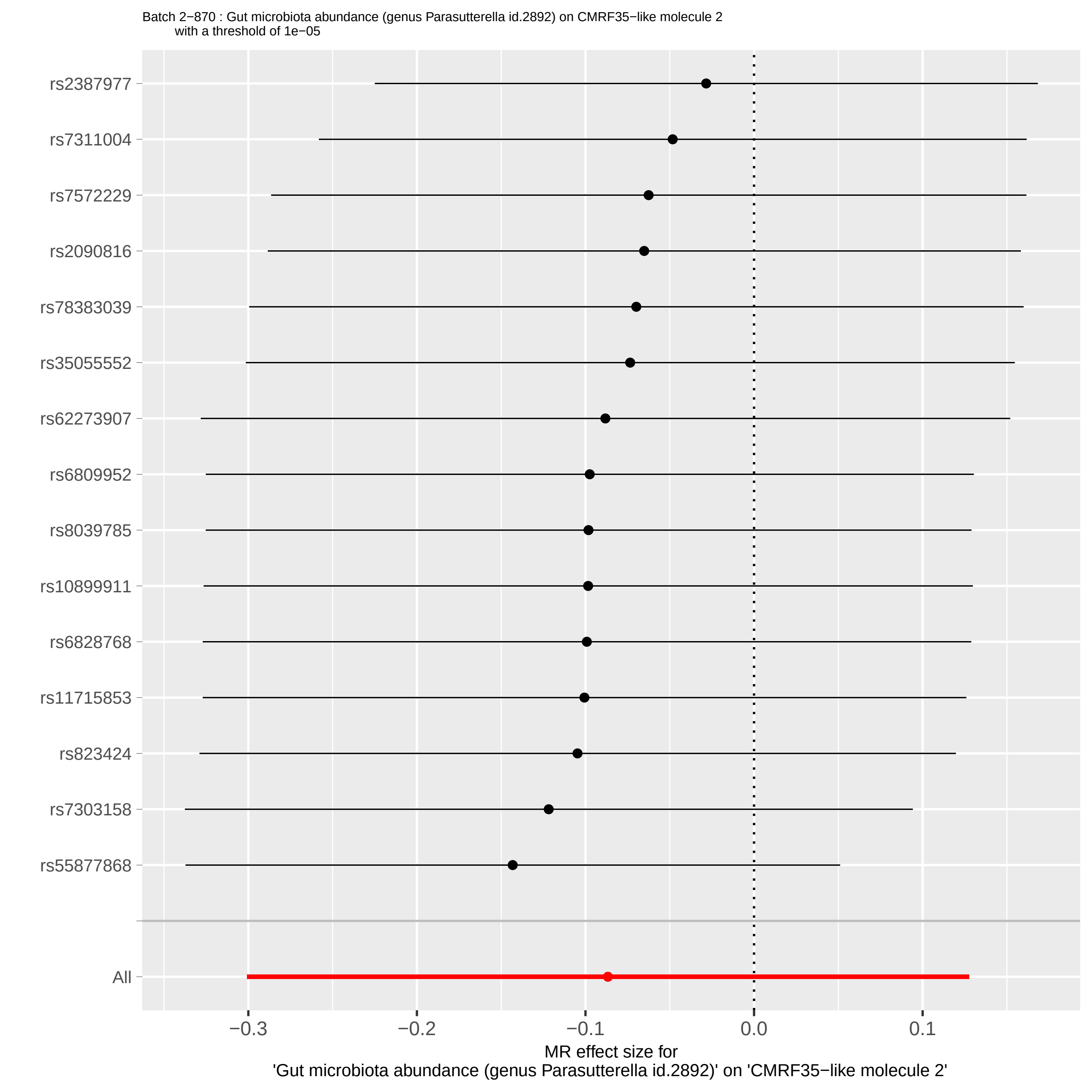

Batch 2−870 : Gut microbiota abundance (genus Parasutterella id.2892) on CMRF35−like molecule 2
with a threshold of 1e−05
rs2387977
rs7311004
rs7572229
rs2090816
rs78383039
rs35055552
rs62273907
rs6809952
rs8039785
rs10899911
rs6828768
rs11715853
rs823424
rs7303158
rs55877868
All
−0.3
−0.2
−0.1
0.0
0.1
MR effect size for
'Gut microbiota abundance (genus Parasutterella id.2892)' on 'CMRF35−like molecule 2'

Batch 2−871 : Gut microbiota abundance (genus Catenibacterium id.2153) on CMRF35−like molecule 7
with a threshold of 1e−05

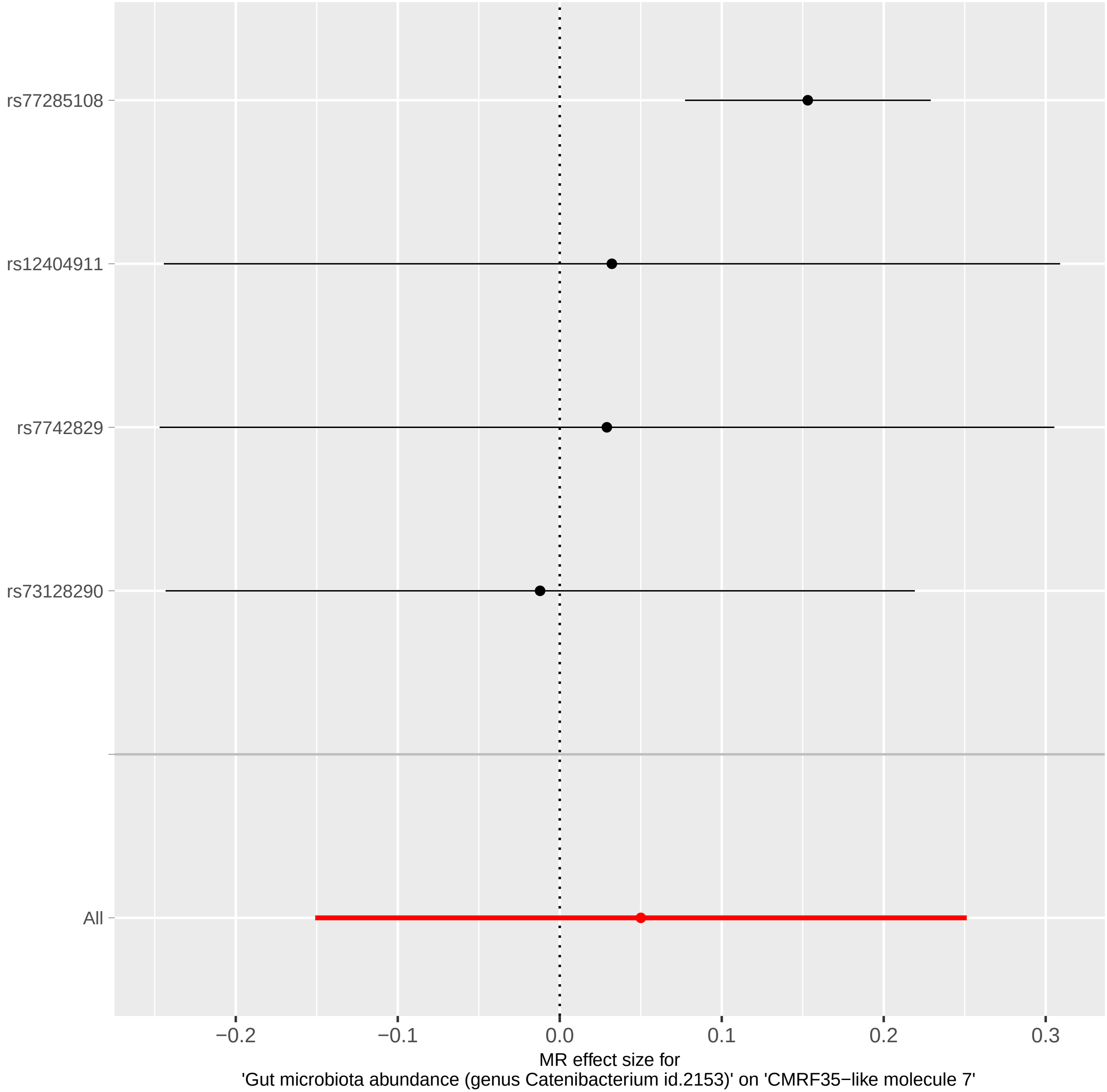

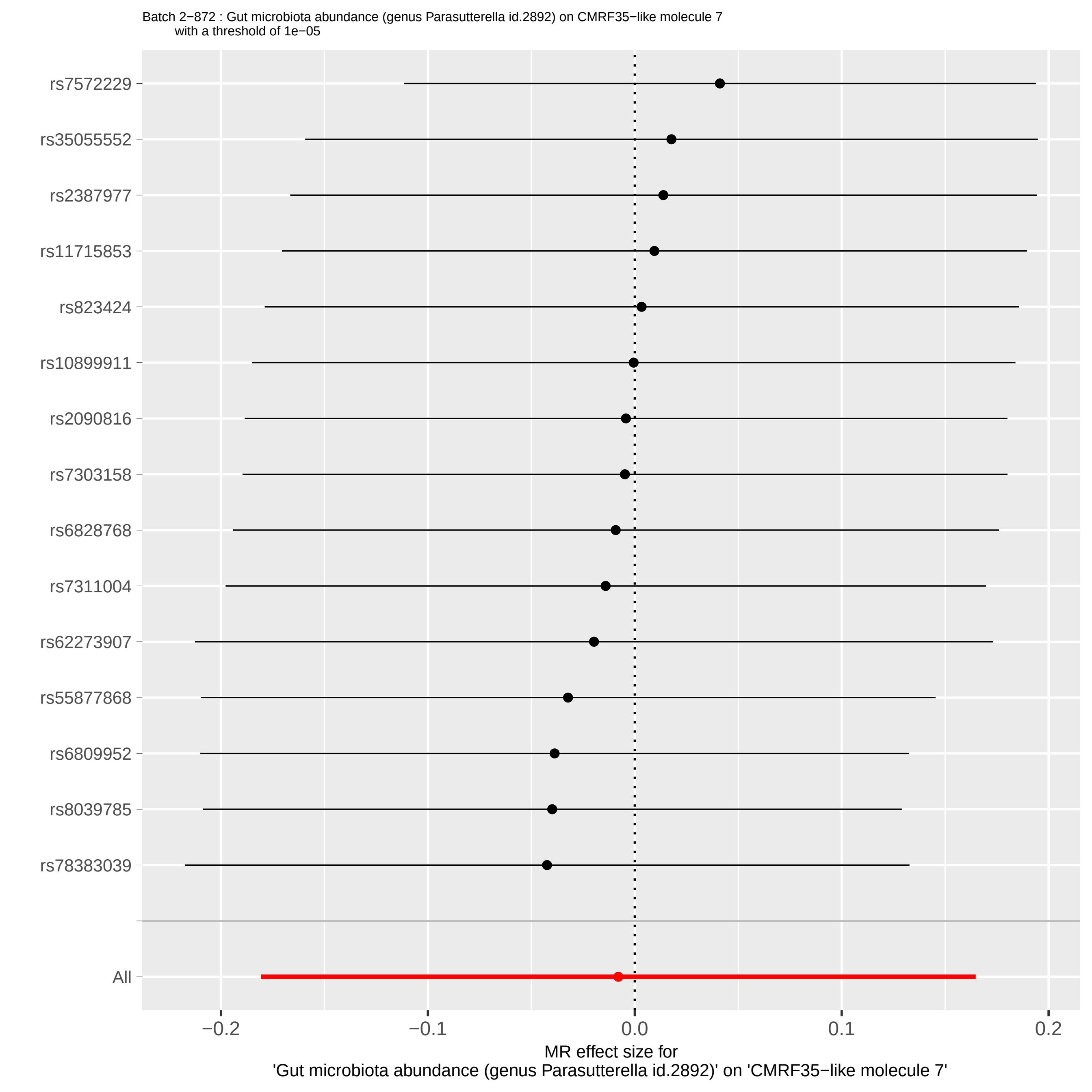

Batch 2−872 : Gut microbiota abundance (genus Parasutterella id.2892) on CMRF35−like molecule 7
with a threshold of 1e−05
rs7572229
rs35055552
rs2387977
rs11715853
rs823424
rs10899911
rs2090816
rs7303158
rs6828768
rs7311004
rs62273907
rs55877868
rs6809952
rs8039785
rs78383039
All
−0.2
−0.1
0.0
0.1
0.2
MR effect size for
'Gut microbiota abundance (genus Parasutterella id.2892)' on 'CMRF35−like molecule 7'

Batch 2−873 : Gut microbiota abundance (genus Catenibacterium id.2153) on CMRF35−like molecule 1
with a threshold of 1e−05

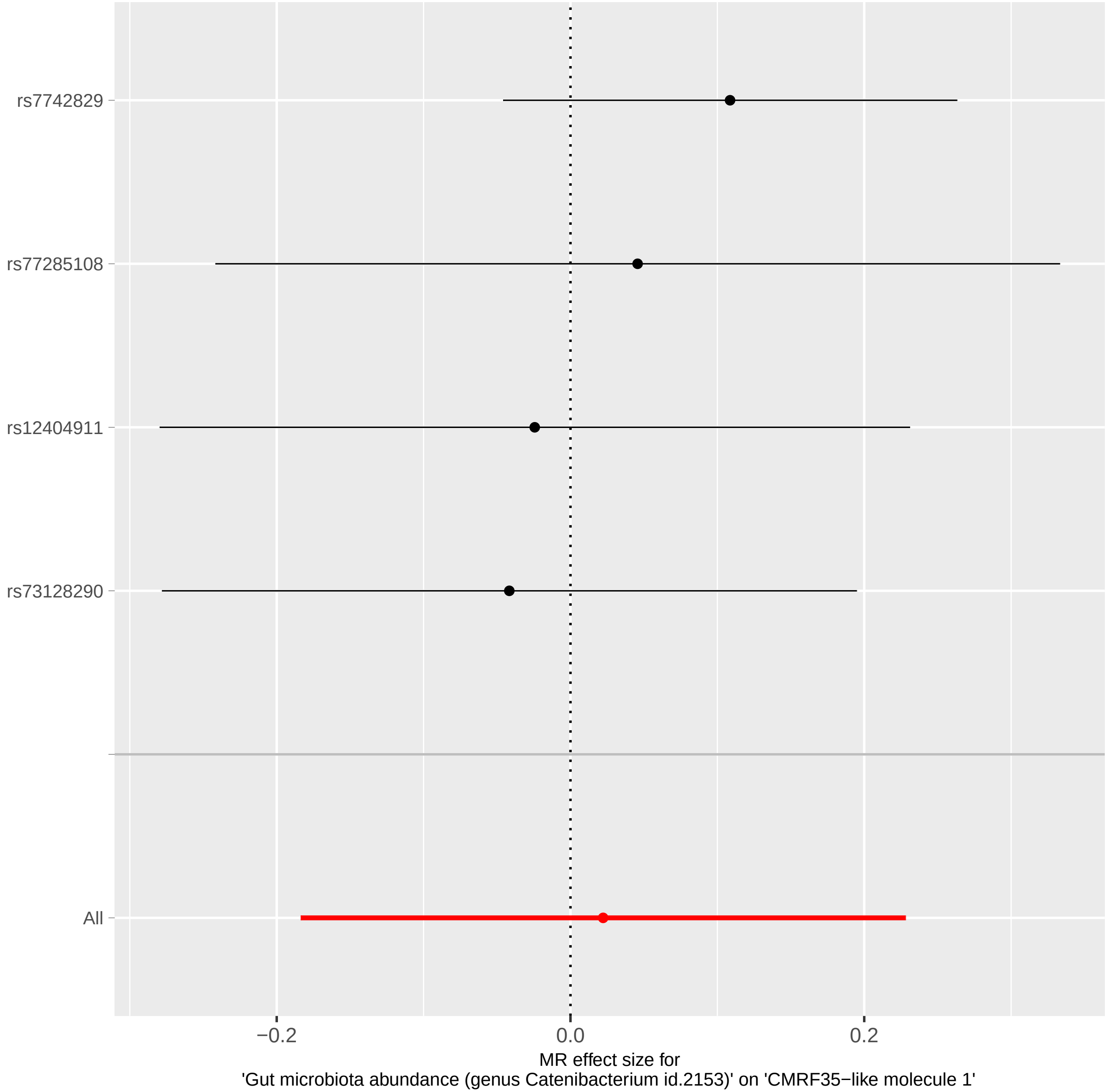

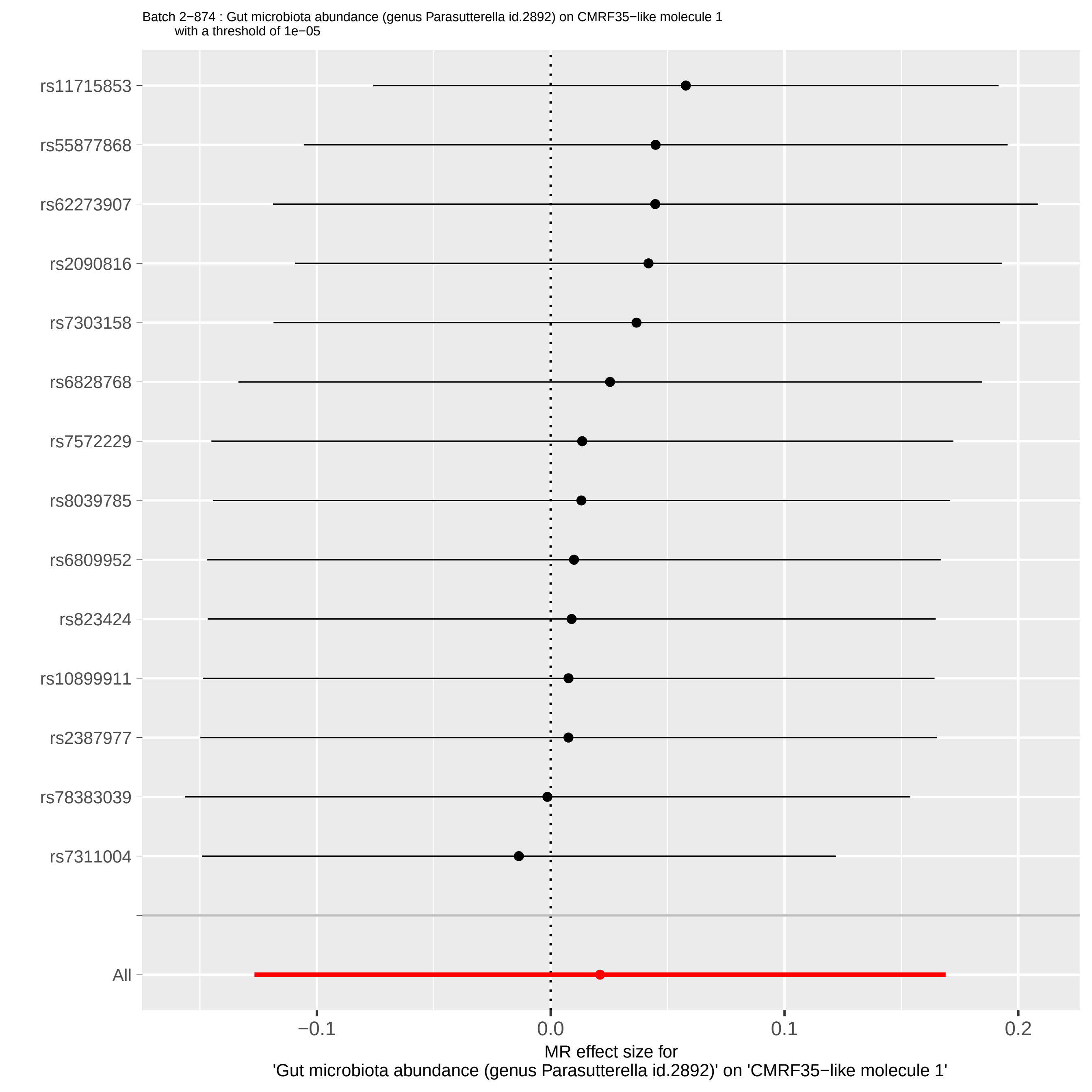

Batch 2−874 : Gut microbiota abundance (genus Parasutterella id.2892) on CMRF35−like molecule 1
with a threshold of 1e−05
rs11715853
rs55877868
rs62273907
rs2090816
rs7303158
rs6828768
rs7572229
rs8039785
rs6809952
rs823424
rs10899911
rs2387977
rs78383039
rs7311004
All
−0.1
0.0
0.1
0.2
MR effect size for
'Gut microbiota abundance (genus Parasutterella id.2892)' on 'CMRF35−like molecule 1'

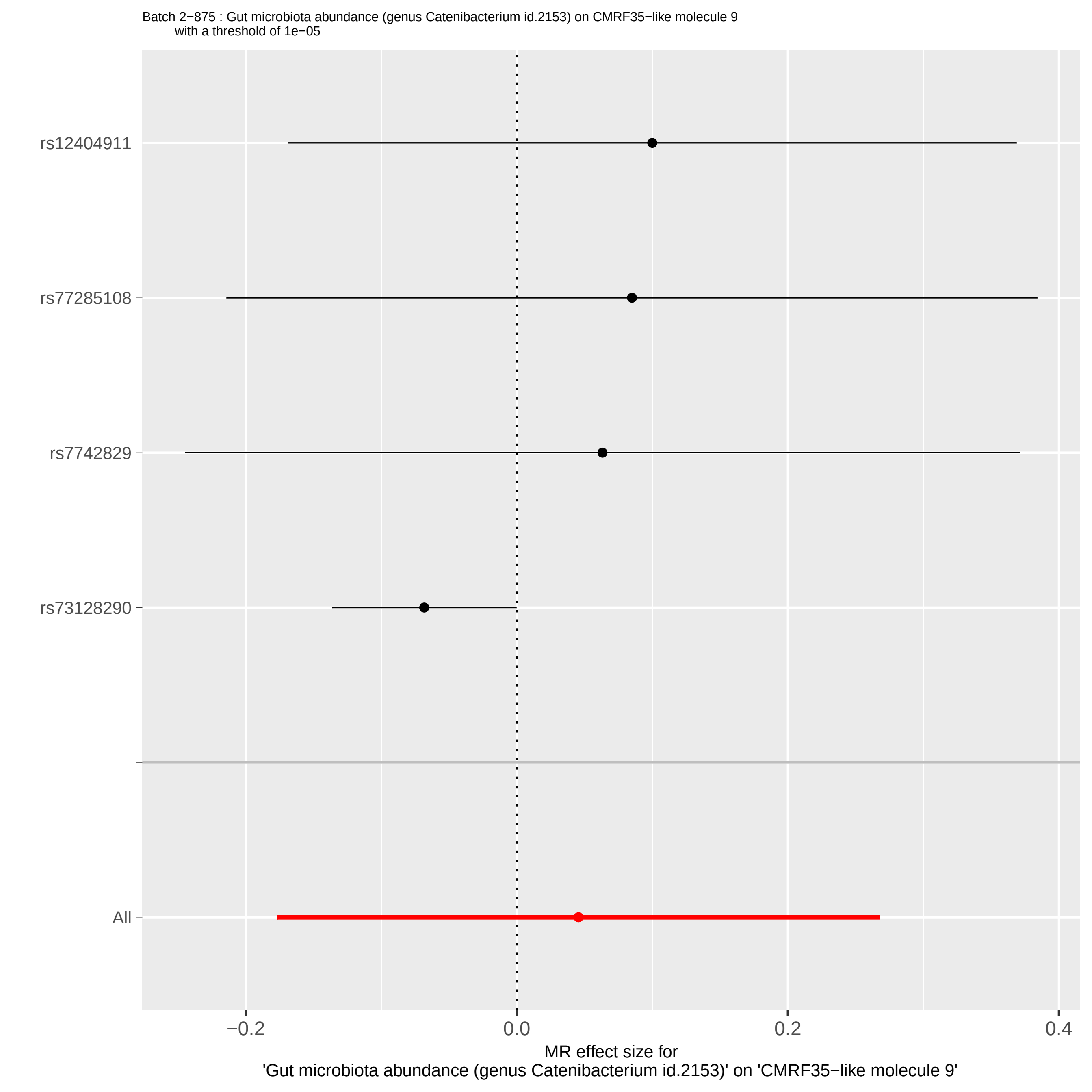

Batch 2−875 : Gut microbiota abundance (genus Catenibacterium id.2153) on CMRF35−like molecule 9
with a threshold of 1e−05
rs12404911
rs77285108
rs7742829
rs73128290
All
−0.2
0.0
0.2
0.4
MR effect size for
'Gut microbiota abundance (genus Catenibacterium id.2153)' on 'CMRF35−like molecule 9'

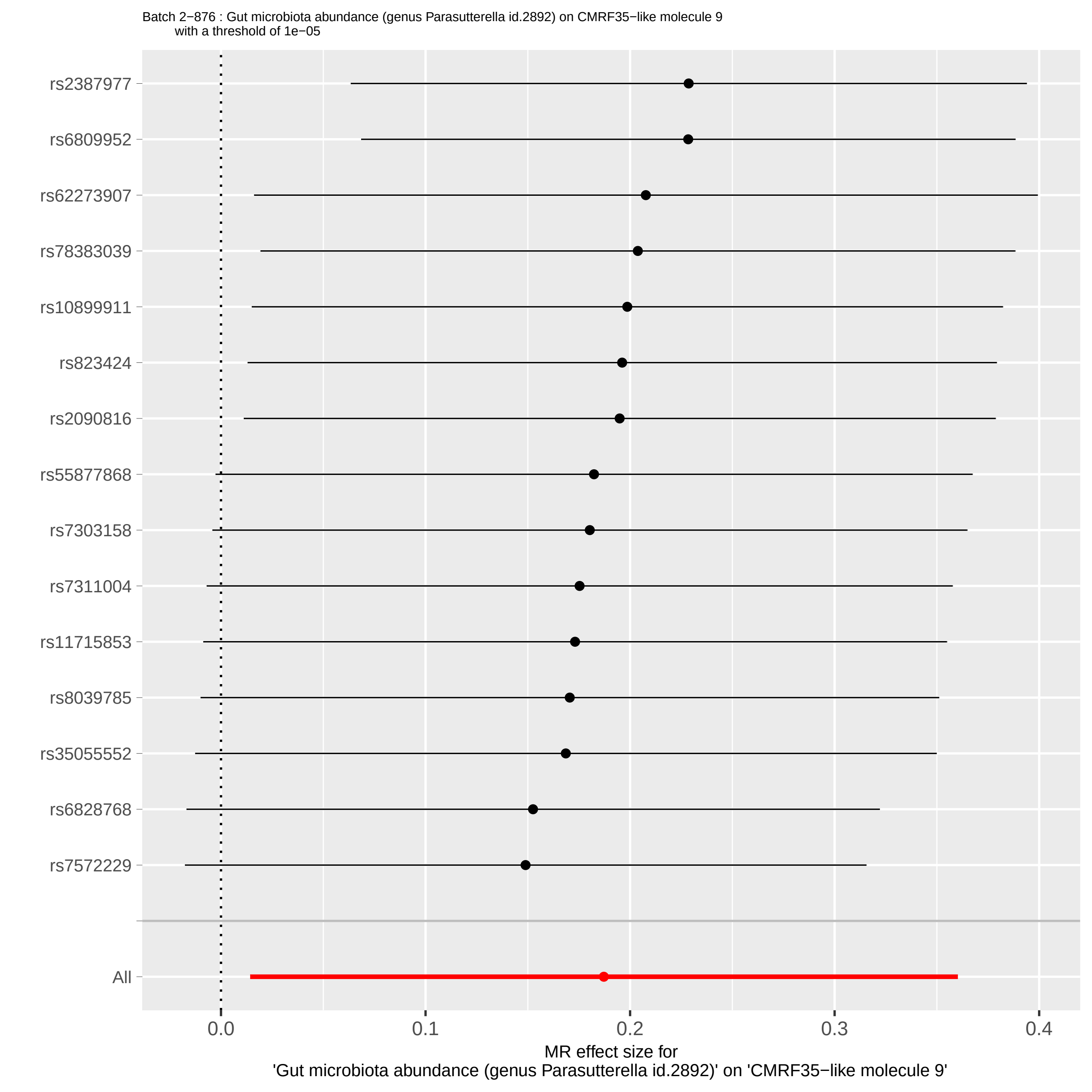

Batch 2−876 : Gut microbiota abundance (genus Parasutterella id.2892) on CMRF35−like molecule 9
with a threshold of 1e−05
rs2387977
rs6809952
rs62273907
rs78383039
rs10899911
rs823424
rs2090816
rs55877868
rs7303158
rs7311004
rs11715853
rs8039785
rs35055552
rs6828768
rs7572229
All
0.0
0.1
0.2
0.3
0.4
MR effect size for
'Gut microbiota abundance (genus Parasutterella id.2892)' on 'CMRF35−like molecule 9'

Batch 2−877 : Gut microbiota abundance (genus Catenibacterium id.2153) on Myeloid cell surface antigen CD33
with a threshold of 1e−05

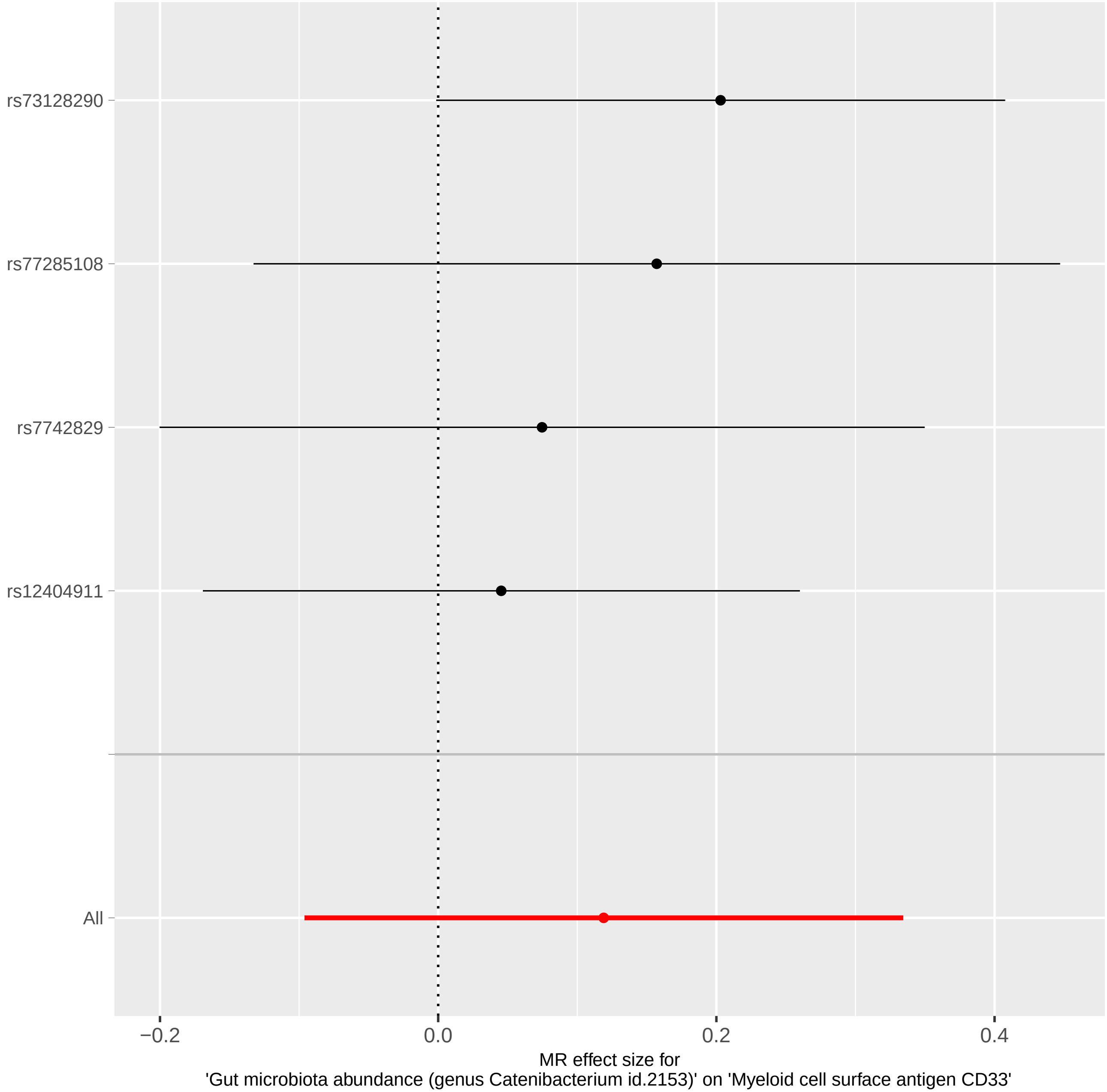

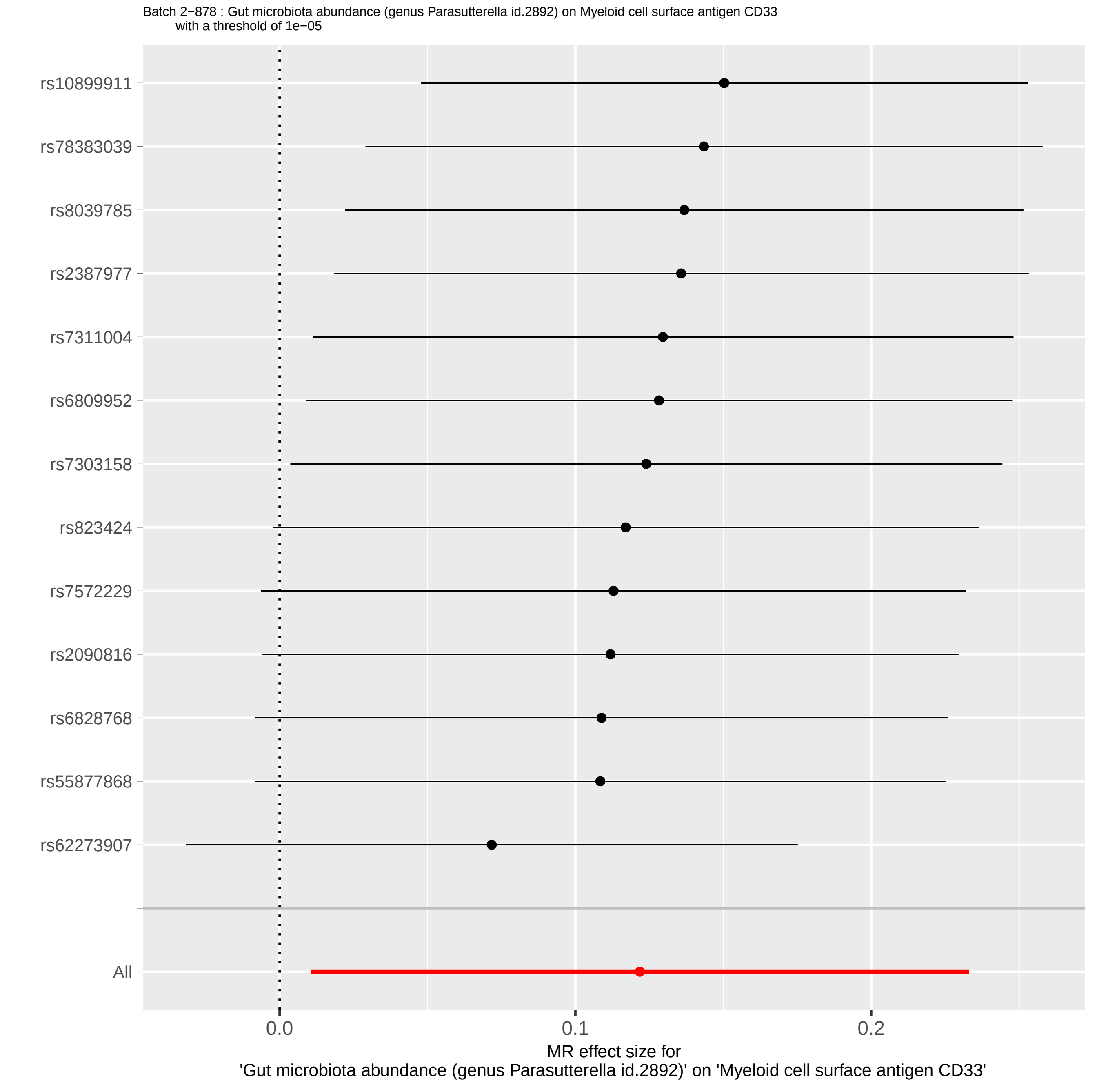
Batch 2−878 : Gut microbiota abundance (genus Parasutterella id.2892) on Myeloid cell surface antigen CD33
with a threshold of 1e−05
rs10899911
rs78383039
rs8039785
rs2387977
rs7311004
rs6809952
rs7303158
rs823424
rs7572229
rs2090816
rs6828768
rs55877868
rs62273907
All
0.0
0.1
0.2
MR effect size for
'Gut microbiota abundance (genus Parasutterella id.2892)' on 'Myeloid cell surface antigen CD33'

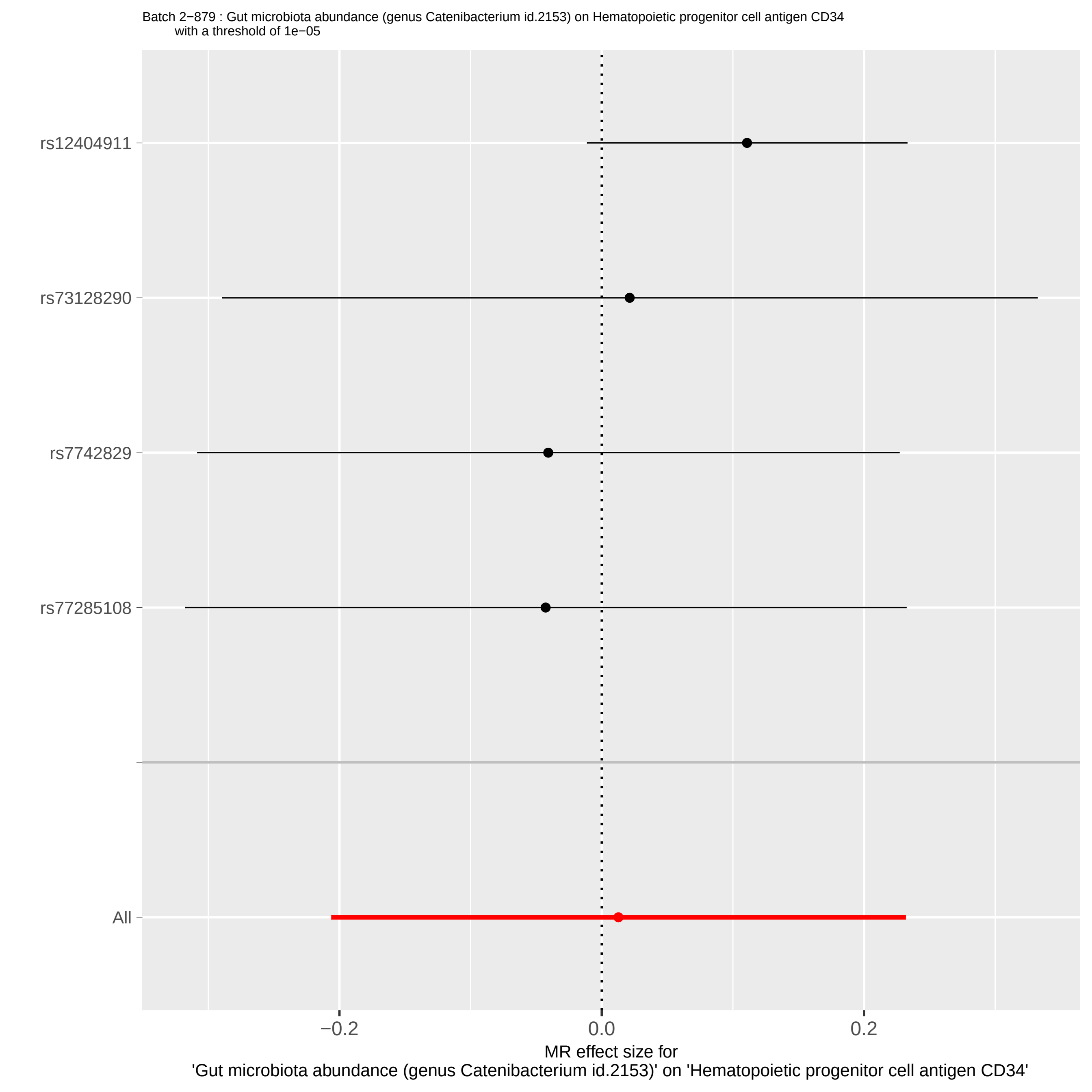

Batch 2−879 : Gut microbiota abundance (genus Catenibacterium id.2153) on Hematopoietic progenitor cell antigen CD34
with a threshold of 1e−05
rs12404911
rs73128290
rs7742829
rs77285108
All
−0.2
0.0
0.2
MR effect size for
'Gut microbiota abundance (genus Catenibacterium id.2153)' on 'Hematopoietic progenitor cell antigen CD34'

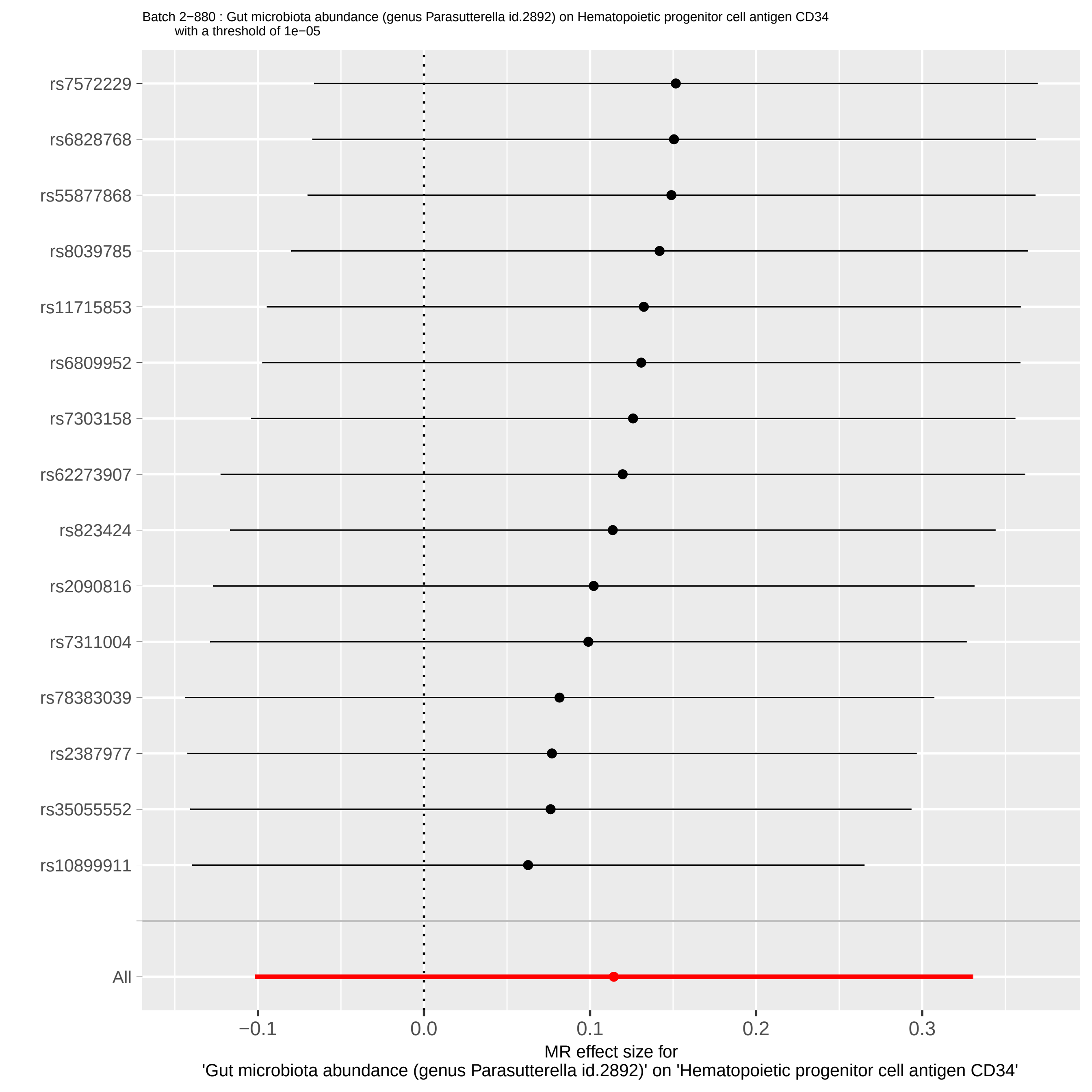

Batch 2−880 : Gut microbiota abundance (genus Parasutterella id.2892) on Hematopoietic progenitor cell antigen CD34
with a threshold of 1e−05
rs7572229
rs6828768
rs55877868
rs8039785
rs11715853
rs6809952
rs7303158
rs62273907
rs823424
rs2090816
rs7311004
rs78383039
rs2387977
rs35055552
rs10899911
All
−0.1
0.0
0.1
0.2
0.3
MR effect size for
'Gut microbiota abundance (genus Parasutterella id.2892)' on 'Hematopoietic progenitor cell antigen CD34'

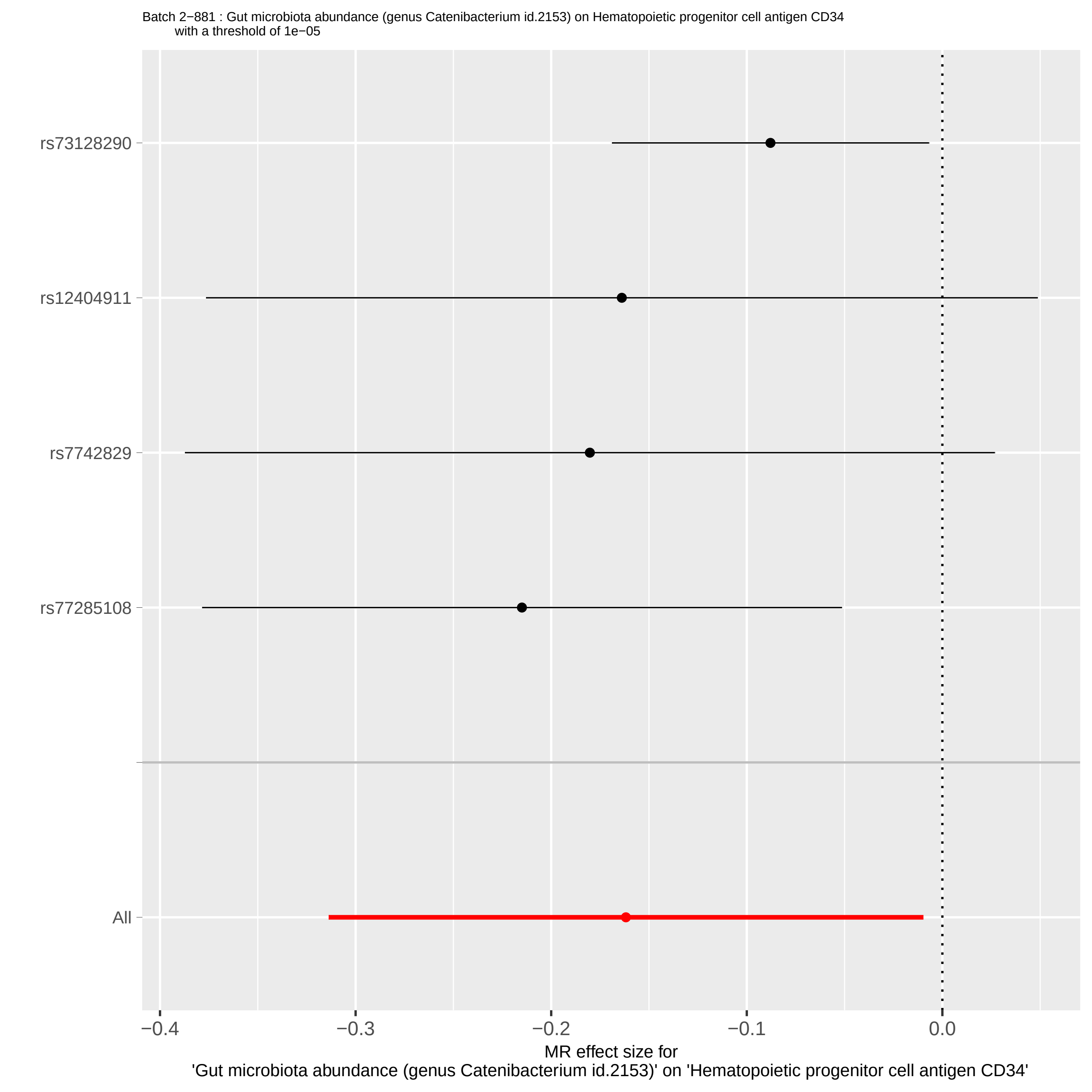

Batch 2−881 : Gut microbiota abundance (genus Catenibacterium id.2153) on Hematopoietic progenitor cell antigen CD34
with a threshold of 1e−05
rs73128290
rs12404911
rs7742829
rs77285108
All
−0.4
−0.3
−0.2
−0.1
0.0
MR effect size for
'Gut microbiota abundance (genus Catenibacterium id.2153)' on 'Hematopoietic progenitor cell antigen CD34'

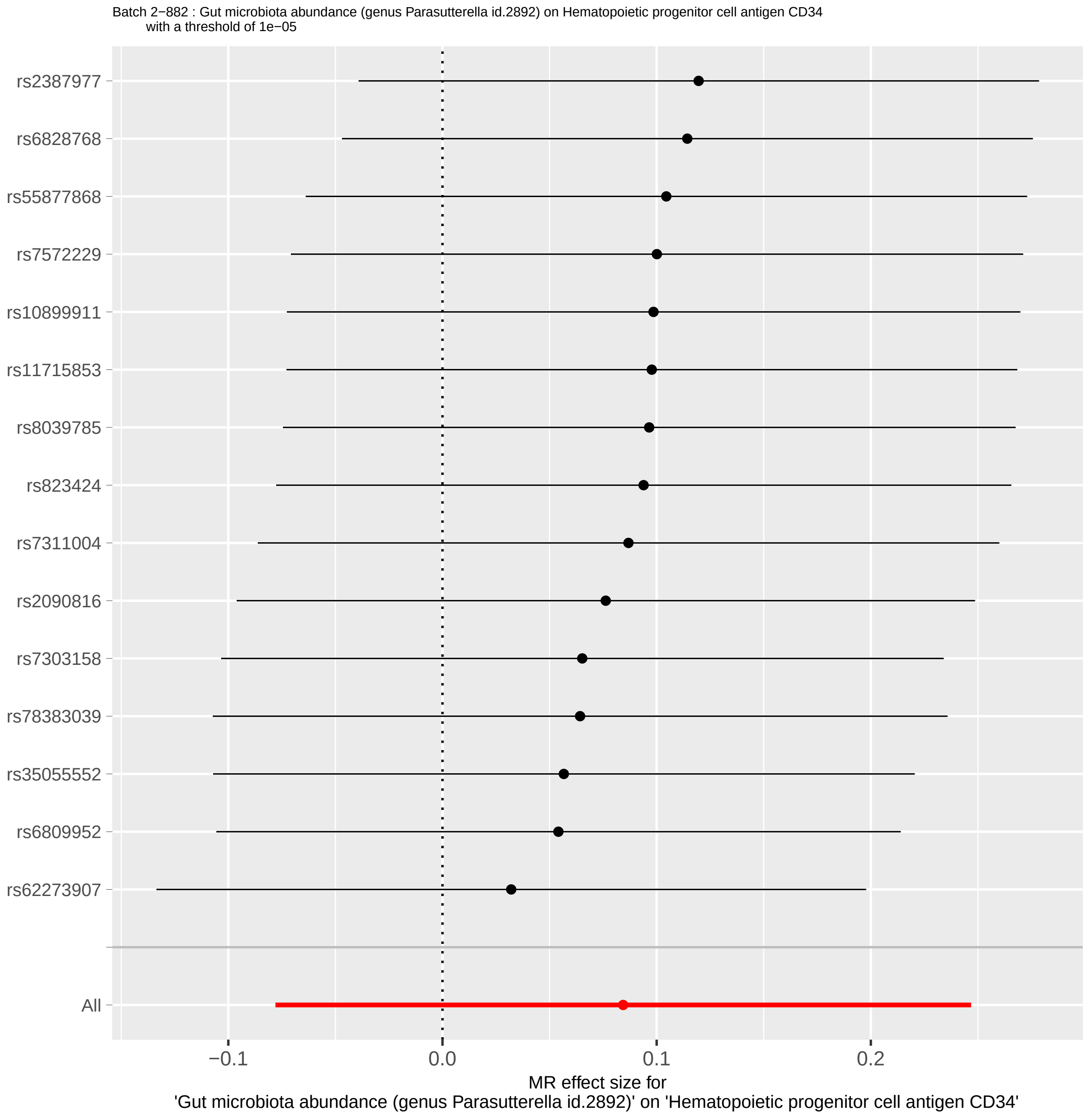

Batch 2−882 : Gut microbiota abundance (genus Parasutterella id.2892) on Hematopoietic progenitor cell antigen CD34
with a threshold of 1e−05
rs2387977
rs6828768
rs55877868
rs7572229
rs10899911
rs11715853
rs8039785
rs823424
rs7311004
rs2090816
rs7303158
rs78383039
rs35055552
rs6809952
rs62273907
All
−0.1
0.0
0.1
0.2
MR effect size for
'Gut microbiota abundance (genus Parasutterella id.2892)' on 'Hematopoietic progenitor cell antigen CD34'

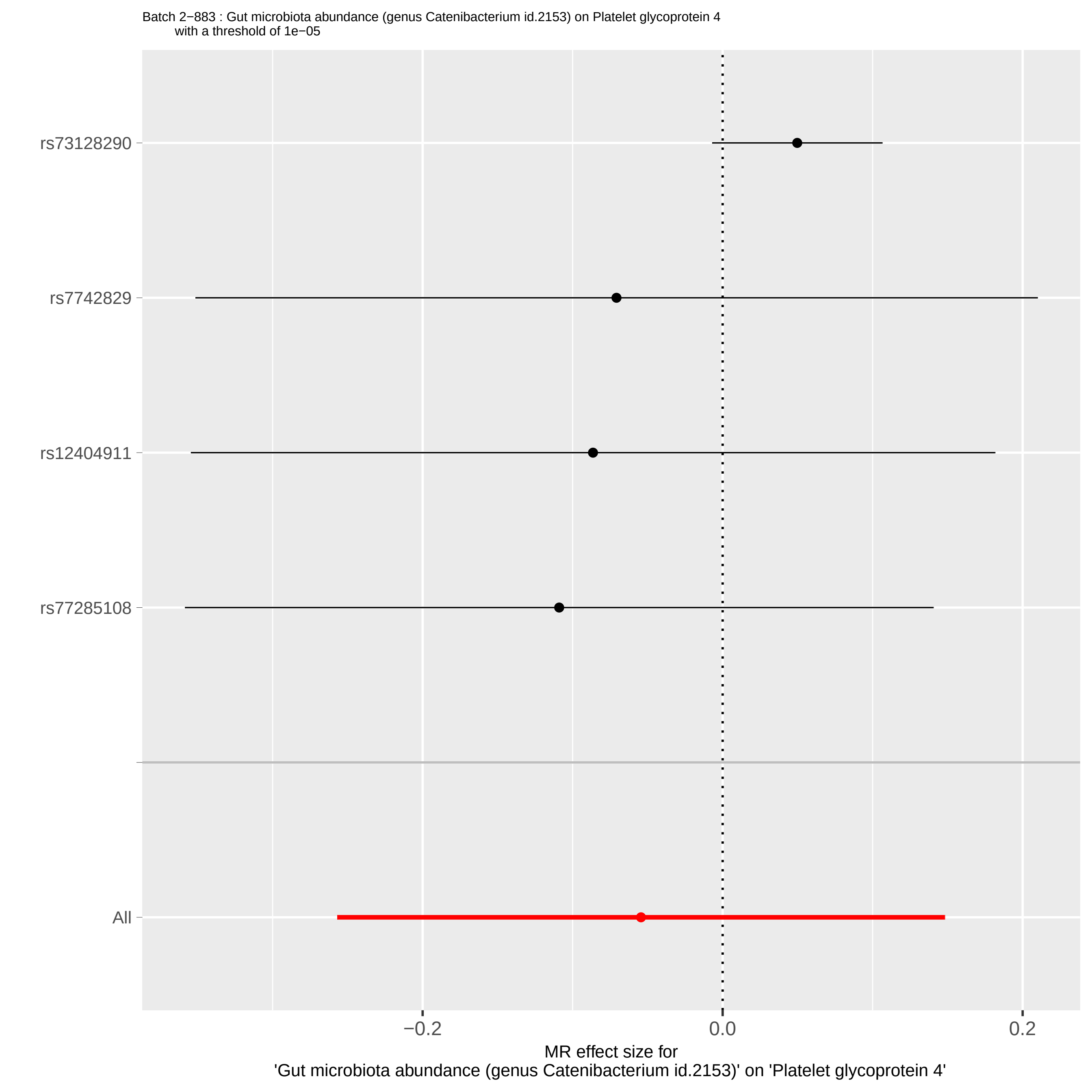
Batch 2−883 : Gut microbiota abundance (genus Catenibacterium id.2153) on Platelet glycoprotein 4
with a threshold of 1e−05
rs73128290
rs7742829
rs12404911
rs77285108
All
−0.2
0.0
0.2
MR effect size for
'Gut microbiota abundance (genus Catenibacterium id.2153)' on 'Platelet glycoprotein 4'

Batch 2−884 : Gut microbiota abundance (genus Parasutterella id.2892) on Platelet glycoprotein 4
with a threshold of 1e−05

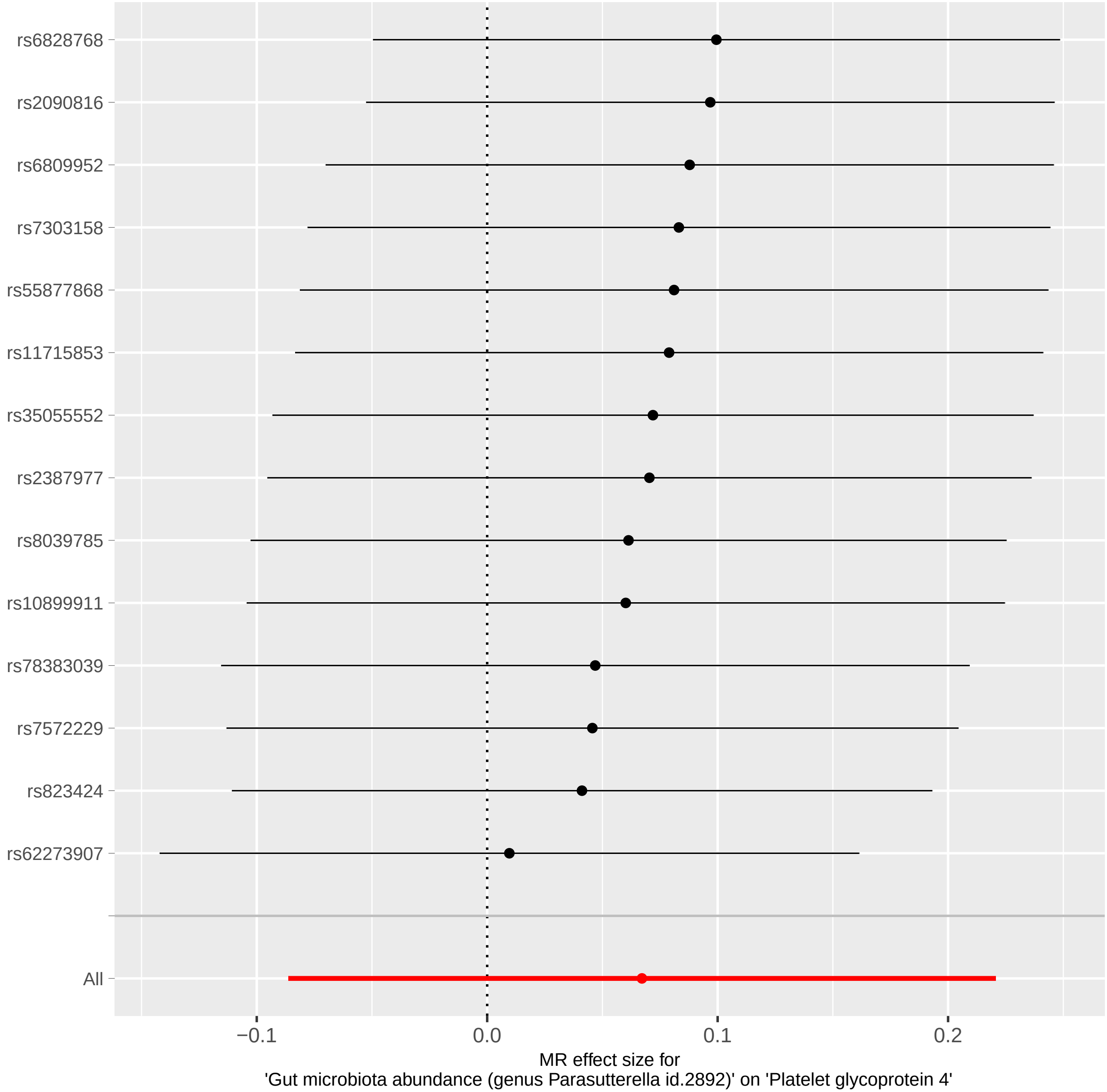

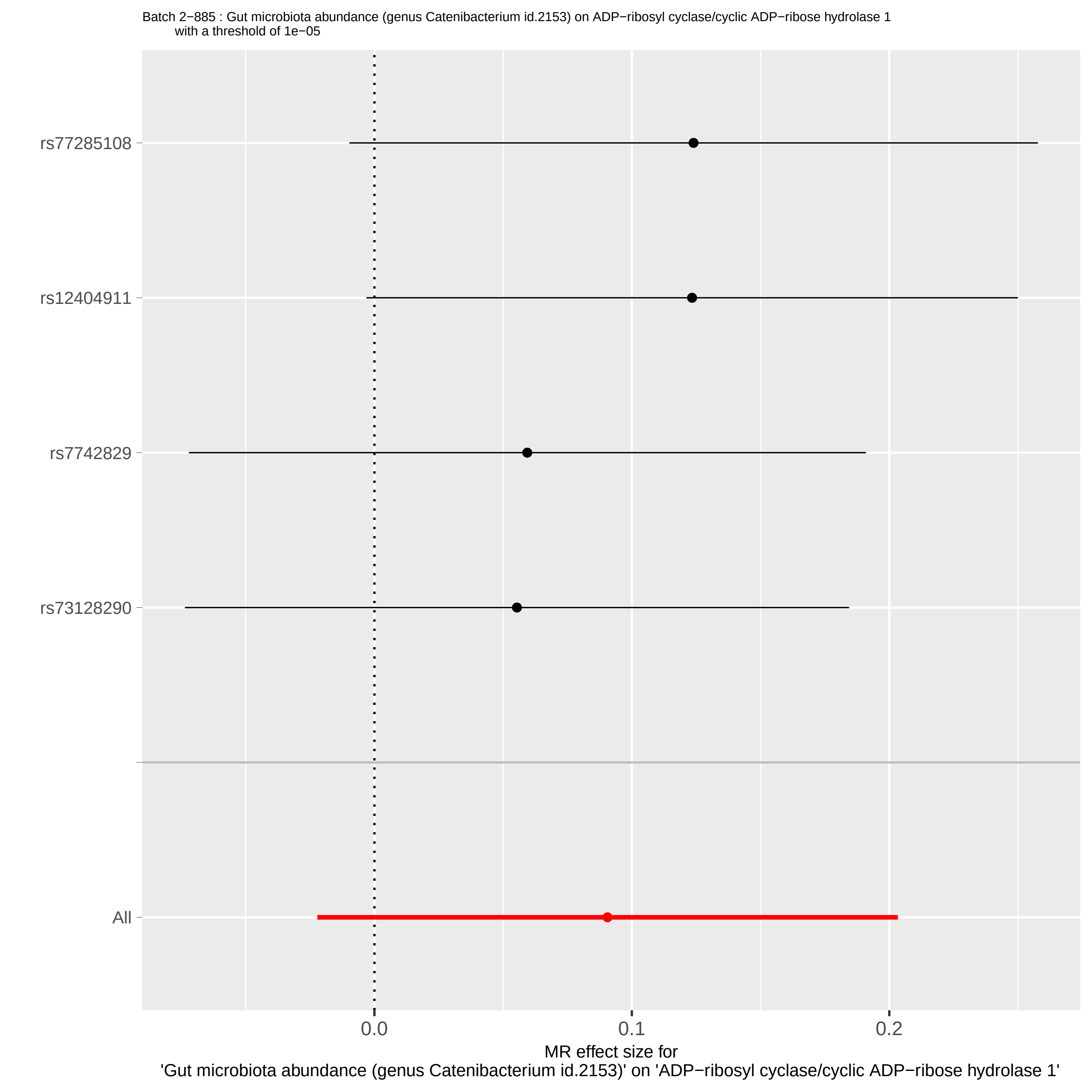
Batch 2−885 : Gut microbiota abundance (genus Catenibacterium id.2153) on ADP−ribosyl cyclase/cyclic ADP−ribose hydrolase 1
with a threshold of 1e−05
rs77285108
rs12404911
rs7742829
rs73128290
All
0.0
0.1
0.2
MR effect size for
'Gut microbiota abundance (genus Catenibacterium id.2153)' on 'ADP−ribosyl cyclase/cyclic ADP−ribose hydrolase 1'

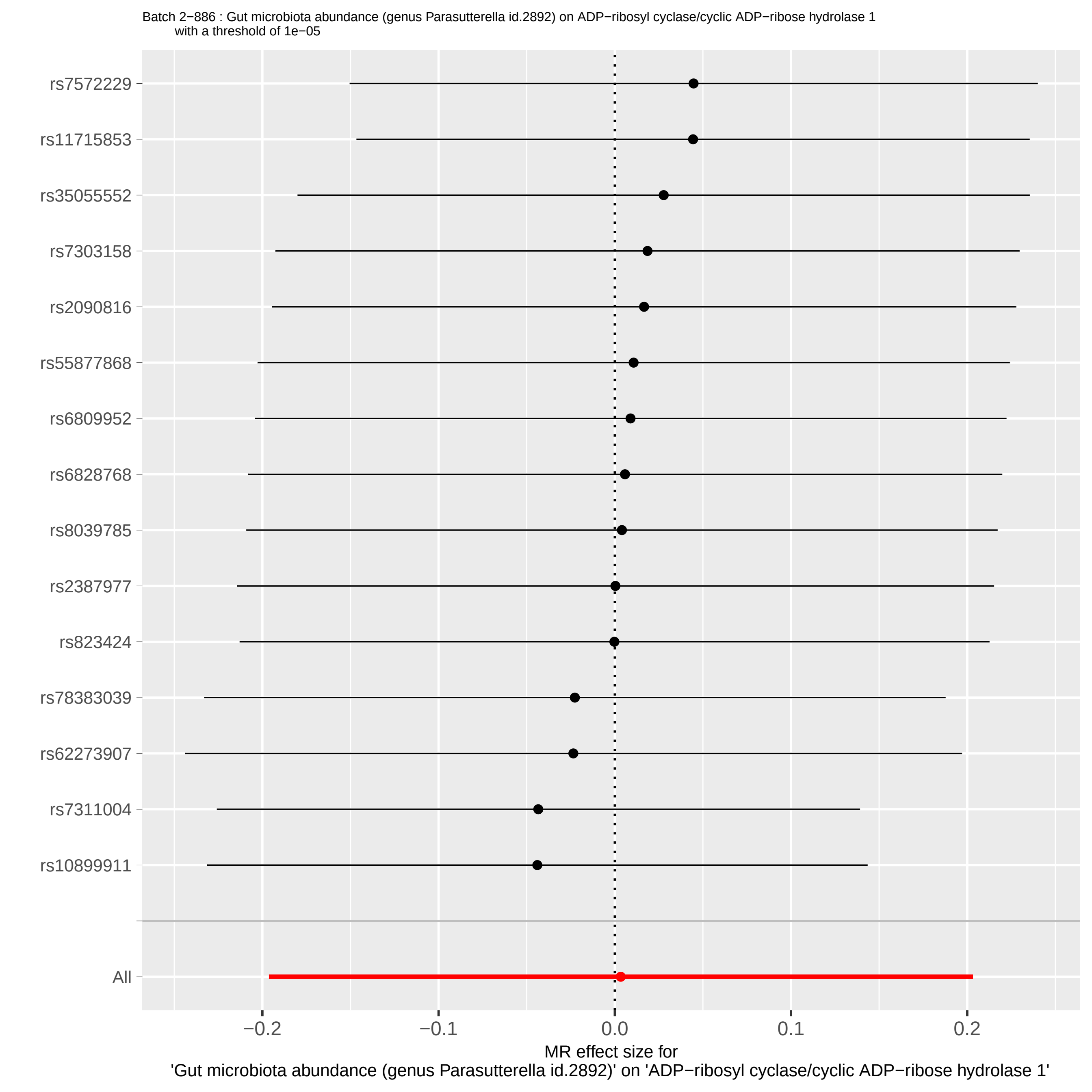

Batch 2−886 : Gut microbiota abundance (genus Parasutterella id.2892) on ADP−ribosyl cyclase/cyclic ADP−ribose hydrolase 1
with a threshold of 1e−05
rs7572229
rs11715853
rs35055552
rs7303158
rs2090816
rs55877868
rs6809952
rs6828768
rs8039785
rs2387977
rs823424
rs78383039
rs62273907
rs7311004
rs10899911
All
−0.2
−0.1
0.0
0.1
0.2
MR effect size for
'Gut microbiota abundance (genus Parasutterella id.2892)' on 'ADP−ribosyl cyclase/cyclic ADP−ribose hydrolase 1'

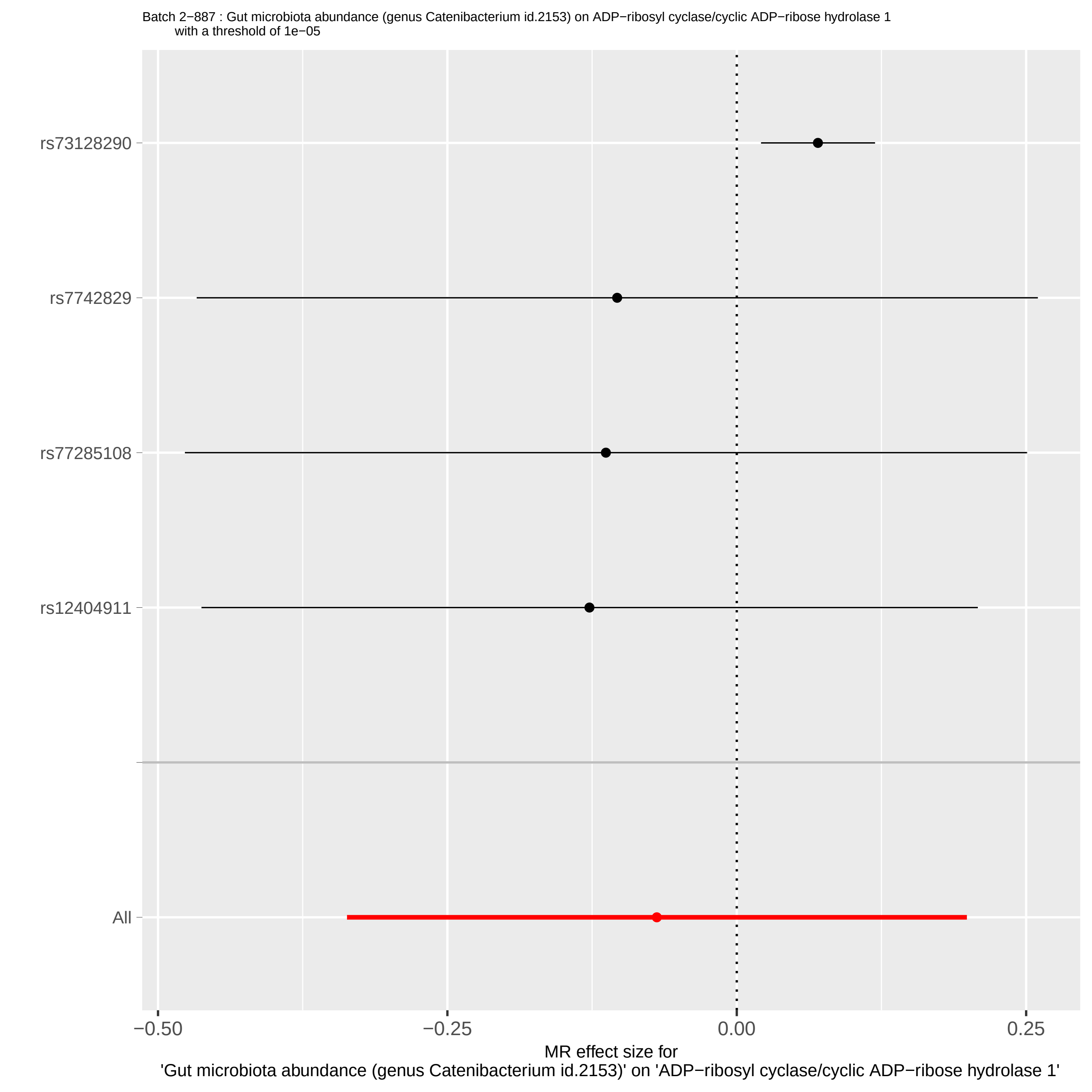

Batch 2−887 : Gut microbiota abundance (genus Catenibacterium id.2153) on ADP−ribosyl cyclase/cyclic ADP−ribose hydrolase 1
with a threshold of 1e−05
rs73128290
rs7742829
rs77285108
rs12404911
All
−0.50
−0.25
0.00
0.25
MR effect size for
'Gut microbiota abundance (genus Catenibacterium id.2153)' on 'ADP−ribosyl cyclase/cyclic ADP−ribose hydrolase 1'

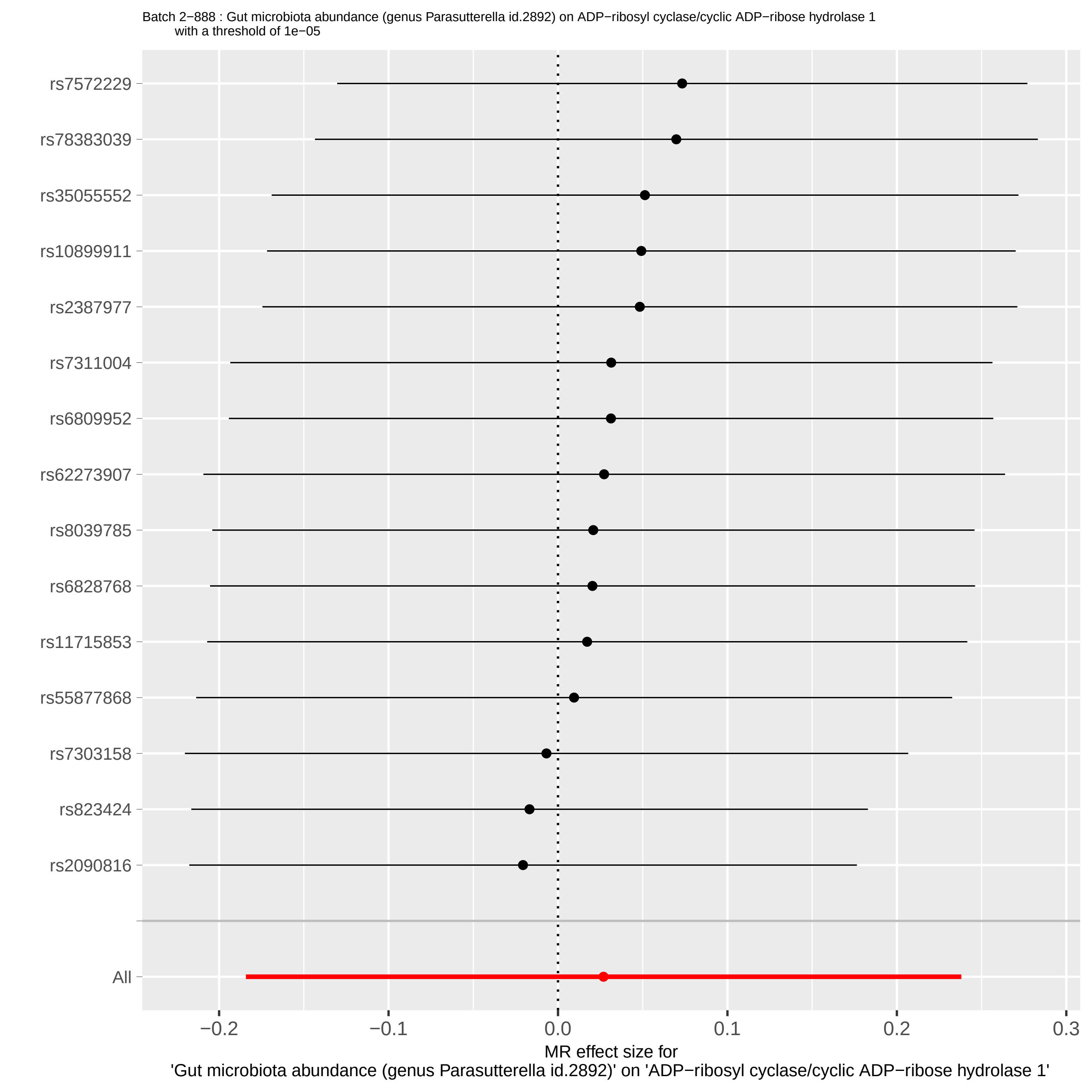

Batch 2−888 : Gut microbiota abundance (genus Parasutterella id.2892) on ADP−ribosyl cyclase/cyclic ADP−ribose hydrolase 1
with a threshold of 1e−05
rs7572229
rs78383039
rs35055552
rs10899911
rs2387977
rs7311004
rs6809952
rs62273907
rs8039785
rs6828768
rs11715853
rs55877868
rs7303158
rs823424
rs2090816
All
−0.2
−0.1
0.0
0.1
0.2
0.3
MR effect size for
'Gut microbiota abundance (genus Parasutterella id.2892)' on 'ADP−ribosyl cyclase/cyclic ADP−ribose hydrolase 1'

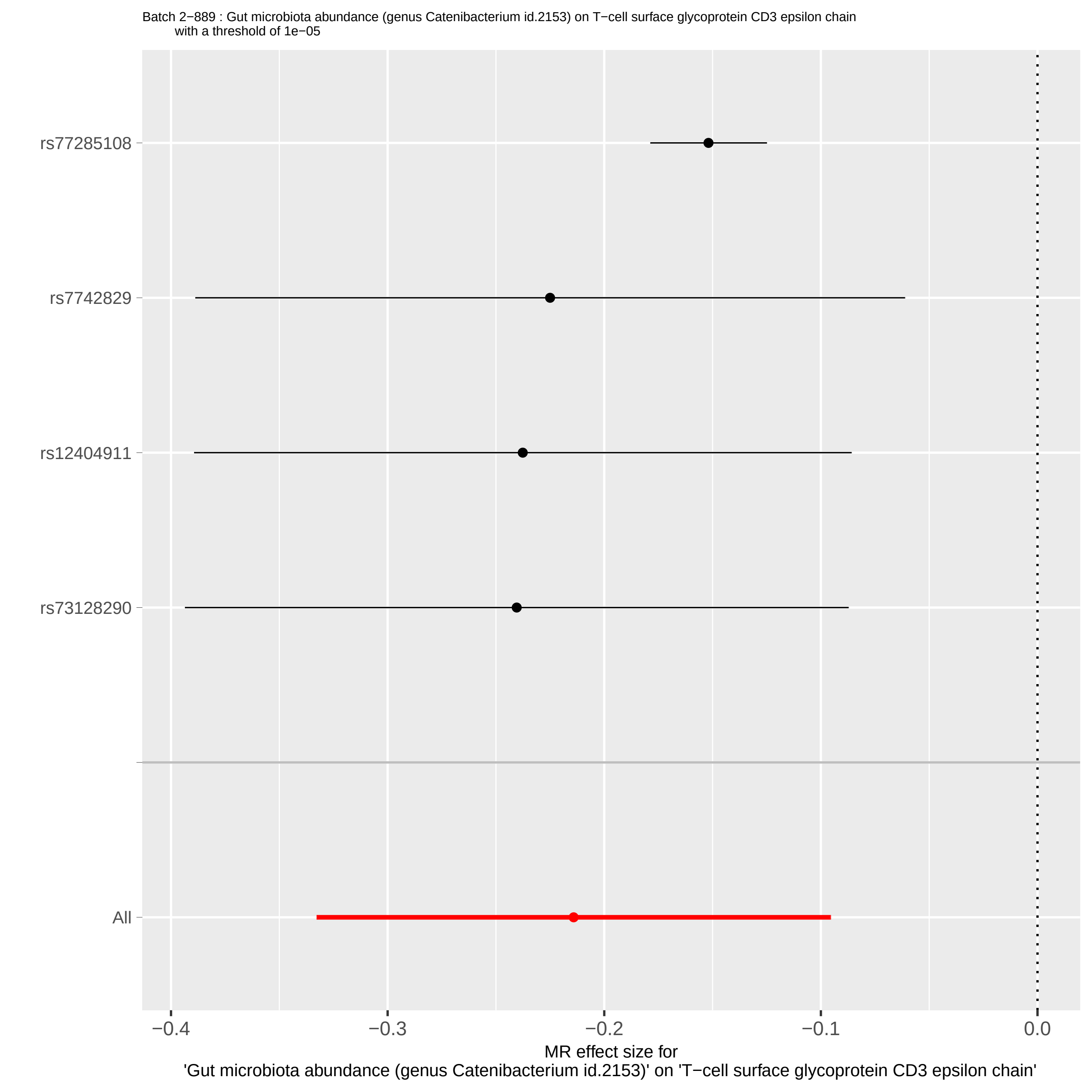
Batch 2−889 : Gut microbiota abundance (genus Catenibacterium id.2153) on T−cell surface glycoprotein CD3 epsilon chain
with a threshold of 1e−05
rs77285108
rs7742829
rs12404911
rs73128290
All
−0.4
−0.3
−0.2
−0.1
0.0
MR effect size for
'Gut microbiota abundance (genus Catenibacterium id.2153)' on 'T−cell surface glycoprotein CD3 epsilon chain'

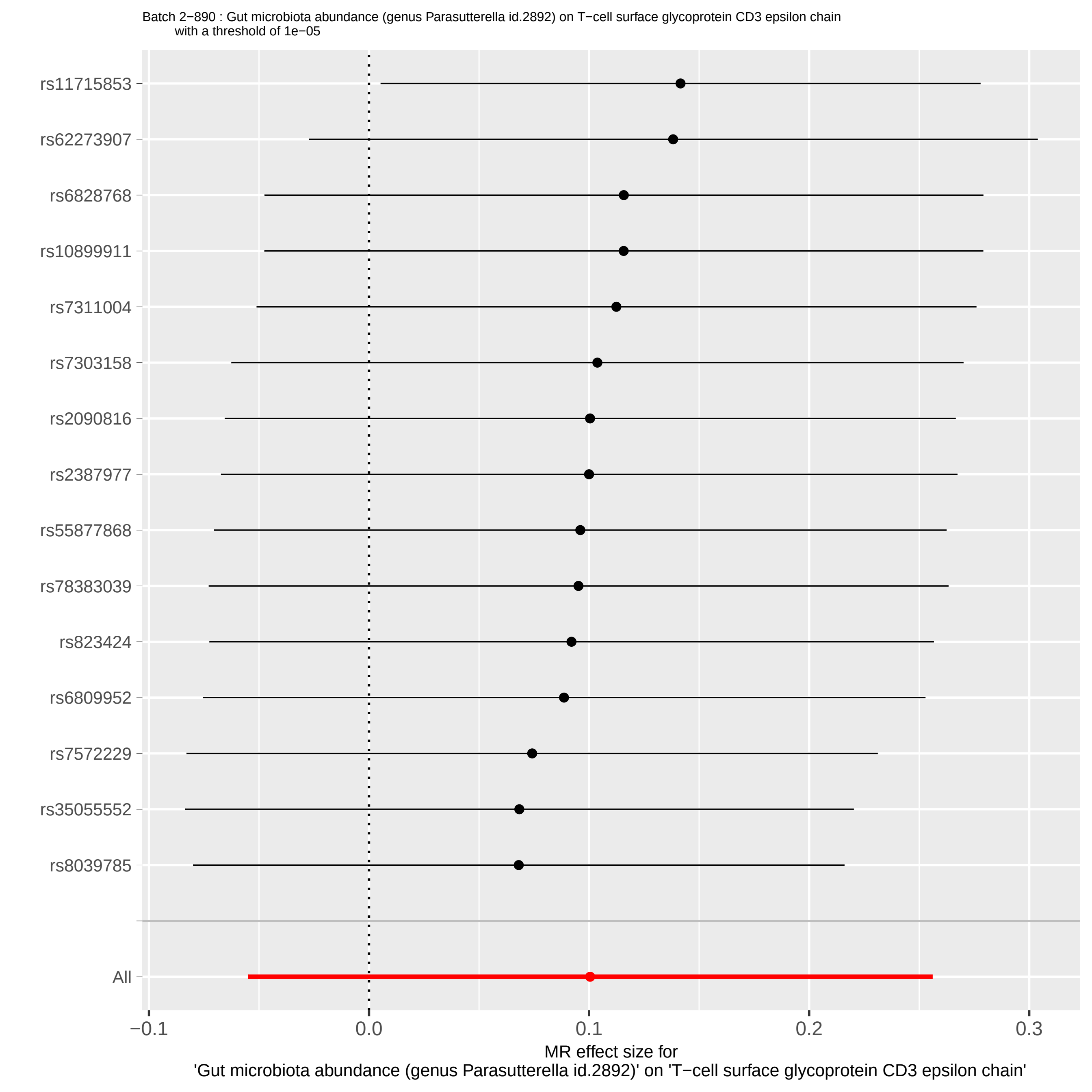

Batch 2−890 : Gut microbiota abundance (genus Parasutterella id.2892) on T−cell surface glycoprotein CD3 epsilon chain
with a threshold of 1e−05
rs11715853
rs62273907
rs6828768
rs10899911
rs7311004
rs7303158
rs2090816
rs2387977
rs55877868
rs78383039
rs823424
rs6809952
rs7572229
rs35055552
rs8039785
All
−0.1
0.0
0.1
0.2
0.3
MR effect size for
'Gut microbiota abundance (genus Parasutterella id.2892)' on 'T−cell surface glycoprotein CD3 epsilon chain'

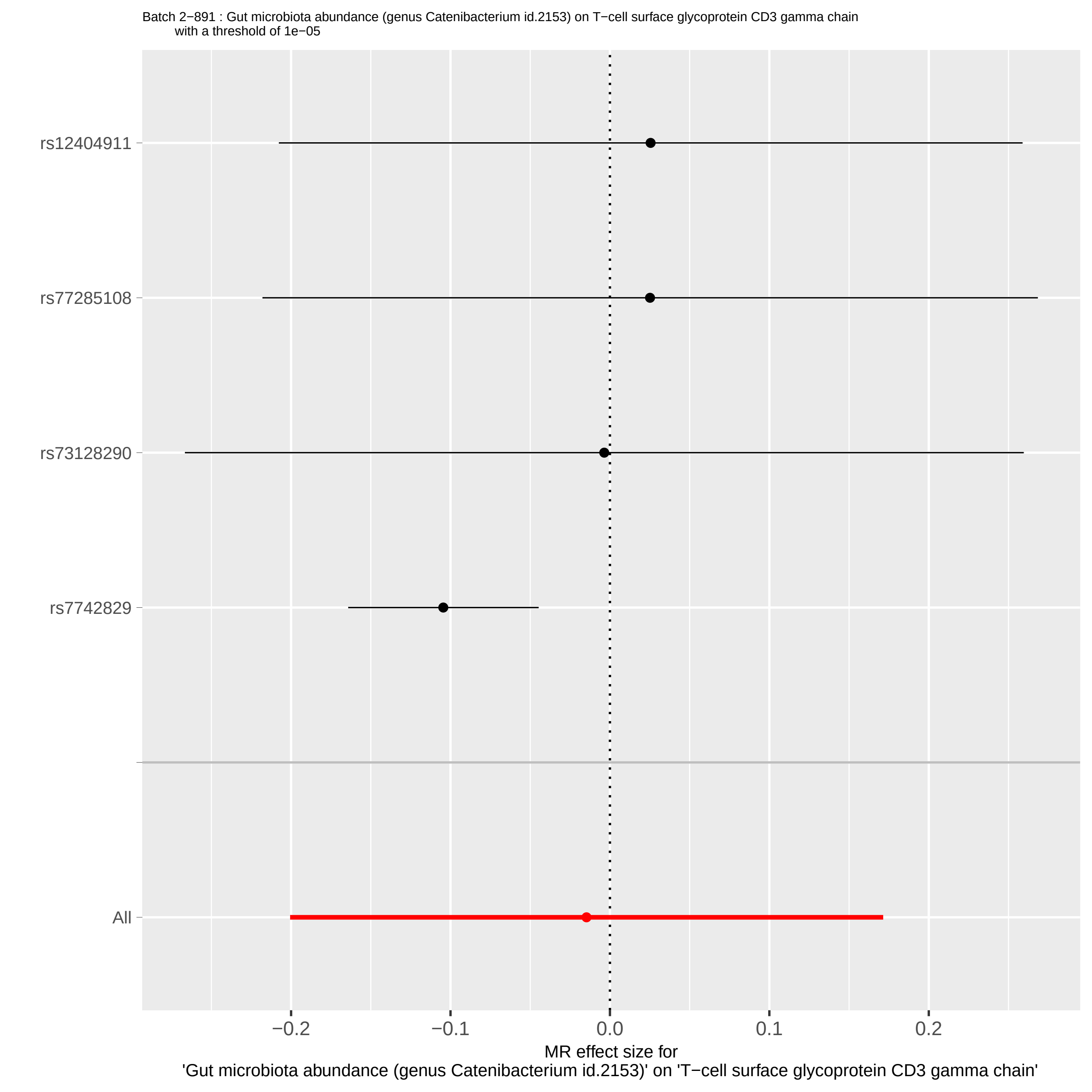
Batch 2−891 : Gut microbiota abundance (genus Catenibacterium id.2153) on T−cell surface glycoprotein CD3 gamma chain
with a threshold of 1e−05
rs12404911
rs77285108
rs73128290
rs7742829
All
−0.2
−0.1
0.0
0.1
0.2
MR effect size for
'Gut microbiota abundance (genus Catenibacterium id.2153)' on 'T−cell surface glycoprotein CD3 gamma chain'

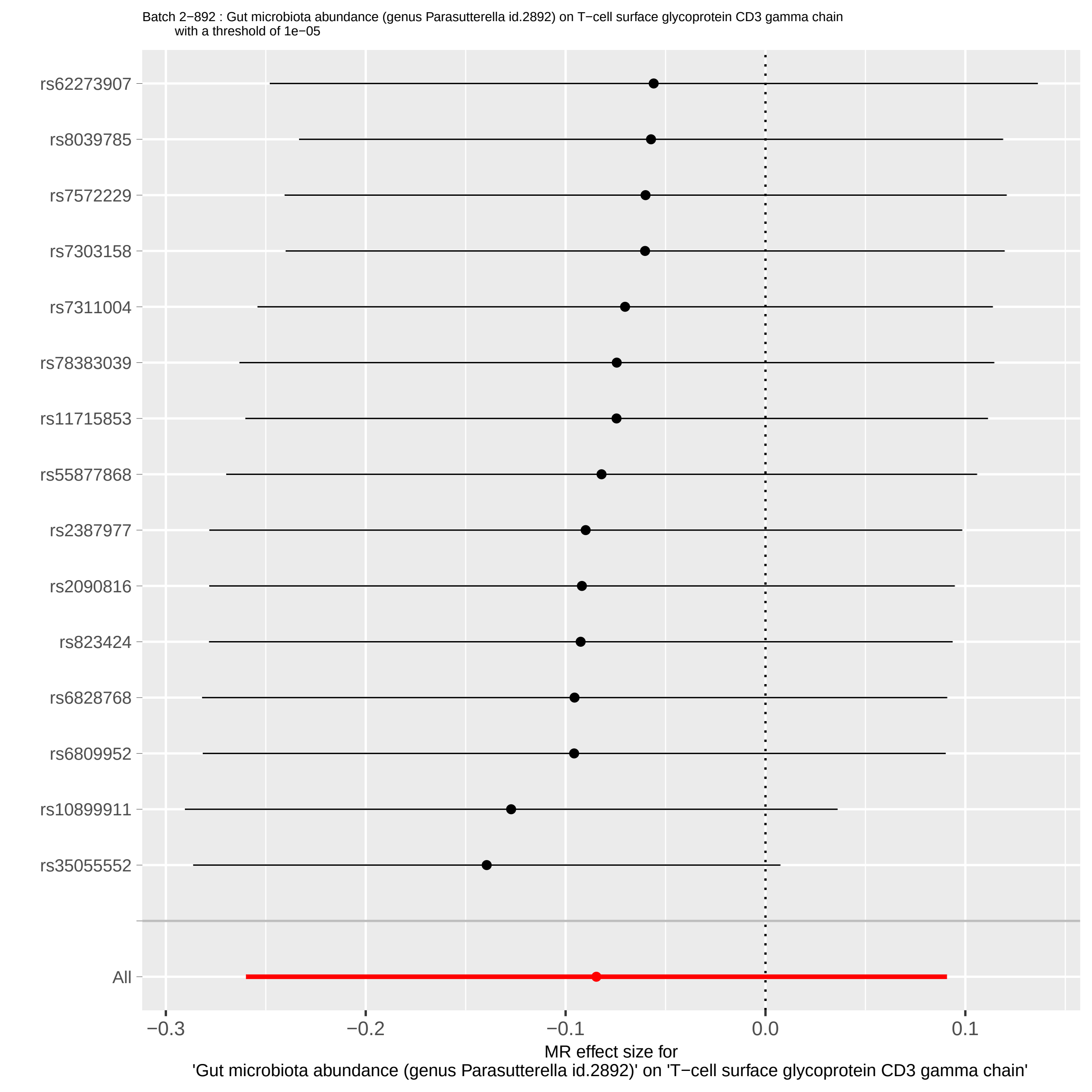

Batch 2−892 : Gut microbiota abundance (genus Parasutterella id.2892) on T−cell surface glycoprotein CD3 gamma chain
with a threshold of 1e−05
rs62273907
rs8039785
rs7572229
rs7303158
rs7311004
rs78383039
rs11715853
rs55877868
rs2387977
rs2090816
rs823424
rs6828768
rs6809952
rs10899911
rs35055552
All
−0.3
−0.2
−0.1
0.0
0.1
MR effect size for
'Gut microbiota abundance (genus Parasutterella id.2892)' on 'T−cell surface glycoprotein CD3 gamma chain'

Batch 2−893 : Gut microbiota abundance (genus Catenibacterium id.2153) on CD40 ligand
with a threshold of 1e−05

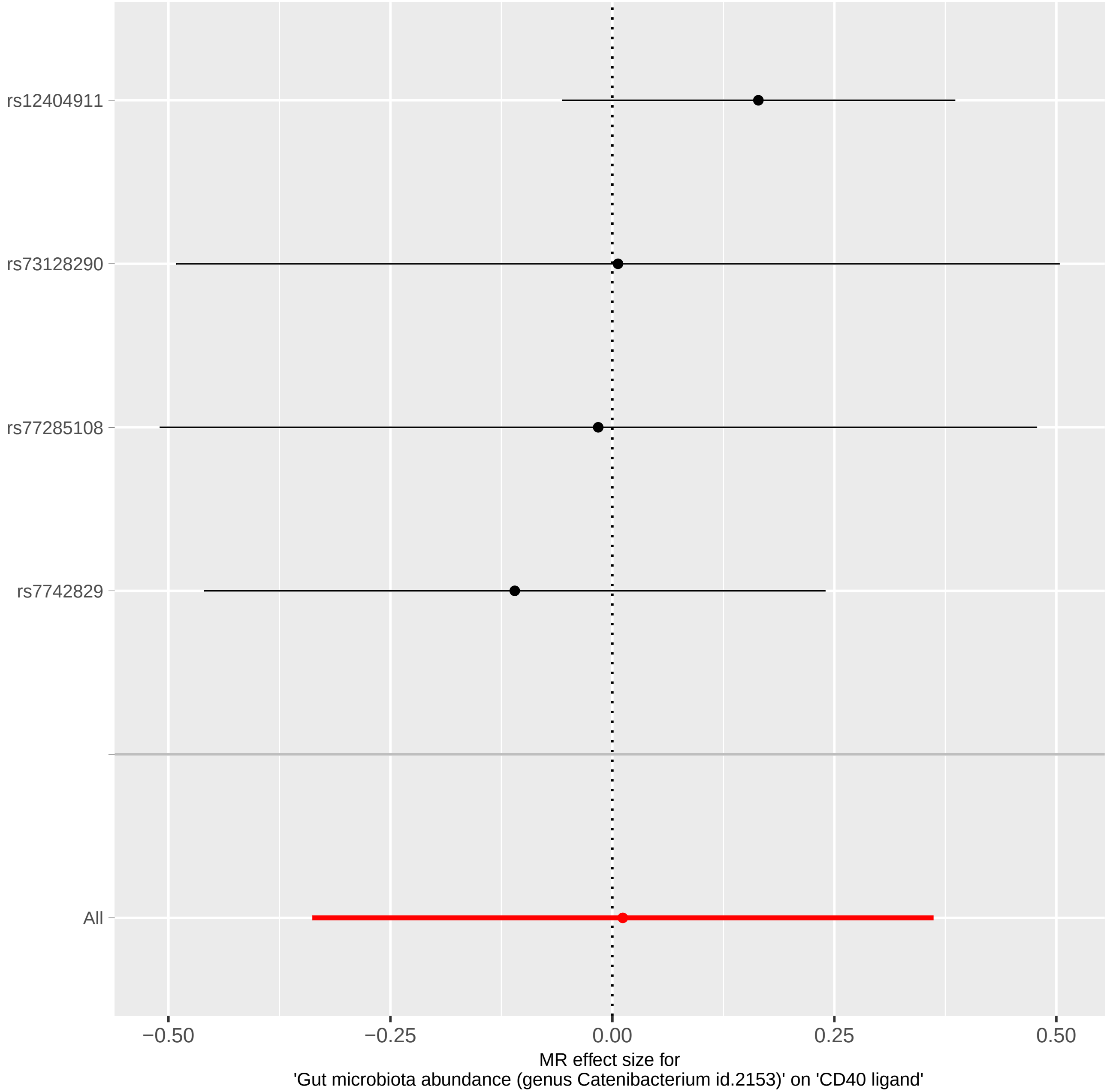

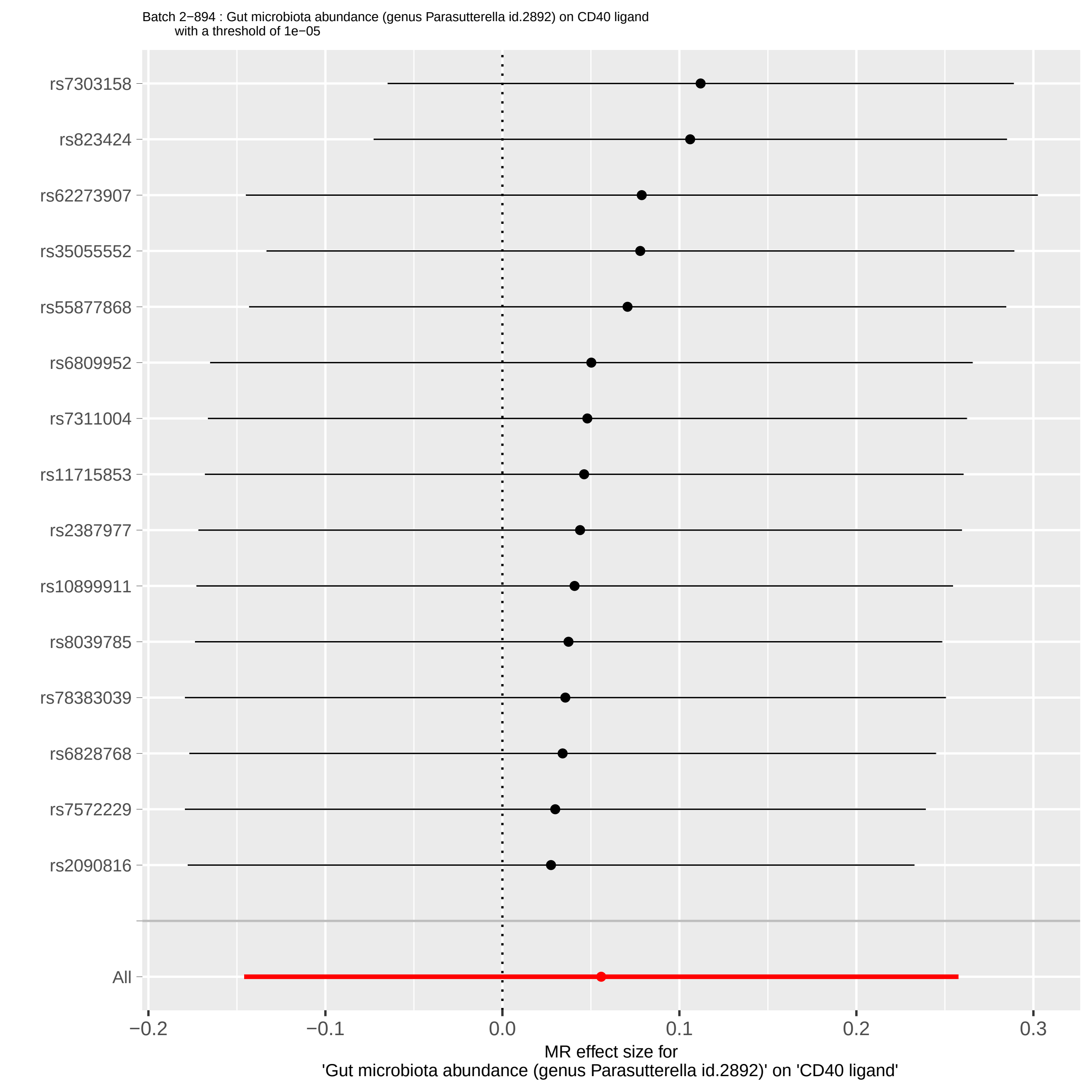
Batch 2−894 : Gut microbiota abundance (genus Parasutterella id.2892) on CD40 ligand
with a threshold of 1e−05
rs7303158
rs823424
rs62273907
rs35055552
rs55877868
rs6809952
rs7311004
rs11715853
rs2387977
rs10899911
rs8039785
rs78383039
rs6828768
rs7572229
rs2090816
All
−0.2
−0.1
0.0
0.1
0.2
0.3
MR effect size for
'Gut microbiota abundance (genus Parasutterella id.2892)' on 'CD40 ligand'

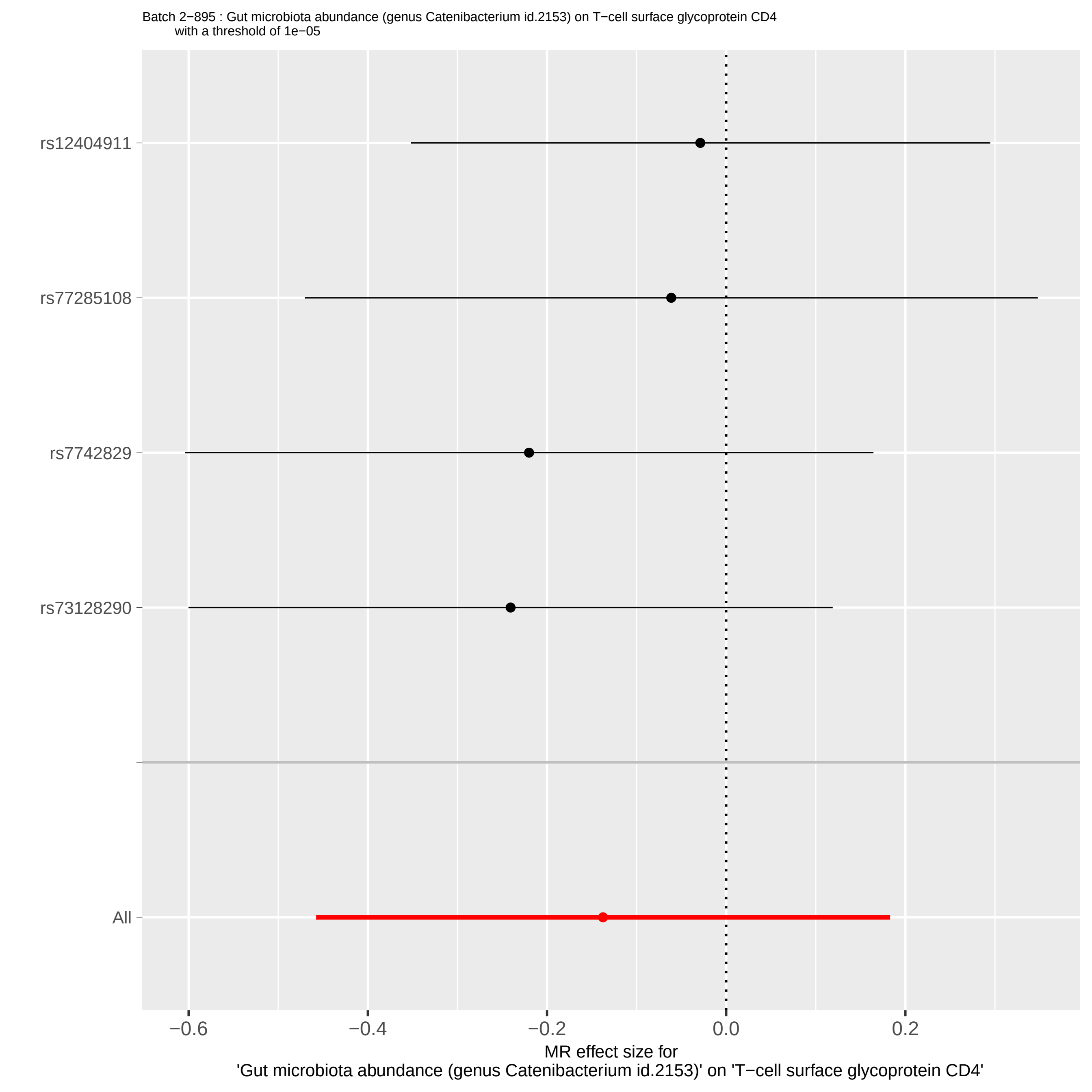
Batch 2−895 : Gut microbiota abundance (genus Catenibacterium id.2153) on T−cell surface glycoprotein CD4
with a threshold of 1e−05
rs12404911
rs77285108
rs7742829
rs73128290
All
−0.6
−0.4
−0.2
0.0
0.2
MR effect size for
'Gut microbiota abundance (genus Catenibacterium id.2153)' on 'T−cell surface glycoprotein CD4'

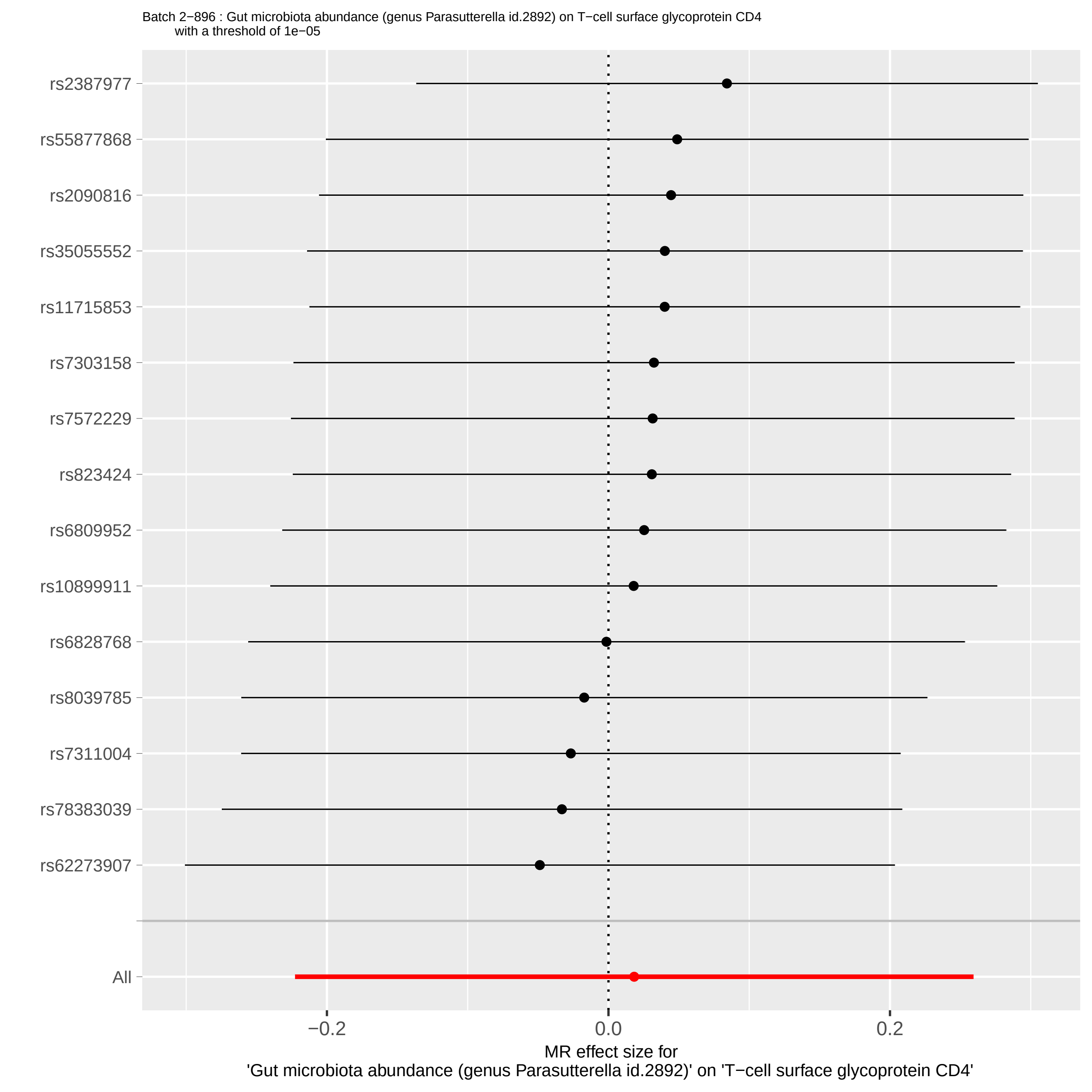

Batch 2−896 : Gut microbiota abundance (genus Parasutterella id.2892) on T−cell surface glycoprotein CD4
with a threshold of 1e−05
rs2387977
rs55877868
rs2090816
rs35055552
rs11715853
rs7303158
rs7572229
rs823424
rs6809952
rs10899911
rs6828768
rs8039785
rs7311004
rs78383039
rs62273907
All
−0.2
0.0
0.2
MR effect size for
'Gut microbiota abundance (genus Parasutterella id.2892)' on 'T−cell surface glycoprotein CD4'

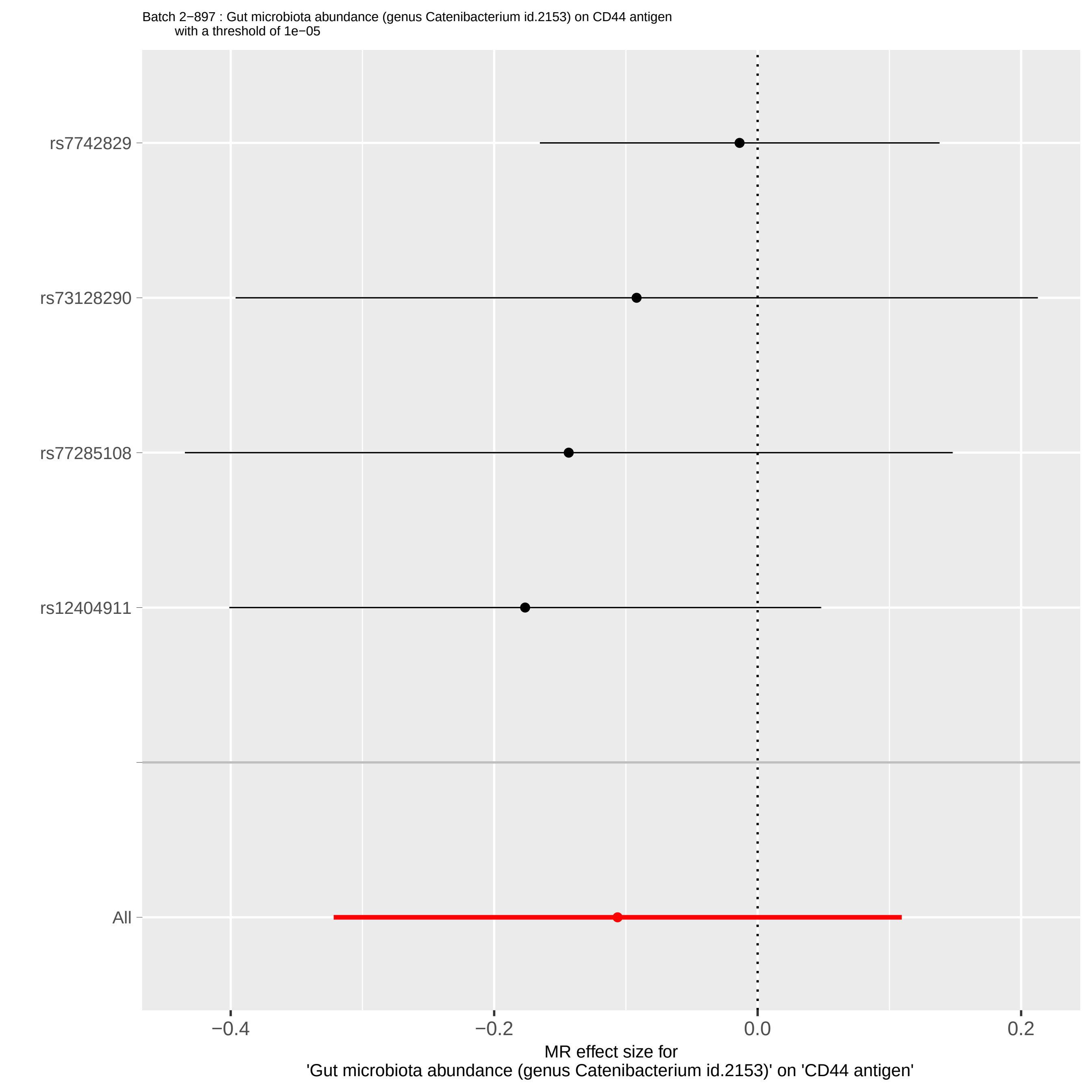

Batch 2−897 : Gut microbiota abundance (genus Catenibacterium id.2153) on CD44 antigen
with a threshold of 1e−05
rs7742829
rs73128290
rs77285108
rs12404911
All
−0.4
−0.2
0.0
0.2
MR effect size for
'Gut microbiota abundance (genus Catenibacterium id.2153)' on 'CD44 antigen'

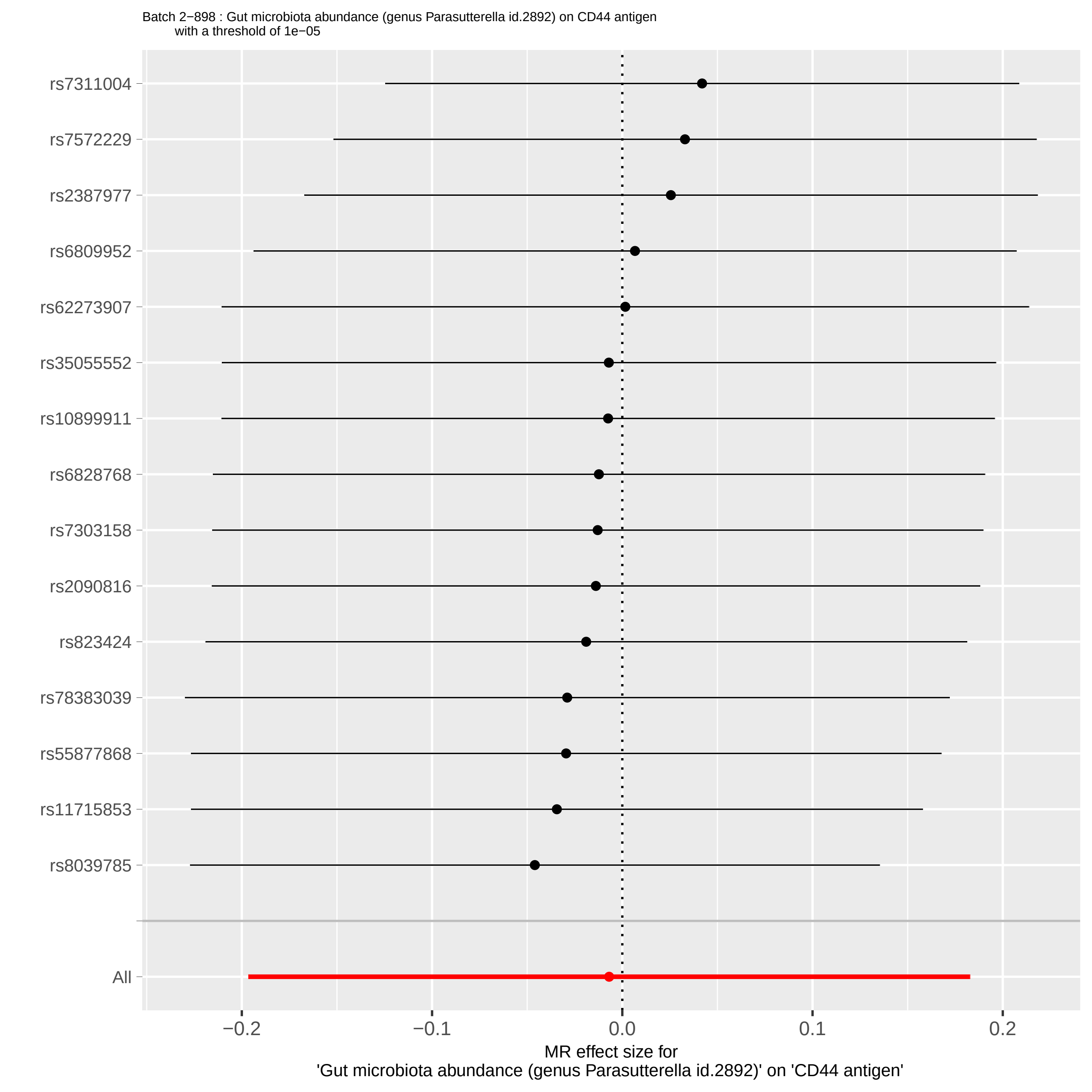

Batch 2−898 : Gut microbiota abundance (genus Parasutterella id.2892) on CD44 antigen
with a threshold of 1e−05
rs7311004
rs7572229
rs2387977
rs6809952
rs62273907
rs35055552
rs10899911
rs6828768
rs7303158
rs2090816
rs823424
rs78383039
rs55877868
rs11715853
rs8039785
All
−0.2
−0.1
0.0
0.1
0.2
MR effect size for
'Gut microbiota abundance (genus Parasutterella id.2892)' on 'CD44 antigen'

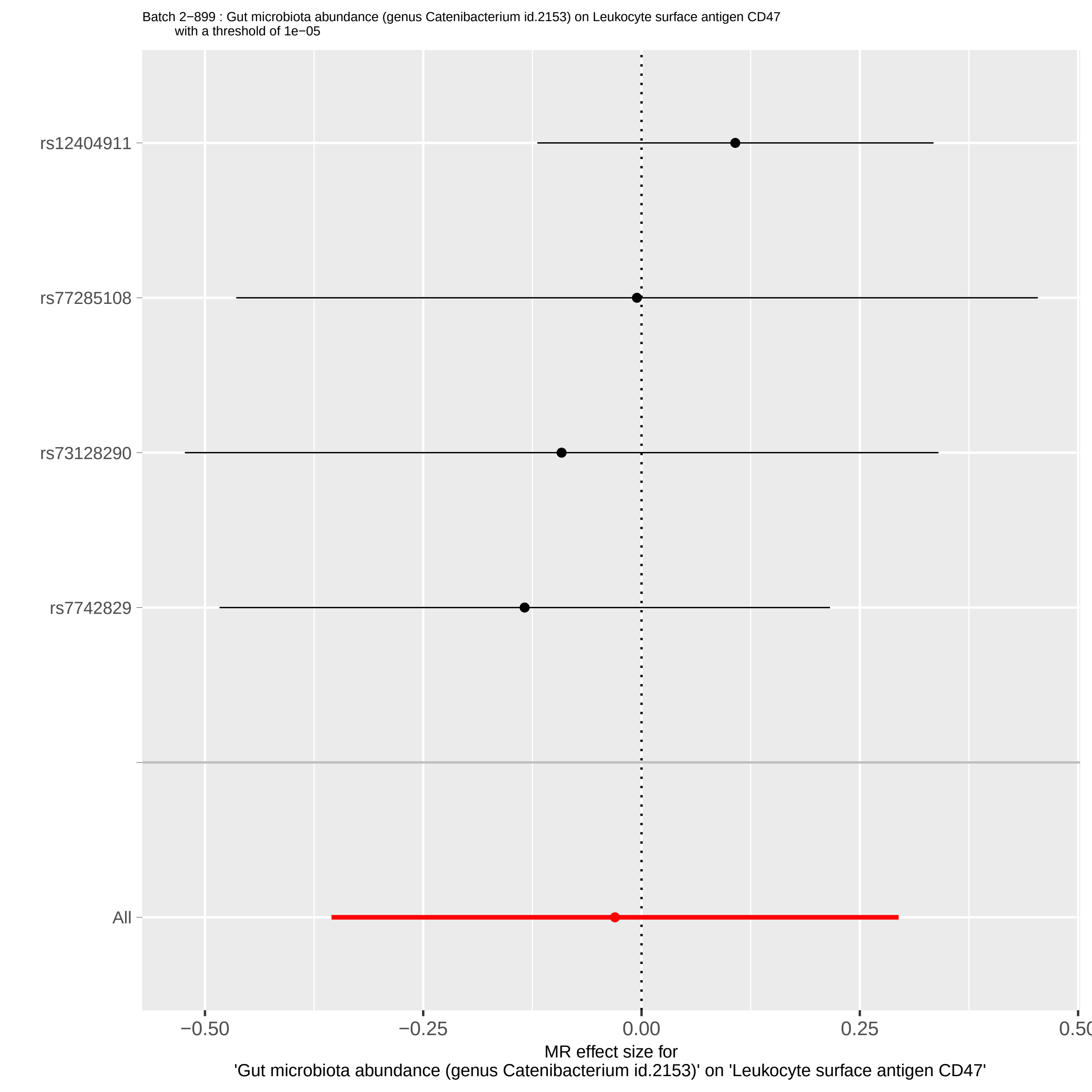

Batch 2−899 : Gut microbiota abundance (genus Catenibacterium id.2153) on Leukocyte surface antigen CD47
with a threshold of 1e−05
rs12404911
rs77285108
rs73128290
rs7742829
All
−0.50
−0.25
0.00
0.25
0.50
MR effect size for
'Gut microbiota abundance (genus Catenibacterium id.2153)' on 'Leukocyte surface antigen CD47'

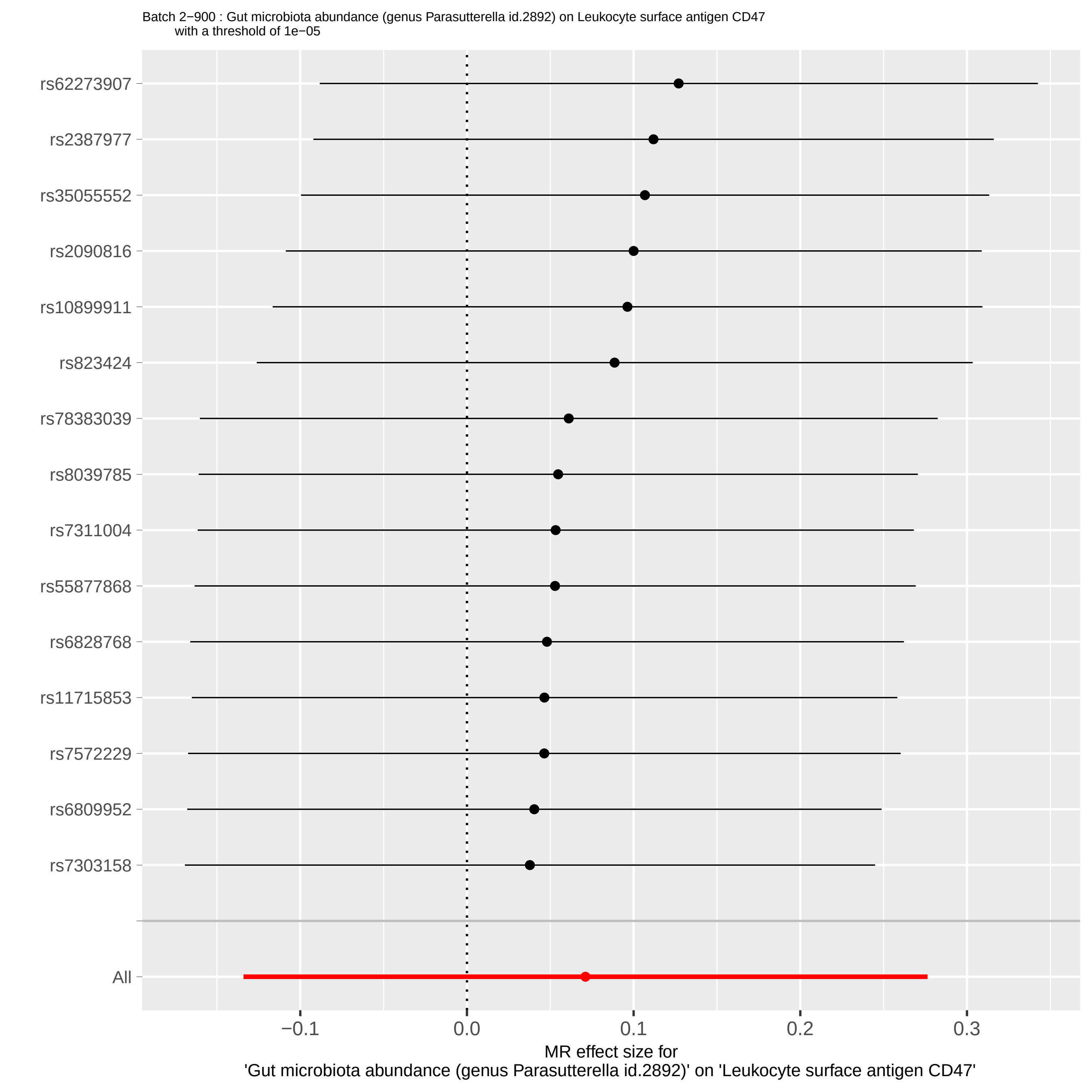
Batch 2−900 : Gut microbiota abundance (genus Parasutterella id.2892) on Leukocyte surface antigen CD47
with a threshold of 1e−05
rs62273907
rs2387977
rs35055552
rs2090816
rs10899911
rs823424
rs78383039
rs8039785
rs7311004
rs55877868
rs6828768
rs11715853
rs7572229
rs6809952
rs7303158
All
−0.1
0.0
0.1
0.2
0.3
MR effect size for
'Gut microbiota abundance (genus Parasutterella id.2892)' on 'Leukocyte surface antigen CD47'

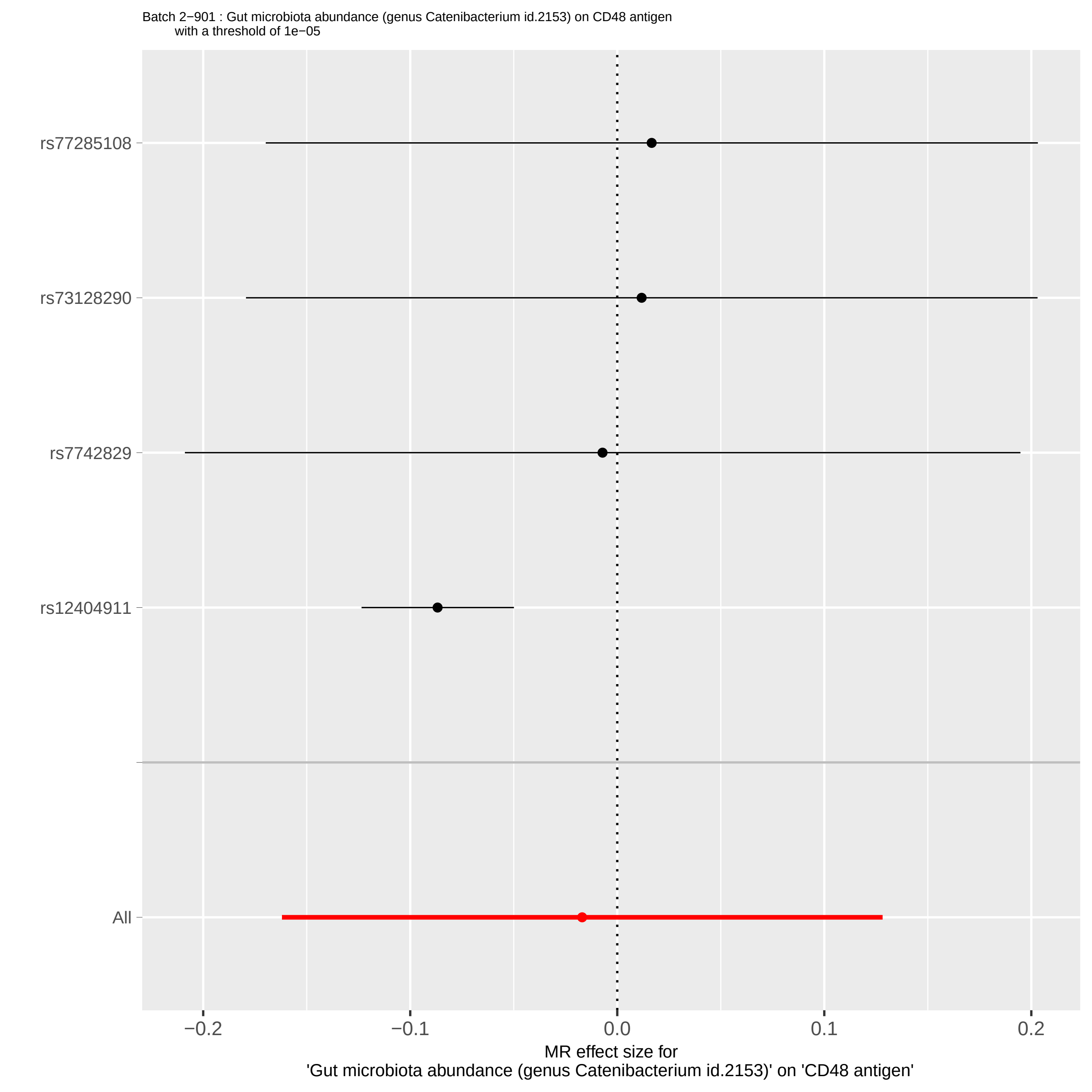

Batch 2−901 : Gut microbiota abundance (genus Catenibacterium id.2153) on CD48 antigen
with a threshold of 1e−05
rs77285108
rs73128290
rs7742829
rs12404911
All
−0.2
−0.1
0.0
0.1
0.2
MR effect size for
'Gut microbiota abundance (genus Catenibacterium id.2153)' on 'CD48 antigen'

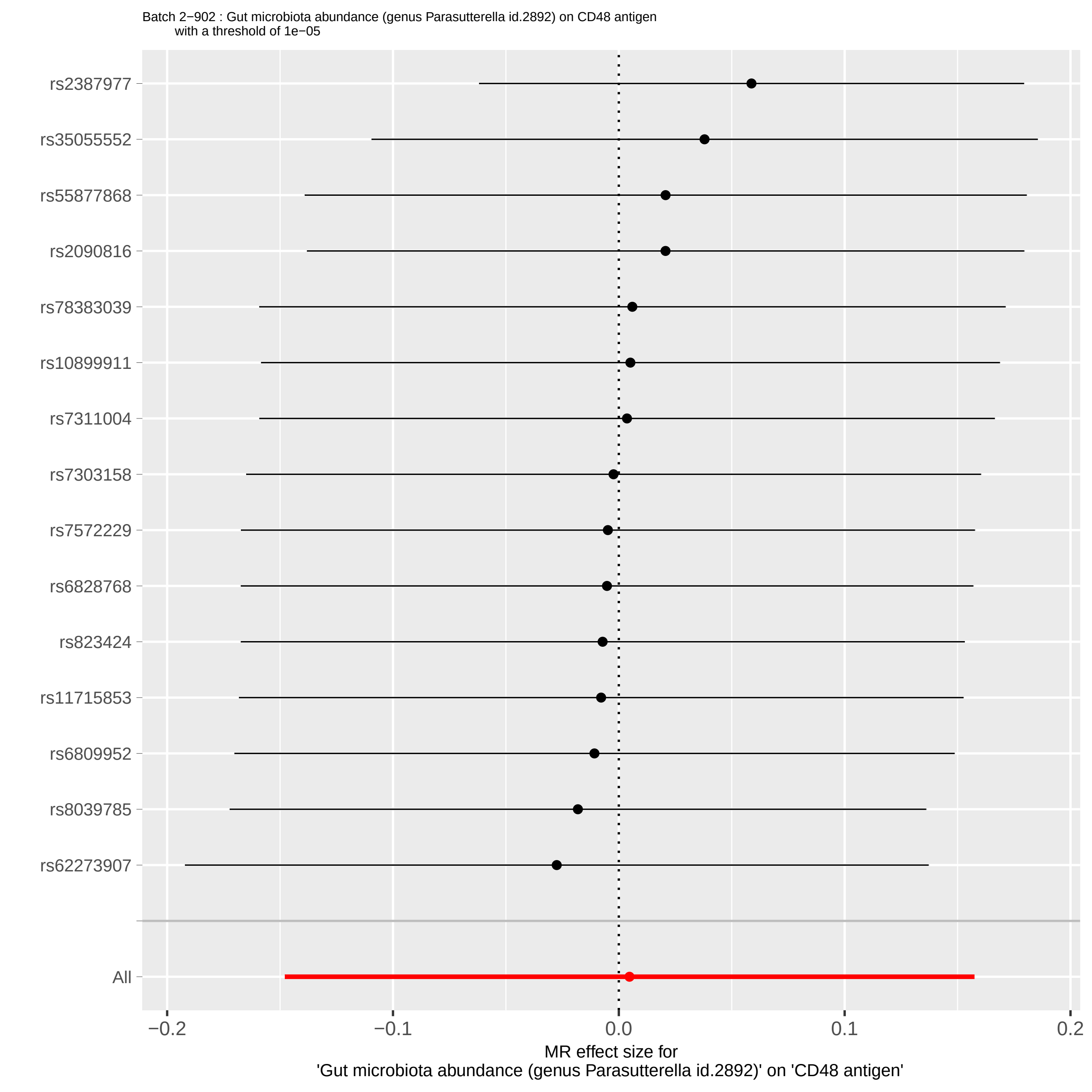
Batch 2−902 : Gut microbiota abundance (genus Parasutterella id.2892) on CD48 antigen
with a threshold of 1e−05
rs2387977
rs35055552
rs55877868
rs2090816
rs78383039
rs10899911
rs7311004
rs7303158
rs7572229
rs6828768
rs823424
rs11715853
rs6809952
rs8039785
rs62273907
All
−0.2
−0.1
0.0
0.1
0.2
MR effect size for
'Gut microbiota abundance (genus Parasutterella id.2892)' on 'CD48 antigen'

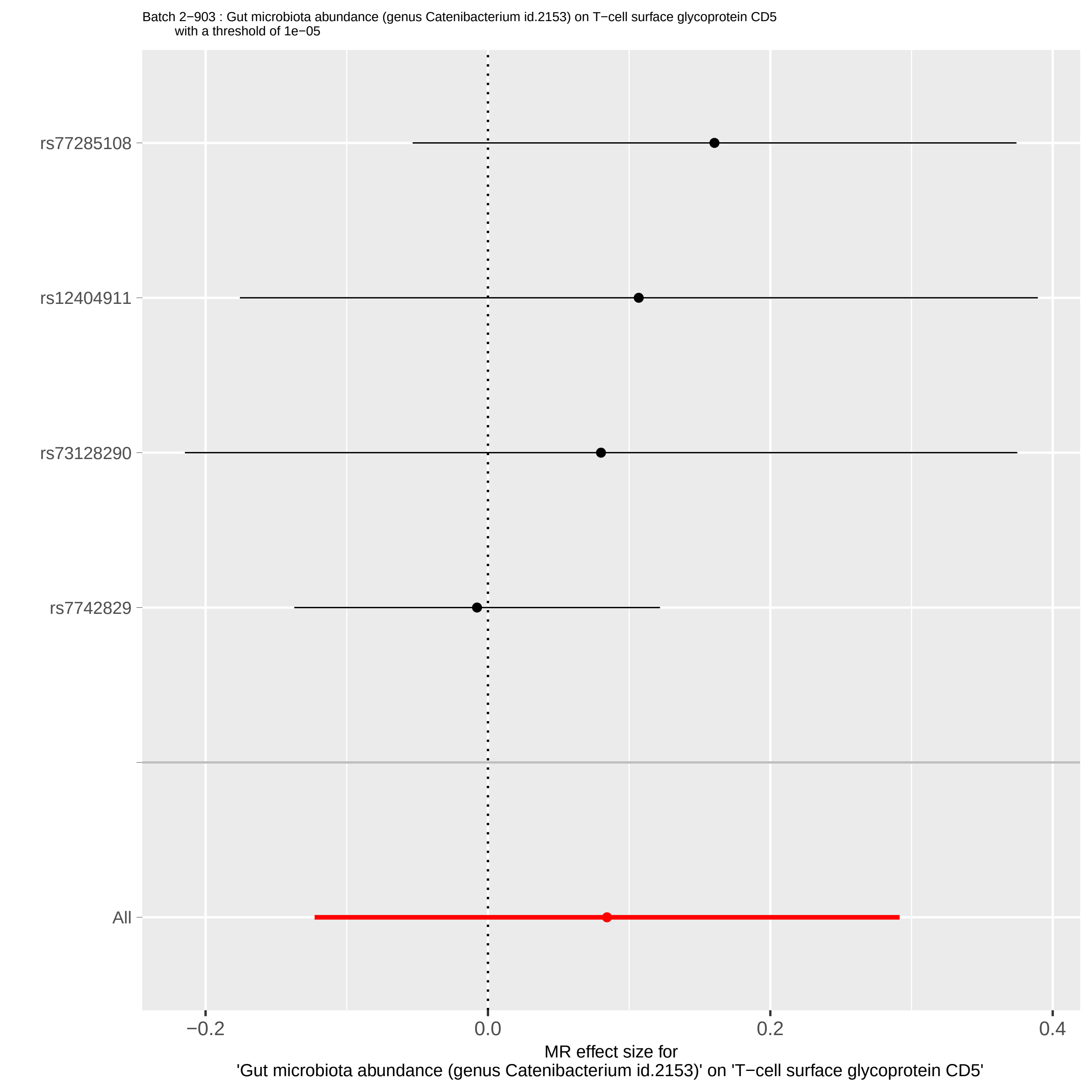

Batch 2−903 : Gut microbiota abundance (genus Catenibacterium id.2153) on T−cell surface glycoprotein CD5
with a threshold of 1e−05
rs77285108
rs12404911
rs73128290
rs7742829
All
−0.2
0.0
0.2
0.4
MR effect size for
'Gut microbiota abundance (genus Catenibacterium id.2153)' on 'T−cell surface glycoprotein CD5'

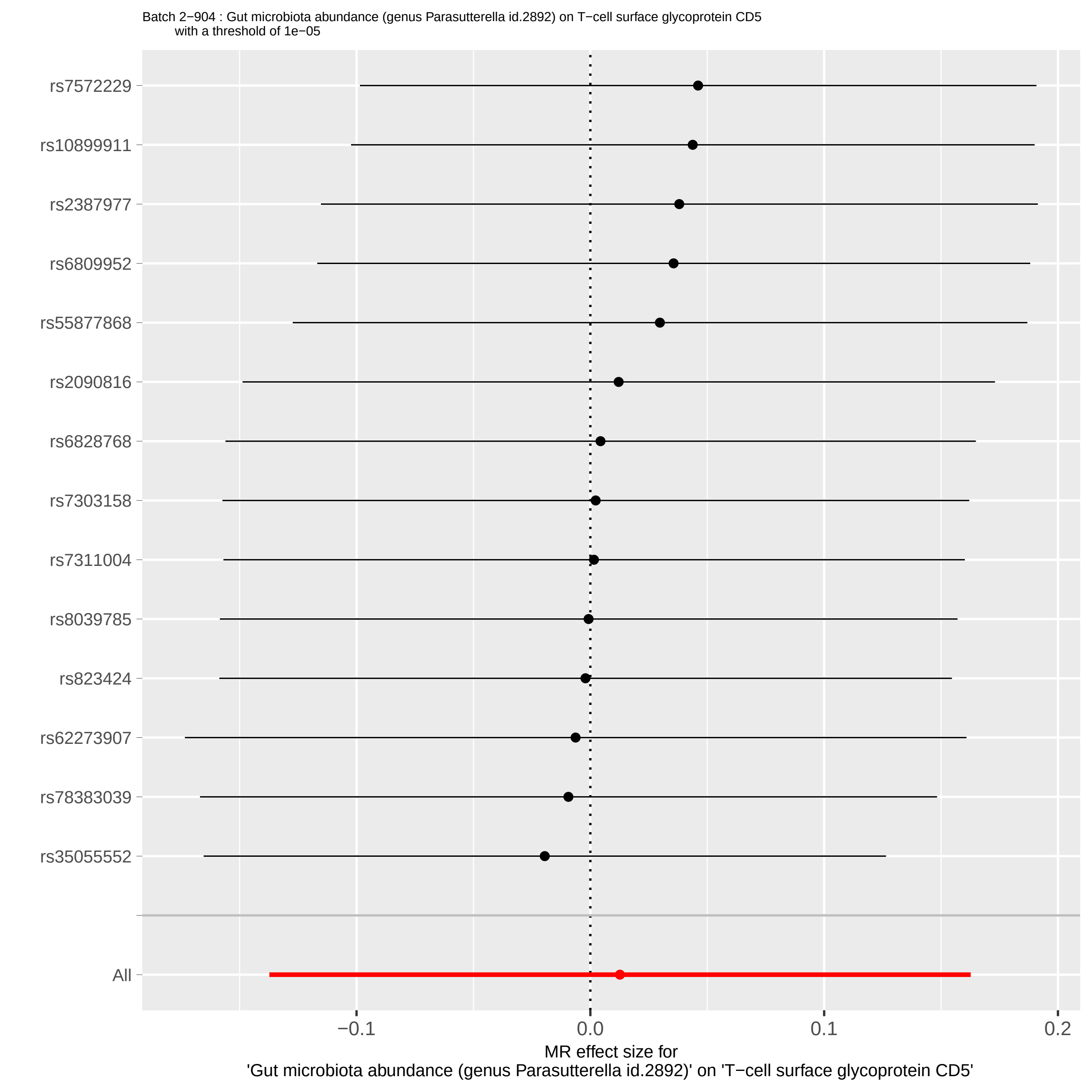
Batch 2−904 : Gut microbiota abundance (genus Parasutterella id.2892) on T−cell surface glycoprotein CD5
with a threshold of 1e−05
rs7572229
rs10899911
rs2387977
rs6809952
rs55877868
rs2090816
rs6828768
rs7303158
rs7311004
rs8039785
rs823424
rs62273907
rs78383039
rs35055552
All
−0.1
0.0
0.1
0.2
MR effect size for
'Gut microbiota abundance (genus Parasutterella id.2892)' on 'T−cell surface glycoprotein CD5'

Batch 2−905 : Gut microbiota abundance (genus Catenibacterium id.2153) on Complement decay−accelerating factor
with a threshold of 1e−05

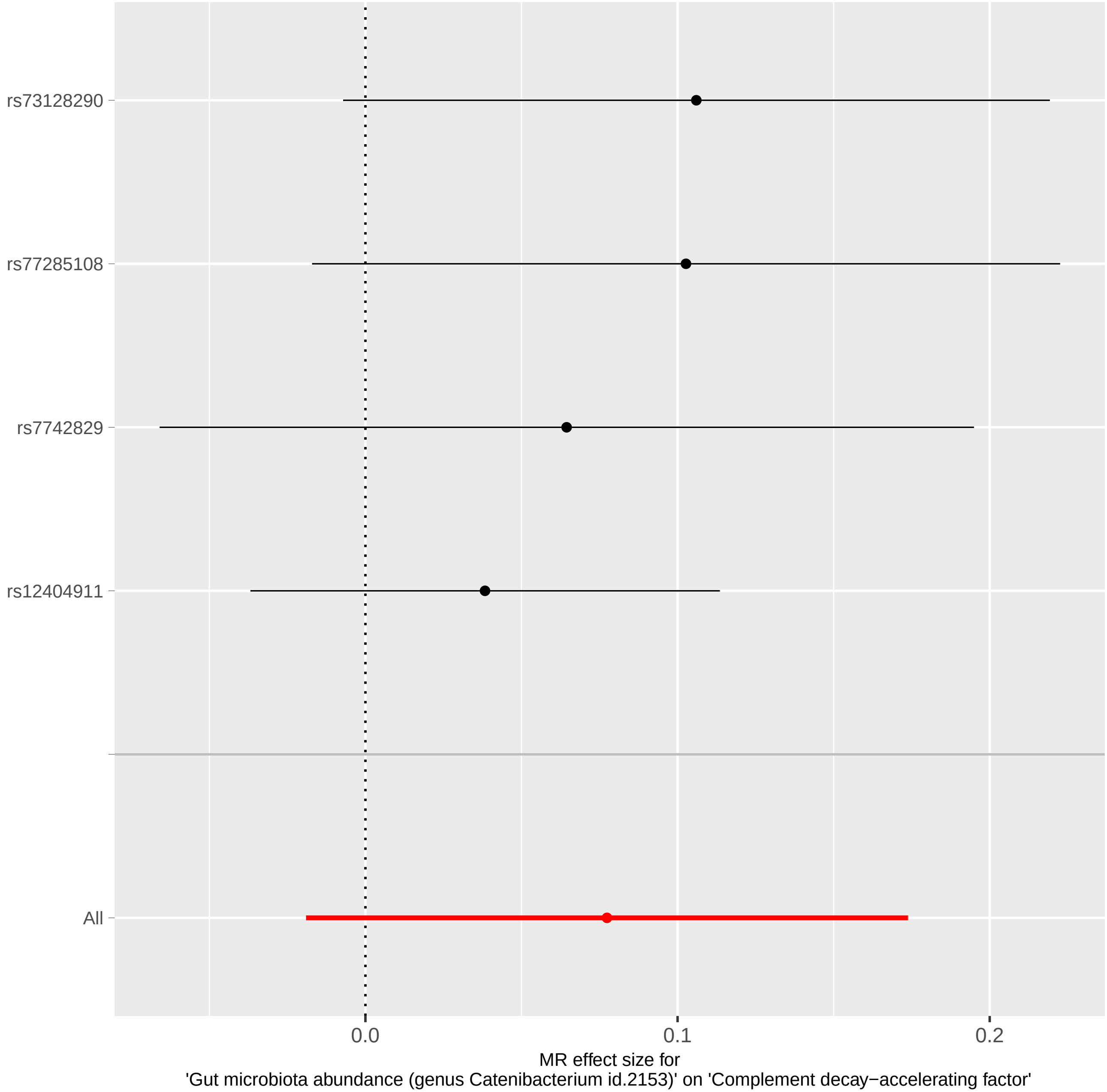

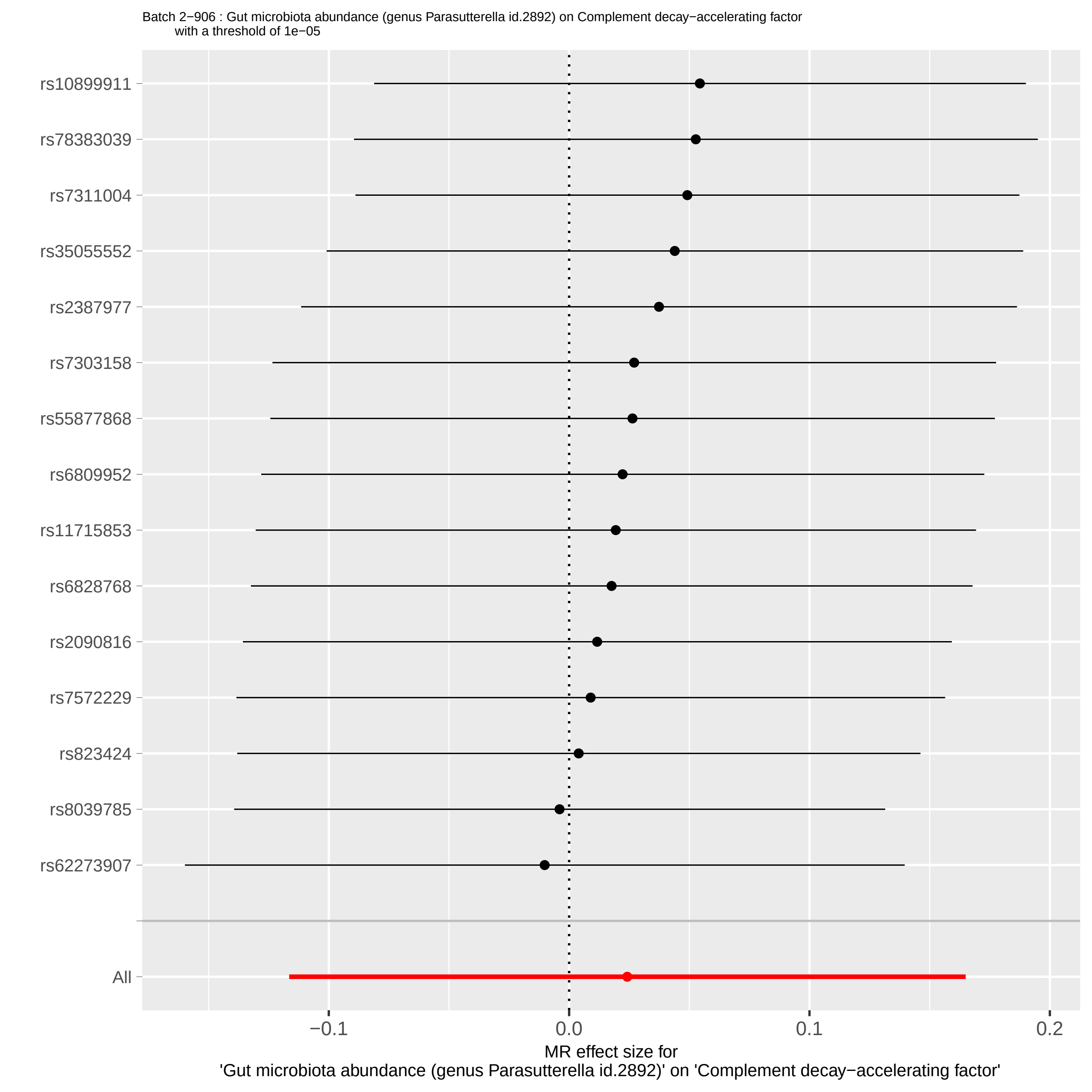
Batch 2−906 : Gut microbiota abundance (genus Parasutterella id.2892) on Complement decay−accelerating factor
with a threshold of 1e−05
rs10899911
rs78383039
rs7311004
rs35055552
rs2387977
rs7303158
rs55877868
rs6809952
rs11715853
rs6828768
rs2090816
rs7572229
rs823424
rs8039785
rs62273907
All
−0.1
0.0
0.1
0.2
MR effect size for
'Gut microbiota abundance (genus Parasutterella id.2892)' on 'Complement decay−accelerating factor'

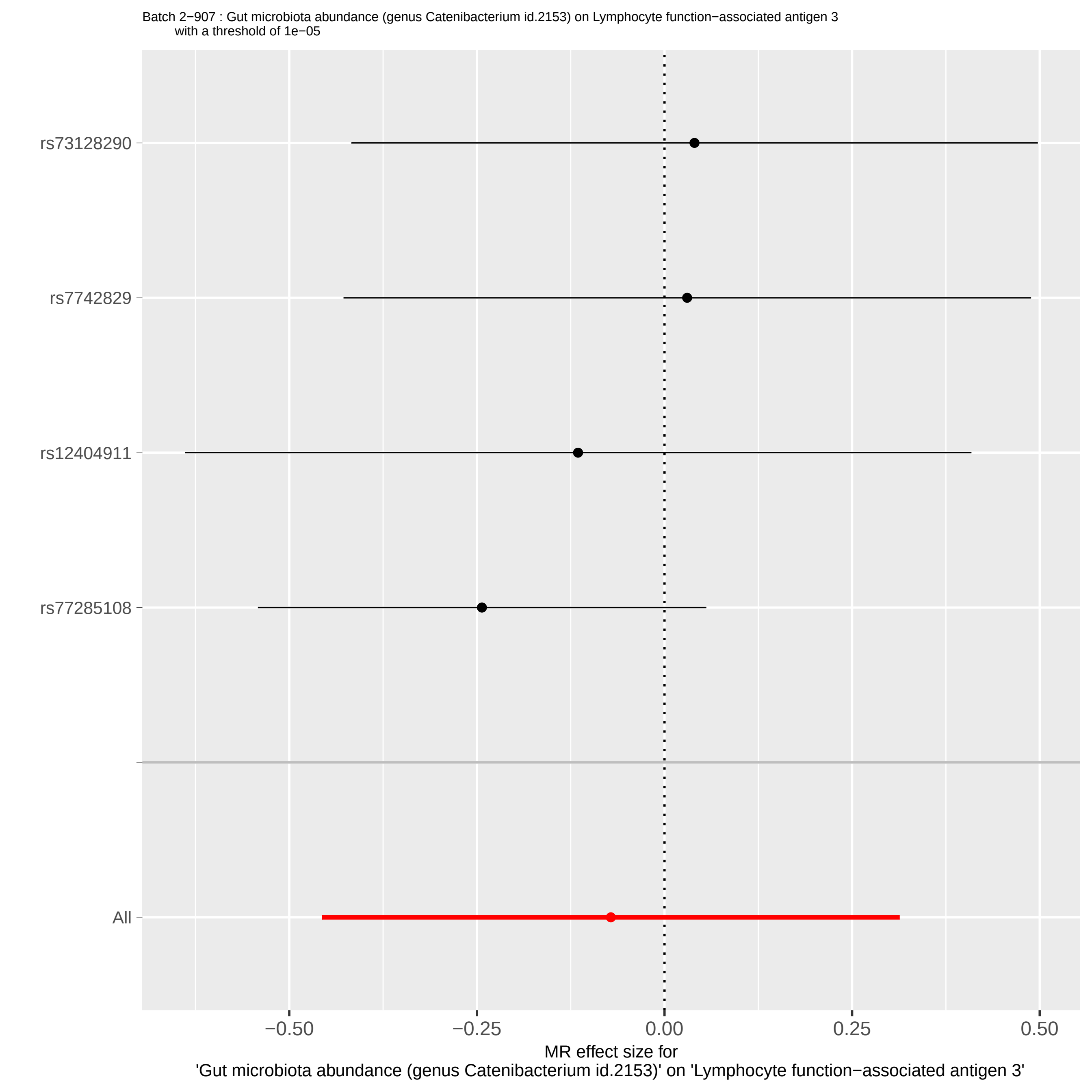
Batch 2−907 : Gut microbiota abundance (genus Catenibacterium id.2153) on Lymphocyte function−associated antigen 3
with a threshold of 1e−05
rs73128290
rs7742829
rs12404911
rs77285108
All
−0.50
−0.25
0.00
0.25
0.50
MR effect size for
'Gut microbiota abundance (genus Catenibacterium id.2153)' on 'Lymphocyte function−associated antigen 3'

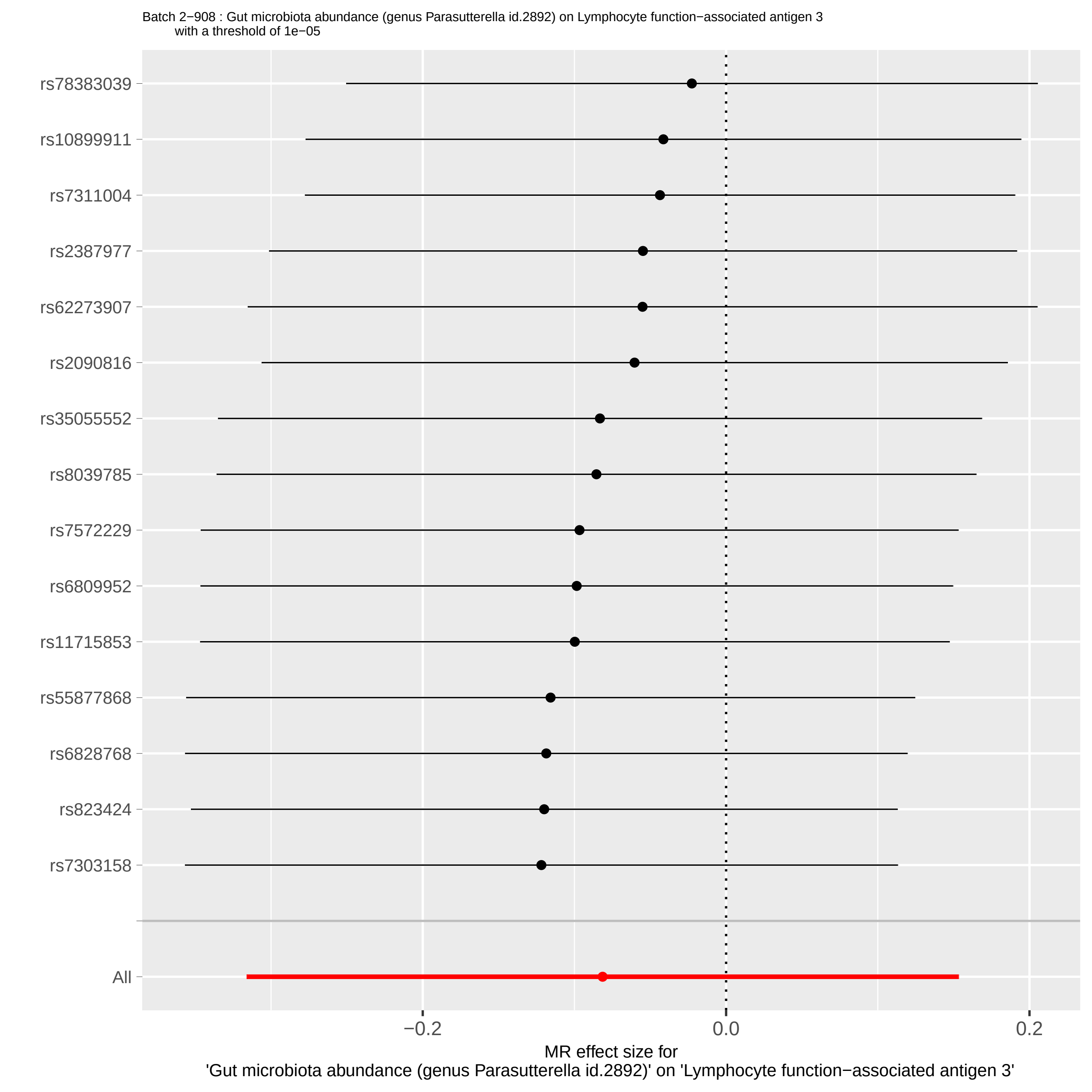

Batch 2−908 : Gut microbiota abundance (genus Parasutterella id.2892) on Lymphocyte function−associated antigen 3
with a threshold of 1e−05
rs78383039
rs10899911
rs7311004
rs2387977
rs62273907
rs2090816
rs35055552
rs8039785
rs7572229
rs6809952
rs11715853
rs55877868
rs6828768
rs823424
rs7303158
All
−0.2
0.0
0.2
MR effect size for
'Gut microbiota abundance (genus Parasutterella id.2892)' on 'Lymphocyte function−associated antigen 3'

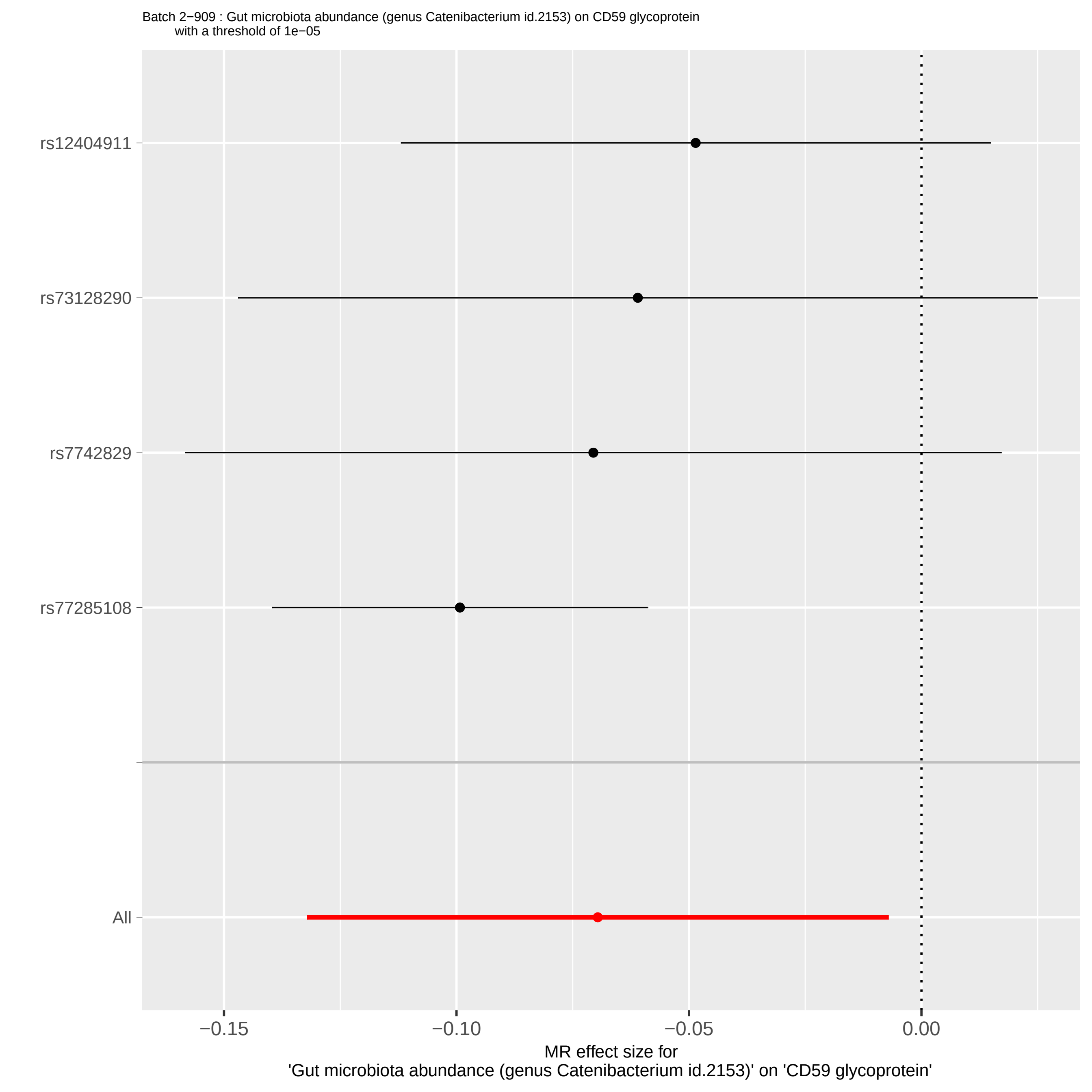

Batch 2−909 : Gut microbiota abundance (genus Catenibacterium id.2153) on CD59 glycoprotein
with a threshold of 1e−05
rs12404911
rs73128290
rs7742829
rs77285108
All
−0.15
−0.10
−0.05
0.00
MR effect size for
'Gut microbiota abundance (genus Catenibacterium id.2153)' on 'CD59 glycoprotein'

Batch 2−910 : Gut microbiota abundance (genus Parasutterella id.2892) on CD59 glycoprotein with a threshold of 1e−05

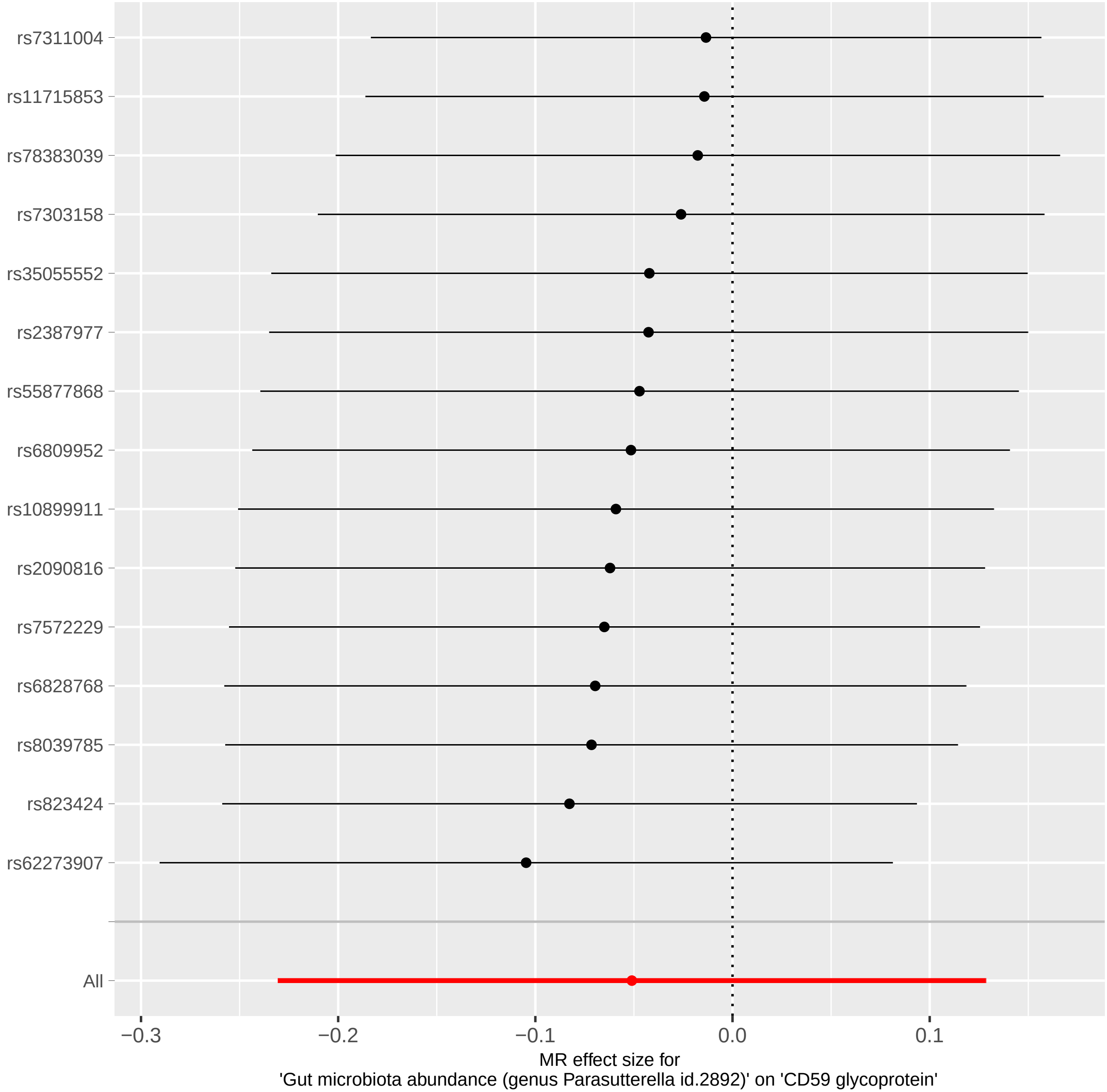

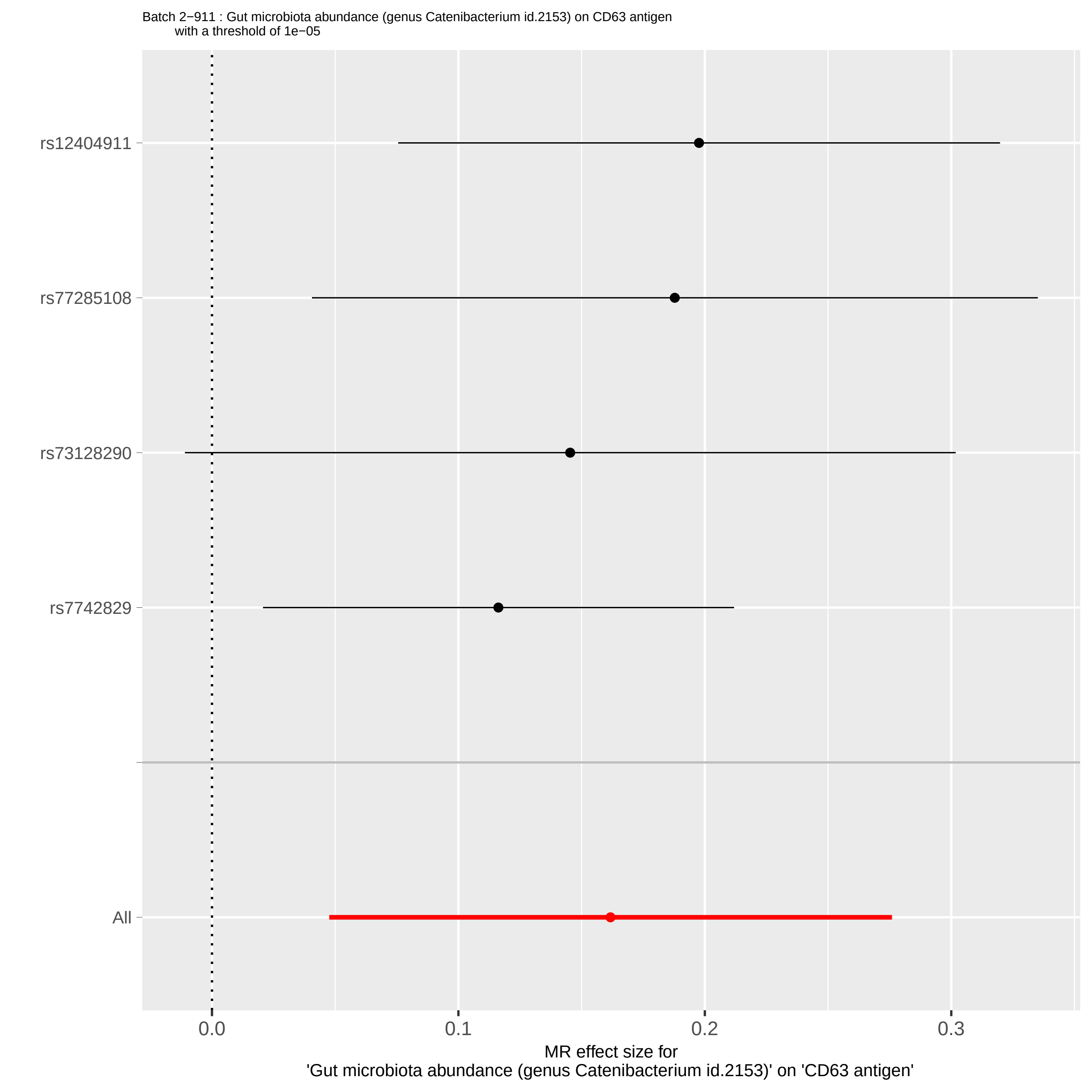
Batch 2−911 : Gut microbiota abundance (genus Catenibacterium id.2153) on CD63 antigen
with a threshold of 1e−05
rs12404911
rs77285108
rs73128290
rs7742829
All
0.0
0.1
0.2
0.3
MR effect size for
'Gut microbiota abundance (genus Catenibacterium id.2153)' on 'CD63 antigen'

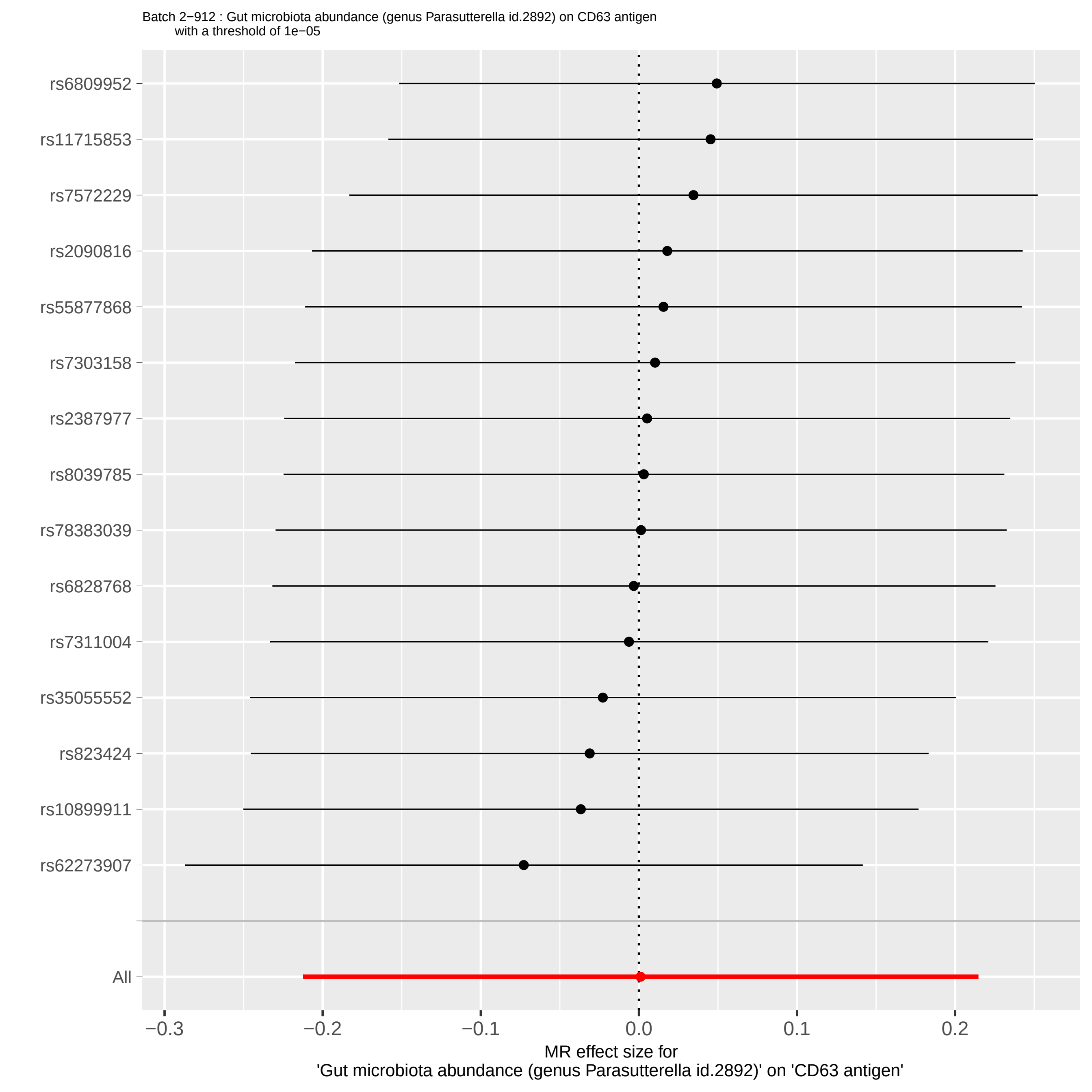
Batch 2−912 : Gut microbiota abundance (genus Parasutterella id.2892) on CD63 antigen
with a threshold of 1e−05
rs6809952
rs11715853
rs7572229
rs2090816
rs55877868
rs7303158
rs2387977
rs8039785
rs78383039
rs6828768
rs7311004
rs35055552
rs823424
rs10899911
rs62273907
All
−0.3
−0.2
−0.1
0.0
0.1
0.2
MR effect size for
'Gut microbiota abundance (genus Parasutterella id.2892)' on 'CD63 antigen'

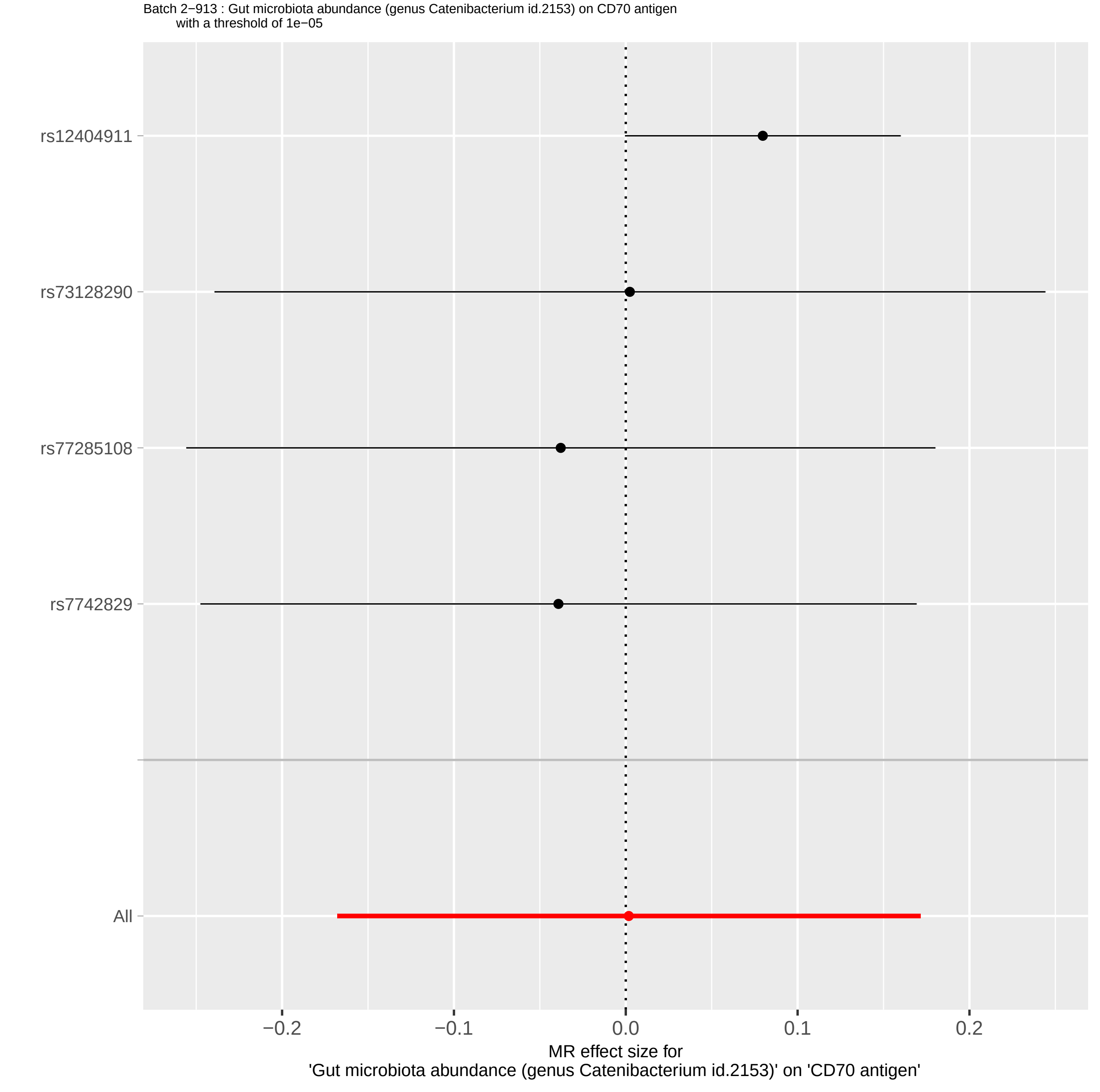
Batch 2−913 : Gut microbiota abundance (genus Catenibacterium id.2153) on CD70 antigen
with a threshold of 1e−05
rs12404911
rs73128290
rs77285108
rs7742829
All
−0.2
−0.1
0.0
0.1
0.2
MR effect size for
'Gut microbiota abundance (genus Catenibacterium id.2153)' on 'CD70 antigen'

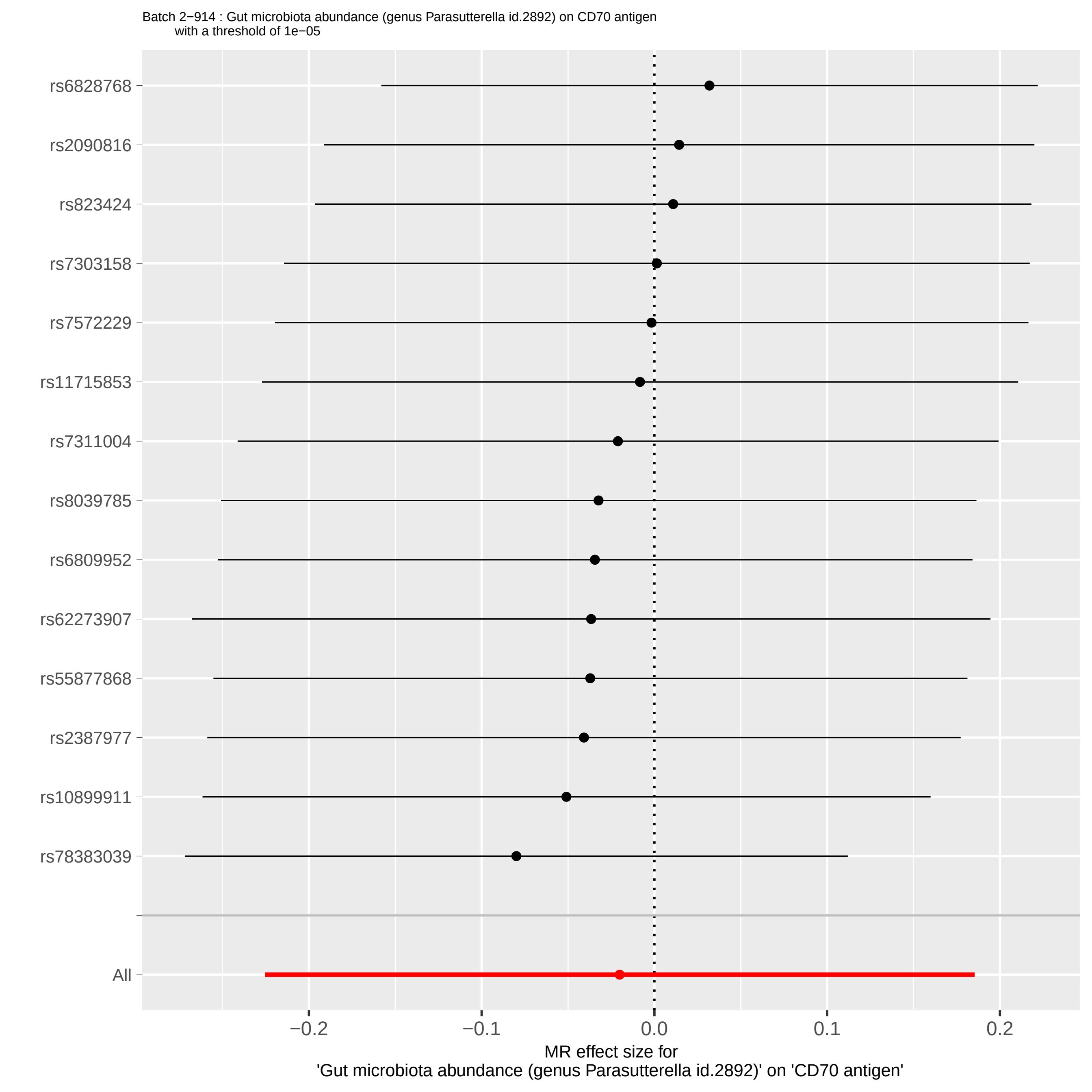

Batch 2−914 : Gut microbiota abundance (genus Parasutterella id.2892) on CD70 antigen
with a threshold of 1e−05
rs6828768
rs2090816
rs823424
rs7303158
rs7572229
rs11715853
rs7311004
rs8039785
rs6809952
rs62273907
rs55877868
rs2387977
rs10899911
rs78383039
All
−0.2
−0.1
0.0
0.1
0.2
MR effect size for
'Gut microbiota abundance (genus Parasutterella id.2892)' on 'CD70 antigen'

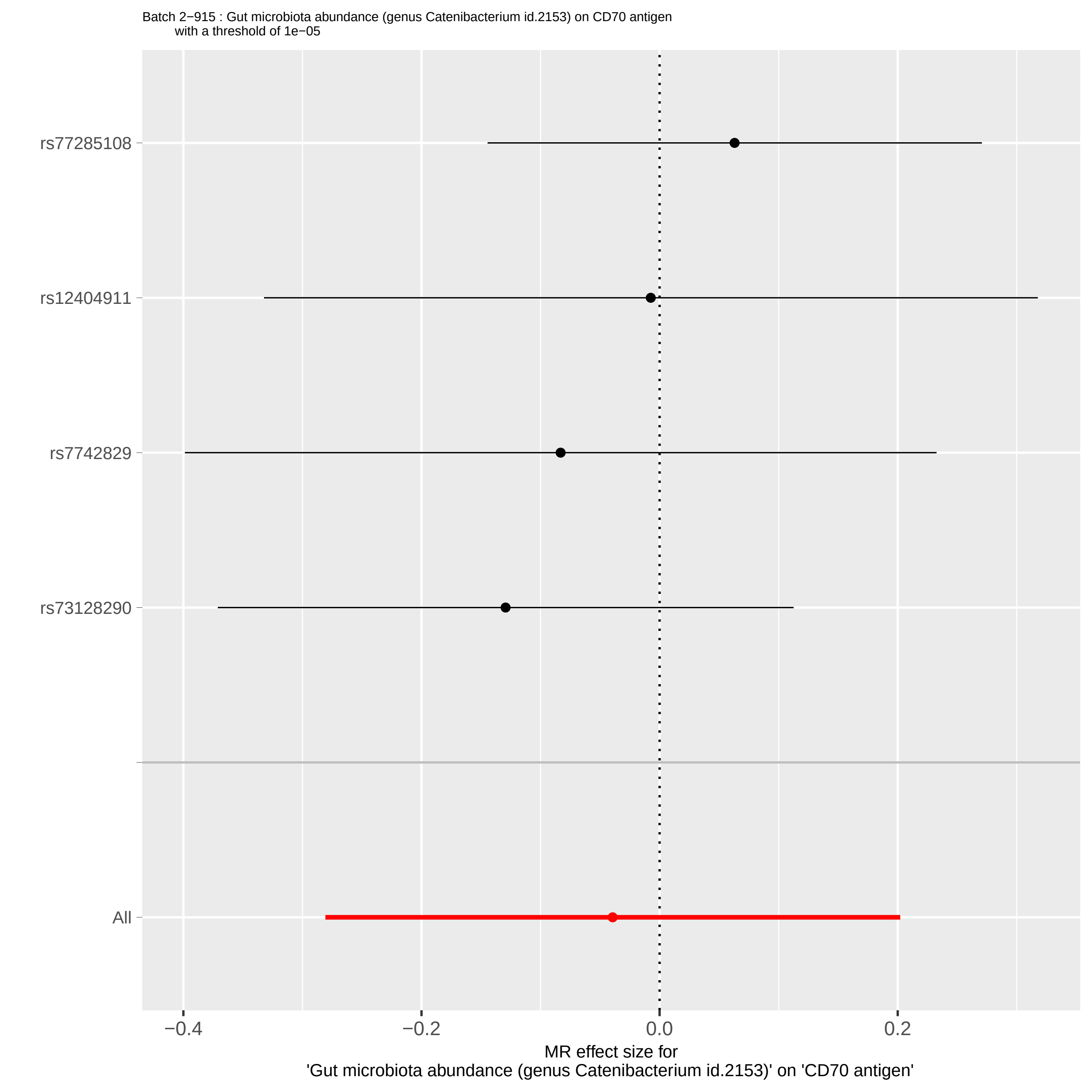
Batch 2−915 : Gut microbiota abundance (genus Catenibacterium id.2153) on CD70 antigen
with a threshold of 1e−05
rs77285108
rs12404911
rs7742829
rs73128290
All
−0.4
−0.2
0.0
0.2
MR effect size for
'Gut microbiota abundance (genus Catenibacterium id.2153)' on 'CD70 antigen'

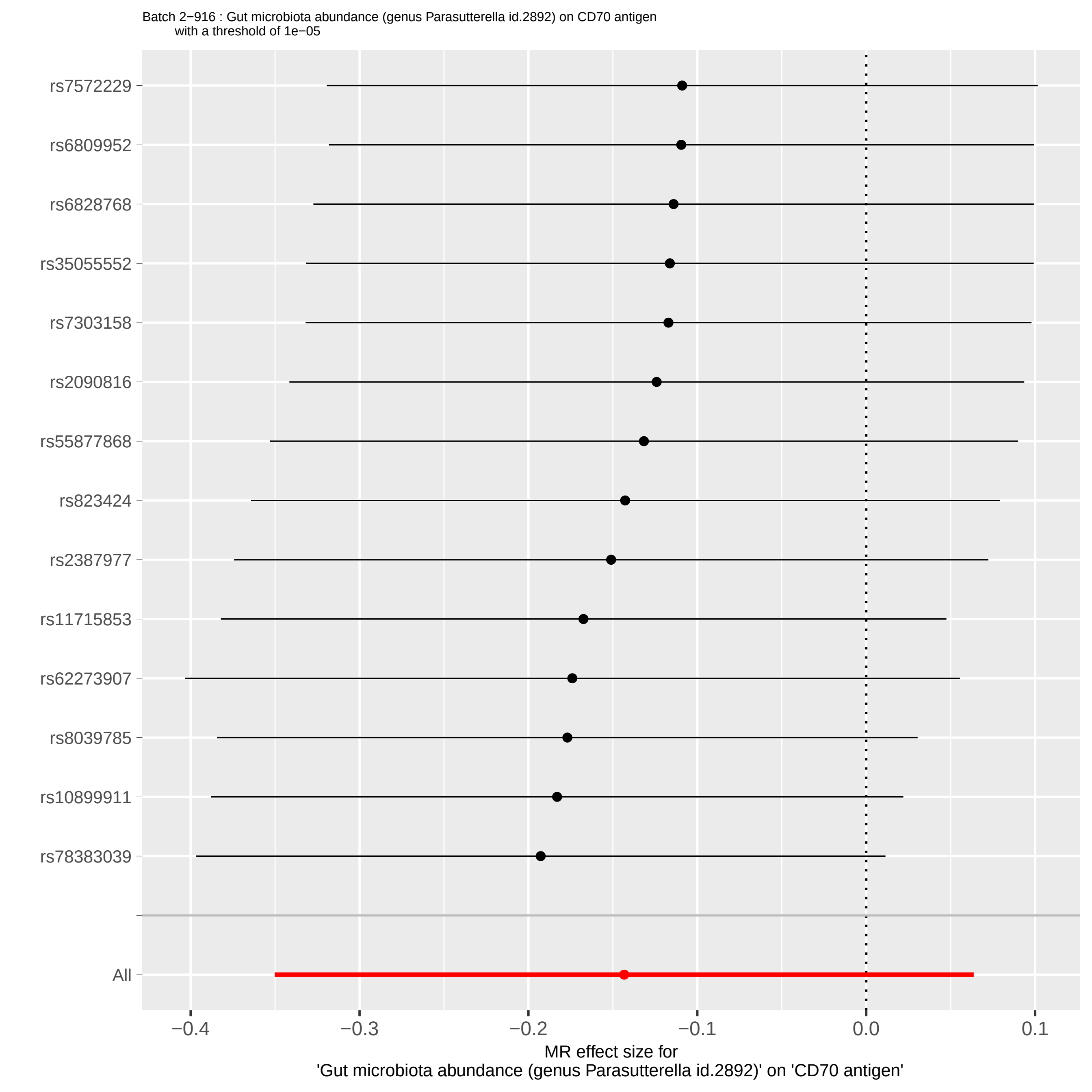

Batch 2−916 : Gut microbiota abundance (genus Parasutterella id.2892) on CD70 antigen
with a threshold of 1e−05
rs7572229
rs6809952
rs6828768
rs35055552
rs7303158
rs2090816
rs55877868
rs823424
rs2387977
rs11715853
rs62273907
rs8039785
rs10899911
rs78383039
All
−0.4
−0.3
−0.2
−0.1
0.0
0.1
MR effect size for
'Gut microbiota abundance (genus Parasutterella id.2892)' on 'CD70 antigen'

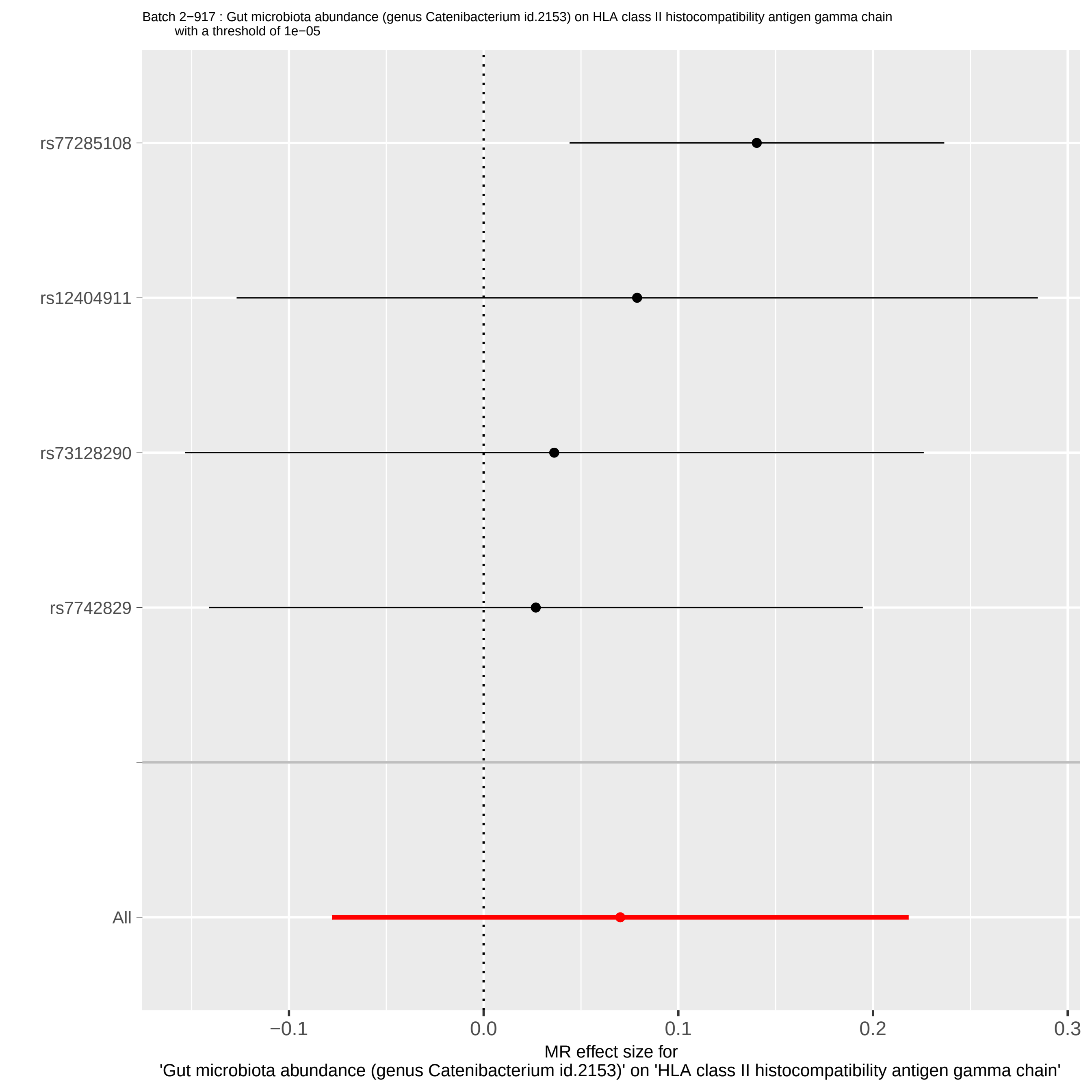
Batch 2−917 : Gut microbiota abundance (genus Catenibacterium id.2153) on HLA class II histocompatibility antigen gamma chain
with a threshold of 1e−05
rs77285108
rs12404911
rs73128290
rs7742829
All
−0.1
0.0
0.1
0.2
0.3
MR effect size for
'Gut microbiota abundance (genus Catenibacterium id.2153)' on 'HLA class II histocompatibility antigen gamma chain'

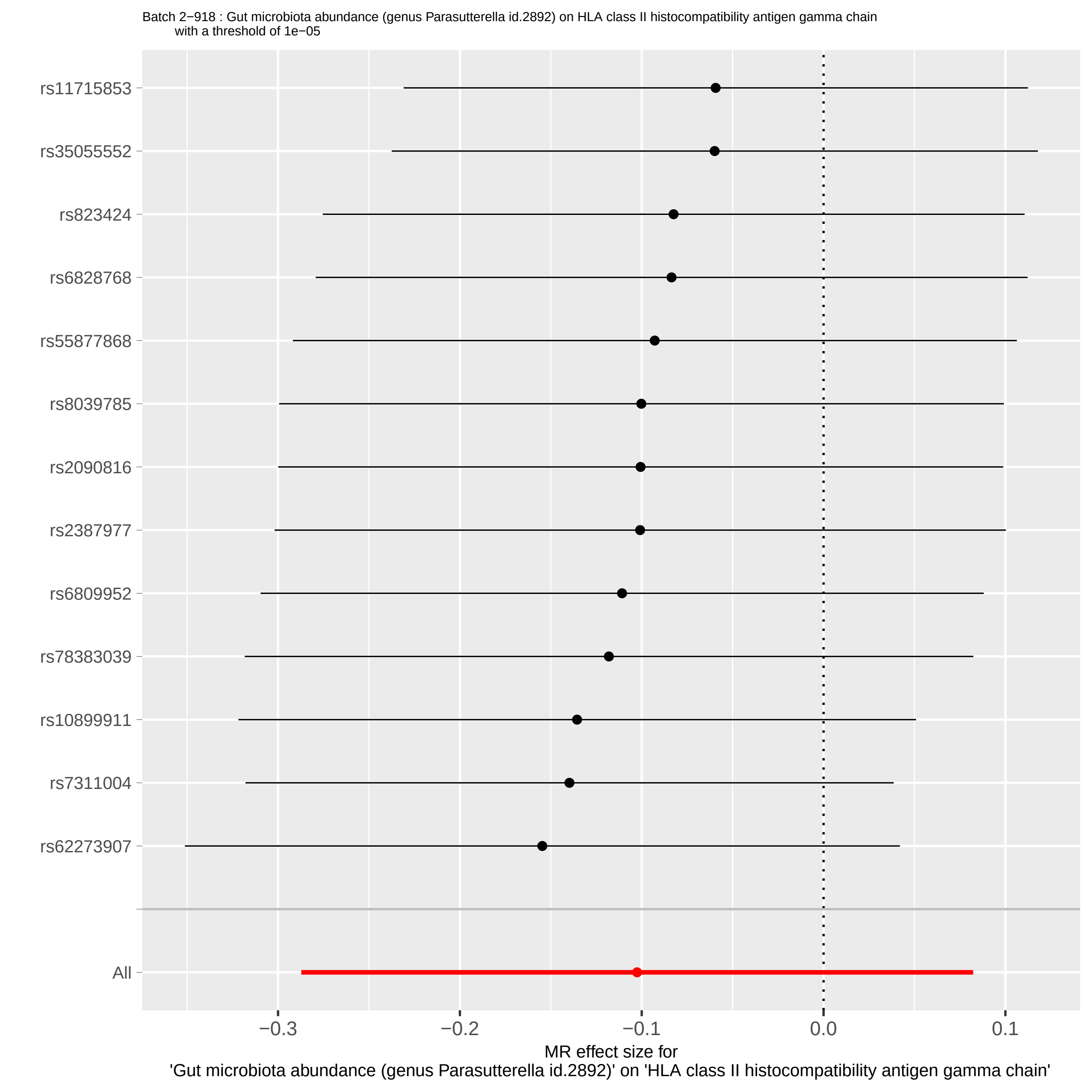
Batch 2−918 : Gut microbiota abundance (genus Parasutterella id.2892) on HLA class II histocompatibility antigen gamma chain
with a threshold of 1e−05
rs11715853
rs35055552
rs823424
rs6828768
rs55877868
rs8039785
rs2090816
rs2387977
rs6809952
rs78383039
rs10899911
rs7311004
rs62273907
All
−0.3
−0.2
−0.1
0.0
0.1
MR effect size for
'Gut microbiota abundance (genus Parasutterella id.2892)' on 'HLA class II histocompatibility antigen gamma chain'

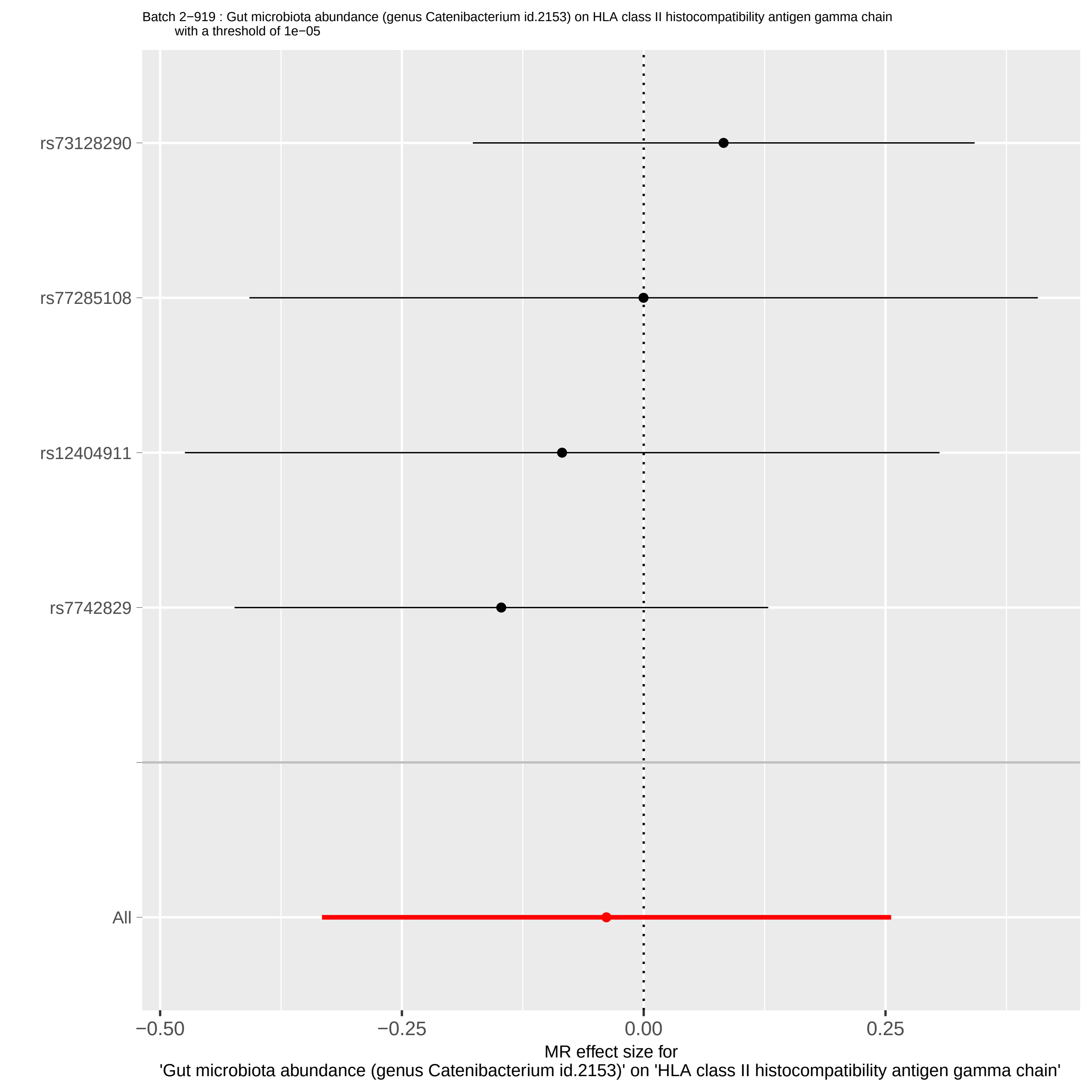
Batch 2−919 : Gut microbiota abundance (genus Catenibacterium id.2153) on HLA class II histocompatibility antigen gamma chain
with a threshold of 1e−05
rs73128290
rs77285108
rs12404911
rs7742829
All
−0.50
−0.25
0.00
0.25
MR effect size for
'Gut microbiota abundance (genus Catenibacterium id.2153)' on 'HLA class II histocompatibility antigen gamma chain'

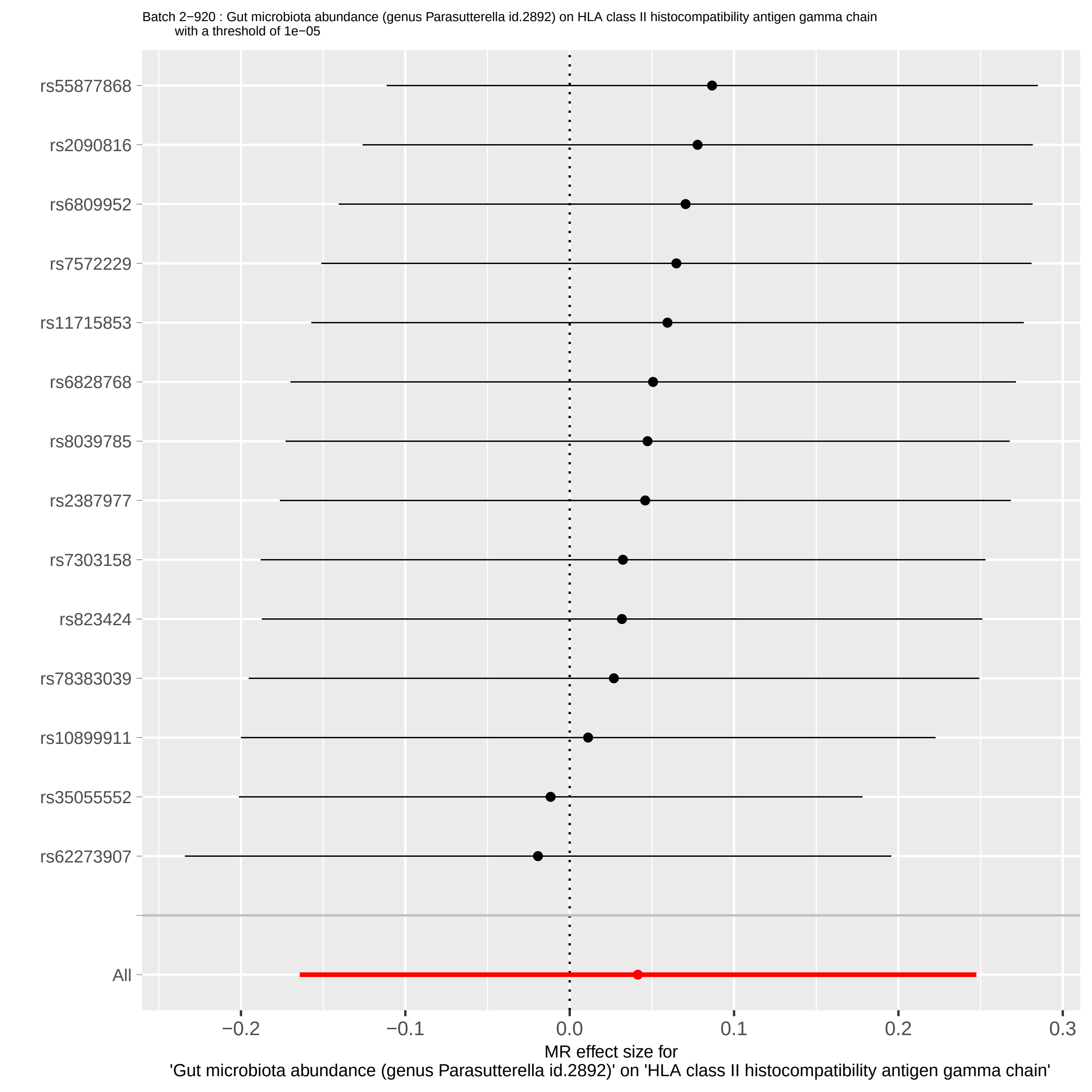

Batch 2−920 : Gut microbiota abundance (genus Parasutterella id.2892) on HLA class II histocompatibility antigen gamma chain
with a threshold of 1e−05
rs55877868
rs2090816
rs6809952
rs7572229
rs11715853
rs6828768
rs8039785
rs2387977
rs7303158
rs823424
rs78383039
rs10899911
rs35055552
rs62273907
All
−0.2
−0.1
0.0
0.1
0.2
0.3
MR effect size for
'Gut microbiota abundance (genus Parasutterella id.2892)' on 'HLA class II histocompatibility antigen gamma chain'

Batch 2−921 : Gut microbiota abundance (genus Catenibacterium id.2153) on B−cell antigen receptor complex−associated protein alpha chain
with a threshold of 1e−05

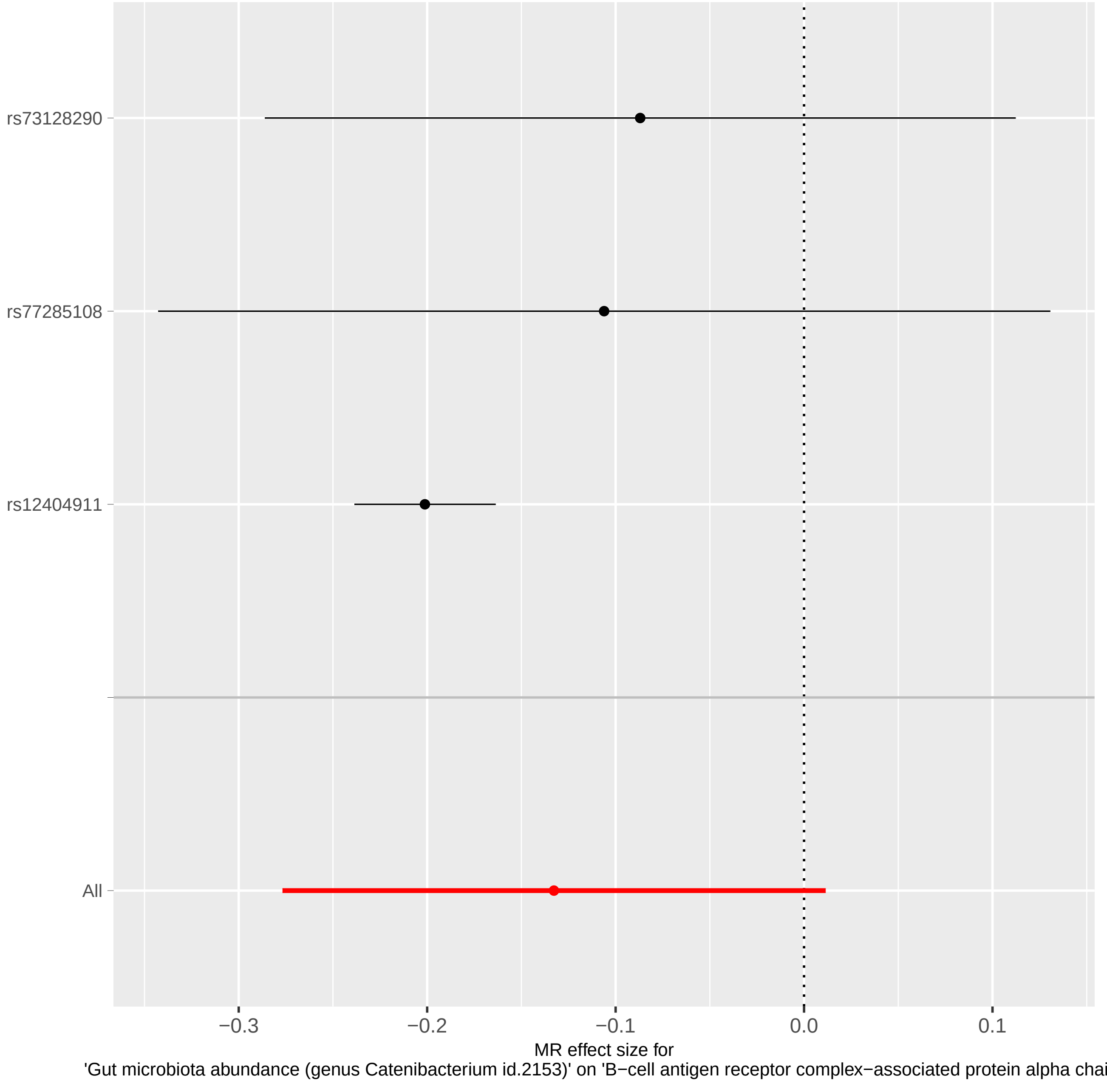

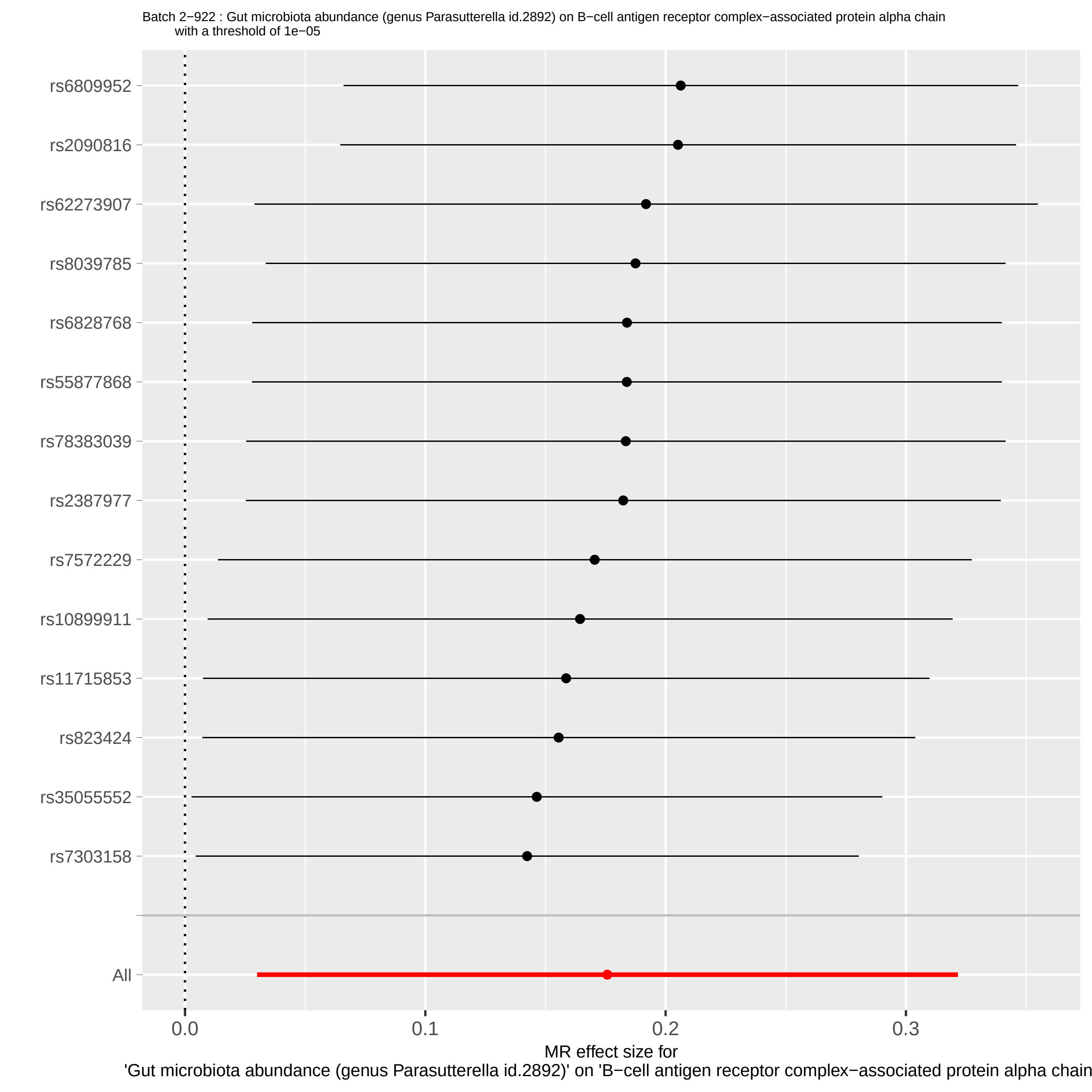
Batch 2−922 : Gut microbiota abundance (genus Parasutterella id.2892) on B−cell antigen receptor complex−associated protein alpha chain with a threshold of 1e−05
rs6809952
rs2090816
rs62273907
rs8039785
rs6828768
rs55877868
rs78383039
rs2387977
rs7572229
rs10899911
rs11715853
rs823424
rs35055552
rs7303158
All
0.0
0.1
0.2
0.3
MR effect size for
'Gut microbiota abundance (genus Parasutterella id.2892)' on 'B−cell antigen receptor complex−associated protein alpha chain

Batch 2−923 : Gut microbiota abundance (genus Catenibacterium id.2153) on B−cell antigen receptor complex−associated protein beta chain with a threshold of 1e−05

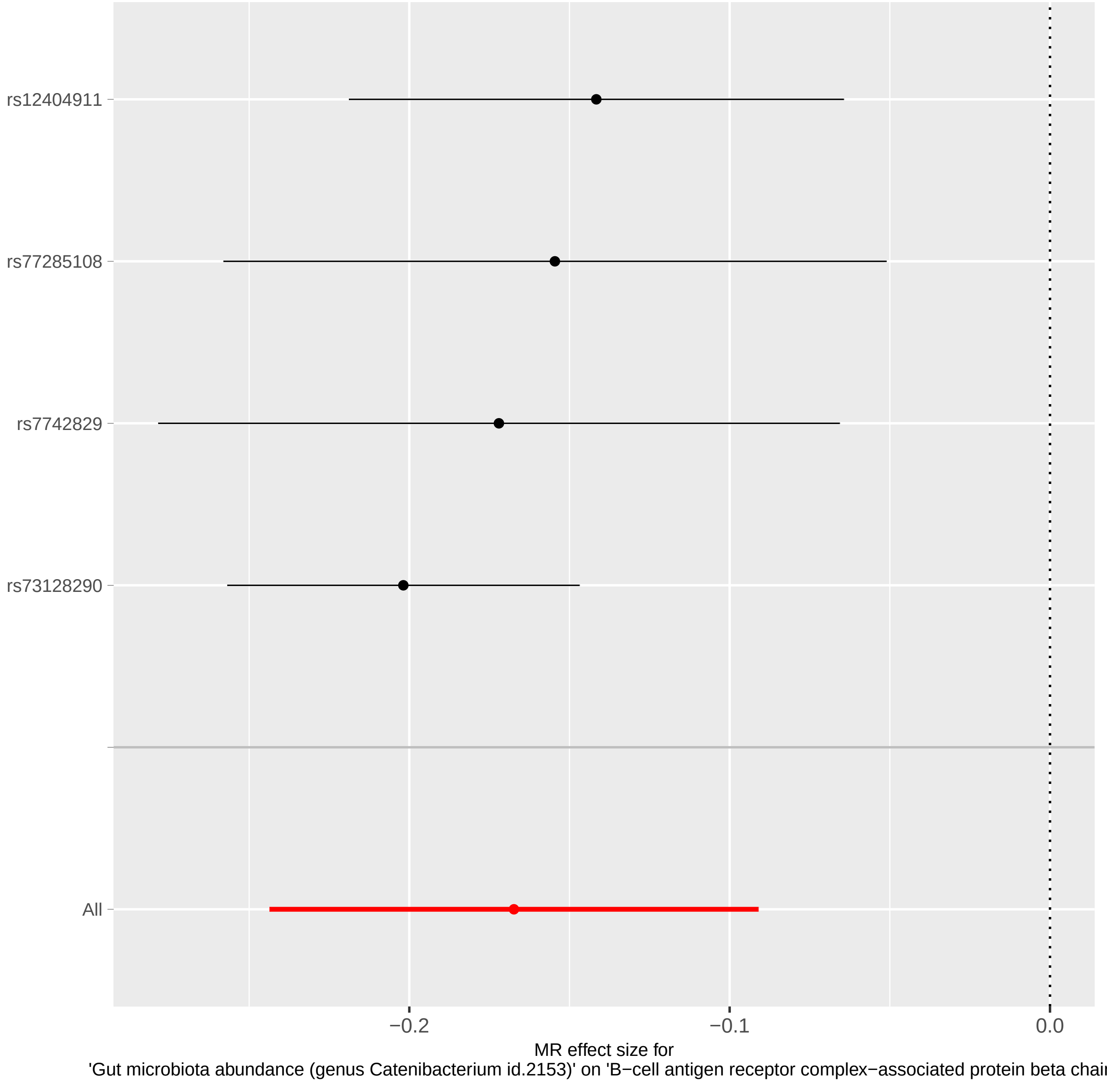

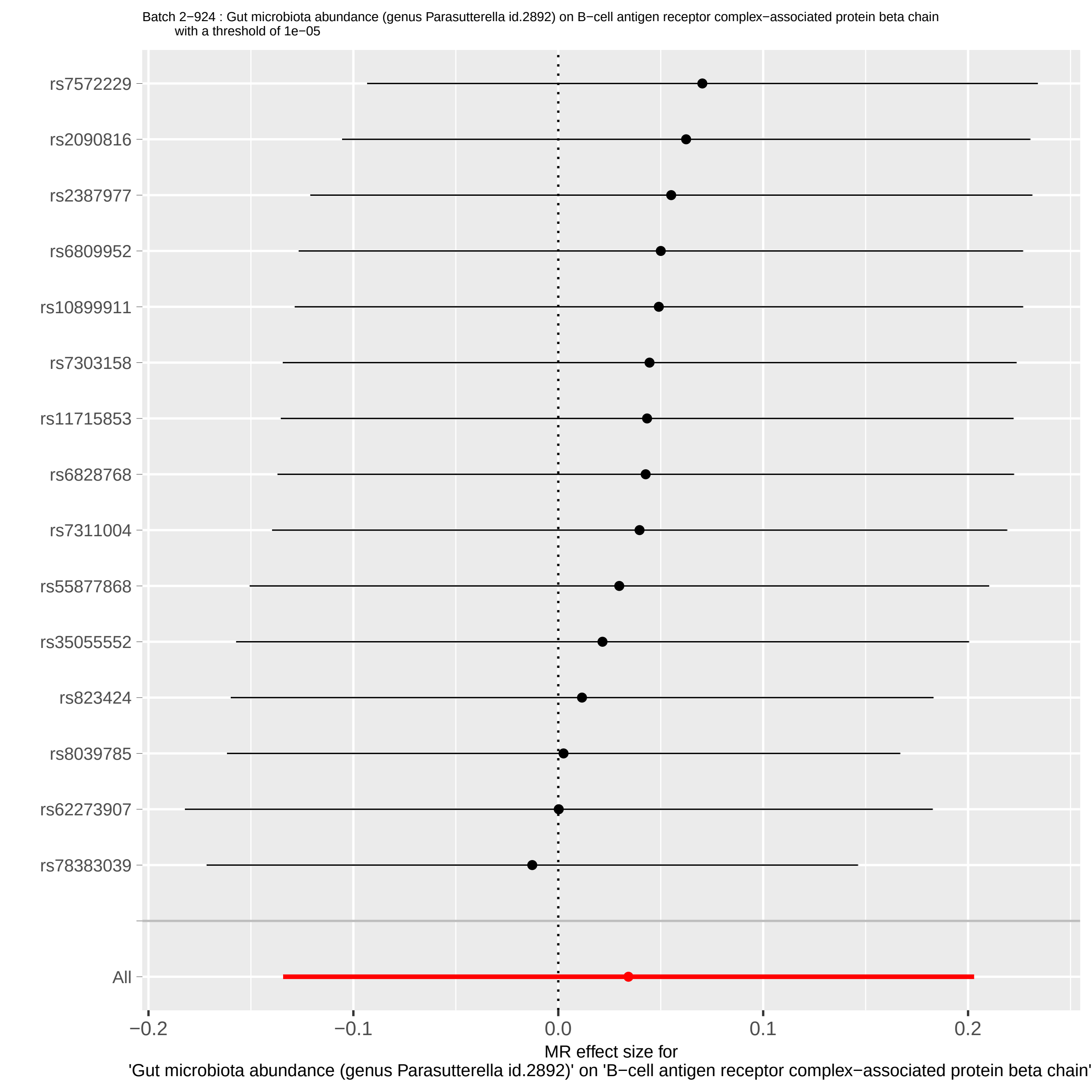

Batch 2−924 : Gut microbiota abundance (genus Parasutterella id.2892) on B−cell antigen receptor complex−associated protein beta chain with a threshold of 1e−05
rs7572229
rs2090816
rs2387977
rs6809952
rs10899911
rs7303158
rs11715853
rs6828768
rs7311004
rs55877868
rs35055552
rs823424
rs8039785
rs62273907
rs78383039
All
−0.2
−0.1
0.0
0.1
0.2
MR effect size for
'Gut microbiota abundance (genus Parasutterella id.2892)' on 'B−cell antigen receptor complex−associated protein beta chain'

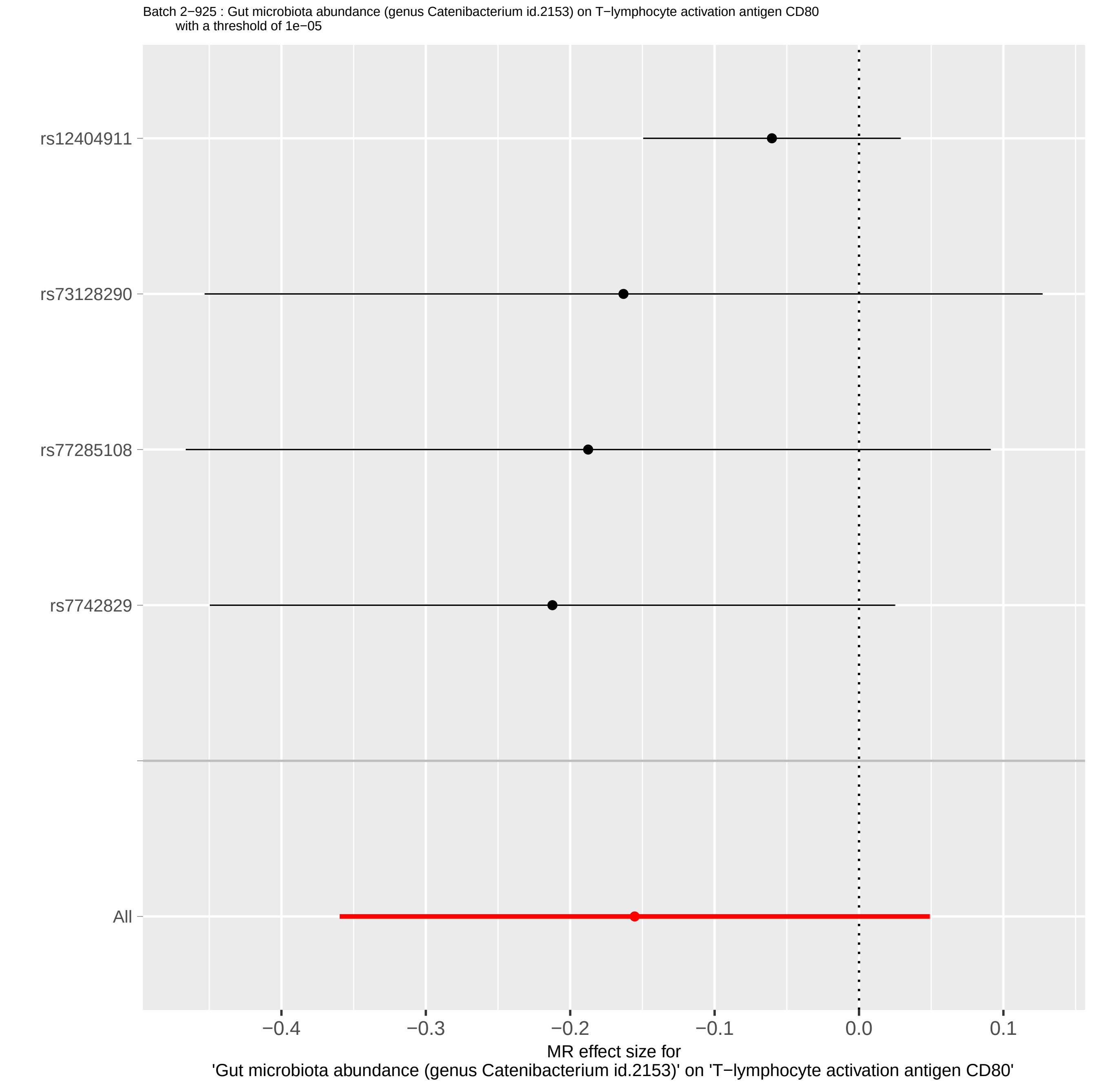
Batch 2−925 : Gut microbiota abundance (genus Catenibacterium id.2153) on T−lymphocyte activation antigen CD80
with a threshold of 1e−05
rs12404911
rs73128290
rs77285108
rs7742829
All
−0.4
−0.3
−0.2
−0.1
0.0
0.1
MR effect size for
'Gut microbiota abundance (genus Catenibacterium id.2153)' on 'T−lymphocyte activation antigen CD80'

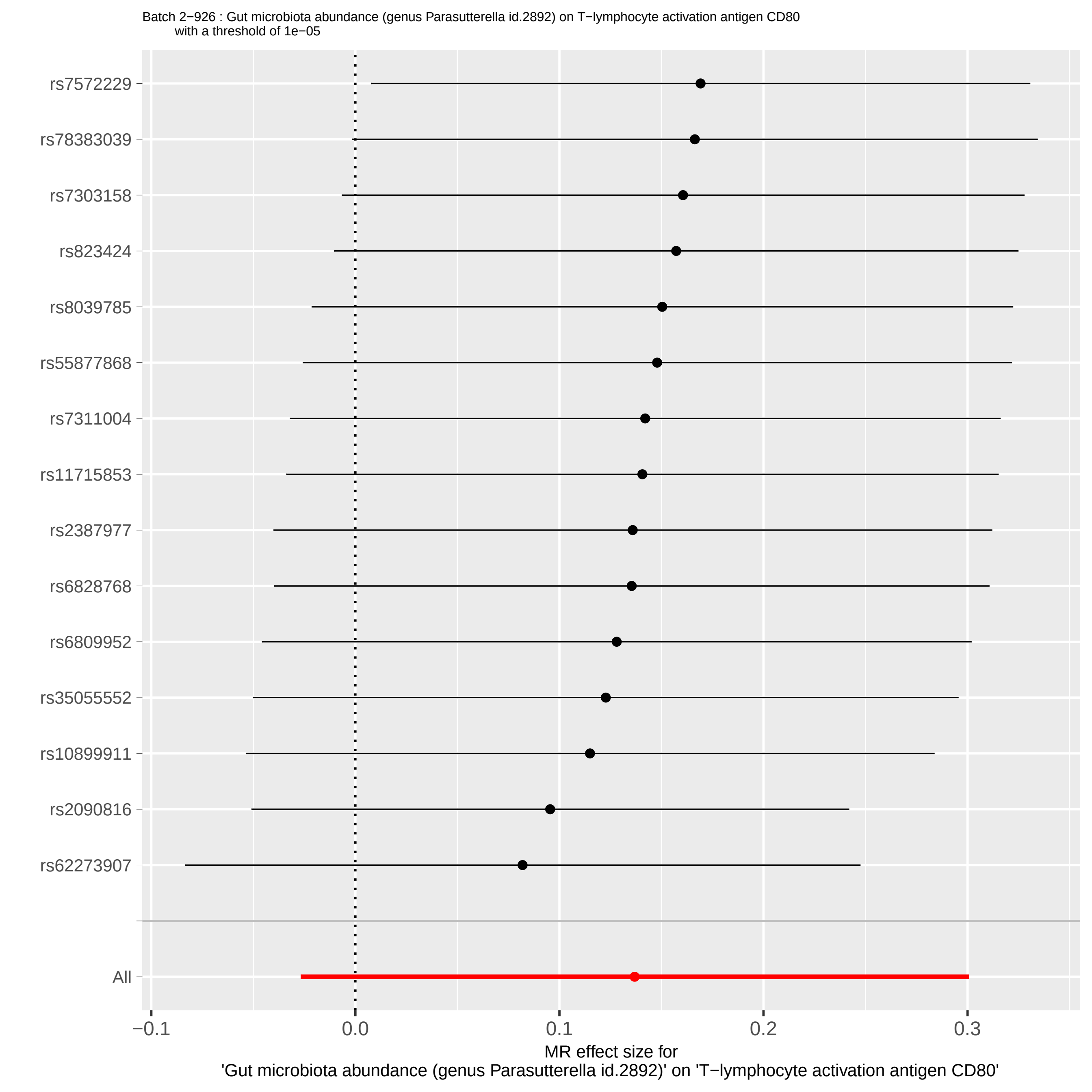

Batch 2−926 : Gut microbiota abundance (genus Parasutterella id.2892) on T−lymphocyte activation antigen CD80
with a threshold of 1e−05
rs7572229
rs78383039
rs7303158
rs823424
rs8039785
rs55877868
rs7311004
rs11715853
rs2387977
rs6828768
rs6809952
rs35055552
rs10899911
rs2090816
rs62273907
All
−0.1
0.0
0.1
0.2
0.3
MR effect size for
'Gut microbiota abundance (genus Parasutterella id.2892)' on 'T−lymphocyte activation antigen CD80'

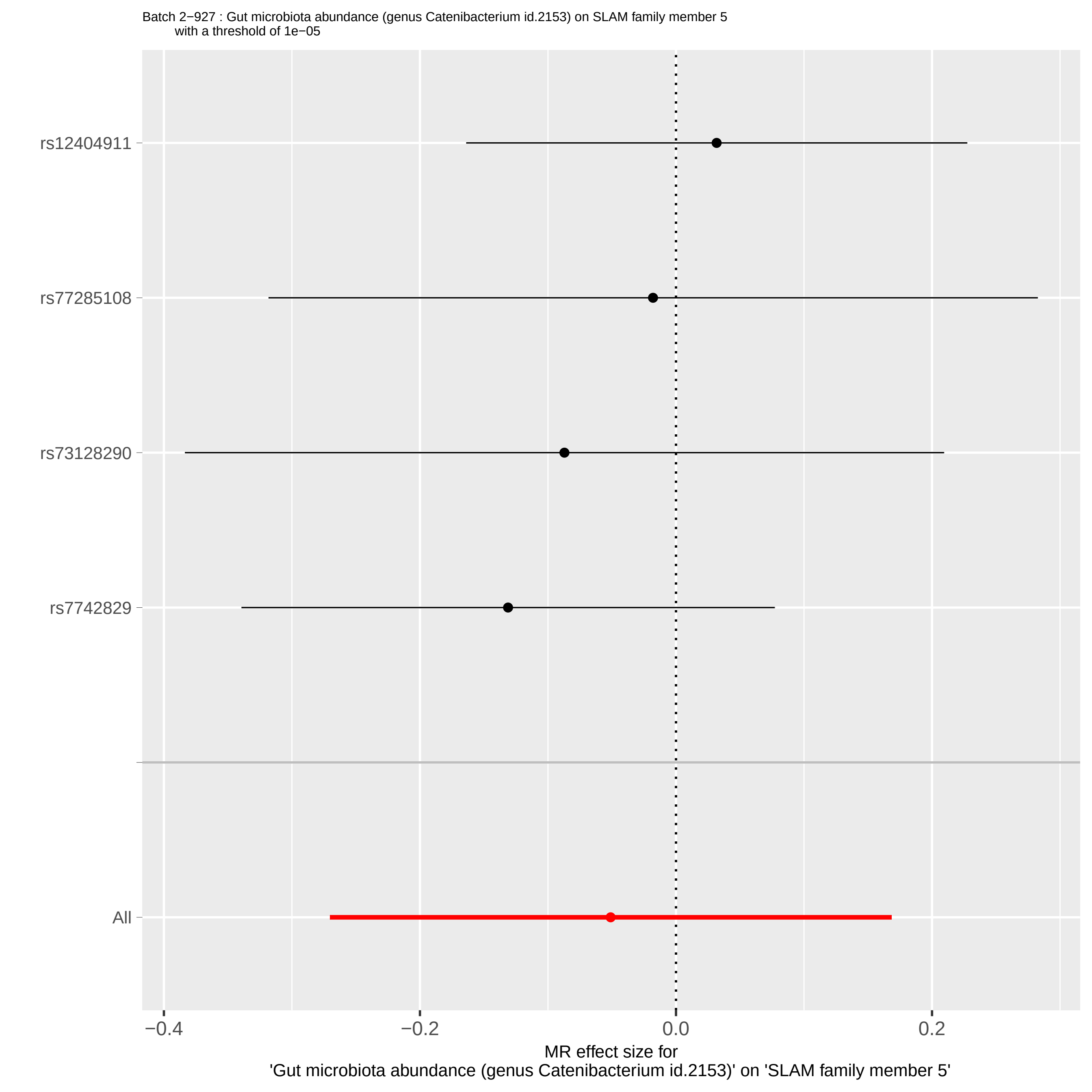
Batch 2−927 : Gut microbiota abundance (genus Catenibacterium id.2153) on SLAM family member 5
with a threshold of 1e−05
rs12404911
rs77285108
rs73128290
rs7742829
All
−0.4
−0.2
0.0
0.2
MR effect size for
'Gut microbiota abundance (genus Catenibacterium id.2153)' on 'SLAM family member 5'

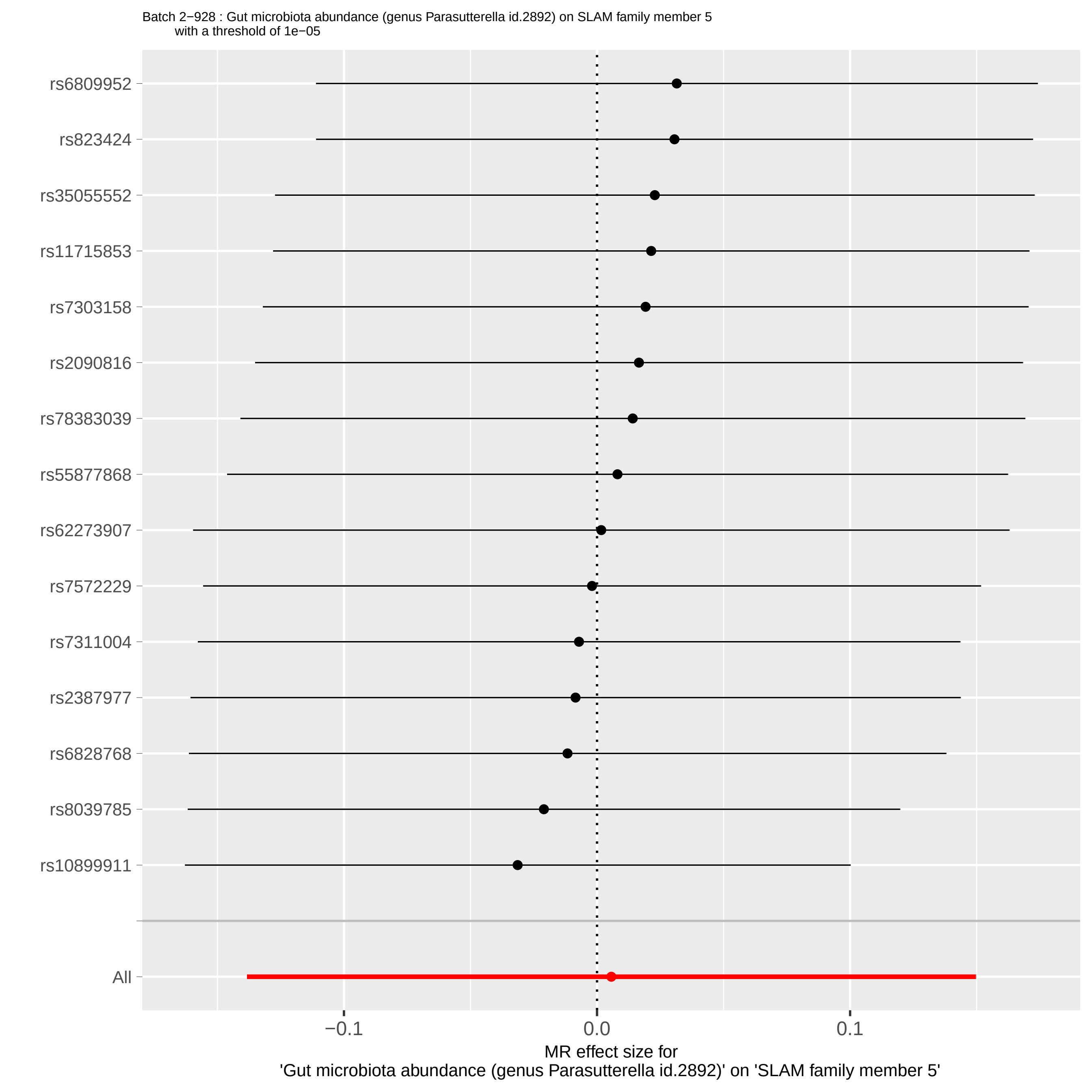
Batch 2−928 : Gut microbiota abundance (genus Parasutterella id.2892) on SLAM family member 5
with a threshold of 1e−05
rs6809952
rs823424
rs35055552
rs11715853
rs7303158
rs2090816
rs78383039
rs55877868
rs62273907
rs7572229
rs7311004
rs2387977
rs6828768
rs8039785
rs10899911
All
−0.1
0.0
0.1
MR effect size for
'Gut microbiota abundance (genus Parasutterella id.2892)' on 'SLAM family member 5'

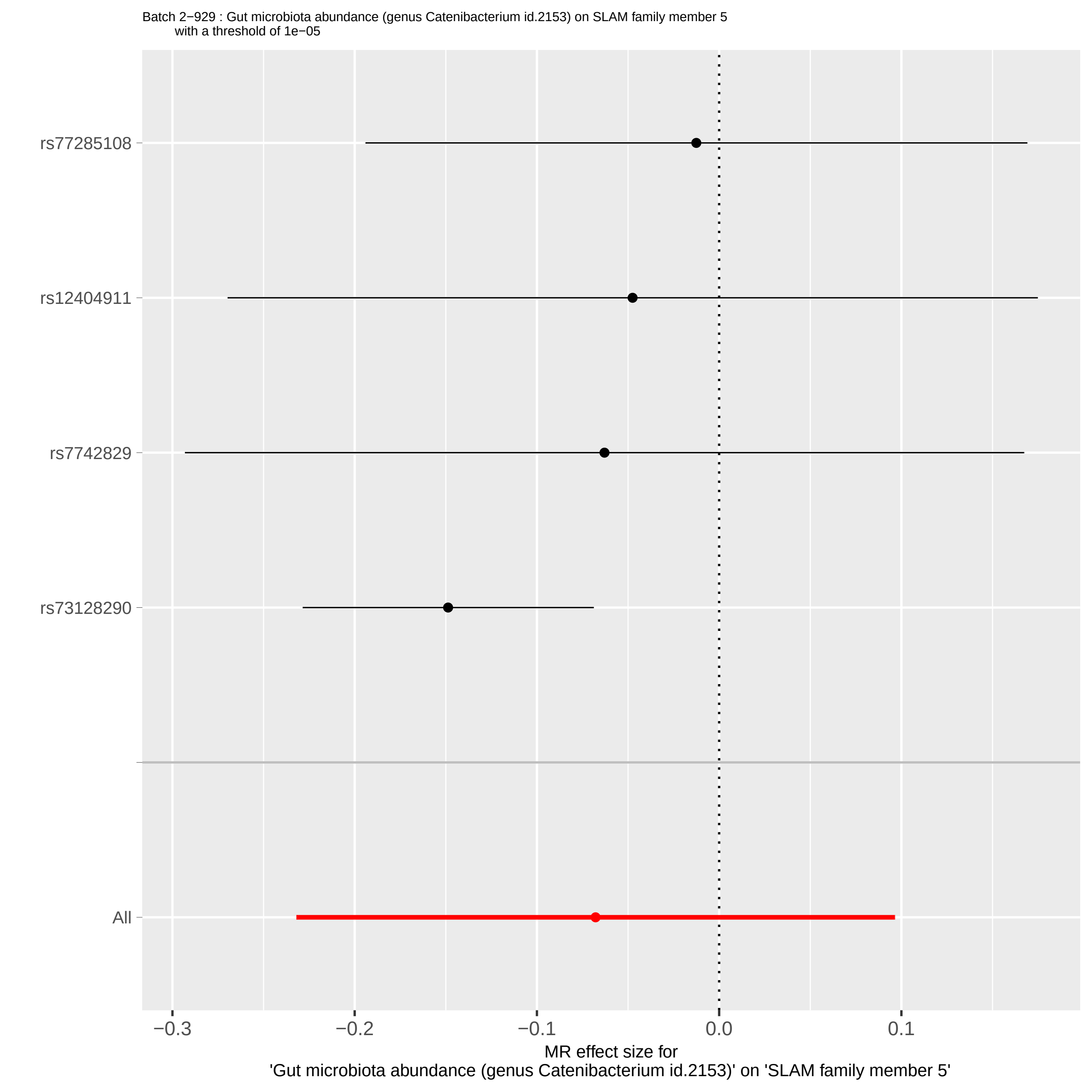

Batch 2−929 : Gut microbiota abundance (genus Catenibacterium id.2153) on SLAM family member 5
with a threshold of 1e−05
rs77285108
rs12404911
rs7742829
rs73128290
All
−0.3
−0.2
−0.1
0.0
0.1
MR effect size for
'Gut microbiota abundance (genus Catenibacterium id.2153)' on 'SLAM family member 5'

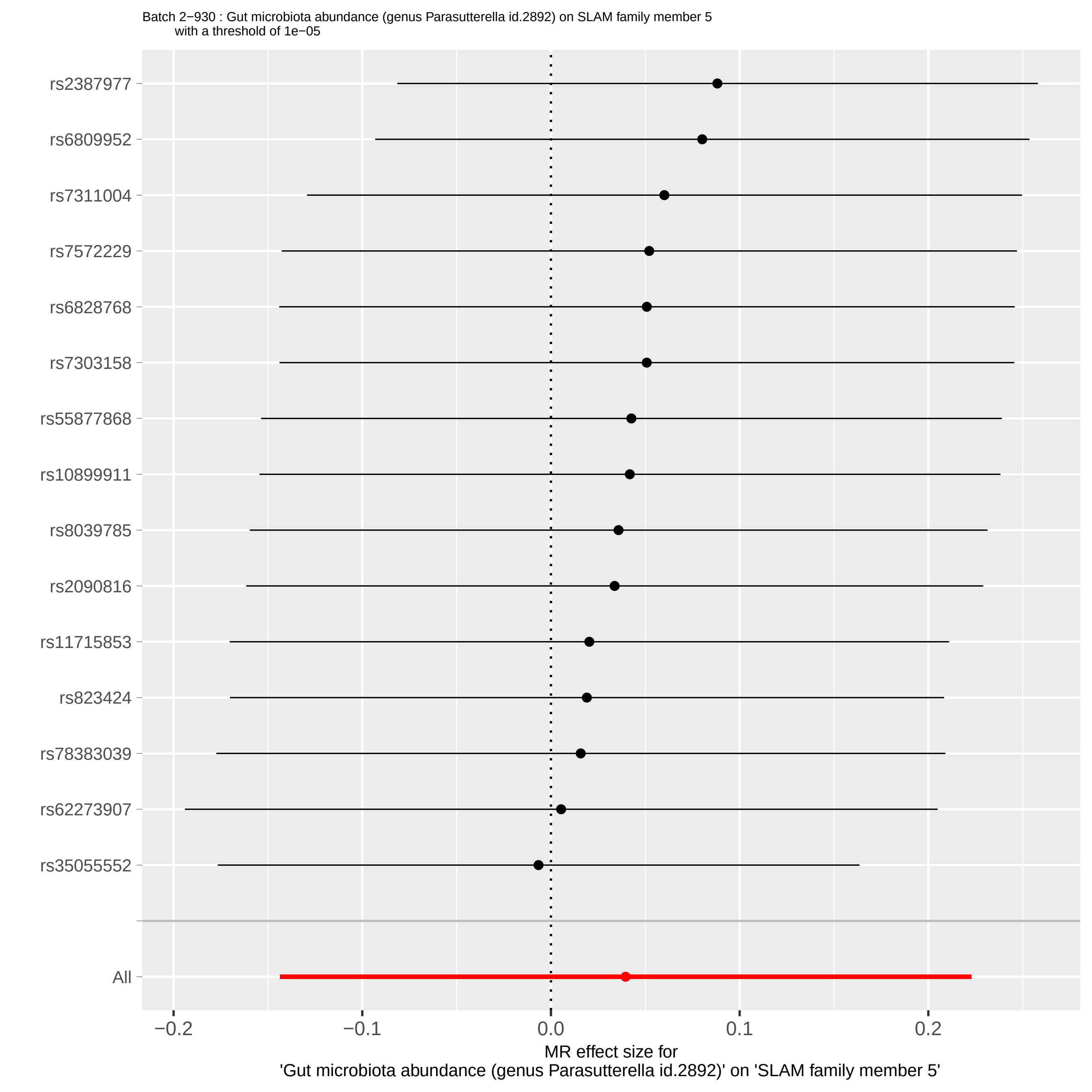
Batch 2−930 : Gut microbiota abundance (genus Parasutterella id.2892) on SLAM family member 5
with a threshold of 1e−05
rs2387977
rs6809952
rs7311004
rs7572229
rs6828768
rs7303158
rs55877868
rs10899911
rs8039785
rs2090816
rs11715853
rs823424
rs78383039
rs62273907
rs35055552
All
−0.2
−0.1
0.0
0.1
0.2
MR effect size for
'Gut microbiota abundance (genus Parasutterella id.2892)' on 'SLAM family member 5'

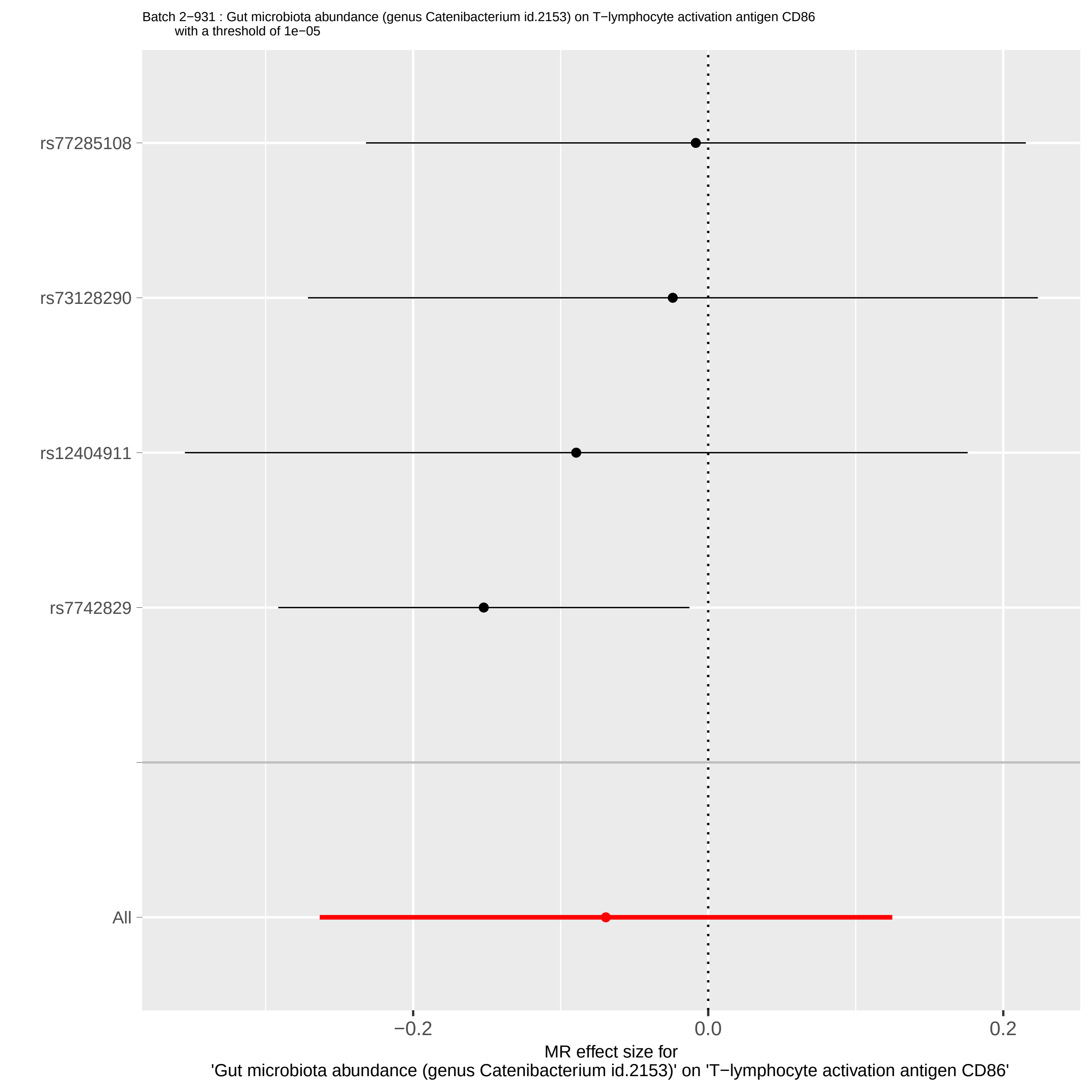

Batch 2−931 : Gut microbiota abundance (genus Catenibacterium id.2153) on T−lymphocyte activation antigen CD86
with a threshold of 1e−05
rs77285108
rs73128290
rs12404911
rs7742829
All
−0.2
0.0
0.2
MR effect size for
'Gut microbiota abundance (genus Catenibacterium id.2153)' on 'T−lymphocyte activation antigen CD86'

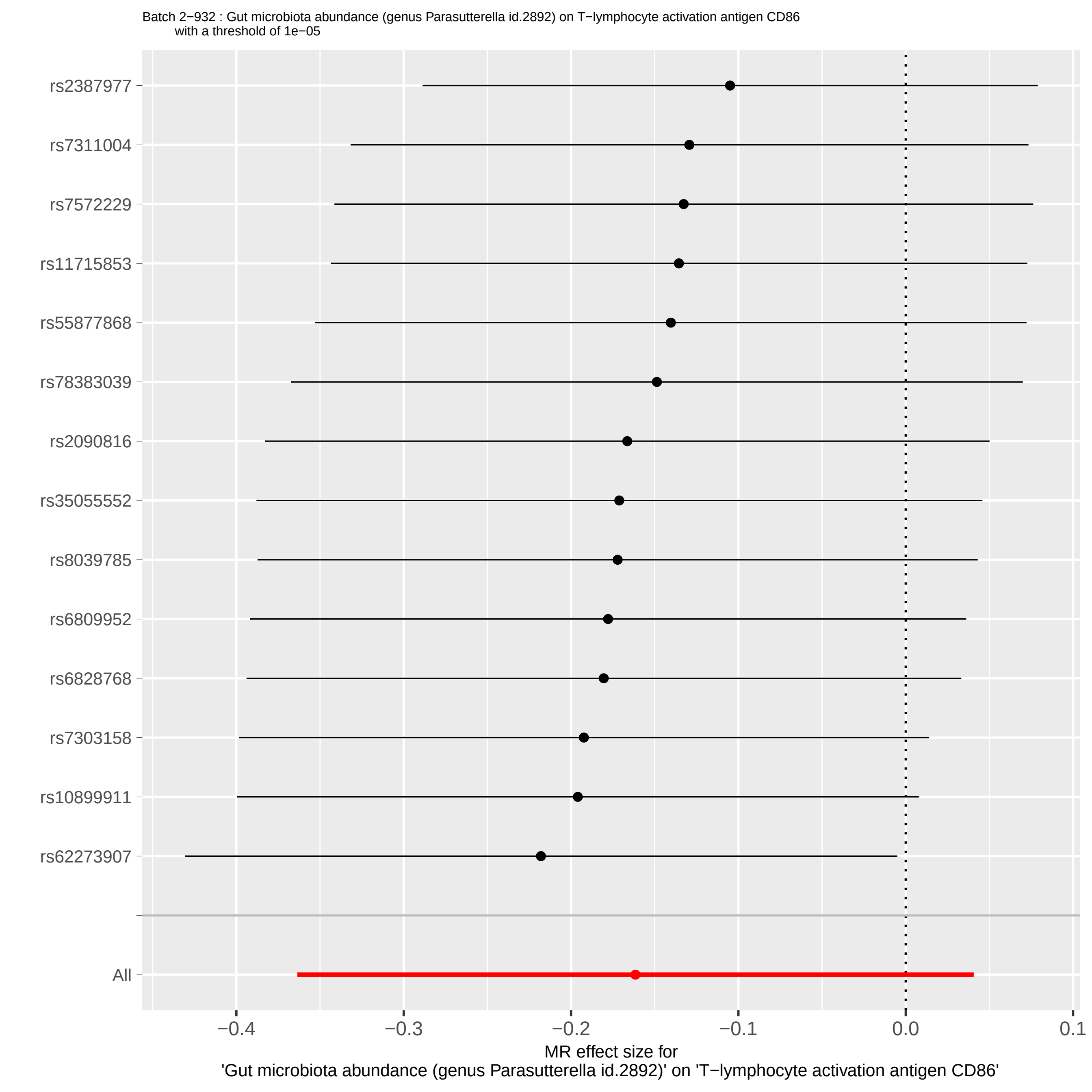

Batch 2−932 : Gut microbiota abundance (genus Parasutterella id.2892) on T−lymphocyte activation antigen CD86
with a threshold of 1e−05
rs2387977
rs7311004
rs7572229
rs11715853
rs55877868
rs78383039
rs2090816
rs35055552
rs8039785
rs6809952
rs6828768
rs7303158
rs10899911
rs62273907
All
−0.4
−0.3
−0.2
−0.1
0.0
0.1
MR effect size for
'Gut microbiota abundance (genus Parasutterella id.2892)' on 'T−lymphocyte activation antigen CD86'

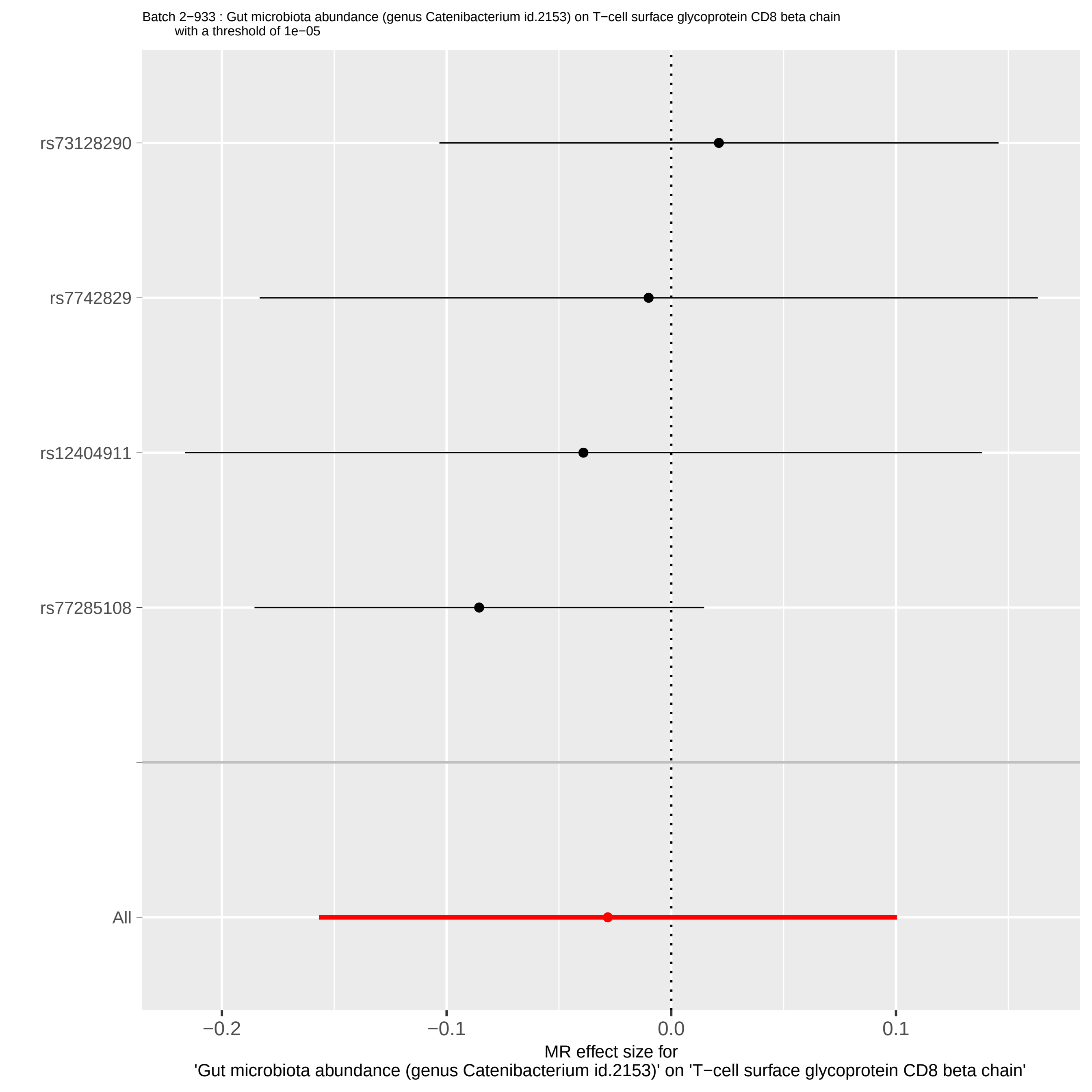
Batch 2−933 : Gut microbiota abundance (genus Catenibacterium id.2153) on T−cell surface glycoprotein CD8 beta chain
with a threshold of 1e−05
rs73128290
rs7742829
rs12404911
rs77285108
All
−0.2
−0.1
0.0
0.1
MR effect size for
'Gut microbiota abundance (genus Catenibacterium id.2153)' on 'T−cell surface glycoprotein CD8 beta chain'

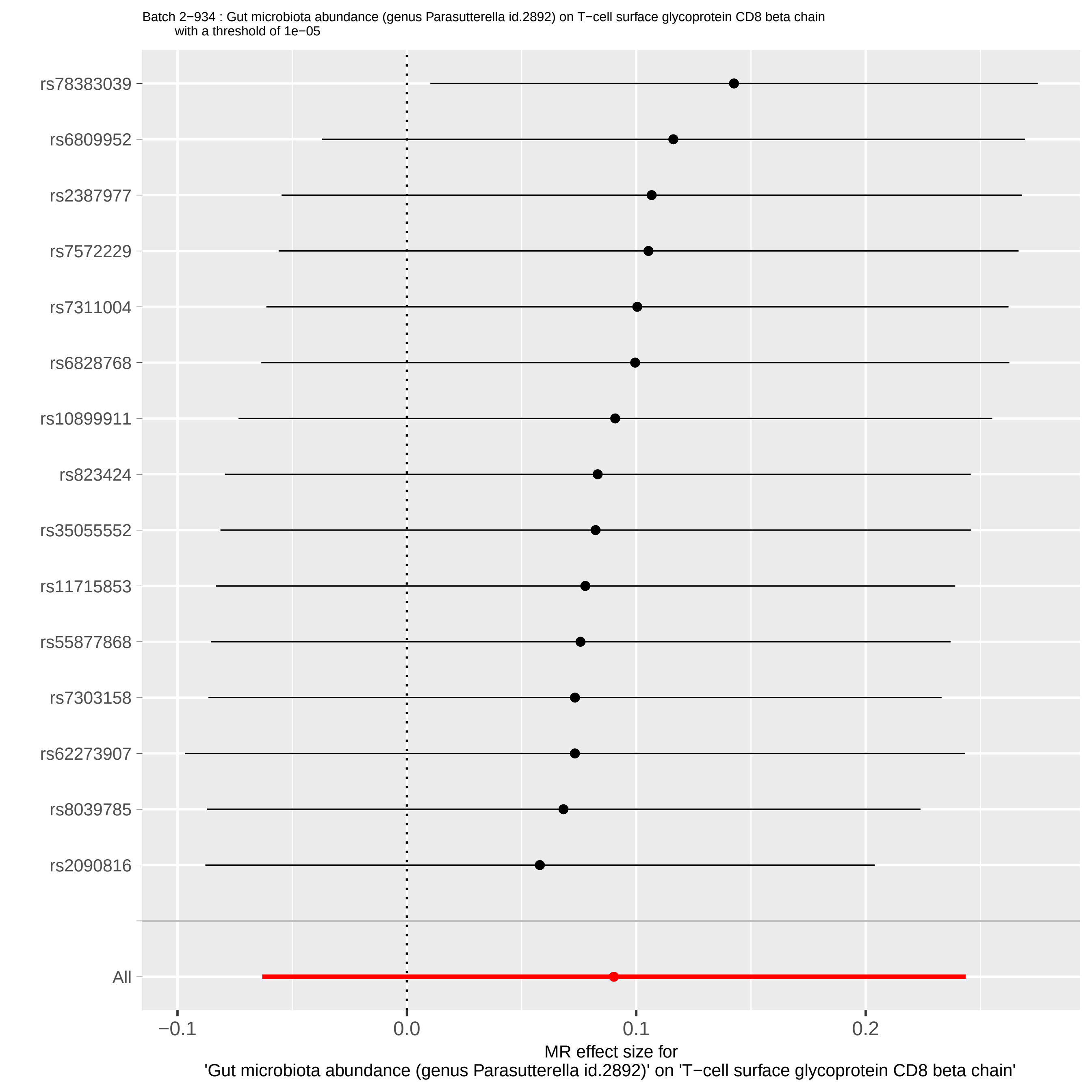
Batch 2−934 : Gut microbiota abundance (genus Parasutterella id.2892) on T−cell surface glycoprotein CD8 beta chain
with a threshold of 1e−05
rs78383039
rs6809952
rs2387977
rs7572229
rs7311004
rs6828768
rs10899911
rs823424
rs35055552
rs11715853
rs55877868
rs7303158
rs62273907
rs8039785
rs2090816
All
−0.1
0.0
0.1
0.2
MR effect size for
'Gut microbiota abundance (genus Parasutterella id.2892)' on 'T−cell surface glycoprotein CD8 beta chain'

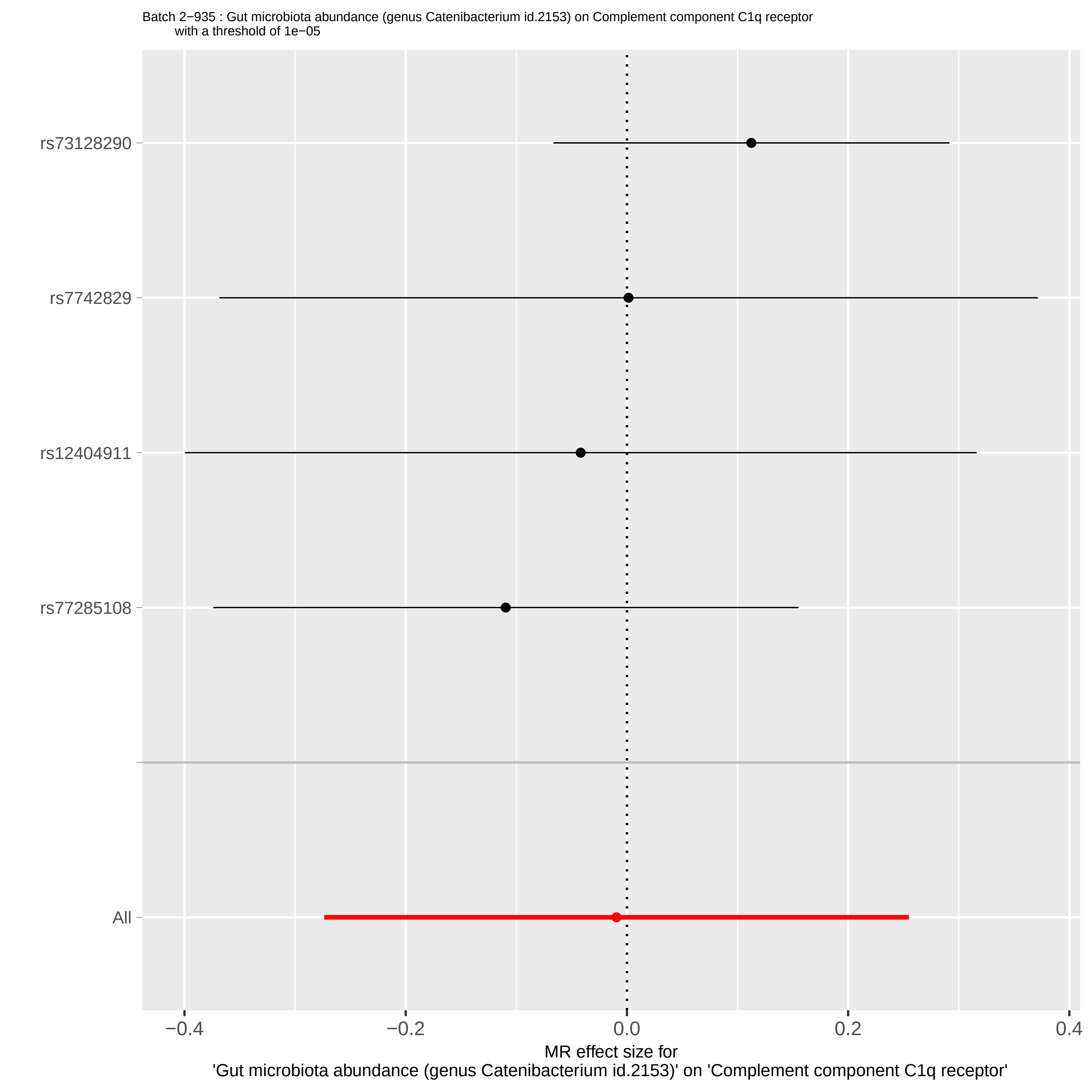
Batch 2−935 : Gut microbiota abundance (genus Catenibacterium id.2153) on Complement component C1q receptor
with a threshold of 1e−05
rs73128290
rs7742829
rs12404911
rs77285108
All
−0.4
−0.2
0.0
0.2
0.4
MR effect size for
'Gut microbiota abundance (genus Catenibacterium id.2153)' on 'Complement component C1q receptor'

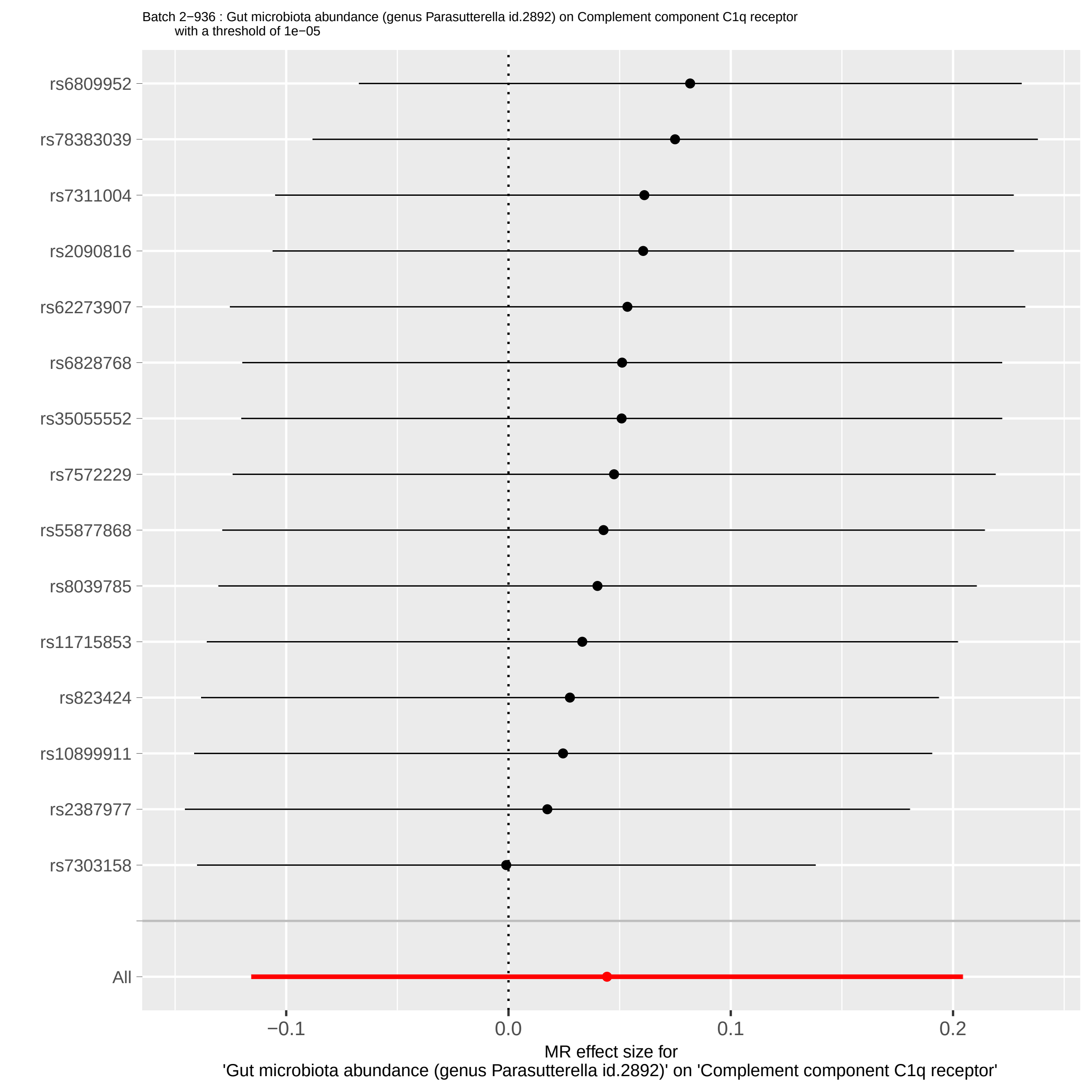

Batch 2−936 : Gut microbiota abundance (genus Parasutterella id.2892) on Complement component C1q receptor
with a threshold of 1e−05
rs6809952
rs78383039
rs7311004
rs2090816
rs62273907
rs6828768
rs35055552
rs7572229
rs55877868
rs8039785
rs11715853
rs823424
rs10899911
rs2387977
rs7303158
All
−0.1
0.0
0.1
0.2
MR effect size for
'Gut microbiota abundance (genus Parasutterella id.2892)' on 'Complement component C1q receptor'

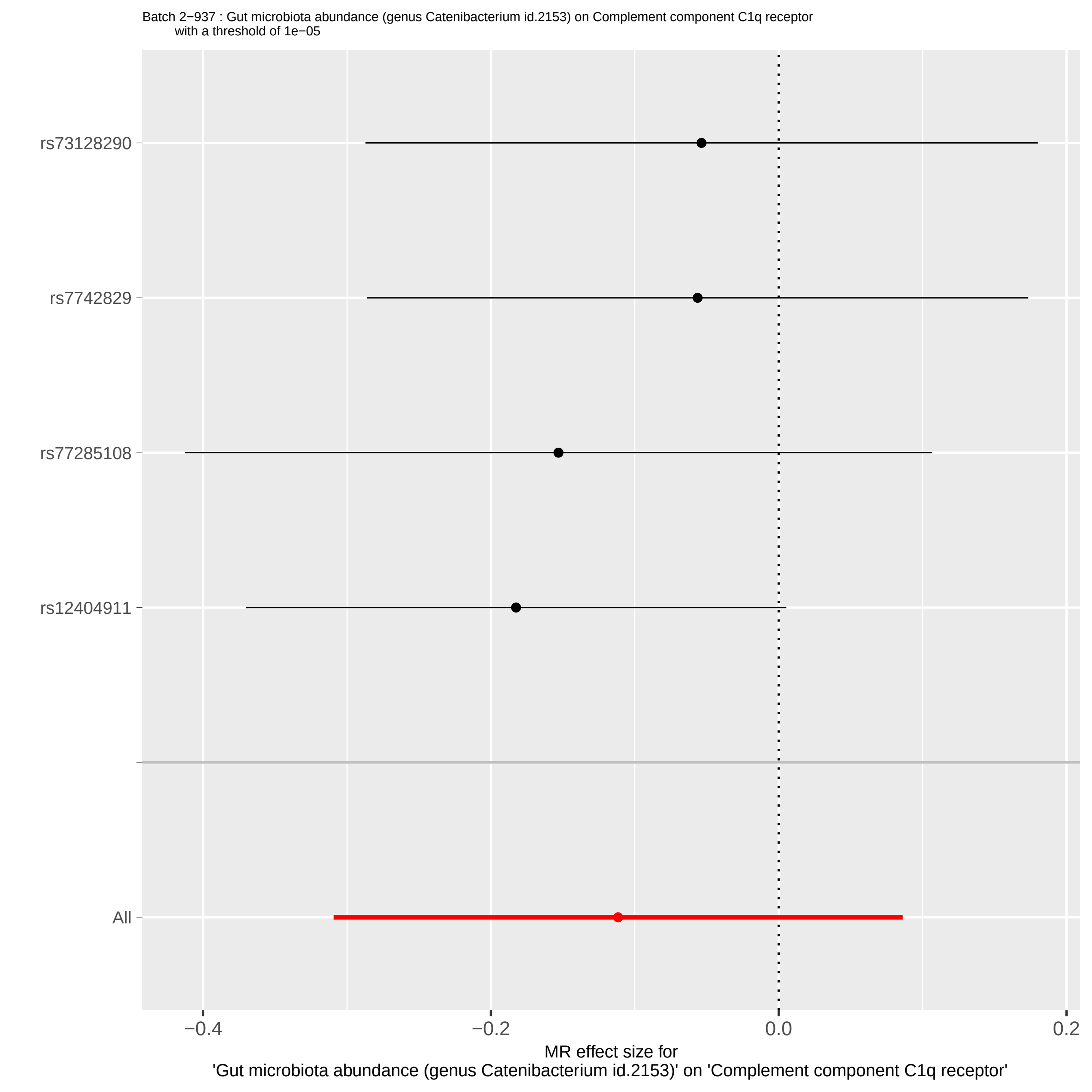
Batch 2−937 : Gut microbiota abundance (genus Catenibacterium id.2153) on Complement component C1q receptor
with a threshold of 1e−05
rs73128290
rs7742829
rs77285108
rs12404911
All
−0.4
−0.2
0.0
0.2
MR effect size for
'Gut microbiota abundance (genus Catenibacterium id.2153)' on 'Complement component C1q receptor'

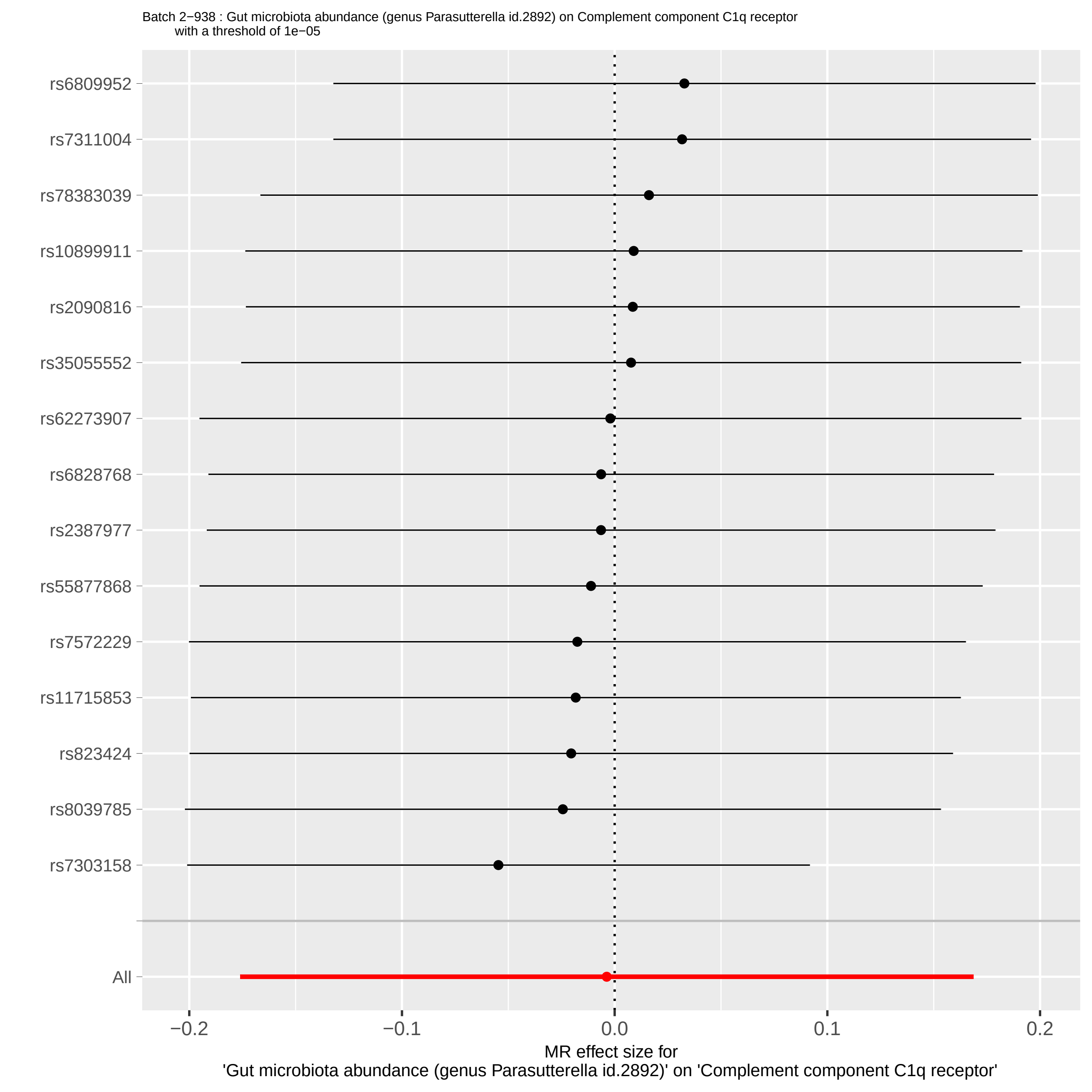

Batch 2−938 : Gut microbiota abundance (genus Parasutterella id.2892) on Complement component C1q receptor with a threshold of 1e−05
rs6809952
rs7311004
rs78383039
rs10899911
rs2090816
rs35055552
rs62273907
rs6828768
rs2387977
rs55877868
rs7572229
rs11715853
rs823424
rs8039785
rs7303158
All
−0.2
−0.1
0.0
0.1
0.2
MR effect size for
'Gut microbiota abundance (genus Parasutterella id.2892)' on 'Complement component C1q receptor'

Batch 2−939 : Gut microbiota abundance (genus Catenibacterium id.2153) on T−cell surface protein tactile
with a threshold of 1e−05

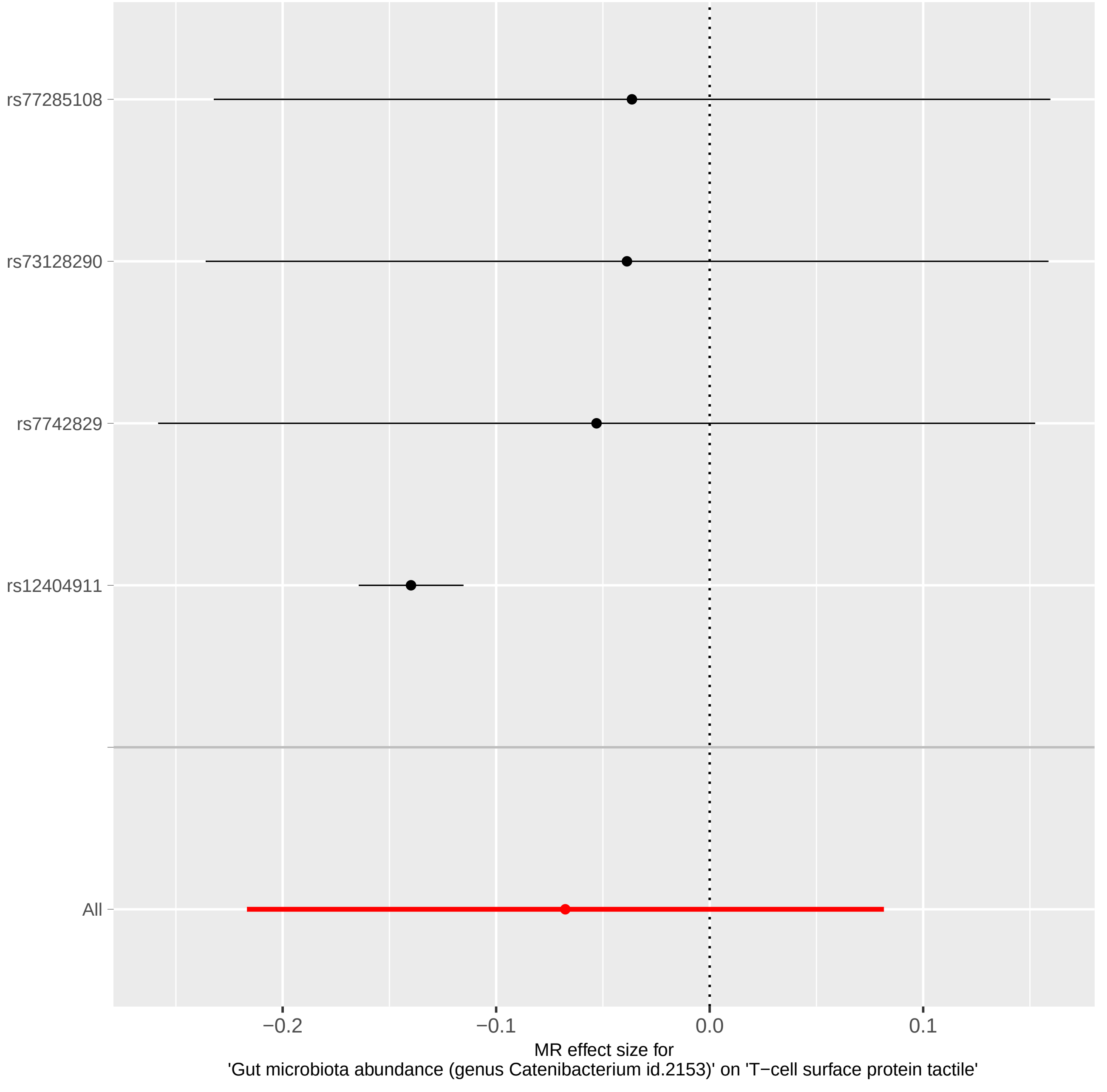

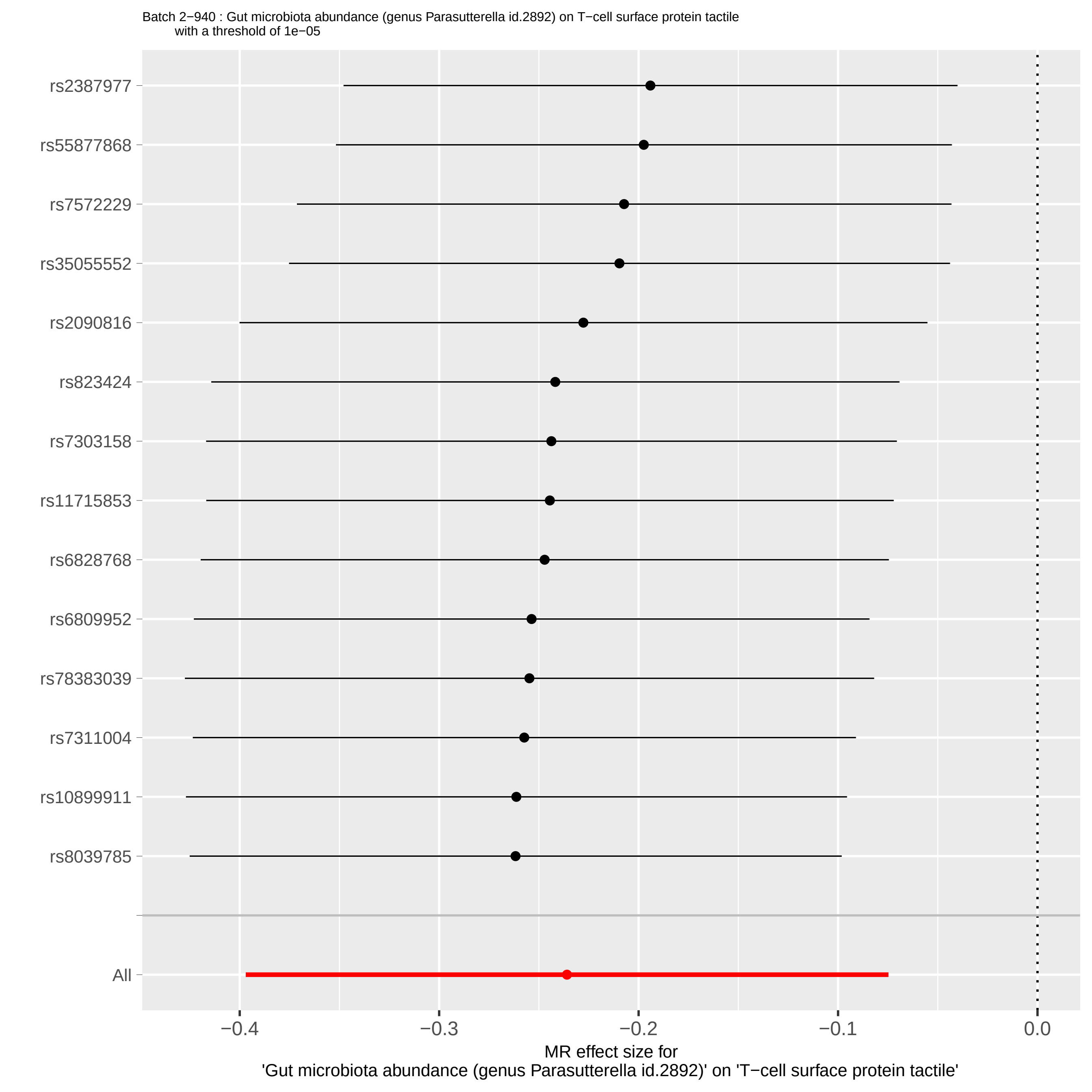

Batch 2−940 : Gut microbiota abundance (genus Parasutterella id.2892) on T−cell surface protein tactile
with a threshold of 1e−05
rs2387977
rs55877868
rs7572229
rs35055552
rs2090816
rs823424
rs7303158
rs11715853
rs6828768
rs6809952
rs78383039
rs7311004
rs10899911
rs8039785
All
−0.4
−0.3
−0.2
−0.1
0.0
MR effect size for
'Gut microbiota abundance (genus Parasutterella id.2892)' on 'T−cell surface protein tactile'

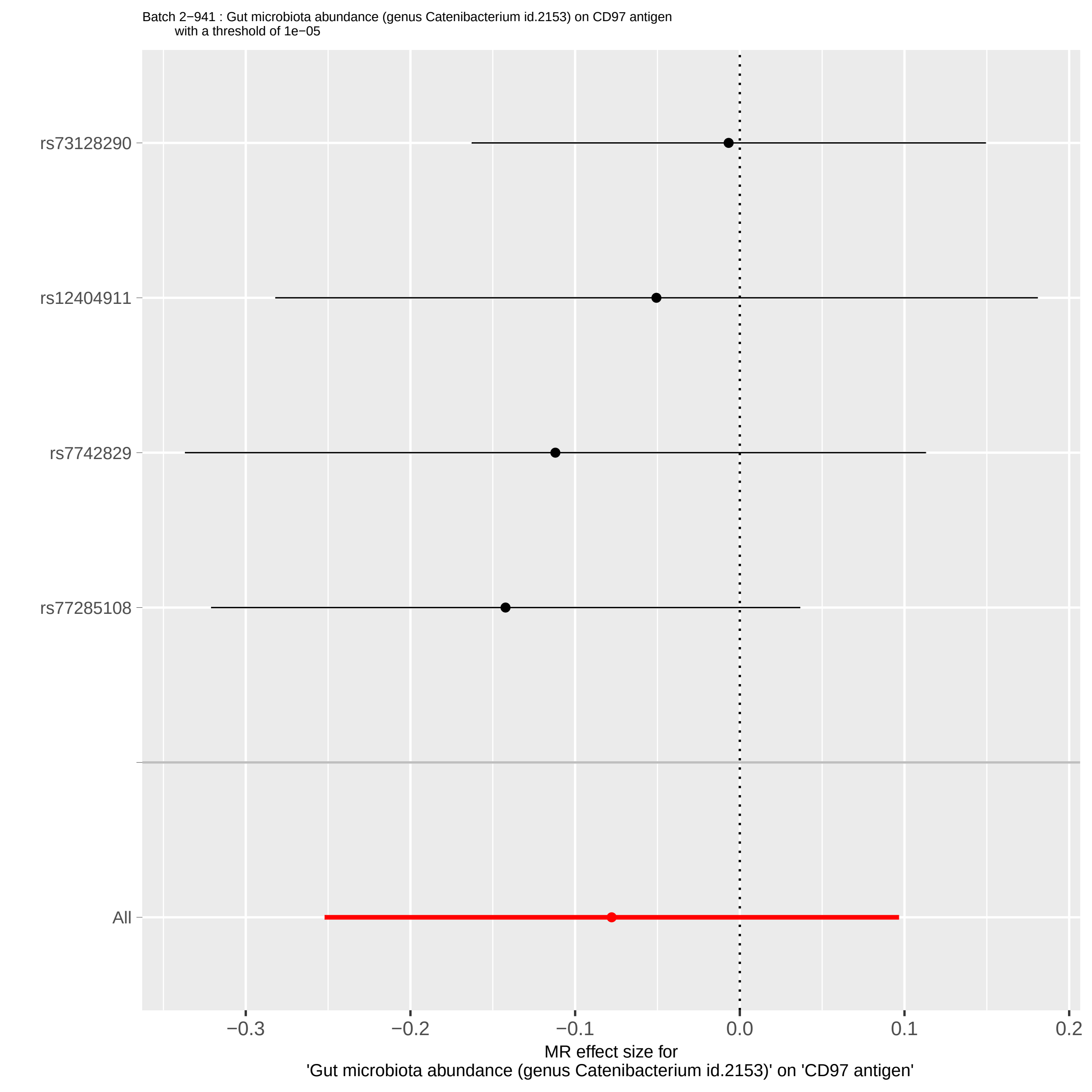
Batch 2−941 : Gut microbiota abundance (genus Catenibacterium id.2153) on CD97 antigen
with a threshold of 1e−05
rs73128290
rs12404911
rs7742829
rs77285108
All
−0.3
−0.2
−0.1
0.0
0.1
0.2
MR effect size for
'Gut microbiota abundance (genus Catenibacterium id.2153)' on 'CD97 antigen'

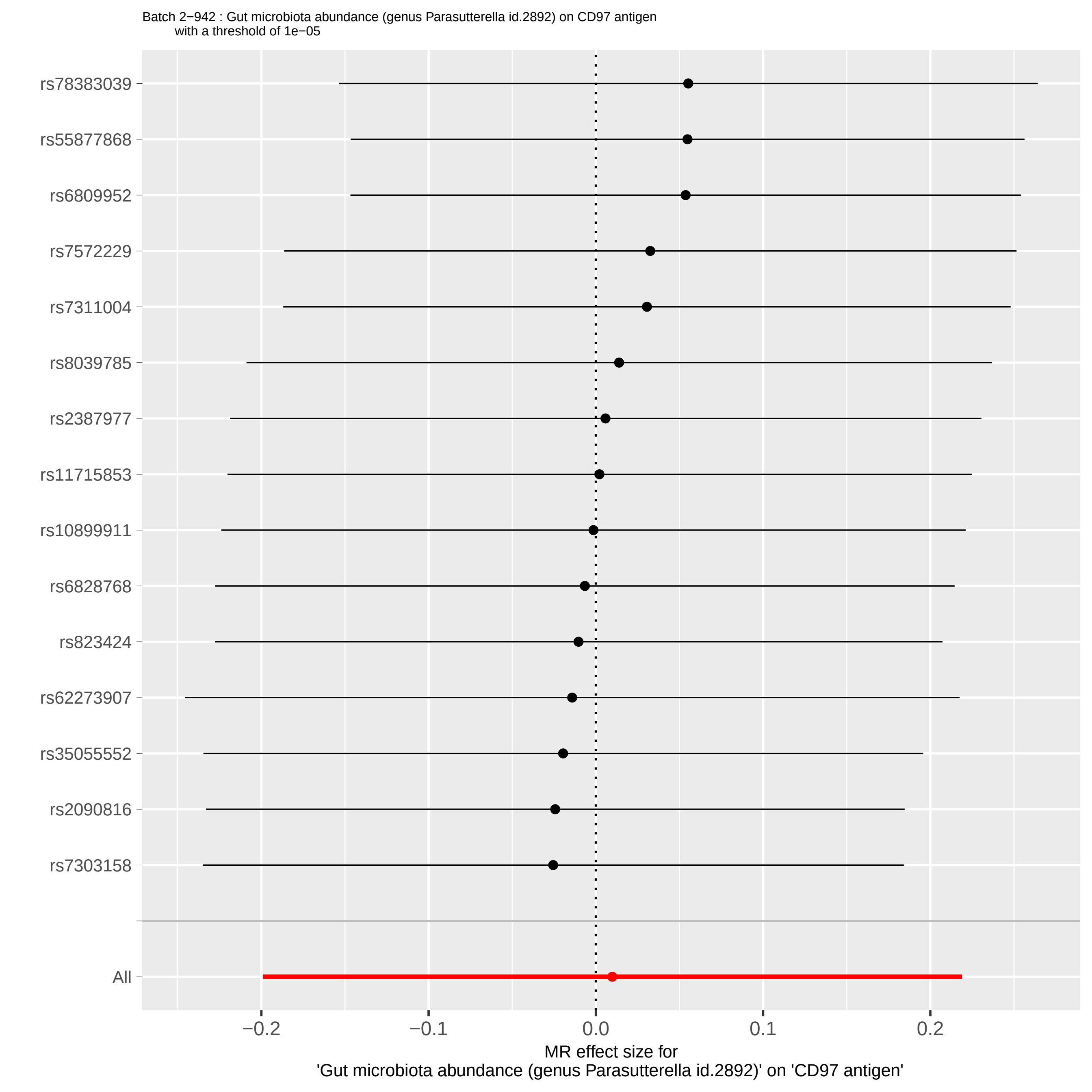

Batch 2−942 : Gut microbiota abundance (genus Parasutterella id.2892) on CD97 antigen
with a threshold of 1e−05
rs78383039
rs55877868
rs6809952
rs7572229
rs7311004
rs8039785
rs2387977
rs11715853
rs10899911
rs6828768
rs823424
rs62273907
rs35055552
rs2090816
rs7303158
All
−0.2
−0.1
0.0
0.1
0.2
MR effect size for
'Gut microbiota abundance (genus Parasutterella id.2892)' on 'CD97 antigen'

Batch 2−943 : Gut microbiota abundance (genus Catenibacterium id.2153) on M−phase inducer phosphatase 2
with a threshold of 1e−05

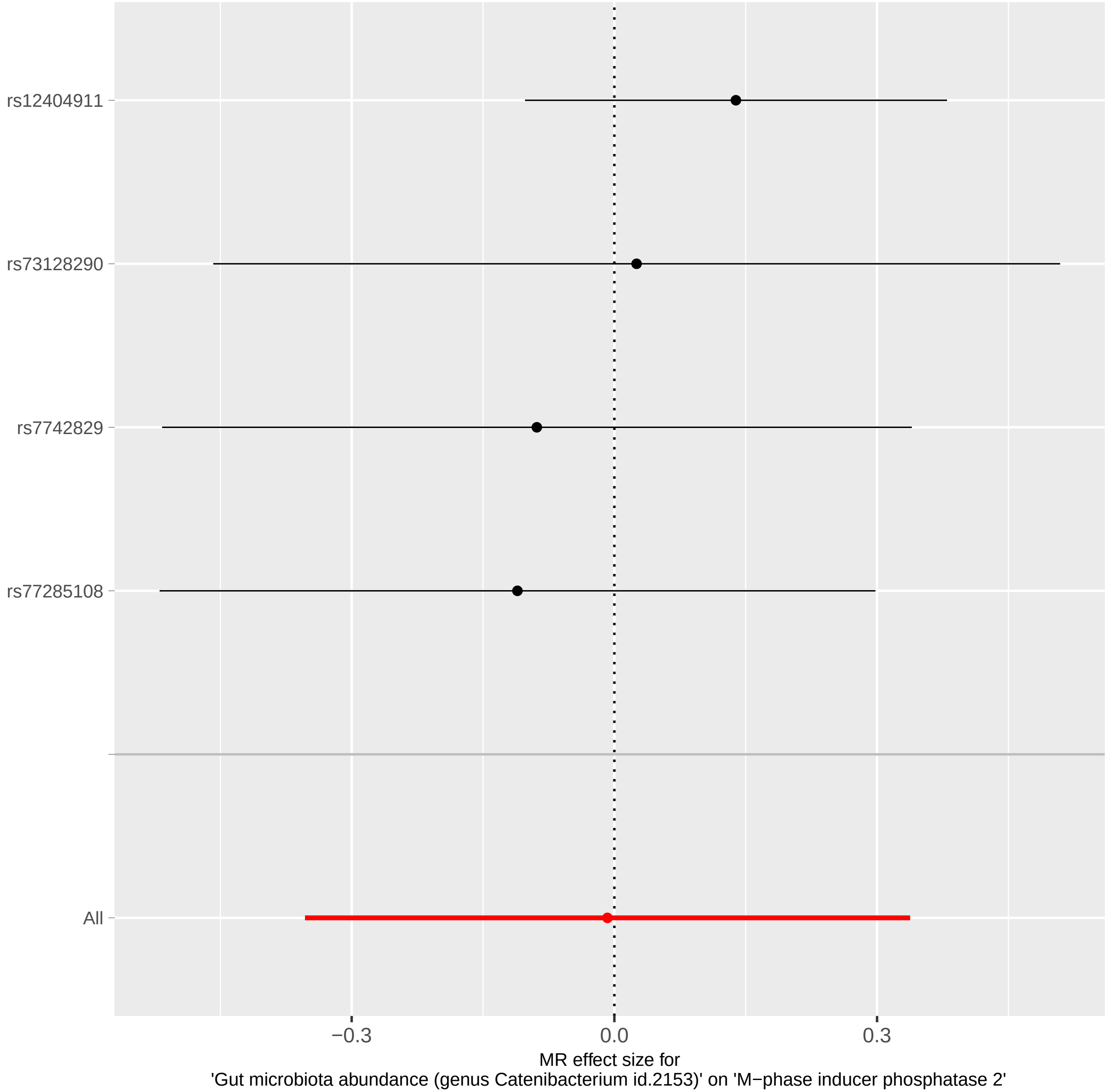

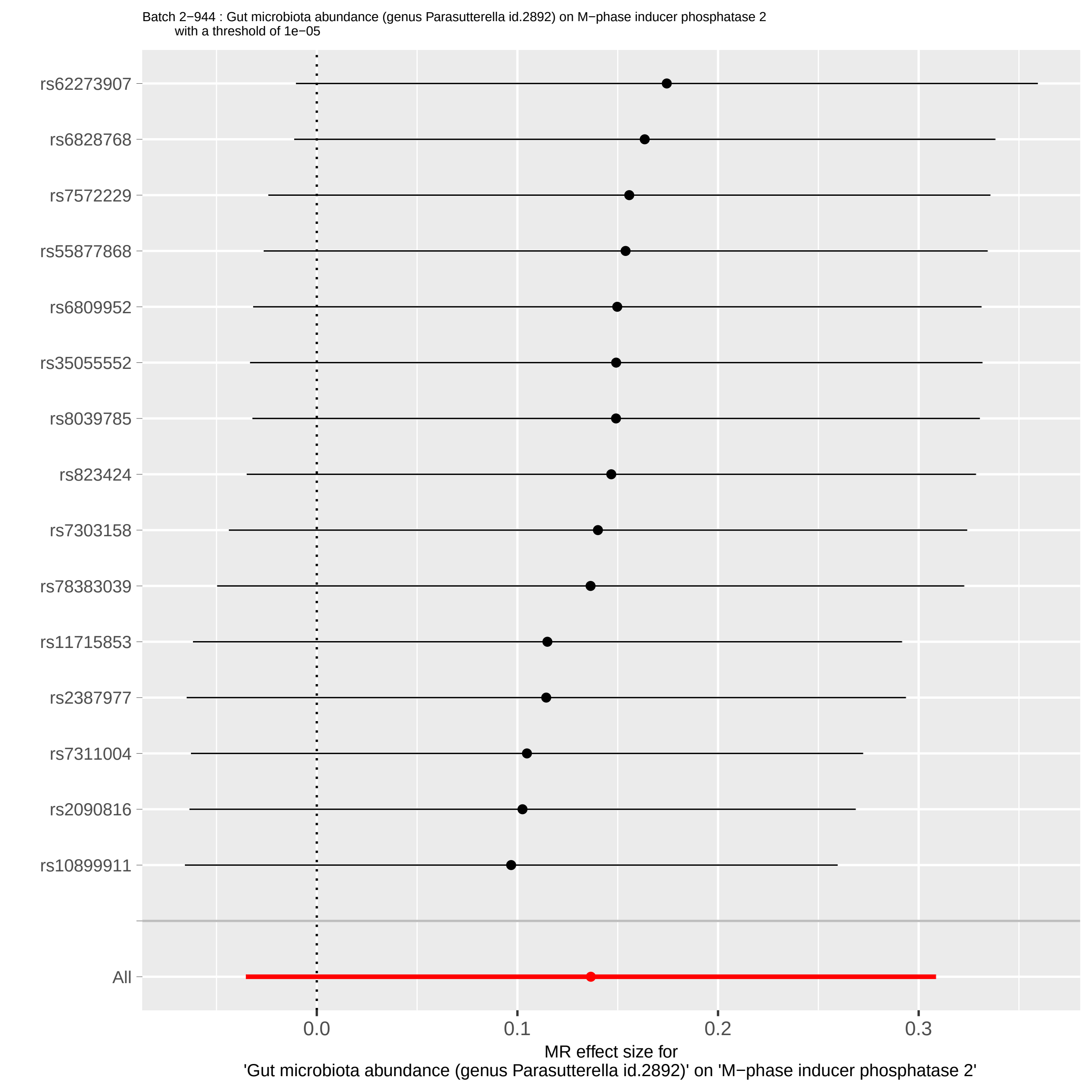
Batch 2−944 : Gut microbiota abundance (genus Parasutterella id.2892) on M−phase inducer phosphatase 2
with a threshold of 1e−05
rs62273907
rs6828768
rs7572229
rs55877868
rs6809952
rs35055552
rs8039785
rs823424
rs7303158
rs78383039
rs11715853
rs2387977
rs7311004
rs2090816
rs10899911
All
0.0
0.1
0.2
0.3
MR effect size for
'Gut microbiota abundance (genus Parasutterella id.2892)' on 'M−phase inducer phosphatase 2'

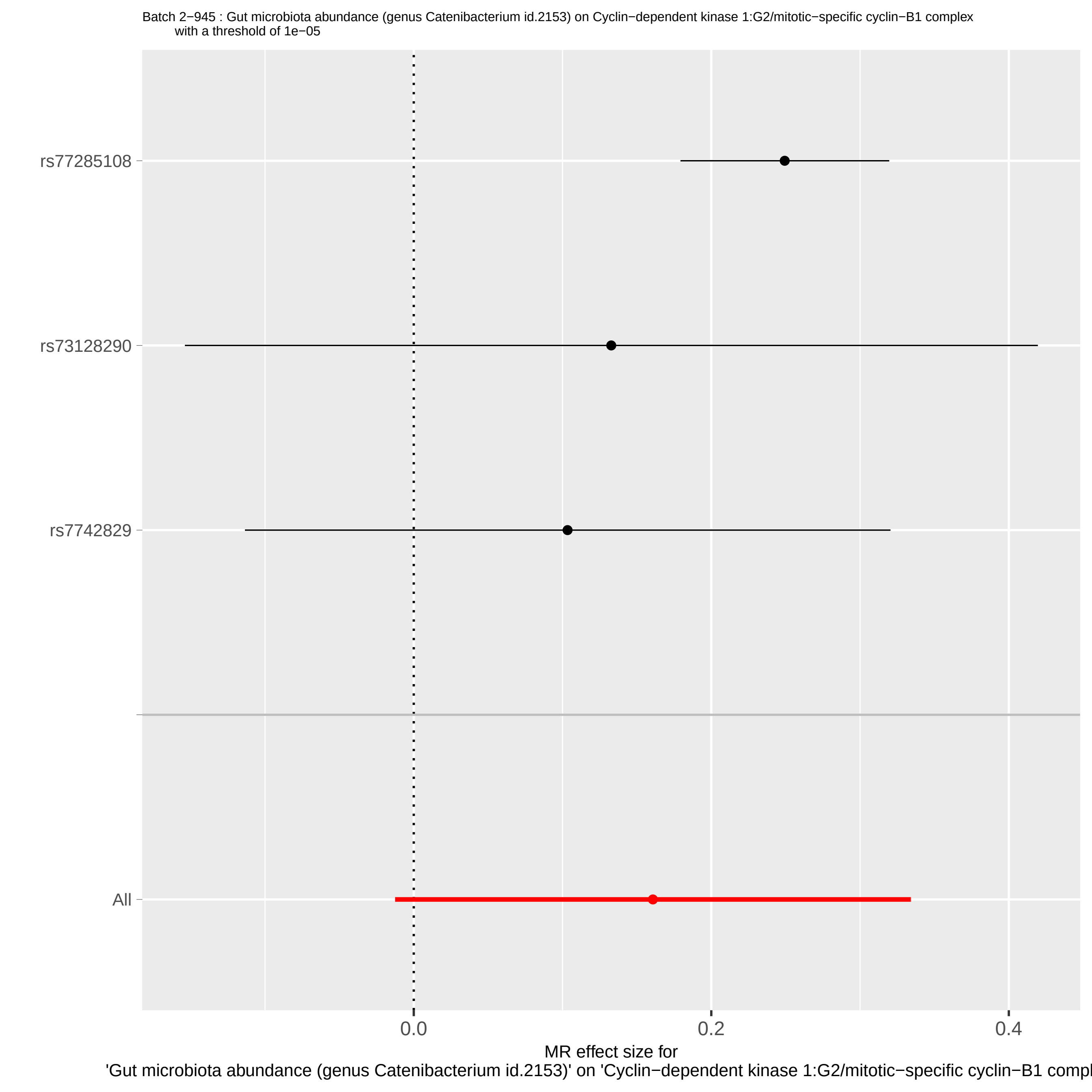
Batch 2−945 : Gut microbiota abundance (genus Catenibacterium id.2153) on Cyclin−dependent kinase 1:G2/mitotic−specific cyclin−B1 complex
with a threshold of 1e−05
rs77285108
rs73128290
rs7742829
All
0.0
0.2
0.4
MR effect size for
'Gut microbiota abundance (genus Catenibacterium id.2153)' on 'Cyclin−dependent kinase 1:G2/mitotic−specific cyclin−B1 compl

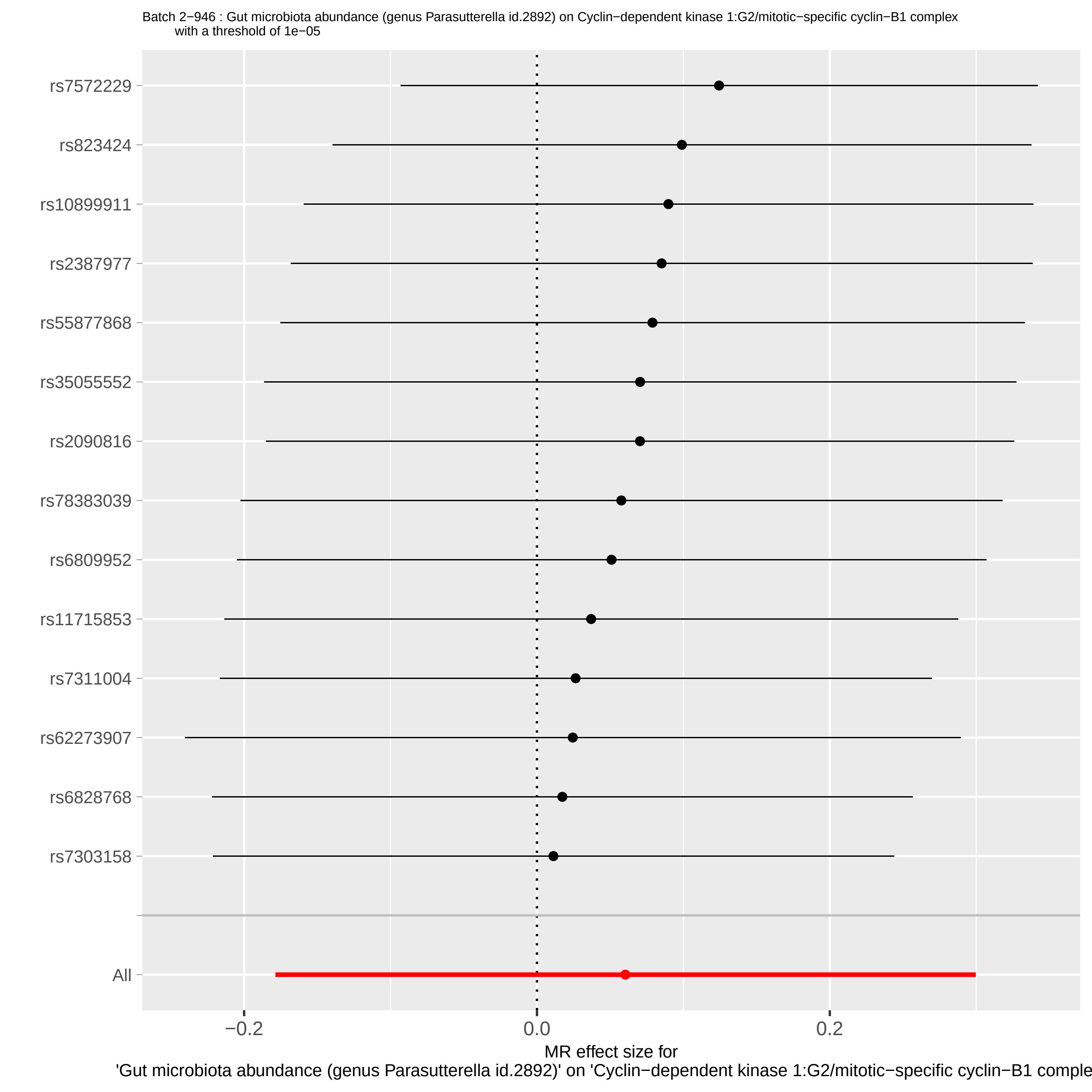
Batch 2−946 : Gut microbiota abundance (genus Parasutterella id.2892) on Cyclin−dependent kinase 1:G2/mitotic−specific cyclin−B1 complex
with a threshold of 1e−05
rs7572229
rs823424
rs10899911
rs2387977
rs55877868
rs35055552
rs2090816
rs78383039
rs6809952
rs11715853
rs7311004
rs62273907
rs6828768
rs7303158
All
−0.2
0.0
0.2
MR effect size for
'Gut microbiota abundance (genus Parasutterella id.2892)' on 'Cyclin−dependent kinase 1:G2/mitotic−specific cyclin−B1 comple

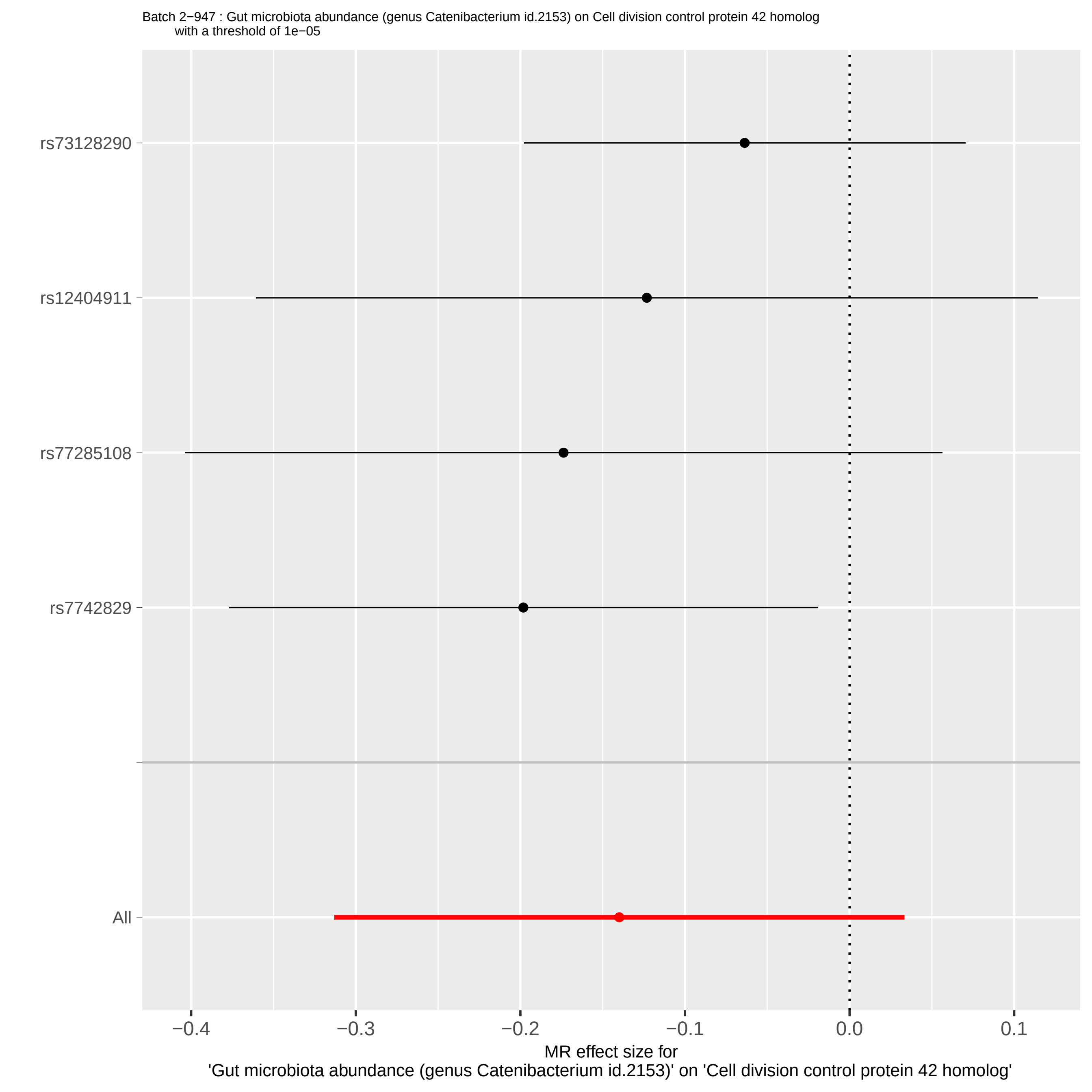

Batch 2−947 : Gut microbiota abundance (genus Catenibacterium id.2153) on Cell division control protein 42 homolog
with a threshold of 1e−05
rs73128290
rs12404911
rs77285108
rs7742829
All
−0.4
−0.3
−0.2
−0.1
0.0
0.1
MR effect size for
'Gut microbiota abundance (genus Catenibacterium id.2153)' on 'Cell division control protein 42 homolog'

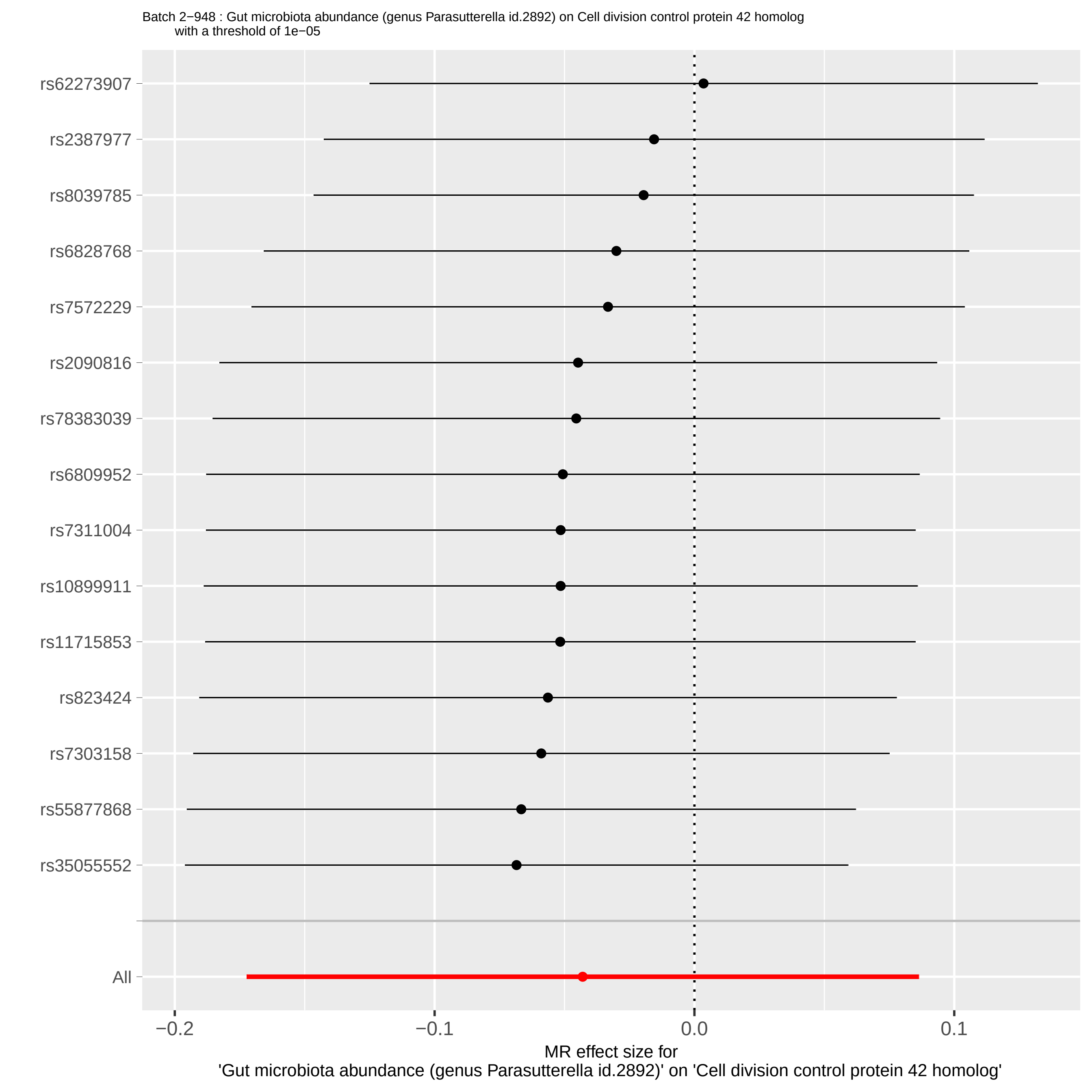
Batch 2−948 : Gut microbiota abundance (genus Parasutterella id.2892) on Cell division control protein 42 homolog
with a threshold of 1e−05
rs62273907
rs2387977
rs8039785
rs6828768
rs7572229
rs2090816
rs78383039
rs6809952
rs7311004
rs10899911
rs11715853
rs823424
rs7303158
rs55877868
rs35055552
All
−0.2
−0.1
0.0
0.1
MR effect size for
'Gut microbiota abundance (genus Parasutterella id.2892)' on 'Cell division control protein 42 homolog'

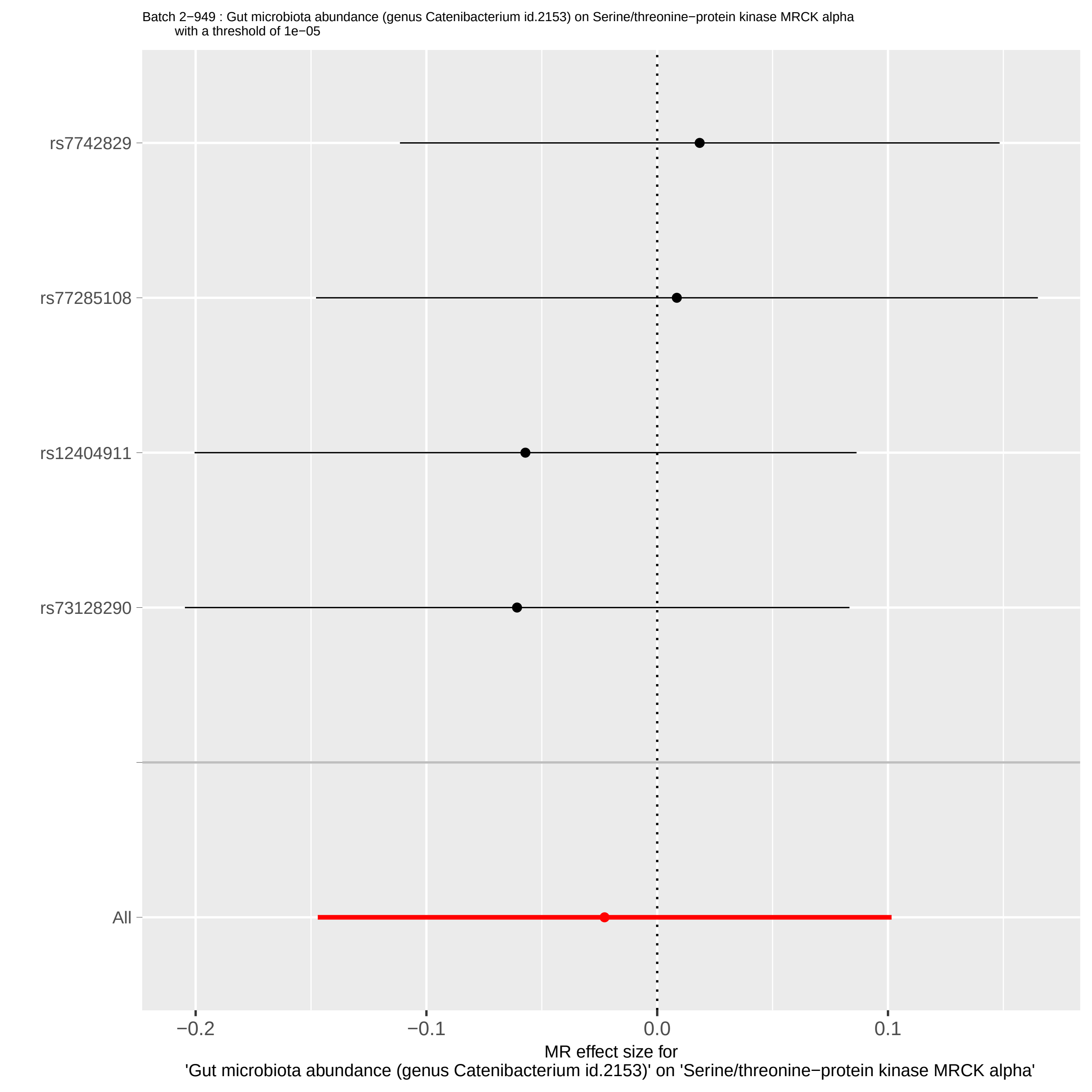
Batch 2−949 : Gut microbiota abundance (genus Catenibacterium id.2153) on Serine/threonine−protein kinase MRCK alpha
with a threshold of 1e−05
rs7742829
rs77285108
rs12404911
rs73128290
All
−0.2
−0.1
0.0
0.1
MR effect size for
'Gut microbiota abundance (genus Catenibacterium id.2153)' on 'Serine/threonine−protein kinase MRCK alpha'

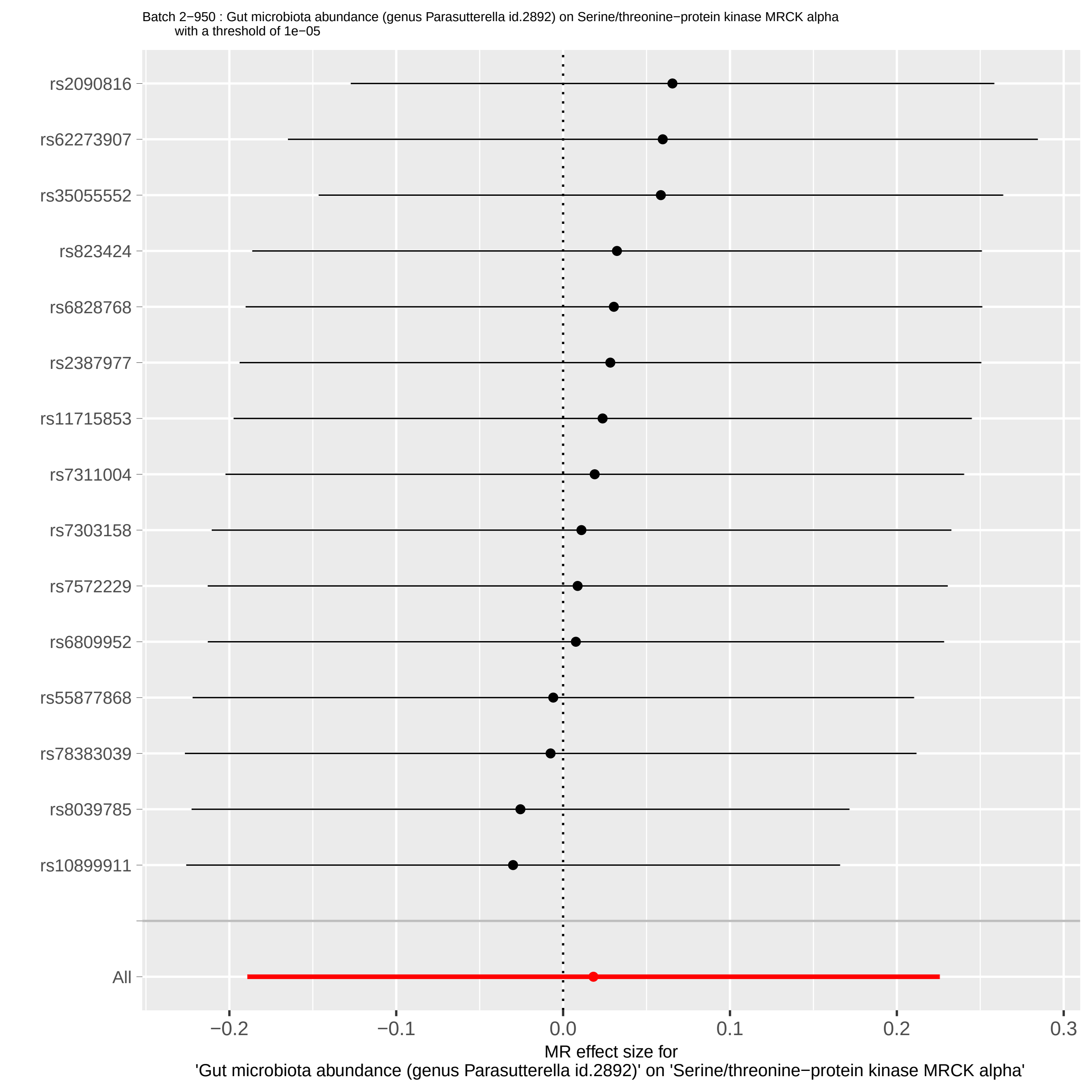
Batch 2−950 : Gut microbiota abundance (genus Parasutterella id.2892) on Serine/threonine−protein kinase MRCK alpha
with a threshold of 1e−05
rs2090816
rs62273907
rs35055552
rs823424
rs6828768
rs2387977
rs11715853
rs7311004
rs7303158
rs7572229
rs6809952
rs55877868
rs78383039
rs8039785
rs10899911
All
−0.2
−0.1
0.0
0.1
0.2
0.3
MR effect size for
'Gut microbiota abundance (genus Parasutterella id.2892)' on 'Serine/threonine−protein kinase MRCK alpha'

Batch 2−951 : Gut microbiota abundance (genus Catenibacterium id.2153) on Serine/threonine−protein kinase MRCK beta
with a threshold of 1e−05

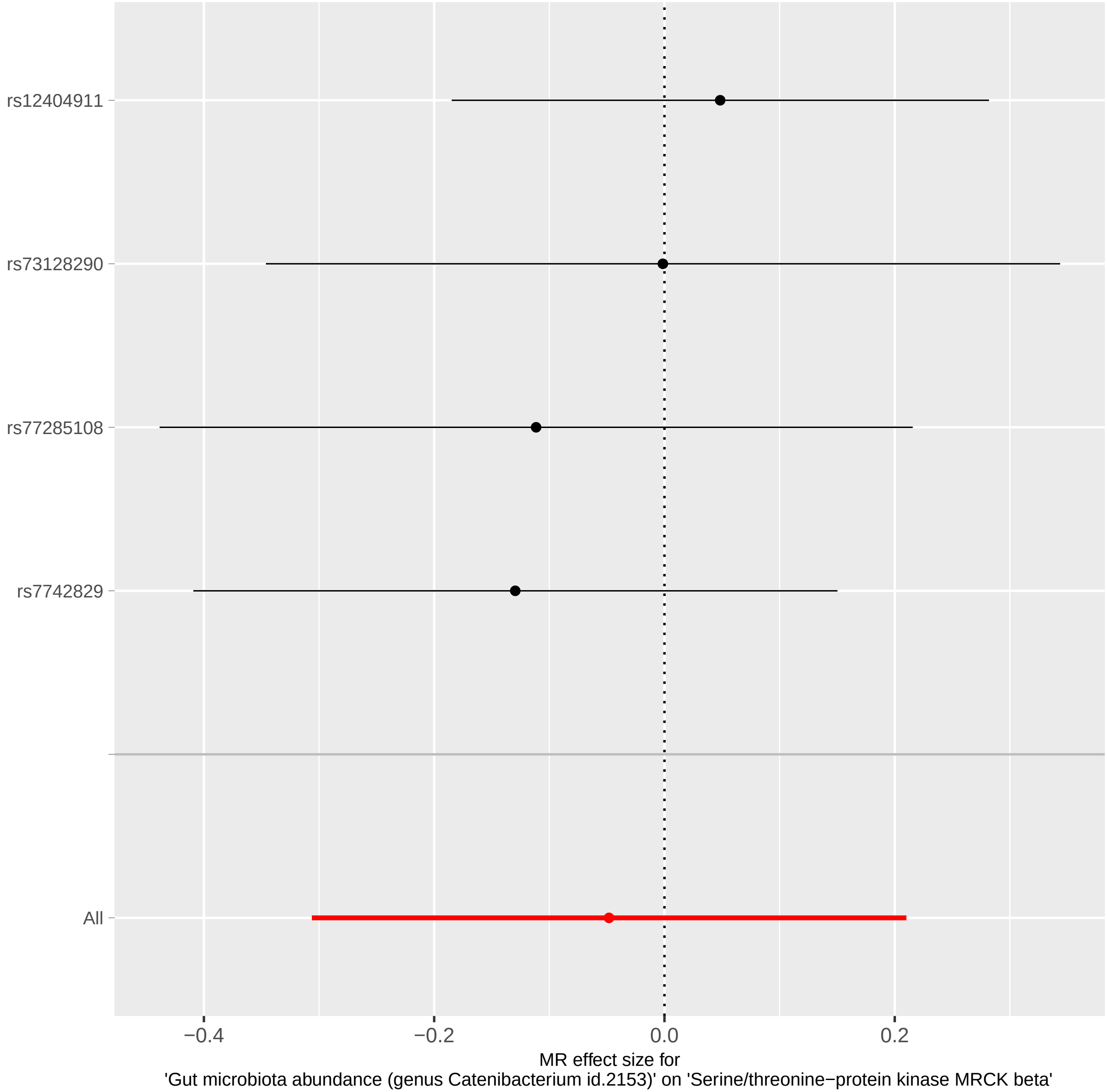

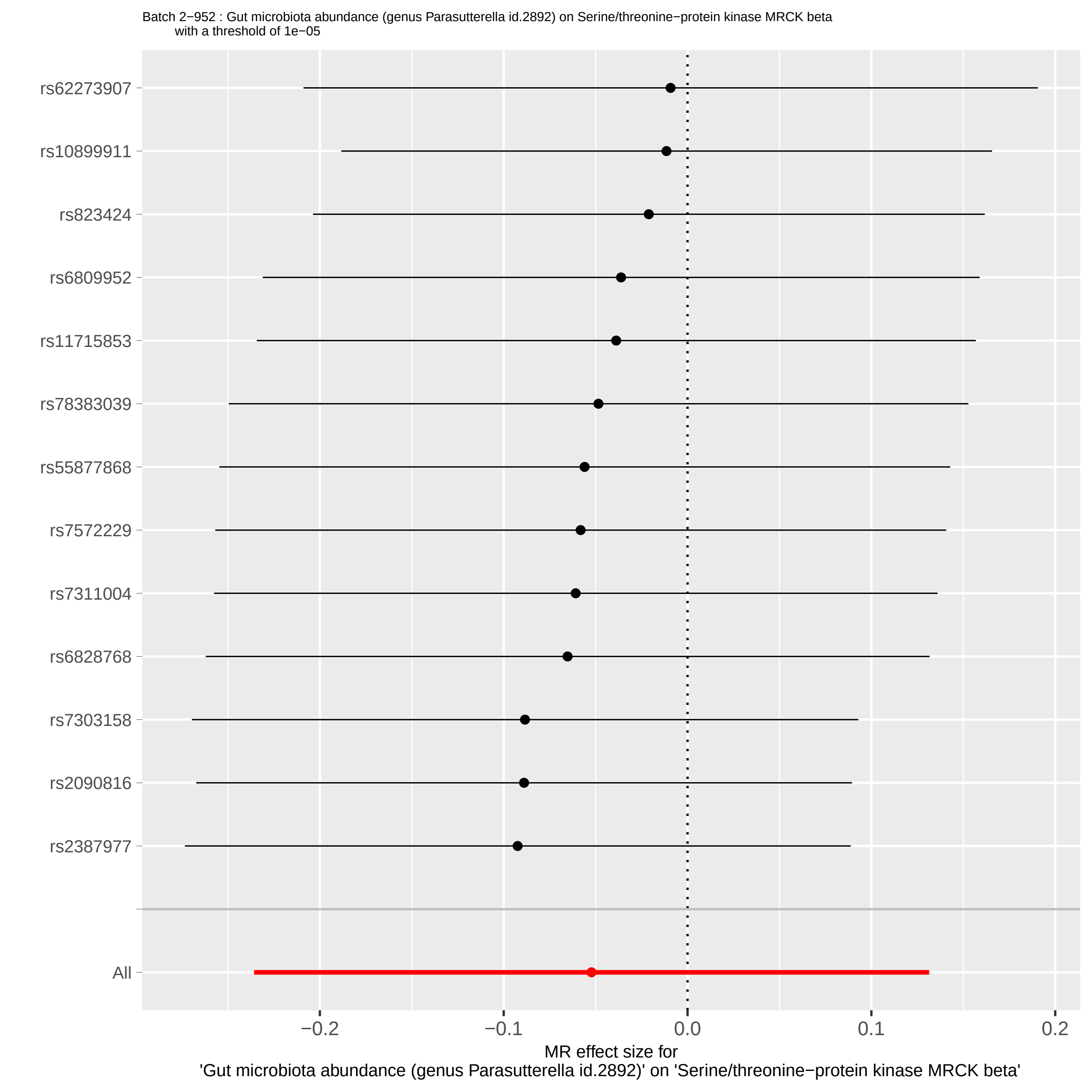

Batch 2−952 : Gut microbiota abundance (genus Parasutterella id.2892) on Serine/threonine−protein kinase MRCK beta
with a threshold of 1e−05
rs62273907
rs10899911
rs823424
rs6809952
rs11715853
rs78383039
rs55877868
rs7572229
rs7311004
rs6828768
rs7303158
rs2090816
rs2387977
All
−0.2
−0.1
0.0
0.1
0.2
MR effect size for
'Gut microbiota abundance (genus Parasutterella id.2892)' on 'Serine/threonine−protein kinase MRCK beta'

Batch 2−953 : Gut microbiota abundance (genus Catenibacterium id.2153) on CUB domain−containing protein 1 with a threshold of 1e−05

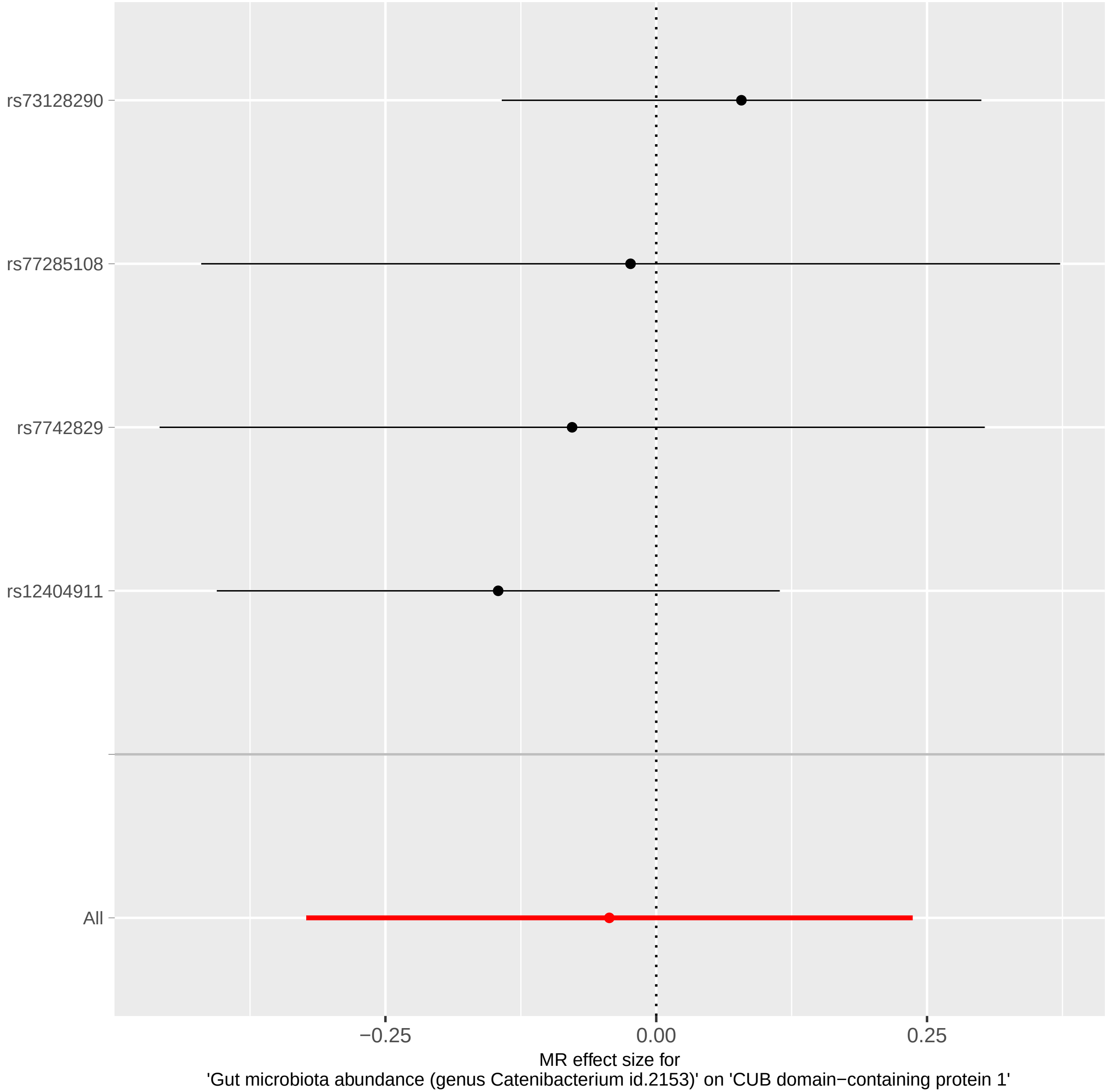

Batch 2−954 : Gut microbiota abundance (genus Parasutterella id.2892) on CUB domain−containing protein 1
with a threshold of 1e−05

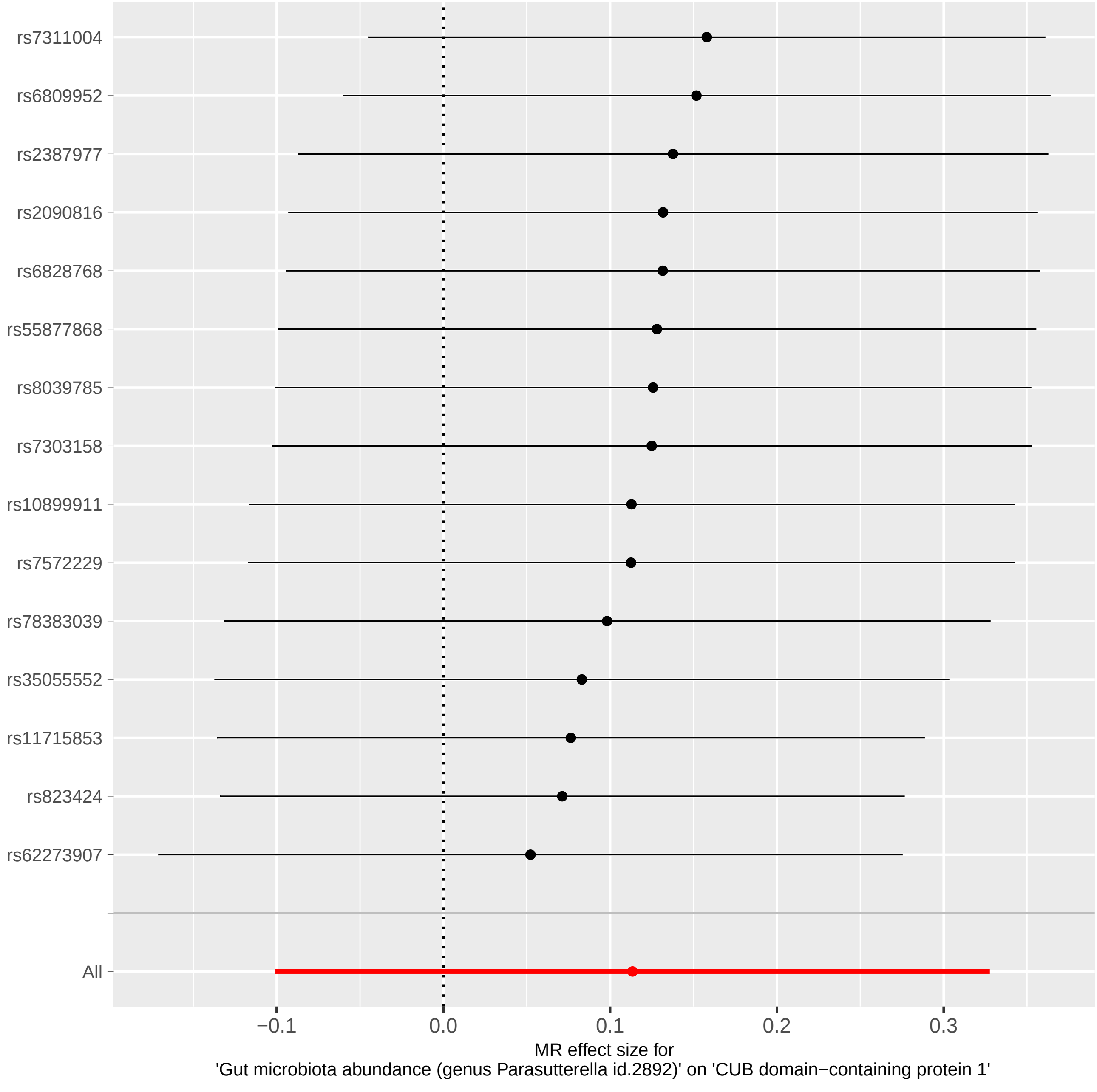

Batch 2−955 : Gut microbiota abundance (genus Catenibacterium id.2153) on Cadherin−12
with a threshold of 1e−05

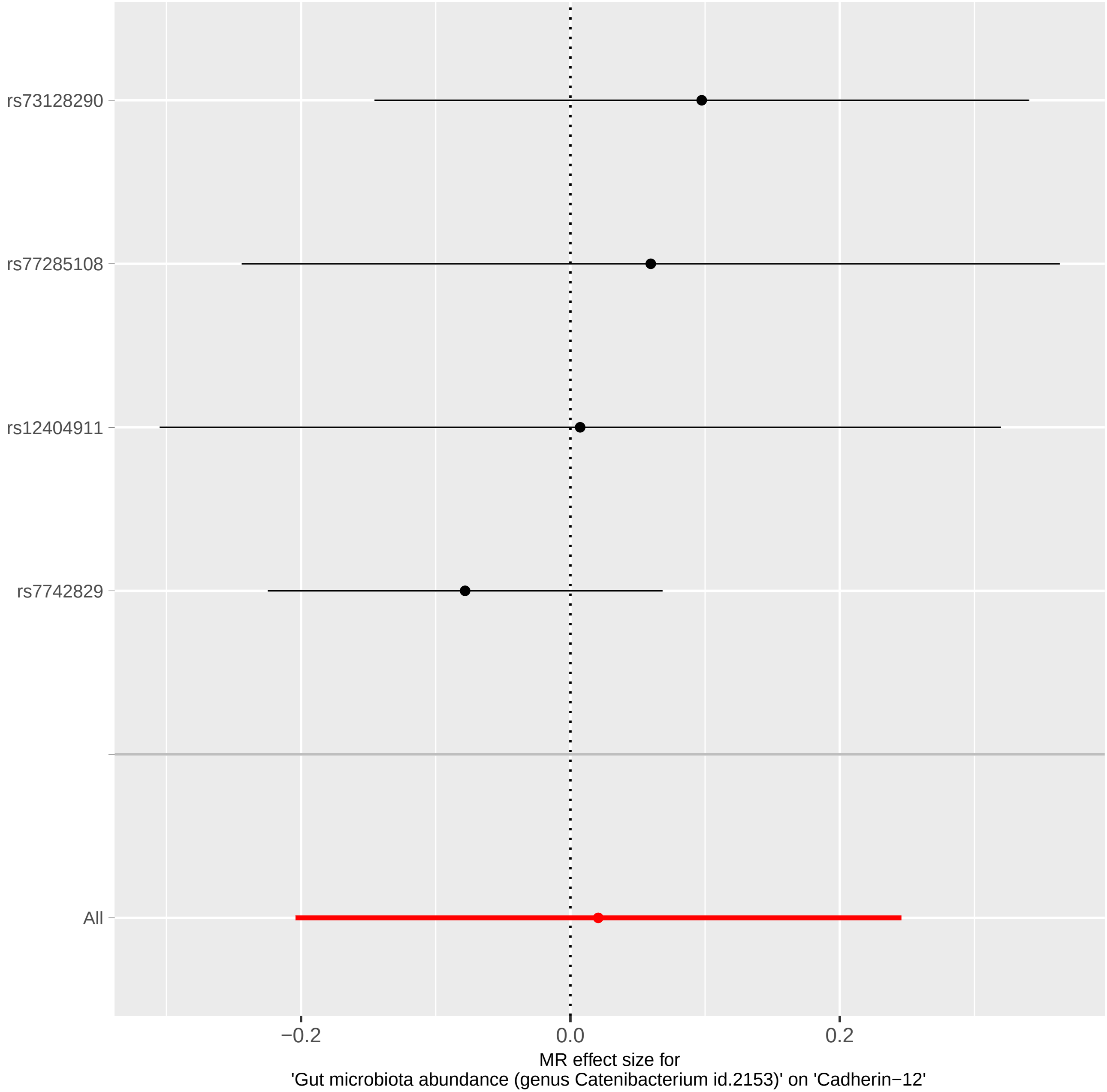

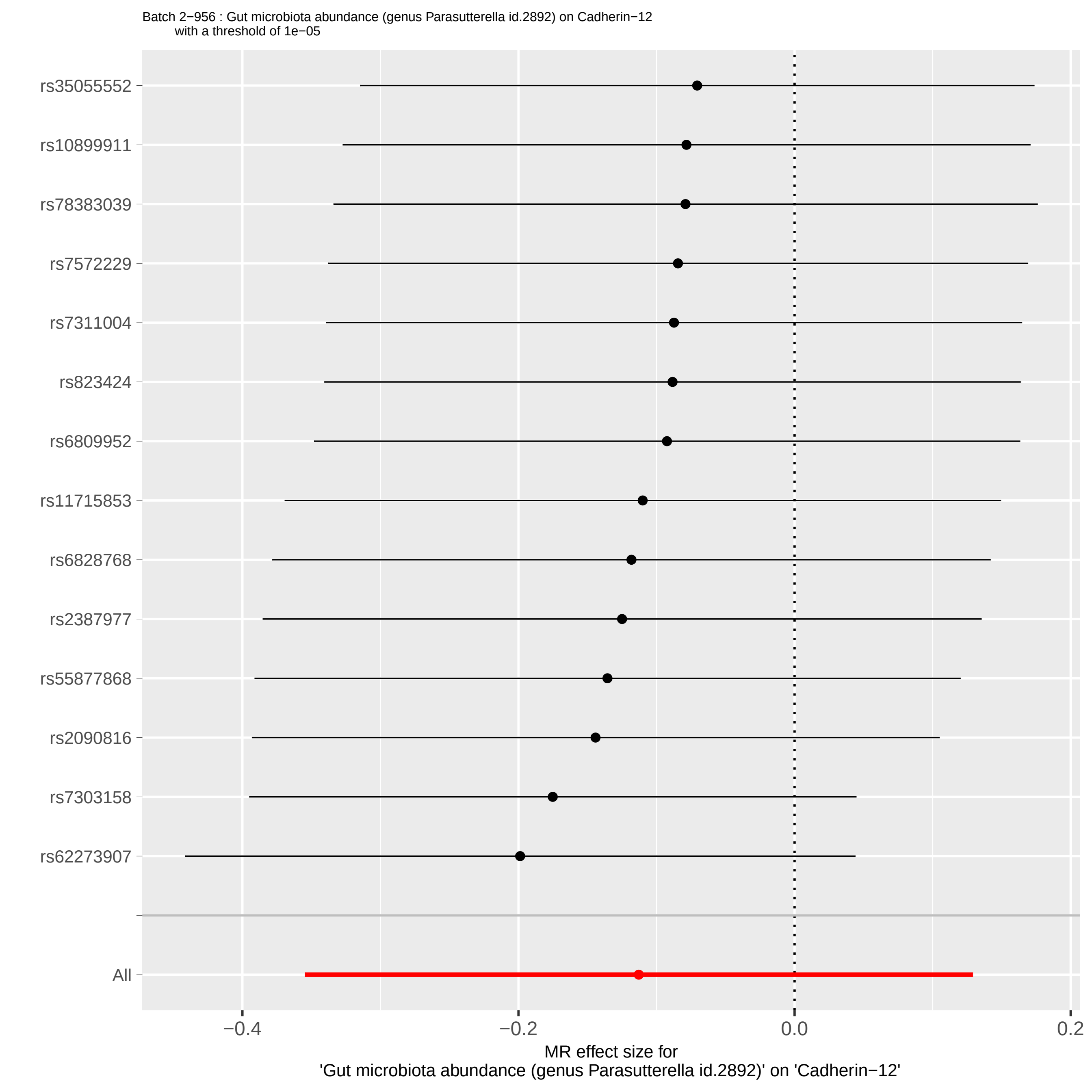
Batch 2−956 : Gut microbiota abundance (genus Parasutterella id.2892) on Cadherin−12
with a threshold of 1e−05
rs35055552
rs10899911
rs78383039
rs7572229
rs7311004
rs823424
rs6809952
rs11715853
rs6828768
rs2387977
rs55877868
rs2090816
rs7303158
rs62273907
All
−0.4
−0.2
0.0
0.2
MR effect size for
'Gut microbiota abundance (genus Parasutterella id.2892)' on 'Cadherin−12'

Batch 2−957 : Gut microbiota abundance (genus Catenibacterium id.2153) on Cadherin−12
with a threshold of 1e−05

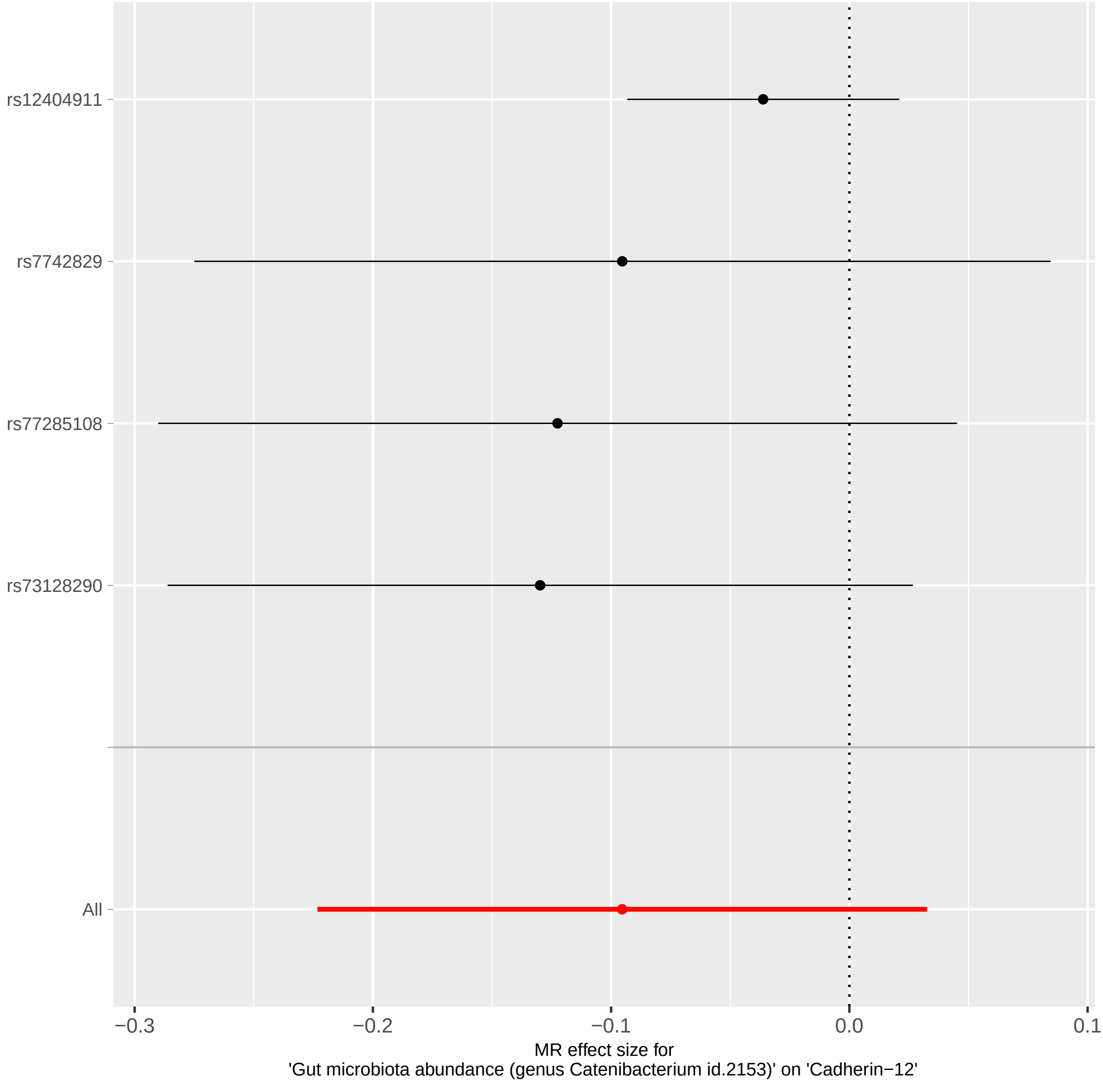

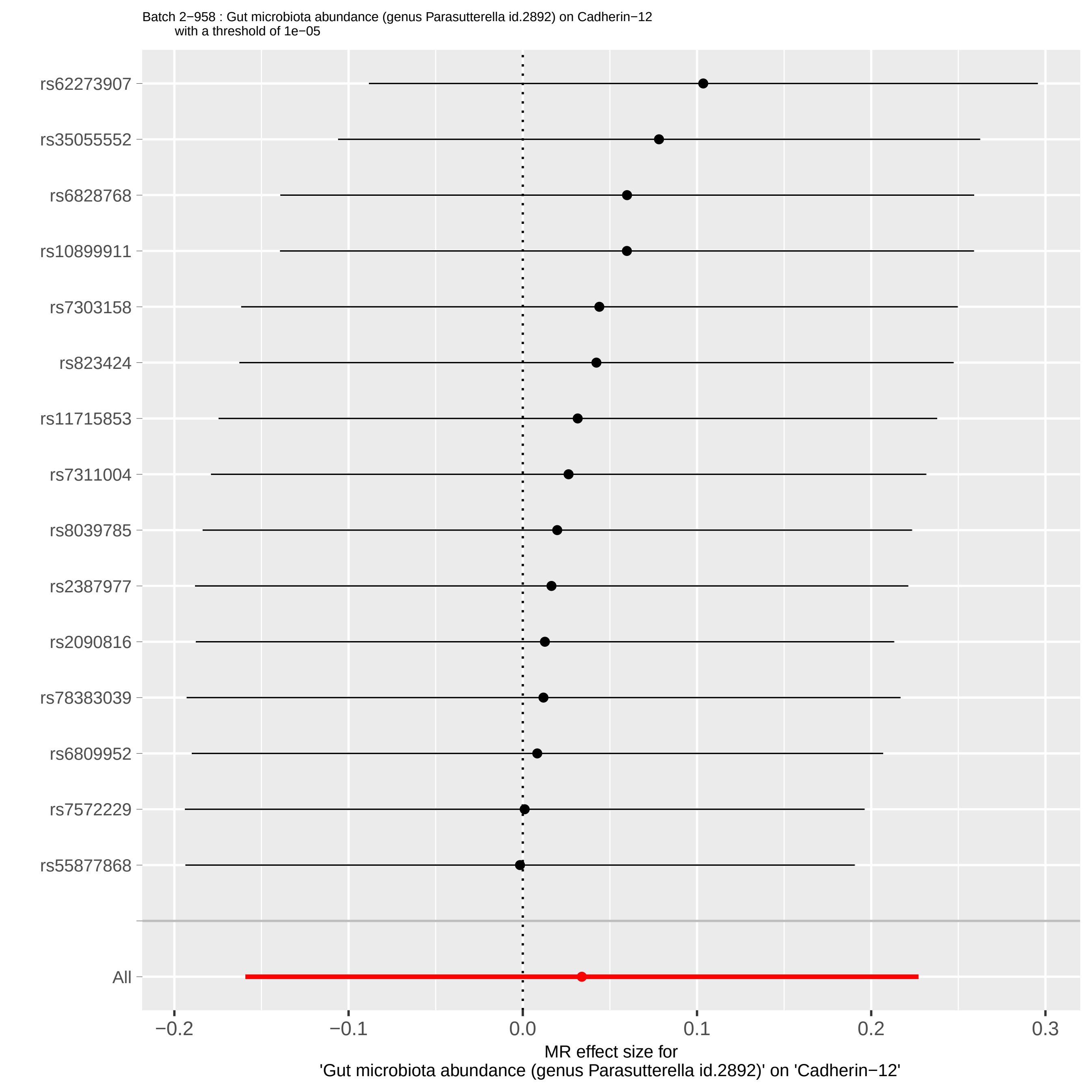

Batch 2−958 : Gut microbiota abundance (genus Parasutterella id.2892) on Cadherin−12
with a threshold of 1e−05
rs62273907
rs35055552
rs6828768
rs10899911
rs7303158
rs823424
rs11715853
rs7311004
rs8039785
rs2387977
rs2090816
rs78383039
rs6809952
rs7572229
rs55877868
All
−0.2
−0.1
0.0
0.1
0.2
0.3
MR effect size for
'Gut microbiota abundance (genus Parasutterella id.2892)' on 'Cadherin−12'

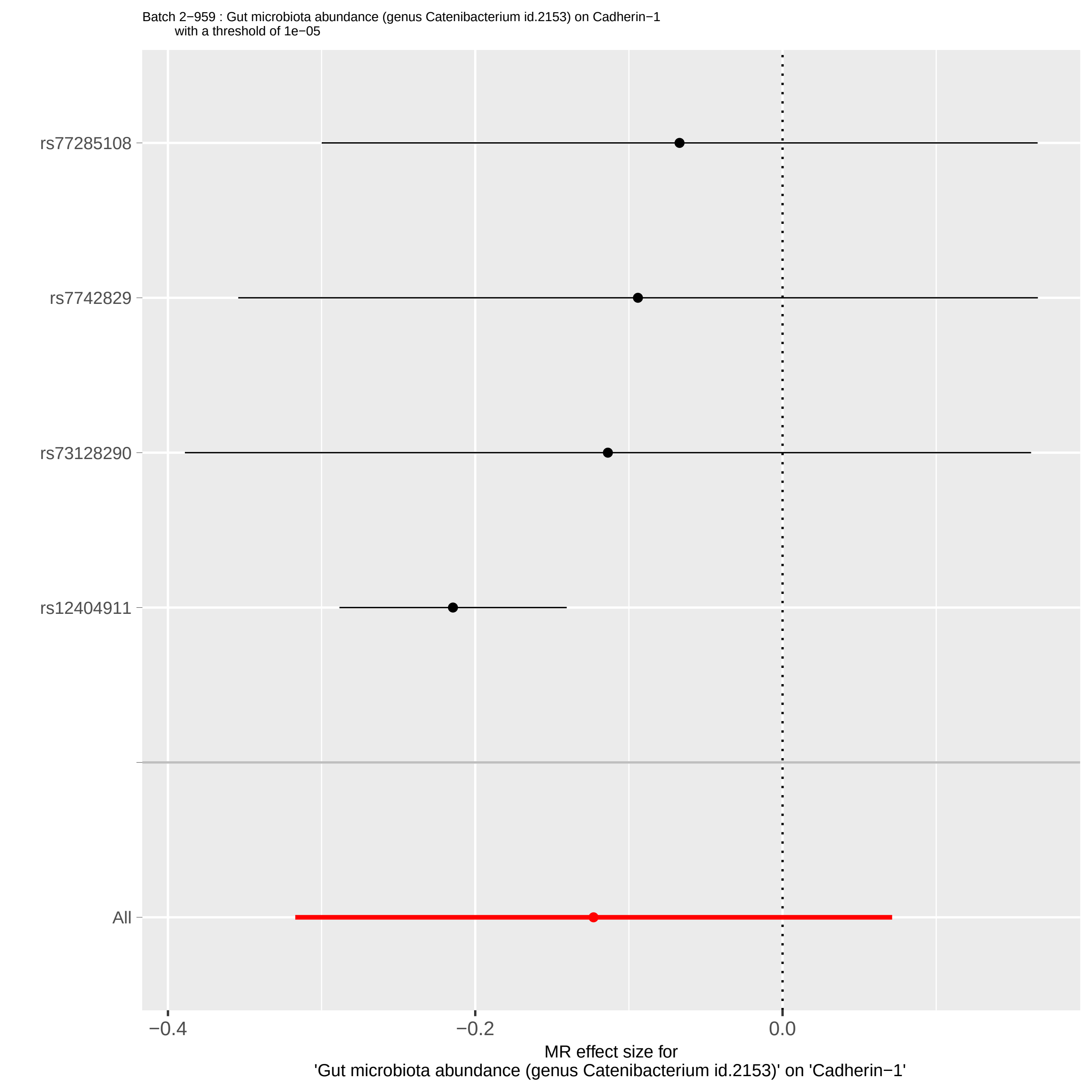
Batch 2−959 : Gut microbiota abundance (genus Catenibacterium id.2153) on Cadherin−1
with a threshold of 1e−05
rs77285108
rs7742829
rs73128290
rs12404911
All
−0.4
−0.2
0.0
MR effect size for
'Gut microbiota abundance (genus Catenibacterium id.2153)' on 'Cadherin−1'

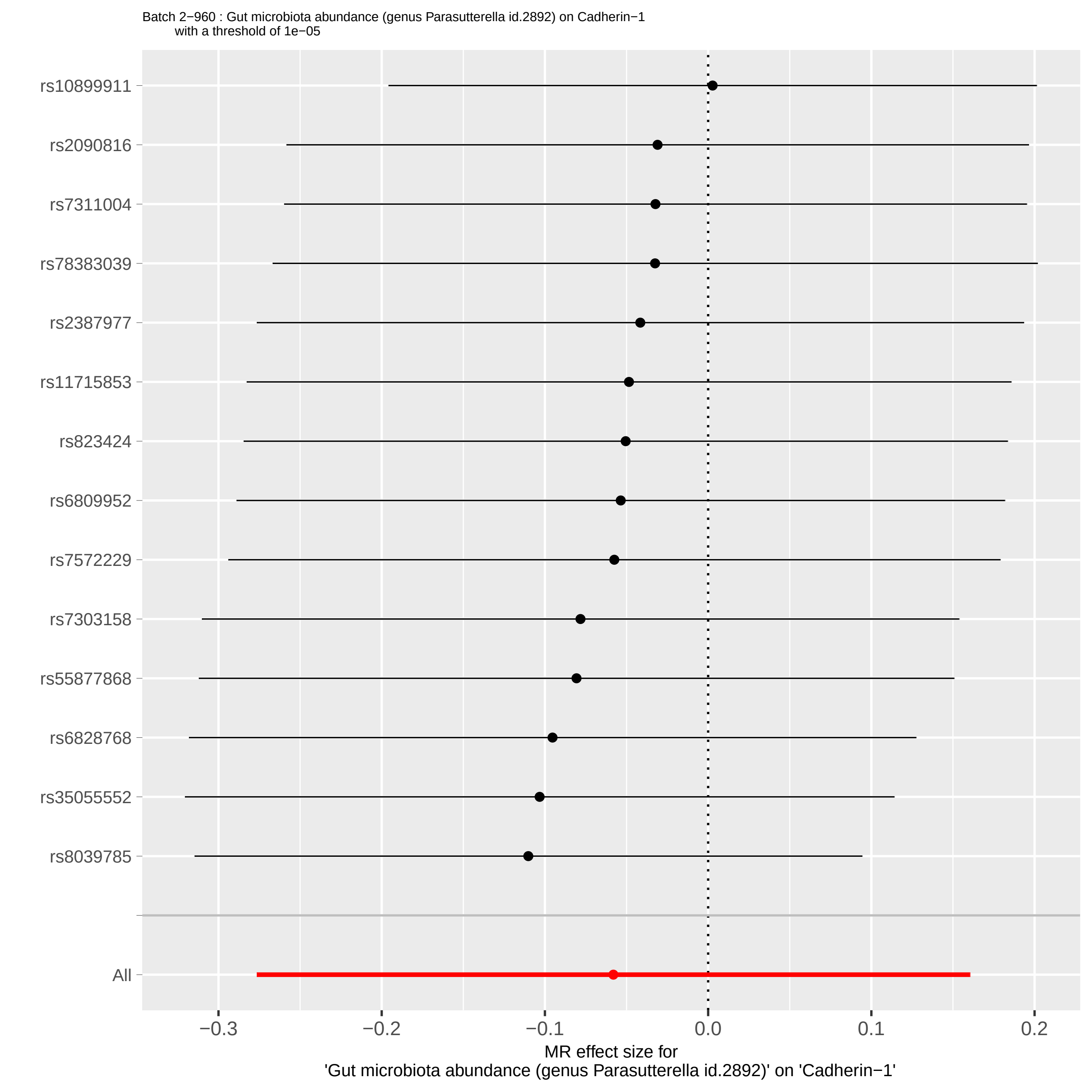
Batch 2−960 : Gut microbiota abundance (genus Parasutterella id.2892) on Cadherin−1
with a threshold of 1e−05
rs10899911
rs2090816
rs7311004
rs78383039
rs2387977
rs11715853
rs823424
rs6809952
rs7572229
rs7303158
rs55877868
rs6828768
rs35055552
rs8039785
All
−0.3
−0.2
−0.1
0.0
0.1
0.2
MR effect size for
'Gut microbiota abundance (genus Parasutterella id.2892)' on 'Cadherin−1'

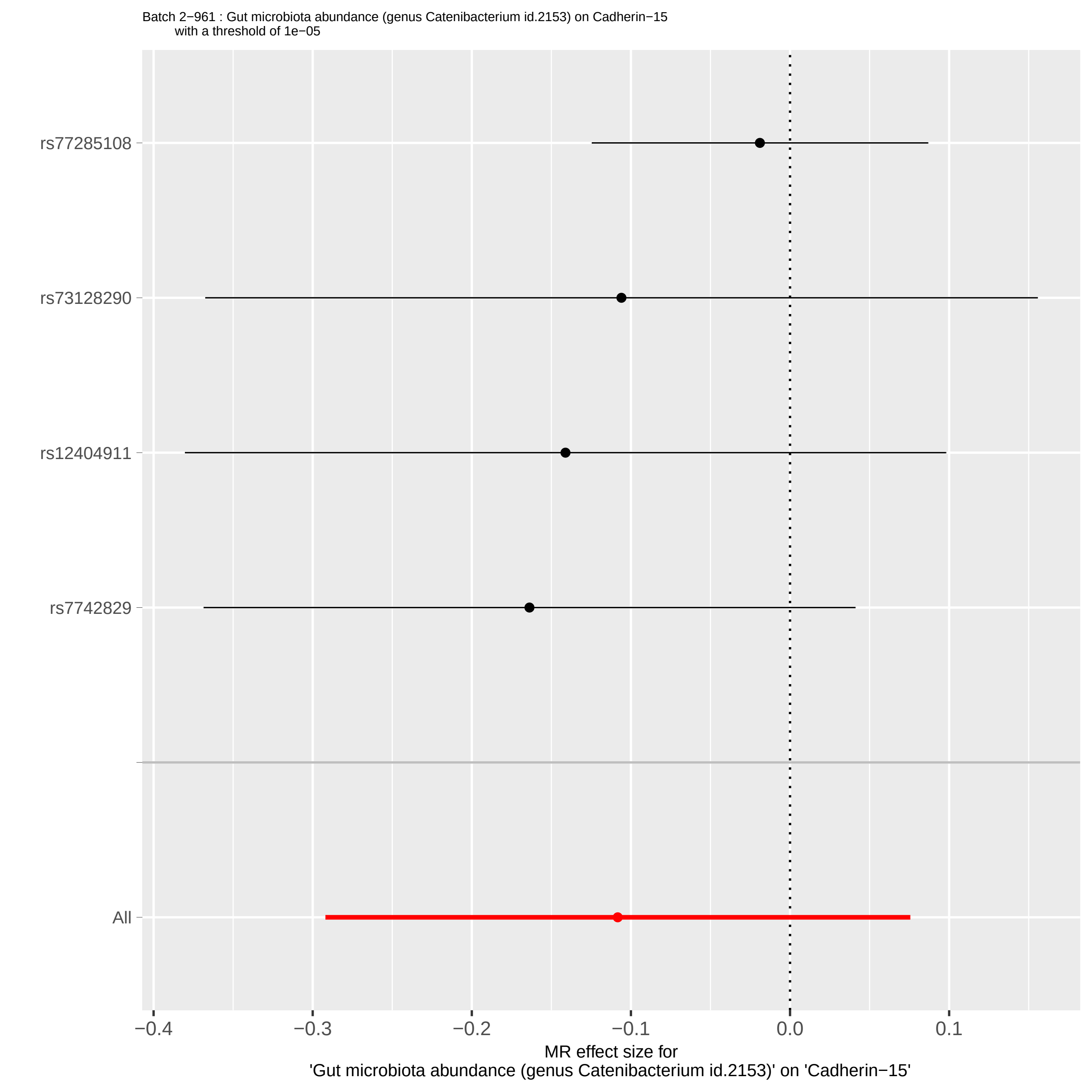
Batch 2−961 : Gut microbiota abundance (genus Catenibacterium id.2153) on Cadherin−15
with a threshold of 1e−05
rs77285108
rs73128290
rs12404911
rs7742829
All
−0.4
−0.3
−0.2
−0.1
0.0
0.1
MR effect size for
'Gut microbiota abundance (genus Catenibacterium id.2153)' on 'Cadherin−15'

Batch 2−962 : Gut microbiota abundance (genus Parasutterella id.2892) on Cadherin−15
with a threshold of 1e−05

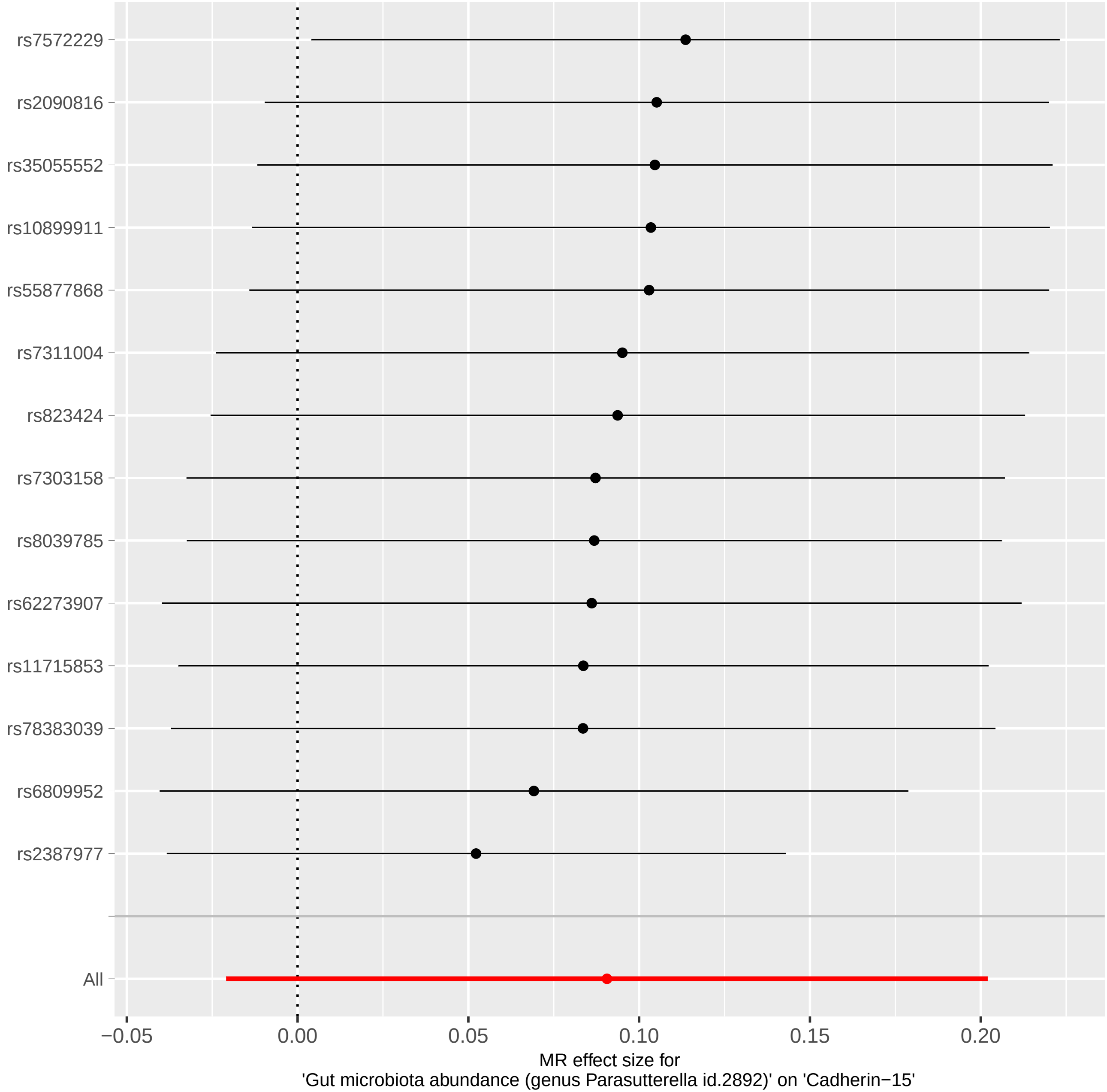

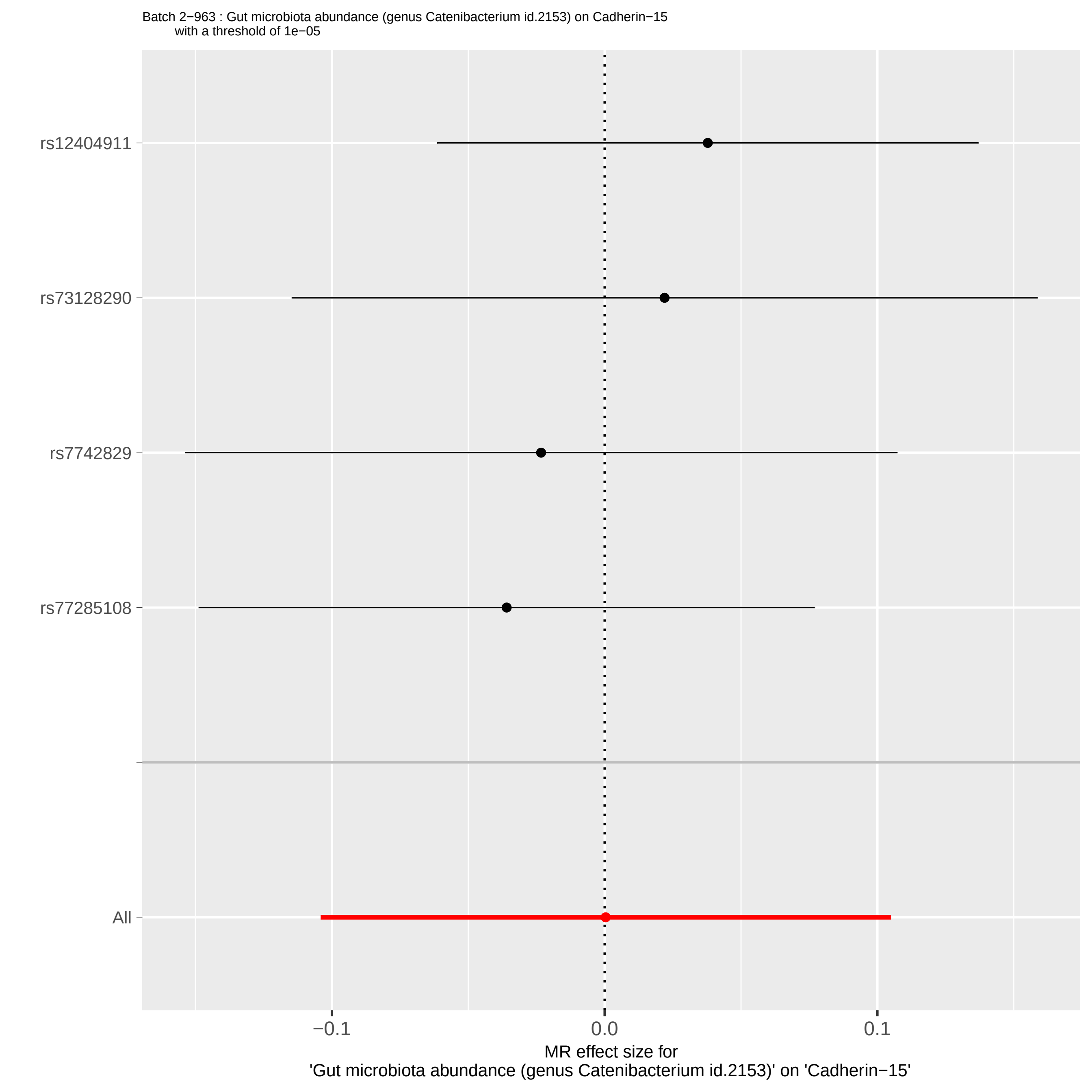
Batch 2−963 : Gut microbiota abundance (genus Catenibacterium id.2153) on Cadherin−15
with a threshold of 1e−05
rs12404911
rs73128290
rs7742829
rs77285108
All
−0.1
0.0
0.1
MR effect size for
'Gut microbiota abundance (genus Catenibacterium id.2153)' on 'Cadherin−15'

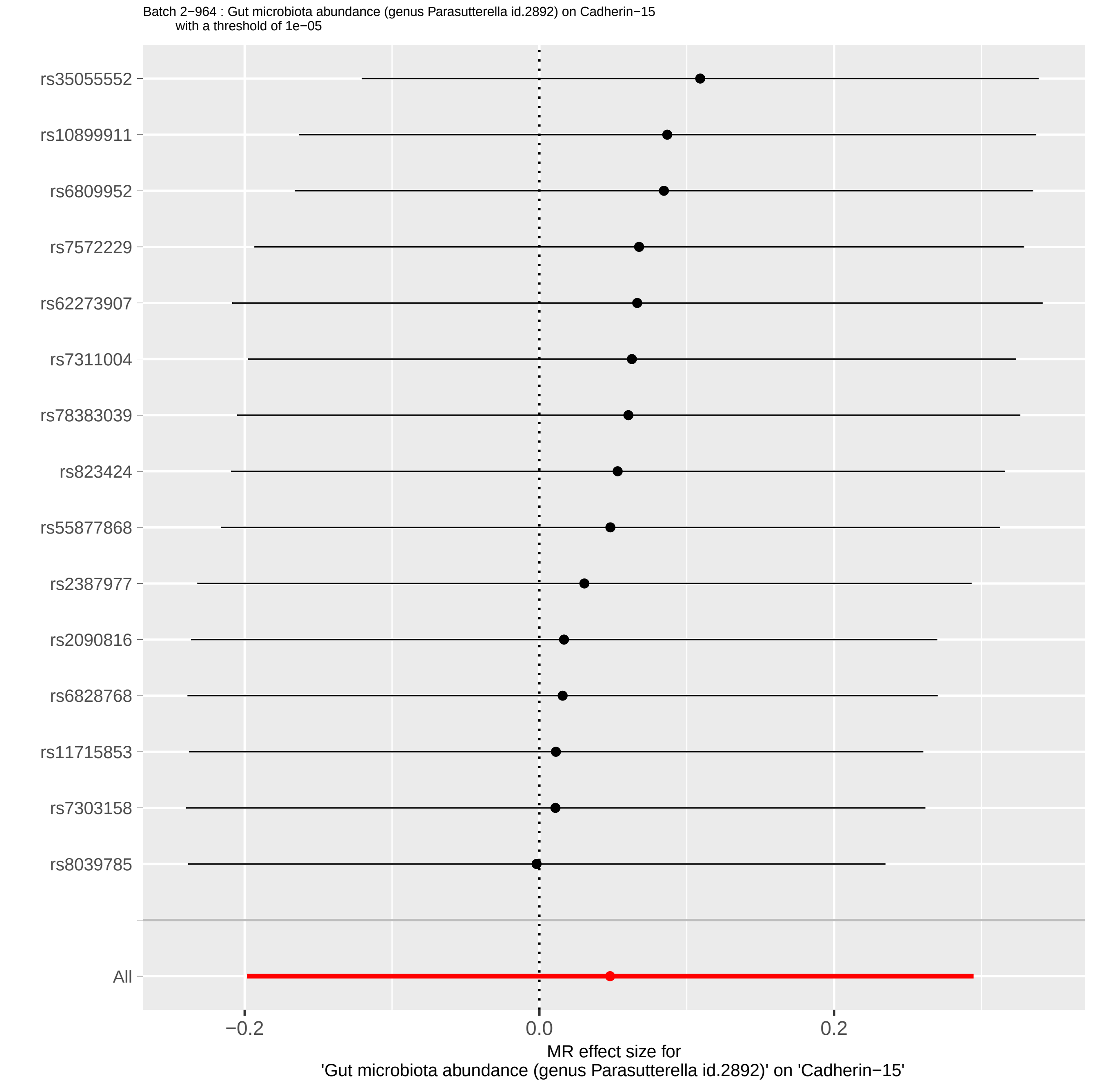

Batch 2−964 : Gut microbiota abundance (genus Parasutterella id.2892) on Cadherin−15
with a threshold of 1e−05
rs35055552
rs10899911
rs6809952
rs7572229
rs62273907
rs7311004
rs78383039
rs823424
rs55877868
rs2387977
rs2090816
rs6828768
rs11715853
rs7303158
rs8039785
All
−0.2
0.0
0.2
MR effect size for
'Gut microbiota abundance (genus Parasutterella id.2892)' on 'Cadherin−15'

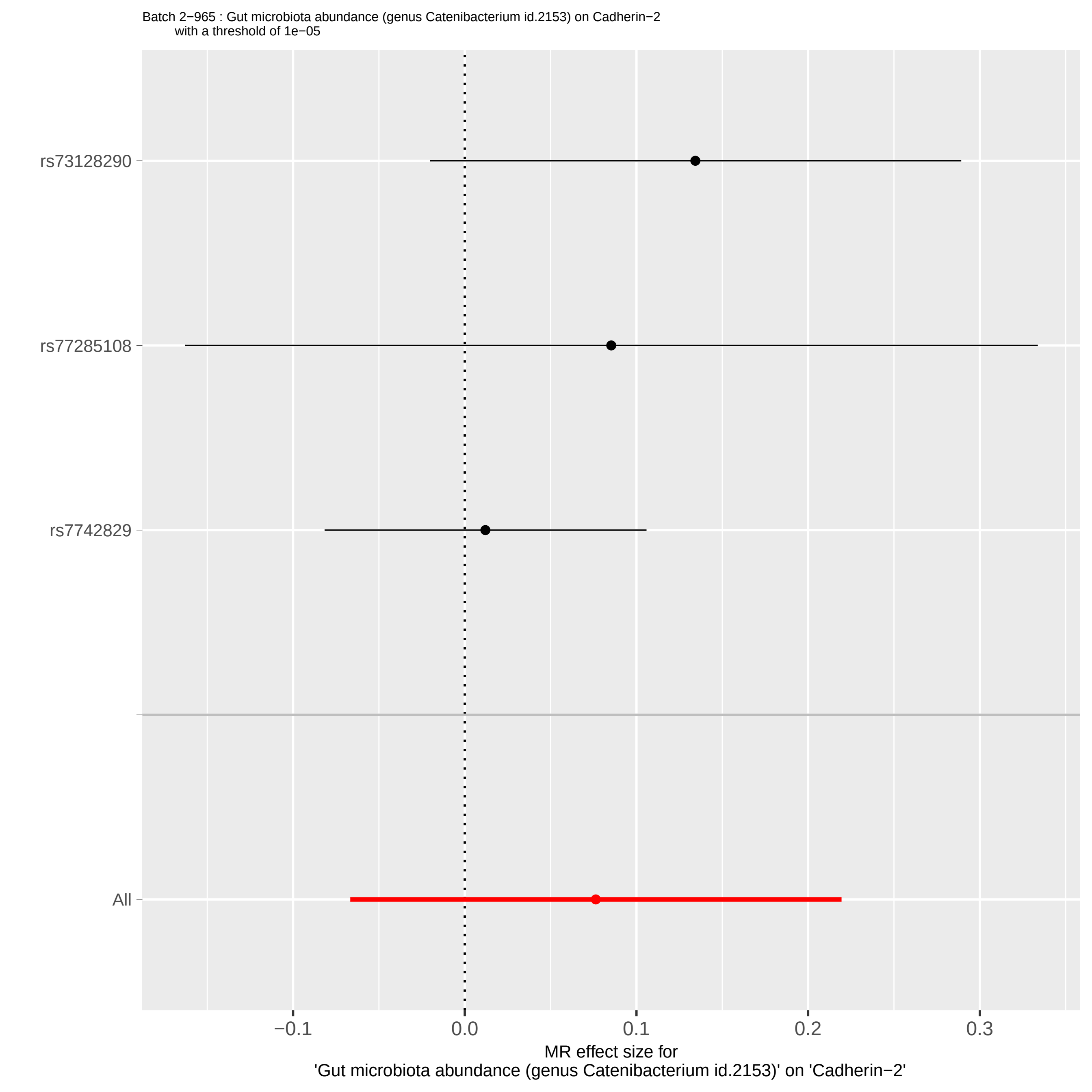

Batch 2−965 : Gut microbiota abundance (genus Catenibacterium id.2153) on Cadherin−2
with a threshold of 1e−05
rs73128290
rs77285108
rs7742829
All
−0.1
0.0
0.1
0.2
0.3
MR effect size for
'Gut microbiota abundance (genus Catenibacterium id.2153)' on 'Cadherin−2'

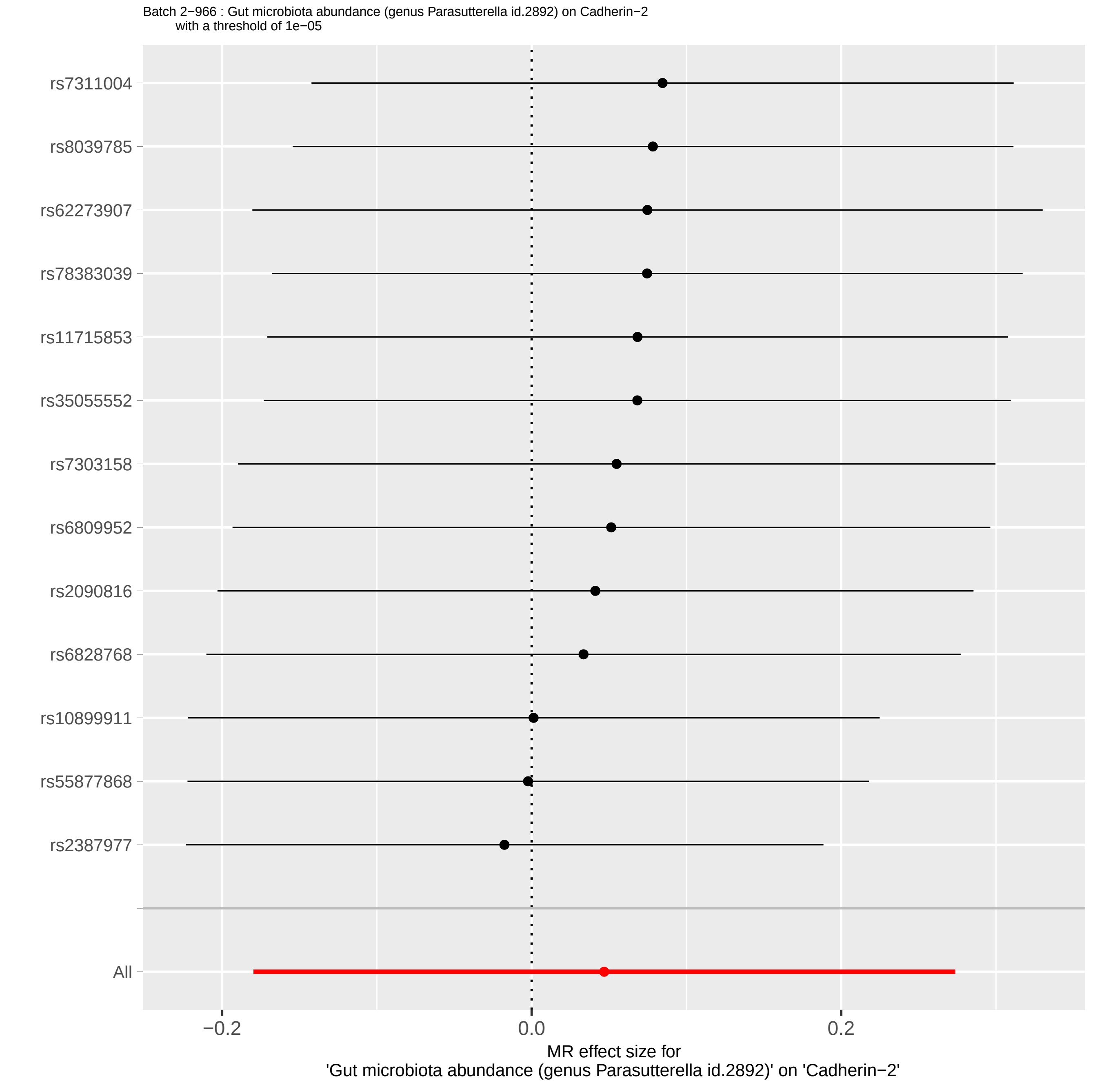

Batch 2−966 : Gut microbiota abundance (genus Parasutterella id.2892) on Cadherin−2
with a threshold of 1e−05
rs7311004
rs8039785
rs62273907
rs78383039
rs11715853
rs35055552
rs7303158
rs6809952
rs2090816
rs6828768
rs10899911
rs55877868
rs2387977
All
−0.2
0.0
0.2
MR effect size for
'Gut microbiota abundance (genus Parasutterella id.2892)' on 'Cadherin−2'

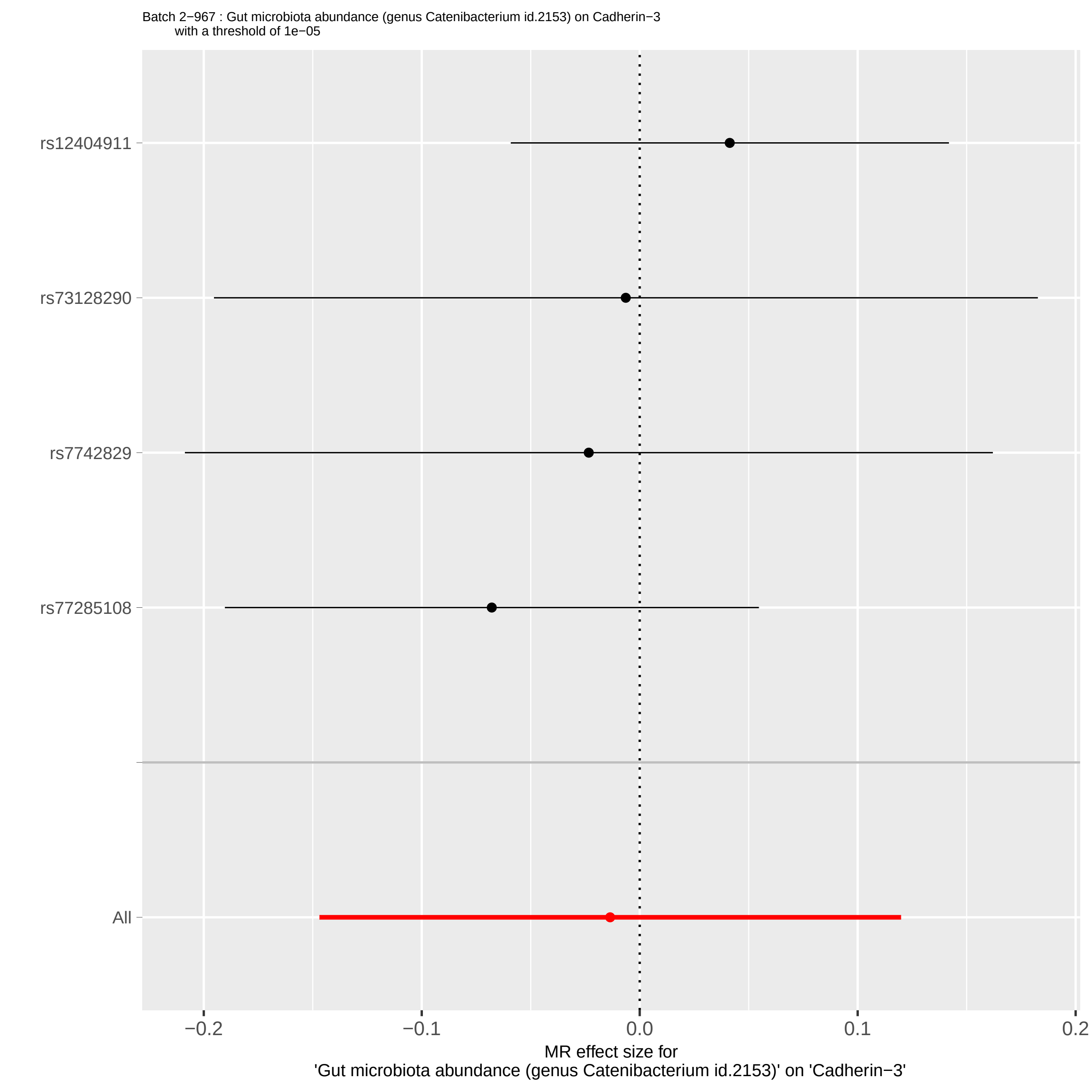
Batch 2−967 : Gut microbiota abundance (genus Catenibacterium id.2153) on Cadherin−3
with a threshold of 1e−05
rs12404911
rs73128290
rs7742829
rs77285108
All
−0.2
−0.1
0.0
0.1
0.2
MR effect size for
'Gut microbiota abundance (genus Catenibacterium id.2153)' on 'Cadherin−3'

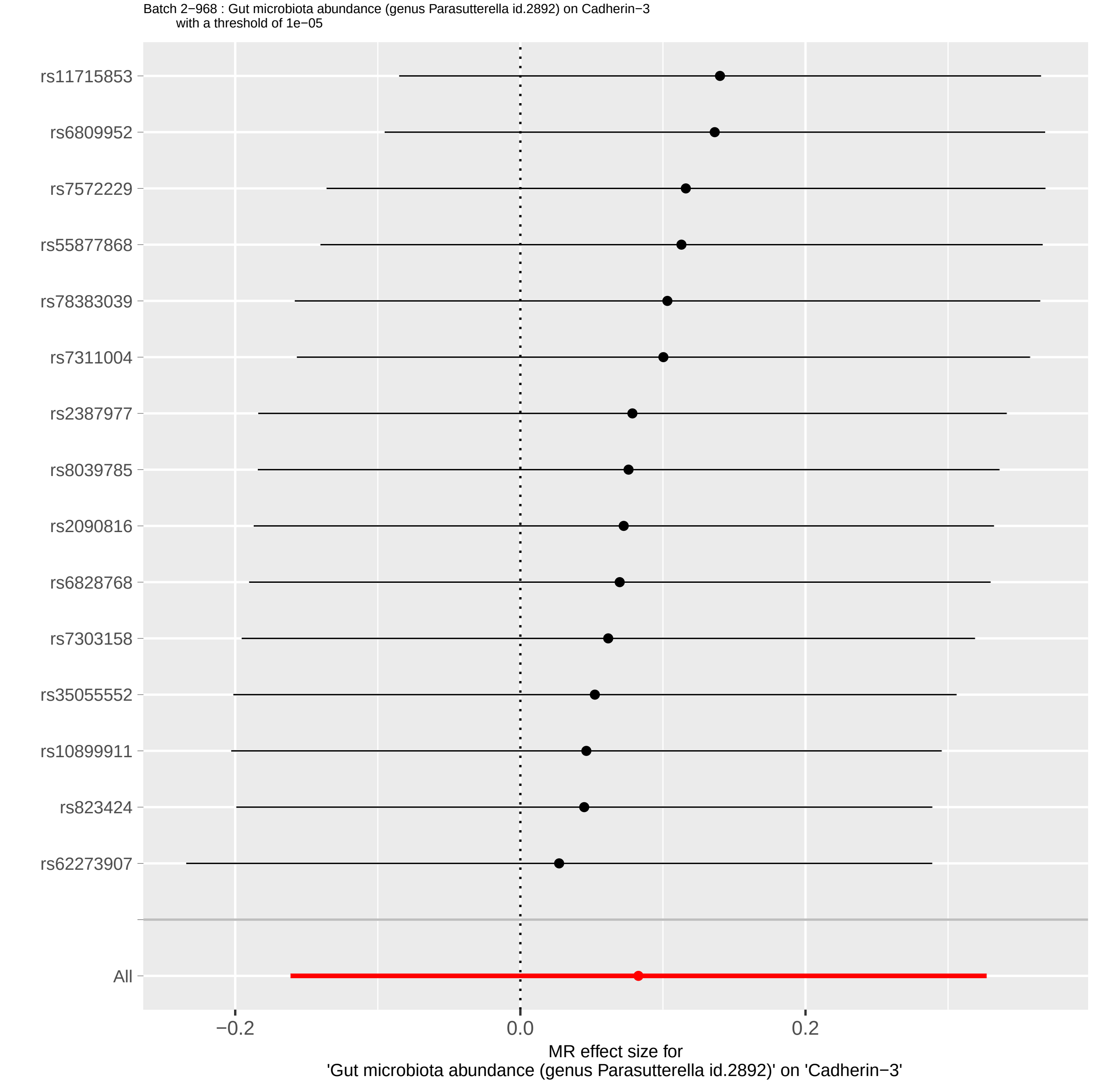

Batch 2−968 : Gut microbiota abundance (genus Parasutterella id.2892) on Cadherin−3
with a threshold of 1e−05
rs11715853
rs6809952
rs7572229
rs55877868
rs78383039
rs7311004
rs2387977
rs8039785
rs2090816
rs6828768
rs7303158
rs35055552
rs10899911
rs823424
rs62273907
All
−0.2
0.0
0.2
MR effect size for
'Gut microbiota abundance (genus Parasutterella id.2892)' on 'Cadherin−3'

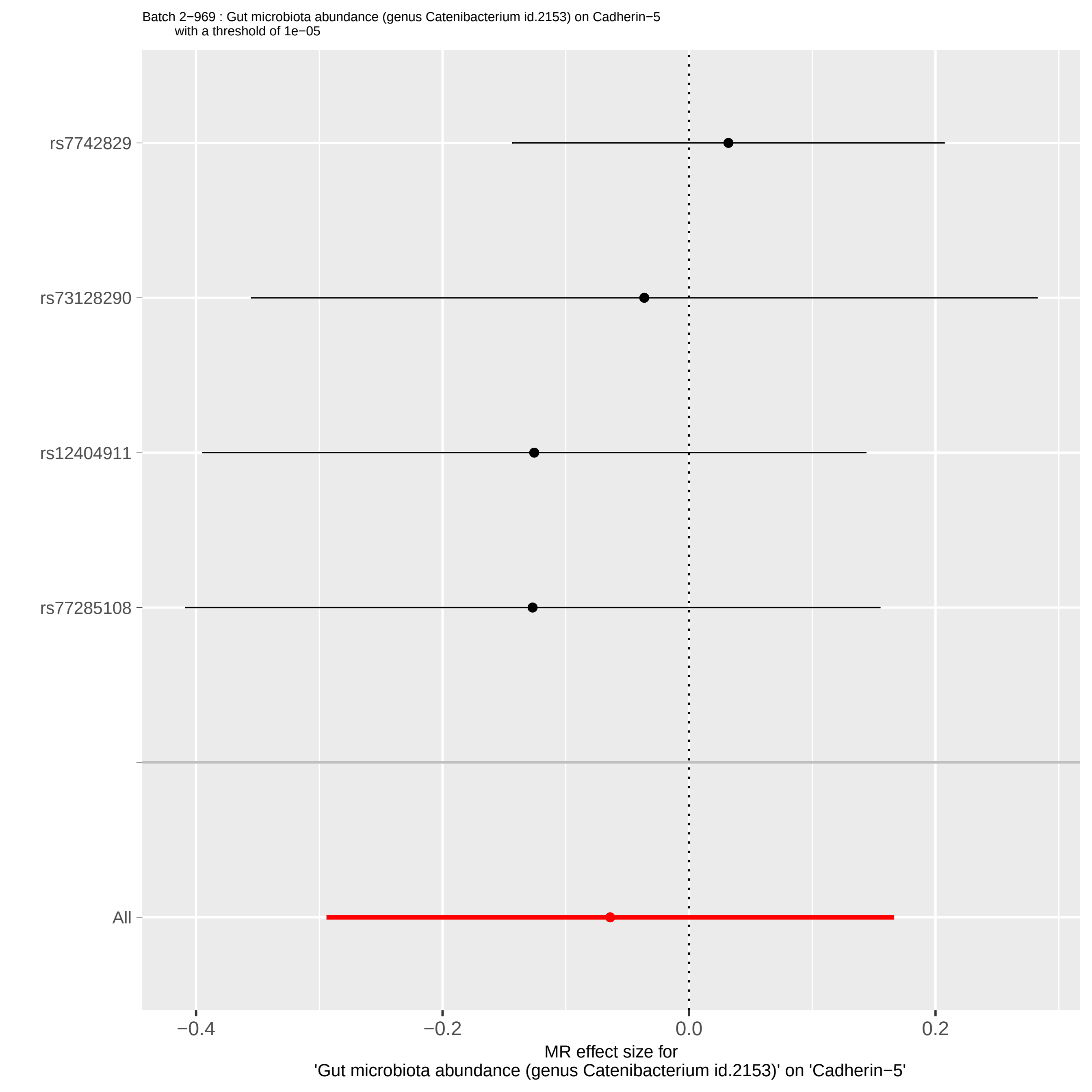
Batch 2−969 : Gut microbiota abundance (genus Catenibacterium id.2153) on Cadherin−5
with a threshold of 1e−05
rs7742829
rs73128290
rs12404911
rs77285108
All
−0.4
−0.2
0.0
0.2
MR effect size for
'Gut microbiota abundance (genus Catenibacterium id.2153)' on 'Cadherin−5'

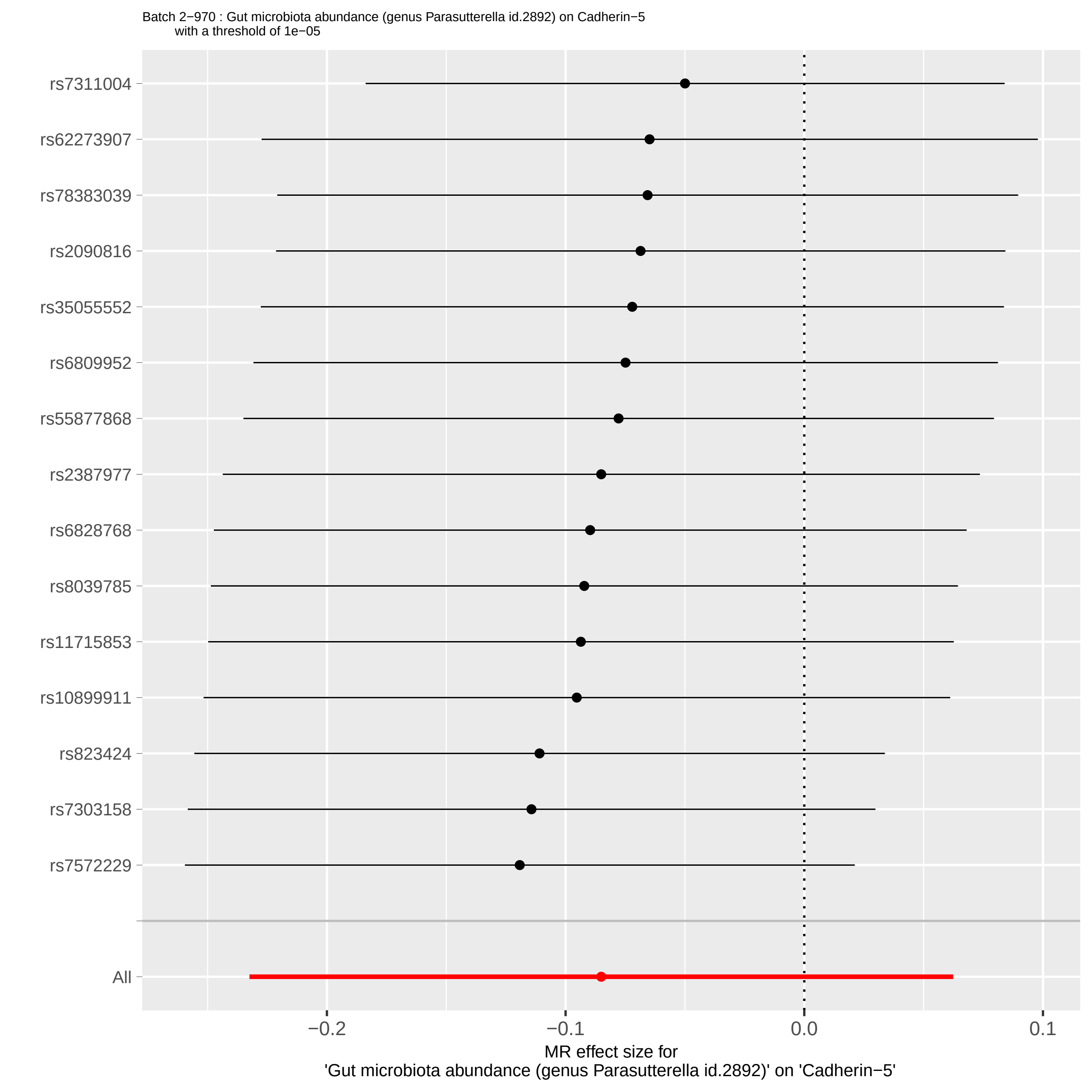
Batch 2−970 : Gut microbiota abundance (genus Parasutterella id.2892) on Cadherin−5
with a threshold of 1e−05
rs7311004
rs62273907
rs78383039
rs2090816
rs35055552
rs6809952
rs55877868
rs2387977
rs6828768
rs8039785
rs11715853
rs10899911
rs823424
rs7303158
rs7572229
All
−0.2
−0.1
0.0
0.1
MR effect size for
'Gut microbiota abundance (genus Parasutterella id.2892)' on 'Cadherin−5'

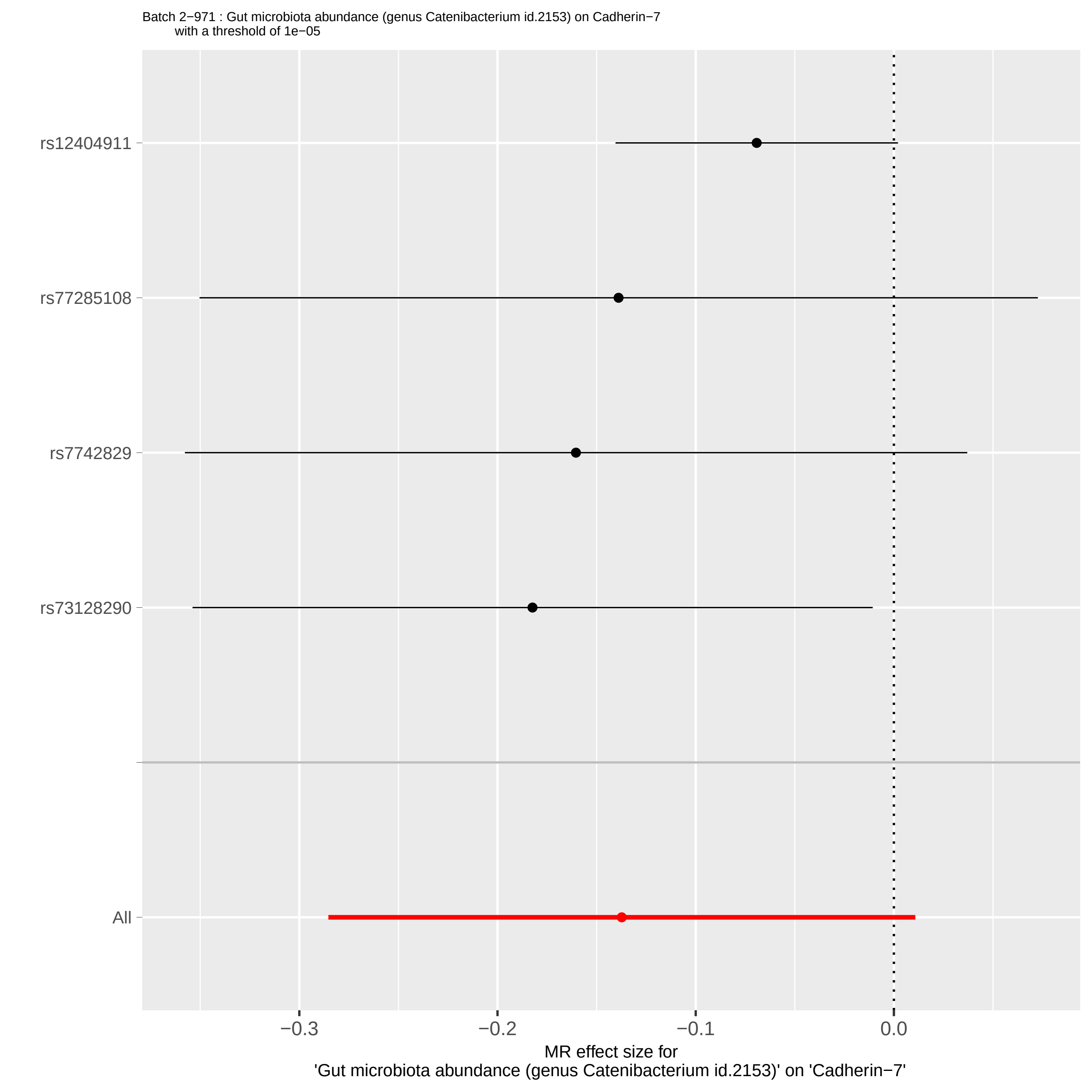
Batch 2−971 : Gut microbiota abundance (genus Catenibacterium id.2153) on Cadherin−7
with a threshold of 1e−05
rs12404911
rs77285108
rs7742829
rs73128290
All
−0.3
−0.2
−0.1
0.0
MR effect size for
'Gut microbiota abundance (genus Catenibacterium id.2153)' on 'Cadherin−7'

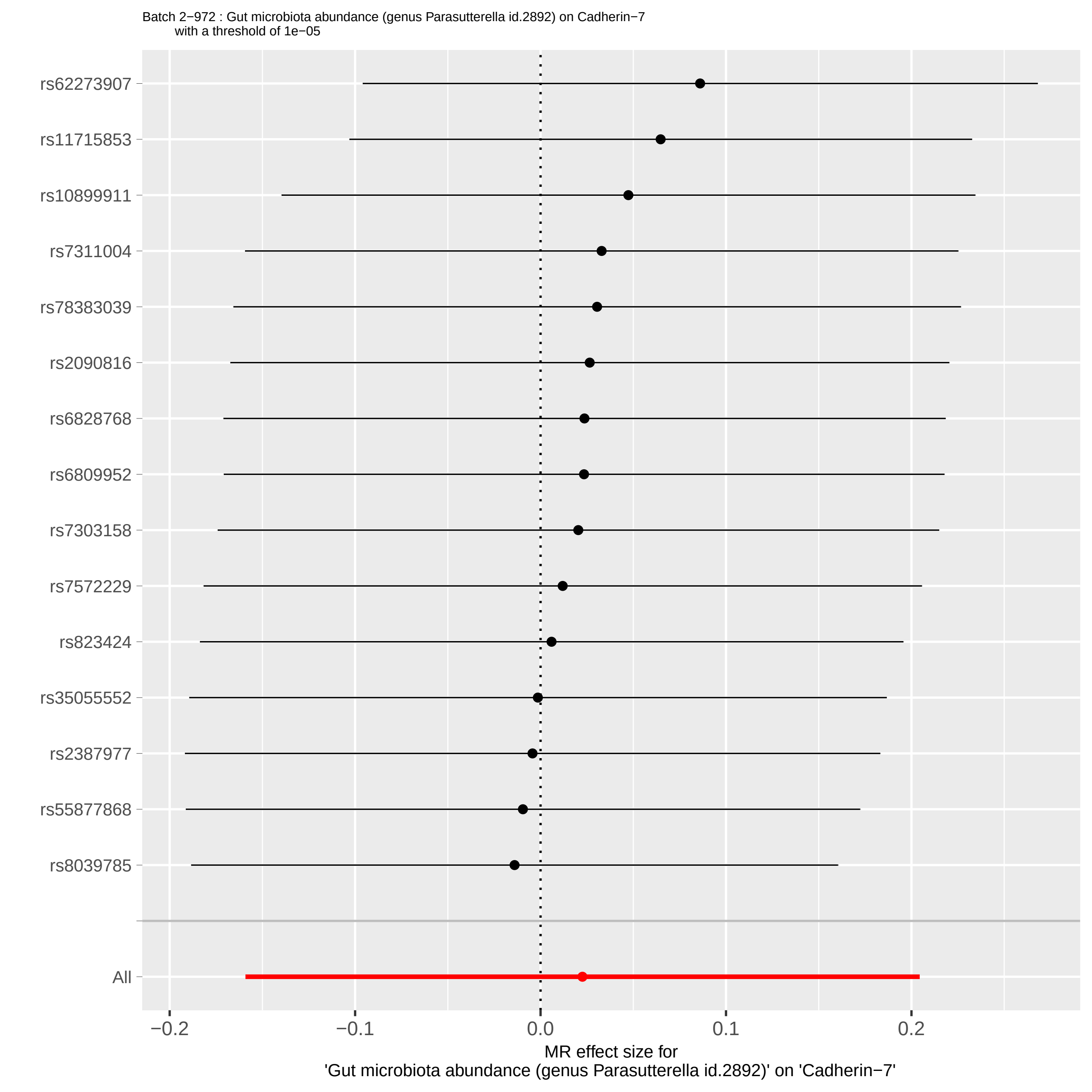

Batch 2−972 : Gut microbiota abundance (genus Parasutterella id.2892) on Cadherin−7
with a threshold of 1e−05
rs62273907
rs11715853
rs10899911
rs7311004
rs78383039
rs2090816
rs6828768
rs6809952
rs7303158
rs7572229
rs823424
rs35055552
rs2387977
rs55877868
rs8039785
All
−0.2
−0.1
0.0
0.1
0.2
MR effect size for
'Gut microbiota abundance (genus Parasutterella id.2892)' on 'Cadherin−7'

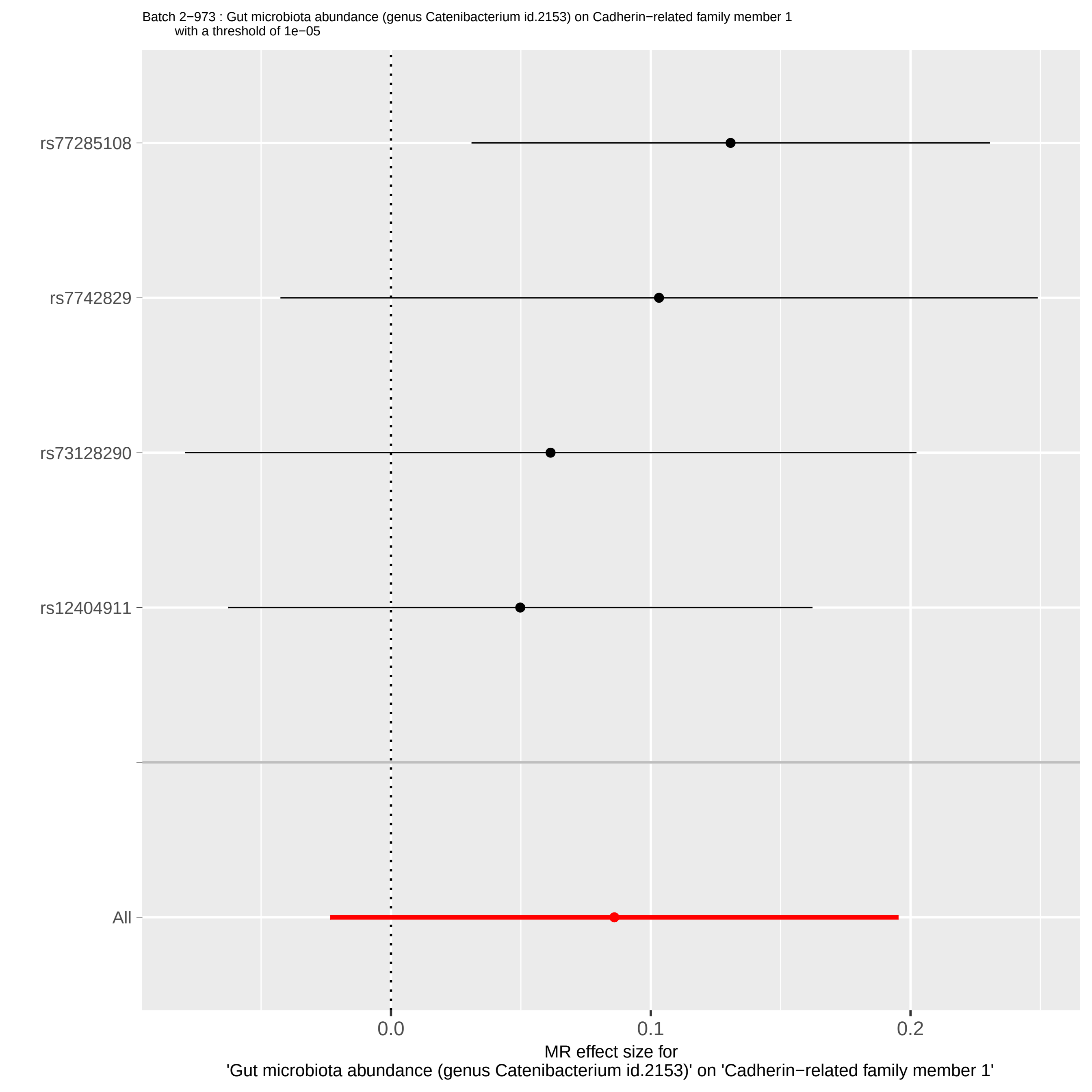

Batch 2−973 : Gut microbiota abundance (genus Catenibacterium id.2153) on Cadherin−related family member 1
with a threshold of 1e−05
rs77285108
rs7742829
rs73128290
rs12404911
All
0.0
0.1
0.2
MR effect size for
'Gut microbiota abundance (genus Catenibacterium id.2153)' on 'Cadherin−related family member 1'

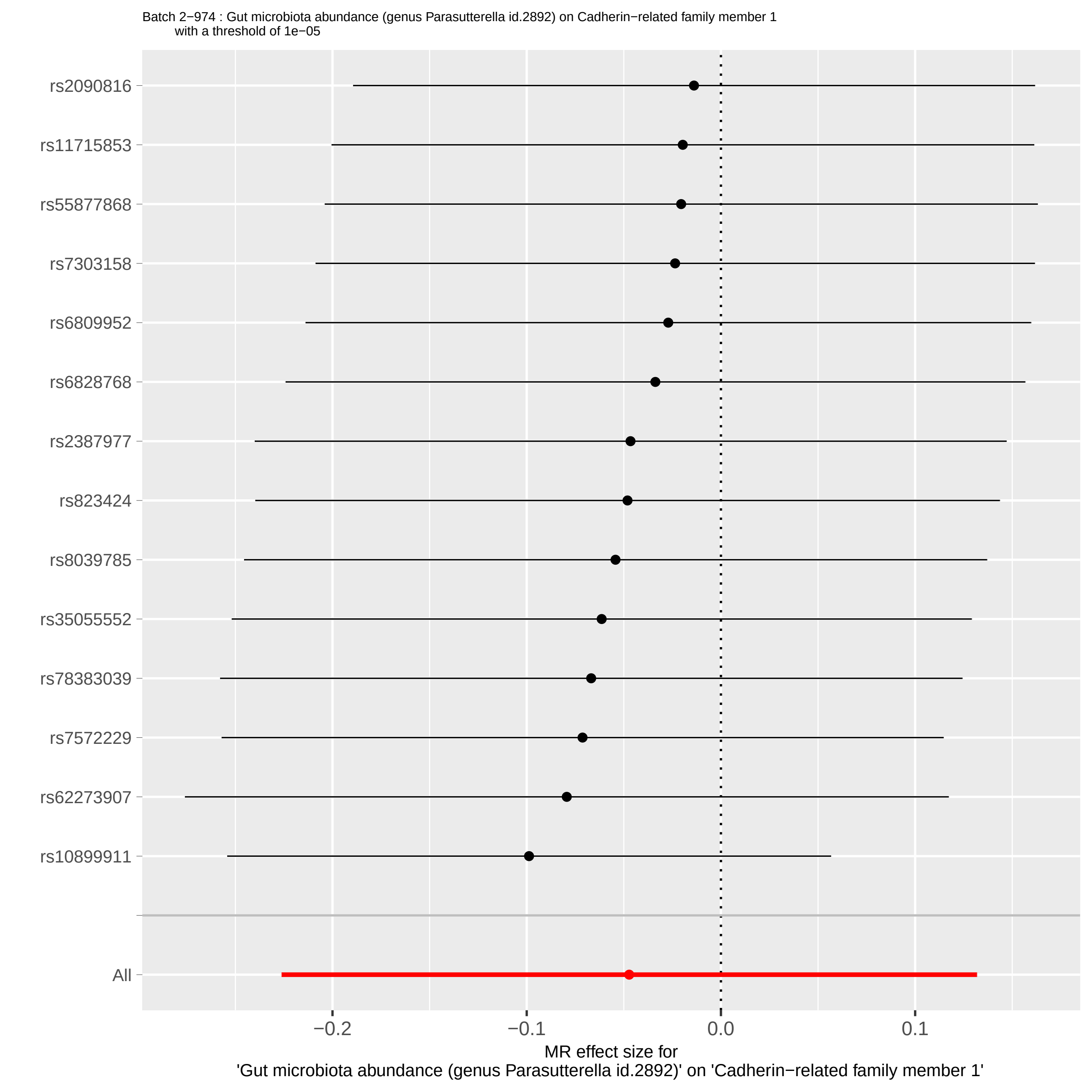
Batch 2−974 : Gut microbiota abundance (genus Parasutterella id.2892) on Cadherin−related family member 1
with a threshold of 1e−05
rs2090816
rs11715853
rs55877868
rs7303158
rs6809952
rs6828768
rs2387977
rs823424
rs8039785
rs35055552
rs78383039
rs7572229
rs62273907
rs10899911
All
−0.2
−0.1
0.0
0.1
MR effect size for
'Gut microbiota abundance (genus Parasutterella id.2892)' on 'Cadherin−related family member 1'

Batch 2−975 : Gut microbiota abundance (genus Catenibacterium id.2153) on Cadherin−related family member 3
with a threshold of 1e−05

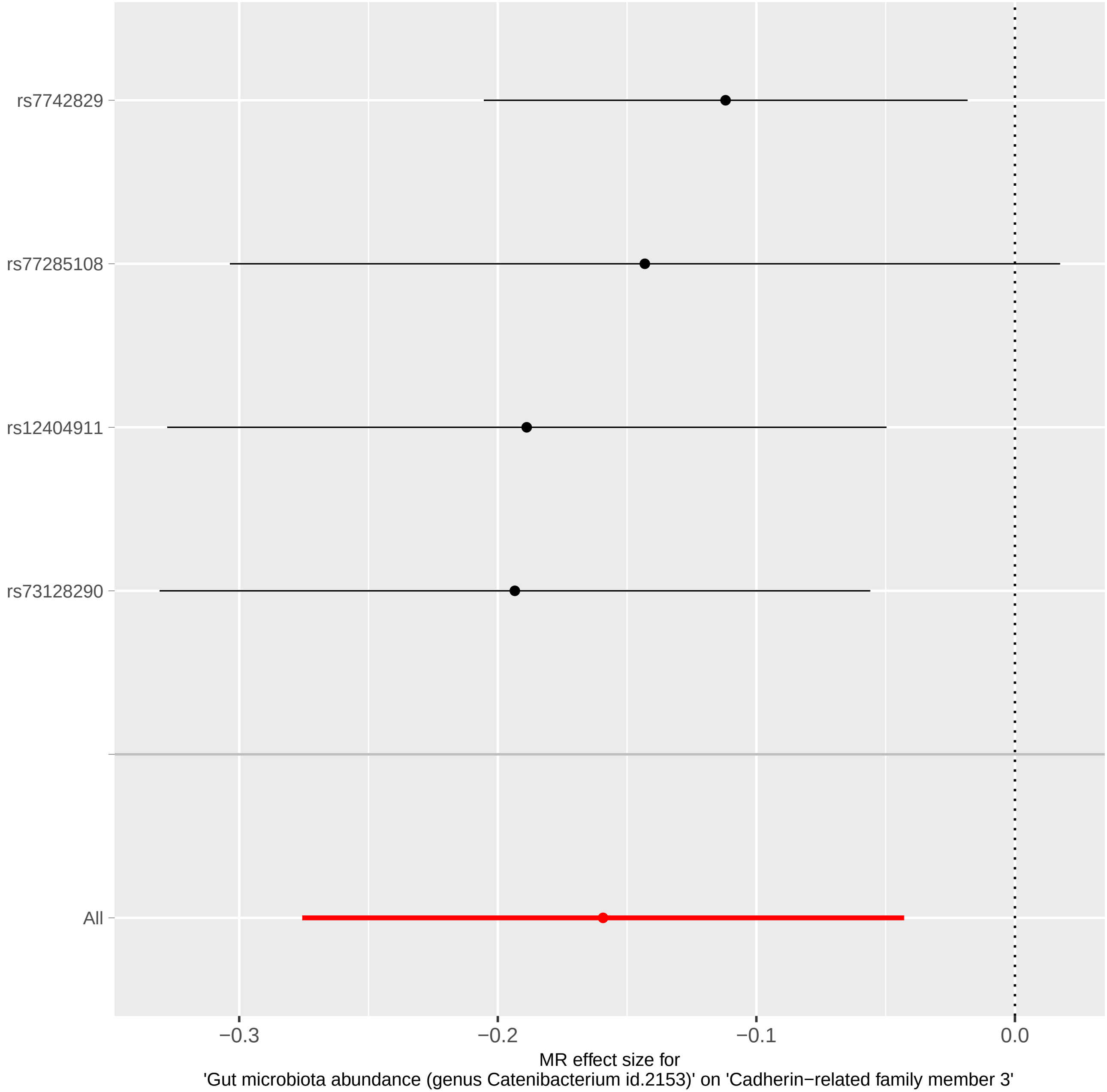

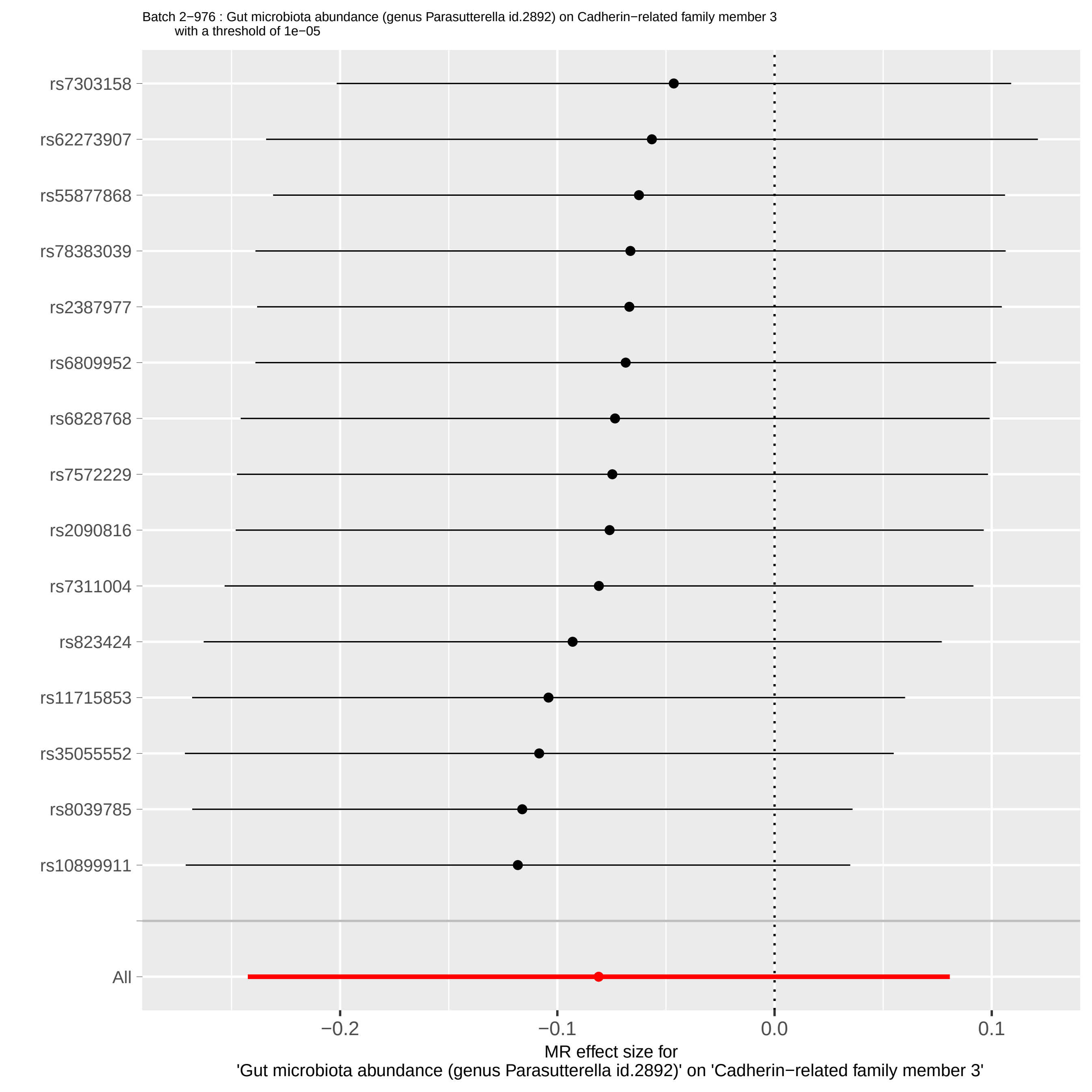

Batch 2−976 : Gut microbiota abundance (genus Parasutterella id.2892) on Cadherin−related family member 3
with a threshold of 1e−05
rs7303158
rs62273907
rs55877868
rs78383039
rs2387977
rs6809952
rs6828768
rs7572229
rs2090816
rs7311004
rs823424
rs11715853
rs35055552
rs8039785
rs10899911
All
−0.2
−0.1
0.0
0.1
MR effect size for
'Gut microbiota abundance (genus Parasutterella id.2892)' on 'Cadherin−related family member 3'

Batch 2−977 : Gut microbiota abundance (genus Catenibacterium id.2153) on Cadherin−related family member 3
with a threshold of 1e−05

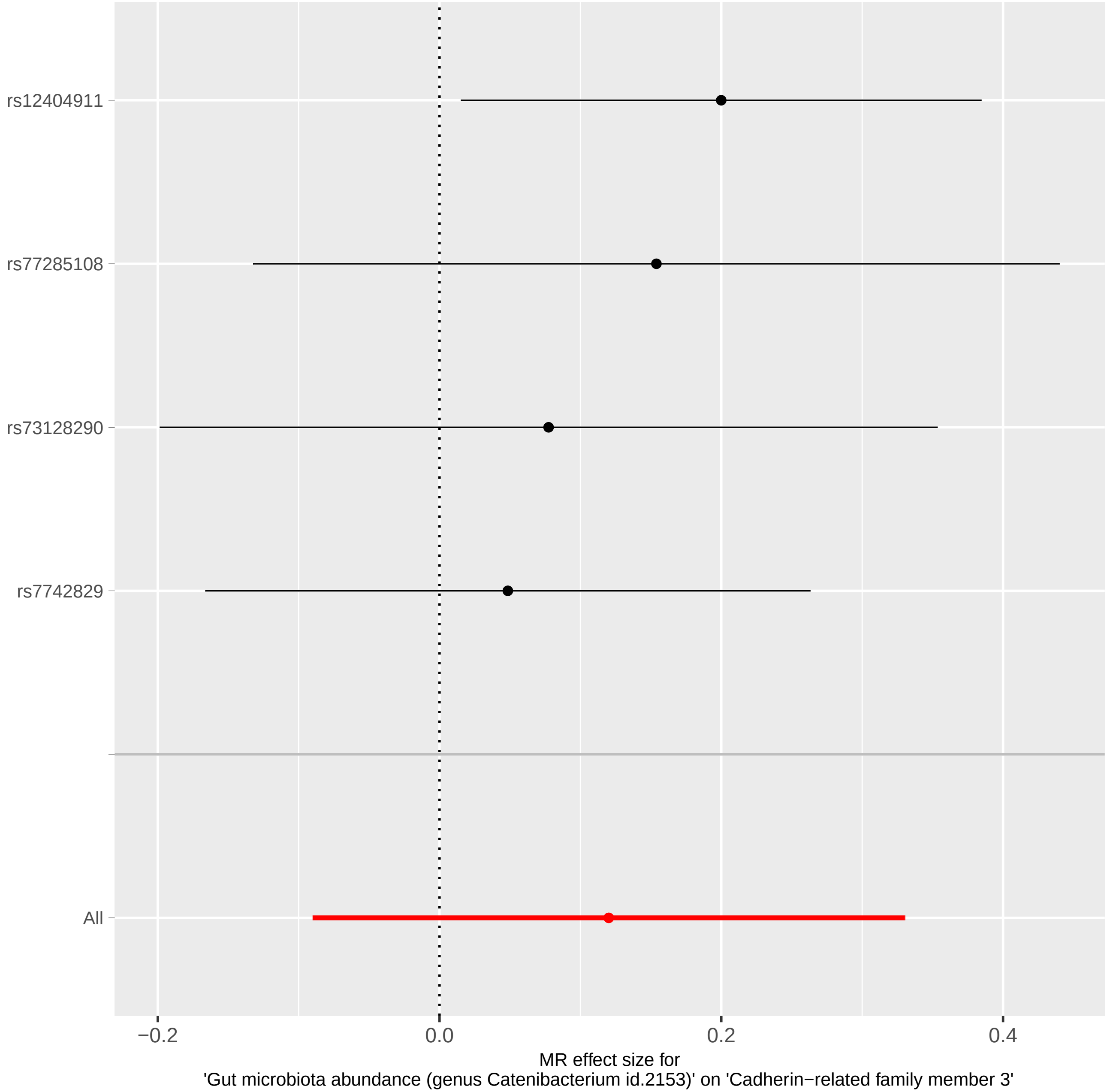

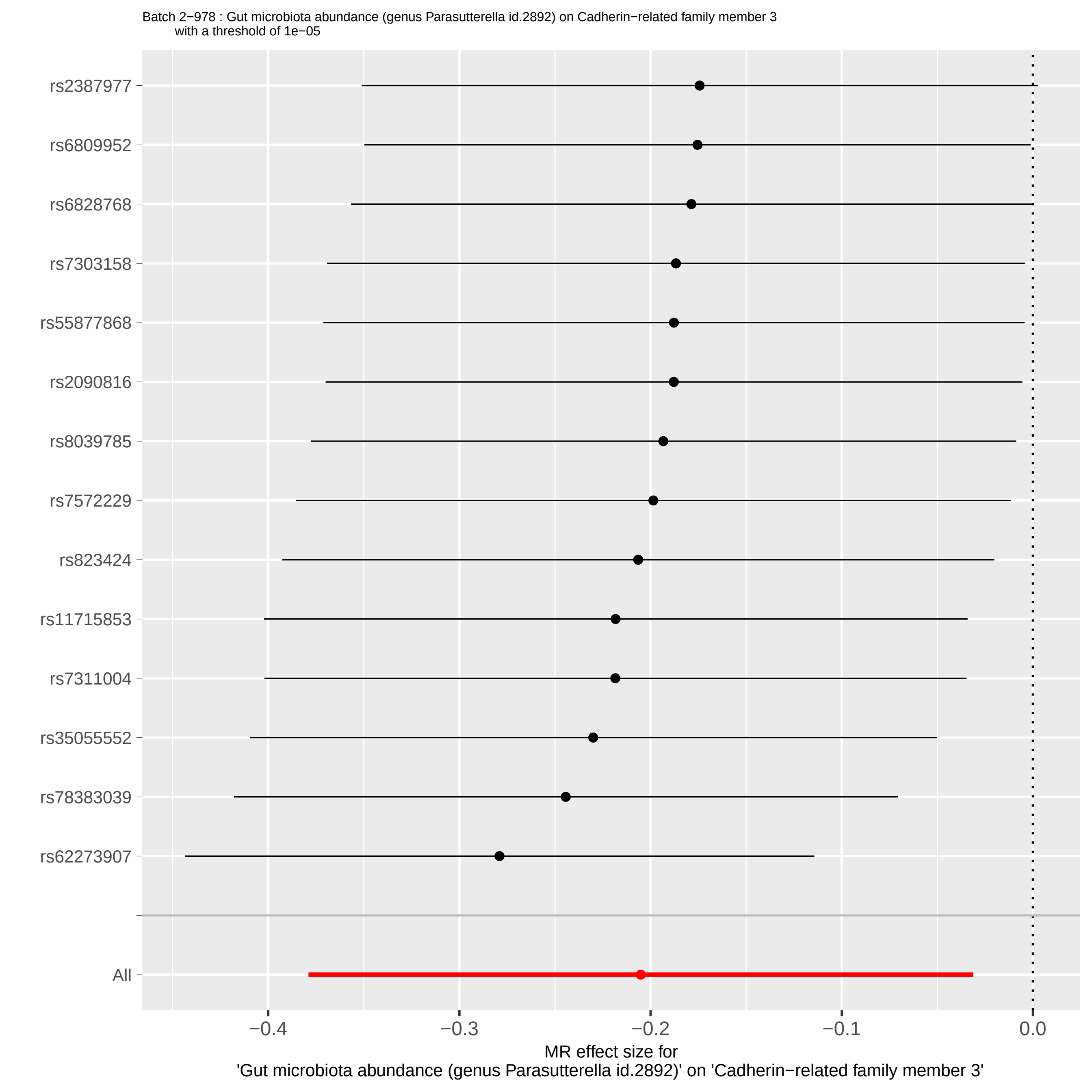
Batch 2−978 : Gut microbiota abundance (genus Parasutterella id.2892) on Cadherin−related family member 3
with a threshold of 1e−05
rs2387977
rs6809952
rs6828768
rs7303158
rs55877868
rs2090816
rs8039785
rs7572229
rs823424
rs11715853
rs7311004
rs35055552
rs78383039
rs62273907
All
−0.4
−0.3
−0.2
−0.1
0.0
MR effect size for
'Gut microbiota abundance (genus Parasutterella id.2892)' on 'Cadherin−related family member 3'

Batch 2−979 : Gut microbiota abundance (genus Catenibacterium id.2153) on Cadherin−related family member 5
with a threshold of 1e−05

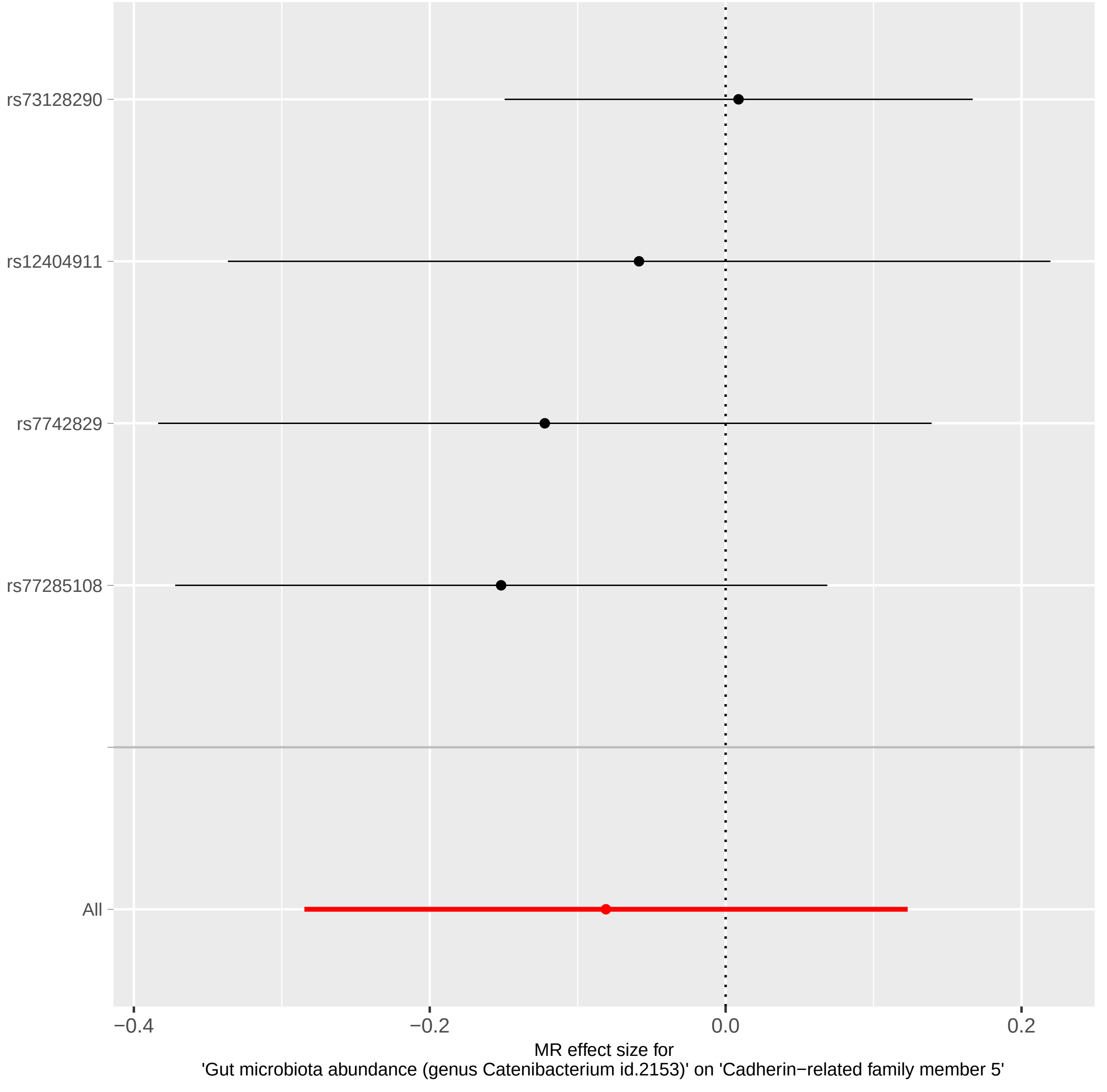

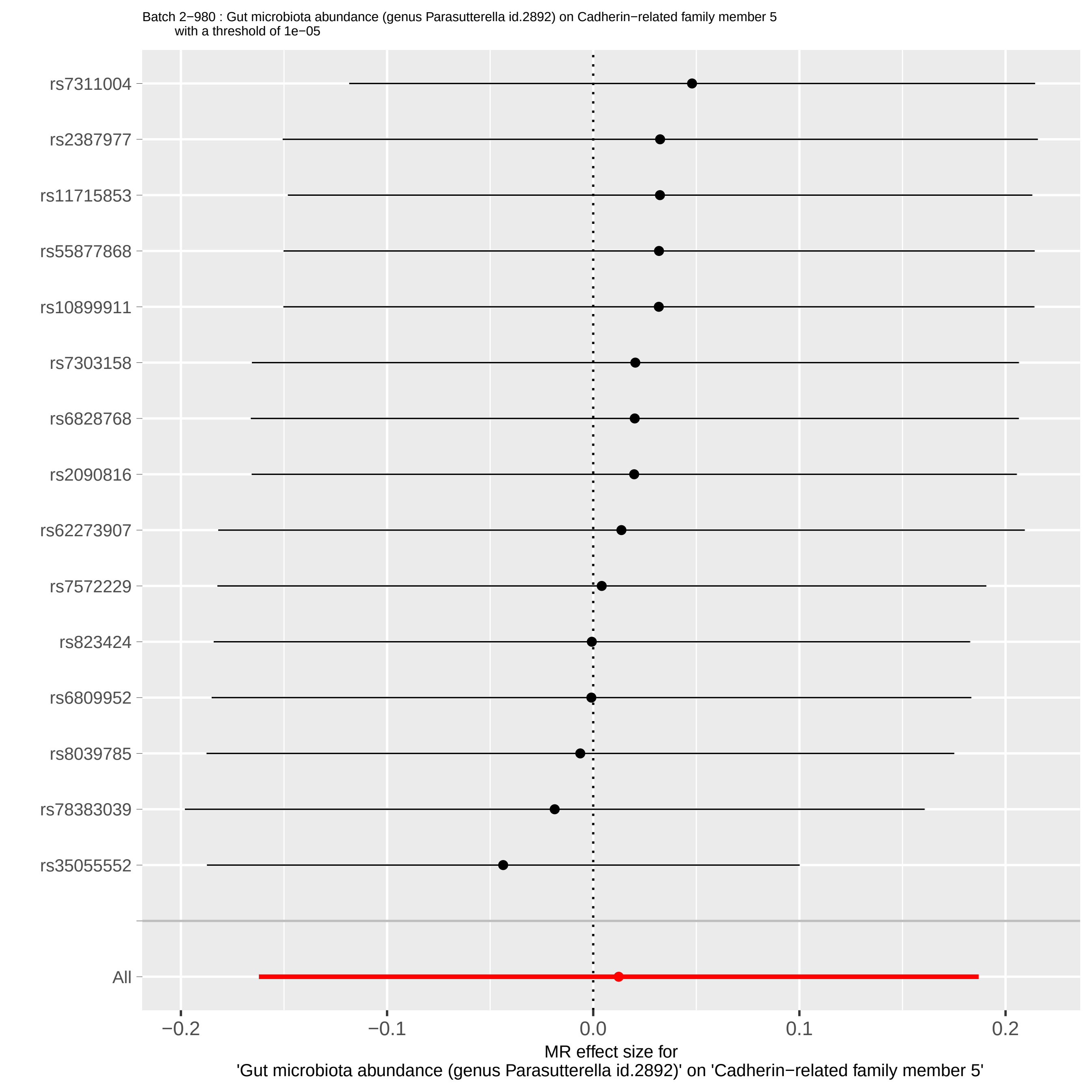
Batch 2−980 : Gut microbiota abundance (genus Parasutterella id.2892) on Cadherin−related family member 5
with a threshold of 1e−05
rs7311004
rs2387977
rs11715853
rs55877868
rs10899911
rs7303158
rs6828768
rs2090816
rs62273907
rs7572229
rs823424
rs6809952
rs8039785
rs78383039
rs35055552
All
−0.2
−0.1
0.0
0.1
0.2
MR effect size for
'Gut microbiota abundance (genus Parasutterella id.2892)' on 'Cadherin−related family member 5'

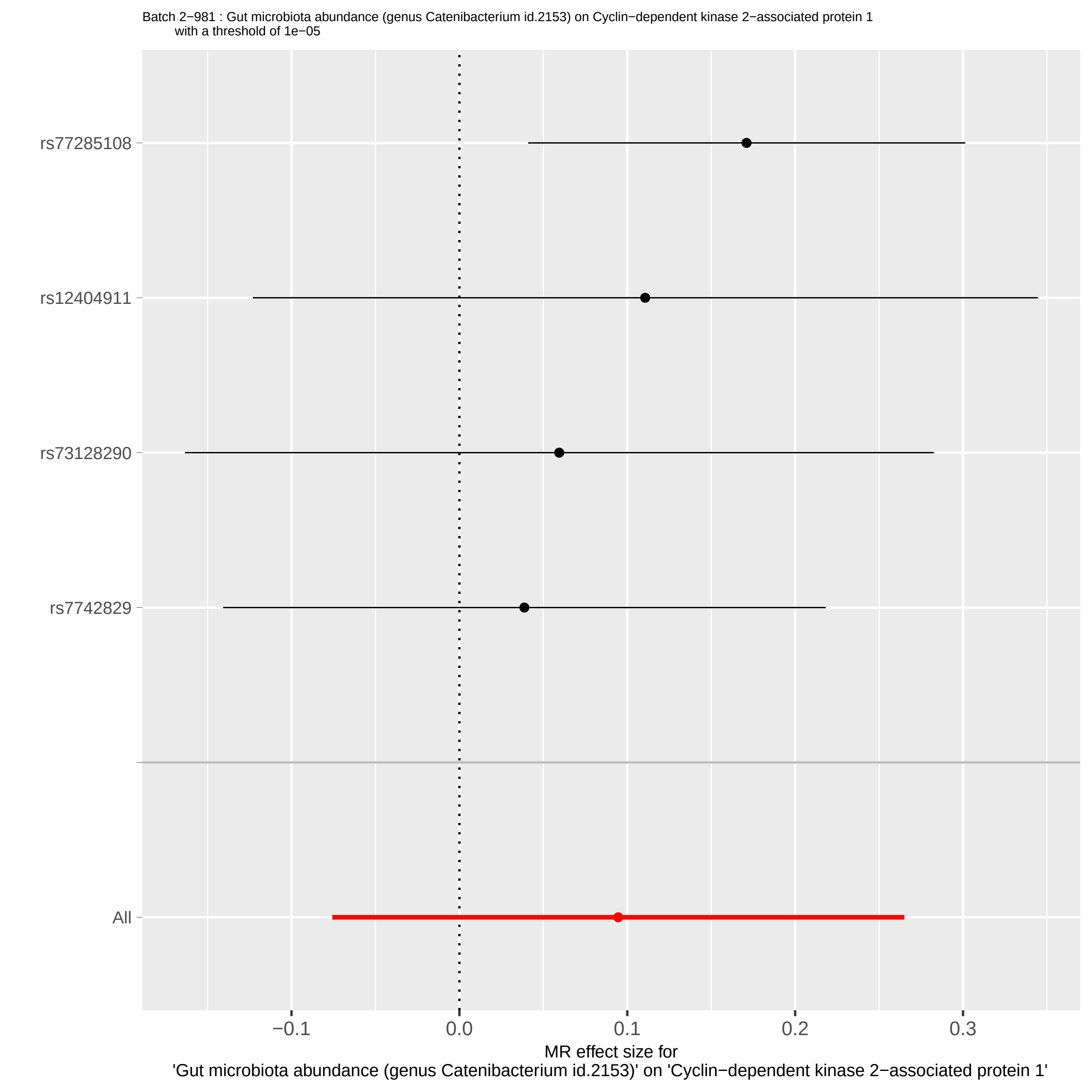
Batch 2−981 : Gut microbiota abundance (genus Catenibacterium id.2153) on Cyclin−dependent kinase 2−associated protein 1
with a threshold of 1e−05
rs77285108
rs12404911
rs73128290
rs7742829
All
−0.1
0.0
0.1
0.2
0.3
MR effect size for
'Gut microbiota abundance (genus Catenibacterium id.2153)' on 'Cyclin−dependent kinase 2−associated protein 1'

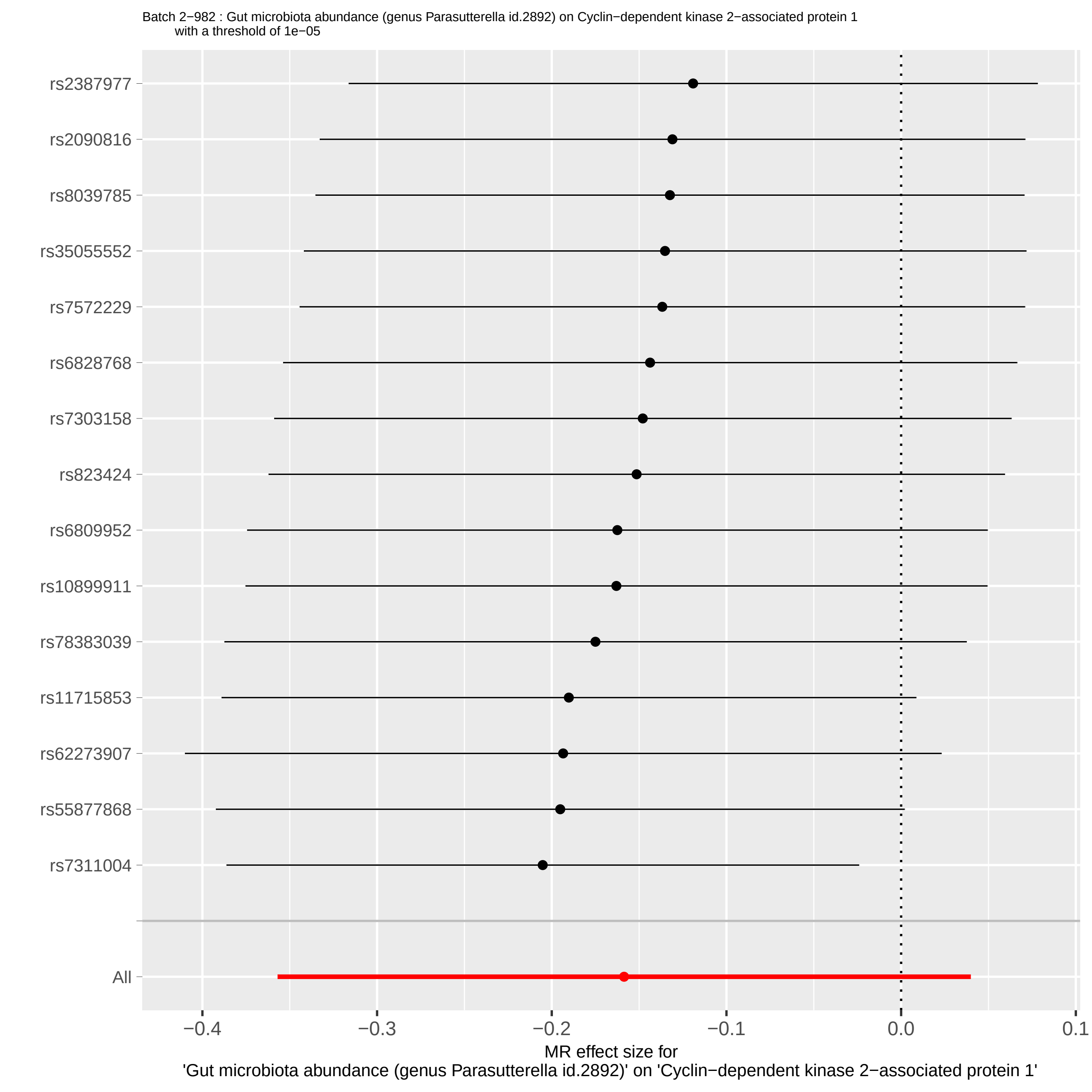
Batch 2−982 : Gut microbiota abundance (genus Parasutterella id.2892) on Cyclin−dependent kinase 2−associated protein 1
with a threshold of 1e−05
rs2387977
rs2090816
rs8039785
rs35055552
rs7572229
rs6828768
rs7303158
rs823424
rs6809952
rs10899911
rs78383039
rs11715853
rs62273907
rs55877868
rs7311004
All
−0.4
−0.3
−0.2
−0.1
0.0
0.1
MR effect size for
'Gut microbiota abundance (genus Parasutterella id.2892)' on 'Cyclin−dependent kinase 2−associated protein 1'

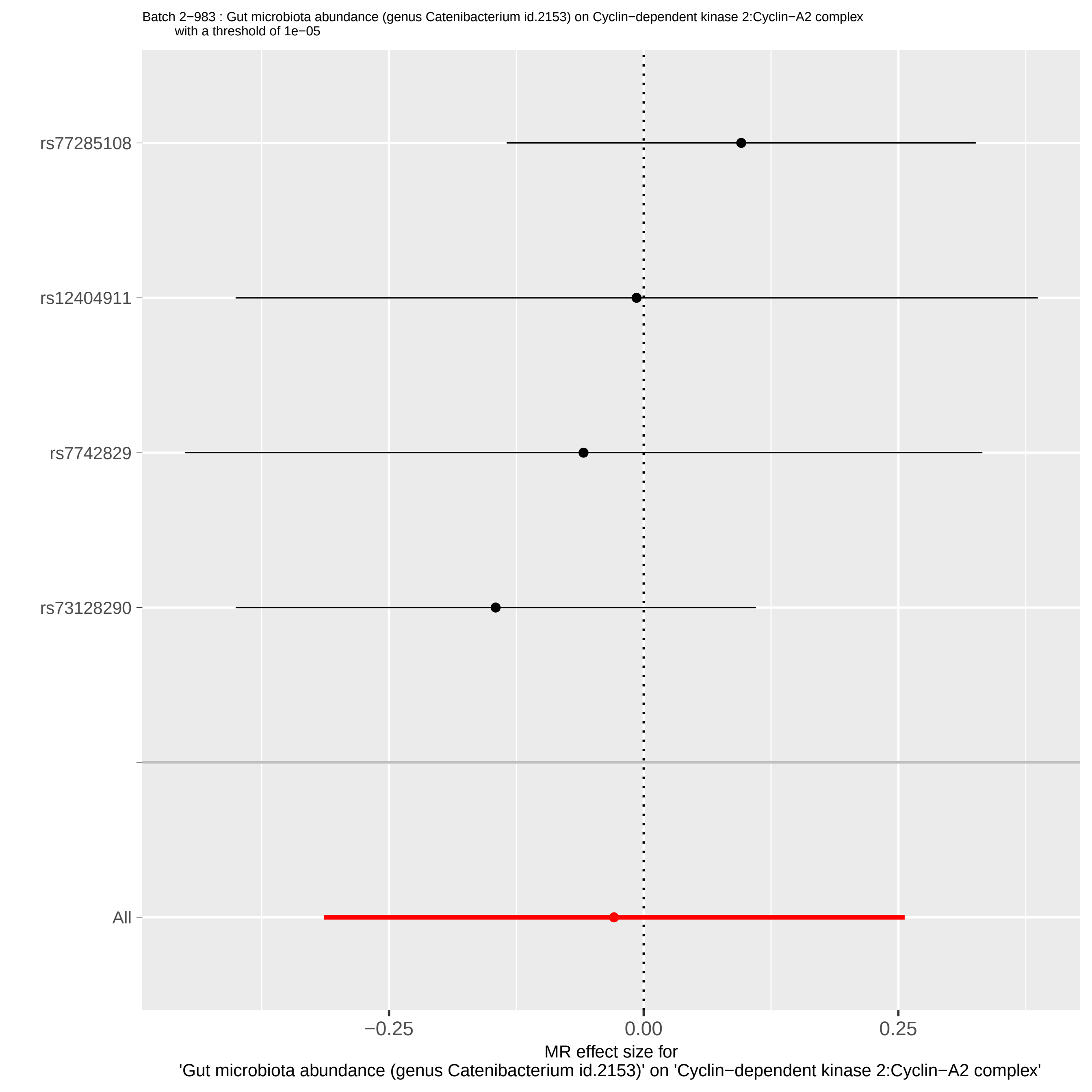

Batch 2−983 : Gut microbiota abundance (genus Catenibacterium id.2153) on Cyclin−dependent kinase 2:Cyclin−A2 complex
with a threshold of 1e−05
rs77285108
rs12404911
rs7742829
rs73128290
All
−0.25
0.00
0.25
MR effect size for
'Gut microbiota abundance (genus Catenibacterium id.2153)' on 'Cyclin−dependent kinase 2:Cyclin−A2 complex'

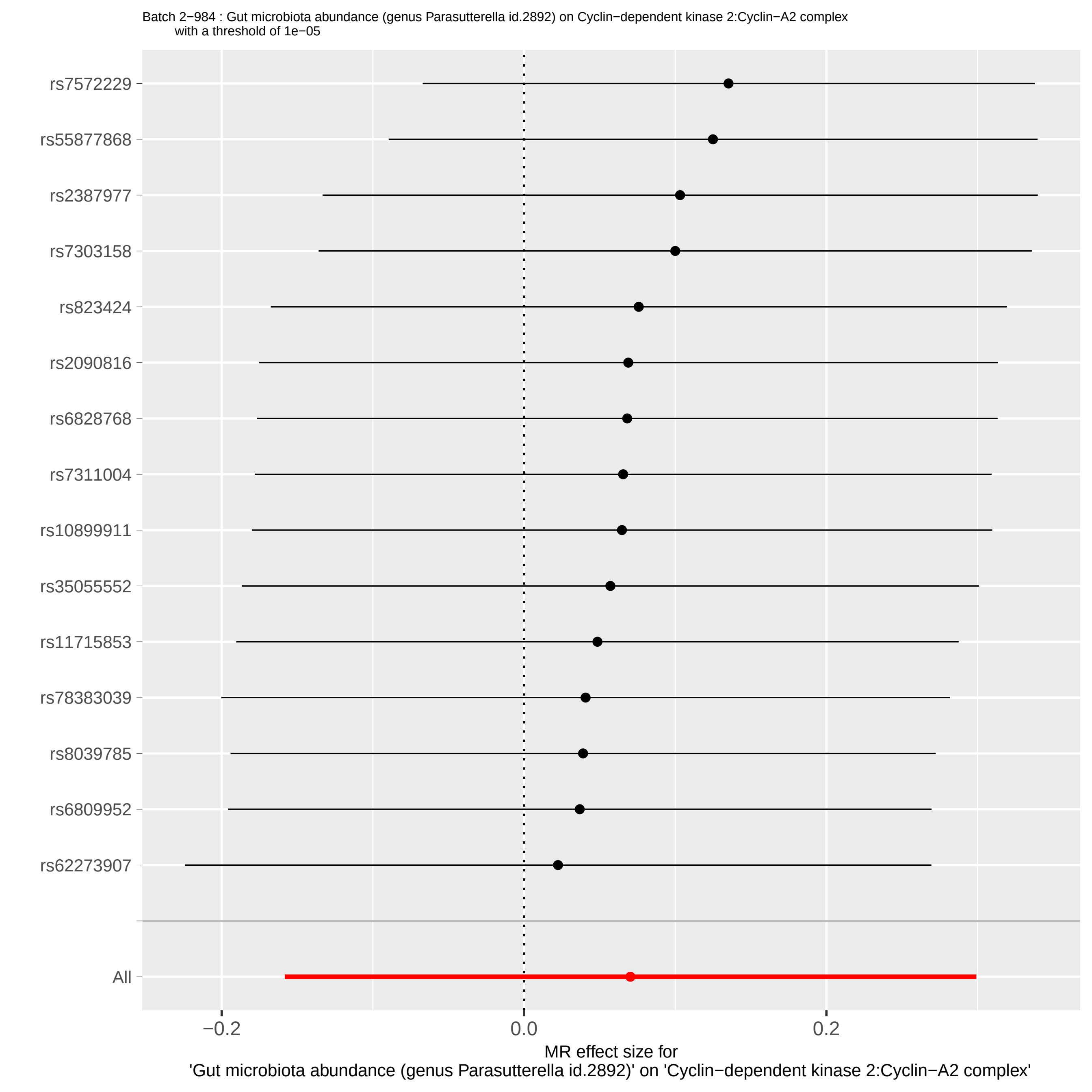
Batch 2−984 : Gut microbiota abundance (genus Parasutterella id.2892) on Cyclin−dependent kinase 2:Cyclin−A2 complex
with a threshold of 1e−05
rs7572229
rs55877868
rs2387977
rs7303158
rs823424
rs2090816
rs6828768
rs7311004
rs10899911
rs35055552
rs11715853
rs78383039
rs8039785
rs6809952
rs62273907
All
−0.2
0.0
0.2
MR effect size for
'Gut microbiota abundance (genus Parasutterella id.2892)' on 'Cyclin−dependent kinase 2:Cyclin−A2 complex'

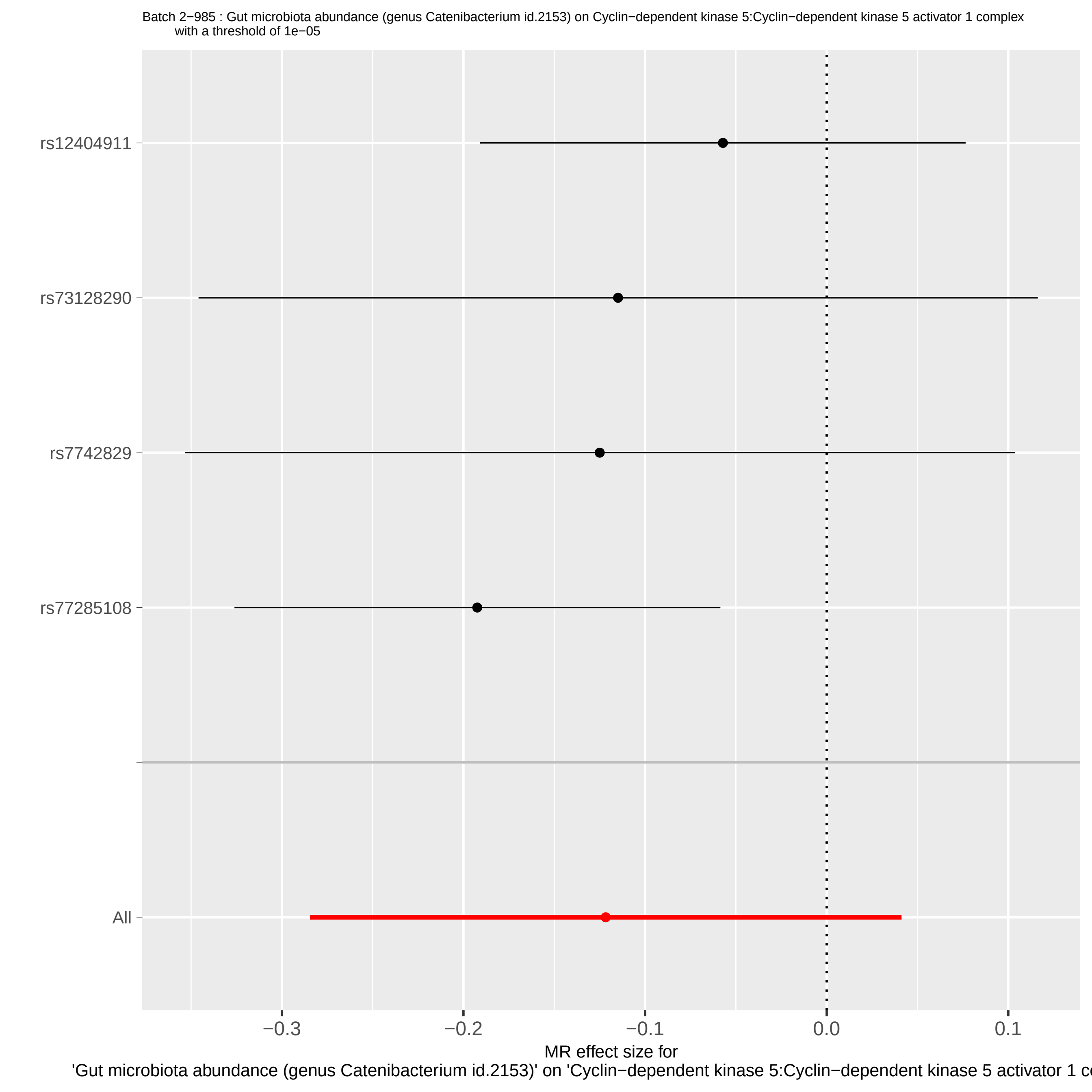
Batch 2−985 : Gut microbiota abundance (genus Catenibacterium id.2153) on Cyclin−dependent kinase 5:Cyclin−dependent kinase 5 activator 1 complex
with a threshold of 1e−05
rs12404911
rs73128290
rs7742829
rs77285108
All
−0.3
−0.2
−0.1
0.0
0.1
MR effect size for
'Gut microbiota abundance (genus Catenibacterium id.2153)' on 'Cyclin−dependent kinase 5:Cyclin−dependent kinase 5 activator 1 co

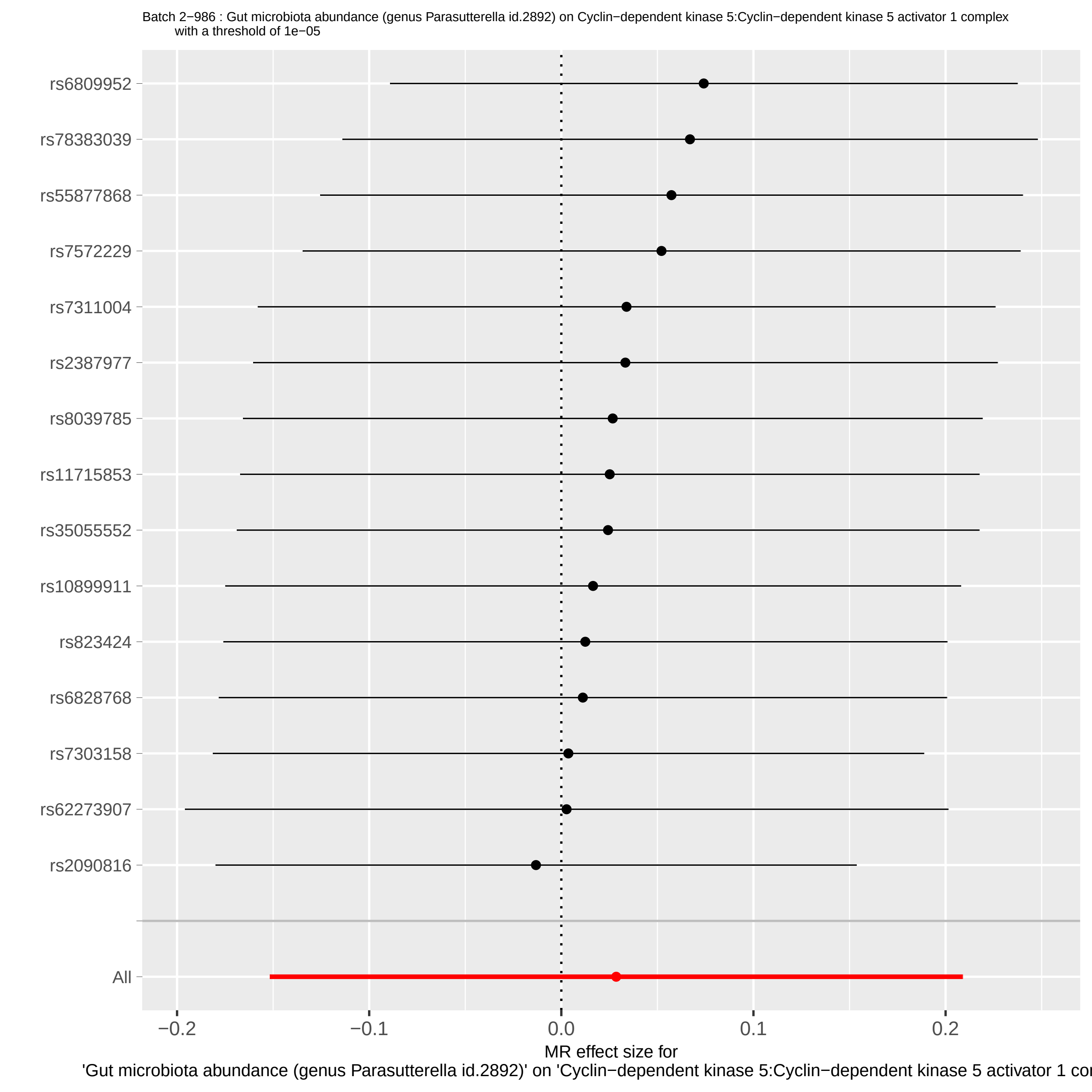

Batch 2−986 : Gut microbiota abundance (genus Parasutterella id.2892) on Cyclin−dependent kinase 5:Cyclin−dependent kinase 5 activator 1 complex
with a threshold of 1e−05
rs6809952
rs78383039
rs55877868
rs7572229
rs7311004
rs2387977
rs8039785
rs11715853
rs35055552
rs10899911
rs823424
rs6828768
rs7303158
rs62273907
rs2090816
All
−0.2
−0.1
0.0
0.1
0.2
MR effect size for
'Gut microbiota abundance (genus Parasutterella id.2892)' on 'Cyclin−dependent kinase 5:Cyclin−dependent kinase 5 activator 1 co

Batch 2−987 : Gut microbiota abundance (genus Catenibacterium id.2153) on Cyclin−dependent kinase 8:Cyclin−C complex
with a threshold of 1e−05

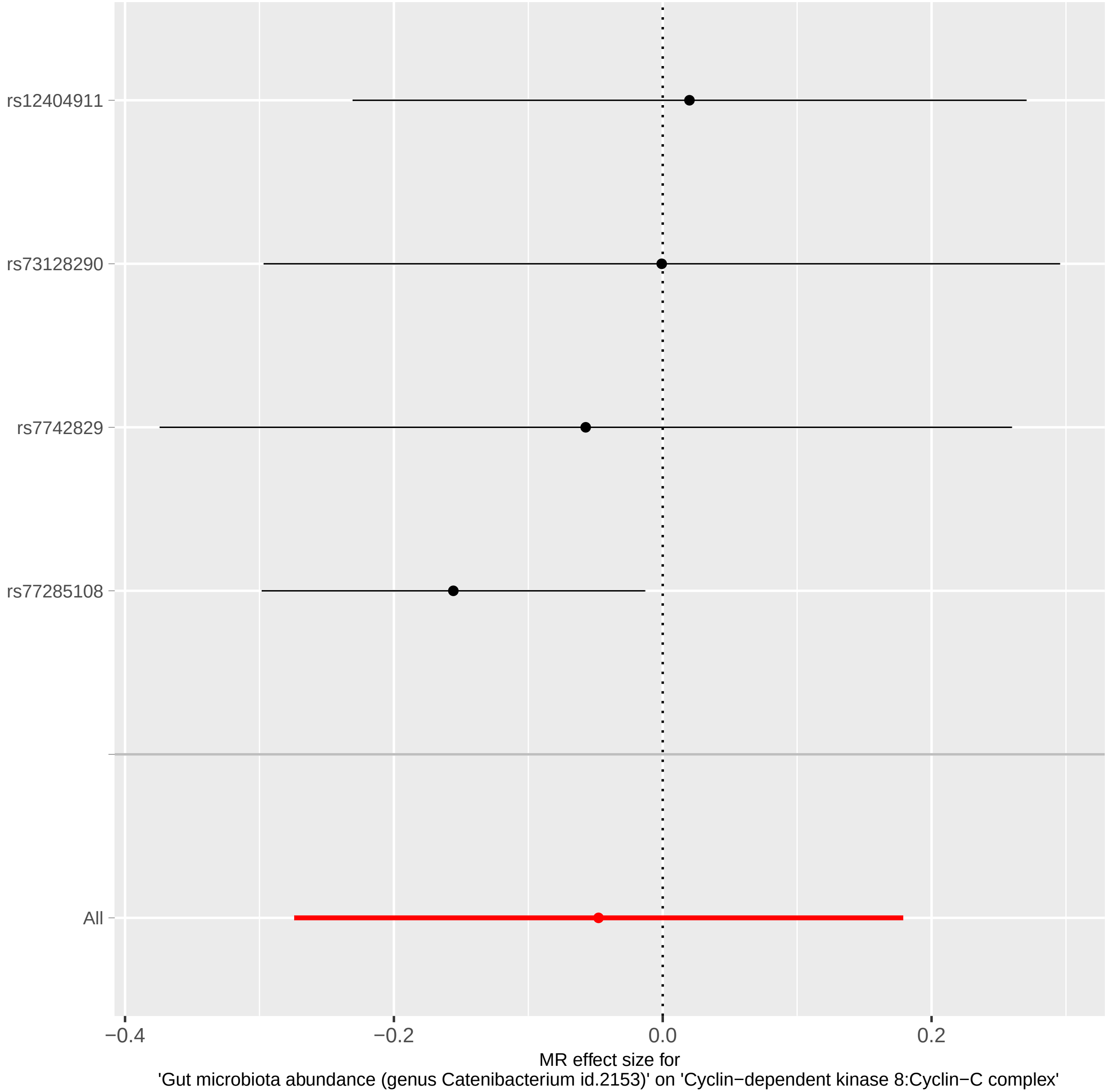

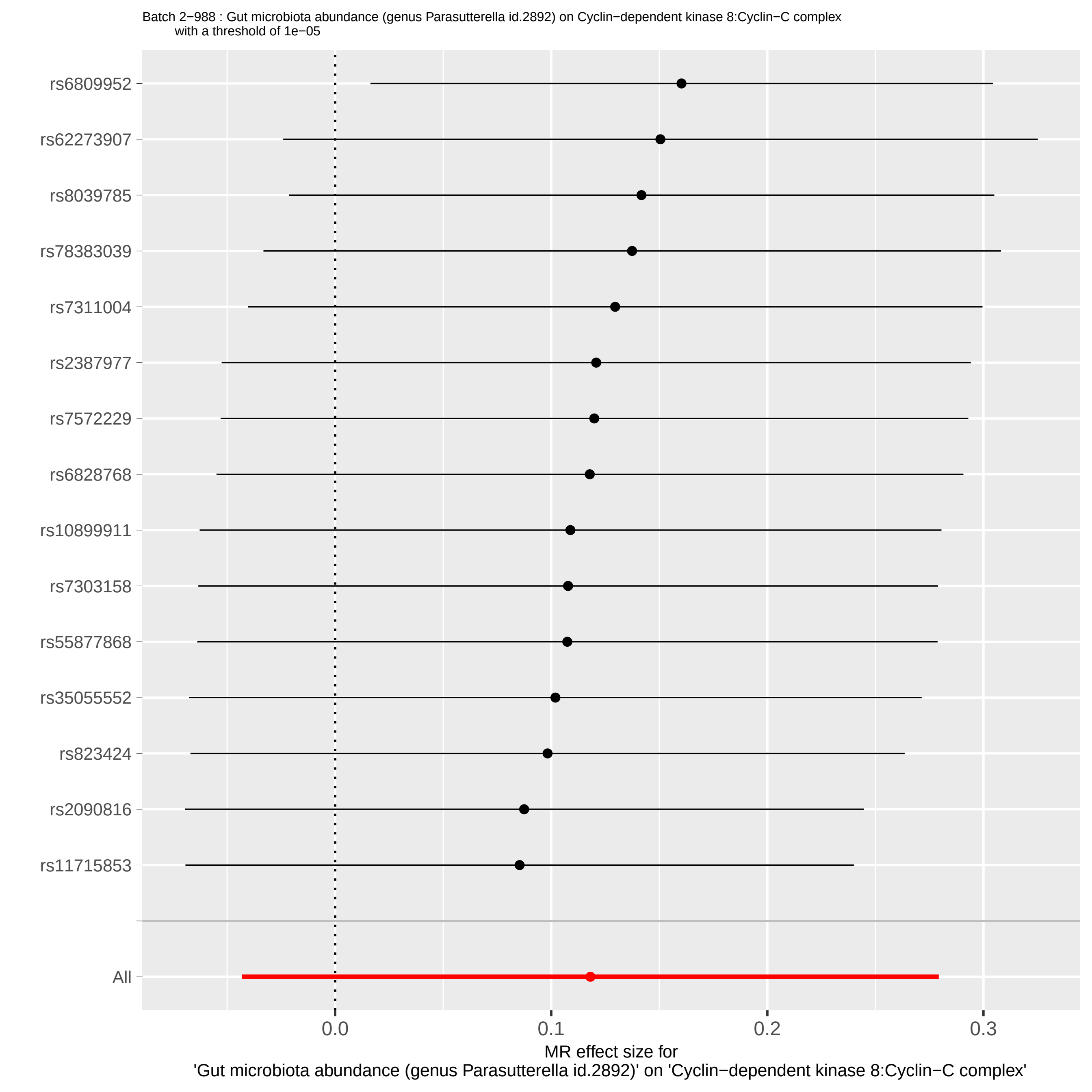

Batch 2−988 : Gut microbiota abundance (genus Parasutterella id.2892) on Cyclin−dependent kinase 8:Cyclin−C complex
with a threshold of 1e−05
rs6809952
rs62273907
rs8039785
rs78383039
rs7311004
rs2387977
rs7572229
rs6828768
rs10899911
rs7303158
rs55877868
rs35055552
rs823424
rs2090816
rs11715853
All
0.0
0.1
0.2
0.3
MR effect size for
'Gut microbiota abundance (genus Parasutterella id.2892)' on 'Cyclin−dependent kinase 8:Cyclin−C complex'

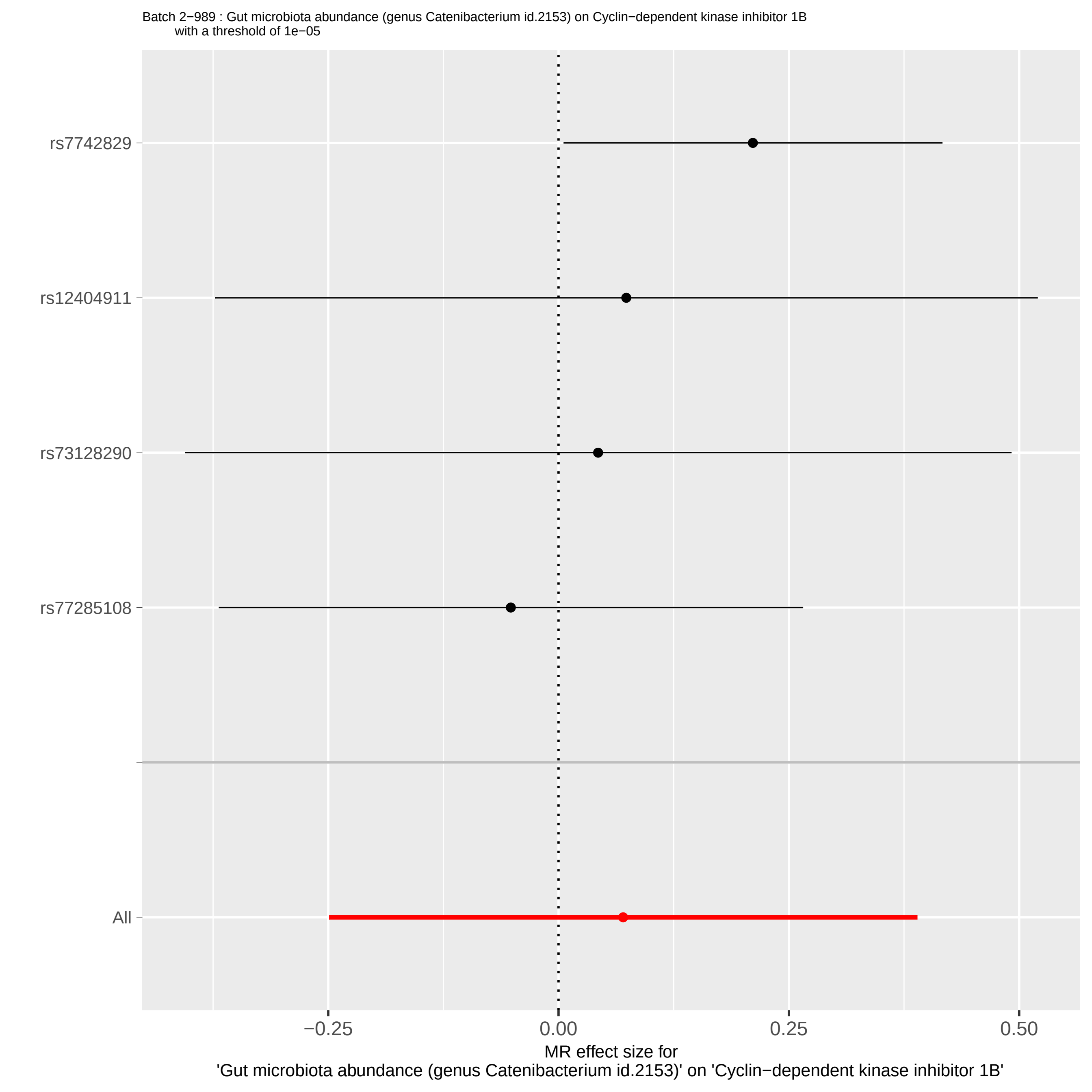
Batch 2−989 : Gut microbiota abundance (genus Catenibacterium id.2153) on Cyclin−dependent kinase inhibitor 1B
with a threshold of 1e−05
rs7742829
rs12404911
rs73128290
rs77285108
All
−0.25
0.00
0.25
0.50
MR effect size for
'Gut microbiota abundance (genus Catenibacterium id.2153)' on 'Cyclin−dependent kinase inhibitor 1B'

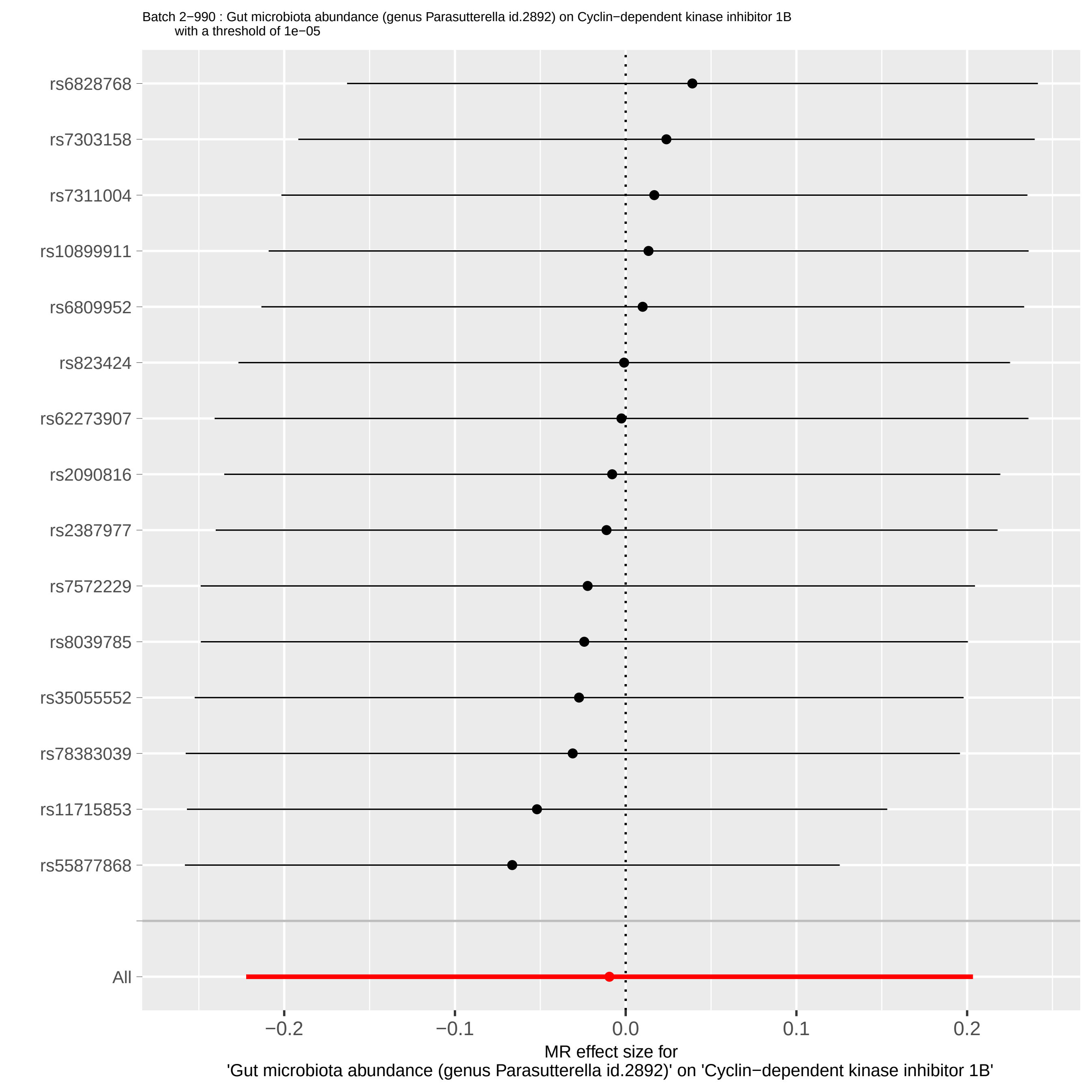
Batch 2−990 : Gut microbiota abundance (genus Parasutterella id.2892) on Cyclin−dependent kinase inhibitor 1B
with a threshold of 1e−05
rs6828768
rs7303158
rs7311004
rs10899911
rs6809952
rs823424
rs62273907
rs2090816
rs2387977
rs7572229
rs8039785
rs35055552
rs78383039
rs11715853
rs55877868
All
−0.2
−0.1
0.0
0.1
0.2
MR effect size for
'Gut microbiota abundance (genus Parasutterella id.2892)' on 'Cyclin−dependent kinase inhibitor 1B'

Batch 2−991 : Gut microbiota abundance (genus Catenibacterium id.2153) on Cyclin−dependent kinase 4 inhibitor B
with a threshold of 1e−05

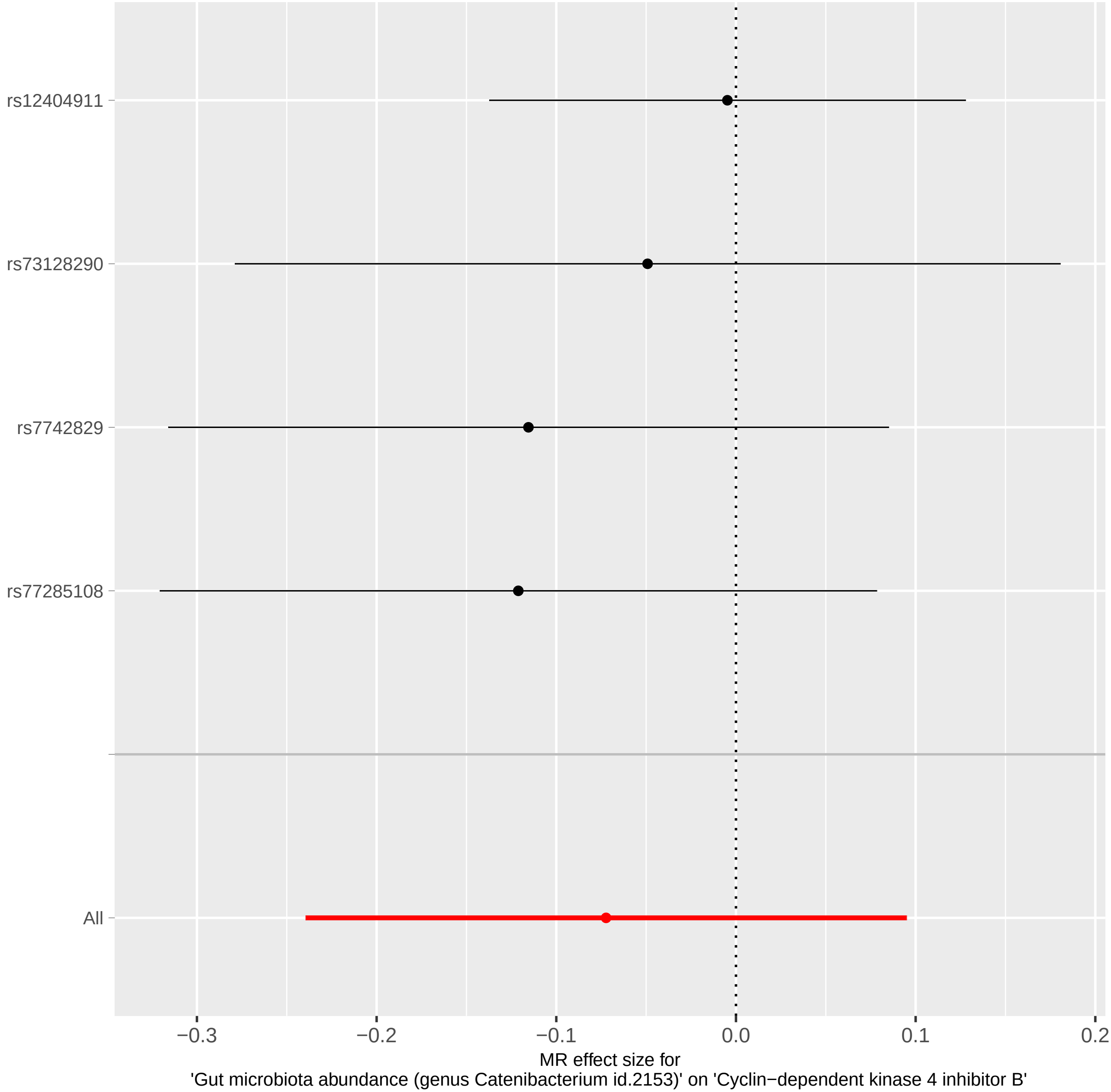

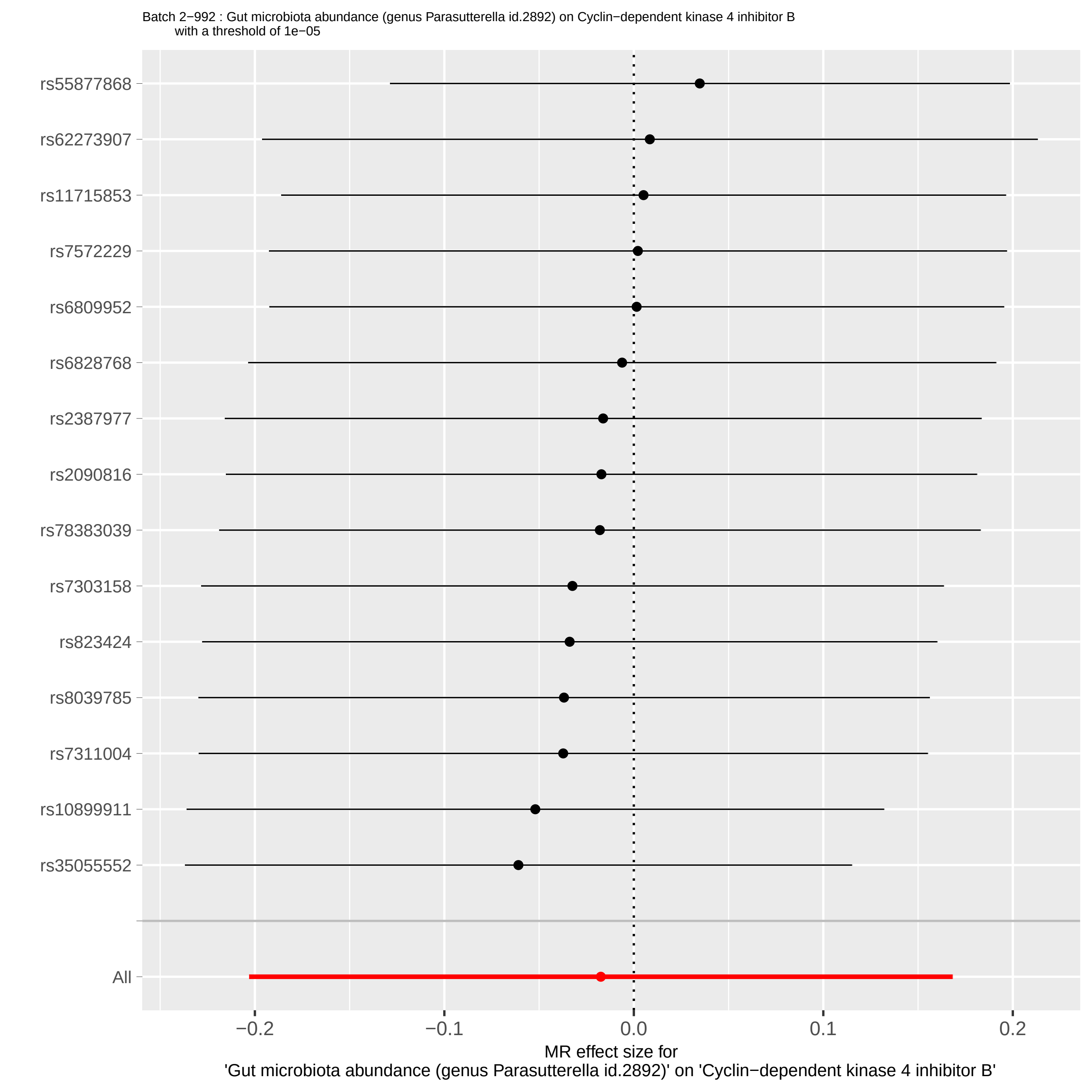

Batch 2−992 : Gut microbiota abundance (genus Parasutterella id.2892) on Cyclin−dependent kinase 4 inhibitor B
with a threshold of 1e−05
rs55877868
rs62273907
rs11715853
rs7572229
rs6809952
rs6828768
rs2387977
rs2090816
rs78383039
rs7303158
rs823424
rs8039785
rs7311004
rs10899911
rs35055552
All
−0.2
−0.1
0.0
0.1
0.2
MR effect size for
'Gut microbiota abundance (genus Parasutterella id.2892)' on 'Cyclin−dependent kinase 4 inhibitor B'

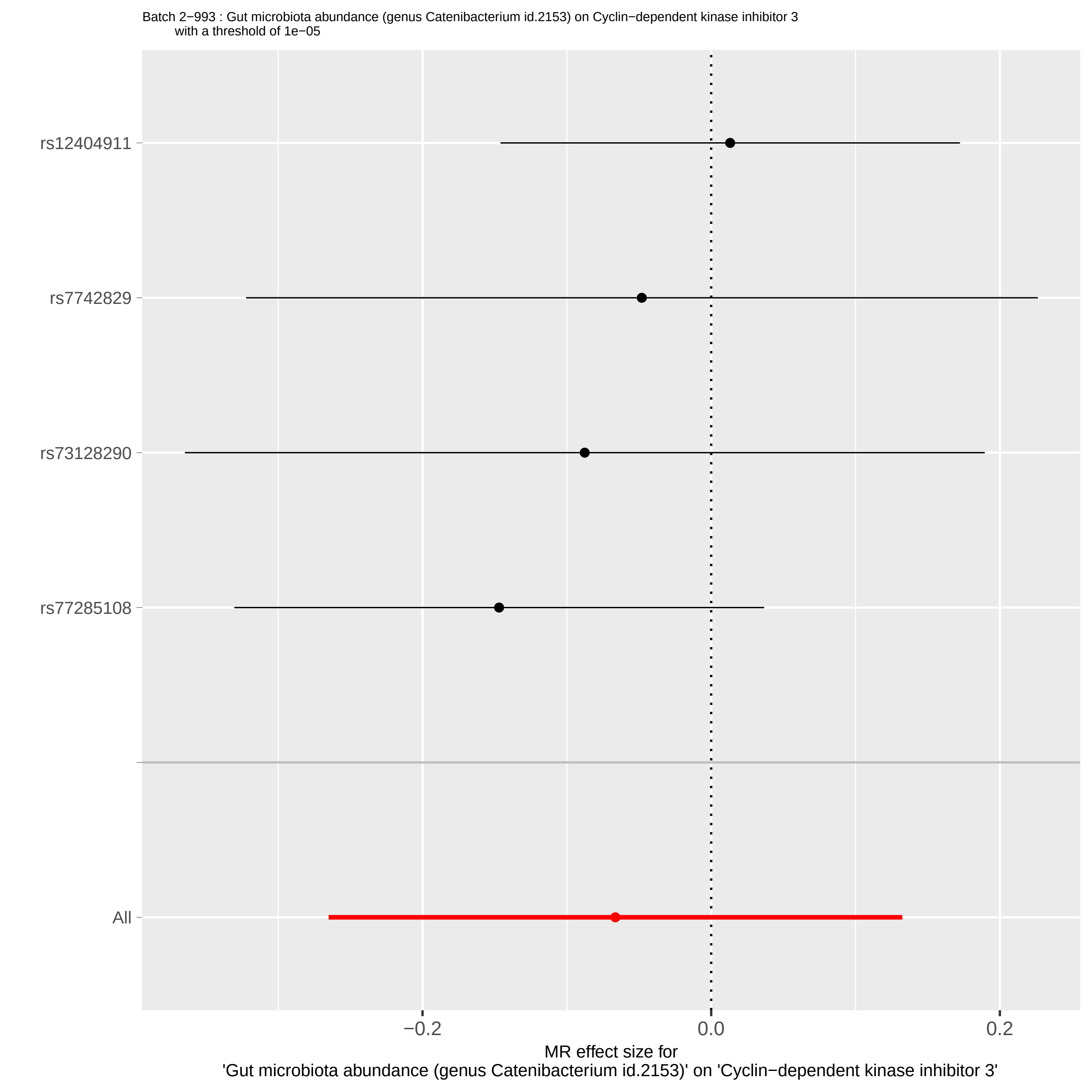

Batch 2−993 : Gut microbiota abundance (genus Catenibacterium id.2153) on Cyclin−dependent kinase inhibitor 3
with a threshold of 1e−05
rs12404911
rs7742829
rs73128290
rs77285108
All
−0.2
0.0
0.2
MR effect size for
'Gut microbiota abundance (genus Catenibacterium id.2153)' on 'Cyclin−dependent kinase inhibitor 3'

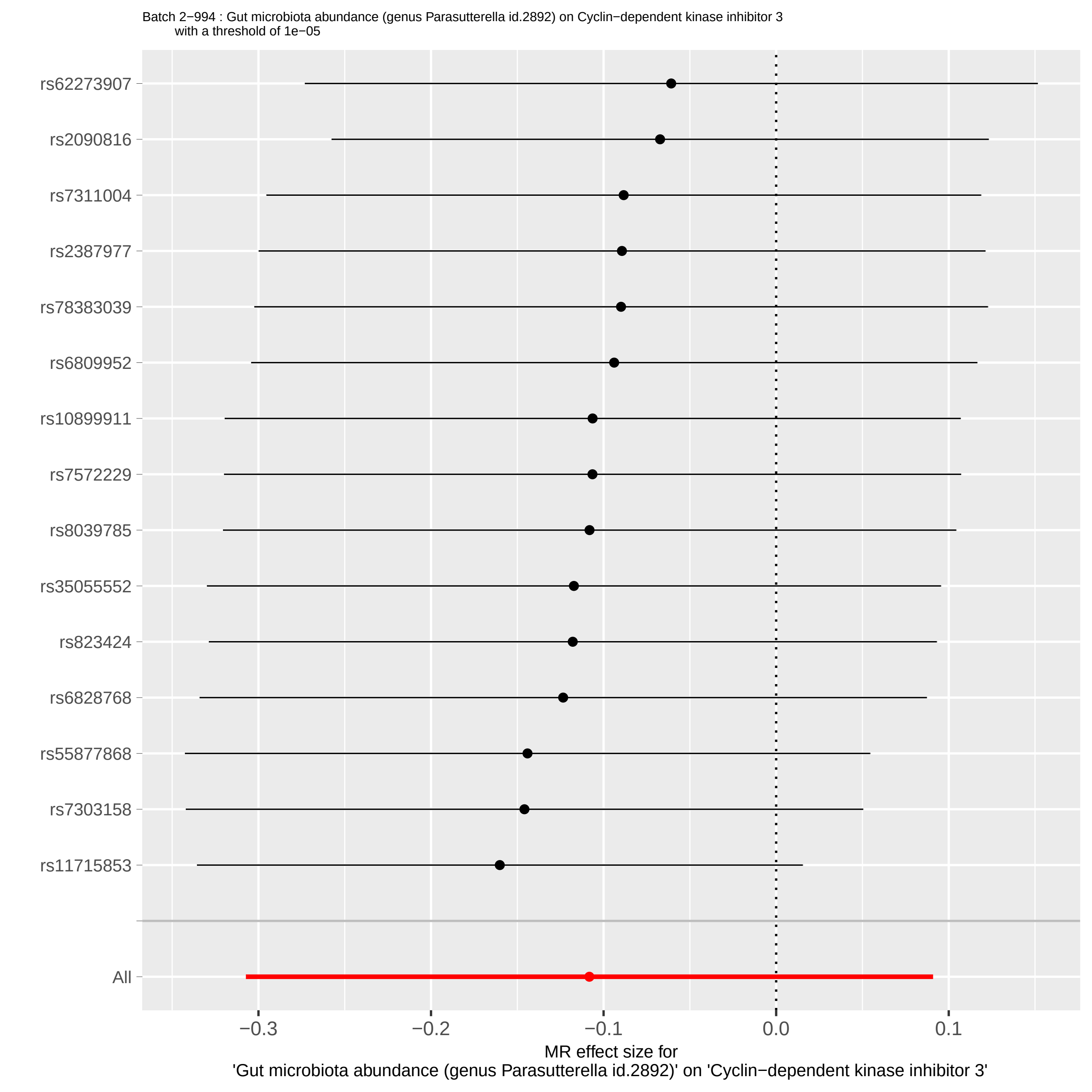

Batch 2−994 : Gut microbiota abundance (genus Parasutterella id.2892) on Cyclin−dependent kinase inhibitor 3
with a threshold of 1e−05
rs62273907
rs2090816
rs7311004
rs2387977
rs78383039
rs6809952
rs10899911
rs7572229
rs8039785
rs35055552
rs823424
rs6828768
rs55877868
rs7303158
rs11715853
All
−0.3
−0.2
−0.1
0.0
0.1
MR effect size for
'Gut microbiota abundance (genus Parasutterella id.2892)' on 'Cyclin−dependent kinase inhibitor 3'

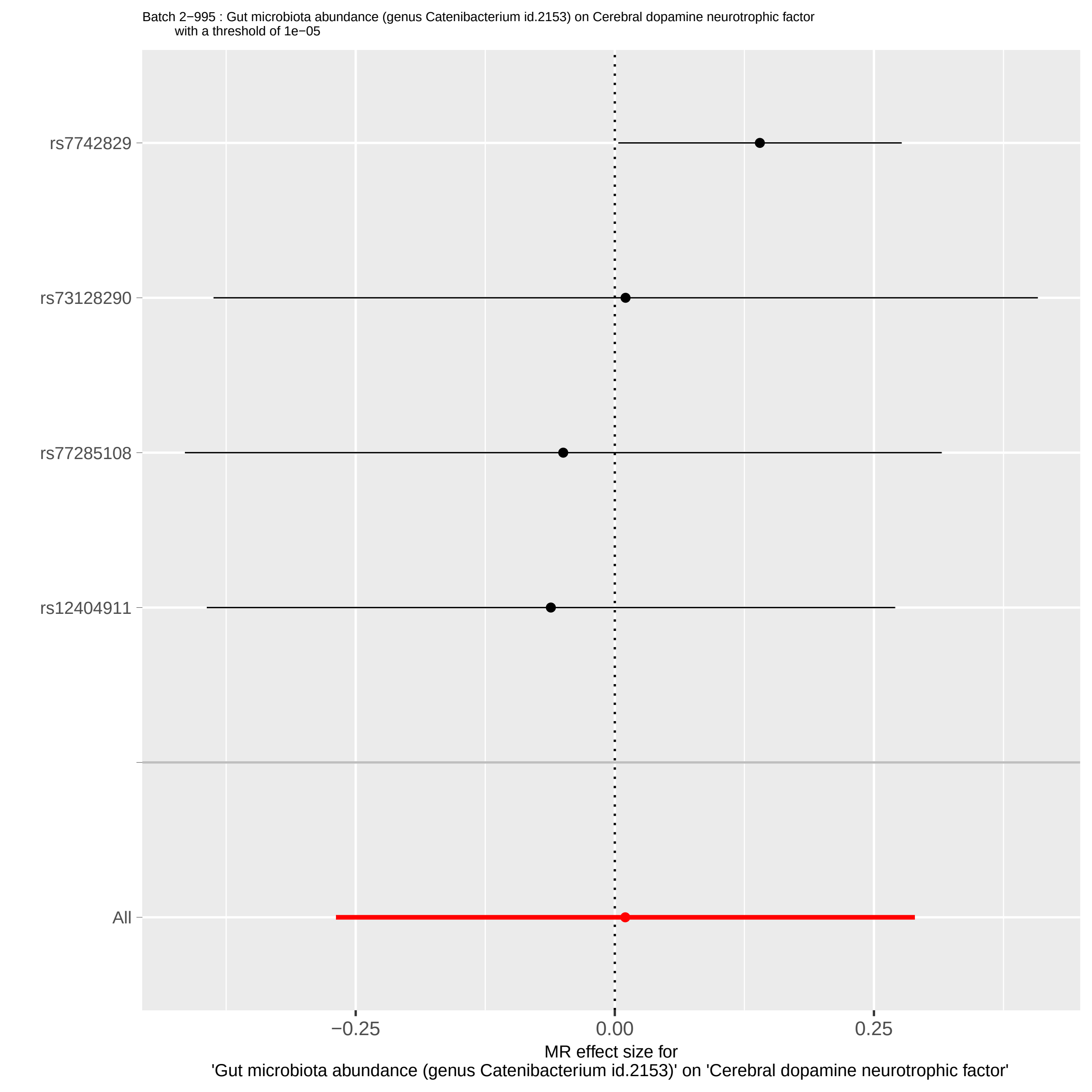

Batch 2−995 : Gut microbiota abundance (genus Catenibacterium id.2153) on Cerebral dopamine neurotrophic factor
with a threshold of 1e−05
rs7742829
rs73128290
rs77285108
rs12404911
All
−0.25
0.00
0.25
MR effect size for
'Gut microbiota abundance (genus Catenibacterium id.2153)' on 'Cerebral dopamine neurotrophic factor'

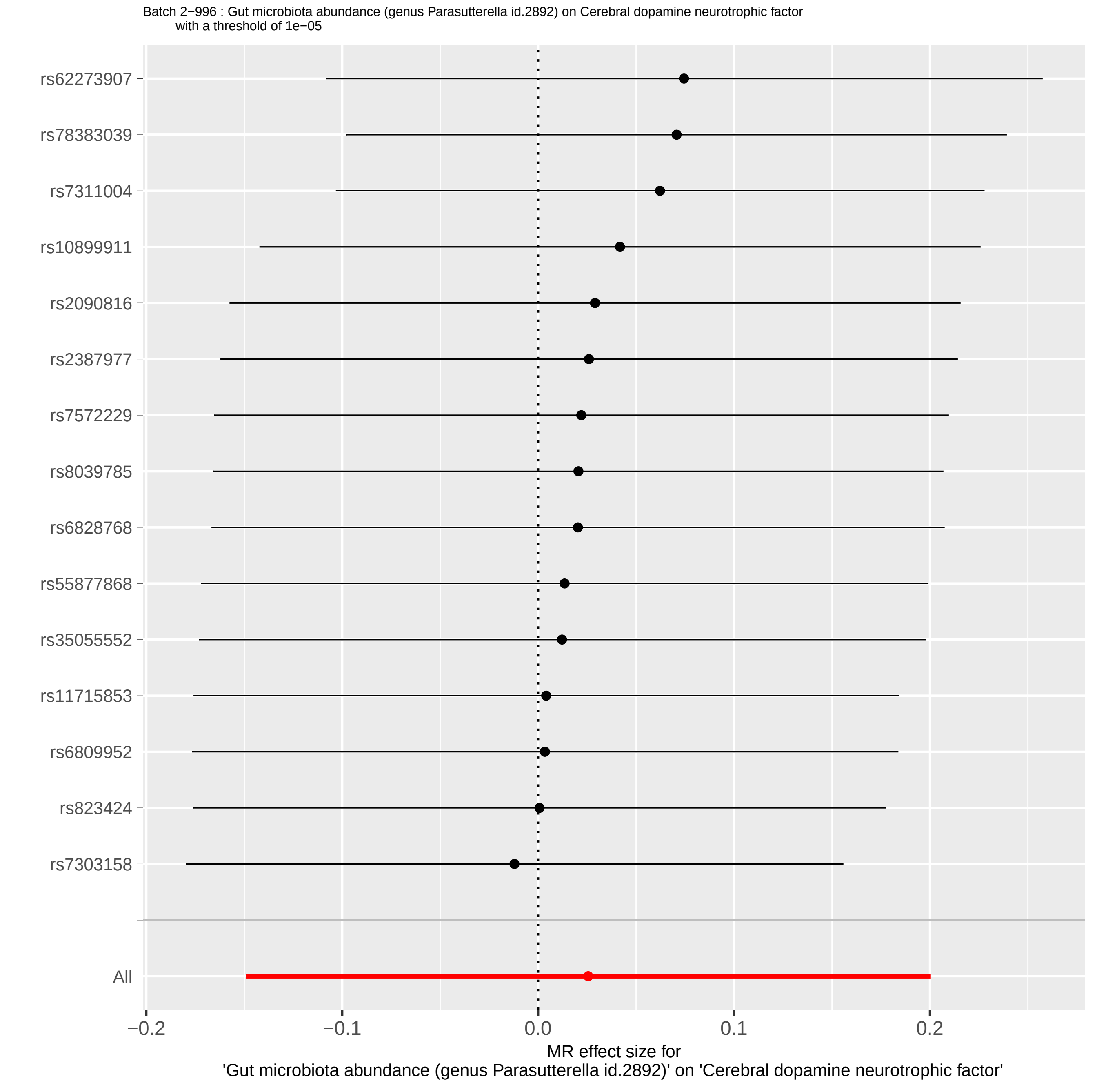

Batch 2−996 : Gut microbiota abundance (genus Parasutterella id.2892) on Cerebral dopamine neurotrophic factor
with a threshold of 1e−05
rs62273907
rs78383039
rs7311004
rs10899911
rs2090816
rs2387977
rs7572229
rs8039785
rs6828768
rs55877868
rs35055552
rs11715853
rs6809952
rs823424
rs7303158
All
−0.2
−0.1
0.0
0.1
0.2
MR effect size for
'Gut microbiota abundance (genus Parasutterella id.2892)' on 'Cerebral dopamine neurotrophic factor'

Batch 2−997 : Gut microbiota abundance (genus Catenibacterium id.2153) on Cell adhesion molecule−related/down−regulated by oncogenes
with a threshold of 1e−05

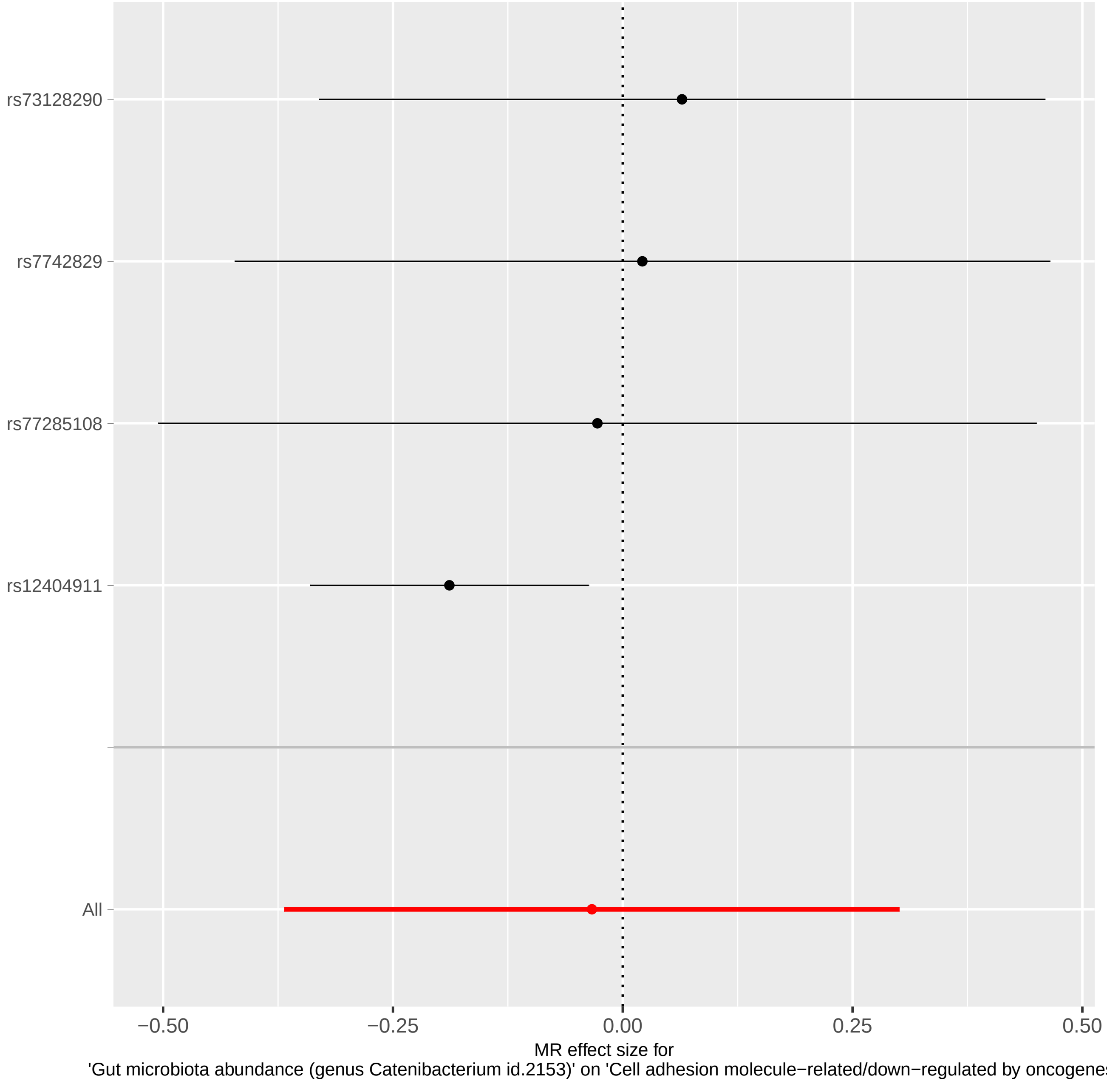

Batch 2−998 : Gut microbiota abundance (genus Parasutterella id.2892) on Cell adhesion molecule−related/down−regulated by oncogenes with a threshold of 1e−05

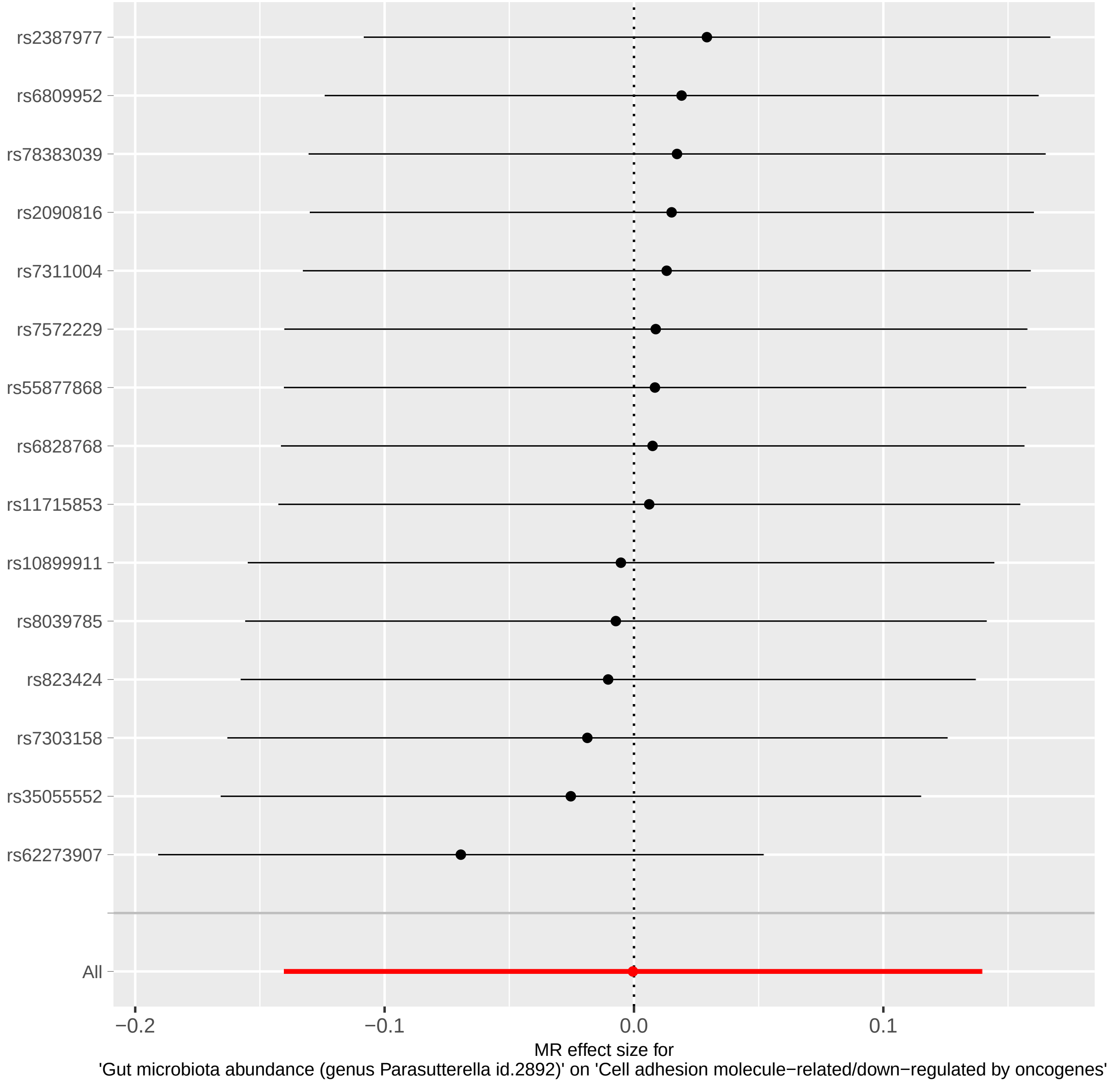

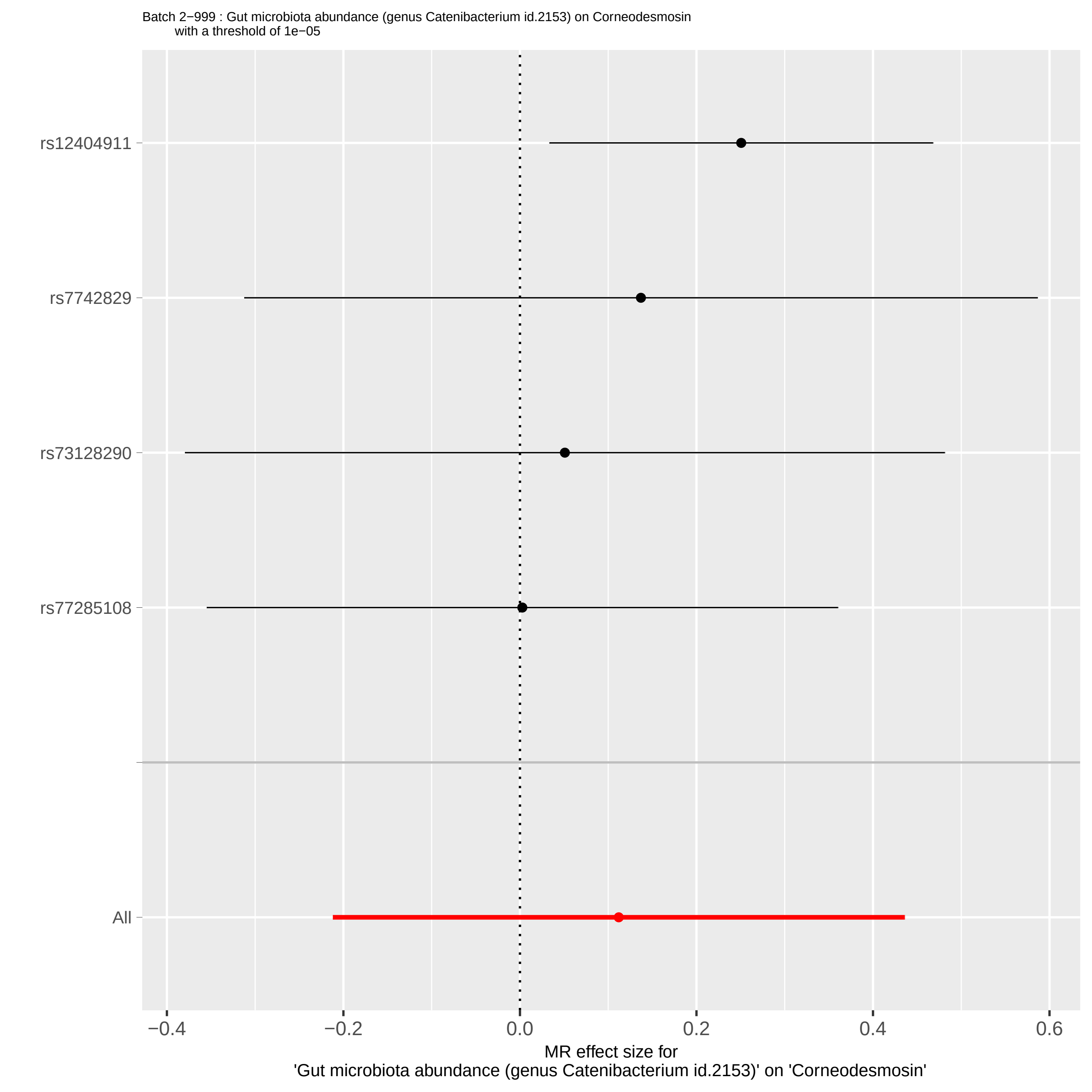

Batch 2−999 : Gut microbiota abundance (genus Catenibacterium id.2153) on Corneodesmosin
with a threshold of 1e−05
rs12404911
rs7742829
rs73128290
rs77285108
All
−0.4
−0.2
0.0
0.2
0.4
0.6
MR effect size for
'Gut microbiota abundance (genus Catenibacterium id.2153)' on 'Corneodesmosin'

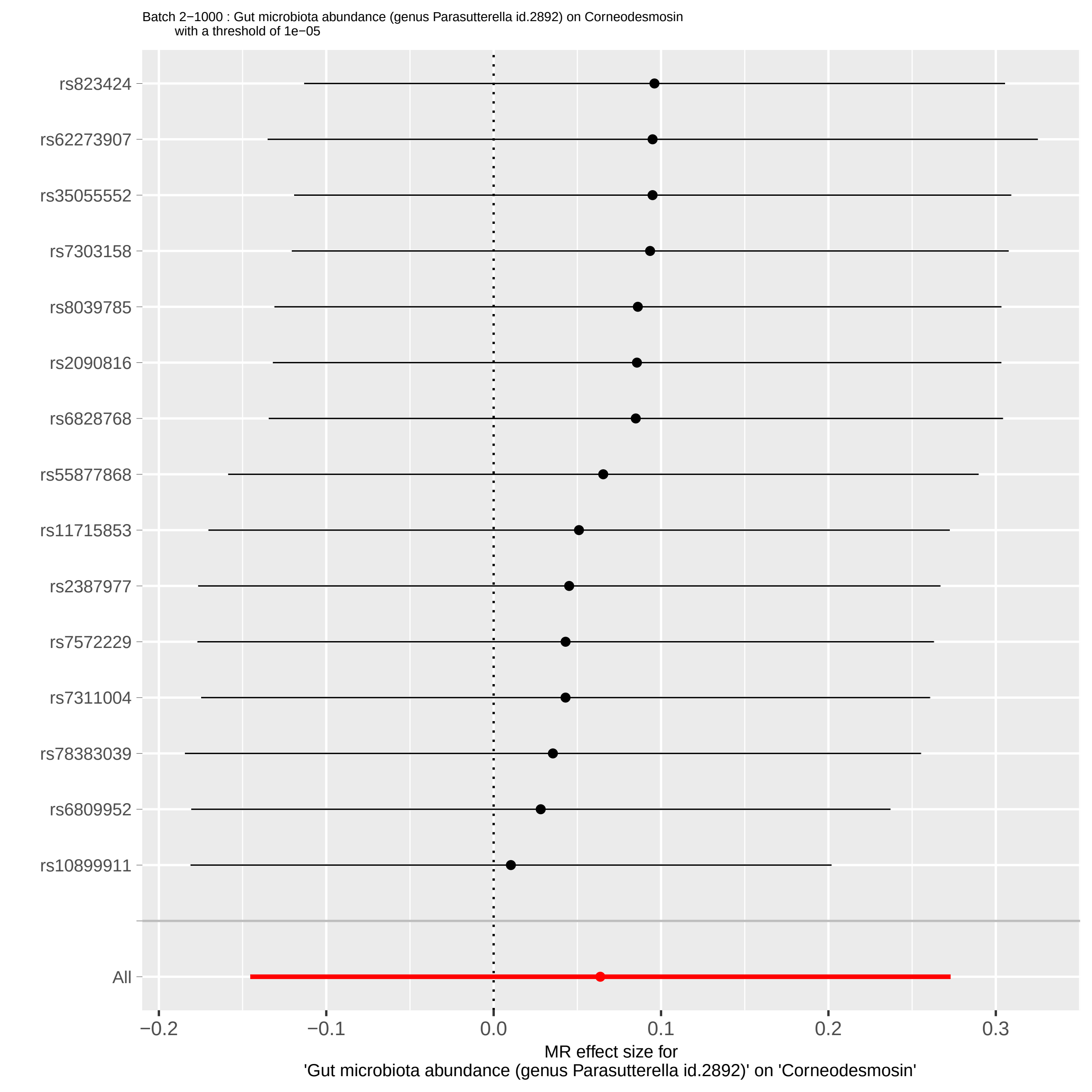

Batch 2−1000 : Gut microbiota abundance (genus Parasutterella id.2892) on Corneodesmosin
with a threshold of 1e−05
rs823424
rs62273907
rs35055552
rs7303158
rs8039785
rs2090816
rs6828768
rs55877868
rs11715853
rs2387977
rs7572229
rs7311004
rs78383039
rs6809952
rs10899911
All
−0.2
−0.1
0.0
0.1
0.2
0.3
MR effect size for
'Gut microbiota abundance (genus Parasutterella id.2892)' on 'Corneodesmosin'

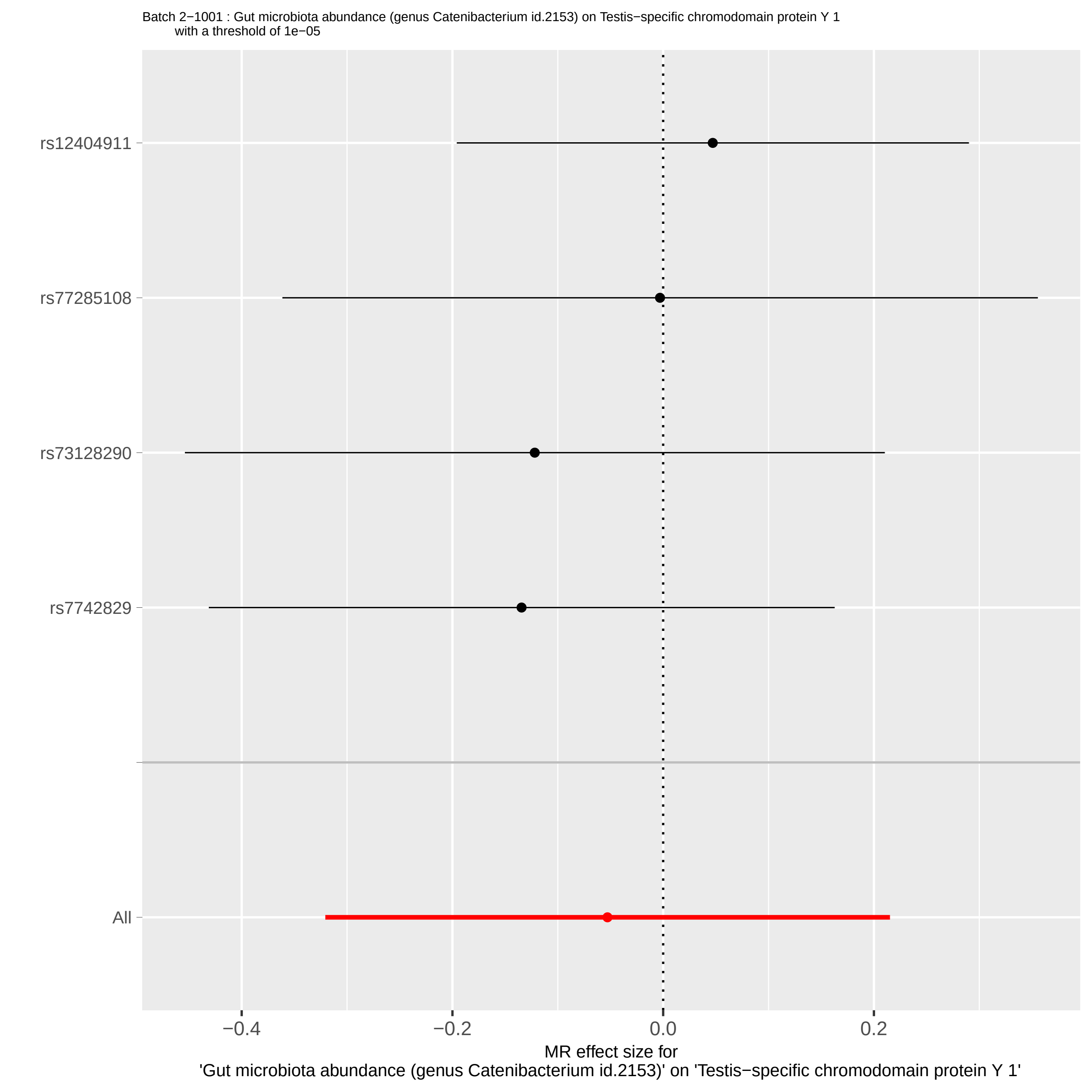
Batch 2−1001 : Gut microbiota abundance (genus Catenibacterium id.2153) on Testis−specific chromodomain protein Y 1
with a threshold of 1e−05
rs12404911
rs77285108
rs73128290
rs7742829
All
−0.4
−0.2
0.0
0.2
MR effect size for
'Gut microbiota abundance (genus Catenibacterium id.2153)' on 'Testis−specific chromodomain protein Y 1'

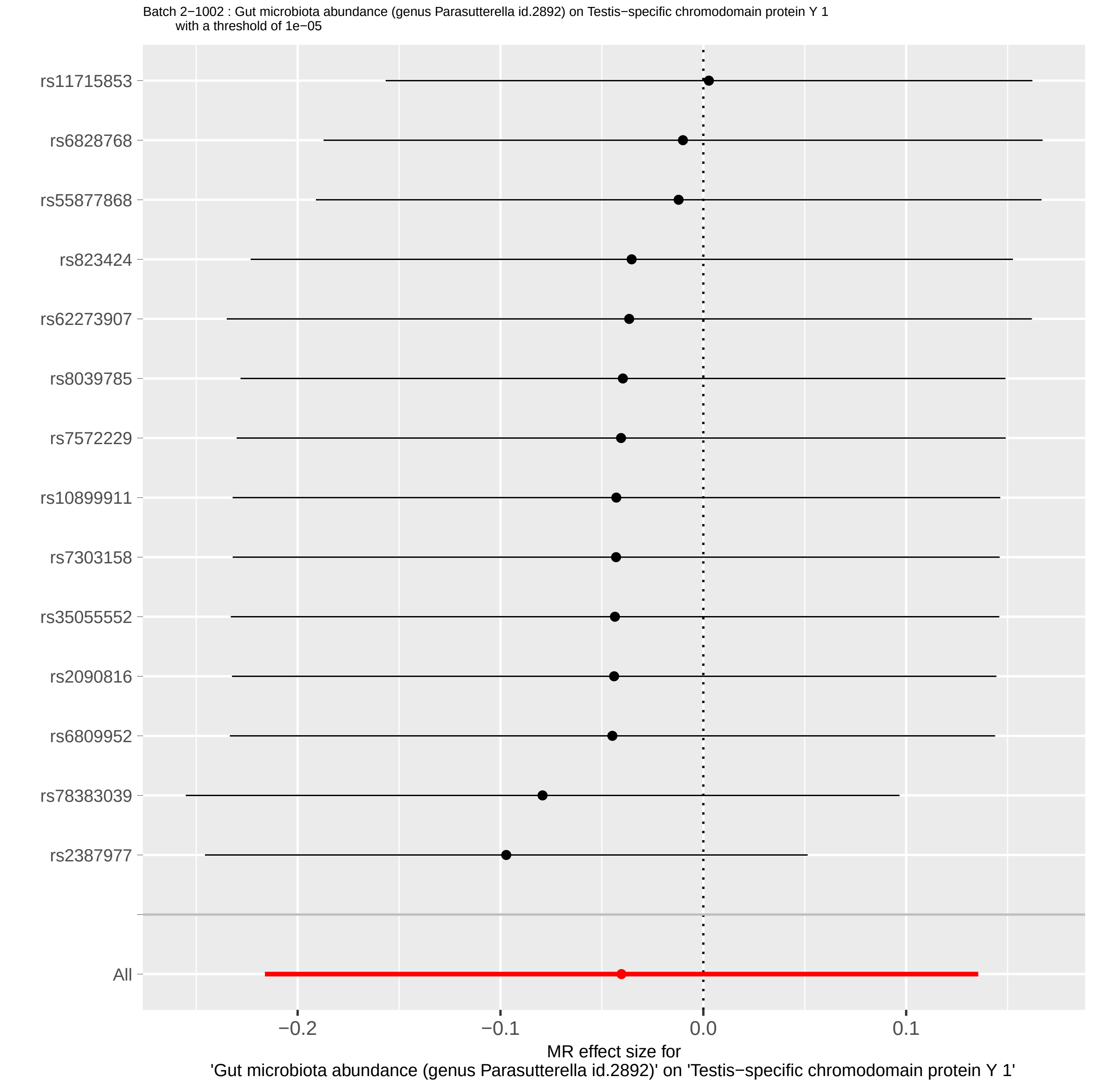
Batch 2−1002 : Gut microbiota abundance (genus Parasutterella id.2892) on Testis−specific chromodomain protein Y 1
with a threshold of 1e−05
rs11715853
rs6828768
rs55877868
rs823424
rs62273907
rs8039785
rs7572229
rs10899911
rs7303158
rs35055552
rs2090816
rs6809952
rs78383039
rs2387977
All
−0.2
−0.1
0.0
0.1
MR effect size for
'Gut microbiota abundance (genus Parasutterella id.2892)' on 'Testis−specific chromodomain protein Y 1'

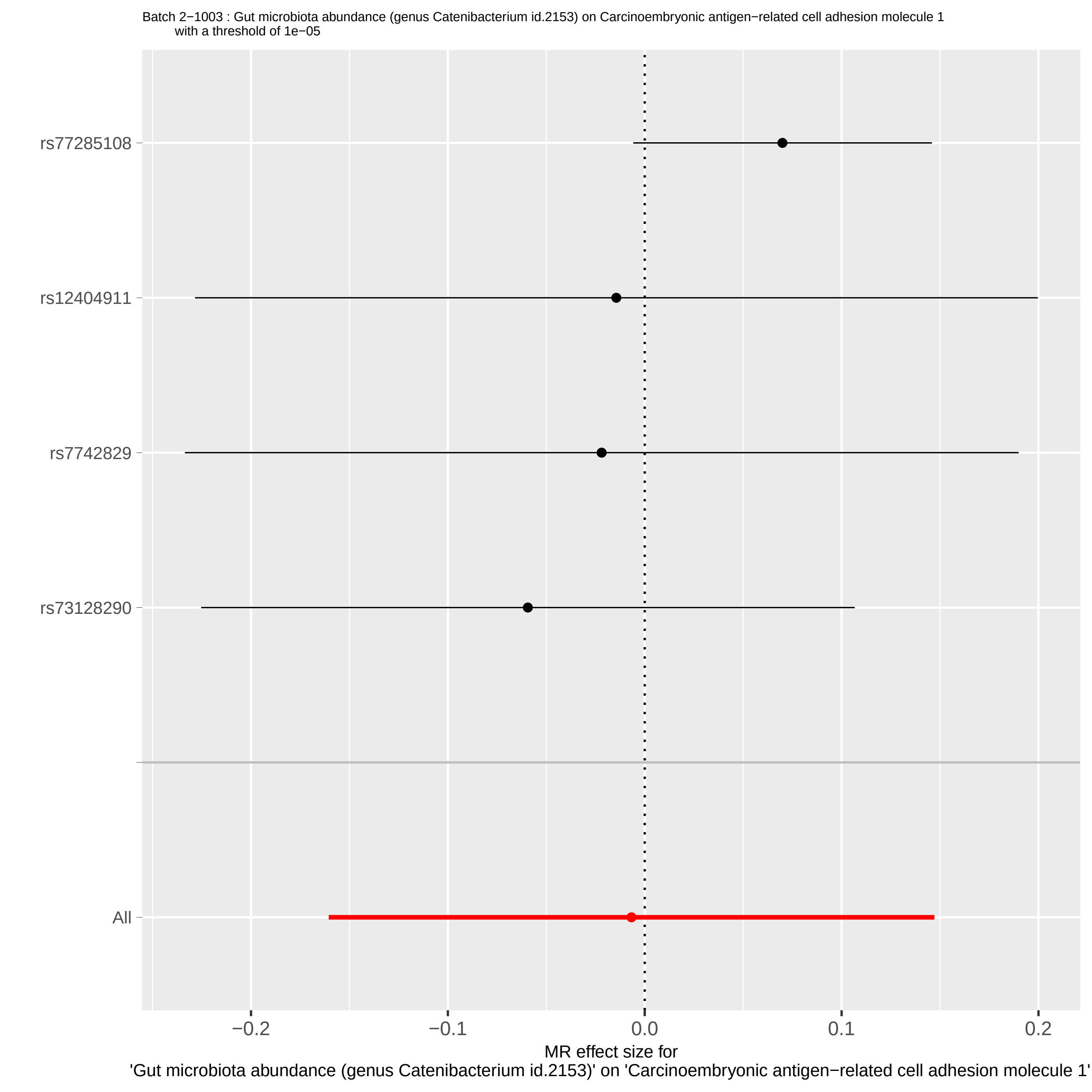
Batch 2−1003 : Gut microbiota abundance (genus Catenibacterium id.2153) on Carcinoembryonic antigen−related cell adhesion molecule 1
with a threshold of 1e−05
rs77285108
rs12404911
rs7742829
rs73128290
All
−0.2
−0.1
0.0
0.1
0.2
MR effect size for
'Gut microbiota abundance (genus Catenibacterium id.2153)' on 'Carcinoembryonic antigen−related cell adhesion molecule 1'

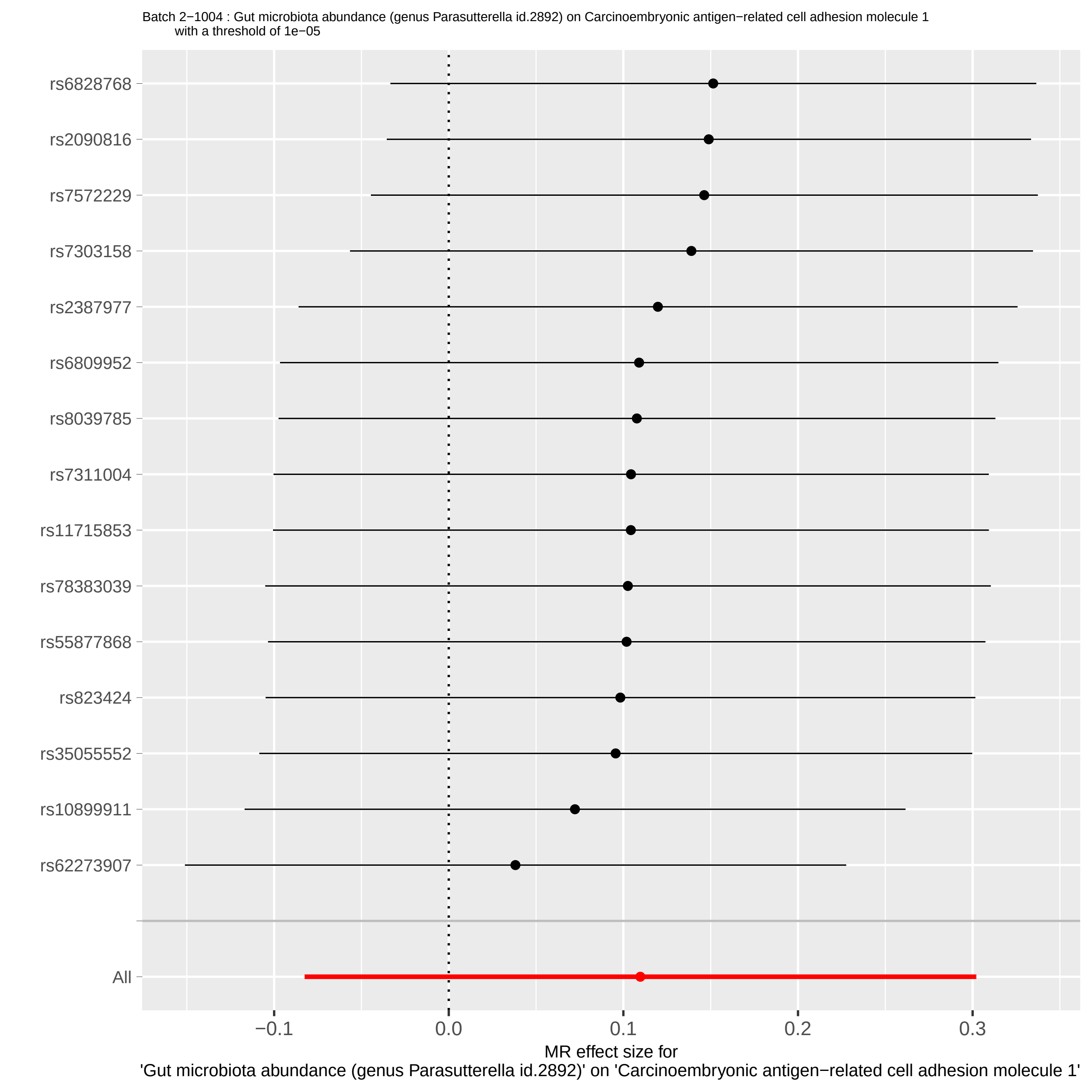

Batch 2−1004 : Gut microbiota abundance (genus Parasutterella id.2892) on Carcinoembryonic antigen−related cell adhesion molecule 1
with a threshold of 1e−05
rs6828768
rs2090816
rs7572229
rs7303158
rs2387977
rs6809952
rs8039785
rs7311004
rs11715853
rs78383039
rs55877868
rs823424
rs35055552
rs10899911
rs62273907
All
−0.1
0.0
0.1
0.2
0.3
MR effect size for
'Gut microbiota abundance (genus Parasutterella id.2892)' on 'Carcinoembryonic antigen−related cell adhesion molecule 1'

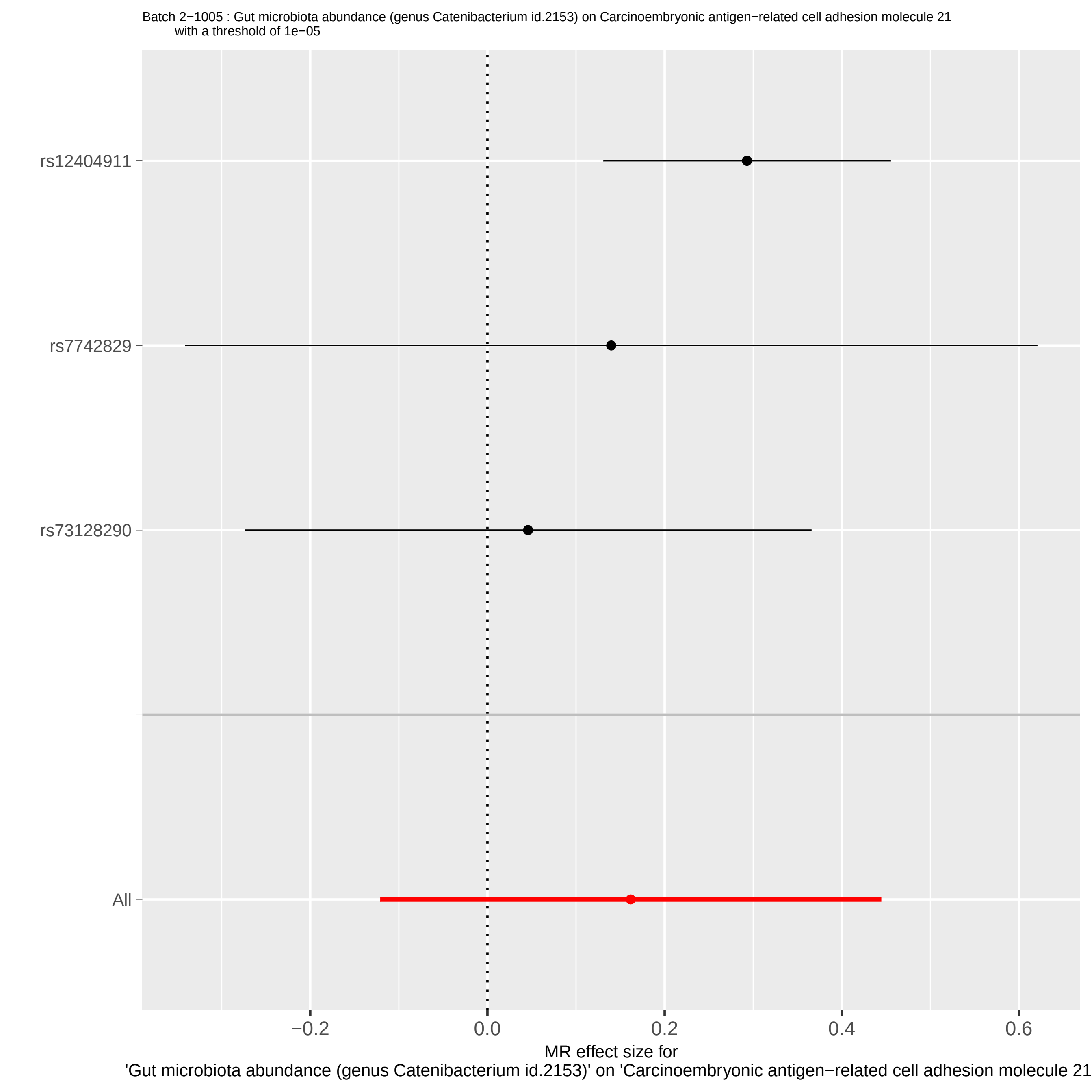

Batch 2−1005 : Gut microbiota abundance (genus Catenibacterium id.2153) on Carcinoembryonic antigen−related cell adhesion molecule 21
with a threshold of 1e−05
rs12404911
rs7742829
rs73128290
All
−0.2
0.0
0.2
0.4
0.6
MR effect size for
'Gut microbiota abundance (genus Catenibacterium id.2153)' on 'Carcinoembryonic antigen−related cell adhesion molecule 21

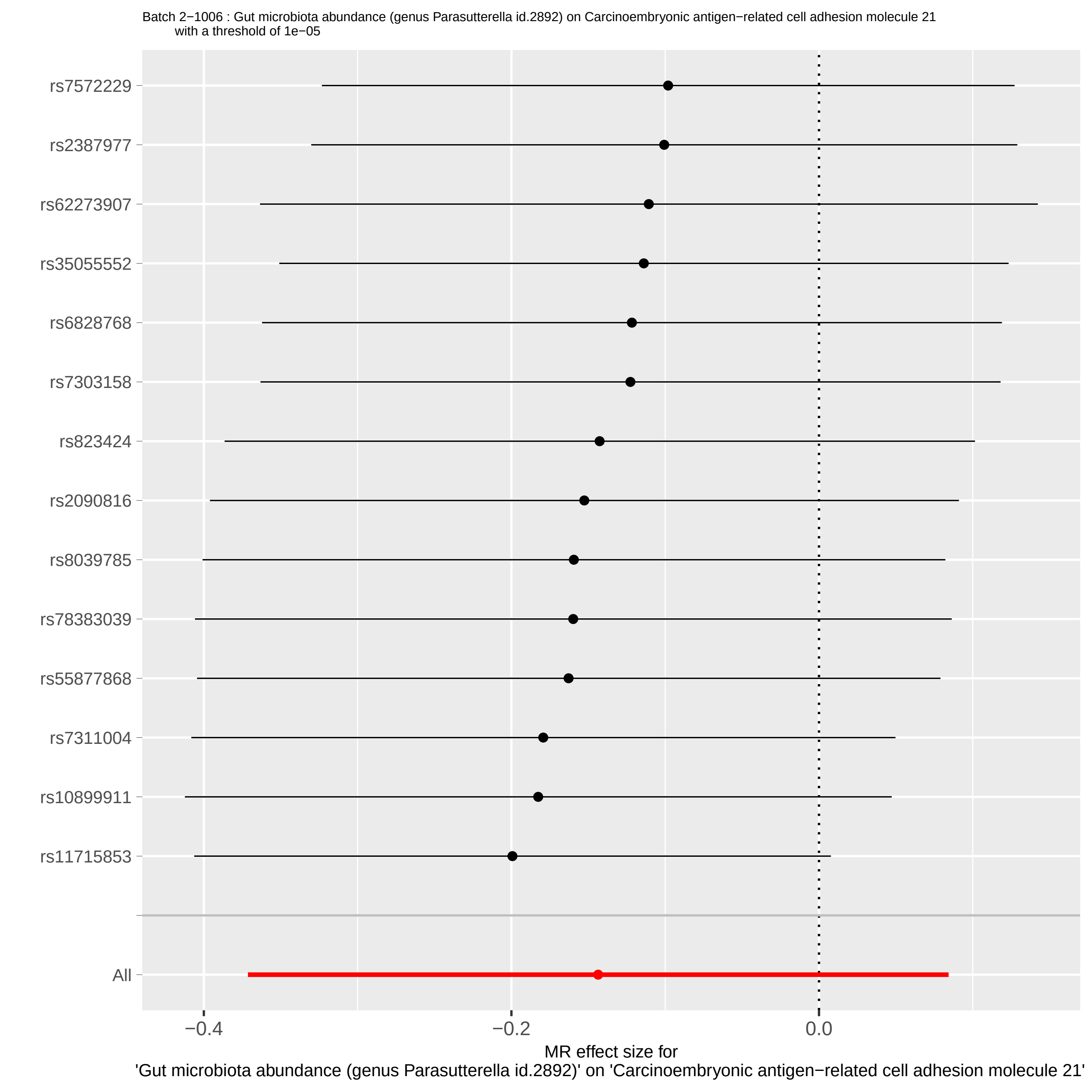

Batch 2−1006 : Gut microbiota abundance (genus Parasutterella id.2892) on Carcinoembryonic antigen−related cell adhesion molecule 21
with a threshold of 1e−05
rs7572229
rs2387977
rs62273907
rs35055552
rs6828768
rs7303158
rs823424
rs2090816
rs8039785
rs78383039
rs55877868
rs7311004
rs10899911
rs11715853
All
−0.4
−0.2
0.0
MR effect size for
'Gut microbiota abundance (genus Parasutterella id.2892)' on 'Carcinoembryonic antigen−related cell adhesion molecule 21'

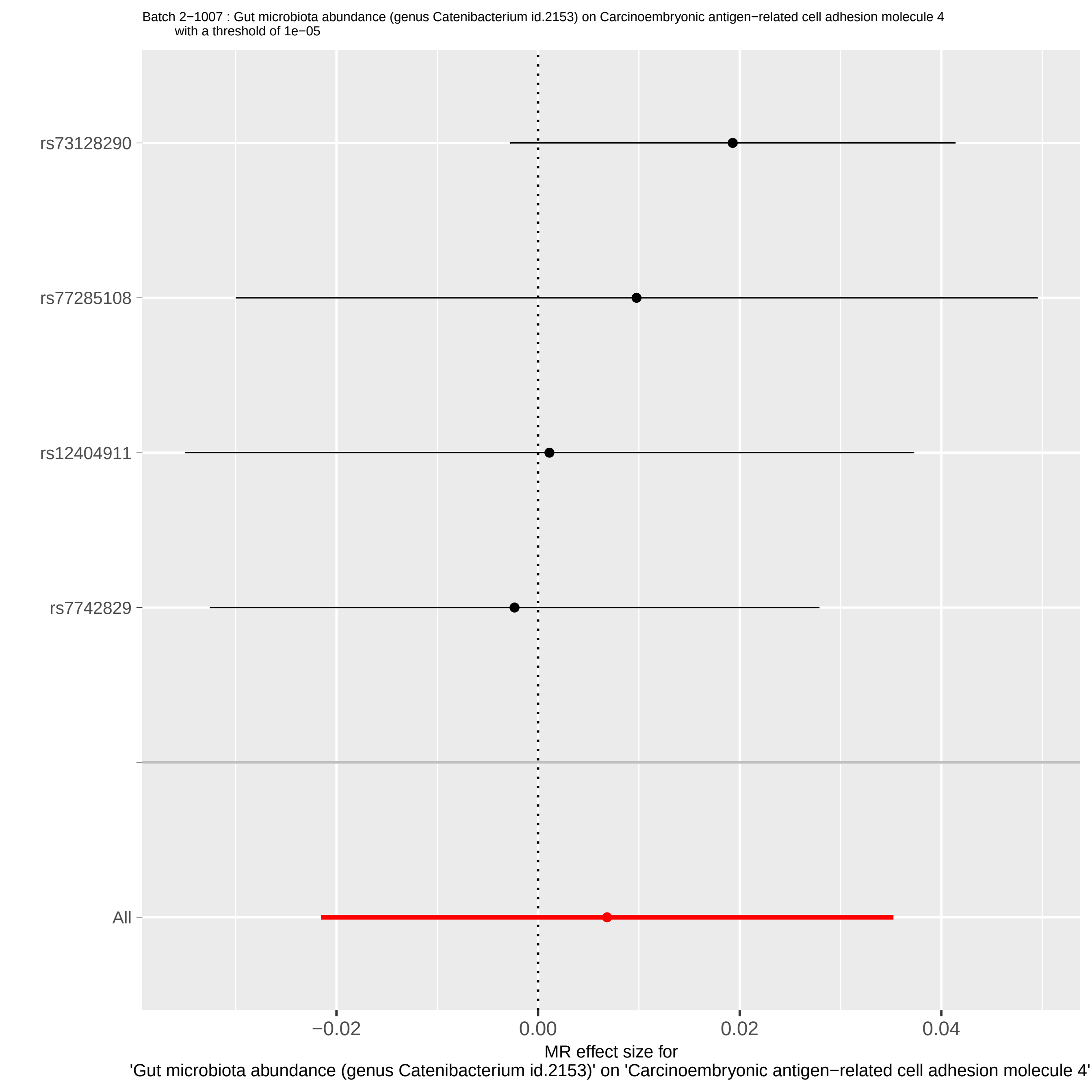
Batch 2−1007 : Gut microbiota abundance (genus Catenibacterium id.2153) on Carcinoembryonic antigen−related cell adhesion molecule 4
with a threshold of 1e−05
rs73128290
rs77285108
rs12404911
rs7742829
All
−0.02
0.00
0.02
0.04
MR effect size for
'Gut microbiota abundance (genus Catenibacterium id.2153)' on 'Carcinoembryonic antigen−related cell adhesion molecule 4'

Batch 2−1008 : Gut microbiota abundance (genus Parasutterella id.2892) on Carcinoembryonic antigen−related cell adhesion molecule 4
with a threshold of 1e−05

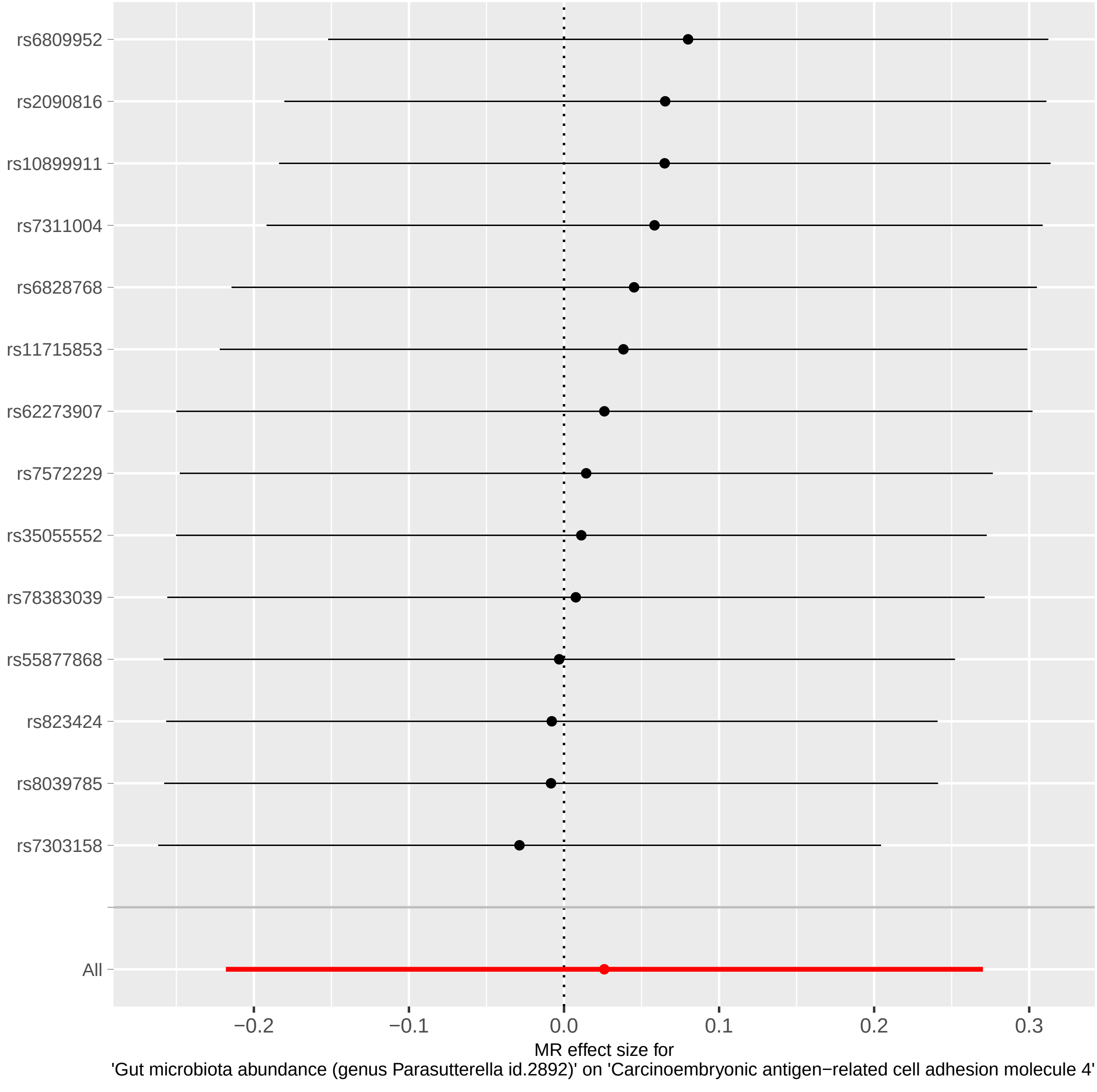

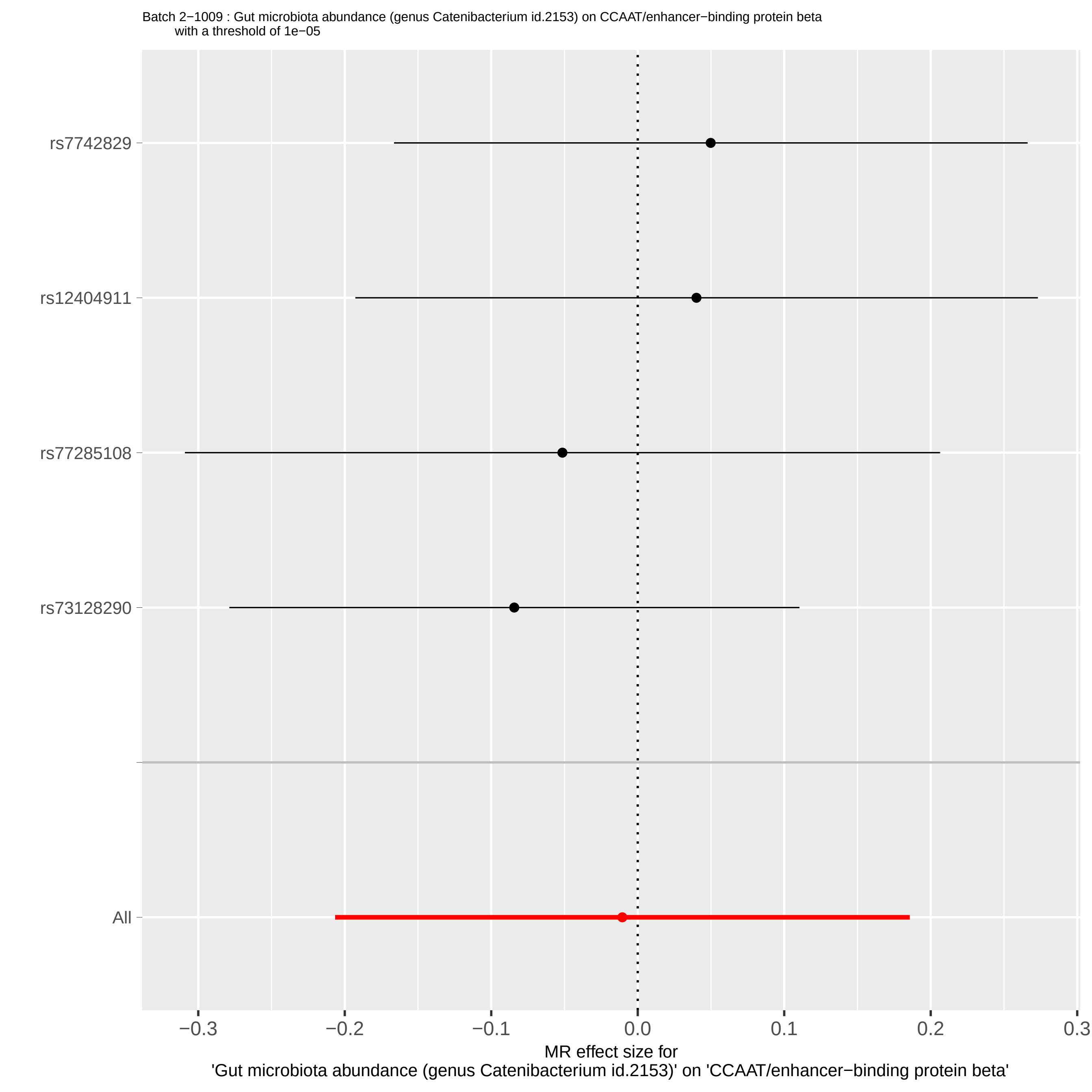
Batch 2−1009 : Gut microbiota abundance (genus Catenibacterium id.2153) on CCAAT/enhancer−binding protein beta
with a threshold of 1e−05
rs7742829
rs12404911
rs77285108
rs73128290
All
−0.3
−0.2
−0.1
0.0
0.1
0.2
0.3
MR effect size for
'Gut microbiota abundance (genus Catenibacterium id.2153)' on 'CCAAT/enhancer−binding protein beta'

Batch 2−1010 : Gut microbiota abundance (genus Parasutterella id.2892) on CCAAT/enhancer−binding protein beta
with a threshold of 1e−05

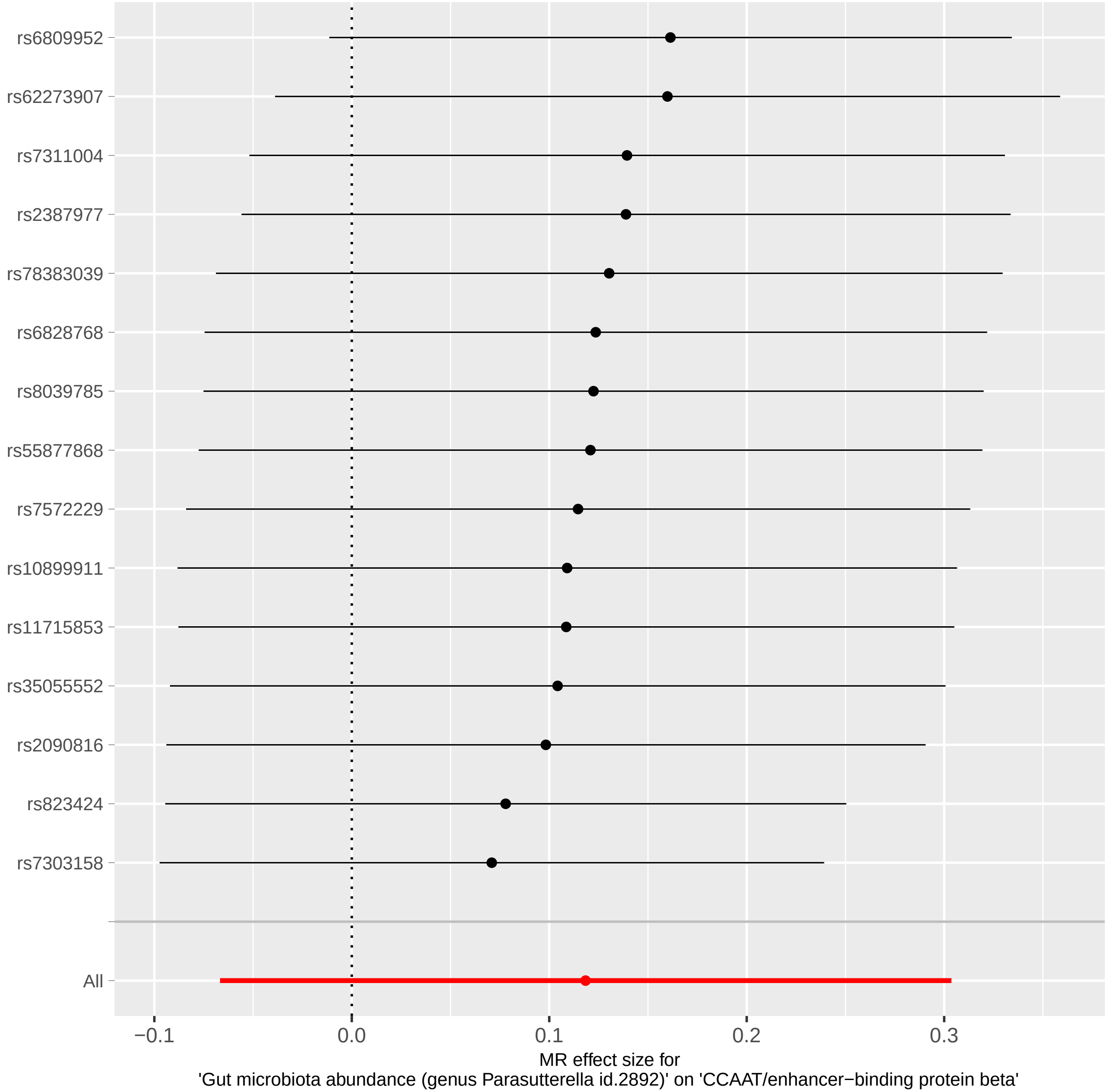

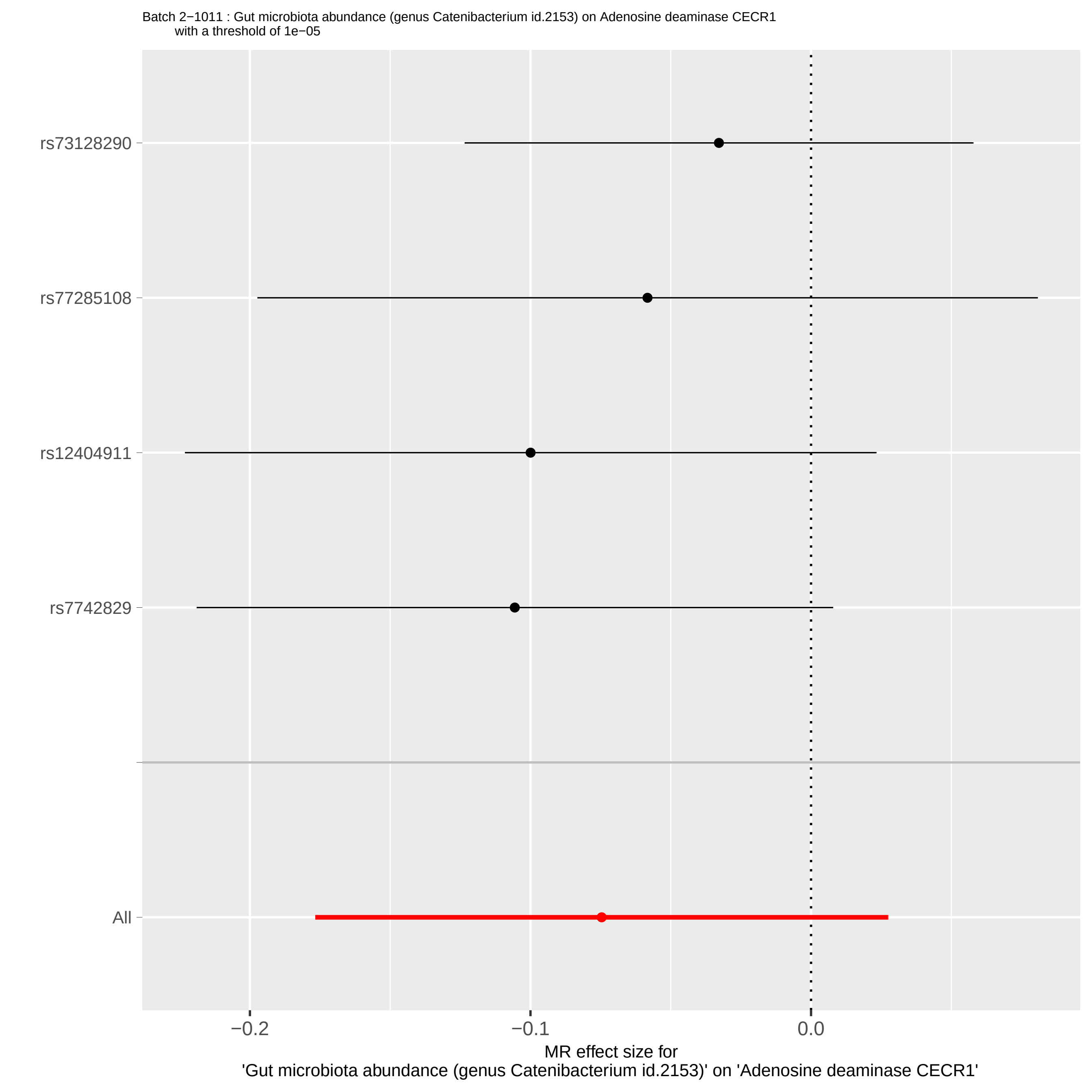

Batch 2−1011 : Gut microbiota abundance (genus Catenibacterium id.2153) on Adenosine deaminase CECR1
with a threshold of 1e−05
rs73128290
rs77285108
rs12404911
rs7742829
All
−0.2
−0.1
0.0
MR effect size for
'Gut microbiota abundance (genus Catenibacterium id.2153)' on 'Adenosine deaminase CECR1'

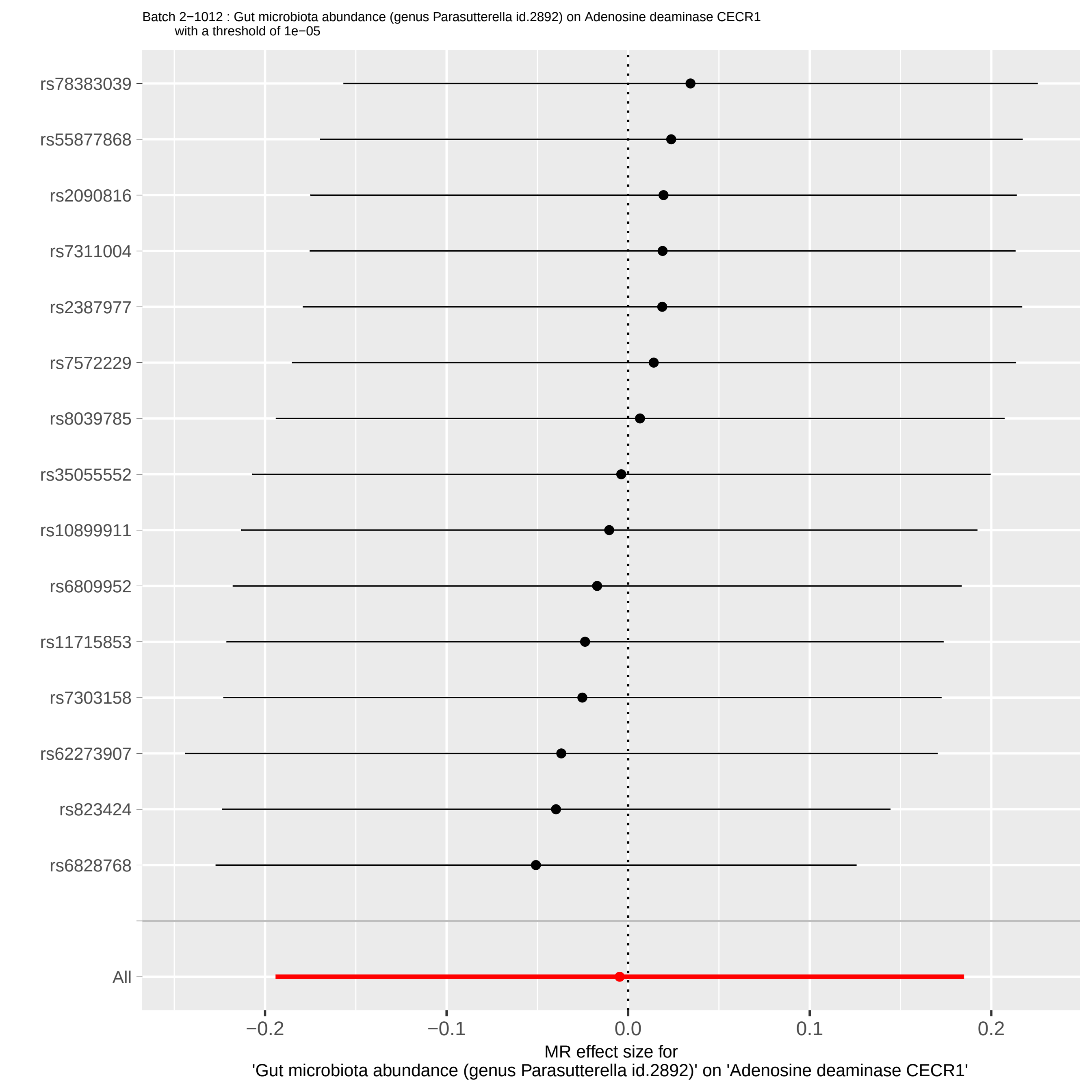

Batch 2−1012 : Gut microbiota abundance (genus Parasutterella id.2892) on Adenosine deaminase CECR1
with a threshold of 1e−05
rs78383039
rs55877868
rs2090816
rs7311004
rs2387977
rs7572229
rs8039785
rs35055552
rs10899911
rs6809952
rs11715853
rs7303158
rs62273907
rs823424
rs6828768
All
−0.2
−0.1
0.0
0.1
0.2
MR effect size for
'Gut microbiota abundance (genus Parasutterella id.2892)' on 'Adenosine deaminase CECR1'

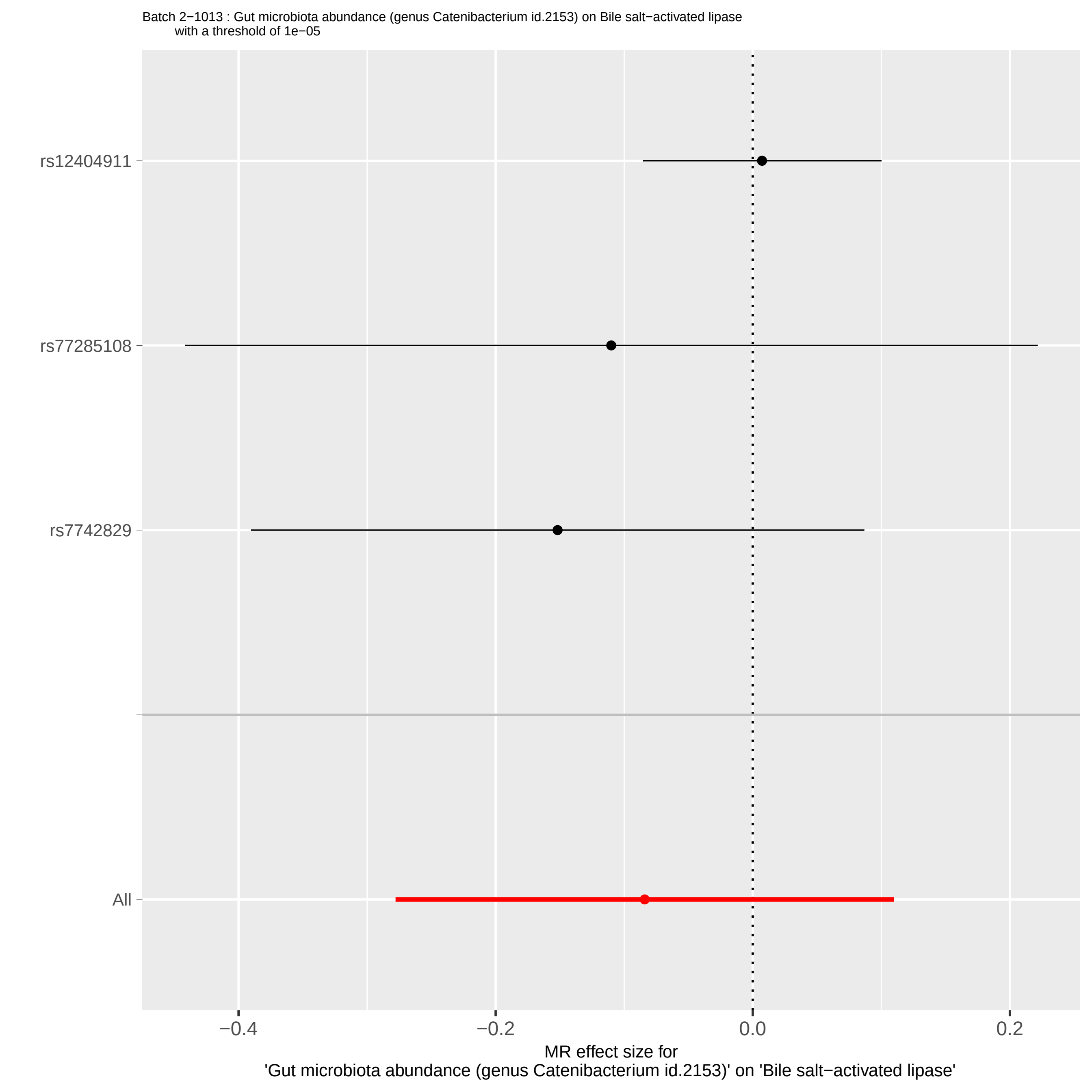
Batch 2−1013 : Gut microbiota abundance (genus Catenibacterium id.2153) on Bile salt−activated lipase
with a threshold of 1e−05
rs12404911
rs77285108
rs7742829
All
−0.4
−0.2
0.0
0.2
MR effect size for
'Gut microbiota abundance (genus Catenibacterium id.2153)' on 'Bile salt−activated lipase'

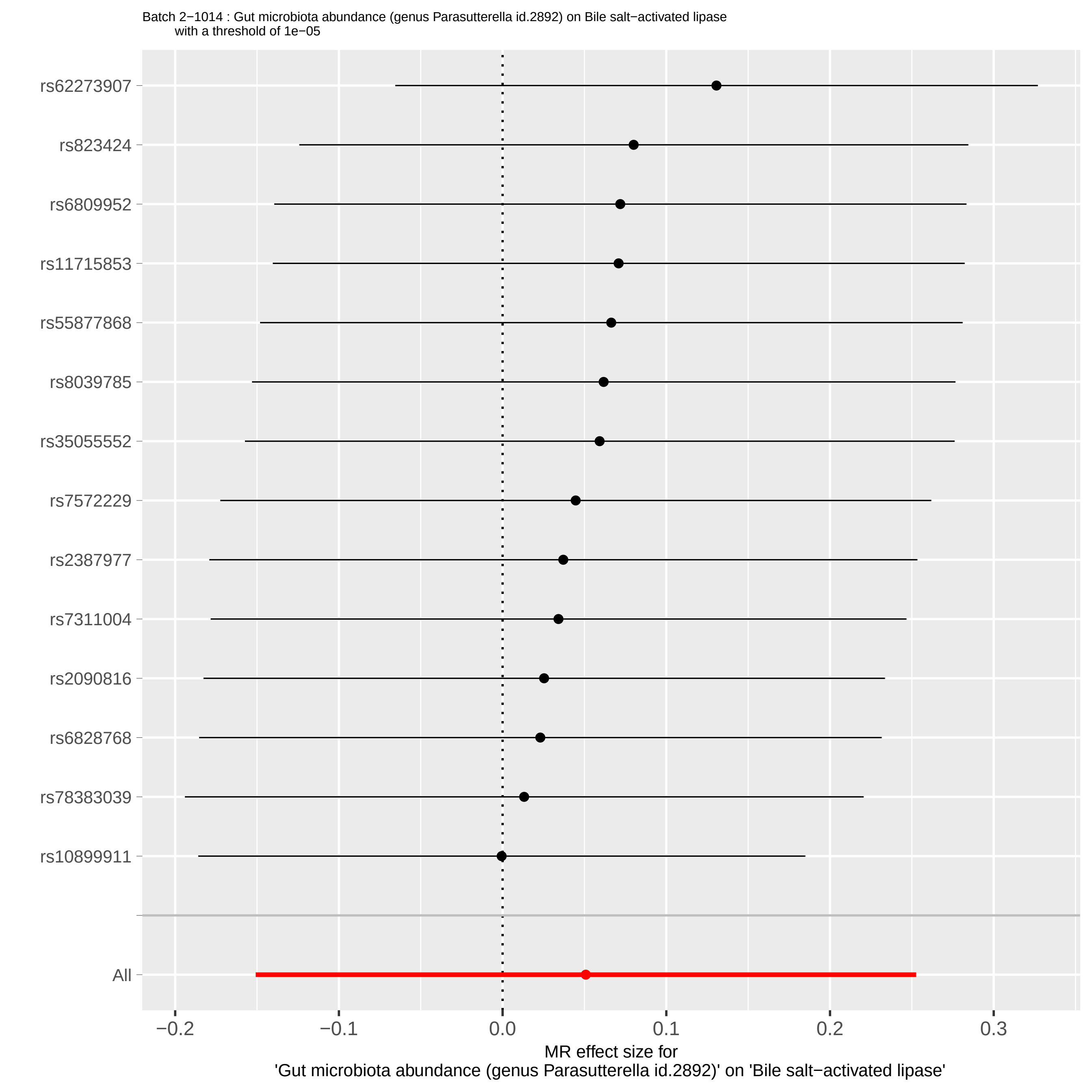

Batch 2−1014 : Gut microbiota abundance (genus Parasutterella id.2892) on Bile salt−activated lipase
with a threshold of 1e−05
rs62273907
rs823424
rs6809952
rs11715853
rs55877868
rs8039785
rs35055552
rs7572229
rs2387977
rs7311004
rs2090816
rs6828768
rs78383039
rs10899911
All
−0.2
−0.1
0.0
0.1
0.2
0.3
MR effect size for
'Gut microbiota abundance (genus Parasutterella id.2892)' on 'Bile salt−activated lipase'

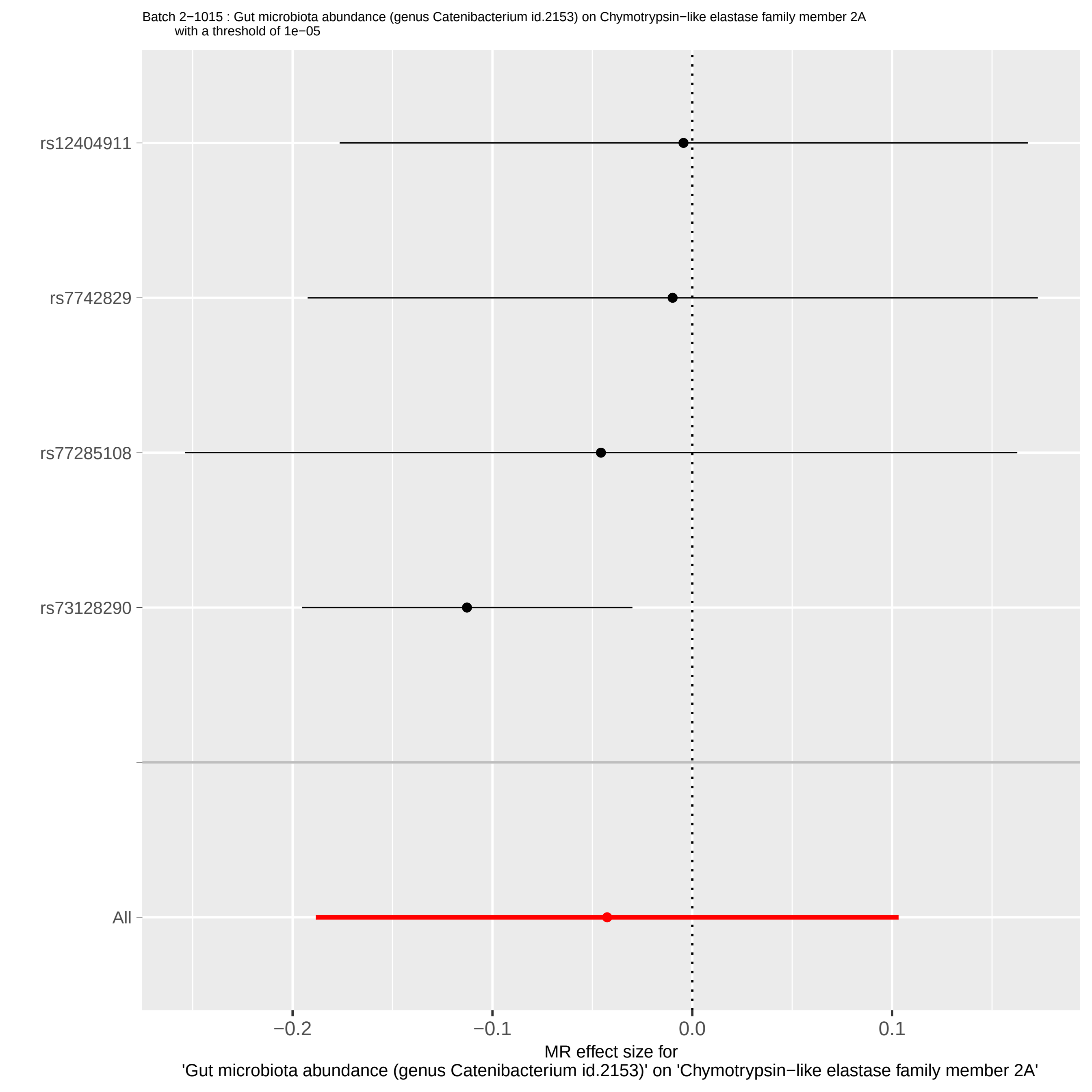

Batch 2−1015 : Gut microbiota abundance (genus Catenibacterium id.2153) on Chymotrypsin−like elastase family member 2A
with a threshold of 1e−05
rs12404911
rs7742829
rs77285108
rs73128290
All
−0.2
−0.1
0.0
0.1
MR effect size for
'Gut microbiota abundance (genus Catenibacterium id.2153)' on 'Chymotrypsin−like elastase family member 2A'

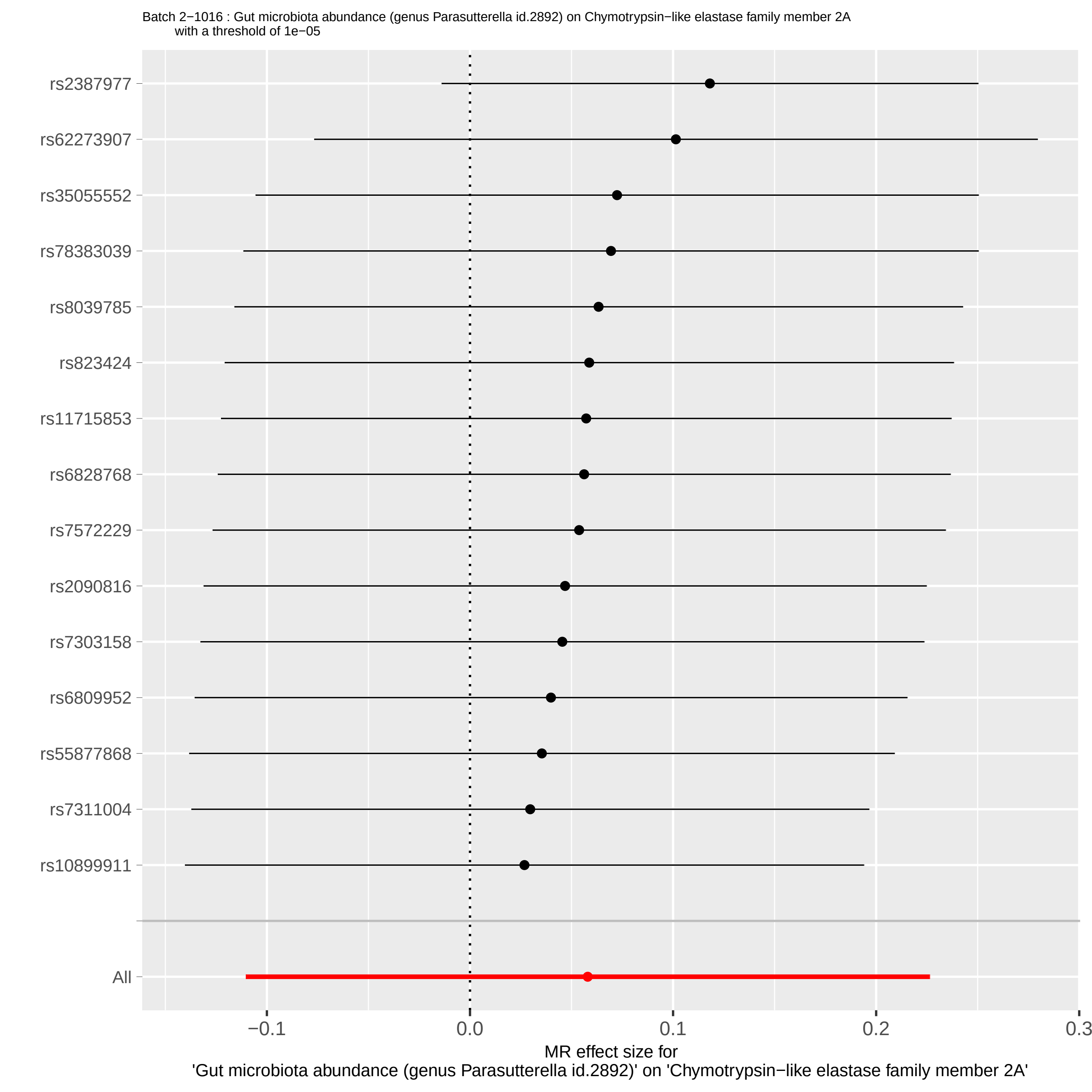

Batch 2−1016 : Gut microbiota abundance (genus Parasutterella id.2892) on Chymotrypsin−like elastase family member 2A
with a threshold of 1e−05
rs2387977
rs62273907
rs35055552
rs78383039
rs8039785
rs823424
rs11715853
rs6828768
rs7572229
rs2090816
rs7303158
rs6809952
rs55877868
rs7311004
rs10899911
All
−0.1
0.0
0.1
0.2
0.3
MR effect size for
'Gut microbiota abundance (genus Parasutterella id.2892)' on 'Chymotrypsin−like elastase family member 2A'

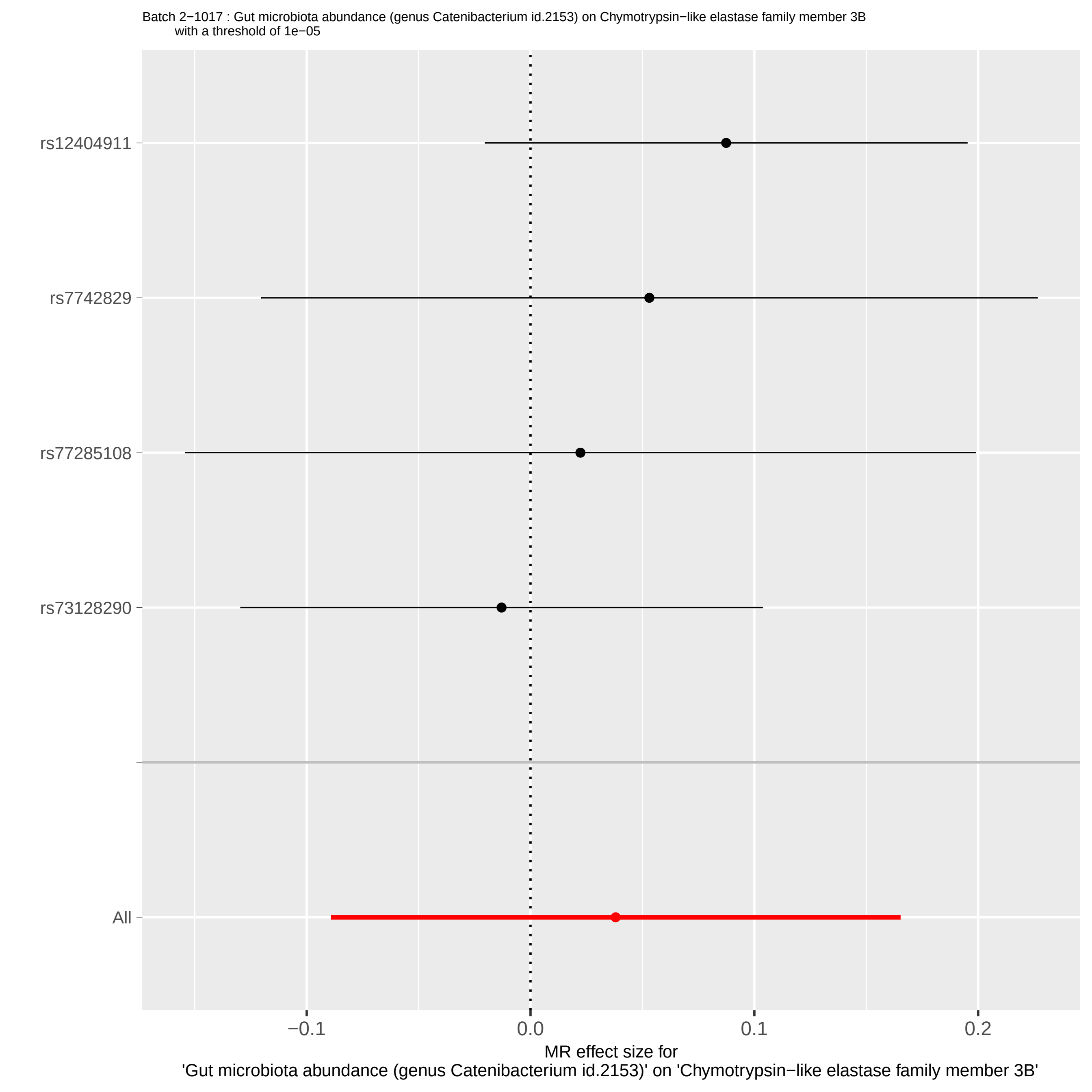
Batch 2−1017 : Gut microbiota abundance (genus Catenibacterium id.2153) on Chymotrypsin−like elastase family member 3B
with a threshold of 1e−05
rs12404911
rs7742829
rs77285108
rs73128290
All
−0.1
0.0
0.1
0.2
MR effect size for
'Gut microbiota abundance (genus Catenibacterium id.2153)' on 'Chymotrypsin−like elastase family member 3B'

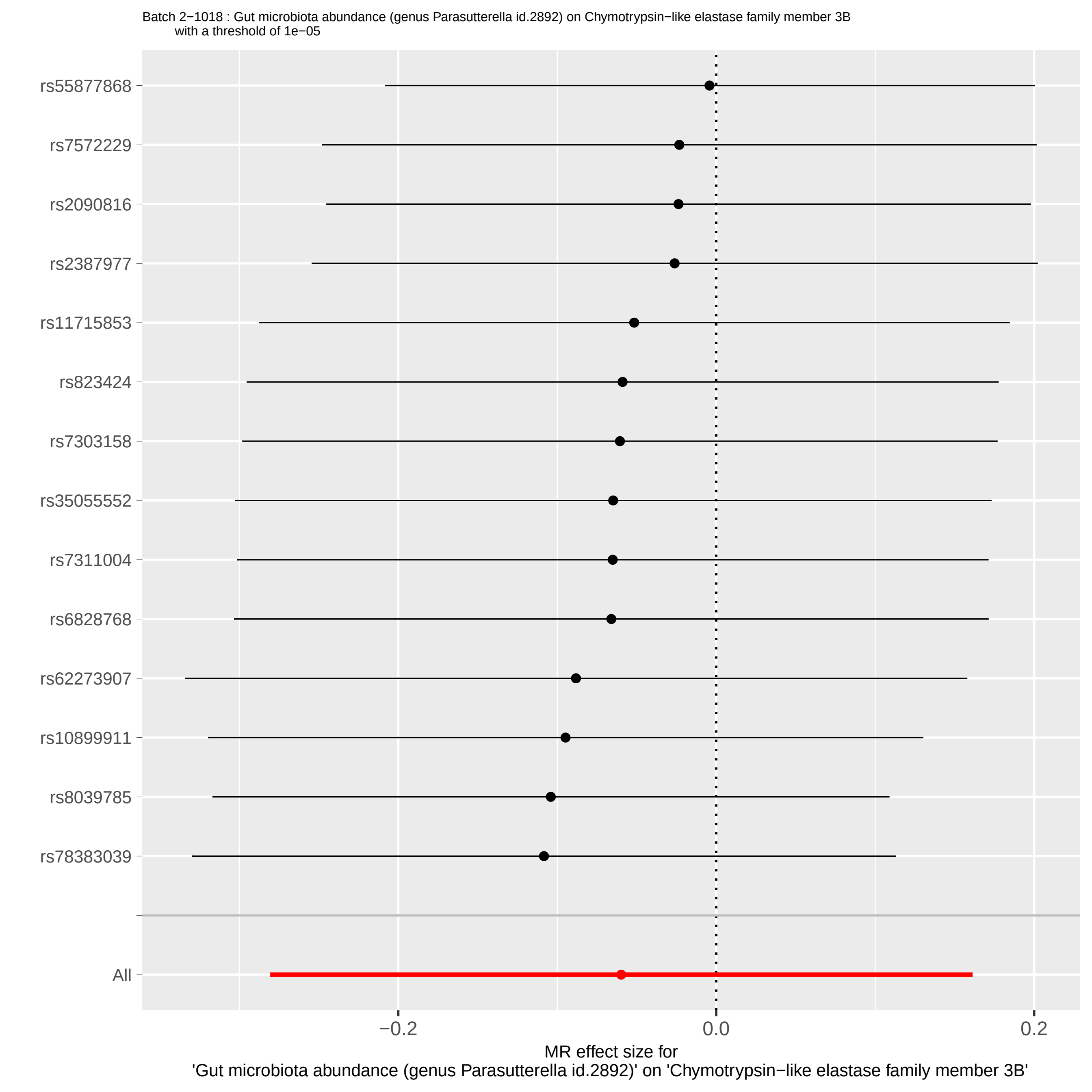
Batch 2−1018 : Gut microbiota abundance (genus Parasutterella id.2892) on Chymotrypsin−like elastase family member 3B
with a threshold of 1e−05
rs55877868
rs7572229
rs2090816
rs2387977
rs11715853
rs823424
rs7303158
rs35055552
rs7311004
rs6828768
rs62273907
rs10899911
rs8039785
rs78383039
All
−0.2
0.0
0.2
MR effect size for
'Gut microbiota abundance (genus Parasutterella id.2892)' on 'Chymotrypsin−like elastase family member 3B'

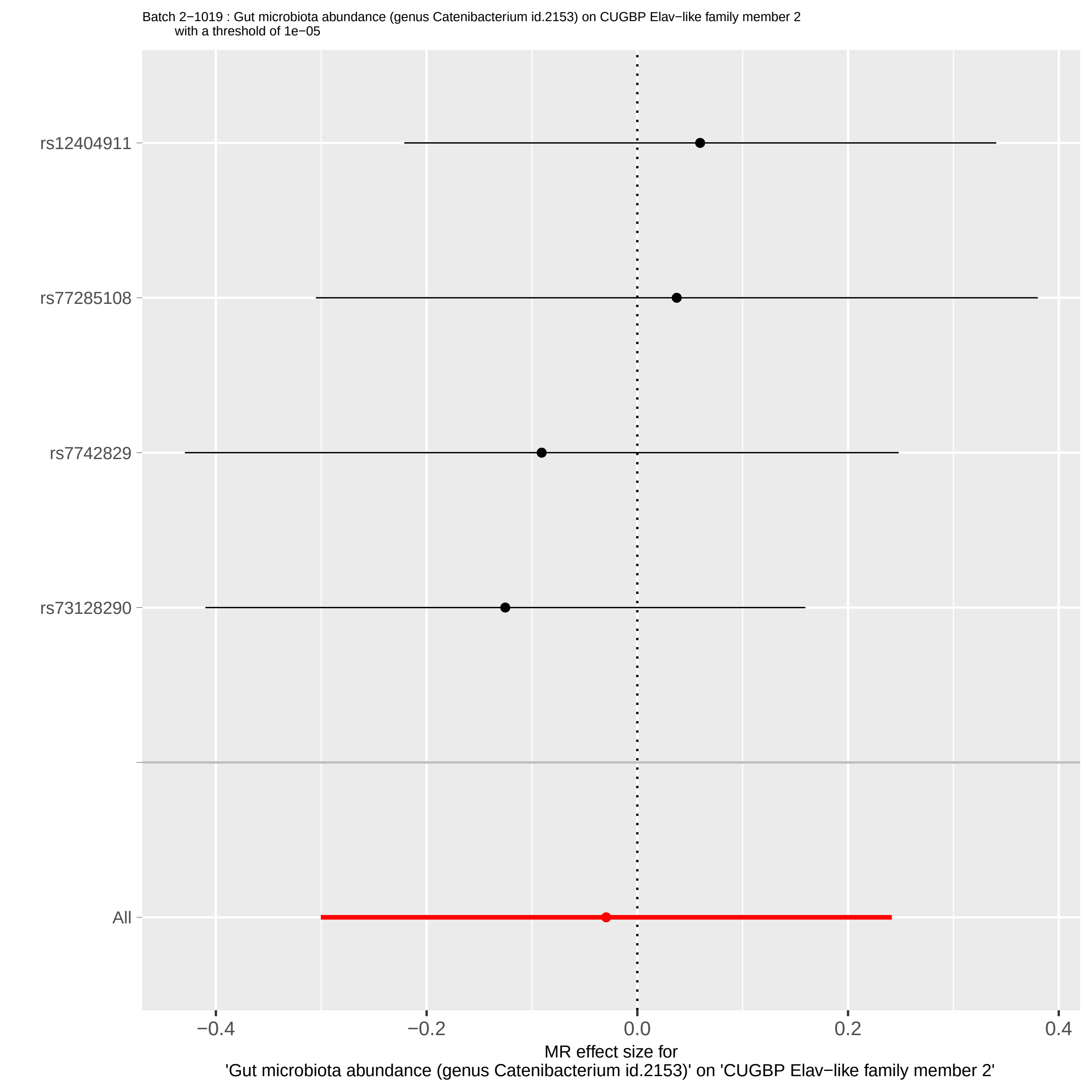

Batch 2−1019 : Gut microbiota abundance (genus Catenibacterium id.2153) on CUGBP Elav−like family member 2
with a threshold of 1e−05
rs12404911
rs77285108
rs7742829
rs73128290
All
−0.4
−0.2
0.0
0.2
0.4
MR effect size for
'Gut microbiota abundance (genus Catenibacterium id.2153)' on 'CUGBP Elav−like family member 2'

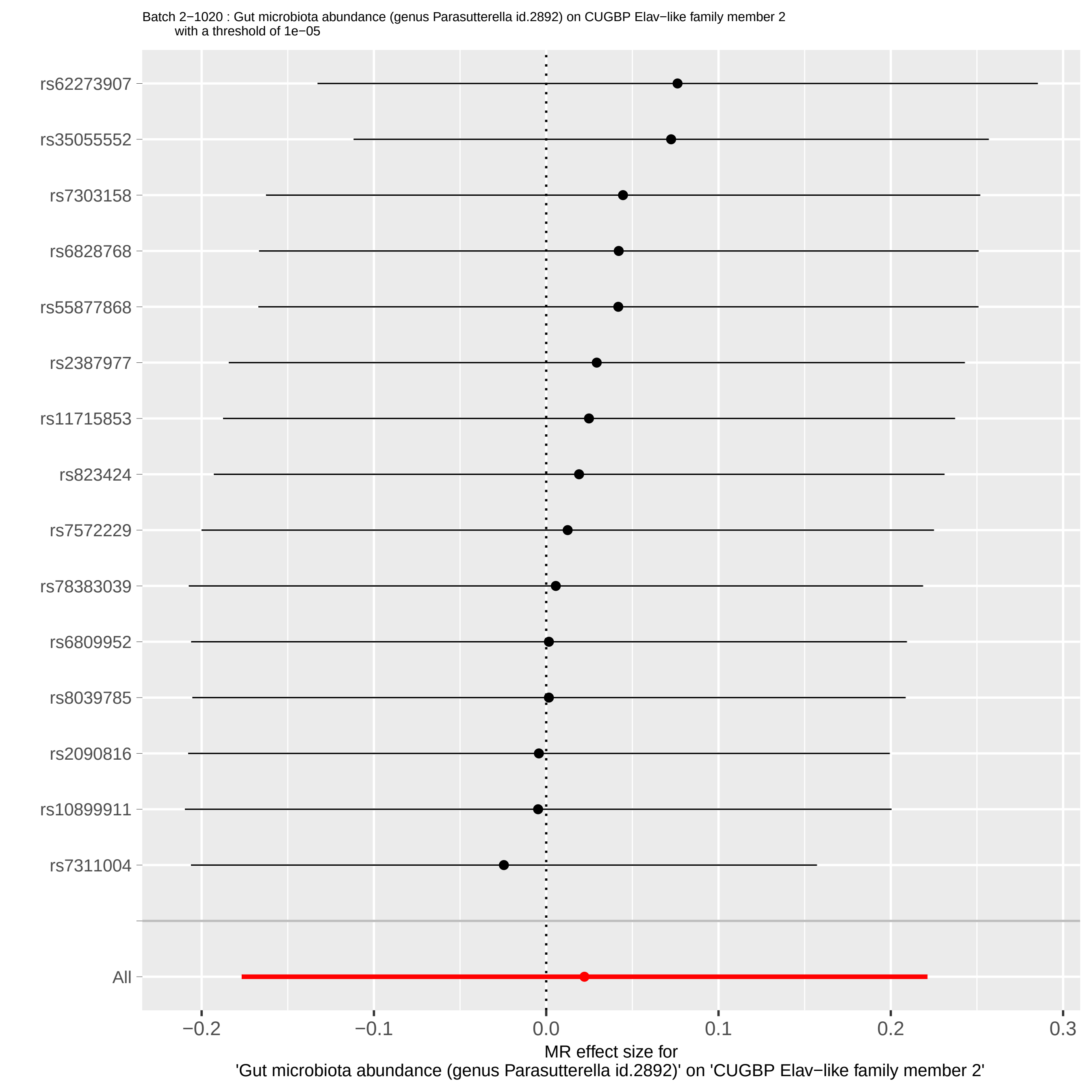
Batch 2−1020 : Gut microbiota abundance (genus Parasutterella id.2892) on CUGBP Elav−like family member 2
with a threshold of 1e−05
rs62273907
rs35055552
rs7303158
rs6828768
rs55877868
rs2387977
rs11715853
rs823424
rs7572229
rs78383039
rs6809952
rs8039785
rs2090816
rs10899911
rs7311004
All
−0.2
−0.1
0.0
0.1
0.2
0.3
MR effect size for
'Gut microbiota abundance (genus Parasutterella id.2892)' on 'CUGBP Elav−like family member 2'

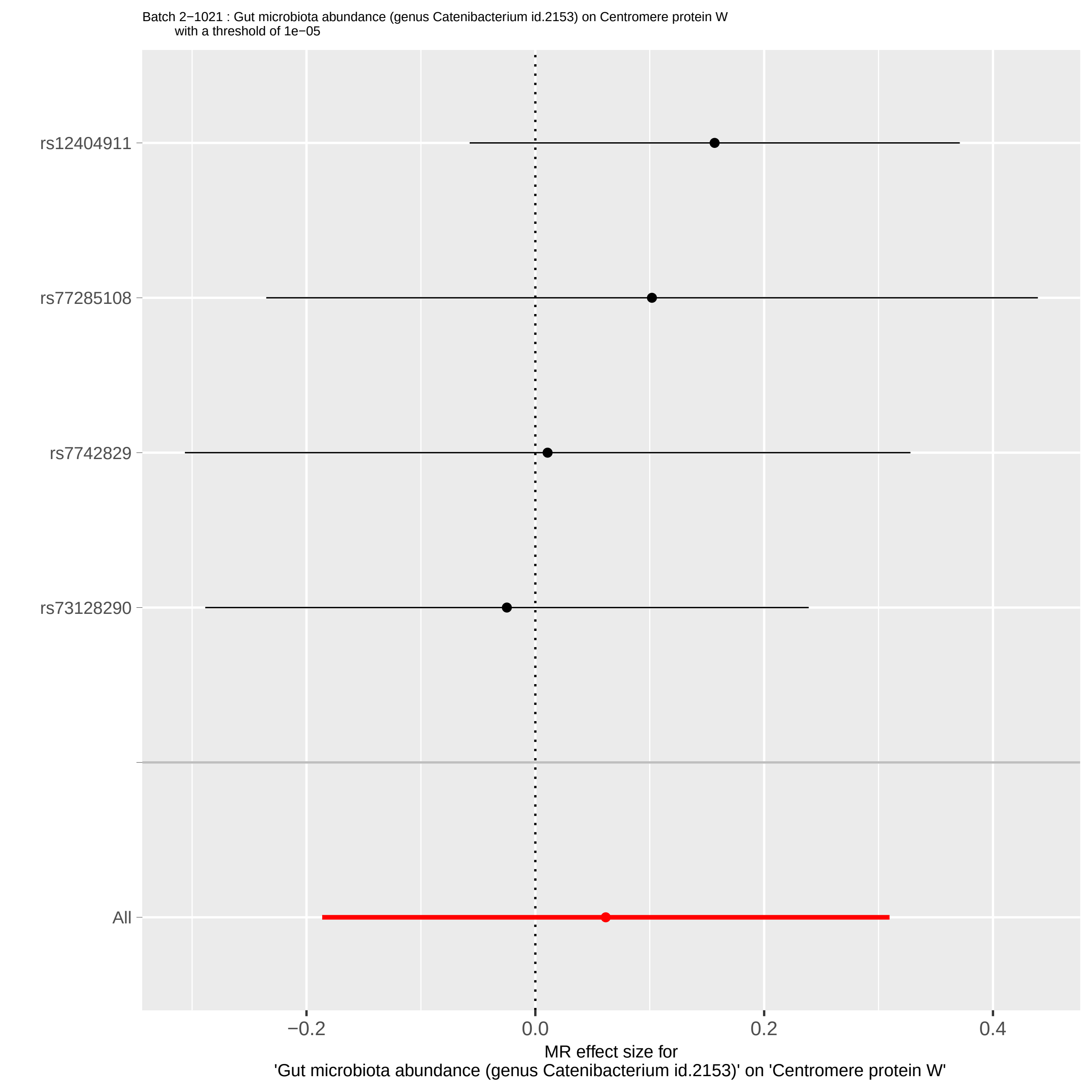
Batch 2−1021 : Gut microbiota abundance (genus Catenibacterium id.2153) on Centromere protein W
with a threshold of 1e−05
rs12404911
rs77285108
rs7742829
rs73128290
All
−0.2
0.0
0.2
0.4
MR effect size for
'Gut microbiota abundance (genus Catenibacterium id.2153)' on 'Centromere protein W'

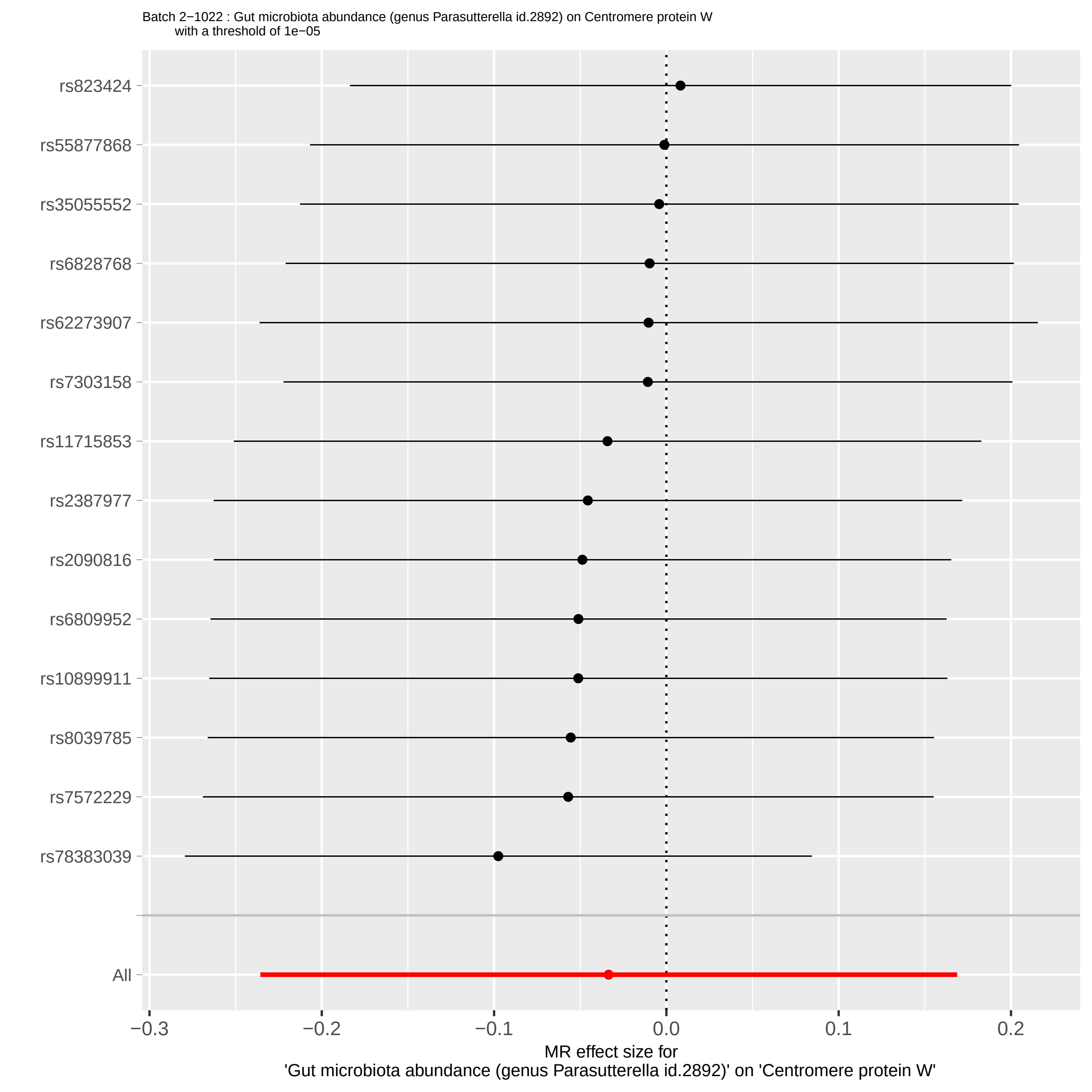

Batch 2−1022 : Gut microbiota abundance (genus Parasutterella id.2892) on Centromere protein W
with a threshold of 1e−05
rs823424
rs55877868
rs35055552
rs6828768
rs62273907
rs7303158
rs11715853
rs2387977
rs2090816
rs6809952
rs10899911
rs8039785
rs7572229
rs78383039
All
−0.3
−0.2
−0.1
0.0
0.1
0.2
MR effect size for
'Gut microbiota abundance (genus Parasutterella id.2892)' on 'Centromere protein W'

Batch 2−1023 : Gut microbiota abundance (genus Catenibacterium id.2153) on Centrosomal protein of 57 kDa
with a threshold of 1e−05

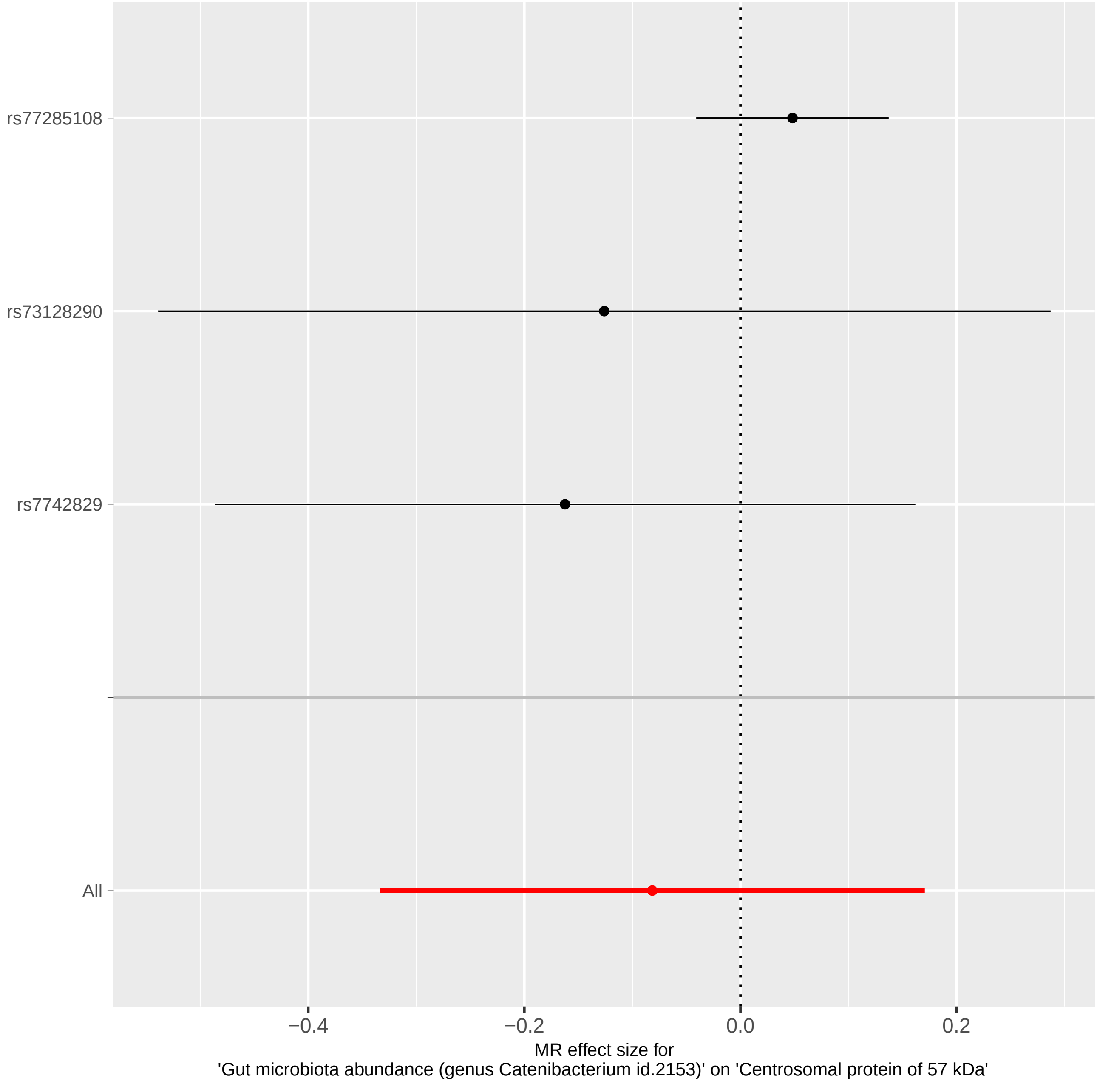

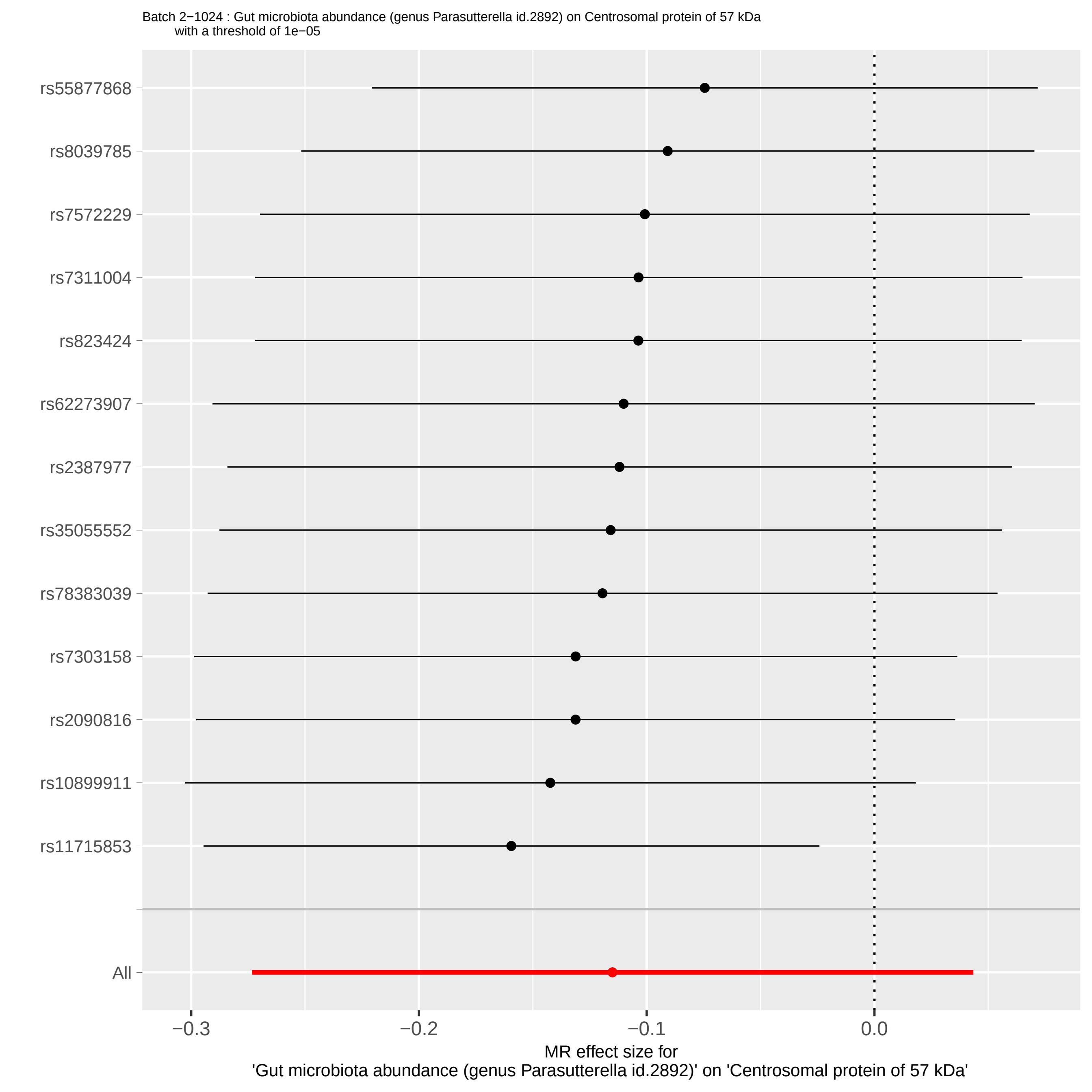

Batch 2−1024 : Gut microbiota abundance (genus Parasutterella id.2892) on Centrosomal protein of 57 kDa
with a threshold of 1e−05
rs55877868
rs8039785
rs7572229
rs7311004
rs823424
rs62273907
rs2387977
rs35055552
rs78383039
rs7303158
rs2090816
rs10899911
rs11715853
All
−0.3
−0.2
−0.1
0.0
MR effect size for
'Gut microbiota abundance (genus Parasutterella id.2892)' on 'Centrosomal protein of 57 kDa'

Batch 2−1025 : Gut microbiota abundance (genus Catenibacterium id.2153) on Centrosomal protein of 57 kDa
with a threshold of 1e−05

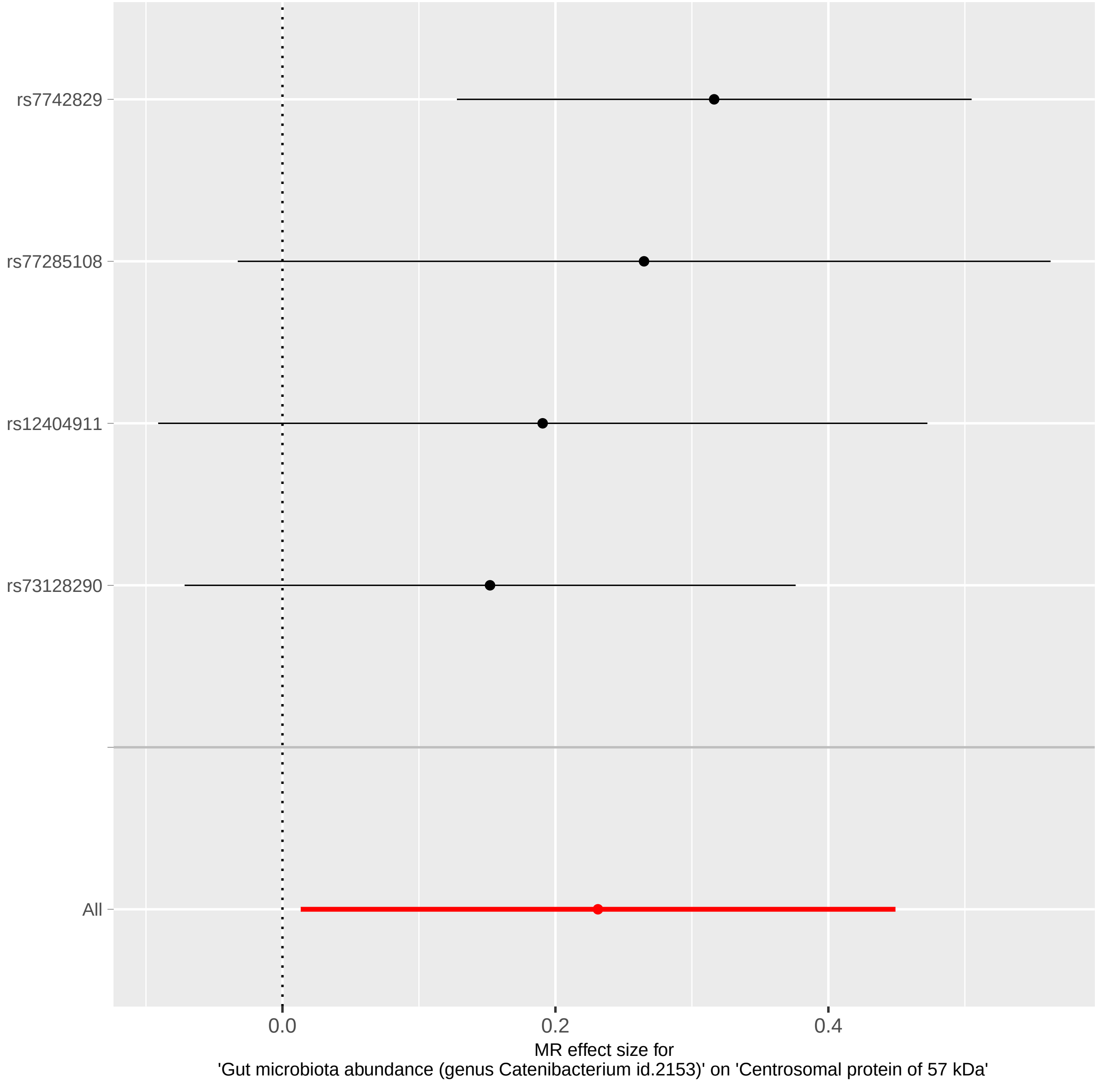

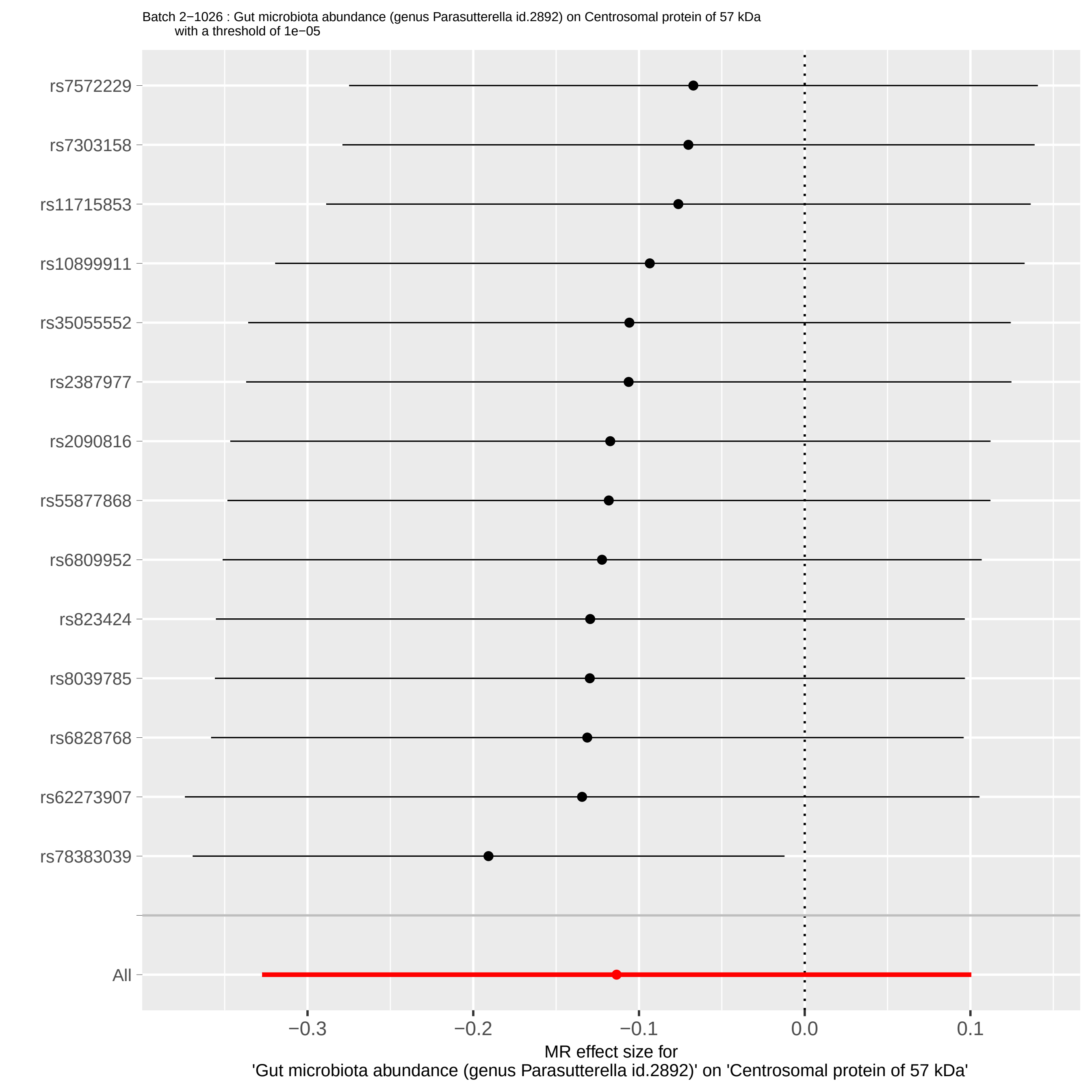

Batch 2−1026 : Gut microbiota abundance (genus Parasutterella id.2892) on Centrosomal protein of 57 kDa
with a threshold of 1e−05
rs7572229
rs7303158
rs11715853
rs10899911
rs35055552
rs2387977
rs2090816
rs55877868
rs6809952
rs823424
rs8039785
rs6828768
rs62273907
rs78383039
All
−0.3
−0.2
−0.1
0.0
0.1
MR effect size for
'Gut microbiota abundance (genus Parasutterella id.2892)' on 'Centrosomal protein of 57 kDa'

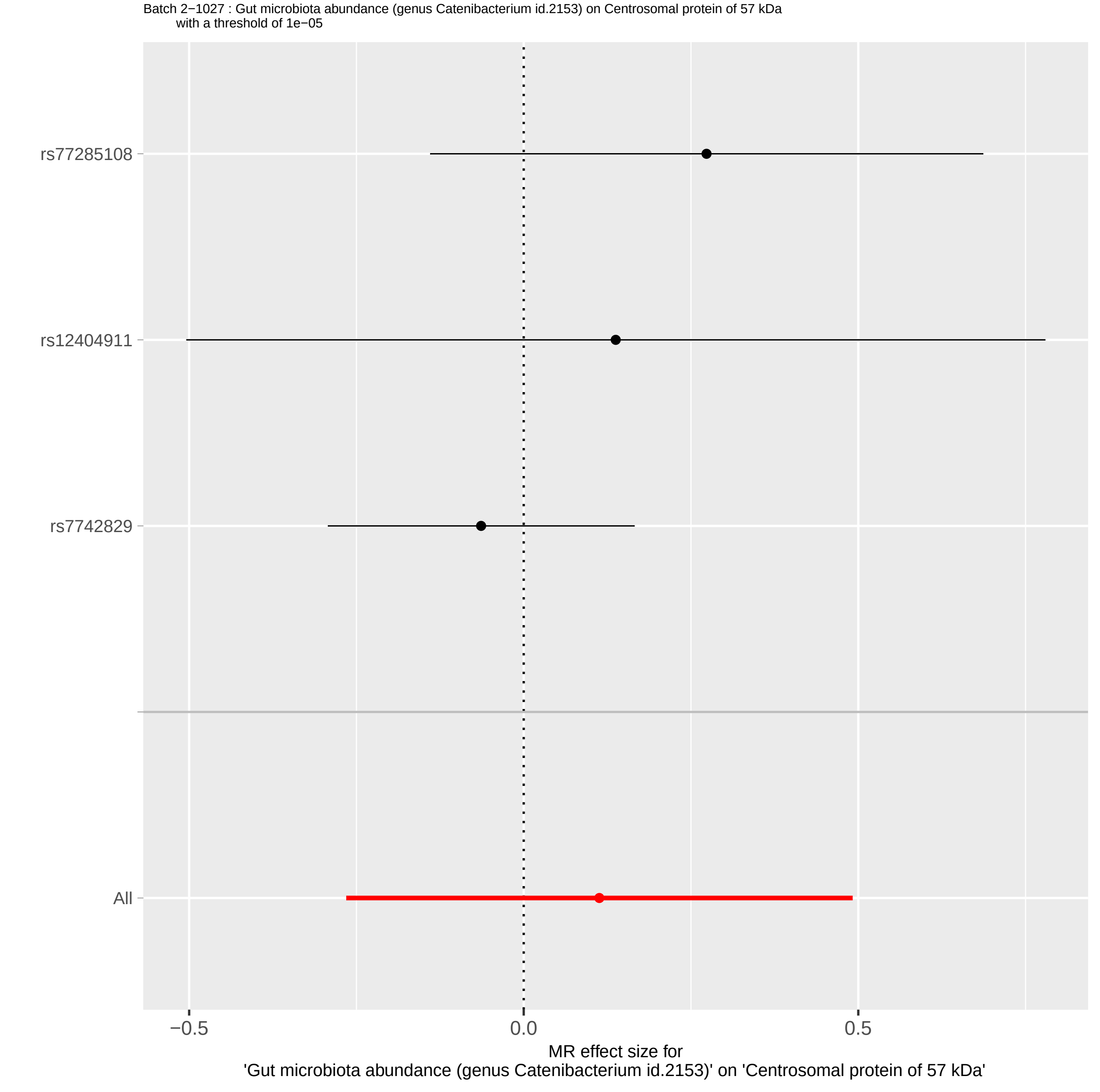

Batch 2−1027 : Gut microbiota abundance (genus Catenibacterium id.2153) on Centrosomal protein of 57 kDa
with a threshold of 1e−05
rs77285108
rs12404911
rs7742829
All
−0.5
0.0
0.5
MR effect size for
'Gut microbiota abundance (genus Catenibacterium id.2153)' on 'Centrosomal protein of 57 kDa'

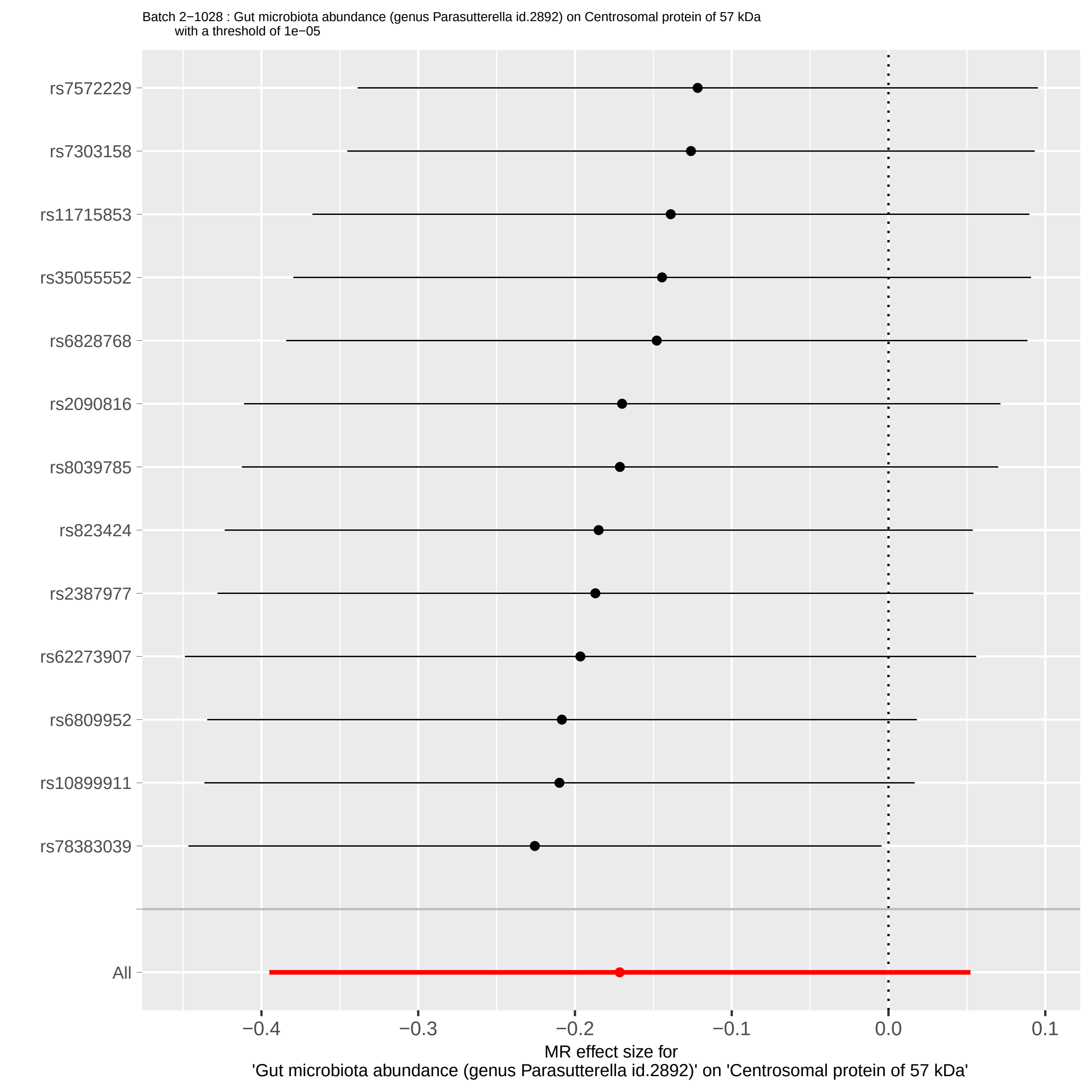

Batch 2−1028 : Gut microbiota abundance (genus Parasutterella id.2892) on Centrosomal protein of 57 kDa
with a threshold of 1e−05
rs7572229
rs7303158
rs11715853
rs35055552
rs6828768
rs2090816
rs8039785
rs823424
rs2387977
rs62273907
rs6809952
rs10899911
rs78383039
All
−0.4
−0.3
−0.2
−0.1
0.0
0.1
MR effect size for
'Gut microbiota abundance (genus Parasutterella id.2892)' on 'Centrosomal protein of 57 kDa'

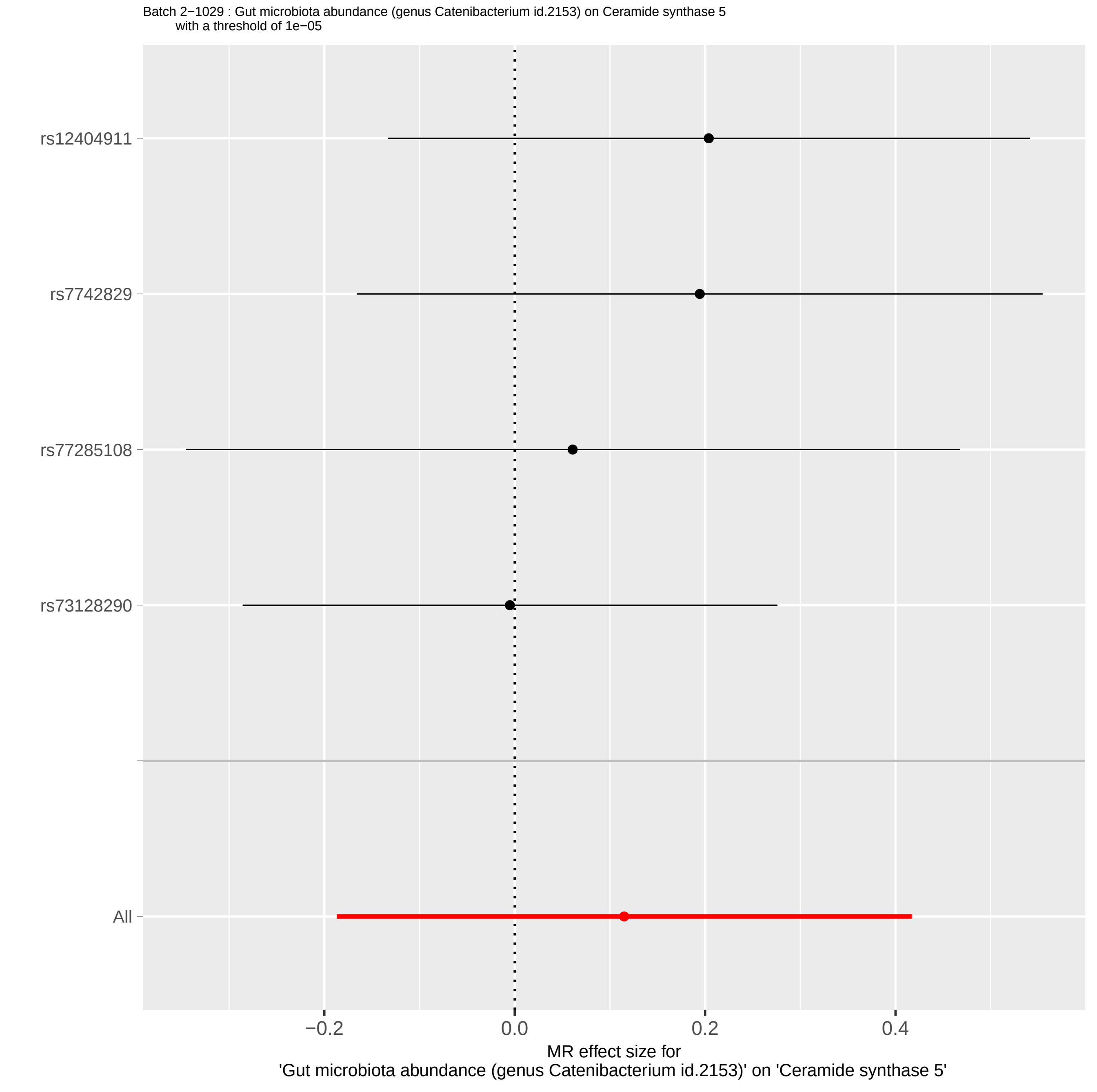
Batch 2−1029 : Gut microbiota abundance (genus Catenibacterium id.2153) on Ceramide synthase 5
with a threshold of 1e−05
rs12404911
rs7742829
rs77285108
rs73128290
All
−0.2
0.0
0.2
0.4
MR effect size for
'Gut microbiota abundance (genus Catenibacterium id.2153)' on 'Ceramide synthase 5'

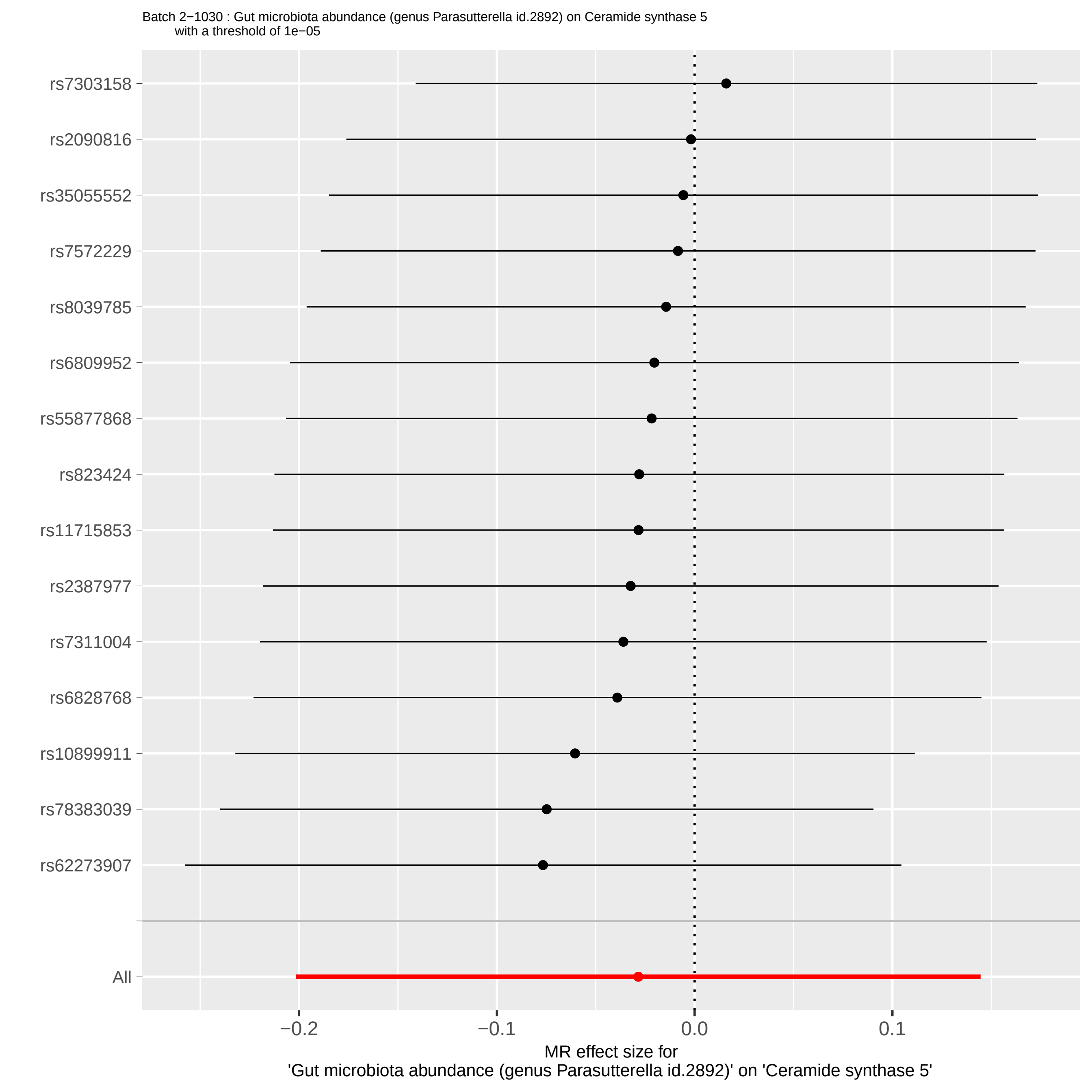
Batch 2−1030 : Gut microbiota abundance (genus Parasutterella id.2892) on Ceramide synthase 5
with a threshold of 1e−05
rs7303158
rs2090816
rs35055552
rs7572229
rs8039785
rs6809952
rs55877868
rs823424
rs11715853
rs2387977
rs7311004
rs6828768
rs10899911
rs78383039
rs62273907
All
−0.2
−0.1
0.0
0.1
MR effect size for
'Gut microbiota abundance (genus Parasutterella id.2892)' on 'Ceramide synthase 5'

Batch 2−1031 : Gut microbiota abundance (genus Catenibacterium id.2153) on Centrin−2
with a threshold of 1e−05

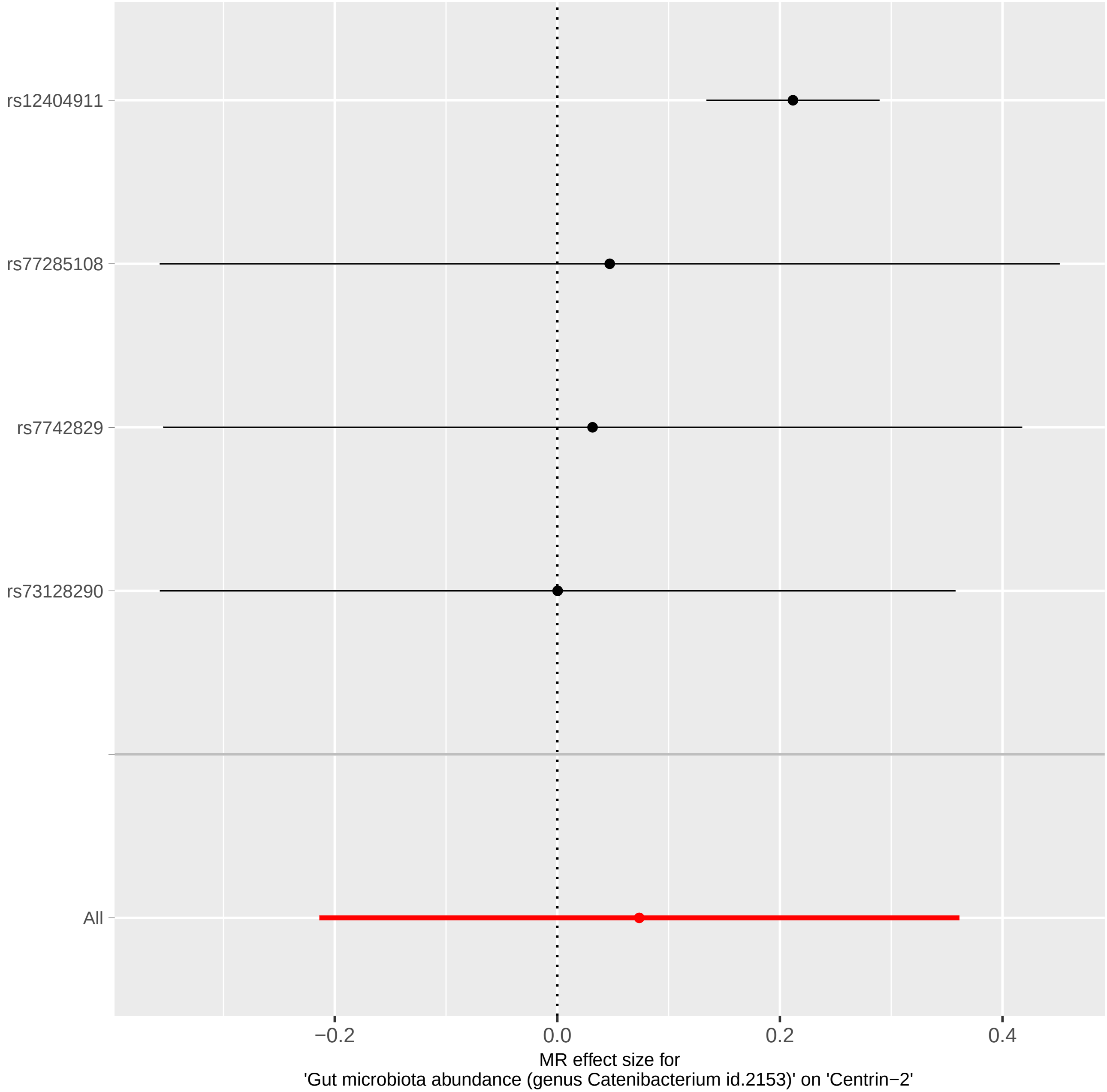

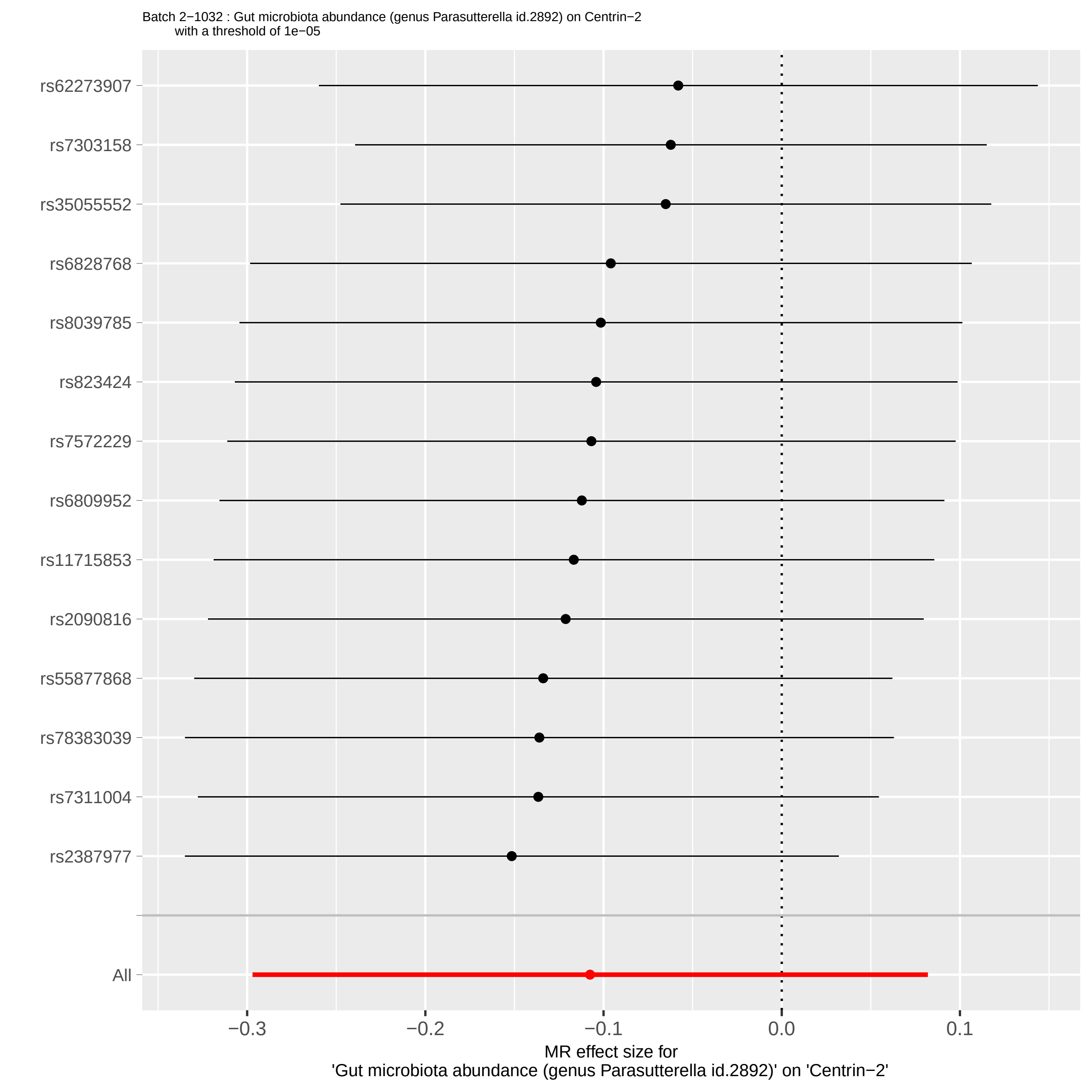

Batch 2−1032 : Gut microbiota abundance (genus Parasutterella id.2892) on Centrin−2
with a threshold of 1e−05
rs62273907
rs7303158
rs35055552
rs6828768
rs8039785
rs823424
rs7572229
rs6809952
rs11715853
rs2090816
rs55877868
rs78383039
rs7311004
rs2387977
All
−0.3
−0.2
−0.1
0.0
0.1
MR effect size for
'Gut microbiota abundance (genus Parasutterella id.2892)' on 'Centrin−2'

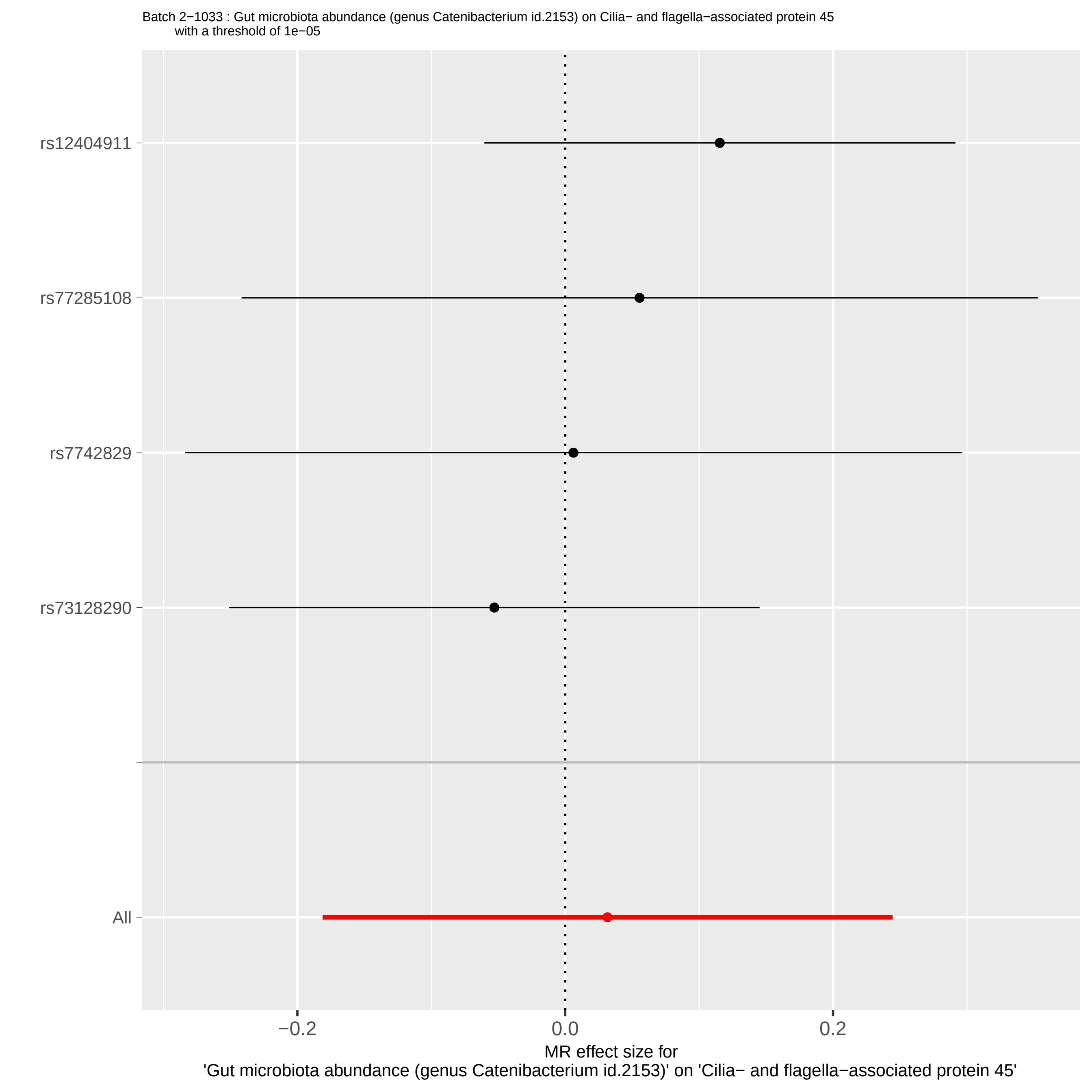

Batch 2−1033 : Gut microbiota abundance (genus Catenibacterium id.2153) on Cilia− and flagella−associated protein 45
with a threshold of 1e−05
rs12404911
rs77285108
rs7742829
rs73128290
All
−0.2
0.0
0.2
MR effect size for
'Gut microbiota abundance (genus Catenibacterium id.2153)' on 'Cilia− and flagella−associated protein 45'

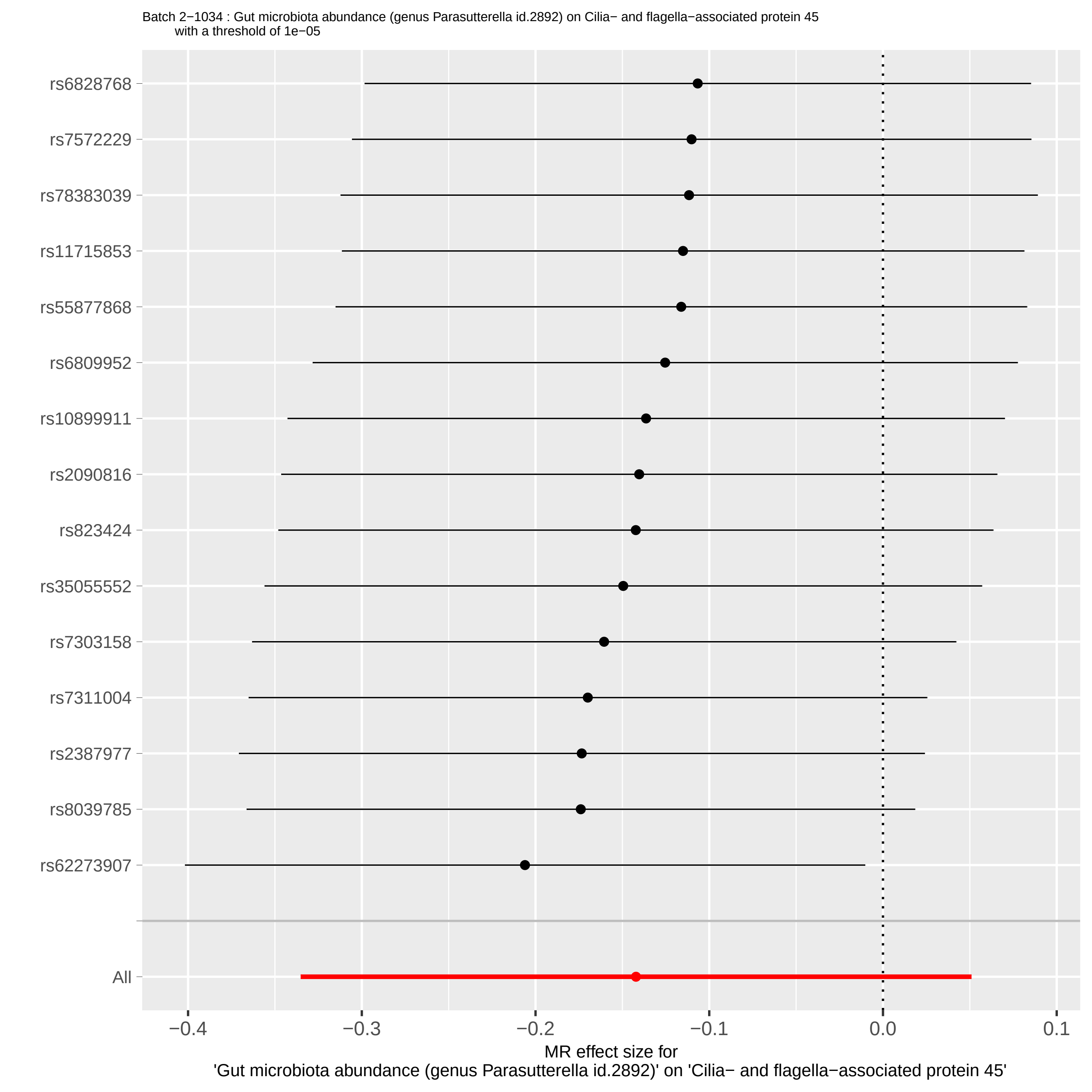
Batch 2−1034 : Gut microbiota abundance (genus Parasutterella id.2892) on Cilia− and flagella−associated protein 45
with a threshold of 1e−05
rs6828768
rs7572229
rs78383039
rs11715853
rs55877868
rs6809952
rs10899911
rs2090816
rs823424
rs35055552
rs7303158
rs7311004
rs2387977
rs8039785
rs62273907
All
−0.4
−0.3
−0.2
−0.1
0.0
0.1
MR effect size for
'Gut microbiota abundance (genus Parasutterella id.2892)' on 'Cilia− and flagella−associated protein 45'

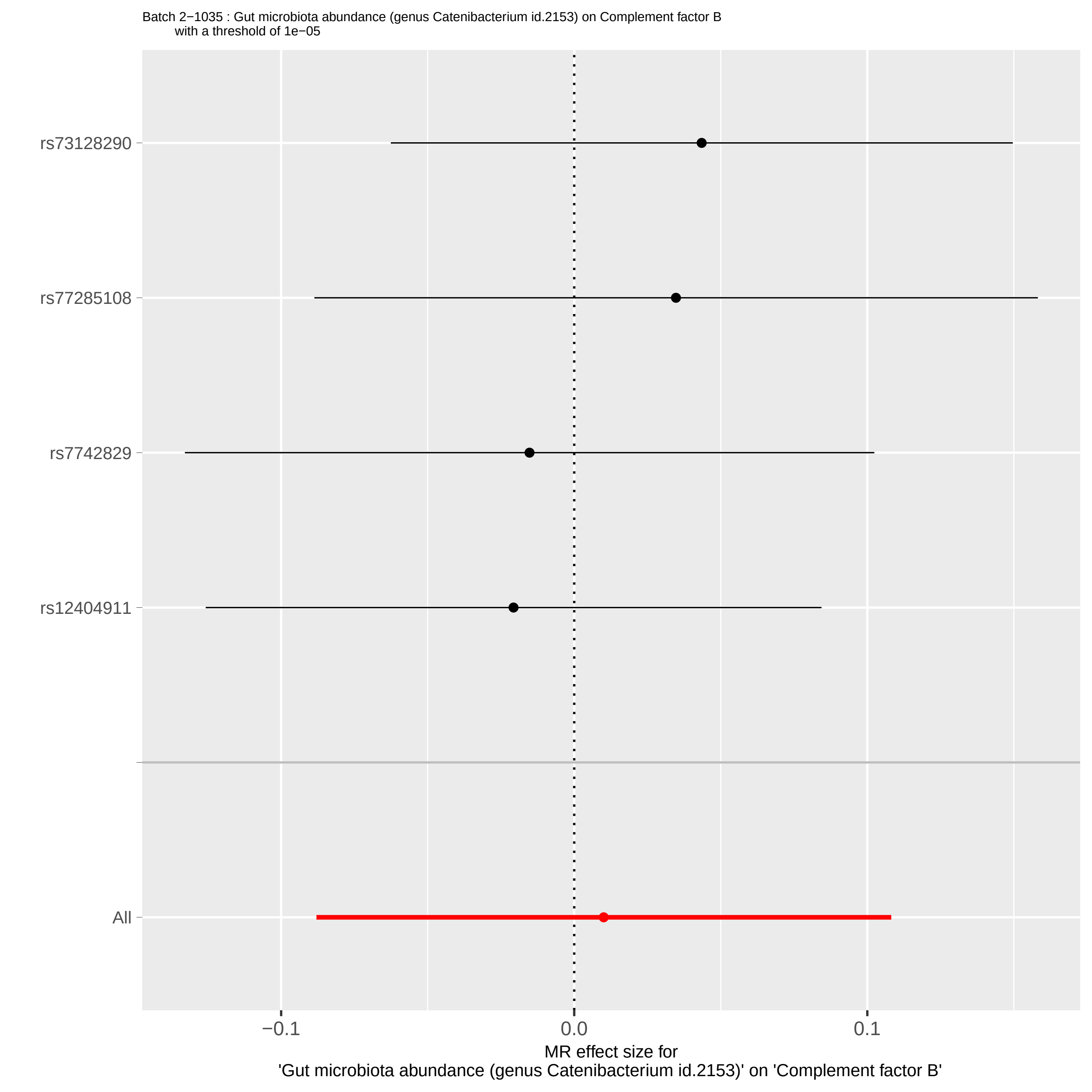

Batch 2−1035 : Gut microbiota abundance (genus Catenibacterium id.2153) on Complement factor B
with a threshold of 1e−05
rs73128290
rs77285108
rs7742829
rs12404911
All
−0.1
0.0
0.1
MR effect size for
'Gut microbiota abundance (genus Catenibacterium id.2153)' on 'Complement factor B'

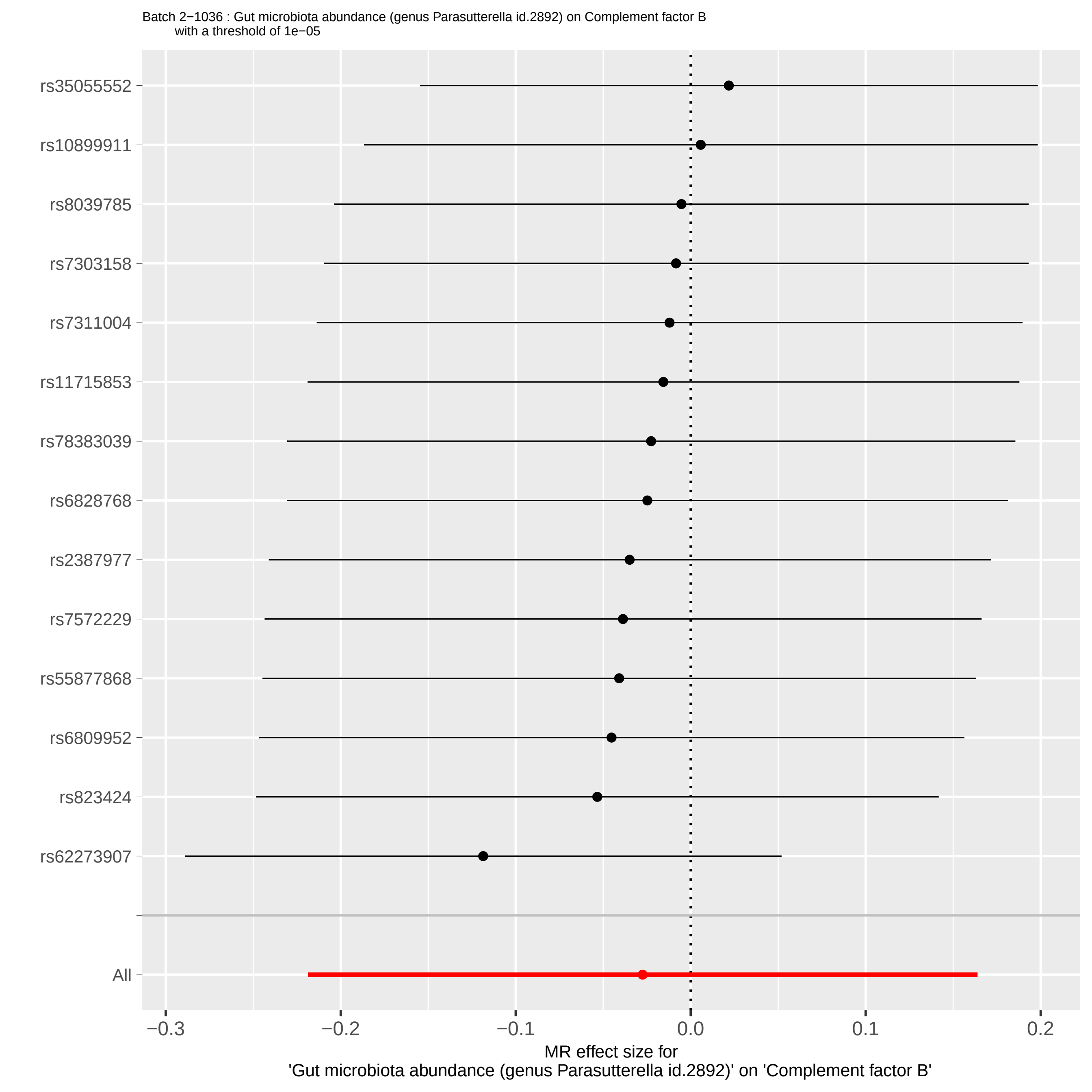
Batch 2−1036 : Gut microbiota abundance (genus Parasutterella id.2892) on Complement factor B
with a threshold of 1e−05
rs35055552
rs10899911
rs8039785
rs7303158
rs7311004
rs11715853
rs78383039
rs6828768
rs2387977
rs7572229
rs55877868
rs6809952
rs823424
rs62273907
All
−0.3
−0.2
−0.1
0.0
0.1
0.2
MR effect size for
'Gut microbiota abundance (genus Parasutterella id.2892)' on 'Complement factor B'

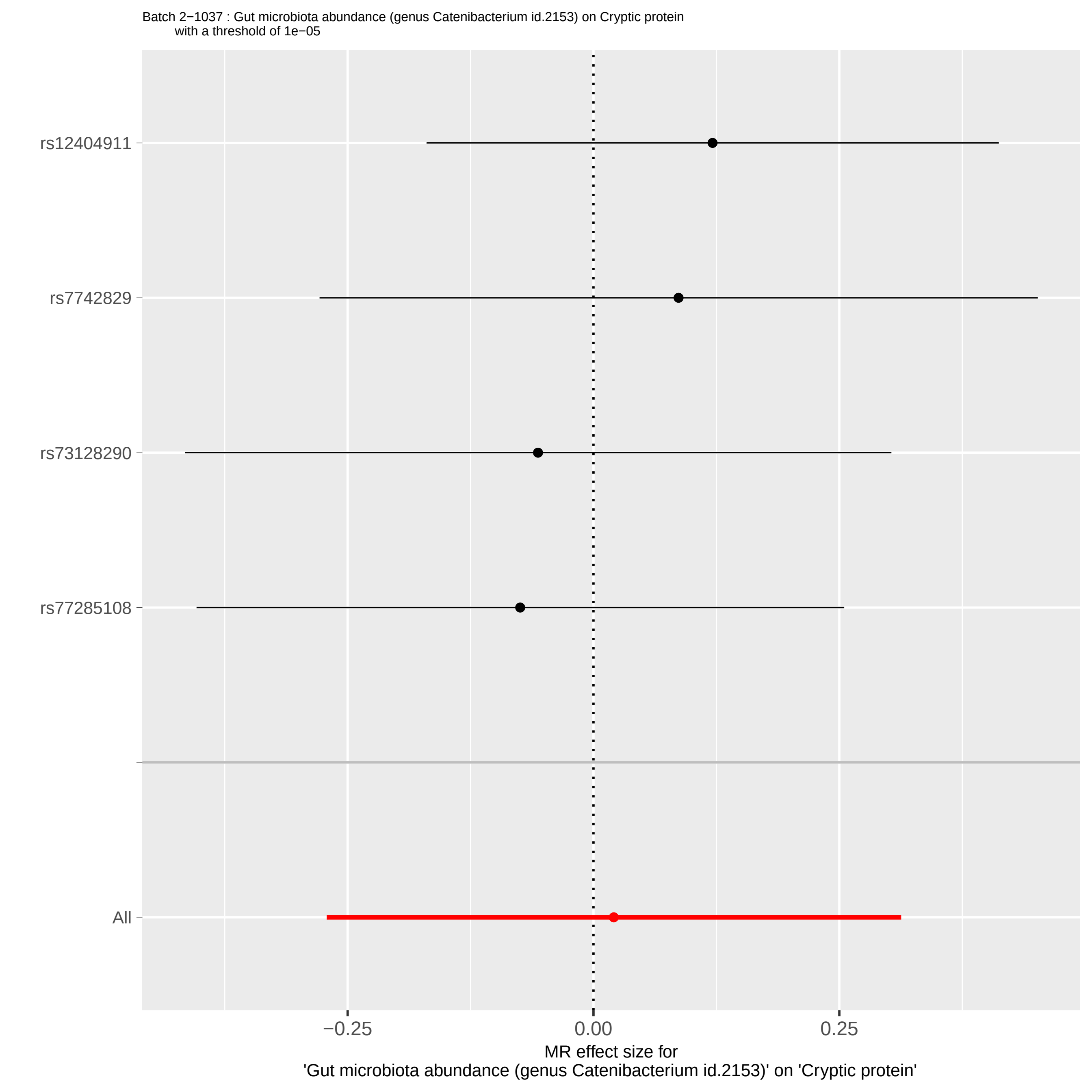
Batch 2−1037 : Gut microbiota abundance (genus Catenibacterium id.2153) on Cryptic protein
with a threshold of 1e−05
rs12404911
rs7742829
rs73128290
rs77285108
All
−0.25
0.00
0.25
MR effect size for
'Gut microbiota abundance (genus Catenibacterium id.2153)' on 'Cryptic protein'

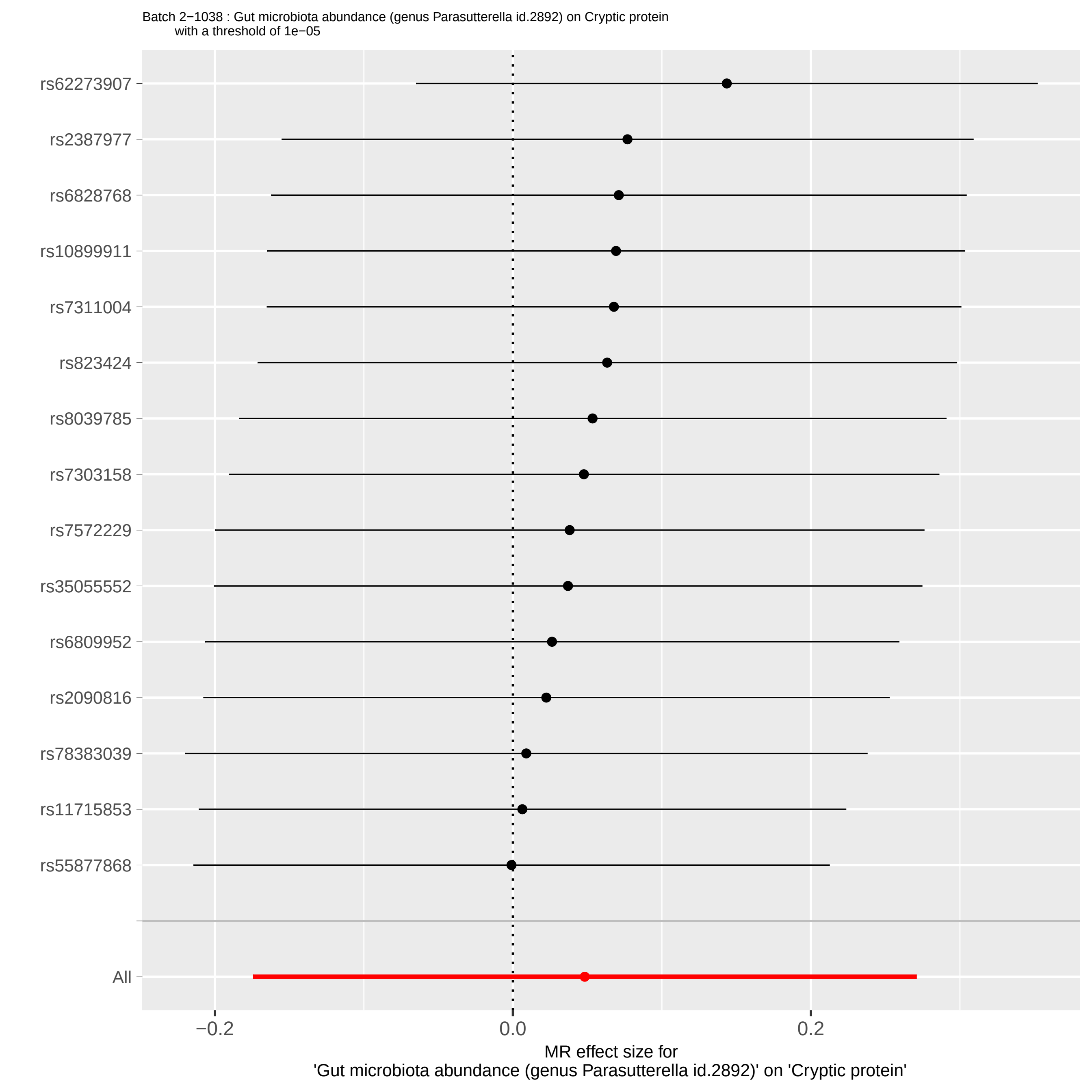

Batch 2−1038 : Gut microbiota abundance (genus Parasutterella id.2892) on Cryptic protein
with a threshold of 1e−05
rs62273907
rs2387977
rs6828768
rs10899911
rs7311004
rs823424
rs8039785
rs7303158
rs7572229
rs35055552
rs6809952
rs2090816
rs78383039
rs11715853
rs55877868
All
−0.2
0.0
0.2
MR effect size for
'Gut microbiota abundance (genus Parasutterella id.2892)' on 'Cryptic protein'

Batch 2−1039 : Gut microbiota abundance (genus Catenibacterium id.2153) on Complement factor H
with a threshold of 1e−05

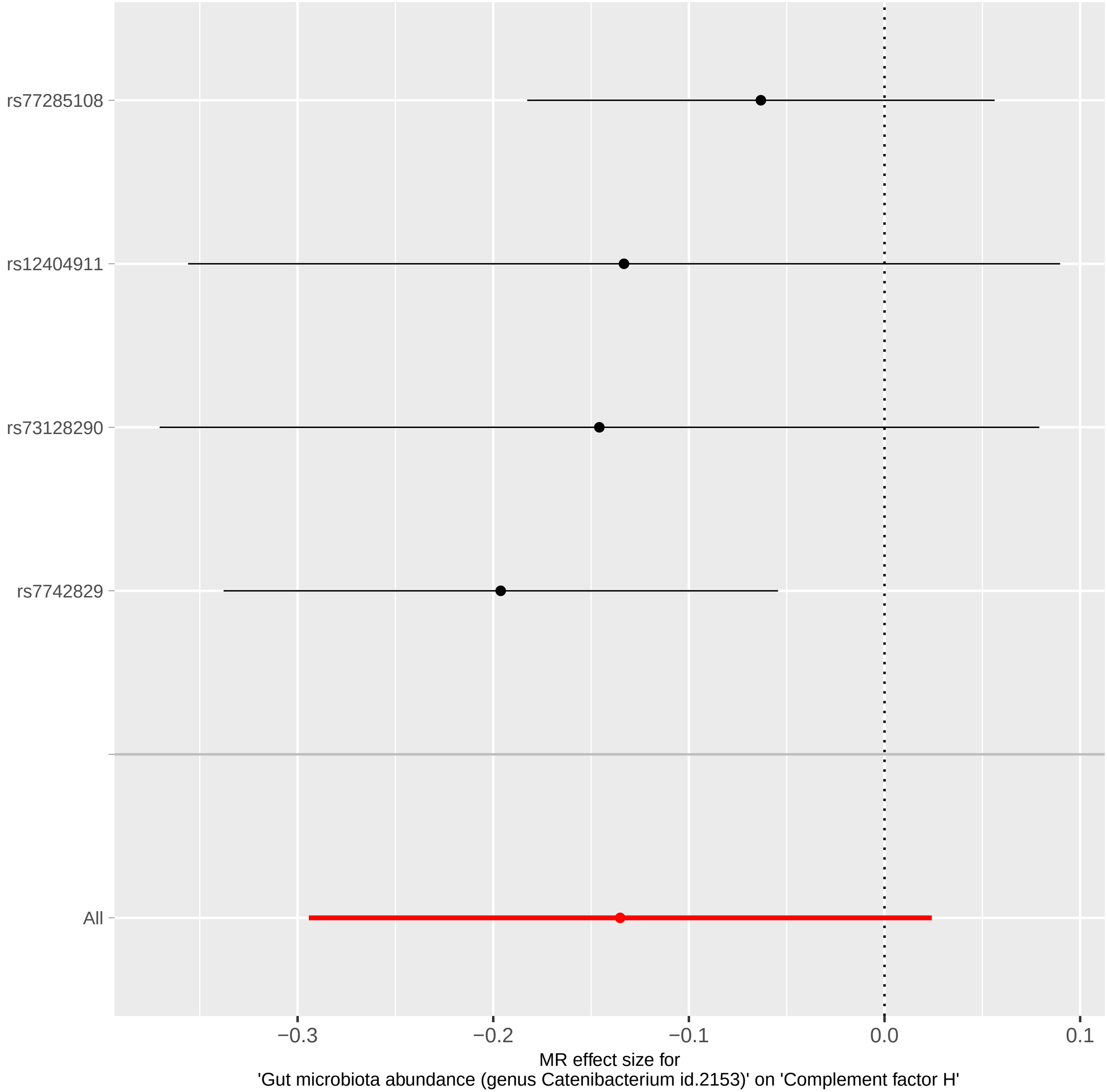

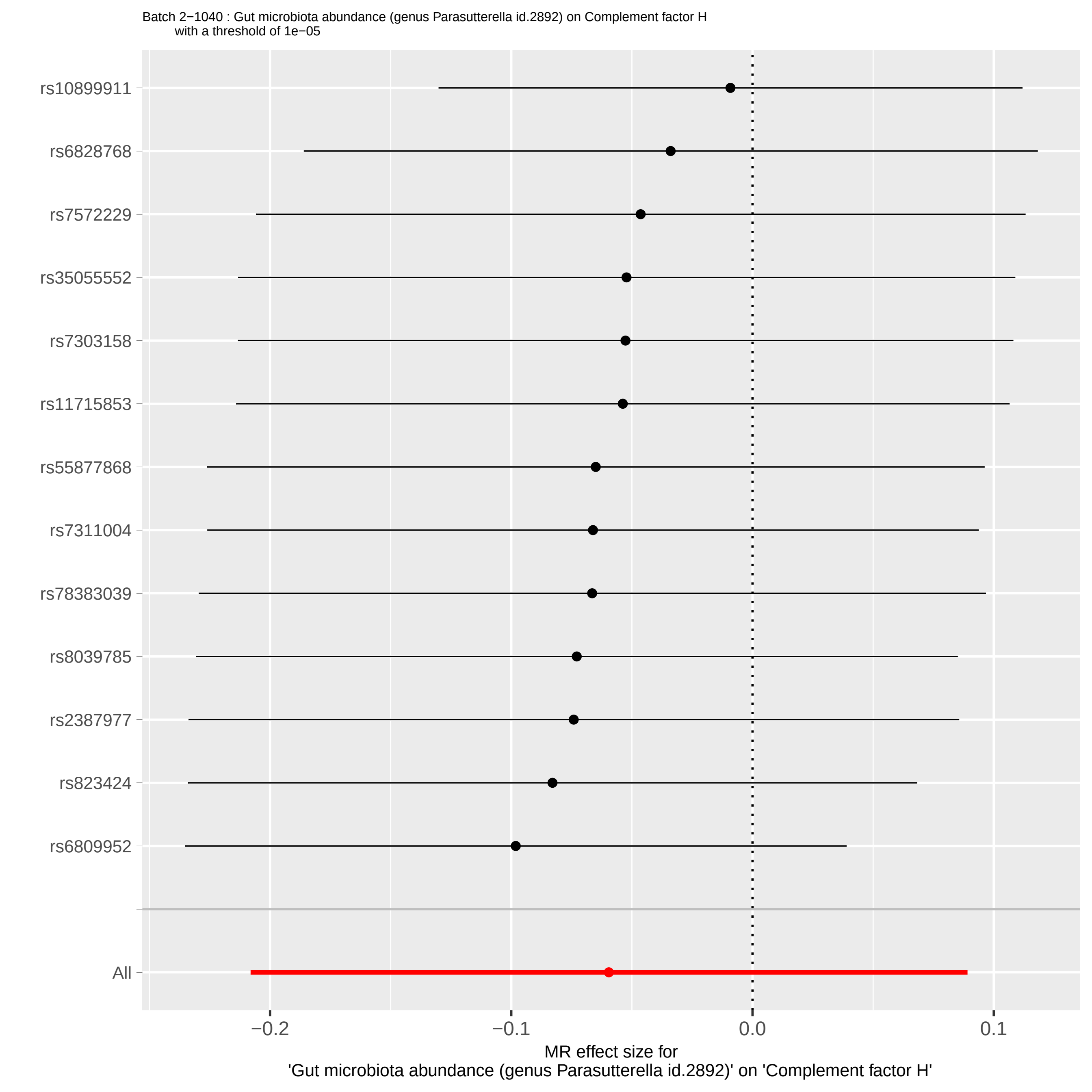
Batch 2−1040 : Gut microbiota abundance (genus Parasutterella id.2892) on Complement factor H
with a threshold of 1e−05
rs10899911
rs6828768
rs7572229
rs35055552
rs7303158
rs11715853
rs55877868
rs7311004
rs78383039
rs8039785
rs2387977
rs823424
rs6809952
All
−0.2
−0.1
0.0
0.1
MR effect size for
'Gut microbiota abundance (genus Parasutterella id.2892)' on 'Complement factor H'

Batch 2−1041 : Gut microbiota abundance (genus Catenibacterium id.2153) on Complement factor H−related protein 5
with a threshold of 1e−05

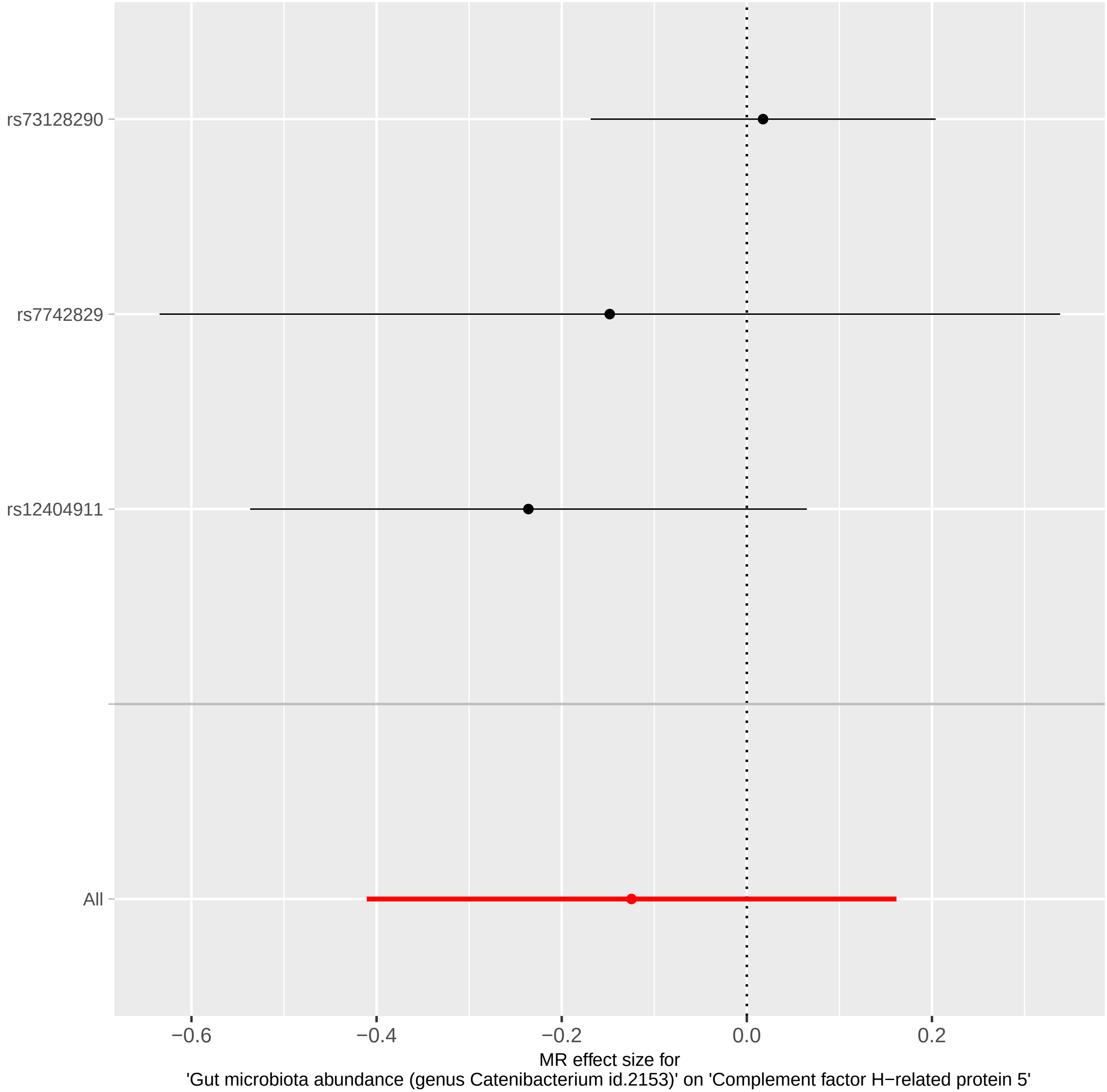

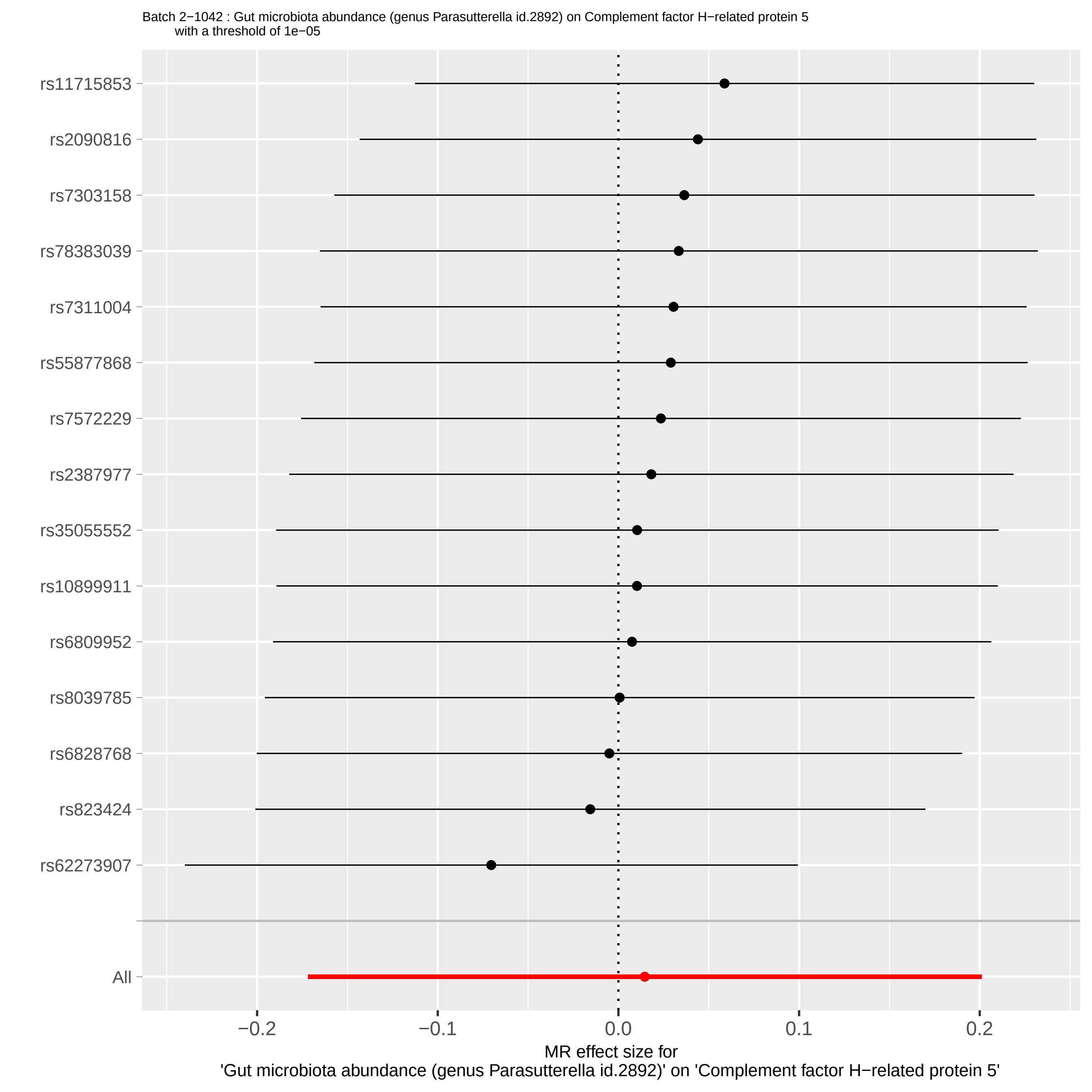
Batch 2−1042 : Gut microbiota abundance (genus Parasutterella id.2892) on Complement factor H−related protein 5
with a threshold of 1e−05
rs11715853
rs2090816
rs7303158
rs78383039
rs7311004
rs55877868
rs7572229
rs2387977
rs35055552
rs10899911
rs6809952
rs8039785
rs6828768
rs823424
rs62273907
All
−0.2
−0.1
0.0
0.1
0.2
MR effect size for
'Gut microbiota abundance (genus Parasutterella id.2892)' on 'Complement factor H−related protein 5'

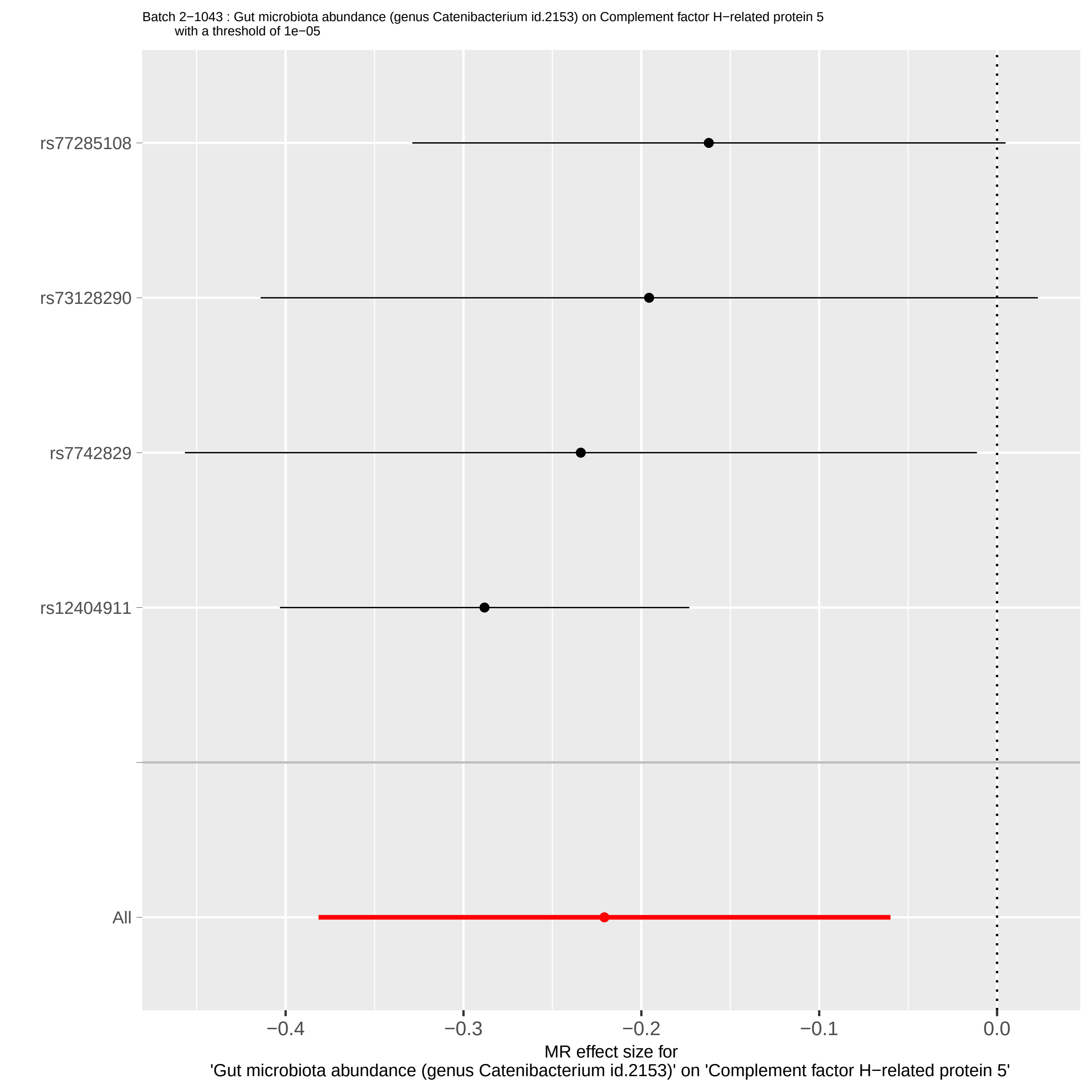

Batch 2−1043 : Gut microbiota abundance (genus Catenibacterium id.2153) on Complement factor H−related protein 5
with a threshold of 1e−05
rs77285108
rs73128290
rs7742829
rs12404911
All
−0.4
−0.3
−0.2
−0.1
0.0
MR effect size for
'Gut microbiota abundance (genus Catenibacterium id.2153)' on 'Complement factor H−related protein 5'

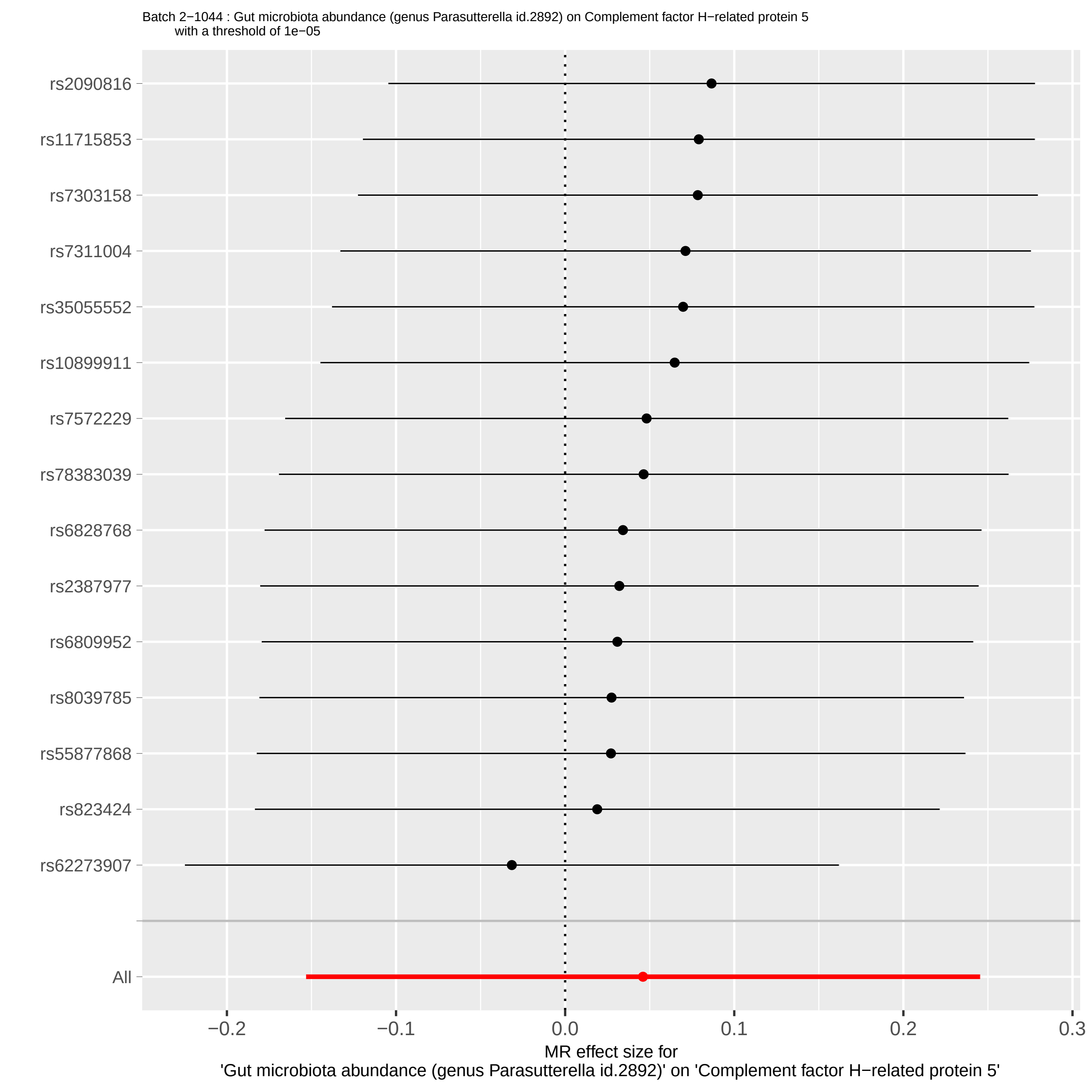

Batch 2−1044 : Gut microbiota abundance (genus Parasutterella id.2892) on Complement factor H−related protein 5
with a threshold of 1e−05
rs2090816
rs11715853
rs7303158
rs7311004
rs35055552
rs10899911
rs7572229
rs78383039
rs6828768
rs2387977
rs6809952
rs8039785
rs55877868
rs823424
rs62273907
All
−0.2
−0.1
0.0
0.1
0.2
0.3
MR effect size for
'Gut microbiota abundance (genus Parasutterella id.2892)' on 'Complement factor H−related protein 5'

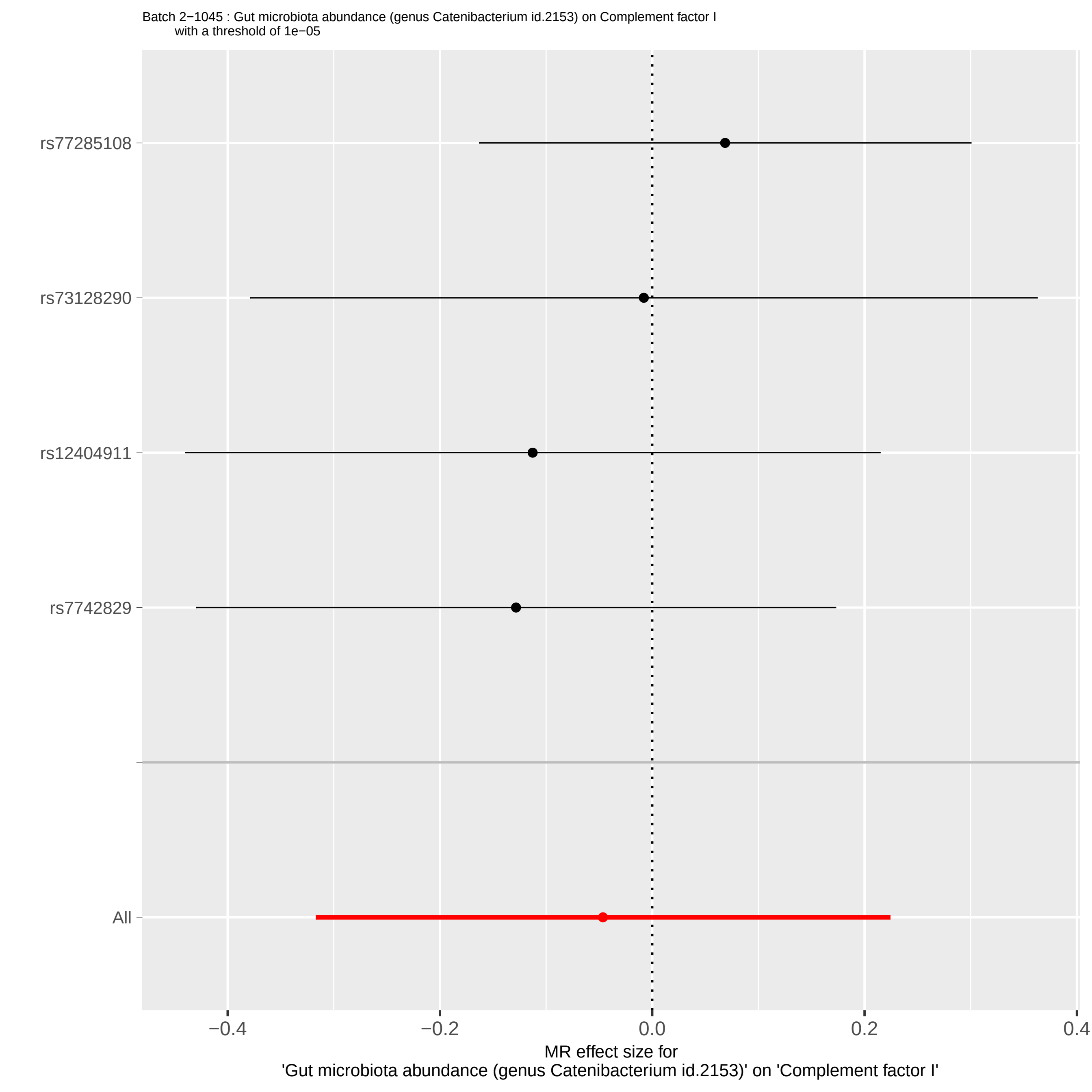

Batch 2−1045 : Gut microbiota abundance (genus Catenibacterium id.2153) on Complement factor I
with a threshold of 1e−05
rs77285108
rs73128290
rs12404911
rs7742829
All
−0.4
−0.2
0.0
0.2
0.4
MR effect size for
'Gut microbiota abundance (genus Catenibacterium id.2153)' on 'Complement factor I'

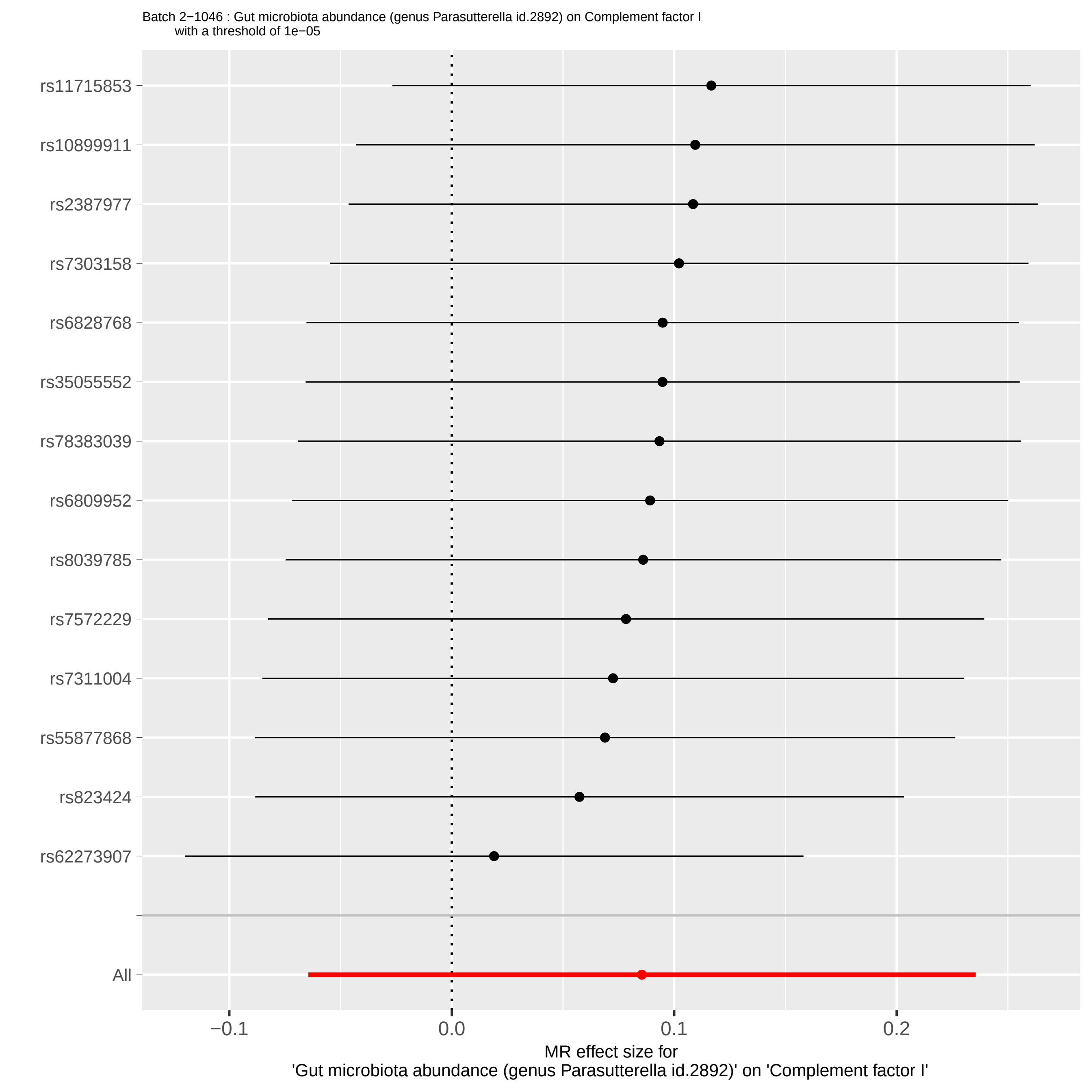

Batch 2−1046 : Gut microbiota abundance (genus Parasutterella id.2892) on Complement factor I
with a threshold of 1e−05
rs11715853
rs10899911
rs2387977
rs7303158
rs6828768
rs35055552
rs78383039
rs6809952
rs8039785
rs7572229
rs7311004
rs55877868
rs823424
rs62273907
All
−0.1
0.0
0.1
0.2
MR effect size for
'Gut microbiota abundance (genus Parasutterella id.2892)' on 'Complement factor I'

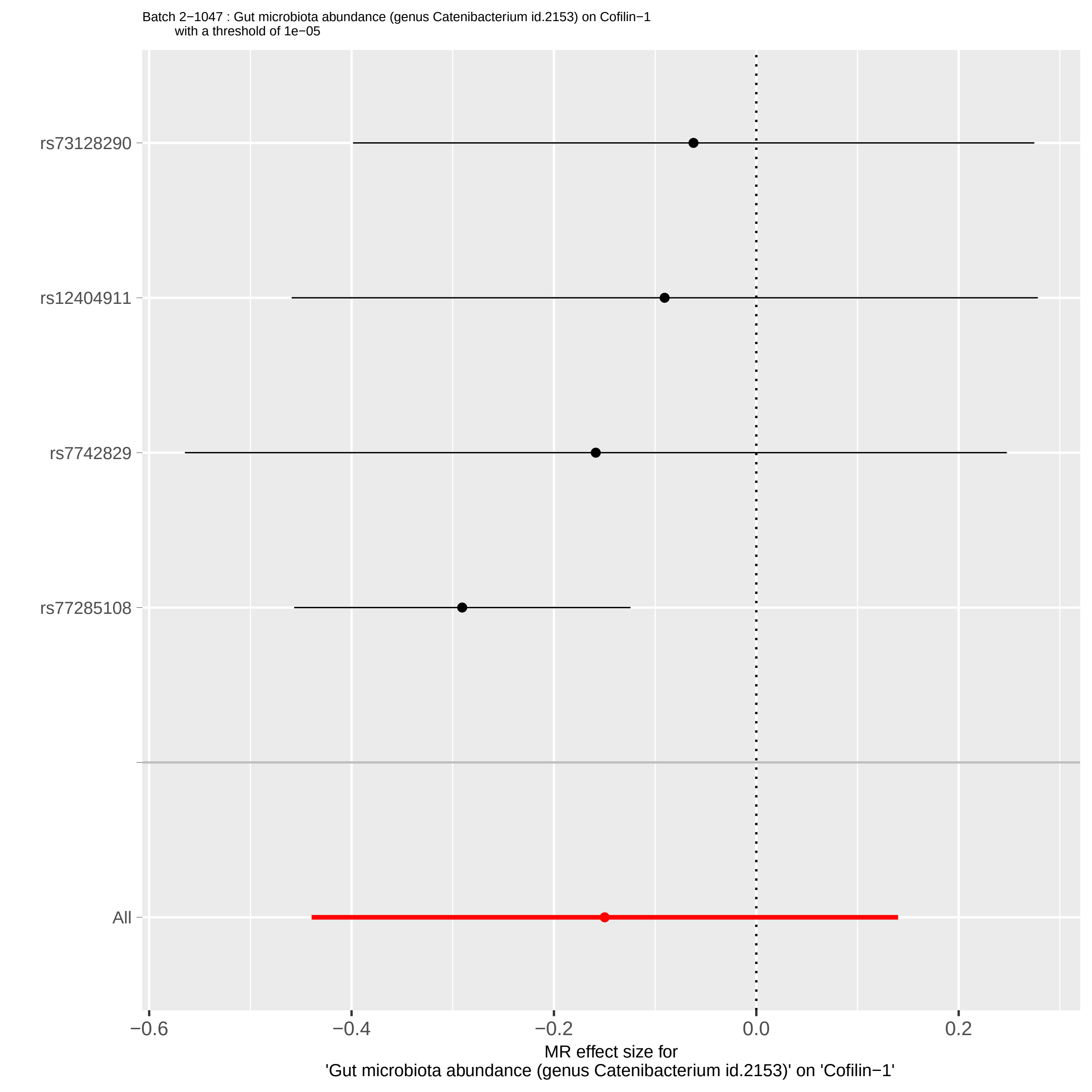
Batch 2−1047 : Gut microbiota abundance (genus Catenibacterium id.2153) on Cofilin−1
with a threshold of 1e−05
rs73128290
rs12404911
rs7742829
rs77285108
All
−0.6
−0.4
−0.2
0.0
0.2
MR effect size for
'Gut microbiota abundance (genus Catenibacterium id.2153)' on 'Cofilin−1'

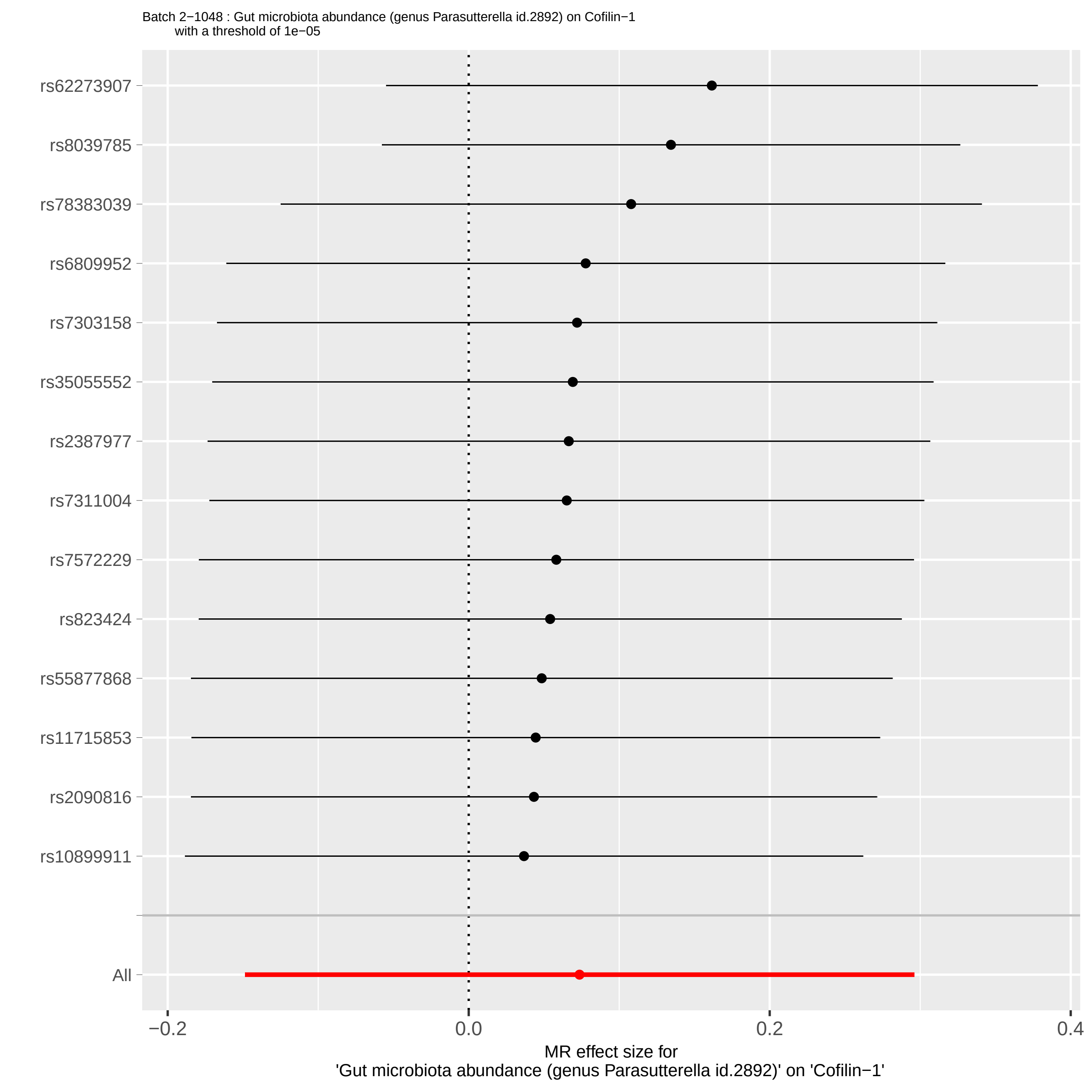
Batch 2−1048 : Gut microbiota abundance (genus Parasutterella id.2892) on Cofilin−1
with a threshold of 1e−05
rs62273907
rs8039785
rs78383039
rs6809952
rs7303158
rs35055552
rs2387977
rs7311004
rs7572229
rs823424
rs55877868
rs11715853
rs2090816
rs10899911
All
−0.2
0.0
0.2
0.4
MR effect size for
'Gut microbiota abundance (genus Parasutterella id.2892)' on 'Cofilin−1'

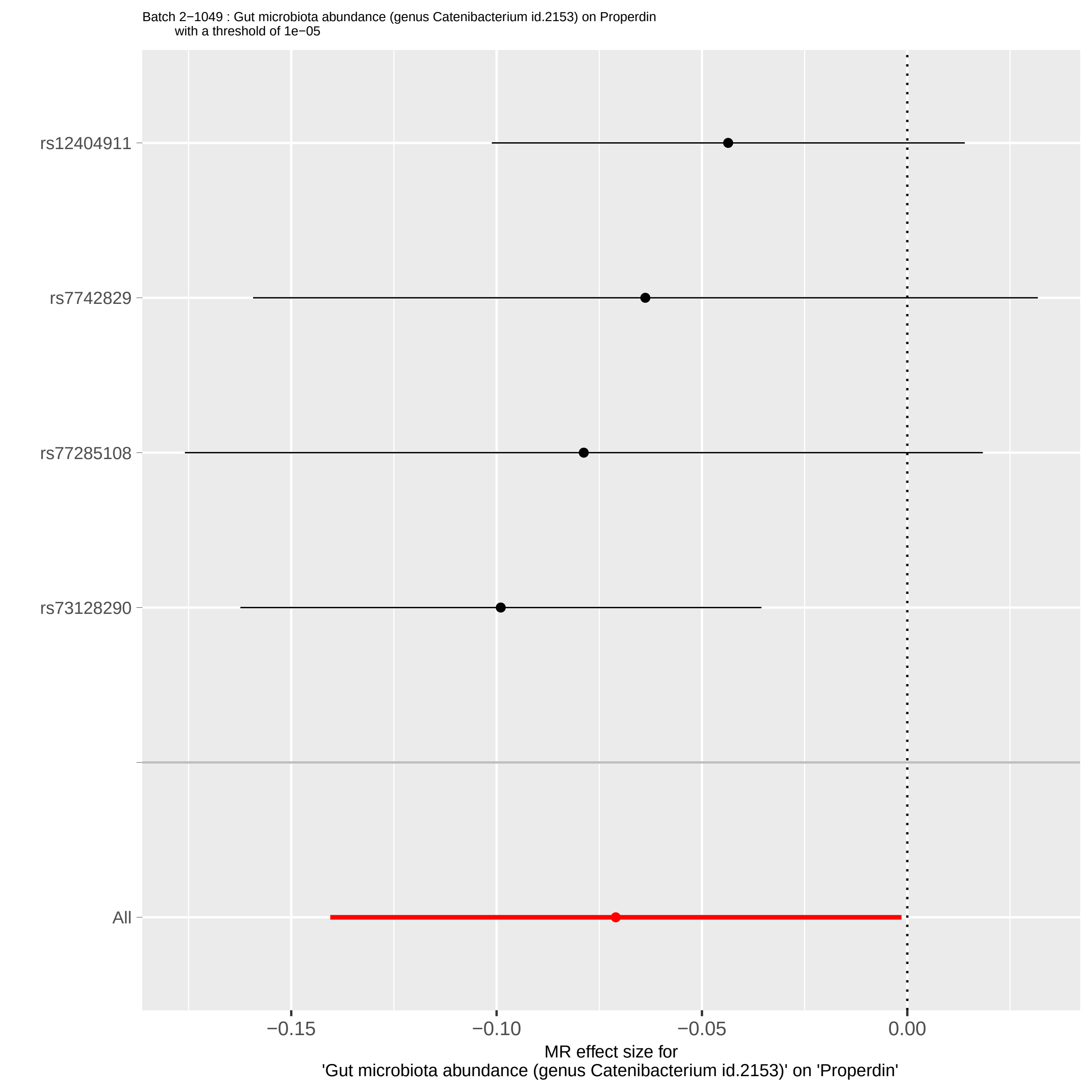
Batch 2−1049 : Gut microbiota abundance (genus Catenibacterium id.2153) on Properdin
with a threshold of 1e−05
rs12404911
rs7742829
rs77285108
rs73128290
All
−0.15
−0.10
−0.05
0.00
MR effect size for
'Gut microbiota abundance (genus Catenibacterium id.2153)' on 'Properdin'

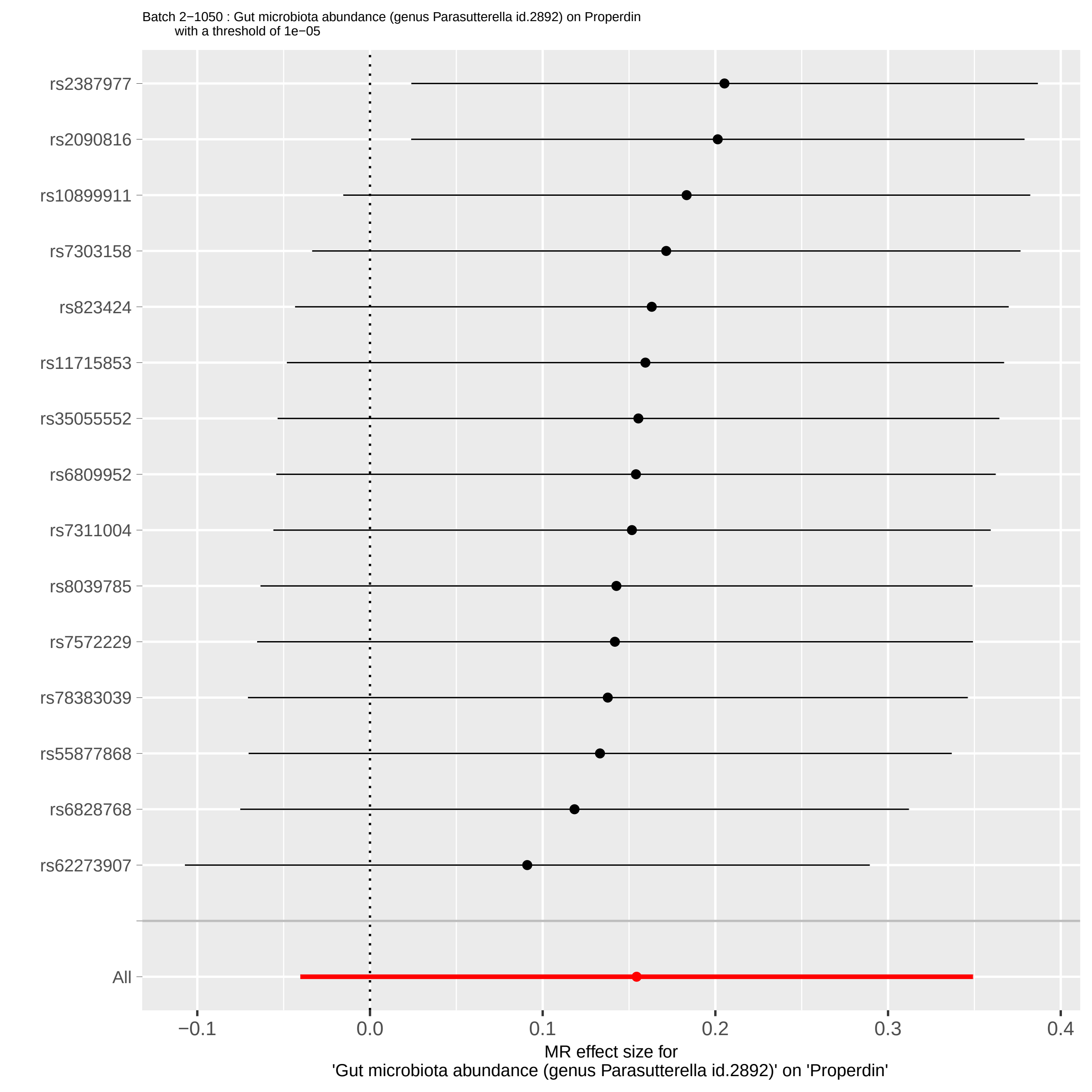

Batch 2−1050 : Gut microbiota abundance (genus Parasutterella id.2892) on Properdin
with a threshold of 1e−05
rs2387977
rs2090816
rs10899911
rs7303158
rs823424
rs11715853
rs35055552
rs6809952
rs7311004
rs8039785
rs7572229
rs78383039
rs55877868
rs6828768
rs62273907
All
−0.1
0.0
0.1
0.2
0.3
0.4
MR effect size for
'Gut microbiota abundance (genus Parasutterella id.2892)' on 'Properdin'

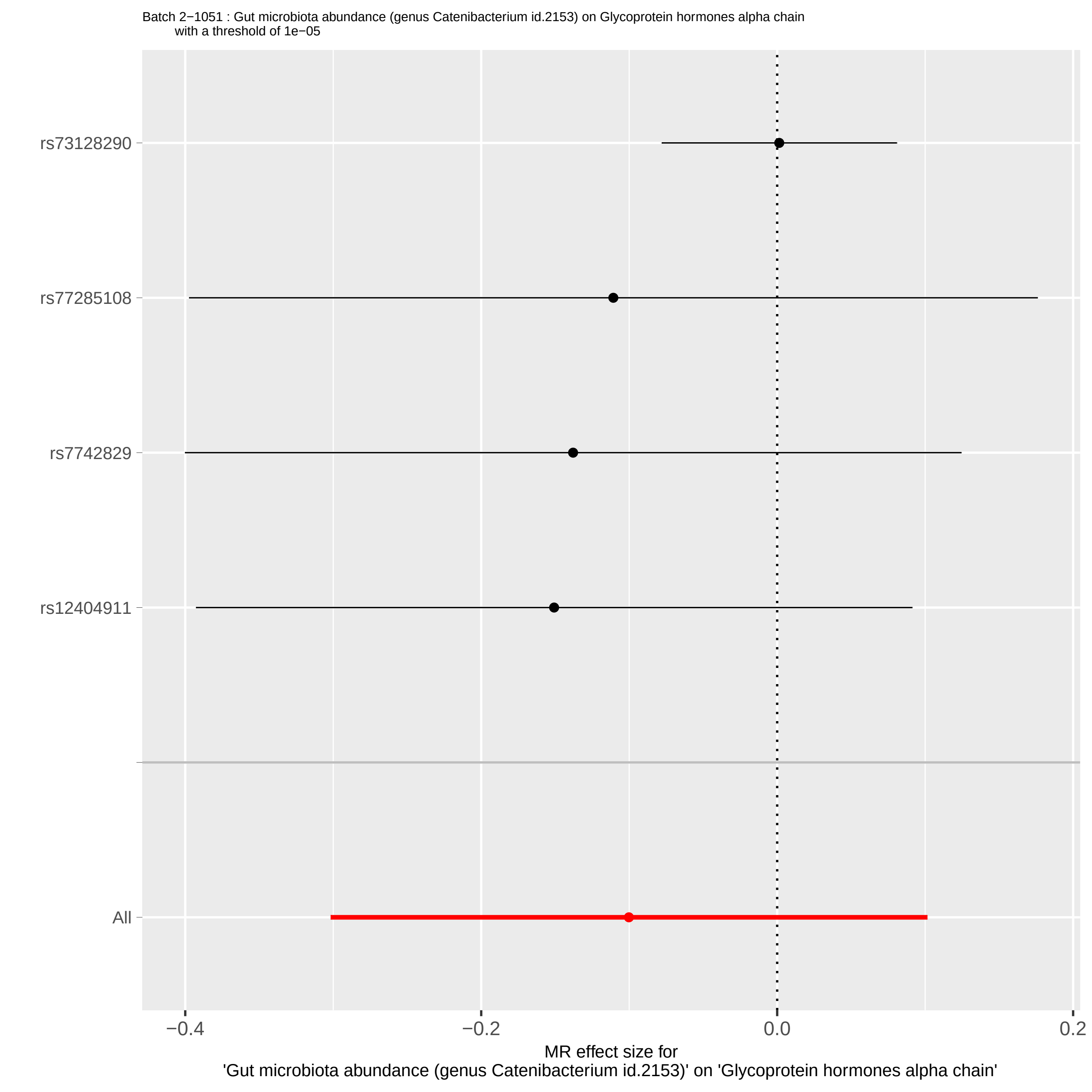
Batch 2−1051 : Gut microbiota abundance (genus Catenibacterium id.2153) on Glycoprotein hormones alpha chain
with a threshold of 1e−05
rs73128290
rs77285108
rs7742829
rs12404911
All
−0.4
−0.2
0.0
0.2
MR effect size for
'Gut microbiota abundance (genus Catenibacterium id.2153)' on 'Glycoprotein hormones alpha chain'

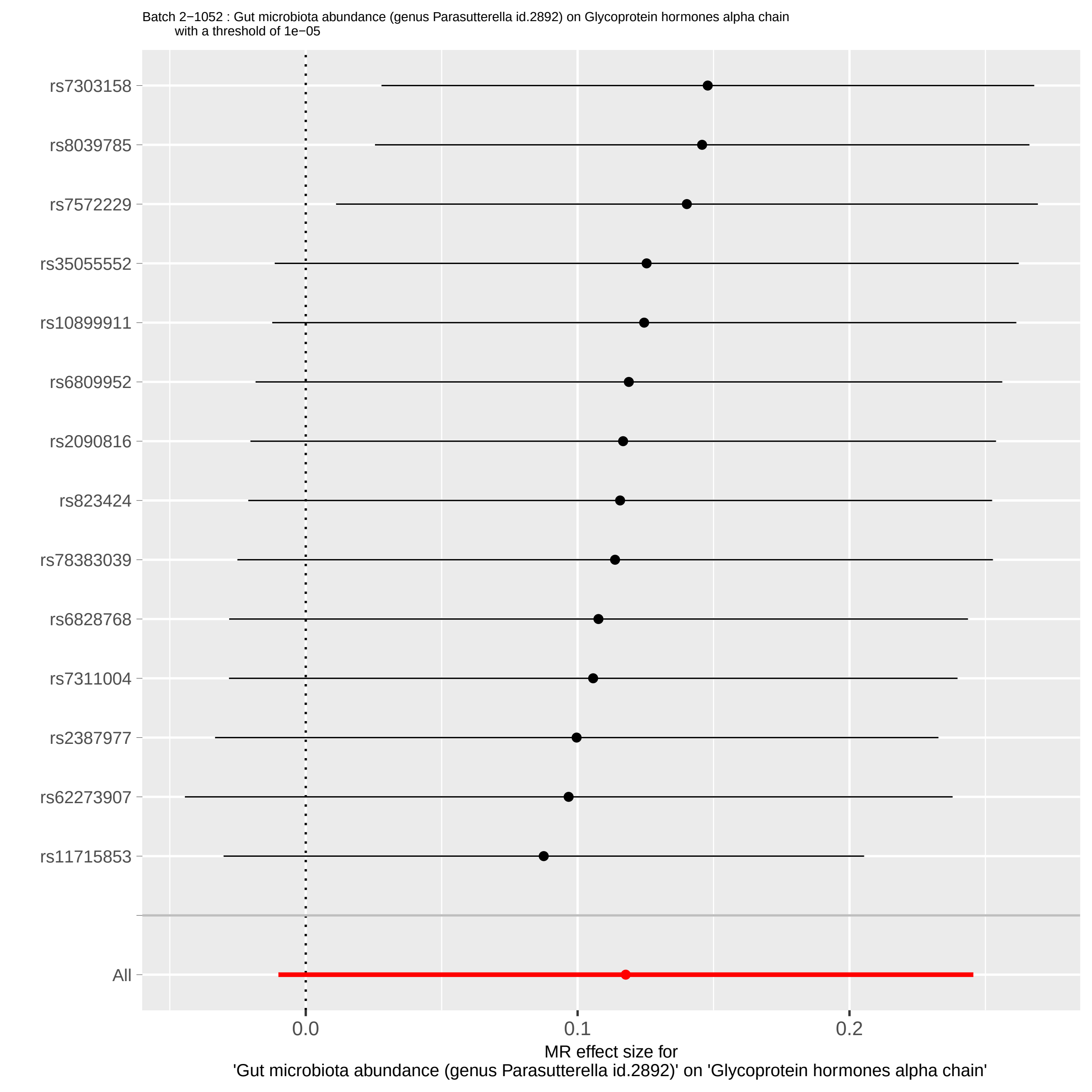
Batch 2−1052 : Gut microbiota abundance (genus Parasutterella id.2892) on Glycoprotein hormones alpha chain
with a threshold of 1e−05
rs7303158
rs8039785
rs7572229
rs35055552
rs10899911
rs6809952
rs2090816
rs823424
rs78383039
rs6828768
rs7311004
rs2387977
rs62273907
rs11715853
All
0.0
0.1
0.2
MR effect size for
'Gut microbiota abundance (genus Parasutterella id.2892)' on 'Glycoprotein hormones alpha chain'

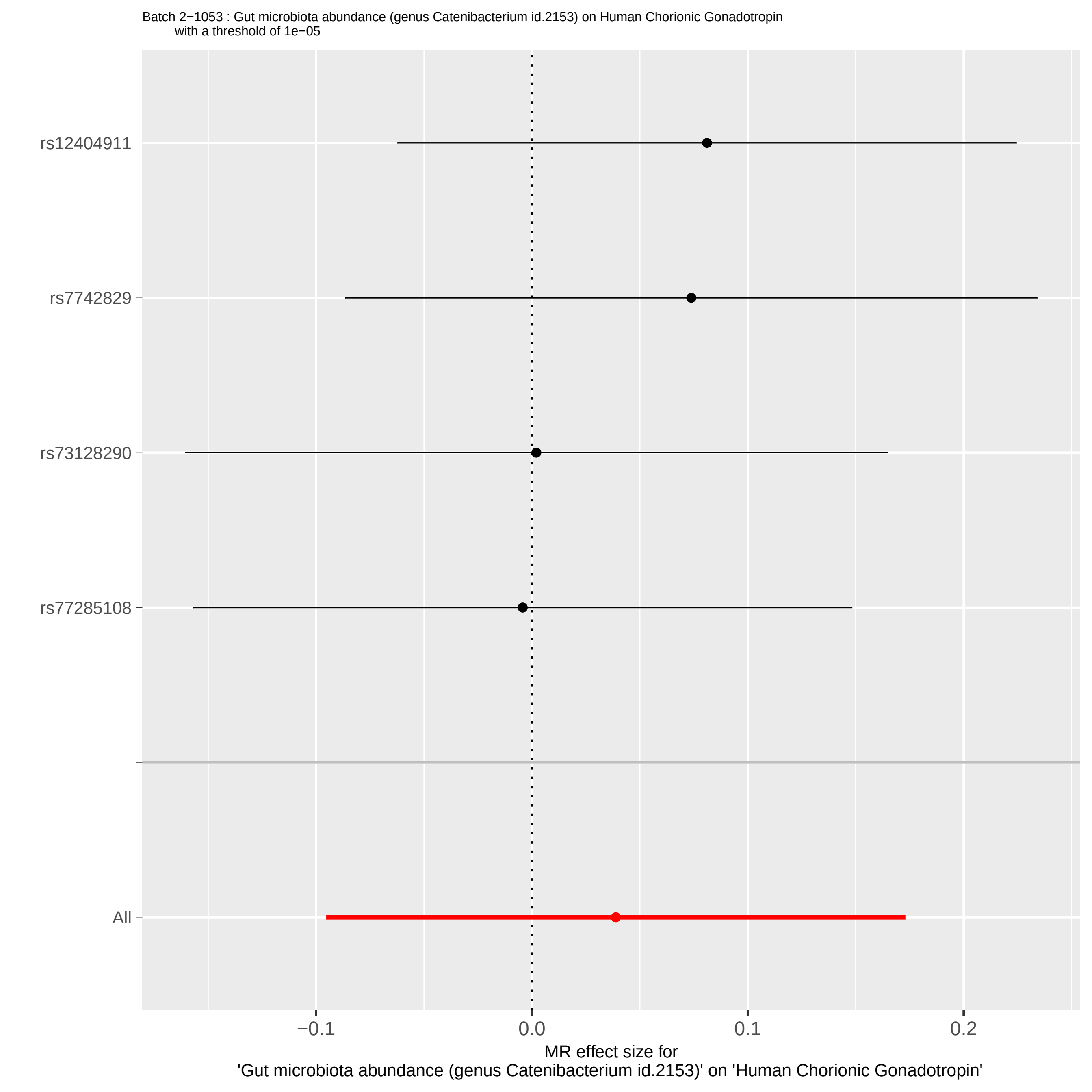
Batch 2−1053 : Gut microbiota abundance (genus Catenibacterium id.2153) on Human Chorionic Gonadotropin
with a threshold of 1e−05
rs12404911
rs7742829
rs73128290
rs77285108
All
−0.1
0.0
0.1
0.2
MR effect size for
'Gut microbiota abundance (genus Catenibacterium id.2153)' on 'Human Chorionic Gonadotropin'

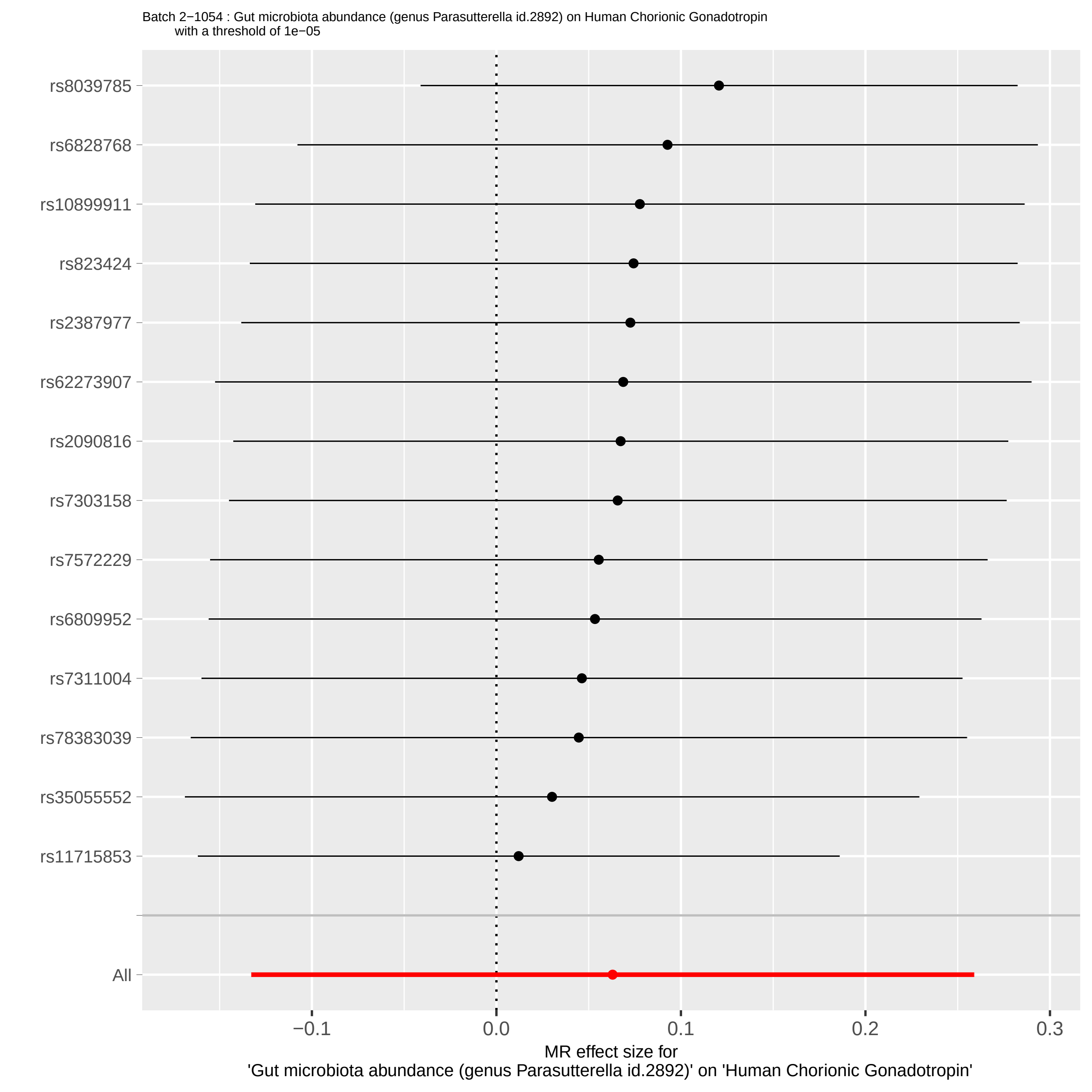
Batch 2−1054 : Gut microbiota abundance (genus Parasutterella id.2892) on Human Chorionic Gonadotropin
with a threshold of 1e−05
rs8039785
rs6828768
rs10899911
rs823424
rs2387977
rs62273907
rs2090816
rs7303158
rs7572229
rs6809952
rs7311004
rs78383039
rs35055552
rs11715853
All
−0.1
0.0
0.1
0.2
0.3
MR effect size for
'Gut microbiota abundance (genus Parasutterella id.2892)' on 'Human Chorionic Gonadotropin'

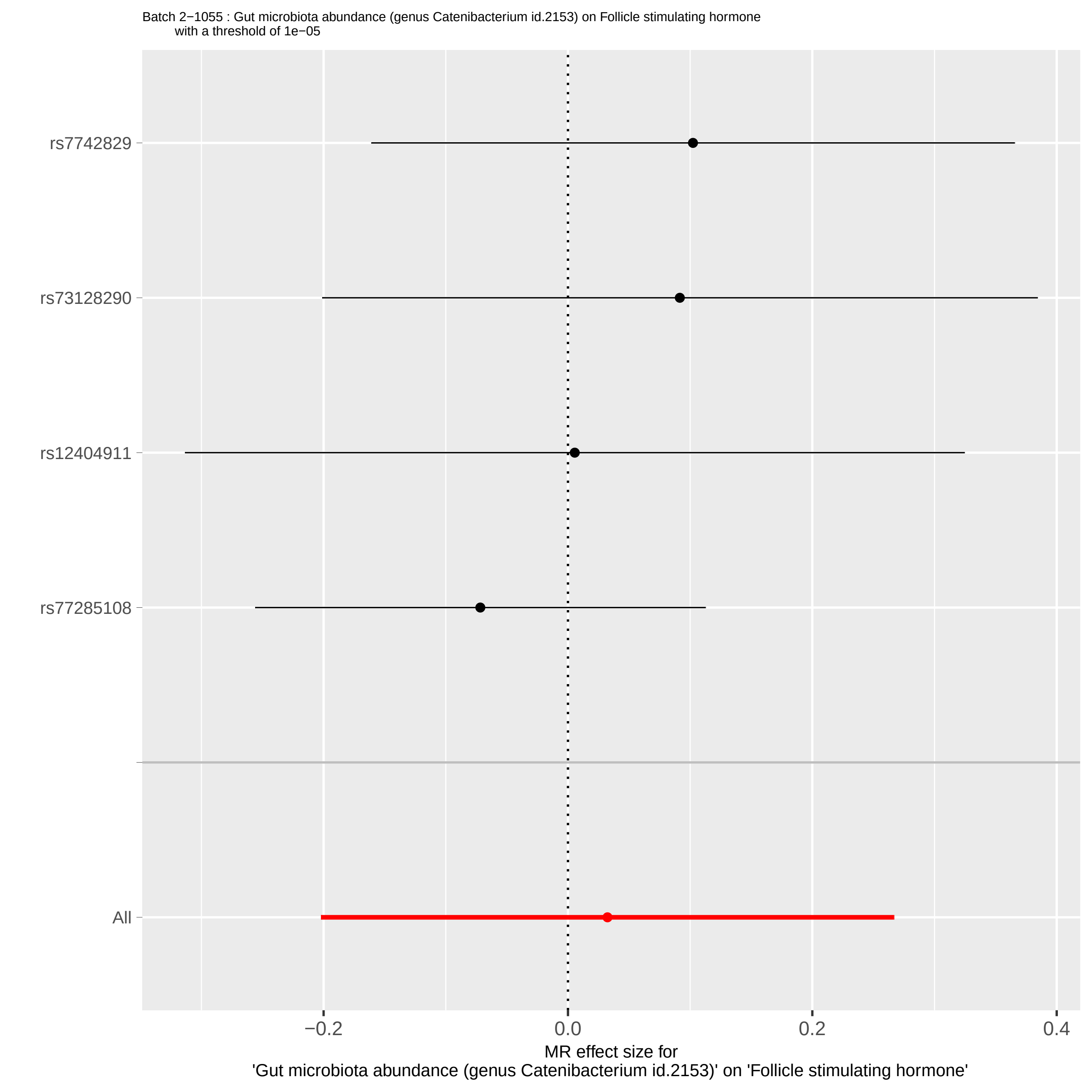
Batch 2−1055 : Gut microbiota abundance (genus Catenibacterium id.2153) on Follicle stimulating hormone
with a threshold of 1e−05
rs7742829
rs73128290
rs12404911
rs77285108
All
−0.2
0.0
0.2
0.4
MR effect size for
'Gut microbiota abundance (genus Catenibacterium id.2153)' on 'Follicle stimulating hormone'

Batch 2−1056 : Gut microbiota abundance (genus Parasutterella id.2892) on Follicle stimulating hormone
with a threshold of 1e−05

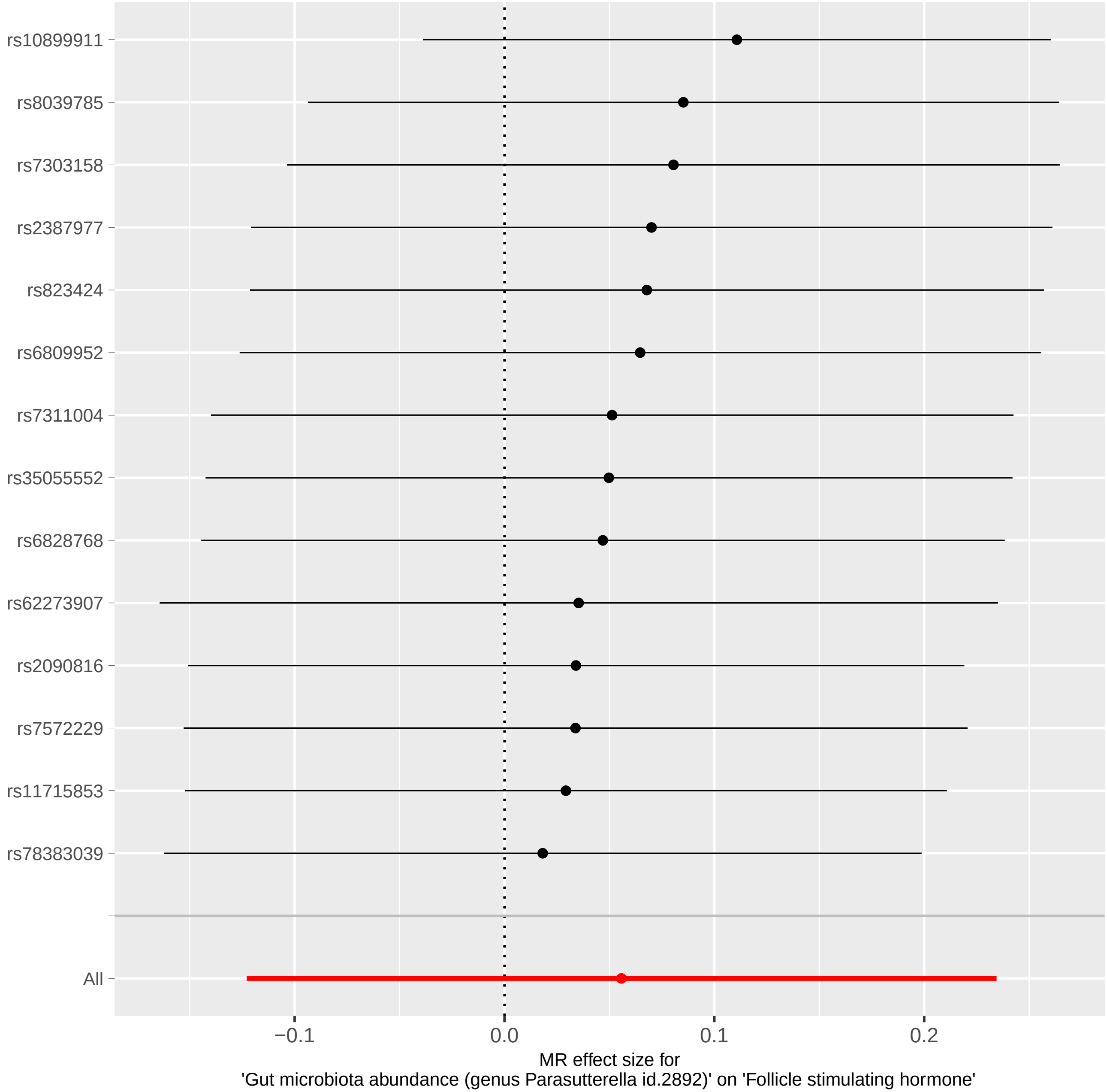

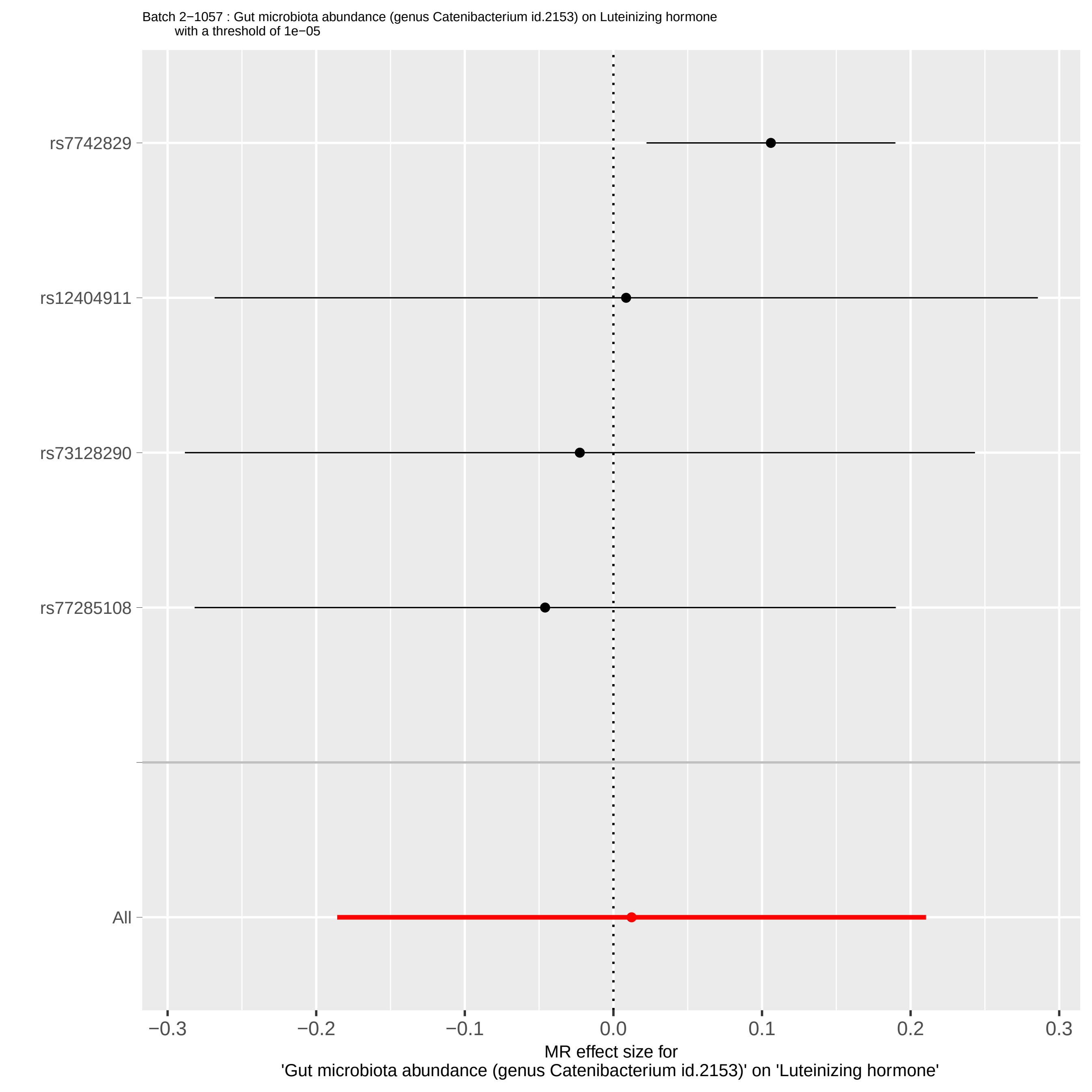
Batch 2−1057 : Gut microbiota abundance (genus Catenibacterium id.2153) on Luteinizing hormone
with a threshold of 1e−05
rs7742829
rs12404911
rs73128290
rs77285108
All
−0.3
−0.2
−0.1
0.0
0.1
0.2
0.3
MR effect size for
'Gut microbiota abundance (genus Catenibacterium id.2153)' on 'Luteinizing hormone'

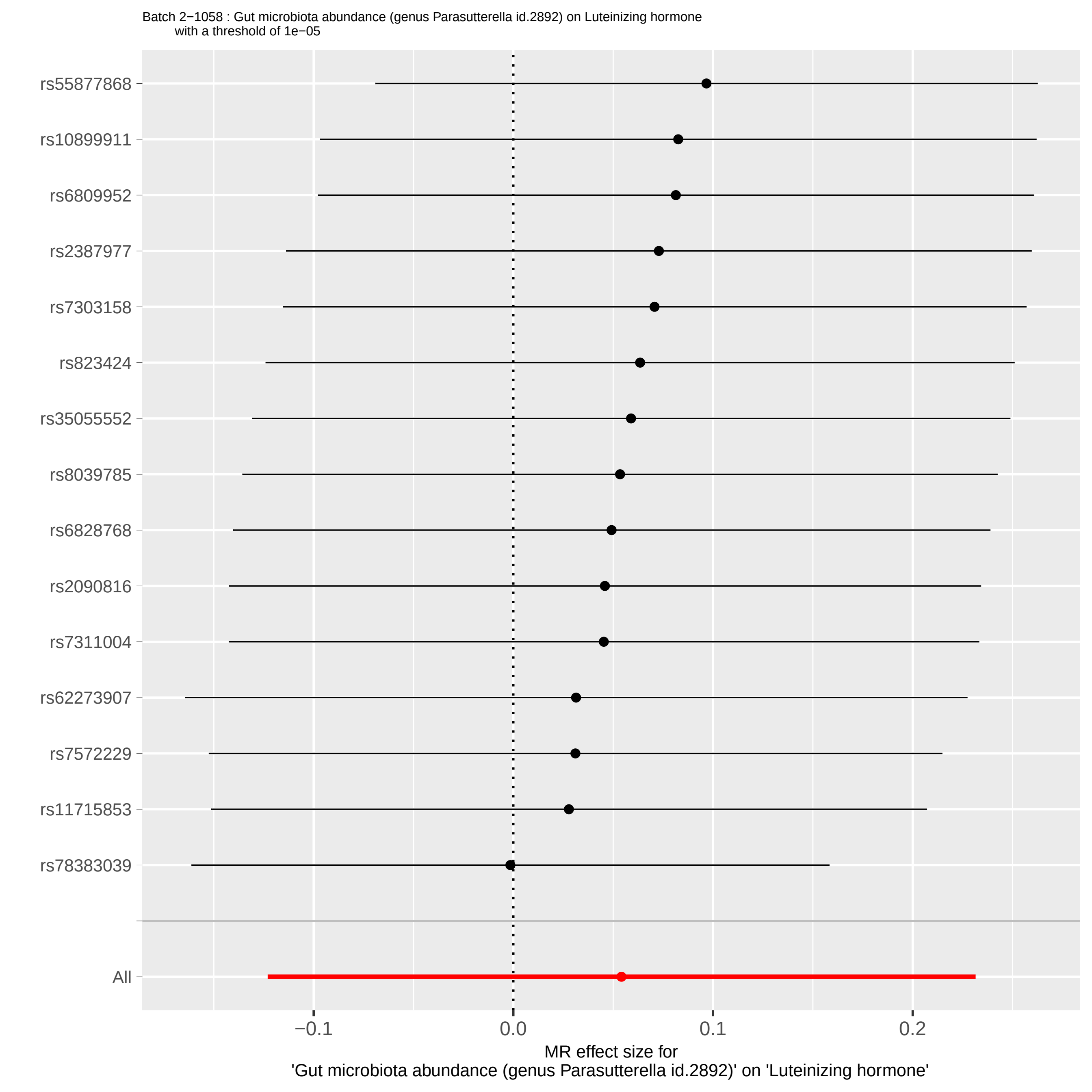
Batch 2−1058 : Gut microbiota abundance (genus Parasutterella id.2892) on Luteinizing hormone
with a threshold of 1e−05
rs55877868
rs10899911
rs6809952
rs2387977
rs7303158
rs823424
rs35055552
rs8039785
rs6828768
rs2090816
rs7311004
rs62273907
rs7572229
rs11715853
rs78383039
All
−0.1
0.0
0.1
0.2
MR effect size for
'Gut microbiota abundance (genus Parasutterella id.2892)' on 'Luteinizing hormone'

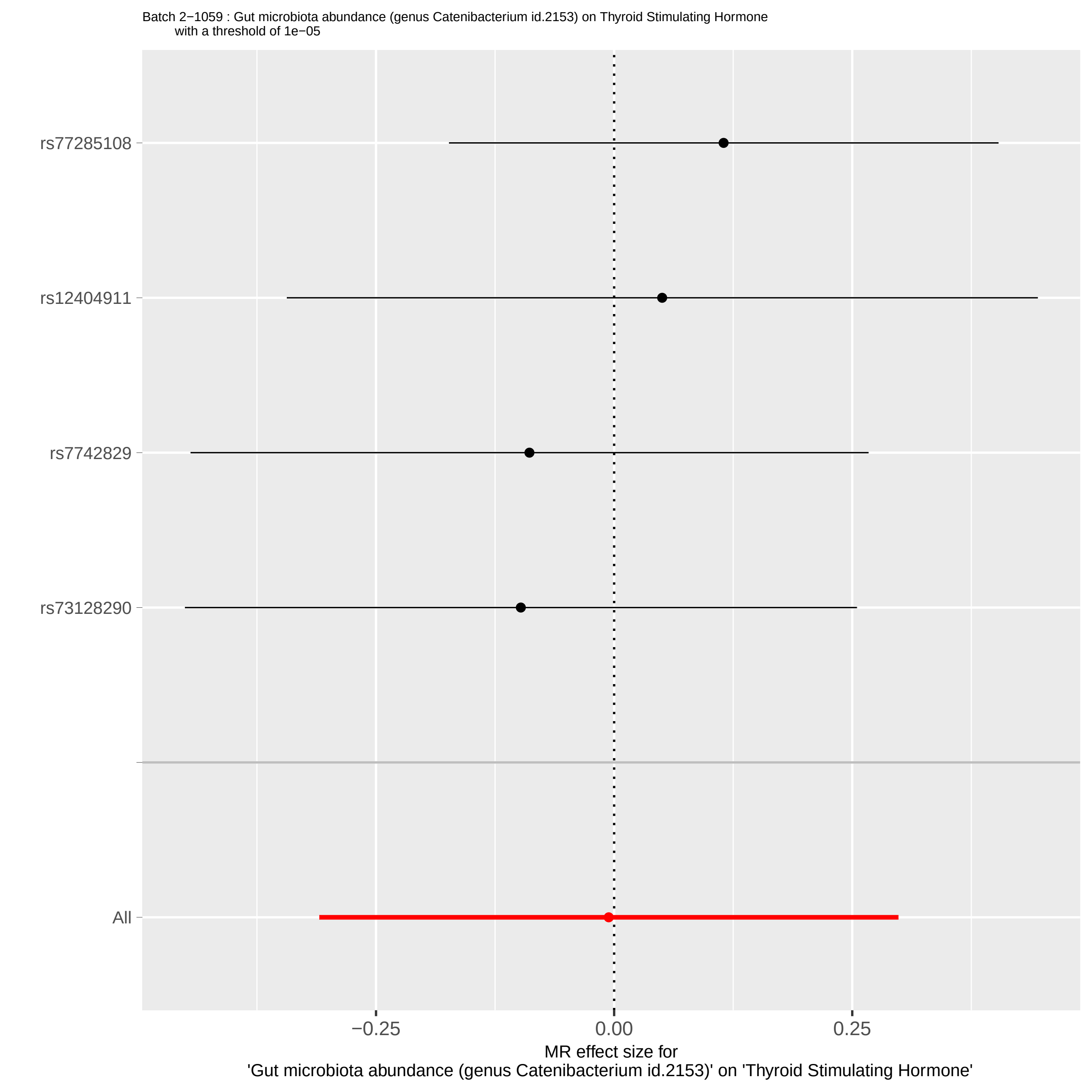
Batch 2−1059 : Gut microbiota abundance (genus Catenibacterium id.2153) on Thyroid Stimulating Hormone
with a threshold of 1e−05
rs77285108
rs12404911
rs7742829
rs73128290
All
−0.25
0.00
0.25
MR effect size for
'Gut microbiota abundance (genus Catenibacterium id.2153)' on 'Thyroid Stimulating Hormone'

Batch 2−1060 : Gut microbiota abundance (genus Parasutterella id.2892) on Thyroid Stimulating Hormone
with a threshold of 1e−05

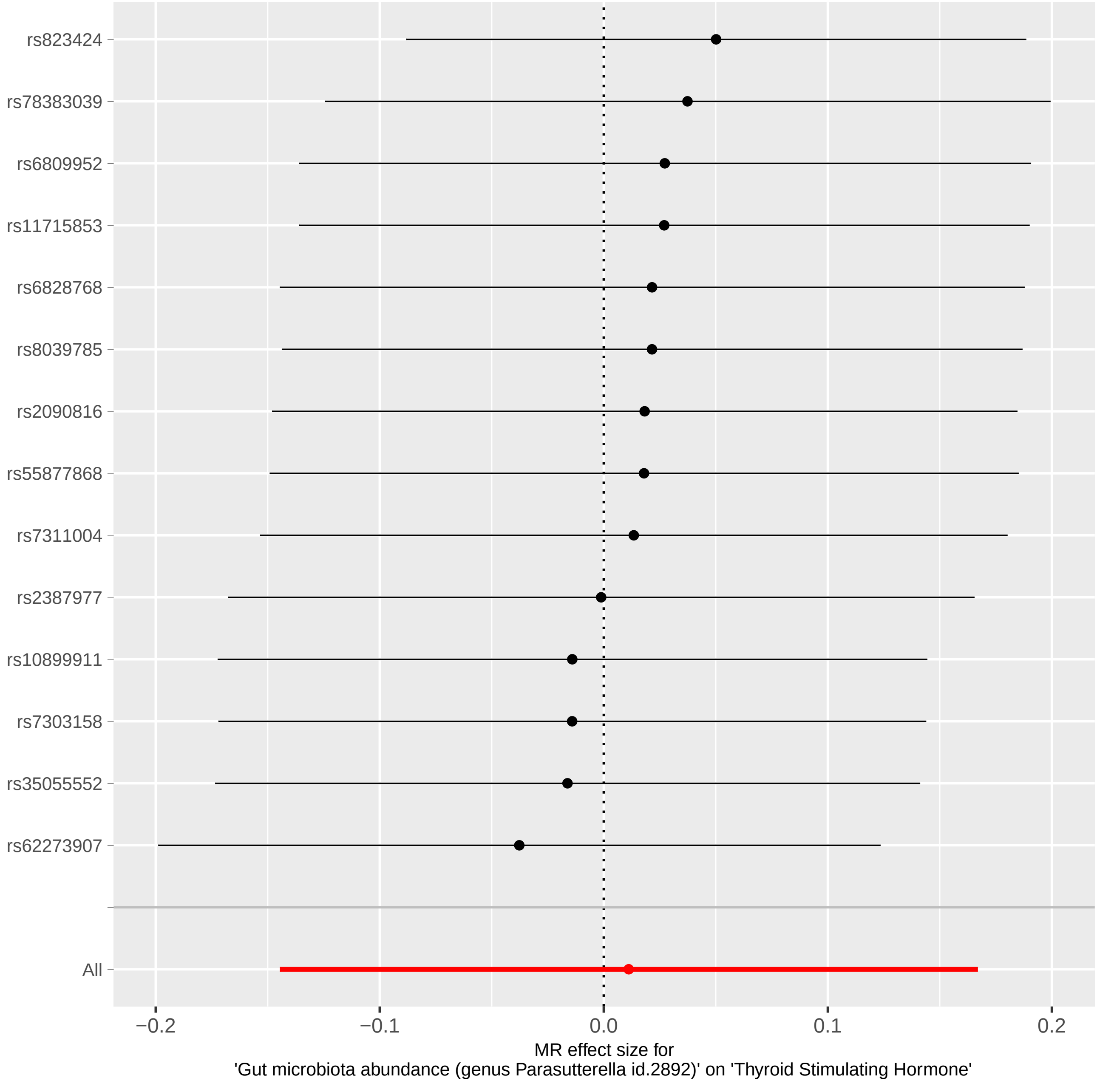

Batch 2−1061 : Gut microbiota abundance (genus Catenibacterium id.2153) on Choriogonadotropin subunit beta variant 2
with a threshold of 1e−05

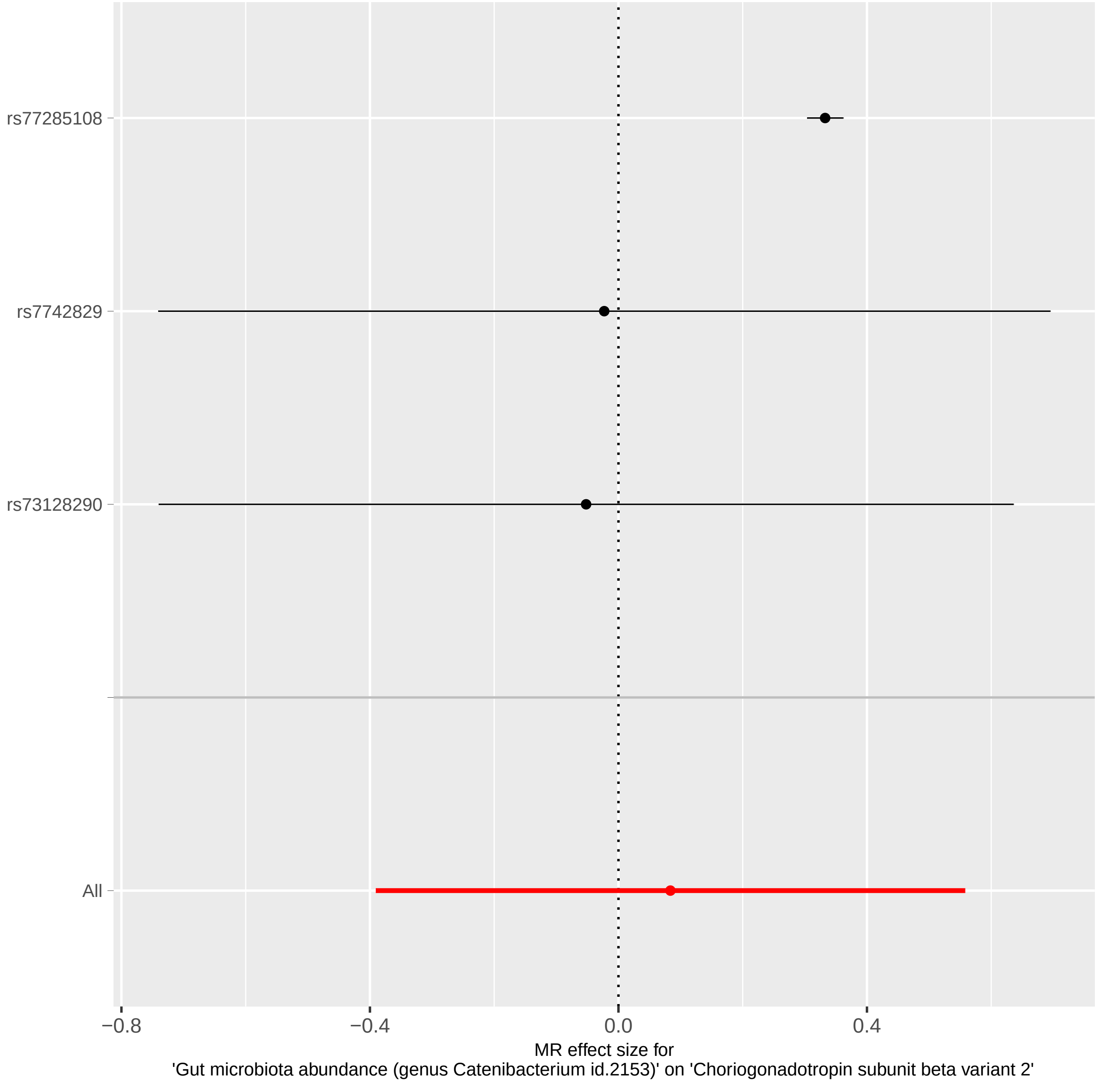

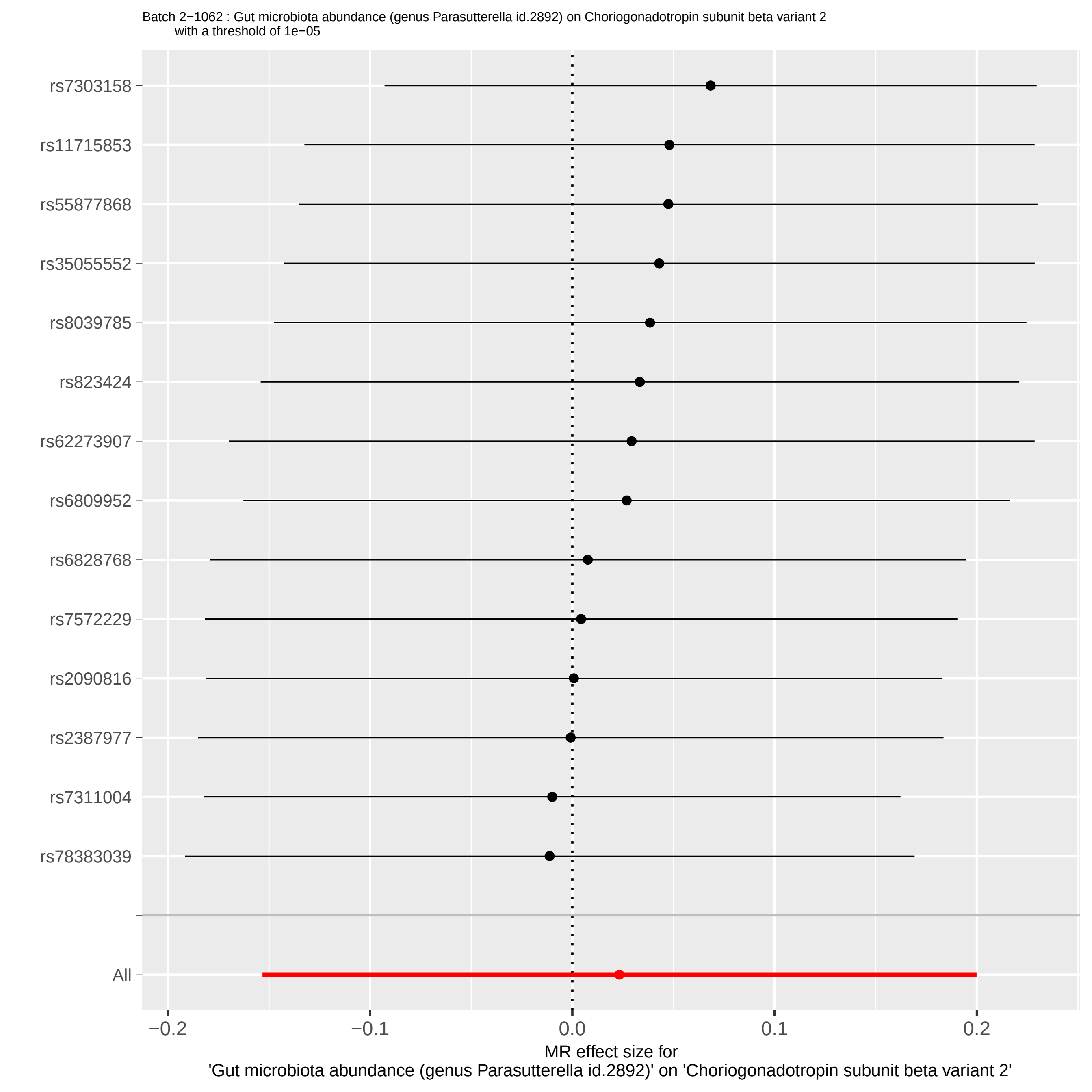
Batch 2−1062 : Gut microbiota abundance (genus Parasutterella id.2892) on Choriogonadotropin subunit beta variant 2
with a threshold of 1e−05
rs7303158
rs11715853
rs55877868
rs35055552
rs8039785
rs823424
rs62273907
rs6809952
rs6828768
rs7572229
rs2090816
rs2387977
rs7311004
rs78383039
All
−0.2
−0.1
0.0
0.1
0.2
MR effect size for
'Gut microbiota abundance (genus Parasutterella id.2892)' on 'Choriogonadotropin subunit beta variant 2'

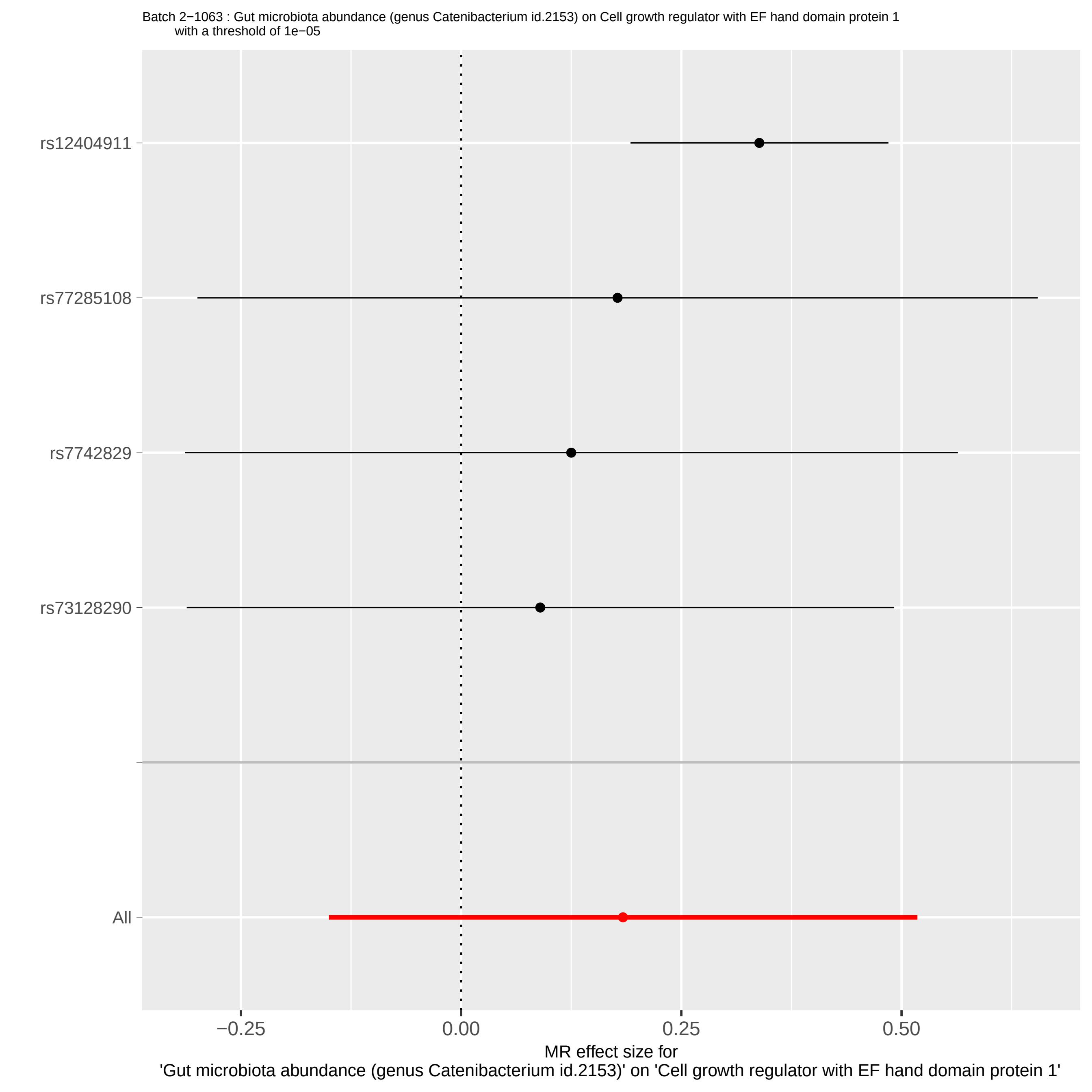
Batch 2−1063 : Gut microbiota abundance (genus Catenibacterium id.2153) on Cell growth regulator with EF hand domain protein 1
with a threshold of 1e−05
rs12404911
rs77285108
rs7742829
rs73128290
All
−0.25
0.00
0.25
0.50
MR effect size for
'Gut microbiota abundance (genus Catenibacterium id.2153)' on 'Cell growth regulator with EF hand domain protein 1'

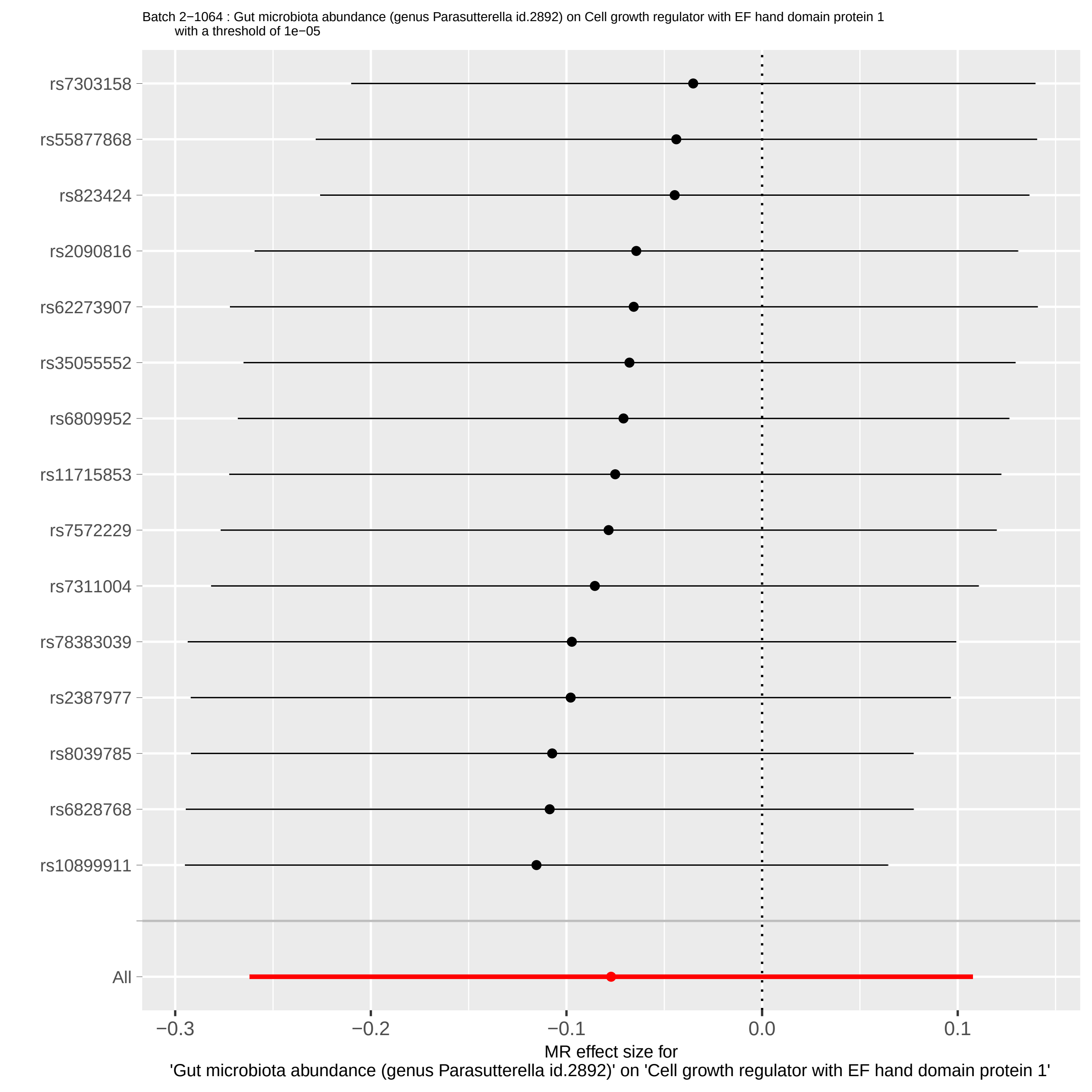

Batch 2−1064 : Gut microbiota abundance (genus Parasutterella id.2892) on Cell growth regulator with EF hand domain protein 1
with a threshold of 1e−05
rs7303158
rs55877868
rs823424
rs2090816
rs62273907
rs35055552
rs6809952
rs11715853
rs7572229
rs7311004
rs78383039
rs2387977
rs8039785
rs6828768
rs10899911
All
−0.3
−0.2
−0.1
0.0
0.1
MR effect size for
'Gut microbiota abundance (genus Parasutterella id.2892)' on 'Cell growth regulator with EF hand domain protein 1'

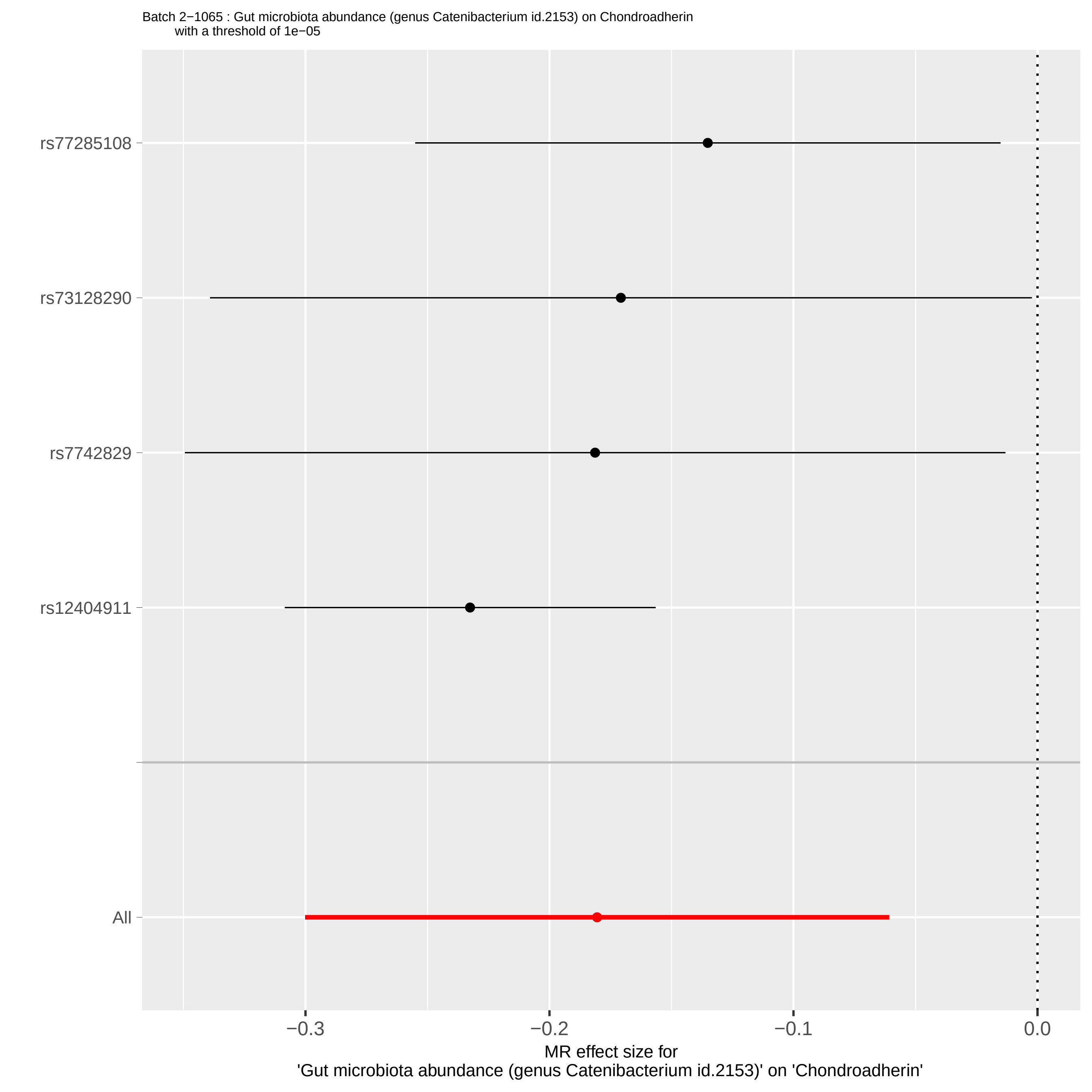
Batch 2−1065 : Gut microbiota abundance (genus Catenibacterium id.2153) on Chondroadherin
with a threshold of 1e−05
rs77285108
rs73128290
rs7742829
rs12404911
All
−0.3
−0.2
−0.1
0.0
MR effect size for
'Gut microbiota abundance (genus Catenibacterium id.2153)' on 'Chondroadherin'

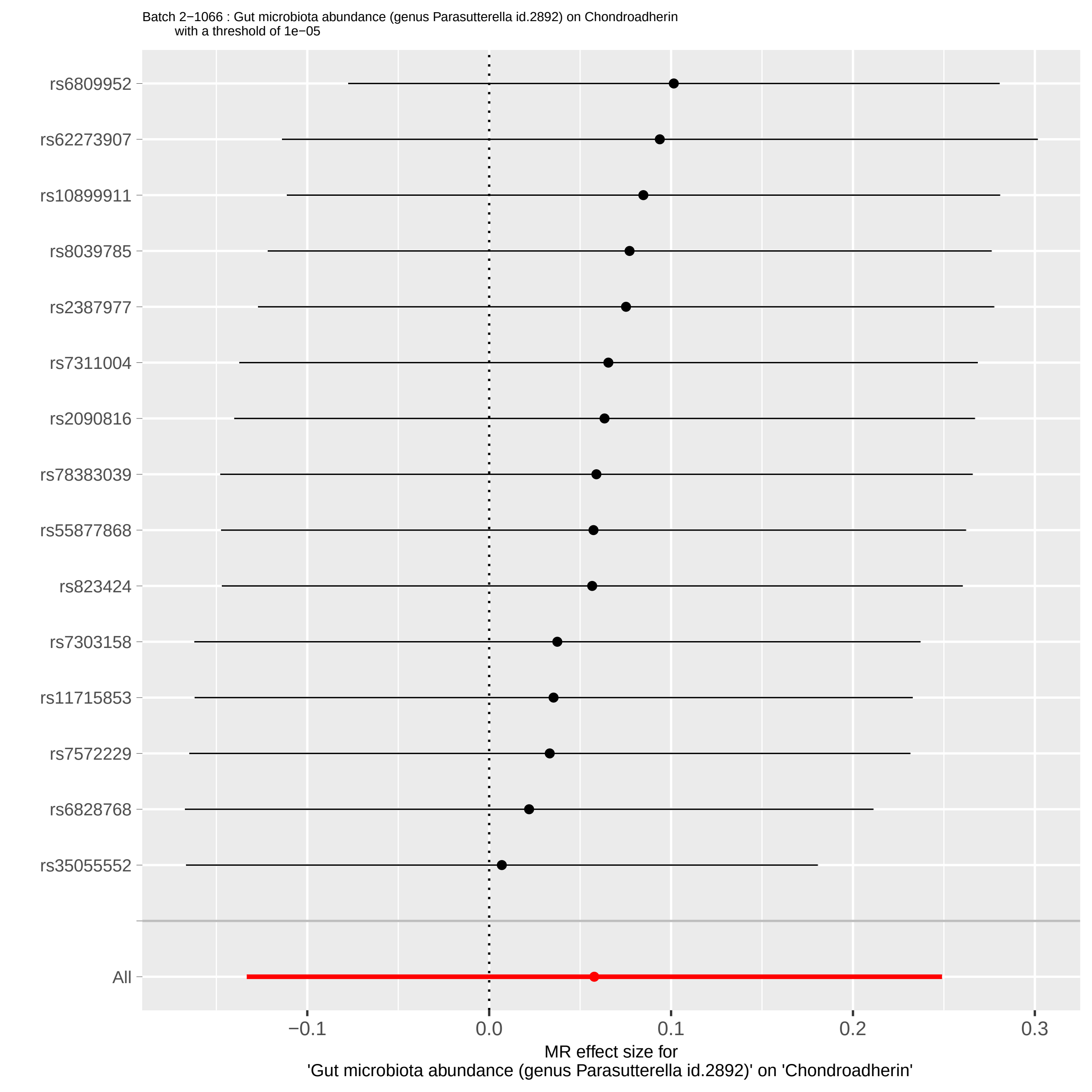

Batch 2−1066 : Gut microbiota abundance (genus Parasutterella id.2892) on Chondroadherin
with a threshold of 1e−05
rs6809952
rs62273907
rs10899911
rs8039785
rs2387977
rs7311004
rs2090816
rs78383039
rs55877868
rs823424
rs7303158
rs11715853
rs7572229
rs6828768
rs35055552
All
−0.1
0.0
0.1
0.2
0.3
MR effect size for
'Gut microbiota abundance (genus Parasutterella id.2892)' on 'Chondroadherin'

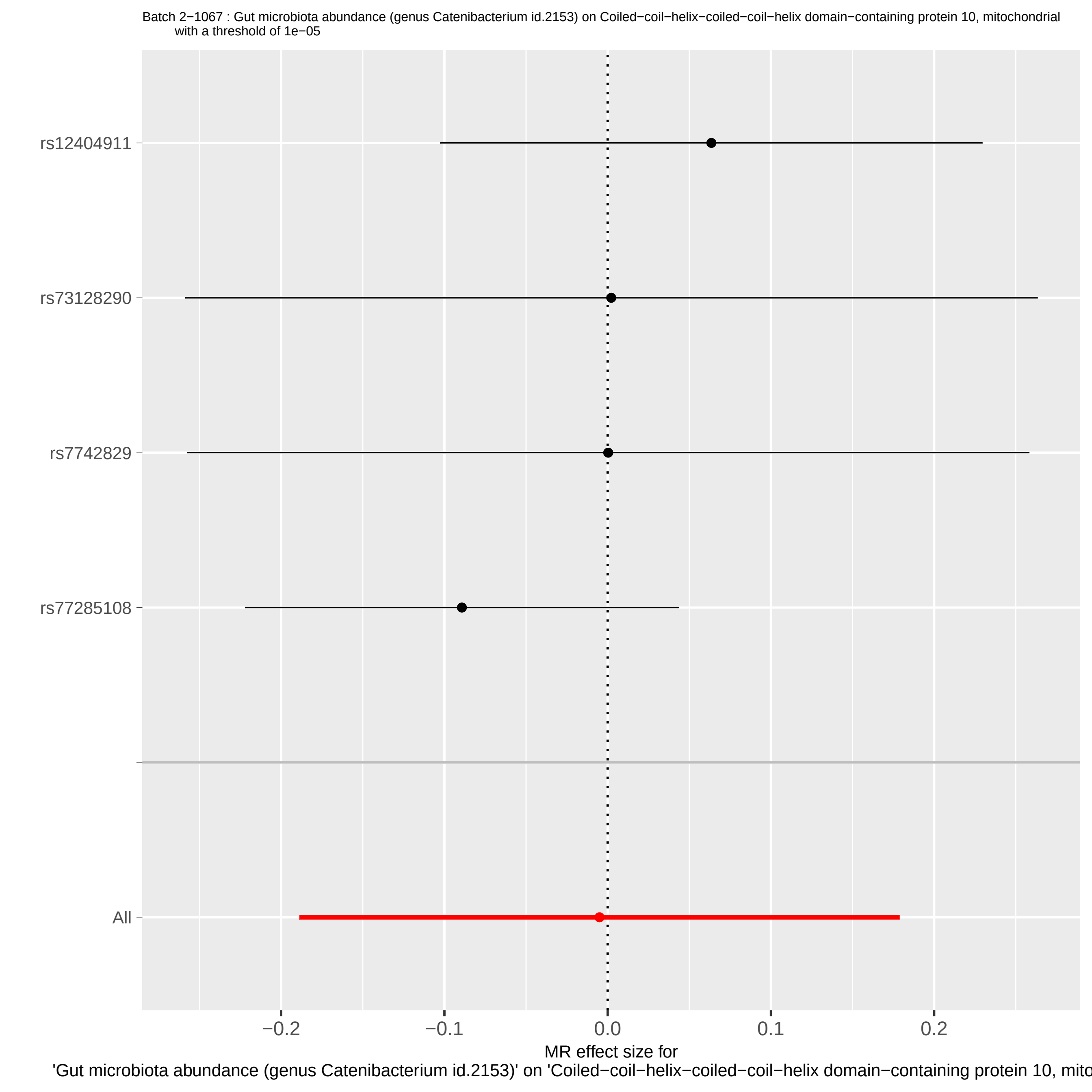

Batch 2−1067 : Gut microbiota abundance (genus Catenibacterium id.2153) on Coiled−coil−helix−coiled−coil−helix domain−containing protein 10, mitochondrial with a threshold of 1e−05
rs12404911
rs73128290
rs7742829
rs77285108
All
−0.2
−0.1
0.0
0.1
0.2
MR effect size for
'Gut microbiota abundance (genus Catenibacterium id.2153)' on 'Coiled−coil−helix−coiled−coil−helix domain−containing protein 10, mito

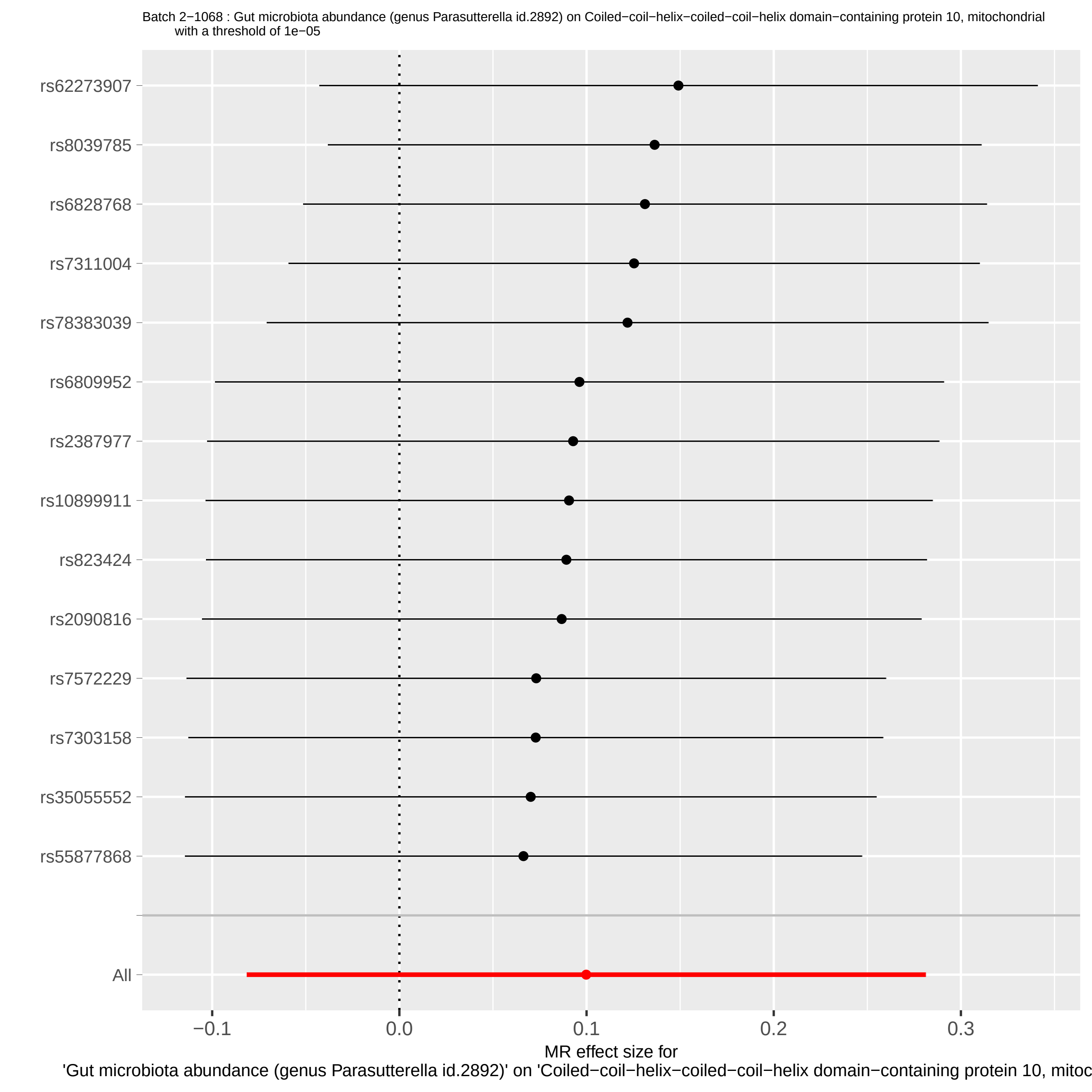
Batch 2−1068 : Gut microbiota abundance (genus Parasutterella id.2892) on Coiled−coil−helix−coiled−coil−helix domain−containing protein 10, mitochondrial
with a threshold of 1e−05
rs62273907
rs8039785
rs6828768
rs7311004
rs78383039
rs6809952
rs2387977
rs10899911
rs823424
rs2090816
rs7572229
rs7303158
rs35055552
rs55877868
All
−0.1
0.0
0.1
0.2
0.3
MR effect size for
'Gut microbiota abundance (genus Parasutterella id.2892)' on 'Coiled−coil−helix−coiled−coil−helix domain−containing protein 10, mitoc

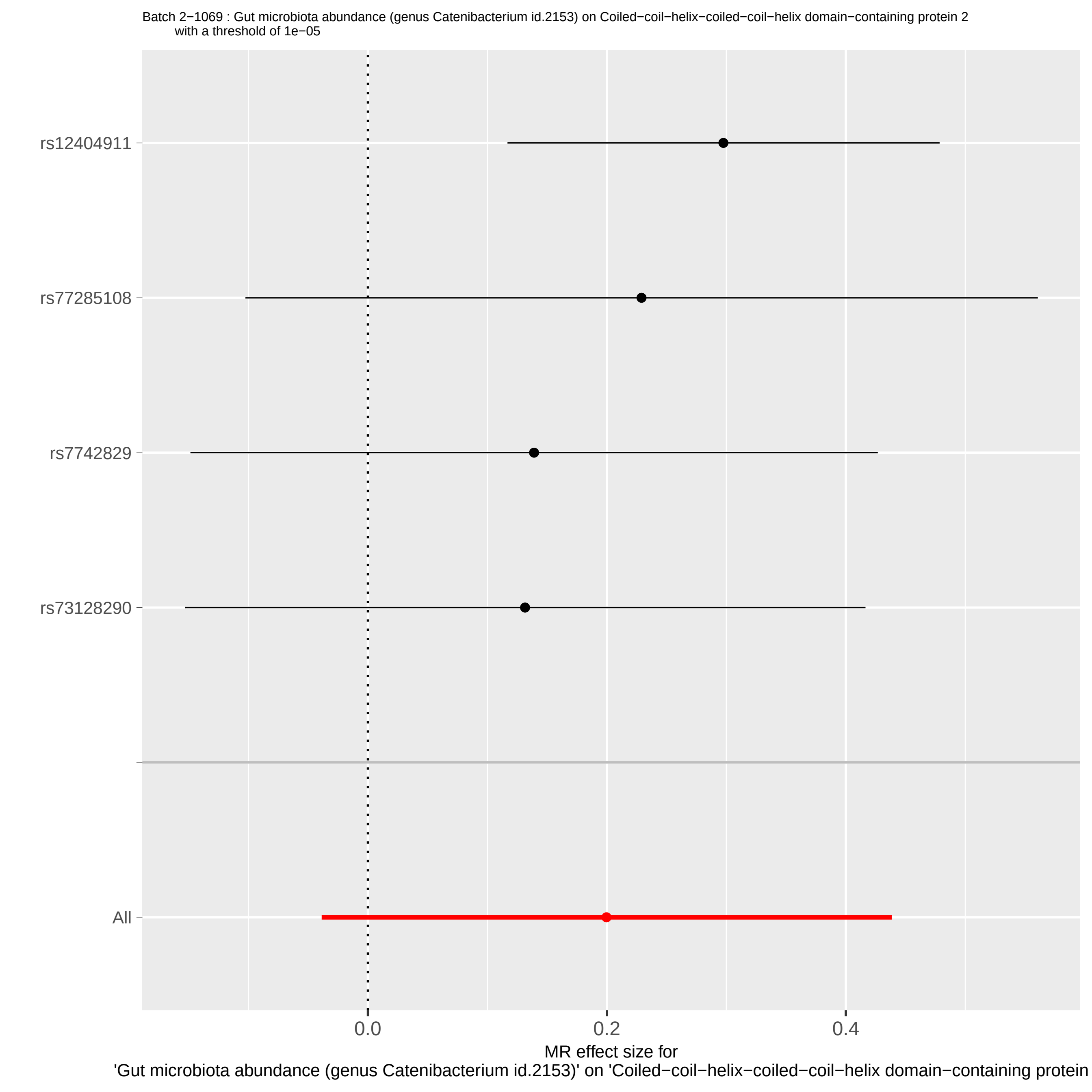

Batch 2−1069 : Gut microbiota abundance (genus Catenibacterium id.2153) on Coiled−coil−helix−coiled−coil−helix domain−containing protein 2
with a threshold of 1e−05
rs12404911
rs77285108
rs7742829
rs73128290
All
0.0
0.2
0.4
MR effect size for
'Gut microbiota abundance (genus Catenibacterium id.2153)' on 'Coiled−coil−helix−coiled−coil−helix domain−containing protein

Batch 2−1070 : Gut microbiota abundance (genus Parasutterella id.2892) on Coiled−coil−helix−coiled−coil−helix domain−containing protein 2 with a threshold of 1e−05

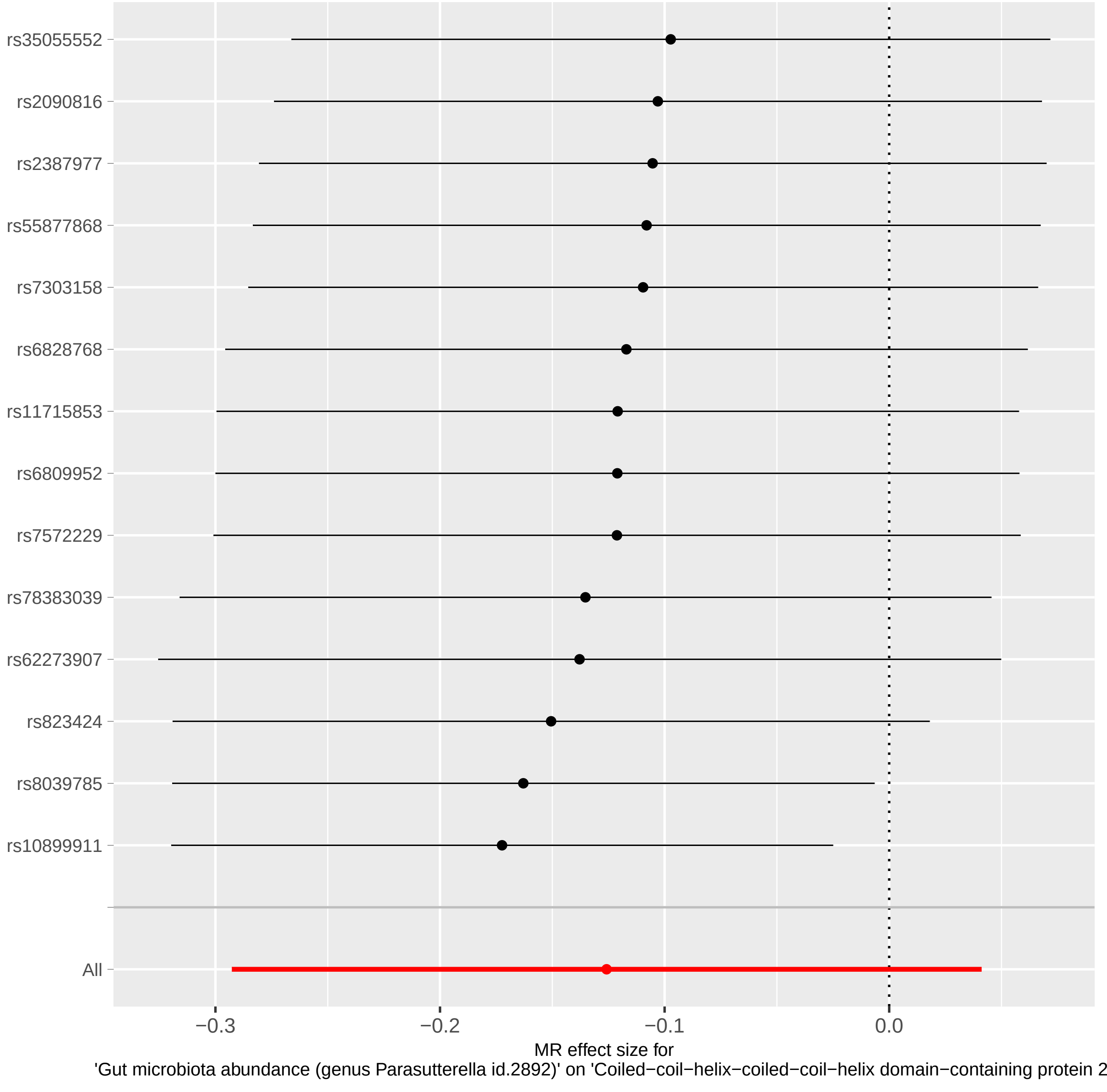

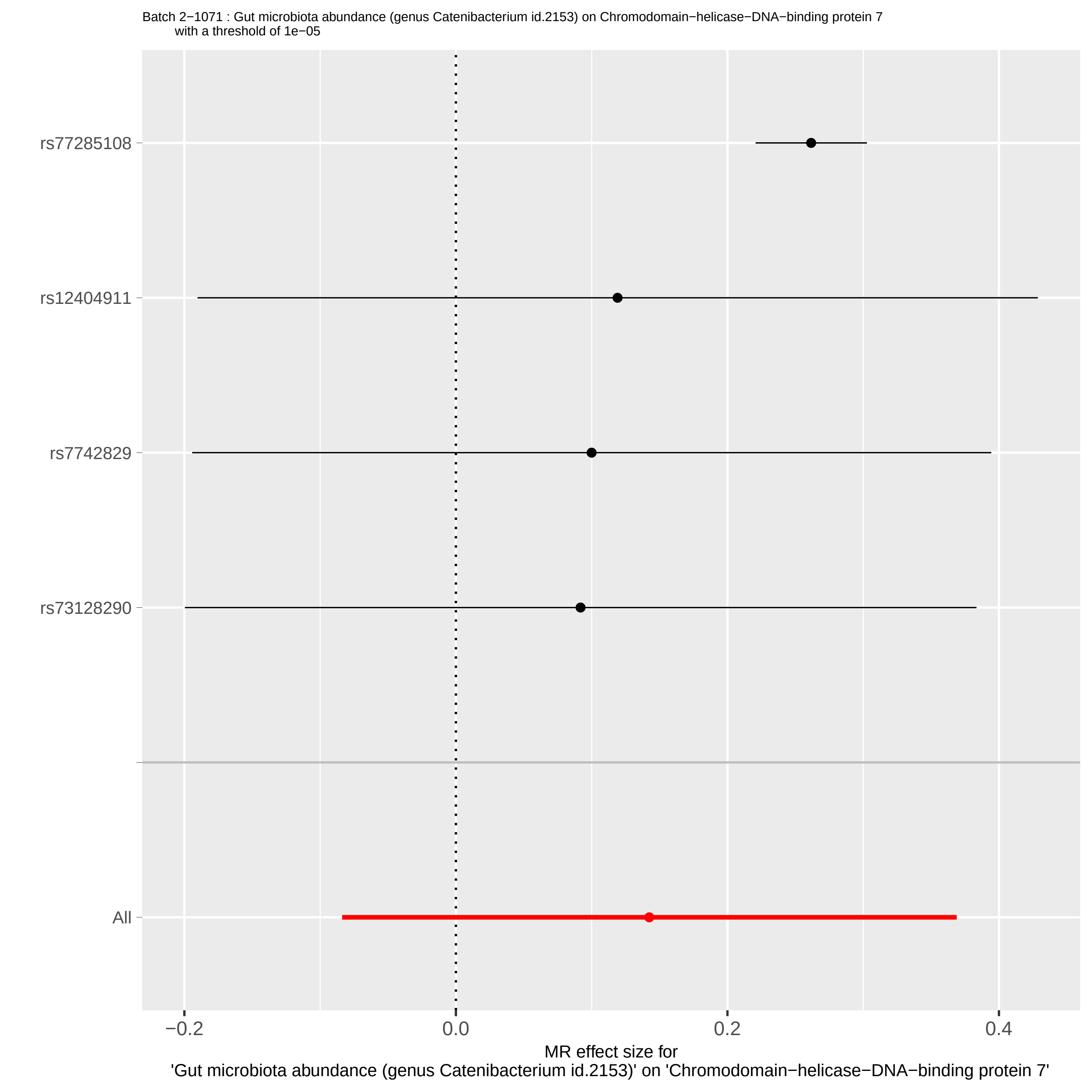
Batch 2−1071 : Gut microbiota abundance (genus Catenibacterium id.2153) on Chromodomain−helicase−DNA−binding protein 7
with a threshold of 1e−05
rs77285108
rs12404911
rs7742829
rs73128290
All
−0.2
0.0
0.2
0.4
MR effect size for
'Gut microbiota abundance (genus Catenibacterium id.2153)' on 'Chromodomain−helicase−DNA−binding protein 7'

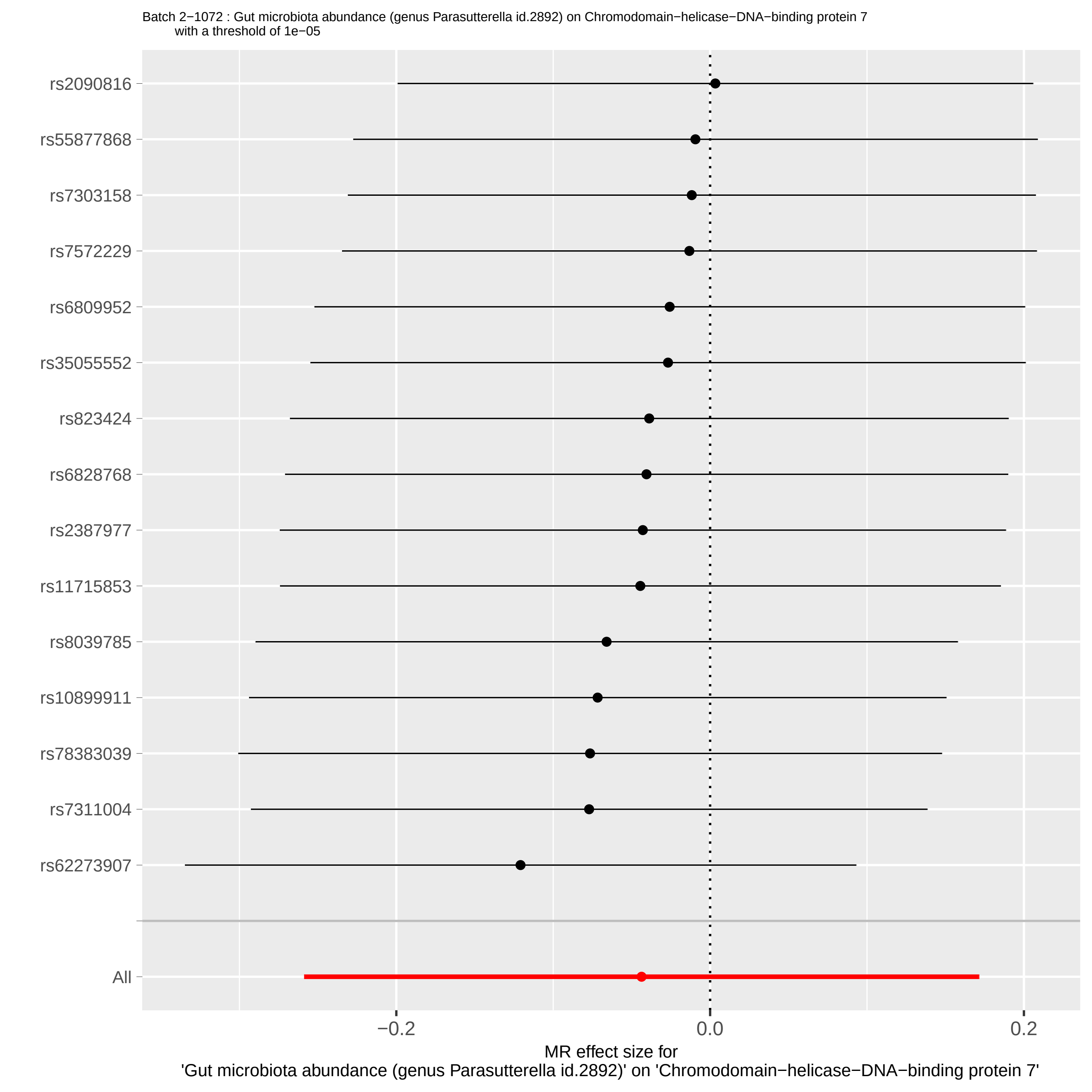
Batch 2−1072 : Gut microbiota abundance (genus Parasutterella id.2892) on Chromodomain−helicase−DNA−binding protein 7
with a threshold of 1e−05
rs2090816
rs55877868
rs7303158
rs7572229
rs6809952
rs35055552
rs823424
rs6828768
rs2387977
rs11715853
rs8039785
rs10899911
rs78383039
rs7311004
rs62273907
All
−0.2
0.0
0.2
MR effect size for
'Gut microbiota abundance (genus Parasutterella id.2892)' on 'Chromodomain−helicase−DNA−binding protein 7'

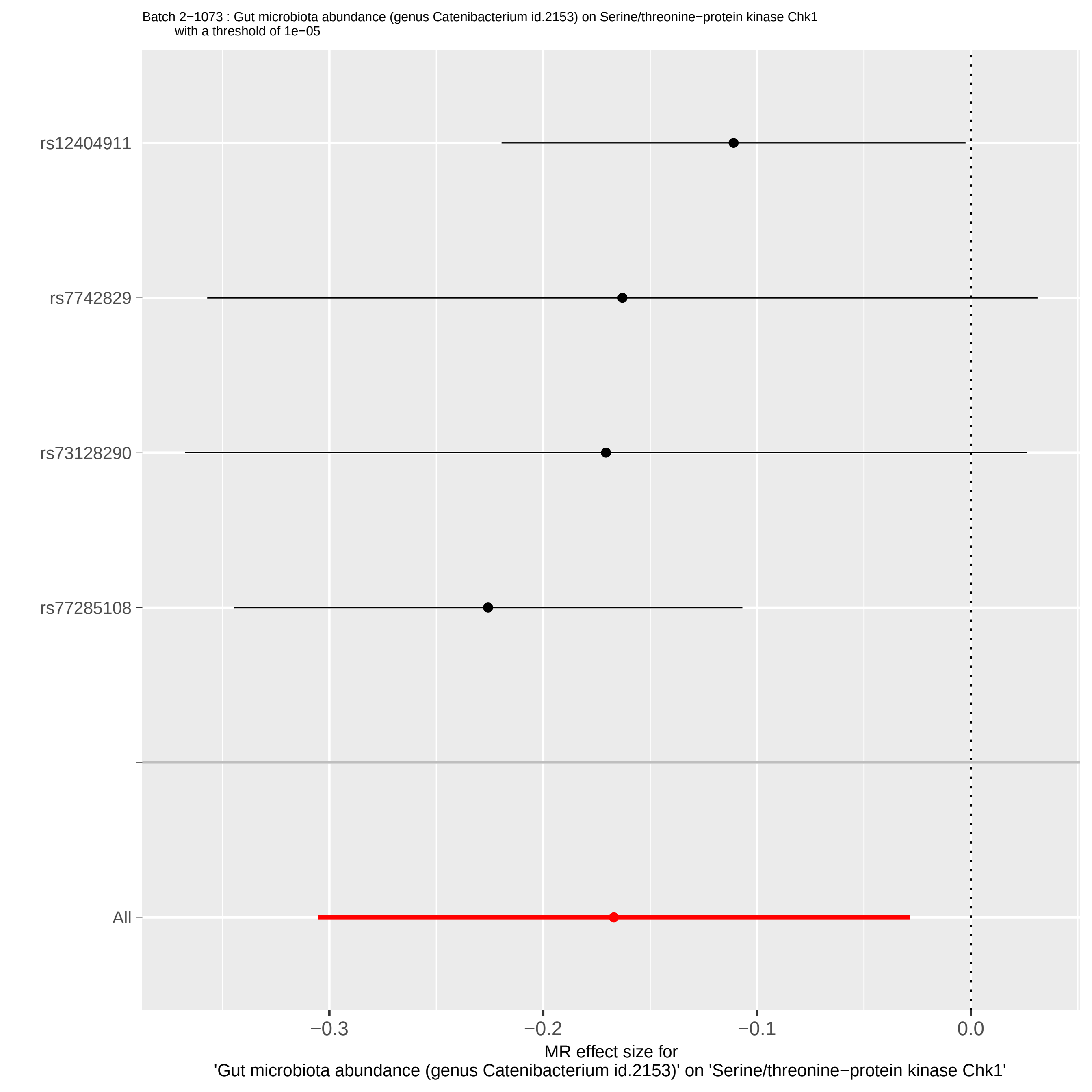
Batch 2−1073 : Gut microbiota abundance (genus Catenibacterium id.2153) on Serine/threonine−protein kinase Chk1
with a threshold of 1e−05
rs12404911
rs7742829
rs73128290
rs77285108
All
−0.3
−0.2
−0.1
0.0
MR effect size for
'Gut microbiota abundance (genus Catenibacterium id.2153)' on 'Serine/threonine−protein kinase Chk1'

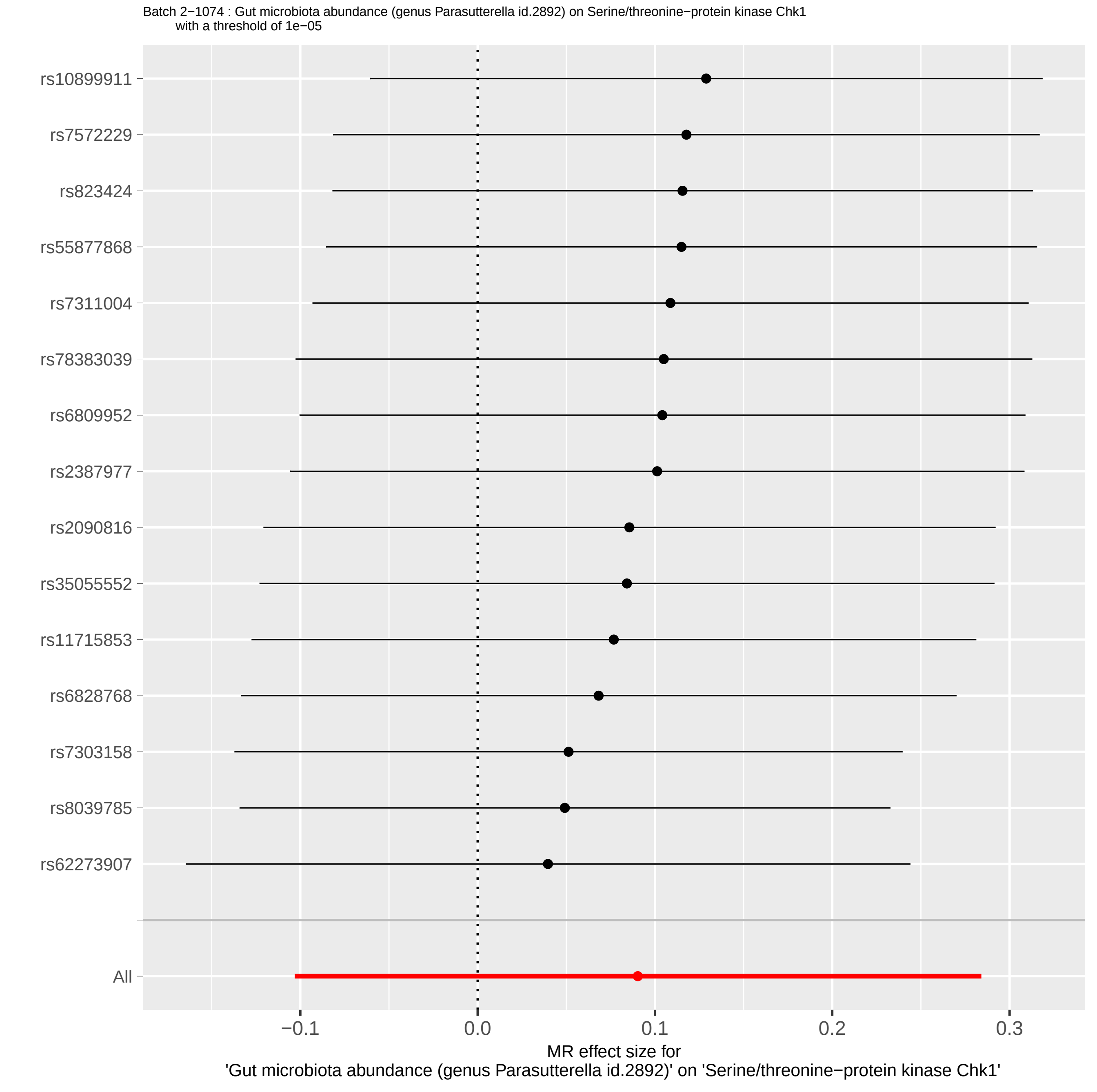

Batch 2−1074 : Gut microbiota abundance (genus Parasutterella id.2892) on Serine/threonine−protein kinase Chk1
with a threshold of 1e−05
rs10899911
rs7572229
rs823424
rs55877868
rs7311004
rs78383039
rs6809952
rs2387977
rs2090816
rs35055552
rs11715853
rs6828768
rs7303158
rs8039785
rs62273907
All
−0.1
0.0
0.1
0.2
0.3
MR effect size for
'Gut microbiota abundance (genus Parasutterella id.2892)' on 'Serine/threonine−protein kinase Chk1'

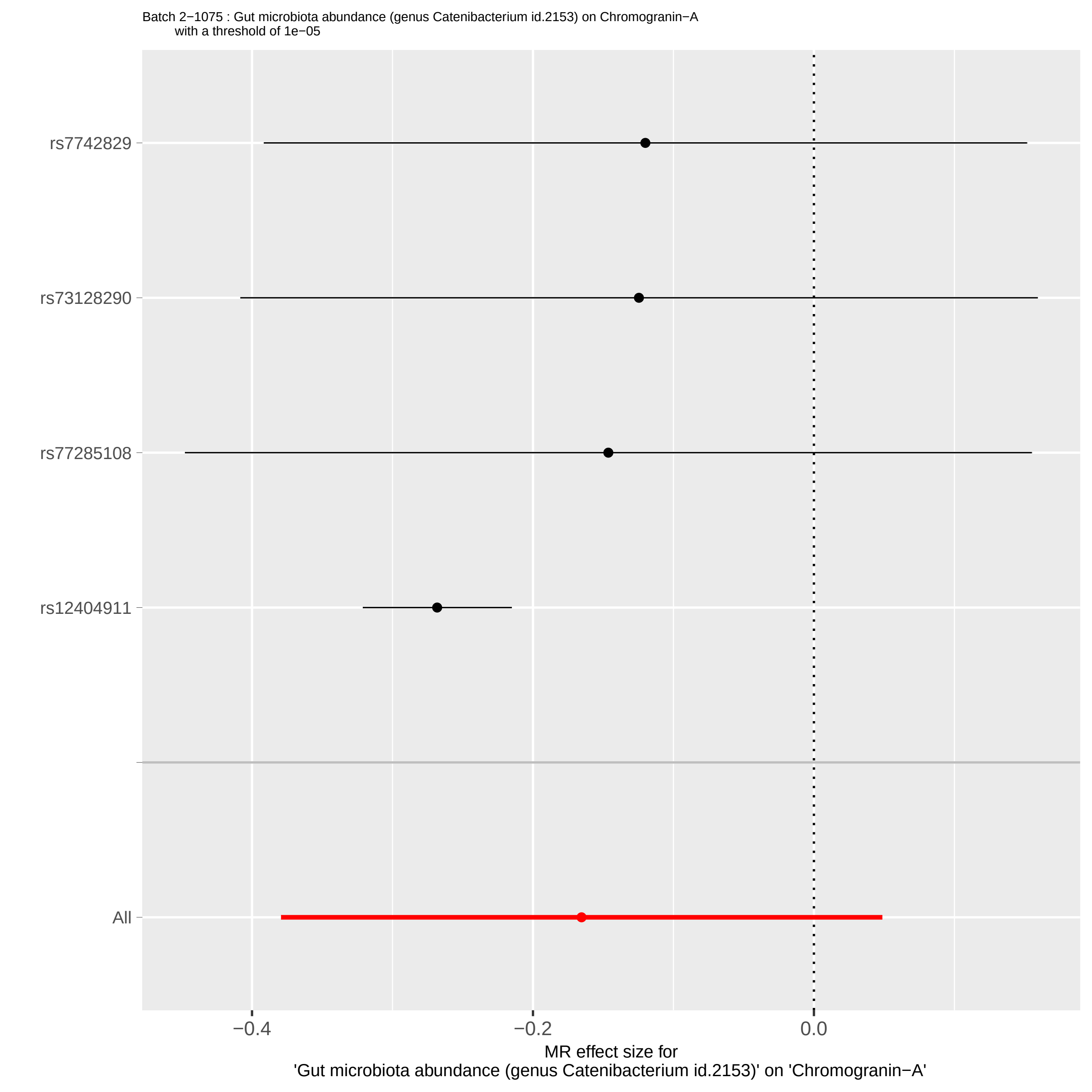
Batch 2−1075 : Gut microbiota abundance (genus Catenibacterium id.2153) on Chromogranin−A
with a threshold of 1e−05
rs7742829
rs73128290
rs77285108
rs12404911
All
−0.4
−0.2
0.0
MR effect size for
'Gut microbiota abundance (genus Catenibacterium id.2153)' on 'Chromogranin−A'

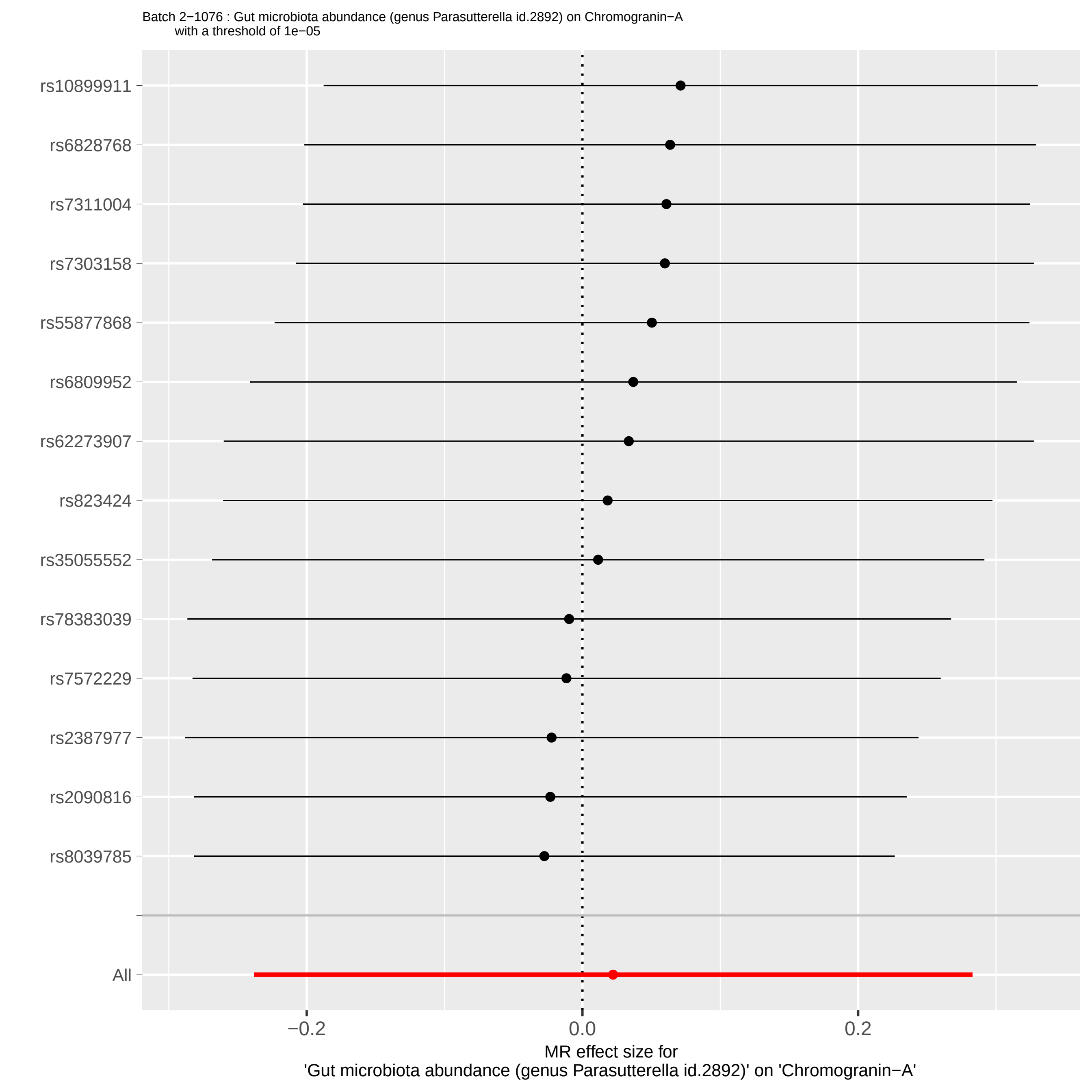

Batch 2−1076 : Gut microbiota abundance (genus Parasutterella id.2892) on Chromogranin−A
with a threshold of 1e−05
rs10899911
rs6828768
rs7311004
rs7303158
rs55877868
rs6809952
rs62273907
rs823424
rs35055552
rs78383039
rs7572229
rs2387977
rs2090816
rs8039785
All
−0.2
0.0
0.2
MR effect size for
'Gut microbiota abundance (genus Parasutterella id.2892)' on 'Chromogranin−A'

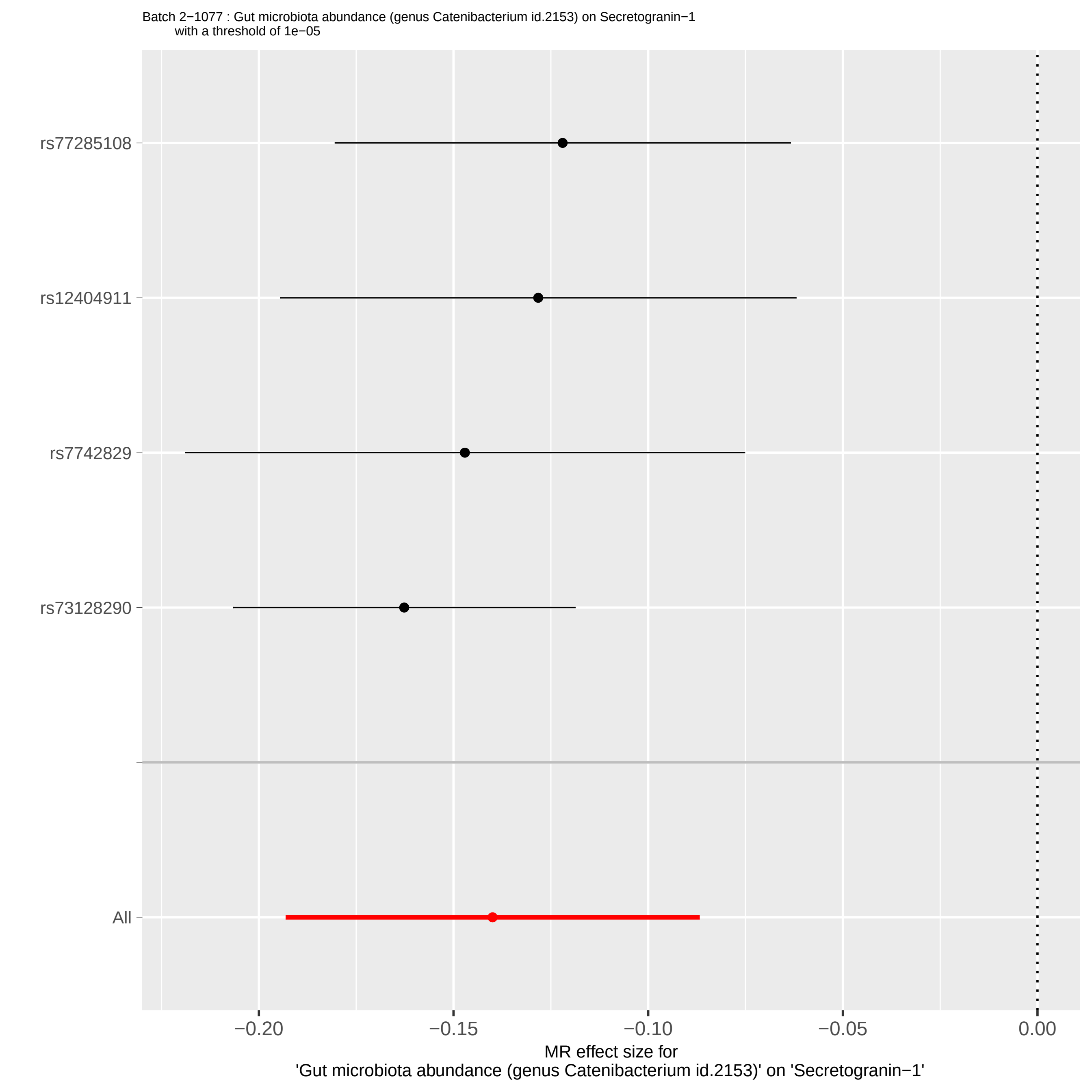
Batch 2−1077 : Gut microbiota abundance (genus Catenibacterium id.2153) on Secretogranin−1
with a threshold of 1e−05
rs77285108
rs12404911
rs7742829
rs73128290
All
−0.20
−0.15
−0.10
−0.05
0.00
MR effect size for
'Gut microbiota abundance (genus Catenibacterium id.2153)' on 'Secretogranin−1'

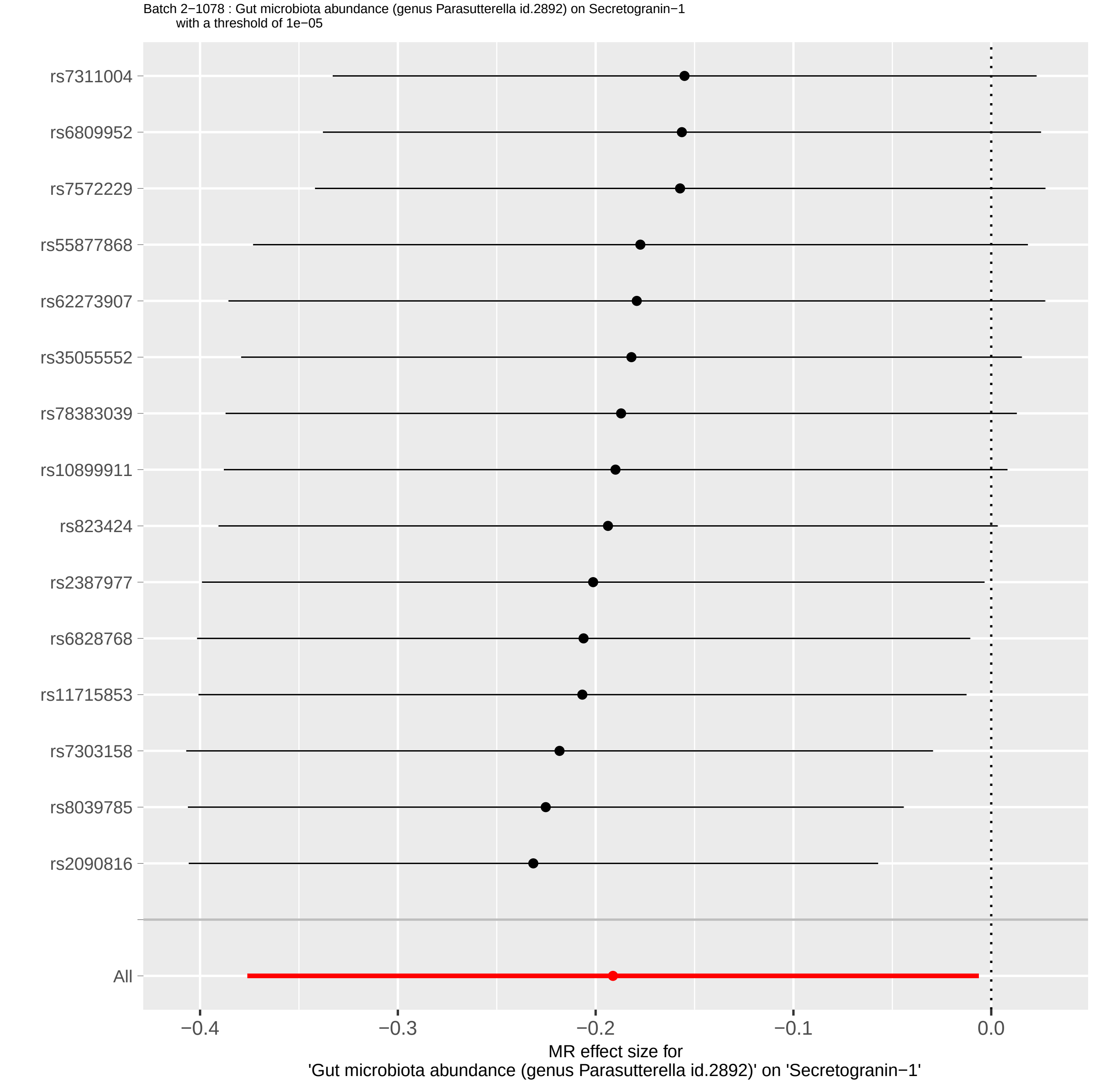
Batch 2−1078 : Gut microbiota abundance (genus Parasutterella id.2892) on Secretogranin−1
with a threshold of 1e−05
rs7311004
rs6809952
rs7572229
rs55877868
rs62273907
rs35055552
rs78383039
rs10899911
rs823424
rs2387977
rs6828768
rs11715853
rs7303158
rs8039785
rs2090816
All
−0.4
−0.3
−0.2
−0.1
0.0
MR effect size for
'Gut microbiota abundance (genus Parasutterella id.2892)' on 'Secretogranin−1'

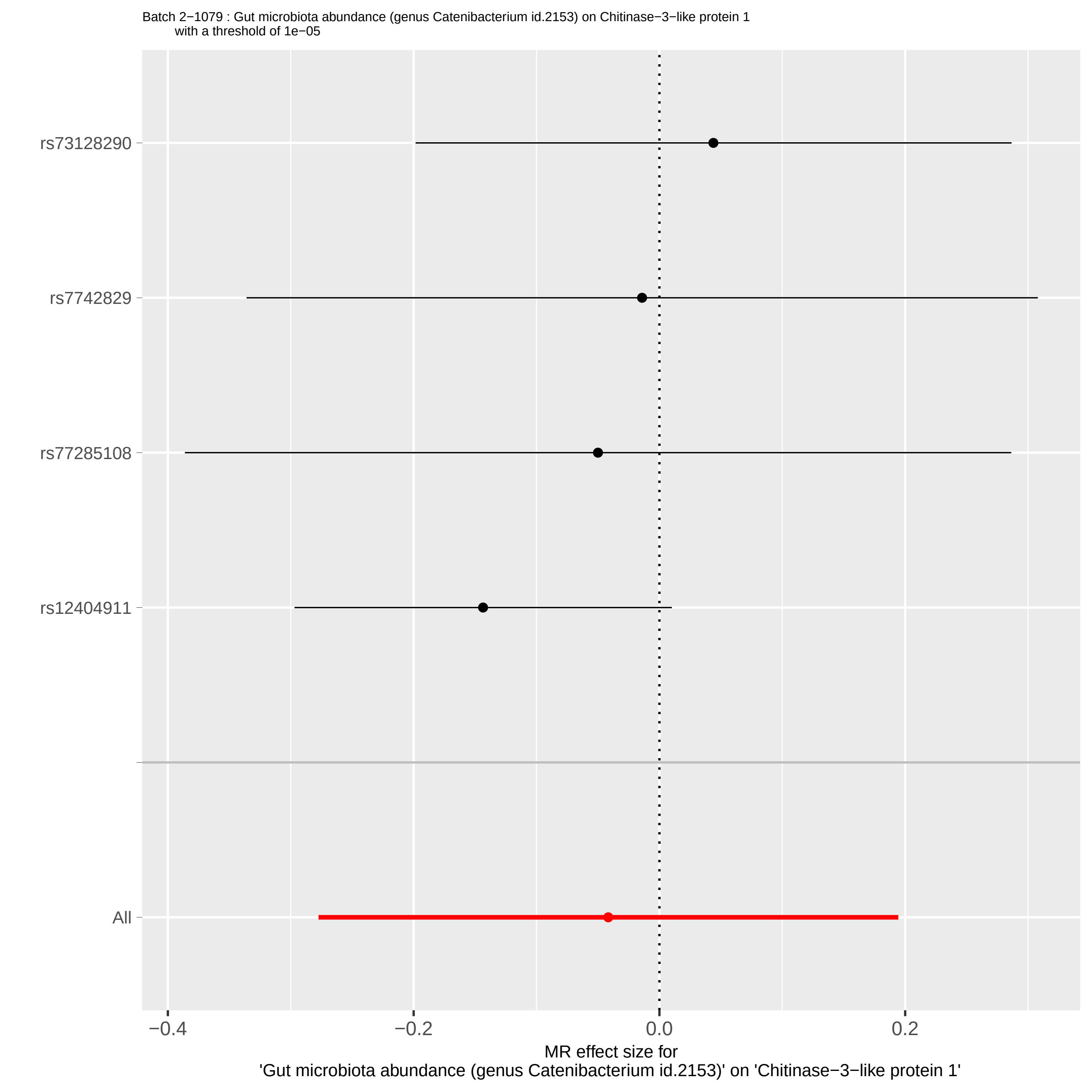
Batch 2−1079 : Gut microbiota abundance (genus Catenibacterium id.2153) on Chitinase−3−like protein 1
with a threshold of 1e−05
rs73128290
rs7742829
rs77285108
rs12404911
All
−0.4
−0.2
0.0
0.2
MR effect size for
'Gut microbiota abundance (genus Catenibacterium id.2153)' on 'Chitinase−3−like protein 1'

Batch 2−1080 : Gut microbiota abundance (genus Parasutterella id.2892) on Chitinase−3−like protein 1
with a threshold of 1e−05

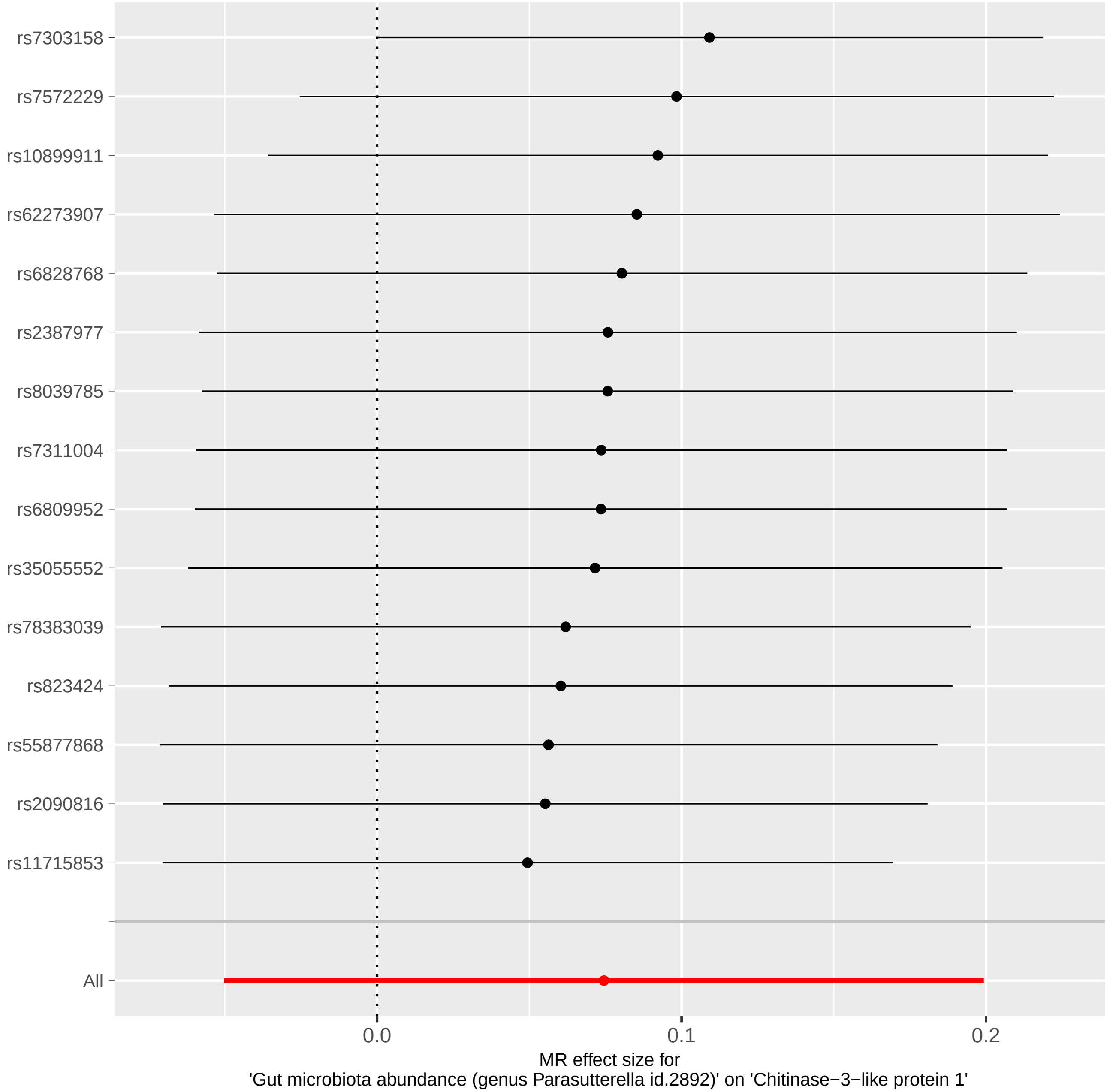

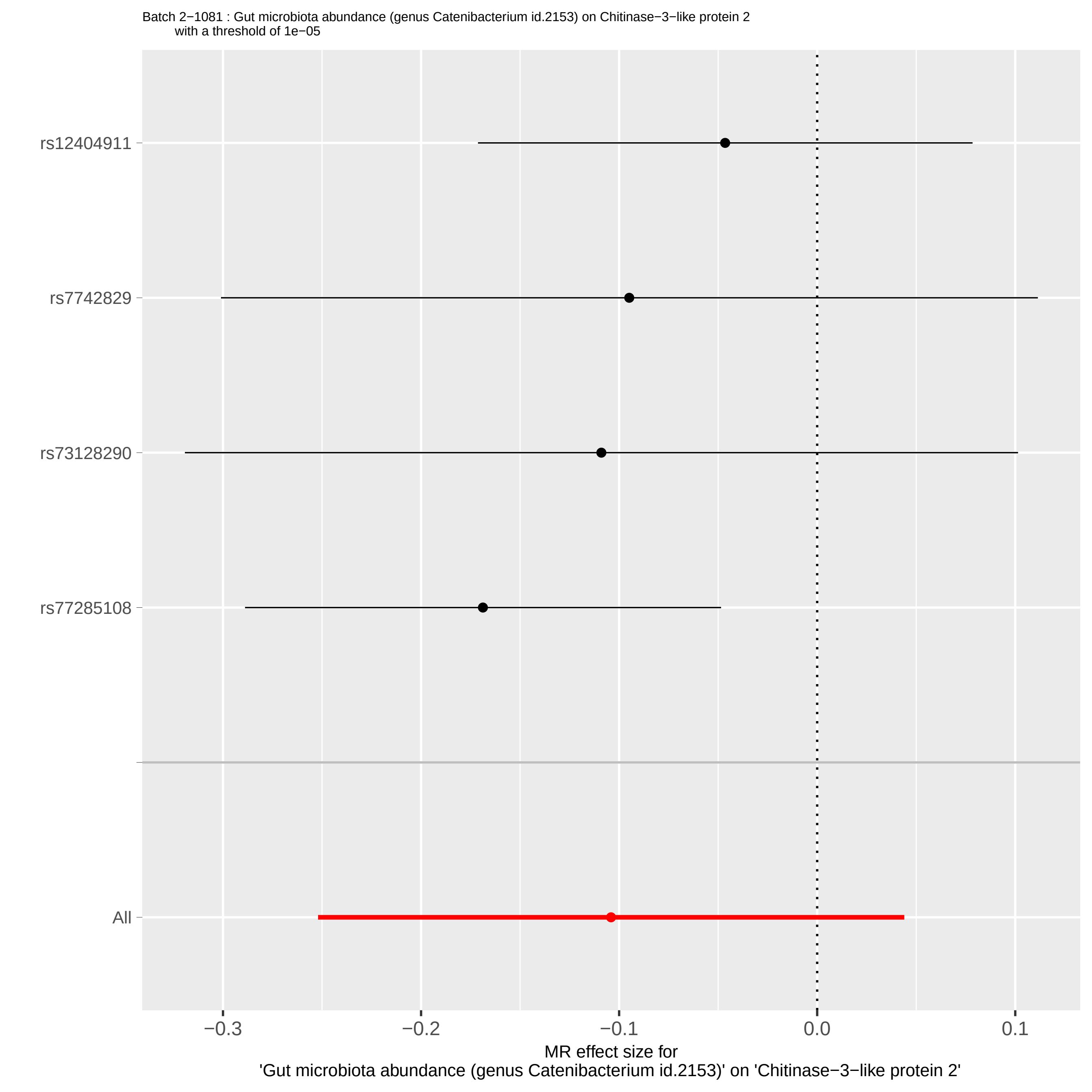
Batch 2−1081 : Gut microbiota abundance (genus Catenibacterium id.2153) on Chitinase−3−like protein 2
with a threshold of 1e−05
rs12404911
rs7742829
rs73128290
rs77285108
All
−0.3
−0.2
−0.1
0.0
0.1
MR effect size for
'Gut microbiota abundance (genus Catenibacterium id.2153)' on 'Chitinase−3−like protein 2'

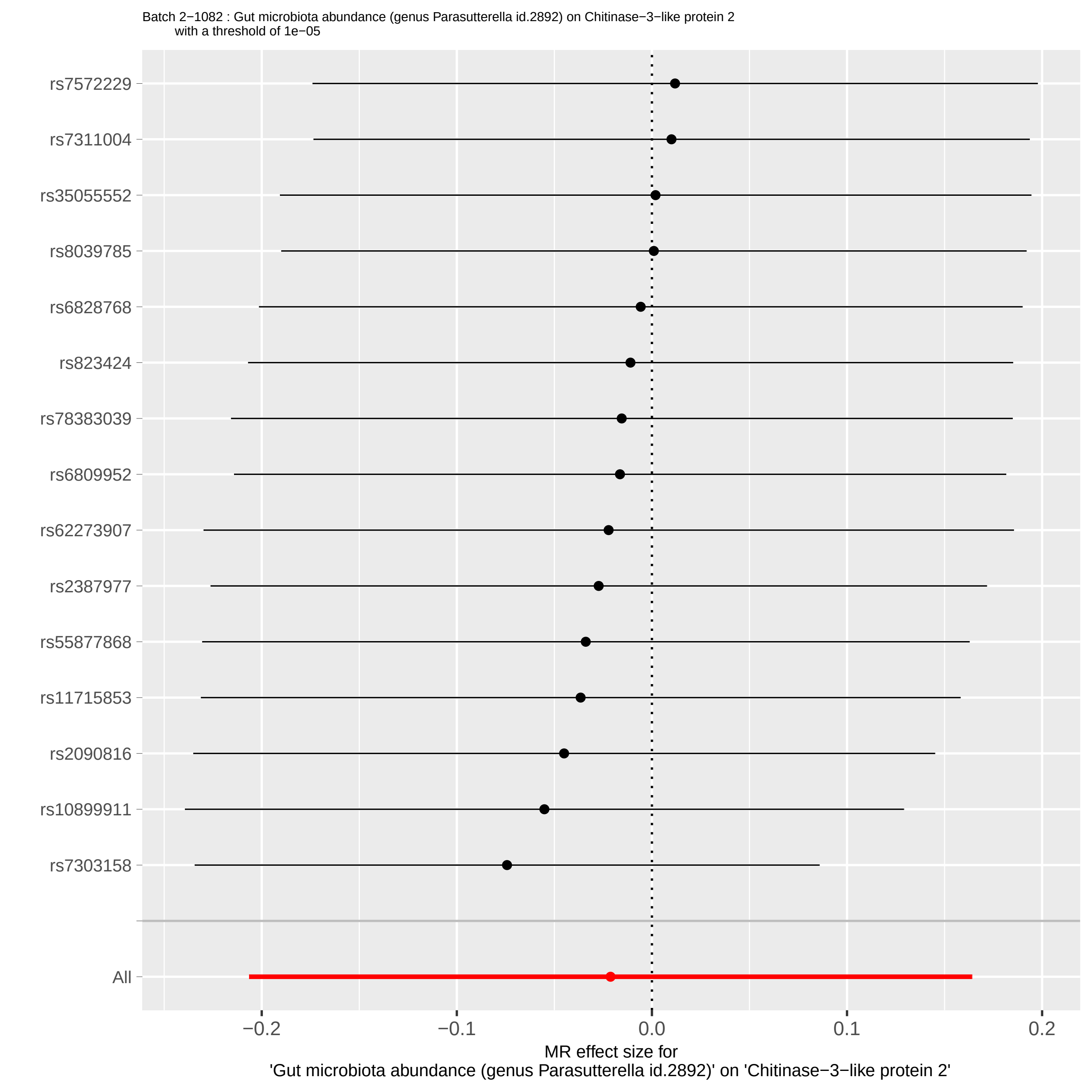

Batch 2−1082 : Gut microbiota abundance (genus Parasutterella id.2892) on Chitinase−3−like protein 2
with a threshold of 1e−05
rs7572229
rs7311004
rs35055552
rs8039785
rs6828768
rs823424
rs78383039
rs6809952
rs62273907
rs2387977
rs55877868
rs11715853
rs2090816
rs10899911
rs7303158
All
−0.2
−0.1
0.0
0.1
0.2
MR effect size for
'Gut microbiota abundance (genus Parasutterella id.2892)' on 'Chitinase−3−like protein 2'

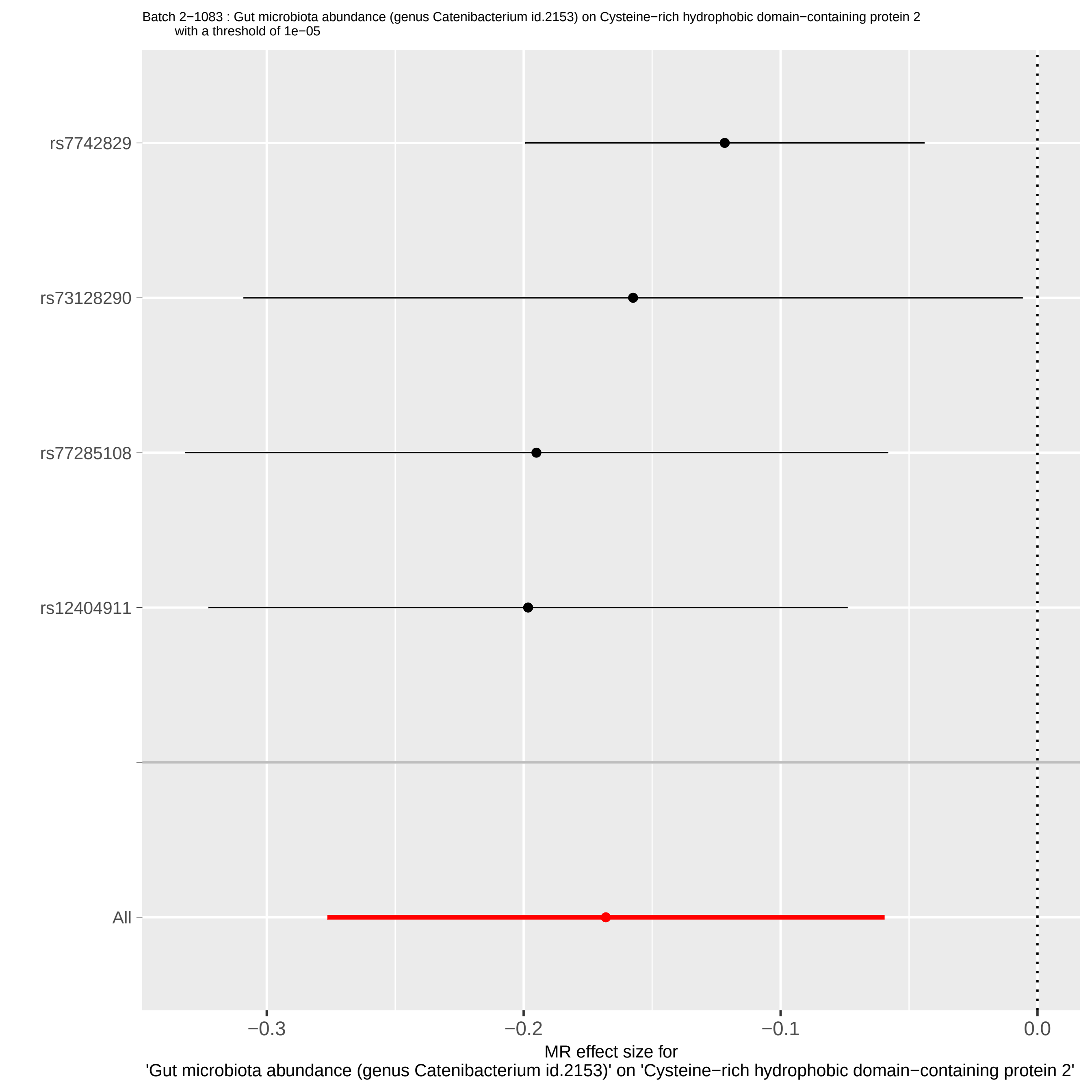

Batch 2−1083 : Gut microbiota abundance (genus Catenibacterium id.2153) on Cysteine−rich hydrophobic domain−containing protein 2
with a threshold of 1e−05
rs7742829
rs73128290
rs77285108
rs12404911
All
−0.3
−0.2
−0.1
0.0
MR effect size for
'Gut microbiota abundance (genus Catenibacterium id.2153)' on 'Cysteine−rich hydrophobic domain−containing protein 2'

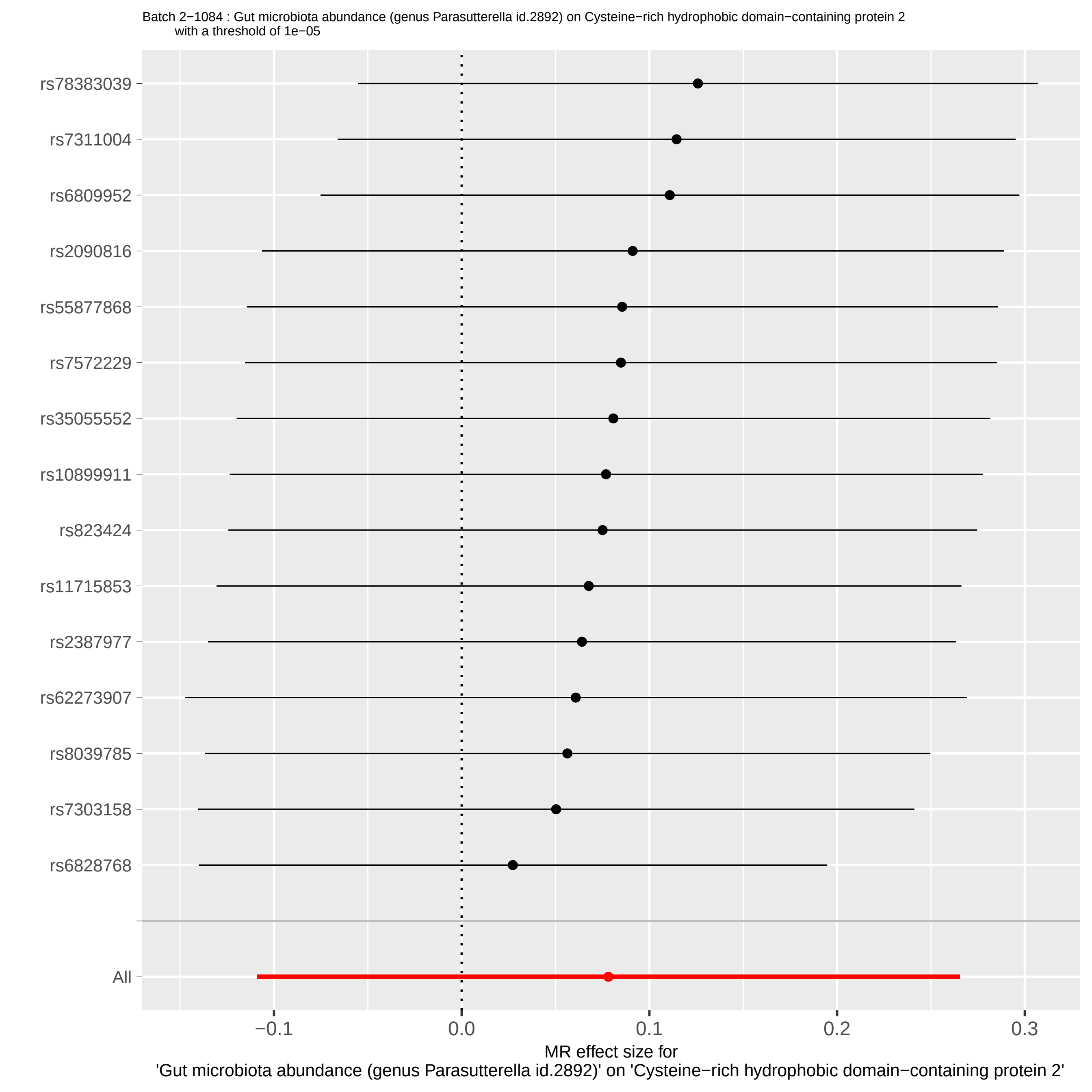

Batch 2−1084 : Gut microbiota abundance (genus Parasutterella id.2892) on Cysteine−rich hydrophobic domain−containing protein 2
with a threshold of 1e−05
rs78383039
rs7311004
rs6809952
rs2090816
rs55877868
rs7572229
rs35055552
rs10899911
rs823424
rs11715853
rs2387977
rs62273907
rs8039785
rs7303158
rs6828768
All
−0.1
0.0
0.1
0.2
0.3
MR effect size for
'Gut microbiota abundance (genus Parasutterella id.2892)' on 'Cysteine−rich hydrophobic domain−containing protein 2'

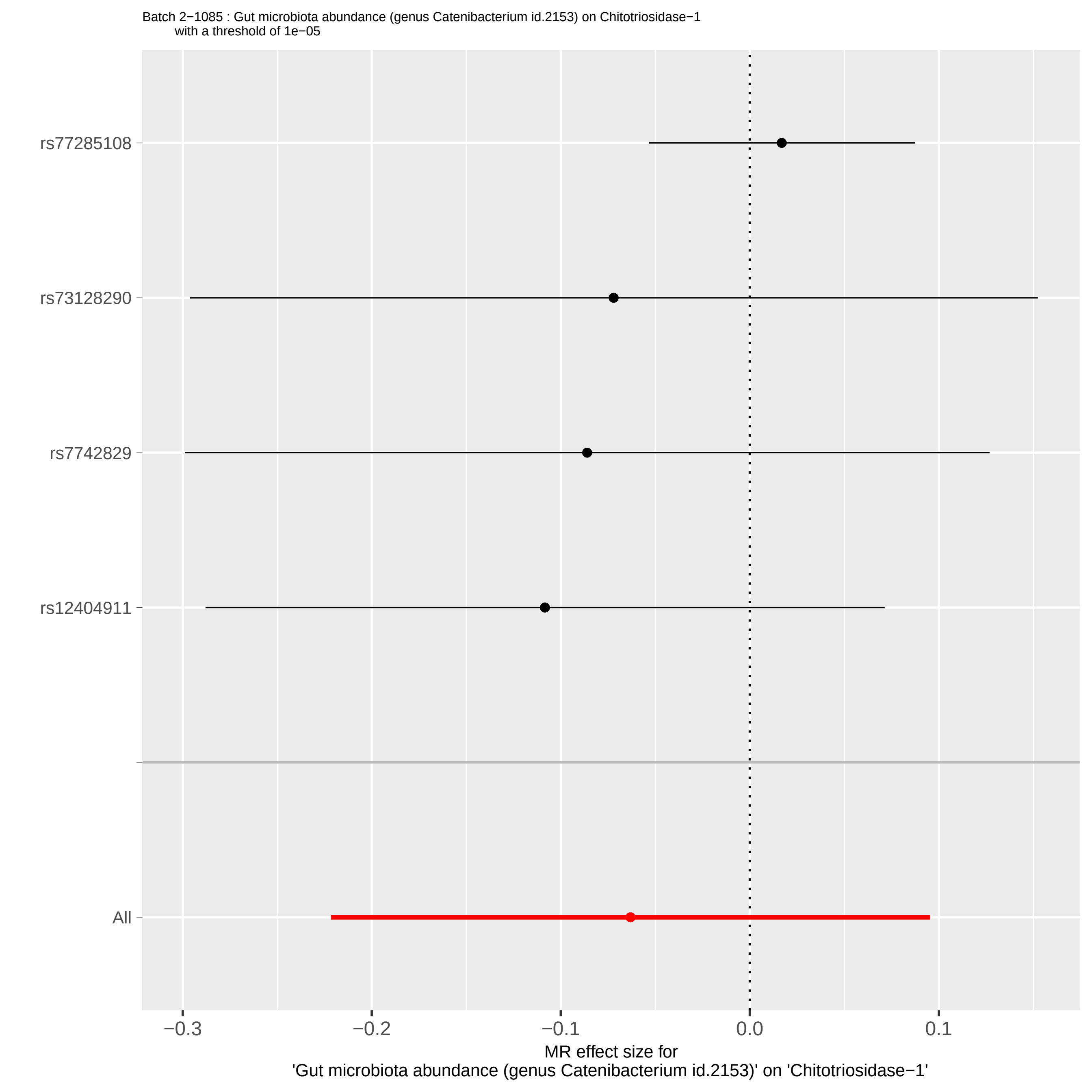

Batch 2−1085 : Gut microbiota abundance (genus Catenibacterium id.2153) on Chitotriosidase−1
with a threshold of 1e−05
rs77285108
rs73128290
rs7742829
rs12404911
All
−0.3
−0.2
−0.1
0.0
0.1
MR effect size for
'Gut microbiota abundance (genus Catenibacterium id.2153)' on 'Chitotriosidase−1'

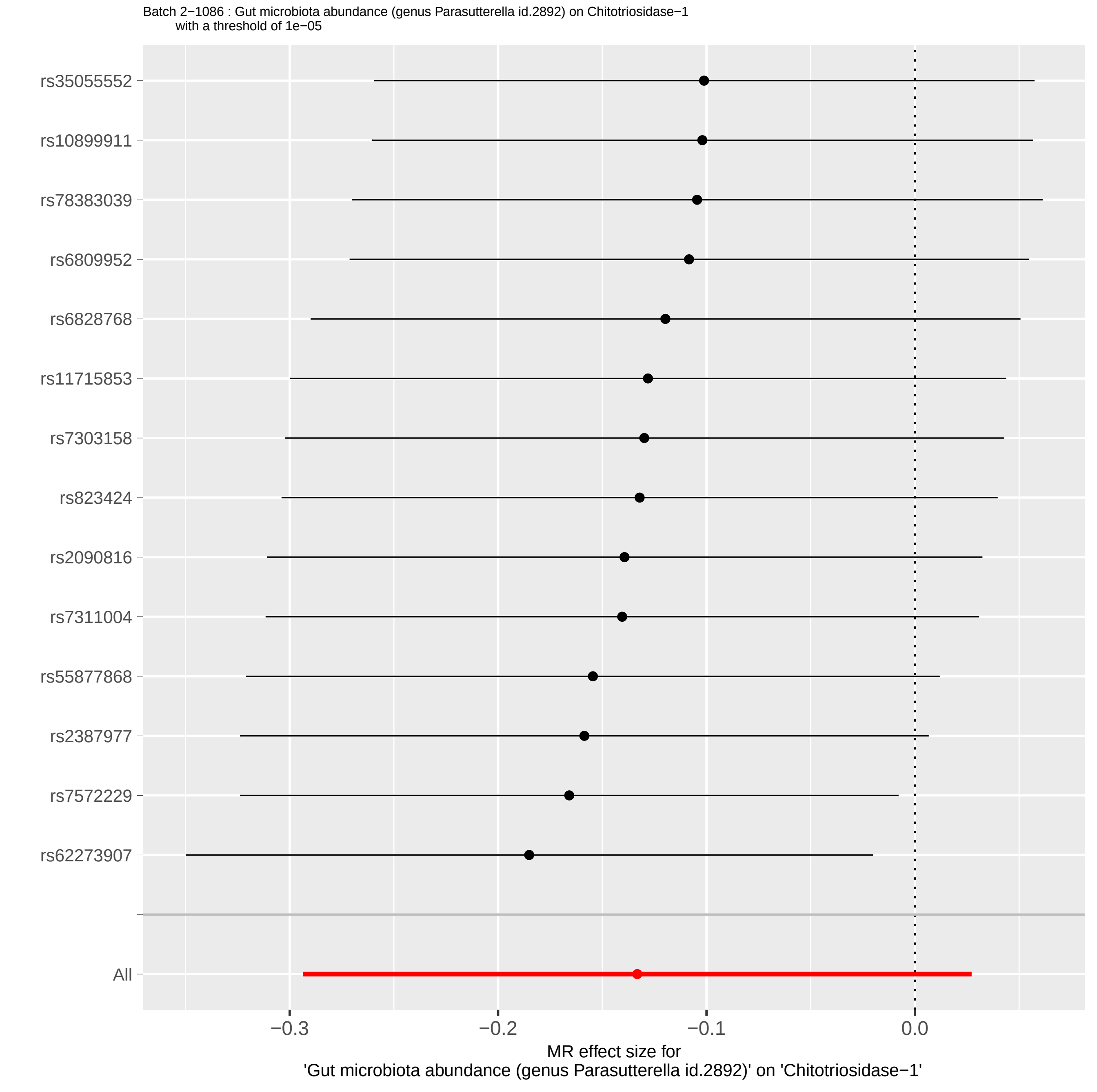

Batch 2−1086 : Gut microbiota abundance (genus Parasutterella id.2892) on Chitotriosidase−1
with a threshold of 1e−05
rs35055552
rs10899911
rs78383039
rs6809952
rs6828768
rs11715853
rs7303158
rs823424
rs2090816
rs7311004
rs55877868
rs2387977
rs7572229
rs62273907
All
−0.3
−0.2
−0.1
0.0
MR effect size for
'Gut microbiota abundance (genus Parasutterella id.2892)' on 'Chitotriosidase−1'

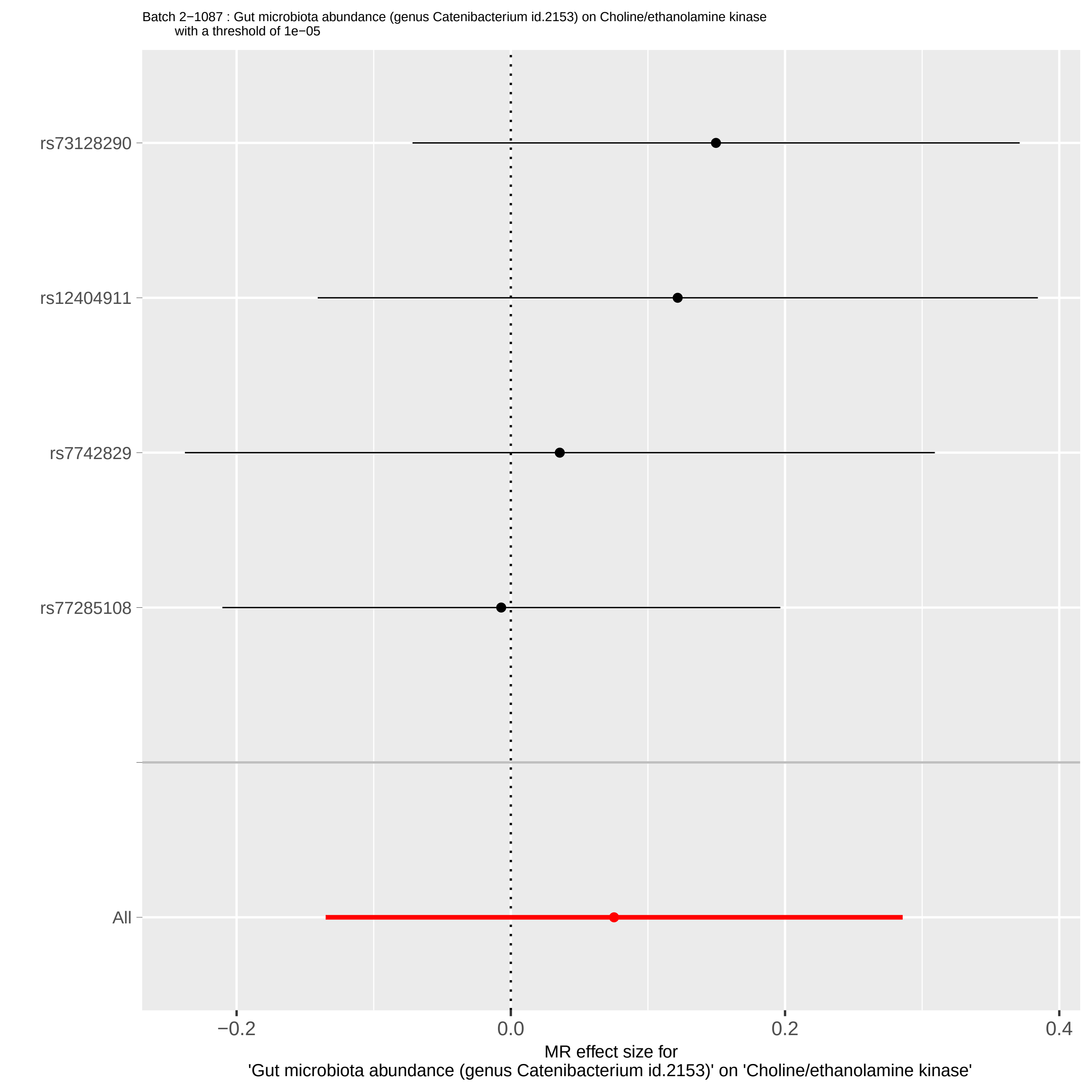
Batch 2−1087 : Gut microbiota abundance (genus Catenibacterium id.2153) on Choline/ethanolamine kinase
with a threshold of 1e−05
rs73128290
rs12404911
rs7742829
rs77285108
All
−0.2
0.0
0.2
0.4
MR effect size for
'Gut microbiota abundance (genus Catenibacterium id.2153)' on 'Choline/ethanolamine kinase'

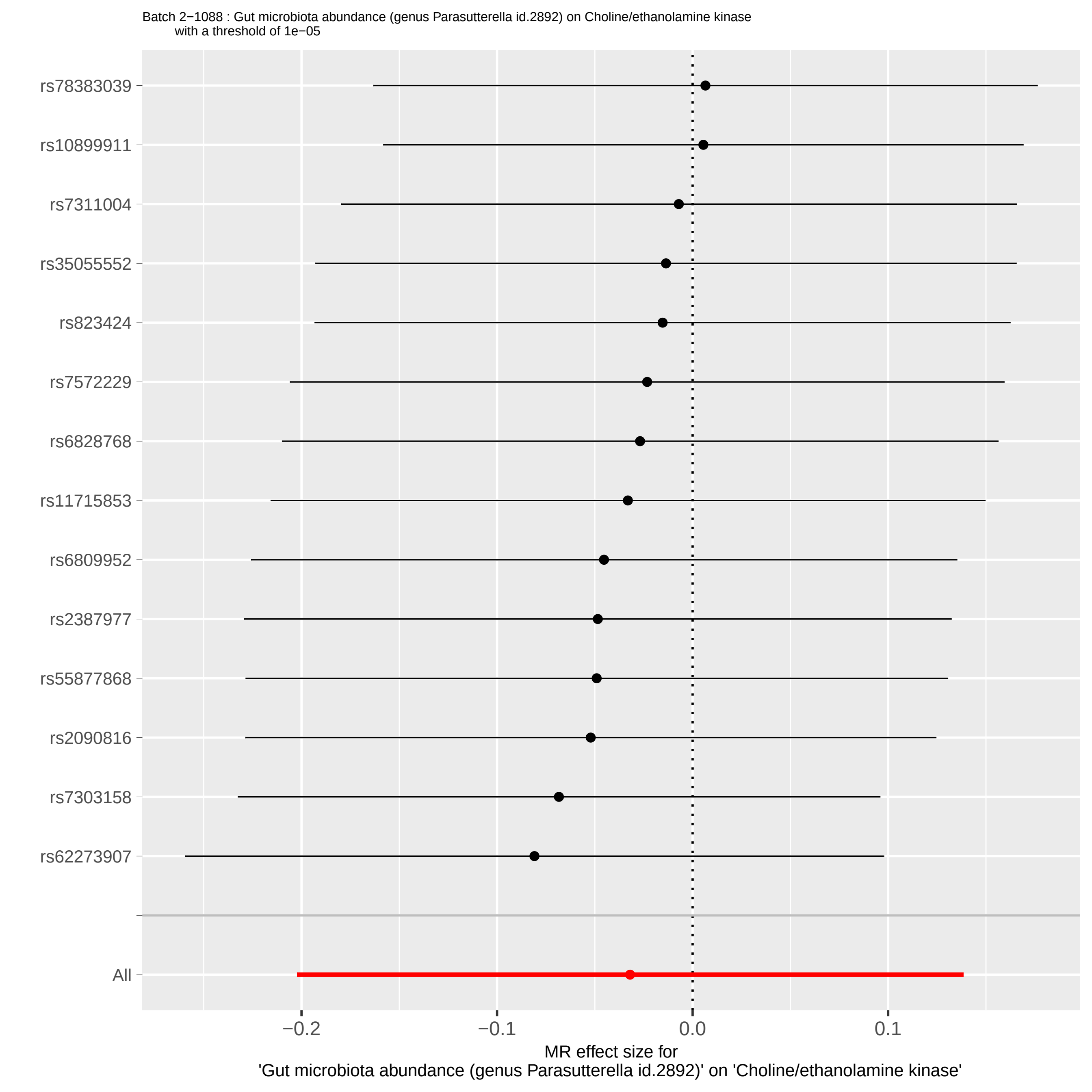
Batch 2−1088 : Gut microbiota abundance (genus Parasutterella id.2892) on Choline/ethanolamine kinase
with a threshold of 1e−05
rs78383039
rs10899911
rs7311004
rs35055552
rs823424
rs7572229
rs6828768
rs11715853
rs6809952
rs2387977
rs55877868
rs2090816
rs7303158
rs62273907
All
−0.2
−0.1
0.0
0.1
MR effect size for
'Gut microbiota abundance (genus Parasutterella id.2892)' on 'Choline/ethanolamine kinase'

Batch 2−1089 : Gut microbiota abundance (genus Catenibacterium id.2153) on Neural cell adhesion molecule L1−like protein
with a threshold of 1e−05

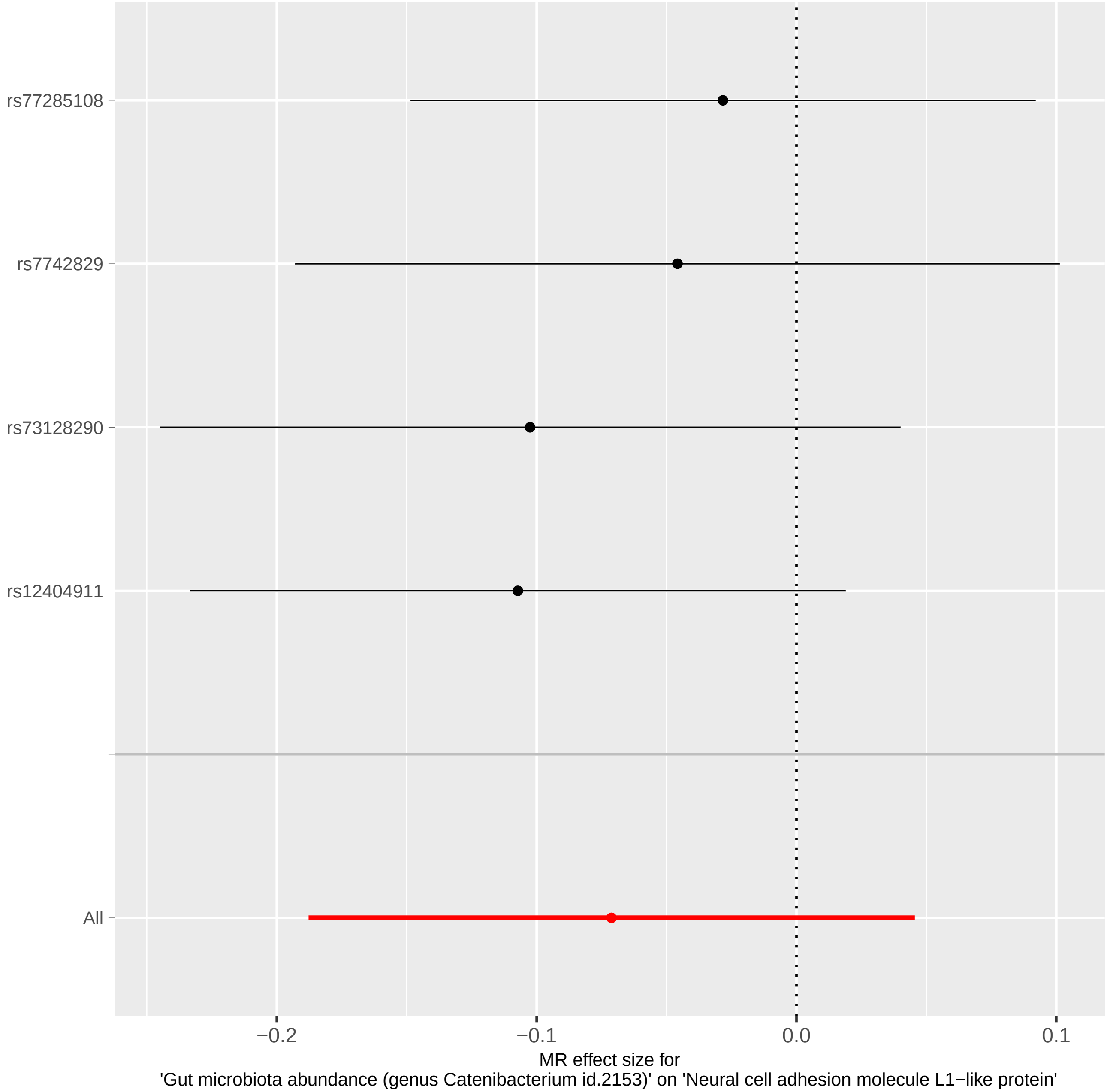

Batch 2−1090 : Gut microbiota abundance (genus Parasutterella id.2892) on Neural cell adhesion molecule L1−like protein
with a threshold of 1e−05

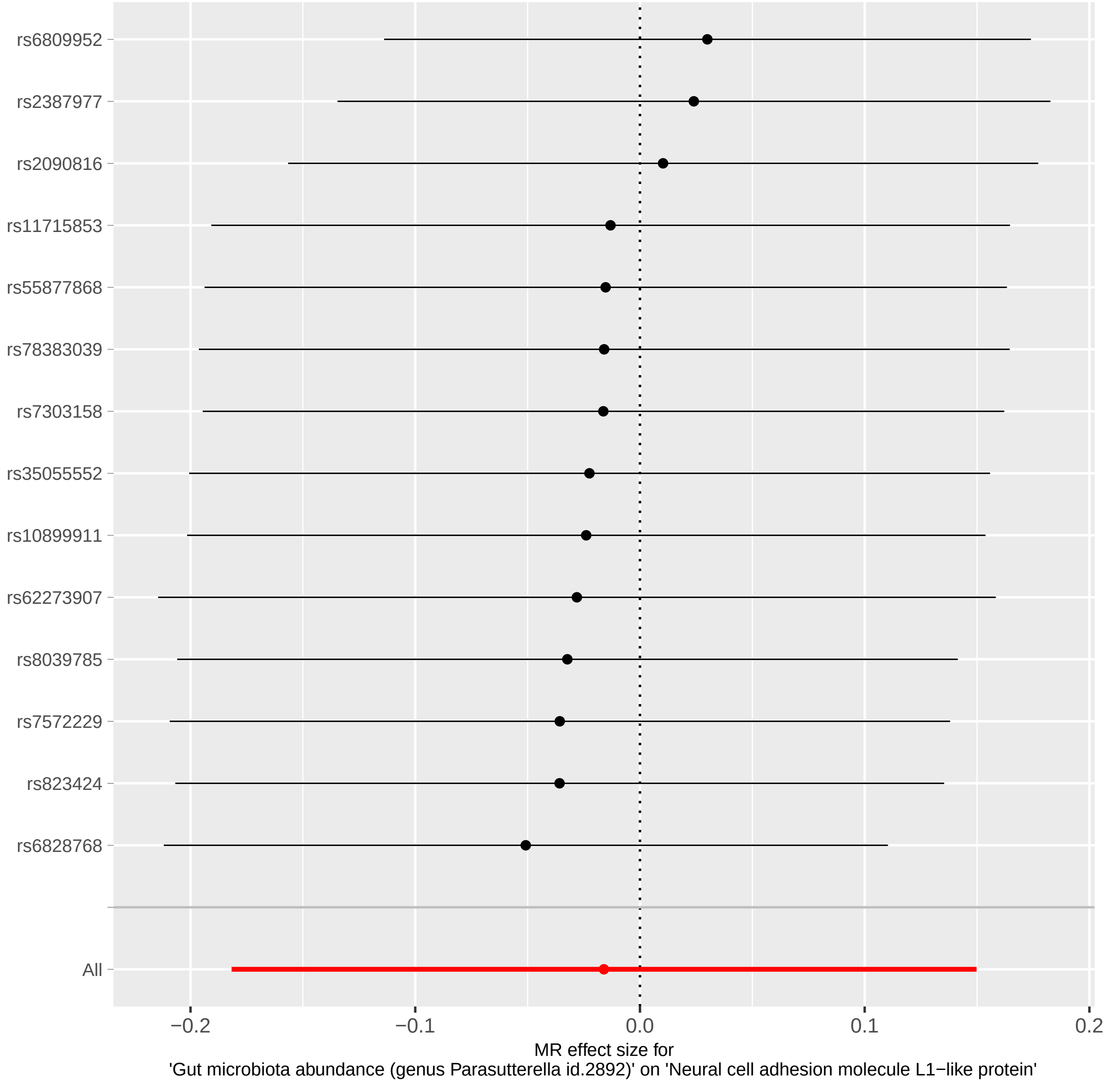

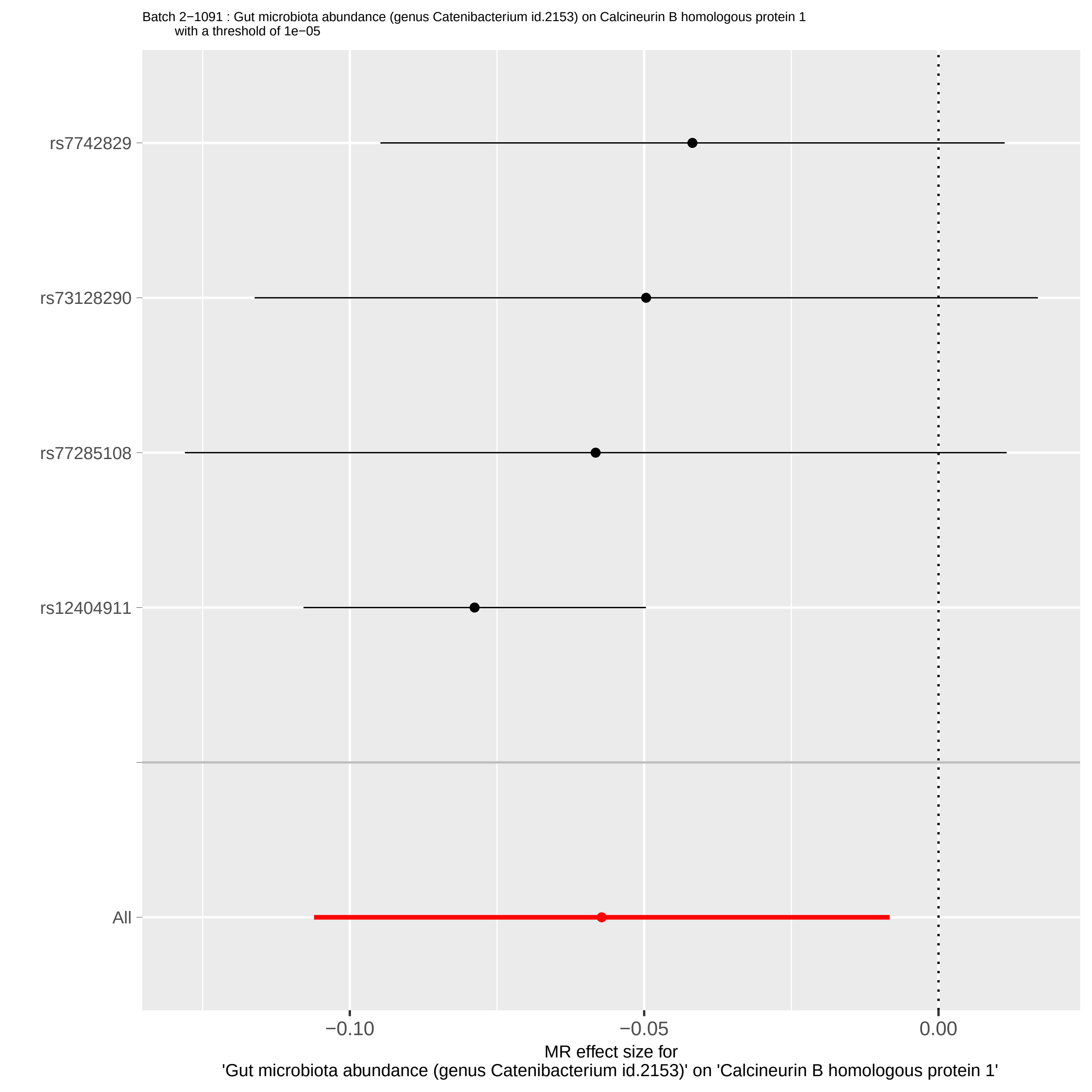

Batch 2−1091 : Gut microbiota abundance (genus Catenibacterium id.2153) on Calcineurin B homologous protein 1
with a threshold of 1e−05
rs7742829
rs73128290
rs77285108
rs12404911
All
−0.10
−0.05
0.00
MR effect size for
'Gut microbiota abundance (genus Catenibacterium id.2153)' on 'Calcineurin B homologous protein 1'

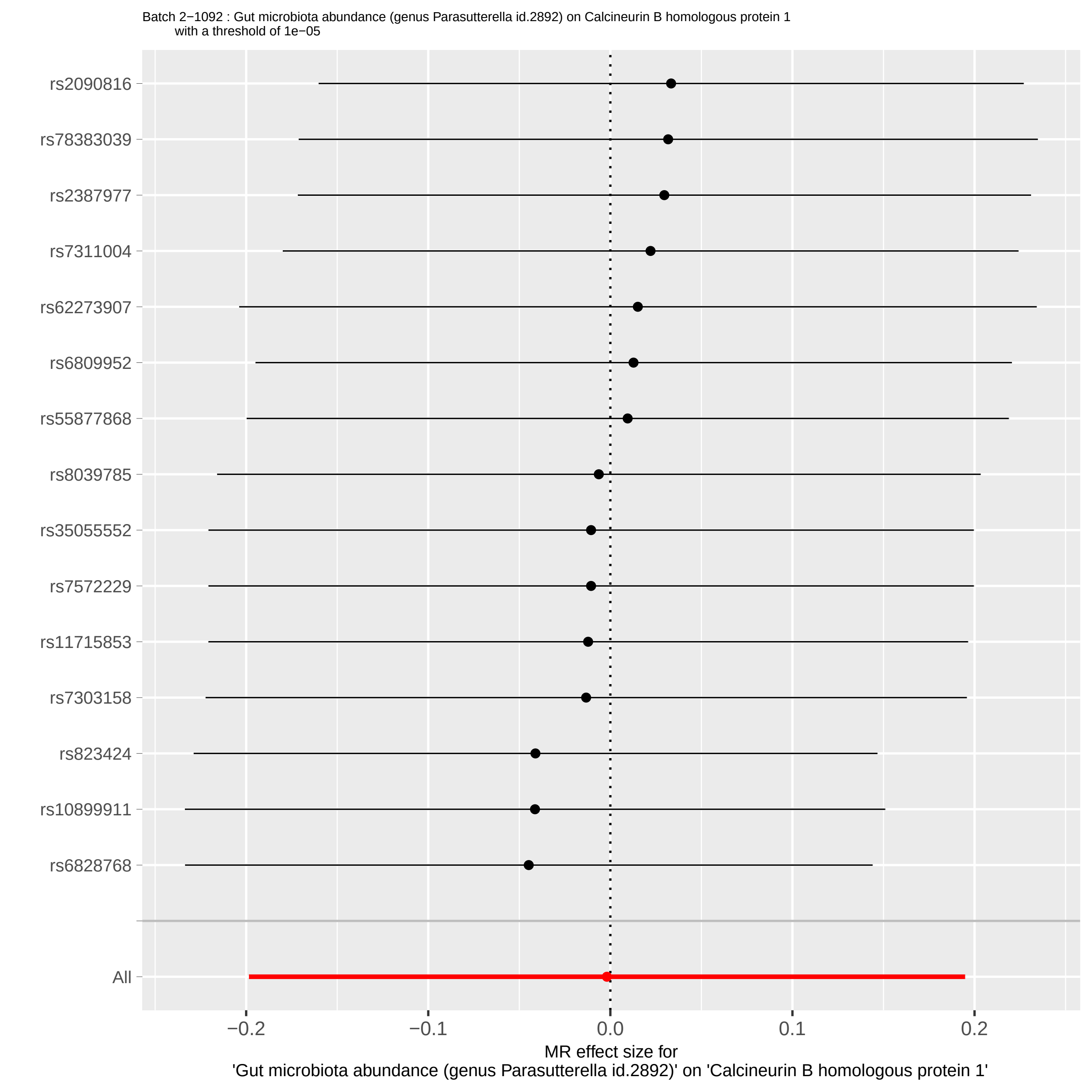

Batch 2−1092 : Gut microbiota abundance (genus Parasutterella id.2892) on Calcineurin B homologous protein 1
with a threshold of 1e−05
rs2090816
rs78383039
rs2387977
rs7311004
rs62273907
rs6809952
rs55877868
rs8039785
rs35055552
rs7572229
rs11715853
rs7303158
rs823424
rs10899911
rs6828768
All
−0.2
−0.1
0.0
0.1
0.2
MR effect size for
'Gut microbiota abundance (genus Parasutterella id.2892)' on 'Calcineurin B homologous protein 1'

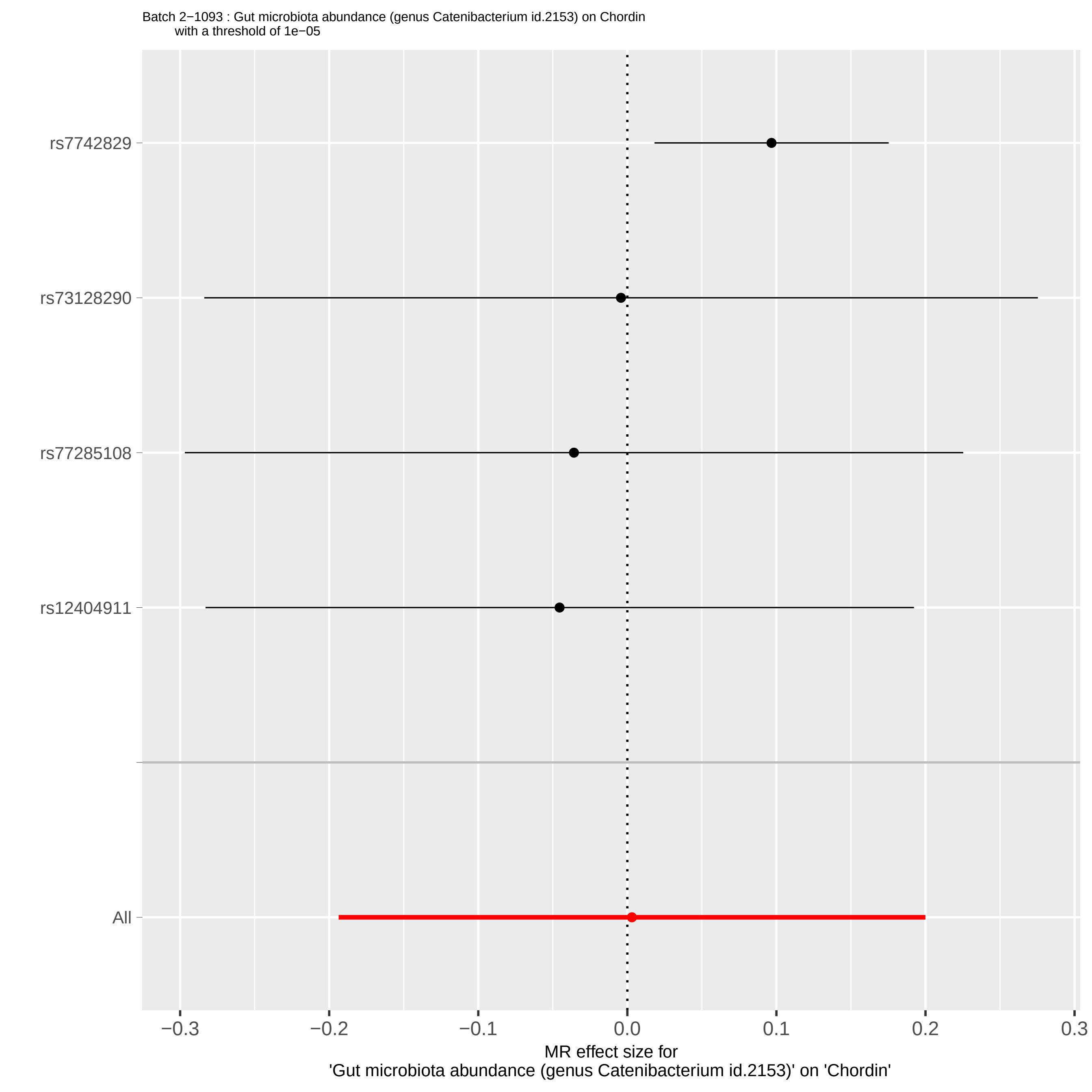

Batch 2−1093 : Gut microbiota abundance (genus Catenibacterium id.2153) on Chordin
with a threshold of 1e−05
rs7742829
rs73128290
rs77285108
rs12404911
All
−0.3
−0.2
−0.1
0.0
0.1
0.2
0.3
MR effect size for
'Gut microbiota abundance (genus Catenibacterium id.2153)' on 'Chordin'

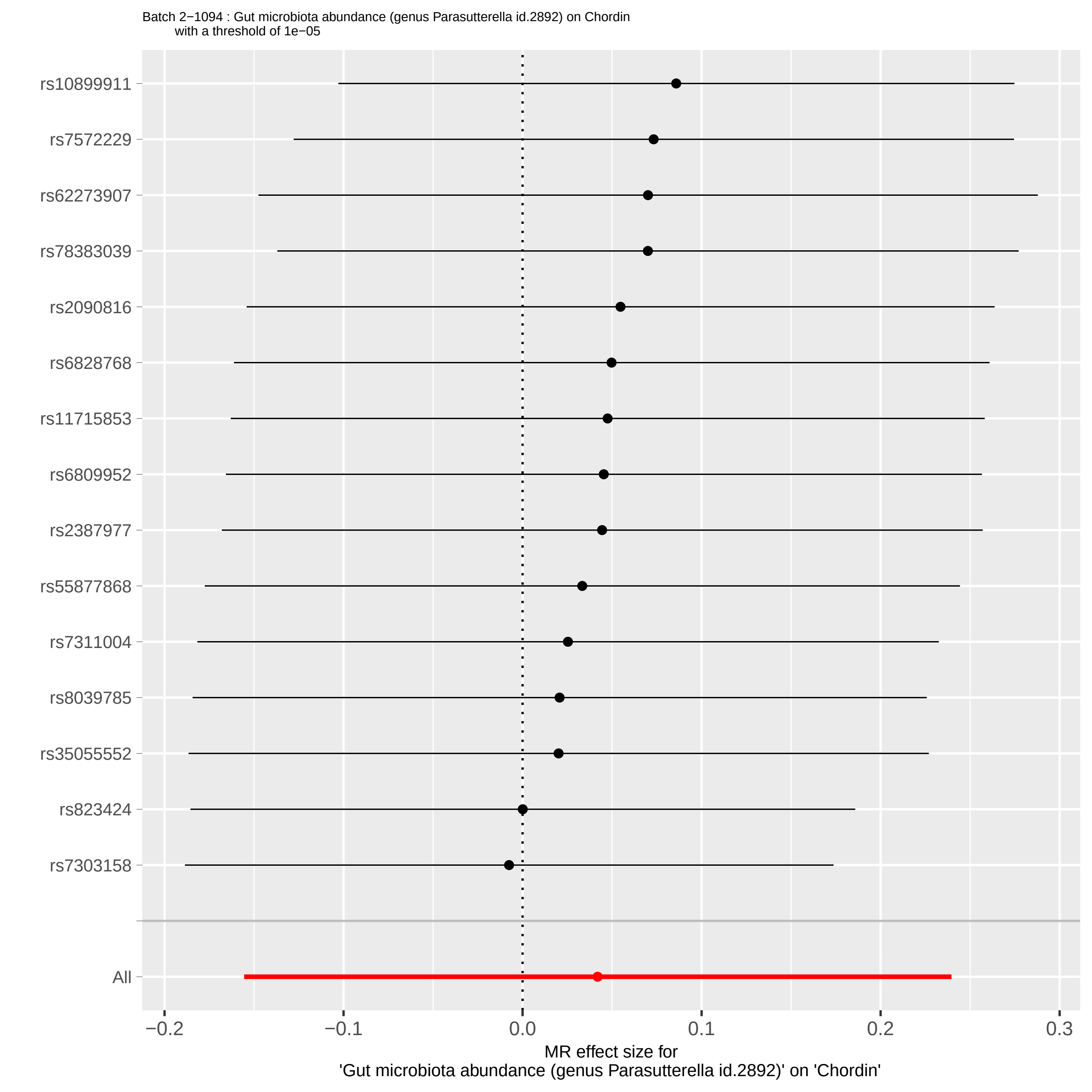
Batch 2−1094 : Gut microbiota abundance (genus Parasutterella id.2892) on Chordin
with a threshold of 1e−05
rs10899911
rs7572229
rs62273907
rs78383039
rs2090816
rs6828768
rs11715853
rs6809952
rs2387977
rs55877868
rs7311004
rs8039785
rs35055552
rs823424
rs7303158
All
−0.2
−0.1
0.0
0.1
0.2
0.3
MR effect size for
'Gut microbiota abundance (genus Parasutterella id.2892)' on 'Chordin'

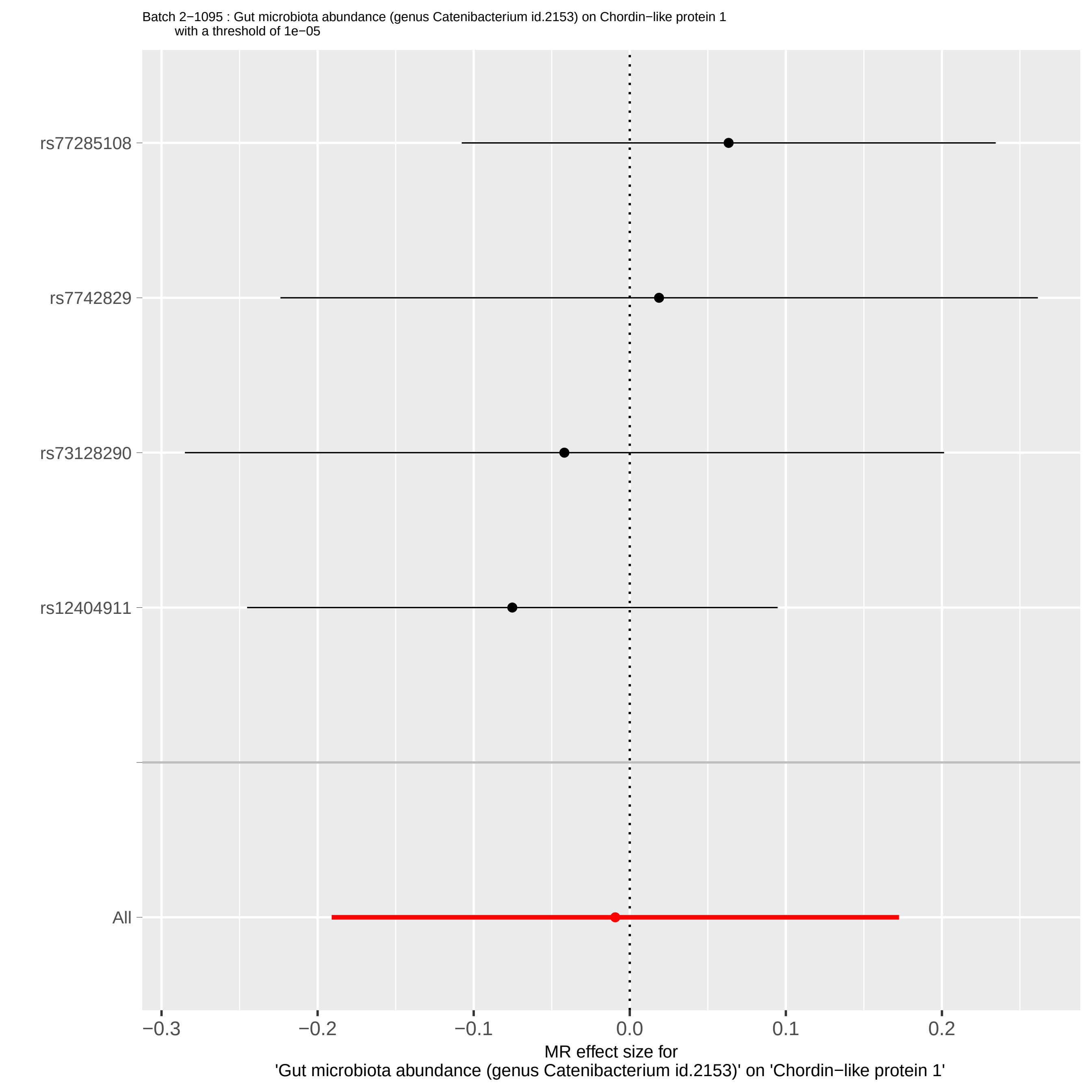
Batch 2−1095 : Gut microbiota abundance (genus Catenibacterium id.2153) on Chordin−like protein 1
with a threshold of 1e−05
rs77285108
rs7742829
rs73128290
rs12404911
All
−0.3
−0.2
−0.1
0.0
0.1
0.2
MR effect size for
'Gut microbiota abundance (genus Catenibacterium id.2153)' on 'Chordin−like protein 1'

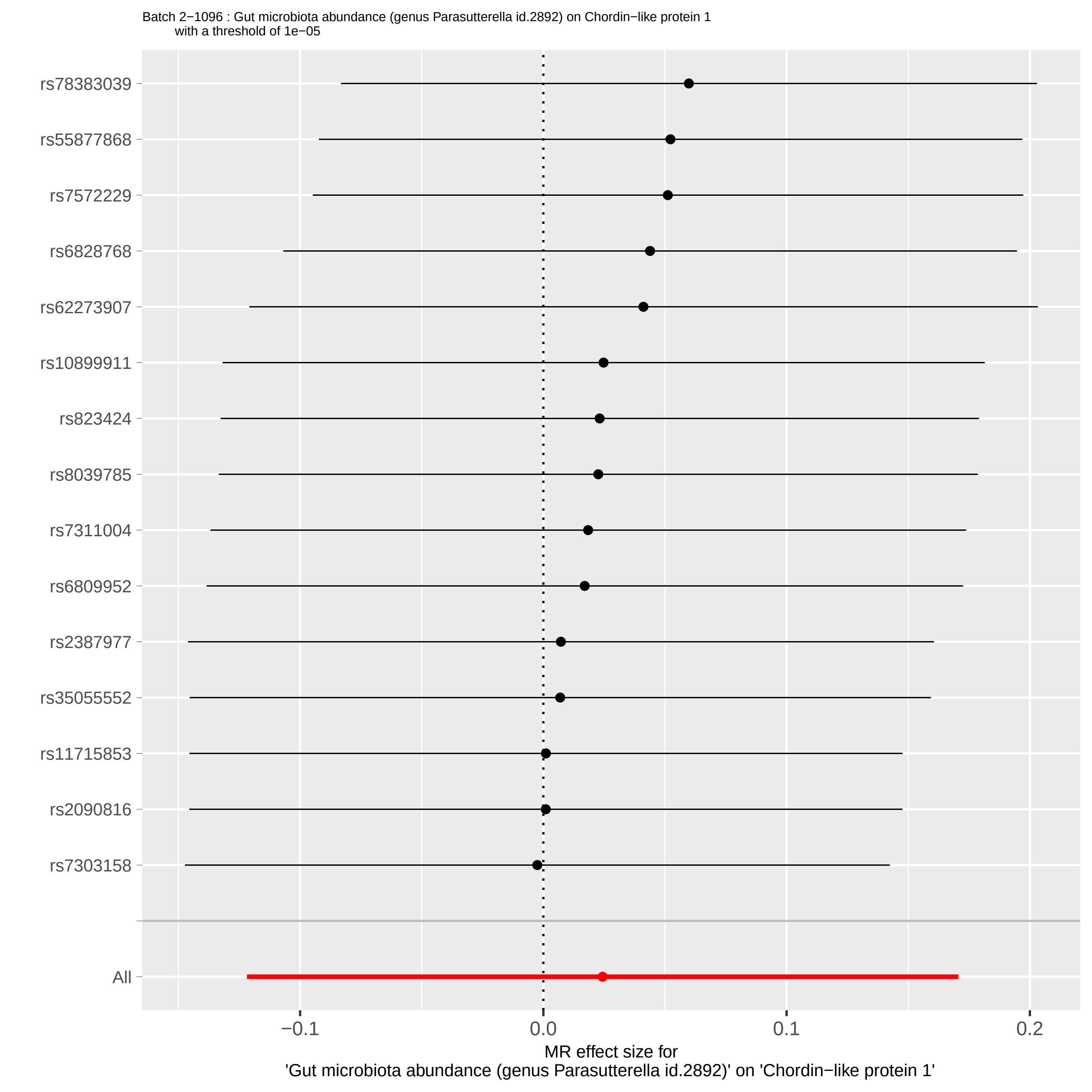
Batch 2−1096 : Gut microbiota abundance (genus Parasutterella id.2892) on Chordin−like protein 1
with a threshold of 1e−05
rs78383039
rs55877868
rs7572229
rs6828768
rs62273907
rs10899911
rs823424
rs8039785
rs7311004
rs6809952
rs2387977
rs35055552
rs11715853
rs2090816
rs7303158
All
−0.1
0.0
0.1
0.2
MR effect size for
'Gut microbiota abundance (genus Parasutterella id.2892)' on 'Chordin−like protein 1'

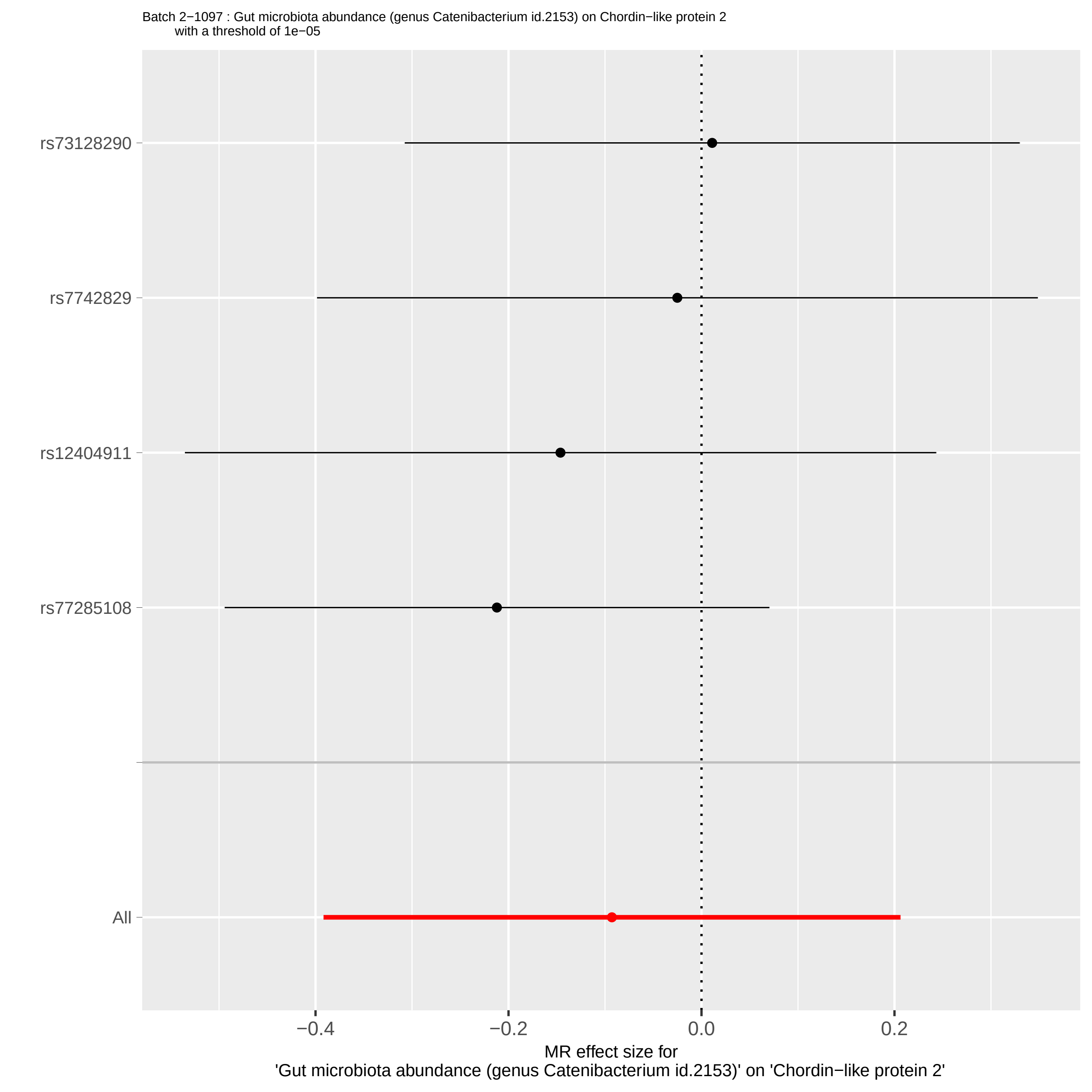
Batch 2−1097 : Gut microbiota abundance (genus Catenibacterium id.2153) on Chordin−like protein 2
with a threshold of 1e−05
rs73128290
rs7742829
rs12404911
rs77285108
All
−0.4
−0.2
0.0
0.2
MR effect size for
'Gut microbiota abundance (genus Catenibacterium id.2153)' on 'Chordin−like protein 2'

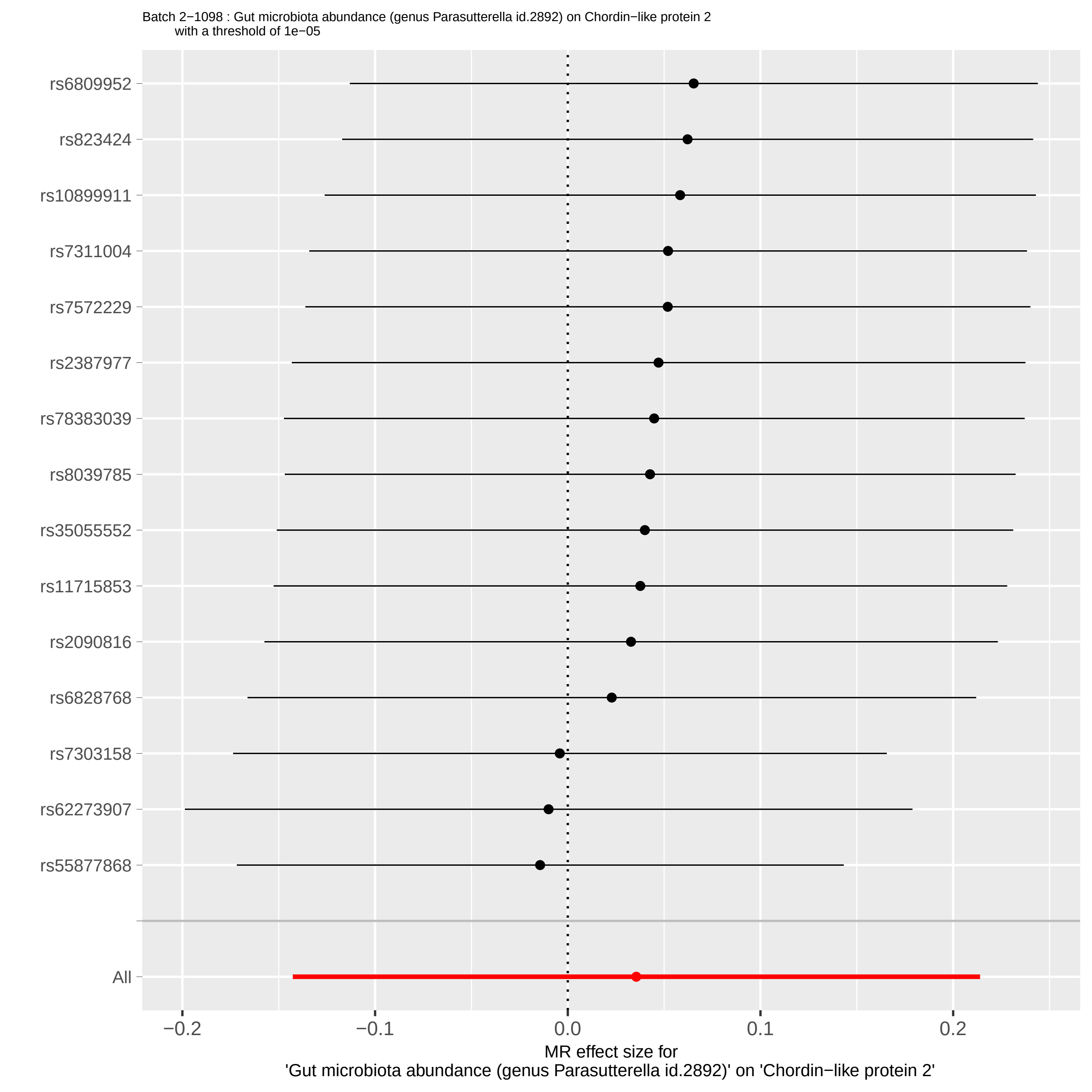
Batch 2−1098 : Gut microbiota abundance (genus Parasutterella id.2892) on Chordin−like protein 2
with a threshold of 1e−05
rs6809952
rs823424
rs10899911
rs7311004
rs7572229
rs2387977
rs78383039
rs8039785
rs35055552
rs11715853
rs2090816
rs6828768
rs7303158
rs62273907
rs55877868
All
−0.2
−0.1
0.0
0.1
0.2
MR effect size for
'Gut microbiota abundance (genus Parasutterella id.2892)' on 'Chordin−like protein 2'

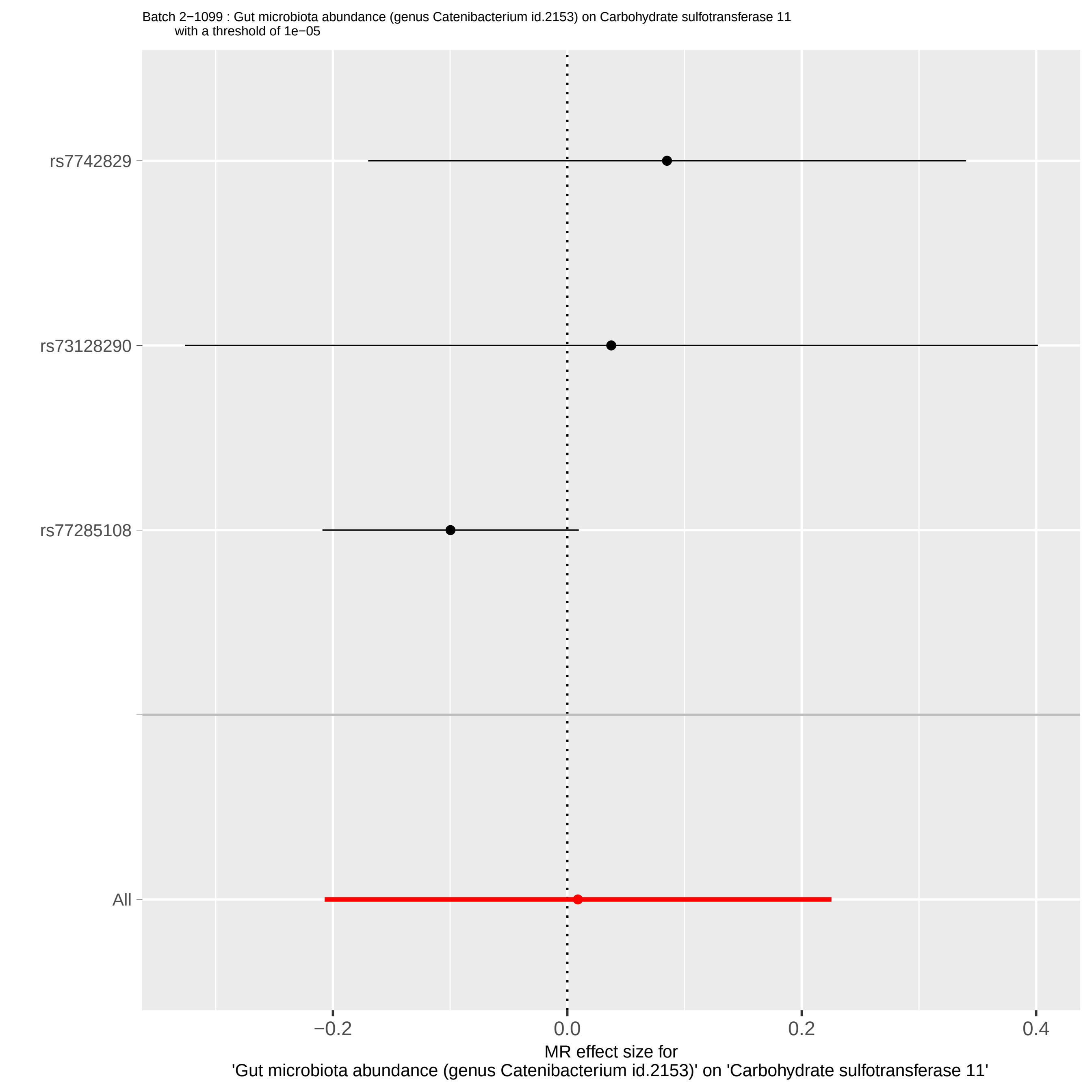
Batch 2−1099 : Gut microbiota abundance (genus Catenibacterium id.2153) on Carbohydrate sulfotransferase 11
with a threshold of 1e−05
rs7742829
rs73128290
rs77285108
All
−0.2
0.0
0.2
0.4
MR effect size for
'Gut microbiota abundance (genus Catenibacterium id.2153)' on 'Carbohydrate sulfotransferase 11'

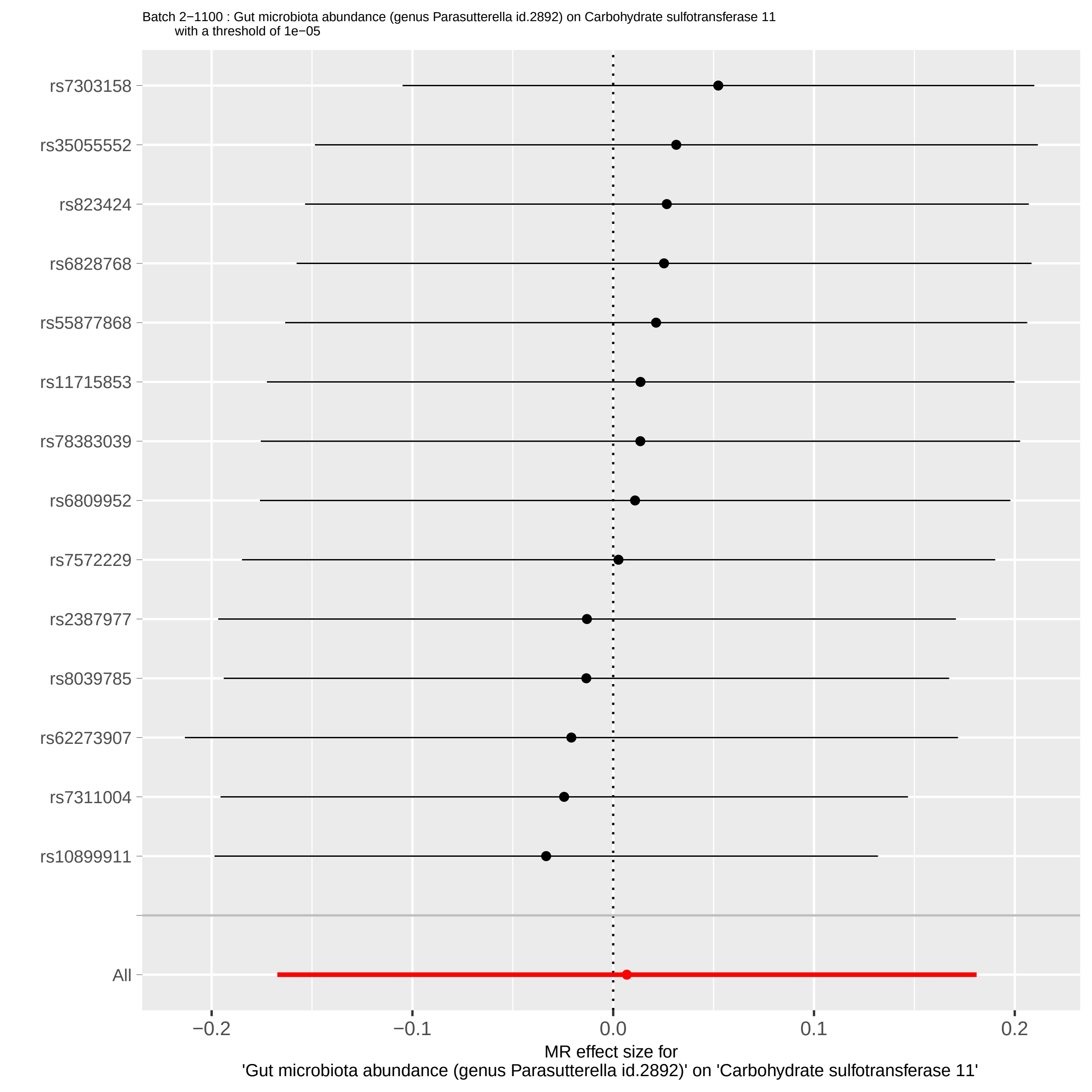

Batch 2−1100 : Gut microbiota abundance (genus Parasutterella id.2892) on Carbohydrate sulfotransferase 11
with a threshold of 1e−05
rs7303158
rs35055552
rs823424
rs6828768
rs55877868
rs11715853
rs78383039
rs6809952
rs7572229
rs2387977
rs8039785
rs62273907
rs7311004
rs10899911
All
−0.2
−0.1
0.0
0.1
0.2
MR effect size for
'Gut microbiota abundance (genus Parasutterella id.2892)' on 'Carbohydrate sulfotransferase 11'

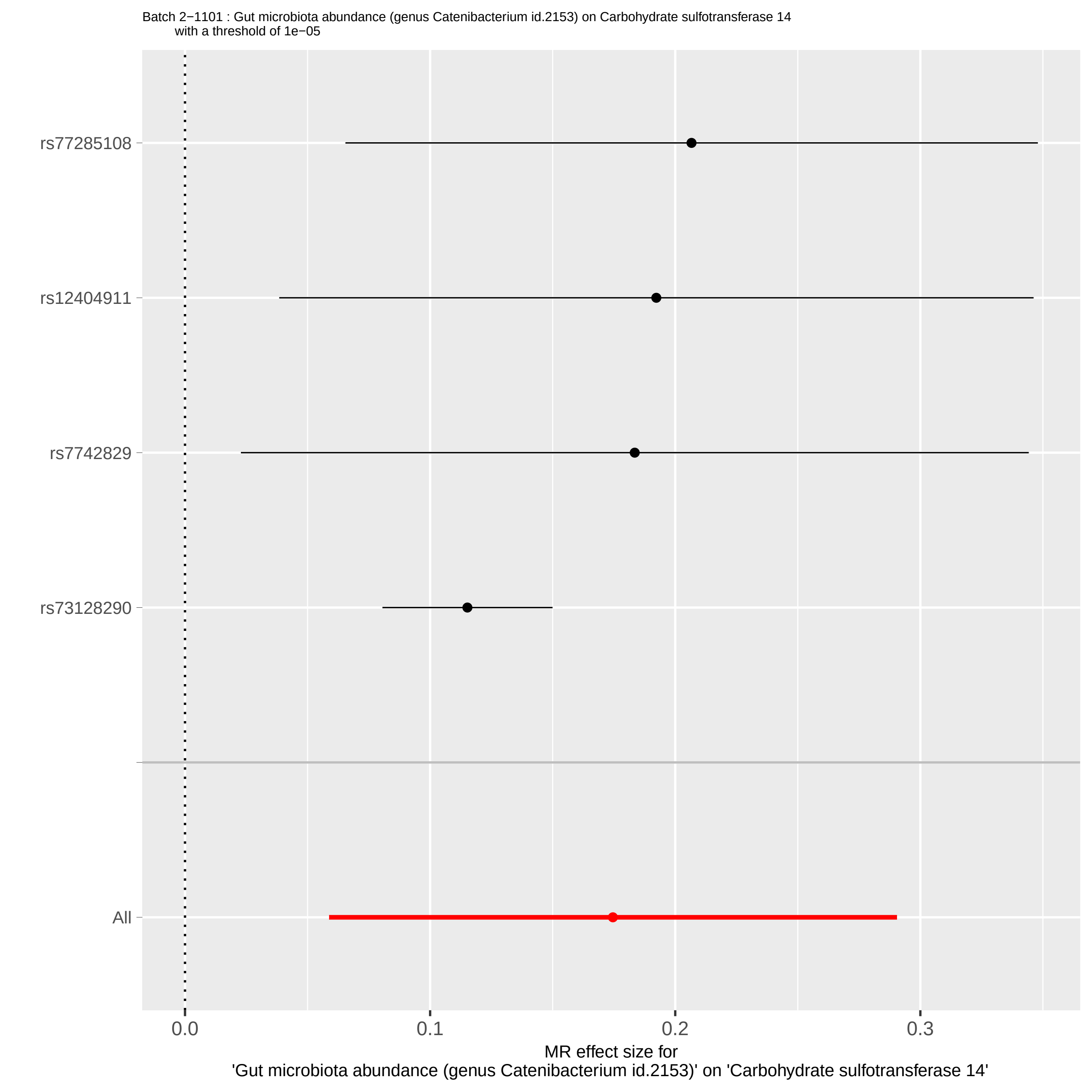
Batch 2−1101 : Gut microbiota abundance (genus Catenibacterium id.2153) on Carbohydrate sulfotransferase 14
with a threshold of 1e−05
rs77285108
rs12404911
rs7742829
rs73128290
All
0.0
0.1
0.2
0.3
MR effect size for
'Gut microbiota abundance (genus Catenibacterium id.2153)' on 'Carbohydrate sulfotransferase 14'

Batch 2−1102 : Gut microbiota abundance (genus Parasutterella id.2892) on Carbohydrate sulfotransferase 14
with a threshold of 1e−05

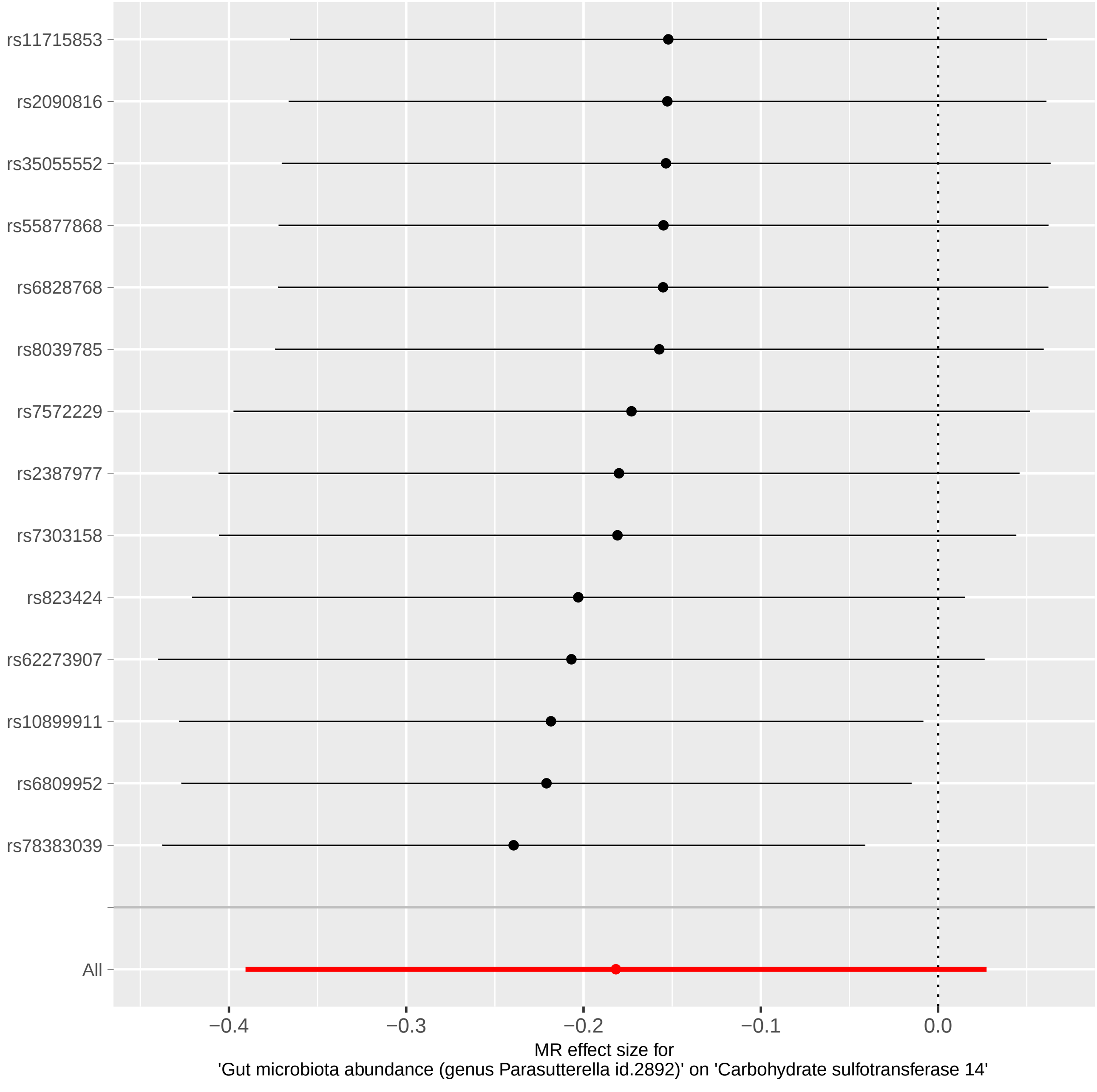

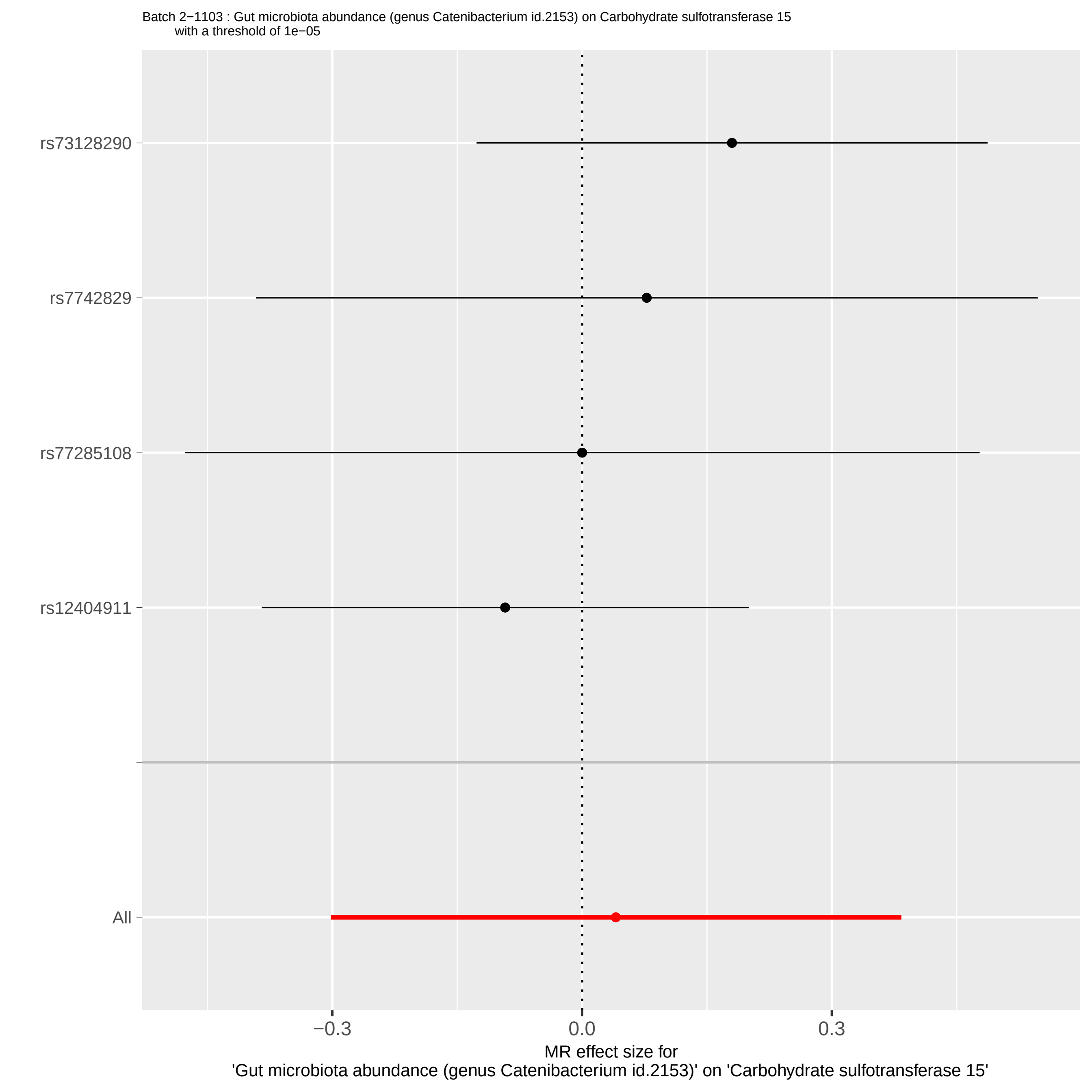
Batch 2−1103 : Gut microbiota abundance (genus Catenibacterium id.2153) on Carbohydrate sulfotransferase 15
with a threshold of 1e−05
rs73128290
rs7742829
rs77285108
rs12404911
All
−0.3
0.0
0.3
MR effect size for
'Gut microbiota abundance (genus Catenibacterium id.2153)' on 'Carbohydrate sulfotransferase 15'

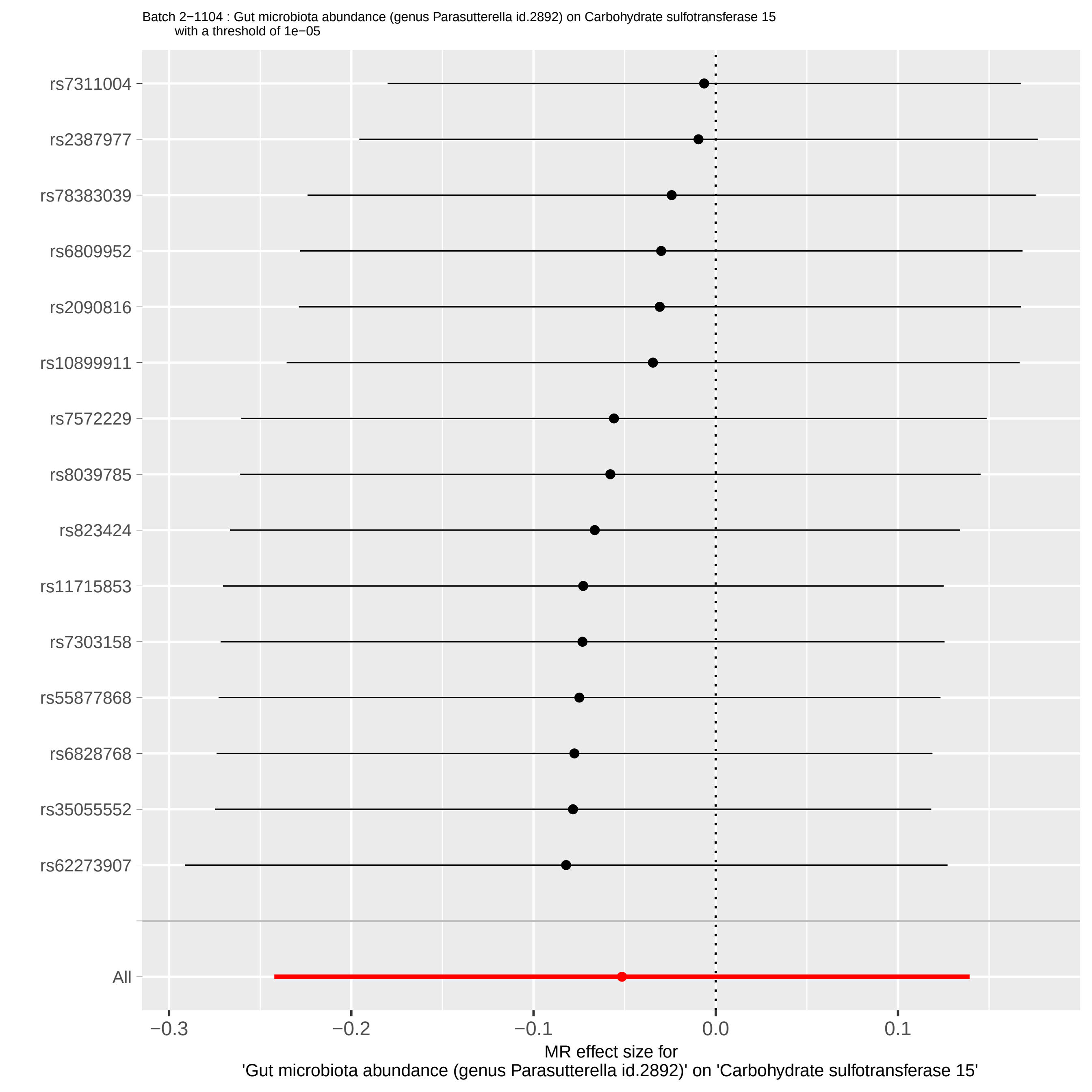
Batch 2−1104 : Gut microbiota abundance (genus Parasutterella id.2892) on Carbohydrate sulfotransferase 15
with a threshold of 1e−05
rs7311004
rs2387977
rs78383039
rs6809952
rs2090816
rs10899911
rs7572229
rs8039785
rs823424
rs11715853
rs7303158
rs55877868
rs6828768
rs35055552
rs62273907
All
−0.3
−0.2
−0.1
0.0
0.1
MR effect size for
'Gut microbiota abundance (genus Parasutterella id.2892)' on 'Carbohydrate sulfotransferase 15'

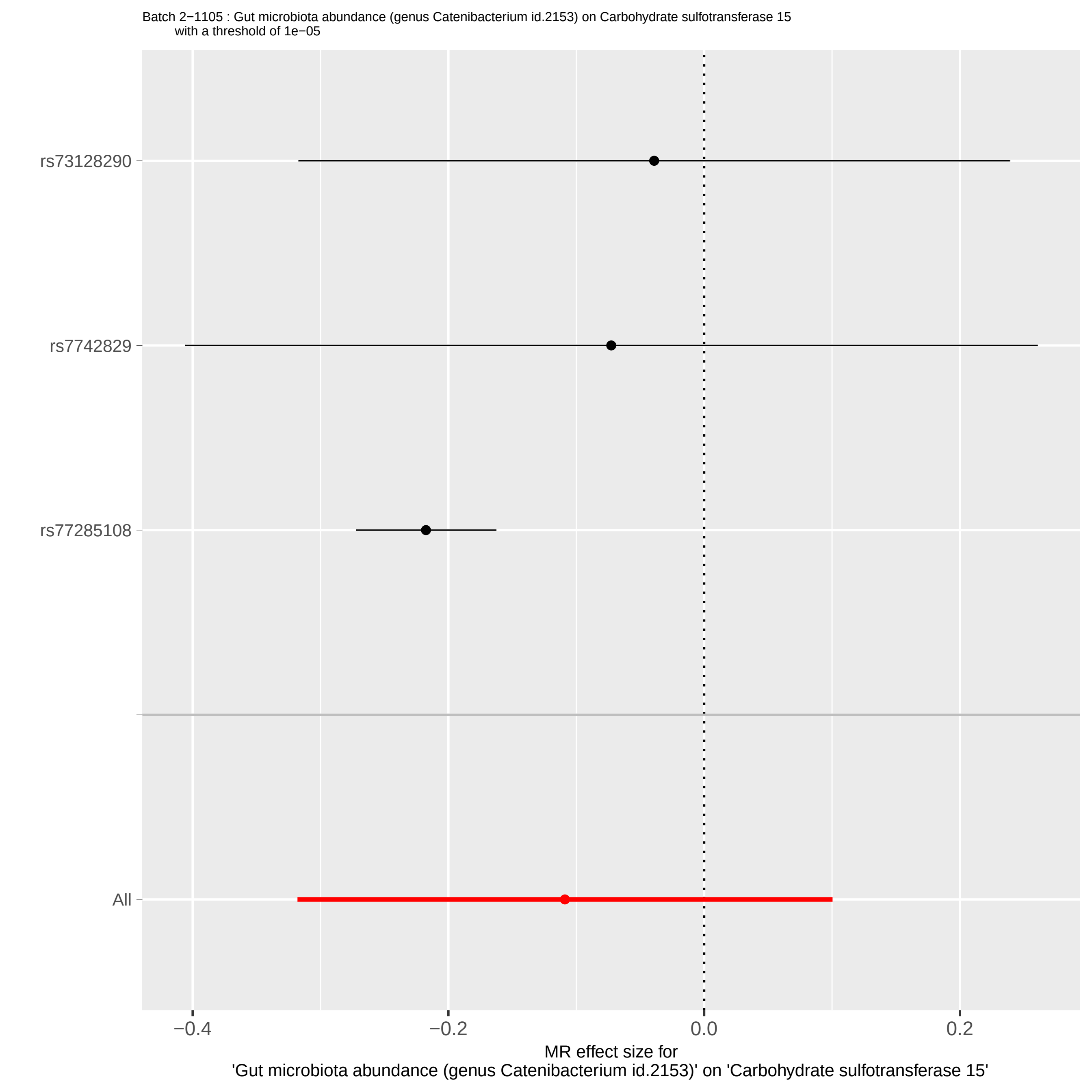

Batch 2−1105 : Gut microbiota abundance (genus Catenibacterium id.2153) on Carbohydrate sulfotransferase 15
with a threshold of 1e−05
rs73128290
rs7742829
rs77285108
All
−0.4
−0.2
0.0
0.2
MR effect size for
'Gut microbiota abundance (genus Catenibacterium id.2153)' on 'Carbohydrate sulfotransferase 15'

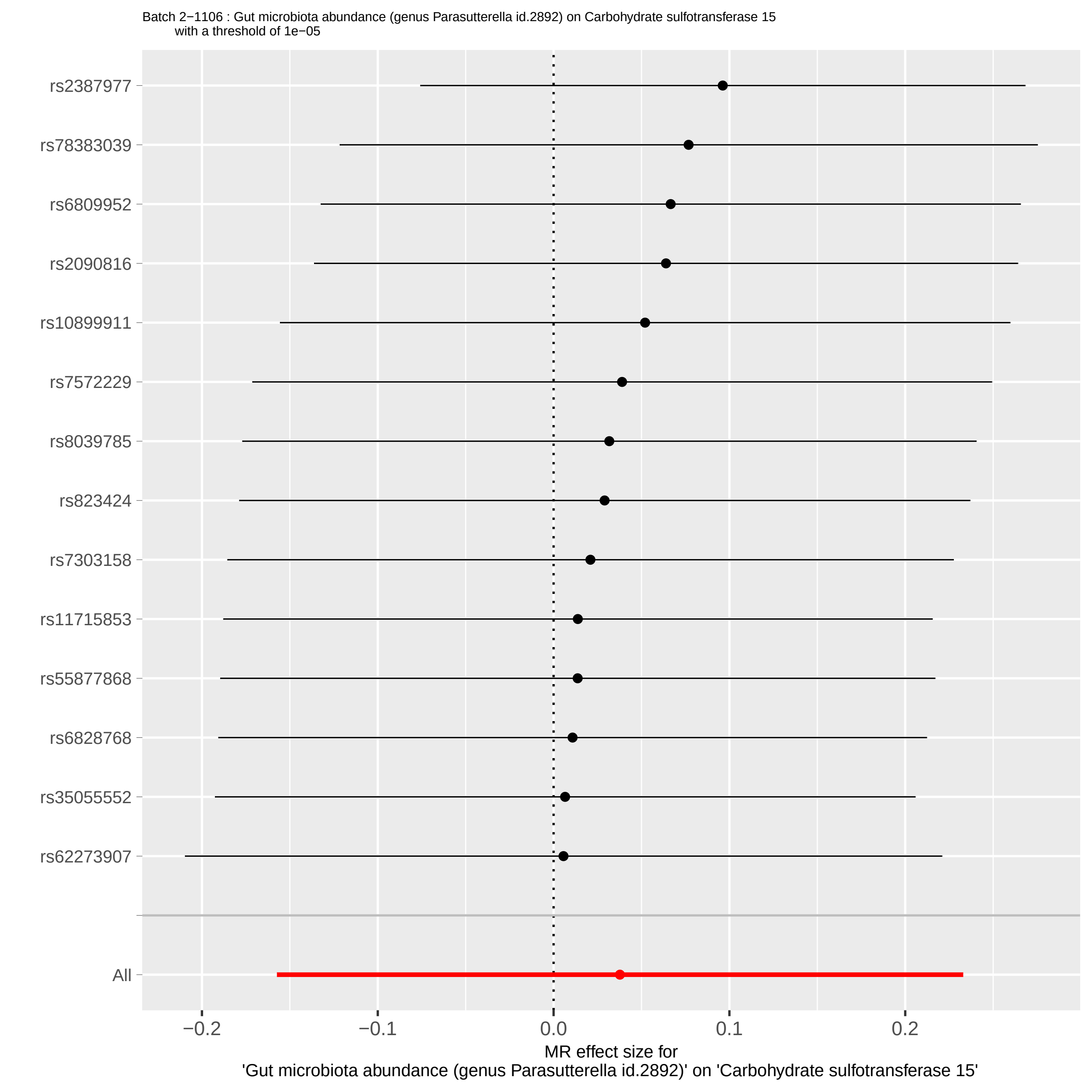

Batch 2−1106 : Gut microbiota abundance (genus Parasutterella id.2892) on Carbohydrate sulfotransferase 15
with a threshold of 1e−05
rs2387977
rs78383039
rs6809952
rs2090816
rs10899911
rs7572229
rs8039785
rs823424
rs7303158
rs11715853
rs55877868
rs6828768
rs35055552
rs62273907
All
−0.2
−0.1
0.0
0.1
0.2
MR effect size for
'Gut microbiota abundance (genus Parasutterella id.2892)' on 'Carbohydrate sulfotransferase 15'

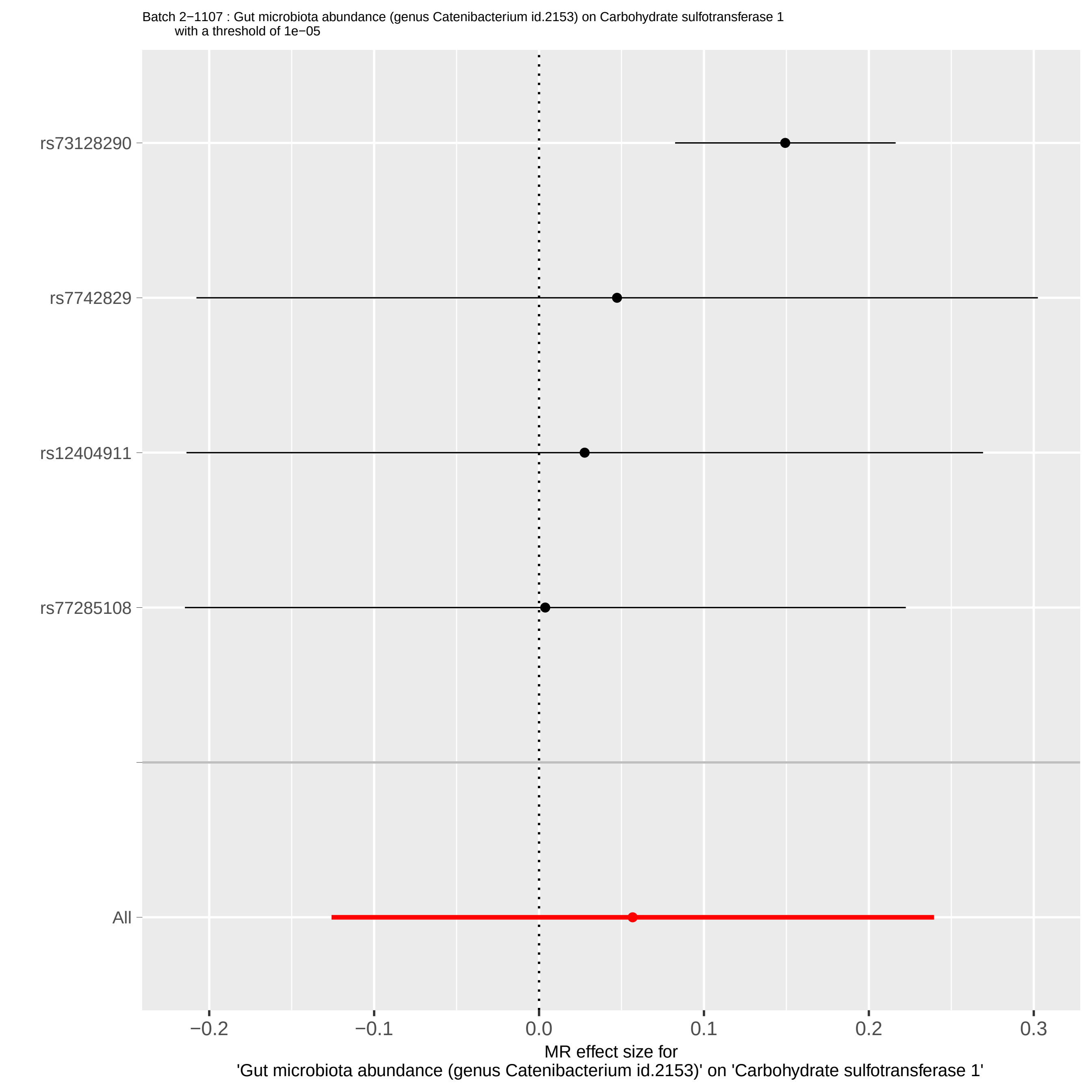

Batch 2−1107 : Gut microbiota abundance (genus Catenibacterium id.2153) on Carbohydrate sulfotransferase 1
with a threshold of 1e−05
rs73128290
rs7742829
rs12404911
rs77285108
All
−0.2
−0.1
0.0
0.1
0.2
0.3
MR effect size for
'Gut microbiota abundance (genus Catenibacterium id.2153)' on 'Carbohydrate sulfotransferase 1'

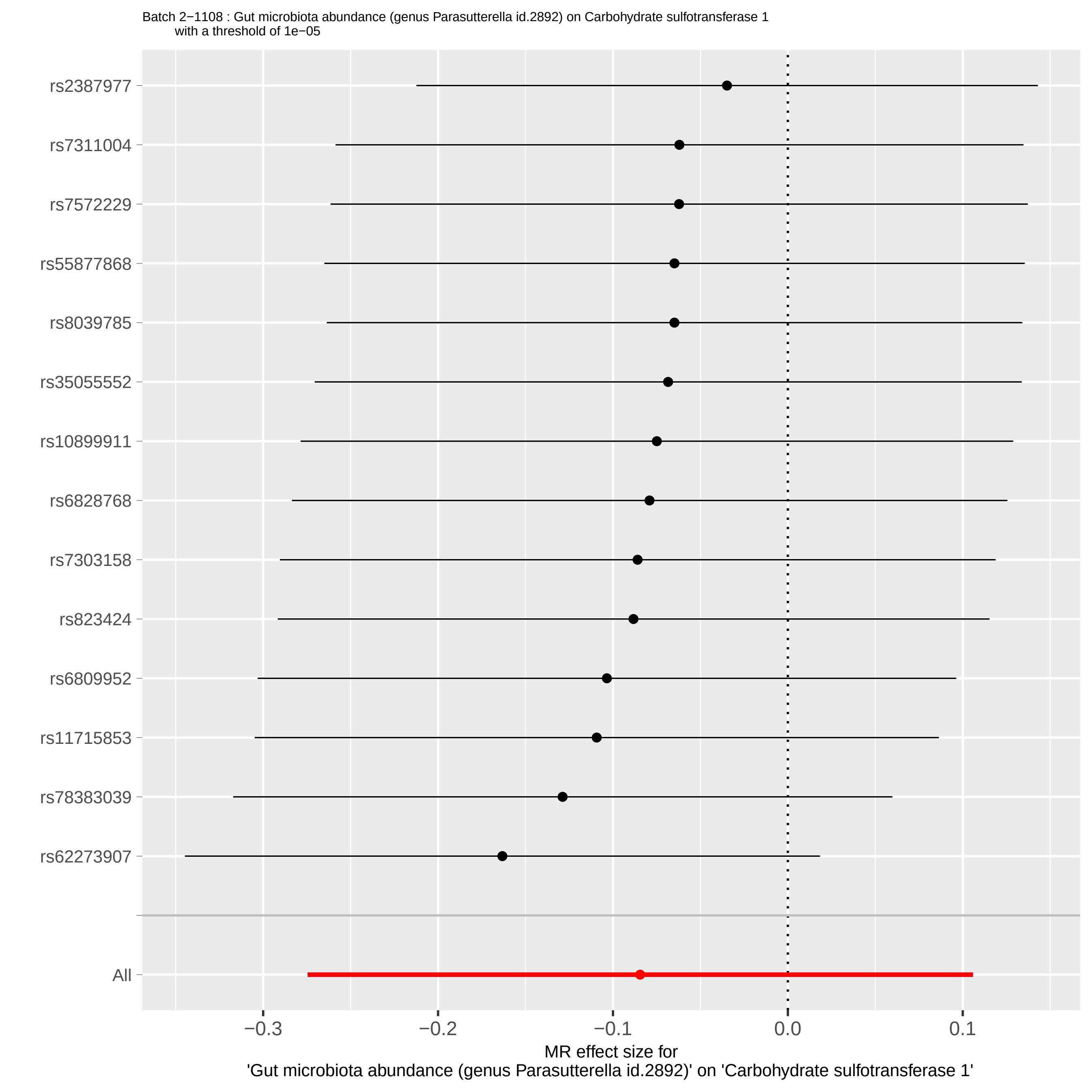

Batch 2−1108 : Gut microbiota abundance (genus Parasutterella id.2892) on Carbohydrate sulfotransferase 1
with a threshold of 1e−05
rs2387977
rs7311004
rs7572229
rs55877868
rs8039785
rs35055552
rs10899911
rs6828768
rs7303158
rs823424
rs6809952
rs11715853
rs78383039
rs62273907
All
−0.3
−0.2
−0.1
0.0
0.1
MR effect size for
'Gut microbiota abundance (genus Parasutterella id.2892)' on 'Carbohydrate sulfotransferase 1'

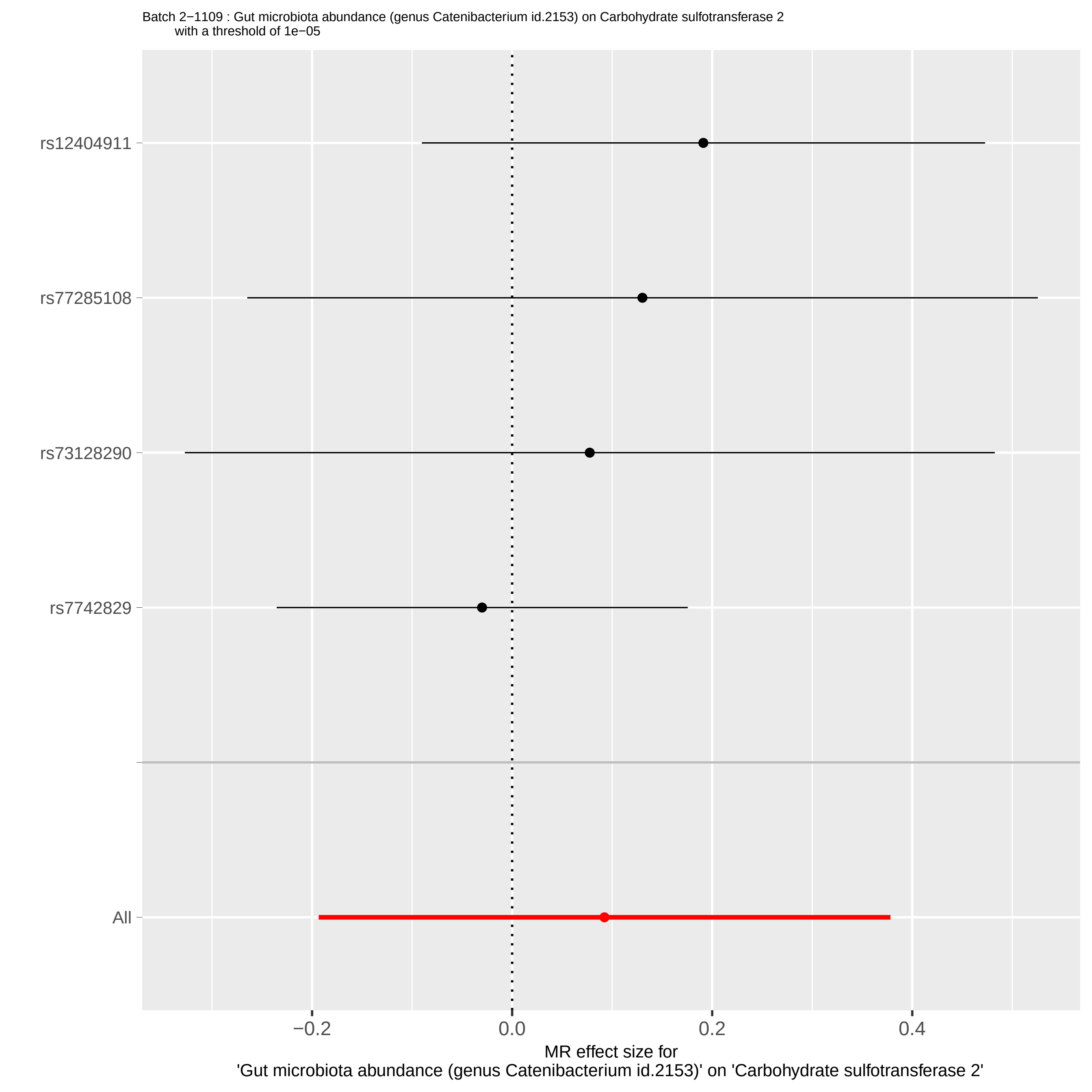

Batch 2−1109 : Gut microbiota abundance (genus Catenibacterium id.2153) on Carbohydrate sulfotransferase 2
with a threshold of 1e−05
rs12404911
rs77285108
rs73128290
rs7742829
All
−0.2
0.0
0.2
0.4
MR effect size for
'Gut microbiota abundance (genus Catenibacterium id.2153)' on 'Carbohydrate sulfotransferase 2'

Batch 2−1110 : Gut microbiota abundance (genus Parasutterella id.2892) on Carbohydrate sulfotransferase 2
with a threshold of 1e−05

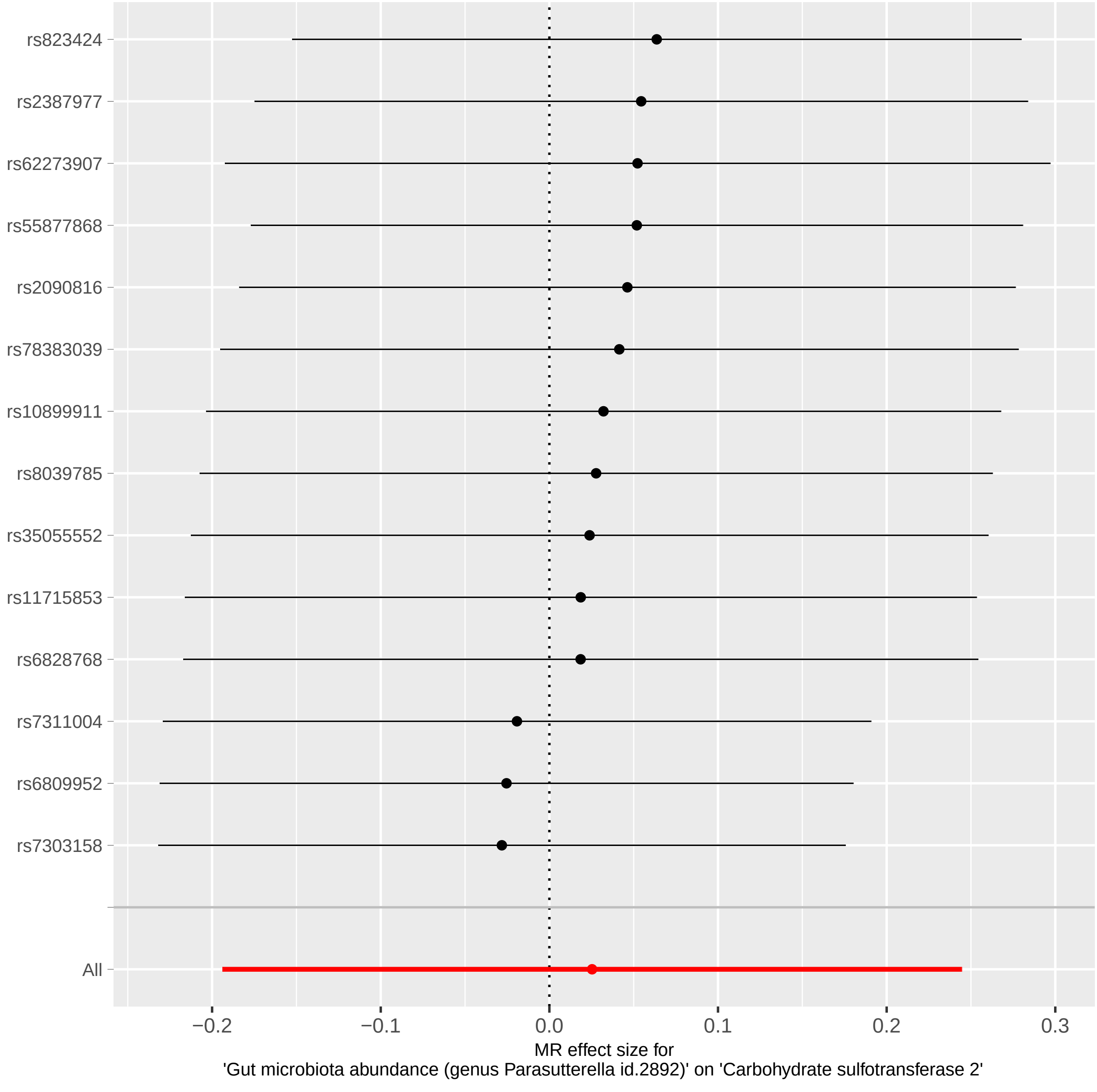

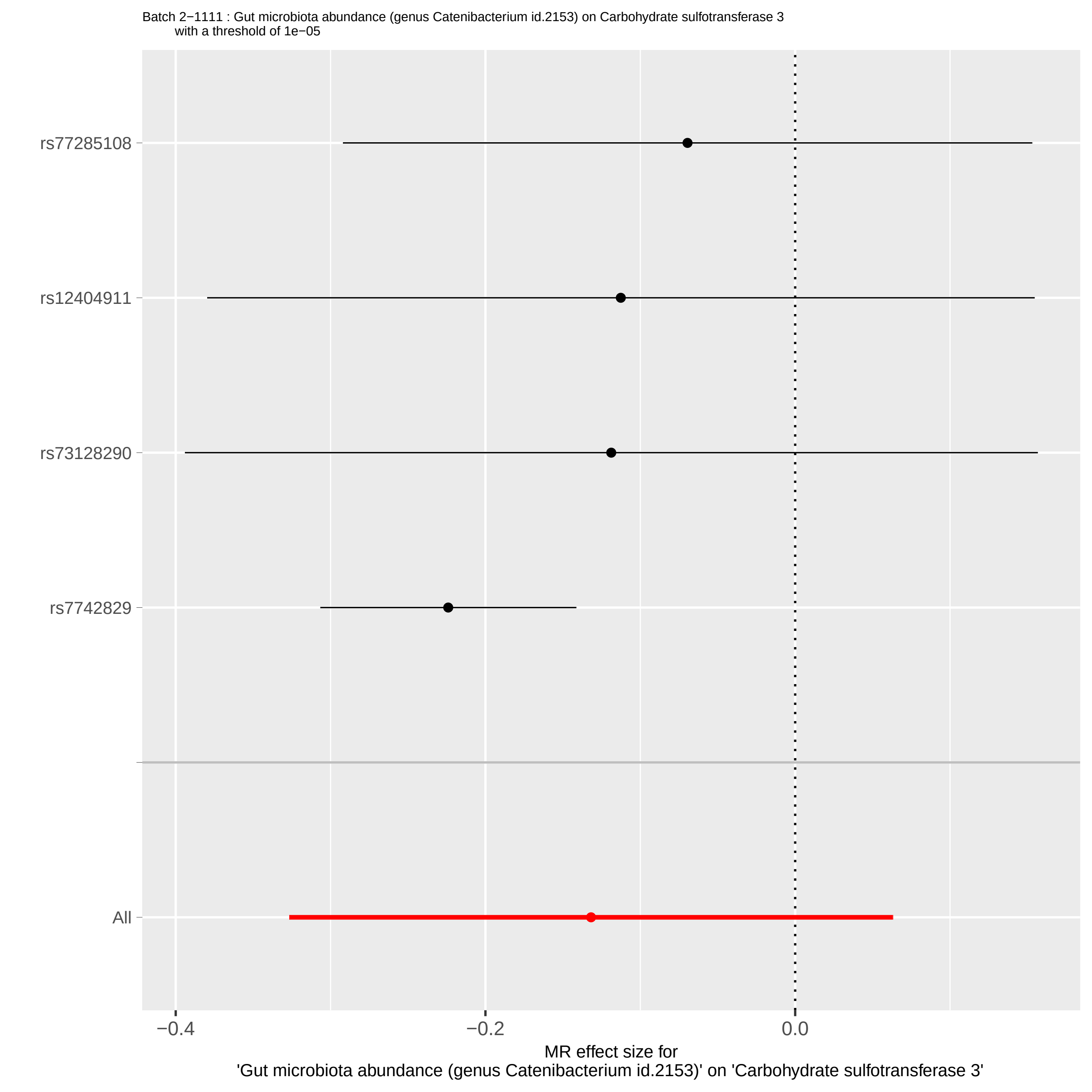
Batch 2−1111 : Gut microbiota abundance (genus Catenibacterium id.2153) on Carbohydrate sulfotransferase 3
with a threshold of 1e−05
rs77285108
rs12404911
rs73128290
rs7742829
All
−0.4
−0.2
0.0
MR effect size for
'Gut microbiota abundance (genus Catenibacterium id.2153)' on 'Carbohydrate sulfotransferase 3'

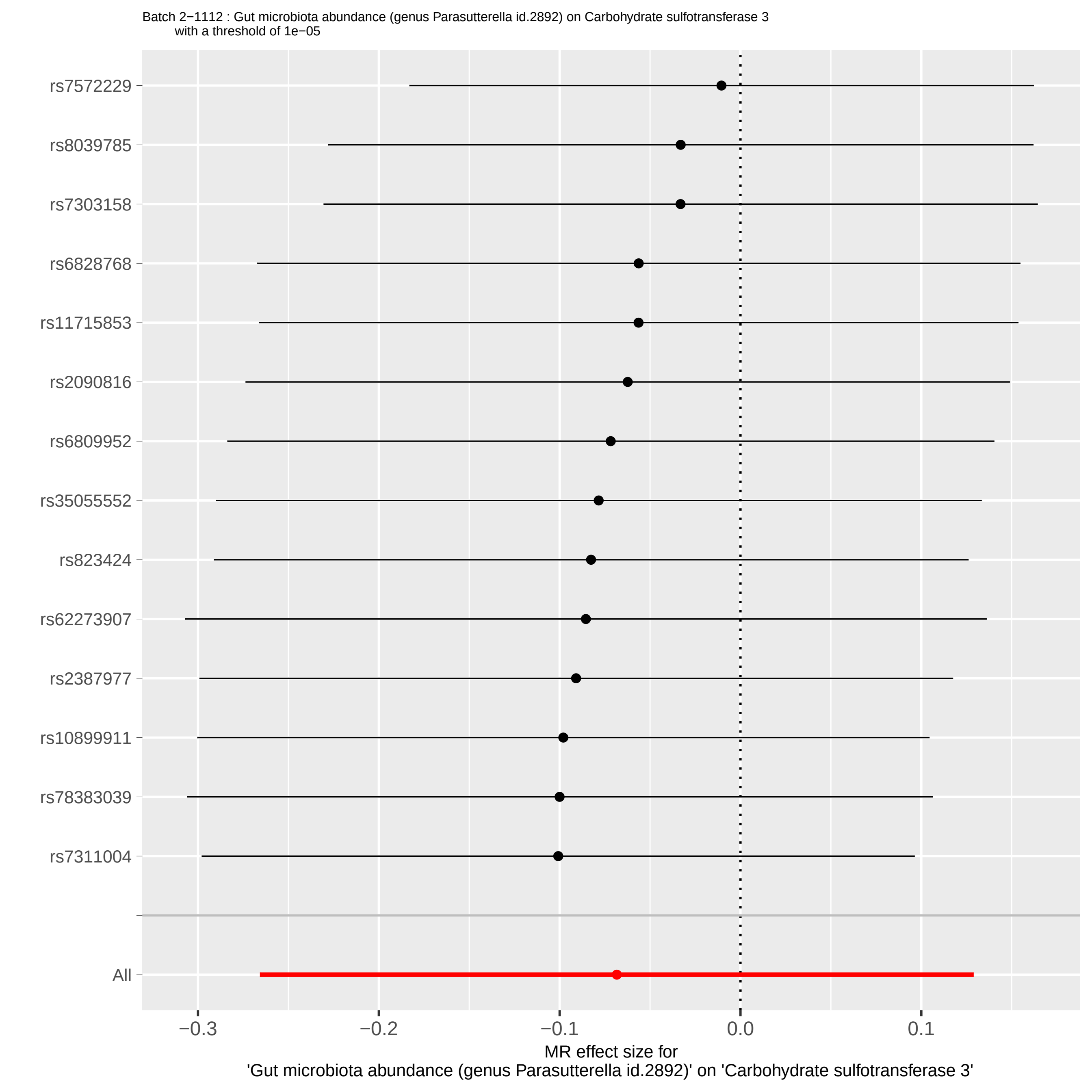

Batch 2−1112 : Gut microbiota abundance (genus Parasutterella id.2892) on Carbohydrate sulfotransferase 3
with a threshold of 1e−05
rs7572229
rs8039785
rs7303158
rs6828768
rs11715853
rs2090816
rs6809952
rs35055552
rs823424
rs62273907
rs2387977
rs10899911
rs78383039
rs7311004
All
−0.3
−0.2
−0.1
0.0
0.1
MR effect size for
'Gut microbiota abundance (genus Parasutterella id.2892)' on 'Carbohydrate sulfotransferase 3'

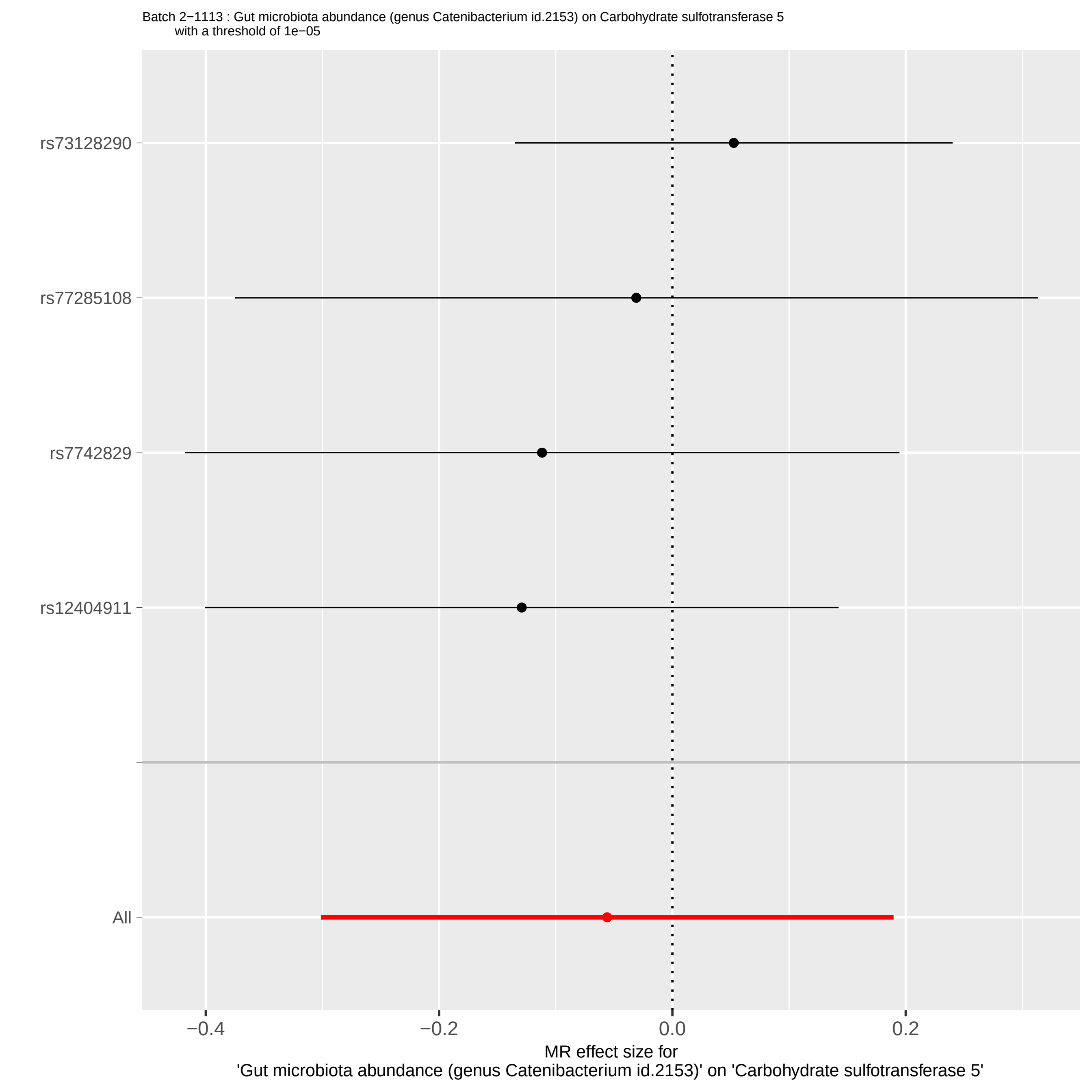
Batch 2−1113 : Gut microbiota abundance (genus Catenibacterium id.2153) on Carbohydrate sulfotransferase 5
with a threshold of 1e−05
rs73128290
rs77285108
rs7742829
rs12404911
All
−0.4
−0.2
0.0
0.2
MR effect size for
'Gut microbiota abundance (genus Catenibacterium id.2153)' on 'Carbohydrate sulfotransferase 5'

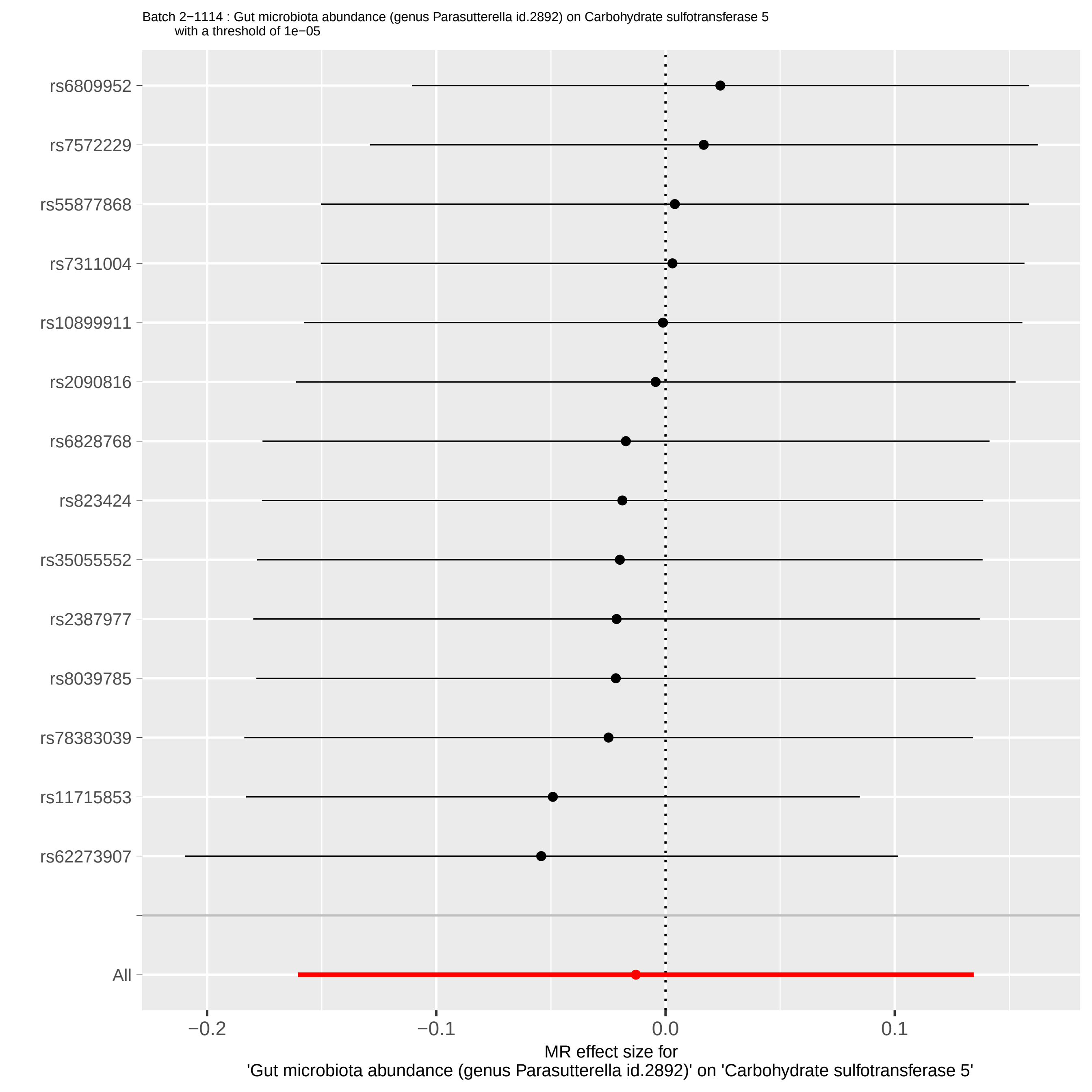
Batch 2−1114 : Gut microbiota abundance (genus Parasutterella id.2892) on Carbohydrate sulfotransferase 5
with a threshold of 1e−05
rs6809952
rs7572229
rs55877868
rs7311004
rs10899911
rs2090816
rs6828768
rs823424
rs35055552
rs2387977
rs8039785
rs78383039
rs11715853
rs62273907
All
−0.2
−0.1
0.0
0.1
MR effect size for
'Gut microbiota abundance (genus Parasutterella id.2892)' on 'Carbohydrate sulfotransferase 5'

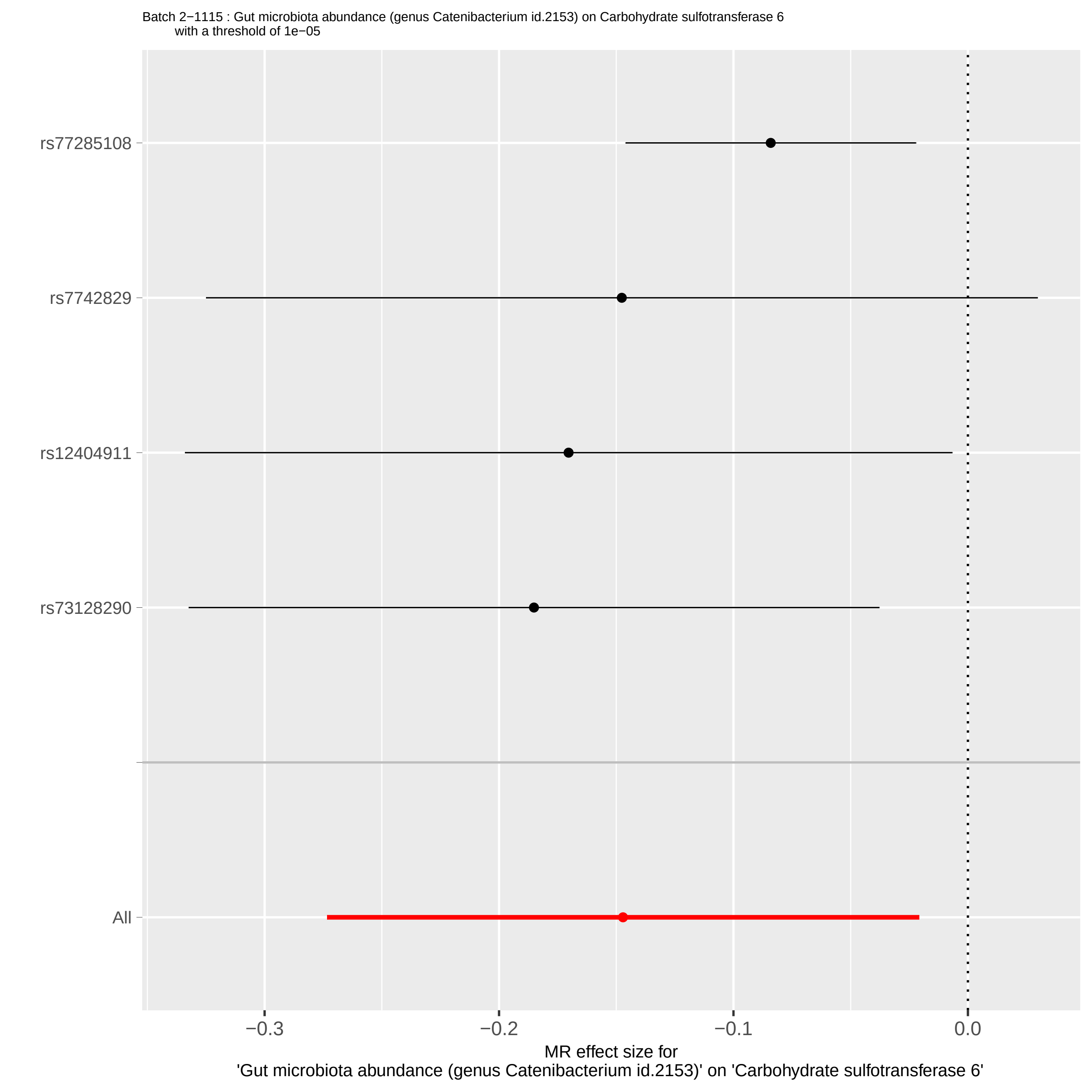
Batch 2−1115 : Gut microbiota abundance (genus Catenibacterium id.2153) on Carbohydrate sulfotransferase 6
with a threshold of 1e−05
rs77285108
rs7742829
rs12404911
rs73128290
All
−0.3
−0.2
−0.1
0.0
MR effect size for
'Gut microbiota abundance (genus Catenibacterium id.2153)' on 'Carbohydrate sulfotransferase 6'

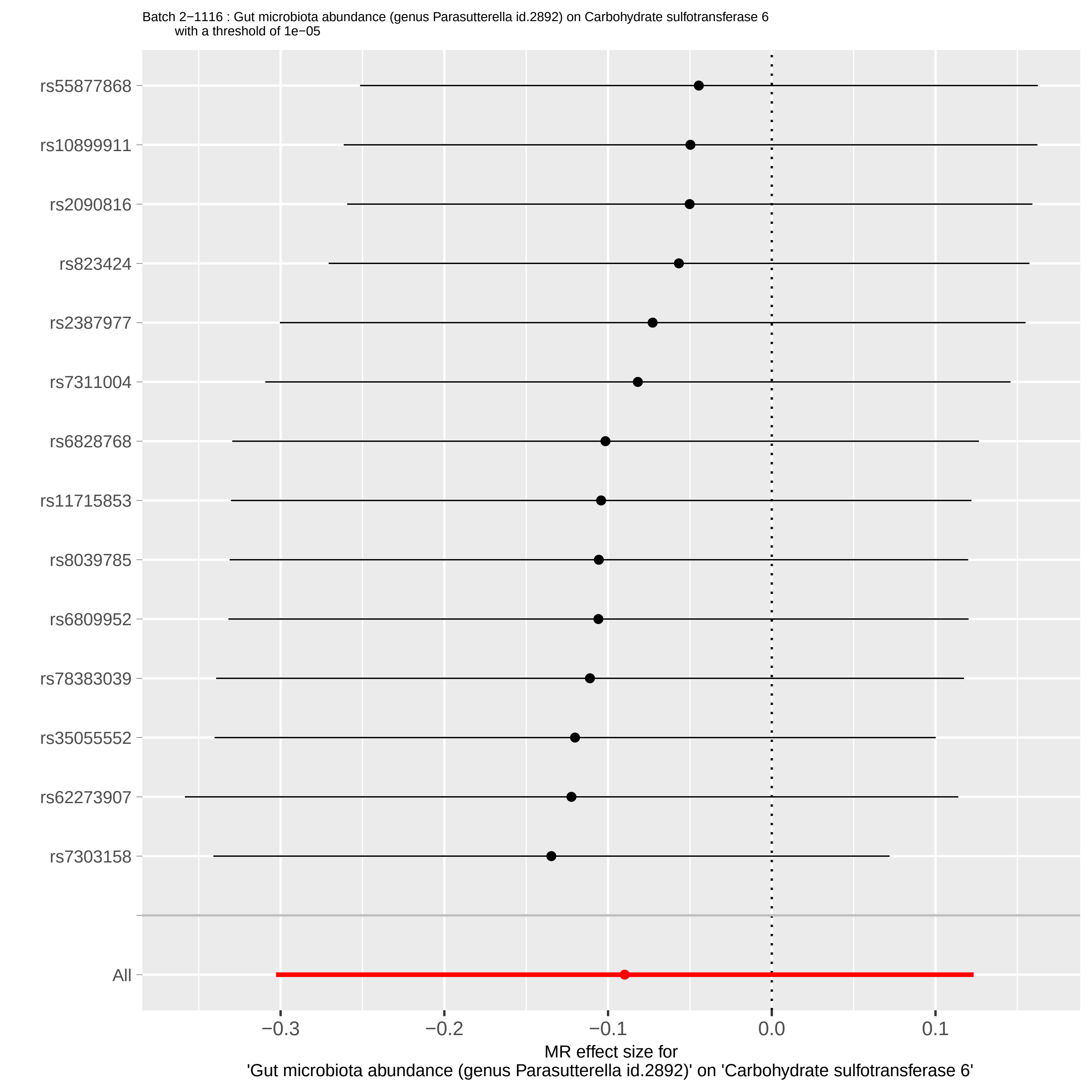

Batch 2−1116 : Gut microbiota abundance (genus Parasutterella id.2892) on Carbohydrate sulfotransferase 6
with a threshold of 1e−05
rs55877868
rs10899911
rs2090816
rs823424
rs2387977
rs7311004
rs6828768
rs11715853
rs8039785
rs6809952
rs78383039
rs35055552
rs62273907
rs7303158
All
−0.3
−0.2
−0.1
0.0
0.1
MR effect size for
'Gut microbiota abundance (genus Parasutterella id.2892)' on 'Carbohydrate sulfotransferase 6'

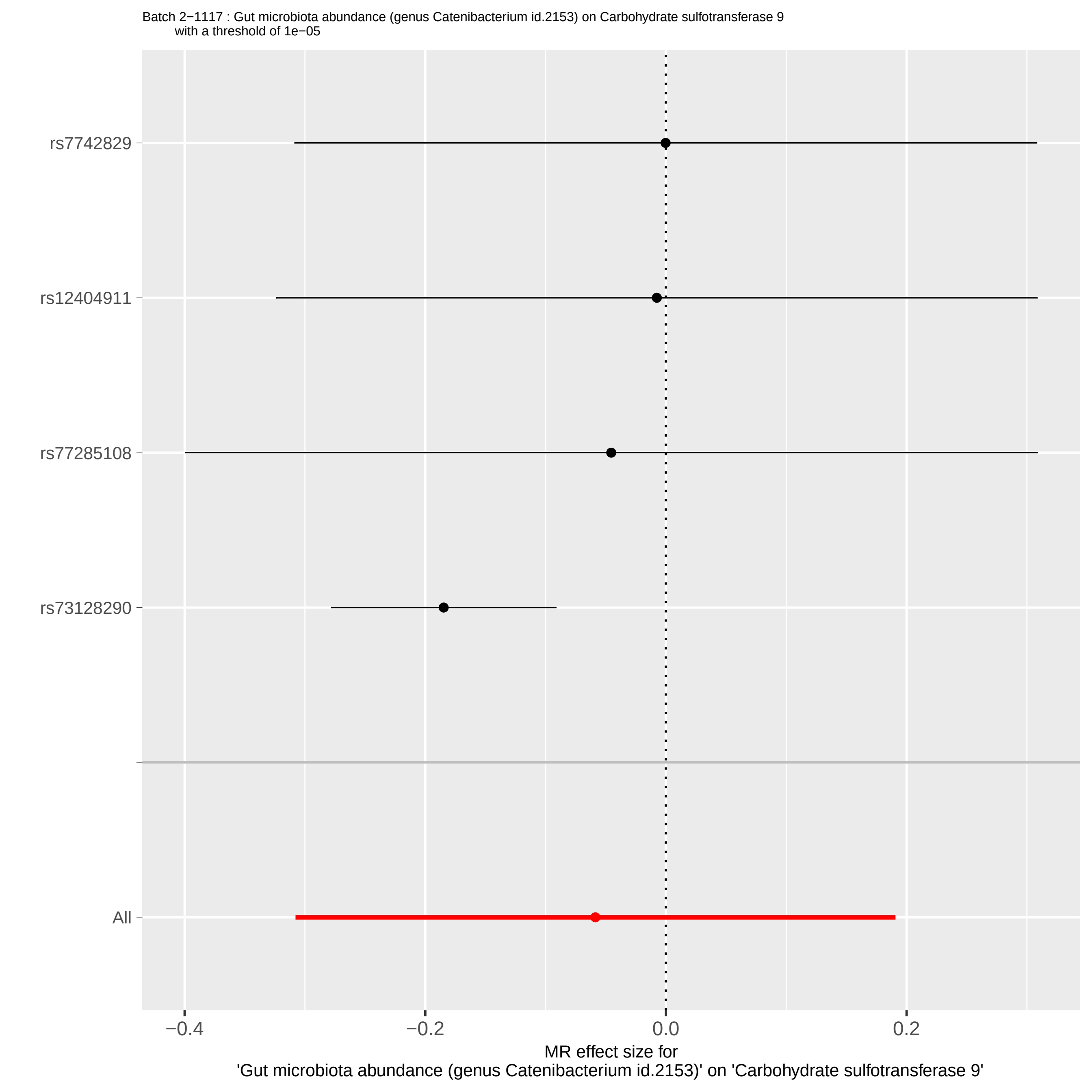

Batch 2−1117 : Gut microbiota abundance (genus Catenibacterium id.2153) on Carbohydrate sulfotransferase 9
with a threshold of 1e−05
rs7742829
rs12404911
rs77285108
rs73128290
All
−0.4
−0.2
0.0
0.2
MR effect size for
'Gut microbiota abundance (genus Catenibacterium id.2153)' on 'Carbohydrate sulfotransferase 9'

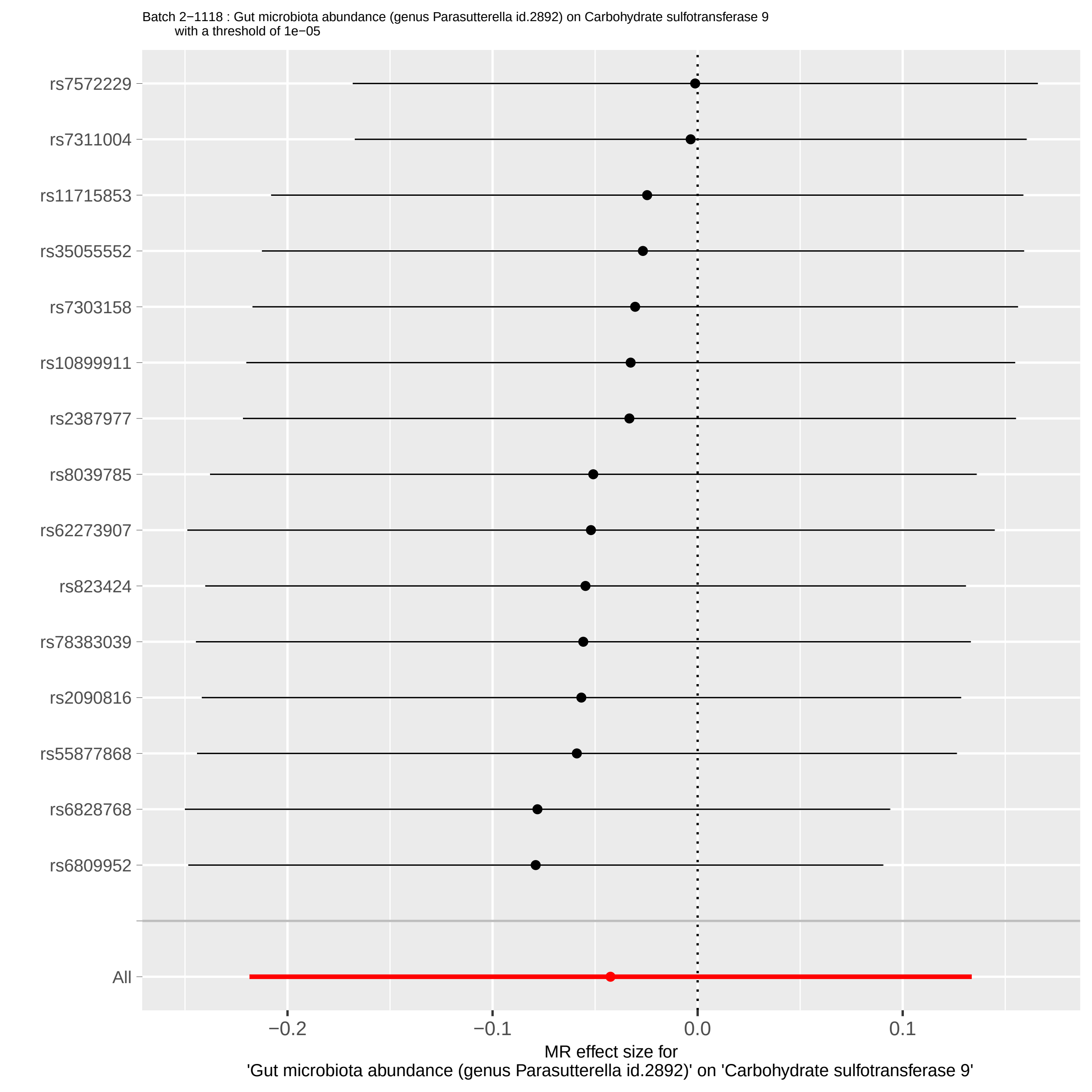
Batch 2−1118 : Gut microbiota abundance (genus Parasutterella id.2892) on Carbohydrate sulfotransferase 9
with a threshold of 1e−05
rs7572229
rs7311004
rs11715853
rs35055552
rs7303158
rs10899911
rs2387977
rs8039785
rs62273907
rs823424
rs78383039
rs2090816
rs55877868
rs6828768
rs6809952
All
−0.2
−0.1
0.0
0.1
MR effect size for
'Gut microbiota abundance (genus Parasutterella id.2892)' on 'Carbohydrate sulfotransferase 9'

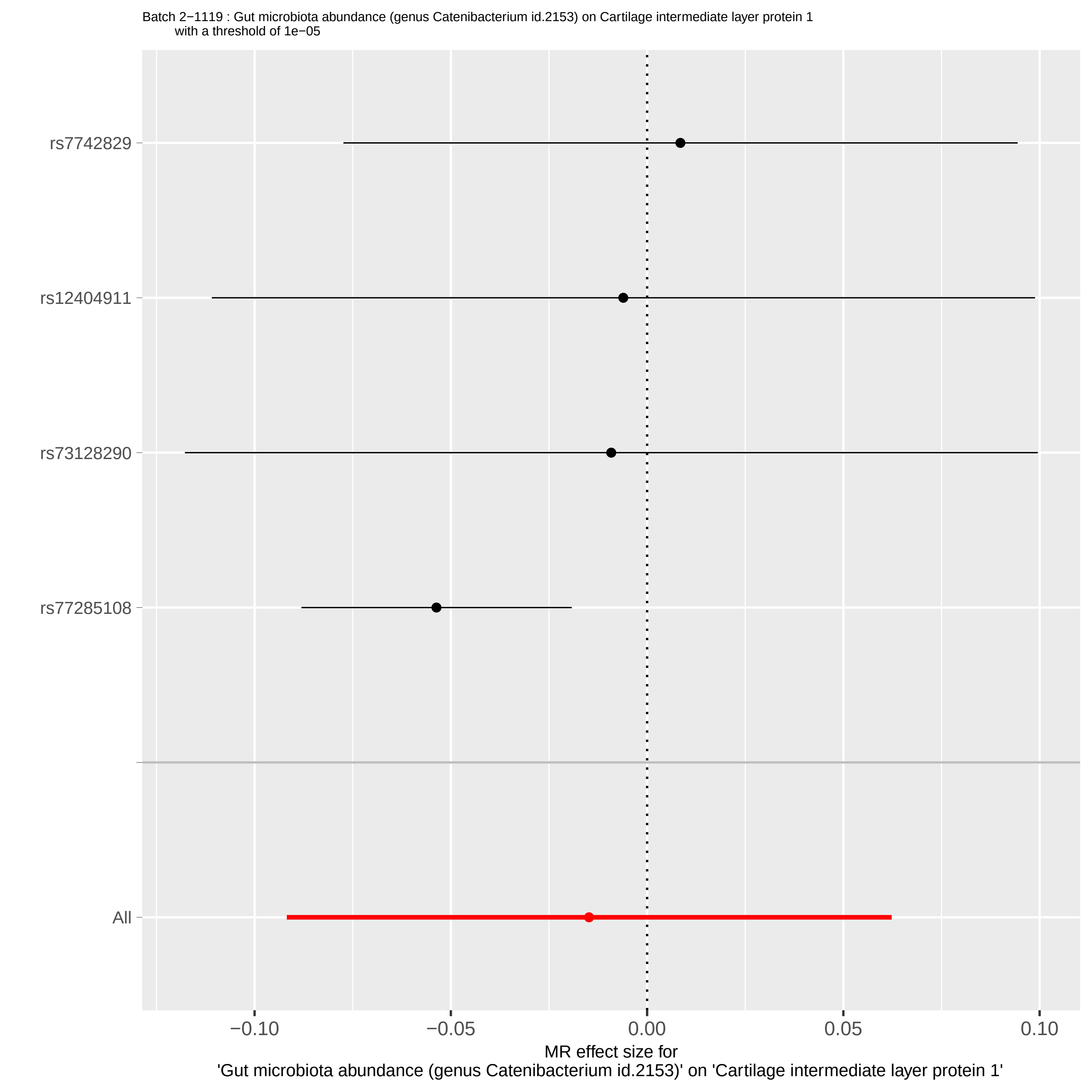

Batch 2−1119 : Gut microbiota abundance (genus Catenibacterium id.2153) on Cartilage intermediate layer protein 1
with a threshold of 1e−05
rs7742829
rs12404911
rs73128290
rs77285108
All
−0.10
−0.05
0.00
0.05
0.10
MR effect size for
'Gut microbiota abundance (genus Catenibacterium id.2153)' on 'Cartilage intermediate layer protein 1'

Batch 2−1120 : Gut microbiota abundance (genus Parasutterella id.2892) on Cartilage intermediate layer protein 1
with a threshold of 1e−05

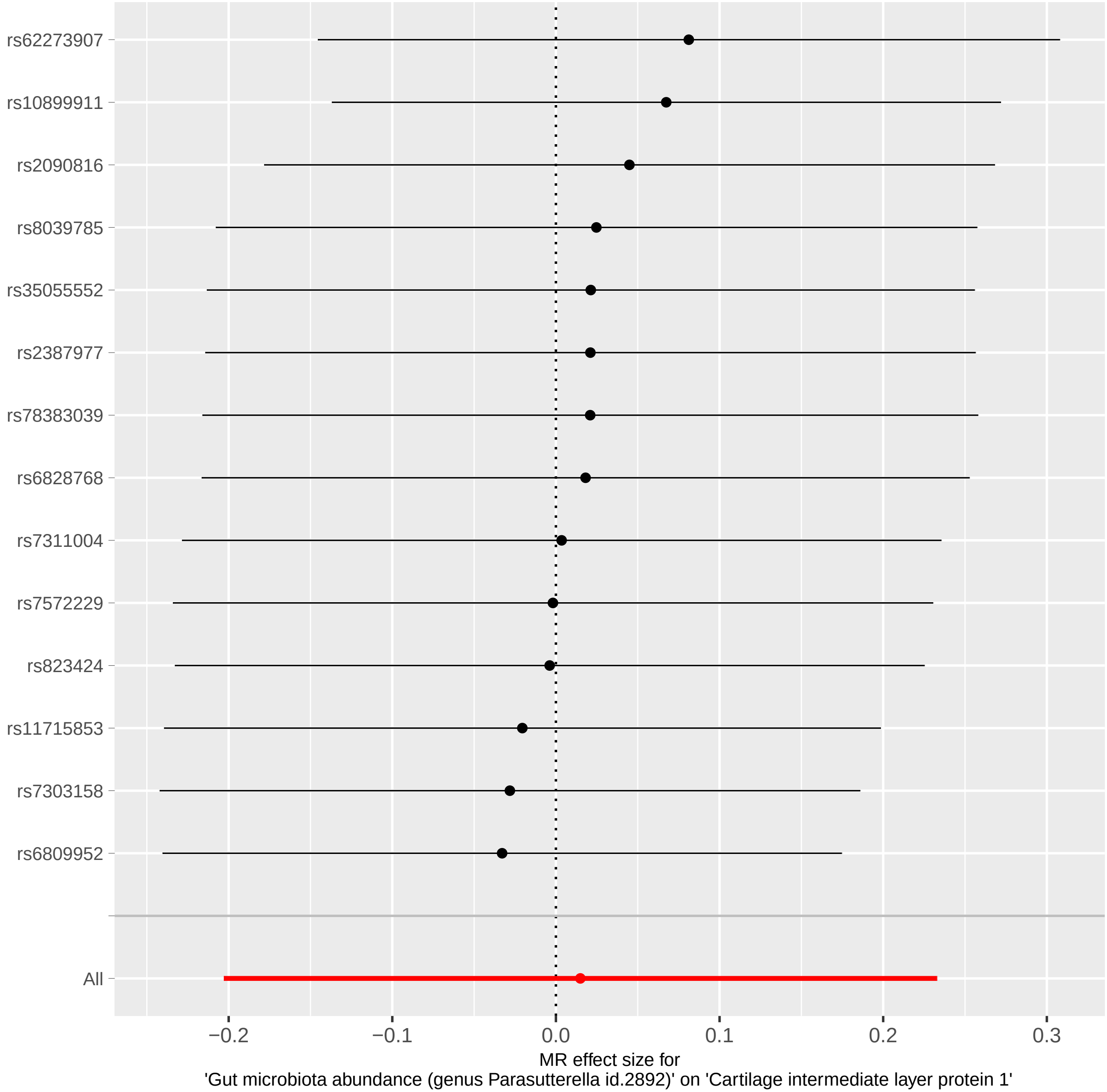

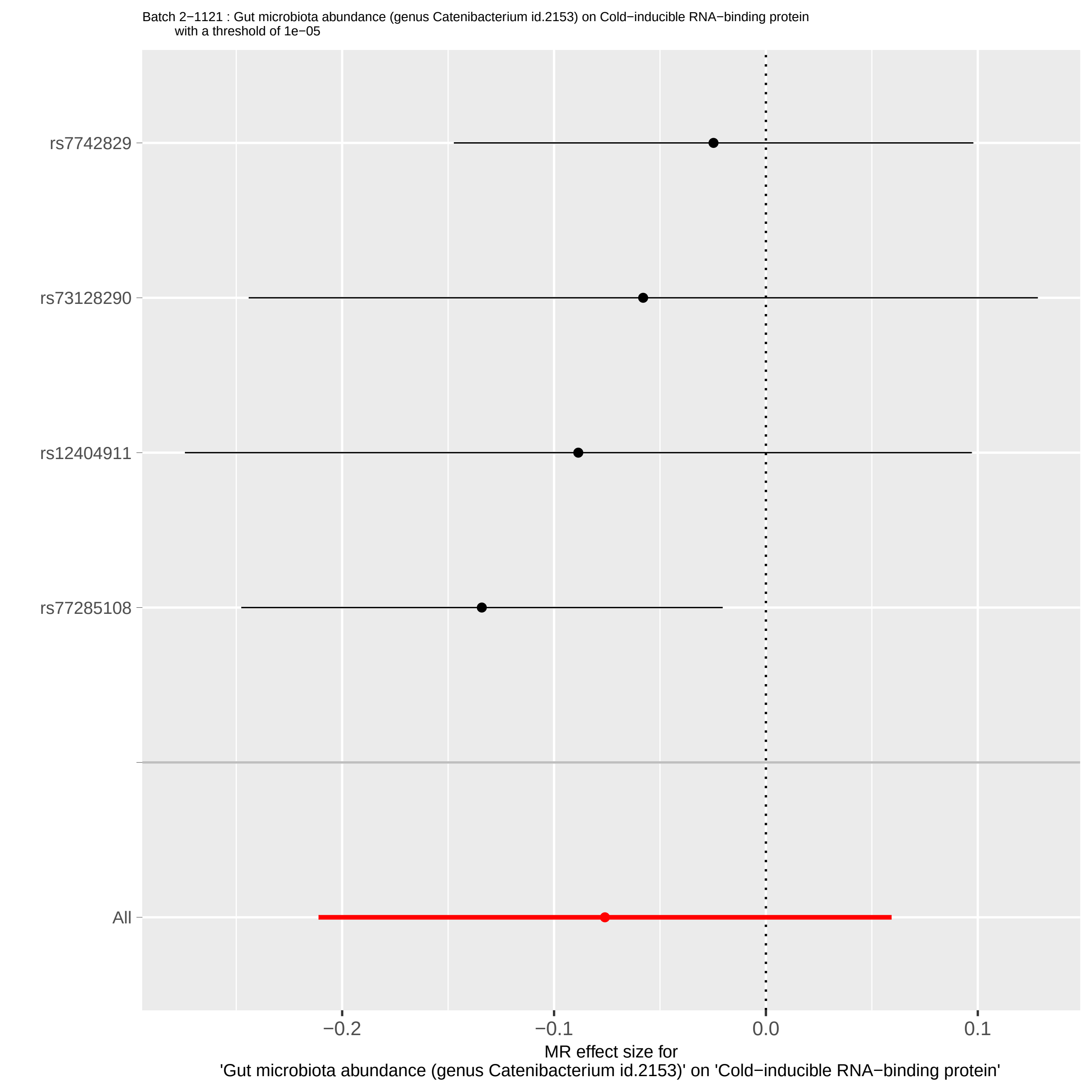
Batch 2−1121 : Gut microbiota abundance (genus Catenibacterium id.2153) on Cold−inducible RNA−binding protein
with a threshold of 1e−05
rs7742829
rs73128290
rs12404911
rs77285108
All
−0.2
−0.1
0.0
0.1
MR effect size for
'Gut microbiota abundance (genus Catenibacterium id.2153)' on 'Cold−inducible RNA−binding protein'

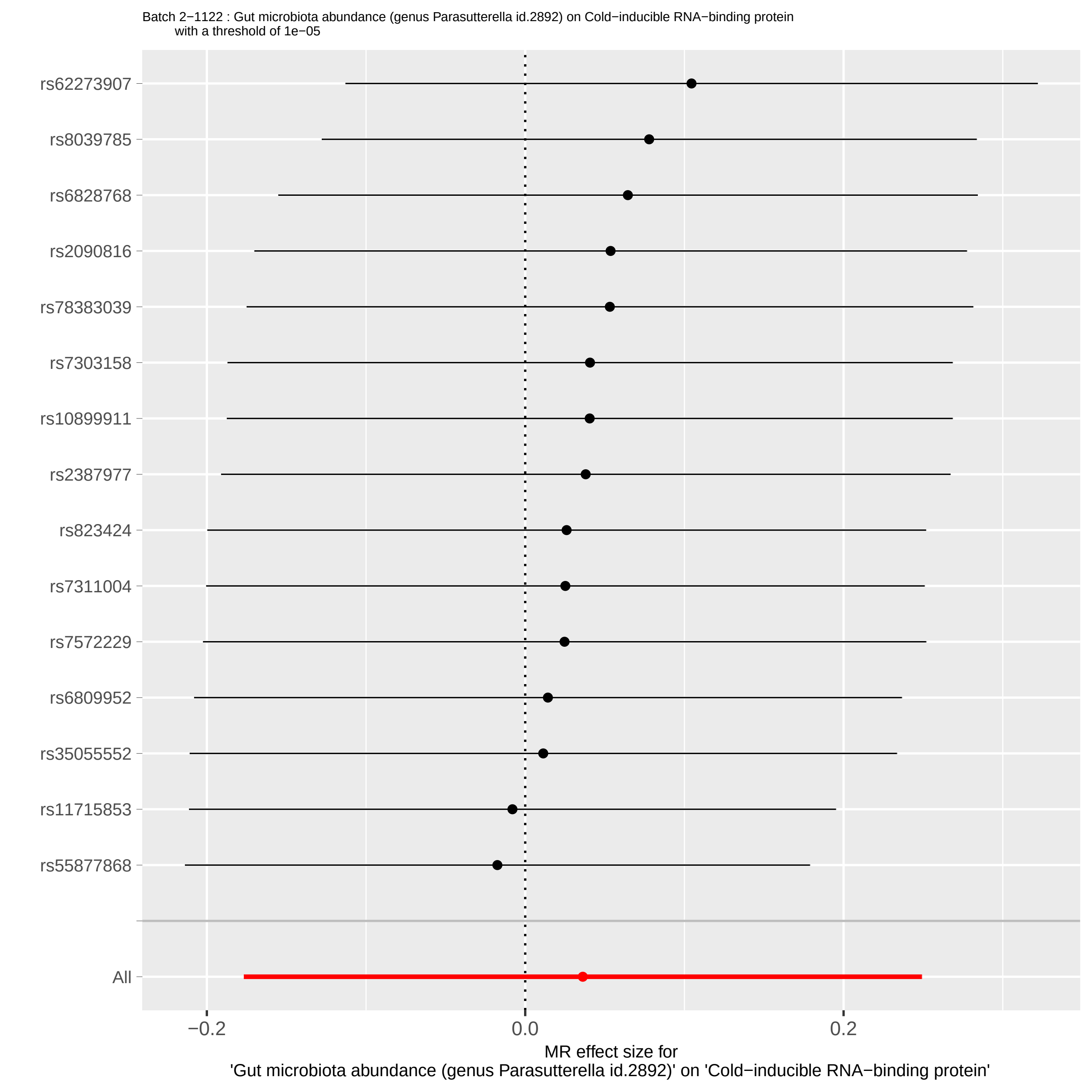

Batch 2−1122 : Gut microbiota abundance (genus Parasutterella id.2892) on Cold−inducible RNA−binding protein
with a threshold of 1e−05
rs62273907
rs8039785
rs6828768
rs2090816
rs78383039
rs7303158
rs10899911
rs2387977
rs823424
rs7311004
rs7572229
rs6809952
rs35055552
rs11715853
rs55877868
All
−0.2
0.0
0.2
MR effect size for
'Gut microbiota abundance (genus Parasutterella id.2892)' on 'Cold−inducible RNA−binding protein'

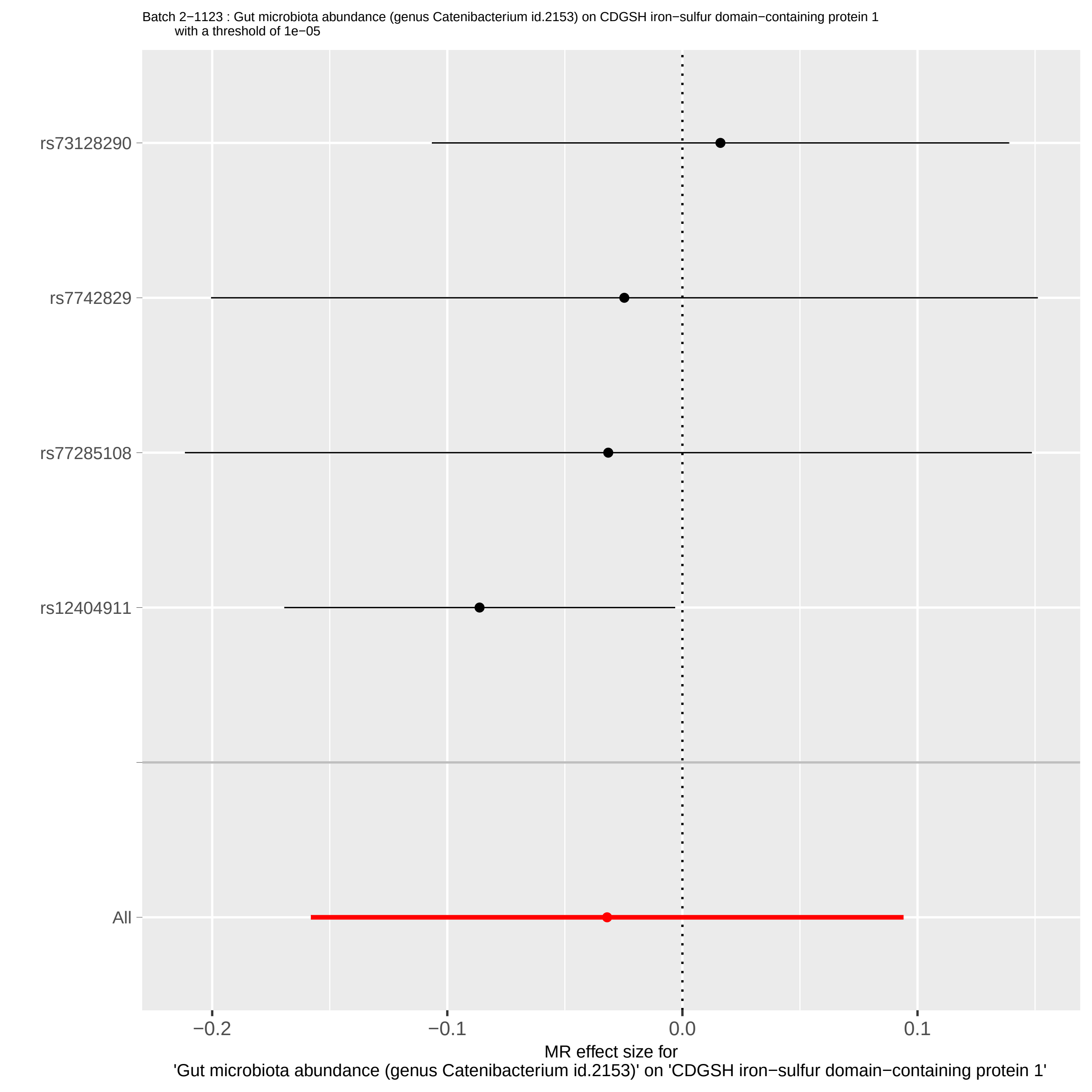

Batch 2−1123 : Gut microbiota abundance (genus Catenibacterium id.2153) on CDGSH iron−sulfur domain−containing protein 1
with a threshold of 1e−05
rs73128290
rs7742829
rs77285108
rs12404911
All
−0.2
−0.1
0.0
0.1
MR effect size for
'Gut microbiota abundance (genus Catenibacterium id.2153)' on 'CDGSH iron−sulfur domain−containing protein 1'

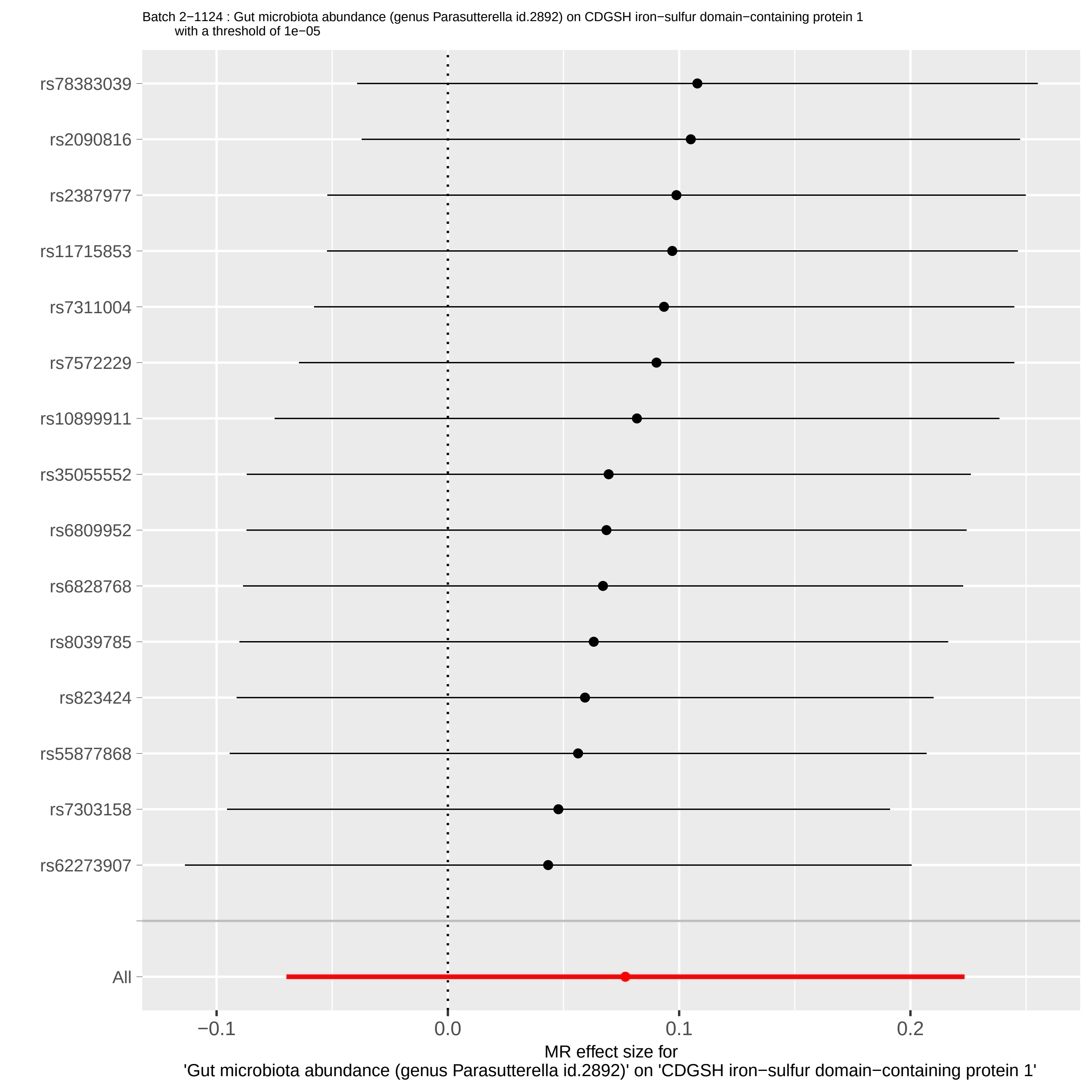

Batch 2−1124 : Gut microbiota abundance (genus Parasutterella id.2892) on CDGSH iron−sulfur domain−containing protein 1
with a threshold of 1e−05
rs78383039
rs2090816
rs2387977
rs11715853
rs7311004
rs7572229
rs10899911
rs35055552
rs6809952
rs6828768
rs8039785
rs823424
rs55877868
rs7303158
rs62273907
All
−0.1
0.0
0.1
0.2
MR effect size for
'Gut microbiota abundance (genus Parasutterella id.2892)' on 'CDGSH iron−sulfur domain−containing protein 1'

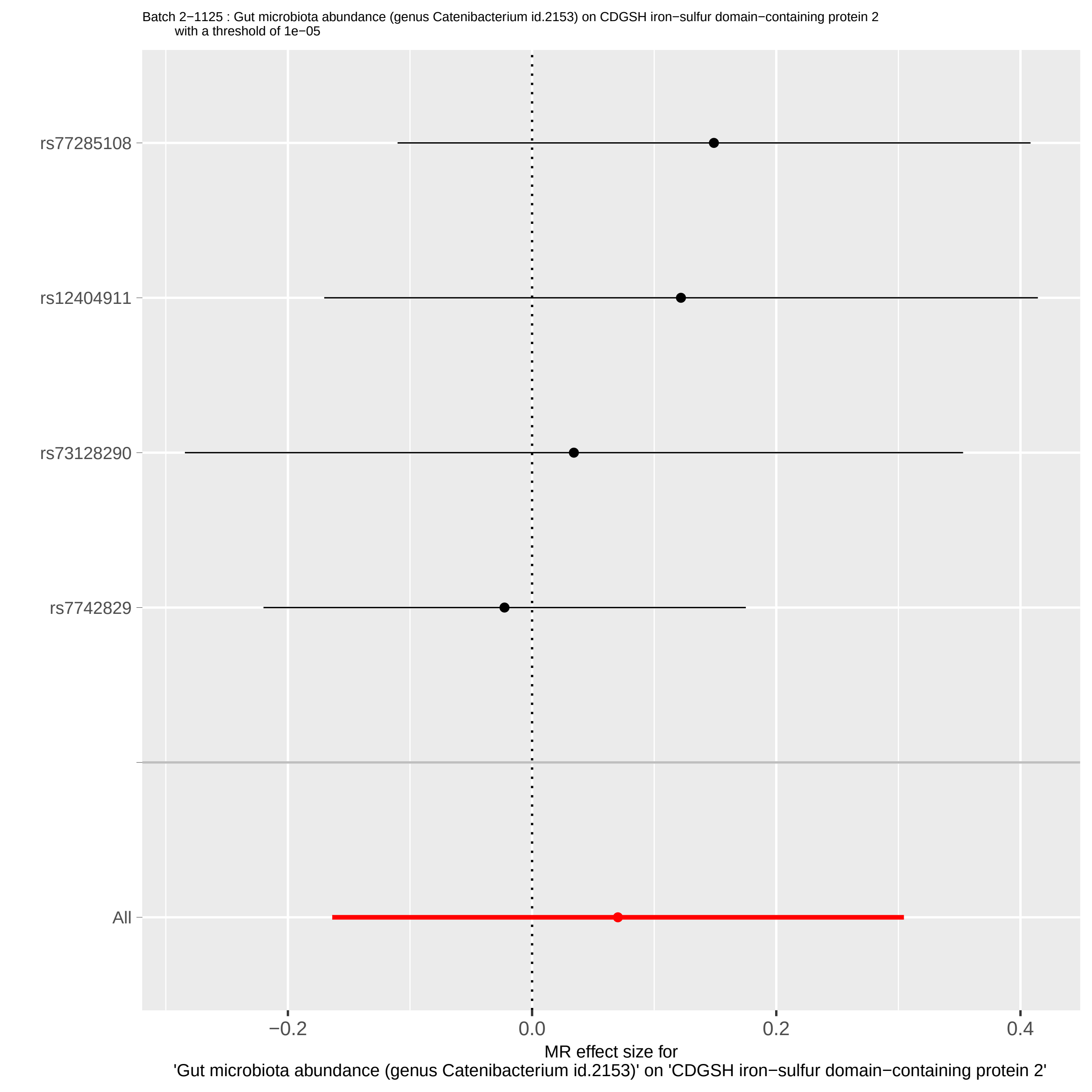

Batch 2−1125 : Gut microbiota abundance (genus Catenibacterium id.2153) on CDGSH iron−sulfur domain−containing protein 2
with a threshold of 1e−05
rs77285108
rs12404911
rs73128290
rs7742829
All
−0.2
0.0
0.2
0.4
MR effect size for
'Gut microbiota abundance (genus Catenibacterium id.2153)' on 'CDGSH iron−sulfur domain−containing protein 2'

Batch 2−1126 : Gut microbiota abundance (genus Parasutterella id.2892) on CDGSH iron−sulfur domain−containing protein 2
with a threshold of 1e−05

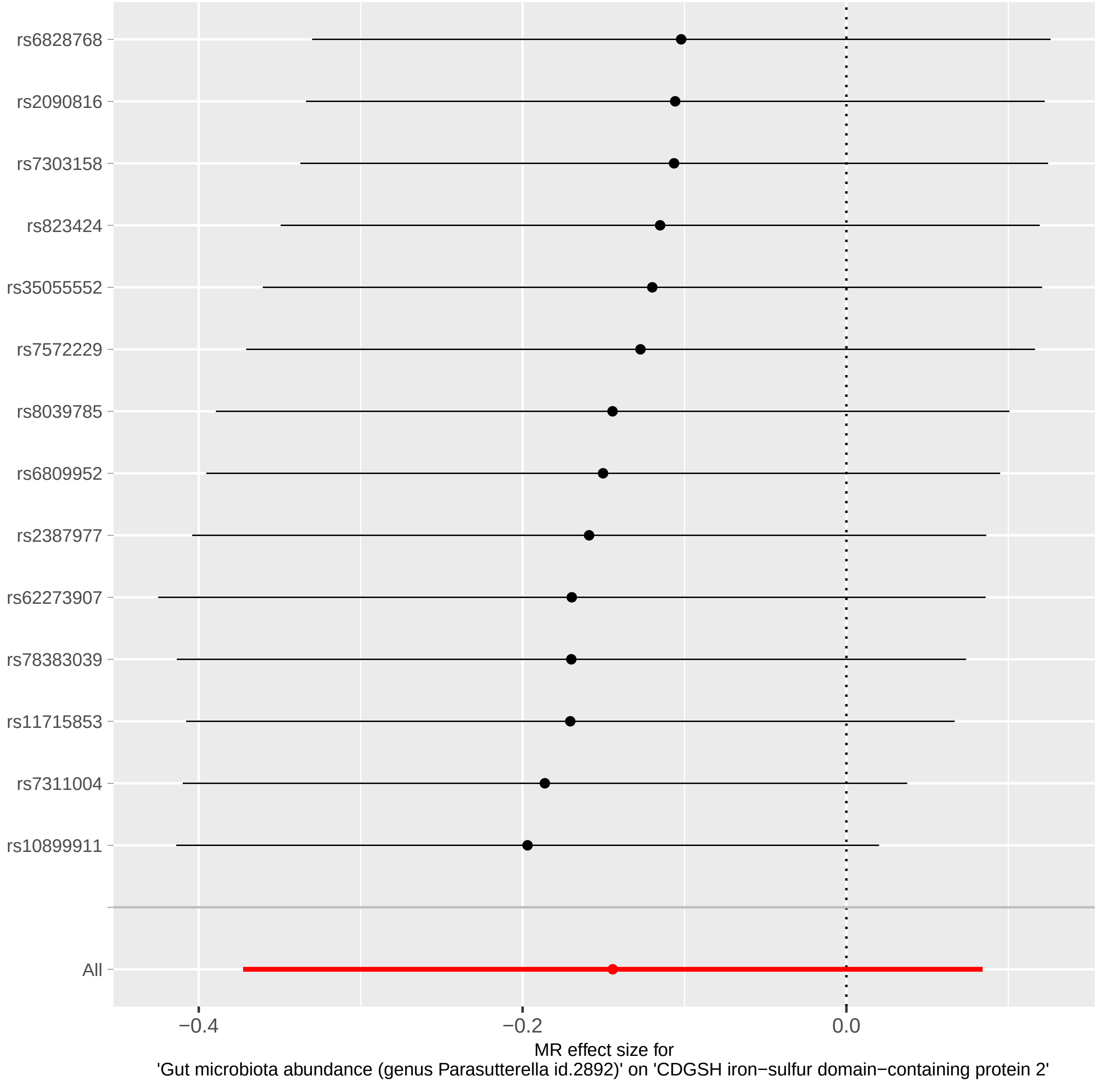

Batch 2−1127 : Gut microbiota abundance (genus Catenibacterium id.2153) on Cytoskeleton−associated protein 2
with a threshold of 1e−05

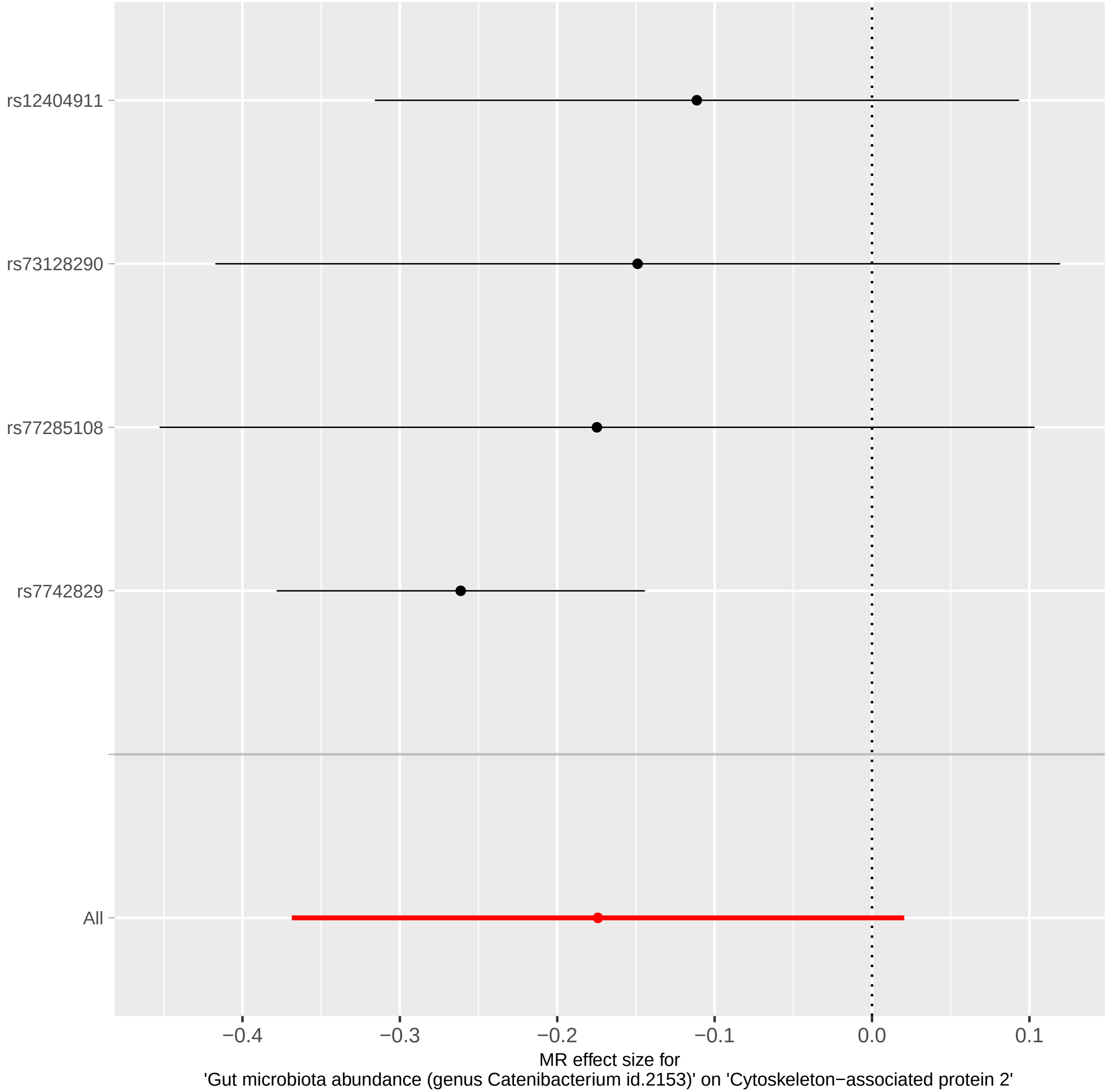

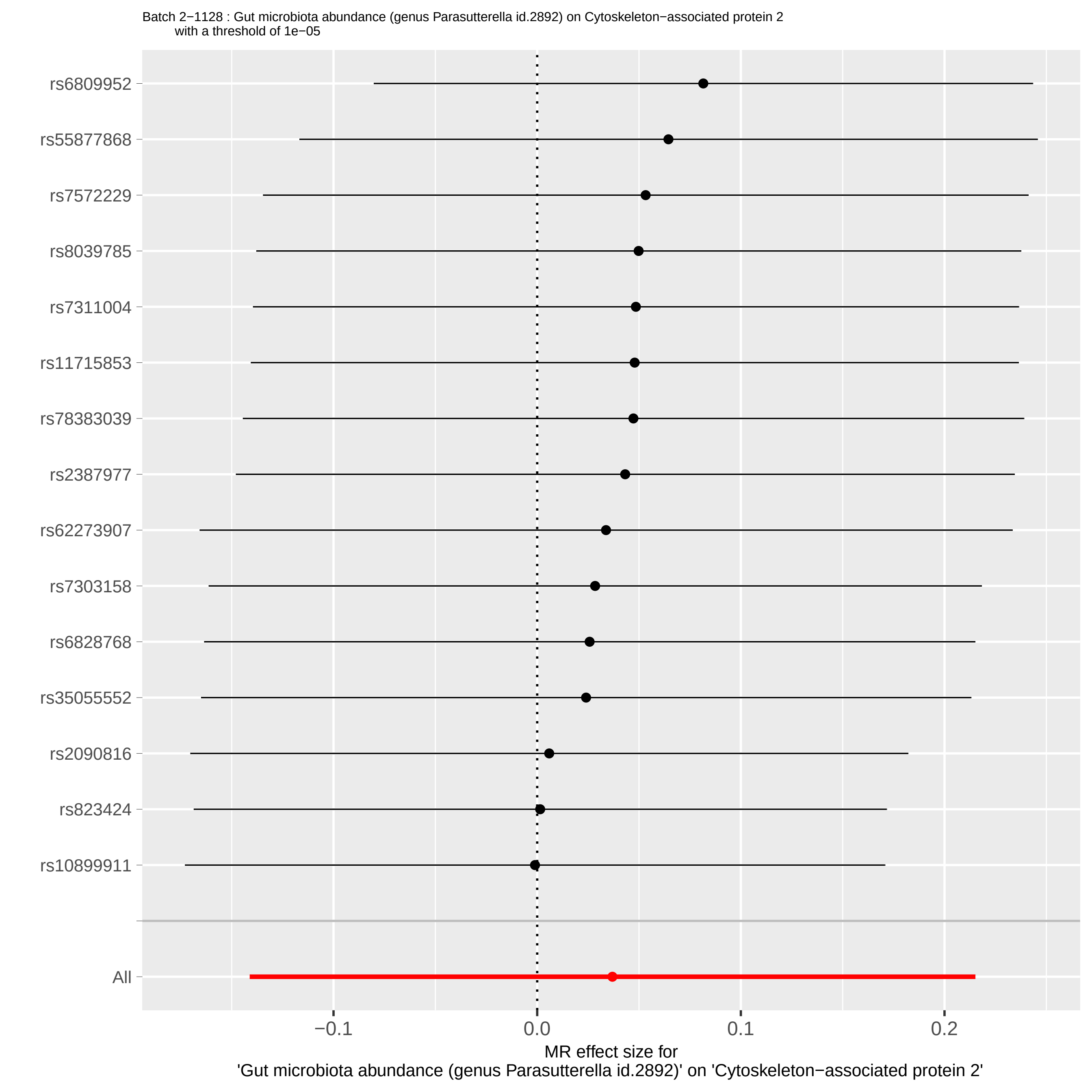
Batch 2−1128 : Gut microbiota abundance (genus Parasutterella id.2892) on Cytoskeleton−associated protein 2
with a threshold of 1e−05
rs6809952
rs55877868
rs7572229
rs8039785
rs7311004
rs11715853
rs78383039
rs2387977
rs62273907
rs7303158
rs6828768
rs35055552
rs2090816
rs823424
rs10899911
All
−0.1
0.0
0.1
0.2
MR effect size for
'Gut microbiota abundance (genus Parasutterella id.2892)' on 'Cytoskeleton−associated protein 2'

Batch 2−1129 : Gut microbiota abundance (genus Catenibacterium id.2153) on Creatine kinase B−type
with a threshold of 1e−05

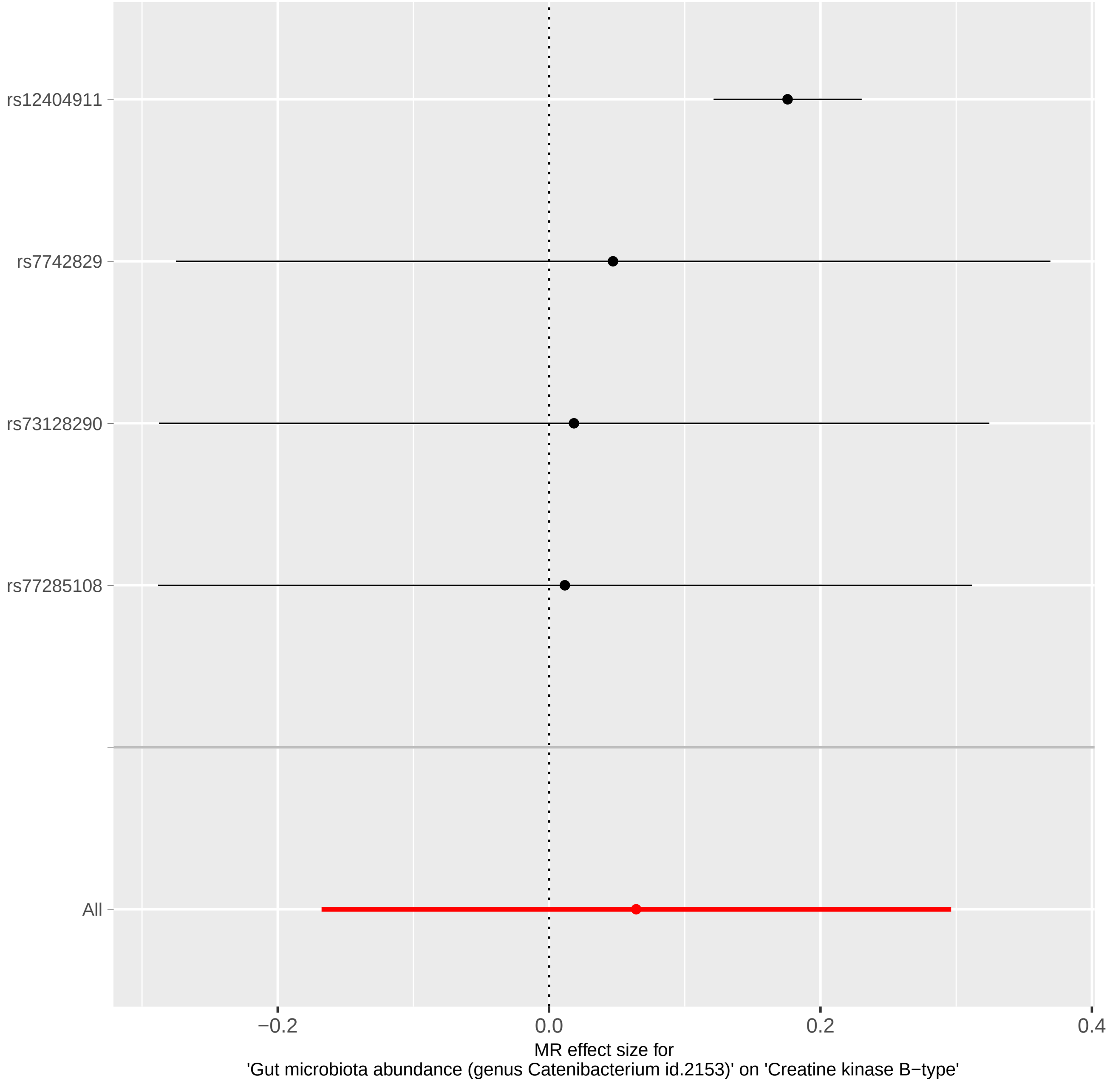

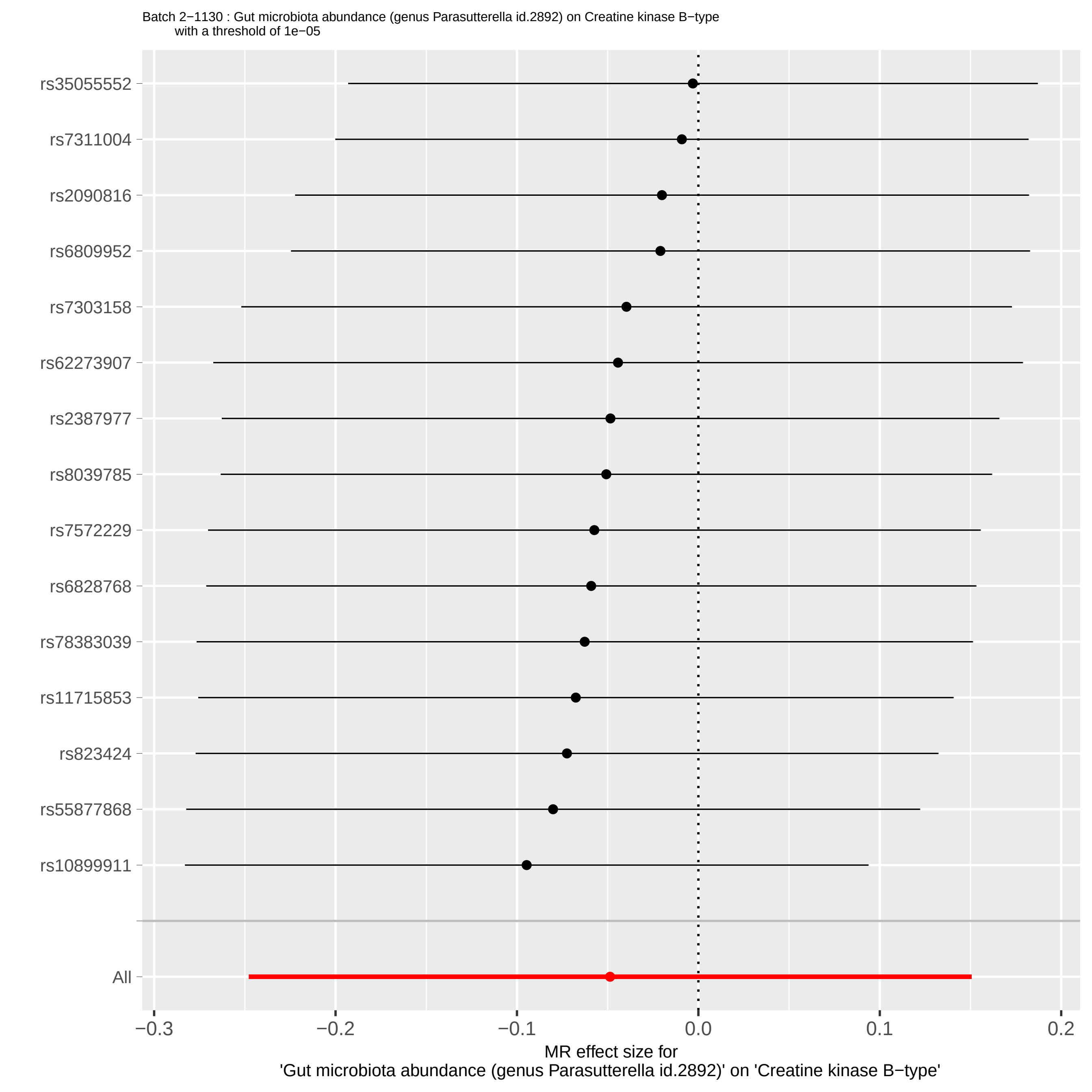
Batch 2−1130 : Gut microbiota abundance (genus Parasutterella id.2892) on Creatine kinase B−type
with a threshold of 1e−05
rs35055552
rs7311004
rs2090816
rs6809952
rs7303158
rs62273907
rs2387977
rs8039785
rs7572229
rs6828768
rs78383039
rs11715853
rs823424
rs55877868
rs10899911
All
−0.3
−0.2
−0.1
0.0
0.1
0.2
MR effect size for
'Gut microbiota abundance (genus Parasutterella id.2892)' on 'Creatine kinase B−type'

Batch 2−1131 : Gut microbiota abundance (genus Catenibacterium id.2153) on Creatine kinase M−type
with a threshold of 1e−05

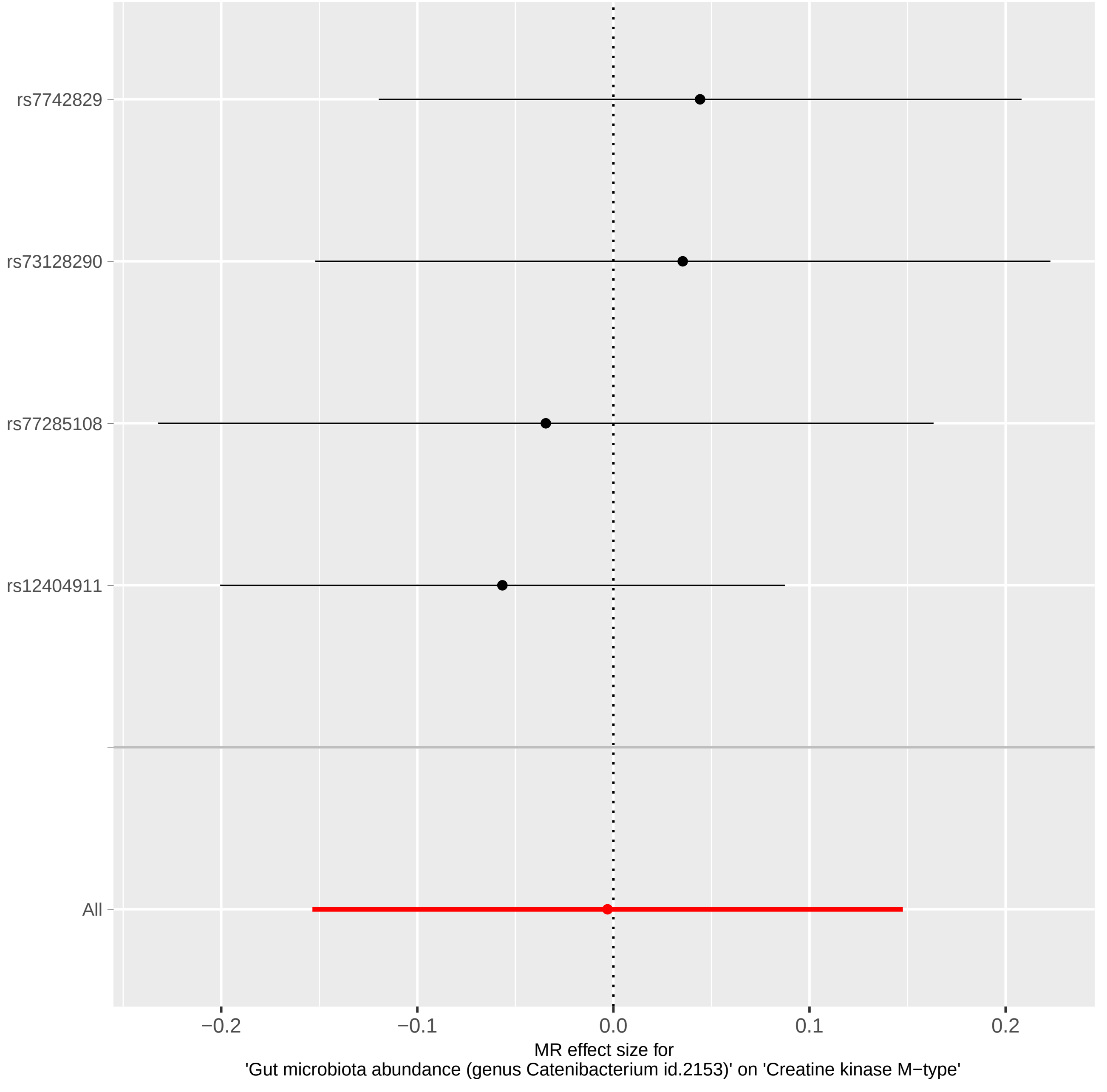

Batch 2−1132 : Gut microbiota abundance (genus Parasutterella id.2892) on Creatine kinase M−type
with a threshold of 1e−05

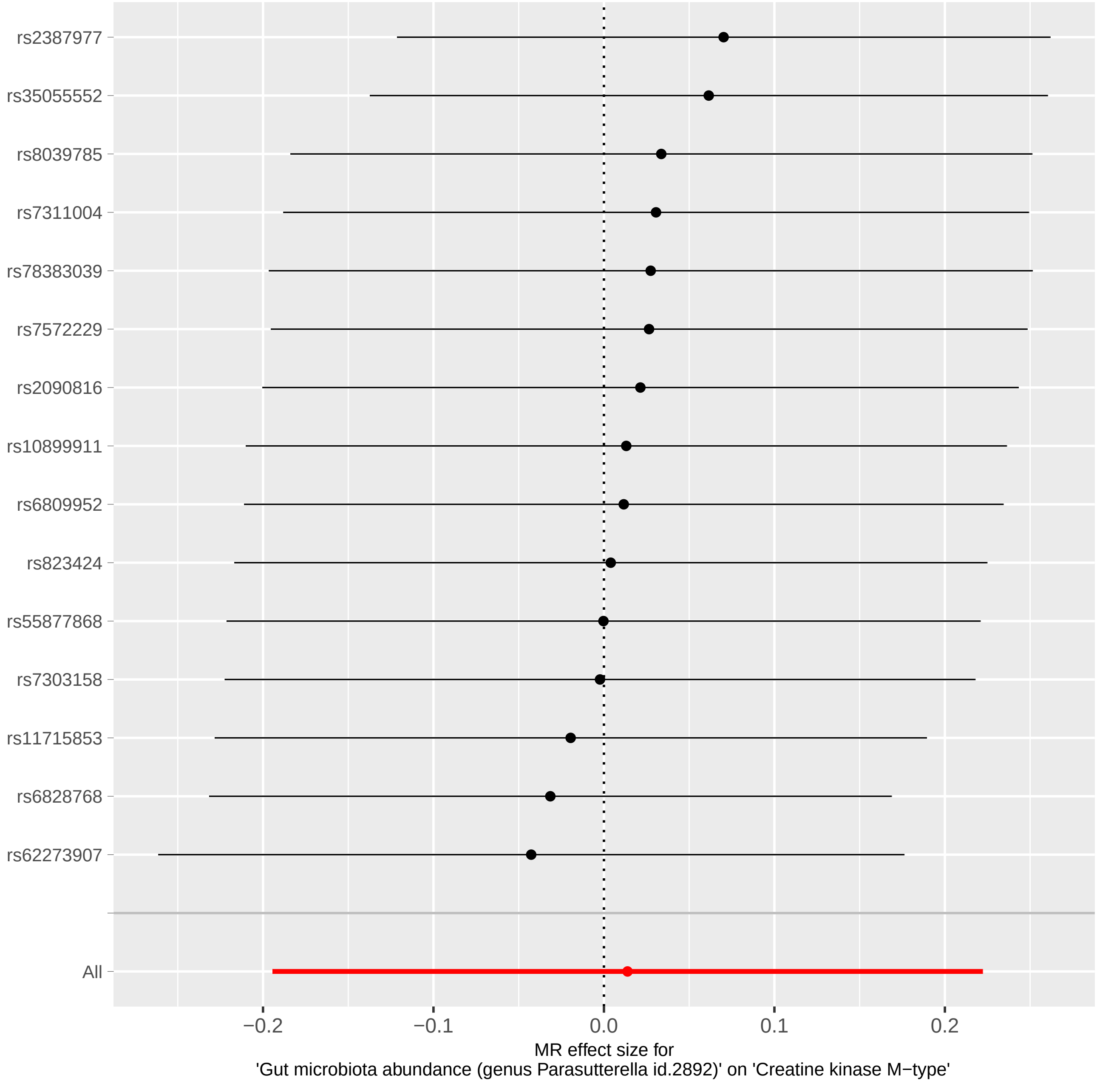

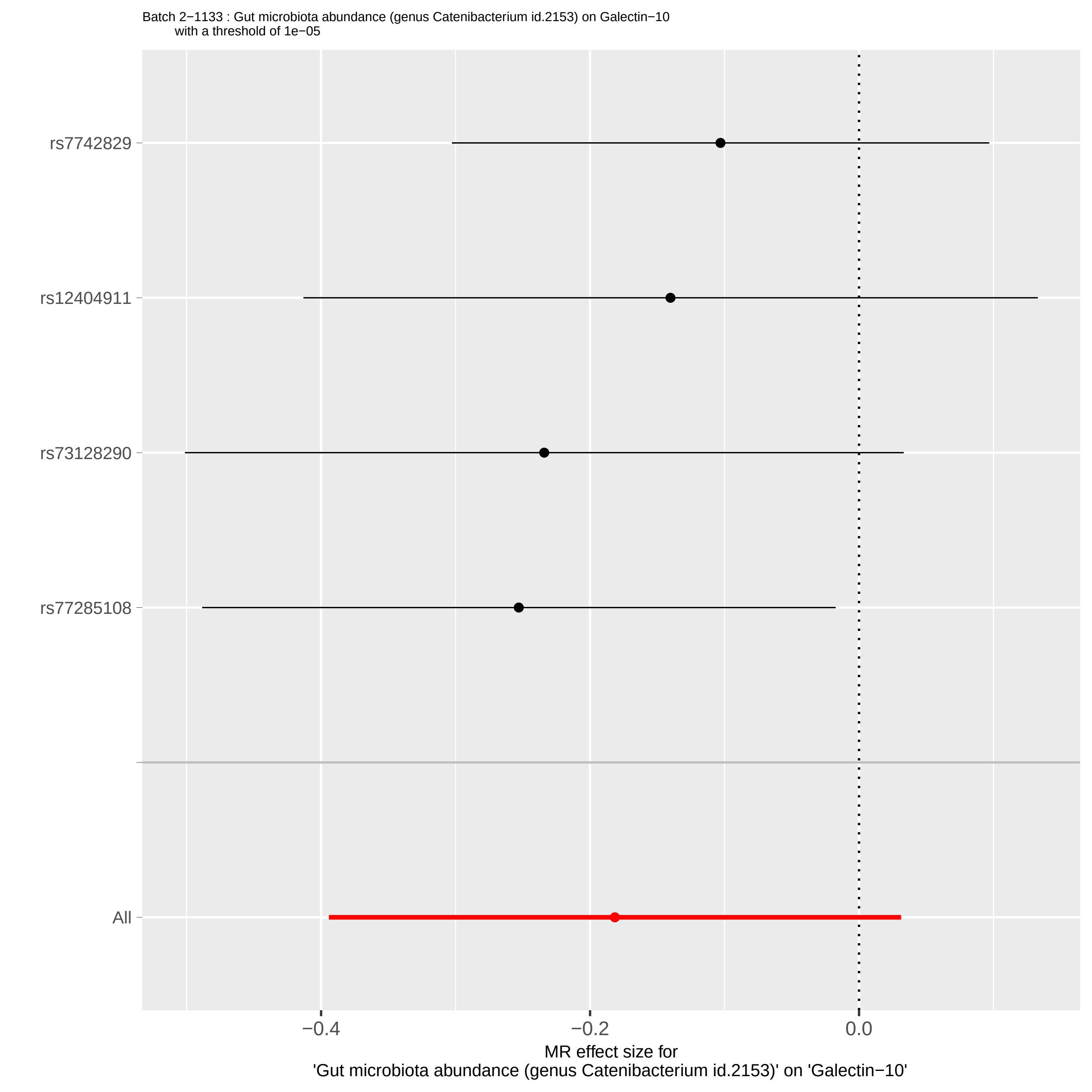

Batch 2−1133 : Gut microbiota abundance (genus Catenibacterium id.2153) on Galectin−10
with a threshold of 1e−05
rs7742829
rs12404911
rs73128290
rs77285108
All
−0.4
−0.2
0.0
MR effect size for
'Gut microbiota abundance (genus Catenibacterium id.2153)' on 'Galectin−10'

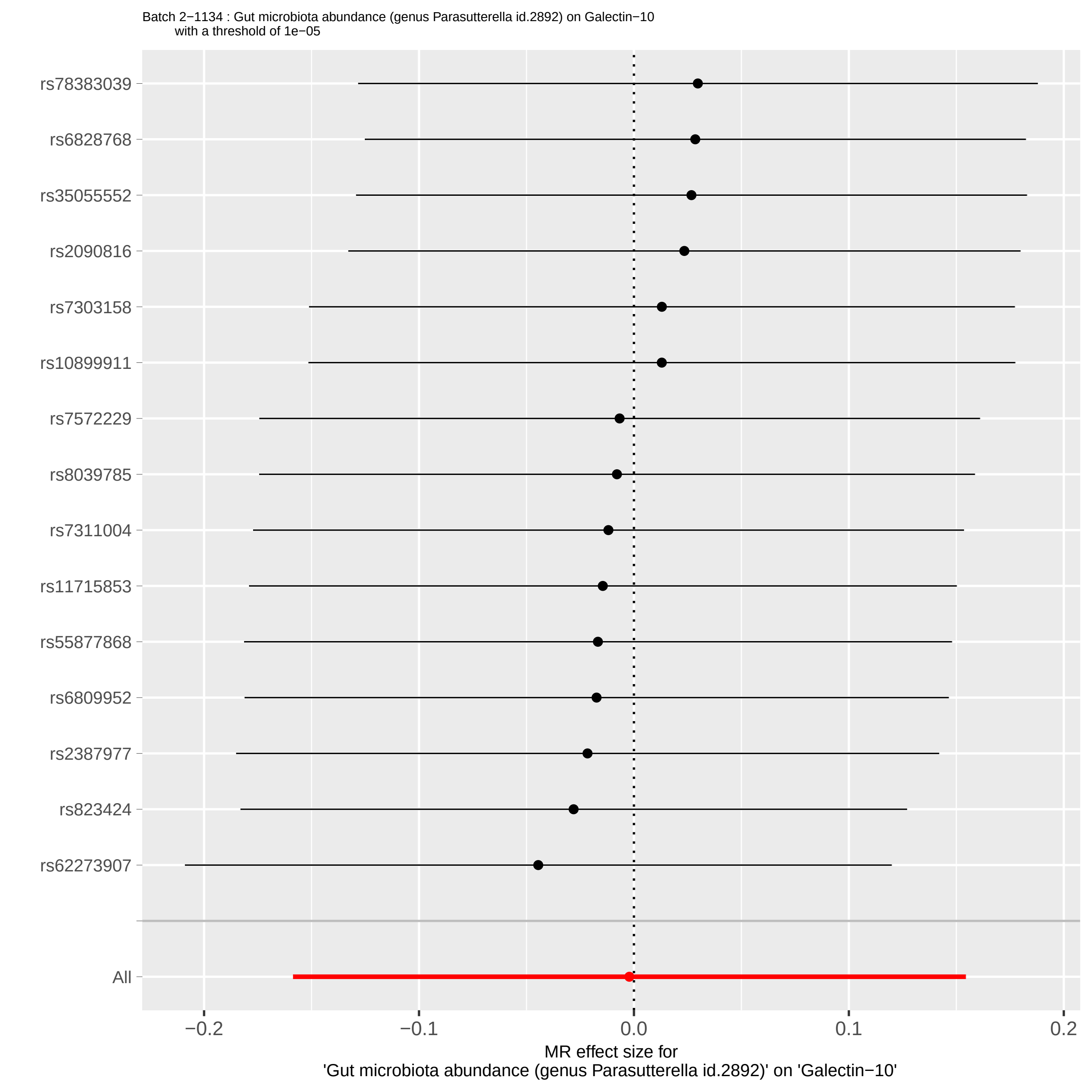

Batch 2−1134 : Gut microbiota abundance (genus Parasutterella id.2892) on Galectin−10
with a threshold of 1e−05
rs78383039
rs6828768
rs35055552
rs2090816
rs7303158
rs10899911
rs7572229
rs8039785
rs7311004
rs11715853
rs55877868
rs6809952
rs2387977
rs823424
rs62273907
All
−0.2
−0.1
0.0
0.1
0.2
MR effect size for
'Gut microbiota abundance (genus Parasutterella id.2892)' on 'Galectin−10'

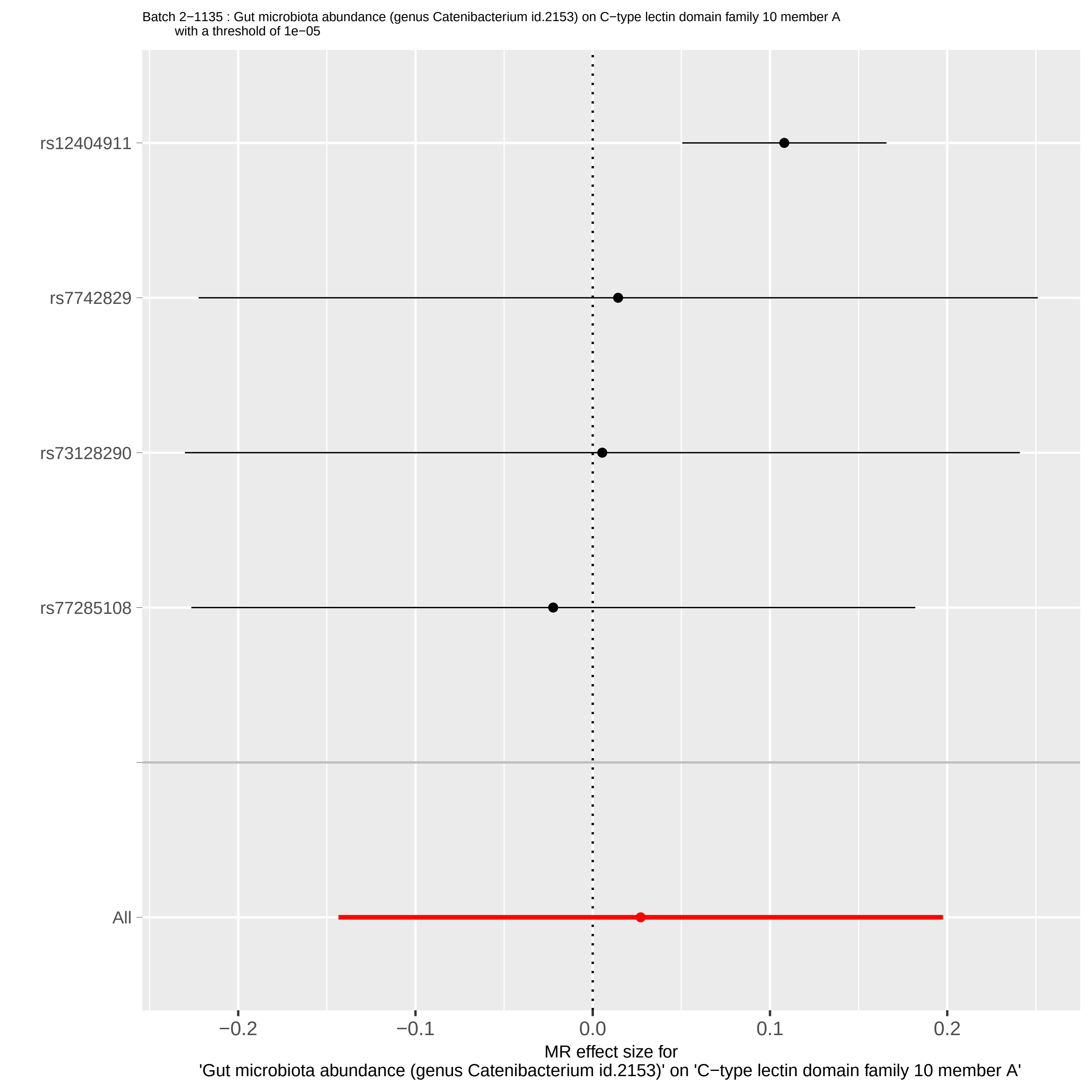

Batch 2−1135 : Gut microbiota abundance (genus Catenibacterium id.2153) on C−type lectin domain family 10 member A
with a threshold of 1e−05
rs12404911
rs7742829
rs73128290
rs77285108
All
−0.2
−0.1
0.0
0.1
0.2
MR effect size for
'Gut microbiota abundance (genus Catenibacterium id.2153)' on 'C−type lectin domain family 10 member A'

Batch 2−1136 : Gut microbiota abundance (genus Parasutterella id.2892) on C−type lectin domain family 10 member A
with a threshold of 1e−05

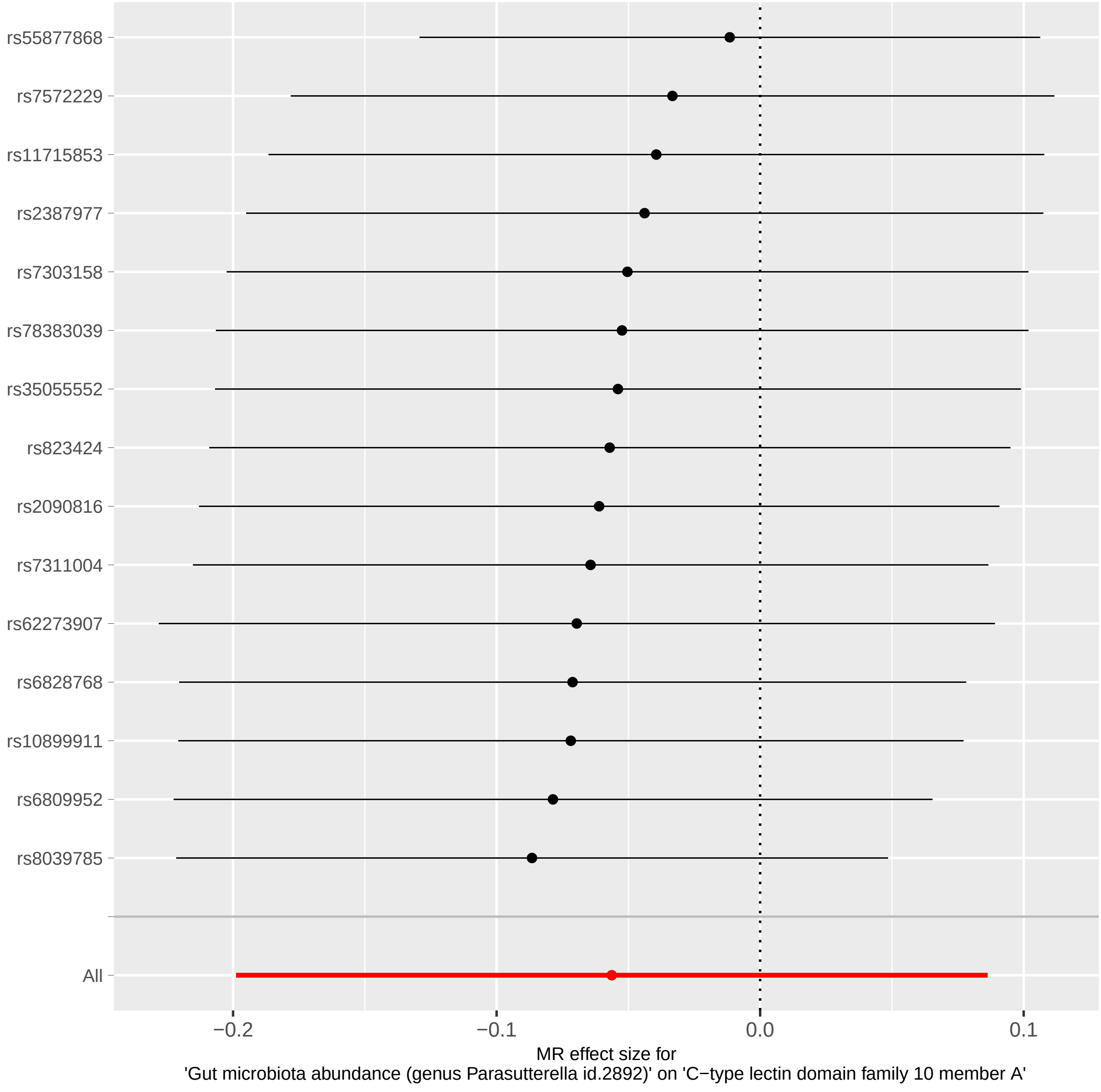

Batch 2−1137 : Gut microbiota abundance (genus Catenibacterium id.2153) on Stem Cell Growth Factor−beta
with a threshold of 1e−05

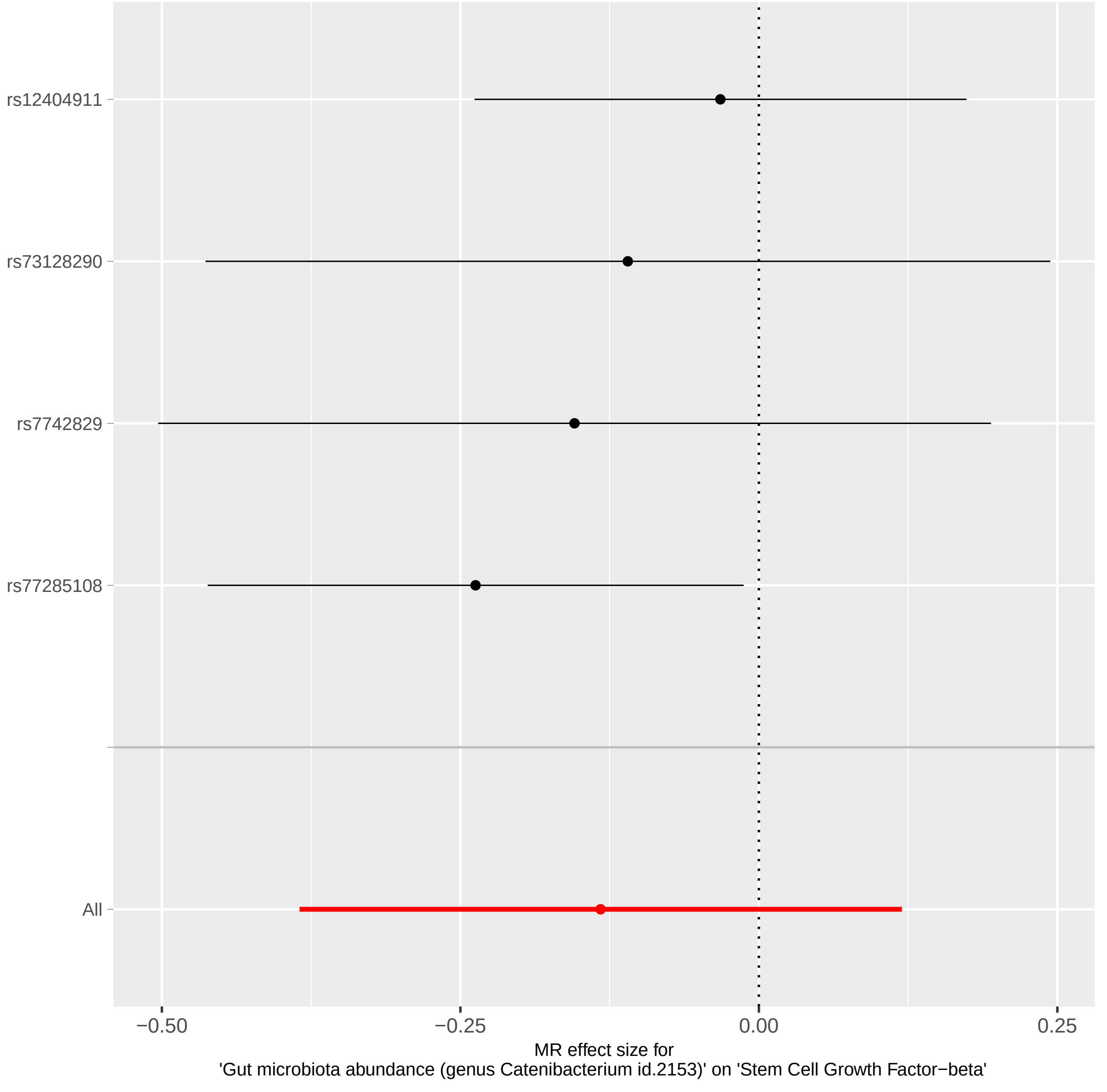

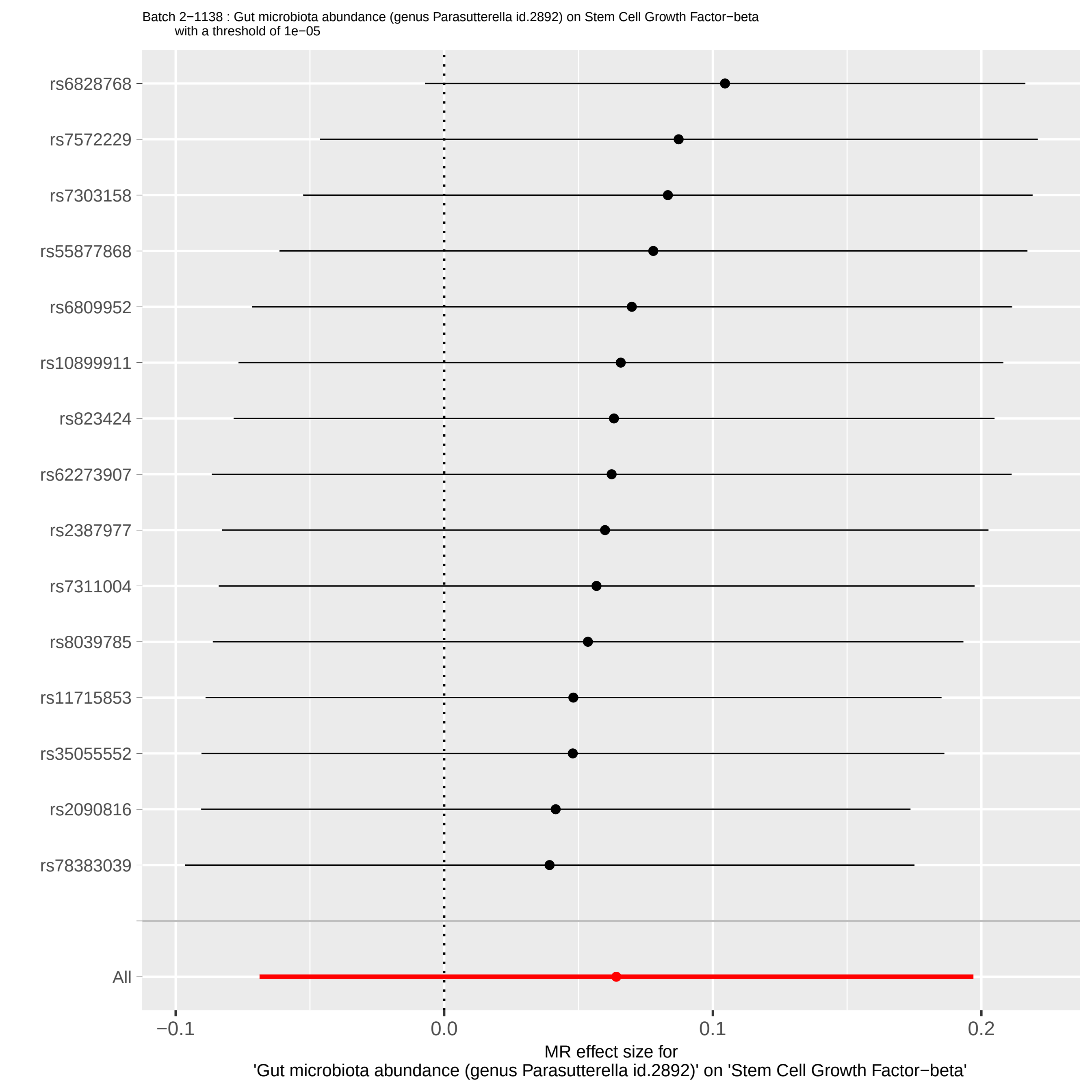
Batch 2−1138 : Gut microbiota abundance (genus Parasutterella id.2892) on Stem Cell Growth Factor−beta
with a threshold of 1e−05
rs6828768
rs7572229
rs7303158
rs55877868
rs6809952
rs10899911
rs823424
rs62273907
rs2387977
rs7311004
rs8039785
rs11715853
rs35055552
rs2090816
rs78383039
All
−0.1
0.0
0.1
0.2
MR effect size for
'Gut microbiota abundance (genus Parasutterella id.2892)' on 'Stem Cell Growth Factor−beta'

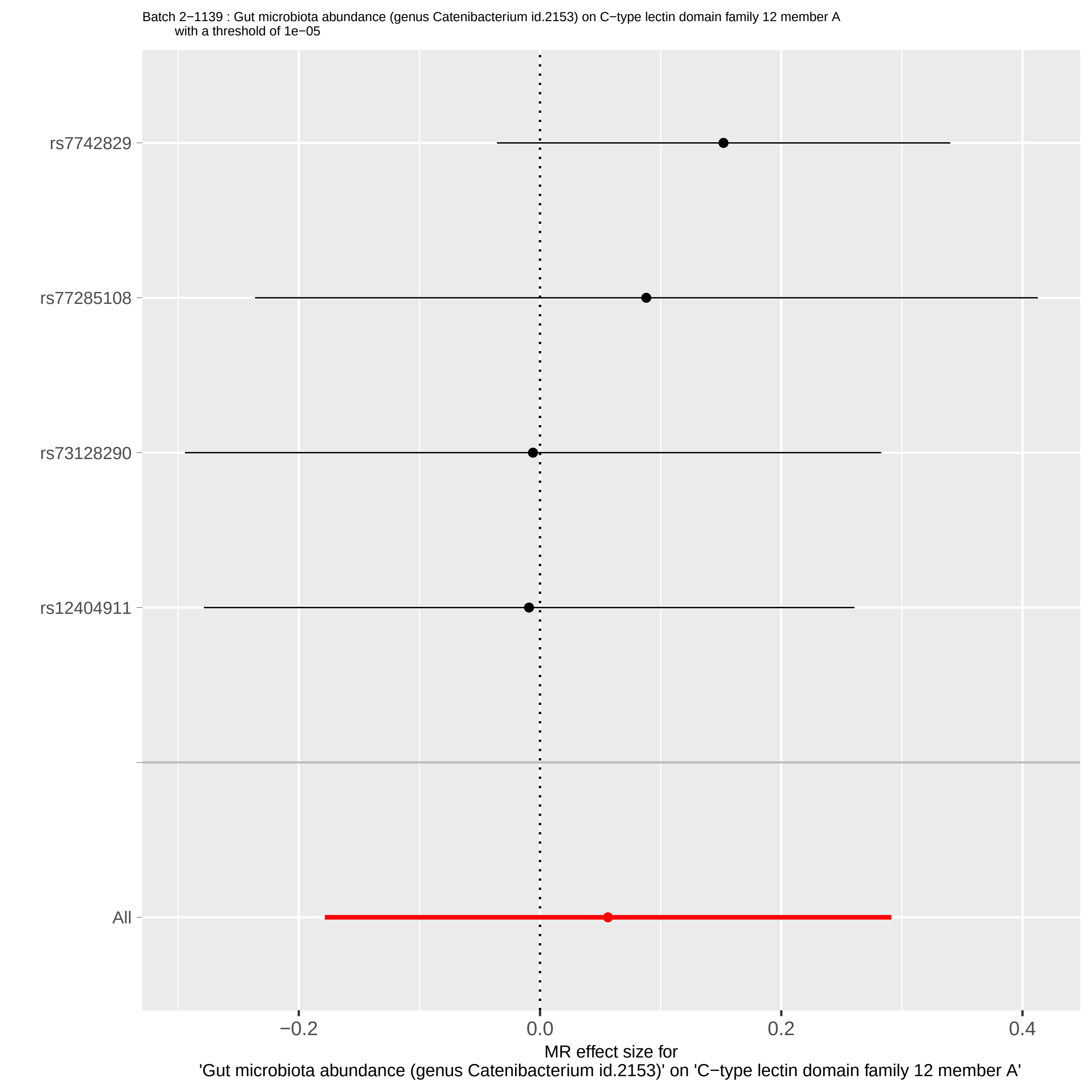
Batch 2−1139 : Gut microbiota abundance (genus Catenibacterium id.2153) on C−type lectin domain family 12 member A
with a threshold of 1e−05
rs7742829
rs77285108
rs73128290
rs12404911
All
−0.2
0.0
0.2
0.4
MR effect size for
'Gut microbiota abundance (genus Catenibacterium id.2153)' on 'C−type lectin domain family 12 member A'

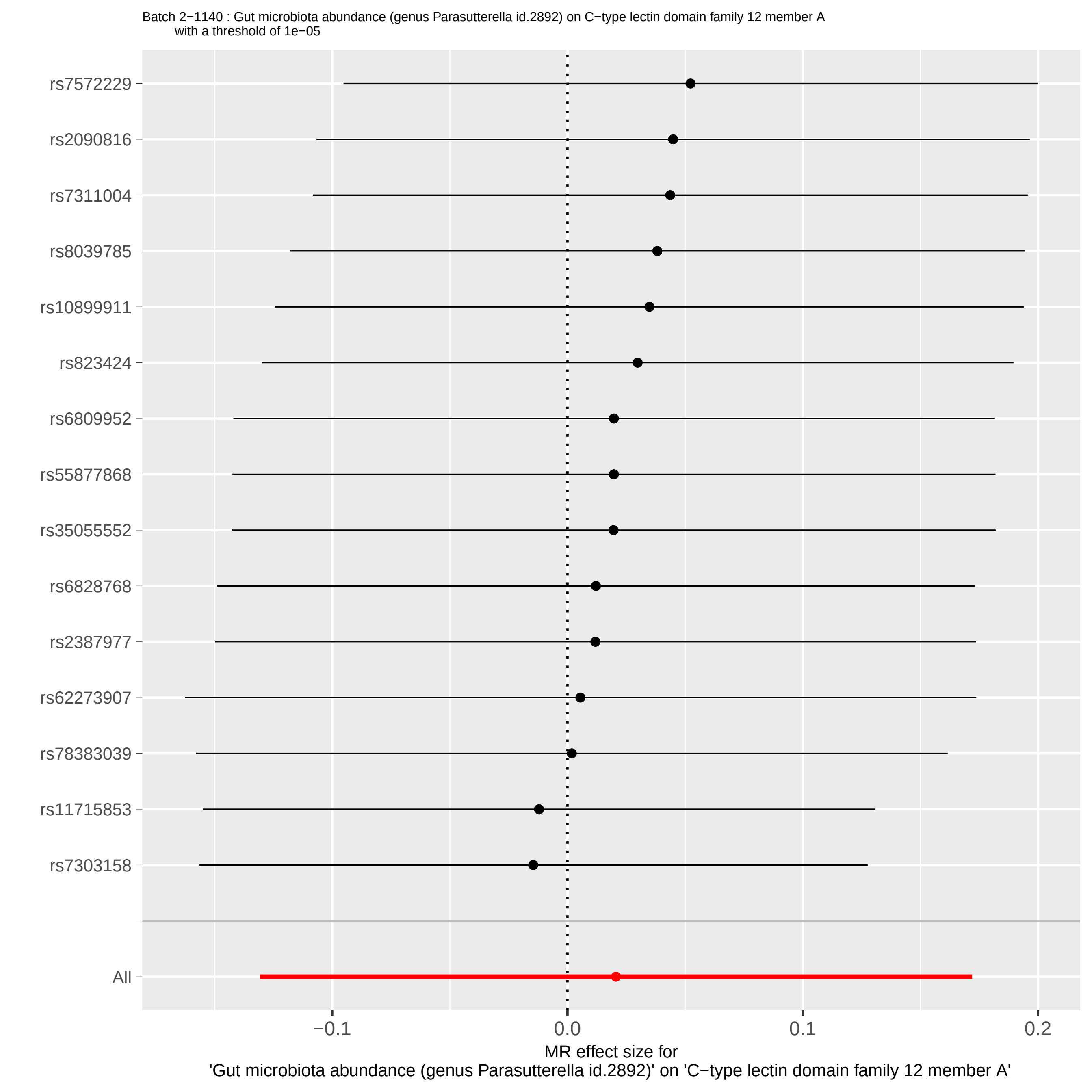

Batch 2−1140 : Gut microbiota abundance (genus Parasutterella id.2892) on C−type lectin domain family 12 member A
with a threshold of 1e−05
rs7572229
rs2090816
rs7311004
rs8039785
rs10899911
rs823424
rs6809952
rs55877868
rs35055552
rs6828768
rs2387977
rs62273907
rs78383039
rs11715853
rs7303158
All
−0.1
0.0
0.1
0.2
MR effect size for
'Gut microbiota abundance (genus Parasutterella id.2892)' on 'C−type lectin domain family 12 member A'

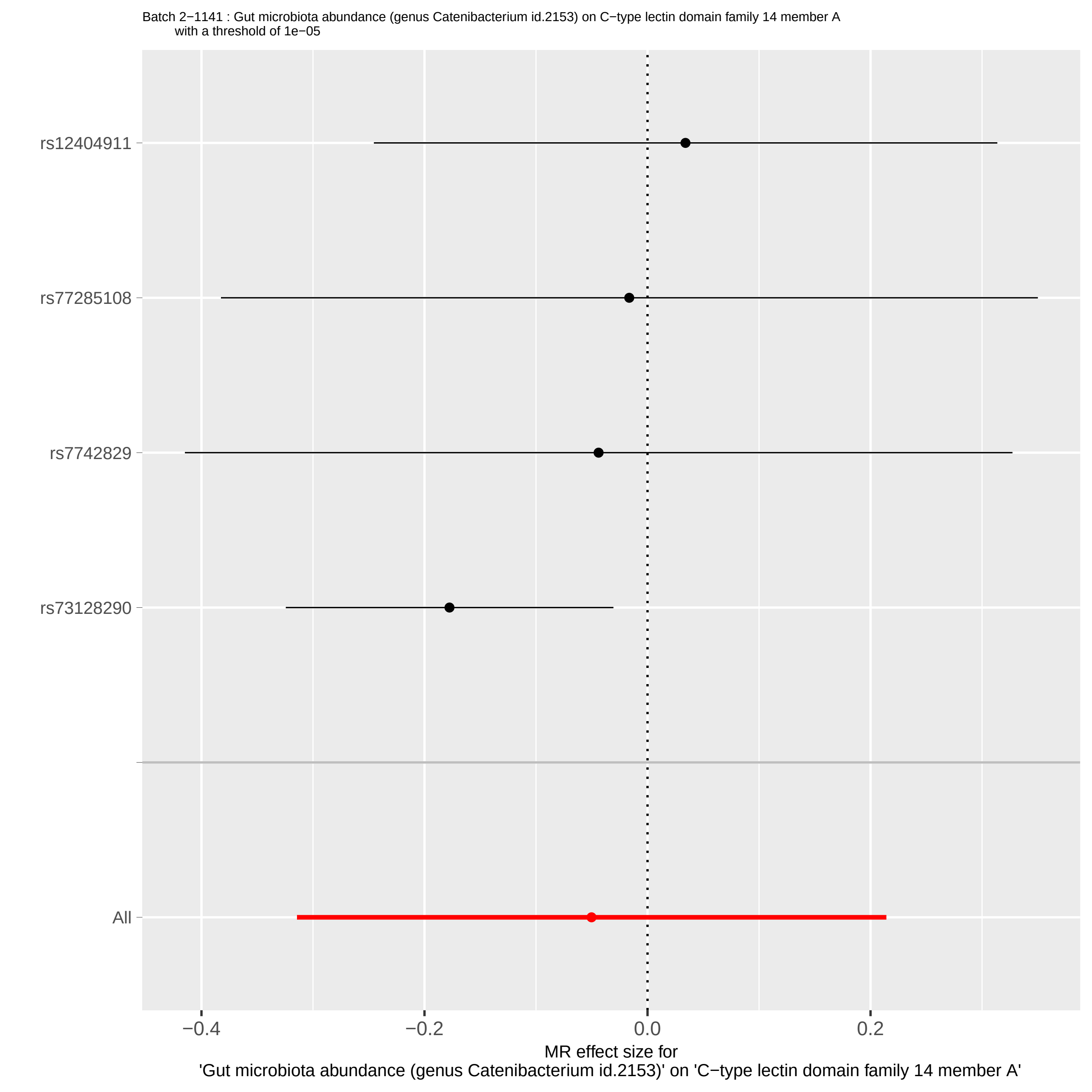
Batch 2−1141 : Gut microbiota abundance (genus Catenibacterium id.2153) on C−type lectin domain family 14 member A
with a threshold of 1e−05
rs12404911
rs77285108
rs7742829
rs73128290
All
−0.4
−0.2
0.0
0.2
MR effect size for
'Gut microbiota abundance (genus Catenibacterium id.2153)' on 'C−type lectin domain family 14 member A'

Batch 2−1142 : Gut microbiota abundance (genus Parasutterella id.2892) on C−type lectin domain family 14 member A
with a threshold of 1e−05

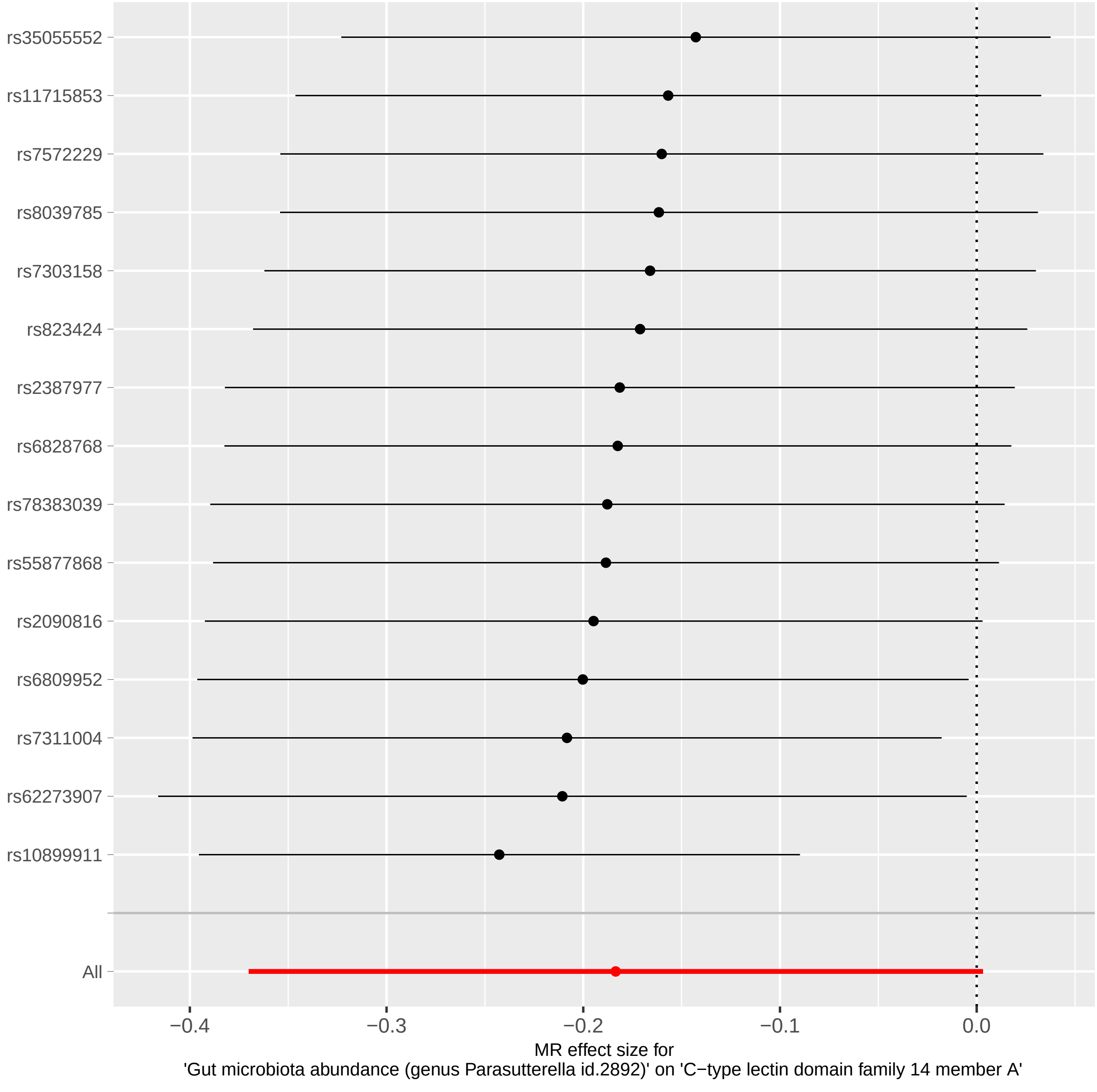

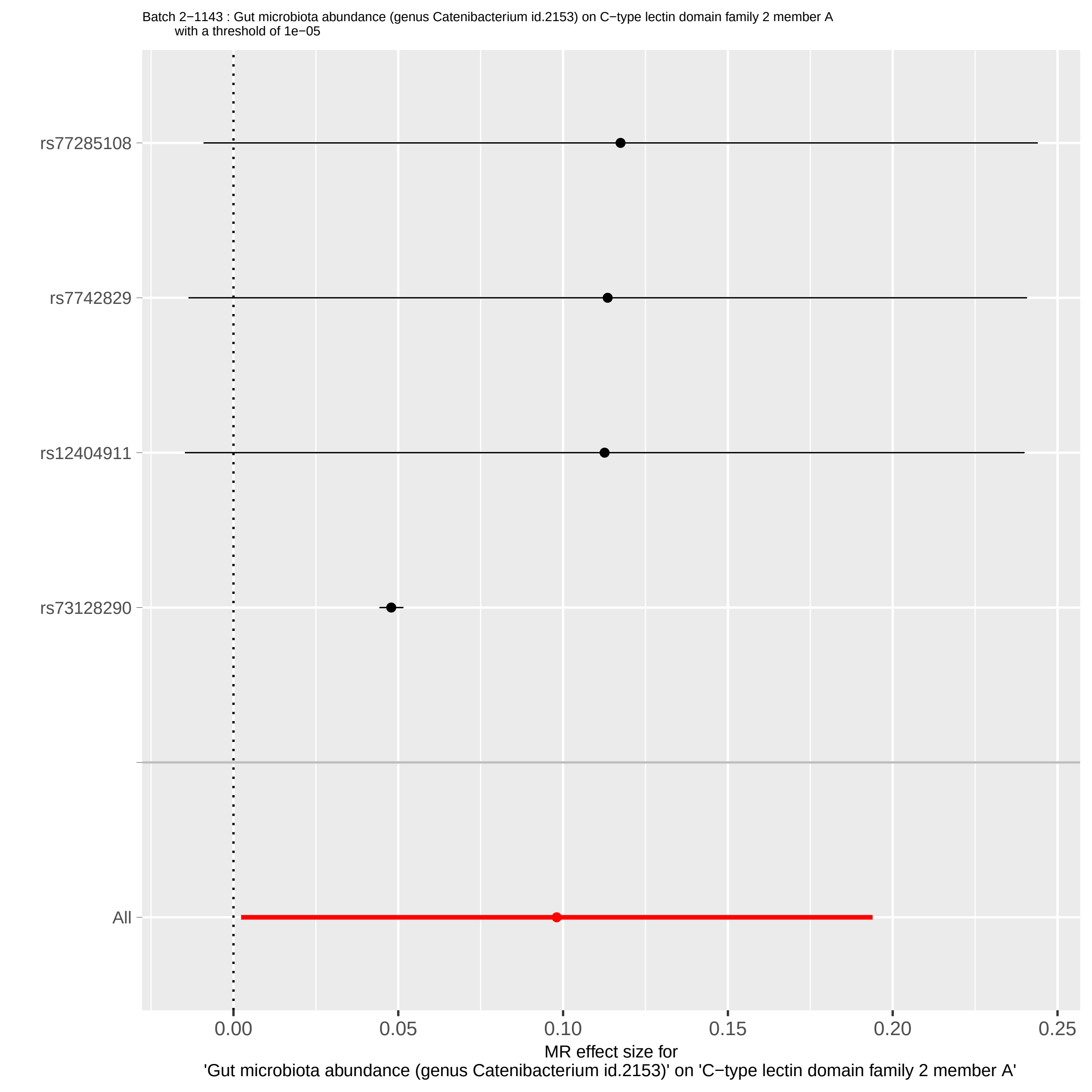

Batch 2−1143 : Gut microbiota abundance (genus Catenibacterium id.2153) on C−type lectin domain family 2 member A
with a threshold of 1e−05
rs77285108
rs7742829
rs12404911
rs73128290
All
0.00
0.05
0.10
0.15
0.20
0.25
MR effect size for
'Gut microbiota abundance (genus Catenibacterium id.2153)' on 'C−type lectin domain family 2 member A'

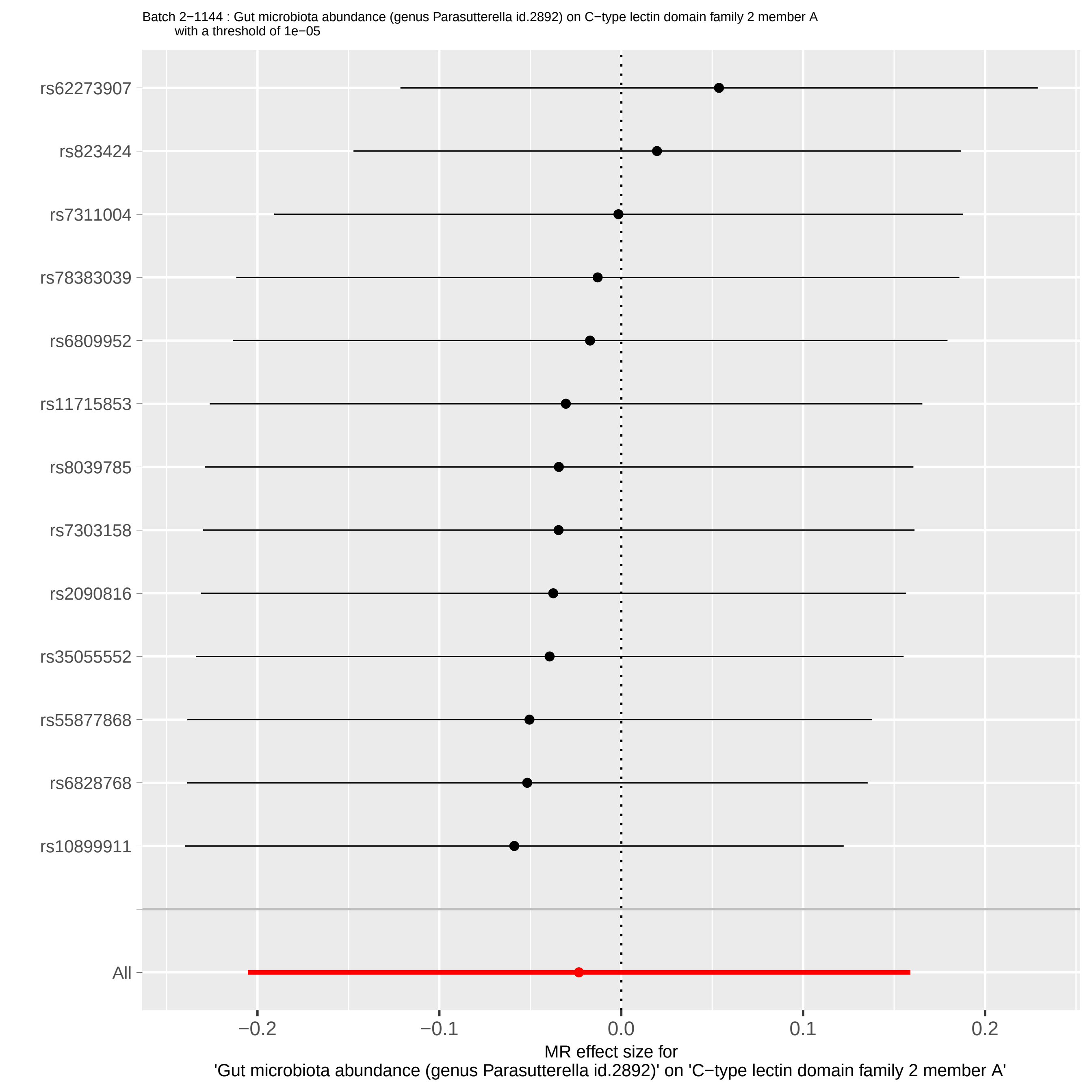
Batch 2−1144 : Gut microbiota abundance (genus Parasutterella id.2892) on C−type lectin domain family 2 member A
with a threshold of 1e−05
rs62273907
rs823424
rs7311004
rs78383039
rs6809952
rs11715853
rs8039785
rs7303158
rs2090816
rs35055552
rs55877868
rs6828768
rs10899911
All
−0.2
−0.1
0.0
0.1
0.2
MR effect size for
'Gut microbiota abundance (genus Parasutterella id.2892)' on 'C−type lectin domain family 2 member A'

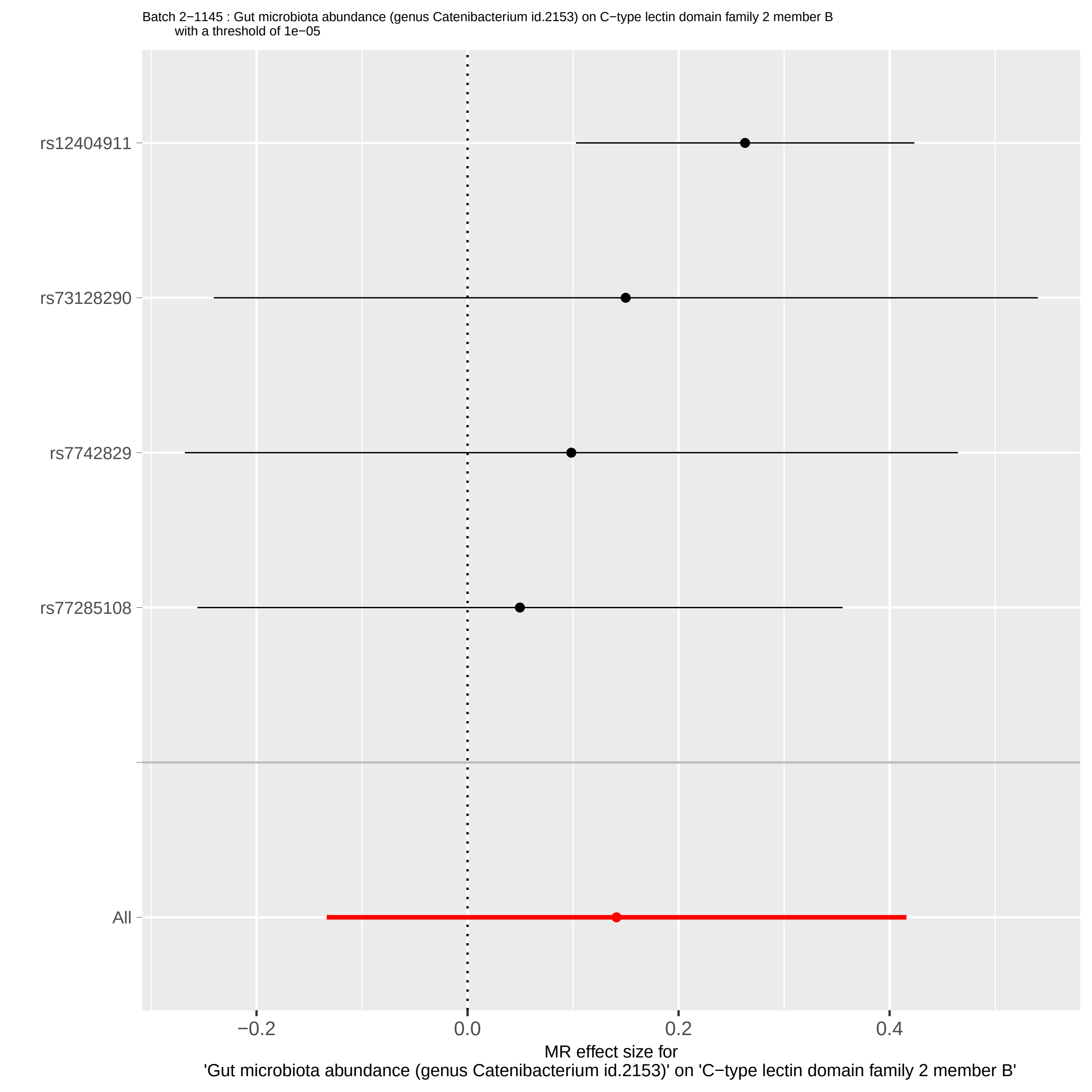
Batch 2−1145 : Gut microbiota abundance (genus Catenibacterium id.2153) on C−type lectin domain family 2 member B
with a threshold of 1e−05
rs12404911
rs73128290
rs7742829
rs77285108
All
−0.2
0.0
0.2
0.4
MR effect size for
'Gut microbiota abundance (genus Catenibacterium id.2153)' on 'C−type lectin domain family 2 member B'

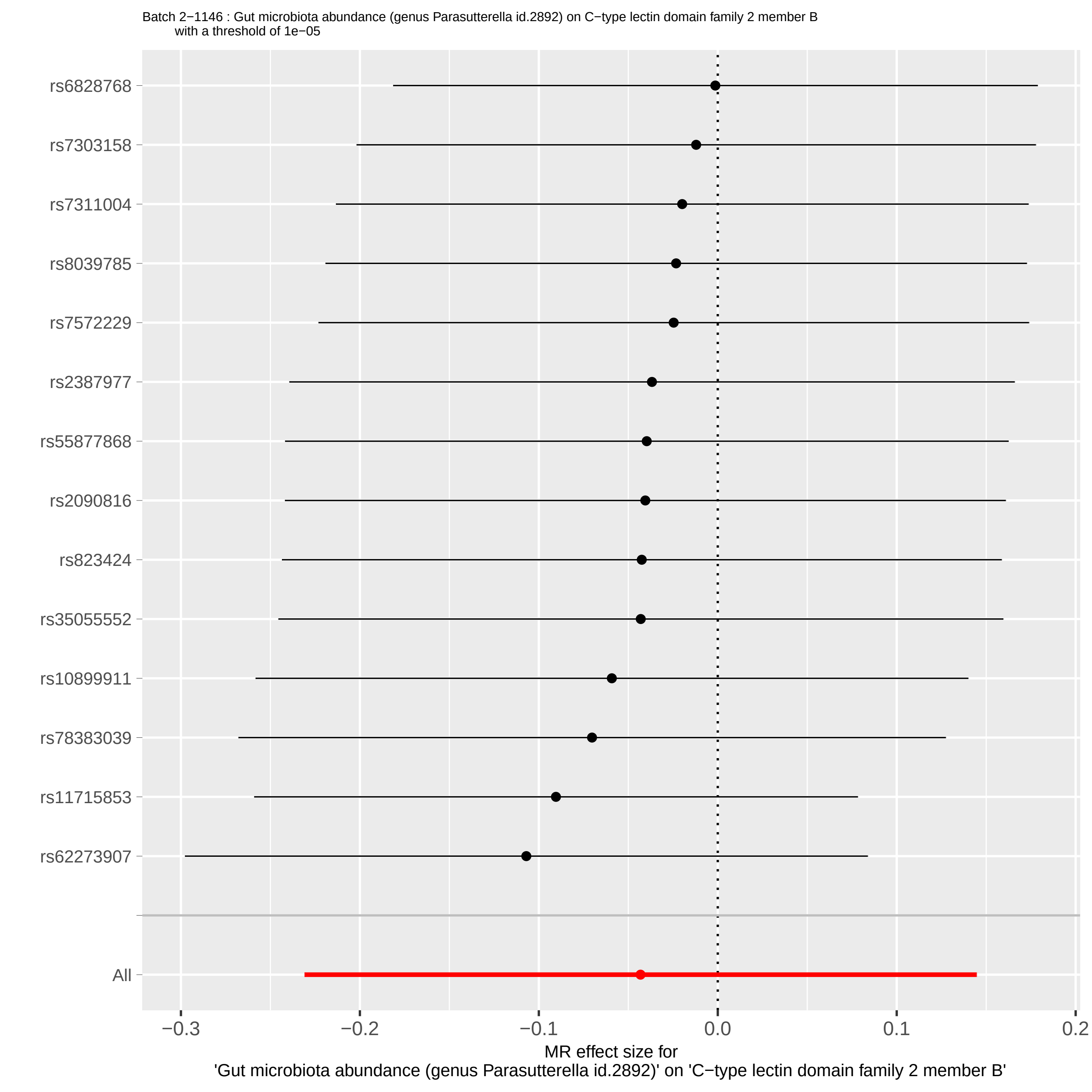

Batch 2−1146 : Gut microbiota abundance (genus Parasutterella id.2892) on C−type lectin domain family 2 member B
with a threshold of 1e−05
rs6828768
rs7303158
rs7311004
rs8039785
rs7572229
rs2387977
rs55877868
rs2090816
rs823424
rs35055552
rs10899911
rs78383039
rs11715853
rs62273907
All
−0.3
−0.2
−0.1
0.0
0.1
0.2
MR effect size for
'Gut microbiota abundance (genus Parasutterella id.2892)' on 'C−type lectin domain family 2 member B'

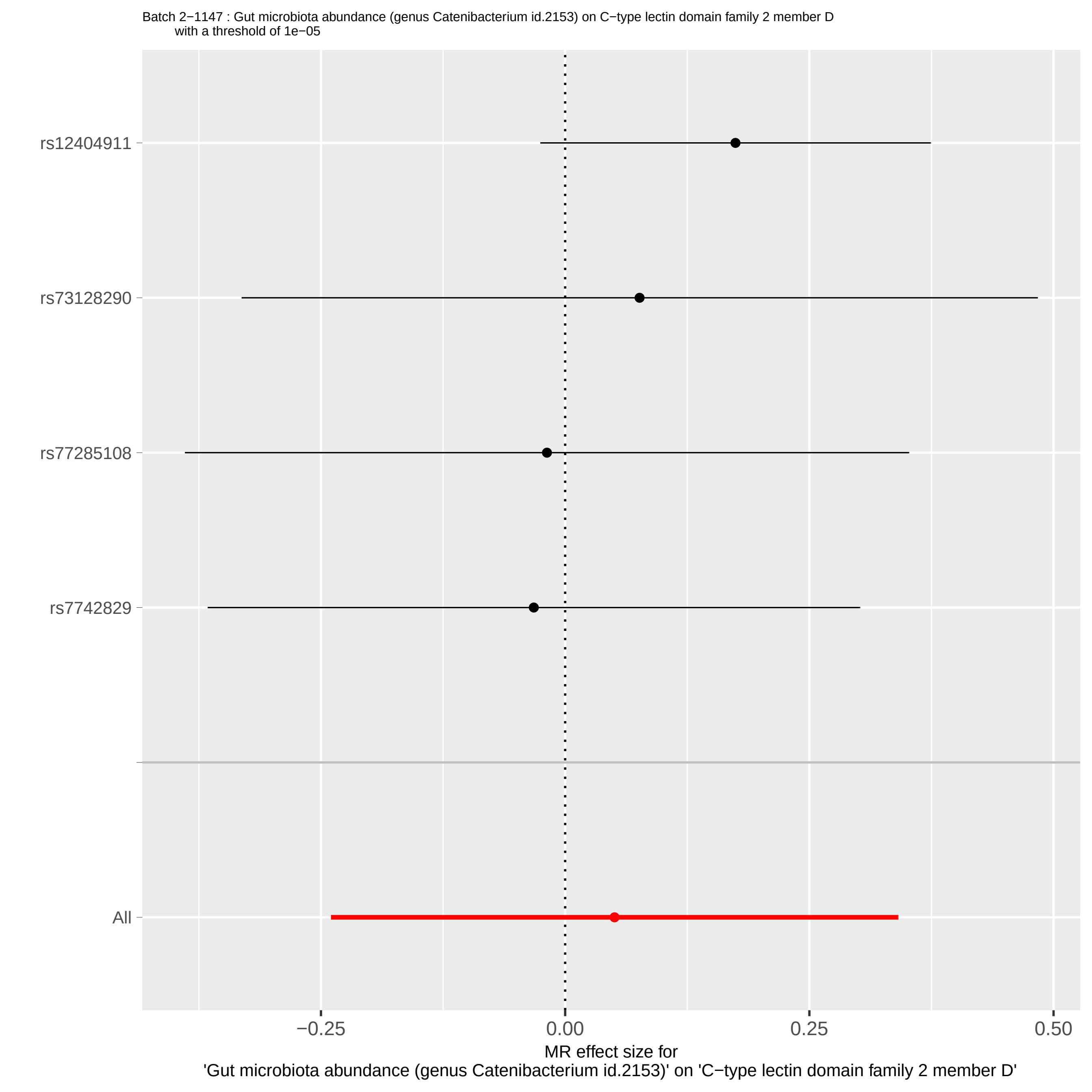
Batch 2−1147 : Gut microbiota abundance (genus Catenibacterium id.2153) on C−type lectin domain family 2 member D
with a threshold of 1e−05
rs12404911
rs73128290
rs77285108
rs7742829
All
−0.25
0.00
0.25
0.50
MR effect size for
'Gut microbiota abundance (genus Catenibacterium id.2153)' on 'C−type lectin domain family 2 member D'

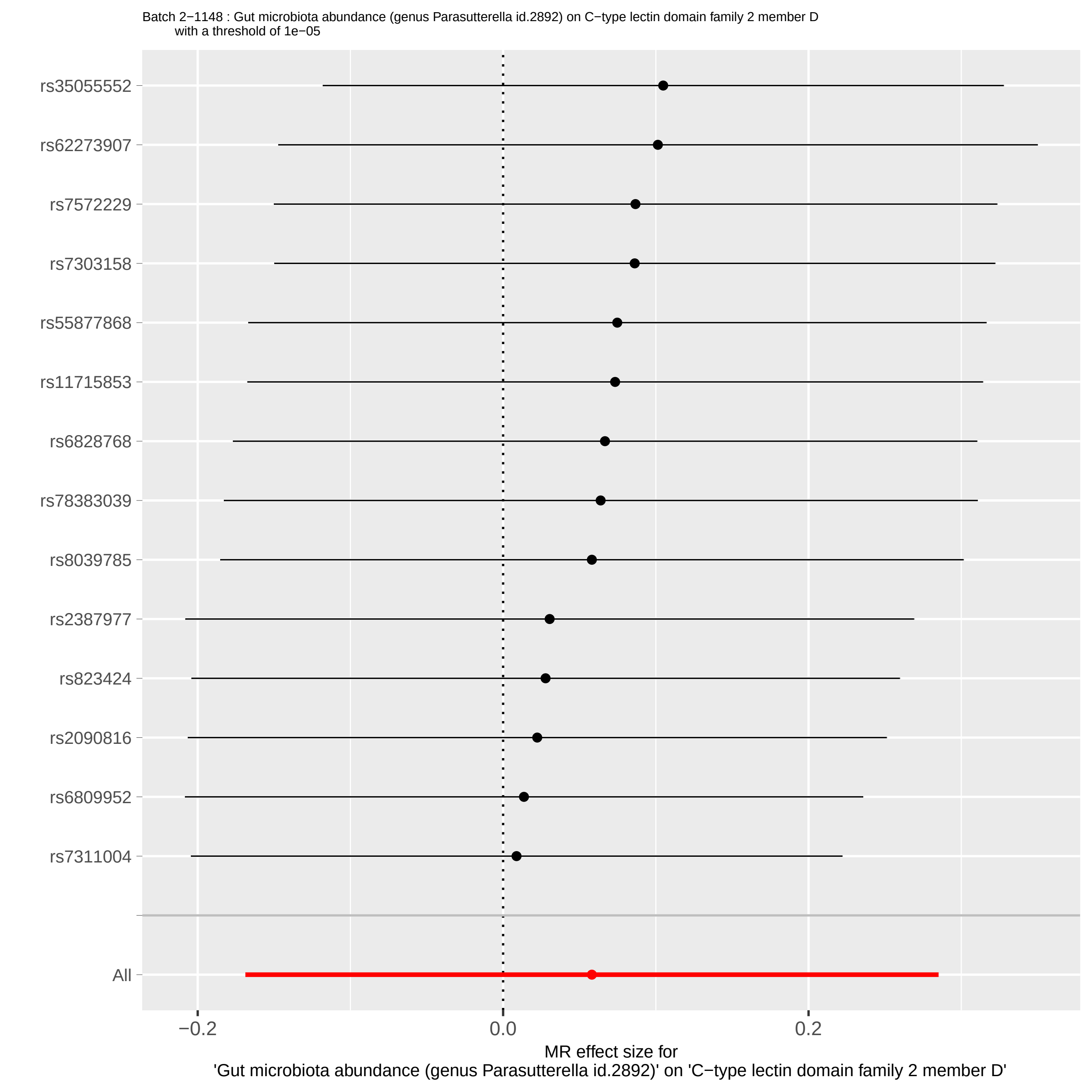
Batch 2−1148 : Gut microbiota abundance (genus Parasutterella id.2892) on C−type lectin domain family 2 member D
with a threshold of 1e−05
rs35055552
rs62273907
rs7572229
rs7303158
rs55877868
rs11715853
rs6828768
rs78383039
rs8039785
rs2387977
rs823424
rs2090816
rs6809952
rs7311004
All
−0.2
0.0
0.2
MR effect size for
'Gut microbiota abundance (genus Parasutterella id.2892)' on 'C−type lectin domain family 2 member D'

Batch 2−1149 : Gut microbiota abundance (genus Catenibacterium id.2153) on C−type lectin domain family 2 member L
with a threshold of 1e−05

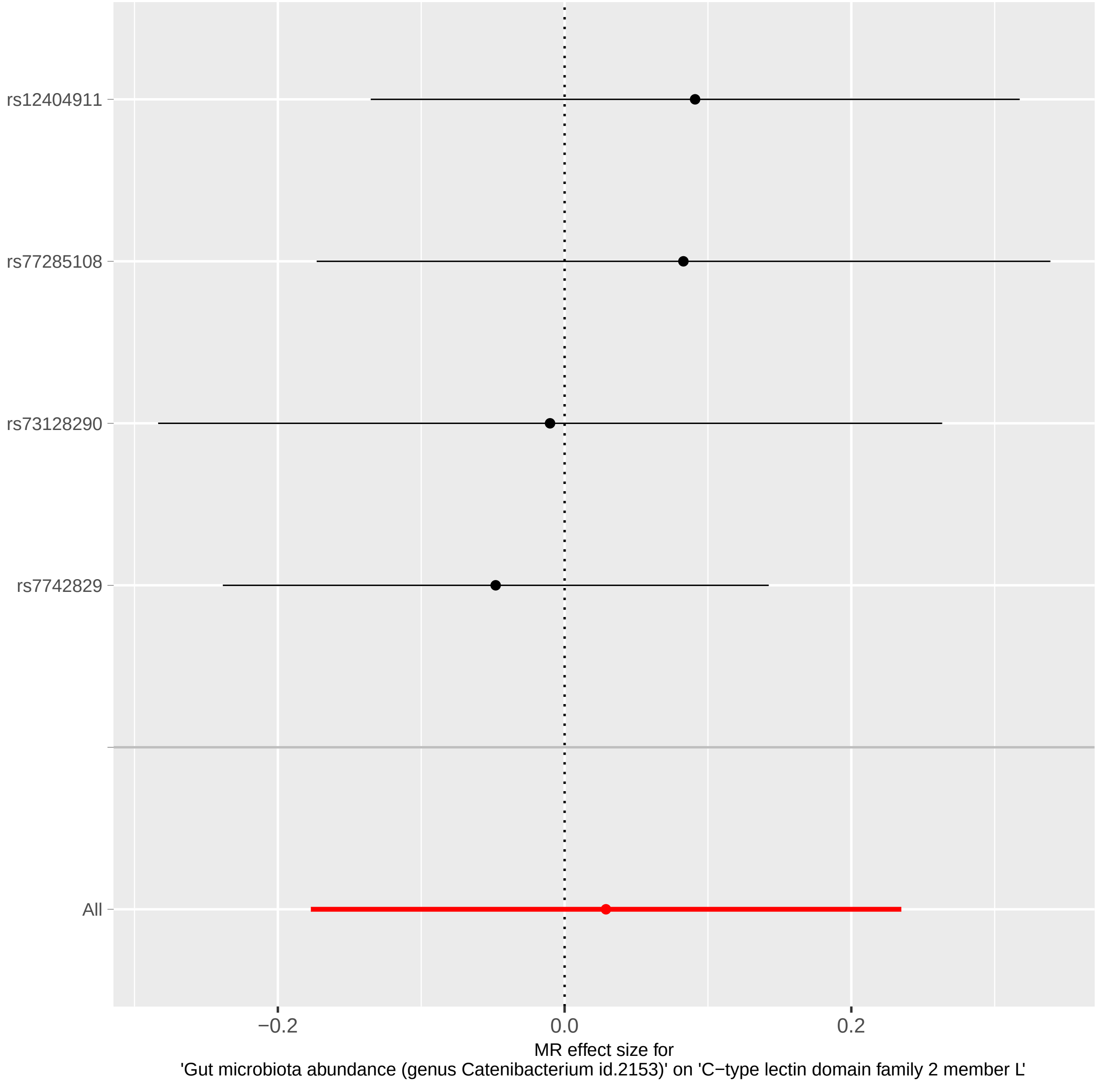

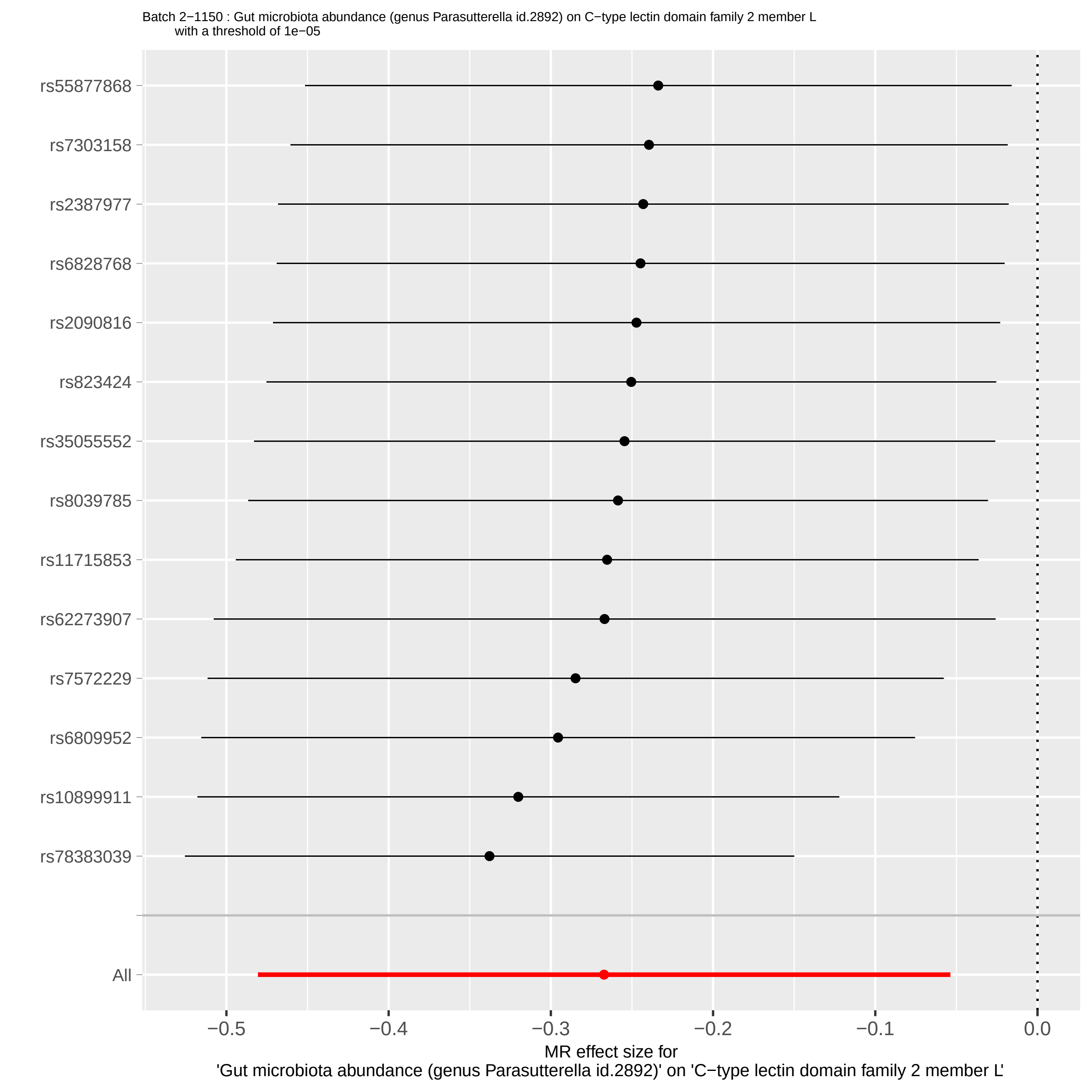
Batch 2−1150 : Gut microbiota abundance (genus Parasutterella id.2892) on C−type lectin domain family 2 member L
with a threshold of 1e−05
rs55877868
rs7303158
rs2387977
rs6828768
rs2090816
rs823424
rs35055552
rs8039785
rs11715853
rs62273907
rs7572229
rs6809952
rs10899911
rs78383039
All
−0.5
−0.4
−0.3
−0.2
−0.1
0.0
MR effect size for
'Gut microbiota abundance (genus Parasutterella id.2892)' on 'C−type lectin domain family 2 member L'

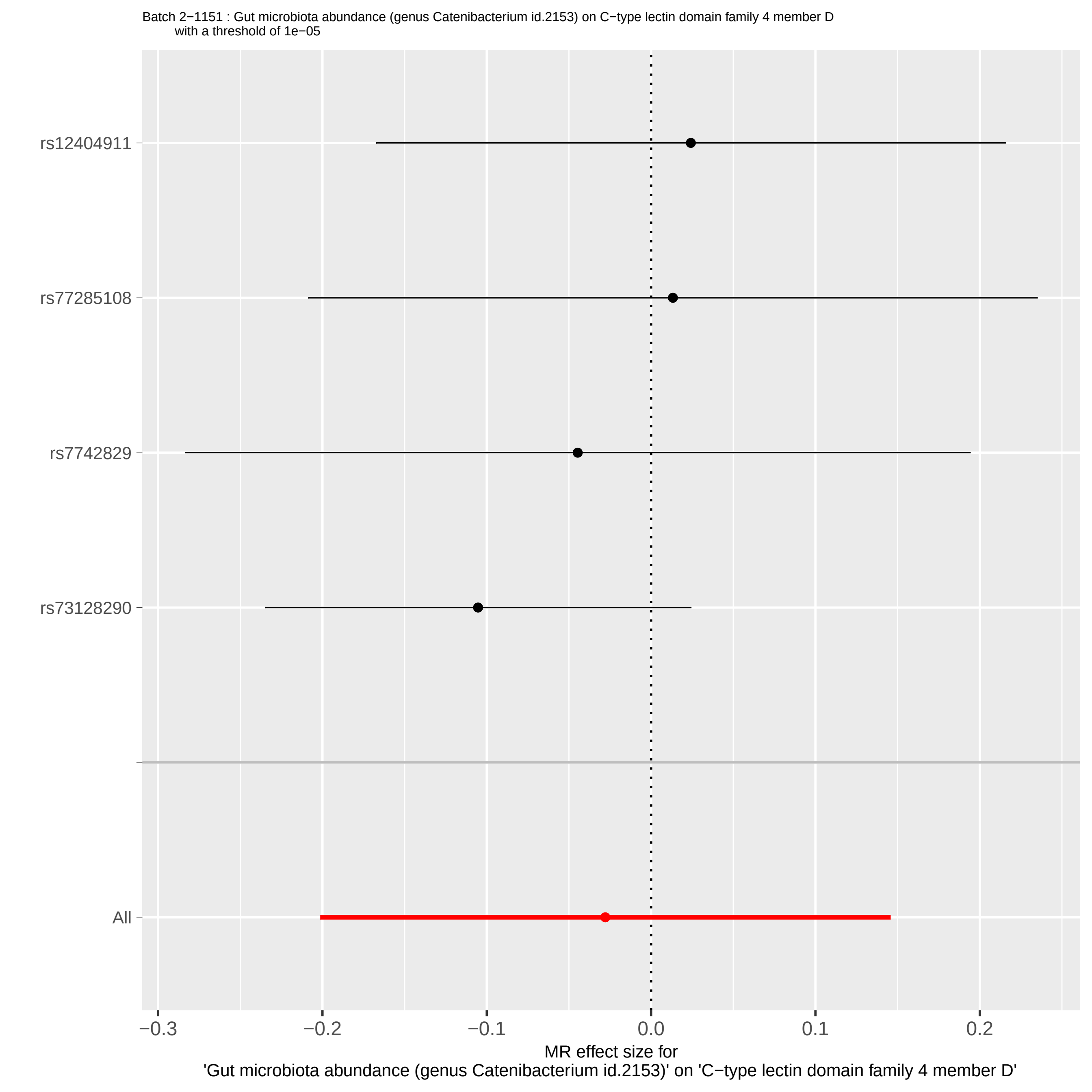
Batch 2−1151 : Gut microbiota abundance (genus Catenibacterium id.2153) on C−type lectin domain family 4 member D
with a threshold of 1e−05
rs12404911
rs77285108
rs7742829
rs73128290
All
−0.3
−0.2
−0.1
0.0
0.1
0.2
MR effect size for
'Gut microbiota abundance (genus Catenibacterium id.2153)' on 'C−type lectin domain family 4 member D'

Batch 2−1152 : Gut microbiota abundance (genus Parasutterella id.2892) on C−type lectin domain family 4 member D
with a threshold of 1e−05

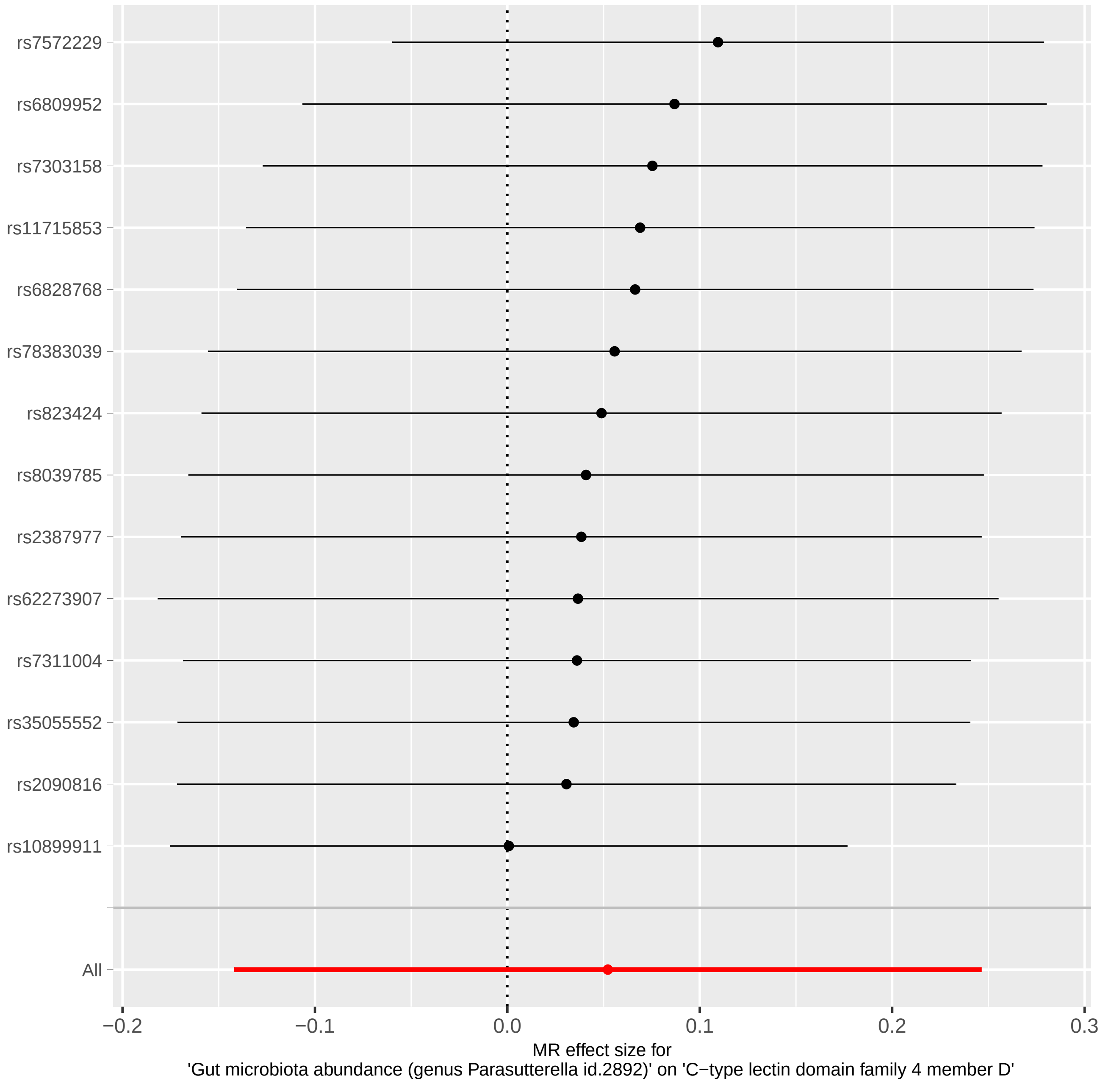

Batch 2−1153 : Gut microbiota abundance (genus Catenibacterium id.2153) on C−type lectin domain family 4 member E
with a threshold of 1e−05

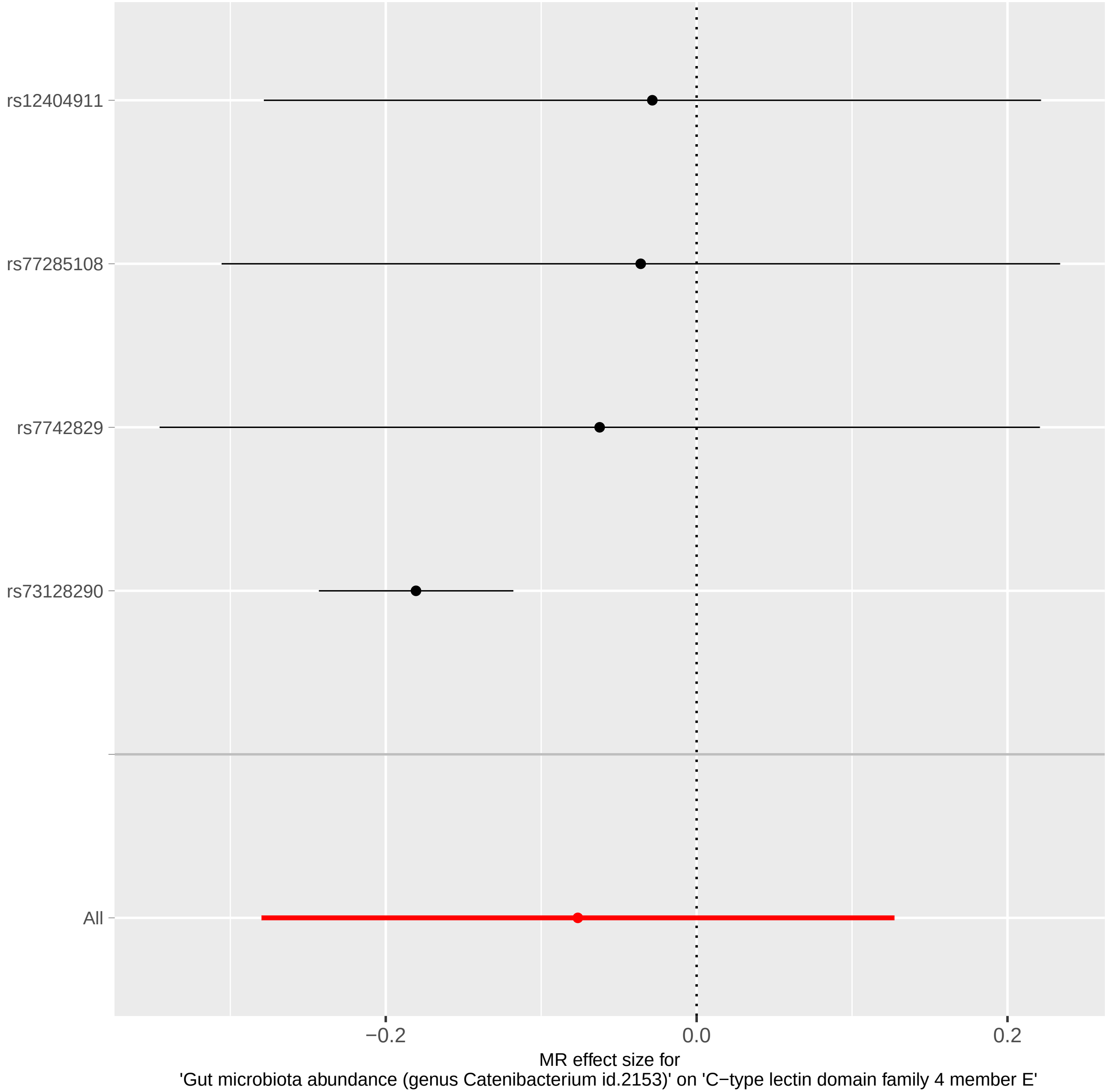

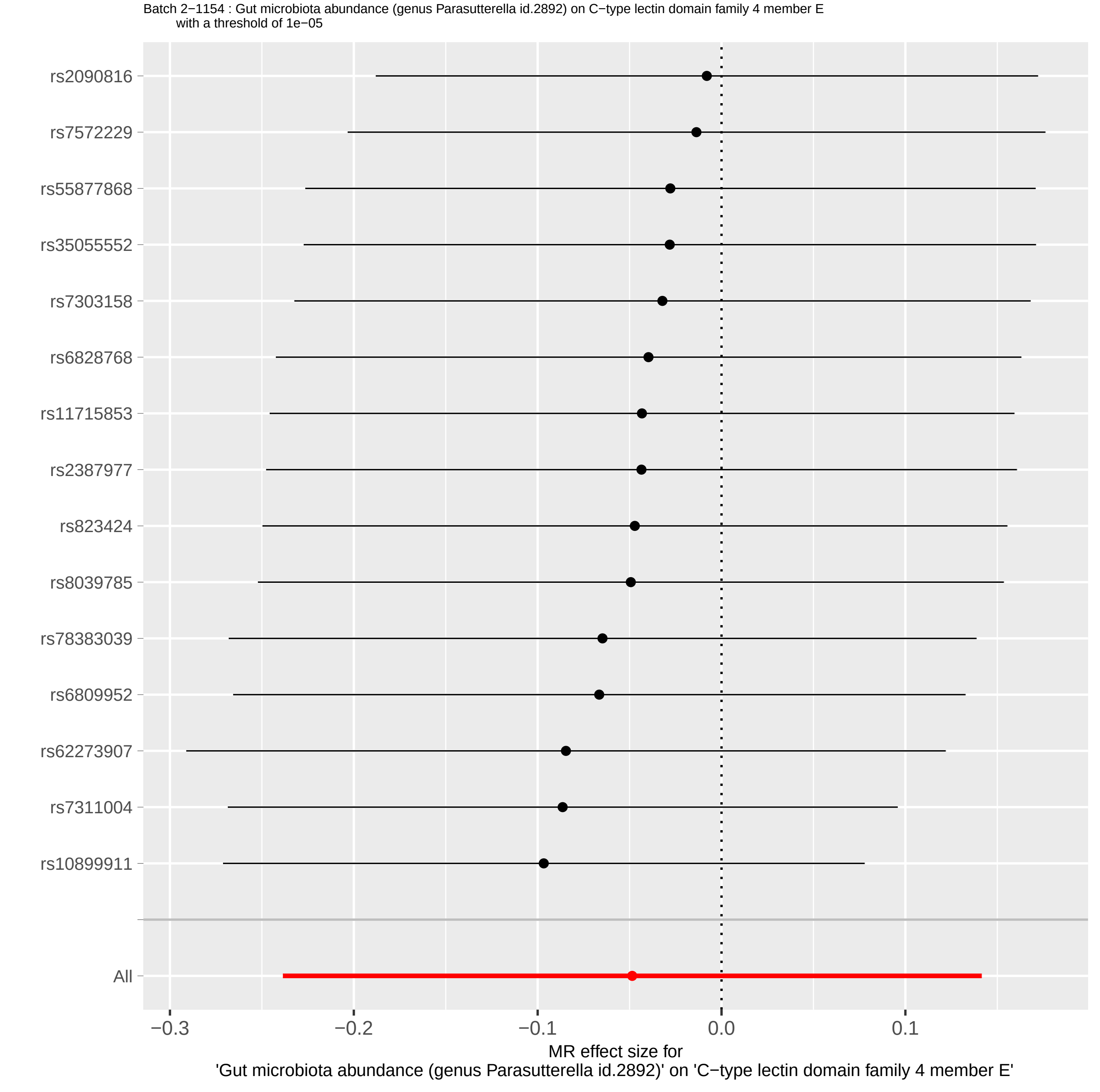

Batch 2−1154 : Gut microbiota abundance (genus Parasutterella id.2892) on C−type lectin domain family 4 member E
with a threshold of 1e−05
rs2090816
rs7572229
rs55877868
rs35055552
rs7303158
rs6828768
rs11715853
rs2387977
rs823424
rs8039785
rs78383039
rs6809952
rs62273907
rs7311004
rs10899911
All
−0.3
−0.2
−0.1
0.0
0.1
MR effect size for
'Gut microbiota abundance (genus Parasutterella id.2892)' on 'C−type lectin domain family 4 member E'

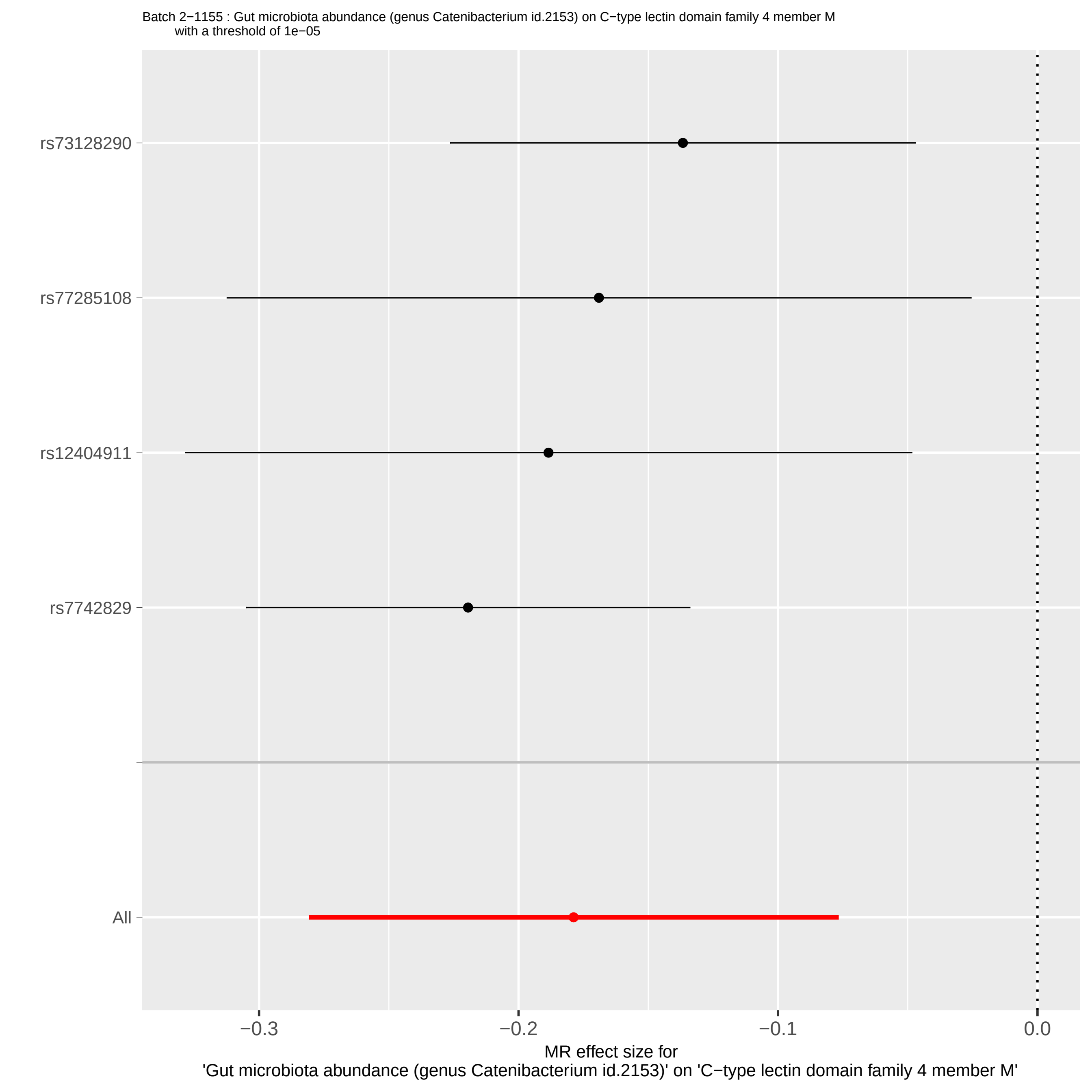

Batch 2−1155 : Gut microbiota abundance (genus Catenibacterium id.2153) on C−type lectin domain family 4 member M
with a threshold of 1e−05
rs73128290
rs77285108
rs12404911
rs7742829
All
−0.3
−0.2
−0.1
0.0
MR effect size for
'Gut microbiota abundance (genus Catenibacterium id.2153)' on 'C−type lectin domain family 4 member M'

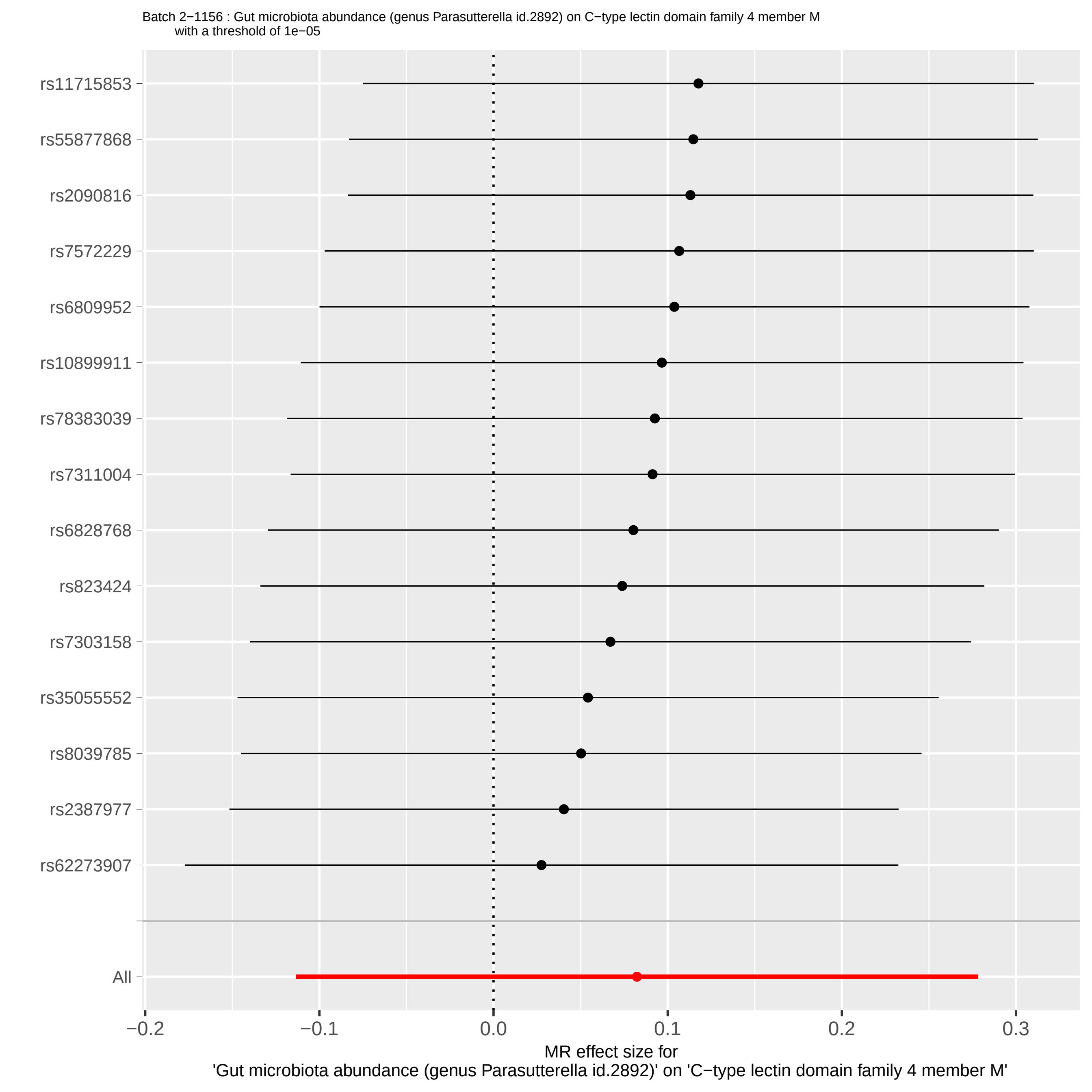

Batch 2−1156 : Gut microbiota abundance (genus Parasutterella id.2892) on C−type lectin domain family 4 member M
with a threshold of 1e−05
rs11715853
rs55877868
rs2090816
rs7572229
rs6809952
rs10899911
rs78383039
rs7311004
rs6828768
rs823424
rs7303158
rs35055552
rs8039785
rs2387977
rs62273907
All
−0.2
−0.1
0.0
0.1
0.2
0.3
MR effect size for
'Gut microbiota abundance (genus Parasutterella id.2892)' on 'C−type lectin domain family 4 member M'

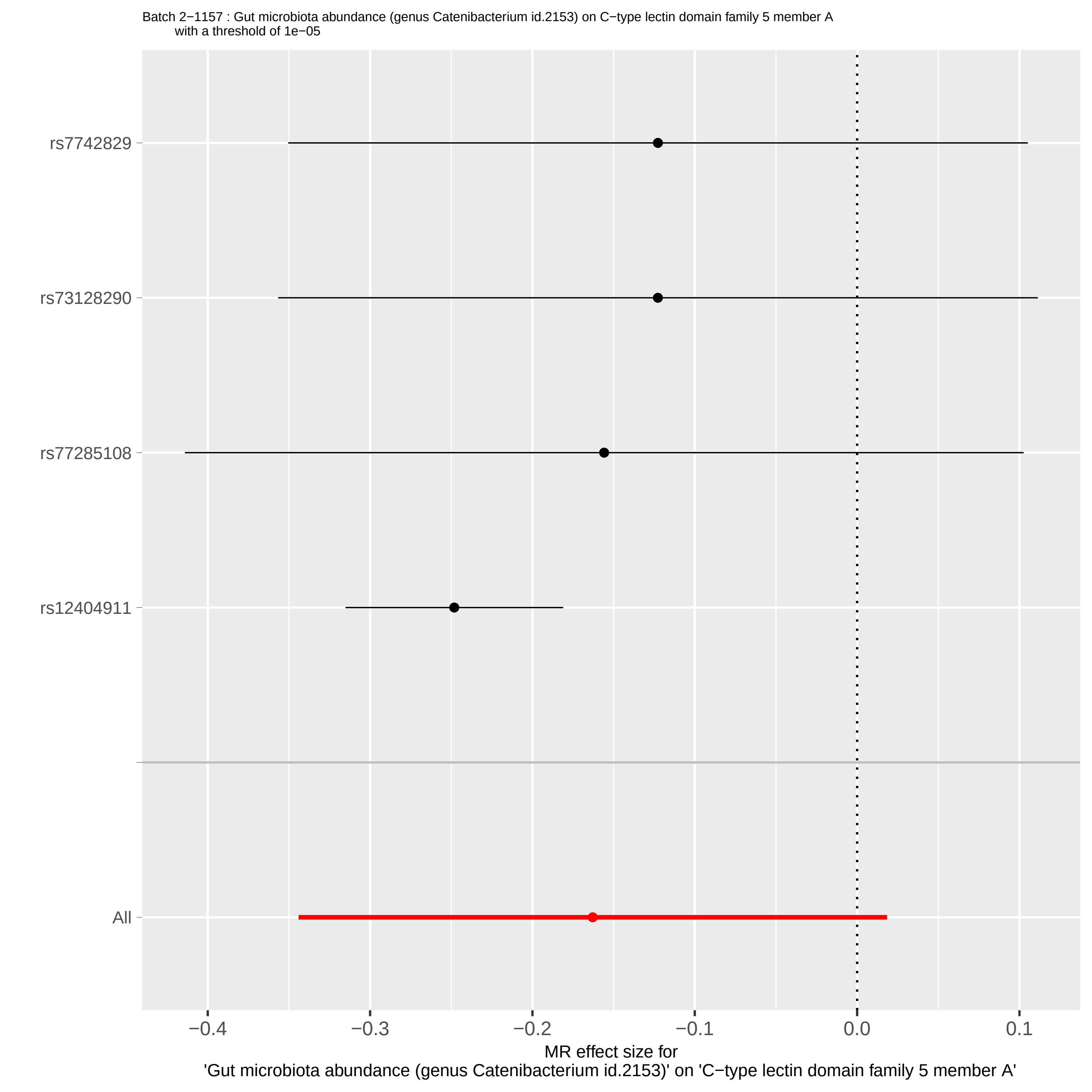

Batch 2−1157 : Gut microbiota abundance (genus Catenibacterium id.2153) on C−type lectin domain family 5 member A
with a threshold of 1e−05
rs7742829
rs73128290
rs77285108
rs12404911
All
−0.4
−0.3
−0.2
−0.1
0.0
0.1
MR effect size for
'Gut microbiota abundance (genus Catenibacterium id.2153)' on 'C−type lectin domain family 5 member A'

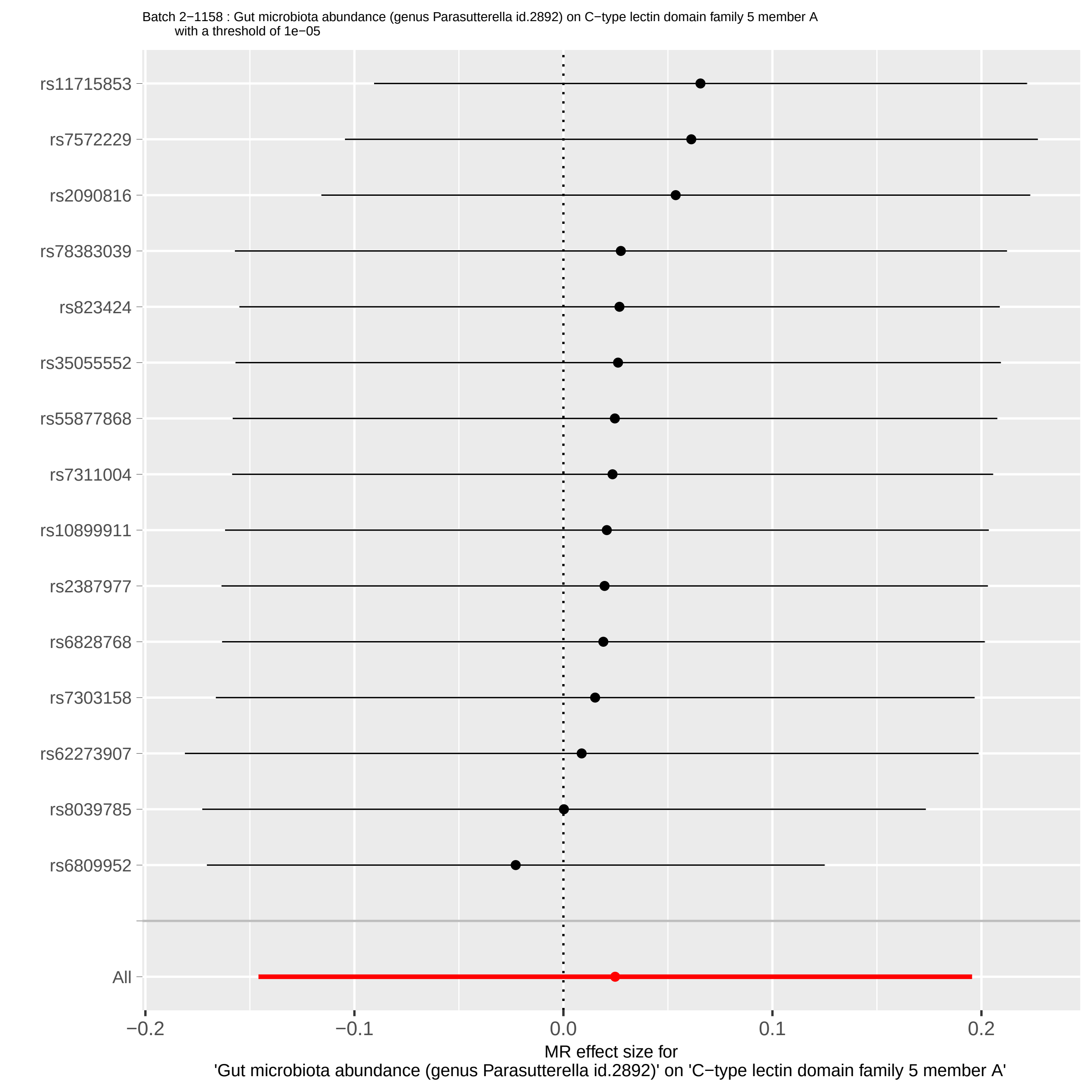
Batch 2−1158 : Gut microbiota abundance (genus Parasutterella id.2892) on C−type lectin domain family 5 member A
with a threshold of 1e−05
rs11715853
rs7572229
rs2090816
rs78383039
rs823424
rs35055552
rs55877868
rs7311004
rs10899911
rs2387977
rs6828768
rs7303158
rs62273907
rs8039785
rs6809952
All
−0.2
−0.1
0.0
0.1
0.2
MR effect size for
'Gut microbiota abundance (genus Parasutterella id.2892)' on 'C−type lectin domain family 5 member A'

Batch 2−1159 : Gut microbiota abundance (genus Catenibacterium id.2153) on C−type lectin domain family 6 member A
with a threshold of 1e−05

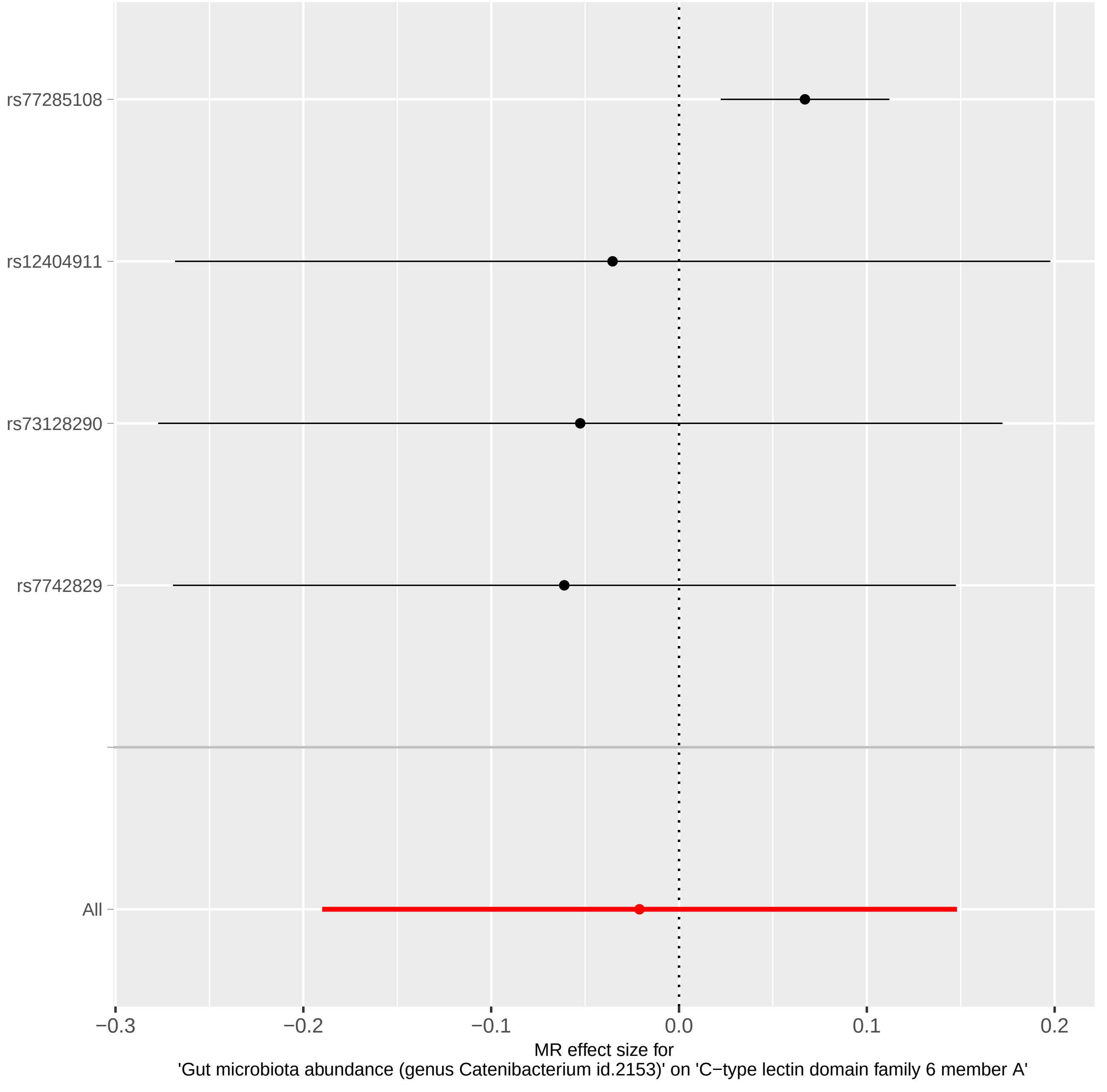

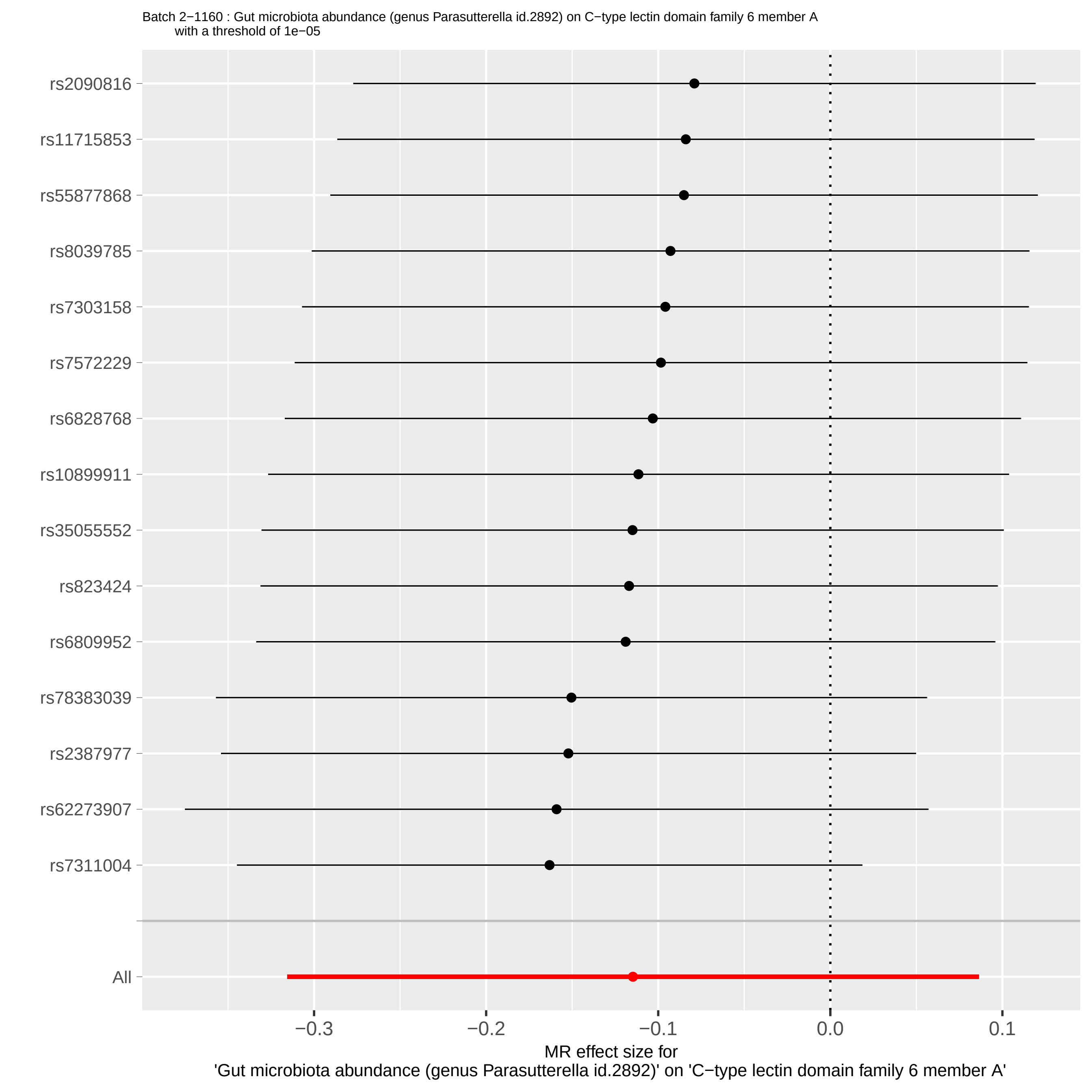
Batch 2−1160 : Gut microbiota abundance (genus Parasutterella id.2892) on C−type lectin domain family 6 member A
with a threshold of 1e−05
rs2090816
rs11715853
rs55877868
rs8039785
rs7303158
rs7572229
rs6828768
rs10899911
rs35055552
rs823424
rs6809952
rs78383039
rs2387977
rs62273907
rs7311004
All
−0.3
−0.2
−0.1
0.0
0.1
MR effect size for
'Gut microbiota abundance (genus Parasutterella id.2892)' on 'C−type lectin domain family 6 member A'

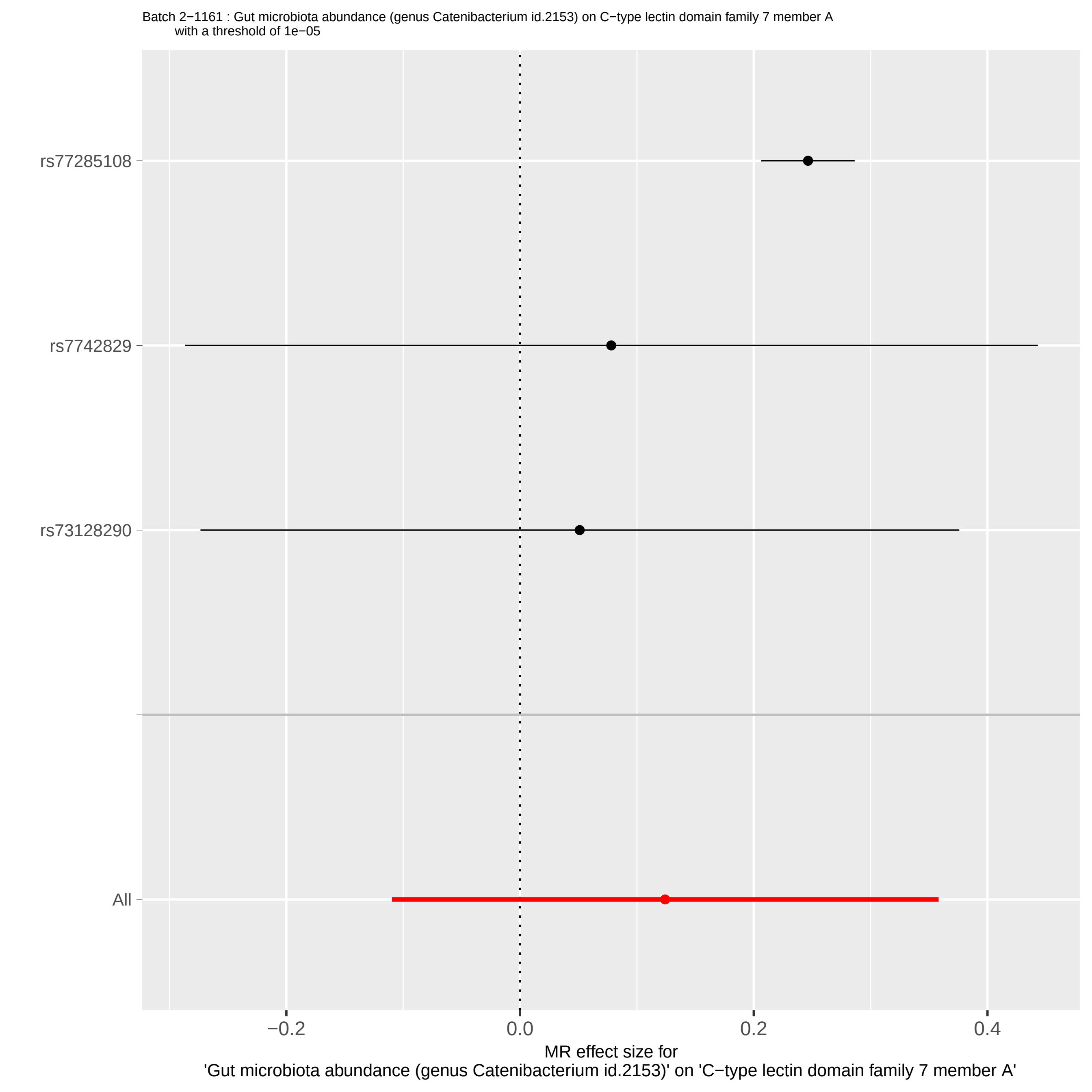

Batch 2−1161 : Gut microbiota abundance (genus Catenibacterium id.2153) on C−type lectin domain family 7 member A
with a threshold of 1e−05
rs77285108
rs7742829
rs73128290
All
−0.2
0.0
0.2
0.4
MR effect size for
'Gut microbiota abundance (genus Catenibacterium id.2153)' on 'C−type lectin domain family 7 member A'

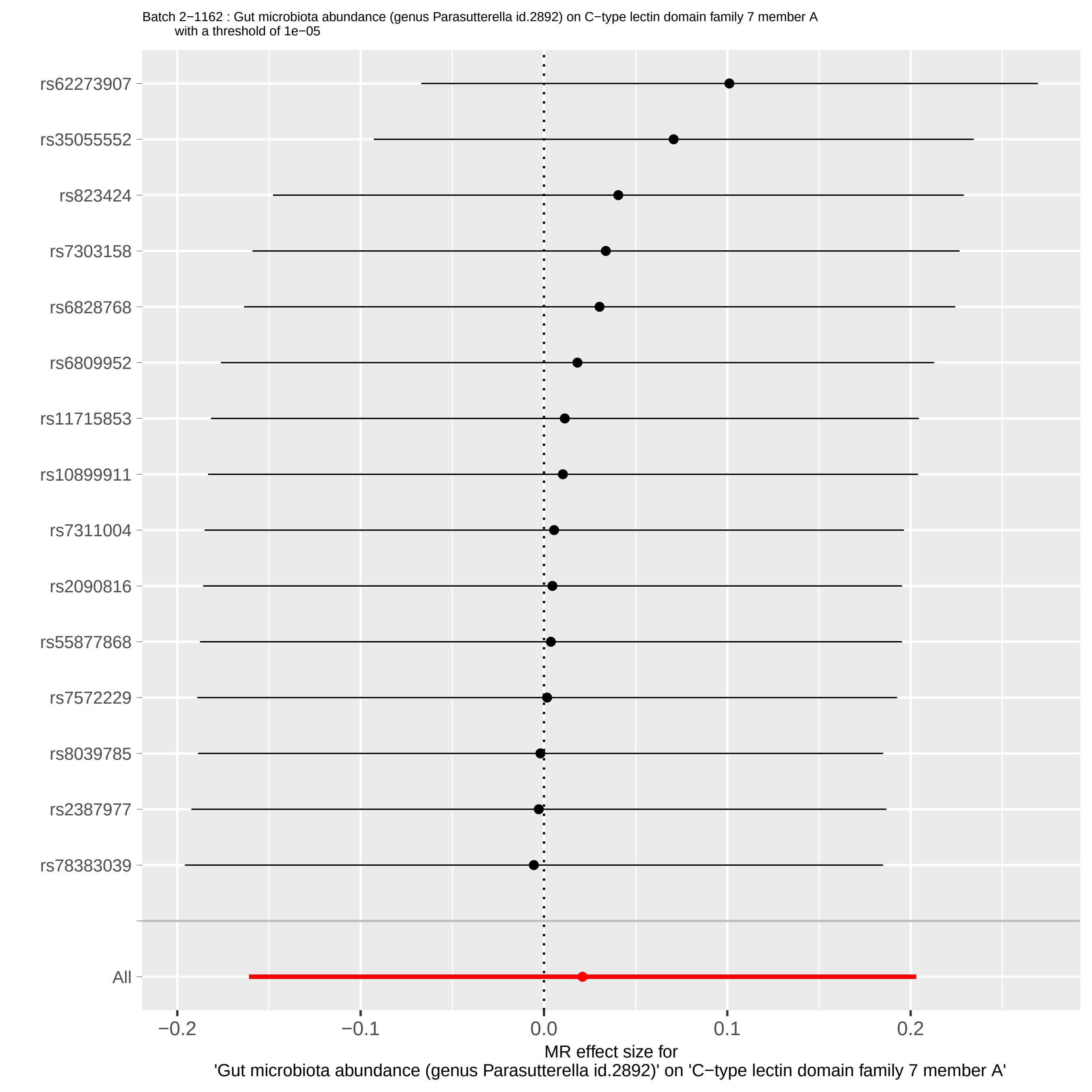

Batch 2−1162 : Gut microbiota abundance (genus Parasutterella id.2892) on C−type lectin domain family 7 member A
with a threshold of 1e−05
rs62273907
rs35055552
rs823424
rs7303158
rs6828768
rs6809952
rs11715853
rs10899911
rs7311004
rs2090816
rs55877868
rs7572229
rs8039785
rs2387977
rs78383039
All
−0.2
−0.1
0.0
0.1
0.2
MR effect size for
'Gut microbiota abundance (genus Parasutterella id.2892)' on 'C−type lectin domain family 7 member A'

Batch 2−1163 : Gut microbiota abundance (genus Catenibacterium id.2153) on C−type lectin−like domain family 1
with a threshold of 1e−05

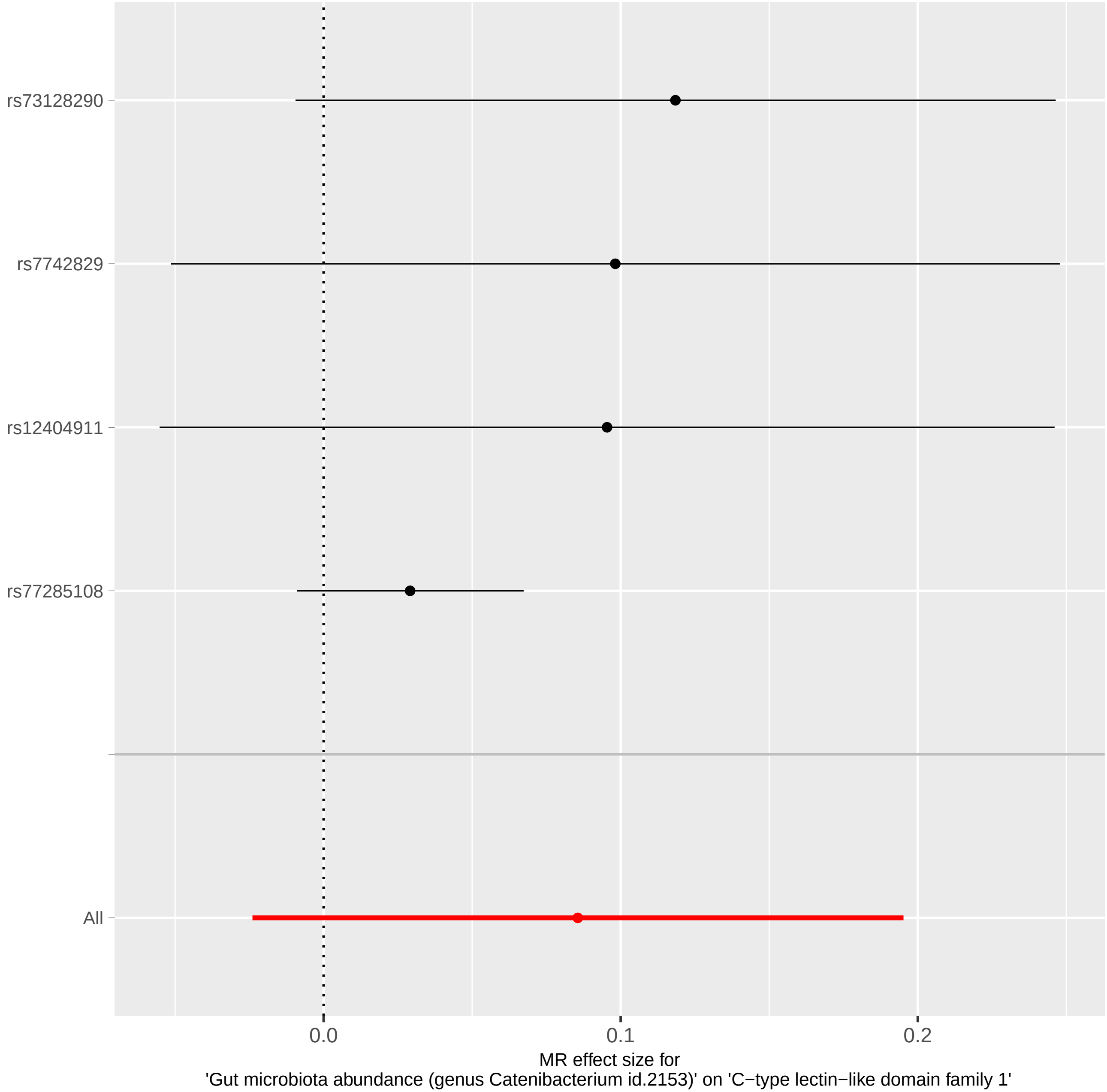

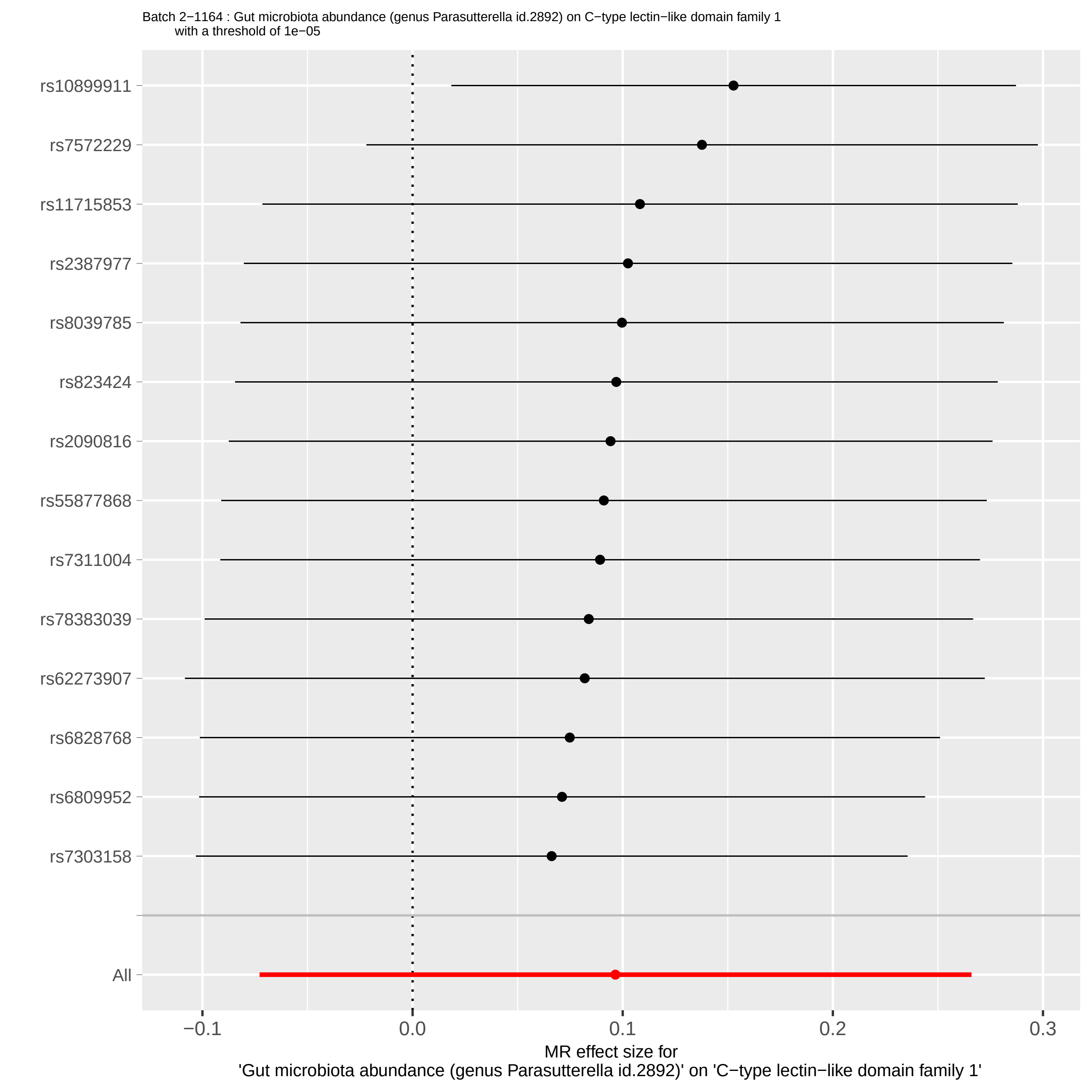
Batch 2−1164 : Gut microbiota abundance (genus Parasutterella id.2892) on C−type lectin−like domain family 1
with a threshold of 1e−05
rs10899911
rs7572229
rs11715853
rs2387977
rs8039785
rs823424
rs2090816
rs55877868
rs7311004
rs78383039
rs62273907
rs6828768
rs6809952
rs7303158
All
−0.1
0.0
0.1
0.2
0.3
MR effect size for
'Gut microbiota abundance (genus Parasutterella id.2892)' on 'C−type lectin−like domain family 1'

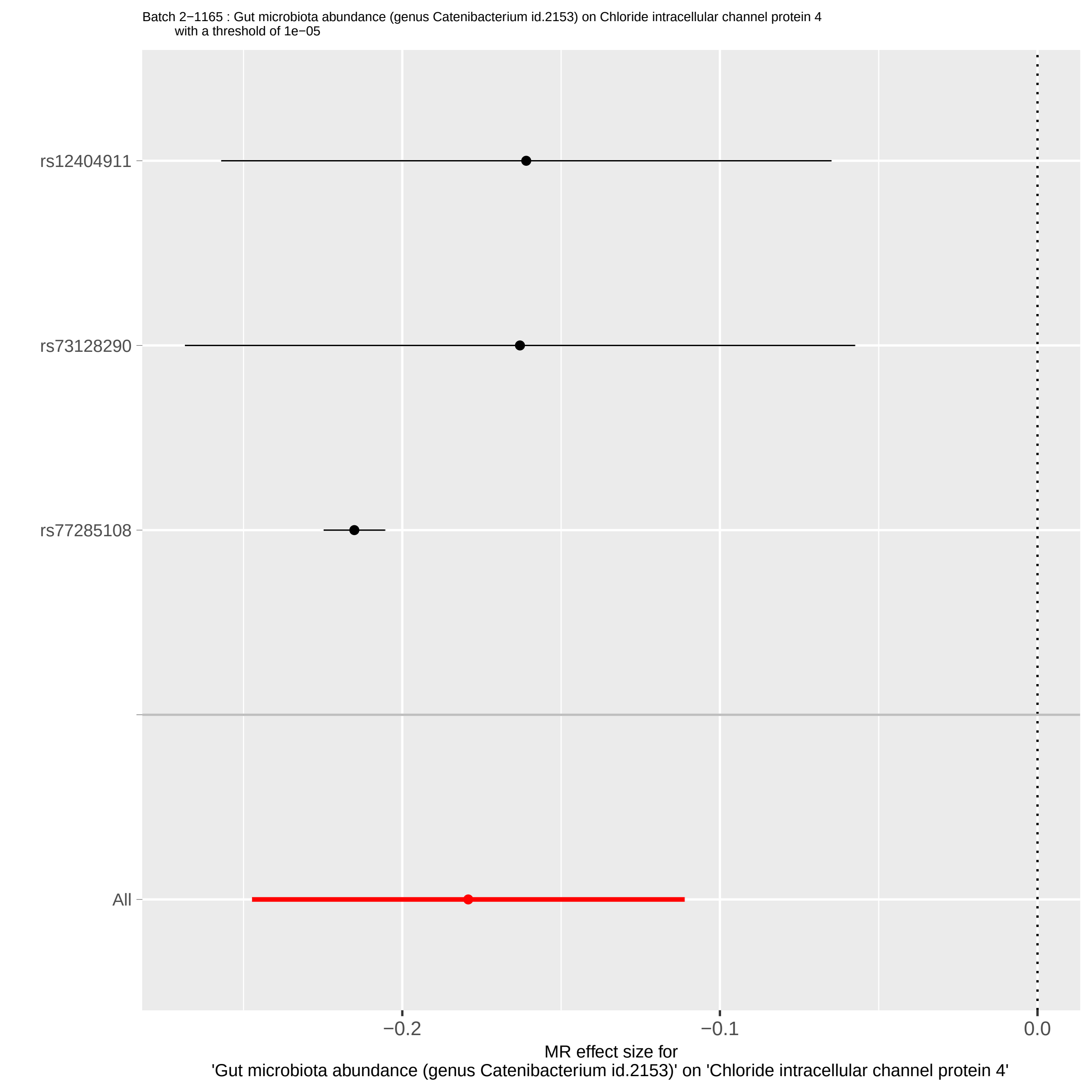
Batch 2−1165 : Gut microbiota abundance (genus Catenibacterium id.2153) on Chloride intracellular channel protein 4
with a threshold of 1e−05
rs12404911
rs73128290
rs77285108
All
−0.2
−0.1
0.0
MR effect size for
'Gut microbiota abundance (genus Catenibacterium id.2153)' on 'Chloride intracellular channel protein 4'

Batch 2−1166 : Gut microbiota abundance (genus Parasutterella id.2892) on Chloride intracellular channel protein 4
with a threshold of 1e−05

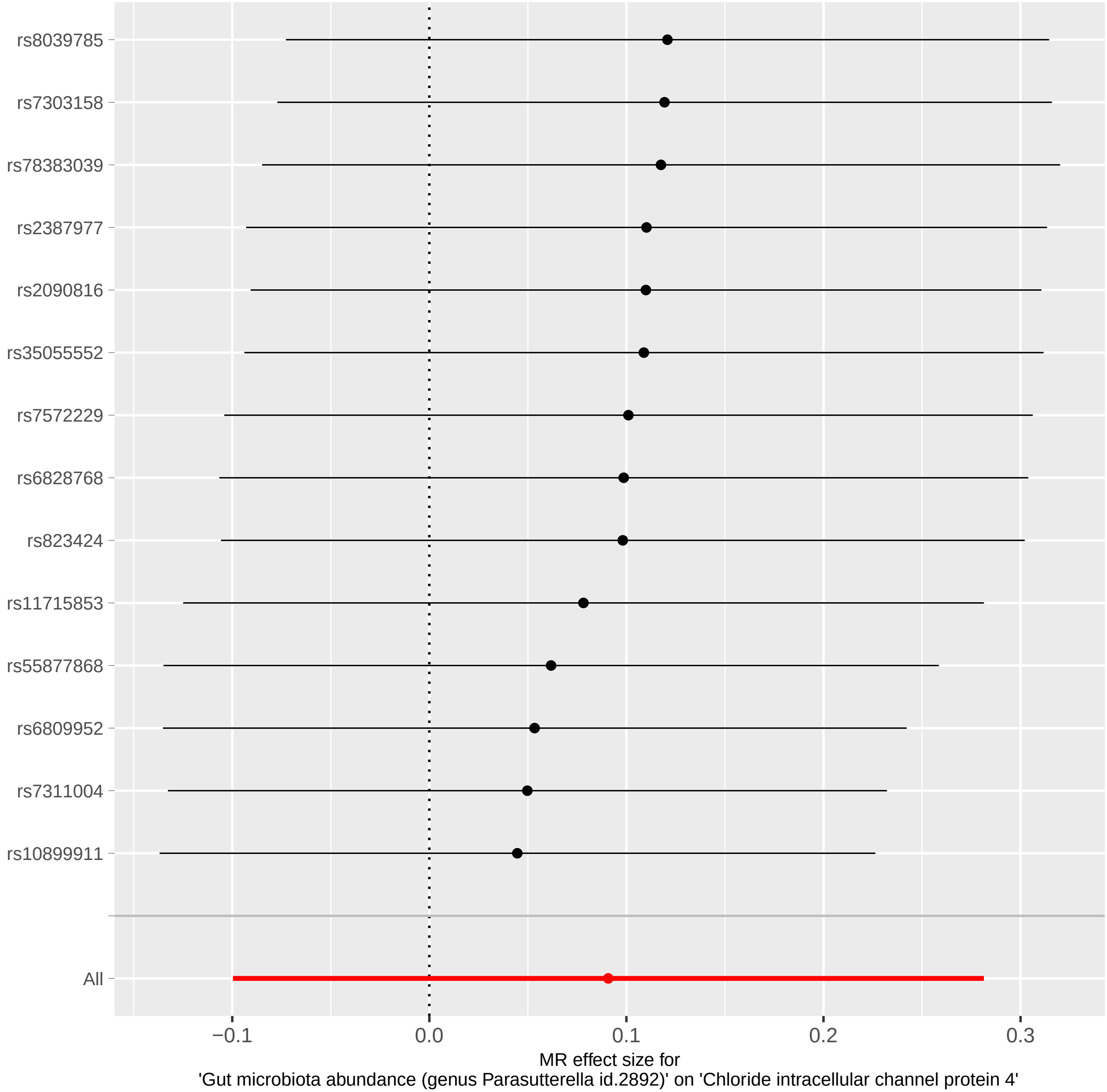

Batch 2−1167 : Gut microbiota abundance (genus Catenibacterium id.2153) on Chloride intracellular channel protein 5
with a threshold of 1e−05

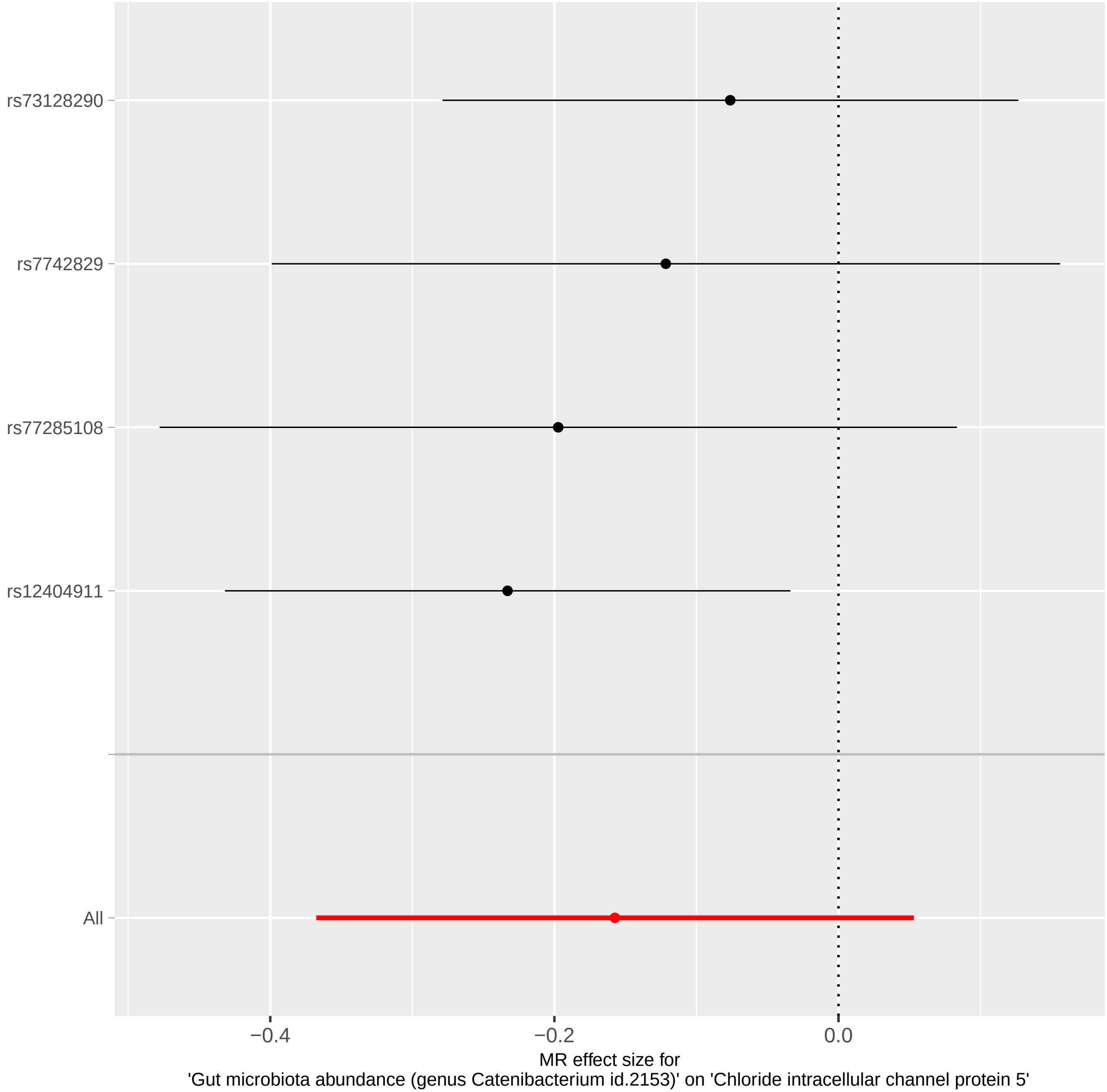

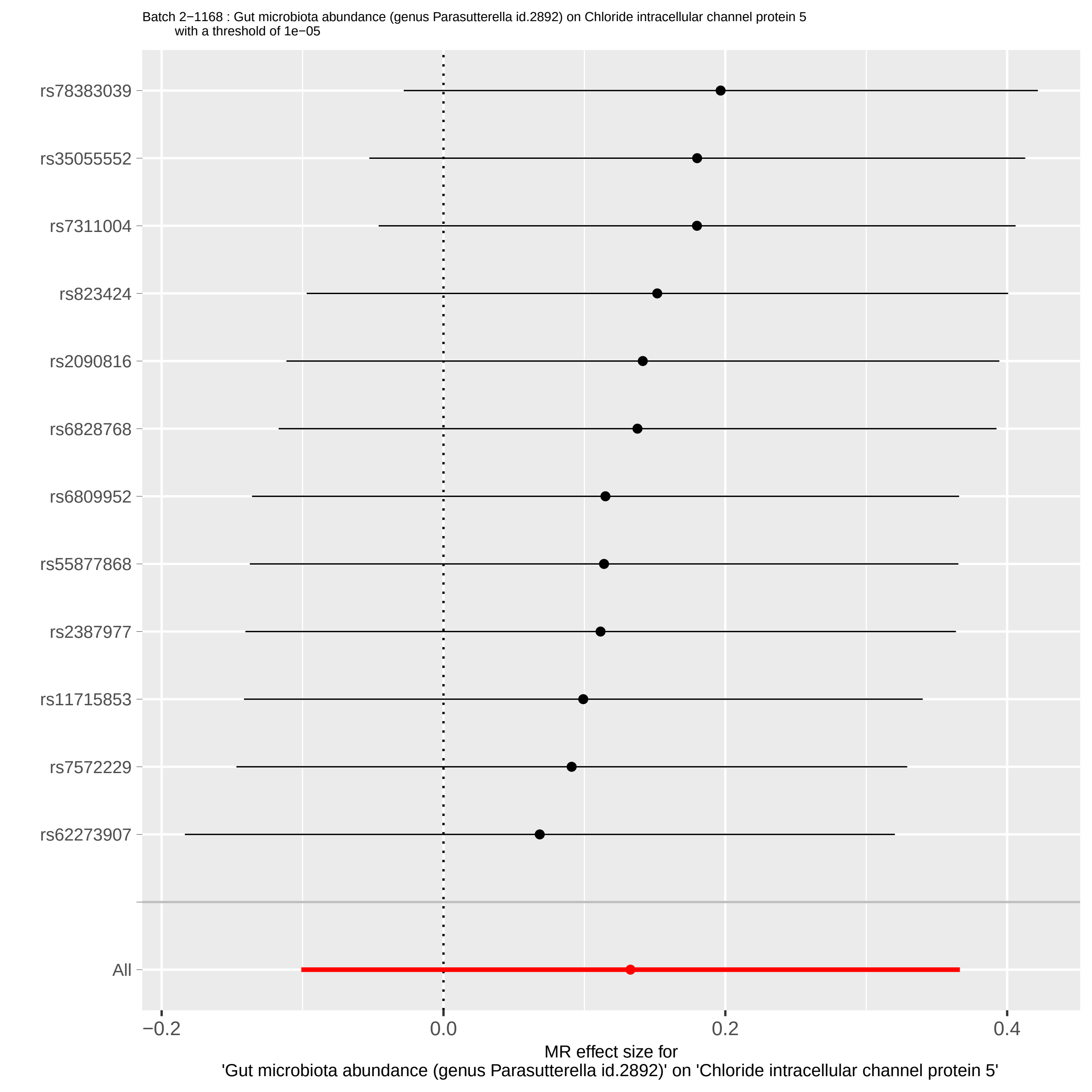

Batch 2−1168 : Gut microbiota abundance (genus Parasutterella id.2892) on Chloride intracellular channel protein 5
with a threshold of 1e−05
rs78383039
rs35055552
rs7311004
rs823424
rs2090816
rs6828768
rs6809952
rs55877868
rs2387977
rs11715853
rs7572229
rs62273907
All
−0.2
0.0
0.2
0.4
MR effect size for
'Gut microbiota abundance (genus Parasutterella id.2892)' on 'Chloride intracellular channel protein 5'

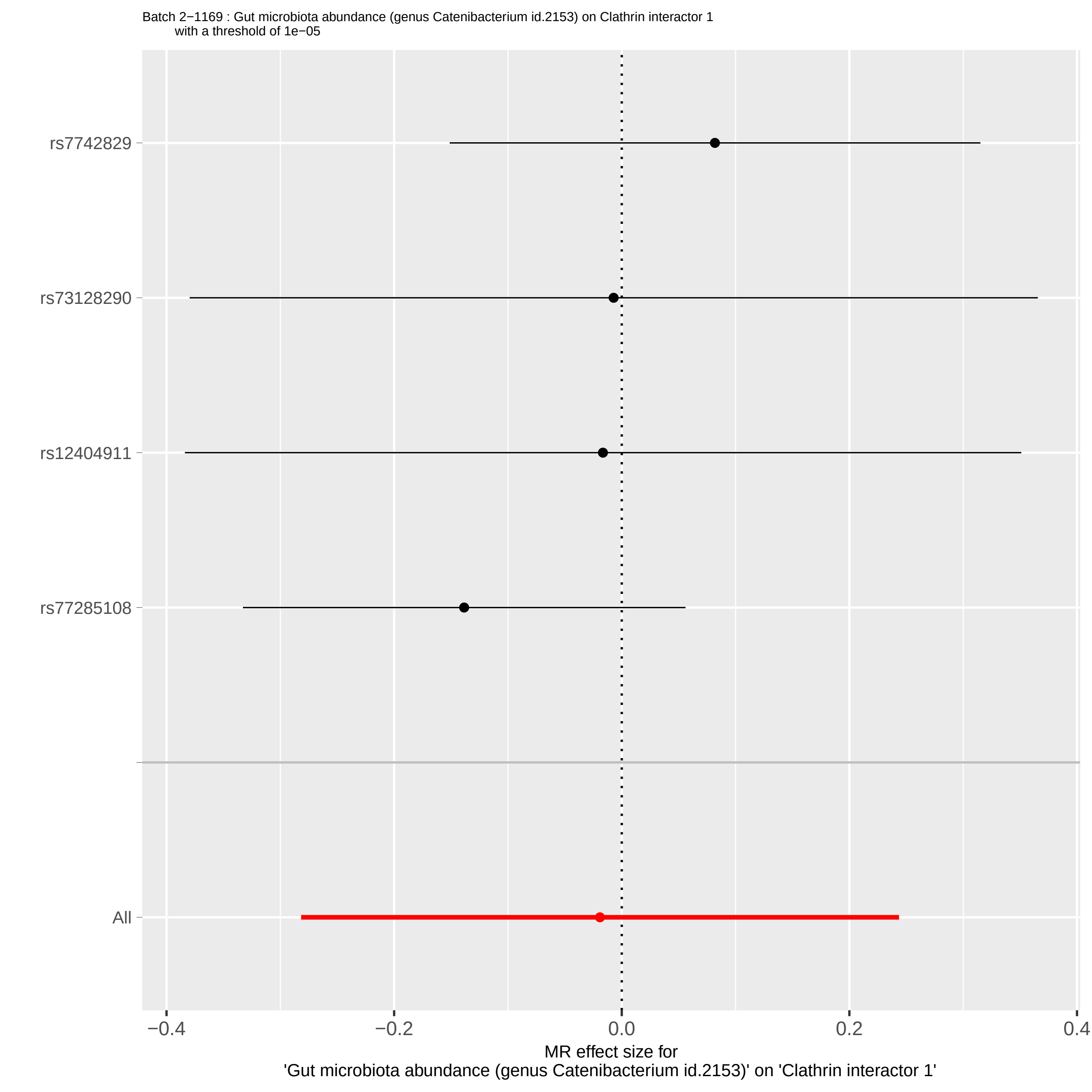
Batch 2−1169 : Gut microbiota abundance (genus Catenibacterium id.2153) on Clathrin interactor 1
with a threshold of 1e−05
rs7742829
rs73128290
rs12404911
rs77285108
All
−0.4
−0.2
0.0
0.2
0.4
MR effect size for
'Gut microbiota abundance (genus Catenibacterium id.2153)' on 'Clathrin interactor 1'

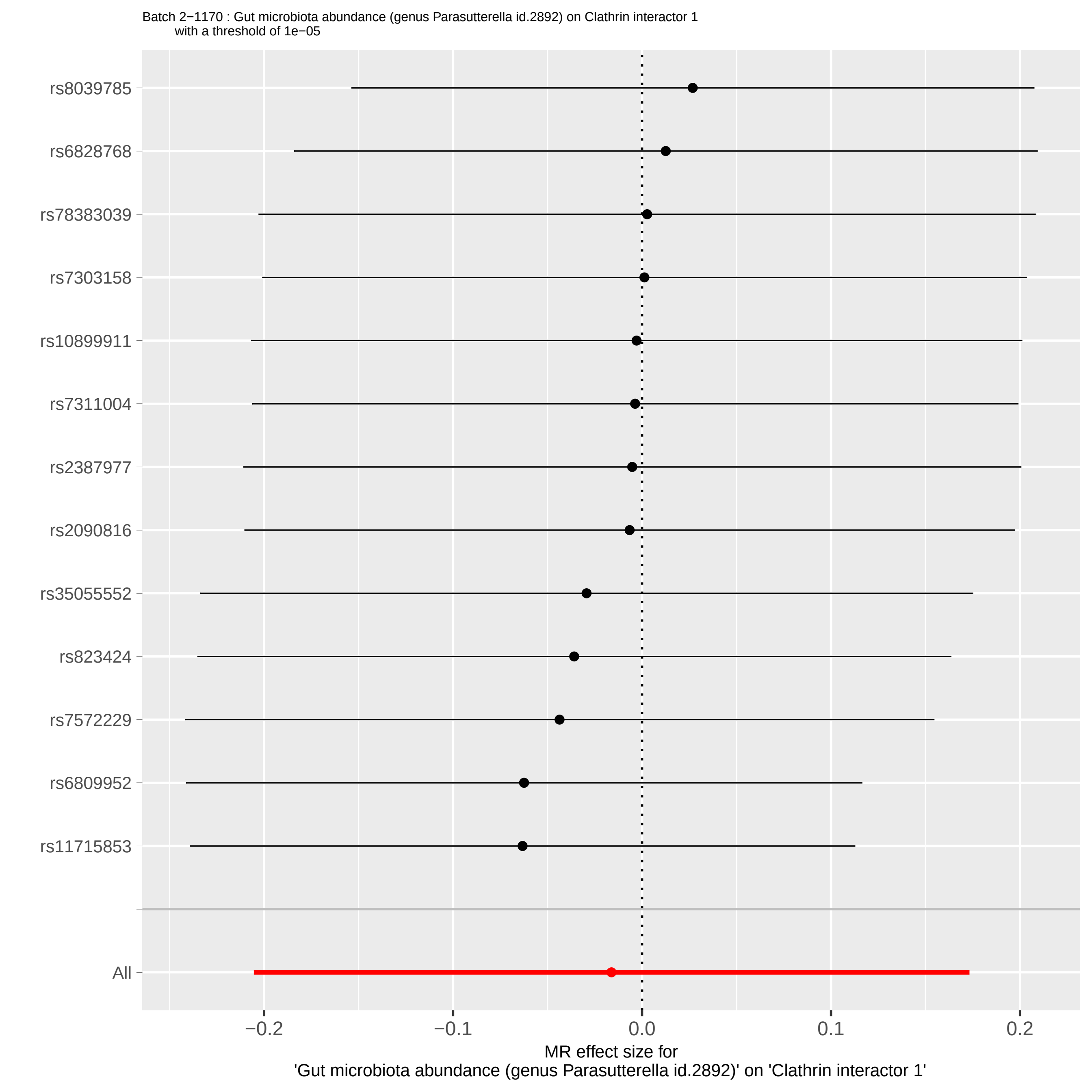
Batch 2−1170 : Gut microbiota abundance (genus Parasutterella id.2892) on Clathrin interactor 1
with a threshold of 1e−05
rs8039785
rs6828768
rs78383039
rs7303158
rs10899911
rs7311004
rs2387977
rs2090816
rs35055552
rs823424
rs7572229
rs6809952
rs11715853
All
−0.2
−0.1
0.0
0.1
0.2
MR effect size for
'Gut microbiota abundance (genus Parasutterella id.2892)' on 'Clathrin interactor 1'

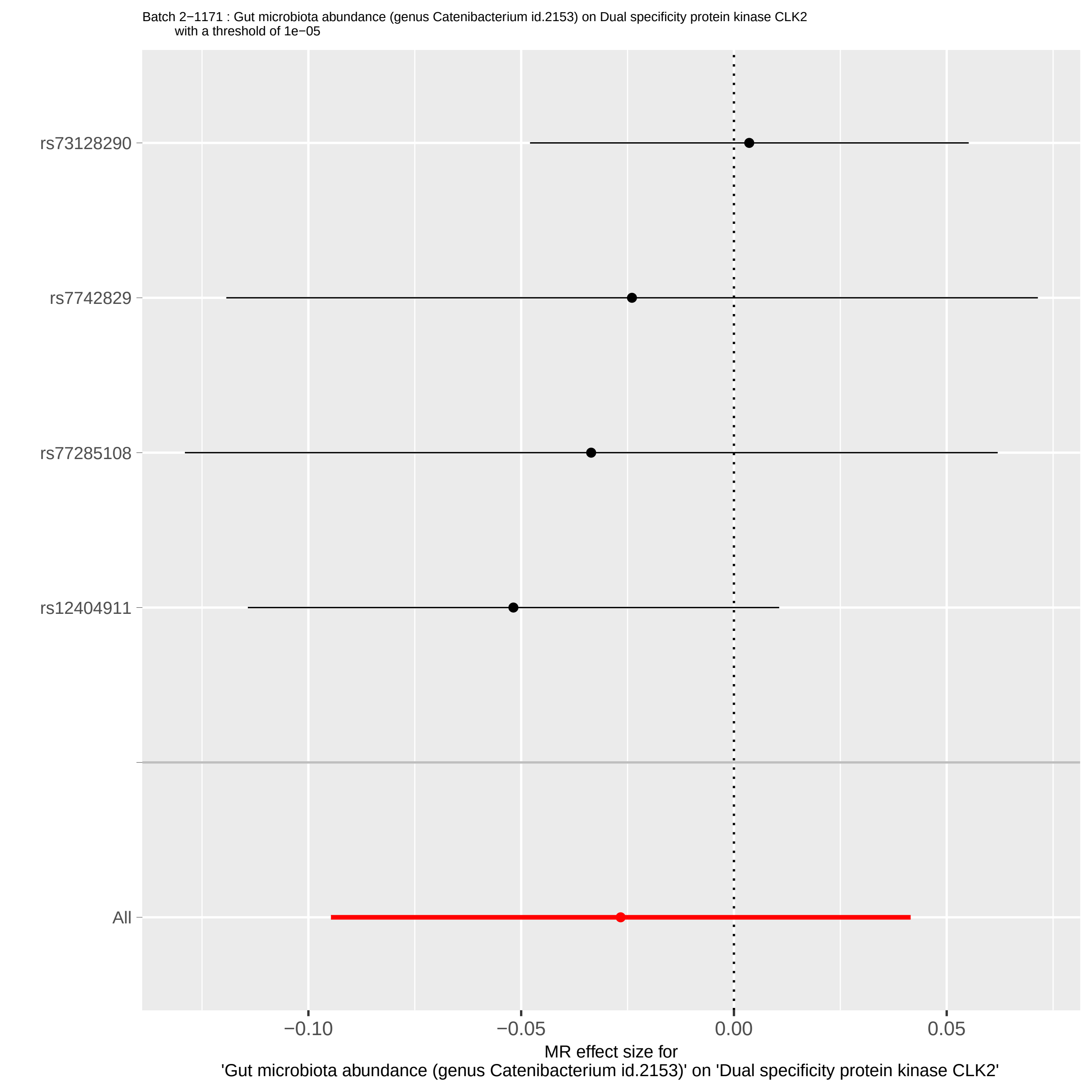
Batch 2−1171 : Gut microbiota abundance (genus Catenibacterium id.2153) on Dual specificity protein kinase CLK2
with a threshold of 1e−05
rs73128290
rs7742829
rs77285108
rs12404911
All
−0.10
−0.05
0.00
0.05
MR effect size for
'Gut microbiota abundance (genus Catenibacterium id.2153)' on 'Dual specificity protein kinase CLK2'

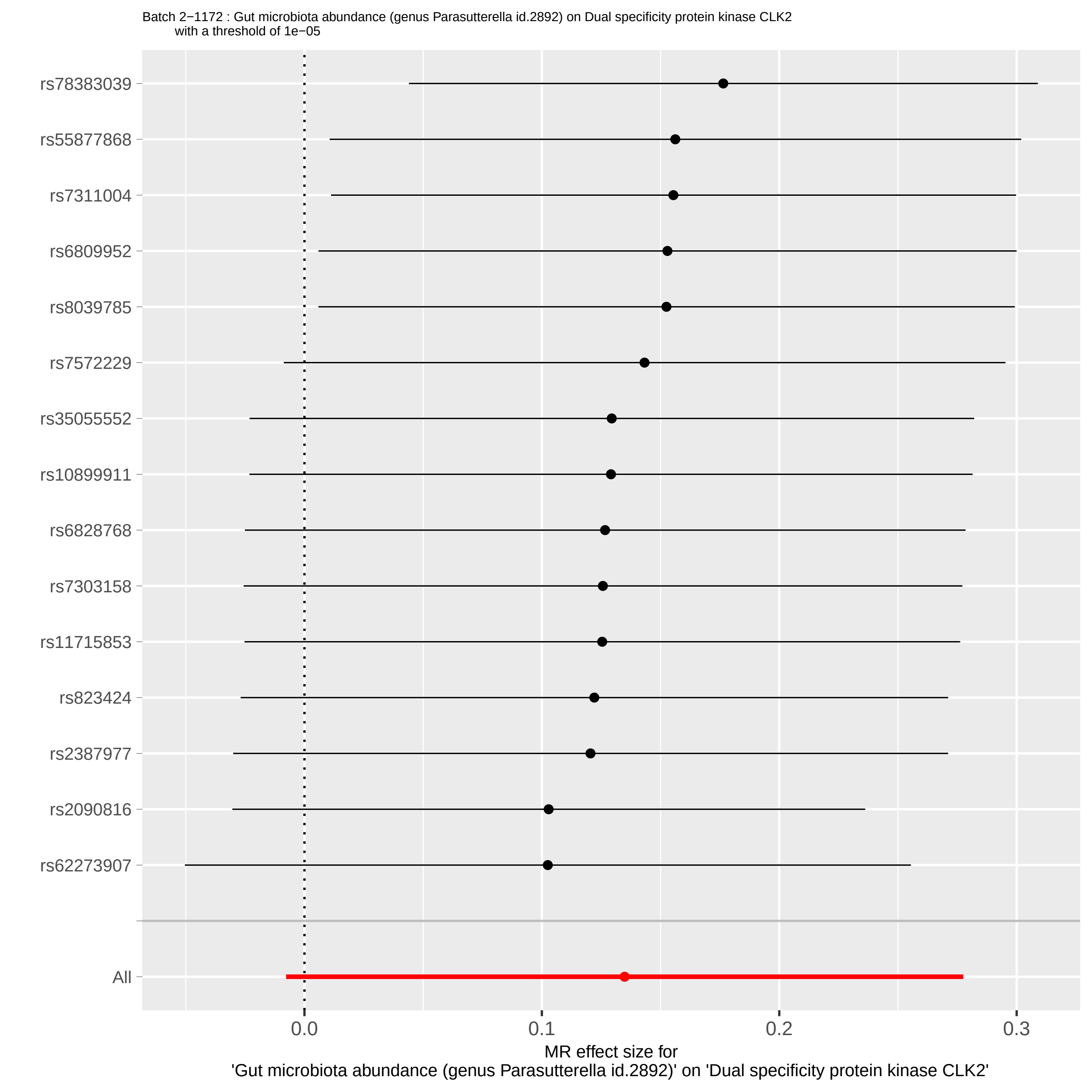

Batch 2−1172 : Gut microbiota abundance (genus Parasutterella id.2892) on Dual specificity protein kinase CLK2
with a threshold of 1e−05
rs78383039
rs55877868
rs7311004
rs6809952
rs8039785
rs7572229
rs35055552
rs10899911
rs6828768
rs7303158
rs11715853
rs823424
rs2387977
rs2090816
rs62273907
All
0.0
0.1
0.2
0.3
MR effect size for
'Gut microbiota abundance (genus Parasutterella id.2892)' on 'Dual specificity protein kinase CLK2'

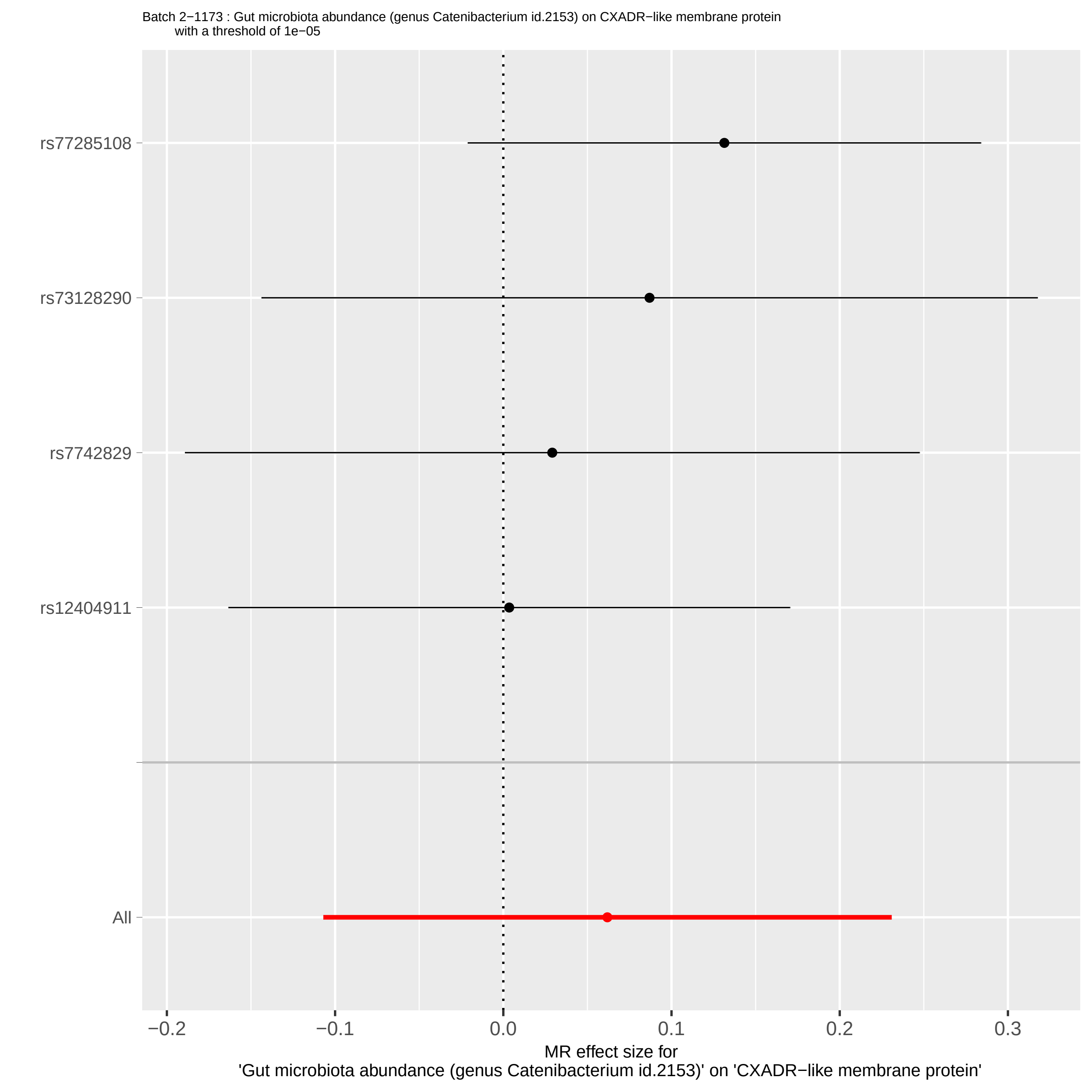

Batch 2−1173 : Gut microbiota abundance (genus Catenibacterium id.2153) on CXADR−like membrane protein
with a threshold of 1e−05
rs77285108
rs73128290
rs7742829
rs12404911
All
−0.2
−0.1
0.0
0.1
0.2
0.3
MR effect size for
'Gut microbiota abundance (genus Catenibacterium id.2153)' on 'CXADR−like membrane protein'

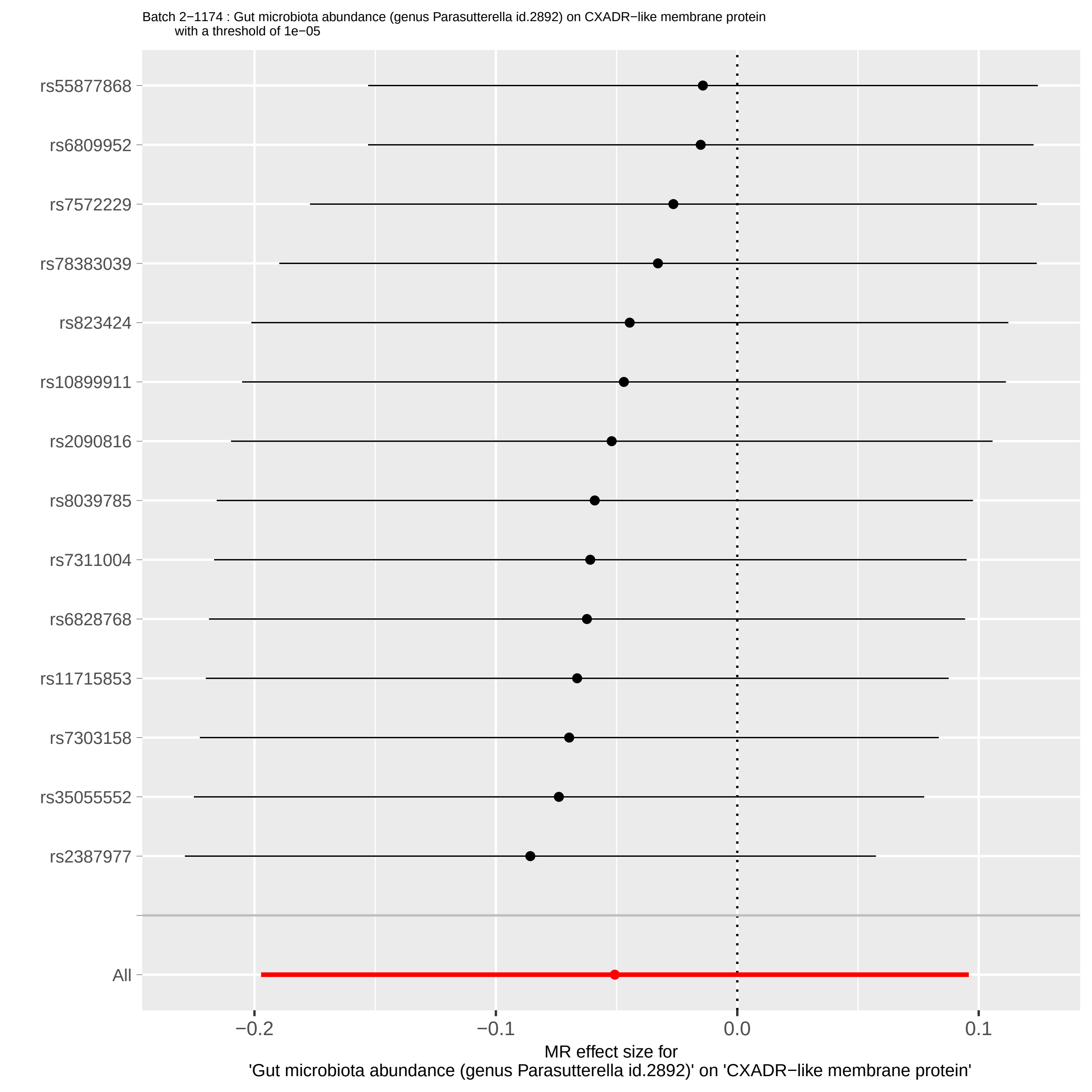
Batch 2−1174 : Gut microbiota abundance (genus Parasutterella id.2892) on CXADR−like membrane protein
with a threshold of 1e−05
rs55877868
rs6809952
rs7572229
rs78383039
rs823424
rs10899911
rs2090816
rs8039785
rs7311004
rs6828768
rs11715853
rs7303158
rs35055552
rs2387977
All
−0.2
−0.1
0.0
0.1
MR effect size for
'Gut microbiota abundance (genus Parasutterella id.2892)' on 'CXADR−like membrane protein'

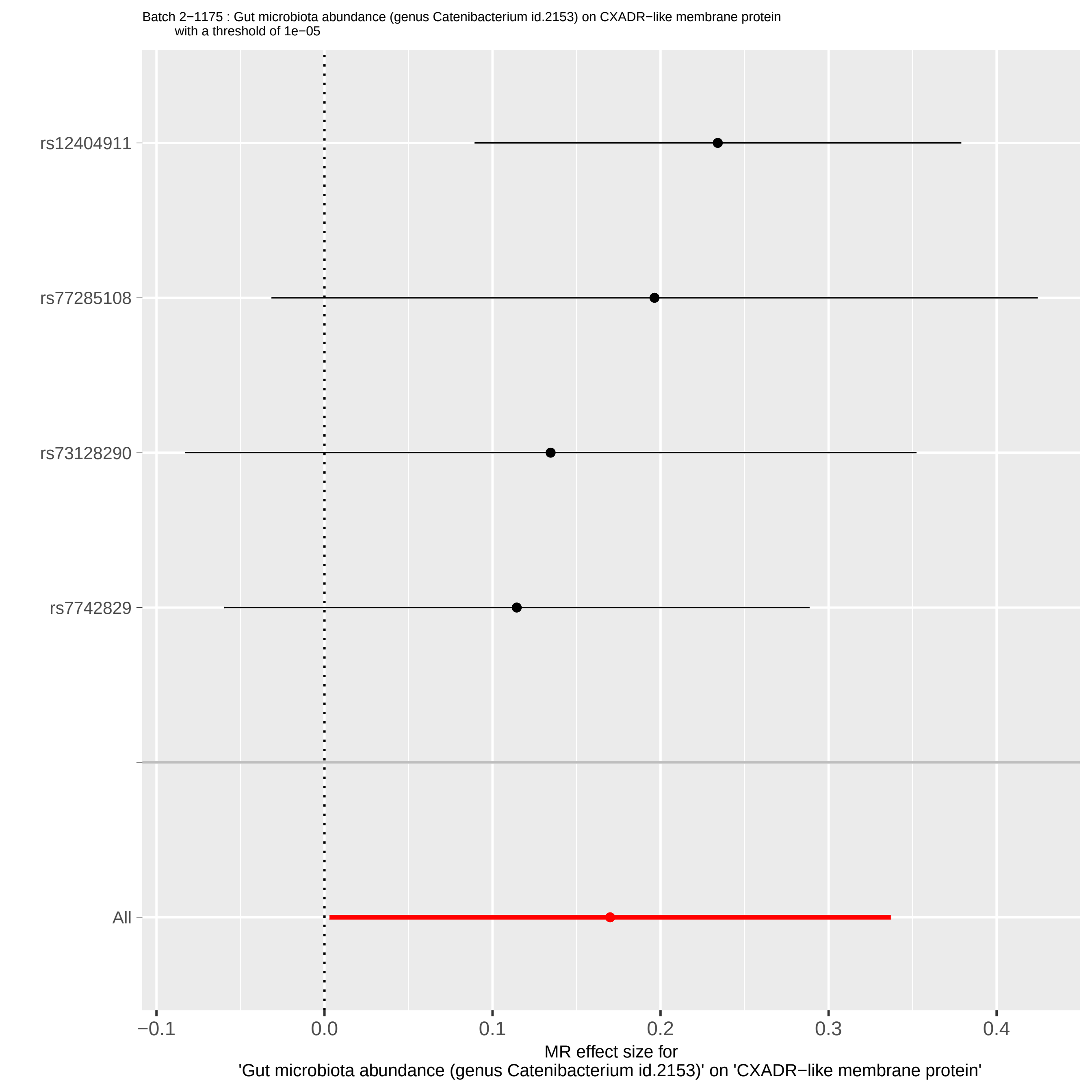

Batch 2−1175 : Gut microbiota abundance (genus Catenibacterium id.2153) on CXADR−like membrane protein
with a threshold of 1e−05
rs12404911
rs77285108
rs73128290
rs7742829
All
−0.1
0.0
0.1
0.2
0.3
0.4
MR effect size for
'Gut microbiota abundance (genus Catenibacterium id.2153)' on 'CXADR−like membrane protein'

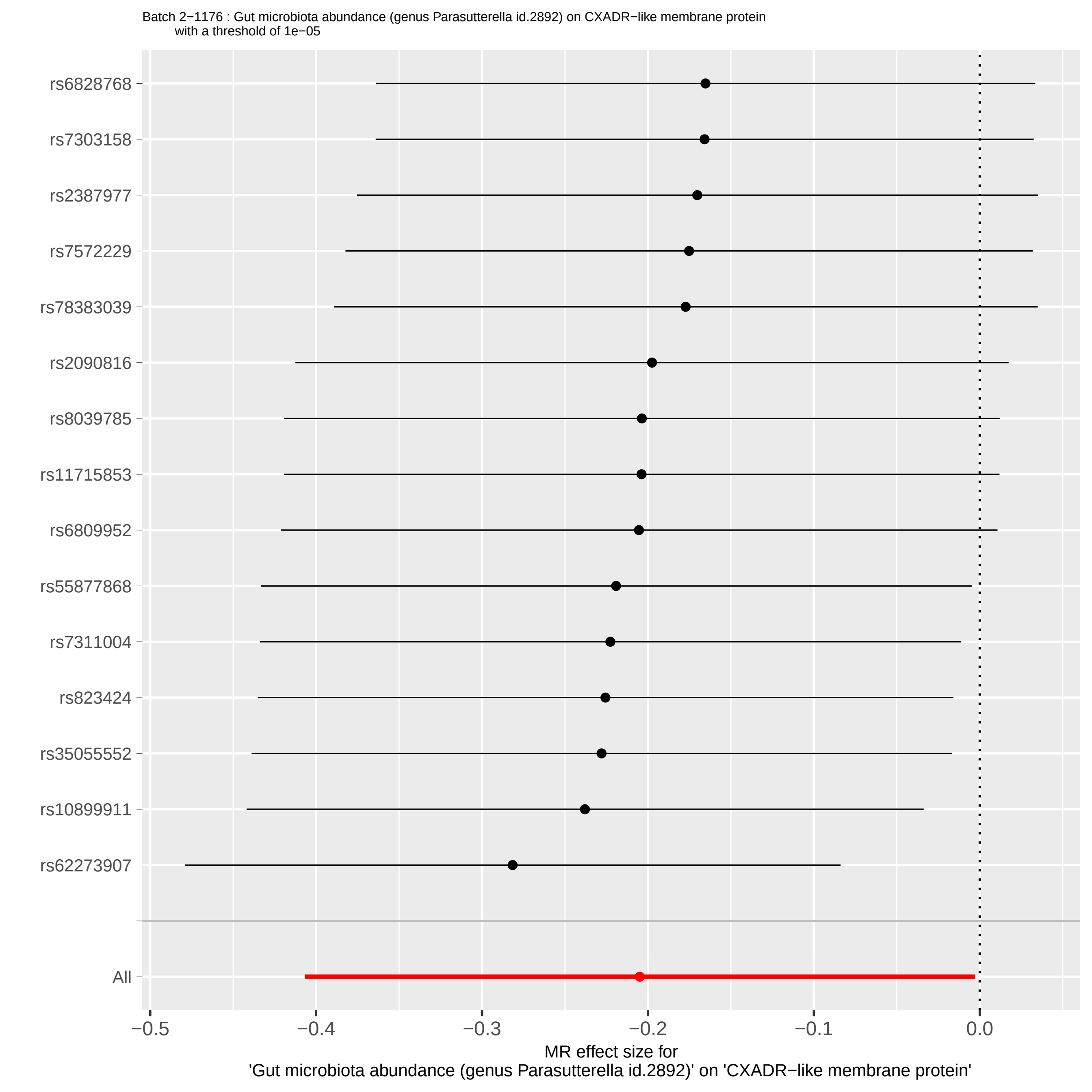
Batch 2−1176 : Gut microbiota abundance (genus Parasutterella id.2892) on CXADR−like membrane protein
with a threshold of 1e−05
rs6828768
rs7303158
rs2387977
rs7572229
rs78383039
rs2090816
rs8039785
rs11715853
rs6809952
rs55877868
rs7311004
rs823424
rs35055552
rs10899911
rs62273907
All
−0.5
−0.4
−0.3
−0.2
−0.1
0.0
MR effect size for
'Gut microbiota abundance (genus Parasutterella id.2892)' on 'CXADR−like membrane protein'

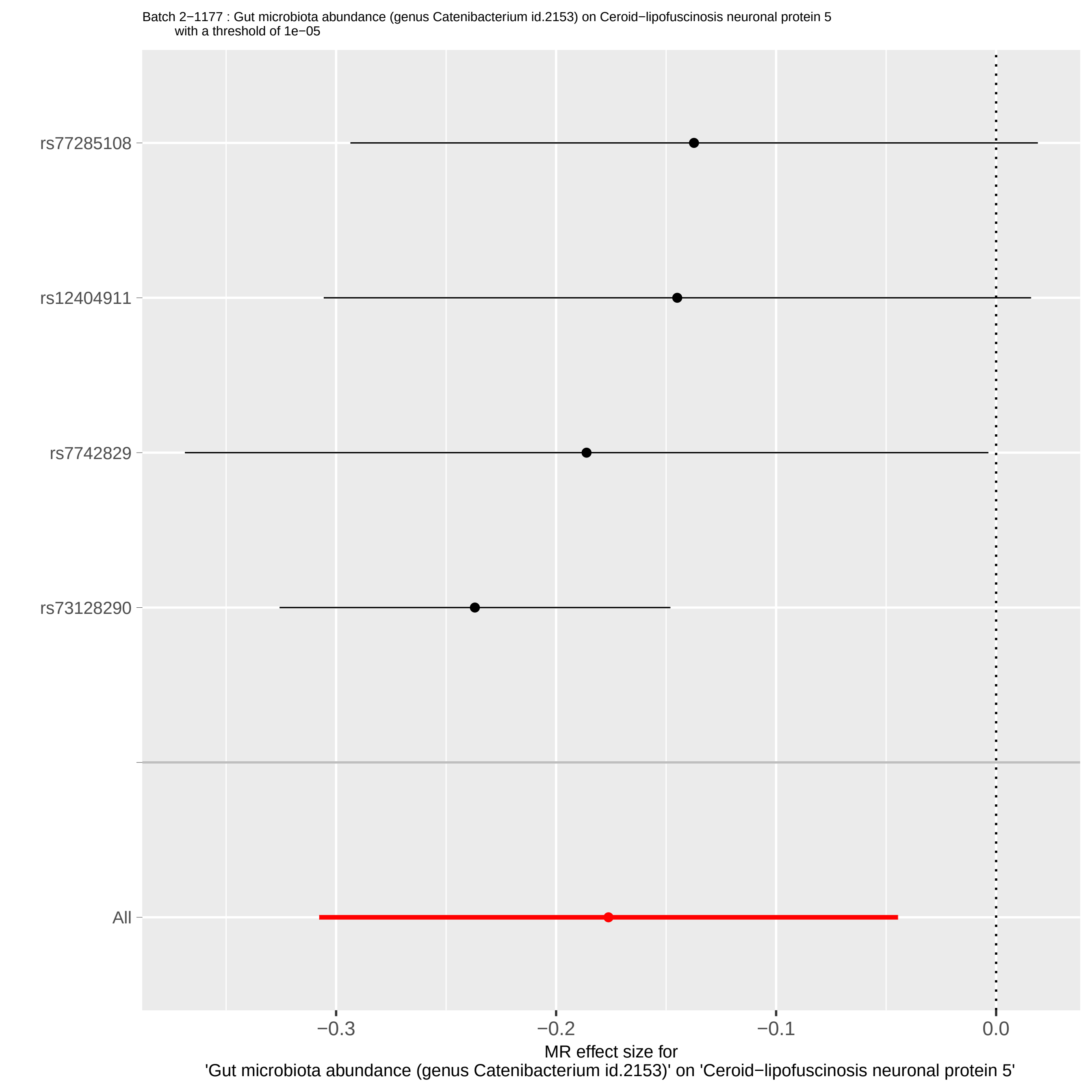
Batch 2−1177 : Gut microbiota abundance (genus Catenibacterium id.2153) on Ceroid−lipofuscinosis neuronal protein 5
with a threshold of 1e−05
rs77285108
rs12404911
rs7742829
rs73128290
All
−0.3
−0.2
−0.1
0.0
MR effect size for
'Gut microbiota abundance (genus Catenibacterium id.2153)' on 'Ceroid−lipofuscinosis neuronal protein 5'

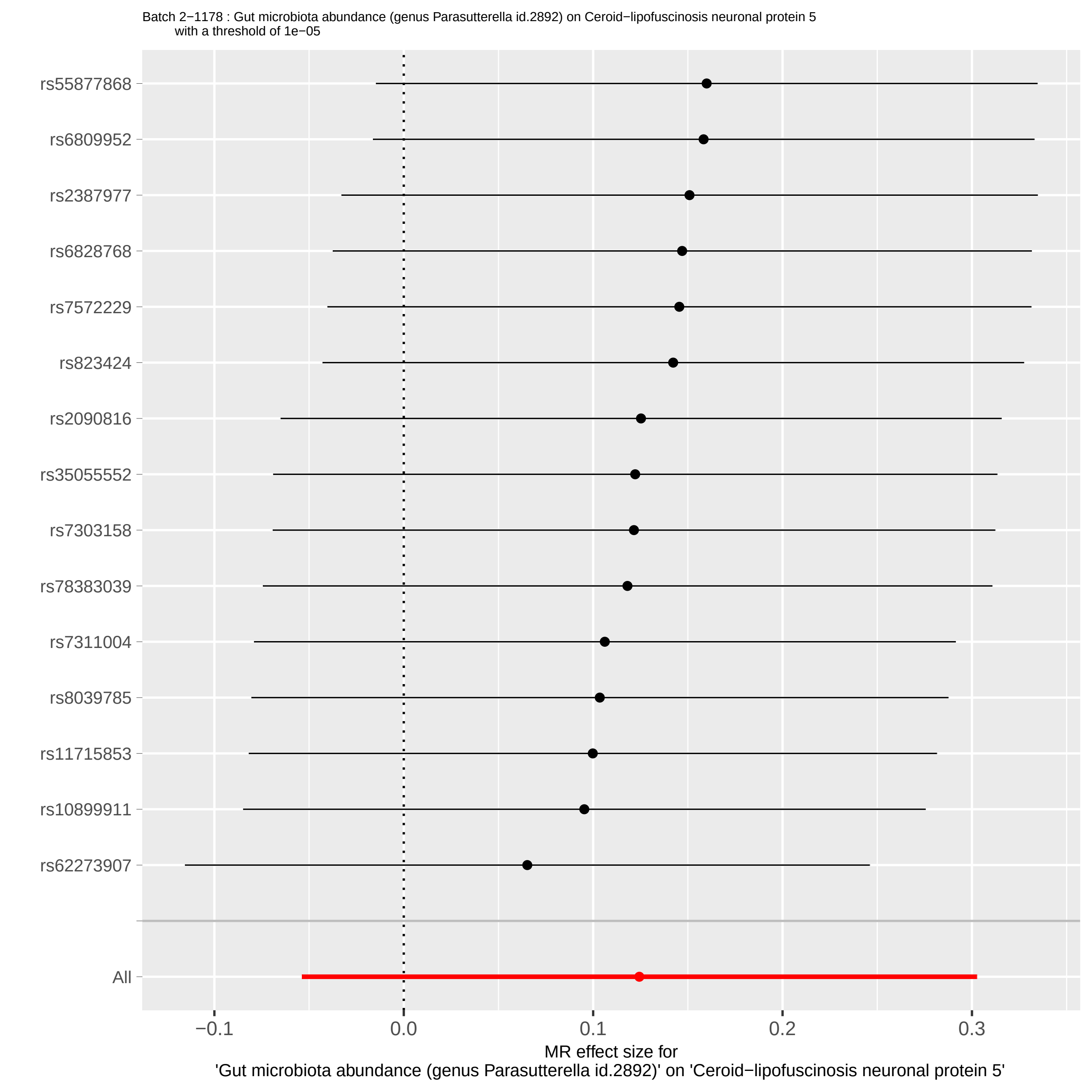
Batch 2−1178 : Gut microbiota abundance (genus Parasutterella id.2892) on Ceroid−lipofuscinosis neuronal protein 5
with a threshold of 1e−05
rs55877868
rs6809952
rs2387977
rs6828768
rs7572229
rs823424
rs2090816
rs35055552
rs7303158
rs78383039
rs7311004
rs8039785
rs11715853
rs10899911
rs62273907
All
−0.1
0.0
0.1
0.2
0.3
MR effect size for
'Gut microbiota abundance (genus Parasutterella id.2892)' on 'Ceroid−lipofuscinosis neuronal protein 5'

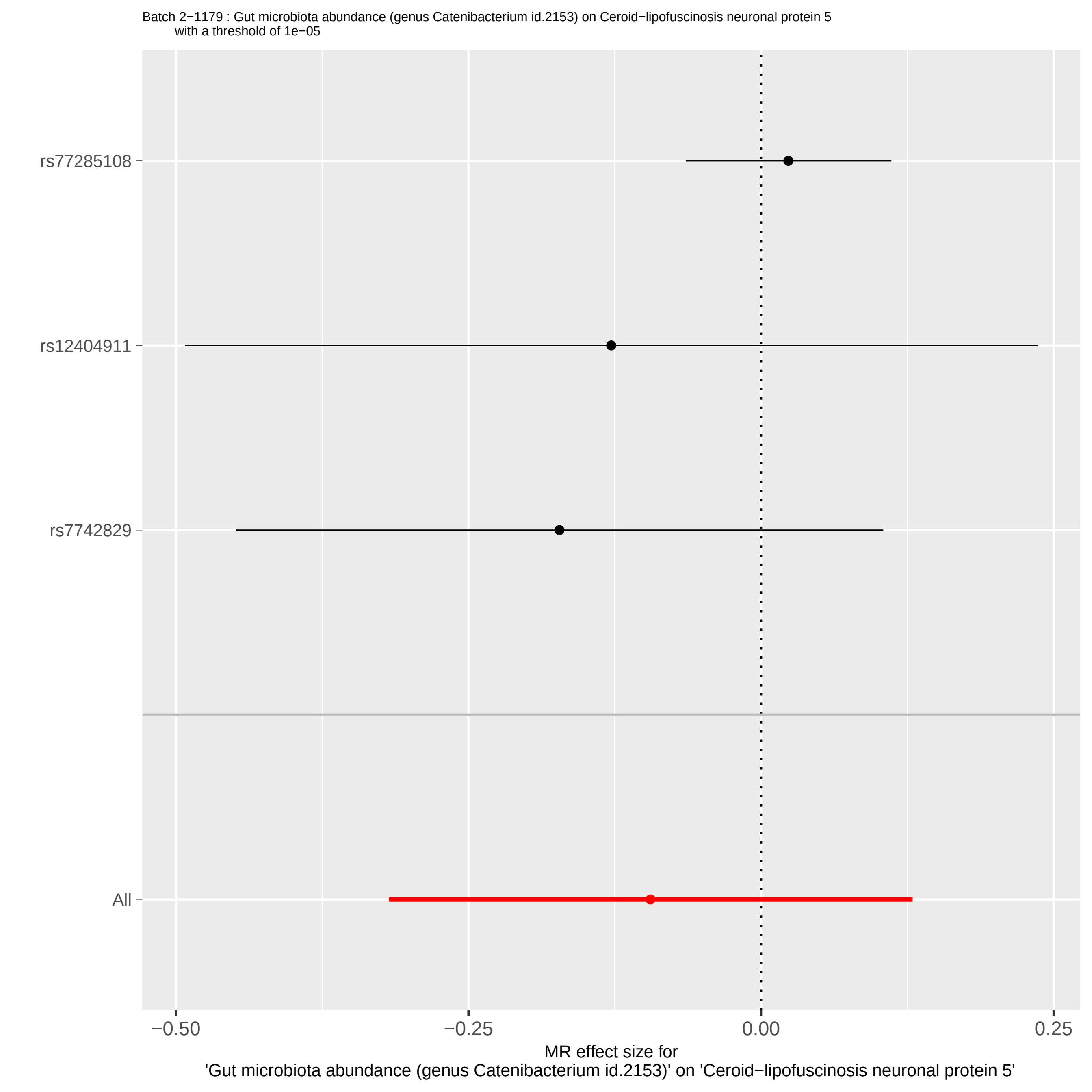
Batch 2−1179 : Gut microbiota abundance (genus Catenibacterium id.2153) on Ceroid−lipofuscinosis neuronal protein 5
with a threshold of 1e−05
rs77285108
rs12404911
rs7742829
All
−0.50
−0.25
0.00
0.25
MR effect size for
'Gut microbiota abundance (genus Catenibacterium id.2153)' on 'Ceroid−lipofuscinosis neuronal protein 5'

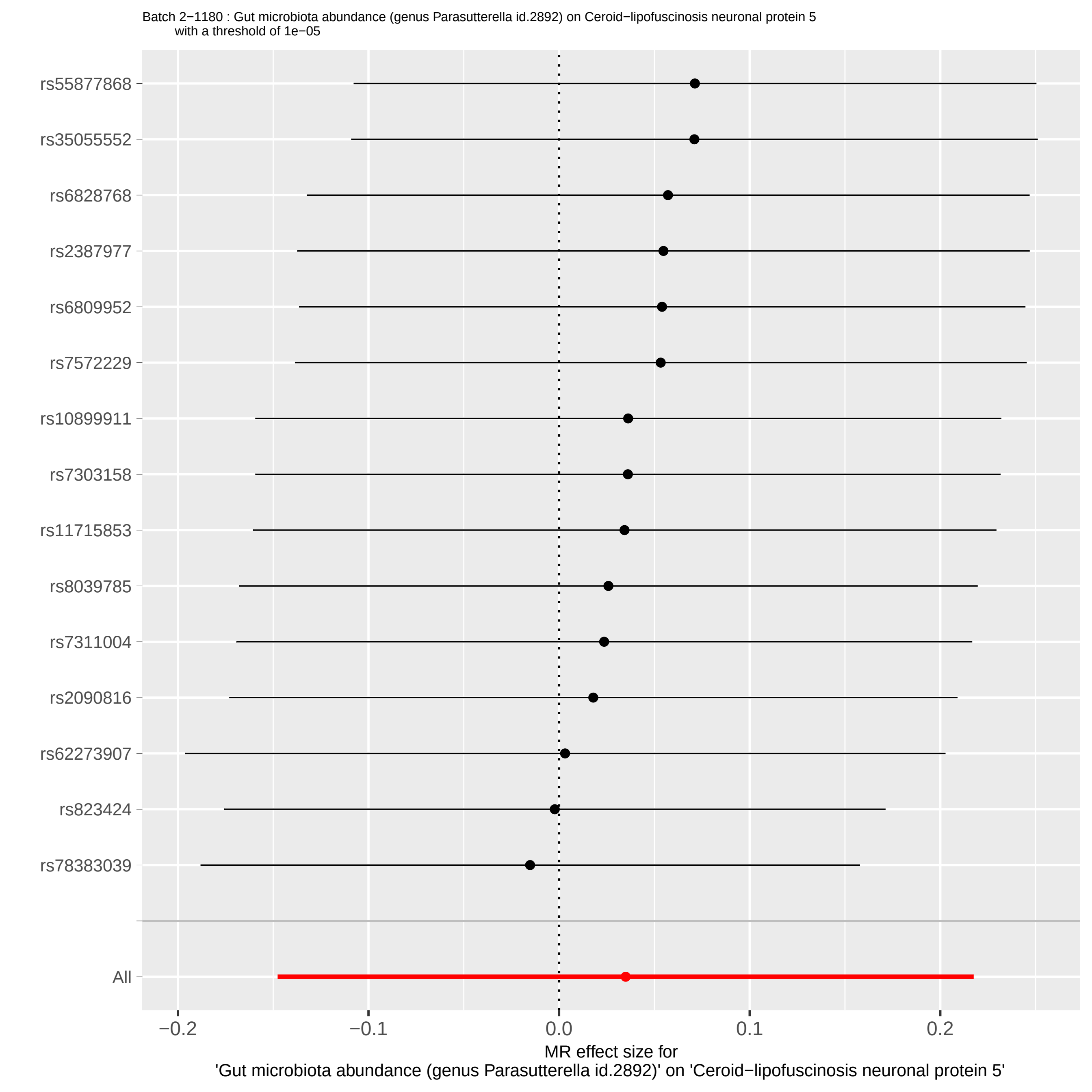
Batch 2−1180 : Gut microbiota abundance (genus Parasutterella id.2892) on Ceroid−lipofuscinosis neuronal protein 5
with a threshold of 1e−05
rs55877868
rs35055552
rs6828768
rs2387977
rs6809952
rs7572229
rs10899911
rs7303158
rs11715853
rs8039785
rs7311004
rs2090816
rs62273907
rs823424
rs78383039
All
−0.2
−0.1
0.0
0.1
0.2
MR effect size for
'Gut microbiota abundance (genus Parasutterella id.2892)' on 'Ceroid−lipofuscinosis neuronal protein 5'

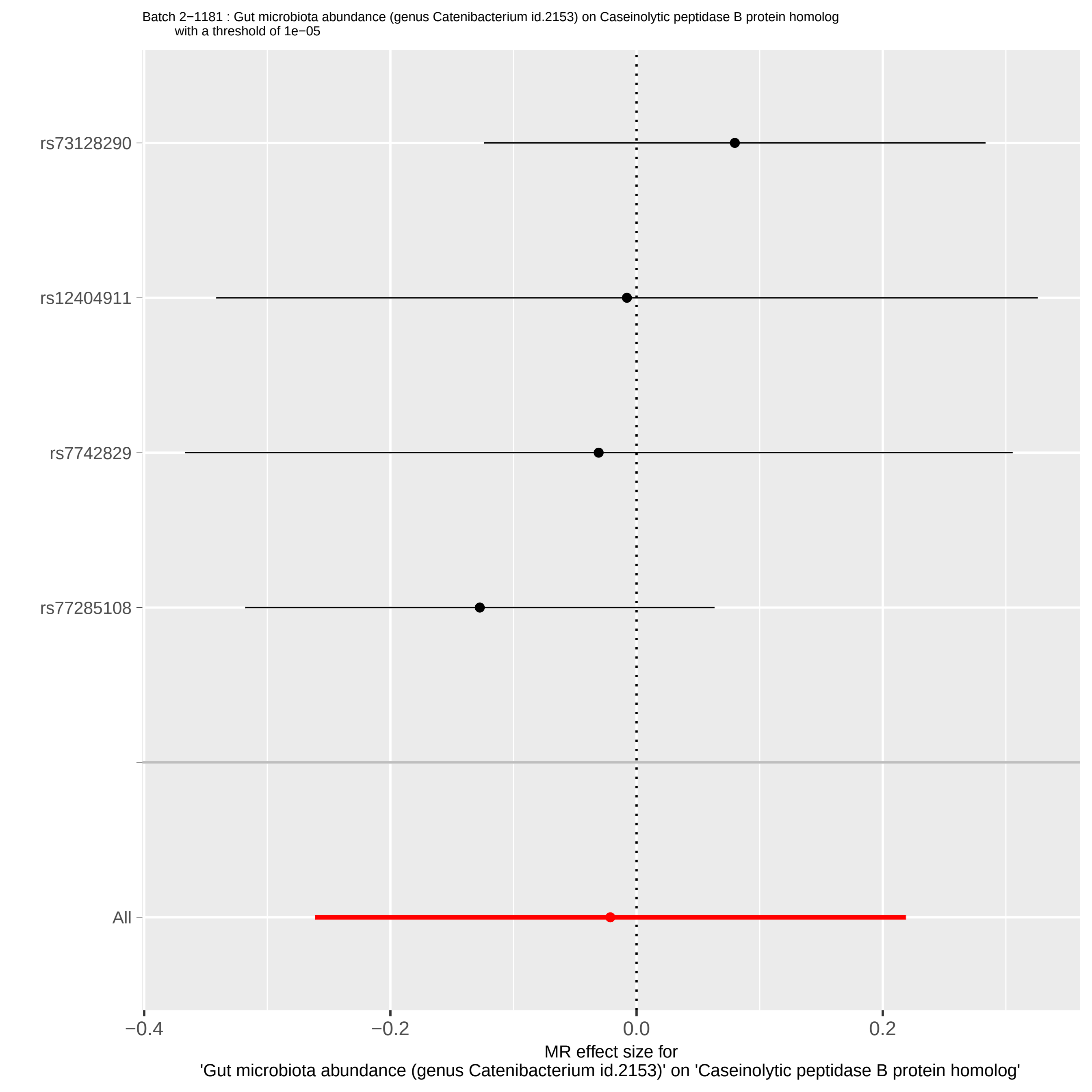
Batch 2−1181 : Gut microbiota abundance (genus Catenibacterium id.2153) on Caseinolytic peptidase B protein homolog
with a threshold of 1e−05
rs73128290
rs12404911
rs7742829
rs77285108
All
−0.4
−0.2
0.0
0.2
MR effect size for
'Gut microbiota abundance (genus Catenibacterium id.2153)' on 'Caseinolytic peptidase B protein homolog'

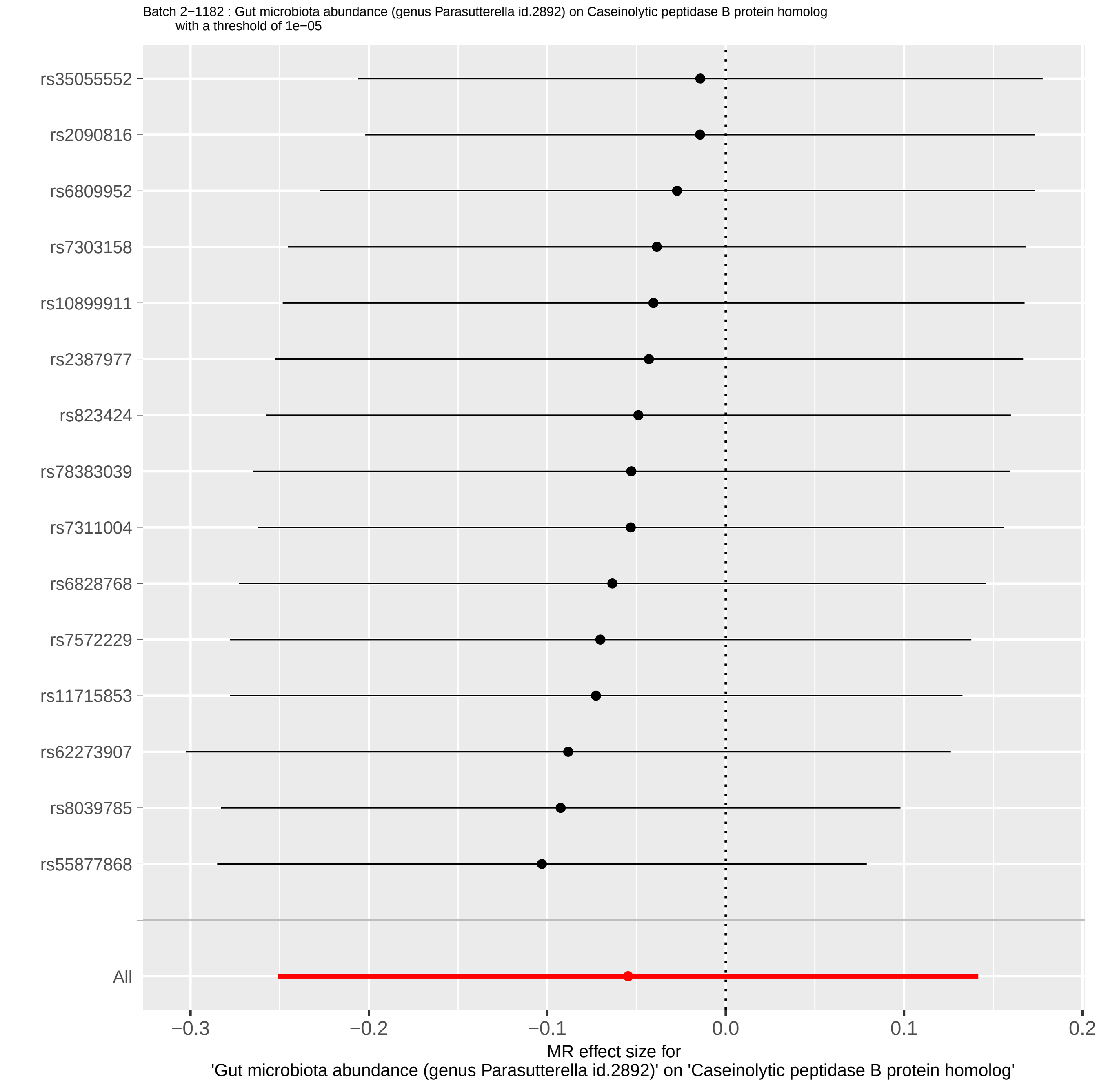
Batch 2−1182 : Gut microbiota abundance (genus Parasutterella id.2892) on Caseinolytic peptidase B protein homolog
with a threshold of 1e−05
rs35055552
rs2090816
rs6809952
rs7303158
rs10899911
rs2387977
rs823424
rs78383039
rs7311004
rs6828768
rs7572229
rs11715853
rs62273907
rs8039785
rs55877868
All
−0.3
−0.2
−0.1
0.0
0.1
0.2
MR effect size for
'Gut microbiota abundance (genus Parasutterella id.2892)' on 'Caseinolytic peptidase B protein homolog'

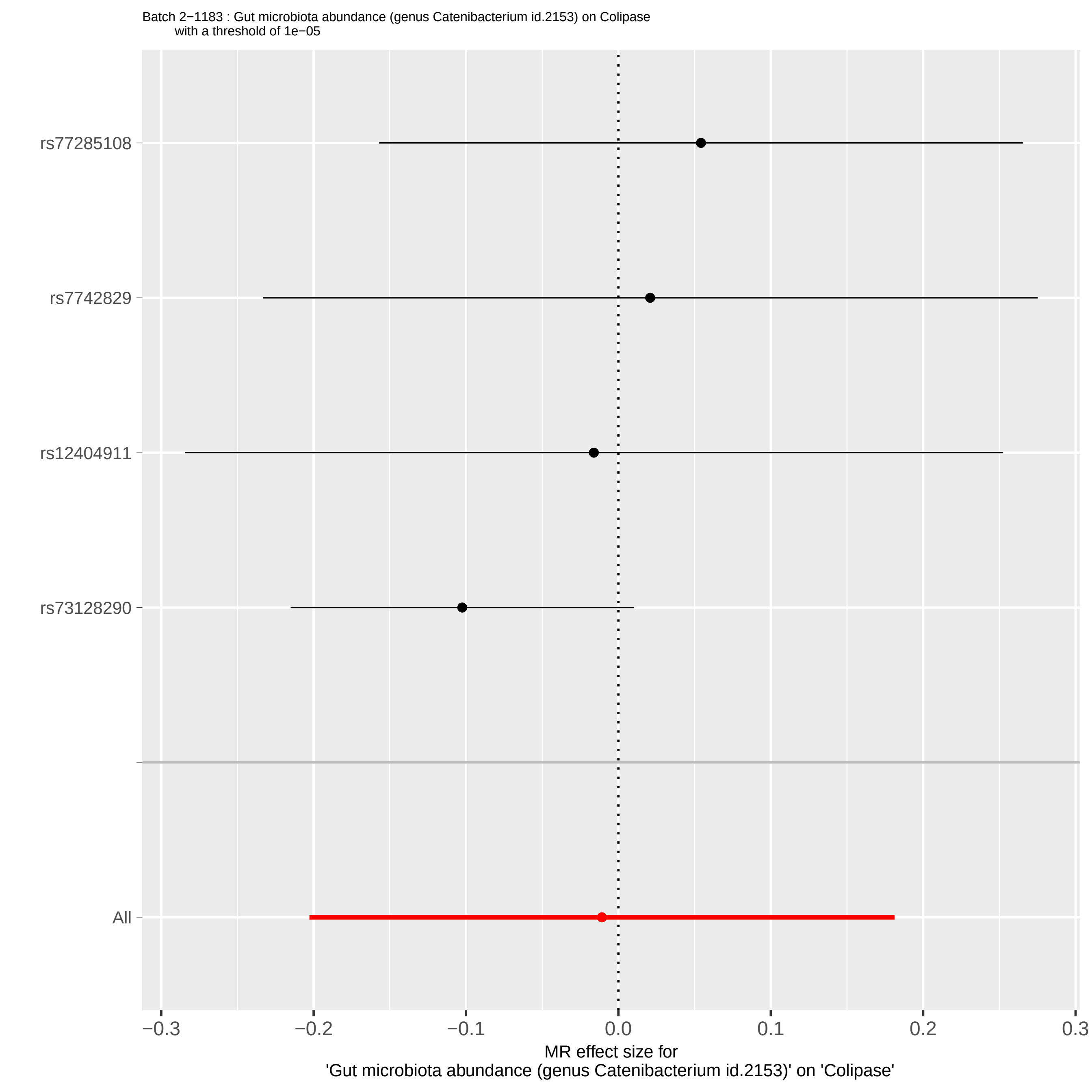
Batch 2−1183 : Gut microbiota abundance (genus Catenibacterium id.2153) on Colipase
with a threshold of 1e−05
rs77285108
rs7742829
rs12404911
rs73128290
All
−0.3
−0.2
−0.1
0.0
0.1
0.2
0.3
MR effect size for
'Gut microbiota abundance (genus Catenibacterium id.2153)' on 'Colipase'

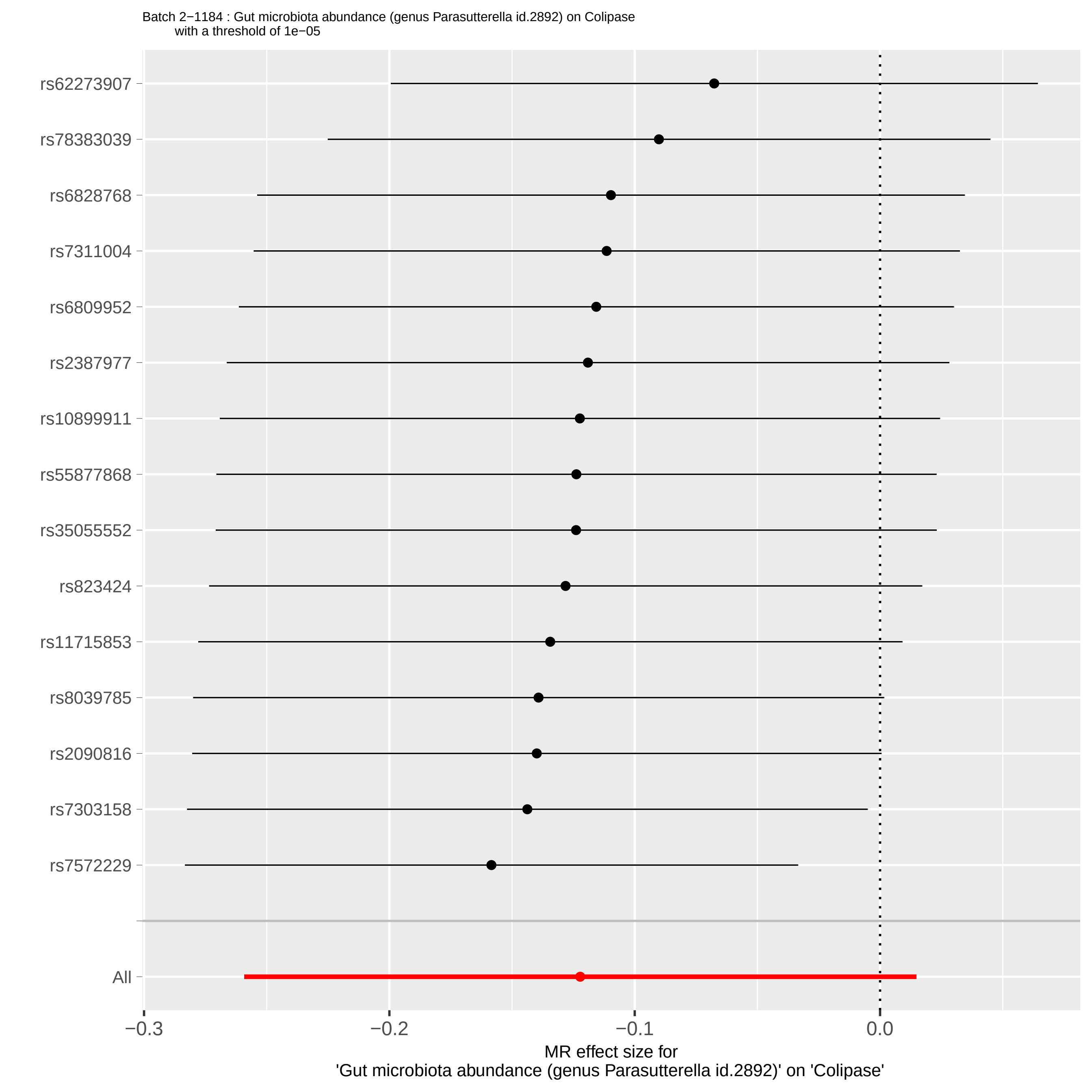

Batch 2−1184 : Gut microbiota abundance (genus Parasutterella id.2892) on Colipase
with a threshold of 1e−05
rs62273907
rs78383039
rs6828768
rs7311004
rs6809952
rs2387977
rs10899911
rs55877868
rs35055552
rs823424
rs11715853
rs8039785
rs2090816
rs7303158
rs7572229
All
−0.3
−0.2
−0.1
0.0
MR effect size for
'Gut microbiota abundance (genus Parasutterella id.2892)' on 'Colipase'

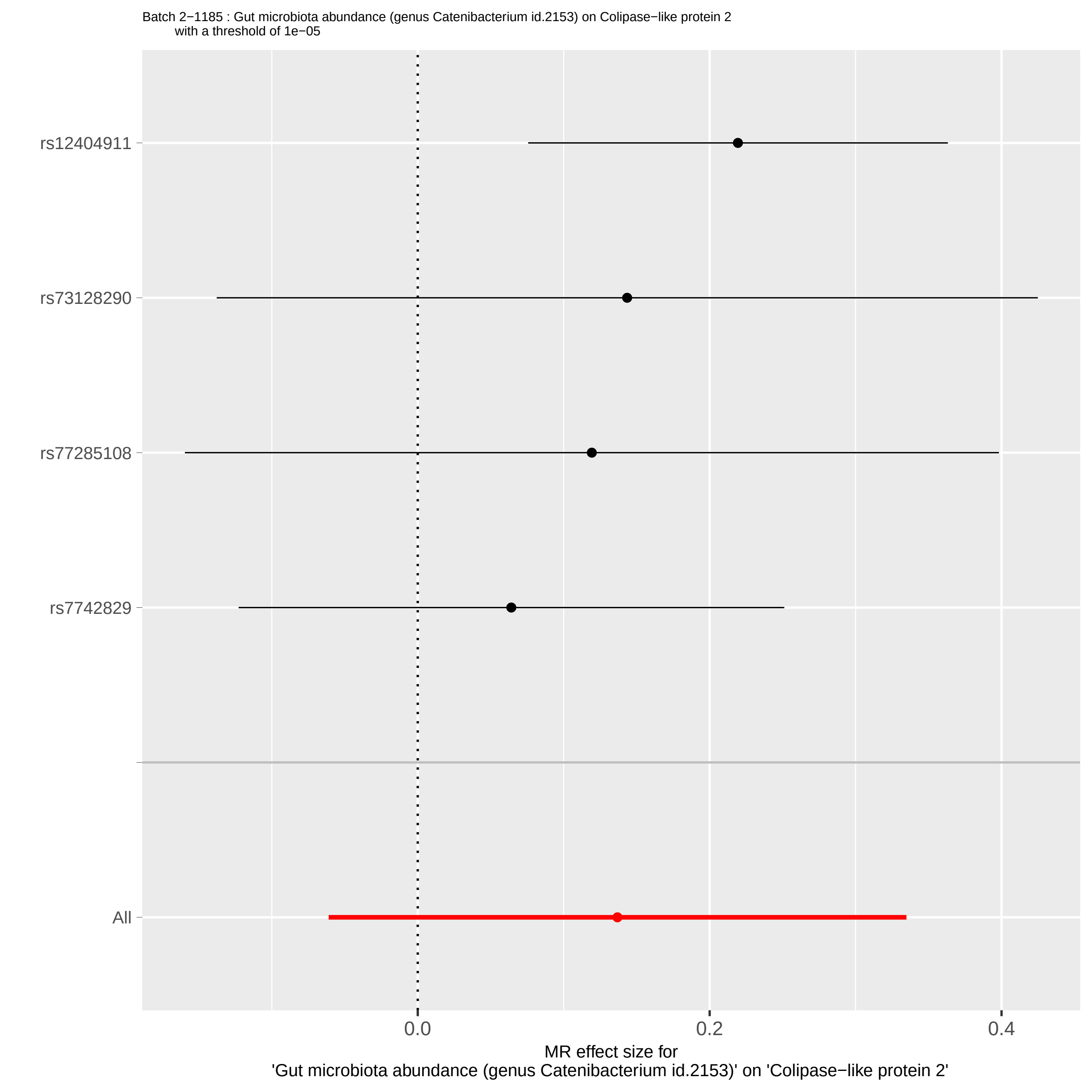
Batch 2−1185 : Gut microbiota abundance (genus Catenibacterium id.2153) on Colipase−like protein 2
with a threshold of 1e−05
rs12404911
rs73128290
rs77285108
rs7742829
All
0.0
0.2
0.4
MR effect size for
'Gut microbiota abundance (genus Catenibacterium id.2153)' on 'Colipase−like protein 2'

Batch 2−1186 : Gut microbiota abundance (genus Parasutterella id.2892) on Colipase−like protein 2
with a threshold of 1e−05

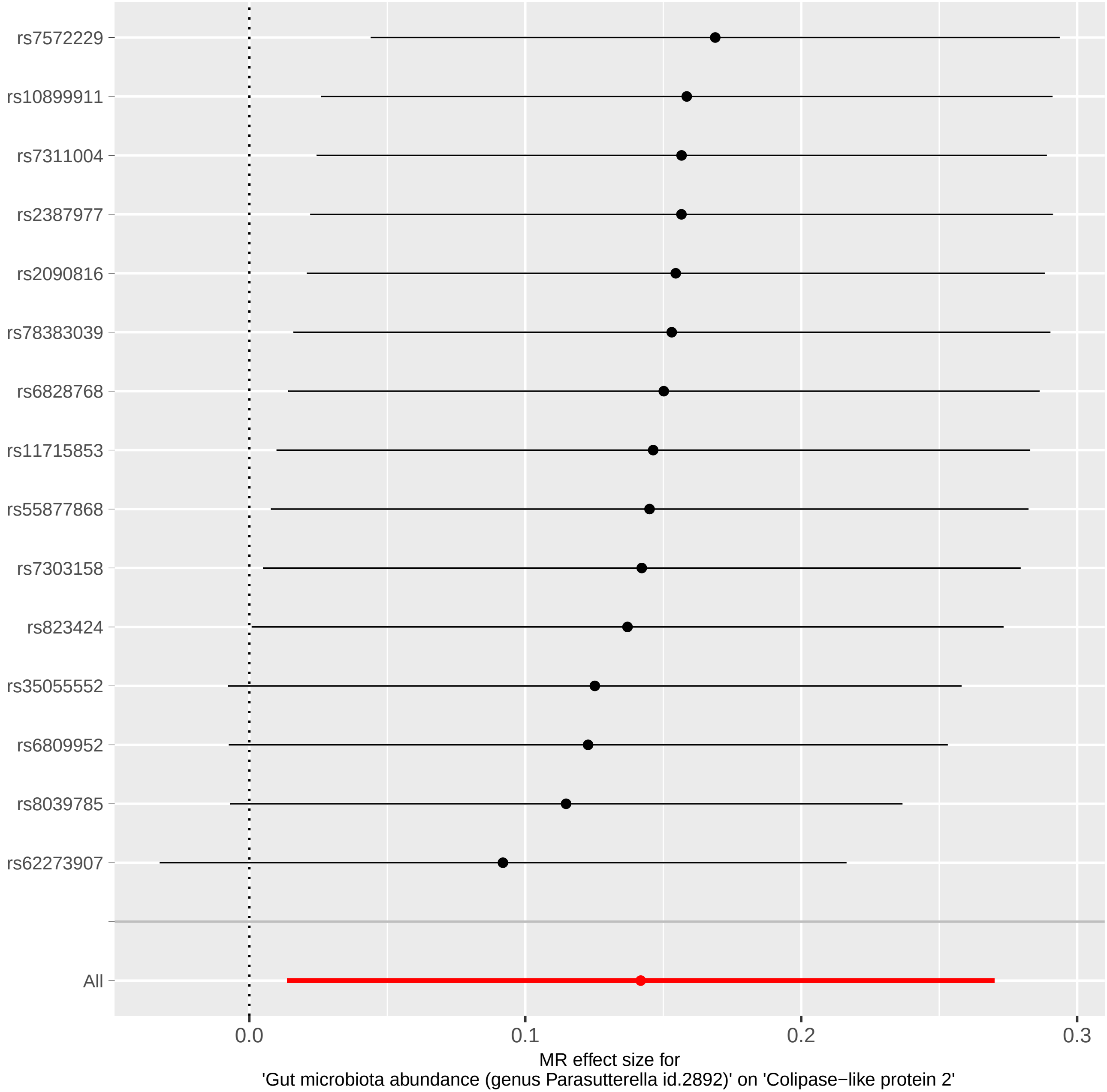

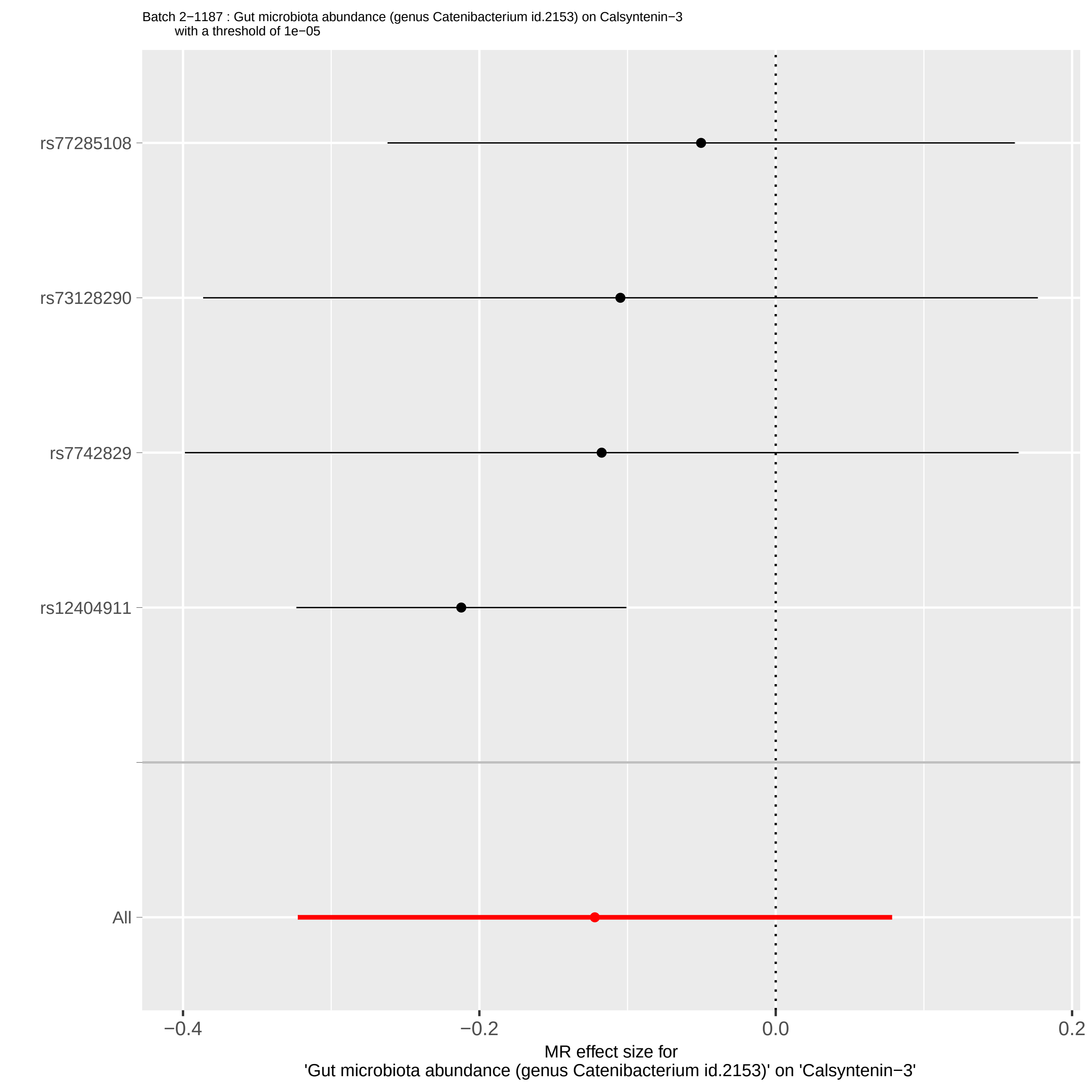
Batch 2−1187 : Gut microbiota abundance (genus Catenibacterium id.2153) on Calsyntenin−3
with a threshold of 1e−05
rs77285108
rs73128290
rs7742829
rs12404911
All
−0.4
−0.2
0.0
0.2
MR effect size for
'Gut microbiota abundance (genus Catenibacterium id.2153)' on 'Calsyntenin−3'

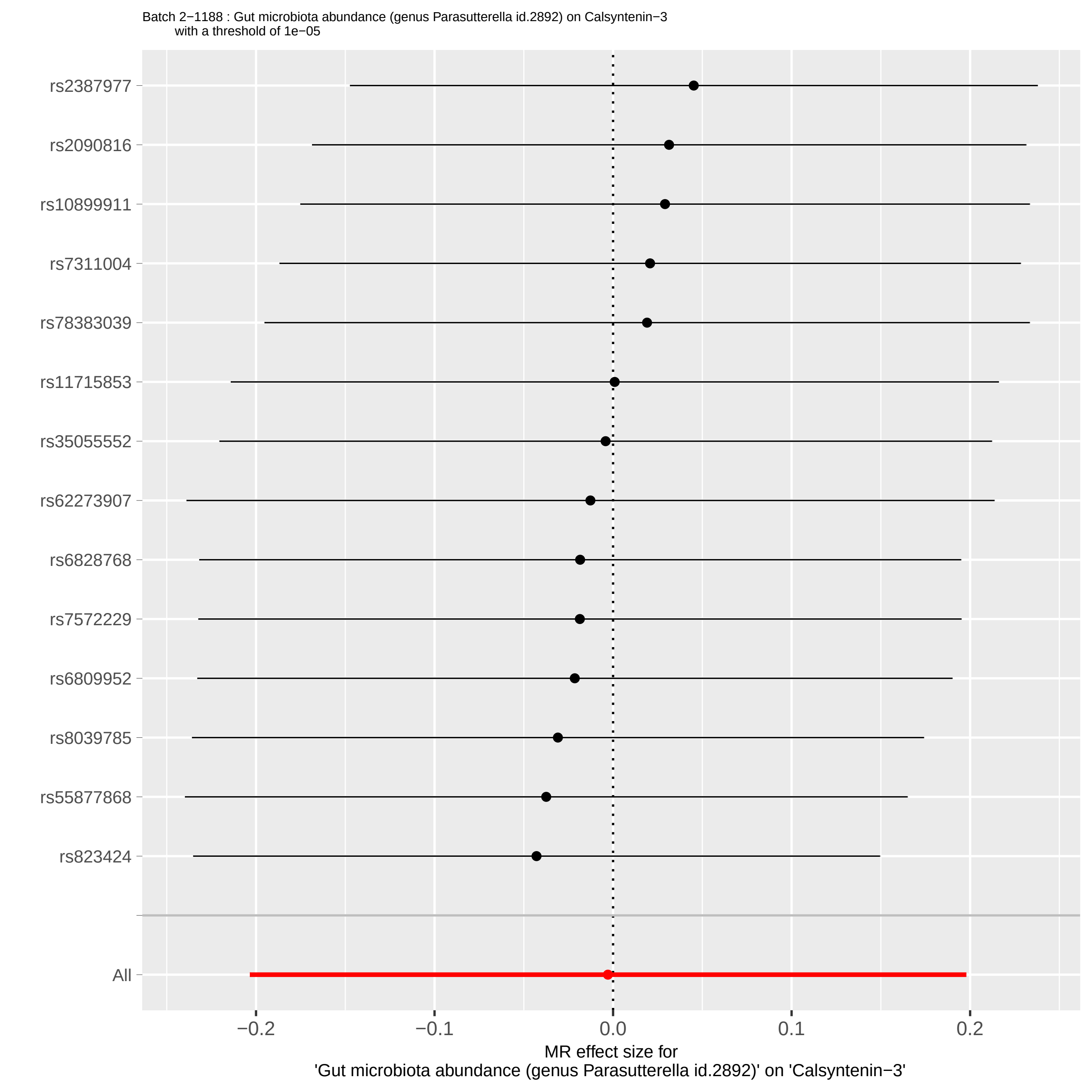
Batch 2−1188 : Gut microbiota abundance (genus Parasutterella id.2892) on Calsyntenin−3
with a threshold of 1e−05
rs2387977
rs2090816
rs10899911
rs7311004
rs78383039
rs11715853
rs35055552
rs62273907
rs6828768
rs7572229
rs6809952
rs8039785
rs55877868
rs823424
All
−0.2
−0.1
0.0
0.1
0.2
MR effect size for
'Gut microbiota abundance (genus Parasutterella id.2892)' on 'Calsyntenin−3'

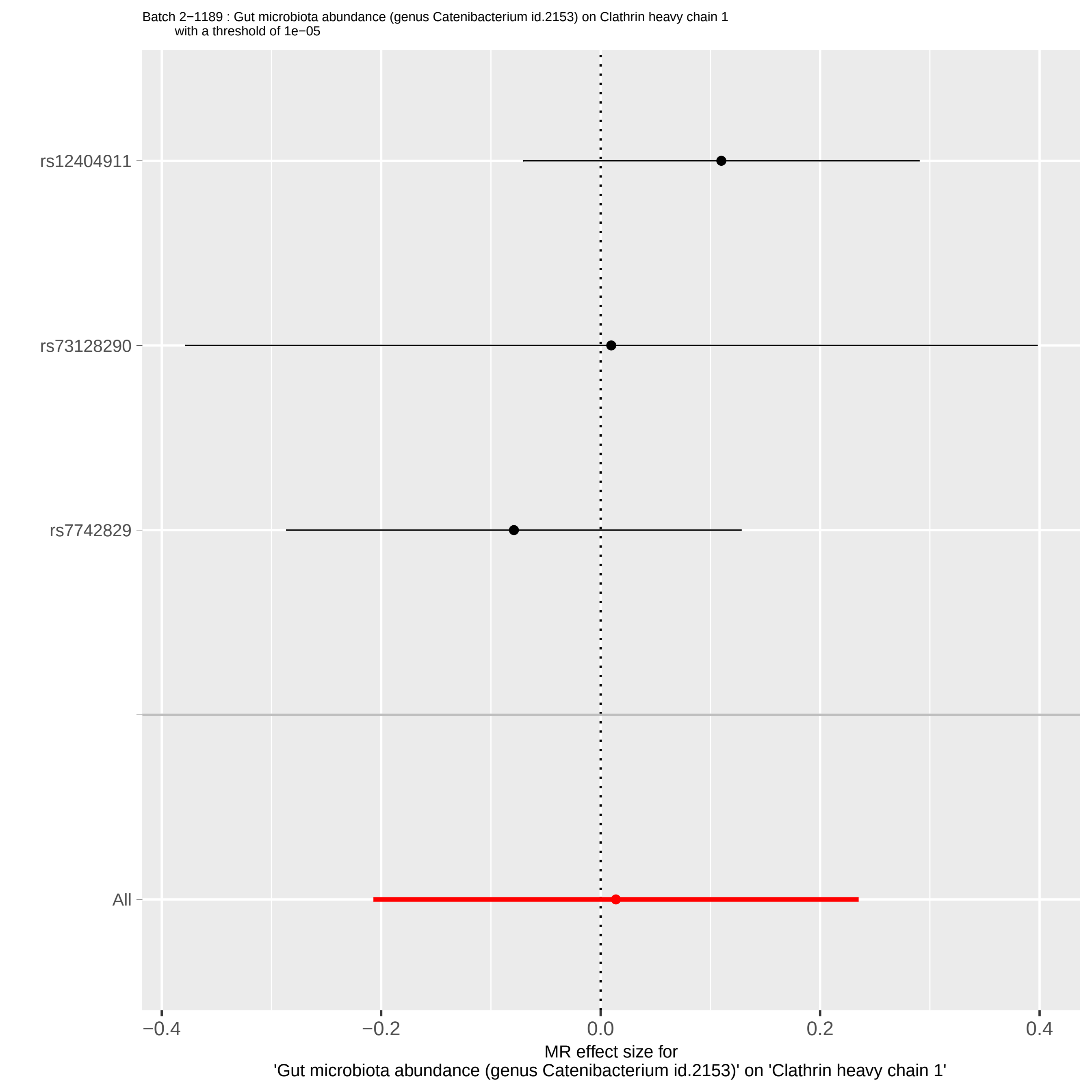

Batch 2−1189 : Gut microbiota abundance (genus Catenibacterium id.2153) on Clathrin heavy chain 1
with a threshold of 1e−05
rs12404911
rs73128290
rs7742829
All
−0.4
−0.2
0.0
0.2
0.4
MR effect size for
'Gut microbiota abundance (genus Catenibacterium id.2153)' on 'Clathrin heavy chain 1'

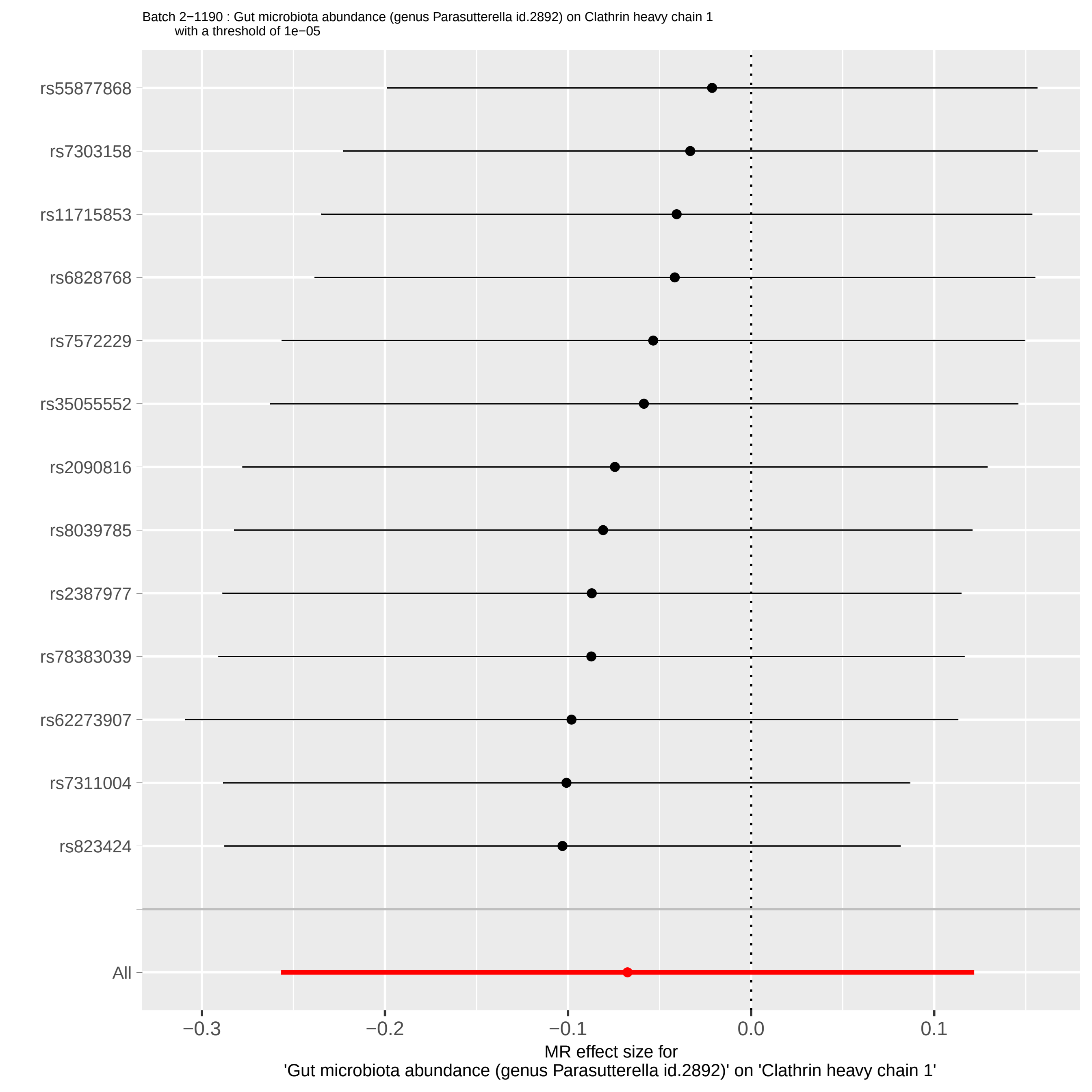
Batch 2−1190 : Gut microbiota abundance (genus Parasutterella id.2892) on Clathrin heavy chain 1
with a threshold of 1e−05
rs55877868
rs7303158
rs11715853
rs6828768
rs7572229
rs35055552
rs2090816
rs8039785
rs2387977
rs78383039
rs62273907
rs7311004
rs823424
All
−0.3
−0.2
−0.1
0.0
0.1
MR effect size for
'Gut microbiota abundance (genus Parasutterella id.2892)' on 'Clathrin heavy chain 1'

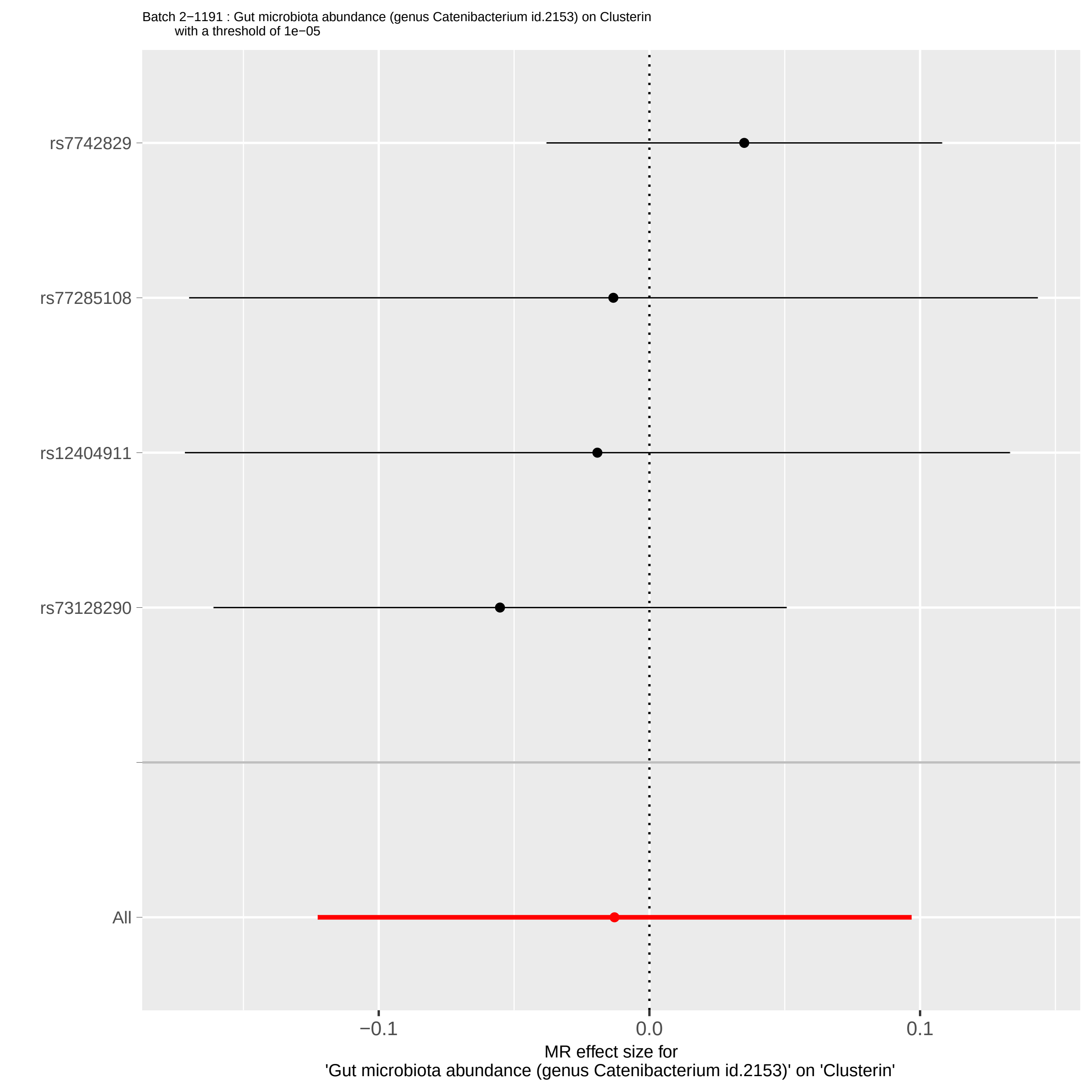
Batch 2−1191 : Gut microbiota abundance (genus Catenibacterium id.2153) on Clusterin
with a threshold of 1e−05
rs7742829
rs77285108
rs12404911
rs73128290
All
−0.1
0.0
0.1
MR effect size for
'Gut microbiota abundance (genus Catenibacterium id.2153)' on 'Clusterin'

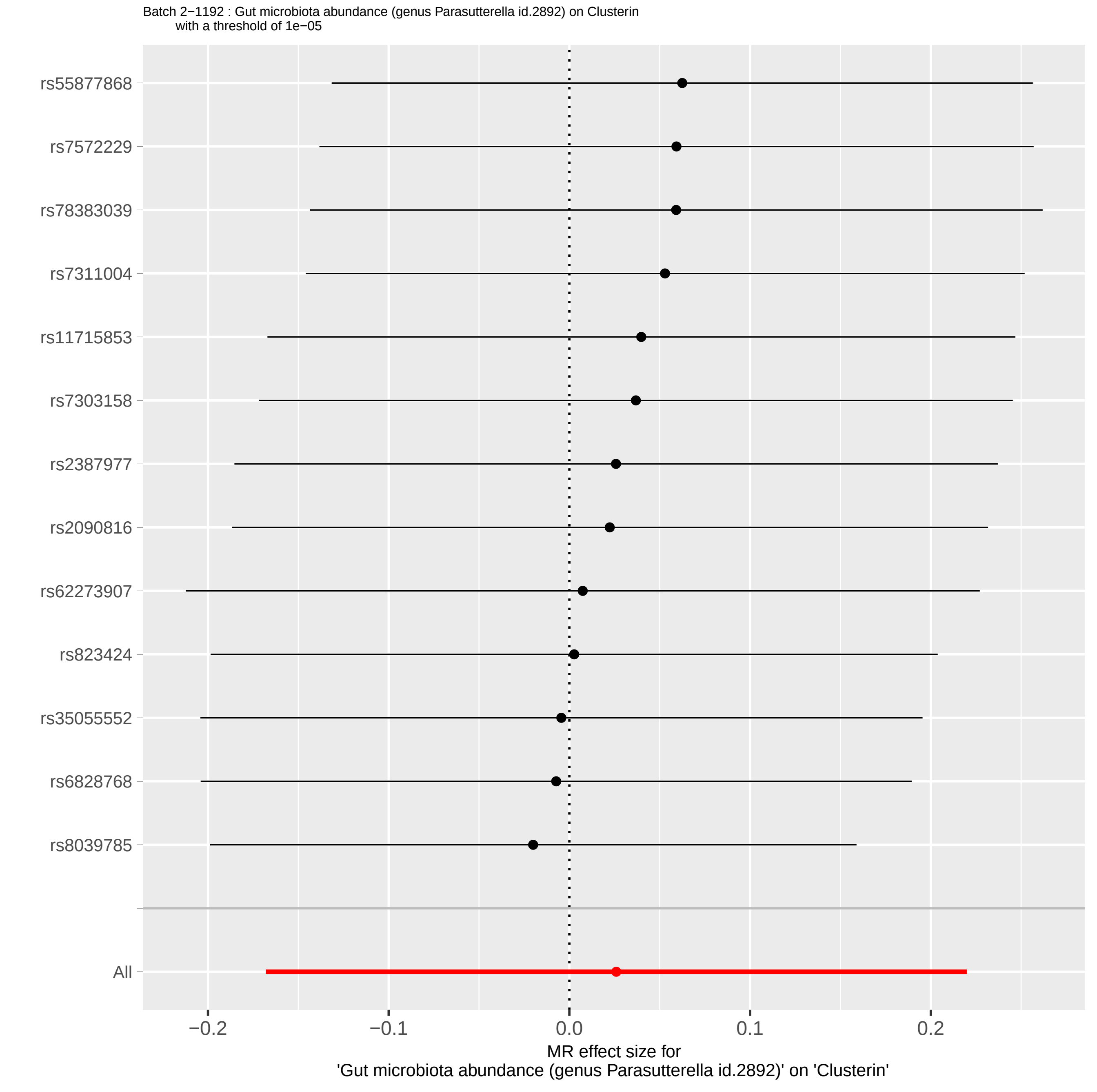
Batch 2−1192 : Gut microbiota abundance (genus Parasutterella id.2892) on Clusterin
with a threshold of 1e−05
rs55877868
rs7572229
rs78383039
rs7311004
rs11715853
rs7303158
rs2387977
rs2090816
rs62273907
rs823424
rs35055552
rs6828768
rs8039785
All
−0.2
−0.1
0.0
0.1
0.2
MR effect size for
'Gut microbiota abundance (genus Parasutterella id.2892)' on 'Clusterin'

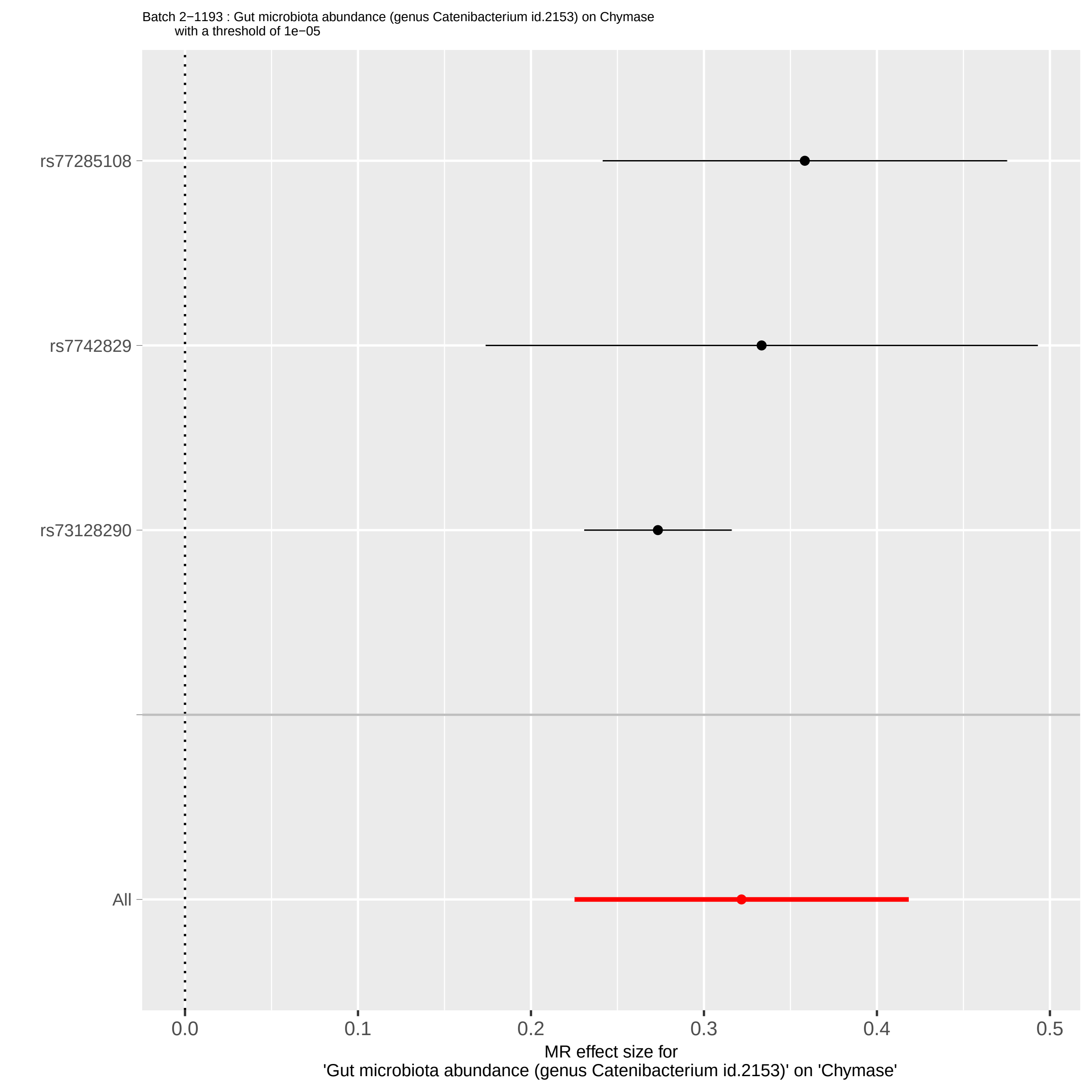
Batch 2−1193 : Gut microbiota abundance (genus Catenibacterium id.2153) on Chymase
with a threshold of 1e−05
rs77285108
rs7742829
rs73128290
All
0.0
0.1
0.2
0.3
0.4
0.5
MR effect size for
'Gut microbiota abundance (genus Catenibacterium id.2153)' on 'Chymase'

Batch 2−1194 : Gut microbiota abundance (genus Parasutterella id.2892) on Chymase
with a threshold of 1e−05

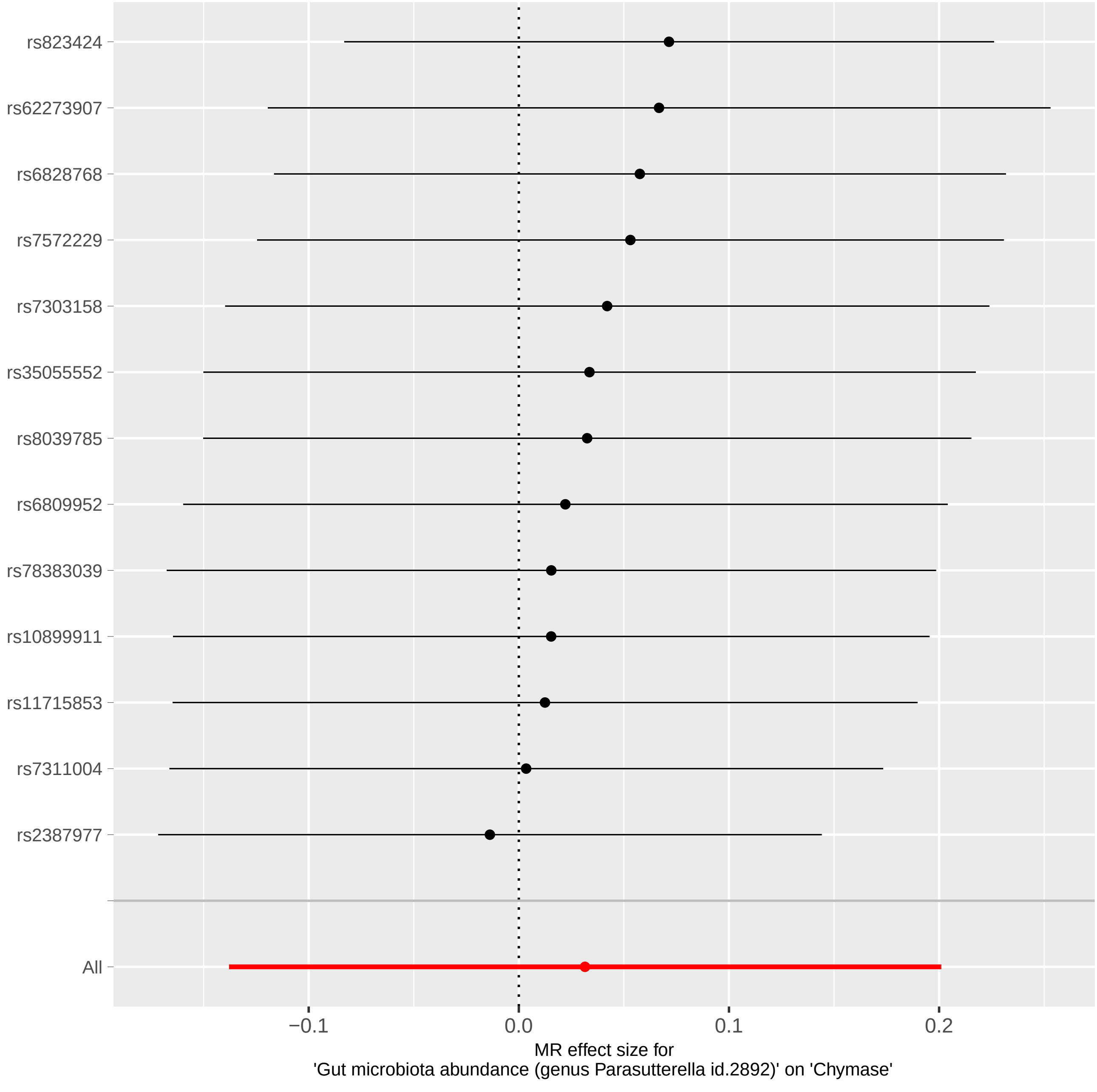

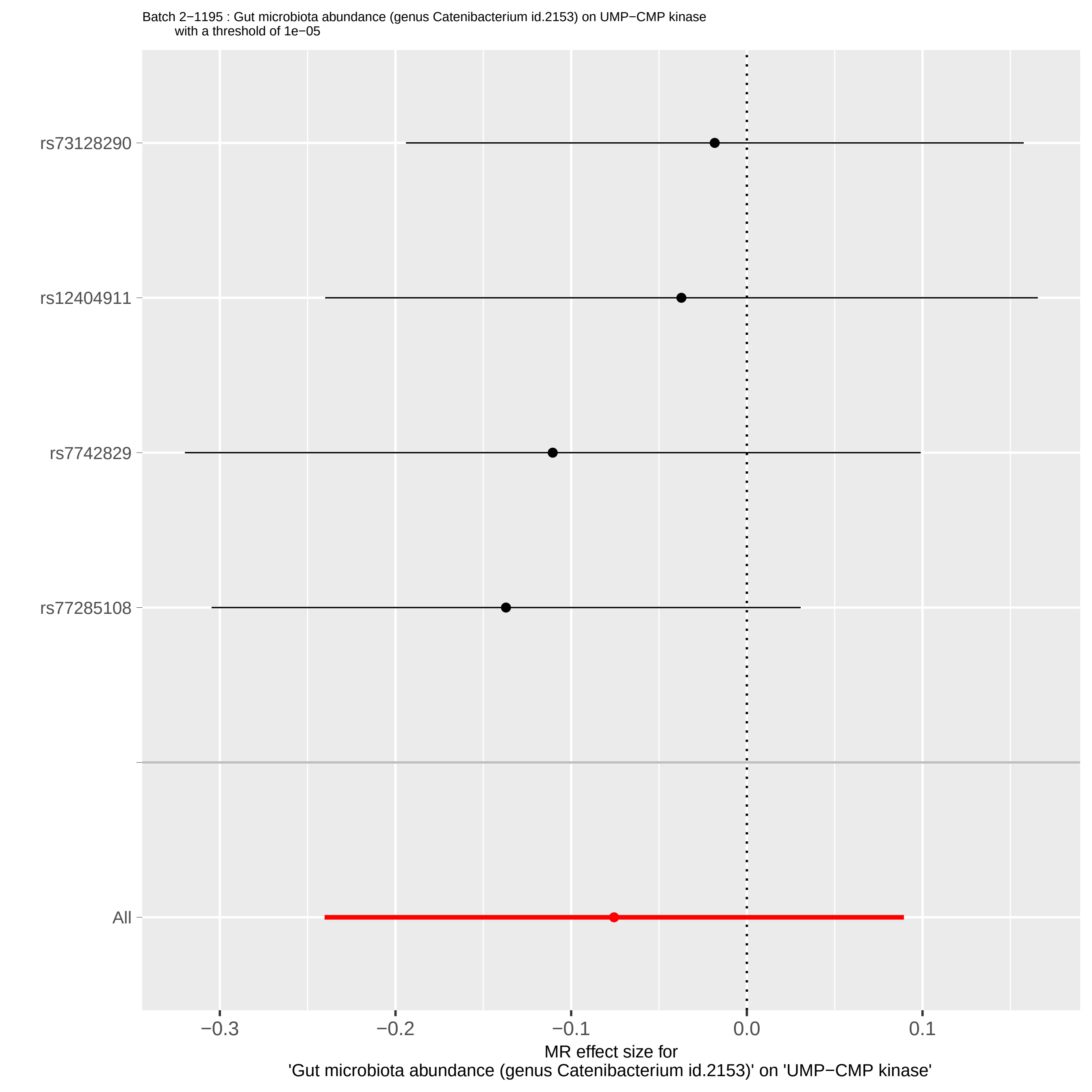

Batch 2−1195 : Gut microbiota abundance (genus Catenibacterium id.2153) on UMP−CMP kinase
with a threshold of 1e−05
rs73128290
rs12404911
rs7742829
rs77285108
All
−0.3
−0.2
−0.1
0.0
0.1
MR effect size for
'Gut microbiota abundance (genus Catenibacterium id.2153)' on 'UMP−CMP kinase'

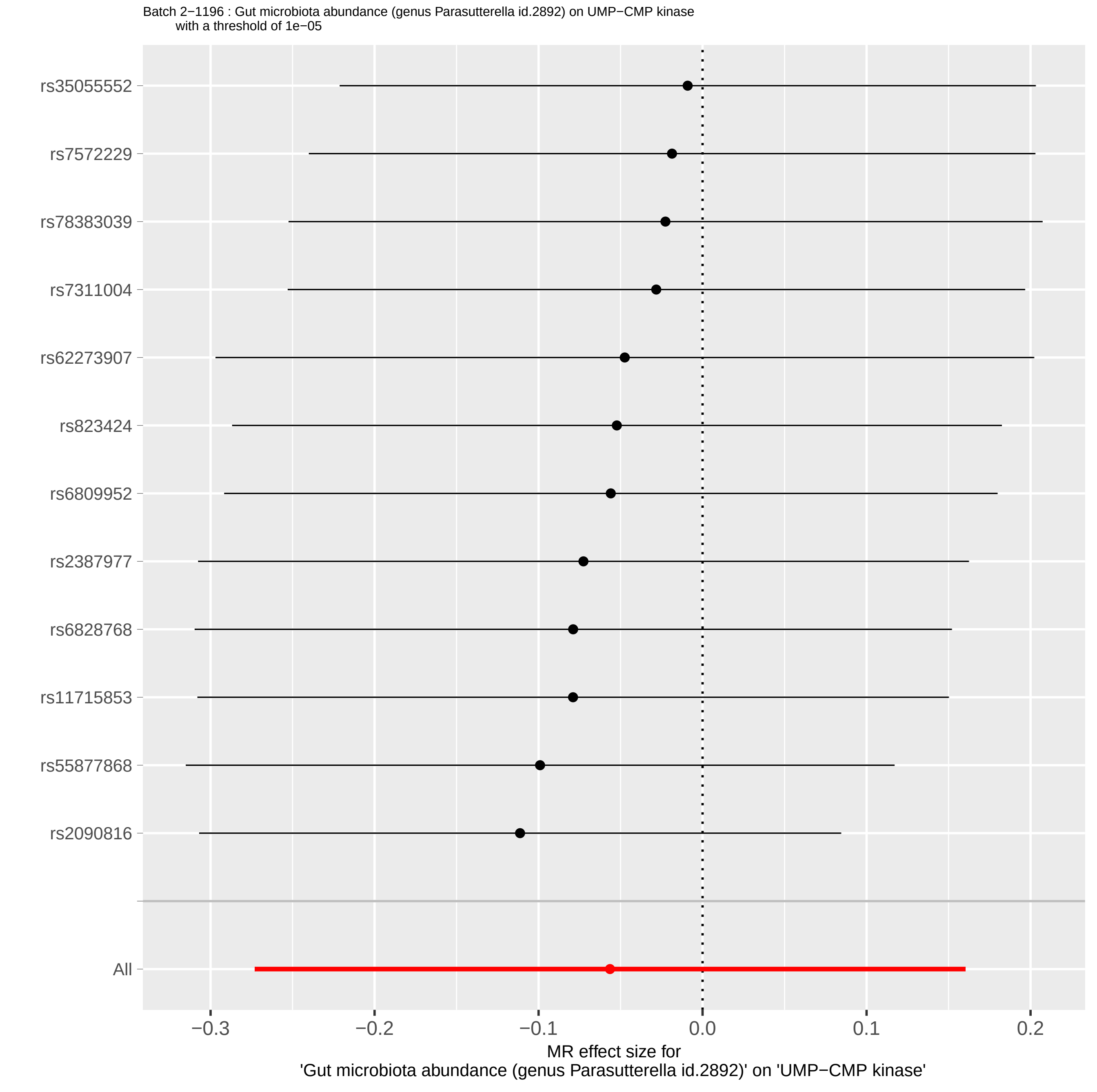

Batch 2−1196 : Gut microbiota abundance (genus Parasutterella id.2892) on UMP−CMP kinase
with a threshold of 1e−05
rs35055552
rs7572229
rs78383039
rs7311004
rs62273907
rs823424
rs6809952
rs2387977
rs6828768
rs11715853
rs55877868
rs2090816
All
−0.3
−0.2
−0.1
0.0
0.1
0.2
MR effect size for
'Gut microbiota abundance (genus Parasutterella id.2892)' on 'UMP−CMP kinase'

Batch 2−1197 : Gut microbiota abundance (genus Catenibacterium id.2153) on UMP−CMP kinase
with a threshold of 1e−05

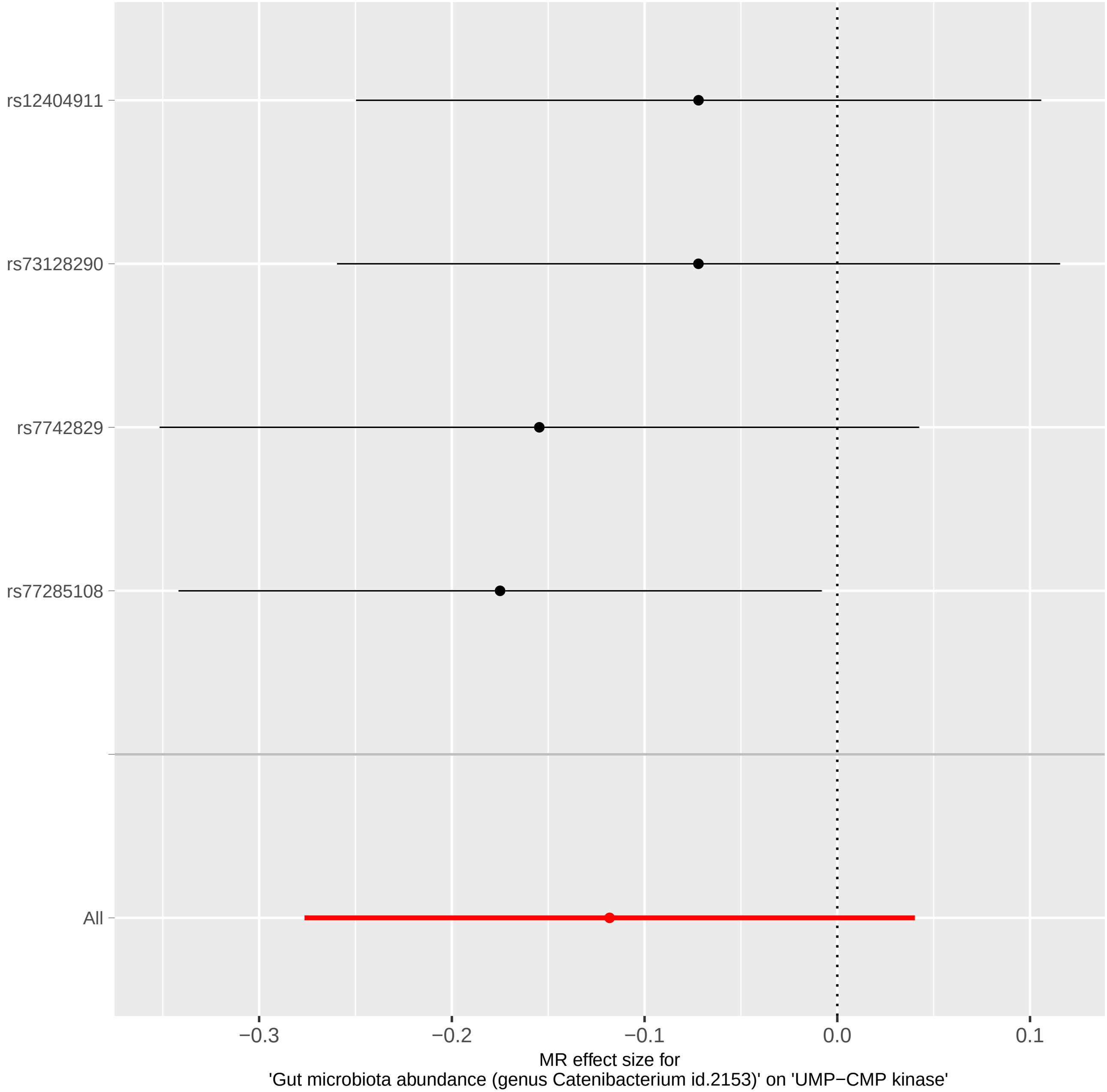

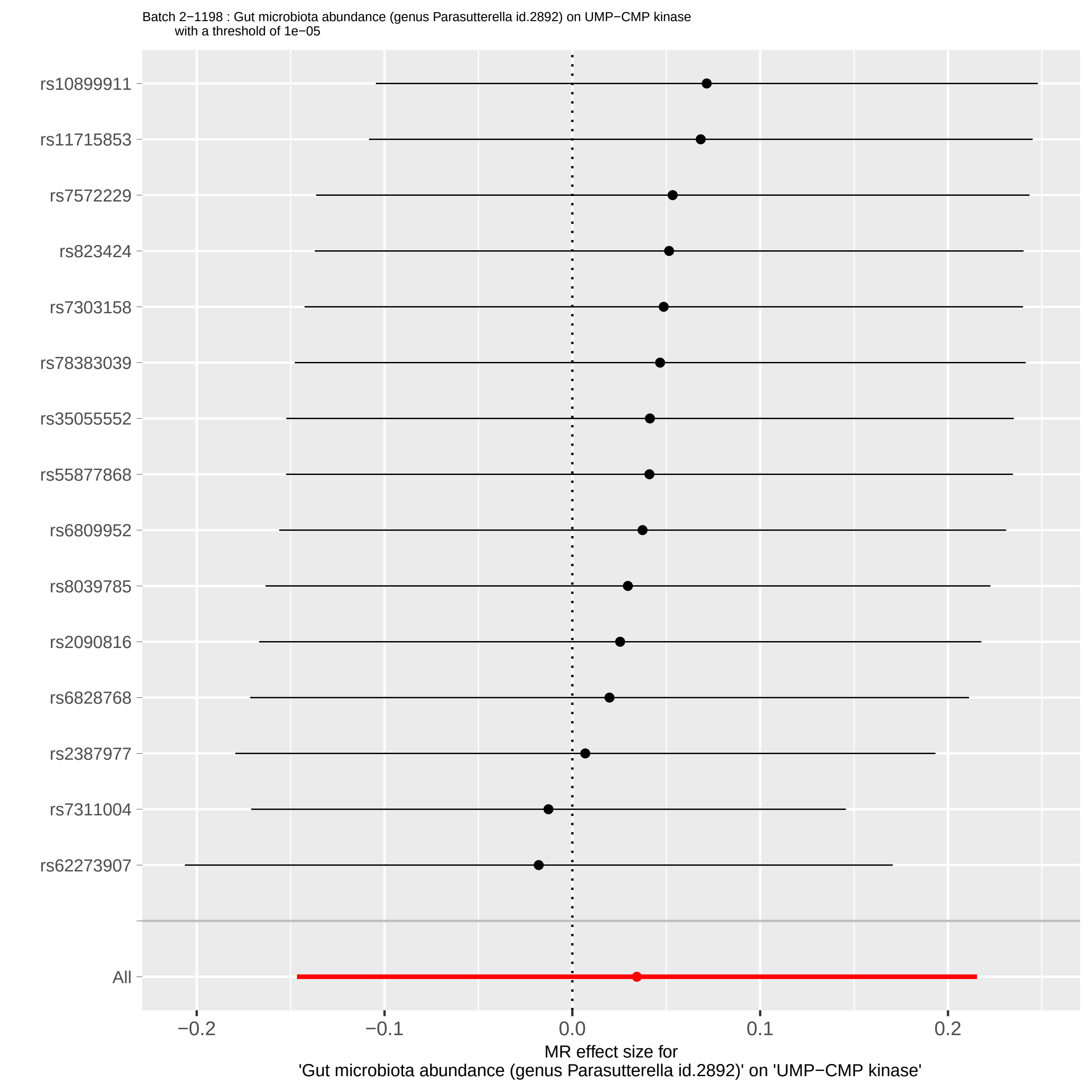
Batch 2−1198 : Gut microbiota abundance (genus Parasutterella id.2892) on UMP−CMP kinase
with a threshold of 1e−05
rs10899911
rs11715853
rs7572229
rs823424
rs7303158
rs78383039
rs35055552
rs55877868
rs6809952
rs8039785
rs2090816
rs6828768
rs2387977
rs7311004
rs62273907
All
−0.2
−0.1
0.0
0.1
0.2
MR effect size for
'Gut microbiota abundance (genus Parasutterella id.2892)' on 'UMP−CMP kinase'

Batch 2−1199 : Gut microbiota abundance (genus Catenibacterium id.2153) on Cytosolic non−specific dipeptidase
with a threshold of 1e−05

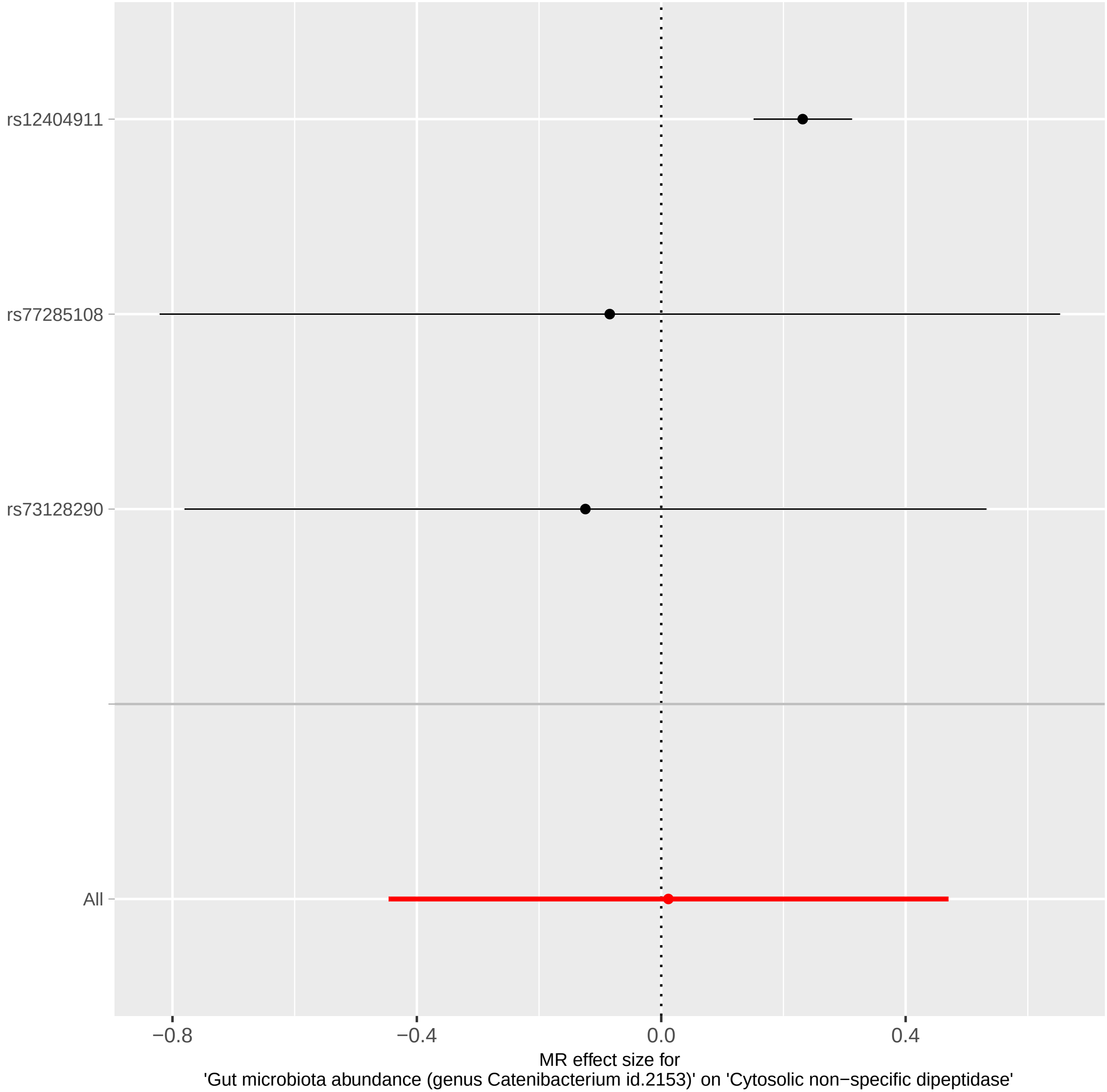

Batch 2−1200 : Gut microbiota abundance (genus Parasutterella id.2892) on Cytosolic non−specific dipeptidase
with a threshold of 1e−05

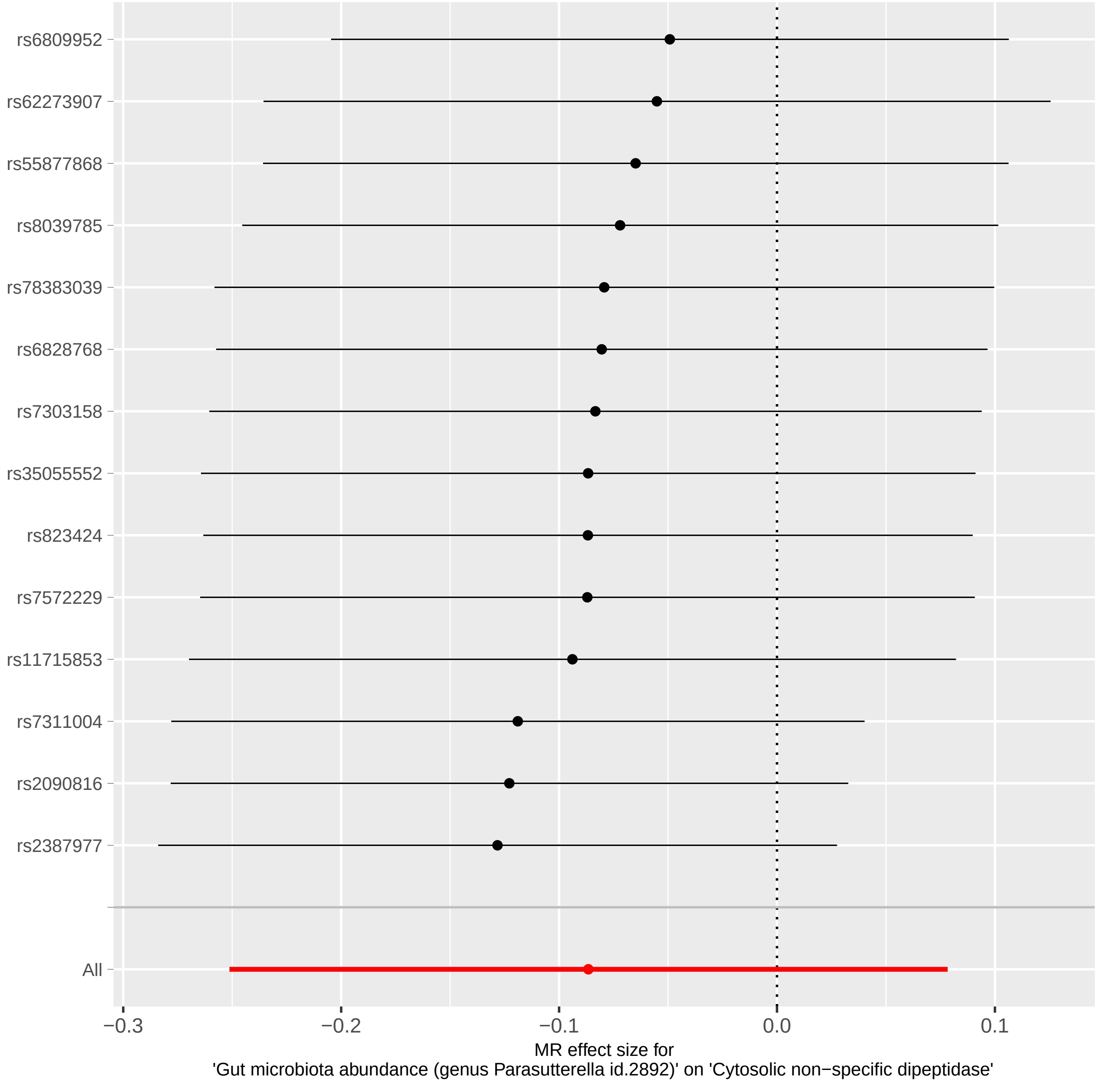

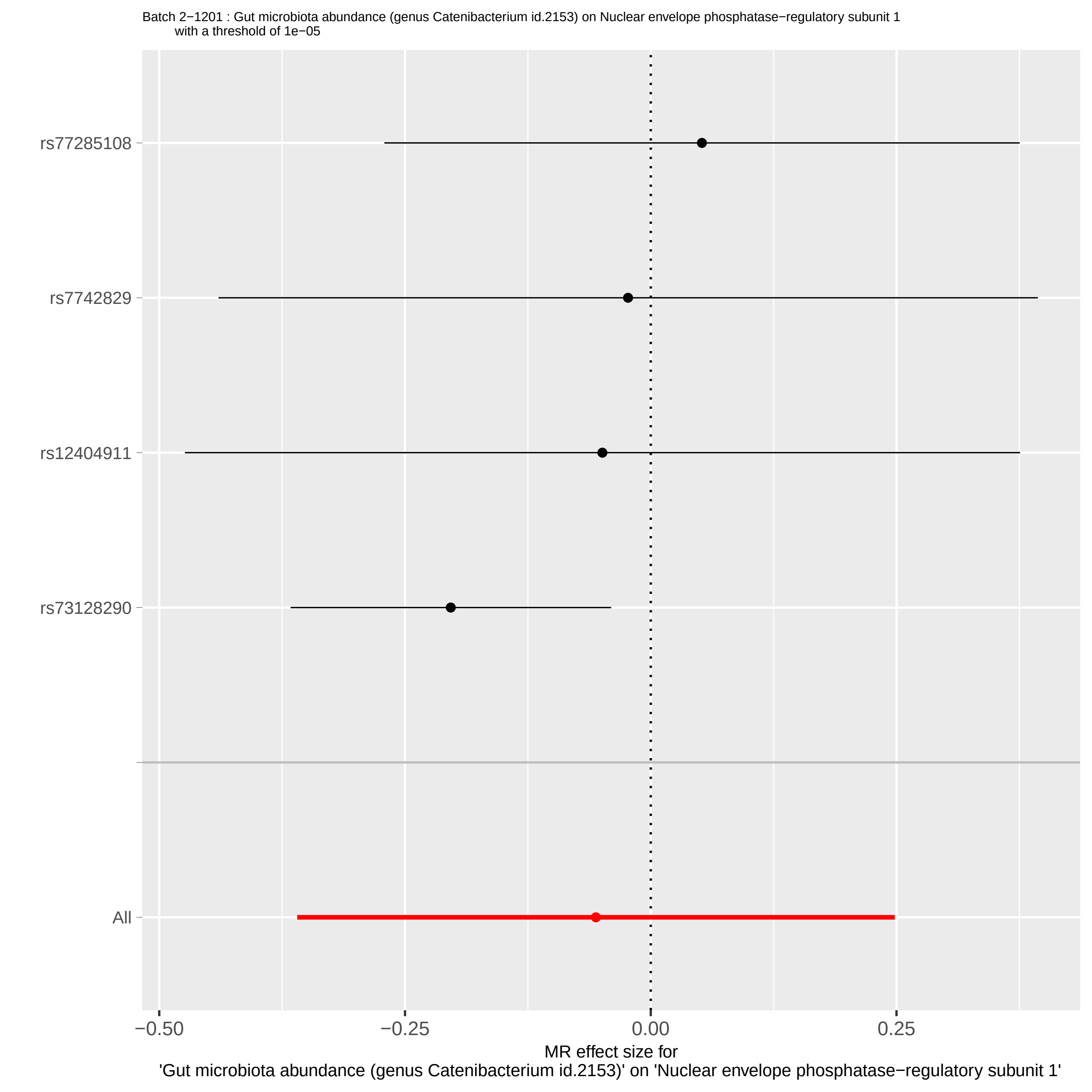
Batch 2−1201 : Gut microbiota abundance (genus Catenibacterium id.2153) on Nuclear envelope phosphatase−regulatory subunit 1
with a threshold of 1e−05
rs77285108
rs7742829
rs12404911
rs73128290
All
−0.50
−0.25
0.00
0.25
MR effect size for
'Gut microbiota abundance (genus Catenibacterium id.2153)' on 'Nuclear envelope phosphatase−regulatory subunit 1'

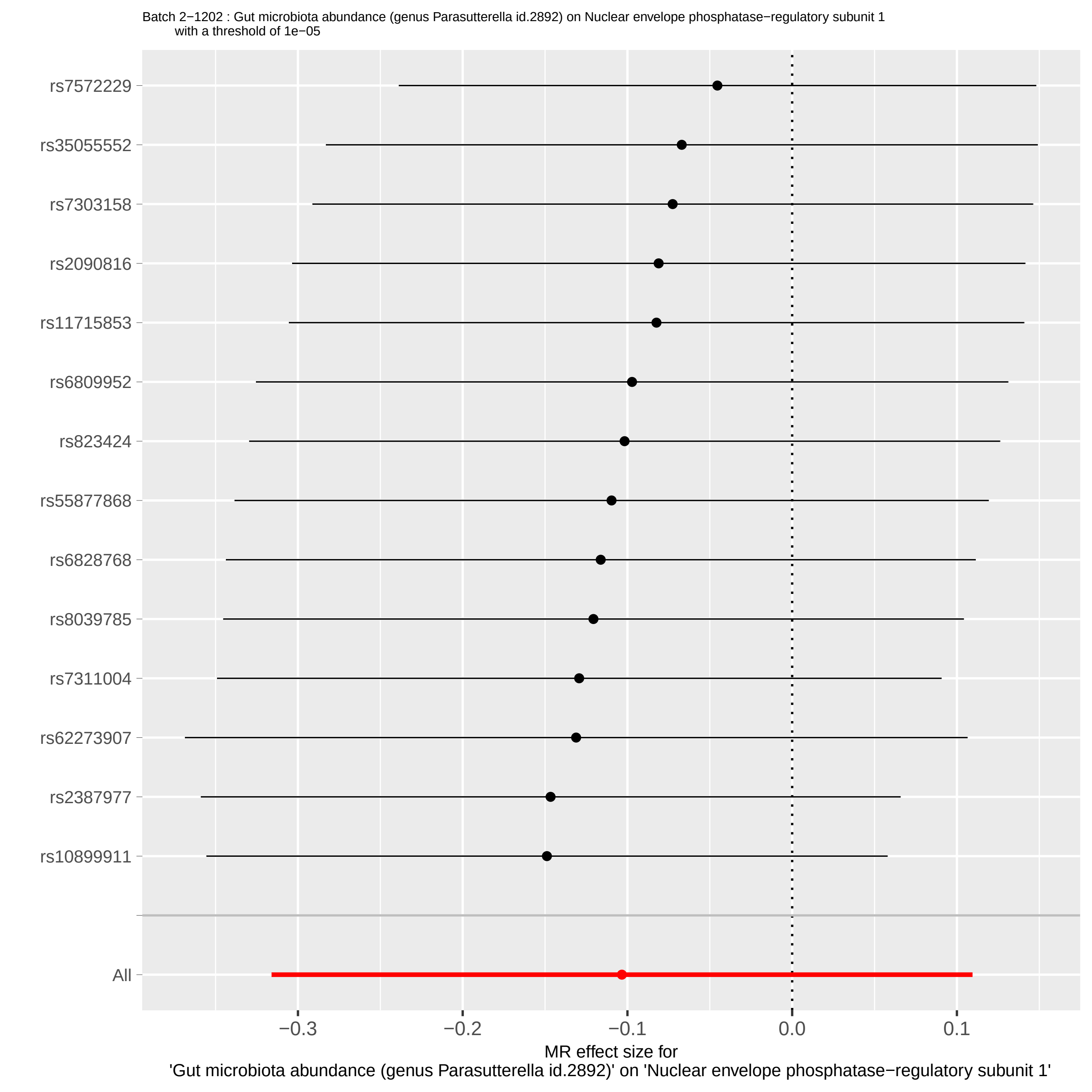
Batch 2−1202 : Gut microbiota abundance (genus Parasutterella id.2892) on Nuclear envelope phosphatase−regulatory subunit 1
with a threshold of 1e−05
rs7572229
rs35055552
rs7303158
rs2090816
rs11715853
rs6809952
rs823424
rs55877868
rs6828768
rs8039785
rs7311004
rs62273907
rs2387977
rs10899911
All
−0.3
−0.2
−0.1
0.0
0.1
MR effect size for
'Gut microbiota abundance (genus Parasutterella id.2892)' on 'Nuclear envelope phosphatase−regulatory subunit 1'

Batch 2−1203 : Gut microbiota abundance (genus Catenibacterium id.2153) on CCR4−NOT transcription complex subunit 1
with a threshold of 1e−05

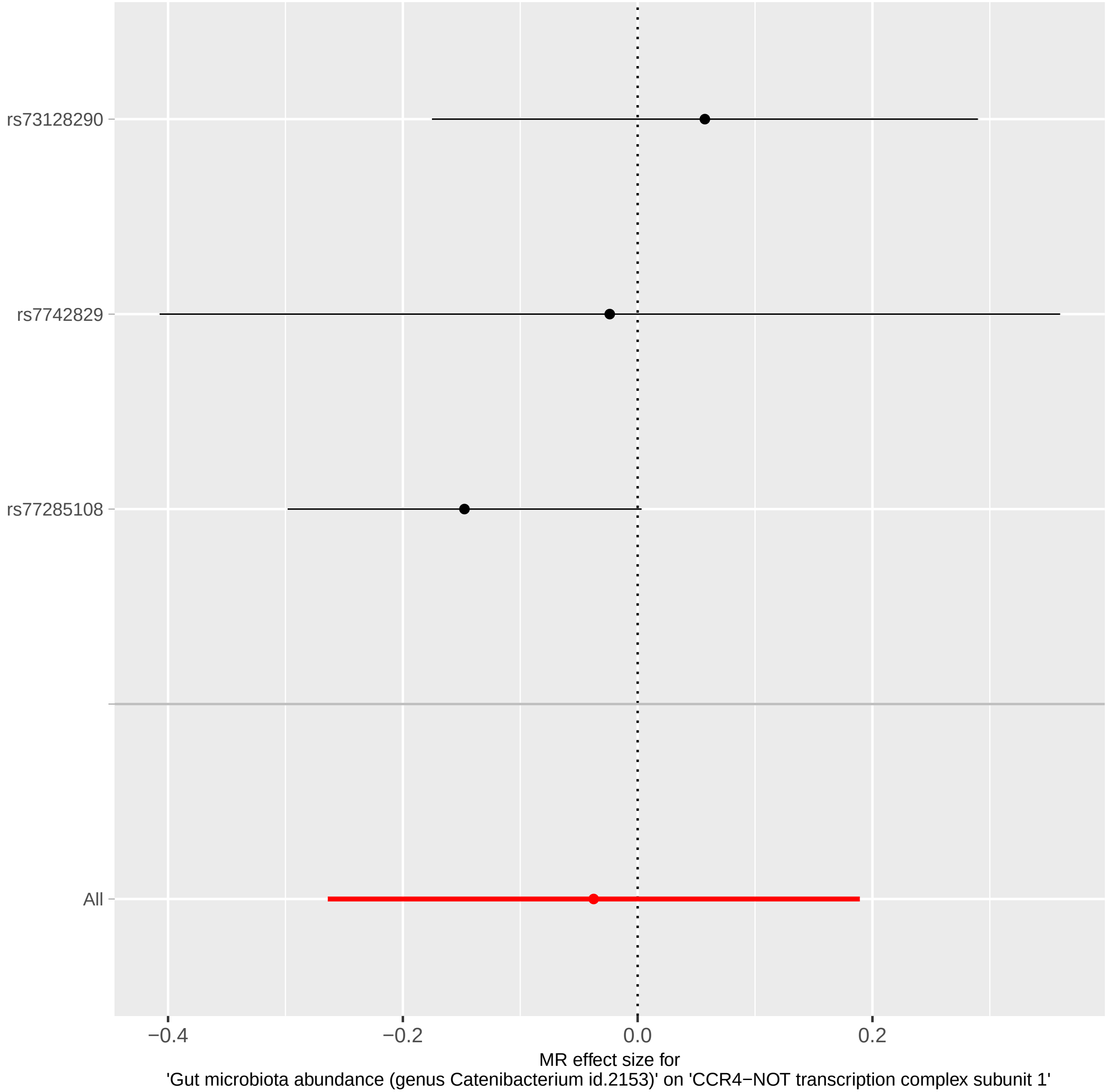

Batch 2−1204 : Gut microbiota abundance (genus Parasutterella id.2892) on CCR4−NOT transcription complex subunit 1
with a threshold of 1e−05

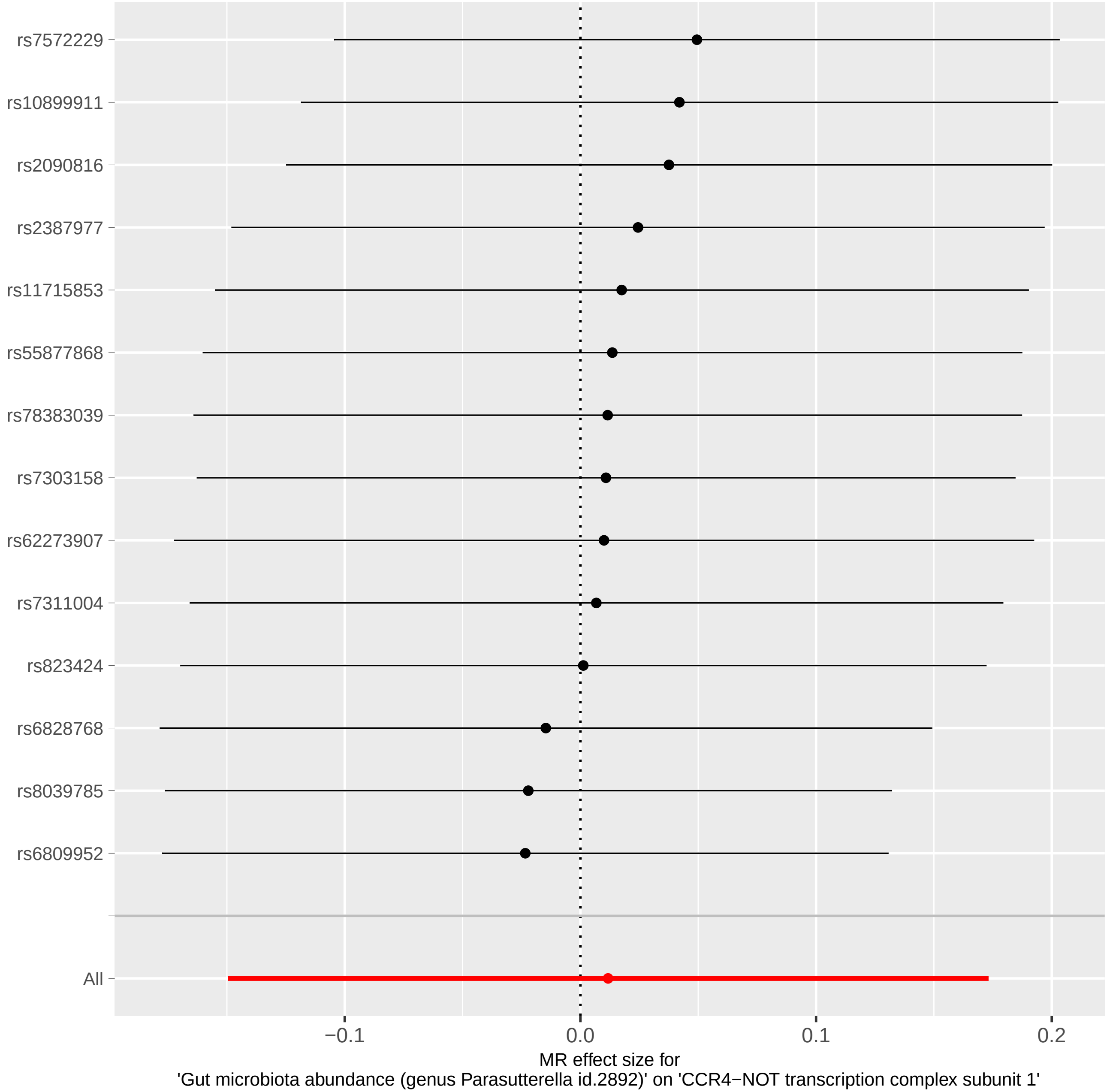

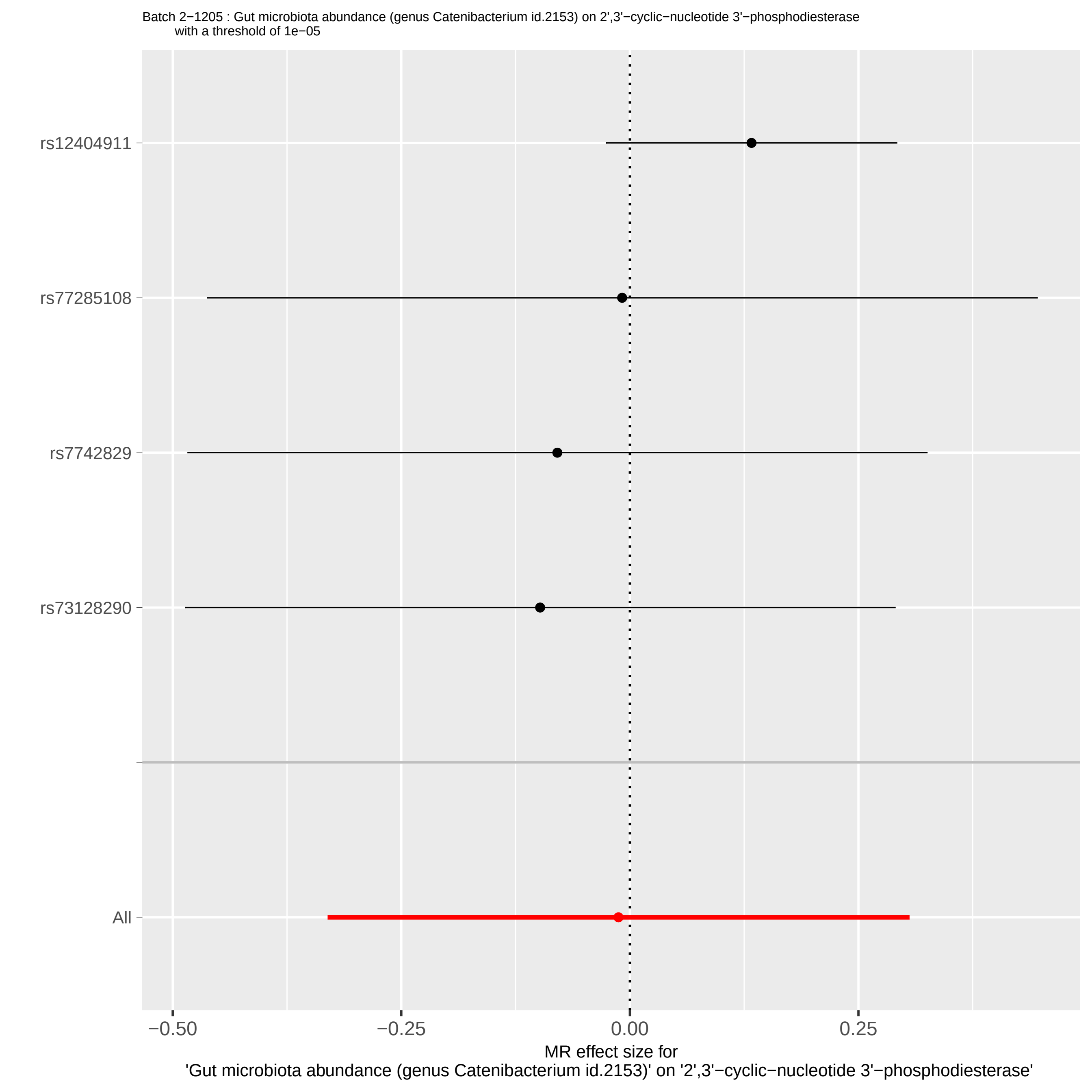
Batch 2−1205 : Gut microbiota abundance (genus Catenibacterium id.2153) on 2',3'−cyclic−nucleotide 3'−phosphodiesterase
with a threshold of 1e−05
rs12404911
rs77285108
rs7742829
rs73128290
All
−0.50
−0.25
0.00
0.25
MR effect size for
'Gut microbiota abundance (genus Catenibacterium id.2153)' on '2',3'−cyclic−nucleotide 3'−phosphodiesterase'

Batch 2−1206 : Gut microbiota abundance (genus Parasutterella id.2892) on 2',3'−cyclic−nucleotide 3'−phosphodiesterase
with a threshold of 1e−05

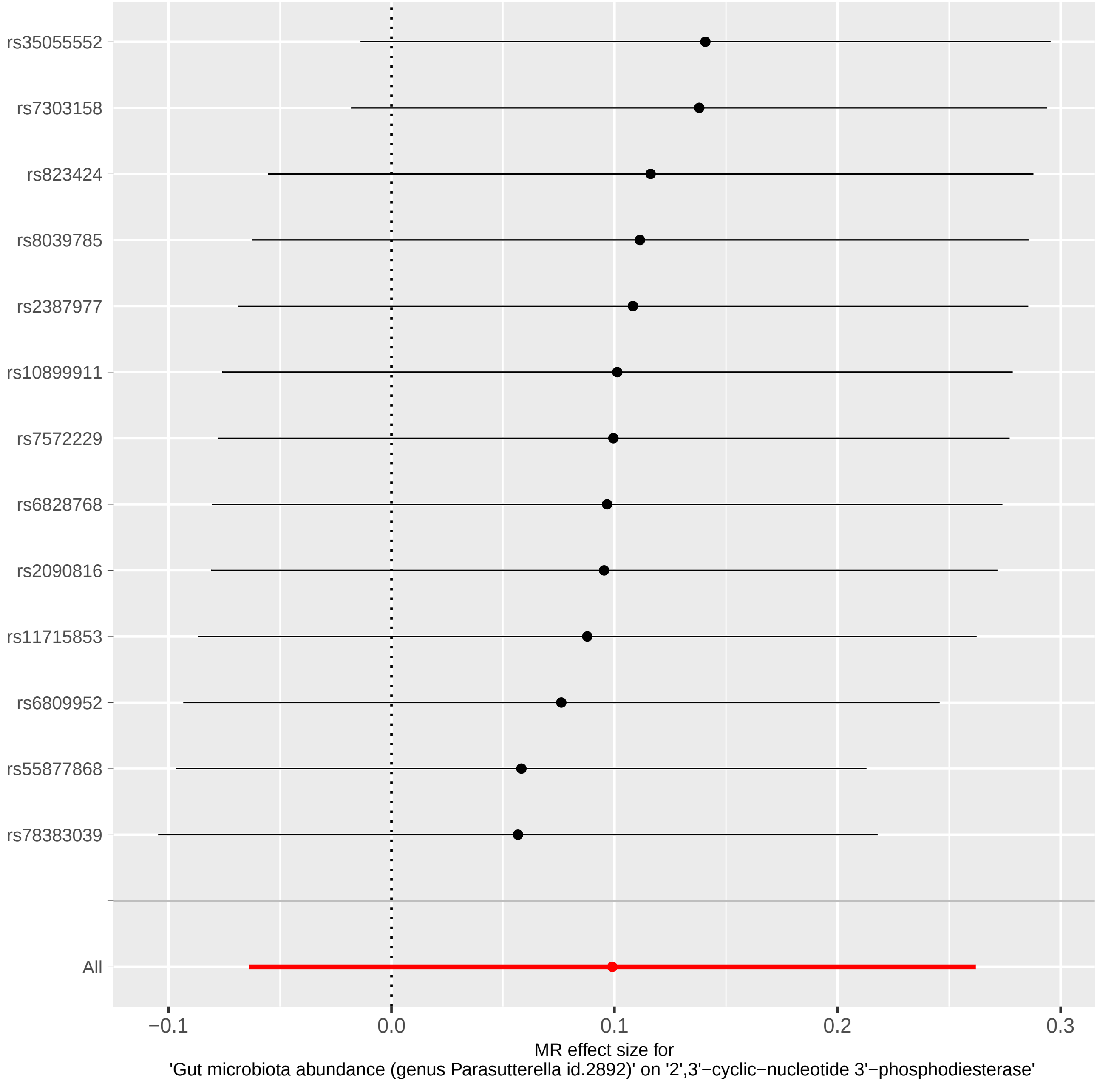

Batch 2−1207 : Gut microbiota abundance (genus Catenibacterium id.2153) on Ciliary neurotrophic factor
with a threshold of 1e−05

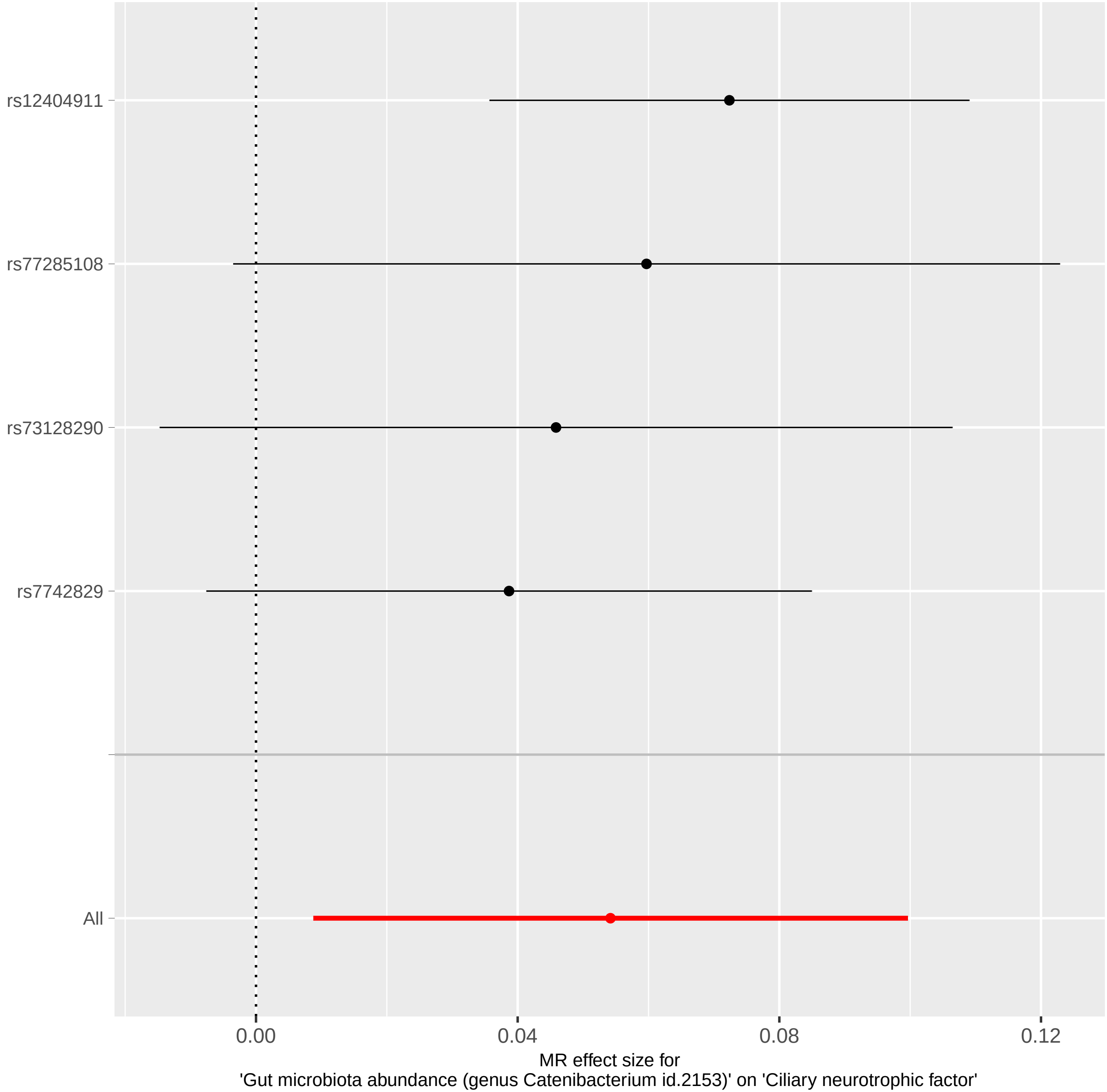

Batch 2−1208 : Gut microbiota abundance (genus Parasutterella id.2892) on Ciliary neurotrophic factor
with a threshold of 1e−05

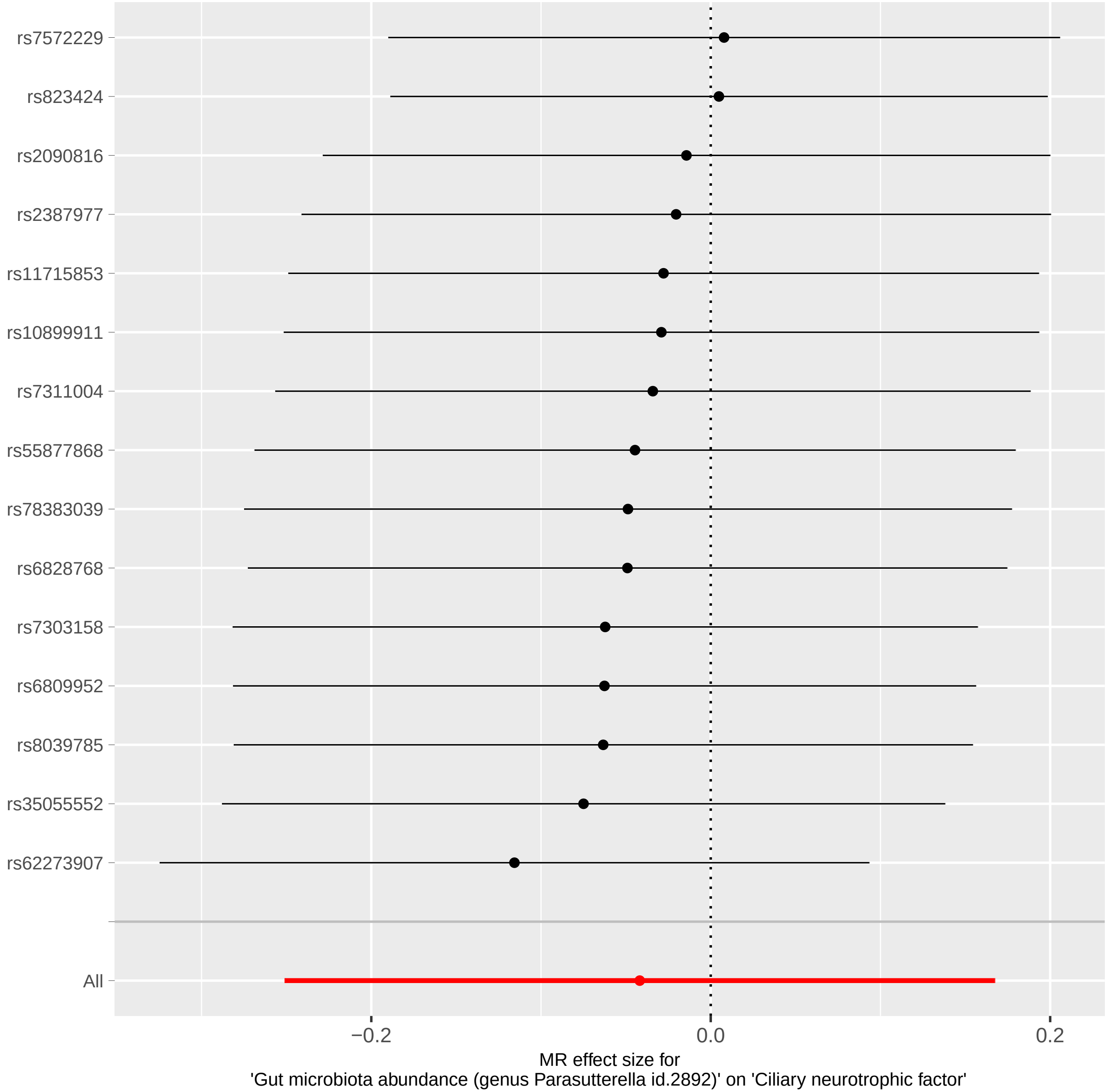

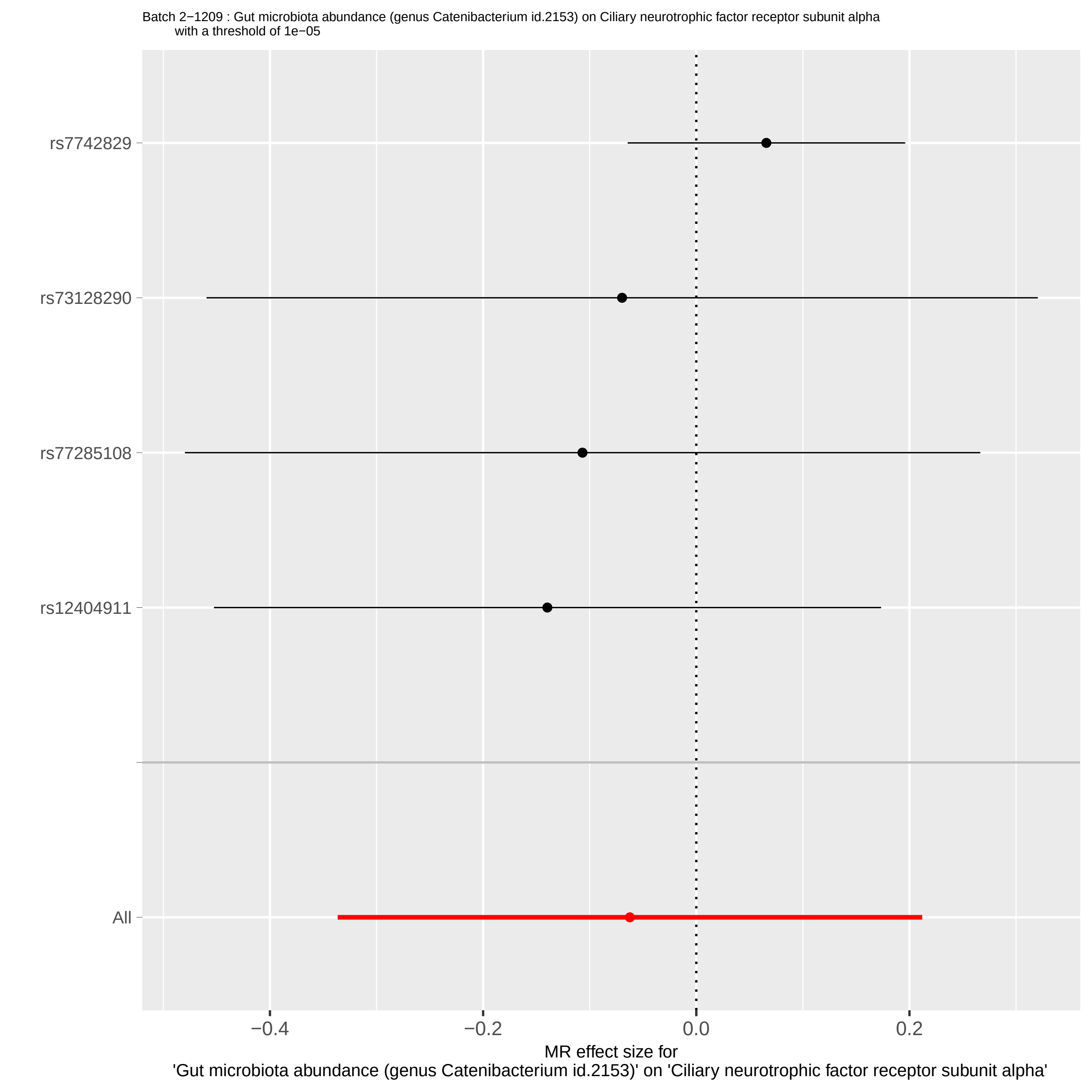
Batch 2−1209 : Gut microbiota abundance (genus Catenibacterium id.2153) on Ciliary neurotrophic factor receptor subunit alpha
with a threshold of 1e−05
rs7742829
rs73128290
rs77285108
rs12404911
All
−0.4
−0.2
0.0
0.2
MR effect size for
'Gut microbiota abundance (genus Catenibacterium id.2153)' on 'Ciliary neurotrophic factor receptor subunit alpha'

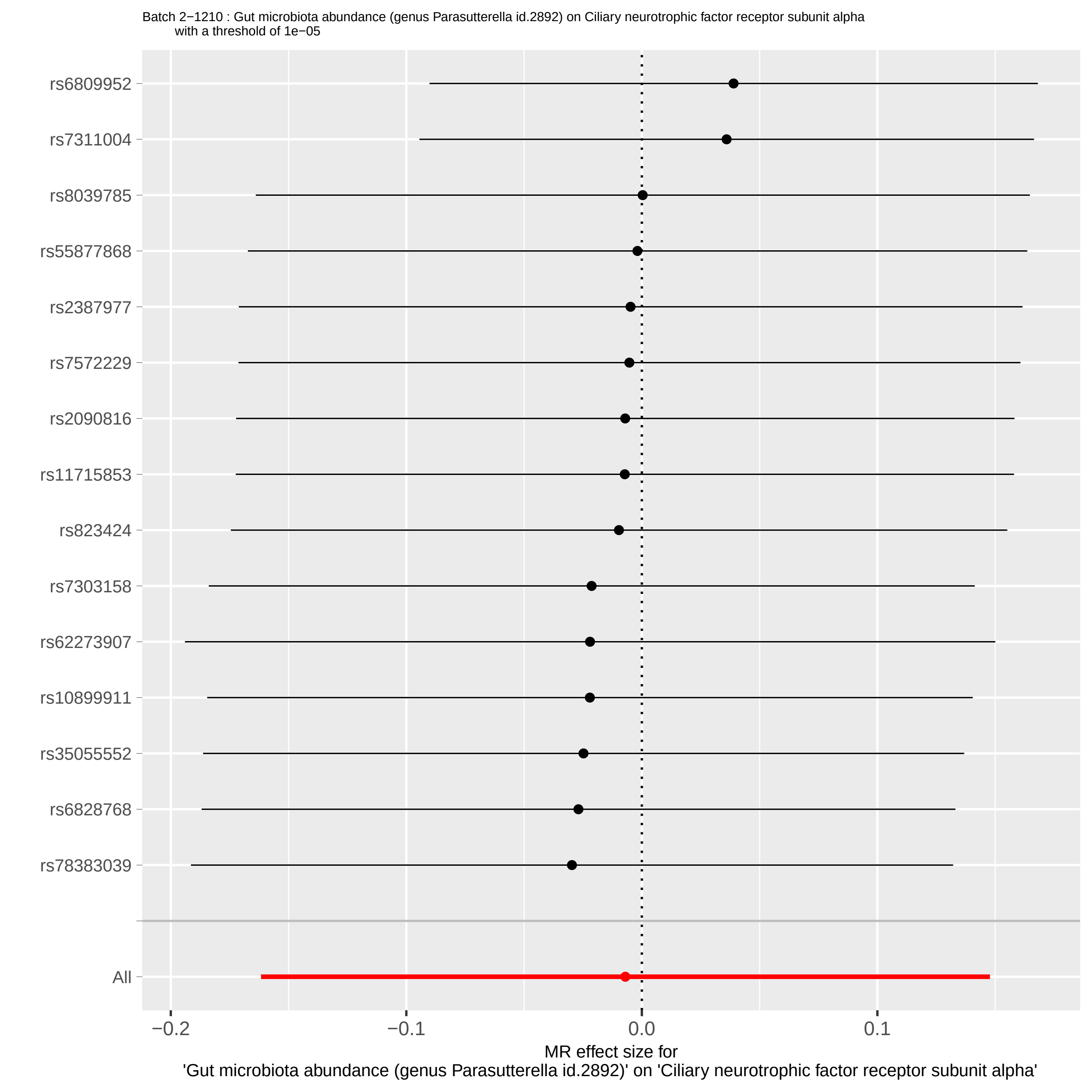
Batch 2−1210 : Gut microbiota abundance (genus Parasutterella id.2892) on Ciliary neurotrophic factor receptor subunit alpha
with a threshold of 1e−05
rs6809952
rs7311004
rs8039785
rs55877868
rs2387977
rs7572229
rs2090816
rs11715853
rs823424
rs7303158
rs62273907
rs10899911
rs35055552
rs6828768
rs78383039
All
−0.2
−0.1
0.0
0.1
MR effect size for
'Gut microbiota abundance (genus Parasutterella id.2892)' on 'Ciliary neurotrophic factor receptor subunit alpha'

Batch 2−1211 : Gut microbiota abundance (genus Catenibacterium id.2153) on Ciliary neurotrophic factor receptor subunit alpha with a threshold of 1e−05

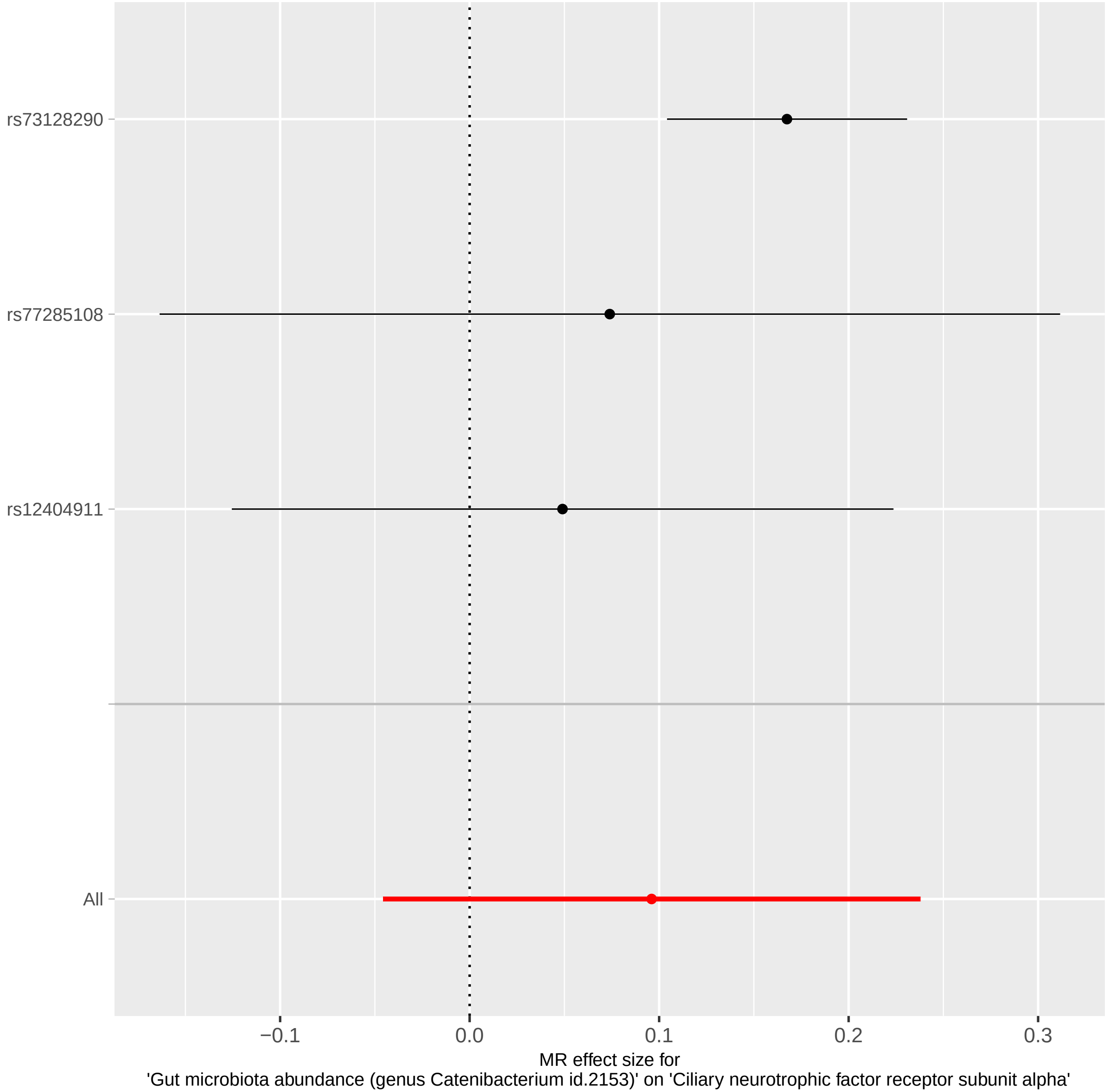

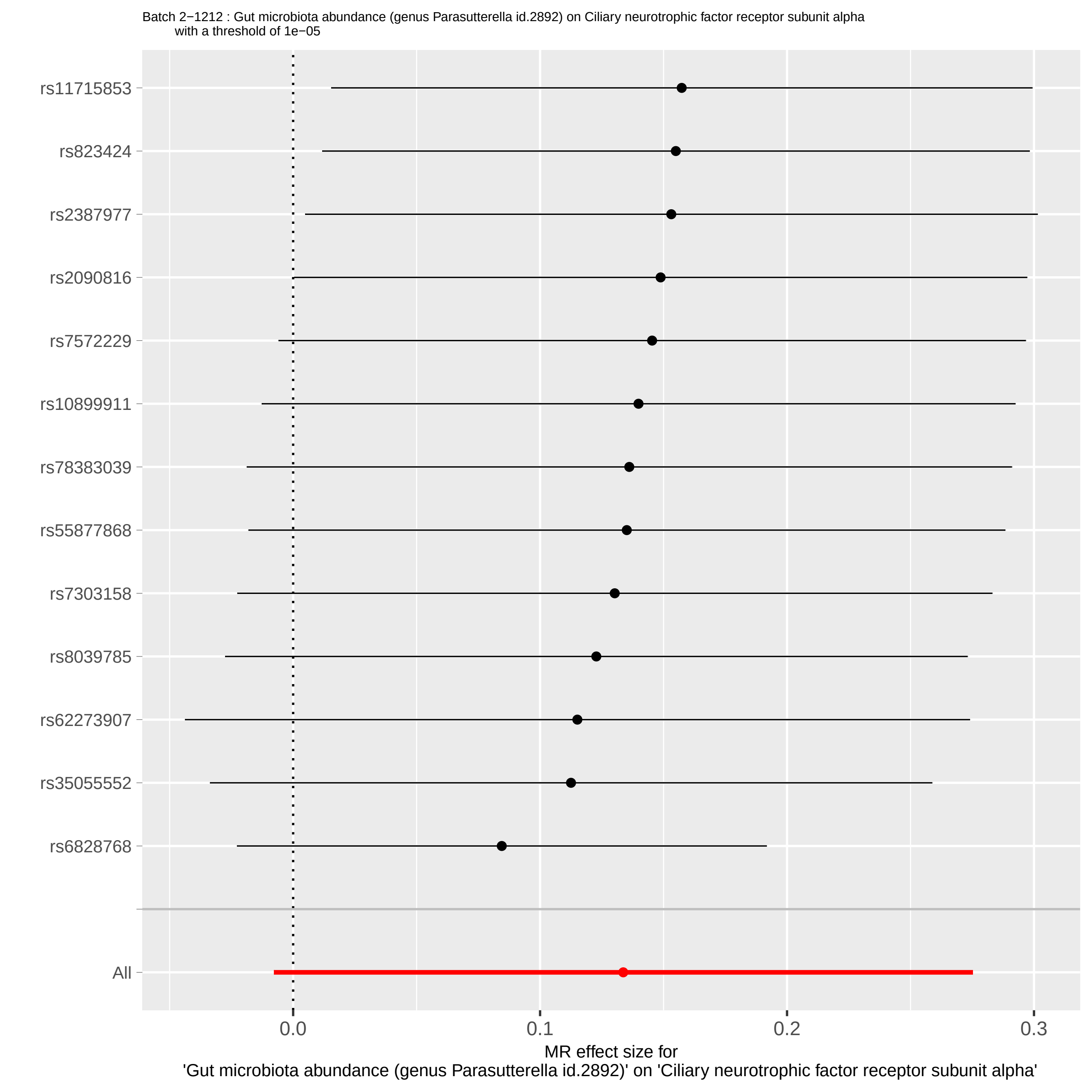
Batch 2−1212 : Gut microbiota abundance (genus Parasutterella id.2892) on Ciliary neurotrophic factor receptor subunit alpha
with a threshold of 1e−05
rs11715853
rs823424
rs2387977
rs2090816
rs7572229
rs10899911
rs78383039
rs55877868
rs7303158
rs8039785
rs62273907
rs35055552
rs6828768
All
0.0
0.1
0.2
0.3
MR effect size for
'Gut microbiota abundance (genus Parasutterella id.2892)' on 'Ciliary neurotrophic factor receptor subunit alpha'

Batch 2−1213 : Gut microbiota abundance (genus Catenibacterium id.2153) on Contactin−2
with a threshold of 1e−05

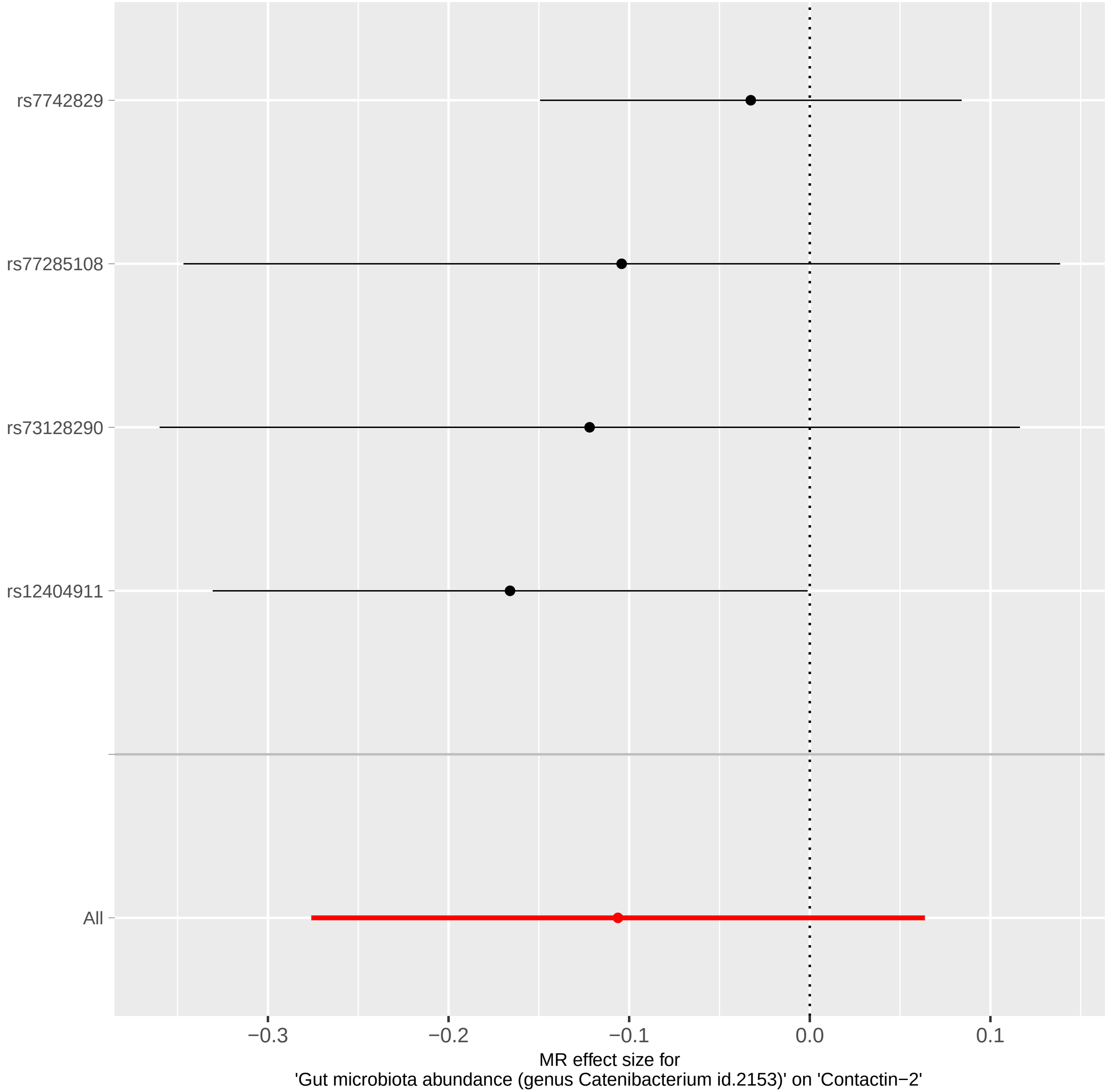

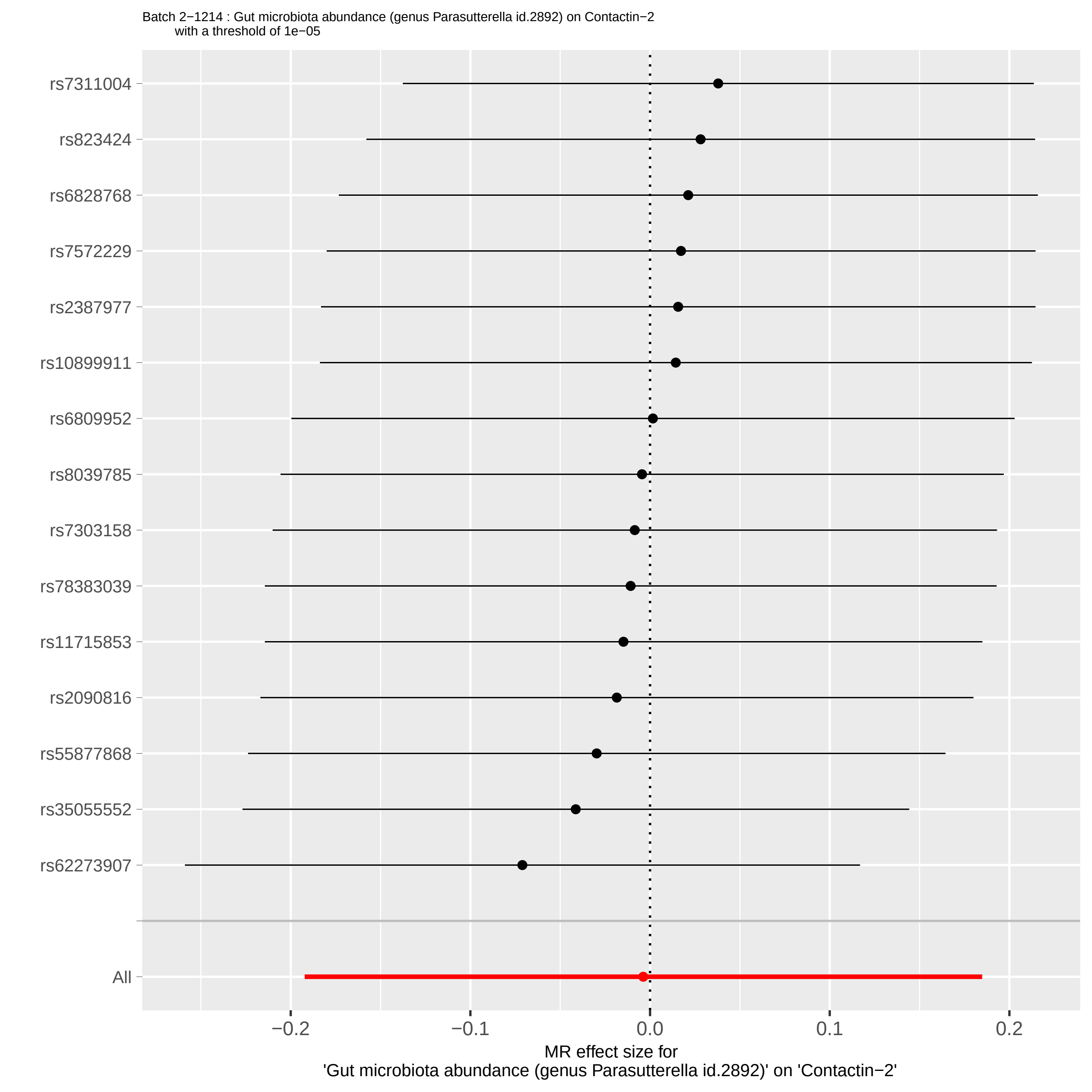
Batch 2−1214 : Gut microbiota abundance (genus Parasutterella id.2892) on Contactin−2
with a threshold of 1e−05
rs7311004
rs823424
rs6828768
rs7572229
rs2387977
rs10899911
rs6809952
rs8039785
rs7303158
rs78383039
rs11715853
rs2090816
rs55877868
rs35055552
rs62273907
All
−0.2
−0.1
0.0
0.1
0.2
MR effect size for
'Gut microbiota abundance (genus Parasutterella id.2892)' on 'Contactin−2'

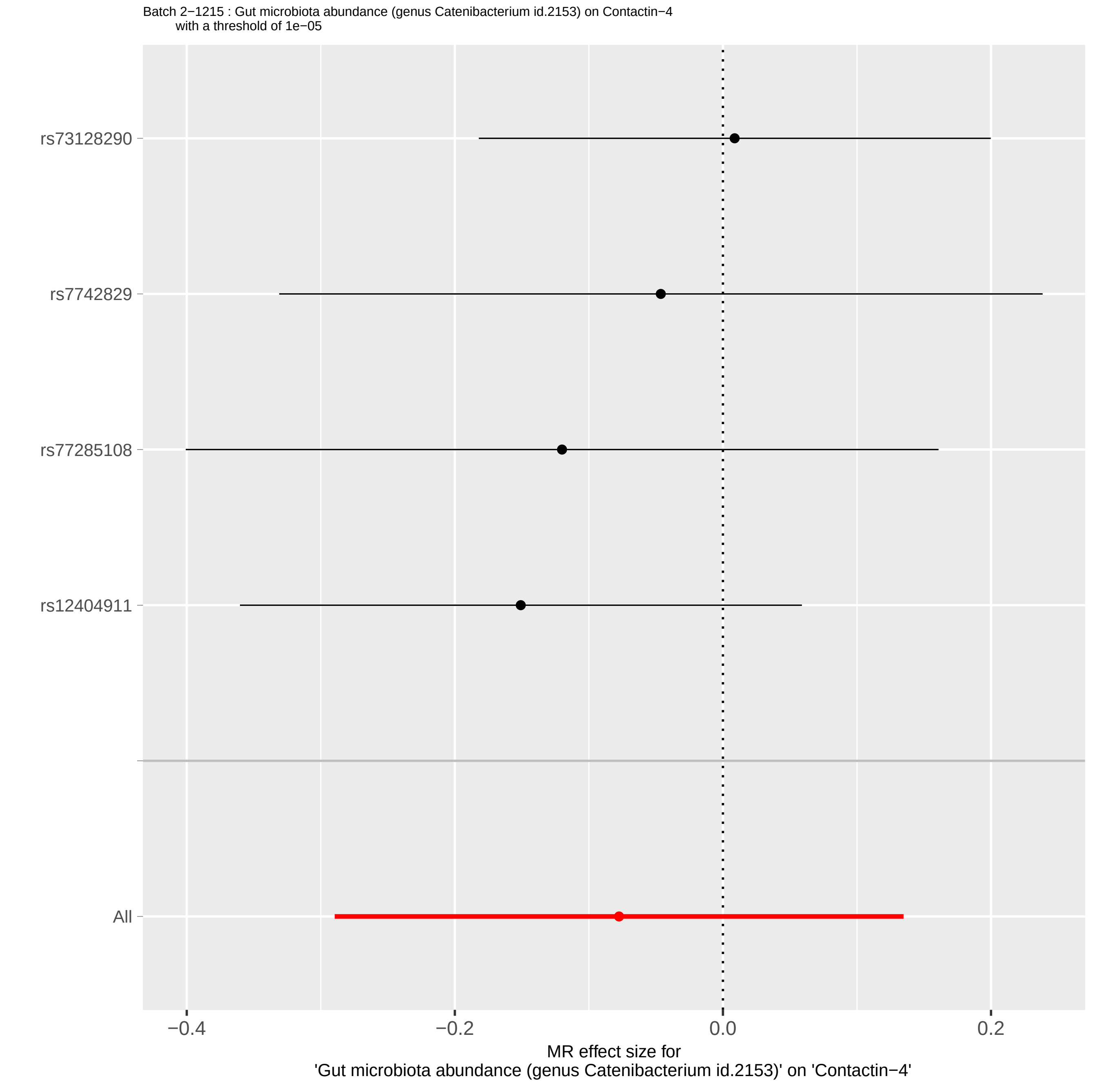

Batch 2−1215 : Gut microbiota abundance (genus Catenibacterium id.2153) on Contactin−4
with a threshold of 1e−05
rs73128290
rs7742829
rs77285108
rs12404911
All
−0.4
−0.2
0.0
0.2
MR effect size for
'Gut microbiota abundance (genus Catenibacterium id.2153)' on 'Contactin−4'

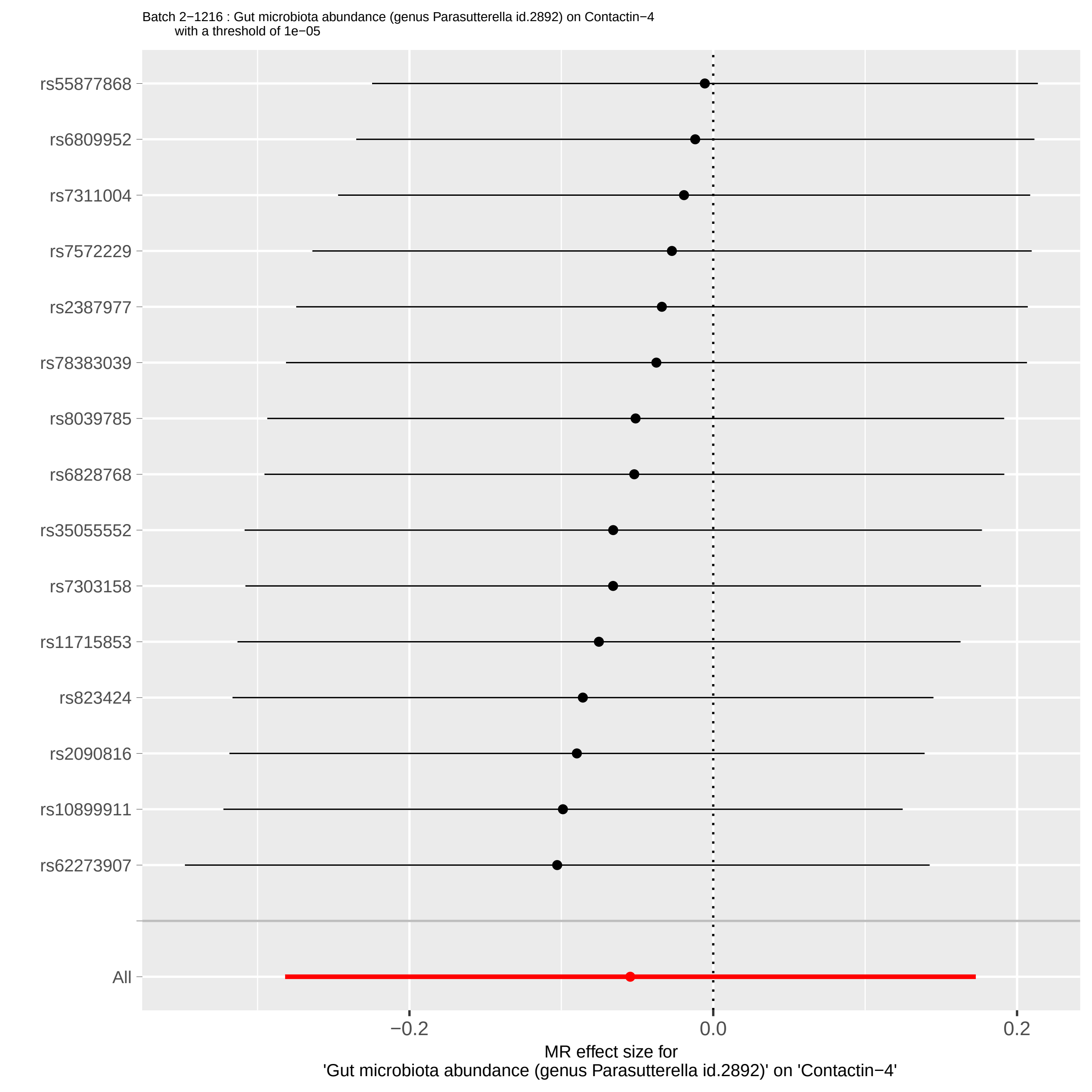
Batch 2−1216 : Gut microbiota abundance (genus Parasutterella id.2892) on Contactin−4
with a threshold of 1e−05
rs55877868
rs6809952
rs7311004
rs7572229
rs2387977
rs78383039
rs8039785
rs6828768
rs35055552
rs7303158
rs11715853
rs823424
rs2090816
rs10899911
rs62273907
All
−0.2
0.0
0.2
MR effect size for
'Gut microbiota abundance (genus Parasutterella id.2892)' on 'Contactin−4'

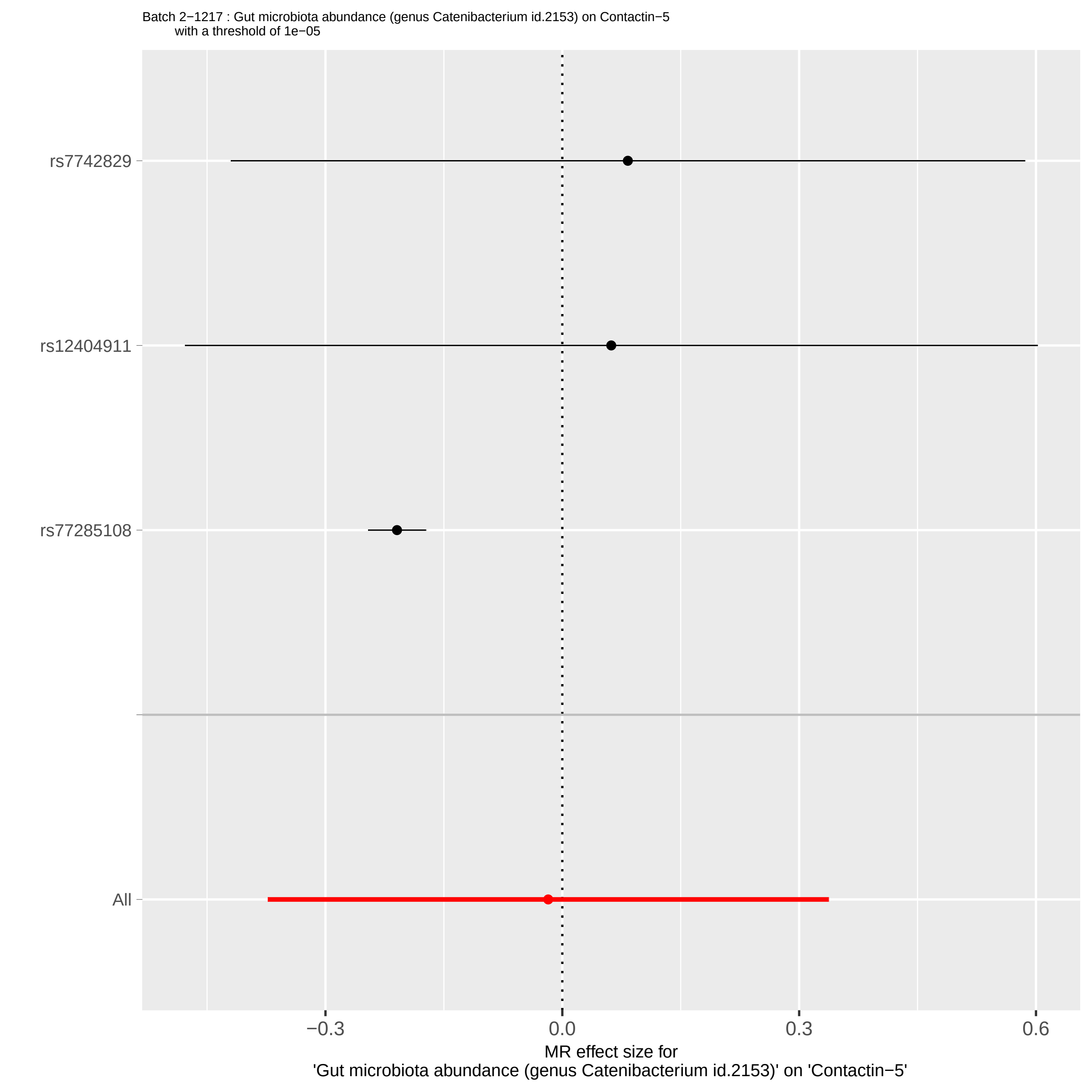
Batch 2−1217 : Gut microbiota abundance (genus Catenibacterium id.2153) on Contactin−5
with a threshold of 1e−05
rs7742829
rs12404911
rs77285108
All
−0.3
0.0
0.3
0.6
MR effect size for
'Gut microbiota abundance (genus Catenibacterium id.2153)' on 'Contactin−5'

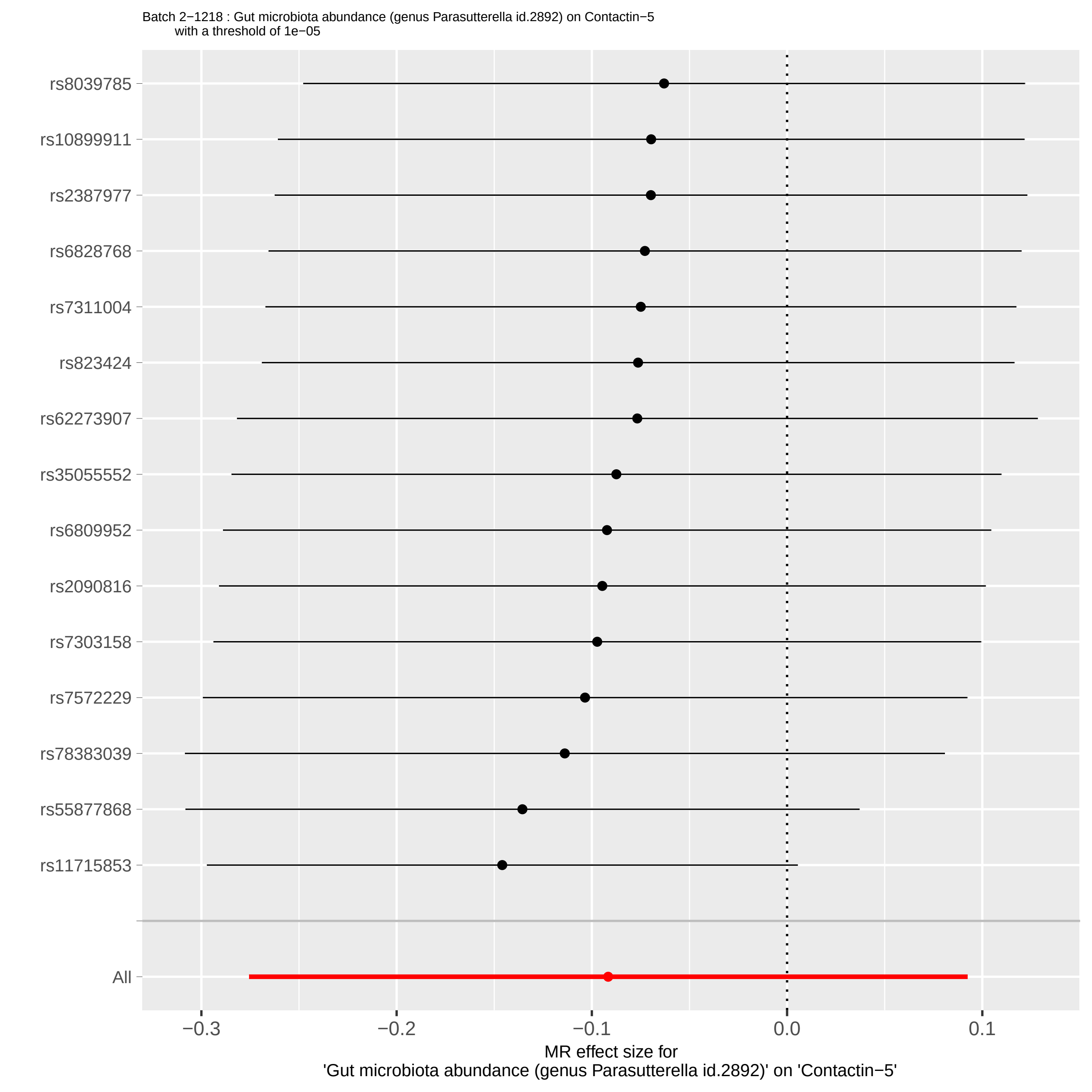
Batch 2−1218 : Gut microbiota abundance (genus Parasutterella id.2892) on Contactin−5
with a threshold of 1e−05
rs8039785
rs10899911
rs2387977
rs6828768
rs7311004
rs823424
rs62273907
rs35055552
rs6809952
rs2090816
rs7303158
rs7572229
rs78383039
rs55877868
rs11715853
All
−0.3
−0.2
−0.1
0.0
0.1
MR effect size for
'Gut microbiota abundance (genus Parasutterella id.2892)' on 'Contactin−5'

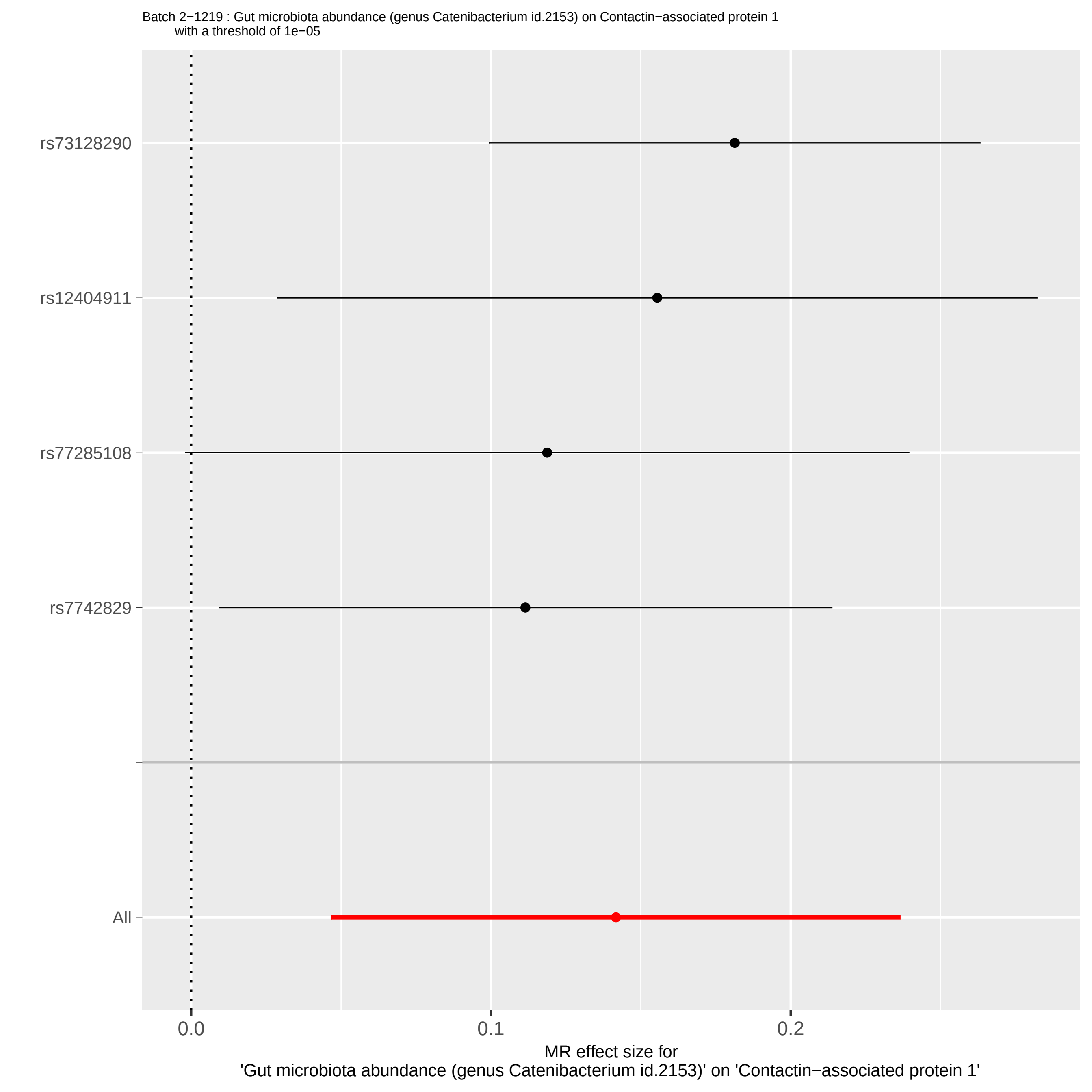
Batch 2−1219 : Gut microbiota abundance (genus Catenibacterium id.2153) on Contactin−associated protein 1
with a threshold of 1e−05
rs73128290
rs12404911
rs77285108
rs7742829
All
0.0
0.1
0.2
MR effect size for
'Gut microbiota abundance (genus Catenibacterium id.2153)' on 'Contactin−associated protein 1'

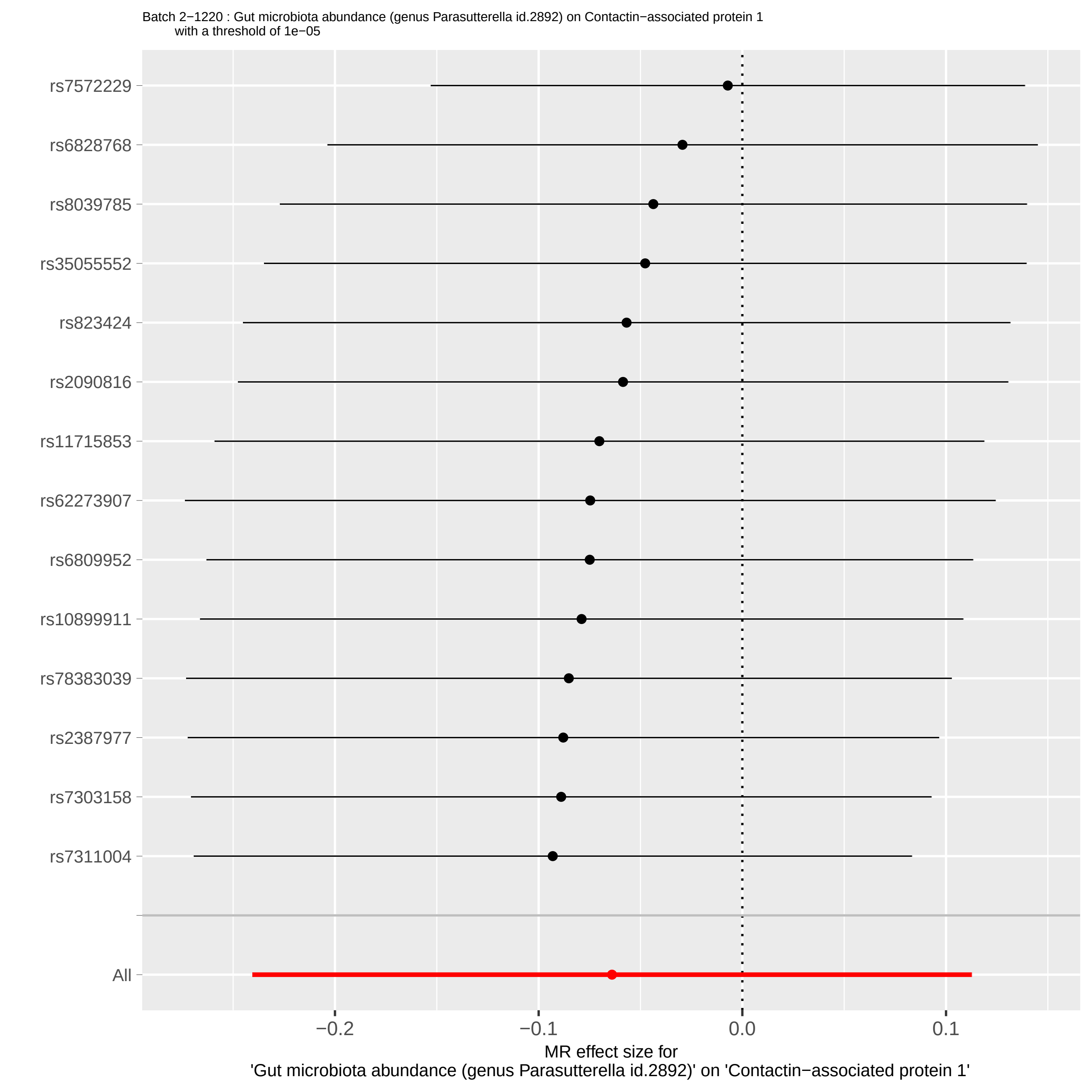

Batch 2−1220 : Gut microbiota abundance (genus Parasutterella id.2892) on Contactin−associated protein 1
with a threshold of 1e−05
rs7572229
rs6828768
rs8039785
rs35055552
rs823424
rs2090816
rs11715853
rs62273907
rs6809952
rs10899911
rs78383039
rs2387977
rs7303158
rs7311004
All
−0.2
−0.1
0.0
0.1
MR effect size for
'Gut microbiota abundance (genus Parasutterella id.2892)' on 'Contactin−associated protein 1'

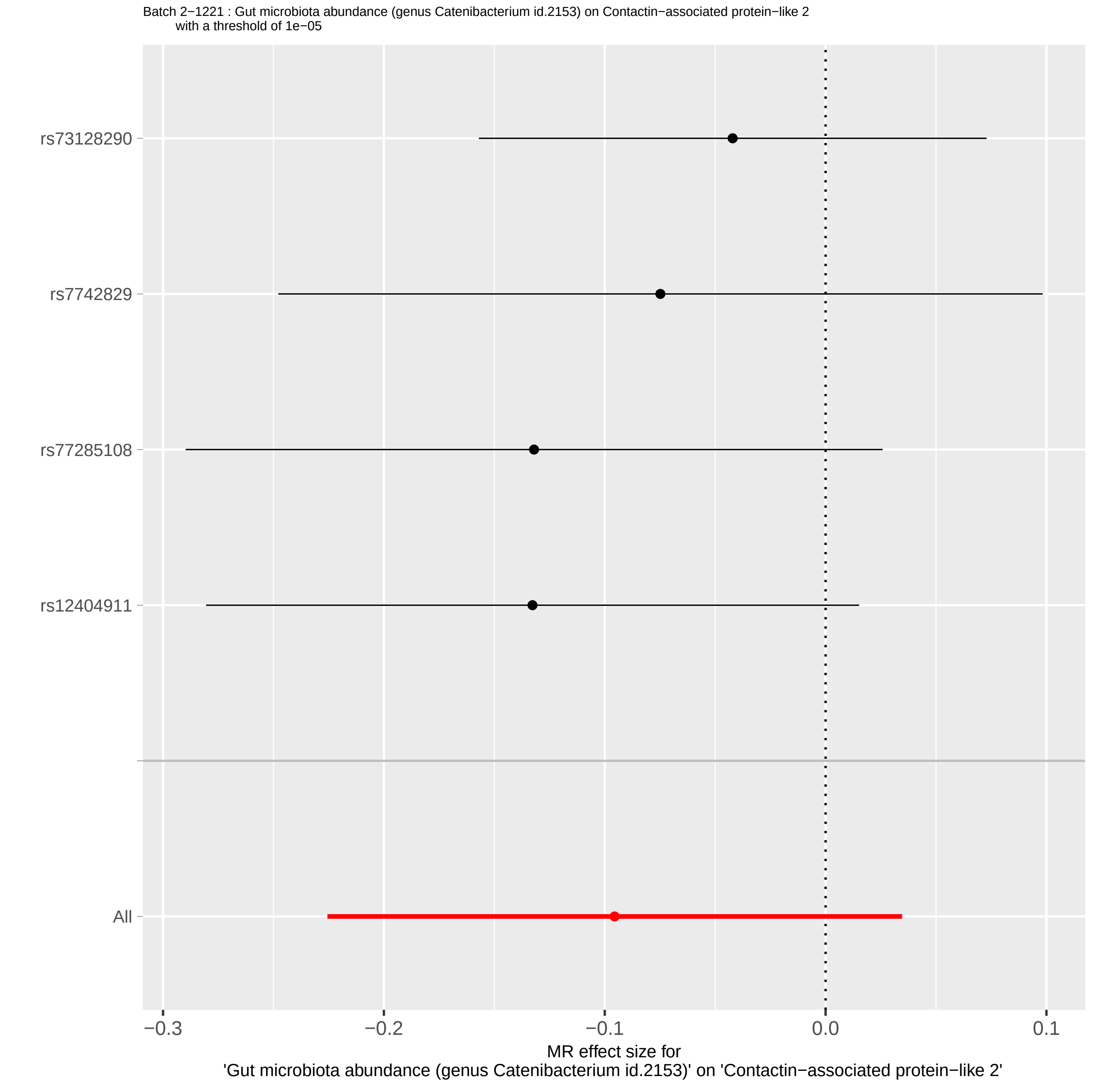

Batch 2−1221 : Gut microbiota abundance (genus Catenibacterium id.2153) on Contactin−associated protein−like 2
with a threshold of 1e−05
rs73128290
rs7742829
rs77285108
rs12404911
All
−0.3
−0.2
−0.1
0.0
0.1
MR effect size for
'Gut microbiota abundance (genus Catenibacterium id.2153)' on 'Contactin−associated protein−like 2'

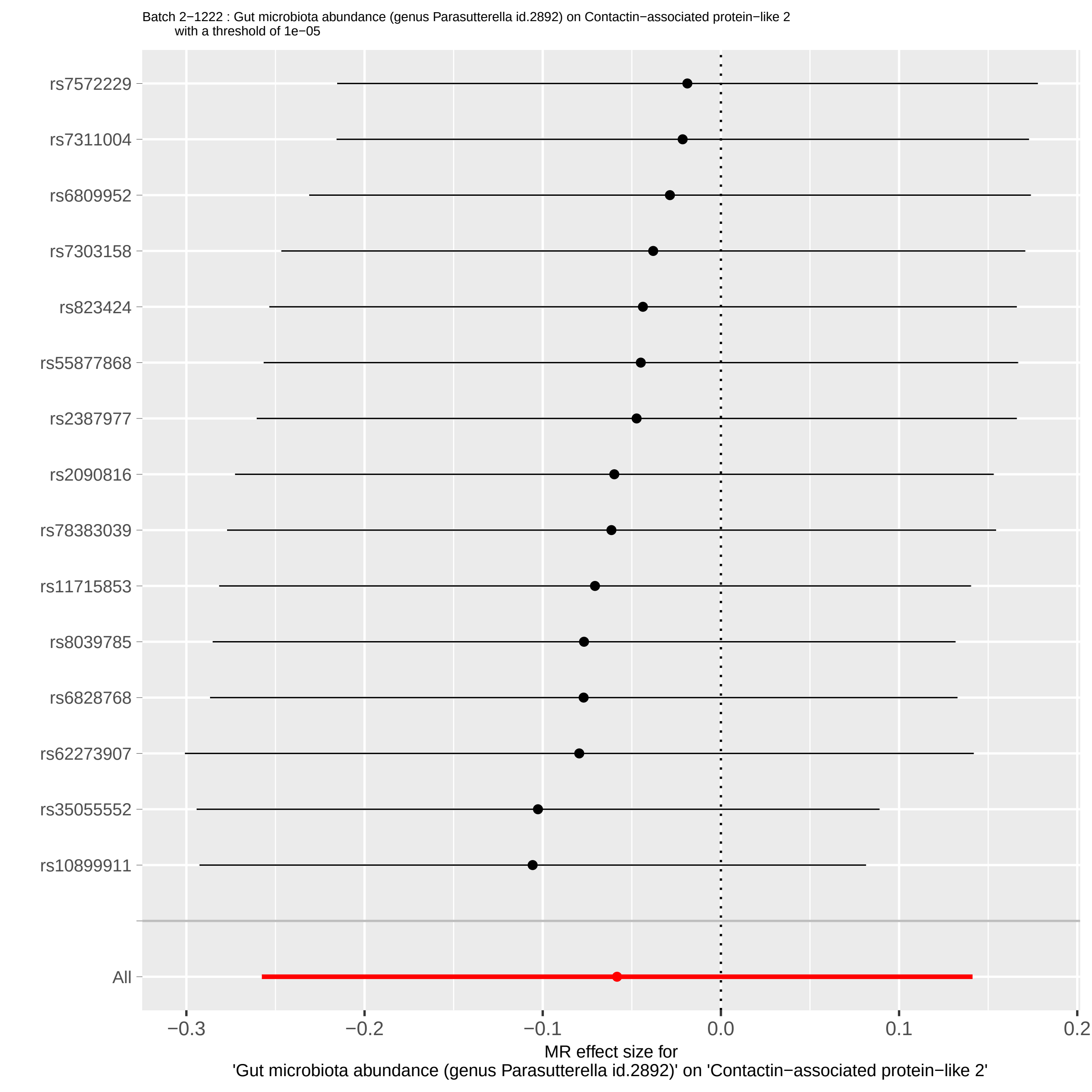

Batch 2−1222 : Gut microbiota abundance (genus Parasutterella id.2892) on Contactin−associated protein−like 2
with a threshold of 1e−05
rs7572229
rs7311004
rs6809952
rs7303158
rs823424
rs55877868
rs2387977
rs2090816
rs78383039
rs11715853
rs8039785
rs6828768
rs62273907
rs35055552
rs10899911
All
−0.3
−0.2
−0.1
0.0
0.1
0.2
MR effect size for
'Gut microbiota abundance (genus Parasutterella id.2892)' on 'Contactin−associated protein−like 2'

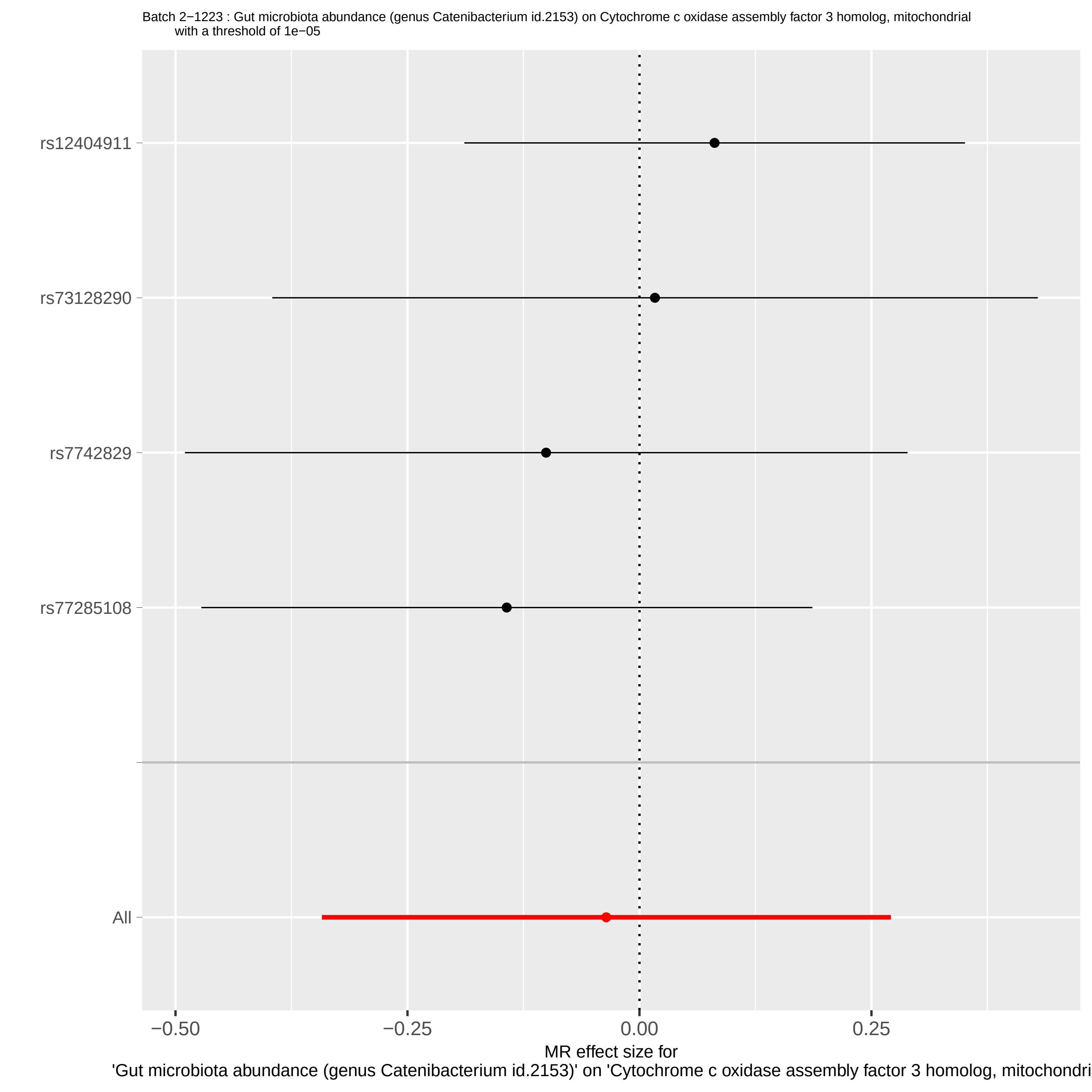
Batch 2−1223 : Gut microbiota abundance (genus Catenibacterium id.2153) on Cytochrome c oxidase assembly factor 3 homolog, mitochondrial
with a threshold of 1e−05
rs12404911
rs73128290
rs7742829
rs77285108
All
−0.50
−0.25
0.00
0.25
MR effect size for
'Gut microbiota abundance (genus Catenibacterium id.2153)' on 'Cytochrome c oxidase assembly factor 3 homolog, mitochondri

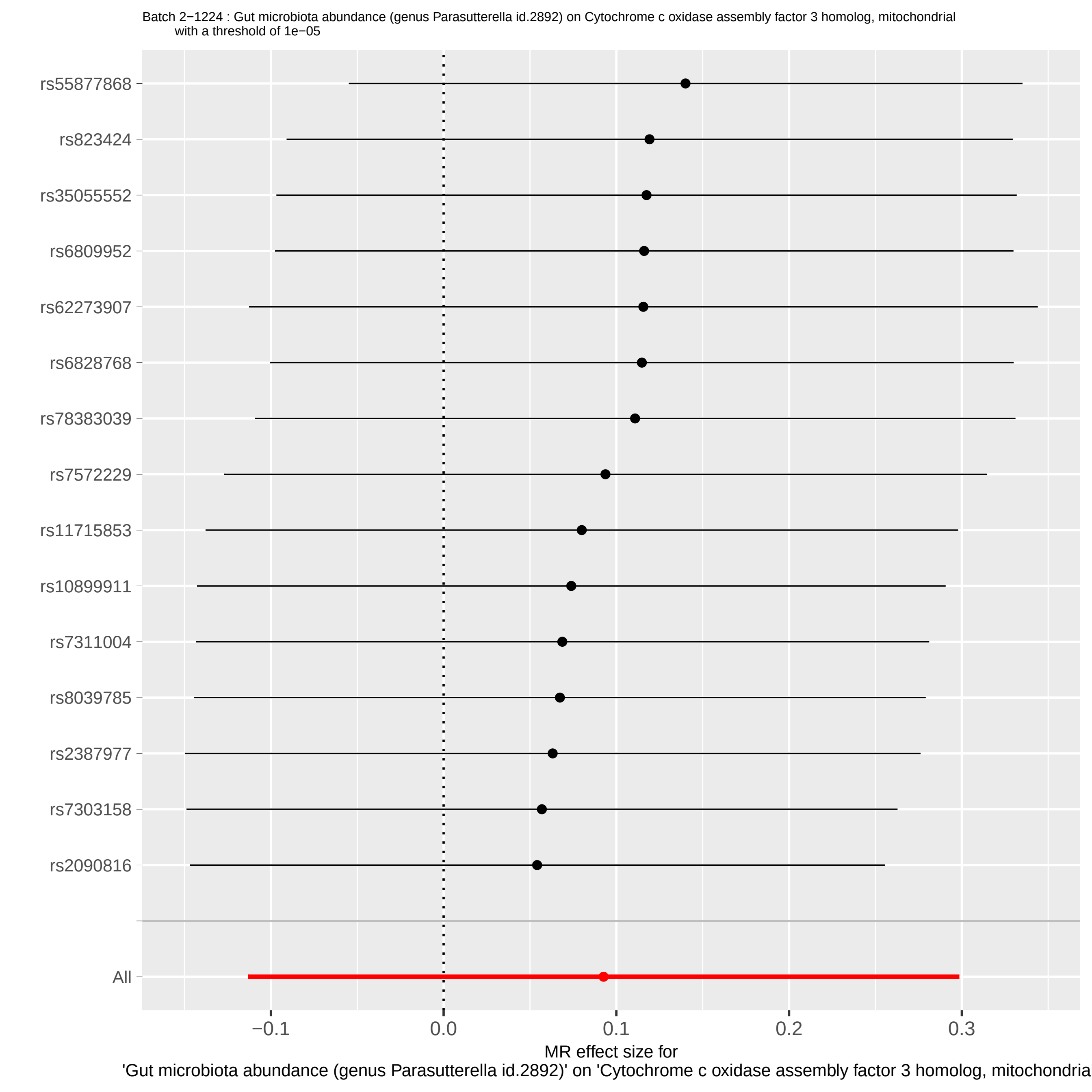
Batch 2−1224 : Gut microbiota abundance (genus Parasutterella id.2892) on Cytochrome c oxidase assembly factor 3 homolog, mitochondrial
with a threshold of 1e−05
rs55877868
rs823424
rs35055552
rs6809952
rs62273907
rs6828768
rs78383039
rs7572229
rs11715853
rs10899911
rs7311004
rs8039785
rs2387977
rs7303158
rs2090816
All
−0.1
0.0
0.1
0.2
0.3
MR effect size for
'Gut microbiota abundance (genus Parasutterella id.2892)' on 'Cytochrome c oxidase assembly factor 3 homolog, mitochondria

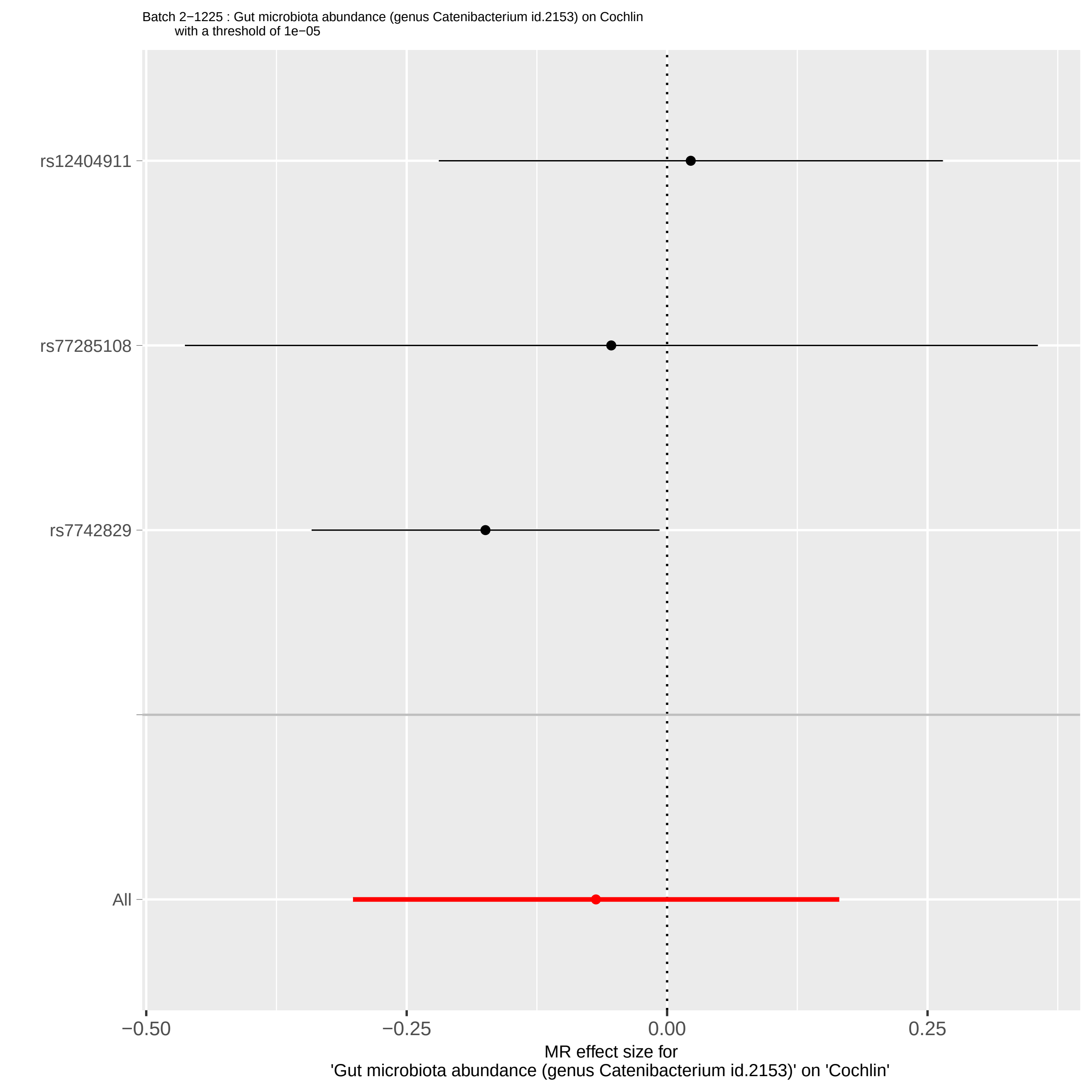

Batch 2−1225 : Gut microbiota abundance (genus Catenibacterium id.2153) on Cochlin
with a threshold of 1e−05
rs12404911
rs77285108
rs7742829
All
−0.50
−0.25
0.00
0.25
MR effect size for
'Gut microbiota abundance (genus Catenibacterium id.2153)' on 'Cochlin'

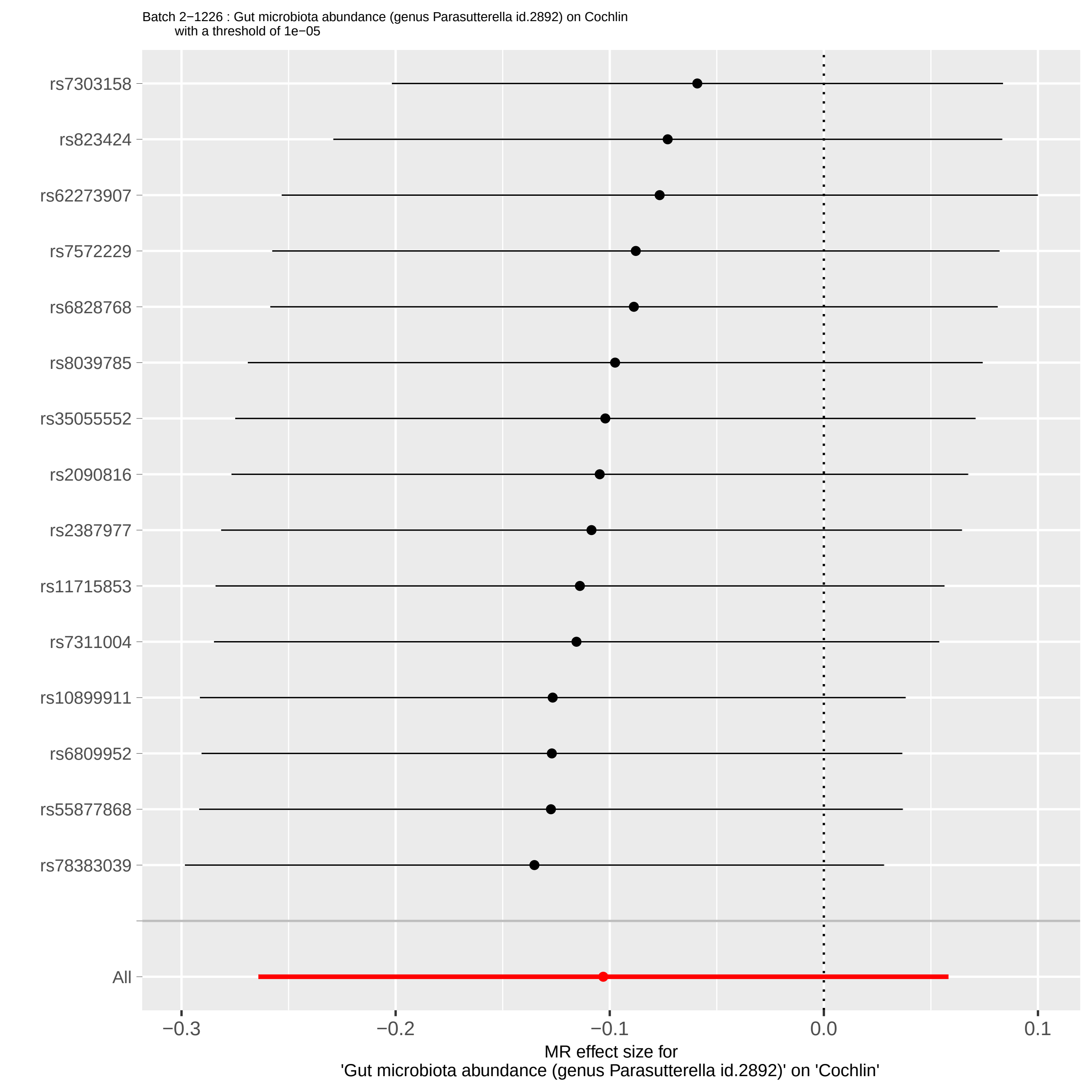
Batch 2−1226 : Gut microbiota abundance (genus Parasutterella id.2892) on Cochlin
with a threshold of 1e−05
rs7303158
rs823424
rs62273907
rs7572229
rs6828768
rs8039785
rs35055552
rs2090816
rs2387977
rs11715853
rs7311004
rs10899911
rs6809952
rs55877868
rs78383039
All
−0.3
−0.2
−0.1
0.0
0.1
MR effect size for
'Gut microbiota abundance (genus Parasutterella id.2892)' on 'Cochlin'

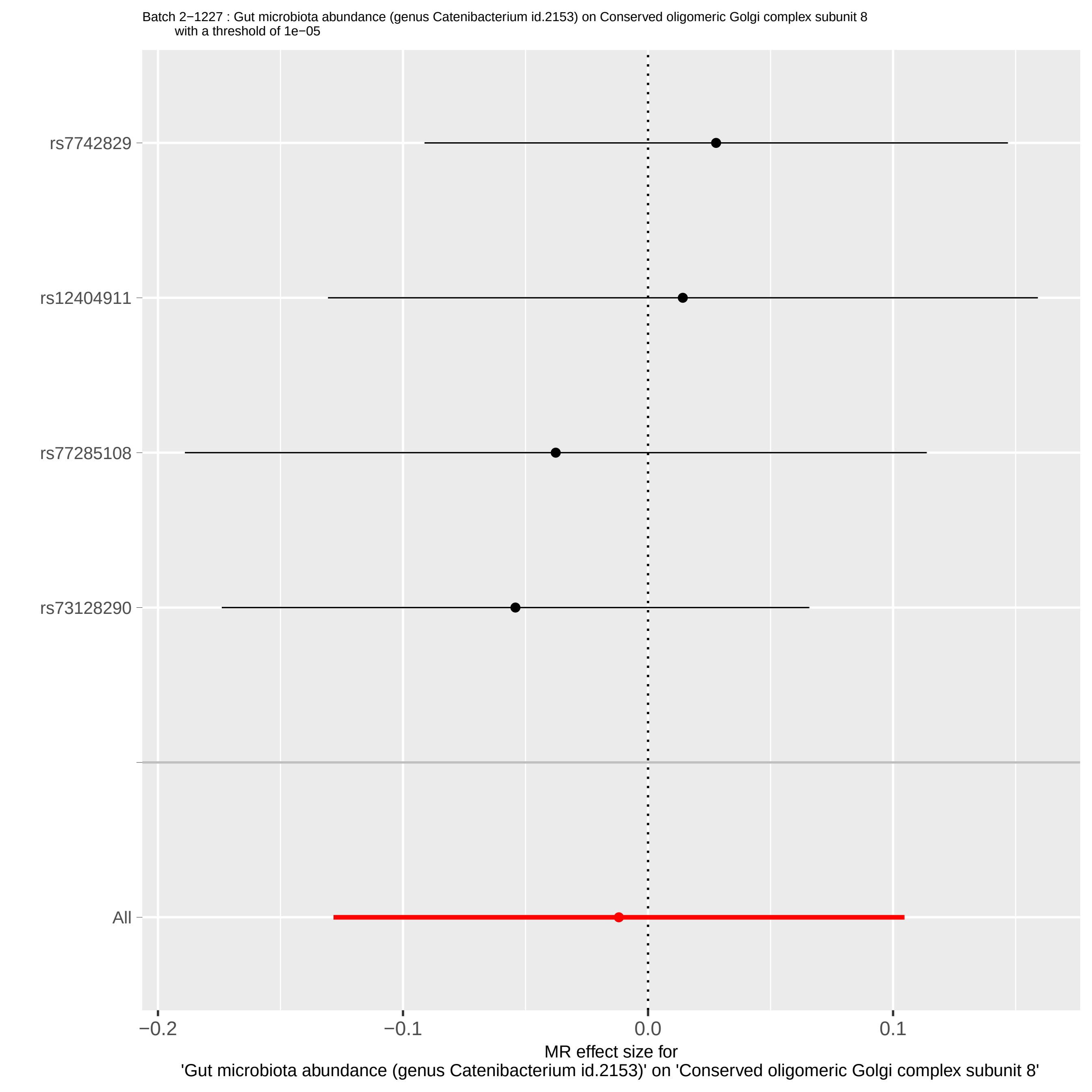
Batch 2−1227 : Gut microbiota abundance (genus Catenibacterium id.2153) on Conserved oligomeric Golgi complex subunit 8
with a threshold of 1e−05
rs7742829
rs12404911
rs77285108
rs73128290
All
−0.2
−0.1
0.0
0.1
MR effect size for
'Gut microbiota abundance (genus Catenibacterium id.2153)' on 'Conserved oligomeric Golgi complex subunit 8'

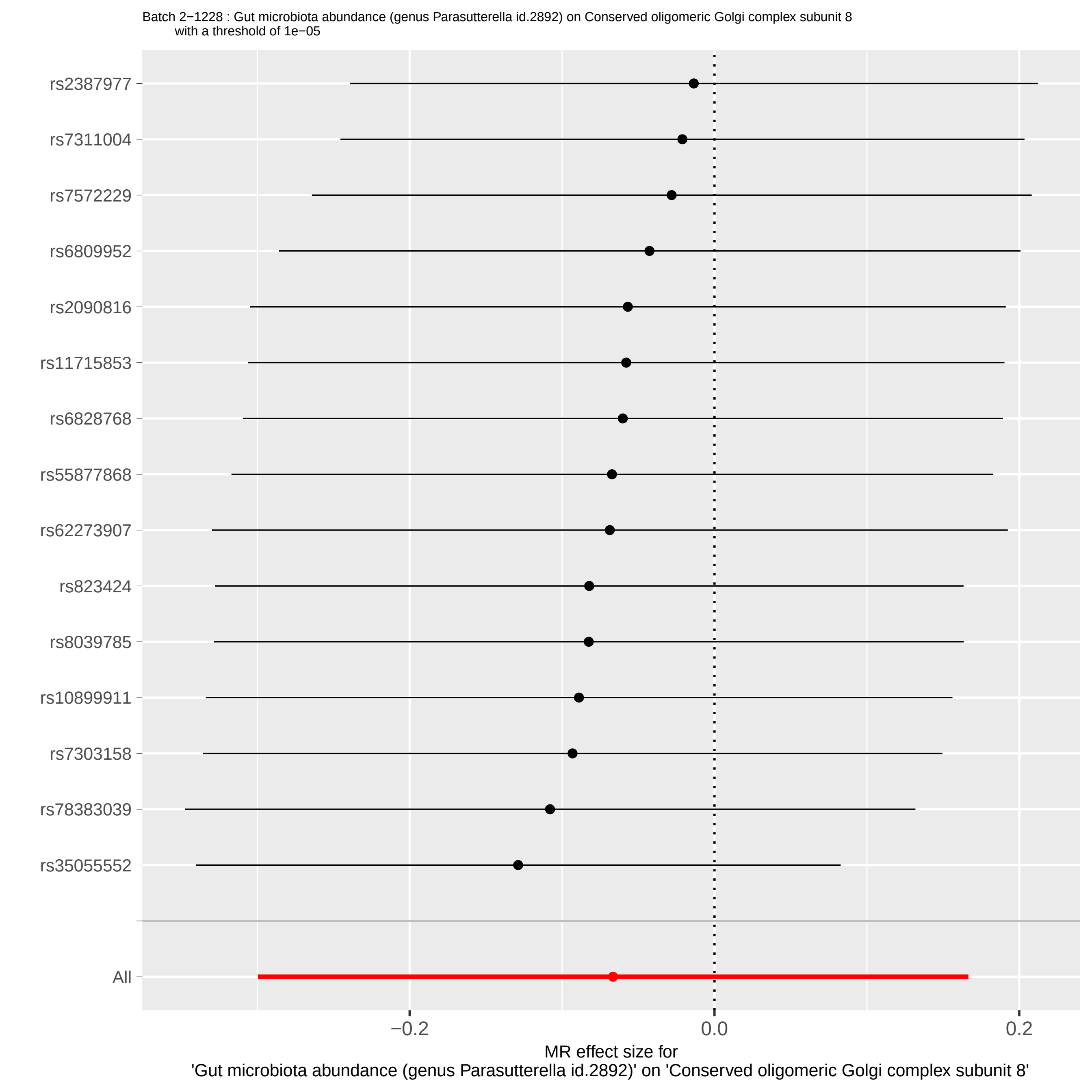
Batch 2−1228 : Gut microbiota abundance (genus Parasutterella id.2892) on Conserved oligomeric Golgi complex subunit 8
with a threshold of 1e−05
rs2387977
rs7311004
rs7572229
rs6809952
rs2090816
rs11715853
rs6828768
rs55877868
rs62273907
rs823424
rs8039785
rs10899911
rs7303158
rs78383039
rs35055552
All
−0.2
0.0
0.2
MR effect size for
'Gut microbiota abundance (genus Parasutterella id.2892)' on 'Conserved oligomeric Golgi complex subunit 8'

Batch 2−1229 : Gut microbiota abundance (genus Catenibacterium id.2153) on Collagen alpha−2(XI) chain
with a threshold of 1e−05

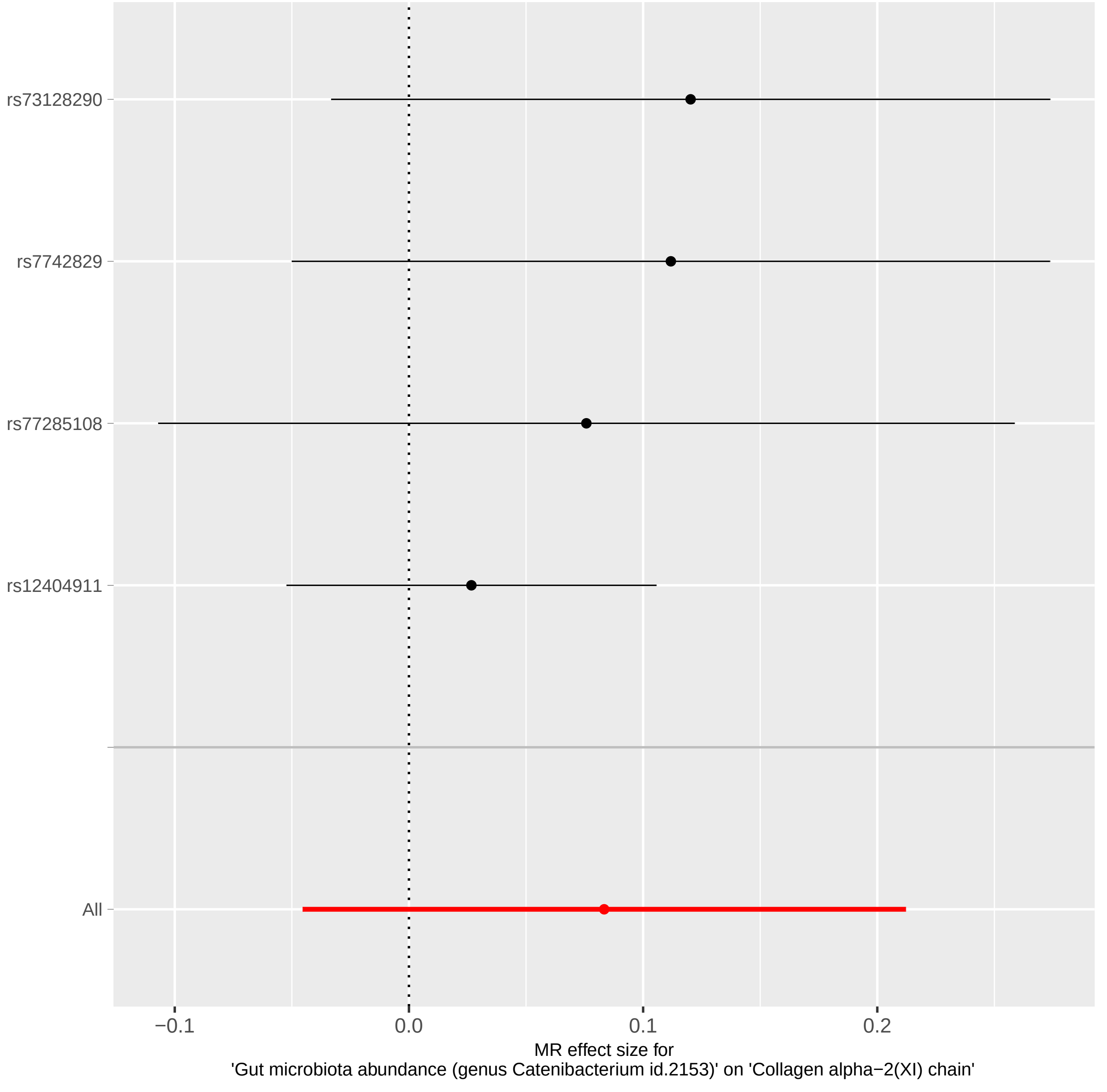

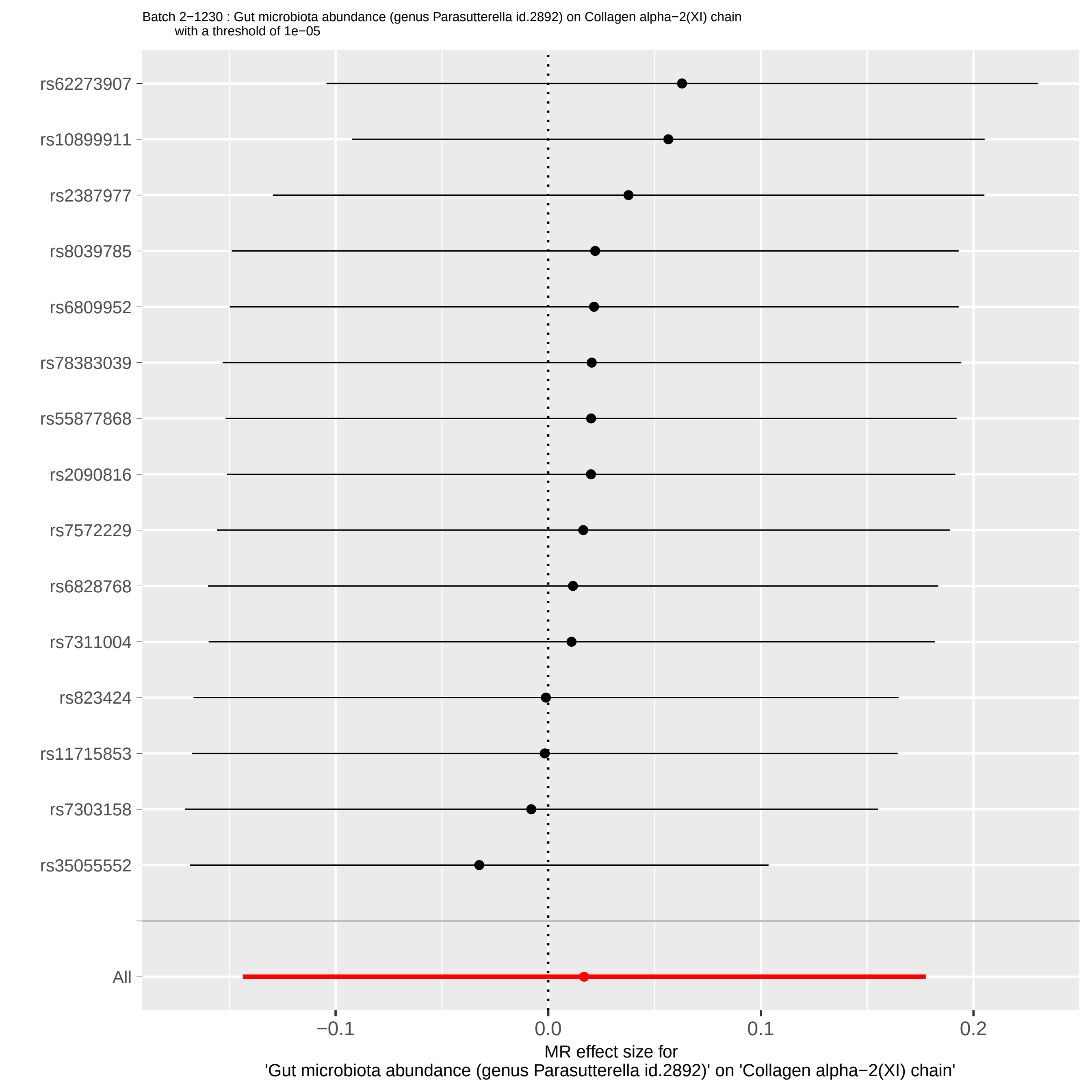
Batch 2−1230 : Gut microbiota abundance (genus Parasutterella id.2892) on Collagen alpha−2(XI) chain
with a threshold of 1e−05
rs62273907
rs10899911
rs2387977
rs8039785
rs6809952
rs78383039
rs55877868
rs2090816
rs7572229
rs6828768
rs7311004
rs823424
rs11715853
rs7303158
rs35055552
All
−0.1
0.0
0.1
0.2
MR effect size for
'Gut microbiota abundance (genus Parasutterella id.2892)' on 'Collagen alpha−2(XI) chain'

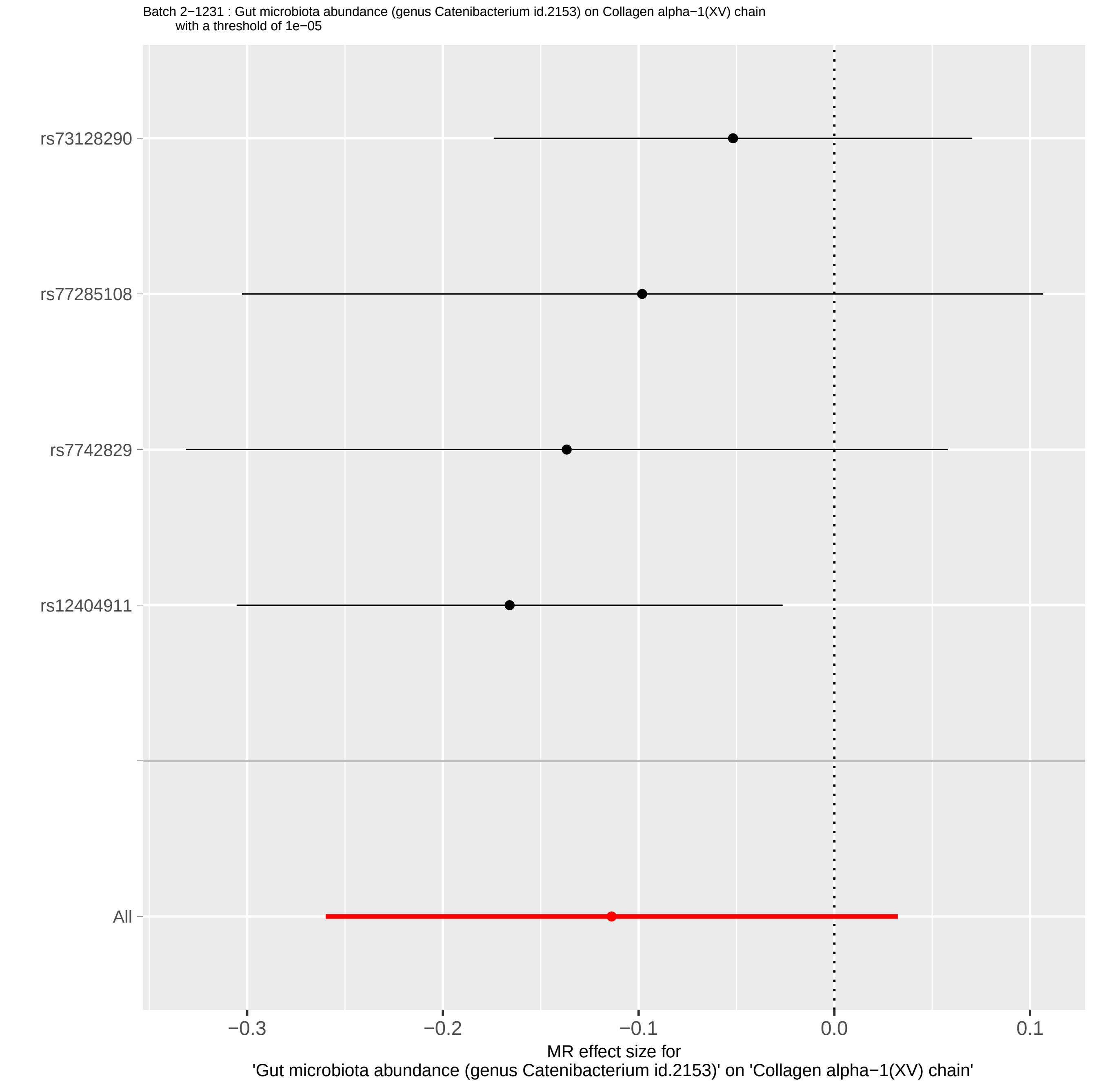
Batch 2−1231 : Gut microbiota abundance (genus Catenibacterium id.2153) on Collagen alpha−1(XV) chain
with a threshold of 1e−05
rs73128290
rs77285108
rs7742829
rs12404911
All
−0.3
−0.2
−0.1
0.0
0.1
MR effect size for
'Gut microbiota abundance (genus Catenibacterium id.2153)' on 'Collagen alpha−1(XV) chain

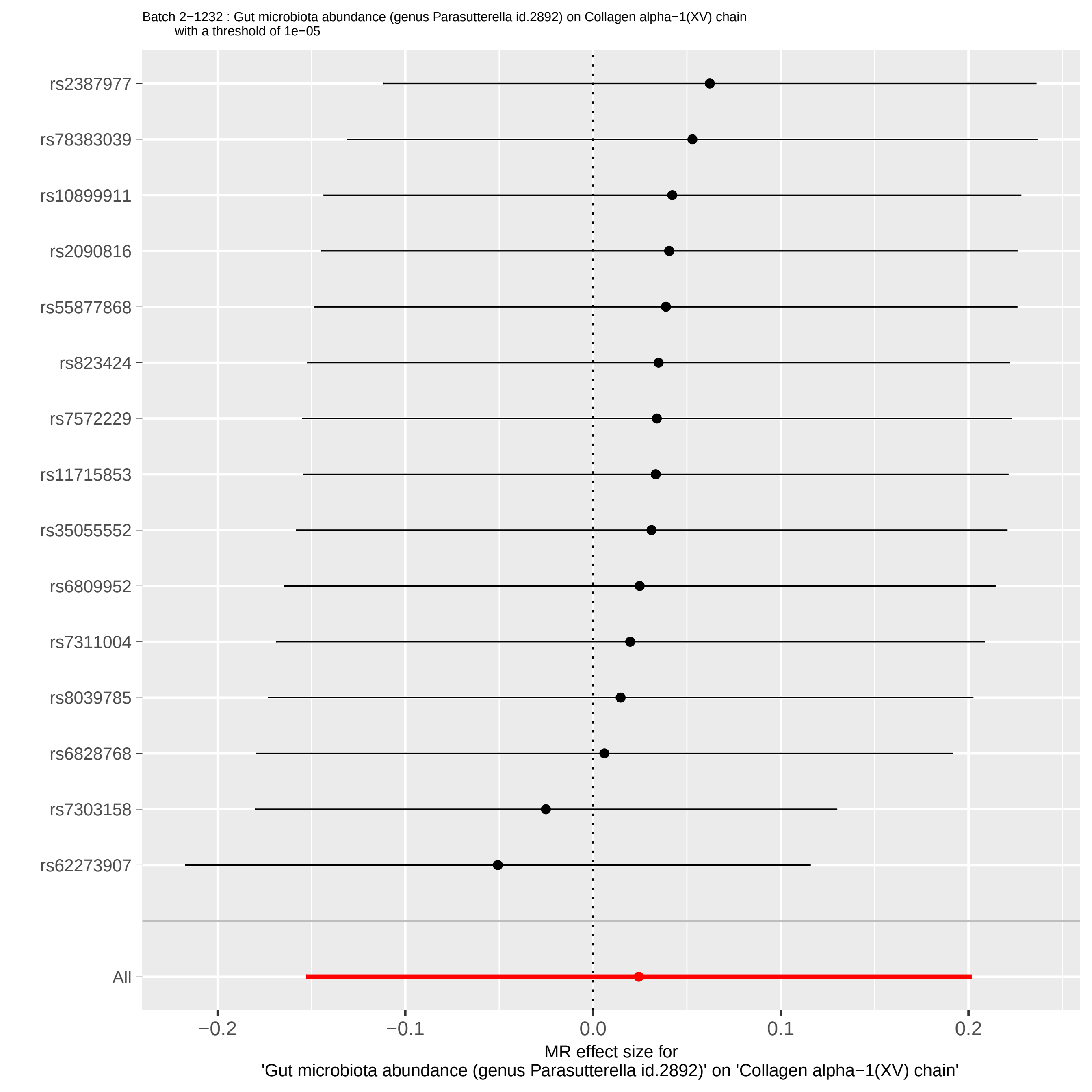

Batch 2−1232 : Gut microbiota abundance (genus Parasutterella id.2892) on Collagen alpha−1(XV) chain
with a threshold of 1e−05
rs2387977
rs78383039
rs10899911
rs2090816
rs55877868
rs823424
rs7572229
rs11715853
rs35055552
rs6809952
rs7311004
rs8039785
rs6828768
rs7303158
rs62273907
All
−0.2
−0.1
0.0
0.1
0.2
MR effect size for
'Gut microbiota abundance (genus Parasutterella id.2892)' on 'Collagen alpha−1(XV) chain'

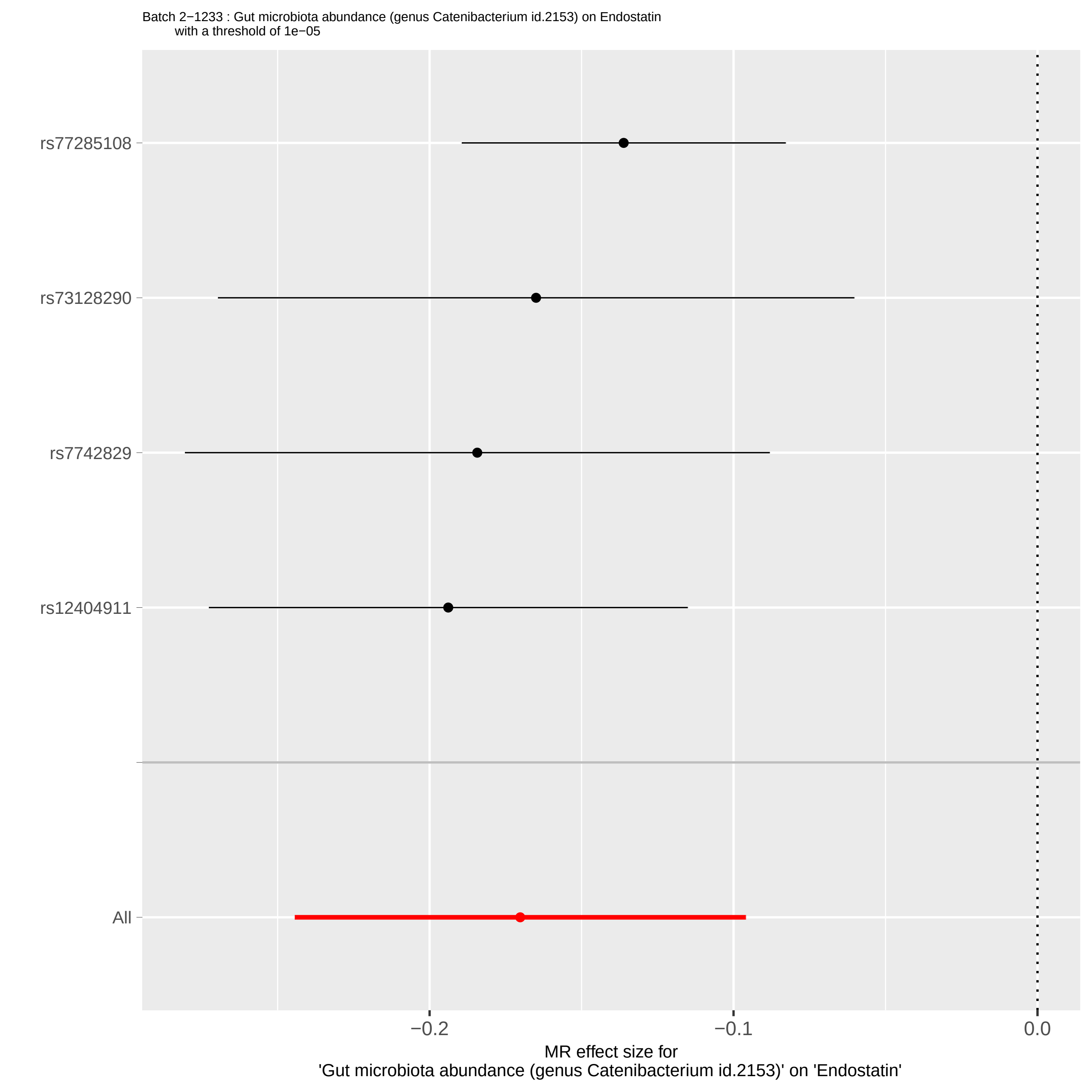
Batch 2−1233 : Gut microbiota abundance (genus Catenibacterium id.2153) on Endostatin
with a threshold of 1e−05
rs77285108
rs73128290
rs7742829
rs12404911
All
−0.2
−0.1
0.0
MR effect size for
'Gut microbiota abundance (genus Catenibacterium id.2153)' on 'Endostatin'

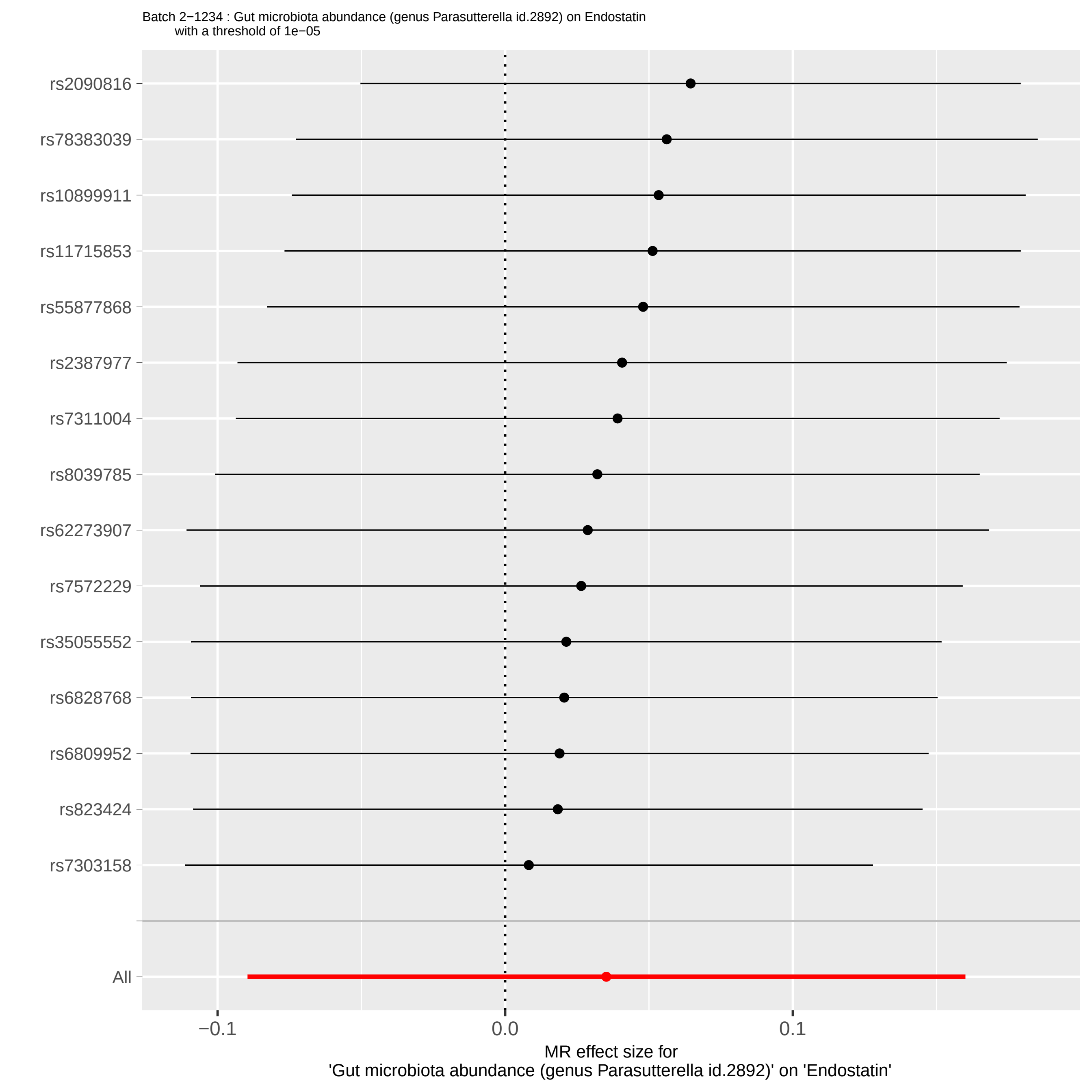

Batch 2−1234 : Gut microbiota abundance (genus Parasutterella id.2892) on Endostatin
with a threshold of 1e−05
rs2090816
rs78383039
rs10899911
rs11715853
rs55877868
rs2387977
rs7311004
rs8039785
rs62273907
rs7572229
rs35055552
rs6828768
rs6809952
rs823424
rs7303158
All
−0.1
0.0
0.1
MR effect size for
'Gut microbiota abundance (genus Parasutterella id.2892)' on 'Endostatin'

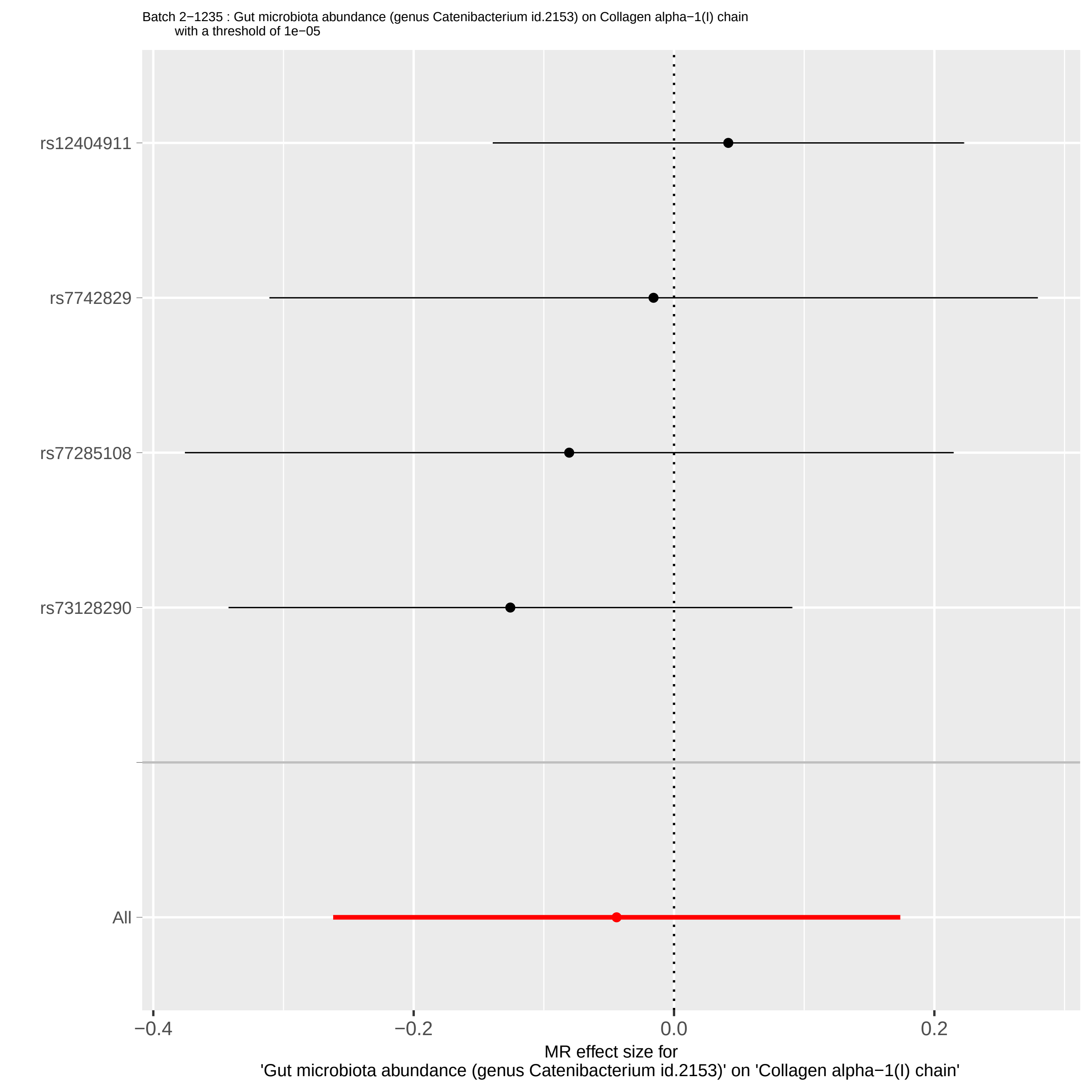

Batch 2−1235 : Gut microbiota abundance (genus Catenibacterium id.2153) on Collagen alpha−1(I) chain
with a threshold of 1e−05
rs12404911
rs7742829
rs77285108
rs73128290
All
−0.4
−0.2
0.0
0.2
MR effect size for
'Gut microbiota abundance (genus Catenibacterium id.2153)' on 'Collagen alpha−1(I) chain'

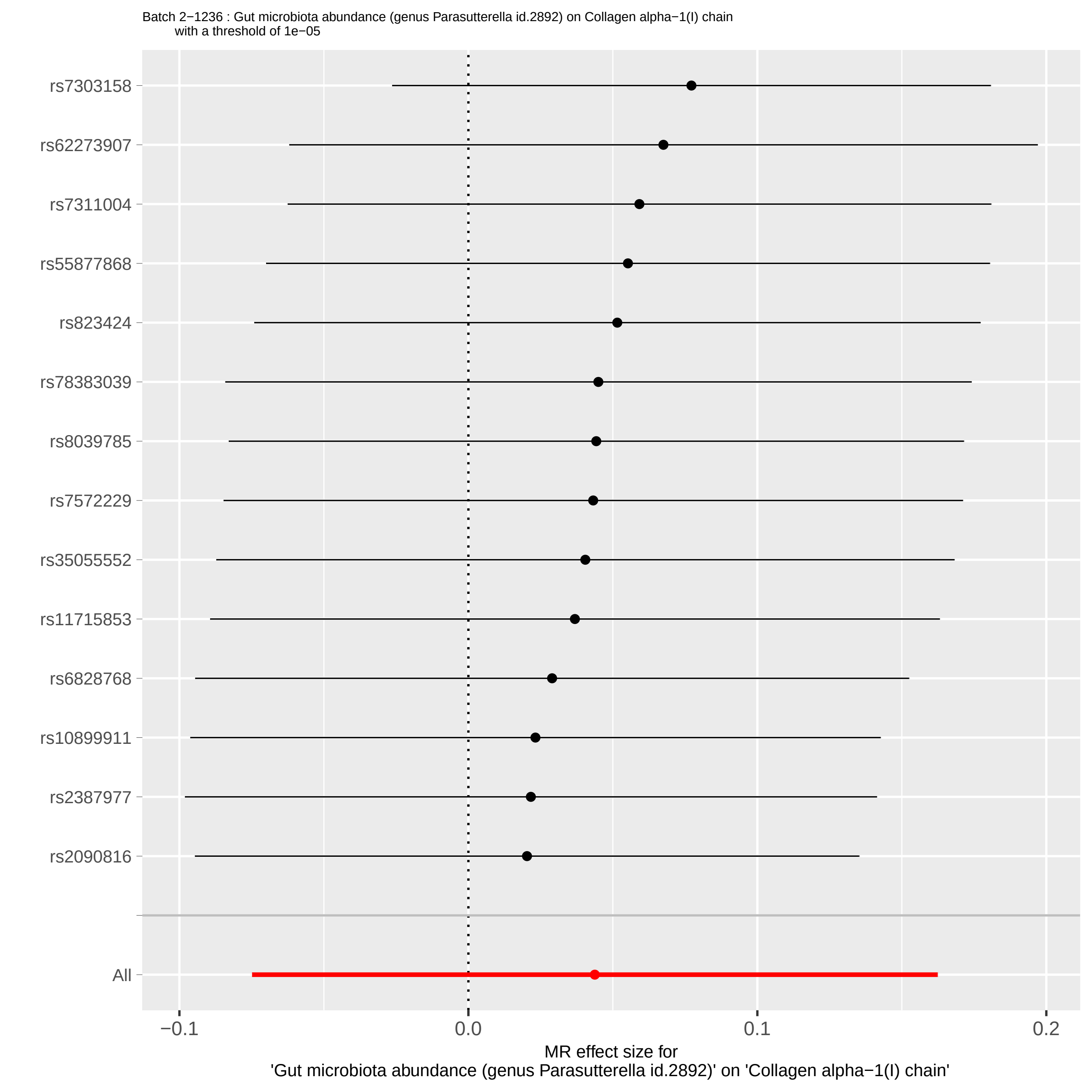
Batch 2−1236 : Gut microbiota abundance (genus Parasutterella id.2892) on Collagen alpha−1(I) chain
with a threshold of 1e−05
rs7303158
rs62273907
rs7311004
rs55877868
rs823424
rs78383039
rs8039785
rs7572229
rs35055552
rs11715853
rs6828768
rs10899911
rs2387977
rs2090816
All
−0.1
0.0
0.1
0.2
MR effect size for
'Gut microbiota abundance (genus Parasutterella id.2892)' on 'Collagen alpha−1(I) chain'

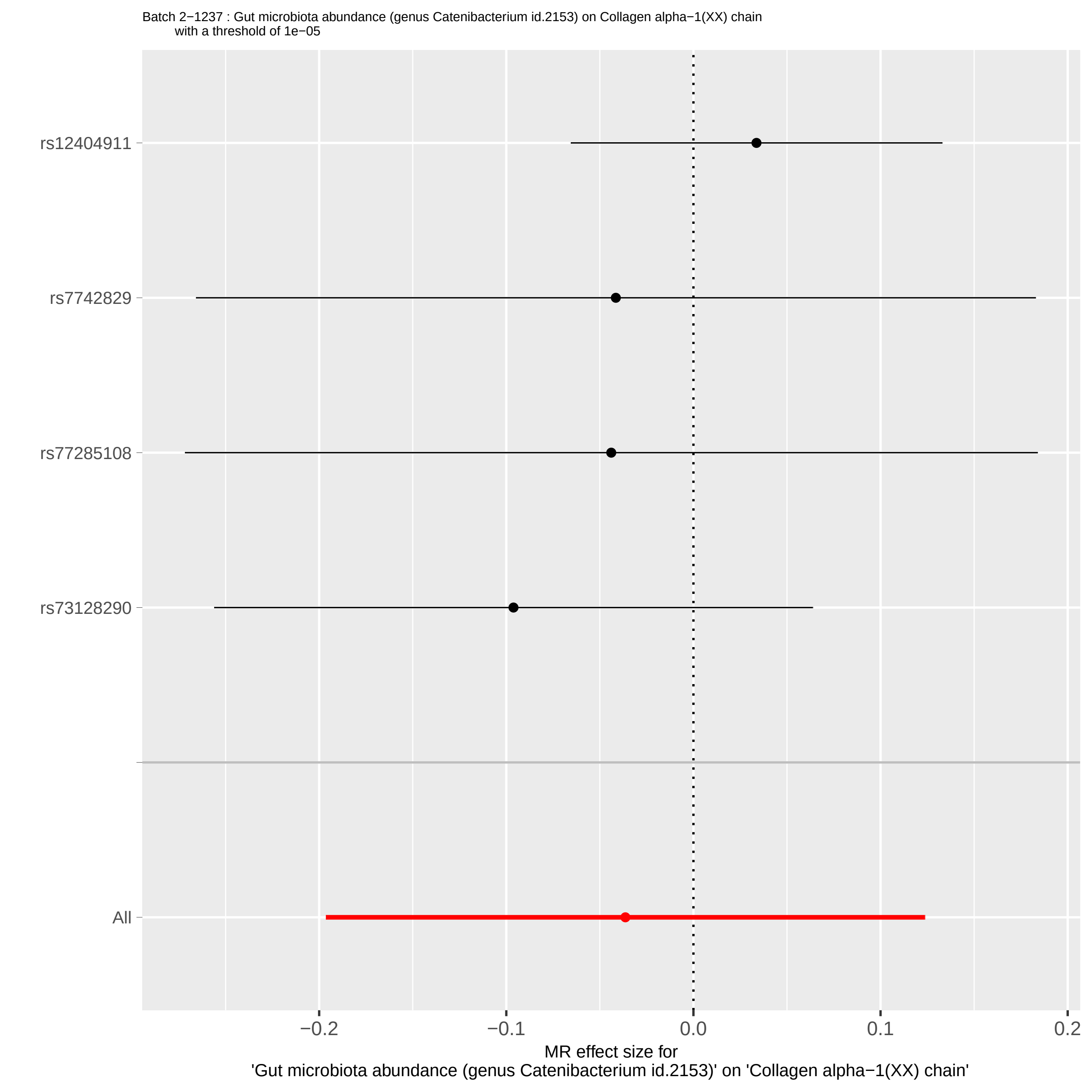
Batch 2−1237 : Gut microbiota abundance (genus Catenibacterium id.2153) on Collagen alpha−1(XX) chain
with a threshold of 1e−05
rs12404911
rs7742829
rs77285108
rs73128290
All
−0.2
−0.1
0.0
0.1
0.2
MR effect size for
'Gut microbiota abundance (genus Catenibacterium id.2153)' on 'Collagen alpha−1(XX) chain'

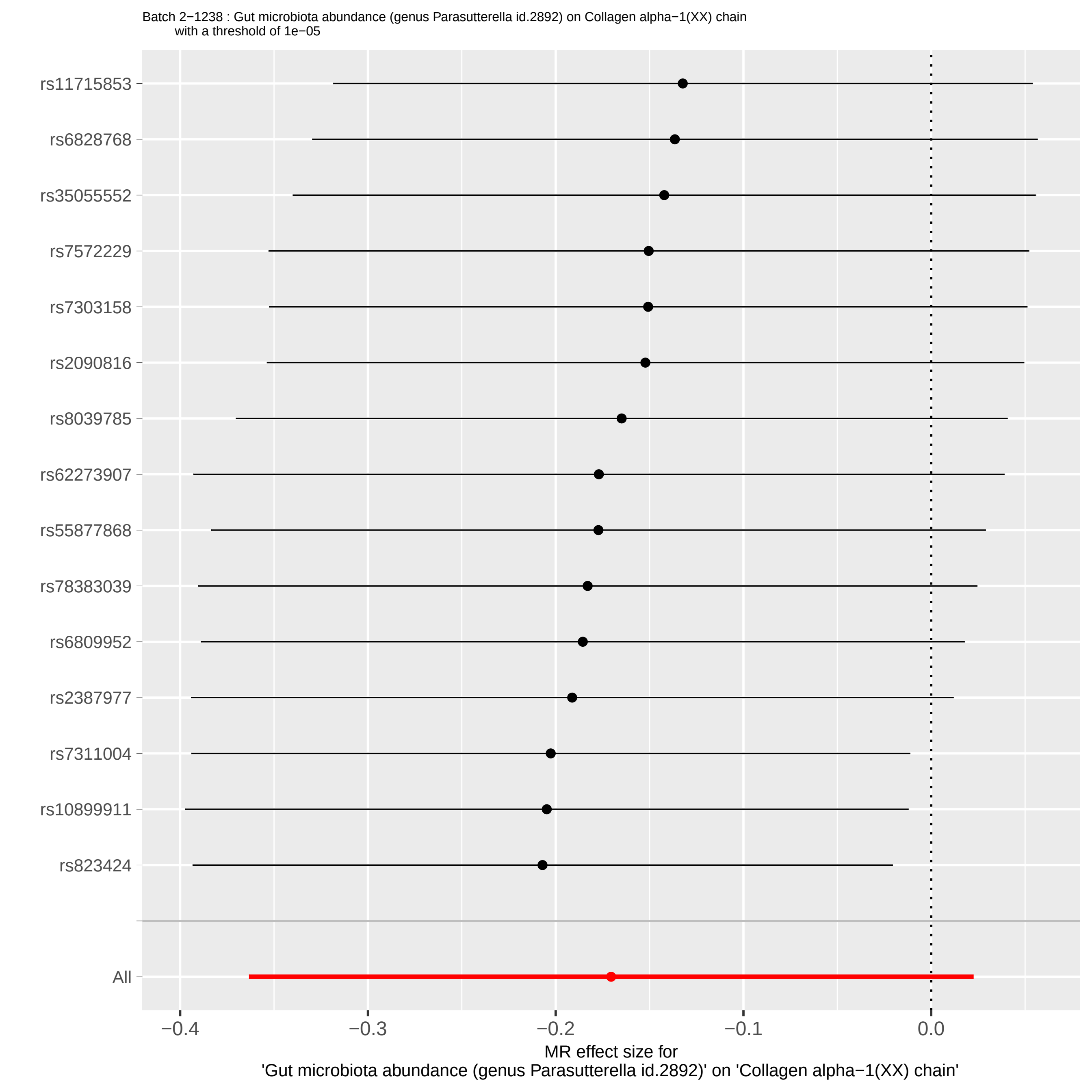
Batch 2−1238 : Gut microbiota abundance (genus Parasutterella id.2892) on Collagen alpha−1(XX) chain
with a threshold of 1e−05
rs11715853
rs6828768
rs35055552
rs7572229
rs7303158
rs2090816
rs8039785
rs62273907
rs55877868
rs78383039
rs6809952
rs2387977
rs7311004
rs10899911
rs823424
All
−0.4
−0.3
−0.2
−0.1
0.0
MR effect size for
'Gut microbiota abundance (genus Parasutterella id.2892)' on 'Collagen alpha−1(XX) chain'

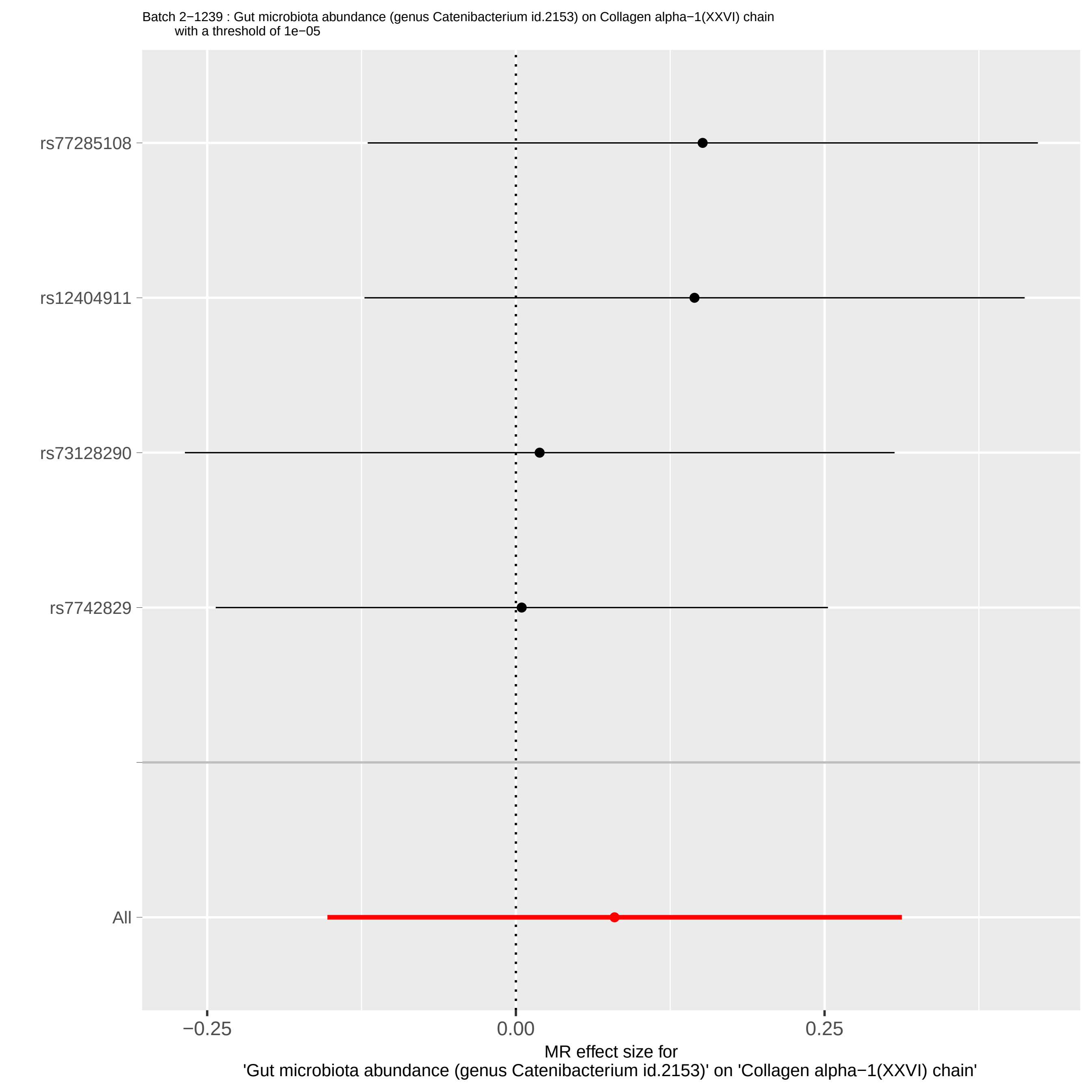
Batch 2−1239 : Gut microbiota abundance (genus Catenibacterium id.2153) on Collagen alpha−1(XXVI) chain
with a threshold of 1e−05
rs77285108
rs12404911
rs73128290
rs7742829
All
−0.25
0.00
0.25
MR effect size for
'Gut microbiota abundance (genus Catenibacterium id.2153)' on 'Collagen alpha−1(XXVI) chain'

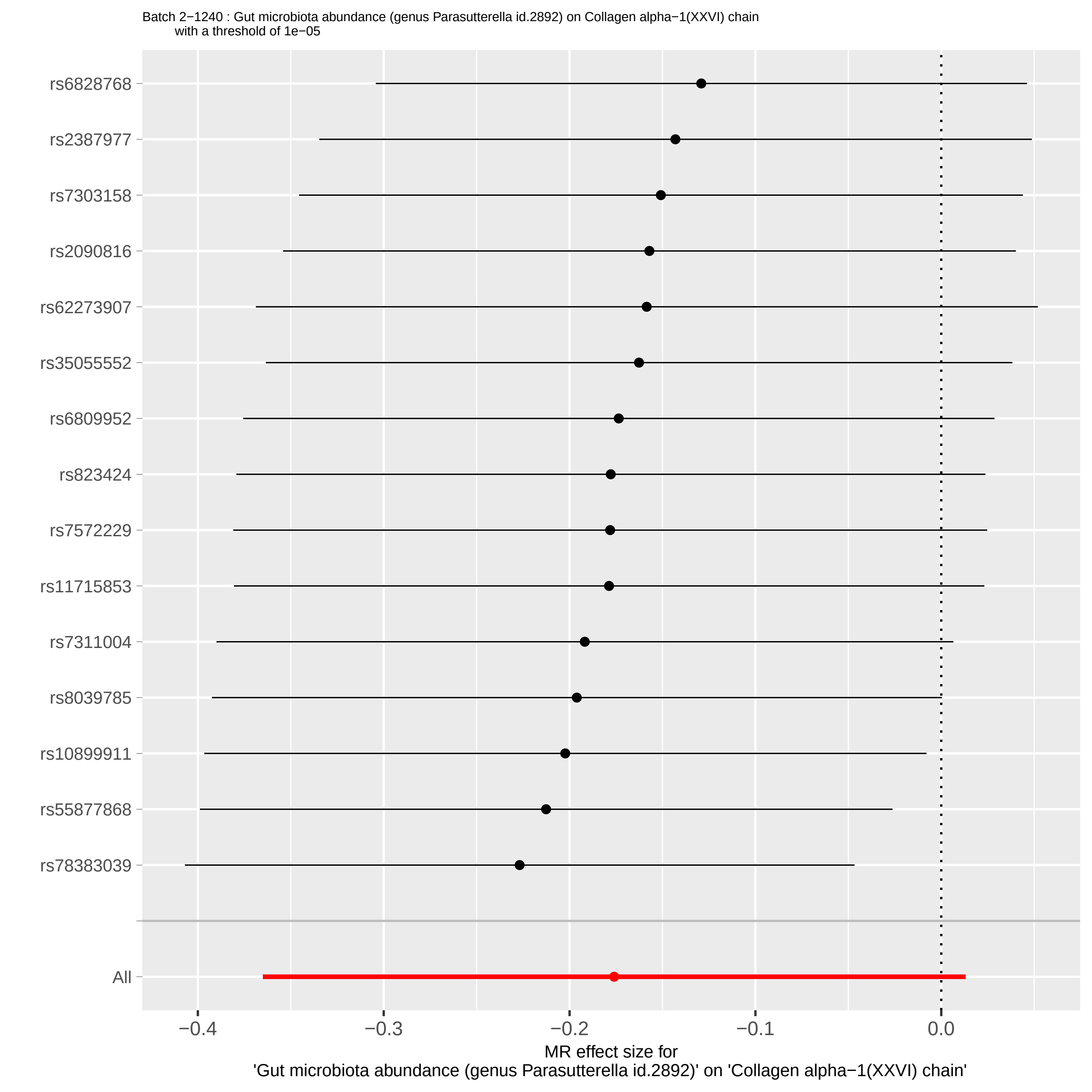

Batch 2−1240 : Gut microbiota abundance (genus Parasutterella id.2892) on Collagen alpha−1(XXVI) chain
with a threshold of 1e−05
rs6828768
rs2387977
rs7303158
rs2090816
rs62273907
rs35055552
rs6809952
rs823424
rs7572229
rs11715853
rs7311004
rs8039785
rs10899911
rs55877868
rs78383039
All
−0.4
−0.3
−0.2
−0.1
0.0
MR effect size for
'Gut microbiota abundance (genus Parasutterella id.2892)' on 'Collagen alpha−1(XXVI) chain'

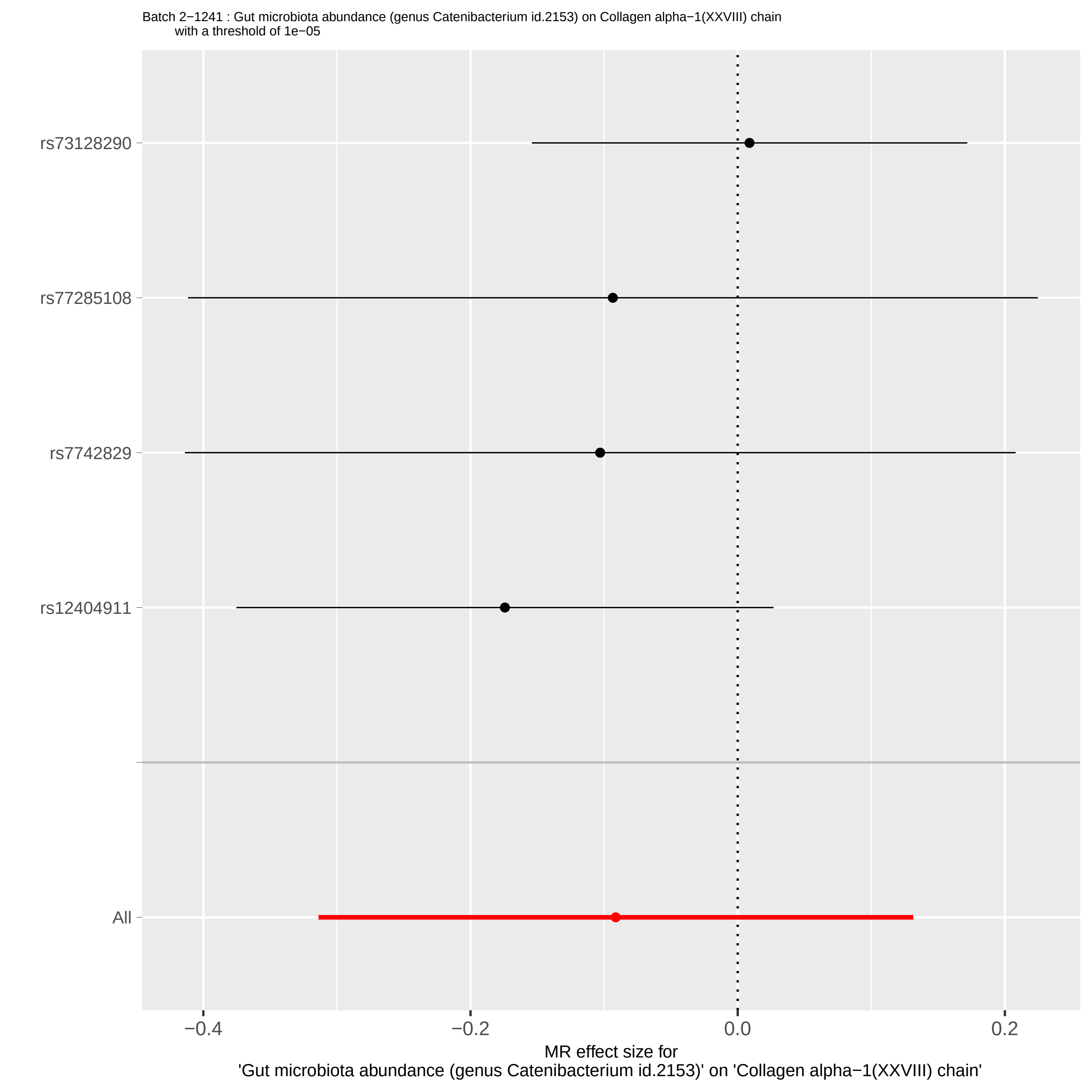
Batch 2−1241 : Gut microbiota abundance (genus Catenibacterium id.2153) on Collagen alpha−1(XXVIII) chain
with a threshold of 1e−05
rs73128290
rs77285108
rs7742829
rs12404911
All
−0.4
−0.2
0.0
0.2
MR effect size for
'Gut microbiota abundance (genus Catenibacterium id.2153)' on 'Collagen alpha−1(XXVIII) chain'

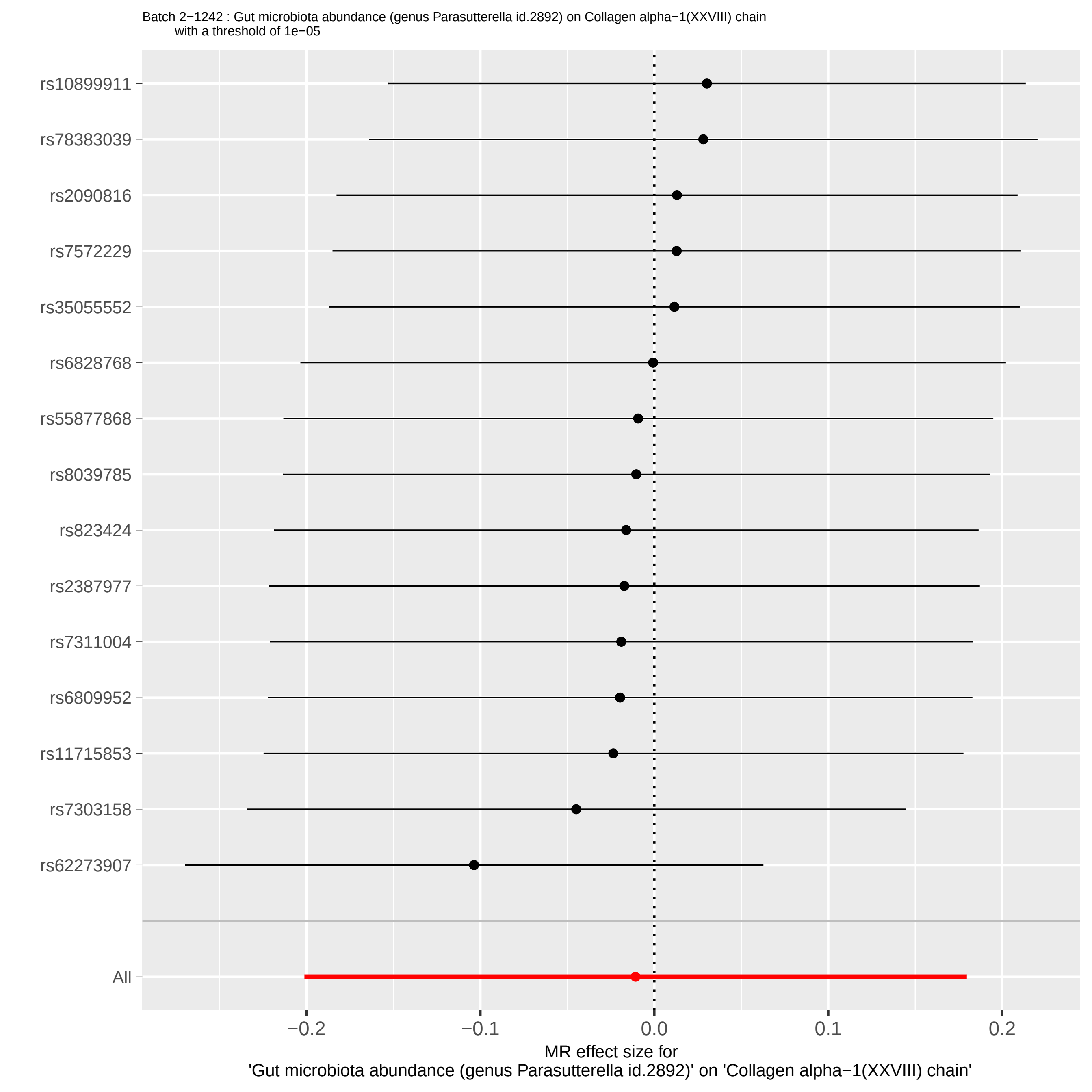

Batch 2−1242 : Gut microbiota abundance (genus Parasutterella id.2892) on Collagen alpha−1(XXVIII) chain
with a threshold of 1e−05
rs10899911
rs78383039
rs2090816
rs7572229
rs35055552
rs6828768
rs55877868
rs8039785
rs823424
rs2387977
rs7311004
rs6809952
rs11715853
rs7303158
rs62273907
All
−0.2
−0.1
0.0
0.1
0.2
MR effect size for
'Gut microbiota abundance (genus Parasutterella id.2892)' on 'Collagen alpha−1(XXVIII) chain'

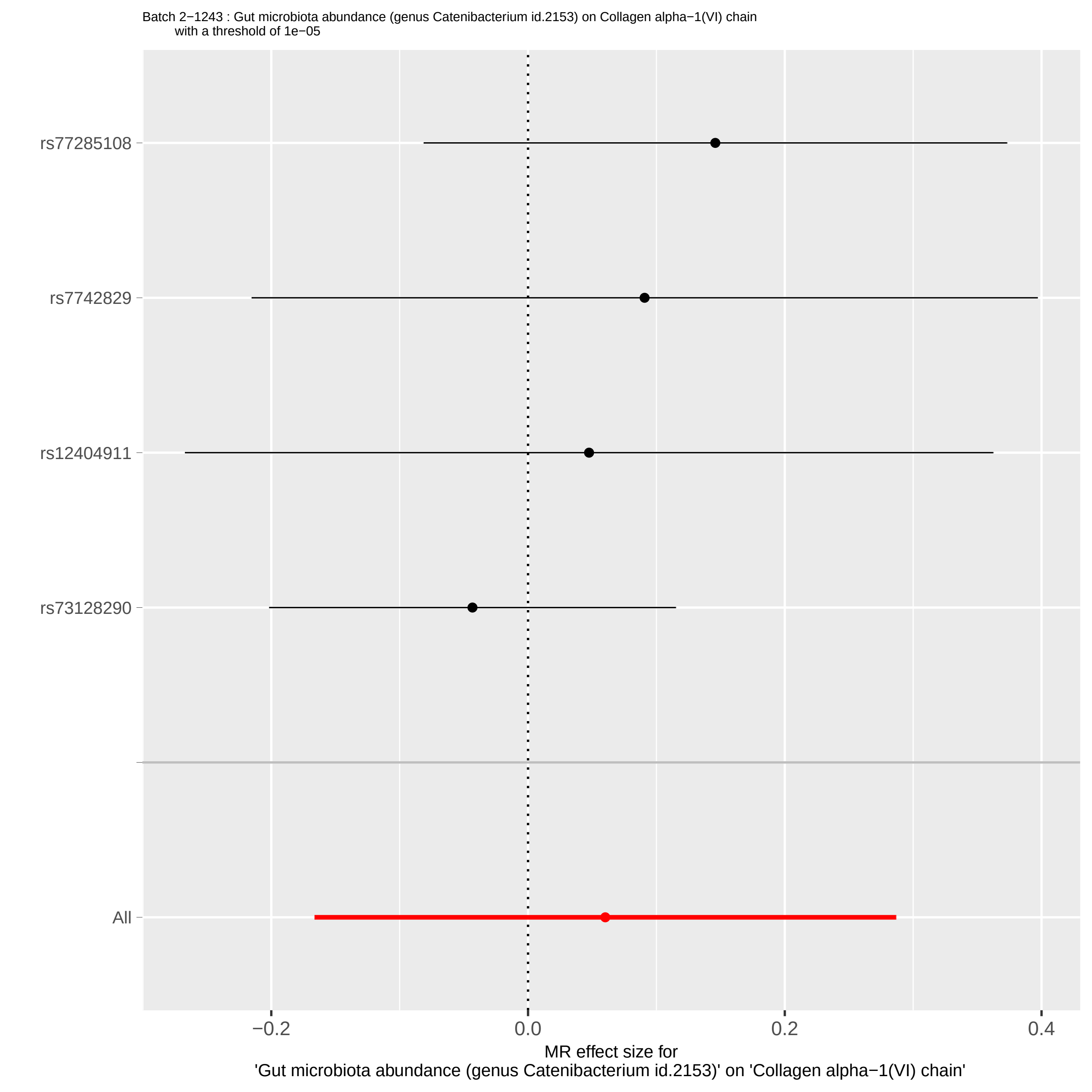
Batch 2−1243 : Gut microbiota abundance (genus Catenibacterium id.2153) on Collagen alpha−1(VI) chain
with a threshold of 1e−05
rs77285108
rs7742829
rs12404911
rs73128290
All
−0.2
0.0
0.2
0.4
MR effect size for
'Gut microbiota abundance (genus Catenibacterium id.2153)' on 'Collagen alpha−1(VI) chain'

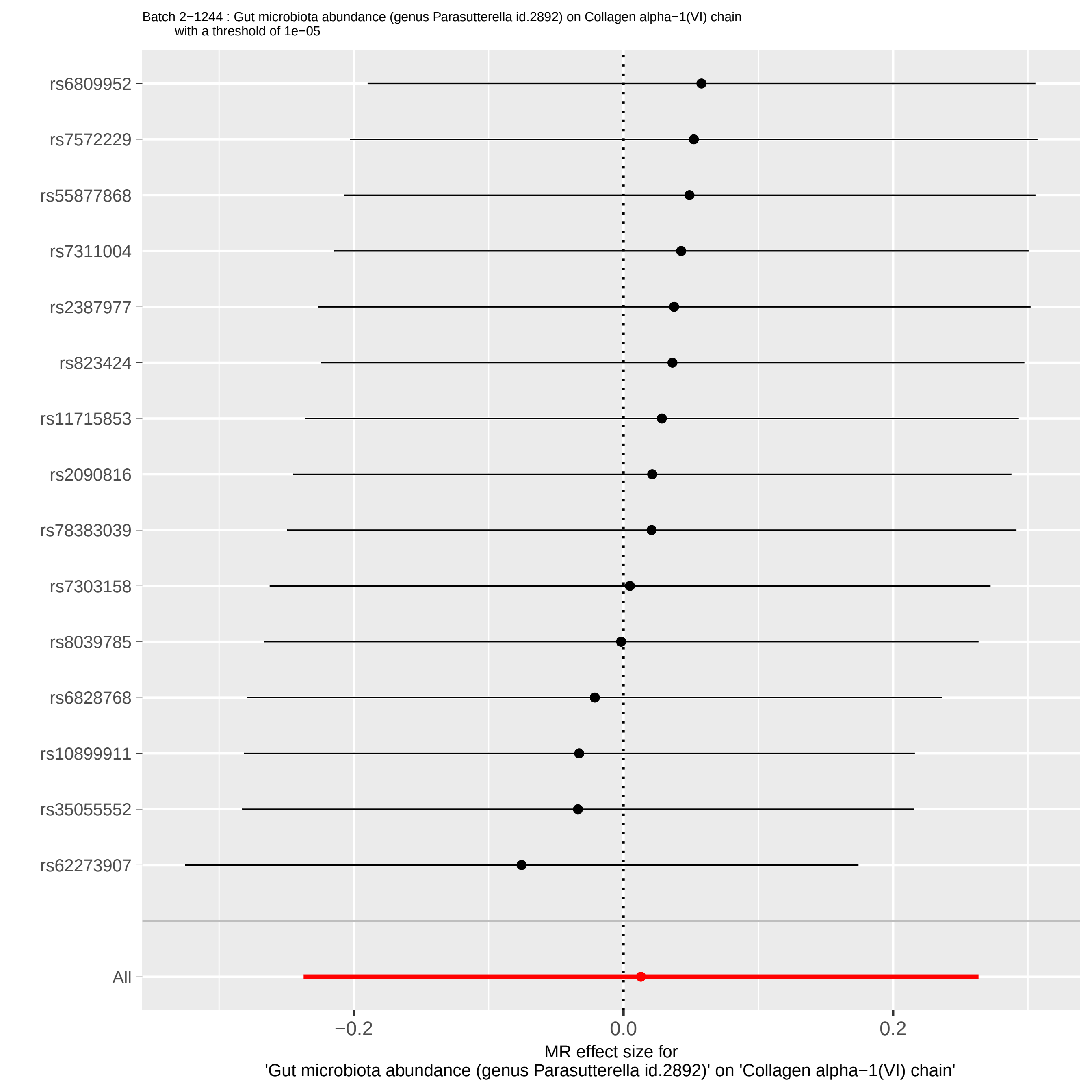

Batch 2−1244 : Gut microbiota abundance (genus Parasutterella id.2892) on Collagen alpha−1(VI) chain
with a threshold of 1e−05
rs6809952
rs7572229
rs55877868
rs7311004
rs2387977
rs823424
rs11715853
rs2090816
rs78383039
rs7303158
rs8039785
rs6828768
rs10899911
rs35055552
rs62273907
All
−0.2
0.0
0.2
MR effect size for
'Gut microbiota abundance (genus Parasutterella id.2892)' on 'Collagen alpha−1(VI) chain'

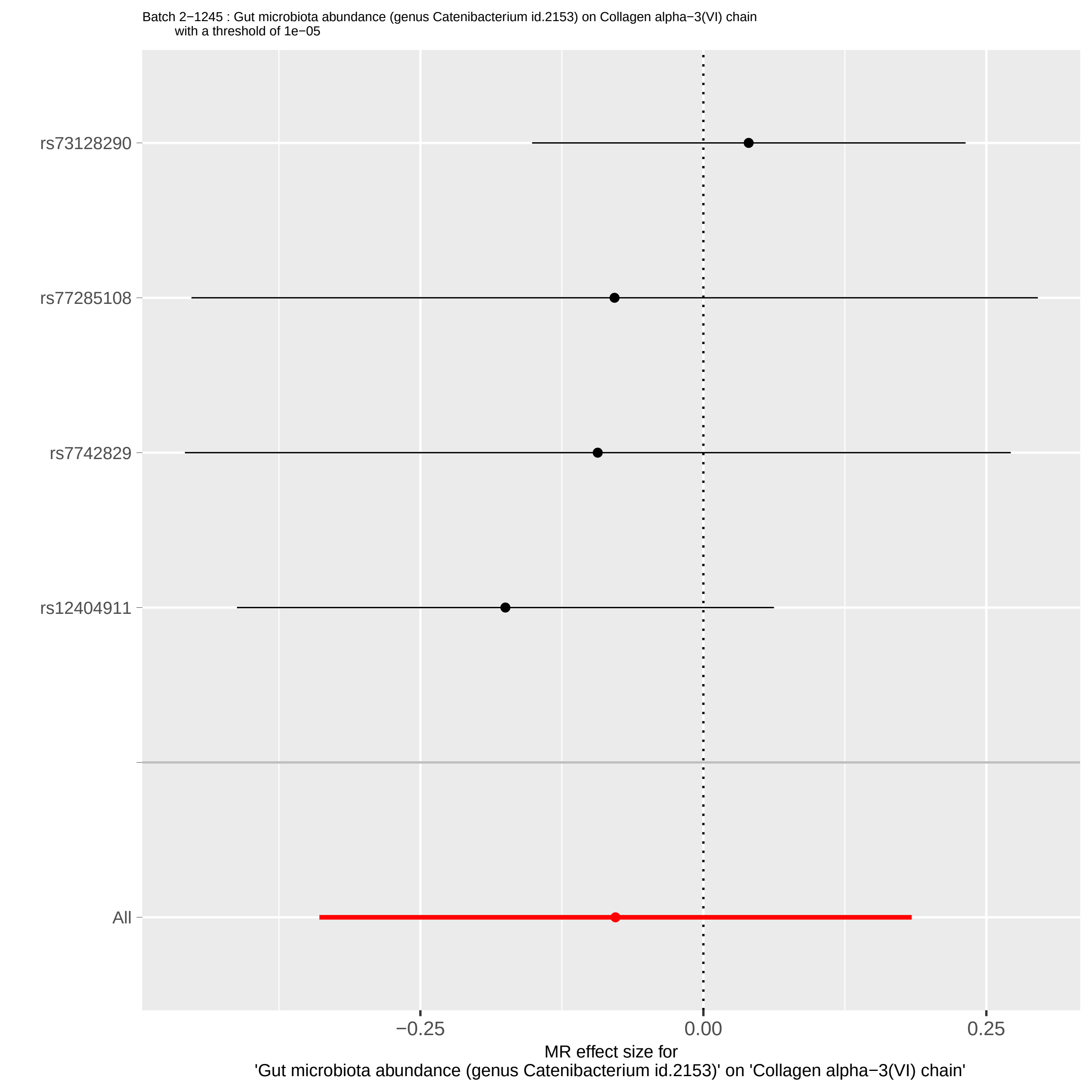

Batch 2−1245 : Gut microbiota abundance (genus Catenibacterium id.2153) on Collagen alpha−3(VI) chain
with a threshold of 1e−05
rs73128290
rs77285108
rs7742829
rs12404911
All
−0.25
0.00
0.25
MR effect size for
'Gut microbiota abundance (genus Catenibacterium id.2153)' on 'Collagen alpha−3(VI) chain'

Batch 2−1246 : Gut microbiota abundance (genus Parasutterella id.2892) on Collagen alpha−3(VI) chain
with a threshold of 1e−05

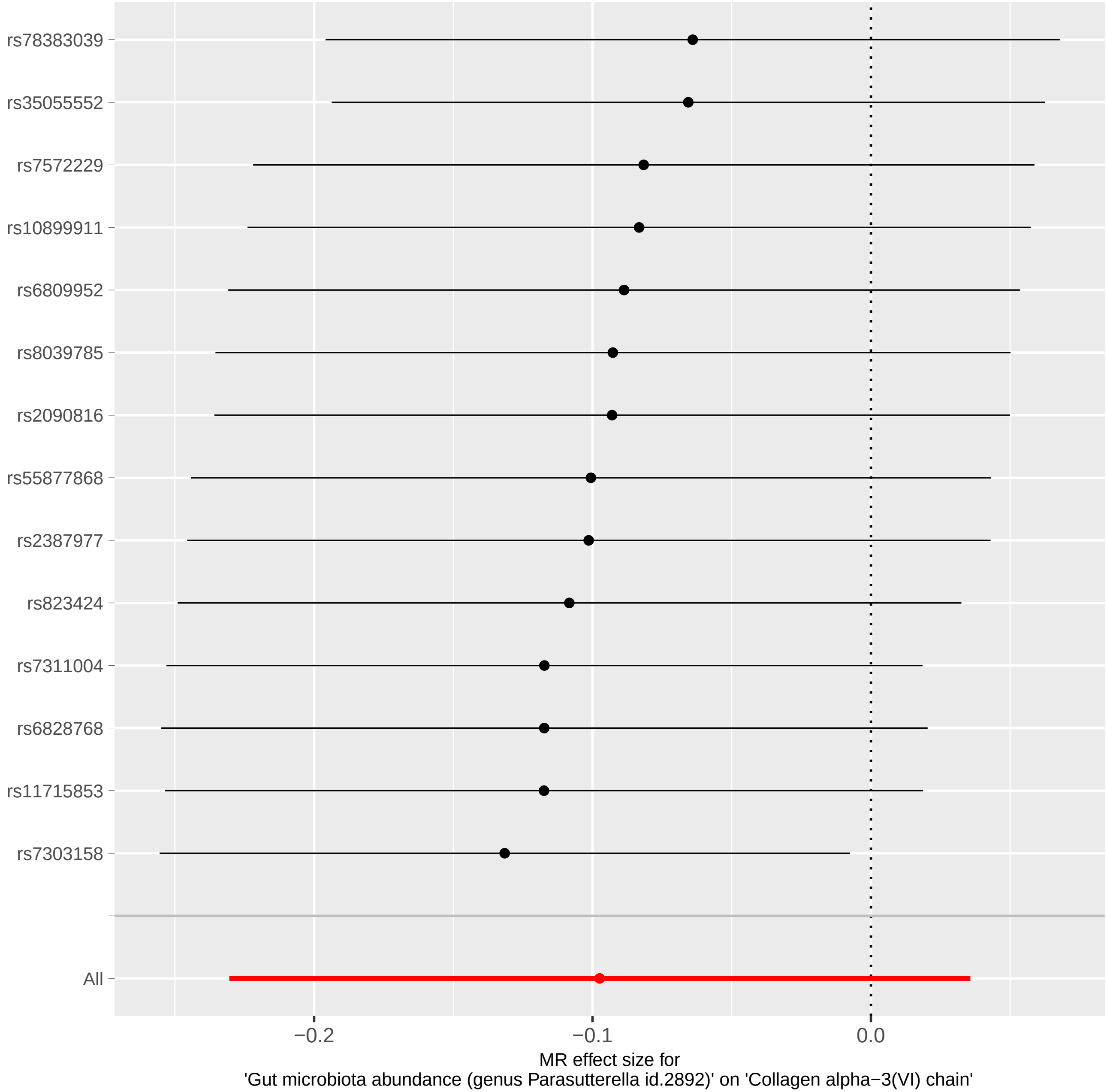

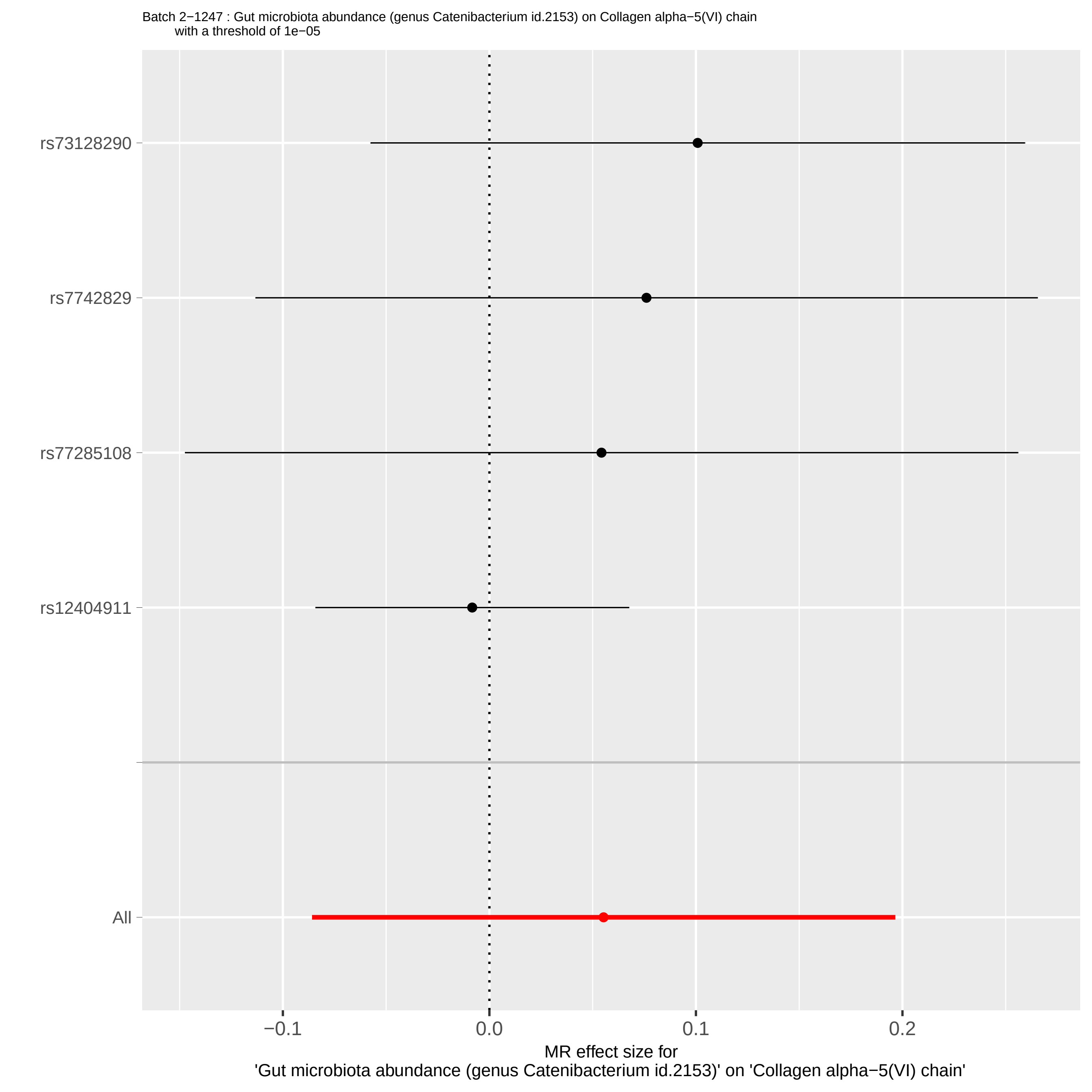
Batch 2−1247 : Gut microbiota abundance (genus Catenibacterium id.2153) on Collagen alpha−5(VI) chain
with a threshold of 1e−05
rs73128290
rs7742829
rs77285108
rs12404911
All
−0.1
0.0
0.1
0.2
MR effect size for
'Gut microbiota abundance (genus Catenibacterium id.2153)' on 'Collagen alpha−5(VI) chain'

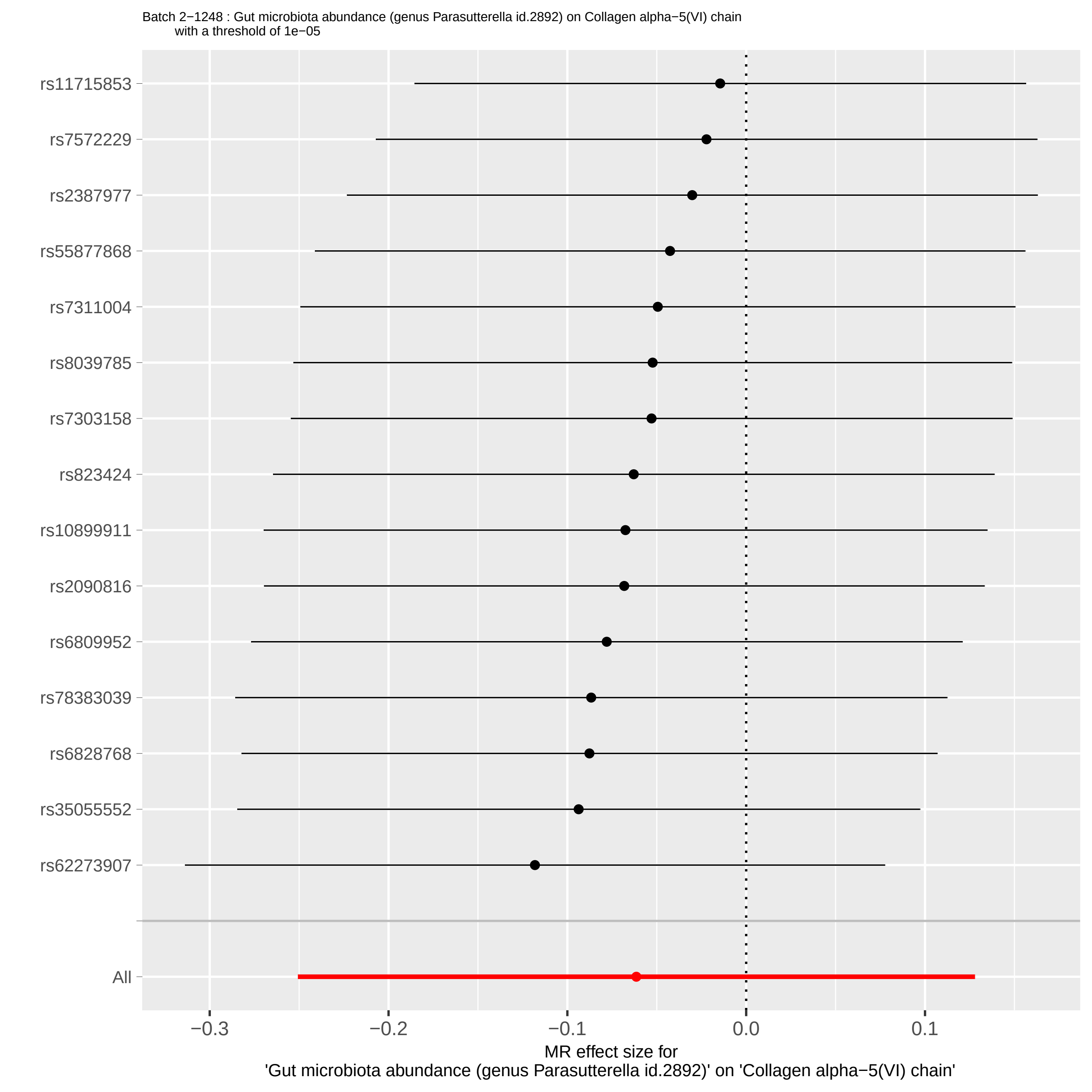
Batch 2−1248 : Gut microbiota abundance (genus Parasutterella id.2892) on Collagen alpha−5(VI) chain
with a threshold of 1e−05
rs11715853
rs7572229
rs2387977
rs55877868
rs7311004
rs8039785
rs7303158
rs823424
rs10899911
rs2090816
rs6809952
rs78383039
rs6828768
rs35055552
rs62273907
All
−0.3
−0.2
−0.1
0.0
0.1
MR effect size for
'Gut microbiota abundance (genus Parasutterella id.2892)' on 'Collagen alpha−5(VI) chain'

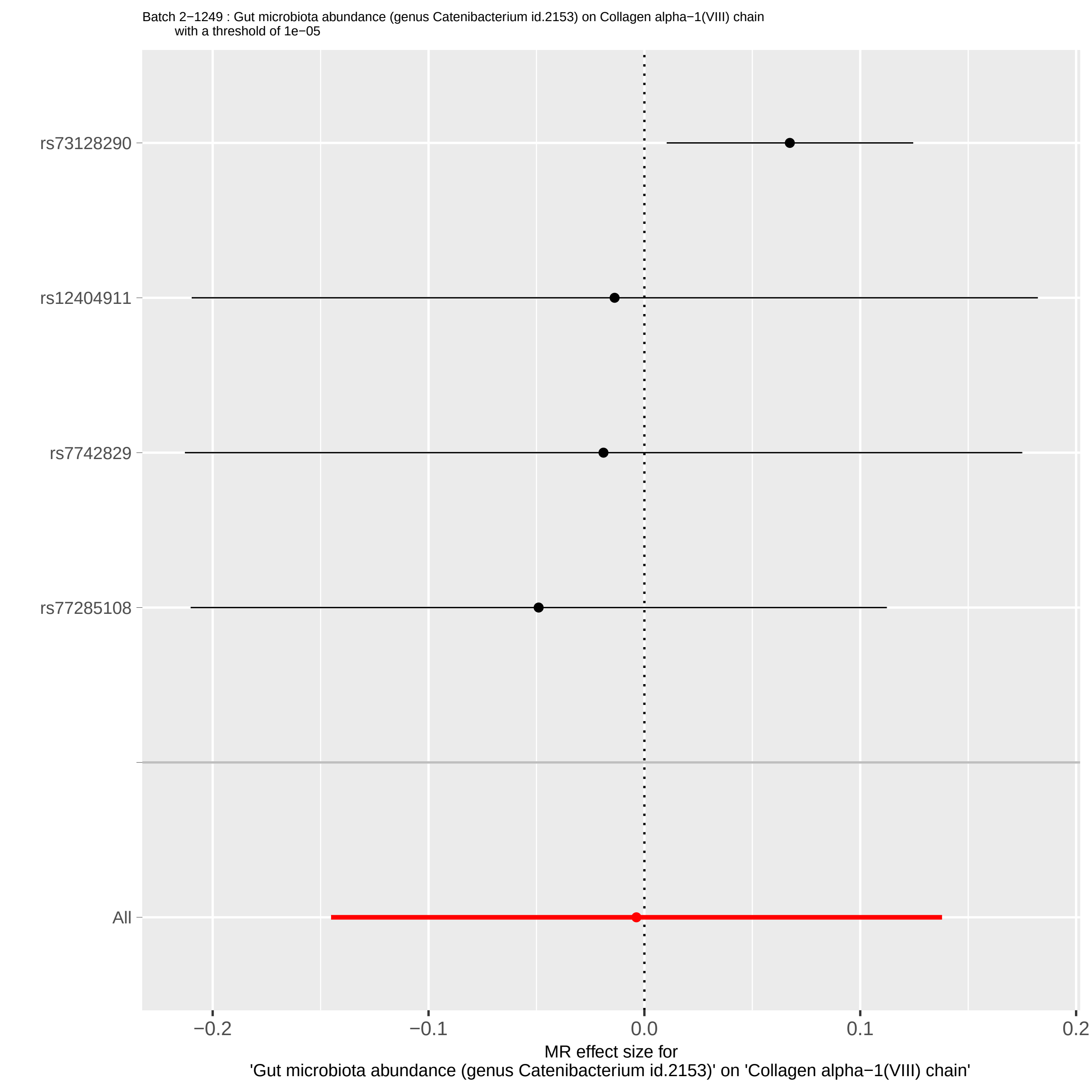
Batch 2−1249 : Gut microbiota abundance (genus Catenibacterium id.2153) on Collagen alpha−1(VIII) chain
with a threshold of 1e−05
rs73128290
rs12404911
rs7742829
rs77285108
All
−0.2
−0.1
0.0
0.1
0.2
MR effect size for
'Gut microbiota abundance (genus Catenibacterium id.2153)' on 'Collagen alpha−1(VIII) chain'

Batch 2−1250 : Gut microbiota abundance (genus Parasutterella id.2892) on Collagen alpha−1(VIII) chain
with a threshold of 1e−05

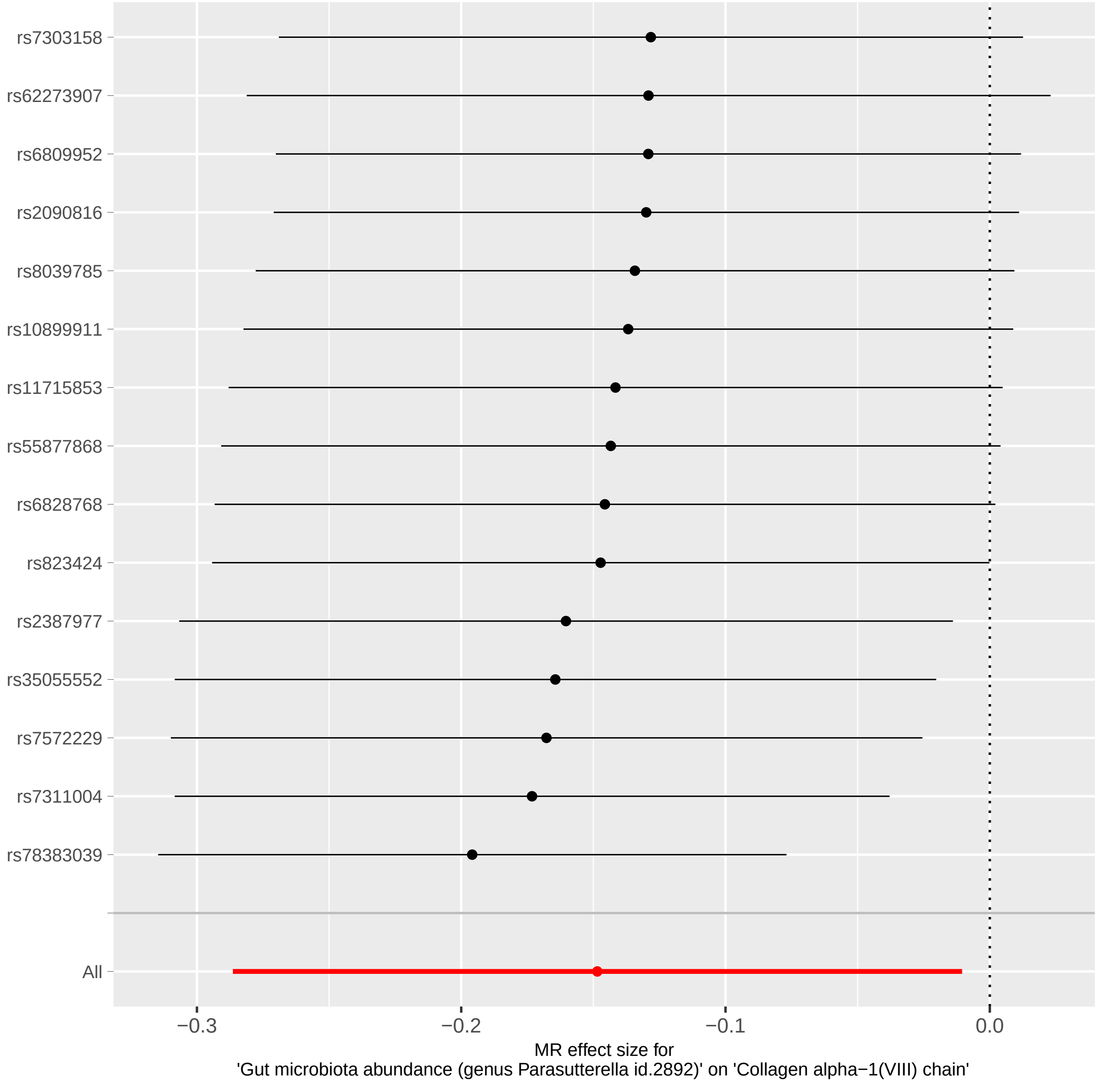

Batch 2−1251 : Gut microbiota abundance (genus Catenibacterium id.2153) on Collagen alpha−2(IX) chain
with a threshold of 1e−05

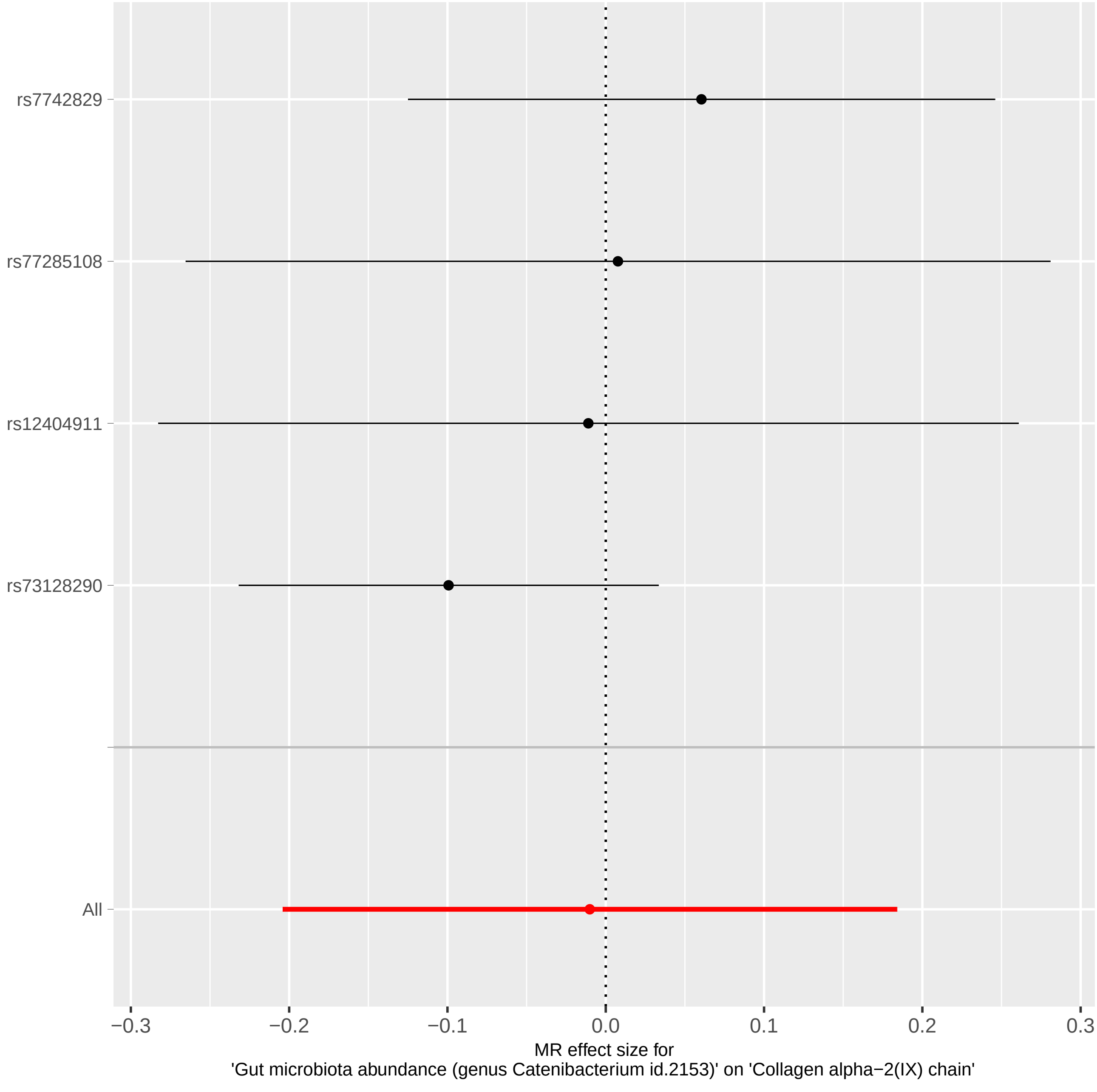

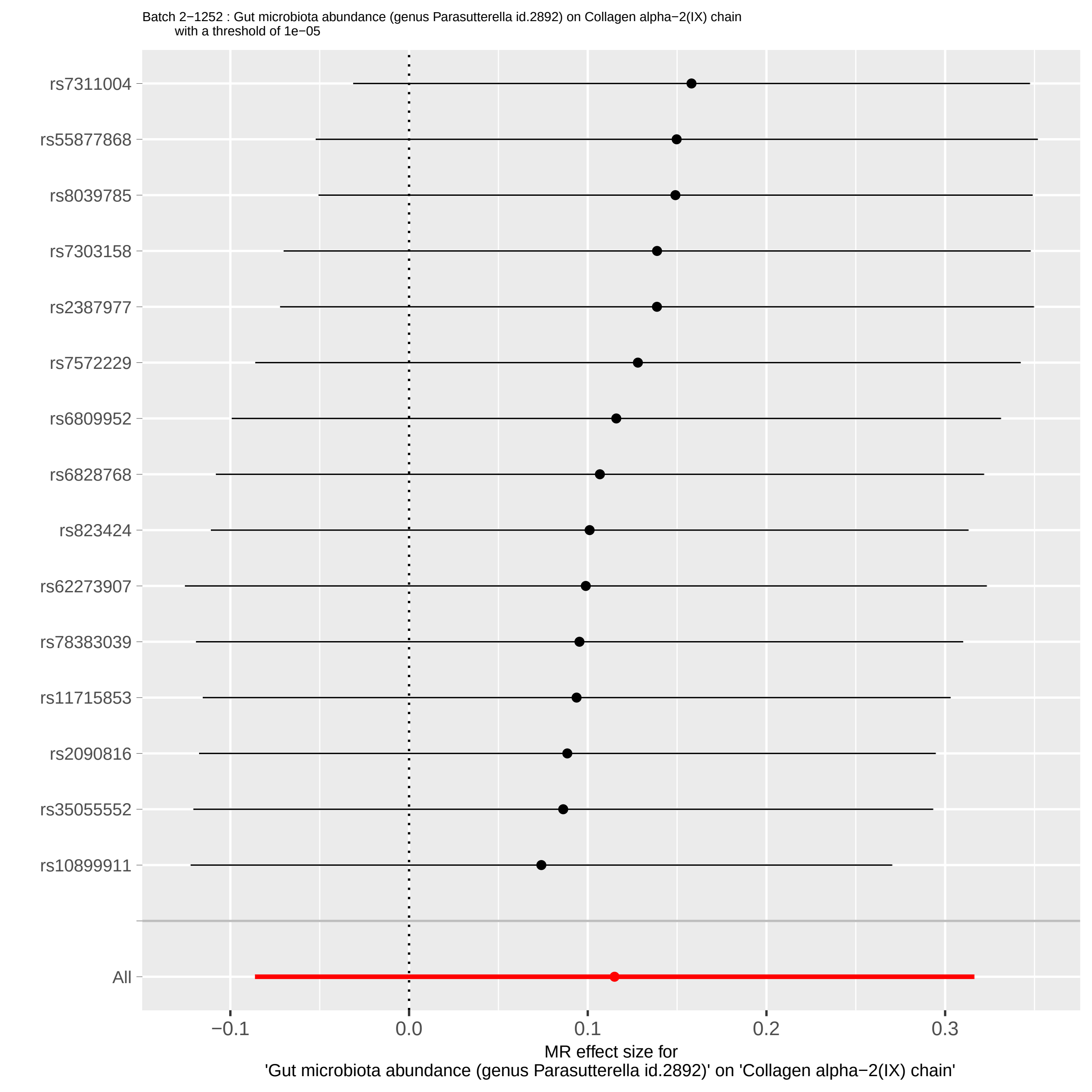
Batch 2−1252 : Gut microbiota abundance (genus Parasutterella id.2892) on Collagen alpha−2(IX) chain
with a threshold of 1e−05
rs7311004
rs55877868
rs8039785
rs7303158
rs2387977
rs7572229
rs6809952
rs6828768
rs823424
rs62273907
rs78383039
rs11715853
rs2090816
rs35055552
rs10899911
All
−0.1
0.0
0.1
0.2
0.3
MR effect size for
'Gut microbiota abundance (genus Parasutterella id.2892)' on 'Collagen alpha−2(IX) chain'

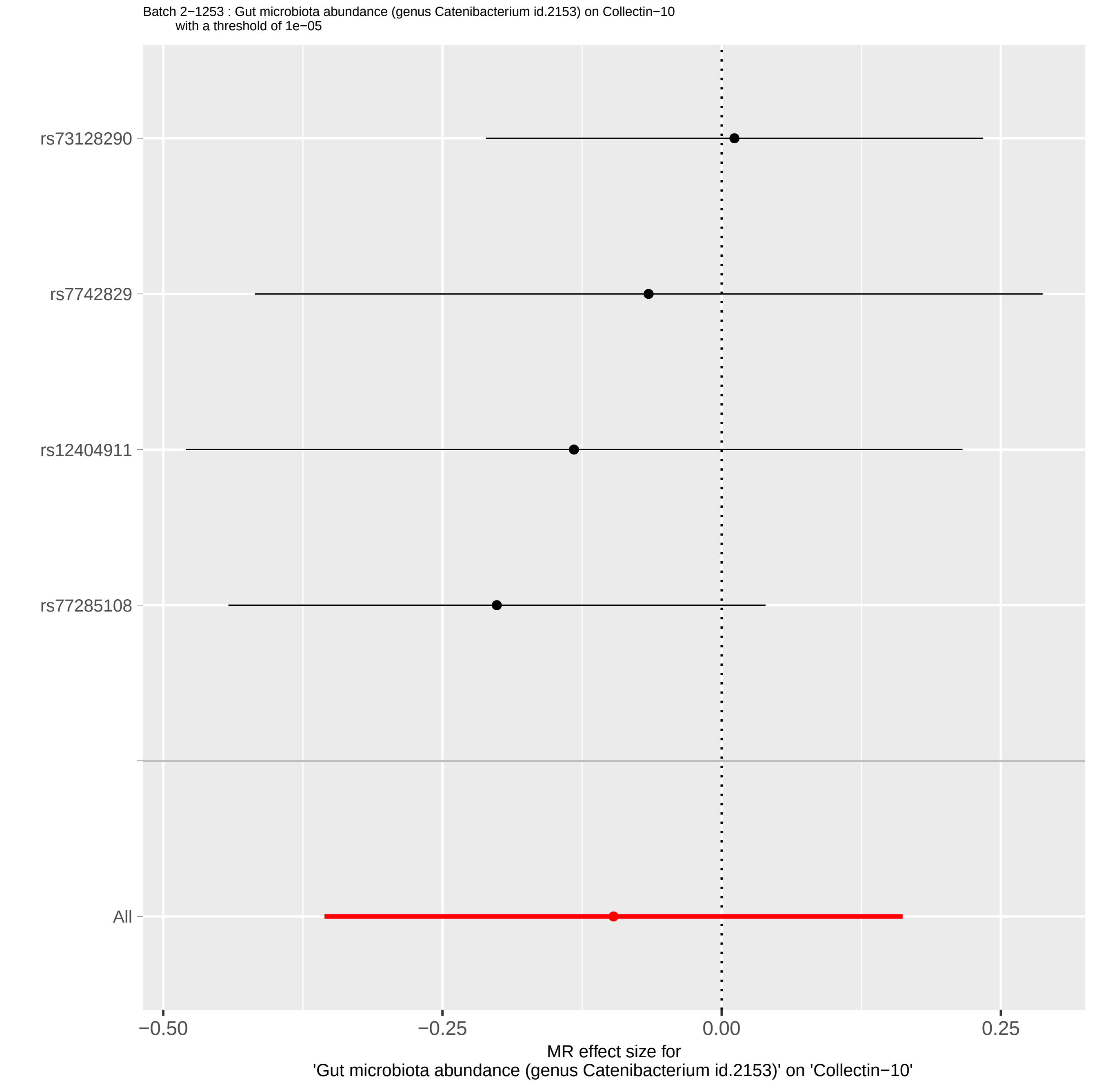
Batch 2−1253 : Gut microbiota abundance (genus Catenibacterium id.2153) on Collectin−10
with a threshold of 1e−05
rs73128290
rs7742829
rs12404911
rs77285108
All
−0.50
−0.25
0.00
0.25
MR effect size for
'Gut microbiota abundance (genus Catenibacterium id.2153)' on 'Collectin−10'

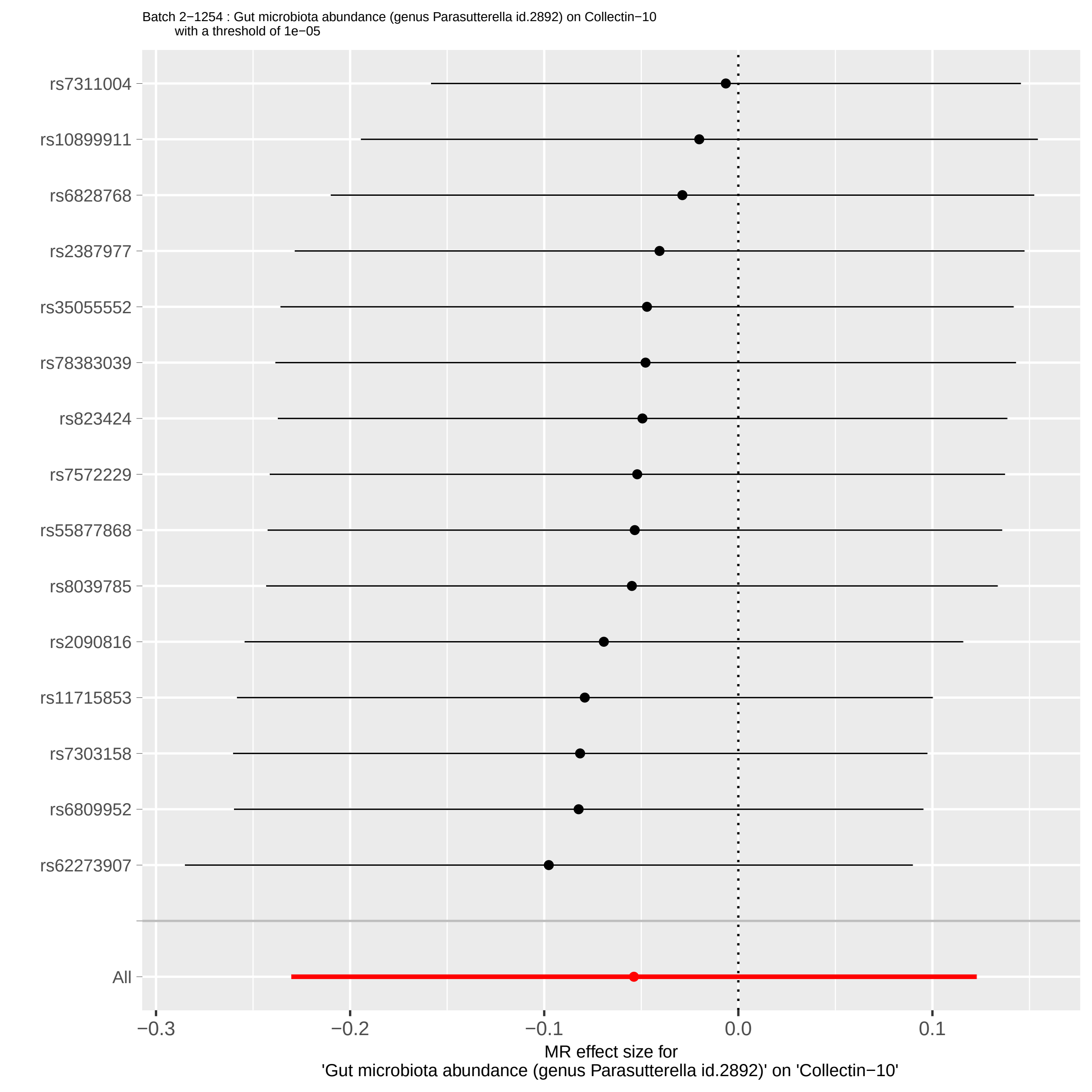

Batch 2−1254 : Gut microbiota abundance (genus Parasutterella id.2892) on Collectin−10
with a threshold of 1e−05
rs7311004
rs10899911
rs6828768
rs2387977
rs35055552
rs78383039
rs823424
rs7572229
rs55877868
rs8039785
rs2090816
rs11715853
rs7303158
rs6809952
rs62273907
All
−0.3
−0.2
−0.1
0.0
0.1
MR effect size for
'Gut microbiota abundance (genus Parasutterella id.2892)' on 'Collectin−10'

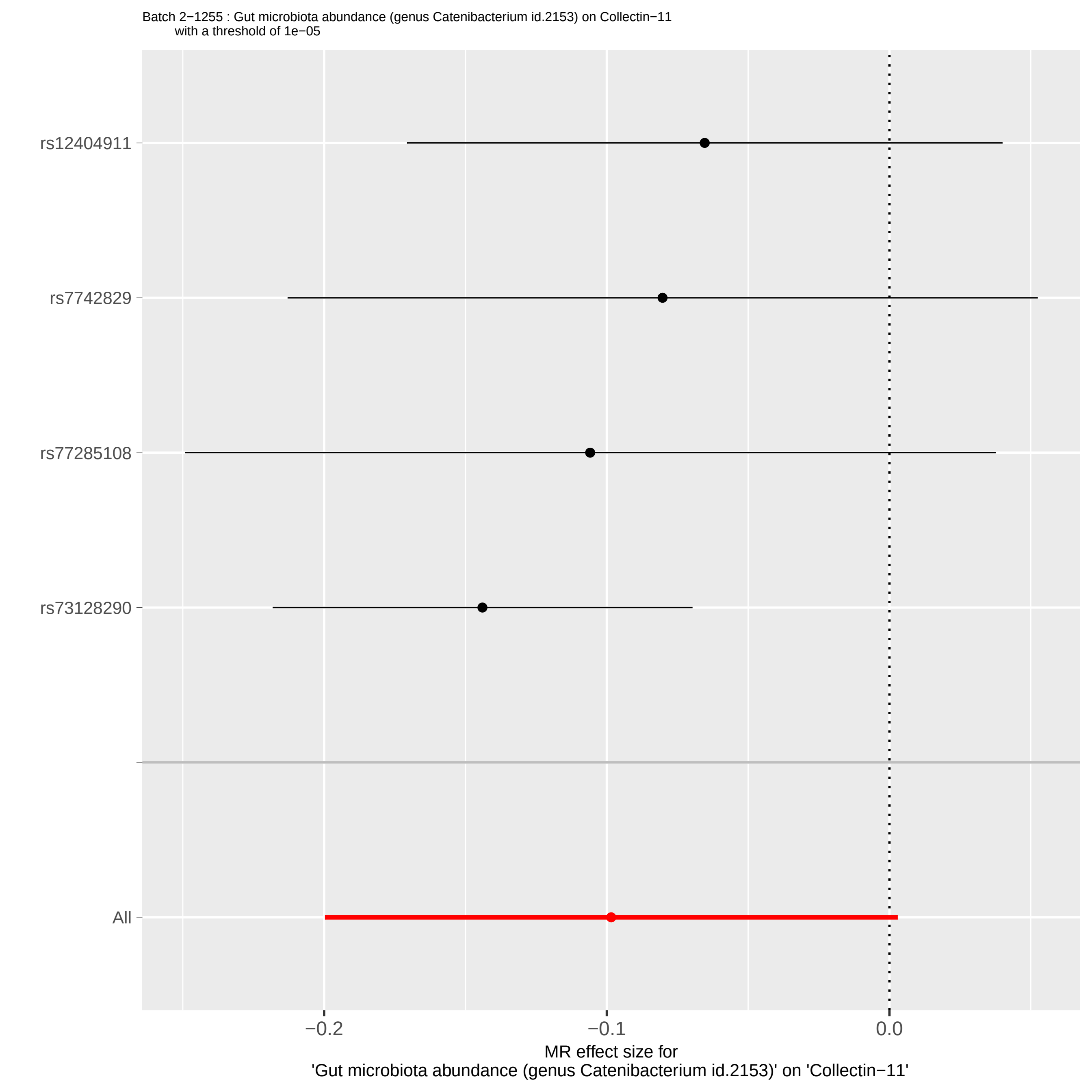
Batch 2−1255 : Gut microbiota abundance (genus Catenibacterium id.2153) on Collectin−11
with a threshold of 1e−05
rs12404911
rs7742829
rs77285108
rs73128290
All
−0.2
−0.1
0.0
MR effect size for
'Gut microbiota abundance (genus Catenibacterium id.2153)' on 'Collectin−11'

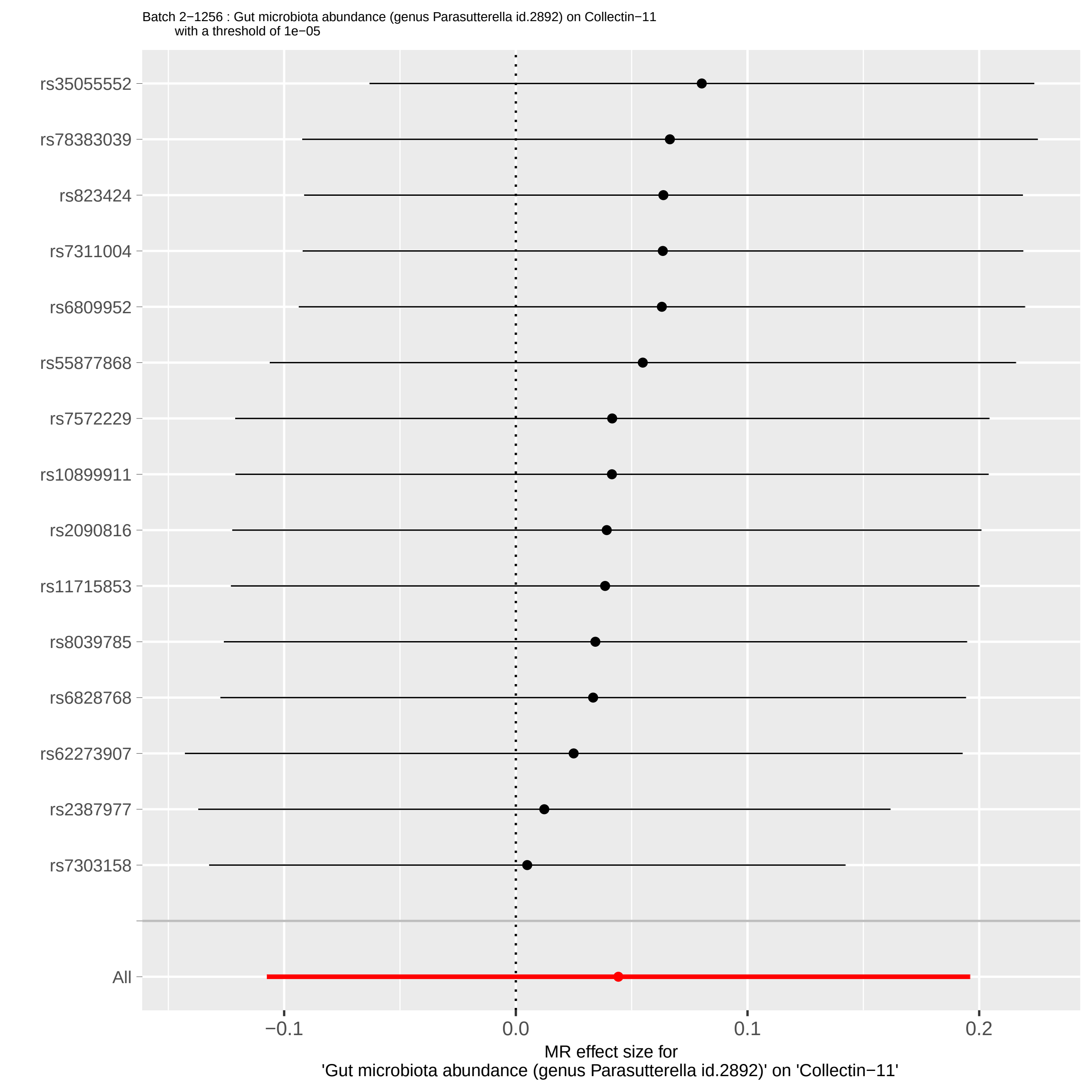

Batch 2−1256 : Gut microbiota abundance (genus Parasutterella id.2892) on Collectin−11
with a threshold of 1e−05
rs35055552
rs78383039
rs823424
rs7311004
rs6809952
rs55877868
rs7572229
rs10899911
rs2090816
rs11715853
rs8039785
rs6828768
rs62273907
rs2387977
rs7303158
All
−0.1
0.0
0.1
0.2
MR effect size for
'Gut microbiota abundance (genus Parasutterella id.2892)' on 'Collectin−11'

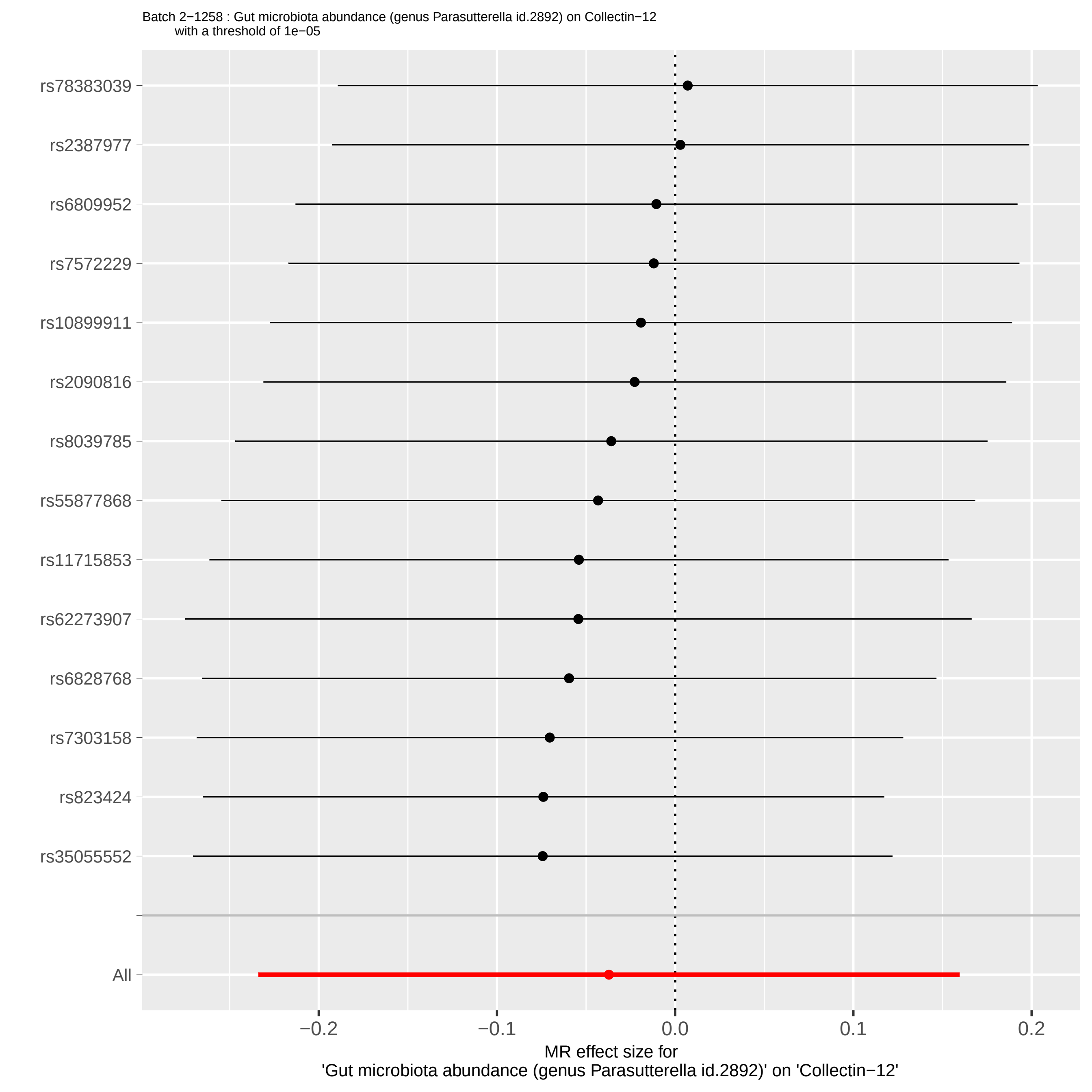

Batch 2−1258 : Gut microbiota abundance (genus Parasutterella id.2892) on Collectin−12
with a threshold of 1e−05
rs78383039
rs2387977
rs6809952
rs7572229
rs10899911
rs2090816
rs8039785
rs55877868
rs11715853
rs62273907
rs6828768
rs7303158
rs823424
rs35055552
All
−0.2
−0.1
0.0
0.1
0.2
MR effect size for
'Gut microbiota abundance (genus Parasutterella id.2892)' on 'Collectin−12'

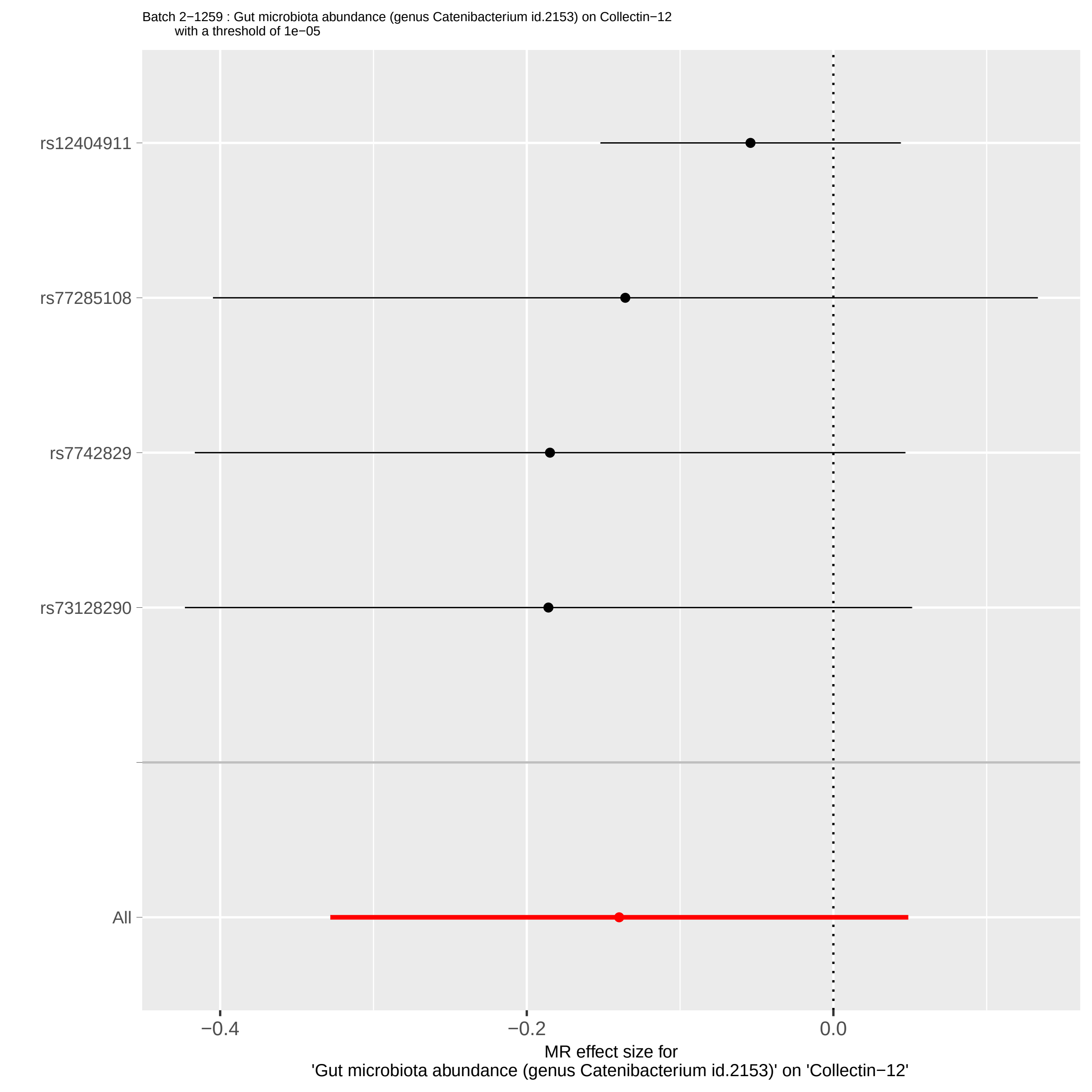
Batch 2−1259 : Gut microbiota abundance (genus Catenibacterium id.2153) on Collectin−12
with a threshold of 1e−05
rs12404911
rs77285108
rs7742829
rs73128290
All
−0.4
−0.2
0.0
MR effect size for
'Gut microbiota abundance (genus Catenibacterium id.2153)' on 'Collectin−12'

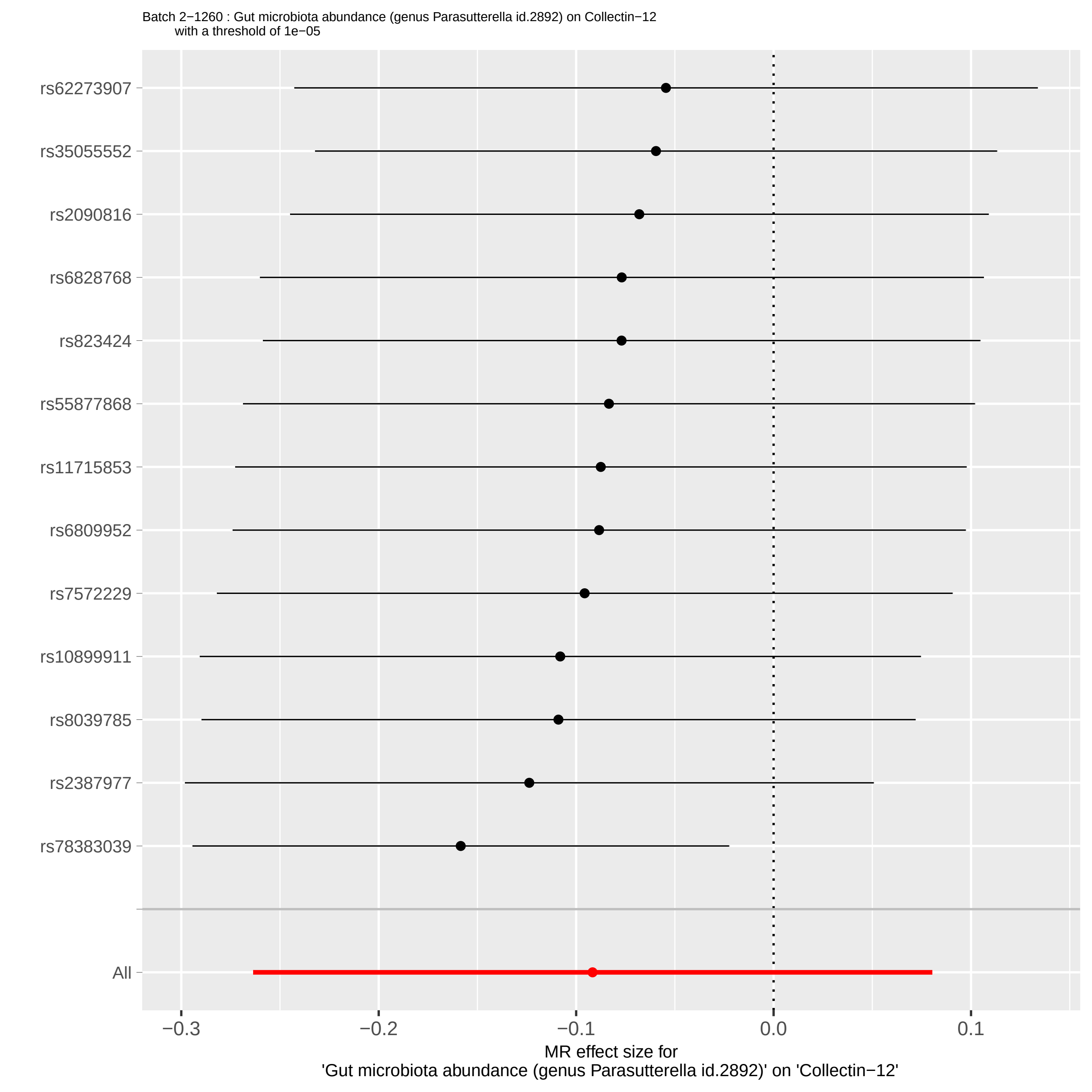
Batch 2−1260 : Gut microbiota abundance (genus Parasutterella id.2892) on Collectin−12
with a threshold of 1e−05
rs62273907
rs35055552
rs2090816
rs6828768
rs823424
rs55877868
rs11715853
rs6809952
rs7572229
rs10899911
rs8039785
rs2387977
rs78383039
All
−0.3
−0.2
−0.1
0.0
0.1
MR effect size for
'Gut microbiota abundance (genus Parasutterella id.2892)' on 'Collectin−12'

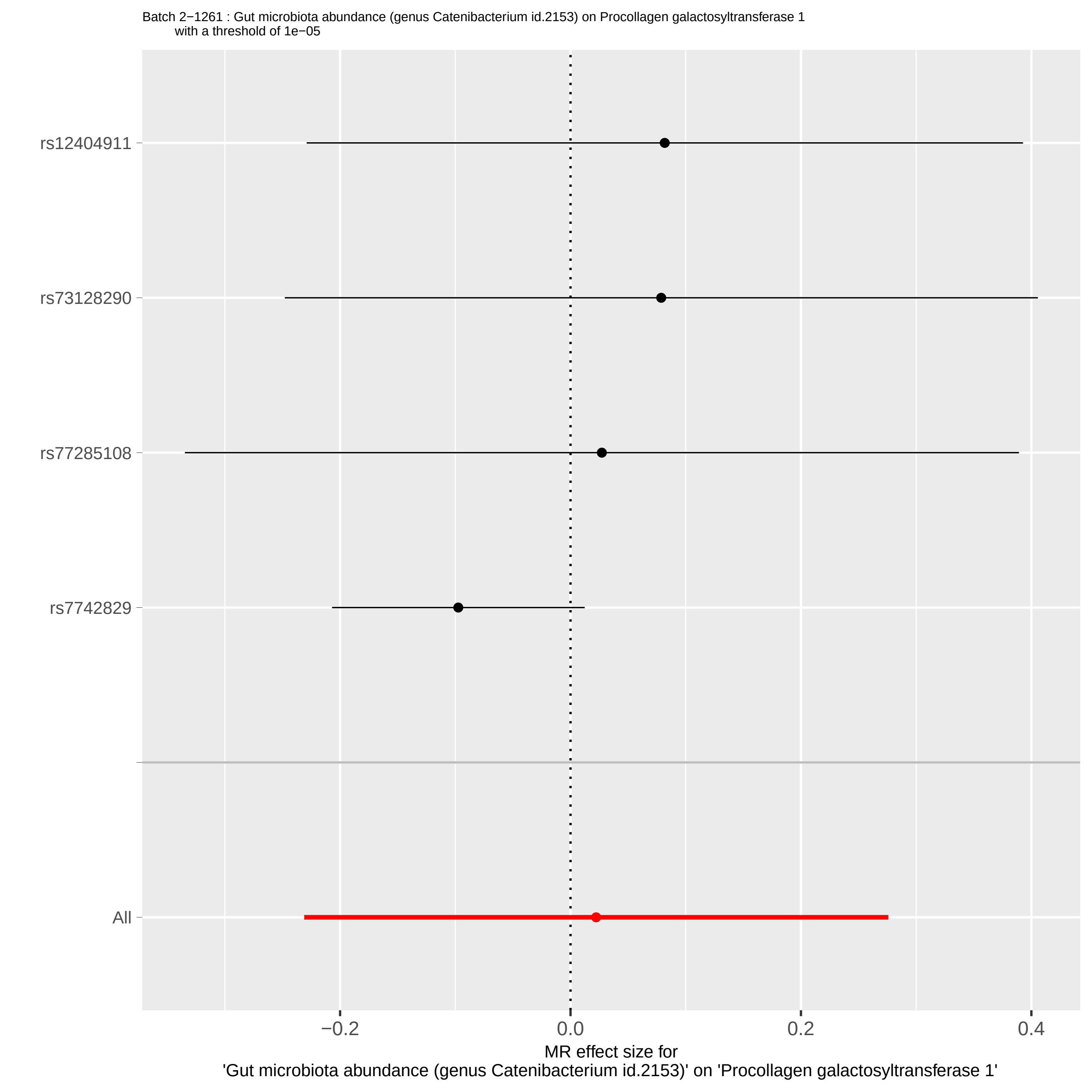
Batch 2−1261 : Gut microbiota abundance (genus Catenibacterium id.2153) on Procollagen galactosyltransferase 1
with a threshold of 1e−05
rs12404911
rs73128290
rs77285108
rs7742829
All
−0.2
0.0
0.2
0.4
MR effect size for
'Gut microbiota abundance (genus Catenibacterium id.2153)' on 'Procollagen galactosyltransferase 1'

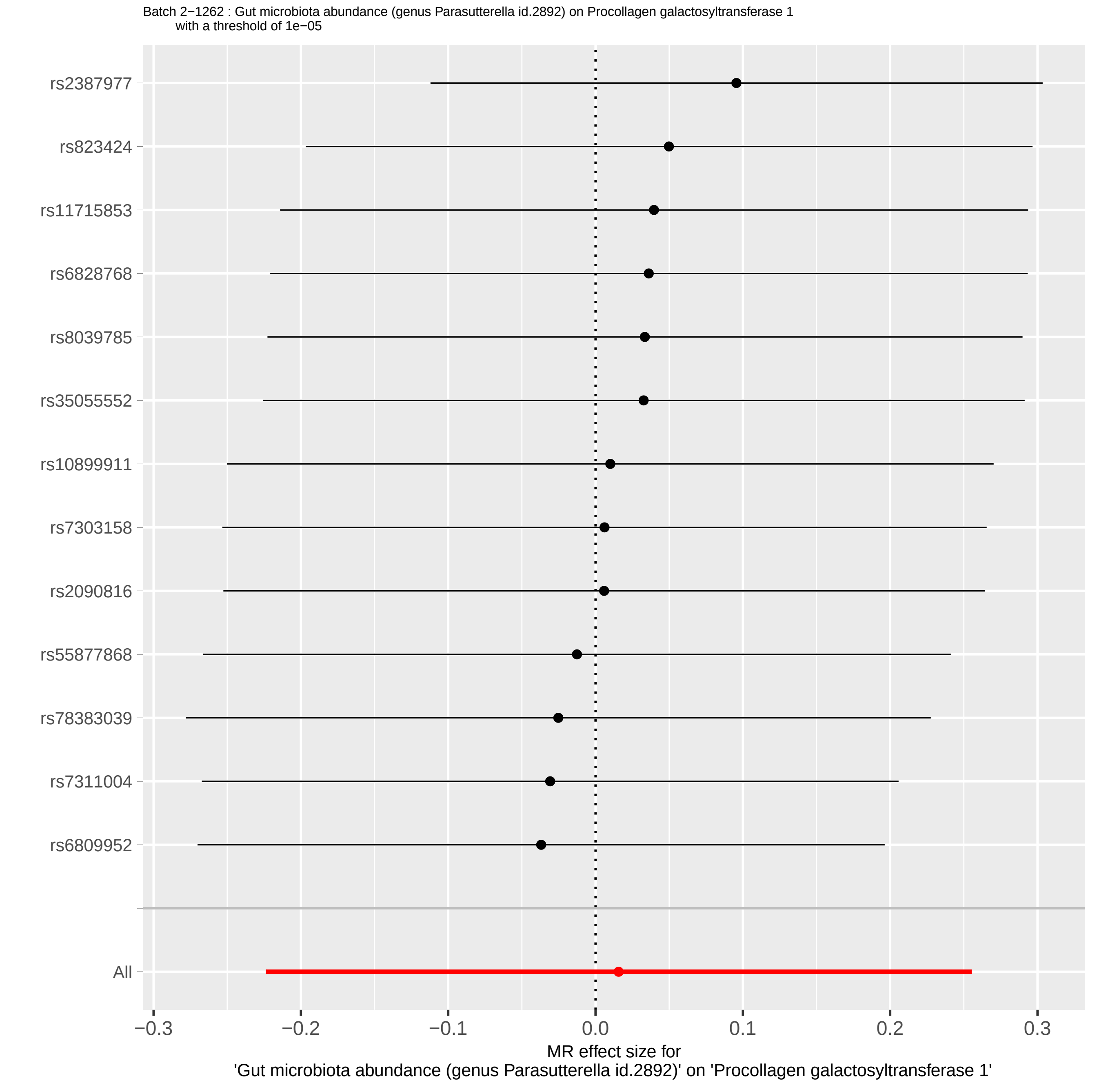

Batch 2−1262 : Gut microbiota abundance (genus Parasutterella id.2892) on Procollagen galactosyltransferase 1
with a threshold of 1e−05
rs2387977
rs823424
rs11715853
rs6828768
rs8039785
rs35055552
rs10899911
rs7303158
rs2090816
rs55877868
rs78383039
rs7311004
rs6809952
All
−0.3
−0.2
−0.1
0.0
0.1
0.2
0.3
MR effect size for
'Gut microbiota abundance (genus Parasutterella id.2892)' on 'Procollagen galactosyltransferase 1'

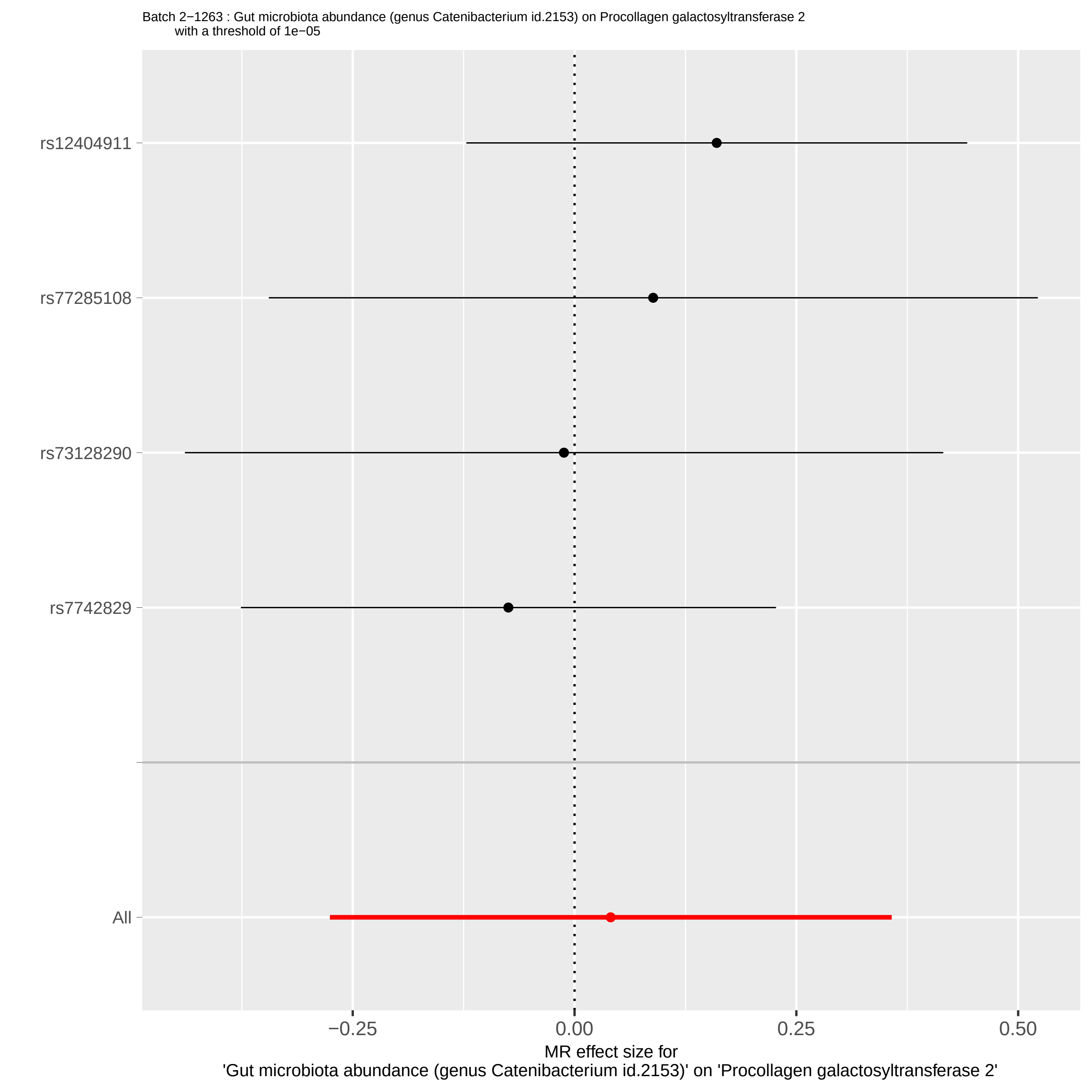

Batch 2−1263 : Gut microbiota abundance (genus Catenibacterium id.2153) on Procollagen galactosyltransferase 2
with a threshold of 1e−05
rs12404911
rs77285108
rs73128290
rs7742829
All
−0.25
0.00
0.25
0.50
MR effect size for
'Gut microbiota abundance (genus Catenibacterium id.2153)' on 'Procollagen galactosyltransferase 2'

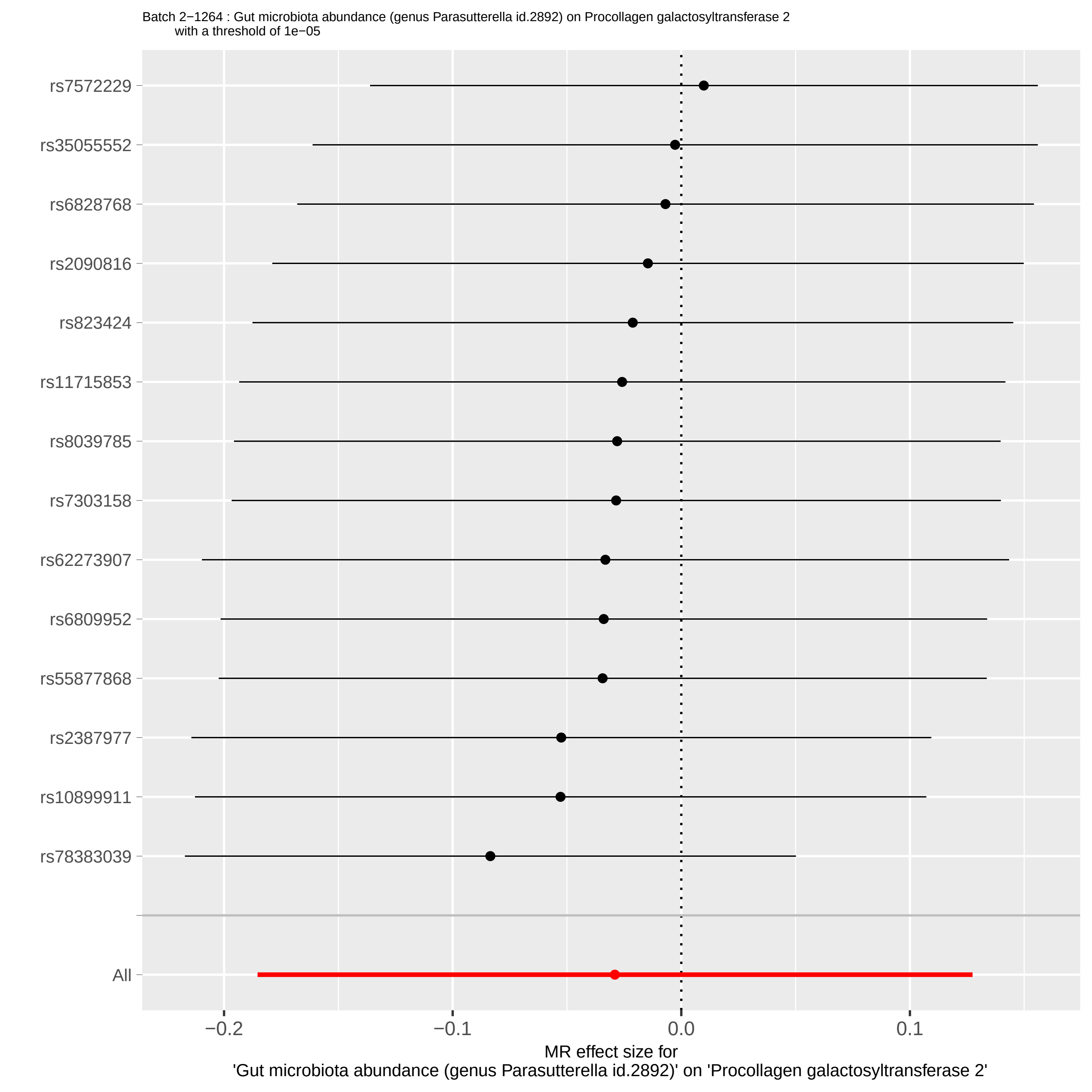
Batch 2−1264 : Gut microbiota abundance (genus Parasutterella id.2892) on Procollagen galactosyltransferase 2
with a threshold of 1e−05
rs7572229
rs35055552
rs6828768
rs2090816
rs823424
rs11715853
rs8039785
rs7303158
rs62273907
rs6809952
rs55877868
rs2387977
rs10899911
rs78383039
All
−0.2
−0.1
0.0
0.1
MR effect size for
'Gut microbiota abundance (genus Parasutterella id.2892)' on 'Procollagen galactosyltransferase 2'

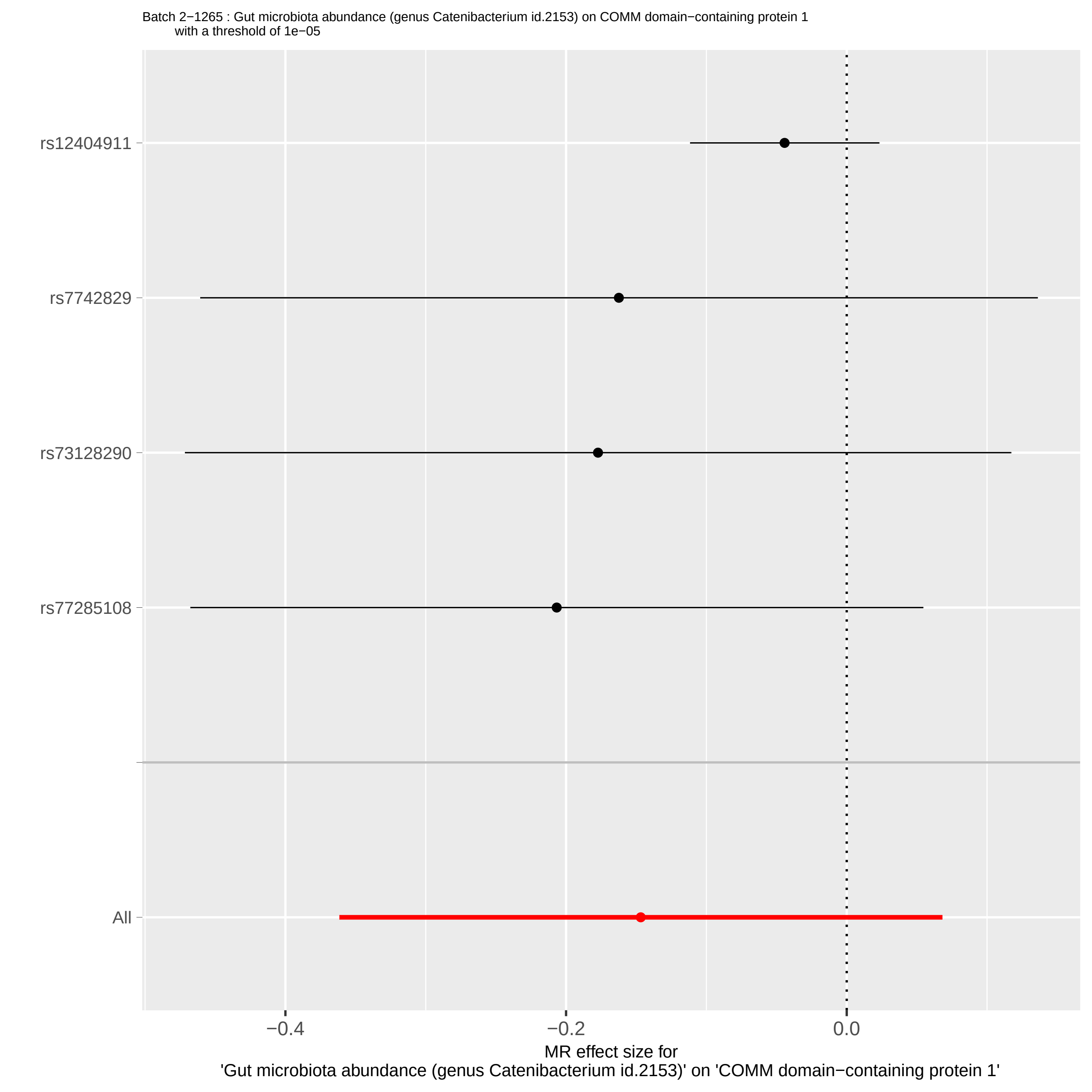
Batch 2−1265 : Gut microbiota abundance (genus Catenibacterium id.2153) on COMM domain−containing protein 1
with a threshold of 1e−05
rs12404911
rs7742829
rs73128290
rs77285108
All
−0.4
−0.2
0.0
MR effect size for
'Gut microbiota abundance (genus Catenibacterium id.2153)' on 'COMM domain−containing protein 1'

Batch 2−1266 : Gut microbiota abundance (genus Parasutterella id.2892) on COMM domain−containing protein 1
with a threshold of 1e−05

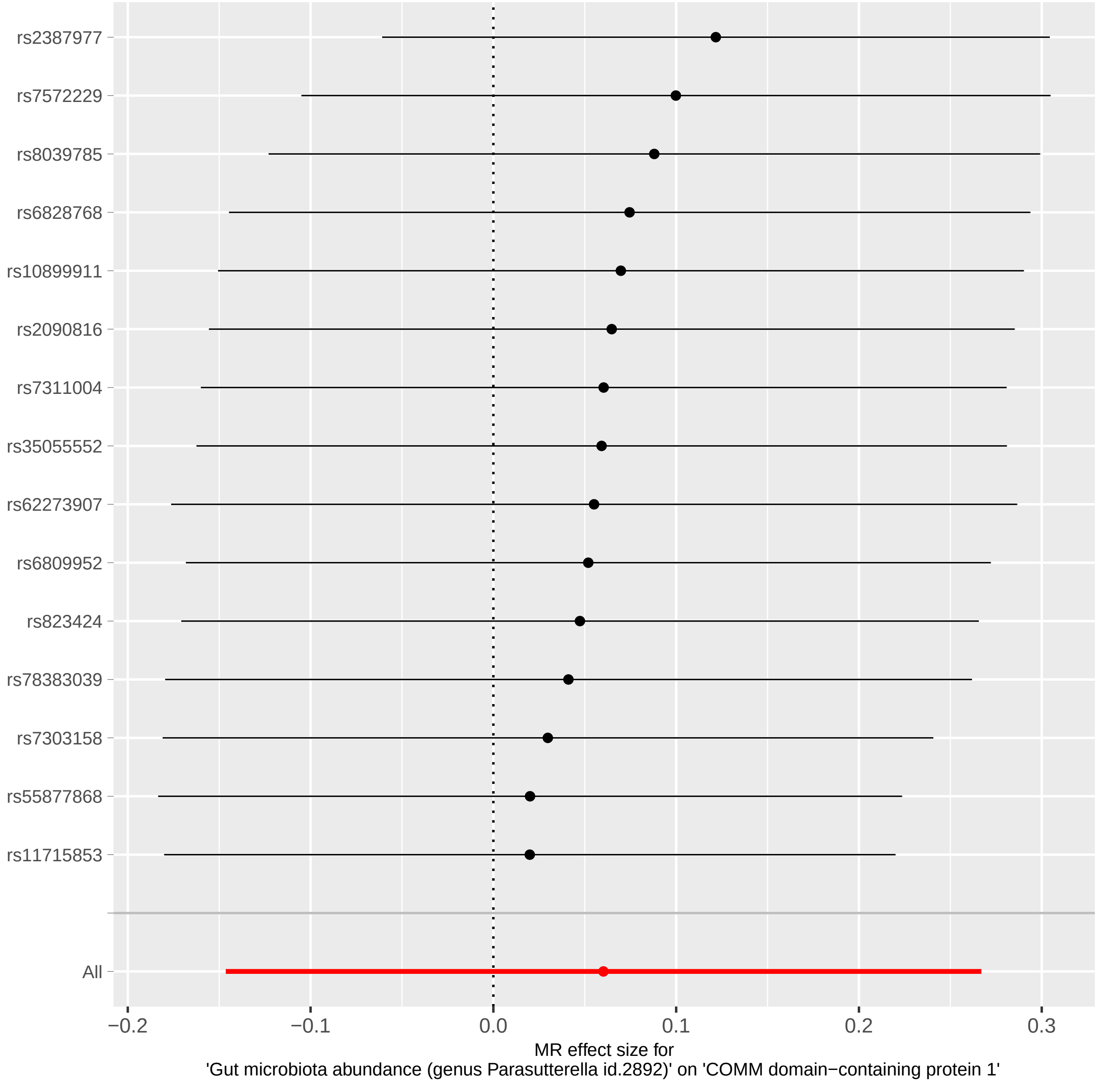

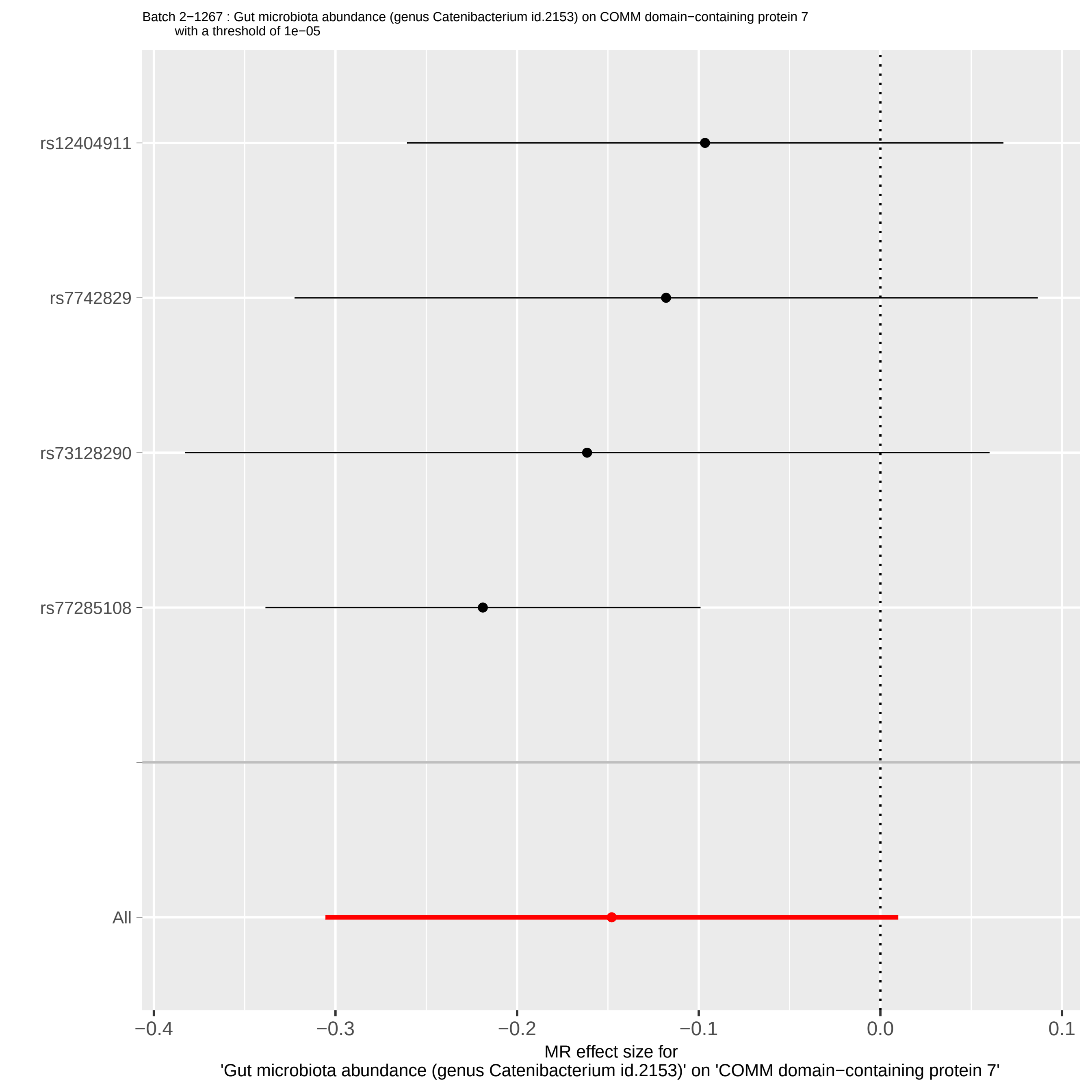

Batch 2−1267 : Gut microbiota abundance (genus Catenibacterium id.2153) on COMM domain−containing protein 7
with a threshold of 1e−05
rs12404911
rs7742829
rs73128290
rs77285108
All
−0.4
−0.3
−0.2
−0.1
0.0
0.1
MR effect size for
'Gut microbiota abundance (genus Catenibacterium id.2153)' on 'COMM domain−containing protein 7'

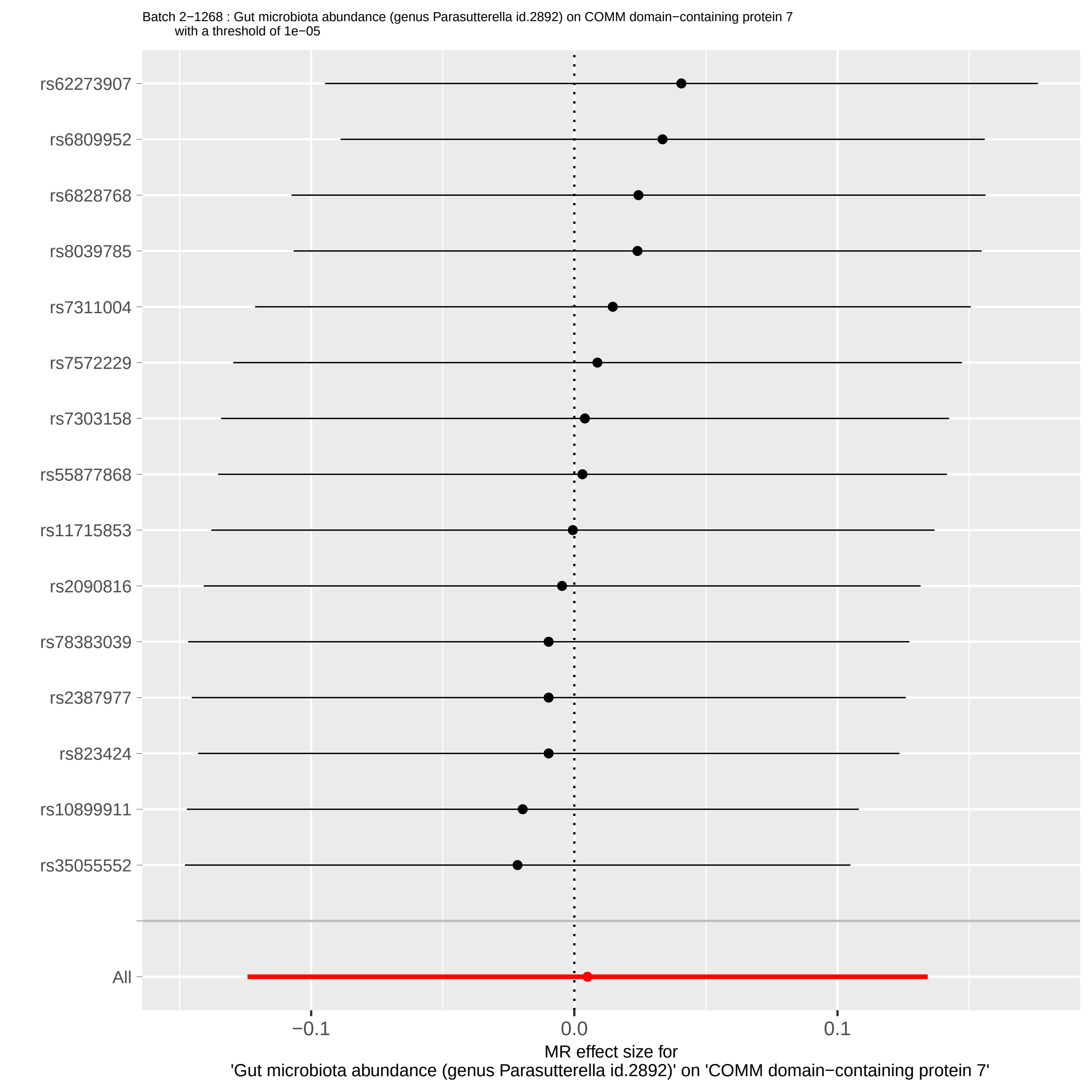

Batch 2−1268 : Gut microbiota abundance (genus Parasutterella id.2892) on COMM domain−containing protein 7
with a threshold of 1e−05
rs62273907
rs6809952
rs6828768
rs8039785
rs7311004
rs7572229
rs7303158
rs55877868
rs11715853
rs2090816
rs78383039
rs2387977
rs823424
rs10899911
rs35055552
All
−0.1
0.0
0.1
MR effect size for
'Gut microbiota abundance (genus Parasutterella id.2892)' on 'COMM domain−containing protein 7'

Batch 2−1269 : Gut microbiota abundance (genus Catenibacterium id.2153) on COP9 signalosome complex subunit 2 with a threshold of 1e−05

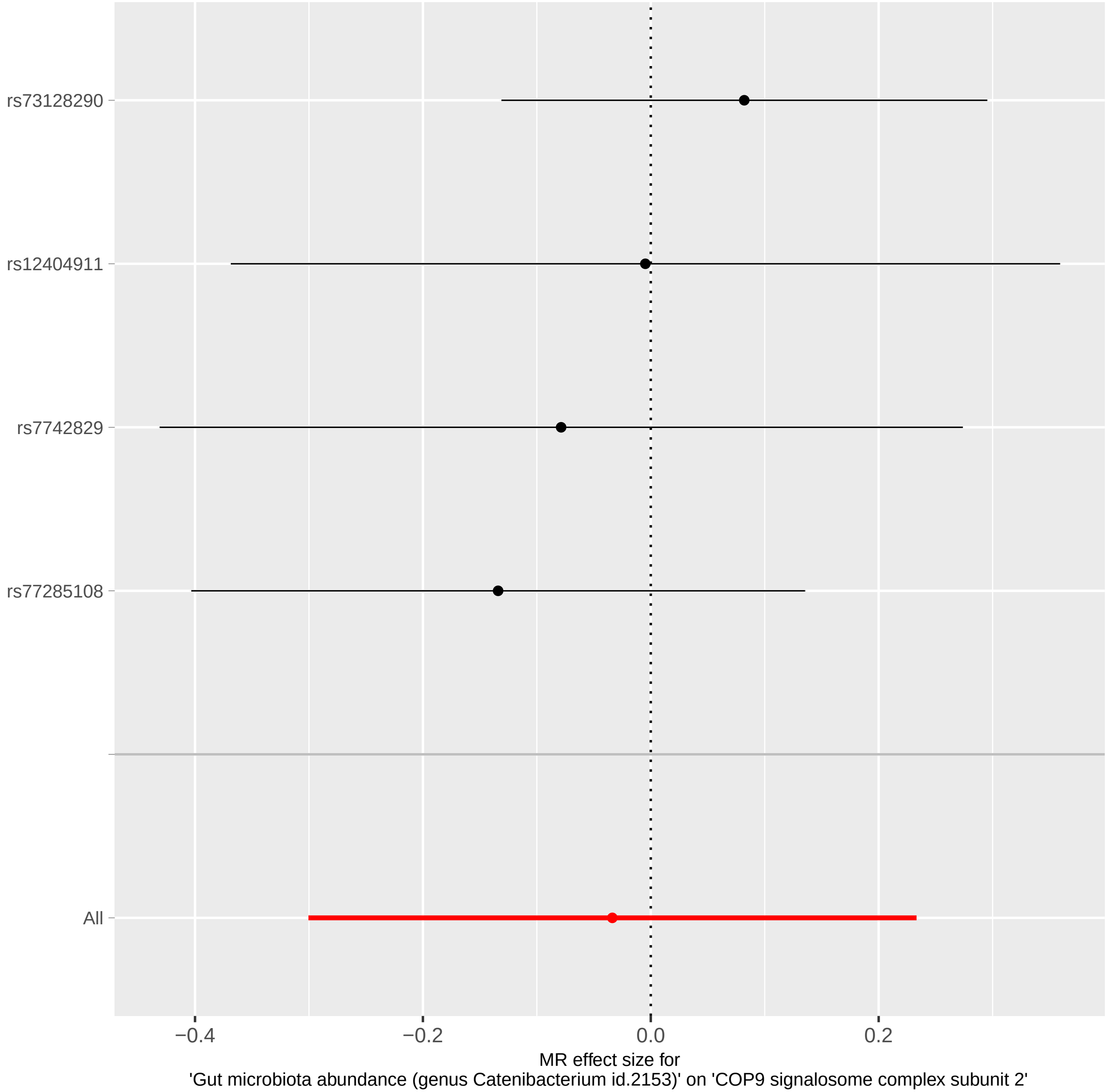

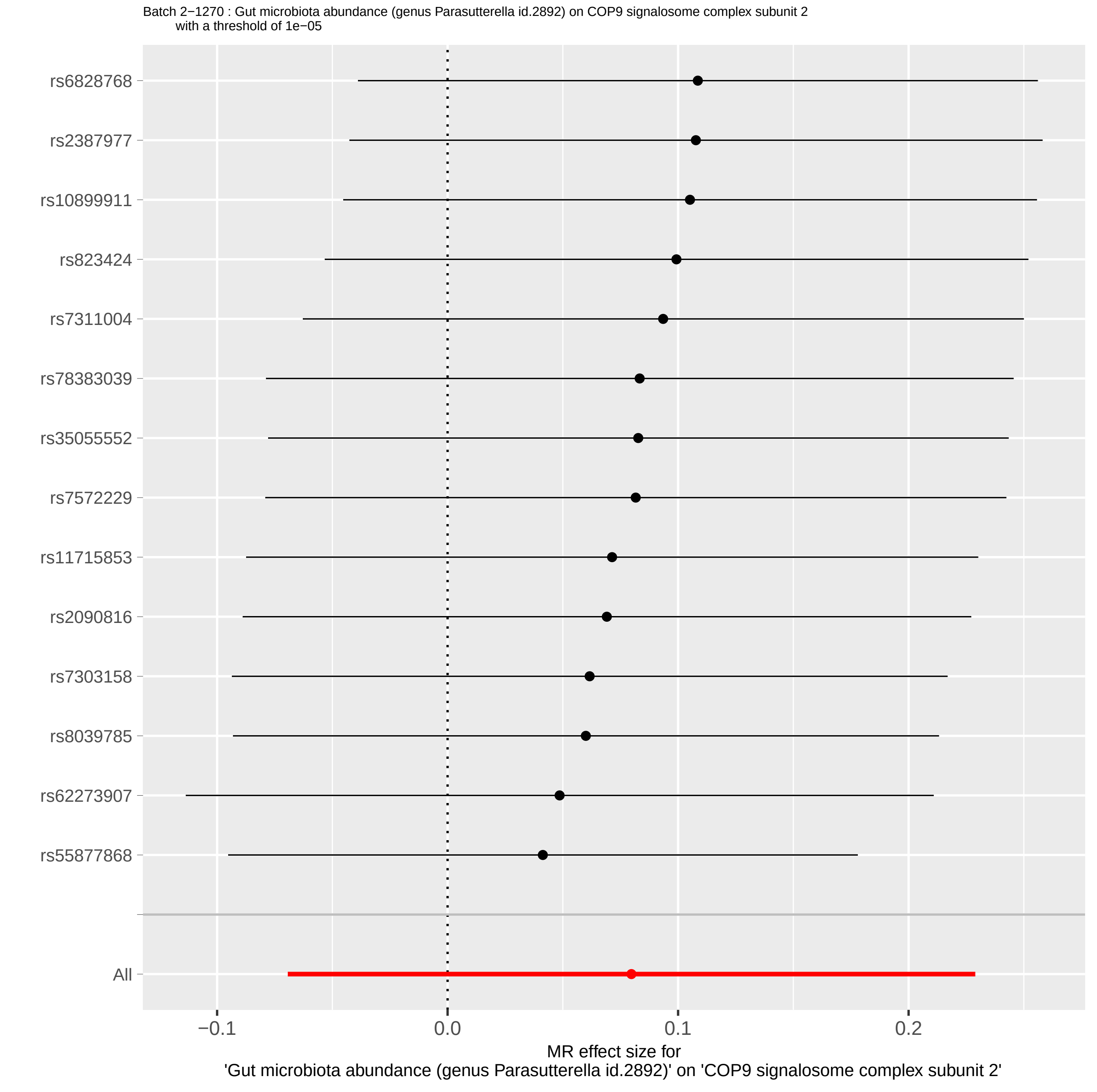

Batch 2−1270 : Gut microbiota abundance (genus Parasutterella id.2892) on COP9 signalosome complex subunit 2
with a threshold of 1e−05
rs6828768
rs2387977
rs10899911
rs823424
rs7311004
rs78383039
rs35055552
rs7572229
rs11715853
rs2090816
rs7303158
rs8039785
rs62273907
rs55877868
All
−0.1
0.0
0.1
0.2
MR effect size for
'Gut microbiota abundance (genus Parasutterella id.2892)' on 'COP9 signalosome complex subunit 2'

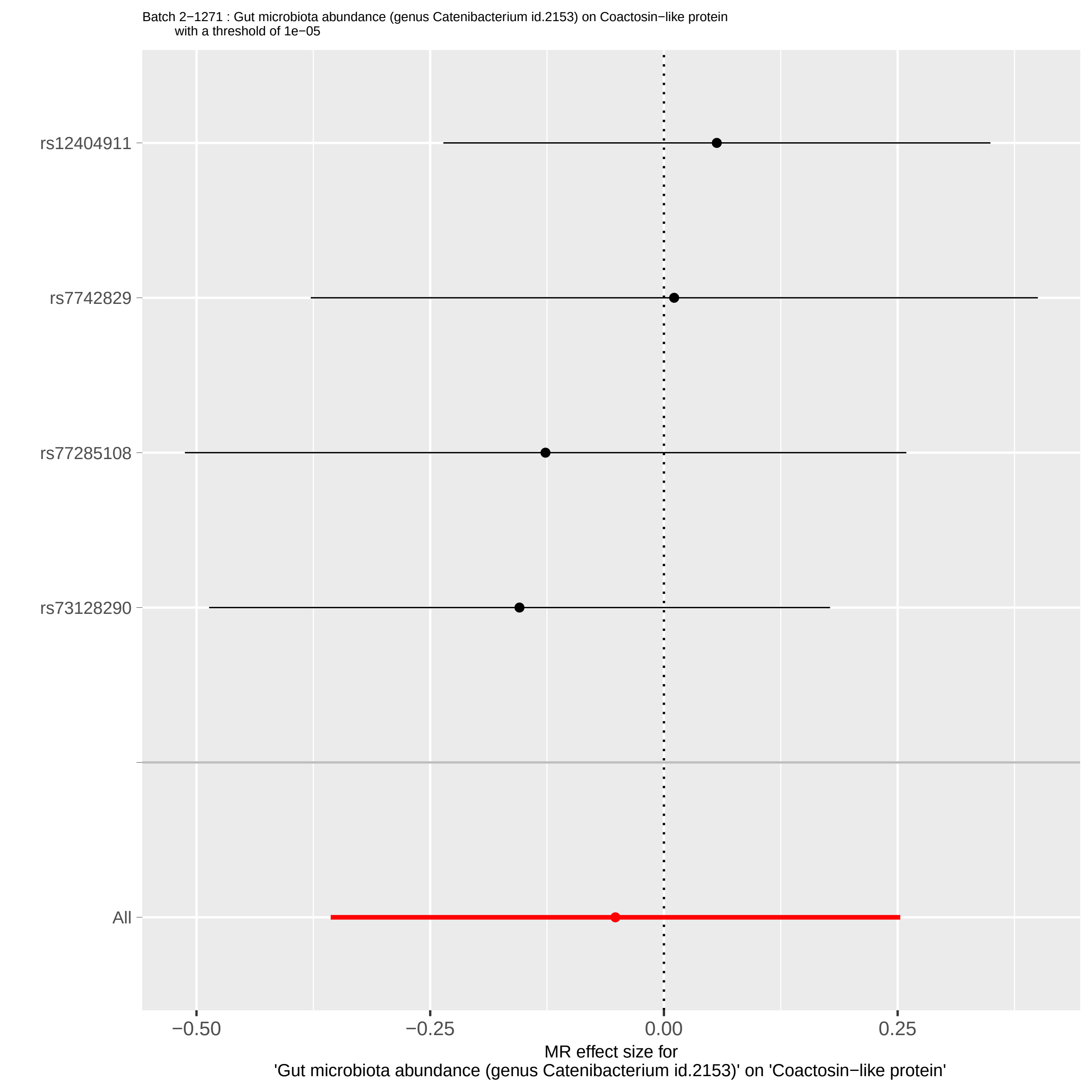
Batch 2−1271 : Gut microbiota abundance (genus Catenibacterium id.2153) on Coactosin−like protein
with a threshold of 1e−05
rs12404911
rs7742829
rs77285108
rs73128290
All
−0.50
−0.25
0.00
0.25
MR effect size for
'Gut microbiota abundance (genus Catenibacterium id.2153)' on 'Coactosin−like protein'

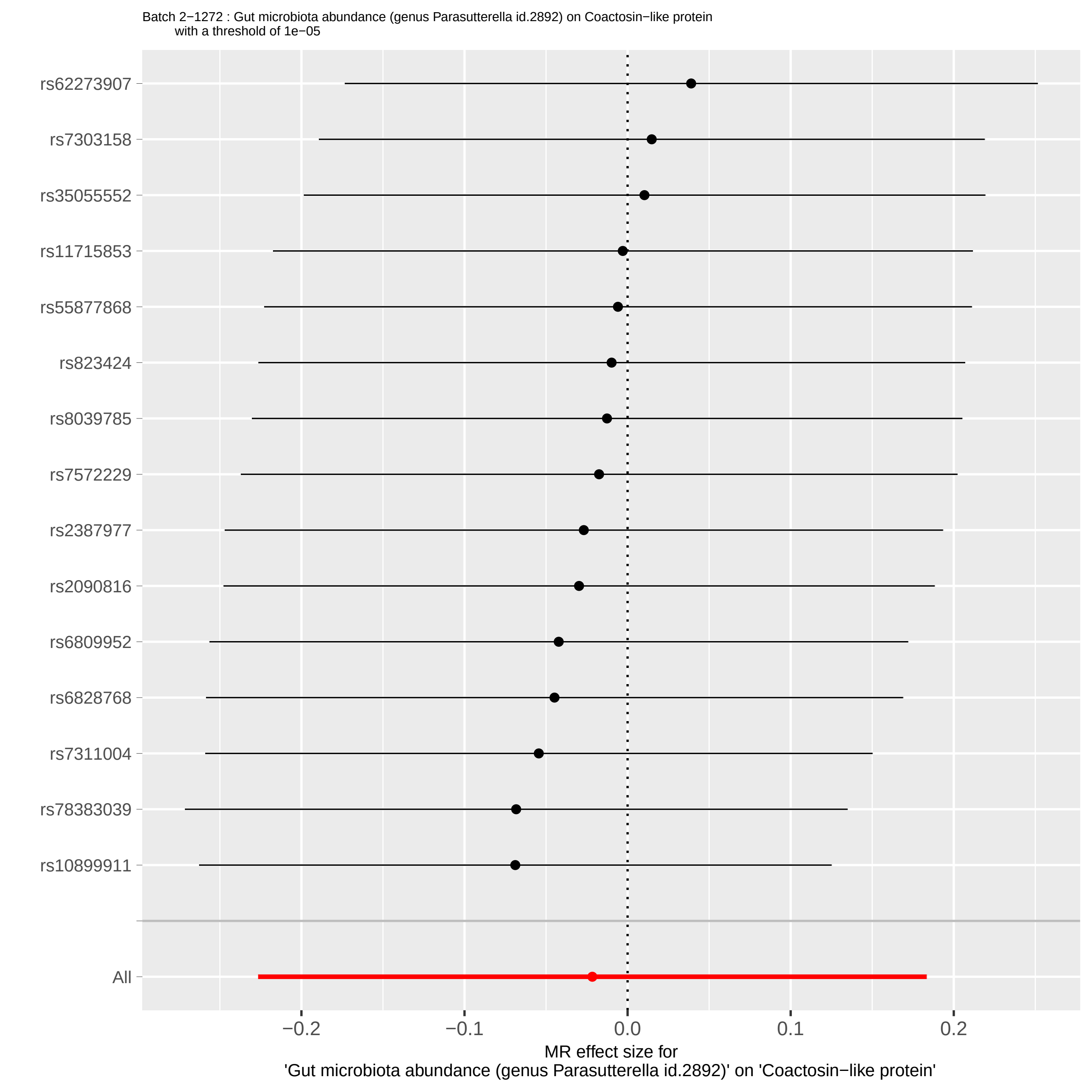

Batch 2−1272 : Gut microbiota abundance (genus Parasutterella id.2892) on Coactosin−like protein
with a threshold of 1e−05
rs62273907
rs7303158
rs35055552
rs11715853
rs55877868
rs823424
rs8039785
rs7572229
rs2387977
rs2090816
rs6809952
rs6828768
rs7311004
rs78383039
rs10899911
All
−0.2
−0.1
0.0
0.1
0.2
MR effect size for
'Gut microbiota abundance (genus Parasutterella id.2892)' on 'Coactosin−like protein'

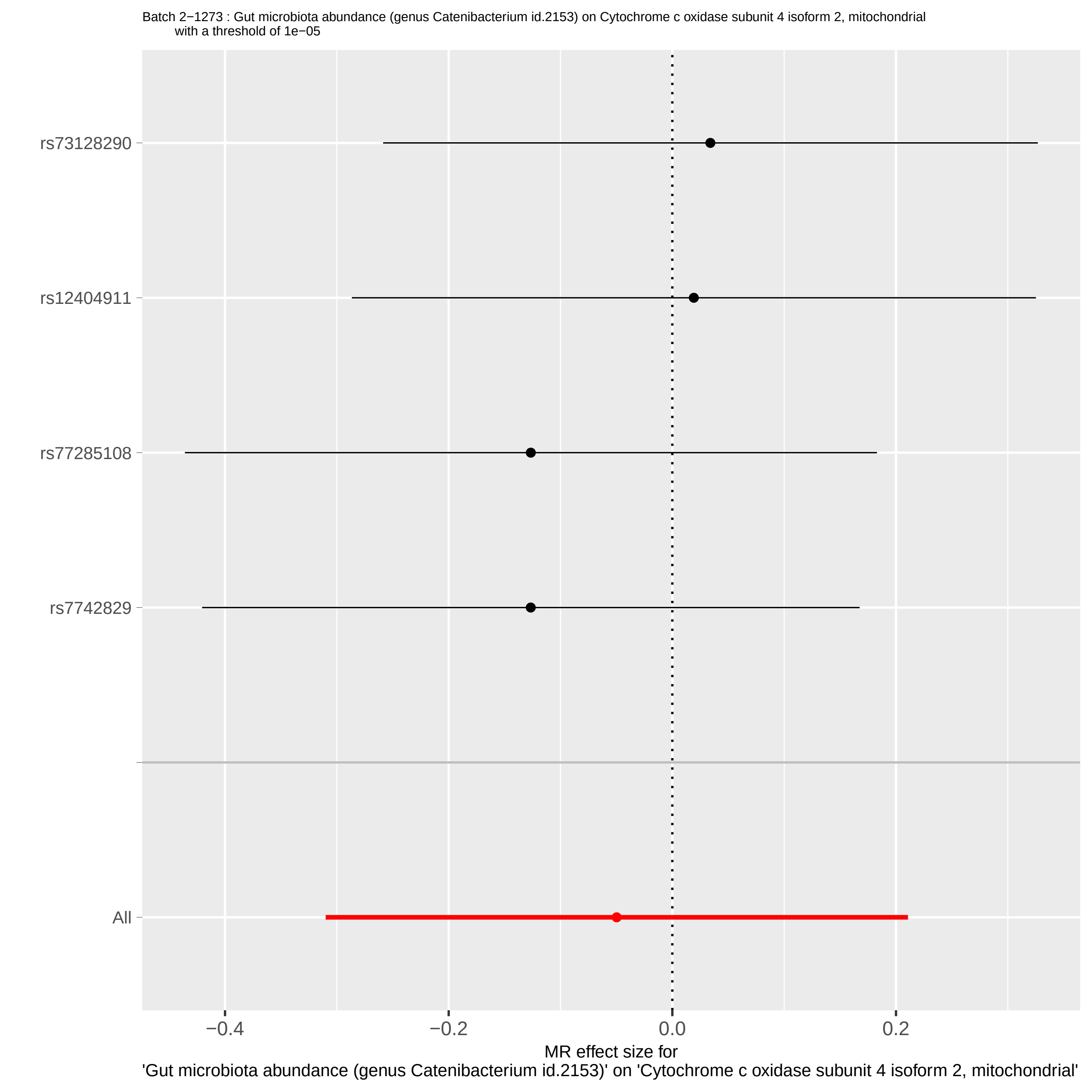
Batch 2−1273 : Gut microbiota abundance (genus Catenibacterium id.2153) on Cytochrome c oxidase subunit 4 isoform 2, mitochondrial
with a threshold of 1e−05
rs73128290
rs12404911
rs77285108
rs7742829
All
−0.4
−0.2
0.0
0.2
MR effect size for
'Gut microbiota abundance (genus Catenibacterium id.2153)' on 'Cytochrome c oxidase subunit 4 isoform 2, mitochondrial'

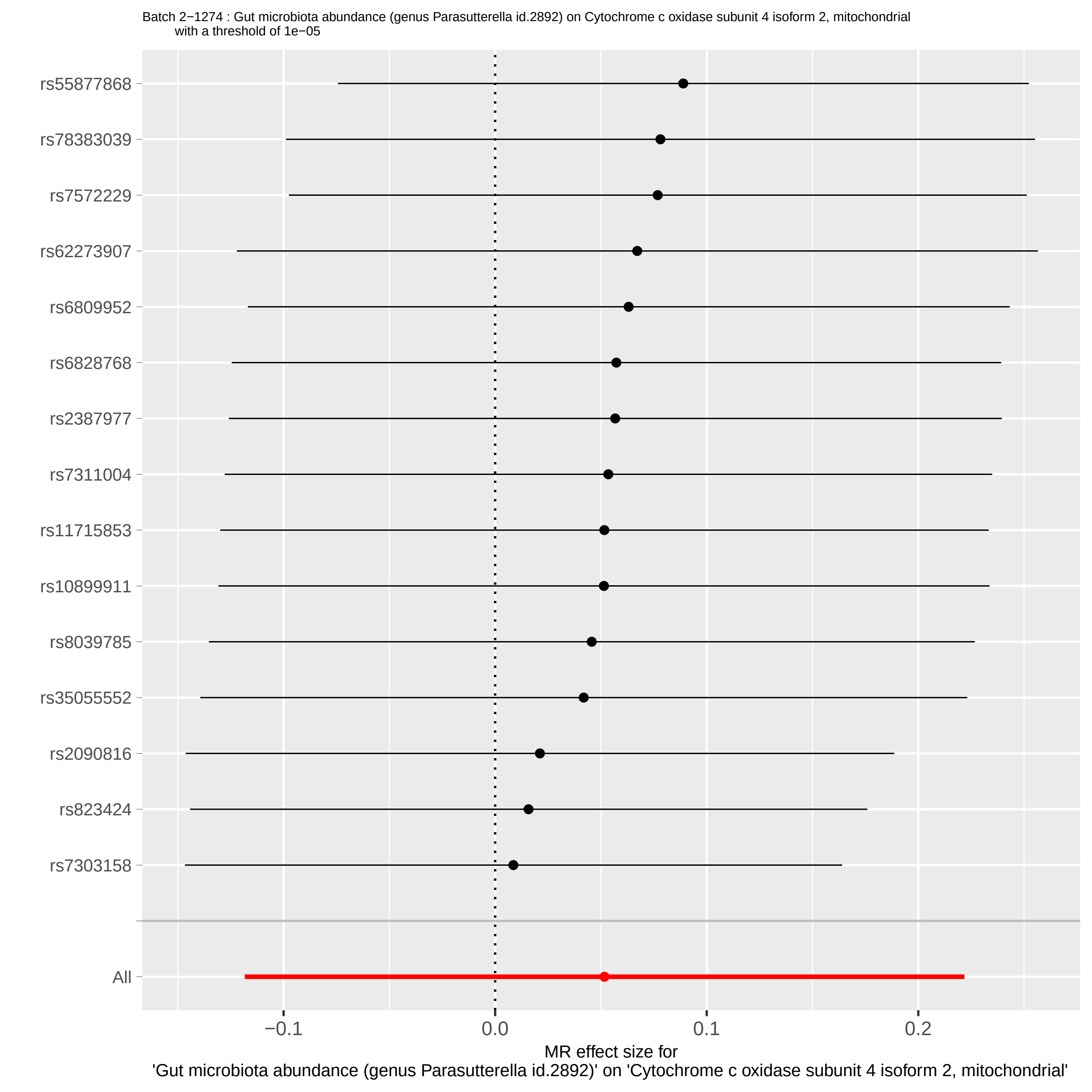
Batch 2−1274 : Gut microbiota abundance (genus Parasutterella id.2892) on Cytochrome c oxidase subunit 4 isoform 2, mitochondrial
with a threshold of 1e−05
rs55877868
rs78383039
rs7572229
rs62273907
rs6809952
rs6828768
rs2387977
rs7311004
rs11715853
rs10899911
rs8039785
rs35055552
rs2090816
rs823424
rs7303158
All
−0.1
0.0
0.1
0.2
MR effect size for
'Gut microbiota abundance (genus Parasutterella id.2892)' on 'Cytochrome c oxidase subunit 4 isoform 2, mitochondrial'

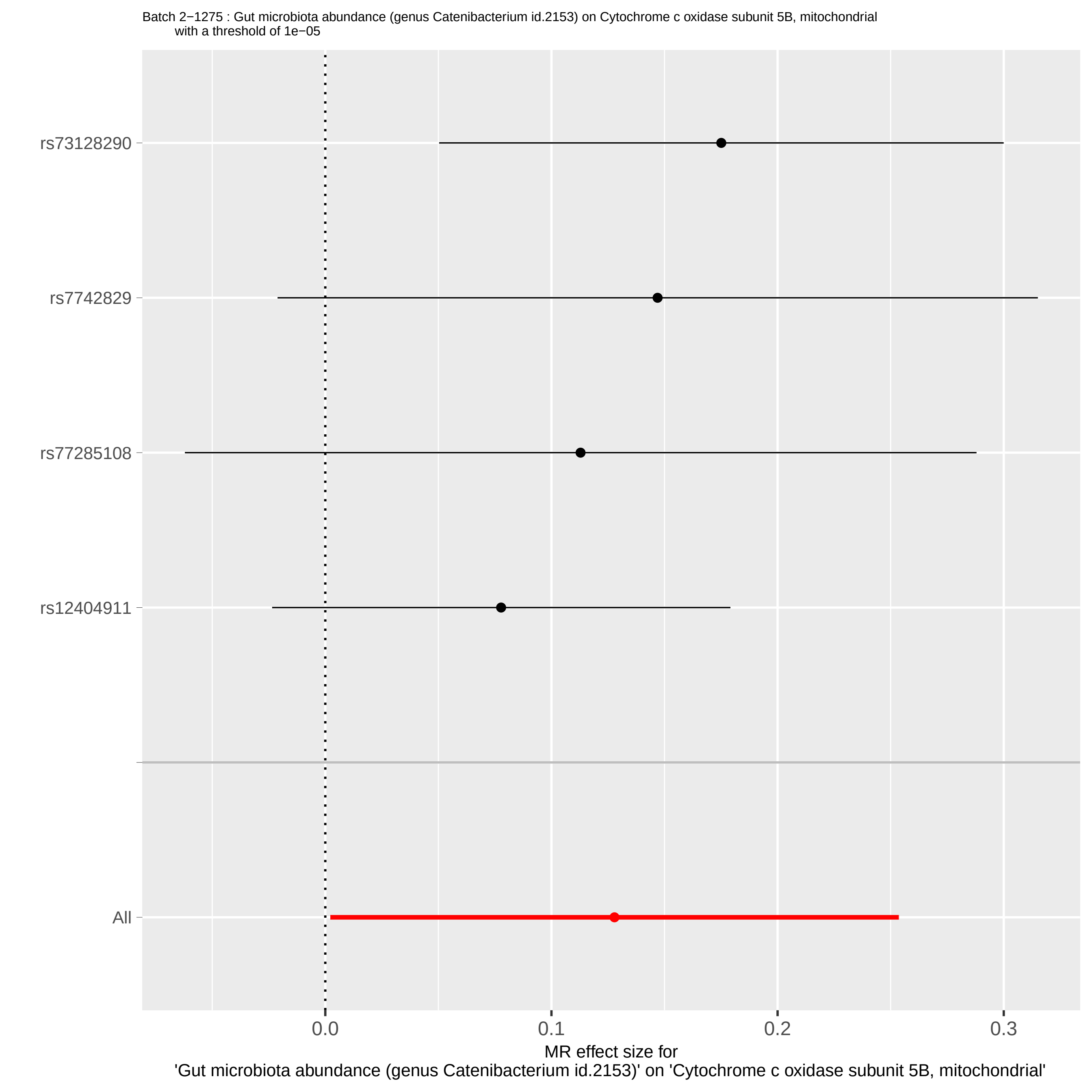

Batch 2−1275 : Gut microbiota abundance (genus Catenibacterium id.2153) on Cytochrome c oxidase subunit 5B, mitochondrial
with a threshold of 1e−05
rs73128290
rs7742829
rs77285108
rs12404911
All
0.0
0.1
0.2
0.3
MR effect size for
'Gut microbiota abundance (genus Catenibacterium id.2153)' on 'Cytochrome c oxidase subunit 5B, mitochondrial'

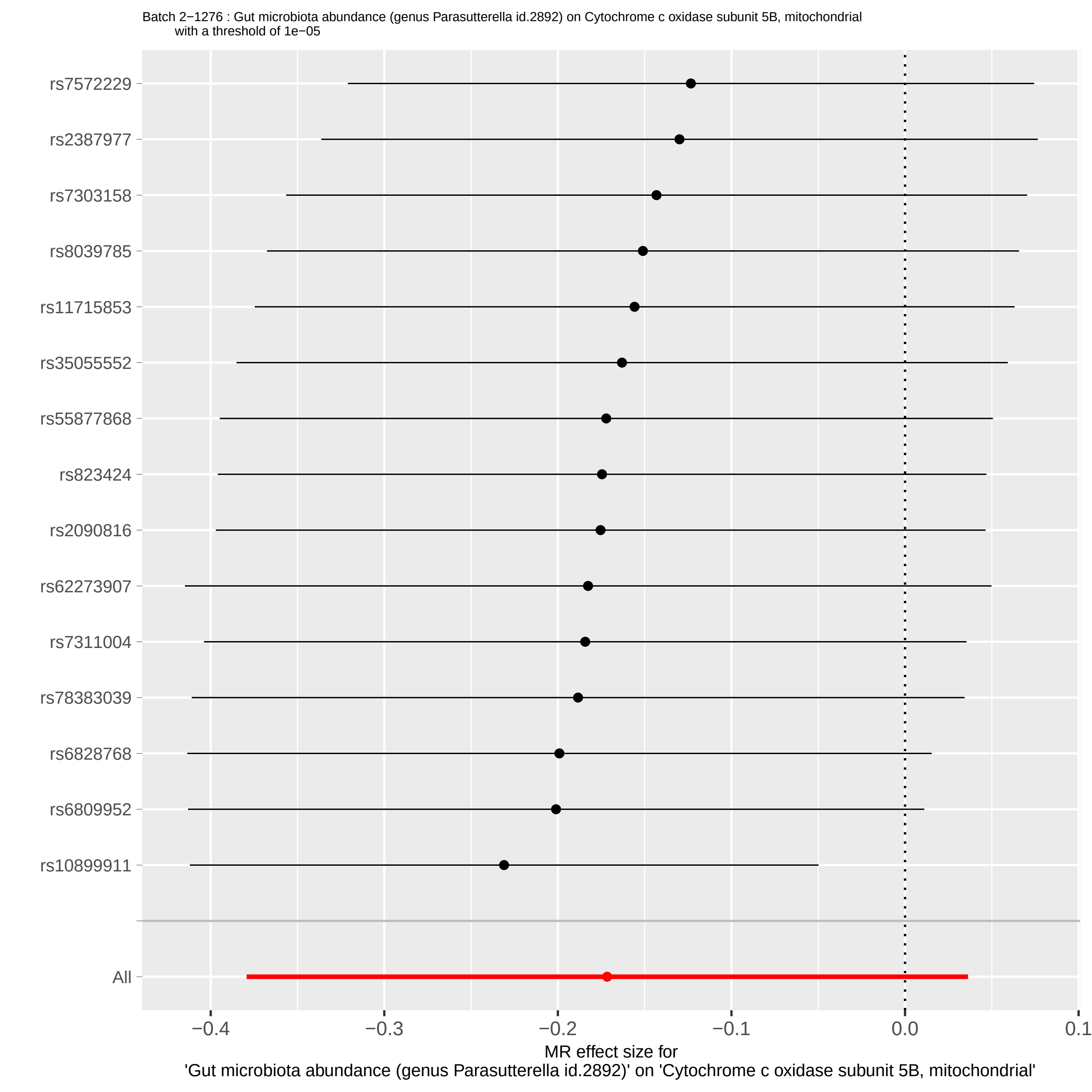
Batch 2−1276 : Gut microbiota abundance (genus Parasutterella id.2892) on Cytochrome c oxidase subunit 5B, mitochondrial
with a threshold of 1e−05
rs7572229
rs2387977
rs7303158
rs8039785
rs11715853
rs35055552
rs55877868
rs823424
rs2090816
rs62273907
rs7311004
rs78383039
rs6828768
rs6809952
rs10899911
All
−0.4
−0.3
−0.2
−0.1
0.0
0.1
MR effect size for
'Gut microbiota abundance (genus Parasutterella id.2892)' on 'Cytochrome c oxidase subunit 5B, mitochondrial'

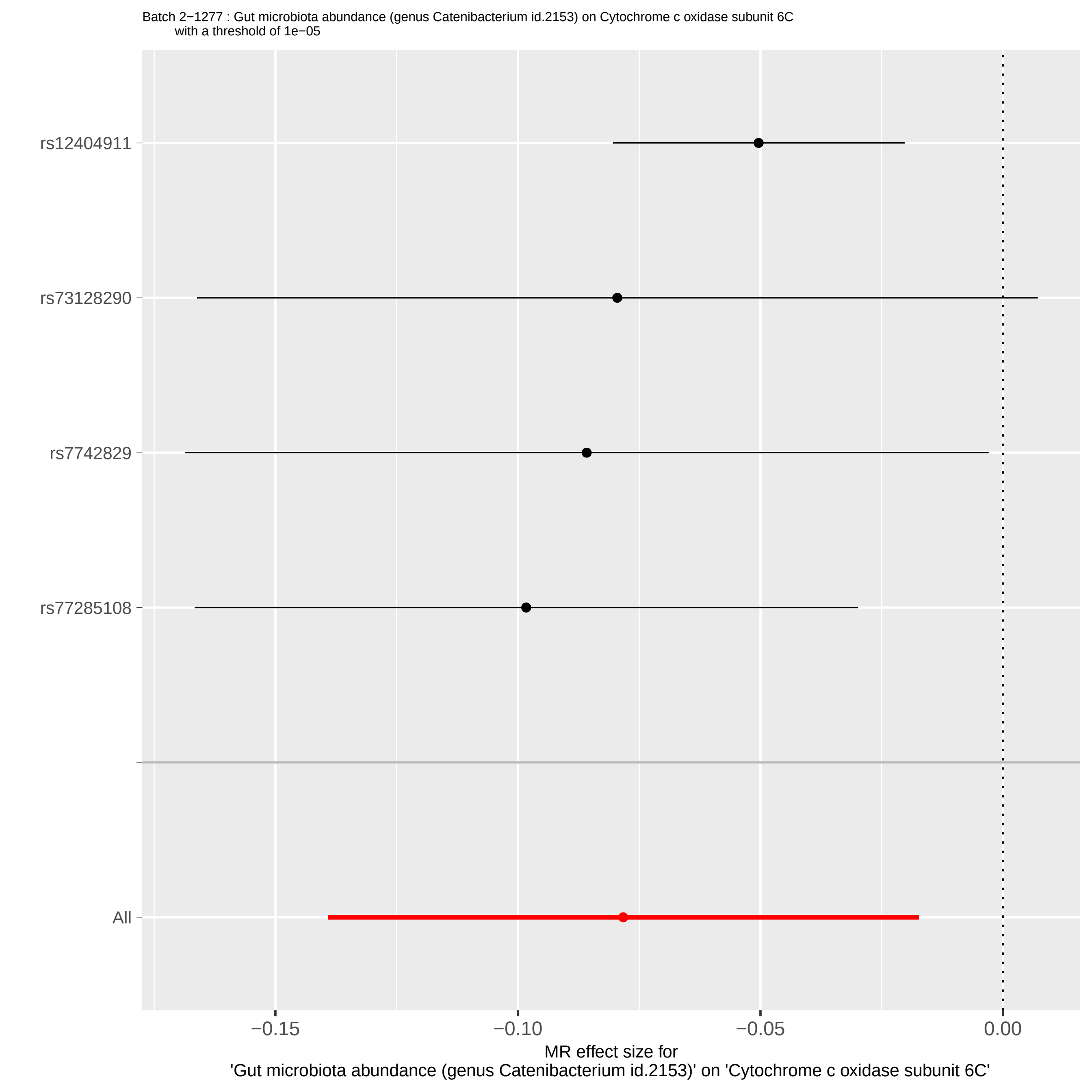
Batch 2−1277 : Gut microbiota abundance (genus Catenibacterium id.2153) on Cytochrome c oxidase subunit 6C
with a threshold of 1e−05
rs12404911
rs73128290
rs7742829
rs77285108
All
−0.15
−0.10
−0.05
0.00
MR effect size for
'Gut microbiota abundance (genus Catenibacterium id.2153)' on 'Cytochrome c oxidase subunit 6C'

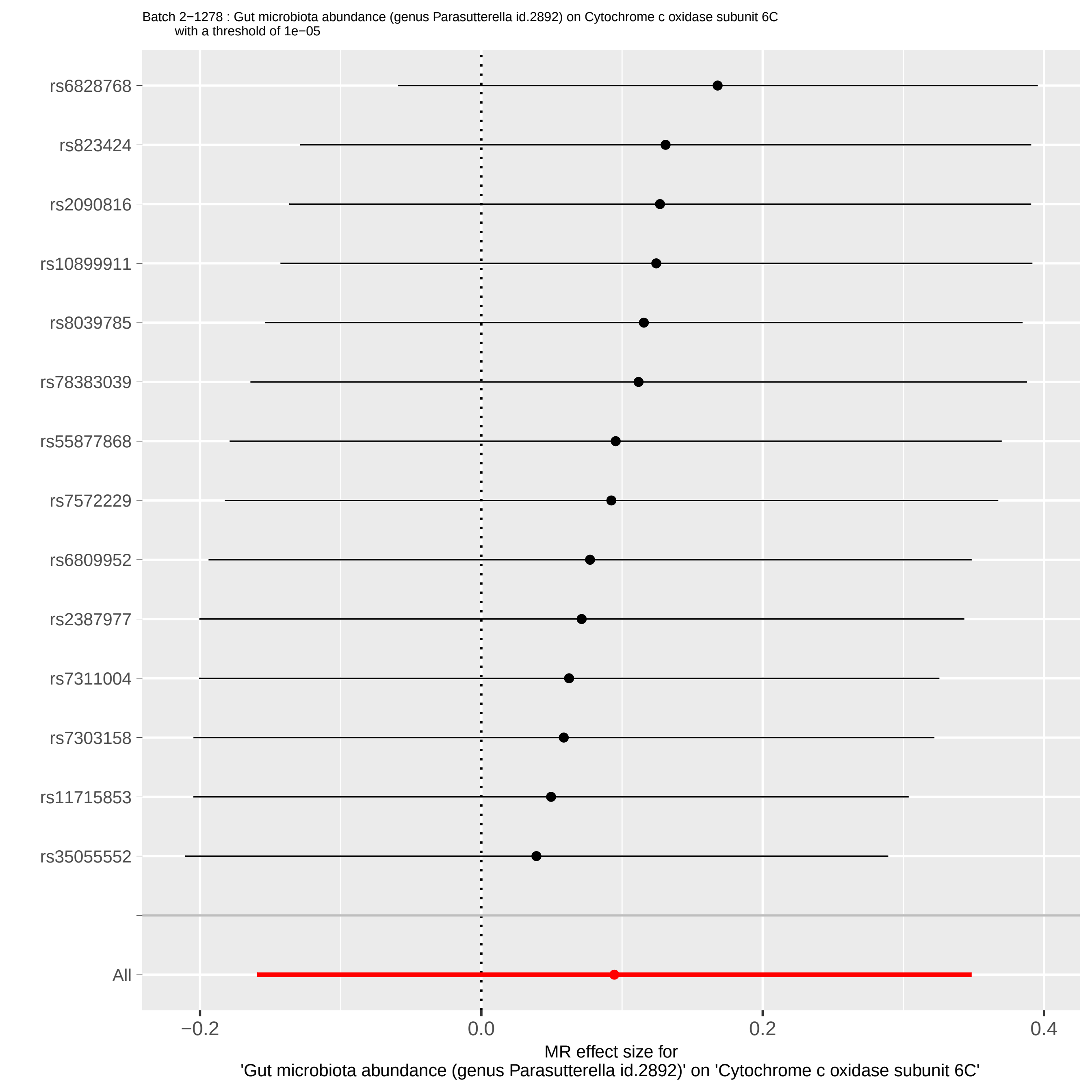

Batch 2−1278 : Gut microbiota abundance (genus Parasutterella id.2892) on Cytochrome c oxidase subunit 6C
with a threshold of 1e−05
rs6828768
rs823424
rs2090816
rs10899911
rs8039785
rs78383039
rs55877868
rs7572229
rs6809952
rs2387977
rs7311004
rs7303158
rs11715853
rs35055552
All
−0.2
0.0
0.2
0.4
MR effect size for
'Gut microbiota abundance (genus Parasutterella id.2892)' on 'Cytochrome c oxidase subunit 6C'

Batch 2−1279 : Gut microbiota abundance (genus Catenibacterium id.2153) on Cytochrome c oxidase subunit 7A1, mitochondrial
with a threshold of 1e−05

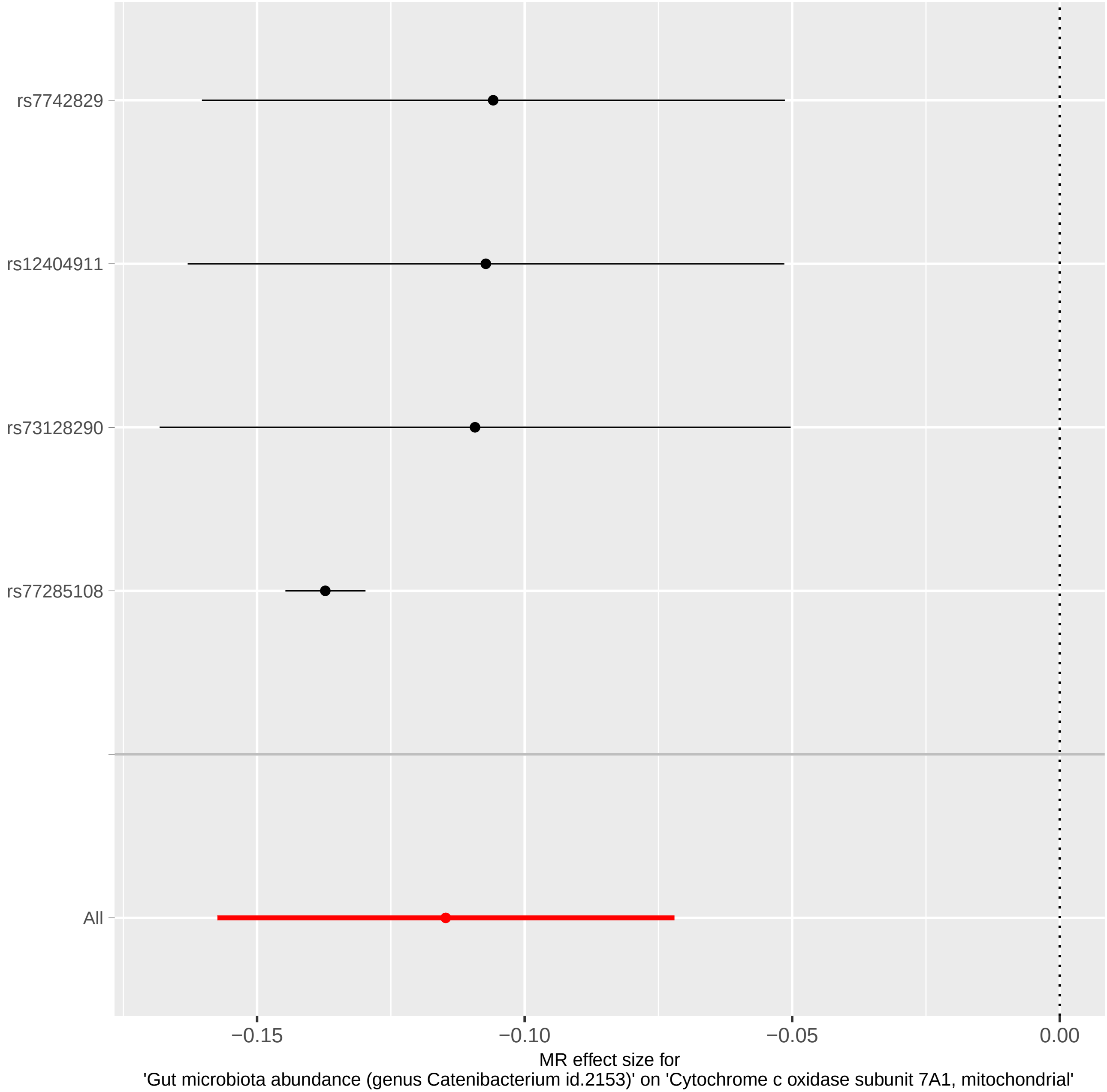

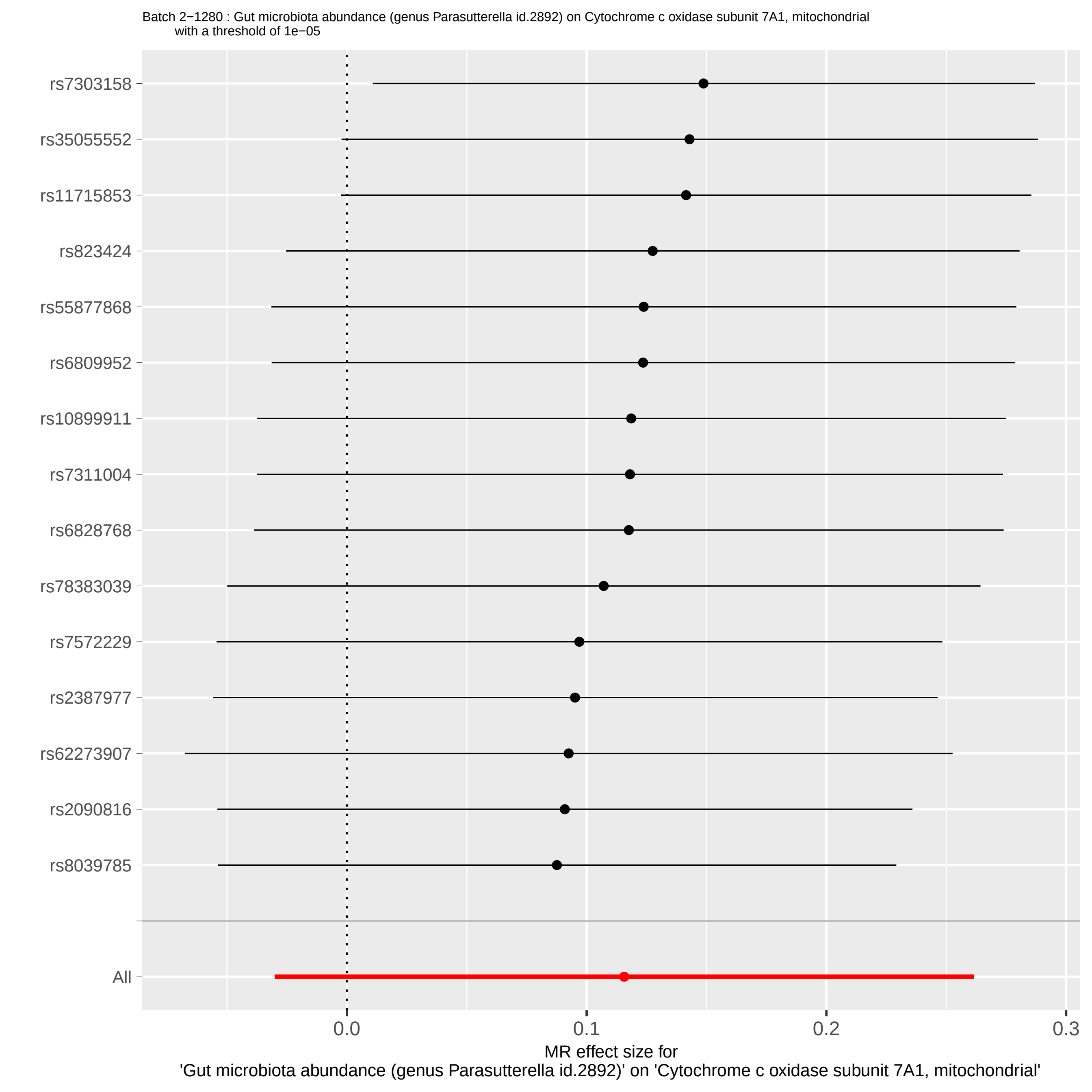

Batch 2−1280 : Gut microbiota abundance (genus Parasutterella id.2892) on Cytochrome c oxidase subunit 7A1, mitochondrial
with a threshold of 1e−05
rs7303158
rs35055552
rs11715853
rs823424
rs55877868
rs6809952
rs10899911
rs7311004
rs6828768
rs78383039
rs7572229
rs2387977
rs62273907
rs2090816
rs8039785
All
0.0
0.1
0.2
0.3
MR effect size for
'Gut microbiota abundance (genus Parasutterella id.2892)' on 'Cytochrome c oxidase subunit 7A1, mitochondrial'

Batch 2−1281 : Gut microbiota abundance (genus Catenibacterium id.2153) on Cytochrome c oxidase subunit 8A, mitochondrial
with a threshold of 1e−05

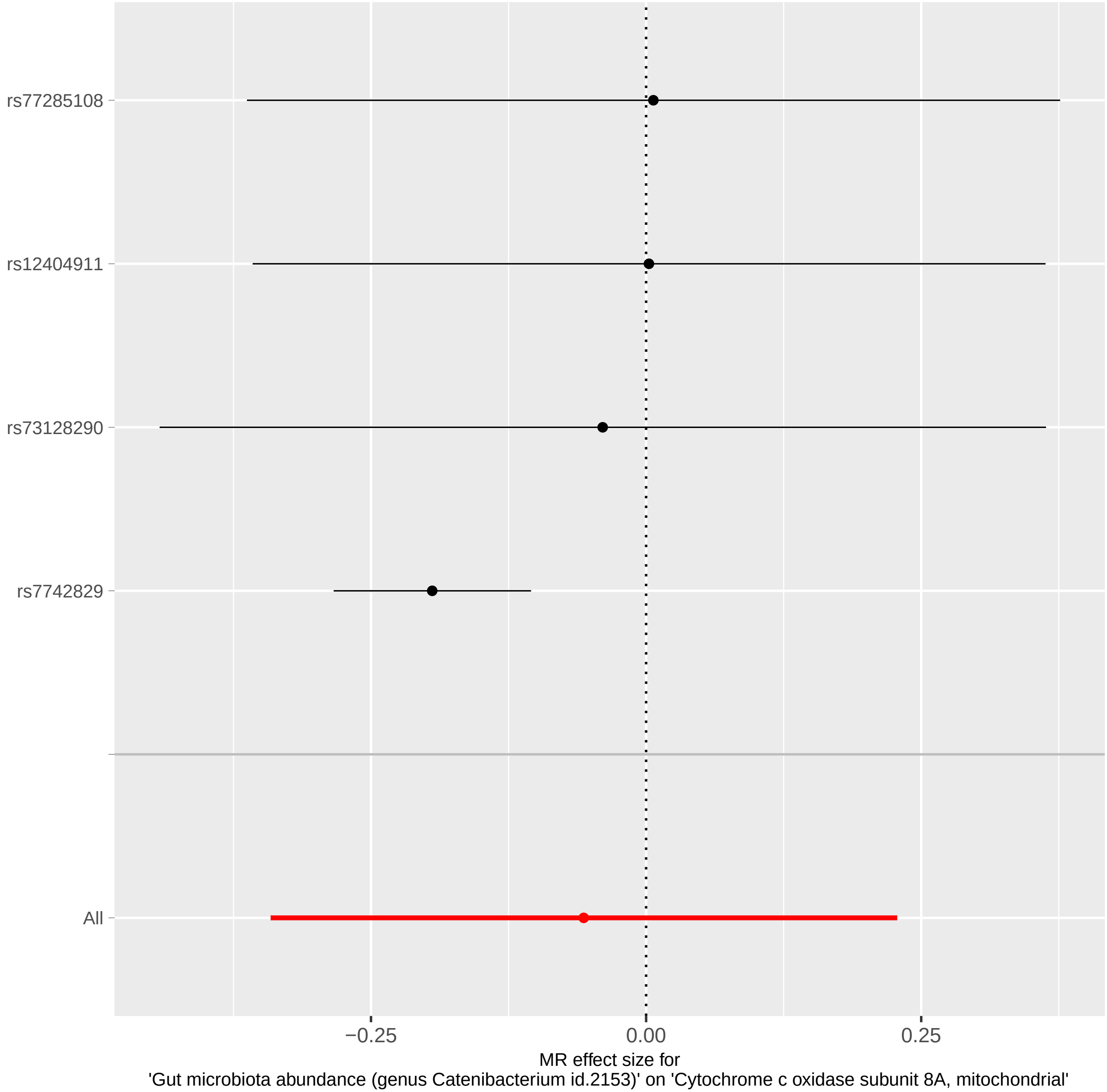

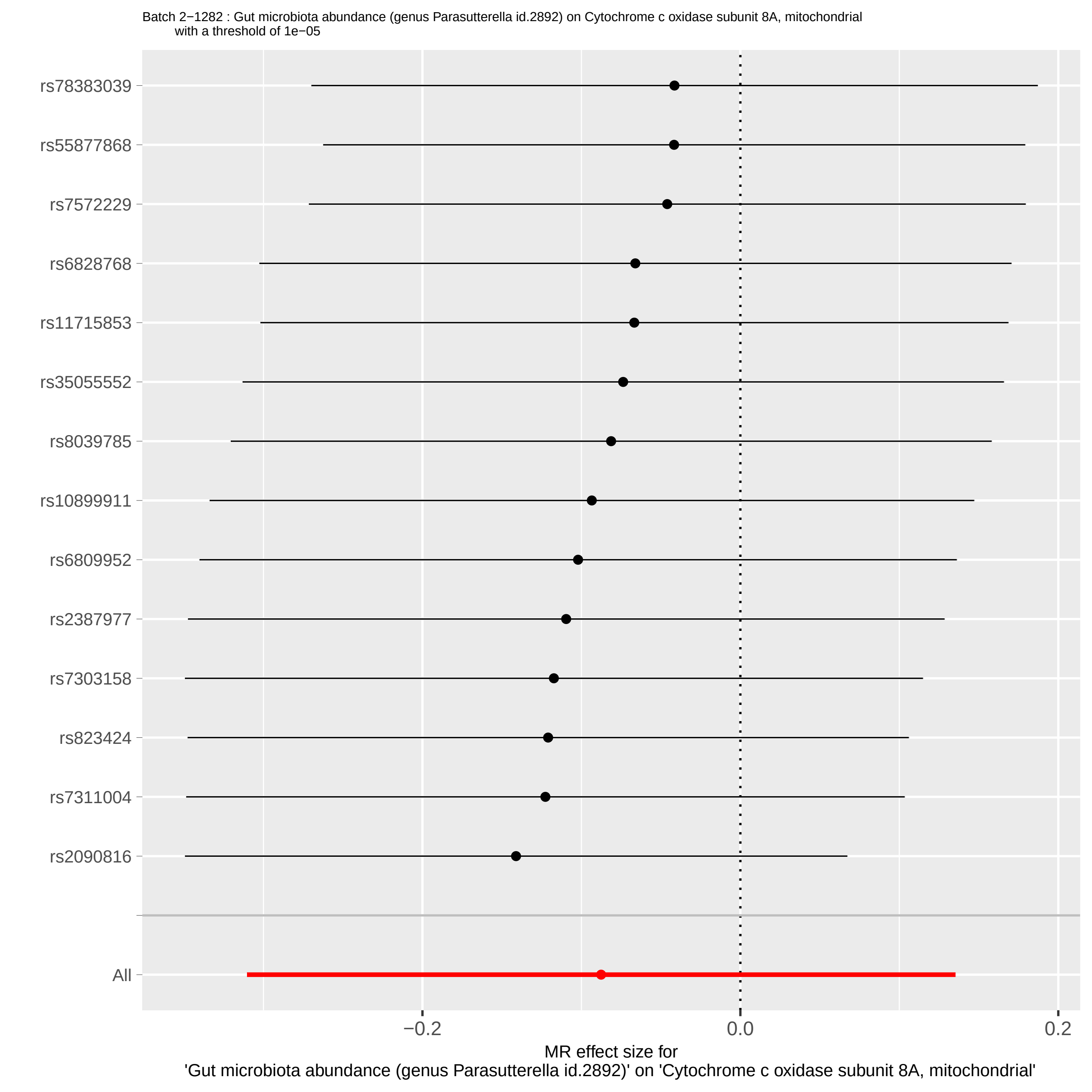
Batch 2−1282 : Gut microbiota abundance (genus Parasutterella id.2892) on Cytochrome c oxidase subunit 8A, mitochondrial
with a threshold of 1e−05
rs78383039
rs55877868
rs7572229
rs6828768
rs11715853
rs35055552
rs8039785
rs10899911
rs6809952
rs2387977
rs7303158
rs823424
rs7311004
rs2090816
All
−0.2
0.0
0.2
MR effect size for
'Gut microbiota abundance (genus Parasutterella id.2892)' on 'Cytochrome c oxidase subunit 8A, mitochondrial'

Batch 2−1283 : Gut microbiota abundance (genus Catenibacterium id.2153) on Carboxypeptidase A2
with a threshold of 1e−05

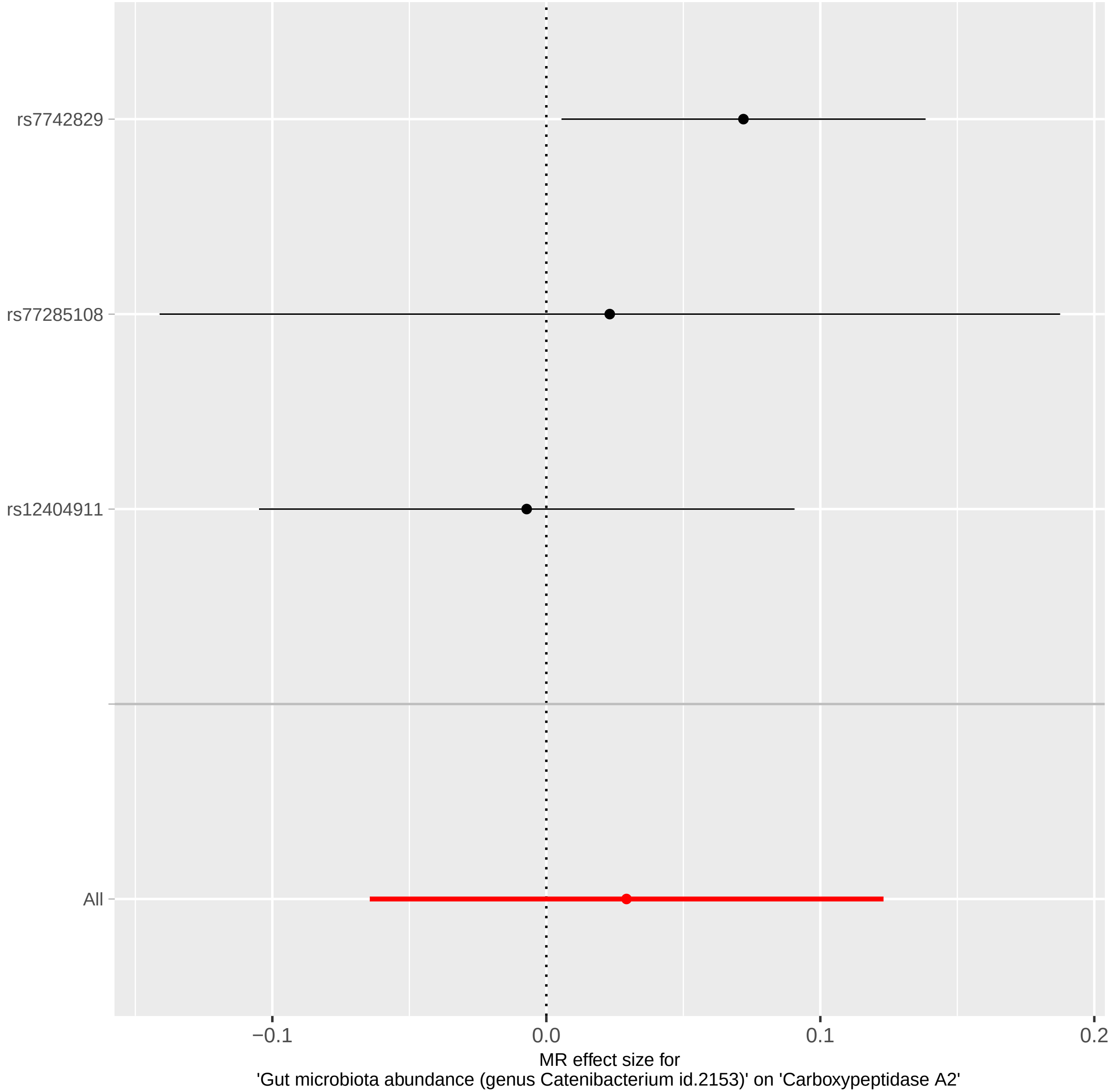

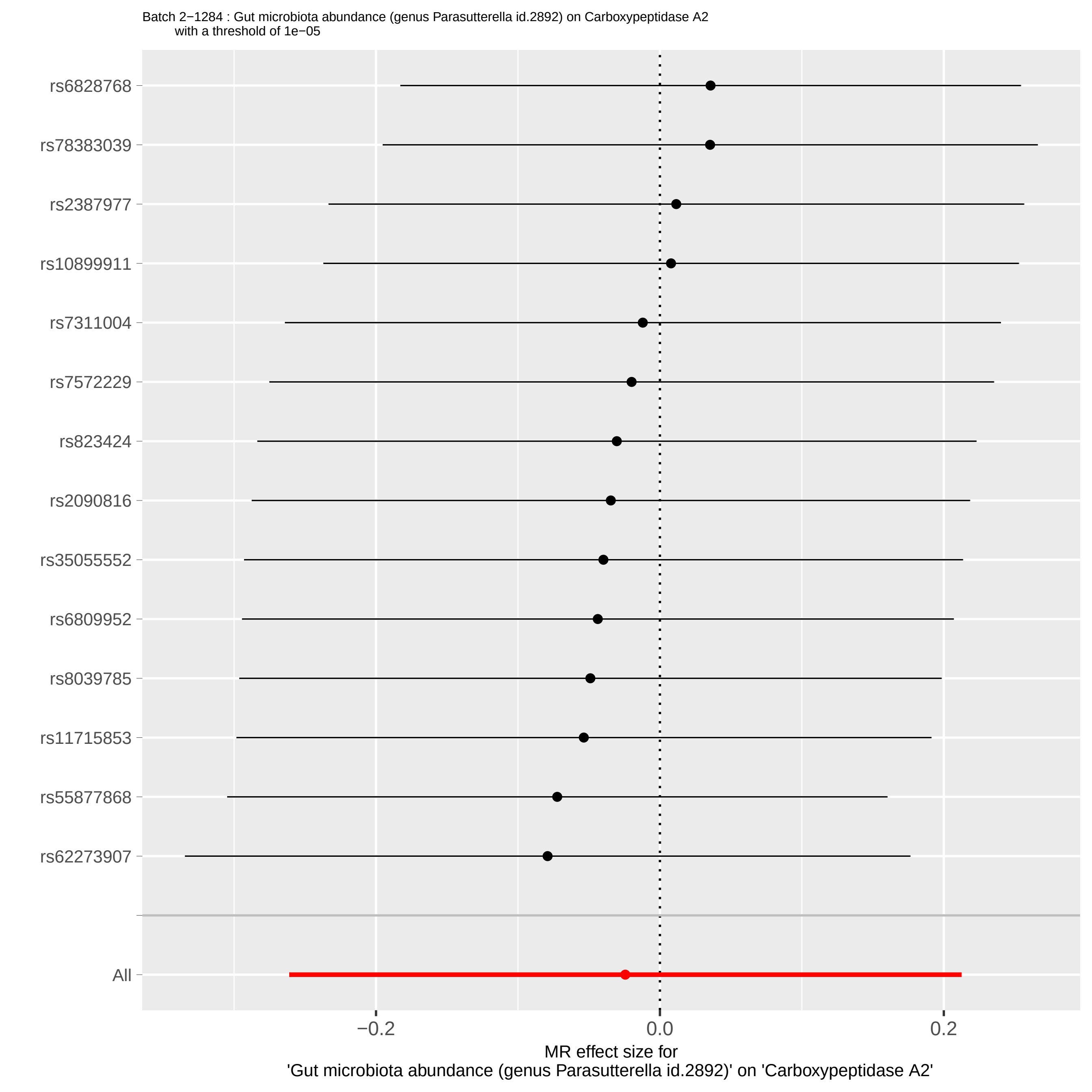

Batch 2−1284 : Gut microbiota abundance (genus Parasutterella id.2892) on Carboxypeptidase A2
with a threshold of 1e−05
rs6828768
rs78383039
rs2387977
rs10899911
rs7311004
rs7572229
rs823424
rs2090816
rs35055552
rs6809952
rs8039785
rs11715853
rs55877868
rs62273907
All
−0.2
0.0
0.2
MR effect size for
'Gut microbiota abundance (genus Parasutterella id.2892)' on 'Carboxypeptidase A2'

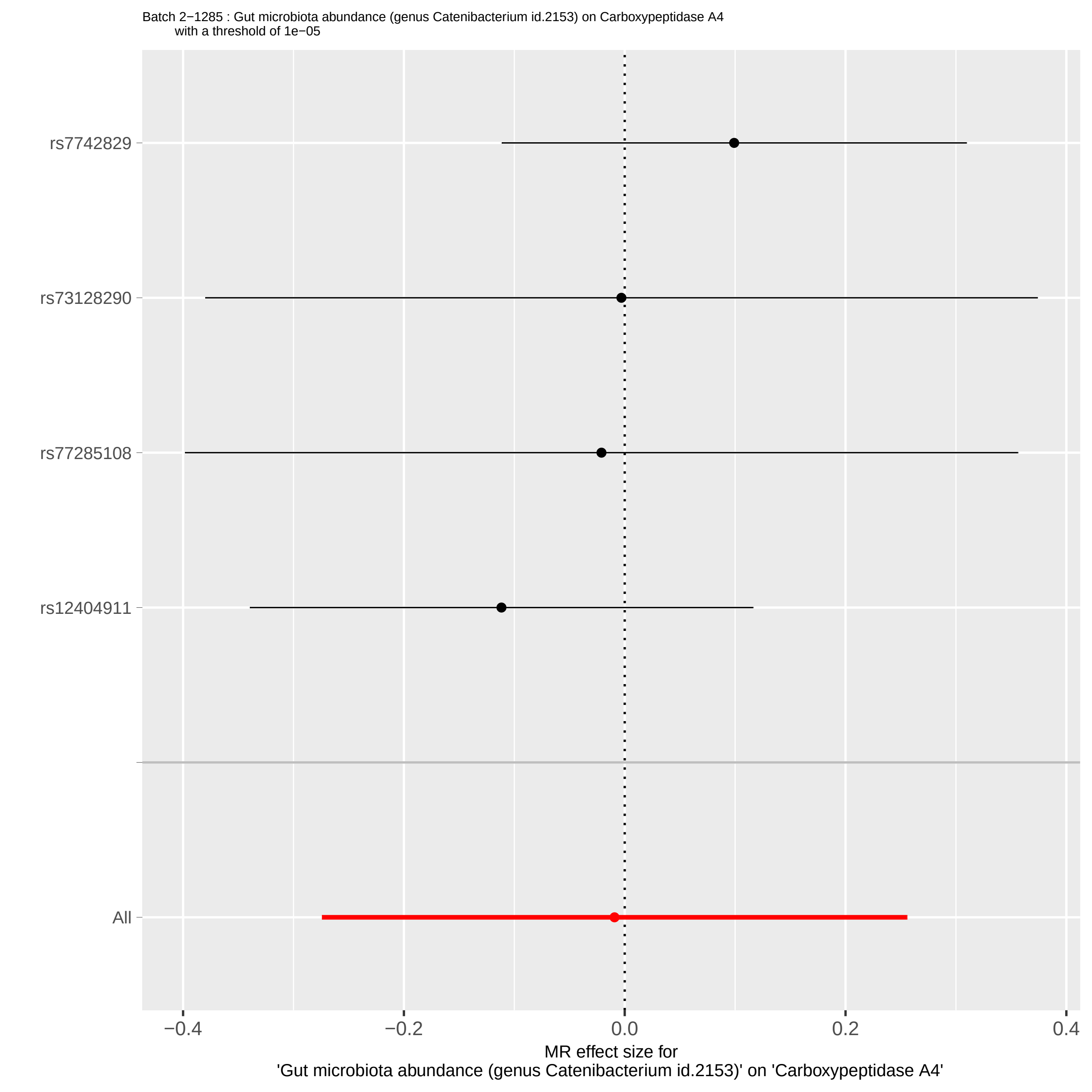
Batch 2−1285 : Gut microbiota abundance (genus Catenibacterium id.2153) on Carboxypeptidase A4
with a threshold of 1e−05
rs7742829
rs73128290
rs77285108
rs12404911
All
−0.4
−0.2
0.0
0.2
0.4
MR effect size for
'Gut microbiota abundance (genus Catenibacterium id.2153)' on 'Carboxypeptidase A4'

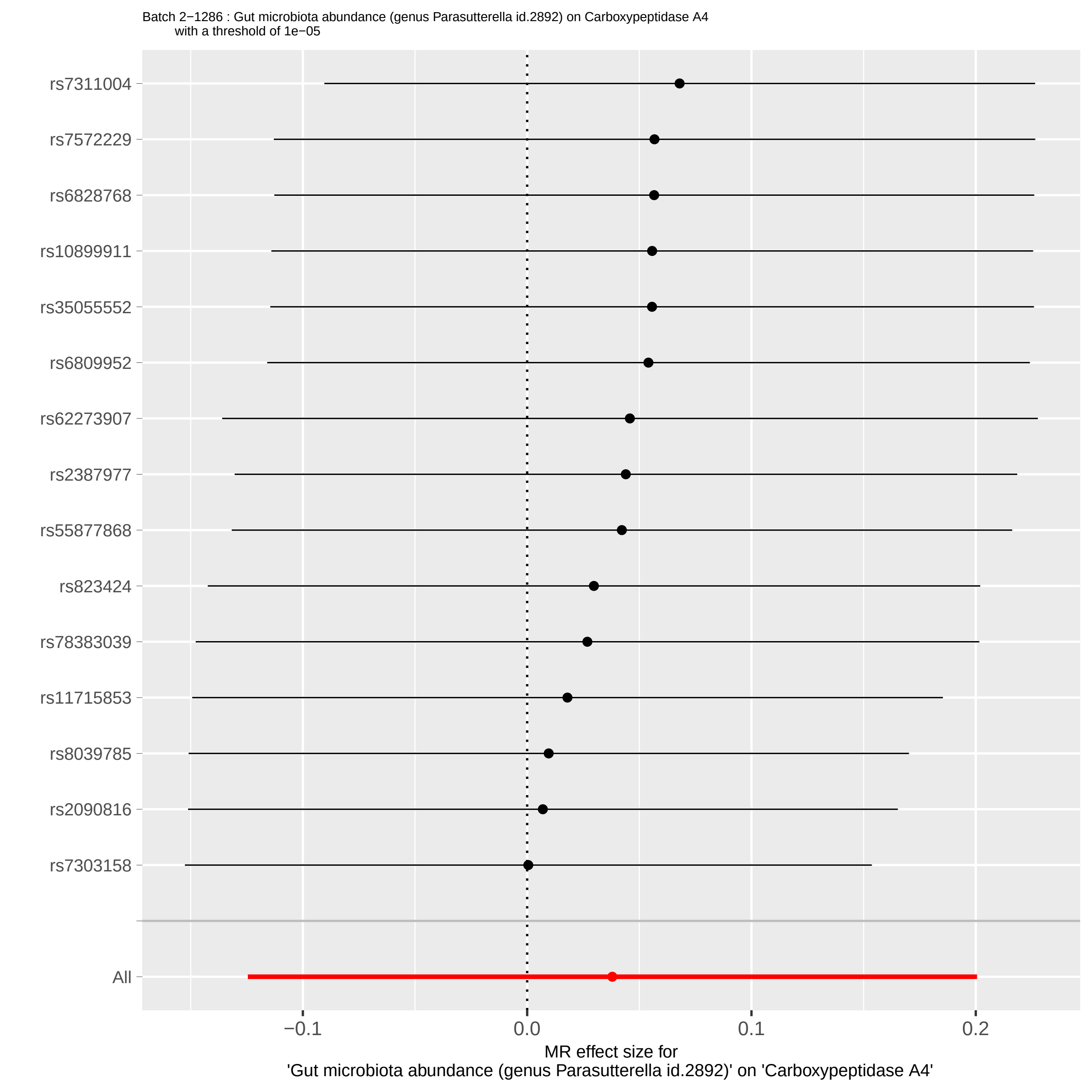

Batch 2−1286 : Gut microbiota abundance (genus Parasutterella id.2892) on Carboxypeptidase A4
with a threshold of 1e−05
rs7311004
rs7572229
rs6828768
rs10899911
rs35055552
rs6809952
rs62273907
rs2387977
rs55877868
rs823424
rs78383039
rs11715853
rs8039785
rs2090816
rs7303158
All
−0.1
0.0
0.1
0.2
MR effect size for
'Gut microbiota abundance (genus Parasutterella id.2892)' on 'Carboxypeptidase A4'

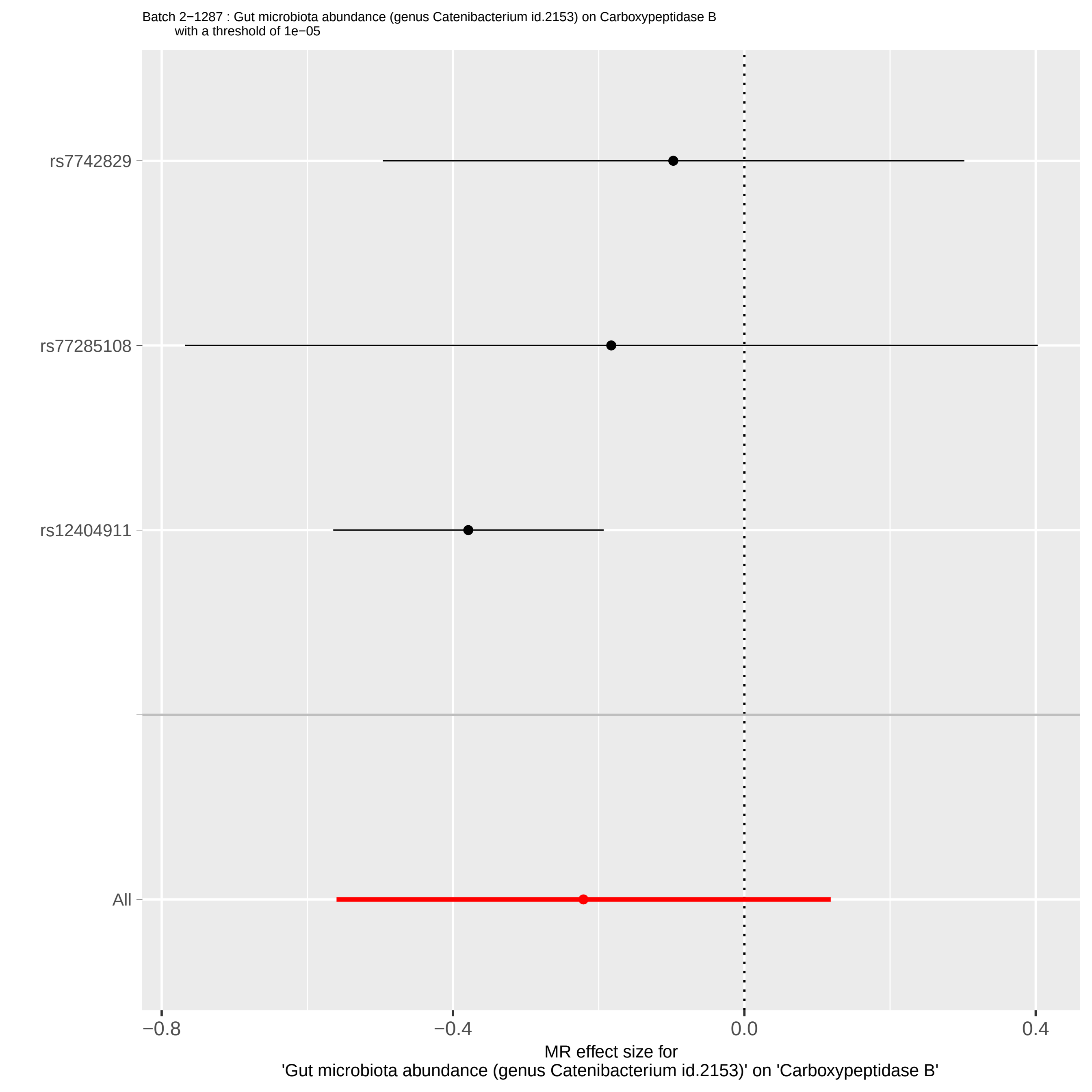

Batch 2−1287 : Gut microbiota abundance (genus Catenibacterium id.2153) on Carboxypeptidase B
with a threshold of 1e−05
rs7742829
rs77285108
rs12404911
All
−0.8
−0.4
0.0
0.4
MR effect size for
'Gut microbiota abundance (genus Catenibacterium id.2153)' on 'Carboxypeptidase B'

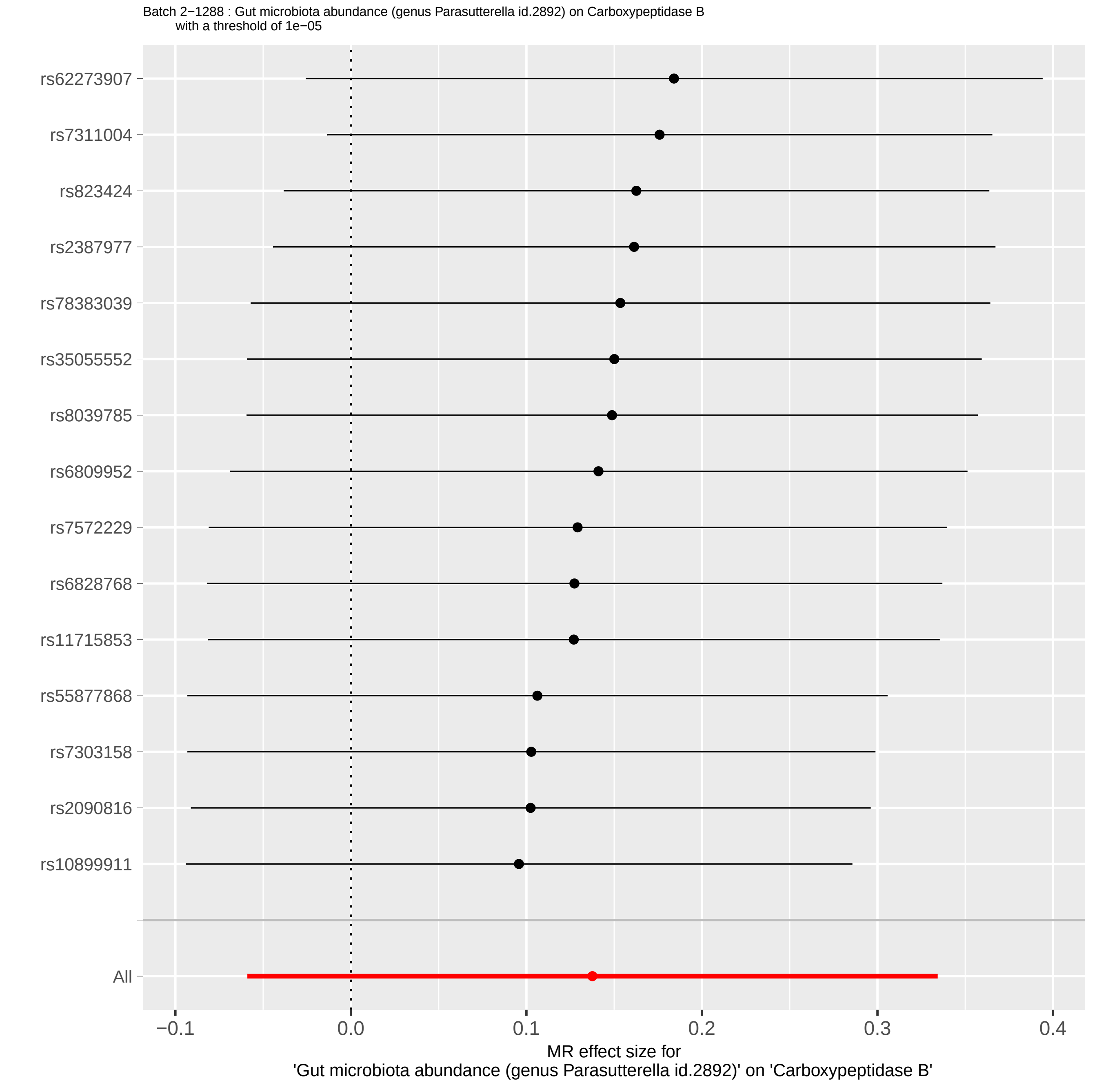

Batch 2−1288 : Gut microbiota abundance (genus Parasutterella id.2892) on Carboxypeptidase B
with a threshold of 1e−05
rs62273907
rs7311004
rs823424
rs2387977
rs78383039
rs35055552
rs8039785
rs6809952
rs7572229
rs6828768
rs11715853
rs55877868
rs7303158
rs2090816
rs10899911
All
−0.1
0.0
0.1
0.2
0.3
0.4
MR effect size for
'Gut microbiota abundance (genus Parasutterella id.2892)' on 'Carboxypeptidase B'

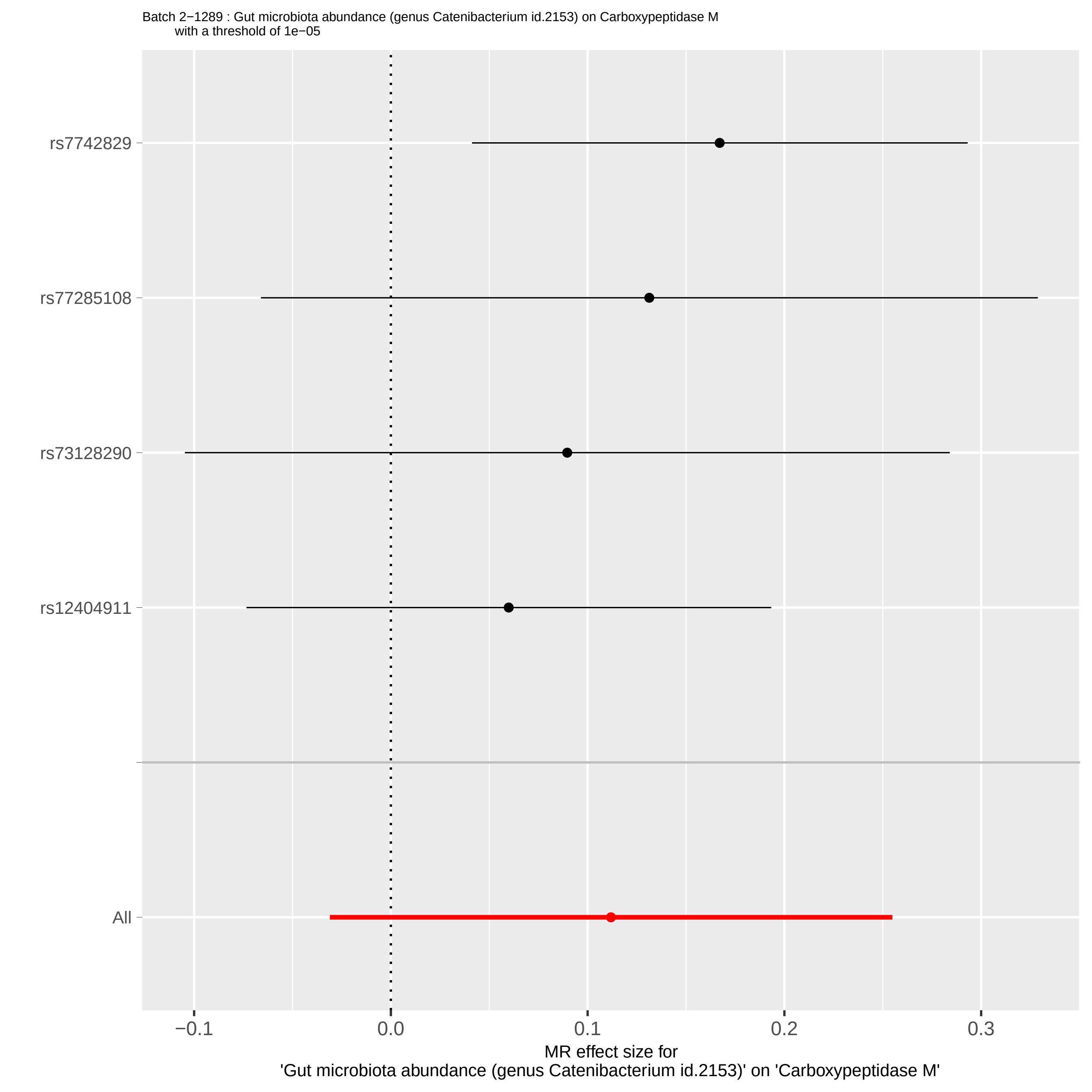

Batch 2−1289 : Gut microbiota abundance (genus Catenibacterium id.2153) on Carboxypeptidase M
with a threshold of 1e−05
rs7742829
rs77285108
rs73128290
rs12404911
All
−0.1
0.0
0.1
0.2
0.3
MR effect size for
'Gut microbiota abundance (genus Catenibacterium id.2153)' on 'Carboxypeptidase M'

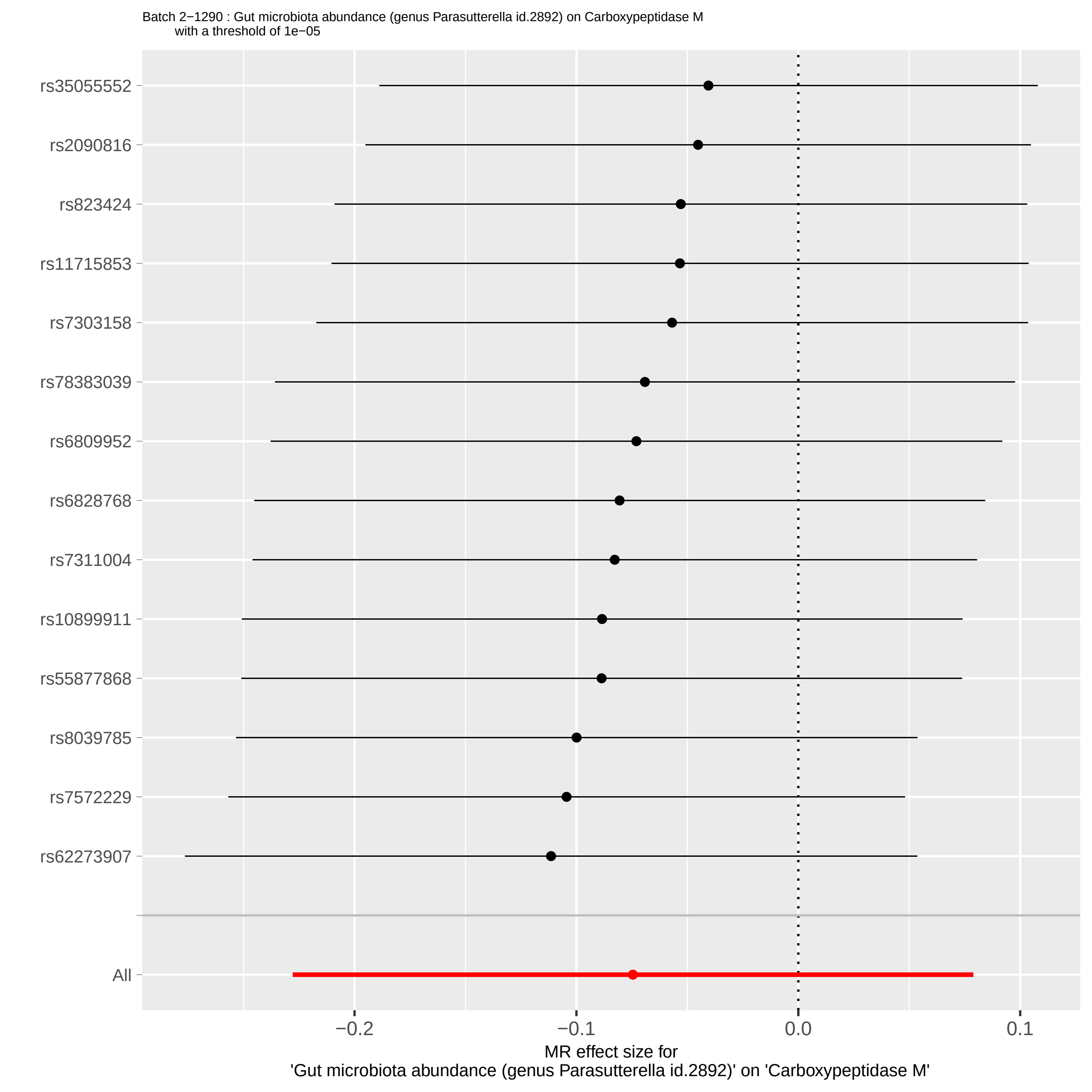
Batch 2−1290 : Gut microbiota abundance (genus Parasutterella id.2892) on Carboxypeptidase M
with a threshold of 1e−05
rs35055552
rs2090816
rs823424
rs11715853
rs7303158
rs78383039
rs6809952
rs6828768
rs7311004
rs10899911
rs55877868
rs8039785
rs7572229
rs62273907
All
−0.2
−0.1
0.0
0.1
MR effect size for
'Gut microbiota abundance (genus Parasutterella id.2892)' on 'Carboxypeptidase M'

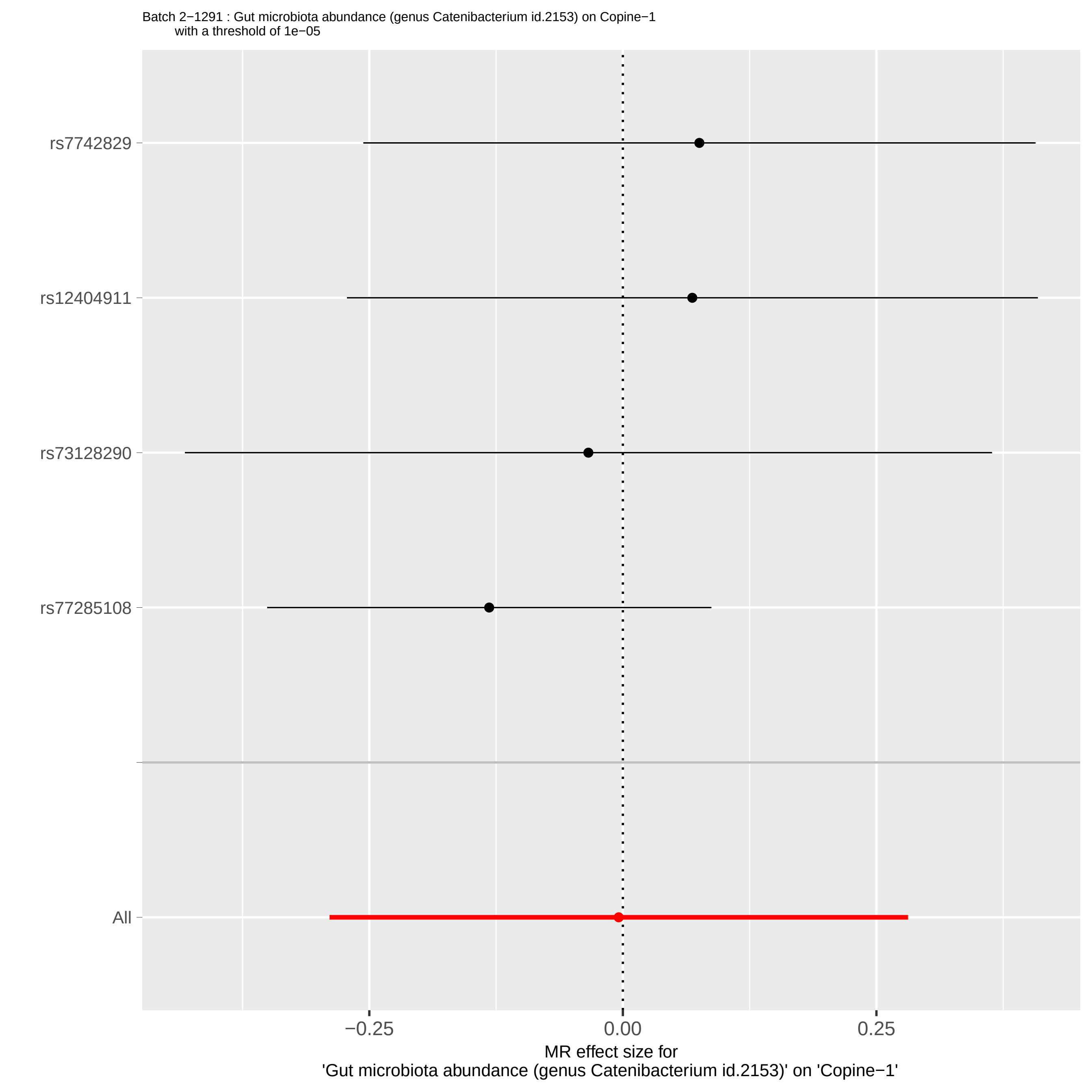

Batch 2−1291 : Gut microbiota abundance (genus Catenibacterium id.2153) on Copine−1
with a threshold of 1e−05
rs7742829
rs12404911
rs73128290
rs77285108
All
−0.25
0.00
0.25
MR effect size for
'Gut microbiota abundance (genus Catenibacterium id.2153)' on 'Copine−1'

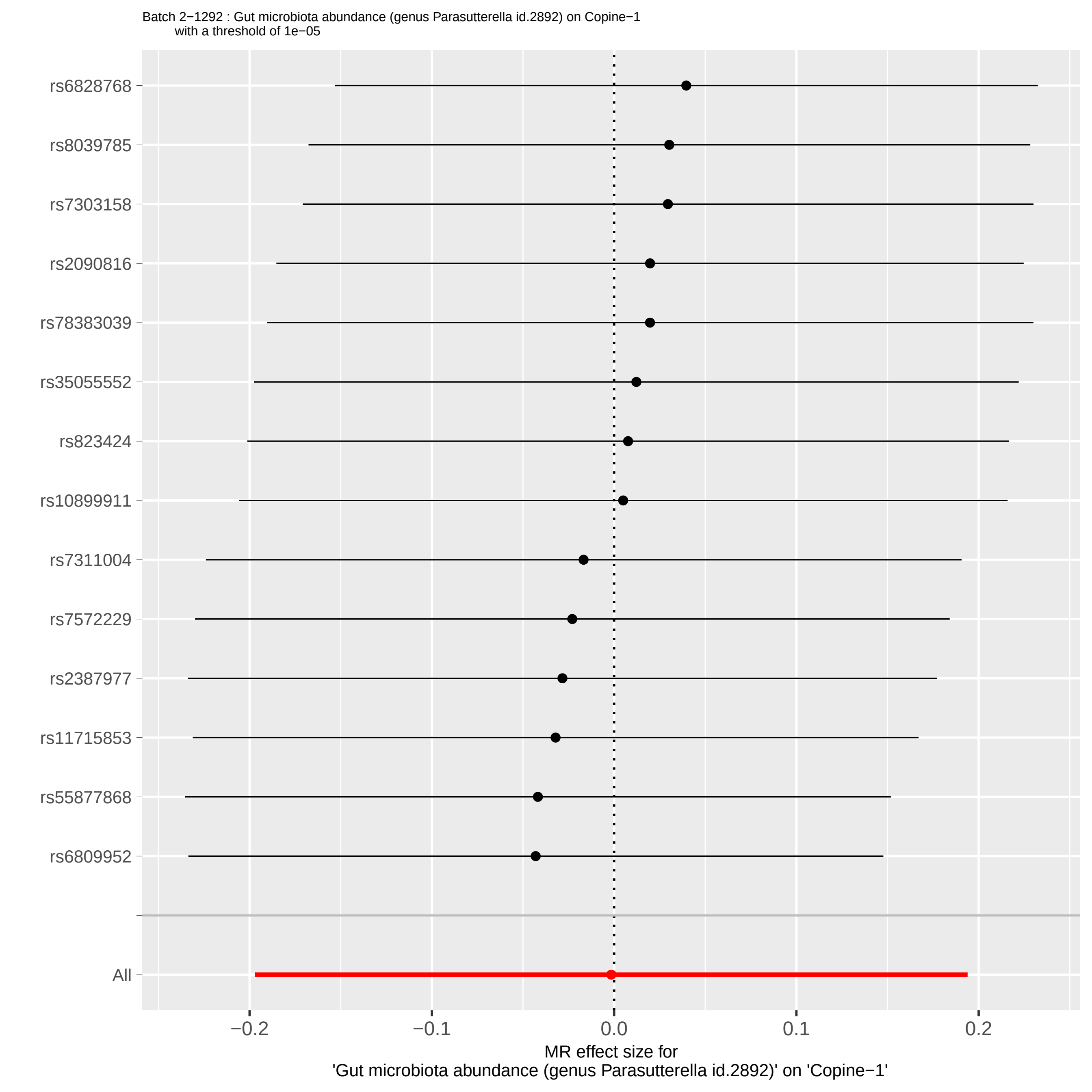

Batch 2−1292 : Gut microbiota abundance (genus Parasutterella id.2892) on Copine−1
with a threshold of 1e−05
rs6828768
rs8039785
rs7303158
rs2090816
rs78383039
rs35055552
rs823424
rs10899911
rs7311004
rs7572229
rs2387977
rs11715853
rs55877868
rs6809952
All
−0.2
−0.1
0.0
0.1
0.2
MR effect size for
'Gut microbiota abundance (genus Parasutterella id.2892)' on 'Copine−1'

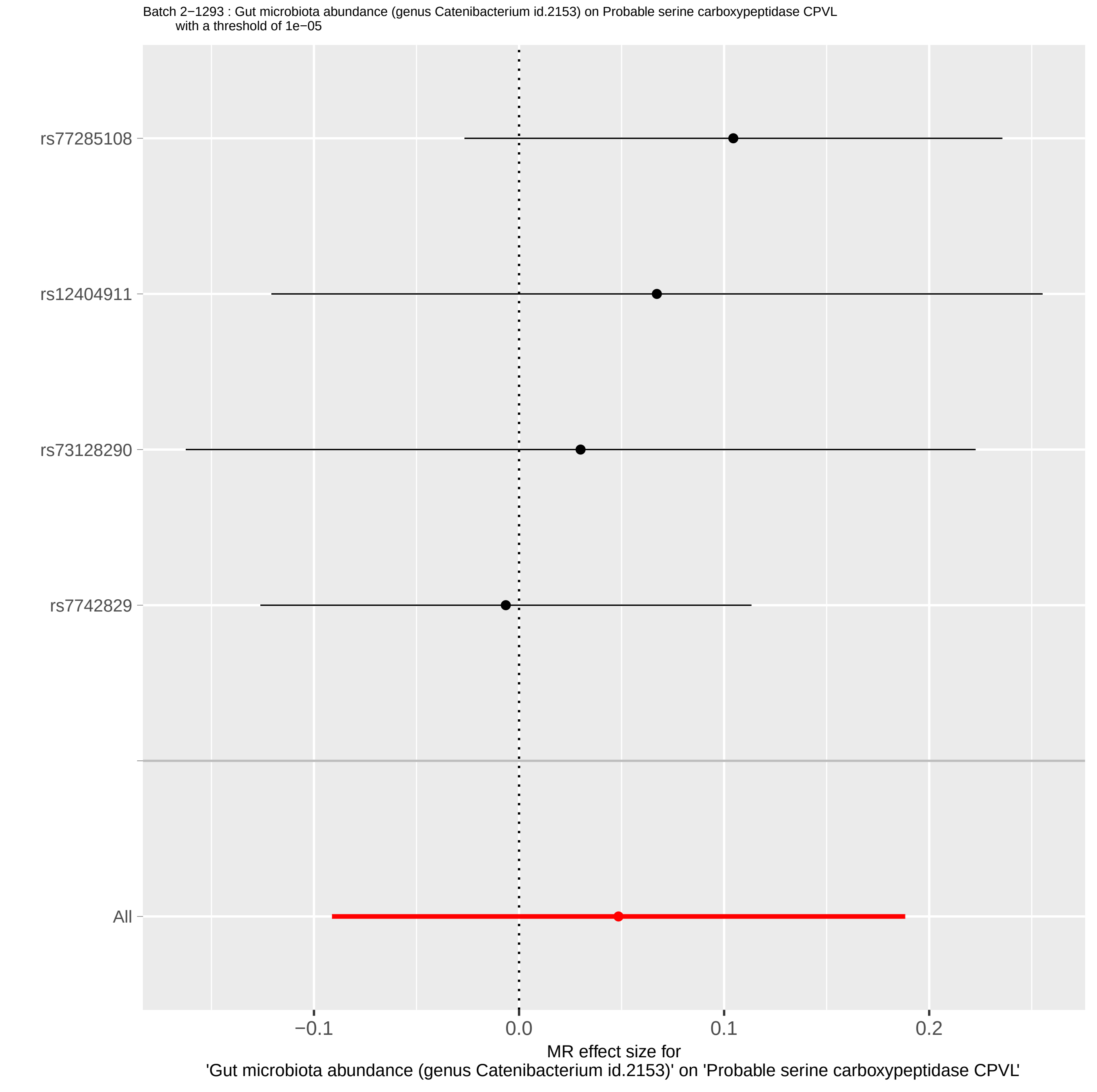
Batch 2−1293 : Gut microbiota abundance (genus Catenibacterium id.2153) on Probable serine carboxypeptidase CPVL
with a threshold of 1e−05
rs77285108
rs12404911
rs73128290
rs7742829
All
−0.1
0.0
0.1
0.2
MR effect size for
'Gut microbiota abundance (genus Catenibacterium id.2153)' on 'Probable serine carboxypeptidase CPVL'

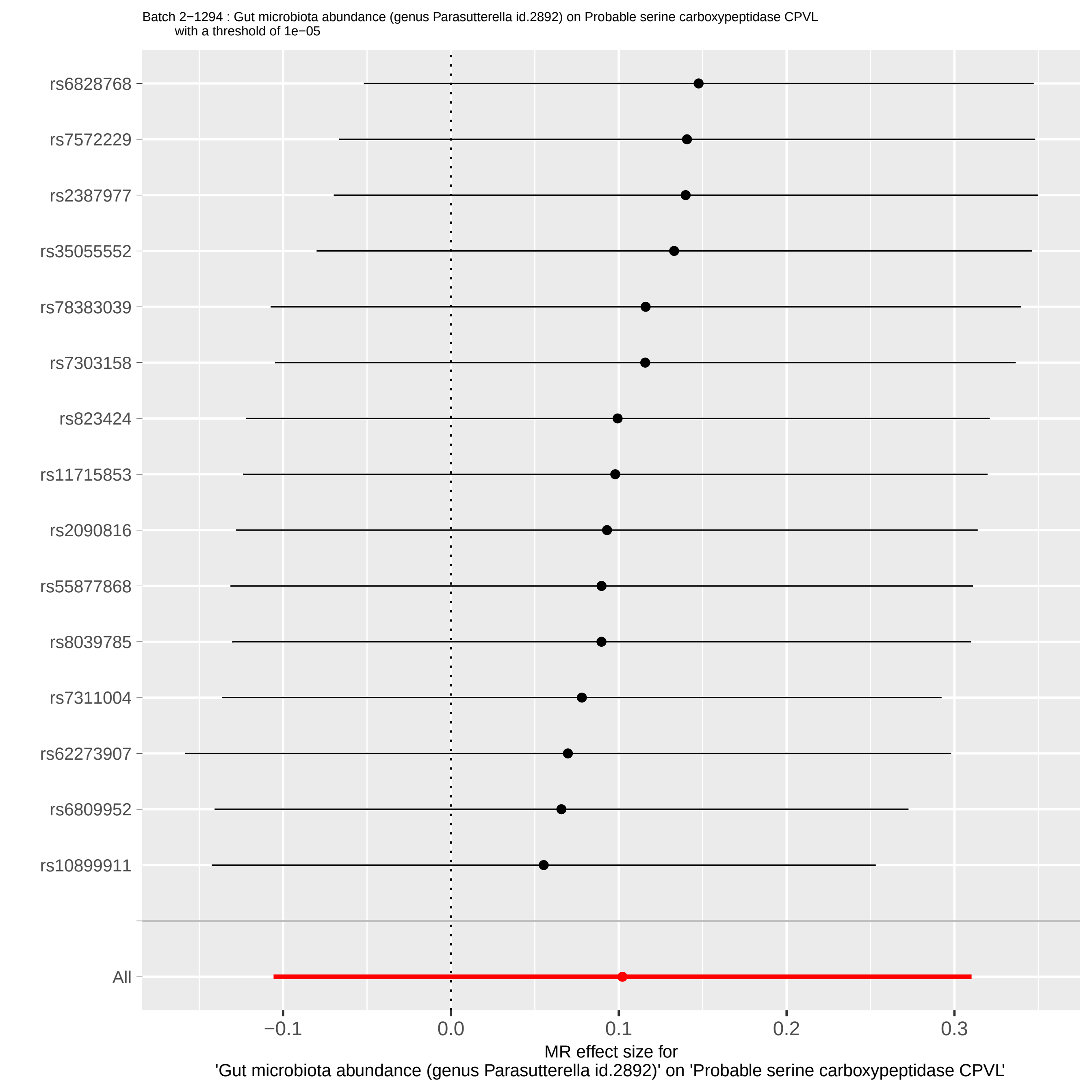
Batch 2−1294 : Gut microbiota abundance (genus Parasutterella id.2892) on Probable serine carboxypeptidase CPVL
with a threshold of 1e−05
rs6828768
rs7572229
rs2387977
rs35055552
rs78383039
rs7303158
rs823424
rs11715853
rs2090816
rs55877868
rs8039785
rs7311004
rs62273907
rs6809952
rs10899911
All
−0.1
0.0
0.1
0.2
0.3
MR effect size for
'Gut microbiota abundance (genus Parasutterella id.2892)' on 'Probable serine carboxypeptidase CPVL'

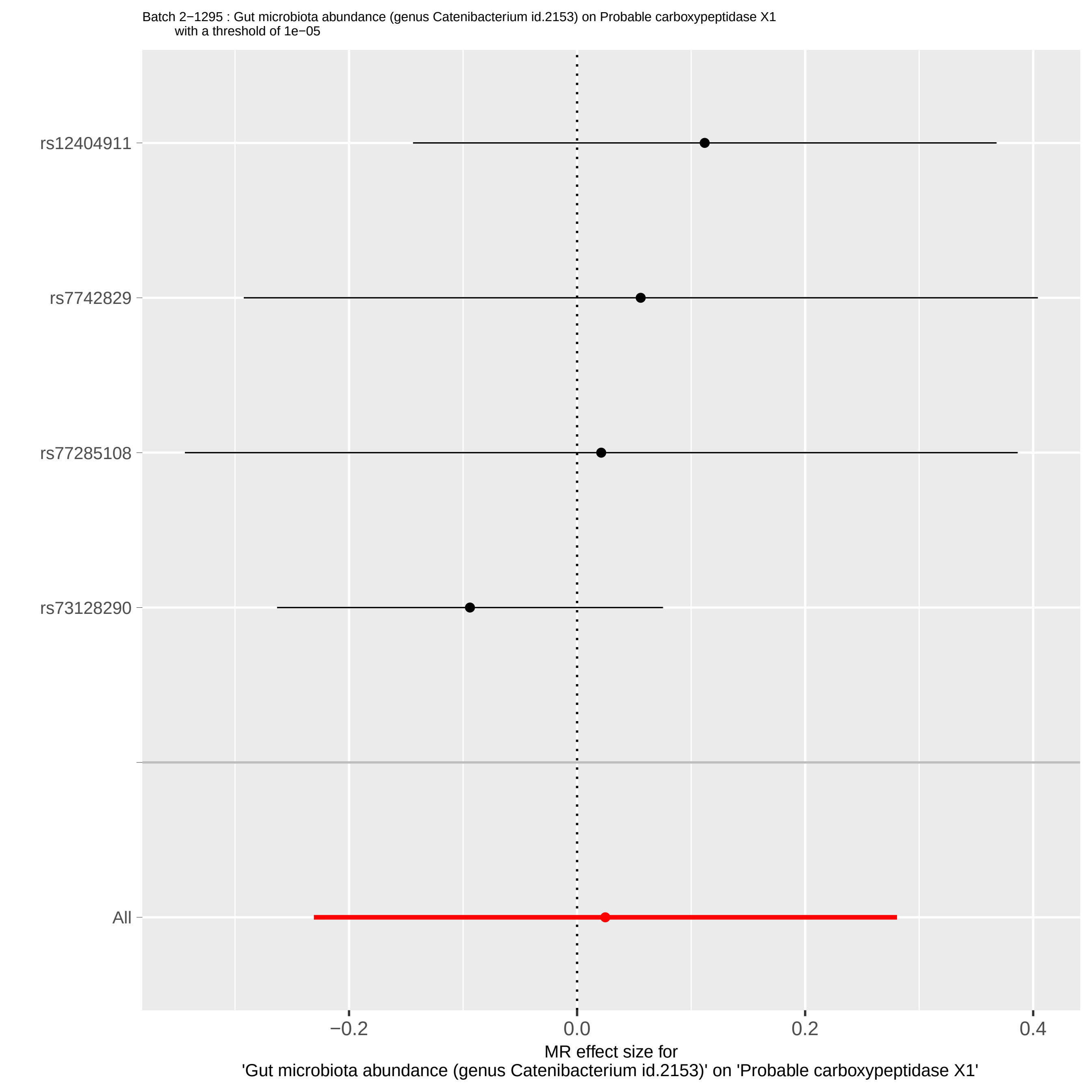
Batch 2−1295 : Gut microbiota abundance (genus Catenibacterium id.2153) on Probable carboxypeptidase X1
with a threshold of 1e−05
rs12404911
rs7742829
rs77285108
rs73128290
All
−0.2
0.0
0.2
0.4
MR effect size for
'Gut microbiota abundance (genus Catenibacterium id.2153)' on 'Probable carboxypeptidase X1'

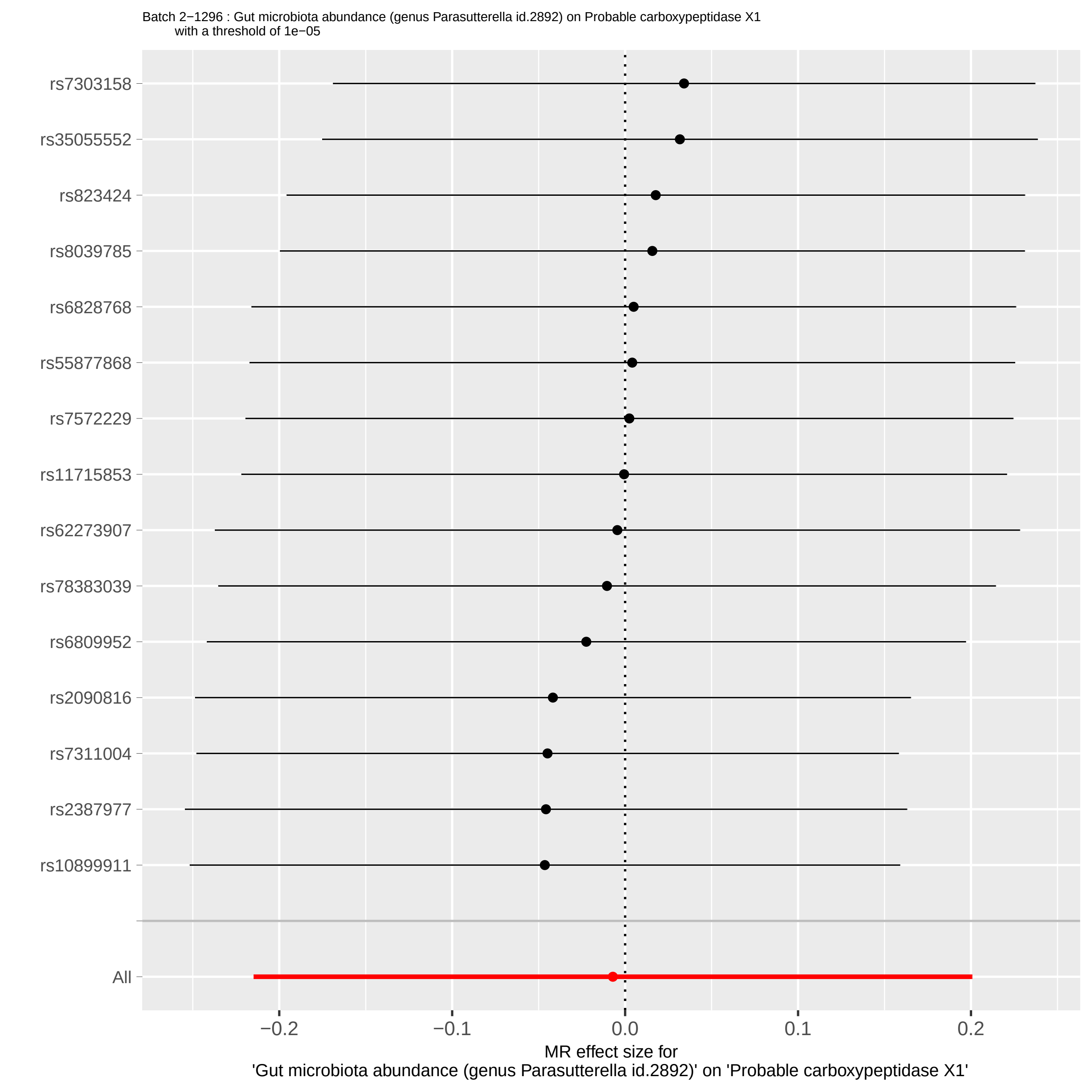
Batch 2−1296 : Gut microbiota abundance (genus Parasutterella id.2892) on Probable carboxypeptidase X1
with a threshold of 1e−05
rs7303158
rs35055552
rs823424
rs8039785
rs6828768
rs55877868
rs7572229
rs11715853
rs62273907
rs78383039
rs6809952
rs2090816
rs7311004
rs2387977
rs10899911
All
−0.2
−0.1
0.0
0.1
0.2
MR effect size for
'Gut microbiota abundance (genus Parasutterella id.2892)' on 'Probable carboxypeptidase X1'

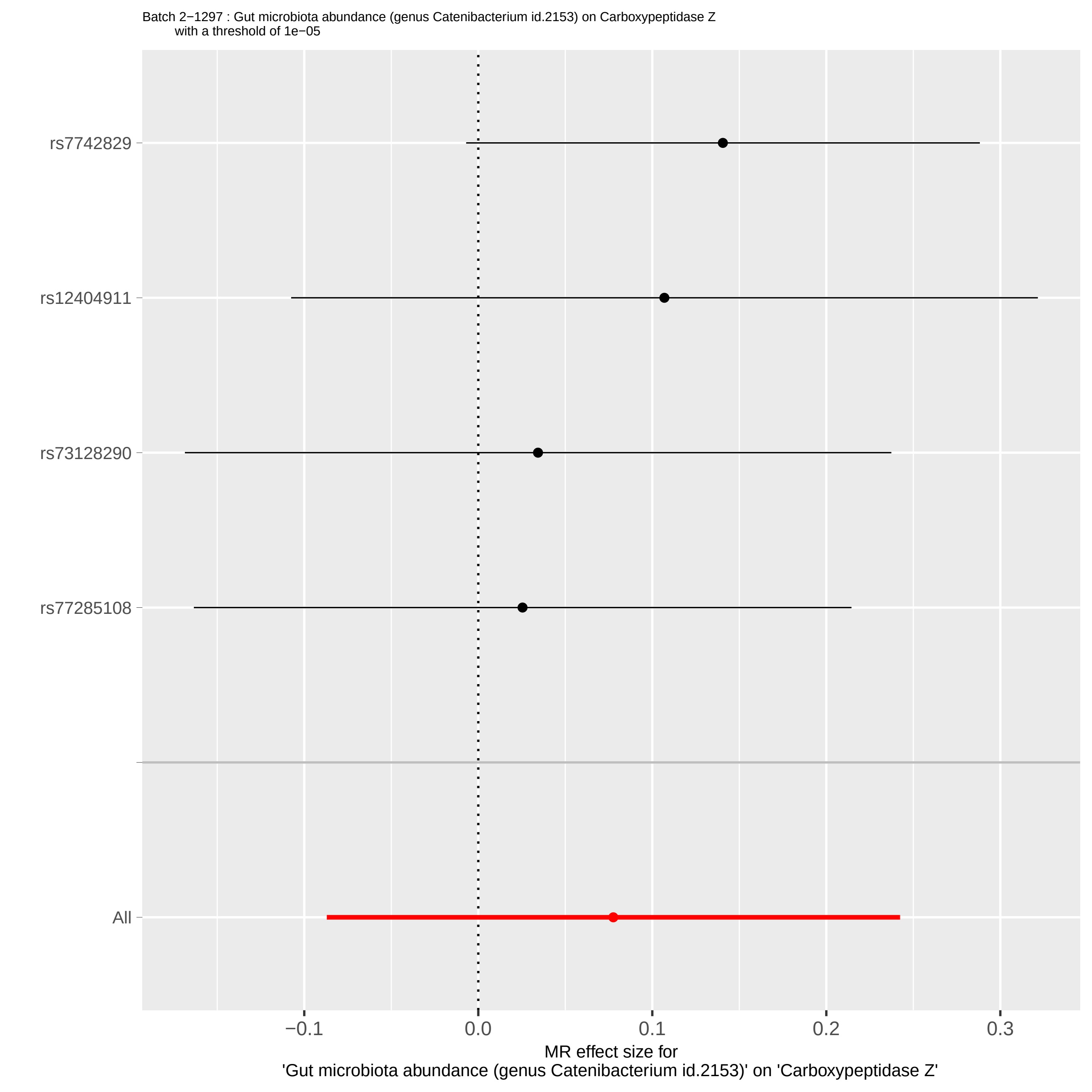
Batch 2−1297 : Gut microbiota abundance (genus Catenibacterium id.2153) on Carboxypeptidase Z
with a threshold of 1e−05
rs7742829
rs12404911
rs73128290
rs77285108
All
−0.1
0.0
0.1
0.2
0.3
MR effect size for
'Gut microbiota abundance (genus Catenibacterium id.2153)' on 'Carboxypeptidase Z'

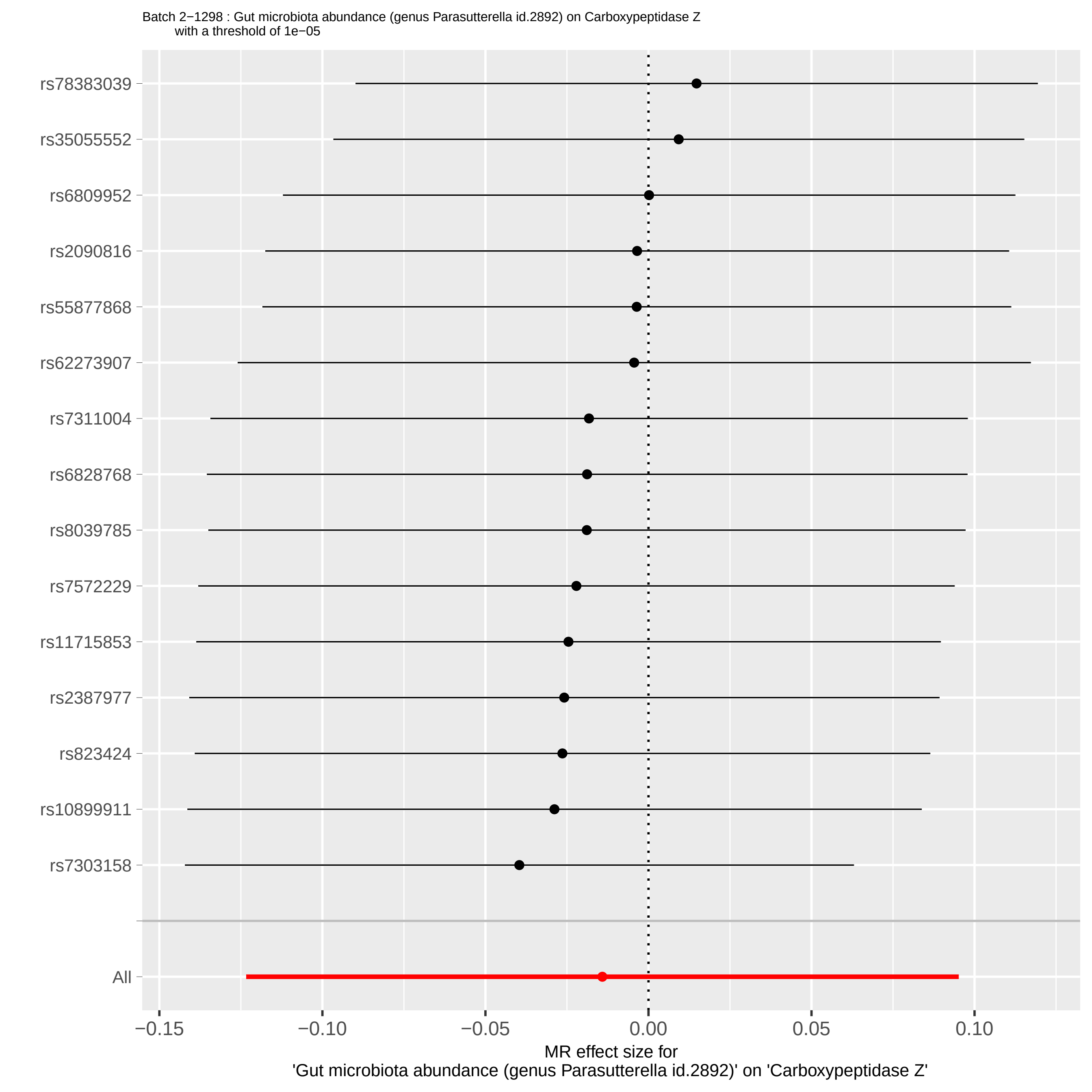
Batch 2−1298 : Gut microbiota abundance (genus Parasutterella id.2892) on Carboxypeptidase Z
with a threshold of 1e−05
rs78383039
rs35055552
rs6809952
rs2090816
rs55877868
rs62273907
rs7311004
rs6828768
rs8039785
rs7572229
rs11715853
rs2387977
rs823424
rs10899911
rs7303158
All
−0.15
−0.10
−0.05
0.00
0.05
0.10
MR effect size for
'Gut microbiota abundance (genus Parasutterella id.2892)' on 'Carboxypeptidase Z'

Batch 2−1299 : Gut microbiota abundance (genus Catenibacterium id.2153) on Cellular retinoic acid−binding protein 1
with a threshold of 1e−05

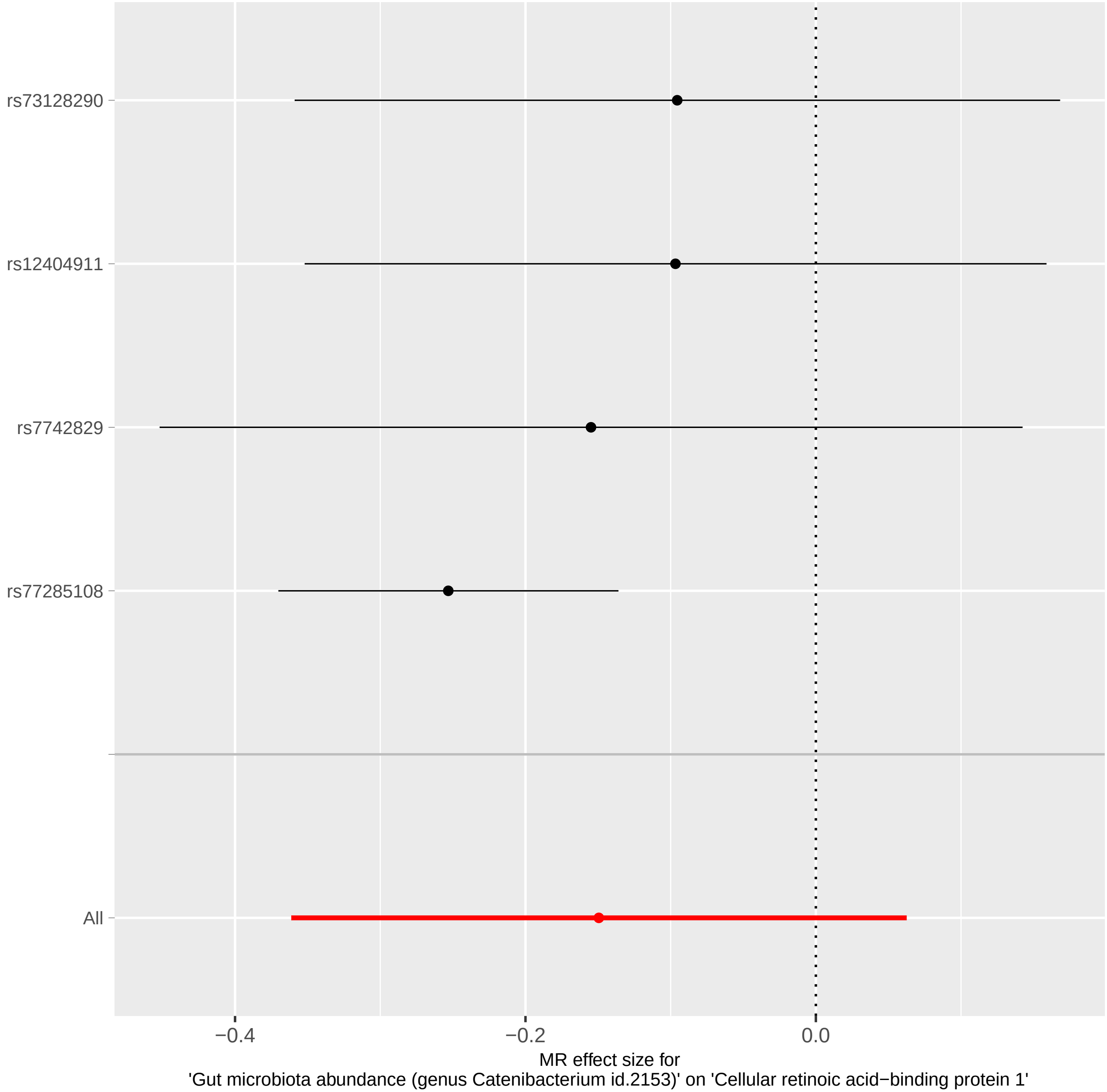

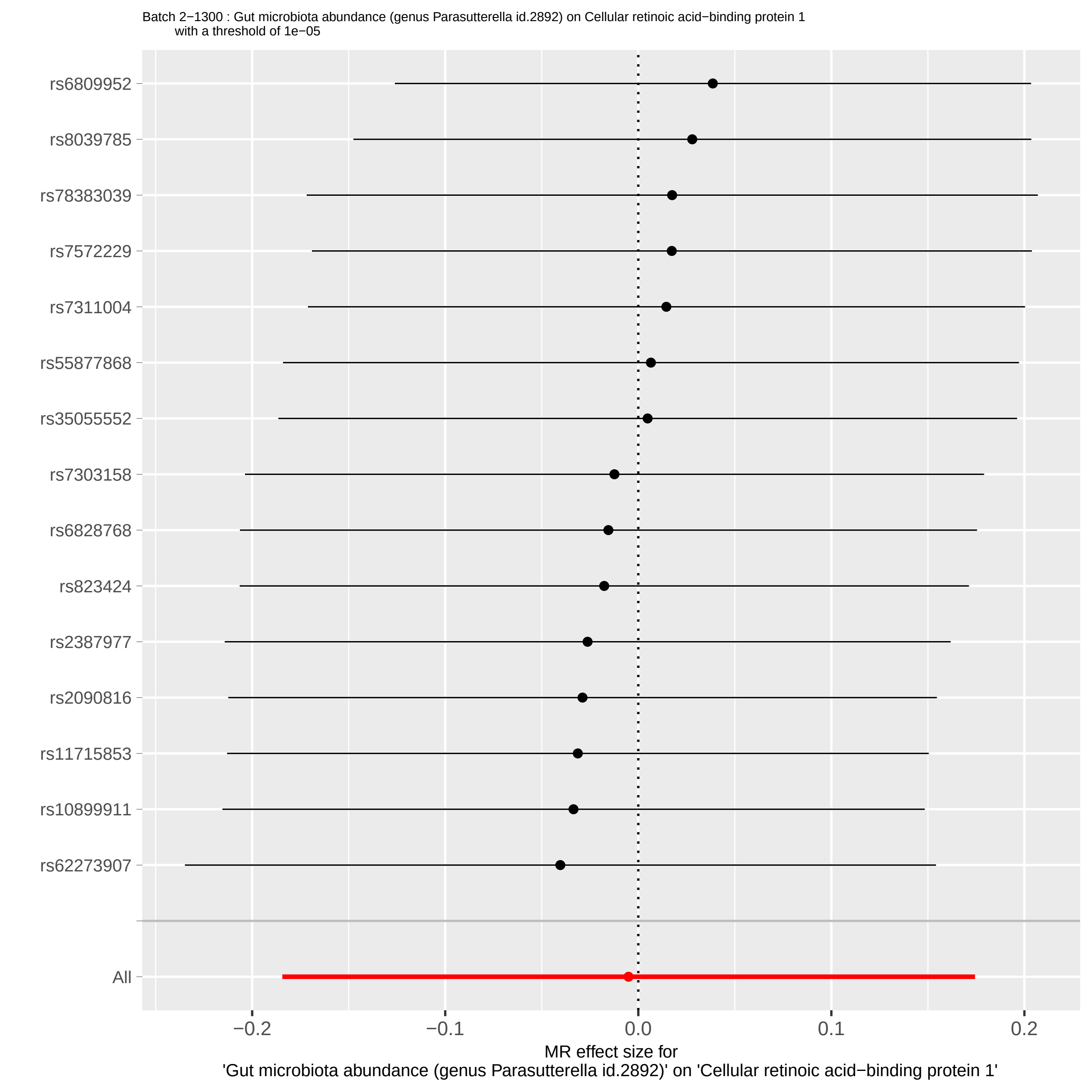

Batch 2−1300 : Gut microbiota abundance (genus Parasutterella id.2892) on Cellular retinoic acid−binding protein 1
with a threshold of 1e−05
rs6809952
rs8039785
rs78383039
rs7572229
rs7311004
rs55877868
rs35055552
rs7303158
rs6828768
rs823424
rs2387977
rs2090816
rs11715853
rs10899911
rs62273907
All
−0.2
−0.1
0.0
0.1
0.2
MR effect size for
'Gut microbiota abundance (genus Parasutterella id.2892)' on 'Cellular retinoic acid−binding protein 1'

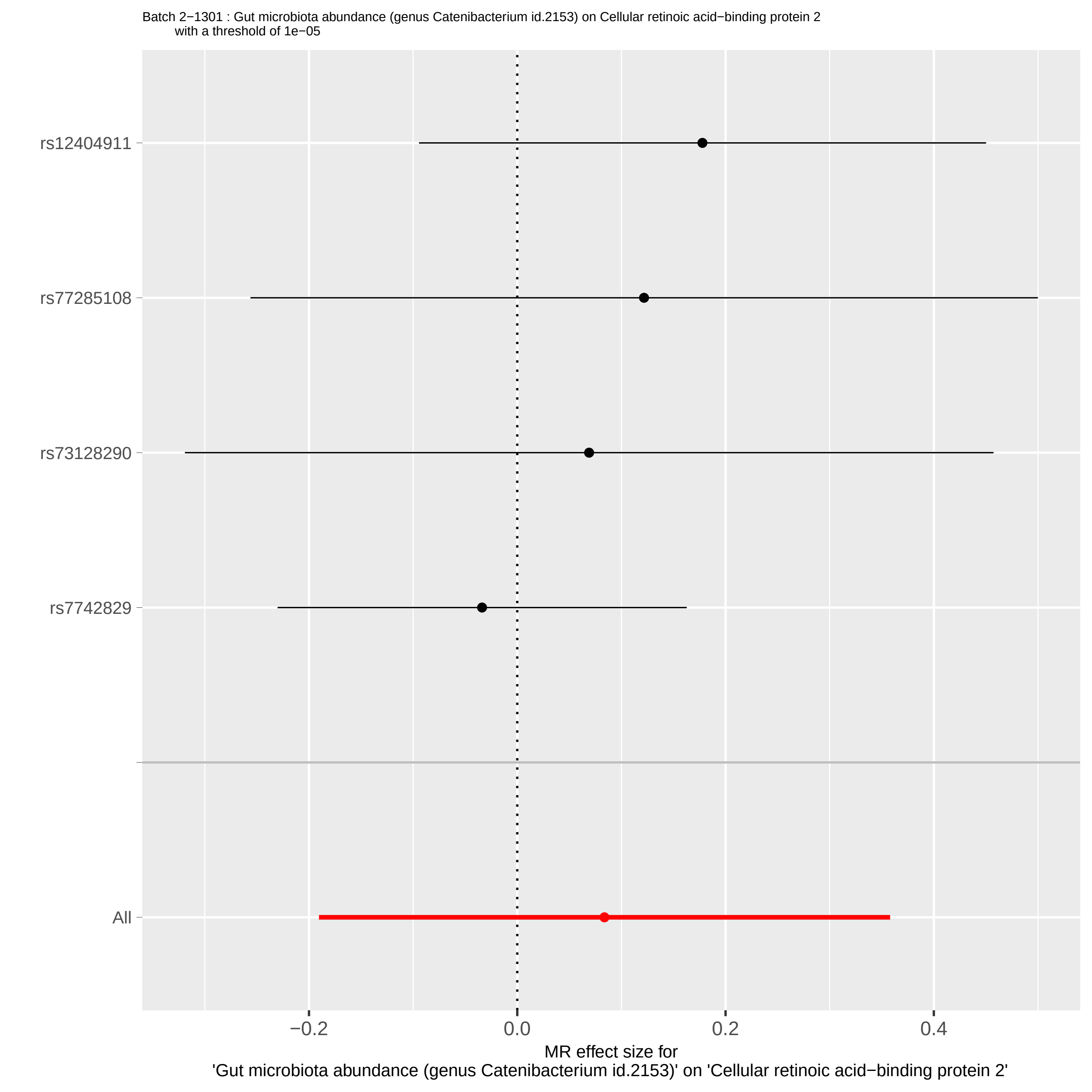

Batch 2−1301 : Gut microbiota abundance (genus Catenibacterium id.2153) on Cellular retinoic acid−binding protein 2
with a threshold of 1e−05
rs12404911
rs77285108
rs73128290
rs7742829
All
−0.2
0.0
0.2
0.4
MR effect size for
'Gut microbiota abundance (genus Catenibacterium id.2153)' on 'Cellular retinoic acid−binding protein 2'

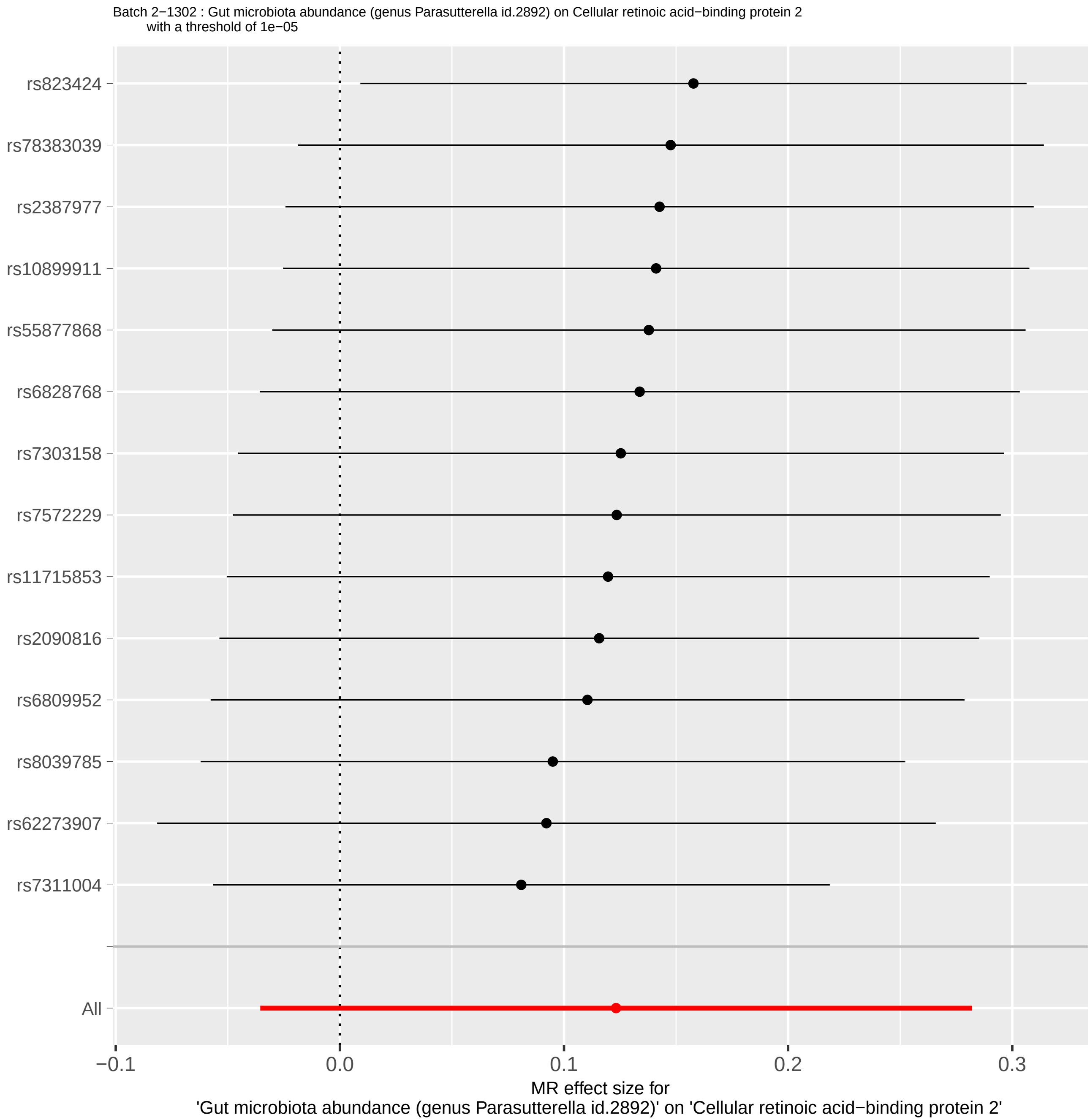

Batch 2−1302 : Gut microbiota abundance (genus Parasutterella id.2892) on Cellular retinoic acid−binding protein 2
with a threshold of 1e−05
rs823424
rs78383039
rs2387977
rs10899911
rs55877868
rs6828768
rs7303158
rs7572229
rs11715853
rs2090816
rs6809952
rs8039785
rs62273907
rs7311004
All
−0.1
0.0
0.1
0.2
0.3
MR effect size for
'Gut microbiota abundance (genus Parasutterella id.2892)' on 'Cellular retinoic acid−binding protein 2'

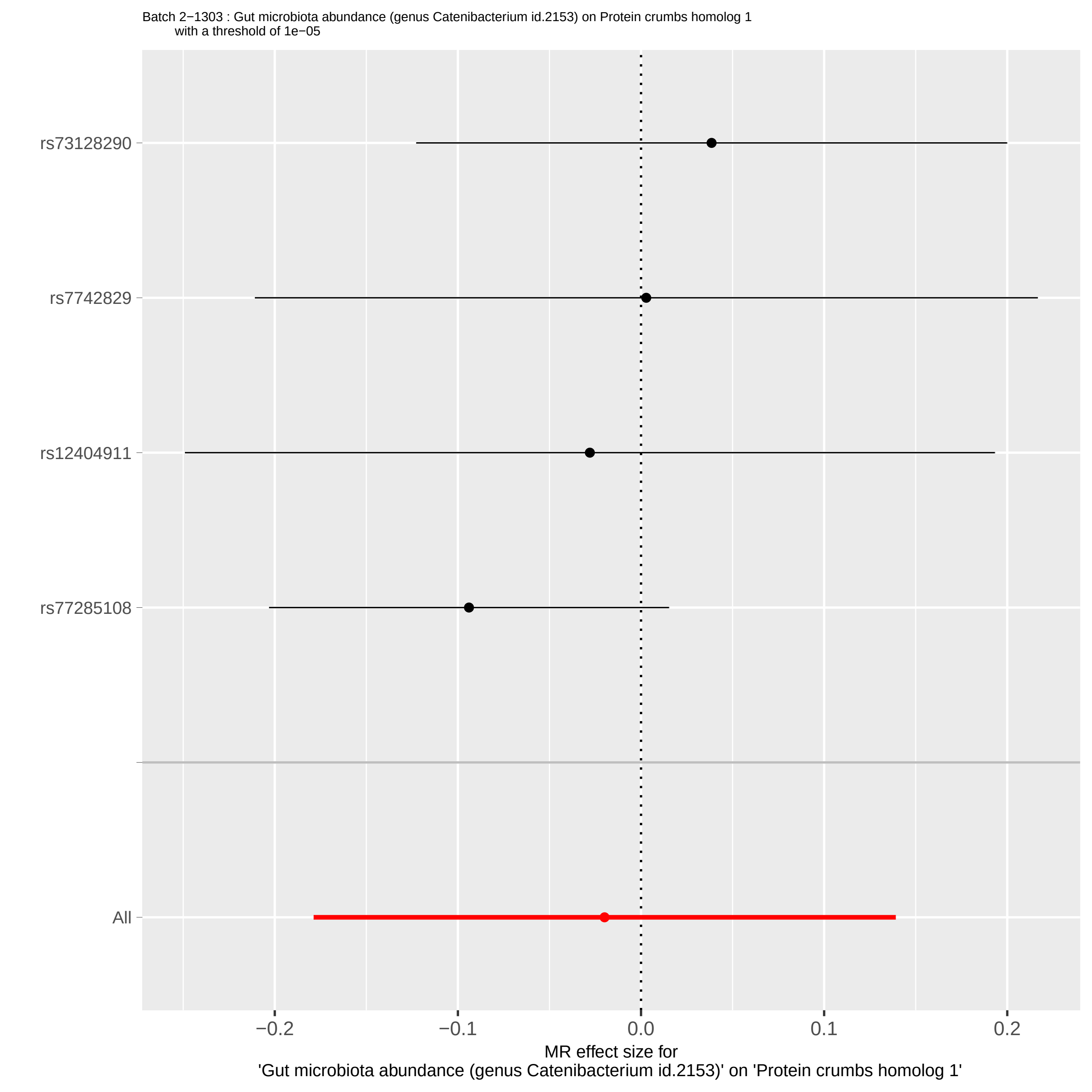
Batch 2−1303 : Gut microbiota abundance (genus Catenibacterium id.2153) on Protein crumbs homolog 1
with a threshold of 1e−05
rs73128290
rs7742829
rs12404911
rs77285108
All
−0.2
−0.1
0.0
0.1
0.2
MR effect size for
'Gut microbiota abundance (genus Catenibacterium id.2153)' on 'Protein crumbs homolog 1'

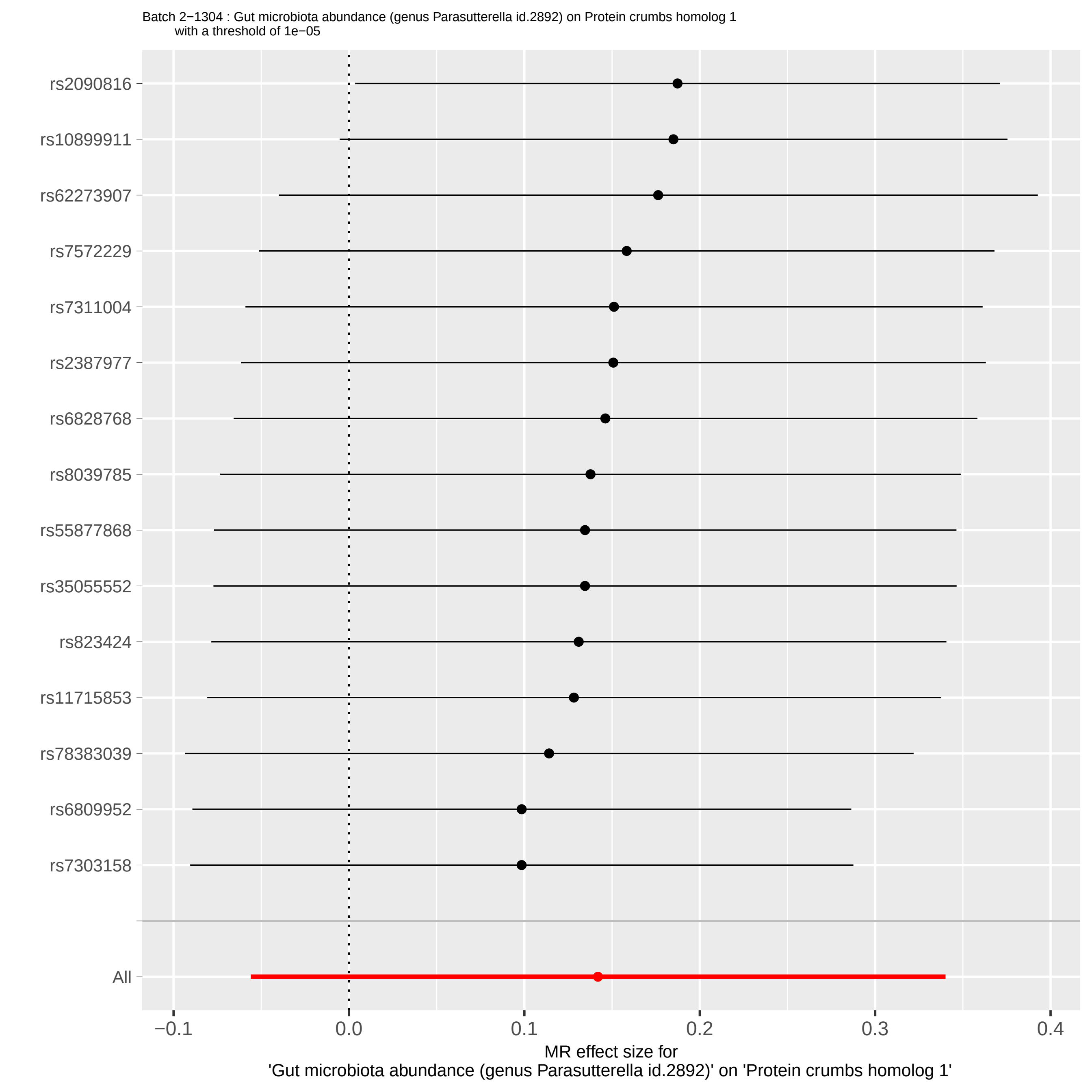
Batch 2−1304 : Gut microbiota abundance (genus Parasutterella id.2892) on Protein crumbs homolog 1
with a threshold of 1e−05
rs2090816
rs10899911
rs62273907
rs7572229
rs7311004
rs2387977
rs6828768
rs8039785
rs55877868
rs35055552
rs823424
rs11715853
rs78383039
rs6809952
rs7303158
All
−0.1
0.0
0.1
0.2
0.3
0.4
MR effect size for
'Gut microbiota abundance (genus Parasutterella id.2892)' on 'Protein crumbs homolog 1'

Batch 2−1305 : Gut microbiota abundance (genus Catenibacterium id.2153) on Cyclic AMP−responsive element−binding protein 3−like protein 1
with a threshold of 1e−05

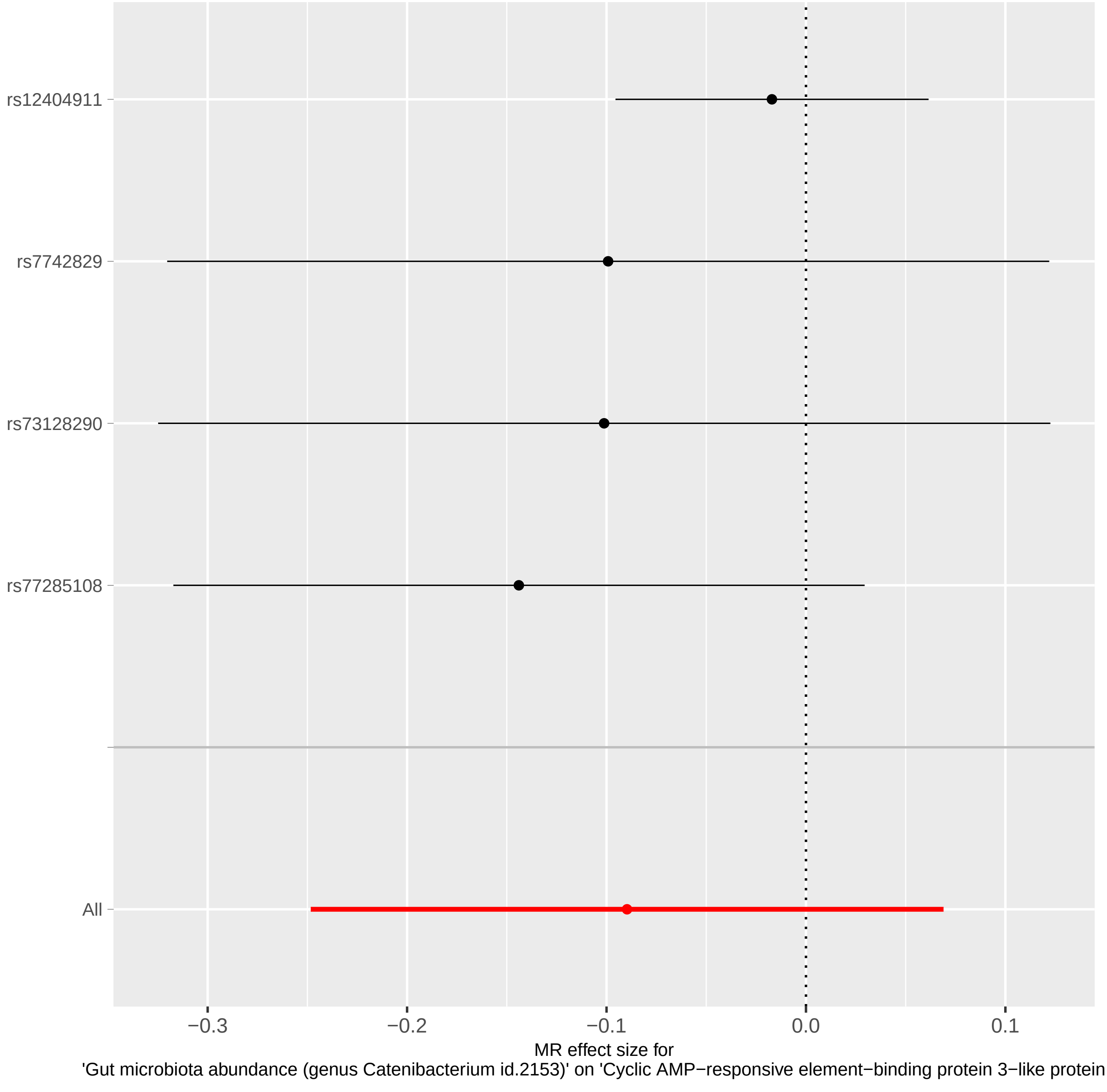

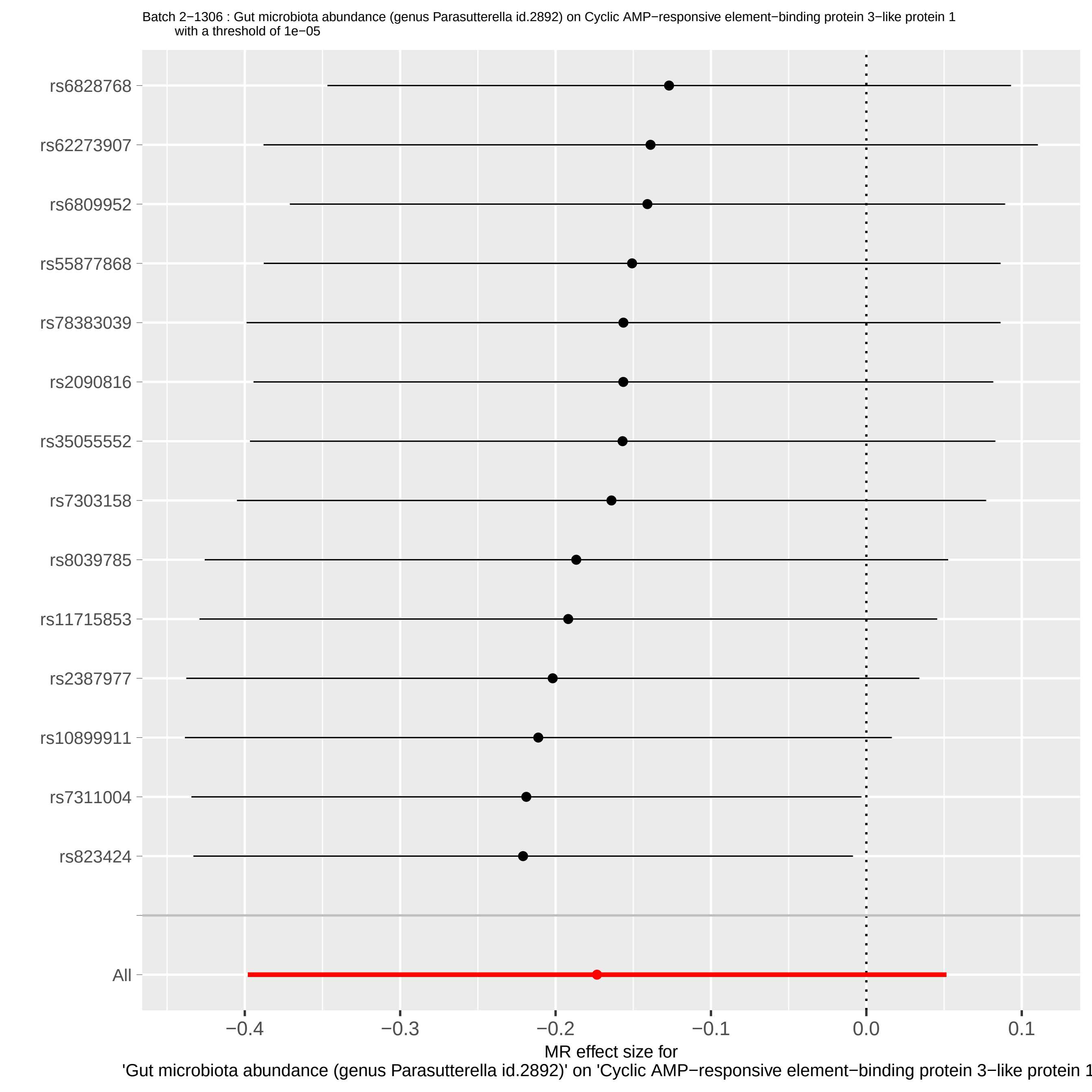
Batch 2−1306 : Gut microbiota abundance (genus Parasutterella id.2892) on Cyclic AMP−responsive element−binding protein 3−like protein 1 with a threshold of 1e−05
rs6828768
rs62273907
rs6809952
rs55877868
rs78383039
rs2090816
rs35055552
rs7303158
rs8039785
rs11715853
rs2387977
rs10899911
rs7311004
rs823424
All
−0.4
−0.3
−0.2
−0.1
0.0
0.1
MR effect size for
'Gut microbiota abundance (genus Parasutterella id.2892)' on 'Cyclic AMP−responsive element−binding protein 3−like protein 1

Batch 2−1307 : Gut microbiota abundance (genus Catenibacterium id.2153) on Cyclic AMP−responsive element−binding protein 3−like protein 4 with a threshold of 1e−05

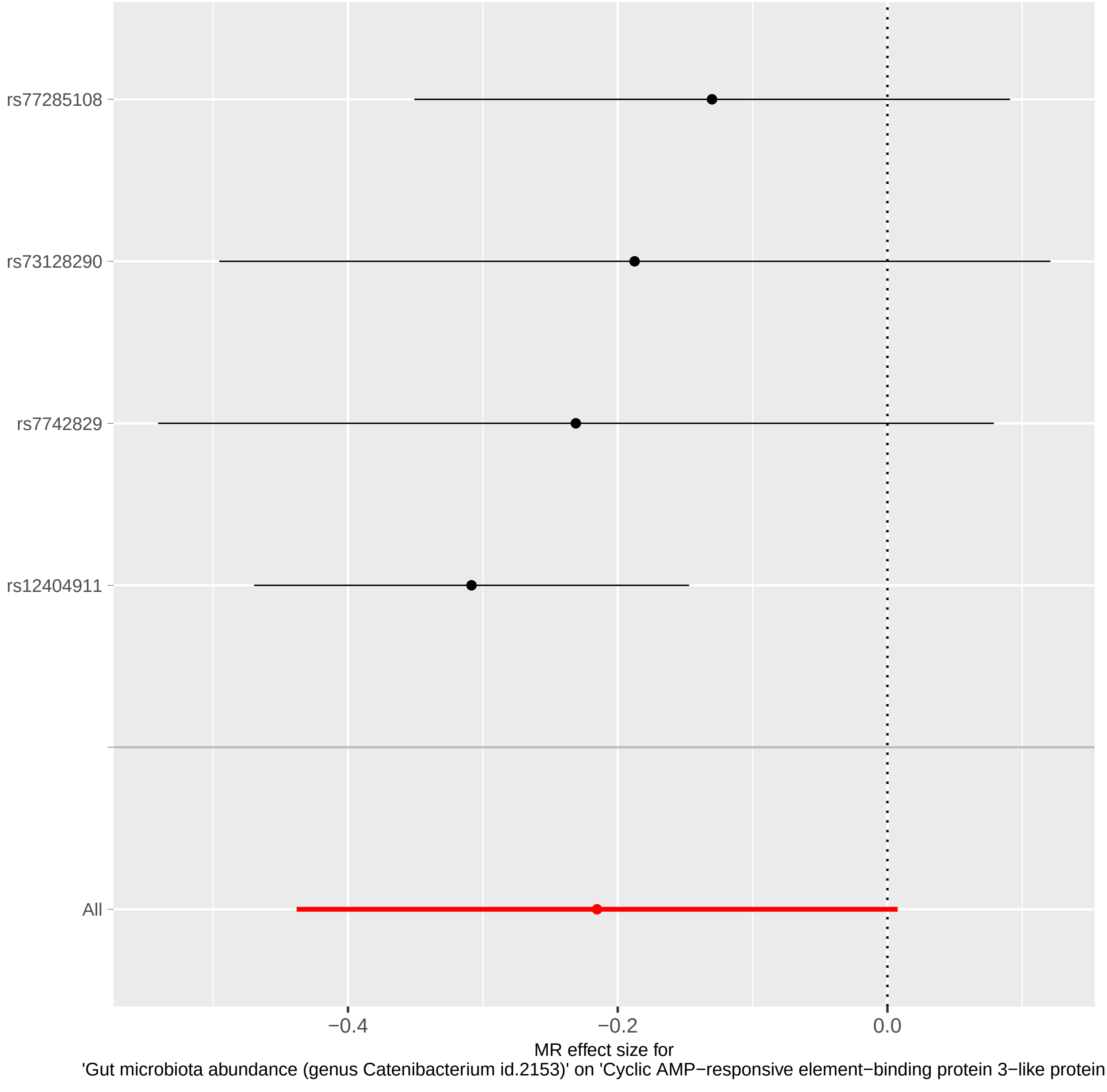

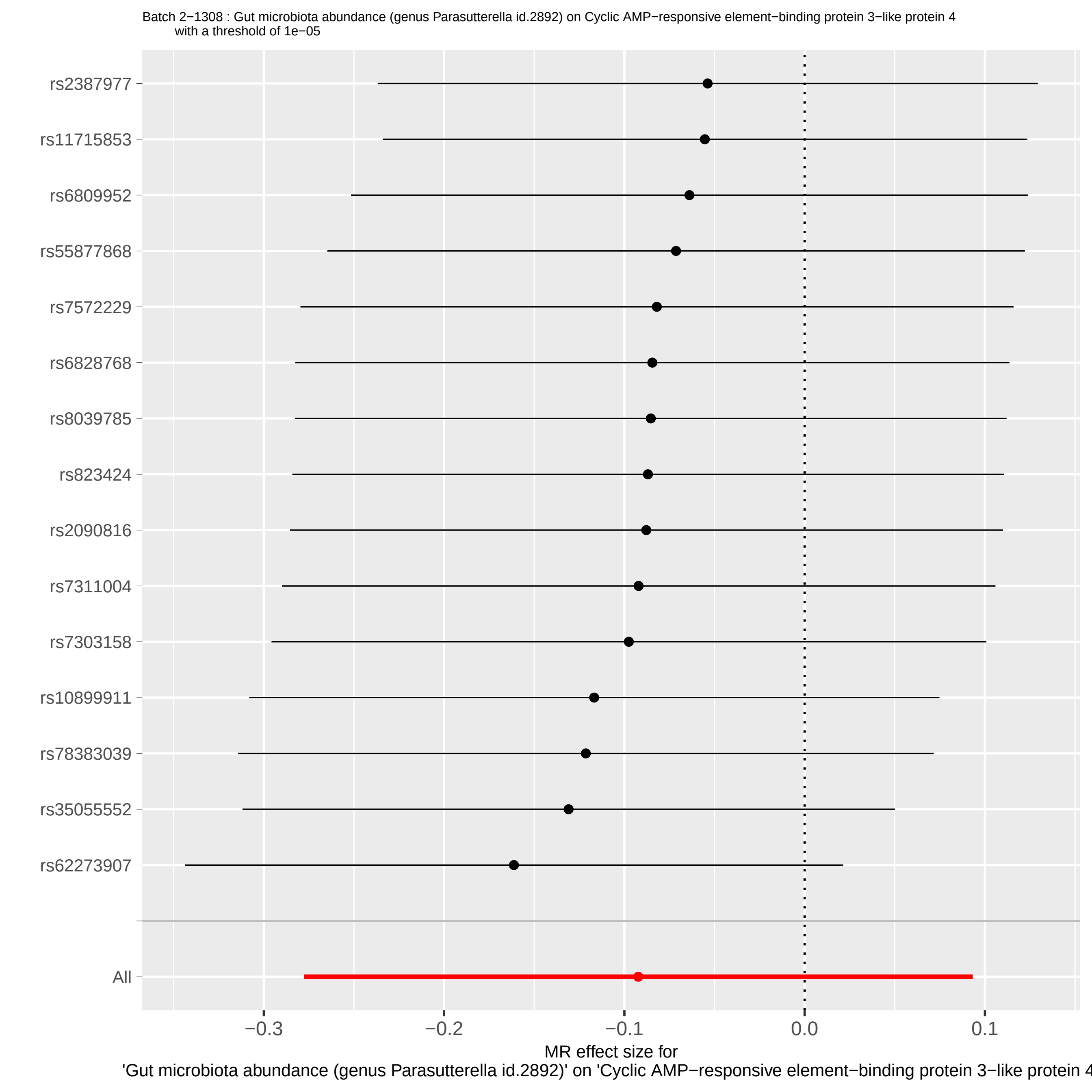
Batch 2−1308 : Gut microbiota abundance (genus Parasutterella id.2892) on Cyclic AMP−responsive element−binding protein 3−like protein 4
with a threshold of 1e−05
rs2387977
rs11715853
rs6809952
rs55877868
rs7572229
rs6828768
rs8039785
rs823424
rs2090816
rs7311004
rs7303158
rs10899911
rs78383039
rs35055552
rs62273907
All
−0.3
−0.2
−0.1
0.0
0.1
MR effect size for
'Gut microbiota abundance (genus Parasutterella id.2892)' on 'Cyclic AMP−responsive element−binding protein 3−like protein 4

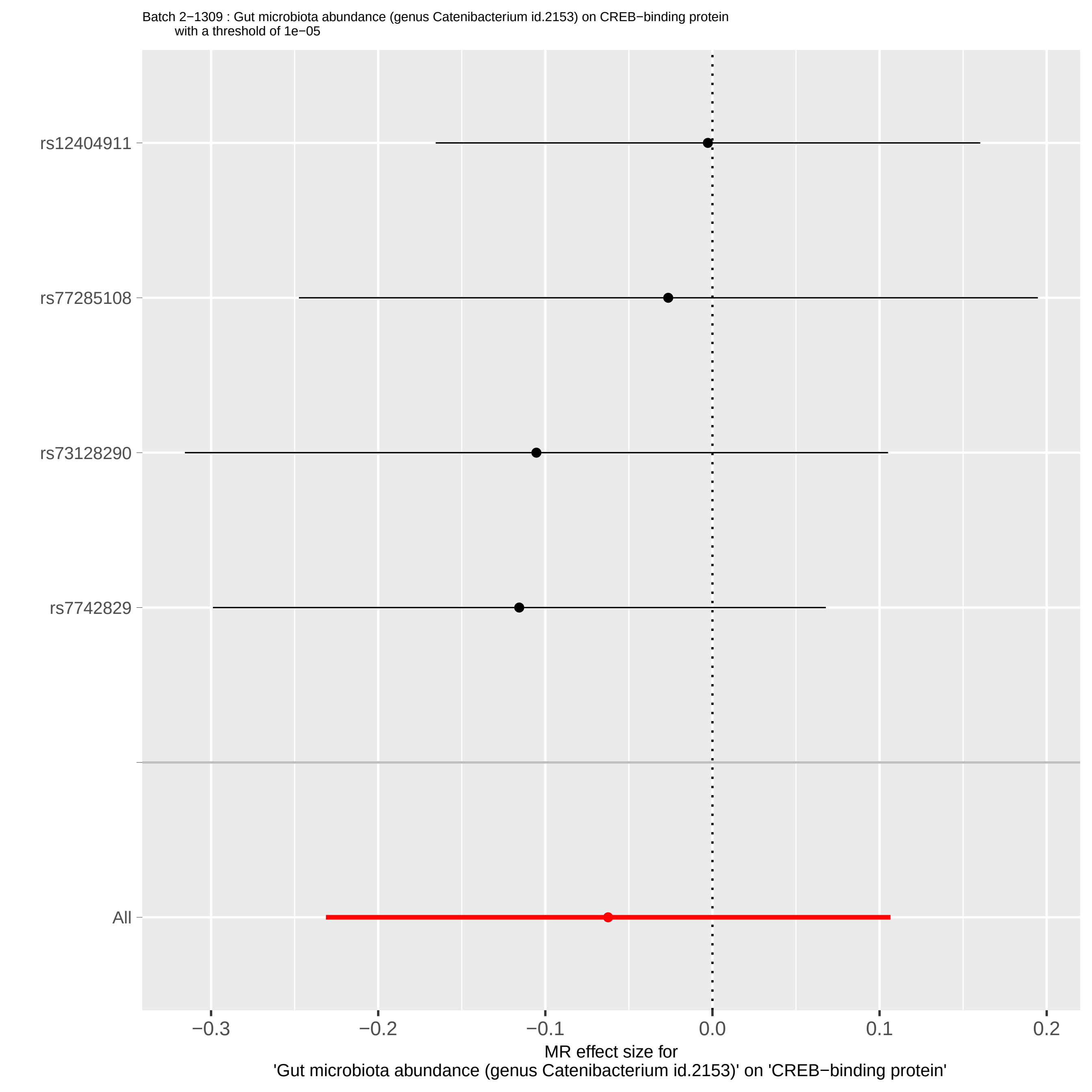

Batch 2−1309 : Gut microbiota abundance (genus Catenibacterium id.2153) on CREB−binding protein
with a threshold of 1e−05
rs12404911
rs77285108
rs73128290
rs7742829
All
−0.3
−0.2
−0.1
0.0
0.1
0.2
MR effect size for
'Gut microbiota abundance (genus Catenibacterium id.2153)' on 'CREB−binding protein'

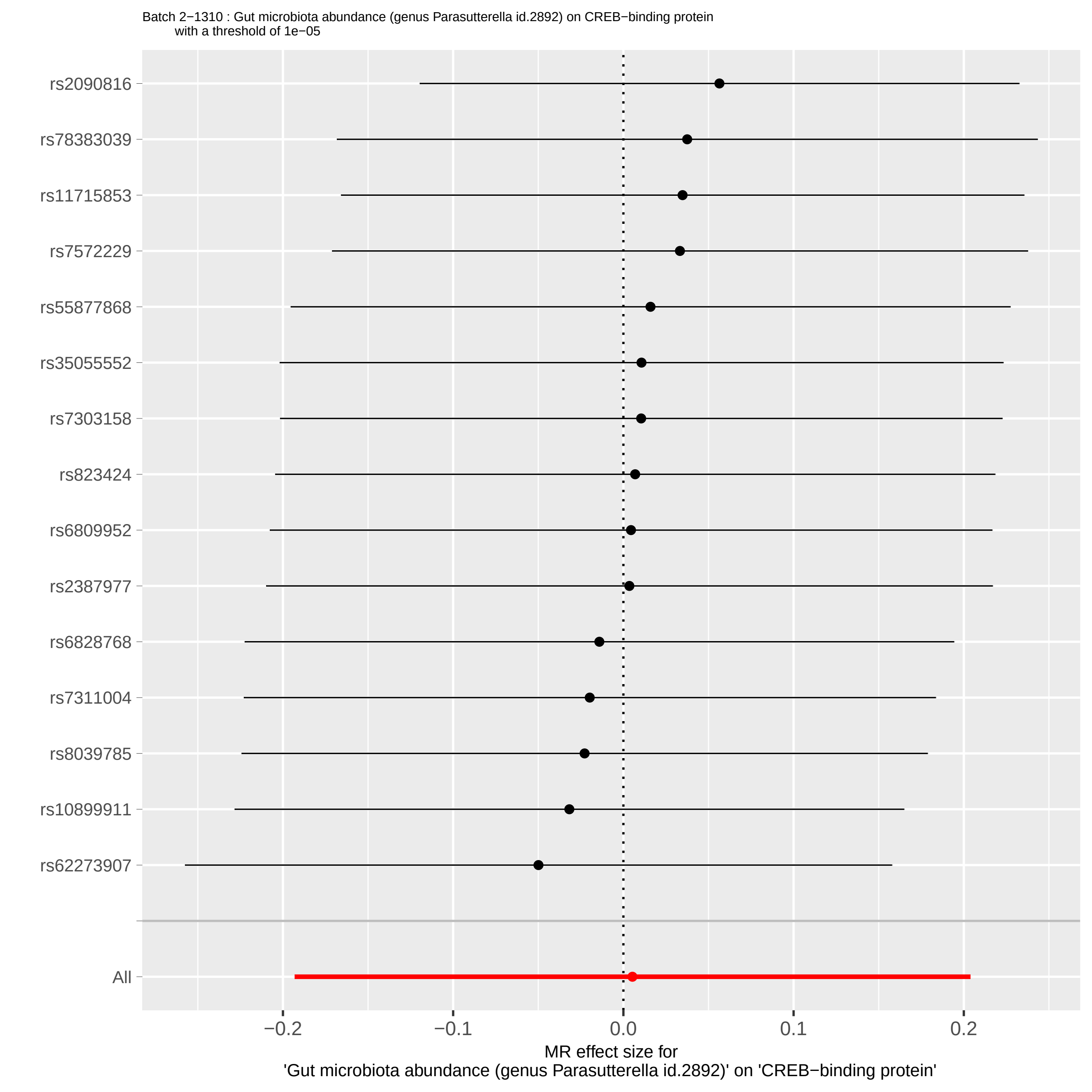
Batch 2−1310 : Gut microbiota abundance (genus Parasutterella id.2892) on CREB−binding protein
with a threshold of 1e−05
rs2090816
rs78383039
rs11715853
rs7572229
rs55877868
rs35055552
rs7303158
rs823424
rs6809952
rs2387977
rs6828768
rs7311004
rs8039785
rs10899911
rs62273907
All
−0.2
−0.1
0.0
0.1
0.2
MR effect size for
'Gut microbiota abundance (genus Parasutterella id.2892)' on 'CREB−binding protein'

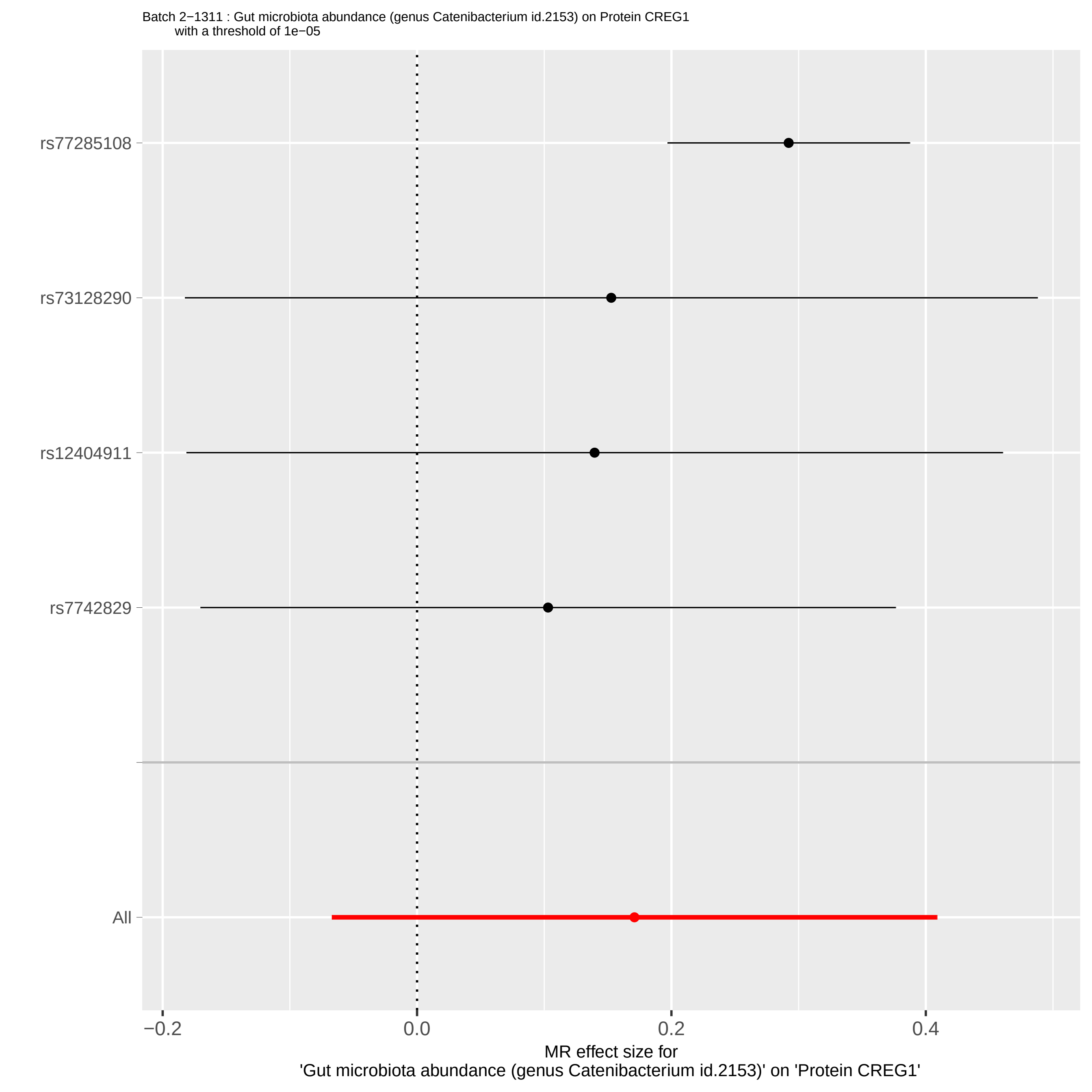

Batch 2−1311 : Gut microbiota abundance (genus Catenibacterium id.2153) on Protein CREG1
with a threshold of 1e−05
rs77285108
rs73128290
rs12404911
rs7742829
All
−0.2
0.0
0.2
0.4
MR effect size for
'Gut microbiota abundance (genus Catenibacterium id.2153)' on 'Protein CREG1'

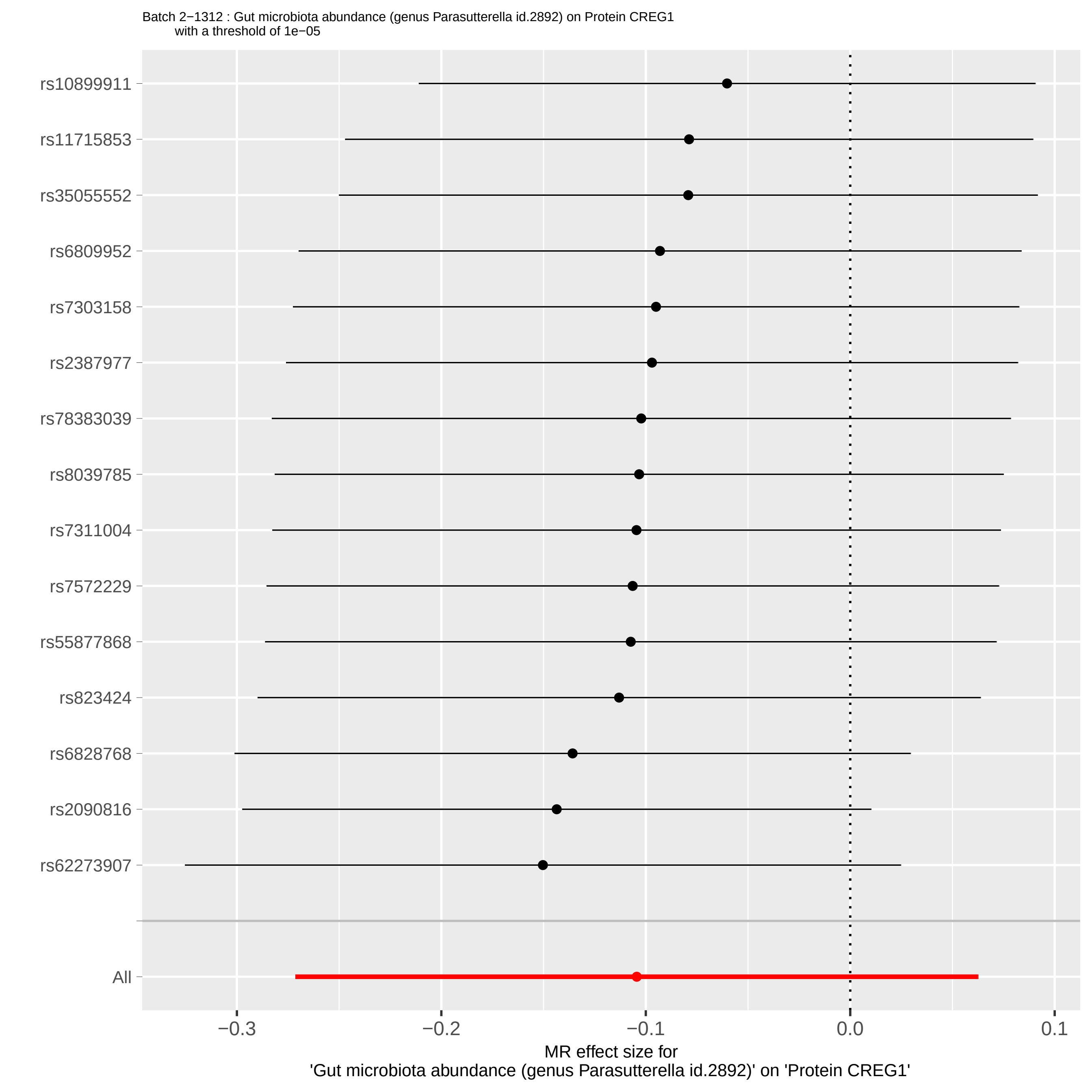
Batch 2−1312 : Gut microbiota abundance (genus Parasutterella id.2892) on Protein CREG1
with a threshold of 1e−05
rs10899911
rs11715853
rs35055552
rs6809952
rs7303158
rs2387977
rs78383039
rs8039785
rs7311004
rs7572229
rs55877868
rs823424
rs6828768
rs2090816
rs62273907
All
−0.3
−0.2
−0.1
0.0
0.1
MR effect size for
'Gut microbiota abundance (genus Parasutterella id.2892)' on 'Protein CREG1'

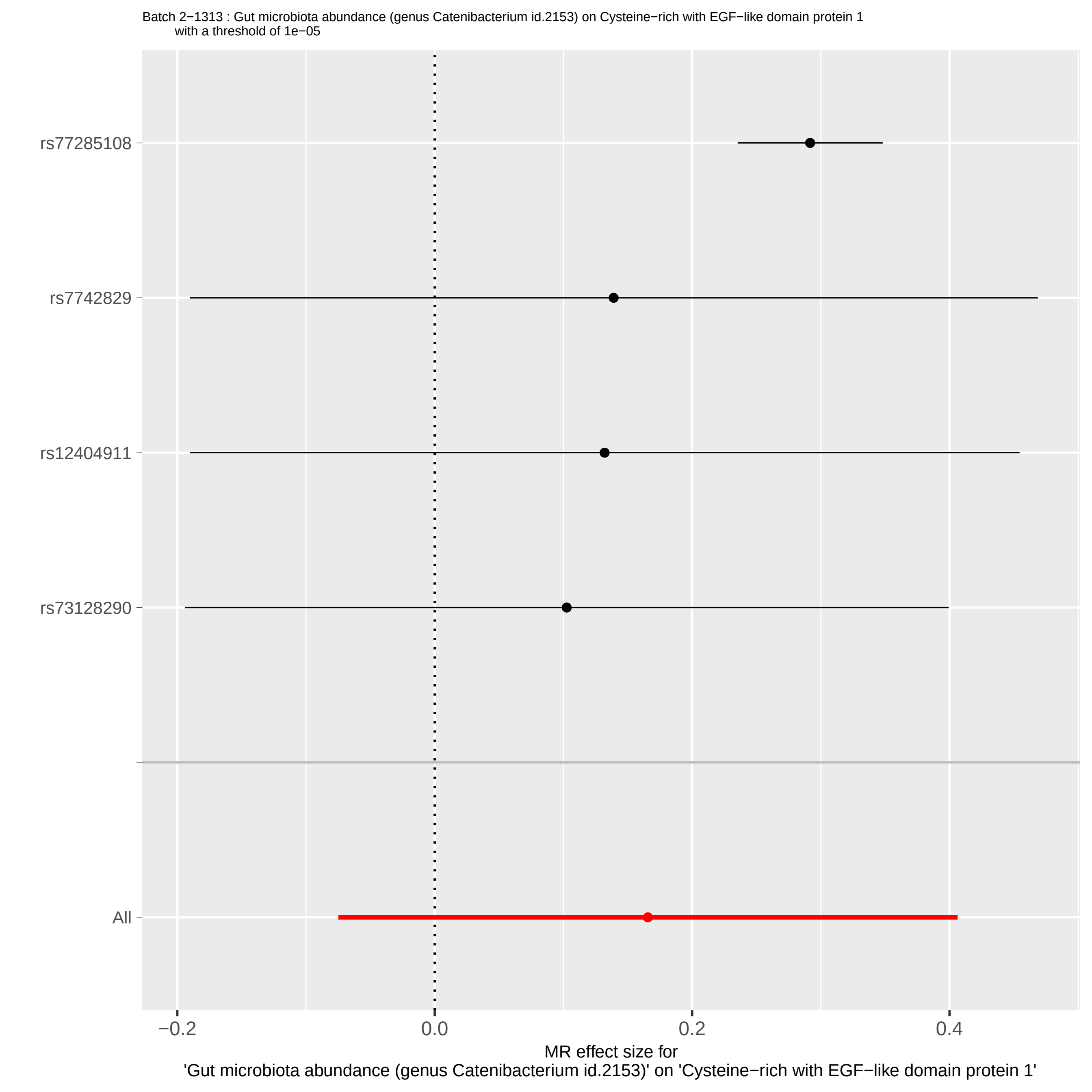
Batch 2−1313 : Gut microbiota abundance (genus Catenibacterium id.2153) on Cysteine−rich with EGF−like domain protein 1
with a threshold of 1e−05
rs77285108
rs7742829
rs12404911
rs73128290
All
−0.2
0.0
0.2
0.4
MR effect size for
'Gut microbiota abundance (genus Catenibacterium id.2153)' on 'Cysteine−rich with EGF−like domain protein 1'

Batch 2−1314 : Gut microbiota abundance (genus Parasutterella id.2892) on Cysteine−rich with EGF−like domain protein 1 with a threshold of 1e−05

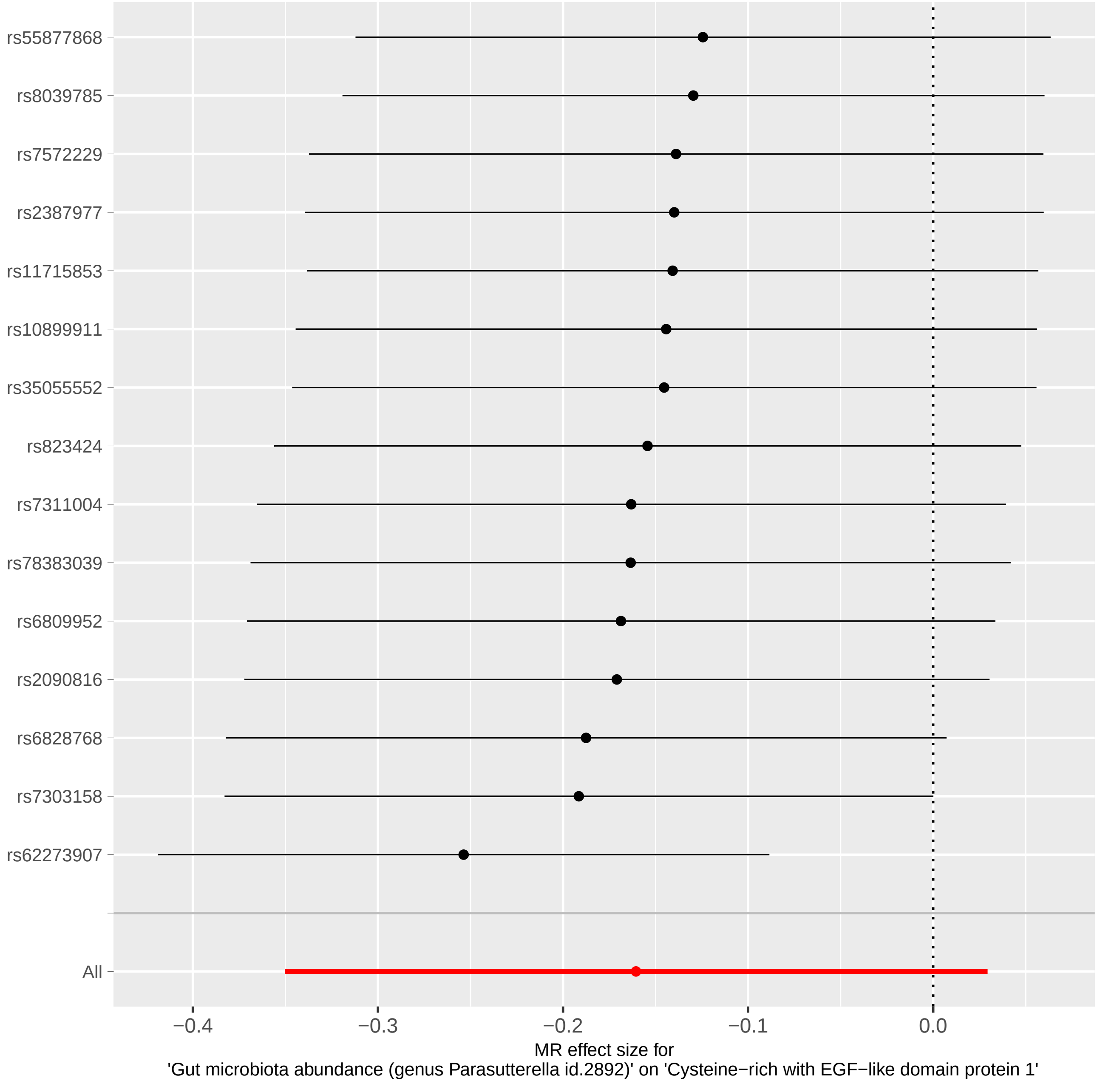

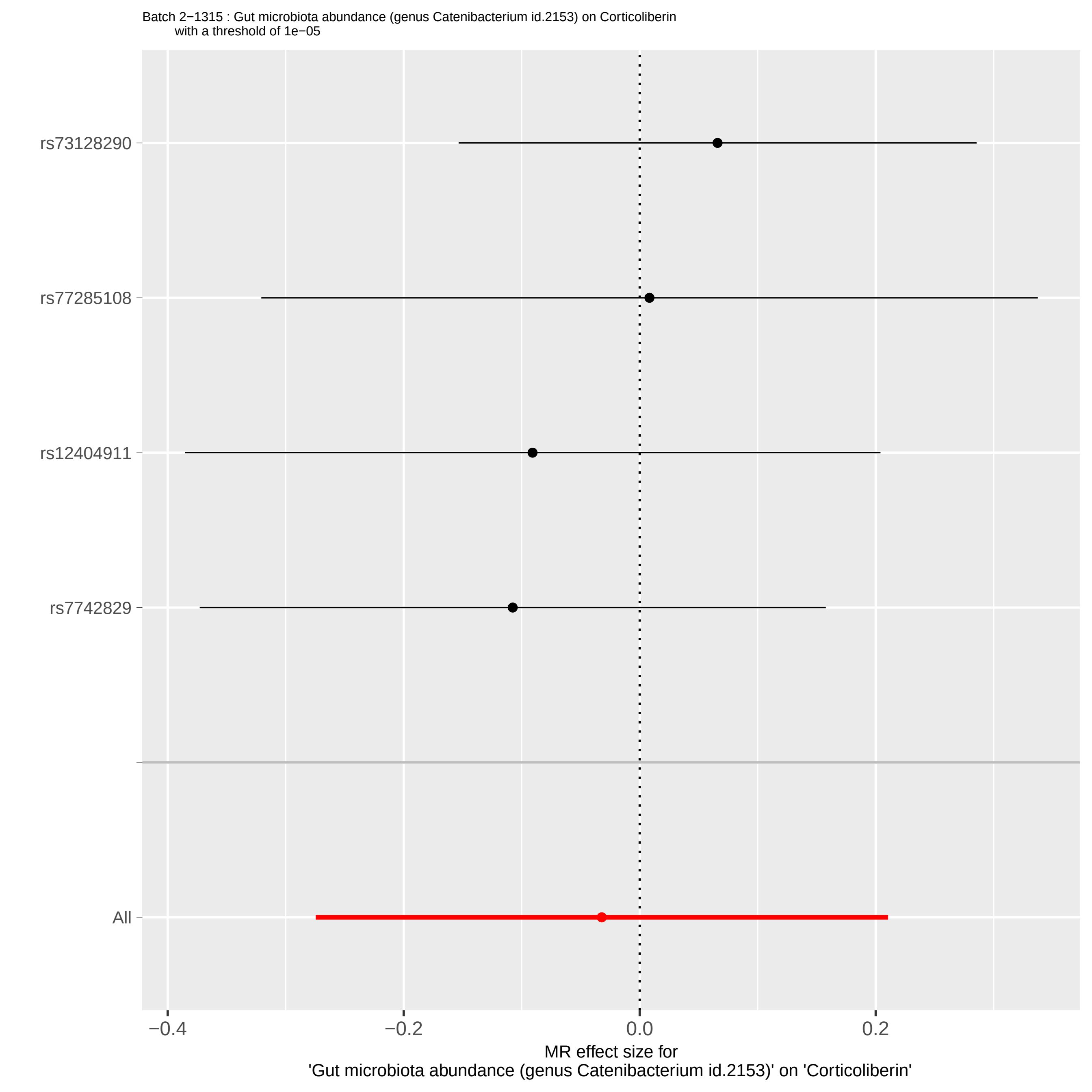
Batch 2−1315 : Gut microbiota abundance (genus Catenibacterium id.2153) on Corticoliberin
with a threshold of 1e−05
rs73128290
rs77285108
rs12404911
rs7742829
All
−0.4
−0.2
0.0
0.2
MR effect size for
'Gut microbiota abundance (genus Catenibacterium id.2153)' on 'Corticoliberin'

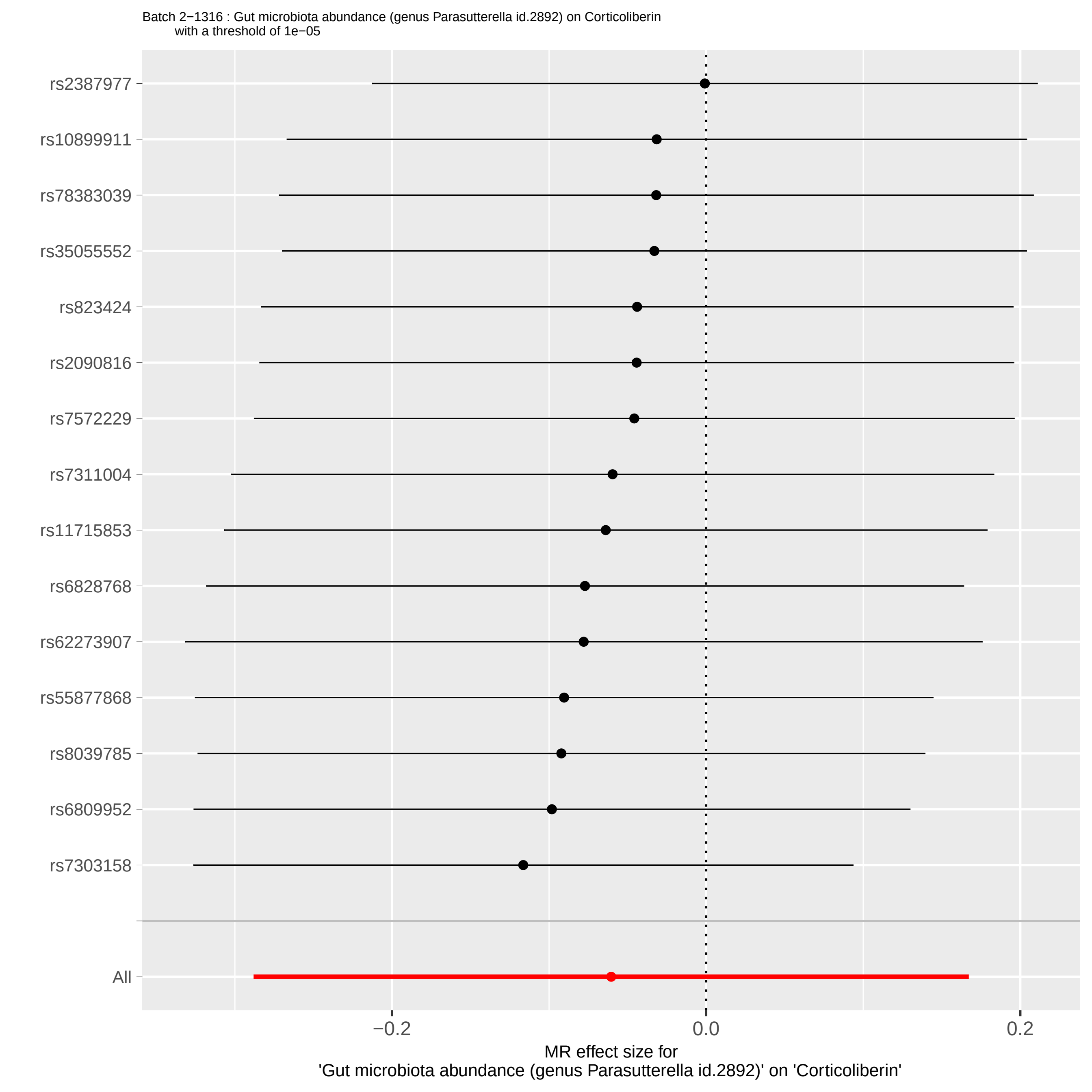

Batch 2−1316 : Gut microbiota abundance (genus Parasutterella id.2892) on Corticoliberin
with a threshold of 1e−05
rs2387977
rs10899911
rs78383039
rs35055552
rs823424
rs2090816
rs7572229
rs7311004
rs11715853
rs6828768
rs62273907
rs55877868
rs8039785
rs6809952
rs7303158
All
−0.2
0.0
0.2
MR effect size for
'Gut microbiota abundance (genus Parasutterella id.2892)' on 'Corticoliberin'

Batch 2−1317 : Gut microbiota abundance (genus Catenibacterium id.2153) on Corticotropin−releasing factor−binding protein
with a threshold of 1e−05

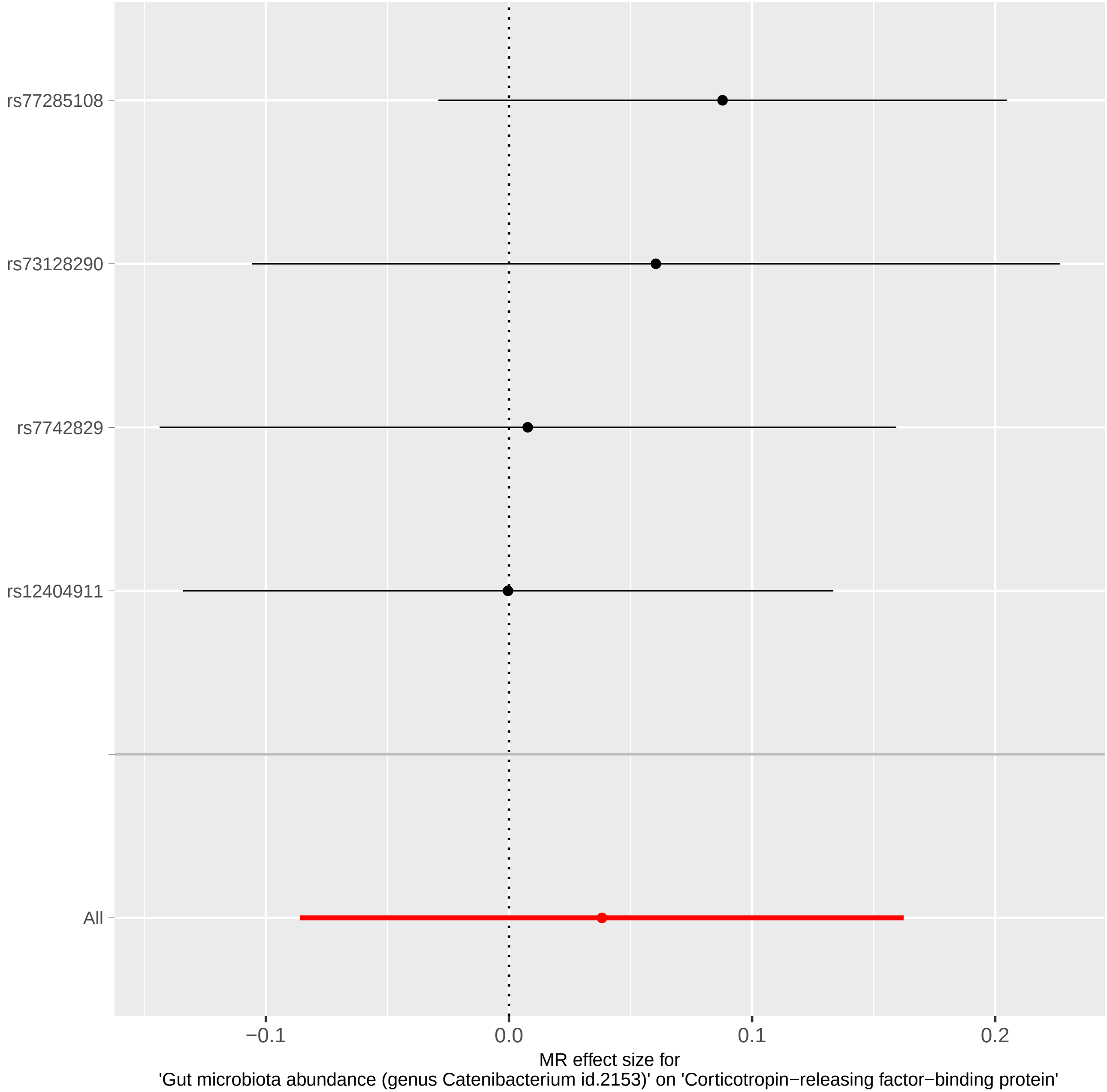

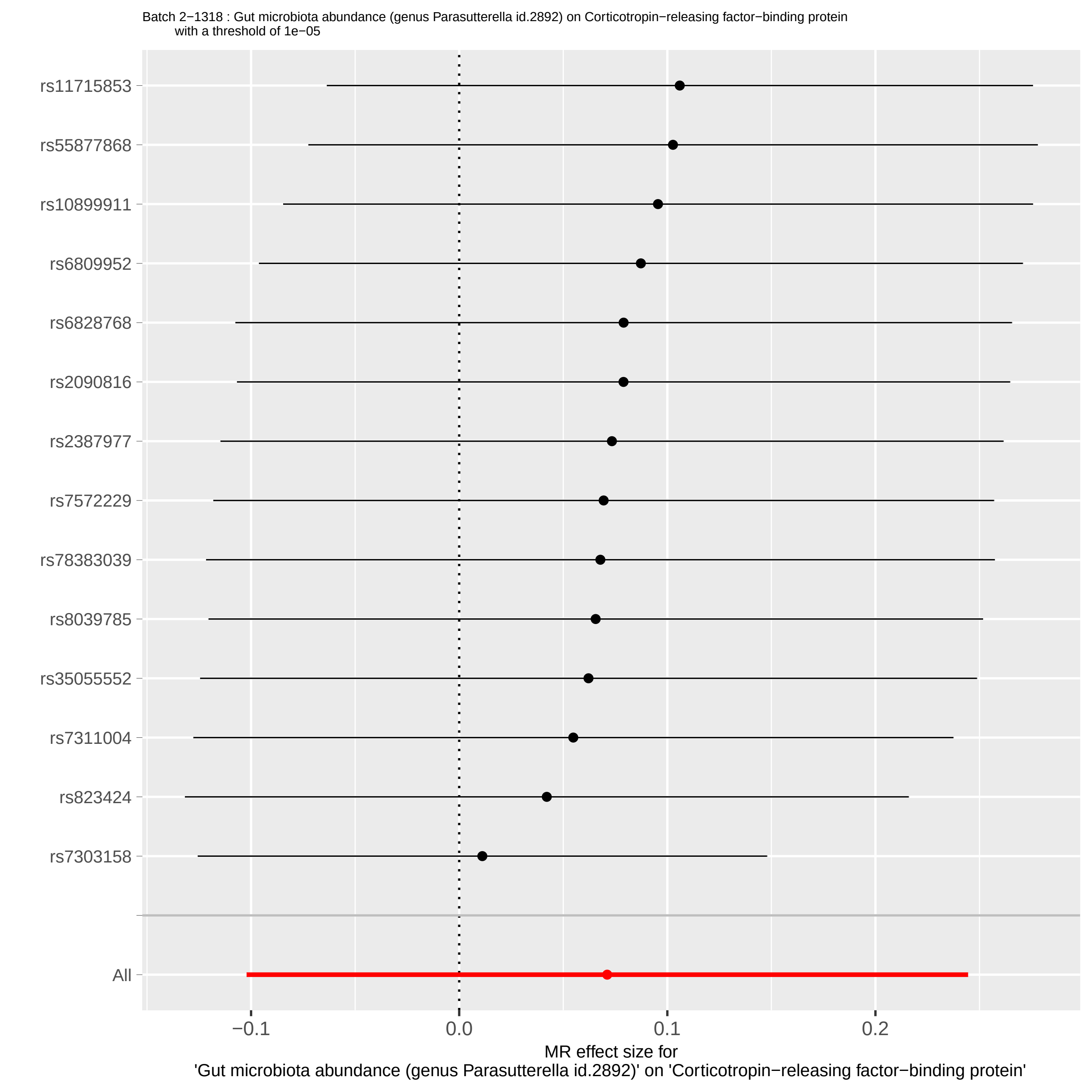
Batch 2−1318 : Gut microbiota abundance (genus Parasutterella id.2892) on Corticotropin−releasing factor−binding protein
with a threshold of 1e−05
rs11715853
rs55877868
rs10899911
rs6809952
rs6828768
rs2090816
rs2387977
rs7572229
rs78383039
rs8039785
rs35055552
rs7311004
rs823424
rs7303158
All
−0.1
0.0
0.1
0.2
MR effect size for
'Gut microbiota abundance (genus Parasutterella id.2892)' on 'Corticotropin−releasing factor−binding protein'

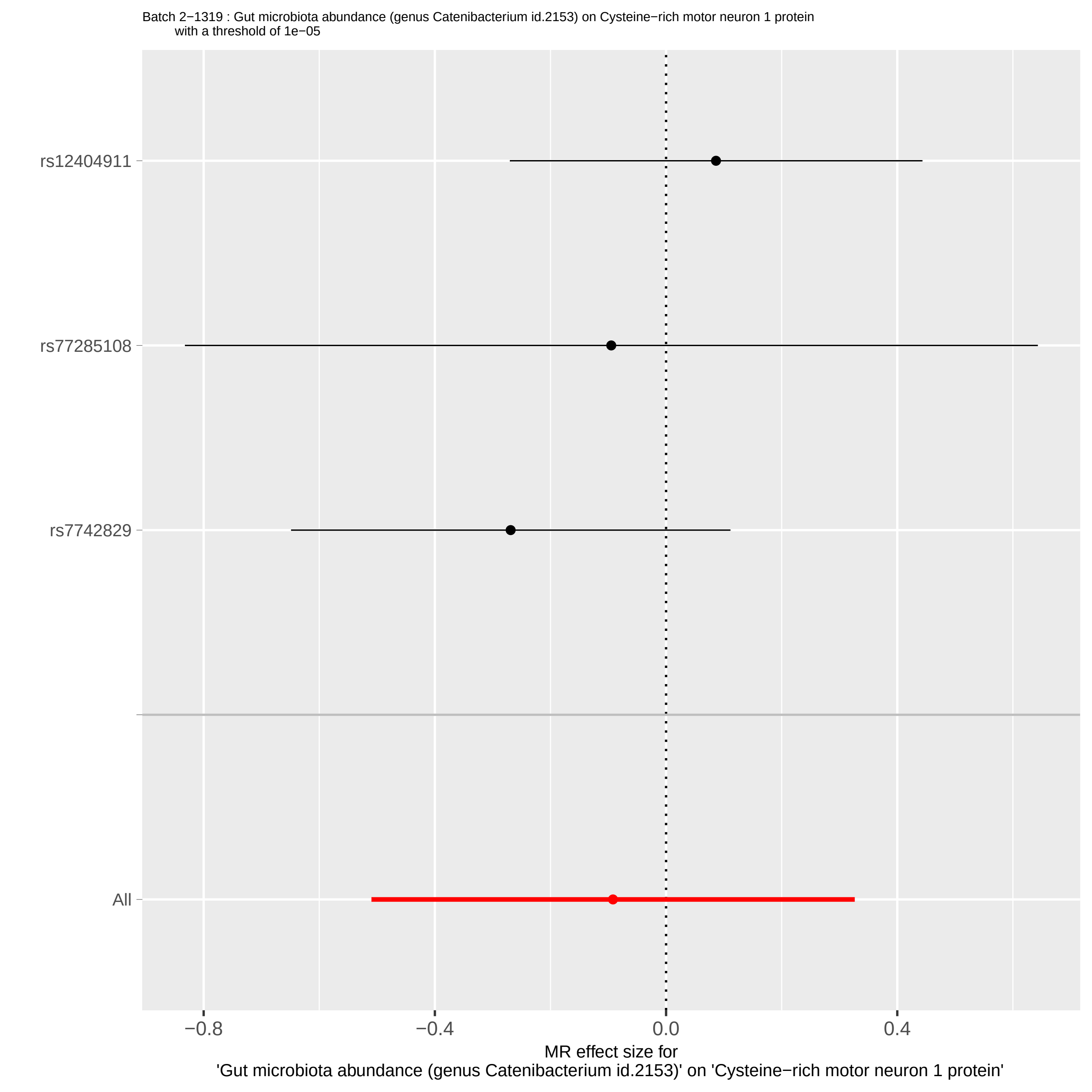
Batch 2−1319 : Gut microbiota abundance (genus Catenibacterium id.2153) on Cysteine−rich motor neuron 1 protein
with a threshold of 1e−05
rs12404911
rs77285108
rs7742829
All
−0.8
−0.4
0.0
0.4
MR effect size for
'Gut microbiota abundance (genus Catenibacterium id.2153)' on 'Cysteine−rich motor neuron 1 protein'

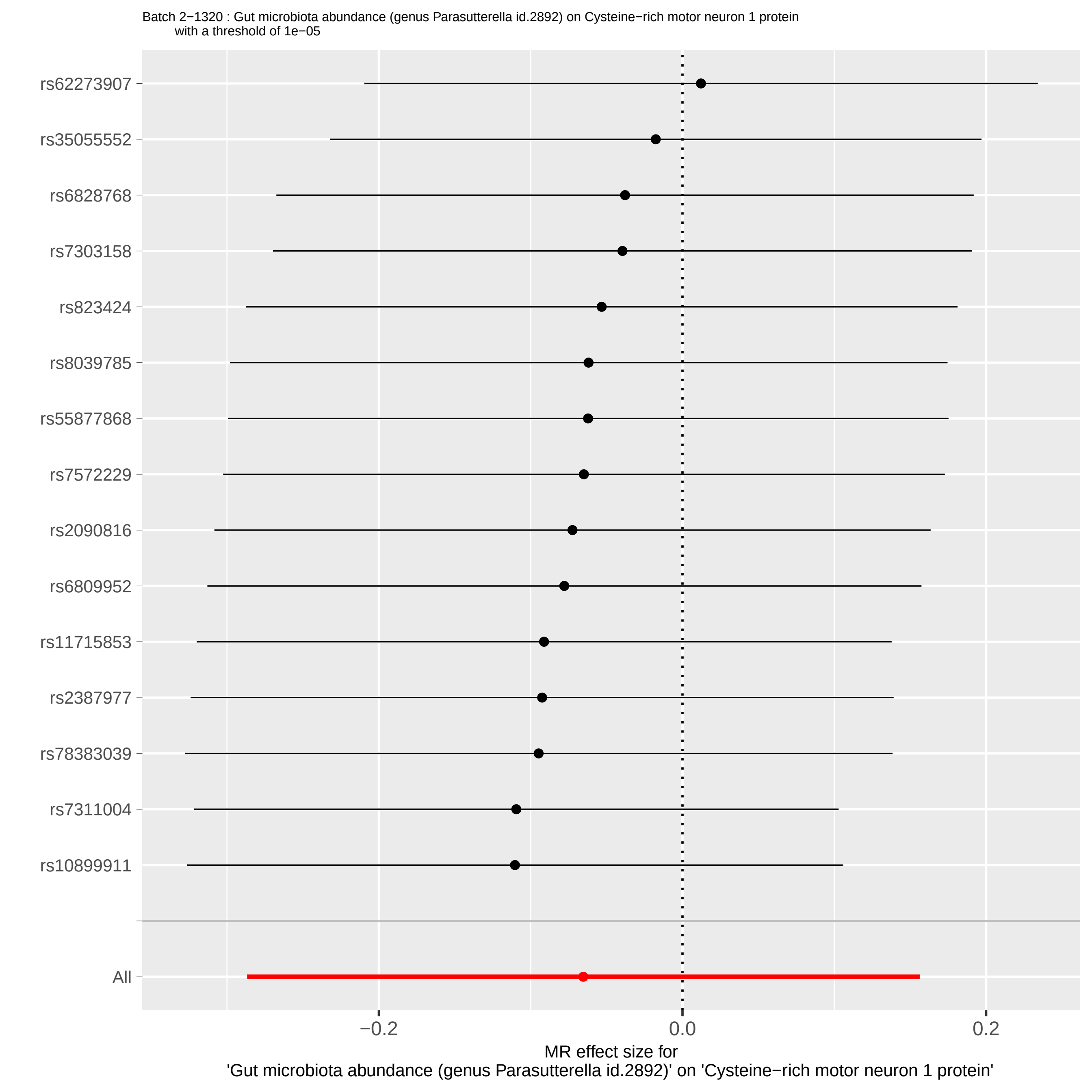

Batch 2−1320 : Gut microbiota abundance (genus Parasutterella id.2892) on Cysteine−rich motor neuron 1 protein
with a threshold of 1e−05
rs62273907
rs35055552
rs6828768
rs7303158
rs823424
rs8039785
rs55877868
rs7572229
rs2090816
rs6809952
rs11715853
rs2387977
rs78383039
rs7311004
rs10899911
All
−0.2
0.0
0.2
MR effect size for
'Gut microbiota abundance (genus Parasutterella id.2892)' on 'Cysteine−rich motor neuron 1 protein'

Batch 2−1321 : Gut microbiota abundance (genus Catenibacterium id.2153) on Cysteine−rich secretory protein 2
with a threshold of 1e−05

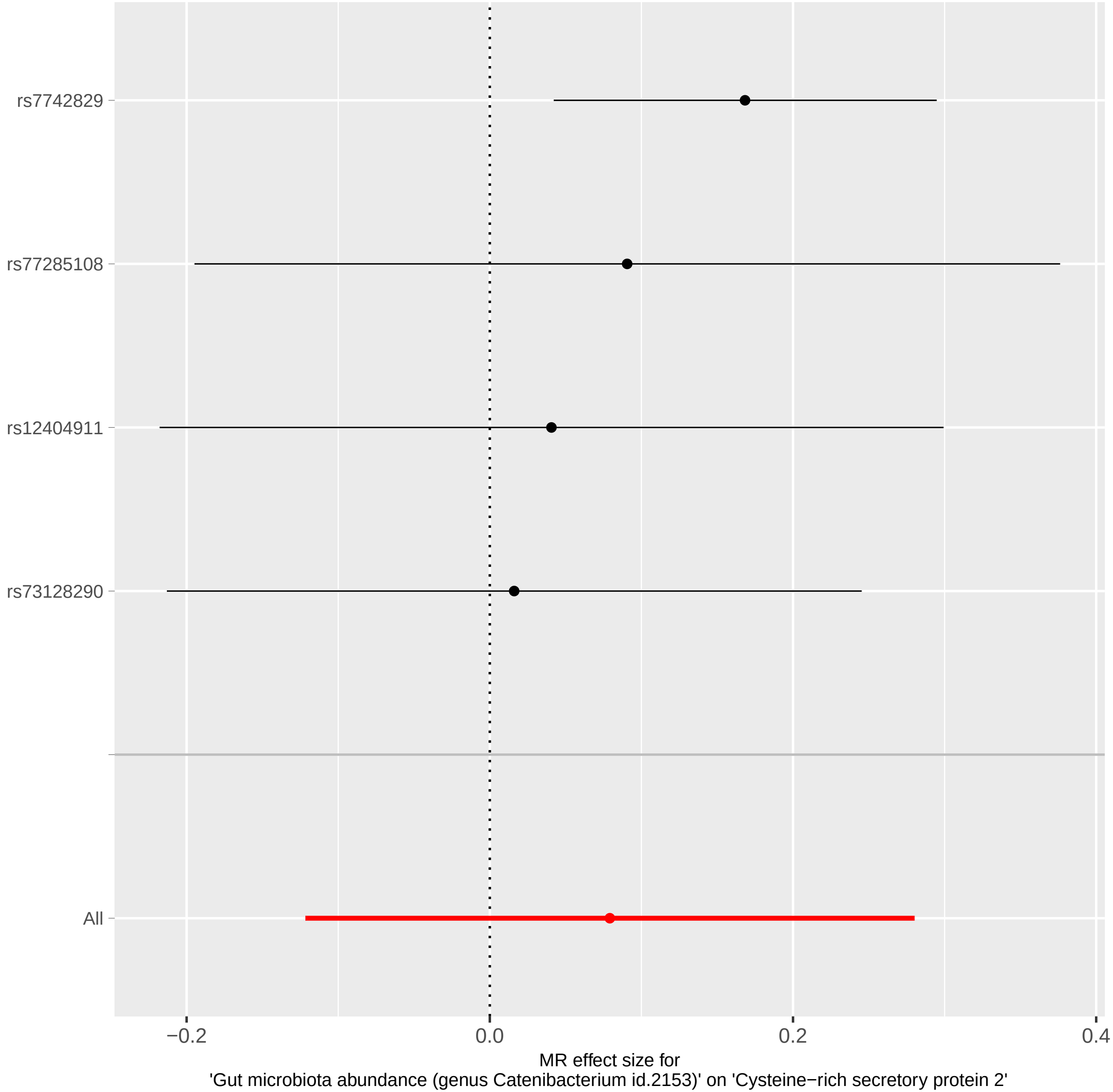

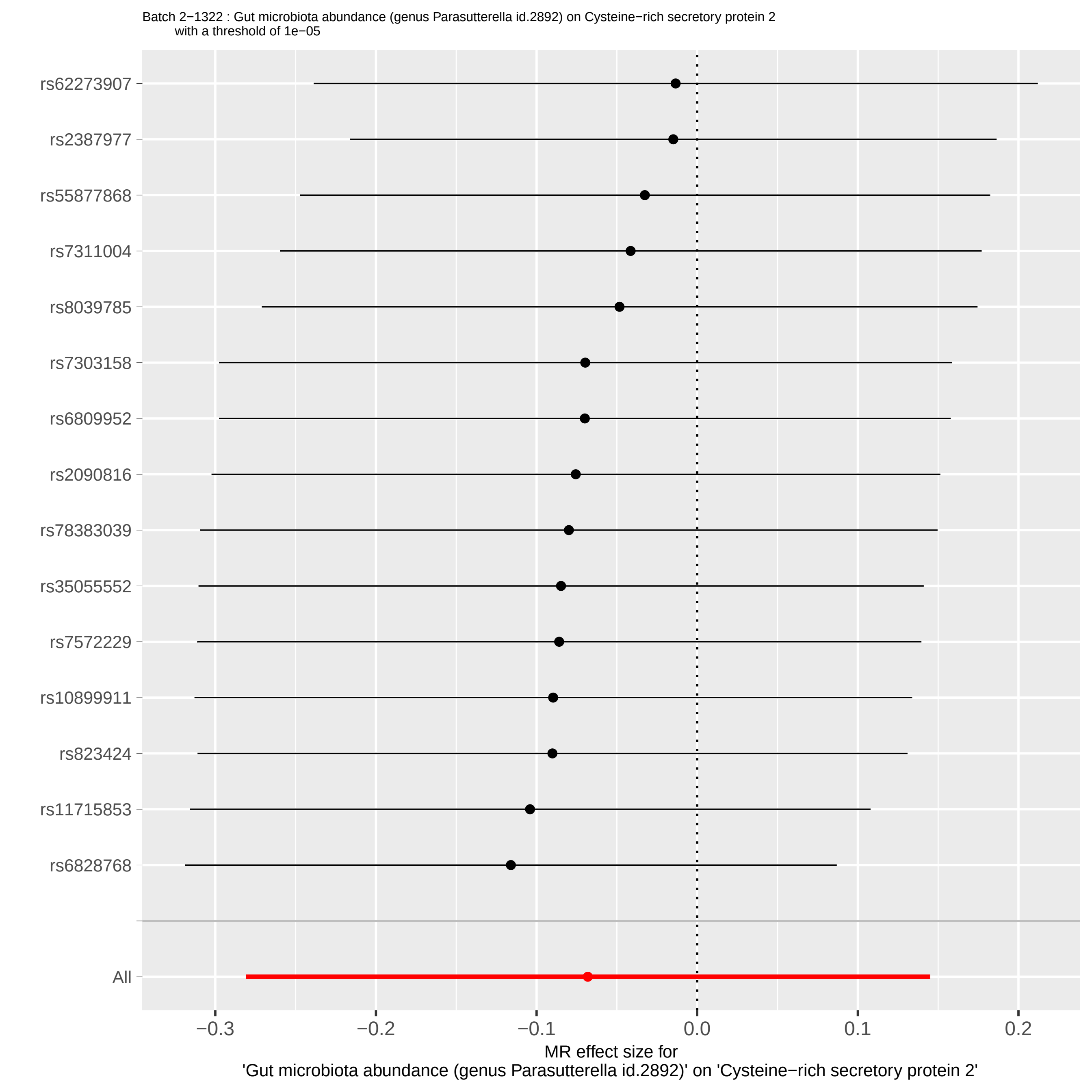

Batch 2−1322 : Gut microbiota abundance (genus Parasutterella id.2892) on Cysteine−rich secretory protein 2
with a threshold of 1e−05
rs62273907
rs2387977
rs55877868
rs7311004
rs8039785
rs7303158
rs6809952
rs2090816
rs78383039
rs35055552
rs7572229
rs10899911
rs823424
rs11715853
rs6828768
All
−0.3
−0.2
−0.1
0.0
0.1
0.2
MR effect size for
'Gut microbiota abundance (genus Parasutterella id.2892)' on 'Cysteine−rich secretory protein 2'

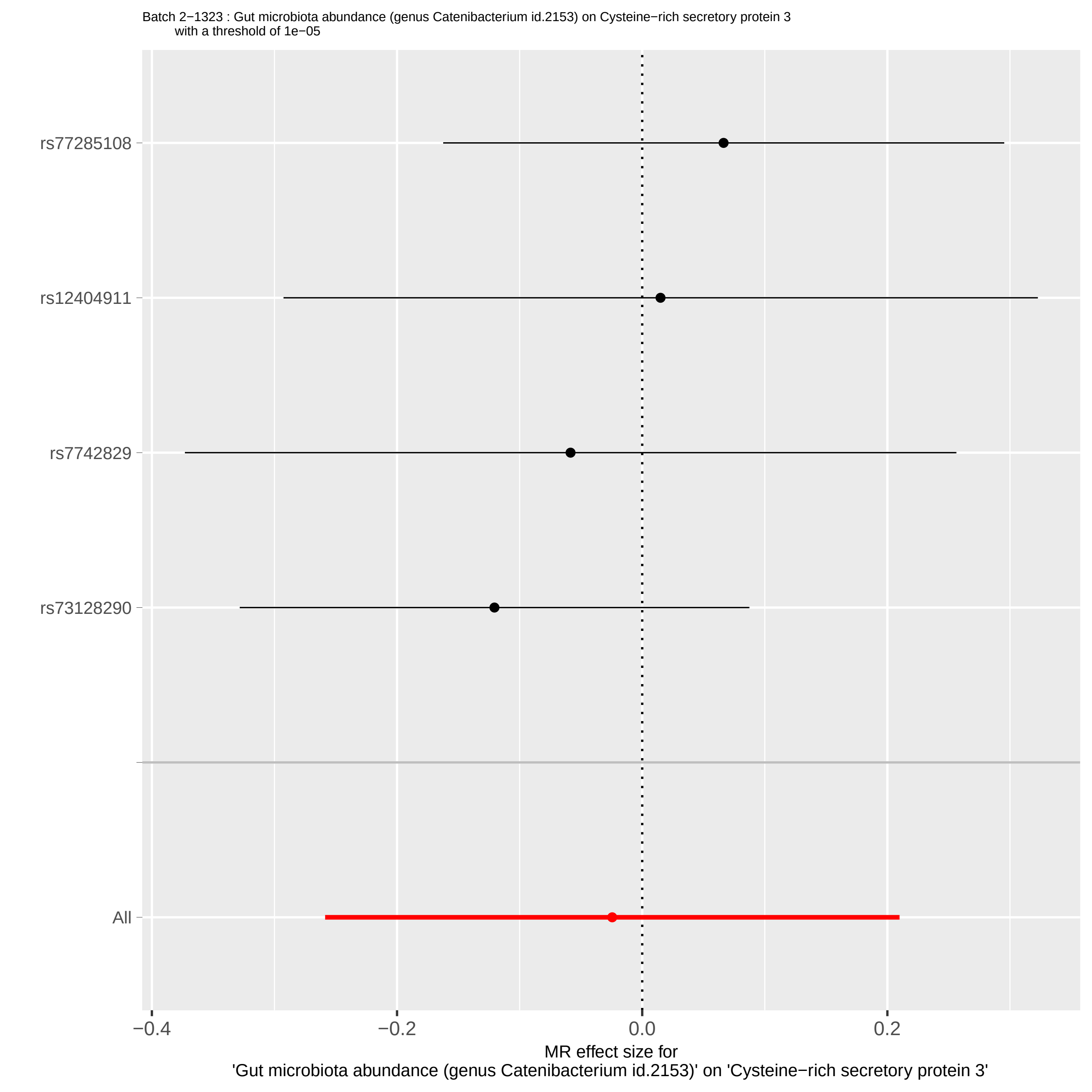

Batch 2−1323 : Gut microbiota abundance (genus Catenibacterium id.2153) on Cysteine−rich secretory protein 3
with a threshold of 1e−05
rs77285108
rs12404911
rs7742829
rs73128290
All
−0.4
−0.2
0.0
0.2
MR effect size for
'Gut microbiota abundance (genus Catenibacterium id.2153)' on 'Cysteine−rich secretory protein 3'

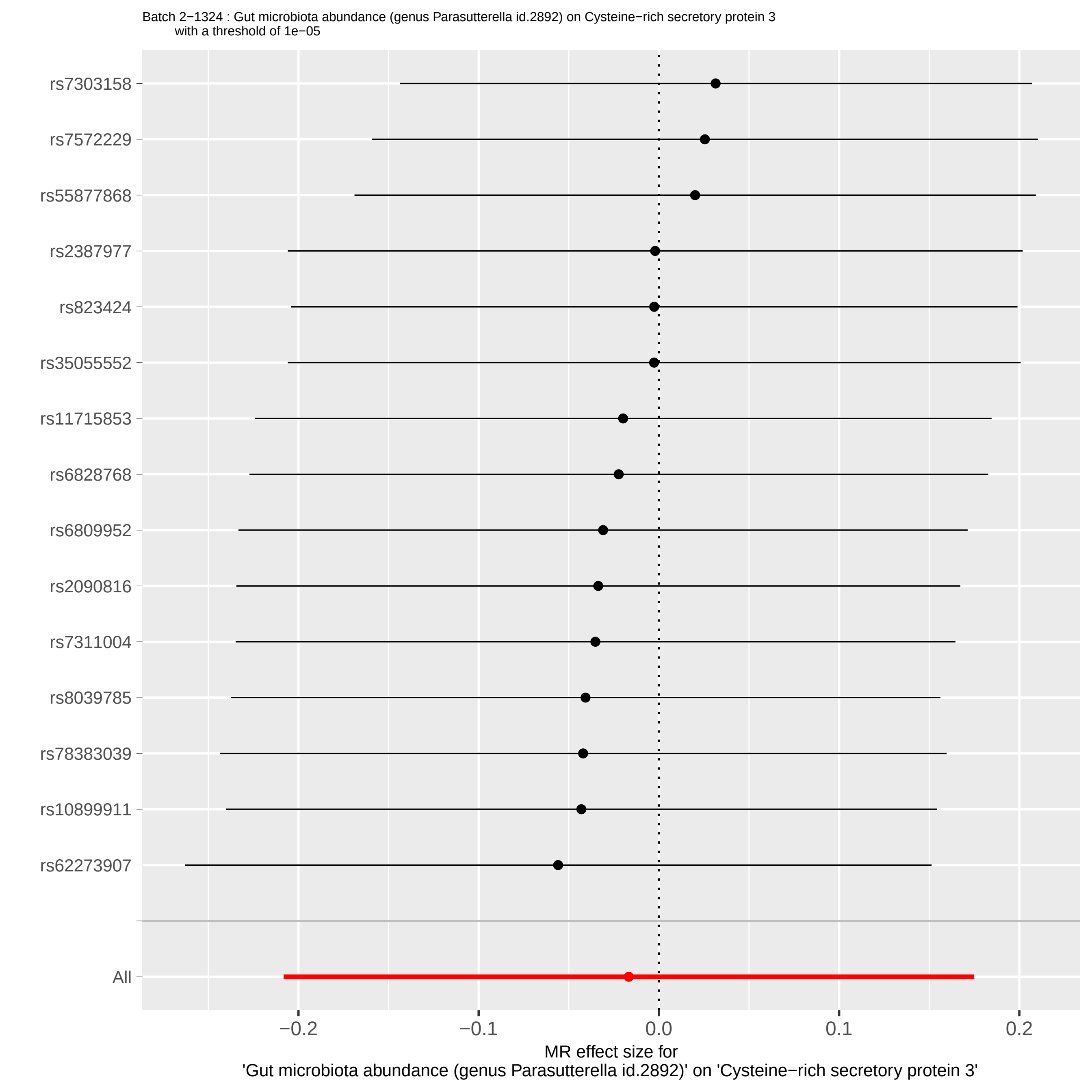
Batch 2−1324 : Gut microbiota abundance (genus Parasutterella id.2892) on Cysteine−rich secretory protein 3
with a threshold of 1e−05
rs7303158
rs7572229
rs55877868
rs2387977
rs823424
rs35055552
rs11715853
rs6828768
rs6809952
rs2090816
rs7311004
rs8039785
rs78383039
rs10899911
rs62273907
All
−0.2
−0.1
0.0
0.1
0.2
MR effect size for
'Gut microbiota abundance (genus Parasutterella id.2892)' on 'Cysteine−rich secretory protein 3'

Batch 2−1325 : Gut microbiota abundance (genus Catenibacterium id.2153) on Cysteine−rich secretory protein LCCL domain−containing 2
with a threshold of 1e−05

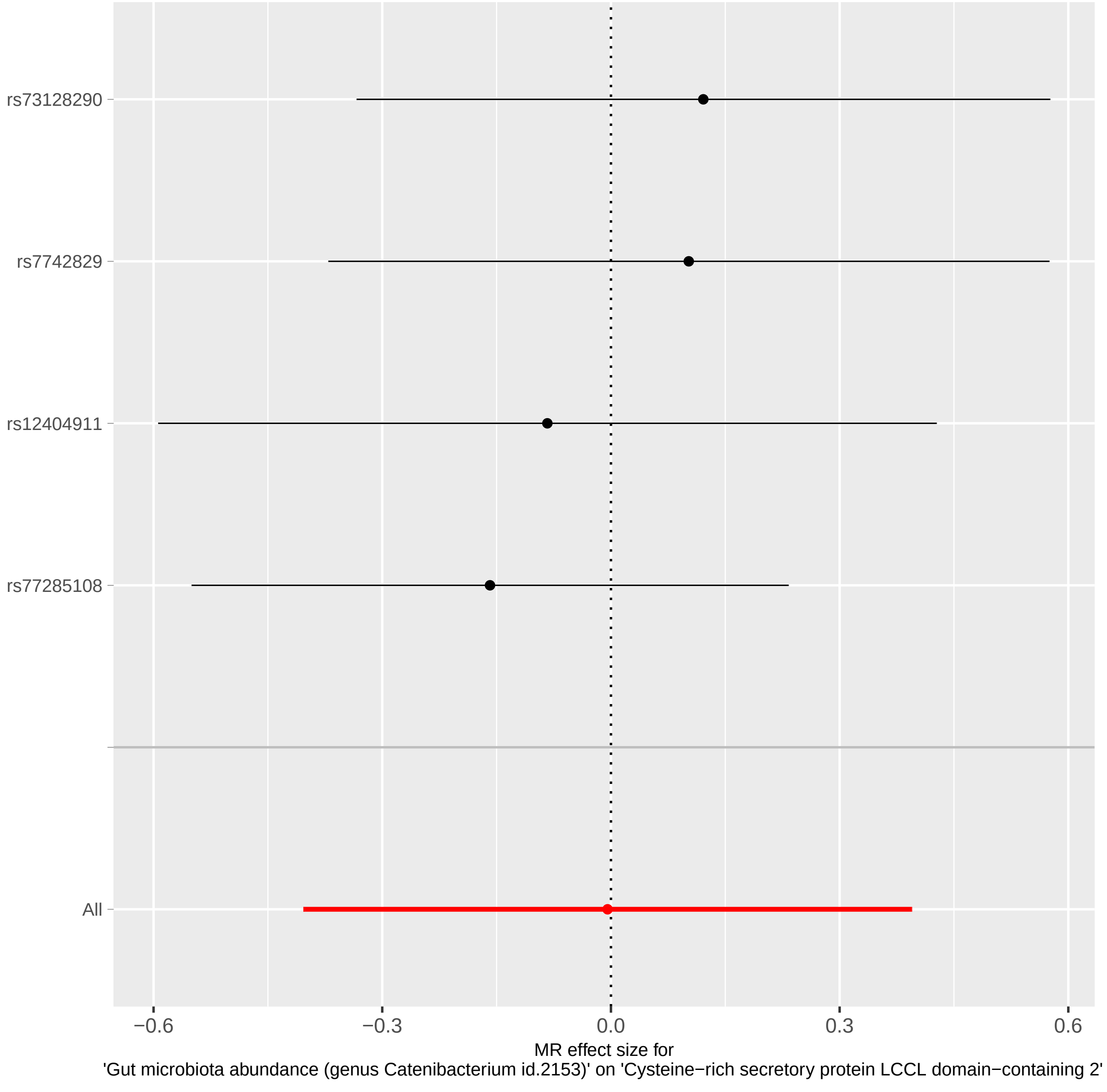

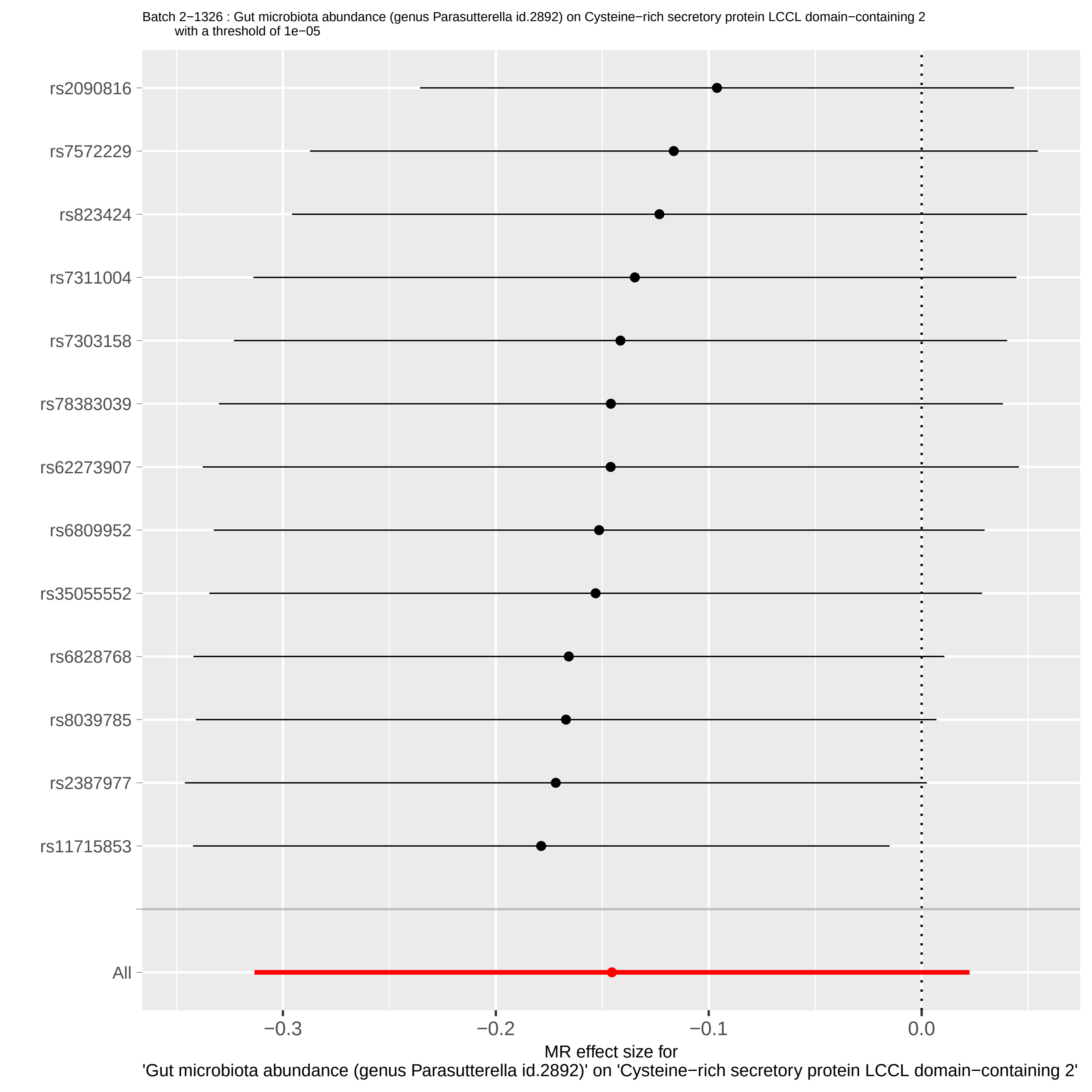
Batch 2−1326 : Gut microbiota abundance (genus Parasutterella id.2892) on Cysteine−rich secretory protein LCCL domain−containing 2
with a threshold of 1e−05
rs2090816
rs7572229
rs823424
rs7311004
rs7303158
rs78383039
rs62273907
rs6809952
rs35055552
rs6828768
rs8039785
rs2387977
rs11715853
All
−0.3
−0.2
−0.1
0.0
MR effect size for
'Gut microbiota abundance (genus Parasutterella id.2892)' on 'Cysteine−rich secretory protein LCCL domain−containing 2'

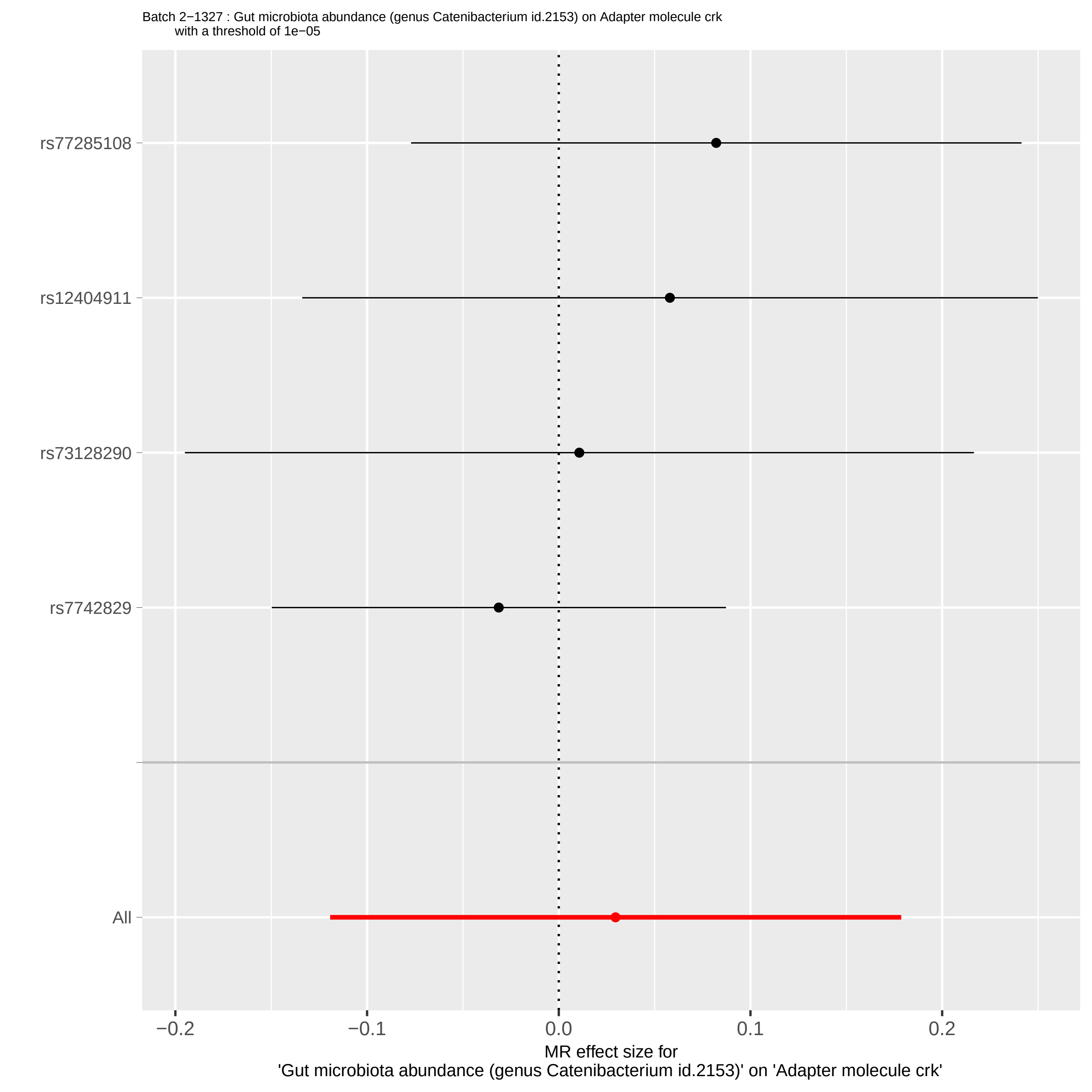
Batch 2−1327 : Gut microbiota abundance (genus Catenibacterium id.2153) on Adapter molecule crk
with a threshold of 1e−05
rs77285108
rs12404911
rs73128290
rs7742829
All
−0.2
−0.1
0.0
0.1
0.2
MR effect size for
'Gut microbiota abundance (genus Catenibacterium id.2153)' on 'Adapter molecule crk'

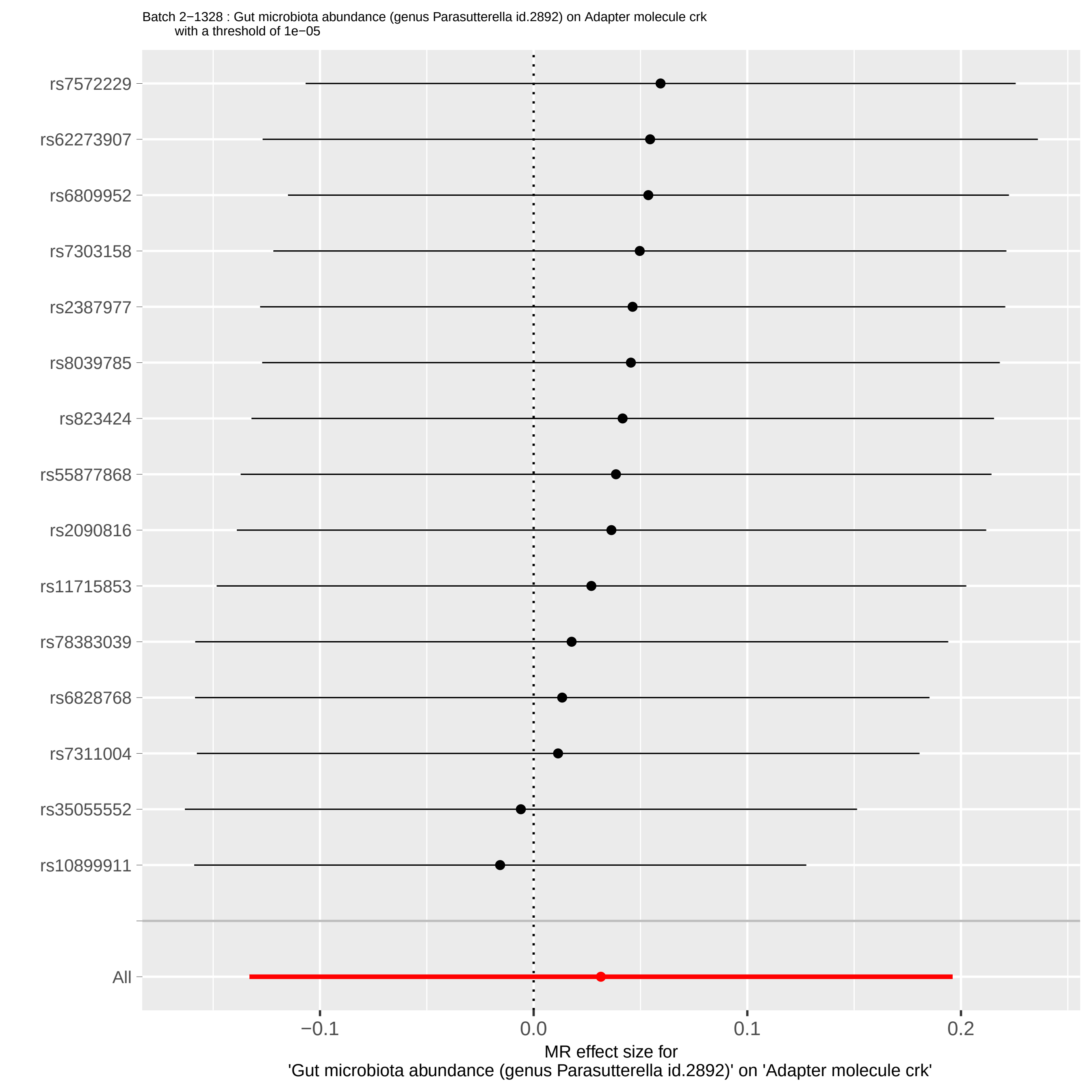

Batch 2−1328 : Gut microbiota abundance (genus Parasutterella id.2892) on Adapter molecule crk
with a threshold of 1e−05
rs7572229
rs62273907
rs6809952
rs7303158
rs2387977
rs8039785
rs823424
rs55877868
rs2090816
rs11715853
rs78383039
rs6828768
rs7311004
rs35055552
rs10899911
All
−0.1
0.0
0.1
0.2
MR effect size for
'Gut microbiota abundance (genus Parasutterella id.2892)' on 'Adapter molecule crk

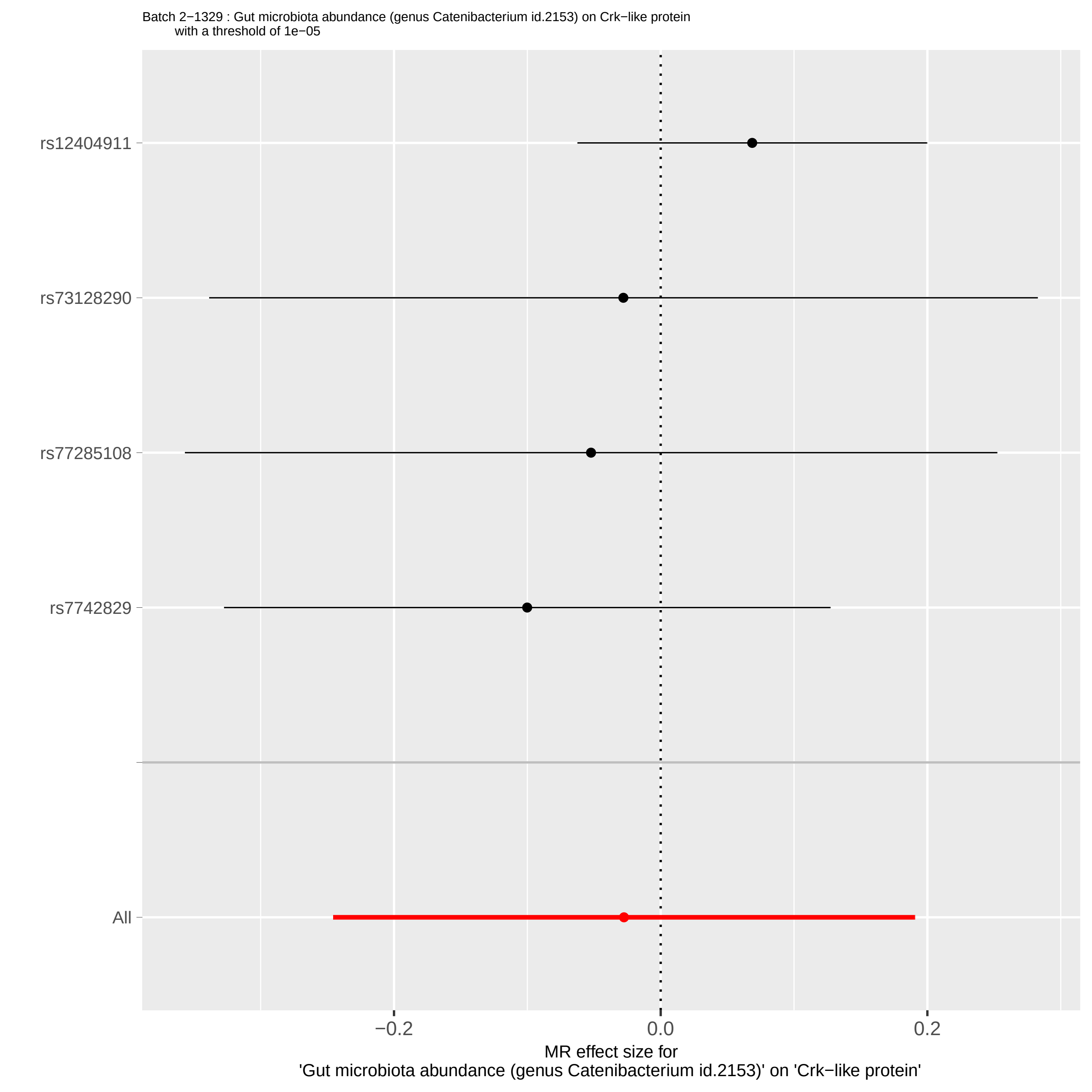
Batch 2−1329 : Gut microbiota abundance (genus Catenibacterium id.2153) on Crk−like protein
with a threshold of 1e−05
rs12404911
rs73128290
rs77285108
rs7742829
All
−0.2
0.0
0.2
MR effect size for
'Gut microbiota abundance (genus Catenibacterium id.2153)' on 'Crk−like protein'

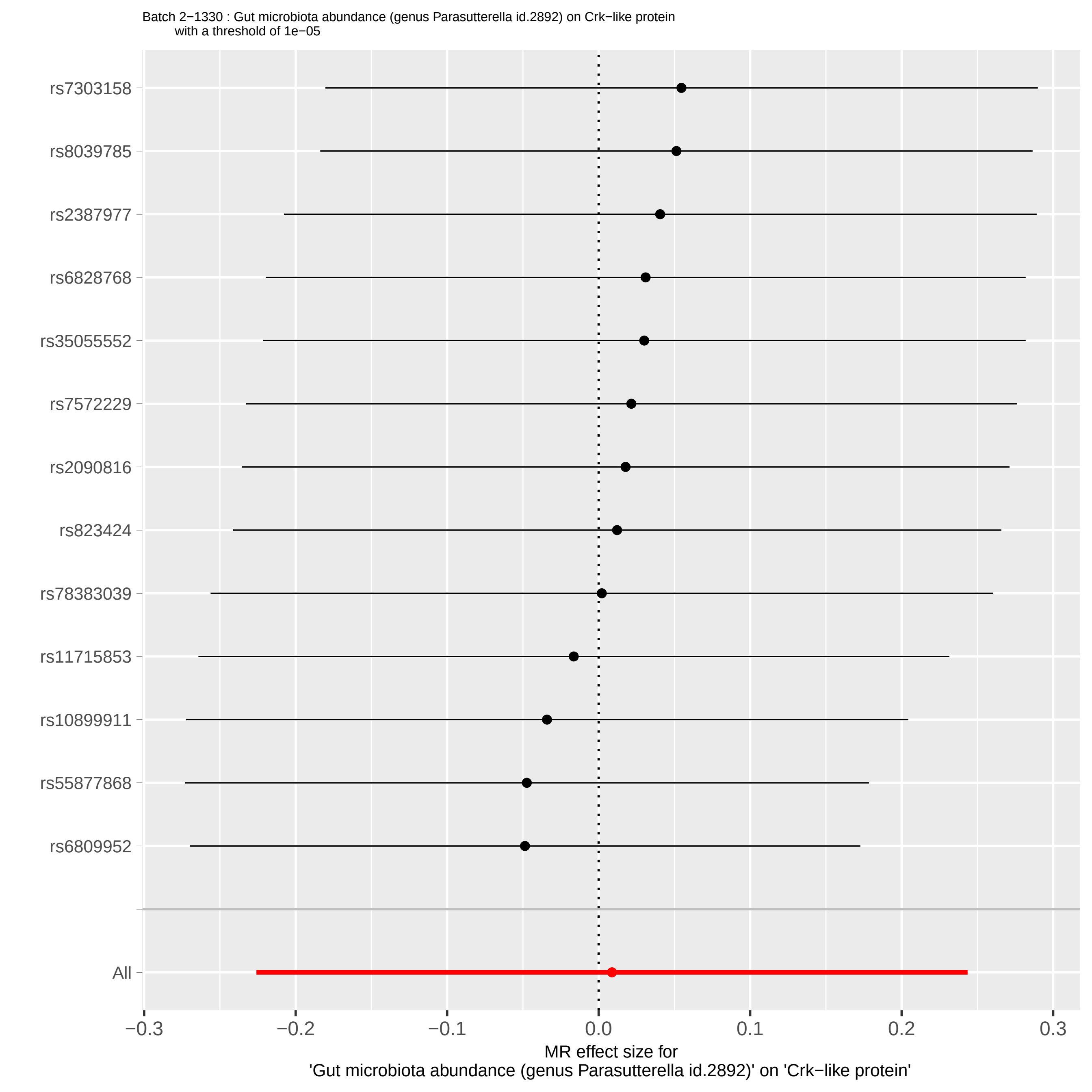
Batch 2−1330 : Gut microbiota abundance (genus Parasutterella id.2892) on Crk−like protein
with a threshold of 1e−05
rs7303158
rs8039785
rs2387977
rs6828768
rs35055552
rs7572229
rs2090816
rs823424
rs78383039
rs11715853
rs10899911
rs55877868
rs6809952
All
−0.3
−0.2
−0.1
0.0
0.1
0.2
0.3
MR effect size for
'Gut microbiota abundance (genus Parasutterella id.2892)' on 'Crk−like protein'

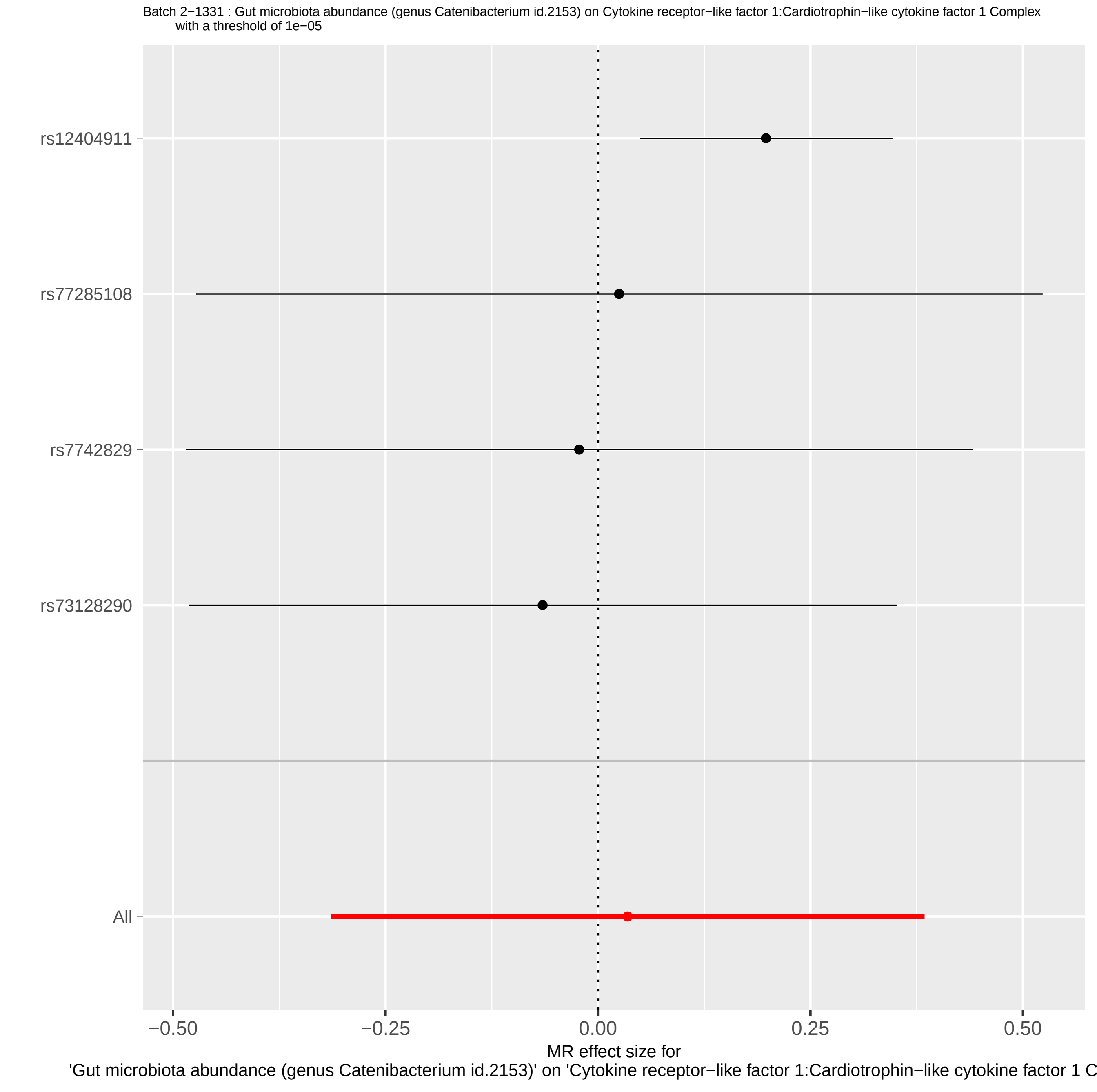
Batch 2−1331 : Gut microbiota abundance (genus Catenibacterium id.2153) on Cytokine receptor−like factor 1:Cardiotrophin−like cytokine factor 1 Complex
with a threshold of 1e−05
rs12404911
rs77285108
rs7742829
rs73128290
All
−0.50
−0.25
0.00
0.25
0.50
MR effect size for
'Gut microbiota abundance (genus Catenibacterium id.2153)' on 'Cytokine receptor−like factor 1:Cardiotrophin−like cytokine factor 1 C

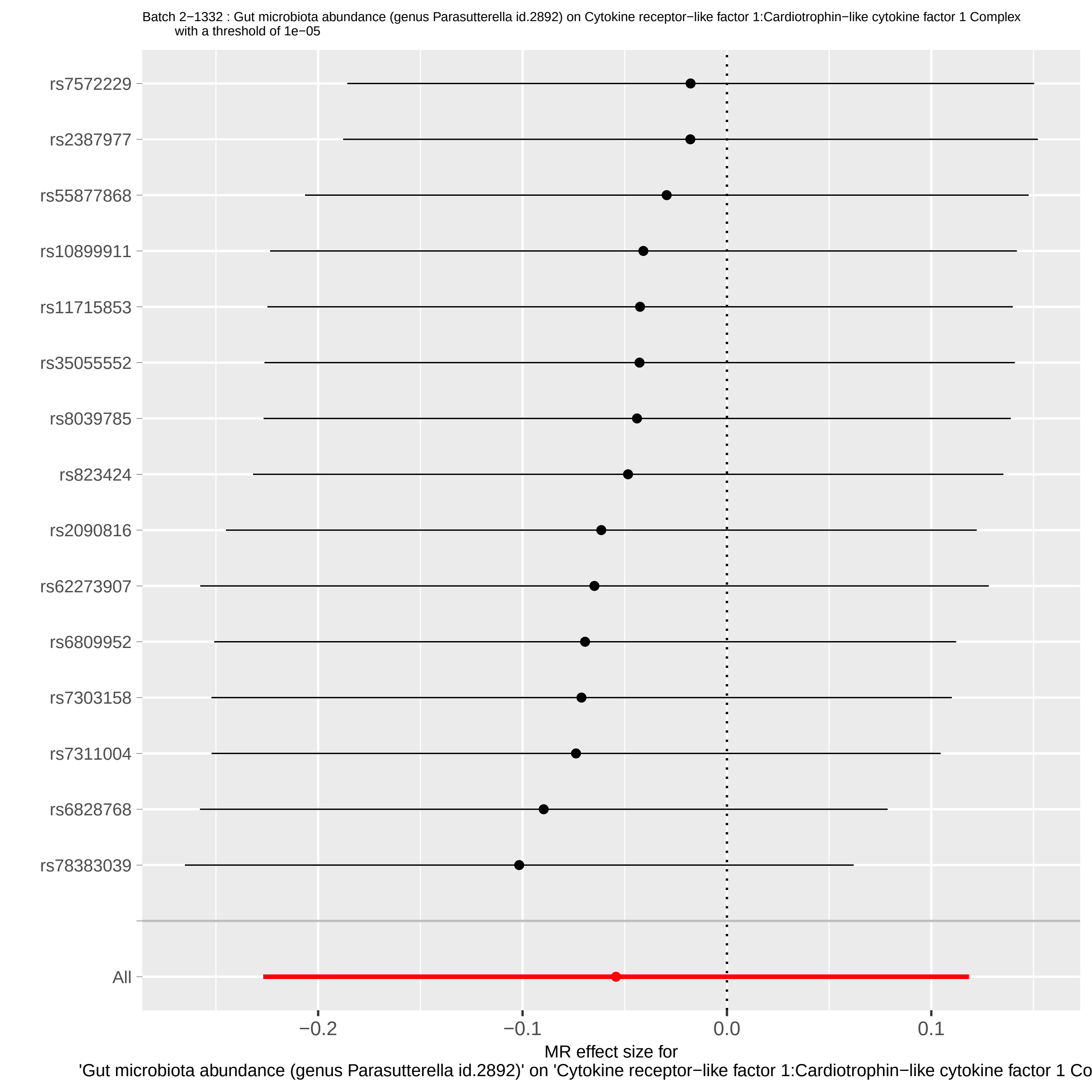
Batch 2−1332 : Gut microbiota abundance (genus Parasutterella id.2892) on Cytokine receptor−like factor 1:Cardiotrophin−like cytokine factor 1 Complex with a threshold of 1e−05
rs7572229
rs2387977
rs55877868
rs10899911
rs11715853
rs35055552
rs8039785
rs823424
rs2090816
rs62273907
rs6809952
rs7303158
rs7311004
rs6828768
rs78383039
All
−0.2
−0.1
0.0
0.1
MR effect size for
'Gut microbiota abundance (genus Parasutterella id.2892)' on 'Cytokine receptor−like factor 1:Cardiotrophin−like cytokine factor 1 Co

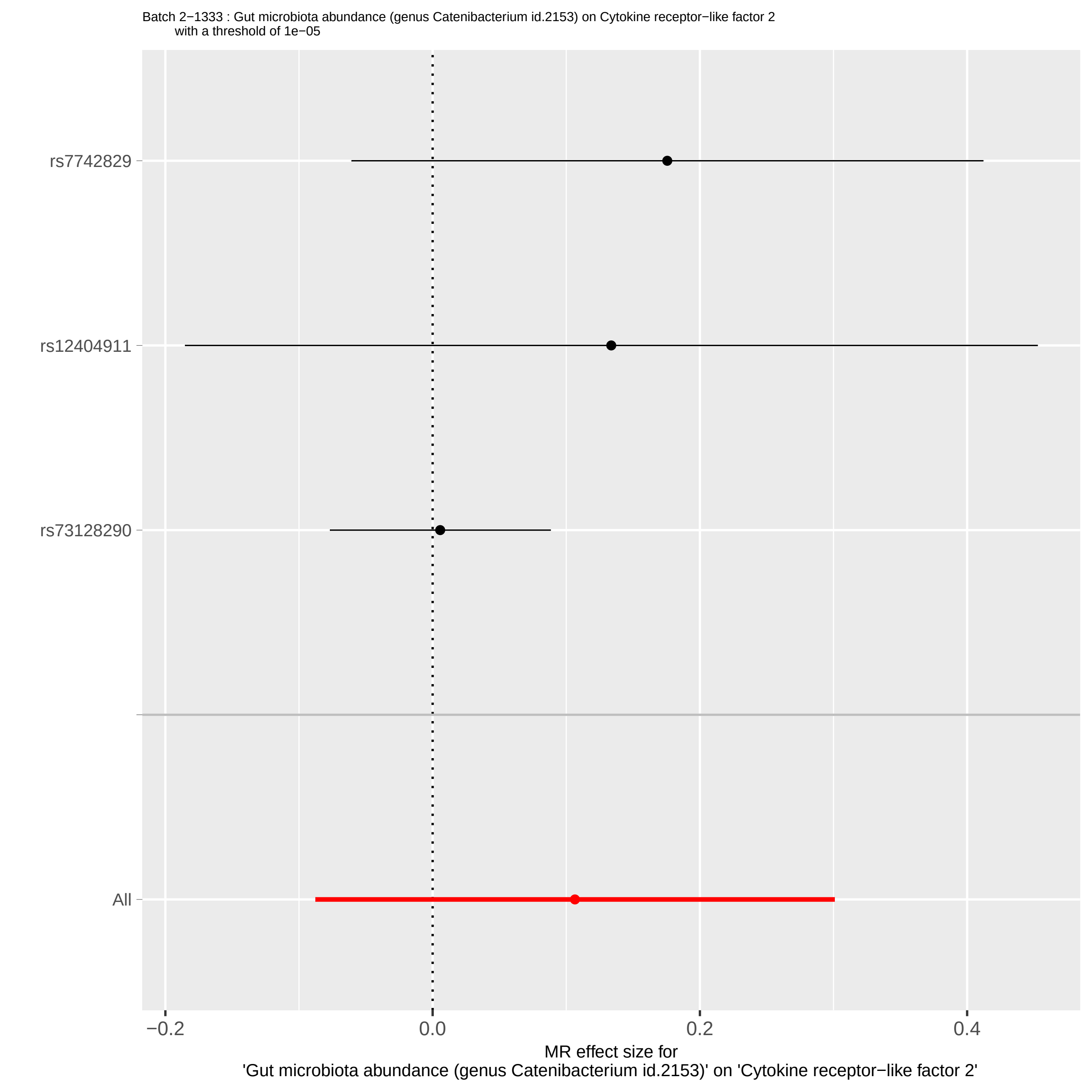

Batch 2−1333 : Gut microbiota abundance (genus Catenibacterium id.2153) on Cytokine receptor−like factor 2
with a threshold of 1e−05
rs7742829
rs12404911
rs73128290
All
−0.2
0.0
0.2
0.4
MR effect size for
'Gut microbiota abundance (genus Catenibacterium id.2153)' on 'Cytokine receptor−like factor 2'

Batch 2−1334 : Gut microbiota abundance (genus Parasutterella id.2892) on Cytokine receptor−like factor 2
with a threshold of 1e−05

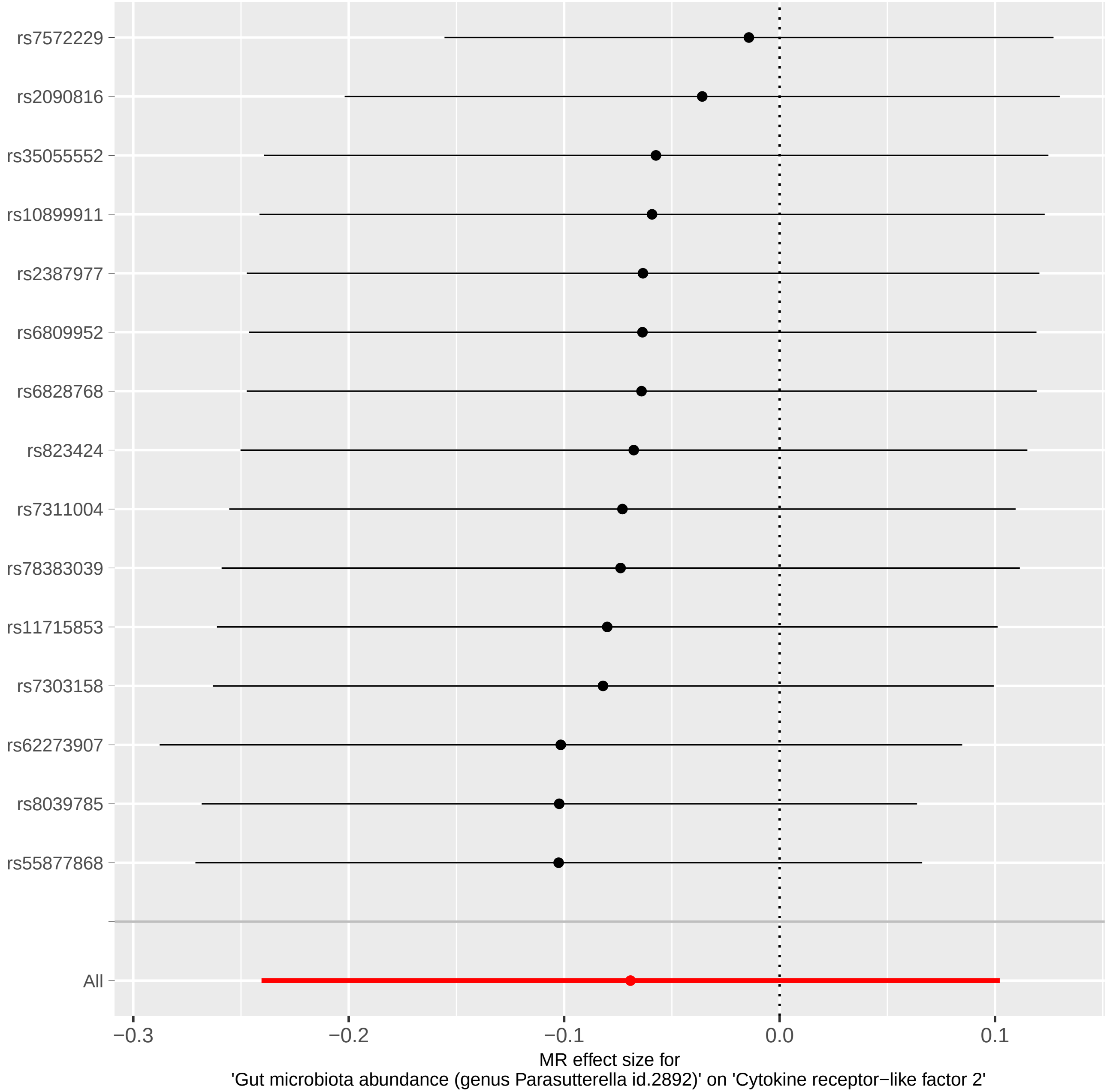

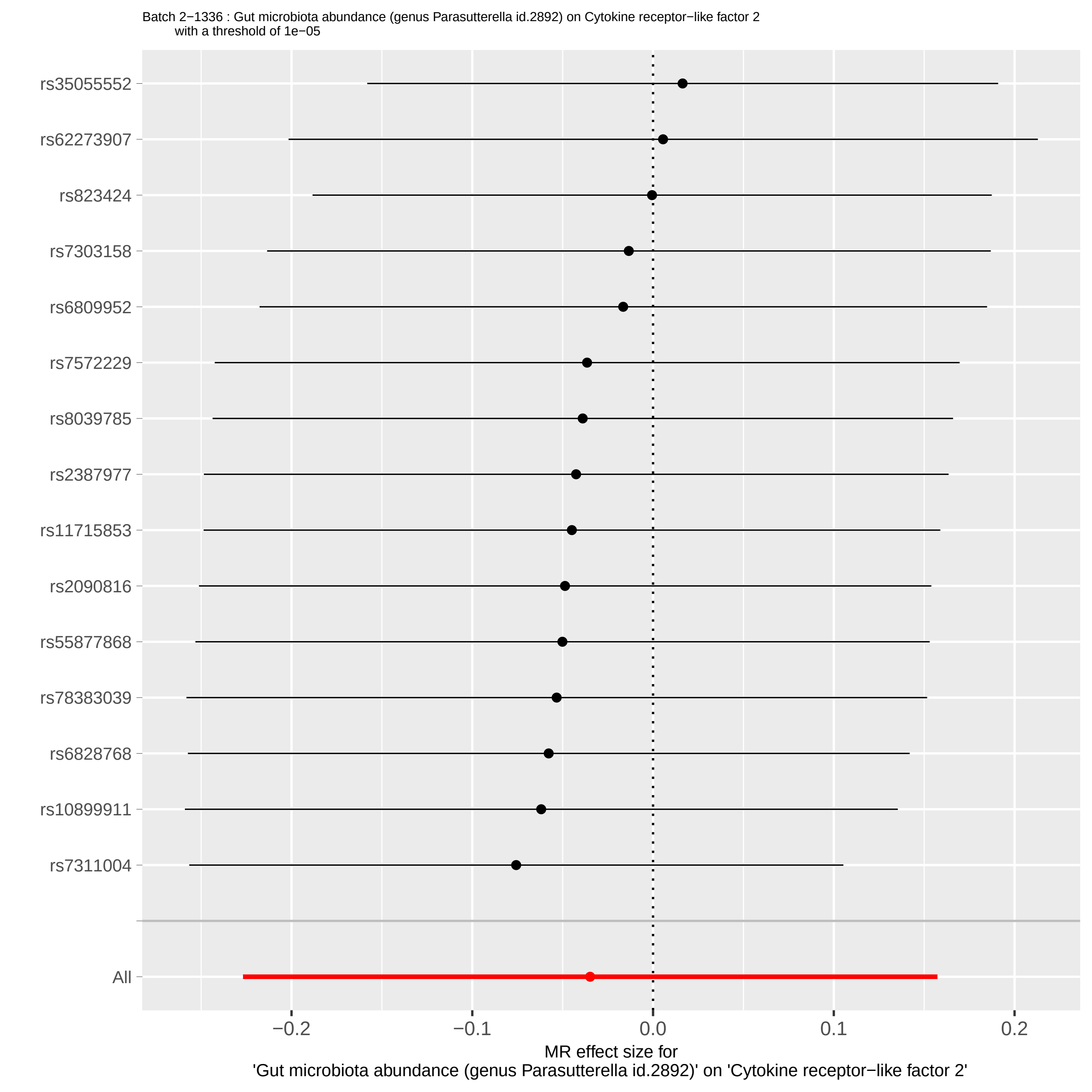
Batch 2−1336 : Gut microbiota abundance (genus Parasutterella id.2892) on Cytokine receptor−like factor 2
with a threshold of 1e−05
rs35055552
rs62273907
rs823424
rs7303158
rs6809952
rs7572229
rs8039785
rs2387977
rs11715853
rs2090816
rs55877868
rs78383039
rs6828768
rs10899911
rs7311004
All
−0.2
−0.1
0.0
0.1
0.2
MR effect size for
'Gut microbiota abundance (genus Parasutterella id.2892)' on 'Cytokine receptor−like factor 2'

Batch 2−1337 : Gut microbiota abundance (genus Catenibacterium id.2153) on Peroxisomal carnitine O−octanoyltransferase
with a threshold of 1e−05

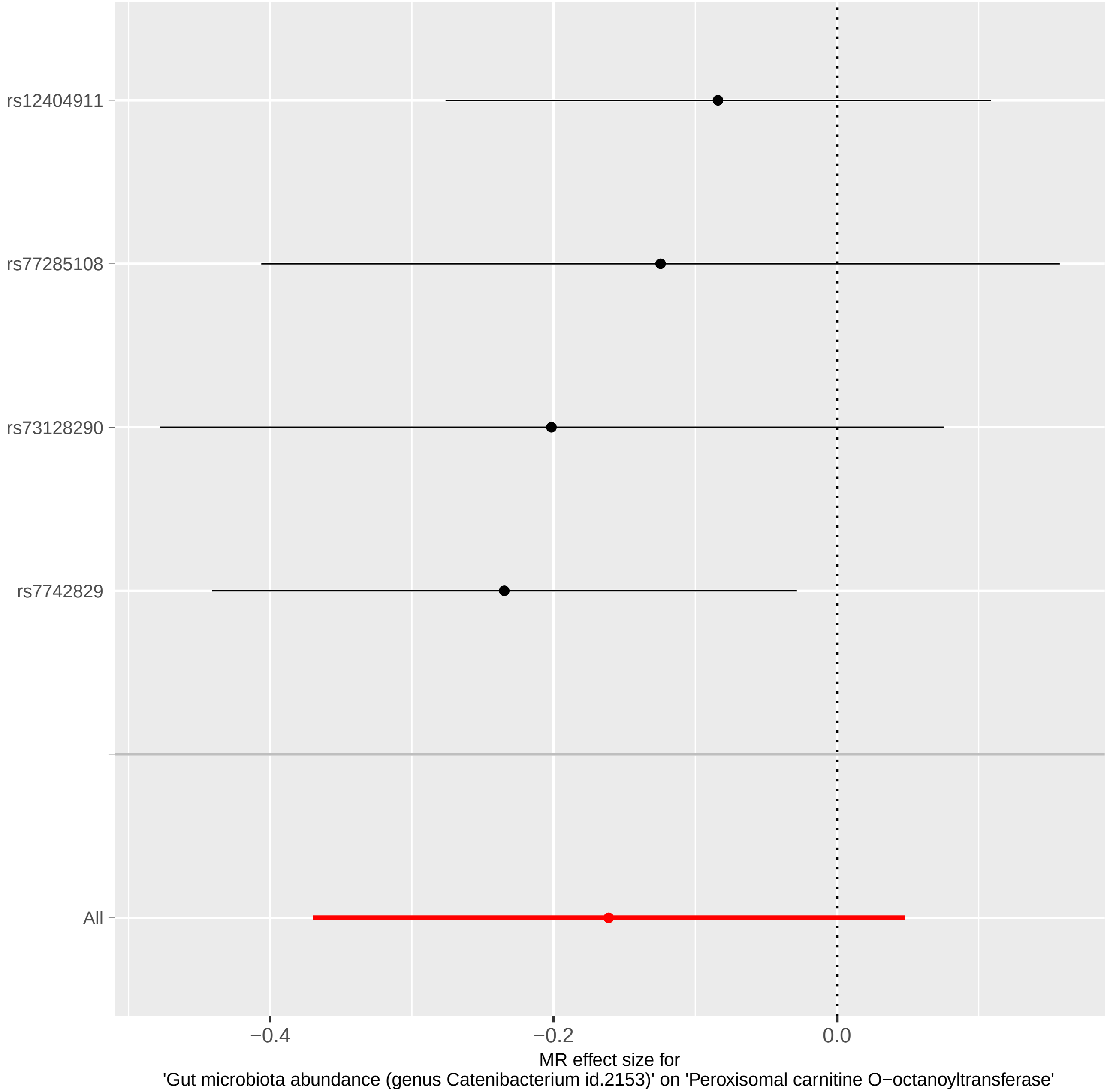

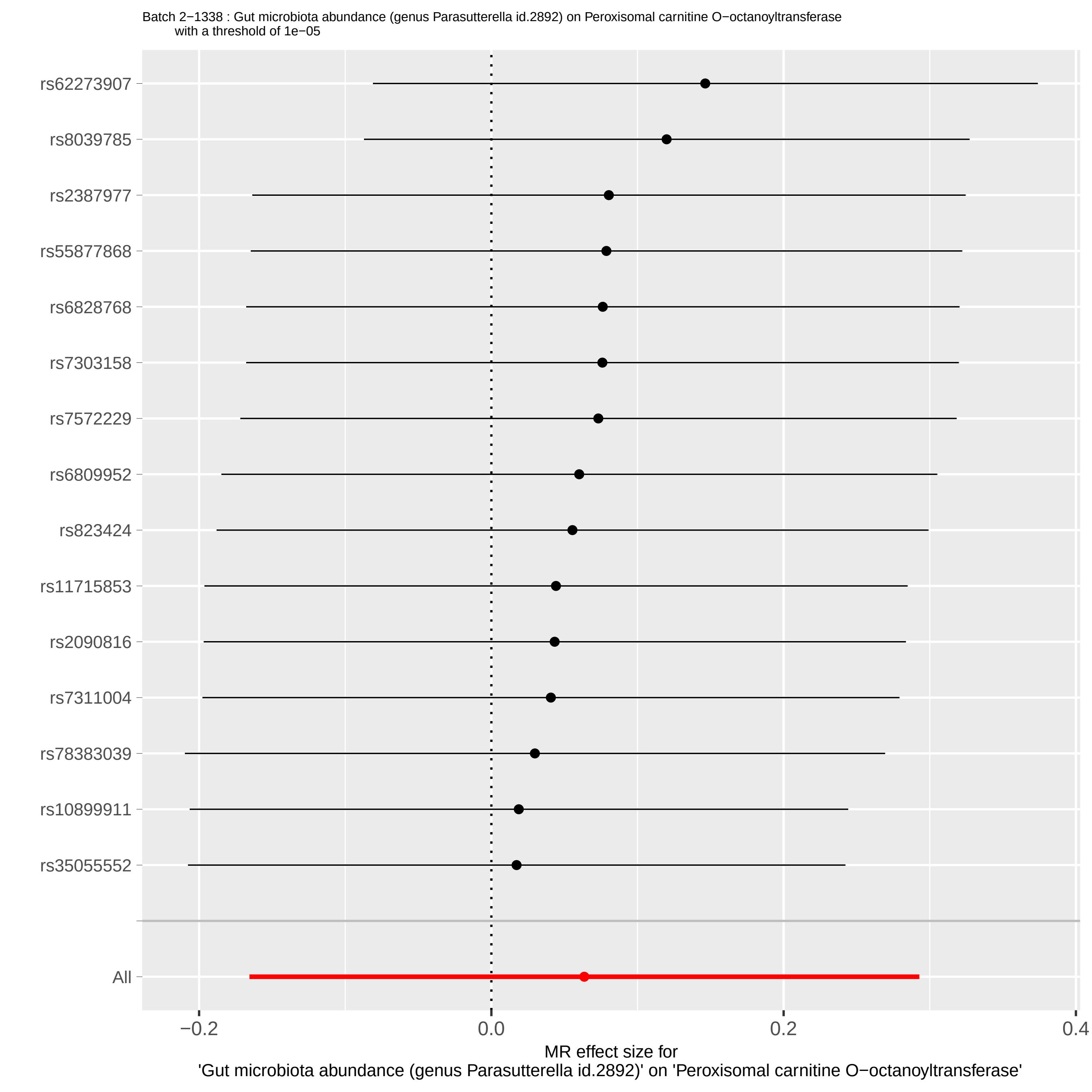
Batch 2−1338 : Gut microbiota abundance (genus Parasutterella id.2892) on Peroxisomal carnitine O−octanoyltransferase
with a threshold of 1e−05
rs62273907
rs8039785
rs2387977
rs55877868
rs6828768
rs7303158
rs7572229
rs6809952
rs823424
rs11715853
rs2090816
rs7311004
rs78383039
rs10899911
rs35055552
All
−0.2
0.0
0.2
0.4
MR effect size for
'Gut microbiota abundance (genus Parasutterella id.2892)' on 'Peroxisomal carnitine O−octanoyltransferase'

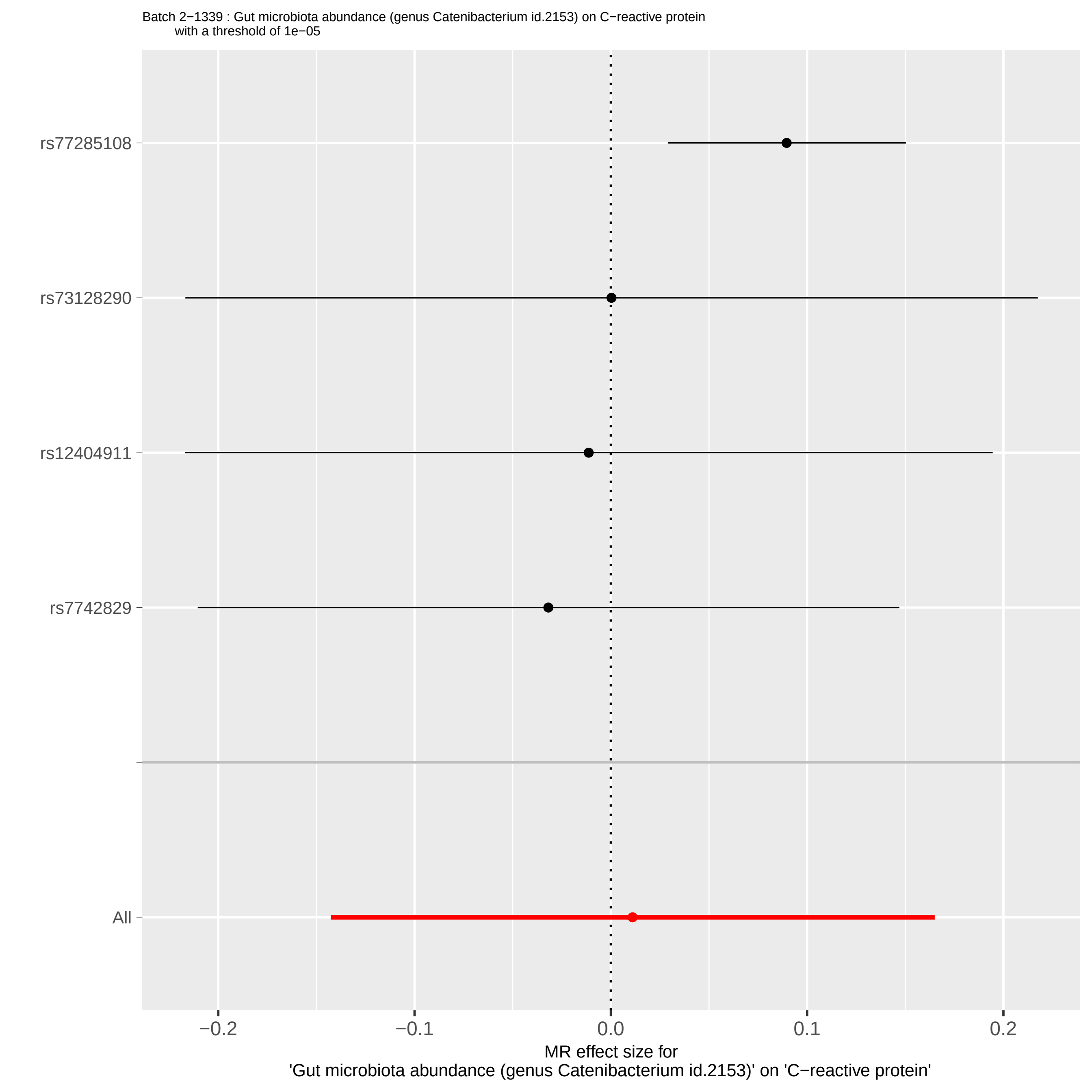
Batch 2−1339 : Gut microbiota abundance (genus Catenibacterium id.2153) on C−reactive protein
with a threshold of 1e−05
rs77285108
rs73128290
rs12404911
rs7742829
All
−0.2
−0.1
0.0
0.1
0.2
MR effect size for
'Gut microbiota abundance (genus Catenibacterium id.2153)' on 'C−reactive protein'

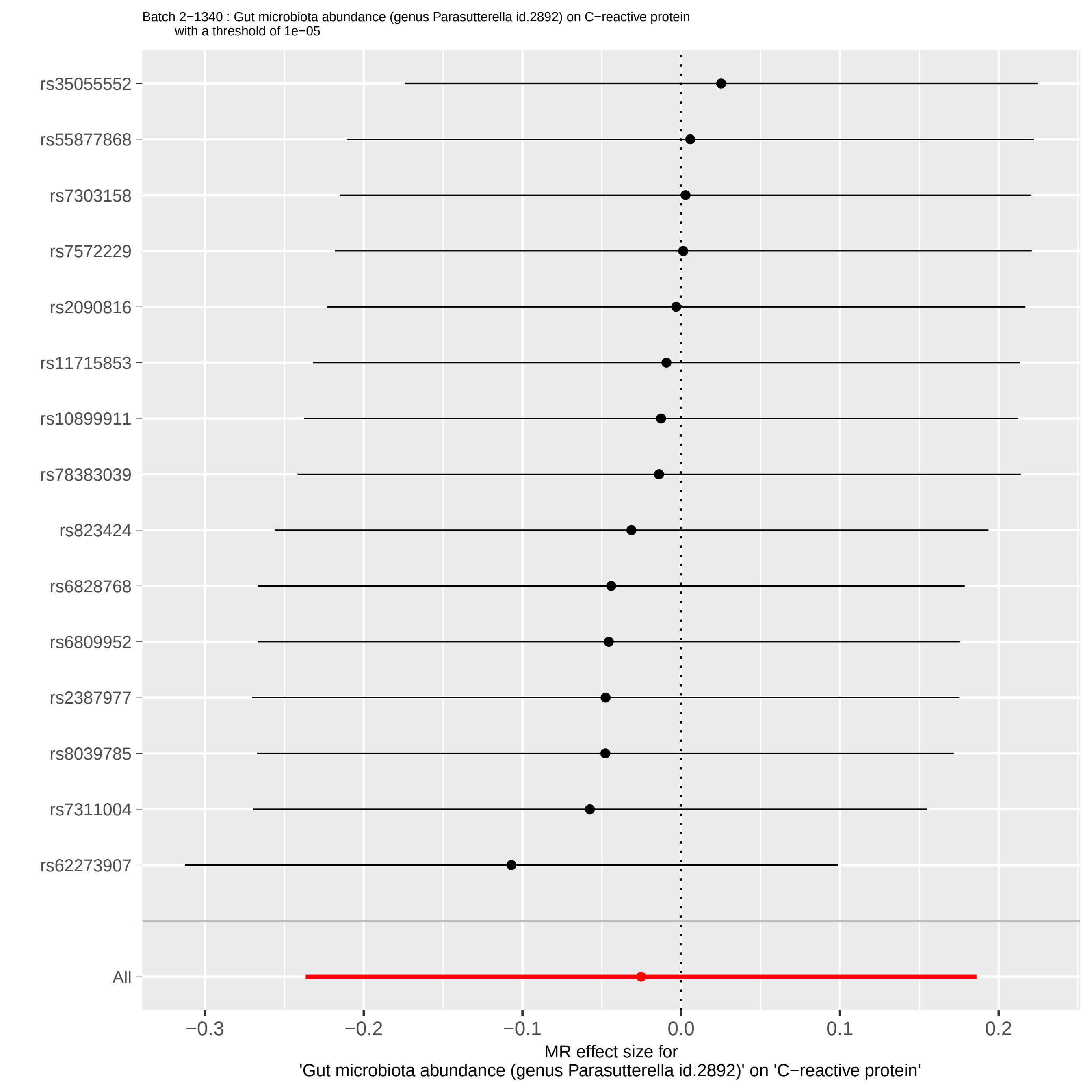

Batch 2−1340 : Gut microbiota abundance (genus Parasutterella id.2892) on C−reactive protein
with a threshold of 1e−05
rs35055552
rs55877868
rs7303158
rs7572229
rs2090816
rs11715853
rs10899911
rs78383039
rs823424
rs6828768
rs6809952
rs2387977
rs8039785
rs7311004
rs62273907
All
−0.3
−0.2
−0.1
0.0
0.1
0.2
MR effect size for
'Gut microbiota abundance (genus Parasutterella id.2892)' on 'C−reactive protein'

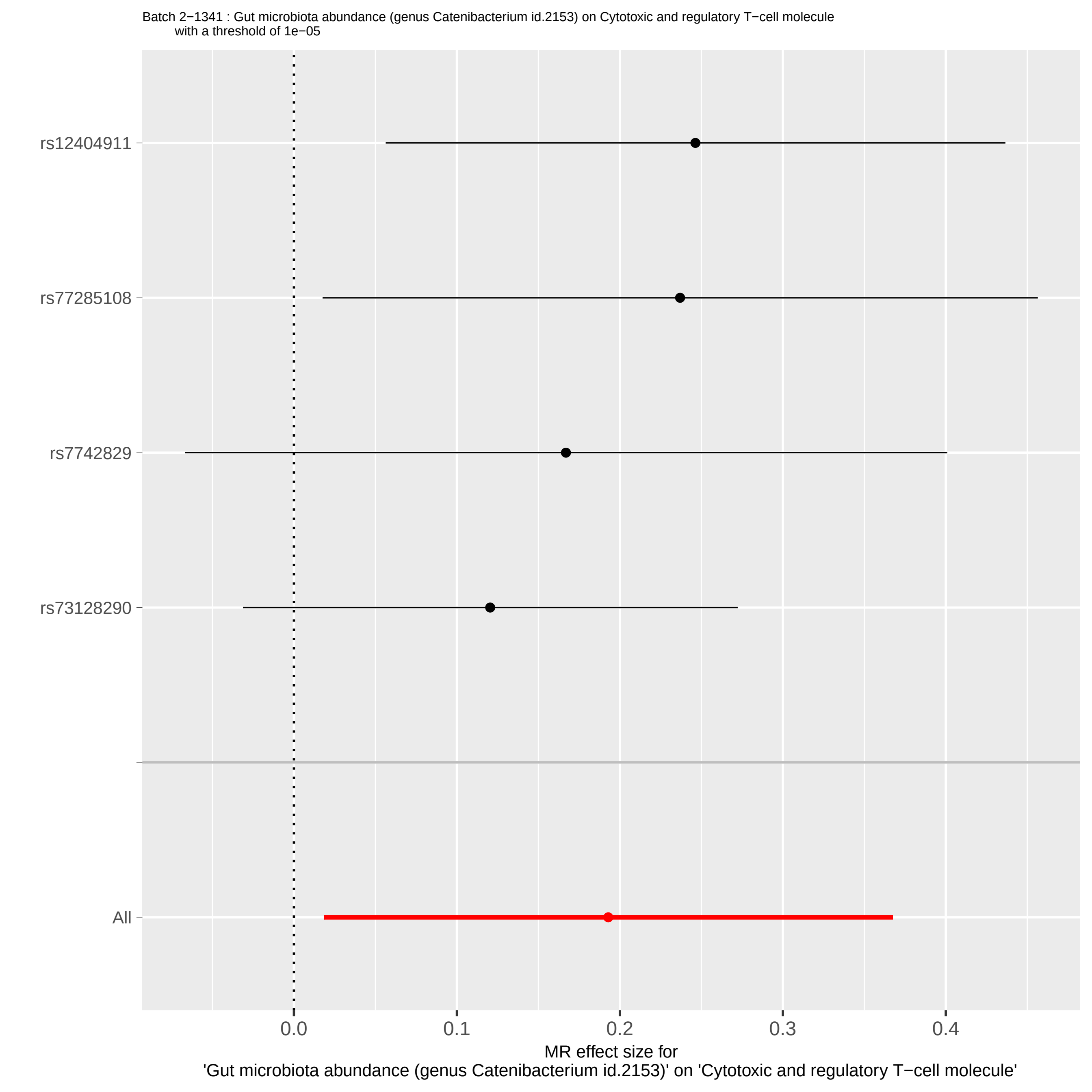

Batch 2−1341 : Gut microbiota abundance (genus Catenibacterium id.2153) on Cytotoxic and regulatory T−cell molecule
with a threshold of 1e−05
rs12404911
rs77285108
rs7742829
rs73128290
All
0.0
0.1
0.2
0.3
0.4
MR effect size for
'Gut microbiota abundance (genus Catenibacterium id.2153)' on 'Cytotoxic and regulatory T−cell molecule'

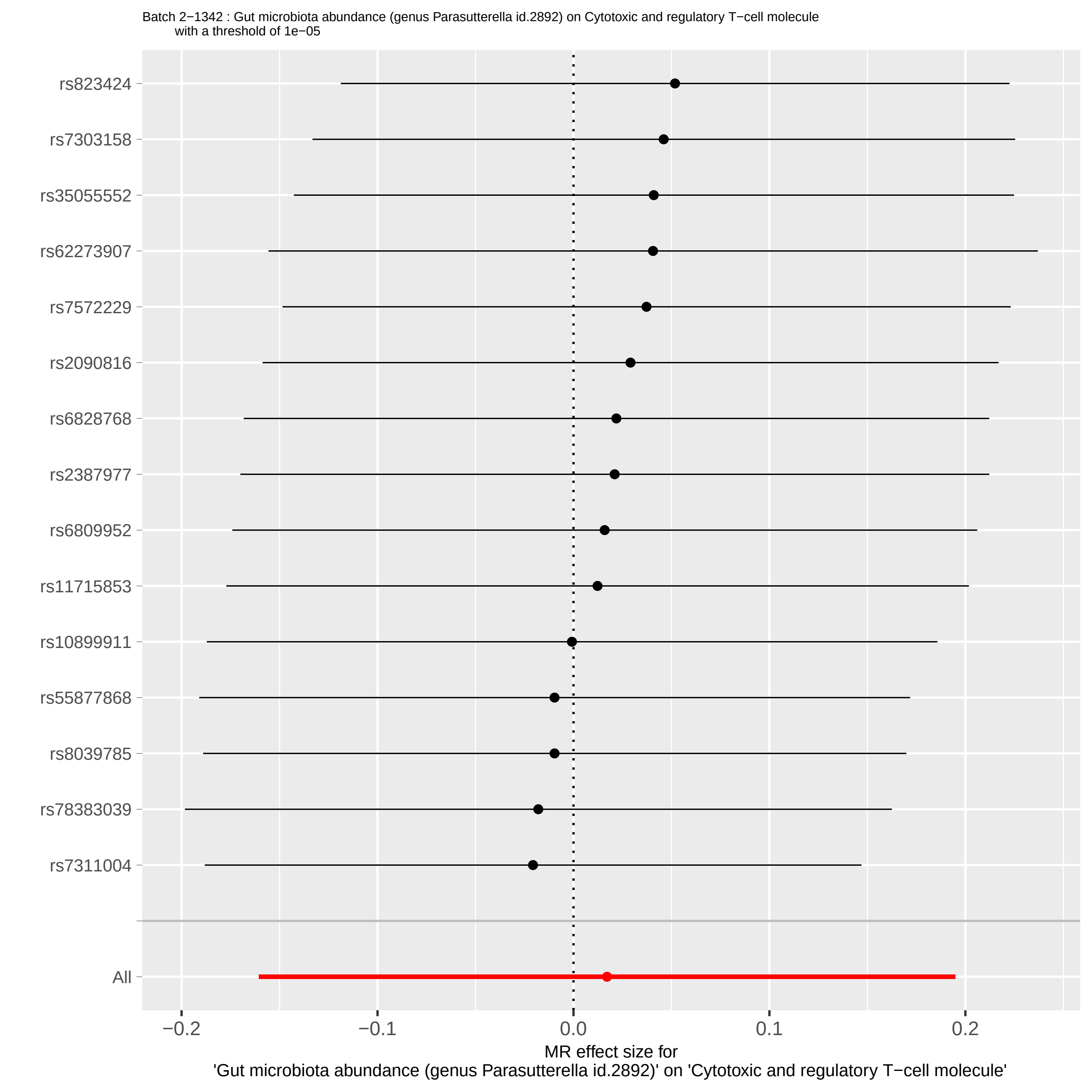

Batch 2−1342 : Gut microbiota abundance (genus Parasutterella id.2892) on Cytotoxic and regulatory T−cell molecule
with a threshold of 1e−05
rs823424
rs7303158
rs35055552
rs62273907
rs7572229
rs2090816
rs6828768
rs2387977
rs6809952
rs11715853
rs10899911
rs55877868
rs8039785
rs78383039
rs7311004
All
−0.2
−0.1
0.0
0.1
0.2
MR effect size for
'Gut microbiota abundance (genus Parasutterella id.2892)' on 'Cytotoxic and regulatory T−cell molecule'

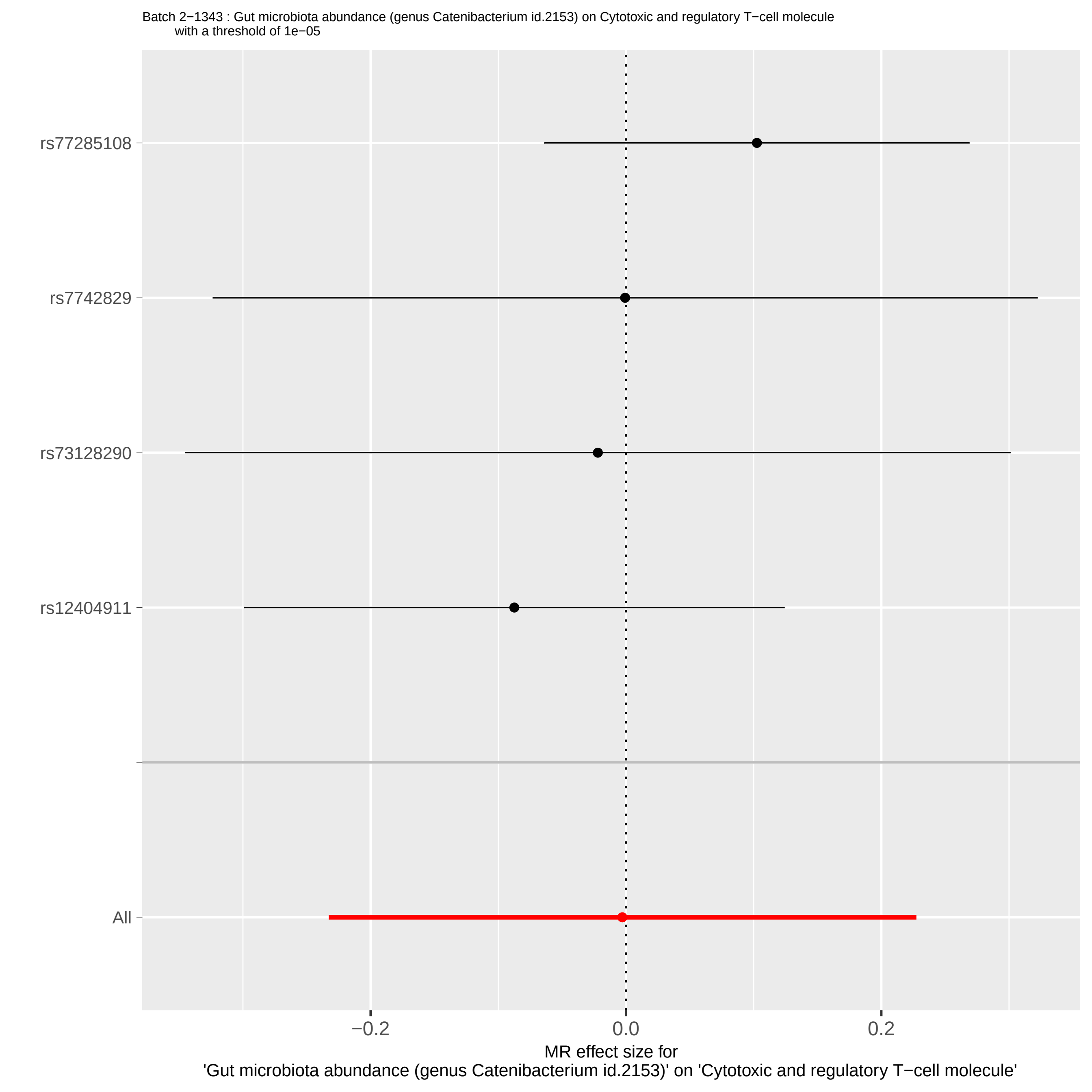
Batch 2−1343 : Gut microbiota abundance (genus Catenibacterium id.2153) on Cytotoxic and regulatory T−cell molecule
with a threshold of 1e−05
rs77285108
rs7742829
rs73128290
rs12404911
All
−0.2
0.0
0.2
MR effect size for
'Gut microbiota abundance (genus Catenibacterium id.2153)' on 'Cytotoxic and regulatory T−cell molecule'

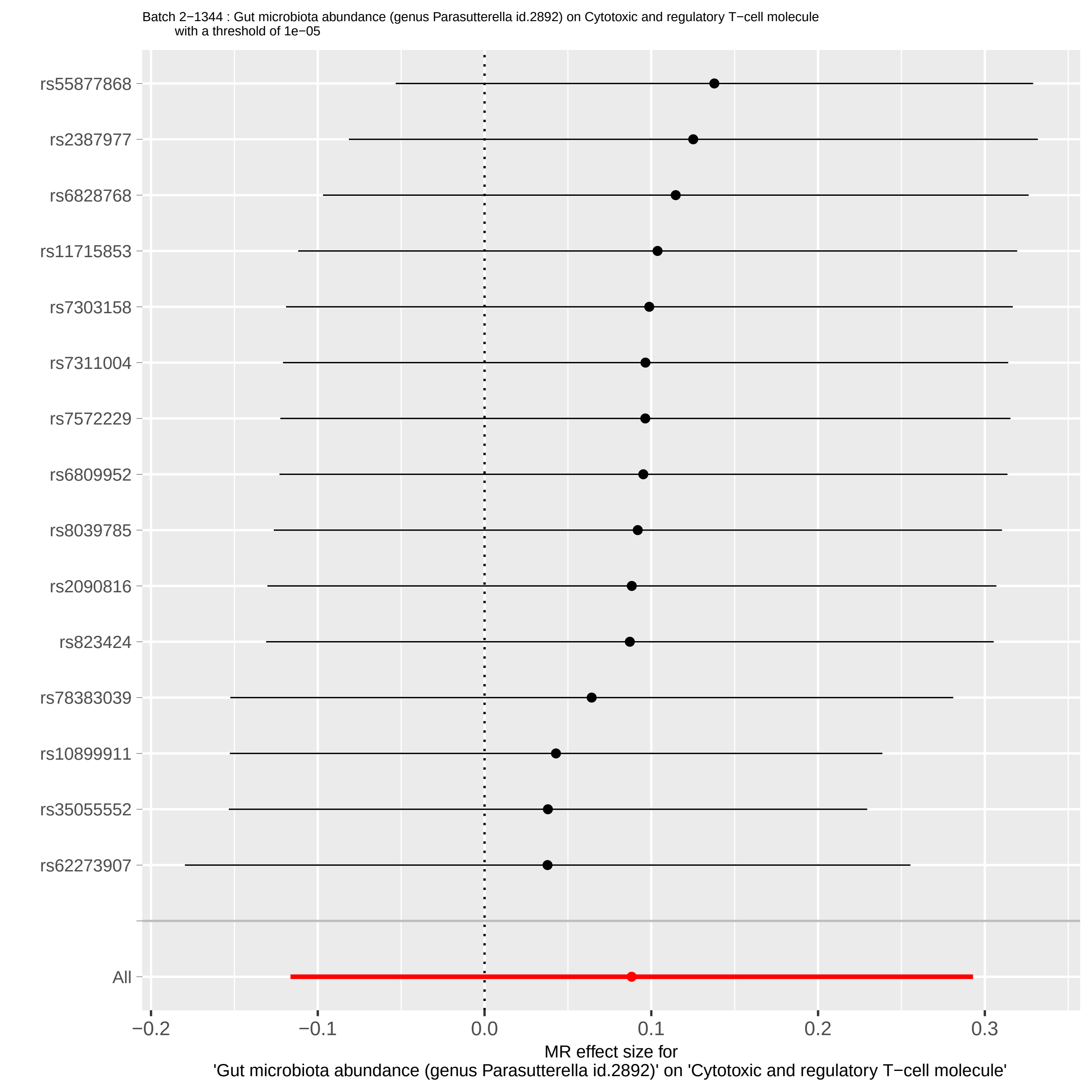
Batch 2−1344 : Gut microbiota abundance (genus Parasutterella id.2892) on Cytotoxic and regulatory T−cell molecule
with a threshold of 1e−05
rs55877868
rs2387977
rs6828768
rs11715853
rs7303158
rs7311004
rs7572229
rs6809952
rs8039785
rs2090816
rs823424
rs78383039
rs10899911
rs35055552
rs62273907
All
−0.2
−0.1
0.0
0.1
0.2
0.3
MR effect size for
'Gut microbiota abundance (genus Parasutterella id.2892)' on 'Cytotoxic and regulatory T−cell molecule'

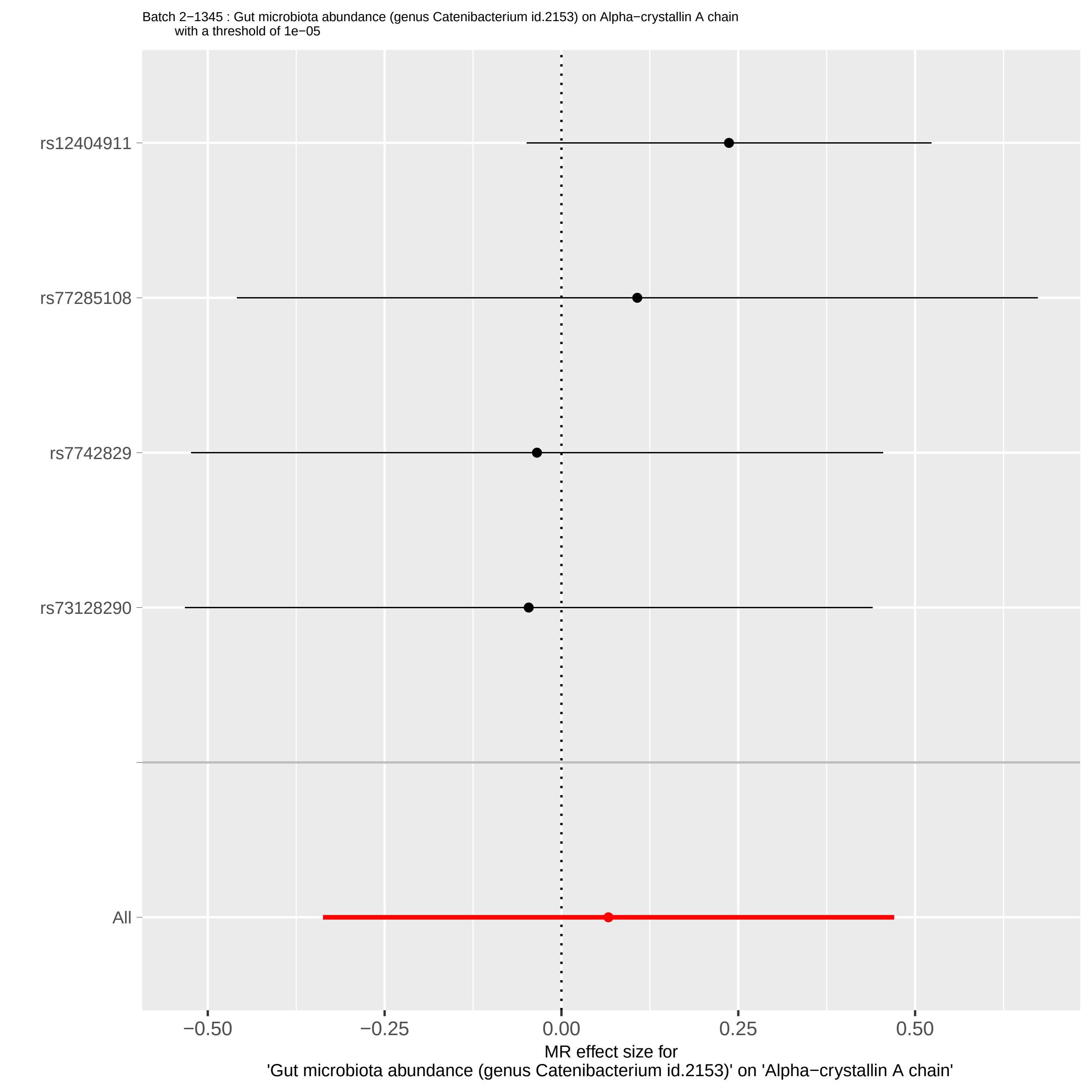

Batch 2−1345 : Gut microbiota abundance (genus Catenibacterium id.2153) on Alpha−crystallin A chain
with a threshold of 1e−05
rs12404911
rs77285108
rs7742829
rs73128290
All
−0.50
−0.25
0.00
0.25
0.50
MR effect size for
'Gut microbiota abundance (genus Catenibacterium id.2153)' on 'Alpha−crystallin A chain'

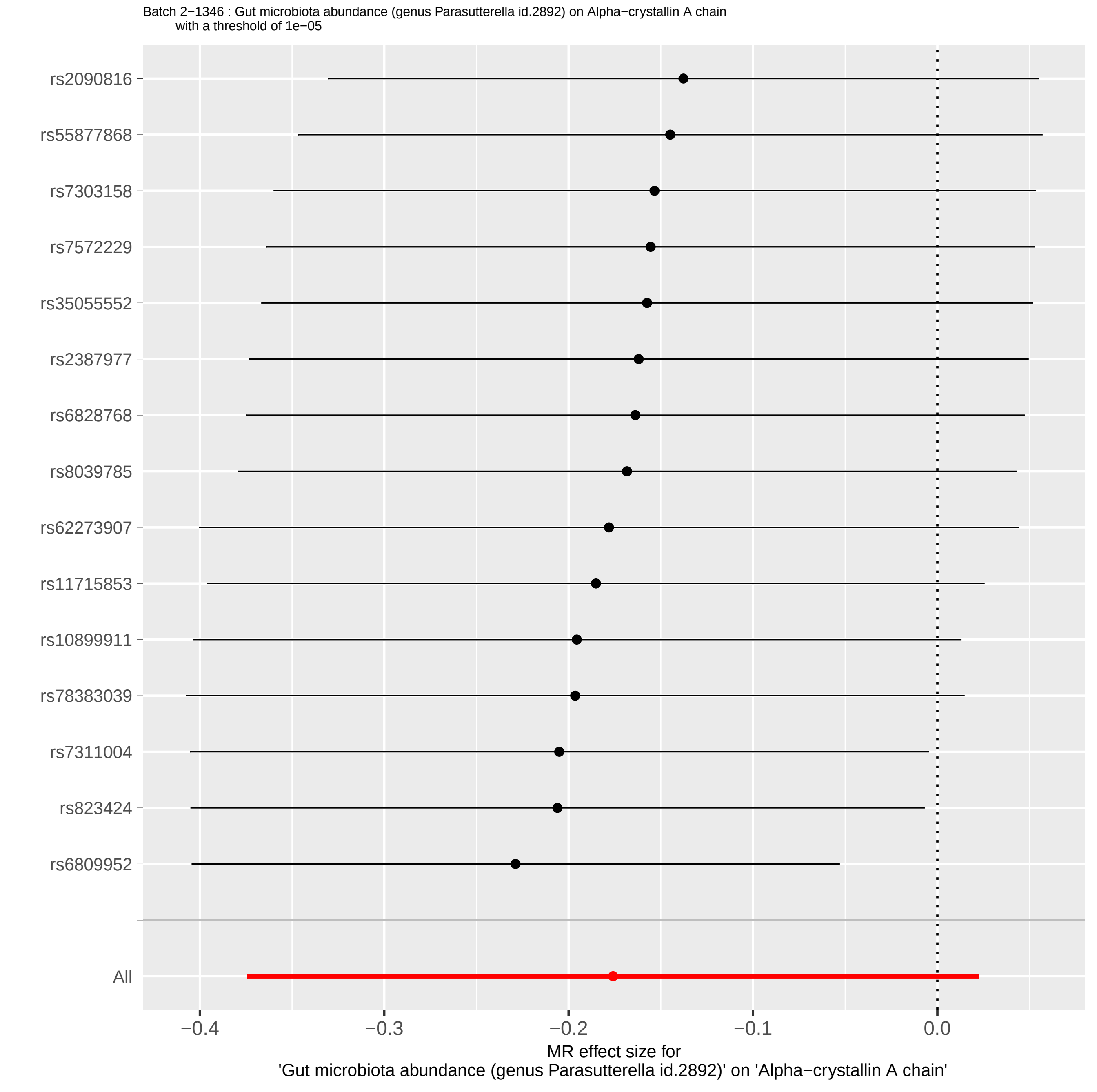
Batch 2−1346 : Gut microbiota abundance (genus Parasutterella id.2892) on Alpha−crystallin A chain
with a threshold of 1e−05
rs2090816
rs55877868
rs7303158
rs7572229
rs35055552
rs2387977
rs6828768
rs8039785
rs62273907
rs11715853
rs10899911
rs78383039
rs7311004
rs823424
rs6809952
All
−0.4
−0.3
−0.2
−0.1
0.0
MR effect size for
'Gut microbiota abundance (genus Parasutterella id.2892)' on 'Alpha−crystallin A chain'

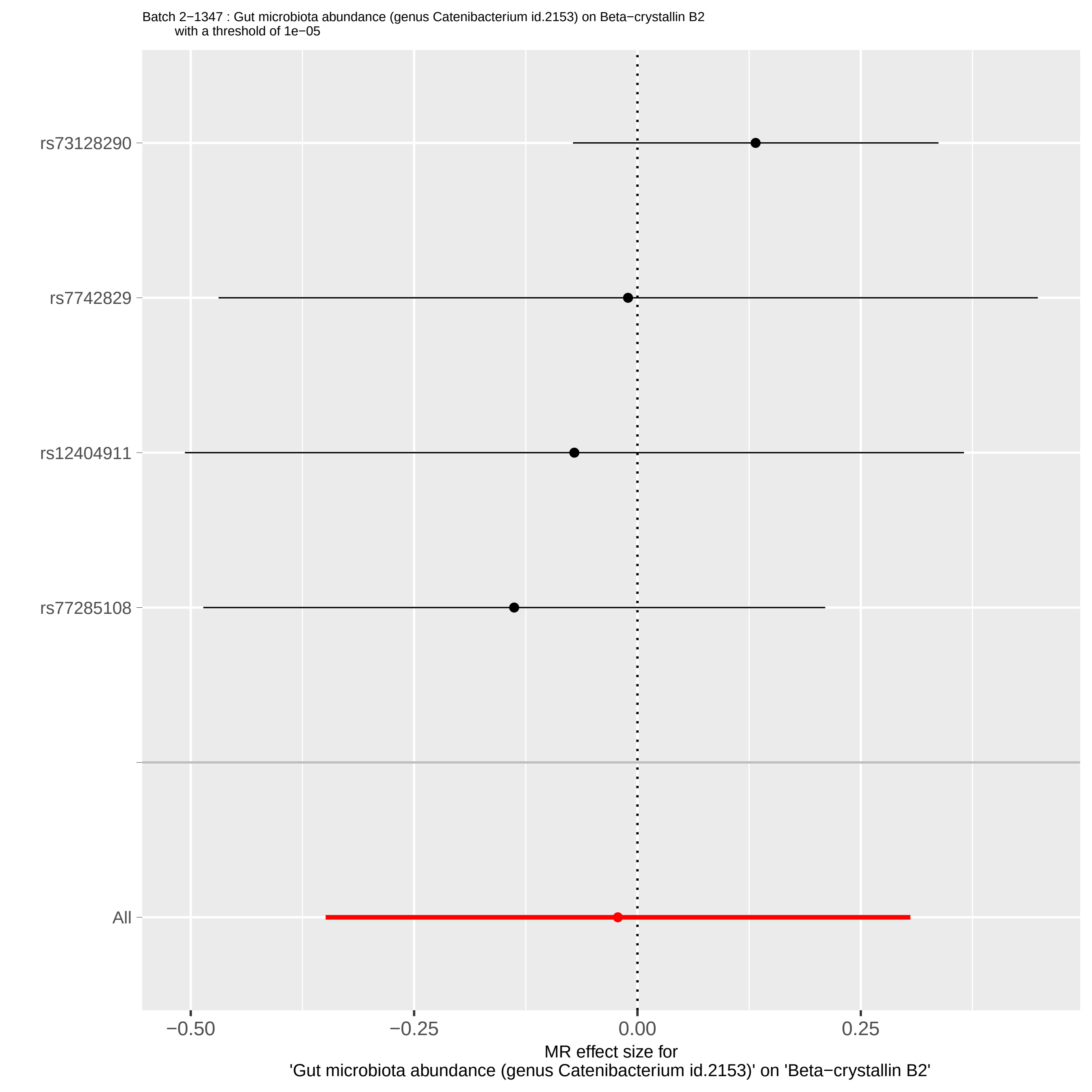
Batch 2−1347 : Gut microbiota abundance (genus Catenibacterium id.2153) on Beta−crystallin B2
with a threshold of 1e−05
rs73128290
rs7742829
rs12404911
rs77285108
All
−0.50
−0.25
0.00
0.25
MR effect size for
'Gut microbiota abundance (genus Catenibacterium id.2153)' on 'Beta−crystallin B2'

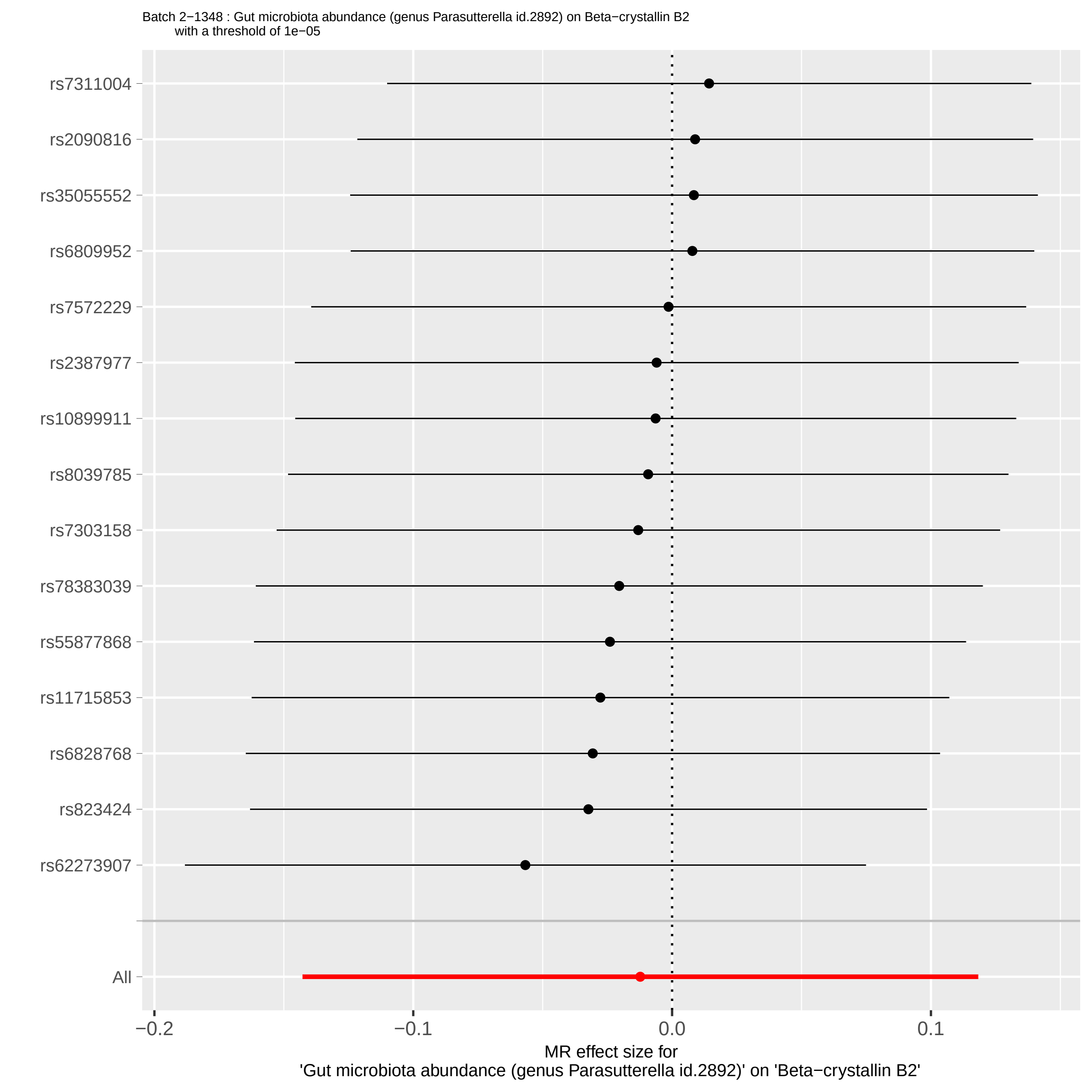
Batch 2−1348 : Gut microbiota abundance (genus Parasutterella id.2892) on Beta−crystallin B2
with a threshold of 1e−05
rs7311004
rs2090816
rs35055552
rs6809952
rs7572229
rs2387977
rs10899911
rs8039785
rs7303158
rs78383039
rs55877868
rs11715853
rs6828768
rs823424
rs62273907
All
−0.2
−0.1
0.0
0.1
MR effect size for
'Gut microbiota abundance (genus Parasutterella id.2892)' on 'Beta−crystallin B2'

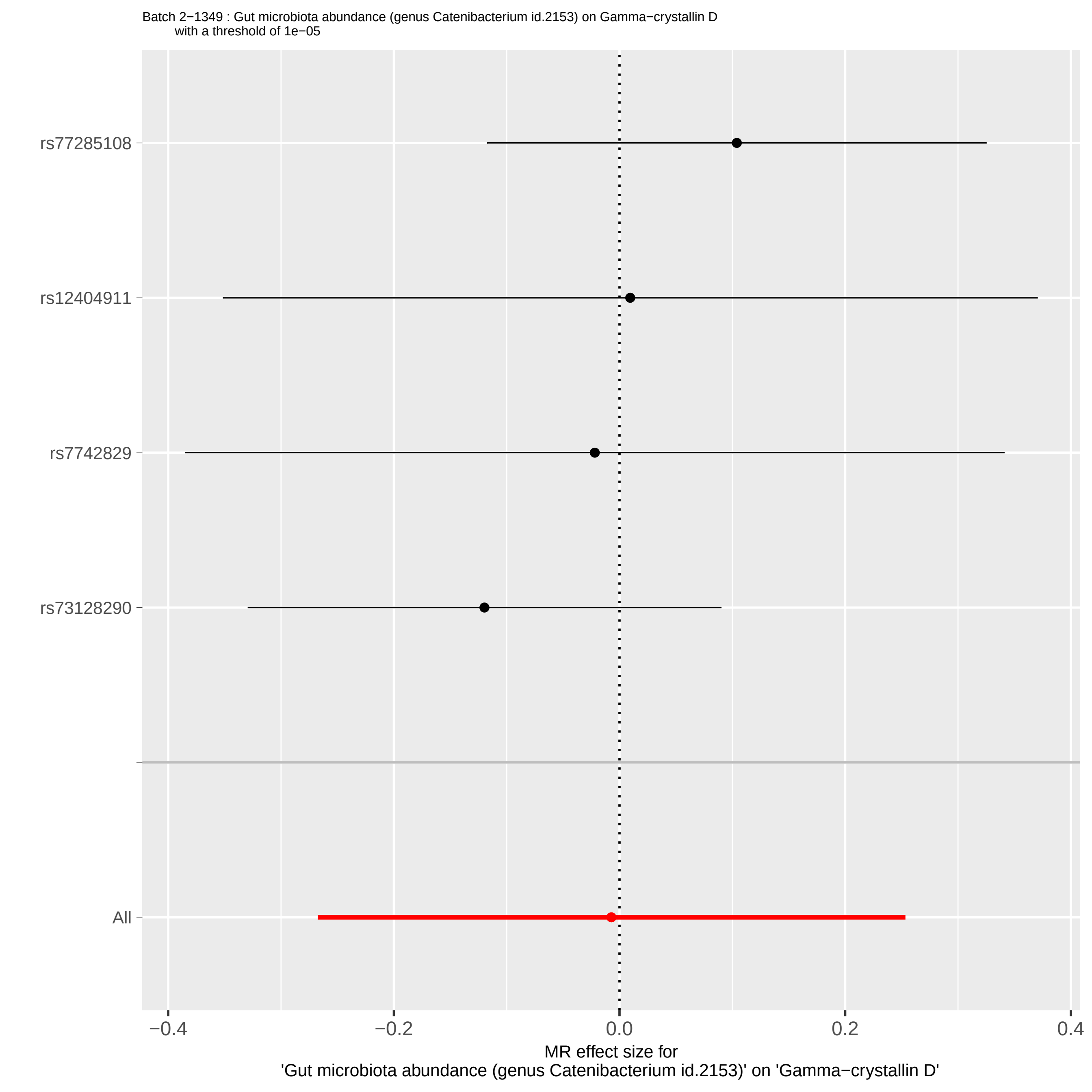

Batch 2−1349 : Gut microbiota abundance (genus Catenibacterium id.2153) on Gamma−crystallin D
with a threshold of 1e−05
rs77285108
rs12404911
rs7742829
rs73128290
All
−0.4
−0.2
0.0
0.2
0.4
MR effect size for
'Gut microbiota abundance (genus Catenibacterium id.2153)' on 'Gamma−crystallin D'

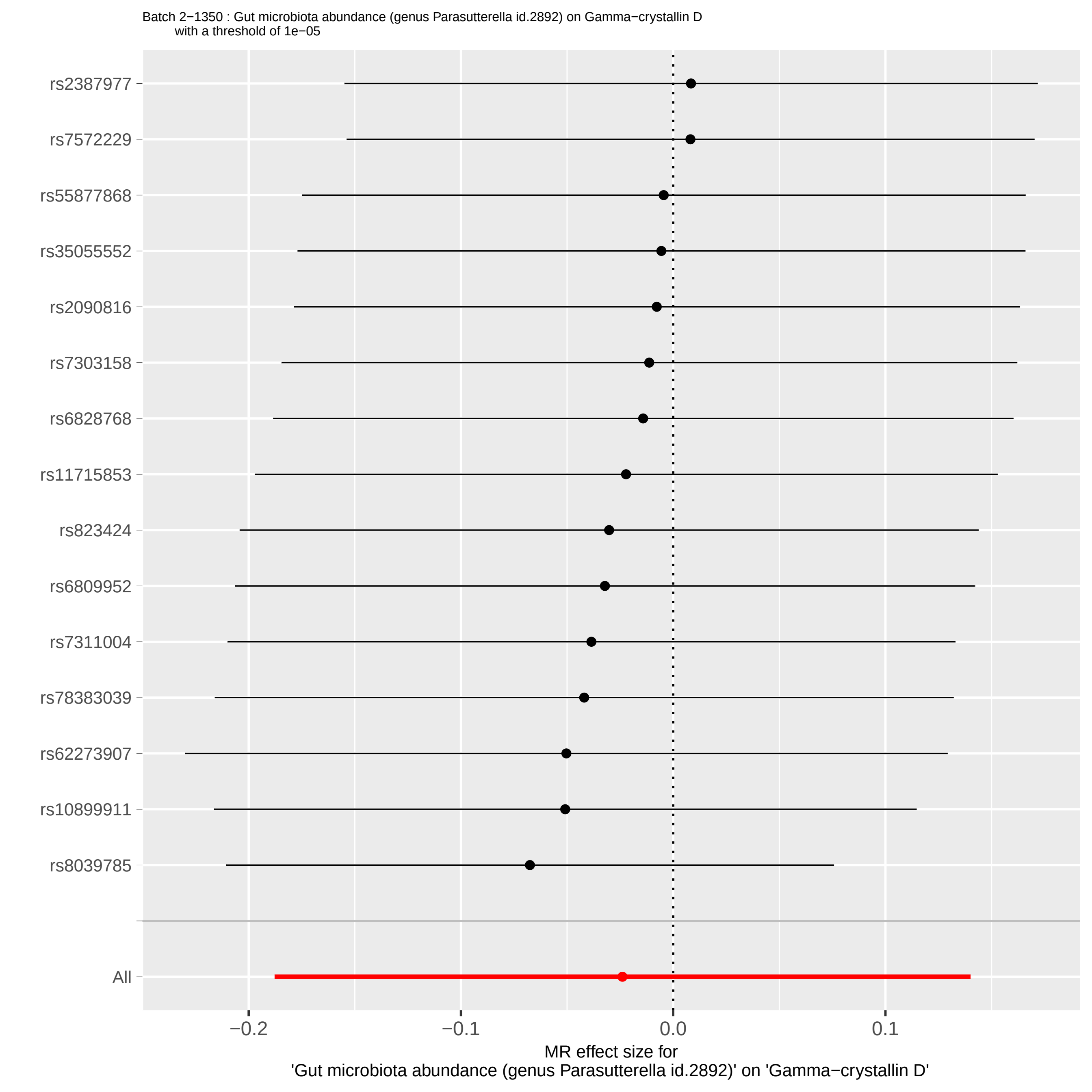
Batch 2−1350 : Gut microbiota abundance (genus Parasutterella id.2892) on Gamma−crystallin D
with a threshold of 1e−05
rs2387977
rs7572229
rs55877868
rs35055552
rs2090816
rs7303158
rs6828768
rs11715853
rs823424
rs6809952
rs7311004
rs78383039
rs62273907
rs10899911
rs8039785
All
−0.2
−0.1
0.0
0.1
MR effect size for
'Gut microbiota abundance (genus Parasutterella id.2892)' on 'Gamma−crystallin D'

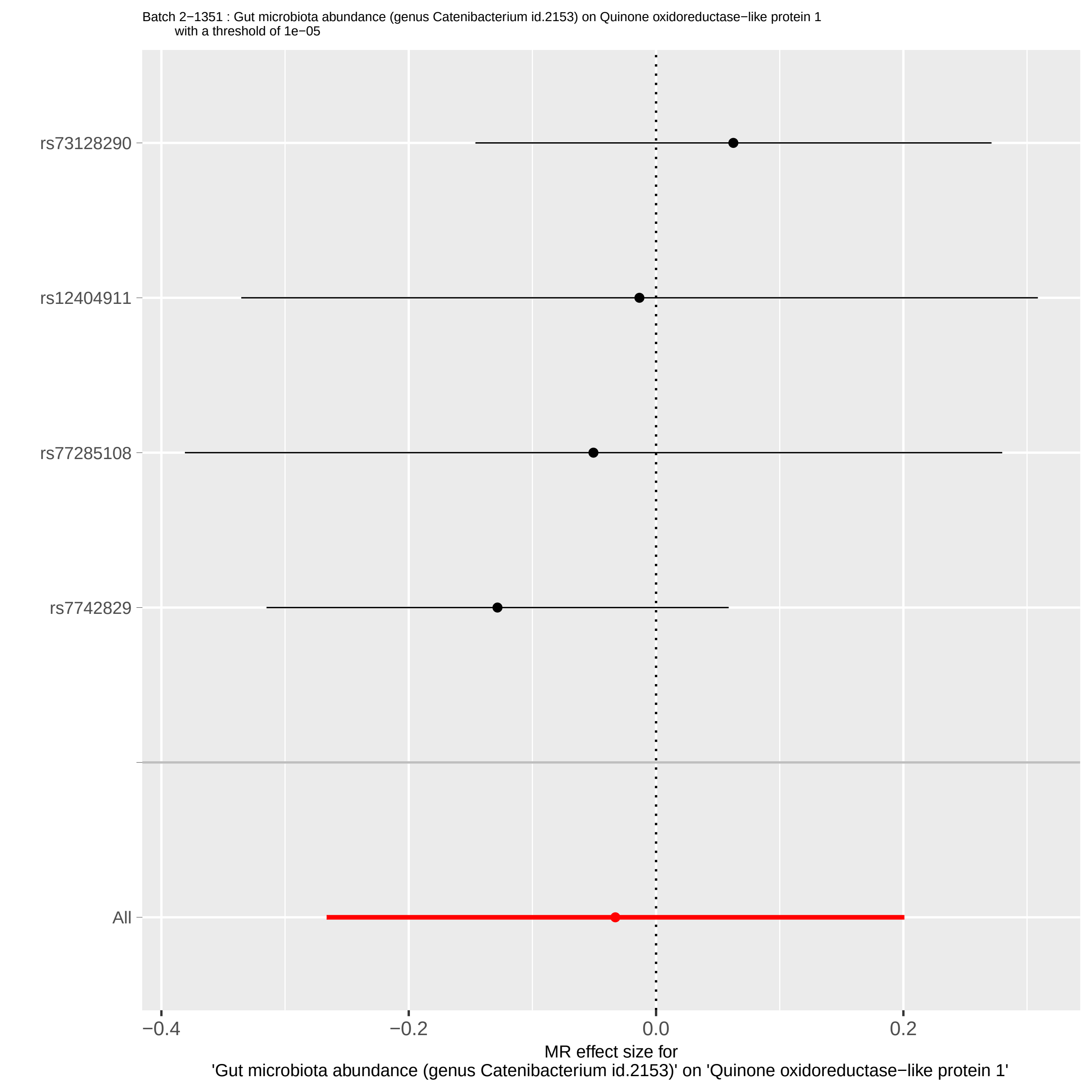

Batch 2−1351 : Gut microbiota abundance (genus Catenibacterium id.2153) on Quinone oxidoreductase−like protein 1
with a threshold of 1e−05
rs73128290
rs12404911
rs77285108
rs7742829
All
−0.4
−0.2
0.0
0.2
MR effect size for
'Gut microbiota abundance (genus Catenibacterium id.2153)' on 'Quinone oxidoreductase−like protein 1'

Batch 2−1352 : Gut microbiota abundance (genus Parasutterella id.2892) on Quinone oxidoreductase−like protein 1
with a threshold of 1e−05

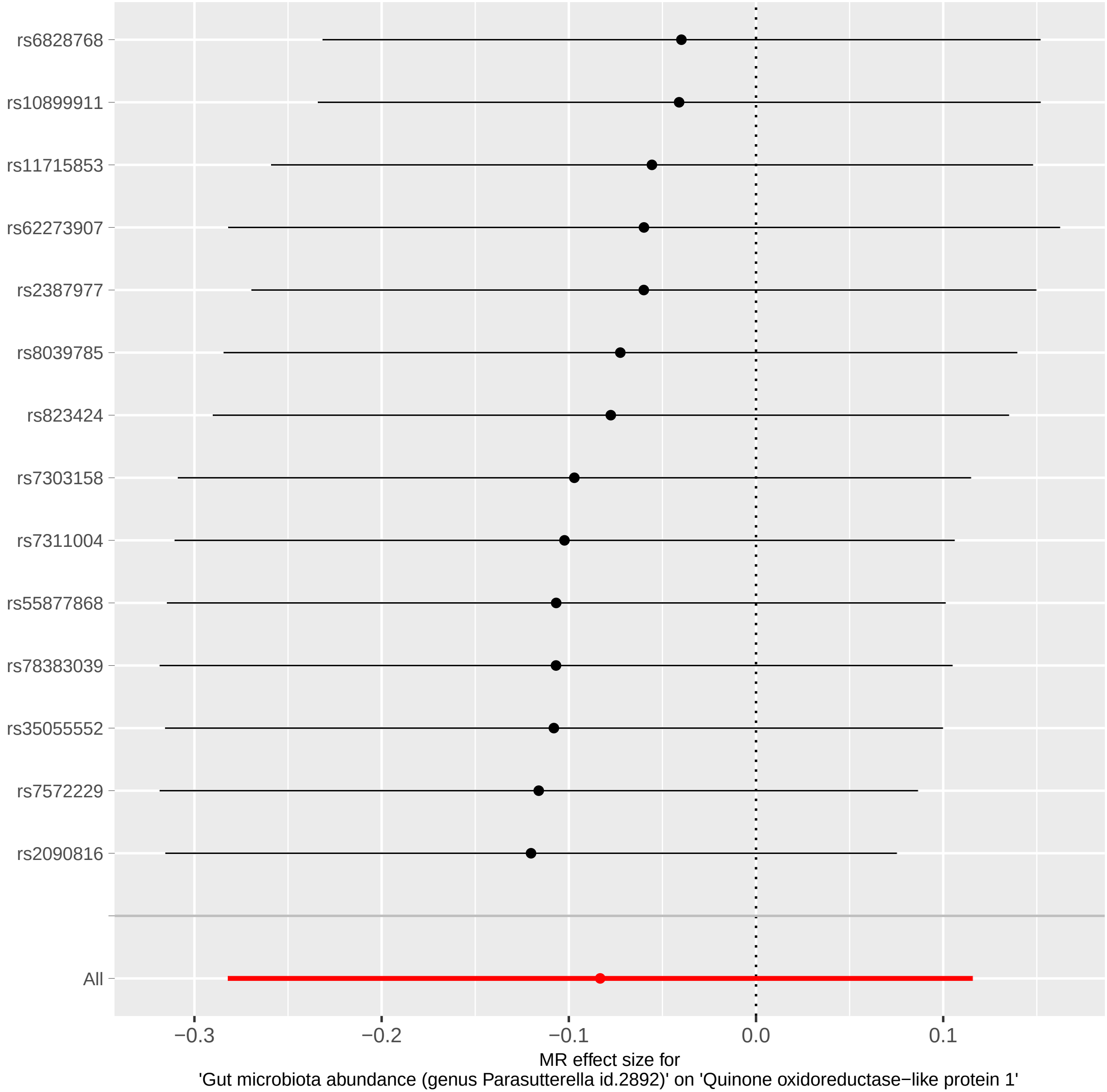

Batch 2−1353 : Gut microbiota abundance (genus Catenibacterium id.2153) on Putative chondrosarcoma−associated gene 1 protein
with a threshold of 1e−05

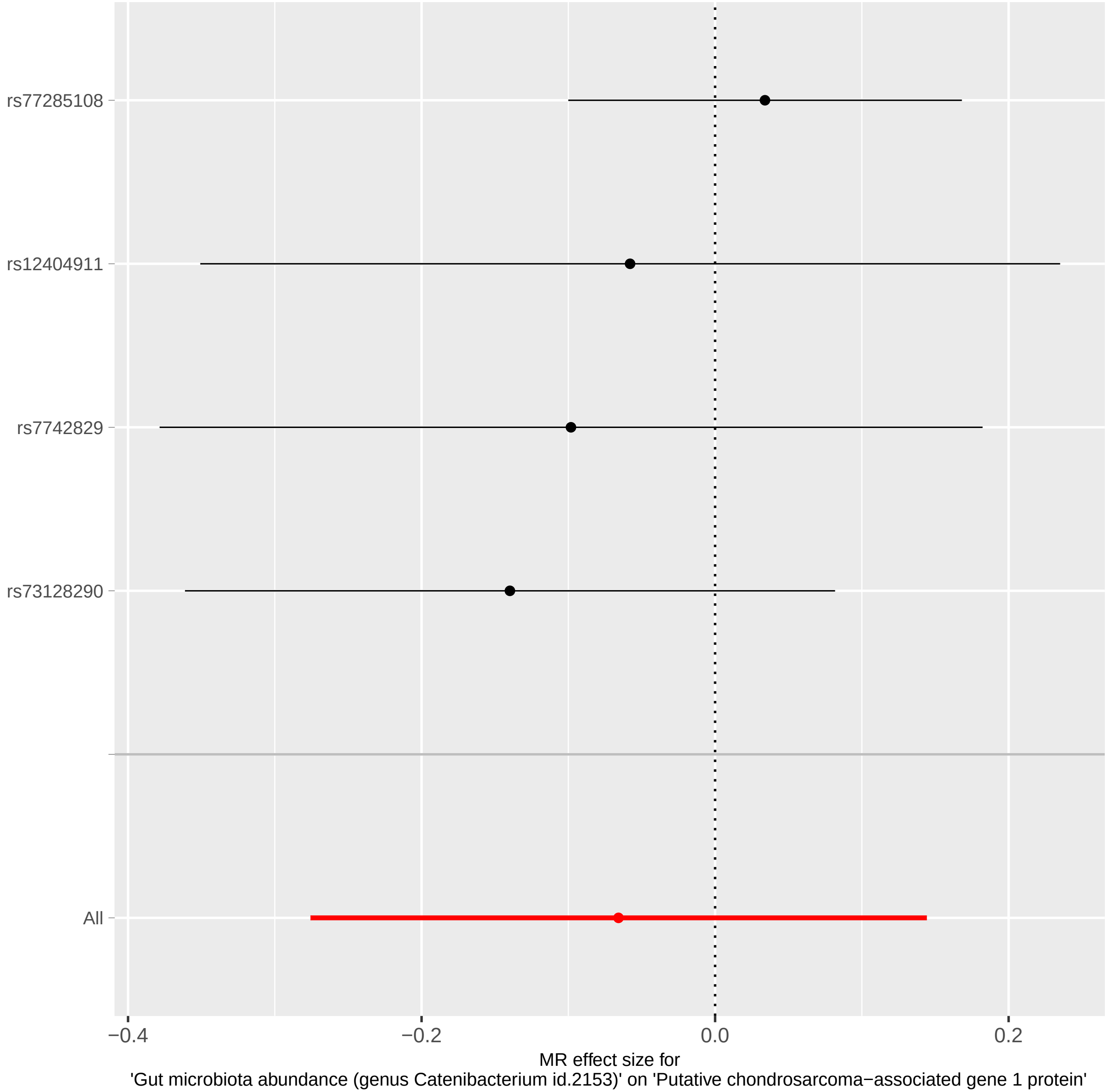

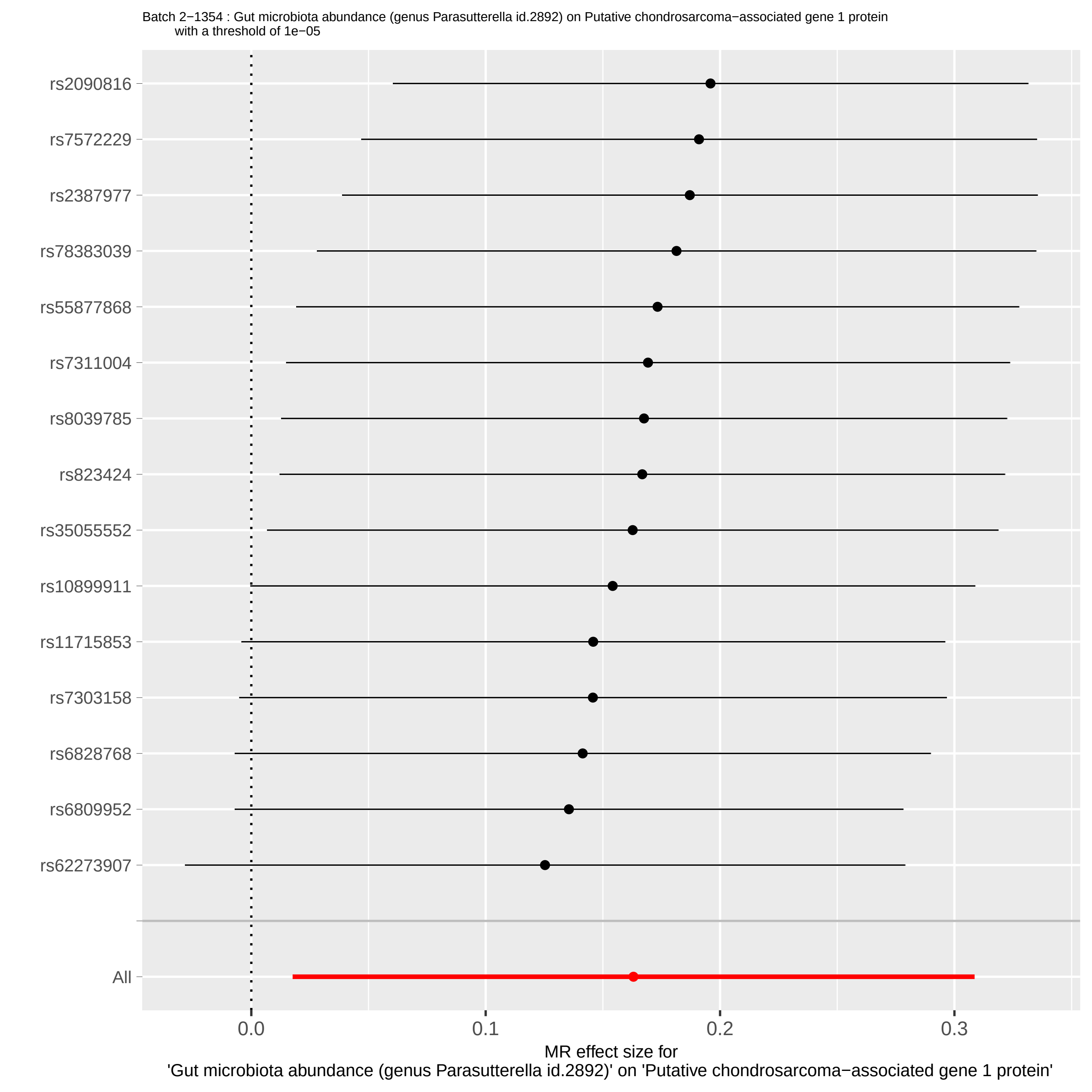

Batch 2−1354 : Gut microbiota abundance (genus Parasutterella id.2892) on Putative chondrosarcoma−associated gene 1 protein
with a threshold of 1e−05
rs2090816
rs7572229
rs2387977
rs78383039
rs55877868
rs7311004
rs8039785
rs823424
rs35055552
rs10899911
rs11715853
rs7303158
rs6828768
rs6809952
rs62273907
All
0.0
0.1
0.2
0.3
MR effect size for
'Gut microbiota abundance (genus Parasutterella id.2892)' on 'Putative chondrosarcoma−associated gene 1 protein'

Batch 2−1355 : Gut microbiota abundance (genus Catenibacterium id.2153) on Cold shock domain−containing protein C2
with a threshold of 1e−05

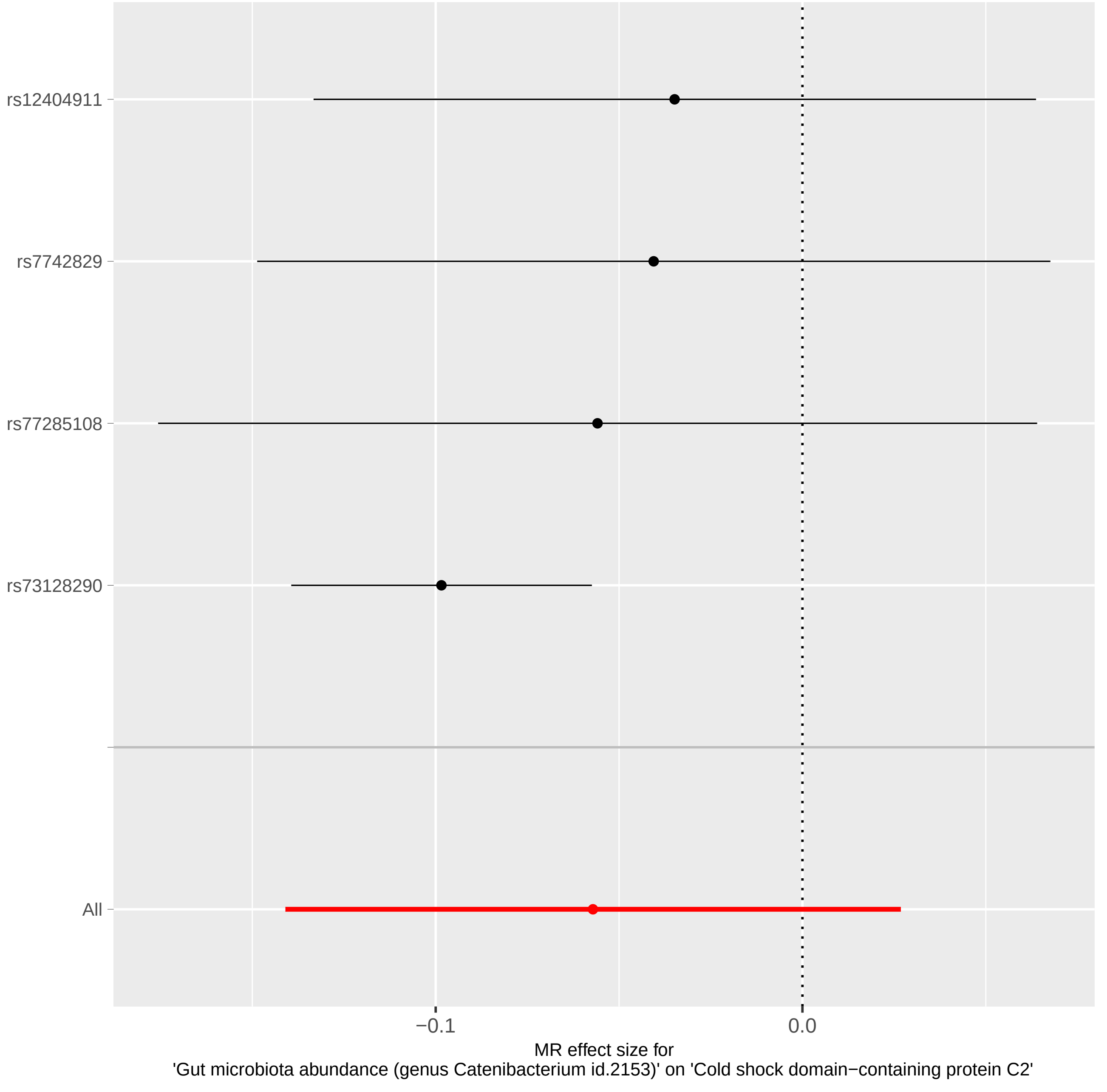

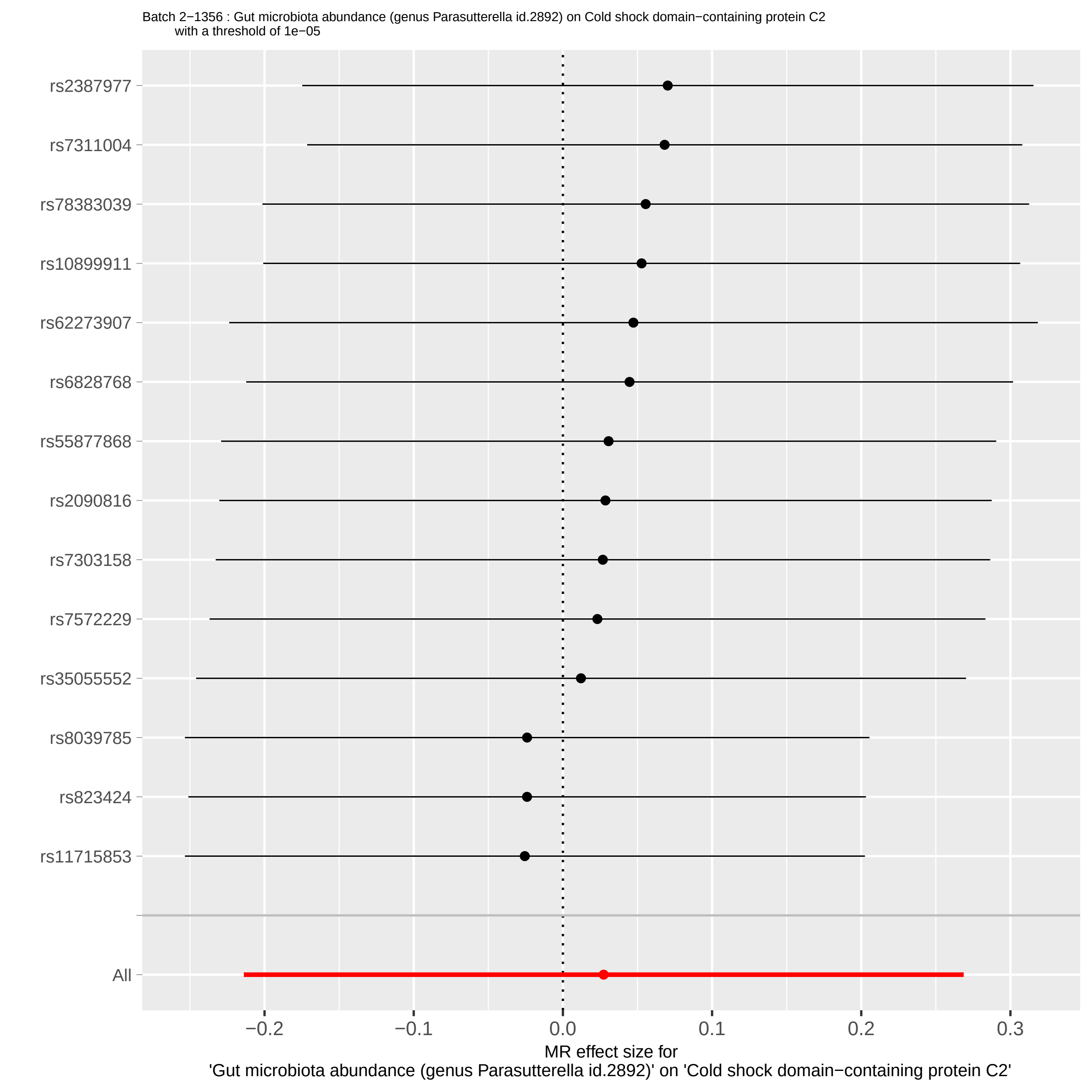
Batch 2−1356 : Gut microbiota abundance (genus Parasutterella id.2892) on Cold shock domain−containing protein C2
with a threshold of 1e−05
rs2387977
rs7311004
rs78383039
rs10899911
rs62273907
rs6828768
rs55877868
rs2090816
rs7303158
rs7572229
rs35055552
rs8039785
rs823424
rs11715853
All
−0.2
−0.1
0.0
0.1
0.2
0.3
MR effect size for
'Gut microbiota abundance (genus Parasutterella id.2892)' on 'Cold shock domain−containing protein C2'

Batch 2−1357 : Gut microbiota abundance (genus Catenibacterium id.2153) on Cold shock domain−containing protein E1
with a threshold of 1e−05

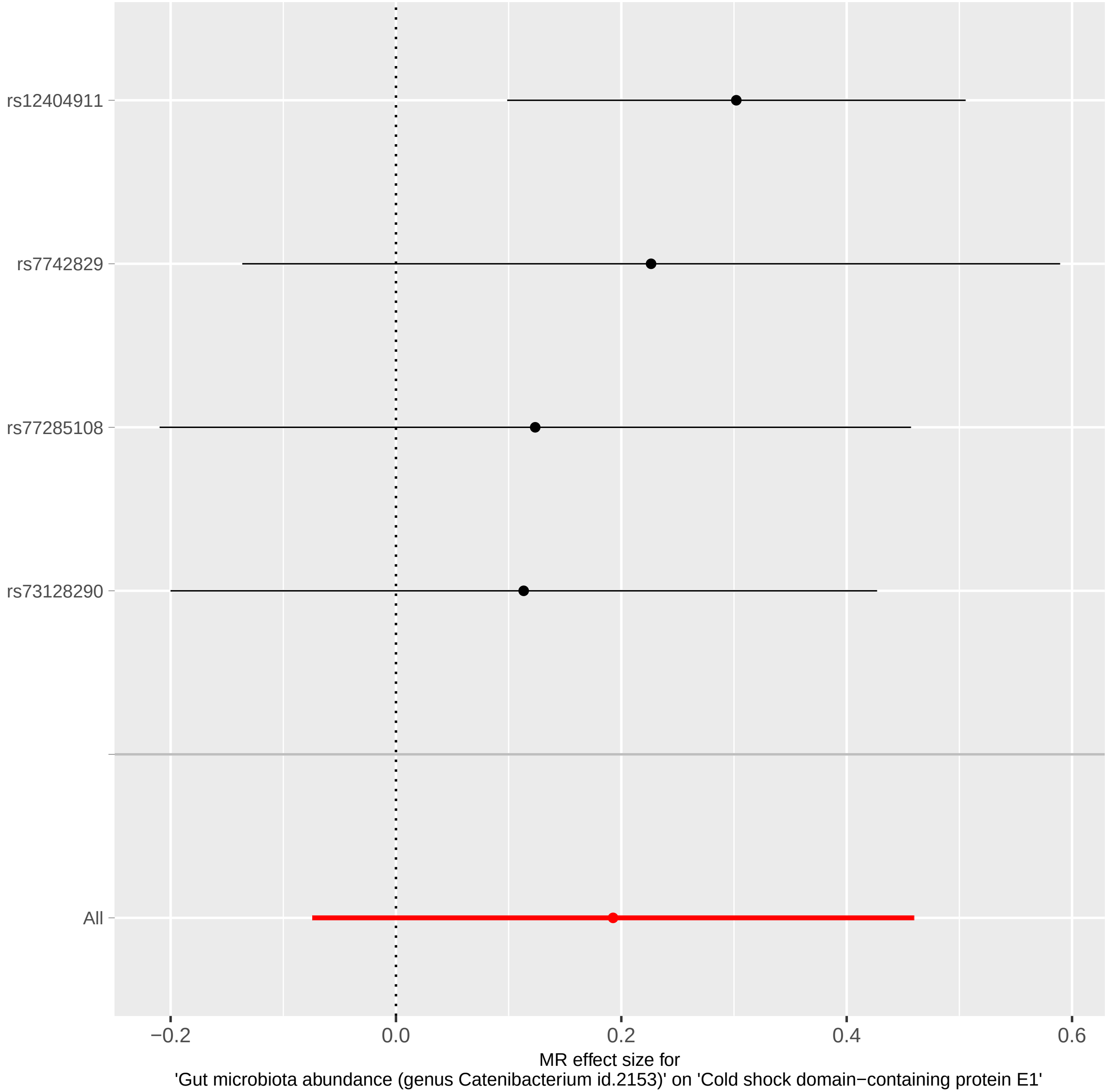

Batch 2−1358 : Gut microbiota abundance (genus Parasutterella id.2892) on Cold shock domain−containing protein E1
with a threshold of 1e−05

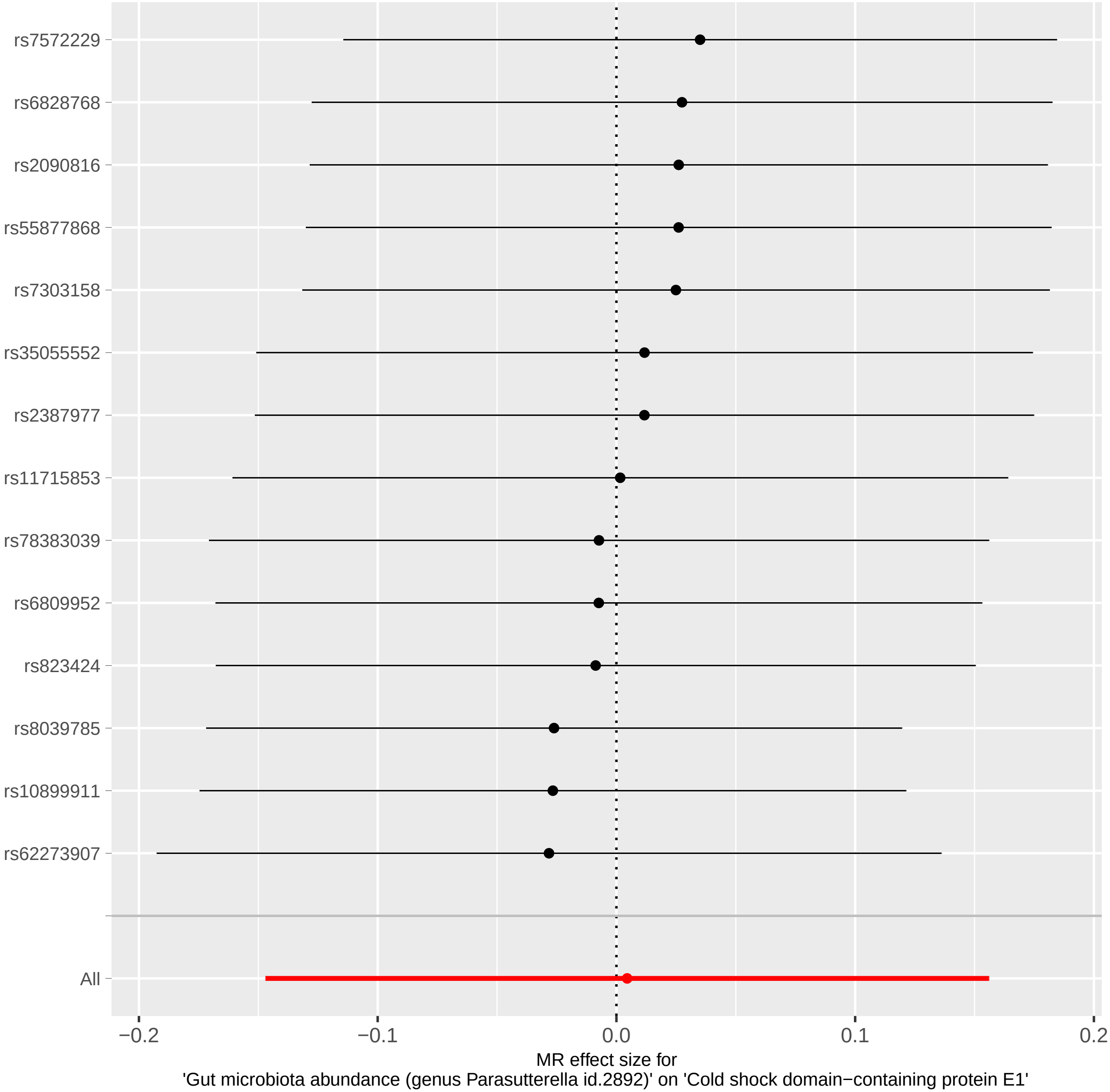

Batch 2−1359 : Gut microbiota abundance (genus Catenibacterium id.2153) on Macrophage colony−stimulating factor 1
with a threshold of 1e−05

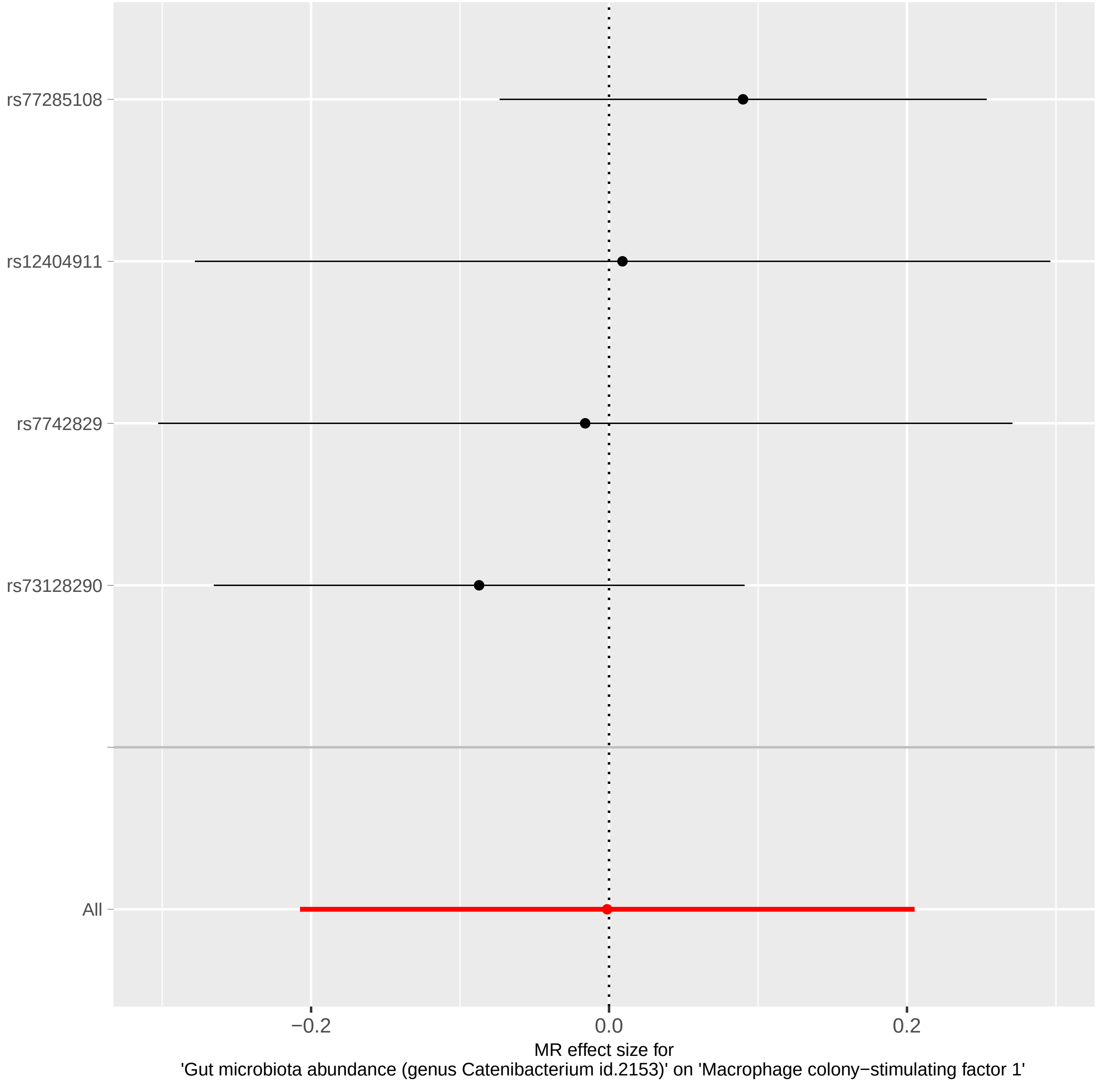

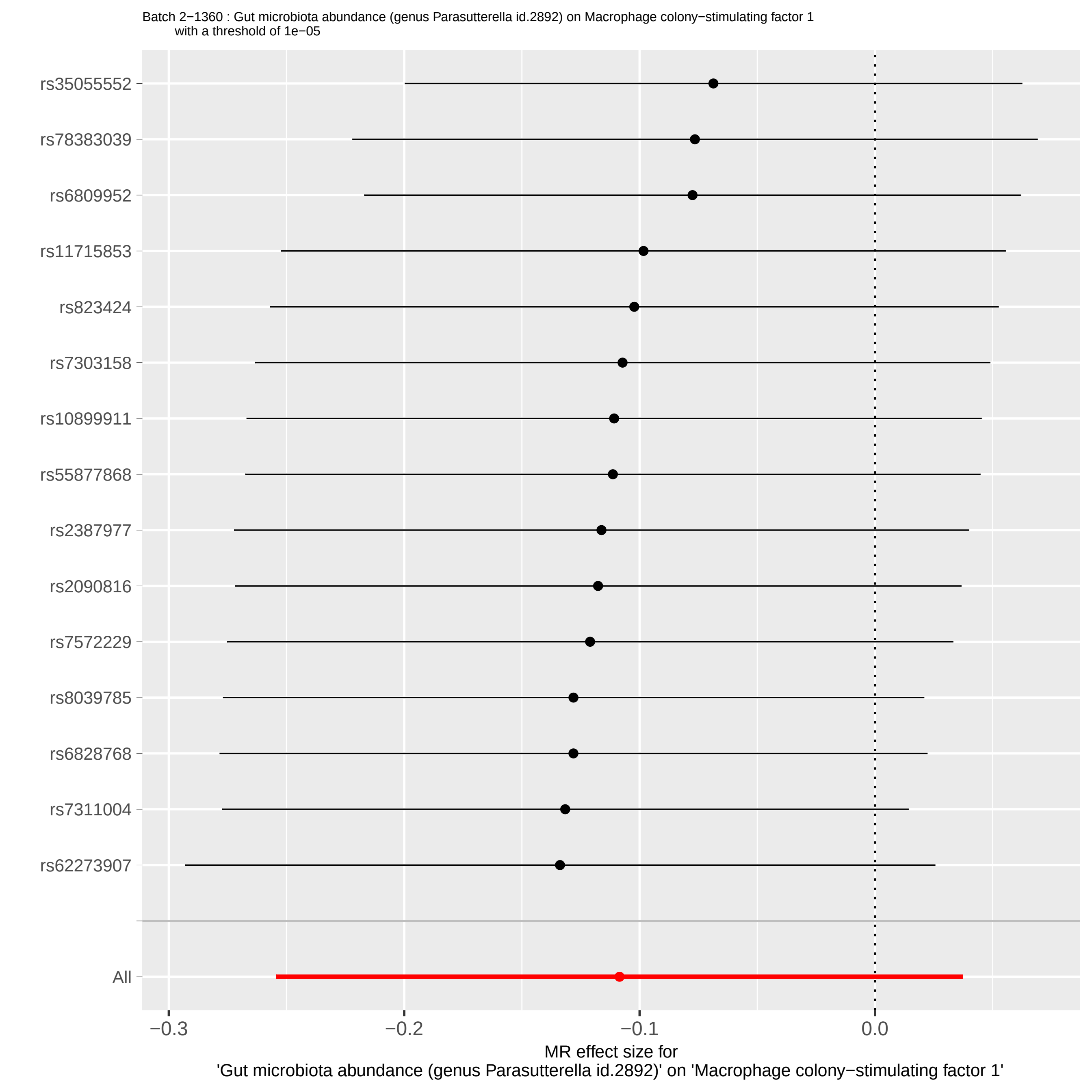
Batch 2−1360 : Gut microbiota abundance (genus Parasutterella id.2892) on Macrophage colony−stimulating factor 1
with a threshold of 1e−05
rs35055552
rs78383039
rs6809952
rs11715853
rs823424
rs7303158
rs10899911
rs55877868
rs2387977
rs2090816
rs7572229
rs8039785
rs6828768
rs7311004
rs62273907
All
−0.3
−0.2
−0.1
0.0
MR effect size for
'Gut microbiota abundance (genus Parasutterella id.2892)' on 'Macrophage colony−stimulating factor 1'

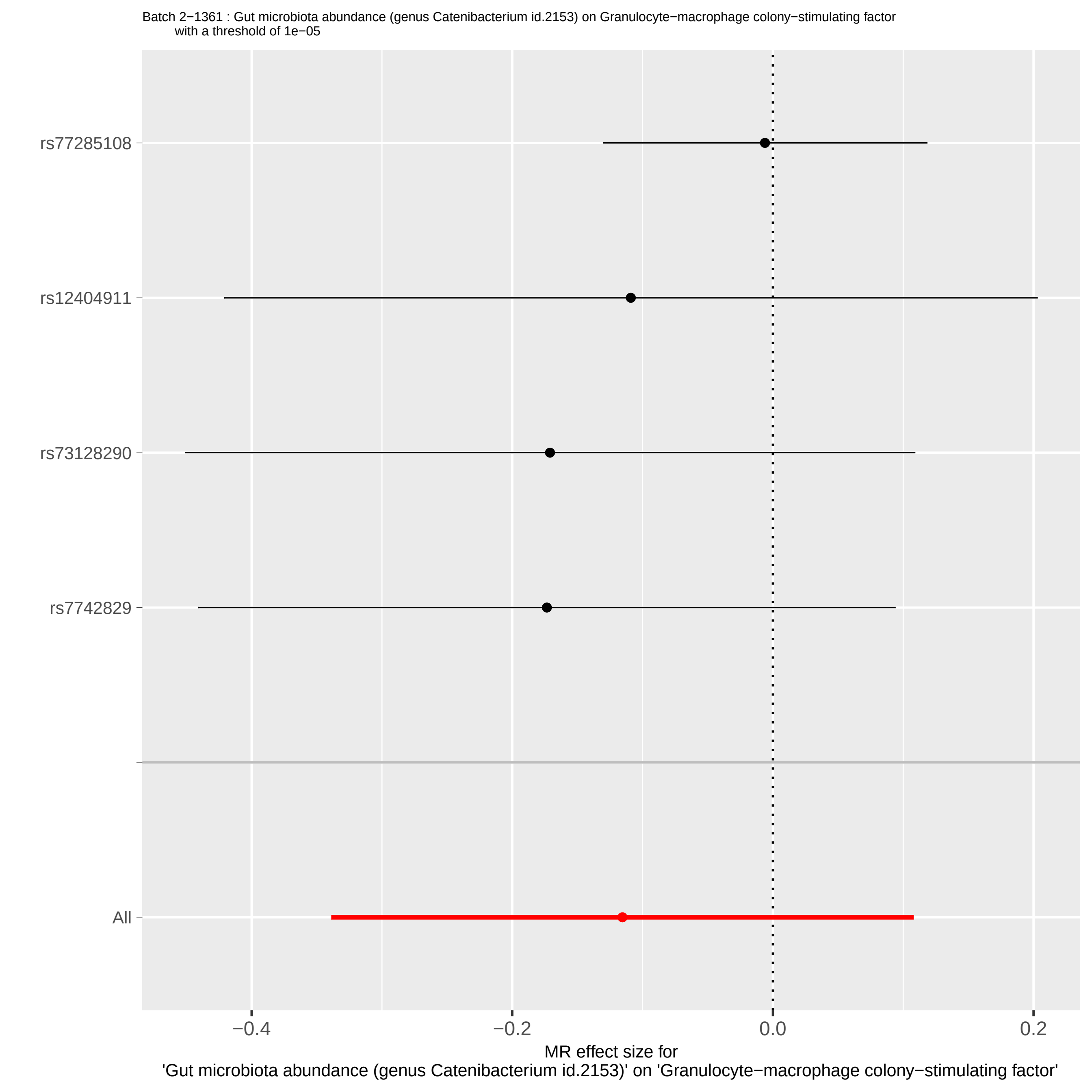
Batch 2−1361 : Gut microbiota abundance (genus Catenibacterium id.2153) on Granulocyte−macrophage colony−stimulating factor
with a threshold of 1e−05
rs77285108
rs12404911
rs73128290
rs7742829
All
−0.4
−0.2
0.0
0.2
MR effect size for
'Gut microbiota abundance (genus Catenibacterium id.2153)' on 'Granulocyte−macrophage colony−stimulating factor'

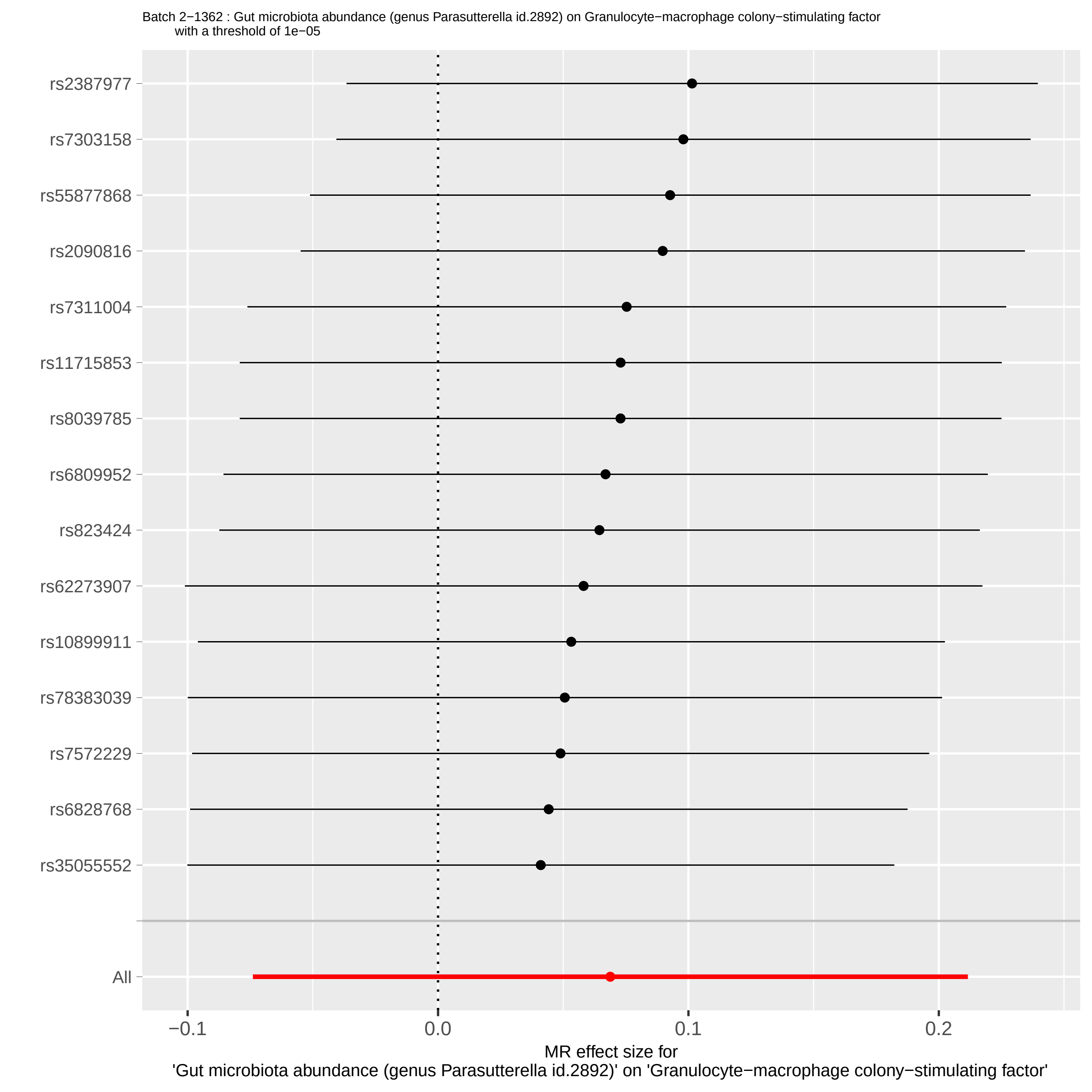

Batch 2−1362 : Gut microbiota abundance (genus Parasutterella id.2892) on Granulocyte−macrophage colony−stimulating factor
with a threshold of 1e−05
rs2387977
rs7303158
rs55877868
rs2090816
rs7311004
rs11715853
rs8039785
rs6809952
rs823424
rs62273907
rs10899911
rs78383039
rs7572229
rs6828768
rs35055552
All
−0.1
0.0
0.1
0.2
MR effect size for
'Gut microbiota abundance (genus Parasutterella id.2892)' on 'Granulocyte−macrophage colony−stimulating factor'

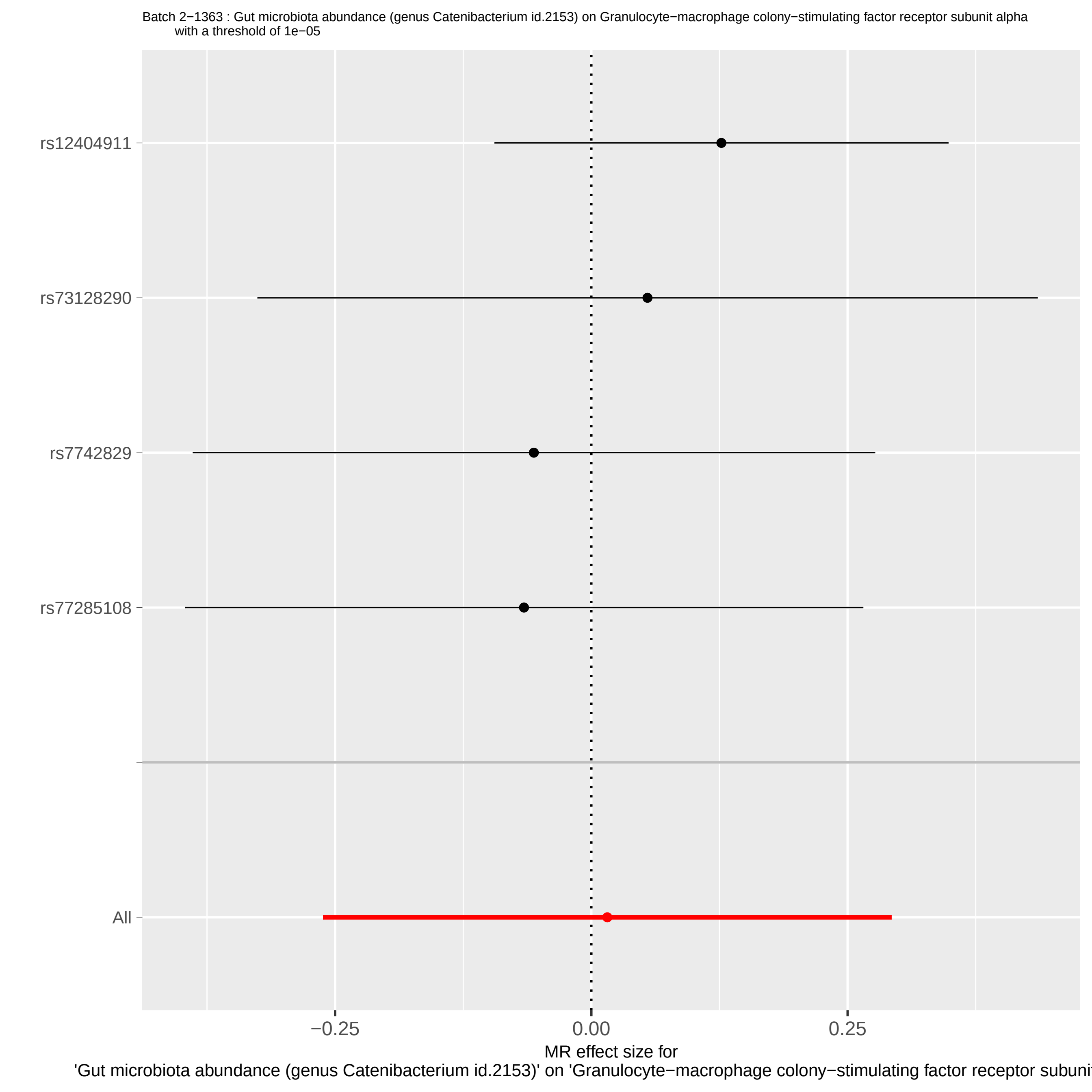
Batch 2−1363 : Gut microbiota abundance (genus Catenibacterium id.2153) on Granulocyte−macrophage colony−stimulating factor receptor subunit alpha with a threshold of 1e−05
rs12404911
rs73128290
rs7742829
rs77285108
All
−0.25
0.00
0.25
MR effect size for
'Gut microbiota abundance (genus Catenibacterium id.2153)' on 'Granulocyte−macrophage colony−stimulating factor receptor subuni

Batch 2−1364 : Gut microbiota abundance (genus Parasutterella id.2892) on Granulocyte−macrophage colony−stimulating factor receptor subunit alpha with a threshold of 1e−05

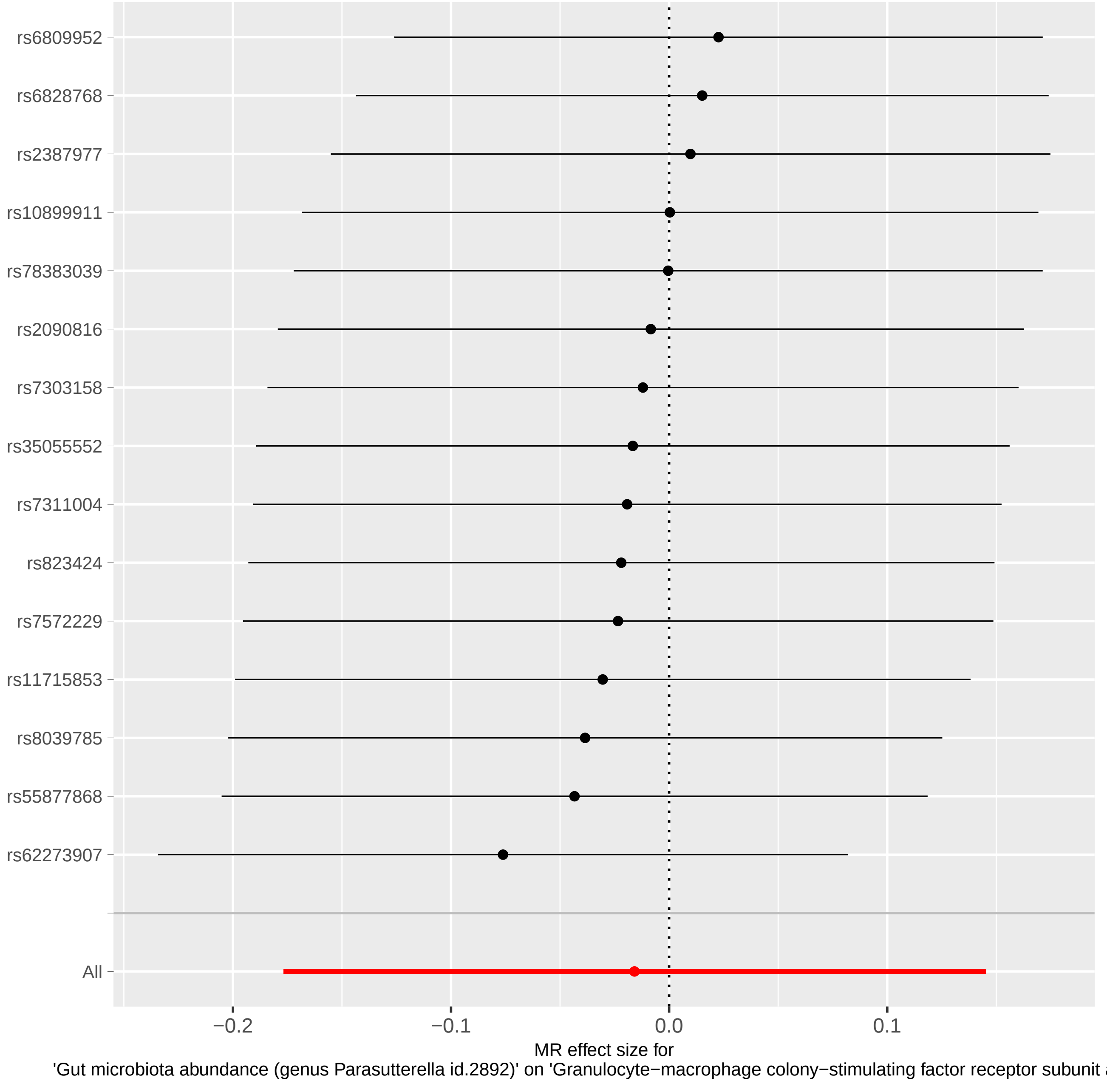

Batch 2−1365 : Gut microbiota abundance (genus Catenibacterium id.2153) on Cytokine receptor common subunit beta
with a threshold of 1e−05

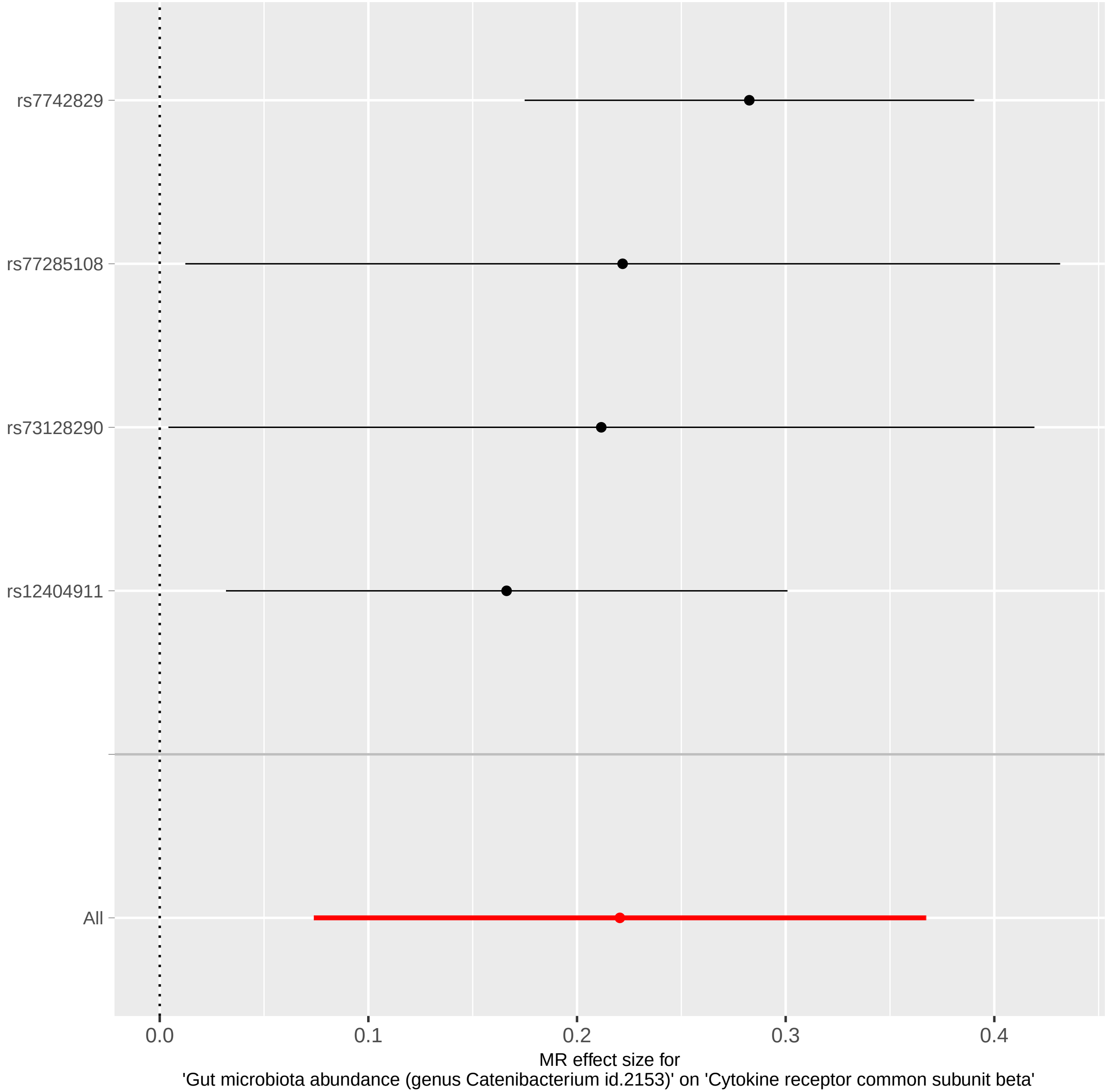

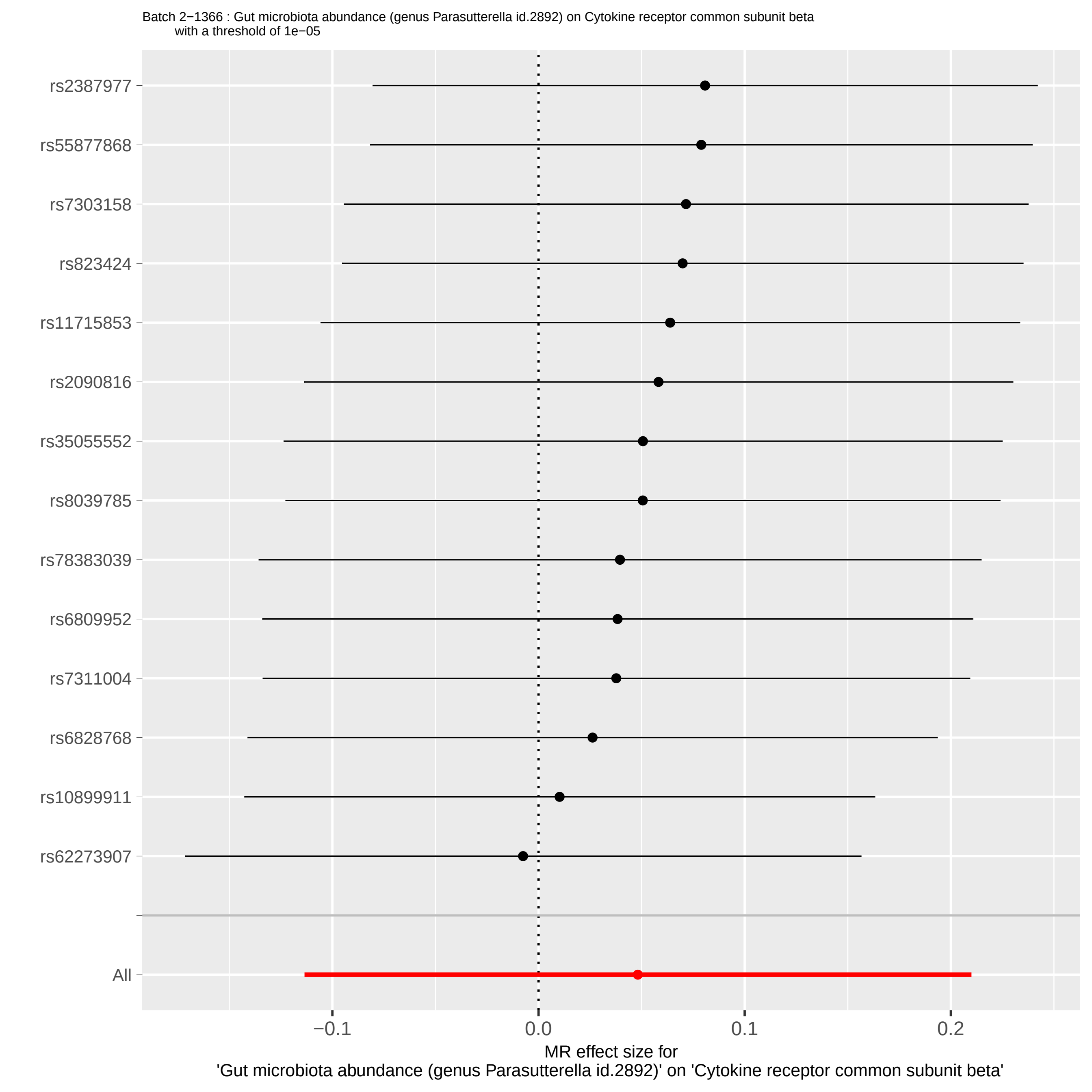

Batch 2−1366 : Gut microbiota abundance (genus Parasutterella id.2892) on Cytokine receptor common subunit beta
with a threshold of 1e−05
rs2387977
rs55877868
rs7303158
rs823424
rs11715853
rs2090816
rs35055552
rs8039785
rs78383039
rs6809952
rs7311004
rs6828768
rs10899911
rs62273907
All
−0.1
0.0
0.1
0.2
MR effect size for
'Gut microbiota abundance (genus Parasutterella id.2892)' on 'Cytokine receptor common subunit beta'

Batch 2−1367 : Gut microbiota abundance (genus Catenibacterium id.2153) on Cytokine receptor common subunit beta
with a threshold of 1e−05

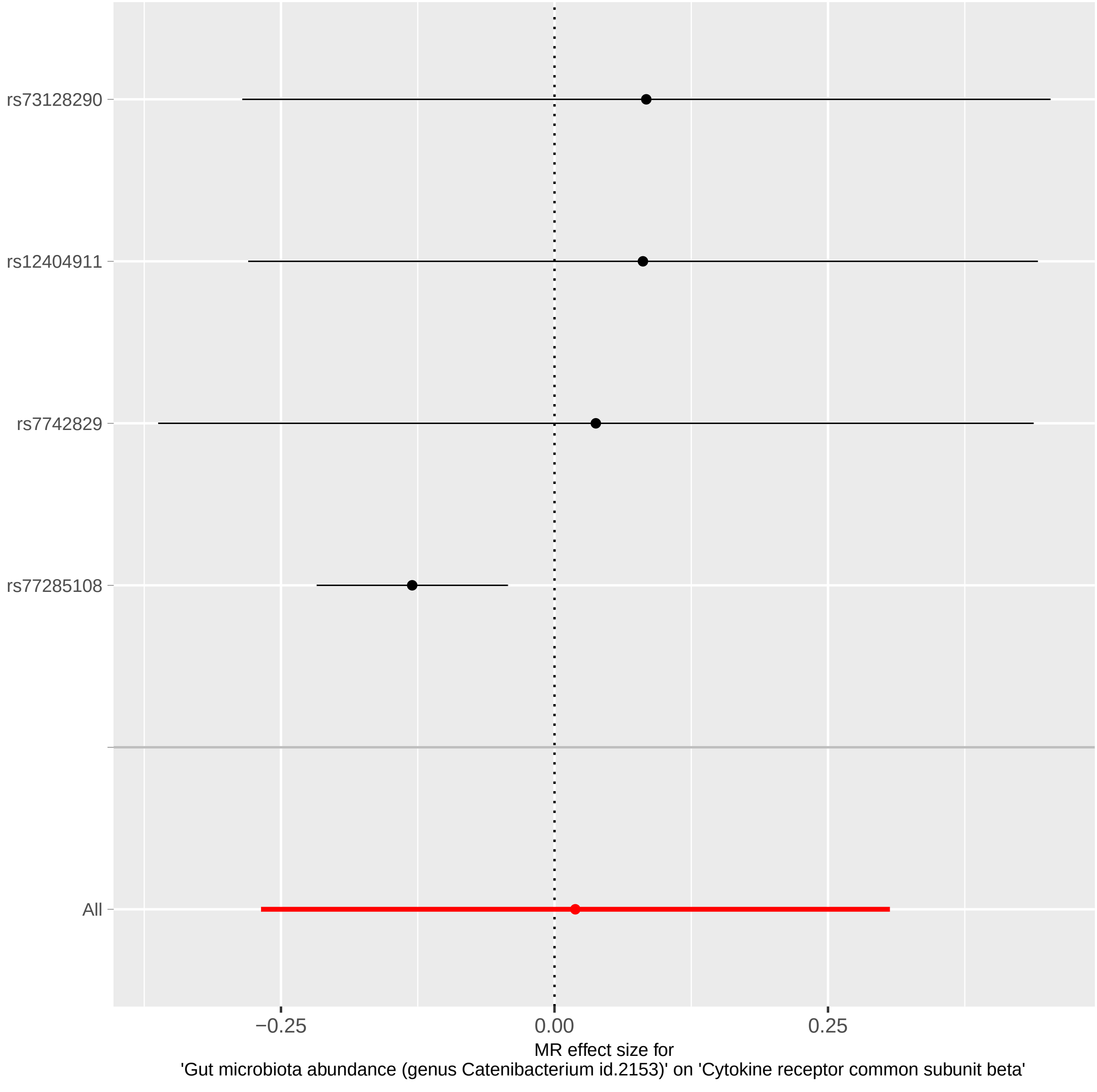

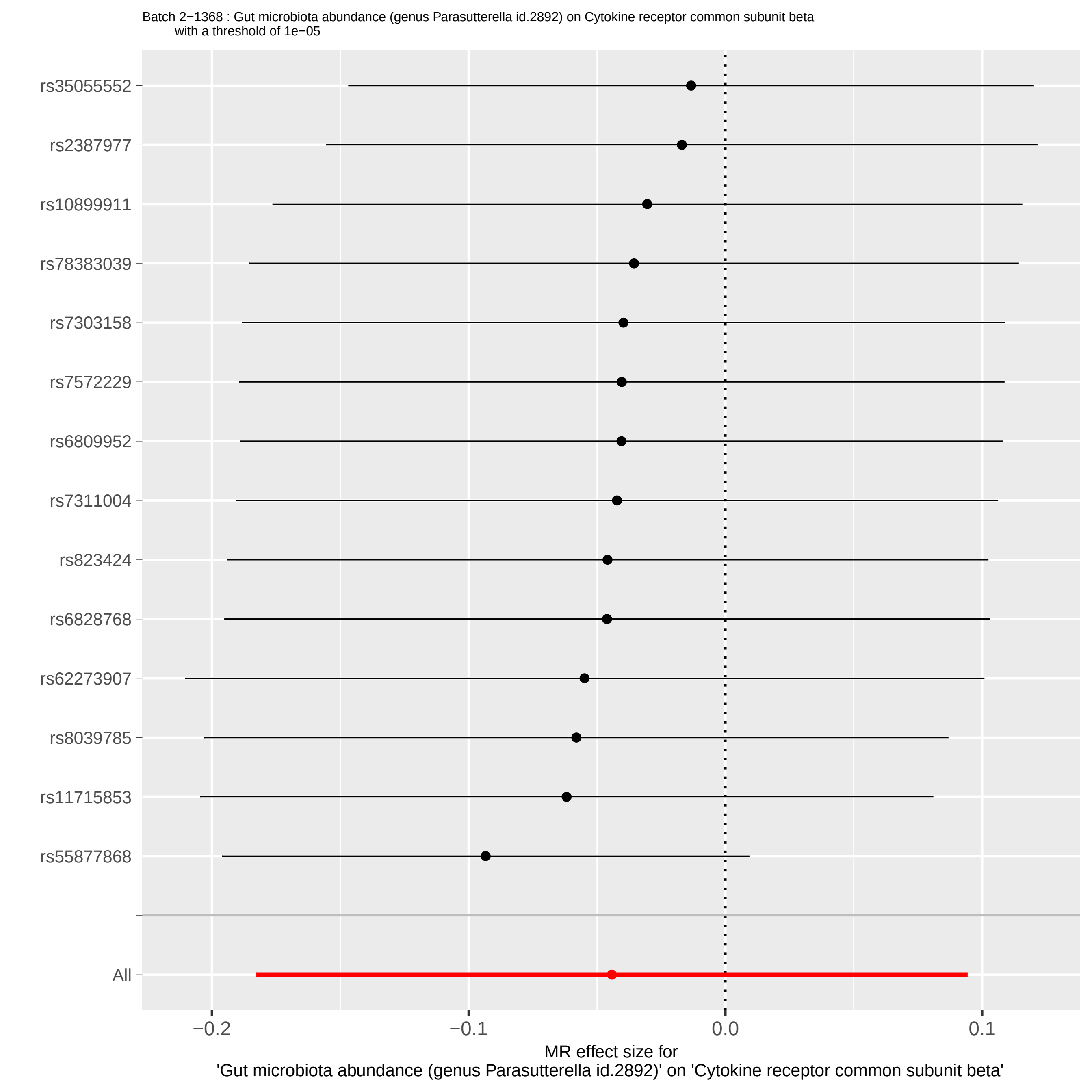
Batch 2−1368 : Gut microbiota abundance (genus Parasutterella id.2892) on Cytokine receptor common subunit beta
with a threshold of 1e−05
rs35055552
rs2387977
rs10899911
rs78383039
rs7303158
rs7572229
rs6809952
rs7311004
rs823424
rs6828768
rs62273907
rs8039785
rs11715853
rs55877868
All
−0.2
−0.1
0.0
0.1
MR effect size for
'Gut microbiota abundance (genus Parasutterella id.2892)' on 'Cytokine receptor common subunit beta'

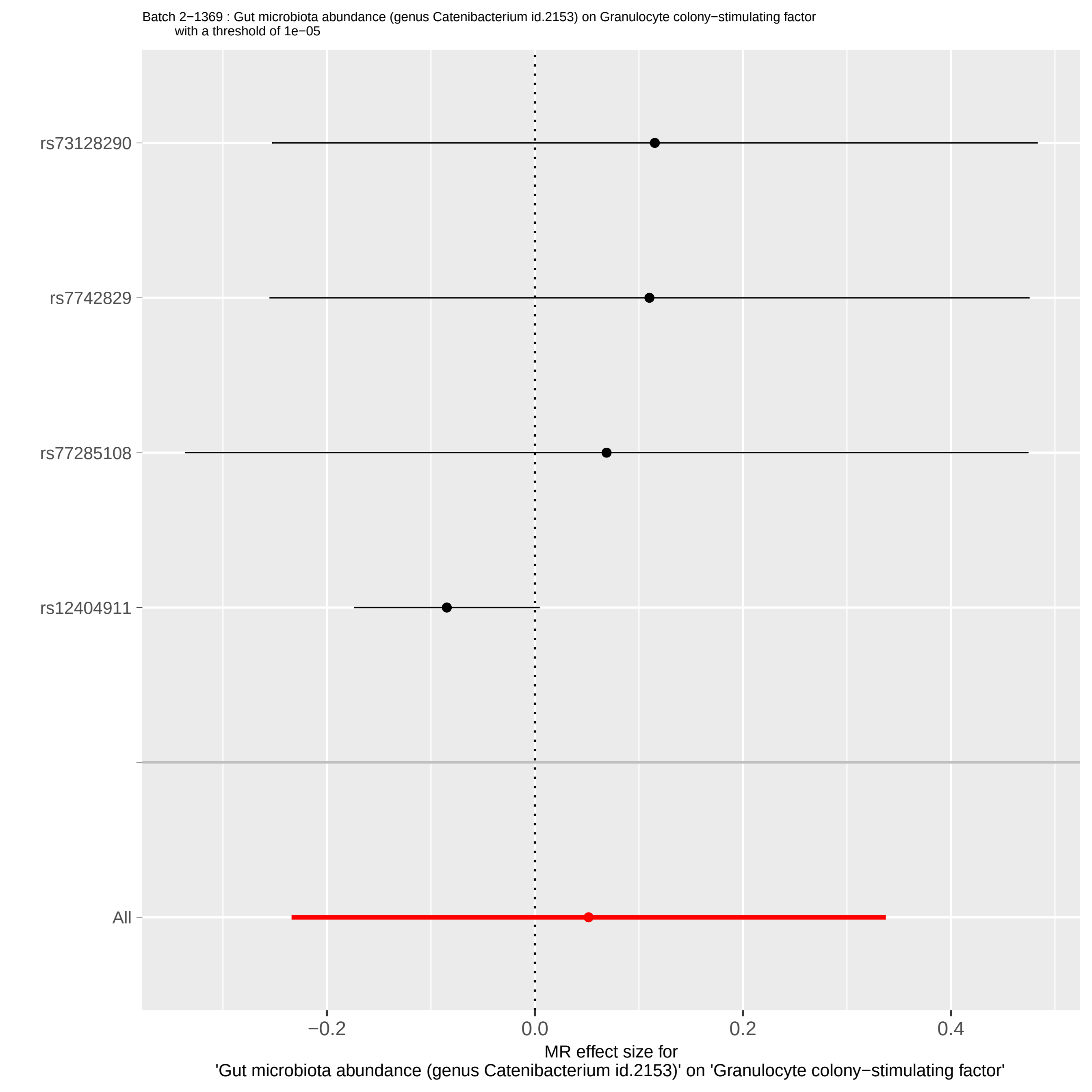
Batch 2−1369 : Gut microbiota abundance (genus Catenibacterium id.2153) on Granulocyte colony−stimulating factor
with a threshold of 1e−05
rs73128290
rs7742829
rs77285108
rs12404911
All
−0.2
0.0
0.2
0.4
MR effect size for
'Gut microbiota abundance (genus Catenibacterium id.2153)' on 'Granulocyte colony−stimulating factor'

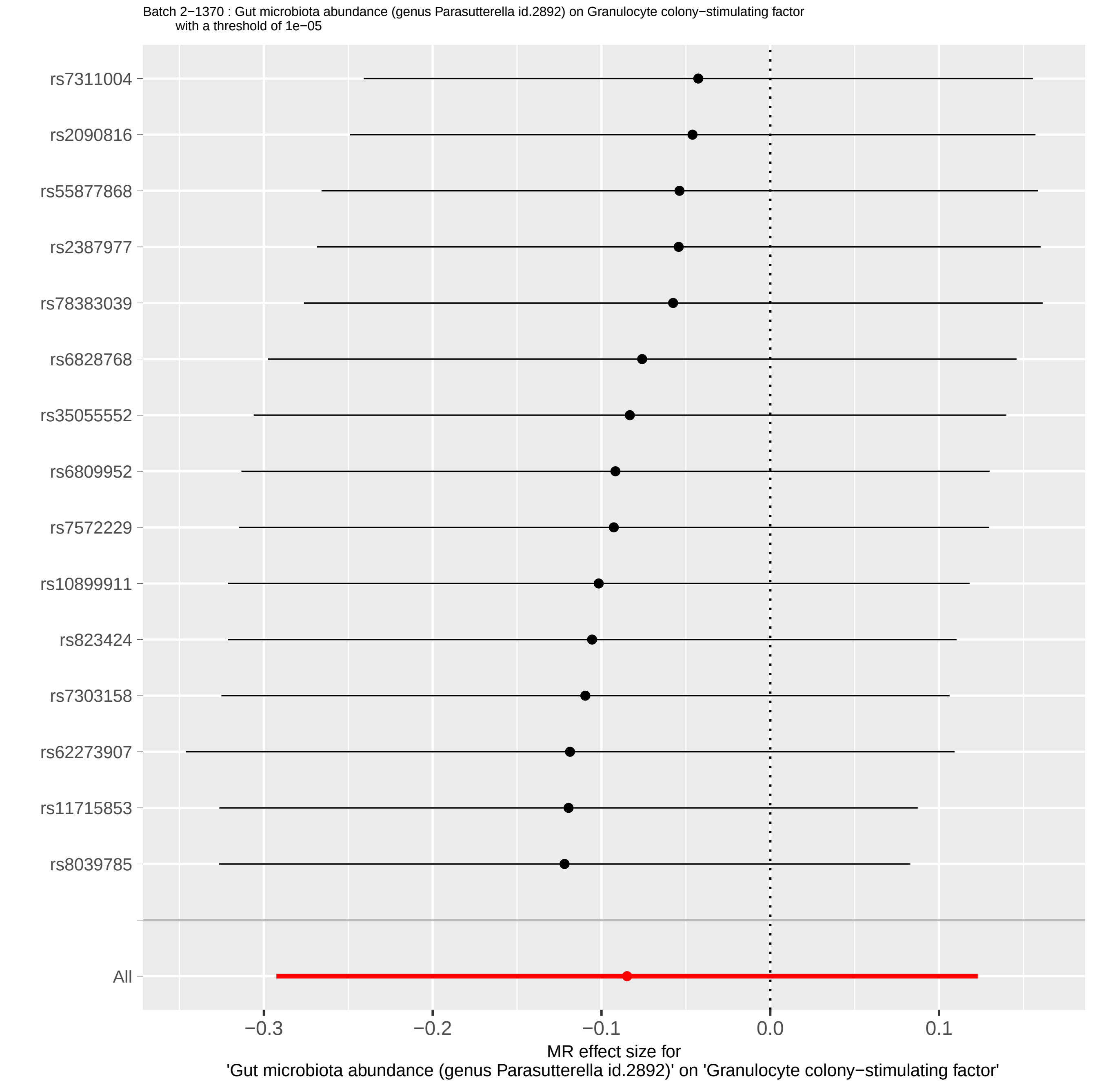

Batch 2−1370 : Gut microbiota abundance (genus Parasutterella id.2892) on Granulocyte colony−stimulating factor
with a threshold of 1e−05
rs7311004
rs2090816
rs55877868
rs2387977
rs78383039
rs6828768
rs35055552
rs6809952
rs7572229
rs10899911
rs823424
rs7303158
rs62273907
rs11715853
rs8039785
All
−0.3
−0.2
−0.1
0.0
0.1
MR effect size for
'Gut microbiota abundance (genus Parasutterella id.2892)' on 'Granulocyte colony−stimulating factor'

Batch 2−1371 : Gut microbiota abundance (genus Catenibacterium id.2153) on Granulocyte colony−stimulating factor receptor
with a threshold of 1e−05

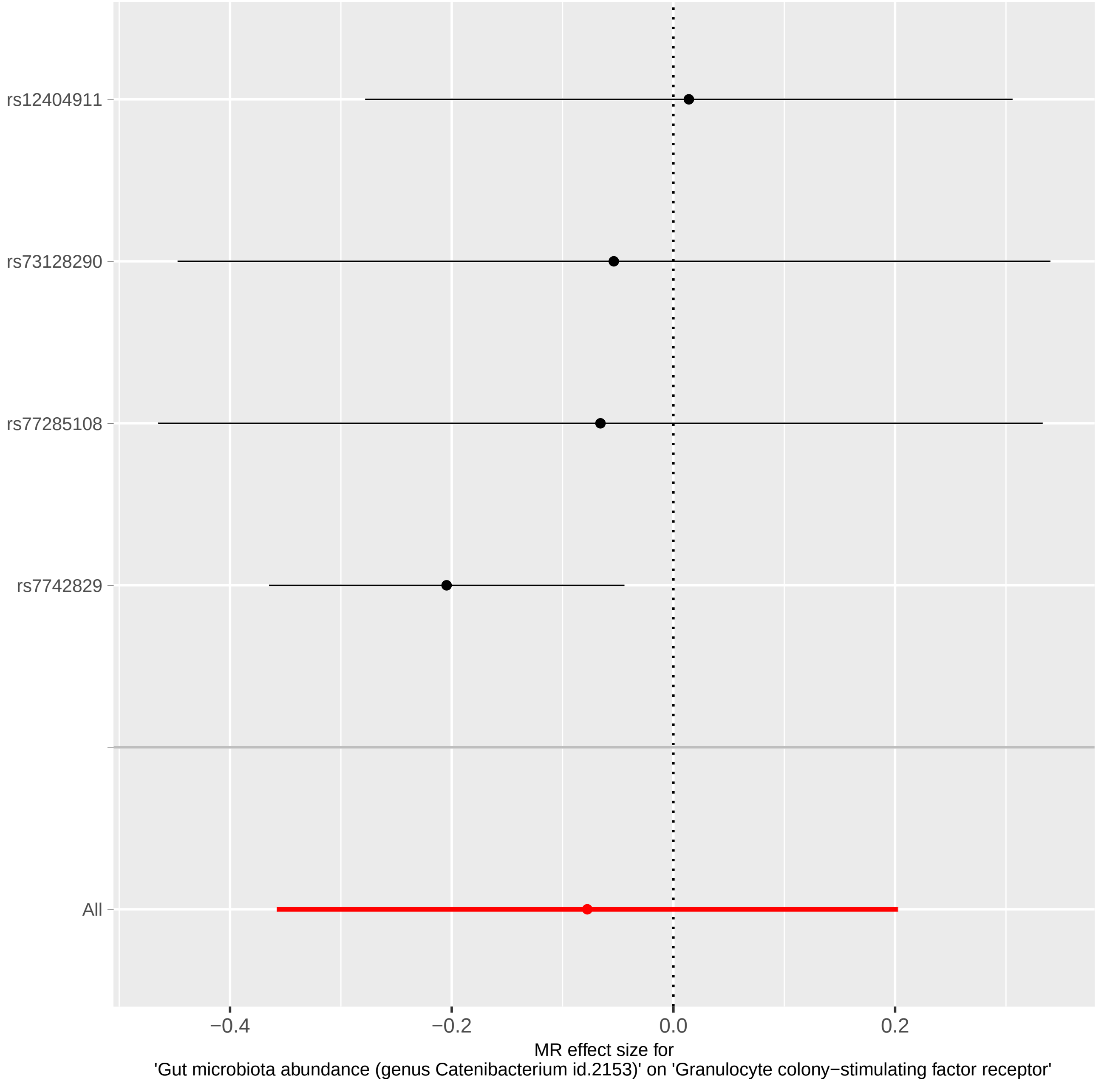

Batch 2−1372 : Gut microbiota abundance (genus Parasutterella id.2892) on Granulocyte colony−stimulating factor receptor
with a threshold of 1e−05

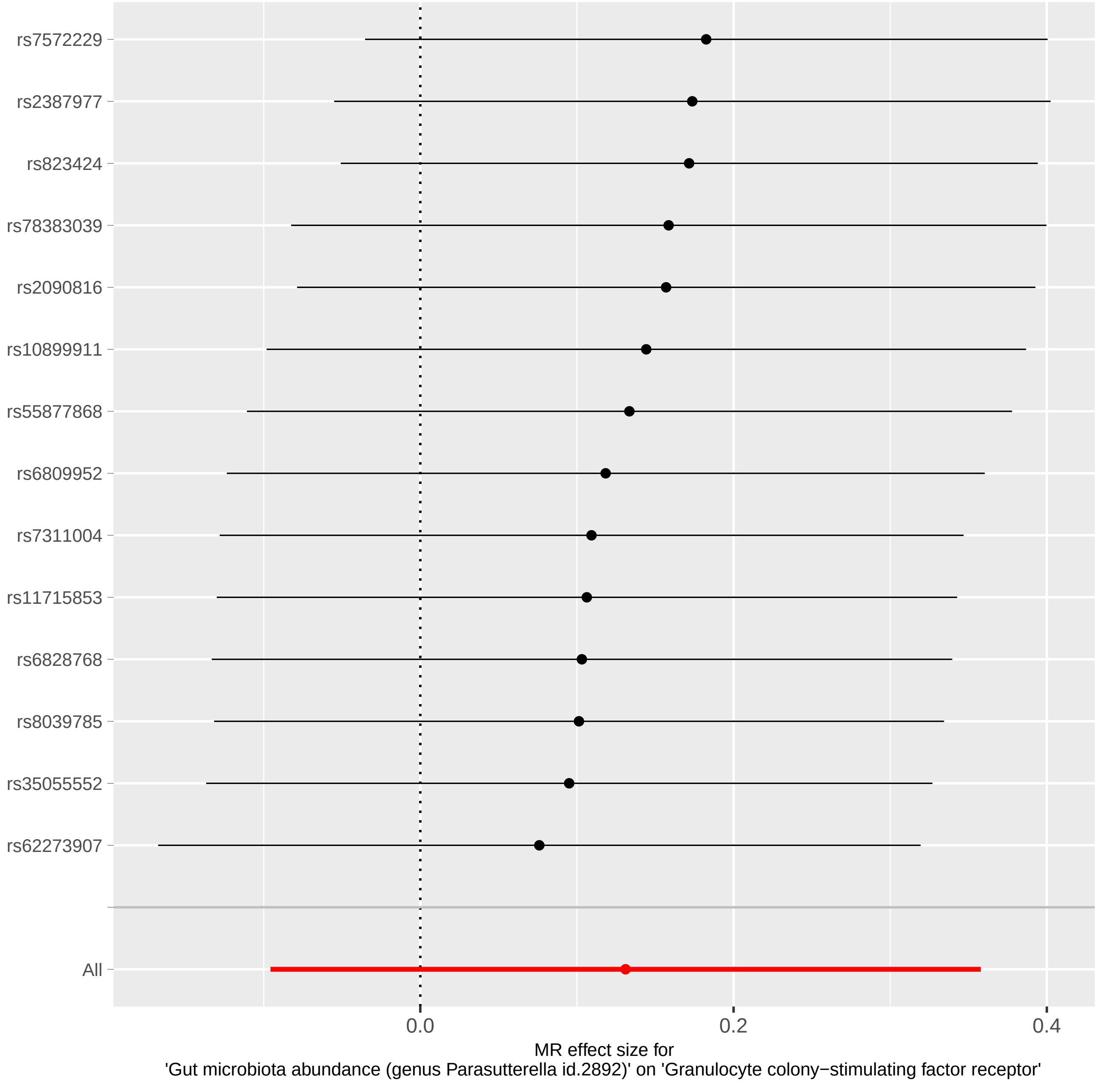

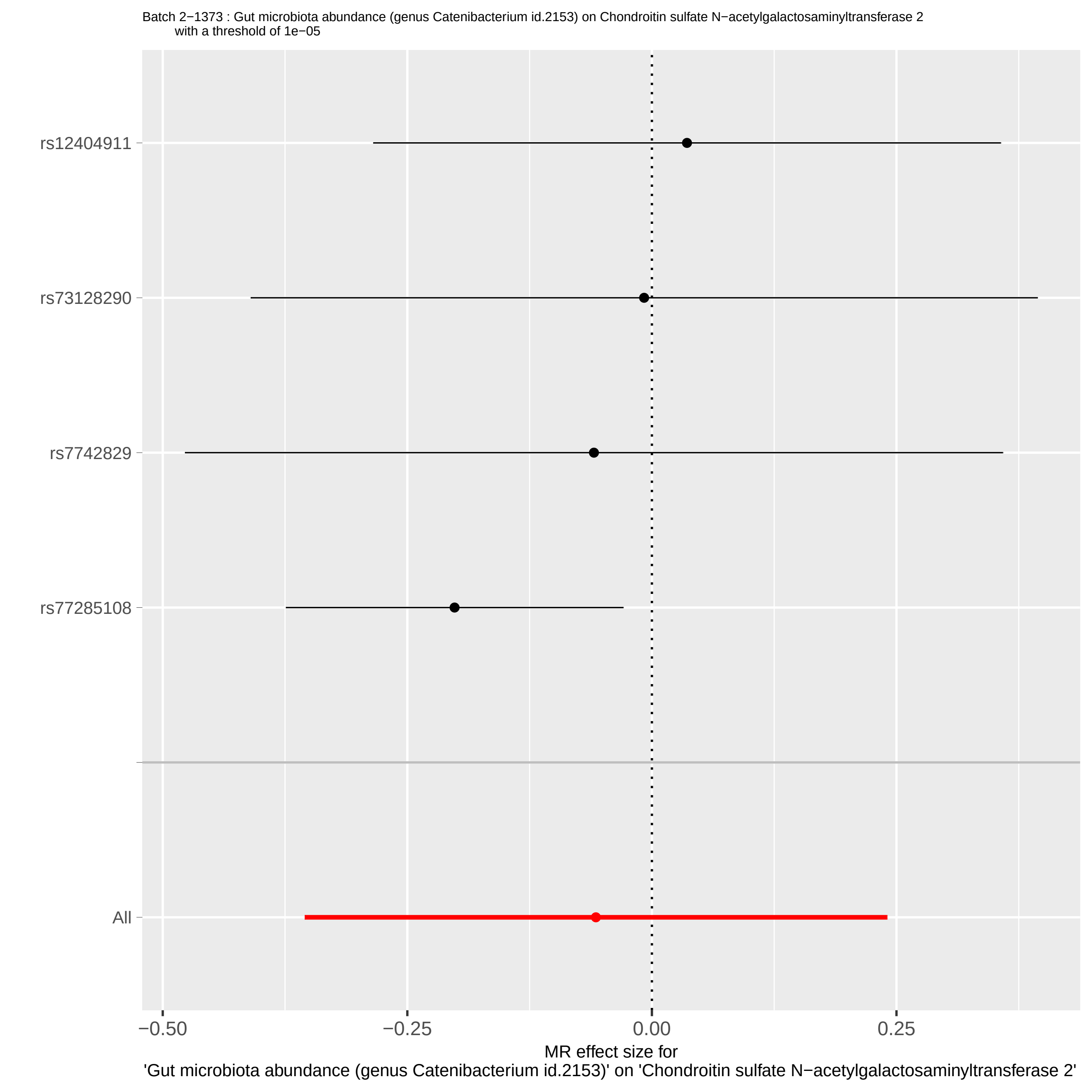

Batch 2−1373 : Gut microbiota abundance (genus Catenibacterium id.2153) on Chondroitin sulfate N−acetylgalactosaminyltransferase 2
with a threshold of 1e−05
rs12404911
rs73128290
rs7742829
rs77285108
All
−0.50
−0.25
0.00
0.25
MR effect size for
'Gut microbiota abundance (genus Catenibacterium id.2153)' on 'Chondroitin sulfate N−acetylgalactosaminyltransferase 2'

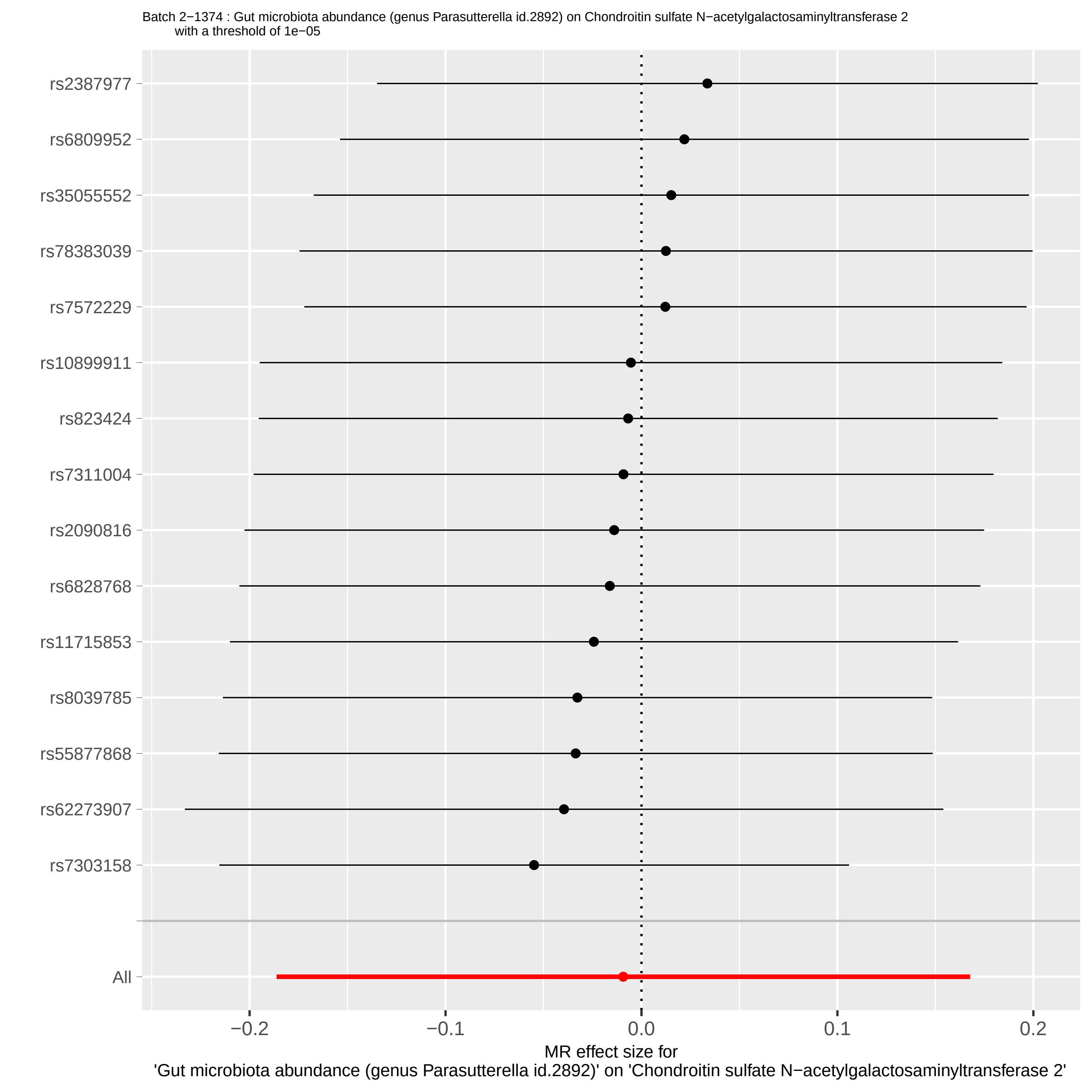

Batch 2−1374 : Gut microbiota abundance (genus Parasutterella id.2892) on Chondroitin sulfate N−acetylgalactosaminyltransferase 2
with a threshold of 1e−05
rs2387977
rs6809952
rs35055552
rs78383039
rs7572229
rs10899911
rs823424
rs7311004
rs2090816
rs6828768
rs11715853
rs8039785
rs55877868
rs62273907
rs7303158
All
−0.2
−0.1
0.0
0.1
0.2
MR effect size for
'Gut microbiota abundance (genus Parasutterella id.2892)' on 'Chondroitin sulfate N−acetylgalactosaminyltransferase 2'

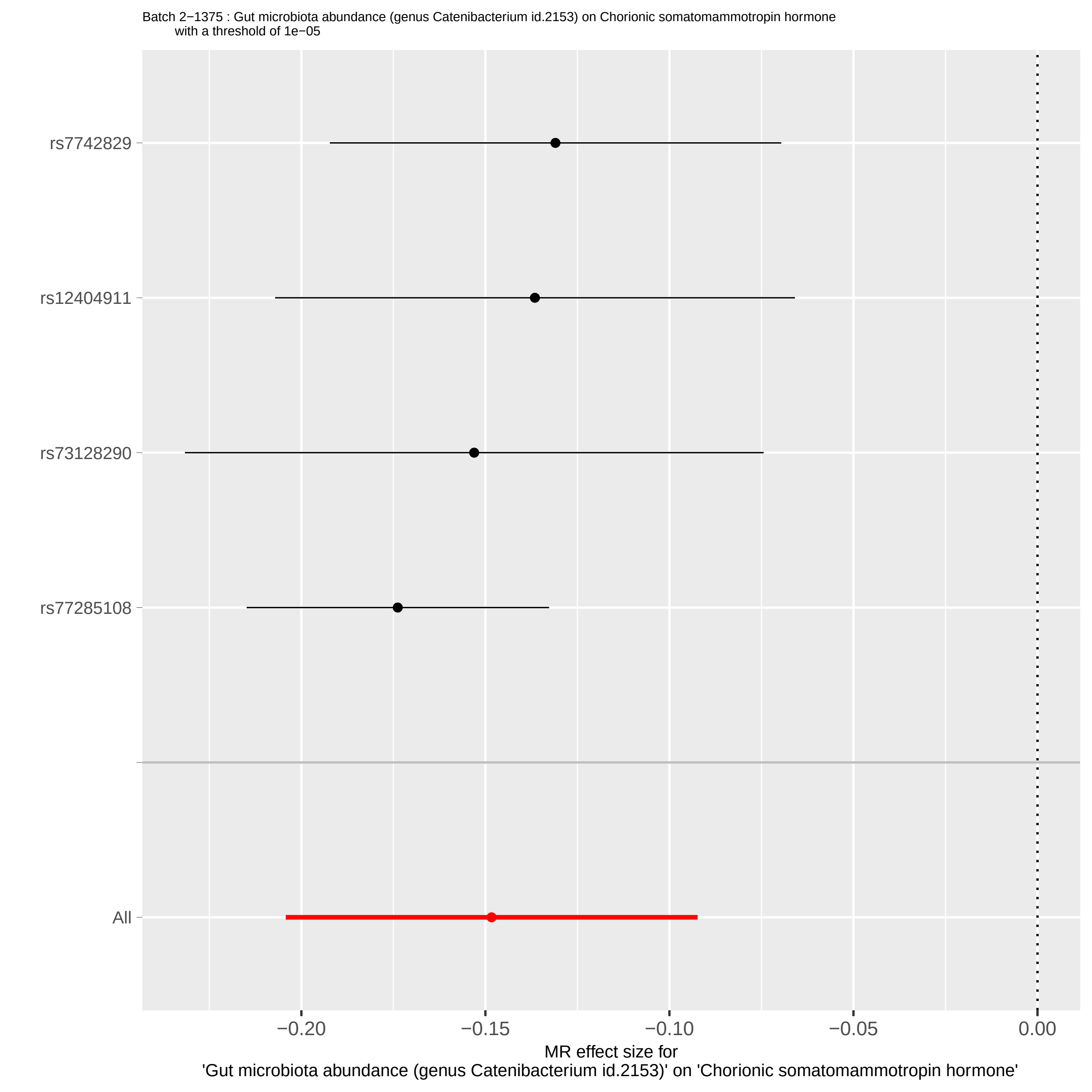
Batch 2−1375 : Gut microbiota abundance (genus Catenibacterium id.2153) on Chorionic somatomammotropin hormone
with a threshold of 1e−05
rs7742829
rs12404911
rs73128290
rs77285108
All
−0.20
−0.15
−0.10
−0.05
0.00
MR effect size for
'Gut microbiota abundance (genus Catenibacterium id.2153)' on 'Chorionic somatomammotropin hormone'

Batch 2−1376 : Gut microbiota abundance (genus Parasutterella id.2892) on Chorionic somatomammotropin hormone
with a threshold of 1e−05

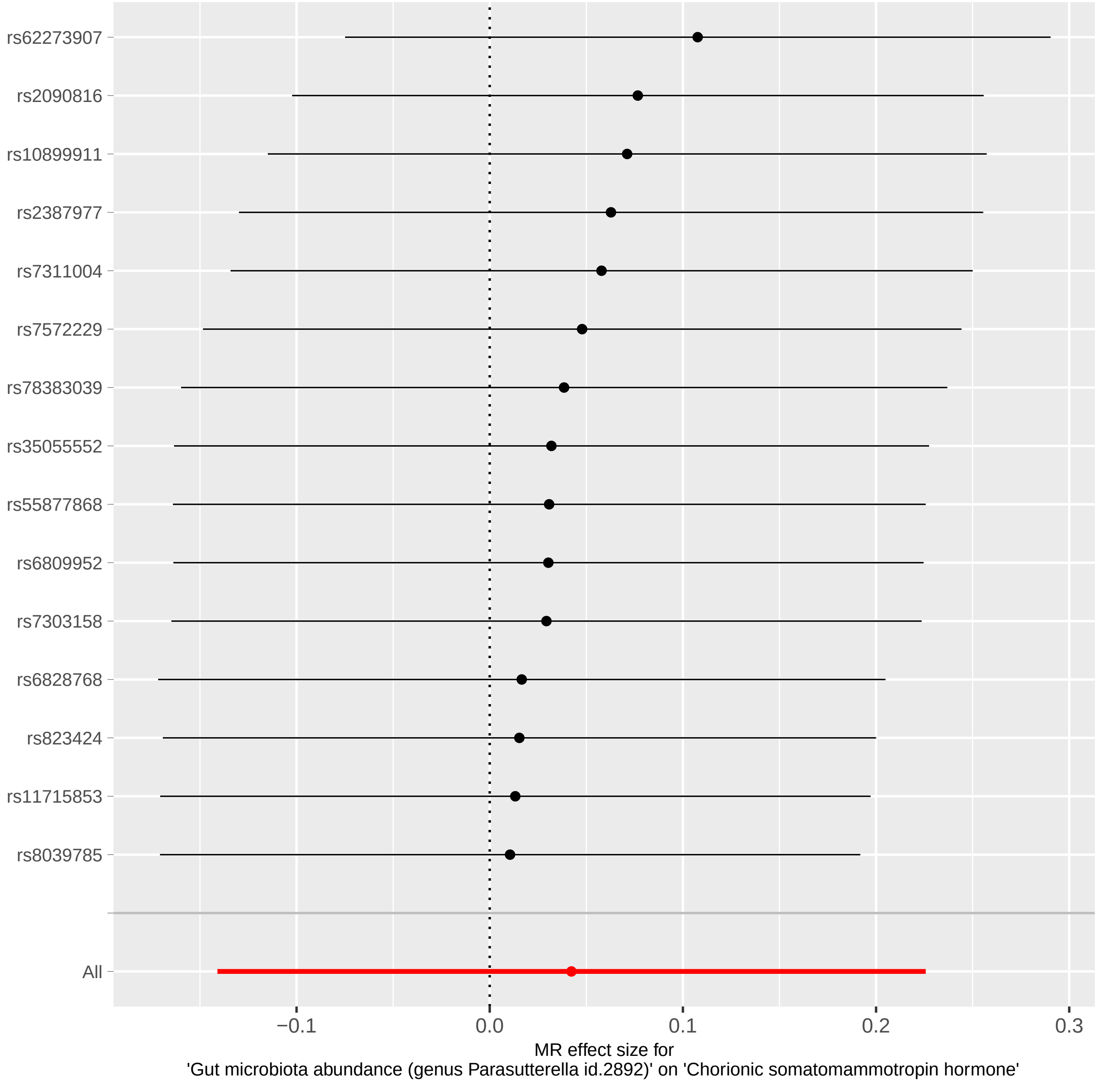

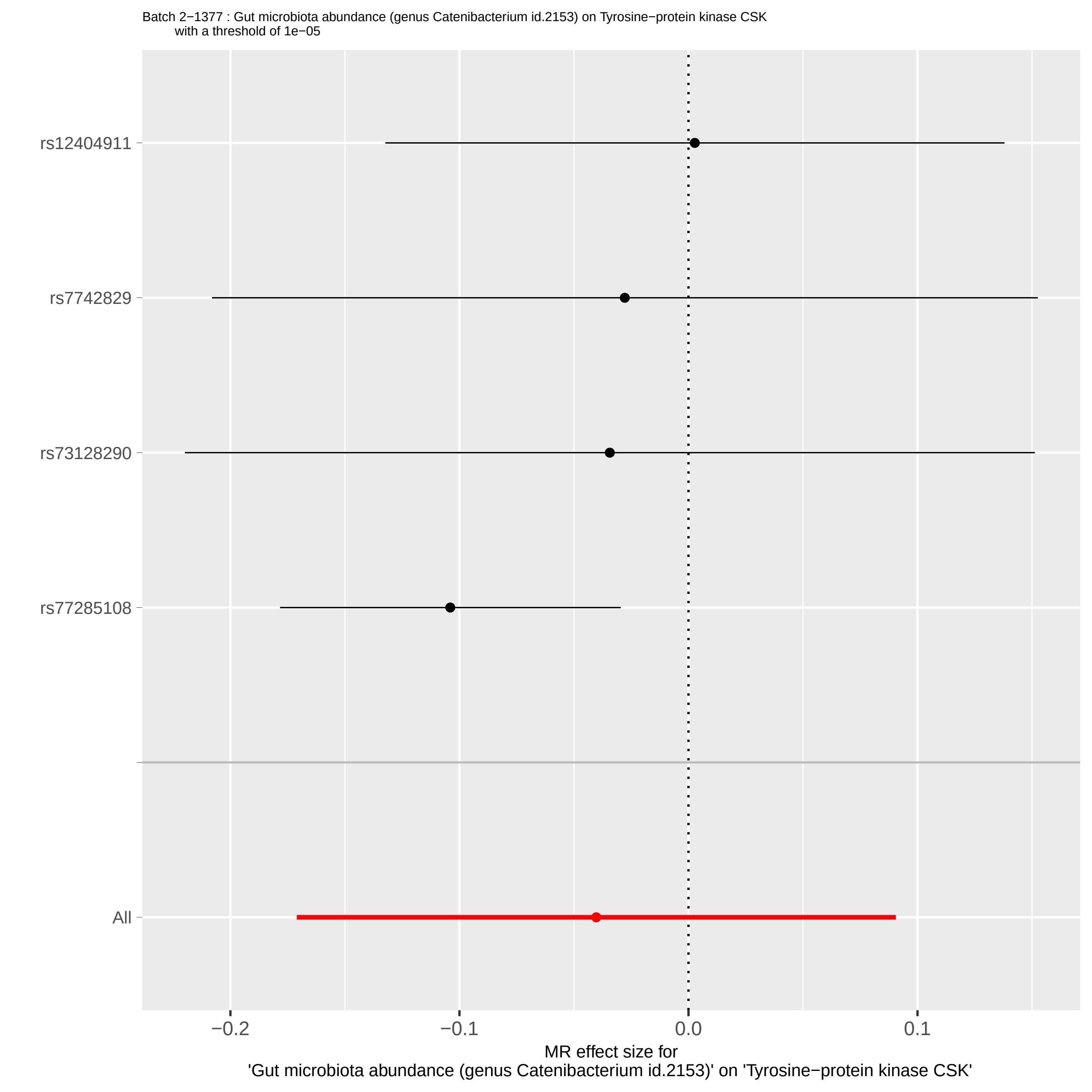

Batch 2−1377 : Gut microbiota abundance (genus Catenibacterium id.2153) on Tyrosine−protein kinase CSK
with a threshold of 1e−05
rs12404911
rs7742829
rs73128290
rs77285108
All
−0.2
−0.1
0.0
0.1
MR effect size for
'Gut microbiota abundance (genus Catenibacterium id.2153)' on 'Tyrosine−protein kinase CSK'

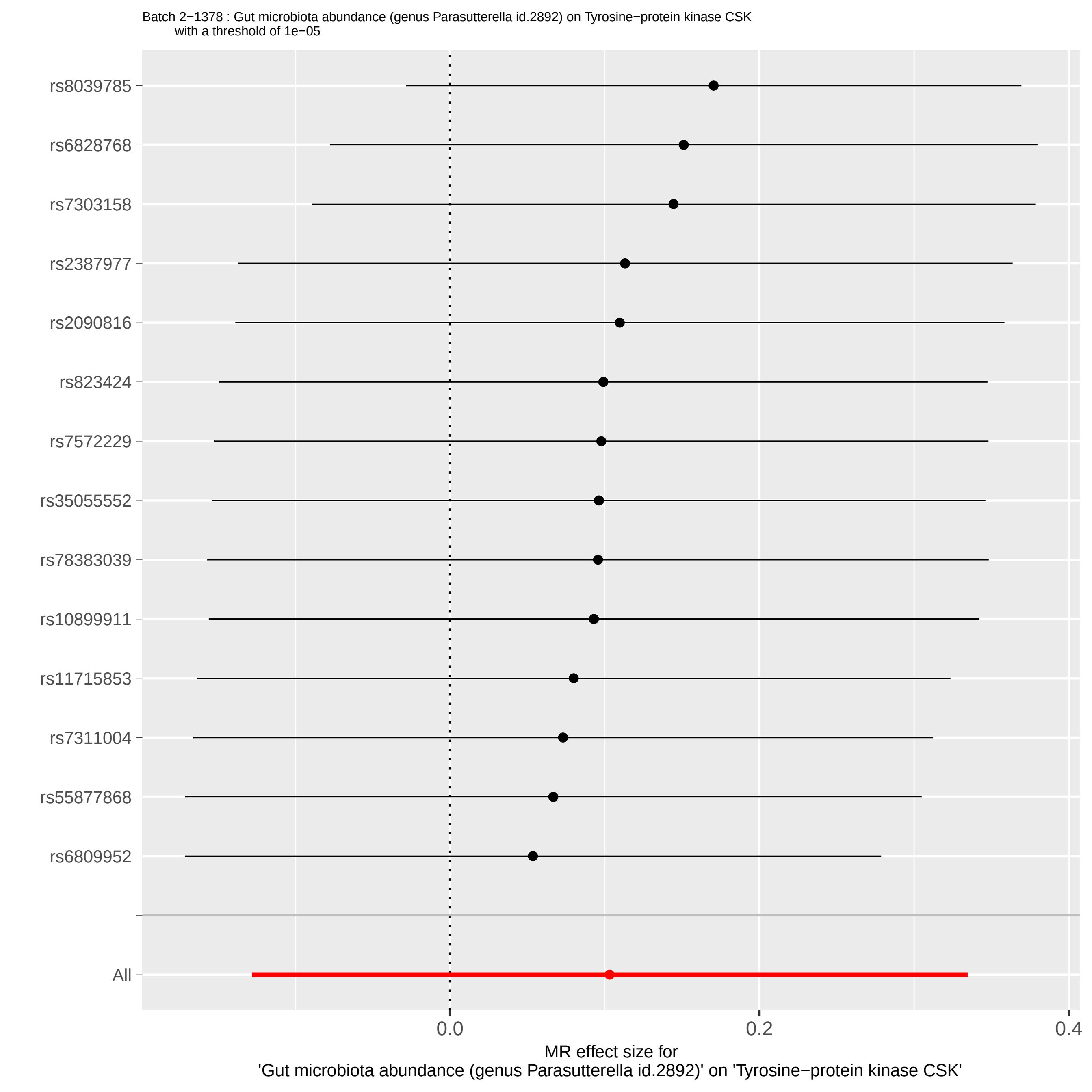
Batch 2−1378 : Gut microbiota abundance (genus Parasutterella id.2892) on Tyrosine−protein kinase CSK
with a threshold of 1e−05
rs8039785
rs6828768
rs7303158
rs2387977
rs2090816
rs823424
rs7572229
rs35055552
rs78383039
rs10899911
rs11715853
rs7311004
rs55877868
rs6809952
All
0.0
0.2
0.4
MR effect size for
'Gut microbiota abundance (genus Parasutterella id.2892)' on 'Tyrosine−protein kinase CSK'

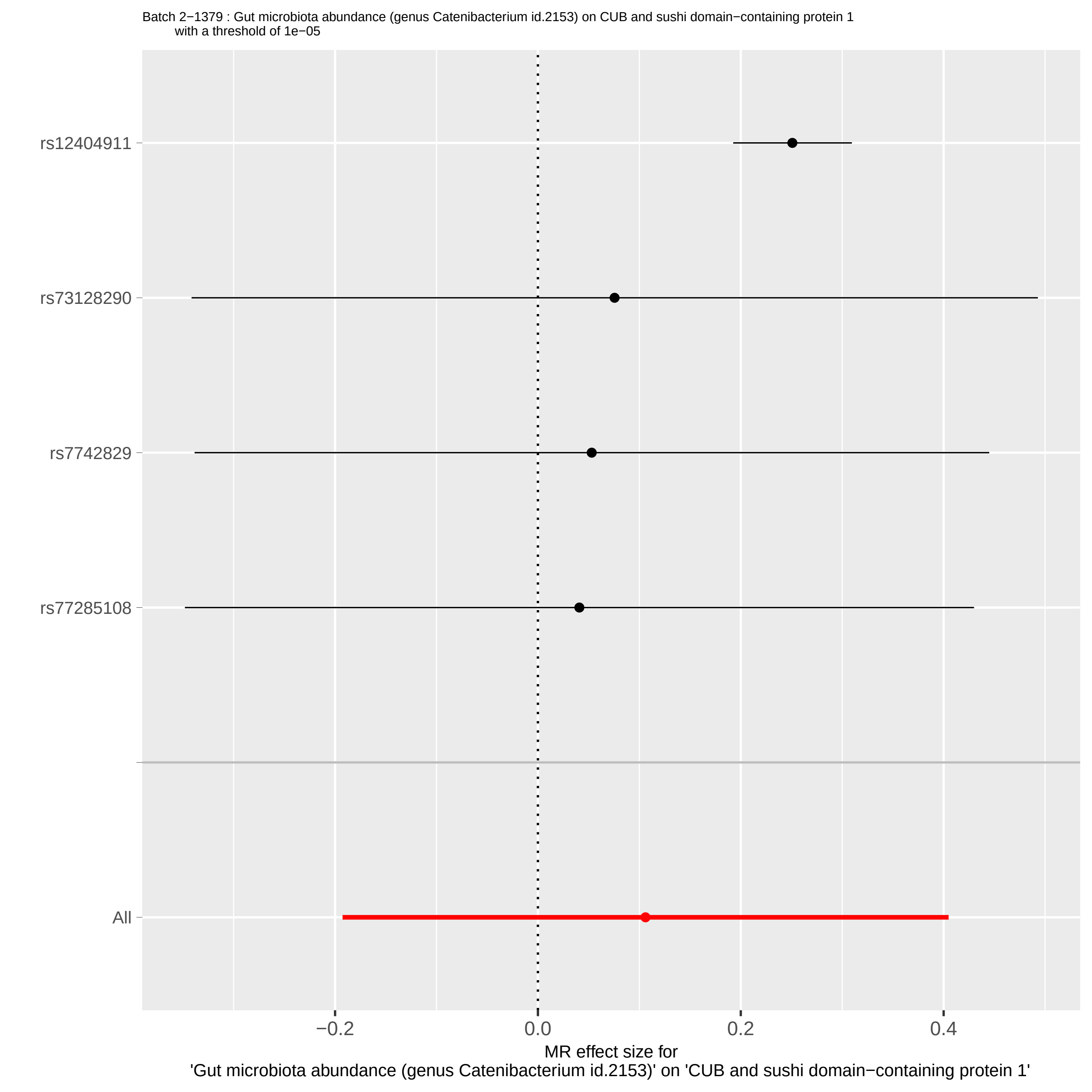
Batch 2−1379 : Gut microbiota abundance (genus Catenibacterium id.2153) on CUB and sushi domain−containing protein 1
with a threshold of 1e−05
rs12404911
rs73128290
rs7742829
rs77285108
All
−0.2
0.0
0.2
0.4
MR effect size for
'Gut microbiota abundance (genus Catenibacterium id.2153)' on 'CUB and sushi domain−containing protein 1'

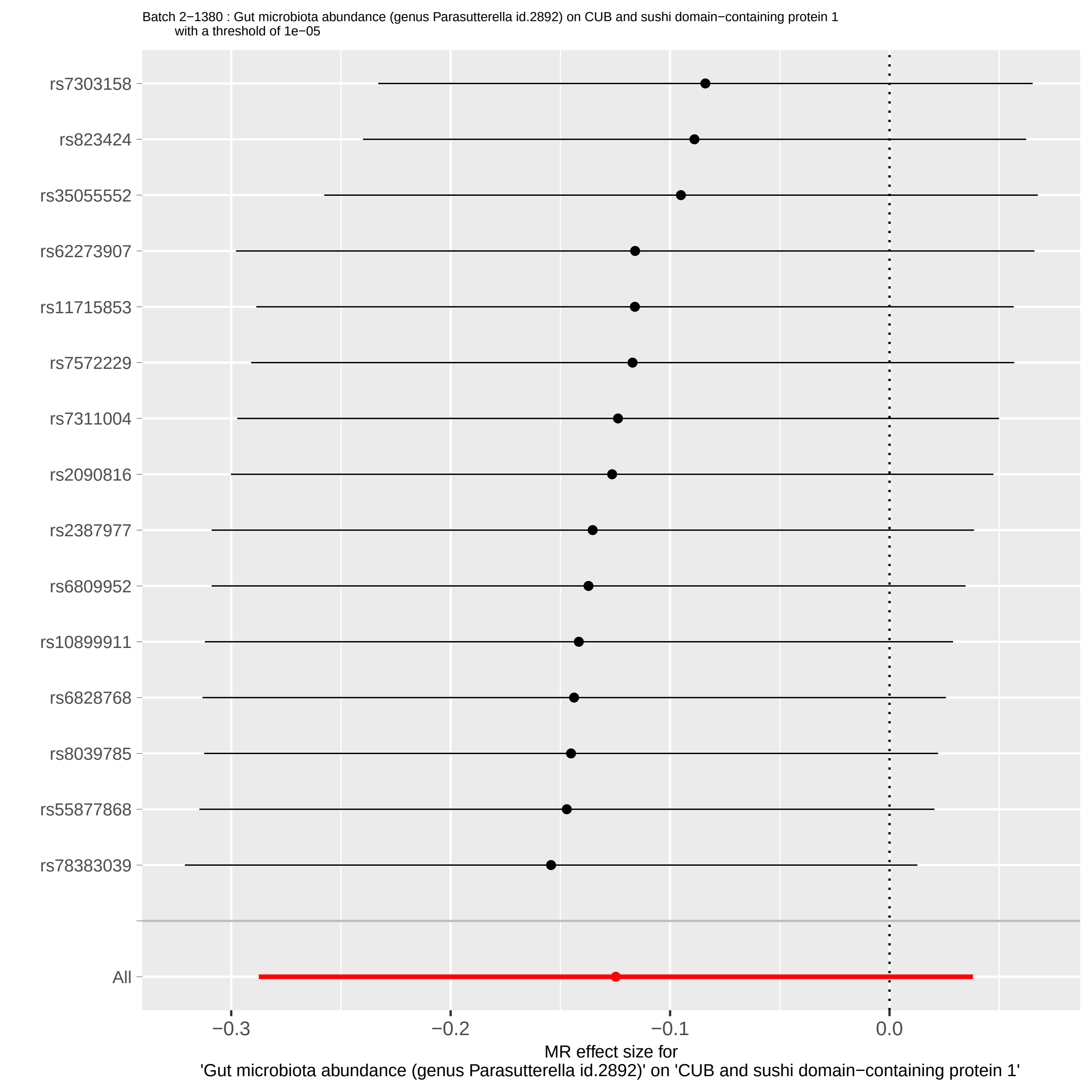

Batch 2−1380 : Gut microbiota abundance (genus Parasutterella id.2892) on CUB and sushi domain−containing protein 1
with a threshold of 1e−05
rs7303158
rs823424
rs35055552
rs62273907
rs11715853
rs7572229
rs7311004
rs2090816
rs2387977
rs6809952
rs10899911
rs6828768
rs8039785
rs55877868
rs78383039
All
−0.3
−0.2
−0.1
0.0
MR effect size for
'Gut microbiota abundance (genus Parasutterella id.2892)' on 'CUB and sushi domain−containing protein 1'

Batch 2−1381 : Gut microbiota abundance (genus Catenibacterium id.2153) on CUB and sushi domain−containing protein 2
with a threshold of 1e−05

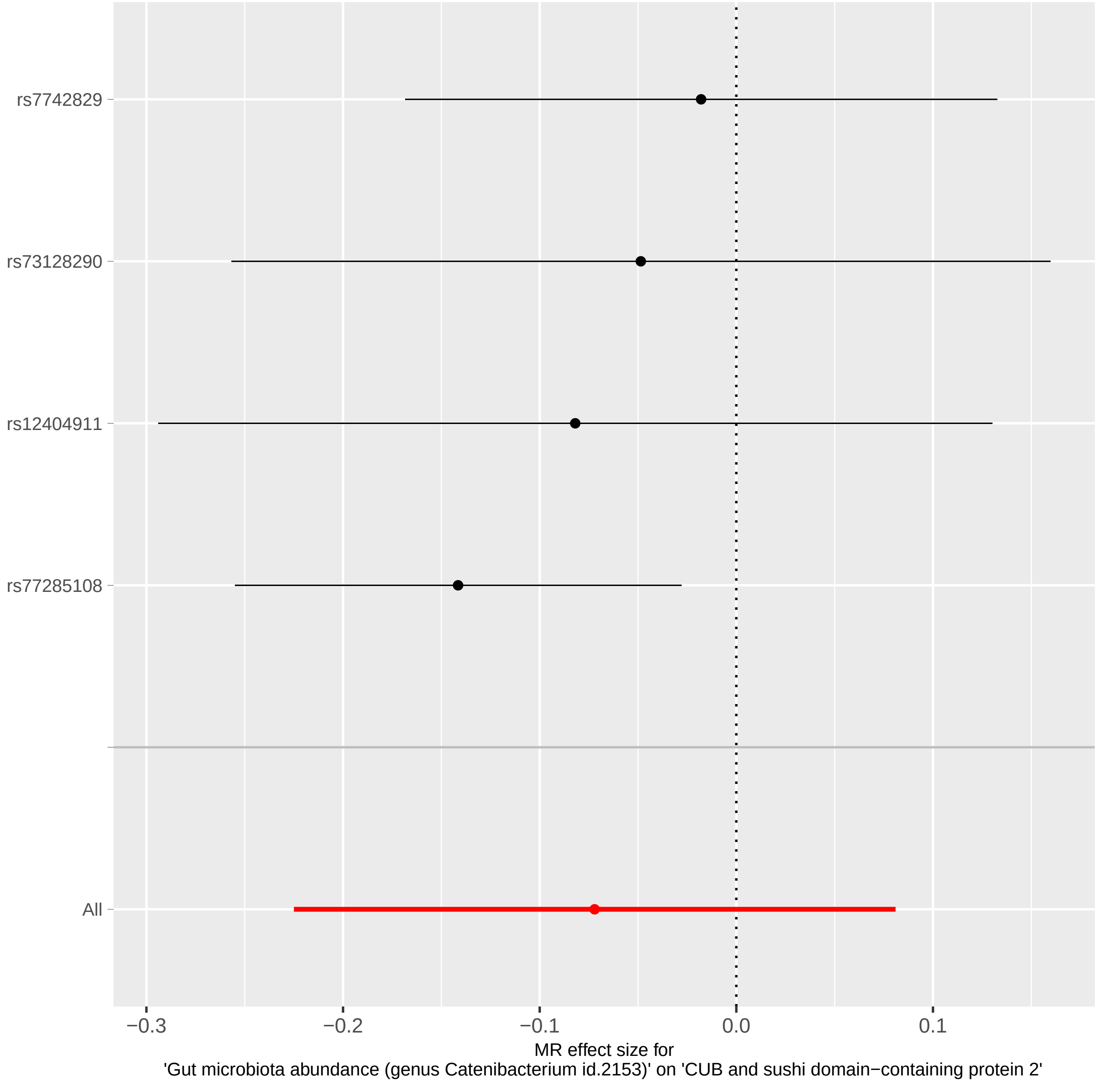

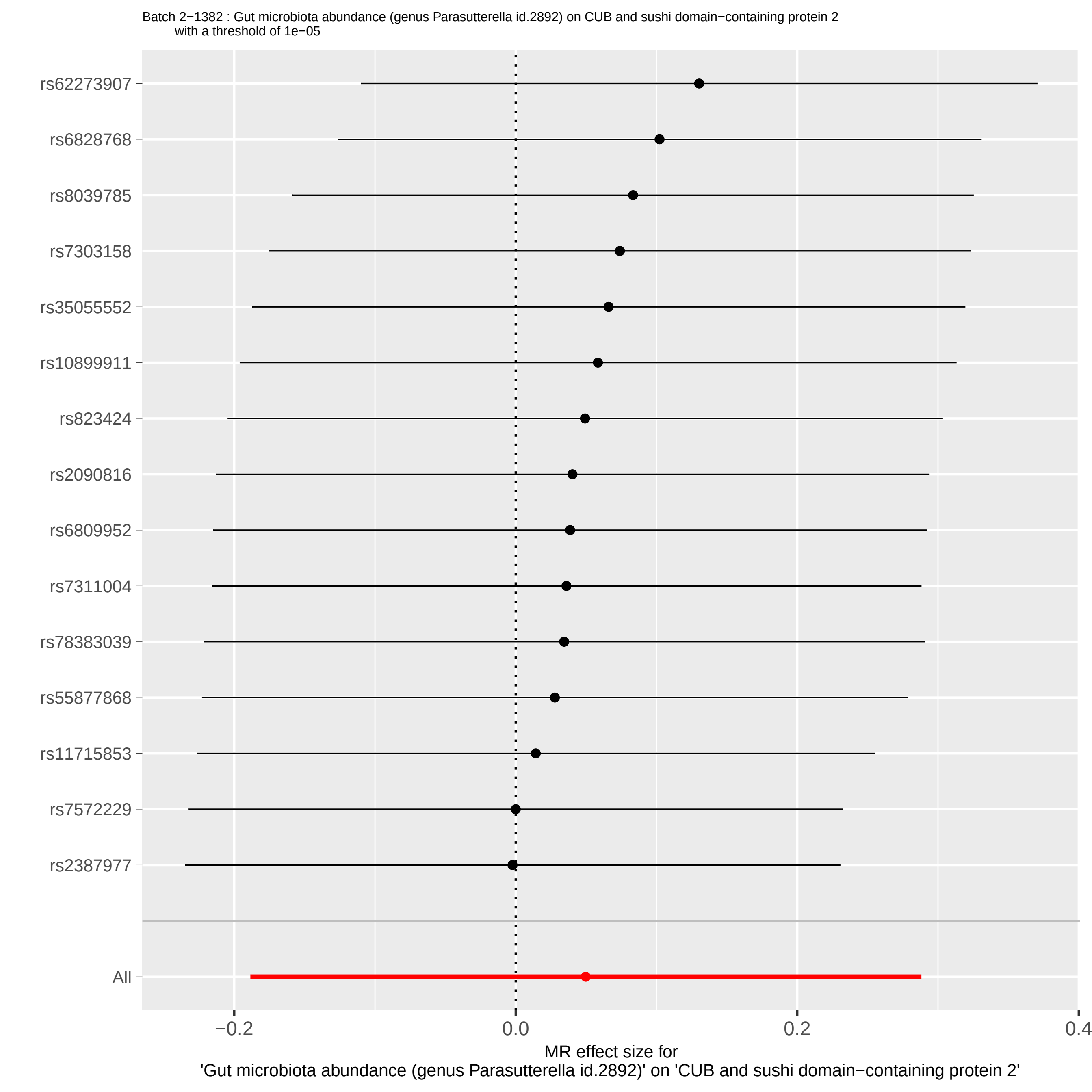

Batch 2−1382 : Gut microbiota abundance (genus Parasutterella id.2892) on CUB and sushi domain−containing protein 2
with a threshold of 1e−05
rs62273907
rs6828768
rs8039785
rs7303158
rs35055552
rs10899911
rs823424
rs2090816
rs6809952
rs7311004
rs78383039
rs55877868
rs11715853
rs7572229
rs2387977
All
−0.2
0.0
0.2
0.4
MR effect size for
'Gut microbiota abundance (genus Parasutterella id.2892)' on 'CUB and sushi domain−containing protein 2'

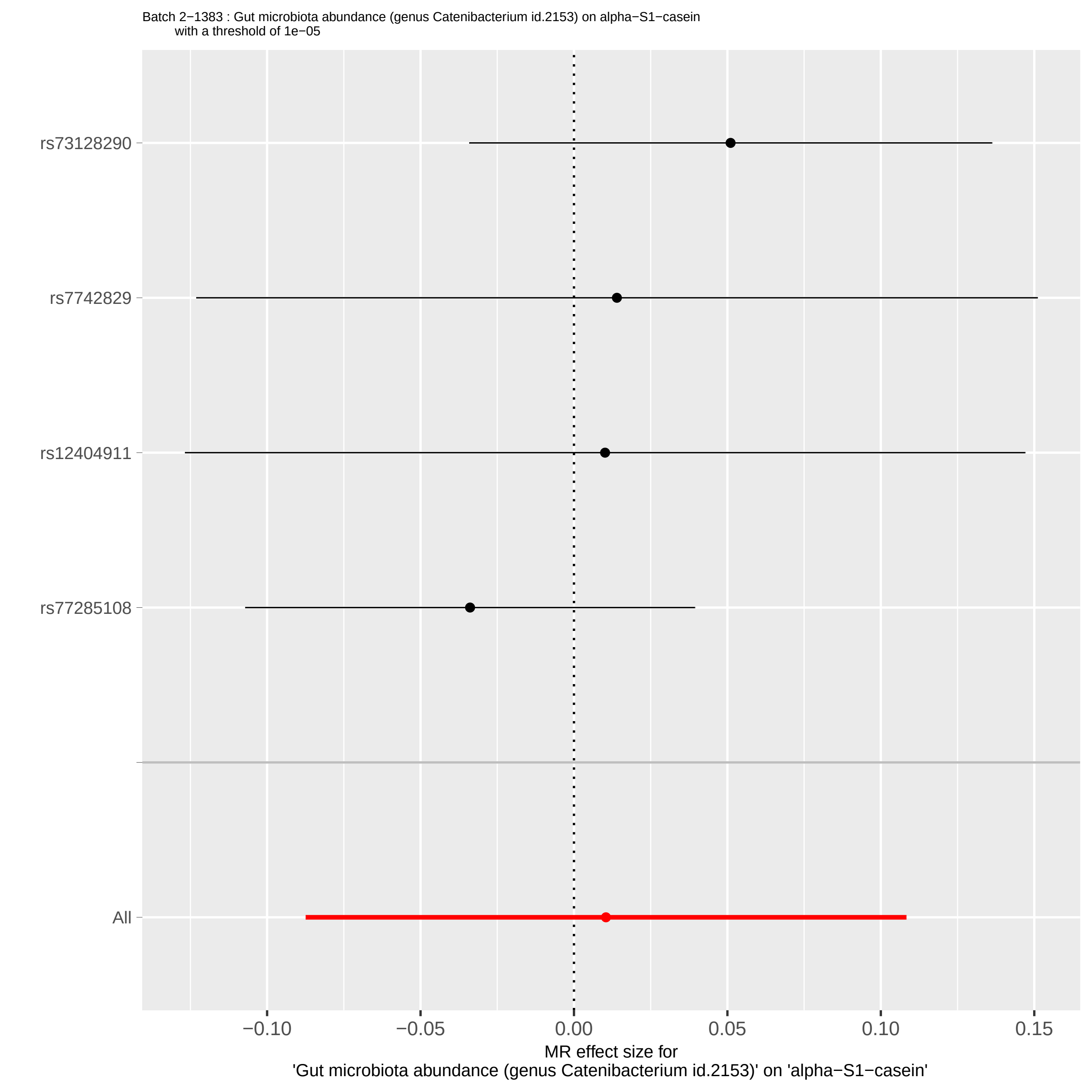
Batch 2−1383 : Gut microbiota abundance (genus Catenibacterium id.2153) on alpha−S1−casein
with a threshold of 1e−05
rs73128290
rs7742829
rs12404911
rs77285108
All
−0.10
−0.05
0.00
0.05
0.10
0.15
MR effect size for
'Gut microbiota abundance (genus Catenibacterium id.2153)' on 'alpha−S1−casein'

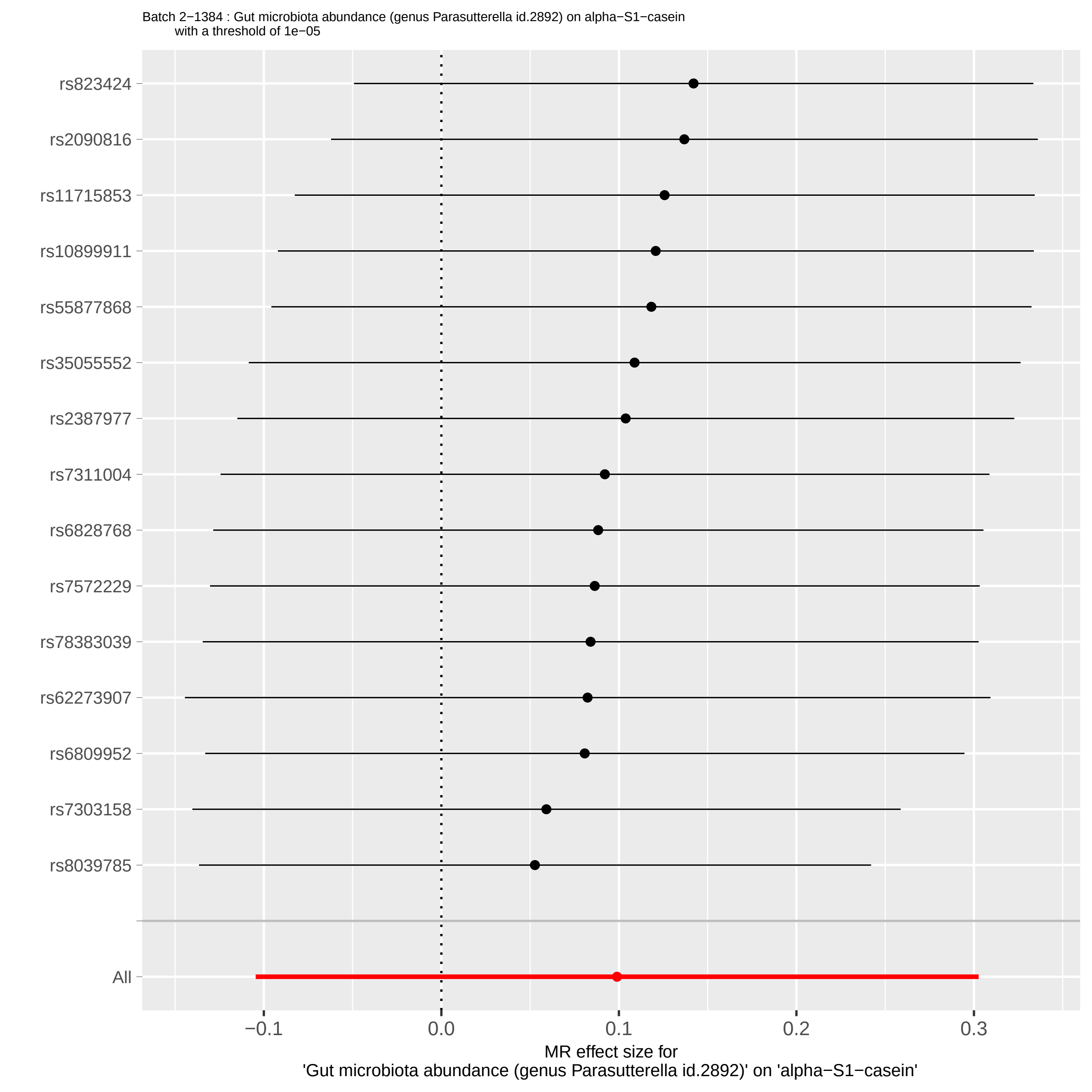

Batch 2−1384 : Gut microbiota abundance (genus Parasutterella id.2892) on alpha−S1−casein
with a threshold of 1e−05
rs823424
rs2090816
rs11715853
rs10899911
rs55877868
rs35055552
rs2387977
rs7311004
rs6828768
rs7572229
rs78383039
rs62273907
rs6809952
rs7303158
rs8039785
All
−0.1
0.0
0.1
0.2
0.3
MR effect size for
'Gut microbiota abundance (genus Parasutterella id.2892)' on 'alpha−S1−casein'

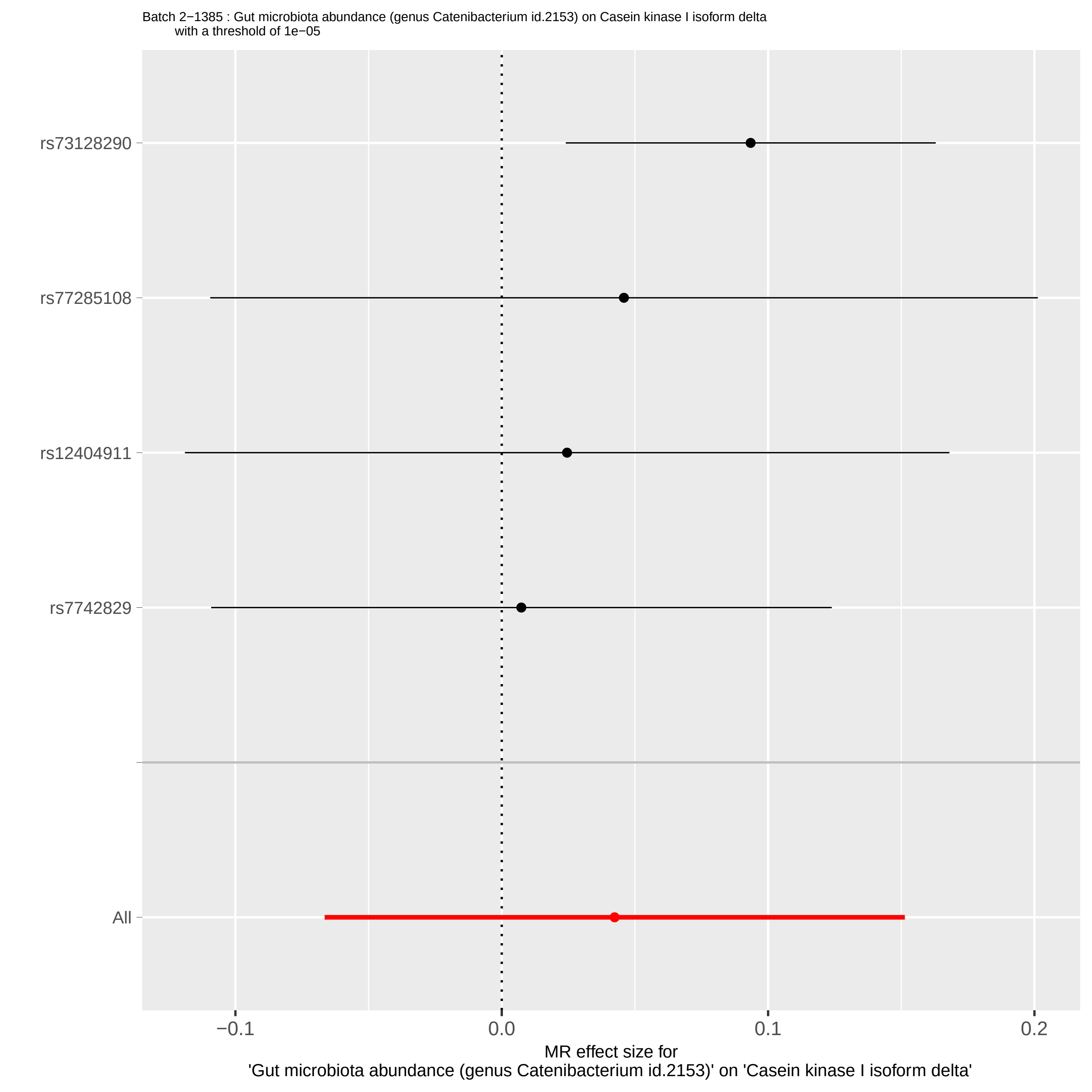
Batch 2−1385 : Gut microbiota abundance (genus Catenibacterium id.2153) on Casein kinase I isoform delta
with a threshold of 1e−05
rs73128290
rs77285108
rs12404911
rs7742829
All
−0.1
0.0
0.1
0.2
MR effect size for
'Gut microbiota abundance (genus Catenibacterium id.2153)' on 'Casein kinase I isoform delta'

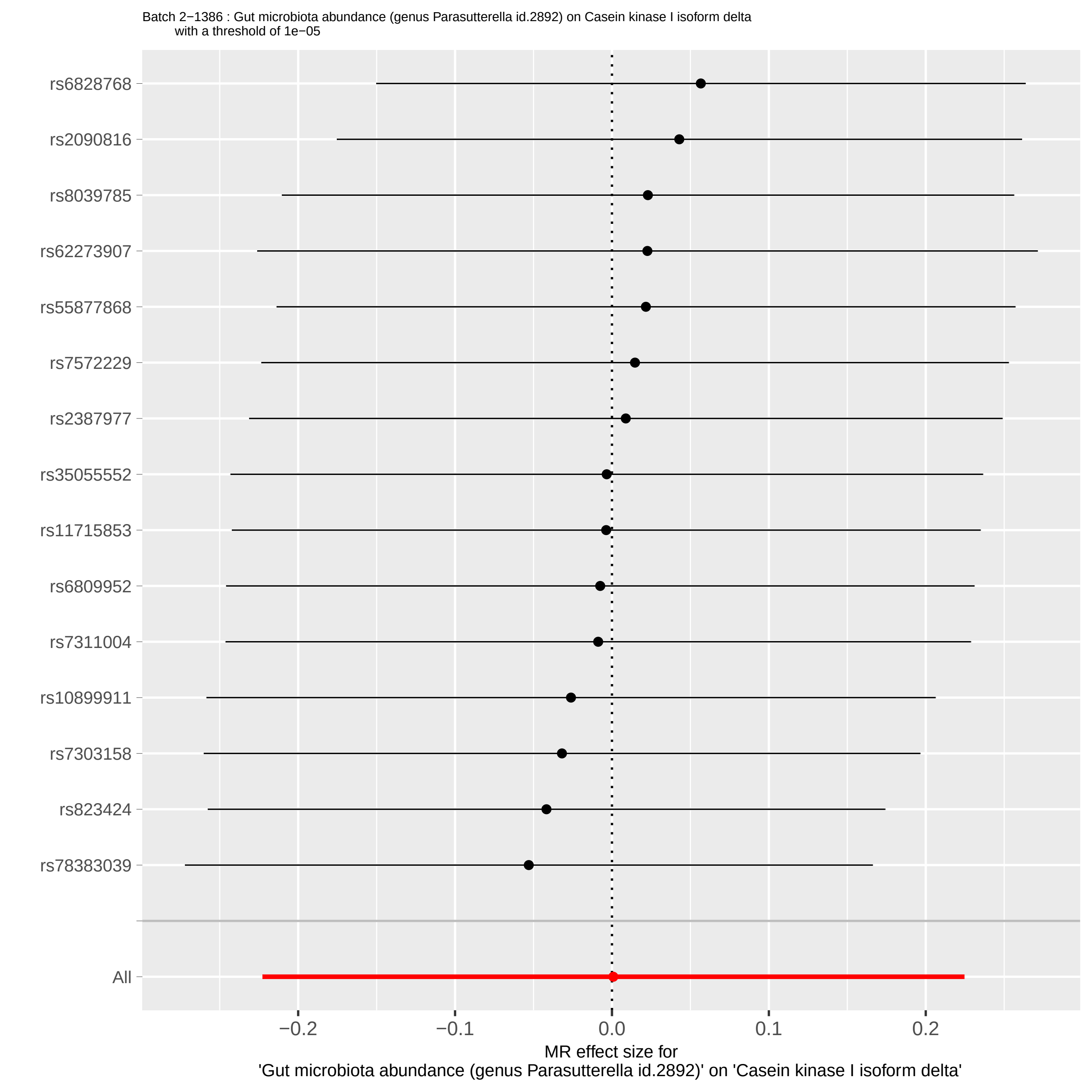
Batch 2−1386 : Gut microbiota abundance (genus Parasutterella id.2892) on Casein kinase I isoform delta
with a threshold of 1e−05
rs6828768
rs2090816
rs8039785
rs62273907
rs55877868
rs7572229
rs2387977
rs35055552
rs11715853
rs6809952
rs7311004
rs10899911
rs7303158
rs823424
rs78383039
All
−0.2
−0.1
0.0
0.1
0.2
MR effect size for
'Gut microbiota abundance (genus Parasutterella id.2892)' on 'Casein kinase I isoform delta'

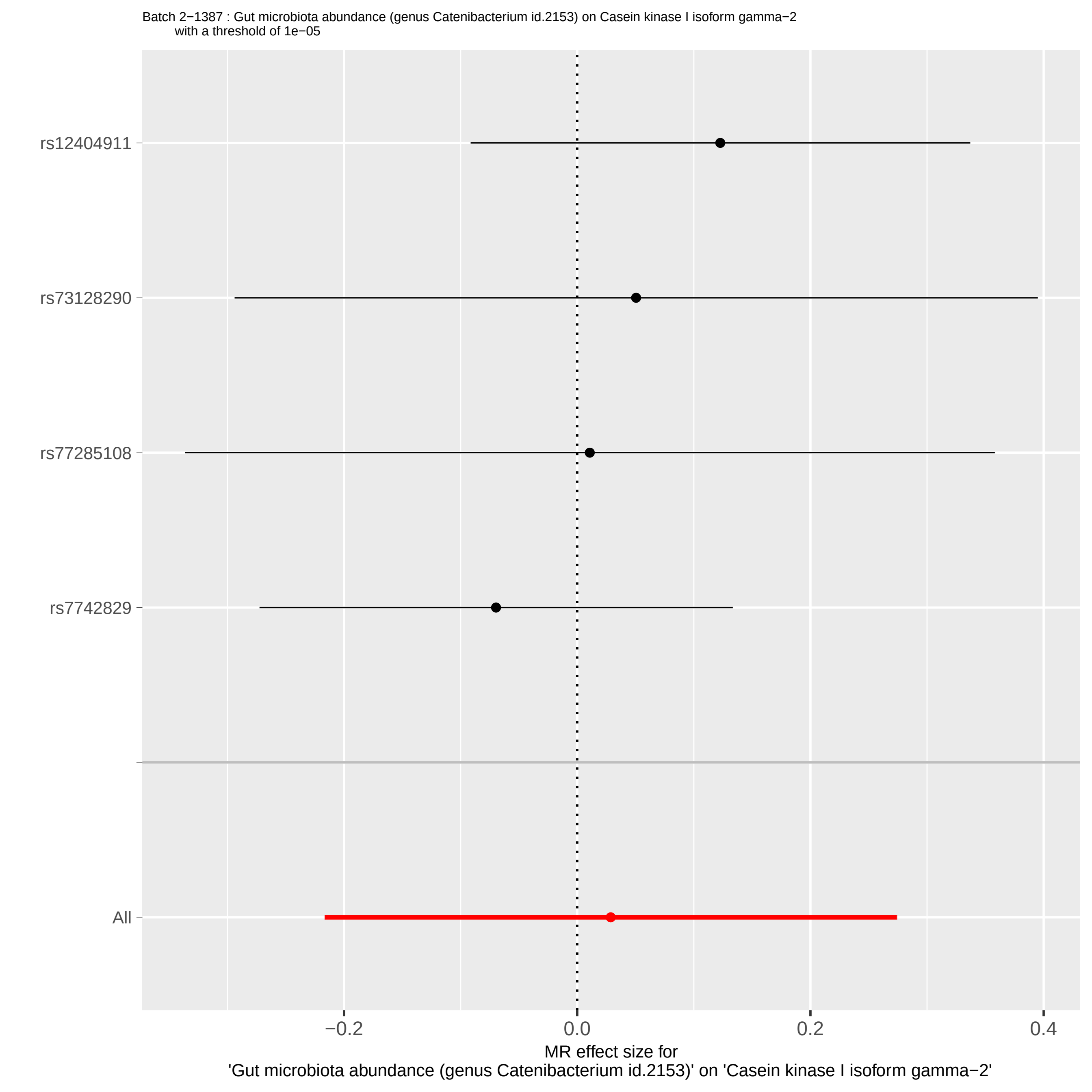

Batch 2−1387 : Gut microbiota abundance (genus Catenibacterium id.2153) on Casein kinase I isoform gamma−2
with a threshold of 1e−05
rs12404911
rs73128290
rs77285108
rs7742829
All
−0.2
0.0
0.2
0.4
MR effect size for
'Gut microbiota abundance (genus Catenibacterium id.2153)' on 'Casein kinase I isoform gamma−2'

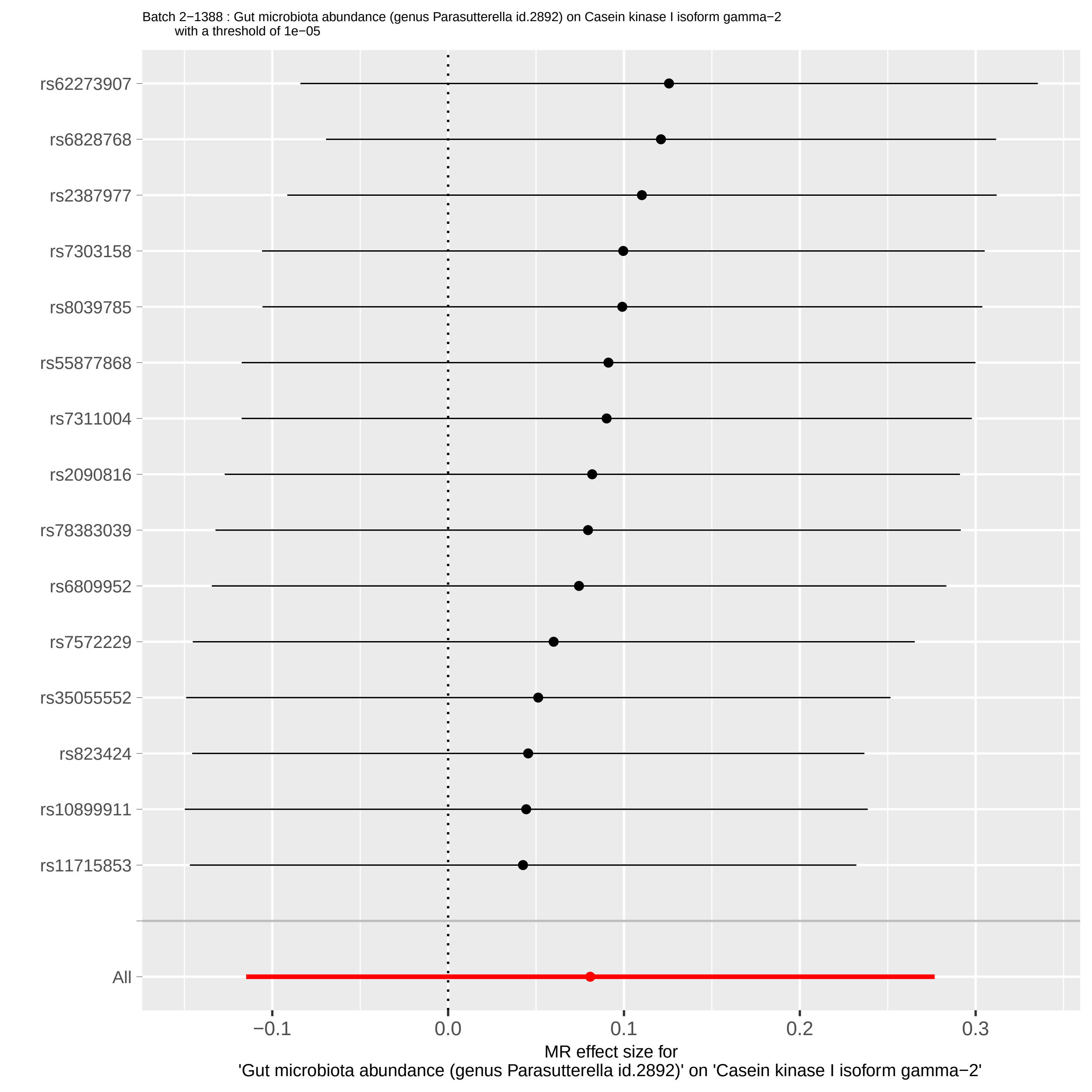
Batch 2−1388 : Gut microbiota abundance (genus Parasutterella id.2892) on Casein kinase I isoform gamma−2
with a threshold of 1e−05
rs62273907
rs6828768
rs2387977
rs7303158
rs8039785
rs55877868
rs7311004
rs2090816
rs78383039
rs6809952
rs7572229
rs35055552
rs823424
rs10899911
rs11715853
All
−0.1
0.0
0.1
0.2
0.3
MR effect size for
'Gut microbiota abundance (genus Parasutterella id.2892)' on 'Casein kinase I isoform gamma−2'

Batch 2−1389 : Gut microbiota abundance (genus Catenibacterium id.2153) on Casein kinase II subunit alpha
with a threshold of 1e−05

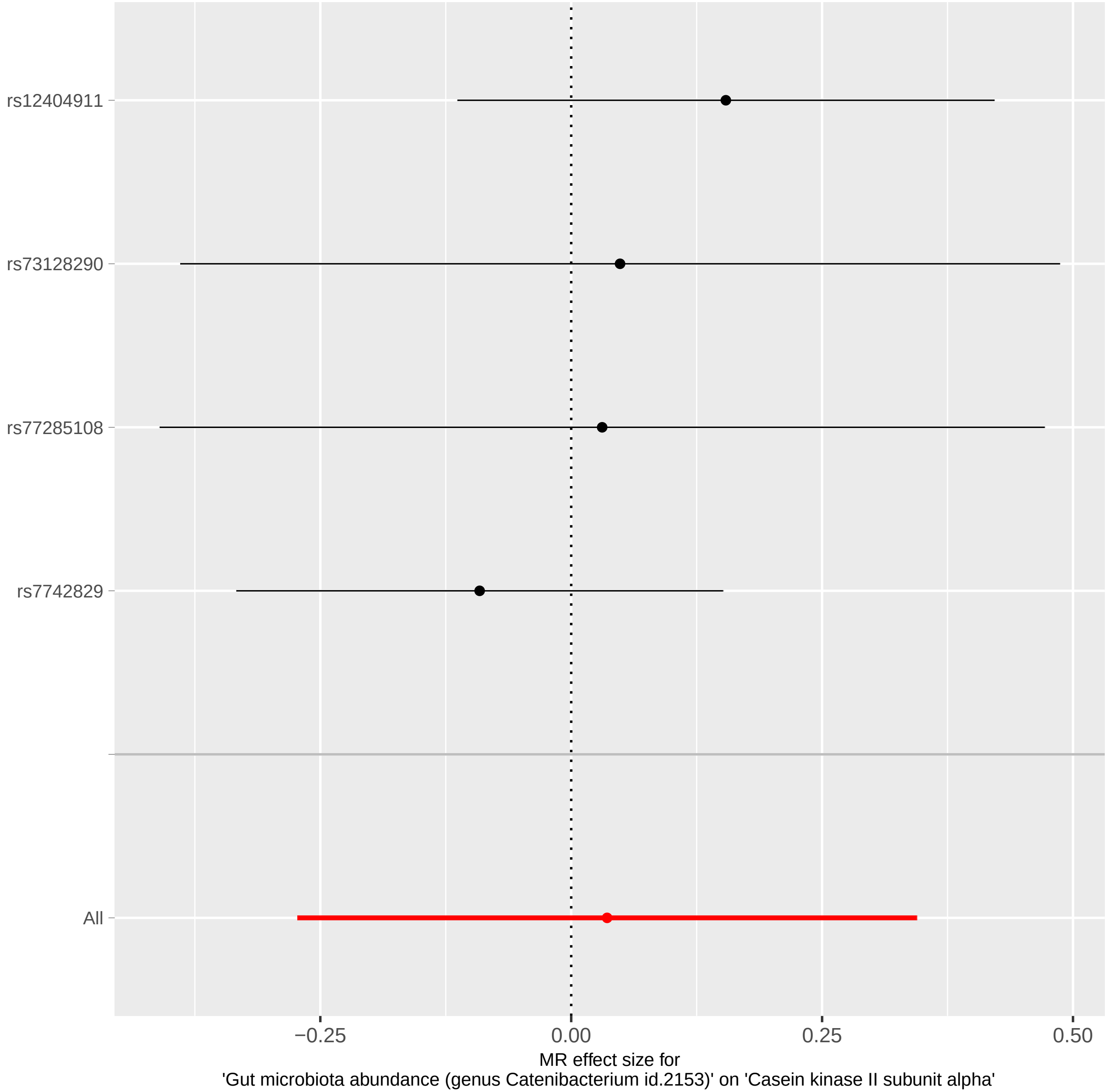

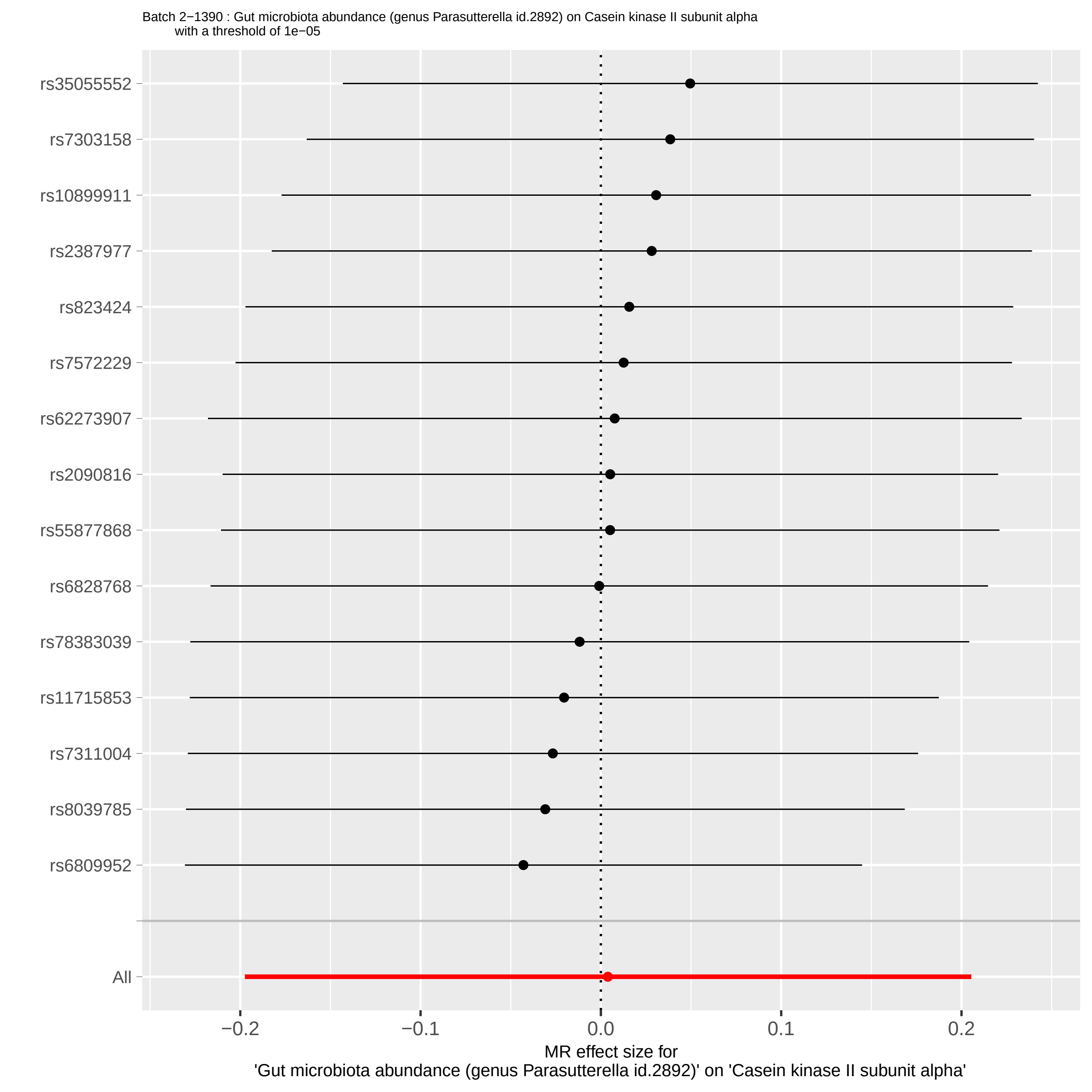
Batch 2−1390 : Gut microbiota abundance (genus Parasutterella id.2892) on Casein kinase II subunit alpha
with a threshold of 1e−05
rs35055552
rs7303158
rs10899911
rs2387977
rs823424
rs7572229
rs62273907
rs2090816
rs55877868
rs6828768
rs78383039
rs11715853
rs7311004
rs8039785
rs6809952
All
−0.2
−0.1
0.0
0.1
0.2
MR effect size for
'Gut microbiota abundance (genus Parasutterella id.2892)' on 'Casein kinase II subunit alpha'

Batch 2−1391 : Gut microbiota abundance (genus Catenibacterium id.2153) on Casein kinase II subunit alpha
with a threshold of 1e−05

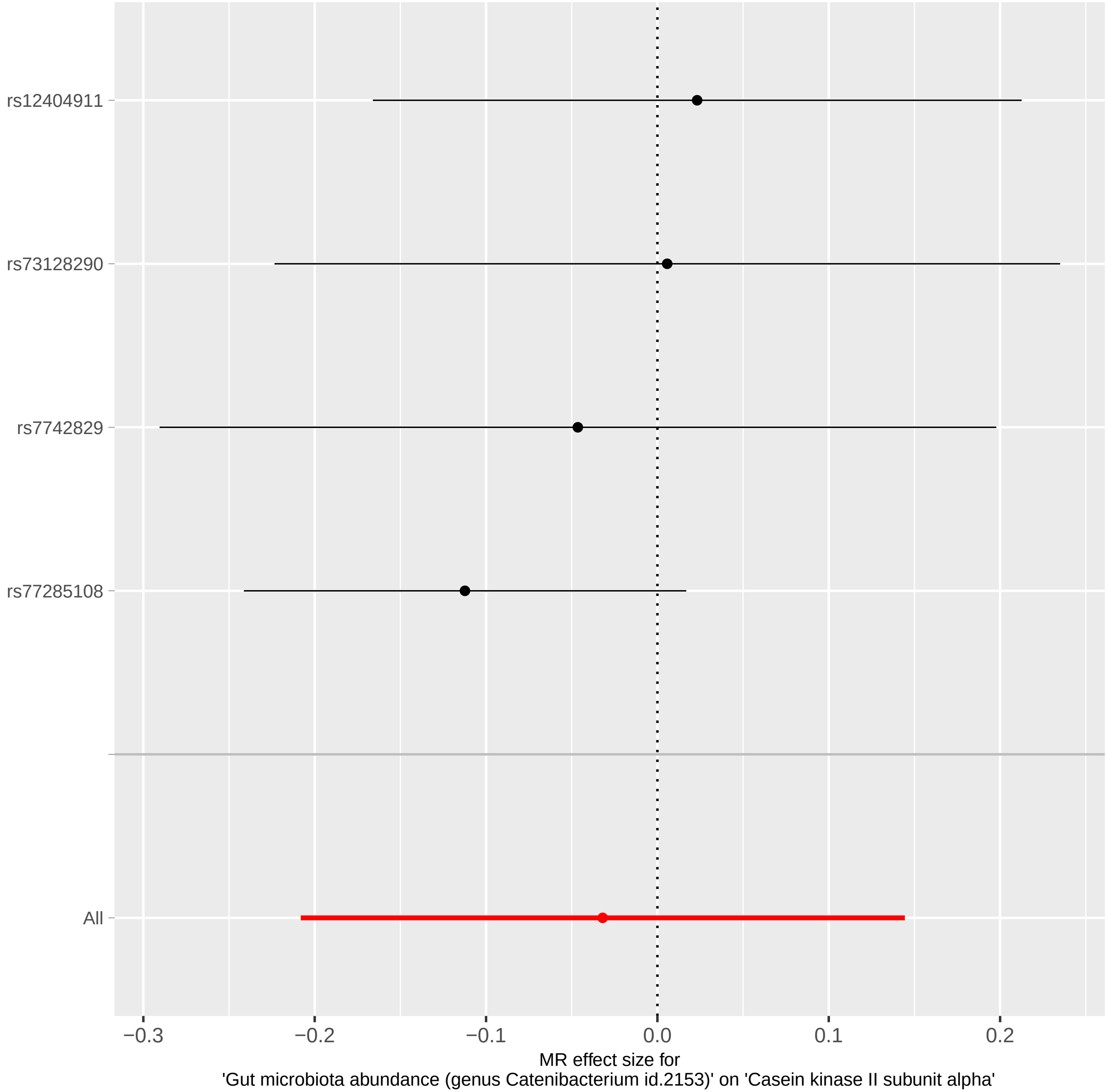

Batch 2−1392 : Gut microbiota abundance (genus Parasutterella id.2892) on Casein kinase II subunit alpha
with a threshold of 1e−05

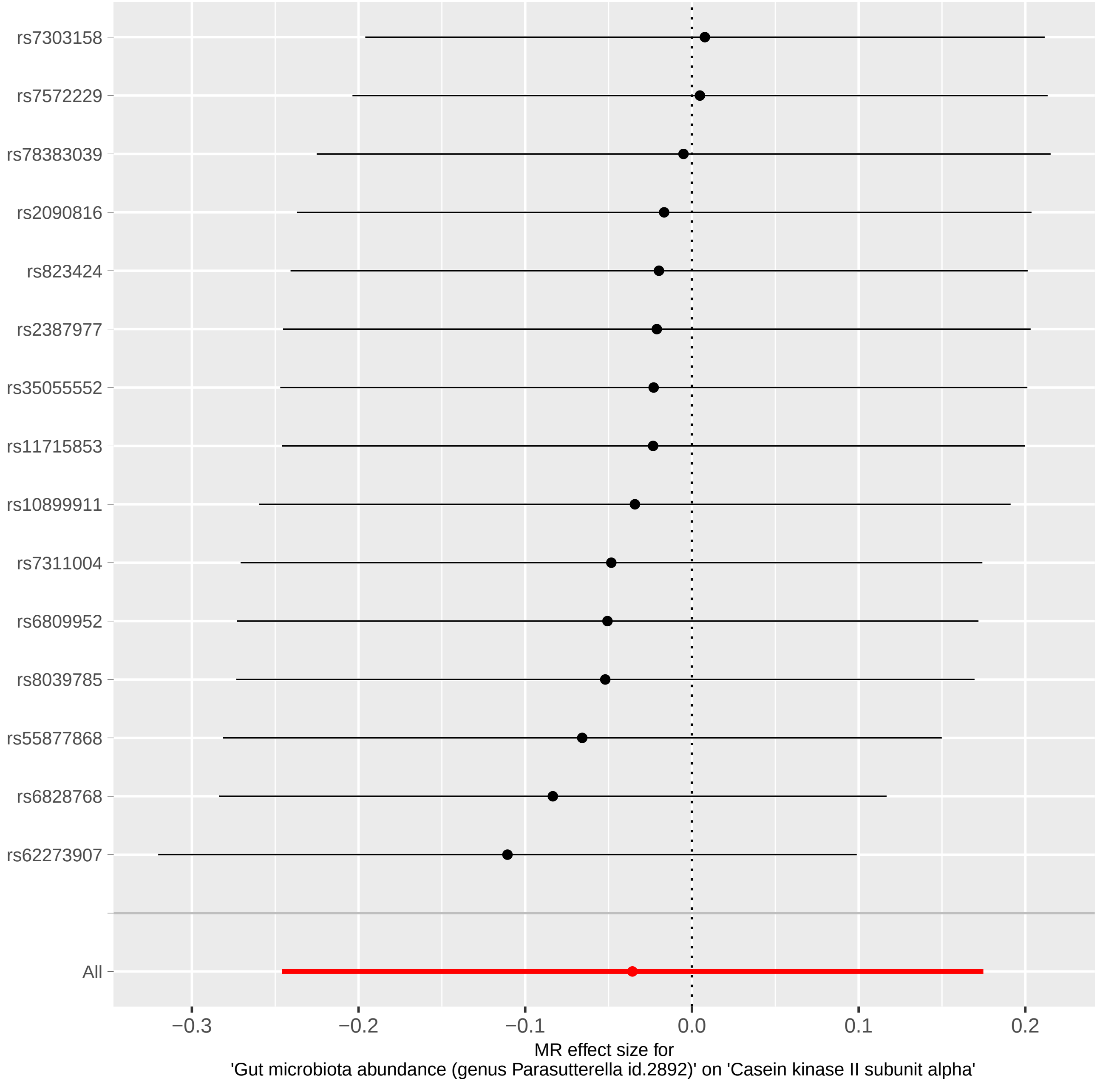

Batch 2−1393 : Gut microbiota abundance (genus Catenibacterium id.2153) on Casein kinase II 2−alpha:2−beta heterotetramer
with a threshold of 1e−05

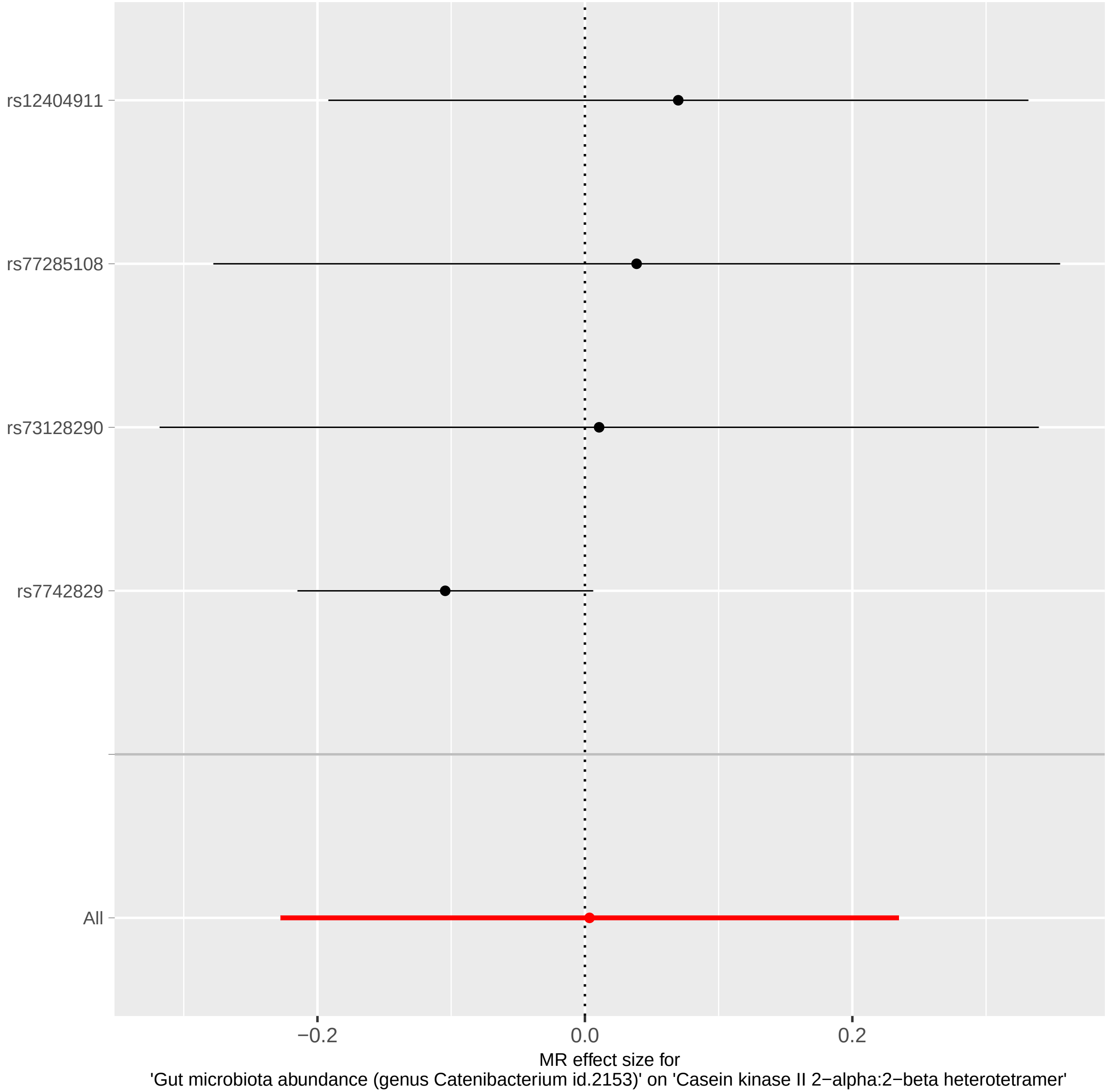

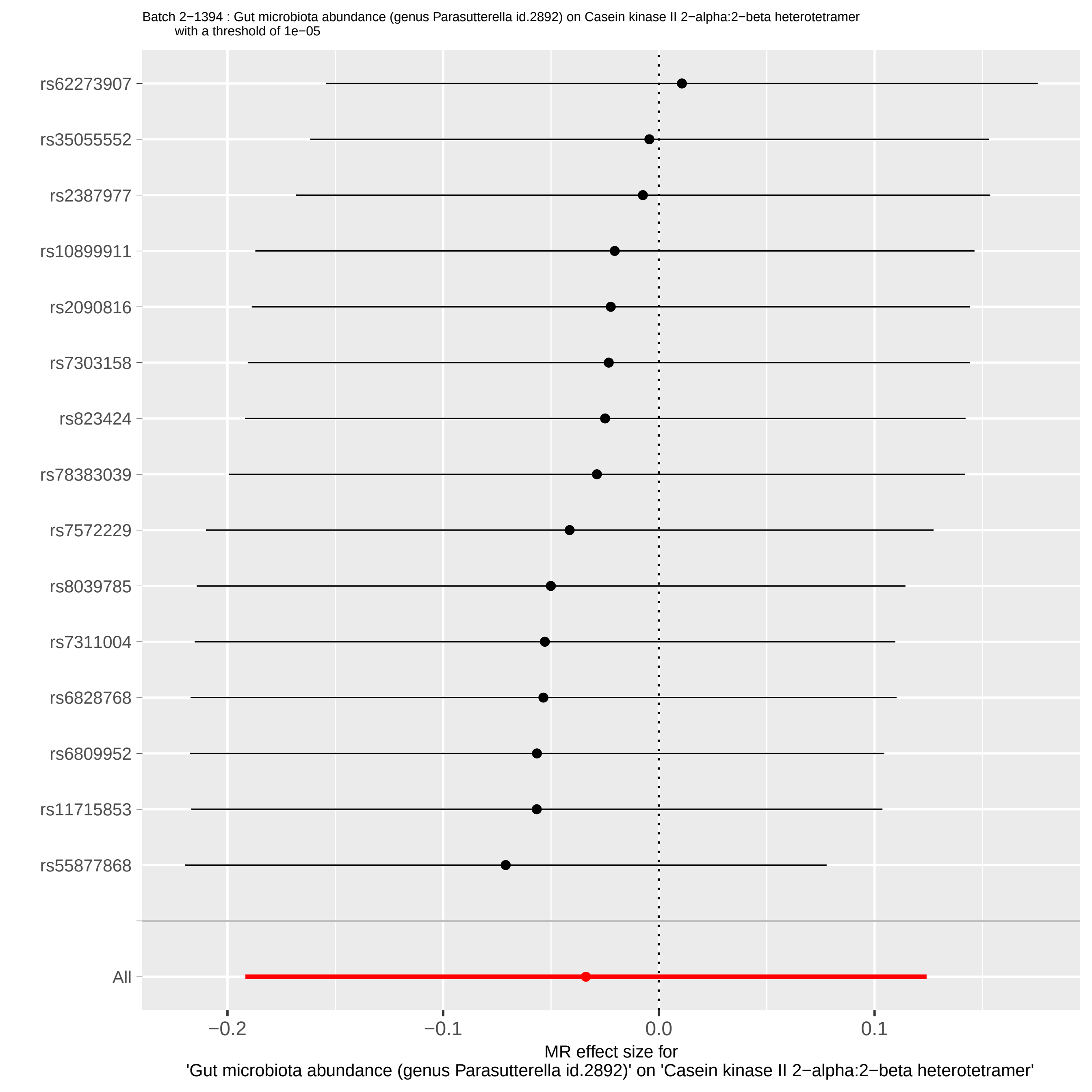
Batch 2−1394 : Gut microbiota abundance (genus Parasutterella id.2892) on Casein kinase II 2−alpha:2−beta heterotetramer
with a threshold of 1e−05
rs62273907
rs35055552
rs2387977
rs10899911
rs2090816
rs7303158
rs823424
rs78383039
rs7572229
rs8039785
rs7311004
rs6828768
rs6809952
rs11715853
rs55877868
All
−0.2
−0.1
0.0
0.1
MR effect size for
'Gut microbiota abundance (genus Parasutterella id.2892)' on 'Casein kinase II 2−alpha:2−beta heterotetramer'

Batch 2−1395 : Gut microbiota abundance (genus Catenibacterium id.2153) on Casein kinase II 2−alpha':2−beta heterotetramer
with a threshold of 1e−05

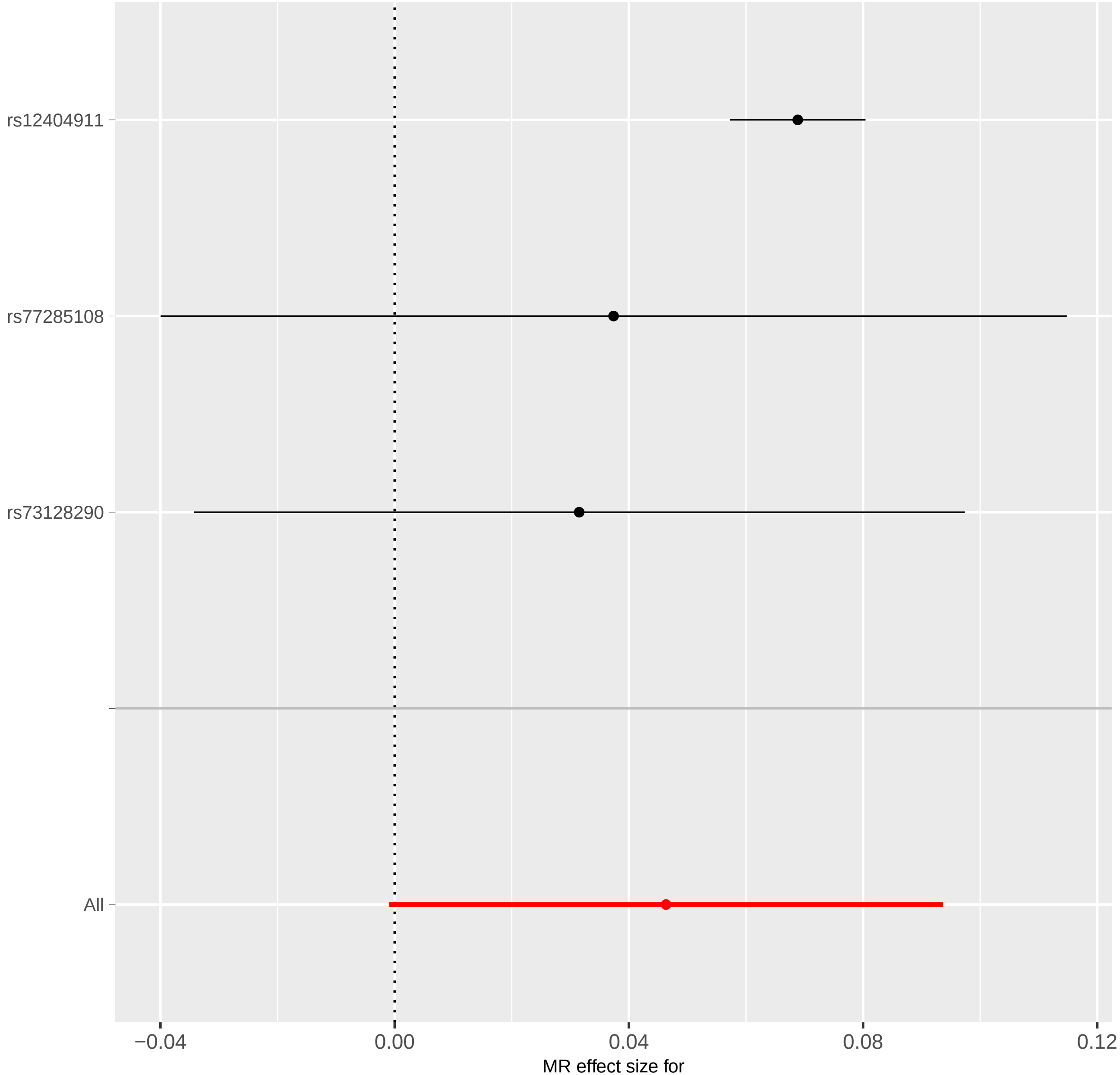

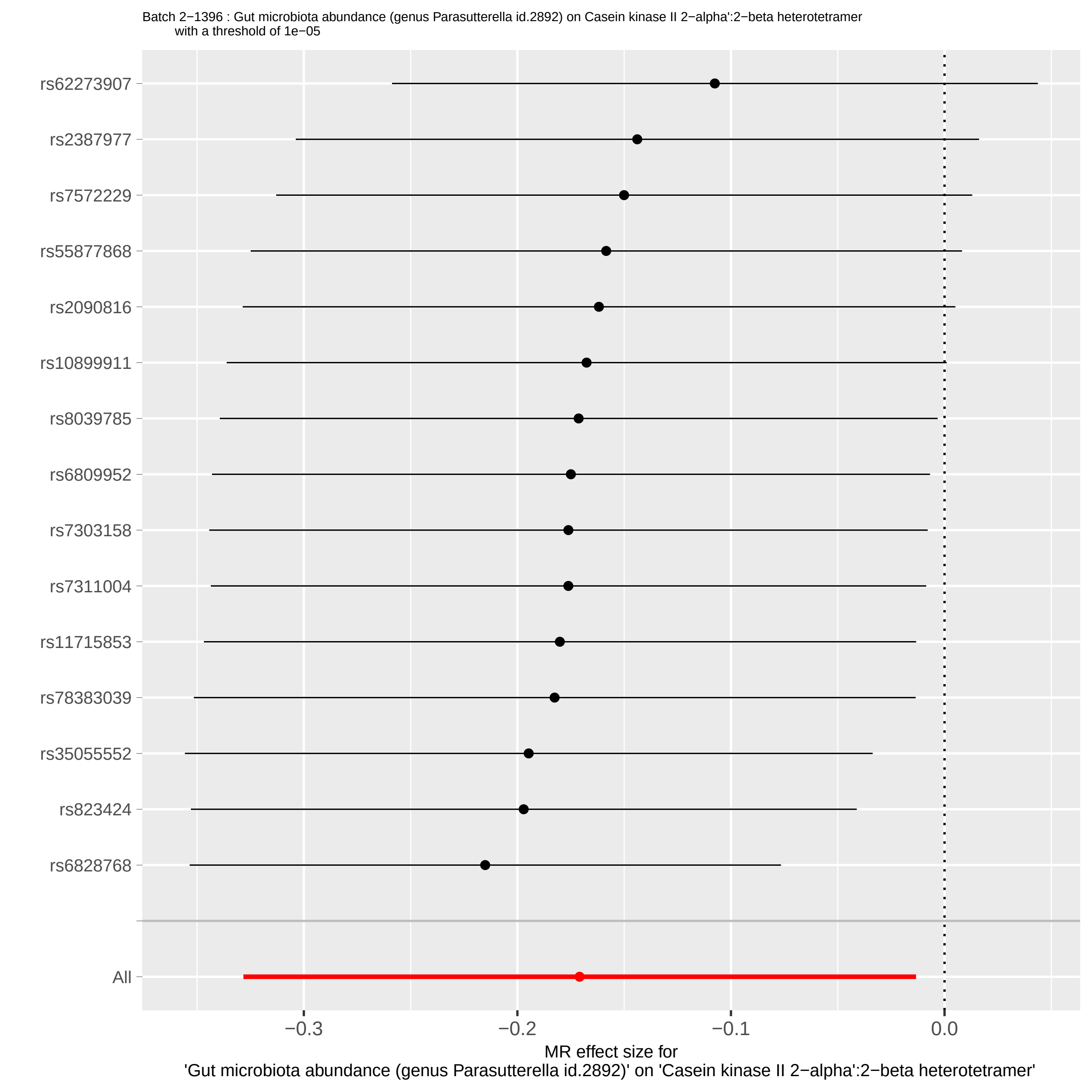

Batch 2−1396 : Gut microbiota abundance (genus Parasutterella id.2892) on Casein kinase II 2−alpha':2−beta heterotetramer
with a threshold of 1e−05
rs62273907
rs2387977
rs7572229
rs55877868
rs2090816
rs10899911
rs8039785
rs6809952
rs7303158
rs7311004
rs11715853
rs78383039
rs35055552
rs823424
rs6828768
All
−0.3
−0.2
−0.1
0.0
MR effect size for
'Gut microbiota abundance (genus Parasutterella id.2892)' on 'Casein kinase II 2−alpha':2−beta heterotetramer'

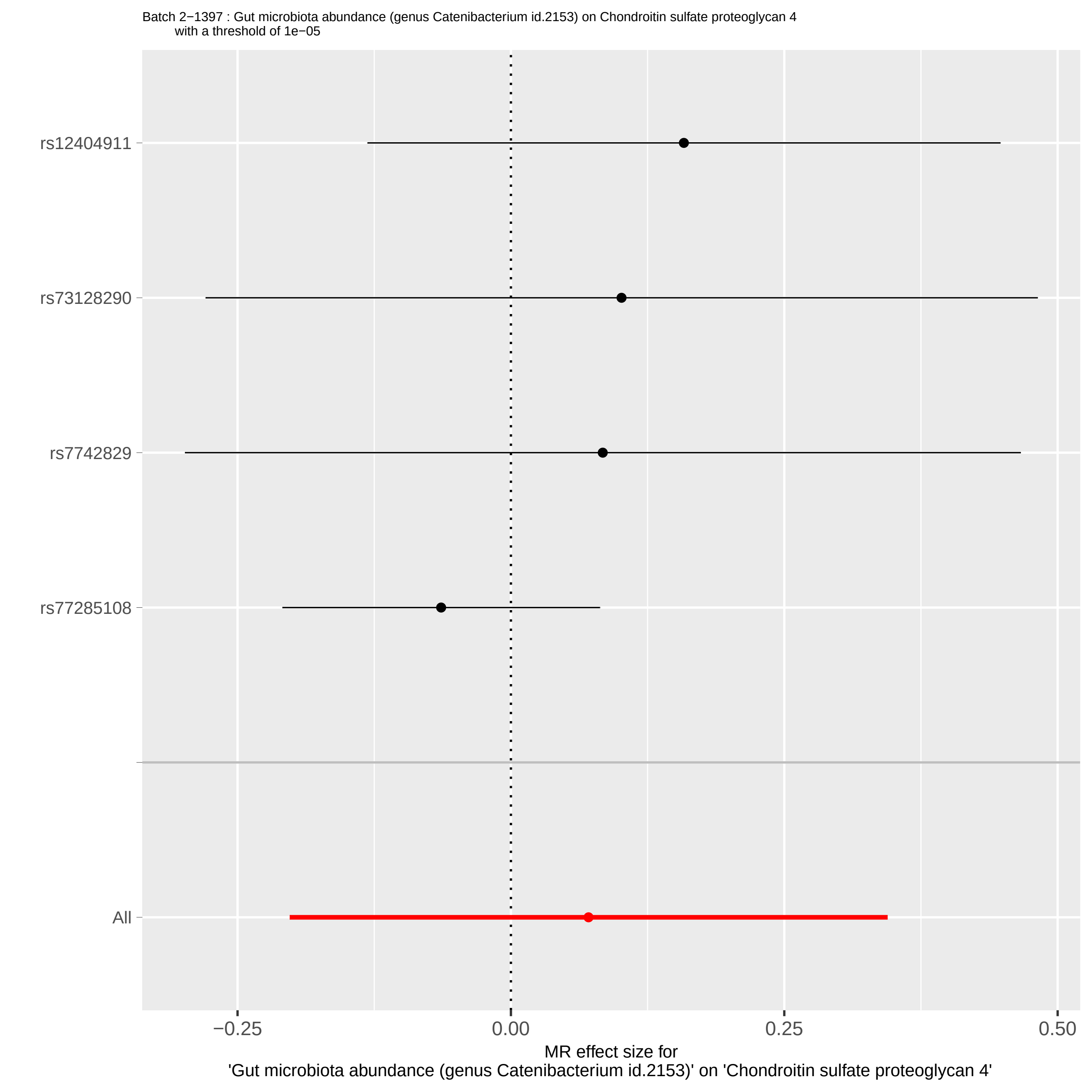
Batch 2−1397 : Gut microbiota abundance (genus Catenibacterium id.2153) on Chondroitin sulfate proteoglycan 4
with a threshold of 1e−05
rs12404911
rs73128290
rs7742829
rs77285108
All
−0.25
0.00
0.25
0.50
MR effect size for
'Gut microbiota abundance (genus Catenibacterium id.2153)' on 'Chondroitin sulfate proteoglycan 4'

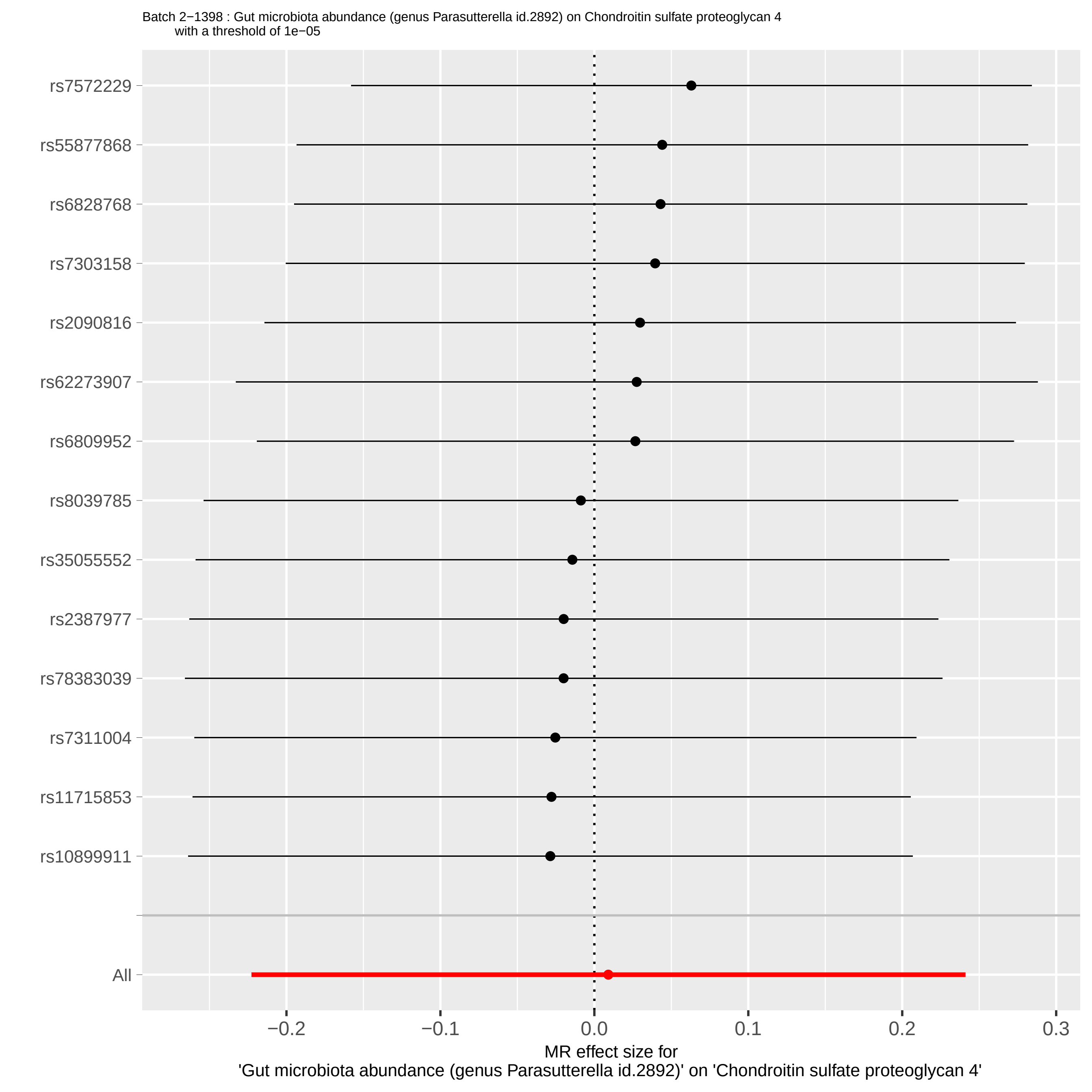
Batch 2−1398 : Gut microbiota abundance (genus Parasutterella id.2892) on Chondroitin sulfate proteoglycan 4
with a threshold of 1e−05
rs7572229
rs55877868
rs6828768
rs7303158
rs2090816
rs62273907
rs6809952
rs8039785
rs35055552
rs2387977
rs78383039
rs7311004
rs11715853
rs10899911
All
−0.2
−0.1
0.0
0.1
0.2
0.3
MR effect size for
'Gut microbiota abundance (genus Parasutterella id.2892)' on 'Chondroitin sulfate proteoglycan 4'

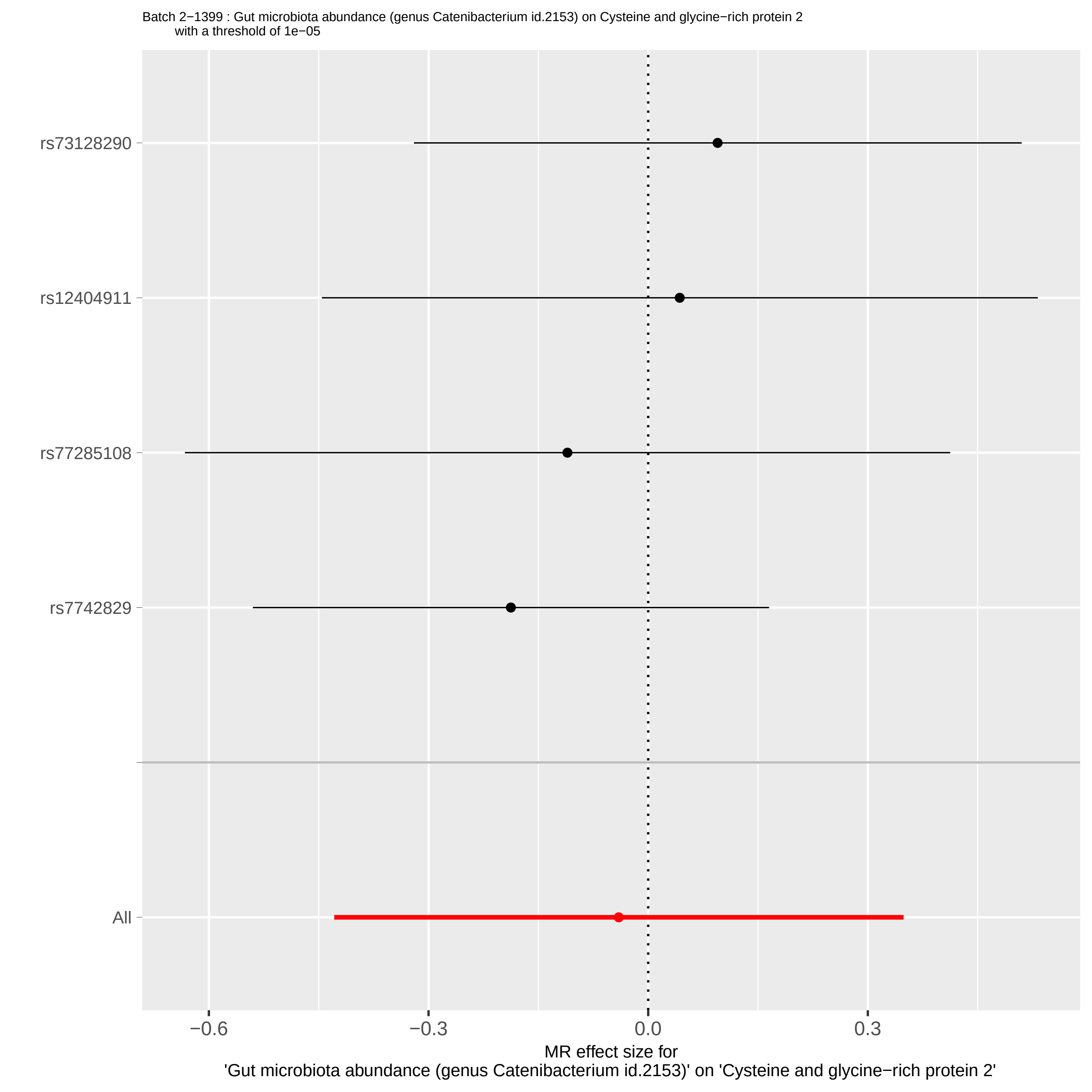

Batch 2−1399 : Gut microbiota abundance (genus Catenibacterium id.2153) on Cysteine and glycine−rich protein 2
with a threshold of 1e−05
rs73128290
rs12404911
rs77285108
rs7742829
All
−0.6
−0.3
0.0
0.3
MR effect size for
'Gut microbiota abundance (genus Catenibacterium id.2153)' on 'Cysteine and glycine−rich protein 2'

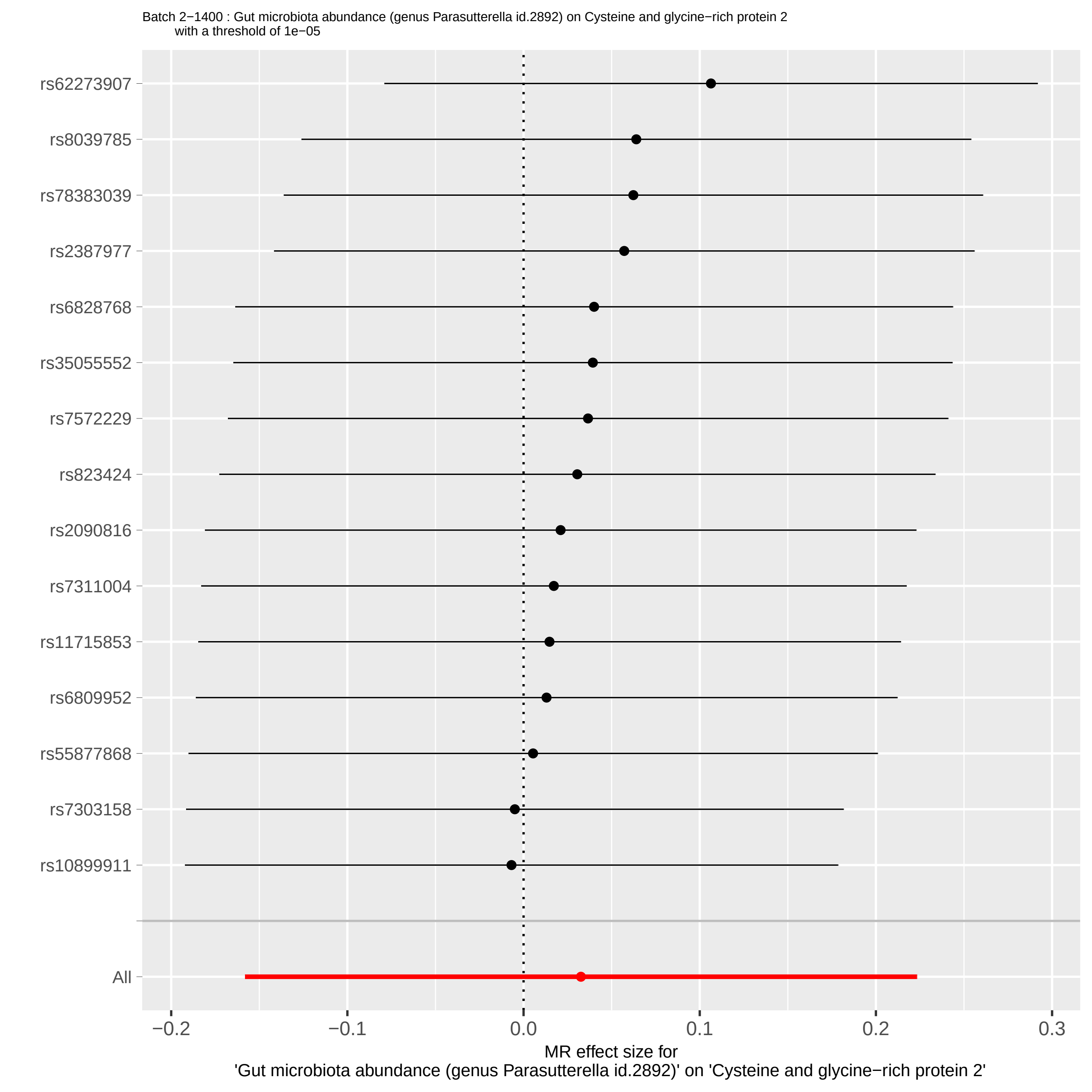

Batch 2−1400 : Gut microbiota abundance (genus Parasutterella id.2892) on Cysteine and glycine−rich protein 2
with a threshold of 1e−05
rs62273907
rs8039785
rs78383039
rs2387977
rs6828768
rs35055552
rs7572229
rs823424
rs2090816
rs7311004
rs11715853
rs6809952
rs55877868
rs7303158
rs10899911
All
−0.2
−0.1
0.0
0.1
0.2
0.3
MR effect size for
'Gut microbiota abundance (genus Parasutterella id.2892)' on 'Cysteine and glycine−rich protein 2'

Batch 2−1401 : Gut microbiota abundance (genus Catenibacterium id.2153) on Cysteine and glycine−rich protein 3
with a threshold of 1e−05

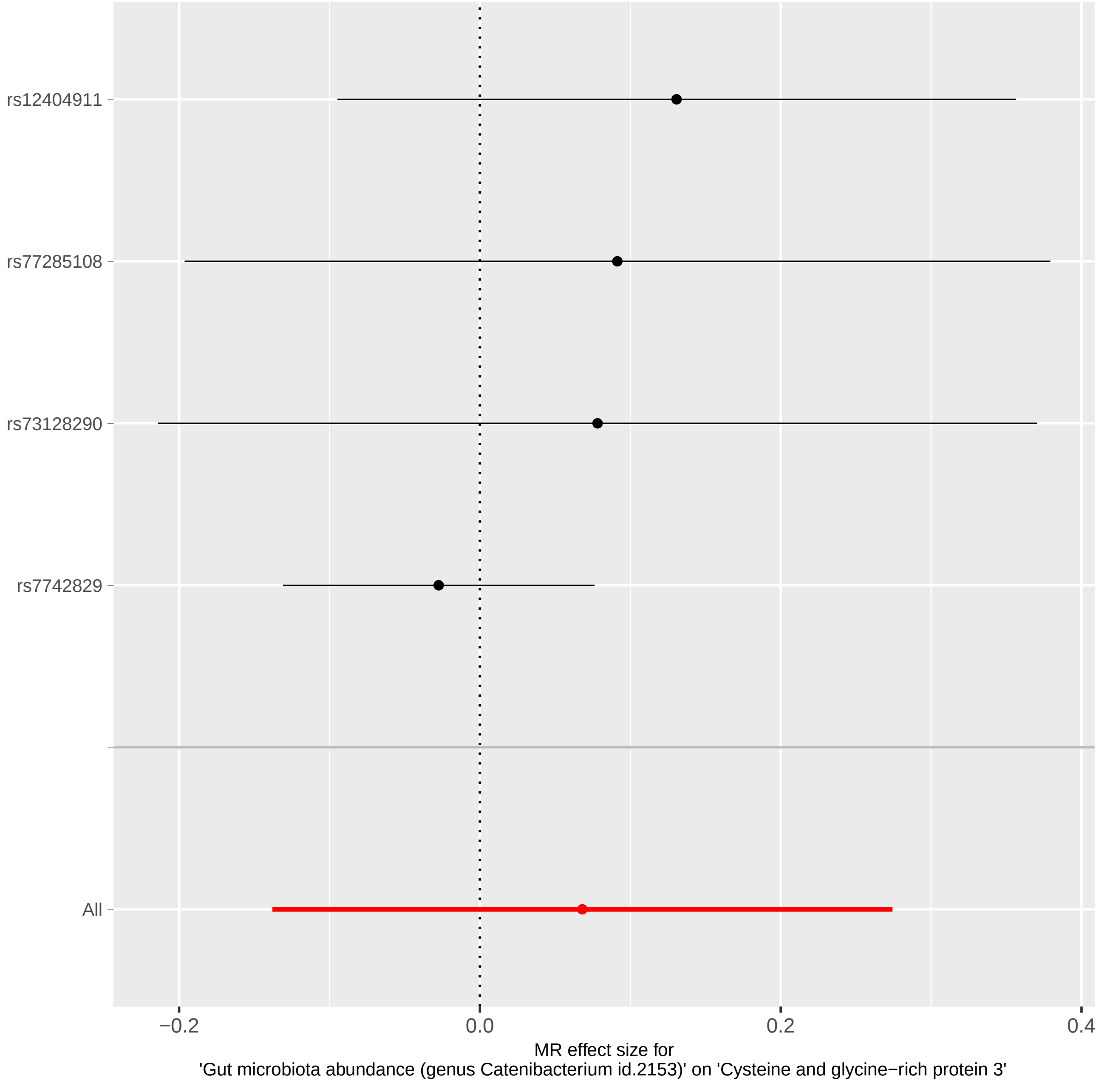

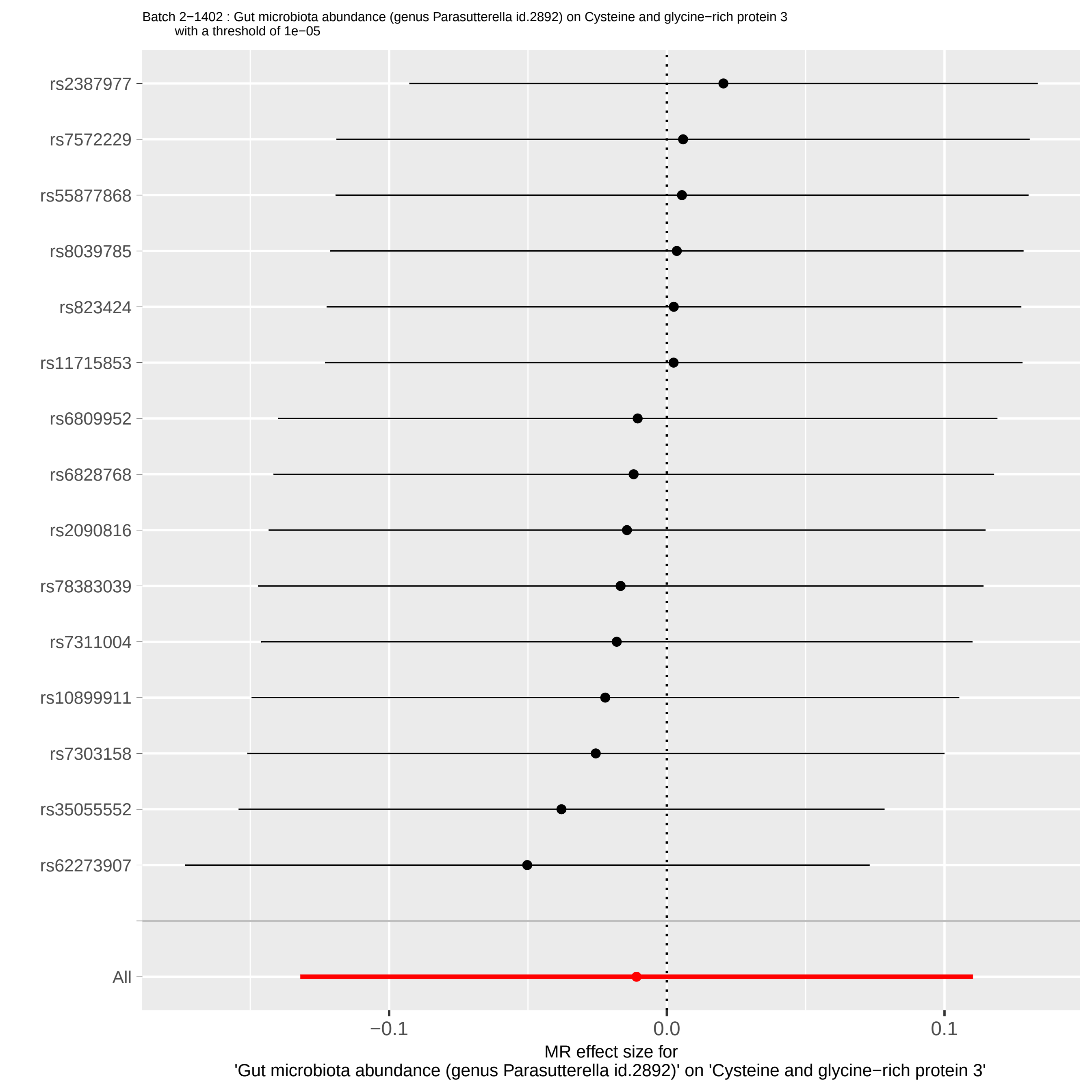
Batch 2−1402 : Gut microbiota abundance (genus Parasutterella id.2892) on Cysteine and glycine−rich protein 3
with a threshold of 1e−05
rs2387977
rs7572229
rs55877868
rs8039785
rs823424
rs11715853
rs6809952
rs6828768
rs2090816
rs78383039
rs7311004
rs10899911
rs7303158
rs35055552
rs62273907
All
−0.1
0.0
0.1
MR effect size for
'Gut microbiota abundance (genus Parasutterella id.2892)' on 'Cysteine and glycine−rich protein 3'

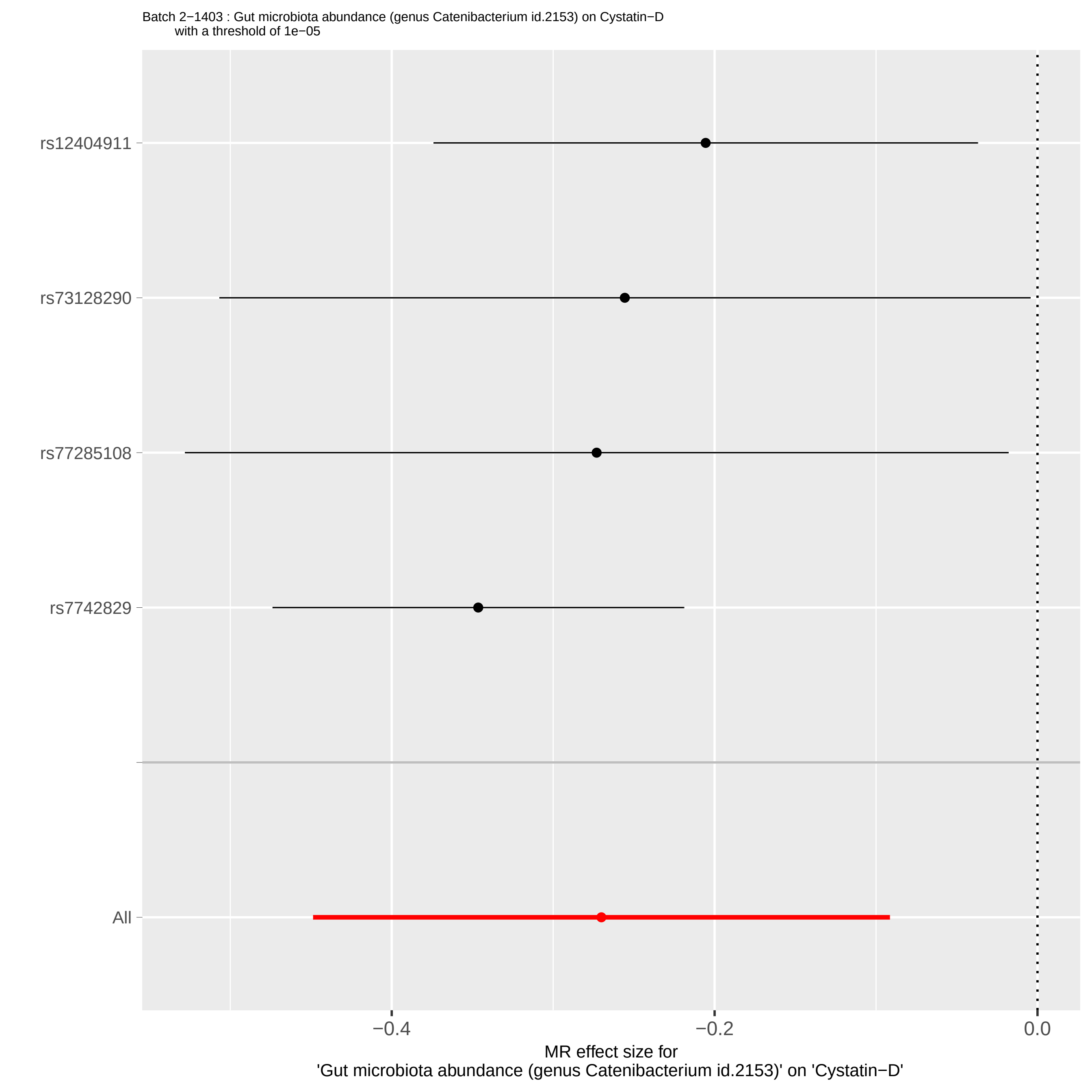

Batch 2−1403 : Gut microbiota abundance (genus Catenibacterium id.2153) on Cystatin−D
with a threshold of 1e−05
rs12404911
rs73128290
rs77285108
rs7742829
All
−0.4
−0.2
0.0
MR effect size for
'Gut microbiota abundance (genus Catenibacterium id.2153)' on 'Cystatin−D'

Batch 2−1404 : Gut microbiota abundance (genus Parasutterella id.2892) on Cystatin−D
with a threshold of 1e−05

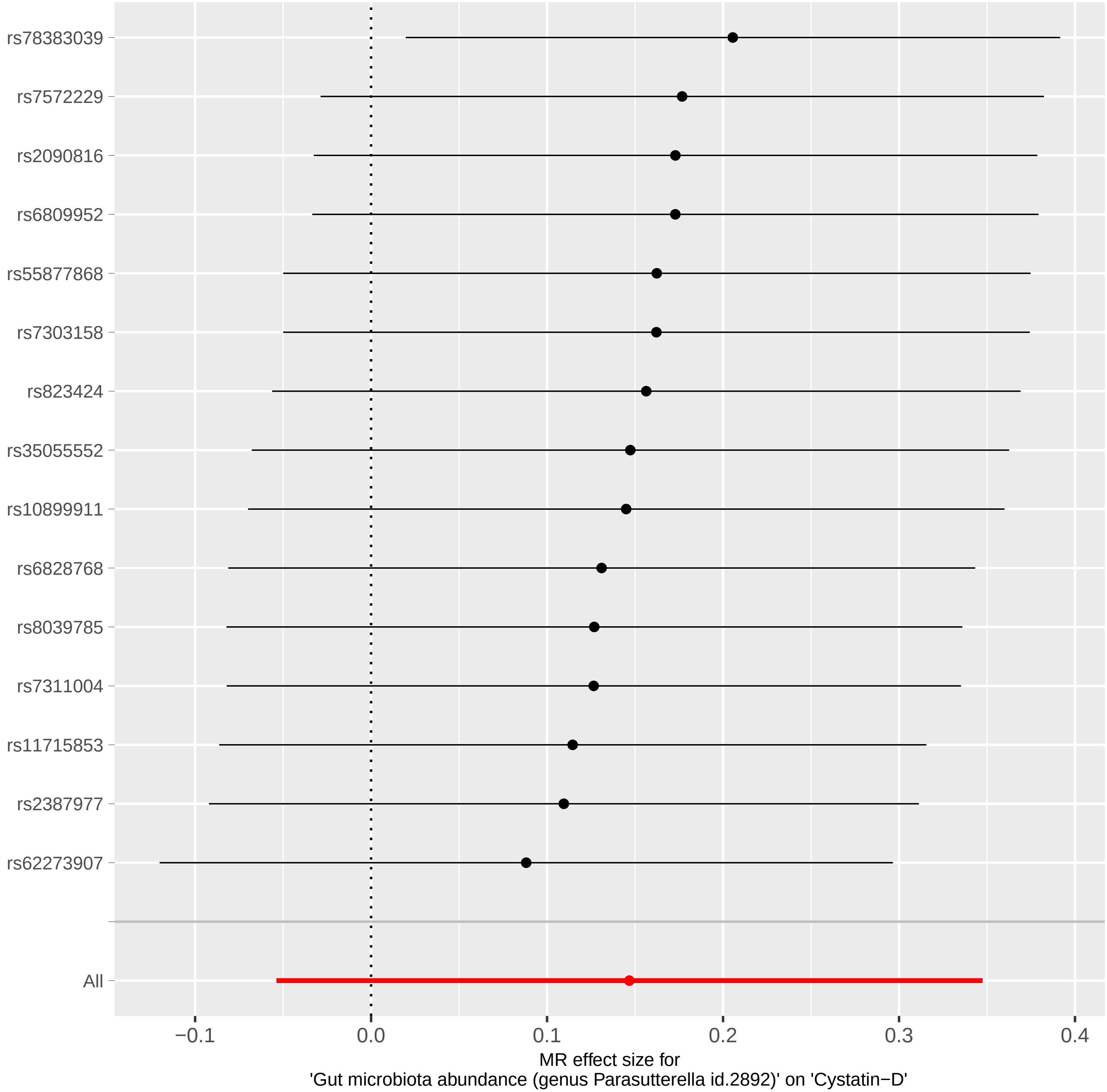

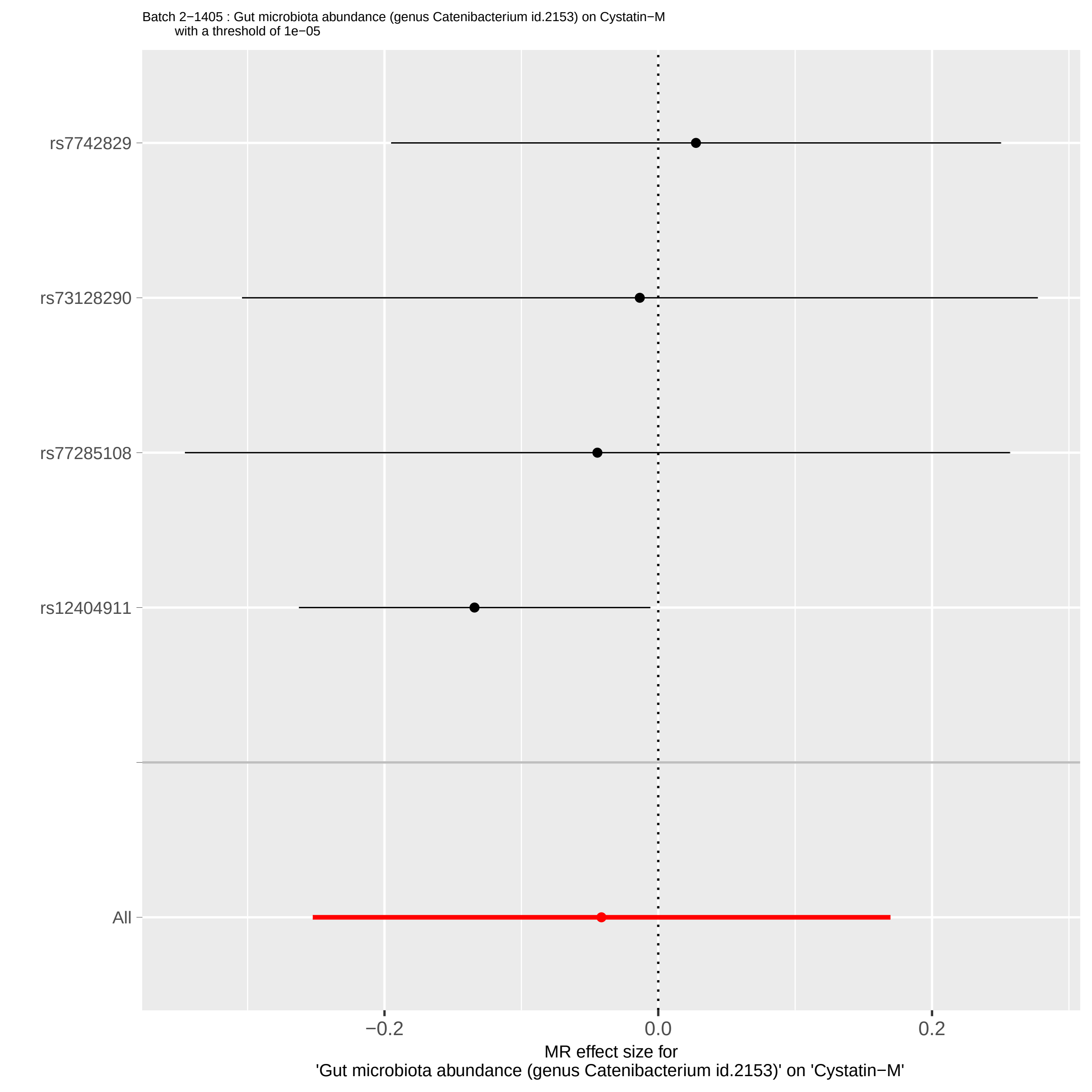

Batch 2−1405 : Gut microbiota abundance (genus Catenibacterium id.2153) on Cystatin−M
with a threshold of 1e−05
rs7742829
rs73128290
rs77285108
rs12404911
All
−0.2
0.0
0.2
MR effect size for
'Gut microbiota abundance (genus Catenibacterium id.2153)' on 'Cystatin−M'

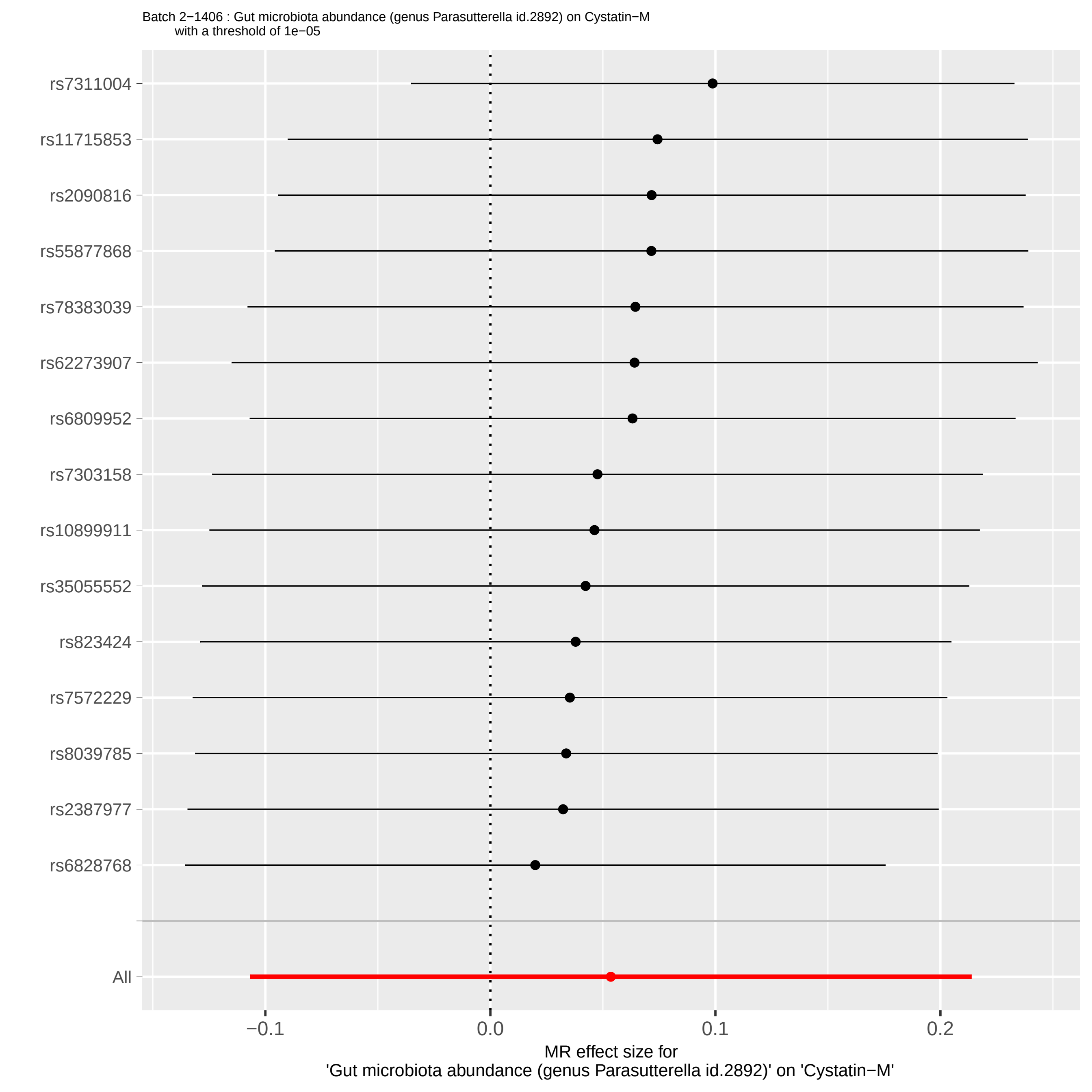

Batch 2−1406 : Gut microbiota abundance (genus Parasutterella id.2892) on Cystatin−M
with a threshold of 1e−05
rs7311004
rs11715853
rs2090816
rs55877868
rs78383039
rs62273907
rs6809952
rs7303158
rs10899911
rs35055552
rs823424
rs7572229
rs8039785
rs2387977
rs6828768
All
−0.1
0.0
0.1
0.2
MR effect size for
'Gut microbiota abundance (genus Parasutterella id.2892)' on 'Cystatin−M'

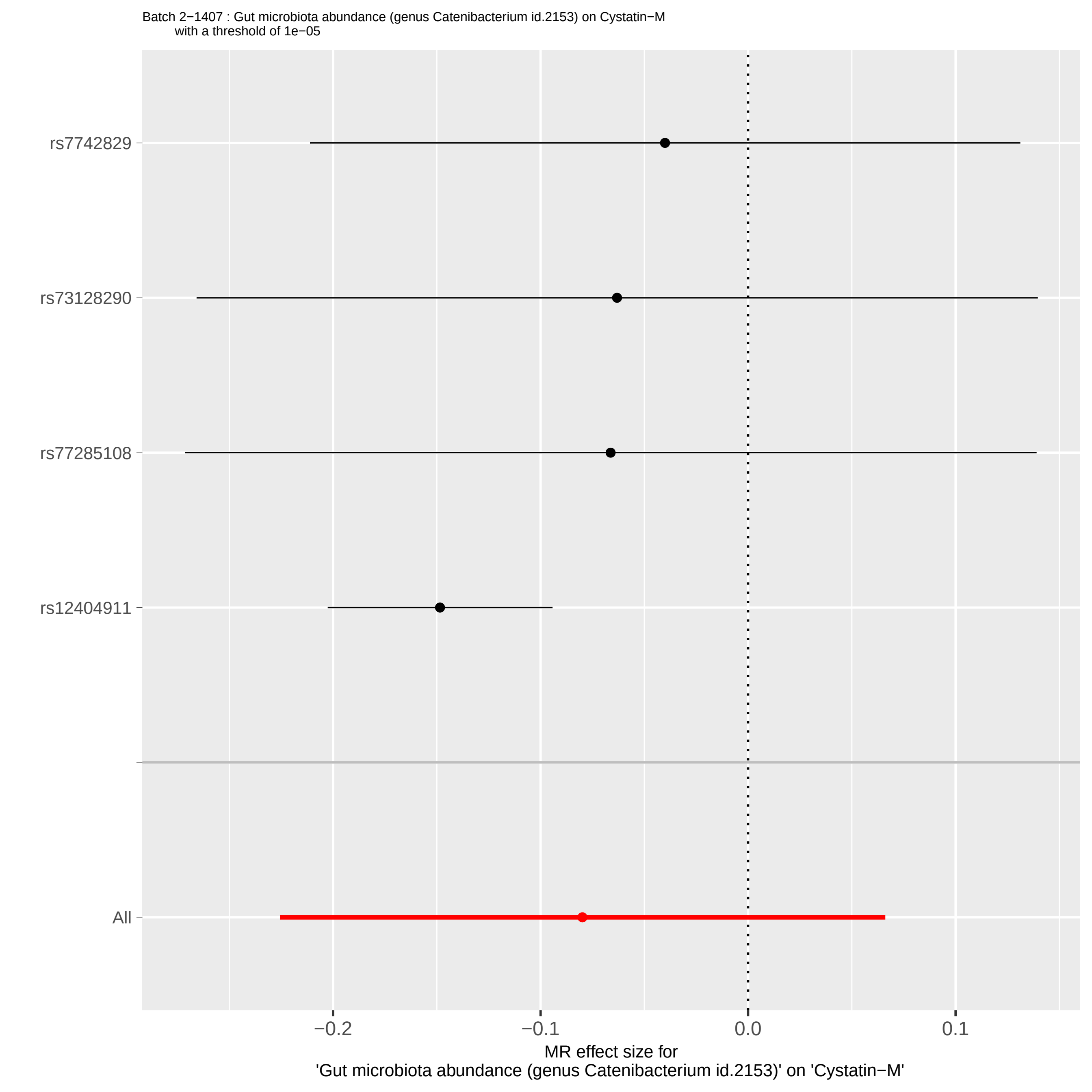
Batch 2−1407 : Gut microbiota abundance (genus Catenibacterium id.2153) on Cystatin−M
with a threshold of 1e−05
rs7742829
rs73128290
rs77285108
rs12404911
All
−0.2
−0.1
0.0
0.1
MR effect size for
'Gut microbiota abundance (genus Catenibacterium id.2153)' on 'Cystatin−M'

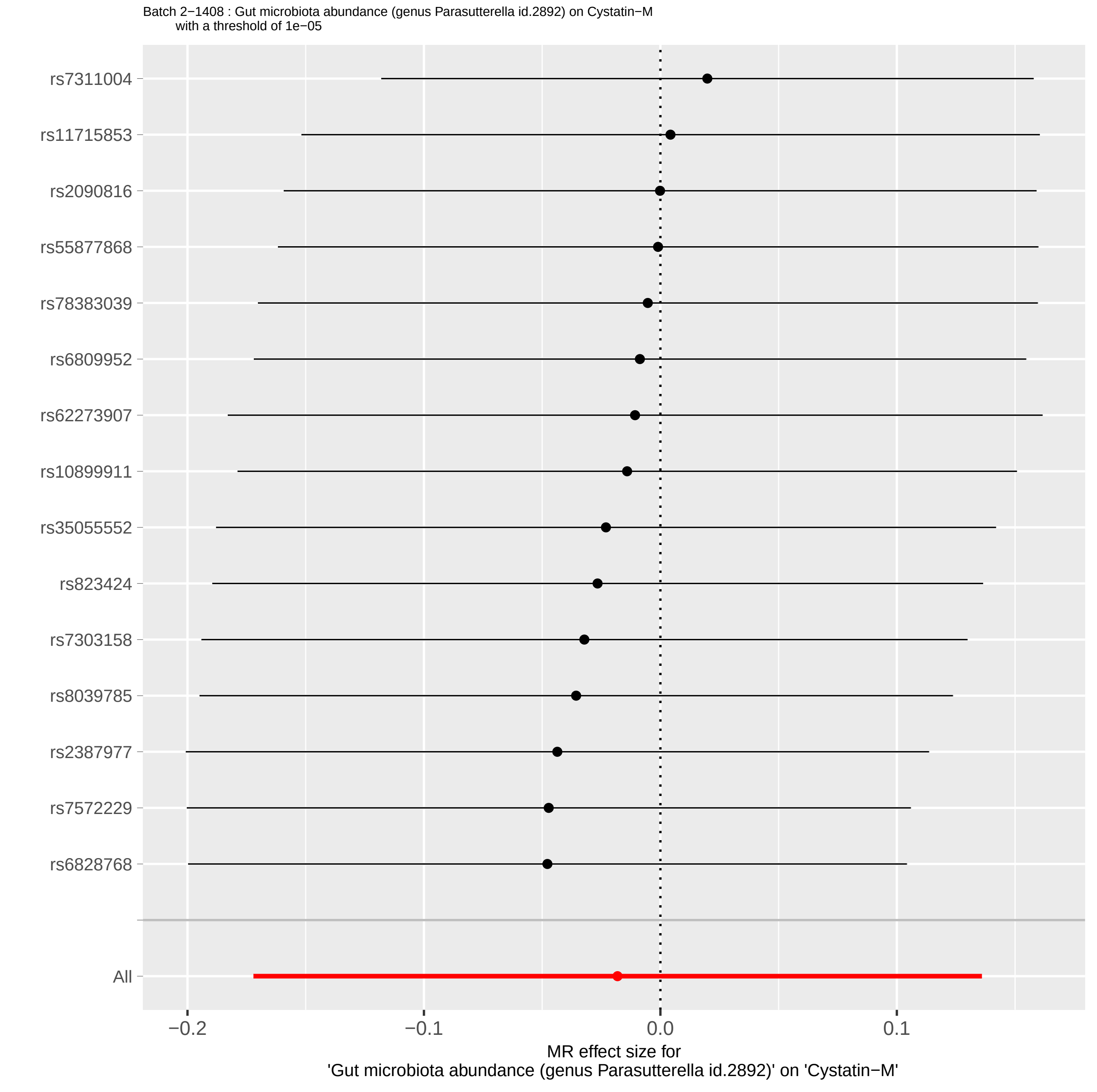
Batch 2−1408 : Gut microbiota abundance (genus Parasutterella id.2892) on Cystatin−M
with a threshold of 1e−05
rs7311004
rs11715853
rs2090816
rs55877868
rs78383039
rs6809952
rs62273907
rs10899911
rs35055552
rs823424
rs7303158
rs8039785
rs2387977
rs7572229
rs6828768
All
−0.2
−0.1
0.0
0.1
MR effect size for
'Gut microbiota abundance (genus Parasutterella id.2892)' on 'Cystatin−M'

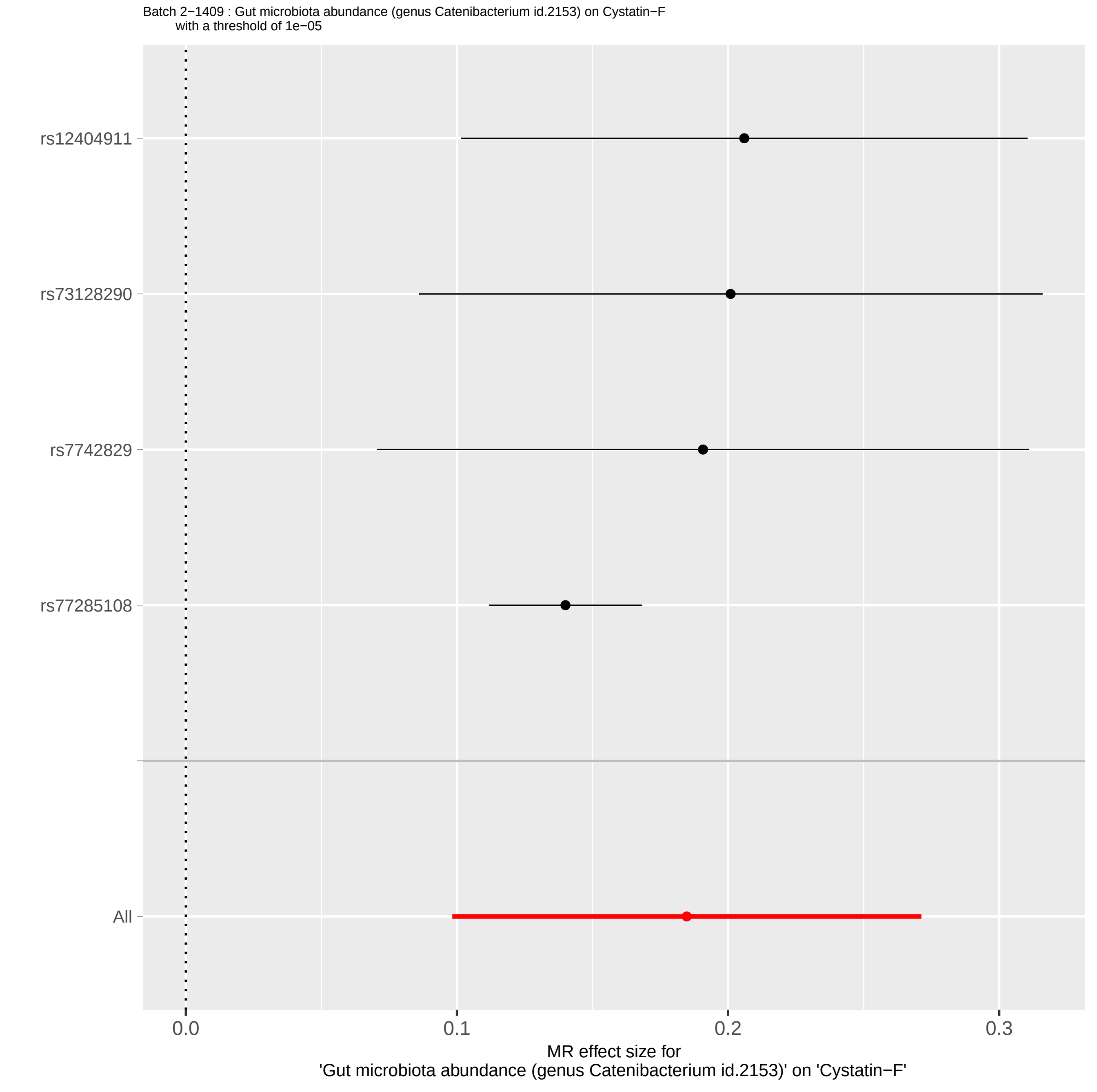
Batch 2−1409 : Gut microbiota abundance (genus Catenibacterium id.2153) on Cystatin−F
with a threshold of 1e−05
rs12404911
rs73128290
rs7742829
rs77285108
All
0.0
0.1
0.2
0.3
MR effect size for
'Gut microbiota abundance (genus Catenibacterium id.2153)' on 'Cystatin−F'

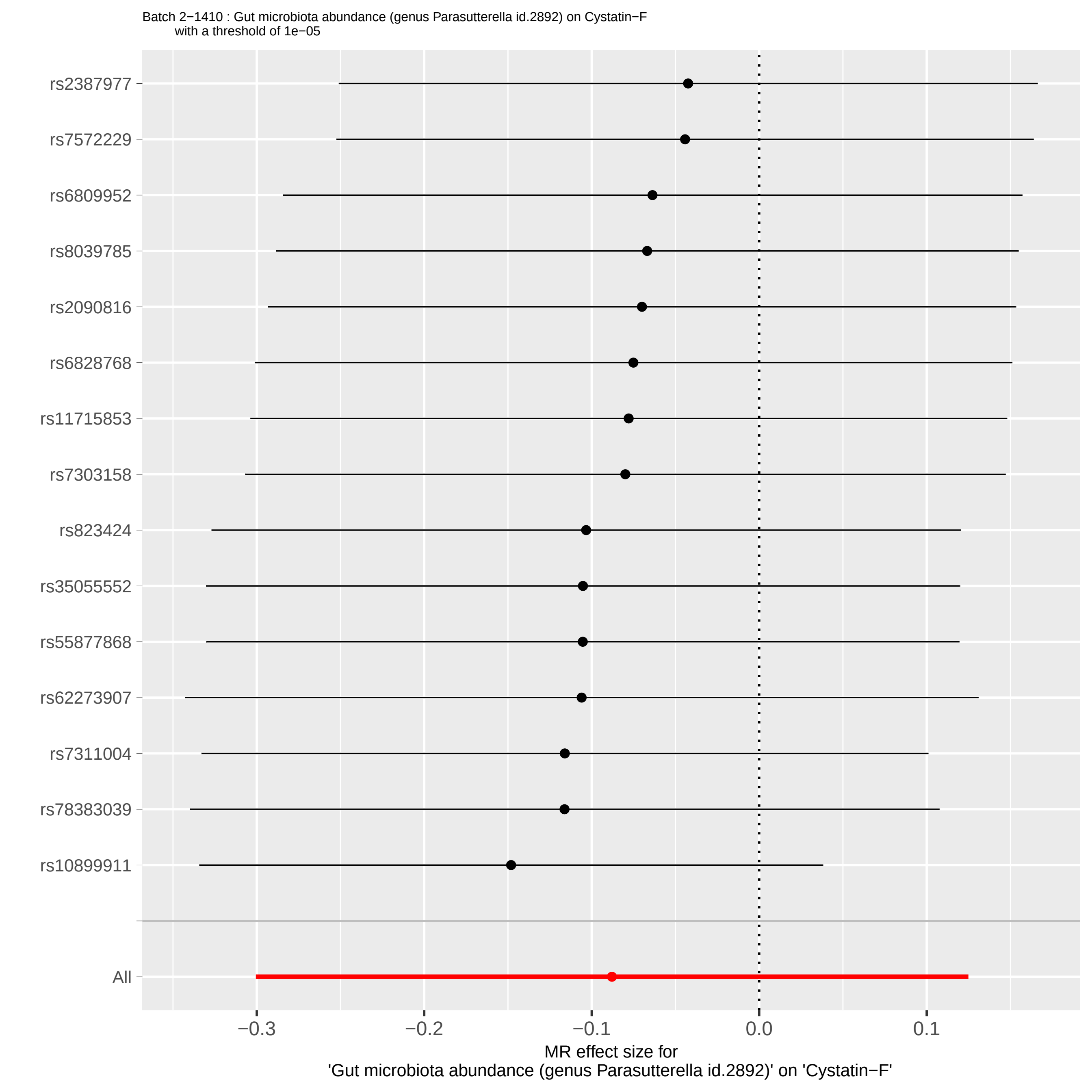
Batch 2−1410 : Gut microbiota abundance (genus Parasutterella id.2892) on Cystatin−F
with a threshold of 1e−05
rs2387977
rs7572229
rs6809952
rs8039785
rs2090816
rs6828768
rs11715853
rs7303158
rs823424
rs35055552
rs55877868
rs62273907
rs7311004
rs78383039
rs10899911
All
−0.3
−0.2
−0.1
0.0
0.1
MR effect size for
'Gut microbiota abundance (genus Parasutterella id.2892)' on 'Cystatin−F'

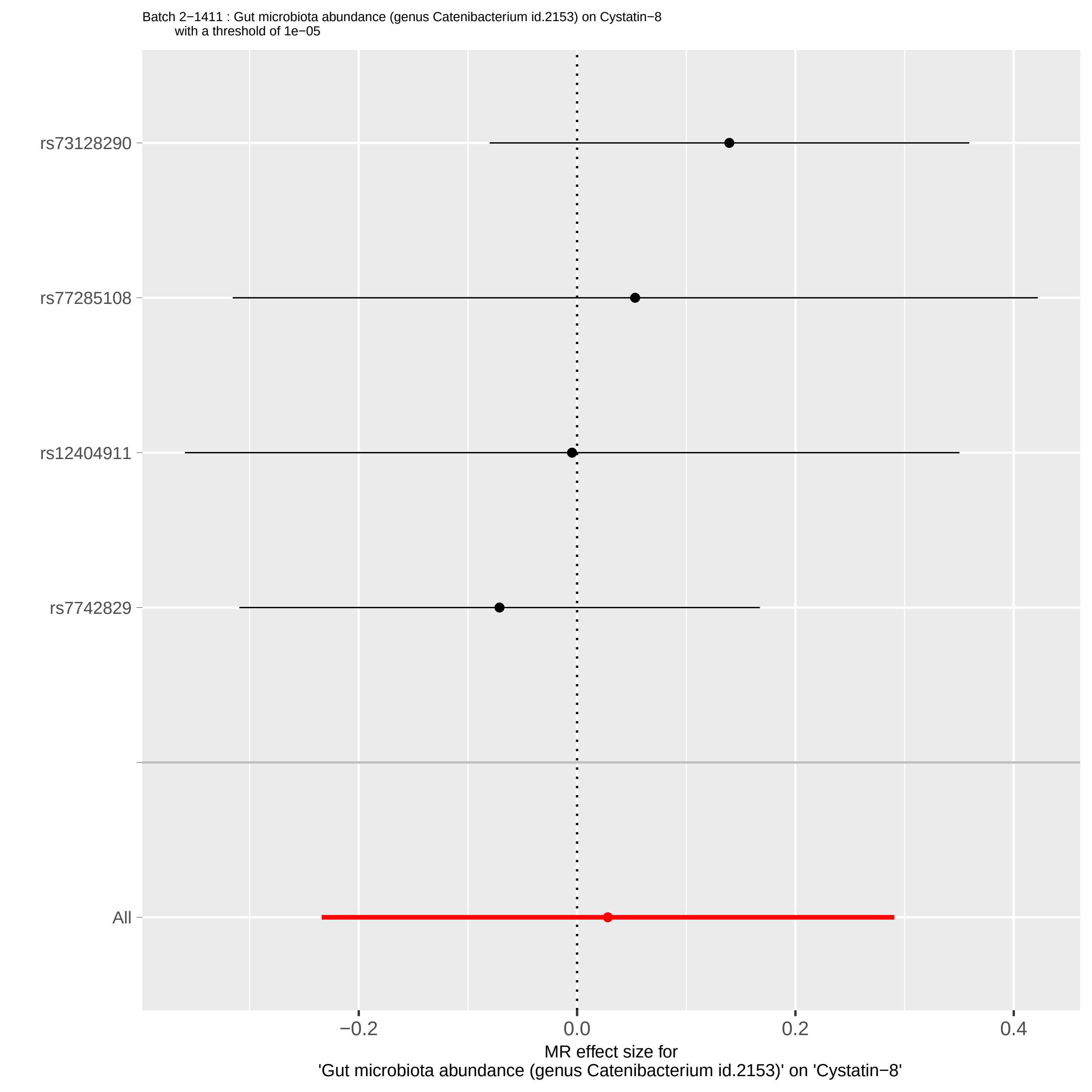
Batch 2−1411 : Gut microbiota abundance (genus Catenibacterium id.2153) on Cystatin−8
with a threshold of 1e−05
rs73128290
rs77285108
rs12404911
rs7742829
All
−0.2
0.0
0.2
0.4
MR effect size for
'Gut microbiota abundance (genus Catenibacterium id.2153)' on 'Cystatin−8'

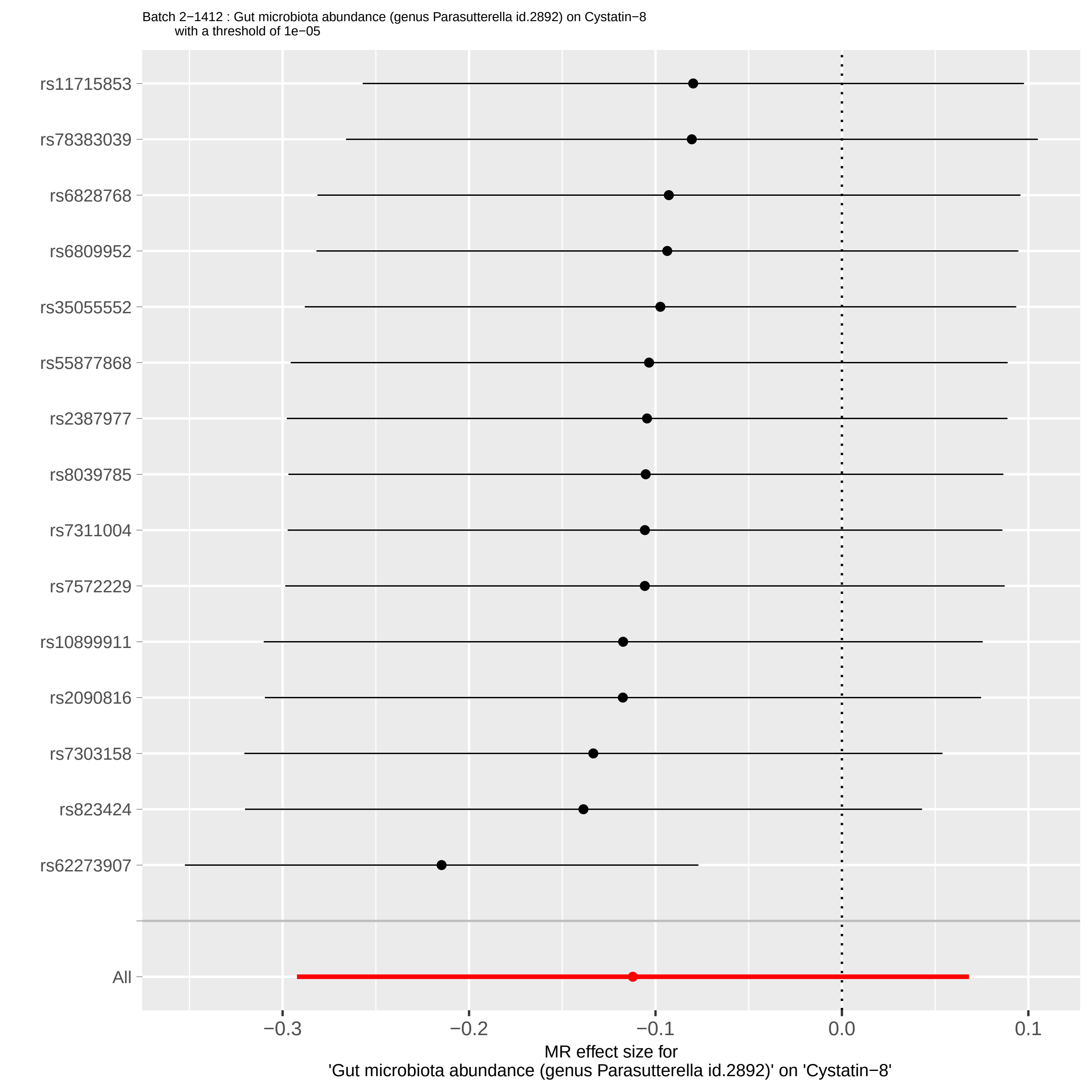

Batch 2−1412 : Gut microbiota abundance (genus Parasutterella id.2892) on Cystatin−8
with a threshold of 1e−05
rs11715853
rs78383039
rs6828768
rs6809952
rs35055552
rs55877868
rs2387977
rs8039785
rs7311004
rs7572229
rs10899911
rs2090816
rs7303158
rs823424
rs62273907
All
−0.3
−0.2
−0.1
0.0
0.1
MR effect size for
'Gut microbiota abundance (genus Parasutterella id.2892)' on 'Cystatin−8'

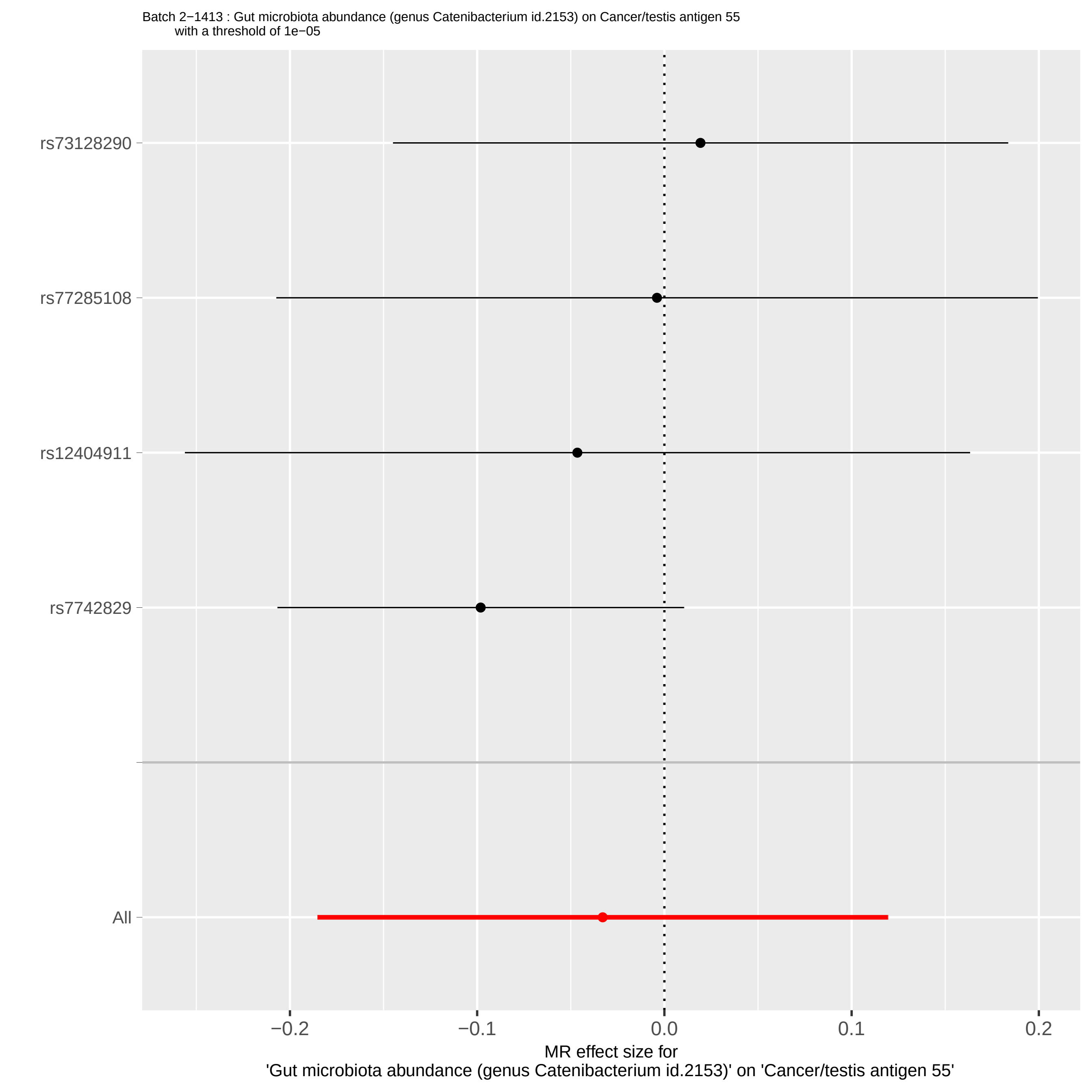
Batch 2−1413 : Gut microbiota abundance (genus Catenibacterium id.2153) on Cancer/testis antigen 55
with a threshold of 1e−05
rs73128290
rs77285108
rs12404911
rs7742829
All
−0.2
−0.1
0.0
0.1
0.2
MR effect size for
'Gut microbiota abundance (genus Catenibacterium id.2153)' on 'Cancer/testis antigen 55'

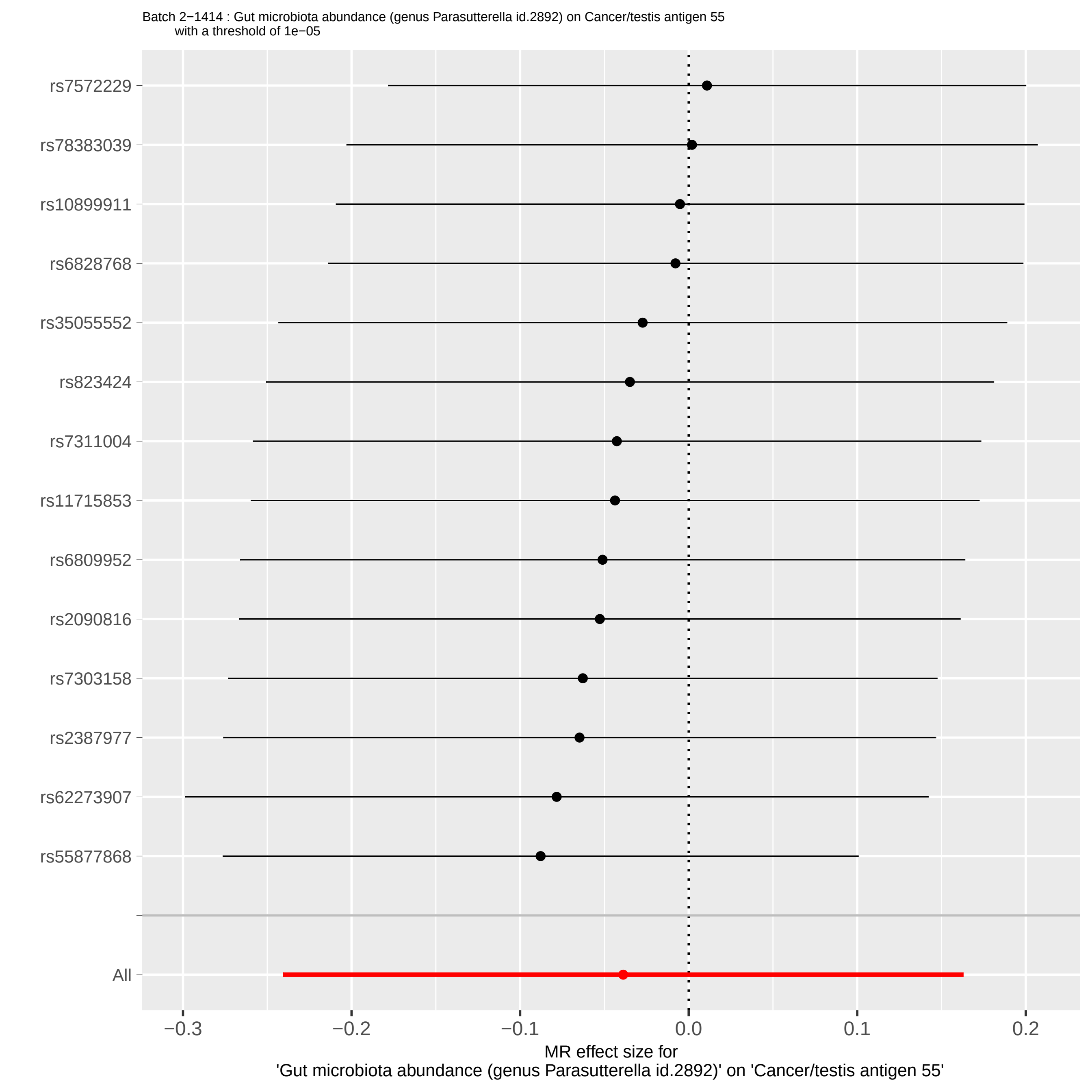
Batch 2−1414 : Gut microbiota abundance (genus Parasutterella id.2892) on Cancer/testis antigen 55
with a threshold of 1e−05
rs7572229
rs78383039
rs10899911
rs6828768
rs35055552
rs823424
rs7311004
rs11715853
rs6809952
rs2090816
rs7303158
rs2387977
rs62273907
rs55877868
All
−0.3
−0.2
−0.1
0.0
0.1
0.2
MR effect size for
'Gut microbiota abundance (genus Parasutterella id.2892)' on 'Cancer/testis antigen 55'

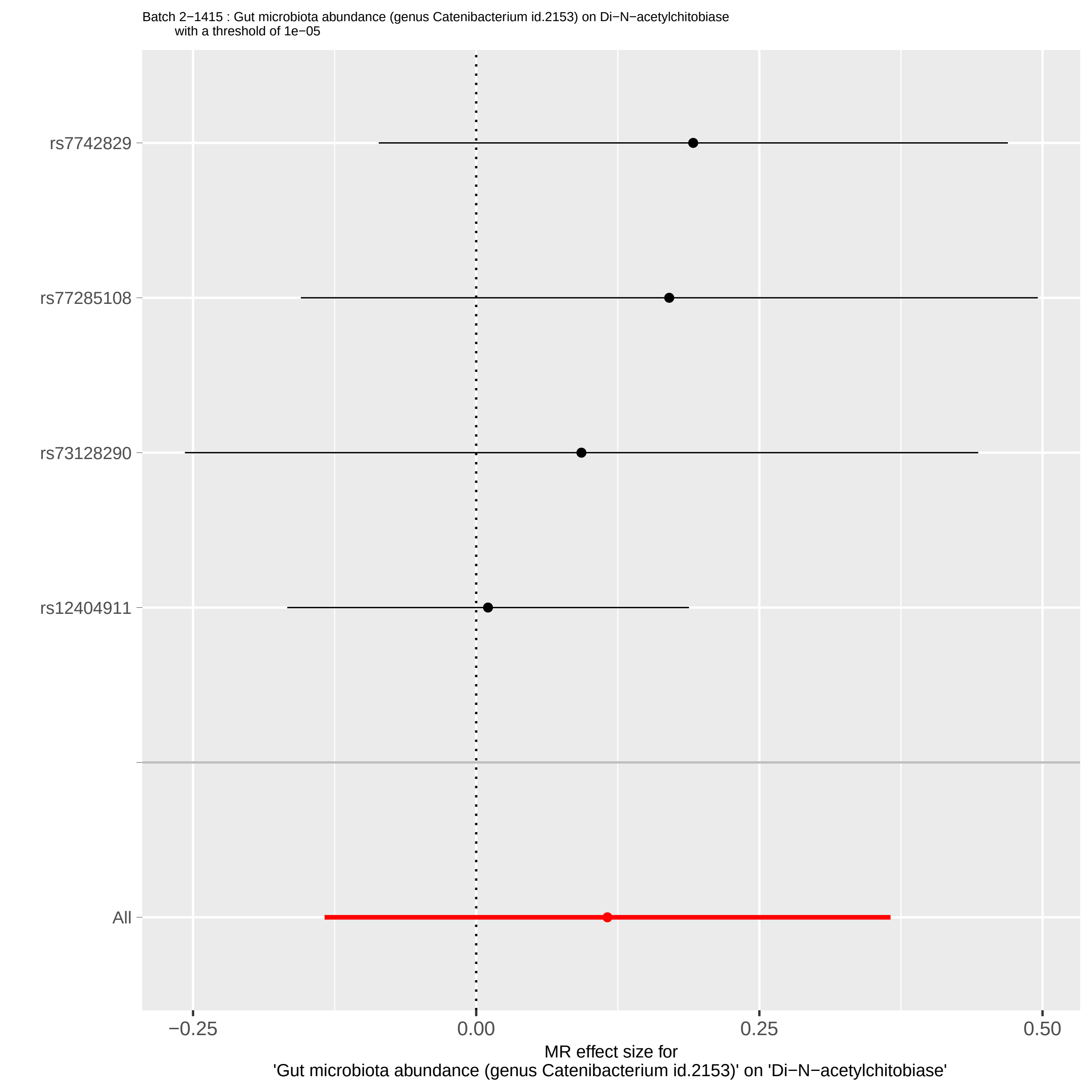

Batch 2−1415 : Gut microbiota abundance (genus Catenibacterium id.2153) on Di−N−acetylchitobiase
with a threshold of 1e−05
rs7742829
rs77285108
rs73128290
rs12404911
All
−0.25
0.00
0.25
0.50
MR effect size for
'Gut microbiota abundance (genus Catenibacterium id.2153)' on 'Di−N−acetylchitobiase'

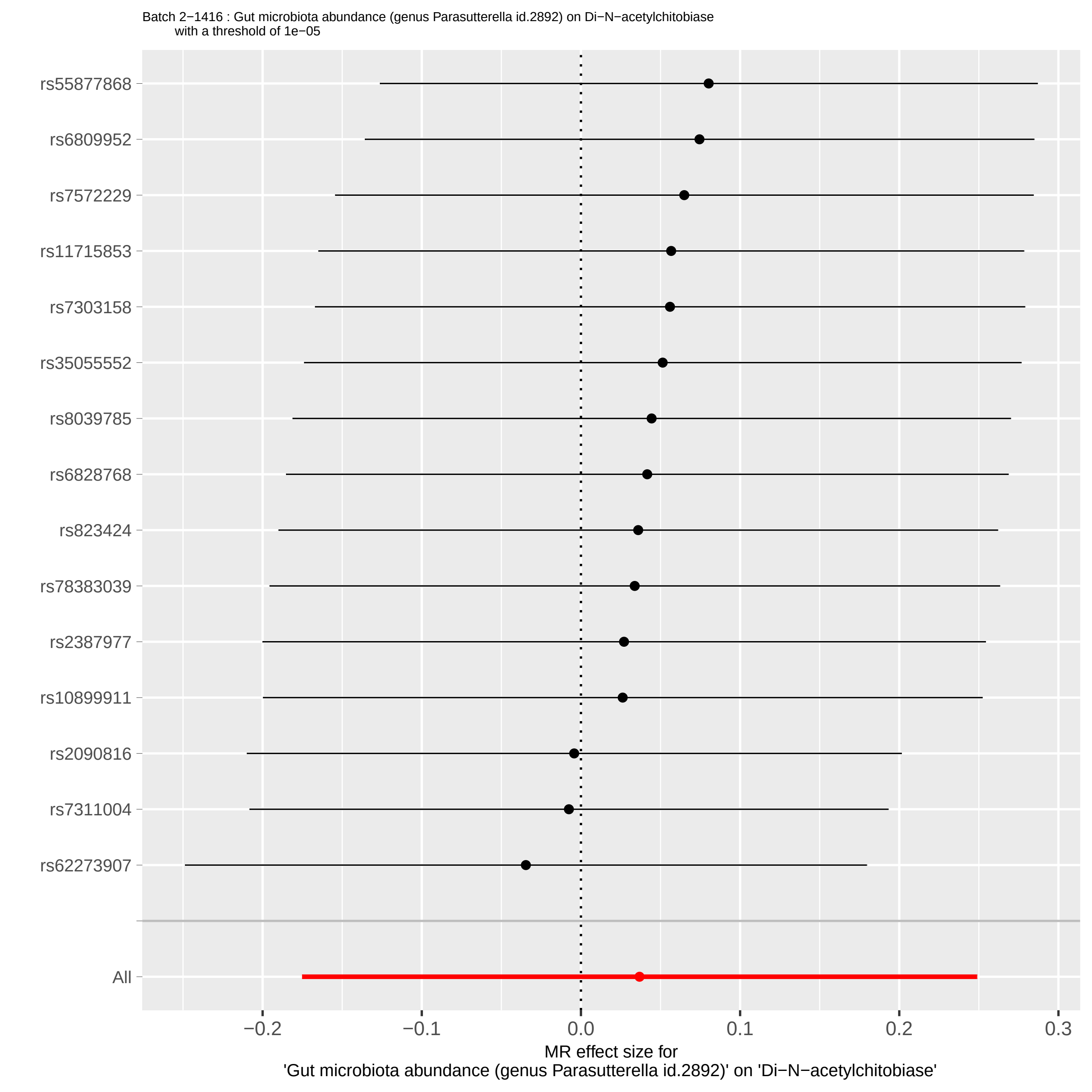
Batch 2−1416 : Gut microbiota abundance (genus Parasutterella id.2892) on Di−N−acetylchitobiase
with a threshold of 1e−05
rs55877868
rs6809952
rs7572229
rs11715853
rs7303158
rs35055552
rs8039785
rs6828768
rs823424
rs78383039
rs2387977
rs10899911
rs2090816
rs7311004
rs62273907
All
−0.2
−0.1
0.0
0.1
0.2
0.3
MR effect size for
'Gut microbiota abundance (genus Parasutterella id.2892)' on 'Di−N−acetylchitobiase'

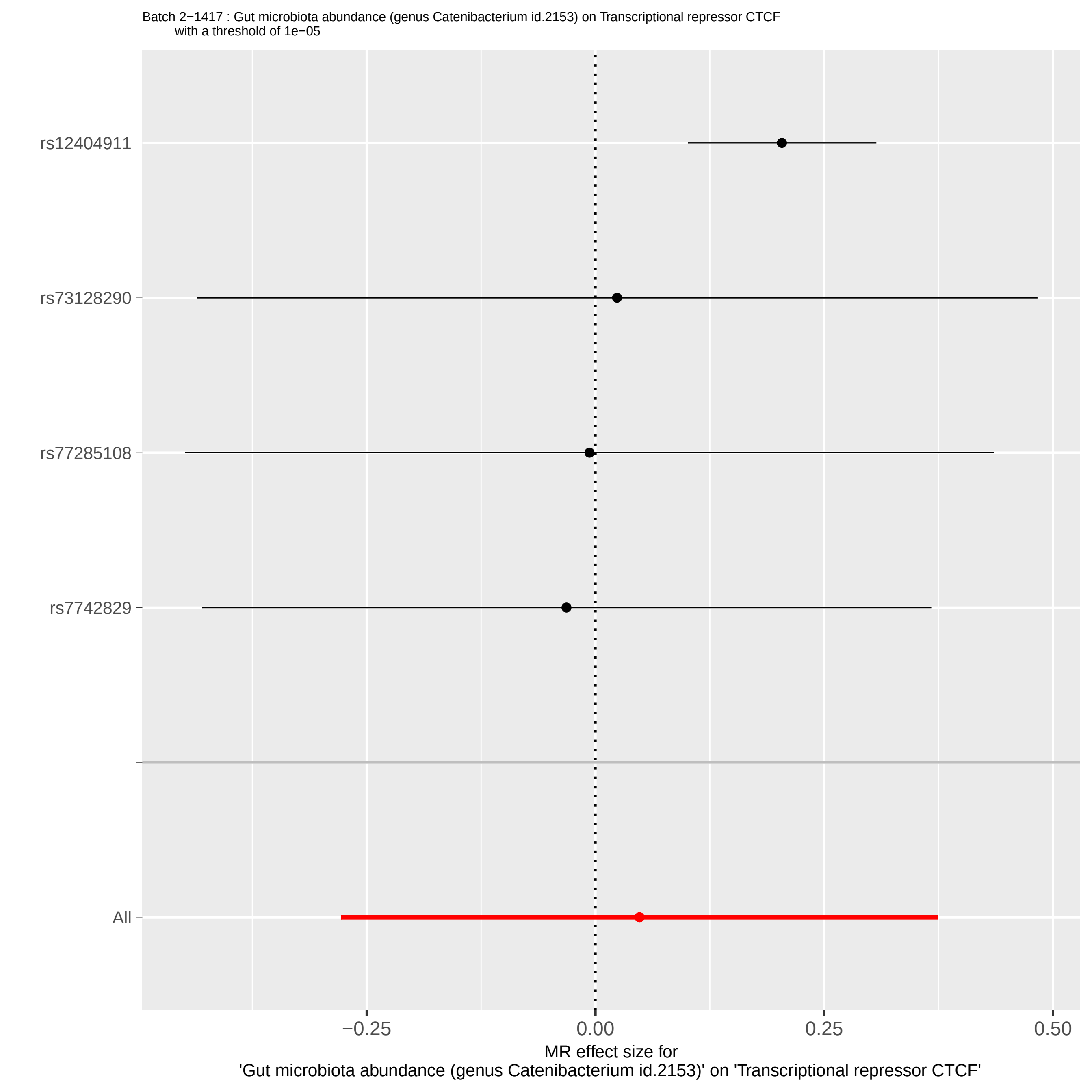
Batch 2−1417 : Gut microbiota abundance (genus Catenibacterium id.2153) on Transcriptional repressor CTCF
with a threshold of 1e−05
rs12404911
rs73128290
rs77285108
rs7742829
All
−0.25
0.00
0.25
0.50
MR effect size for
'Gut microbiota abundance (genus Catenibacterium id.2153)' on 'Transcriptional repressor CTCF'

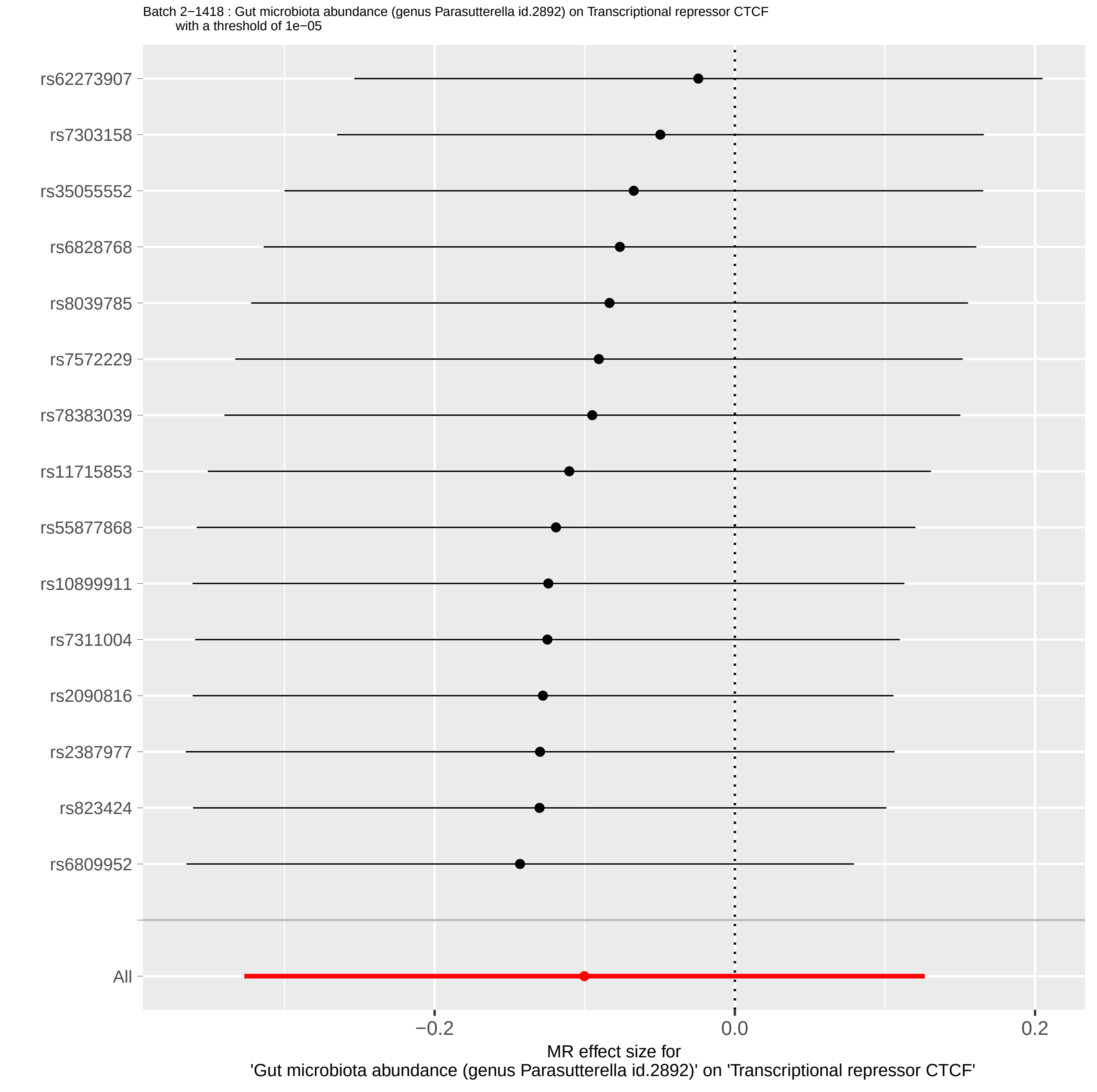
Batch 2−1418 : Gut microbiota abundance (genus Parasutterella id.2892) on Transcriptional repressor CTCF
with a threshold of 1e−05
rs62273907
rs7303158
rs35055552
rs6828768
rs8039785
rs7572229
rs78383039
rs11715853
rs55877868
rs10899911
rs7311004
rs2090816
rs2387977
rs823424
rs6809952
All
−0.2
0.0
0.2
MR effect size for
'Gut microbiota abundance (genus Parasutterella id.2892)' on 'Transcriptional repressor CTCF'

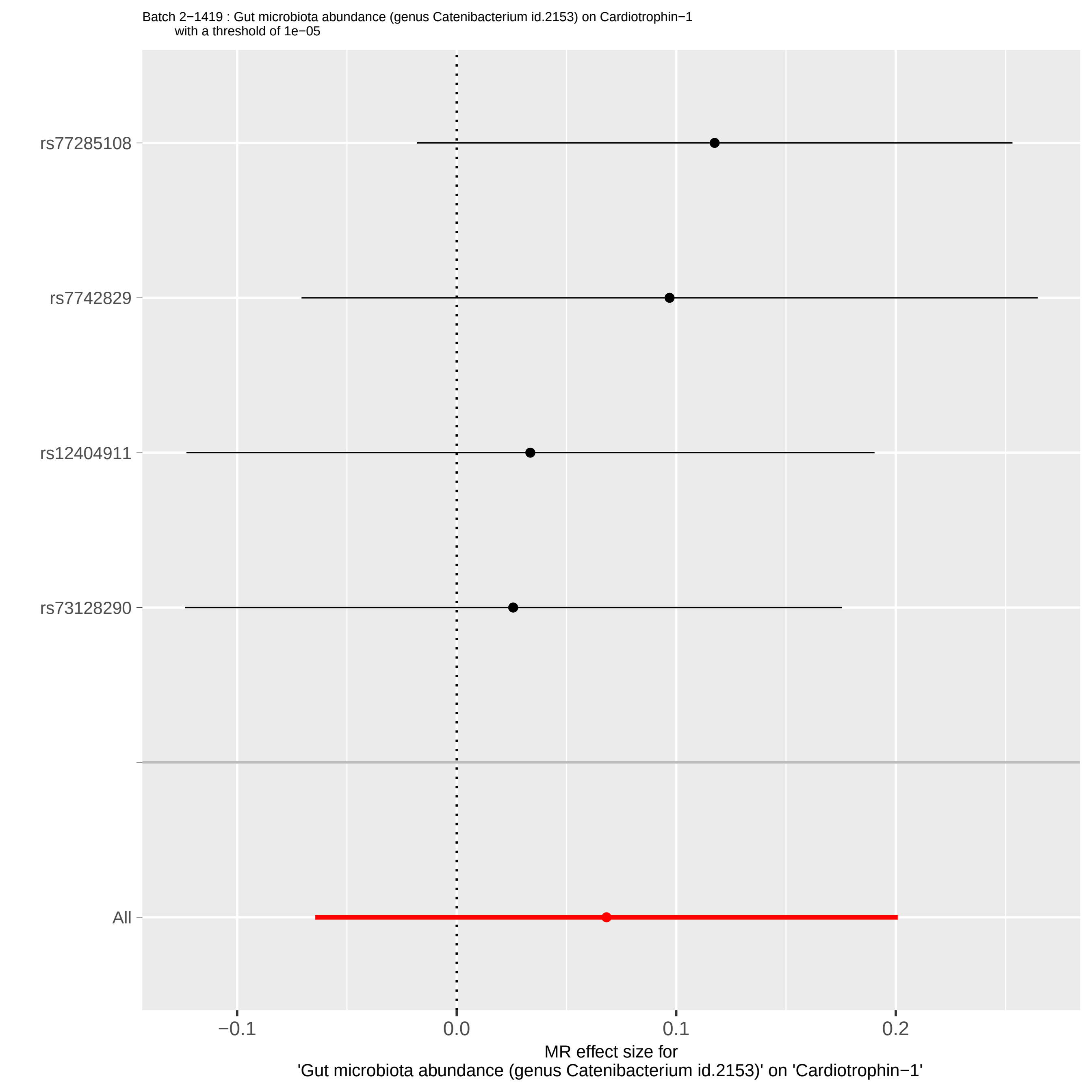

Batch 2−1419 : Gut microbiota abundance (genus Catenibacterium id.2153) on Cardiotrophin−1
with a threshold of 1e−05
rs77285108
rs7742829
rs12404911
rs73128290
All
−0.1
0.0
0.1
0.2
MR effect size for
'Gut microbiota abundance (genus Catenibacterium id.2153)' on 'Cardiotrophin−1'

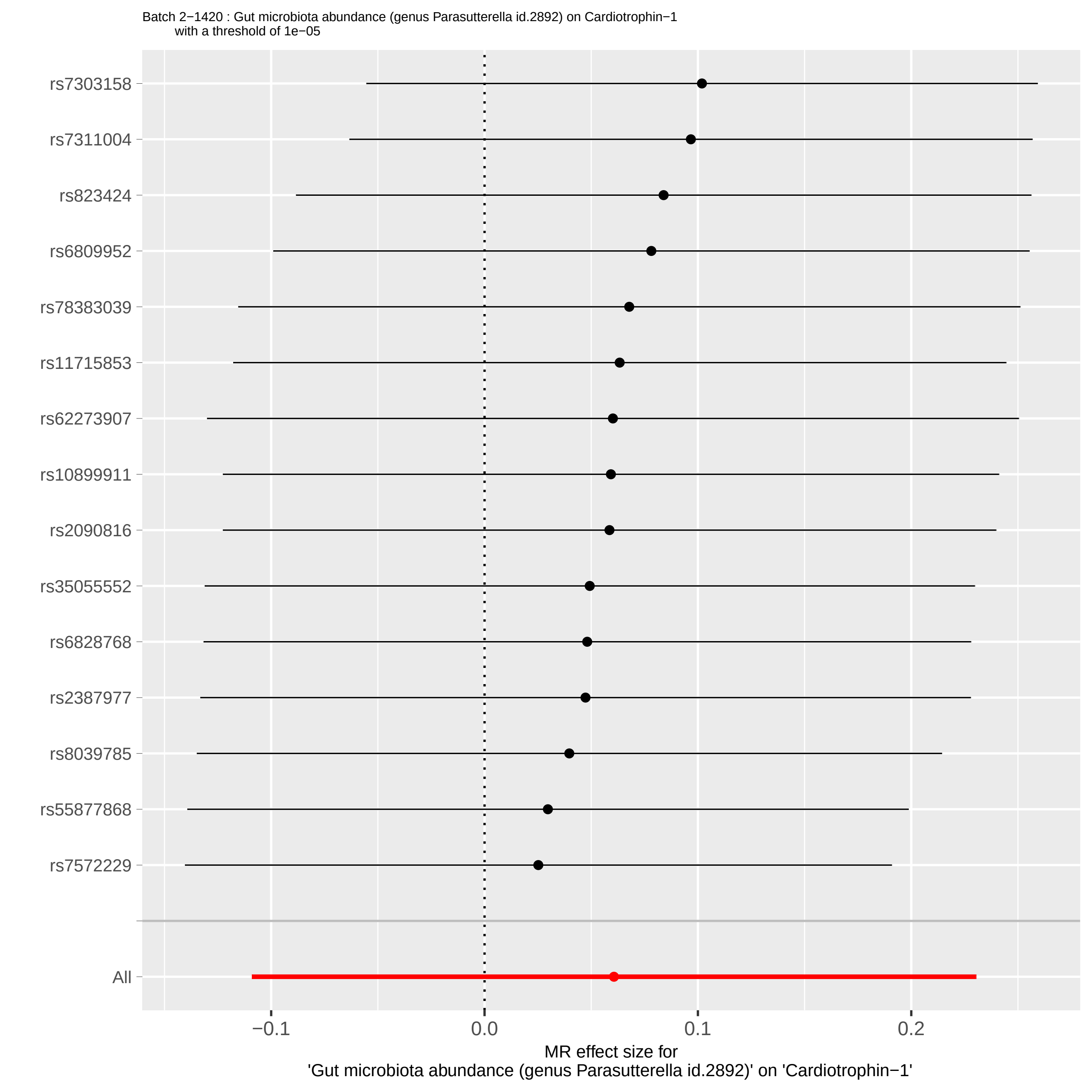

Batch 2−1420 : Gut microbiota abundance (genus Parasutterella id.2892) on Cardiotrophin−1
with a threshold of 1e−05
rs7303158
rs7311004
rs823424
rs6809952
rs78383039
rs11715853
rs62273907
rs10899911
rs2090816
rs35055552
rs6828768
rs2387977
rs8039785
rs55877868
rs7572229
All
−0.1
0.0
0.1
0.2
MR effect size for
'Gut microbiota abundance (genus Parasutterella id.2892)' on 'Cardiotrophin−1'

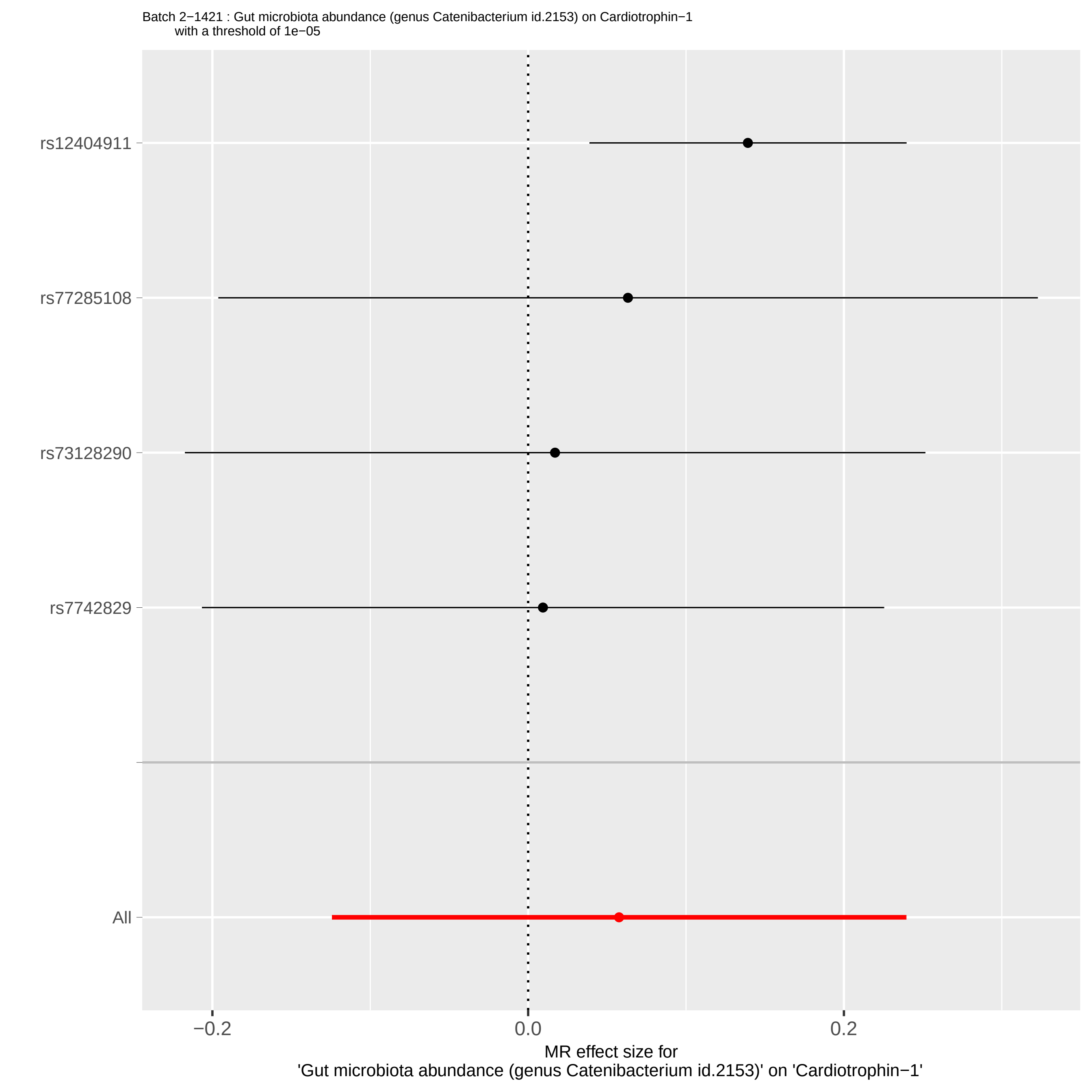
Batch 2−1421 : Gut microbiota abundance (genus Catenibacterium id.2153) on Cardiotrophin−1
with a threshold of 1e−05
rs12404911
rs77285108
rs73128290
rs7742829
All
−0.2
0.0
0.2
MR effect size for
'Gut microbiota abundance (genus Catenibacterium id.2153)' on 'Cardiotrophin−1'

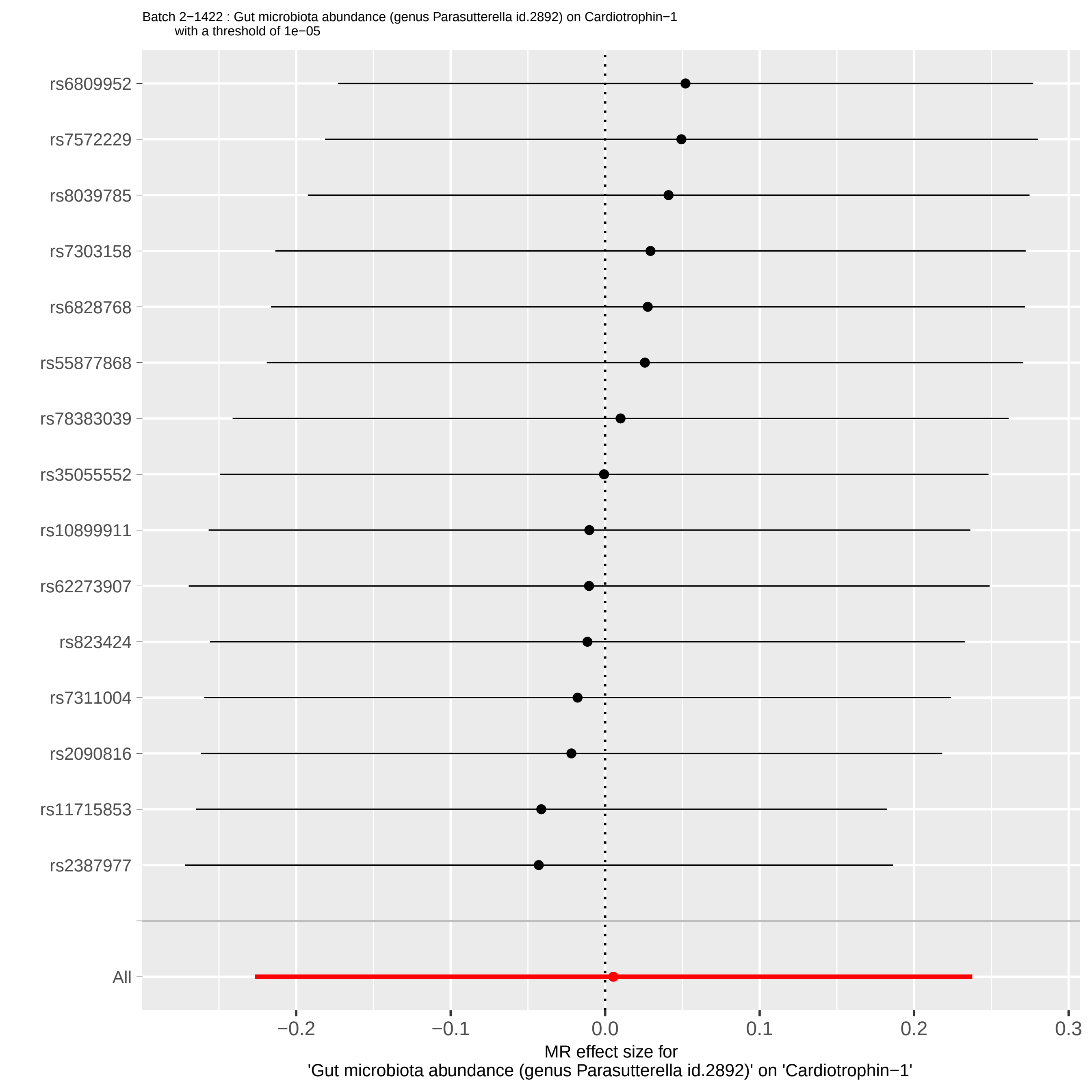
Batch 2−1422 : Gut microbiota abundance (genus Parasutterella id.2892) on Cardiotrophin−1
with a threshold of 1e−05
rs6809952
rs7572229
rs8039785
rs7303158
rs6828768
rs55877868
rs78383039
rs35055552
rs10899911
rs62273907
rs823424
rs7311004
rs2090816
rs11715853
rs2387977
All
−0.2
−0.1
0.0
0.1
0.2
0.3
MR effect size for
'Gut microbiota abundance (genus Parasutterella id.2892)' on 'Cardiotrophin−1'

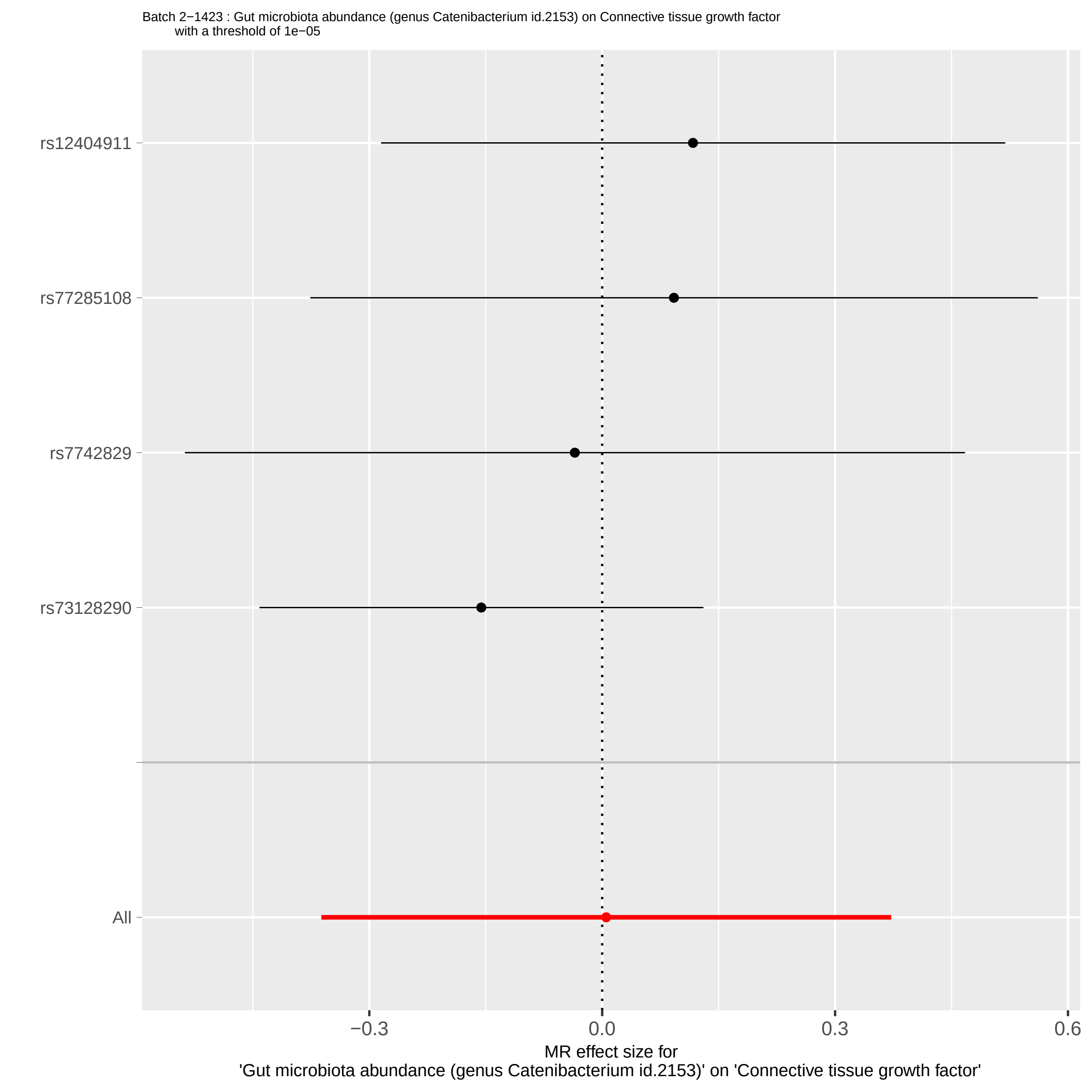
Batch 2−1423 : Gut microbiota abundance (genus Catenibacterium id.2153) on Connective tissue growth factor
with a threshold of 1e−05
rs12404911
rs77285108
rs7742829
rs73128290
All
−0.3
0.0
0.3
0.6
MR effect size for
'Gut microbiota abundance (genus Catenibacterium id.2153)' on 'Connective tissue growth factor'

Batch 2−1424 : Gut microbiota abundance (genus Parasutterella id.2892) on Connective tissue growth factor
with a threshold of 1e−05

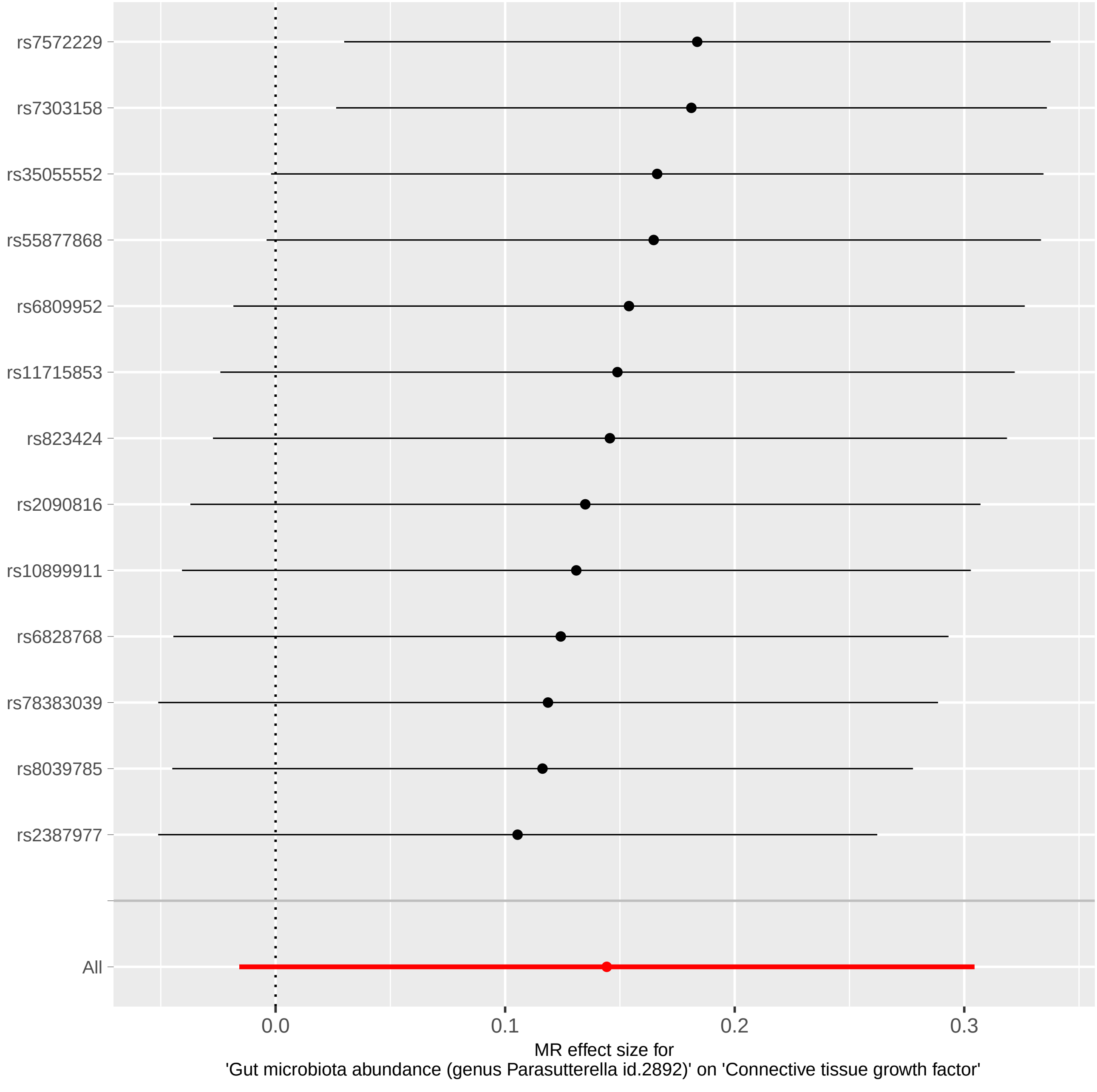

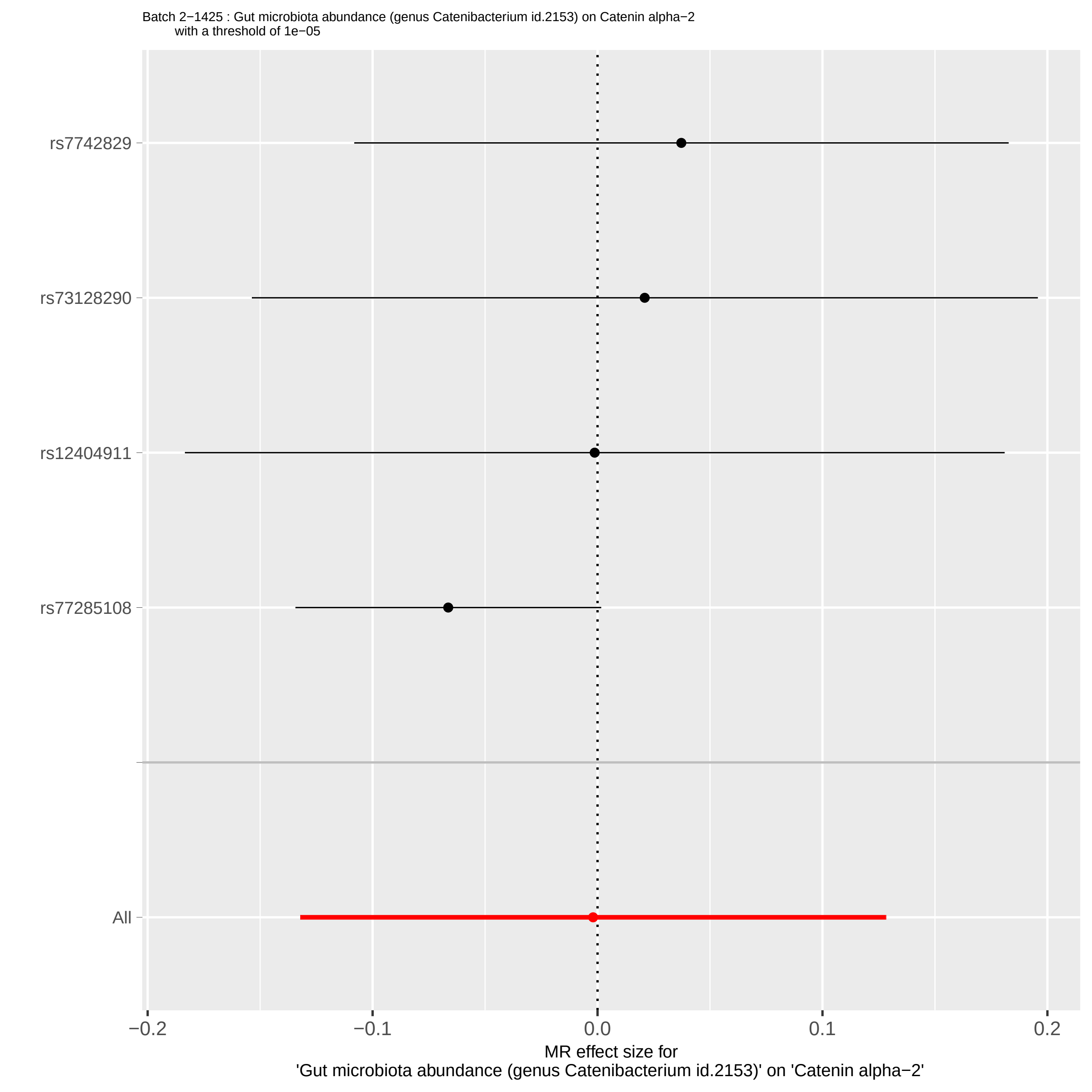

Batch 2−1425 : Gut microbiota abundance (genus Catenibacterium id.2153) on Catenin alpha−2
with a threshold of 1e−05
rs7742829
rs73128290
rs12404911
rs77285108
All
−0.2
−0.1
0.0
0.1
0.2
MR effect size for
'Gut microbiota abundance (genus Catenibacterium id.2153)' on 'Catenin alpha−2'

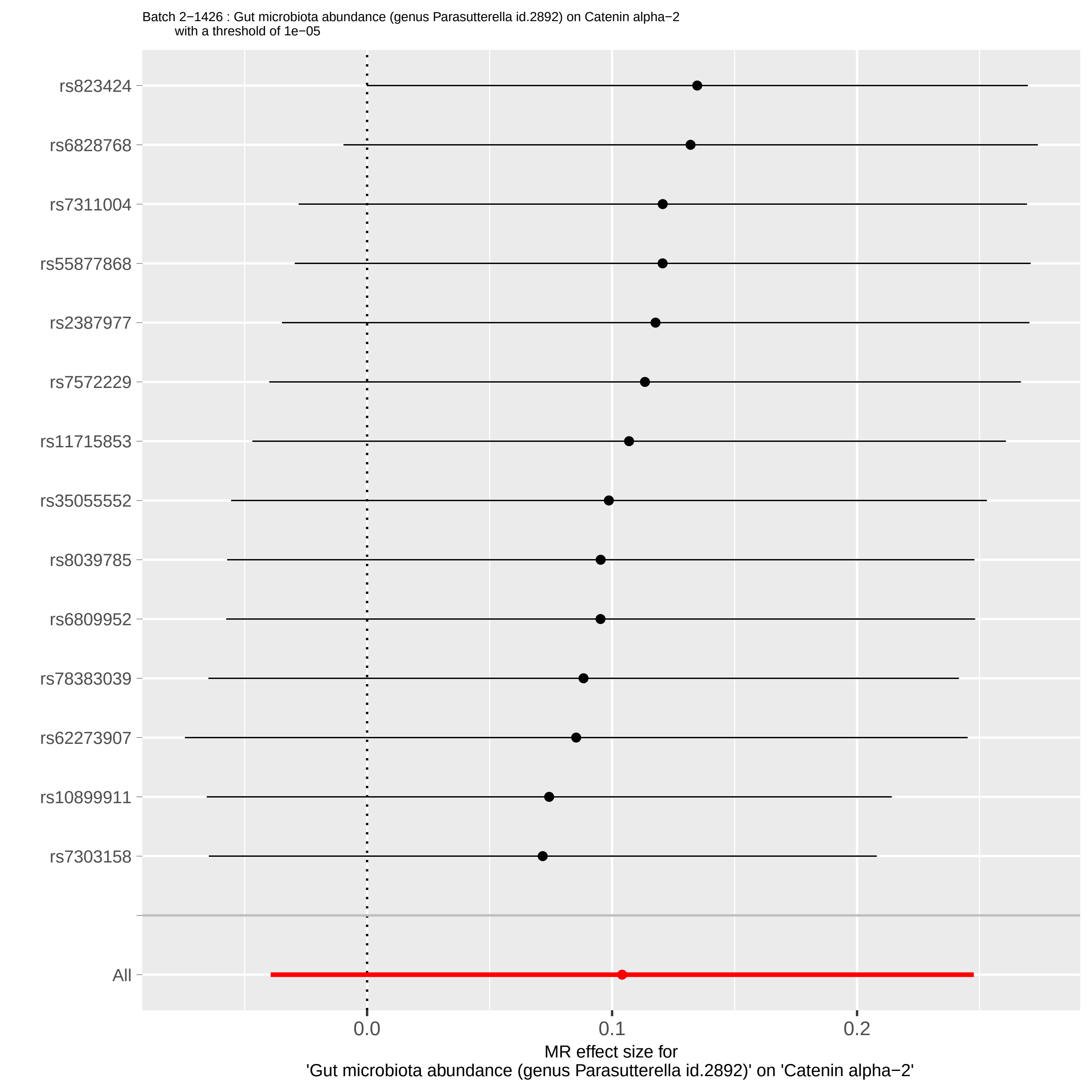

Batch 2−1426 : Gut microbiota abundance (genus Parasutterella id.2892) on Catenin alpha−2
with a threshold of 1e−05
rs823424
rs6828768
rs7311004
rs55877868
rs2387977
rs7572229
rs11715853
rs35055552
rs8039785
rs6809952
rs78383039
rs62273907
rs10899911
rs7303158
All
0.0
0.1
0.2
MR effect size for
'Gut microbiota abundance (genus Parasutterella id.2892)' on 'Catenin alpha−2'

Batch 2−1427 : Gut microbiota abundance (genus Catenibacterium id.2153) on Chymotrypsinogen B
with a threshold of 1e−05

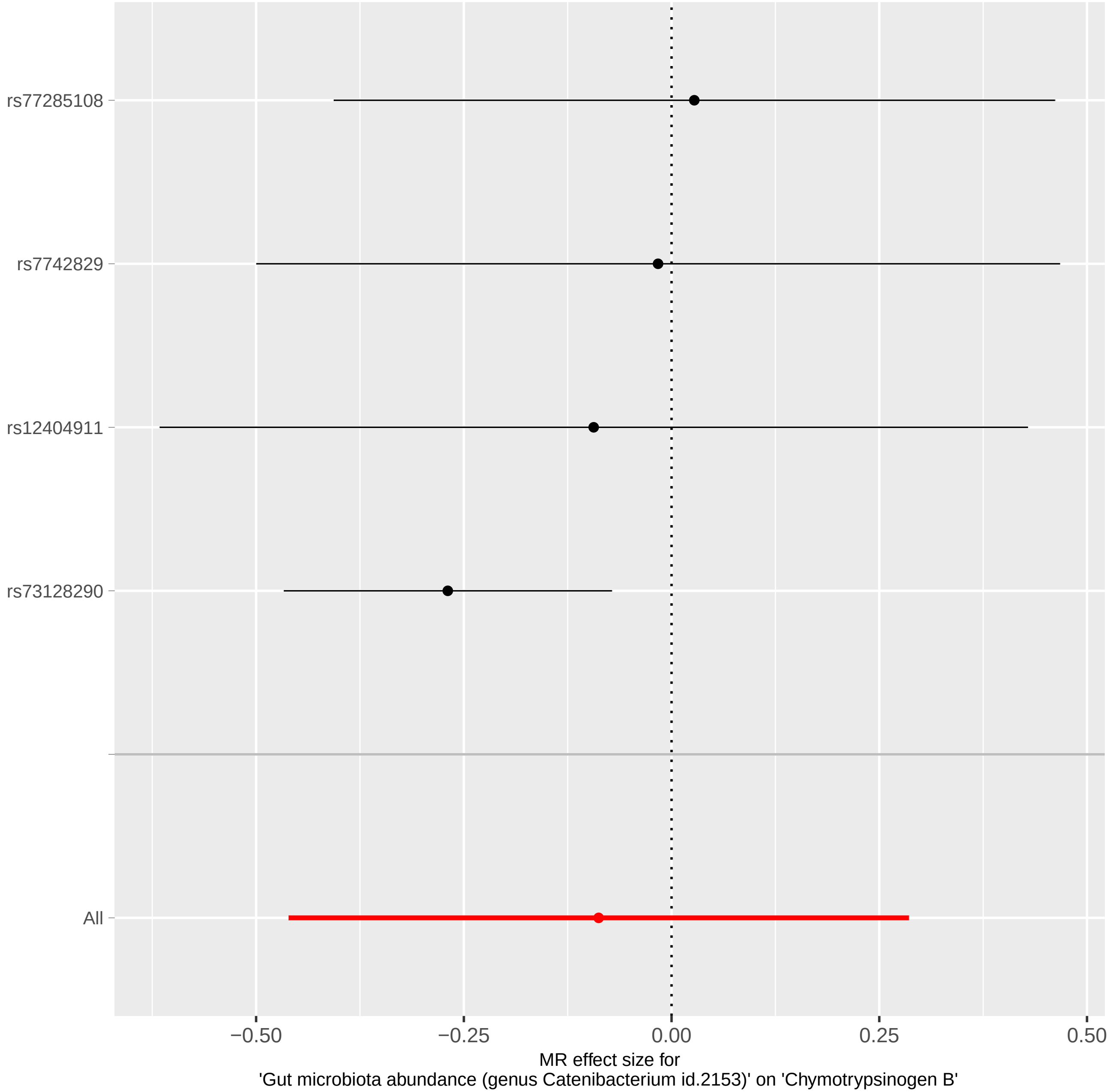

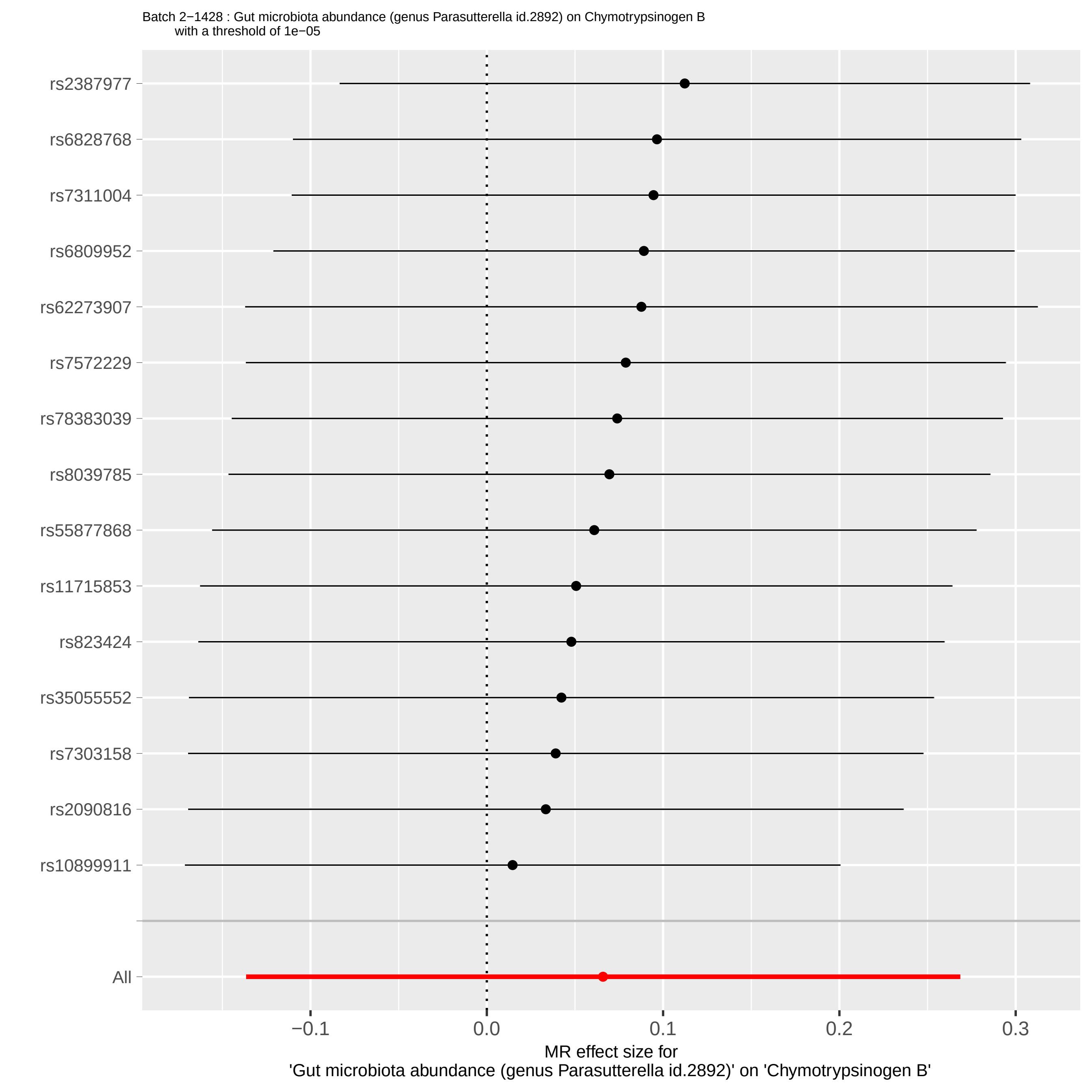

Batch 2−1428 : Gut microbiota abundance (genus Parasutterella id.2892) on Chymotrypsinogen B
with a threshold of 1e−05
rs2387977
rs6828768
rs7311004
rs6809952
rs62273907
rs7572229
rs78383039
rs8039785
rs55877868
rs11715853
rs823424
rs35055552
rs7303158
rs2090816
rs10899911
All
−0.1
0.0
0.1
0.2
0.3
MR effect size for
'Gut microbiota abundance (genus Parasutterella id.2892)' on 'Chymotrypsinogen B'

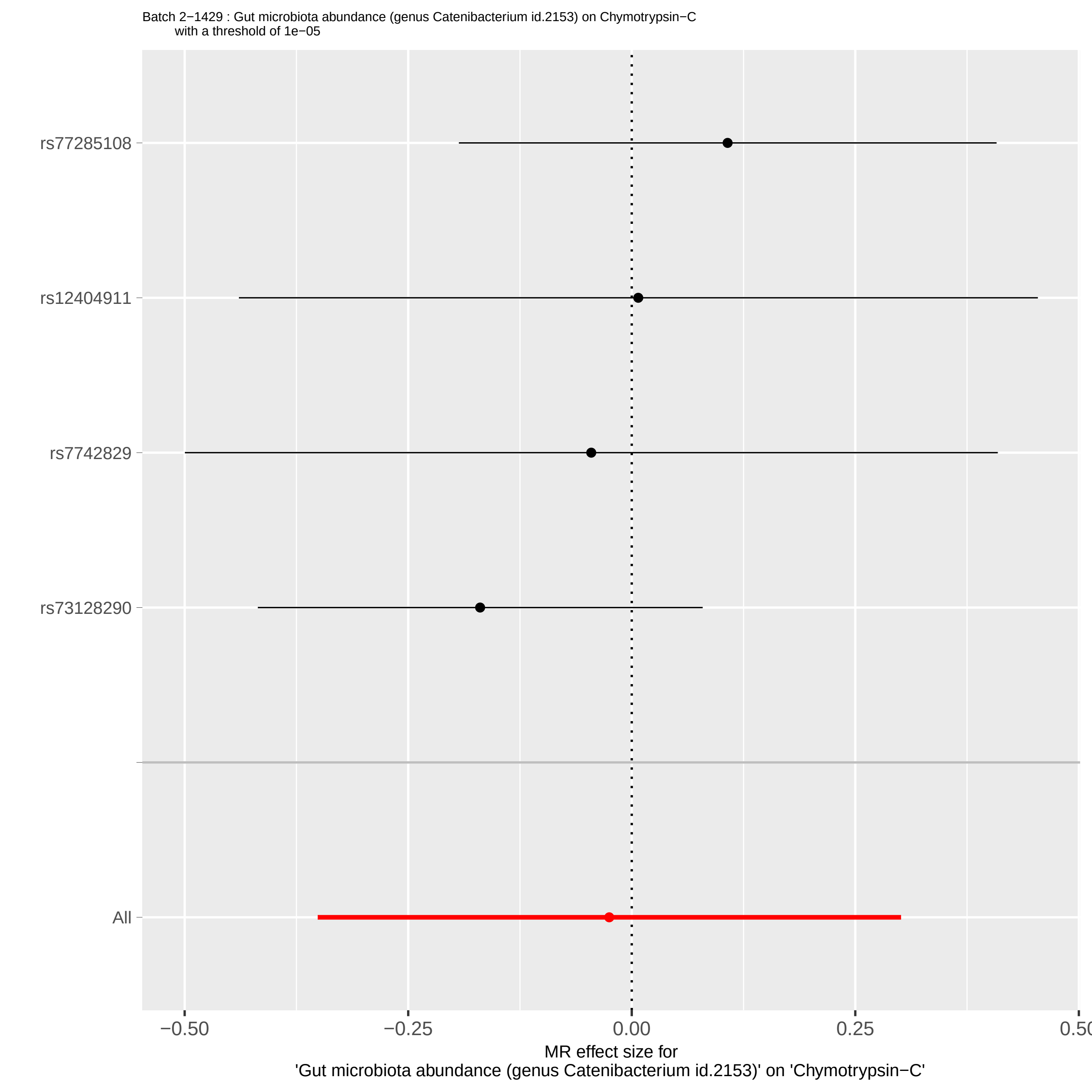

Batch 2−1429 : Gut microbiota abundance (genus Catenibacterium id.2153) on Chymotrypsin−C
with a threshold of 1e−05
rs77285108
rs12404911
rs7742829
rs73128290
All
−0.50
−0.25
0.00
0.25
0.50
MR effect size for
'Gut microbiota abundance (genus Catenibacterium id.2153)' on 'Chymotrypsin−C'

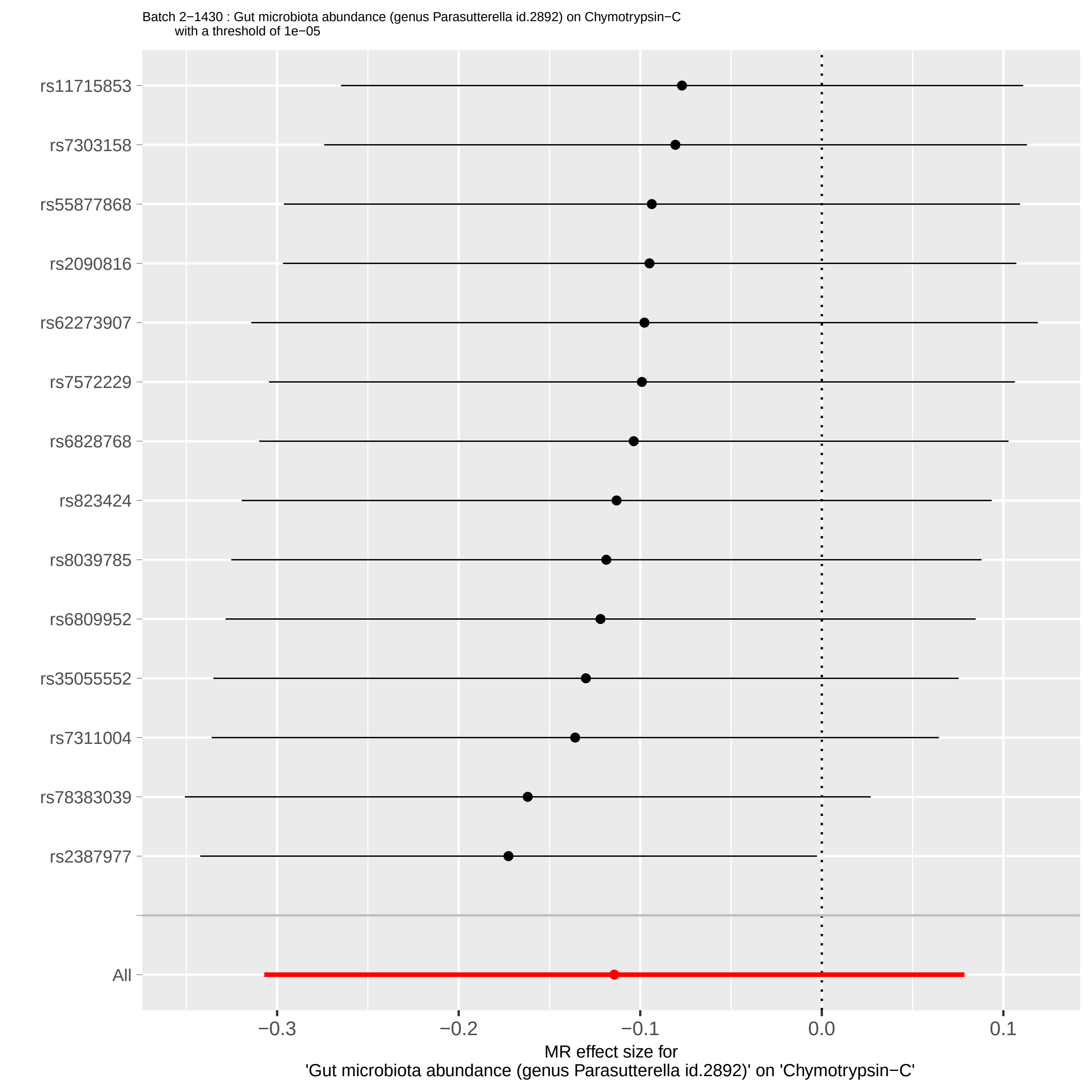

Batch 2−1430 : Gut microbiota abundance (genus Parasutterella id.2892) on Chymotrypsin−C
with a threshold of 1e−05
rs11715853
rs7303158
rs55877868
rs2090816
rs62273907
rs7572229
rs6828768
rs823424
rs8039785
rs6809952
rs35055552
rs7311004
rs78383039
rs2387977
All
−0.3
−0.2
−0.1
0.0
0.1
MR effect size for
'Gut microbiota abundance (genus Parasutterella id.2892)' on 'Chymotrypsin−C'

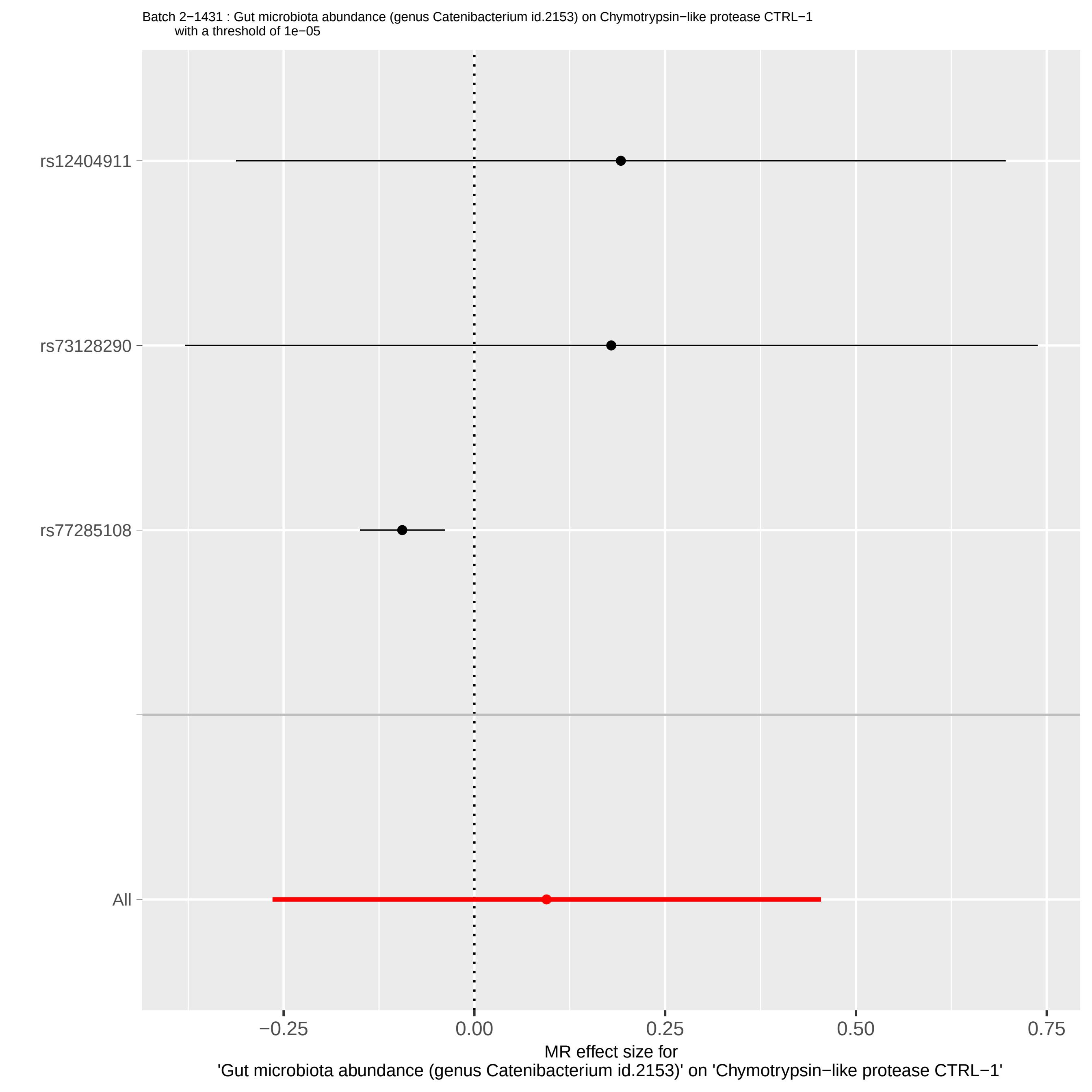

Batch 2−1431 : Gut microbiota abundance (genus Catenibacterium id.2153) on Chymotrypsin−like protease CTRL−1
with a threshold of 1e−05
rs12404911
rs73128290
rs77285108
All
−0.25
0.00
0.25
0.50
0.75
MR effect size for
'Gut microbiota abundance (genus Catenibacterium id.2153)' on 'Chymotrypsin−like protease CTRL−1'

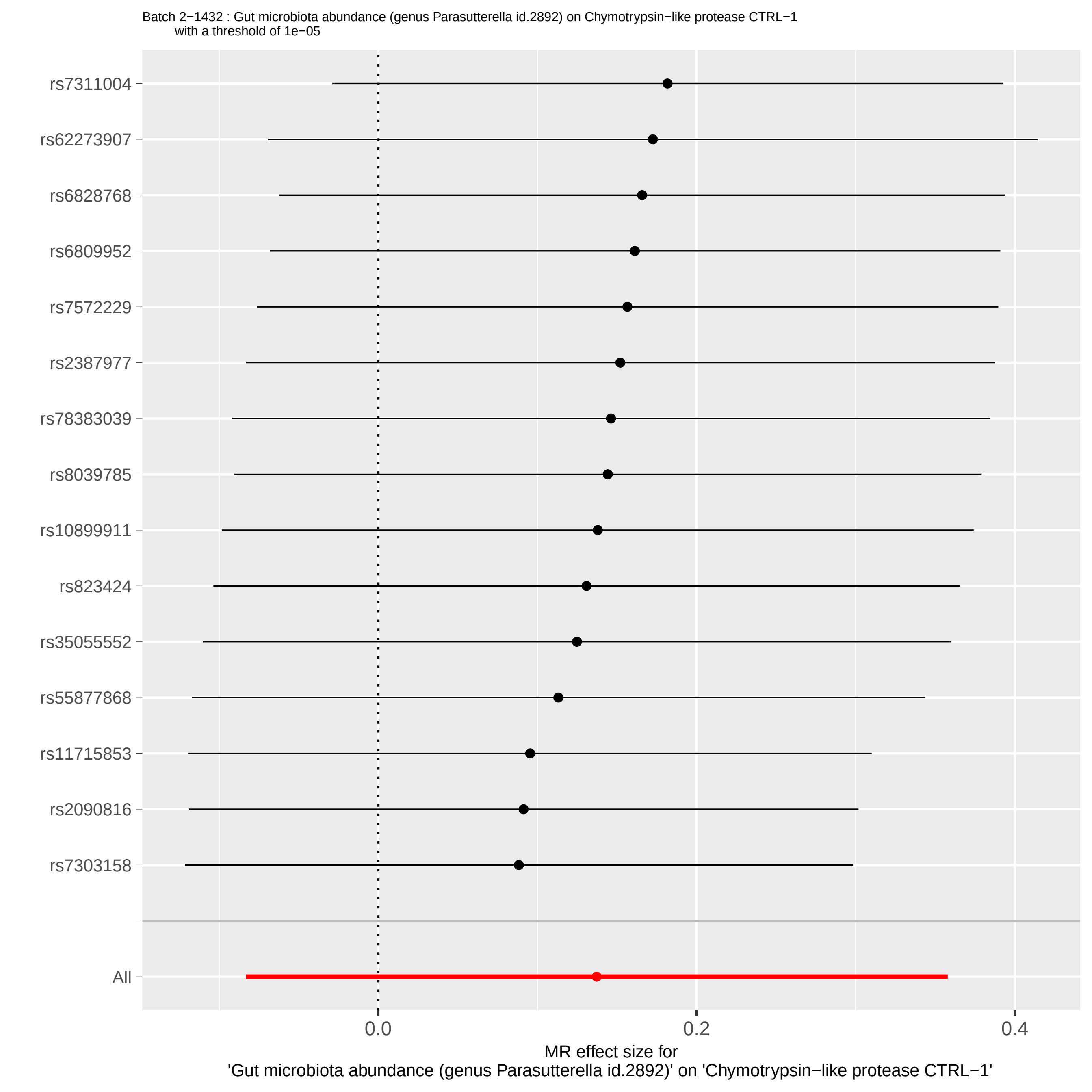

Batch 2−1432 : Gut microbiota abundance (genus Parasutterella id.2892) on Chymotrypsin−like protease CTRL−1
with a threshold of 1e−05
rs7311004
rs62273907
rs6828768
rs6809952
rs7572229
rs2387977
rs78383039
rs8039785
rs10899911
rs823424
rs35055552
rs55877868
rs11715853
rs2090816
rs7303158
All
0.0
0.2
0.4
MR effect size for
'Gut microbiota abundance (genus Parasutterella id.2892)' on 'Chymotrypsin−like protease CTRL−1'

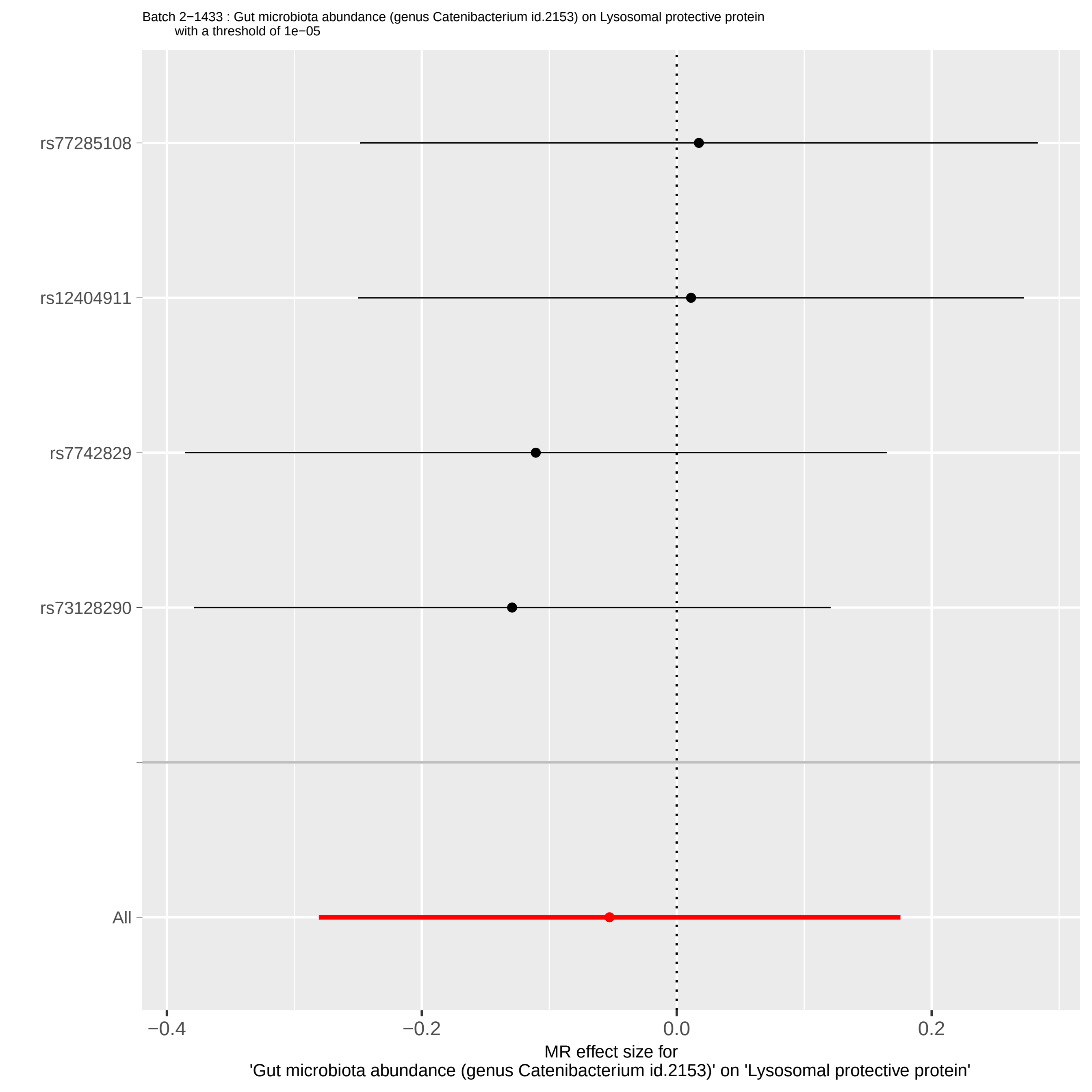
Batch 2−1433 : Gut microbiota abundance (genus Catenibacterium id.2153) on Lysosomal protective protein
with a threshold of 1e−05
rs77285108
rs12404911
rs7742829
rs73128290
All
−0.4
−0.2
0.0
0.2
MR effect size for
'Gut microbiota abundance (genus Catenibacterium id.2153)' on 'Lysosomal protective protein'

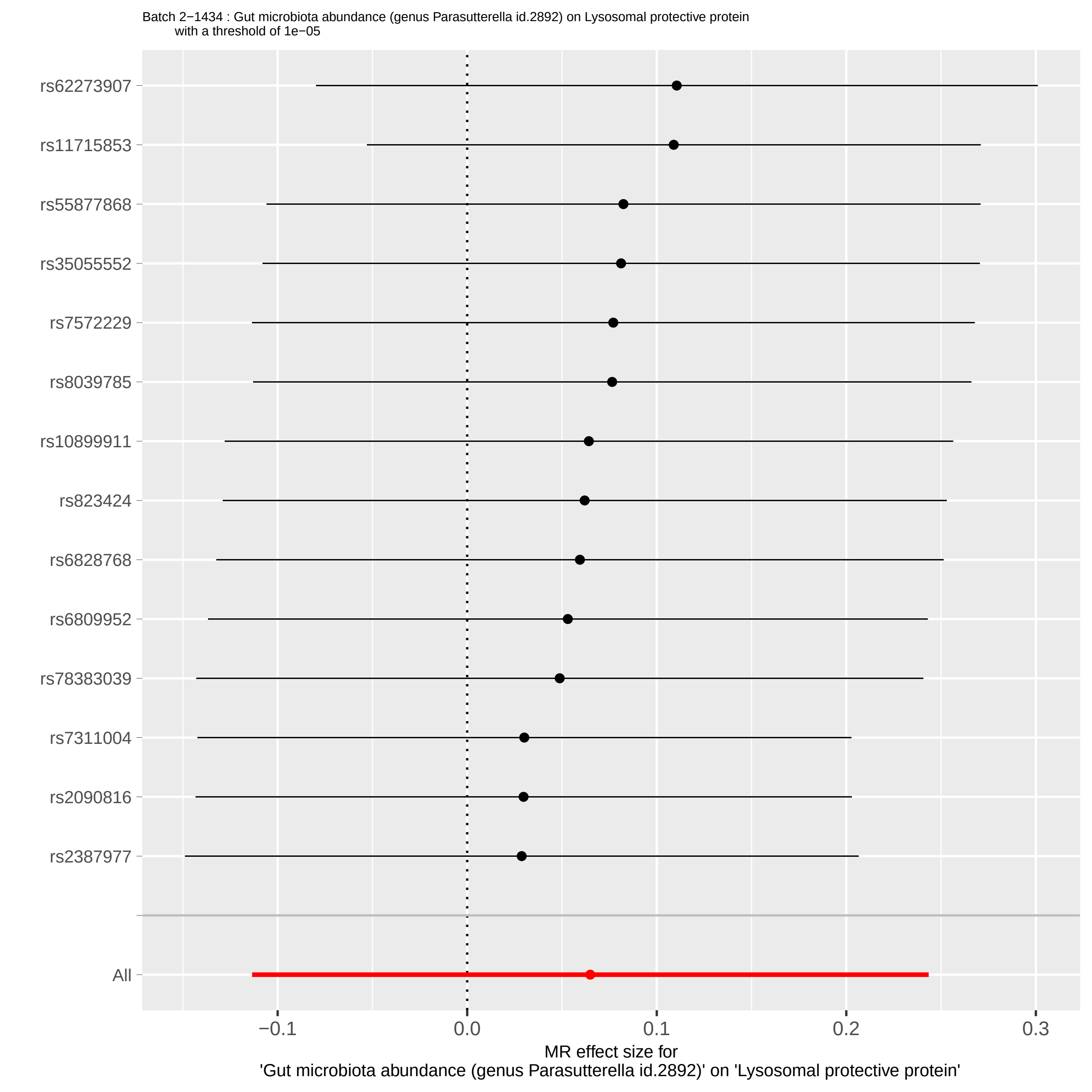
Batch 2−1434 : Gut microbiota abundance (genus Parasutterella id.2892) on Lysosomal protective protein
with a threshold of 1e−05
rs62273907
rs11715853
rs55877868
rs35055552
rs7572229
rs8039785
rs10899911
rs823424
rs6828768
rs6809952
rs78383039
rs7311004
rs2090816
rs2387977
All
−0.1
0.0
0.1
0.2
0.3
MR effect size for
'Gut microbiota abundance (genus Parasutterella id.2892)' on 'Lysosomal protective protein'

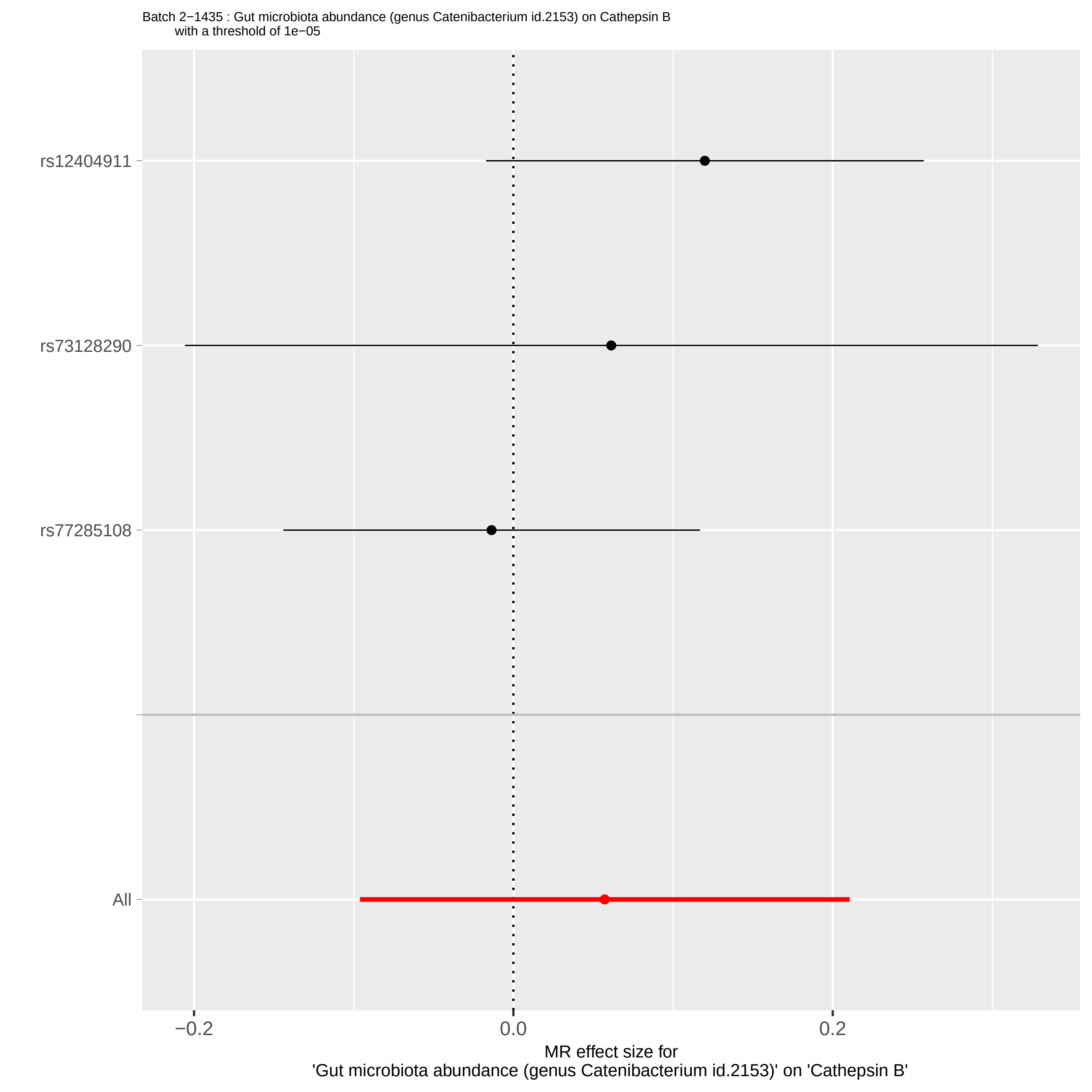
Batch 2−1435 : Gut microbiota abundance (genus Catenibacterium id.2153) on Cathepsin B
with a threshold of 1e−05
rs12404911
rs73128290
rs77285108
All
−0.2
0.0
0.2
MR effect size for
'Gut microbiota abundance (genus Catenibacterium id.2153)' on 'Cathepsin B'

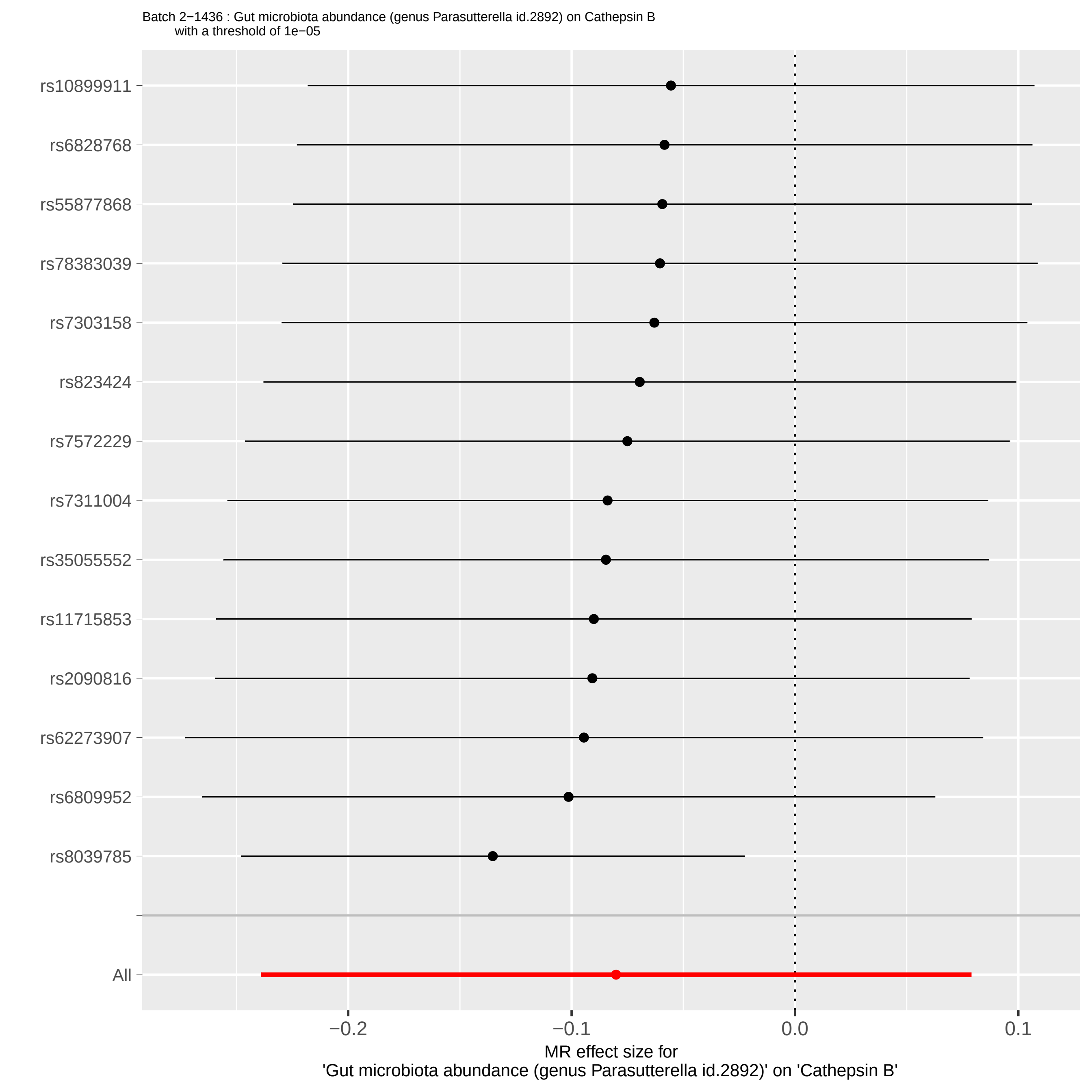

Batch 2−1436 : Gut microbiota abundance (genus Parasutterella id.2892) on Cathepsin B
with a threshold of 1e−05
rs10899911
rs6828768
rs55877868
rs78383039
rs7303158
rs823424
rs7572229
rs7311004
rs35055552
rs11715853
rs2090816
rs62273907
rs6809952
rs8039785
All
−0.2
−0.1
0.0
0.1
MR effect size for
'Gut microbiota abundance (genus Parasutterella id.2892)' on 'Cathepsin B'

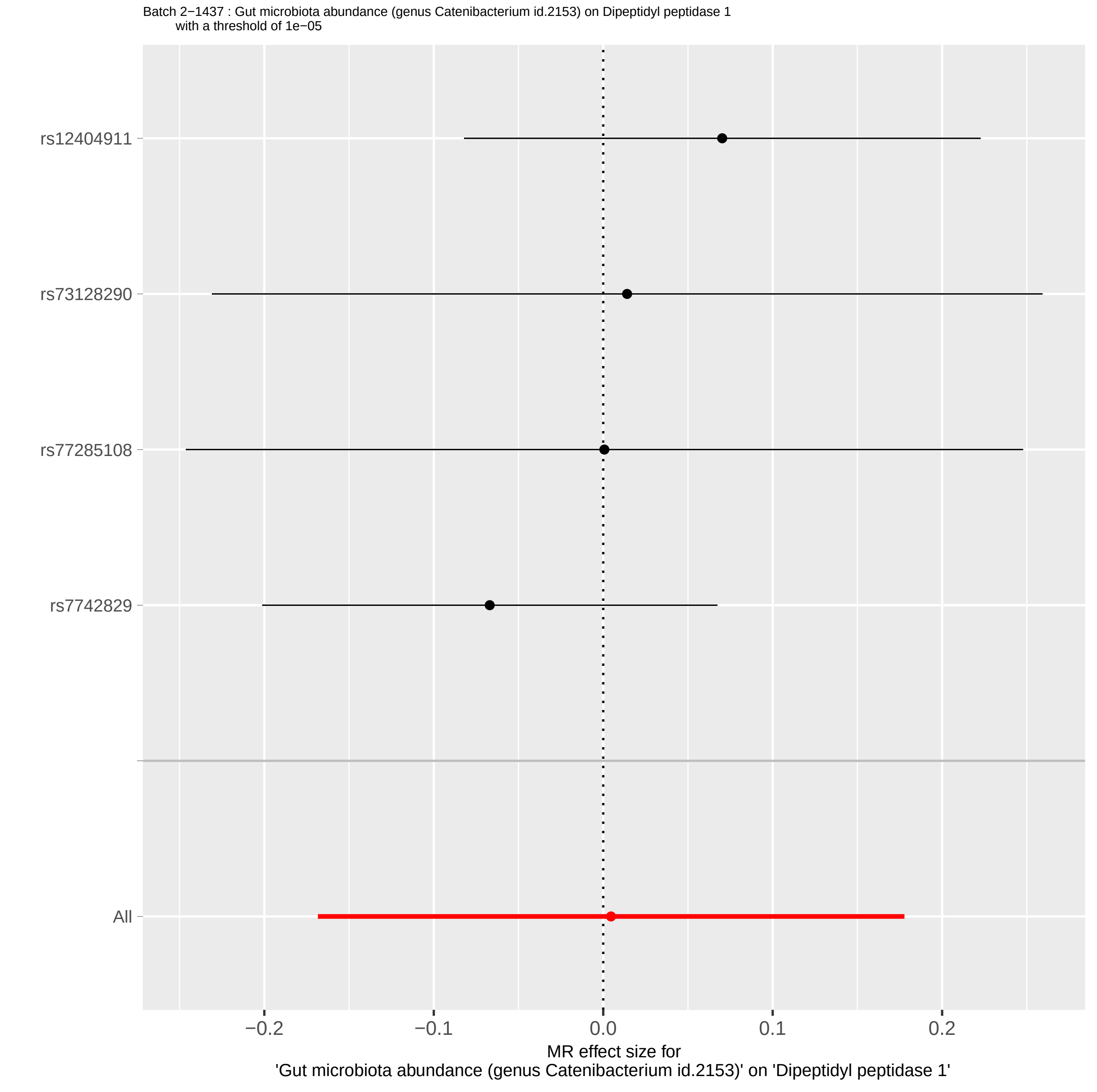

Batch 2−1437 : Gut microbiota abundance (genus Catenibacterium id.2153) on Dipeptidyl peptidase 1
with a threshold of 1e−05
rs12404911
rs73128290
rs77285108
rs7742829
All
−0.2
−0.1
0.0
0.1
0.2
MR effect size for
'Gut microbiota abundance (genus Catenibacterium id.2153)' on 'Dipeptidyl peptidase 1'

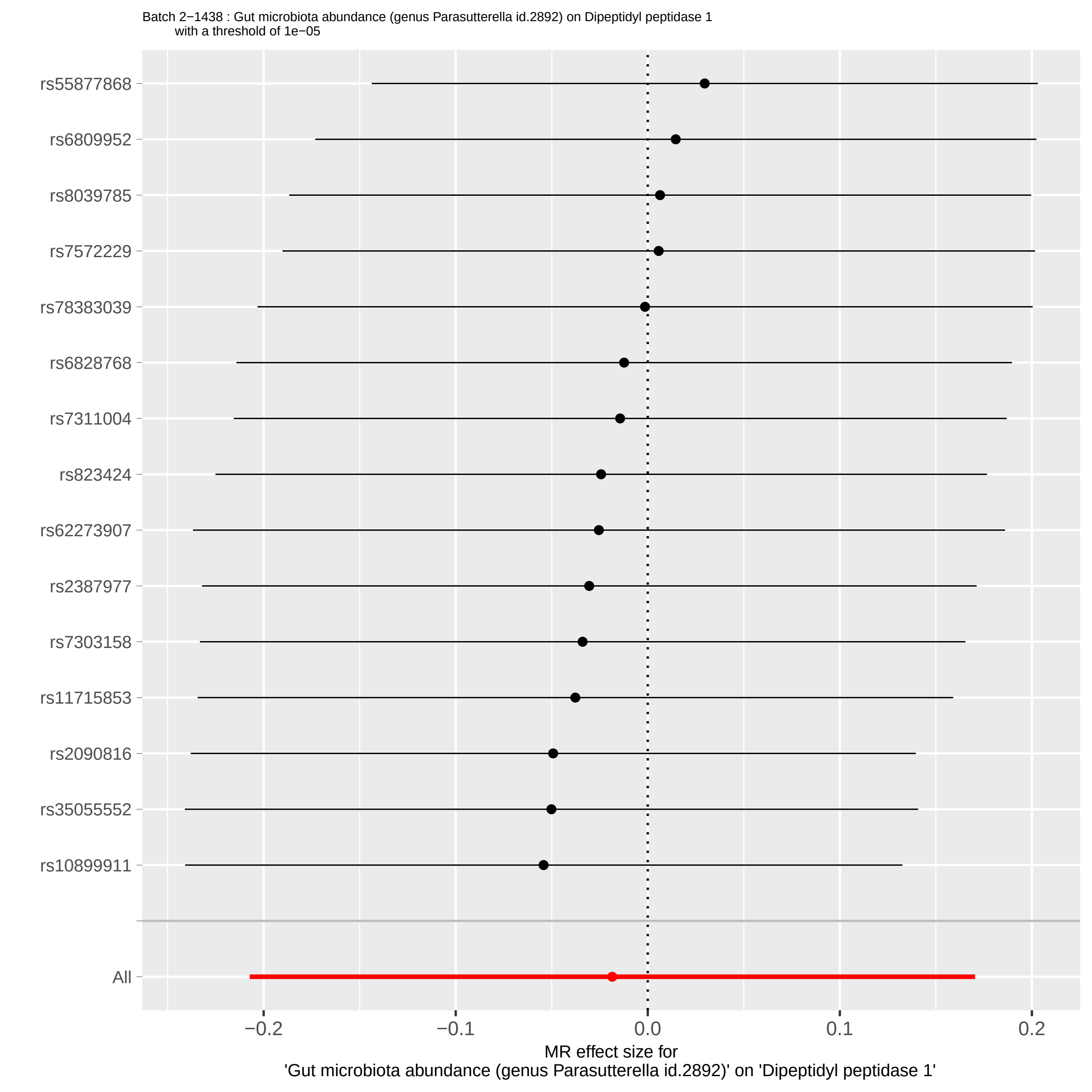
Batch 2−1438 : Gut microbiota abundance (genus Parasutterella id.2892) on Dipeptidyl peptidase 1
with a threshold of 1e−05
rs55877868
rs6809952
rs8039785
rs7572229
rs78383039
rs6828768
rs7311004
rs823424
rs62273907
rs2387977
rs7303158
rs11715853
rs2090816
rs35055552
rs10899911
All
−0.2
−0.1
0.0
0.1
0.2
MR effect size for
'Gut microbiota abundance (genus Parasutterella id.2892)' on 'Dipeptidyl peptidase 1'

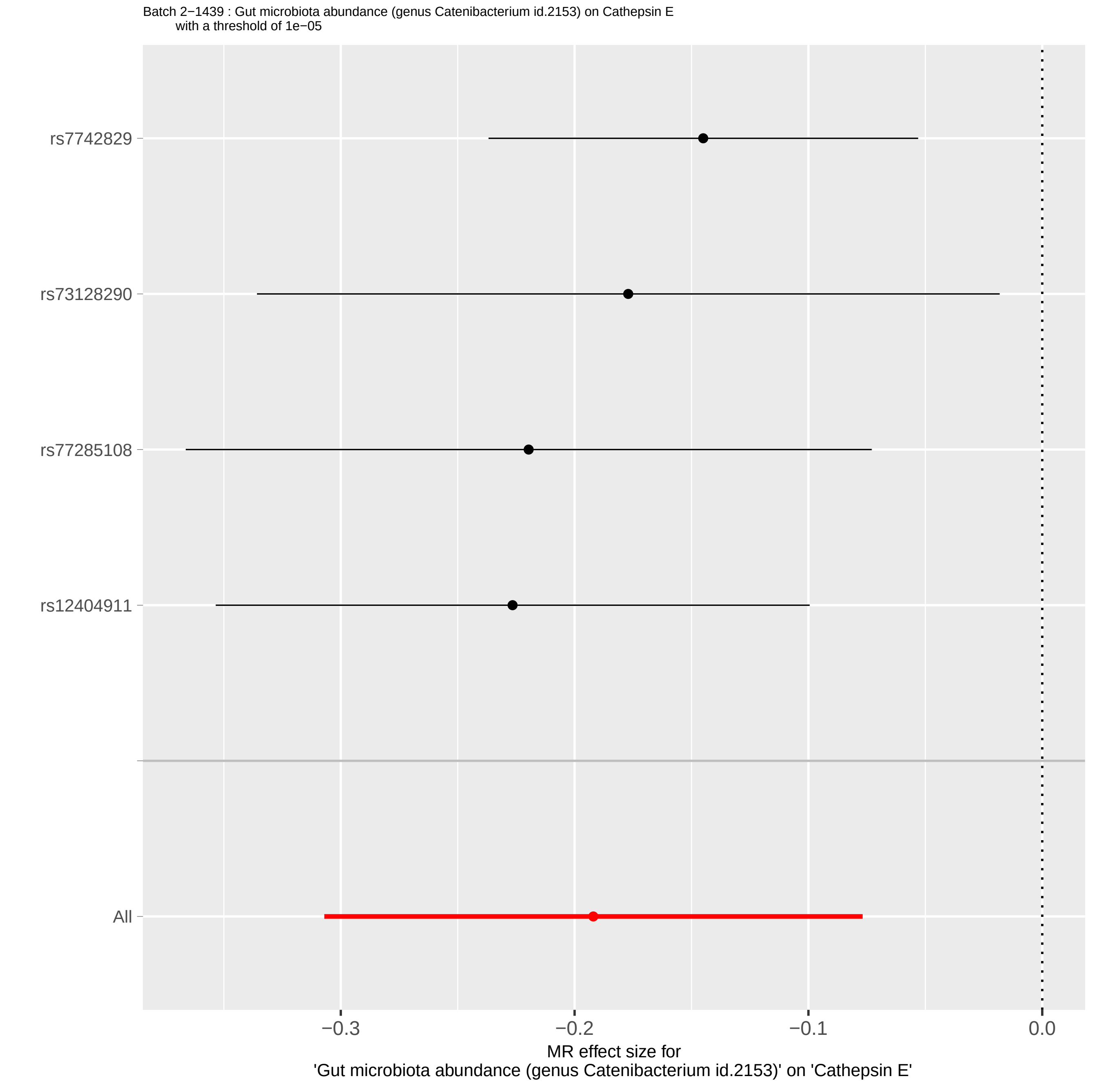

Batch 2−1439 : Gut microbiota abundance (genus Catenibacterium id.2153) on Cathepsin E
with a threshold of 1e−05
rs7742829
rs73128290
rs77285108
rs12404911
All
−0.3
−0.2
−0.1
0.0
MR effect size for
'Gut microbiota abundance (genus Catenibacterium id.2153)' on 'Cathepsin E'

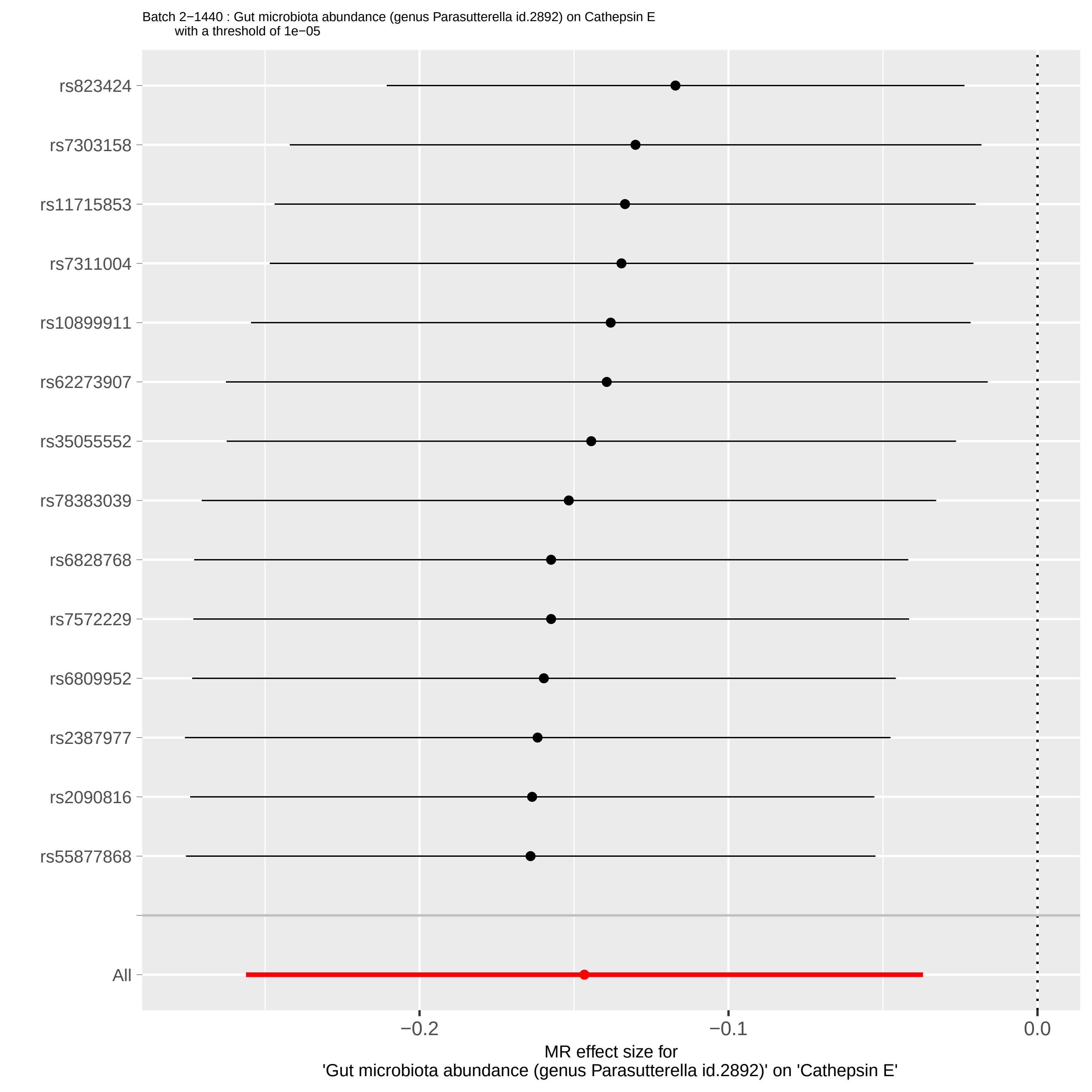
Batch 2−1440 : Gut microbiota abundance (genus Parasutterella id.2892) on Cathepsin E
with a threshold of 1e−05
rs823424
rs7303158
rs11715853
rs7311004
rs10899911
rs62273907
rs35055552
rs78383039
rs6828768
rs7572229
rs6809952
rs2387977
rs2090816
rs55877868
All
−0.2
−0.1
0.0
MR effect size for
'Gut microbiota abundance (genus Parasutterella id.2892)' on 'Cathepsin E'

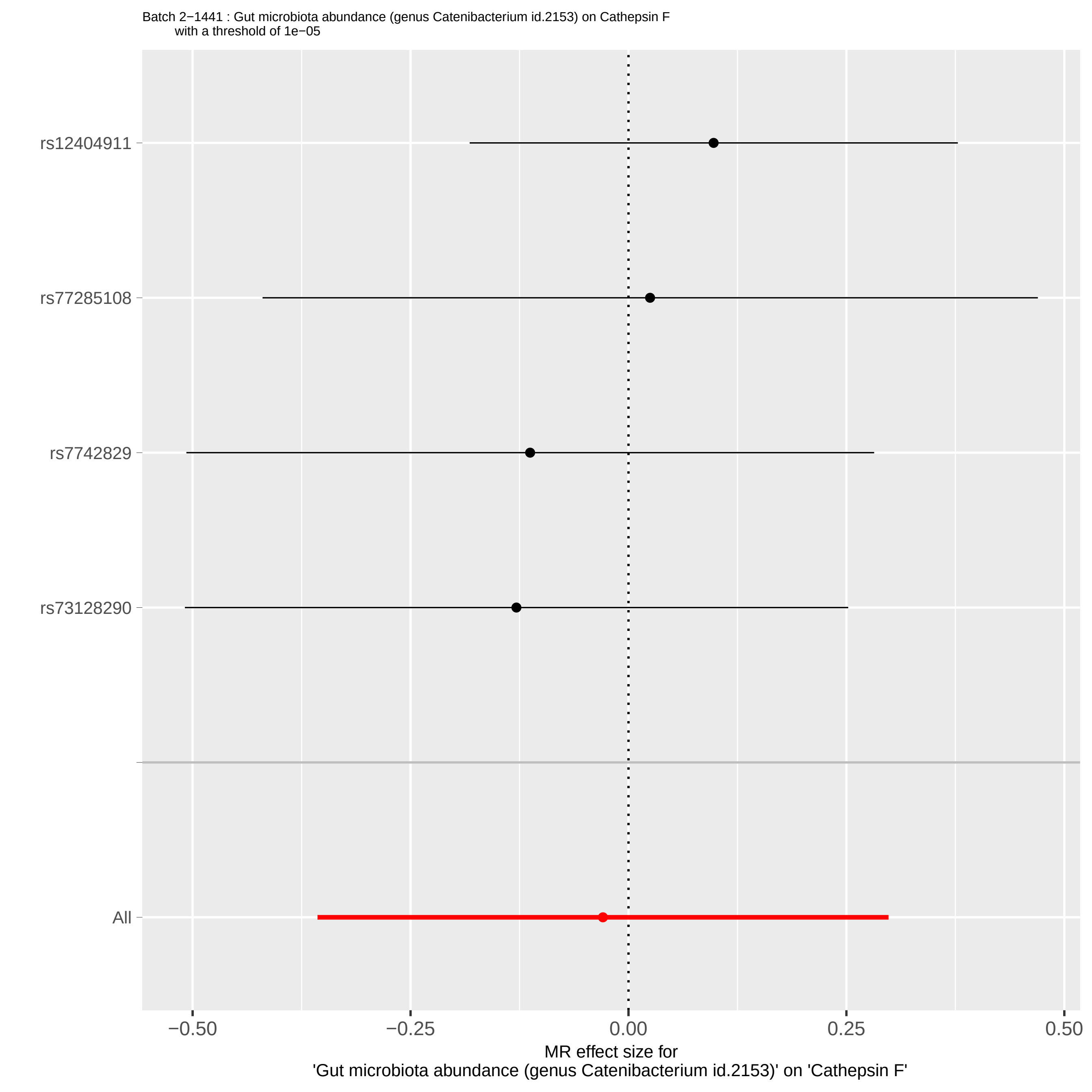
Batch 2−1441 : Gut microbiota abundance (genus Catenibacterium id.2153) on Cathepsin F
with a threshold of 1e−05
rs12404911
rs77285108
rs7742829
rs73128290
All
−0.50
−0.25
0.00
0.25
0.50
MR effect size for
'Gut microbiota abundance (genus Catenibacterium id.2153)' on 'Cathepsin F'

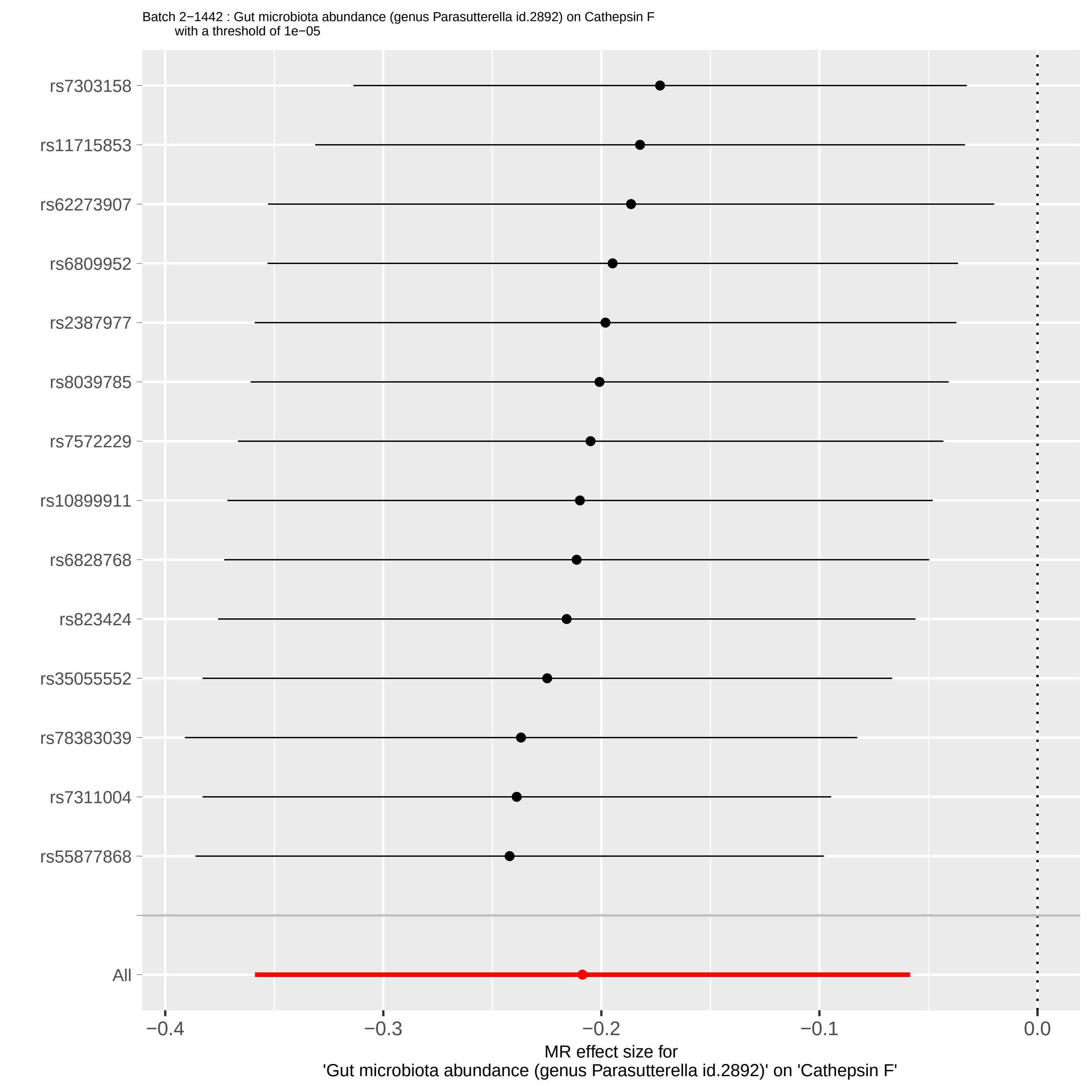
Batch 2−1442 : Gut microbiota abundance (genus Parasutterella id.2892) on Cathepsin F
with a threshold of 1e−05
rs7303158
rs11715853
rs62273907
rs6809952
rs2387977
rs8039785
rs7572229
rs10899911
rs6828768
rs823424
rs35055552
rs78383039
rs7311004
rs55877868
All
−0.4
−0.3
−0.2
−0.1
0.0
MR effect size for
'Gut microbiota abundance (genus Parasutterella id.2892)' on 'Cathepsin F'

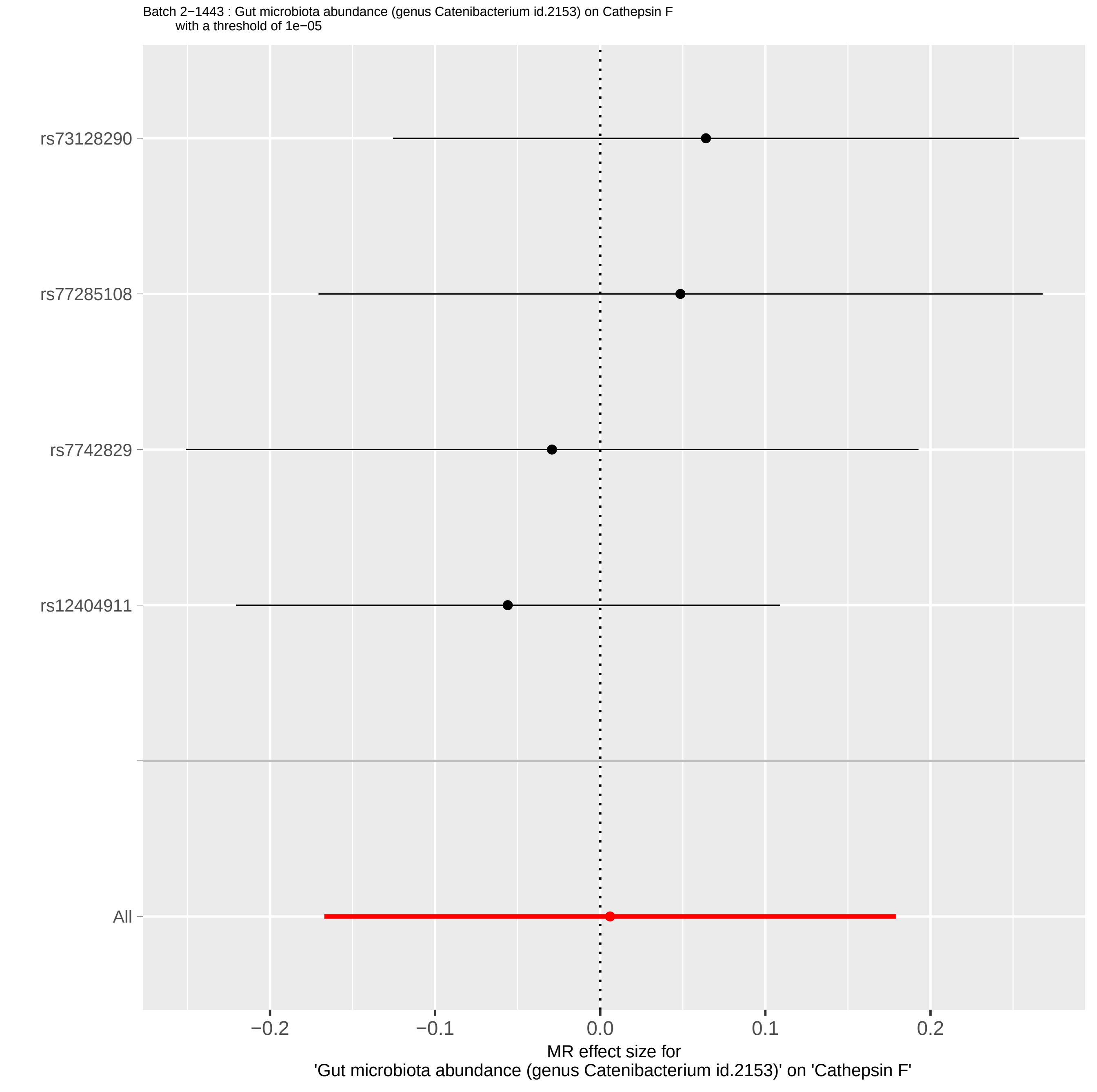
Batch 2−1443 : Gut microbiota abundance (genus Catenibacterium id.2153) on Cathepsin F
with a threshold of 1e−05
rs73128290
rs77285108
rs7742829
rs12404911
All
−0.2
−0.1
0.0
0.1
0.2
MR effect size for
'Gut microbiota abundance (genus Catenibacterium id.2153)' on 'Cathepsin F'

Batch 2−1444 : Gut microbiota abundance (genus Parasutterella id.2892) on Cathepsin F
with a threshold of 1e−05

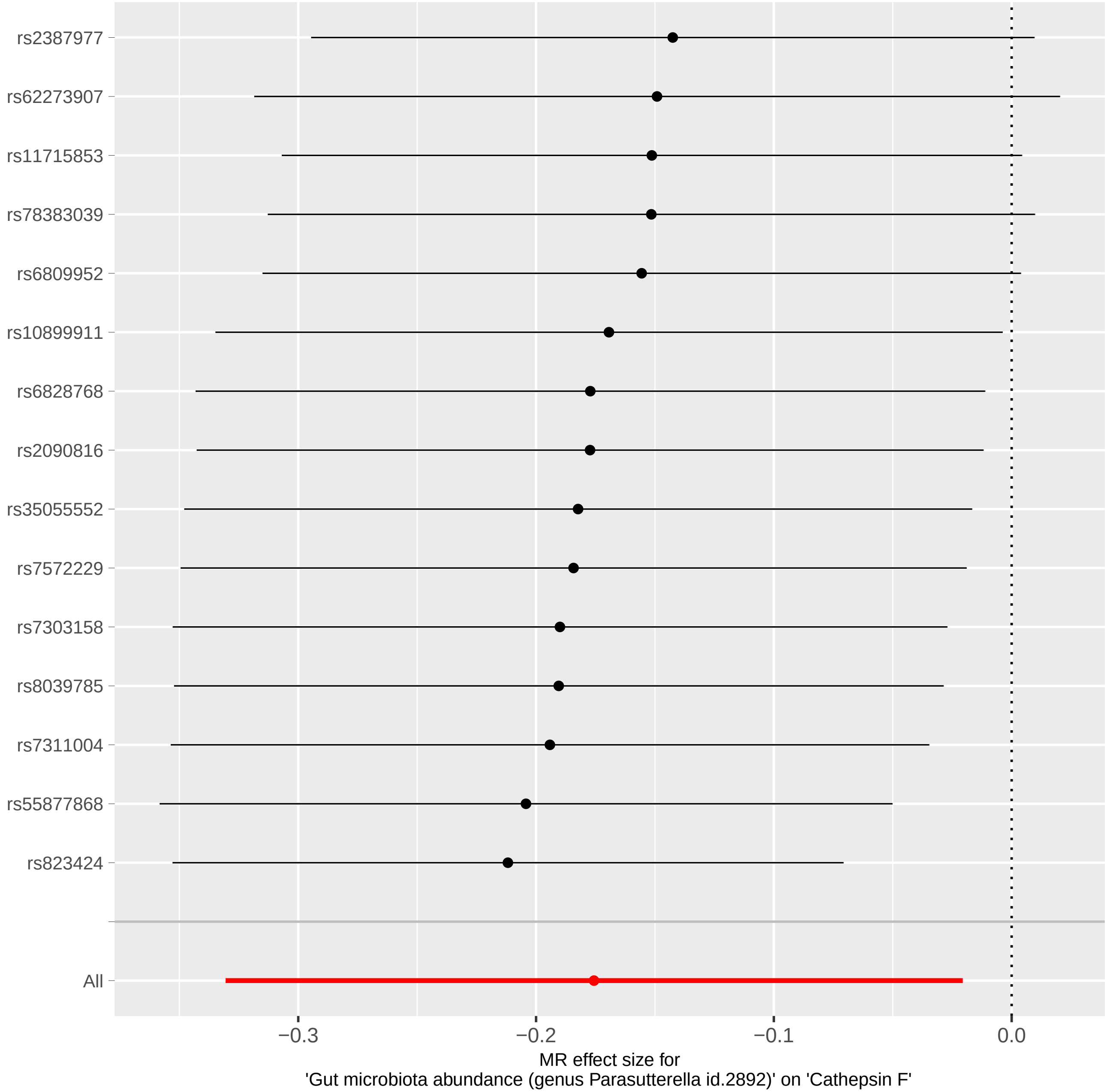

Batch 2−1445 : Gut microbiota abundance (genus Catenibacterium id.2153) on Cathepsin G
with a threshold of 1e−05

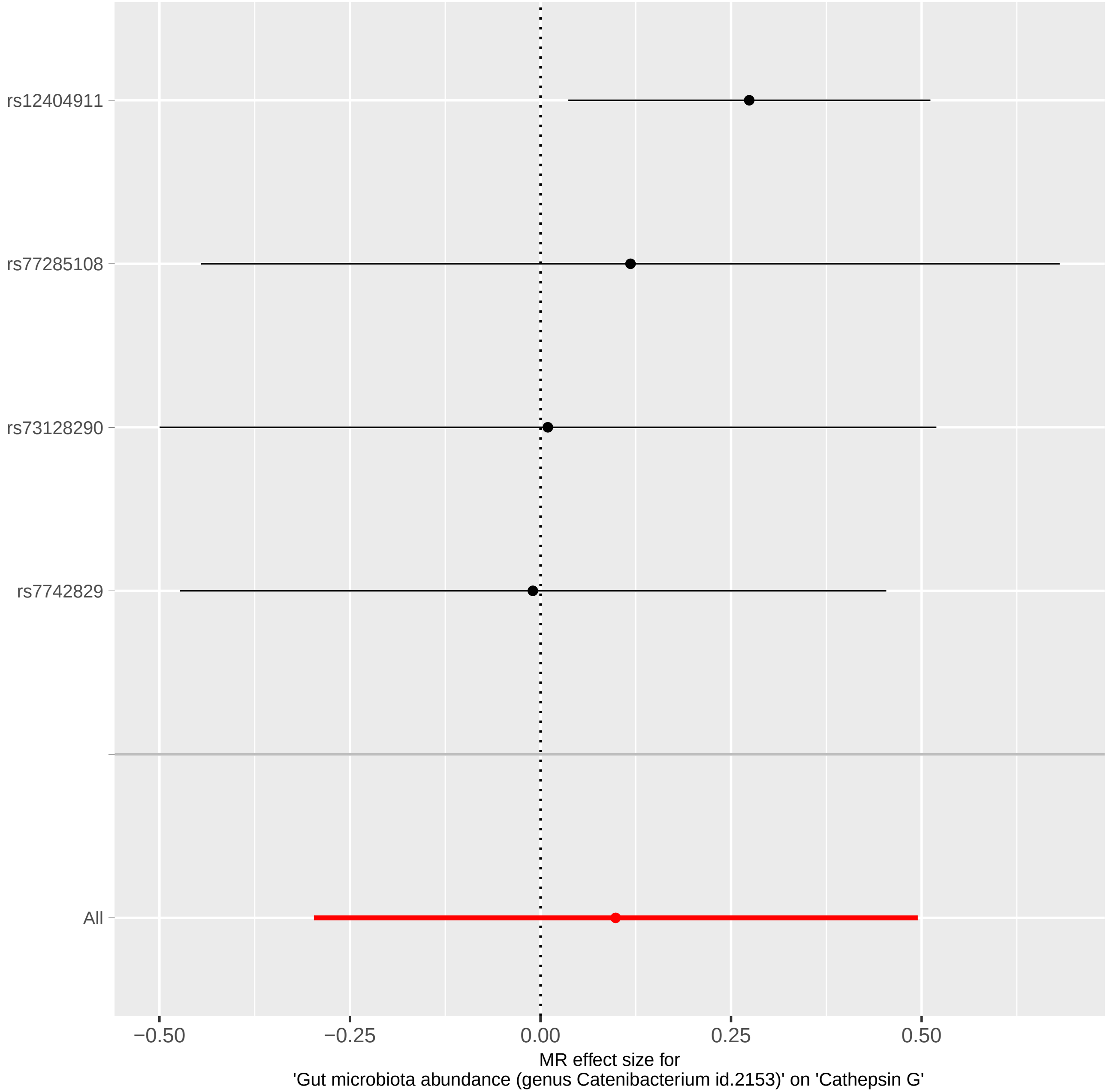

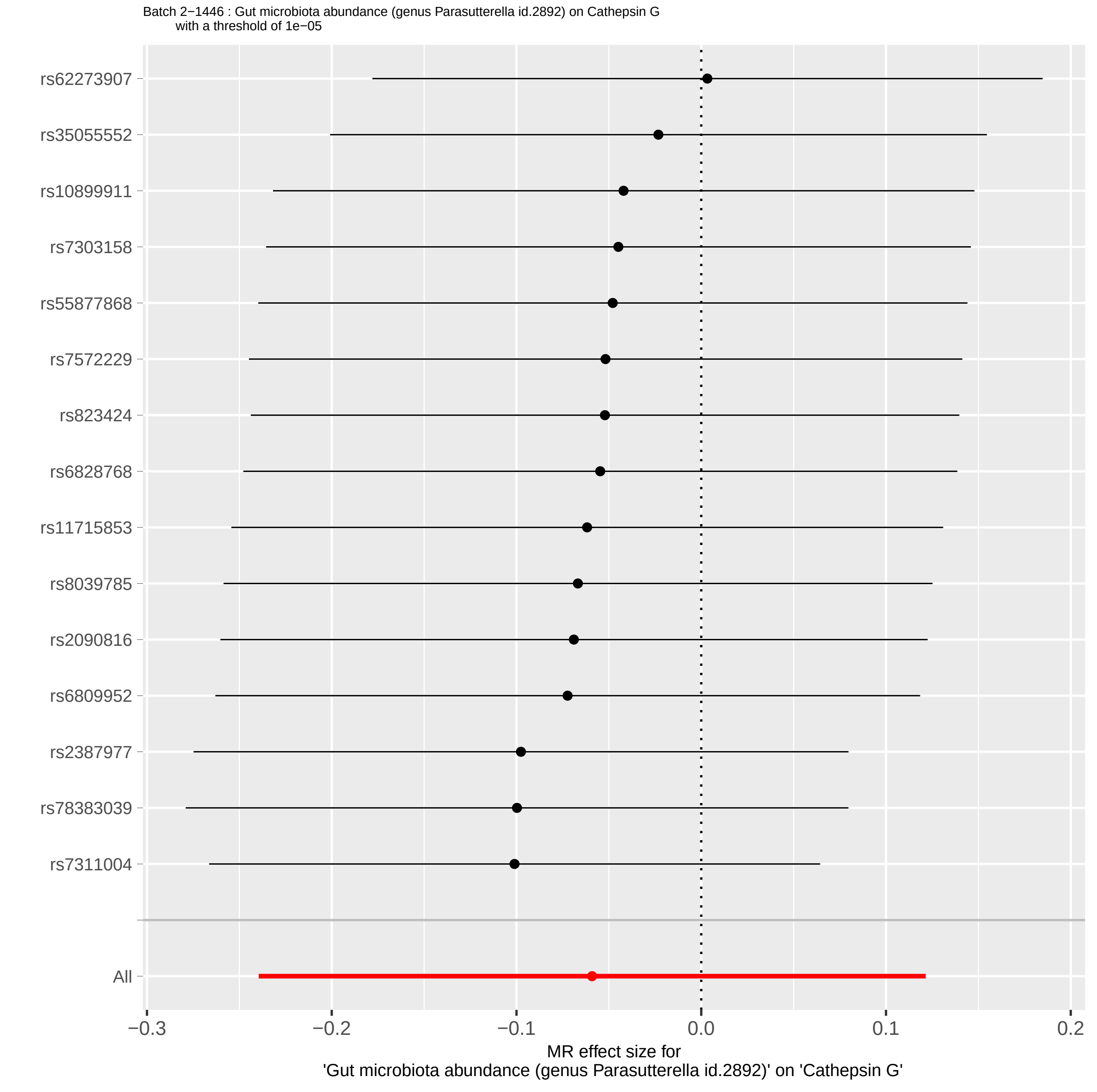
Batch 2−1446 : Gut microbiota abundance (genus Parasutterella id.2892) on Cathepsin G
with a threshold of 1e−05
rs62273907
rs35055552
rs10899911
rs7303158
rs55877868
rs7572229
rs823424
rs6828768
rs11715853
rs8039785
rs2090816
rs6809952
rs2387977
rs78383039
rs7311004
All
−0.3
−0.2
−0.1
0.0
0.1
0.2
MR effect size for
'Gut microbiota abundance (genus Parasutterella id.2892)' on 'Cathepsin G'

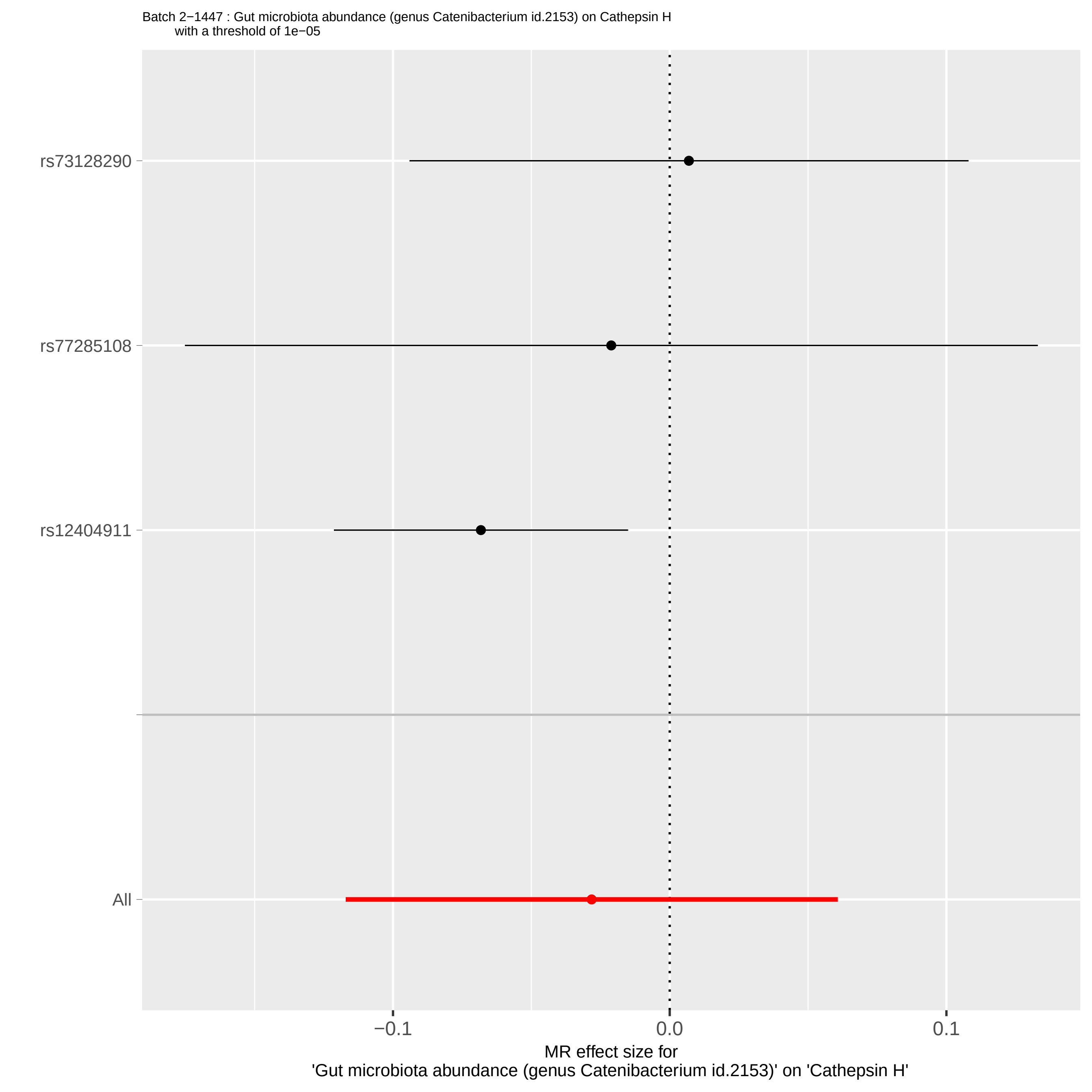

Batch 2−1447 : Gut microbiota abundance (genus Catenibacterium id.2153) on Cathepsin H
with a threshold of 1e−05
rs73128290
rs77285108
rs12404911
All
−0.1
0.0
0.1
MR effect size for
'Gut microbiota abundance (genus Catenibacterium id.2153)' on 'Cathepsin H'

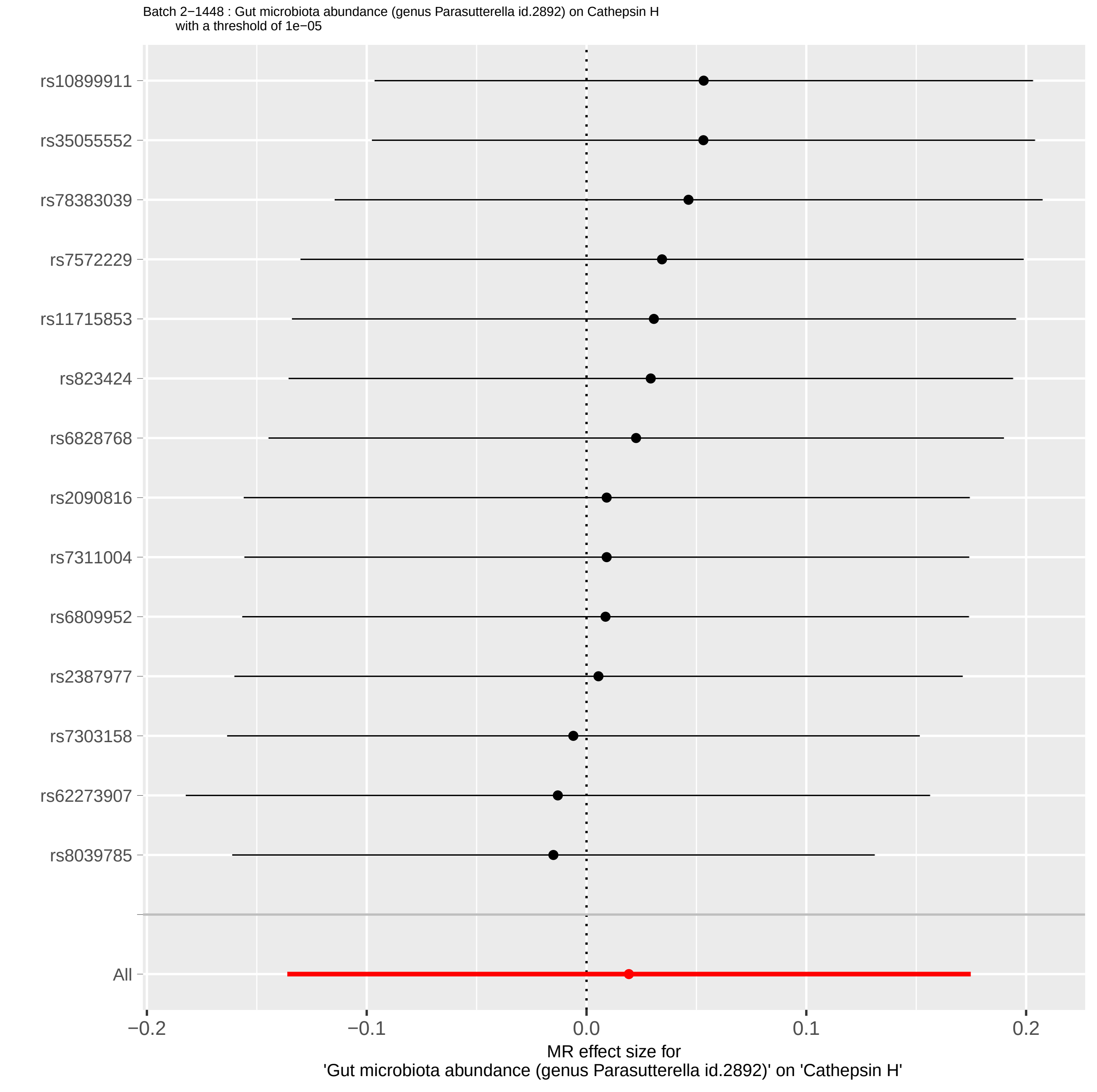

Batch 2−1448 : Gut microbiota abundance (genus Parasutterella id.2892) on Cathepsin H
with a threshold of 1e−05
rs10899911
rs35055552
rs78383039
rs7572229
rs11715853
rs823424
rs6828768
rs2090816
rs7311004
rs6809952
rs2387977
rs7303158
rs62273907
rs8039785
All
−0.2
−0.1
0.0
0.1
0.2
MR effect size for
'Gut microbiota abundance (genus Parasutterella id.2892)' on 'Cathepsin H'

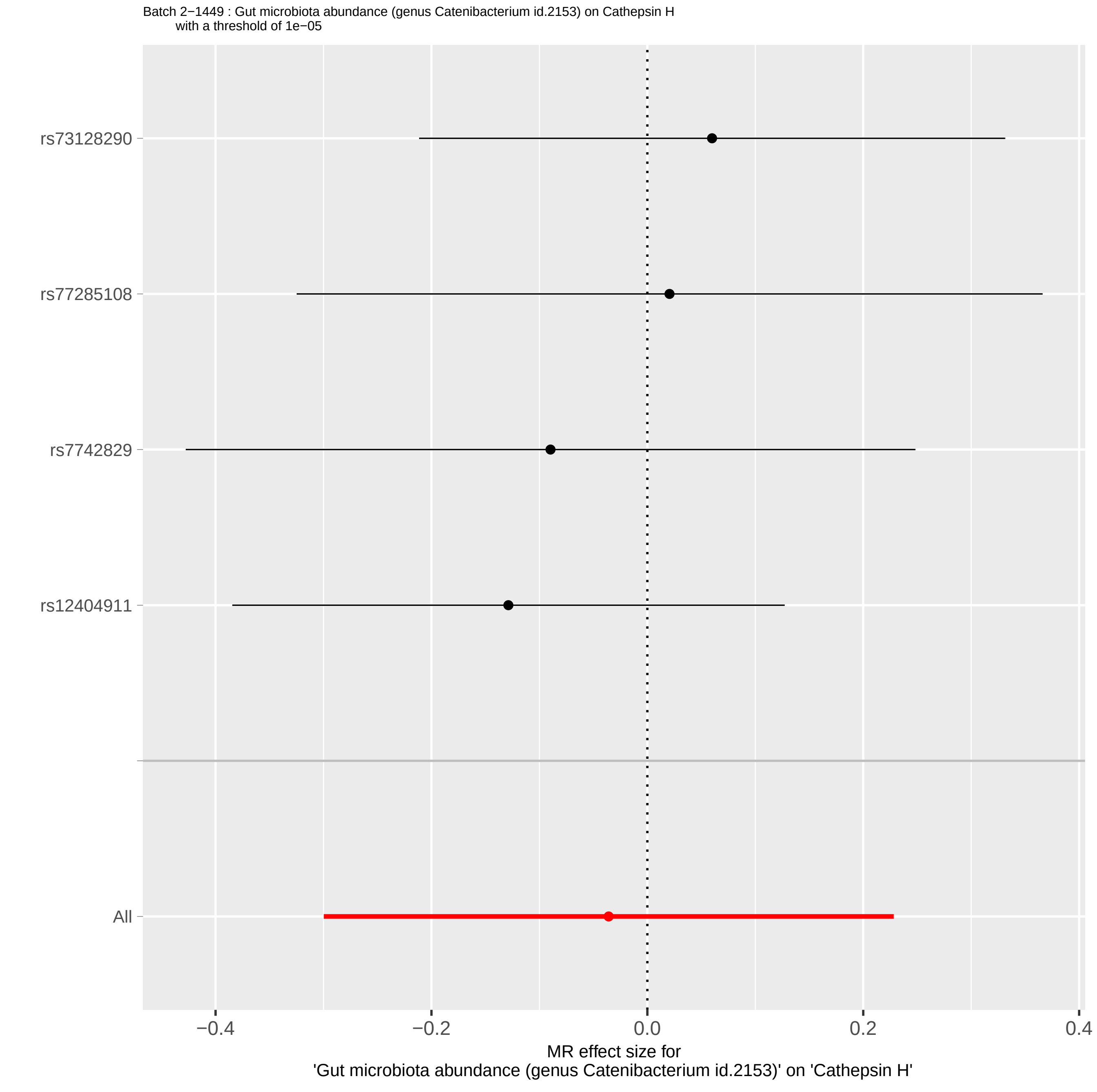
Batch 2−1449 : Gut microbiota abundance (genus Catenibacterium id.2153) on Cathepsin H
with a threshold of 1e−05
rs73128290
rs77285108
rs7742829
rs12404911
All
−0.4
−0.2
0.0
0.2
0.4
MR effect size for
'Gut microbiota abundance (genus Catenibacterium id.2153)' on 'Cathepsin H'

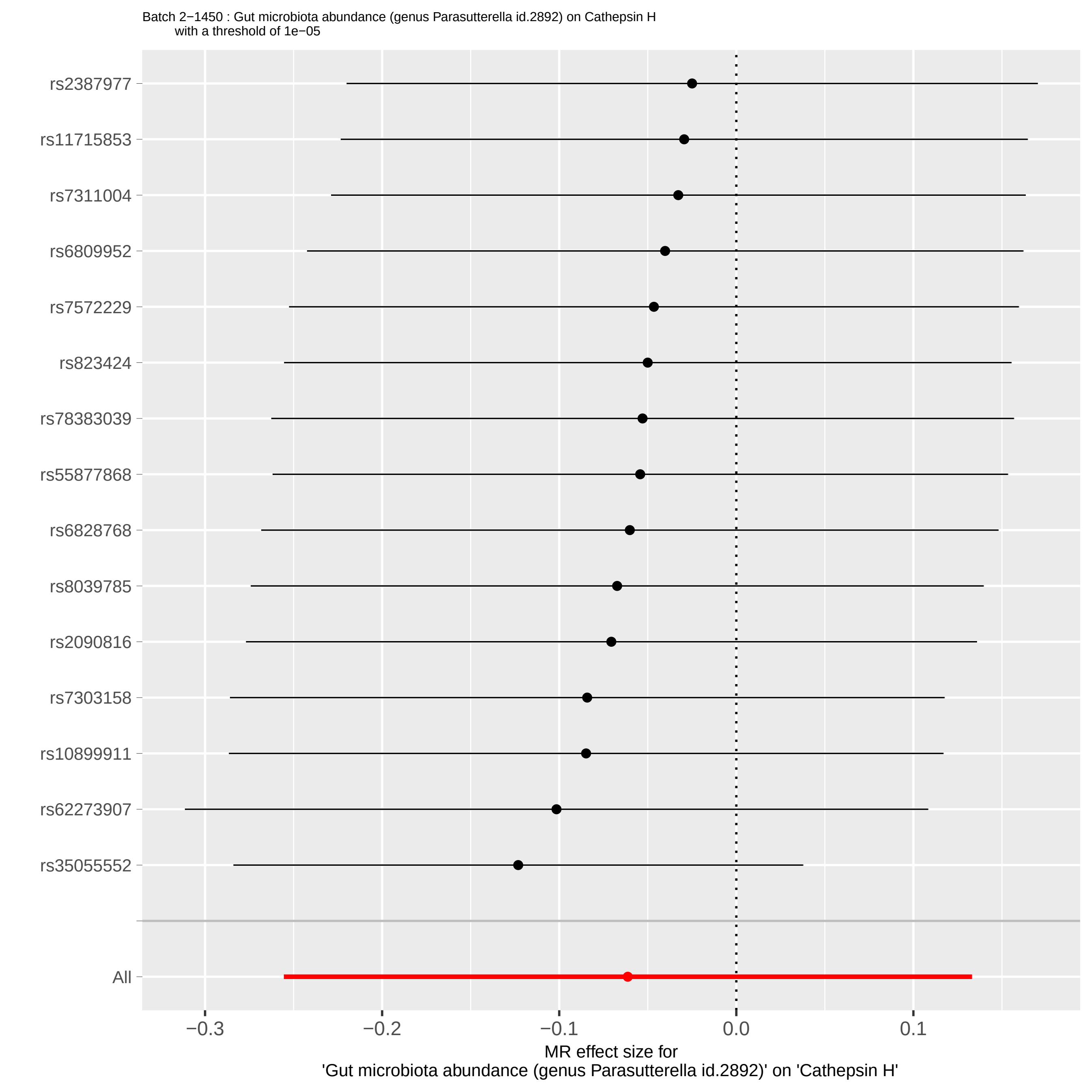
Batch 2−1450 : Gut microbiota abundance (genus Parasutterella id.2892) on Cathepsin H
with a threshold of 1e−05
rs2387977
rs11715853
rs7311004
rs6809952
rs7572229
rs823424
rs78383039
rs55877868
rs6828768
rs8039785
rs2090816
rs7303158
rs10899911
rs62273907
rs35055552
All
−0.3
−0.2
−0.1
0.0
0.1
MR effect size for
'Gut microbiota abundance (genus Parasutterella id.2892)' on 'Cathepsin H'

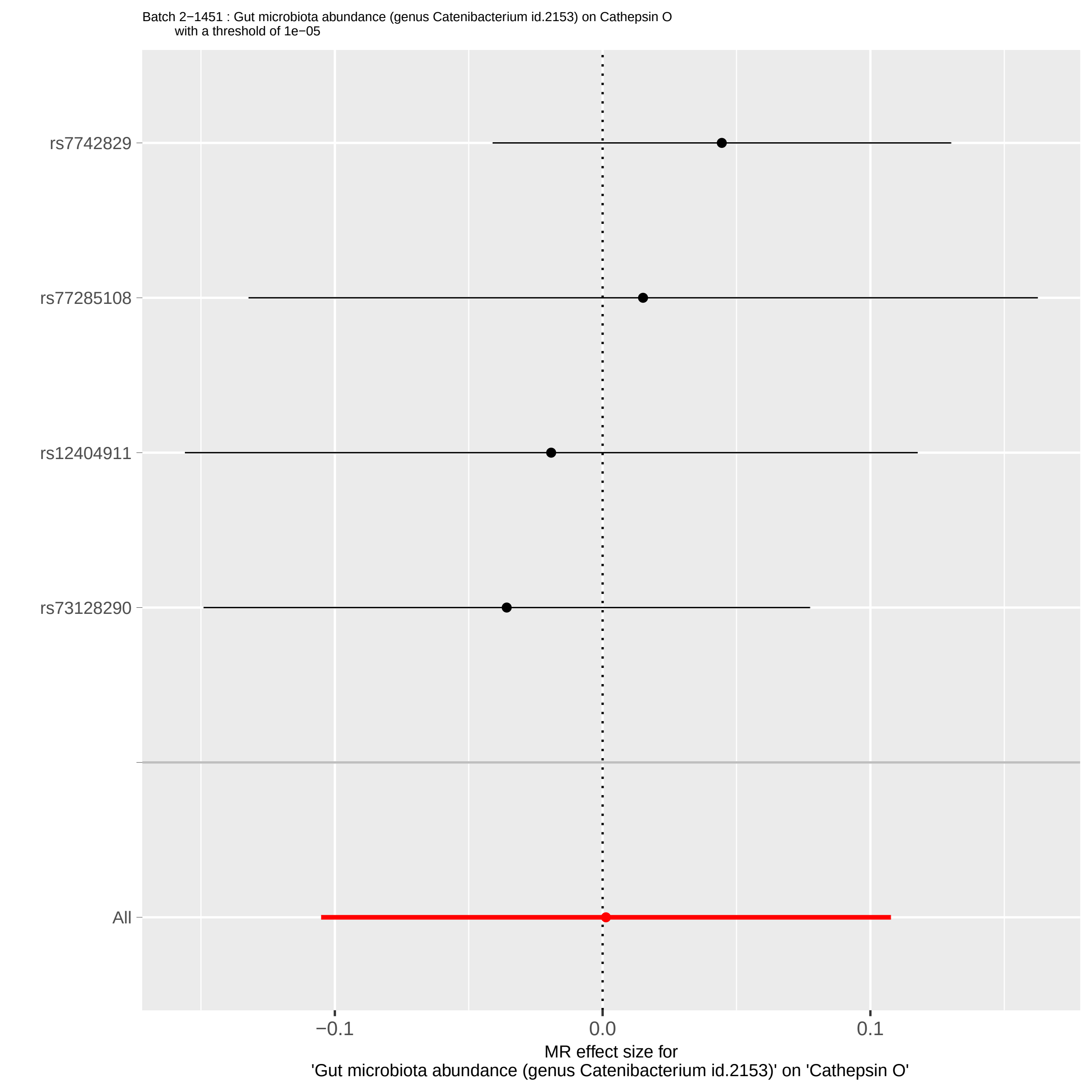
Batch 2−1451 : Gut microbiota abundance (genus Catenibacterium id.2153) on Cathepsin O
with a threshold of 1e−05
rs7742829
rs77285108
rs12404911
rs73128290
All
−0.1
0.0
0.1
MR effect size for
'Gut microbiota abundance (genus Catenibacterium id.2153)' on 'Cathepsin O'

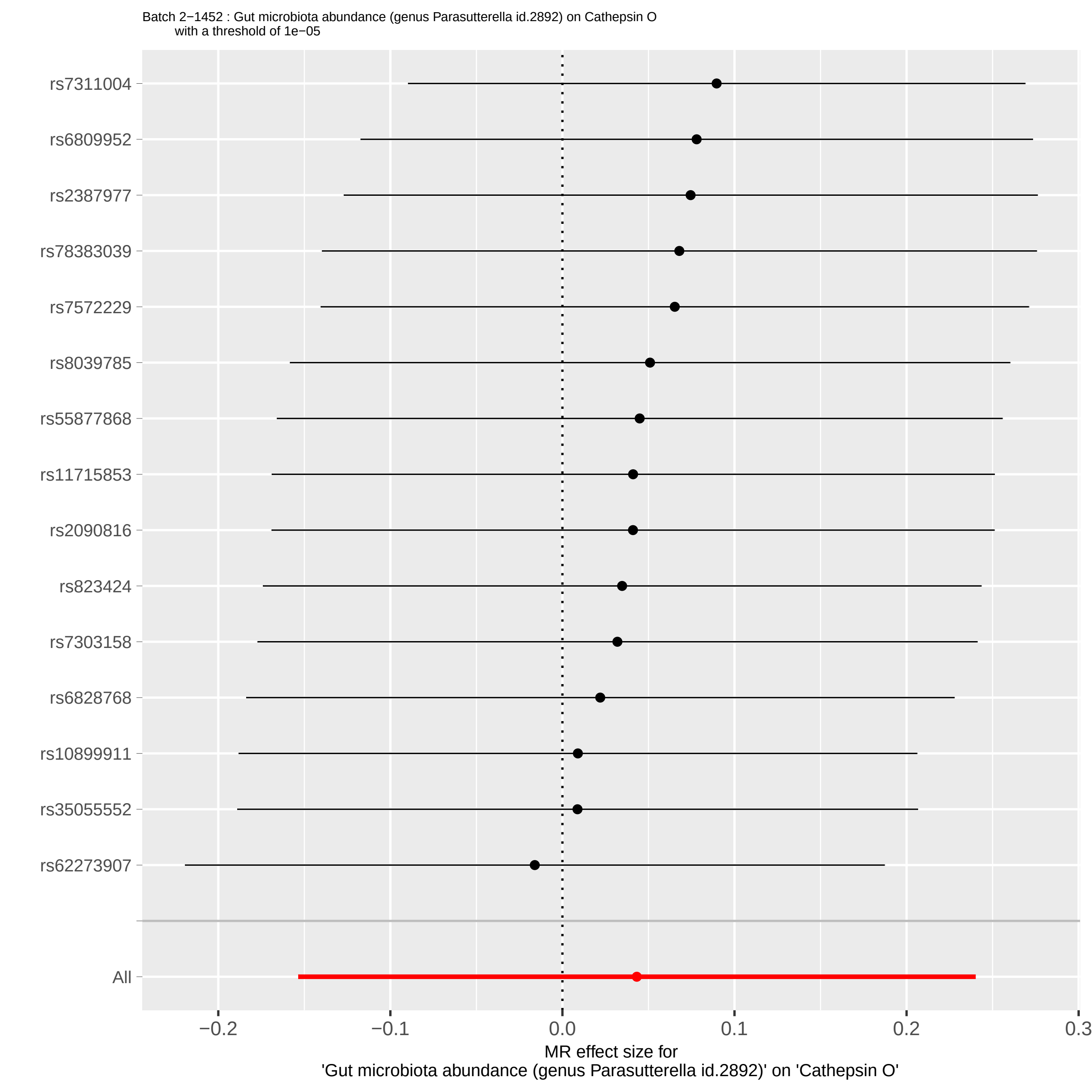
Batch 2−1452 : Gut microbiota abundance (genus Parasutterella id.2892) on Cathepsin O
with a threshold of 1e−05
rs7311004
rs6809952
rs2387977
rs78383039
rs7572229
rs8039785
rs55877868
rs11715853
rs2090816
rs823424
rs7303158
rs6828768
rs10899911
rs35055552
rs62273907
All
−0.2
−0.1
0.0
0.1
0.2
0.3
MR effect size for
'Gut microbiota abundance (genus Parasutterella id.2892)' on 'Cathepsin O'

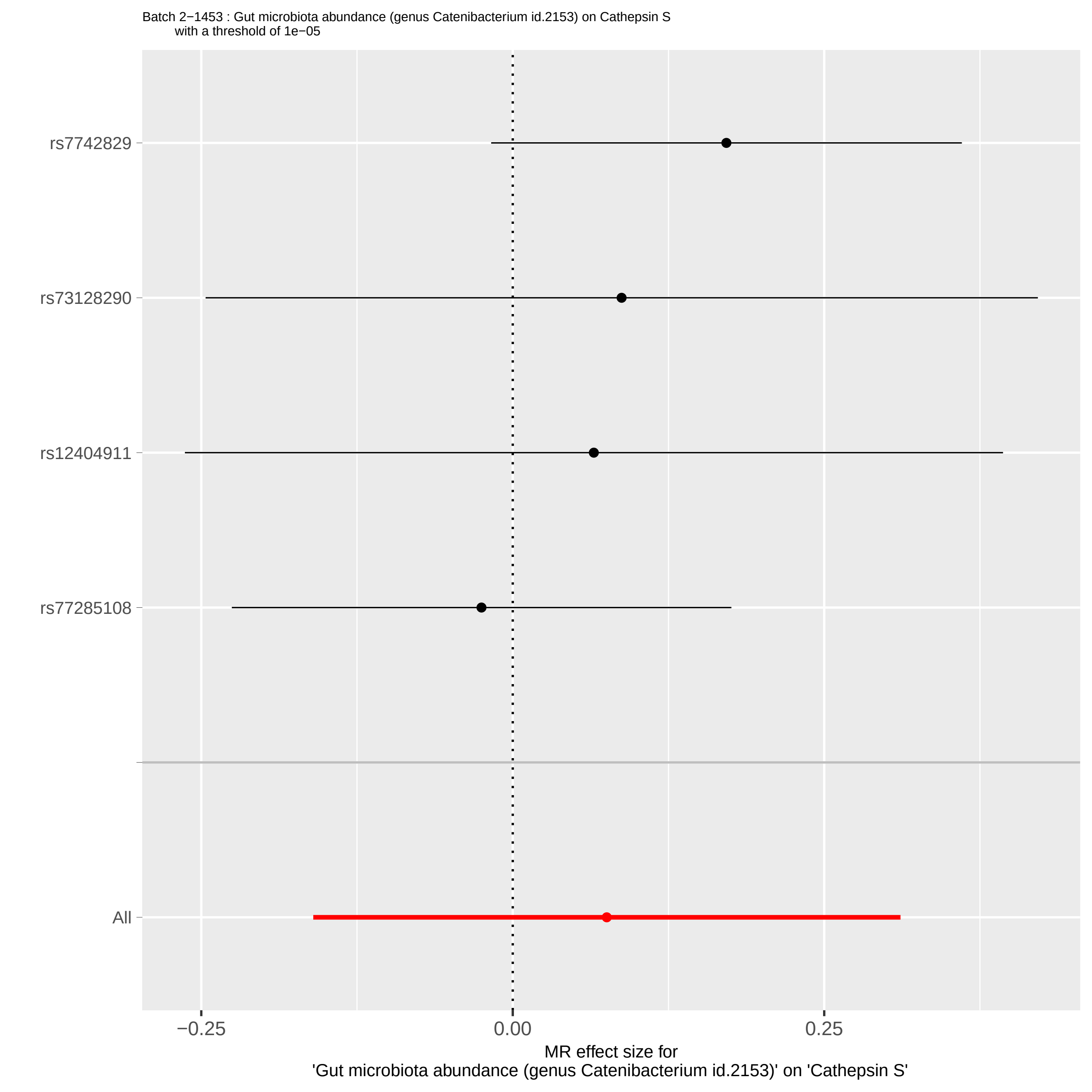

Batch 2−1453 : Gut microbiota abundance (genus Catenibacterium id.2153) on Cathepsin S
with a threshold of 1e−05
rs7742829
rs73128290
rs12404911
rs77285108
All
−0.25
0.00
0.25
MR effect size for
'Gut microbiota abundance (genus Catenibacterium id.2153)' on 'Cathepsin S'

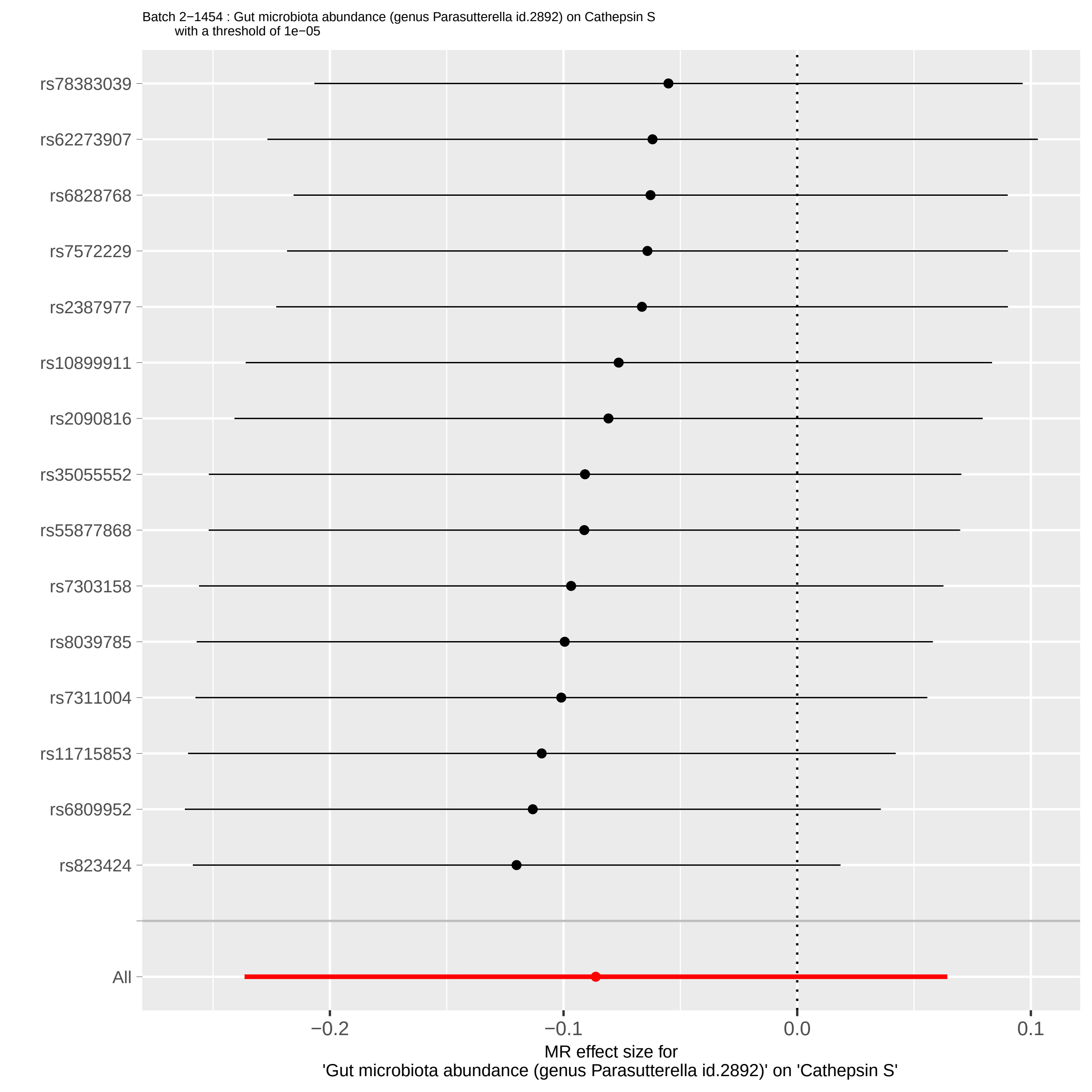

Batch 2−1454 : Gut microbiota abundance (genus Parasutterella id.2892) on Cathepsin S
with a threshold of 1e−05
rs78383039
rs62273907
rs6828768
rs7572229
rs2387977
rs10899911
rs2090816
rs35055552
rs55877868
rs7303158
rs8039785
rs7311004
rs11715853
rs6809952
rs823424
All
−0.2
−0.1
0.0
0.1
MR effect size for
'Gut microbiota abundance (genus Parasutterella id.2892)' on 'Cathepsin S'

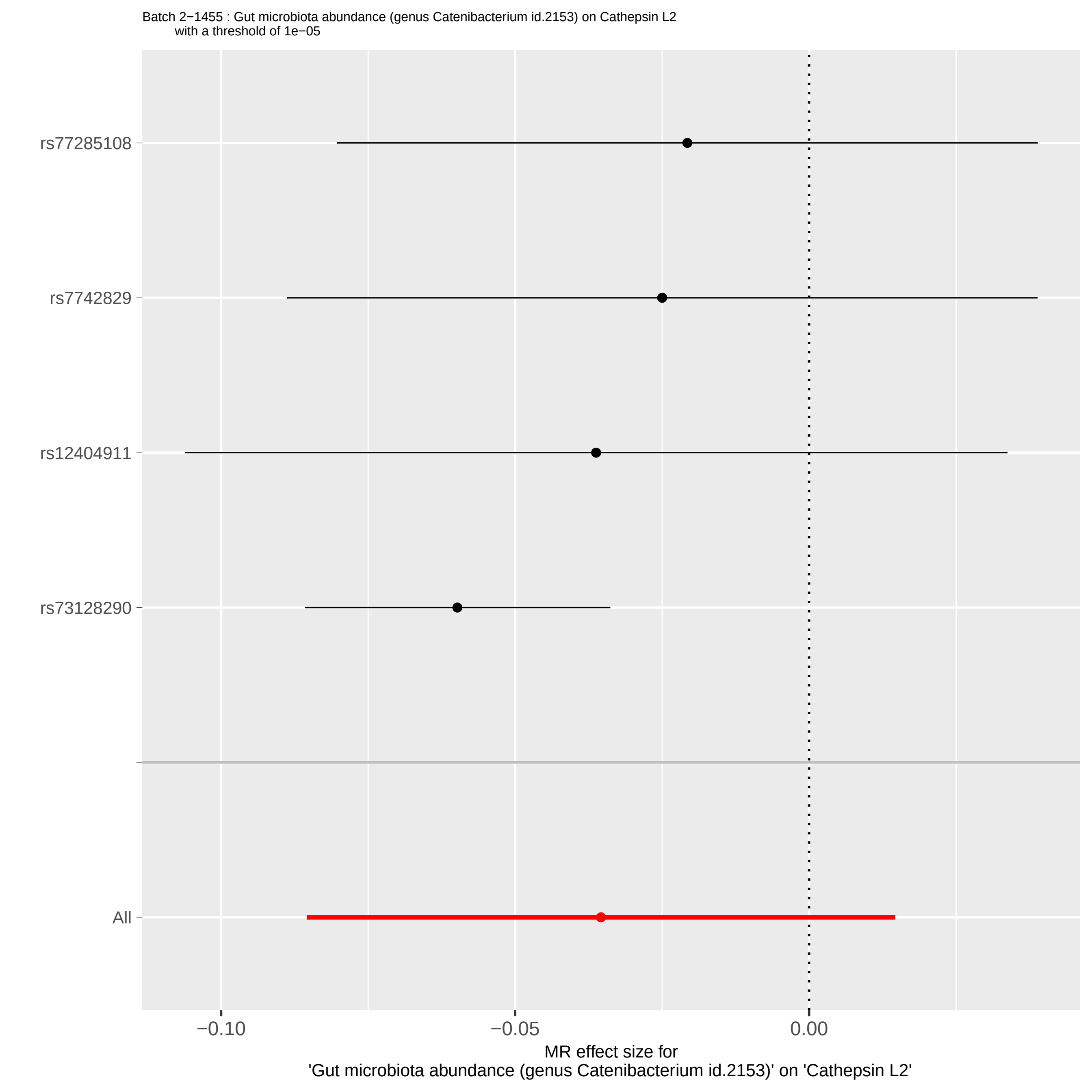
Batch 2−1455 : Gut microbiota abundance (genus Catenibacterium id.2153) on Cathepsin L2
with a threshold of 1e−05
rs77285108
rs7742829
rs12404911
rs73128290
All
−0.10
−0.05
0.00
MR effect size for
'Gut microbiota abundance (genus Catenibacterium id.2153)' on 'Cathepsin L2'

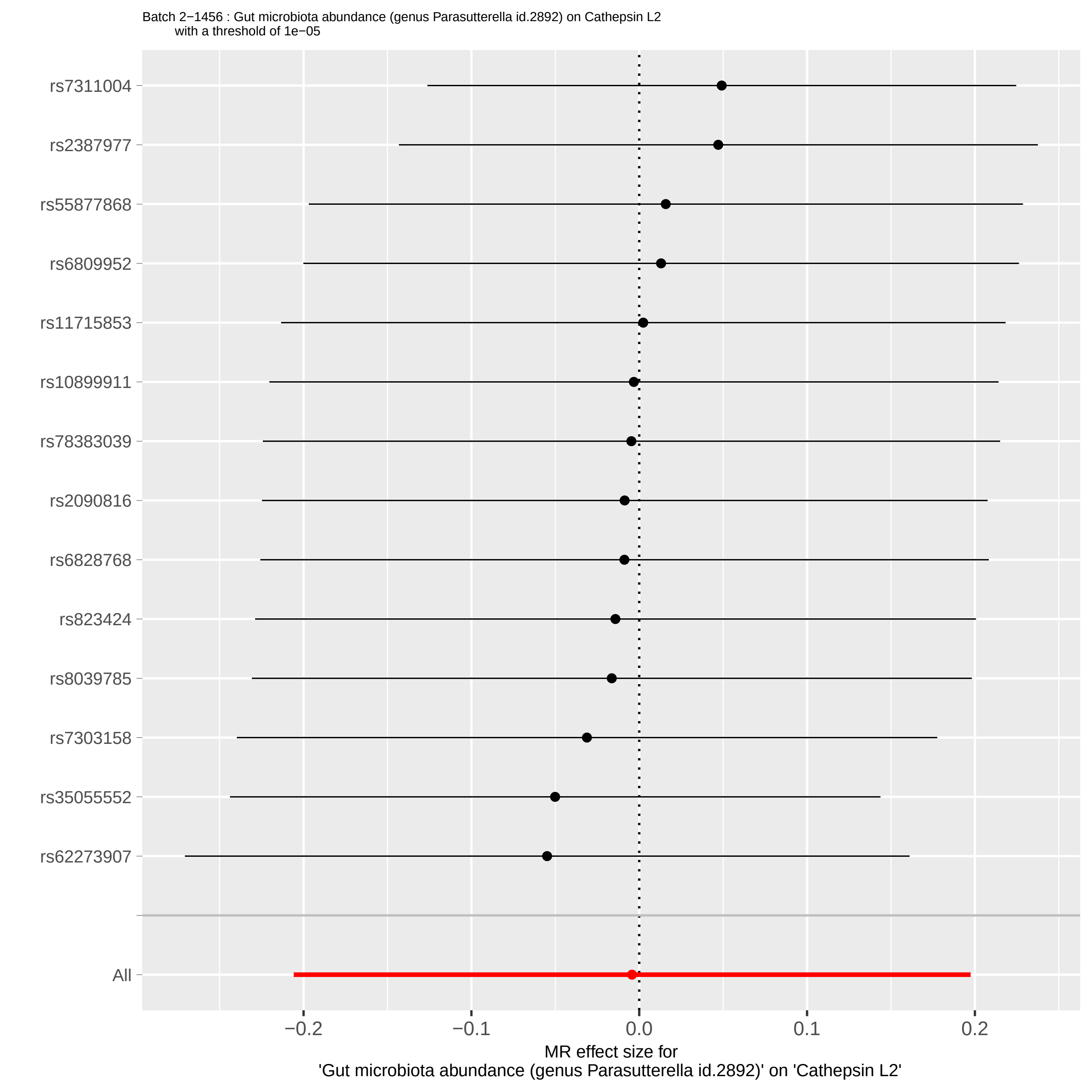
Batch 2−1456 : Gut microbiota abundance (genus Parasutterella id.2892) on Cathepsin L2
with a threshold of 1e−05
rs7311004
rs2387977
rs55877868
rs6809952
rs11715853
rs10899911
rs78383039
rs2090816
rs6828768
rs823424
rs8039785
rs7303158
rs35055552
rs62273907
All
−0.2
−0.1
0.0
0.1
0.2
MR effect size for
'Gut microbiota abundance (genus Parasutterella id.2892)' on 'Cathepsin L2'

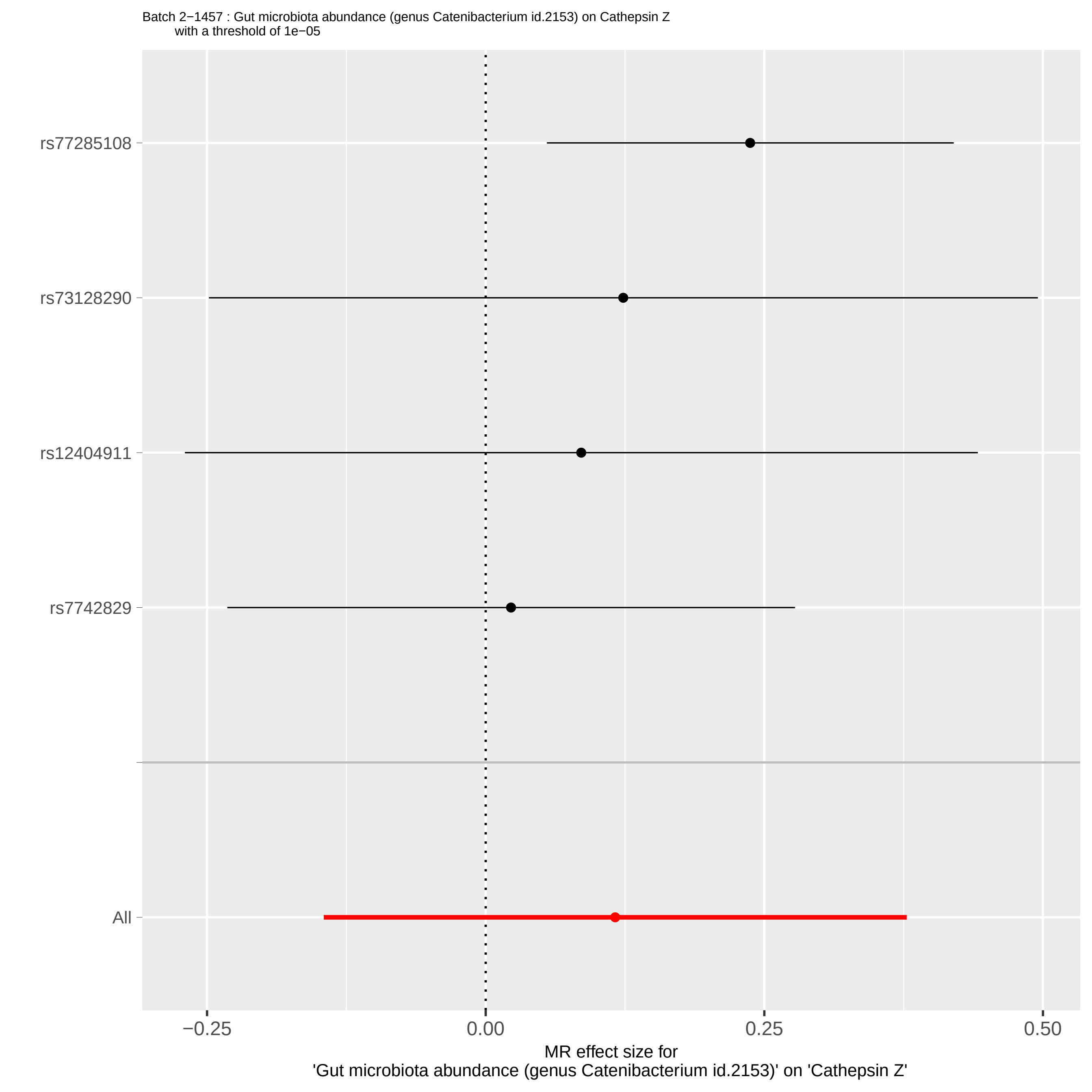

Batch 2−1457 : Gut microbiota abundance (genus Catenibacterium id.2153) on Cathepsin Z
with a threshold of 1e−05
rs77285108
rs73128290
rs12404911
rs7742829
All
−0.25
0.00
0.25
0.50
MR effect size for
'Gut microbiota abundance (genus Catenibacterium id.2153)' on 'Cathepsin Z'

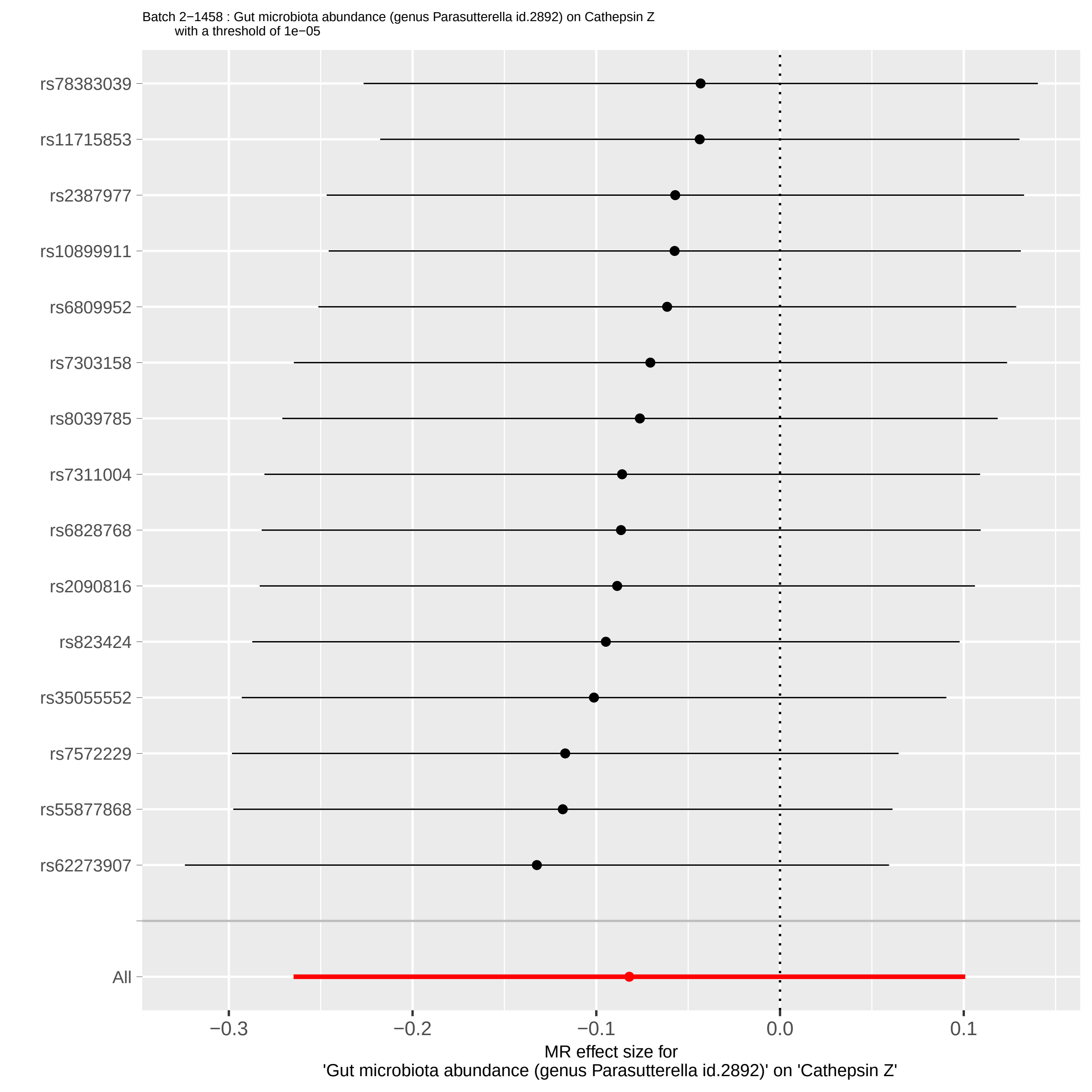
Batch 2−1458 : Gut microbiota abundance (genus Parasutterella id.2892) on Cathepsin Z
with a threshold of 1e−05
rs78383039
rs11715853
rs2387977
rs10899911
rs6809952
rs7303158
rs8039785
rs7311004
rs6828768
rs2090816
rs823424
rs35055552
rs7572229
rs55877868
rs62273907
All
−0.3
−0.2
−0.1
0.0
0.1
MR effect size for
'Gut microbiota abundance (genus Parasutterella id.2892)' on 'Cathepsin Z'

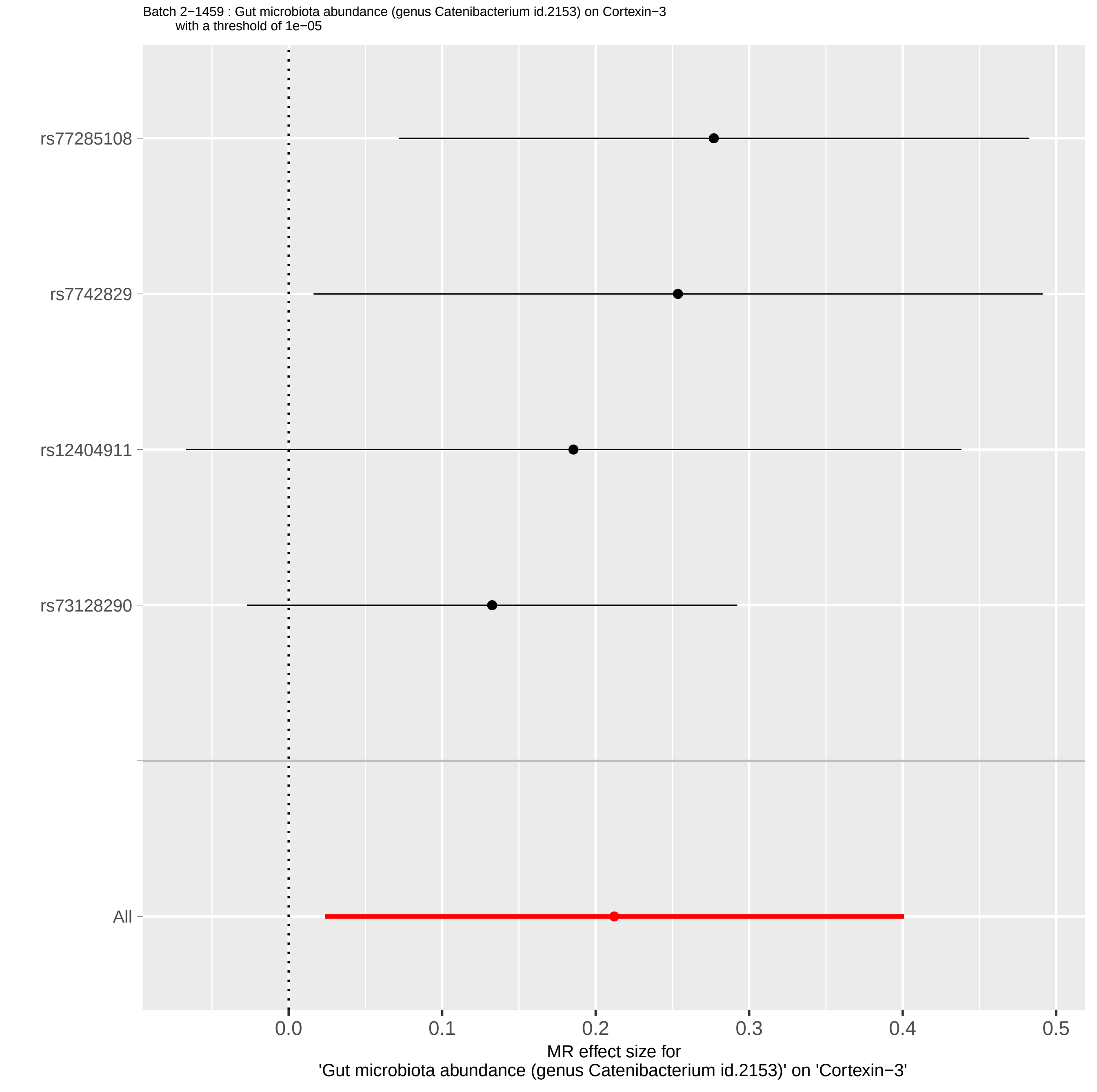
Batch 2−1459 : Gut microbiota abundance (genus Catenibacterium id.2153) on Cortexin−3
with a threshold of 1e−05
rs77285108
rs7742829
rs12404911
rs73128290
All
0.0
0.1
0.2
0.3
0.4
0.5
MR effect size for
'Gut microbiota abundance (genus Catenibacterium id.2153)' on 'Cortexin−3'

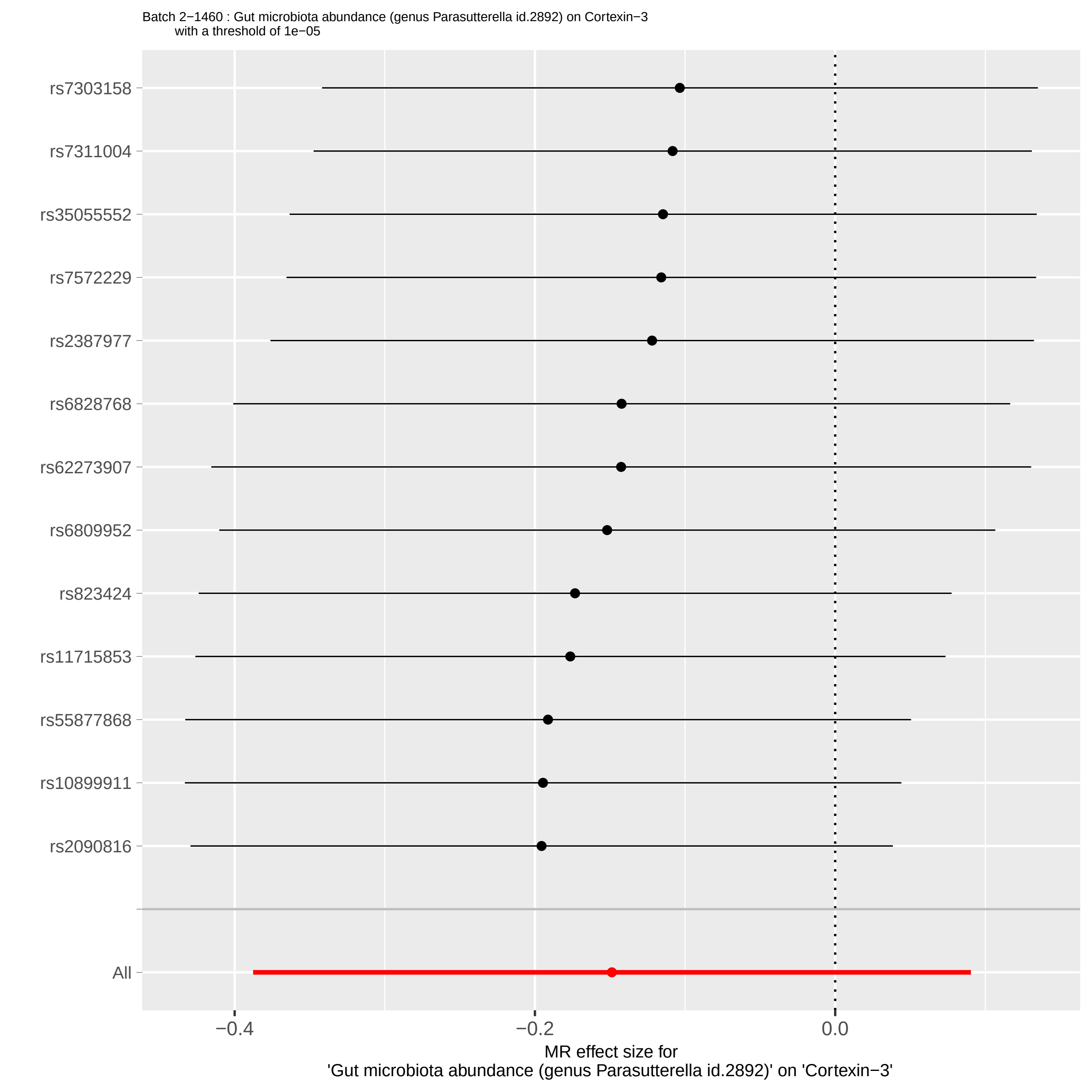

Batch 2−1460 : Gut microbiota abundance (genus Parasutterella id.2892) on Cortexin−3
with a threshold of 1e−05
rs7303158
rs7311004
rs35055552
rs7572229
rs2387977
rs6828768
rs62273907
rs6809952
rs823424
rs11715853
rs55877868
rs10899911
rs2090816
All
−0.4
−0.2
0.0
MR effect size for
'Gut microbiota abundance (genus Parasutterella id.2892)' on 'Cortexin−3'

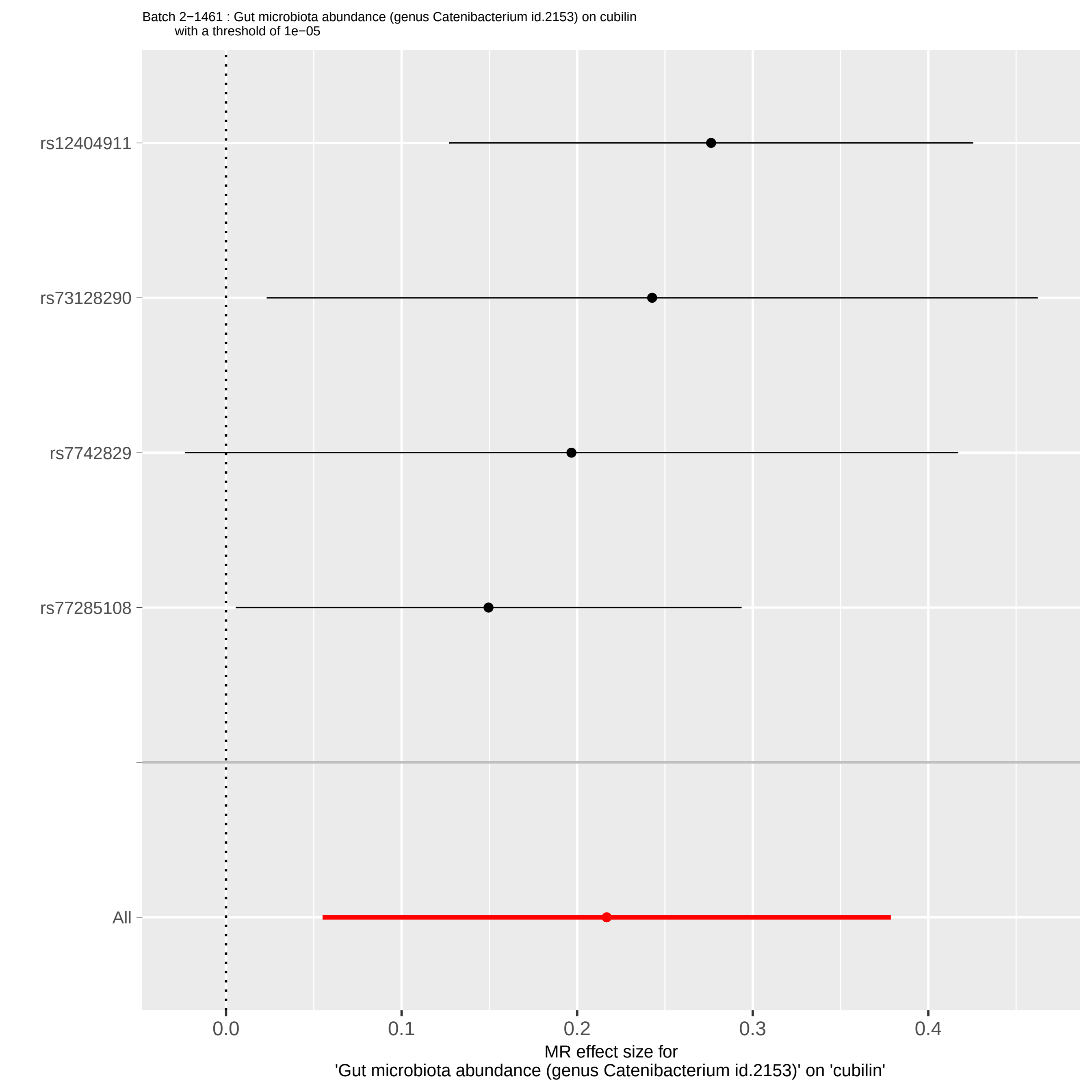
Batch 2−1461 : Gut microbiota abundance (genus Catenibacterium id.2153) on cubilin
with a threshold of 1e−05
rs12404911
rs73128290
rs7742829
rs77285108
All
0.0
0.1
0.2
0.3
0.4
MR effect size for
'Gut microbiota abundance (genus Catenibacterium id.2153)' on 'cubilin'

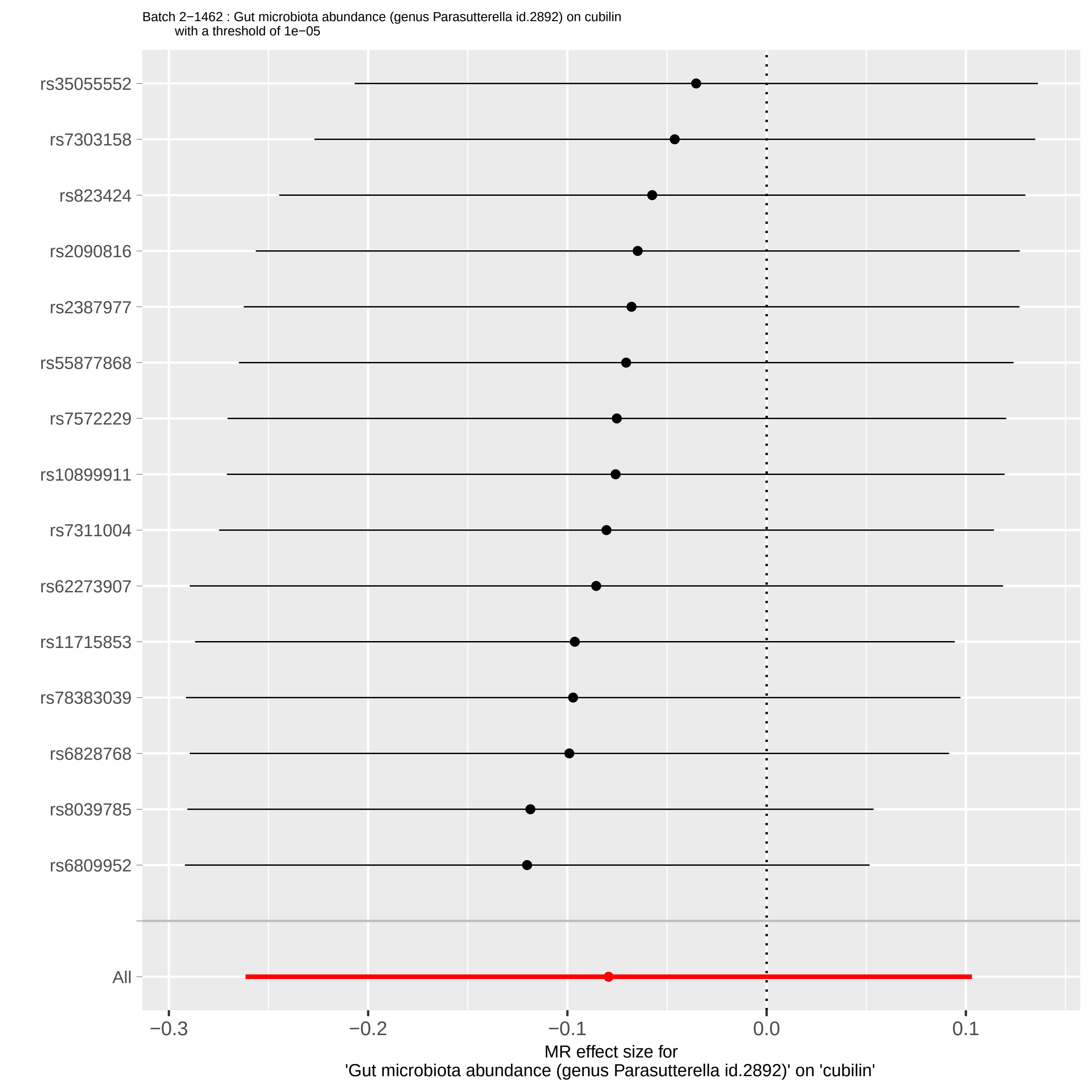
Batch 2−1462 : Gut microbiota abundance (genus Parasutterella id.2892) on cubilin
with a threshold of 1e−05
rs35055552
rs7303158
rs823424
rs2090816
rs2387977
rs55877868
rs7572229
rs10899911
rs7311004
rs62273907
rs11715853
rs78383039
rs6828768
rs8039785
rs6809952
All
−0.3
−0.2
−0.1
0.0
0.1
MR effect size for
'Gut microbiota abundance (genus Parasutterella id.2892)' on 'cubilin'

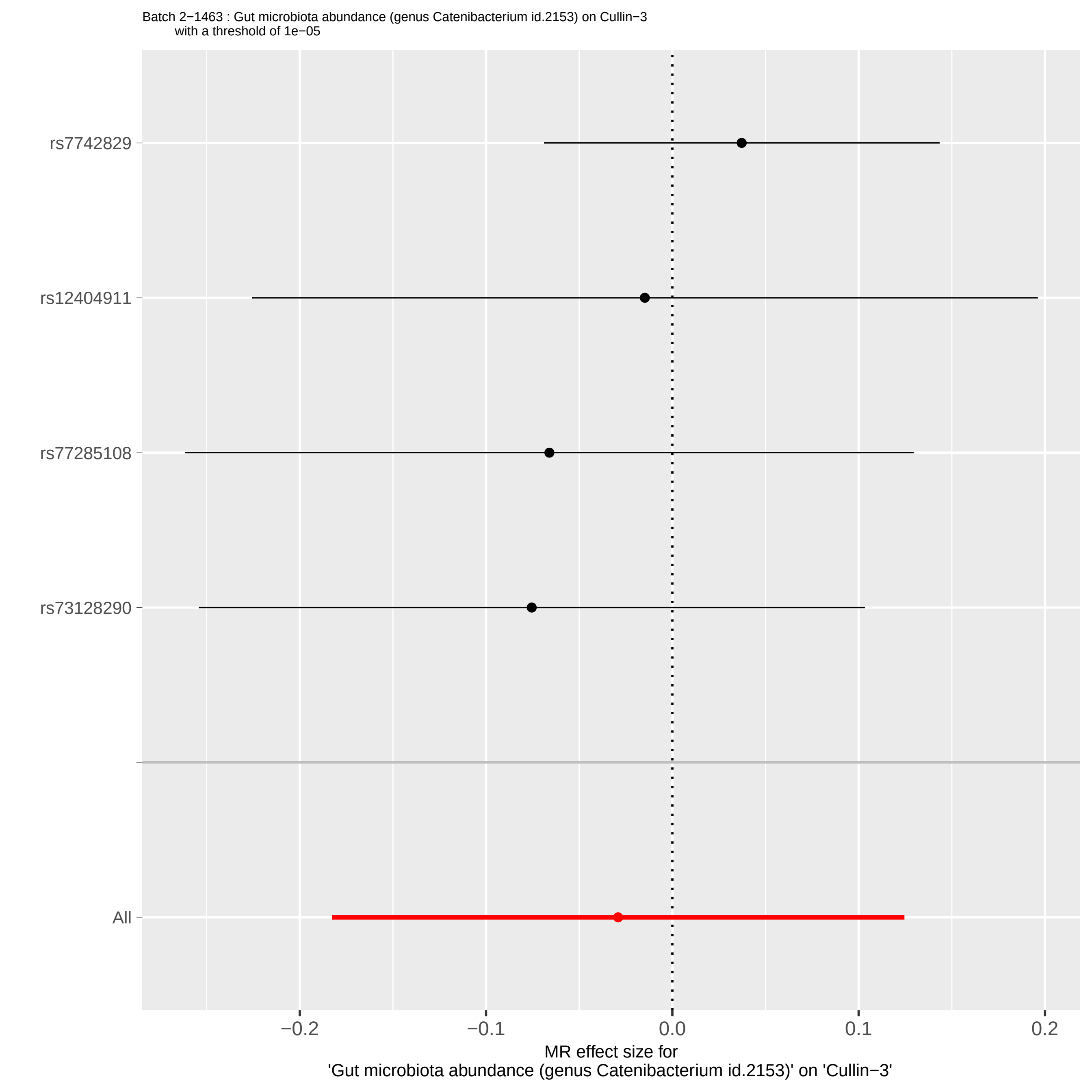

Batch 2−1463 : Gut microbiota abundance (genus Catenibacterium id.2153) on Cullin−3
with a threshold of 1e−05
rs7742829
rs12404911
rs77285108
rs73128290
All
−0.2
−0.1
0.0
0.1
0.2
MR effect size for
'Gut microbiota abundance (genus Catenibacterium id.2153)' on 'Cullin−3'

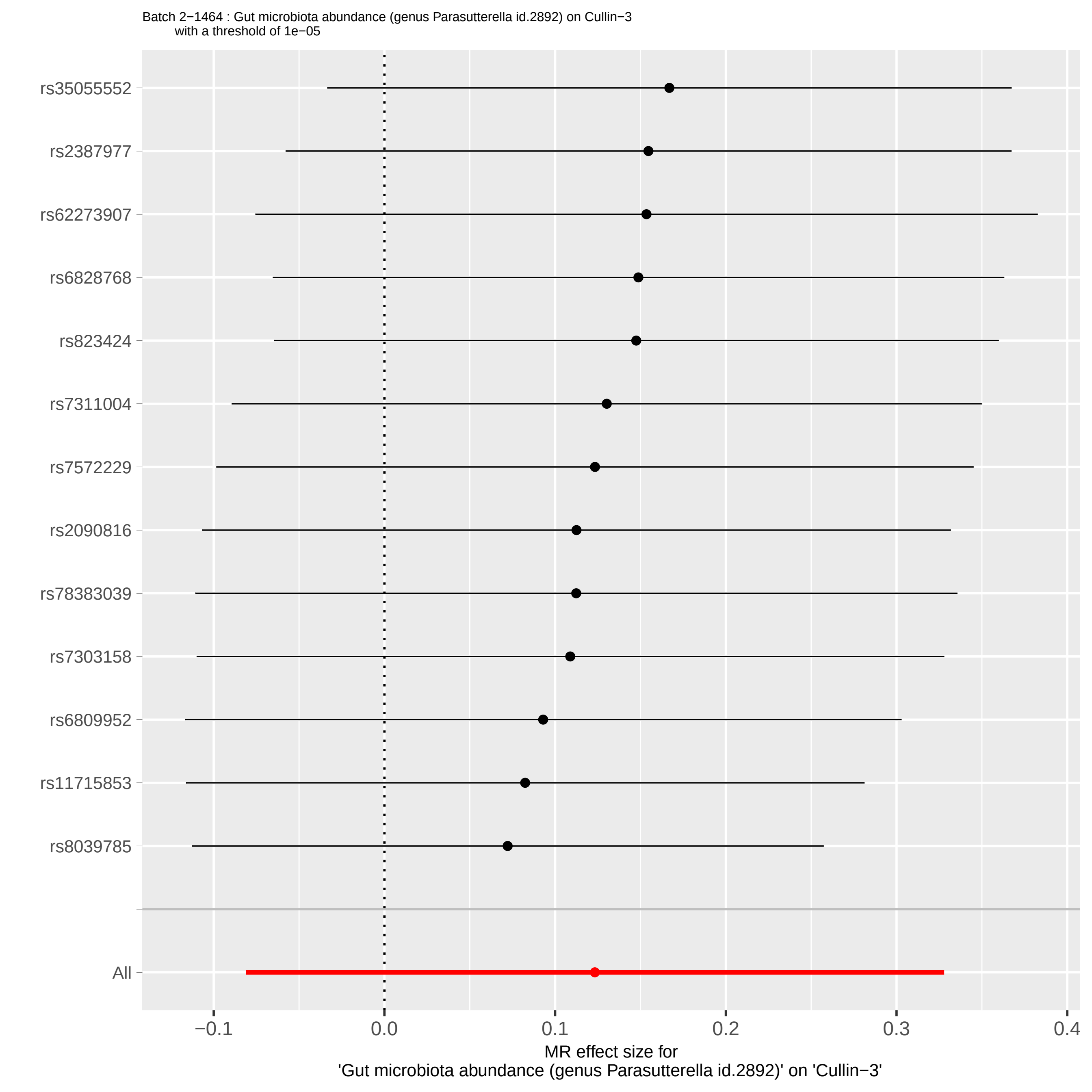
Batch 2−1464 : Gut microbiota abundance (genus Parasutterella id.2892) on Cullin−3
with a threshold of 1e−05
rs35055552
rs2387977
rs62273907
rs6828768
rs823424
rs7311004
rs7572229
rs2090816
rs78383039
rs7303158
rs6809952
rs11715853
rs8039785
All
−0.1
0.0
0.1
0.2
0.3
0.4
MR effect size for
'Gut microbiota abundance (genus Parasutterella id.2892)' on 'Cullin−3

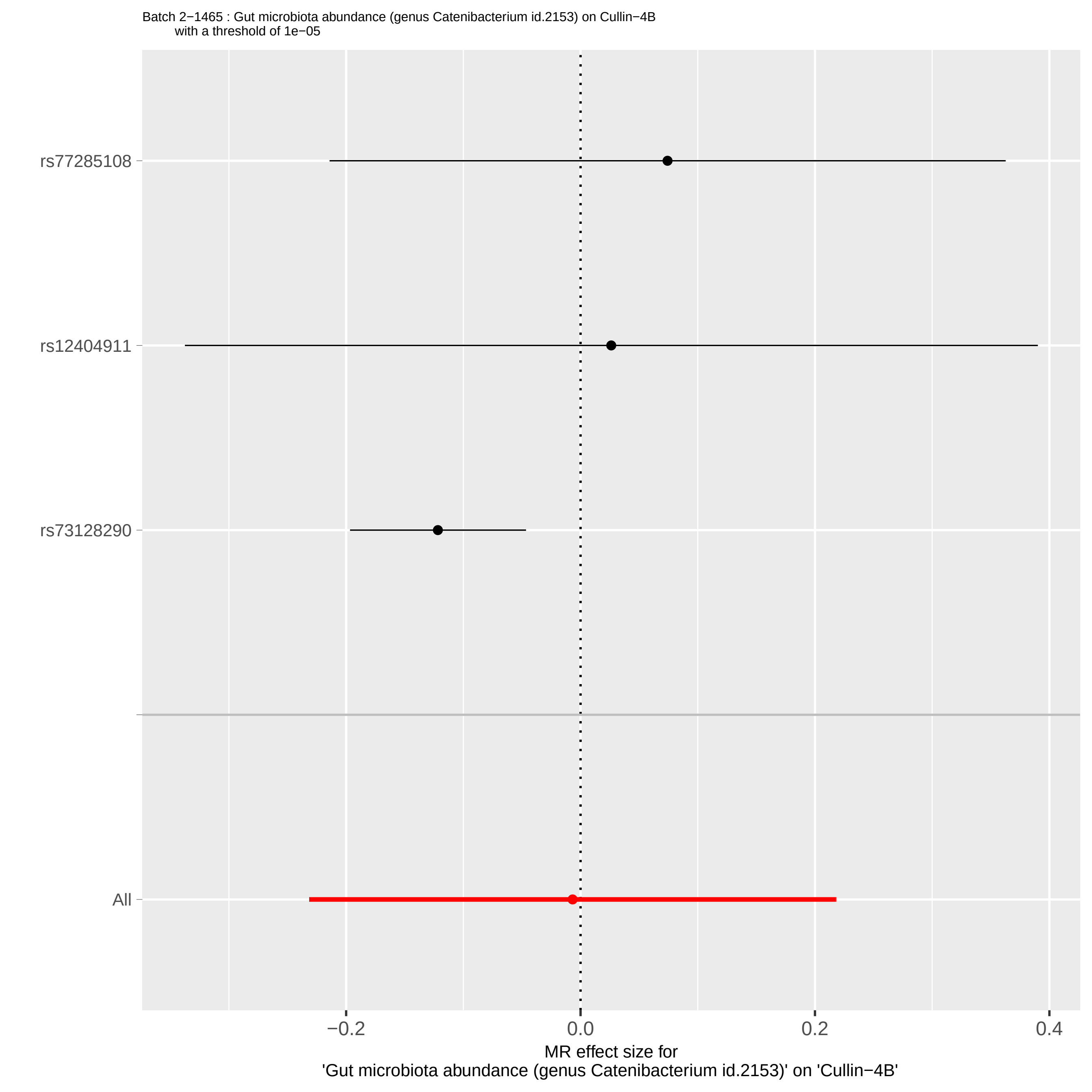

Batch 2−1465 : Gut microbiota abundance (genus Catenibacterium id.2153) on Cullin−4B
with a threshold of 1e−05
rs77285108
rs12404911
rs73128290
All
−0.2
0.0
0.2
0.4
MR effect size for
'Gut microbiota abundance (genus Catenibacterium id.2153)' on 'Cullin−4B'

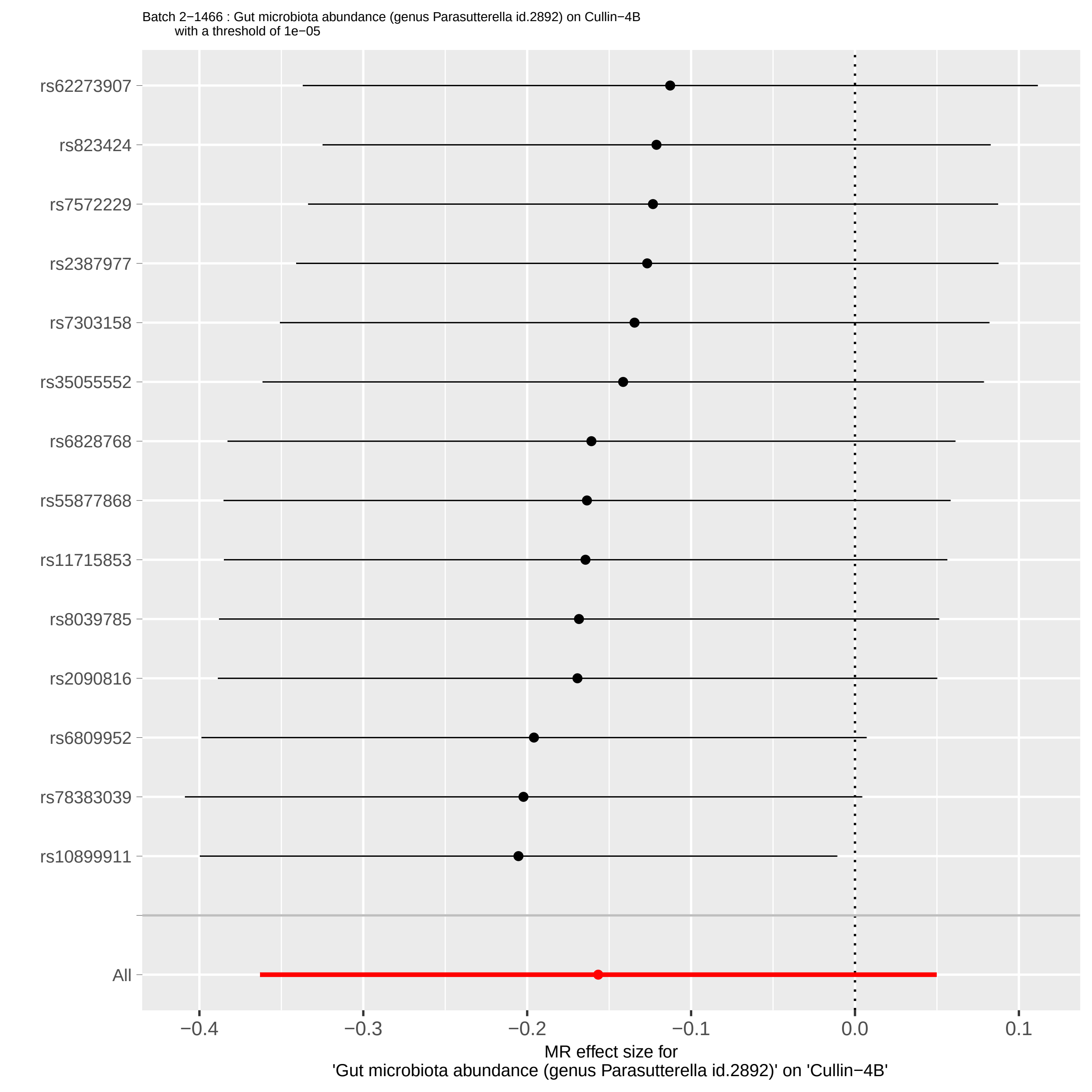
Batch 2−1466 : Gut microbiota abundance (genus Parasutterella id.2892) on Cullin−4B
with a threshold of 1e−05
rs62273907
rs823424
rs7572229
rs2387977
rs7303158
rs35055552
rs6828768
rs55877868
rs11715853
rs8039785
rs2090816
rs6809952
rs78383039
rs10899911
All
−0.4
−0.3
−0.2
−0.1
0.0
0.1
MR effect size for
'Gut microbiota abundance (genus Parasutterella id.2892)' on 'Cullin−4B'

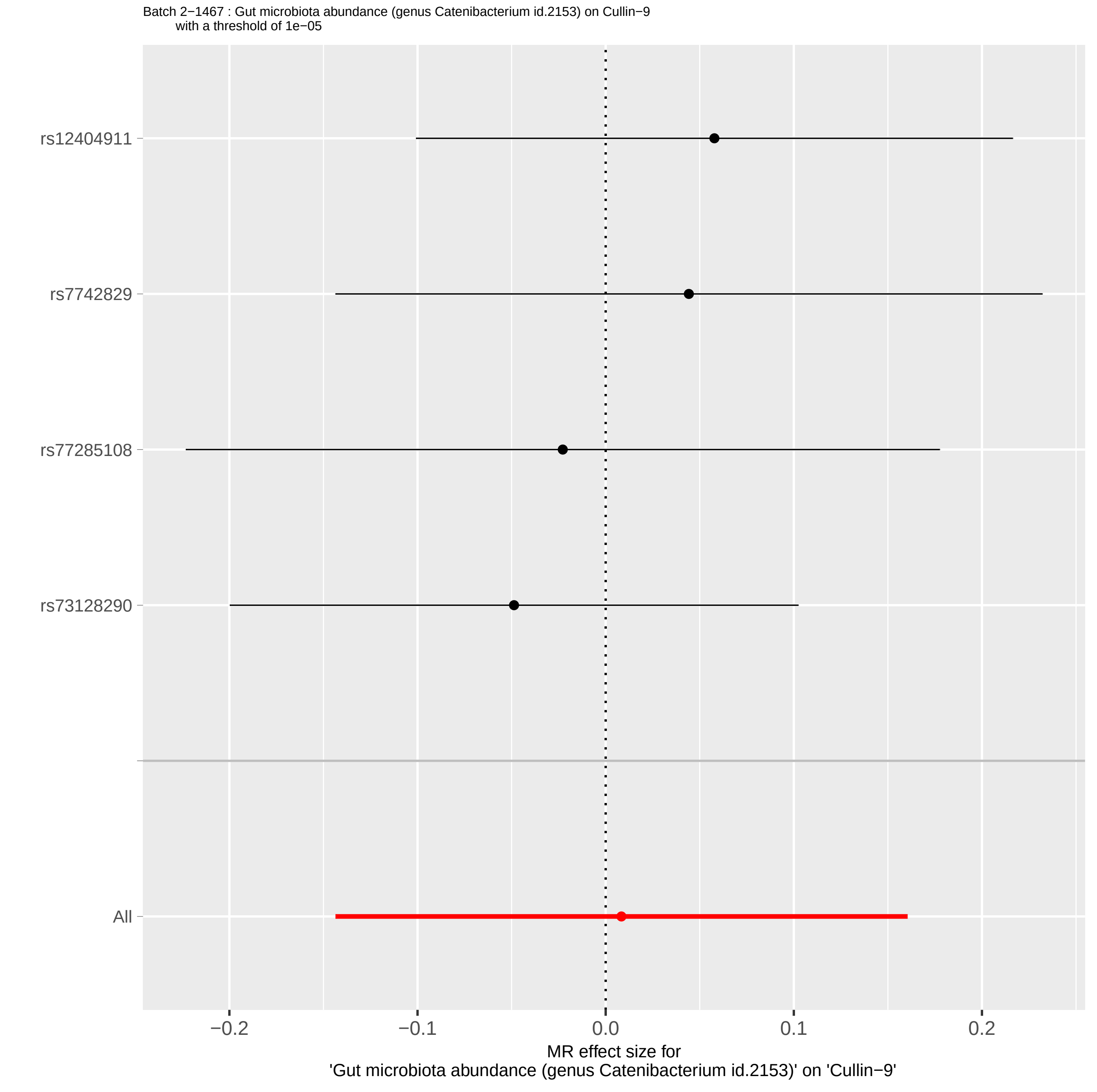

Batch 2−1467 : Gut microbiota abundance (genus Catenibacterium id.2153) on Cullin−9
with a threshold of 1e−05
rs12404911
rs7742829
rs77285108
rs73128290
All
−0.2
−0.1
0.0
0.1
0.2
MR effect size for
'Gut microbiota abundance (genus Catenibacterium id.2153)' on 'Cullin−9'

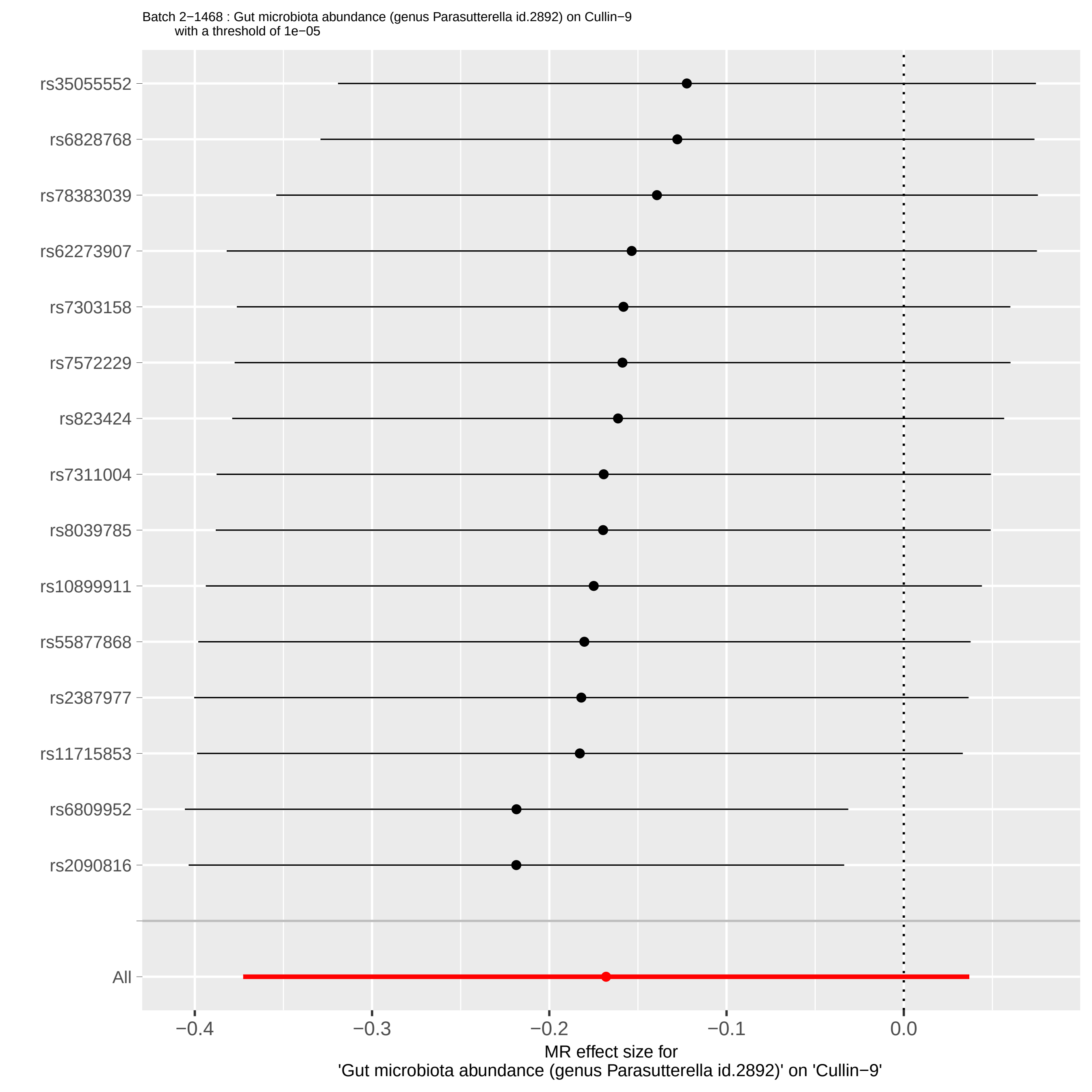
Batch 2−1468 : Gut microbiota abundance (genus Parasutterella id.2892) on Cullin−9
with a threshold of 1e−05
rs35055552
rs6828768
rs78383039
rs62273907
rs7303158
rs7572229
rs823424
rs7311004
rs8039785
rs10899911
rs55877868
rs2387977
rs11715853
rs6809952
rs2090816
All
−0.4
−0.3
−0.2
−0.1
0.0
MR effect size for
'Gut microbiota abundance (genus Parasutterella id.2892)' on 'Cullin−9

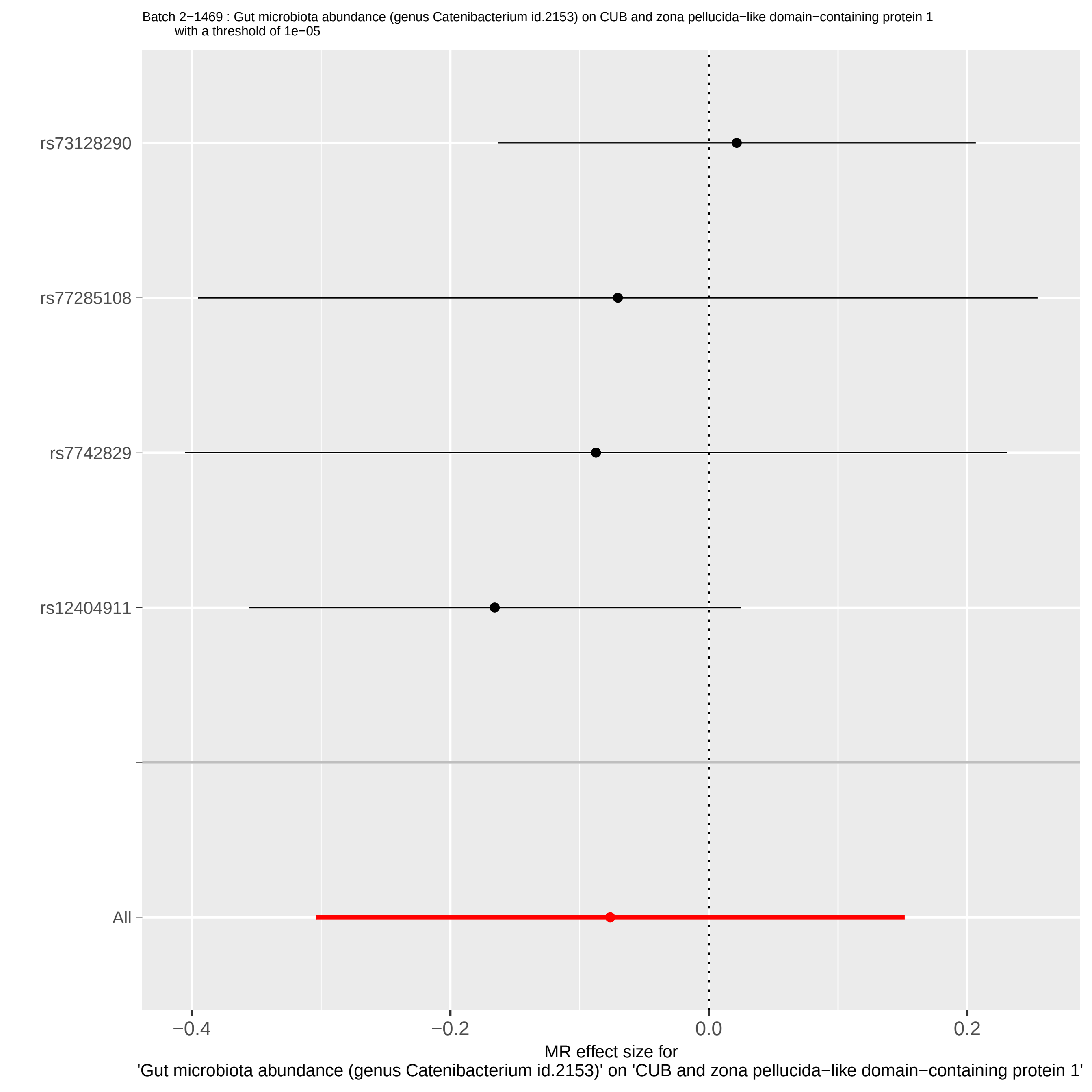

Batch 2−1469 : Gut microbiota abundance (genus Catenibacterium id.2153) on CUB and zona pellucida−like domain−containing protein 1
with a threshold of 1e−05
rs73128290
rs77285108
rs7742829
rs12404911
All
−0.4
−0.2
0.0
0.2
MR effect size for
'Gut microbiota abundance (genus Catenibacterium id.2153)' on 'CUB and zona pellucida−like domain−containing protein 1'

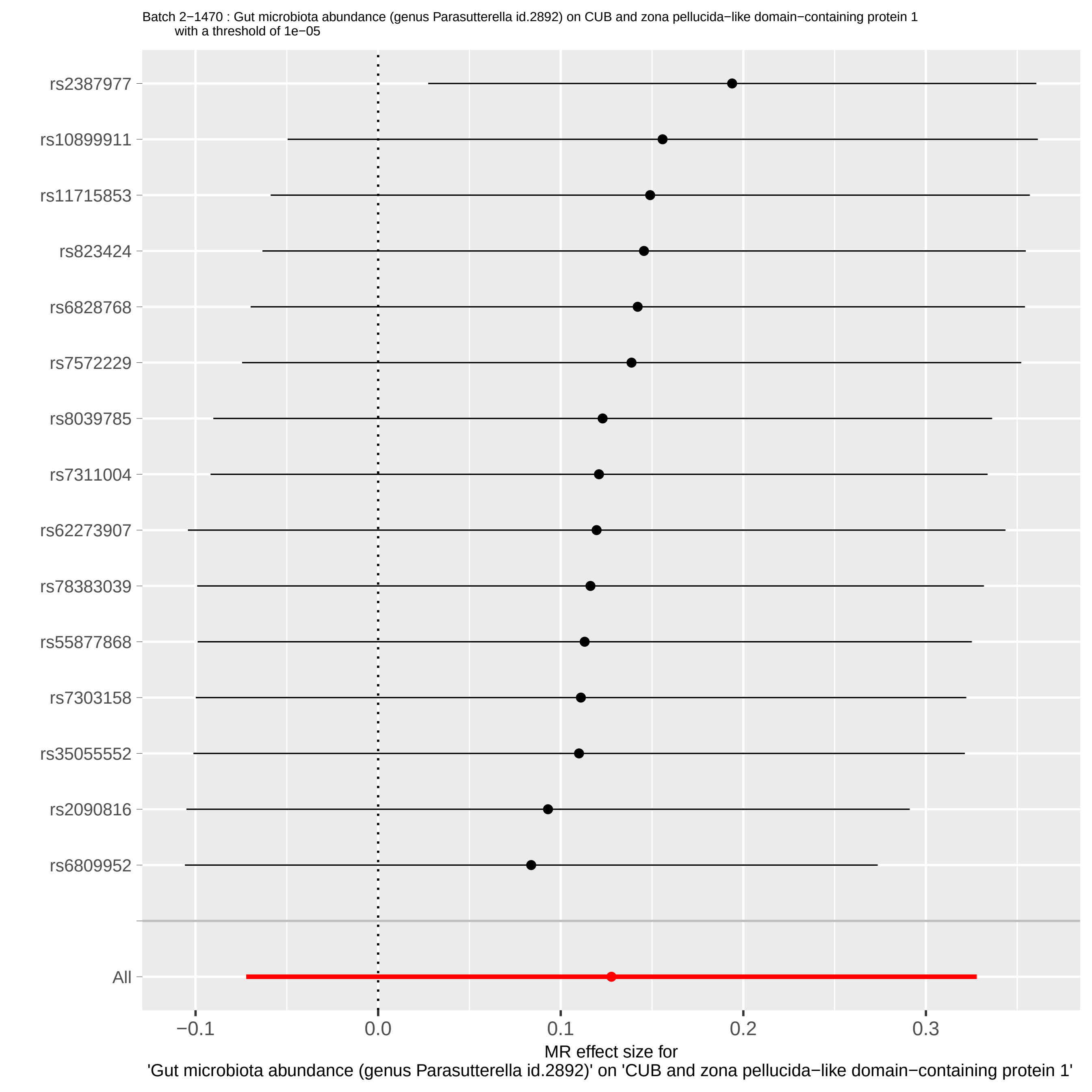
Batch 2−1470 : Gut microbiota abundance (genus Parasutterella id.2892) on CUB and zona pellucida−like domain−containing protein 1
with a threshold of 1e−05
rs2387977
rs10899911
rs11715853
rs823424
rs6828768
rs7572229
rs8039785
rs7311004
rs62273907
rs78383039
rs55877868
rs7303158
rs35055552
rs2090816
rs6809952
All
−0.1
0.0
0.1
0.2
0.3
MR effect size for
'Gut microbiota abundance (genus Parasutterella id.2892)' on 'CUB and zona pellucida−like domain−containing protein 1'

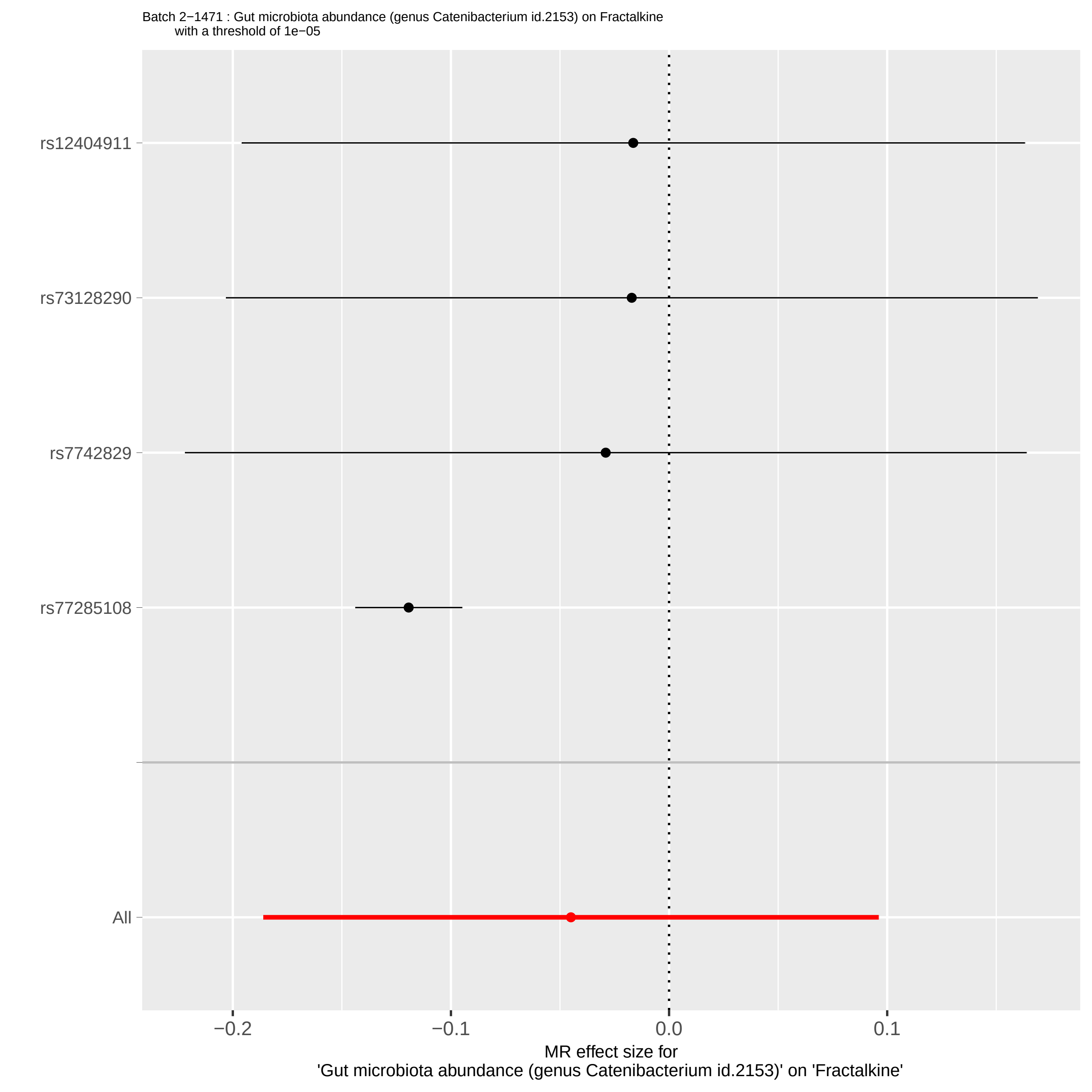

Batch 2−1471 : Gut microbiota abundance (genus Catenibacterium id.2153) on Fractalkine
with a threshold of 1e−05
rs12404911
rs73128290
rs7742829
rs77285108
All
−0.2
−0.1
0.0
0.1
MR effect size for
'Gut microbiota abundance (genus Catenibacterium id.2153)' on 'Fractalkine'

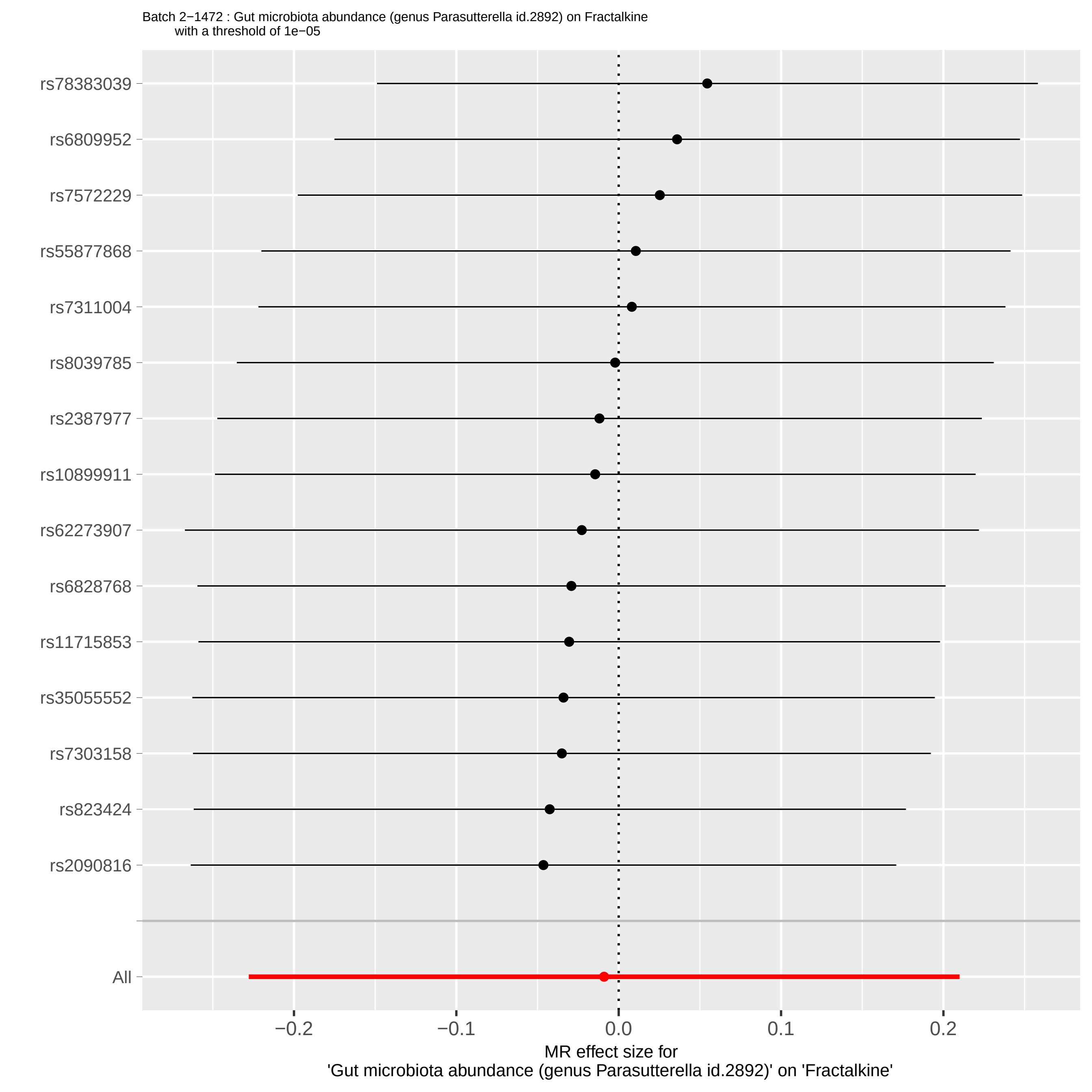

Batch 2−1472 : Gut microbiota abundance (genus Parasutterella id.2892) on Fractalkine
with a threshold of 1e−05
rs78383039
rs6809952
rs7572229
rs55877868
rs7311004
rs8039785
rs2387977
rs10899911
rs62273907
rs6828768
rs11715853
rs35055552
rs7303158
rs823424
rs2090816
All
−0.2
−0.1
0.0
0.1
0.2
MR effect size for
'Gut microbiota abundance (genus Parasutterella id.2892)' on 'Fractalkine'

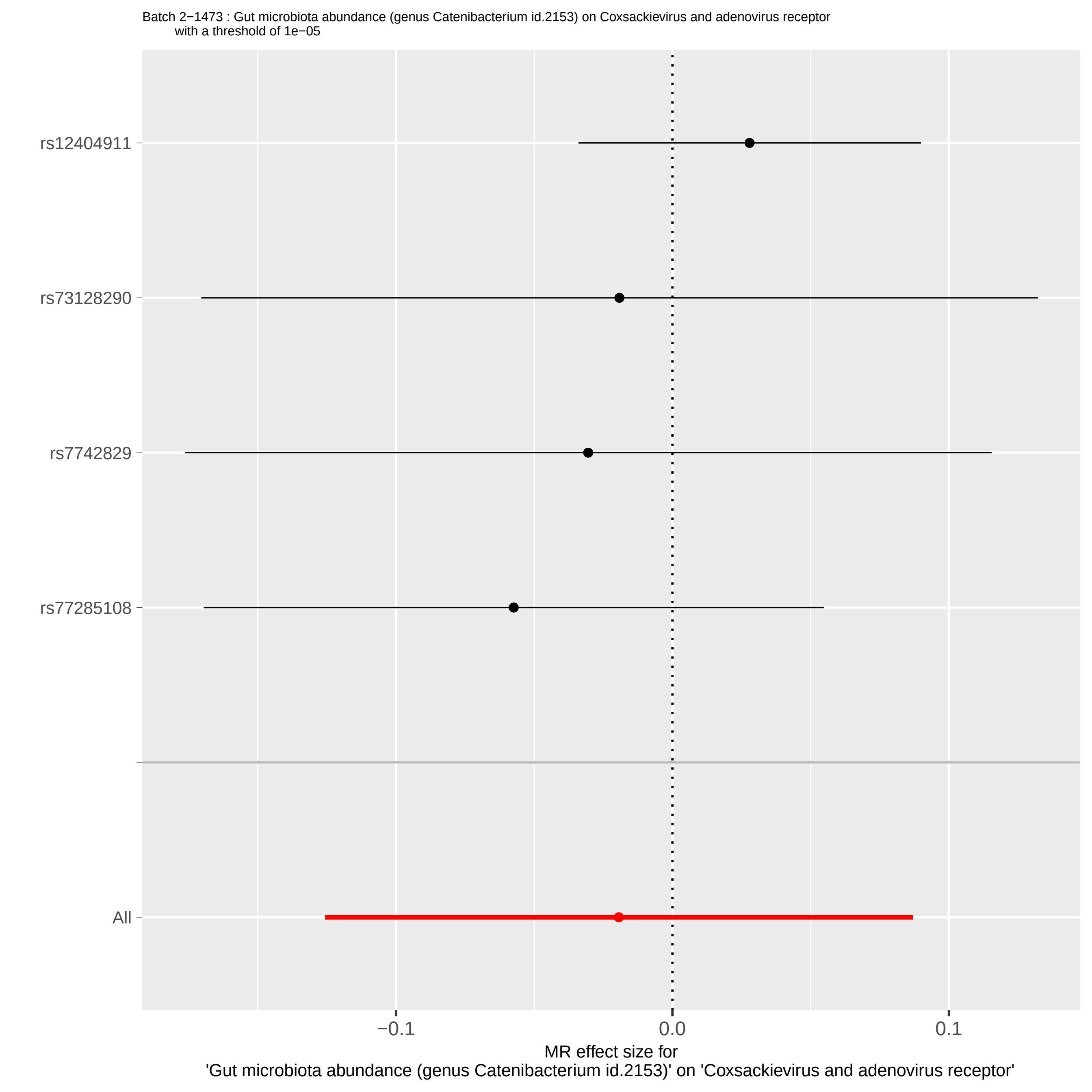
Batch 2−1473 : Gut microbiota abundance (genus Catenibacterium id.2153) on Coxsackievirus and adenovirus receptor
with a threshold of 1e−05
rs12404911
rs73128290
rs7742829
rs77285108
All
−0.1
0.0
0.1
MR effect size for
'Gut microbiota abundance (genus Catenibacterium id.2153)' on 'Coxsackievirus and adenovirus receptor'

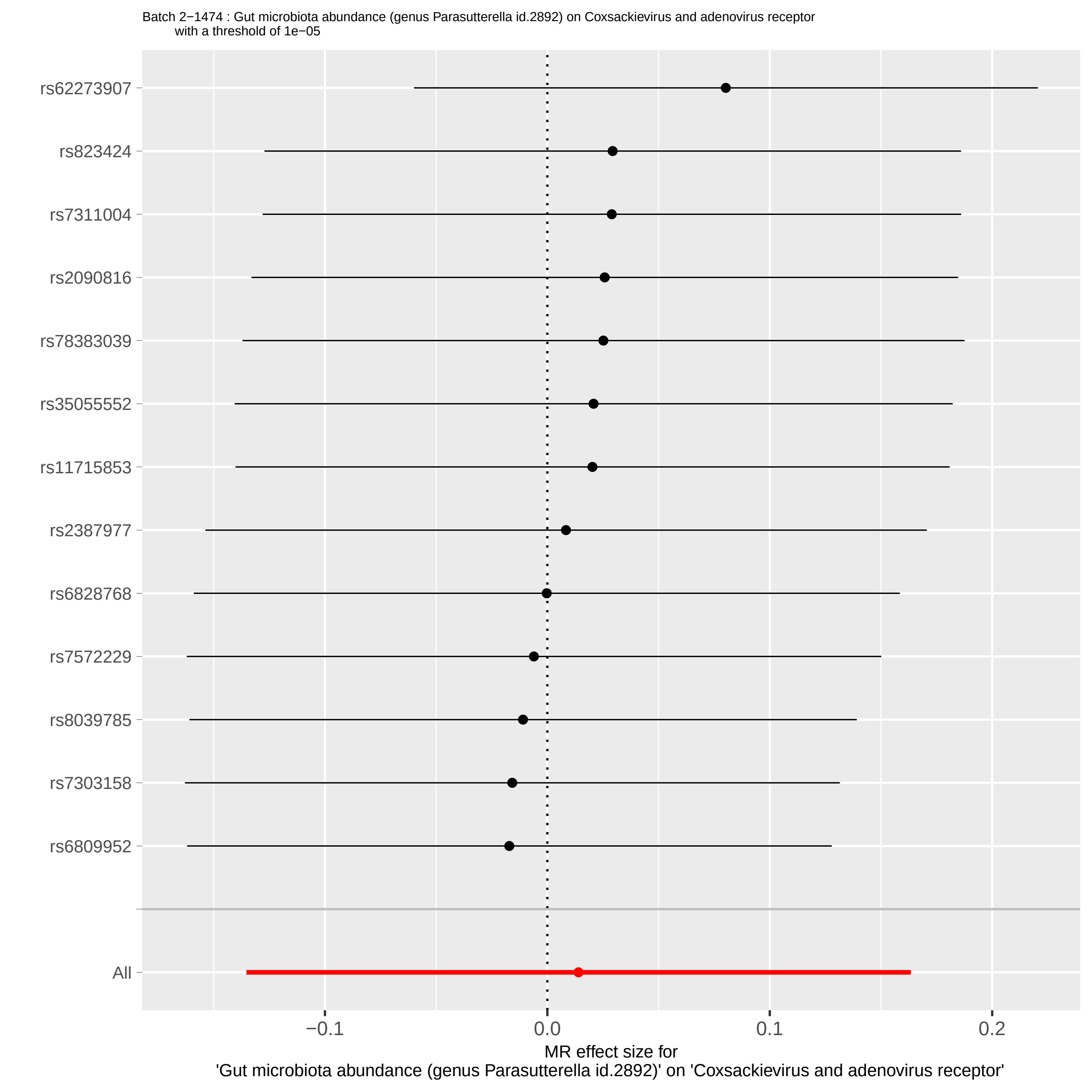
Batch 2−1474 : Gut microbiota abundance (genus Parasutterella id.2892) on Coxsackievirus and adenovirus receptor
with a threshold of 1e−05
rs62273907
rs823424
rs7311004
rs2090816
rs78383039
rs35055552
rs11715853
rs2387977
rs6828768
rs7572229
rs8039785
rs7303158
rs6809952
All
−0.1
0.0
0.1
0.2
MR effect size for
'Gut microbiota abundance (genus Parasutterella id.2892)' on 'Coxsackievirus and adenovirus receptor'

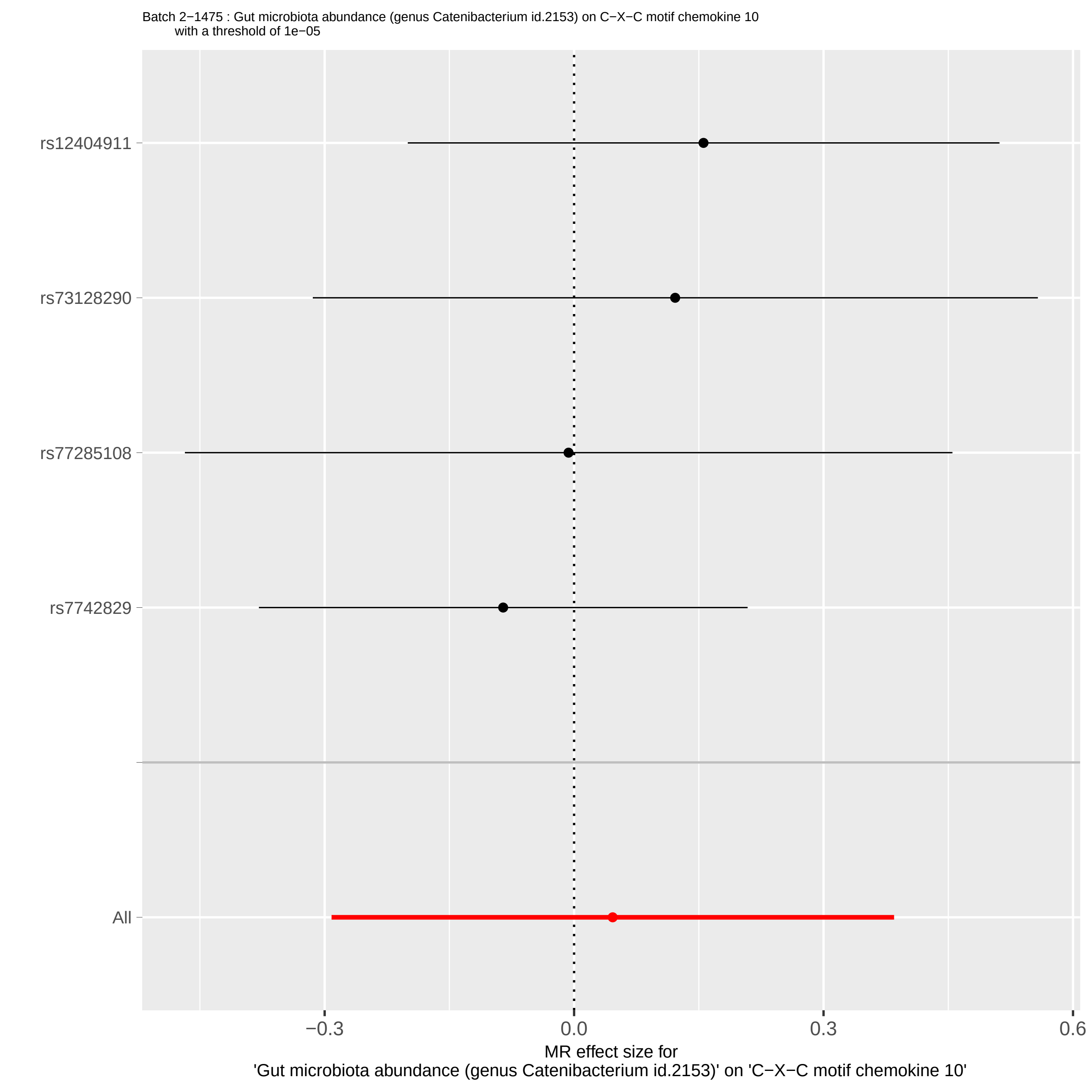
Batch 2−1475 : Gut microbiota abundance (genus Catenibacterium id.2153) on C−X−C motif chemokine 10
with a threshold of 1e−05
rs12404911
rs73128290
rs77285108
rs7742829
All
−0.3
0.0
0.3
0.6
MR effect size for
'Gut microbiota abundance (genus Catenibacterium id.2153)' on 'C−X−C motif chemokine 10'

Batch 2−1476 : Gut microbiota abundance (genus Parasutterella id.2892) on C−X−C motif chemokine 10
with a threshold of 1e−05

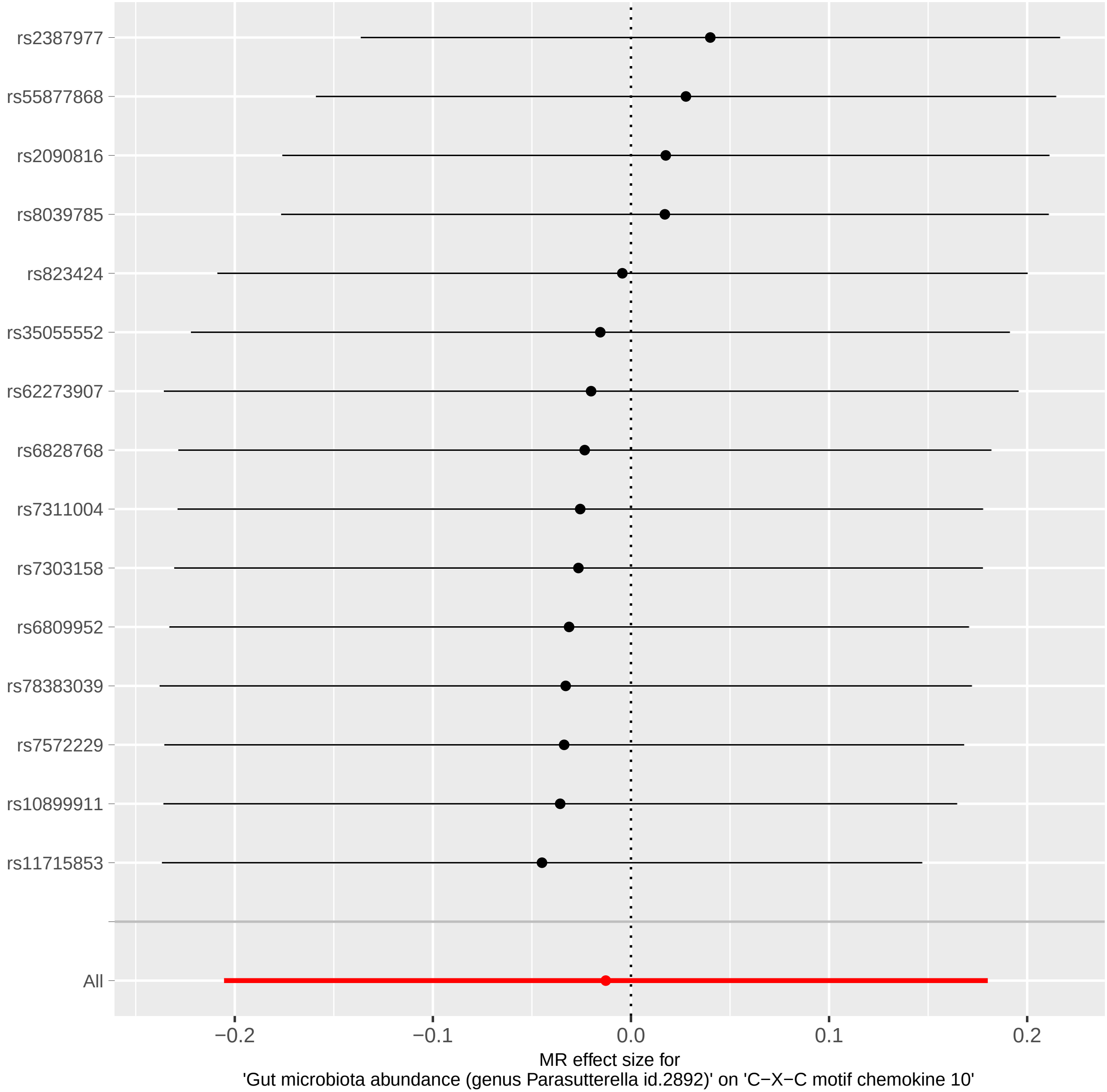

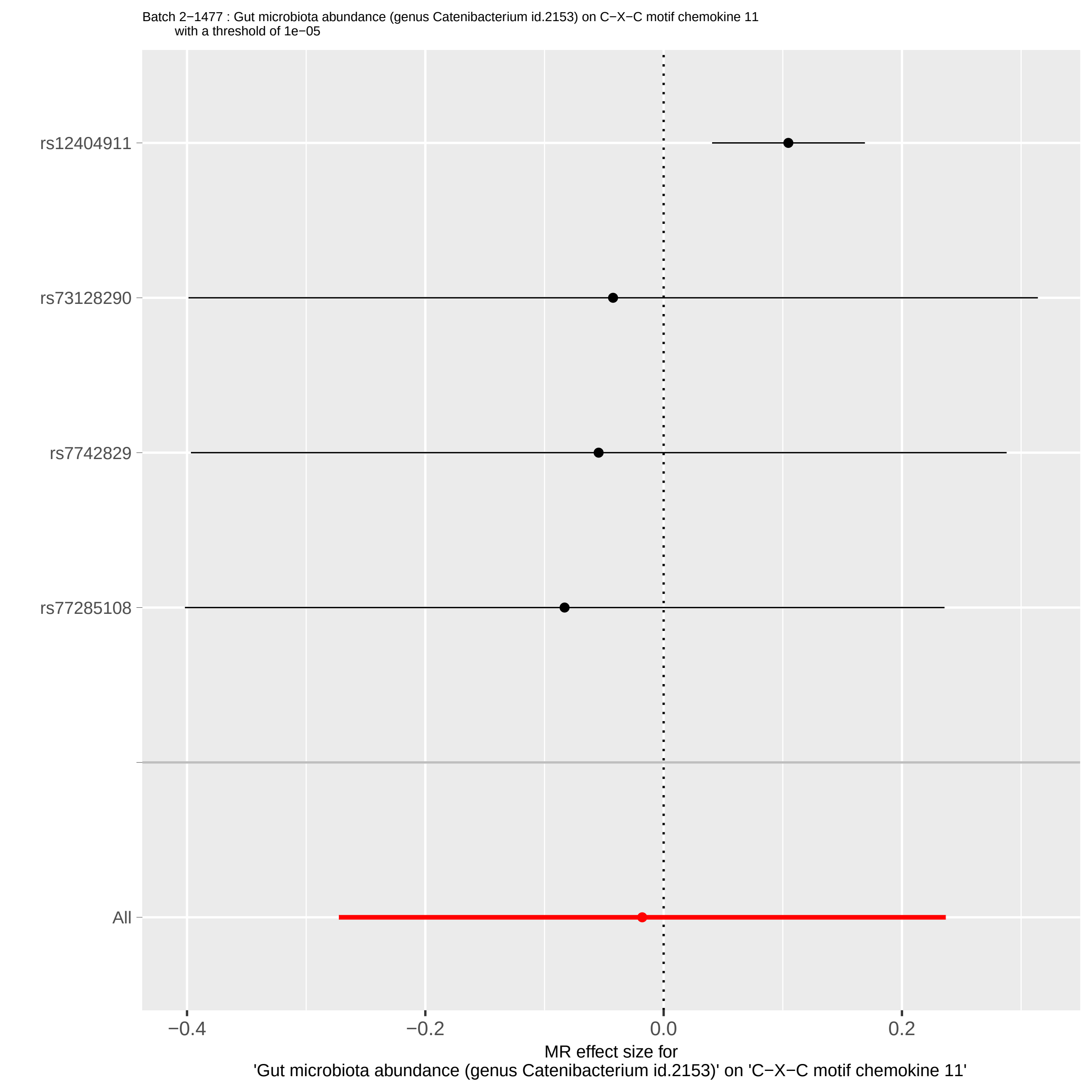
Batch 2−1477 : Gut microbiota abundance (genus Catenibacterium id.2153) on C−X−C motif chemokine 11
with a threshold of 1e−05
rs12404911
rs73128290
rs7742829
rs77285108
All
−0.4
−0.2
0.0
0.2
MR effect size for
'Gut microbiota abundance (genus Catenibacterium id.2153)' on 'C−X−C motif chemokine 11'

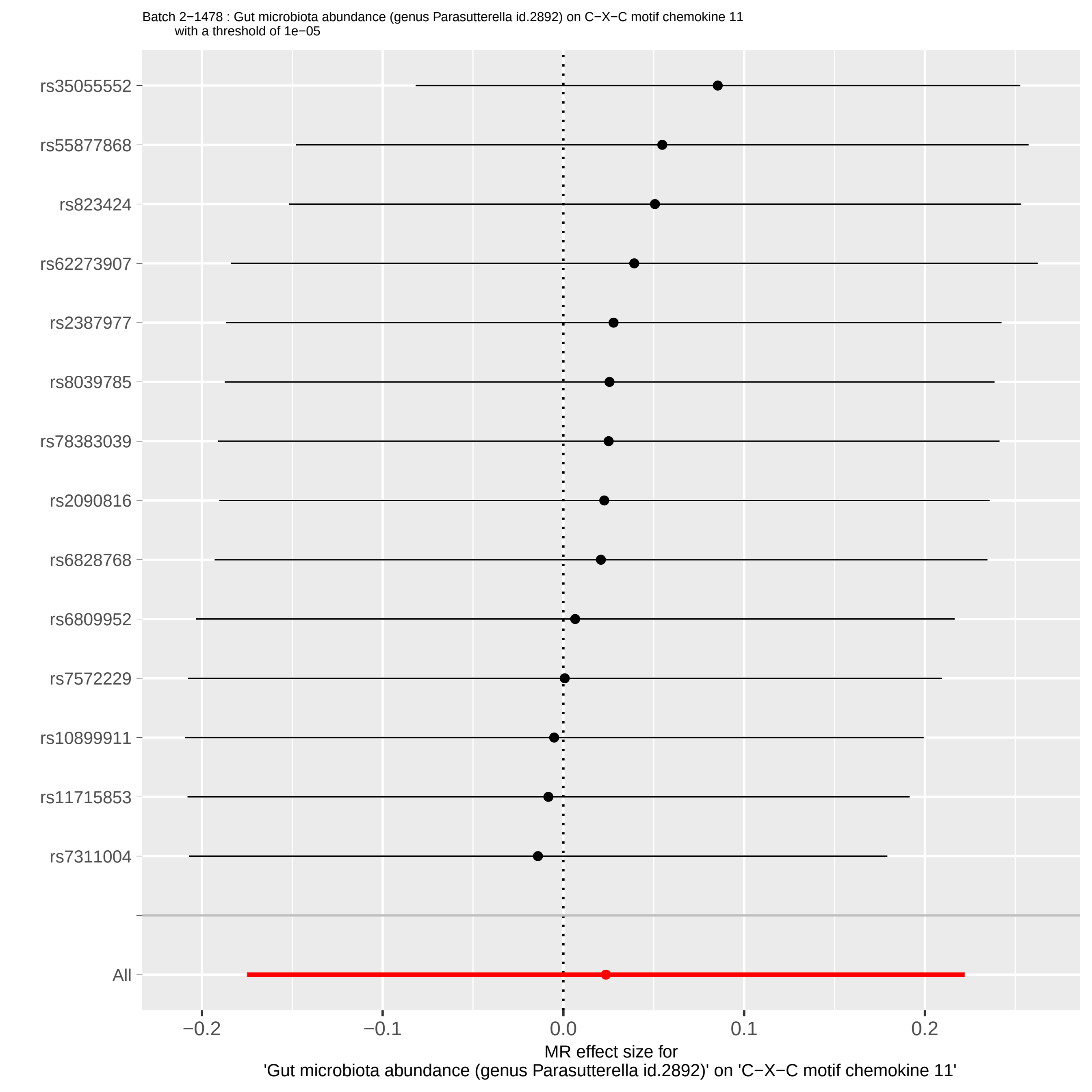
Batch 2−1478 : Gut microbiota abundance (genus Parasutterella id.2892) on C−X−C motif chemokine 11
with a threshold of 1e−05
rs35055552
rs55877868
rs823424
rs62273907
rs2387977
rs8039785
rs78383039
rs2090816
rs6828768
rs6809952
rs7572229
rs10899911
rs11715853
rs7311004
All
−0.2
−0.1
0.0
0.1
0.2
MR effect size for
'Gut microbiota abundance (genus Parasutterella id.2892)' on 'C−X−C motif chemokine 11'

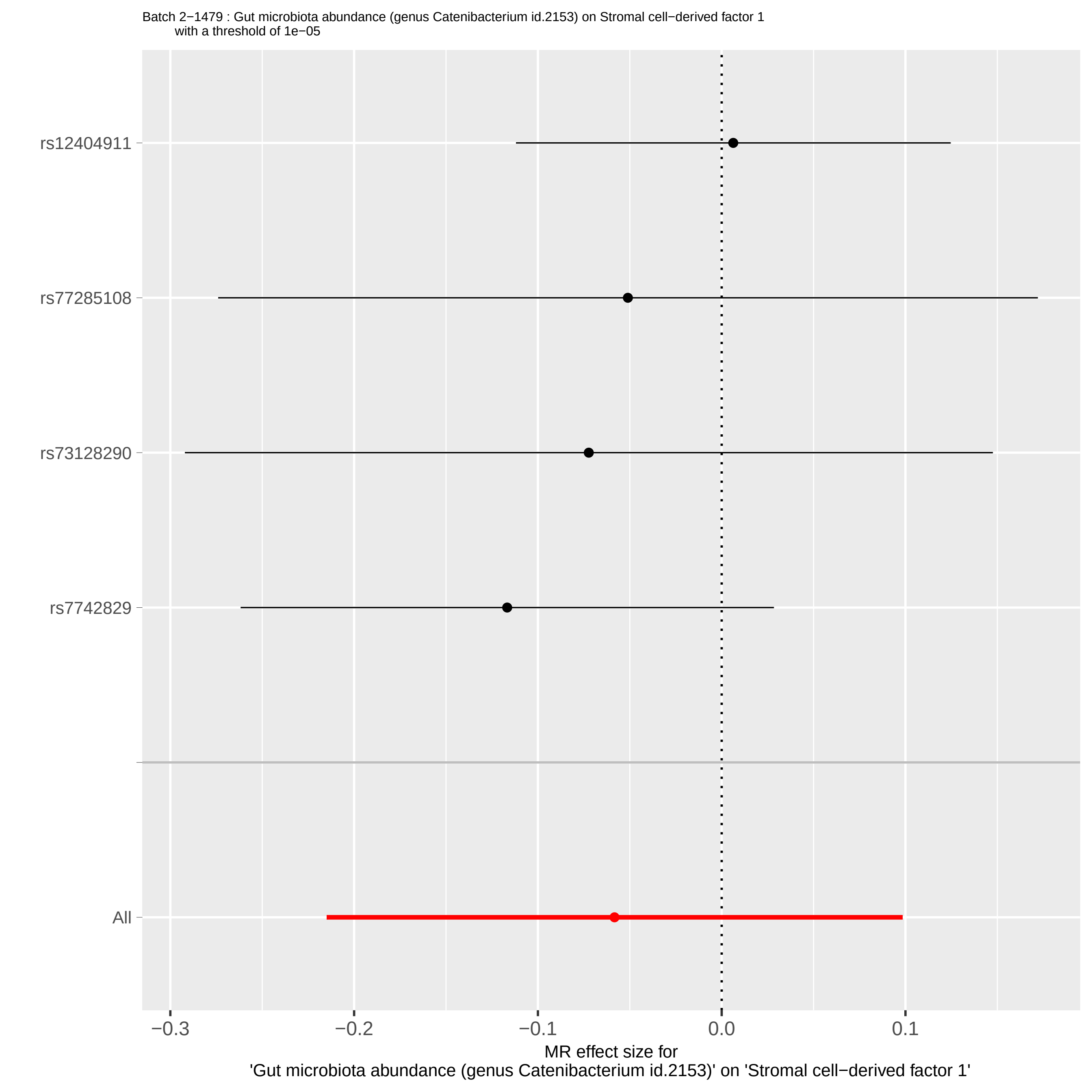

Batch 2−1479 : Gut microbiota abundance (genus Catenibacterium id.2153) on Stromal cell−derived factor 1
with a threshold of 1e−05
rs12404911
rs77285108
rs73128290
rs7742829
All
−0.3
−0.2
−0.1
0.0
0.1
MR effect size for
'Gut microbiota abundance (genus Catenibacterium id.2153)' on 'Stromal cell−derived factor 1'

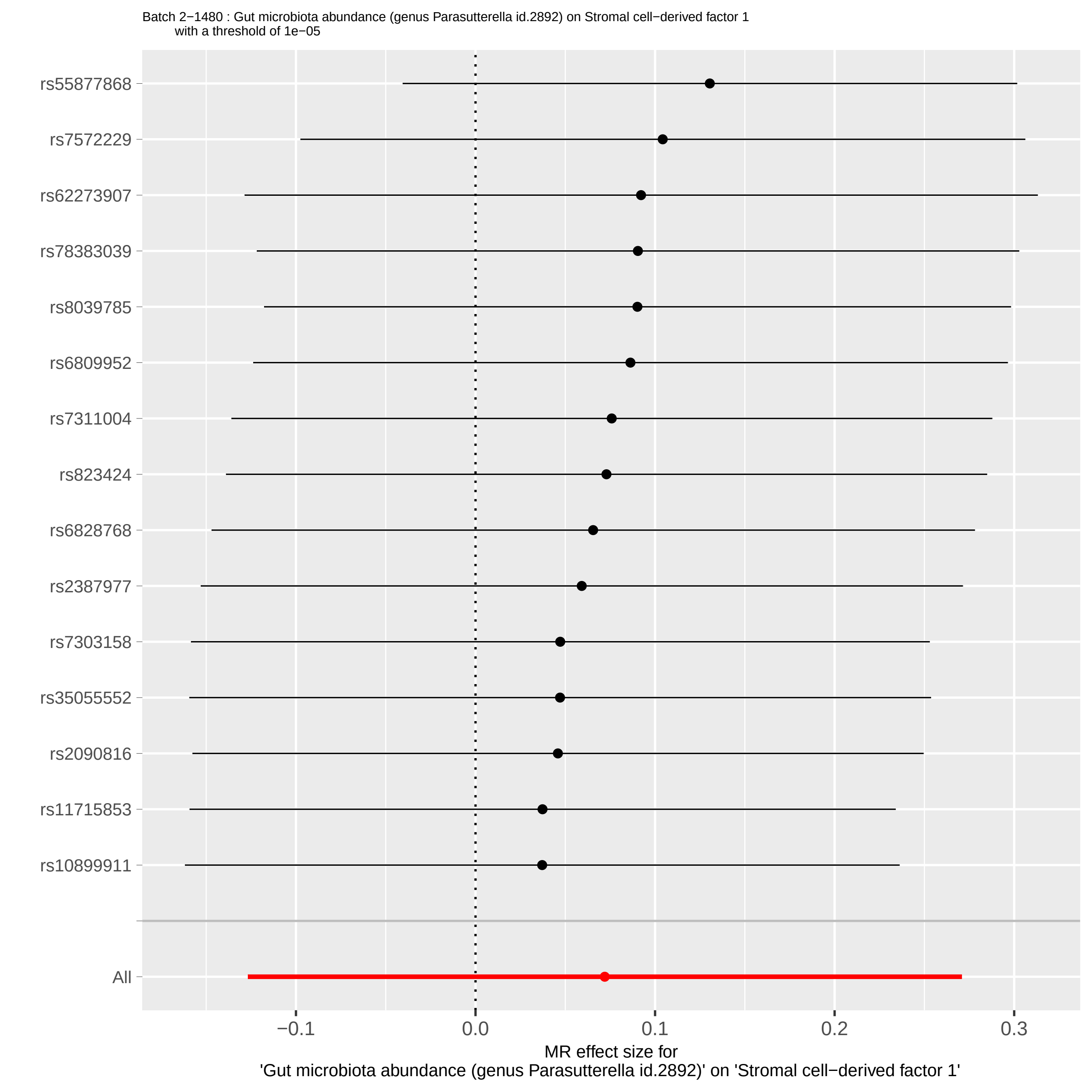
Batch 2−1480 : Gut microbiota abundance (genus Parasutterella id.2892) on Stromal cell−derived factor 1
with a threshold of 1e−05
rs55877868
rs7572229
rs62273907
rs78383039
rs8039785
rs6809952
rs7311004
rs823424
rs6828768
rs2387977
rs7303158
rs35055552
rs2090816
rs11715853
rs10899911
All
−0.1
0.0
0.1
0.2
0.3
MR effect size for
'Gut microbiota abundance (genus Parasutterella id.2892)' on 'Stromal cell−derived factor 1'

Batch 2−1481 : Gut microbiota abundance (genus Catenibacterium id.2153) on Stromal cell−derived factor 1
with a threshold of 1e−05

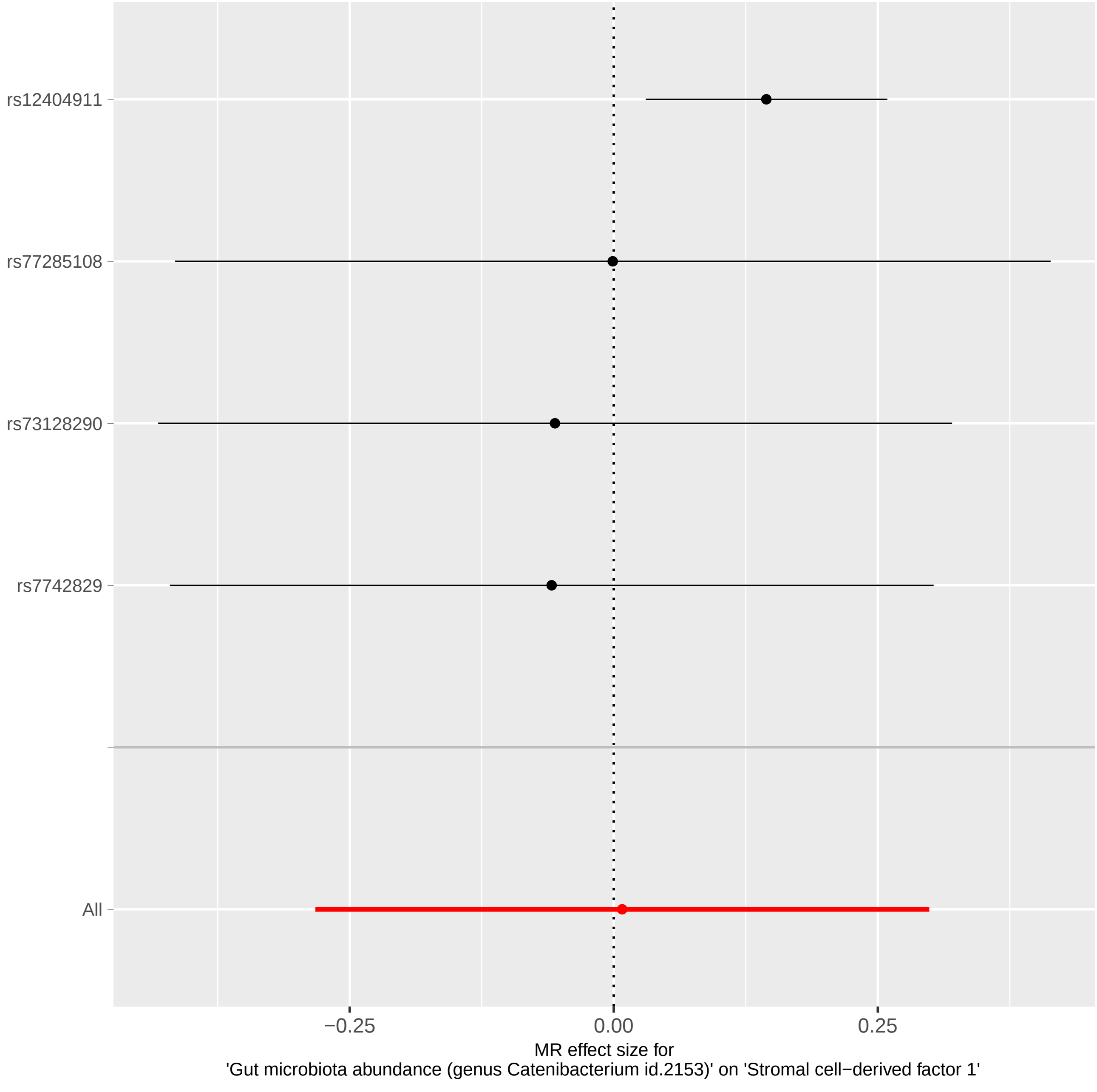

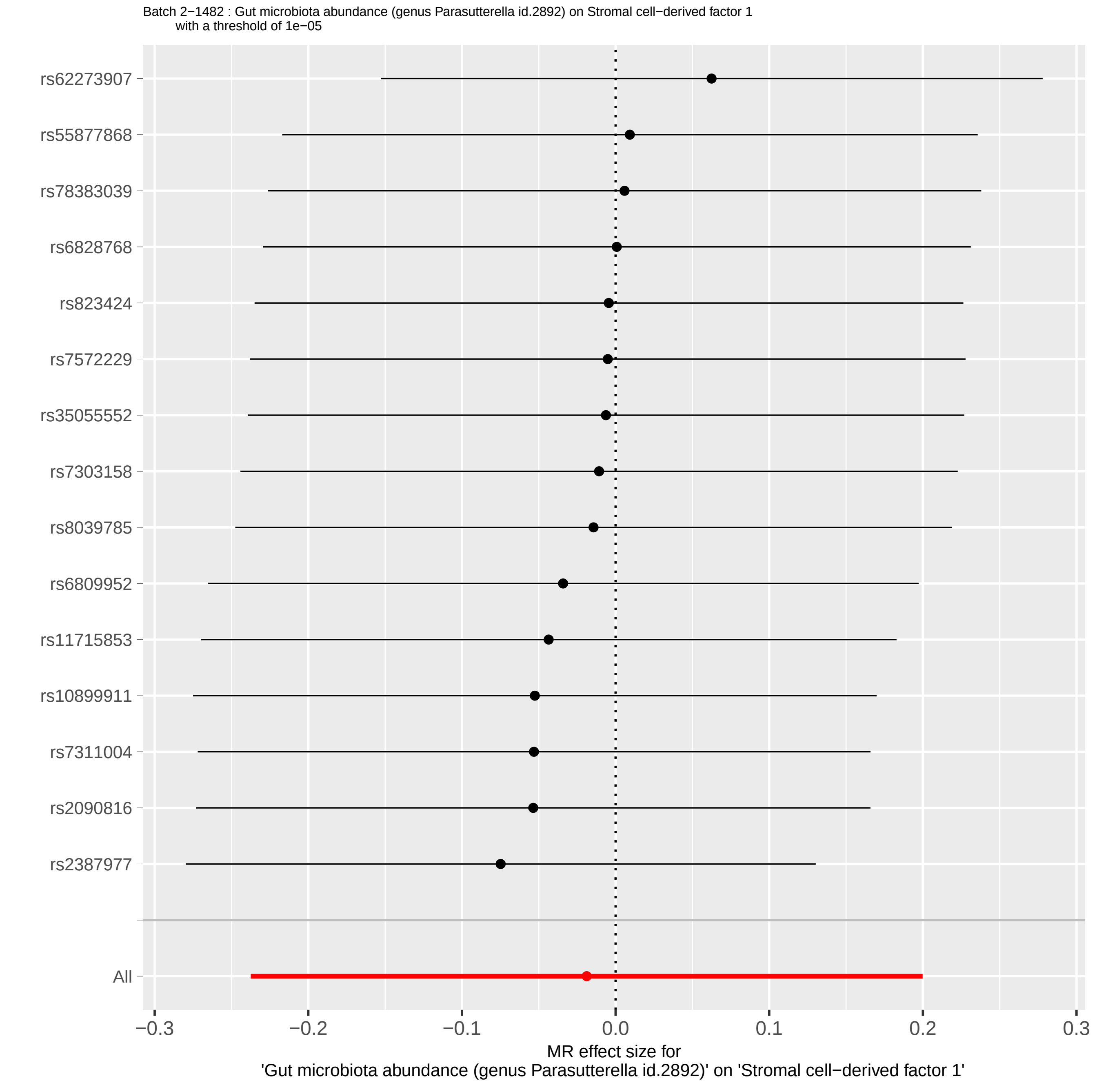
Batch 2−1482 : Gut microbiota abundance (genus Parasutterella id.2892) on Stromal cell−derived factor 1
with a threshold of 1e−05
rs62273907
rs55877868
rs78383039
rs6828768
rs823424
rs7572229
rs35055552
rs7303158
rs8039785
rs6809952
rs11715853
rs10899911
rs7311004
rs2090816
rs2387977
All
−0.3
−0.2
−0.1
0.0
0.1
0.2
0.3
MR effect size for
'Gut microbiota abundance (genus Parasutterella id.2892)' on 'Stromal cell−derived factor 1'

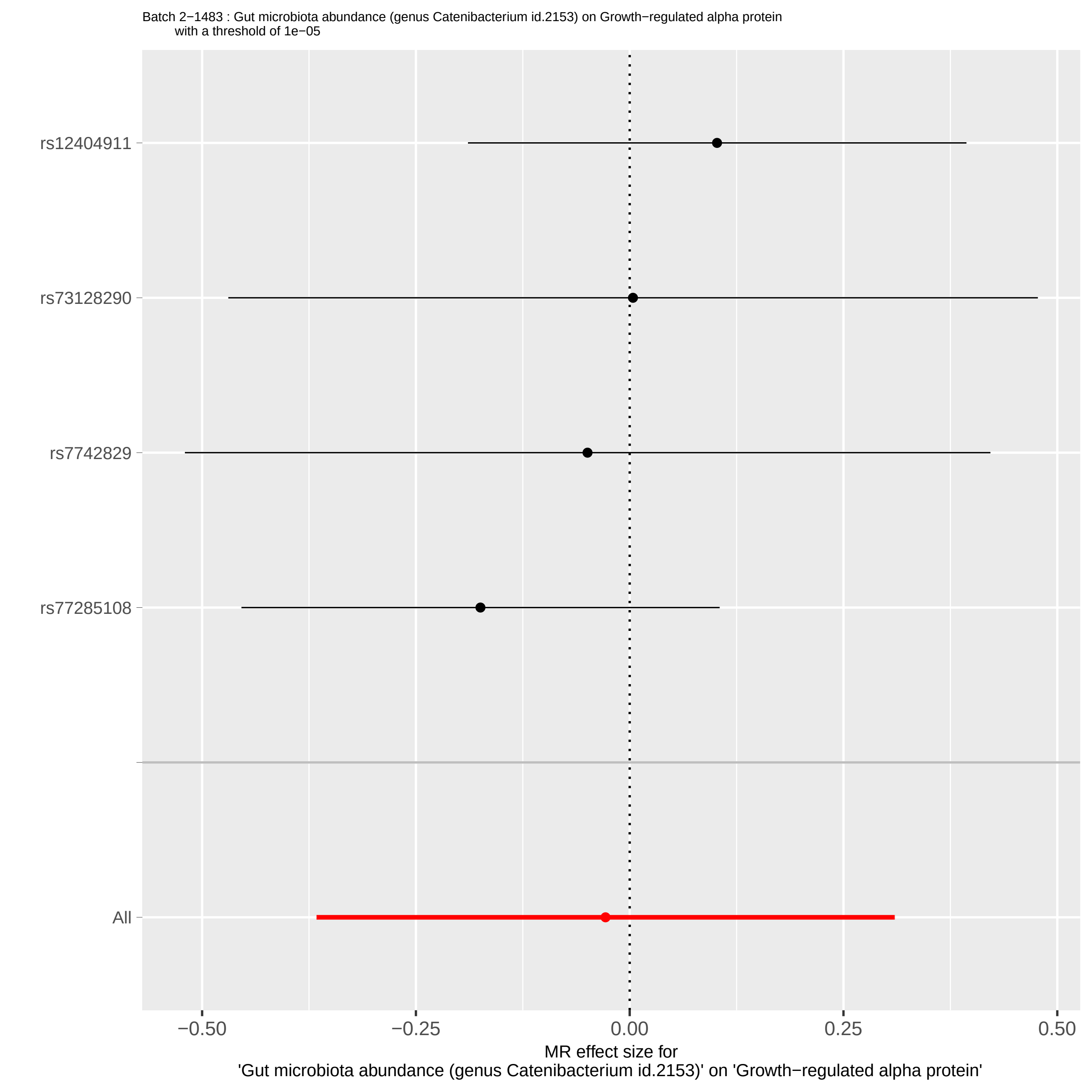
Batch 2−1483 : Gut microbiota abundance (genus Catenibacterium id.2153) on Growth−regulated alpha protein
with a threshold of 1e−05
rs12404911
rs73128290
rs7742829
rs77285108
All
−0.50
−0.25
0.00
0.25
0.50
MR effect size for
'Gut microbiota abundance (genus Catenibacterium id.2153)' on 'Growth−regulated alpha protein'

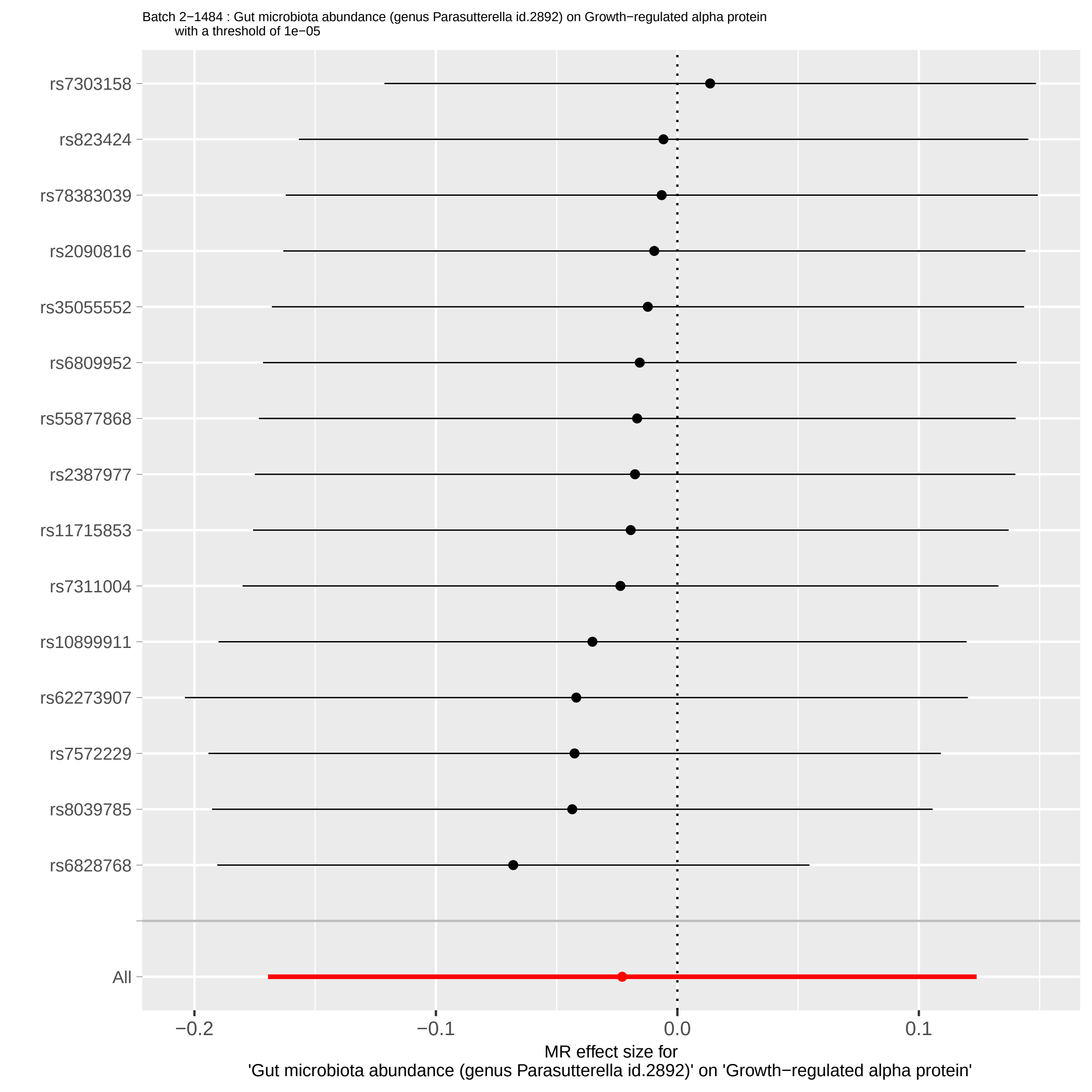
Batch 2−1484 : Gut microbiota abundance (genus Parasutterella id.2892) on Growth−regulated alpha protein
with a threshold of 1e−05
rs7303158
rs823424
rs78383039
rs2090816
rs35055552
rs6809952
rs55877868
rs2387977
rs11715853
rs7311004
rs10899911
rs62273907
rs7572229
rs8039785
rs6828768
All
−0.2
−0.1
0.0
0.1
MR effect size for
'Gut microbiota abundance (genus Parasutterella id.2892)' on 'Growth−regulated alpha protein'

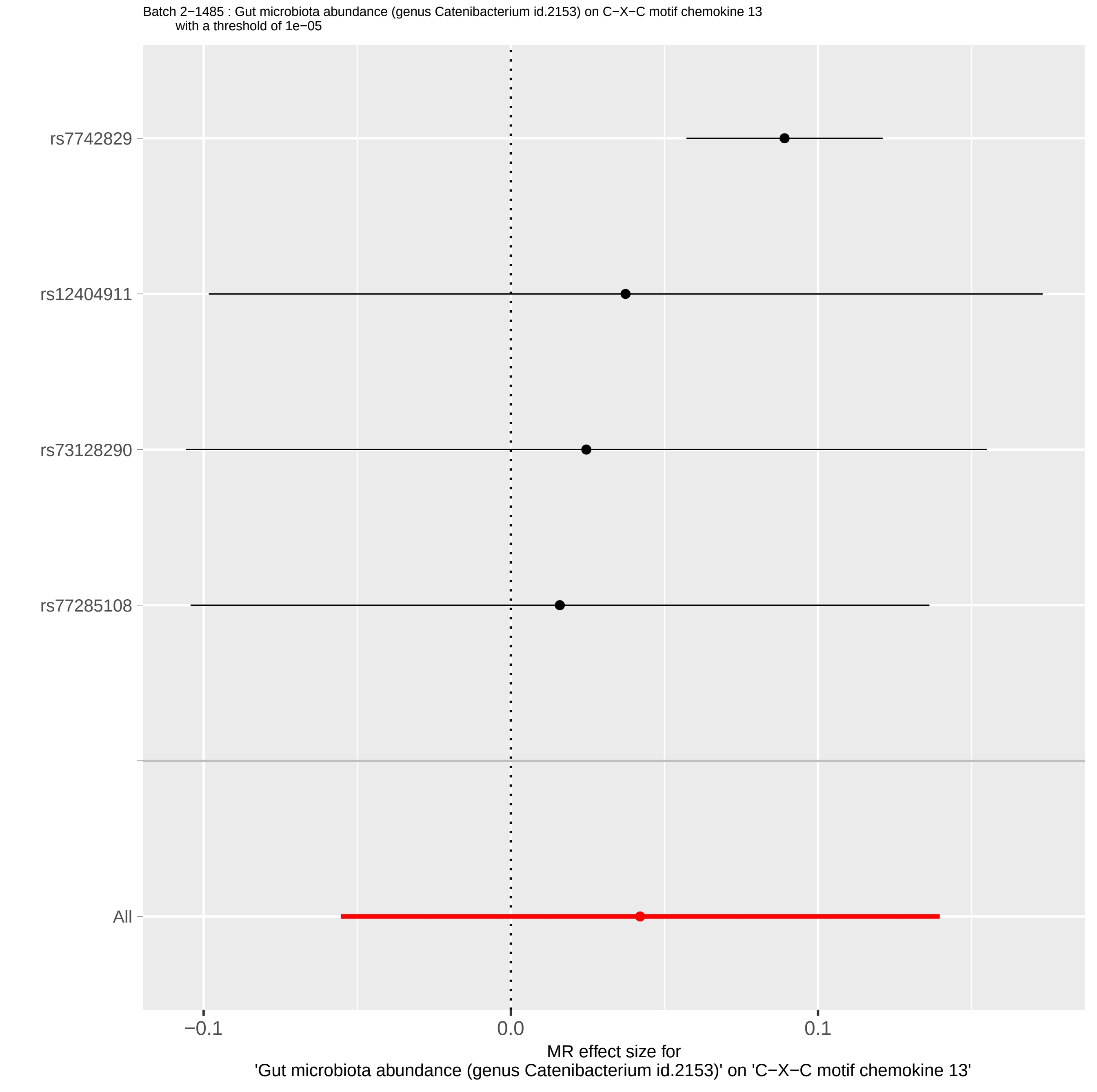
Batch 2−1485 : Gut microbiota abundance (genus Catenibacterium id.2153) on C−X−C motif chemokine 13
with a threshold of 1e−05
rs7742829
rs12404911
rs73128290
rs77285108
All
−0.1
0.0
0.1
MR effect size for
'Gut microbiota abundance (genus Catenibacterium id.2153)' on 'C−X−C motif chemokine 13'

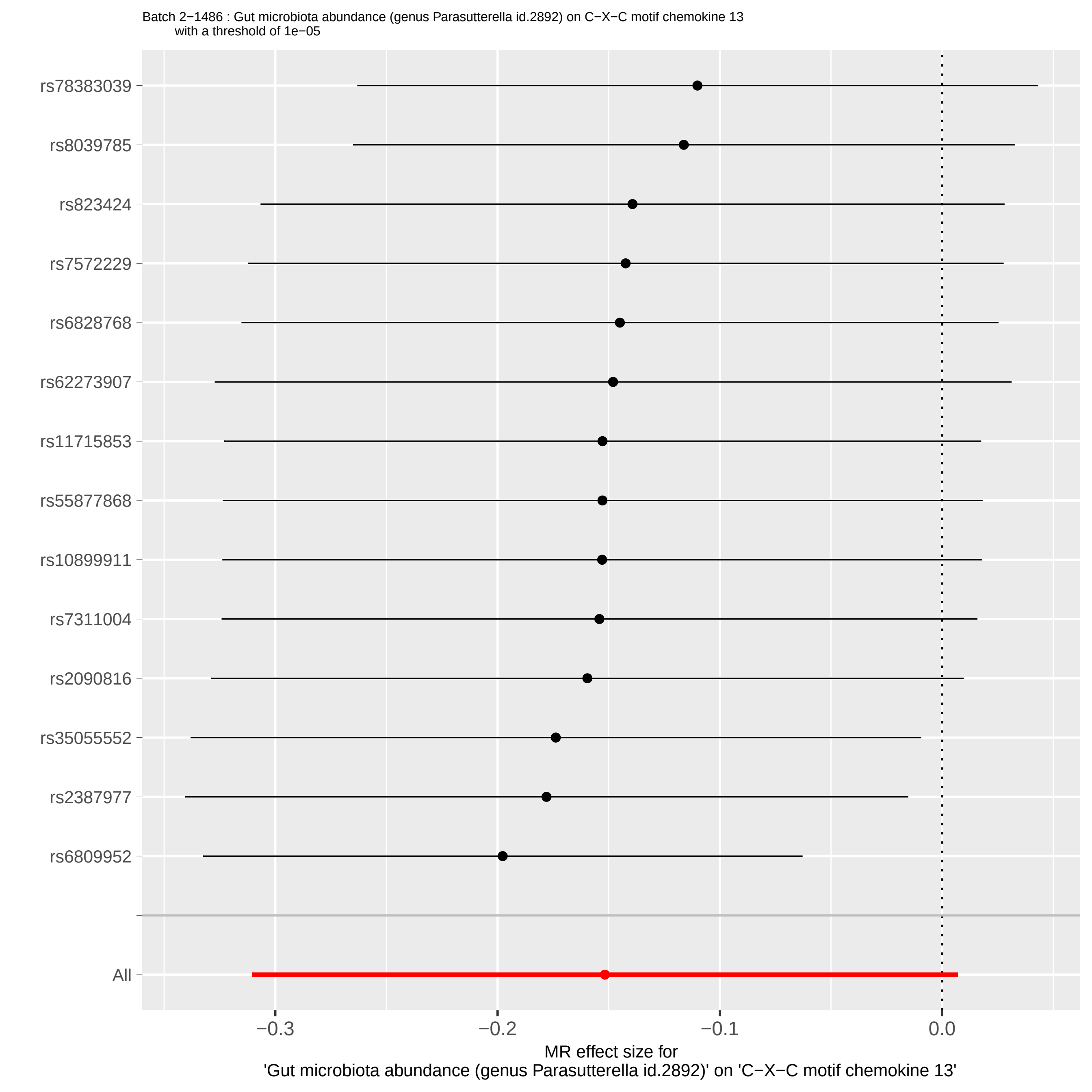
Batch 2−1486 : Gut microbiota abundance (genus Parasutterella id.2892) on C−X−C motif chemokine 13
with a threshold of 1e−05
rs78383039
rs8039785
rs823424
rs7572229
rs6828768
rs62273907
rs11715853
rs55877868
rs10899911
rs7311004
rs2090816
rs35055552
rs2387977
rs6809952
All
−0.3
−0.2
−0.1
0.0
MR effect size for
'Gut microbiota abundance (genus Parasutterella id.2892)' on 'C−X−C motif chemokine 13'

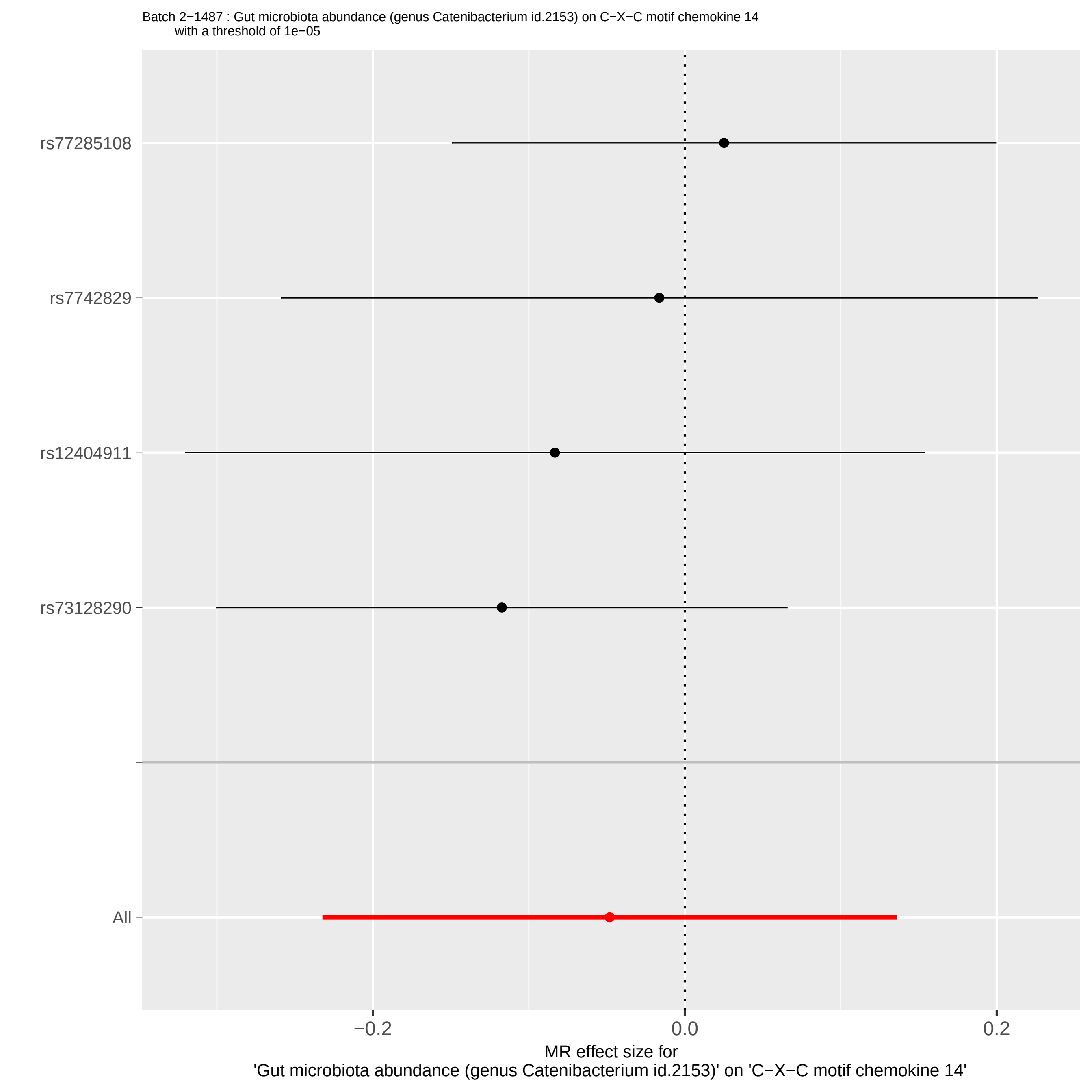

Batch 2−1487 : Gut microbiota abundance (genus Catenibacterium id.2153) on C−X−C motif chemokine 14
with a threshold of 1e−05
rs77285108
rs7742829
rs12404911
rs73128290
All
−0.2
0.0
0.2
MR effect size for
'Gut microbiota abundance (genus Catenibacterium id.2153)' on 'C−X−C motif chemokine 14'

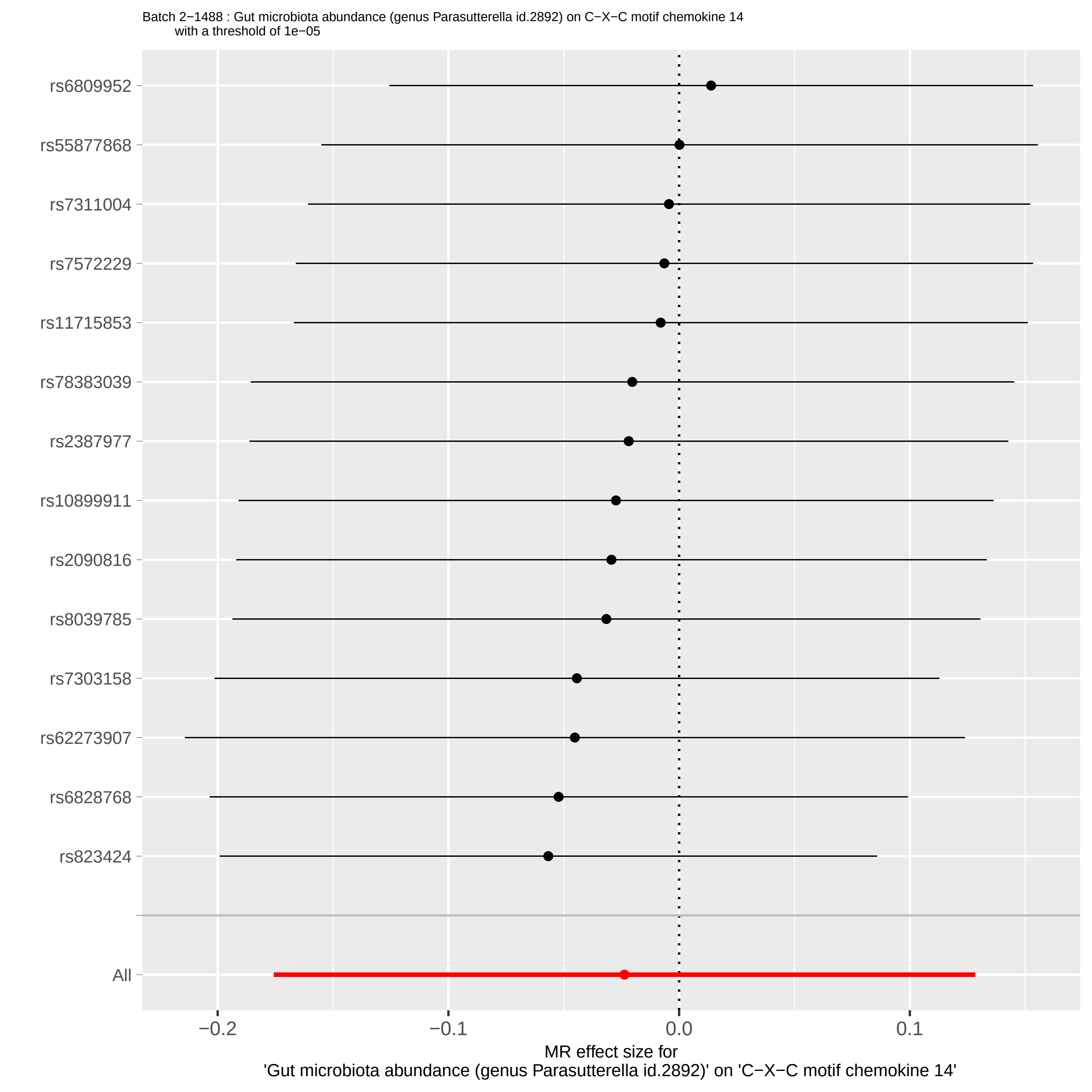
Batch 2−1488 : Gut microbiota abundance (genus Parasutterella id.2892) on C−X−C motif chemokine 14
with a threshold of 1e−05
rs6809952
rs55877868
rs7311004
rs7572229
rs11715853
rs78383039
rs2387977
rs10899911
rs2090816
rs8039785
rs7303158
rs62273907
rs6828768
rs823424
All
−0.2
−0.1
0.0
0.1
MR effect size for
'Gut microbiota abundance (genus Parasutterella id.2892)' on 'C−X−C motif chemokine 14'

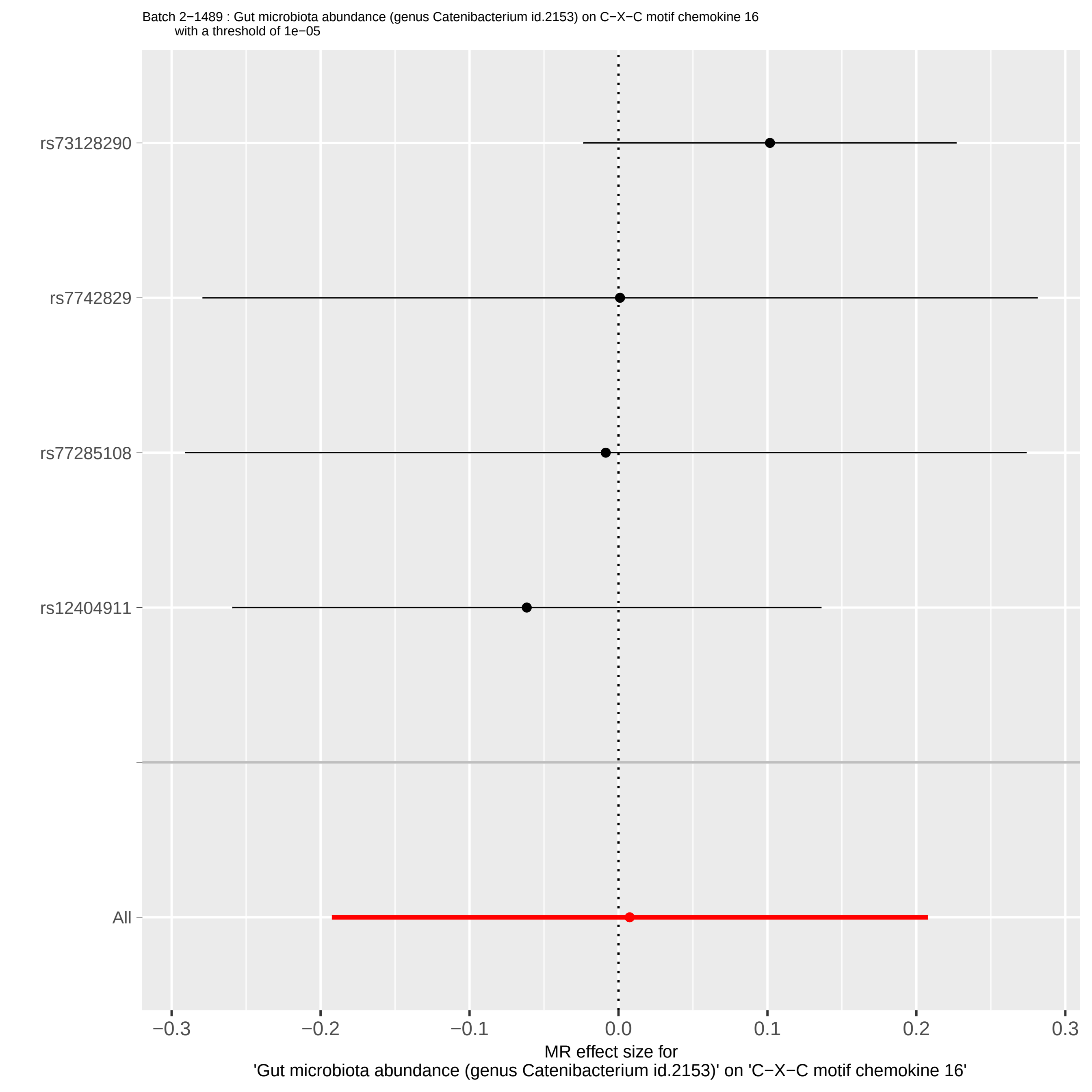
Batch 2−1489 : Gut microbiota abundance (genus Catenibacterium id.2153) on C−X−C motif chemokine 16
with a threshold of 1e−05
rs73128290
rs7742829
rs77285108
rs12404911
All
−0.3
−0.2
−0.1
0.0
0.1
0.2
0.3
MR effect size for
'Gut microbiota abundance (genus Catenibacterium id.2153)' on 'C−X−C motif chemokine 16'

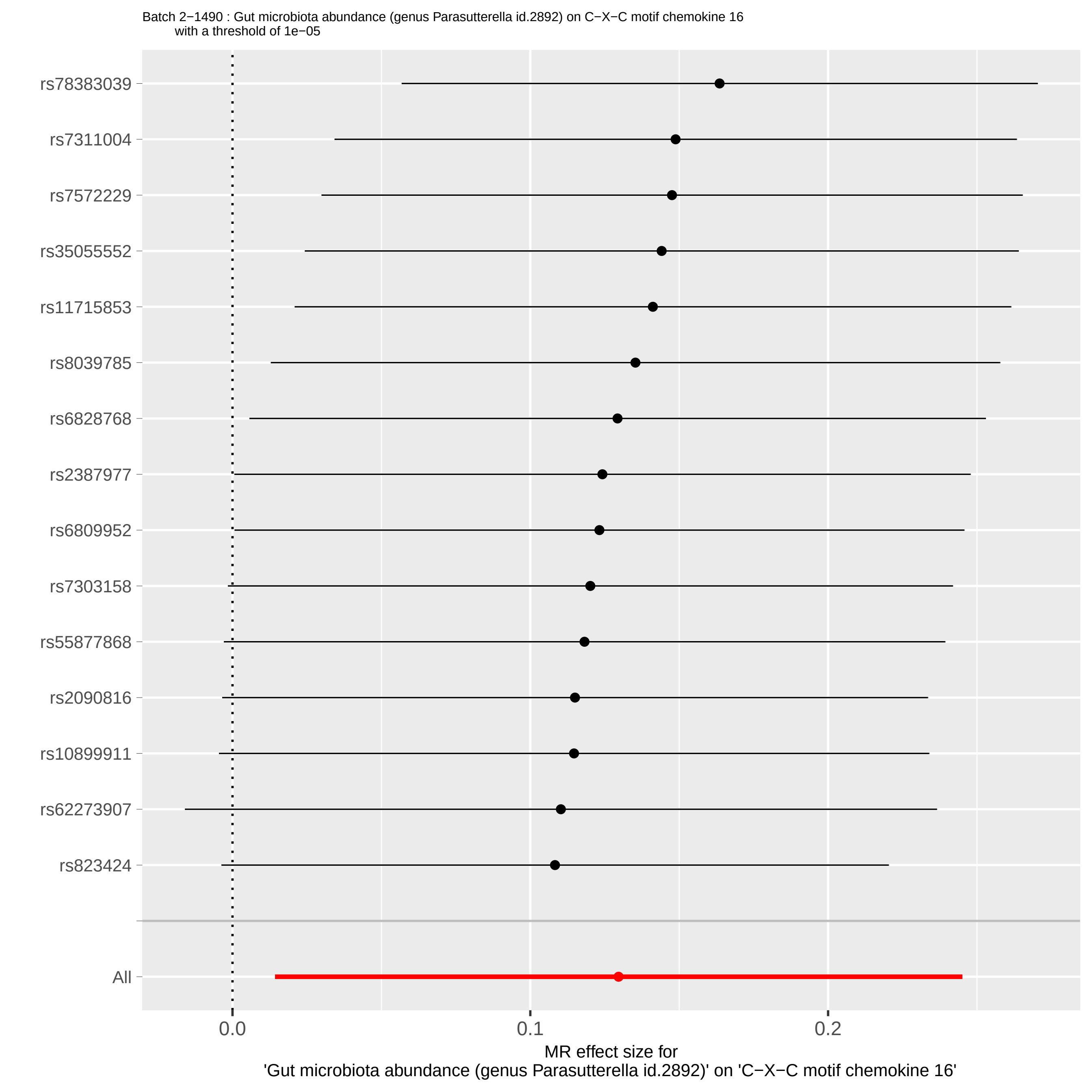

Batch 2−1490 : Gut microbiota abundance (genus Parasutterella id.2892) on C−X−C motif chemokine 16
with a threshold of 1e−05
rs78383039
rs7311004
rs7572229
rs35055552
rs11715853
rs8039785
rs6828768
rs2387977
rs6809952
rs7303158
rs55877868
rs2090816
rs10899911
rs62273907
rs823424
All
0.0
0.1
0.2
MR effect size for
'Gut microbiota abundance (genus Parasutterella id.2892)' on 'C−X−C motif chemokine 16'

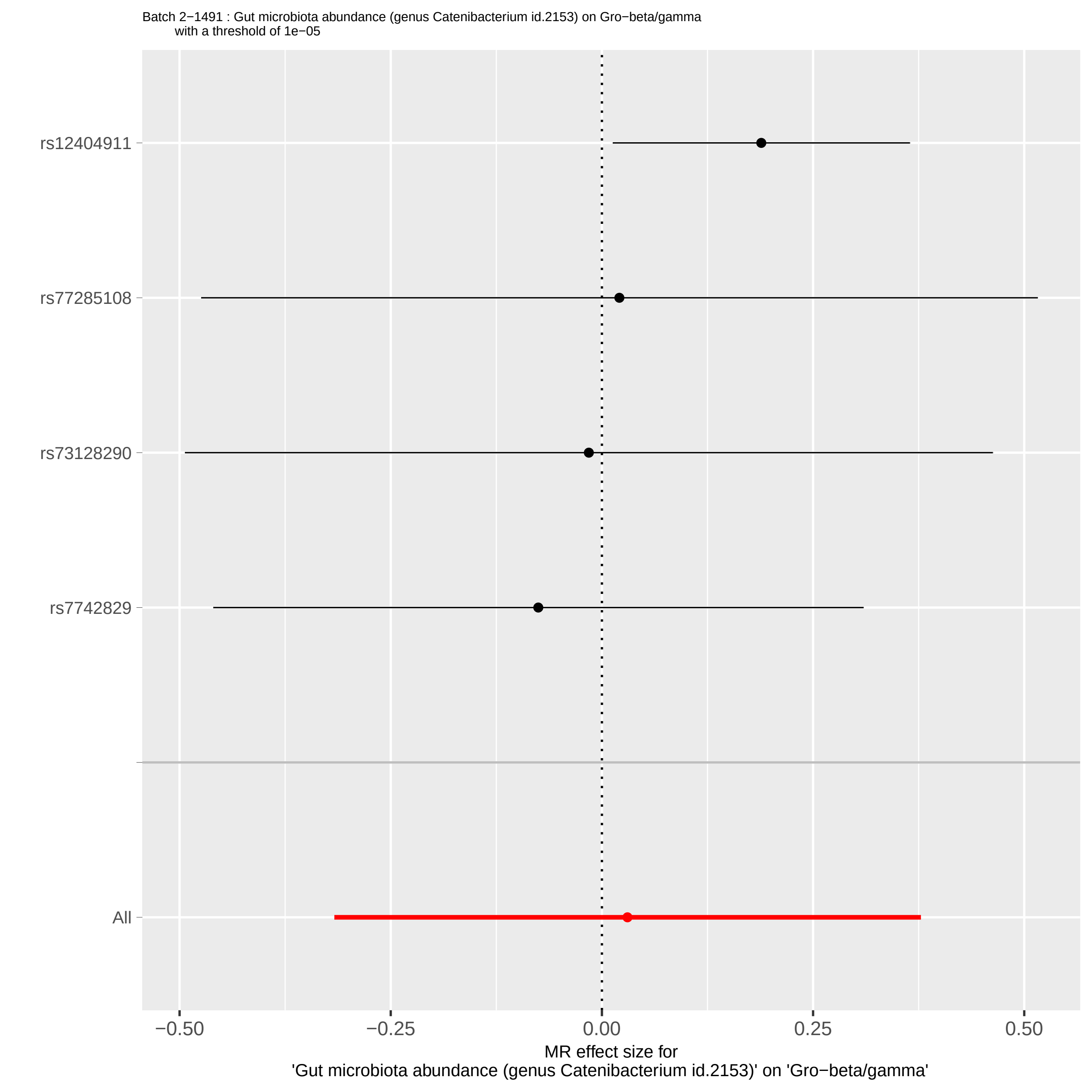
Batch 2−1491 : Gut microbiota abundance (genus Catenibacterium id.2153) on Gro−beta/gamma
with a threshold of 1e−05
rs12404911
rs77285108
rs73128290
rs7742829
All
−0.50
−0.25
0.00
0.25
0.50
MR effect size for
'Gut microbiota abundance (genus Catenibacterium id.2153)' on 'Gro−beta/gamma'

Batch 2−1492 : Gut microbiota abundance (genus Parasutterella id.2892) on Gro−beta/gamma
with a threshold of 1e−05

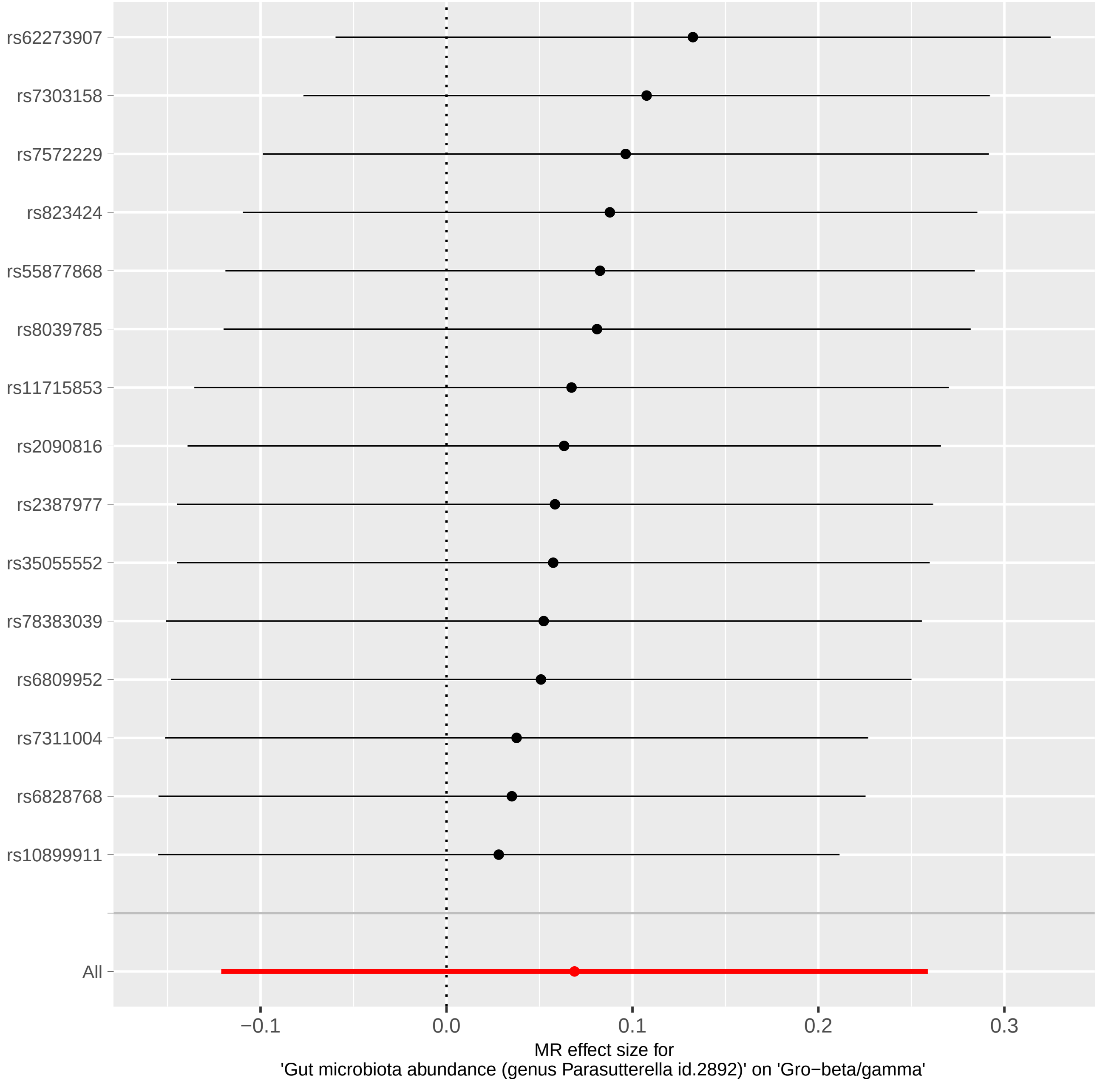

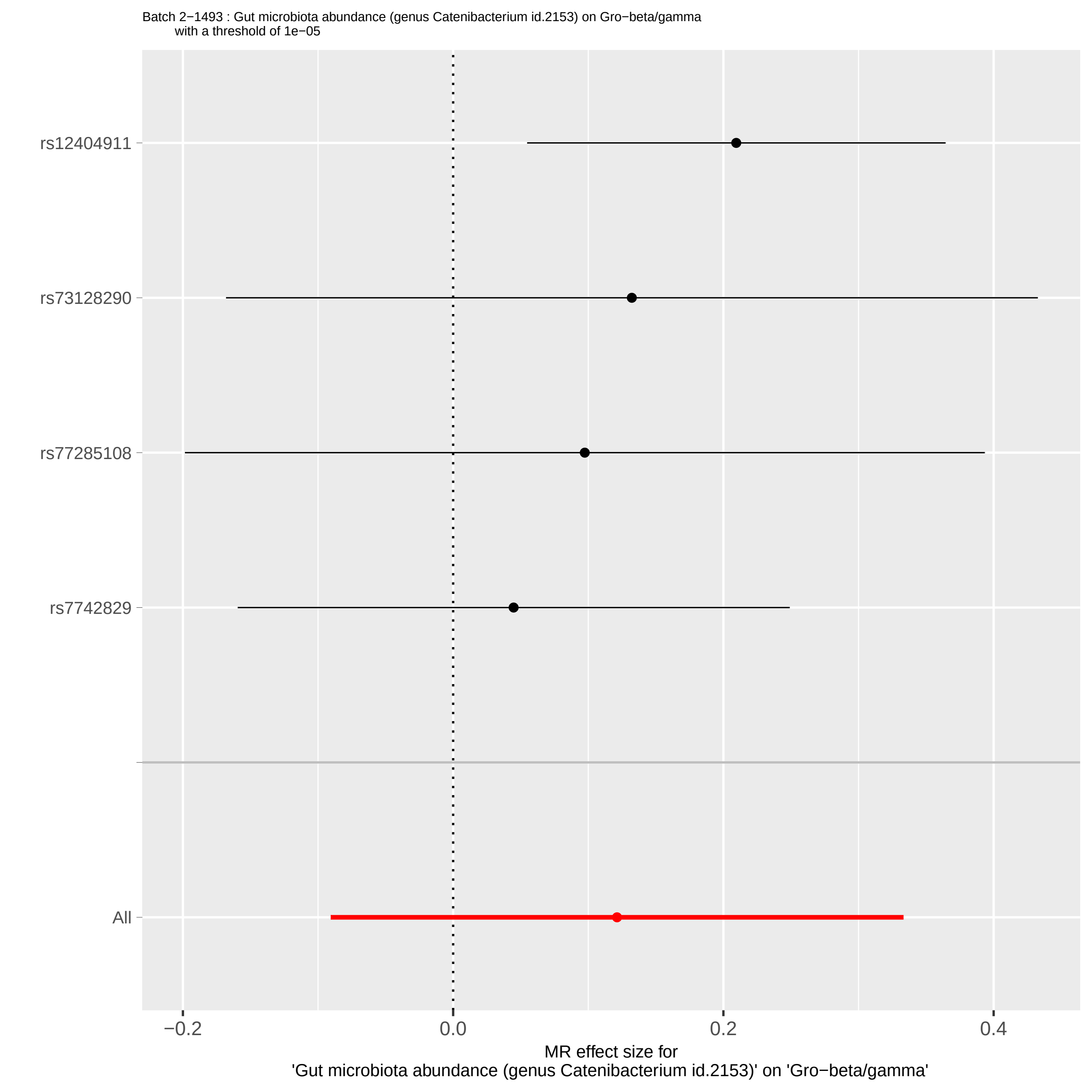
Batch 2−1493 : Gut microbiota abundance (genus Catenibacterium id.2153) on Gro−beta/gamma
with a threshold of 1e−05
rs12404911
rs73128290
rs77285108
rs7742829
All
−0.2
0.0
0.2
0.4
MR effect size for
'Gut microbiota abundance (genus Catenibacterium id.2153)' on 'Gro−beta/gamma'

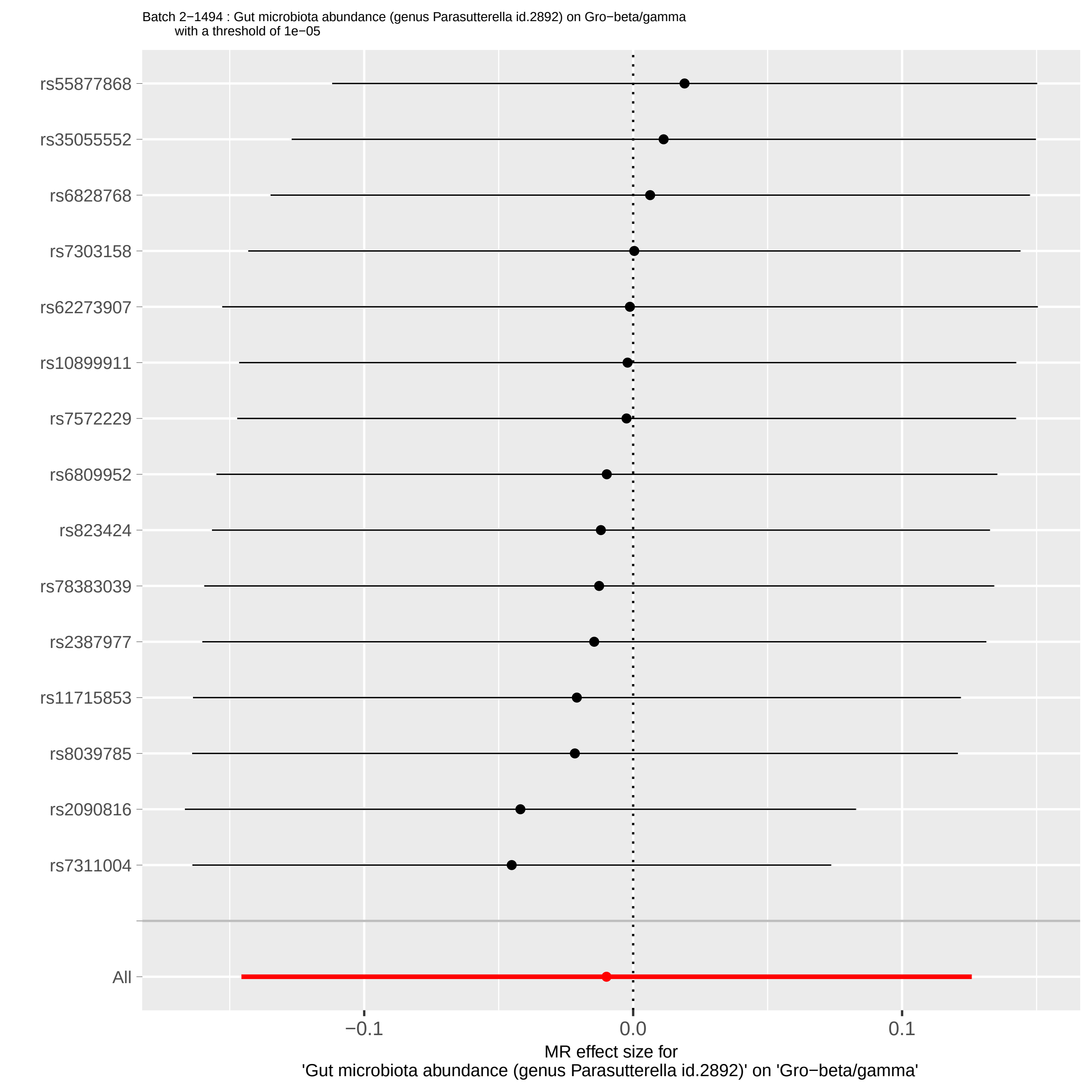

Batch 2−1494 : Gut microbiota abundance (genus Parasutterella id.2892) on Gro−beta/gamma
with a threshold of 1e−05
rs55877868
rs35055552
rs6828768
rs7303158
rs62273907
rs10899911
rs7572229
rs6809952
rs823424
rs78383039
rs2387977
rs11715853
rs8039785
rs2090816
rs7311004
All
−0.1
0.0
0.1
MR effect size for
'Gut microbiota abundance (genus Parasutterella id.2892)' on 'Gro−beta/gamma'

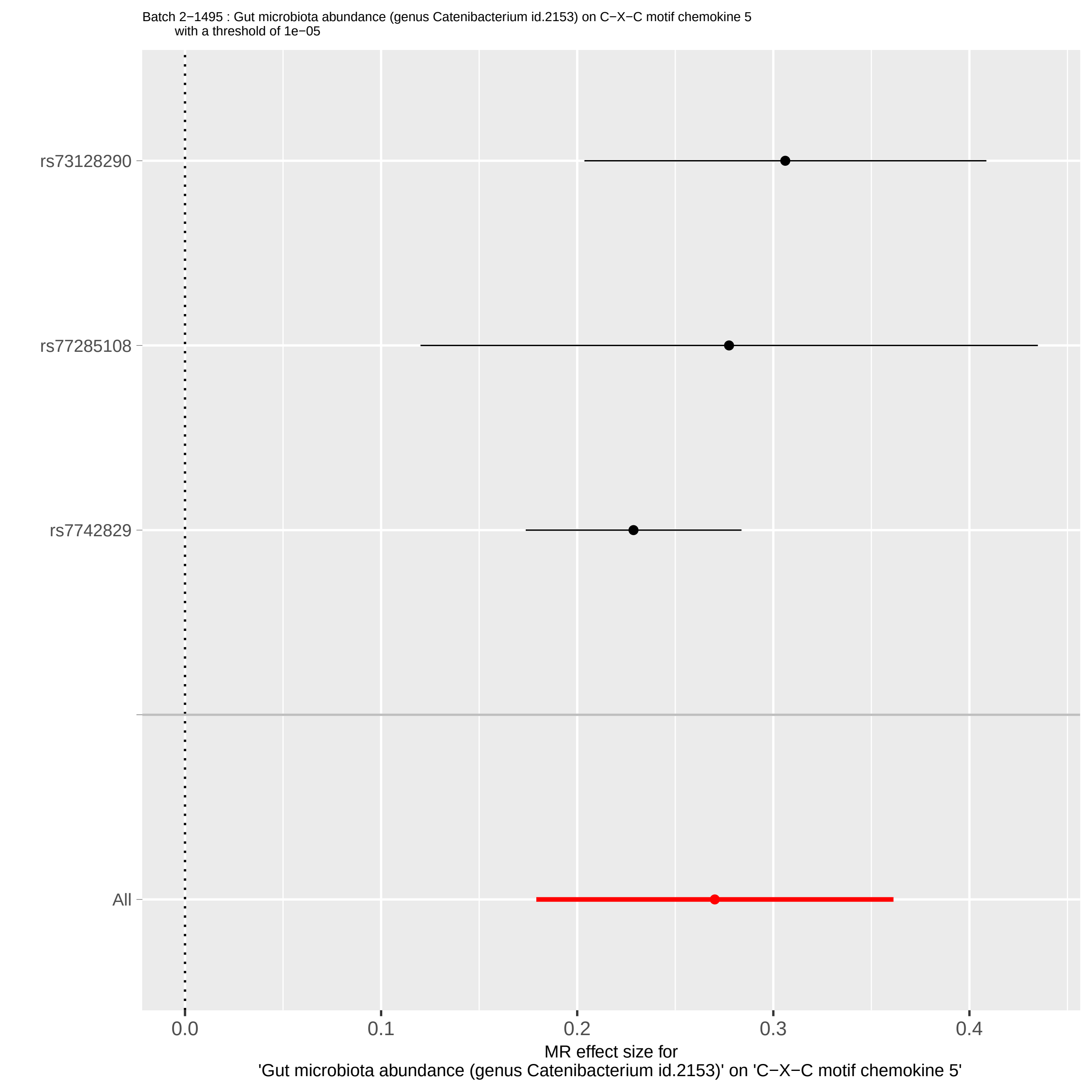
Batch 2−1495 : Gut microbiota abundance (genus Catenibacterium id.2153) on C−X−C motif chemokine 5
with a threshold of 1e−05
rs73128290
rs77285108
rs7742829
All
0.0
0.1
0.2
0.3
0.4
MR effect size for
'Gut microbiota abundance (genus Catenibacterium id.2153)' on 'C−X−C motif chemokine 5'

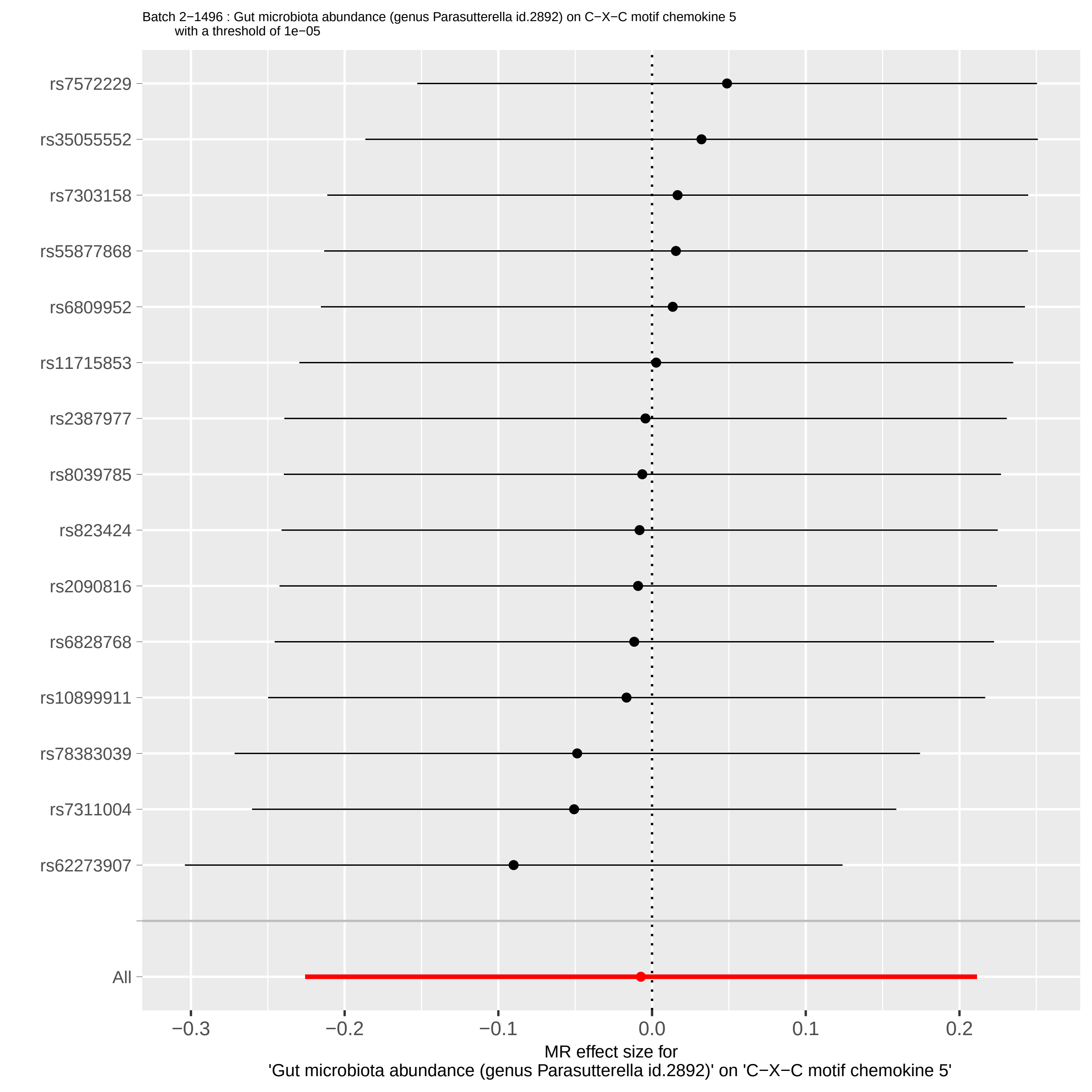

Batch 2−1496 : Gut microbiota abundance (genus Parasutterella id.2892) on C−X−C motif chemokine 5
with a threshold of 1e−05
rs7572229
rs35055552
rs7303158
rs55877868
rs6809952
rs11715853
rs2387977
rs8039785
rs823424
rs2090816
rs6828768
rs10899911
rs78383039
rs7311004
rs62273907
All
−0.3
−0.2
−0.1
0.0
0.1
0.2
MR effect size for
'Gut microbiota abundance (genus Parasutterella id.2892)' on 'C−X−C motif chemokine 5'

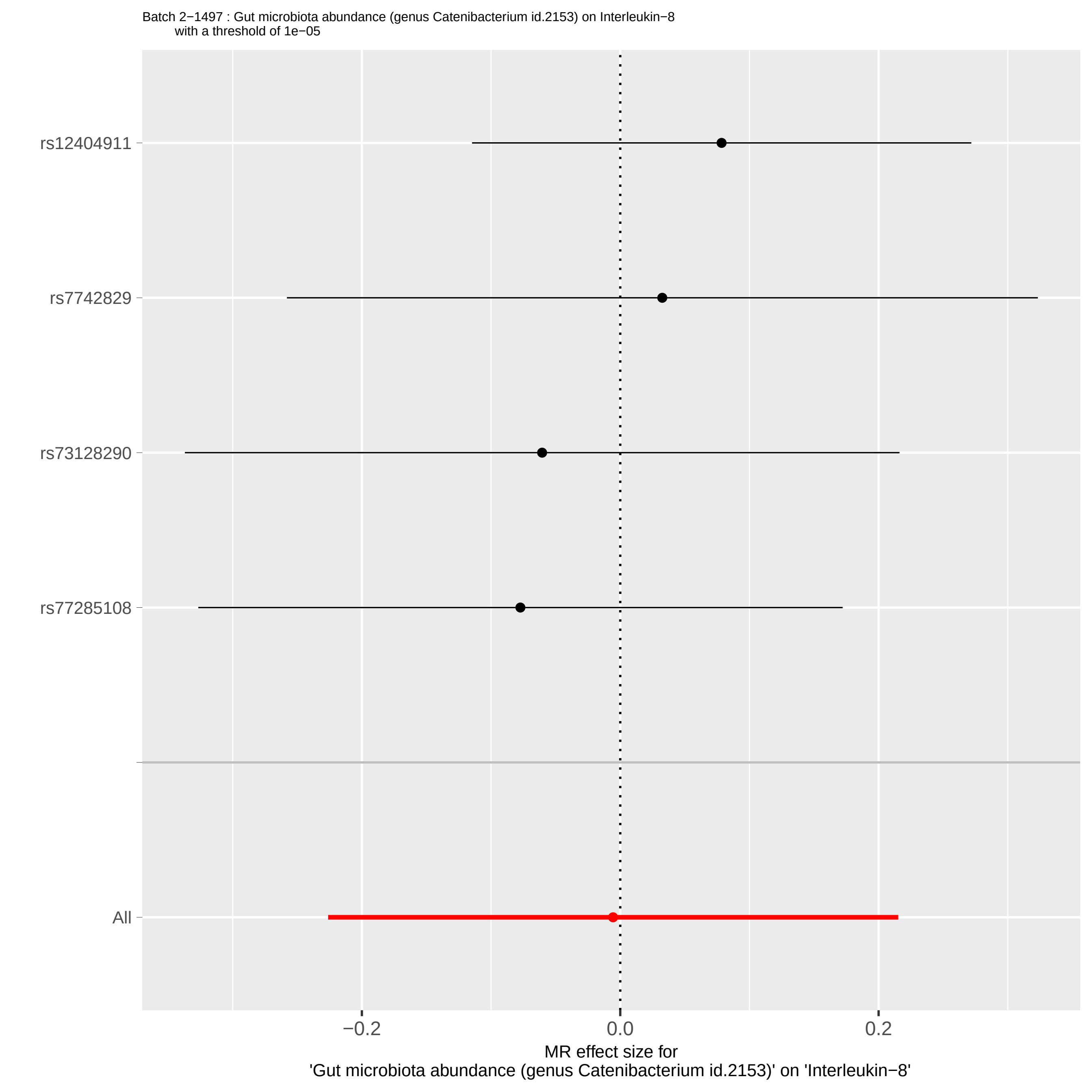
Batch 2−1497 : Gut microbiota abundance (genus Catenibacterium id.2153) on Interleukin−8
with a threshold of 1e−05
rs12404911
rs7742829
rs73128290
rs77285108
All
−0.2
0.0
0.2
MR effect size for
'Gut microbiota abundance (genus Catenibacterium id.2153)' on 'Interleukin−8'

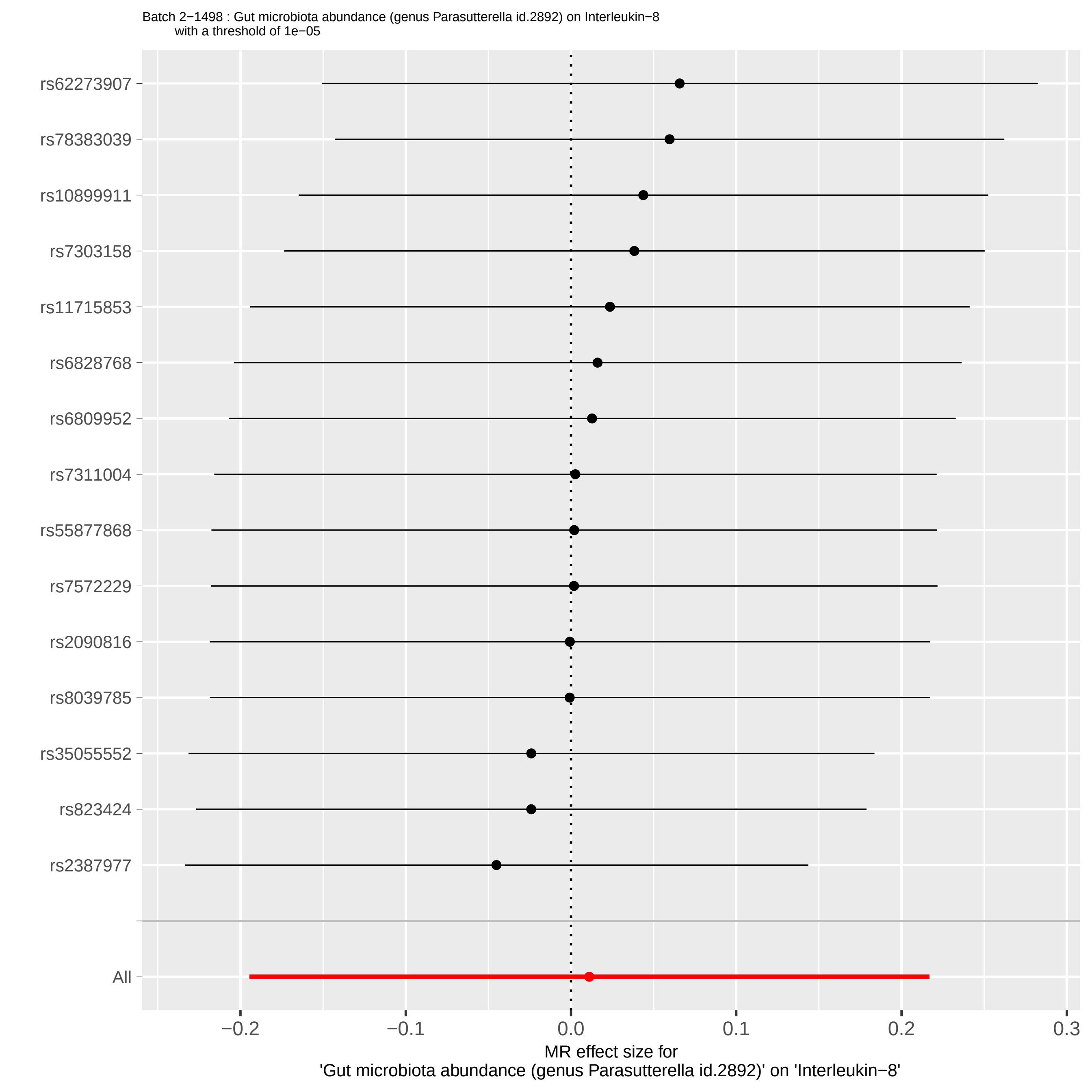
Batch 2−1498 : Gut microbiota abundance (genus Parasutterella id.2892) on Interleukin−8
with a threshold of 1e−05
rs62273907
rs78383039
rs10899911
rs7303158
rs11715853
rs6828768
rs6809952
rs7311004
rs55877868
rs7572229
rs2090816
rs8039785
rs35055552
rs823424
rs2387977
All
−0.2
−0.1
0.0
0.1
0.2
0.3
MR effect size for
'Gut microbiota abundance (genus Parasutterella id.2892)' on 'Interleukin−8'

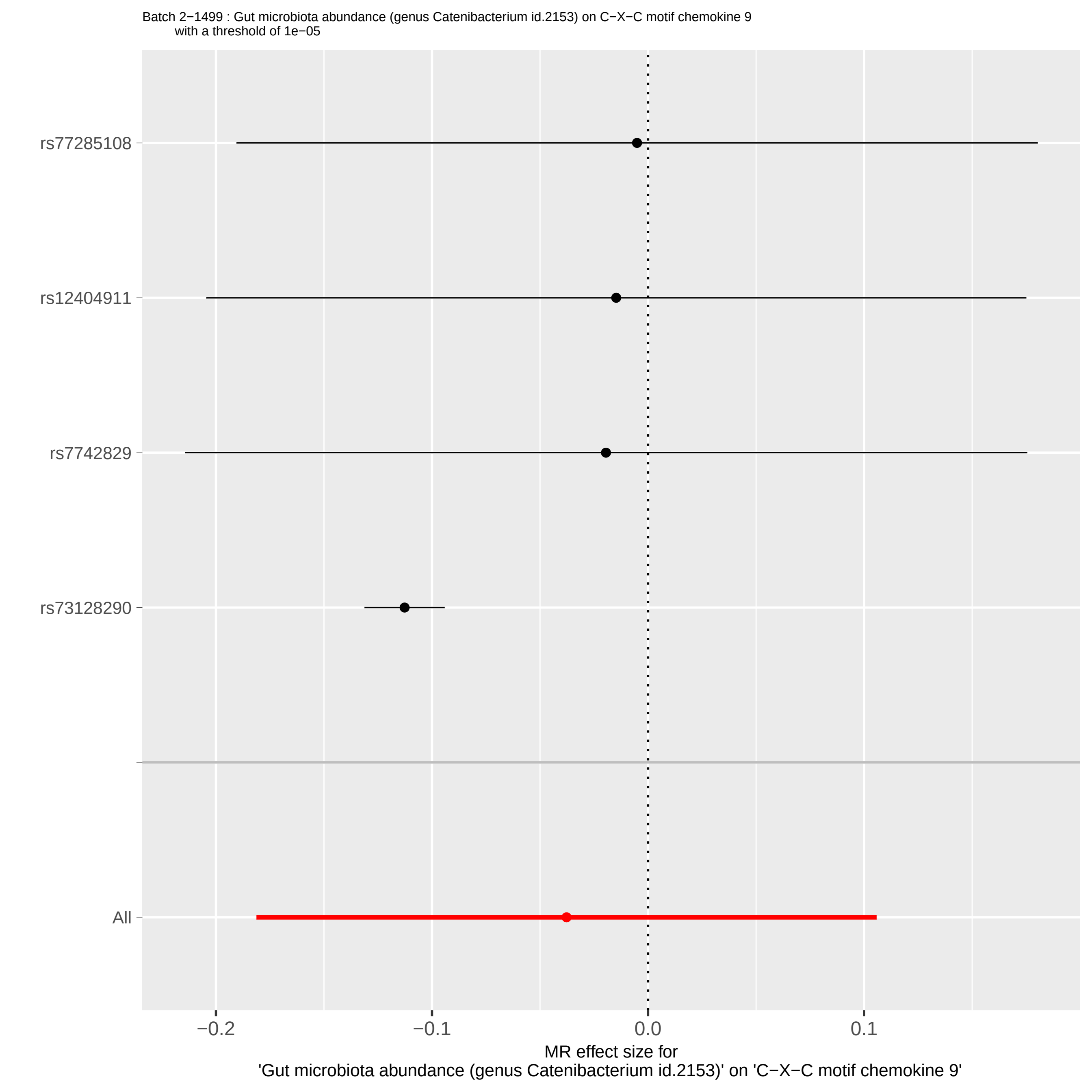

Batch 2−1499 : Gut microbiota abundance (genus Catenibacterium id.2153) on C−X−C motif chemokine 9
with a threshold of 1e−05
rs77285108
rs12404911
rs7742829
rs73128290
All
−0.2
−0.1
0.0
0.1
MR effect size for
'Gut microbiota abundance (genus Catenibacterium id.2153)' on 'C−X−C motif chemokine 9'

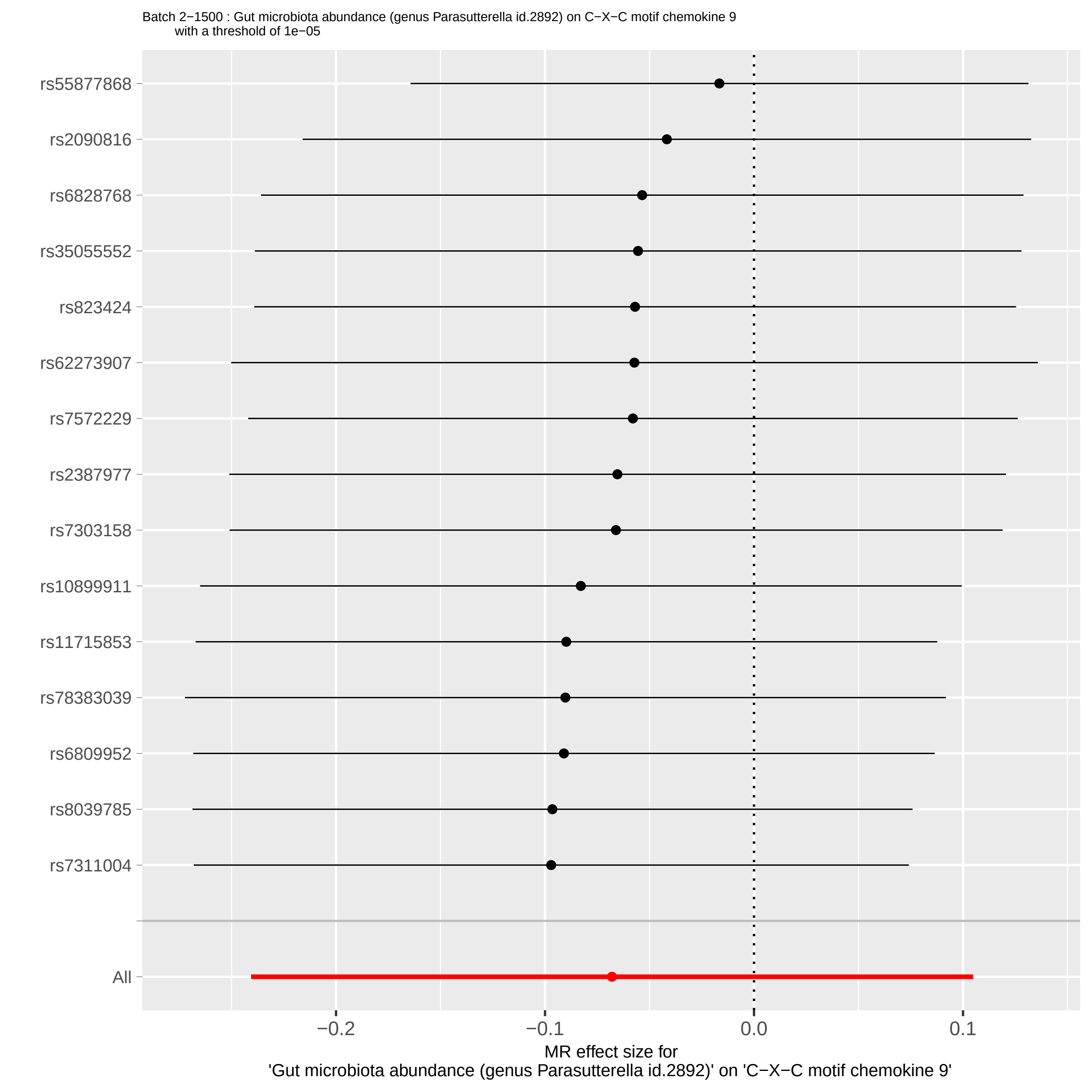

Batch 2−1500 : Gut microbiota abundance (genus Parasutterella id.2892) on C−X−C motif chemokine 9
with a threshold of 1e−05
rs55877868
rs2090816
rs6828768
rs35055552
rs823424
rs62273907
rs7572229
rs2387977
rs7303158
rs10899911
rs11715853
rs78383039
rs6809952
rs8039785
rs7311004
All
−0.2
−0.1
0.0
0.1
MR effect size for
'Gut microbiota abundance (genus Parasutterella id.2892)' on 'C−X−C motif chemokine 9'

Batch 2−1501 : Gut microbiota abundance (genus Catenibacterium id.2153) on C−X−C motif chemokine 9
with a threshold of 1e−05

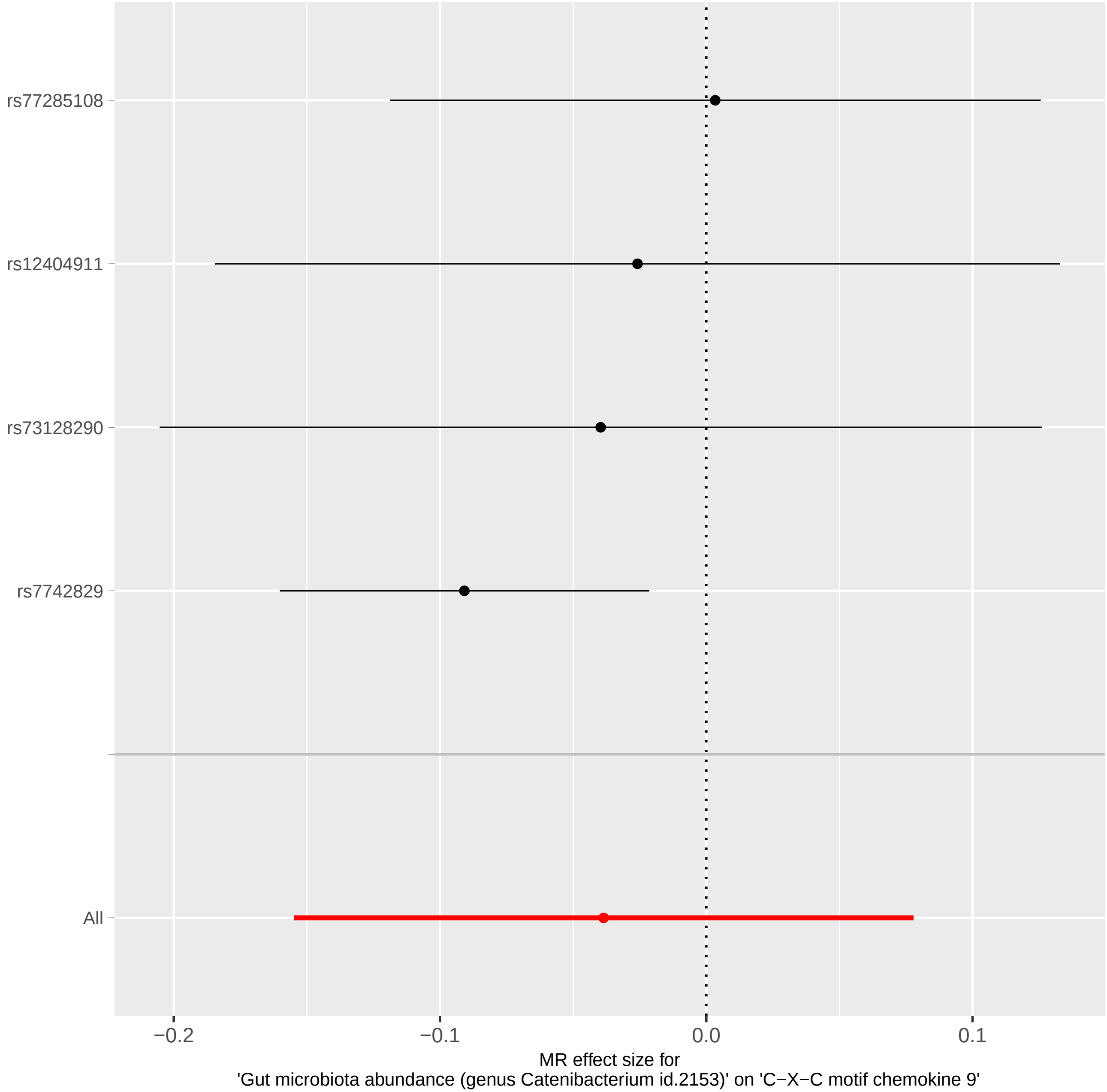

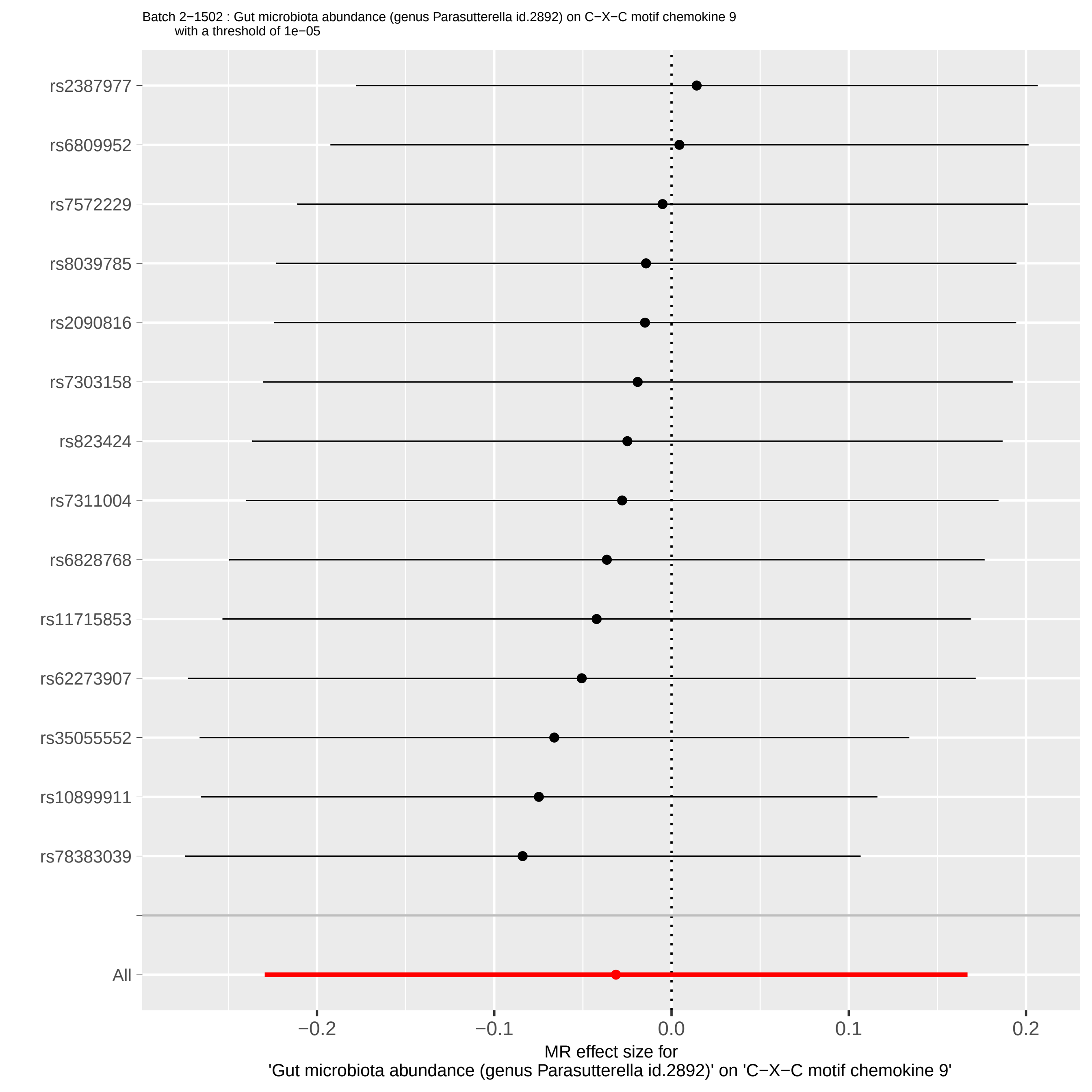

Batch 2−1502 : Gut microbiota abundance (genus Parasutterella id.2892) on C−X−C motif chemokine 9
with a threshold of 1e−05
rs2387977
rs6809952
rs7572229
rs8039785
rs2090816
rs7303158
rs823424
rs7311004
rs6828768
rs11715853
rs62273907
rs35055552
rs10899911
rs78383039
All
−0.2
−0.1
0.0
0.1
0.2
MR effect size for
'Gut microbiota abundance (genus Parasutterella id.2892)' on 'C−X−C motif chemokine 9'

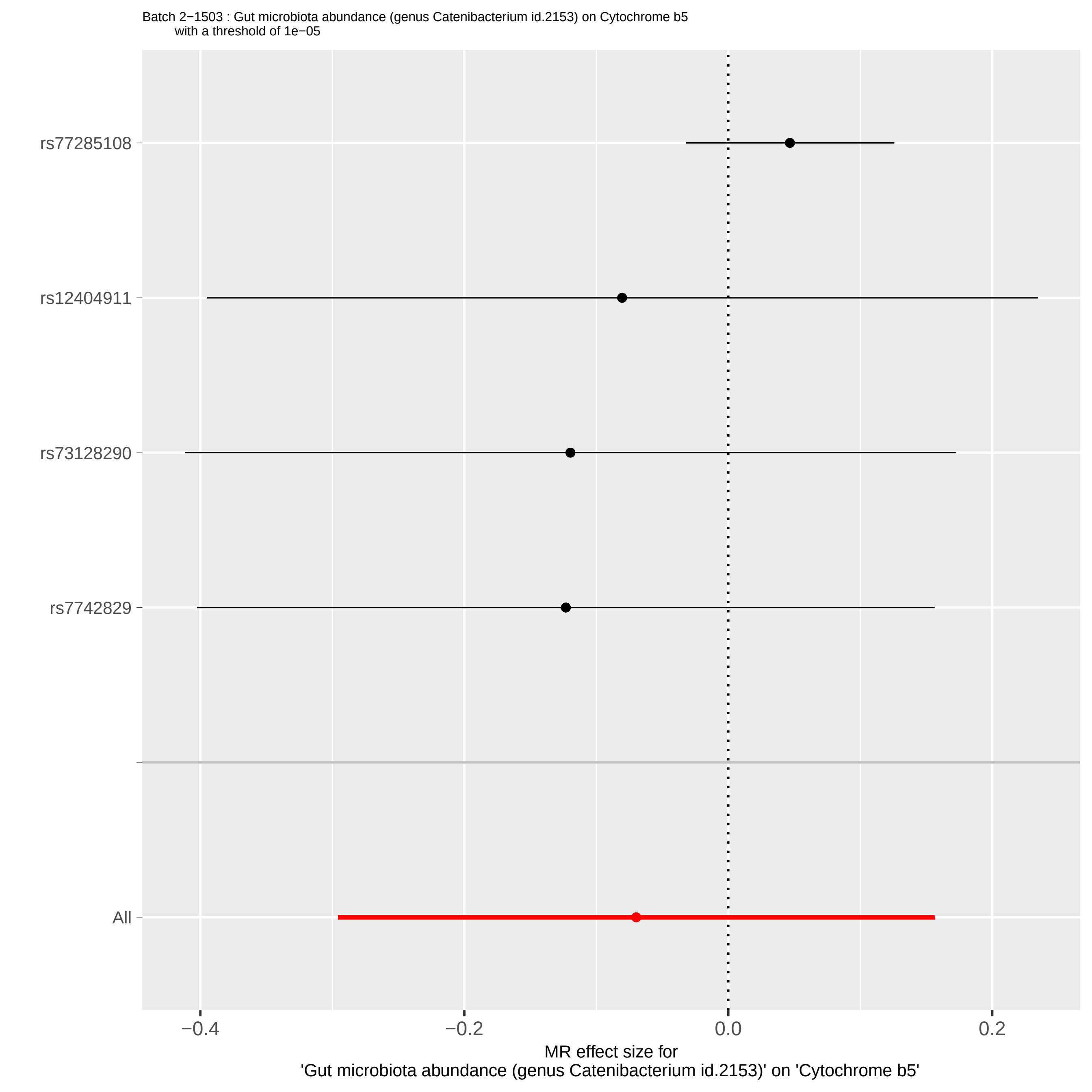
Batch 2−1503 : Gut microbiota abundance (genus Catenibacterium id.2153) on Cytochrome b5
with a threshold of 1e−05
rs77285108
rs12404911
rs73128290
rs7742829
All
−0.4
−0.2
0.0
0.2
MR effect size for
'Gut microbiota abundance (genus Catenibacterium id.2153)' on 'Cytochrome b5'

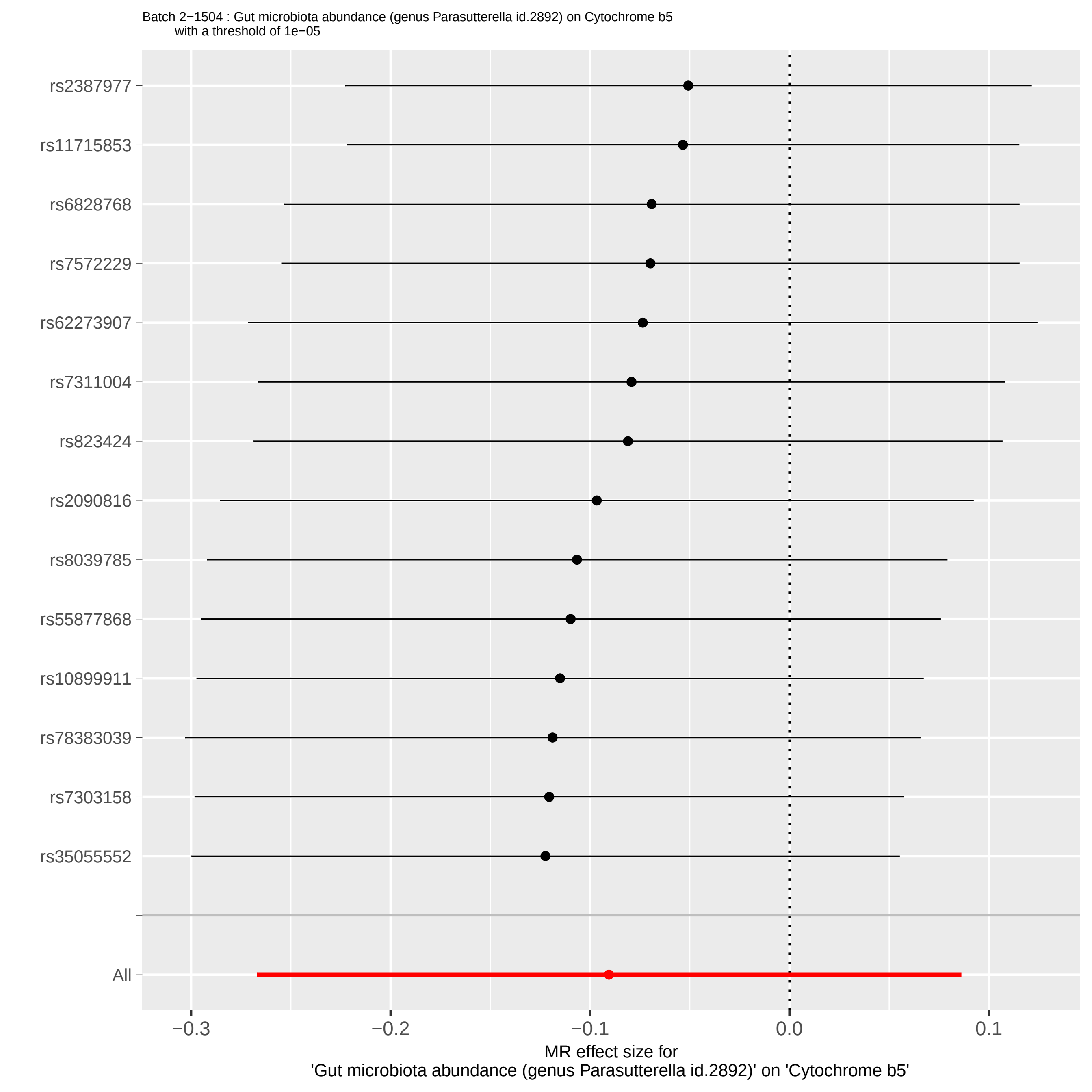

Batch 2−1504 : Gut microbiota abundance (genus Parasutterella id.2892) on Cytochrome b5
with a threshold of 1e−05
rs2387977
rs11715853
rs6828768
rs7572229
rs62273907
rs7311004
rs823424
rs2090816
rs8039785
rs55877868
rs10899911
rs78383039
rs7303158
rs35055552
All
−0.3
−0.2
−0.1
0.0
0.1
MR effect size for
'Gut microbiota abundance (genus Parasutterella id.2892)' on 'Cytochrome b5'

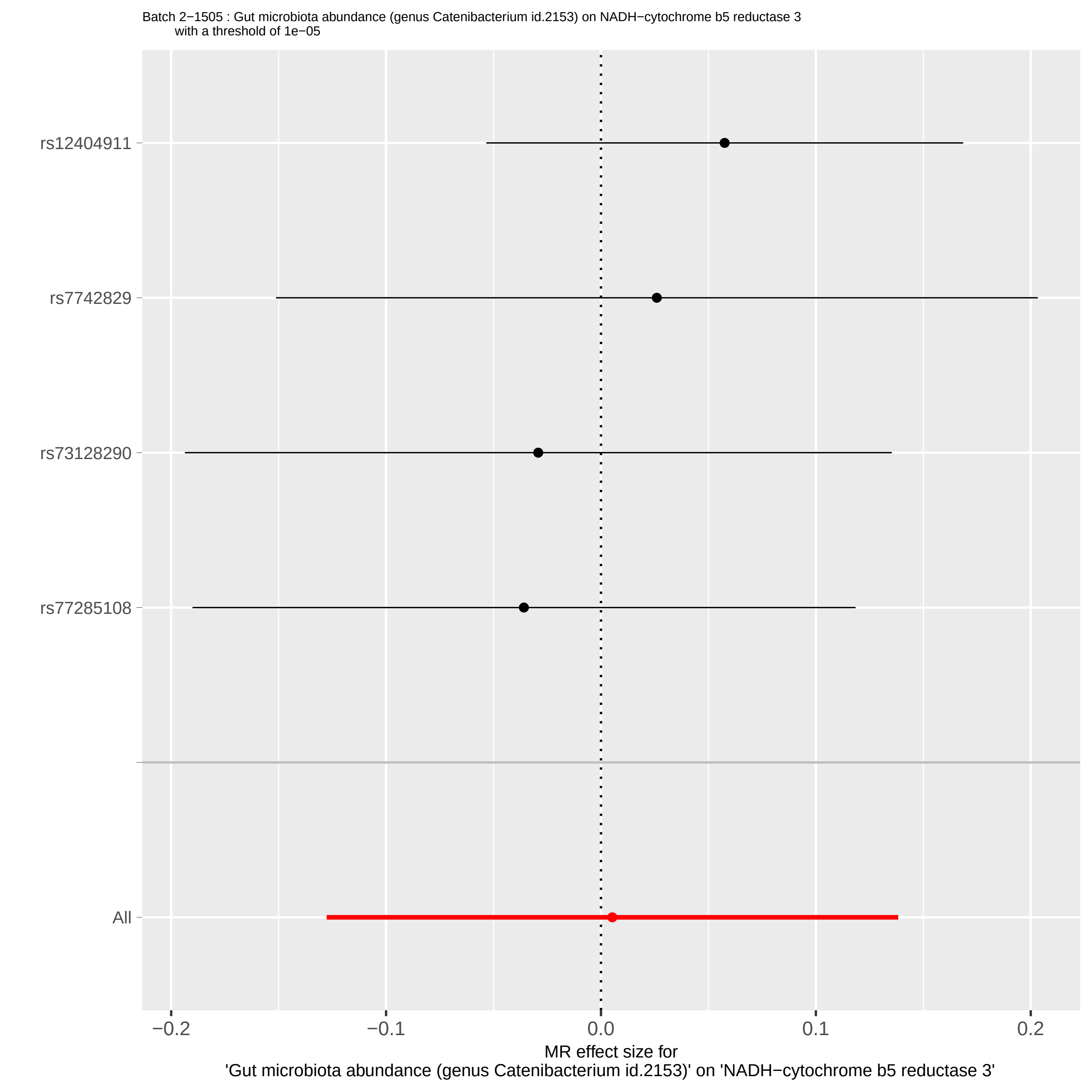

Batch 2−1505 : Gut microbiota abundance (genus Catenibacterium id.2153) on NADH−cytochrome b5 reductase 3
with a threshold of 1e−05
rs12404911
rs7742829
rs73128290
rs77285108
All
−0.2
−0.1
0.0
0.1
0.2
MR effect size for
'Gut microbiota abundance (genus Catenibacterium id.2153)' on 'NADH−cytochrome b5 reductase 3'

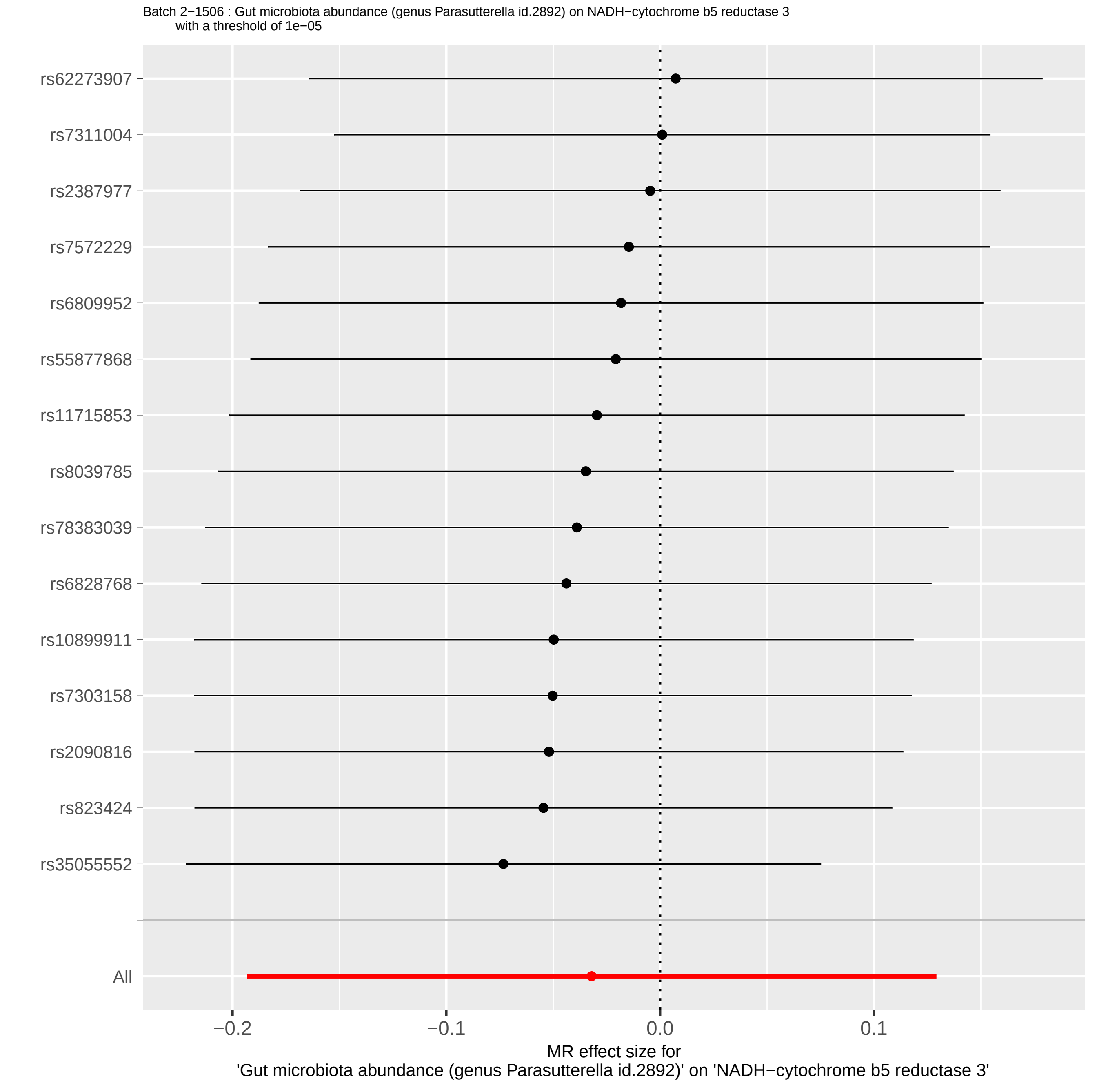
Batch 2−1506 : Gut microbiota abundance (genus Parasutterella id.2892) on NADH−cytochrome b5 reductase 3
with a threshold of 1e−05
rs62273907
rs7311004
rs2387977
rs7572229
rs6809952
rs55877868
rs11715853
rs8039785
rs78383039
rs6828768
rs10899911
rs7303158
rs2090816
rs823424
rs35055552
All
−0.2
−0.1
0.0
0.1
MR effect size for
'Gut microbiota abundance (genus Parasutterella id.2892)' on 'NADH−cytochrome b5 reductase 3'

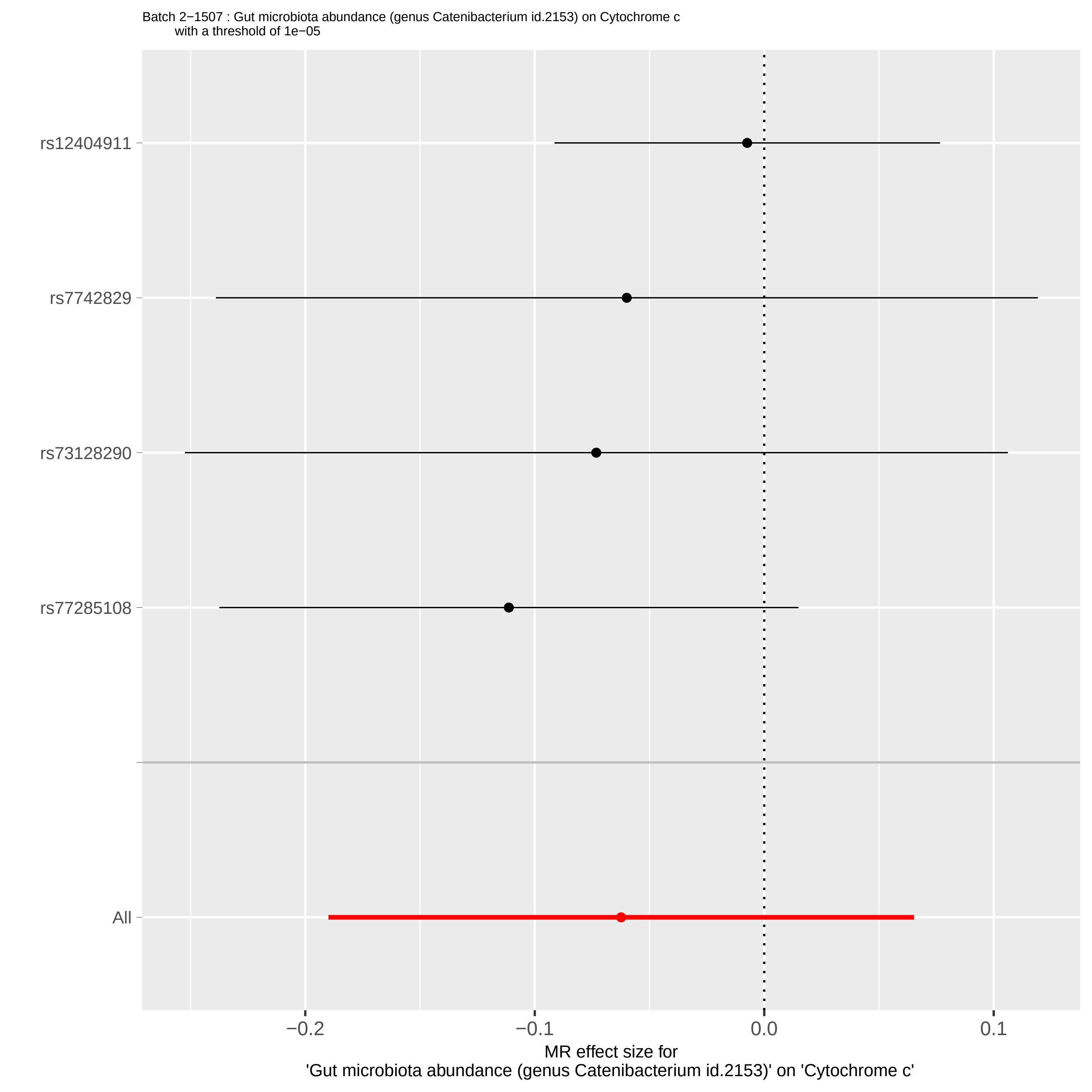
Batch 2−1507 : Gut microbiota abundance (genus Catenibacterium id.2153) on Cytochrome c
with a threshold of 1e−05
rs12404911
rs7742829
rs73128290
rs77285108
All
−0.2
−0.1
0.0
0.1
MR effect size for
'Gut microbiota abundance (genus Catenibacterium id.2153)' on 'Cytochrome c'

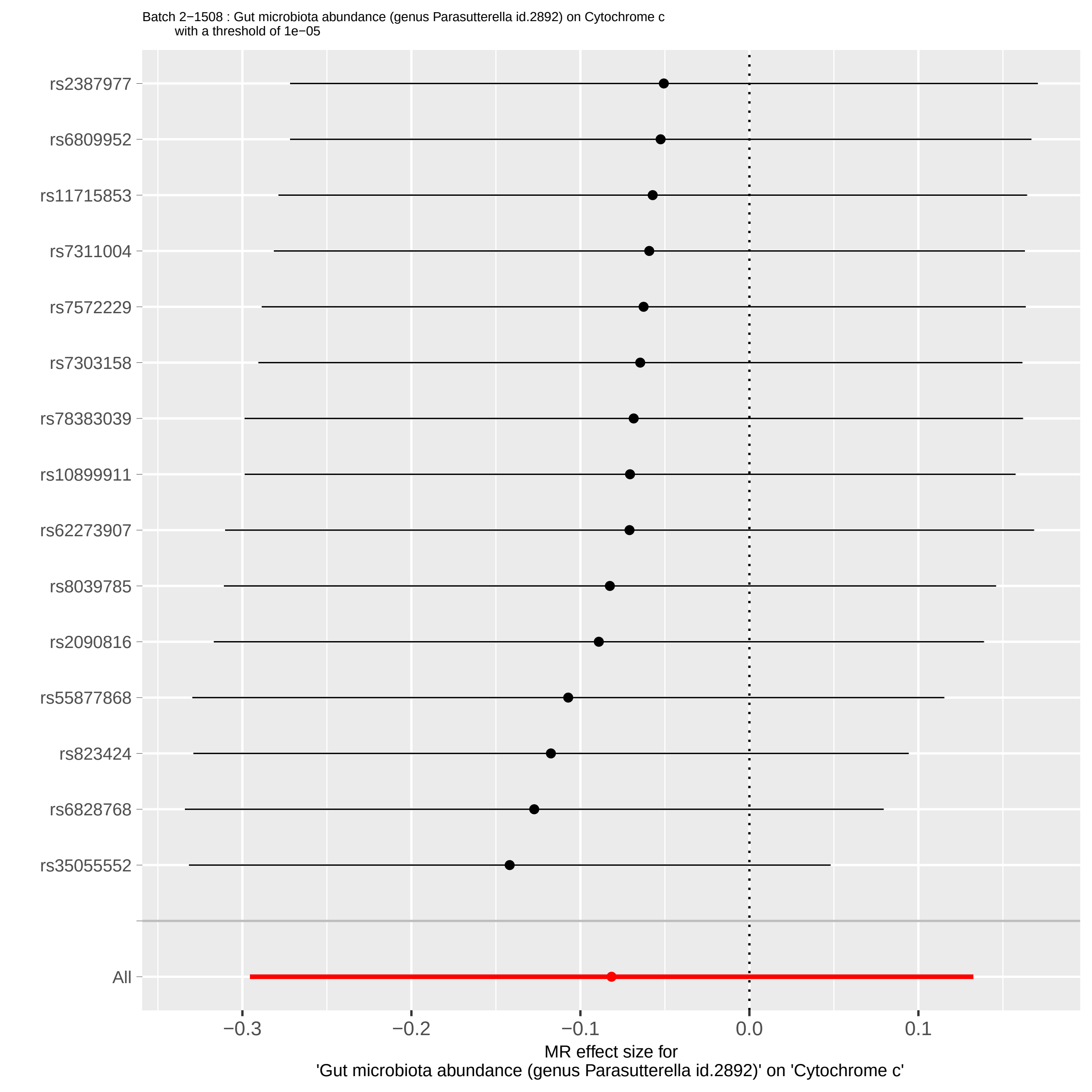

Batch 2−1508 : Gut microbiota abundance (genus Parasutterella id.2892) on Cytochrome c
with a threshold of 1e−05
rs2387977
rs6809952
rs11715853
rs7311004
rs7572229
rs7303158
rs78383039
rs10899911
rs62273907
rs8039785
rs2090816
rs55877868
rs823424
rs6828768
rs35055552
All
−0.3
−0.2
−0.1
0.0
0.1
MR effect size for
'Gut microbiota abundance (genus Parasutterella id.2892)' on 'Cytochrome c'

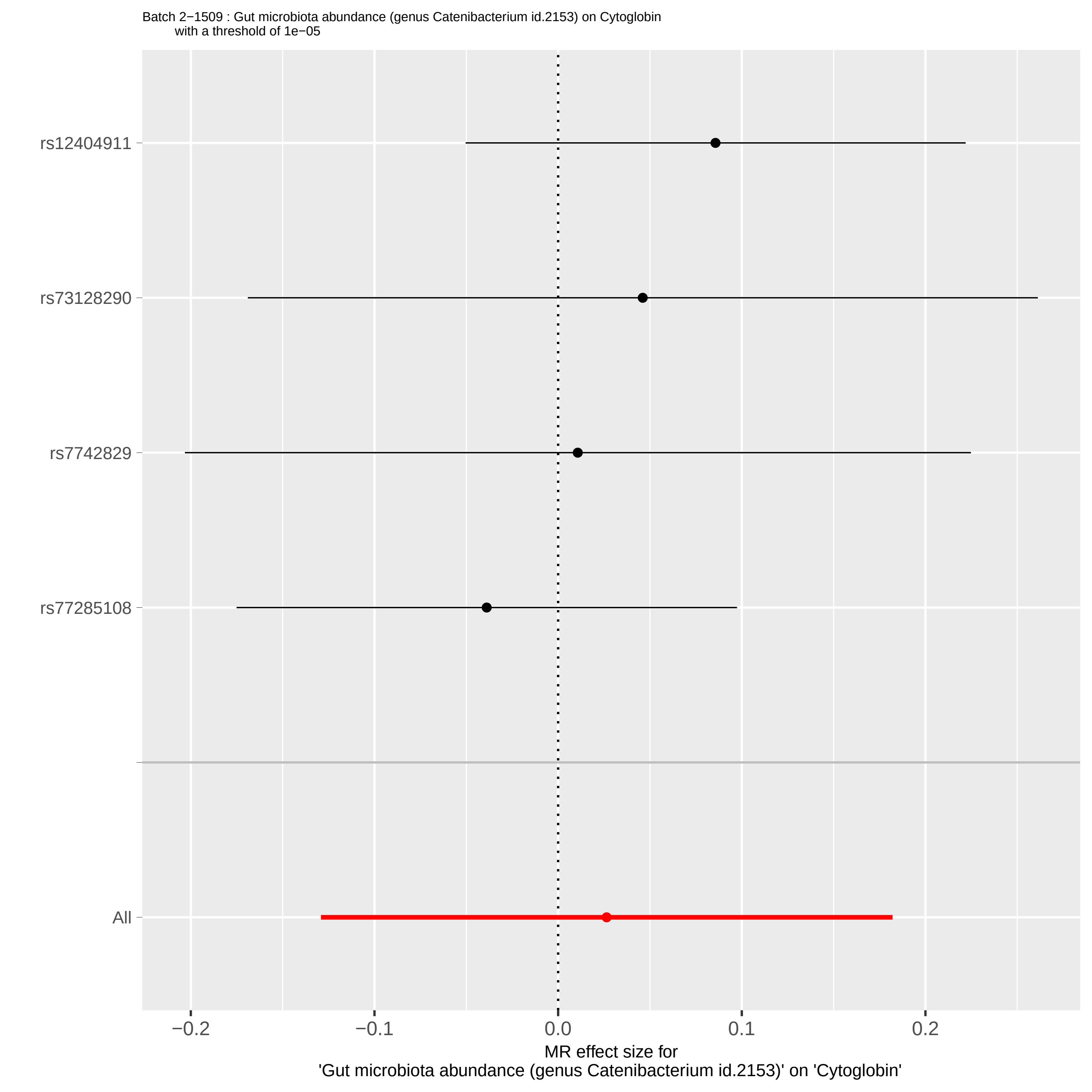

Batch 2−1509 : Gut microbiota abundance (genus Catenibacterium id.2153) on Cytoglobin
with a threshold of 1e−05
rs12404911
rs73128290
rs7742829
rs77285108
All
−0.2
−0.1
0.0
0.1
0.2
MR effect size for
'Gut microbiota abundance (genus Catenibacterium id.2153)' on 'Cytoglobin'

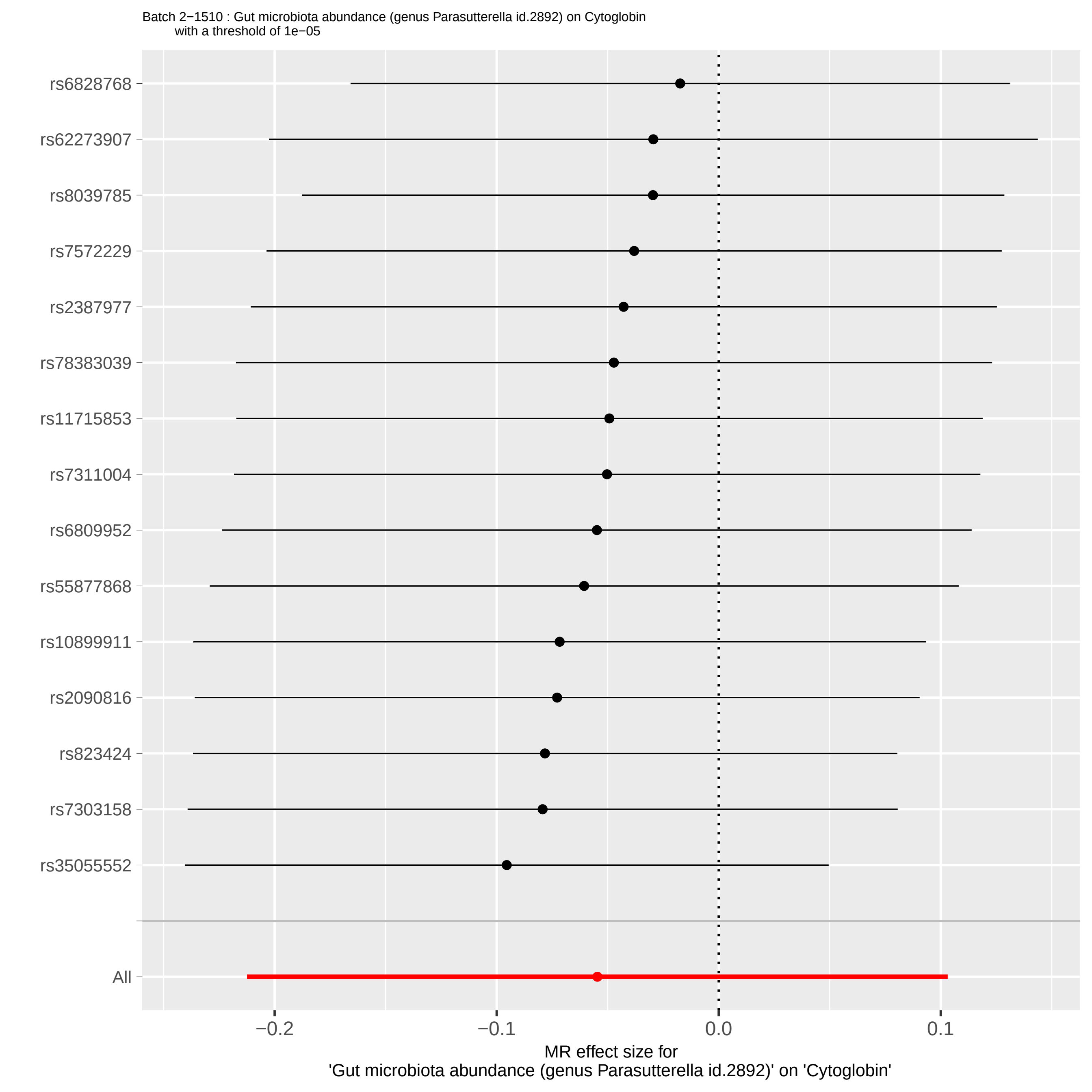
Batch 2−1510 : Gut microbiota abundance (genus Parasutterella id.2892) on Cytoglobin
with a threshold of 1e−05
rs6828768
rs62273907
rs8039785
rs7572229
rs2387977
rs78383039
rs11715853
rs7311004
rs6809952
rs55877868
rs10899911
rs2090816
rs823424
rs7303158
rs35055552
All
−0.2
−0.1
0.0
0.1
MR effect size for
'Gut microbiota abundance (genus Parasutterella id.2892)' on 'Cytoglobin'

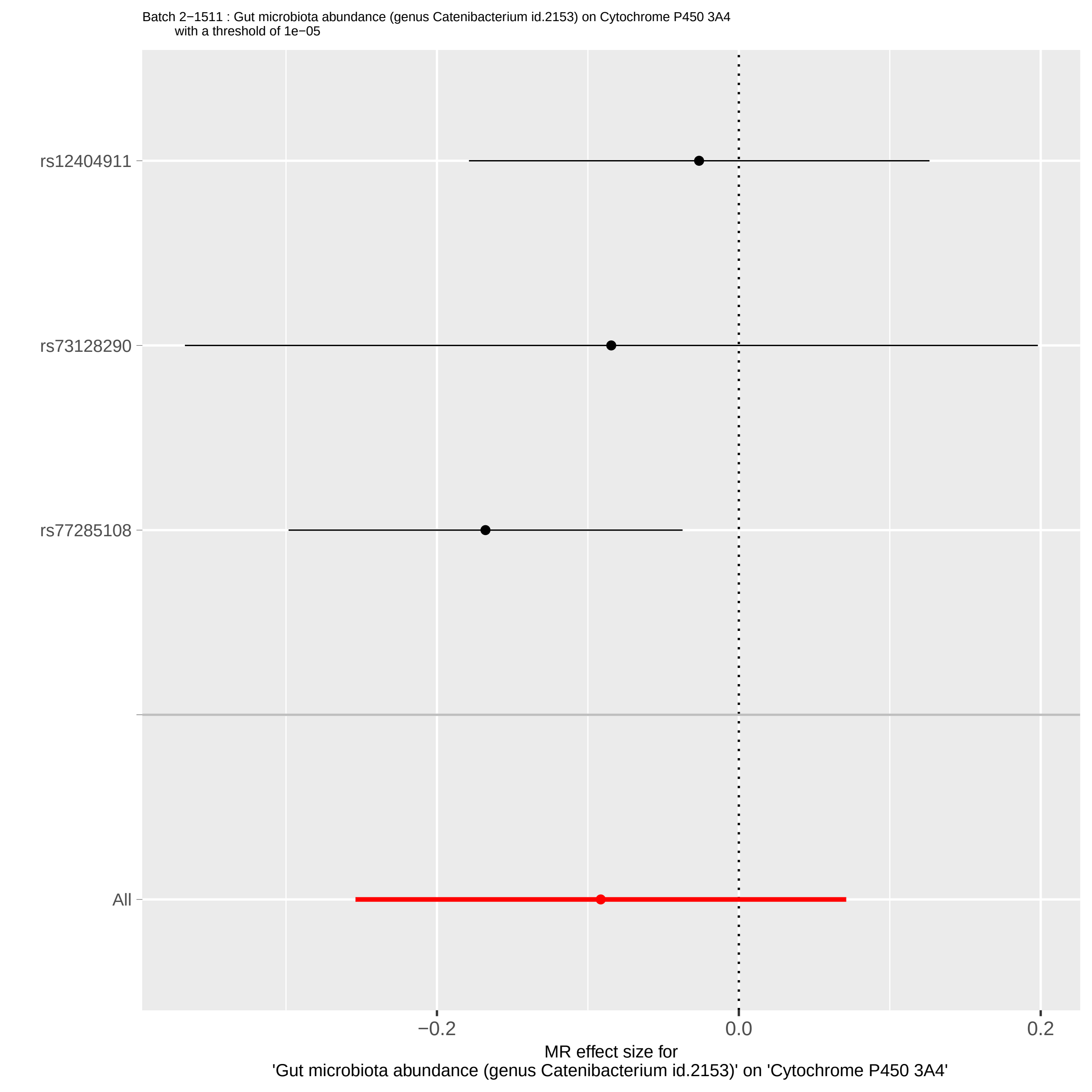
Batch 2−1511 : Gut microbiota abundance (genus Catenibacterium id.2153) on Cytochrome P450 3A4
with a threshold of 1e−05
rs12404911
rs73128290
rs77285108
All
−0.2
0.0
0.2
MR effect size for
'Gut microbiota abundance (genus Catenibacterium id.2153)' on 'Cytochrome P450 3A4'

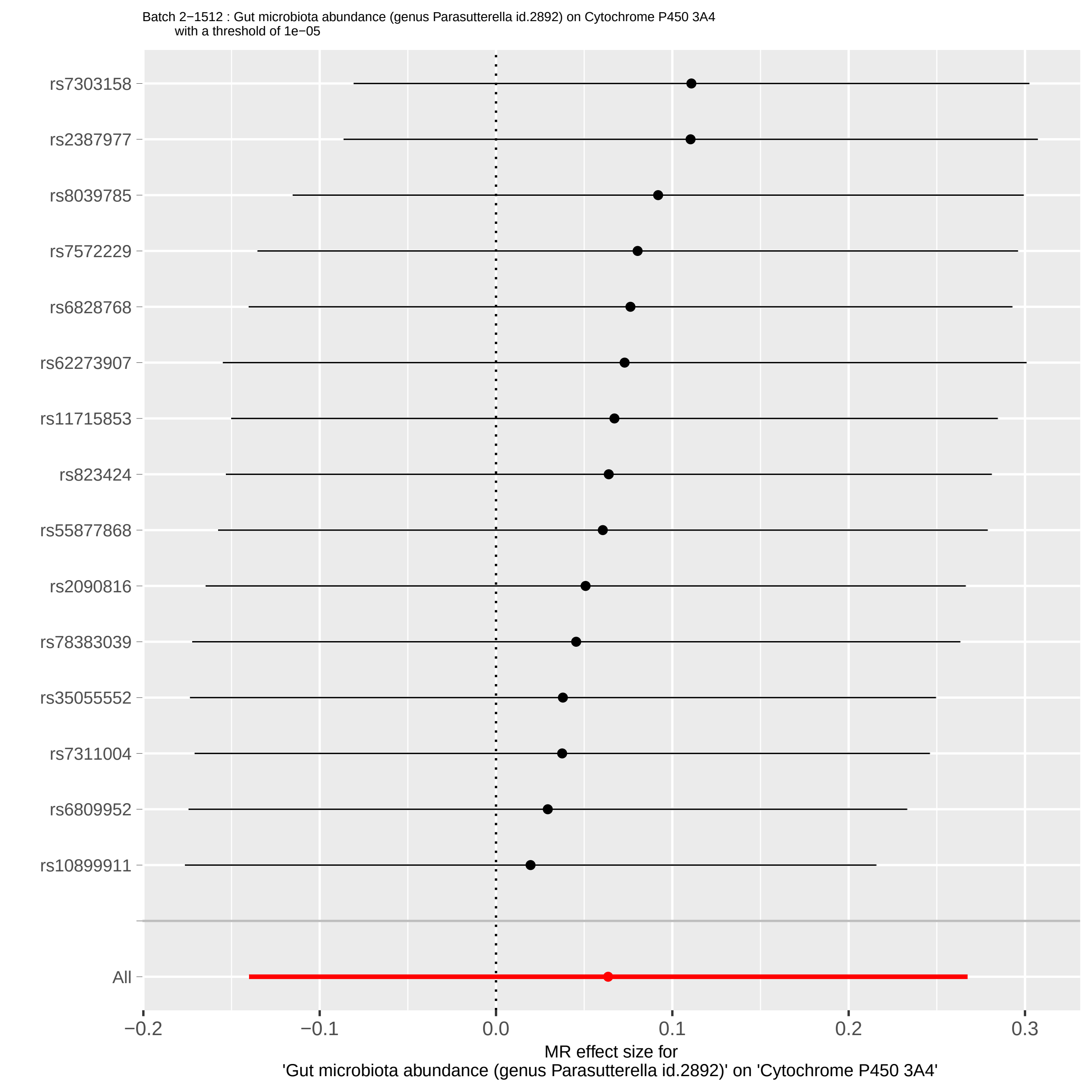

Batch 2−1512 : Gut microbiota abundance (genus Parasutterella id.2892) on Cytochrome P450 3A4
with a threshold of 1e−05
rs7303158
rs2387977
rs8039785
rs7572229
rs6828768
rs62273907
rs11715853
rs823424
rs55877868
rs2090816
rs78383039
rs35055552
rs7311004
rs6809952
rs10899911
All
−0.2
−0.1
0.0
0.1
0.2
0.3
MR effect size for
'Gut microbiota abundance (genus Parasutterella id.2892)' on 'Cytochrome P450 3A4'

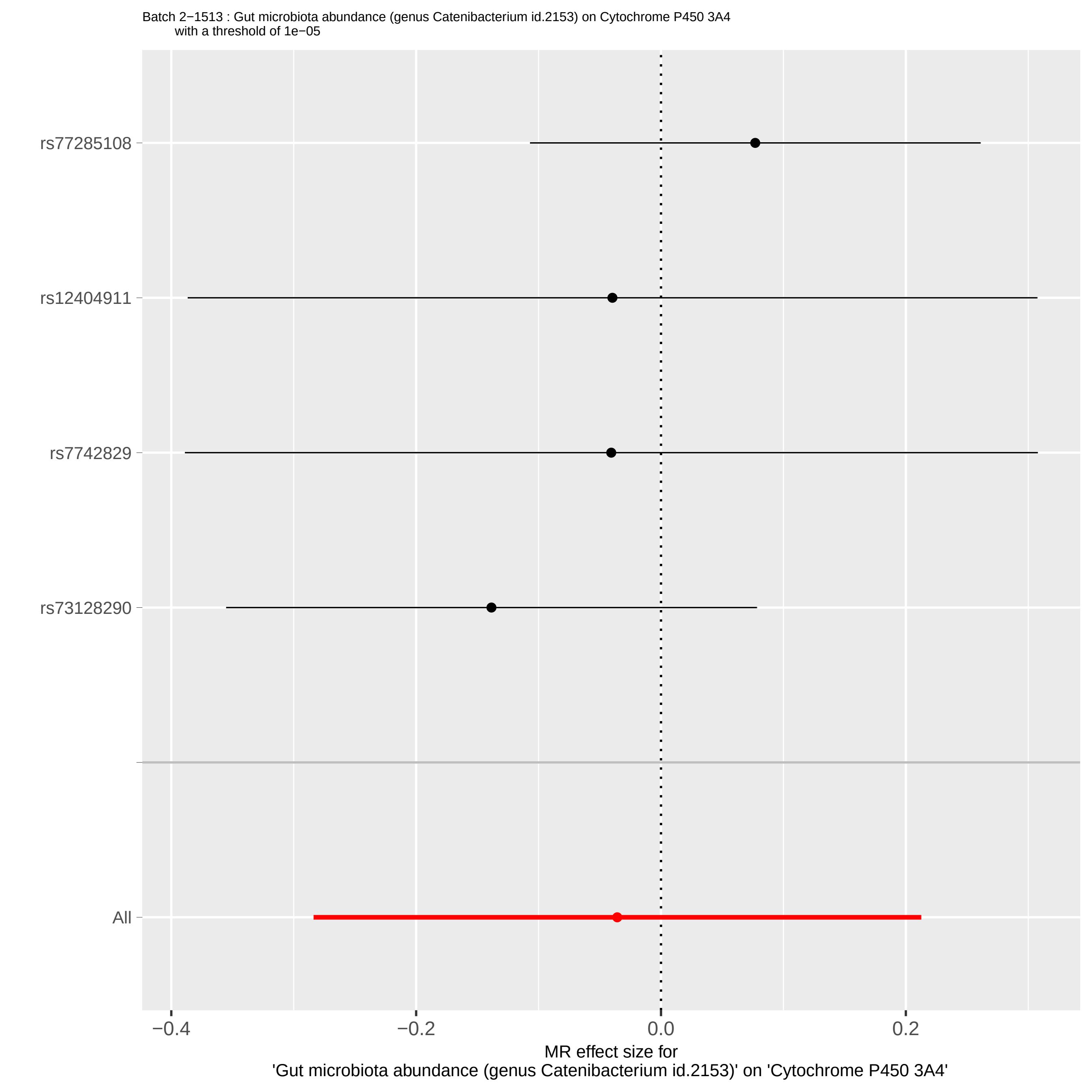

Batch 2−1513 : Gut microbiota abundance (genus Catenibacterium id.2153) on Cytochrome P450 3A4
with a threshold of 1e−05
rs77285108
rs12404911
rs7742829
rs73128290
All
−0.4
−0.2
0.0
0.2
MR effect size for
'Gut microbiota abundance (genus Catenibacterium id.2153)' on 'Cytochrome P450 3A4'

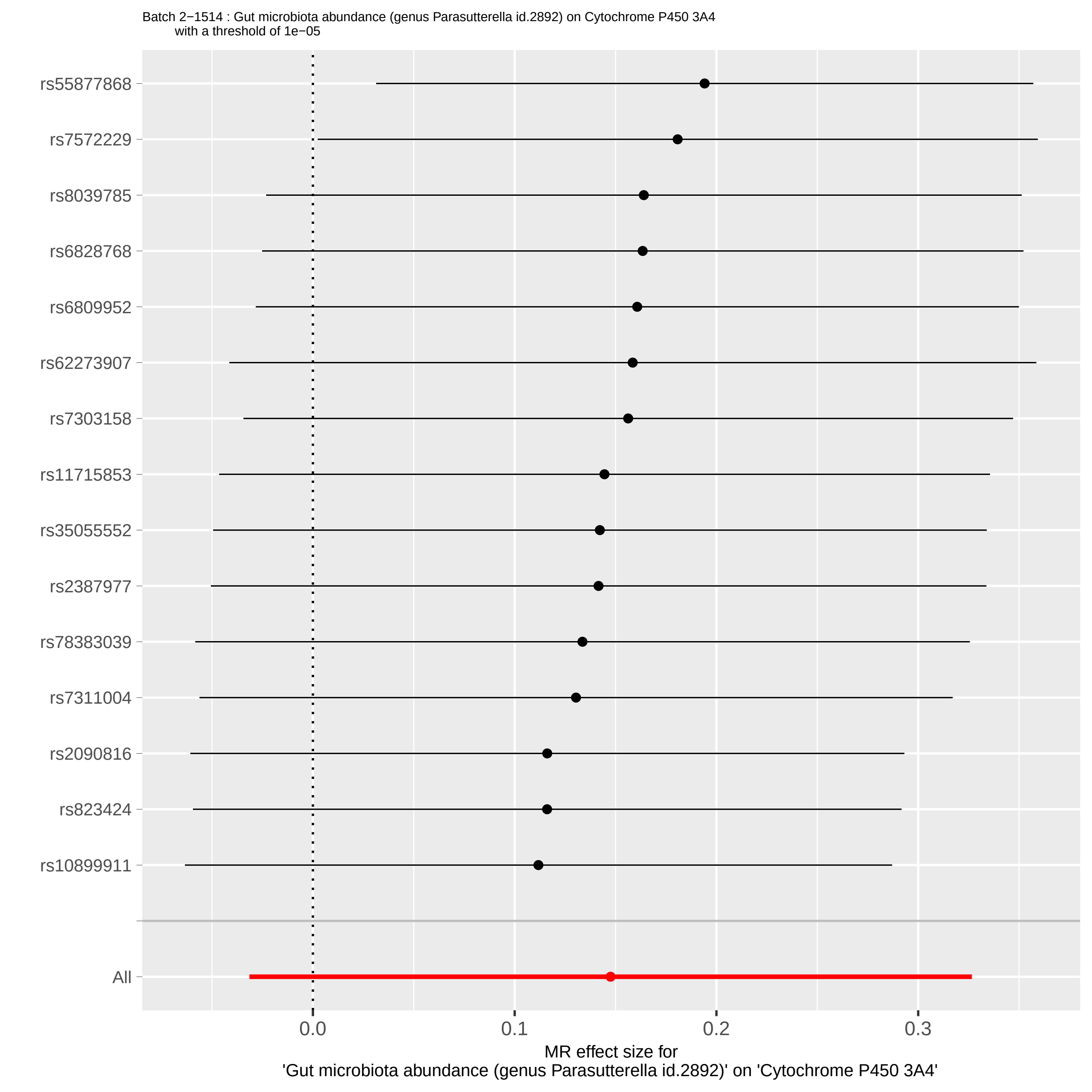

Batch 2−1514 : Gut microbiota abundance (genus Parasutterella id.2892) on Cytochrome P450 3A4
with a threshold of 1e−05
rs55877868
rs7572229
rs8039785
rs6828768
rs6809952
rs62273907
rs7303158
rs11715853
rs35055552
rs2387977
rs78383039
rs7311004
rs2090816
rs823424
rs10899911
All
0.0
0.1
0.2
0.3
MR effect size for
'Gut microbiota abundance (genus Parasutterella id.2892)' on 'Cytochrome P450 3A4'

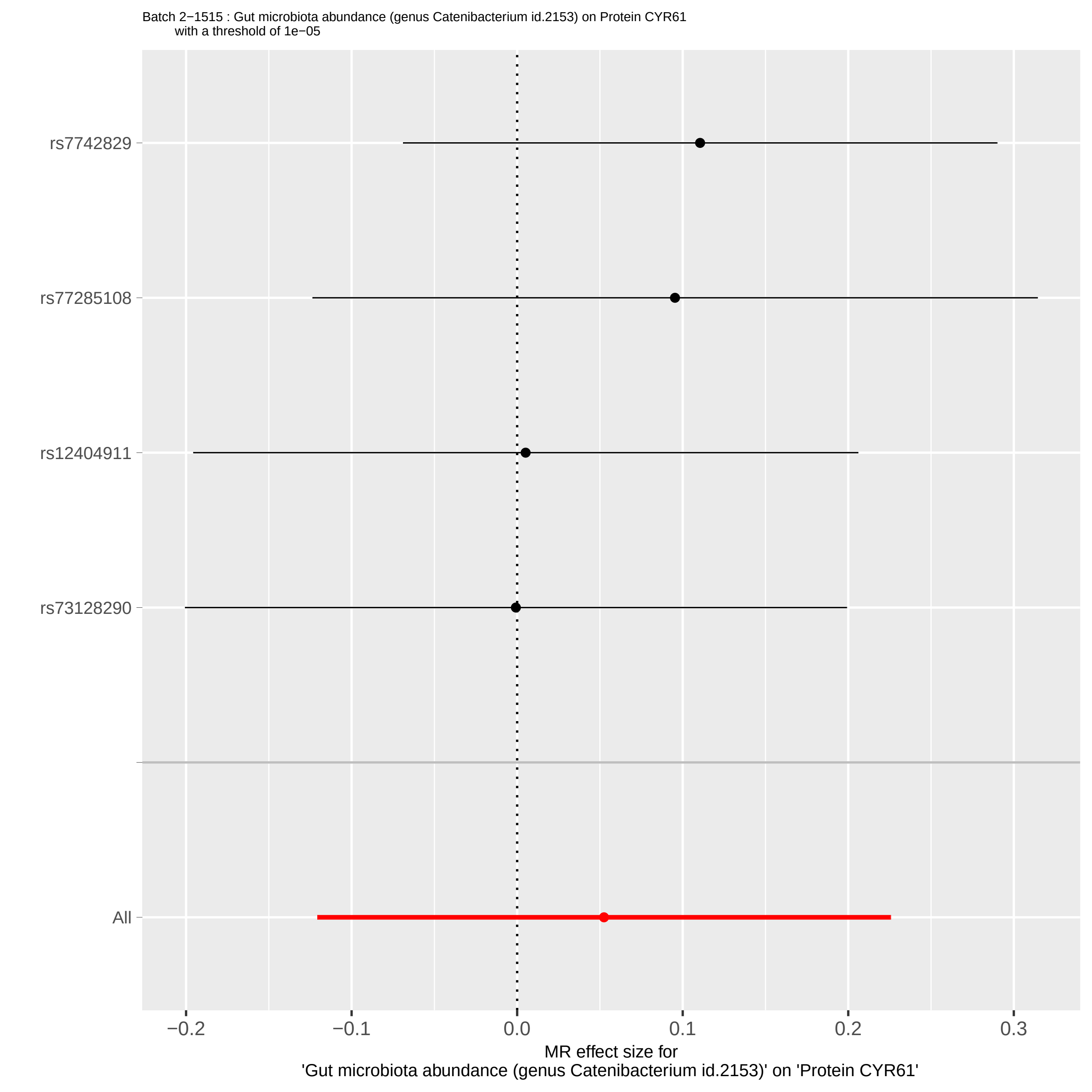

Batch 2−1515 : Gut microbiota abundance (genus Catenibacterium id.2153) on Protein CYR61
with a threshold of 1e−05
rs7742829
rs77285108
rs12404911
rs73128290
All
−0.2
−0.1
0.0
0.1
0.2
0.3
MR effect size for
'Gut microbiota abundance (genus Catenibacterium id.2153)' on 'Protein CYR61'

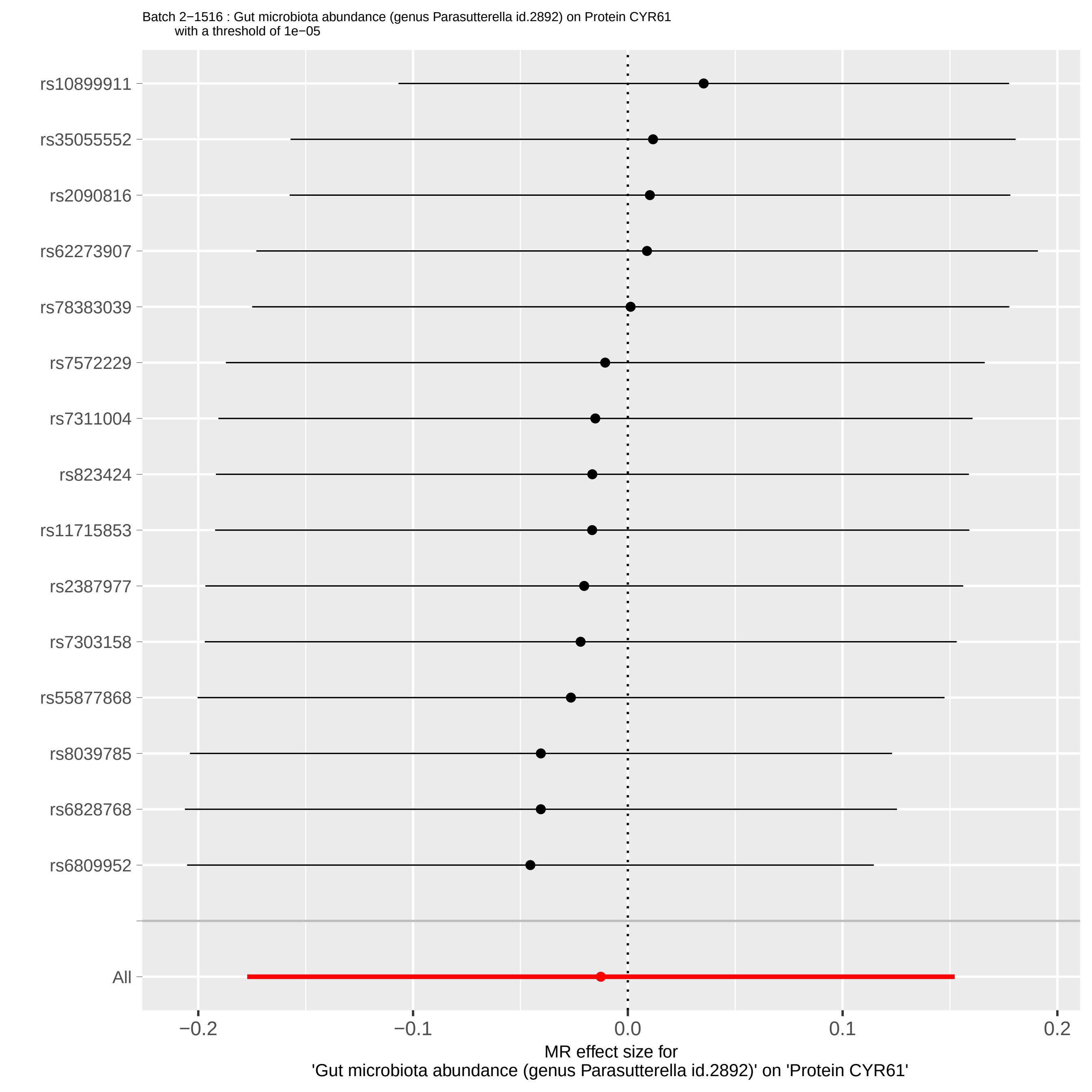
Batch 2−1516 : Gut microbiota abundance (genus Parasutterella id.2892) on Protein CYR61
with a threshold of 1e−05
rs10899911
rs35055552
rs2090816
rs62273907
rs78383039
rs7572229
rs7311004
rs823424
rs11715853
rs2387977
rs7303158
rs55877868
rs8039785
rs6828768
rs6809952
All
−0.2
−0.1
0.0
0.1
0.2
MR effect size for
'Gut microbiota abundance (genus Parasutterella id.2892)' on 'Protein CYR61'

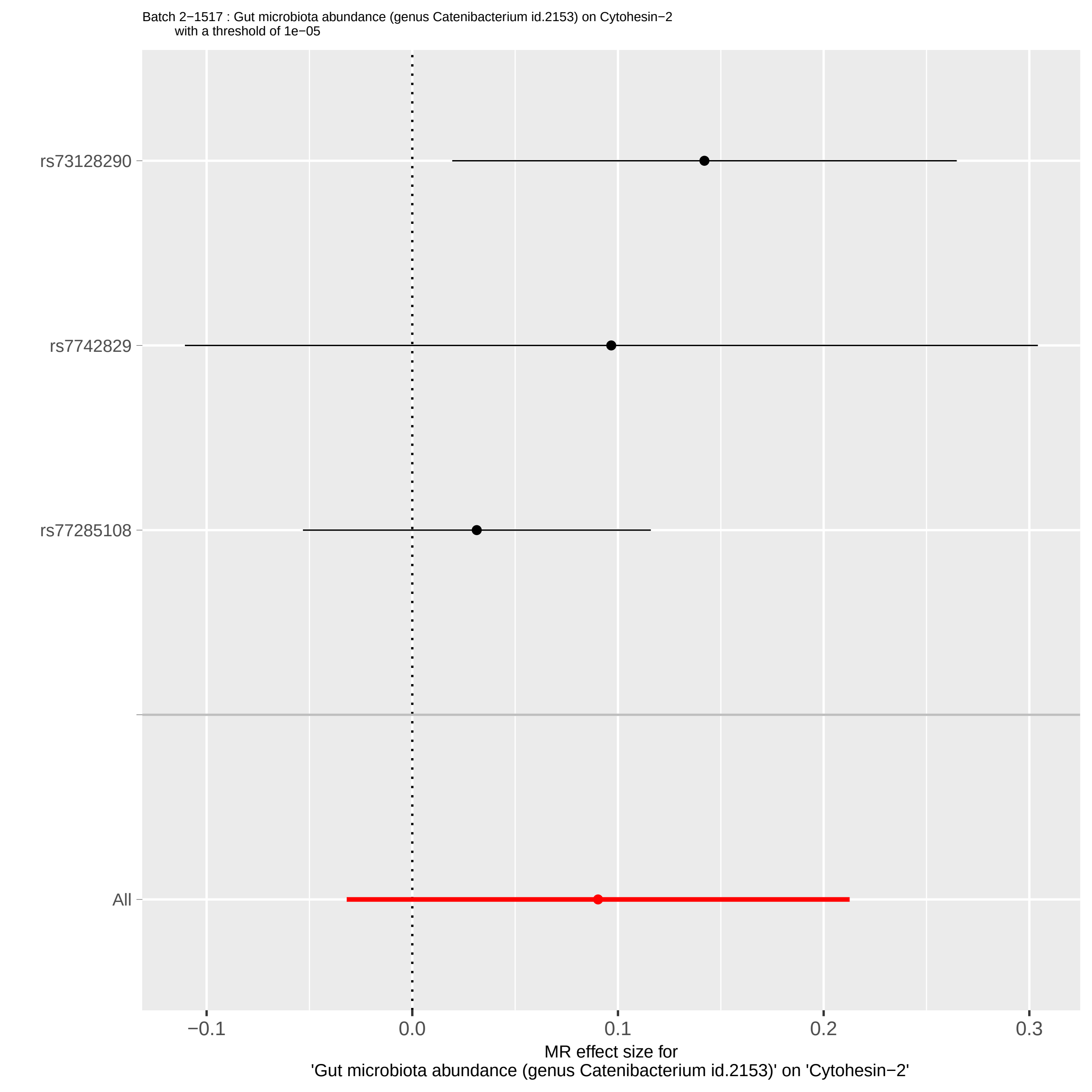

Batch 2−1517 : Gut microbiota abundance (genus Catenibacterium id.2153) on Cytohesin−2
with a threshold of 1e−05
rs73128290
rs7742829
rs77285108
All
−0.1
0.0
0.1
0.2
0.3
MR effect size for
'Gut microbiota abundance (genus Catenibacterium id.2153)' on 'Cytohesin−2'

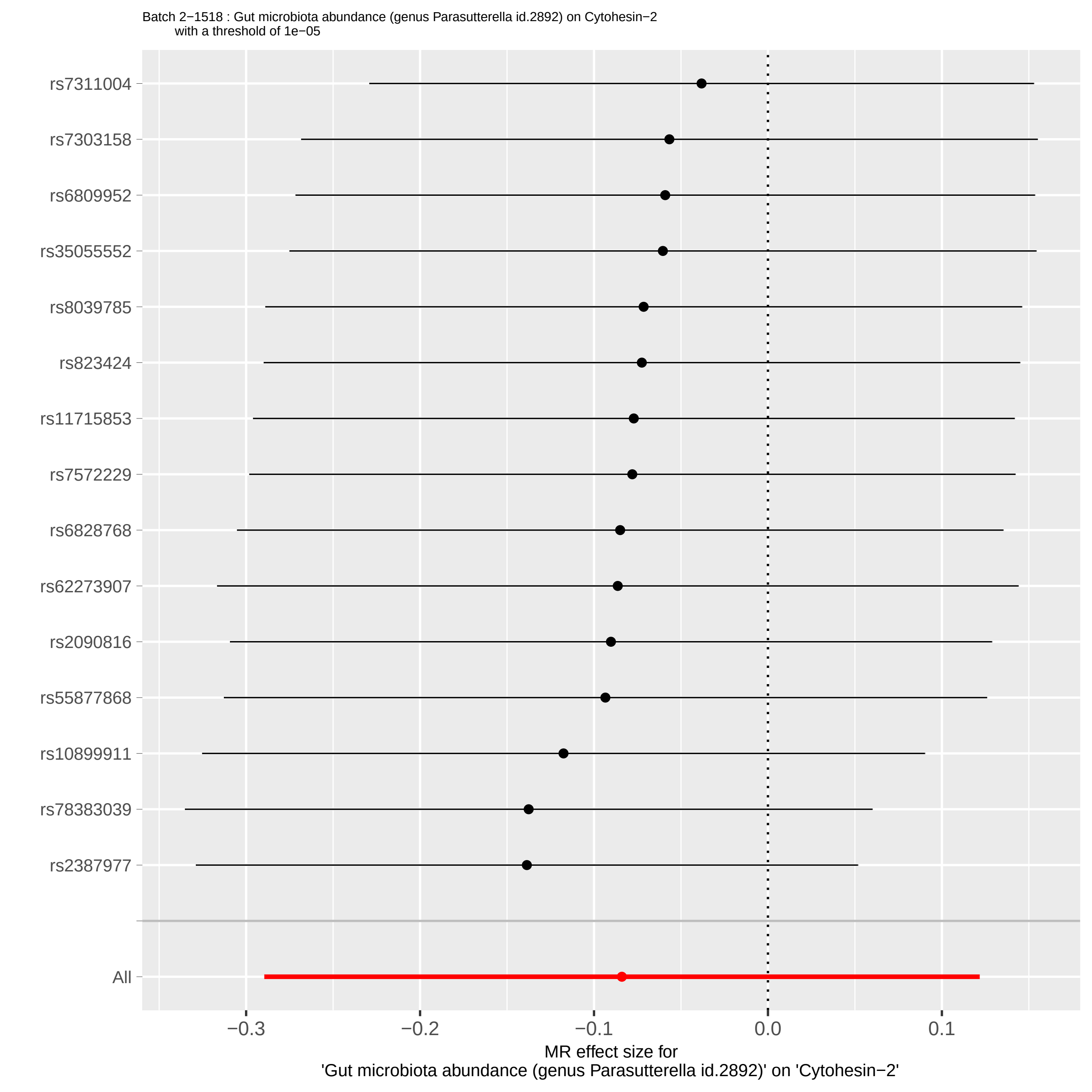
Batch 2−1518 : Gut microbiota abundance (genus Parasutterella id.2892) on Cytohesin−2
with a threshold of 1e−05
rs7311004
rs7303158
rs6809952
rs35055552
rs8039785
rs823424
rs11715853
rs7572229
rs6828768
rs62273907
rs2090816
rs55877868
rs10899911
rs78383039
rs2387977
All
−0.3
−0.2
−0.1
0.0
0.1
MR effect size for
'Gut microbiota abundance (genus Parasutterella id.2892)' on 'Cytohesin−2'

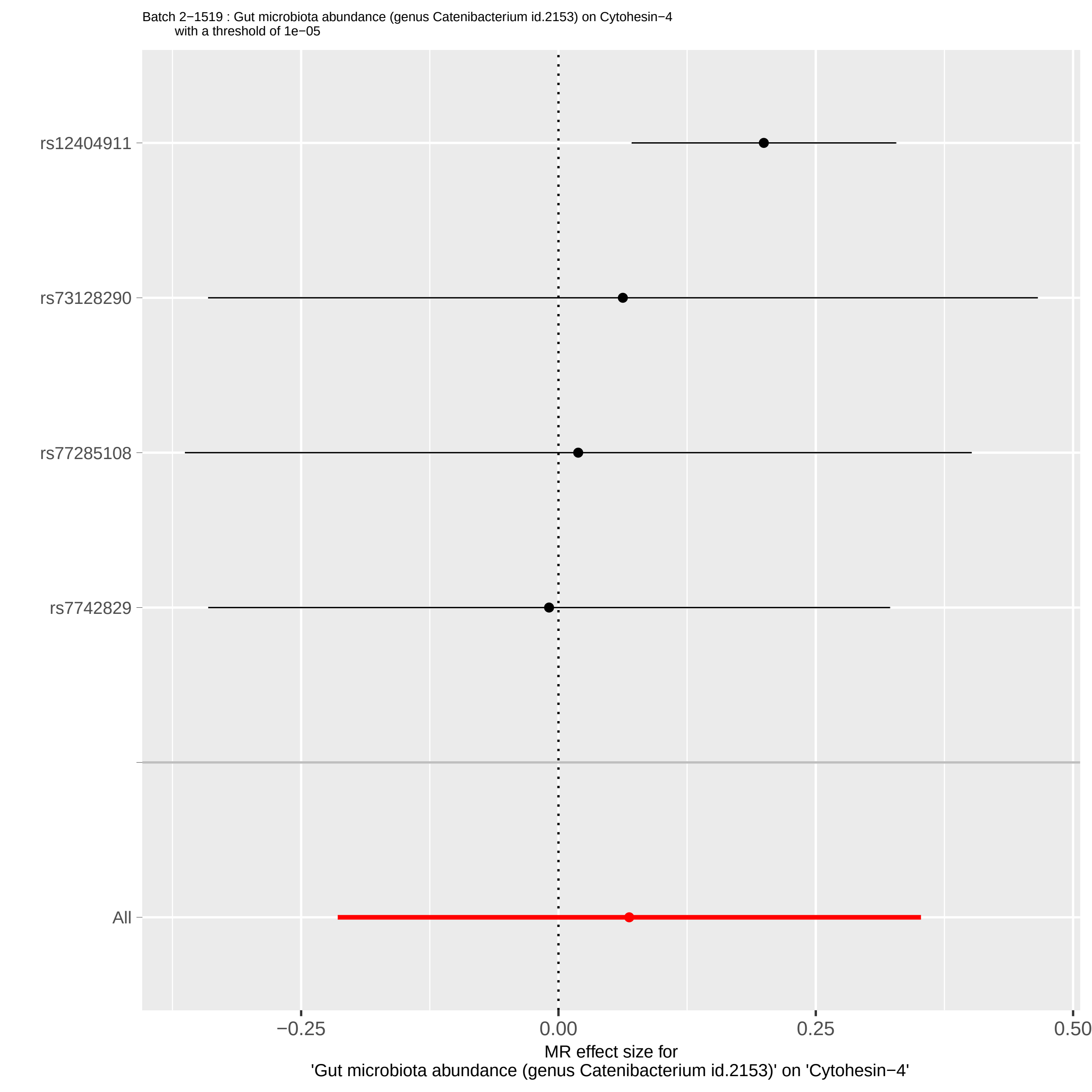

Batch 2−1519 : Gut microbiota abundance (genus Catenibacterium id.2153) on Cytohesin−4
with a threshold of 1e−05
rs12404911
rs73128290
rs77285108
rs7742829
All
−0.25
0.00
0.25
0.50
MR effect size for
'Gut microbiota abundance (genus Catenibacterium id.2153)' on 'Cytohesin−4'

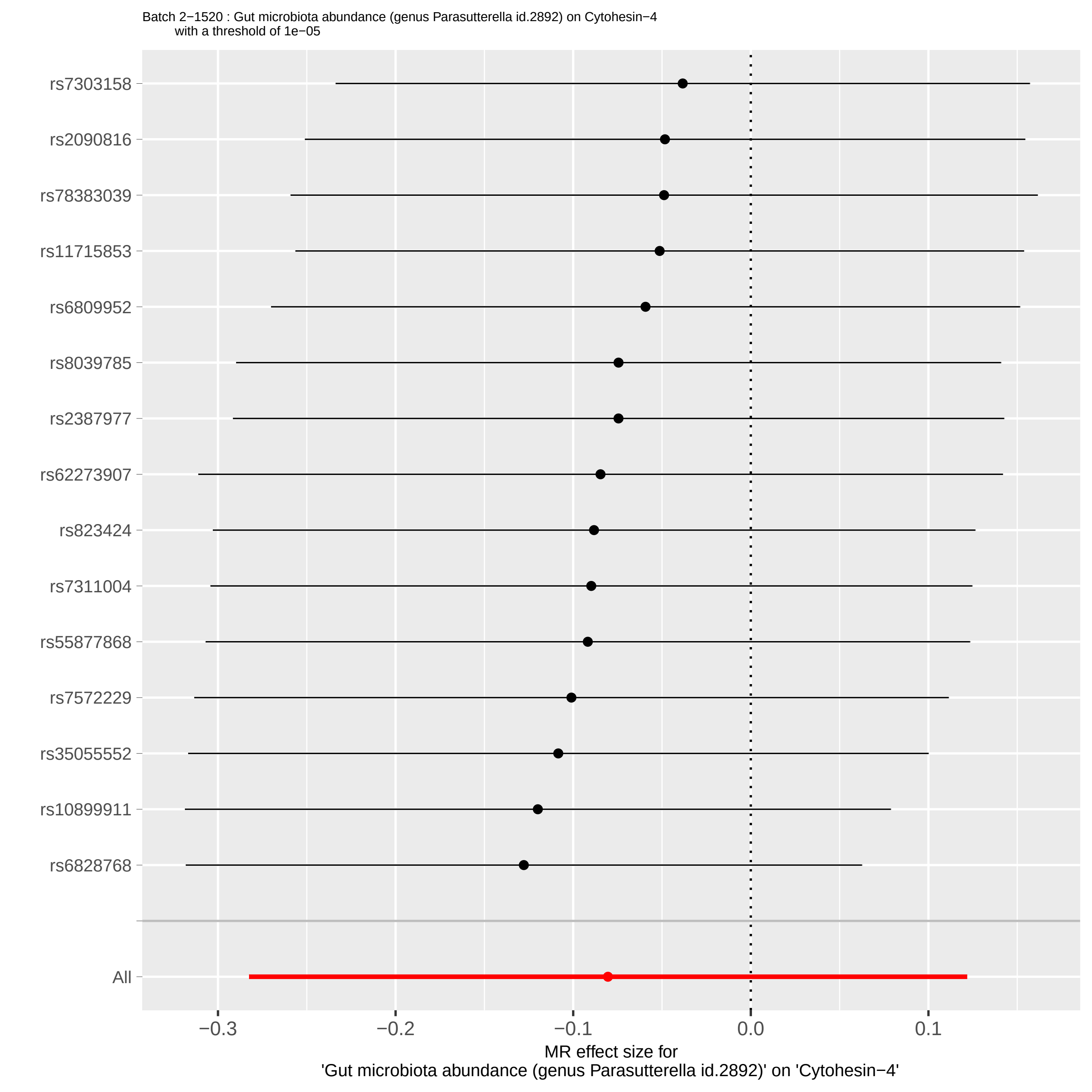

Batch 2−1520 : Gut microbiota abundance (genus Parasutterella id.2892) on Cytohesin−4
with a threshold of 1e−05
rs7303158
rs2090816
rs78383039
rs11715853
rs6809952
rs8039785
rs2387977
rs62273907
rs823424
rs7311004
rs55877868
rs7572229
rs35055552
rs10899911
rs6828768
All
−0.3
−0.2
−0.1
0.0
0.1
MR effect size for
'Gut microbiota abundance (genus Parasutterella id.2892)' on 'Cytohesin−4'

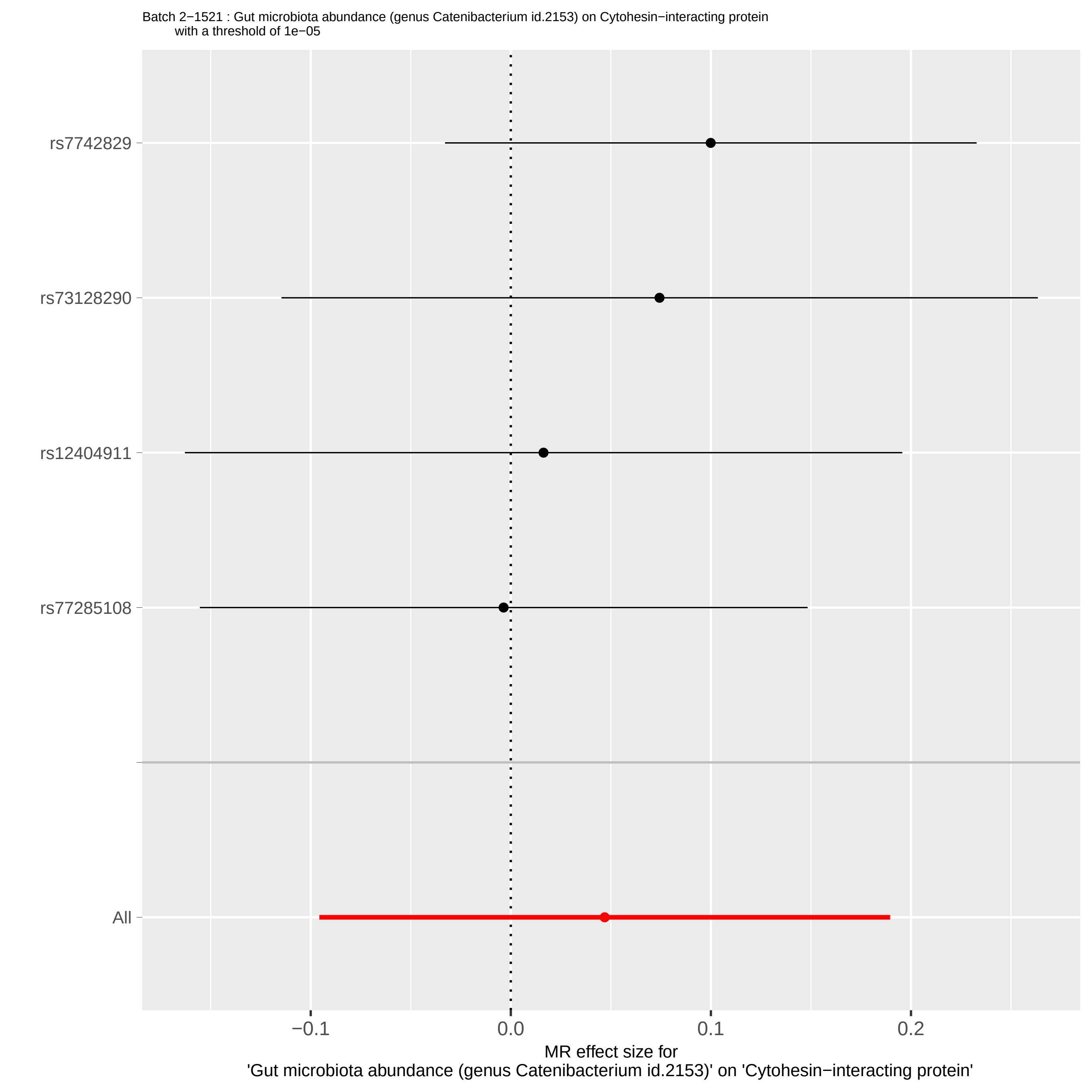

Batch 2−1521 : Gut microbiota abundance (genus Catenibacterium id.2153) on Cytohesin−interacting protein
with a threshold of 1e−05
rs7742829
rs73128290
rs12404911
rs77285108
All
−0.1
0.0
0.1
0.2
MR effect size for
'Gut microbiota abundance (genus Catenibacterium id.2153)' on 'Cytohesin−interacting protein'

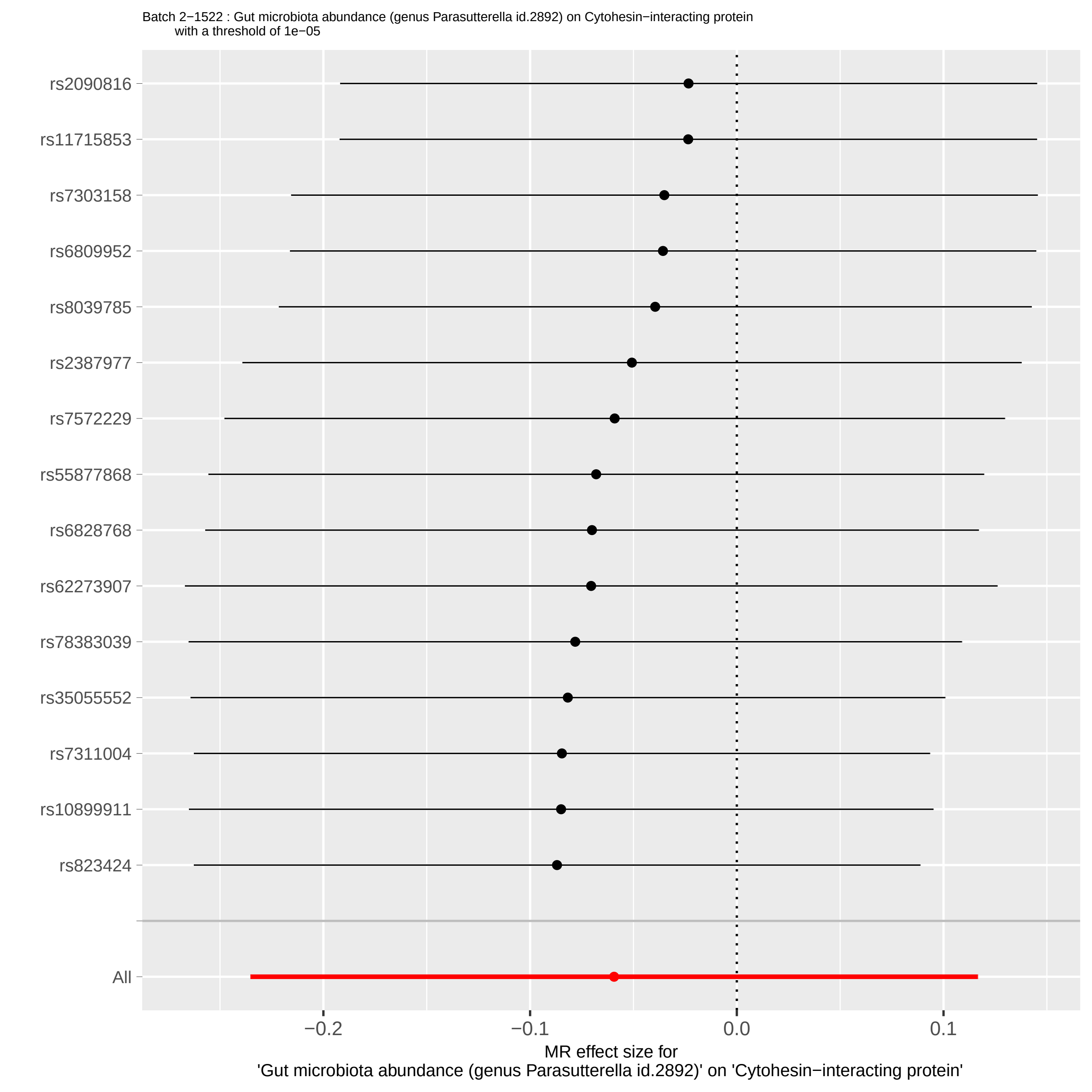
Batch 2−1522 : Gut microbiota abundance (genus Parasutterella id.2892) on Cytohesin−interacting protein
with a threshold of 1e−05
rs2090816
rs11715853
rs7303158
rs6809952
rs8039785
rs2387977
rs7572229
rs55877868
rs6828768
rs62273907
rs78383039
rs35055552
rs7311004
rs10899911
rs823424
All
−0.2
−0.1
0.0
0.1
MR effect size for
'Gut microbiota abundance (genus Parasutterella id.2892)' on 'Cytohesin−interacting protein'

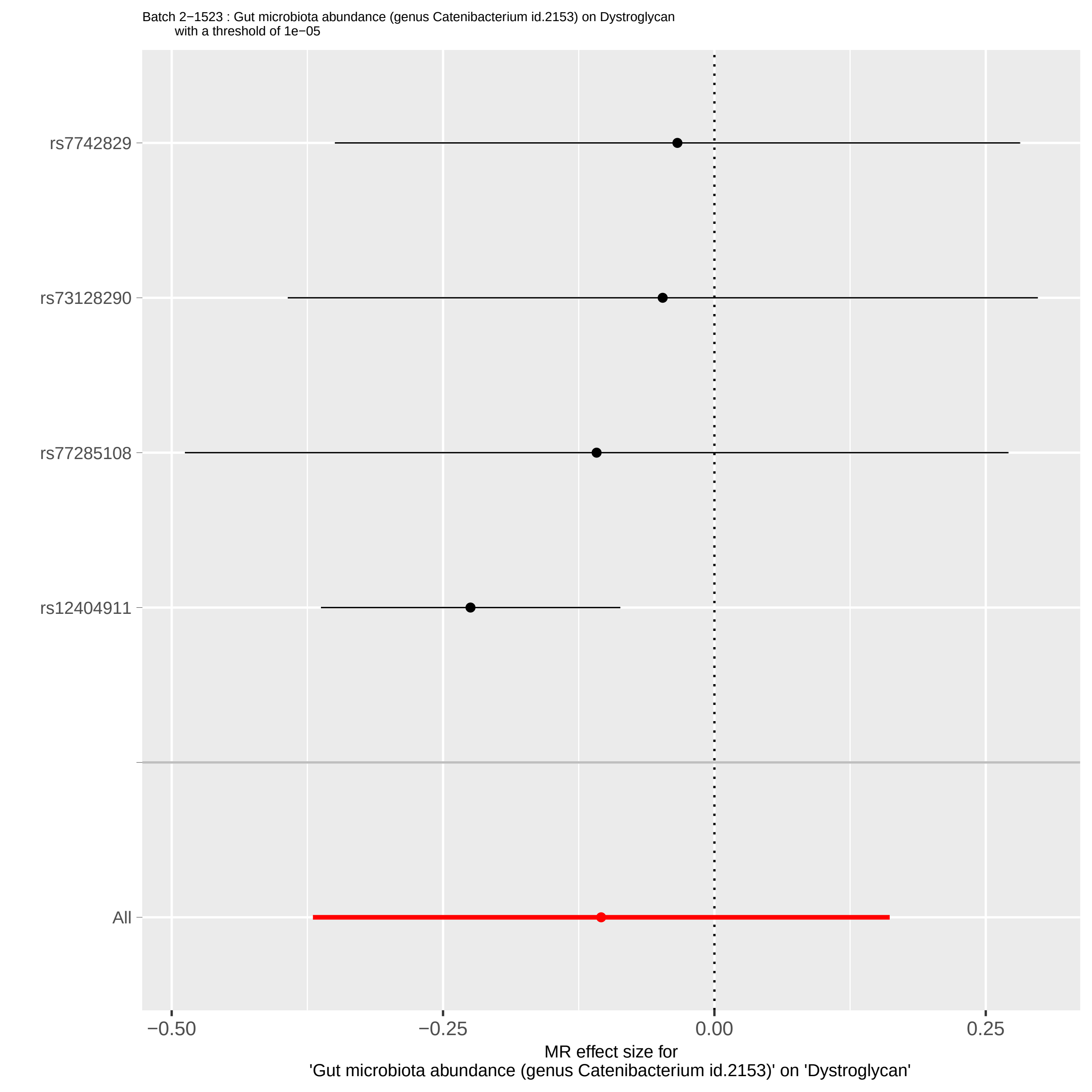

Batch 2−1523 : Gut microbiota abundance (genus Catenibacterium id.2153) on Dystroglycan
with a threshold of 1e−05
rs7742829
rs73128290
rs77285108
rs12404911
All
−0.50
−0.25
0.00
0.25
MR effect size for
'Gut microbiota abundance (genus Catenibacterium id.2153)' on 'Dystroglycan'

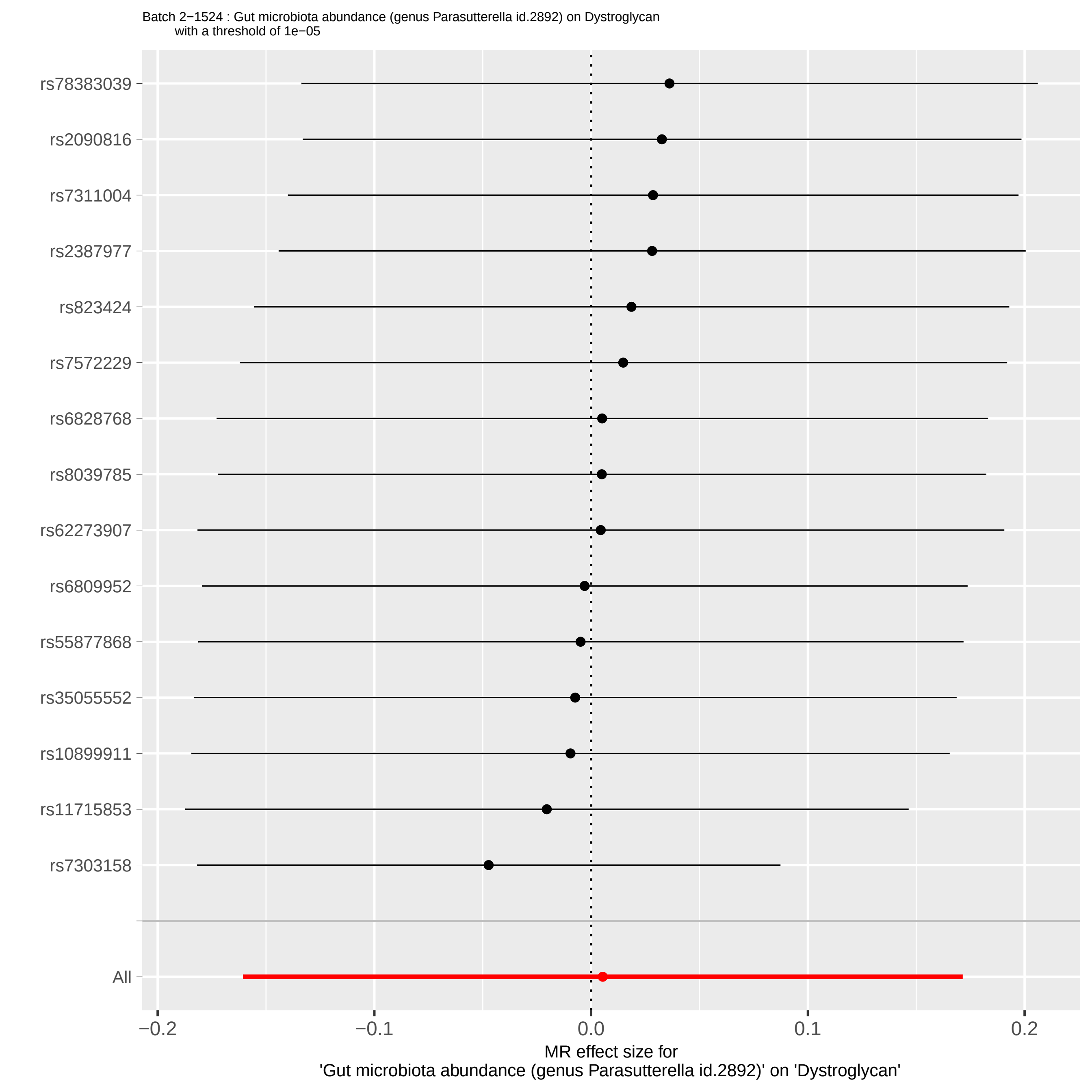
Batch 2−1524 : Gut microbiota abundance (genus Parasutterella id.2892) on Dystroglycan
with a threshold of 1e−05
rs78383039
rs2090816
rs7311004
rs2387977
rs823424
rs7572229
rs6828768
rs8039785
rs62273907
rs6809952
rs55877868
rs35055552
rs10899911
rs11715853
rs7303158
All
−0.2
−0.1
0.0
0.1
0.2
MR effect size for
'Gut microbiota abundance (genus Parasutterella id.2892)' on 'Dystroglycan'

Batch 2−1525 : Gut microbiota abundance (genus Catenibacterium id.2153) on Death−associated protein kinase 2
with a threshold of 1e−05

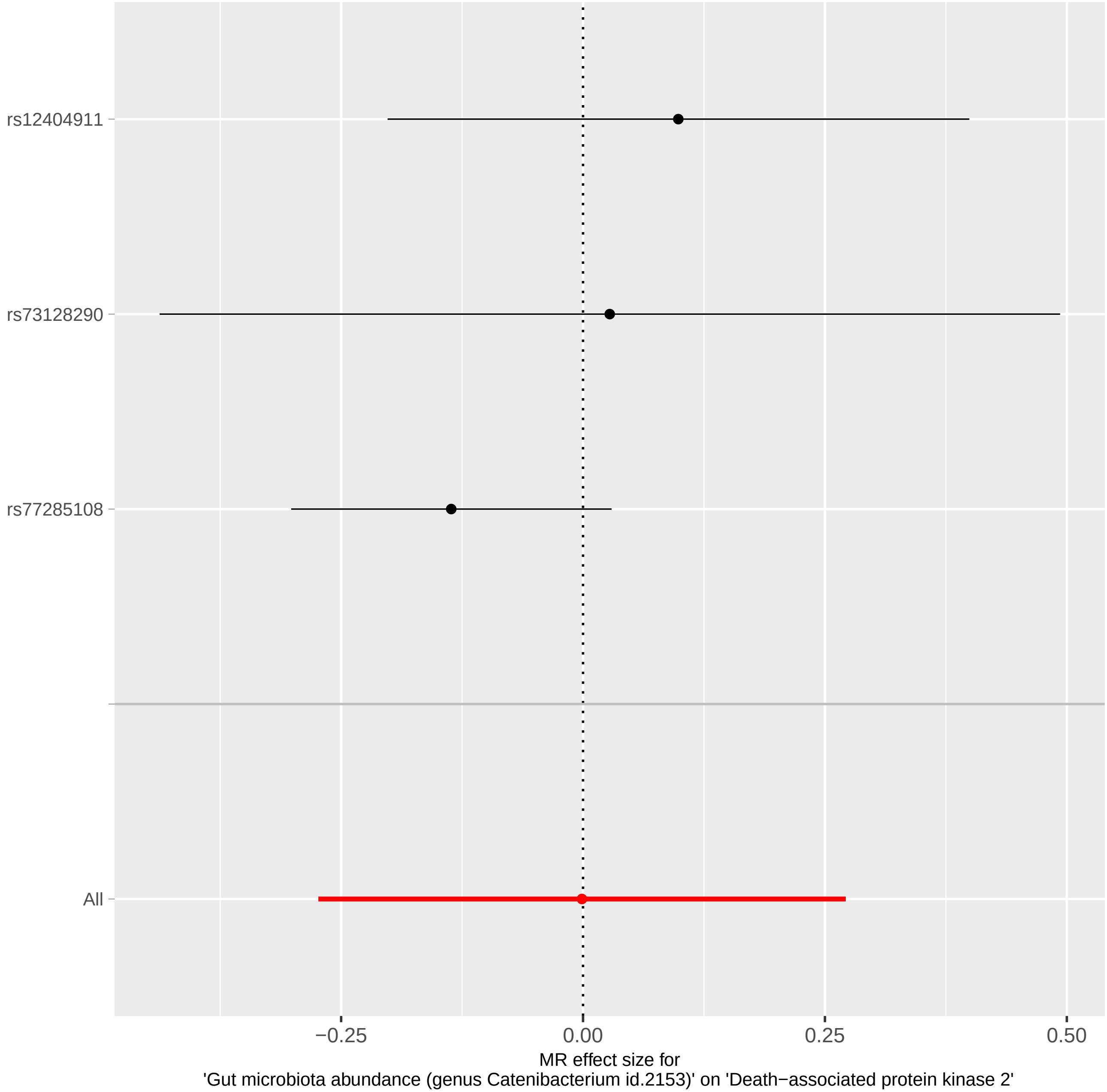

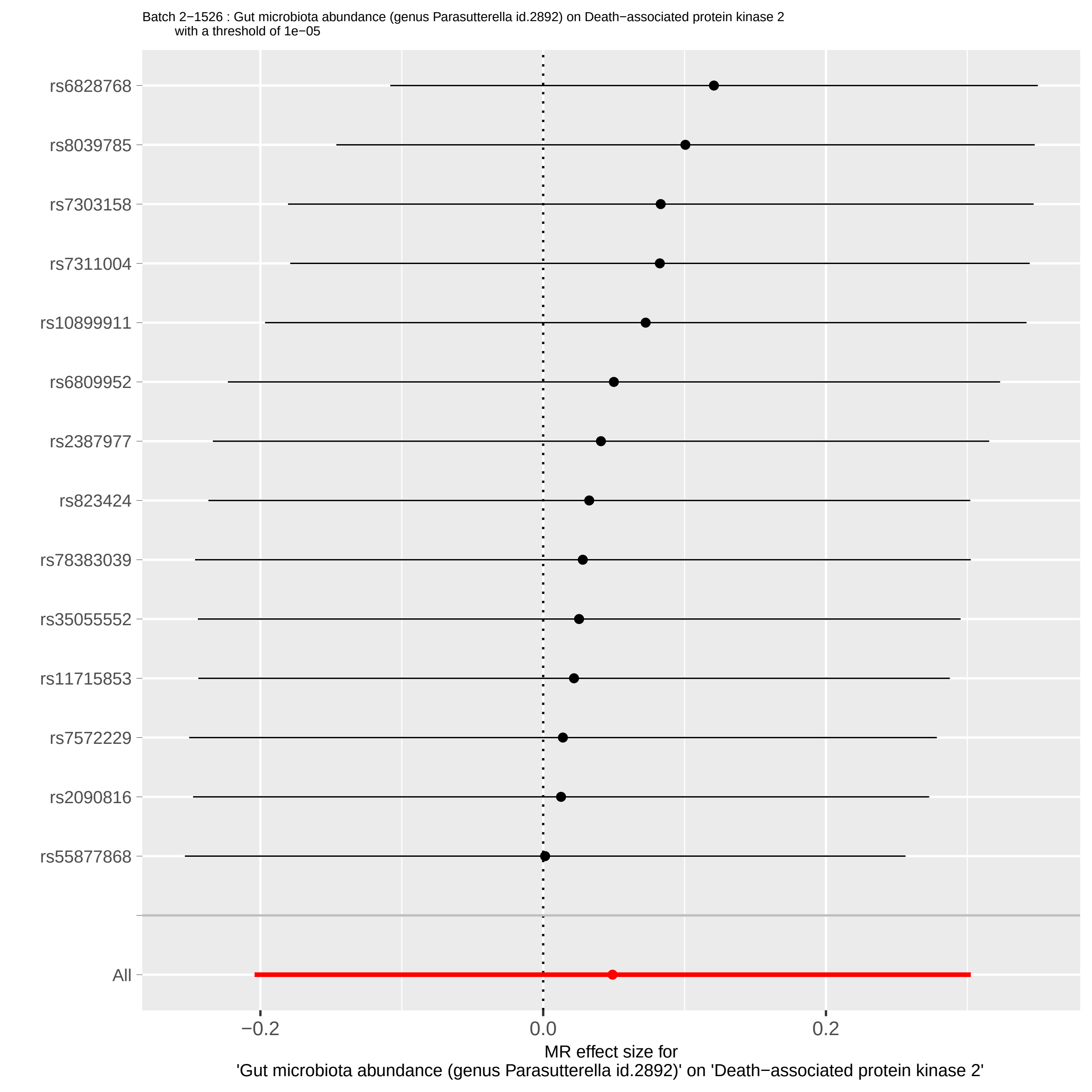
Batch 2−1526 : Gut microbiota abundance (genus Parasutterella id.2892) on Death−associated protein kinase 2
with a threshold of 1e−05
rs6828768
rs8039785
rs7303158
rs7311004
rs10899911
rs6809952
rs2387977
rs823424
rs78383039
rs35055552
rs11715853
rs7572229
rs2090816
rs55877868
All
−0.2
0.0
0.2
MR effect size for
'Gut microbiota abundance (genus Parasutterella id.2892)' on 'Death−associated protein kinase 2'

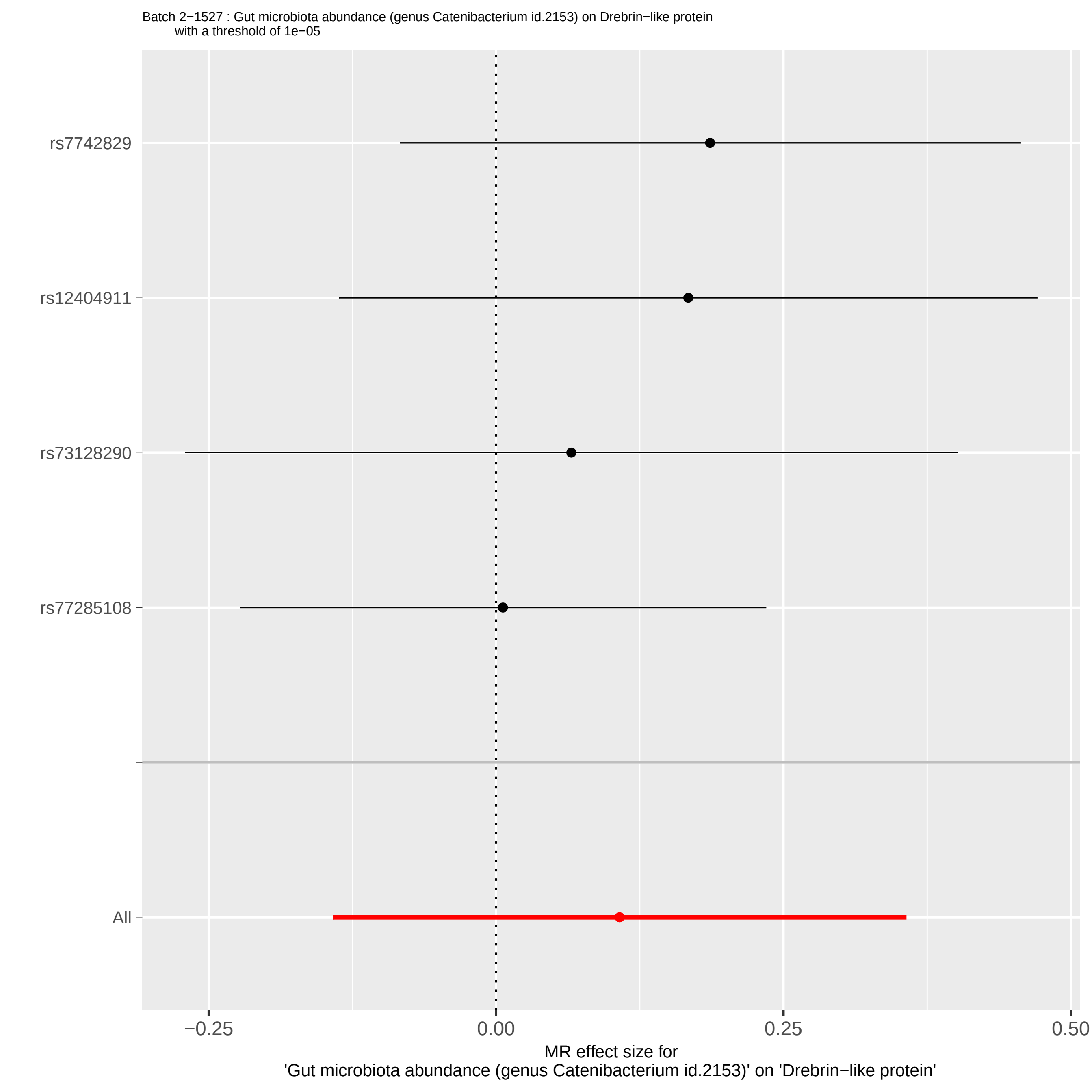
Batch 2−1527 : Gut microbiota abundance (genus Catenibacterium id.2153) on Drebrin−like protein
with a threshold of 1e−05
rs7742829
rs12404911
rs73128290
rs77285108
All
−0.25
0.00
0.25
0.50
MR effect size for
'Gut microbiota abundance (genus Catenibacterium id.2153)' on 'Drebrin−like protein'

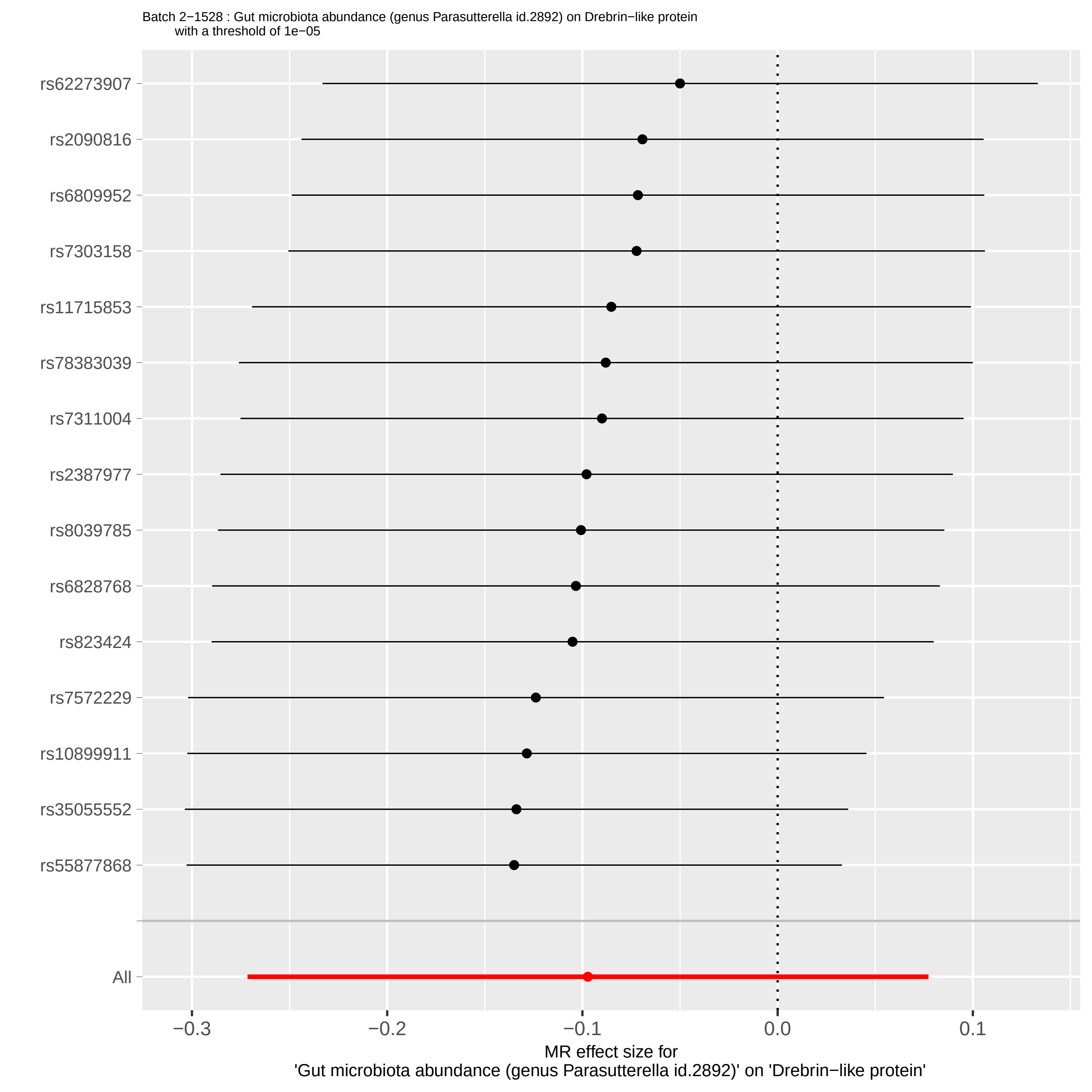

Batch 2−1528 : Gut microbiota abundance (genus Parasutterella id.2892) on Drebrin−like protein
with a threshold of 1e−05
rs62273907
rs2090816
rs6809952
rs7303158
rs11715853
rs78383039
rs7311004
rs2387977
rs8039785
rs6828768
rs823424
rs7572229
rs10899911
rs35055552
rs55877868
All
−0.3
−0.2
−0.1
0.0
0.1
MR effect size for
'Gut microbiota abundance (genus Parasutterella id.2892)' on 'Drebrin−like protein'

Batch 2−1529 : Gut microbiota abundance (genus Catenibacterium id.2153) on DDB1− and CUL4−associated factor 5
with a threshold of 1e−05

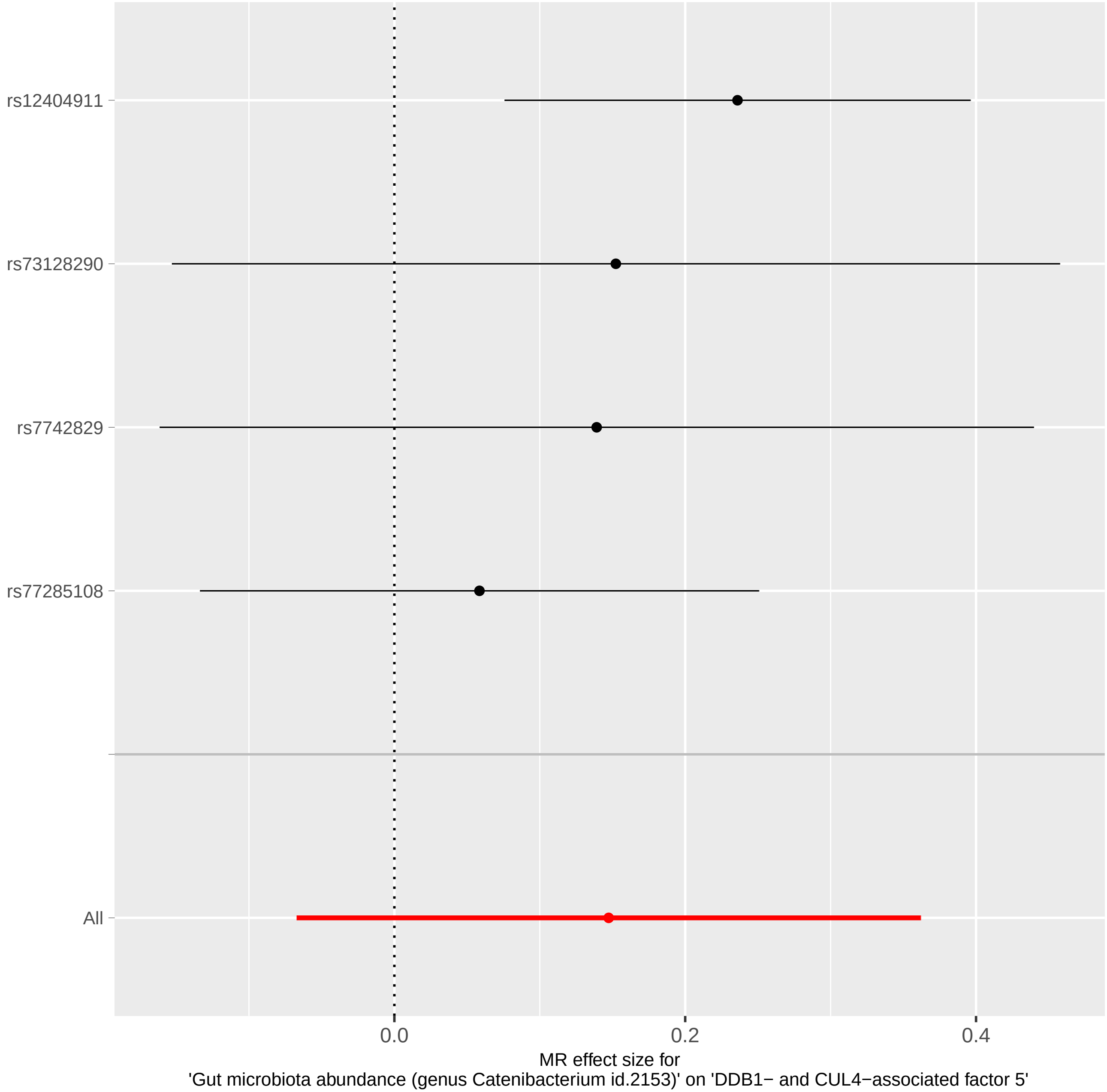

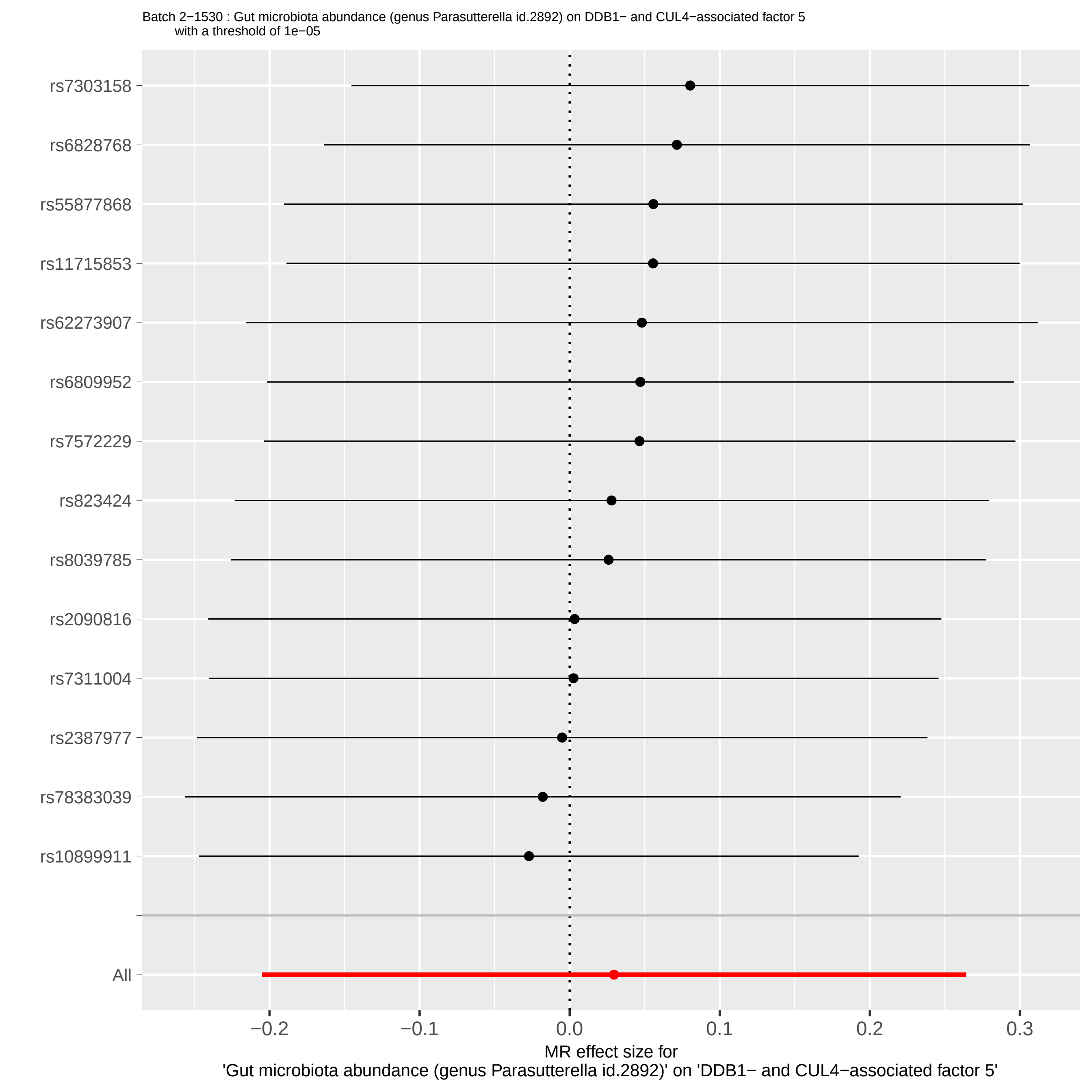

Batch 2−1530 : Gut microbiota abundance (genus Parasutterella id.2892) on DDB1− and CUL4−associated factor 5
with a threshold of 1e−05
rs7303158
rs6828768
rs55877868
rs11715853
rs62273907
rs6809952
rs7572229
rs823424
rs8039785
rs2090816
rs7311004
rs2387977
rs78383039
rs10899911
All
−0.2
−0.1
0.0
0.1
0.2
0.3
MR effect size for
'Gut microbiota abundance (genus Parasutterella id.2892)' on 'DDB1− and CUL4−associated factor 5'

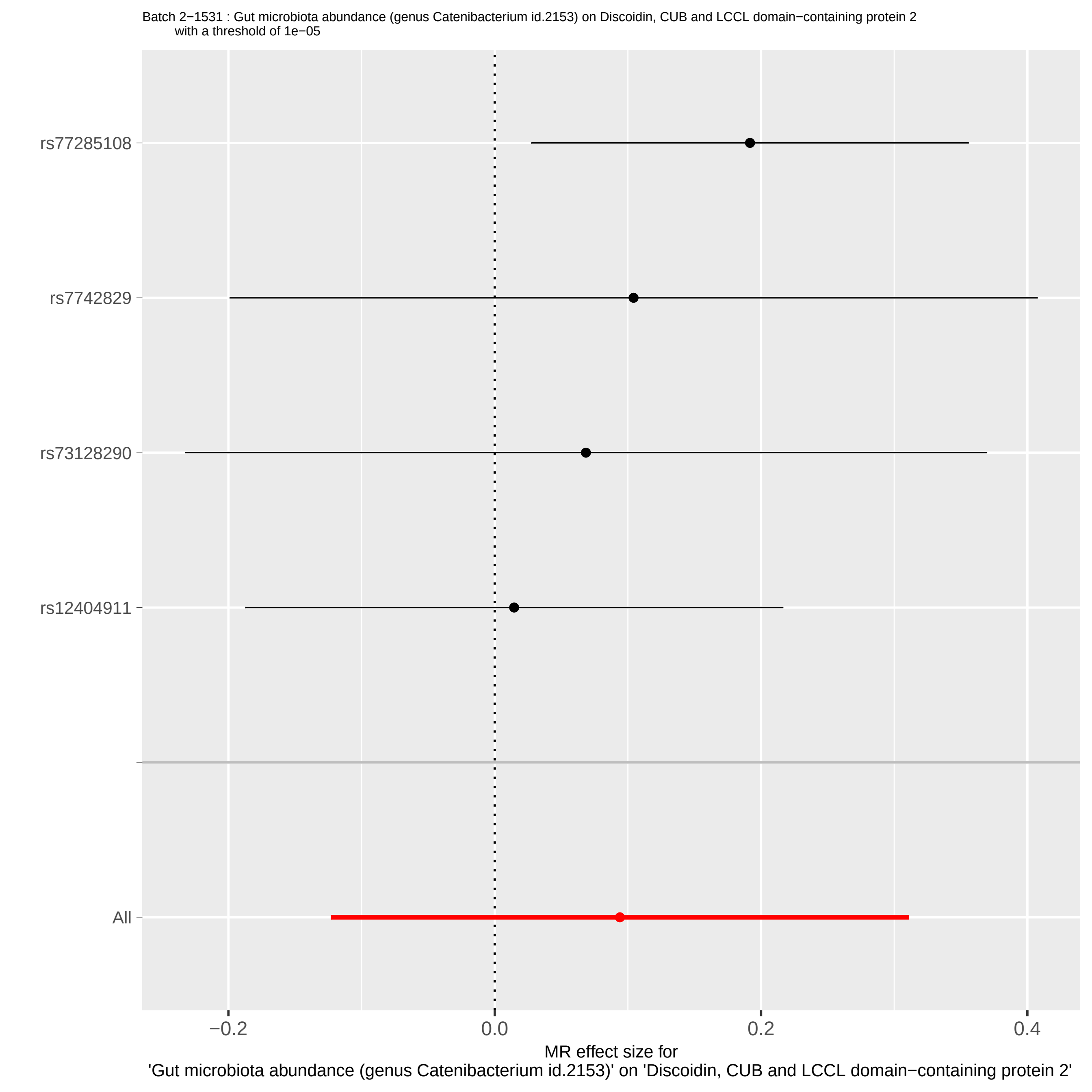
Batch 2−1531 : Gut microbiota abundance (genus Catenibacterium id.2153) on Discoidin, CUB and LCCL domain−containing protein 2
with a threshold of 1e−05
rs77285108
rs7742829
rs73128290
rs12404911
All
−0.2
0.0
0.2
0.4
MR effect size for
'Gut microbiota abundance (genus Catenibacterium id.2153)' on 'Discoidin, CUB and LCCL domain−containing protein 2'

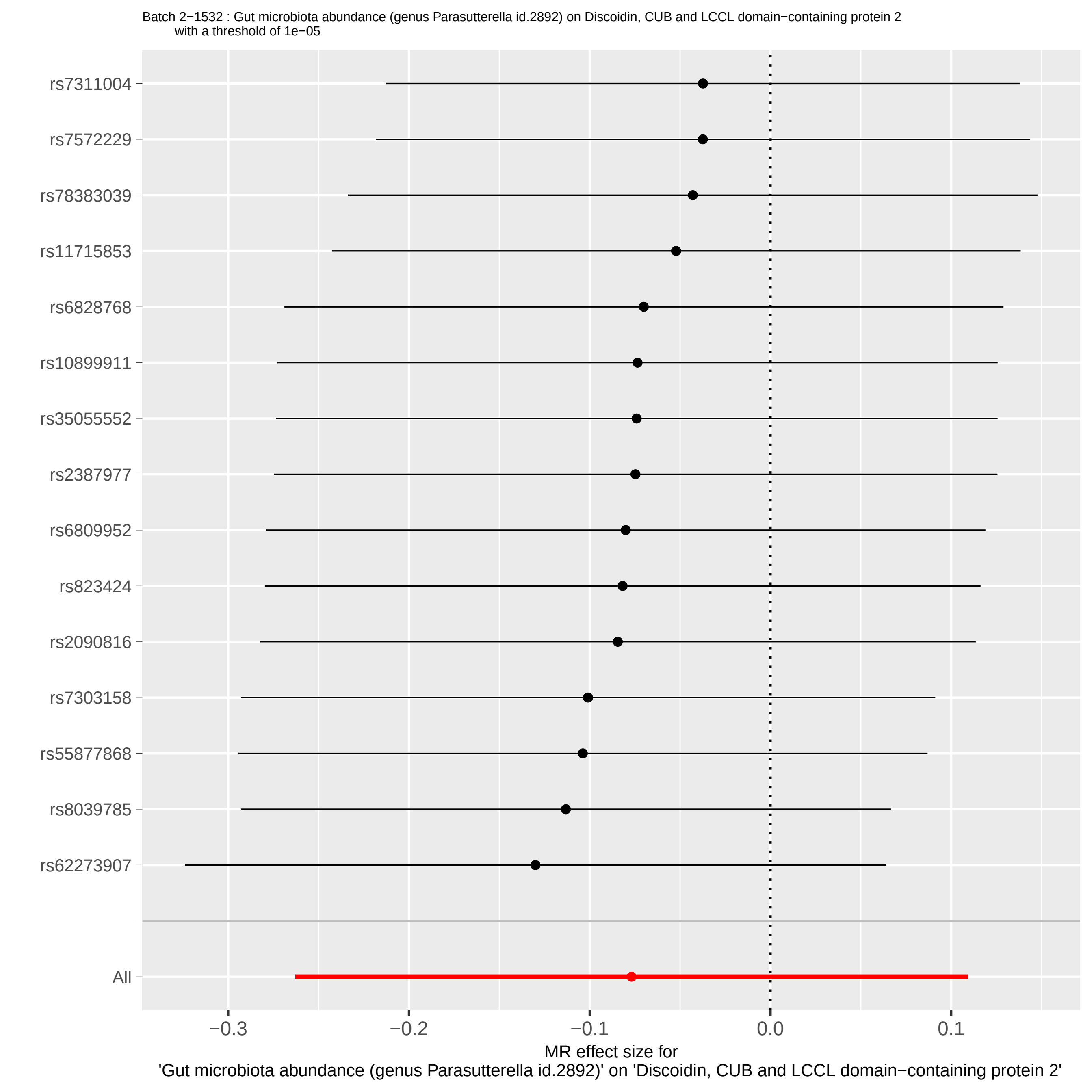
Batch 2−1532 : Gut microbiota abundance (genus Parasutterella id.2892) on Discoidin, CUB and LCCL domain−containing protein 2
with a threshold of 1e−05
rs7311004
rs7572229
rs78383039
rs11715853
rs6828768
rs10899911
rs35055552
rs2387977
rs6809952
rs823424
rs2090816
rs7303158
rs55877868
rs8039785
rs62273907
All
−0.3
−0.2
−0.1
0.0
0.1
MR effect size for
'Gut microbiota abundance (genus Parasutterella id.2892)' on 'Discoidin, CUB and LCCL domain−containing protein 2'

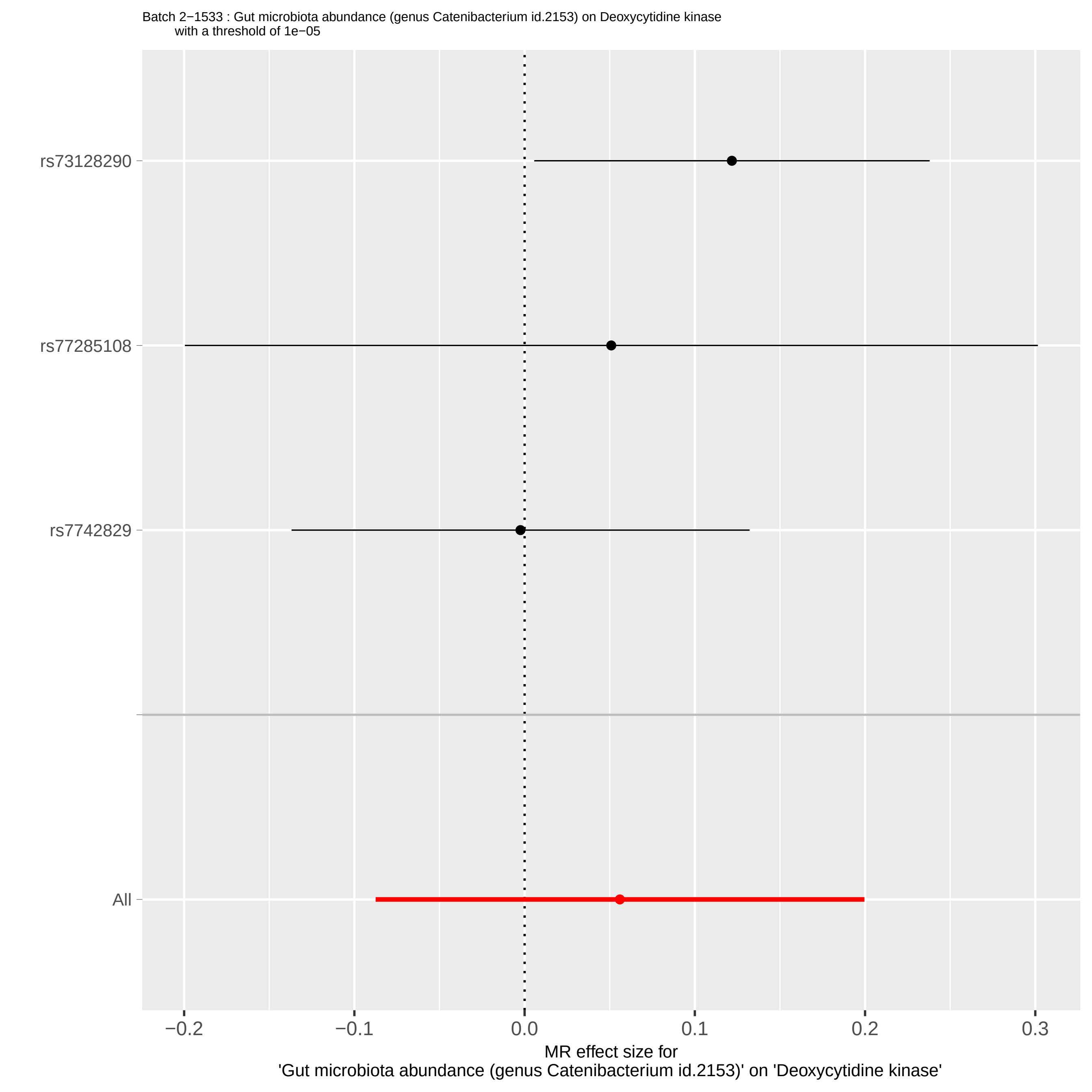
Batch 2−1533 : Gut microbiota abundance (genus Catenibacterium id.2153) on Deoxycytidine kinase
with a threshold of 1e−05
rs73128290
rs77285108
rs7742829
All
−0.2
−0.1
0.0
0.1
0.2
0.3
MR effect size for
'Gut microbiota abundance (genus Catenibacterium id.2153)' on 'Deoxycytidine kinase'

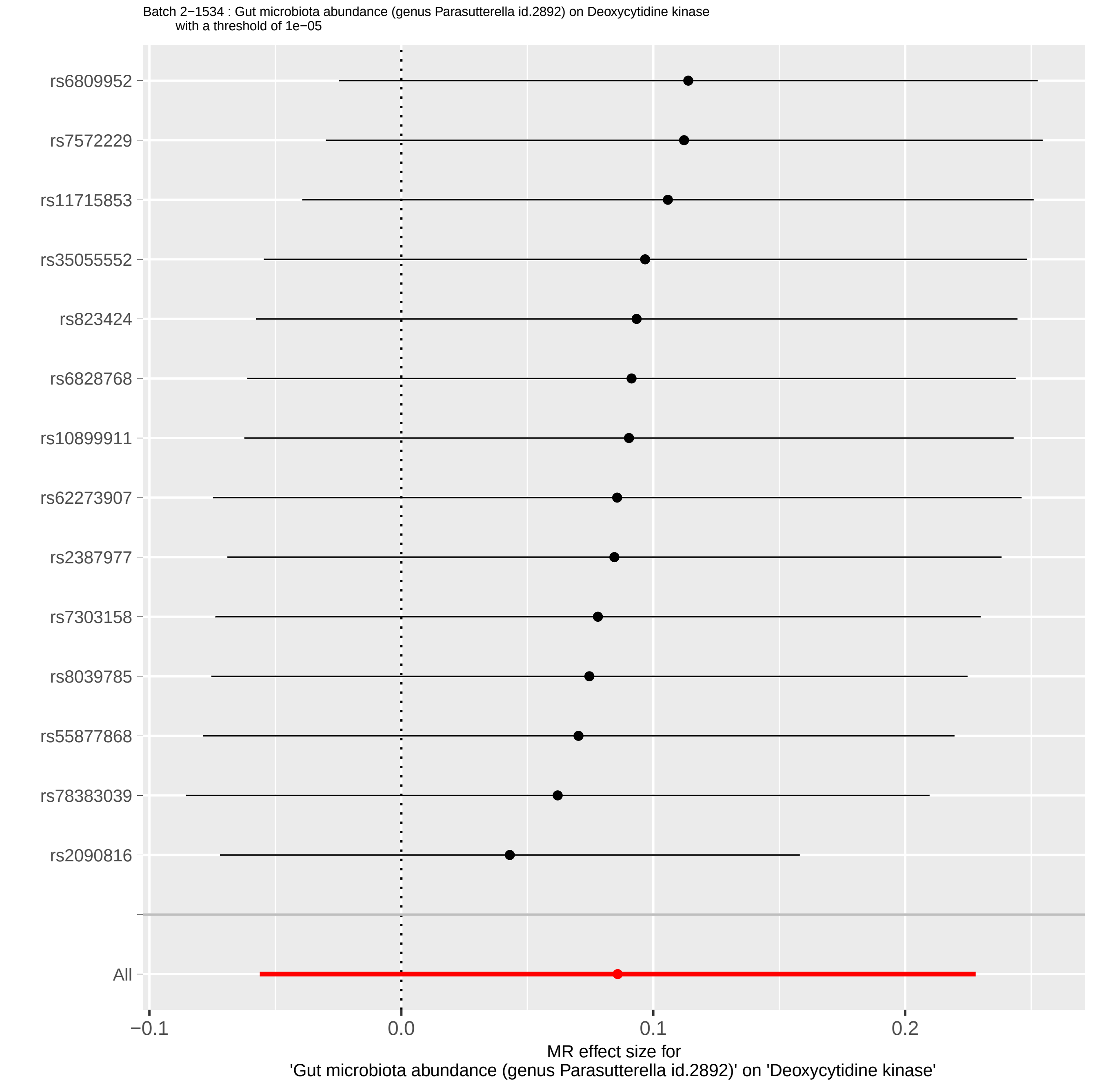

Batch 2−1534 : Gut microbiota abundance (genus Parasutterella id.2892) on Deoxycytidine kinase
with a threshold of 1e−05
rs6809952
rs7572229
rs11715853
rs35055552
rs823424
rs6828768
rs10899911
rs62273907
rs2387977
rs7303158
rs8039785
rs55877868
rs78383039
rs2090816
All
−0.1
0.0
0.1
0.2
MR effect size for
'Gut microbiota abundance (genus Parasutterella id.2892)' on 'Deoxycytidine kinase'

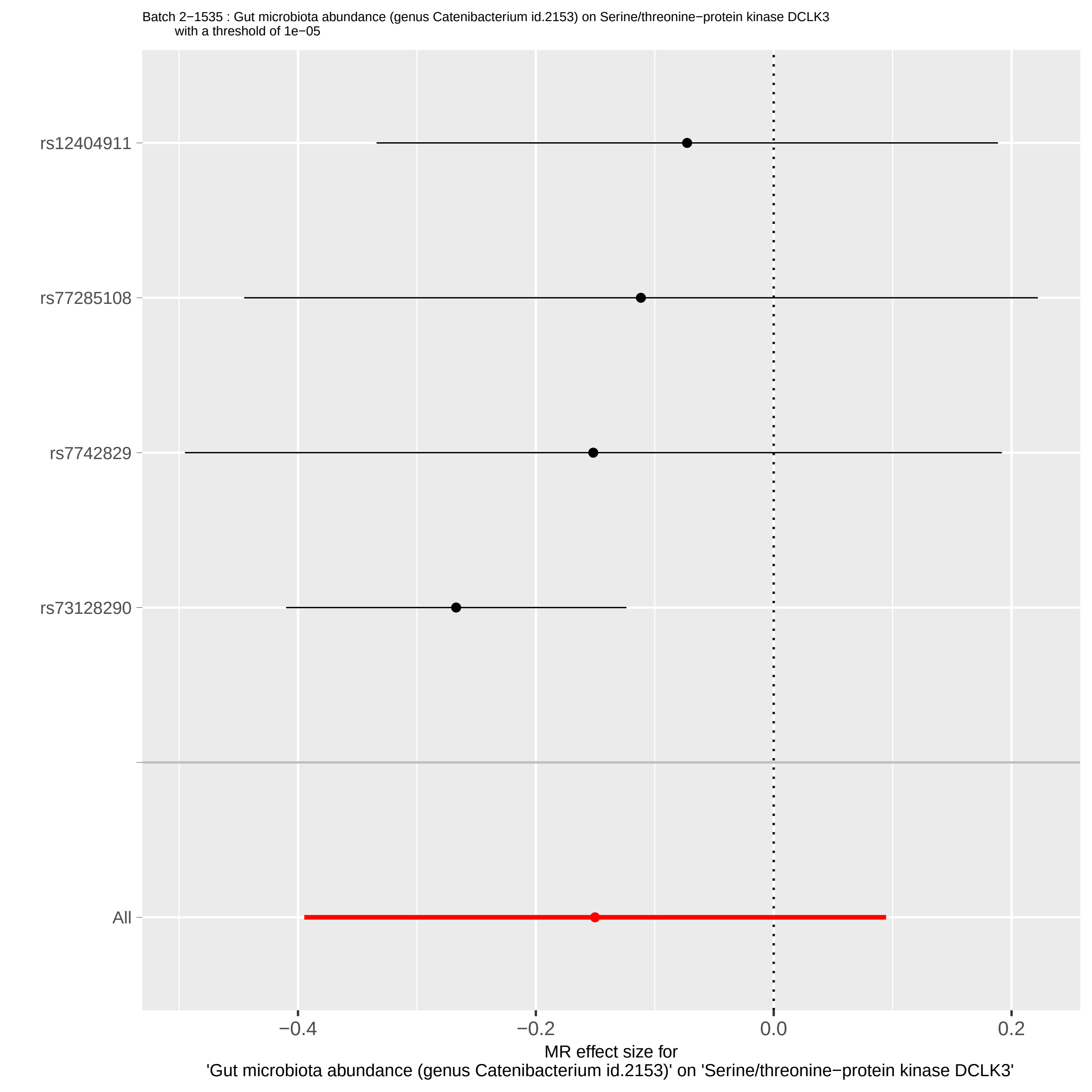

Batch 2−1535 : Gut microbiota abundance (genus Catenibacterium id.2153) on Serine/threonine−protein kinase DCLK3
with a threshold of 1e−05
rs12404911
rs77285108
rs7742829
rs73128290
All
−0.4
−0.2
0.0
0.2
MR effect size for
'Gut microbiota abundance (genus Catenibacterium id.2153)' on 'Serine/threonine−protein kinase DCLK3'

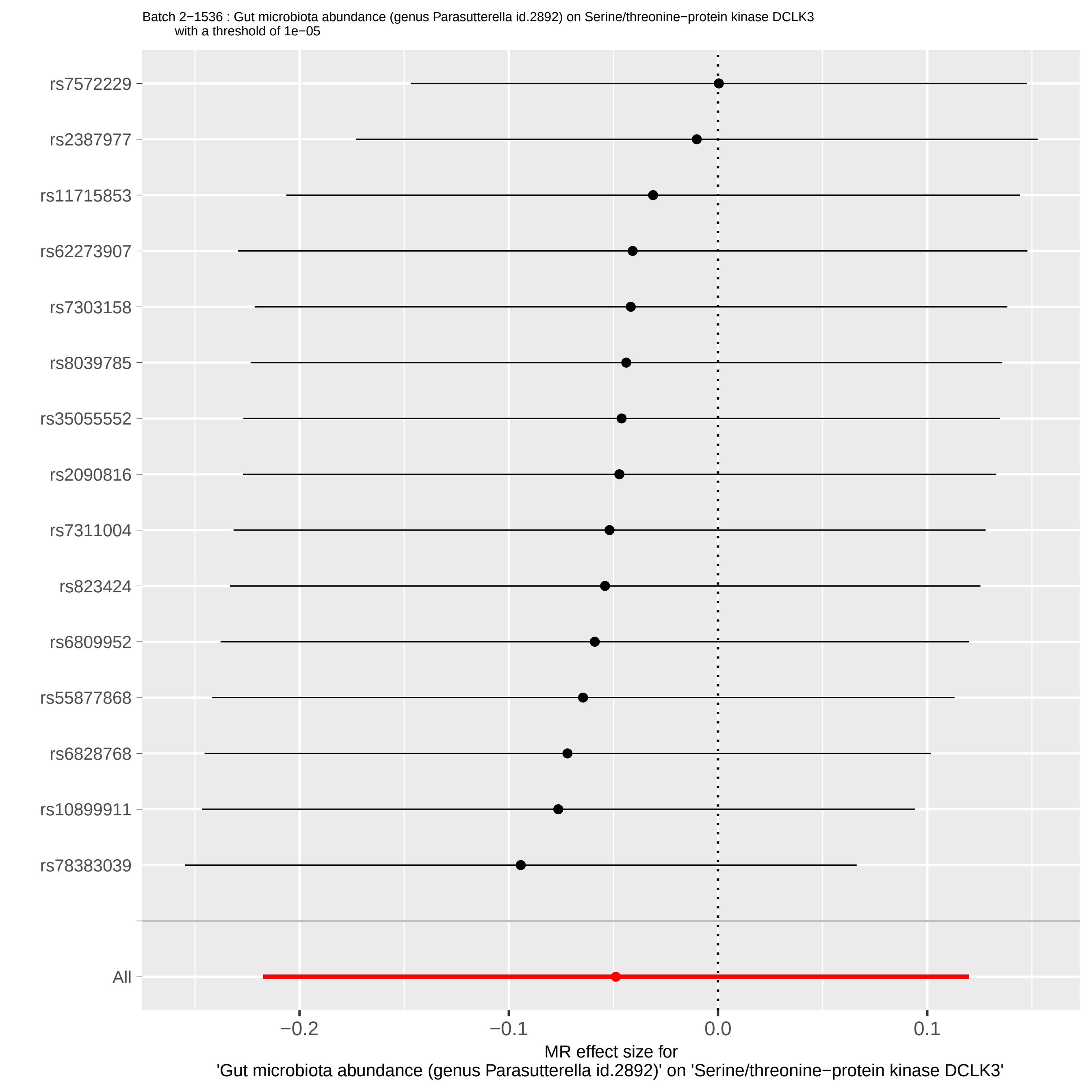
Batch 2−1536 : Gut microbiota abundance (genus Parasutterella id.2892) on Serine/threonine−protein kinase DCLK3
with a threshold of 1e−05
rs7572229
rs2387977
rs11715853
rs62273907
rs7303158
rs8039785
rs35055552
rs2090816
rs7311004
rs823424
rs6809952
rs55877868
rs6828768
rs10899911
rs78383039
All
−0.2
−0.1
0.0
0.1
MR effect size for
'Gut microbiota abundance (genus Parasutterella id.2892)' on 'Serine/threonine−protein kinase DCLK3'

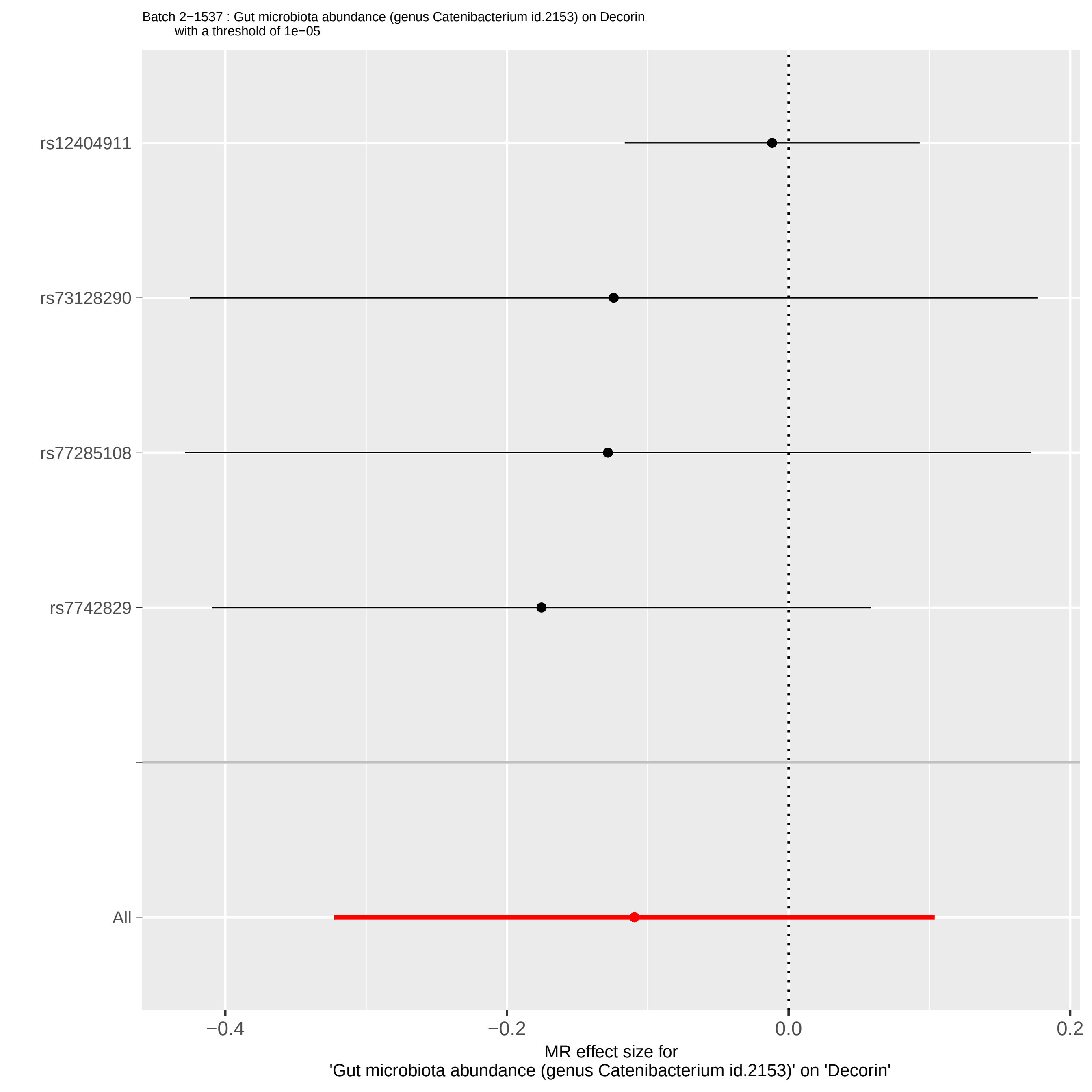

Batch 2−1537 : Gut microbiota abundance (genus Catenibacterium id.2153) on Decorin
with a threshold of 1e−05
rs12404911
rs73128290
rs77285108
rs7742829
All
−0.4
−0.2
0.0
0.2
MR effect size for
'Gut microbiota abundance (genus Catenibacterium id.2153)' on 'Decorin'

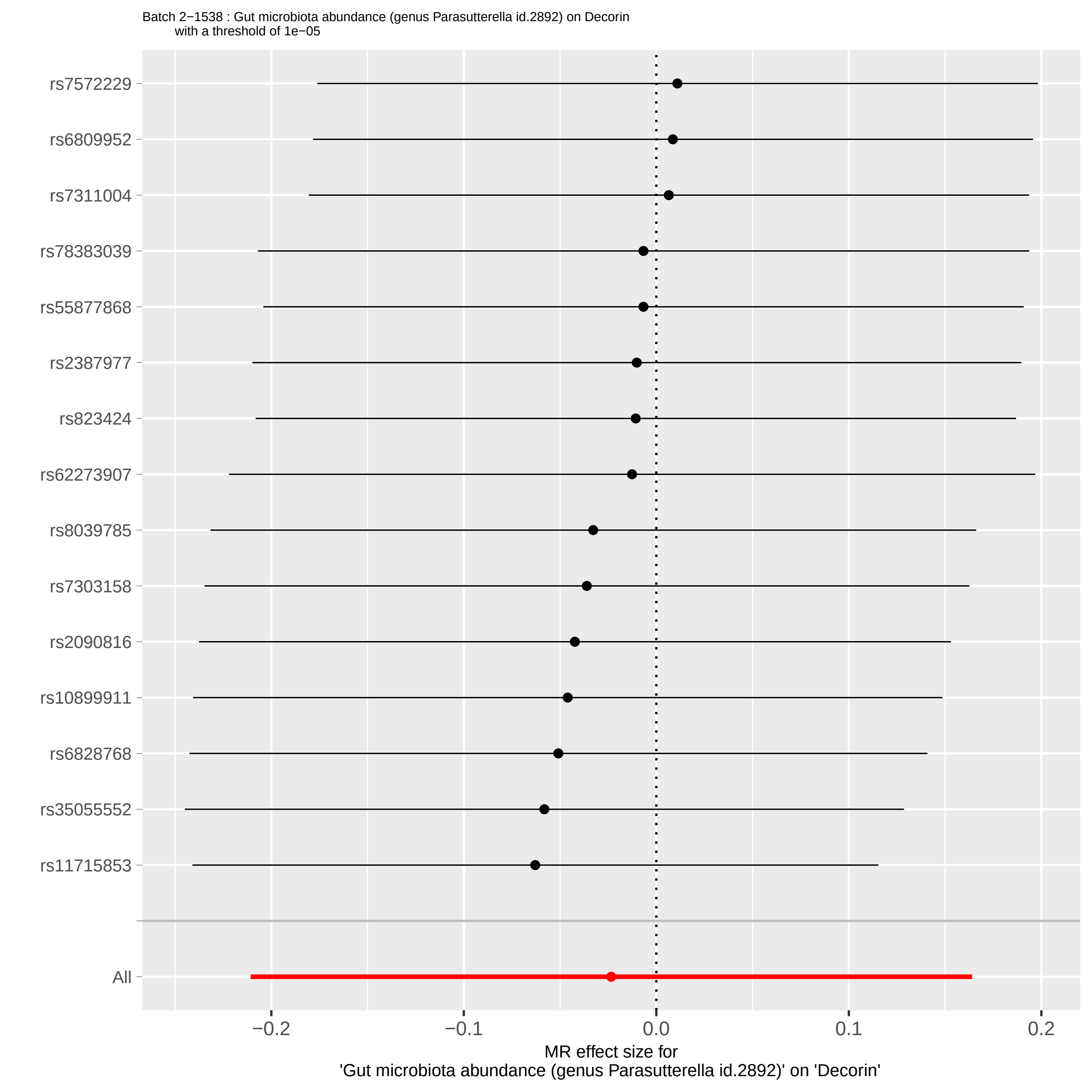

Batch 2−1538 : Gut microbiota abundance (genus Parasutterella id.2892) on Decorin
with a threshold of 1e−05
rs7572229
rs6809952
rs7311004
rs78383039
rs55877868
rs2387977
rs823424
rs62273907
rs8039785
rs7303158
rs2090816
rs10899911
rs6828768
rs35055552
rs11715853
All
−0.2
−0.1
0.0
0.1
0.2
MR effect size for
'Gut microbiota abundance (genus Parasutterella id.2892)' on 'Decorin'

Batch 2−1539 : Gut microbiota abundance (genus Catenibacterium id.2153) on mRNA−decapping enzyme 1A
with a threshold of 1e−05

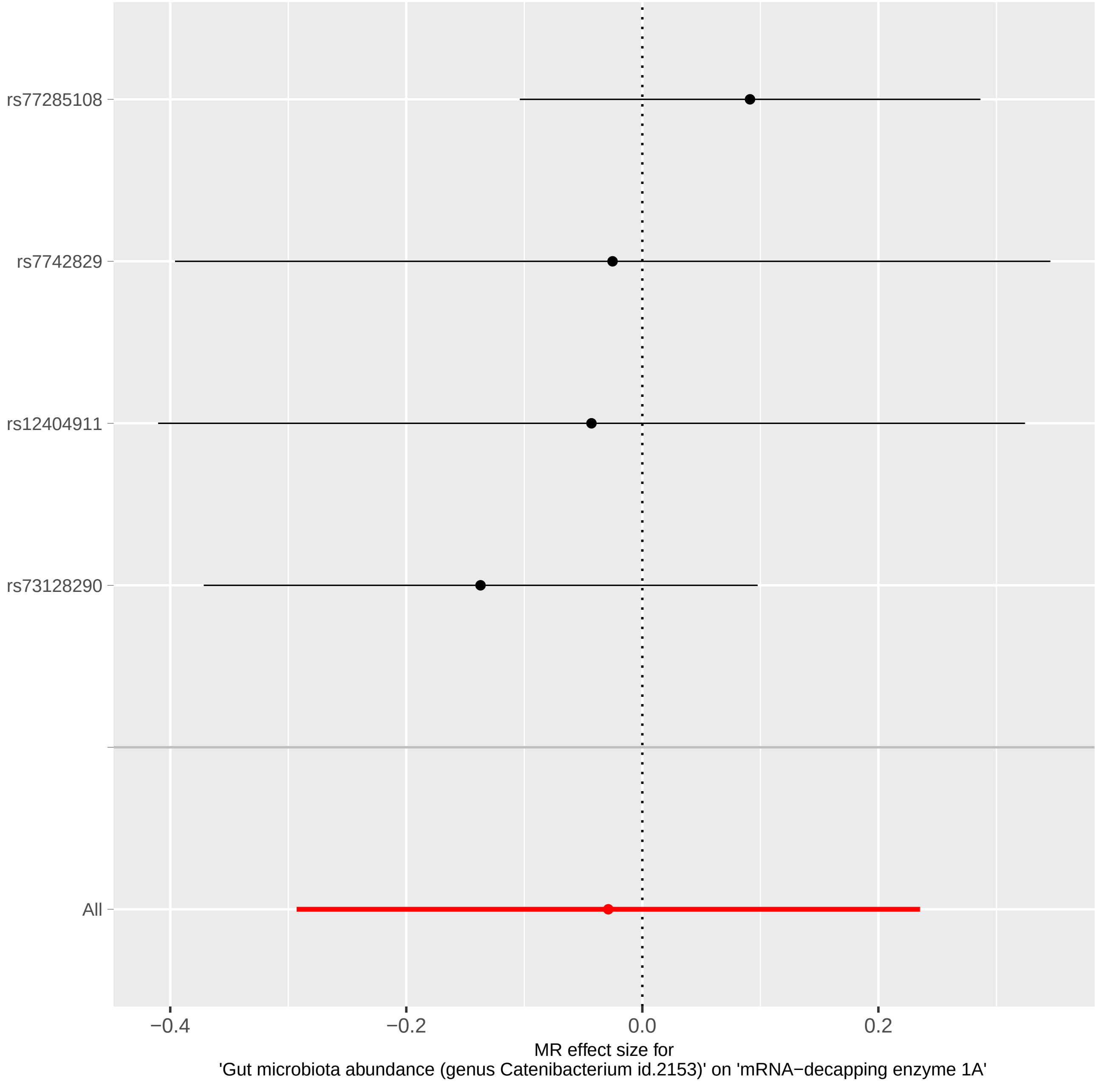

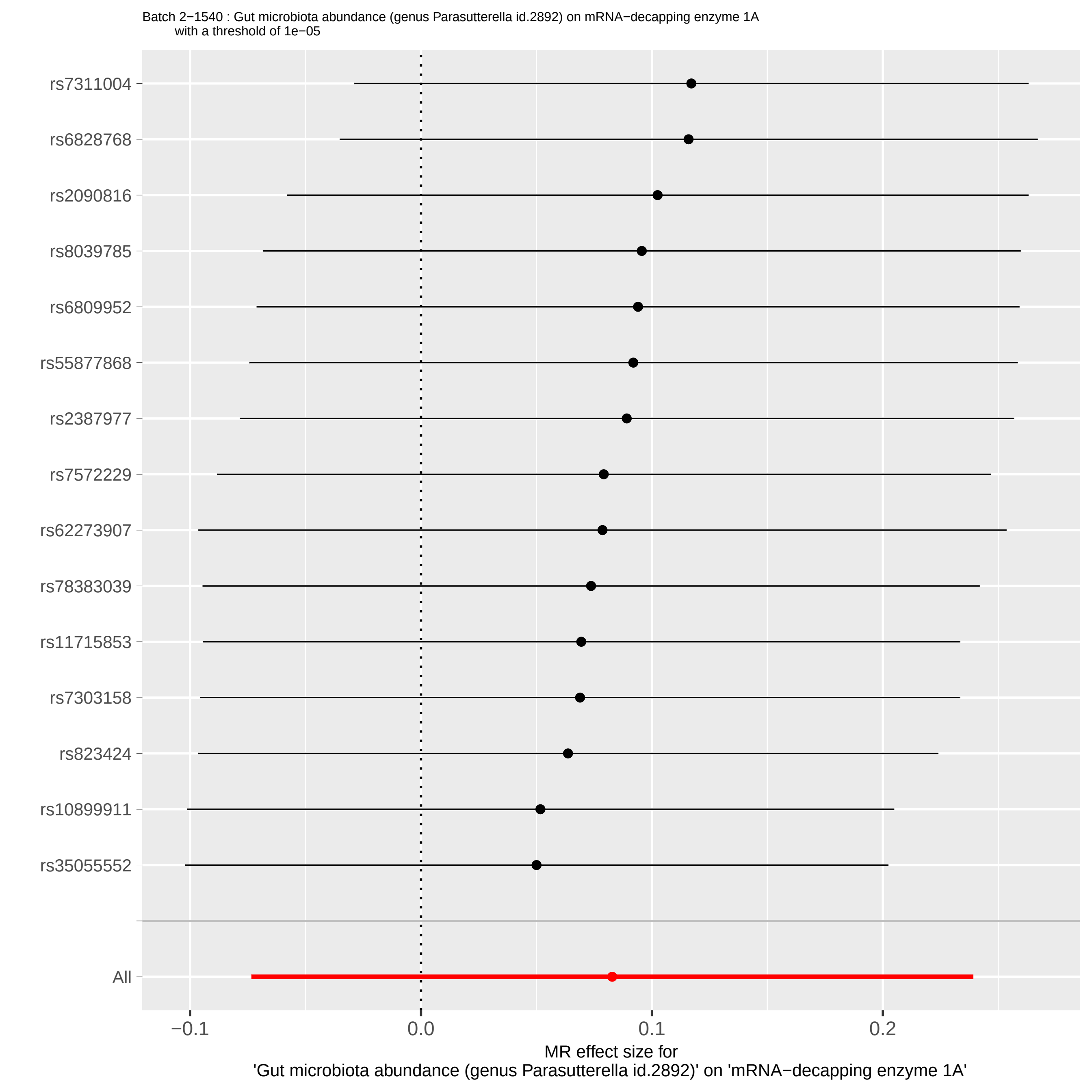
Batch 2−1540 : Gut microbiota abundance (genus Parasutterella id.2892) on mRNA−decapping enzyme 1A
with a threshold of 1e−05
rs7311004
rs6828768
rs2090816
rs8039785
rs6809952
rs55877868
rs2387977
rs7572229
rs62273907
rs78383039
rs11715853
rs7303158
rs823424
rs10899911
rs35055552
All
−0.1
0.0
0.1
0.2
MR effect size for
'Gut microbiota abundance (genus Parasutterella id.2892)' on 'mRNA−decapping enzyme 1A'

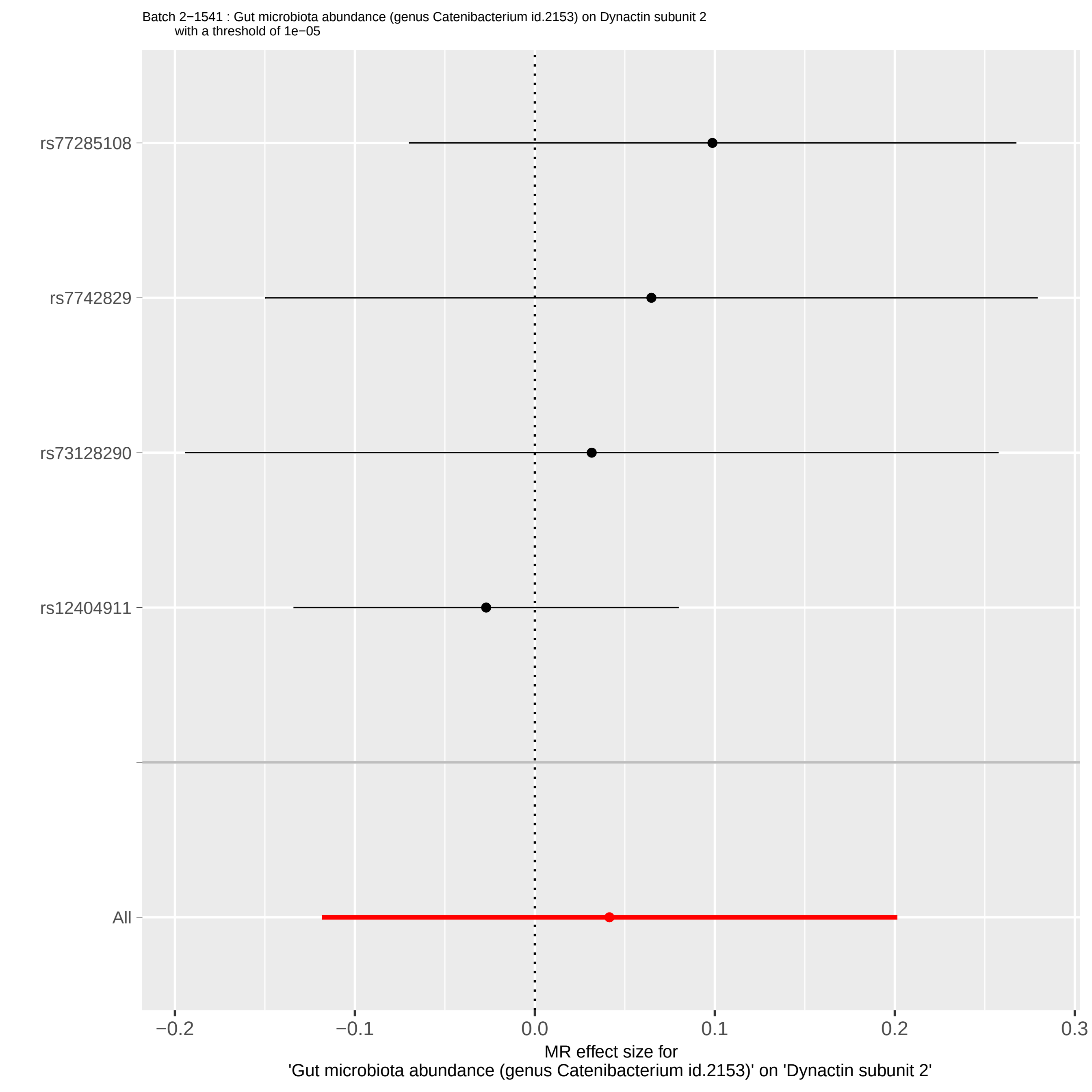
Batch 2−1541 : Gut microbiota abundance (genus Catenibacterium id.2153) on Dynactin subunit 2
with a threshold of 1e−05
rs77285108
rs7742829
rs73128290
rs12404911
All
−0.2
−0.1
0.0
0.1
0.2
0.3
MR effect size for
'Gut microbiota abundance (genus Catenibacterium id.2153)' on 'Dynactin subunit 2'

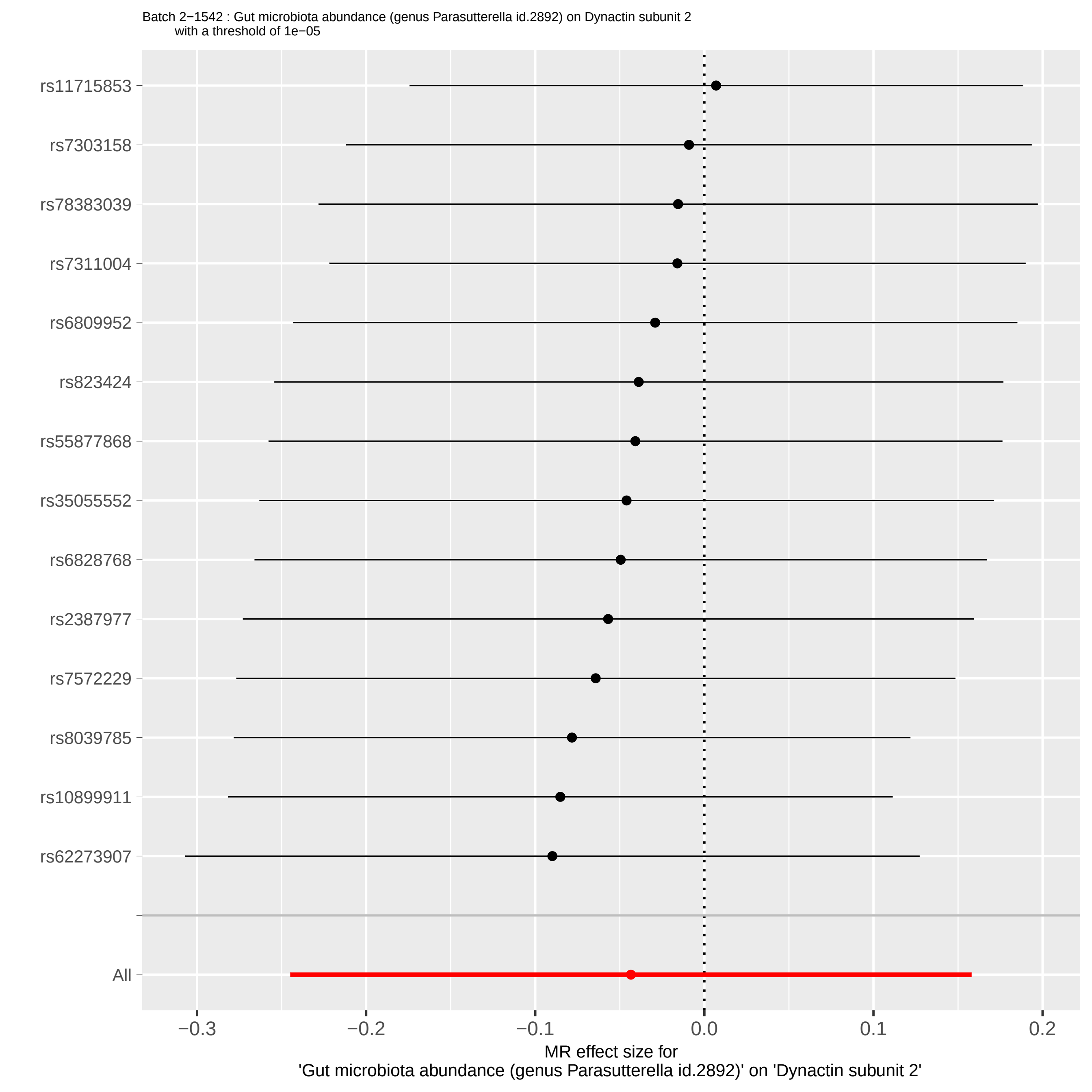

Batch 2−1542 : Gut microbiota abundance (genus Parasutterella id.2892) on Dynactin subunit 2
with a threshold of 1e−05
rs11715853
rs7303158
rs78383039
rs7311004
rs6809952
rs823424
rs55877868
rs35055552
rs6828768
rs2387977
rs7572229
rs8039785
rs10899911
rs62273907
All
−0.3
−0.2
−0.1
0.0
0.1
0.2
MR effect size for
'Gut microbiota abundance (genus Parasutterella id.2892)' on 'Dynactin subunit 2'

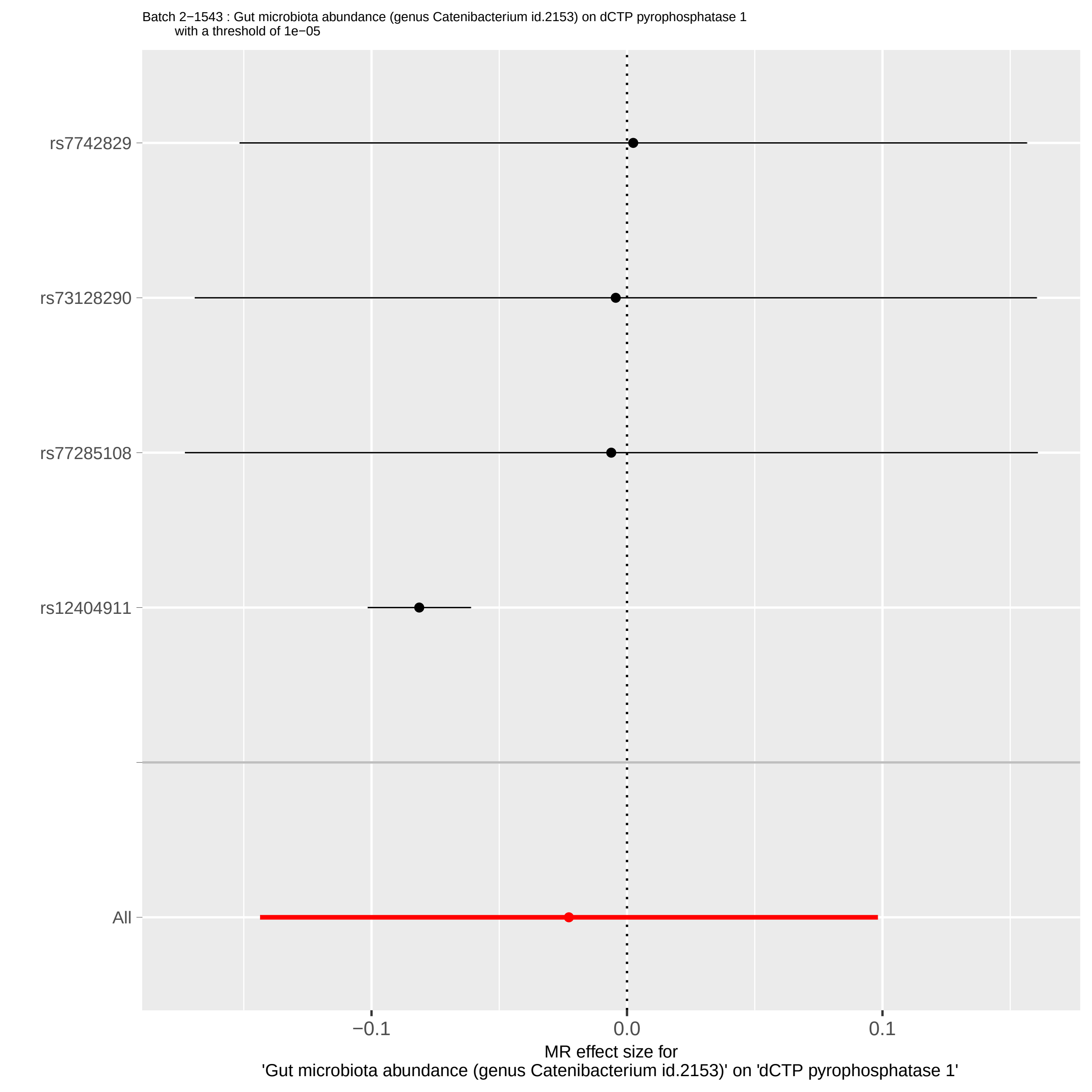
Batch 2−1543 : Gut microbiota abundance (genus Catenibacterium id.2153) on dCTP pyrophosphatase 1
with a threshold of 1e−05
rs7742829
rs73128290
rs77285108
rs12404911
All
−0.1
0.0
0.1
MR effect size for
'Gut microbiota abundance (genus Catenibacterium id.2153)' on 'dCTP pyrophosphatase 1'

Batch 2−1544 : Gut microbiota abundance (genus Parasutterella id.2892) on dCTP pyrophosphatase 1
with a threshold of 1e−05

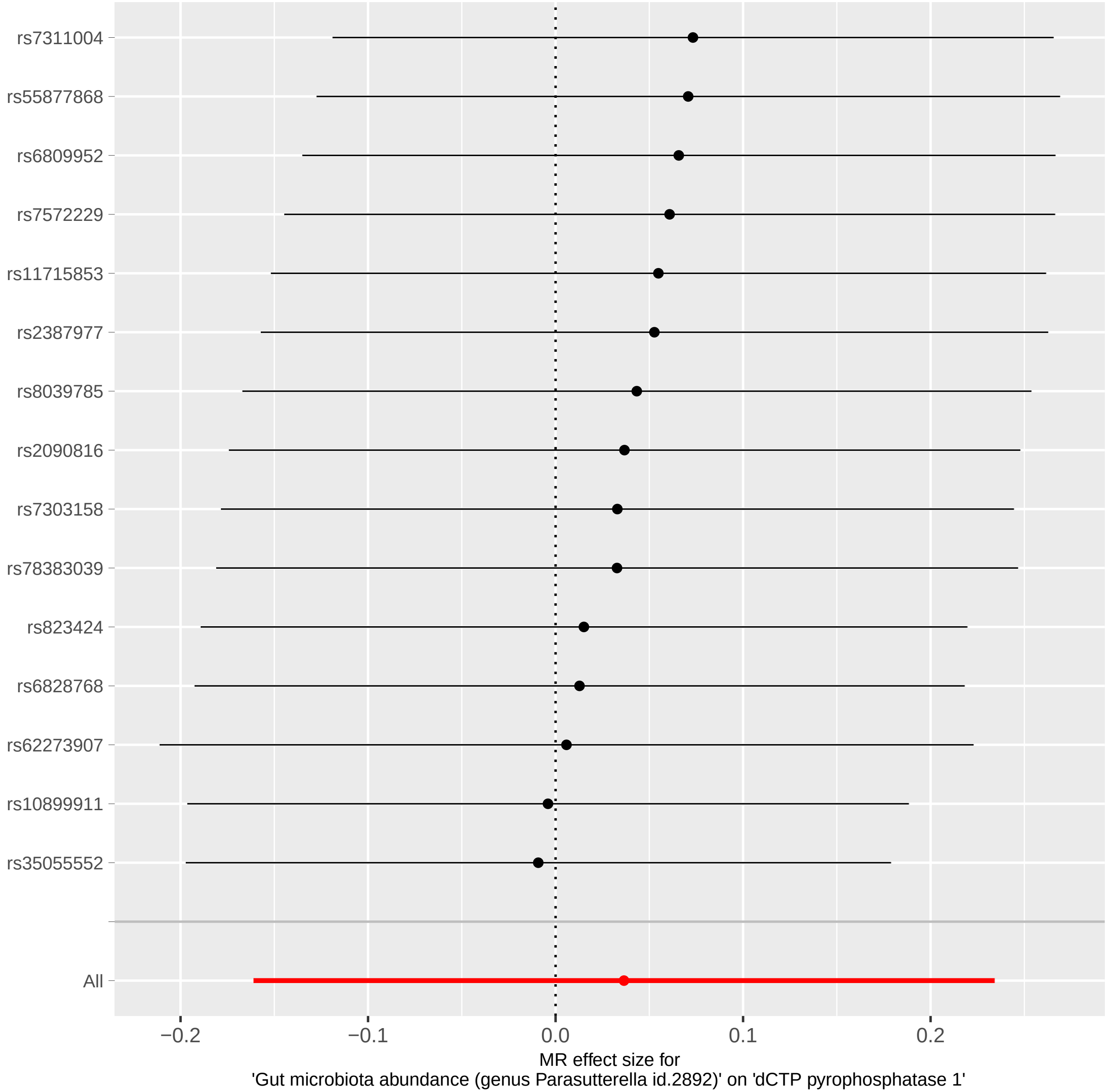

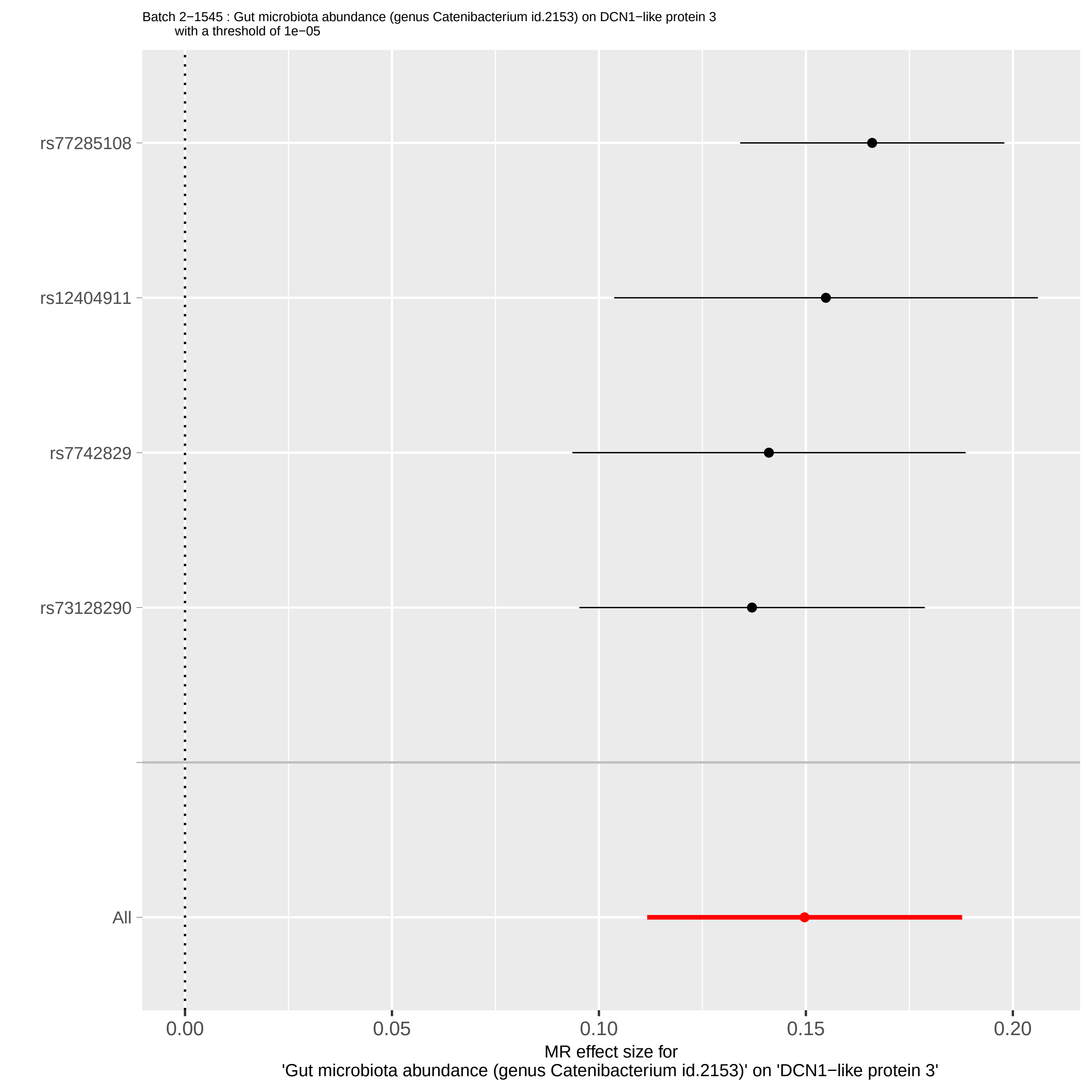
Batch 2−1545 : Gut microbiota abundance (genus Catenibacterium id.2153) on DCN1−like protein 3
with a threshold of 1e−05
rs77285108
rs12404911
rs7742829
rs73128290
All
0.00
0.05
0.10
0.15
0.20
MR effect size for
'Gut microbiota abundance (genus Catenibacterium id.2153)' on 'DCN1−like protein 3'

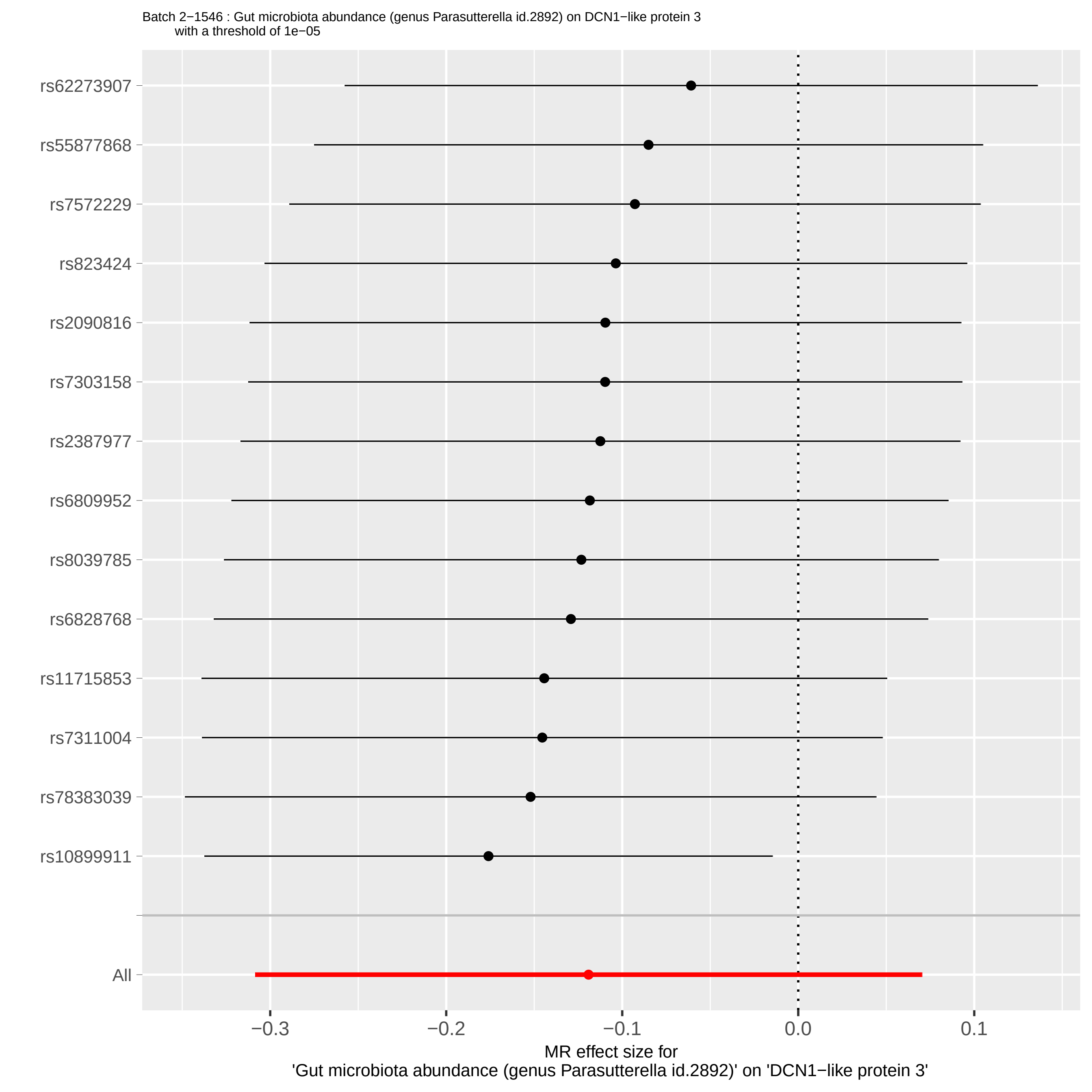

Batch 2−1546 : Gut microbiota abundance (genus Parasutterella id.2892) on DCN1−like protein 3
with a threshold of 1e−05
rs62273907
rs55877868
rs7572229
rs823424
rs2090816
rs7303158
rs2387977
rs6809952
rs8039785
rs6828768
rs11715853
rs7311004
rs78383039
rs10899911
All
−0.3
−0.2
−0.1
0.0
0.1
MR effect size for
'Gut microbiota abundance (genus Parasutterella id.2892)' on 'DCN1−like protein 3'

Batch 2−1547 : Gut microbiota abundance (genus Catenibacterium id.2153) on DCN1−like protein 5
with a threshold of 1e−05

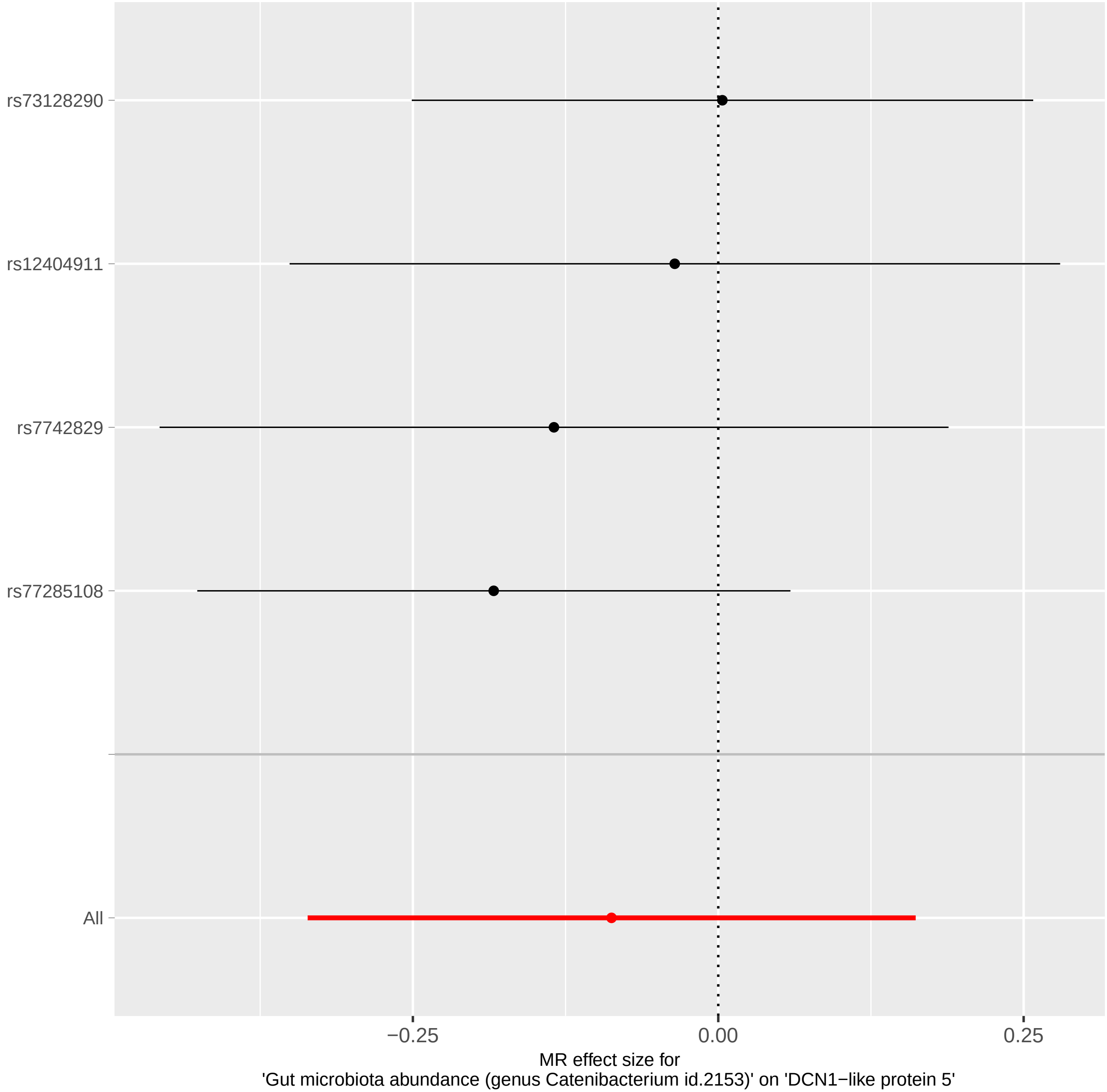

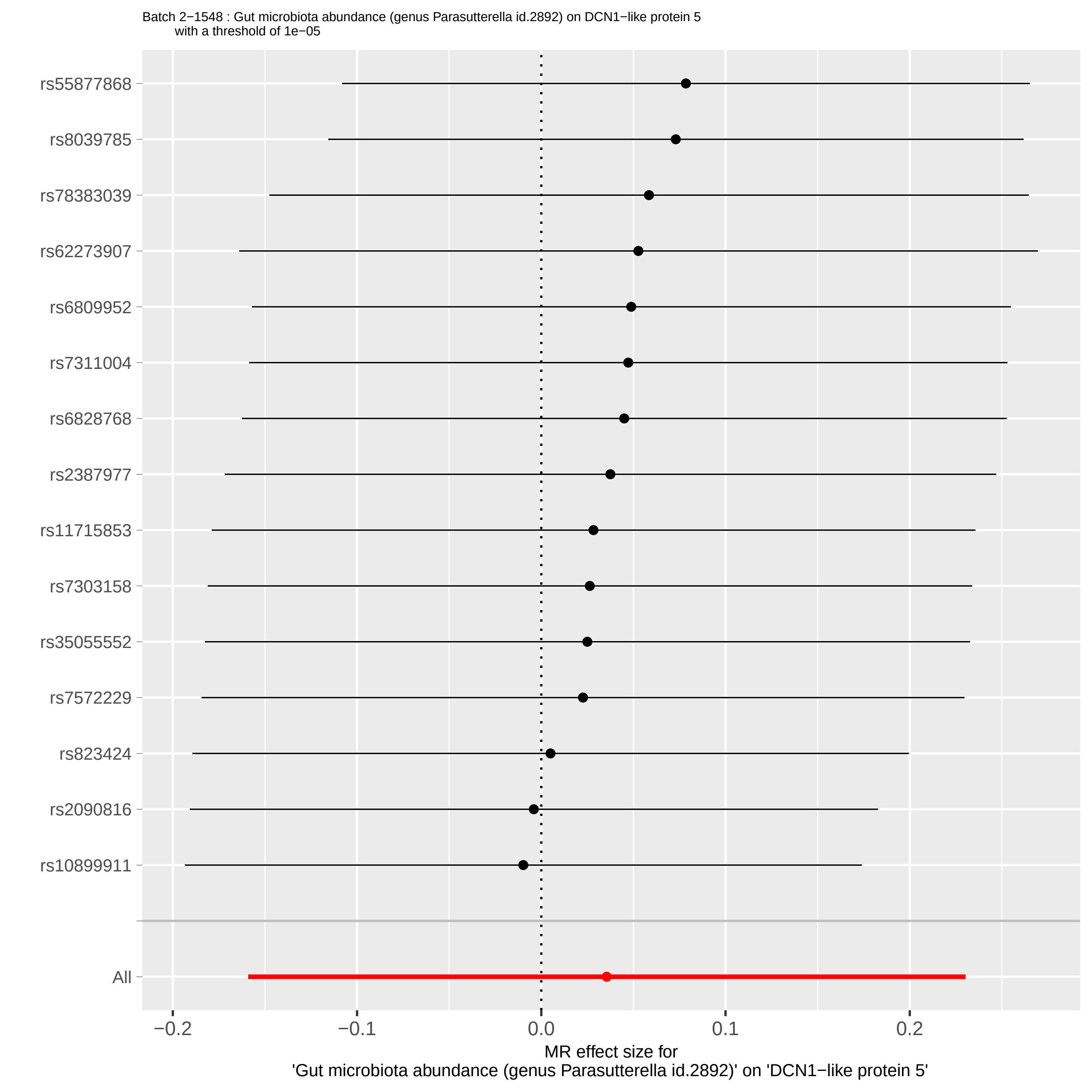
Batch 2−1548 : Gut microbiota abundance (genus Parasutterella id.2892) on DCN1−like protein 5
with a threshold of 1e−05
rs55877868
rs8039785
rs78383039
rs62273907
rs6809952
rs7311004
rs6828768
rs2387977
rs11715853
rs7303158
rs35055552
rs7572229
rs823424
rs2090816
rs10899911
All
−0.2
−0.1
0.0
0.1
0.2
MR effect size for
'Gut microbiota abundance (genus Parasutterella id.2892)' on 'DCN1−like protein 5'

Batch 2−1549 : Gut microbiota abundance (genus Catenibacterium id.2153) on Aromatic−L−amino−acid decarboxylase
with a threshold of 1e−05

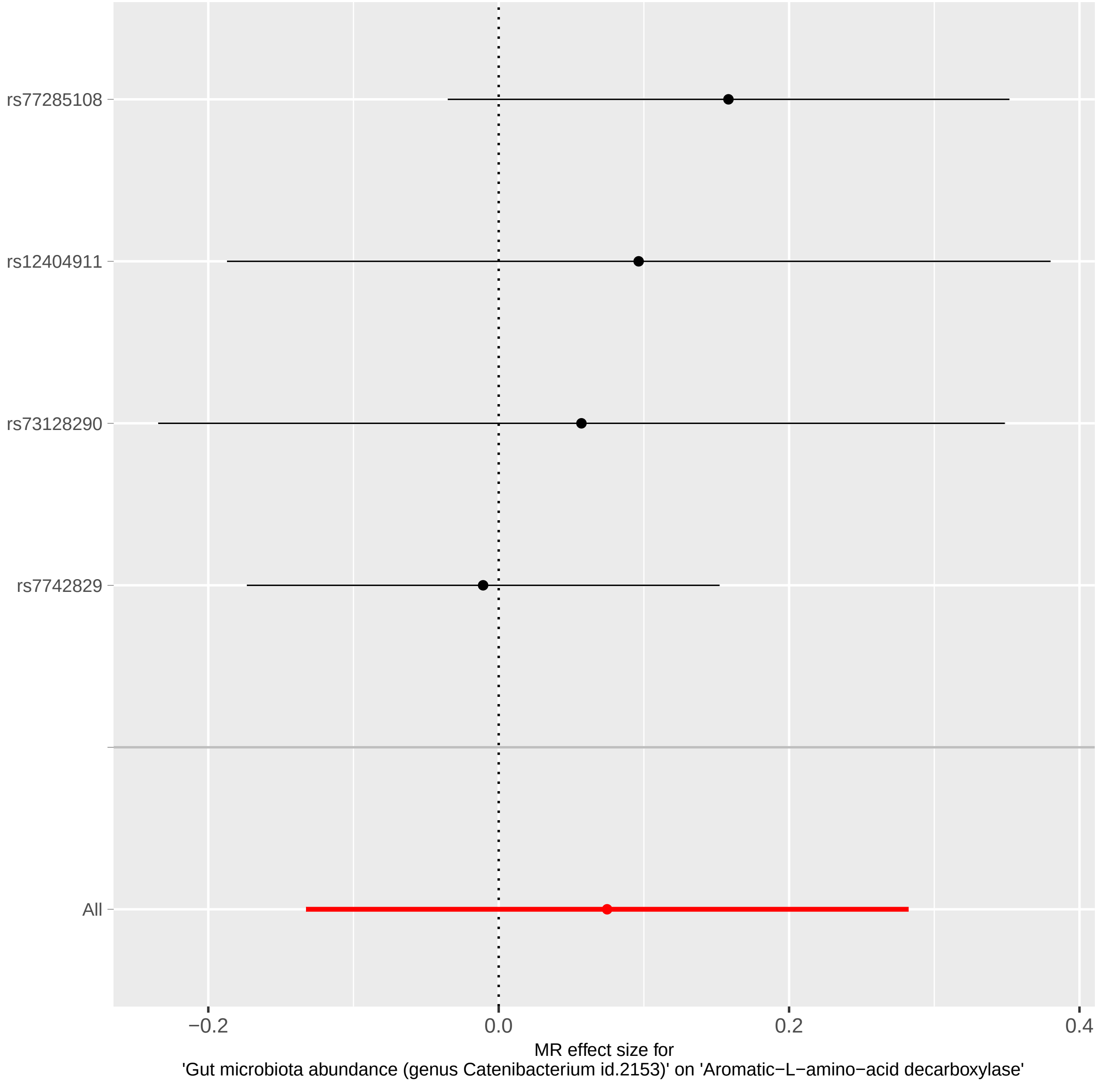

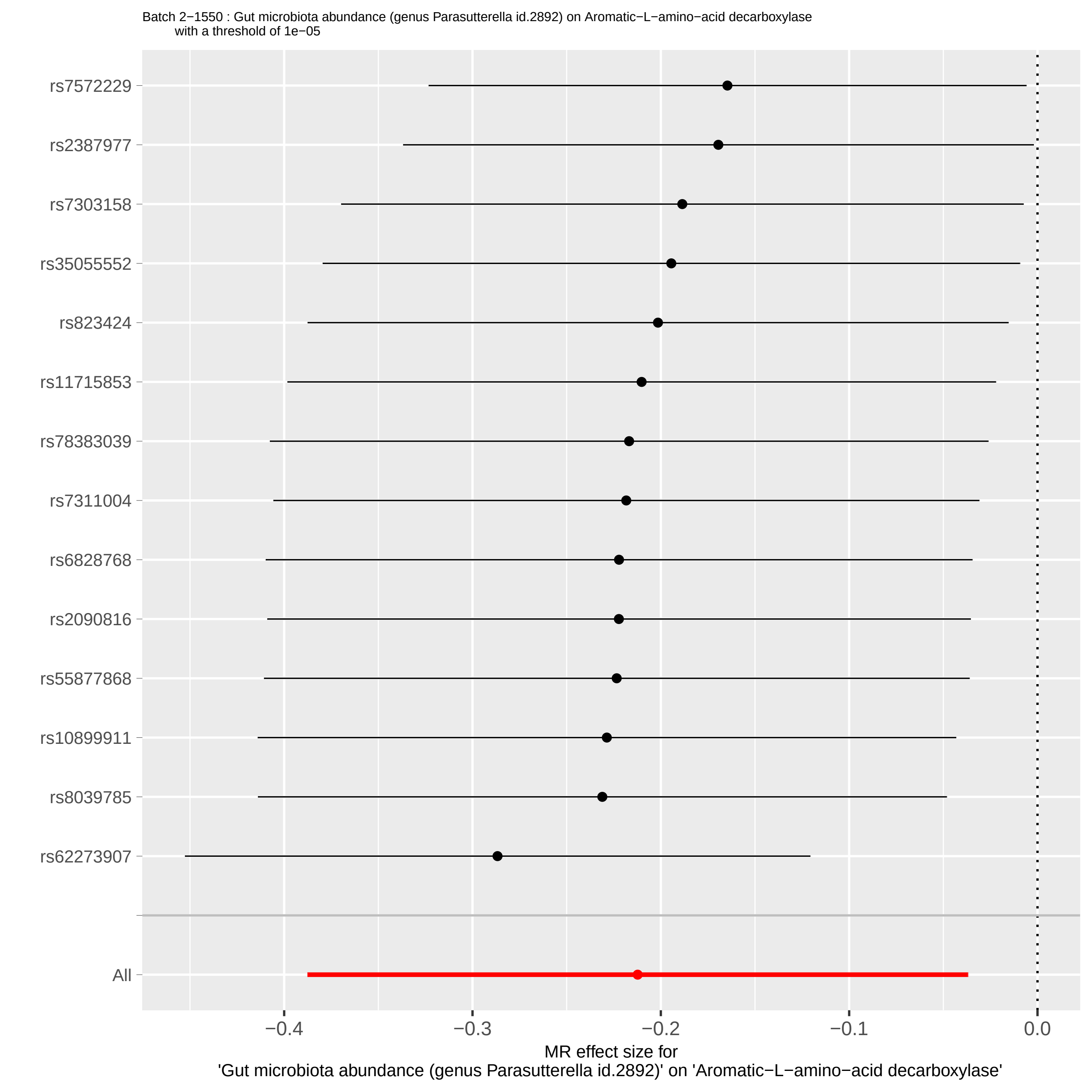
Batch 2−1550 : Gut microbiota abundance (genus Parasutterella id.2892) on Aromatic−L−amino−acid decarboxylase
with a threshold of 1e−05
rs7572229
rs2387977
rs7303158
rs35055552
rs823424
rs11715853
rs78383039
rs7311004
rs6828768
rs2090816
rs55877868
rs10899911
rs8039785
rs62273907
All
−0.4
−0.3
−0.2
−0.1
0.0
MR effect size for
'Gut microbiota abundance (genus Parasutterella id.2892)' on 'Aromatic−L−amino−acid decarboxylase'

Batch 2−1551 : Gut microbiota abundance (genus Catenibacterium id.2153) on Discoidin domain−containing receptor 2
with a threshold of 1e−05

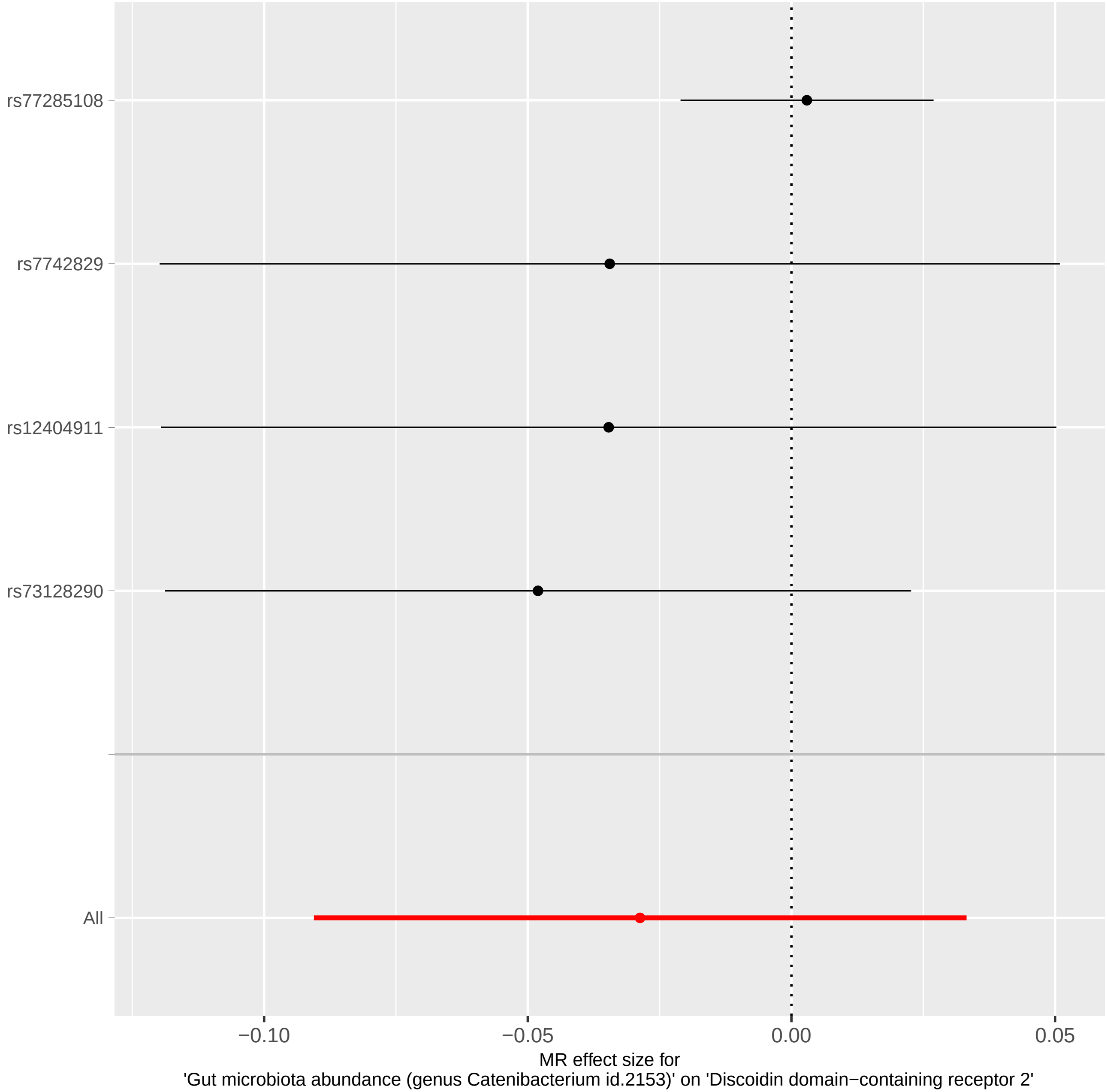

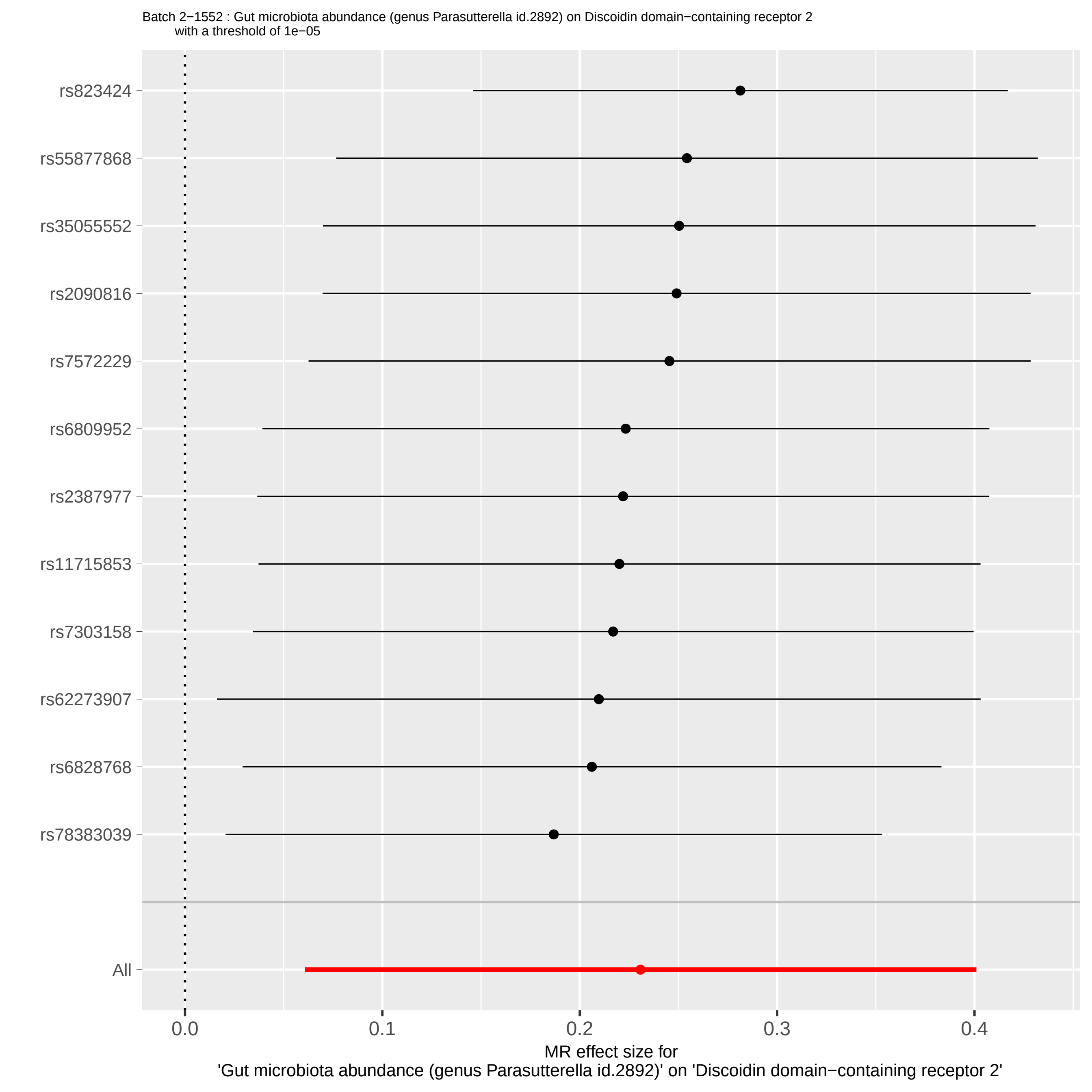
Batch 2−1552 : Gut microbiota abundance (genus Parasutterella id.2892) on Discoidin domain−containing receptor 2
with a threshold of 1e−05
rs823424
rs55877868
rs35055552
rs2090816
rs7572229
rs6809952
rs2387977
rs11715853
rs7303158
rs62273907
rs6828768
rs78383039
All
0.0
0.1
0.2
0.3
0.4
MR effect size for
'Gut microbiota abundance (genus Parasutterella id.2892)' on 'Discoidin domain−containing receptor 2'

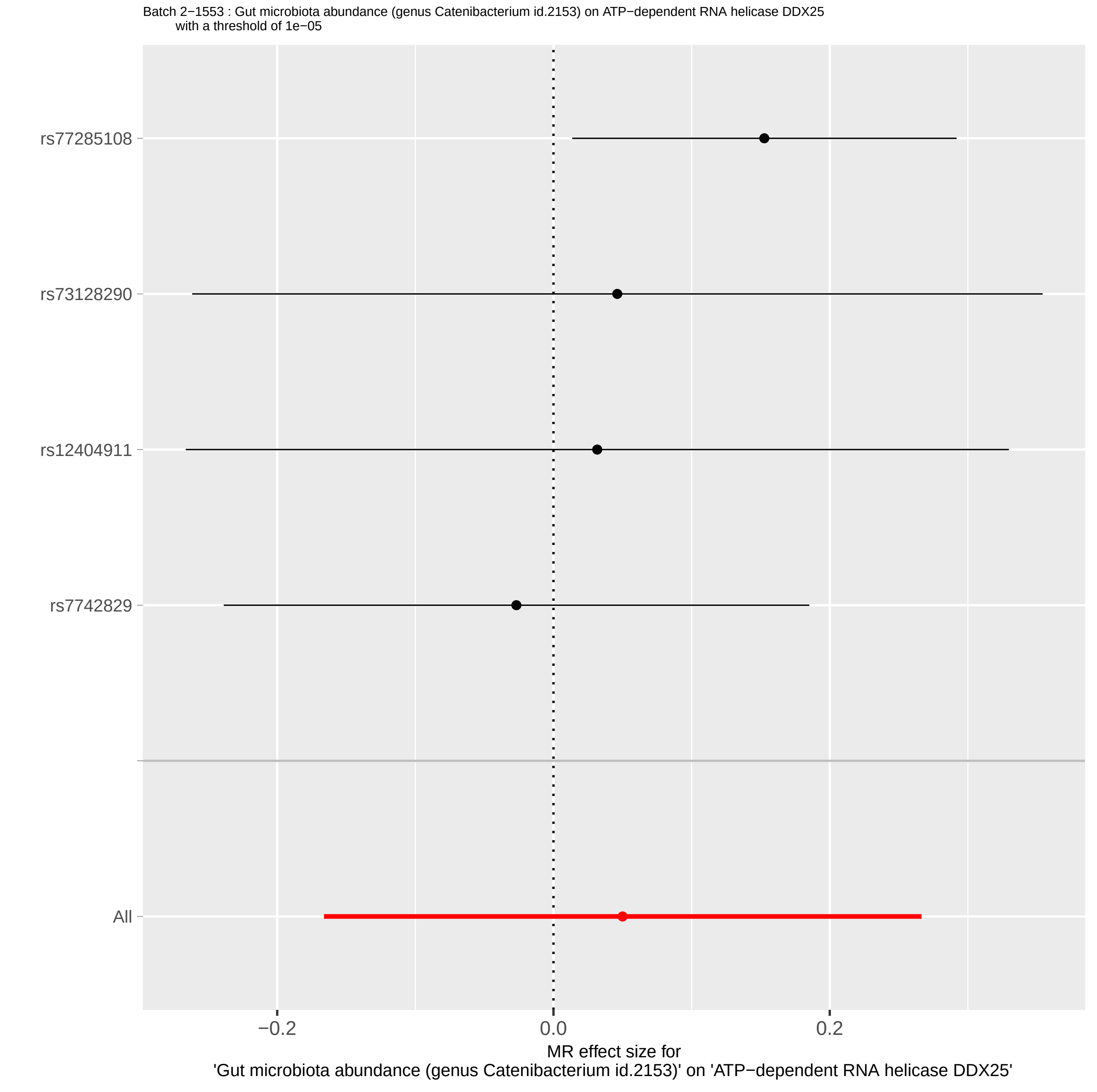

Batch 2−1553 : Gut microbiota abundance (genus Catenibacterium id.2153) on ATP−dependent RNA helicase DDX25
with a threshold of 1e−05
rs77285108
rs73128290
rs12404911
rs7742829
All
−0.2
0.0
0.2
MR effect size for
'Gut microbiota abundance (genus Catenibacterium id.2153)' on 'ATP−dependent RNA helicase DDX25'

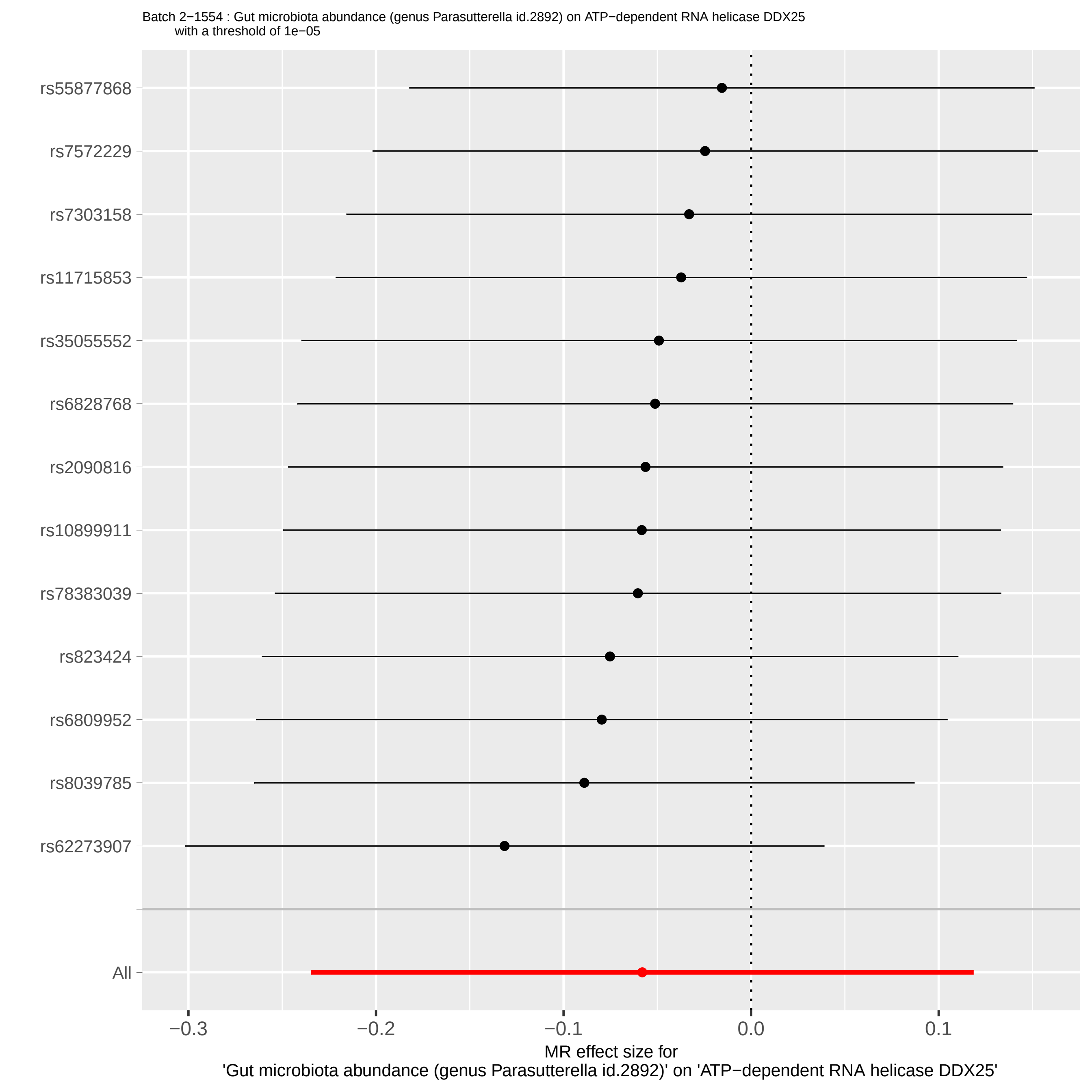

Batch 2−1554 : Gut microbiota abundance (genus Parasutterella id.2892) on ATP−dependent RNA helicase DDX25
with a threshold of 1e−05
rs55877868
rs7572229
rs7303158
rs11715853
rs35055552
rs6828768
rs2090816
rs10899911
rs78383039
rs823424
rs6809952
rs8039785
rs62273907
All
−0.3
−0.2
−0.1
0.0
0.1
MR effect size for
'Gut microbiota abundance (genus Parasutterella id.2892)' on 'ATP−dependent RNA helicase DDX25'

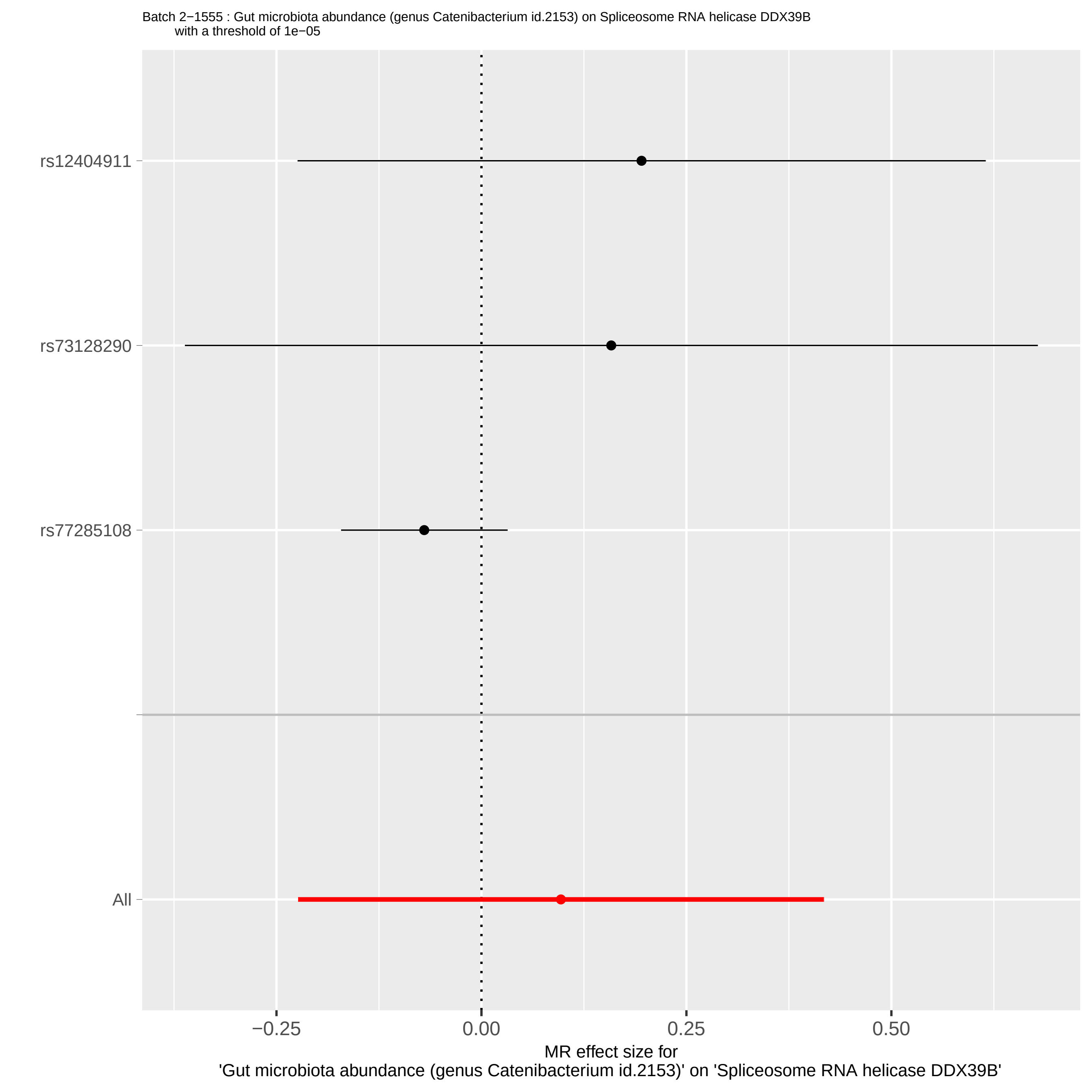
Batch 2−1555 : Gut microbiota abundance (genus Catenibacterium id.2153) on Spliceosome RNA helicase DDX39B
with a threshold of 1e−05
rs12404911
rs73128290
rs77285108
All
−0.25
0.00
0.25
0.50
MR effect size for
'Gut microbiota abundance (genus Catenibacterium id.2153)' on 'Spliceosome RNA helicase DDX39B'

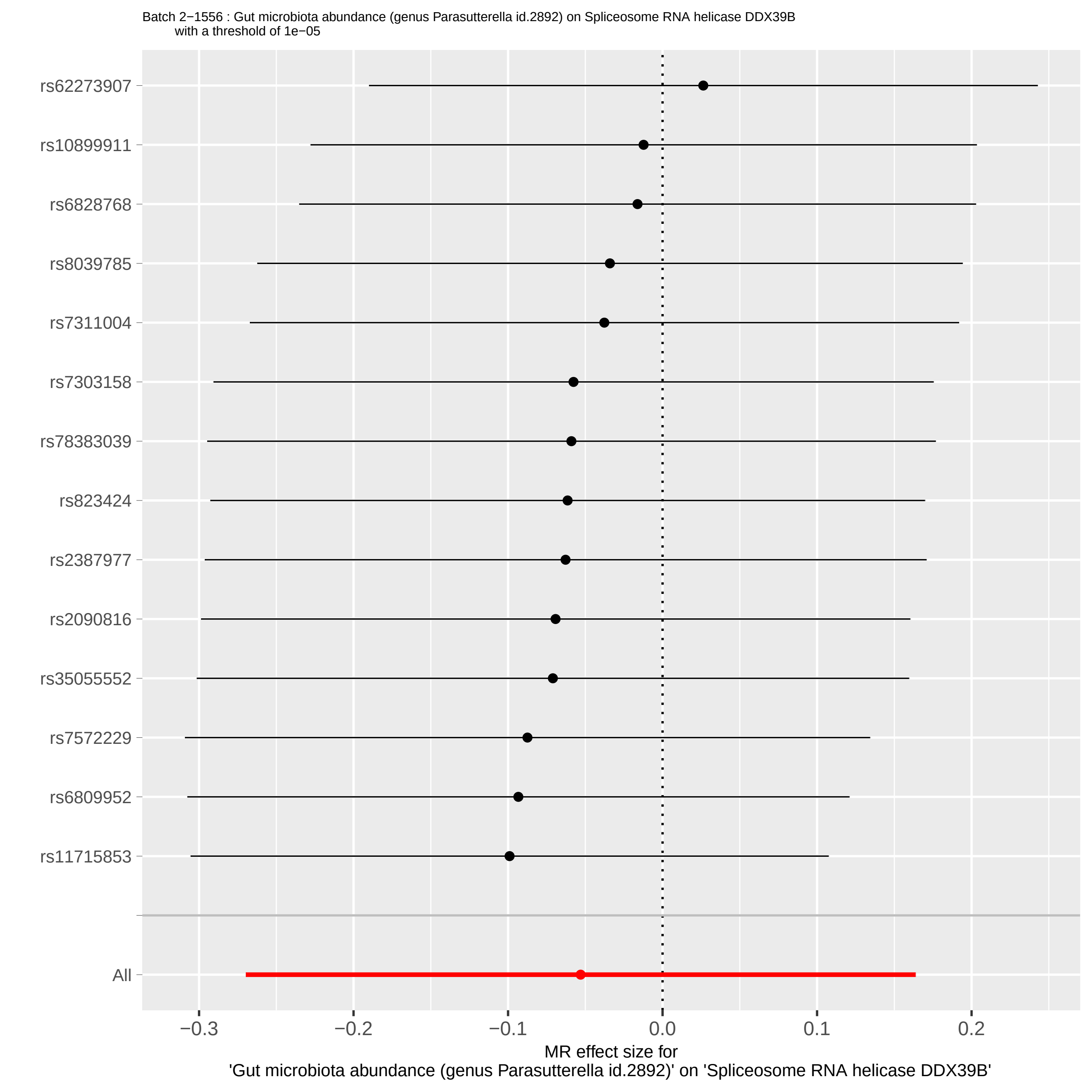

Batch 2−1556 : Gut microbiota abundance (genus Parasutterella id.2892) on Spliceosome RNA helicase DDX39B
with a threshold of 1e−05
rs62273907
rs10899911
rs6828768
rs8039785
rs7311004
rs7303158
rs78383039
rs823424
rs2387977
rs2090816
rs35055552
rs7572229
rs6809952
rs11715853
All
−0.3
−0.2
−0.1
0.0
0.1
0.2
MR effect size for
'Gut microbiota abundance (genus Parasutterella id.2892)' on 'Spliceosome RNA helicase DDX39B'

Batch 2−1557 : Gut microbiota abundance (genus Catenibacterium id.2153) on Probable ATP−dependent RNA helicase DDX58
with a threshold of 1e−05

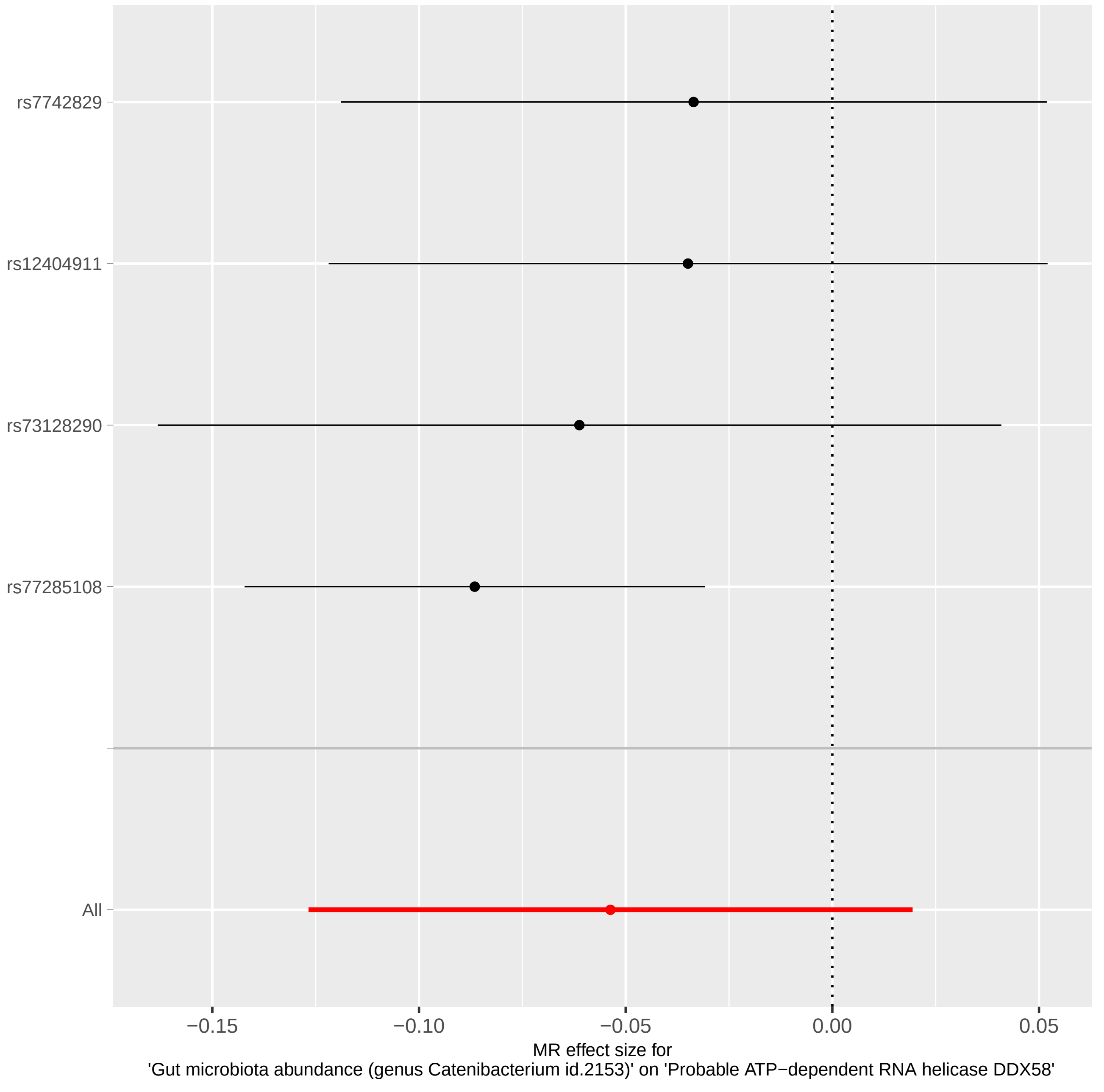

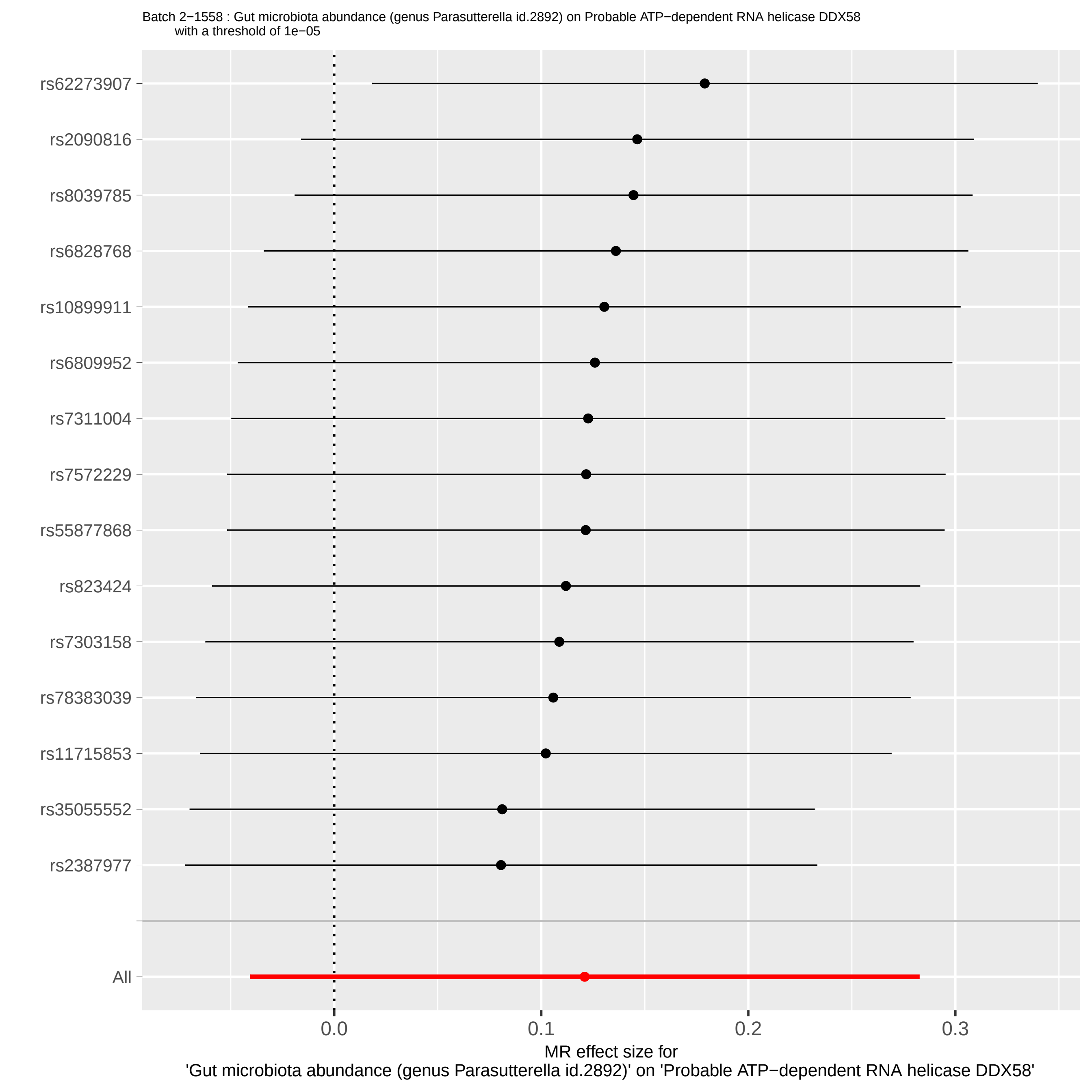
Batch 2−1558 : Gut microbiota abundance (genus Parasutterella id.2892) on Probable ATP−dependent RNA helicase DDX58
with a threshold of 1e−05
rs62273907
rs2090816
rs8039785
rs6828768
rs10899911
rs6809952
rs7311004
rs7572229
rs55877868
rs823424
rs7303158
rs78383039
rs11715853
rs35055552
rs2387977
All
0.0
0.1
0.2
0.3
MR effect size for
'Gut microbiota abundance (genus Parasutterella id.2892)' on 'Probable ATP−dependent RNA helicase DDX58'

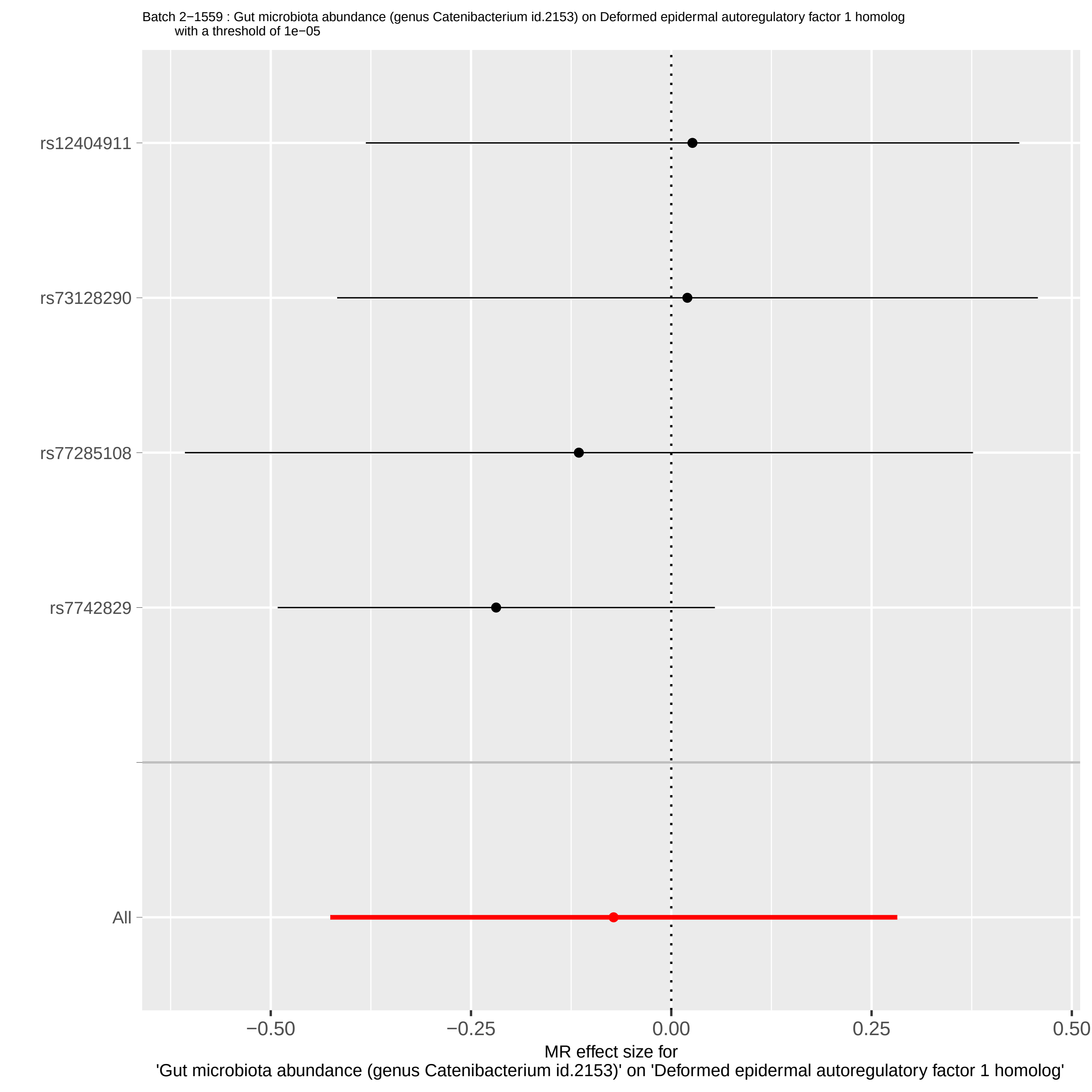

Batch 2−1559 : Gut microbiota abundance (genus Catenibacterium id.2153) on Deformed epidermal autoregulatory factor 1 homolog
with a threshold of 1e−05
rs12404911
rs73128290
rs77285108
rs7742829
All
−0.50
−0.25
0.00
0.25
0.50
MR effect size for
'Gut microbiota abundance (genus Catenibacterium id.2153)' on 'Deformed epidermal autoregulatory factor 1 homolog'

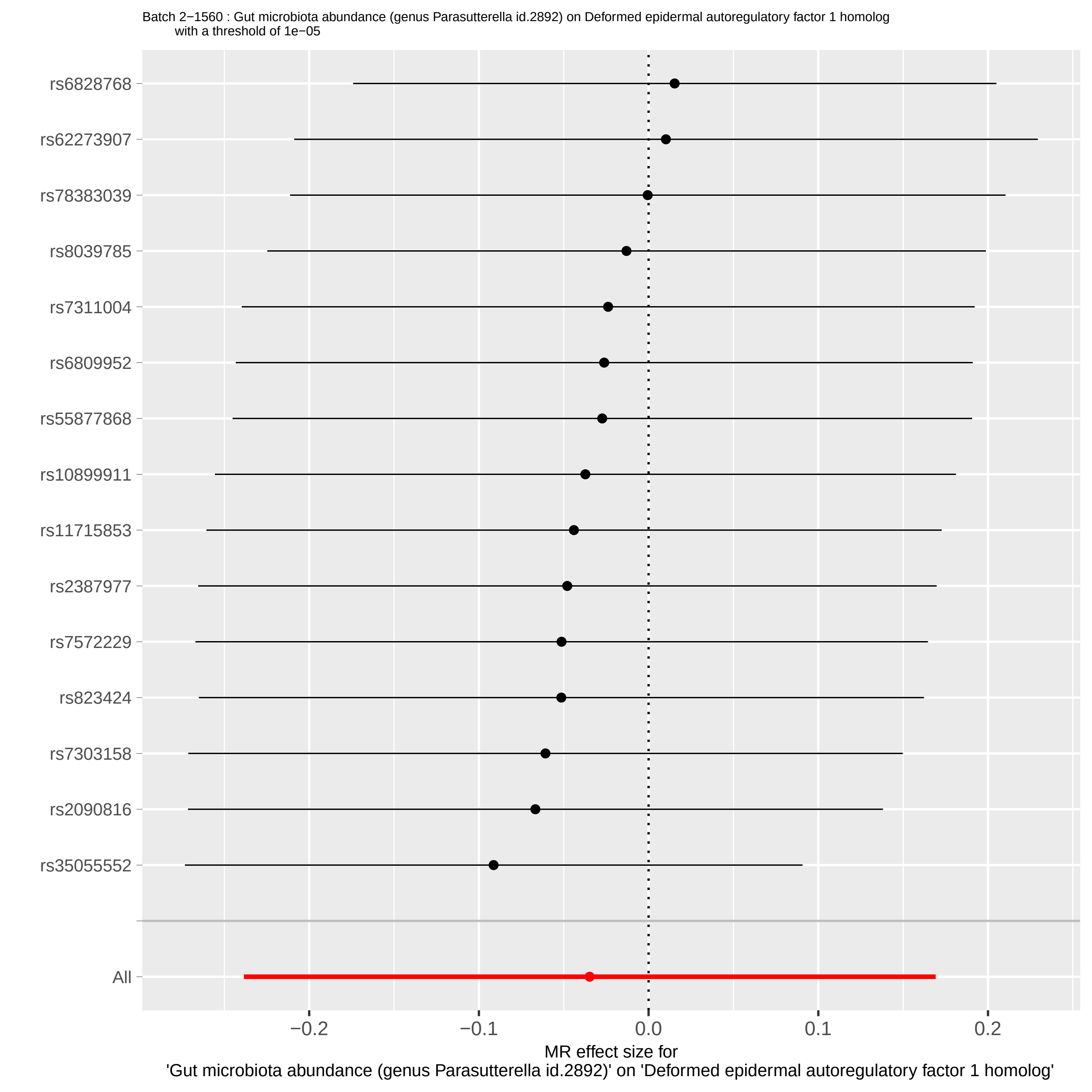

Batch 2−1560 : Gut microbiota abundance (genus Parasutterella id.2892) on Deformed epidermal autoregulatory factor 1 homolog
with a threshold of 1e−05
rs6828768
rs62273907
rs78383039
rs8039785
rs7311004
rs6809952
rs55877868
rs10899911
rs11715853
rs2387977
rs7572229
rs823424
rs7303158
rs2090816
rs35055552
All
−0.2
−0.1
0.0
0.1
0.2
MR effect size for
'Gut microbiota abundance (genus Parasutterella id.2892)' on 'Deformed epidermal autoregulatory factor 1 homolog'

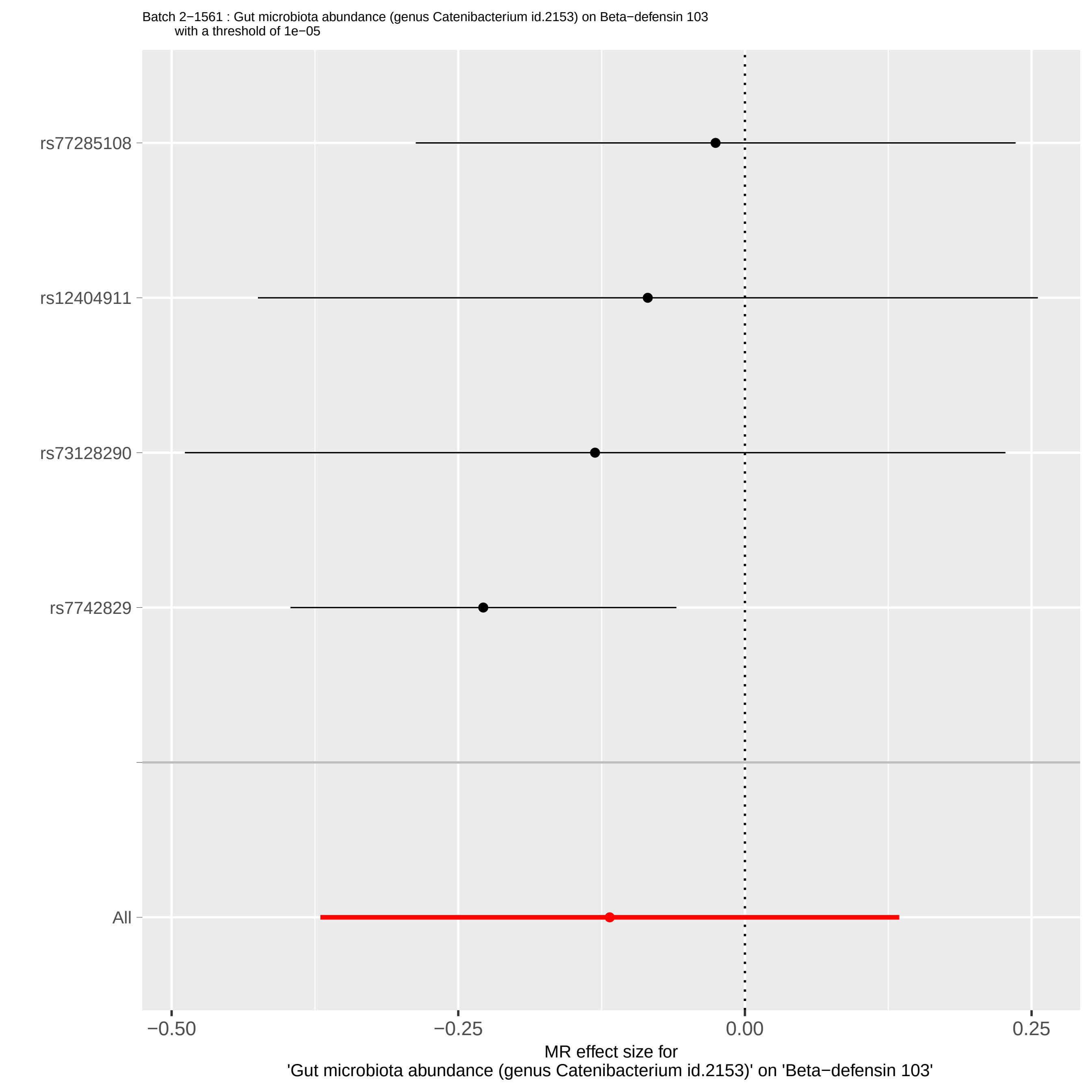
Batch 2−1561 : Gut microbiota abundance (genus Catenibacterium id.2153) on Beta−defensin 103
with a threshold of 1e−05
rs77285108
rs12404911
rs73128290
rs7742829
All
−0.50
−0.25
0.00
0.25
MR effect size for
'Gut microbiota abundance (genus Catenibacterium id.2153)' on 'Beta−defensin 103'

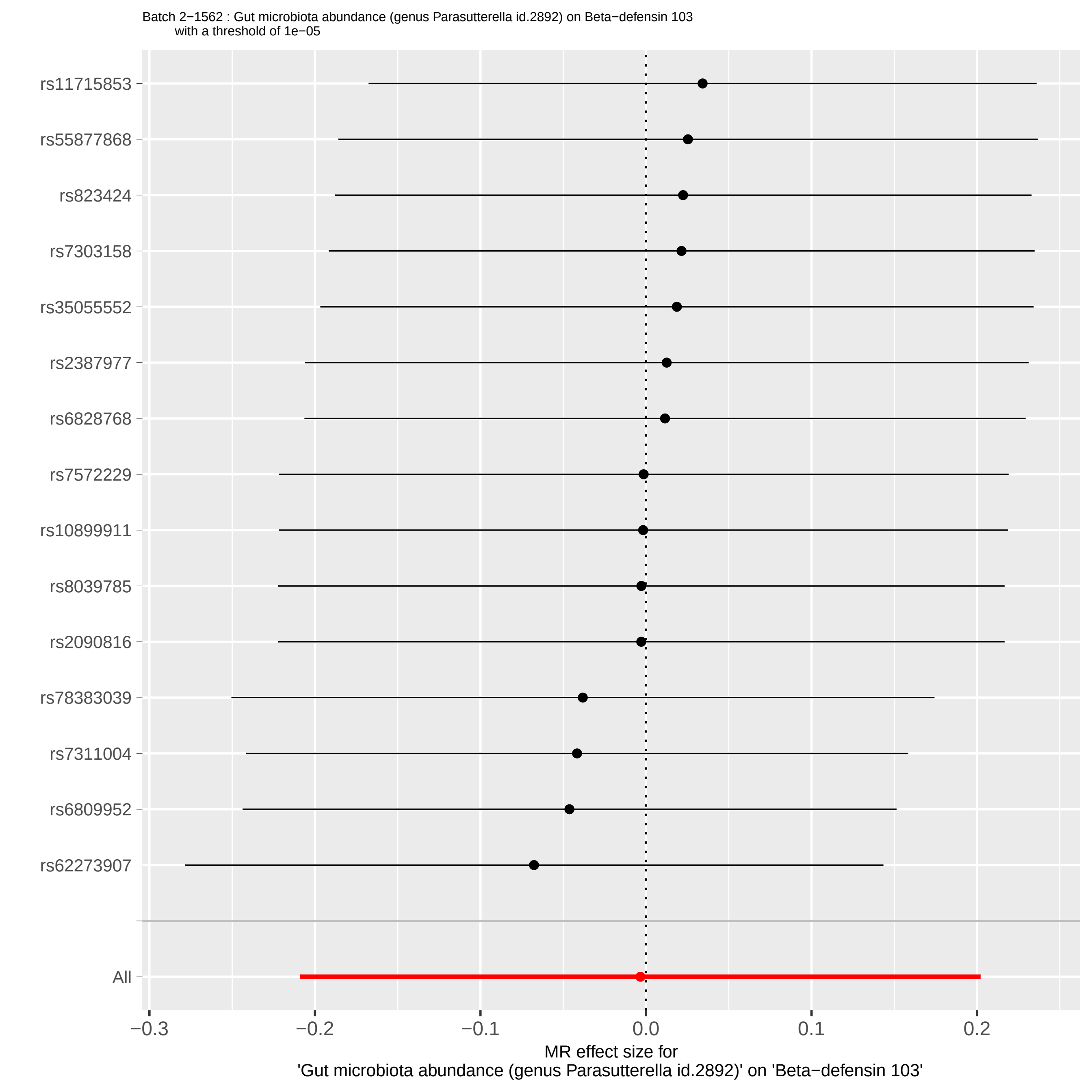
Batch 2−1562 : Gut microbiota abundance (genus Parasutterella id.2892) on Beta−defensin 103
with a threshold of 1e−05
rs11715853
rs55877868
rs823424
rs7303158
rs35055552
rs2387977
rs6828768
rs7572229
rs10899911
rs8039785
rs2090816
rs78383039
rs7311004
rs6809952
rs62273907
All
−0.3
−0.2
−0.1
0.0
0.1
0.2
MR effect size for
'Gut microbiota abundance (genus Parasutterella id.2892)' on 'Beta−defensin 103'

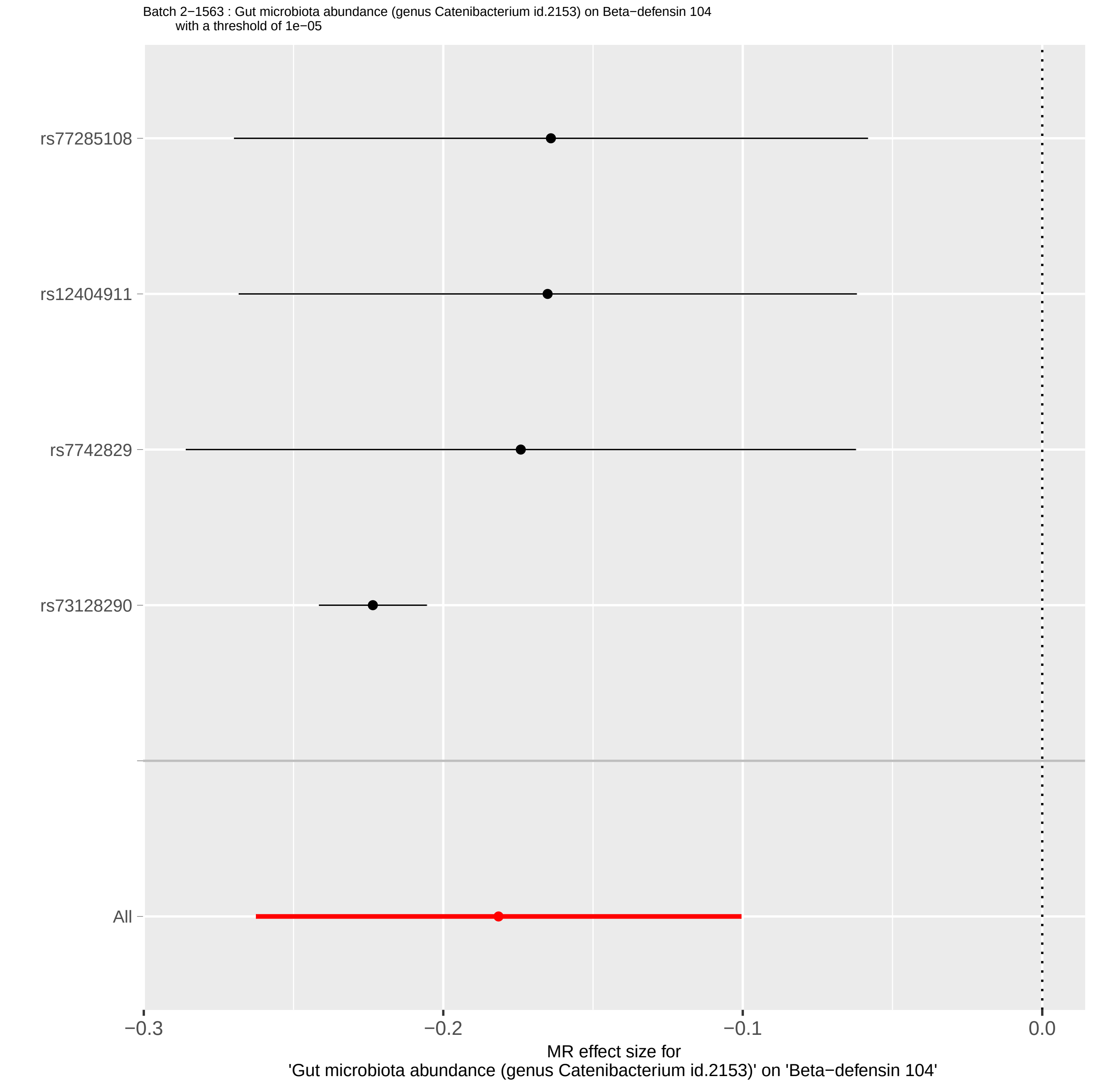
Batch 2−1563 : Gut microbiota abundance (genus Catenibacterium id.2153) on Beta−defensin 104
with a threshold of 1e−05
rs77285108
rs12404911
rs7742829
rs73128290
All
−0.3
−0.2
−0.1
0.0
MR effect size for
'Gut microbiota abundance (genus Catenibacterium id.2153)' on 'Beta−defensin 104'

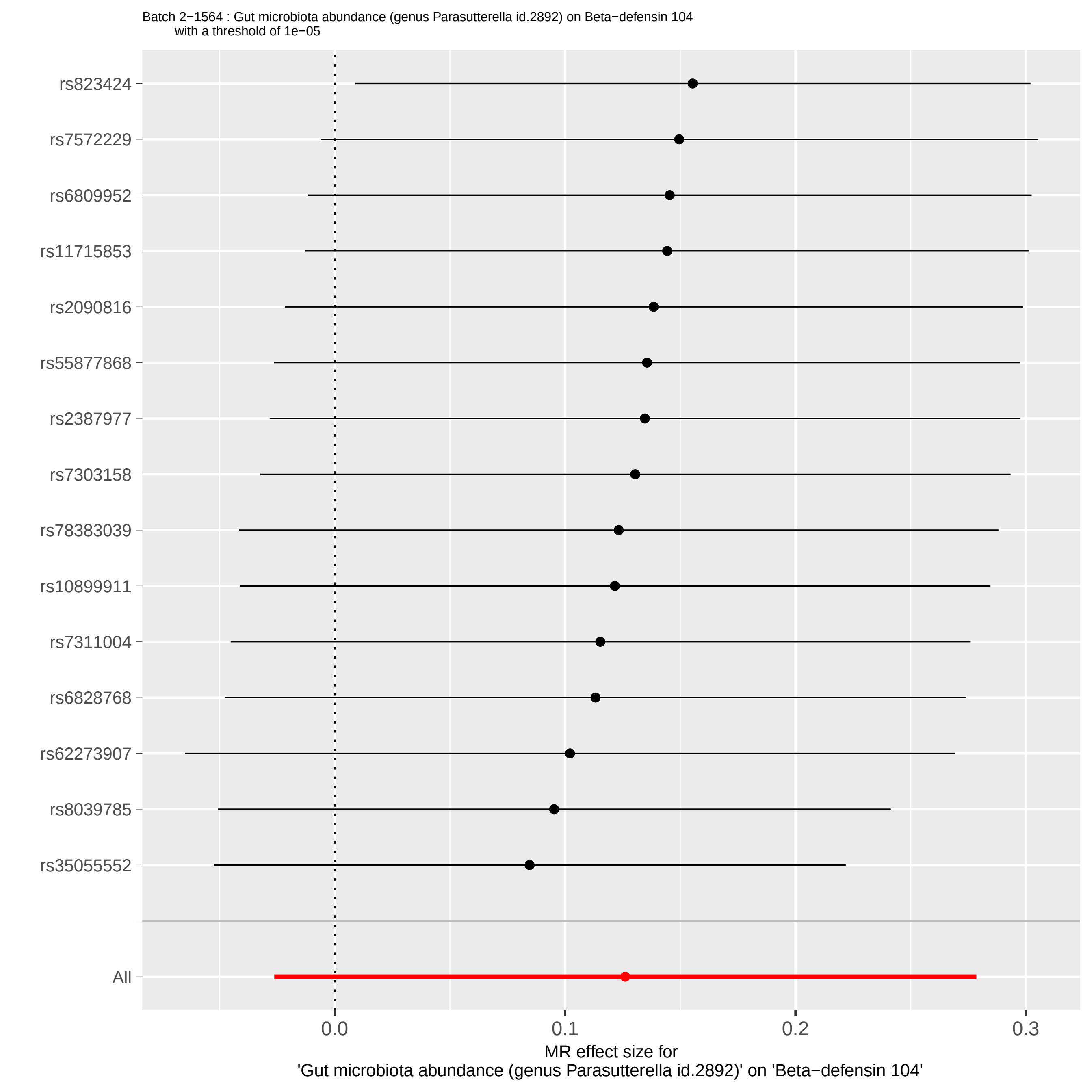

Batch 2−1564 : Gut microbiota abundance (genus Parasutterella id.2892) on Beta−defensin 104
with a threshold of 1e−05
rs823424
rs7572229
rs6809952
rs11715853
rs2090816
rs55877868
rs2387977
rs7303158
rs78383039
rs10899911
rs7311004
rs6828768
rs62273907
rs8039785
rs35055552
All
0.0
0.1
0.2
0.3
MR effect size for
'Gut microbiota abundance (genus Parasutterella id.2892)' on 'Beta−defensin 104'

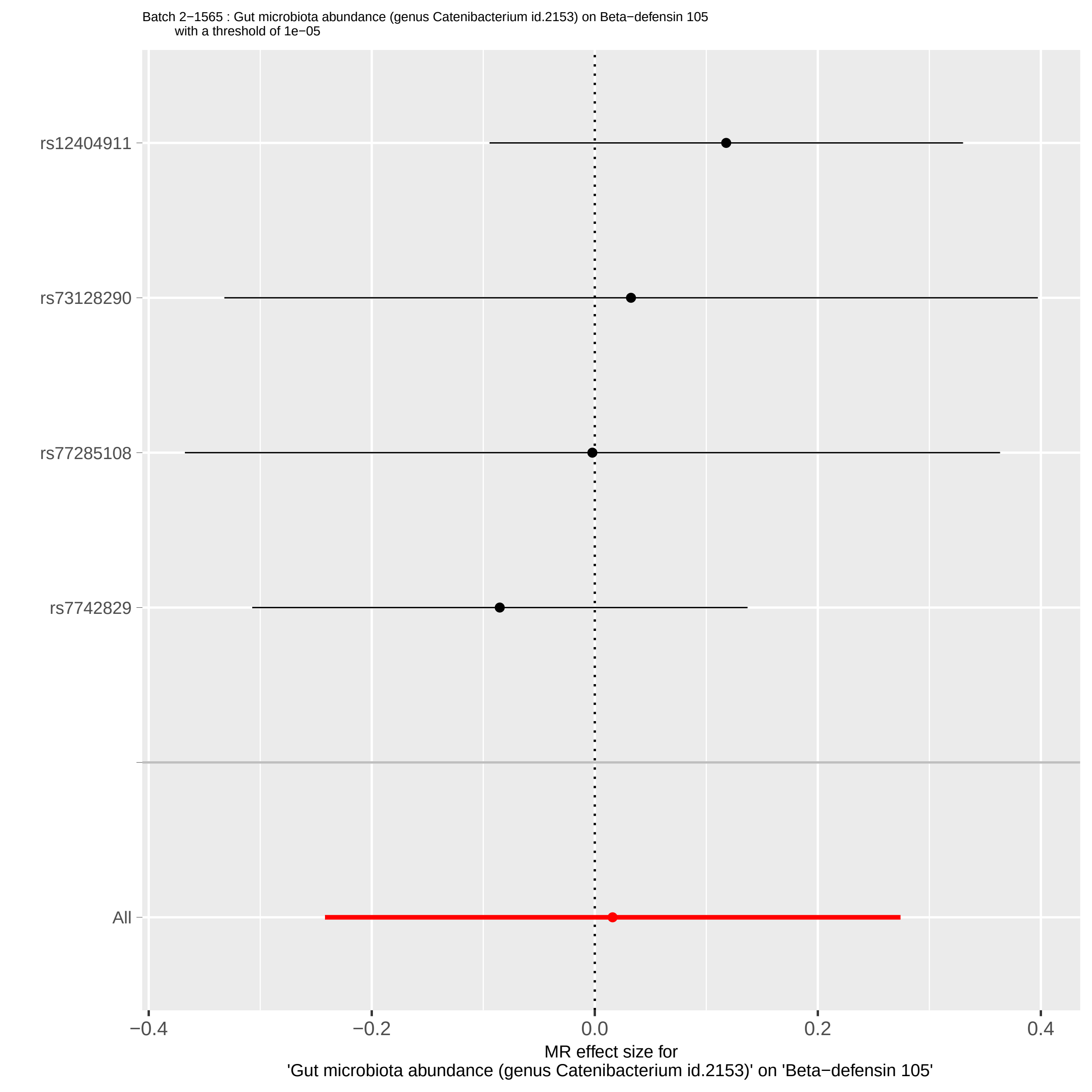
Batch 2−1565 : Gut microbiota abundance (genus Catenibacterium id.2153) on Beta−defensin 105
with a threshold of 1e−05
rs12404911
rs73128290
rs77285108
rs7742829
All
−0.4
−0.2
0.0
0.2
0.4
MR effect size for
'Gut microbiota abundance (genus Catenibacterium id.2153)' on 'Beta−defensin 105'

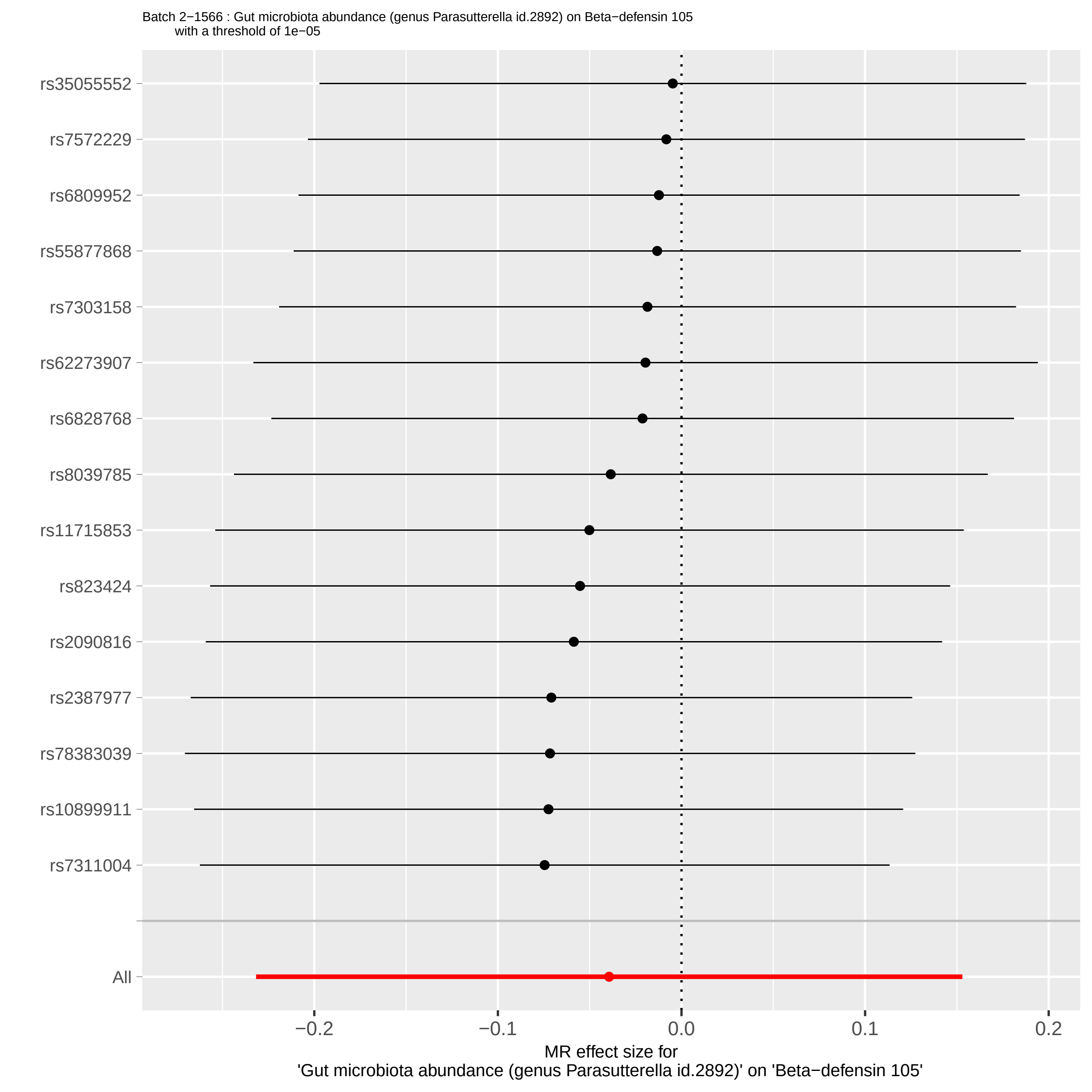

Batch 2−1566 : Gut microbiota abundance (genus Parasutterella id.2892) on Beta−defensin 105
with a threshold of 1e−05
rs35055552
rs7572229
rs6809952
rs55877868
rs7303158
rs62273907
rs6828768
rs8039785
rs11715853
rs823424
rs2090816
rs2387977
rs78383039
rs10899911
rs7311004
All
−0.2
−0.1
0.0
0.1
0.2
MR effect size for
'Gut microbiota abundance (genus Parasutterella id.2892)' on 'Beta−defensin 105'

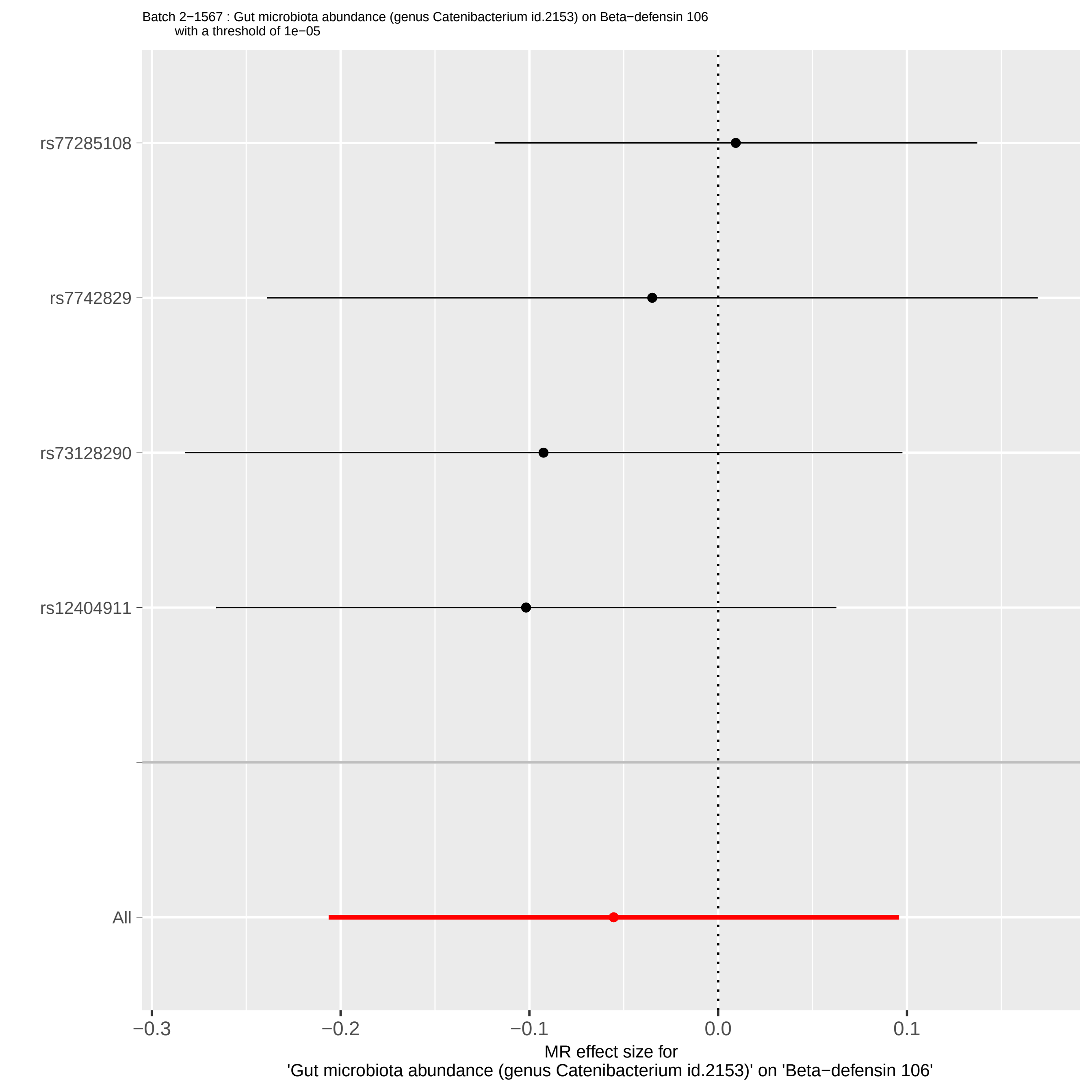
Batch 2−1567 : Gut microbiota abundance (genus Catenibacterium id.2153) on Beta−defensin 106
with a threshold of 1e−05
rs77285108
rs7742829
rs73128290
rs12404911
All
−0.3
−0.2
−0.1
0.0
0.1
MR effect size for
'Gut microbiota abundance (genus Catenibacterium id.2153)' on 'Beta−defensin 106'

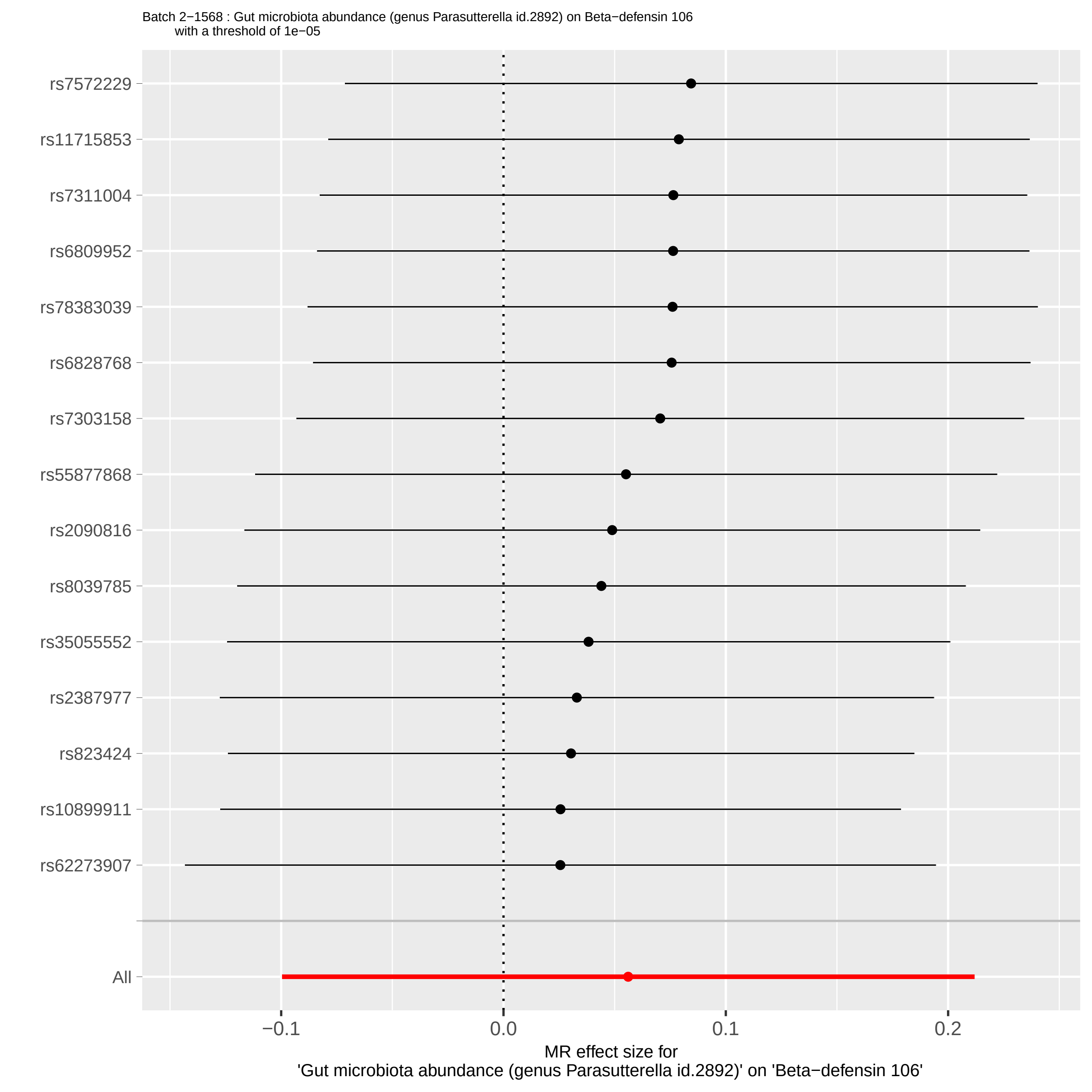
Batch 2−1568 : Gut microbiota abundance (genus Parasutterella id.2892) on Beta−defensin 106
with a threshold of 1e−05
rs7572229
rs11715853
rs7311004
rs6809952
rs78383039
rs6828768
rs7303158
rs55877868
rs2090816
rs8039785
rs35055552
rs2387977
rs823424
rs10899911
rs62273907
All
−0.1
0.0
0.1
0.2
MR effect size for
'Gut microbiota abundance (genus Parasutterella id.2892)' on 'Beta−defensin 106'

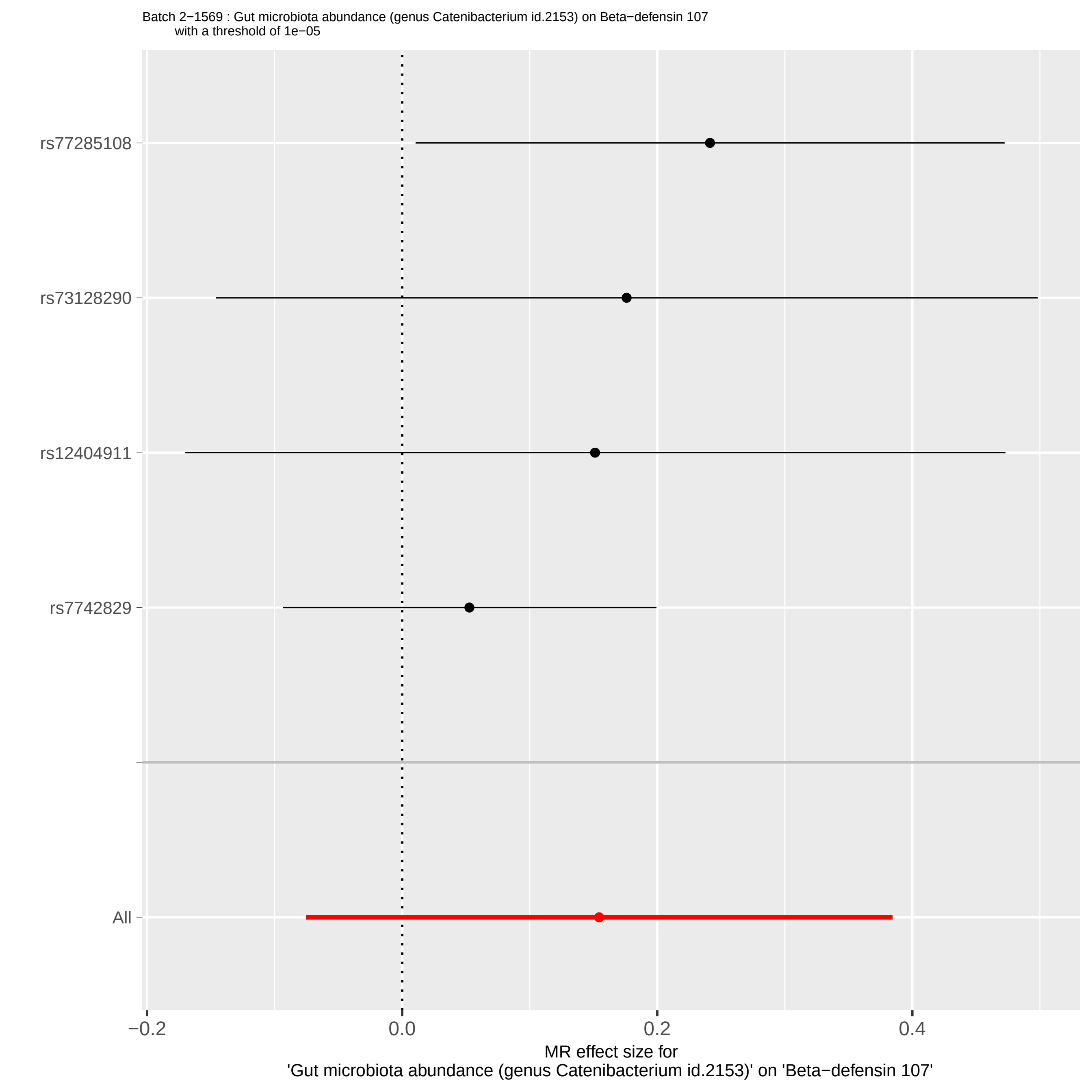
Batch 2−1569 : Gut microbiota abundance (genus Catenibacterium id.2153) on Beta−defensin 107
with a threshold of 1e−05
rs77285108
rs73128290
rs12404911
rs7742829
All
−0.2
0.0
0.2
0.4
MR effect size for
'Gut microbiota abundance (genus Catenibacterium id.2153)' on 'Beta−defensin 107'

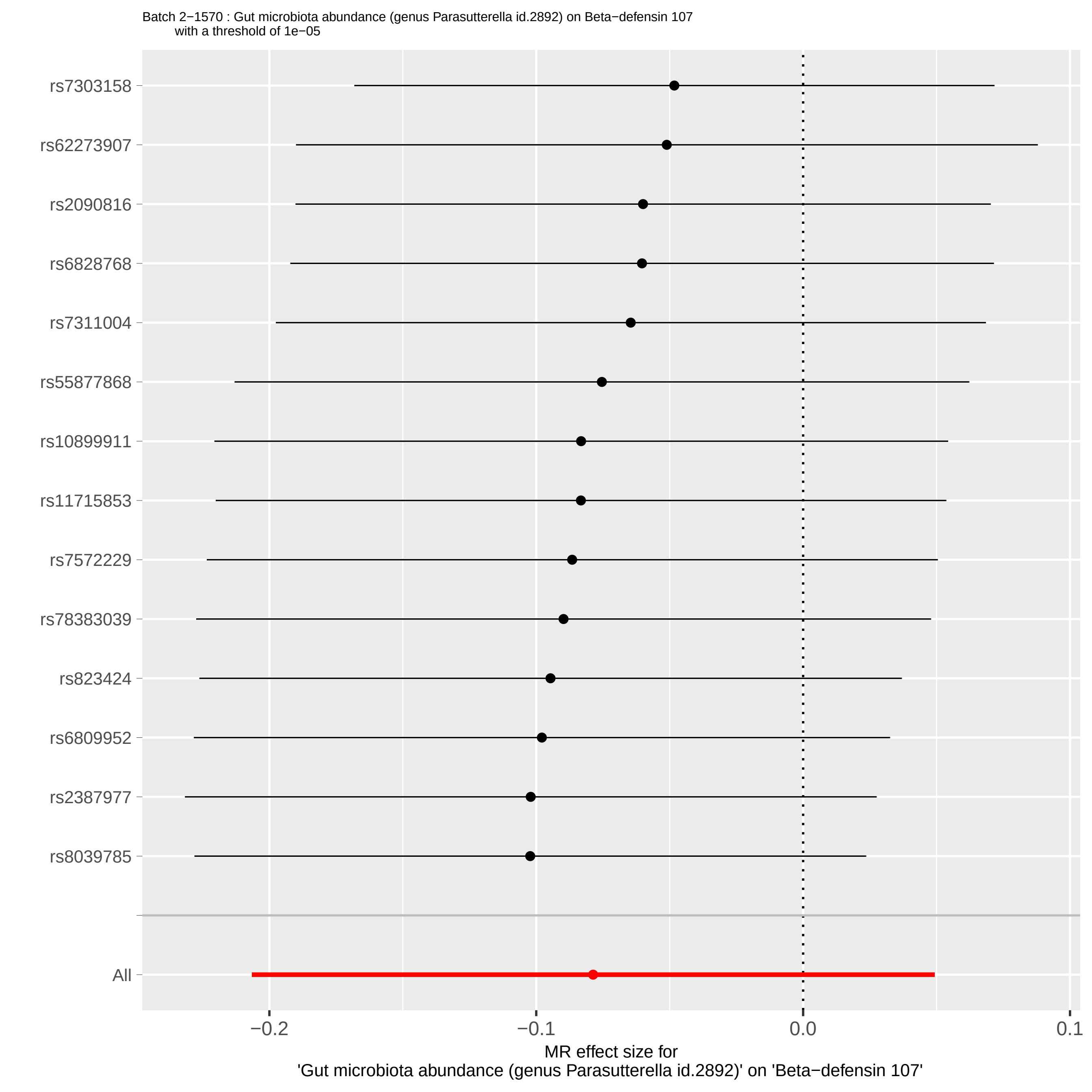
Batch 2−1570 : Gut microbiota abundance (genus Parasutterella id.2892) on Beta−defensin 107
with a threshold of 1e−05
rs7303158
rs62273907
rs2090816
rs6828768
rs7311004
rs55877868
rs10899911
rs11715853
rs7572229
rs78383039
rs823424
rs6809952
rs2387977
rs8039785
All
−0.2
−0.1
0.0
0.1
MR effect size for
'Gut microbiota abundance (genus Parasutterella id.2892)' on 'Beta−defensin 107'

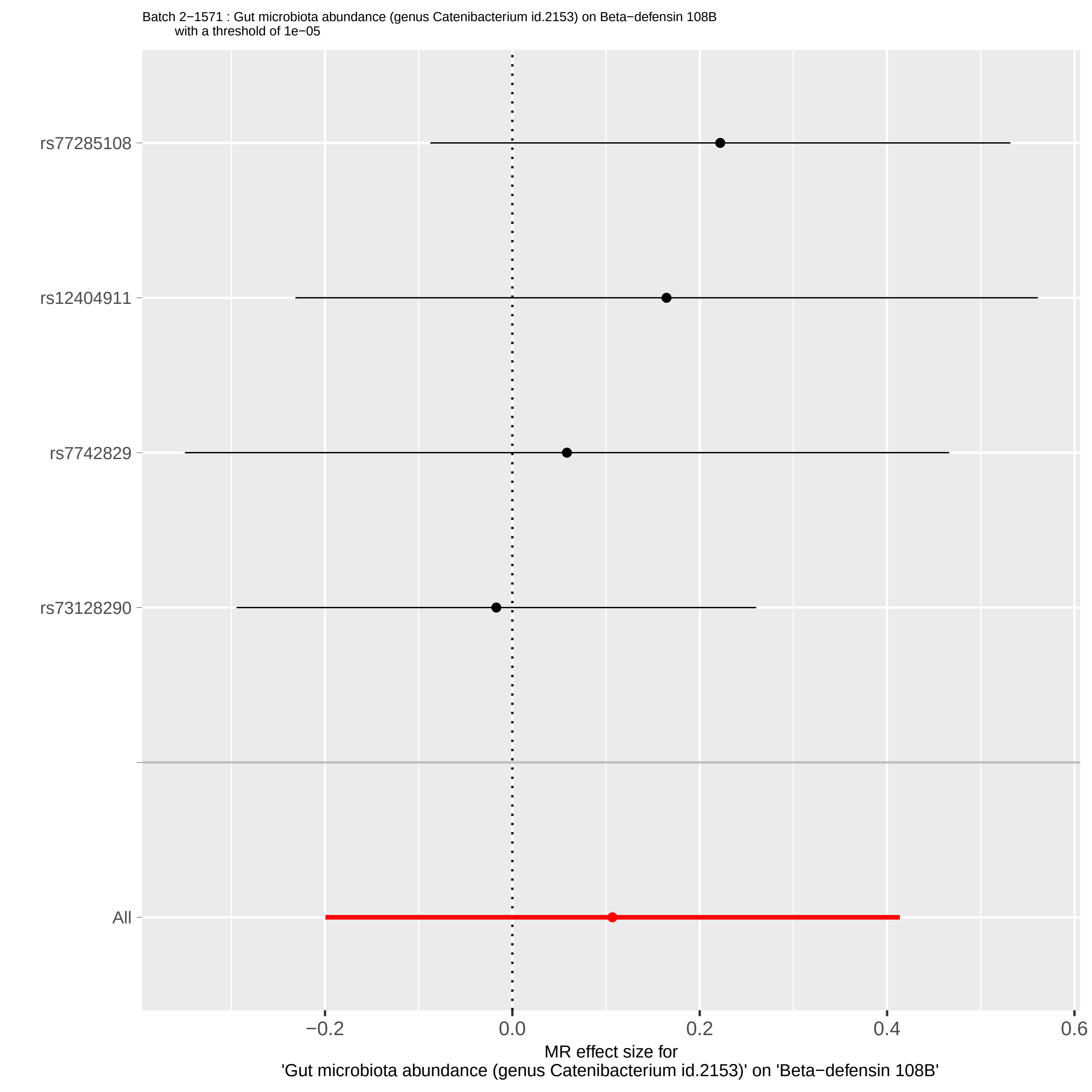

Batch 2−1571 : Gut microbiota abundance (genus Catenibacterium id.2153) on Beta−defensin 108B
with a threshold of 1e−05
rs77285108
rs12404911
rs7742829
rs73128290
All
−0.2
0.0
0.2
0.4
0.6
MR effect size for
'Gut microbiota abundance (genus Catenibacterium id.2153)' on 'Beta−defensin 108B'

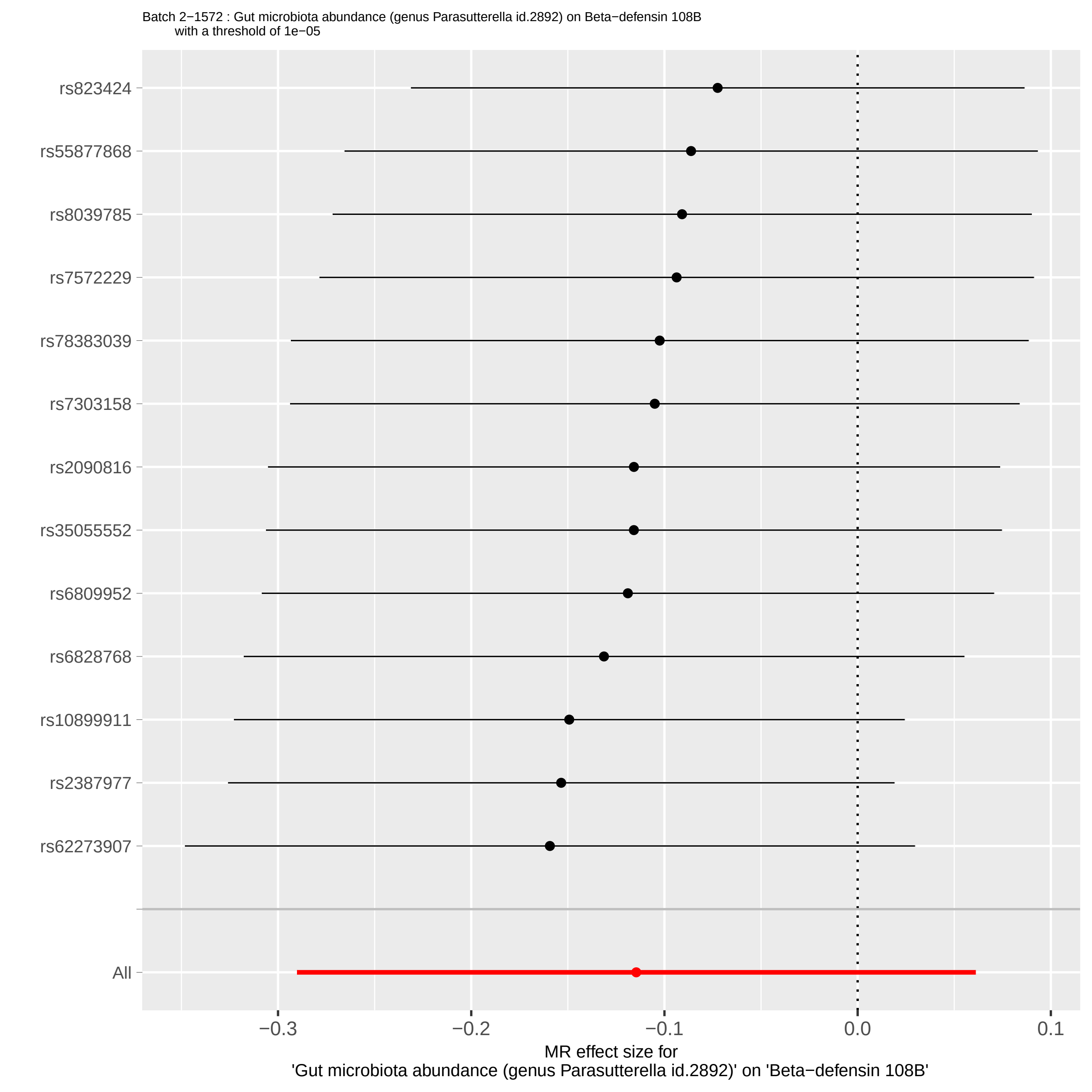

Batch 2−1572 : Gut microbiota abundance (genus Parasutterella id.2892) on Beta−defensin 108B
with a threshold of 1e−05
rs823424
rs55877868
rs8039785
rs7572229
rs78383039
rs7303158
rs2090816
rs35055552
rs6809952
rs6828768
rs10899911
rs2387977
rs62273907
All
−0.3
−0.2
−0.1
0.0
0.1
MR effect size for
'Gut microbiota abundance (genus Parasutterella id.2892)' on 'Beta−defensin 108B'

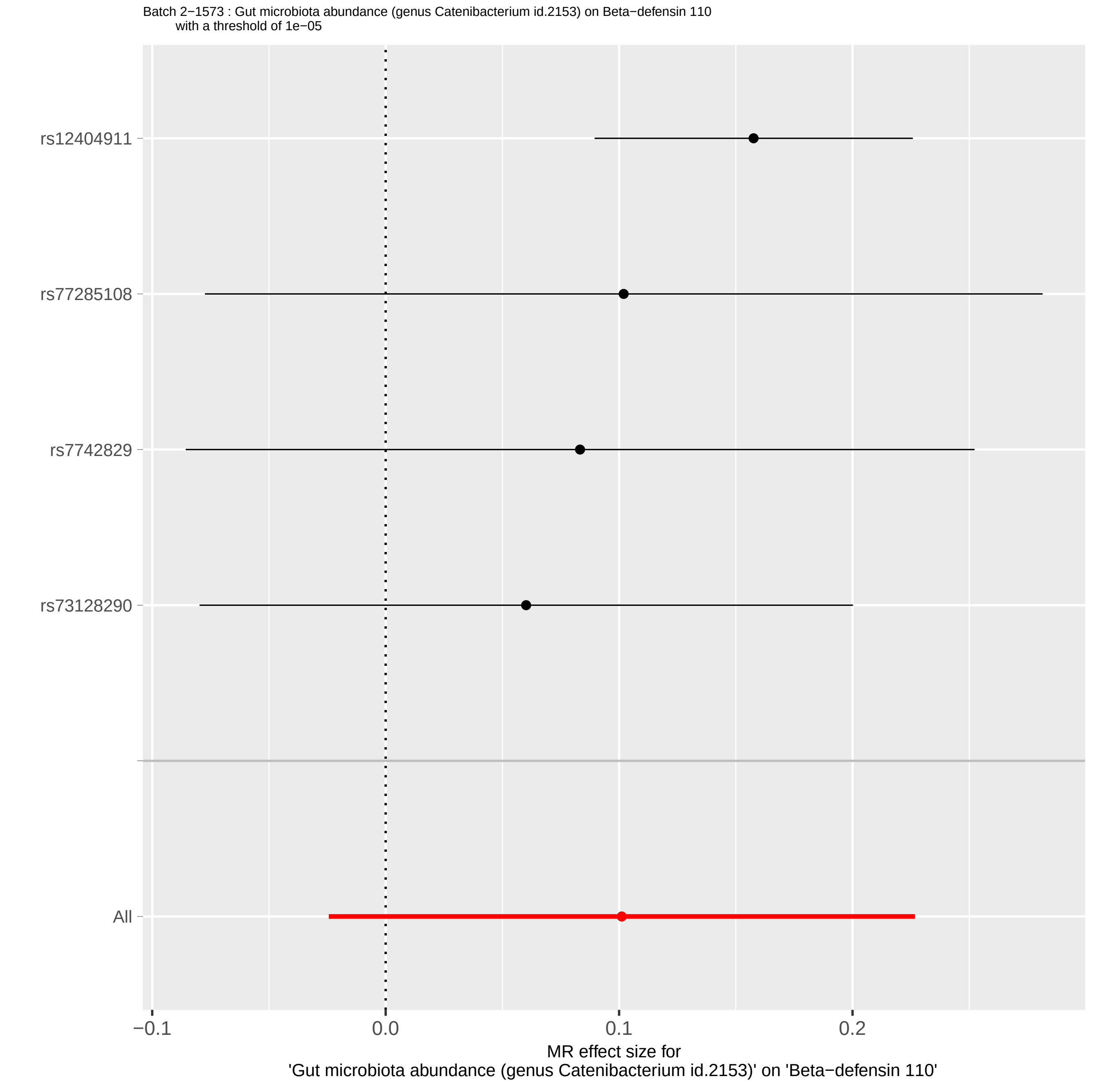

Batch 2−1573 : Gut microbiota abundance (genus Catenibacterium id.2153) on Beta−defensin 110
with a threshold of 1e−05
rs12404911
rs77285108
rs7742829
rs73128290
All
−0.1
0.0
0.1
0.2
MR effect size for
'Gut microbiota abundance (genus Catenibacterium id.2153)' on 'Beta−defensin 110'

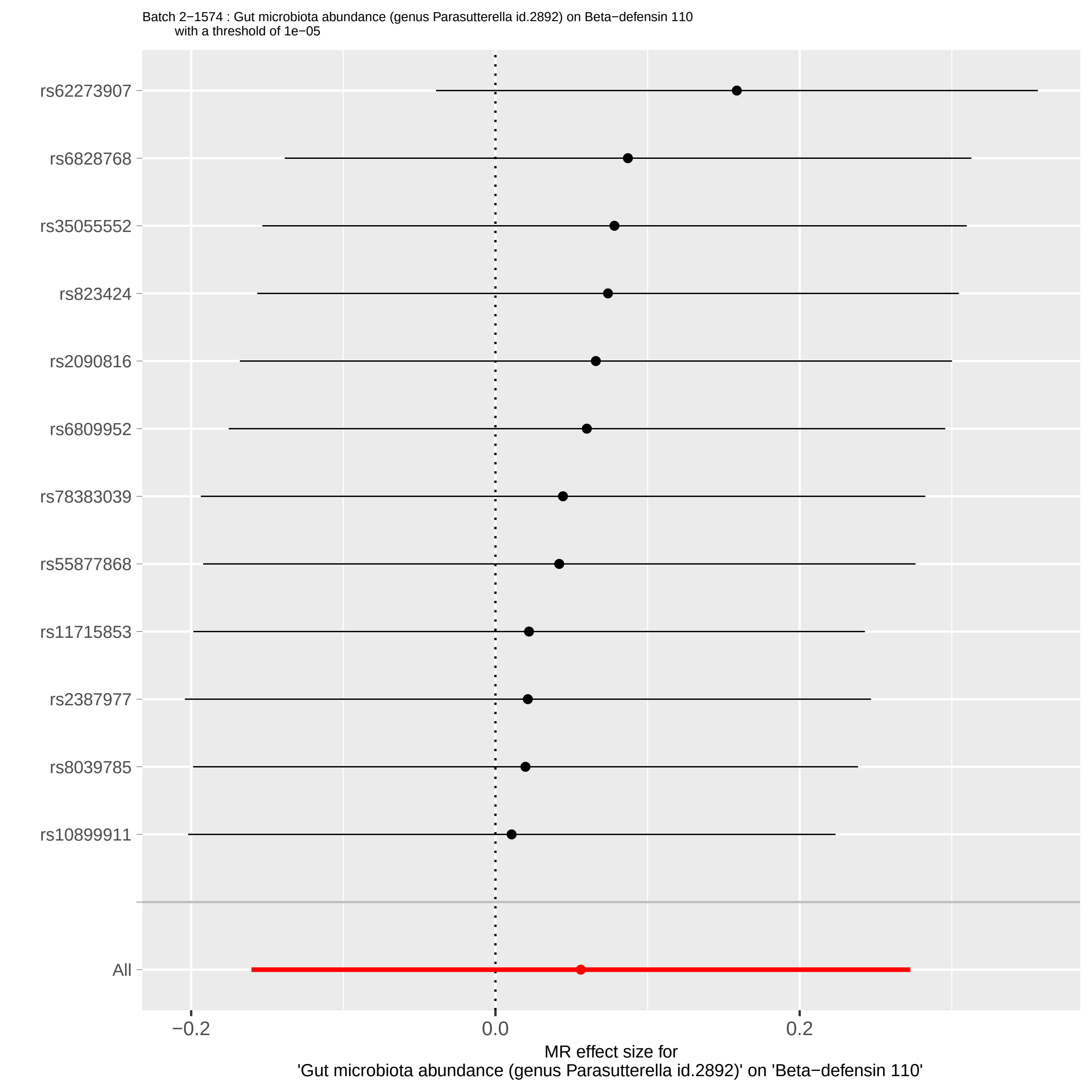

Batch 2−1574 : Gut microbiota abundance (genus Parasutterella id.2892) on Beta−defensin 110
with a threshold of 1e−05
rs62273907
rs6828768
rs35055552
rs823424
rs2090816
rs6809952
rs78383039
rs55877868
rs11715853
rs2387977
rs8039785
rs10899911
All
−0.2
0.0
0.2
MR effect size for
'Gut microbiota abundance (genus Parasutterella id.2892)' on 'Beta−defensin 110'

Batch 2−1575 : Gut microbiota abundance (genus Catenibacterium id.2153) on Beta−defensin 112
with a threshold of 1e−05

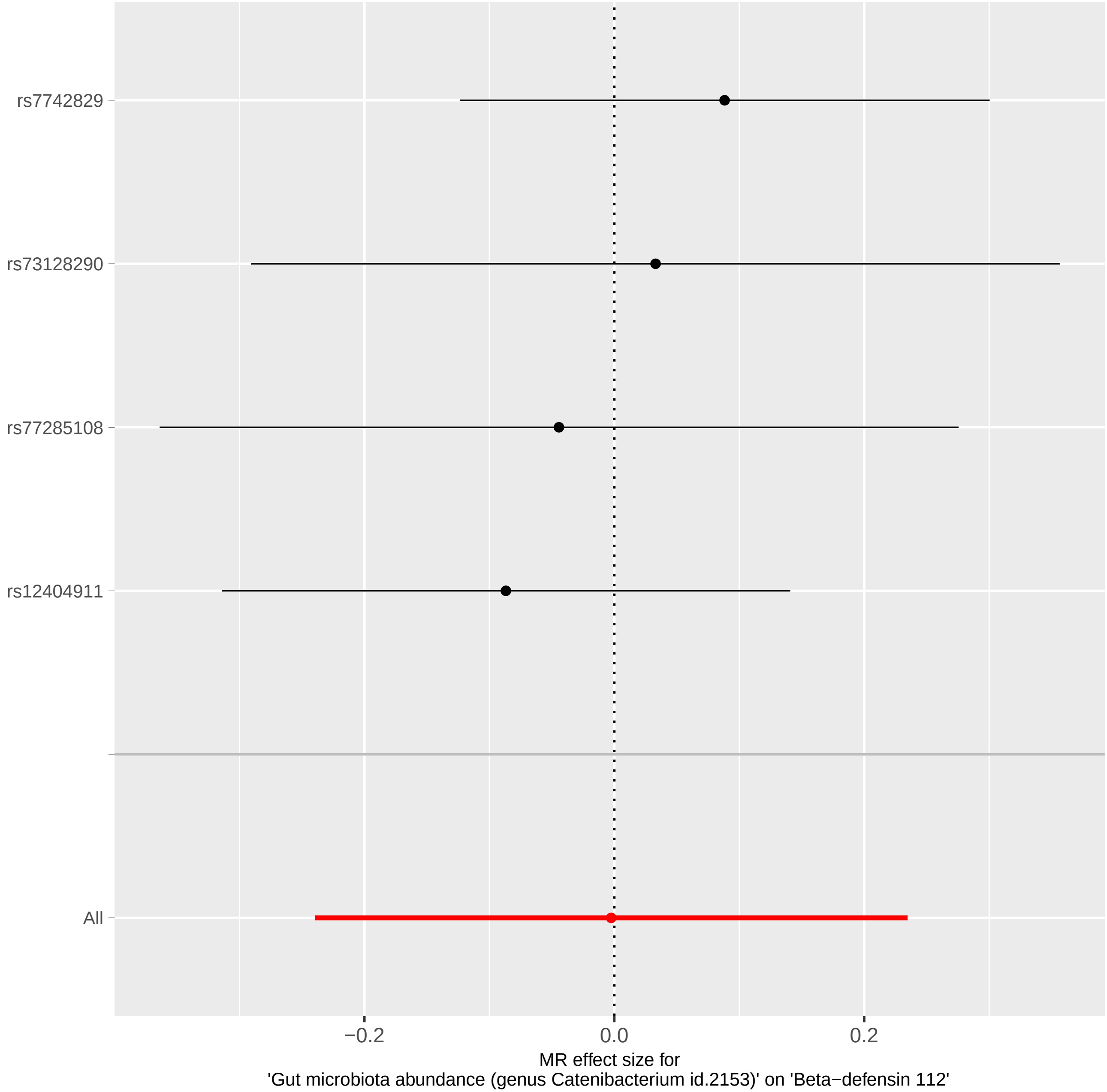

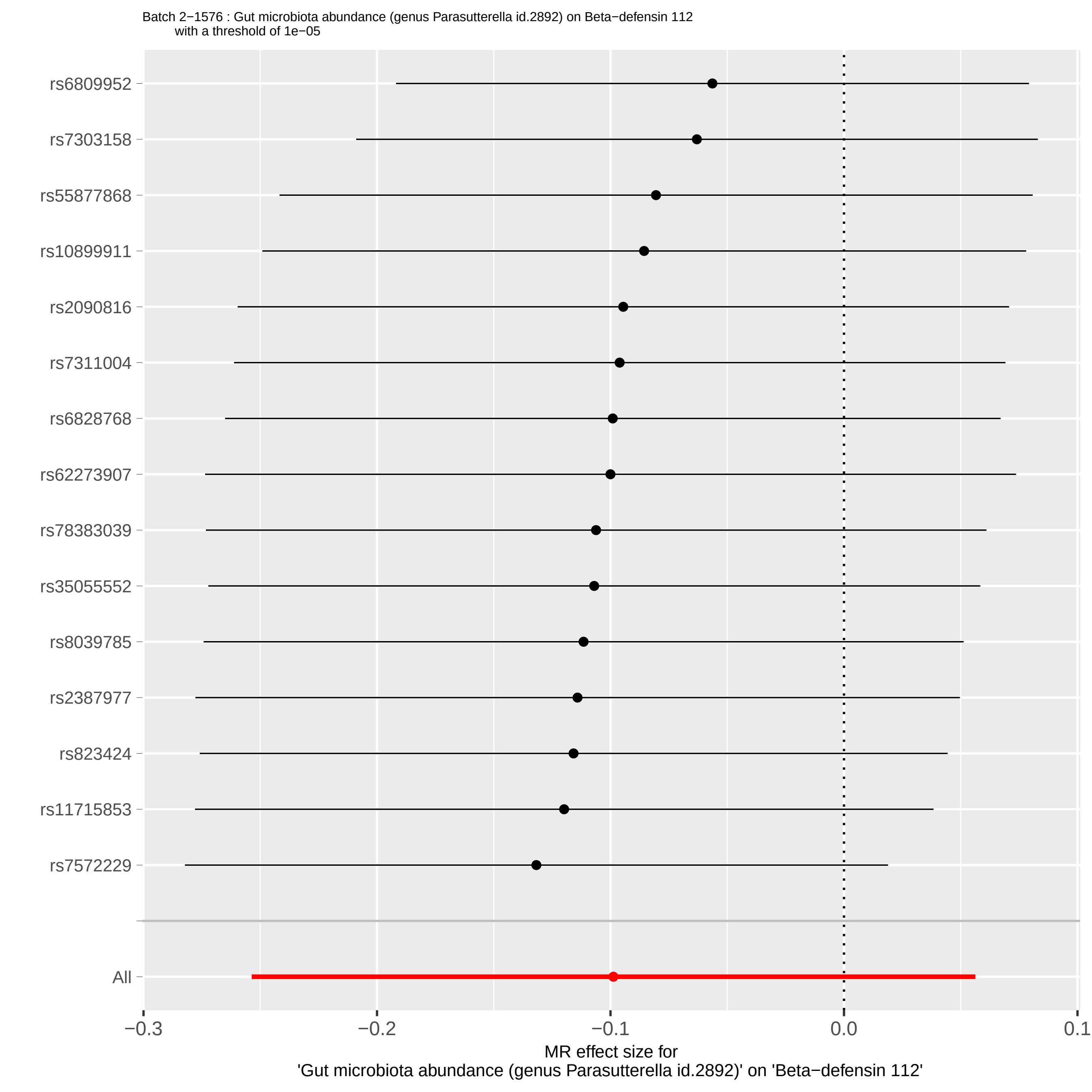
Batch 2−1576 : Gut microbiota abundance (genus Parasutterella id.2892) on Beta−defensin 112
with a threshold of 1e−05
rs6809952
rs7303158
rs55877868
rs10899911
rs2090816
rs7311004
rs6828768
rs62273907
rs78383039
rs35055552
rs8039785
rs2387977
rs823424
rs11715853
rs7572229
All
−0.3
−0.2
−0.1
0.0
0.1
MR effect size for
'Gut microbiota abundance (genus Parasutterella id.2892)' on 'Beta−defensin 112'

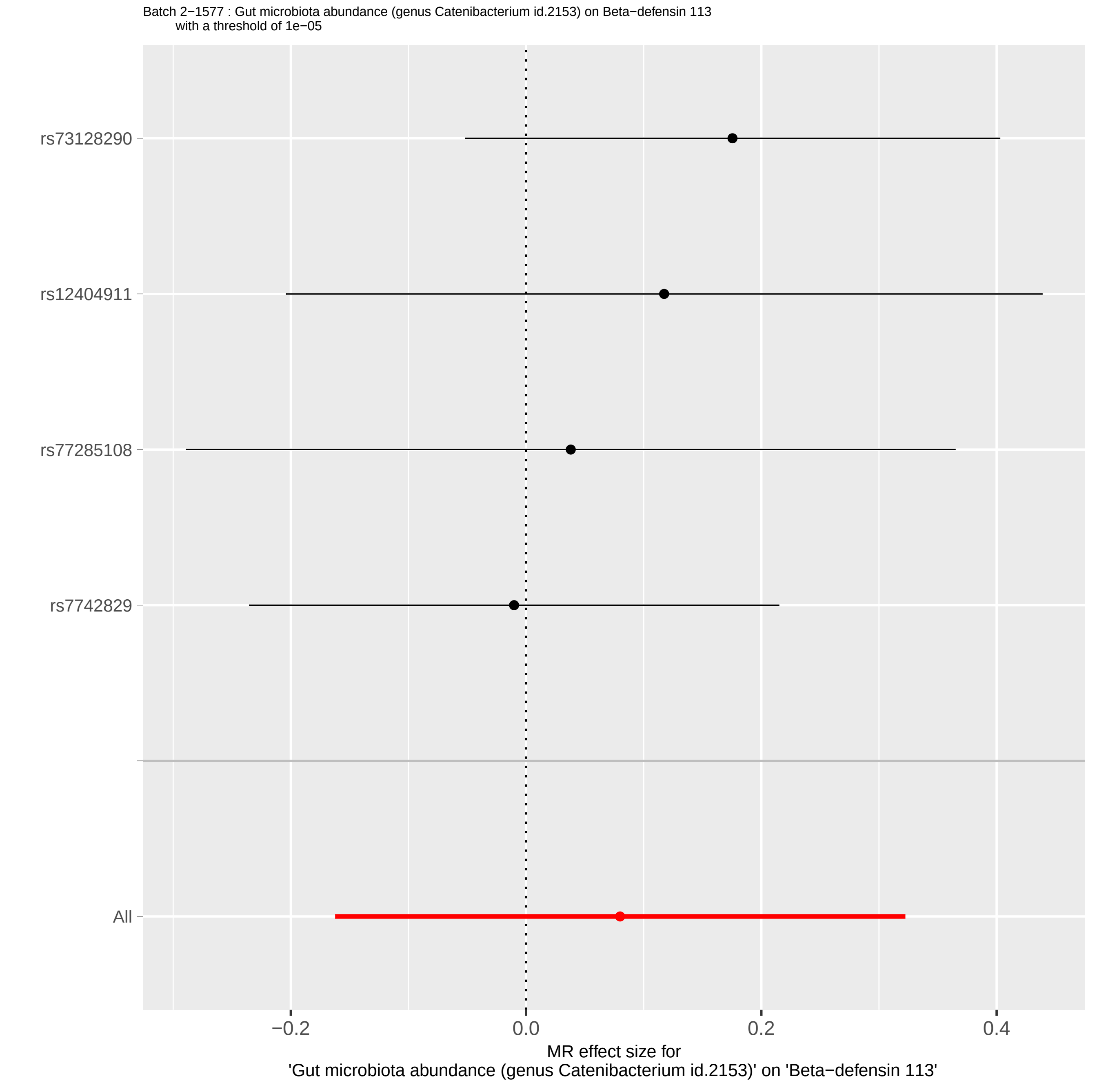

Batch 2−1577 : Gut microbiota abundance (genus Catenibacterium id.2153) on Beta−defensin 113
with a threshold of 1e−05
rs73128290
rs12404911
rs77285108
rs7742829
All
−0.2
0.0
0.2
0.4
MR effect size for
'Gut microbiota abundance (genus Catenibacterium id.2153)' on 'Beta−defensin 113'

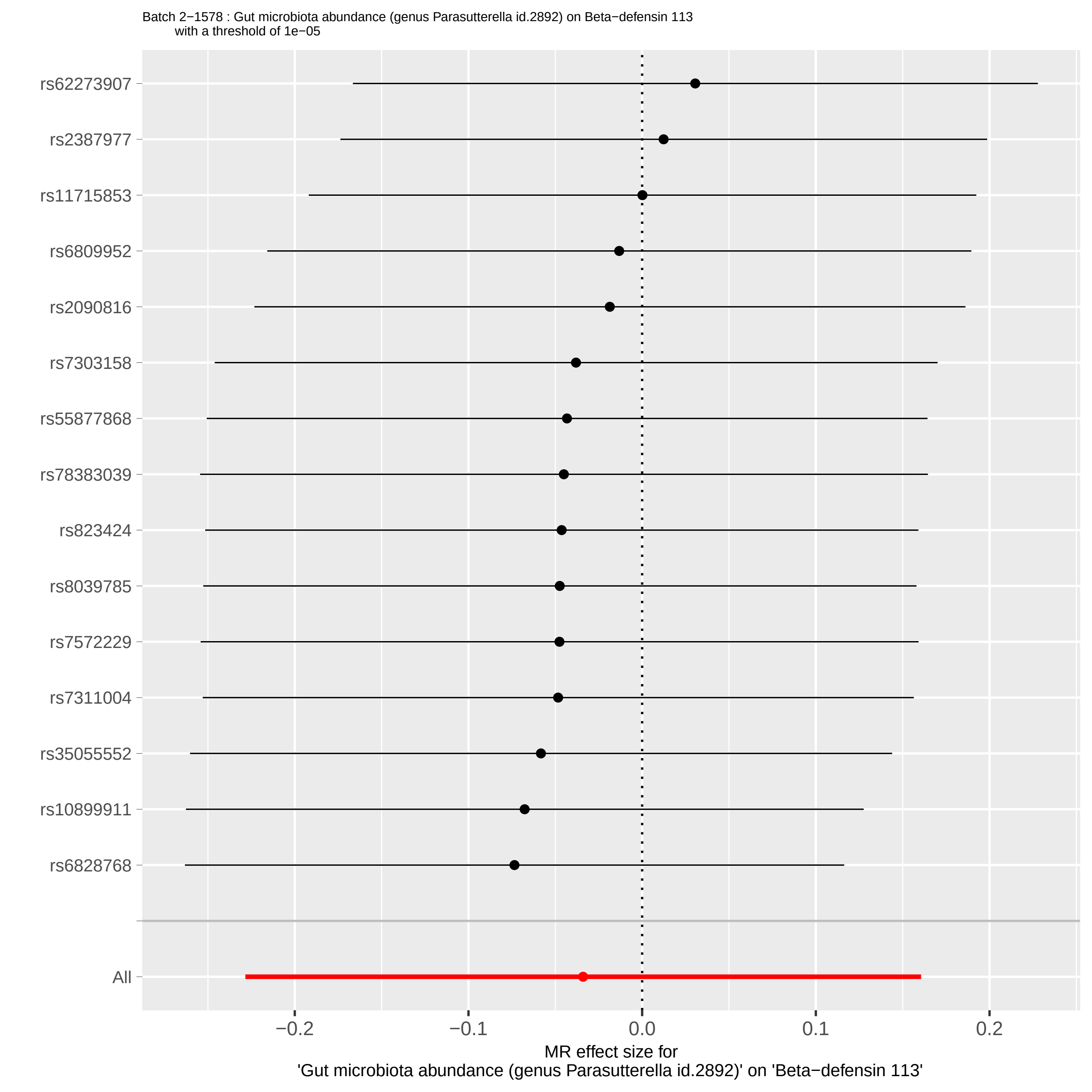
Batch 2−1578 : Gut microbiota abundance (genus Parasutterella id.2892) on Beta−defensin 113
with a threshold of 1e−05
rs62273907
rs2387977
rs11715853
rs6809952
rs2090816
rs7303158
rs55877868
rs78383039
rs823424
rs8039785
rs7572229
rs7311004
rs35055552
rs10899911
rs6828768
All
−0.2
−0.1
0.0
0.1
0.2
MR effect size for
'Gut microbiota abundance (genus Parasutterella id.2892)' on 'Beta−defensin 113'

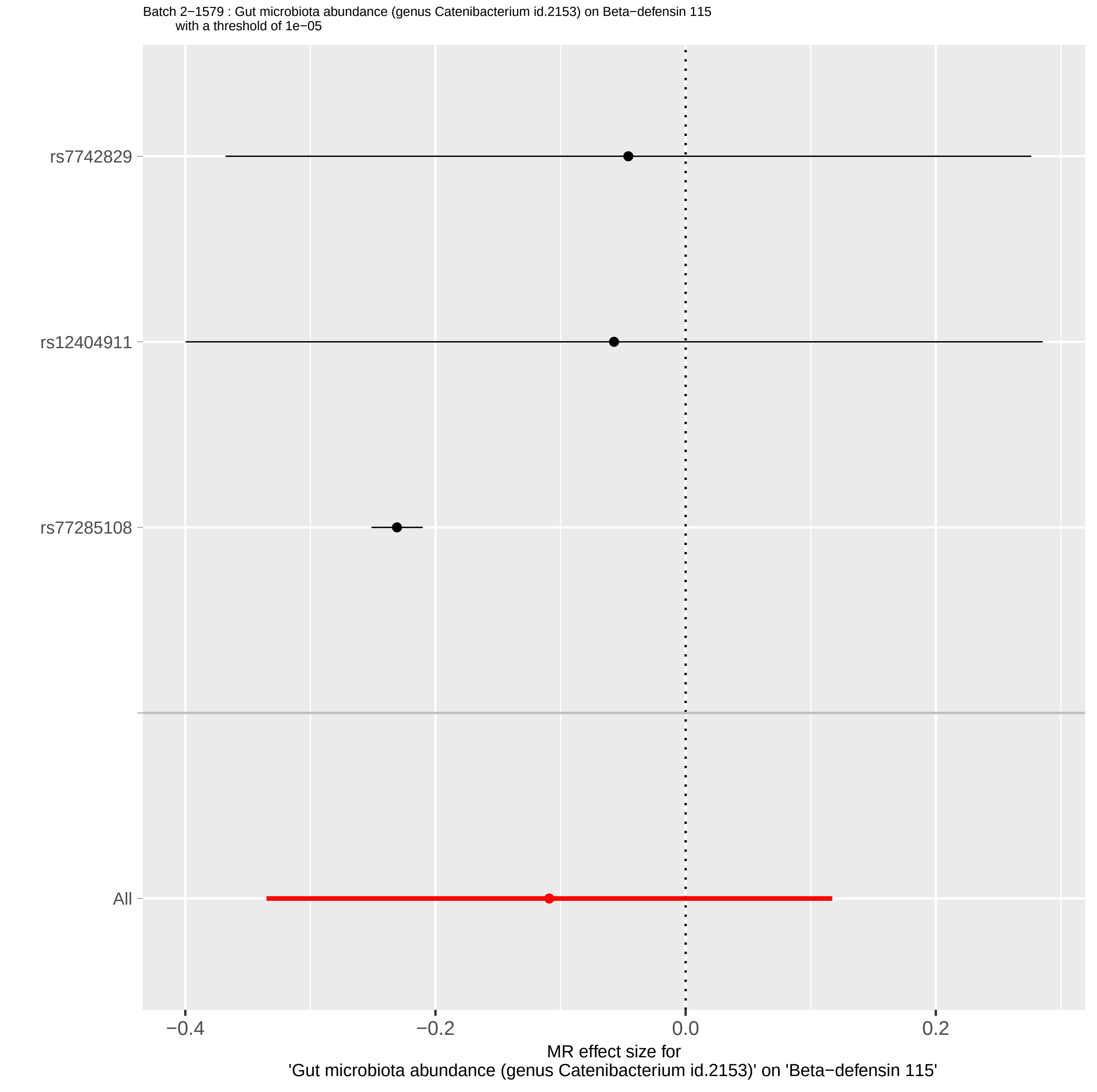

Batch 2−1579 : Gut microbiota abundance (genus Catenibacterium id.2153) on Beta−defensin 115
with a threshold of 1e−05
rs7742829
rs12404911
rs77285108
All
−0.4
−0.2
0.0
0.2
MR effect size for
'Gut microbiota abundance (genus Catenibacterium id.2153)' on 'Beta−defensin 115'

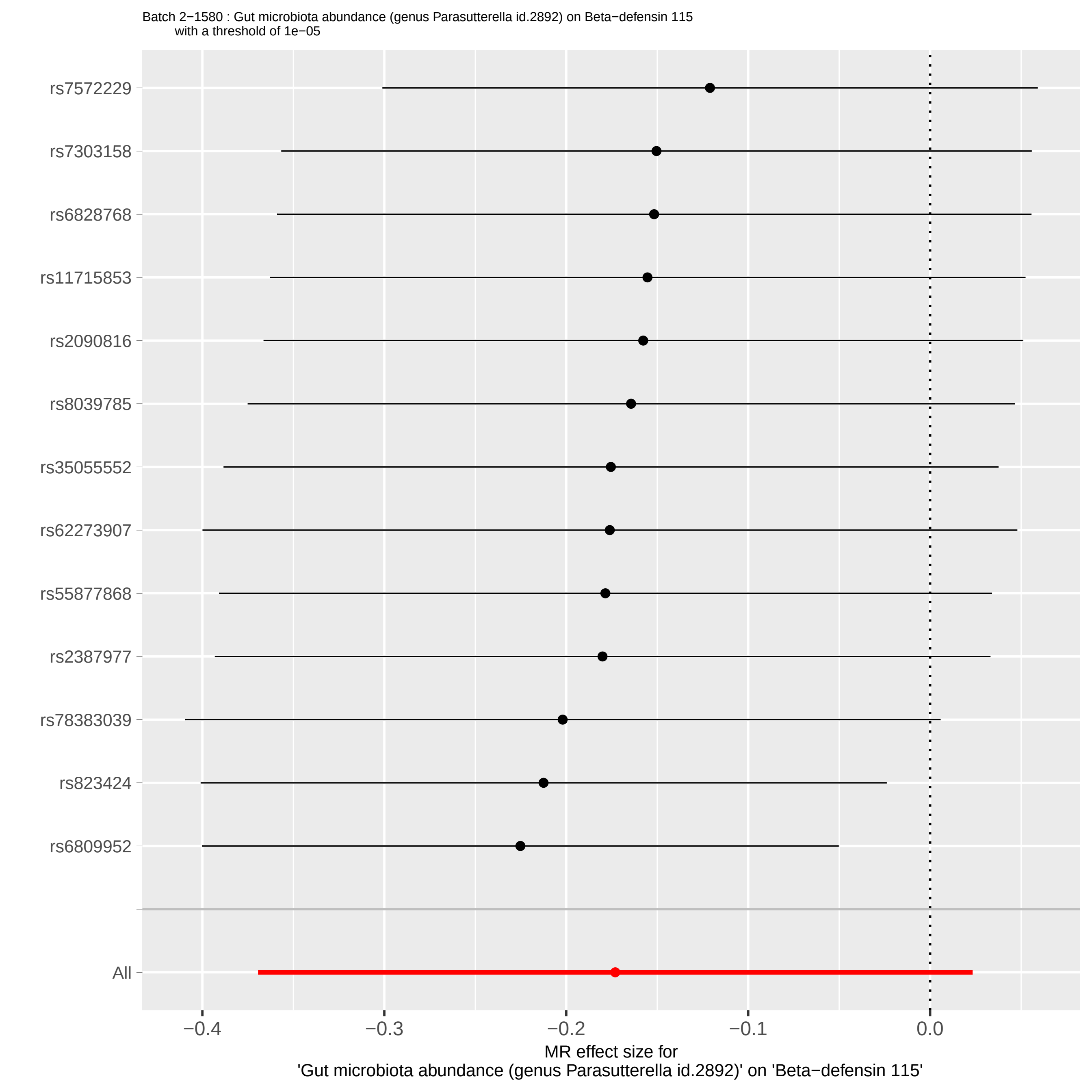
Batch 2−1580 : Gut microbiota abundance (genus Parasutterella id.2892) on Beta−defensin 115
with a threshold of 1e−05
rs7572229
rs7303158
rs6828768
rs11715853
rs2090816
rs8039785
rs35055552
rs62273907
rs55877868
rs2387977
rs78383039
rs823424
rs6809952
All
−0.4
−0.3
−0.2
−0.1
0.0
MR effect size for
'Gut microbiota abundance (genus Parasutterella id.2892)' on 'Beta−defensin 115'

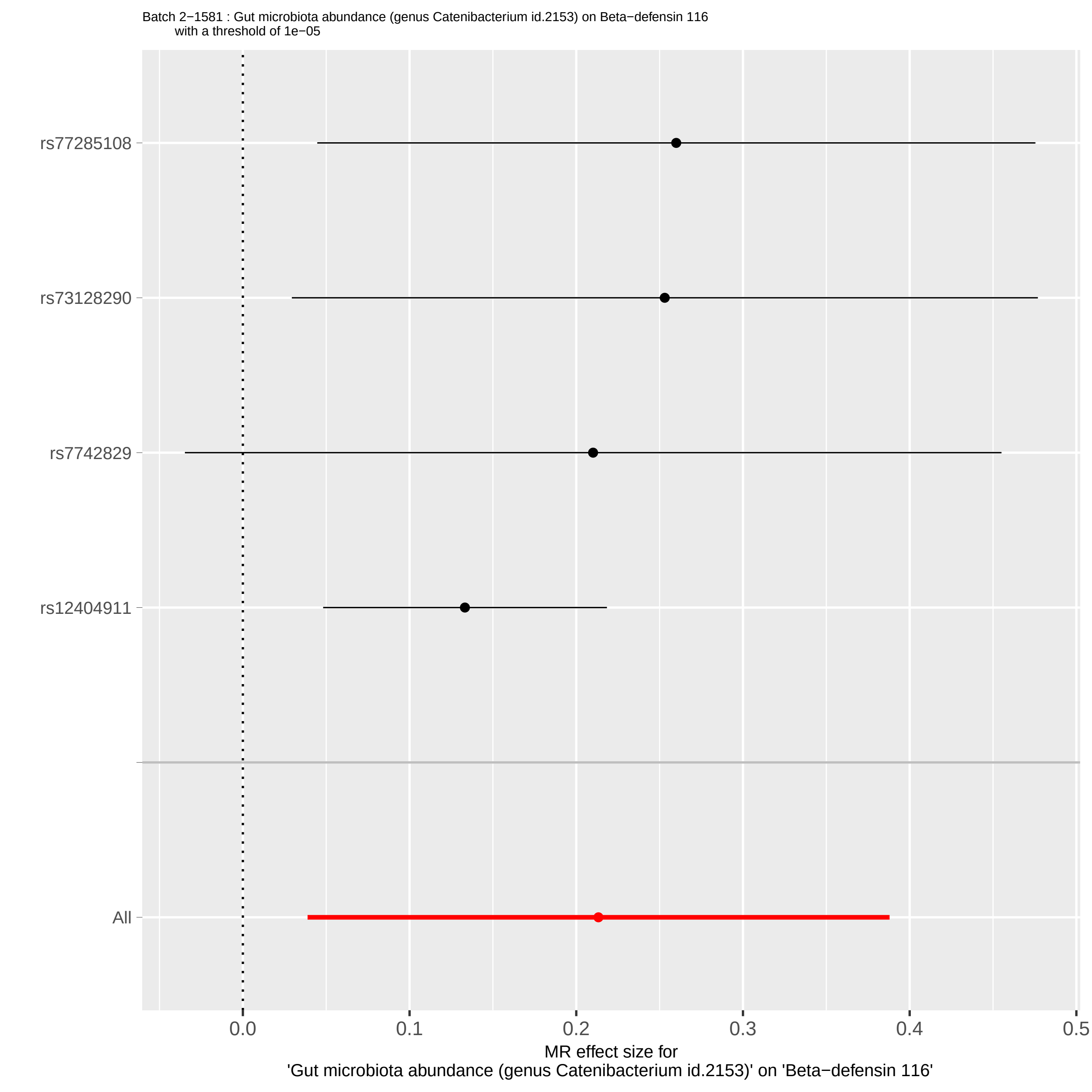
Batch 2−1581 : Gut microbiota abundance (genus Catenibacterium id.2153) on Beta−defensin 116
with a threshold of 1e−05
rs77285108
rs73128290
rs7742829
rs12404911
All
0.0
0.1
0.2
0.3
0.4
0.5
MR effect size for
'Gut microbiota abundance (genus Catenibacterium id.2153)' on 'Beta−defensin 116'

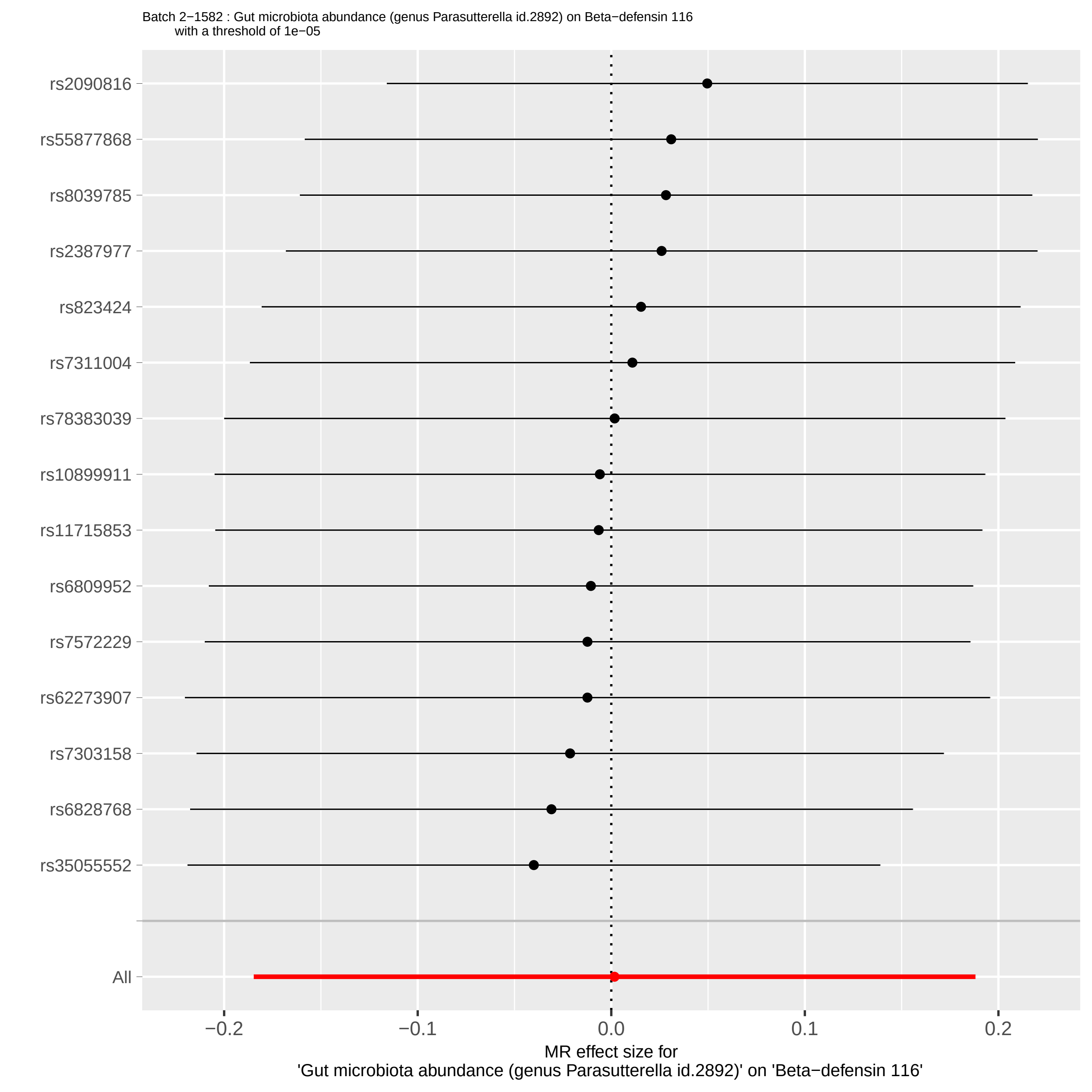
Batch 2−1582 : Gut microbiota abundance (genus Parasutterella id.2892) on Beta−defensin 116
with a threshold of 1e−05
rs2090816
rs55877868
rs8039785
rs2387977
rs823424
rs7311004
rs78383039
rs10899911
rs11715853
rs6809952
rs7572229
rs62273907
rs7303158
rs6828768
rs35055552
All
−0.2
−0.1
0.0
0.1
0.2
MR effect size for
'Gut microbiota abundance (genus Parasutterella id.2892)' on 'Beta−defensin 116'

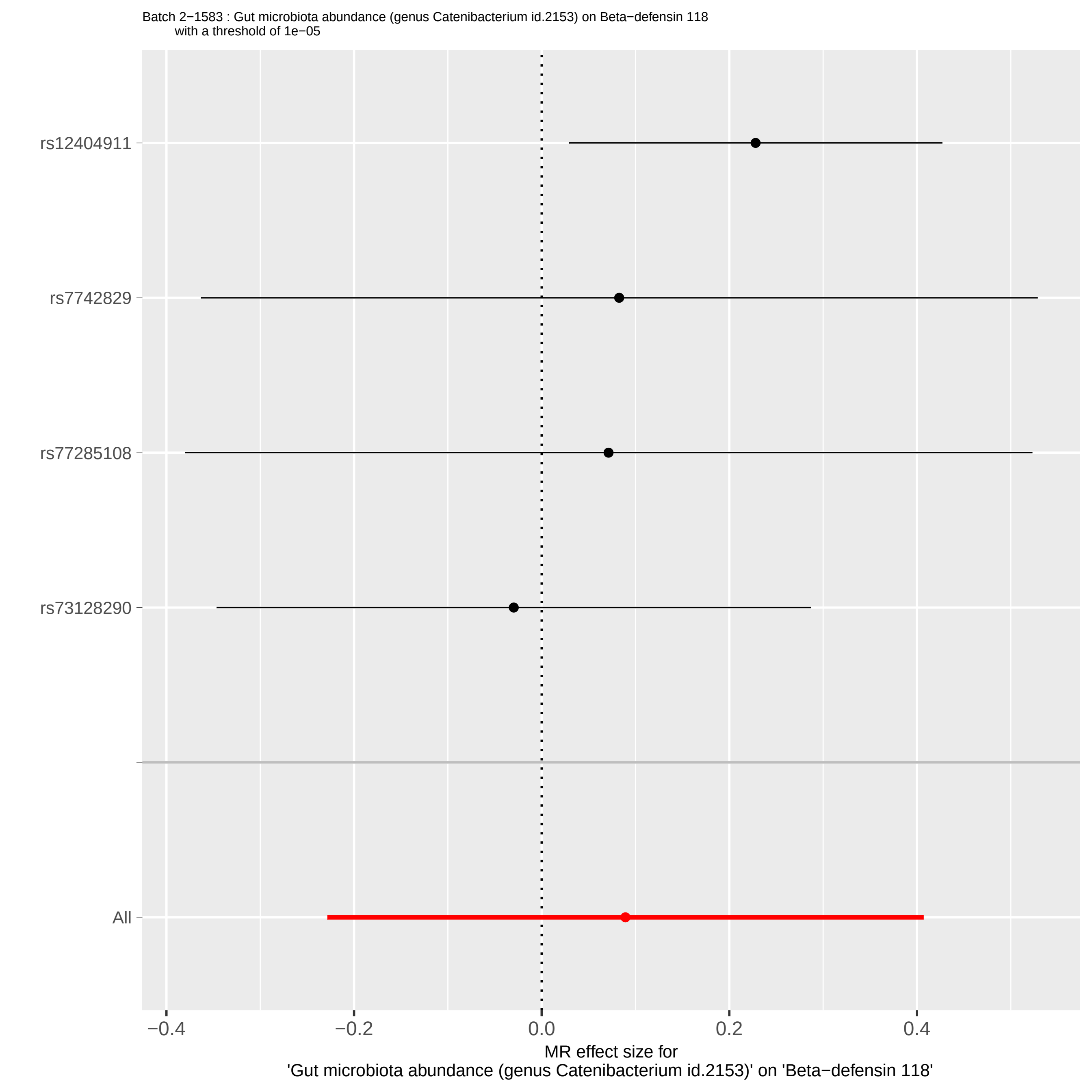
Batch 2−1583 : Gut microbiota abundance (genus Catenibacterium id.2153) on Beta−defensin 118
with a threshold of 1e−05
rs12404911
rs7742829
rs77285108
rs73128290
All
−0.4
−0.2
0.0
0.2
0.4
MR effect size for
'Gut microbiota abundance (genus Catenibacterium id.2153)' on 'Beta−defensin 118'

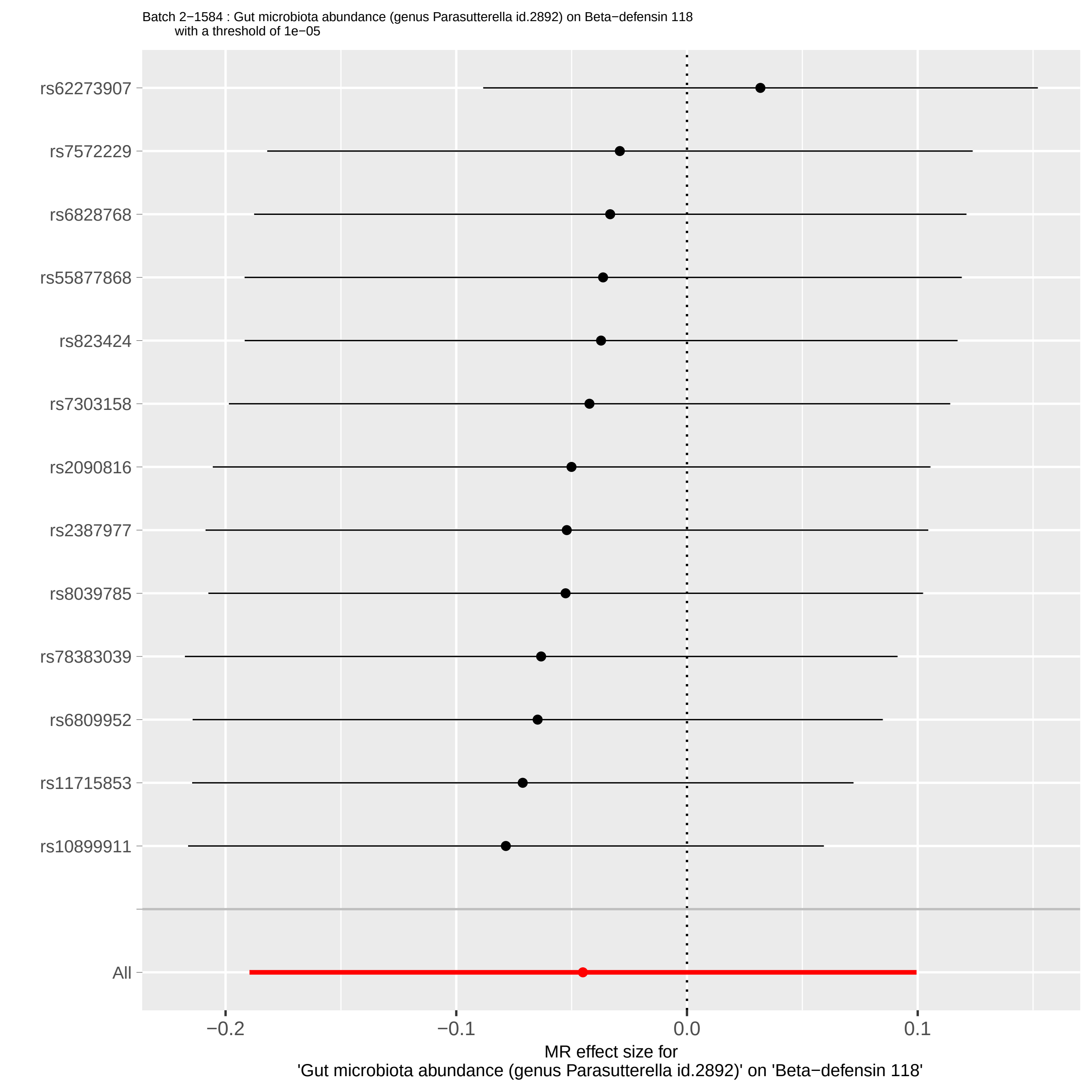
Batch 2−1584 : Gut microbiota abundance (genus Parasutterella id.2892) on Beta−defensin 118
with a threshold of 1e−05
rs62273907
rs7572229
rs6828768
rs55877868
rs823424
rs7303158
rs2090816
rs2387977
rs8039785
rs78383039
rs6809952
rs11715853
rs10899911
All
−0.2
−0.1
0.0
0.1
MR effect size for
'Gut microbiota abundance (genus Parasutterella id.2892)' on 'Beta−defensin 118'

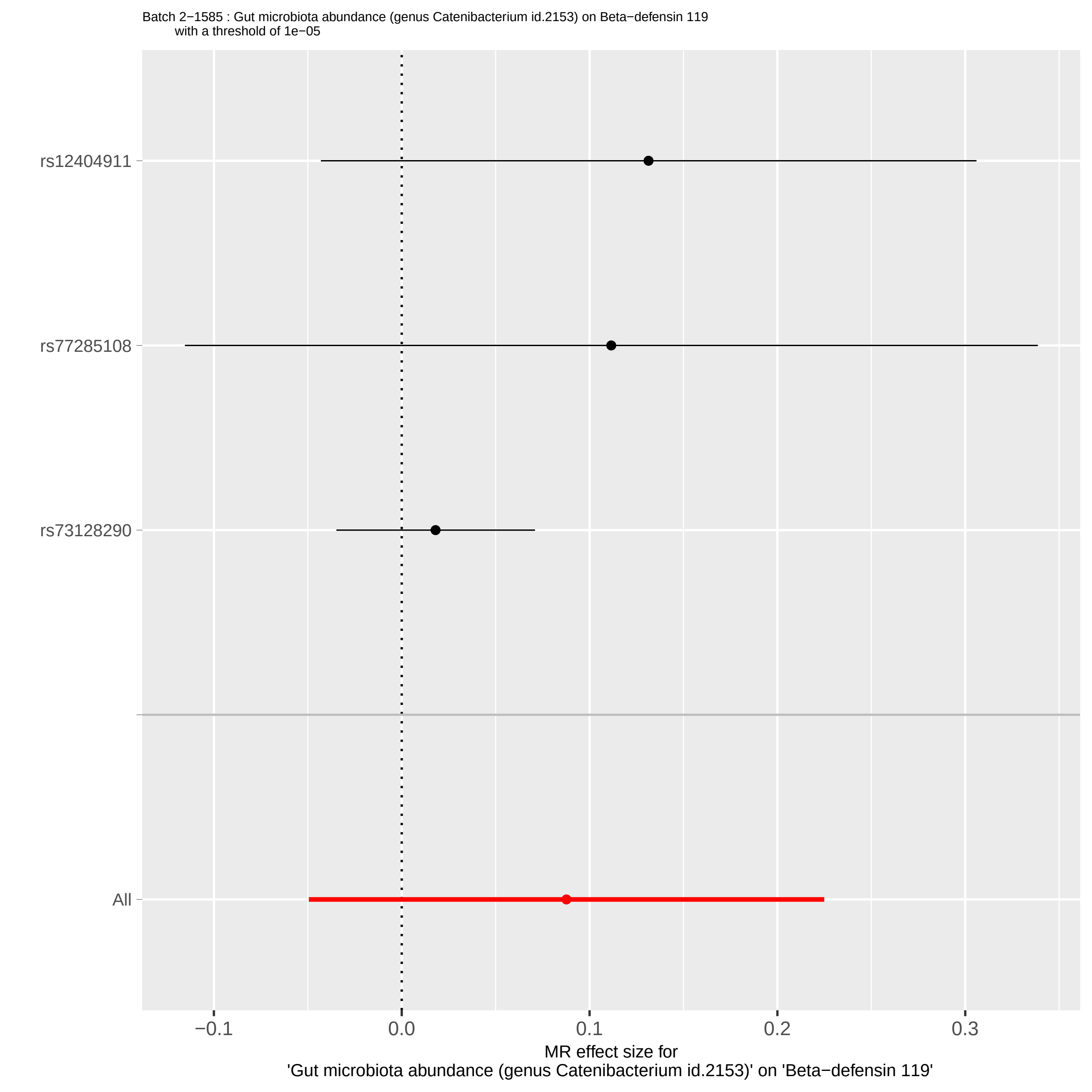

Batch 2−1585 : Gut microbiota abundance (genus Catenibacterium id.2153) on Beta−defensin 119
with a threshold of 1e−05
rs12404911
rs77285108
rs73128290
All
−0.1
0.0
0.1
0.2
0.3
MR effect size for
'Gut microbiota abundance (genus Catenibacterium id.2153)' on 'Beta−defensin 119'

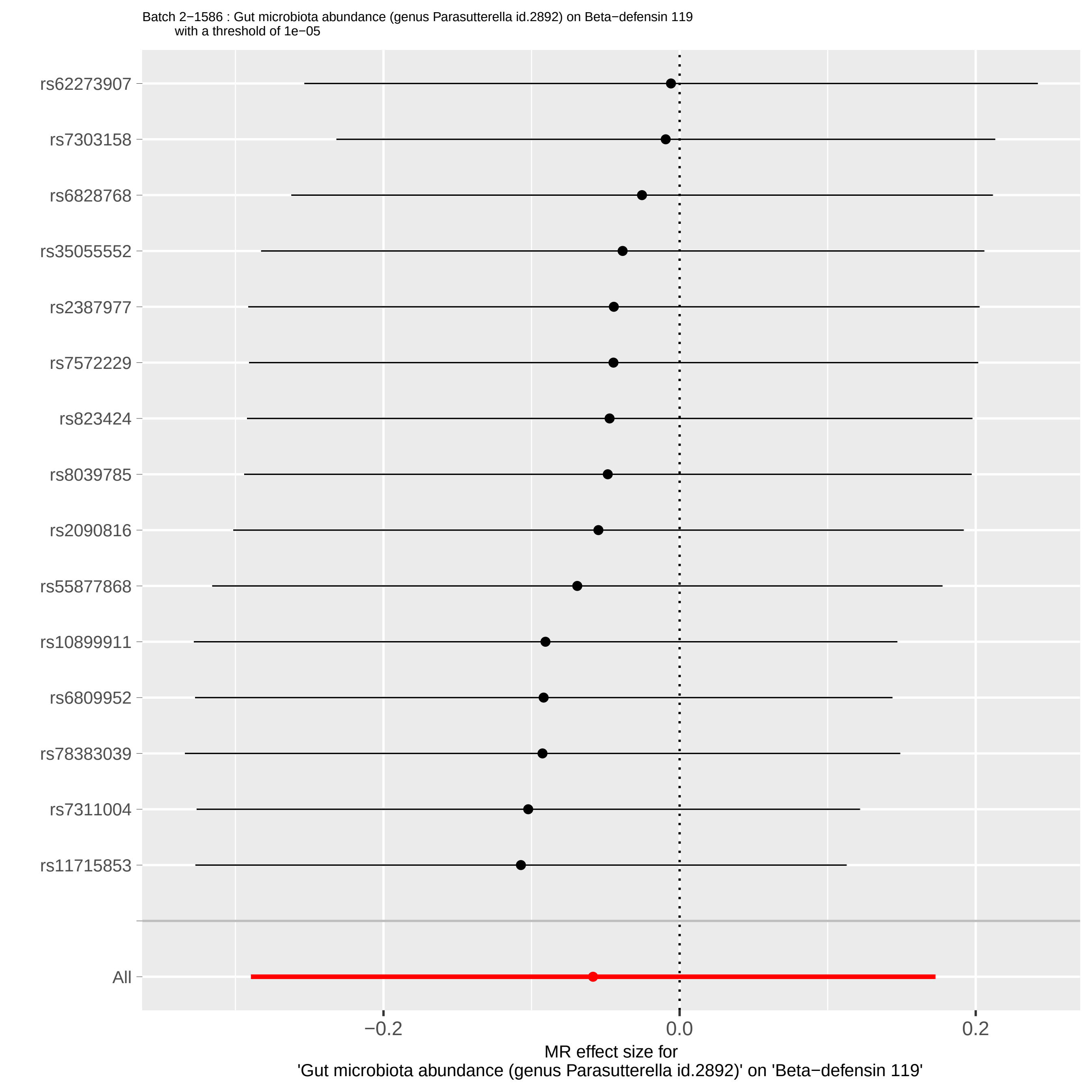
Batch 2−1586 : Gut microbiota abundance (genus Parasutterella id.2892) on Beta−defensin 119
with a threshold of 1e−05
rs62273907
rs7303158
rs6828768
rs35055552
rs2387977
rs7572229
rs823424
rs8039785
rs2090816
rs55877868
rs10899911
rs6809952
rs78383039
rs7311004
rs11715853
All
−0.2
0.0
0.2
MR effect size for
'Gut microbiota abundance (genus Parasutterella id.2892)' on 'Beta−defensin 119'

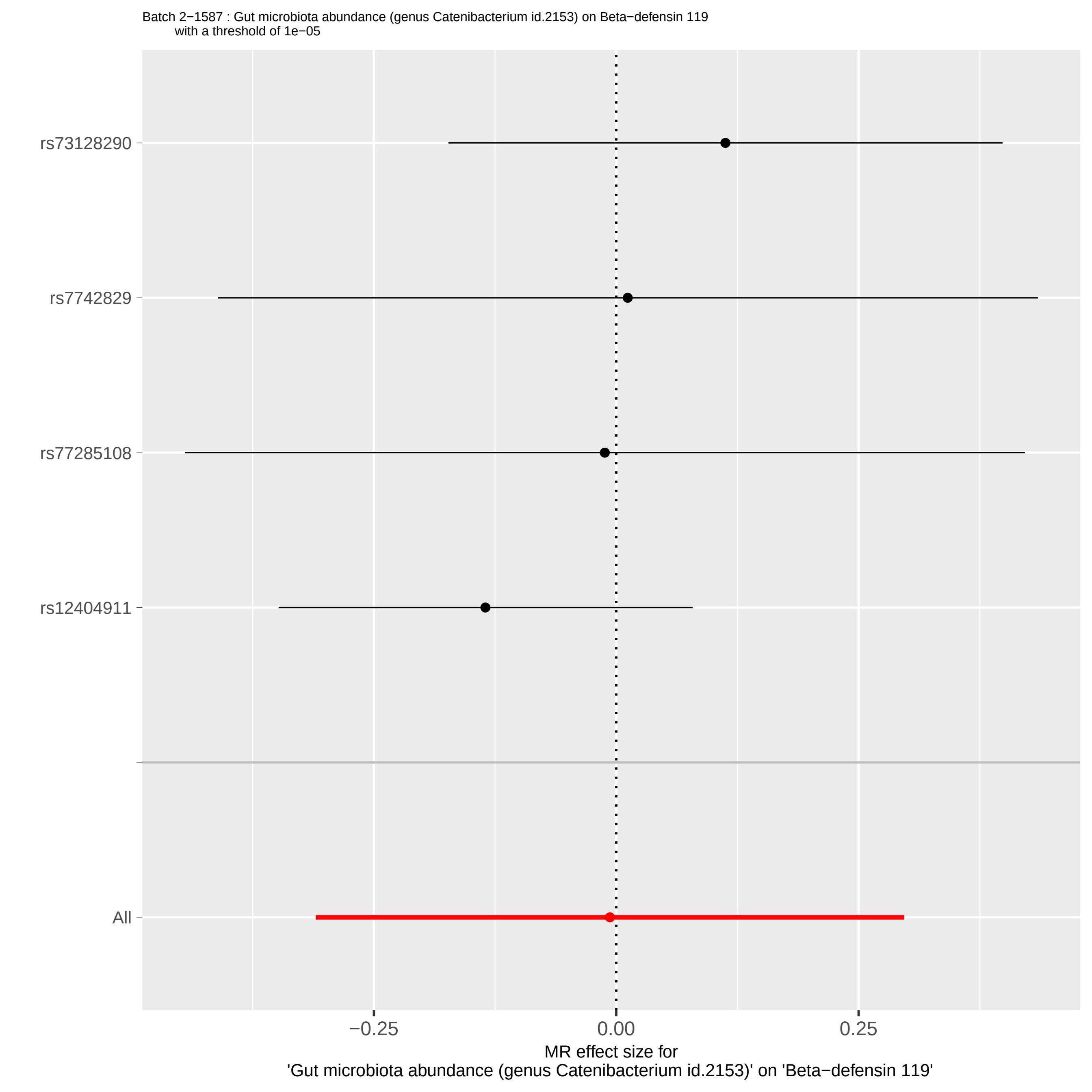

Batch 2−1587 : Gut microbiota abundance (genus Catenibacterium id.2153) on Beta−defensin 119
with a threshold of 1e−05
rs73128290
rs7742829
rs77285108
rs12404911
All
−0.25
0.00
0.25
MR effect size for
'Gut microbiota abundance (genus Catenibacterium id.2153)' on 'Beta−defensin 119'

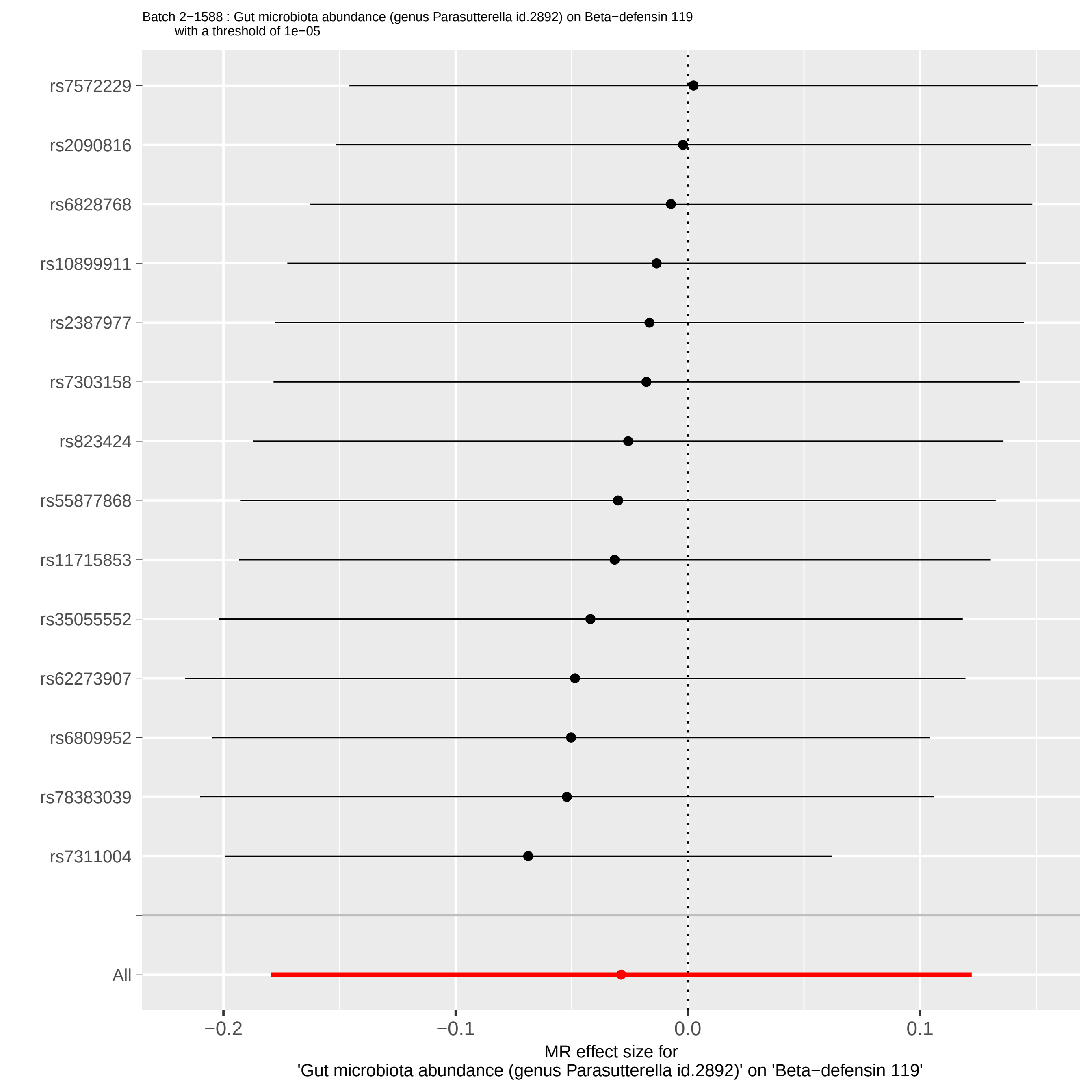

Batch 2−1588 : Gut microbiota abundance (genus Parasutterella id.2892) on Beta−defensin 119
with a threshold of 1e−05
rs7572229
rs2090816
rs6828768
rs10899911
rs2387977
rs7303158
rs823424
rs55877868
rs11715853
rs35055552
rs62273907
rs6809952
rs78383039
rs7311004
All
−0.2
−0.1
0.0
0.1
MR effect size for
'Gut microbiota abundance (genus Parasutterella id.2892)' on 'Beta−defensin 119'

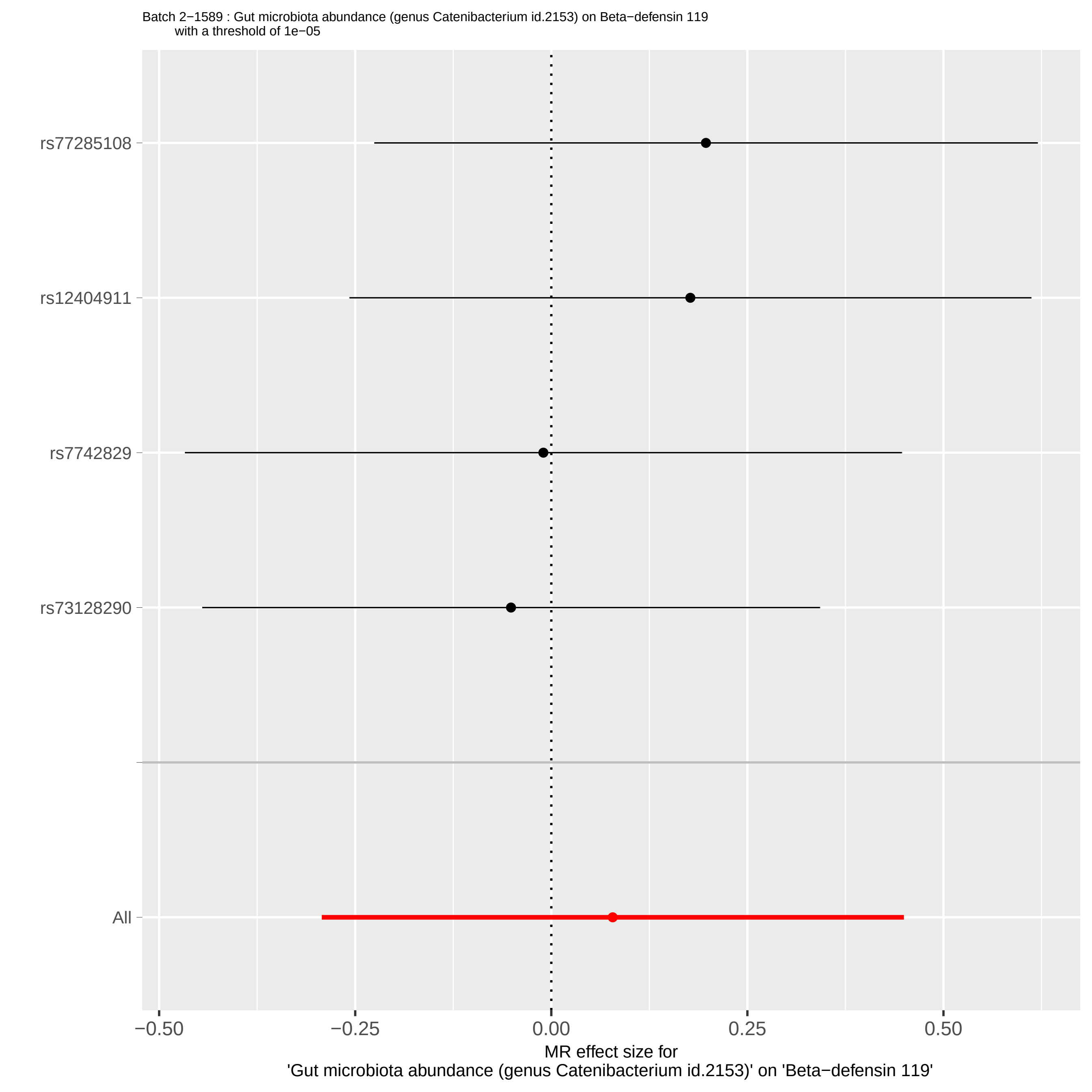

Batch 2−1589 : Gut microbiota abundance (genus Catenibacterium id.2153) on Beta−defensin 119
with a threshold of 1e−05
rs77285108
rs12404911
rs7742829
rs73128290
All
−0.50
−0.25
0.00
0.25
0.50
MR effect size for
'Gut microbiota abundance (genus Catenibacterium id.2153)' on 'Beta−defensin 119'

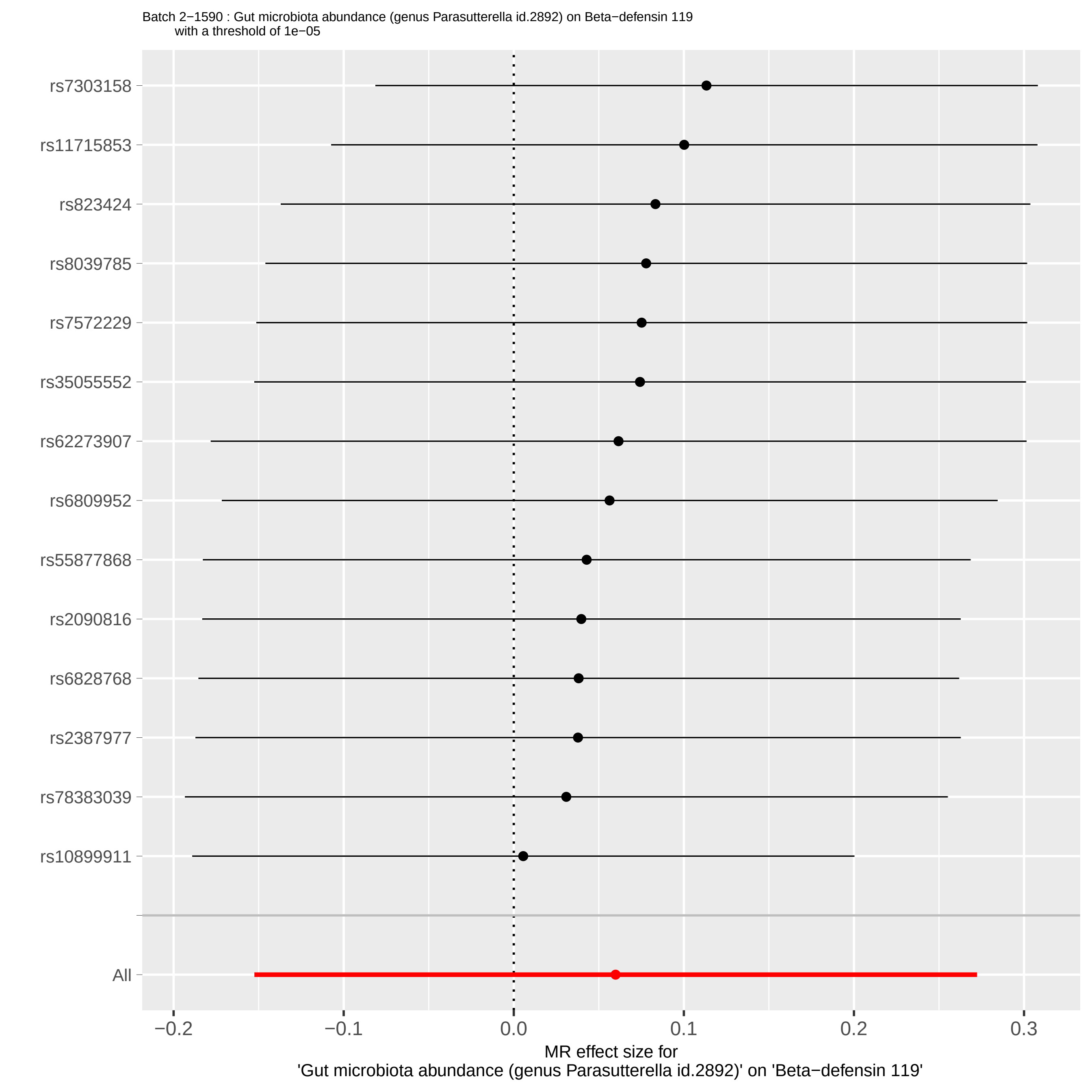
Batch 2−1590 : Gut microbiota abundance (genus Parasutterella id.2892) on Beta−defensin 119
with a threshold of 1e−05
rs7303158
rs11715853
rs823424
rs8039785
rs7572229
rs35055552
rs62273907
rs6809952
rs55877868
rs2090816
rs6828768
rs2387977
rs78383039
rs10899911
All
−0.2
−0.1
0.0
0.1
0.2
0.3
MR effect size for
'Gut microbiota abundance (genus Parasutterella id.2892)' on 'Beta−defensin 119'

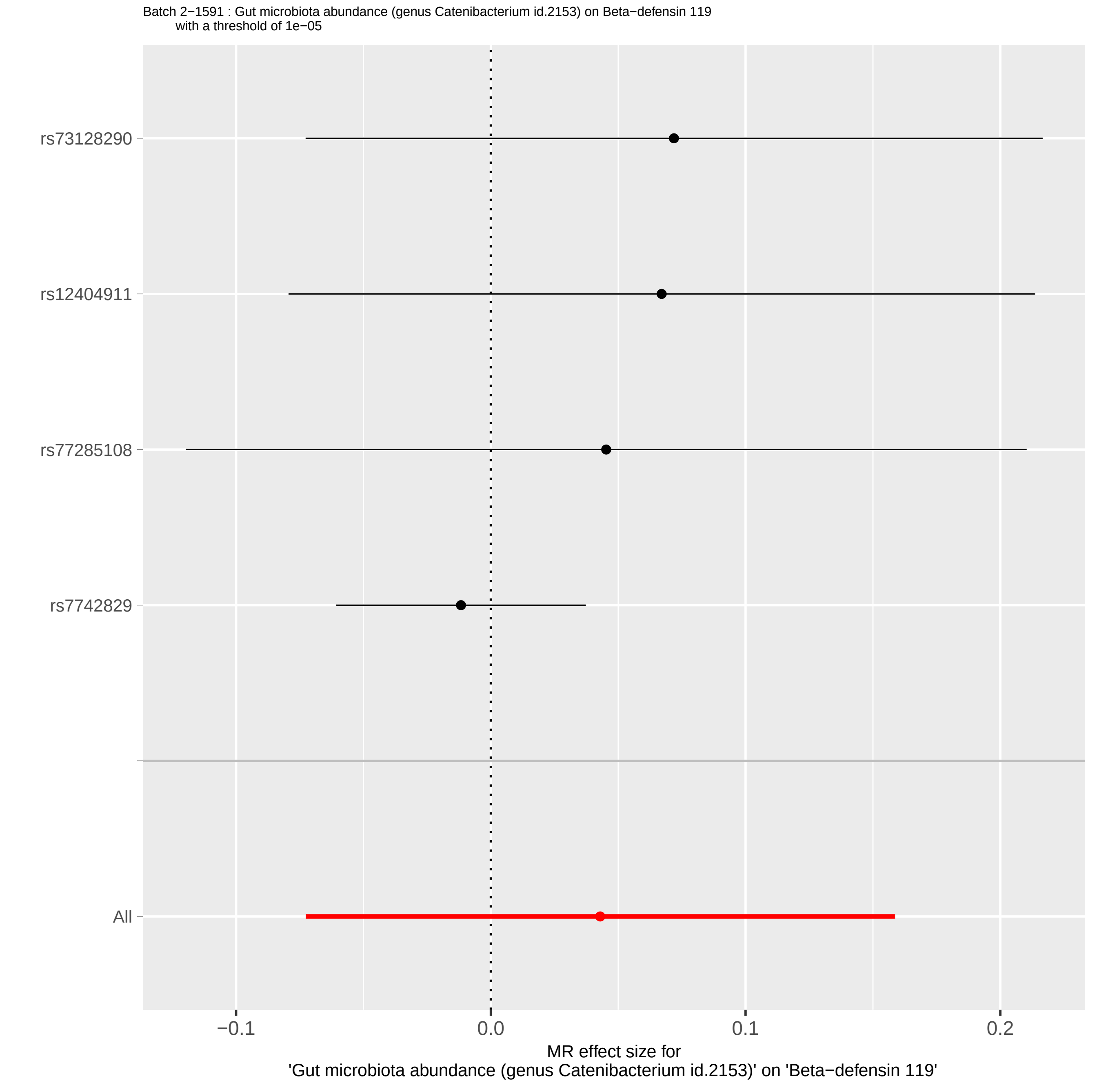
Batch 2−1591 : Gut microbiota abundance (genus Catenibacterium id.2153) on Beta−defensin 119
with a threshold of 1e−05
rs73128290
rs12404911
rs77285108
rs7742829
All
−0.1
0.0
0.1
0.2
MR effect size for
'Gut microbiota abundance (genus Catenibacterium id.2153)' on 'Beta−defensin 119'

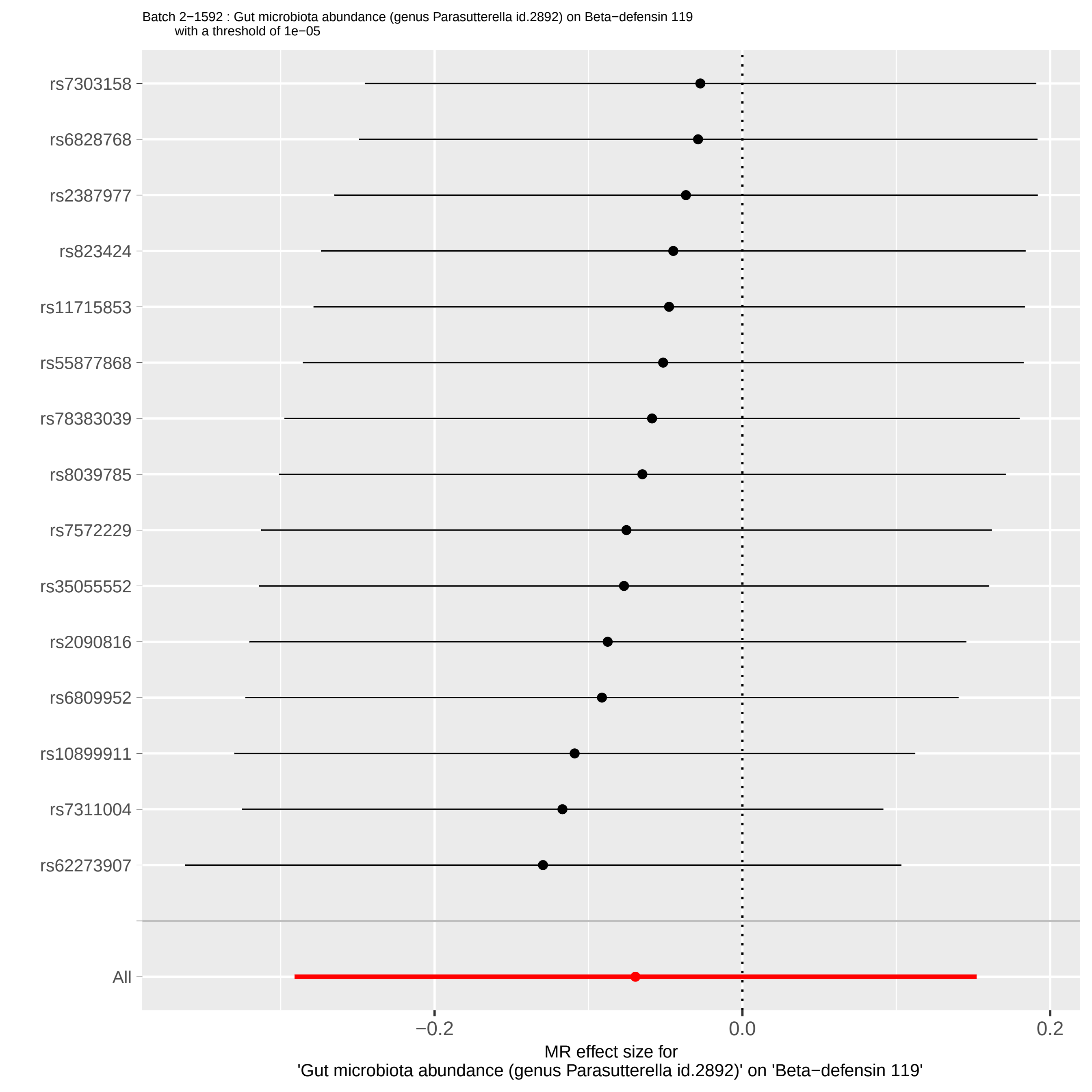
Batch 2−1592 : Gut microbiota abundance (genus Parasutterella id.2892) on Beta−defensin 119
with a threshold of 1e−05
rs7303158
rs6828768
rs2387977
rs823424
rs11715853
rs55877868
rs78383039
rs8039785
rs7572229
rs35055552
rs2090816
rs6809952
rs10899911
rs7311004
rs62273907
All
−0.2
0.0
0.2
MR effect size for
'Gut microbiota abundance (genus Parasutterella id.2892)' on 'Beta−defensin 119'

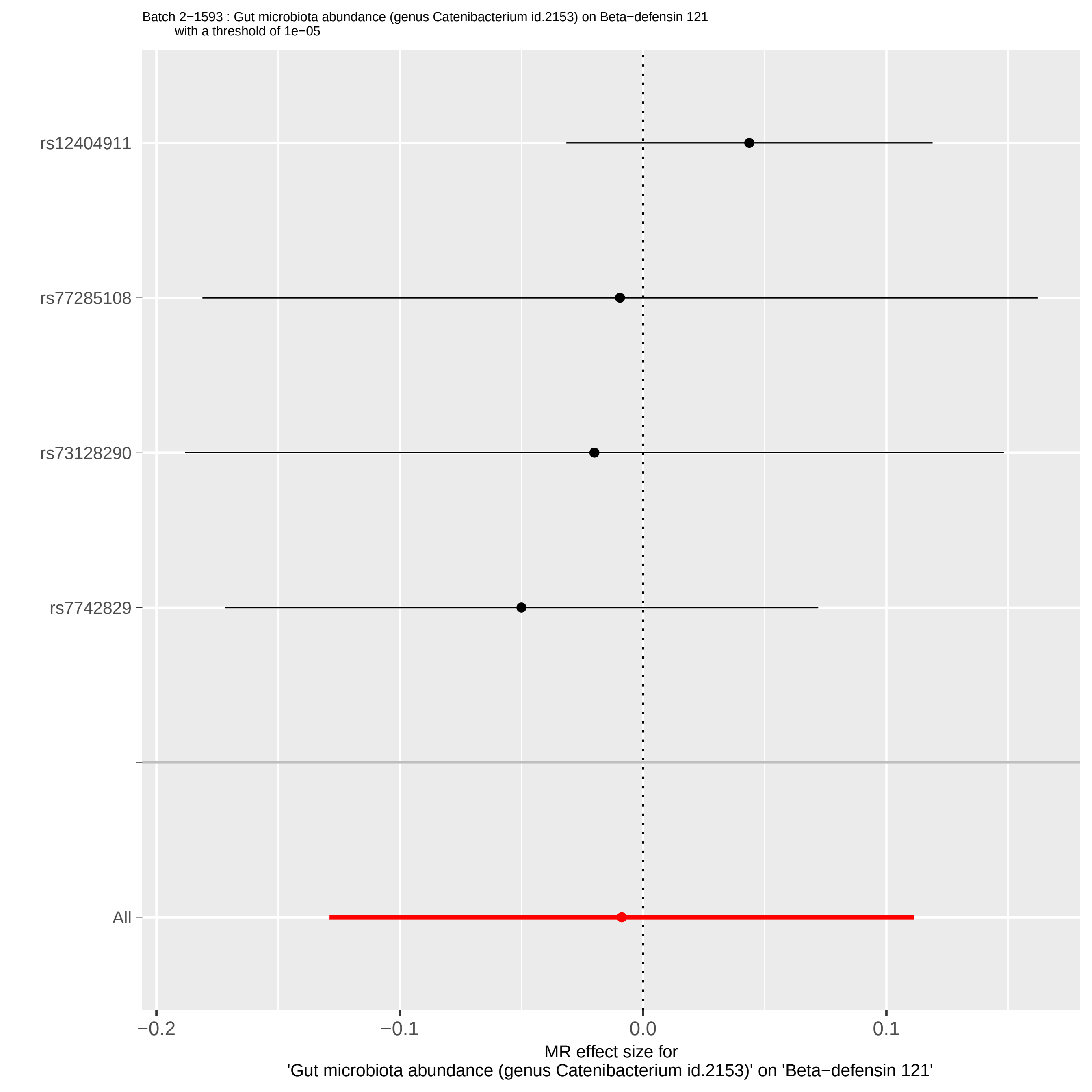
Batch 2−1593 : Gut microbiota abundance (genus Catenibacterium id.2153) on Beta−defensin 121
with a threshold of 1e−05
rs12404911
rs77285108
rs73128290
rs7742829
All
−0.2
−0.1
0.0
0.1
MR effect size for
'Gut microbiota abundance (genus Catenibacterium id.2153)' on 'Beta−defensin 121'

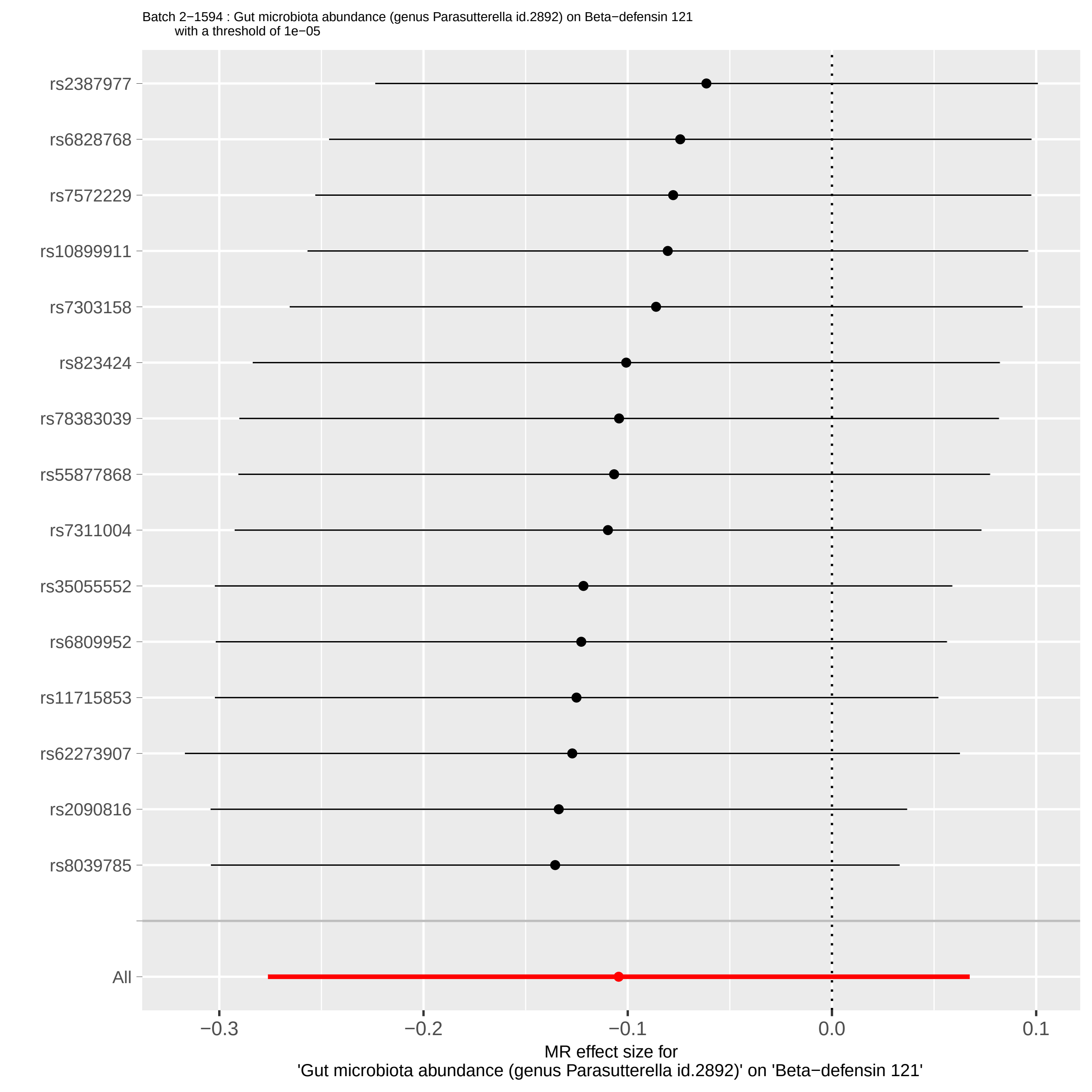

Batch 2−1594 : Gut microbiota abundance (genus Parasutterella id.2892) on Beta−defensin 121
with a threshold of 1e−05
rs2387977
rs6828768
rs7572229
rs10899911
rs7303158
rs823424
rs78383039
rs55877868
rs7311004
rs35055552
rs6809952
rs11715853
rs62273907
rs2090816
rs8039785
All
−0.3
−0.2
−0.1
0.0
0.1
MR effect size for
'Gut microbiota abundance (genus Parasutterella id.2892)' on 'Beta−defensin 121'

Batch 2−1595 : Gut microbiota abundance (genus Catenibacterium id.2153) on Beta−defensin 123
with a threshold of 1e−05

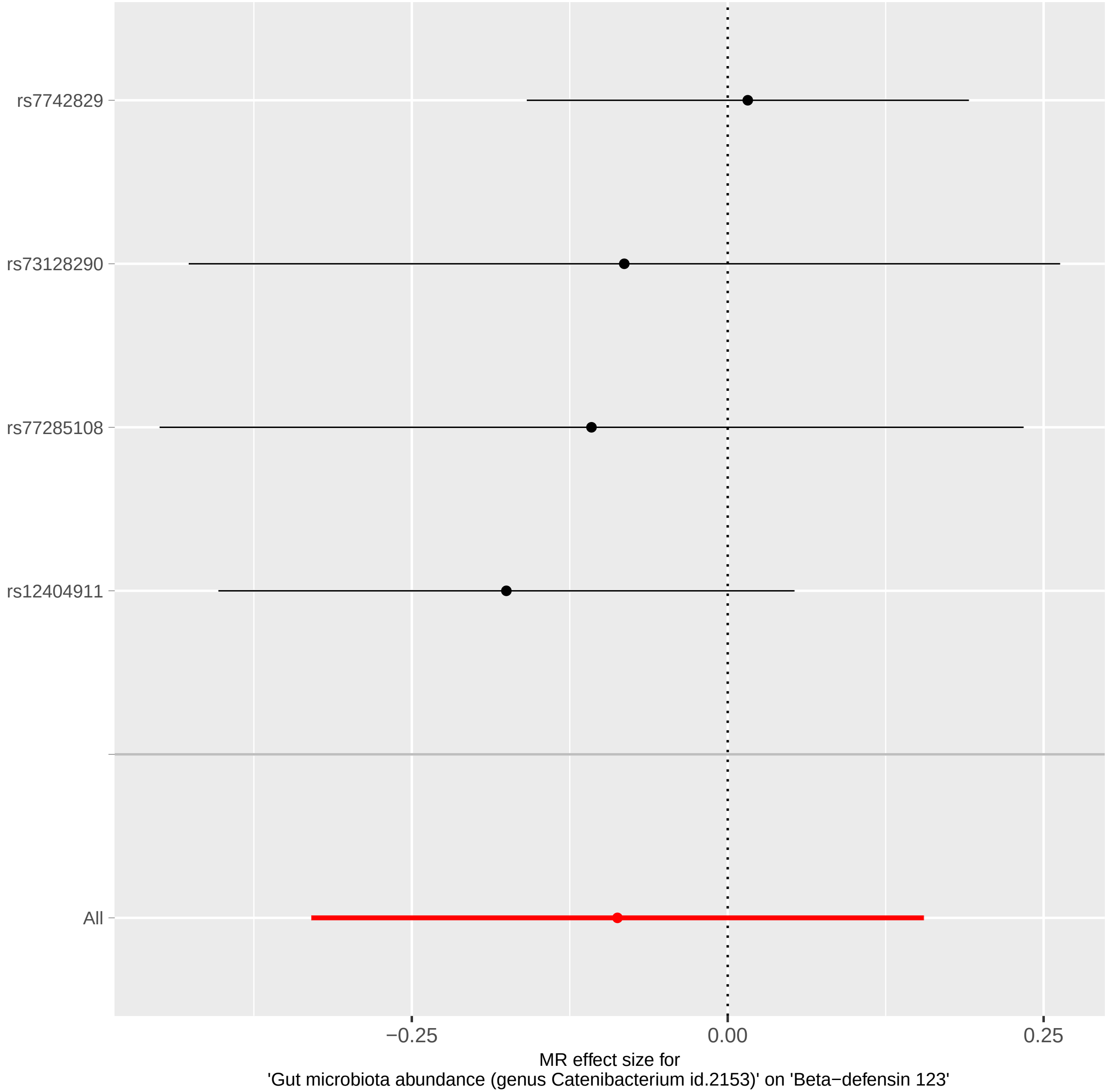

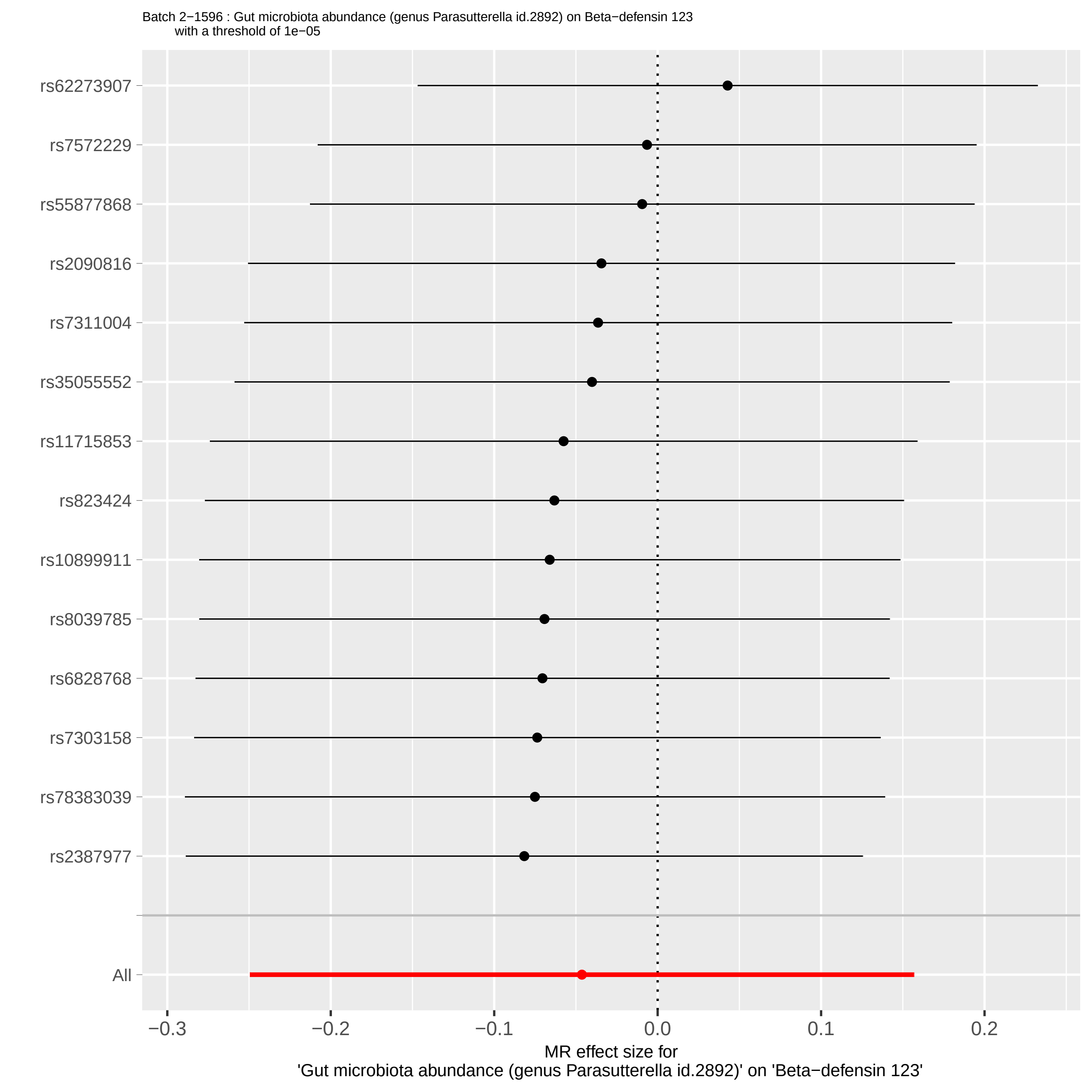

Batch 2−1596 : Gut microbiota abundance (genus Parasutterella id.2892) on Beta−defensin 123
with a threshold of 1e−05
rs62273907
rs7572229
rs55877868
rs2090816
rs7311004
rs35055552
rs11715853
rs823424
rs10899911
rs8039785
rs6828768
rs7303158
rs78383039
rs2387977
All
−0.3
−0.2
−0.1
0.0
0.1
0.2
MR effect size for
'Gut microbiota abundance (genus Parasutterella id.2892)' on 'Beta−defensin 123'

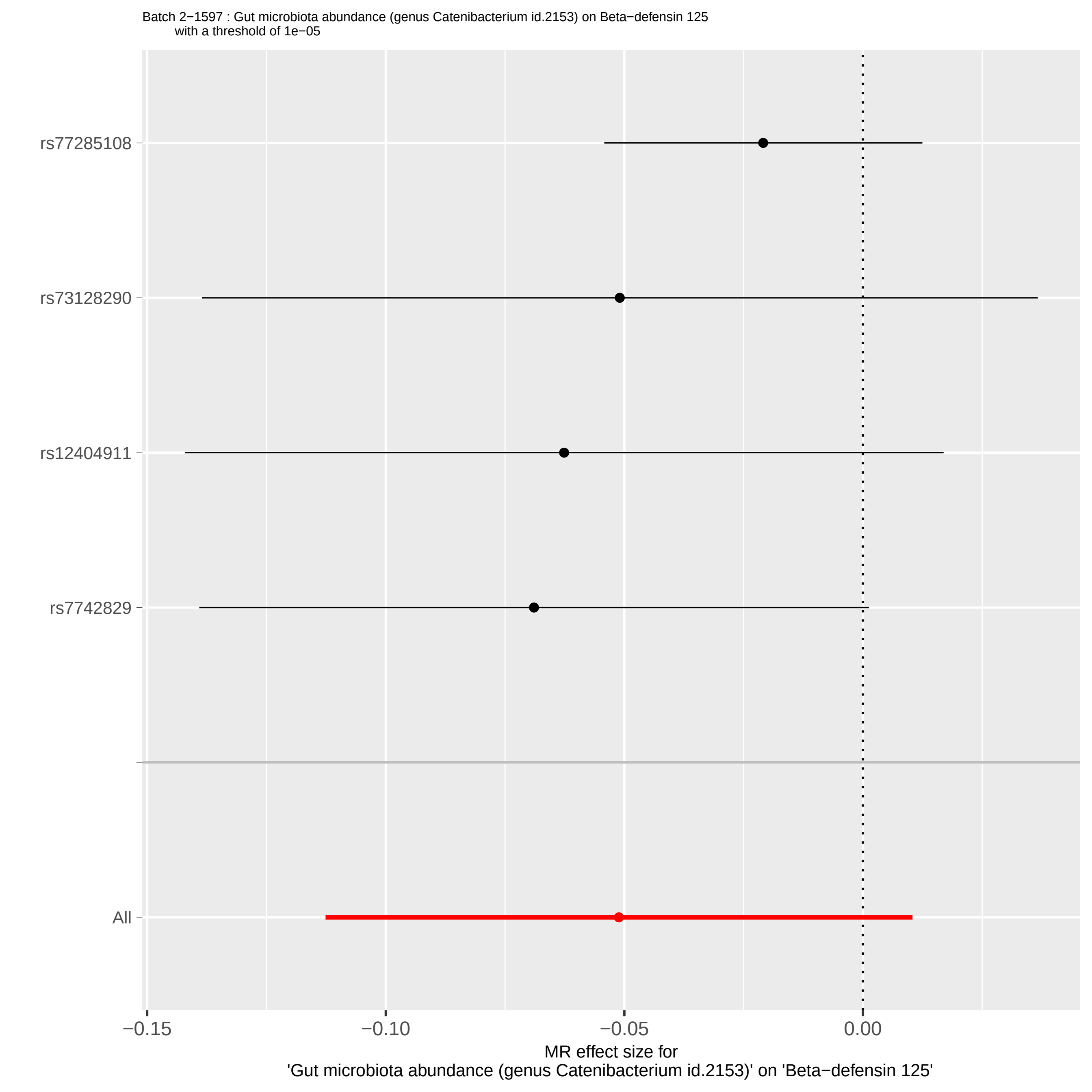

Batch 2−1597 : Gut microbiota abundance (genus Catenibacterium id.2153) on Beta−defensin 125
with a threshold of 1e−05
rs77285108
rs73128290
rs12404911
rs7742829
All
−0.15
−0.10
−0.05
0.00
MR effect size for
'Gut microbiota abundance (genus Catenibacterium id.2153)' on 'Beta−defensin 125'

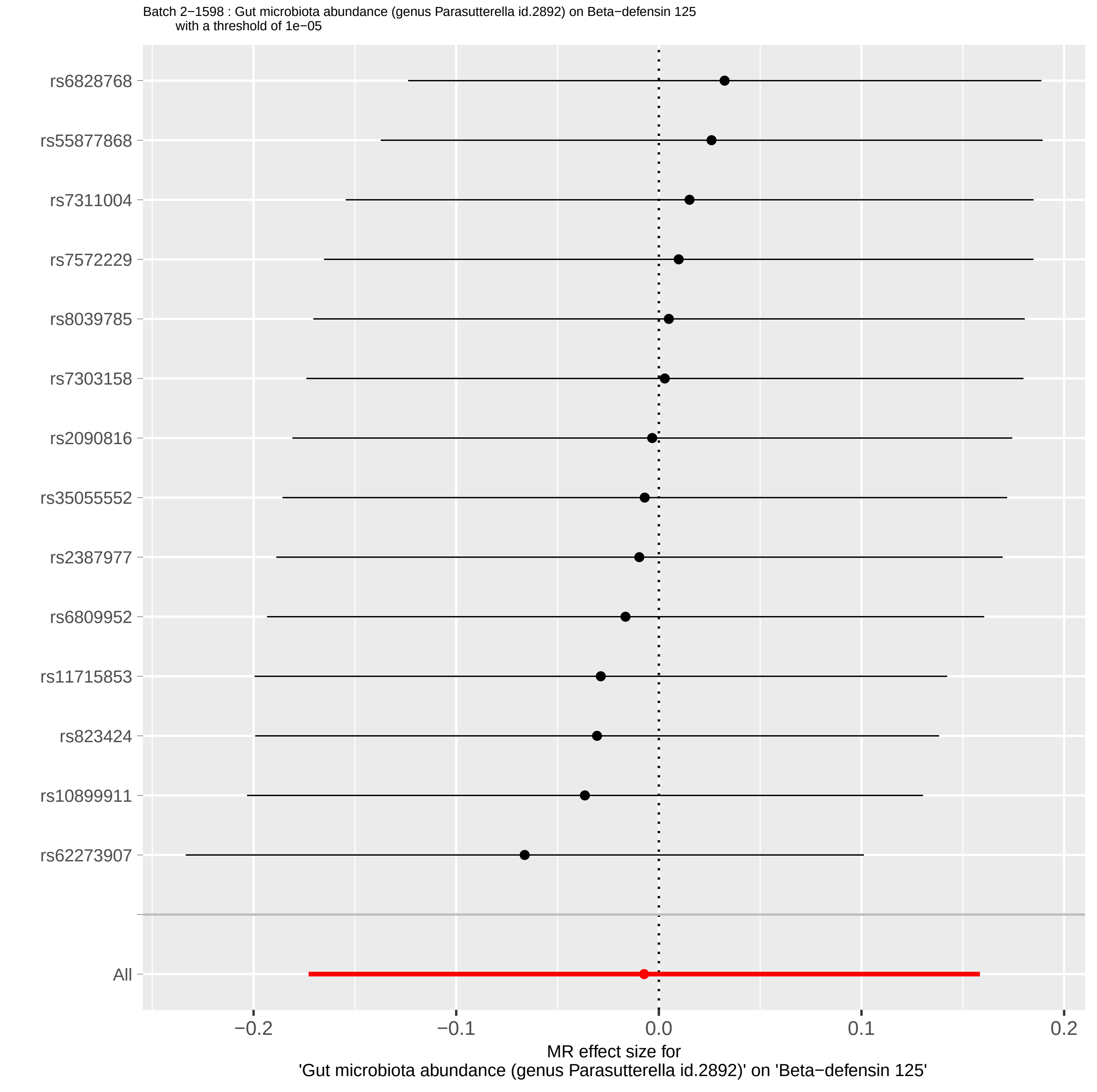

Batch 2−1598 : Gut microbiota abundance (genus Parasutterella id.2892) on Beta−defensin 125
with a threshold of 1e−05
rs6828768
rs55877868
rs7311004
rs7572229
rs8039785
rs7303158
rs2090816
rs35055552
rs2387977
rs6809952
rs11715853
rs823424
rs10899911
rs62273907
All
−0.2
−0.1
0.0
0.1
0.2
MR effect size for
'Gut microbiota abundance (genus Parasutterella id.2892)' on 'Beta−defensin 125'

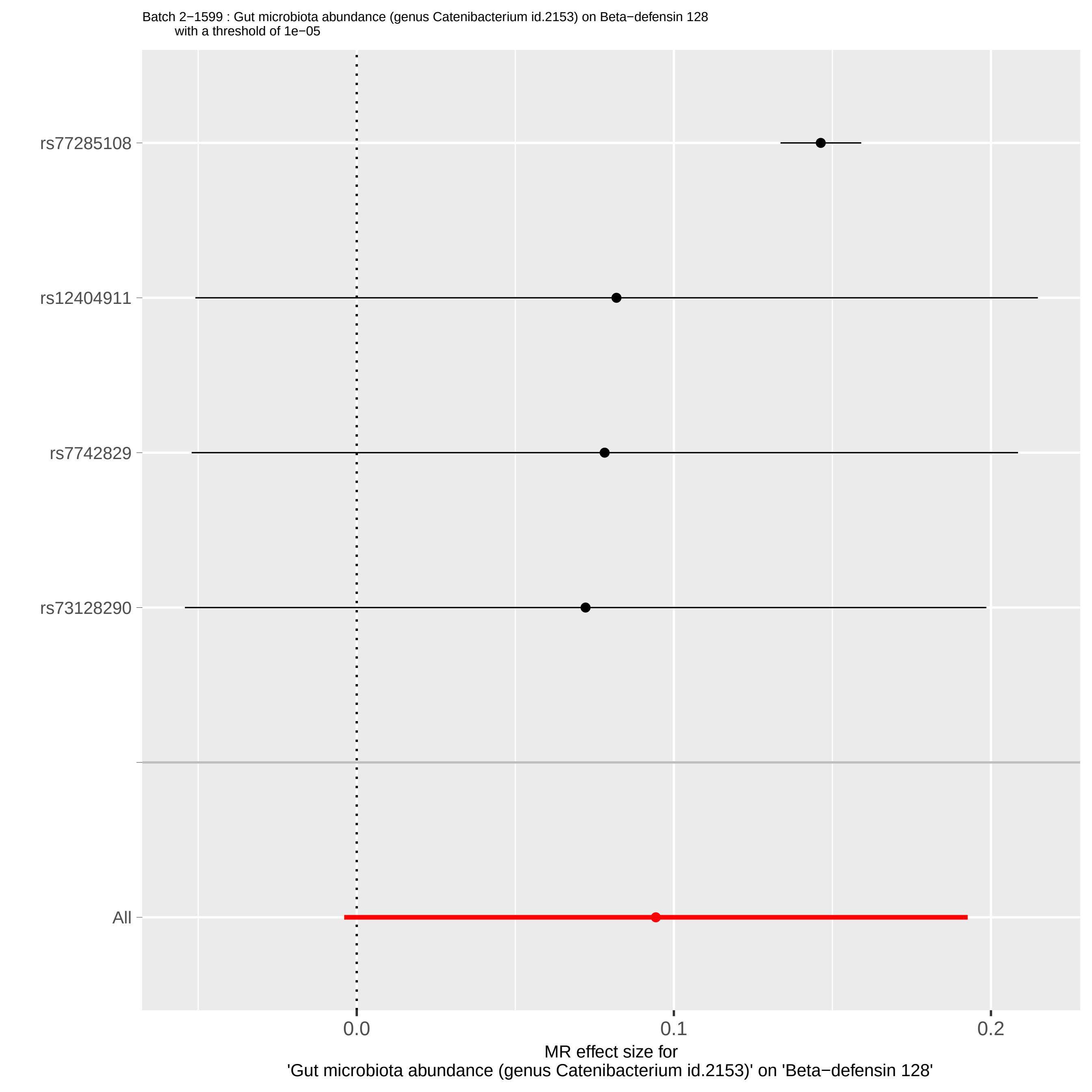
Batch 2−1599 : Gut microbiota abundance (genus Catenibacterium id.2153) on Beta−defensin 128
with a threshold of 1e−05
rs77285108
rs12404911
rs7742829
rs73128290
All
0.0
0.1
0.2
MR effect size for
'Gut microbiota abundance (genus Catenibacterium id.2153)' on 'Beta−defensin 128'

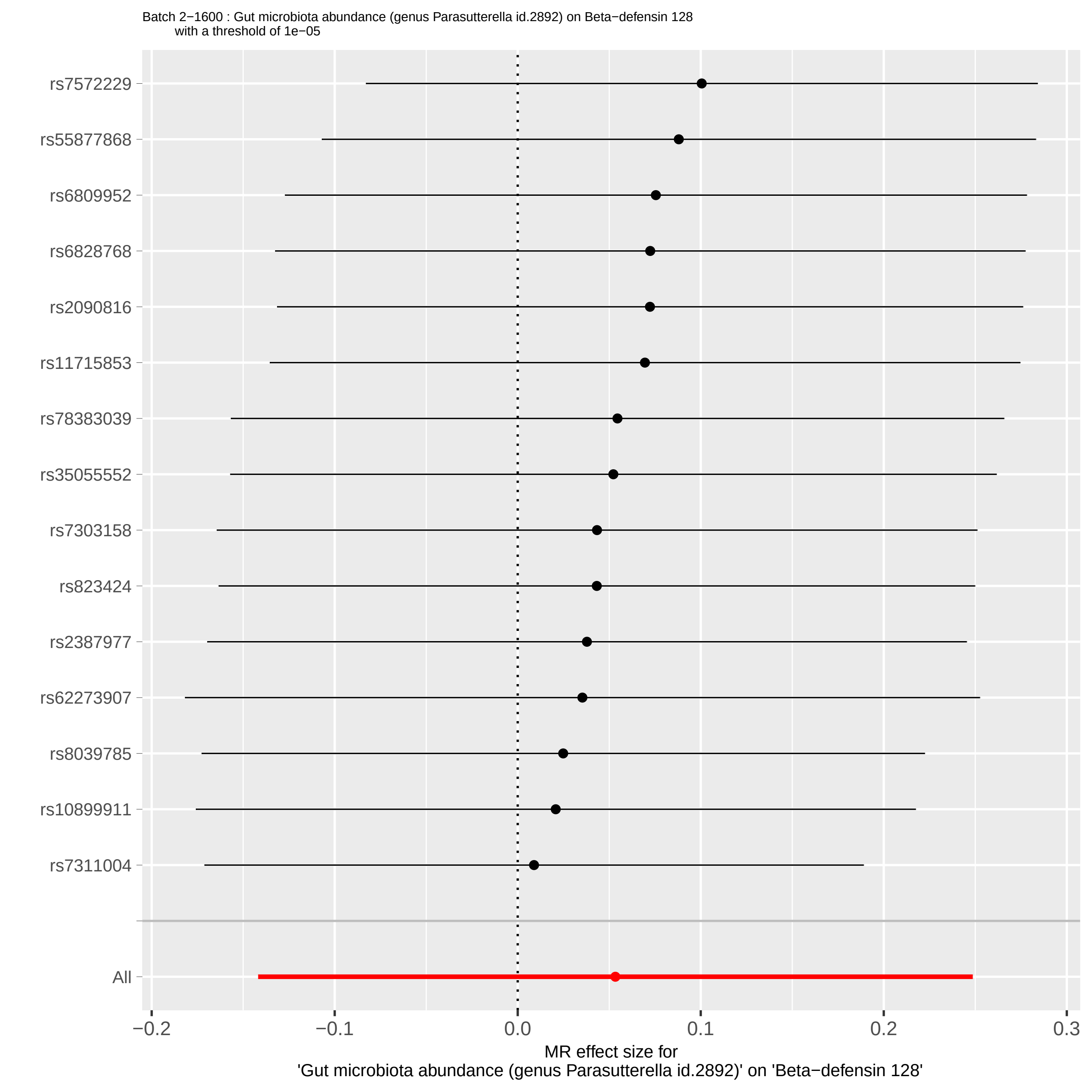

Batch 2−1600 : Gut microbiota abundance (genus Parasutterella id.2892) on Beta−defensin 128
with a threshold of 1e−05
rs7572229
rs55877868
rs6809952
rs6828768
rs2090816
rs11715853
rs78383039
rs35055552
rs7303158
rs823424
rs2387977
rs62273907
rs8039785
rs10899911
rs7311004
All
−0.2
−0.1
0.0
0.1
0.2
0.3
MR effect size for
'Gut microbiota abundance (genus Parasutterella id.2892)' on 'Beta−defensin 128'

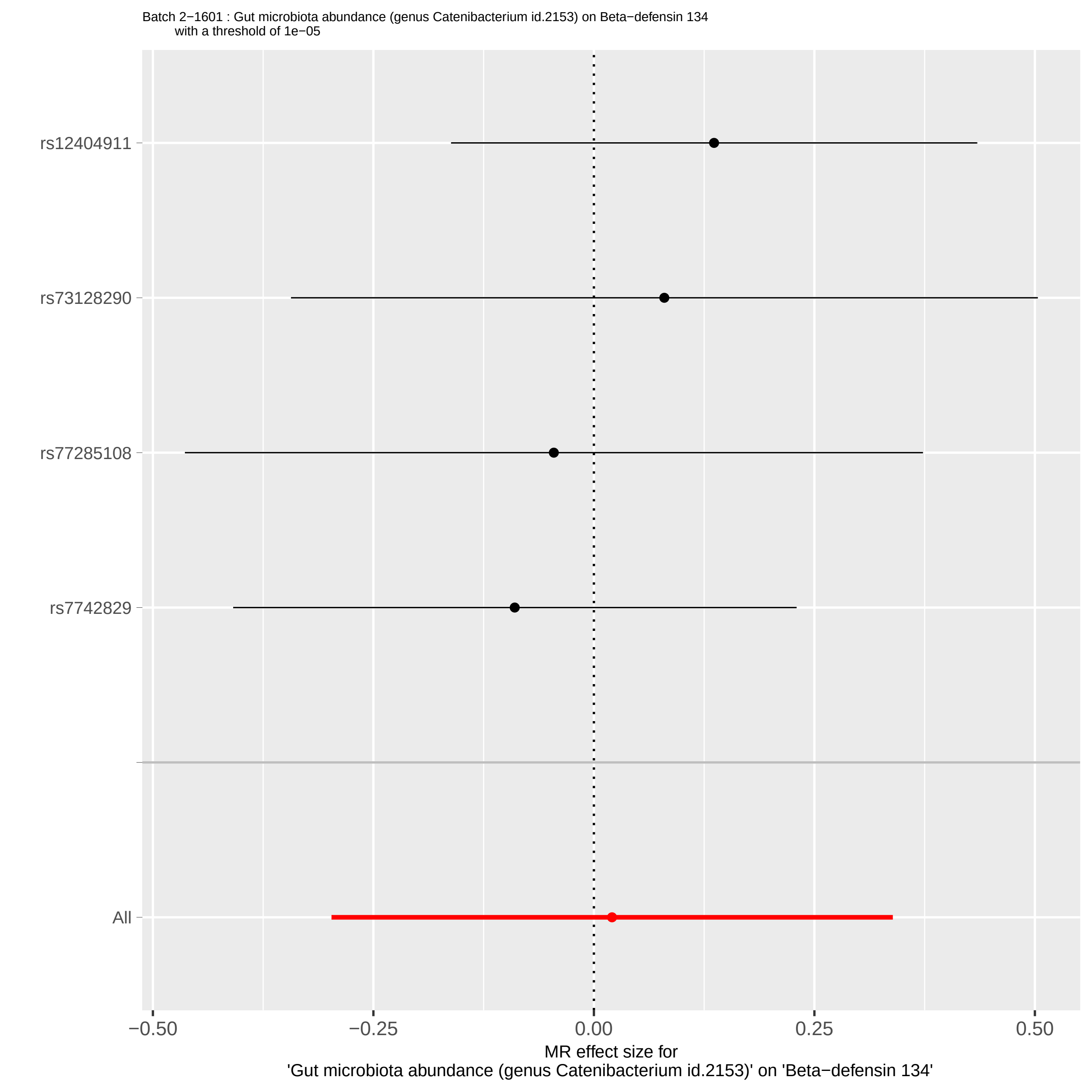
Batch 2−1601 : Gut microbiota abundance (genus Catenibacterium id.2153) on Beta−defensin 134
with a threshold of 1e−05
rs12404911
rs73128290
rs77285108
rs7742829
All
−0.50
−0.25
0.00
0.25
0.50
MR effect size for
'Gut microbiota abundance (genus Catenibacterium id.2153)' on 'Beta−defensin 134'

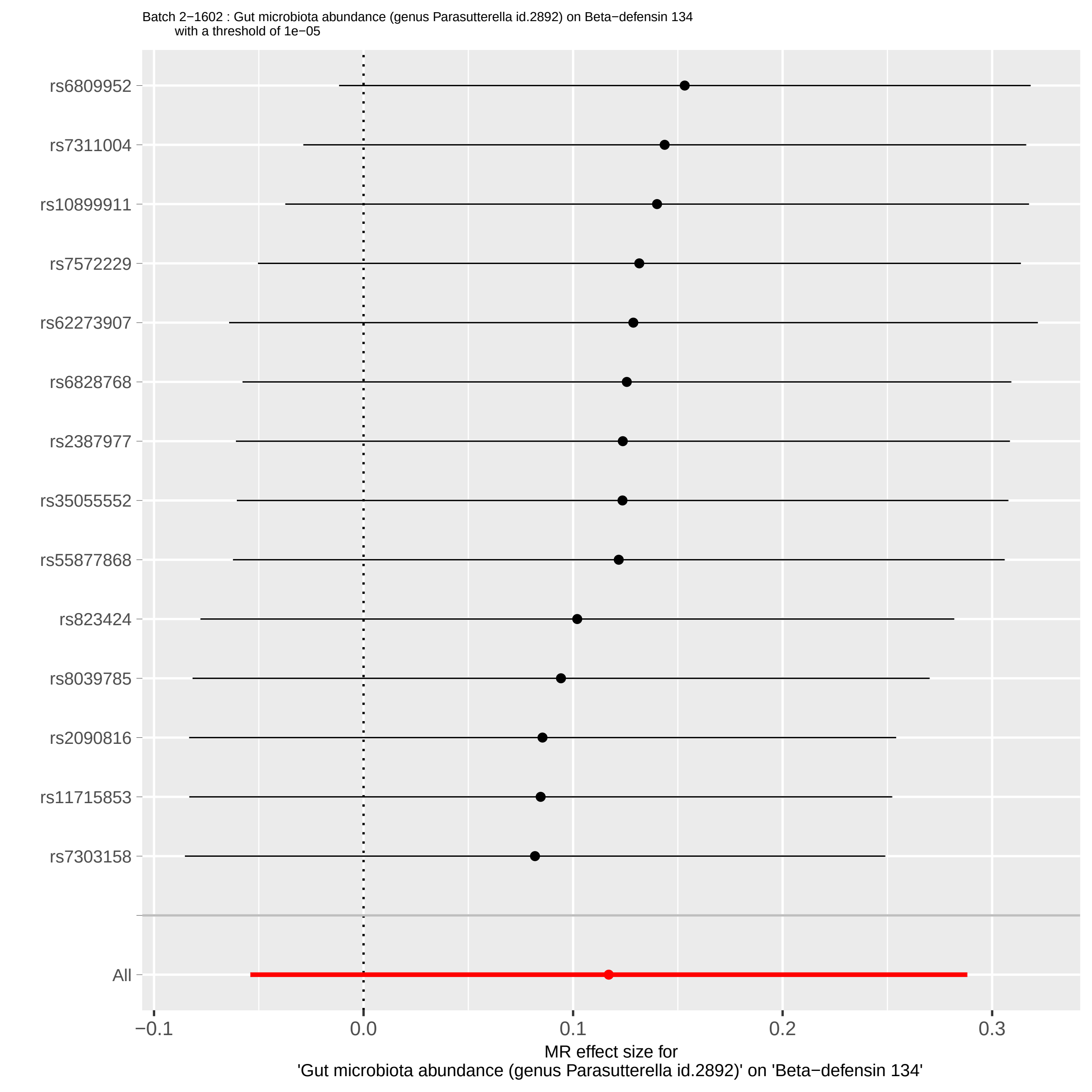

Batch 2−1602 : Gut microbiota abundance (genus Parasutterella id.2892) on Beta−defensin 134
with a threshold of 1e−05
rs6809952
rs7311004
rs10899911
rs7572229
rs62273907
rs6828768
rs2387977
rs35055552
rs55877868
rs823424
rs8039785
rs2090816
rs11715853
rs7303158
All
−0.1
0.0
0.1
0.2
0.3
MR effect size for
'Gut microbiota abundance (genus Parasutterella id.2892)' on 'Beta−defensin 134'

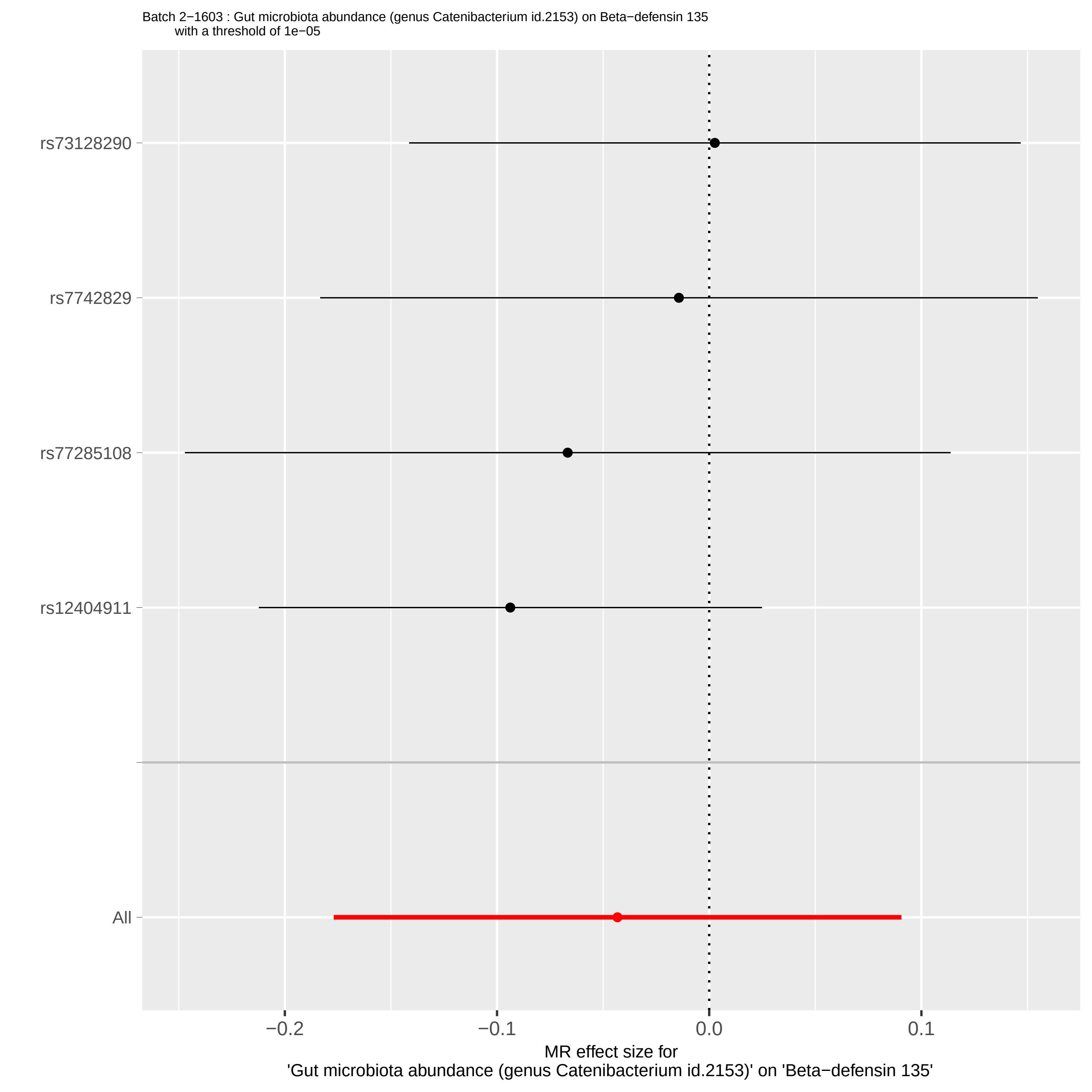
Batch 2−1603 : Gut microbiota abundance (genus Catenibacterium id.2153) on Beta−defensin 135
with a threshold of 1e−05
rs73128290
rs7742829
rs77285108
rs12404911
All
−0.2
−0.1
0.0
0.1
MR effect size for
'Gut microbiota abundance (genus Catenibacterium id.2153)' on 'Beta−defensin 135'

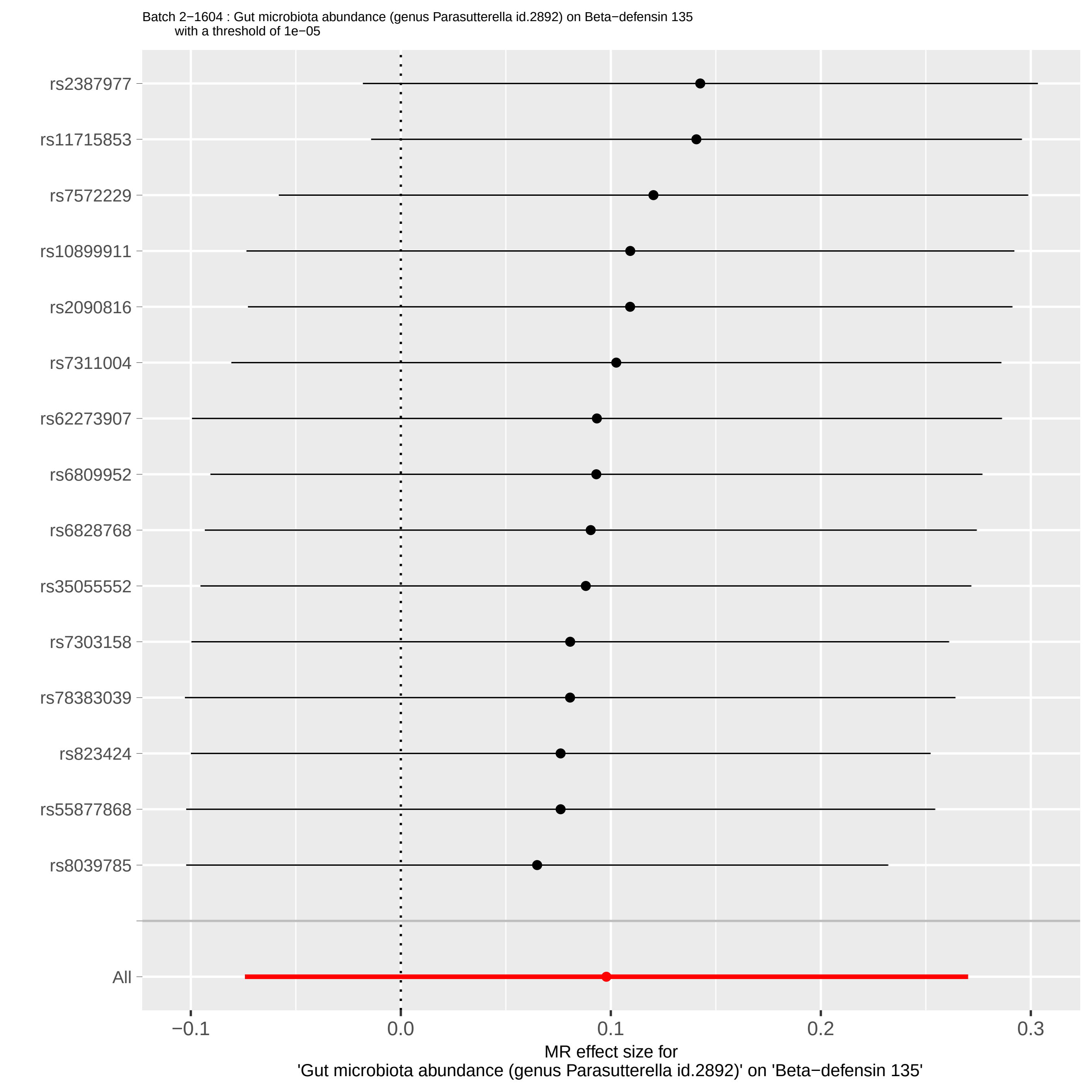

Batch 2−1604 : Gut microbiota abundance (genus Parasutterella id.2892) on Beta−defensin 135
with a threshold of 1e−05
rs2387977
rs11715853
rs7572229
rs10899911
rs2090816
rs7311004
rs62273907
rs6809952
rs6828768
rs35055552
rs7303158
rs78383039
rs823424
rs55877868
rs8039785
All
−0.1
0.0
0.1
0.2
0.3
MR effect size for
'Gut microbiota abundance (genus Parasutterella id.2892)' on 'Beta−defensin 135'

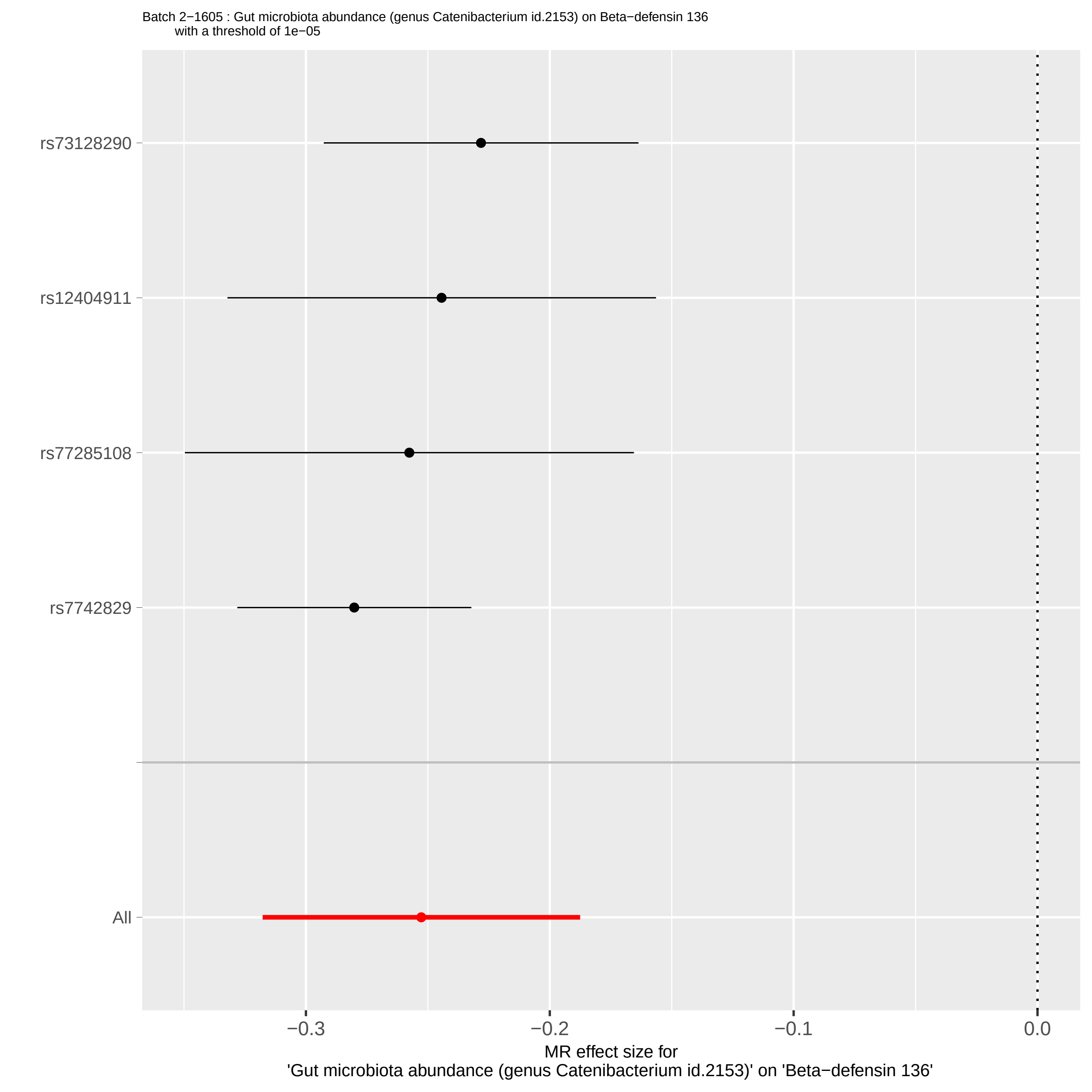
Batch 2−1605 : Gut microbiota abundance (genus Catenibacterium id.2153) on Beta−defensin 136
with a threshold of 1e−05
rs73128290
rs12404911
rs77285108
rs7742829
All
−0.3
−0.2
−0.1
0.0
MR effect size for
'Gut microbiota abundance (genus Catenibacterium id.2153)' on 'Beta−defensin 136'

Batch 2−1606 : Gut microbiota abundance (genus Parasutterella id.2892) on Beta−defensin 136
with a threshold of 1e−05

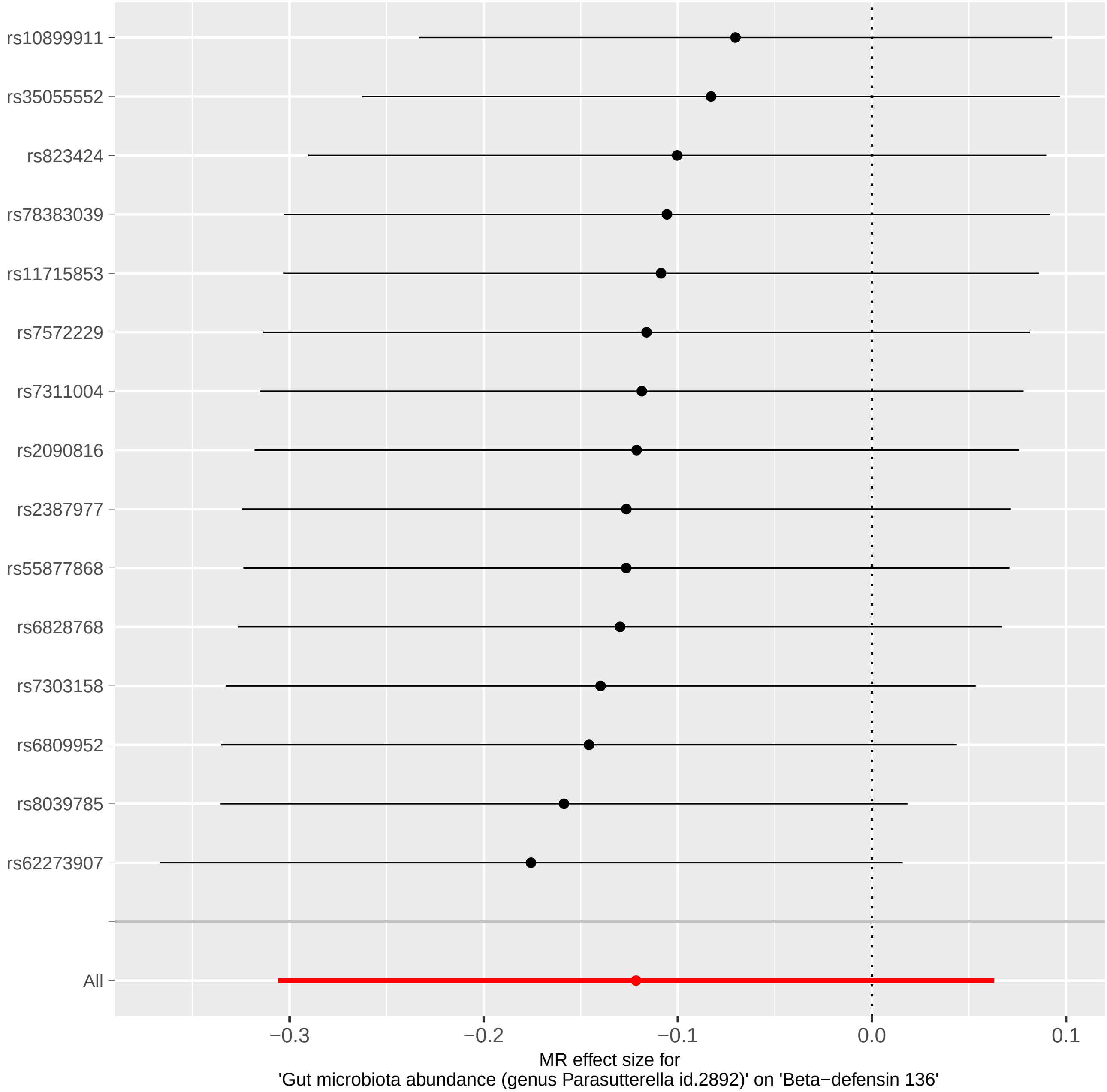

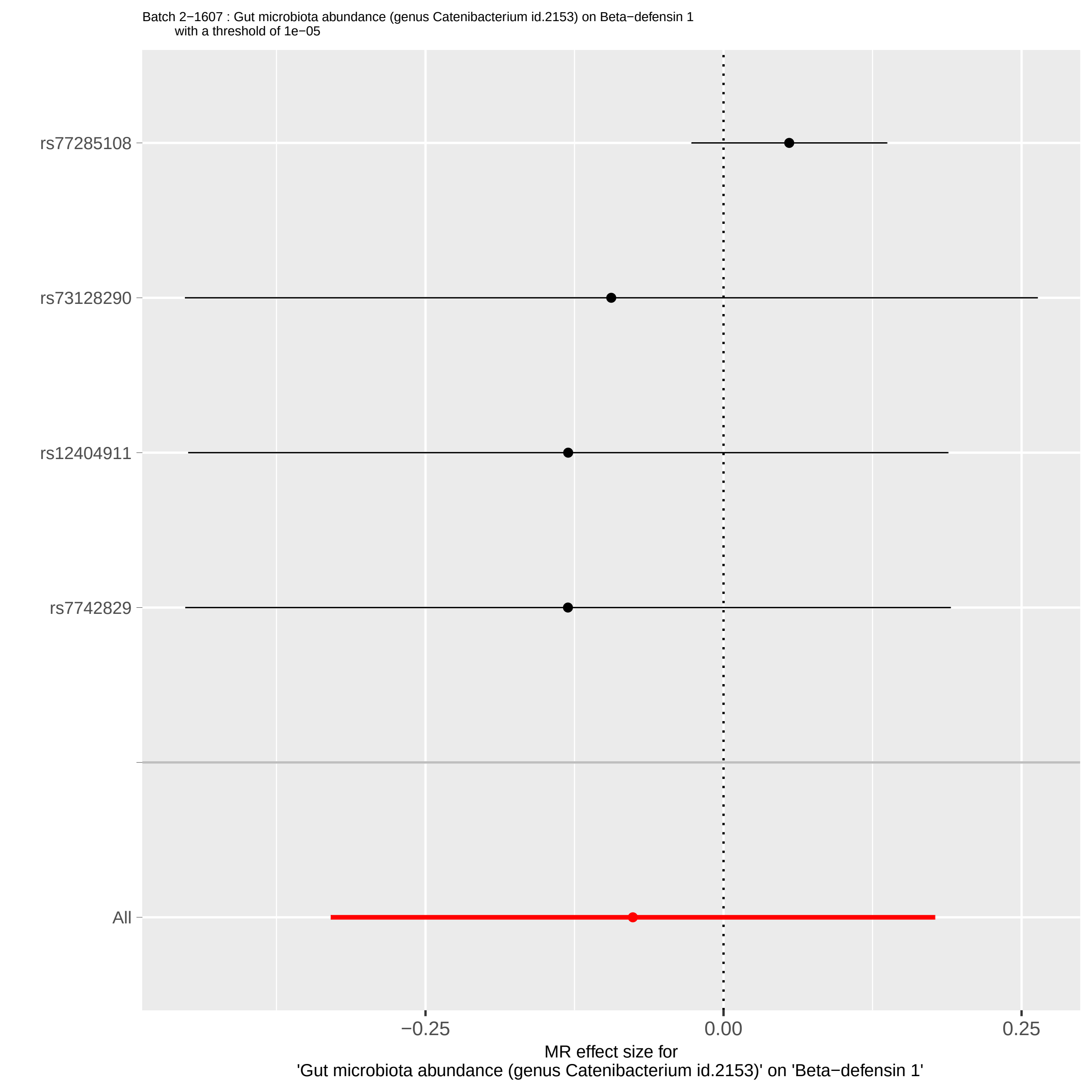

Batch 2−1607 : Gut microbiota abundance (genus Catenibacterium id.2153) on Beta−defensin 1
with a threshold of 1e−05
rs77285108
rs73128290
rs12404911
rs7742829
All
−0.25
0.00
0.25
MR effect size for
'Gut microbiota abundance (genus Catenibacterium id.2153)' on 'Beta−defensin 1'

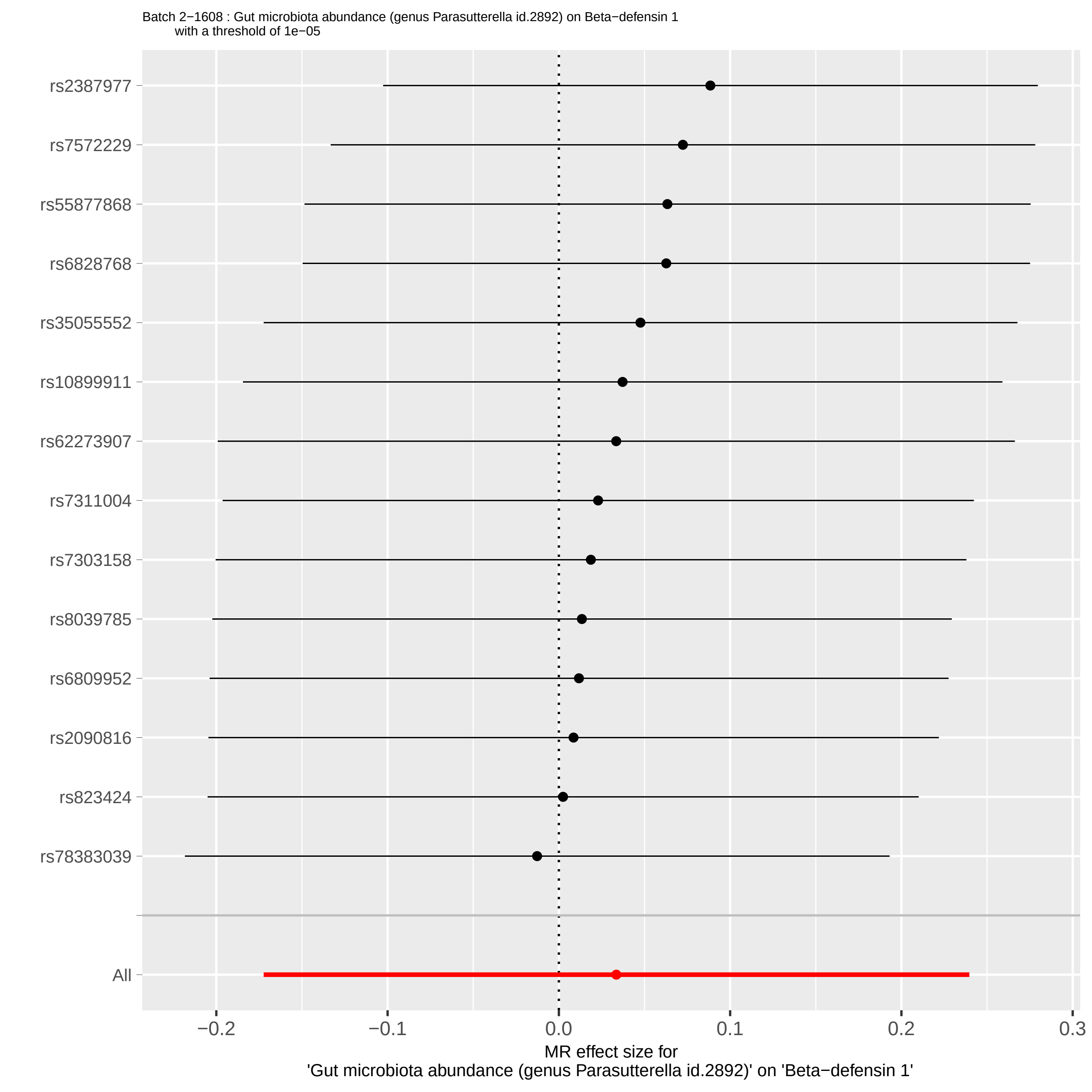
Batch 2−1608 : Gut microbiota abundance (genus Parasutterella id.2892) on Beta−defensin 1
with a threshold of 1e−05
rs2387977
rs7572229
rs55877868
rs6828768
rs35055552
rs10899911
rs62273907
rs7311004
rs7303158
rs8039785
rs6809952
rs2090816
rs823424
rs78383039
All
−0.2
−0.1
0.0
0.1
0.2
0.3
MR effect size for
'Gut microbiota abundance (genus Parasutterella id.2892)' on 'Beta−defensin 1'

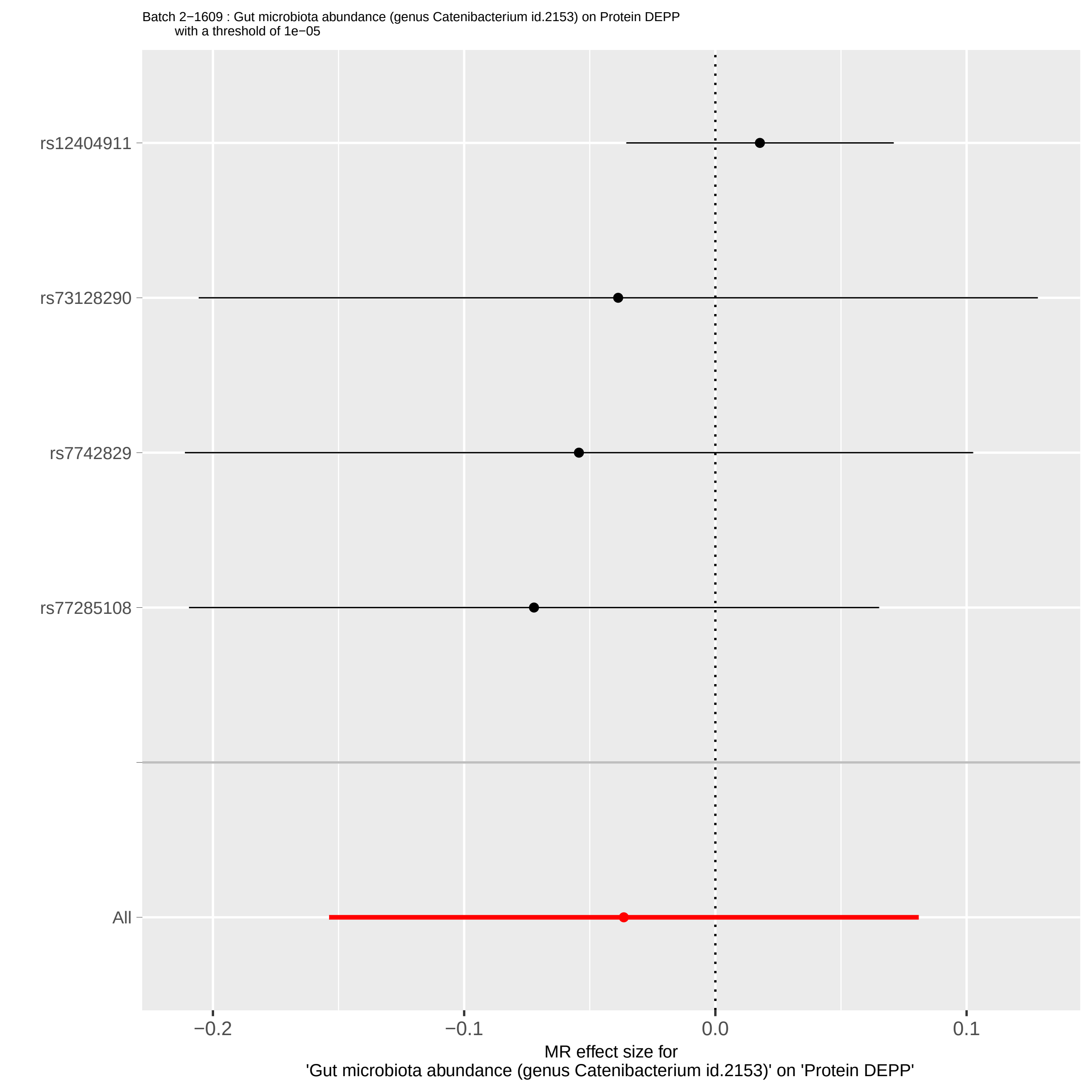

Batch 2−1609 : Gut microbiota abundance (genus Catenibacterium id.2153) on Protein DEPP
with a threshold of 1e−05
rs12404911
rs73128290
rs7742829
rs77285108
All
−0.2
−0.1
0.0
0.1
MR effect size for
'Gut microbiota abundance (genus Catenibacterium id.2153)' on 'Protein DEPP'

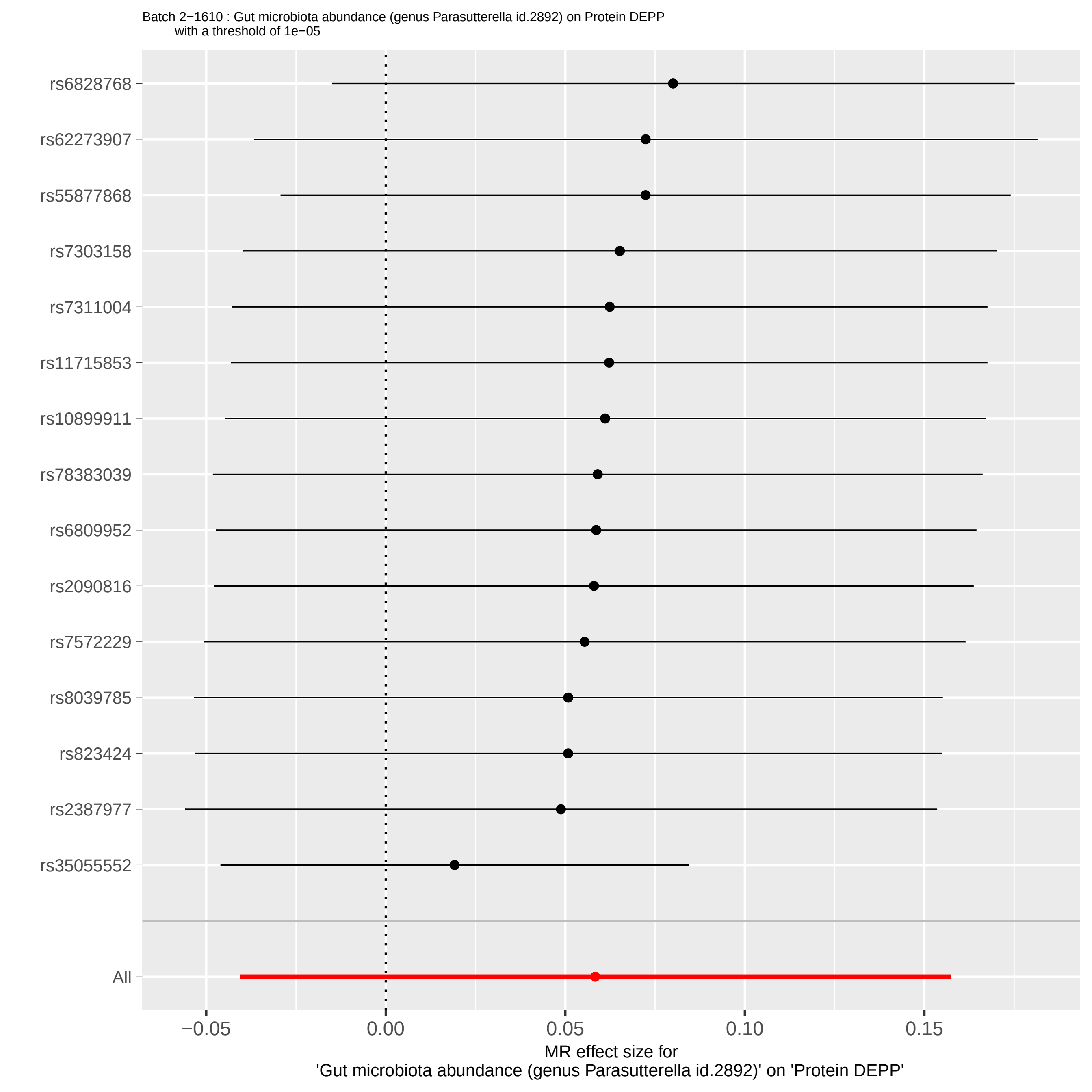
Batch 2−1610 : Gut microbiota abundance (genus Parasutterella id.2892) on Protein DEPP
with a threshold of 1e−05
rs6828768
rs62273907
rs55877868
rs7303158
rs7311004
rs11715853
rs10899911
rs78383039
rs6809952
rs2090816
rs7572229
rs8039785
rs823424
rs2387977
rs35055552
All
−0.05
0.00
0.05
0.10
0.15
MR effect size for
'Gut microbiota abundance (genus Parasutterella id.2892)' on 'Protein DEPP'

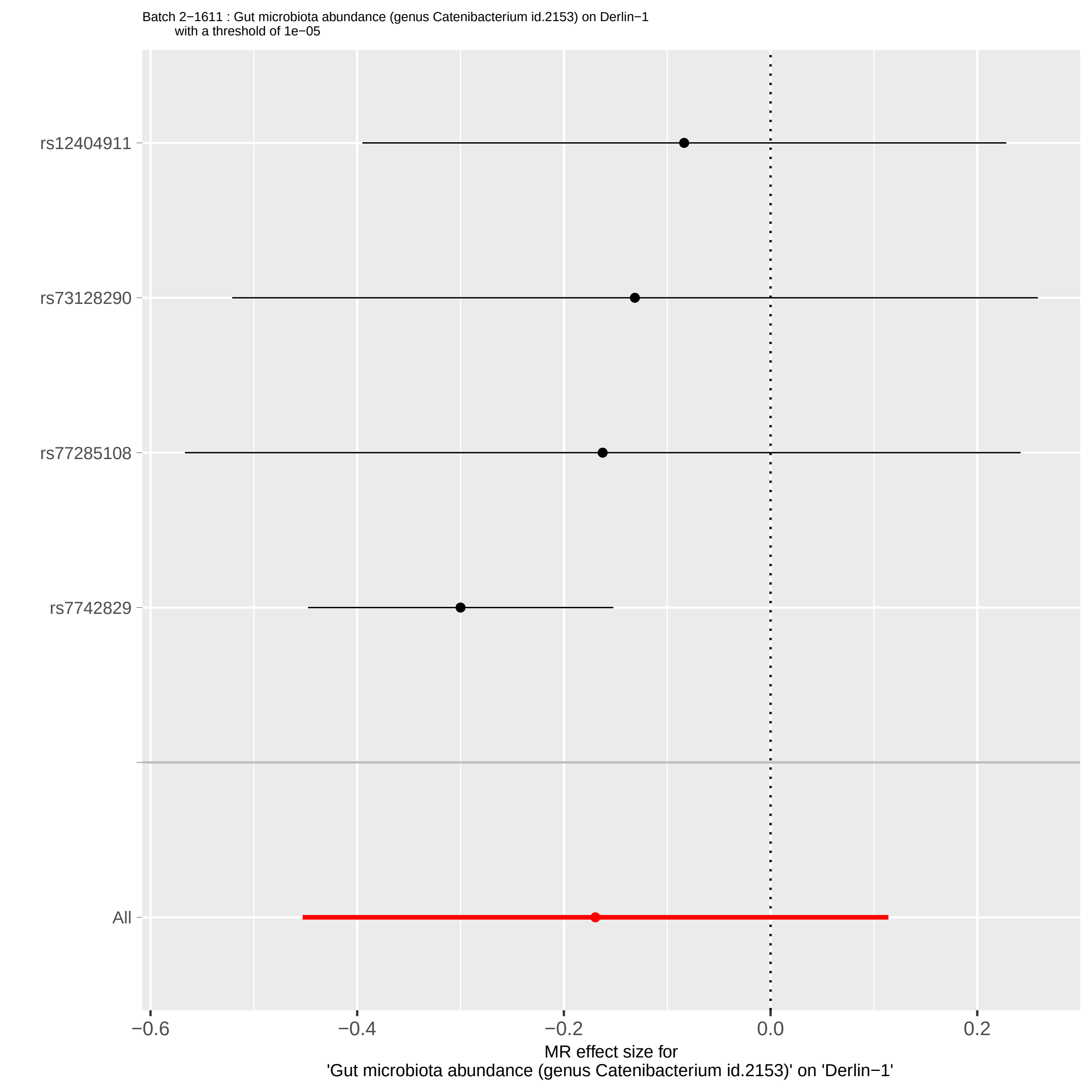
Batch 2−1611 : Gut microbiota abundance (genus Catenibacterium id.2153) on Derlin−1
with a threshold of 1e−05
rs12404911
rs73128290
rs77285108
rs7742829
All
−0.6
−0.4
−0.2
0.0
0.2
MR effect size for
'Gut microbiota abundance (genus Catenibacterium id.2153)' on 'Derlin−1'

Batch 2−1612 : Gut microbiota abundance (genus Parasutterella id.2892) on Derlin−1
with a threshold of 1e−05

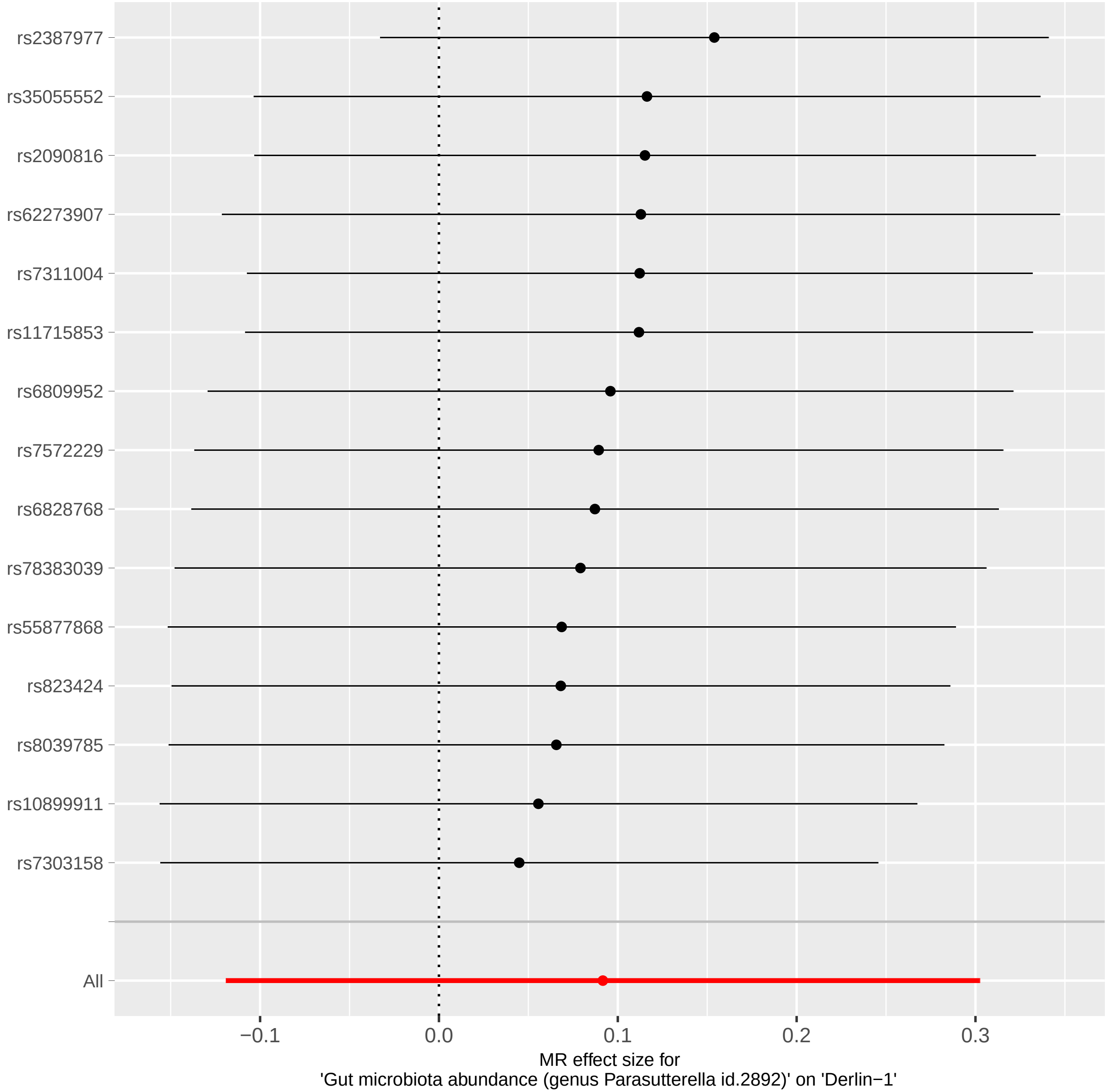

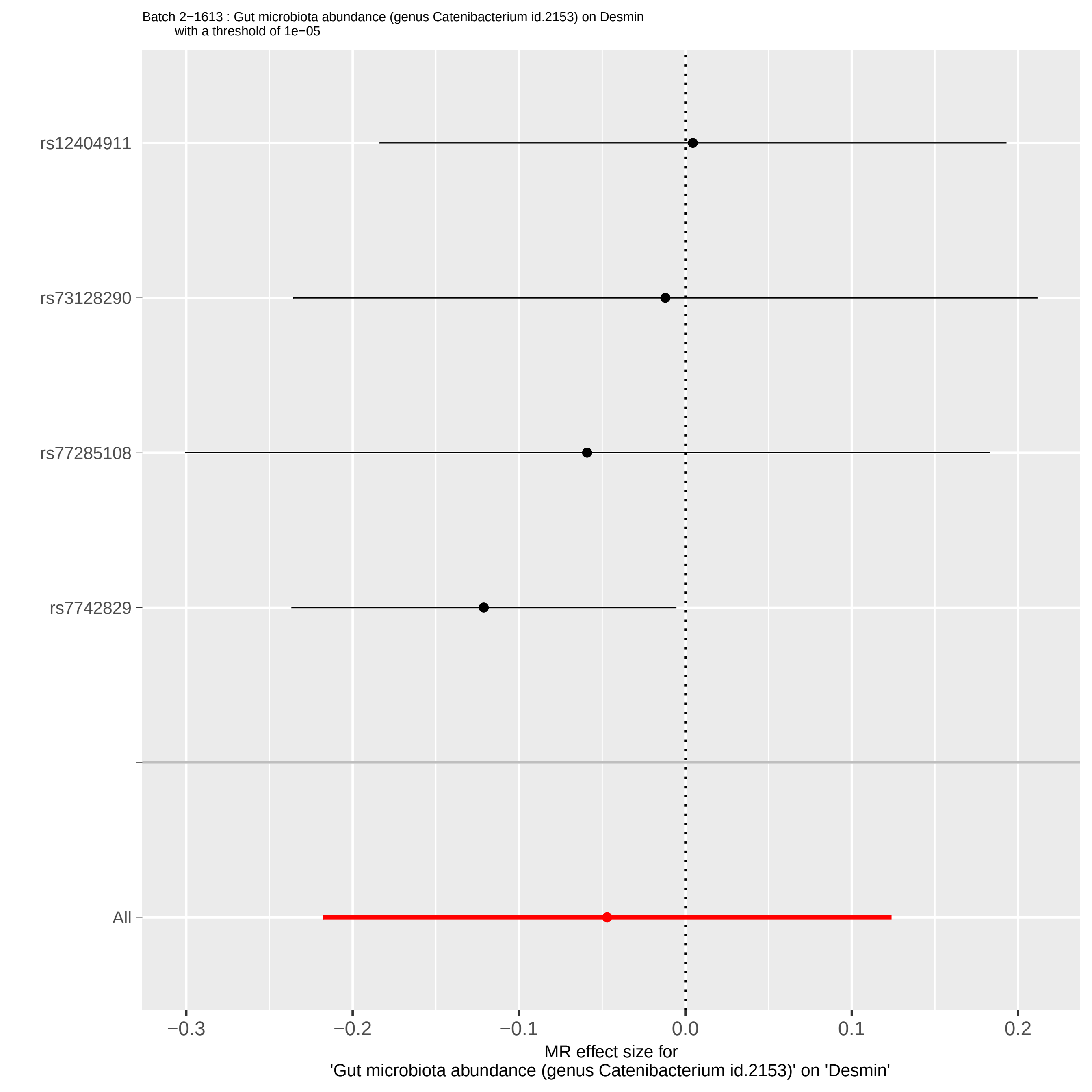

Batch 2−1613 : Gut microbiota abundance (genus Catenibacterium id.2153) on Desmin
with a threshold of 1e−05
rs12404911
rs73128290
rs77285108
rs7742829
All
−0.3
−0.2
−0.1
0.0
0.1
0.2
MR effect size for
'Gut microbiota abundance (genus Catenibacterium id.2153)' on 'Desmin'

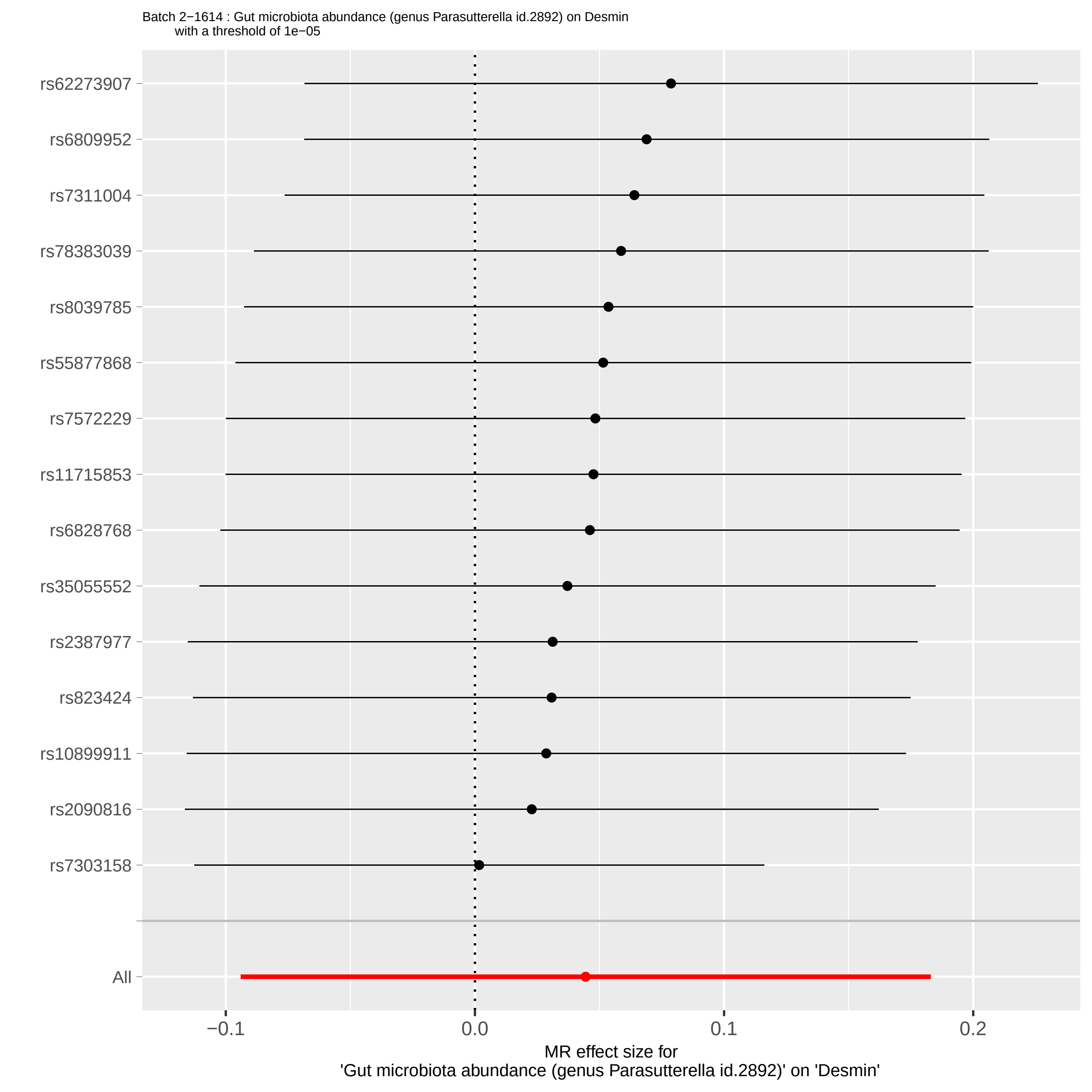

Batch 2−1614 : Gut microbiota abundance (genus Parasutterella id.2892) on Desmin
with a threshold of 1e−05
rs62273907
rs6809952
rs7311004
rs78383039
rs8039785
rs55877868
rs7572229
rs11715853
rs6828768
rs35055552
rs2387977
rs823424
rs10899911
rs2090816
rs7303158
All
−0.1
0.0
0.1
0.2
MR effect size for
'Gut microbiota abundance (genus Parasutterella id.2892)' on 'Desmin'

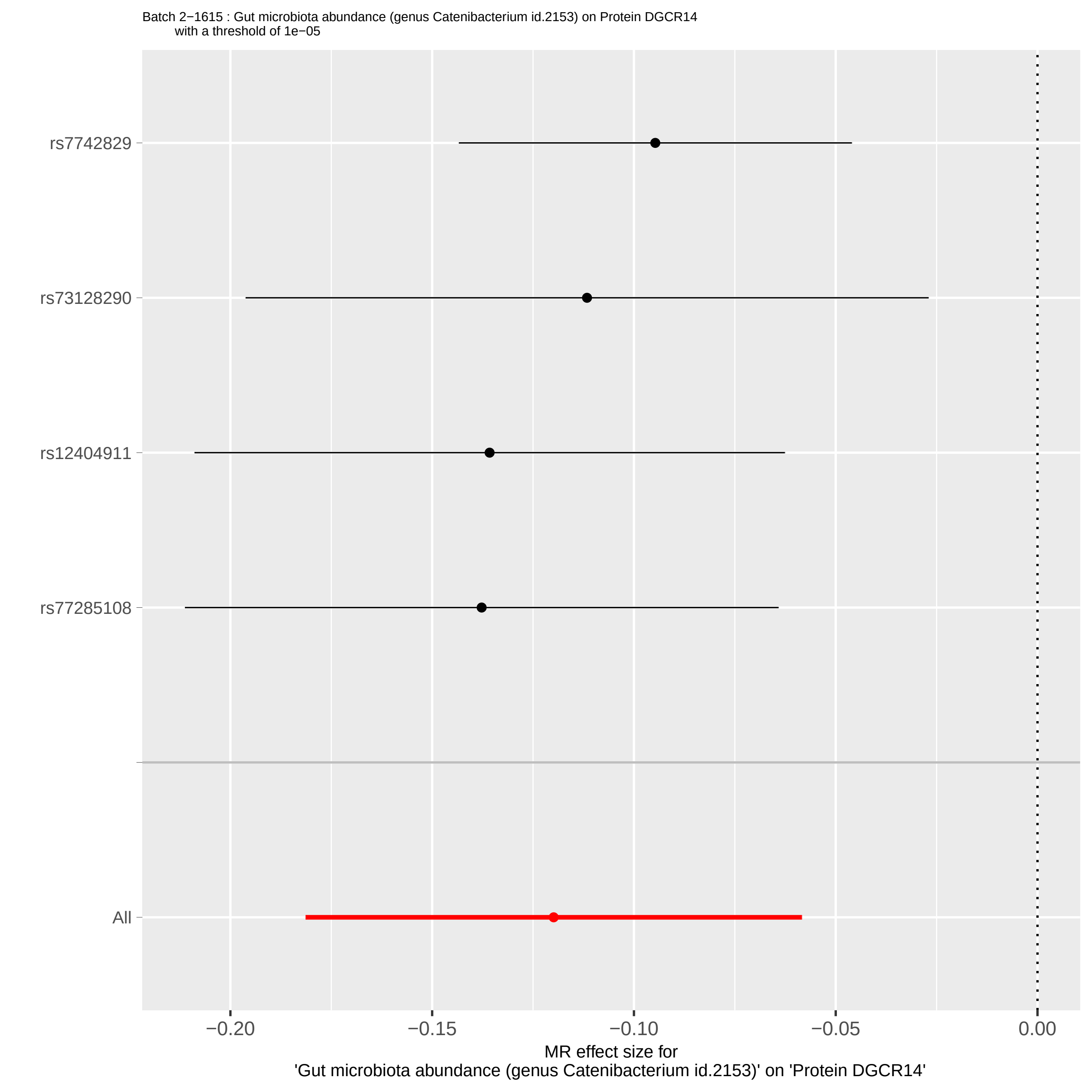

Batch 2−1615 : Gut microbiota abundance (genus Catenibacterium id.2153) on Protein DGCR14
with a threshold of 1e−05
rs7742829
rs73128290
rs12404911
rs77285108
All
−0.20
−0.15
−0.10
−0.05
0.00
MR effect size for
'Gut microbiota abundance (genus Catenibacterium id.2153)' on 'Protein DGCR14'

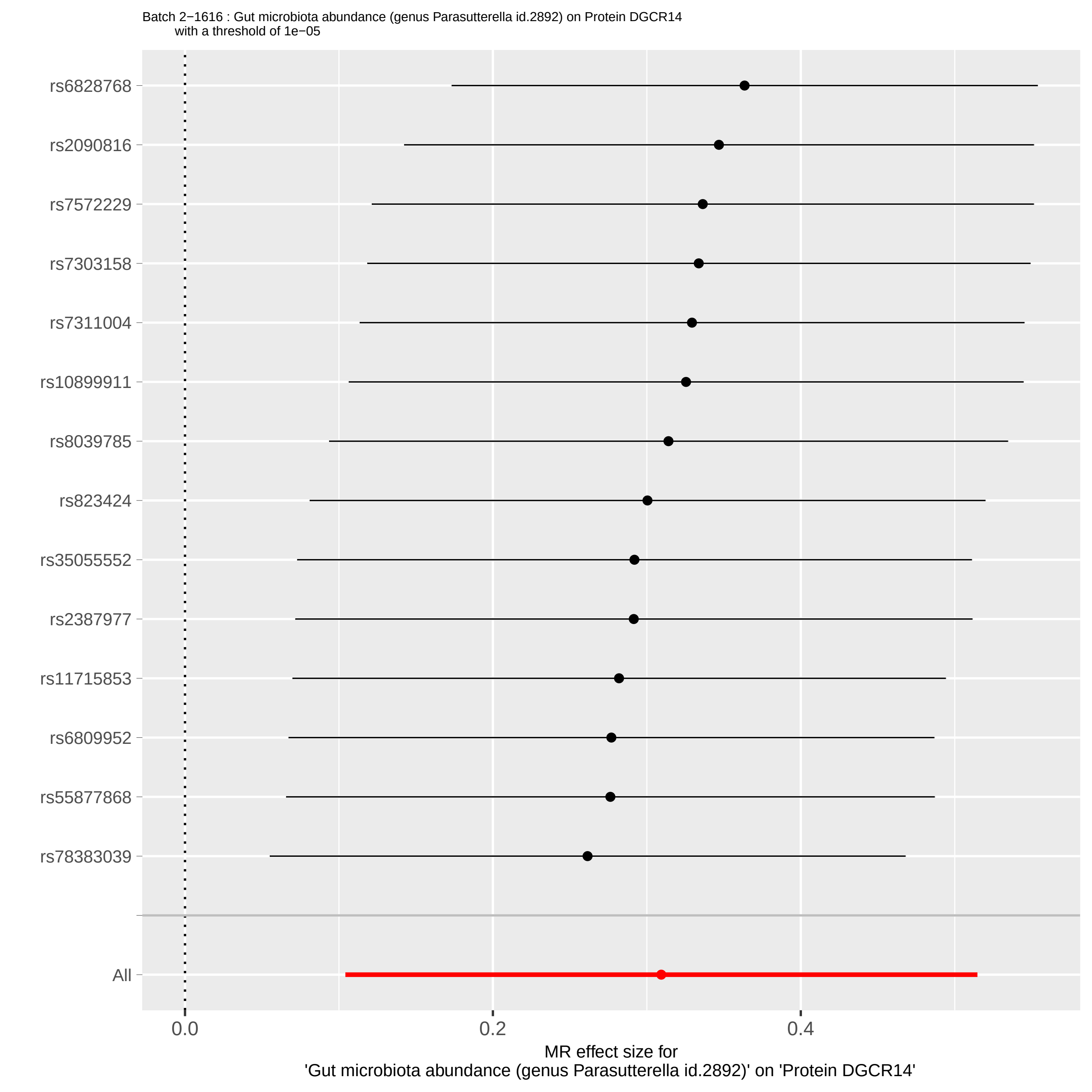

Batch 2−1616 : Gut microbiota abundance (genus Parasutterella id.2892) on Protein DGCR14
with a threshold of 1e−05
rs6828768
rs2090816
rs7572229
rs7303158
rs7311004
rs10899911
rs8039785
rs823424
rs35055552
rs2387977
rs11715853
rs6809952
rs55877868
rs78383039
All
0.0
0.2
0.4
MR effect size for
'Gut microbiota abundance (genus Parasutterella id.2892)' on 'Protein DGCR14'

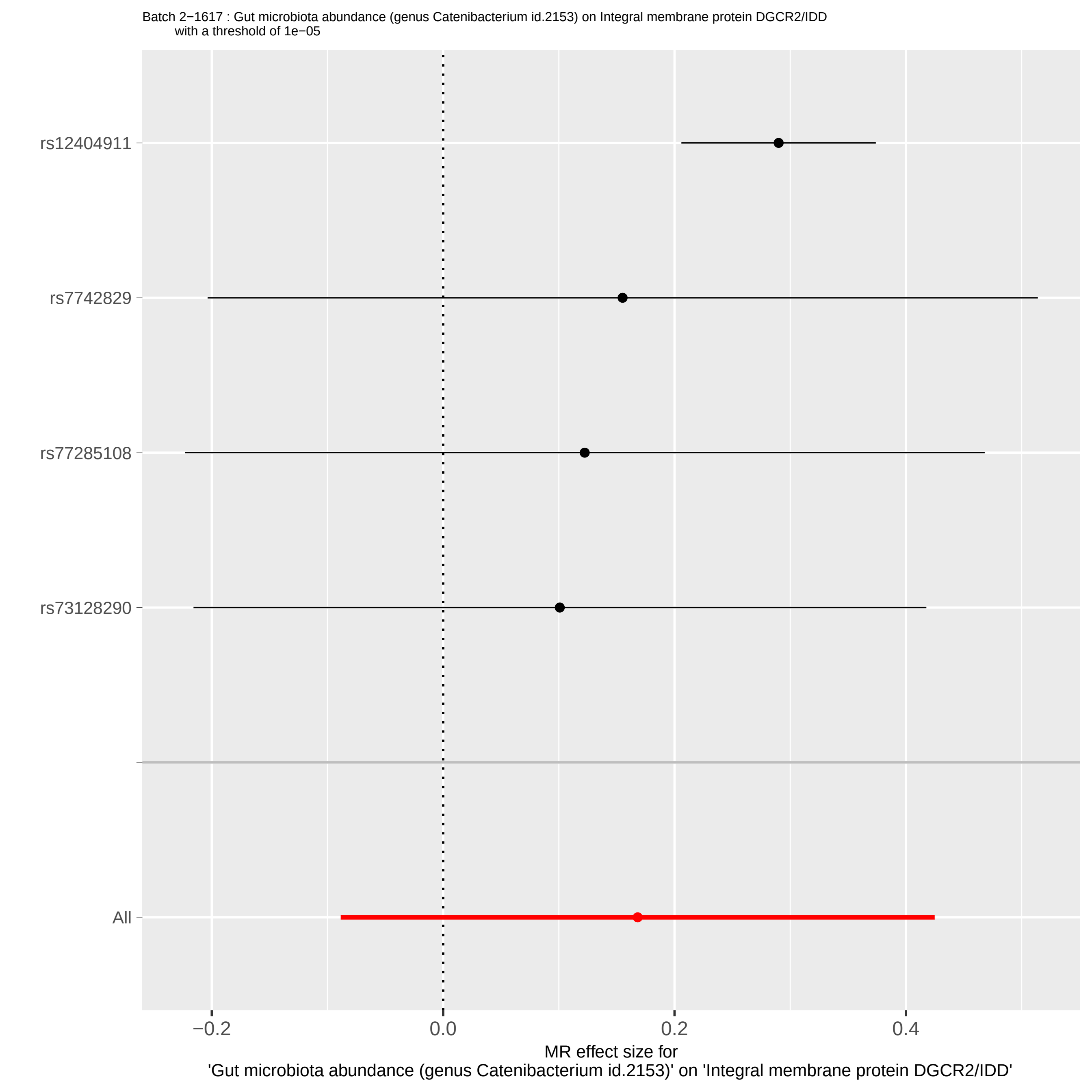

Batch 2−1617 : Gut microbiota abundance (genus Catenibacterium id.2153) on Integral membrane protein DGCR2/IDD
with a threshold of 1e−05
rs12404911
rs7742829
rs77285108
rs73128290
All
−0.2
0.0
0.2
0.4
MR effect size for
'Gut microbiota abundance (genus Catenibacterium id.2153)' on 'Integral membrane protein DGCR2/IDD'

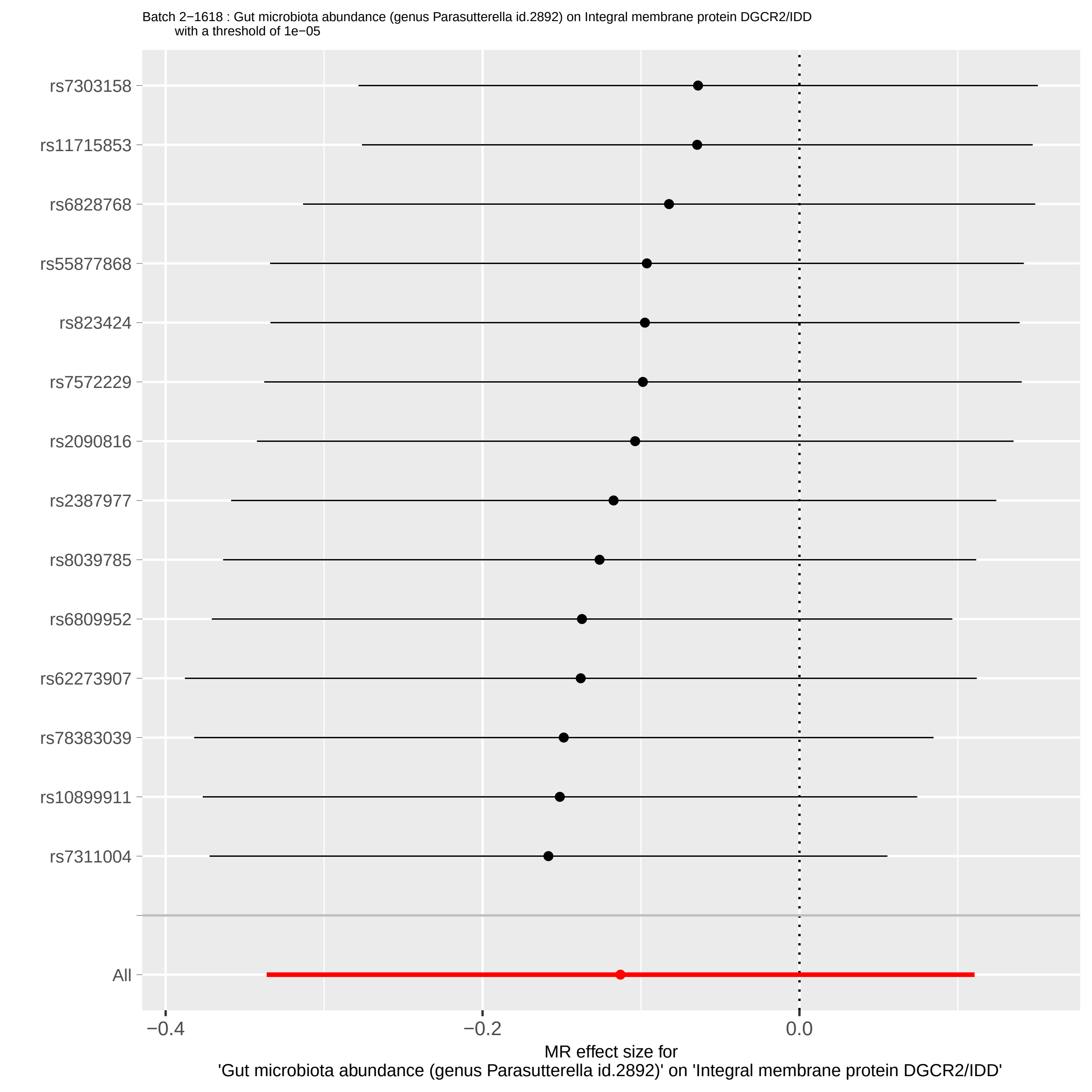

Batch 2−1618 : Gut microbiota abundance (genus Parasutterella id.2892) on Integral membrane protein DGCR2/IDD
with a threshold of 1e−05
rs7303158
rs11715853
rs6828768
rs55877868
rs823424
rs7572229
rs2090816
rs2387977
rs8039785
rs6809952
rs62273907
rs78383039
rs10899911
rs7311004
All
−0.4
−0.2
0.0
MR effect size for
'Gut microbiota abundance (genus Parasutterella id.2892)' on 'Integral membrane protein DGCR2/IDD'

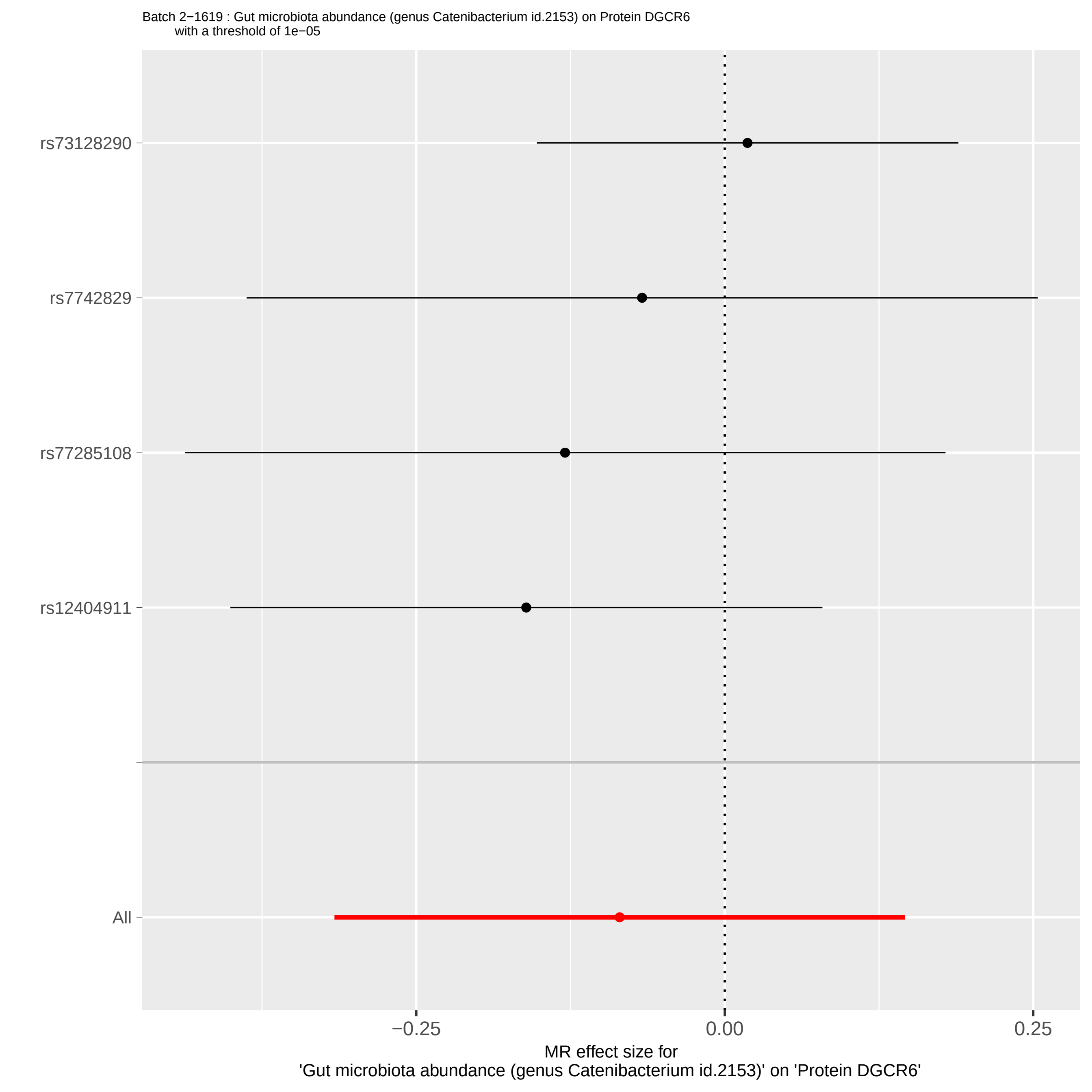

Batch 2−1619 : Gut microbiota abundance (genus Catenibacterium id.2153) on Protein DGCR6
with a threshold of 1e−05
rs73128290
rs7742829
rs77285108
rs12404911
All
−0.25
0.00
0.25
MR effect size for
'Gut microbiota abundance (genus Catenibacterium id.2153)' on 'Protein DGCR6'

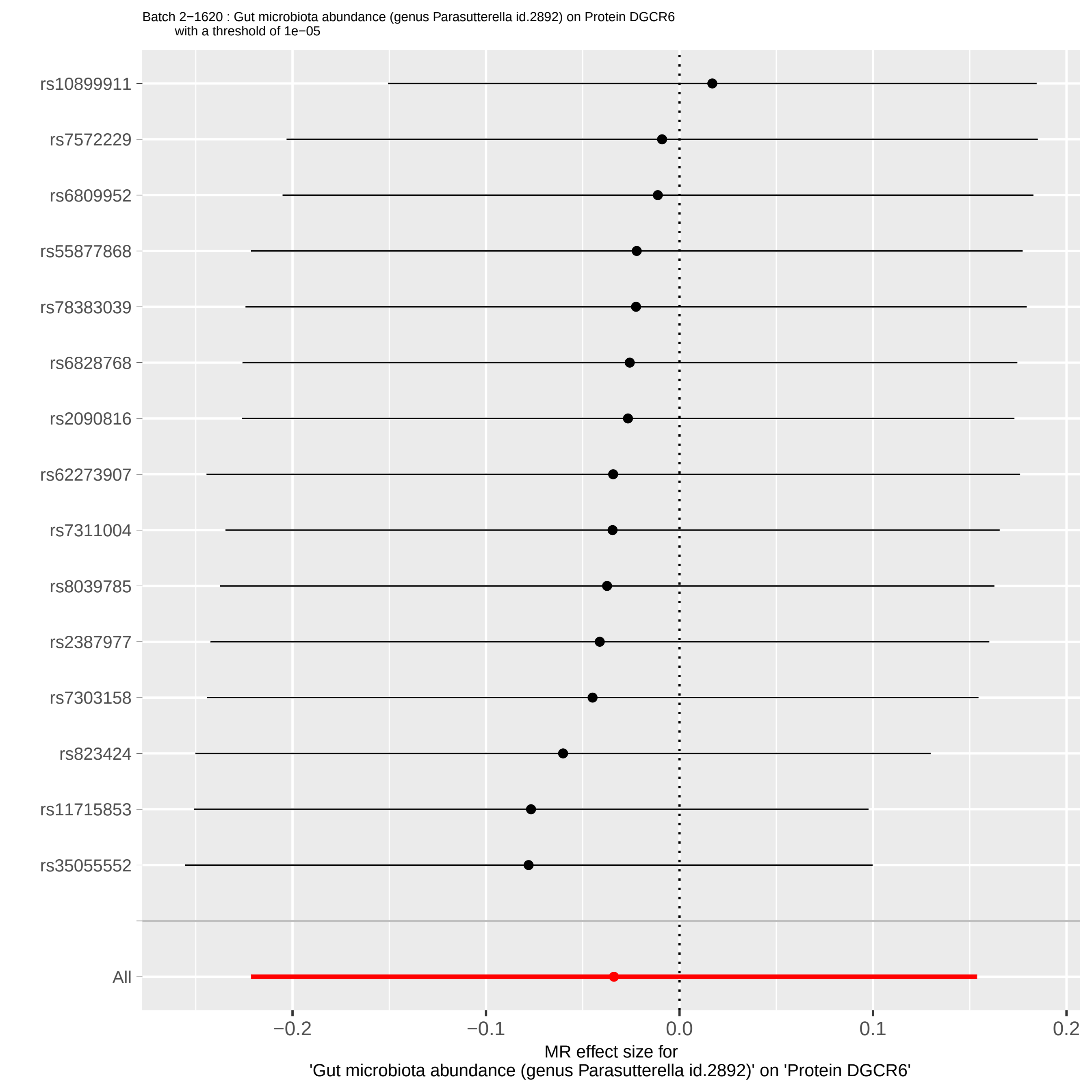
Batch 2−1620 : Gut microbiota abundance (genus Parasutterella id.2892) on Protein DGCR6
with a threshold of 1e−05
rs10899911
rs7572229
rs6809952
rs55877868
rs78383039
rs6828768
rs2090816
rs62273907
rs7311004
rs8039785
rs2387977
rs7303158
rs823424
rs11715853
rs35055552
All
−0.2
−0.1
0.0
0.1
0.2
MR effect size for
'Gut microbiota abundance (genus Parasutterella id.2892)' on 'Protein DGCR6'

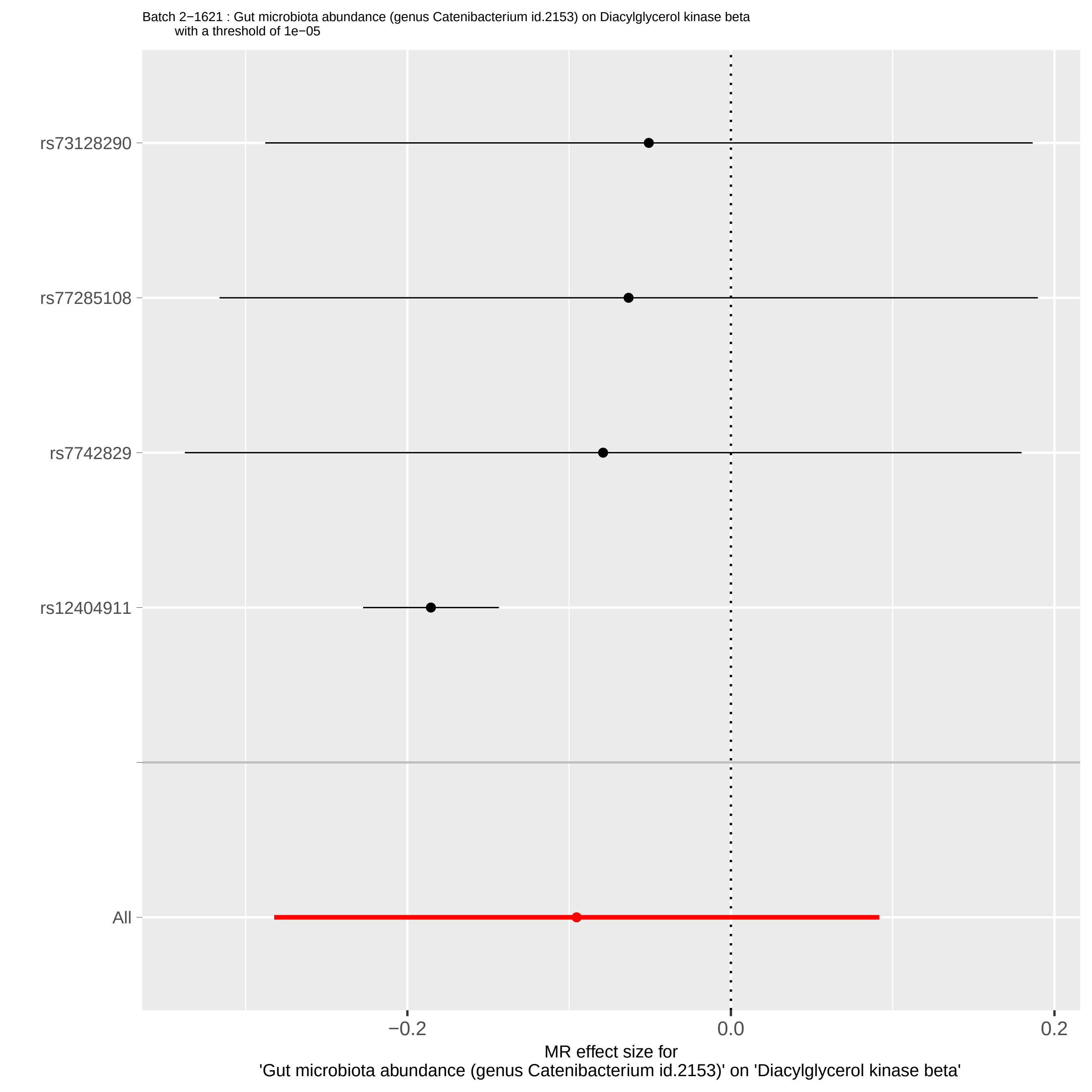
Batch 2−1621 : Gut microbiota abundance (genus Catenibacterium id.2153) on Diacylglycerol kinase beta
with a threshold of 1e−05
rs73128290
rs77285108
rs7742829
rs12404911
All
−0.2
0.0
0.2
MR effect size for
'Gut microbiota abundance (genus Catenibacterium id.2153)' on 'Diacylglycerol kinase beta'

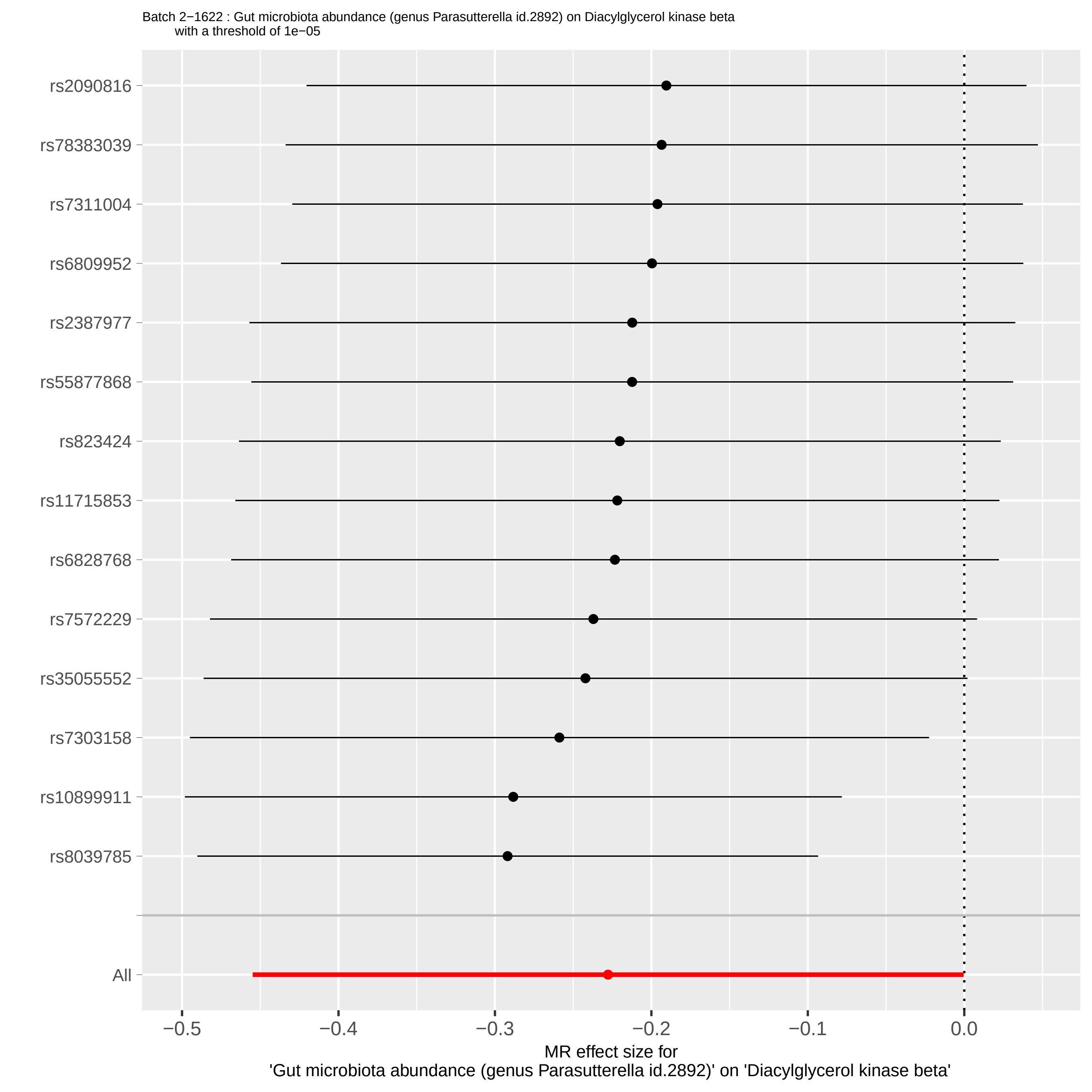
Batch 2−1622 : Gut microbiota abundance (genus Parasutterella id.2892) on Diacylglycerol kinase beta
with a threshold of 1e−05
rs2090816
rs78383039
rs7311004
rs6809952
rs2387977
rs55877868
rs823424
rs11715853
rs6828768
rs7572229
rs35055552
rs7303158
rs10899911
rs8039785
All
−0.5
−0.4
−0.3
−0.2
−0.1
0.0
MR effect size for
'Gut microbiota abundance (genus Parasutterella id.2892)' on 'Diacylglycerol kinase beta'

Batch 2−1623 : Gut microbiota abundance (genus Catenibacterium id.2153) on Dihydrofolate reductase
with a threshold of 1e−05

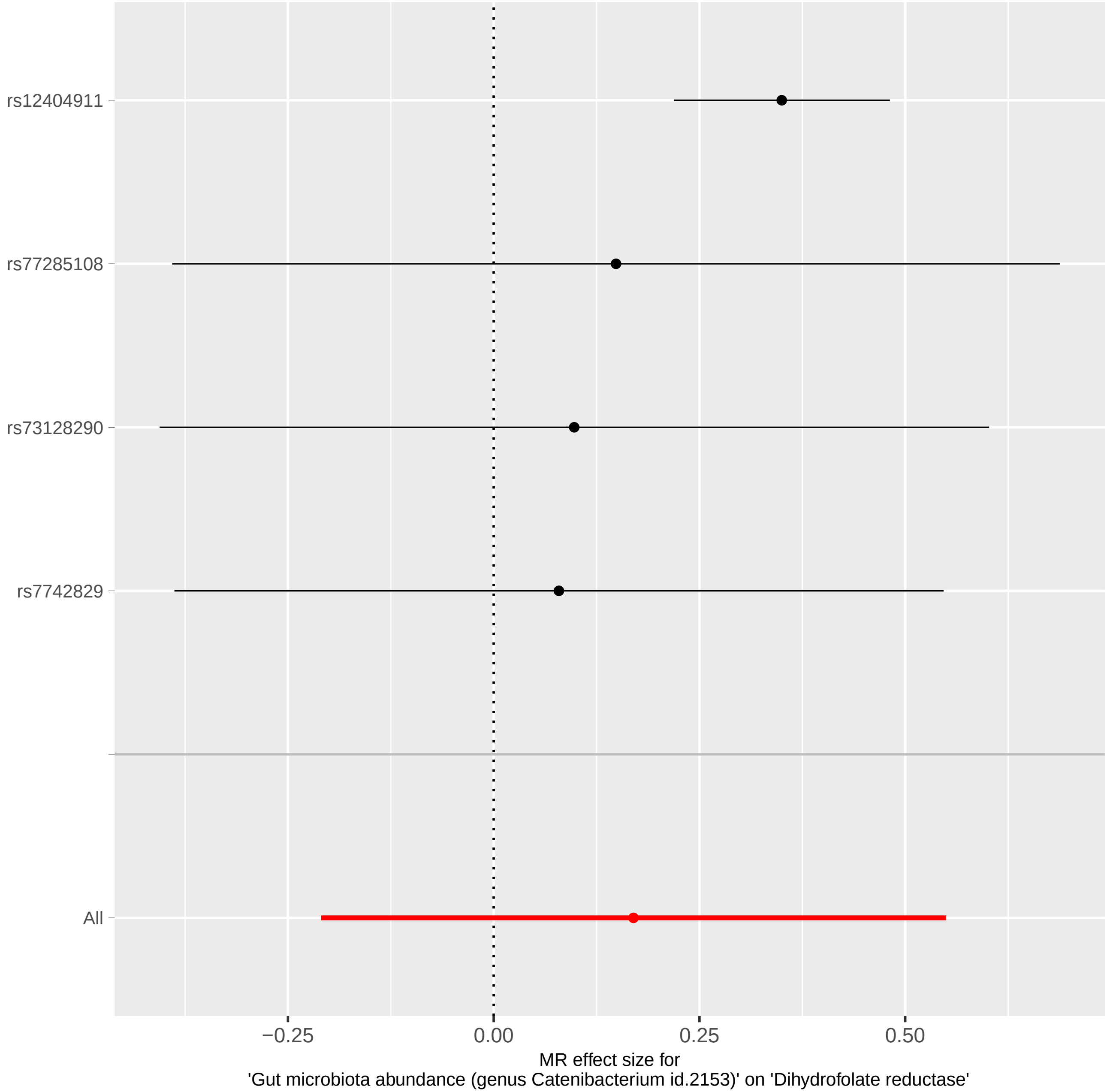

Batch 2−1624 : Gut microbiota abundance (genus Parasutterella id.2892) on Dihydrofolate reductase
with a threshold of 1e−05

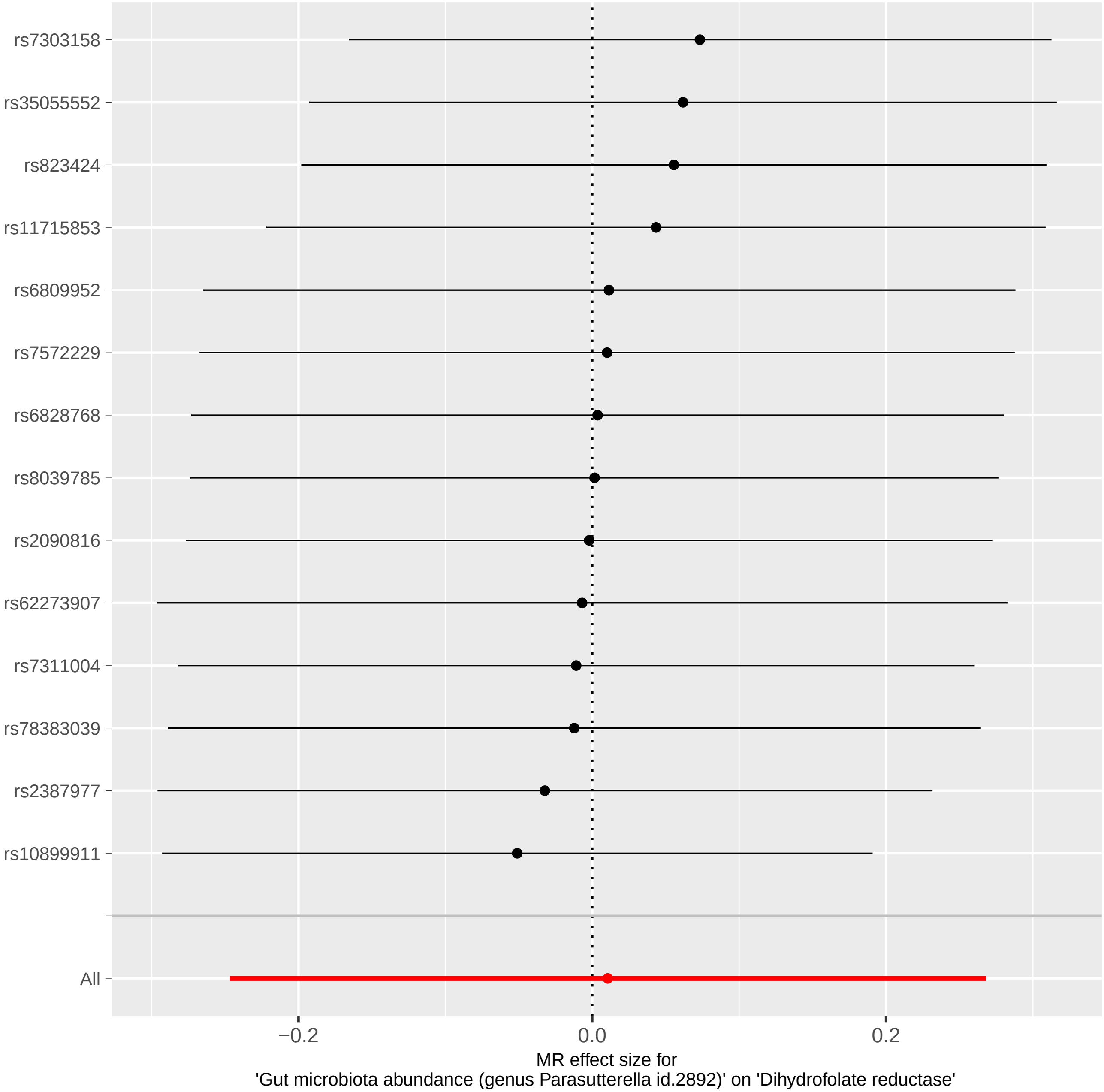

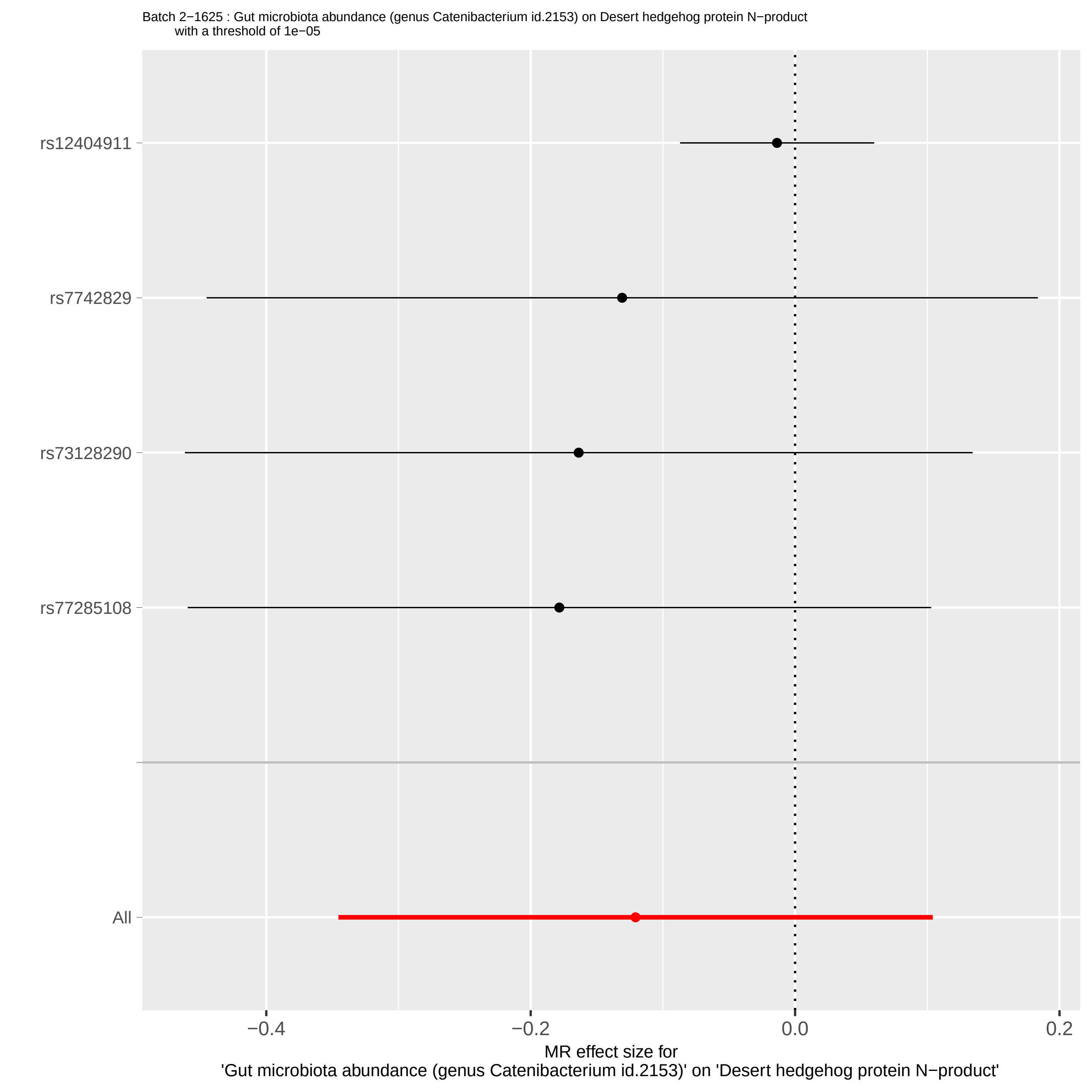
Batch 2−1625 : Gut microbiota abundance (genus Catenibacterium id.2153) on Desert hedgehog protein N−product
with a threshold of 1e−05
rs12404911
rs7742829
rs73128290
rs77285108
All
−0.4
−0.2
0.0
0.2
MR effect size for
'Gut microbiota abundance (genus Catenibacterium id.2153)' on 'Desert hedgehog protein N−product'

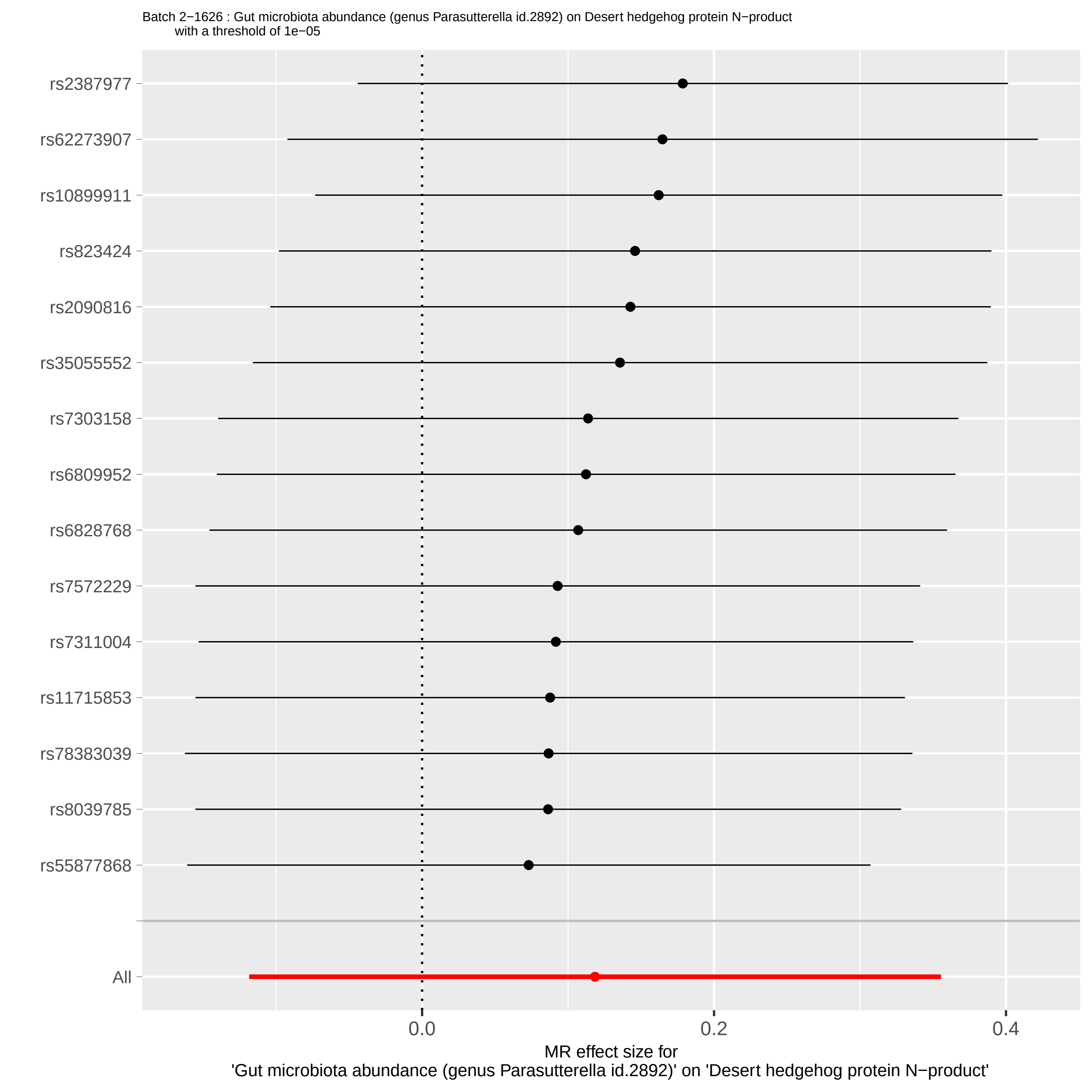

Batch 2−1626 : Gut microbiota abundance (genus Parasutterella id.2892) on Desert hedgehog protein N−product
with a threshold of 1e−05
rs2387977
rs62273907
rs10899911
rs823424
rs2090816
rs35055552
rs7303158
rs6809952
rs6828768
rs7572229
rs7311004
rs11715853
rs78383039
rs8039785
rs55877868
All
0.0
0.2
0.4
MR effect size for
'Gut microbiota abundance (genus Parasutterella id.2892)' on 'Desert hedgehog protein N−product'

Batch 2−1627 : Gut microbiota abundance (genus Catenibacterium id.2153) on Pre−mRNA−splicing factor ATP−dependent RNA helicase PRP16 with a threshold of 1e−05

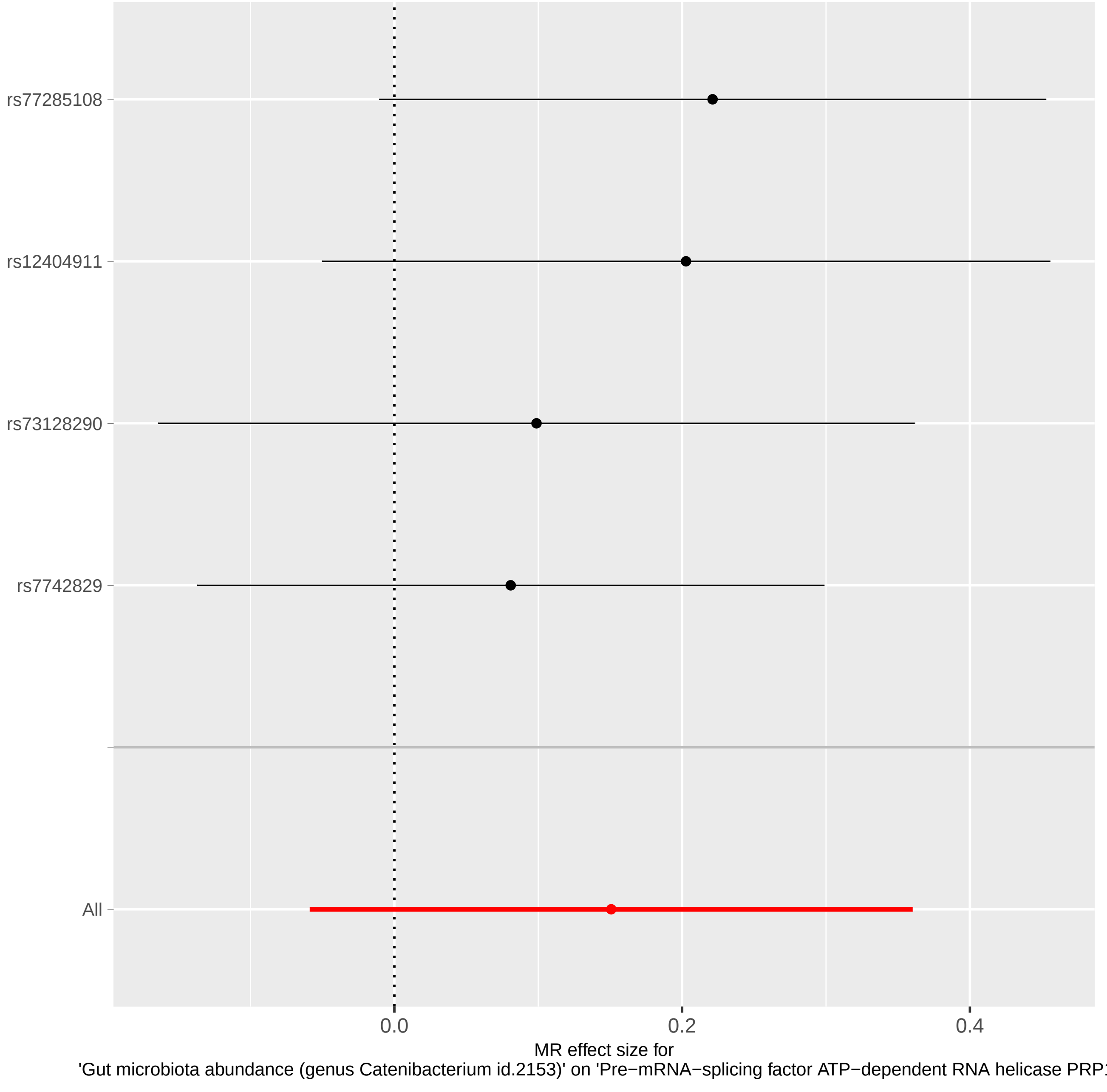

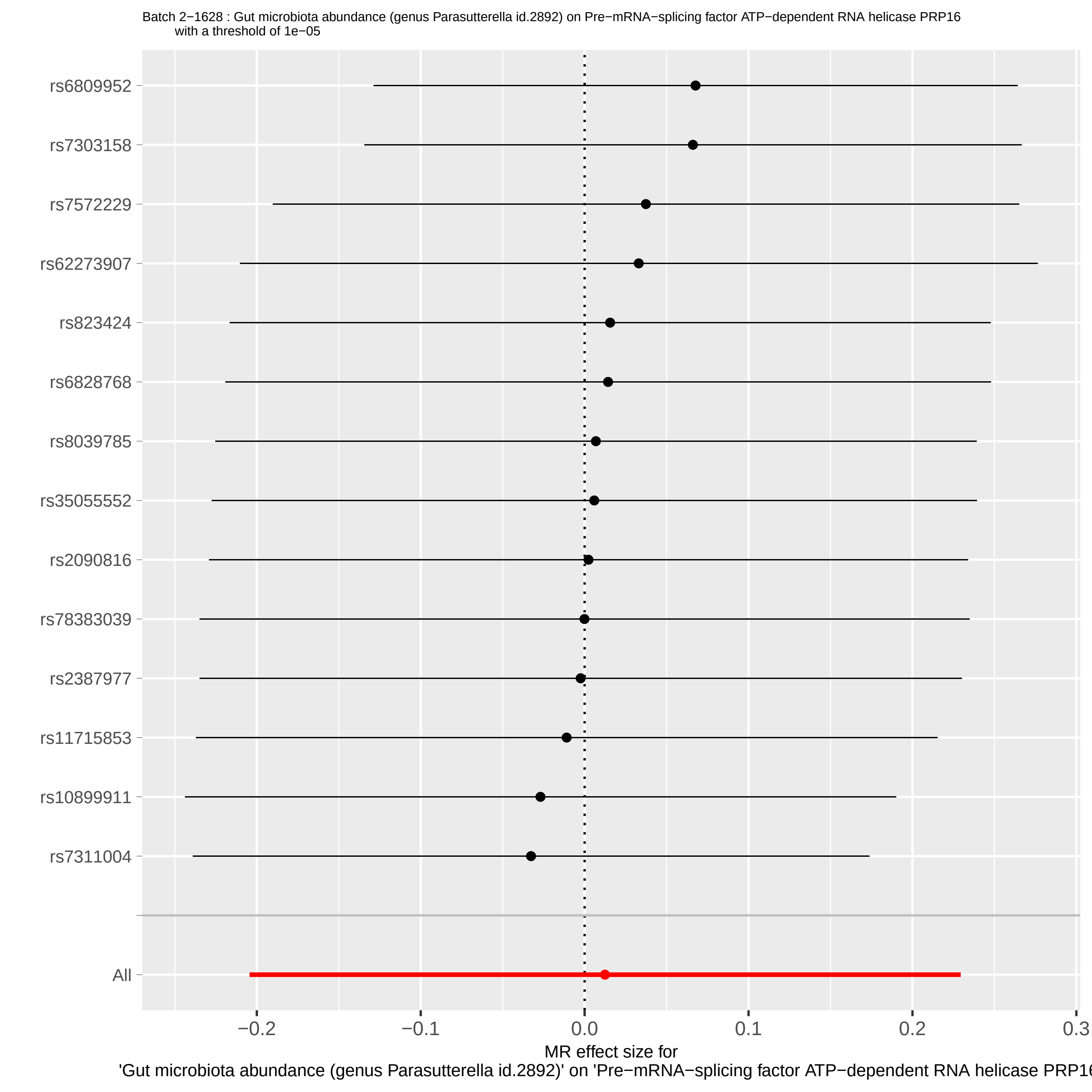

Batch 2−1628 : Gut microbiota abundance (genus Parasutterella id.2892) on Pre−mRNA−splicing factor ATP−dependent RNA helicase PRP16
with a threshold of 1e−05
rs6809952
rs7303158
rs7572229
rs62273907
rs823424
rs6828768
rs8039785
rs35055552
rs2090816
rs78383039
rs2387977
rs11715853
rs10899911
rs7311004
All
−0.2
−0.1
0.0
0.1
0.2
0.3
MR effect size for
'Gut microbiota abundance (genus Parasutterella id.2892)' on 'Pre−mRNA−splicing factor ATP−dependent RNA helicase PRP16

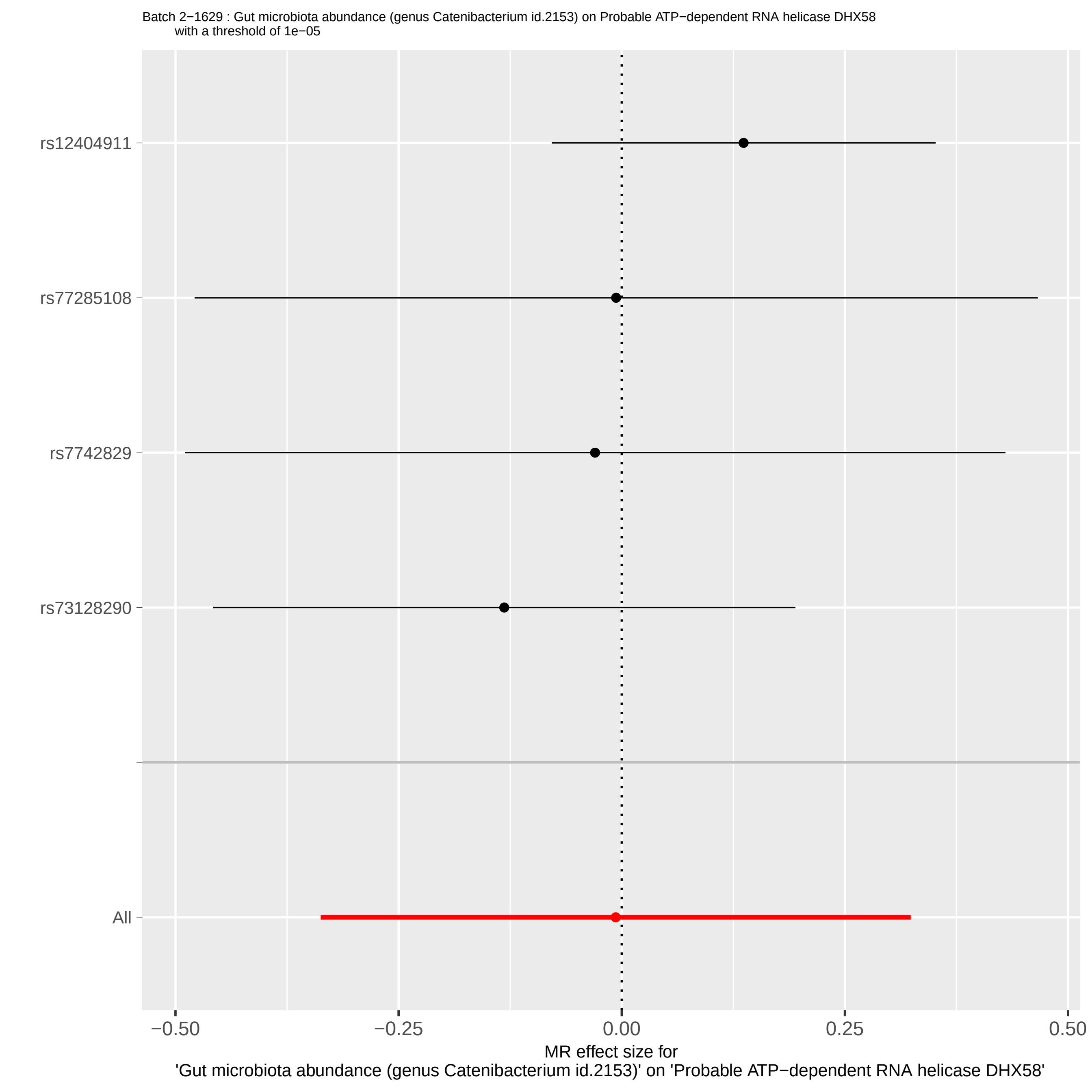

Batch 2−1629 : Gut microbiota abundance (genus Catenibacterium id.2153) on Probable ATP−dependent RNA helicase DHX58
with a threshold of 1e−05
rs12404911
rs77285108
rs7742829
rs73128290
All
−0.50
−0.25
0.00
0.25
0.50
MR effect size for
'Gut microbiota abundance (genus Catenibacterium id.2153)' on 'Probable ATP−dependent RNA helicase DHX58'

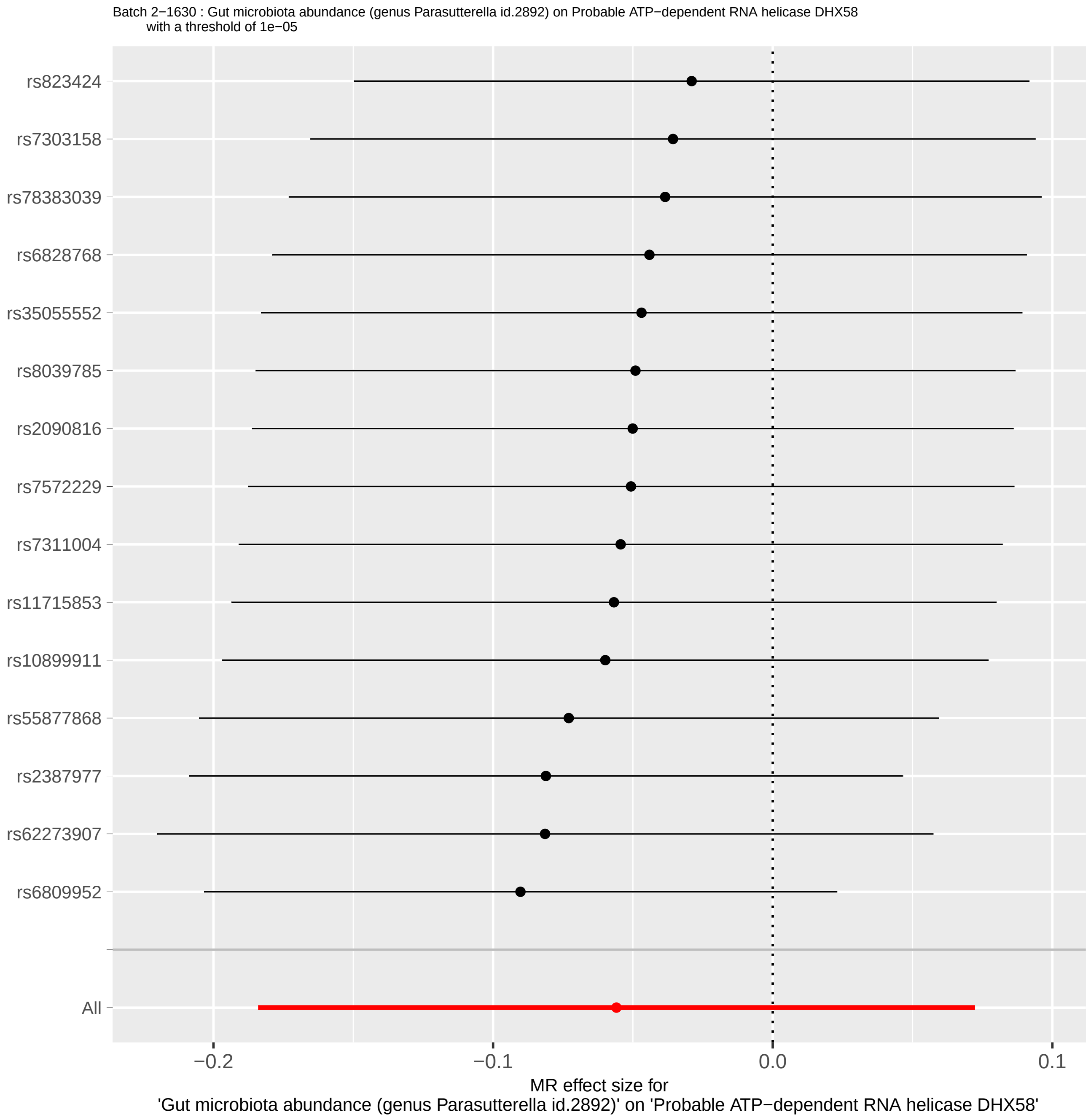
Batch 2−1630 : Gut microbiota abundance (genus Parasutterella id.2892) on Probable ATP−dependent RNA helicase DHX58
with a threshold of 1e−05
rs823424
rs7303158
rs78383039
rs6828768
rs35055552
rs8039785
rs2090816
rs7572229
rs7311004
rs11715853
rs10899911
rs55877868
rs2387977
rs62273907
rs6809952
All
−0.2
−0.1
0.0
0.1
MR effect size for
'Gut microbiota abundance (genus Parasutterella id.2892)' on 'Probable ATP−dependent RNA helicase DHX58'

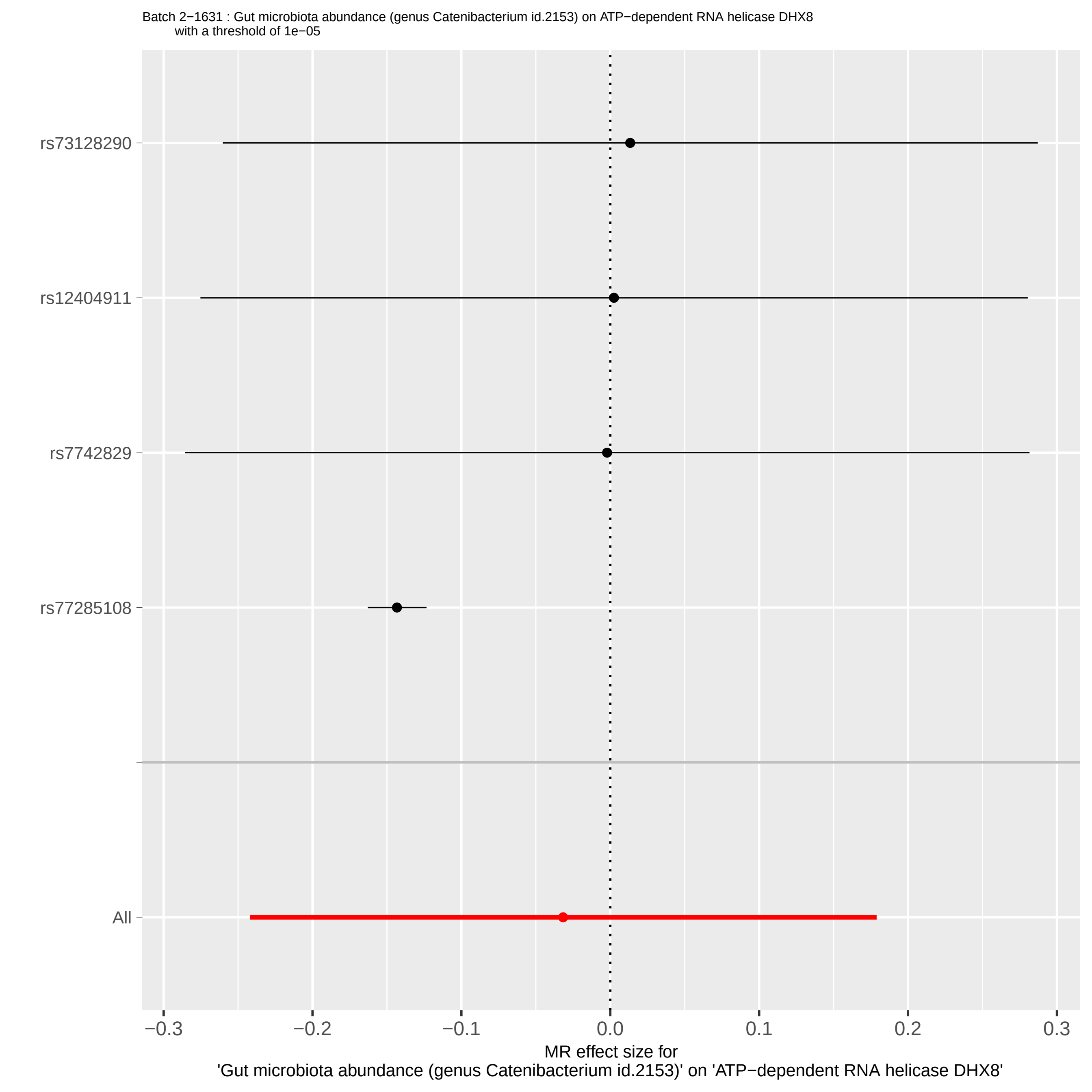
Batch 2−1631 : Gut microbiota abundance (genus Catenibacterium id.2153) on ATP−dependent RNA helicase DHX8
with a threshold of 1e−05
rs73128290
rs12404911
rs7742829
rs77285108
All
−0.3
−0.2
−0.1
0.0
0.1
0.2
0.3
MR effect size for
'Gut microbiota abundance (genus Catenibacterium id.2153)' on 'ATP−dependent RNA helicase DHX8'

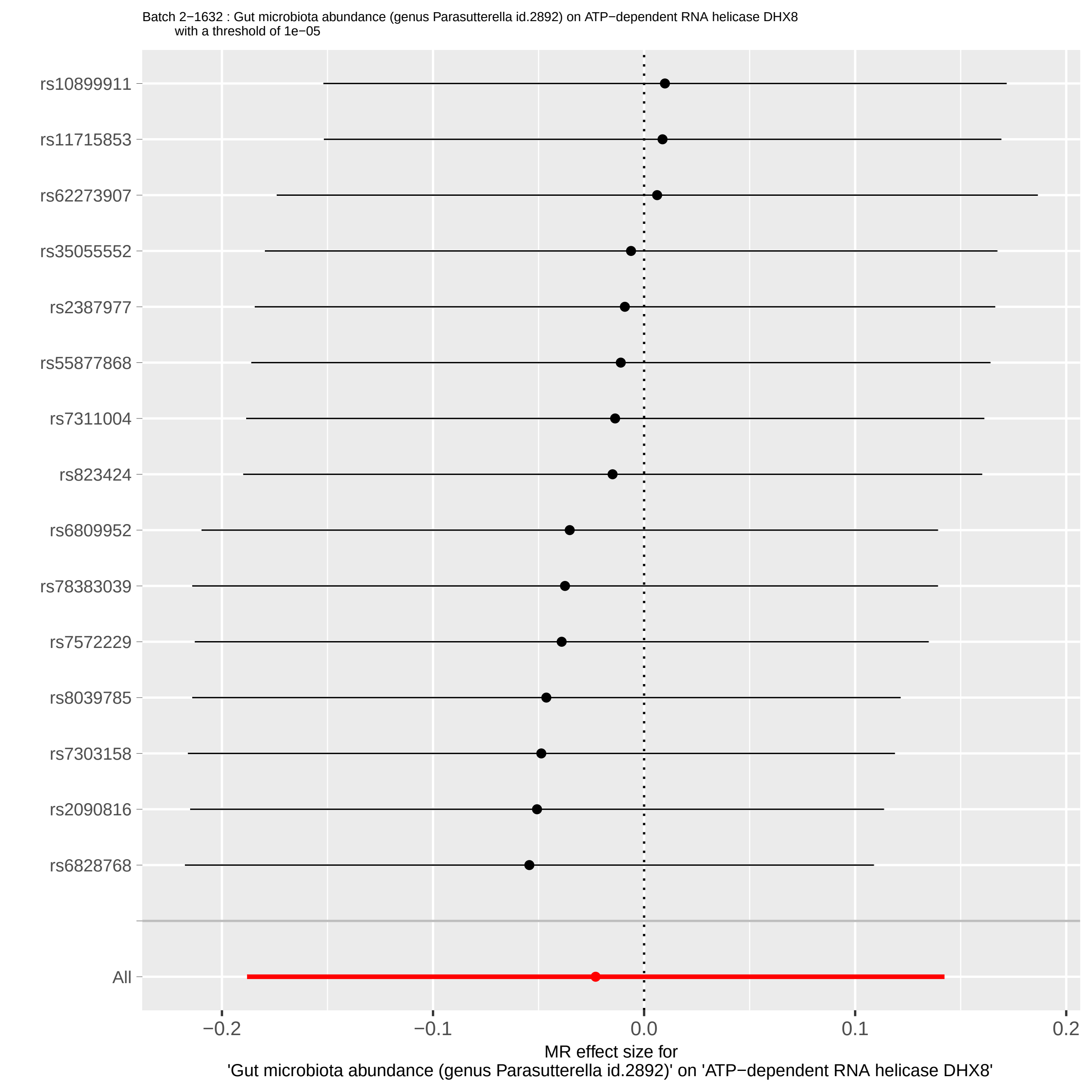

Batch 2−1632 : Gut microbiota abundance (genus Parasutterella id.2892) on ATP−dependent RNA helicase DHX8
with a threshold of 1e−05
rs10899911
rs11715853
rs62273907
rs35055552
rs2387977
rs55877868
rs7311004
rs823424
rs6809952
rs78383039
rs7572229
rs8039785
rs7303158
rs2090816
rs6828768
All
−0.2
−0.1
0.0
0.1
0.2
MR effect size for
'Gut microbiota abundance (genus Parasutterella id.2892)' on 'ATP−dependent RNA helicase DHX8'

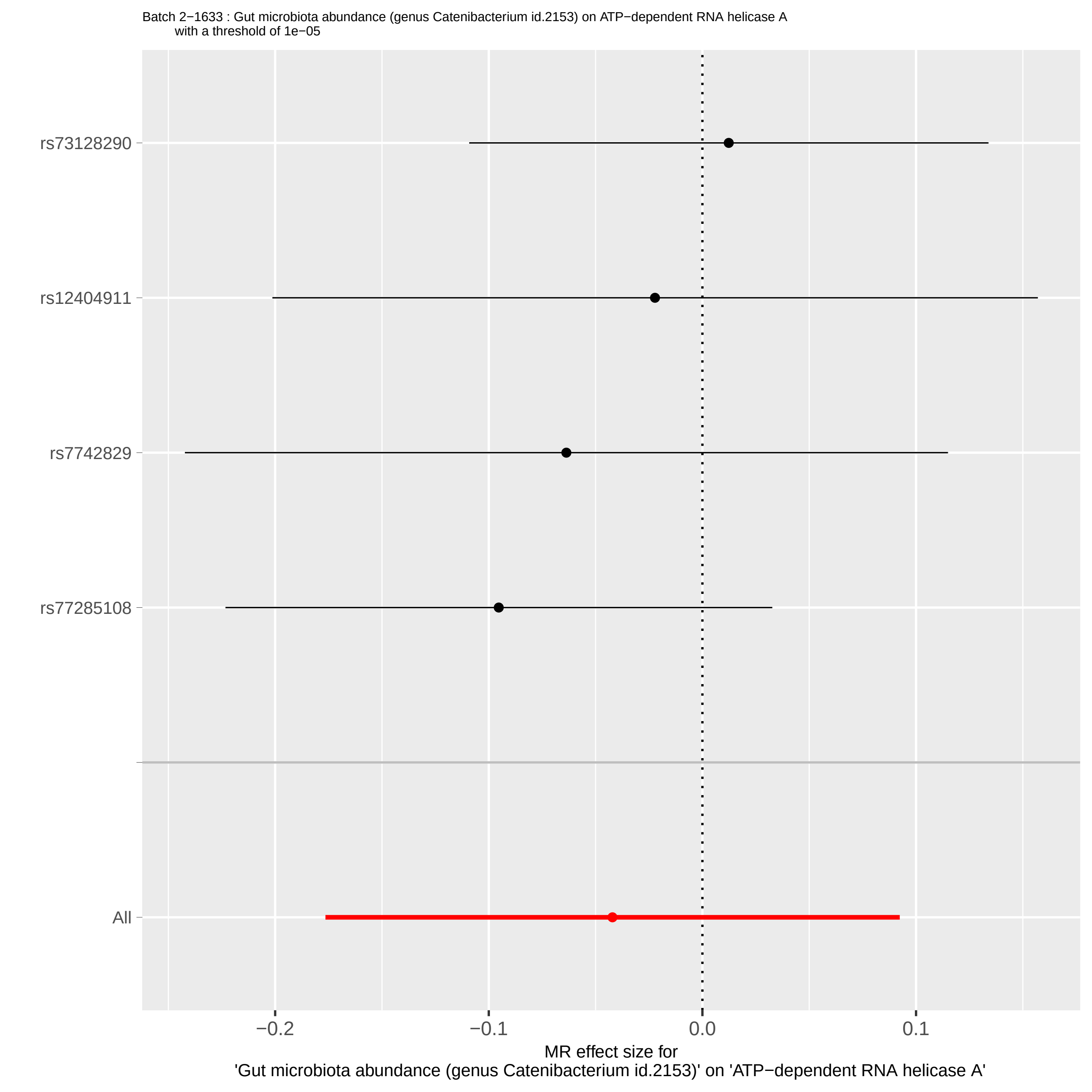
Batch 2−1633 : Gut microbiota abundance (genus Catenibacterium id.2153) on ATP−dependent RNA helicase A
with a threshold of 1e−05
rs73128290
rs12404911
rs7742829
rs77285108
All
−0.2
−0.1
0.0
0.1
MR effect size for
'Gut microbiota abundance (genus Catenibacterium id.2153)' on 'ATP−dependent RNA helicase A'

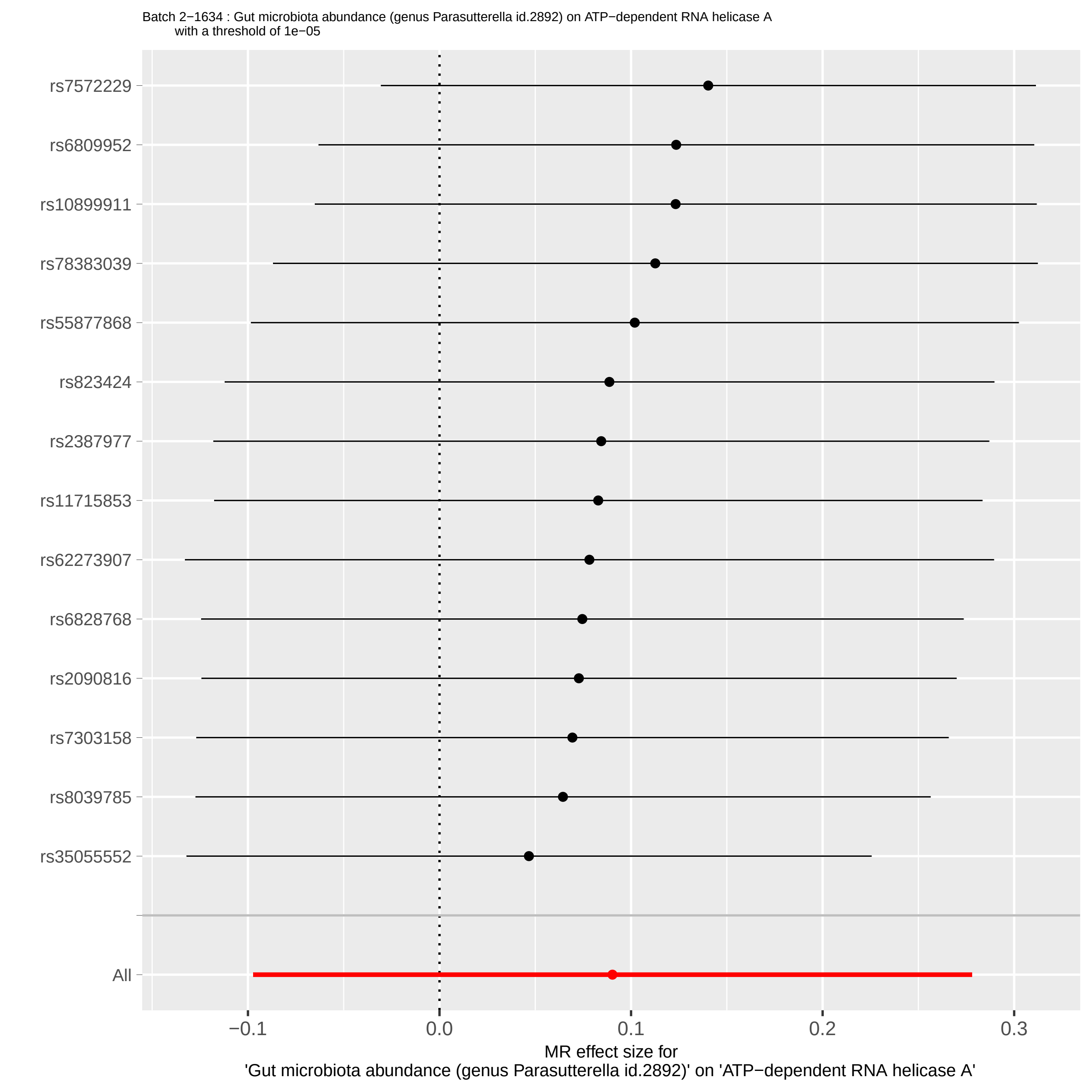
Batch 2−1634 : Gut microbiota abundance (genus Parasutterella id.2892) on ATP−dependent RNA helicase A
with a threshold of 1e−05
rs7572229
rs6809952
rs10899911
rs78383039
rs55877868
rs823424
rs2387977
rs11715853
rs62273907
rs6828768
rs2090816
rs7303158
rs8039785
rs35055552
All
−0.1
0.0
0.1
0.2
0.3
MR effect size for
'Gut microbiota abundance (genus Parasutterella id.2892)' on 'ATP−dependent RNA helicase A'

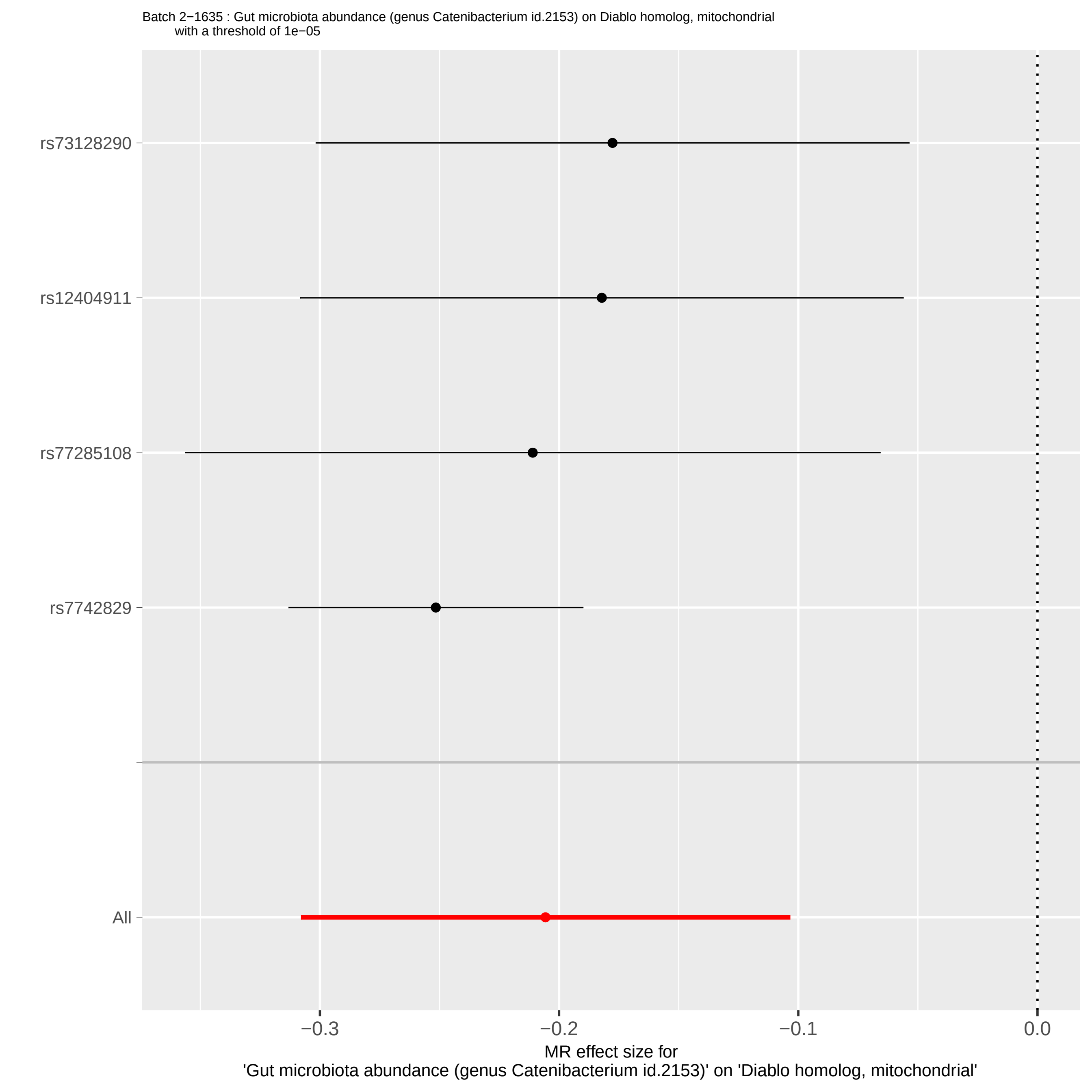
Batch 2−1635 : Gut microbiota abundance (genus Catenibacterium id.2153) on Diablo homolog, mitochondrial
with a threshold of 1e−05
rs73128290
rs12404911
rs77285108
rs7742829
All
−0.3
−0.2
−0.1
0.0
MR effect size for
'Gut microbiota abundance (genus Catenibacterium id.2153)' on 'Diablo homolog, mitochondrial'

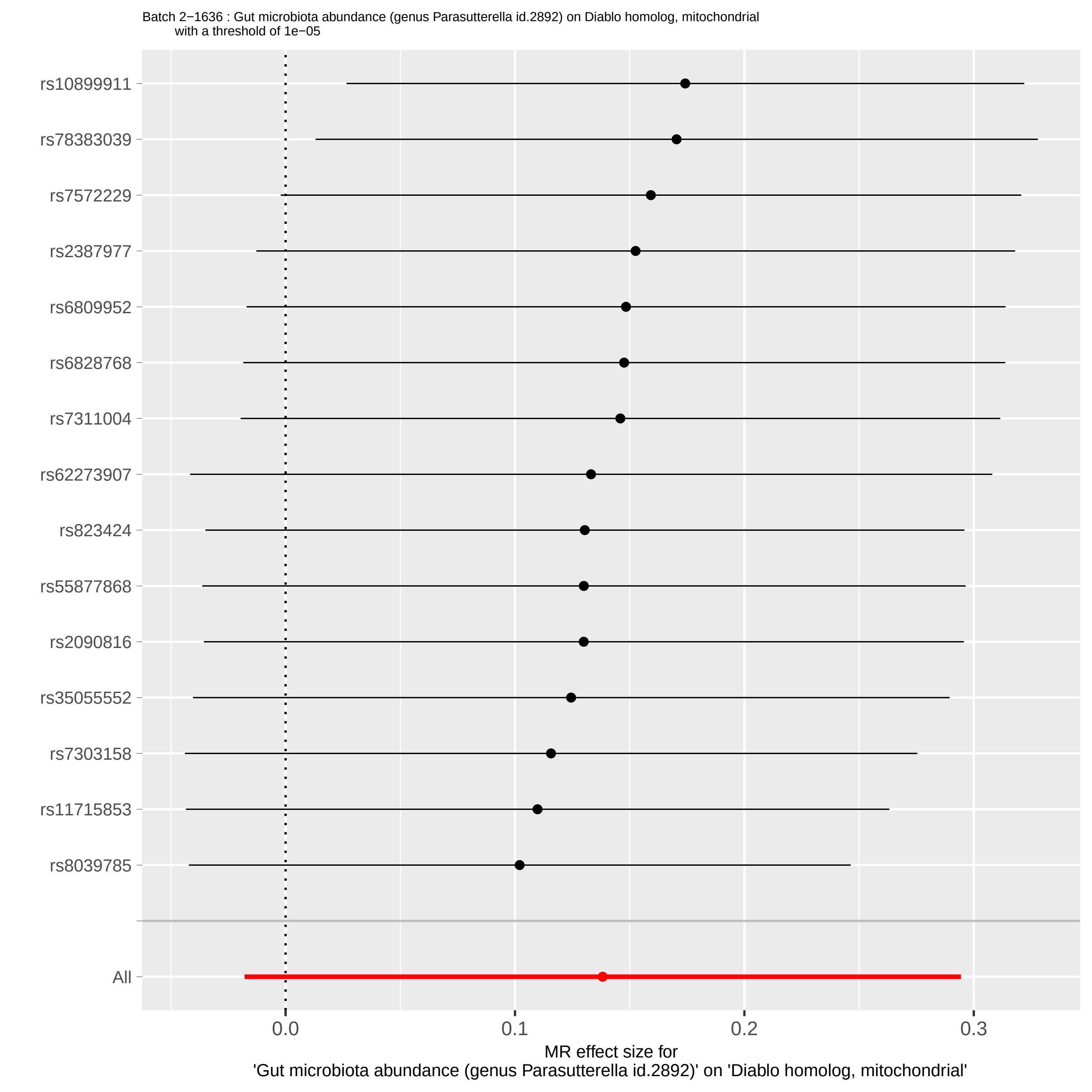

Batch 2−1636 : Gut microbiota abundance (genus Parasutterella id.2892) on Diablo homolog, mitochondrial
with a threshold of 1e−05
rs10899911
rs78383039
rs7572229
rs2387977
rs6809952
rs6828768
rs7311004
rs62273907
rs823424
rs55877868
rs2090816
rs35055552
rs7303158
rs11715853
rs8039785
All
0.0
0.1
0.2
0.3
MR effect size for
'Gut microbiota abundance (genus Parasutterella id.2892)' on 'Diablo homolog, mitochondrial'

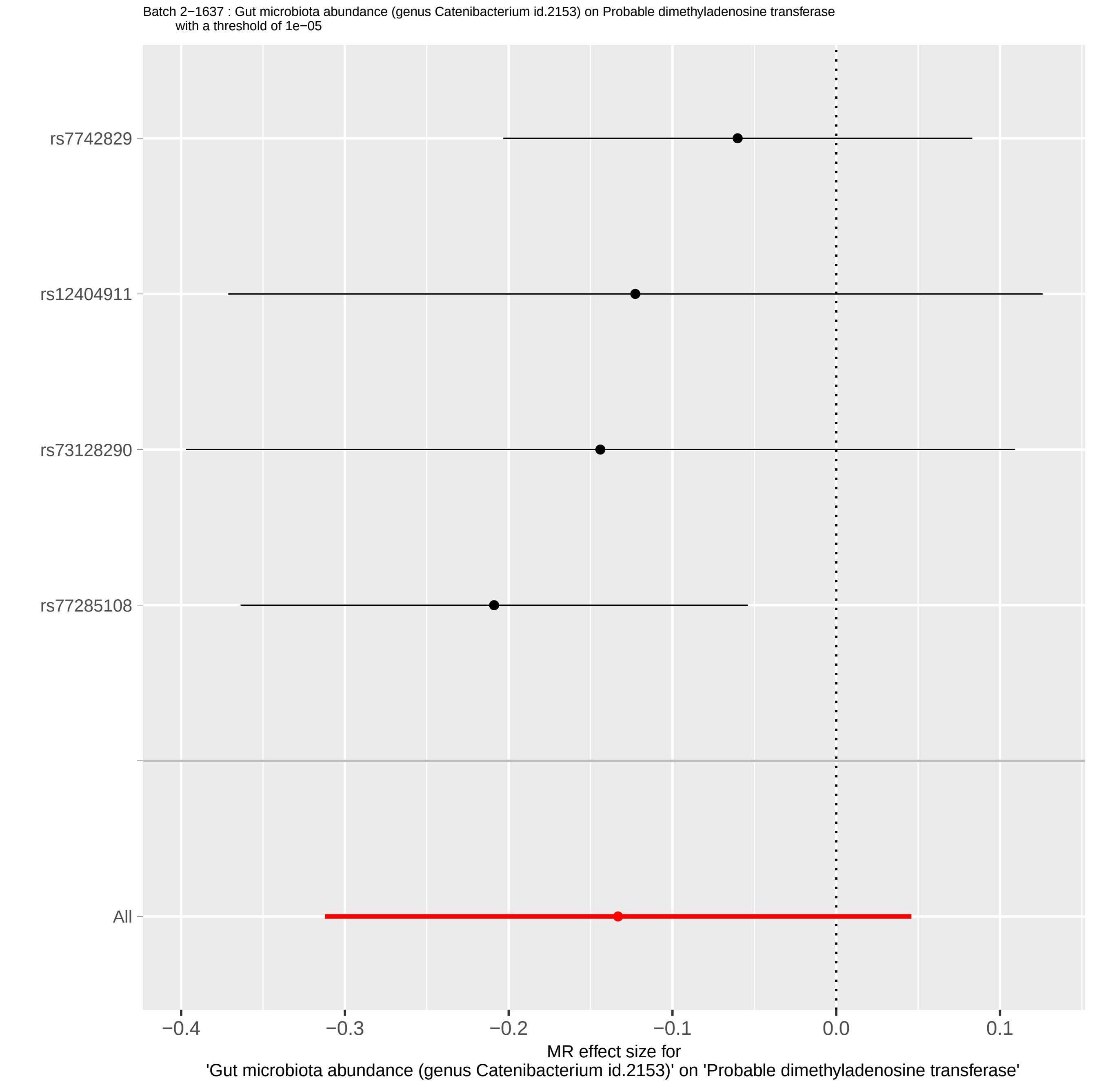

Batch 2−1637 : Gut microbiota abundance (genus Catenibacterium id.2153) on Probable dimethyladenosine transferase
with a threshold of 1e−05
rs7742829
rs12404911
rs73128290
rs77285108
All
−0.4
−0.3
−0.2
−0.1
0.0
0.1
MR effect size for
'Gut microbiota abundance (genus Catenibacterium id.2153)' on 'Probable dimethyladenosine transferase'

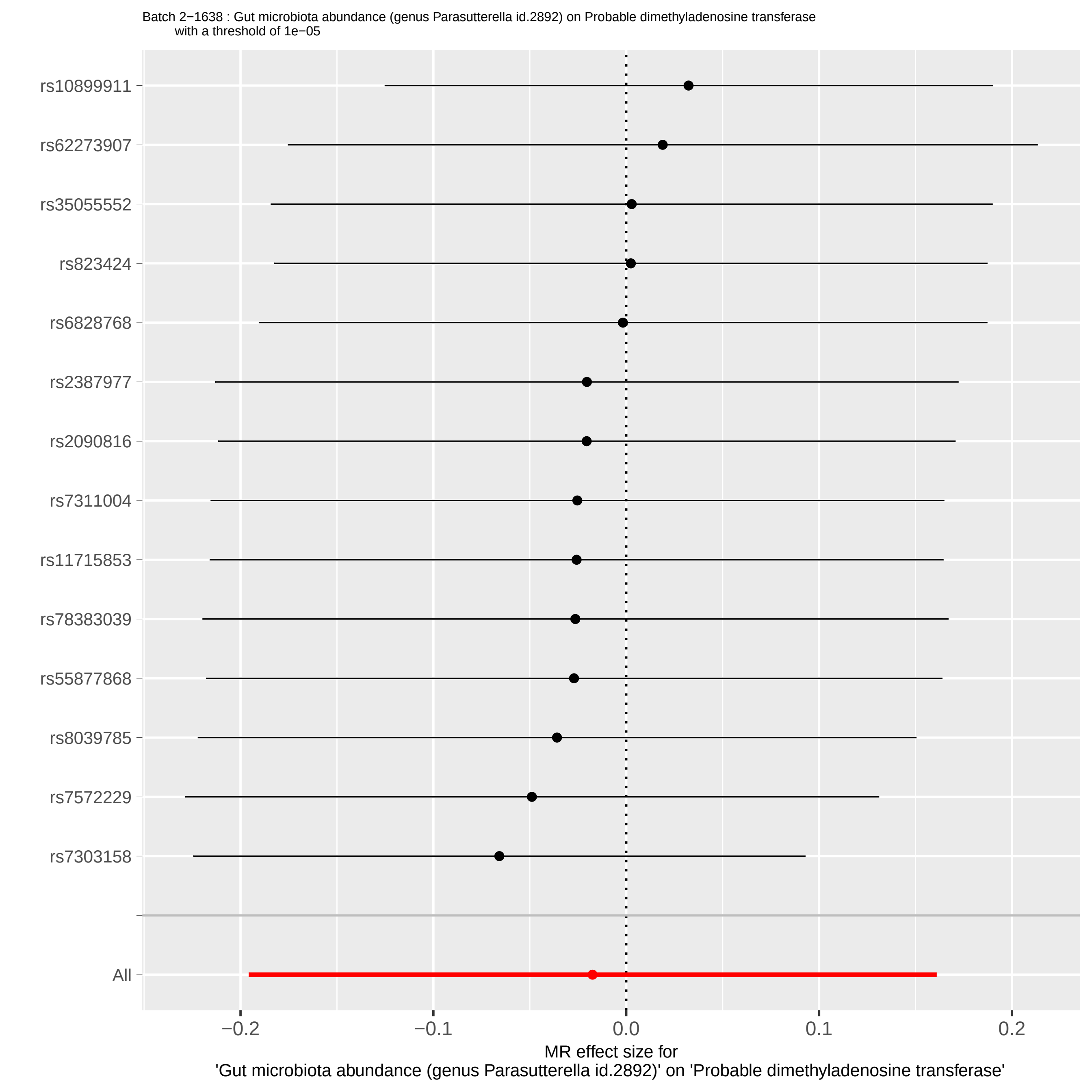
Batch 2−1638 : Gut microbiota abundance (genus Parasutterella id.2892) on Probable dimethyladenosine transferase
with a threshold of 1e−05
rs10899911
rs62273907
rs35055552
rs823424
rs6828768
rs2387977
rs2090816
rs7311004
rs11715853
rs78383039
rs55877868
rs8039785
rs7572229
rs7303158
All
−0.2
−0.1
0.0
0.1
0.2
MR effect size for
'Gut microbiota abundance (genus Parasutterella id.2892)' on 'Probable dimethyladenosine transferase'

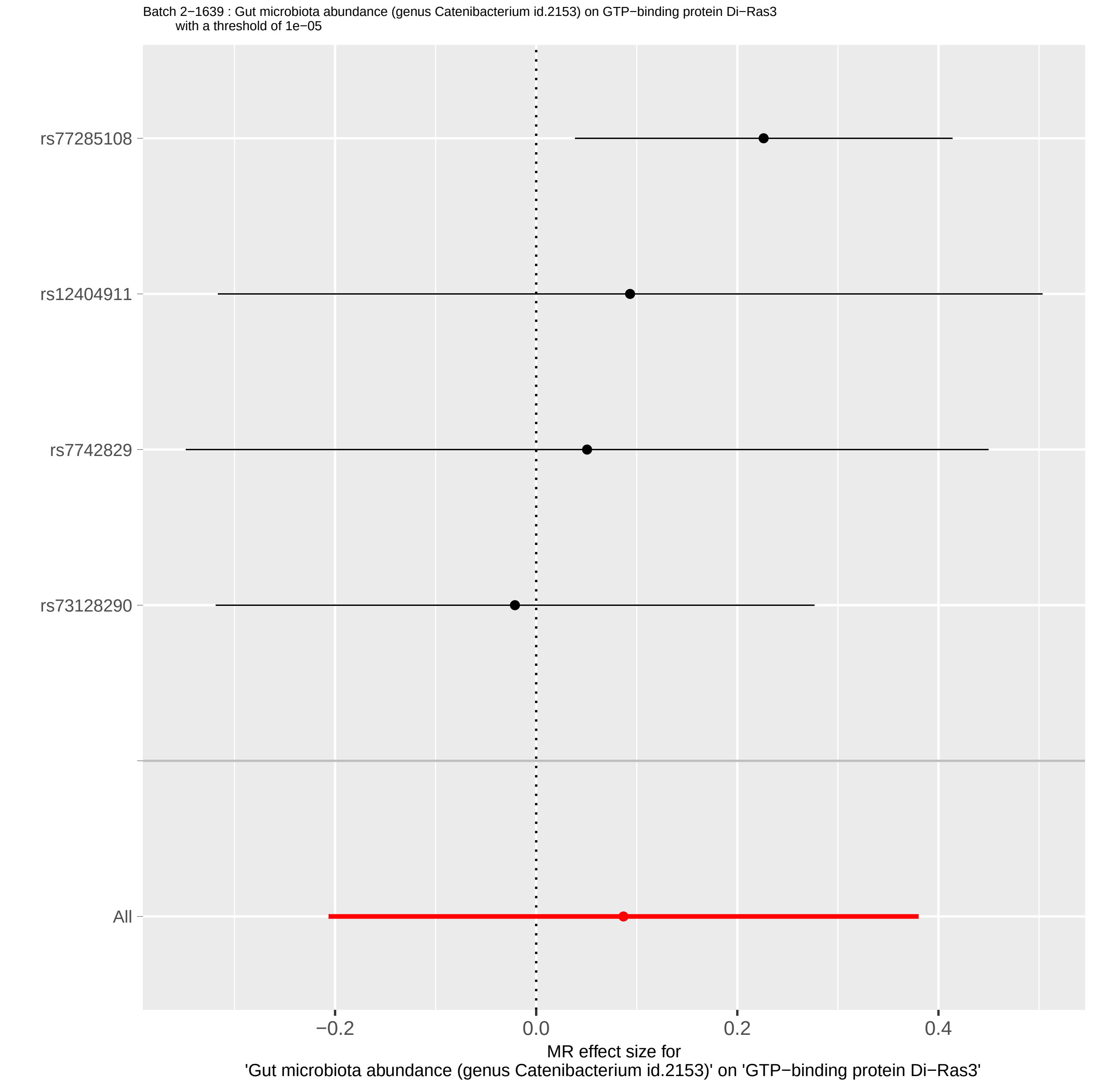
Batch 2−1639 : Gut microbiota abundance (genus Catenibacterium id.2153) on GTP−binding protein Di−Ras3
with a threshold of 1e−05
rs77285108
rs12404911
rs7742829
rs73128290
All
−0.2
0.0
0.2
0.4
MR effect size for
'Gut microbiota abundance (genus Catenibacterium id.2153)' on 'GTP−binding protein Di−Ras3'

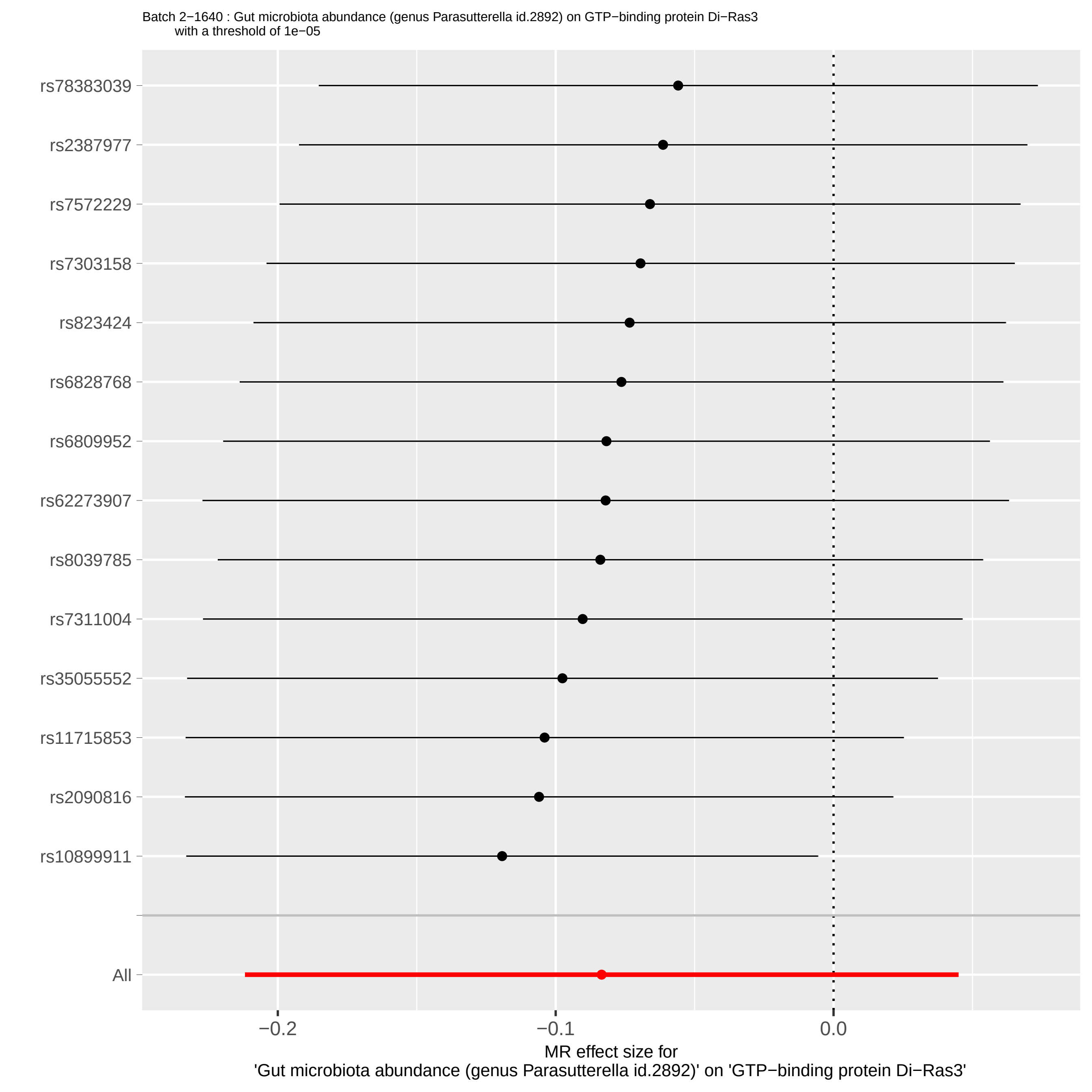

Batch 2−1640 : Gut microbiota abundance (genus Parasutterella id.2892) on GTP−binding protein Di−Ras3
with a threshold of 1e−05
rs78383039
rs2387977
rs7572229
rs7303158
rs823424
rs6828768
rs6809952
rs62273907
rs8039785
rs7311004
rs35055552
rs11715853
rs2090816
rs10899911
All
−0.2
−0.1
0.0
MR effect size for
'Gut microbiota abundance (genus Parasutterella id.2892)' on 'GTP−binding protein Di−Ras3'

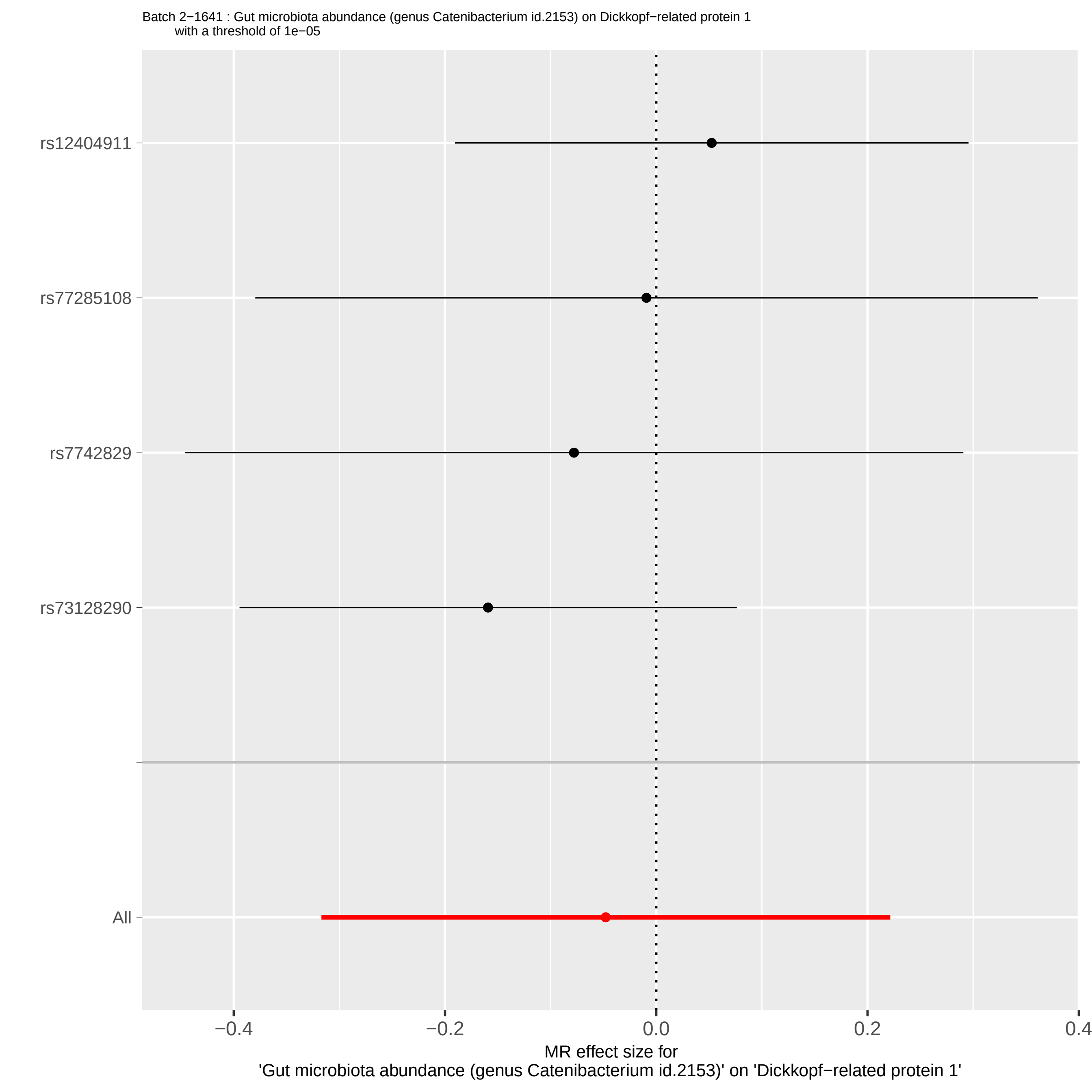
Batch 2−1641 : Gut microbiota abundance (genus Catenibacterium id.2153) on Dickkopf−related protein 1
with a threshold of 1e−05
rs12404911
rs77285108
rs7742829
rs73128290
All
−0.4
−0.2
0.0
0.2
0.4
MR effect size for
'Gut microbiota abundance (genus Catenibacterium id.2153)' on 'Dickkopf−related protein 1'

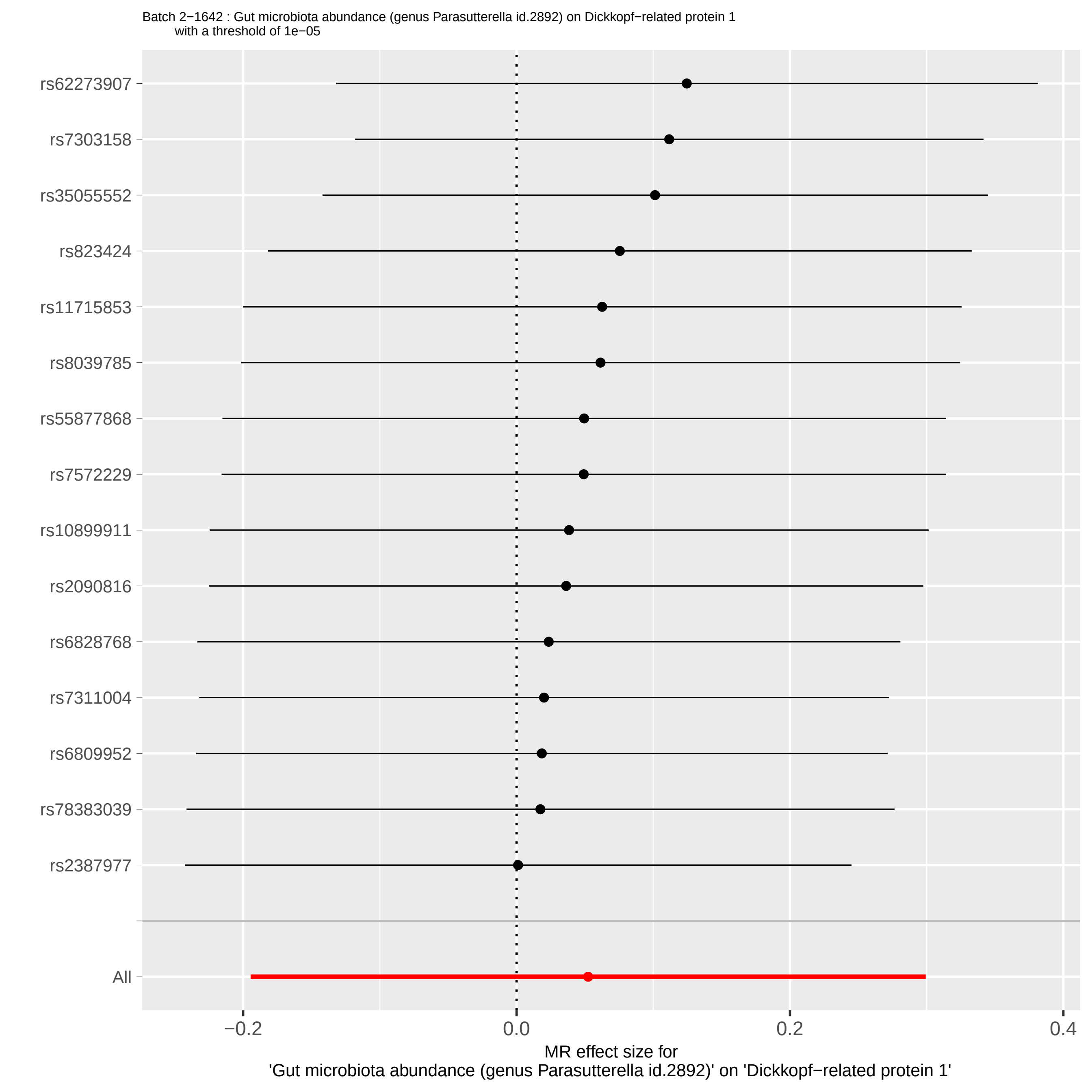
Batch 2−1642 : Gut microbiota abundance (genus Parasutterella id.2892) on Dickkopf−related protein 1
with a threshold of 1e−05
rs62273907
rs7303158
rs35055552
rs823424
rs11715853
rs8039785
rs55877868
rs7572229
rs10899911
rs2090816
rs6828768
rs7311004
rs6809952
rs78383039
rs2387977
All
−0.2
0.0
0.2
0.4
MR effect size for
'Gut microbiota abundance (genus Parasutterella id.2892)' on 'Dickkopf−related protein 1'

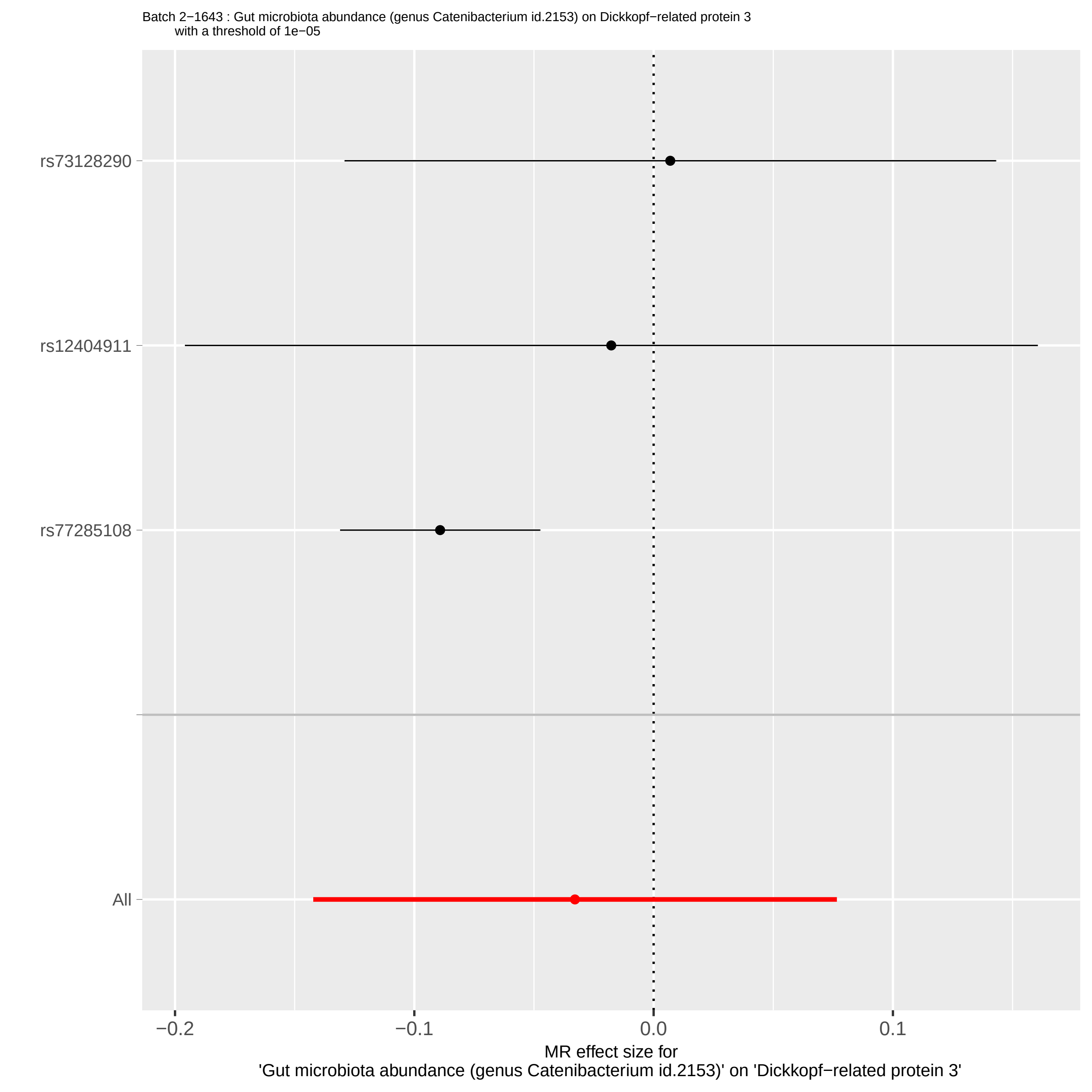
Batch 2−1643 : Gut microbiota abundance (genus Catenibacterium id.2153) on Dickkopf−related protein 3
with a threshold of 1e−05
rs73128290
rs12404911
rs77285108
All
−0.2
−0.1
0.0
0.1
MR effect size for
'Gut microbiota abundance (genus Catenibacterium id.2153)' on 'Dickkopf−related protein 3'

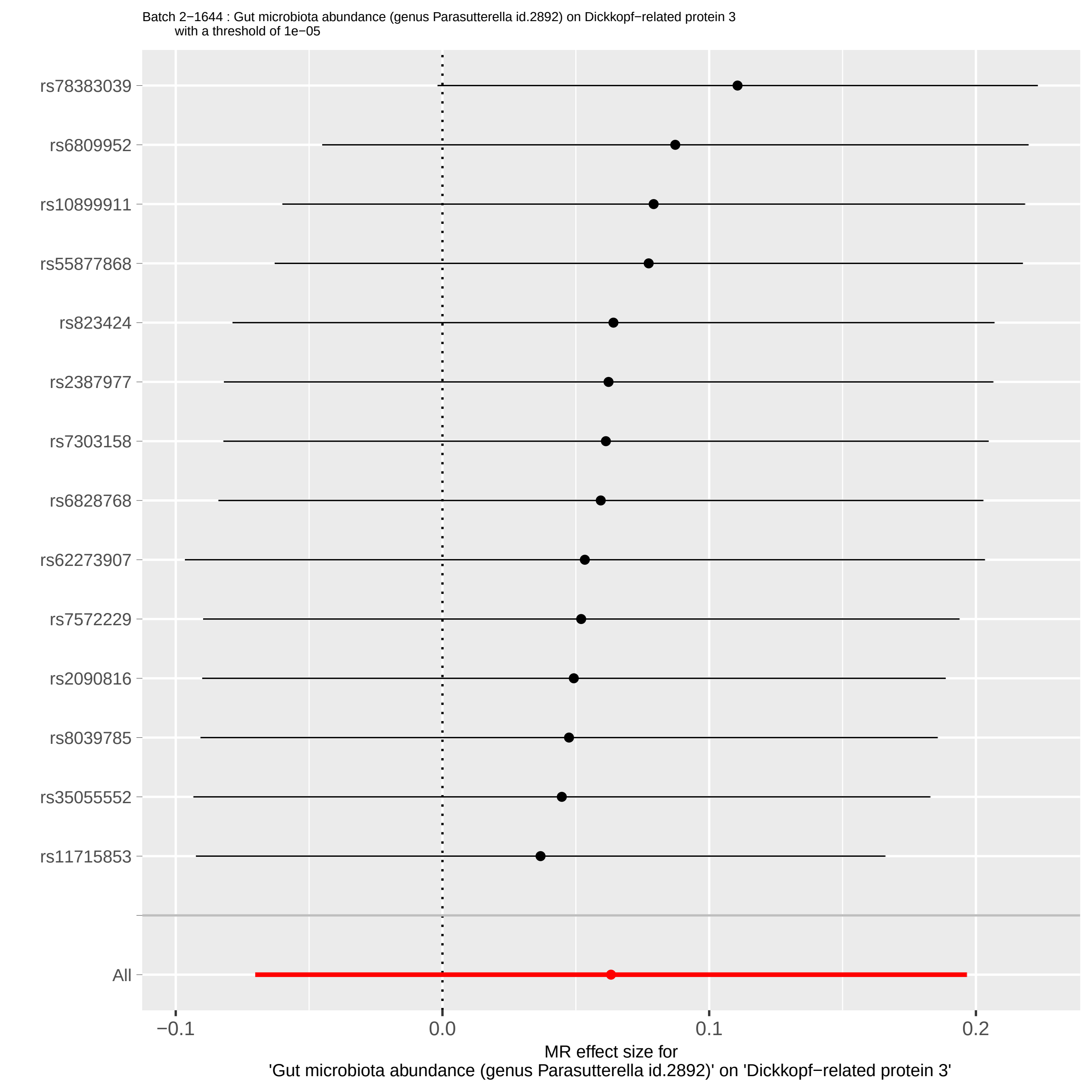

Batch 2−1644 : Gut microbiota abundance (genus Parasutterella id.2892) on Dickkopf−related protein 3
with a threshold of 1e−05
rs78383039
rs6809952
rs10899911
rs55877868
rs823424
rs2387977
rs7303158
rs6828768
rs62273907
rs7572229
rs2090816
rs8039785
rs35055552
rs11715853
All
−0.1
0.0
0.1
0.2
MR effect size for
'Gut microbiota abundance (genus Parasutterella id.2892)' on 'Dickkopf−related protein 3'

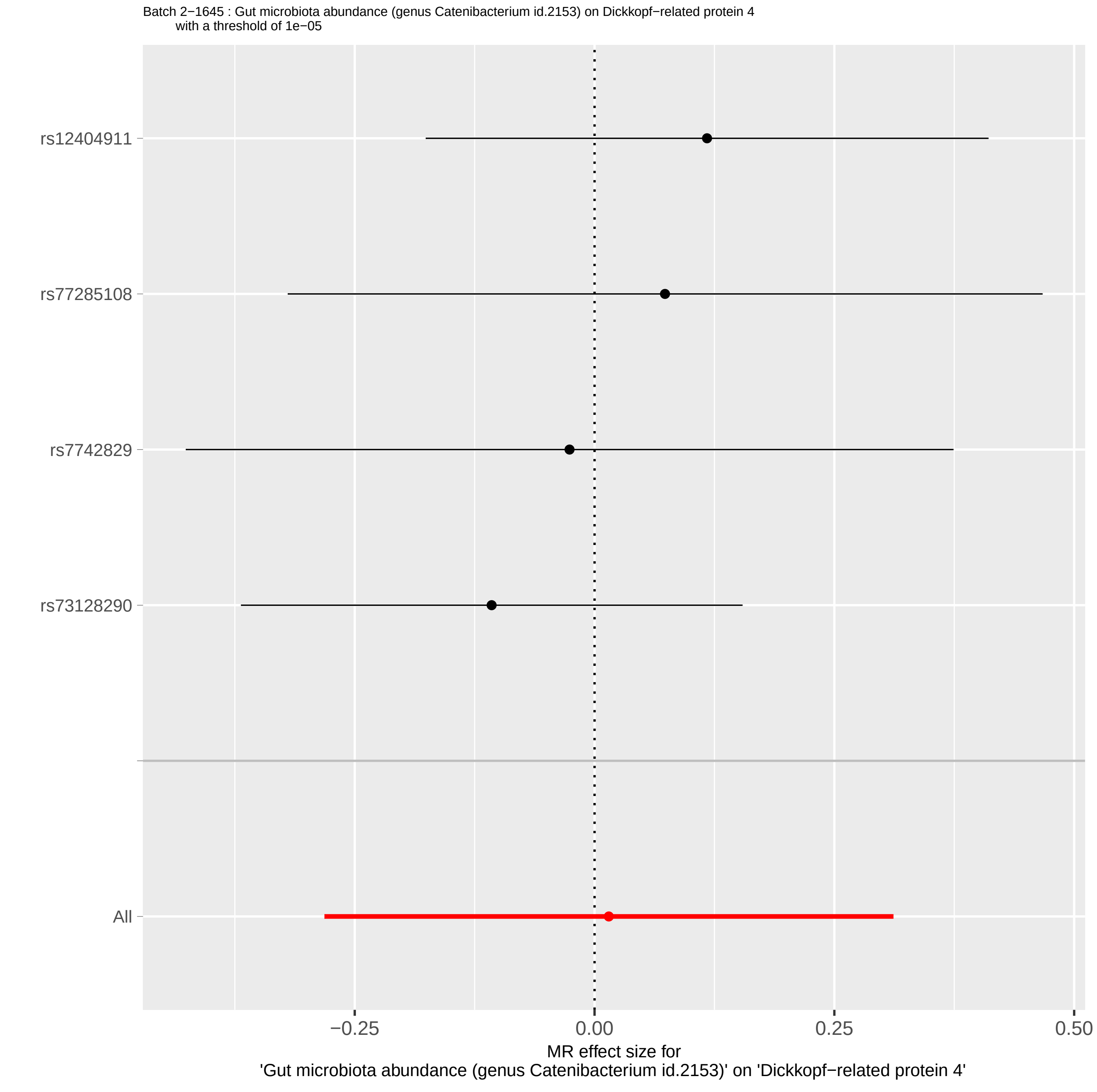
Batch 2−1645 : Gut microbiota abundance (genus Catenibacterium id.2153) on Dickkopf−related protein 4
with a threshold of 1e−05
rs12404911
rs77285108
rs7742829
rs73128290
All
−0.25
0.00
0.25
0.50
MR effect size for
'Gut microbiota abundance (genus Catenibacterium id.2153)' on 'Dickkopf−related protein 4'

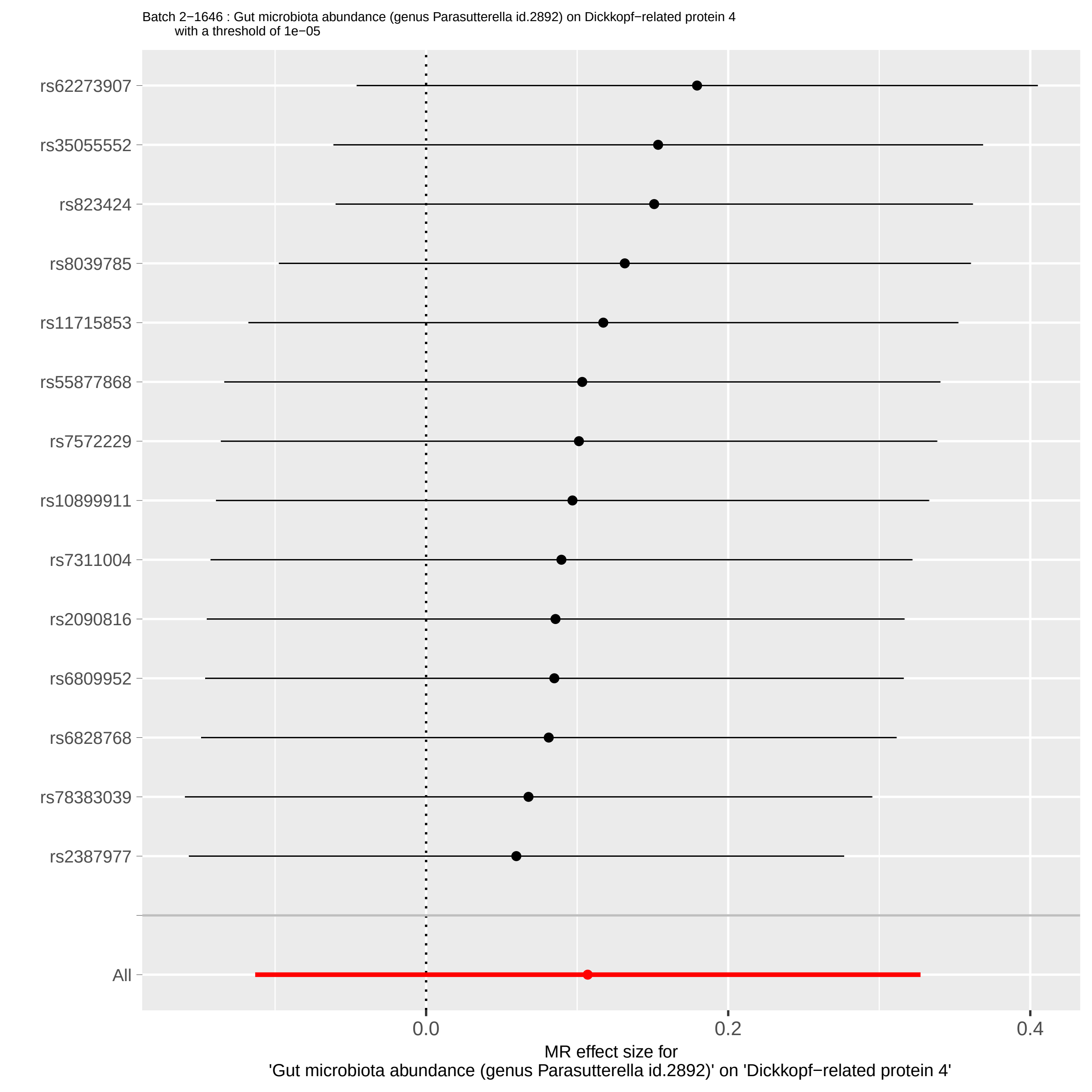
Batch 2−1646 : Gut microbiota abundance (genus Parasutterella id.2892) on Dickkopf−related protein 4
with a threshold of 1e−05
rs62273907
rs35055552
rs823424
rs8039785
rs11715853
rs55877868
rs7572229
rs10899911
rs7311004
rs2090816
rs6809952
rs6828768
rs78383039
rs2387977
All
0.0
0.2
0.4
MR effect size for
'Gut microbiota abundance (genus Parasutterella id.2892)' on 'Dickkopf−related protein 4'

Batch 2−1647 : Gut microbiota abundance (genus Catenibacterium id.2153) on Dickkopf−like protein 1
with a threshold of 1e−05

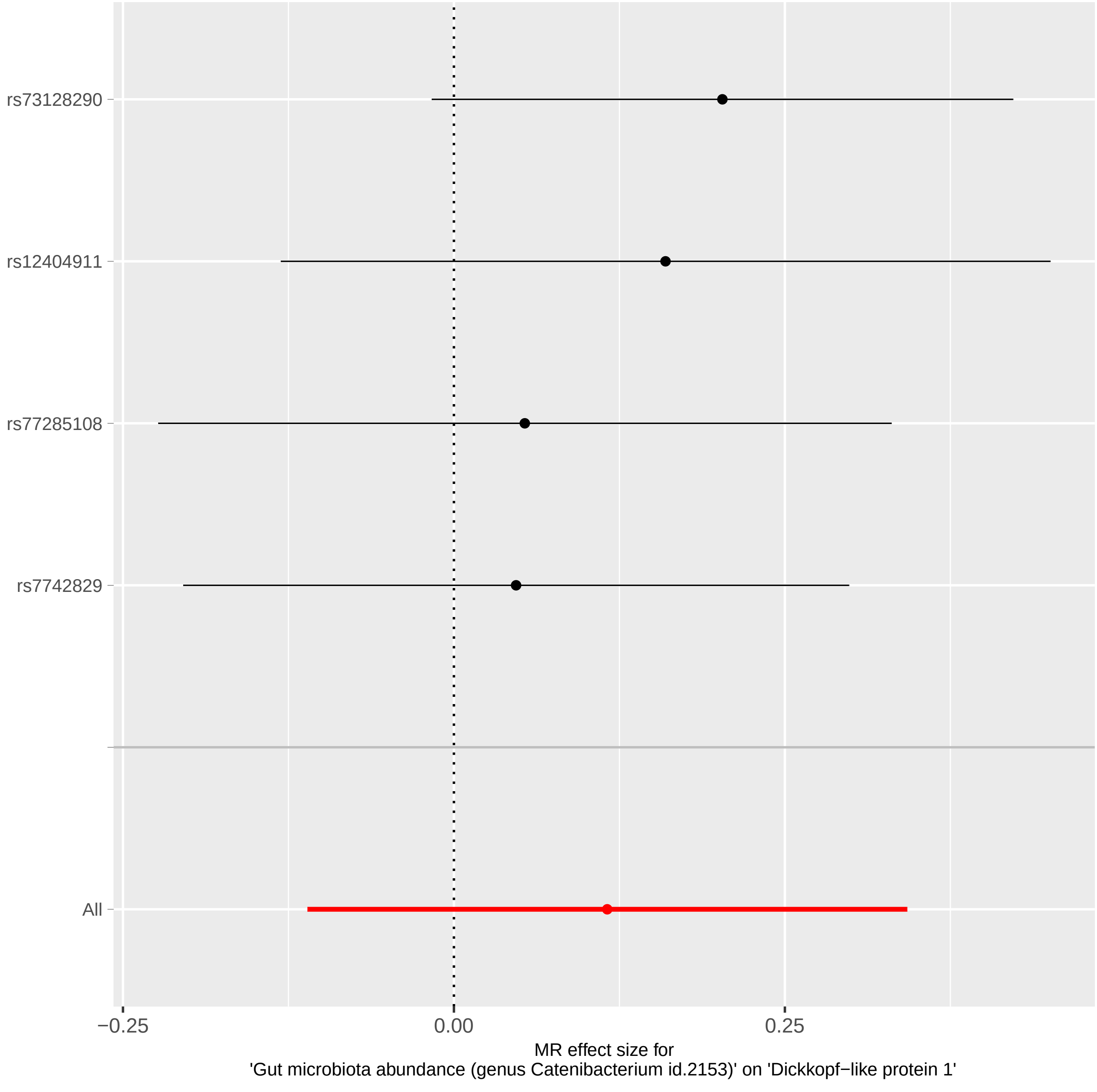

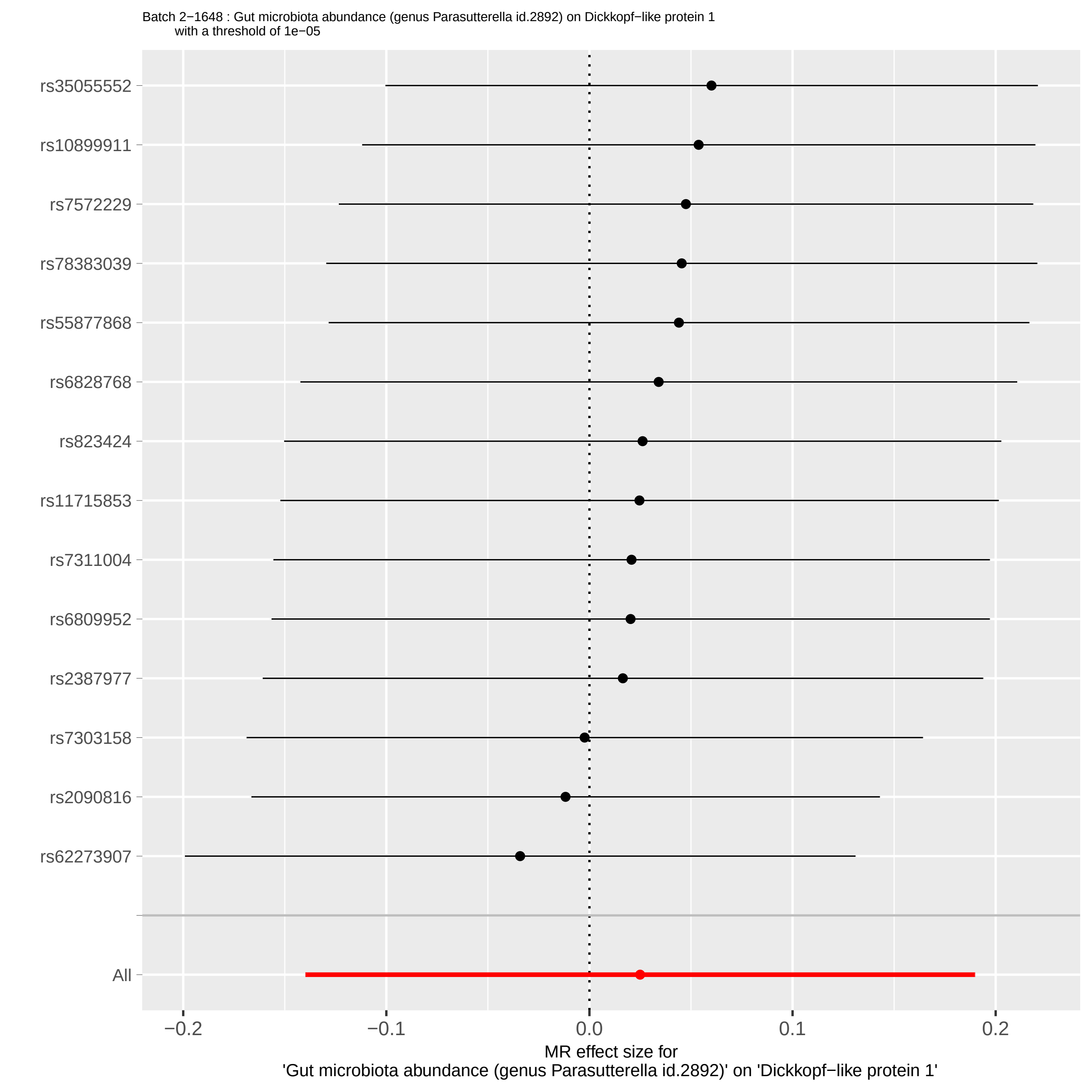
Batch 2−1648 : Gut microbiota abundance (genus Parasutterella id.2892) on Dickkopf−like protein 1
with a threshold of 1e−05
rs35055552
rs10899911
rs7572229
rs78383039
rs55877868
rs6828768
rs823424
rs11715853
rs7311004
rs6809952
rs2387977
rs7303158
rs2090816
rs62273907
All
−0.2
−0.1
0.0
0.1
0.2
MR effect size for
'Gut microbiota abundance (genus Parasutterella id.2892)' on 'Dickkopf−like protein 1'

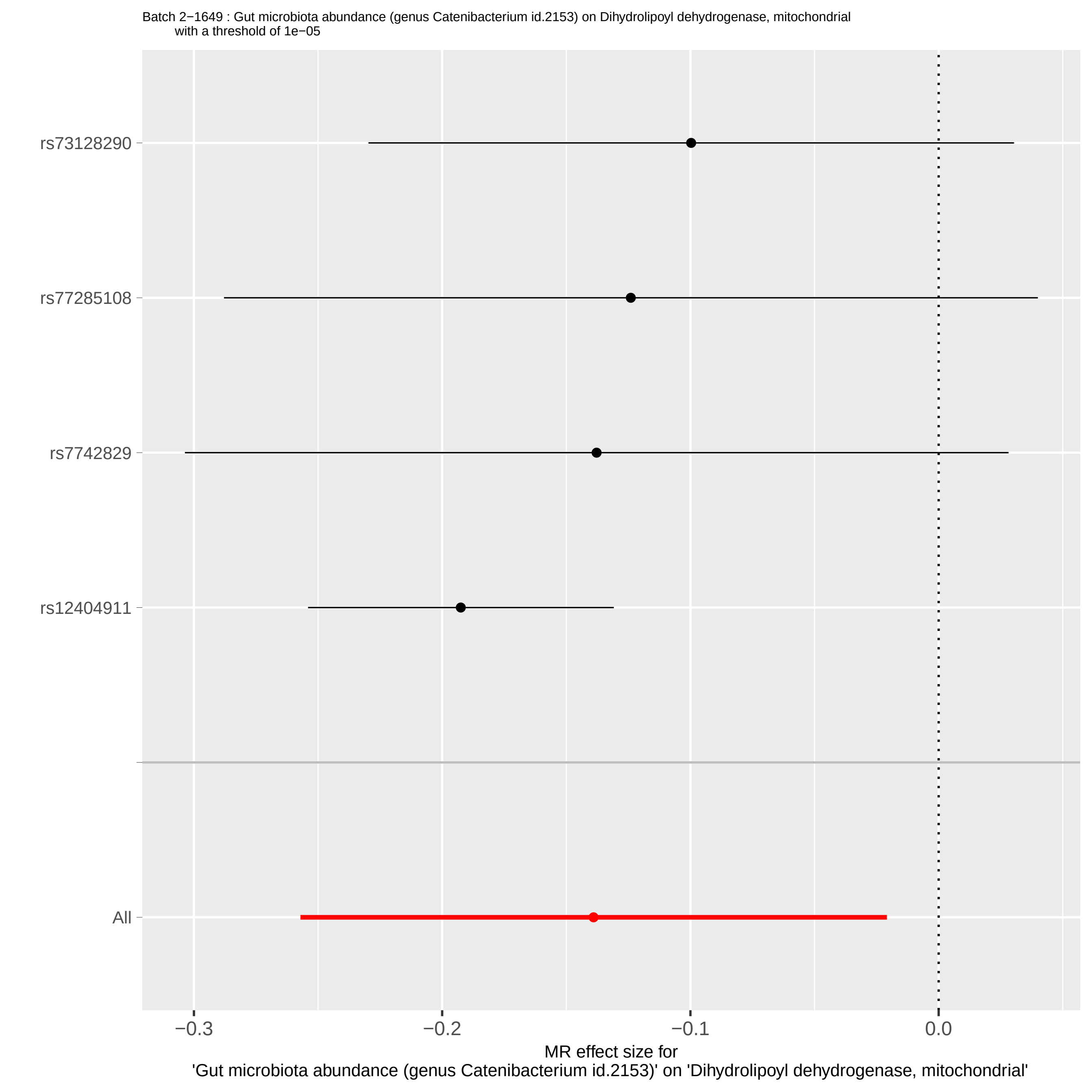

Batch 2−1649 : Gut microbiota abundance (genus Catenibacterium id.2153) on Dihydrolipoyl dehydrogenase, mitochondrial
with a threshold of 1e−05
rs73128290
rs77285108
rs7742829
rs12404911
All
−0.3
−0.2
−0.1
0.0
MR effect size for
'Gut microbiota abundance (genus Catenibacterium id.2153)' on 'Dihydrolipoyl dehydrogenase, mitochondrial'

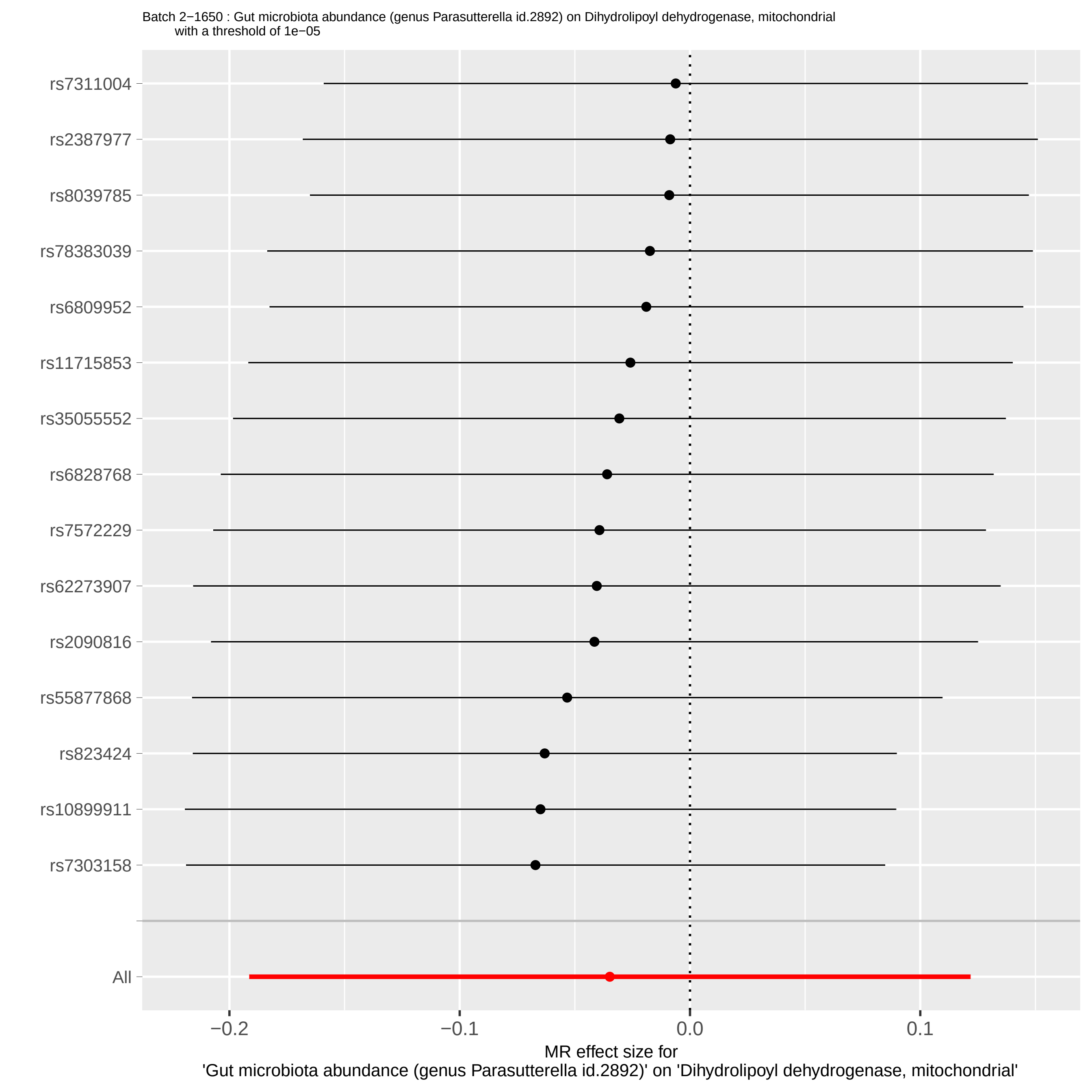

Batch 2−1650 : Gut microbiota abundance (genus Parasutterella id.2892) on Dihydrolipoyl dehydrogenase, mitochondrial
with a threshold of 1e−05
rs7311004
rs2387977
rs8039785
rs78383039
rs6809952
rs11715853
rs35055552
rs6828768
rs7572229
rs62273907
rs2090816
rs55877868
rs823424
rs10899911
rs7303158
All
−0.2
−0.1
0.0
0.1
MR effect size for
'Gut microbiota abundance (genus Parasutterella id.2892)' on 'Dihydrolipoyl dehydrogenase, mitochondrial'

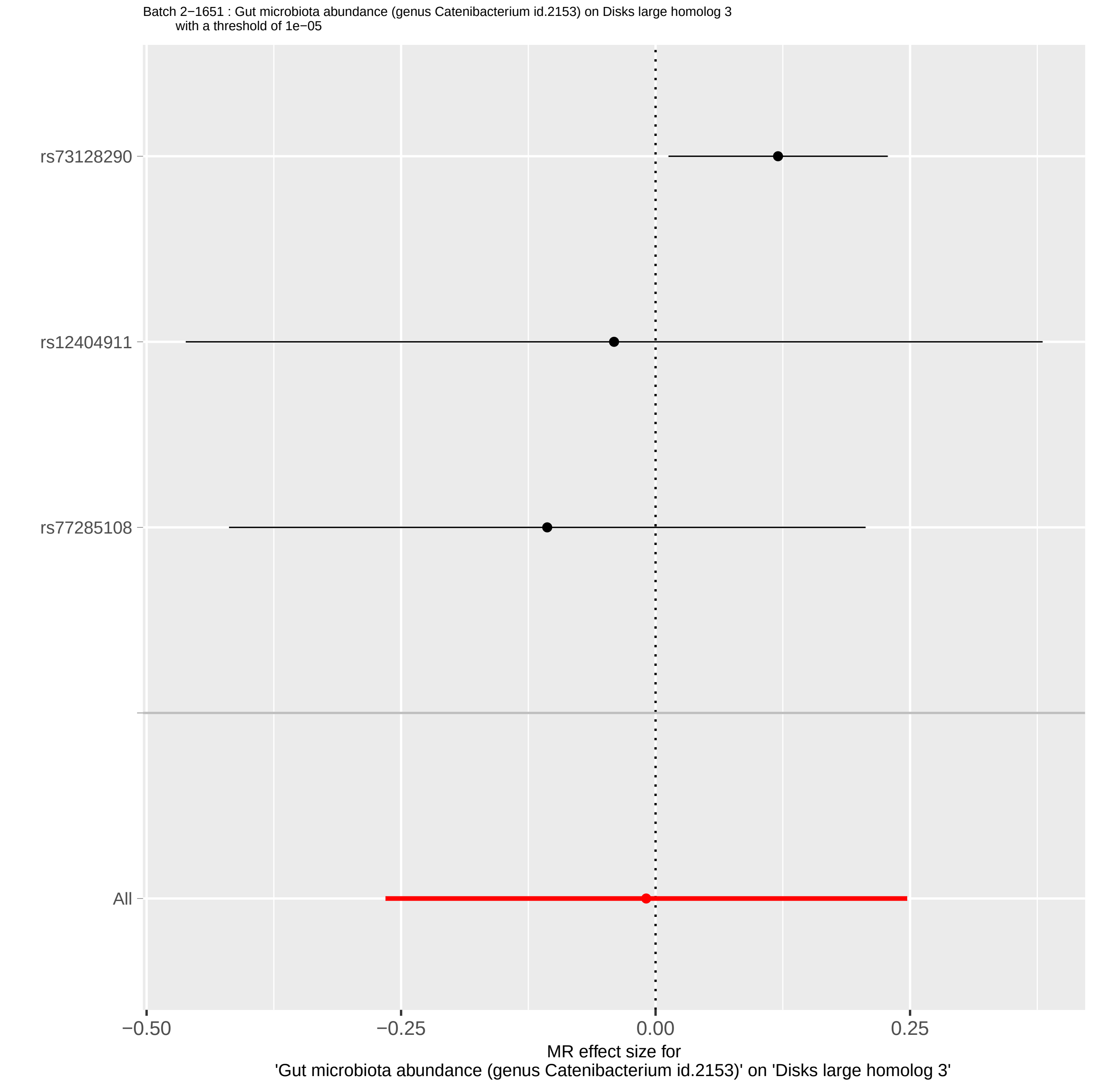

Batch 2−1651 : Gut microbiota abundance (genus Catenibacterium id.2153) on Disks large homolog 3
with a threshold of 1e−05
rs73128290
rs12404911
rs77285108
All
−0.50
−0.25
0.00
0.25
MR effect size for
'Gut microbiota abundance (genus Catenibacterium id.2153)' on 'Disks large homolog 3'

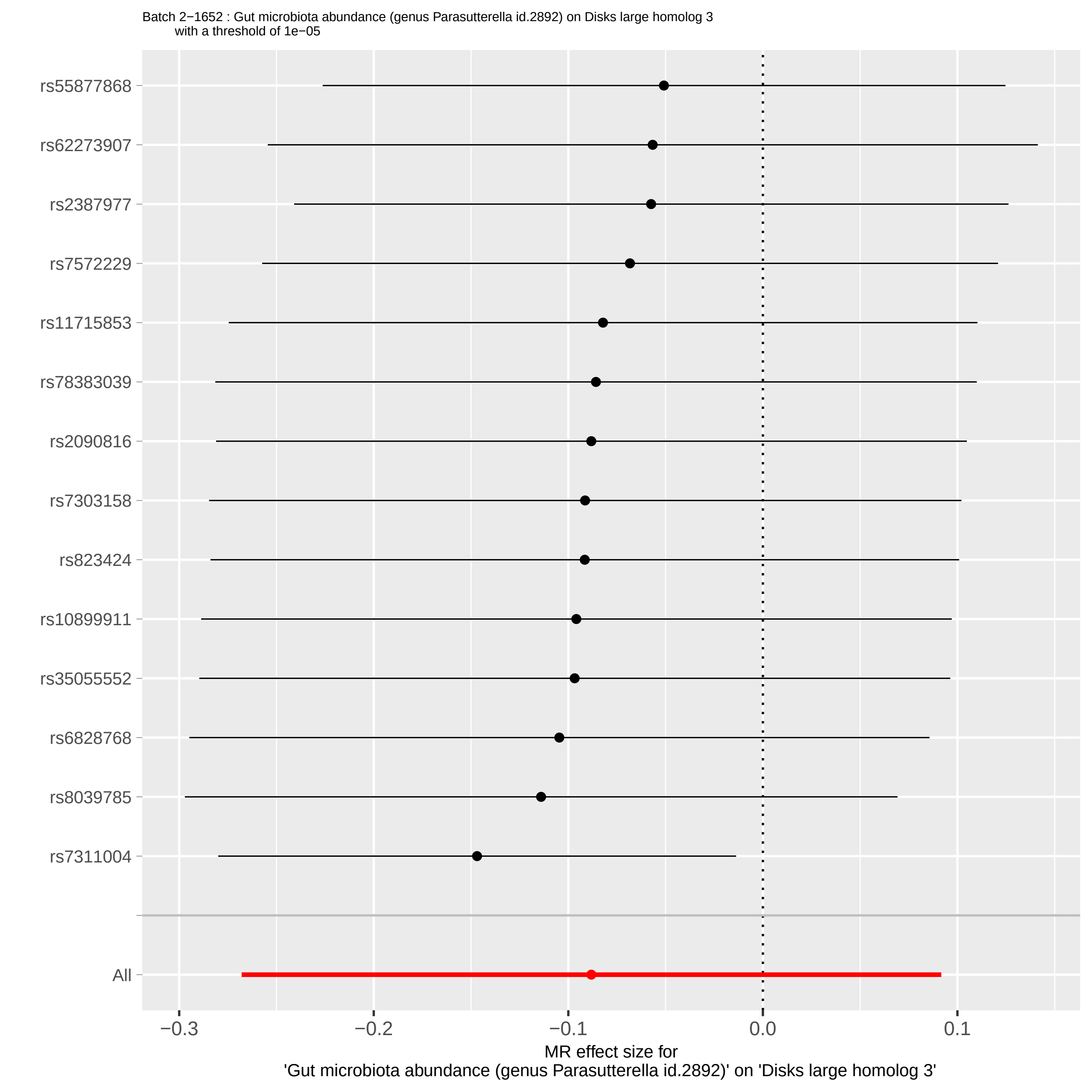

Batch 2−1652 : Gut microbiota abundance (genus Parasutterella id.2892) on Disks large homolog 3
with a threshold of 1e−05
rs55877868
rs62273907
rs2387977
rs7572229
rs11715853
rs78383039
rs2090816
rs7303158
rs823424
rs10899911
rs35055552
rs6828768
rs8039785
rs7311004
All
−0.3
−0.2
−0.1
0.0
0.1
MR effect size for
'Gut microbiota abundance (genus Parasutterella id.2892)' on 'Disks large homolog 3'

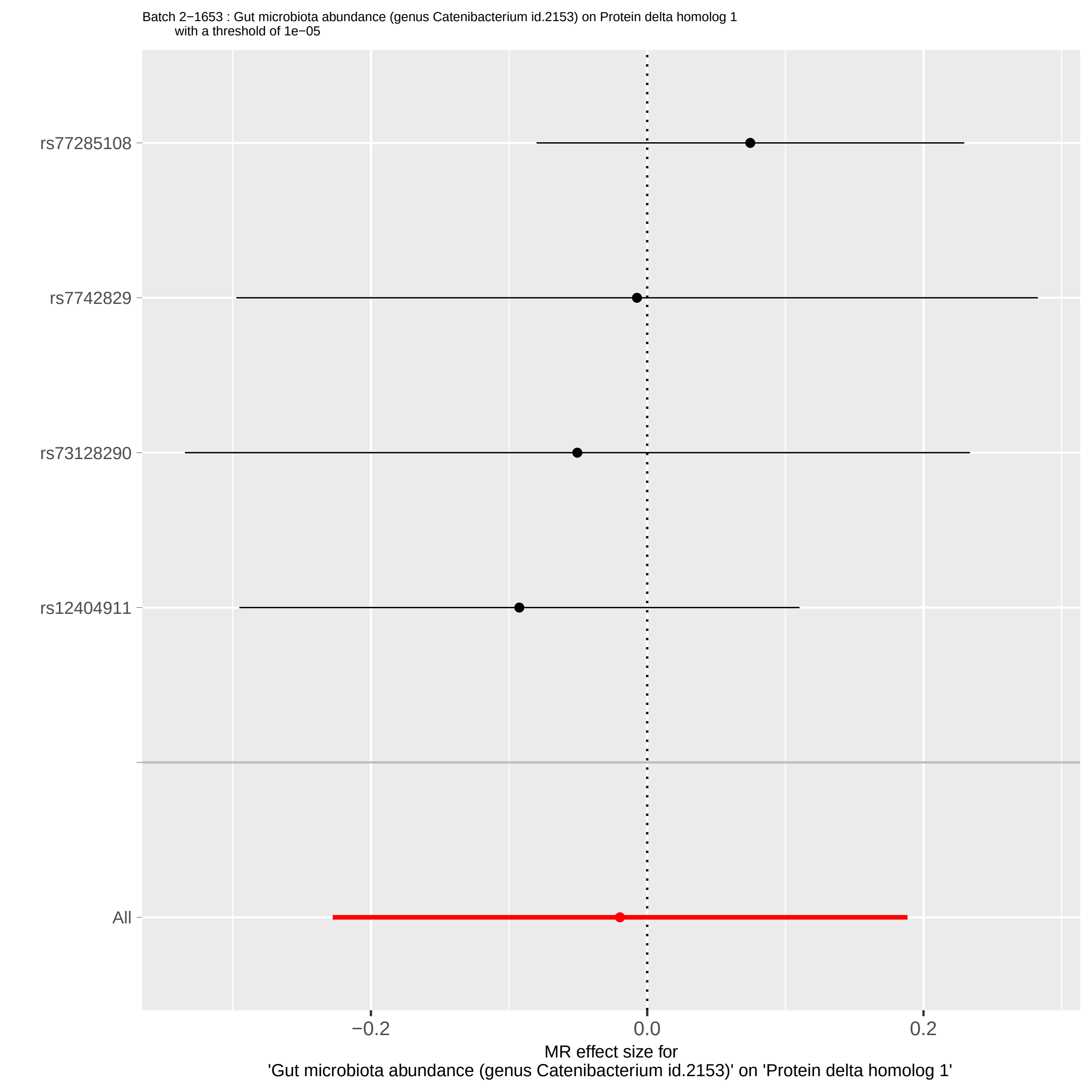
Batch 2−1653 : Gut microbiota abundance (genus Catenibacterium id.2153) on Protein delta homolog 1
with a threshold of 1e−05
rs77285108
rs7742829
rs73128290
rs12404911
All
−0.2
0.0
0.2
MR effect size for
'Gut microbiota abundance (genus Catenibacterium id.2153)' on 'Protein delta homolog 1'

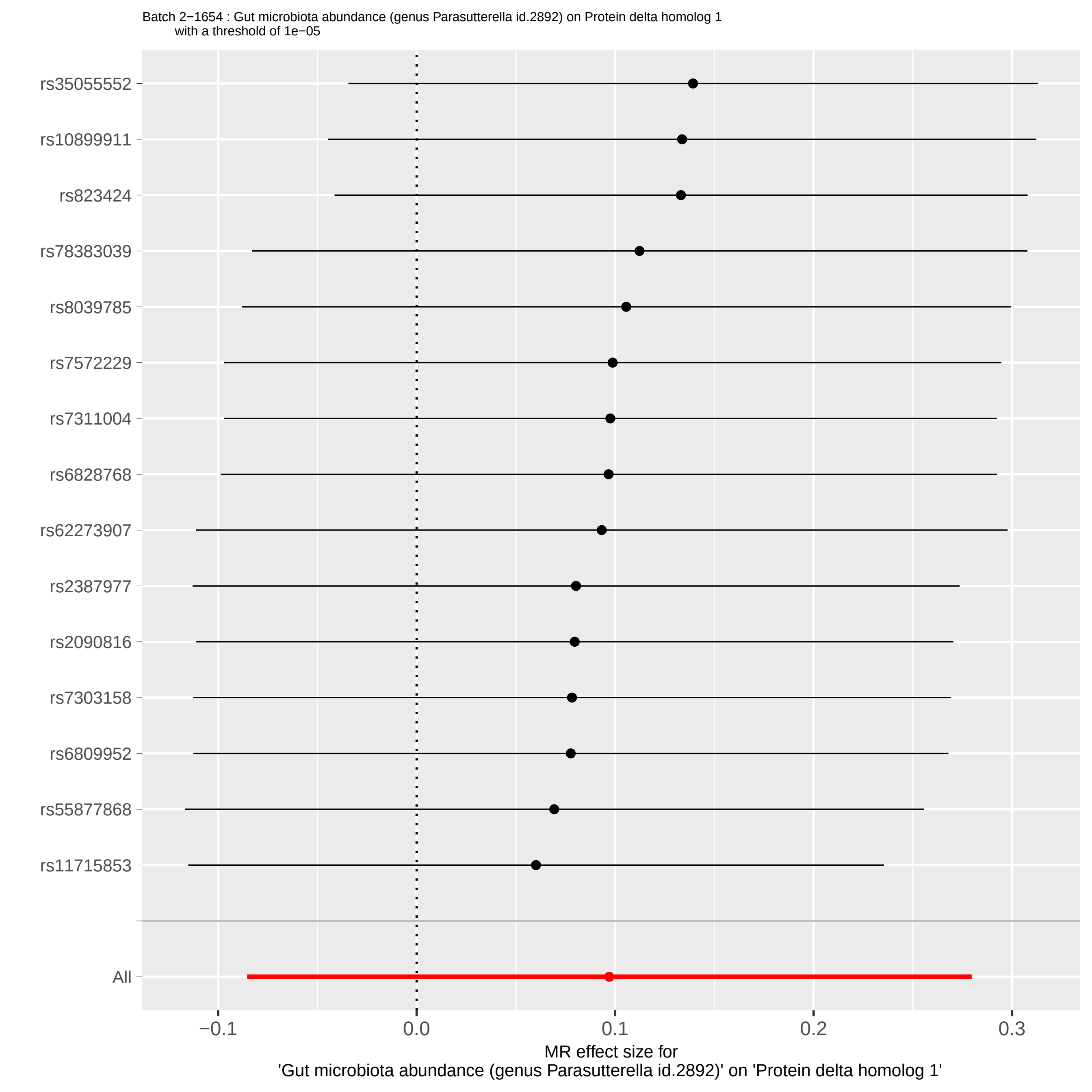

Batch 2−1654 : Gut microbiota abundance (genus Parasutterella id.2892) on Protein delta homolog 1
with a threshold of 1e−05
rs35055552
rs10899911
rs823424
rs78383039
rs8039785
rs7572229
rs7311004
rs6828768
rs62273907
rs2387977
rs2090816
rs7303158
rs6809952
rs55877868
rs11715853
All
−0.1
0.0
0.1
0.2
0.3
MR effect size for
'Gut microbiota abundance (genus Parasutterella id.2892)' on 'Protein delta homolog 1'

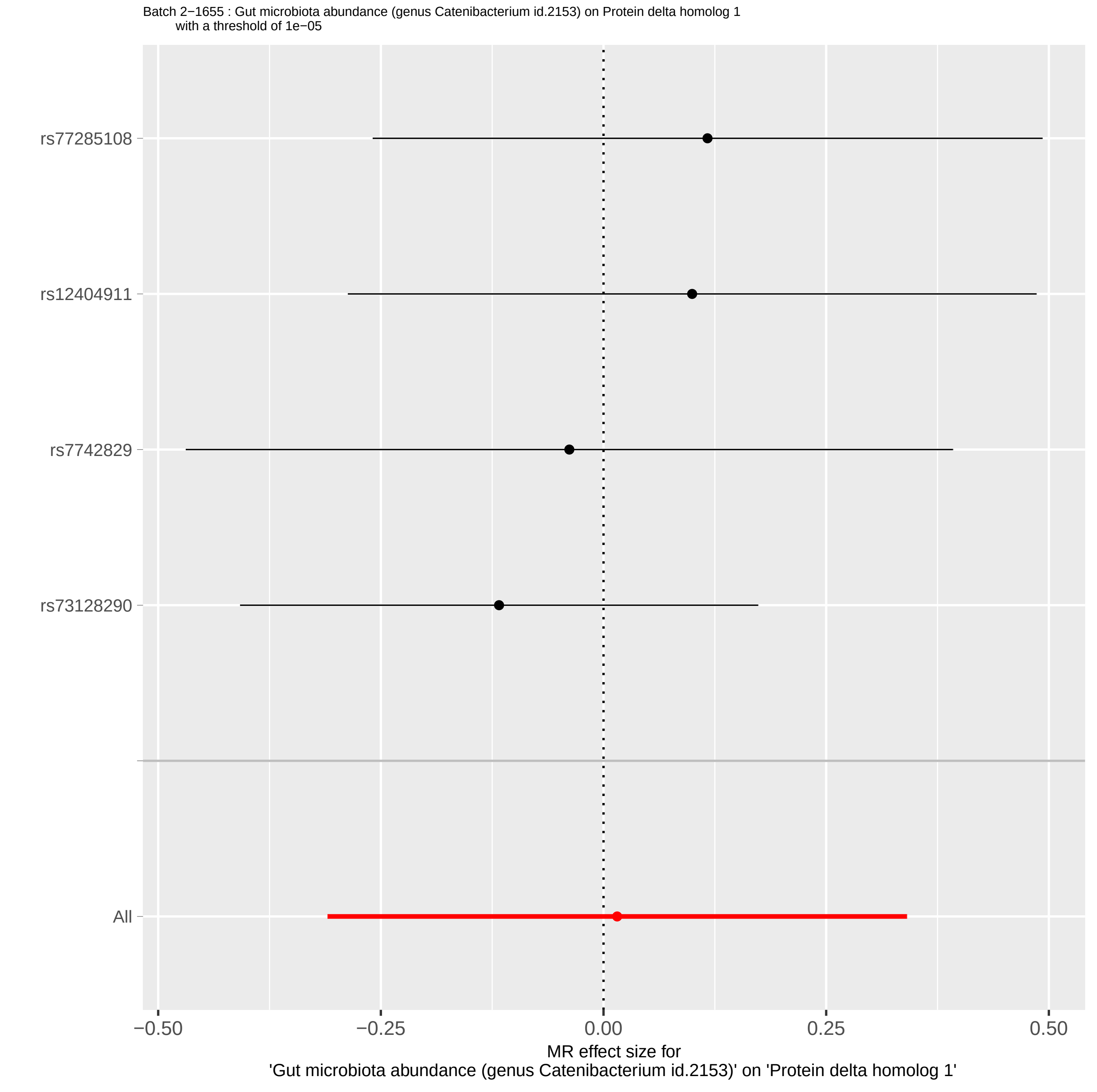
Batch 2−1655 : Gut microbiota abundance (genus Catenibacterium id.2153) on Protein delta homolog 1
with a threshold of 1e−05
rs77285108
rs12404911
rs7742829
rs73128290
All
−0.50
−0.25
0.00
0.25
0.50
MR effect size for
'Gut microbiota abundance (genus Catenibacterium id.2153)' on 'Protein delta homolog 1'

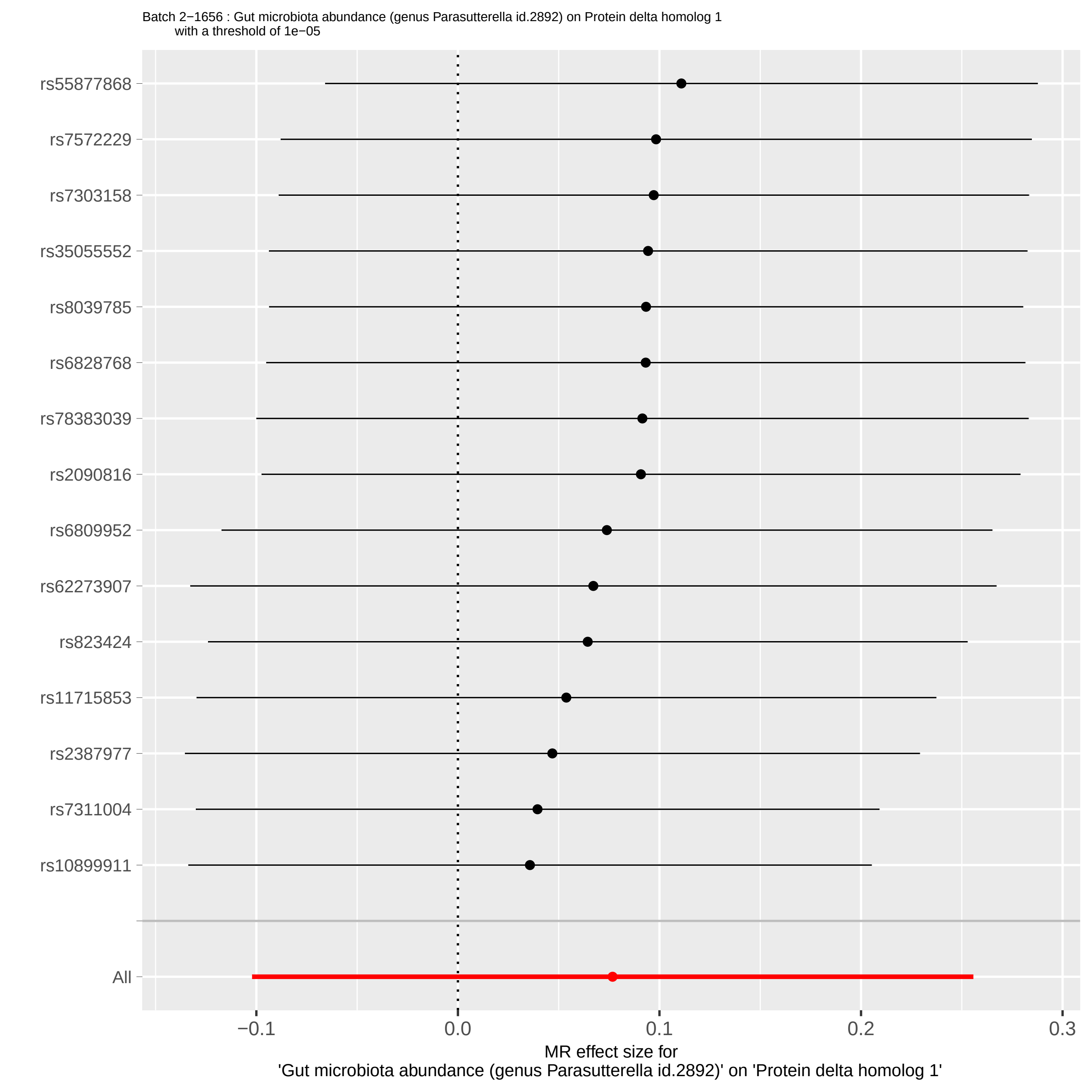
Batch 2−1656 : Gut microbiota abundance (genus Parasutterella id.2892) on Protein delta homolog 1
with a threshold of 1e−05
rs55877868
rs7572229
rs7303158
rs35055552
rs8039785
rs6828768
rs78383039
rs2090816
rs6809952
rs62273907
rs823424
rs11715853
rs2387977
rs7311004
rs10899911
All
−0.1
0.0
0.1
0.2
0.3
MR effect size for
'Gut microbiota abundance (genus Parasutterella id.2892)' on 'Protein delta homolog 1'

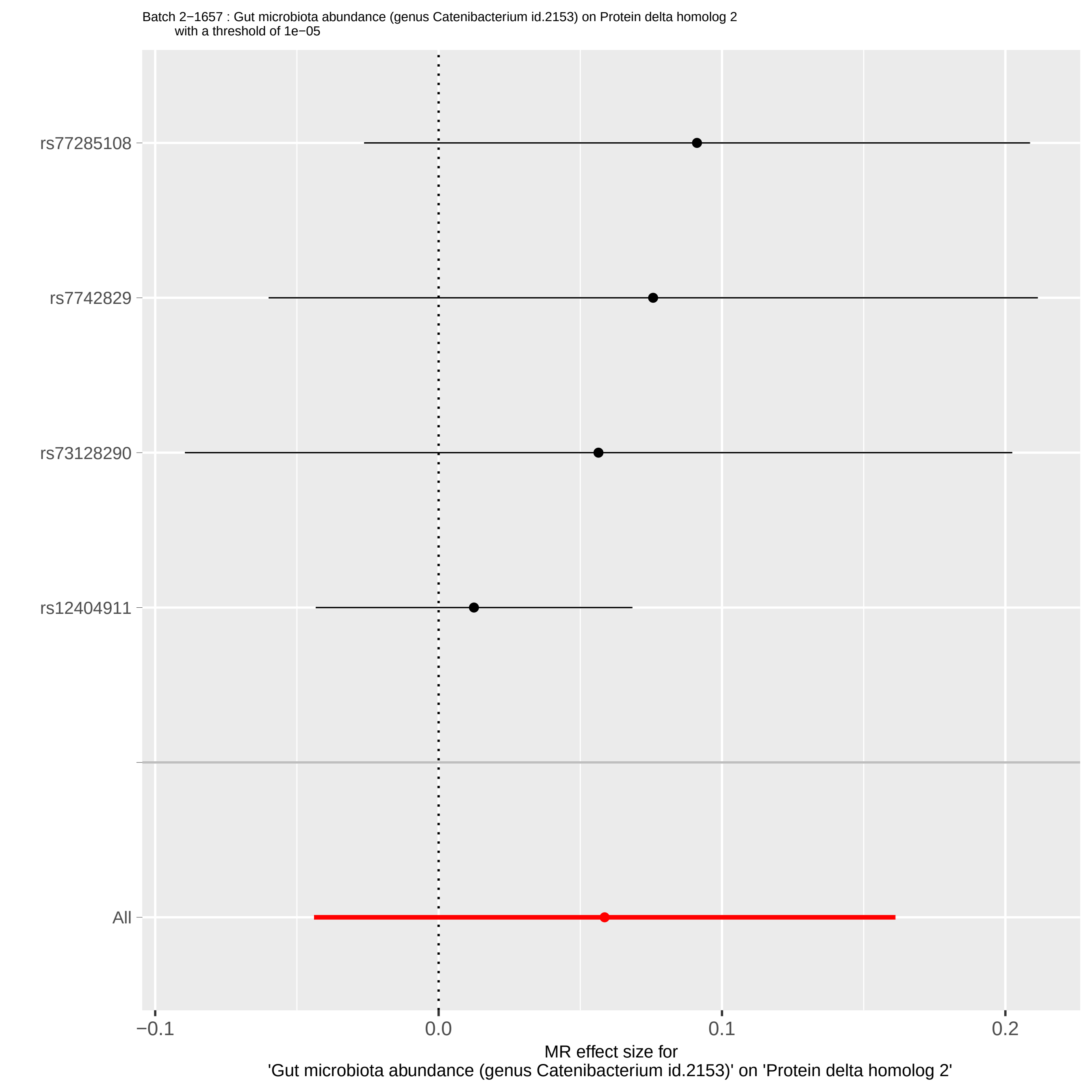
Batch 2−1657 : Gut microbiota abundance (genus Catenibacterium id.2153) on Protein delta homolog 2
with a threshold of 1e−05
rs77285108
rs7742829
rs73128290
rs12404911
All
−0.1
0.0
0.1
0.2
MR effect size for
'Gut microbiota abundance (genus Catenibacterium id.2153)' on 'Protein delta homolog 2'

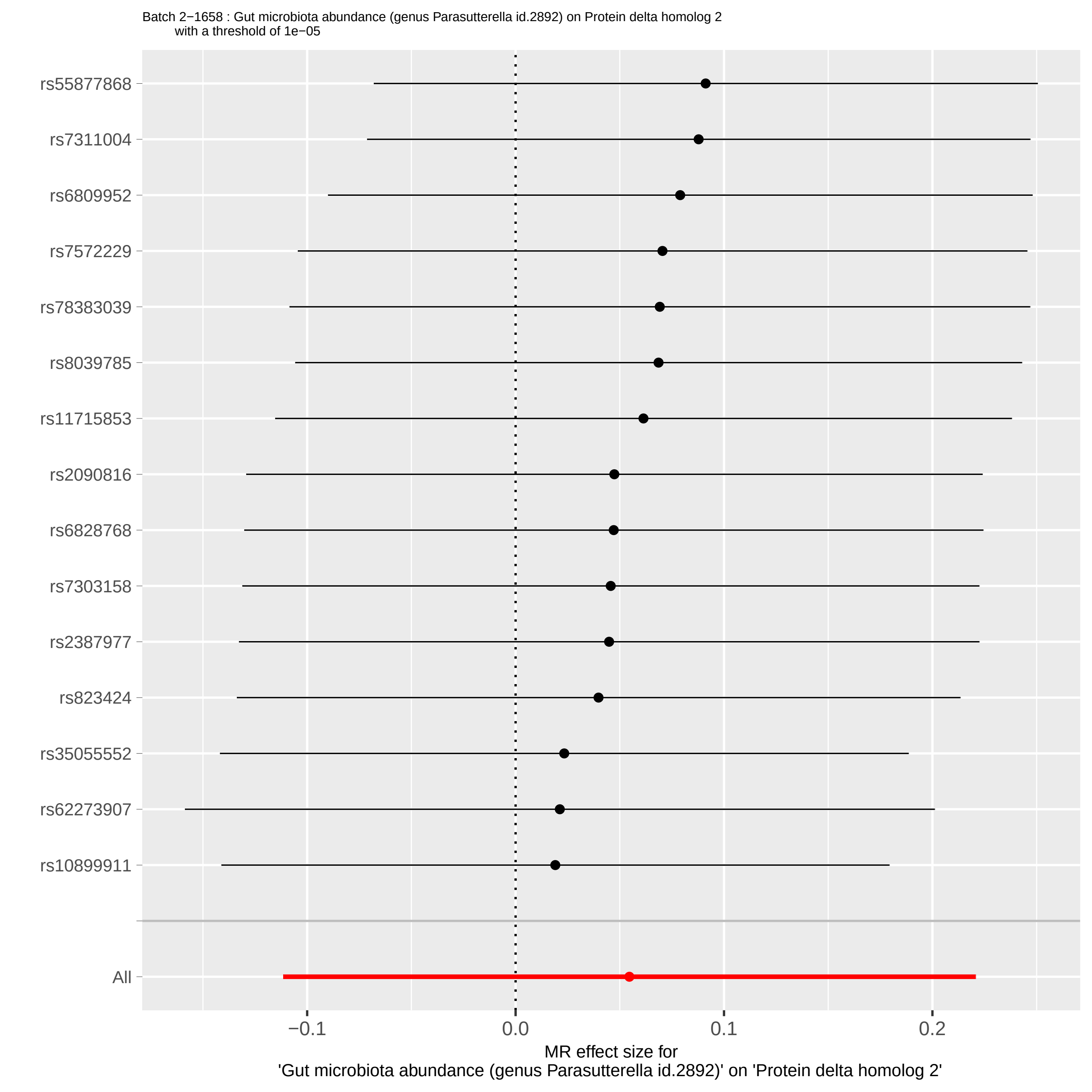

Batch 2−1658 : Gut microbiota abundance (genus Parasutterella id.2892) on Protein delta homolog 2
with a threshold of 1e−05
rs55877868
rs7311004
rs6809952
rs7572229
rs78383039
rs8039785
rs11715853
rs2090816
rs6828768
rs7303158
rs2387977
rs823424
rs35055552
rs62273907
rs10899911
All
−0.1
0.0
0.1
0.2
MR effect size for
'Gut microbiota abundance (genus Parasutterella id.2892)' on 'Protein delta homolog 2'

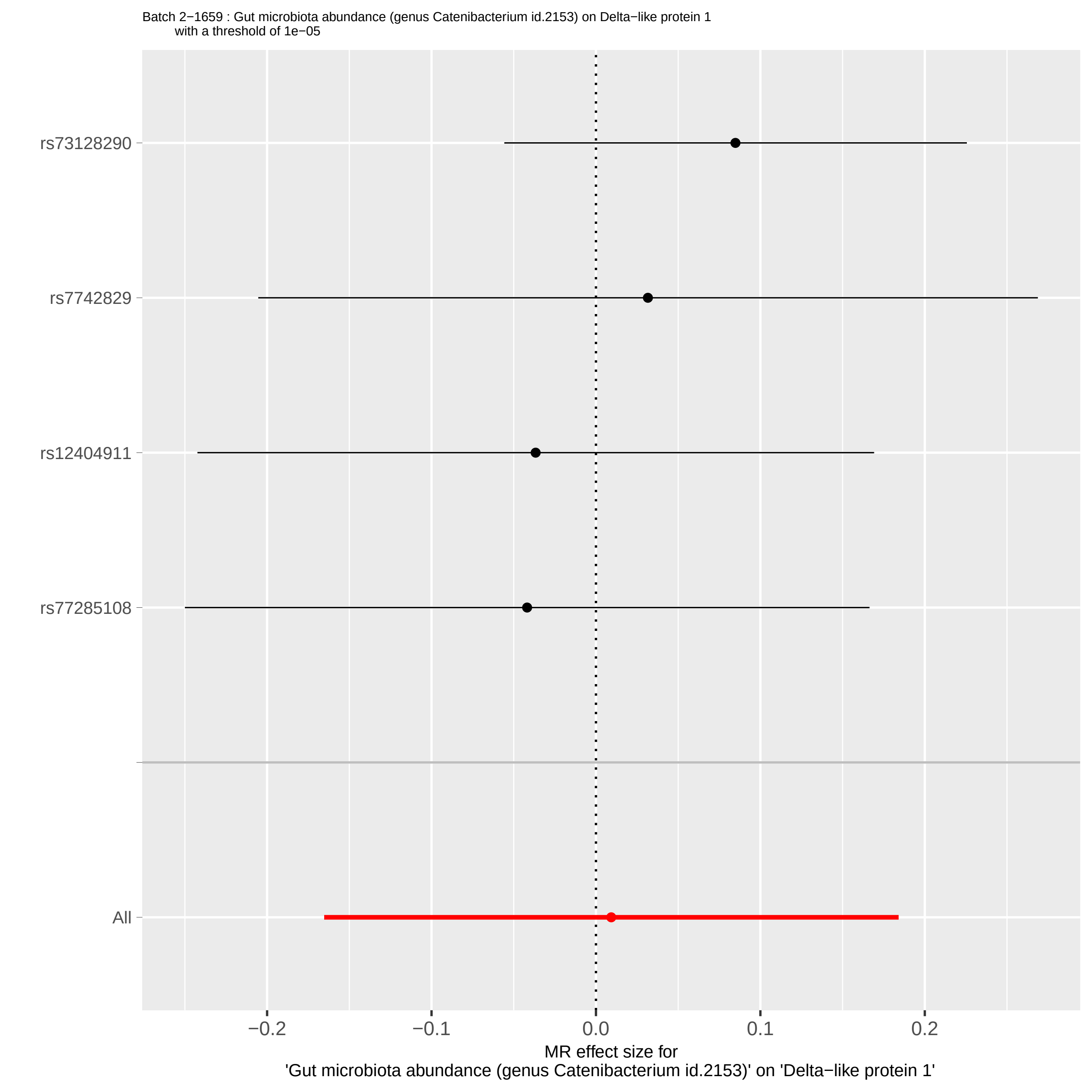
Batch 2−1659 : Gut microbiota abundance (genus Catenibacterium id.2153) on Delta−like protein 1
with a threshold of 1e−05
rs73128290
rs7742829
rs12404911
rs77285108
All
−0.2
−0.1
0.0
0.1
0.2
MR effect size for
'Gut microbiota abundance (genus Catenibacterium id.2153)' on 'Delta−like protein 1'

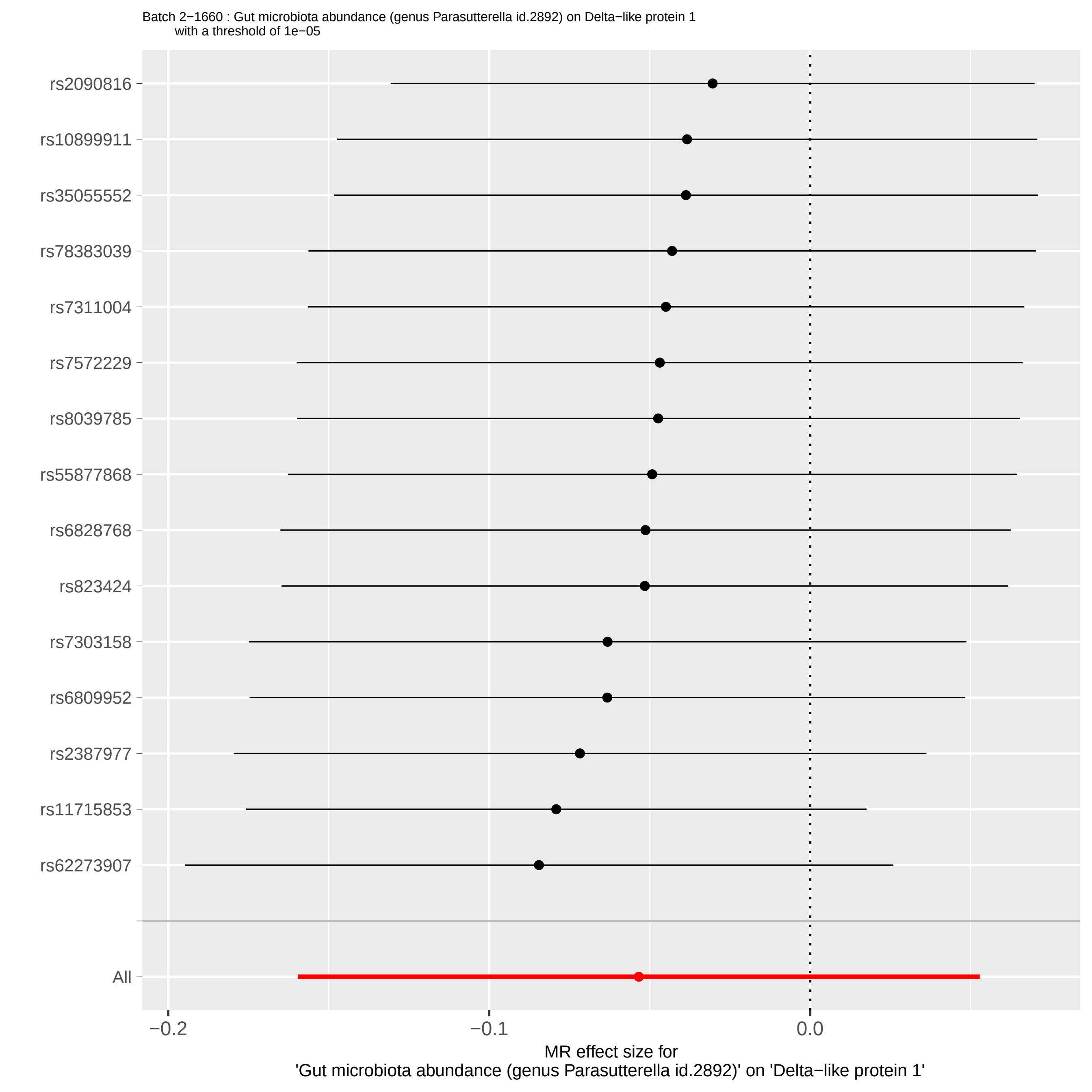
Batch 2−1660 : Gut microbiota abundance (genus Parasutterella id.2892) on Delta−like protein 1
with a threshold of 1e−05
rs2090816
rs10899911
rs35055552
rs78383039
rs7311004
rs7572229
rs8039785
rs55877868
rs6828768
rs823424
rs7303158
rs6809952
rs2387977
rs11715853
rs62273907
All
−0.2
−0.1
0.0
MR effect size for
'Gut microbiota abundance (genus Parasutterella id.2892)' on 'Delta−like protein 1'

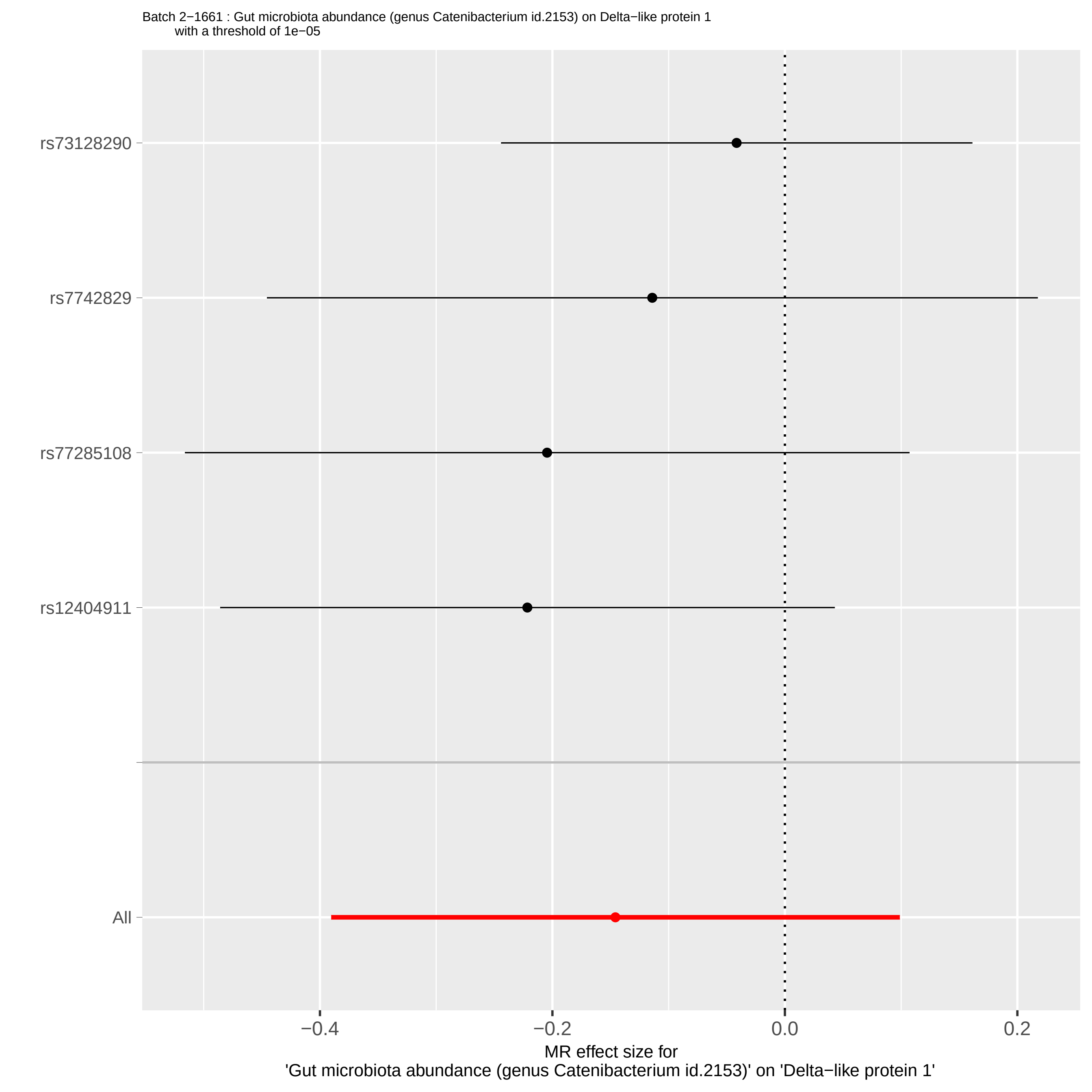

Batch 2−1661 : Gut microbiota abundance (genus Catenibacterium id.2153) on Delta−like protein 1
with a threshold of 1e−05
rs73128290
rs7742829
rs77285108
rs12404911
All
−0.4
−0.2
0.0
0.2
MR effect size for
'Gut microbiota abundance (genus Catenibacterium id.2153)' on 'Delta−like protein 1'

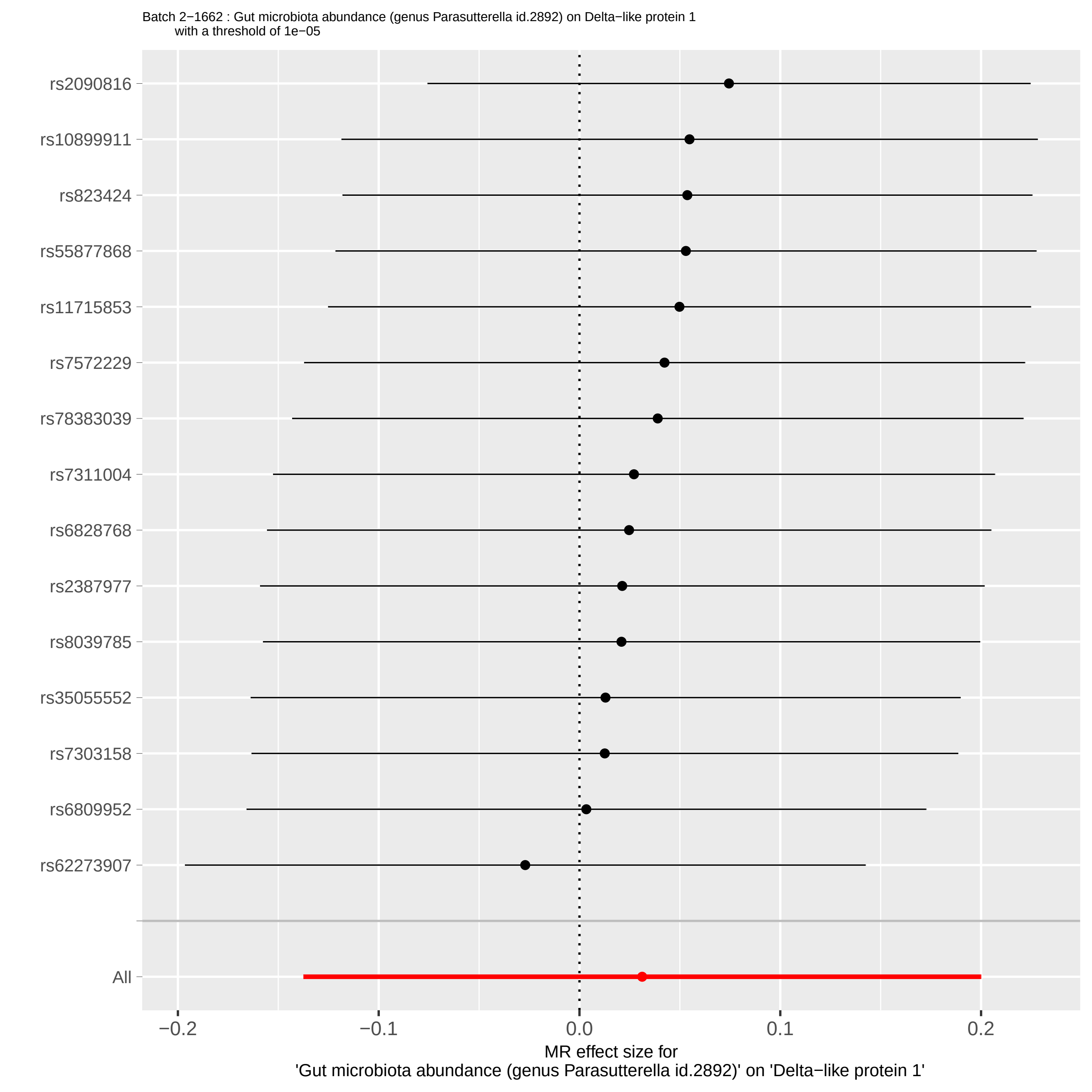

Batch 2−1662 : Gut microbiota abundance (genus Parasutterella id.2892) on Delta−like protein 1
with a threshold of 1e−05
rs2090816
rs10899911
rs823424
rs55877868
rs11715853
rs7572229
rs78383039
rs7311004
rs6828768
rs2387977
rs8039785
rs35055552
rs7303158
rs6809952
rs62273907
All
−0.2
−0.1
0.0
0.1
0.2
MR effect size for
'Gut microbiota abundance (genus Parasutterella id.2892)' on 'Delta−like protein 1'

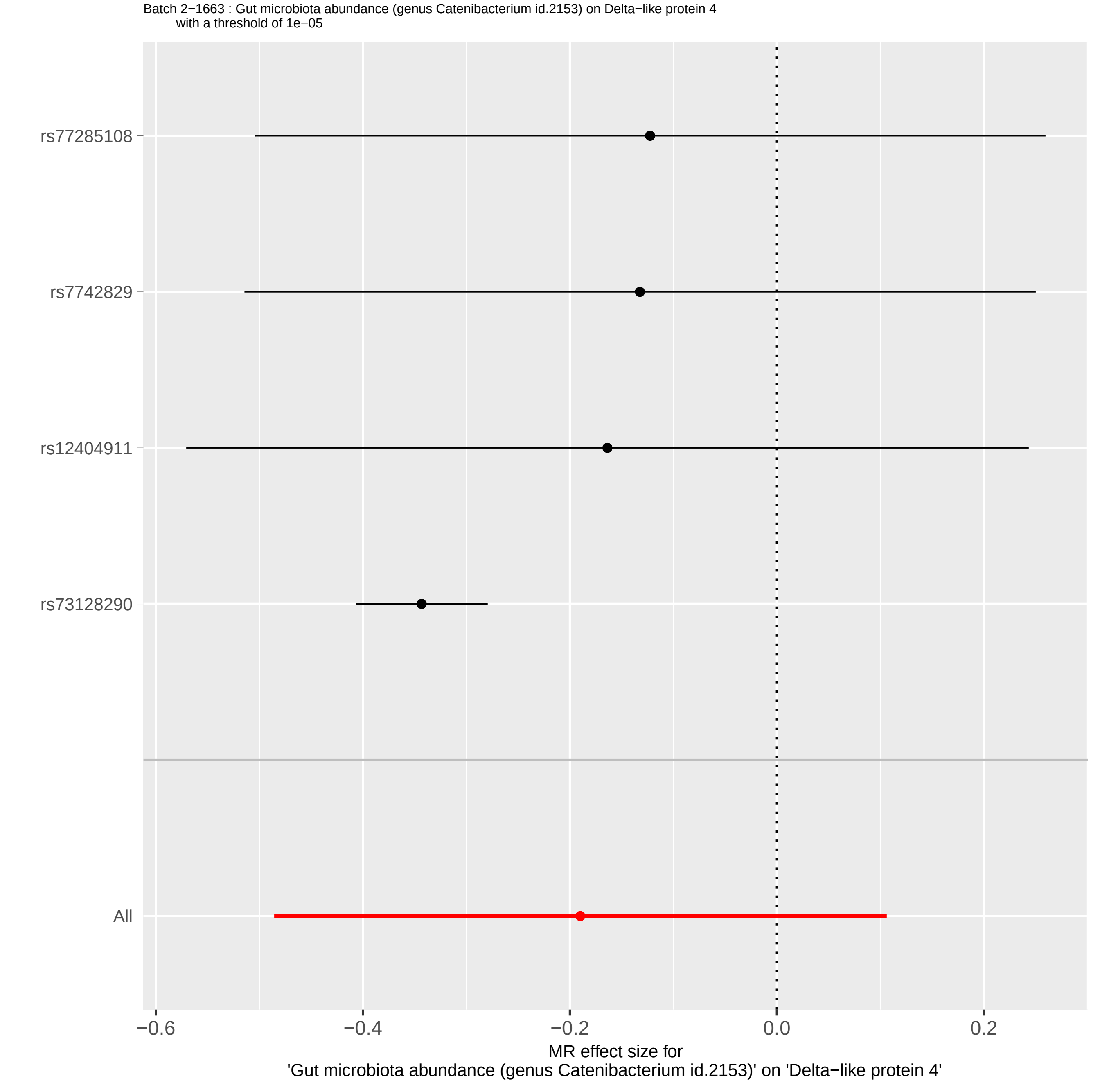
Batch 2−1663 : Gut microbiota abundance (genus Catenibacterium id.2153) on Delta−like protein 4
with a threshold of 1e−05
rs77285108
rs7742829
rs12404911
rs73128290
All
−0.6
−0.4
−0.2
0.0
0.2
MR effect size for
'Gut microbiota abundance (genus Catenibacterium id.2153)' on 'Delta−like protein 4'

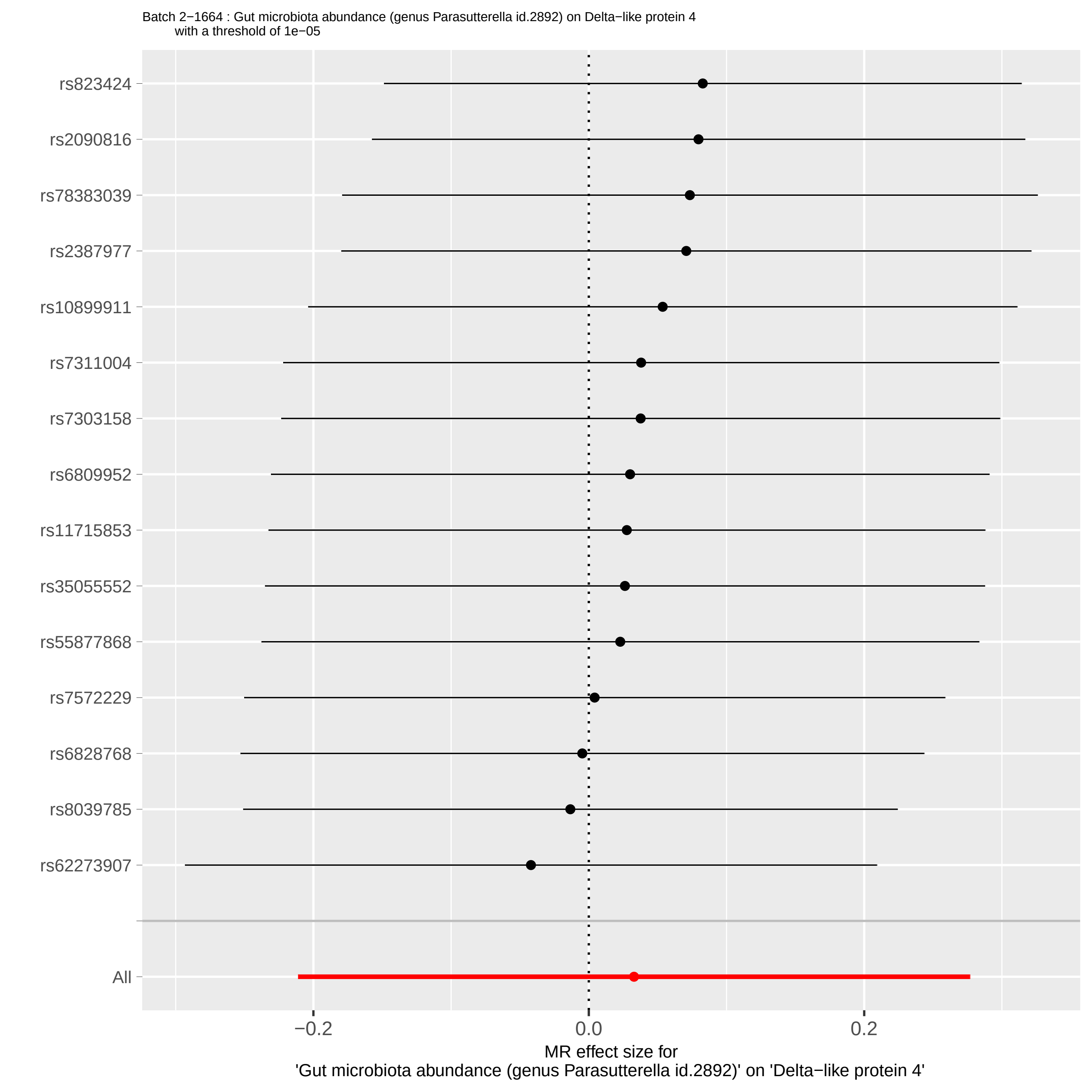

Batch 2−1664 : Gut microbiota abundance (genus Parasutterella id.2892) on Delta−like protein 4
with a threshold of 1e−05
rs823424
rs2090816
rs78383039
rs2387977
rs10899911
rs7311004
rs7303158
rs6809952
rs11715853
rs35055552
rs55877868
rs7572229
rs6828768
rs8039785
rs62273907
All
−0.2
0.0
0.2
MR effect size for
'Gut microbiota abundance (genus Parasutterella id.2892)' on 'Delta−like protein 4'

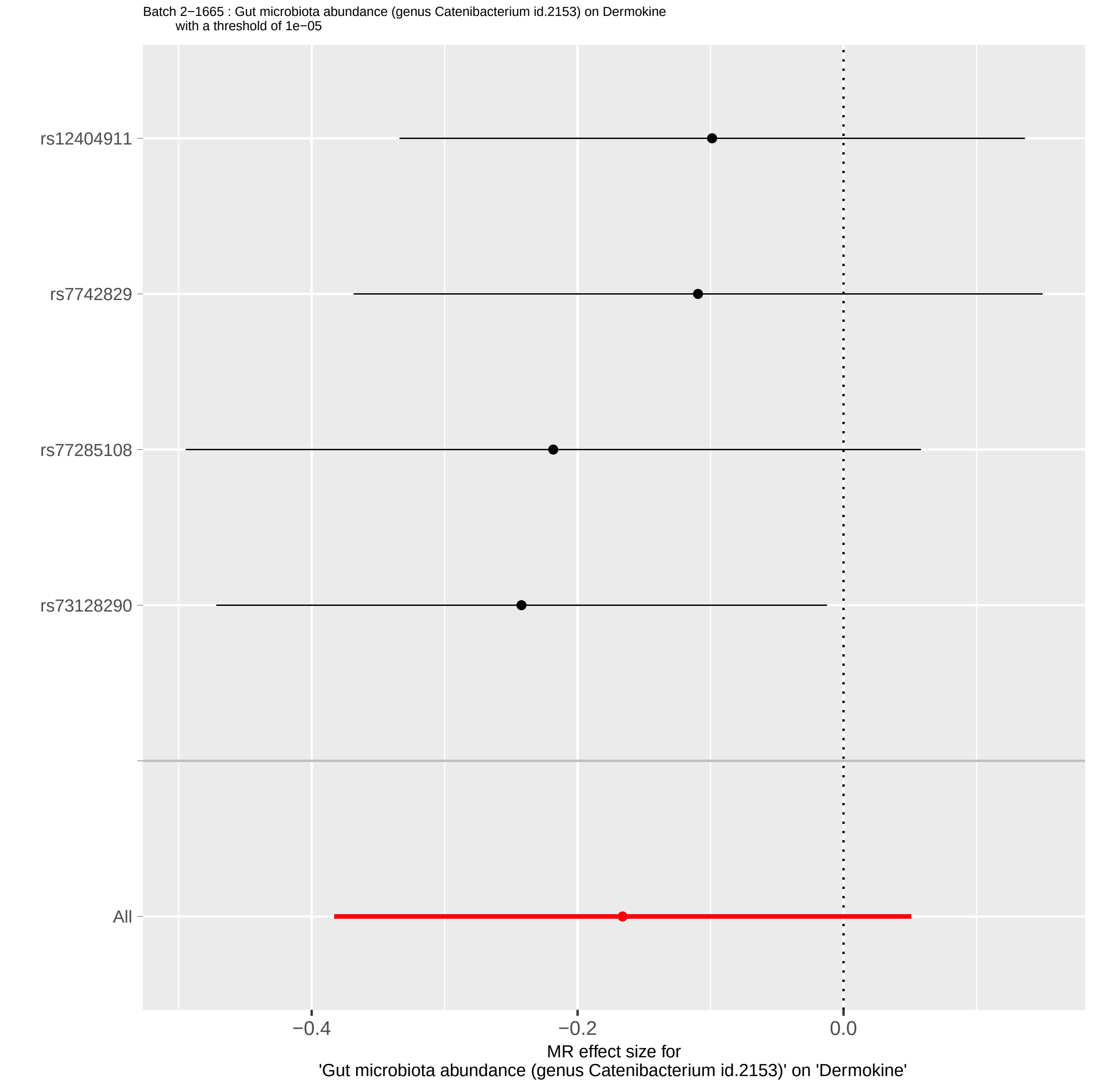

Batch 2−1665 : Gut microbiota abundance (genus Catenibacterium id.2153) on Dermokine
with a threshold of 1e−05
rs12404911
rs7742829
rs77285108
rs73128290
All
−0.4
−0.2
0.0
MR effect size for
'Gut microbiota abundance (genus Catenibacterium id.2153)' on 'Dermokine'

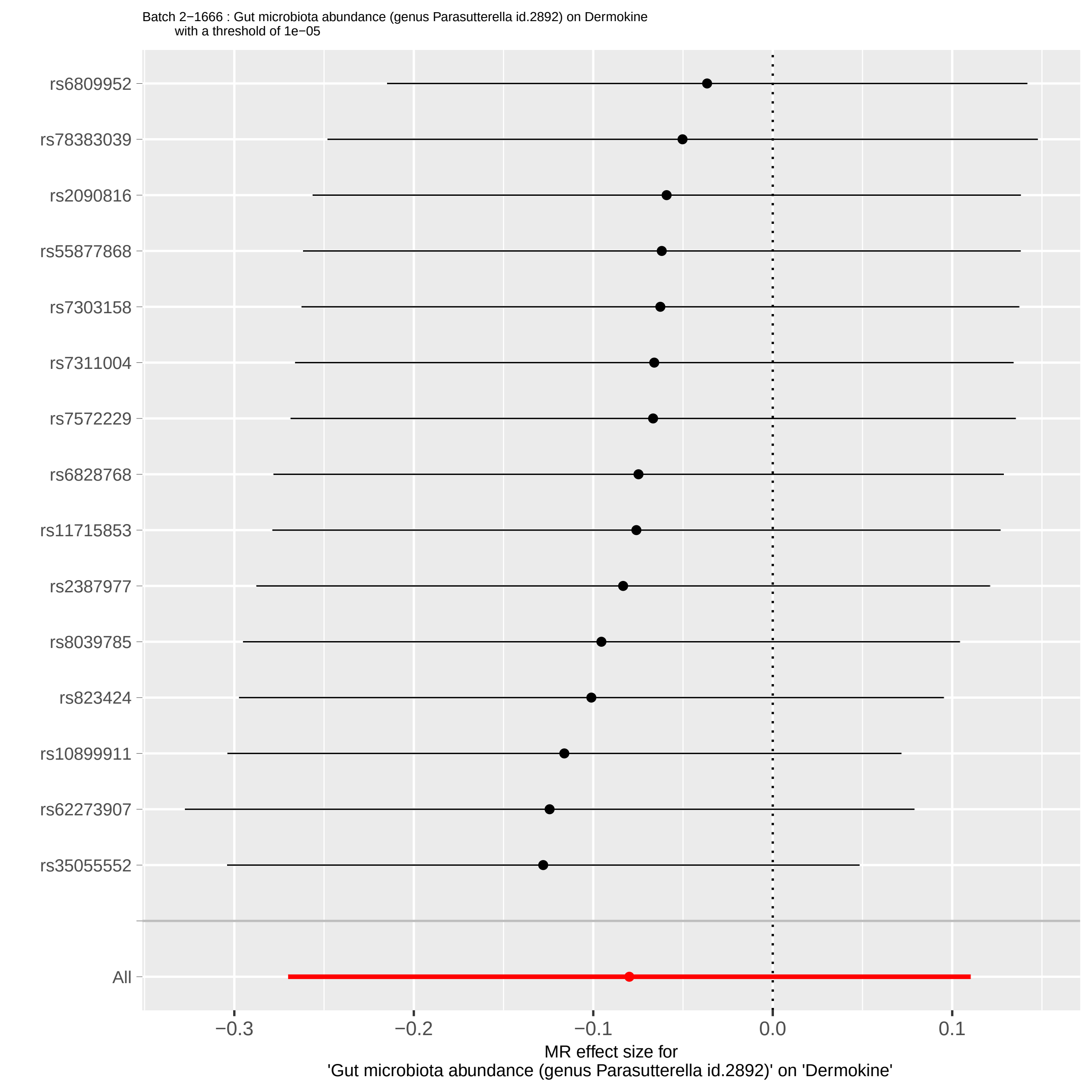

Batch 2−1666 : Gut microbiota abundance (genus Parasutterella id.2892) on Dermokine
with a threshold of 1e−05
rs6809952
rs78383039
rs2090816
rs55877868
rs7303158
rs7311004
rs7572229
rs6828768
rs11715853
rs2387977
rs8039785
rs823424
rs10899911
rs62273907
rs35055552
All
−0.3
−0.2
−0.1
0.0
0.1
MR effect size for
'Gut microbiota abundance (genus Parasutterella id.2892)' on 'Dermokine'

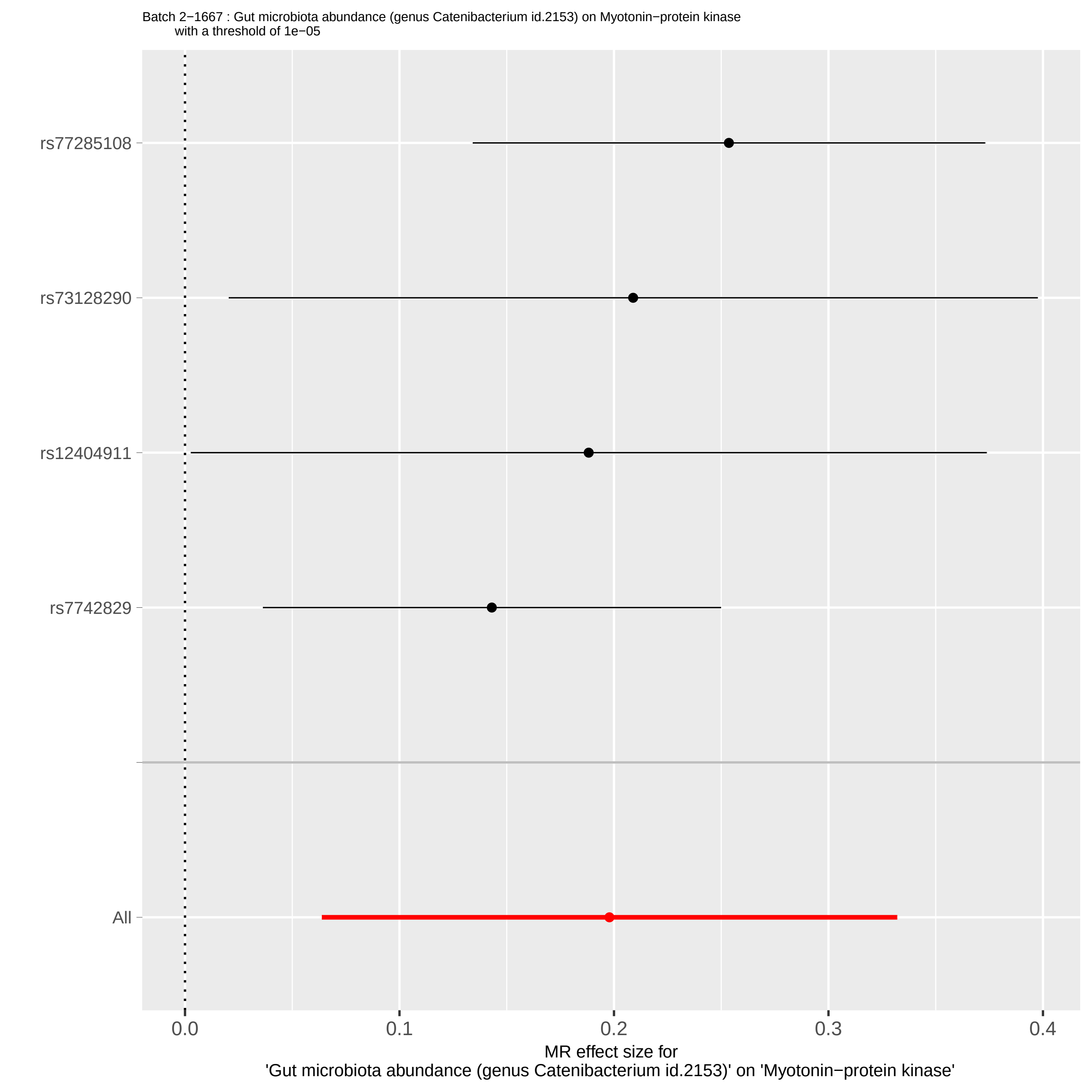
Batch 2−1667 : Gut microbiota abundance (genus Catenibacterium id.2153) on Myotonin−protein kinase
with a threshold of 1e−05
rs77285108
rs73128290
rs12404911
rs7742829
All
0.0
0.1
0.2
0.3
0.4
MR effect size for
'Gut microbiota abundance (genus Catenibacterium id.2153)' on 'Myotonin−protein kinase'

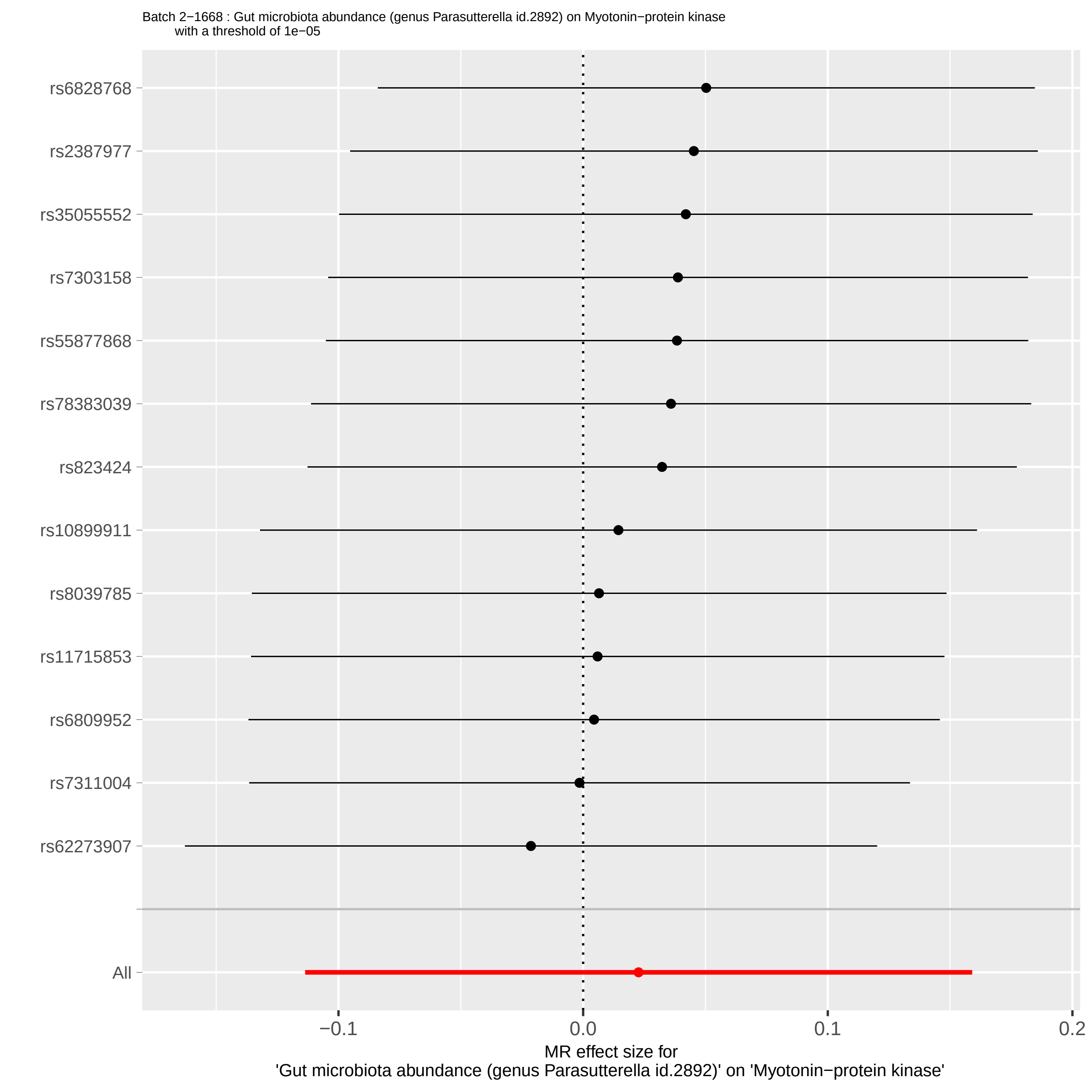

Batch 2−1668 : Gut microbiota abundance (genus Parasutterella id.2892) on Myotonin−protein kinase
with a threshold of 1e−05
rs6828768
rs2387977
rs35055552
rs7303158
rs55877868
rs78383039
rs823424
rs10899911
rs8039785
rs11715853
rs6809952
rs7311004
rs62273907
All
−0.1
0.0
0.1
0.2
MR effect size for
'Gut microbiota abundance (genus Parasutterella id.2892)' on 'Myotonin−protein kinase'

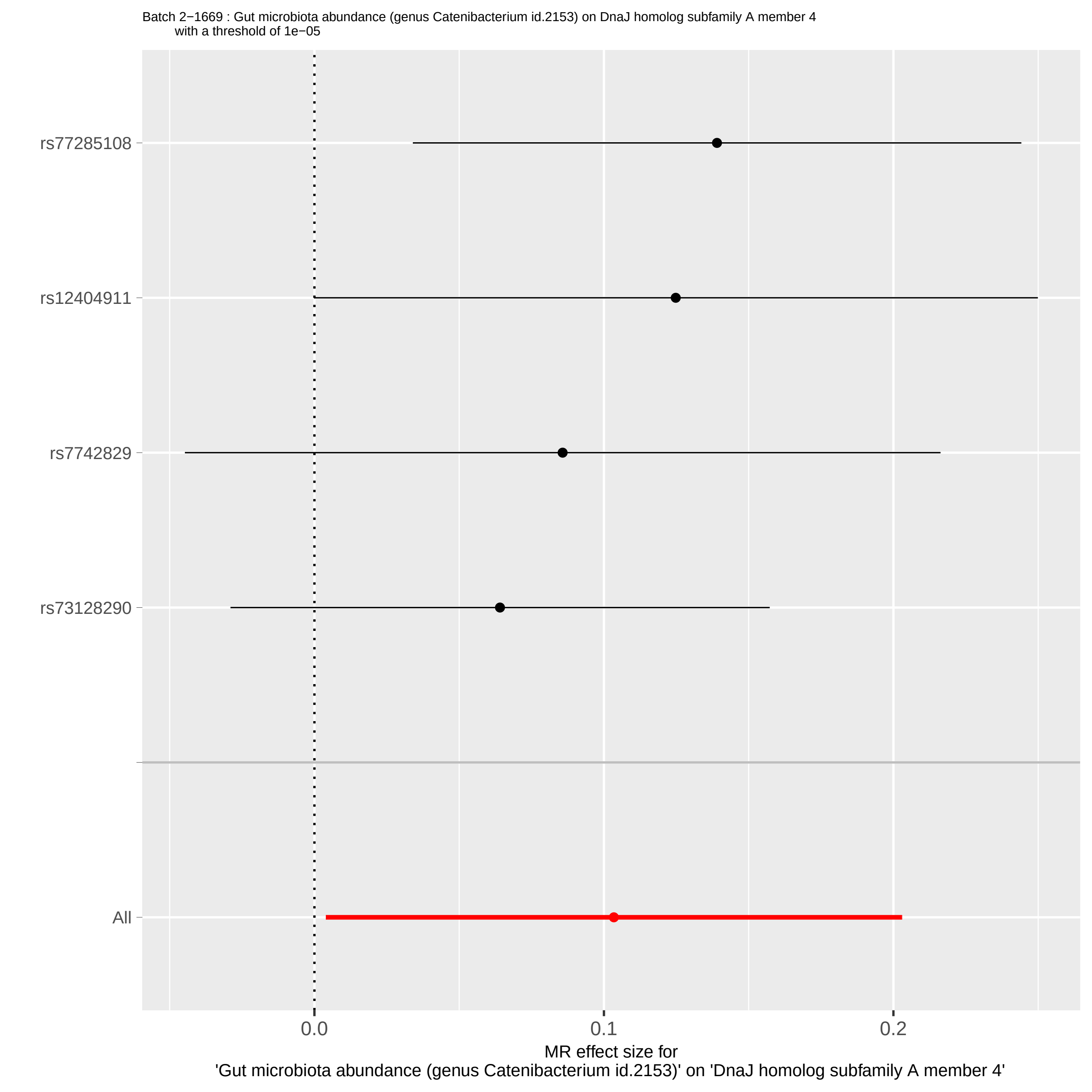

Batch 2−1669 : Gut microbiota abundance (genus Catenibacterium id.2153) on DnaJ homolog subfamily A member 4
with a threshold of 1e−05
rs77285108
rs12404911
rs7742829
rs73128290
All
0.0
0.1
0.2
MR effect size for
'Gut microbiota abundance (genus Catenibacterium id.2153)' on 'DnaJ homolog subfamily A member 4'

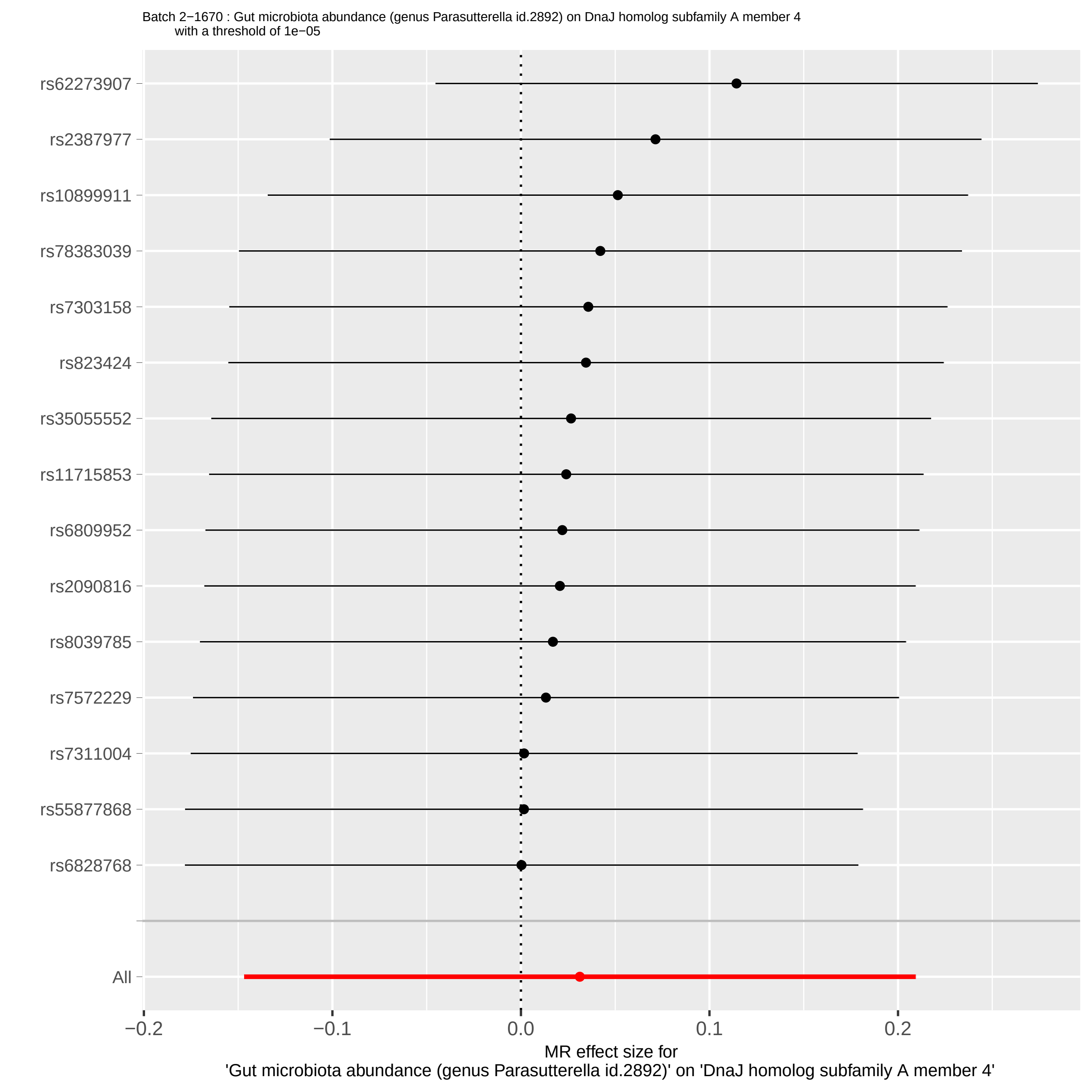
Batch 2−1670 : Gut microbiota abundance (genus Parasutterella id.2892) on DnaJ homolog subfamily A member 4
with a threshold of 1e−05
rs62273907
rs2387977
rs10899911
rs78383039
rs7303158
rs823424
rs35055552
rs11715853
rs6809952
rs2090816
rs8039785
rs7572229
rs7311004
rs55877868
rs6828768
All
−0.2
−0.1
0.0
0.1
0.2
MR effect size for
'Gut microbiota abundance (genus Parasutterella id.2892)' on 'DnaJ homolog subfamily A member 4'

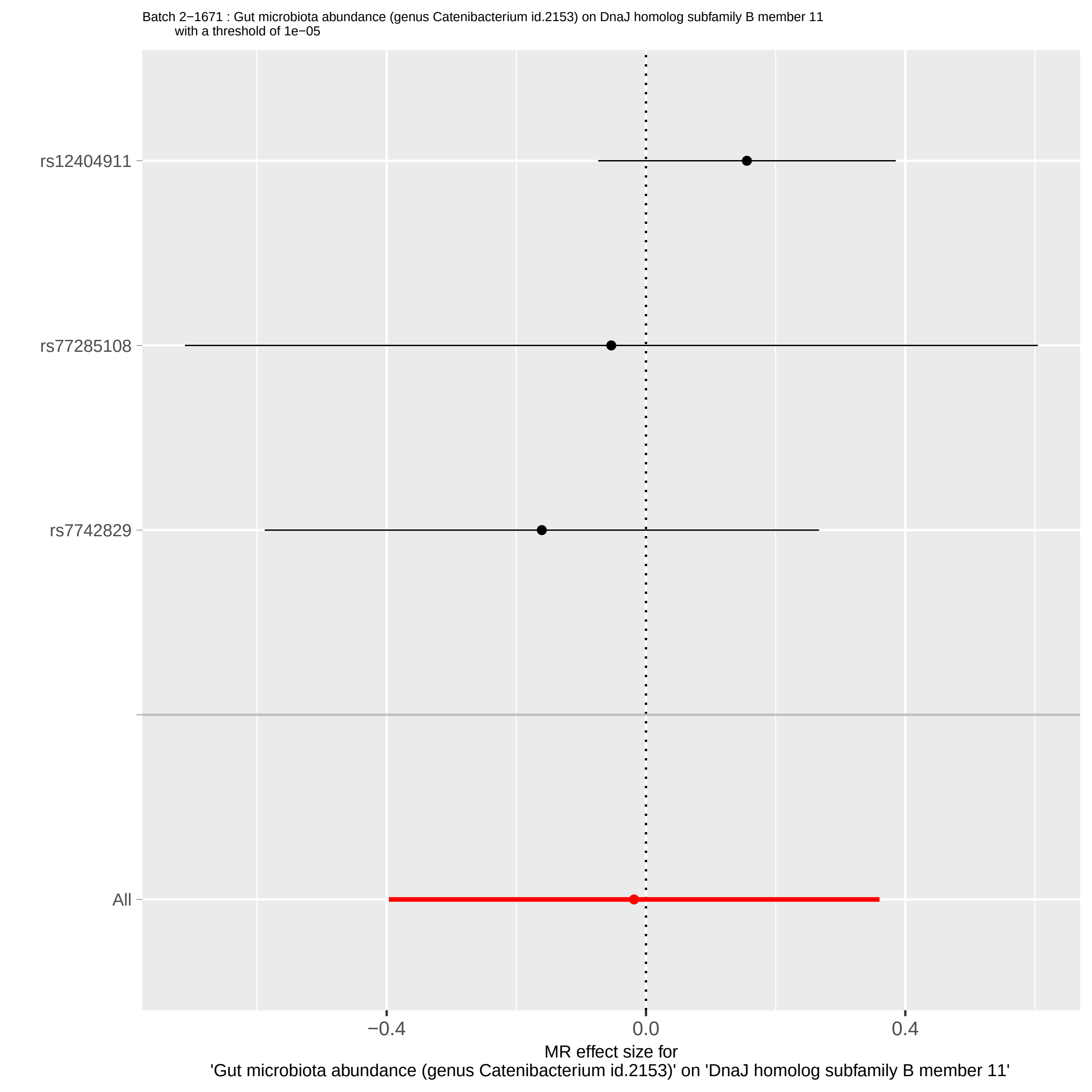
Batch 2−1671 : Gut microbiota abundance (genus Catenibacterium id.2153) on DnaJ homolog subfamily B member 11
with a threshold of 1e−05
rs12404911
rs77285108
rs7742829
All
−0.4
0.0
0.4
MR effect size for
'Gut microbiota abundance (genus Catenibacterium id.2153)' on 'DnaJ homolog subfamily B member 11'

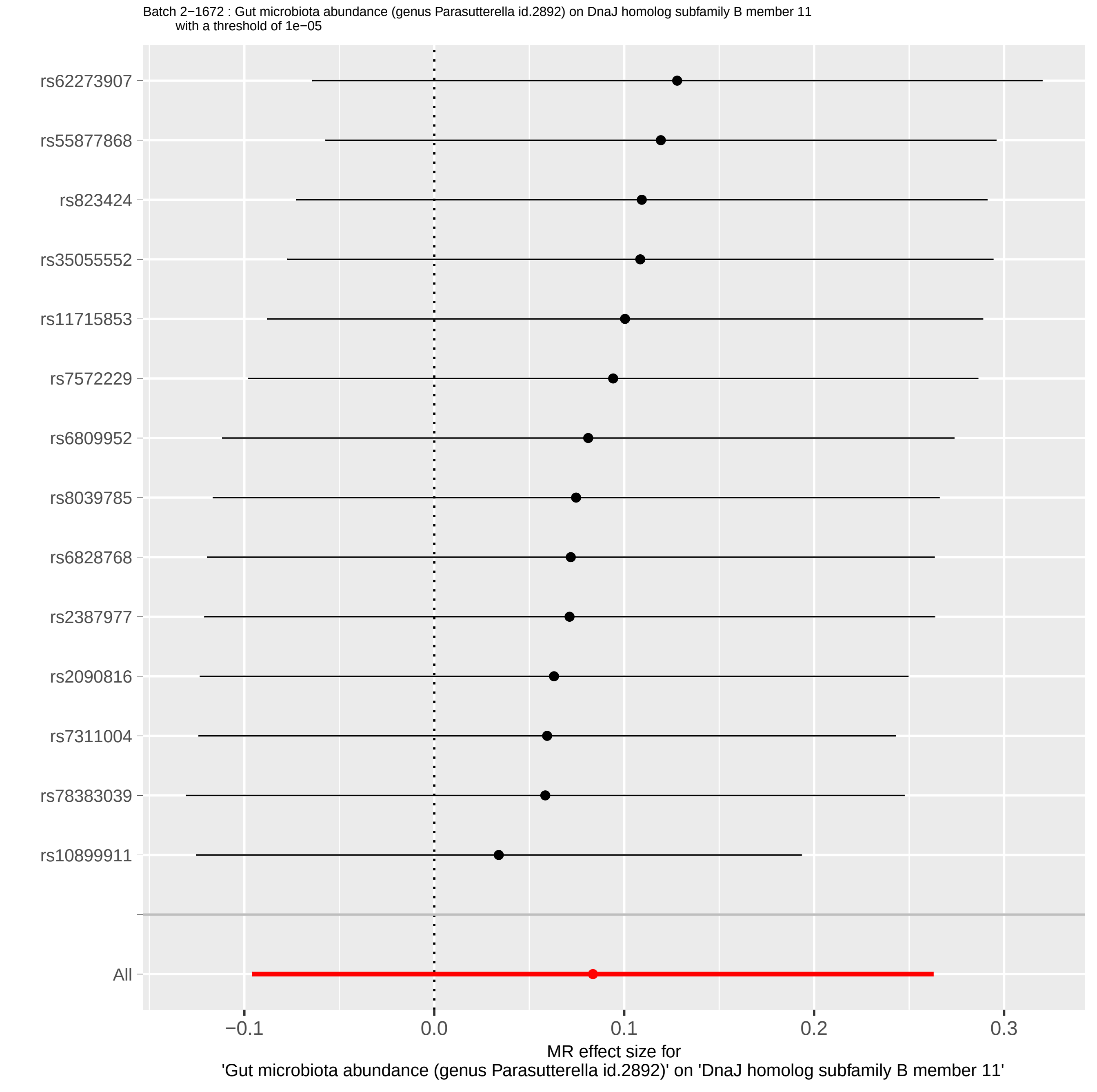

Batch 2−1672 : Gut microbiota abundance (genus Parasutterella id.2892) on DnaJ homolog subfamily B member 11
with a threshold of 1e−05
rs62273907
rs55877868
rs823424
rs35055552
rs11715853
rs7572229
rs6809952
rs8039785
rs6828768
rs2387977
rs2090816
rs7311004
rs78383039
rs10899911
All
−0.1
0.0
0.1
0.2
0.3
MR effect size for
'Gut microbiota abundance (genus Parasutterella id.2892)' on 'DnaJ homolog subfamily B member 11'

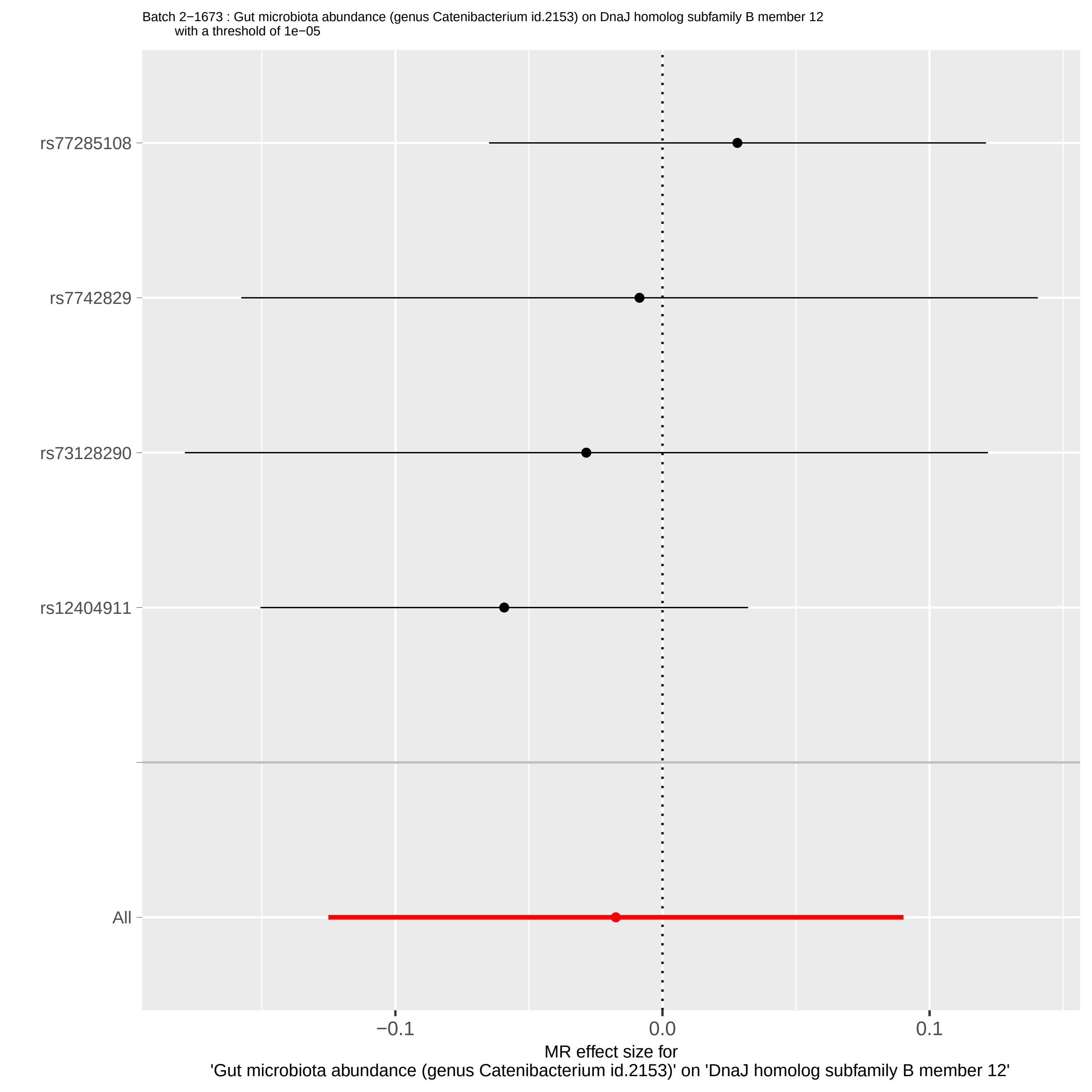
Batch 2−1673 : Gut microbiota abundance (genus Catenibacterium id.2153) on DnaJ homolog subfamily B member 12
with a threshold of 1e−05
rs77285108
rs7742829
rs73128290
rs12404911
All
−0.1
0.0
0.1
MR effect size for
'Gut microbiota abundance (genus Catenibacterium id.2153)' on 'DnaJ homolog subfamily B member 12'

Batch 2−1674 : Gut microbiota abundance (genus Parasutterella id.2892) on DnaJ homolog subfamily B member 12
with a threshold of 1e−05

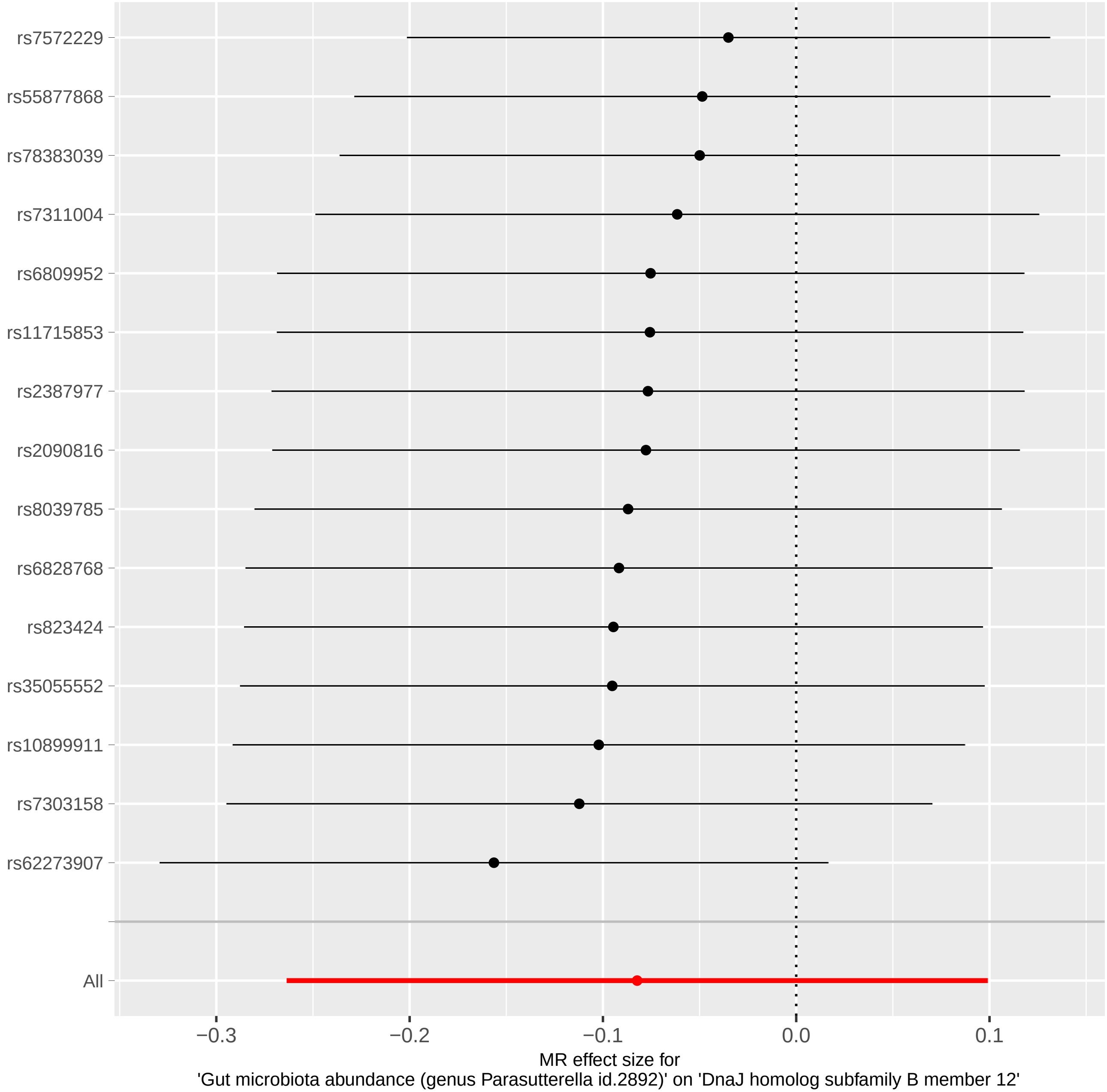

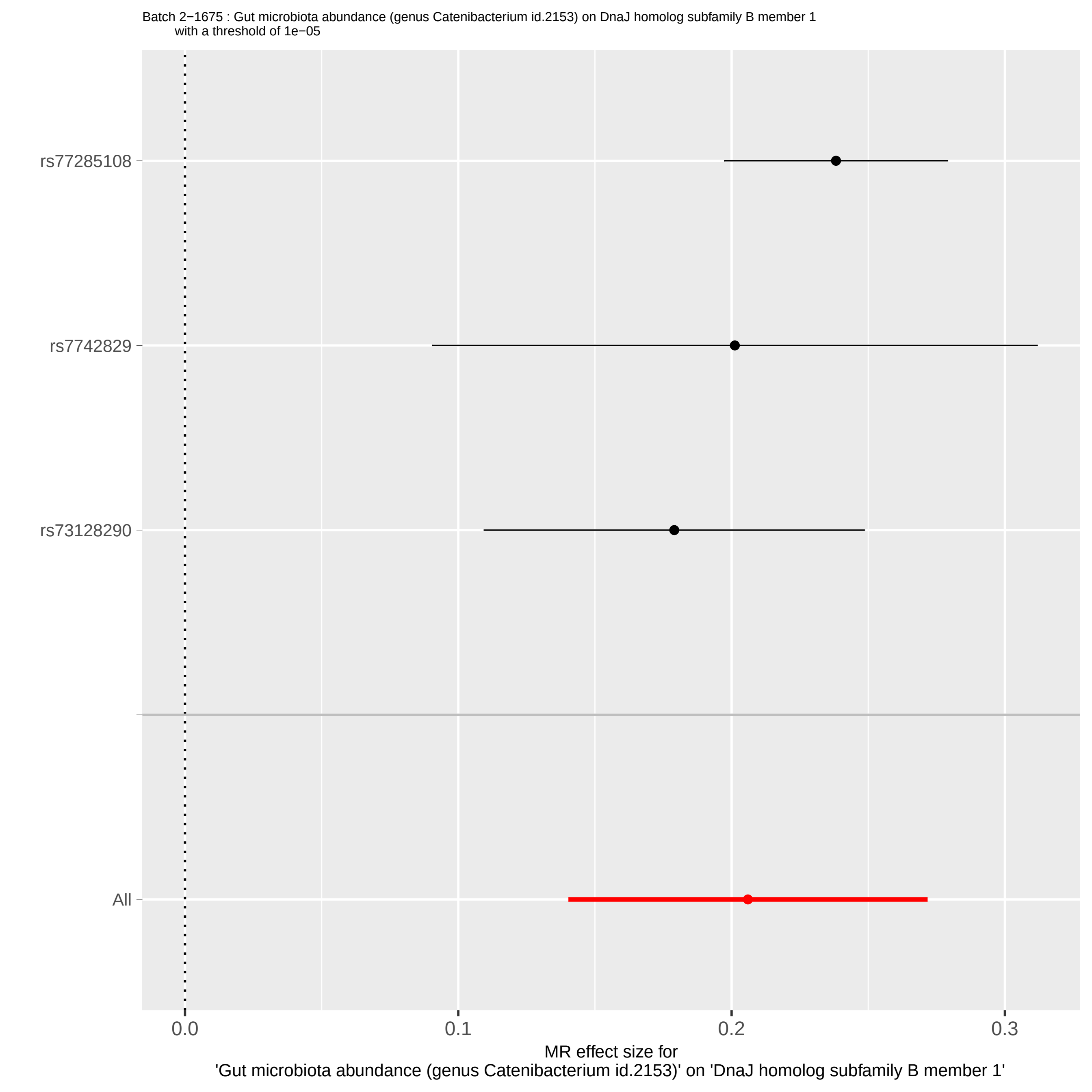

Batch 2−1675 : Gut microbiota abundance (genus Catenibacterium id.2153) on DnaJ homolog subfamily B member 1
with a threshold of 1e−05
rs77285108
rs7742829
rs73128290
All
0.0
0.1
0.2
0.3
MR effect size for
'Gut microbiota abundance (genus Catenibacterium id.2153)' on 'DnaJ homolog subfamily B member 1'

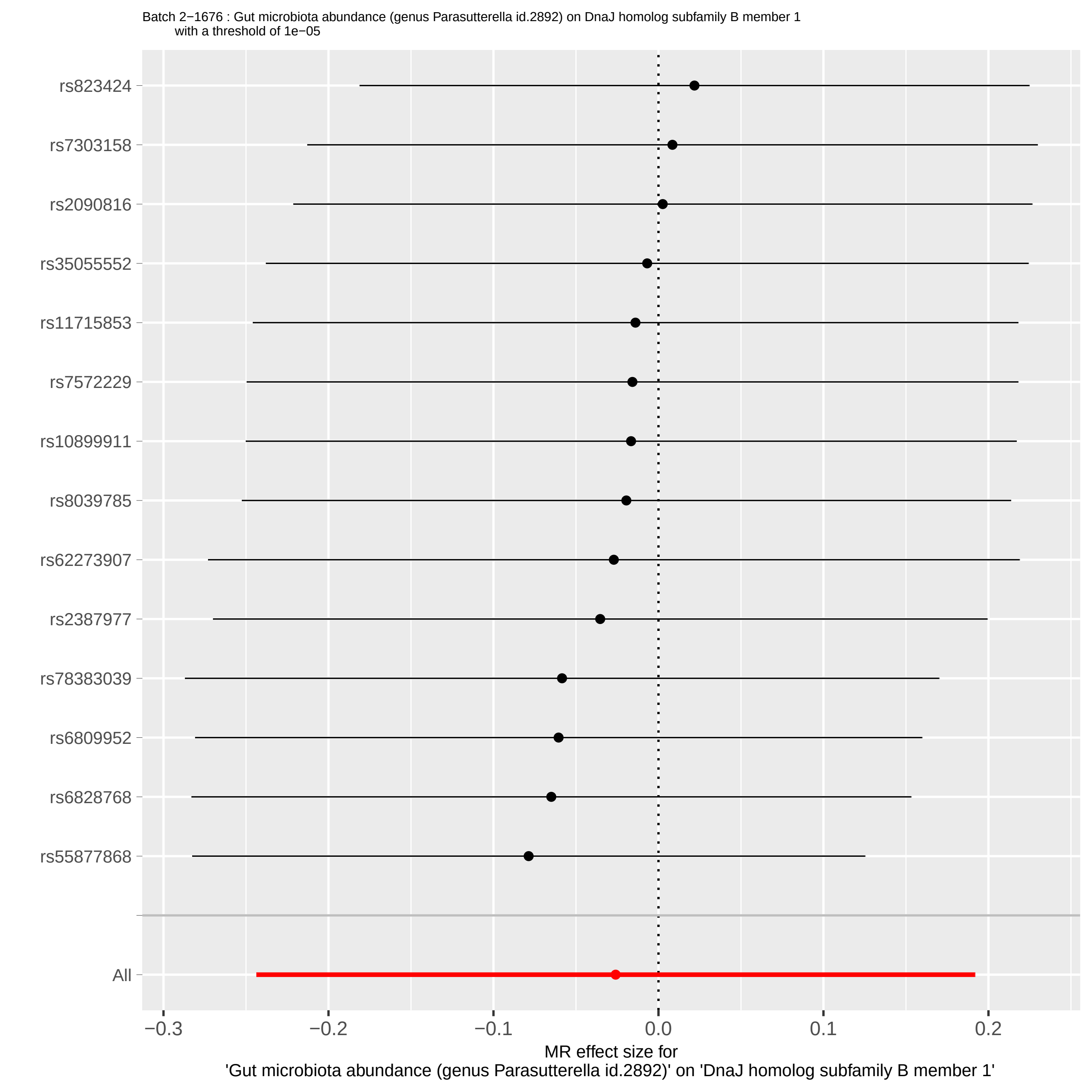
Batch 2−1676 : Gut microbiota abundance (genus Parasutterella id.2892) on DnaJ homolog subfamily B member 1
with a threshold of 1e−05
rs823424
rs7303158
rs2090816
rs35055552
rs11715853
rs7572229
rs10899911
rs8039785
rs62273907
rs2387977
rs78383039
rs6809952
rs6828768
rs55877868
All
−0.3
−0.2
−0.1
0.0
0.1
0.2
MR effect size for
'Gut microbiota abundance (genus Parasutterella id.2892)' on 'DnaJ homolog subfamily B member 1'

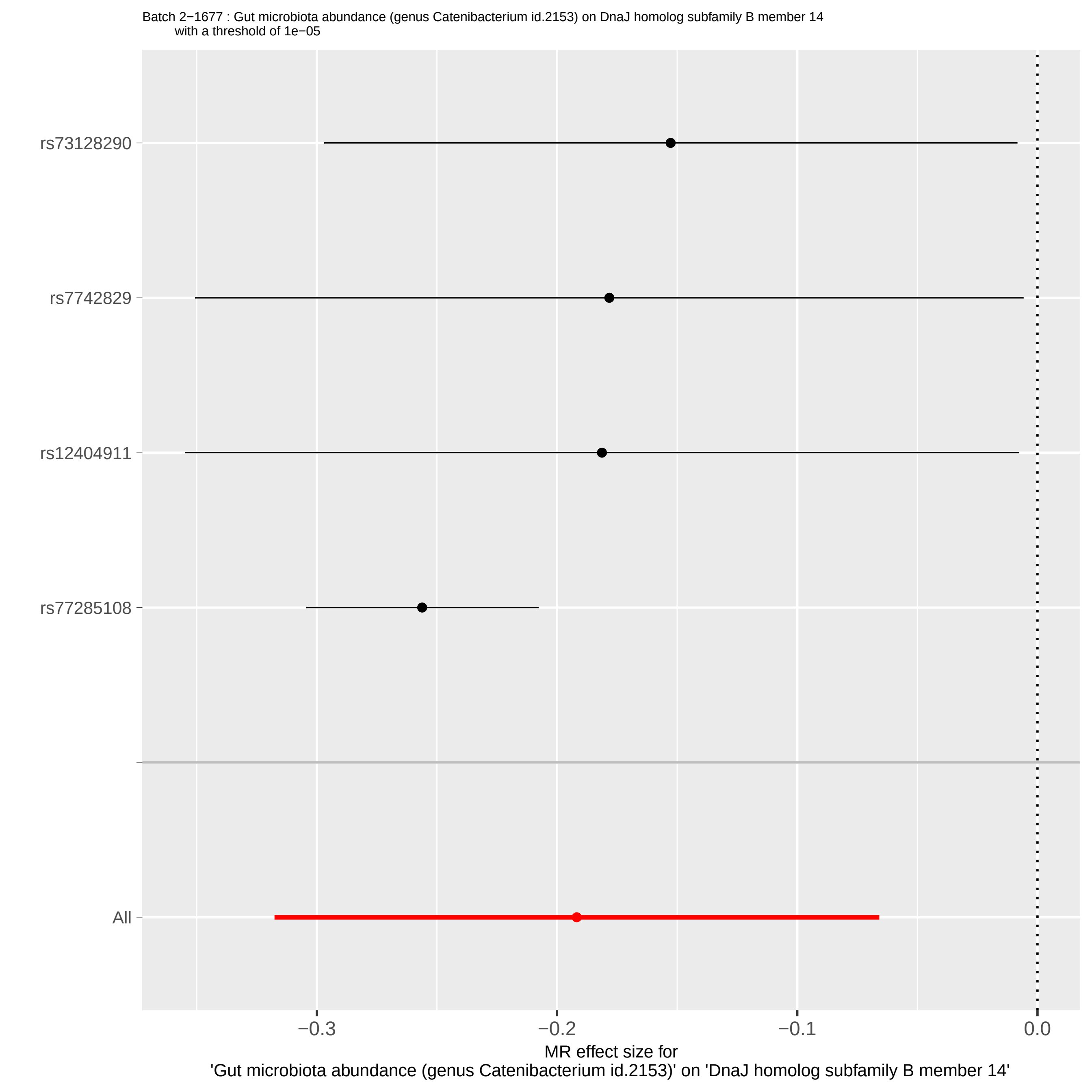
Batch 2−1677 : Gut microbiota abundance (genus Catenibacterium id.2153) on DnaJ homolog subfamily B member 14
with a threshold of 1e−05
rs73128290
rs7742829
rs12404911
rs77285108
All
−0.3
−0.2
−0.1
0.0
MR effect size for
'Gut microbiota abundance (genus Catenibacterium id.2153)' on 'DnaJ homolog subfamily B member 14'

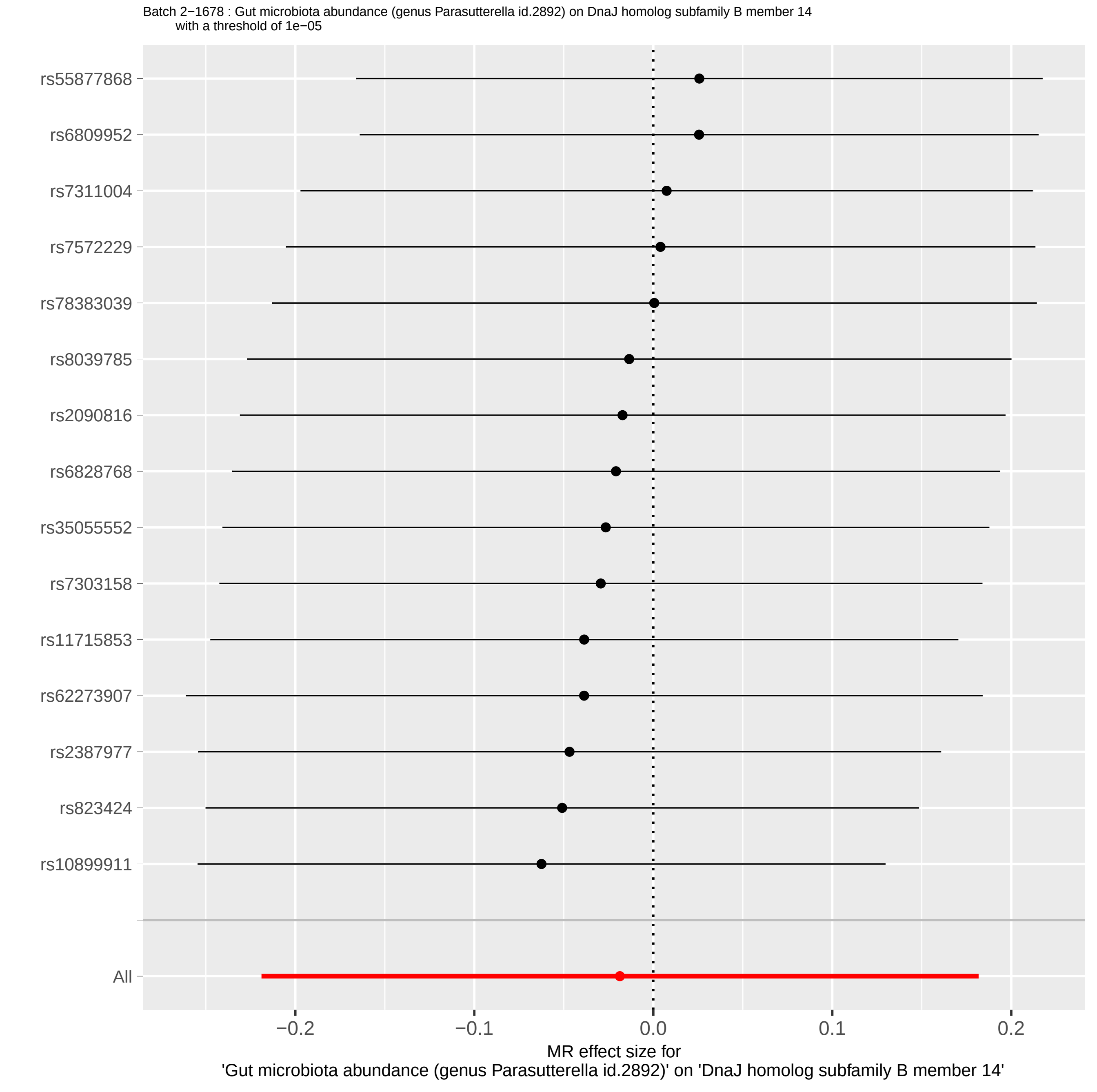

Batch 2−1678 : Gut microbiota abundance (genus Parasutterella id.2892) on DnaJ homolog subfamily B member 14
with a threshold of 1e−05
rs55877868
rs6809952
rs7311004
rs7572229
rs78383039
rs8039785
rs2090816
rs6828768
rs35055552
rs7303158
rs11715853
rs62273907
rs2387977
rs823424
rs10899911
All
−0.2
−0.1
0.0
0.1
0.2
MR effect size for
'Gut microbiota abundance (genus Parasutterella id.2892)' on 'DnaJ homolog subfamily B member 14'

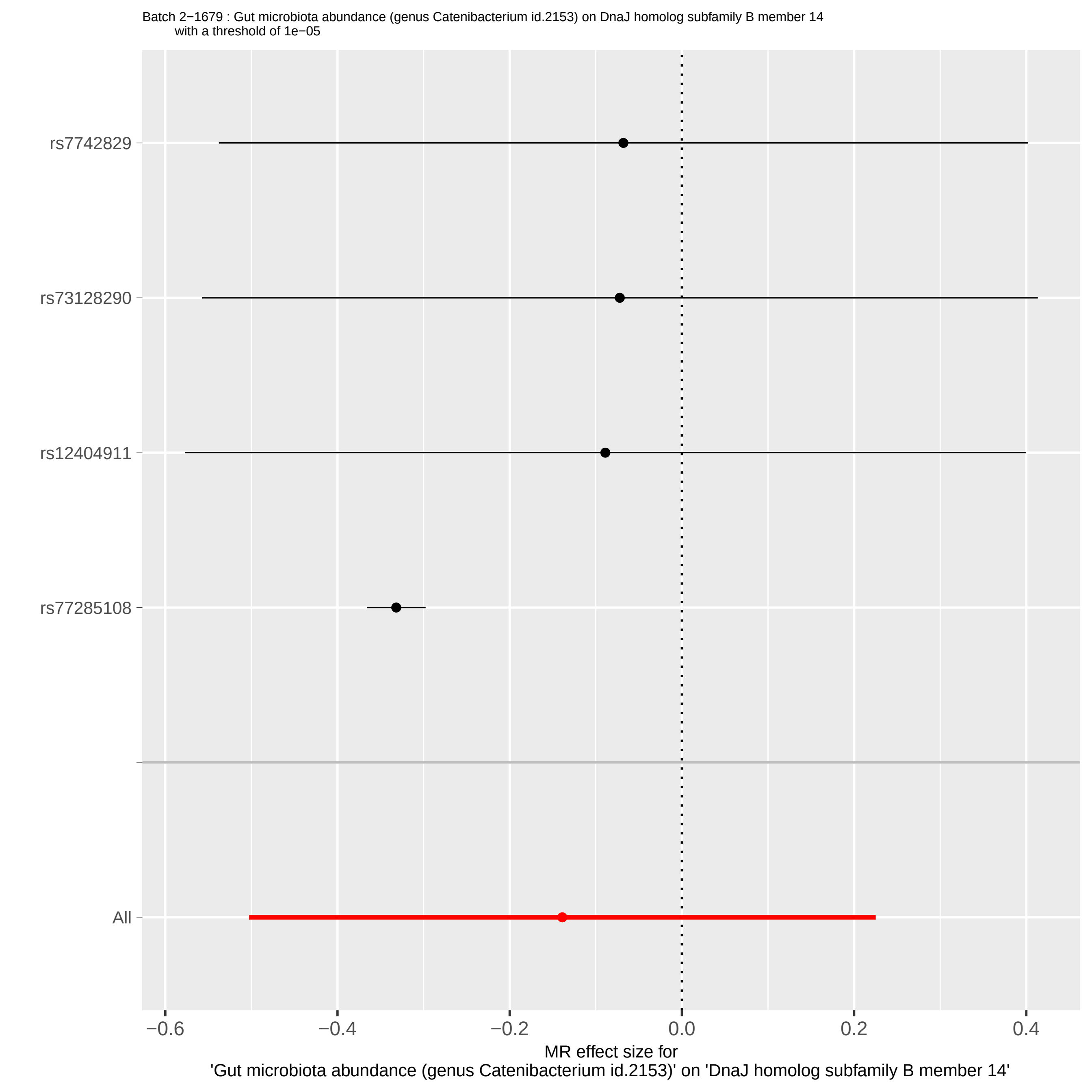
Batch 2−1679 : Gut microbiota abundance (genus Catenibacterium id.2153) on DnaJ homolog subfamily B member 14
with a threshold of 1e−05
rs7742829
rs73128290
rs12404911
rs77285108
All
−0.6
−0.4
−0.2
0.0
0.2
0.4
MR effect size for
'Gut microbiota abundance (genus Catenibacterium id.2153)' on 'DnaJ homolog subfamily B member 14'

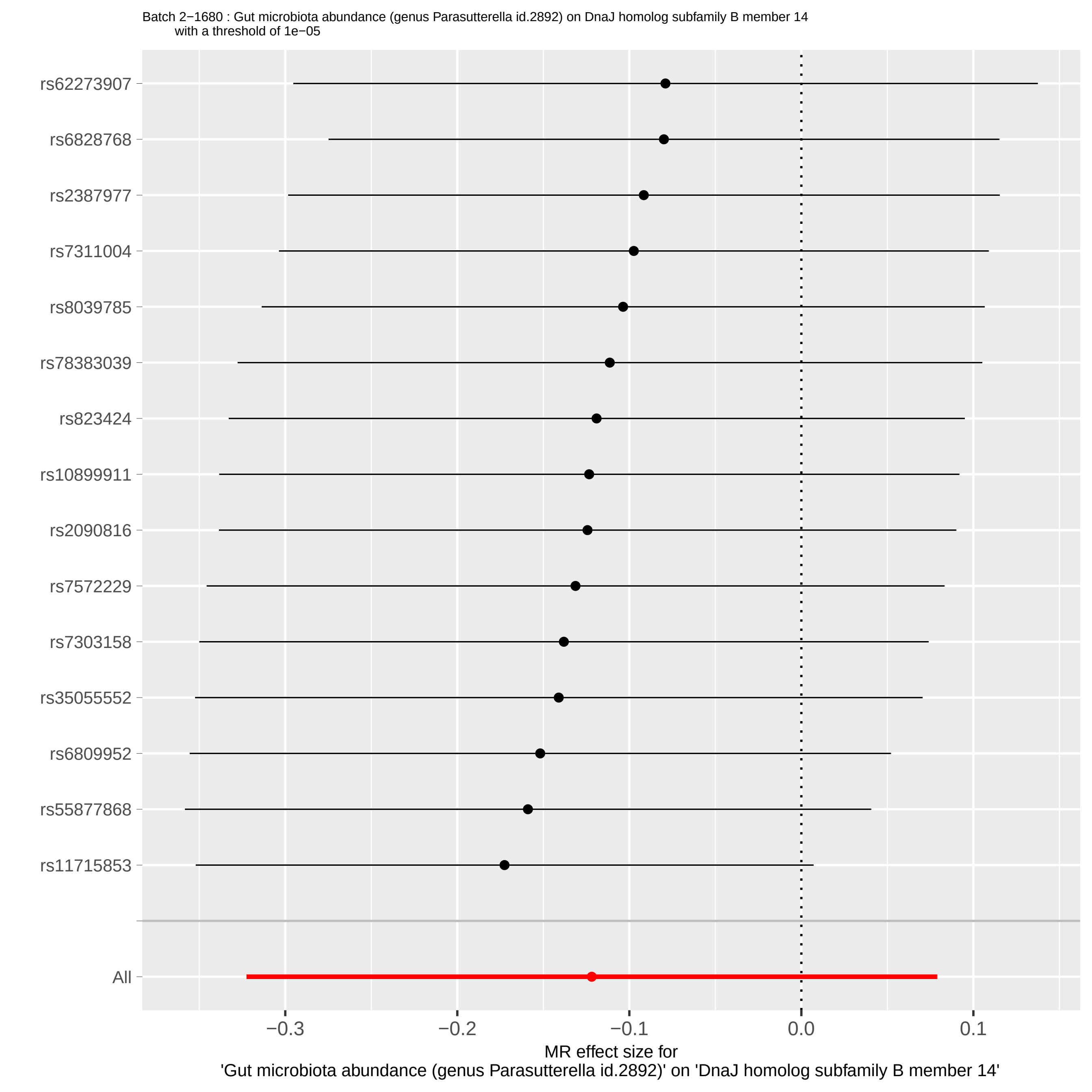
Batch 2−1680 : Gut microbiota abundance (genus Parasutterella id.2892) on DnaJ homolog subfamily B member 14
with a threshold of 1e−05
rs62273907
rs6828768
rs2387977
rs7311004
rs8039785
rs78383039
rs823424
rs10899911
rs2090816
rs7572229
rs7303158
rs35055552
rs6809952
rs55877868
rs11715853
All
−0.3
−0.2
−0.1
0.0
0.1
MR effect size for
'Gut microbiota abundance (genus Parasutterella id.2892)' on 'DnaJ homolog subfamily B member 14'

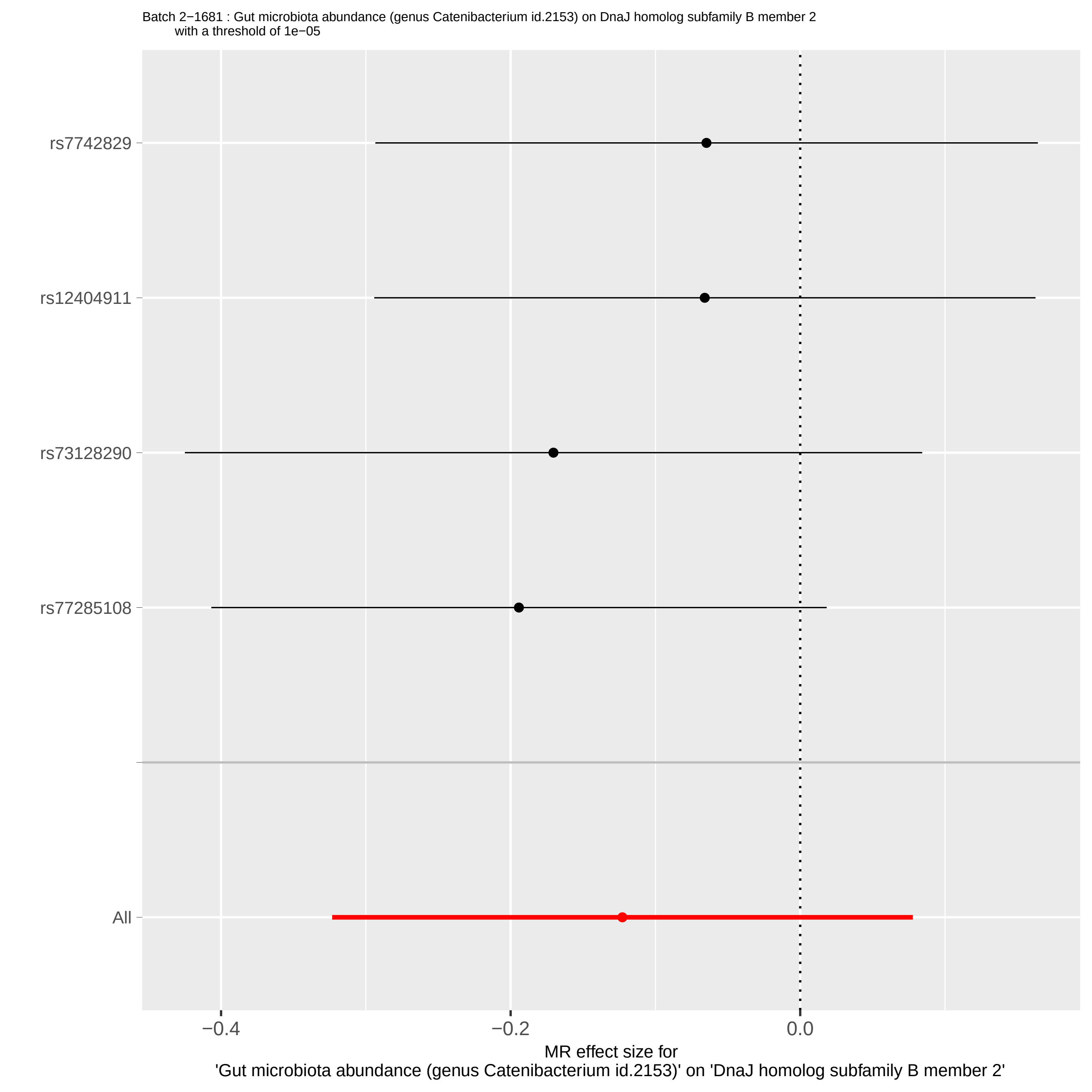
Batch 2−1681 : Gut microbiota abundance (genus Catenibacterium id.2153) on DnaJ homolog subfamily B member 2
with a threshold of 1e−05
rs7742829
rs12404911
rs73128290
rs77285108
All
−0.4
−0.2
0.0
MR effect size for
'Gut microbiota abundance (genus Catenibacterium id.2153)' on 'DnaJ homolog subfamily B member 2'

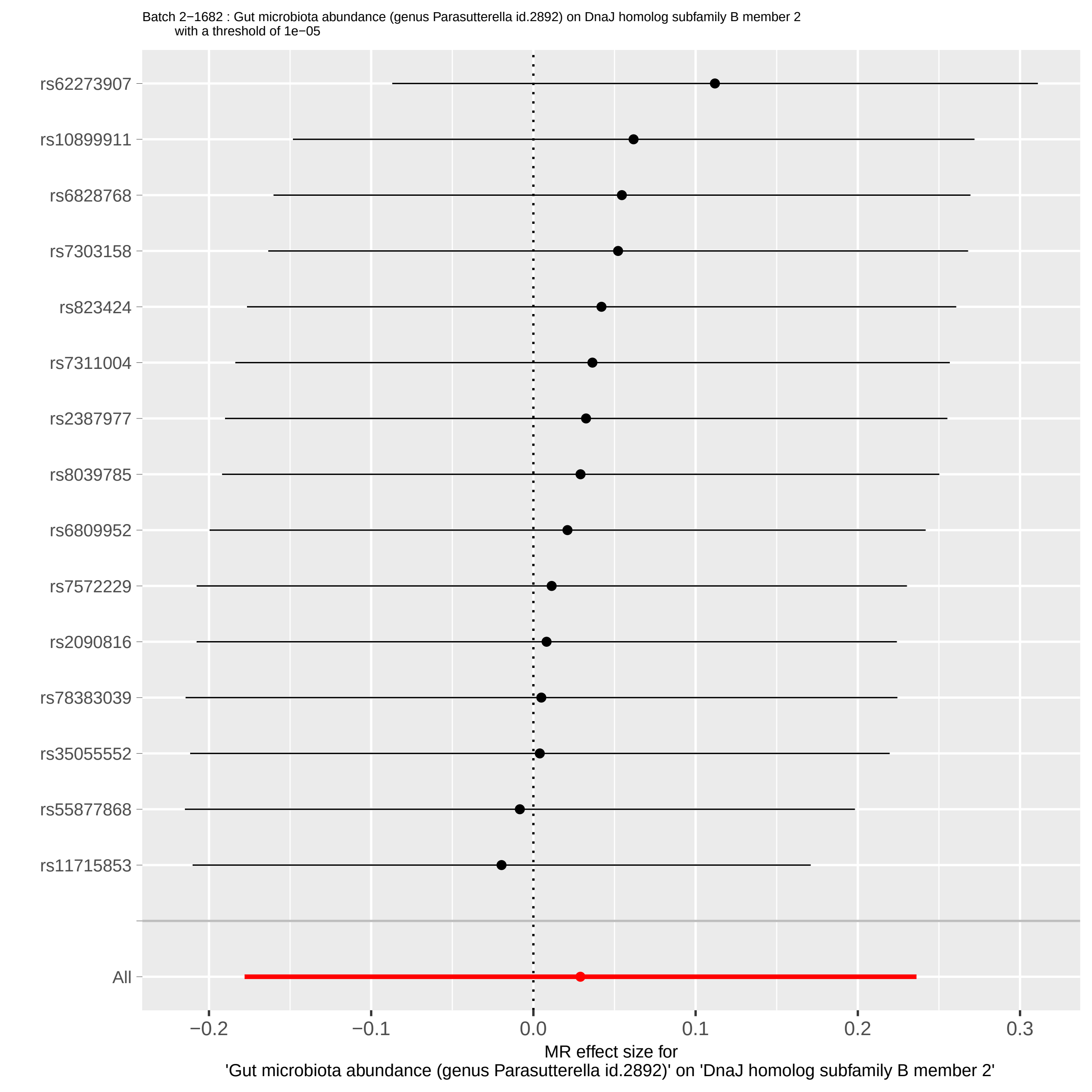
Batch 2−1682 : Gut microbiota abundance (genus Parasutterella id.2892) on DnaJ homolog subfamily B member 2
with a threshold of 1e−05
rs62273907
rs10899911
rs6828768
rs7303158
rs823424
rs7311004
rs2387977
rs8039785
rs6809952
rs7572229
rs2090816
rs78383039
rs35055552
rs55877868
rs11715853
All
−0.2
−0.1
0.0
0.1
0.2
0.3
MR effect size for
'Gut microbiota abundance (genus Parasutterella id.2892)' on 'DnaJ homolog subfamily B member 2'

Batch 2−1683 : Gut microbiota abundance (genus Catenibacterium id.2153) on DnaJ homolog subfamily B member 9
with a threshold of 1e−05

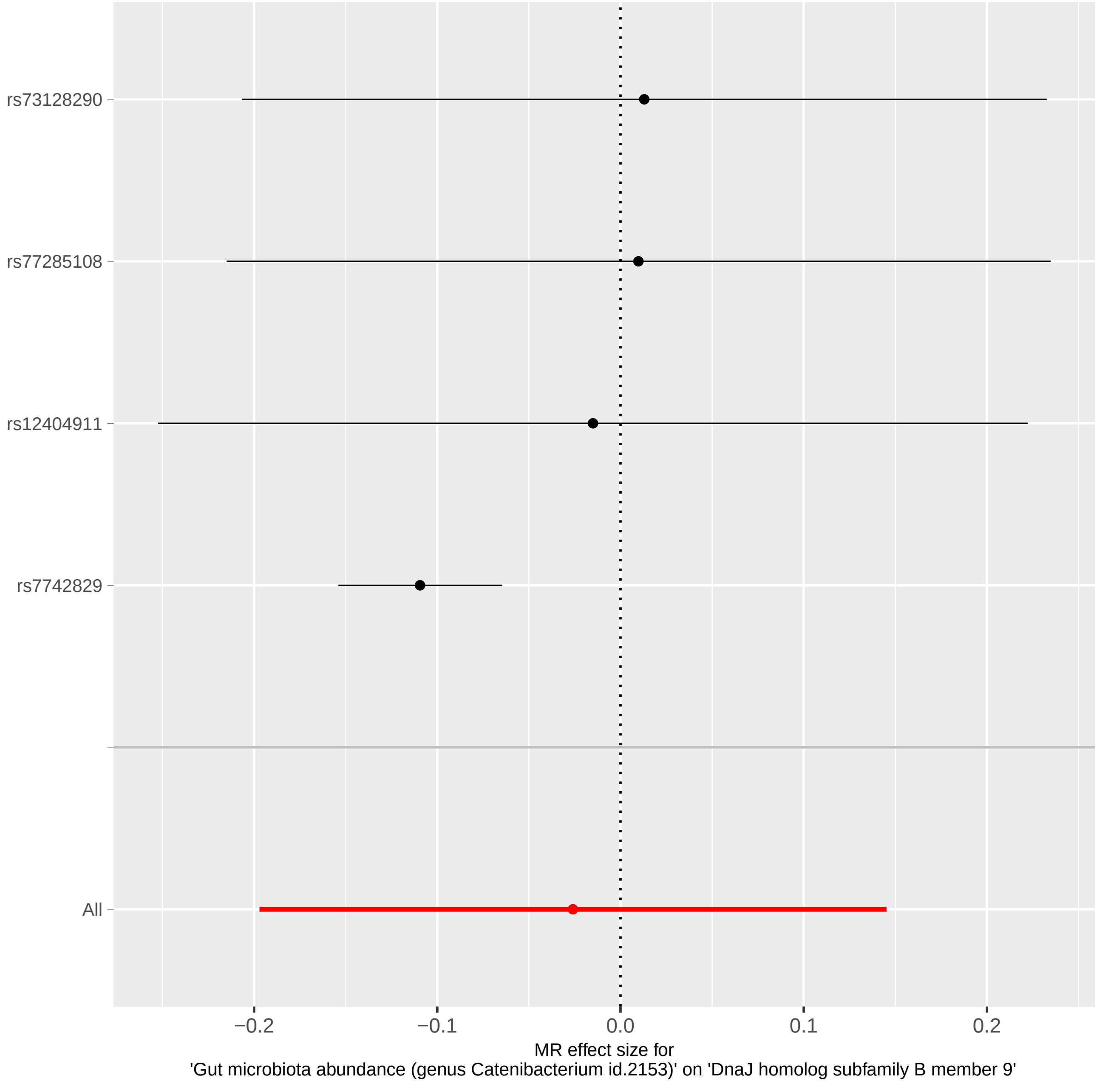

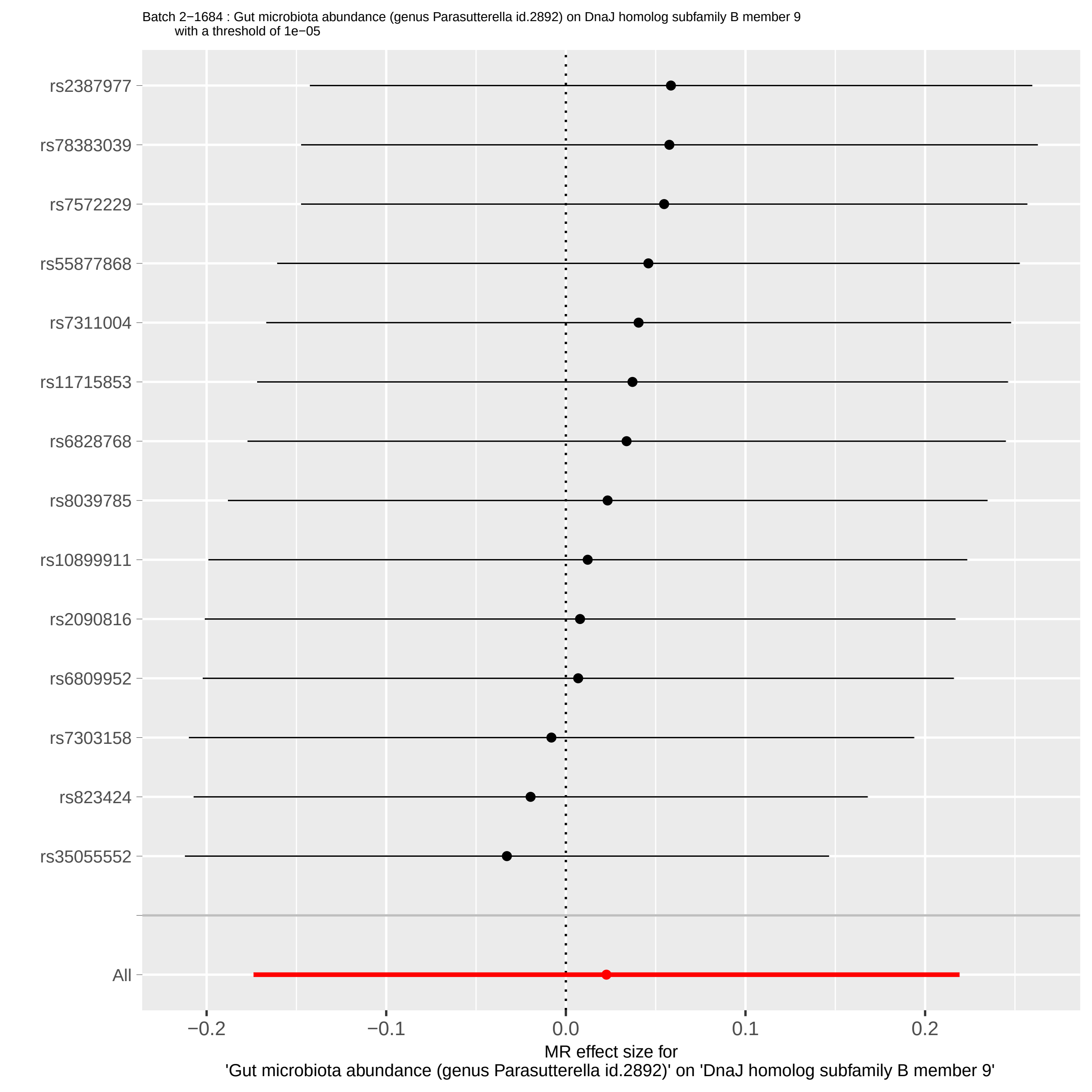

Batch 2−1684 : Gut microbiota abundance (genus Parasutterella id.2892) on DnaJ homolog subfamily B member 9
with a threshold of 1e−05
rs2387977
rs78383039
rs7572229
rs55877868
rs7311004
rs11715853
rs6828768
rs8039785
rs10899911
rs2090816
rs6809952
rs7303158
rs823424
rs35055552
All
−0.2
−0.1
0.0
0.1
0.2
MR effect size for
'Gut microbiota abundance (genus Parasutterella id.2892)' on 'DnaJ homolog subfamily B member 9'

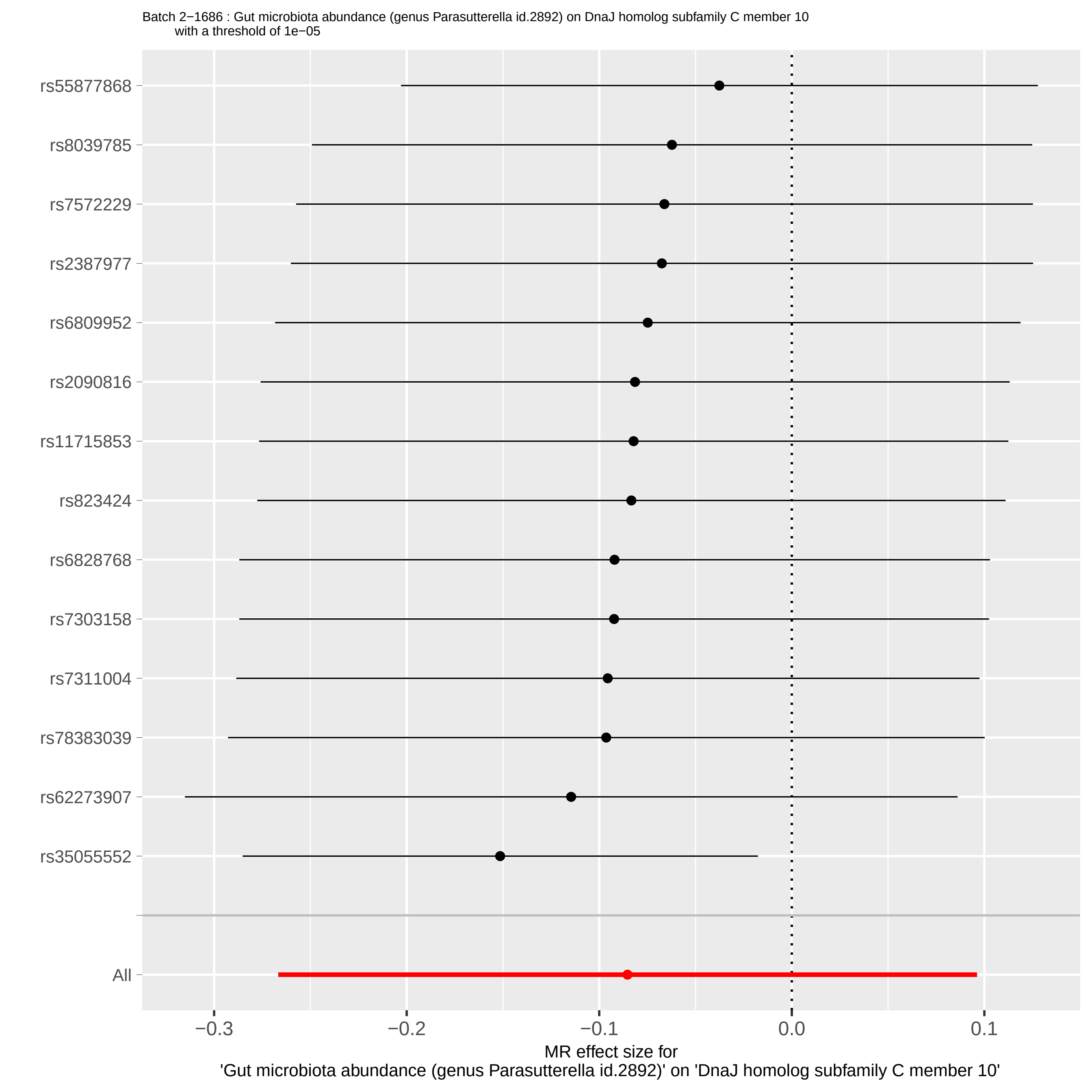

Batch 2−1686 : Gut microbiota abundance (genus Parasutterella id.2892) on DnaJ homolog subfamily C member 10
with a threshold of 1e−05
rs55877868
rs8039785
rs7572229
rs2387977
rs6809952
rs2090816
rs11715853
rs823424
rs6828768
rs7303158
rs7311004
rs78383039
rs62273907
rs35055552
All
−0.3
−0.2
−0.1
0.0
0.1
MR effect size for
'Gut microbiota abundance (genus Parasutterella id.2892)' on 'DnaJ homolog subfamily C member 10'

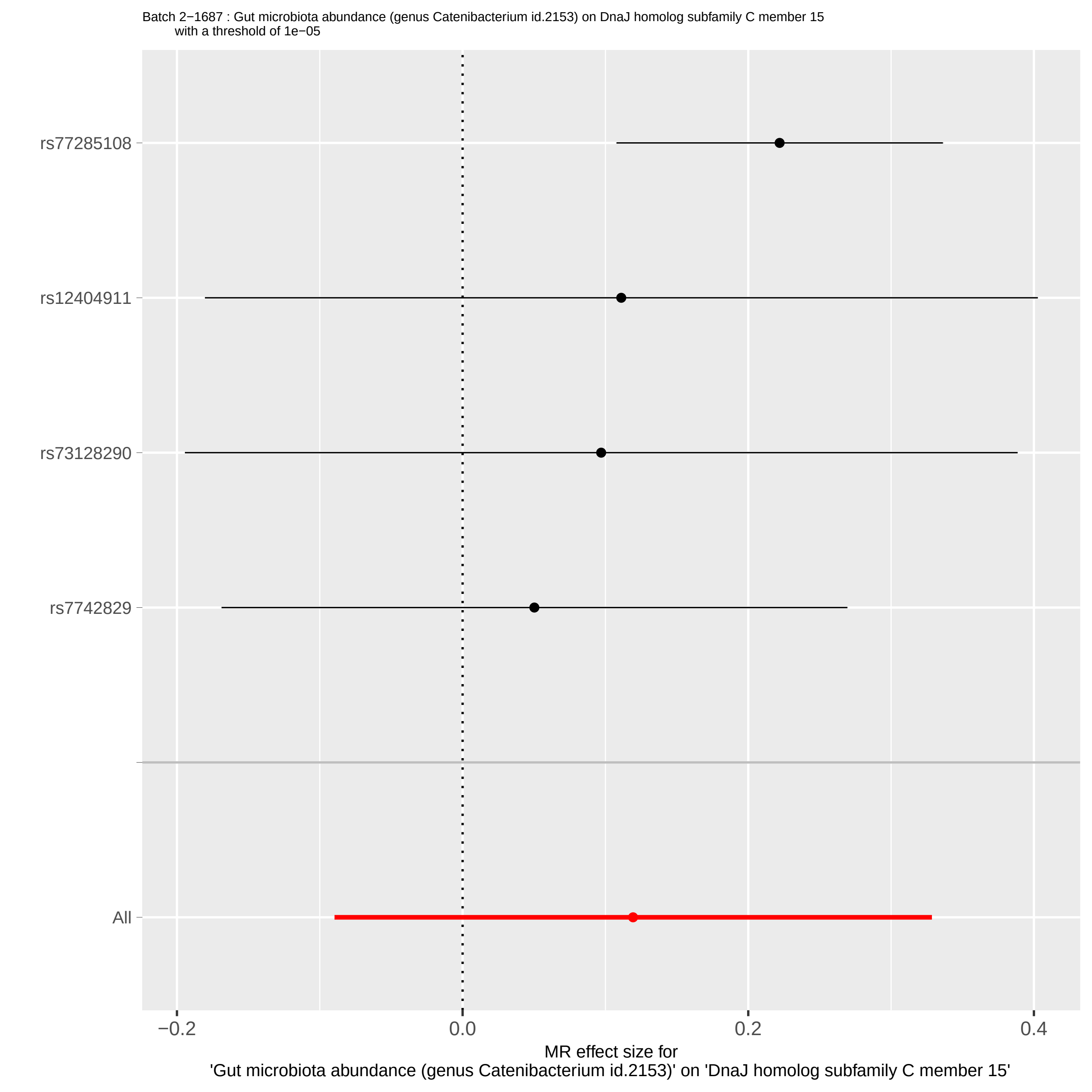
Batch 2−1687 : Gut microbiota abundance (genus Catenibacterium id.2153) on DnaJ homolog subfamily C member 15
with a threshold of 1e−05
rs77285108
rs12404911
rs73128290
rs7742829
All
−0.2
0.0
0.2
0.4
MR effect size for
'Gut microbiota abundance (genus Catenibacterium id.2153)' on 'DnaJ homolog subfamily C member 15'

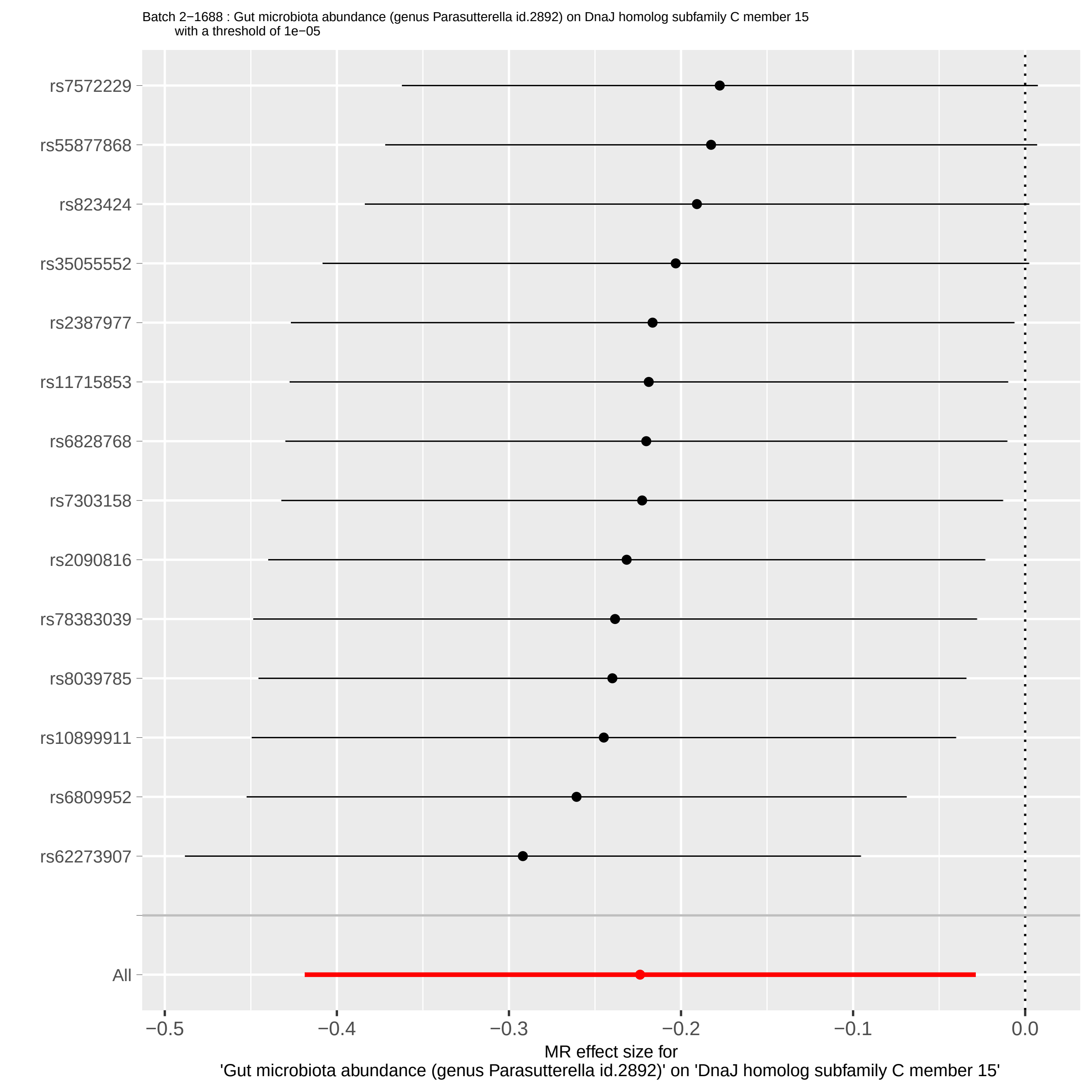

Batch 2−1688 : Gut microbiota abundance (genus Parasutterella id.2892) on DnaJ homolog subfamily C member 15
with a threshold of 1e−05
rs7572229
rs55877868
rs823424
rs35055552
rs2387977
rs11715853
rs6828768
rs7303158
rs2090816
rs78383039
rs8039785
rs10899911
rs6809952
rs62273907
All
−0.5
−0.4
−0.3
−0.2
−0.1
0.0
MR effect size for
'Gut microbiota abundance (genus Parasutterella id.2892)' on 'DnaJ homolog subfamily C member 15'

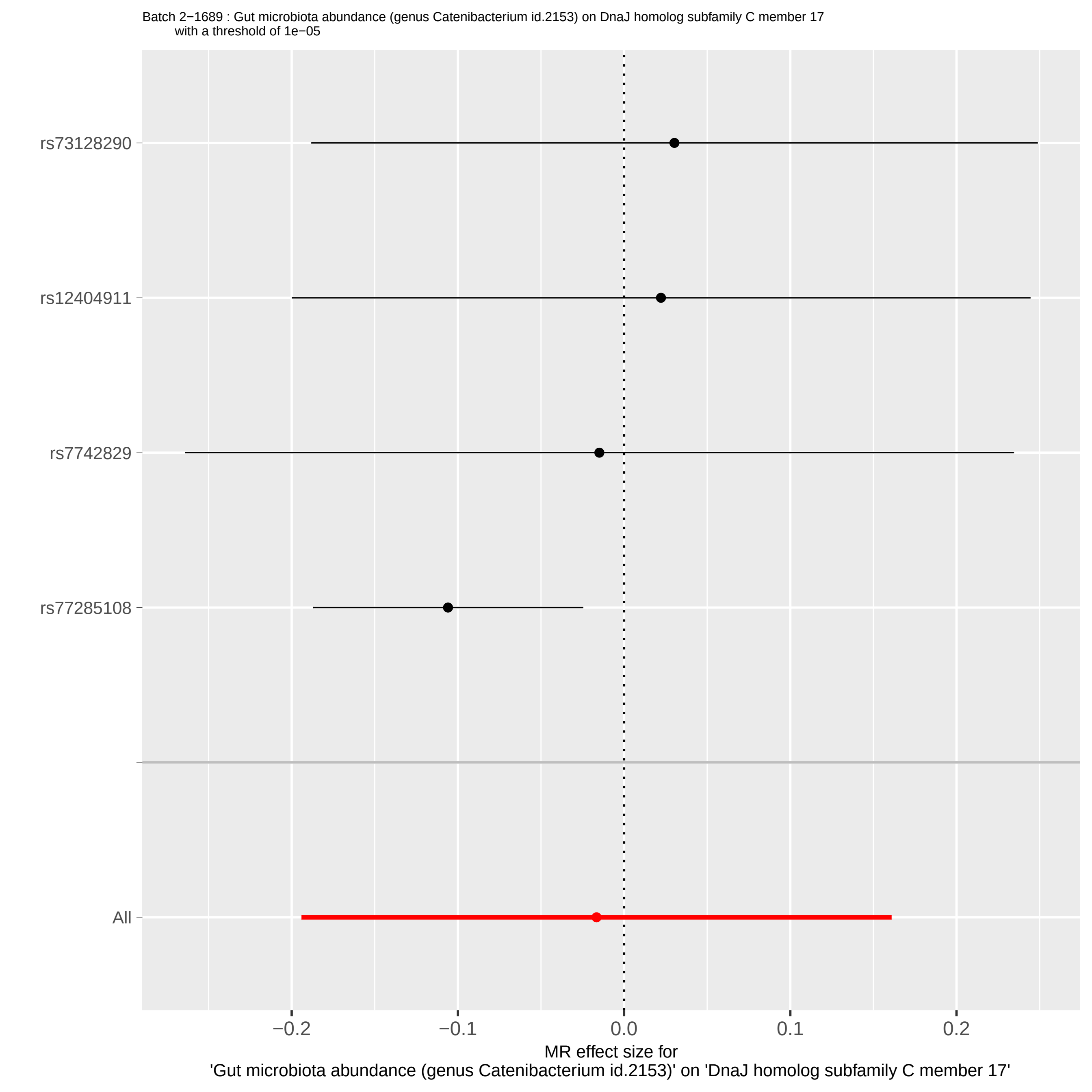

Batch 2−1689 : Gut microbiota abundance (genus Catenibacterium id.2153) on DnaJ homolog subfamily C member 17
with a threshold of 1e−05
rs73128290
rs12404911
rs7742829
rs77285108
All
−0.2
−0.1
0.0
0.1
0.2
MR effect size for
'Gut microbiota abundance (genus Catenibacterium id.2153)' on 'DnaJ homolog subfamily C member 17'

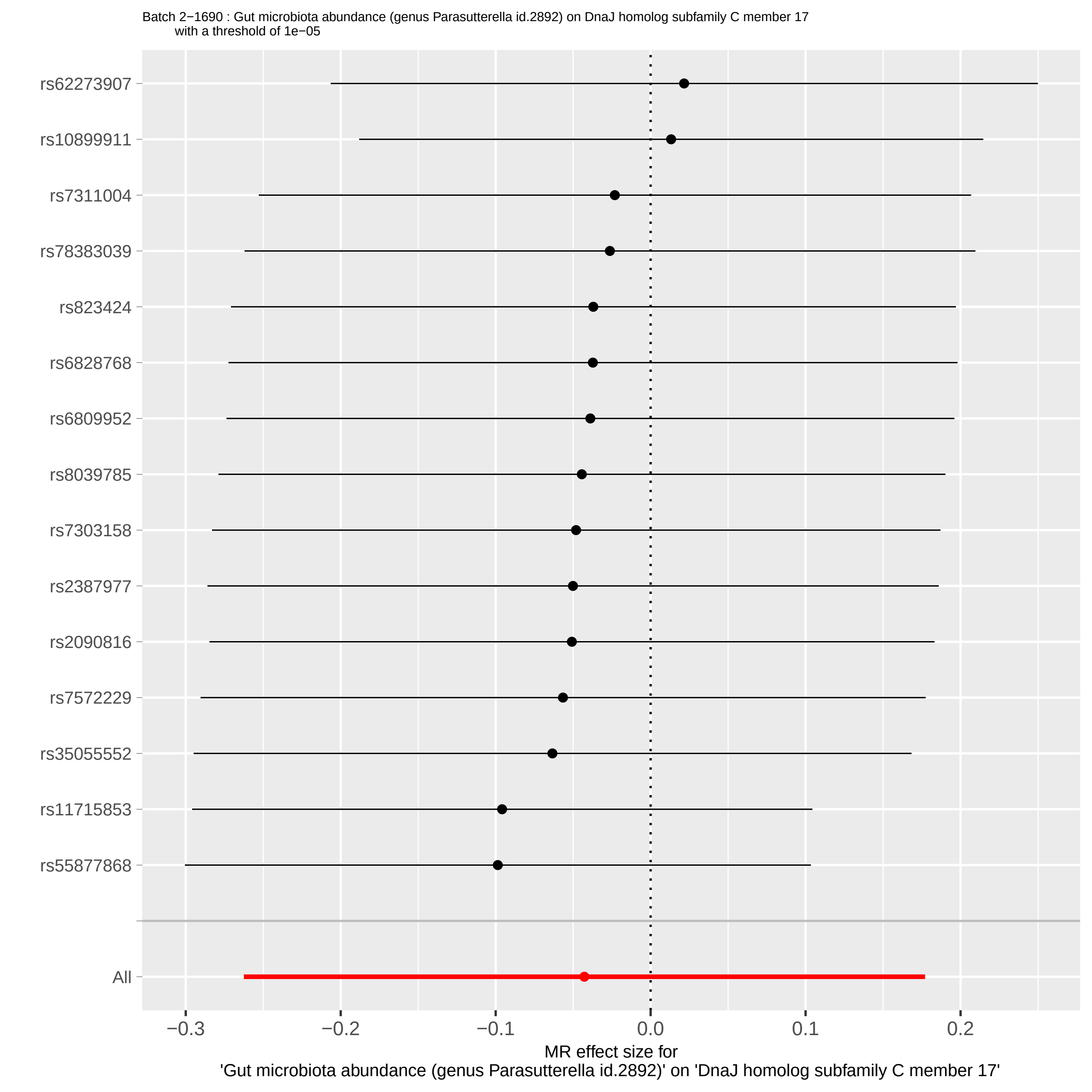
Batch 2−1690 : Gut microbiota abundance (genus Parasutterella id.2892) on DnaJ homolog subfamily C member 17
with a threshold of 1e−05
rs62273907
rs10899911
rs7311004
rs78383039
rs823424
rs6828768
rs6809952
rs8039785
rs7303158
rs2387977
rs2090816
rs7572229
rs35055552
rs11715853
rs55877868
All
−0.3
−0.2
−0.1
0.0
0.1
0.2
MR effect size for
'Gut microbiota abundance (genus Parasutterella id.2892)' on 'DnaJ homolog subfamily C member 17'

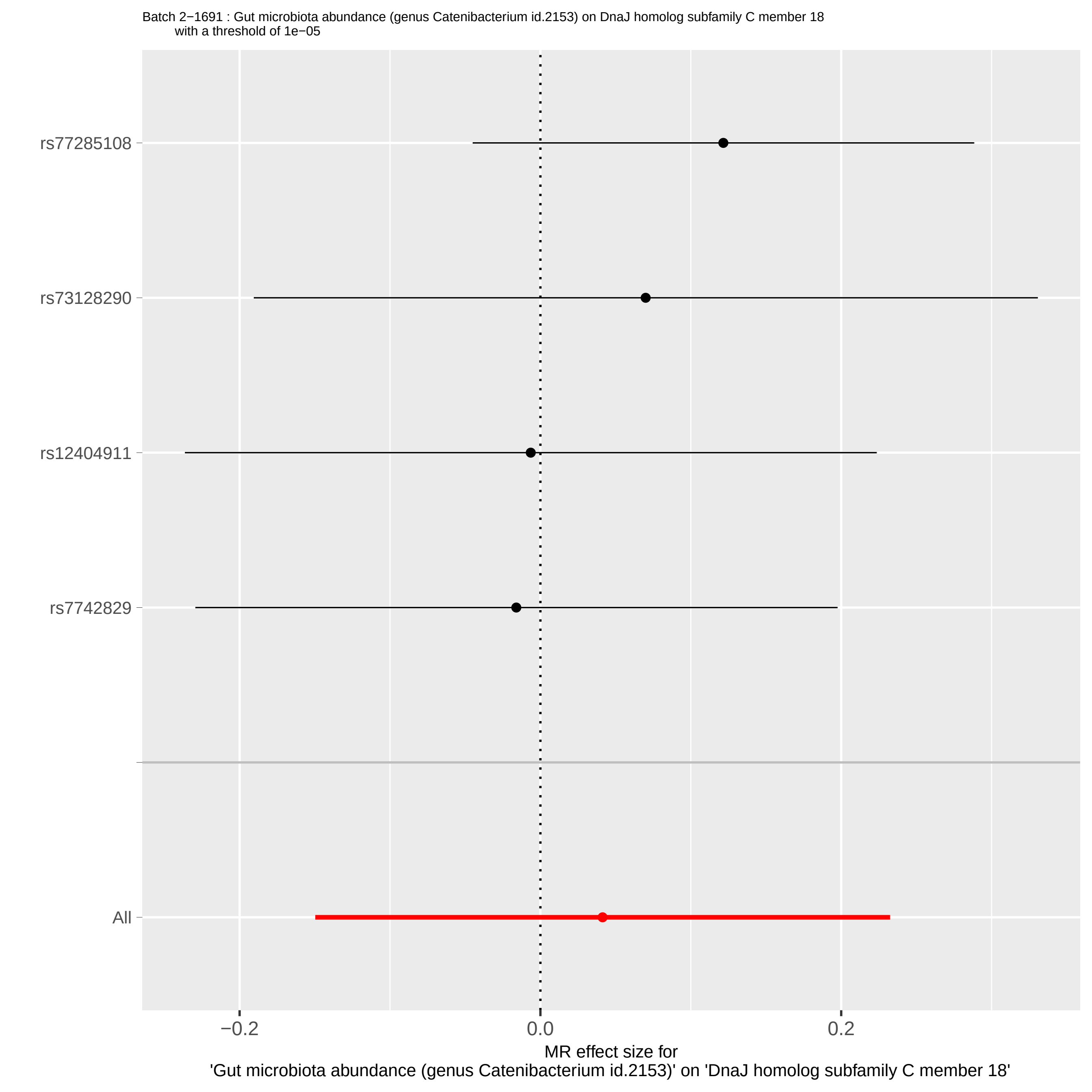
Batch 2−1691 : Gut microbiota abundance (genus Catenibacterium id.2153) on DnaJ homolog subfamily C member 18
with a threshold of 1e−05
rs77285108
rs73128290
rs12404911
rs7742829
All
−0.2
0.0
0.2
MR effect size for
'Gut microbiota abundance (genus Catenibacterium id.2153)' on 'DnaJ homolog subfamily C member 18'

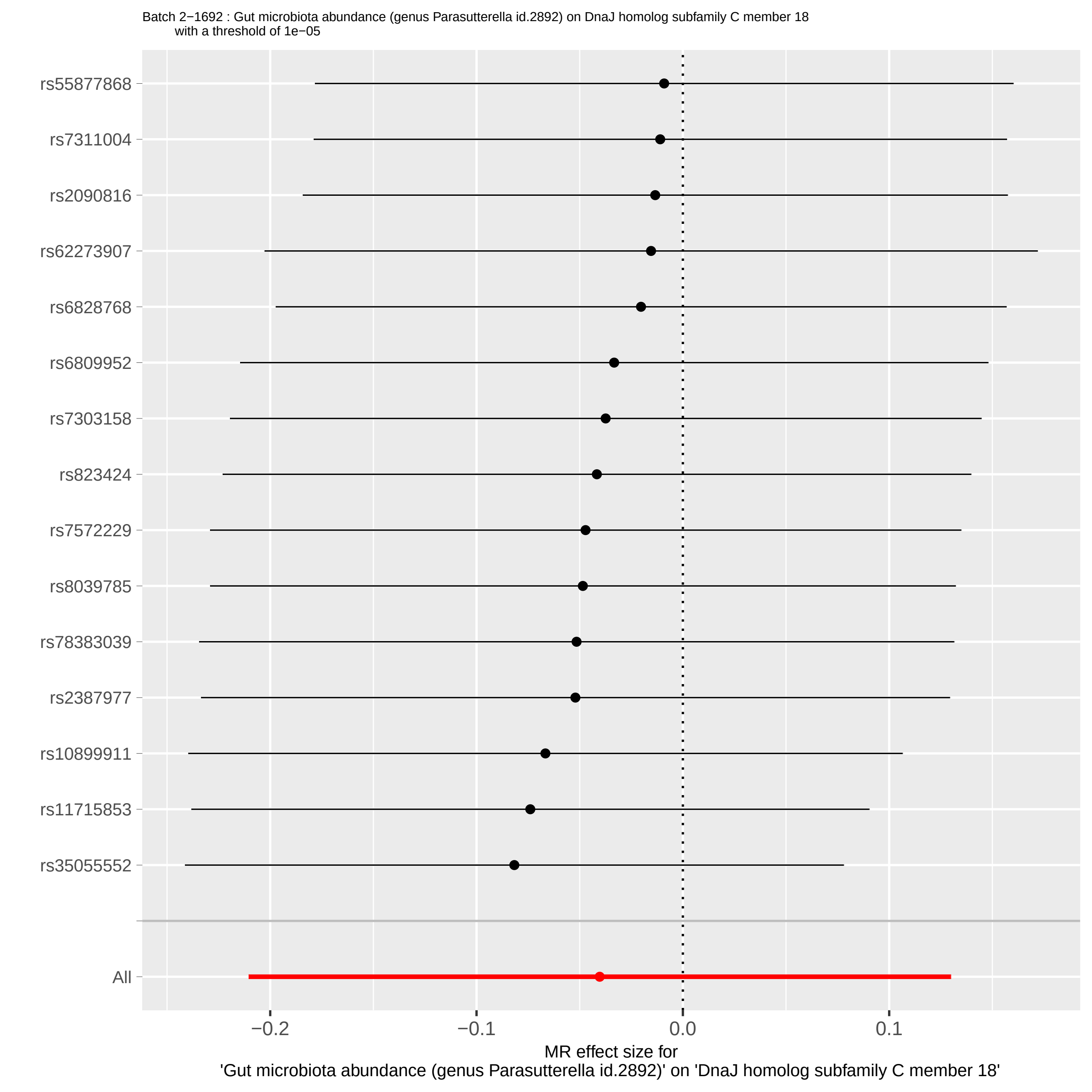

Batch 2−1692 : Gut microbiota abundance (genus Parasutterella id.2892) on DnaJ homolog subfamily C member 18
with a threshold of 1e−05
rs55877868
rs7311004
rs2090816
rs62273907
rs6828768
rs6809952
rs7303158
rs823424
rs7572229
rs8039785
rs78383039
rs2387977
rs10899911
rs11715853
rs35055552
All
−0.2
−0.1
0.0
0.1
MR effect size for
'Gut microbiota abundance (genus Parasutterella id.2892)' on 'DnaJ homolog subfamily C member 18'

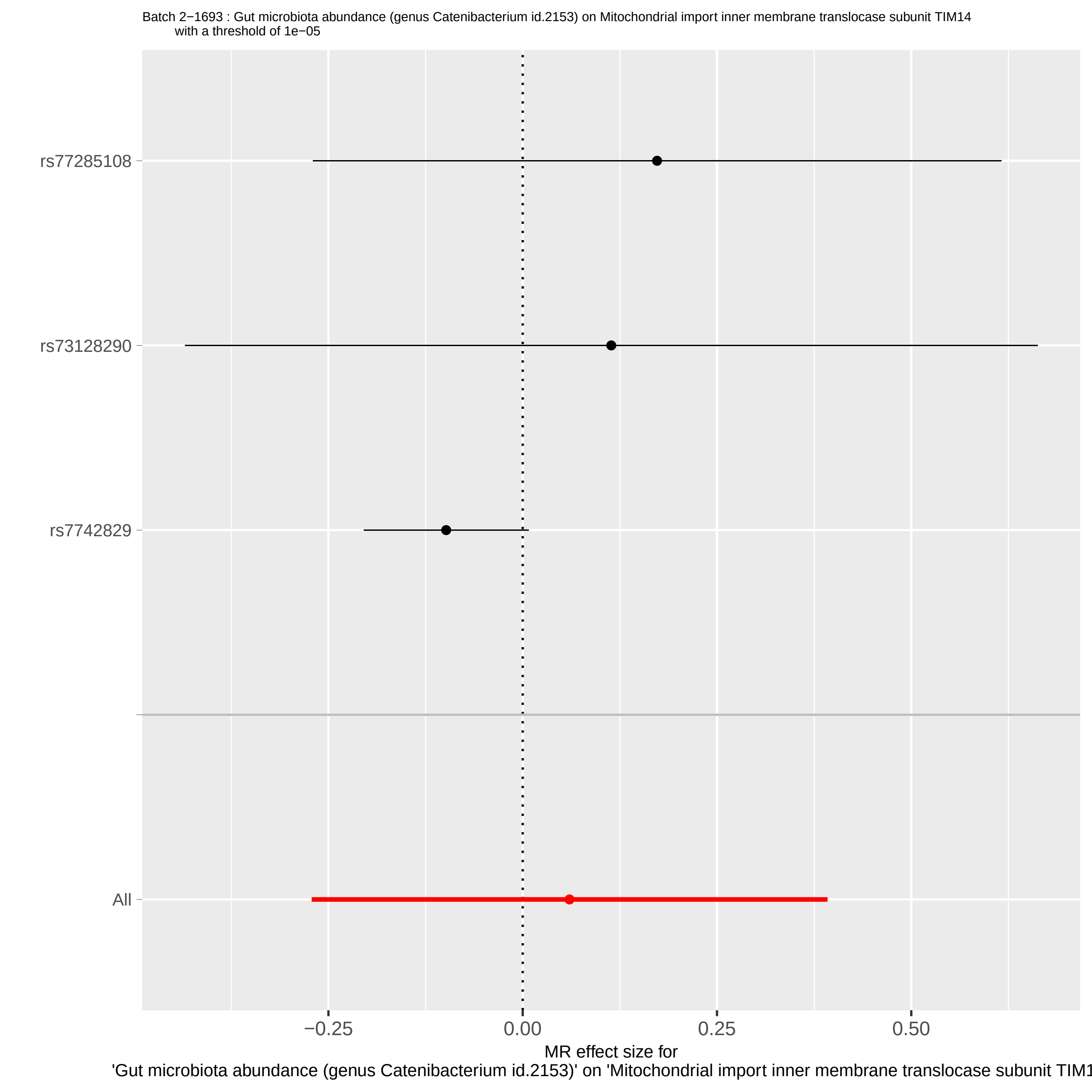

Batch 2−1693 : Gut microbiota abundance (genus Catenibacterium id.2153) on Mitochondrial import inner membrane translocase subunit TIM14
with a threshold of 1e−05
rs77285108
rs73128290
rs7742829
All
−0.25
0.00
0.25
0.50
MR effect size for
'Gut microbiota abundance (genus Catenibacterium id.2153)' on 'Mitochondrial import inner membrane translocase subunit TIM1

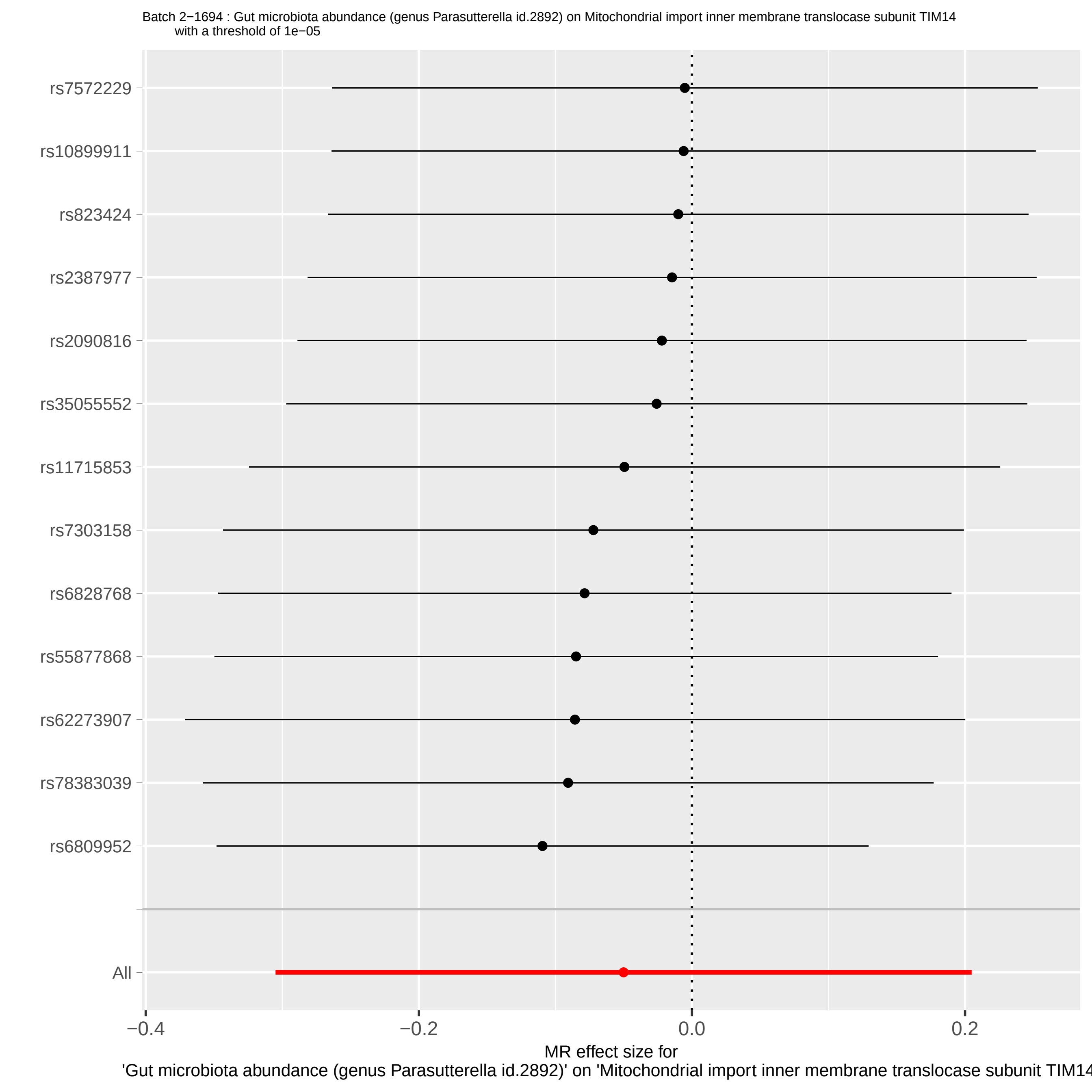
Batch 2−1694 : Gut microbiota abundance (genus Parasutterella id.2892) on Mitochondrial import inner membrane translocase subunit TIM14
with a threshold of 1e−05
rs7572229
rs10899911
rs823424
rs2387977
rs2090816
rs35055552
rs11715853
rs7303158
rs6828768
rs55877868
rs62273907
rs78383039
rs6809952
All
−0.4
−0.2
0.0
0.2
MR effect size for
'Gut microbiota abundance (genus Parasutterella id.2892)' on 'Mitochondrial import inner membrane translocase subunit TIM14

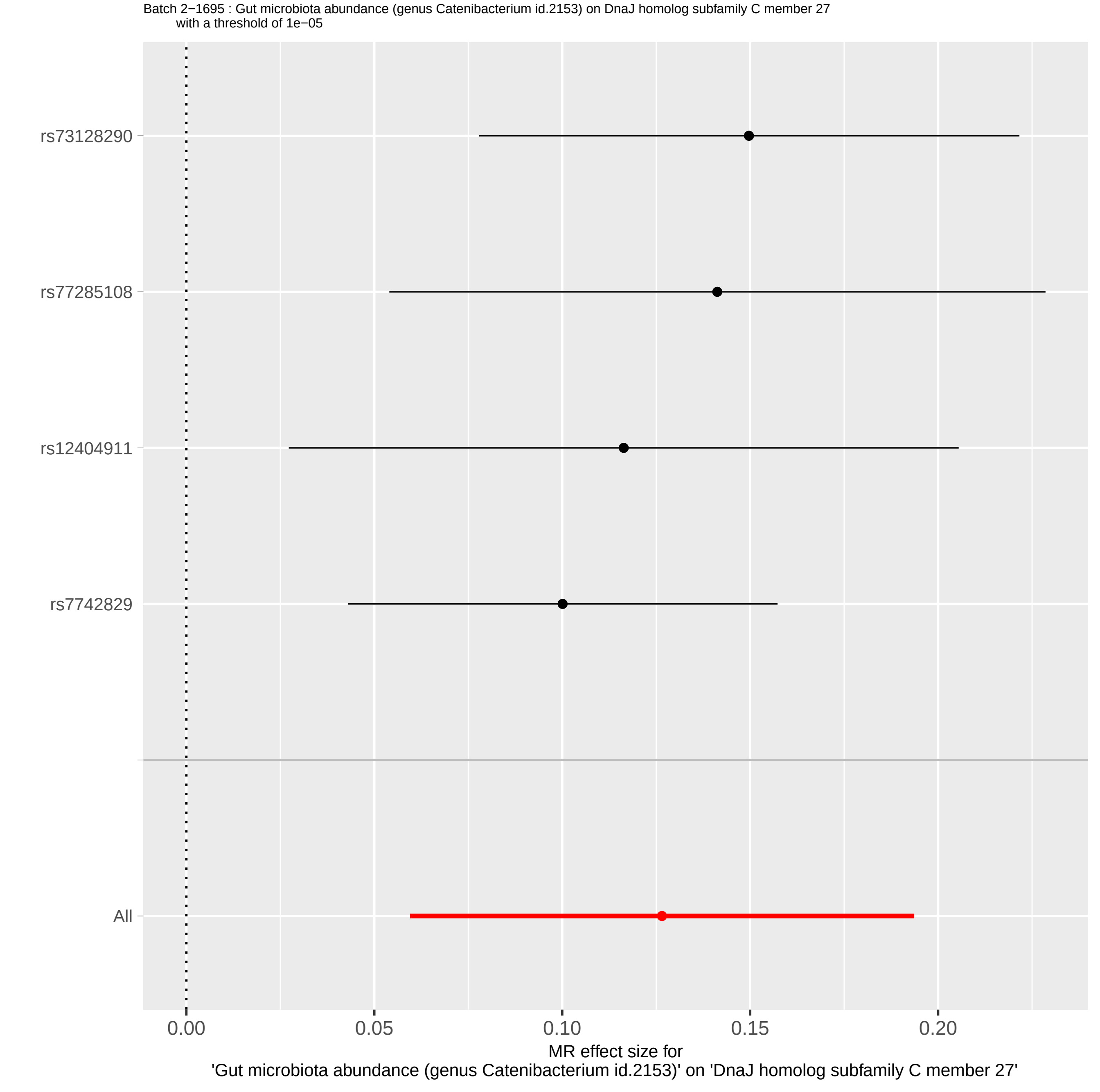

Batch 2−1695 : Gut microbiota abundance (genus Catenibacterium id.2153) on DnaJ homolog subfamily C member 27
with a threshold of 1e−05
rs73128290
rs77285108
rs12404911
rs7742829
All
0.00
0.05
0.10
0.15
0.20
MR effect size for
'Gut microbiota abundance (genus Catenibacterium id.2153)' on 'DnaJ homolog subfamily C member 27'

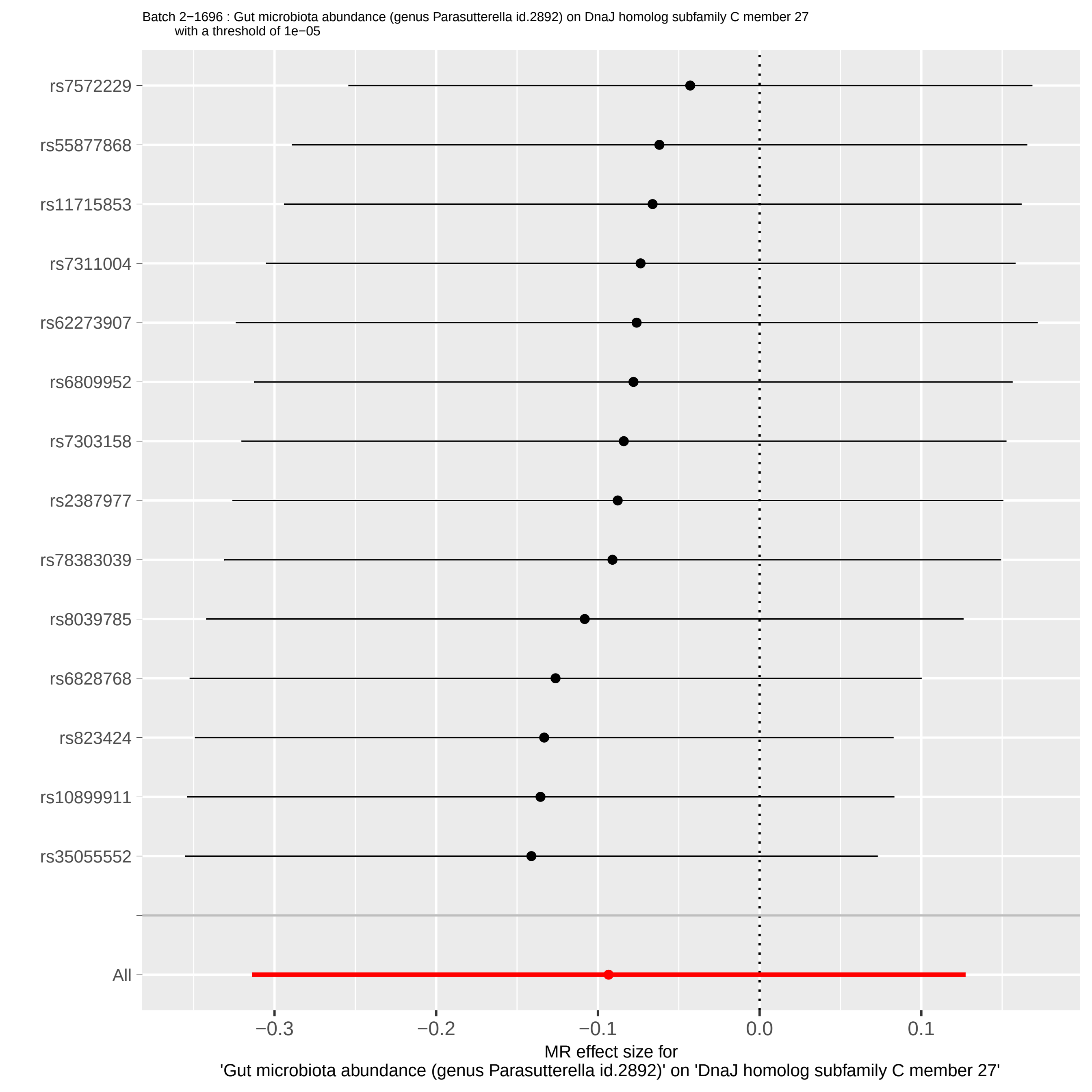

Batch 2−1696 : Gut microbiota abundance (genus Parasutterella id.2892) on DnaJ homolog subfamily C member 27
with a threshold of 1e−05
rs7572229
rs55877868
rs11715853
rs7311004
rs62273907
rs6809952
rs7303158
rs2387977
rs78383039
rs8039785
rs6828768
rs823424
rs10899911
rs35055552
All
−0.3
−0.2
−0.1
0.0
0.1
MR effect size for
'Gut microbiota abundance (genus Parasutterella id.2892)' on 'DnaJ homolog subfamily C member 27'

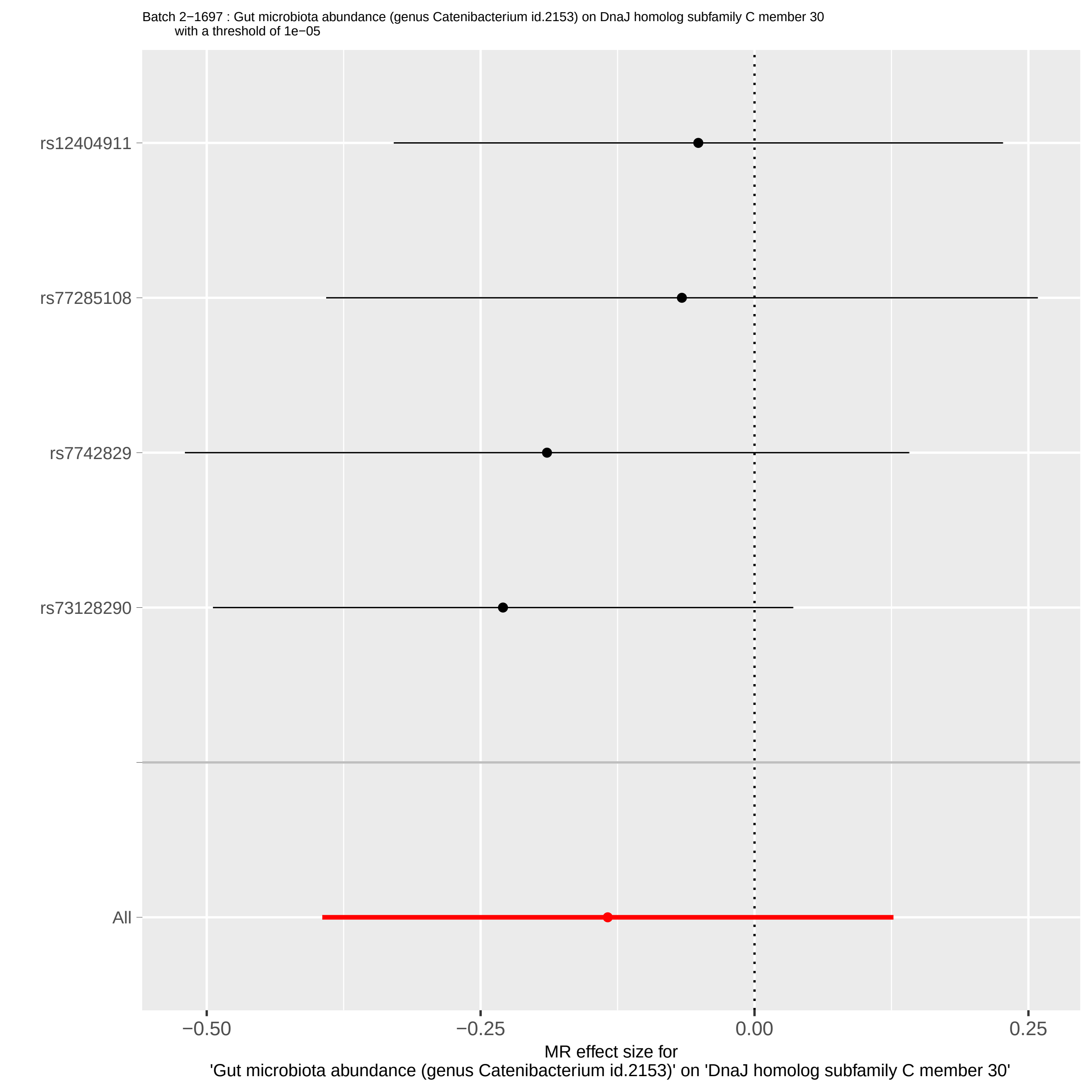

Batch 2−1697 : Gut microbiota abundance (genus Catenibacterium id.2153) on DnaJ homolog subfamily C member 30
with a threshold of 1e−05
rs12404911
rs77285108
rs7742829
rs73128290
All
−0.50
−0.25
0.00
0.25
MR effect size for
'Gut microbiota abundance (genus Catenibacterium id.2153)' on 'DnaJ homolog subfamily C member 30'

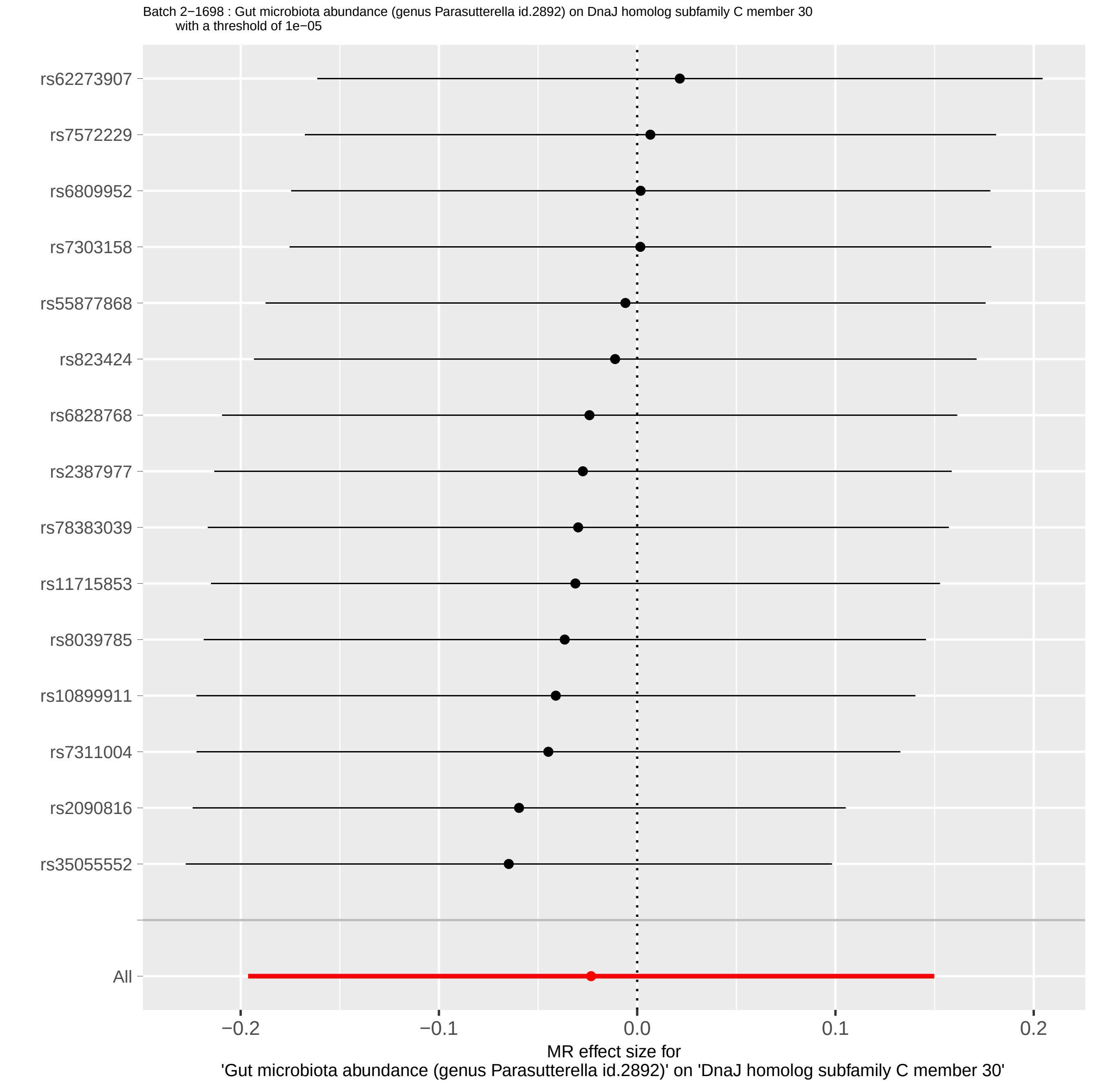
Batch 2−1698 : Gut microbiota abundance (genus Parasutterella id.2892) on DnaJ homolog subfamily C member 30
with a threshold of 1e−05
rs62273907
rs7572229
rs6809952
rs7303158
rs55877868
rs823424
rs6828768
rs2387977
rs78383039
rs11715853
rs8039785
rs10899911
rs7311004
rs2090816
rs35055552
All
−0.2
−0.1
0.0
0.1
0.2
MR effect size for
'Gut microbiota abundance (genus Parasutterella id.2892)' on 'DnaJ homolog subfamily C member 30'

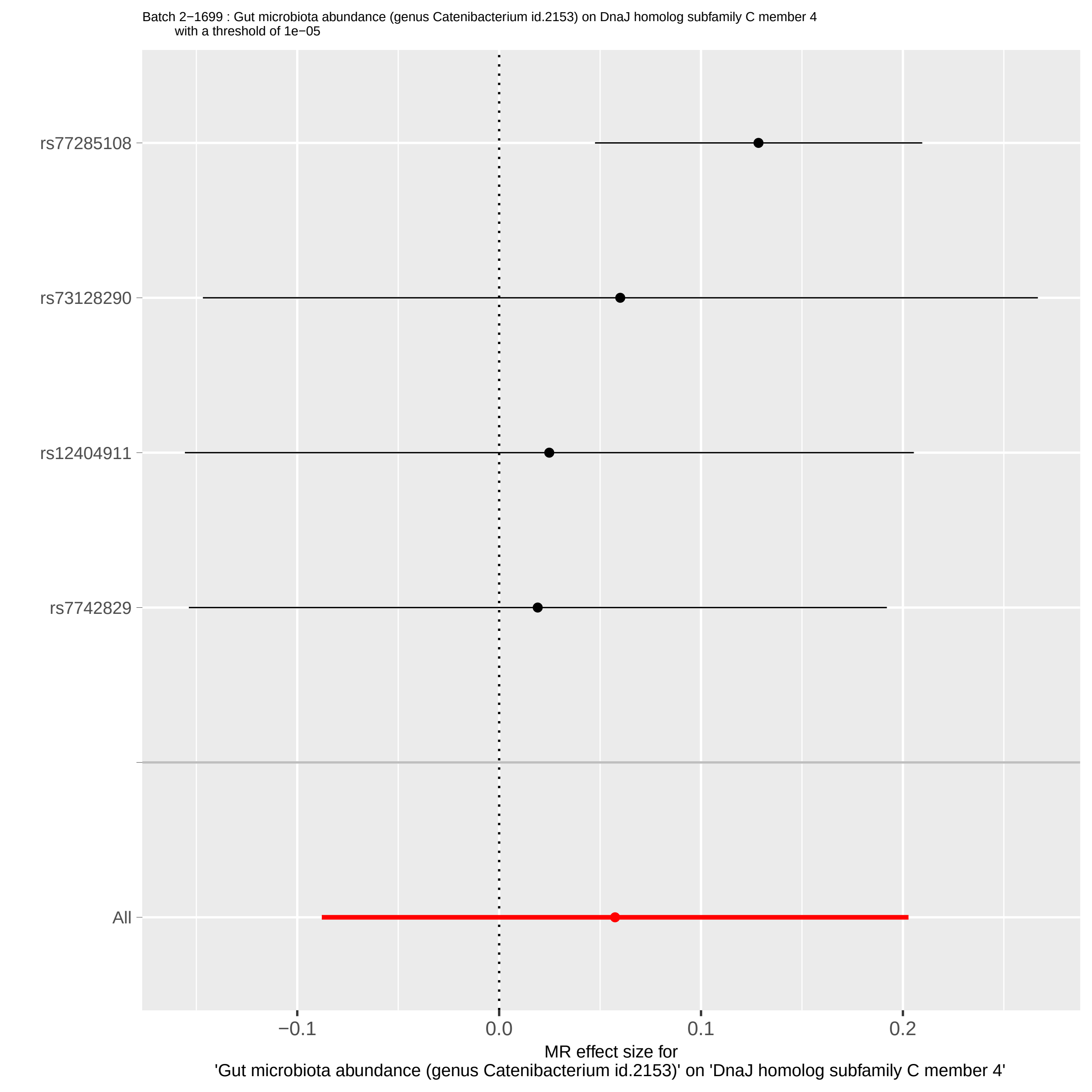
Batch 2−1699 : Gut microbiota abundance (genus Catenibacterium id.2153) on DnaJ homolog subfamily C member 4
with a threshold of 1e−05
rs77285108
rs73128290
rs12404911
rs7742829
All
−0.1
0.0
0.1
0.2
MR effect size for
'Gut microbiota abundance (genus Catenibacterium id.2153)' on 'DnaJ homolog subfamily C member 4'

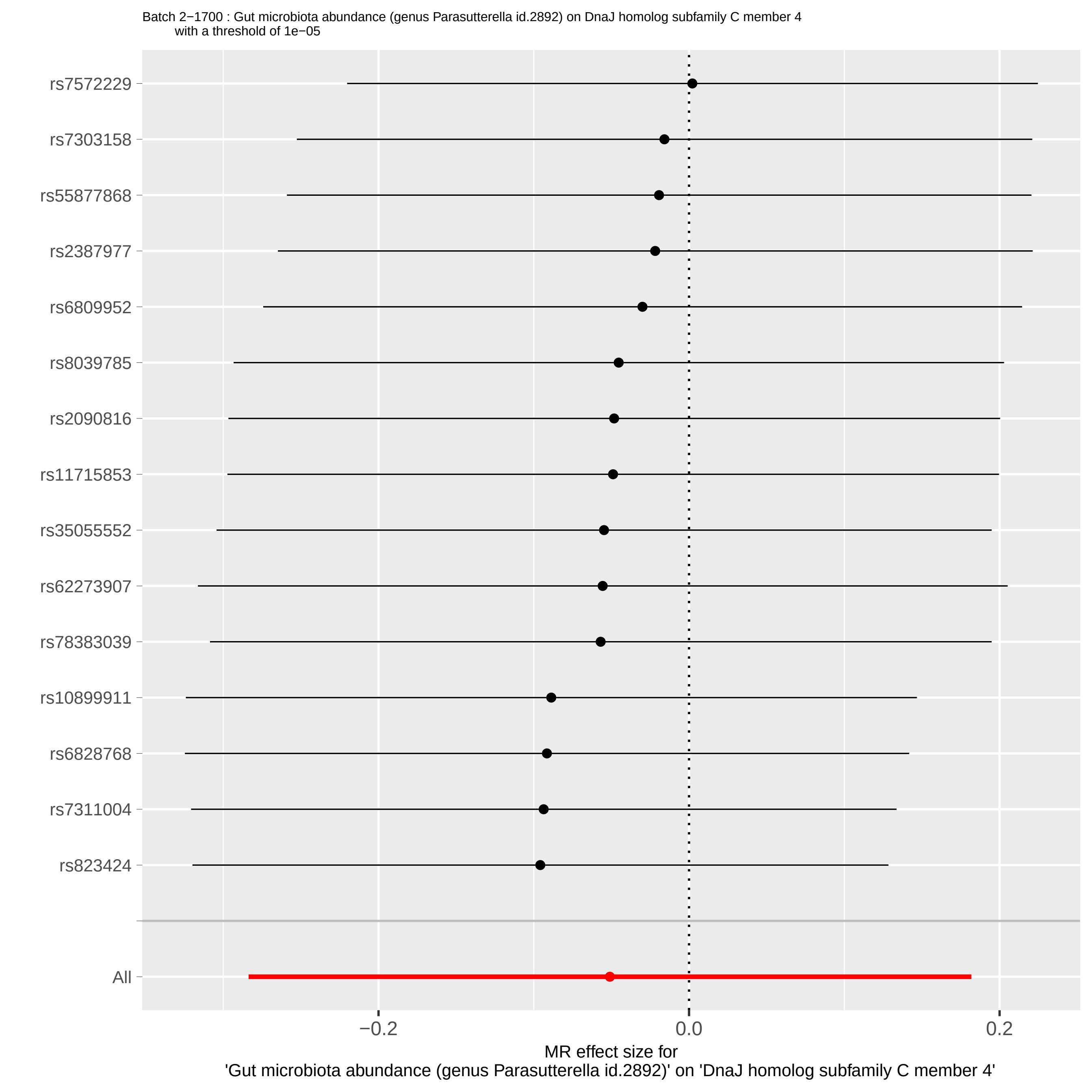
Batch 2−1700 : Gut microbiota abundance (genus Parasutterella id.2892) on DnaJ homolog subfamily C member 4
with a threshold of 1e−05
rs7572229
rs7303158
rs55877868
rs2387977
rs6809952
rs8039785
rs2090816
rs11715853
rs35055552
rs62273907
rs78383039
rs10899911
rs6828768
rs7311004
rs823424
All
−0.2
0.0
0.2
MR effect size for
'Gut microbiota abundance (genus Parasutterella id.2892)' on 'DnaJ homolog subfamily C member 4'

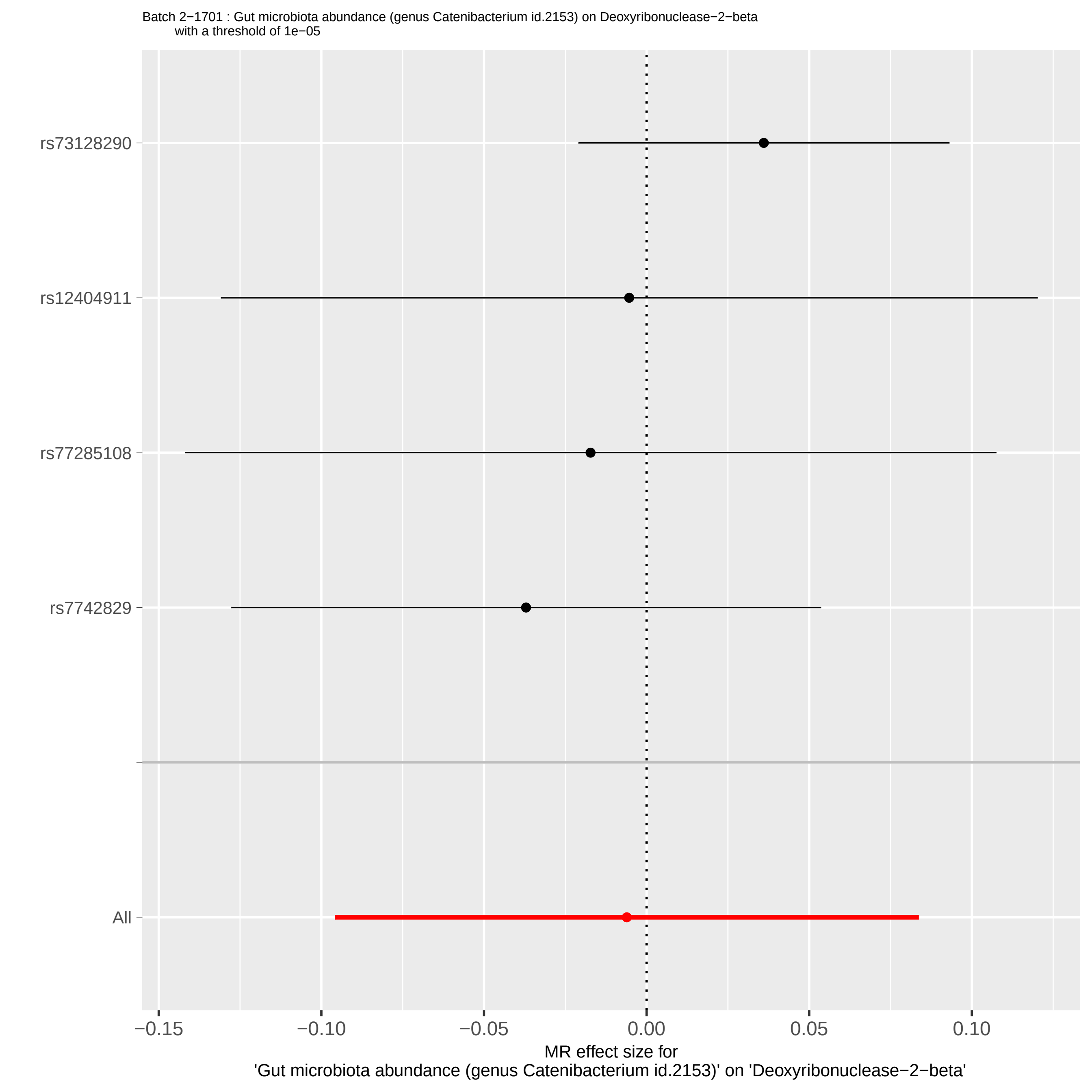
Batch 2−1701 : Gut microbiota abundance (genus Catenibacterium id.2153) on Deoxyribonuclease−2−beta
with a threshold of 1e−05
rs73128290
rs12404911
rs77285108
rs7742829
All
−0.15
−0.10
−0.05
0.00
0.05
0.10
MR effect size for
'Gut microbiota abundance (genus Catenibacterium id.2153)' on 'Deoxyribonuclease−2−beta'

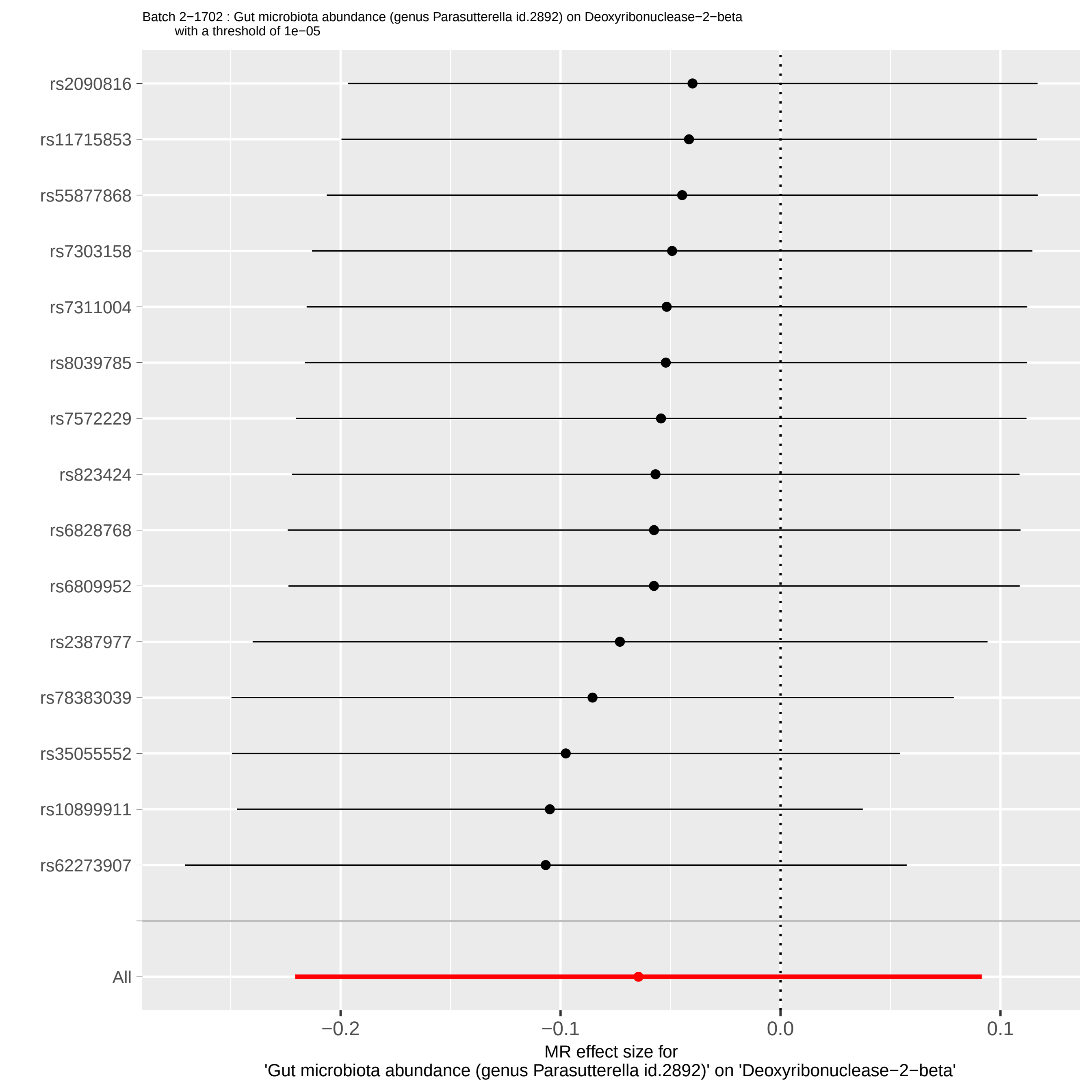

Batch 2−1702 : Gut microbiota abundance (genus Parasutterella id.2892) on Deoxyribonuclease−2−beta
with a threshold of 1e−05
rs2090816
rs11715853
rs55877868
rs7303158
rs7311004
rs8039785
rs7572229
rs823424
rs6828768
rs6809952
rs2387977
rs78383039
rs35055552
rs10899911
rs62273907
All
−0.2
−0.1
0.0
0.1
MR effect size for
'Gut microbiota abundance (genus Parasutterella id.2892)' on 'Deoxyribonuclease−2−beta'

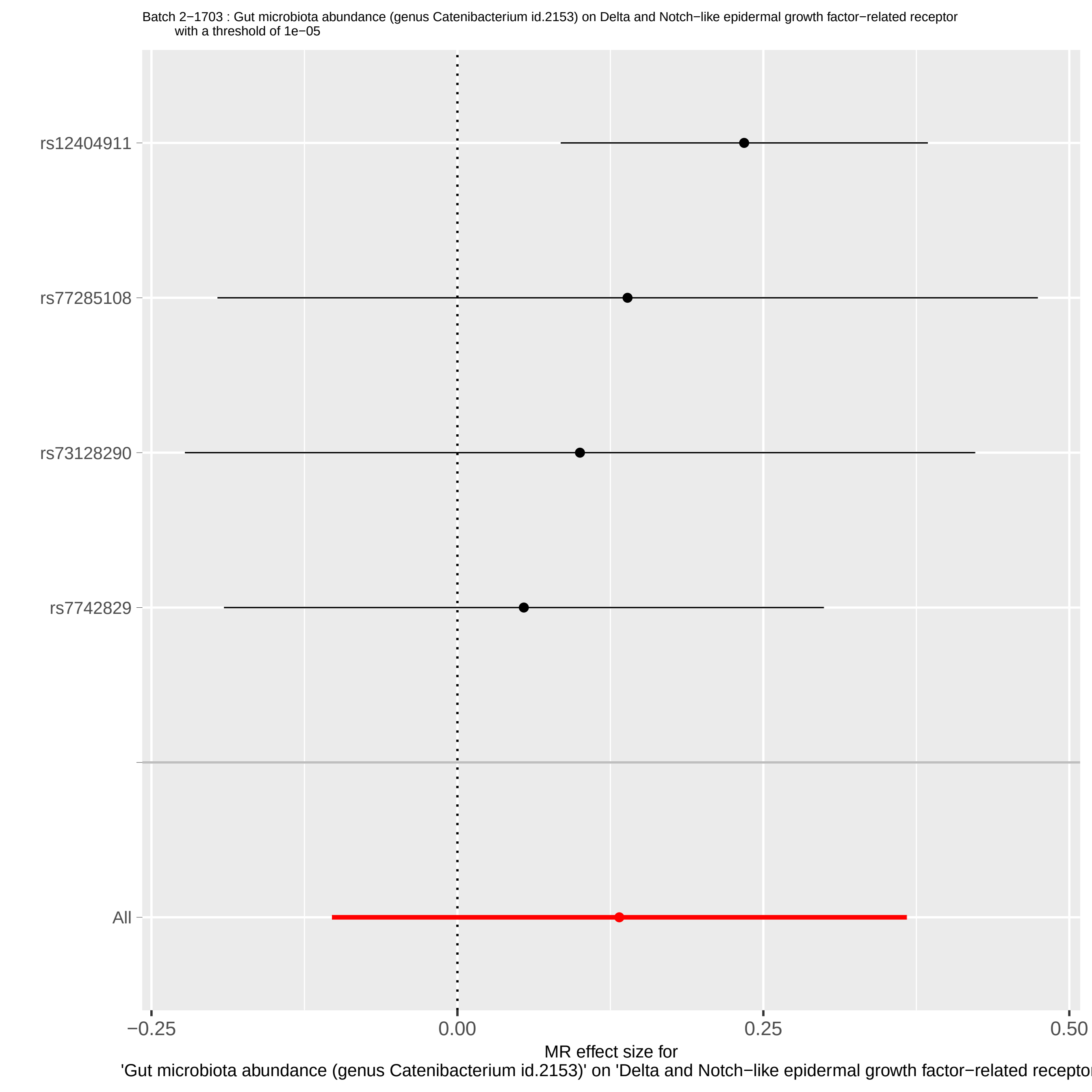
Batch 2−1703 : Gut microbiota abundance (genus Catenibacterium id.2153) on Delta and Notch−like epidermal growth factor−related receptor
with a threshold of 1e−05
rs12404911
rs77285108
rs73128290
rs7742829
All
−0.25
0.00
0.25
0.50
MR effect size for
'Gut microbiota abundance (genus Catenibacterium id.2153)' on 'Delta and Notch−like epidermal growth factor−related receptor'

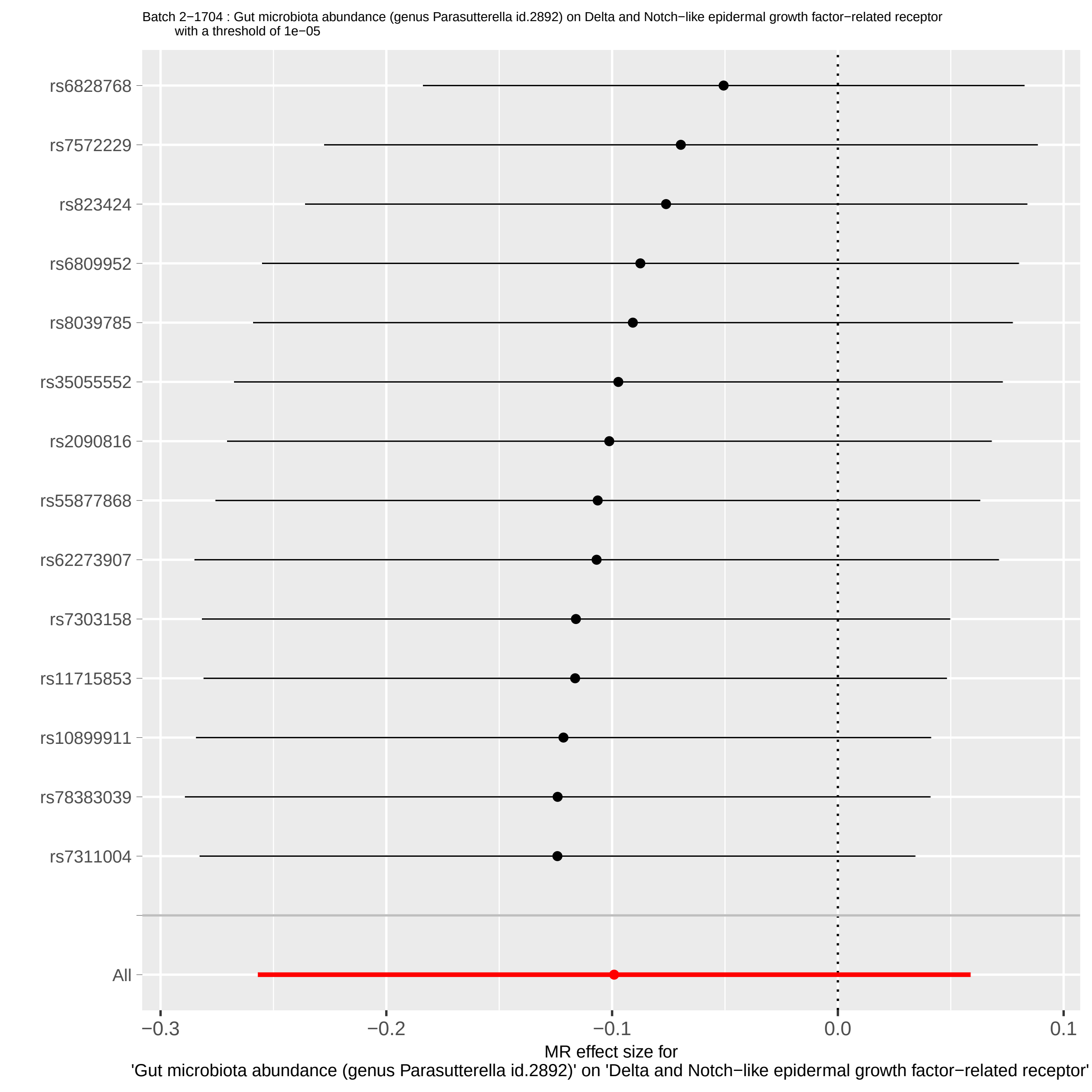
Batch 2−1704 : Gut microbiota abundance (genus Parasutterella id.2892) on Delta and Notch−like epidermal growth factor−related receptor
with a threshold of 1e−05
rs6828768
rs7572229
rs823424
rs6809952
rs8039785
rs35055552
rs2090816
rs55877868
rs62273907
rs7303158
rs11715853
rs10899911
rs78383039
rs7311004
All
−0.3
−0.2
−0.1
0.0
0.1
MR effect size for
'Gut microbiota abundance (genus Parasutterella id.2892)' on 'Delta and Notch−like epidermal growth factor−related receptor'

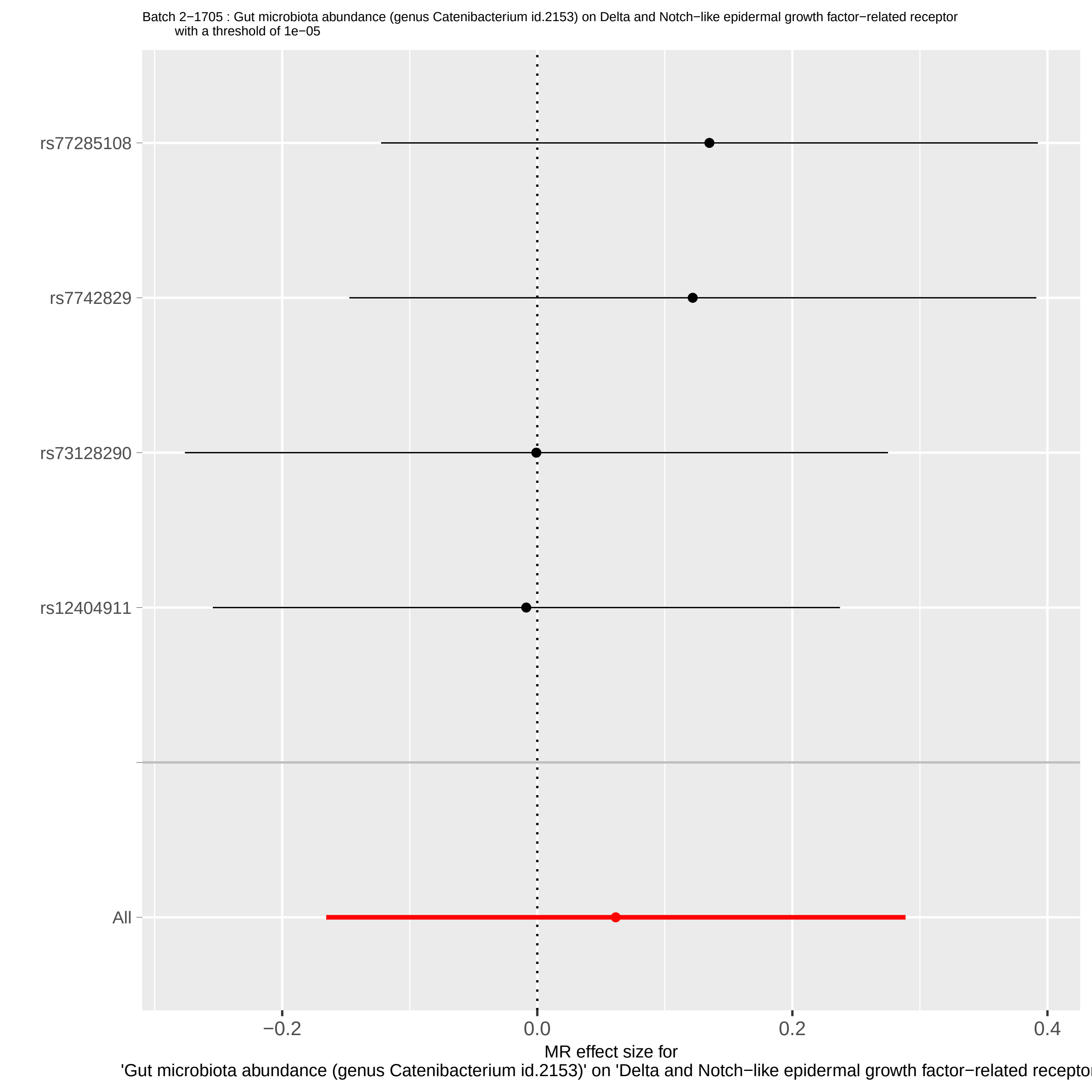
Batch 2−1705 : Gut microbiota abundance (genus Catenibacterium id.2153) on Delta and Notch−like epidermal growth factor−related receptor
with a threshold of 1e−05
rs77285108
rs7742829
rs73128290
rs12404911
All
−0.2
0.0
0.2
0.4
MR effect size for
'Gut microbiota abundance (genus Catenibacterium id.2153)' on 'Delta and Notch−like epidermal growth factor−related recepto

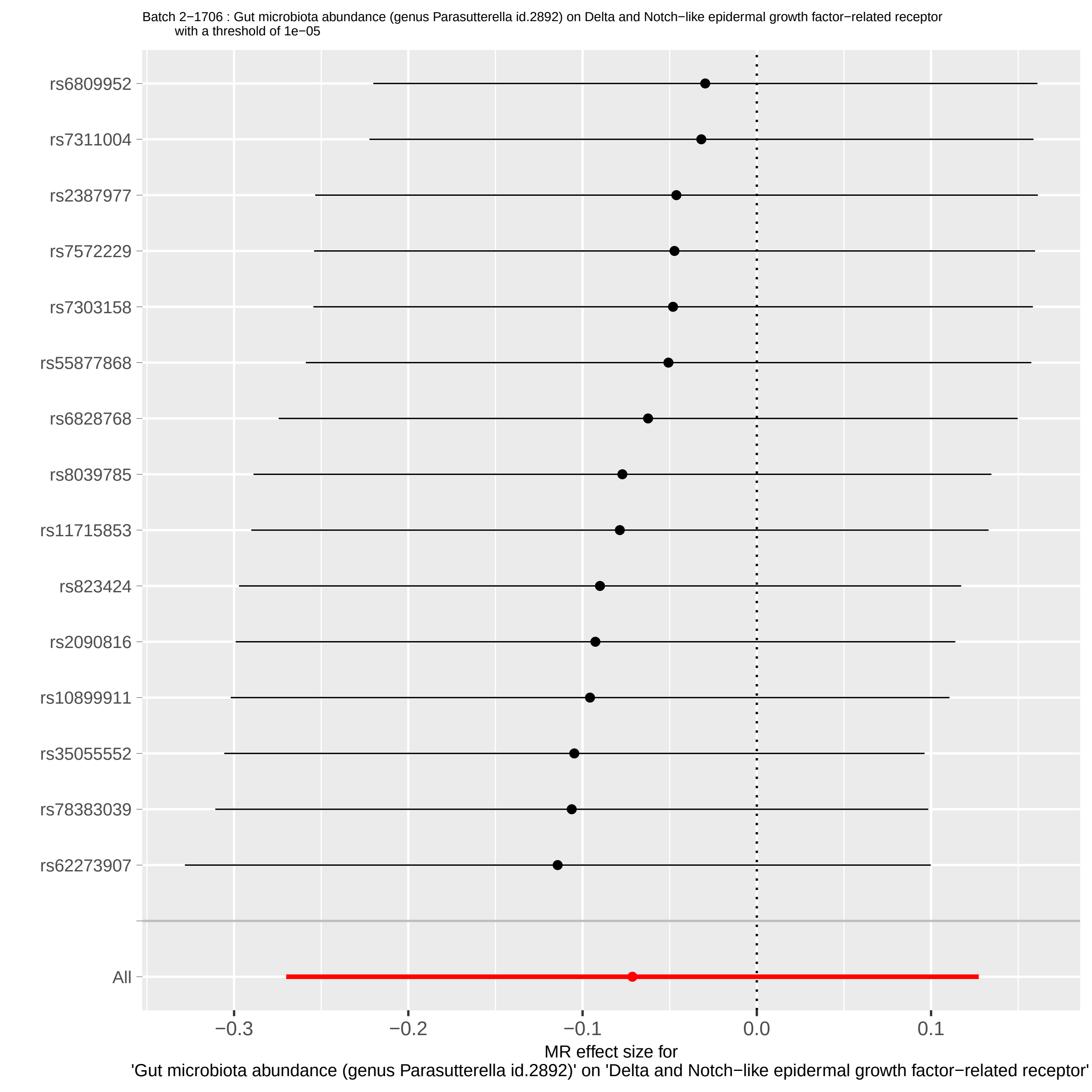

Batch 2−1706 : Gut microbiota abundance (genus Parasutterella id.2892) on Delta and Notch−like epidermal growth factor−related receptor
with a threshold of 1e−05
rs6809952
rs7311004
rs2387977
rs7572229
rs7303158
rs55877868
rs6828768
rs8039785
rs11715853
rs823424
rs2090816
rs10899911
rs35055552
rs78383039
rs62273907
All
−0.3
−0.2
−0.1
0.0
0.1
MR effect size for
'Gut microbiota abundance (genus Parasutterella id.2892)' on 'Delta and Notch−like epidermal growth factor−related receptor'

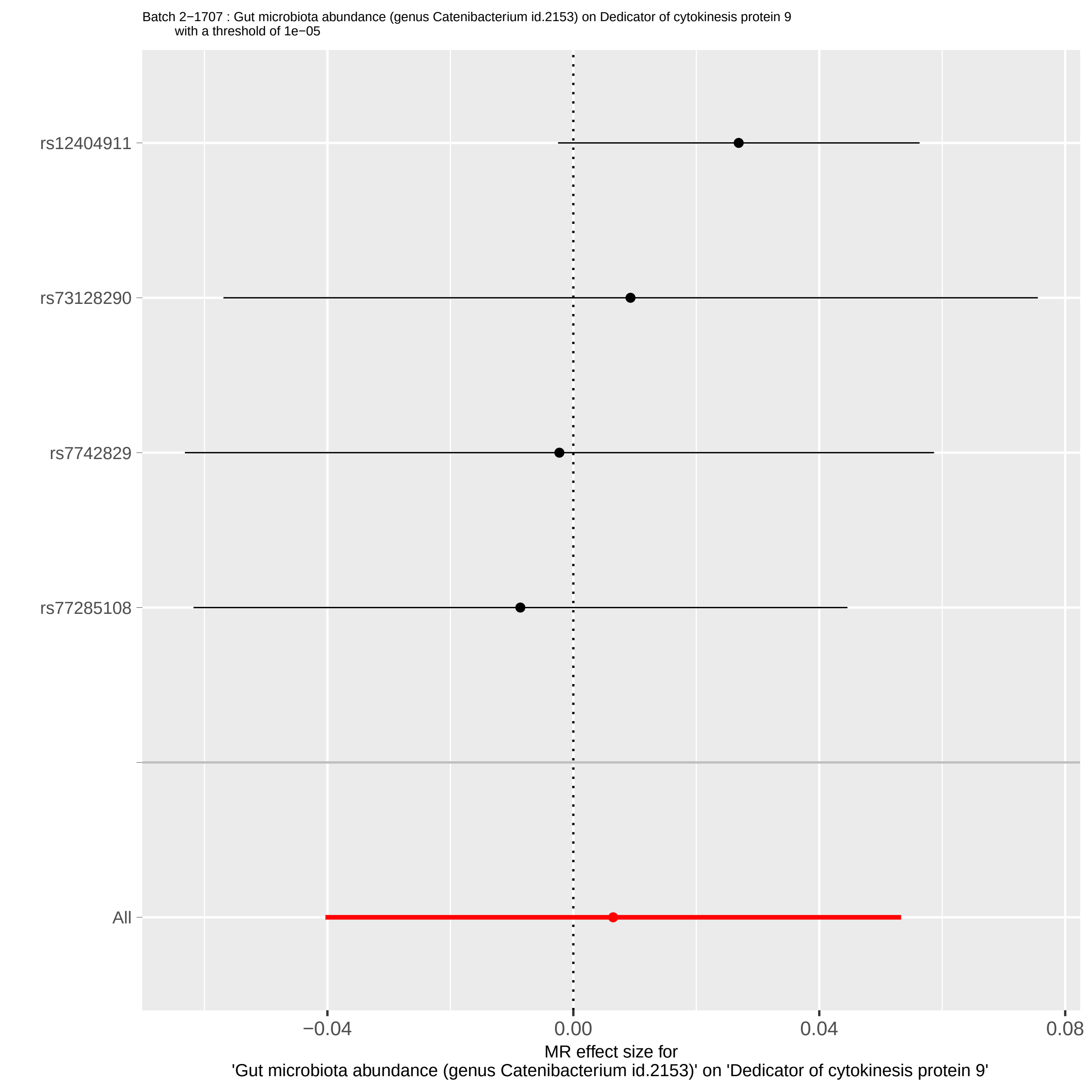

Batch 2−1707 : Gut microbiota abundance (genus Catenibacterium id.2153) on Dedicator of cytokinesis protein 9
with a threshold of 1e−05
rs12404911
rs73128290
rs7742829
rs77285108
All
−0.04
0.00
0.04
0.08
MR effect size for
'Gut microbiota abundance (genus Catenibacterium id.2153)' on 'Dedicator of cytokinesis protein 9'

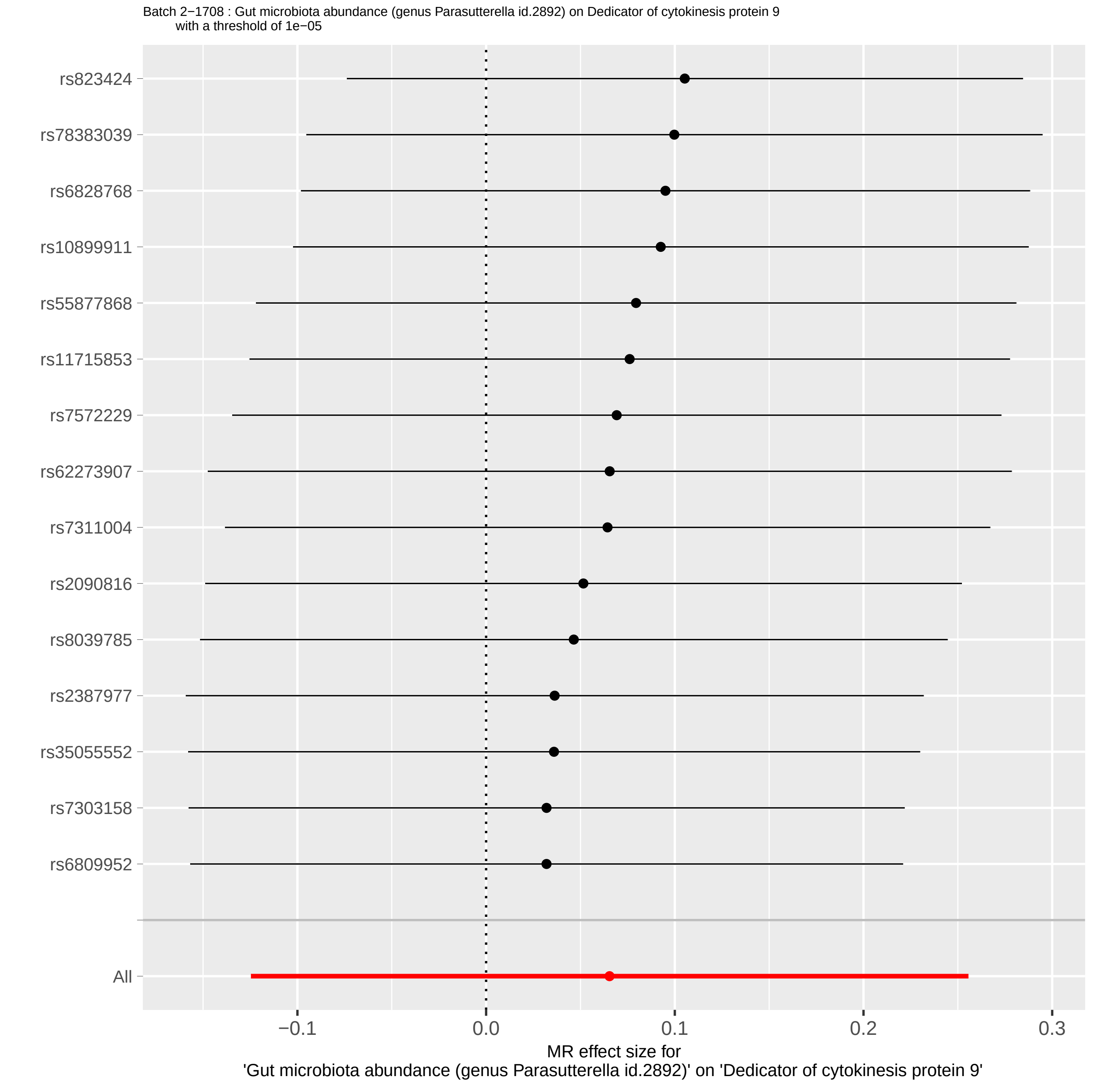

Batch 2−1708 : Gut microbiota abundance (genus Parasutterella id.2892) on Dedicator of cytokinesis protein 9
with a threshold of 1e−05
rs823424
rs78383039
rs6828768
rs10899911
rs55877868
rs11715853
rs7572229
rs62273907
rs7311004
rs2090816
rs8039785
rs2387977
rs35055552
rs7303158
rs6809952
All
−0.1
0.0
0.1
0.2
0.3
MR effect size for
'Gut microbiota abundance (genus Parasutterella id.2892)' on 'Dedicator of cytokinesis protein 9'

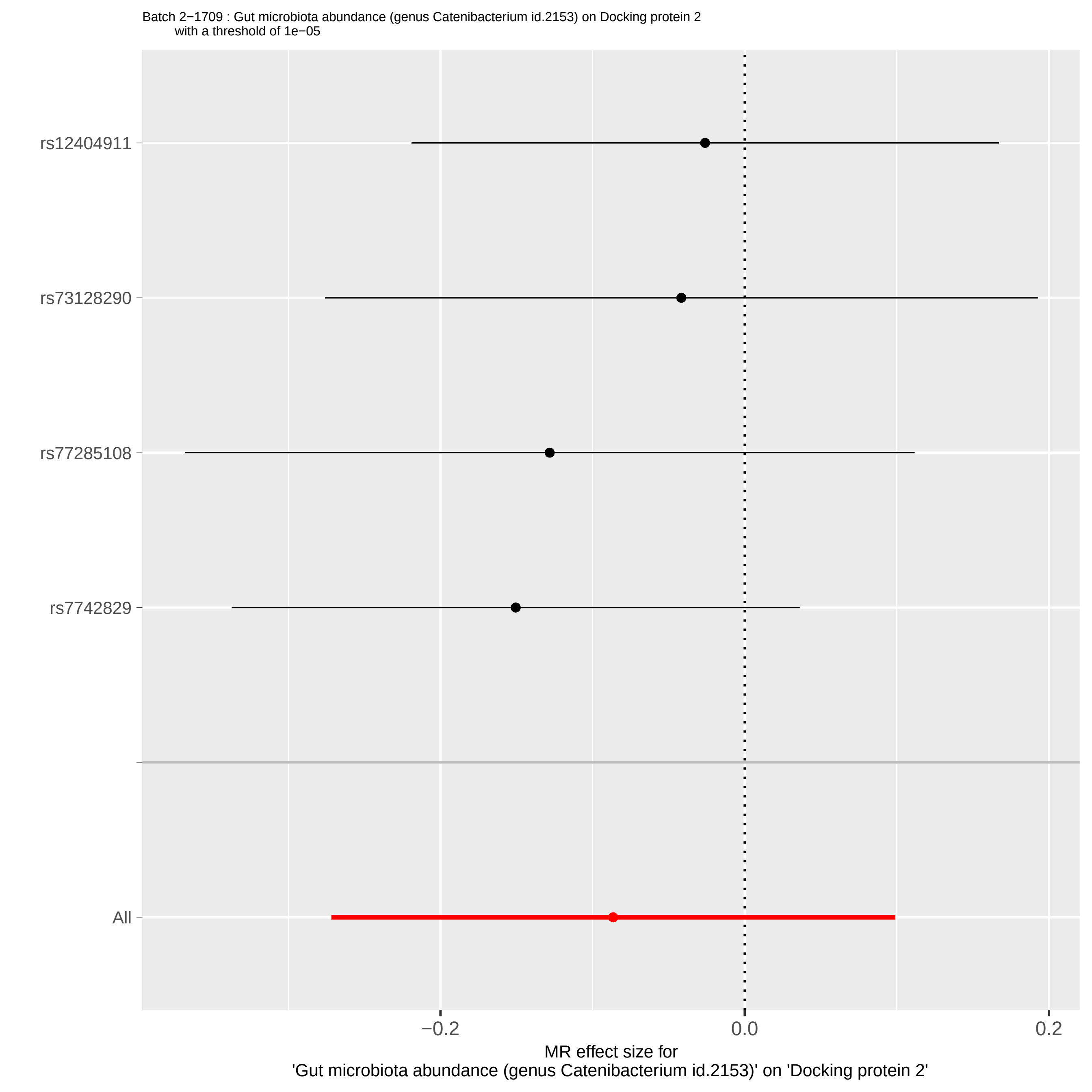
Batch 2−1709 : Gut microbiota abundance (genus Catenibacterium id.2153) on Docking protein 2
with a threshold of 1e−05
rs12404911
rs73128290
rs77285108
rs7742829
All
−0.2
0.0
0.2
MR effect size for
'Gut microbiota abundance (genus Catenibacterium id.2153)' on 'Docking protein 2'

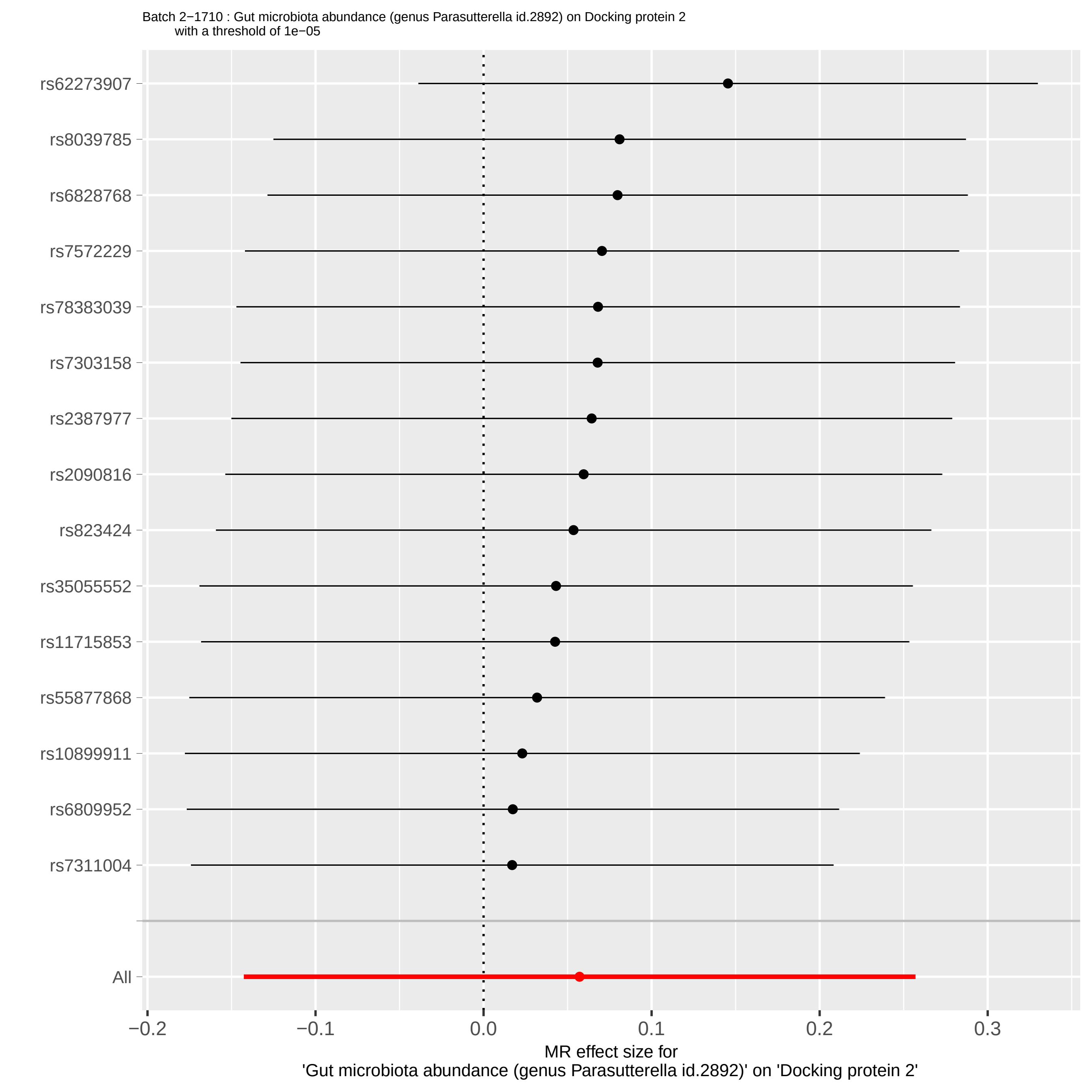

Batch 2−1710 : Gut microbiota abundance (genus Parasutterella id.2892) on Docking protein 2
with a threshold of 1e−05
rs62273907
rs8039785
rs6828768
rs7572229
rs78383039
rs7303158
rs2387977
rs2090816
rs823424
rs35055552
rs11715853
rs55877868
rs10899911
rs6809952
rs7311004
All
−0.2
−0.1
0.0
0.1
0.2
0.3
MR effect size for
'Gut microbiota abundance (genus Parasutterella id.2892)' on 'Docking protein 2'

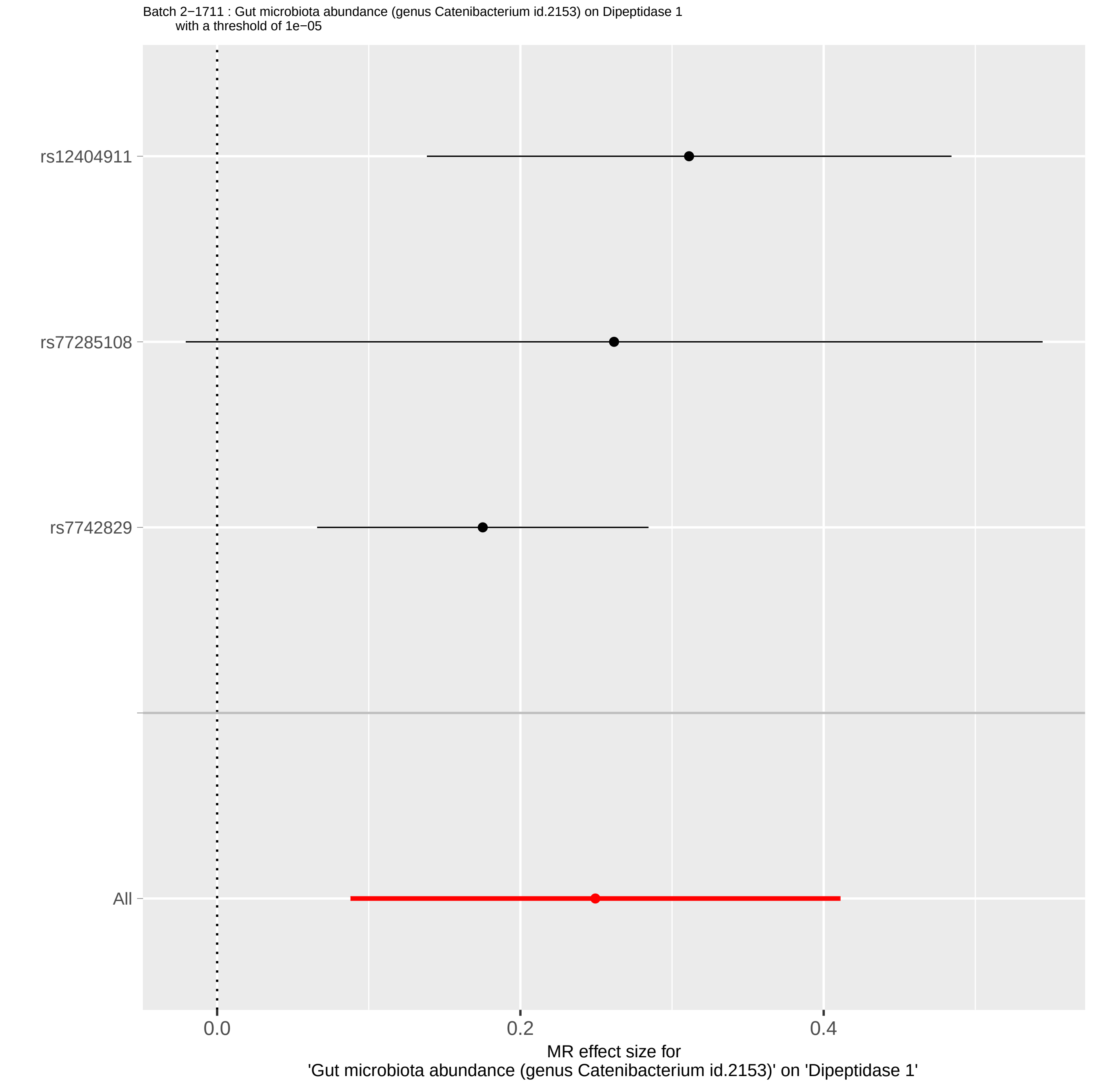
Batch 2−1711 : Gut microbiota abundance (genus Catenibacterium id.2153) on Dipeptidase 1
with a threshold of 1e−05
rs12404911
rs77285108
rs7742829
All
0.0
0.2
0.4
MR effect size for
'Gut microbiota abundance (genus Catenibacterium id.2153)' on 'Dipeptidase 1'

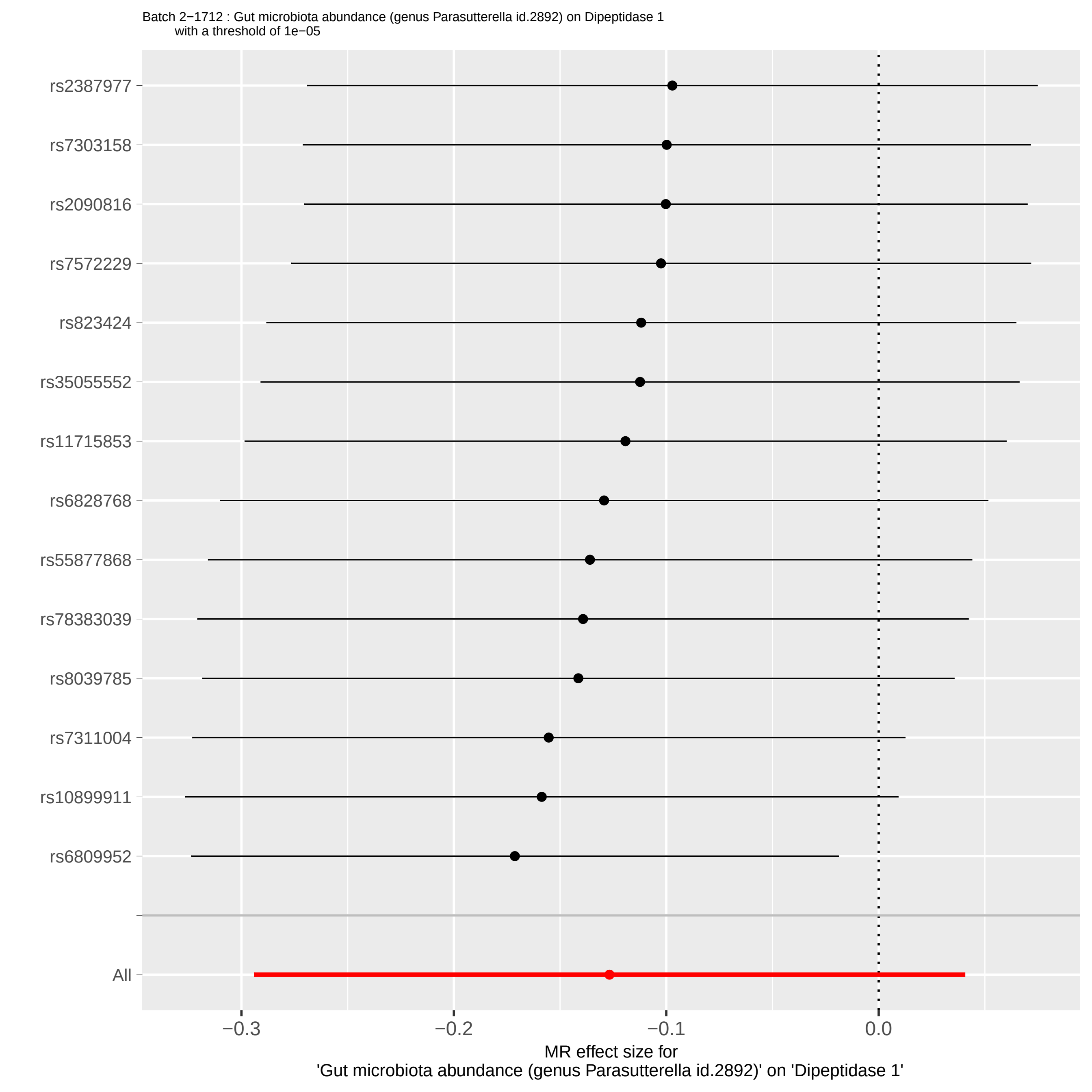
Batch 2−1712 : Gut microbiota abundance (genus Parasutterella id.2892) on Dipeptidase 1
with a threshold of 1e−05
rs2387977
rs7303158
rs2090816
rs7572229
rs823424
rs35055552
rs11715853
rs6828768
rs55877868
rs78383039
rs8039785
rs7311004
rs10899911
rs6809952
All
−0.3
−0.2
−0.1
0.0
MR effect size for
'Gut microbiota abundance (genus Parasutterella id.2892)' on 'Dipeptidase 1'

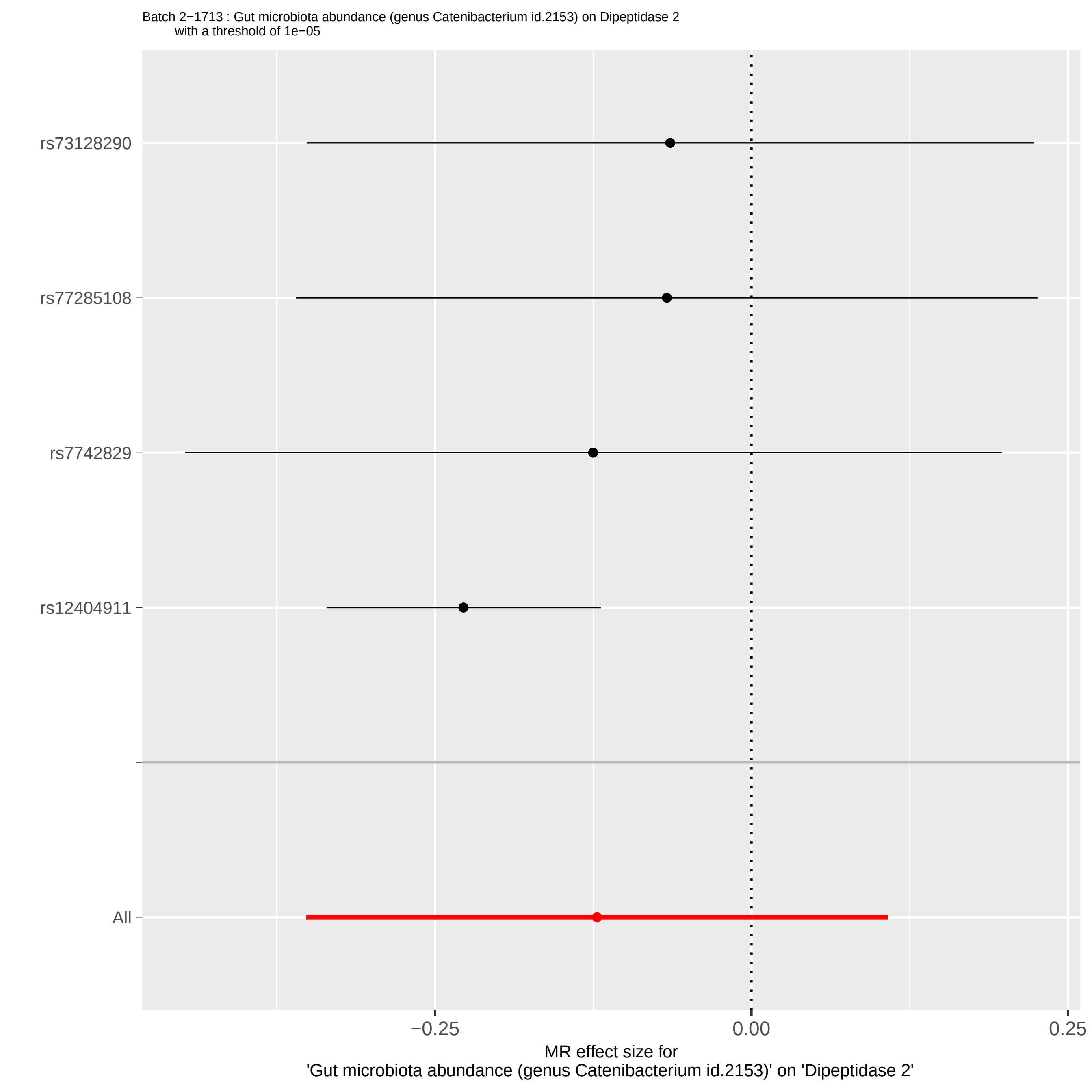

Batch 2−1713 : Gut microbiota abundance (genus Catenibacterium id.2153) on Dipeptidase 2
with a threshold of 1e−05
rs73128290
rs77285108
rs7742829
rs12404911
All
−0.25
0.00
0.25
MR effect size for
'Gut microbiota abundance (genus Catenibacterium id.2153)' on 'Dipeptidase 2'

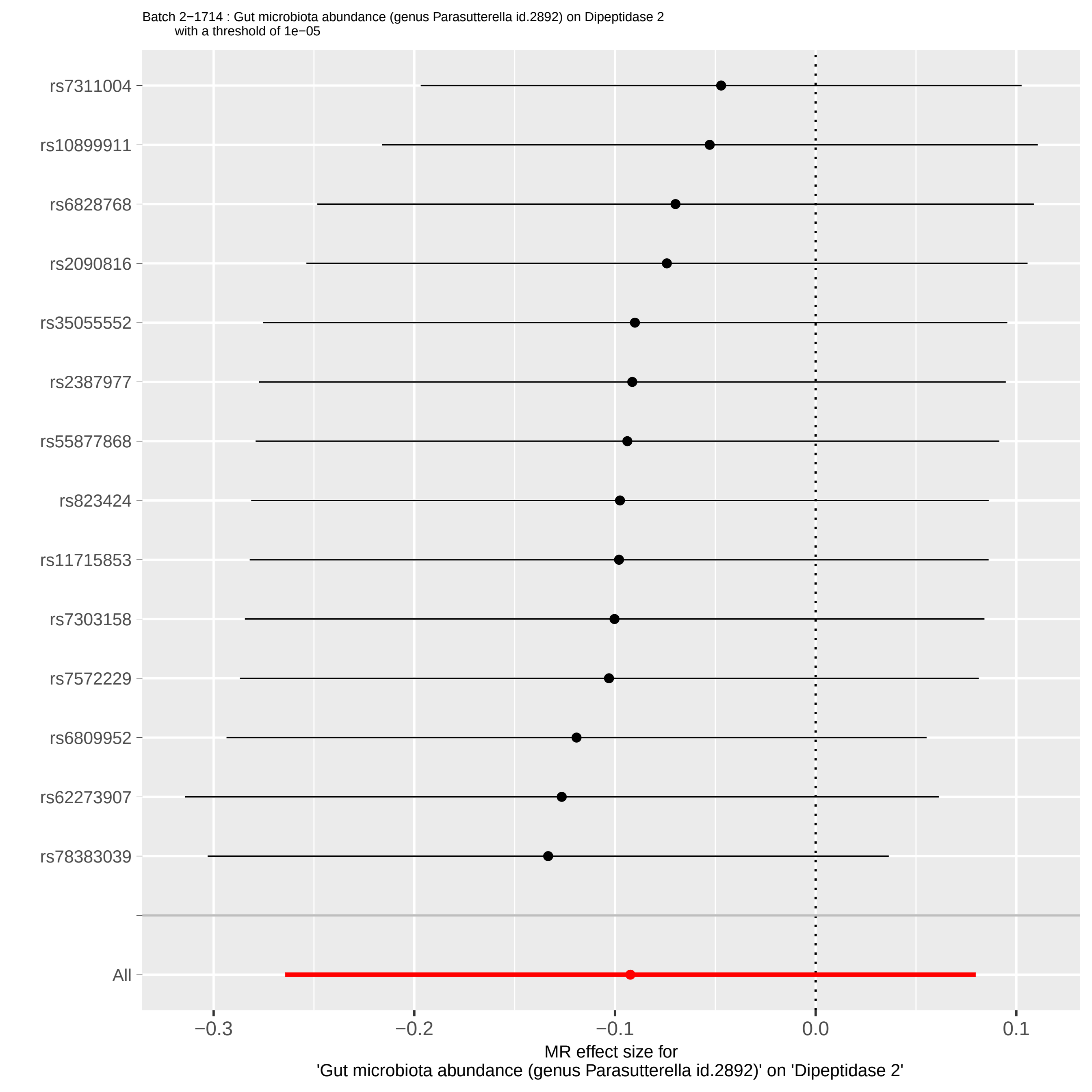

Batch 2−1714 : Gut microbiota abundance (genus Parasutterella id.2892) on Dipeptidase 2
with a threshold of 1e−05
rs7311004
rs10899911
rs6828768
rs2090816
rs35055552
rs2387977
rs55877868
rs823424
rs11715853
rs7303158
rs7572229
rs6809952
rs62273907
rs78383039
All
−0.3
−0.2
−0.1
0.0
0.1
MR effect size for
'Gut microbiota abundance (genus Parasutterella id.2892)' on 'Dipeptidase 2'

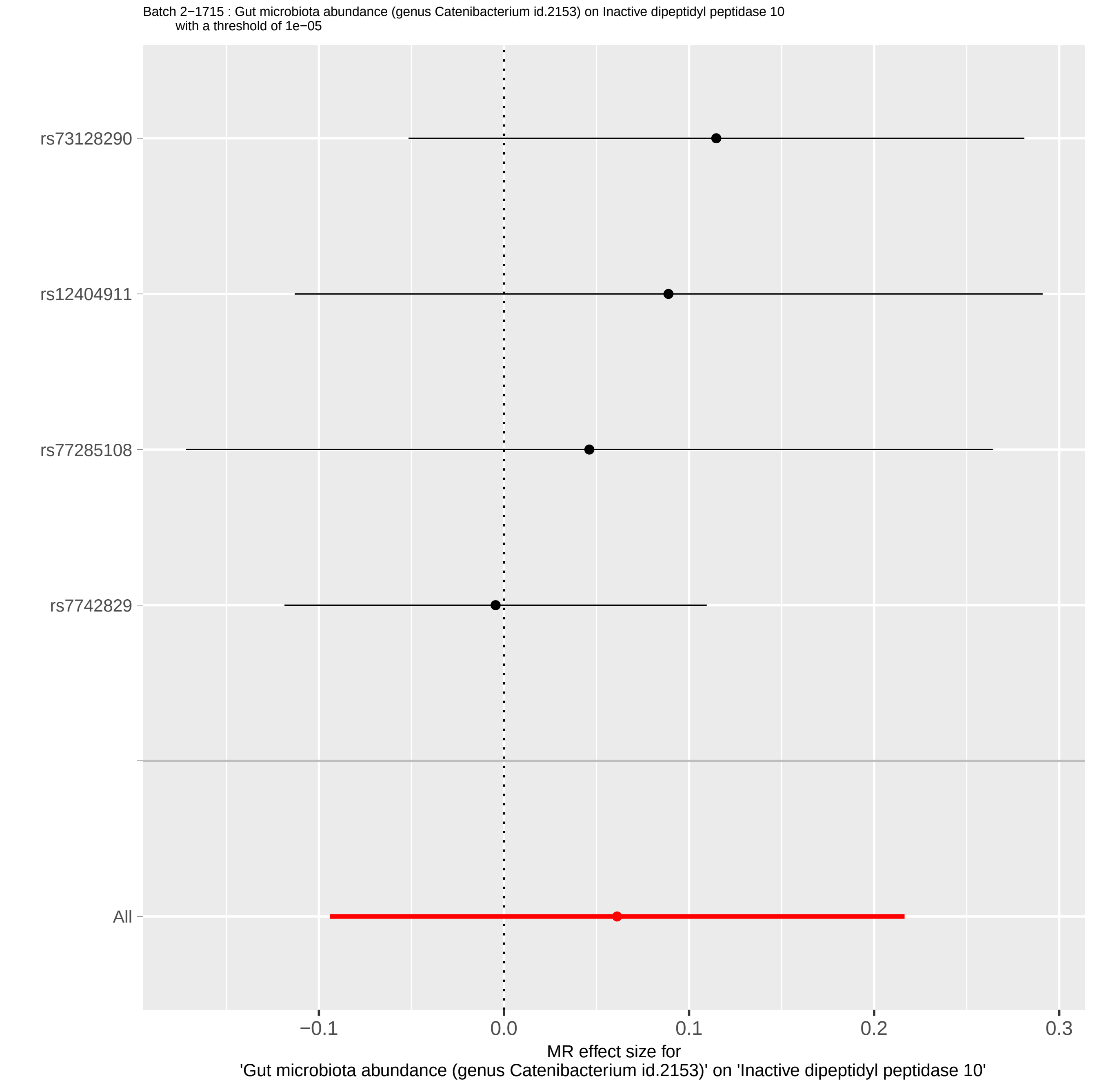

Batch 2−1715 : Gut microbiota abundance (genus Catenibacterium id.2153) on Inactive dipeptidyl peptidase 10
with a threshold of 1e−05
rs73128290
rs12404911
rs77285108
rs7742829
All
−0.1
0.0
0.1
0.2
0.3
MR effect size for
'Gut microbiota abundance (genus Catenibacterium id.2153)' on 'Inactive dipeptidyl peptidase 10'

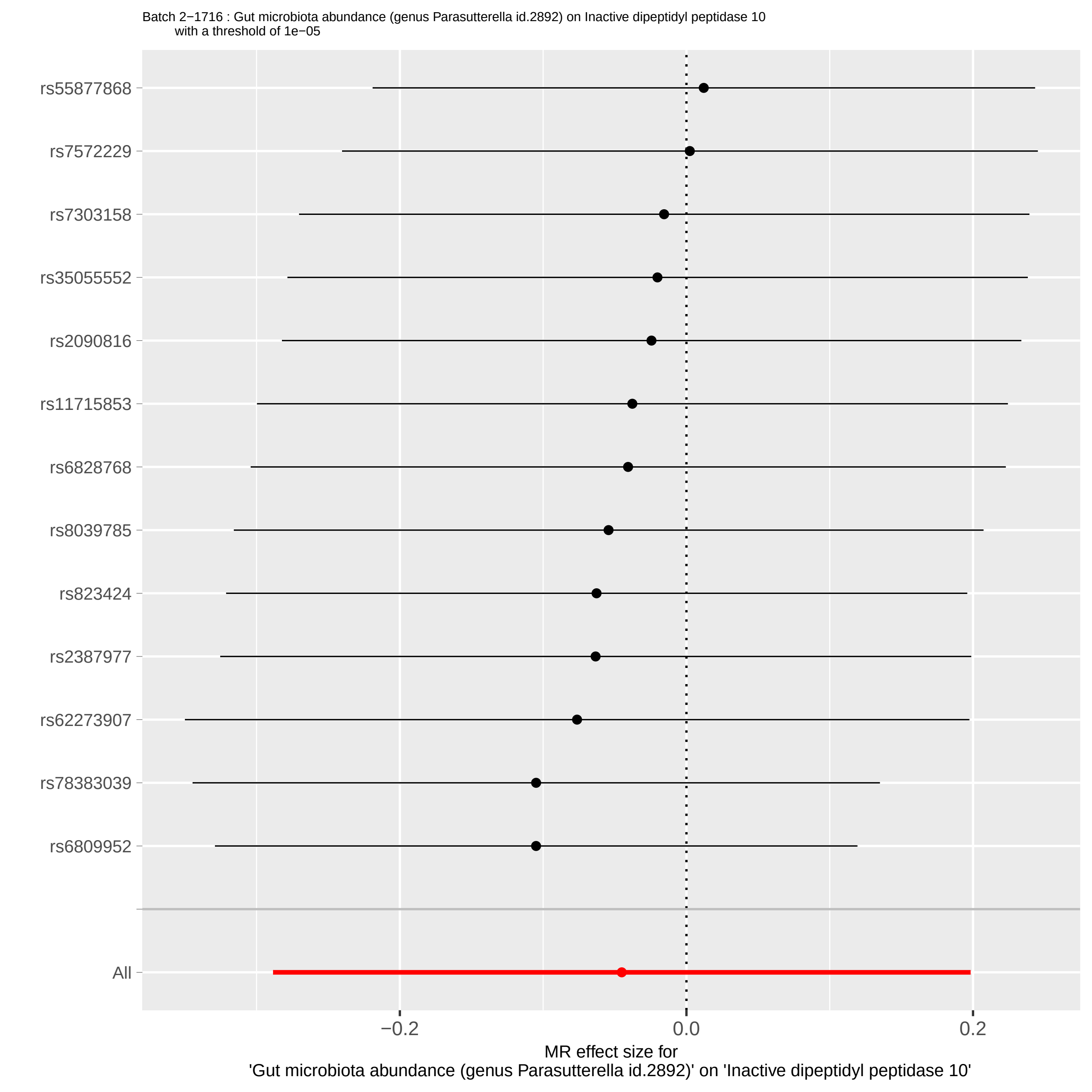
Batch 2−1716 : Gut microbiota abundance (genus Parasutterella id.2892) on Inactive dipeptidyl peptidase 10
with a threshold of 1e−05
rs55877868
rs7572229
rs7303158
rs35055552
rs2090816
rs11715853
rs6828768
rs8039785
rs823424
rs2387977
rs62273907
rs78383039
rs6809952
All
−0.2
0.0
0.2
MR effect size for
'Gut microbiota abundance (genus Parasutterella id.2892)' on 'Inactive dipeptidyl peptidase 10'

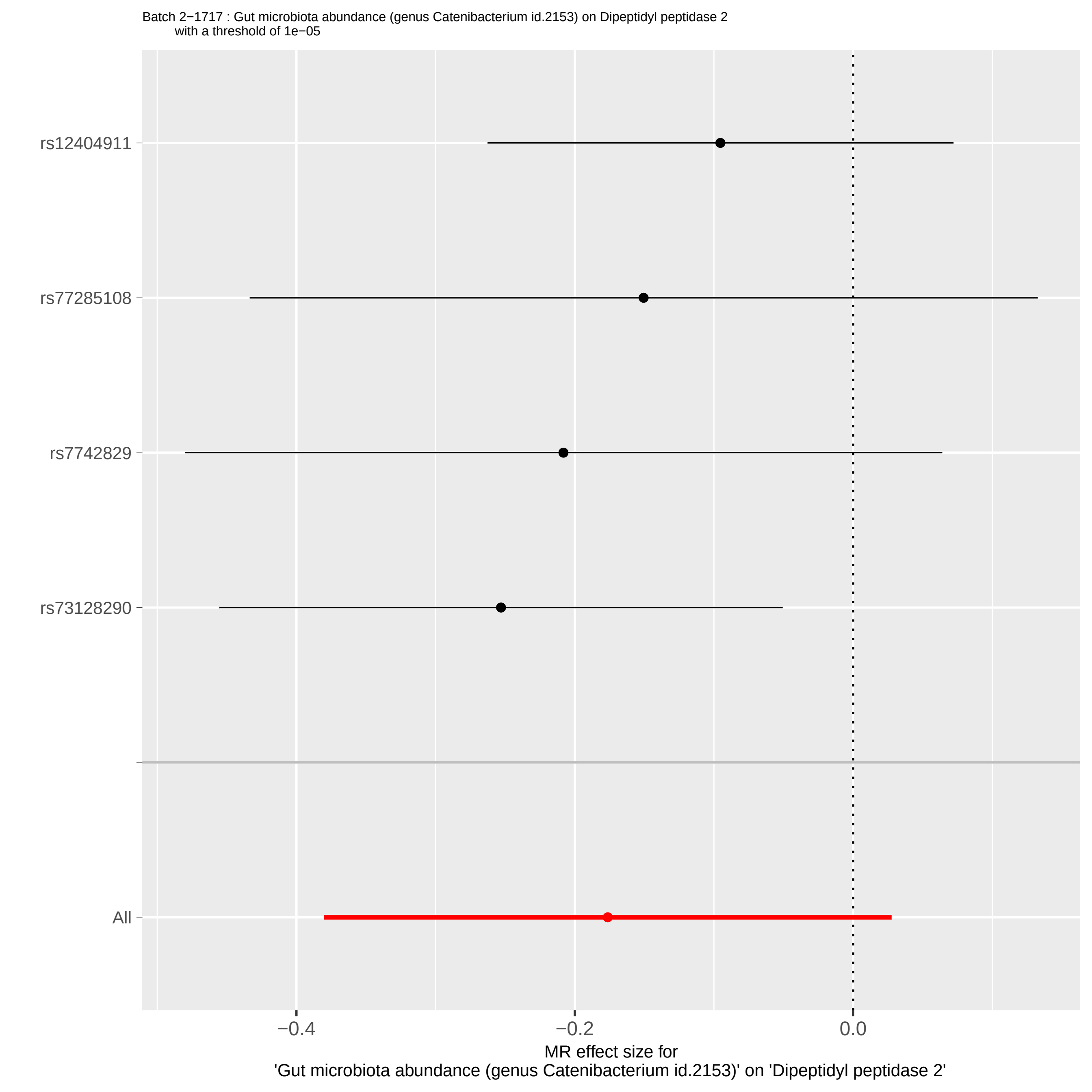

Batch 2−1717 : Gut microbiota abundance (genus Catenibacterium id.2153) on Dipeptidyl peptidase 2
with a threshold of 1e−05
rs12404911
rs77285108
rs7742829
rs73128290
All
−0.4
−0.2
0.0
MR effect size for
'Gut microbiota abundance (genus Catenibacterium id.2153)' on 'Dipeptidyl peptidase 2'

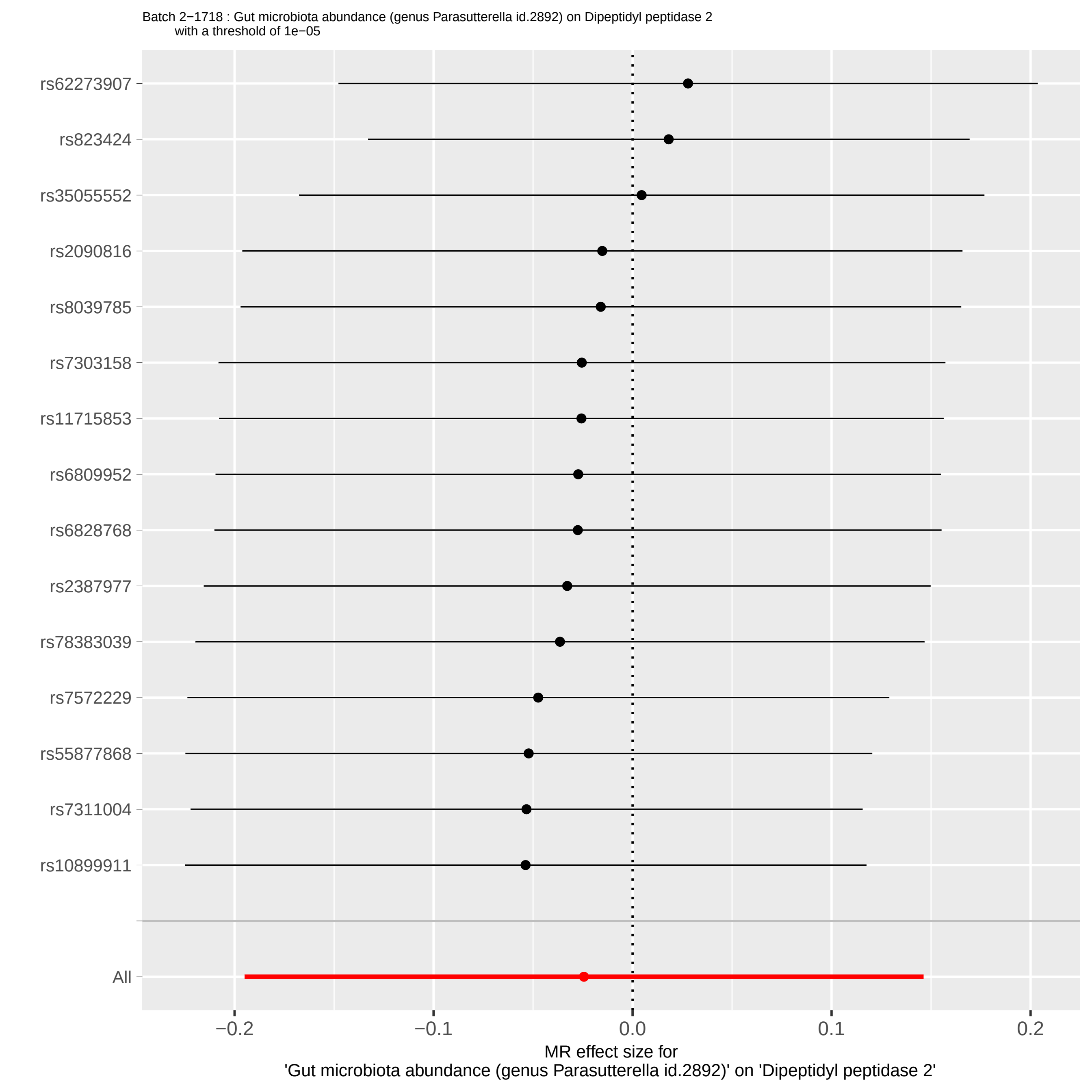
Batch 2−1718 : Gut microbiota abundance (genus Parasutterella id.2892) on Dipeptidyl peptidase 2
with a threshold of 1e−05
rs62273907
rs823424
rs35055552
rs2090816
rs8039785
rs7303158
rs11715853
rs6809952
rs6828768
rs2387977
rs78383039
rs7572229
rs55877868
rs7311004
rs10899911
All
−0.2
−0.1
0.0
0.1
0.2
MR effect size for
'Gut microbiota abundance (genus Parasutterella id.2892)' on 'Dipeptidyl peptidase 2'

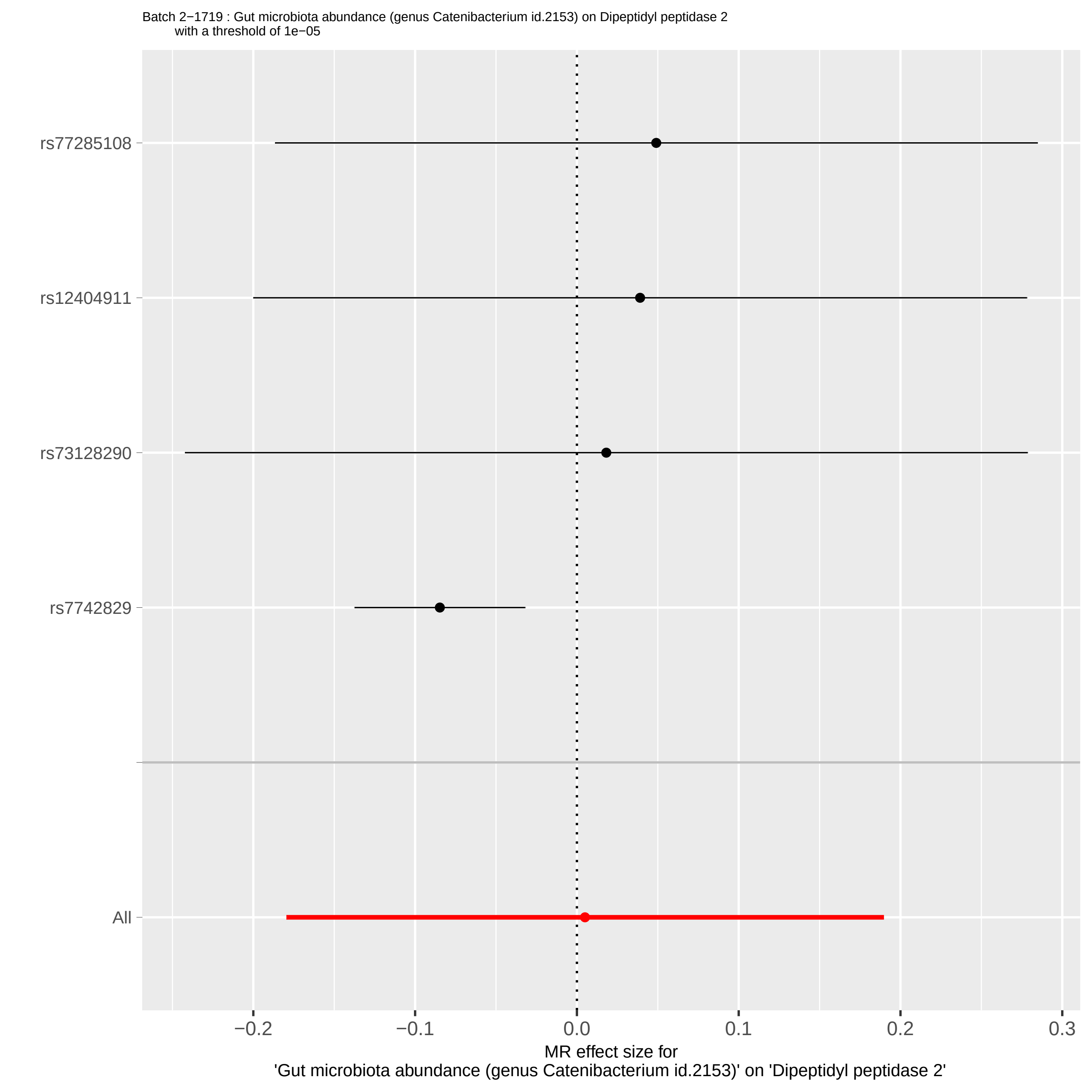
Batch 2−1719 : Gut microbiota abundance (genus Catenibacterium id.2153) on Dipeptidyl peptidase 2
with a threshold of 1e−05
rs77285108
rs12404911
rs73128290
rs7742829
All
−0.2
−0.1
0.0
0.1
0.2
0.3
MR effect size for
'Gut microbiota abundance (genus Catenibacterium id.2153)' on 'Dipeptidyl peptidase 2'

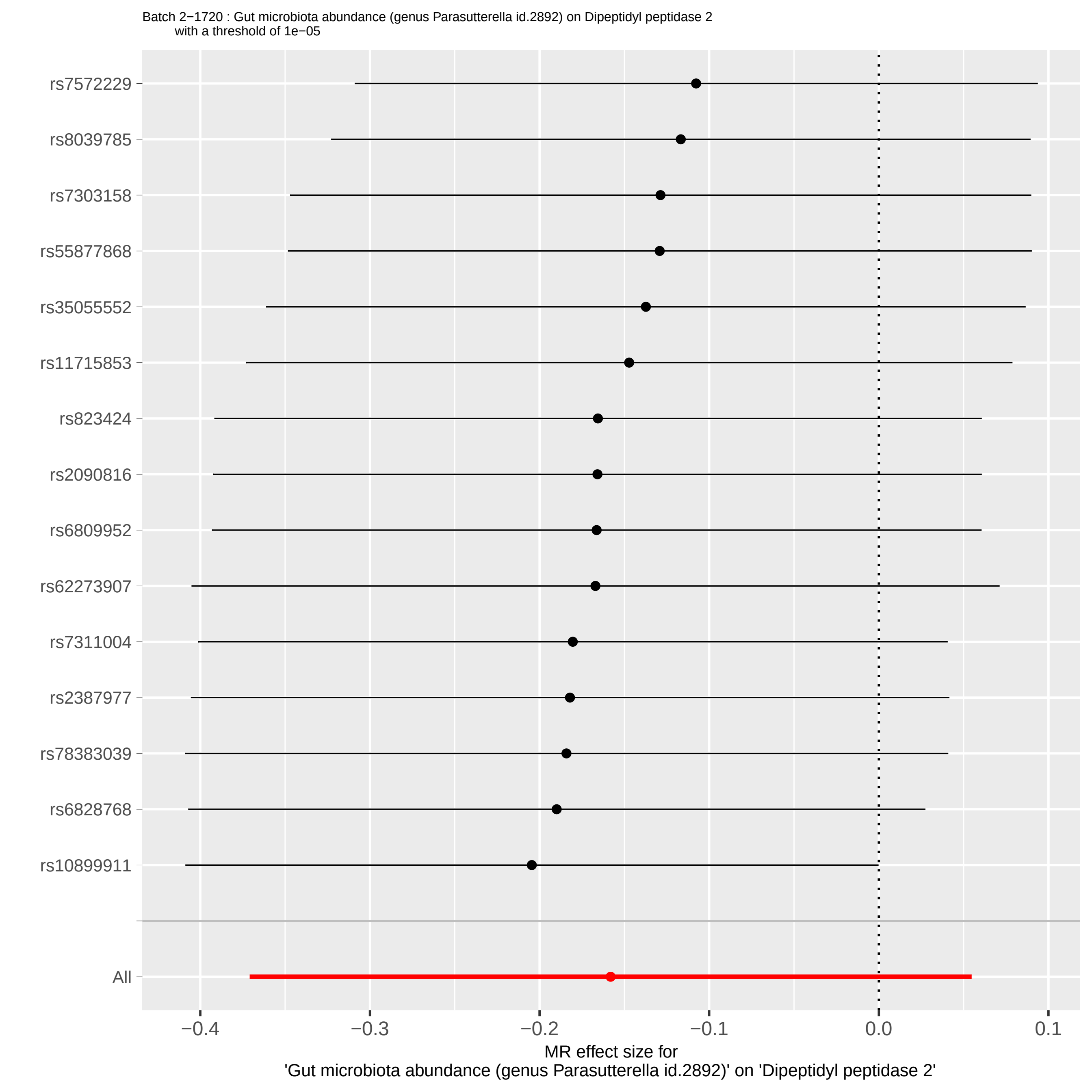
Batch 2−1720 : Gut microbiota abundance (genus Parasutterella id.2892) on Dipeptidyl peptidase 2
with a threshold of 1e−05
rs7572229
rs8039785
rs7303158
rs55877868
rs35055552
rs11715853
rs823424
rs2090816
rs6809952
rs62273907
rs7311004
rs2387977
rs78383039
rs6828768
rs10899911
All
−0.4
−0.3
−0.2
−0.1
0.0
0.1
MR effect size for
'Gut microbiota abundance (genus Parasutterella id.2892)' on 'Dipeptidyl peptidase 2'

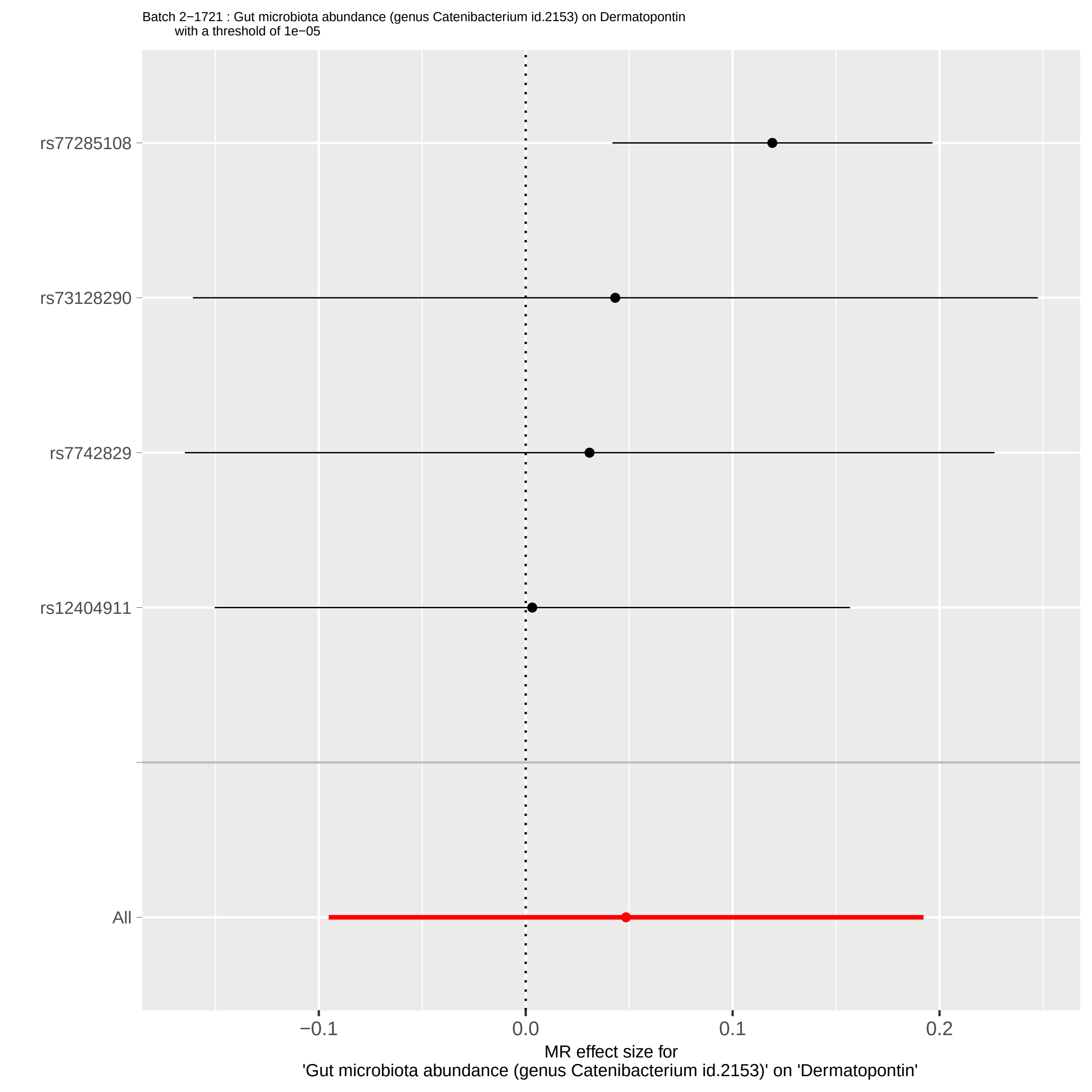

Batch 2−1721 : Gut microbiota abundance (genus Catenibacterium id.2153) on Dermatopontin
with a threshold of 1e−05
rs77285108
rs73128290
rs7742829
rs12404911
All
−0.1
0.0
0.1
0.2
MR effect size for
'Gut microbiota abundance (genus Catenibacterium id.2153)' on 'Dermatopontin'

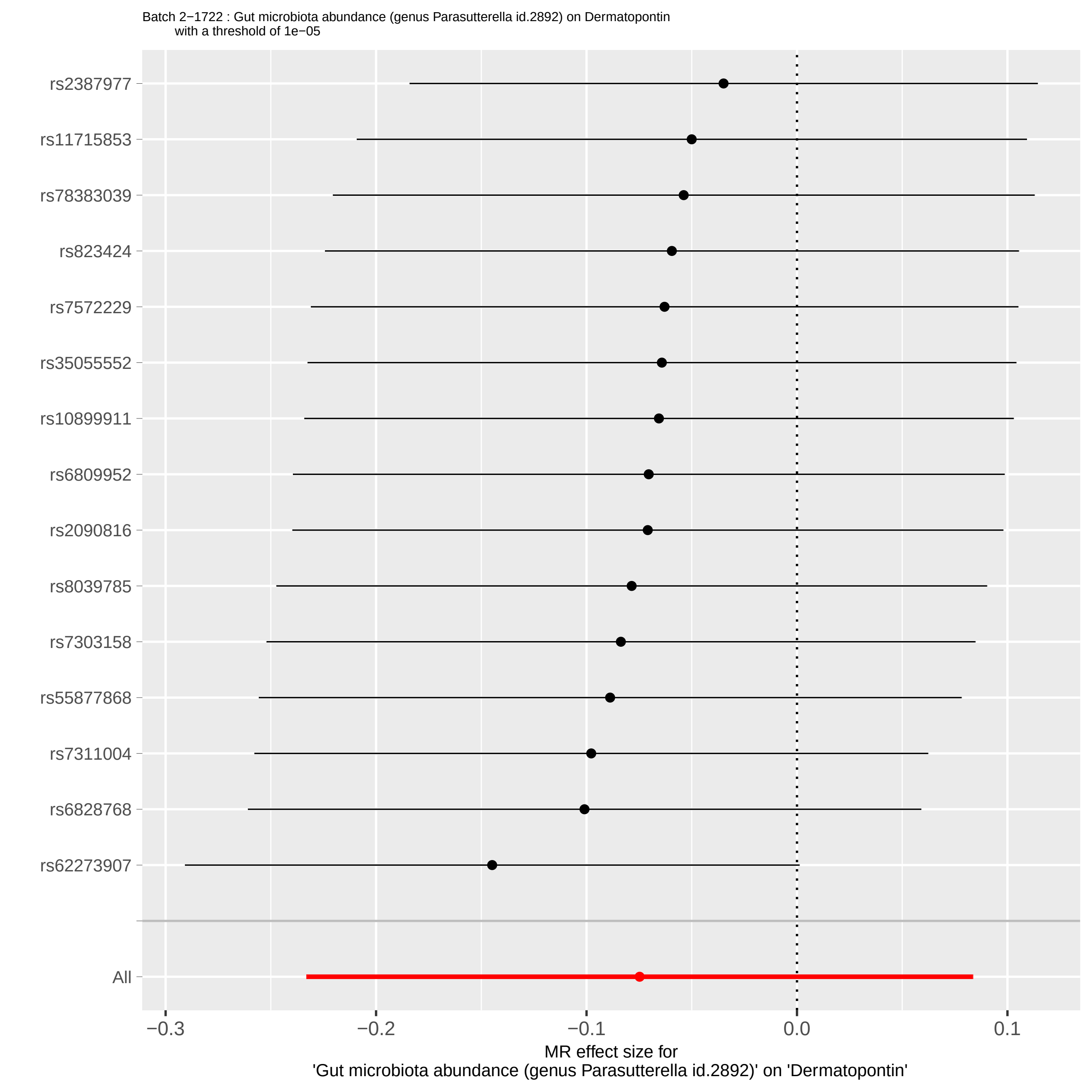

Batch 2−1722 : Gut microbiota abundance (genus Parasutterella id.2892) on Dermatopontin
with a threshold of 1e−05
rs2387977
rs11715853
rs78383039
rs823424
rs7572229
rs35055552
rs10899911
rs6809952
rs2090816
rs8039785
rs7303158
rs55877868
rs7311004
rs6828768
rs62273907
All
−0.3
−0.2
−0.1
0.0
0.1
MR effect size for
'Gut microbiota abundance (genus Parasutterella id.2892)' on 'Dermatopontin'

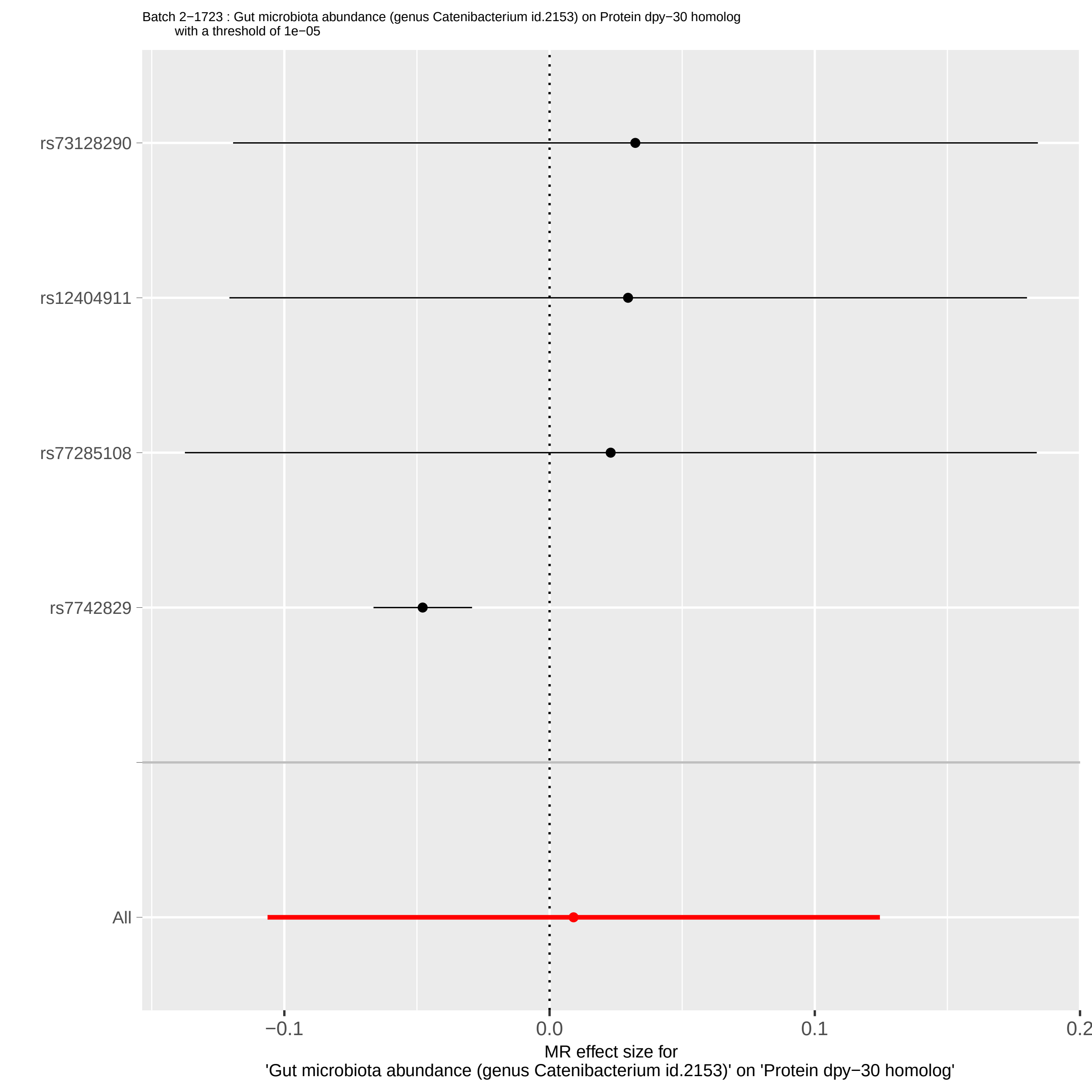

Batch 2−1723 : Gut microbiota abundance (genus Catenibacterium id.2153) on Protein dpy−30 homolog
with a threshold of 1e−05
rs73128290
rs12404911
rs77285108
rs7742829
All
−0.1
0.0
0.1
0.2
MR effect size for
'Gut microbiota abundance (genus Catenibacterium id.2153)' on 'Protein dpy−30 homolog'

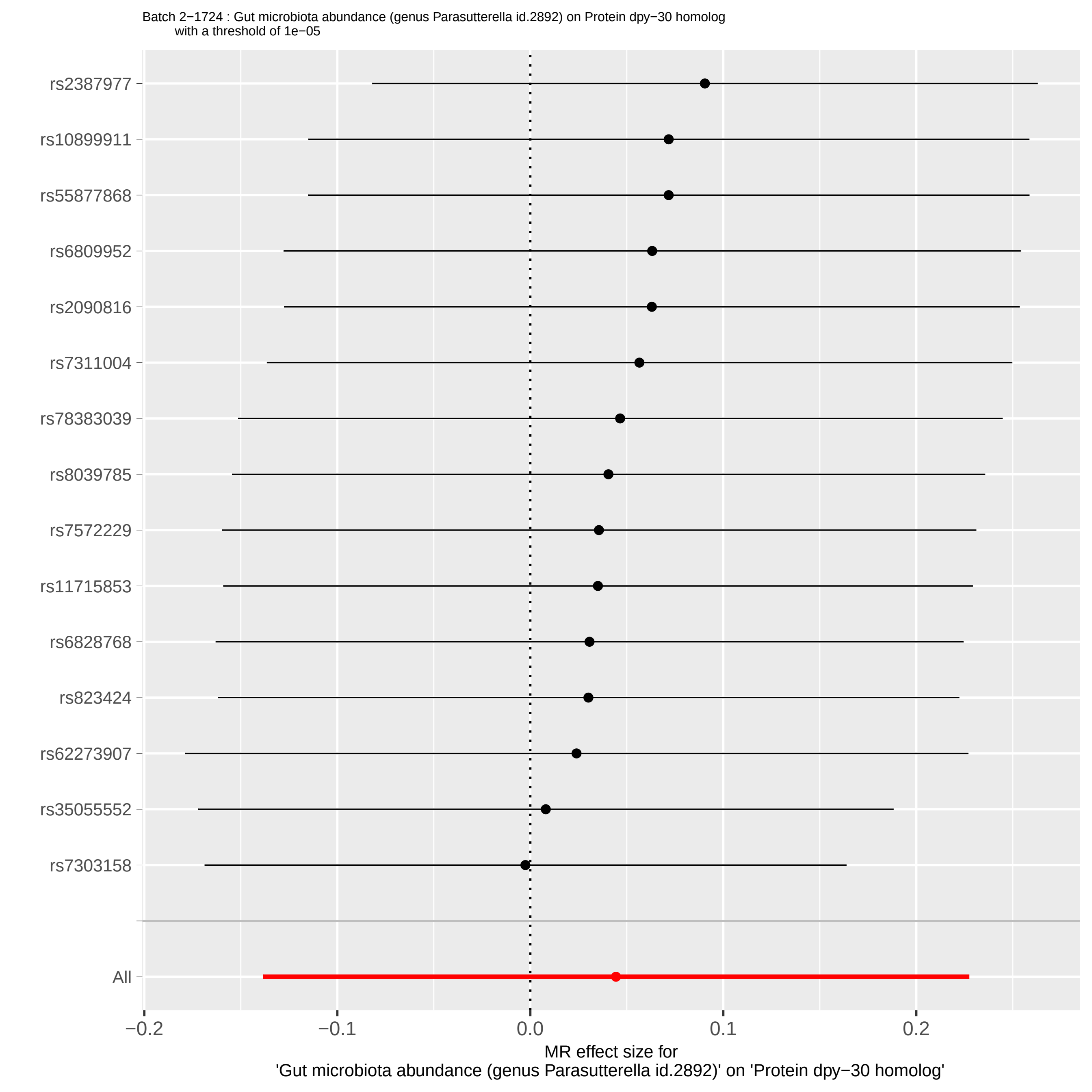

Batch 2−1724 : Gut microbiota abundance (genus Parasutterella id.2892) on Protein dpy−30 homolog
with a threshold of 1e−05
rs2387977
rs10899911
rs55877868
rs6809952
rs2090816
rs7311004
rs78383039
rs8039785
rs7572229
rs11715853
rs6828768
rs823424
rs62273907
rs35055552
rs7303158
All
−0.2
−0.1
0.0
0.1
0.2
MR effect size for
'Gut microbiota abundance (genus Parasutterella id.2892)' on 'Protein dpy−30 homolog'

Batch 2−1725 : Gut microbiota abundance (genus Catenibacterium id.2153) on Dihydropyrimidinase−related protein 3
with a threshold of 1e−05

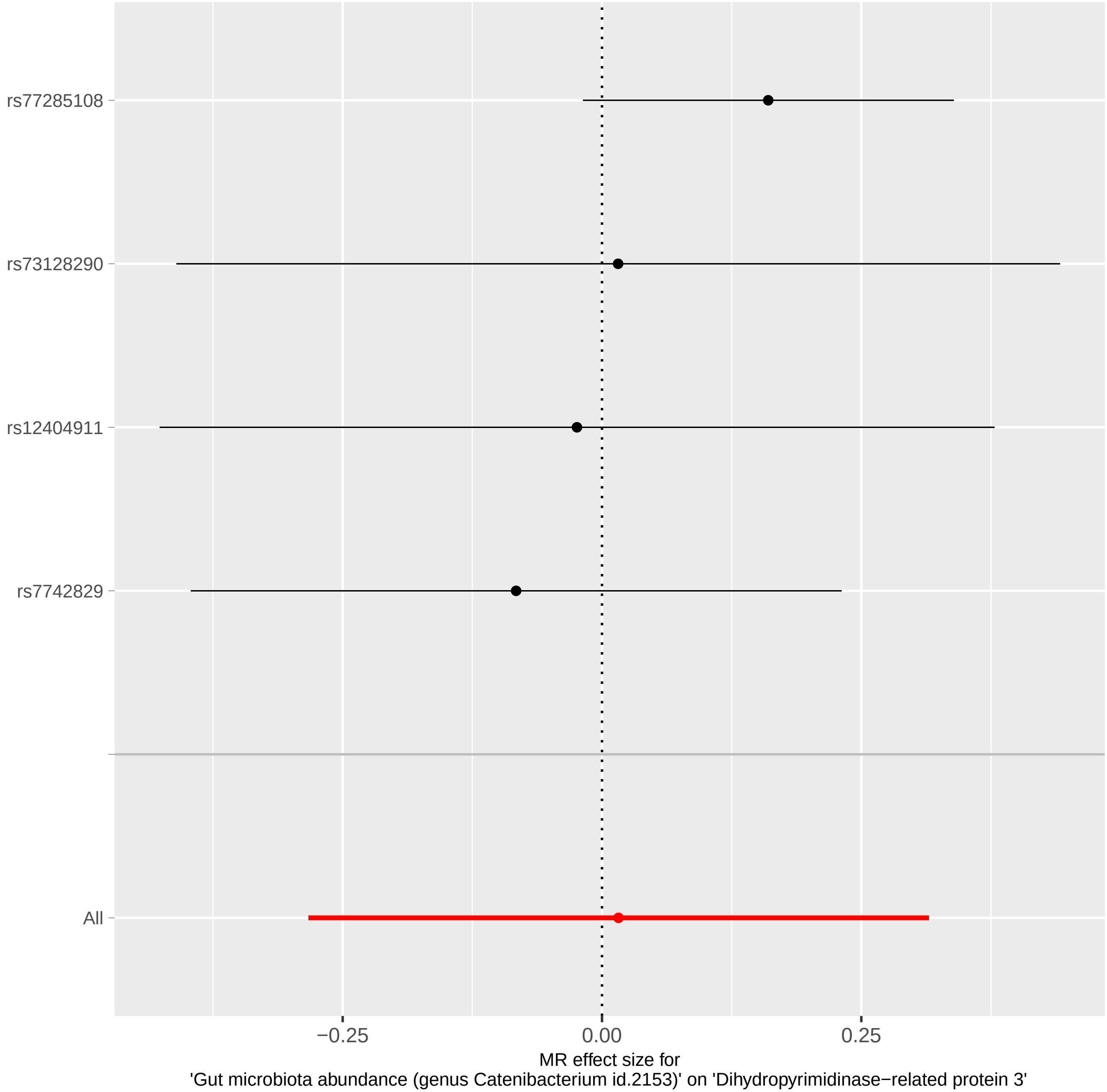

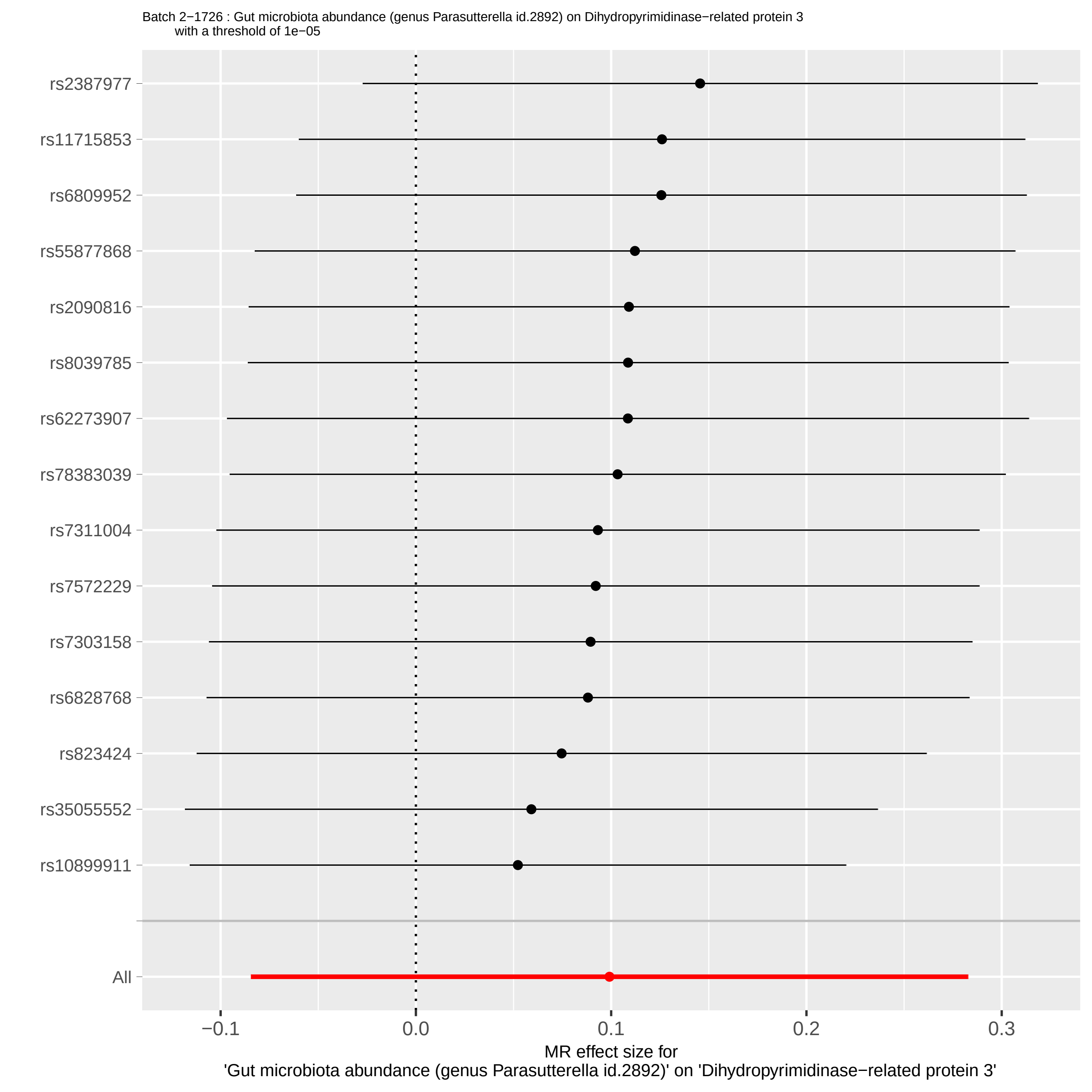
Batch 2−1726 : Gut microbiota abundance (genus Parasutterella id.2892) on Dihydropyrimidinase−related protein 3
with a threshold of 1e−05
rs2387977
rs11715853
rs6809952
rs55877868
rs2090816
rs8039785
rs62273907
rs78383039
rs7311004
rs7572229
rs7303158
rs6828768
rs823424
rs35055552
rs10899911
All
−0.1
0.0
0.1
0.2
0.3
MR effect size for
'Gut microbiota abundance (genus Parasutterella id.2892)' on 'Dihydropyrimidinase−related protein 3'

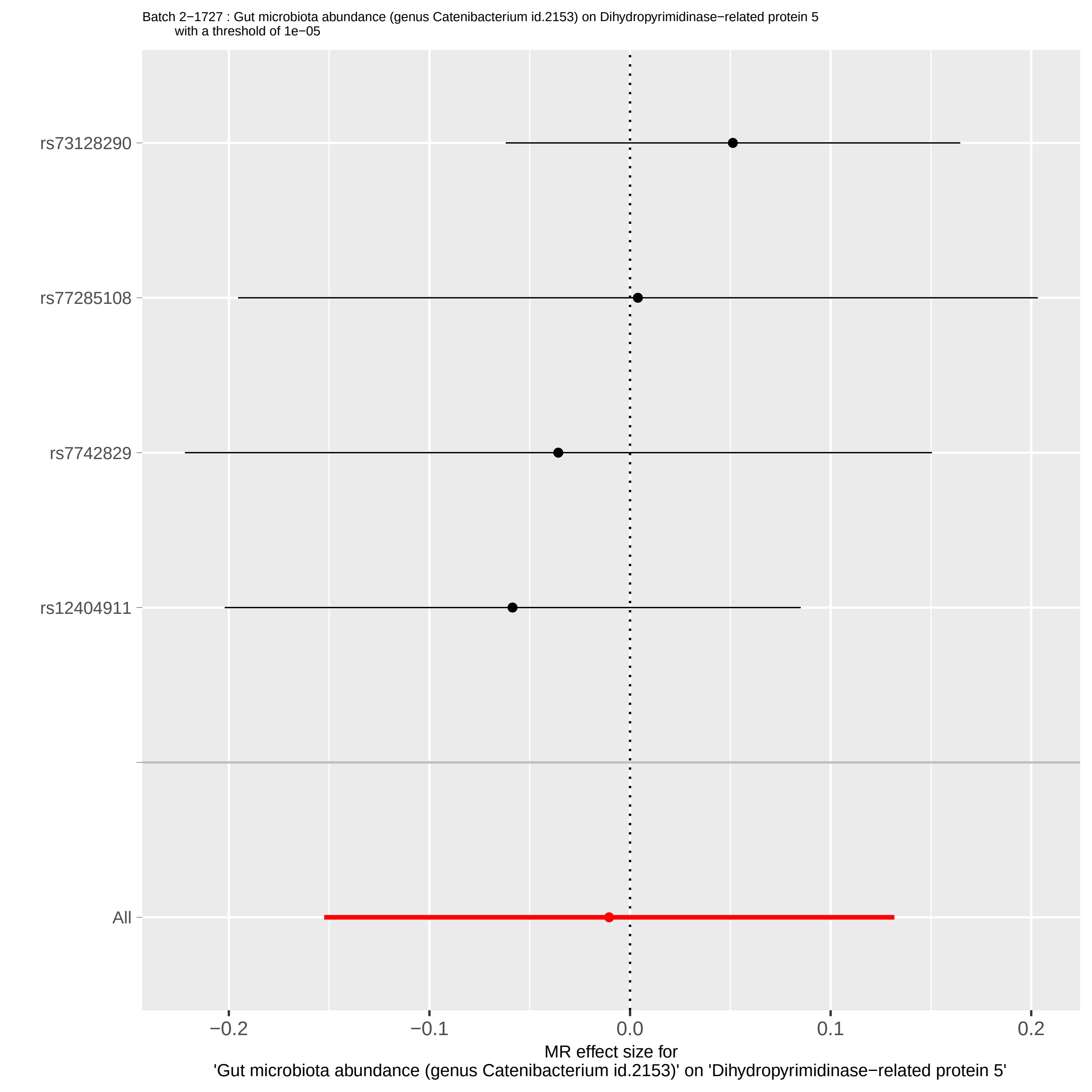
Batch 2−1727 : Gut microbiota abundance (genus Catenibacterium id.2153) on Dihydropyrimidinase−related protein 5
with a threshold of 1e−05
rs73128290
rs77285108
rs7742829
rs12404911
All
−0.2
−0.1
0.0
0.1
0.2
MR effect size for
'Gut microbiota abundance (genus Catenibacterium id.2153)' on 'Dihydropyrimidinase−related protein 5'

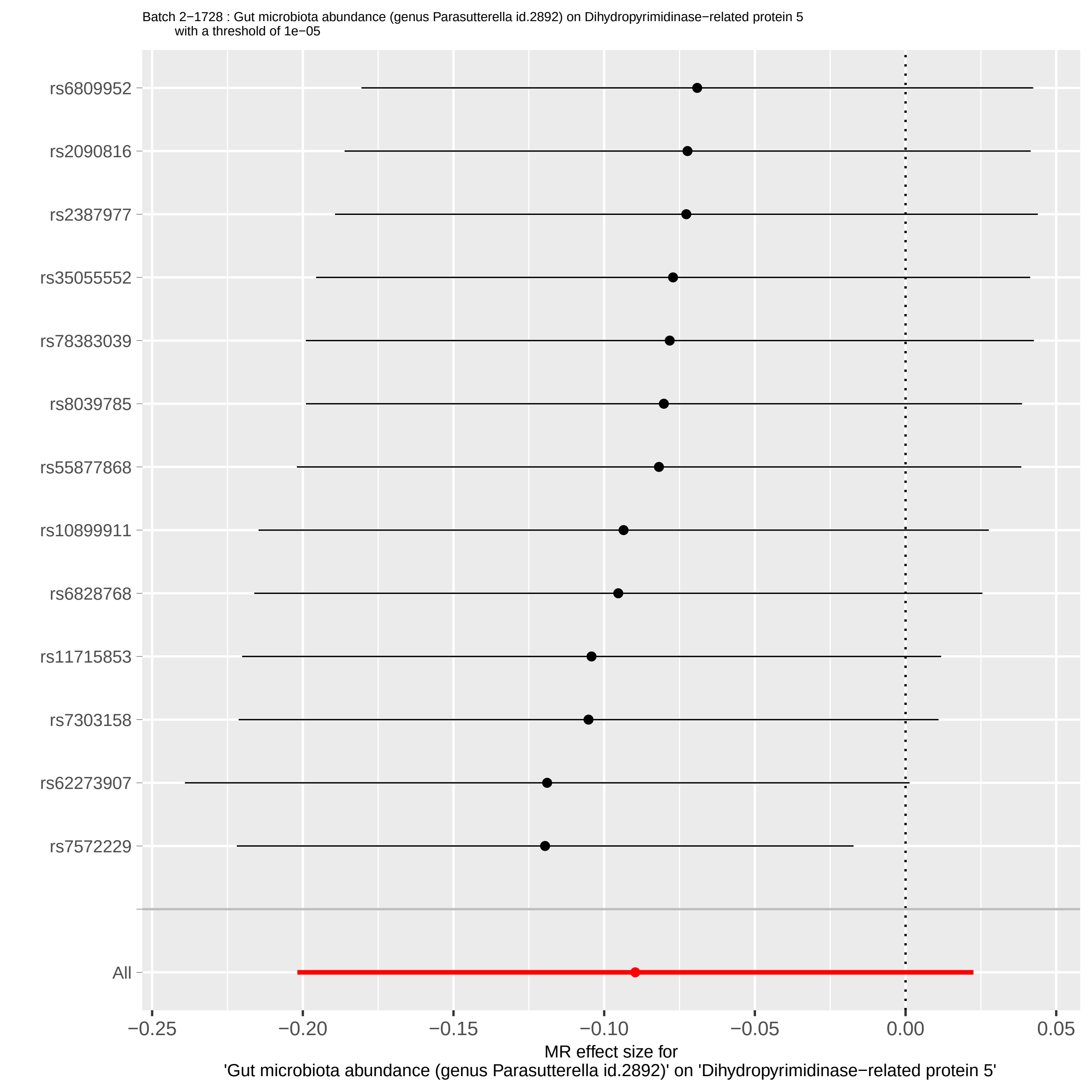
Batch 2−1728 : Gut microbiota abundance (genus Parasutterella id.2892) on Dihydropyrimidinase−related protein 5
with a threshold of 1e−05
rs6809952
rs2090816
rs2387977
rs35055552
rs78383039
rs8039785
rs55877868
rs10899911
rs6828768
rs11715853
rs7303158
rs62273907
rs7572229
All
−0.25
−0.20
−0.15
−0.10
−0.05
0.00
0.05
MR effect size for
'Gut microbiota abundance (genus Parasutterella id.2892)' on 'Dihydropyrimidinase−related protein 5'

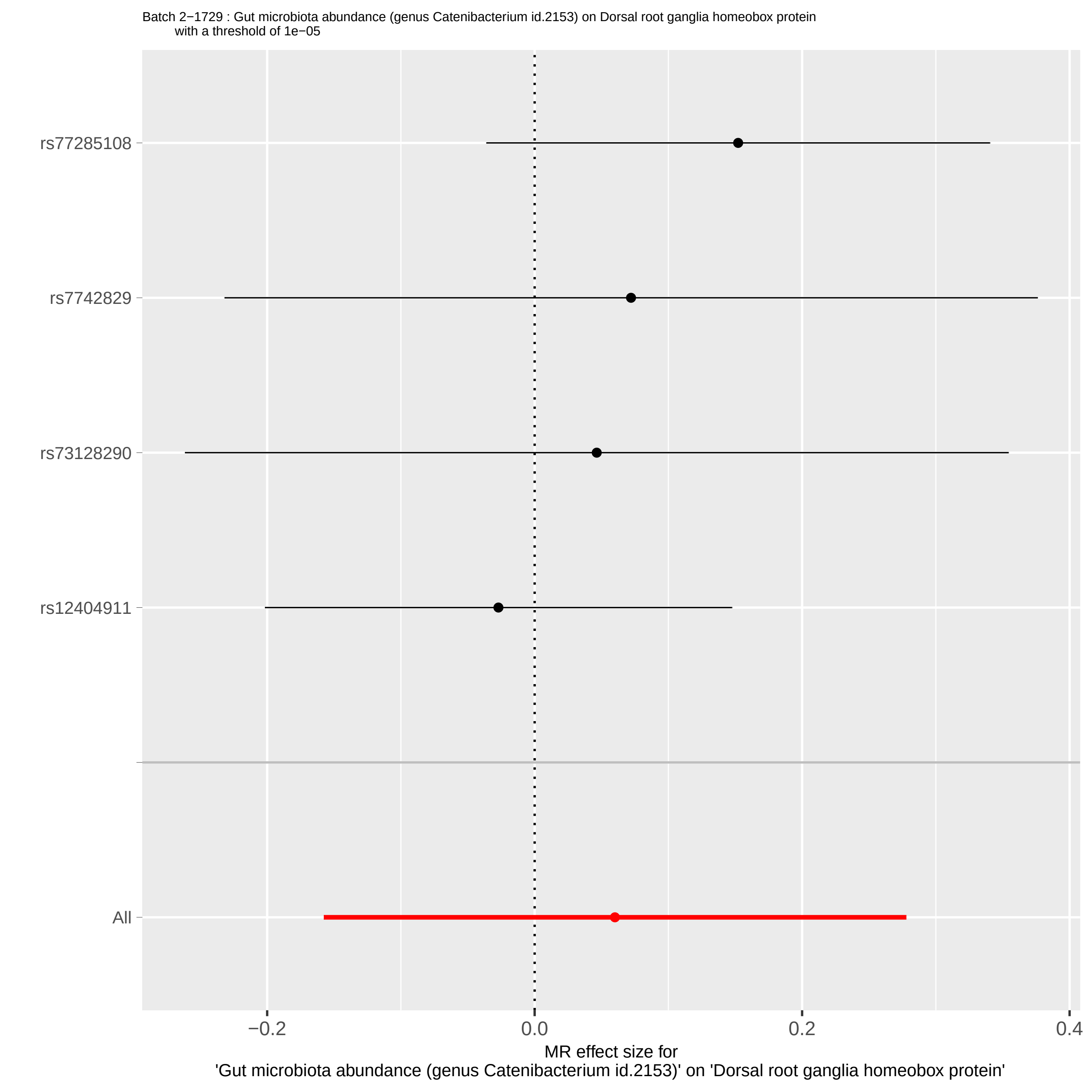
Batch 2−1729 : Gut microbiota abundance (genus Catenibacterium id.2153) on Dorsal root ganglia homeobox protein
with a threshold of 1e−05
rs77285108
rs7742829
rs73128290
rs12404911
All
−0.2
0.0
0.2
0.4
MR effect size for
'Gut microbiota abundance (genus Catenibacterium id.2153)' on 'Dorsal root ganglia homeobox protein'

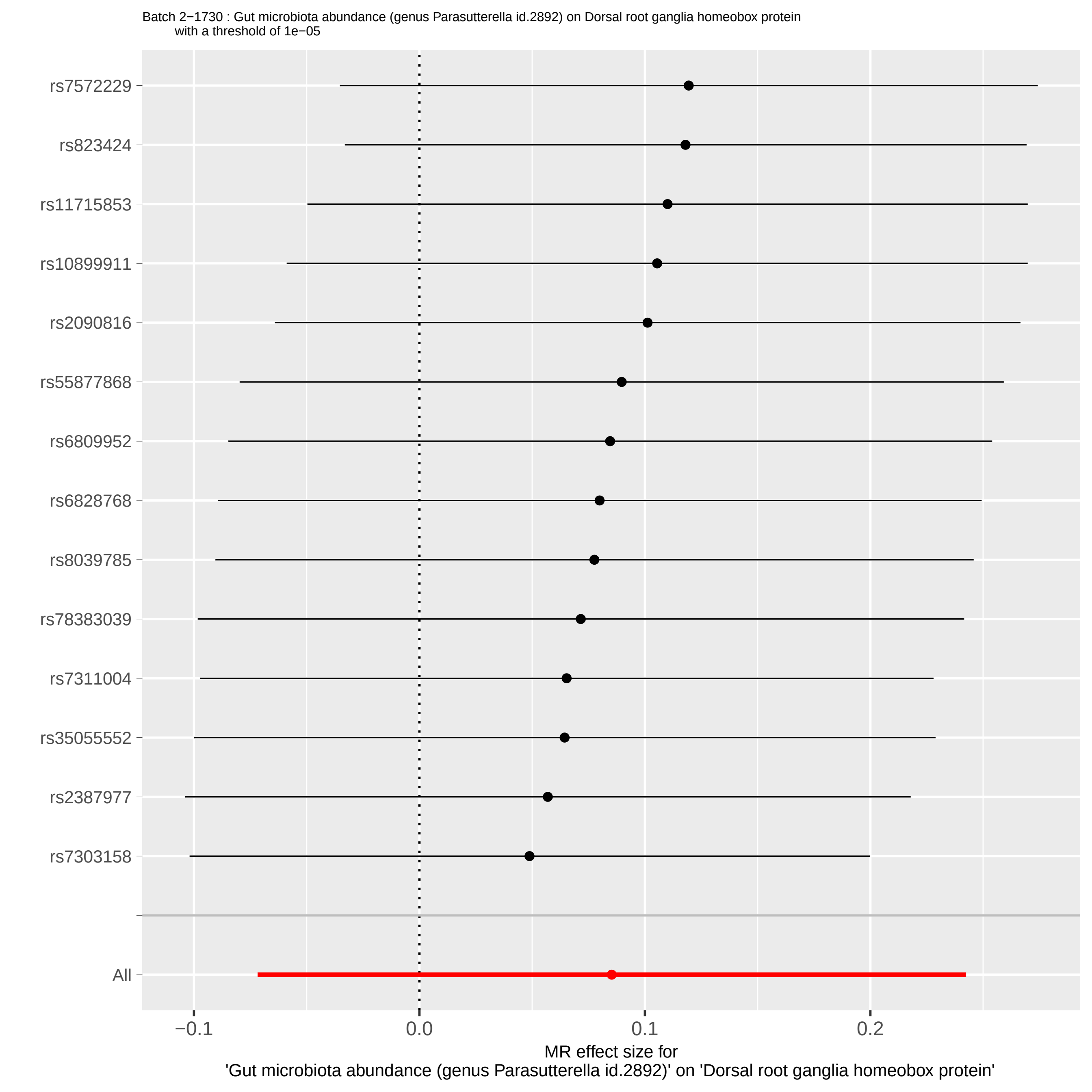

Batch 2−1730 : Gut microbiota abundance (genus Parasutterella id.2892) on Dorsal root ganglia homeobox protein
with a threshold of 1e−05
rs7572229
rs823424
rs11715853
rs10899911
rs2090816
rs55877868
rs6809952
rs6828768
rs8039785
rs78383039
rs7311004
rs35055552
rs2387977
rs7303158
All
−0.1
0.0
0.1
0.2
MR effect size for
'Gut microbiota abundance (genus Parasutterella id.2892)' on 'Dorsal root ganglia homeobox protein'

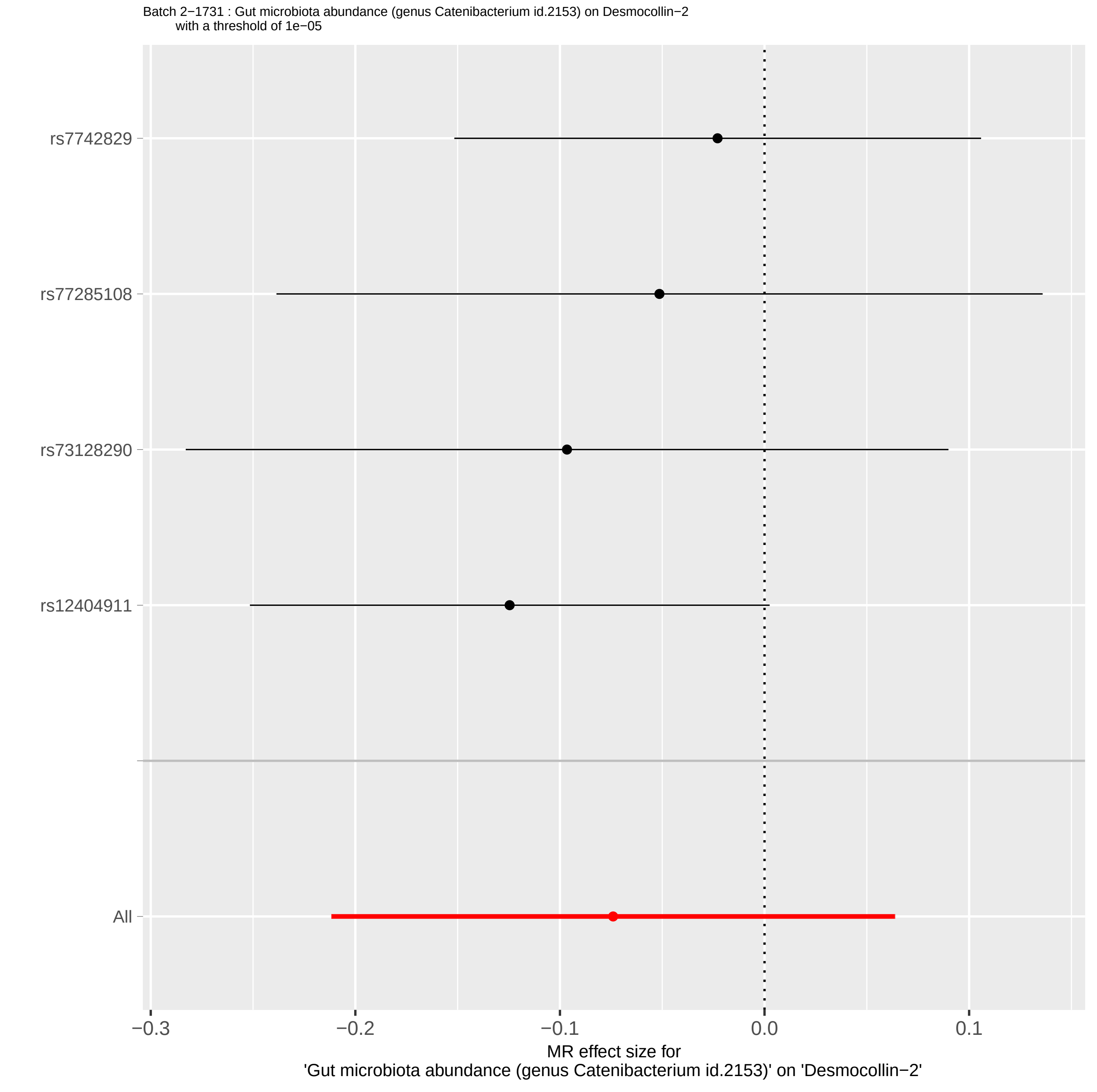
Batch 2−1731 : Gut microbiota abundance (genus Catenibacterium id.2153) on Desmocollin−2
with a threshold of 1e−05
rs7742829
rs77285108
rs73128290
rs12404911
All
−0.3
−0.2
−0.1
0.0
0.1
MR effect size for
'Gut microbiota abundance (genus Catenibacterium id.2153)' on 'Desmocollin−2'

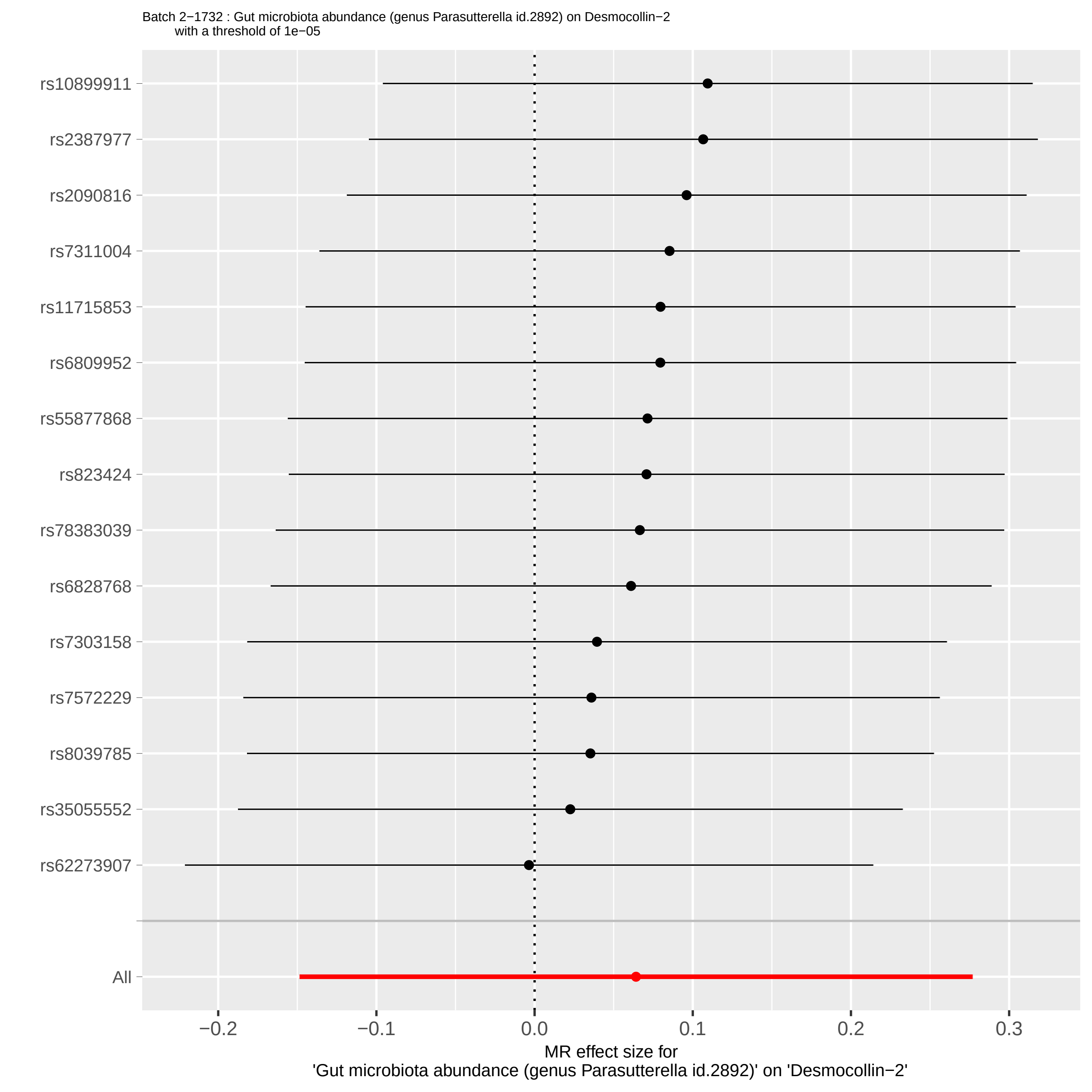

Batch 2−1732 : Gut microbiota abundance (genus Parasutterella id.2892) on Desmocollin−2
with a threshold of 1e−05
rs10899911
rs2387977
rs2090816
rs7311004
rs11715853
rs6809952
rs55877868
rs823424
rs78383039
rs6828768
rs7303158
rs7572229
rs8039785
rs35055552
rs62273907
All
−0.2
−0.1
0.0
0.1
0.2
0.3
MR effect size for
'Gut microbiota abundance (genus Parasutterella id.2892)' on 'Desmocollin−2'

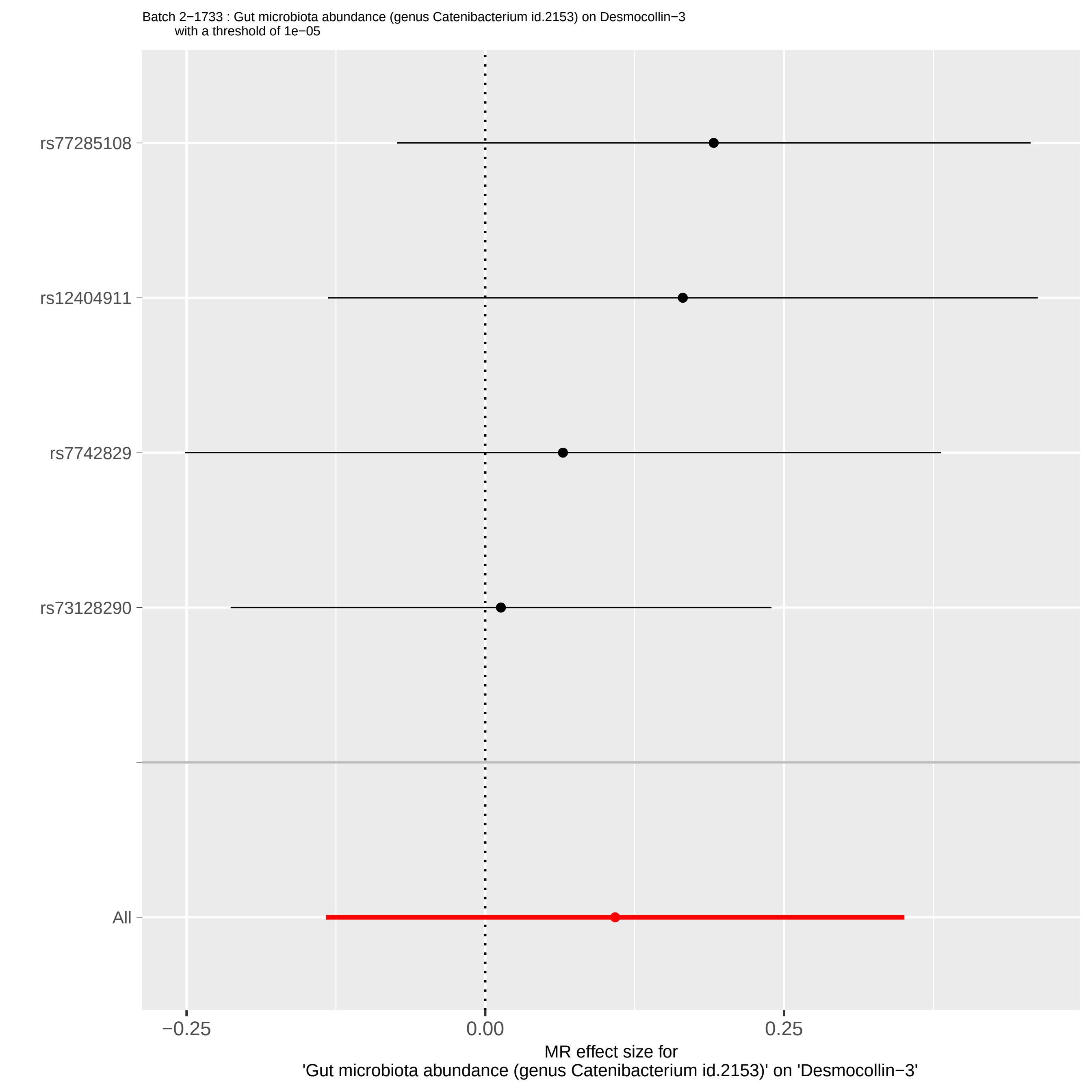
Batch 2−1733 : Gut microbiota abundance (genus Catenibacterium id.2153) on Desmocollin−3
with a threshold of 1e−05
rs77285108
rs12404911
rs7742829
rs73128290
All
−0.25
0.00
0.25
MR effect size for
'Gut microbiota abundance (genus Catenibacterium id.2153)' on 'Desmocollin−3'

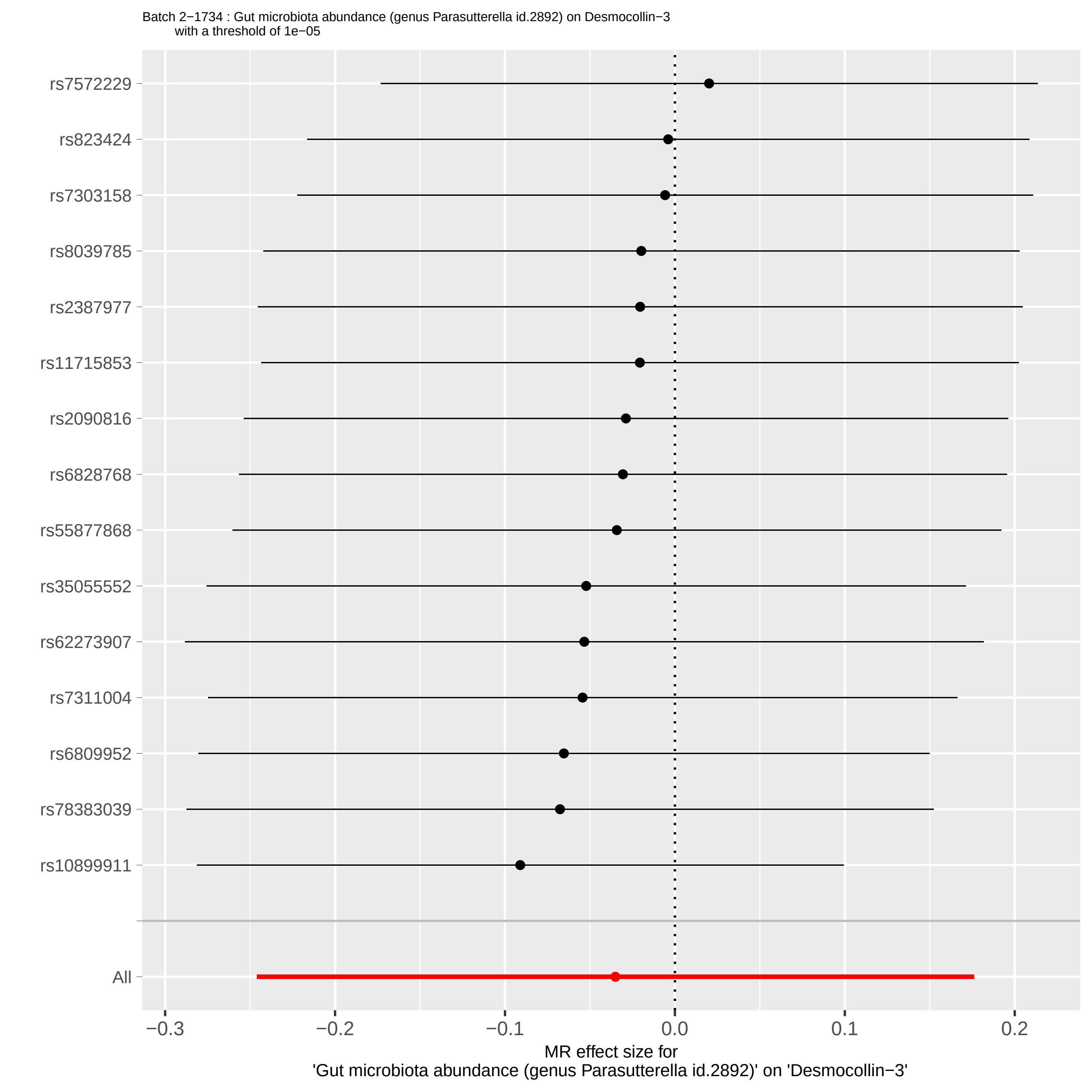
Batch 2−1734 : Gut microbiota abundance (genus Parasutterella id.2892) on Desmocollin−3
with a threshold of 1e−05
rs7572229
rs823424
rs7303158
rs8039785
rs2387977
rs11715853
rs2090816
rs6828768
rs55877868
rs35055552
rs62273907
rs7311004
rs6809952
rs78383039
rs10899911
All
−0.3
−0.2
−0.1
0.0
0.1
0.2
MR effect size for
'Gut microbiota abundance (genus Parasutterella id.2892)' on 'Desmocollin−3'

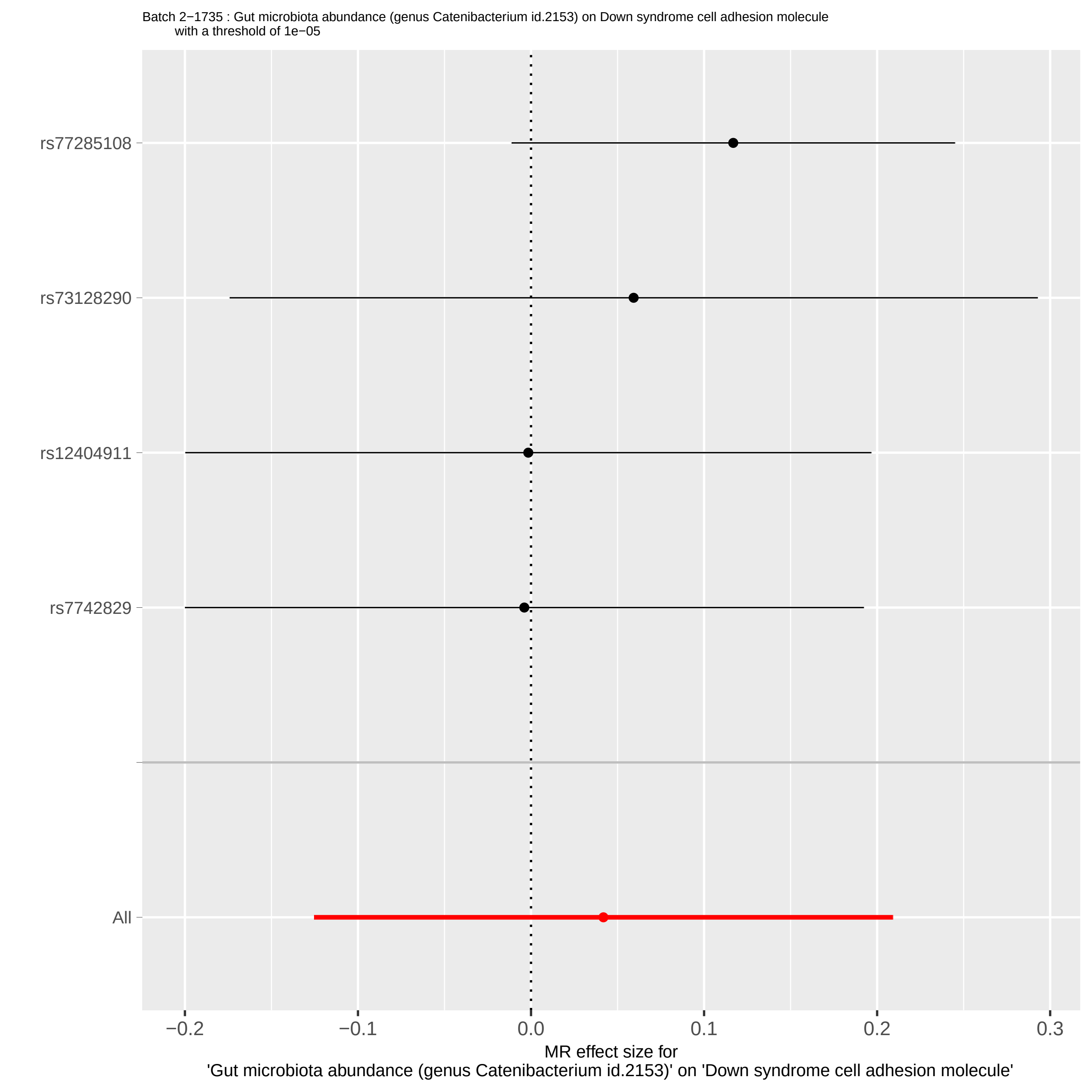
Batch 2−1735 : Gut microbiota abundance (genus Catenibacterium id.2153) on Down syndrome cell adhesion molecule
with a threshold of 1e−05
rs77285108
rs73128290
rs12404911
rs7742829
All
−0.2
−0.1
0.0
0.1
0.2
0.3
MR effect size for
'Gut microbiota abundance (genus Catenibacterium id.2153)' on 'Down syndrome cell adhesion molecule'

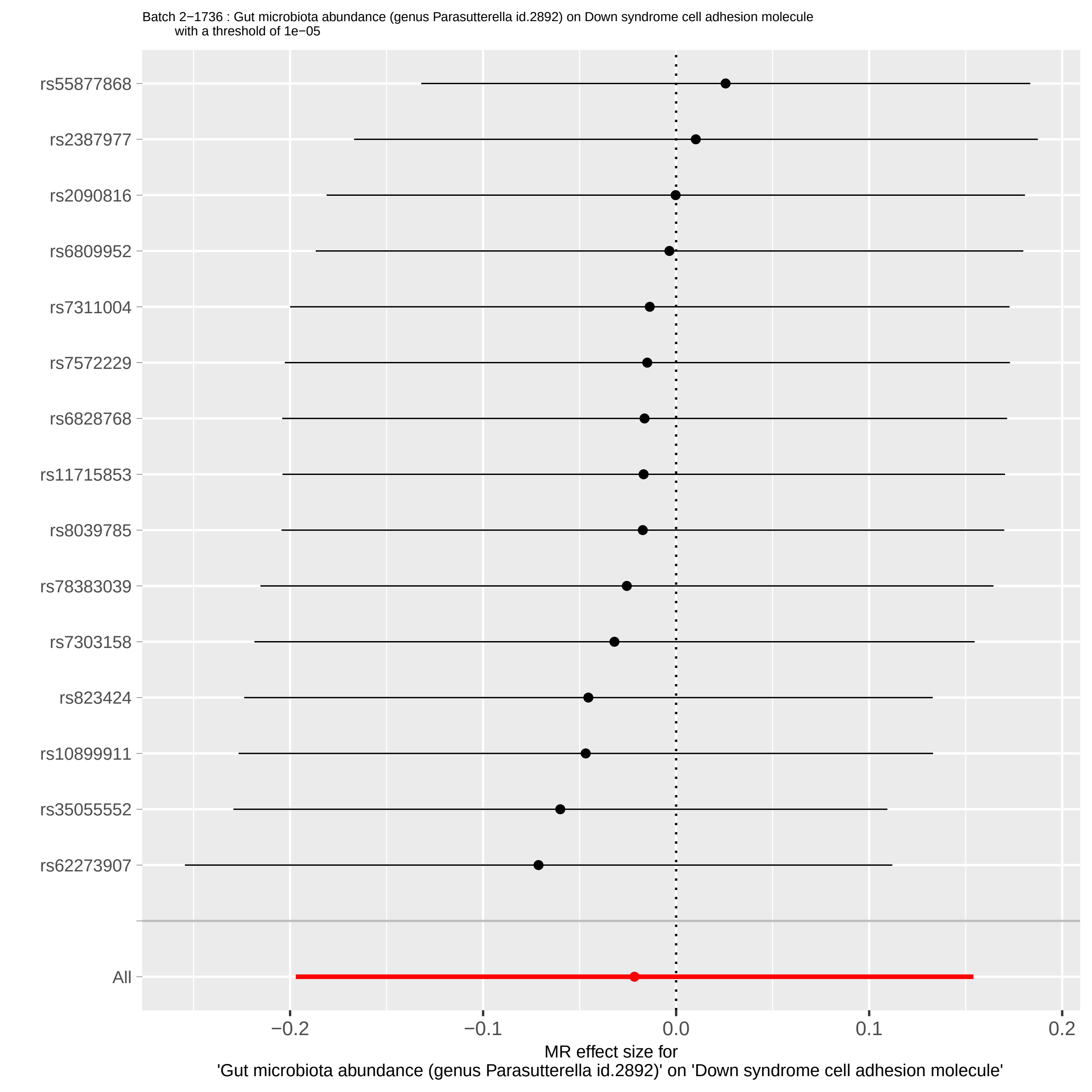
Batch 2−1736 : Gut microbiota abundance (genus Parasutterella id.2892) on Down syndrome cell adhesion molecule
with a threshold of 1e−05
rs55877868
rs2387977
rs2090816
rs6809952
rs7311004
rs7572229
rs6828768
rs11715853
rs8039785
rs78383039
rs7303158
rs823424
rs10899911
rs35055552
rs62273907
All
−0.2
−0.1
0.0
0.1
0.2
MR effect size for
'Gut microbiota abundance (genus Parasutterella id.2892)' on 'Down syndrome cell adhesion molecule'

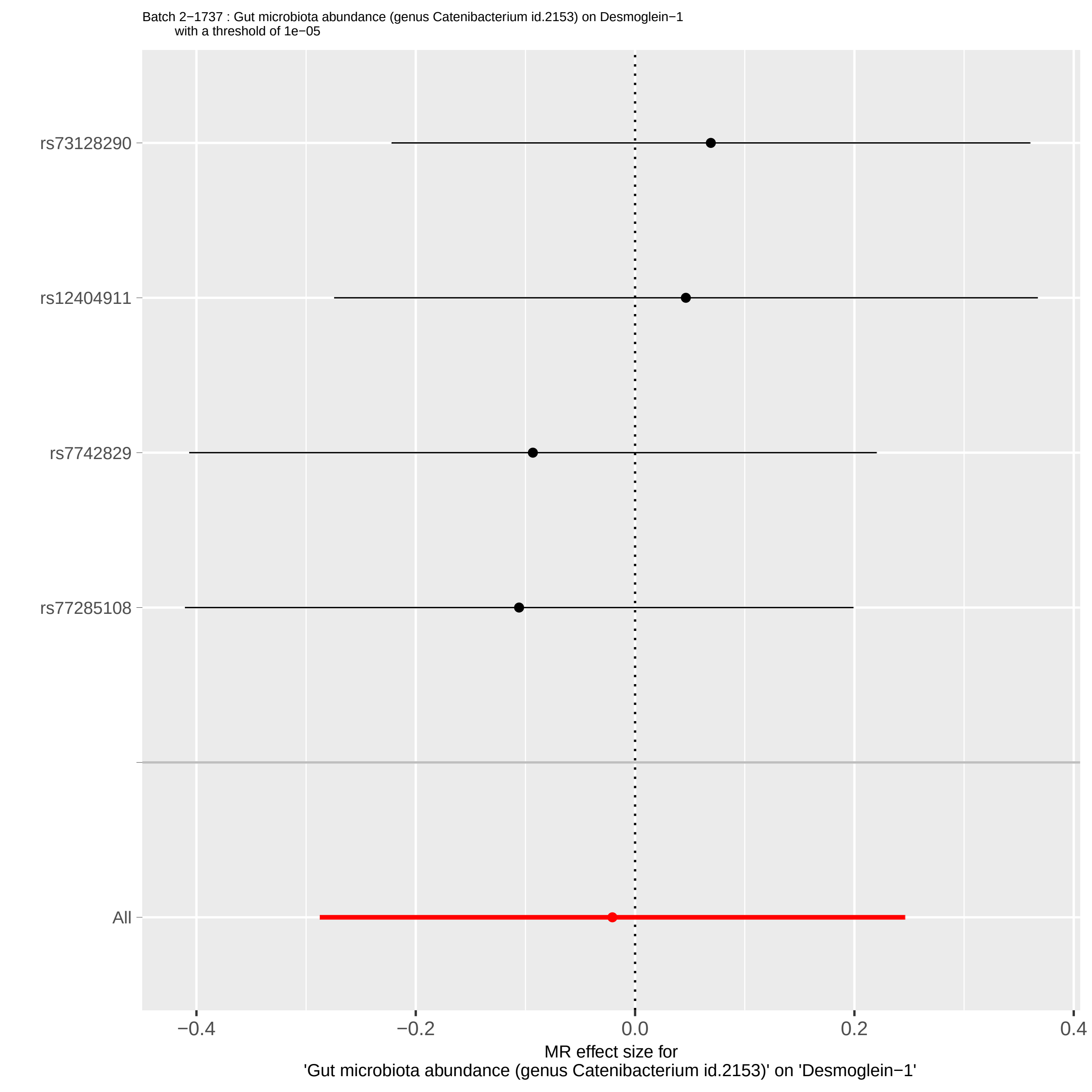
Batch 2−1737 : Gut microbiota abundance (genus Catenibacterium id.2153) on Desmoglein−1
with a threshold of 1e−05
rs73128290
rs12404911
rs7742829
rs77285108
All
−0.4
−0.2
0.0
0.2
0.4
MR effect size for
'Gut microbiota abundance (genus Catenibacterium id.2153)' on 'Desmoglein−1'

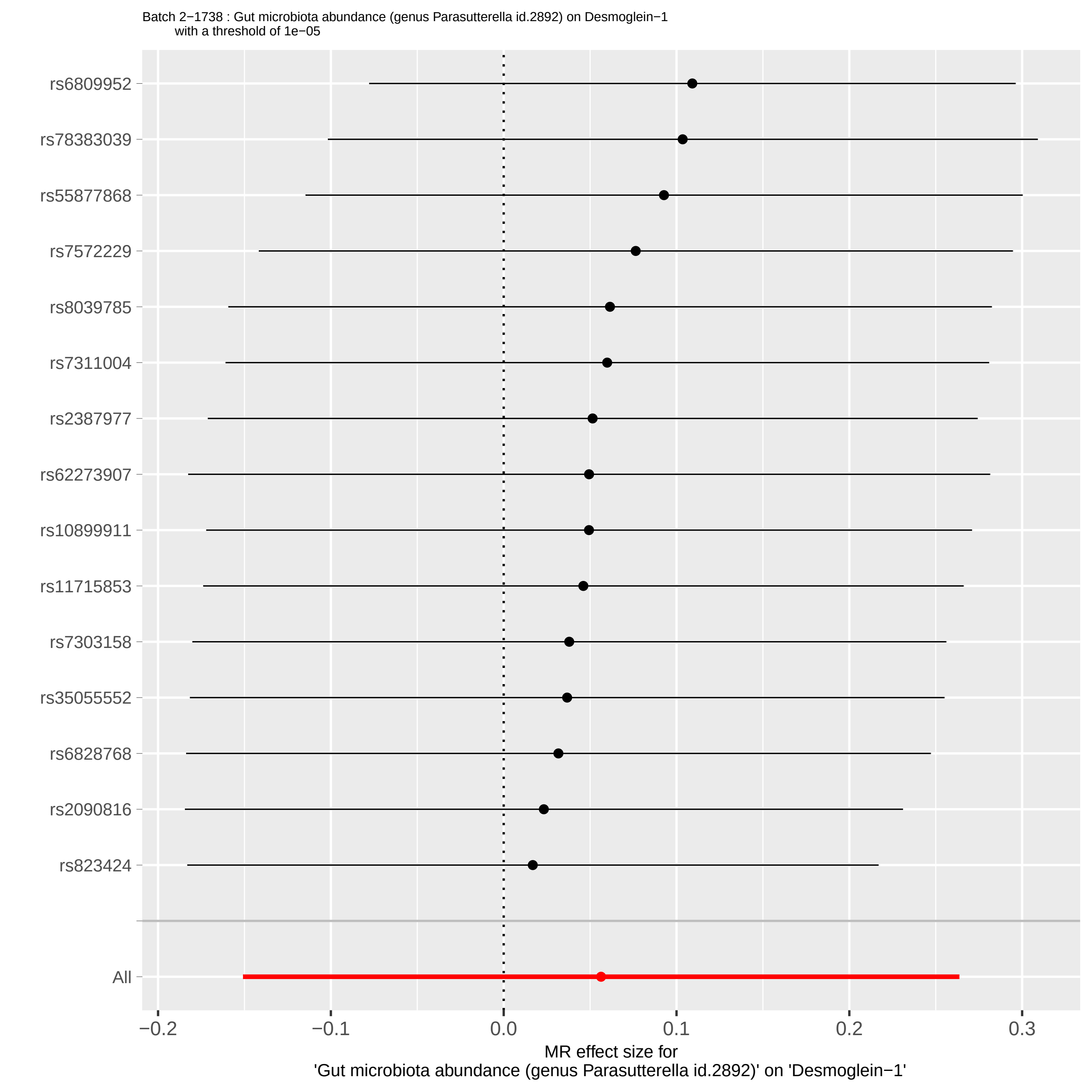
Batch 2−1738 : Gut microbiota abundance (genus Parasutterella id.2892) on Desmoglein−1
with a threshold of 1e−05
rs6809952
rs78383039
rs55877868
rs7572229
rs8039785
rs7311004
rs2387977
rs62273907
rs10899911
rs11715853
rs7303158
rs35055552
rs6828768
rs2090816
rs823424
All
−0.2
−0.1
0.0
0.1
0.2
0.3
MR effect size for
'Gut microbiota abundance (genus Parasutterella id.2892)' on 'Desmoglein−1'

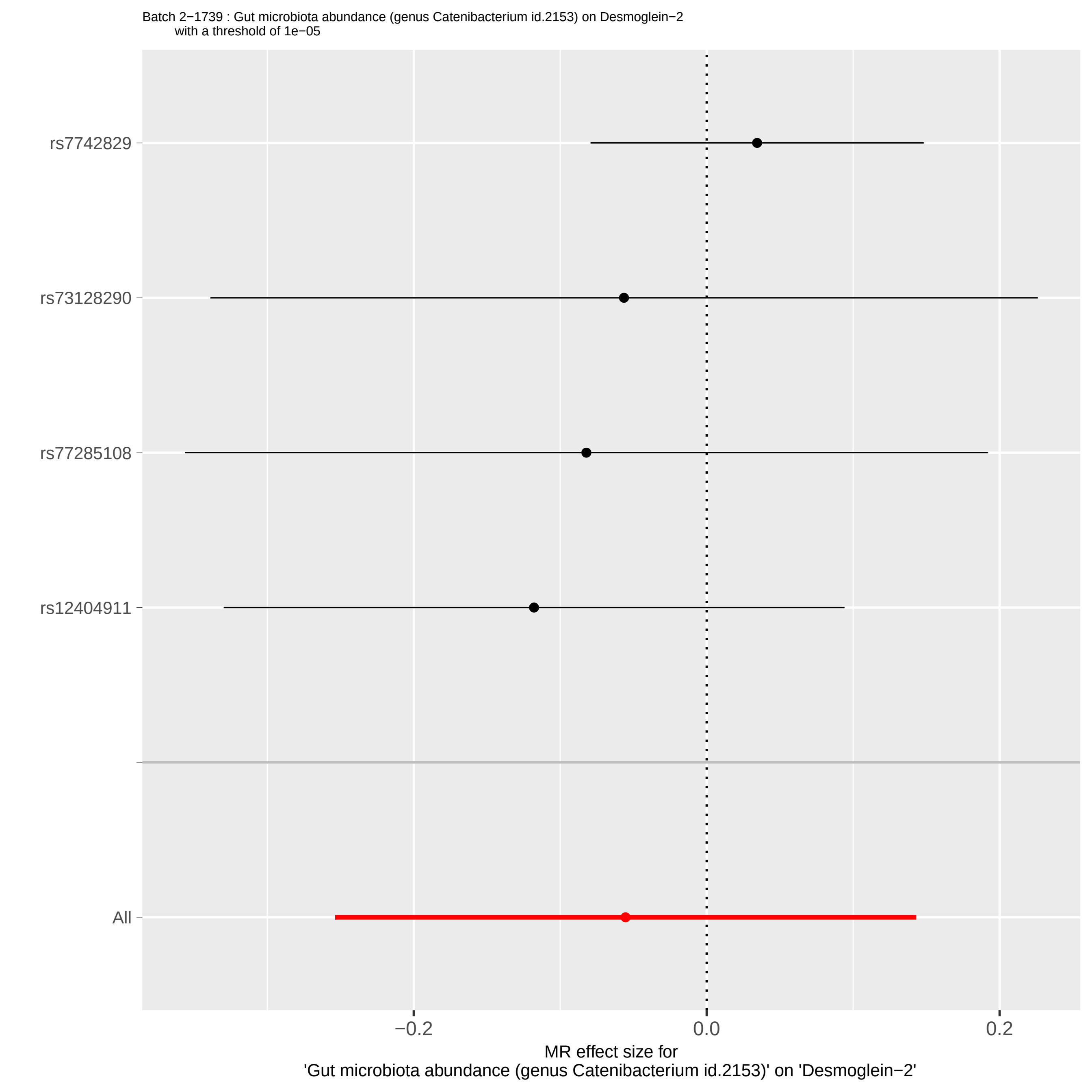

Batch 2−1739 : Gut microbiota abundance (genus Catenibacterium id.2153) on Desmoglein−2
with a threshold of 1e−05
rs7742829
rs73128290
rs77285108
rs12404911
All
−0.2
0.0
0.2
MR effect size for
'Gut microbiota abundance (genus Catenibacterium id.2153)' on 'Desmoglein−2'

Batch 2−1740 : Gut microbiota abundance (genus Parasutterella id.2892) on Desmoglein−2
with a threshold of 1e−05

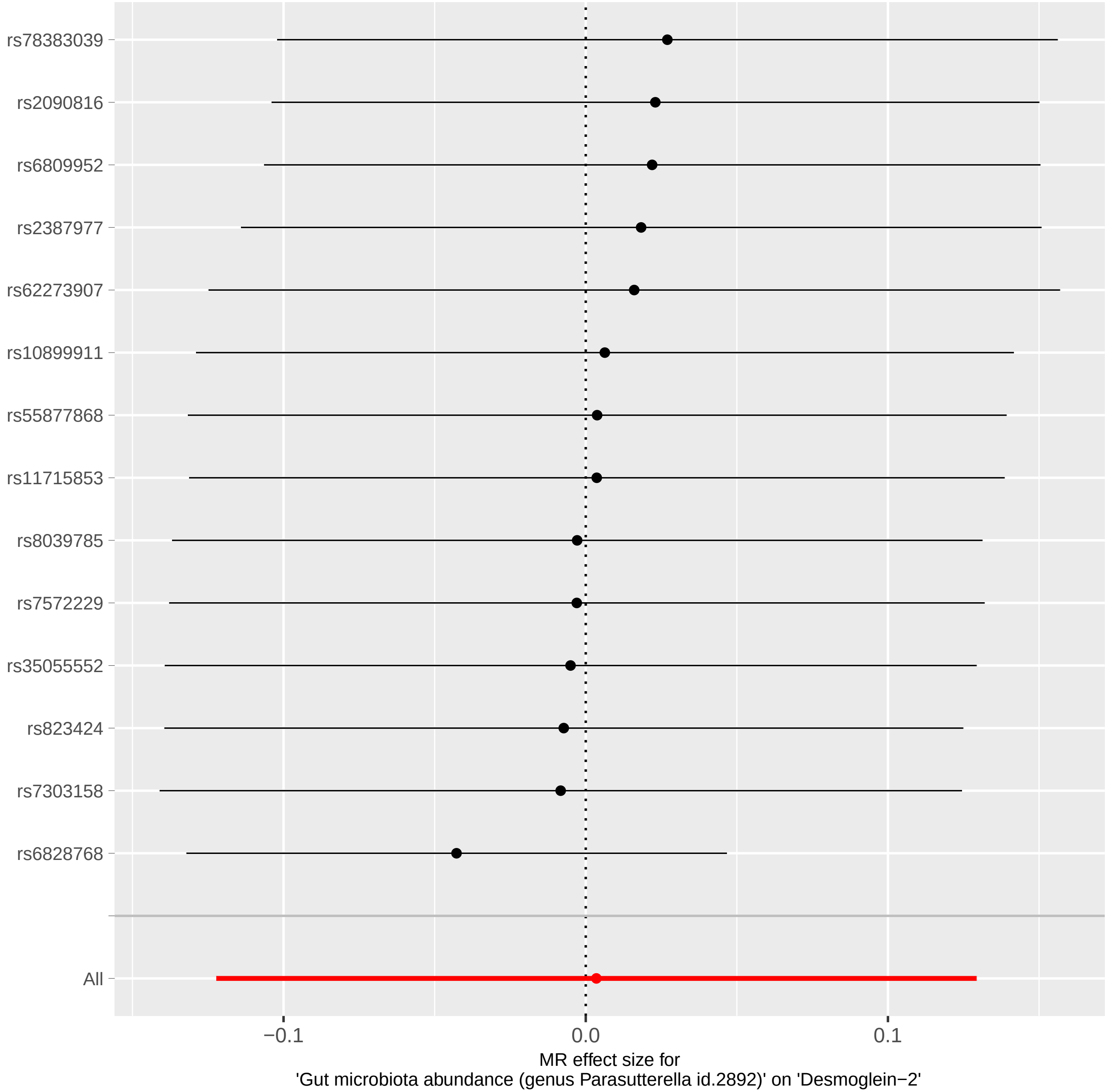

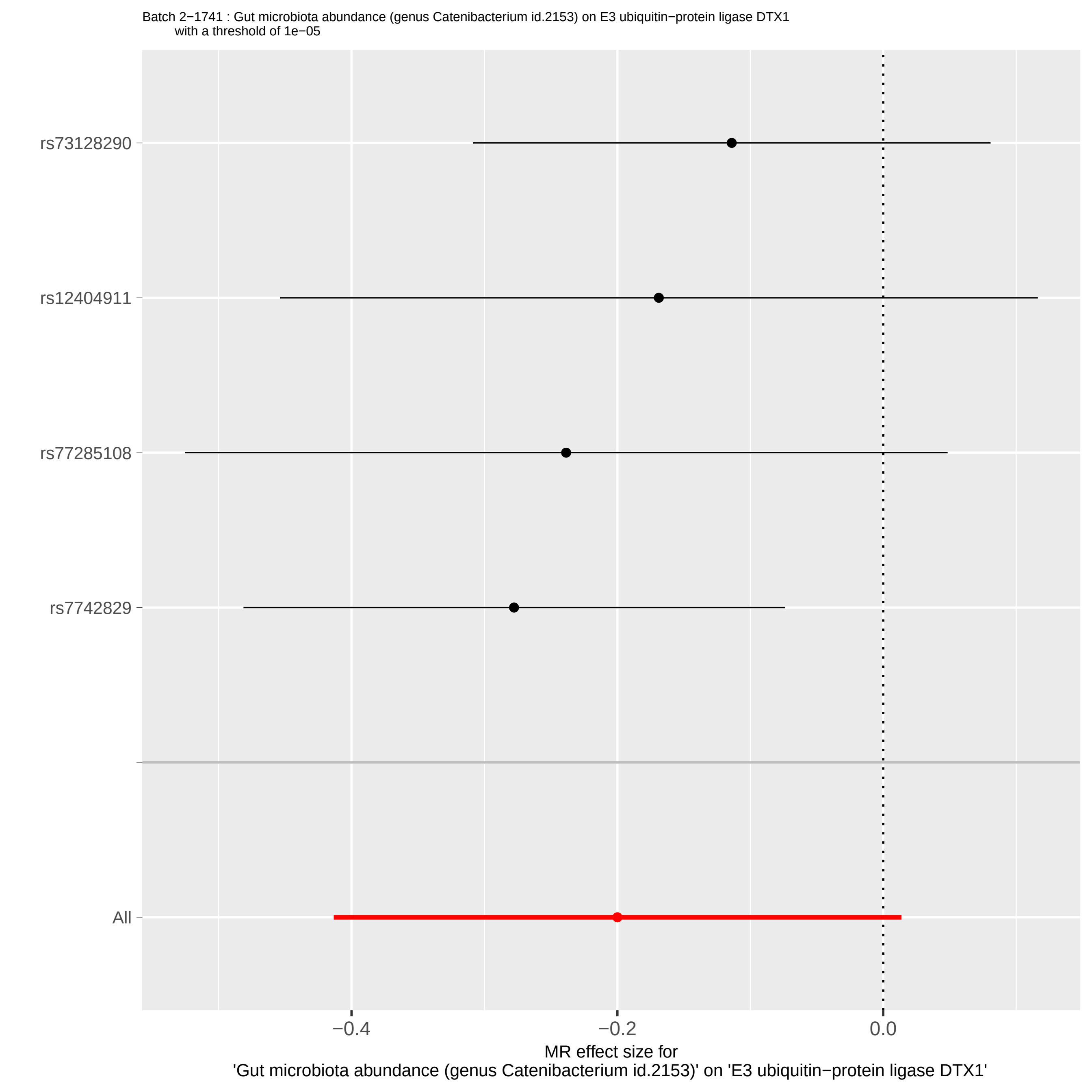
Batch 2−1741 : Gut microbiota abundance (genus Catenibacterium id.2153) on E3 ubiquitin−protein ligase DTX1
with a threshold of 1e−05
rs73128290
rs12404911
rs77285108
rs7742829
All
−0.4
−0.2
0.0
MR effect size for
'Gut microbiota abundance (genus Catenibacterium id.2153)' on 'E3 ubiquitin−protein ligase DTX1'

Batch 2−1742 : Gut microbiota abundance (genus Parasutterella id.2892) on E3 ubiquitin−protein ligase DTX1
with a threshold of 1e−05

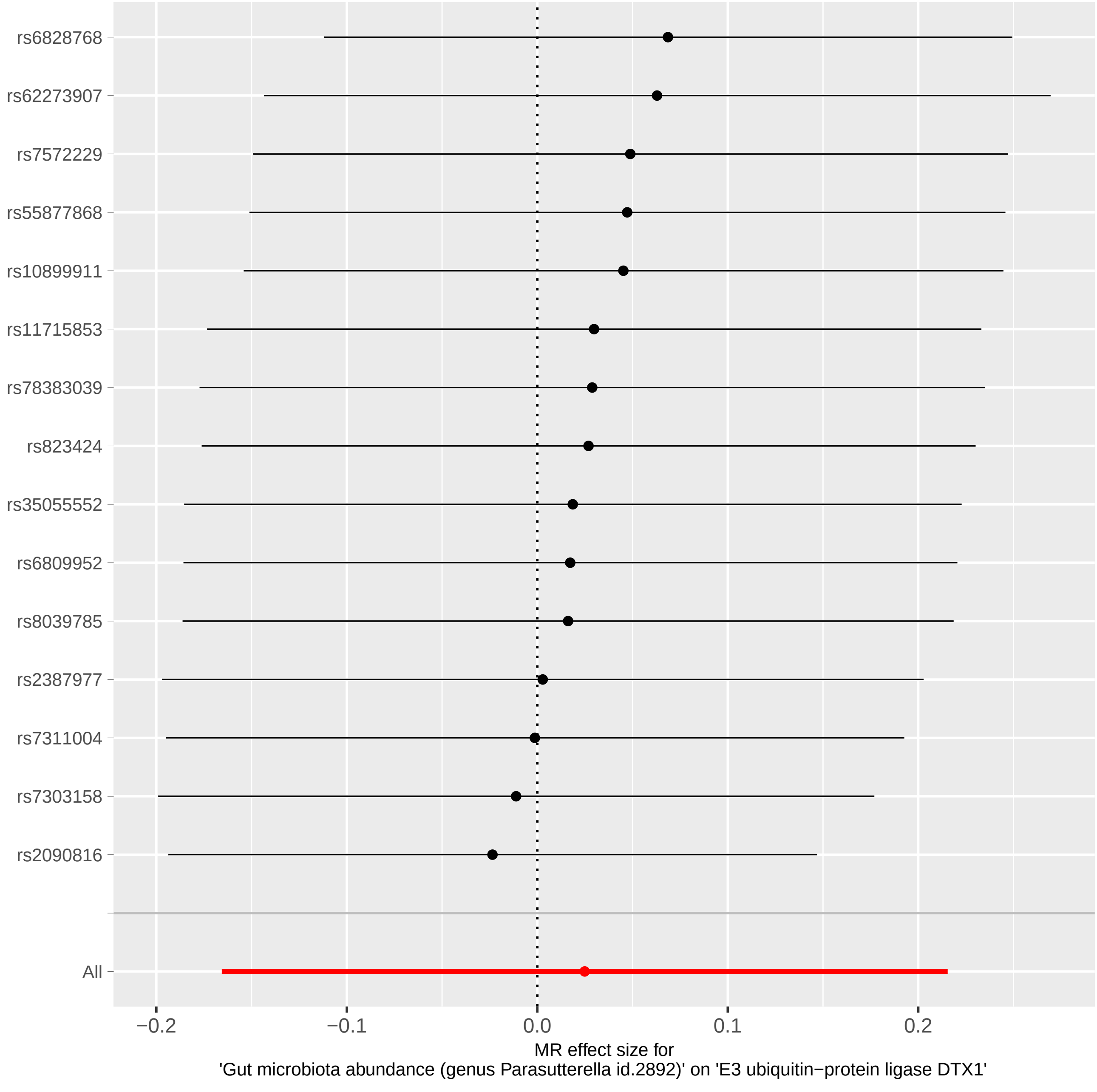

Batch 2−1743 : Gut microbiota abundance (genus Catenibacterium id.2153) on E3 ubiquitin−protein ligase DTX3L
with a threshold of 1e−05

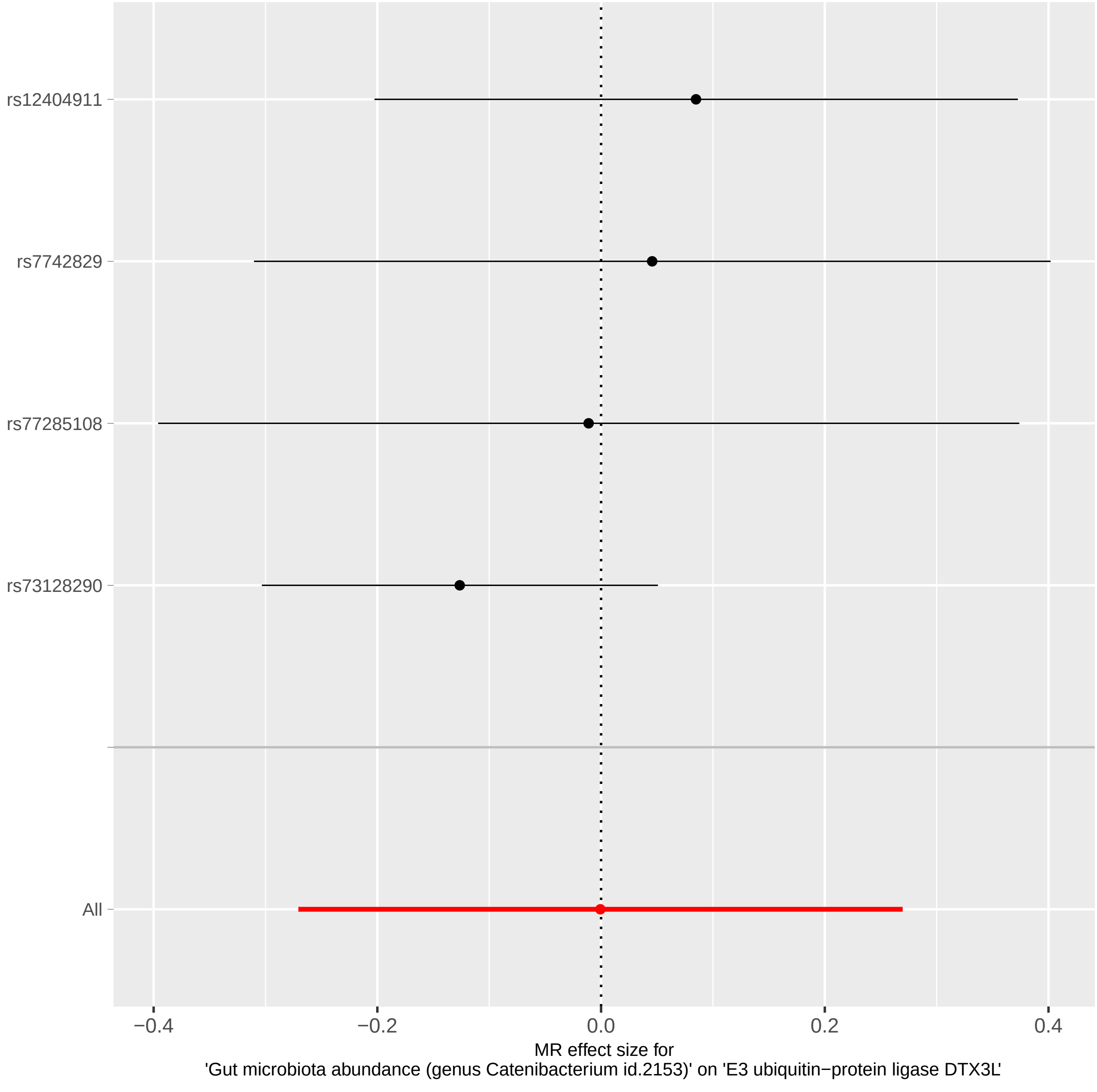

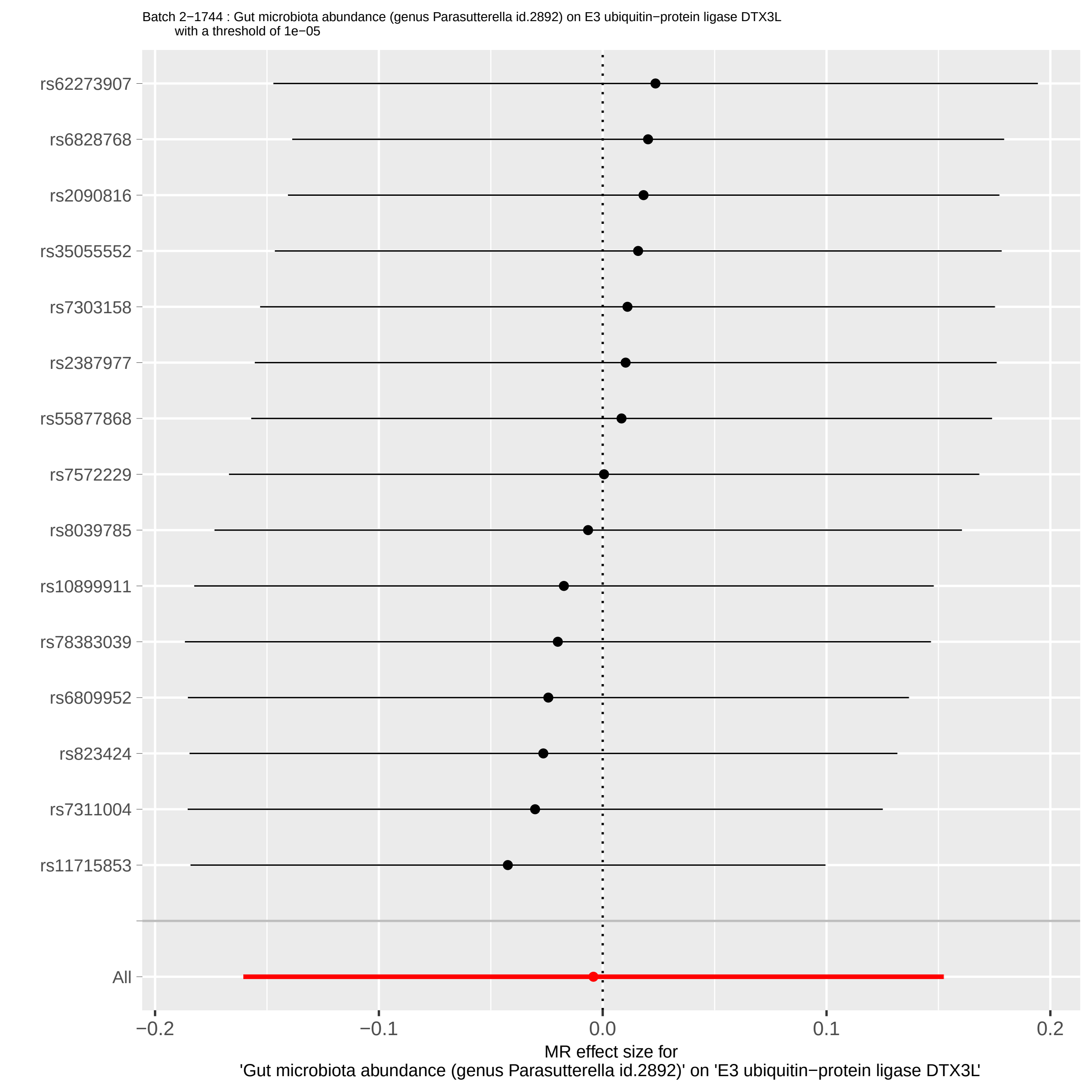
Batch 2−1744 : Gut microbiota abundance (genus Parasutterella id.2892) on E3 ubiquitin−protein ligase DTX3L
with a threshold of 1e−05
rs62273907
rs6828768
rs2090816
rs35055552
rs7303158
rs2387977
rs55877868
rs7572229
rs8039785
rs10899911
rs78383039
rs6809952
rs823424
rs7311004
rs11715853
All
−0.2
−0.1
0.0
0.1
0.2
MR effect size for
'Gut microbiota abundance (genus Parasutterella id.2892)' on 'E3 ubiquitin−protein ligase DTX3L'

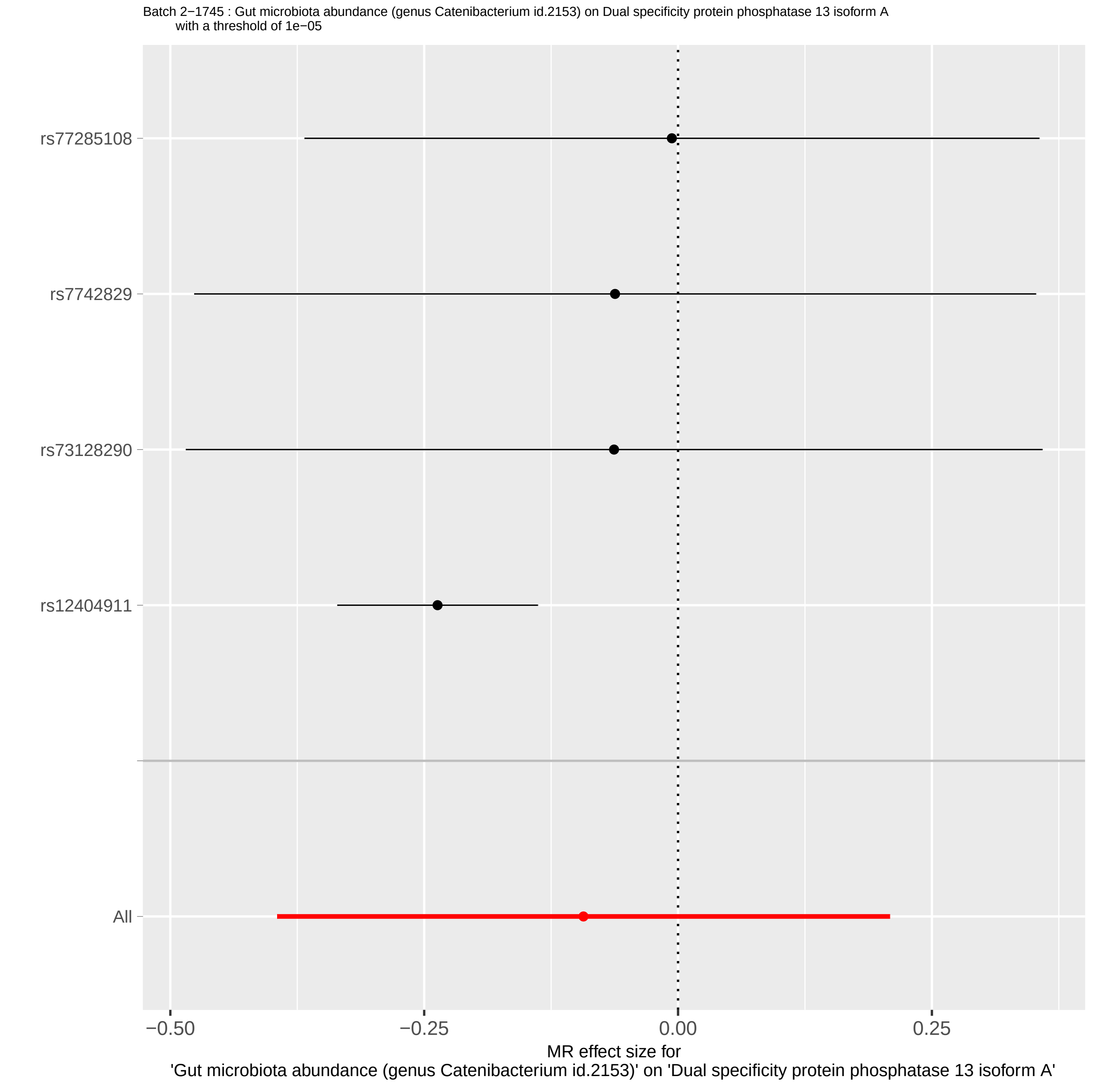
Batch 2−1745 : Gut microbiota abundance (genus Catenibacterium id.2153) on Dual specificity protein phosphatase 13 isoform A
with a threshold of 1e−05
rs77285108
rs7742829
rs73128290
rs12404911
All
−0.50
−0.25
0.00
0.25
MR effect size for
'Gut microbiota abundance (genus Catenibacterium id.2153)' on 'Dual specificity protein phosphatase 13 isoform A'

Batch 2−1746 : Gut microbiota abundance (genus Parasutterella id.2892) on Dual specificity protein phosphatase 13 isoform A
with a threshold of 1e−05

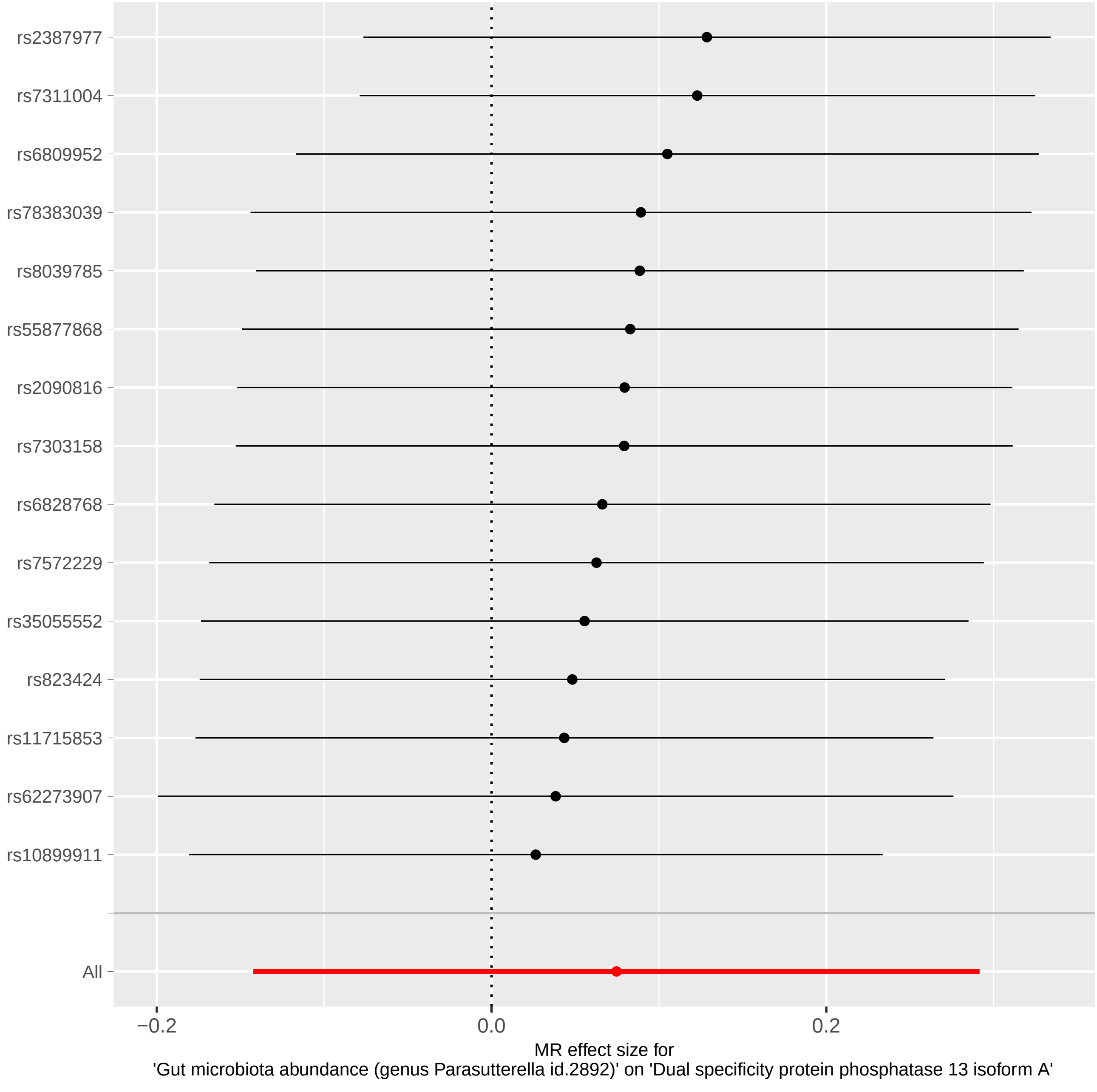

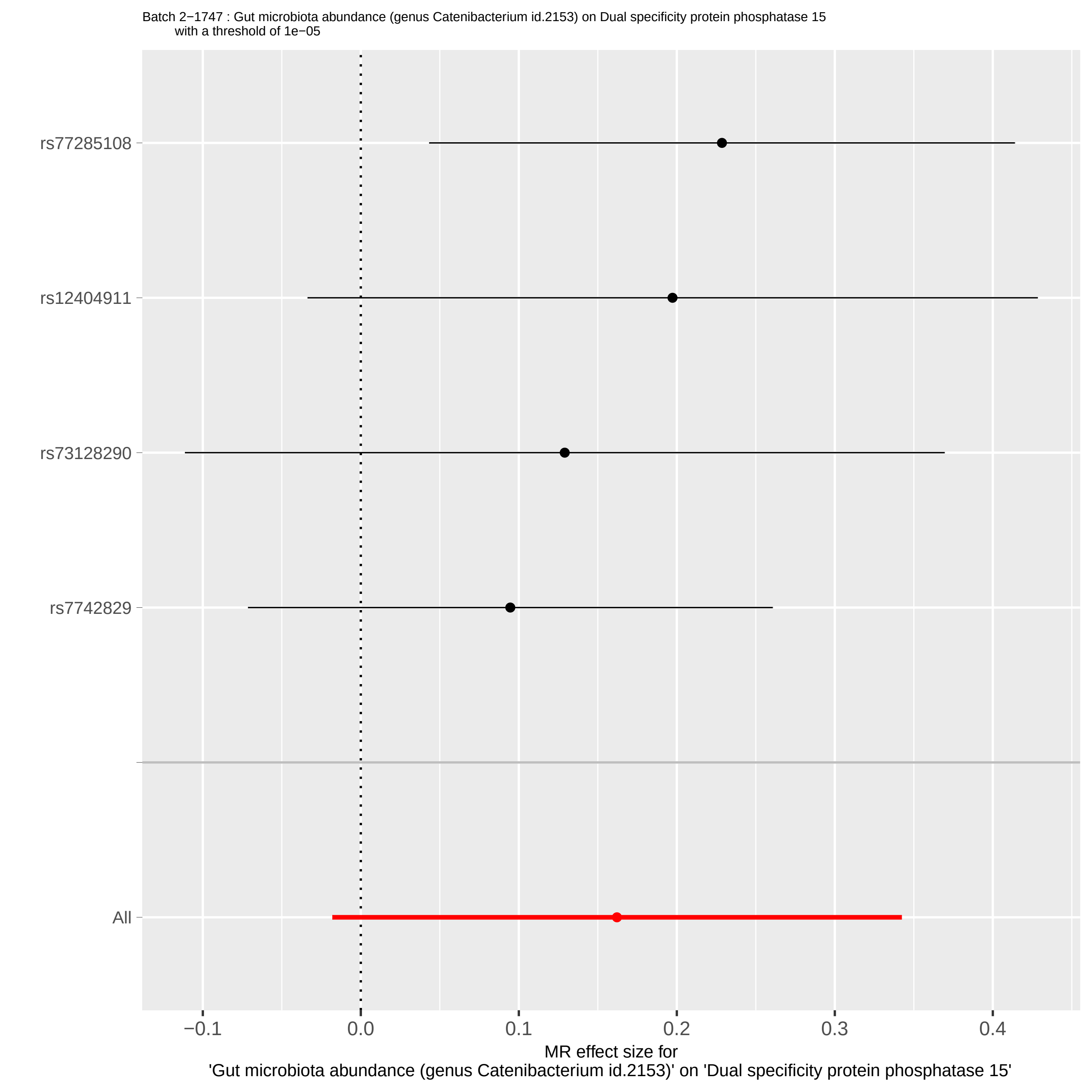
Batch 2−1747 : Gut microbiota abundance (genus Catenibacterium id.2153) on Dual specificity protein phosphatase 15
with a threshold of 1e−05
rs77285108
rs12404911
rs73128290
rs7742829
All
−0.1
0.0
0.1
0.2
0.3
0.4
MR effect size for
'Gut microbiota abundance (genus Catenibacterium id.2153)' on 'Dual specificity protein phosphatase 15'

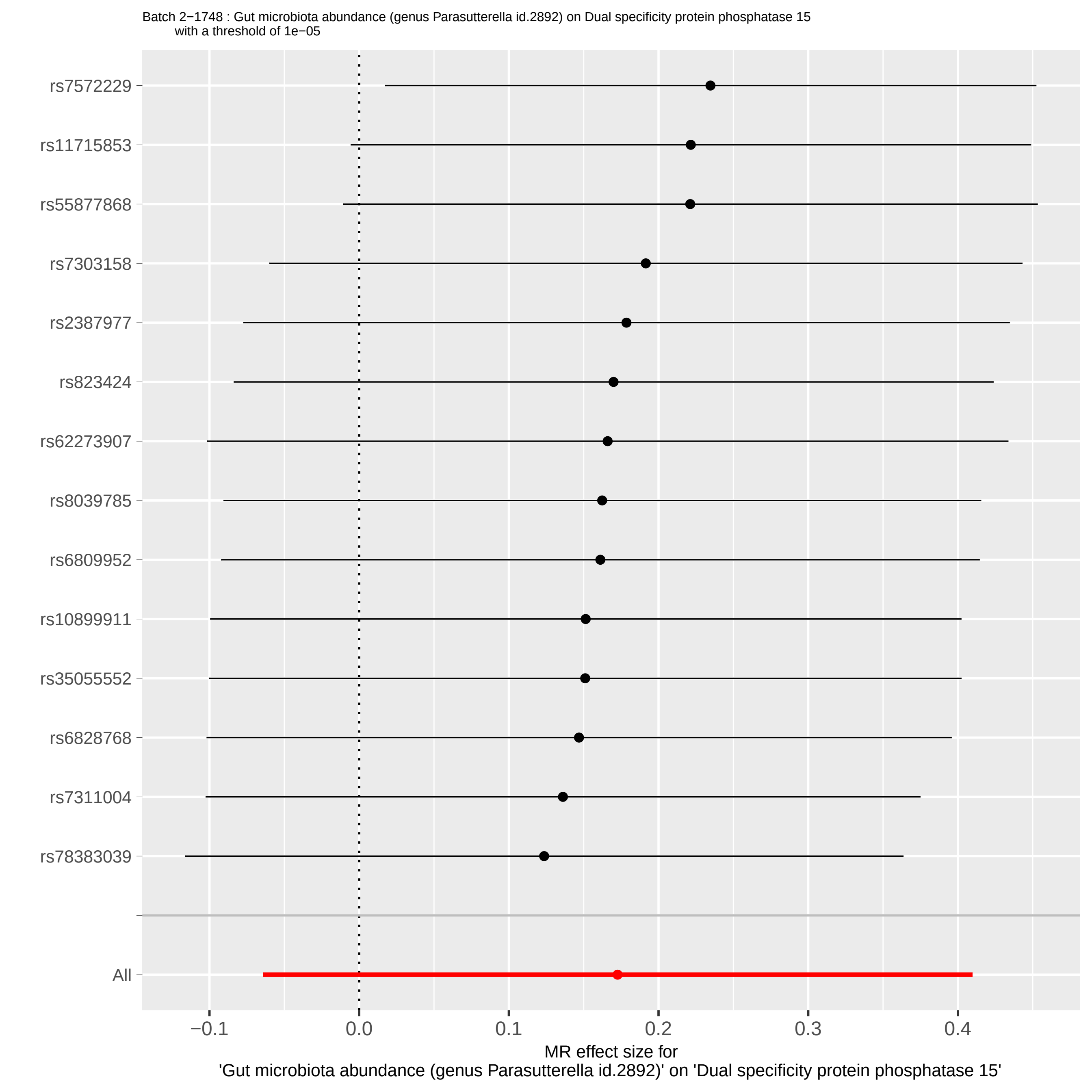
Batch 2−1748 : Gut microbiota abundance (genus Parasutterella id.2892) on Dual specificity protein phosphatase 15
with a threshold of 1e−05
rs7572229
rs11715853
rs55877868
rs7303158
rs2387977
rs823424
rs62273907
rs8039785
rs6809952
rs10899911
rs35055552
rs6828768
rs7311004
rs78383039
All
−0.1
0.0
0.1
0.2
0.3
0.4
MR effect size for
'Gut microbiota abundance (genus Parasutterella id.2892)' on 'Dual specificity protein phosphatase 15'

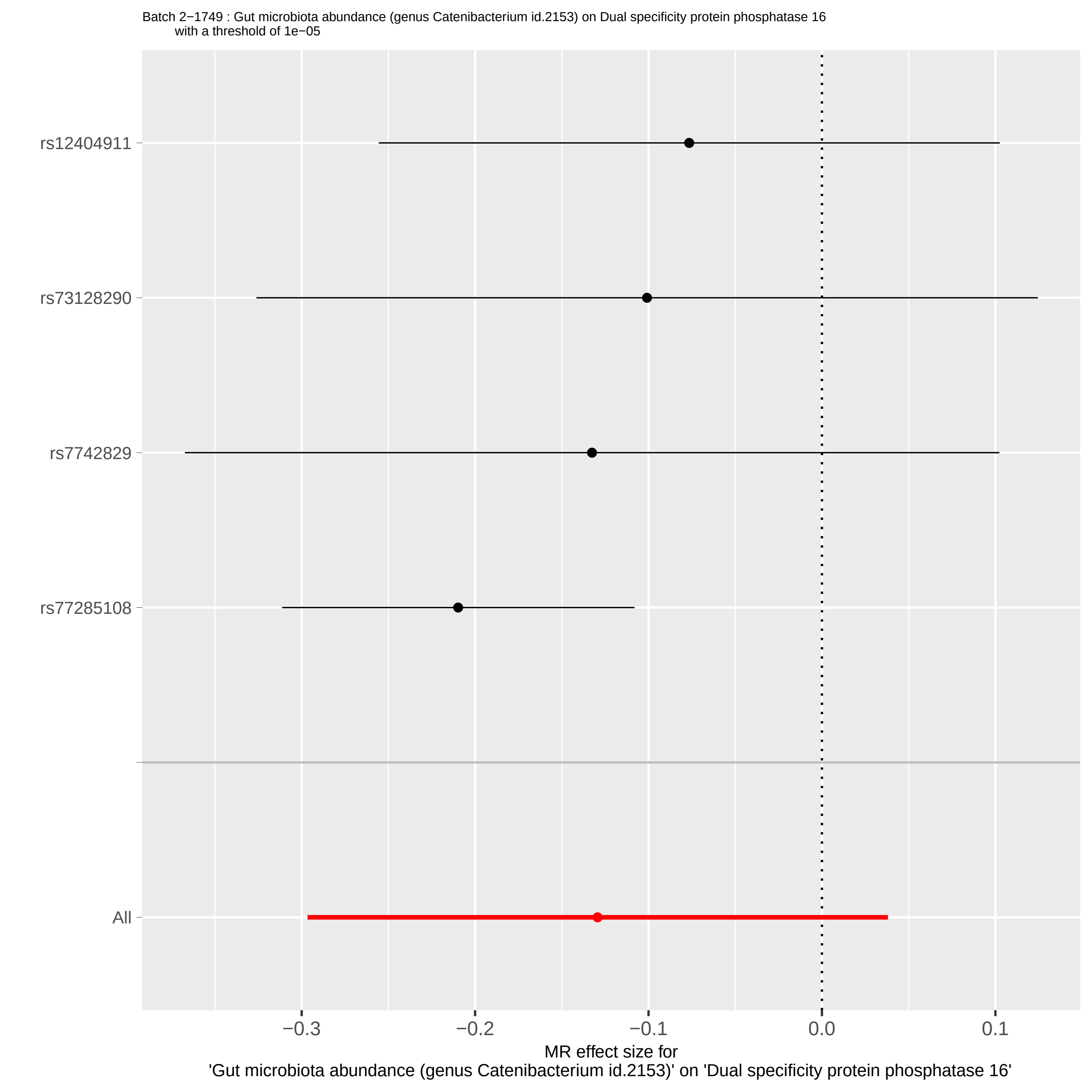

Batch 2−1749 : Gut microbiota abundance (genus Catenibacterium id.2153) on Dual specificity protein phosphatase 16
with a threshold of 1e−05
rs12404911
rs73128290
rs7742829
rs77285108
All
−0.3
−0.2
−0.1
0.0
0.1
MR effect size for
'Gut microbiota abundance (genus Catenibacterium id.2153)' on 'Dual specificity protein phosphatase 16'

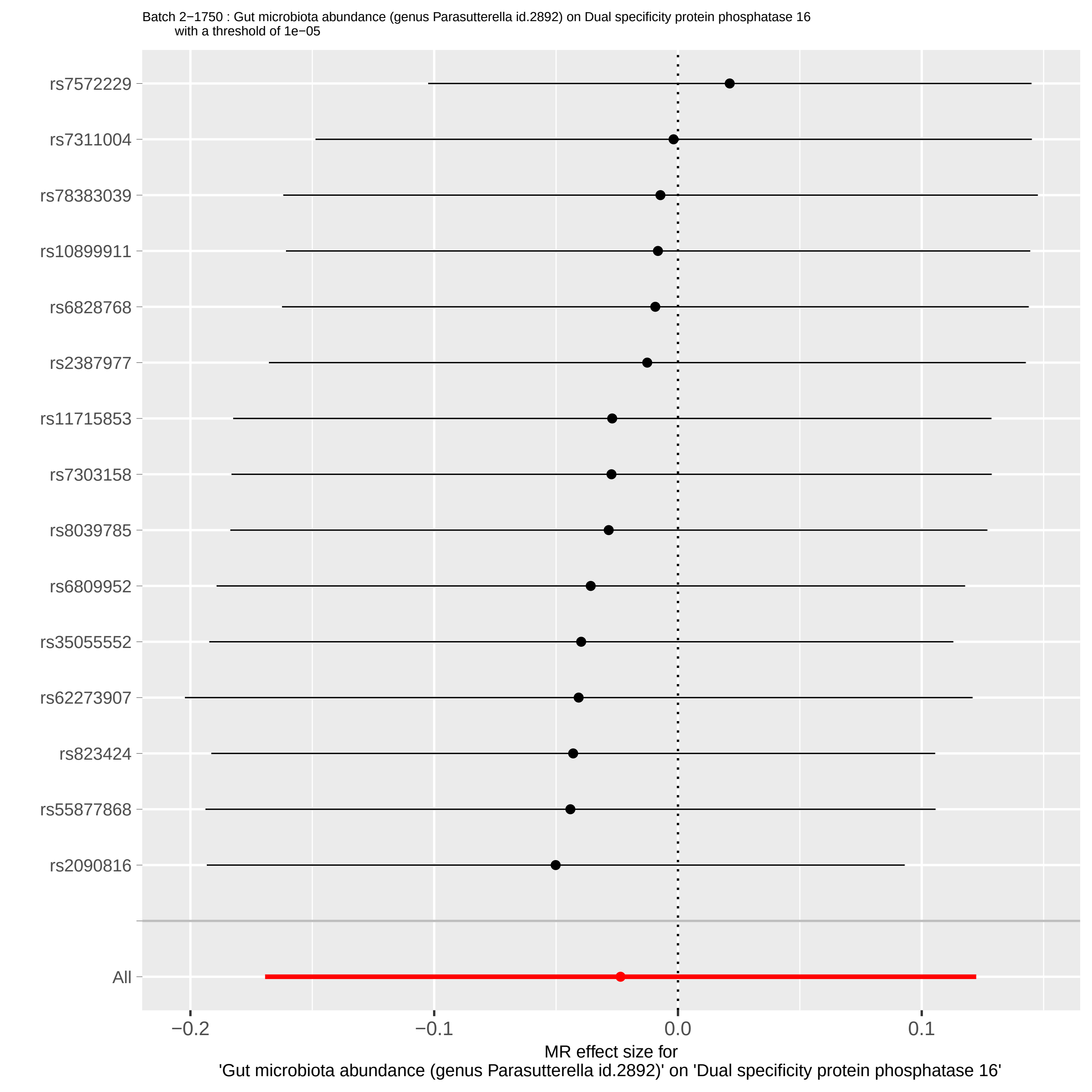

Batch 2−1750 : Gut microbiota abundance (genus Parasutterella id.2892) on Dual specificity protein phosphatase 16
with a threshold of 1e−05
rs7572229
rs7311004
rs78383039
rs10899911
rs6828768
rs2387977
rs11715853
rs7303158
rs8039785
rs6809952
rs35055552
rs62273907
rs823424
rs55877868
rs2090816
All
−0.2
−0.1
0.0
0.1
MR effect size for
'Gut microbiota abundance (genus Parasutterella id.2892)' on 'Dual specificity protein phosphatase 16'

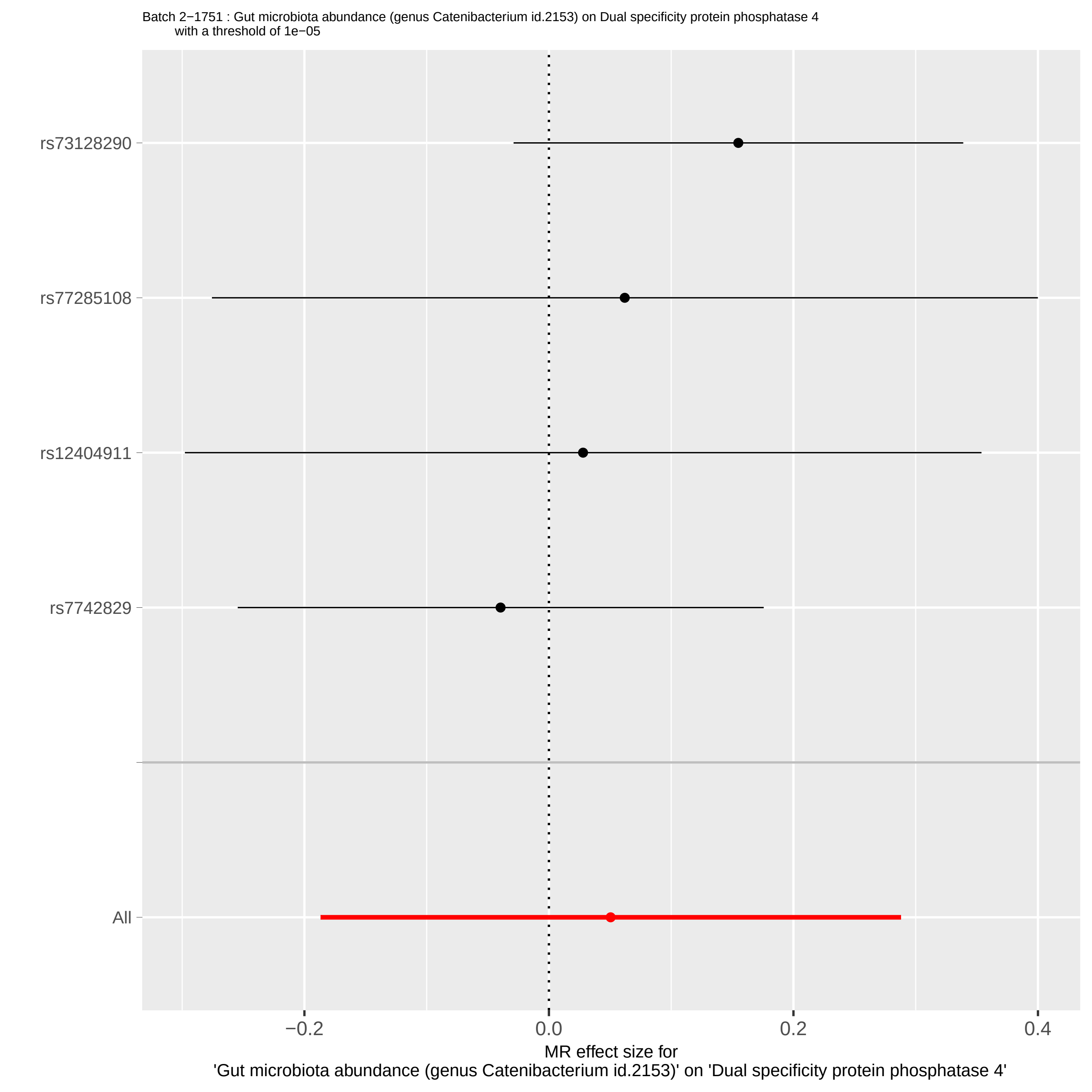
Batch 2−1751 : Gut microbiota abundance (genus Catenibacterium id.2153) on Dual specificity protein phosphatase 4
with a threshold of 1e−05
rs73128290
rs77285108
rs12404911
rs7742829
All
−0.2
0.0
0.2
0.4
MR effect size for
'Gut microbiota abundance (genus Catenibacterium id.2153)' on 'Dual specificity protein phosphatase 4'

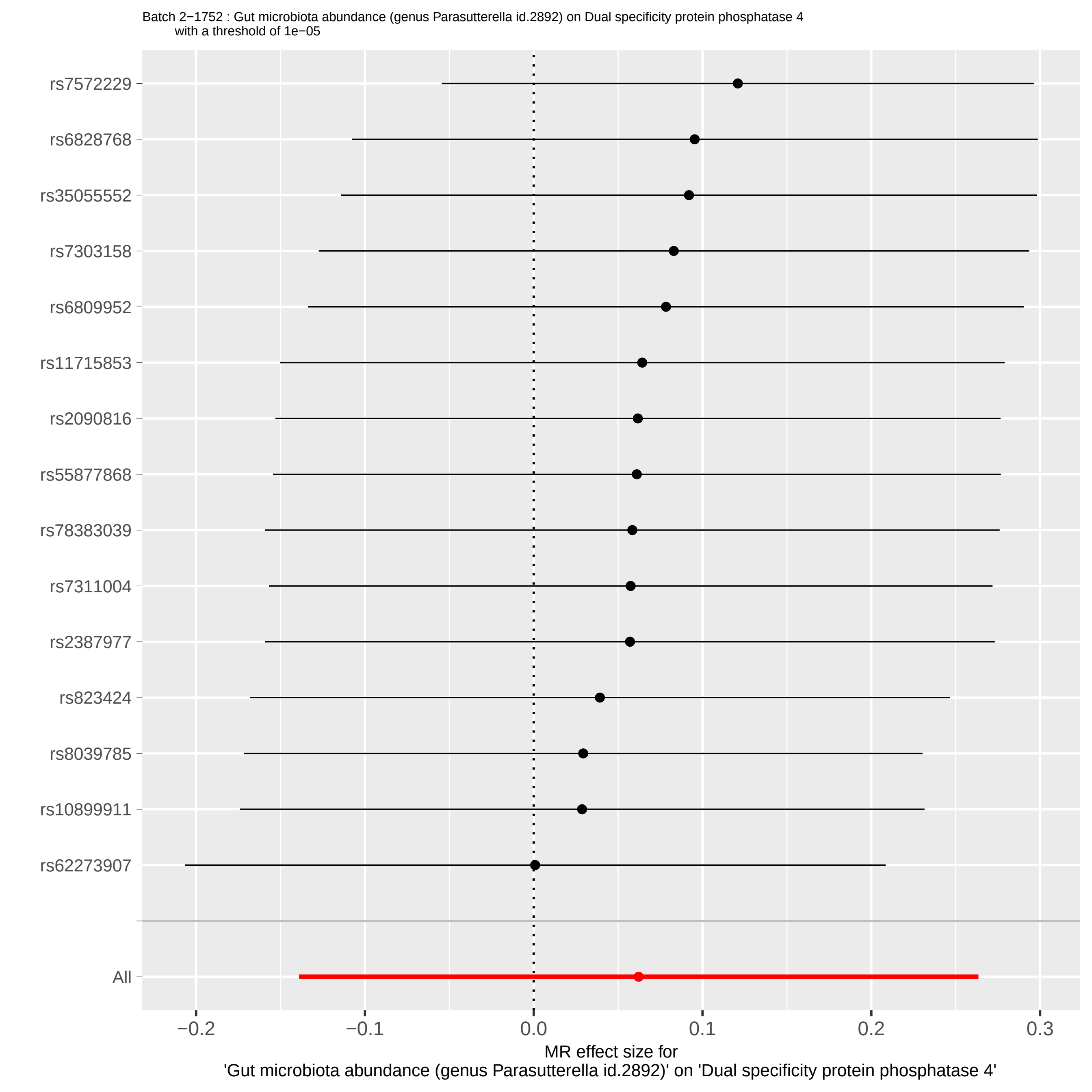
Batch 2−1752 : Gut microbiota abundance (genus Parasutterella id.2892) on Dual specificity protein phosphatase 4
with a threshold of 1e−05
rs7572229
rs6828768
rs35055552
rs7303158
rs6809952
rs11715853
rs2090816
rs55877868
rs78383039
rs7311004
rs2387977
rs823424
rs8039785
rs10899911
rs62273907
All
−0.2
−0.1
0.0
0.1
0.2
0.3
MR effect size for
'Gut microbiota abundance (genus Parasutterella id.2892)' on 'Dual specificity protein phosphatase 4'

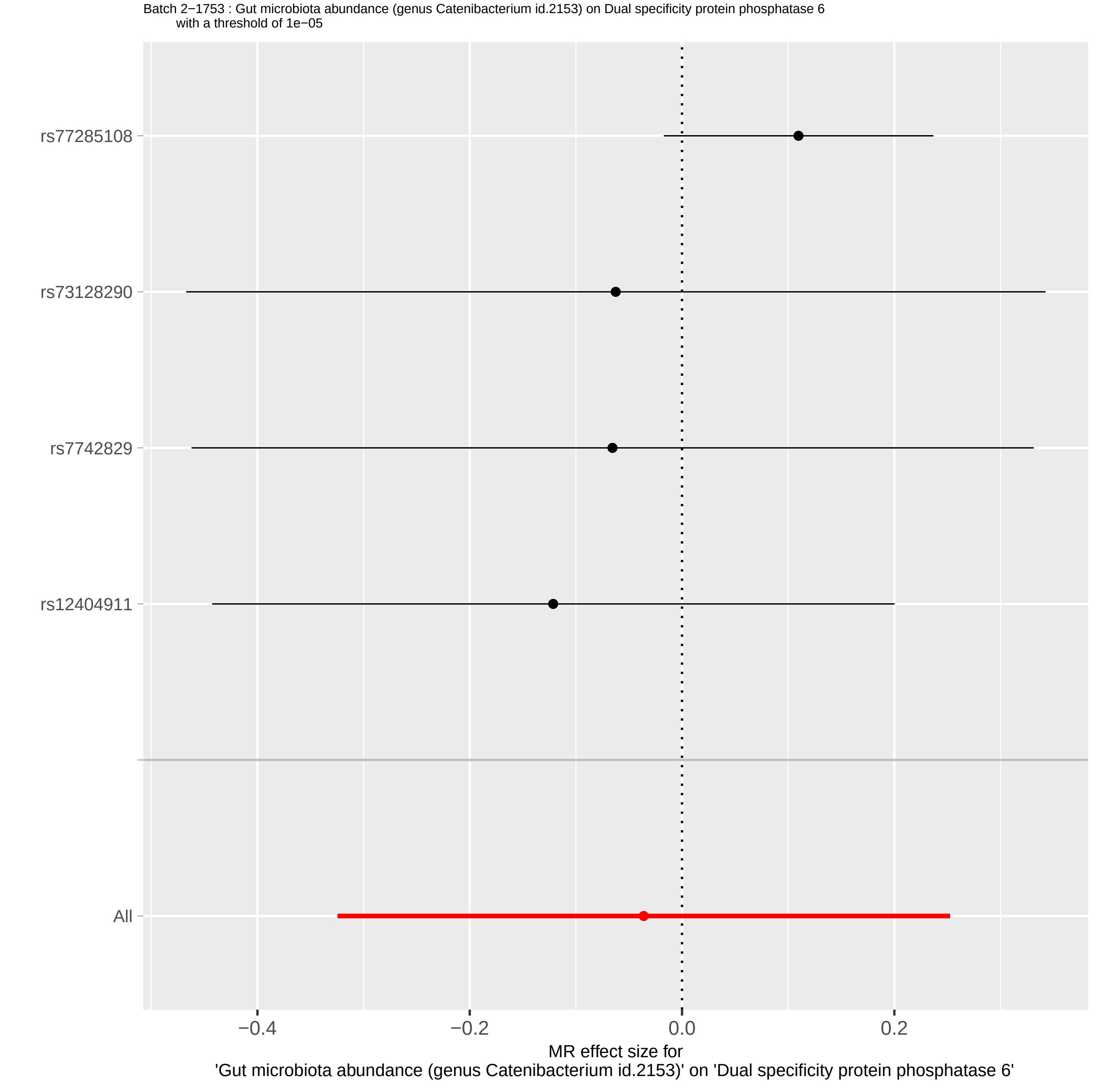
Batch 2−1753 : Gut microbiota abundance (genus Catenibacterium id.2153) on Dual specificity protein phosphatase 6
with a threshold of 1e−05
rs77285108
rs73128290
rs7742829
rs12404911
All
−0.4
−0.2
0.0
0.2
MR effect size for
'Gut microbiota abundance (genus Catenibacterium id.2153)' on 'Dual specificity protein phosphatase 6'

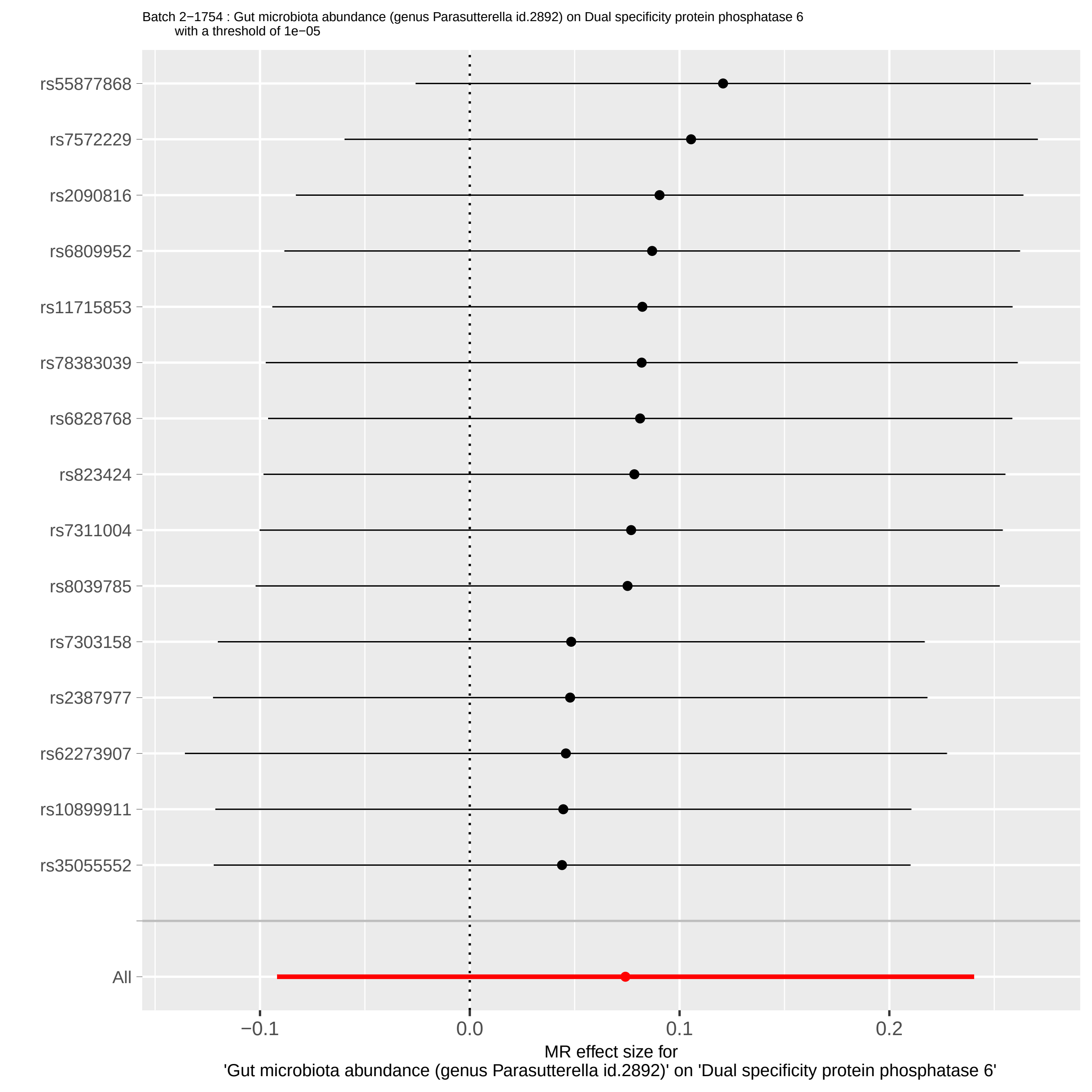
Batch 2−1754 : Gut microbiota abundance (genus Parasutterella id.2892) on Dual specificity protein phosphatase 6
with a threshold of 1e−05
rs55877868
rs7572229
rs2090816
rs6809952
rs11715853
rs78383039
rs6828768
rs823424
rs7311004
rs8039785
rs7303158
rs2387977
rs62273907
rs10899911
rs35055552
All
−0.1
0.0
0.1
0.2
MR effect size for
'Gut microbiota abundance (genus Parasutterella id.2892)' on 'Dual specificity protein phosphatase 6'

Batch 2−1755 : Gut microbiota abundance (genus Catenibacterium id.2153) on Segment polarity protein dishevelled homolog DVL−2
with a threshold of 1e−05

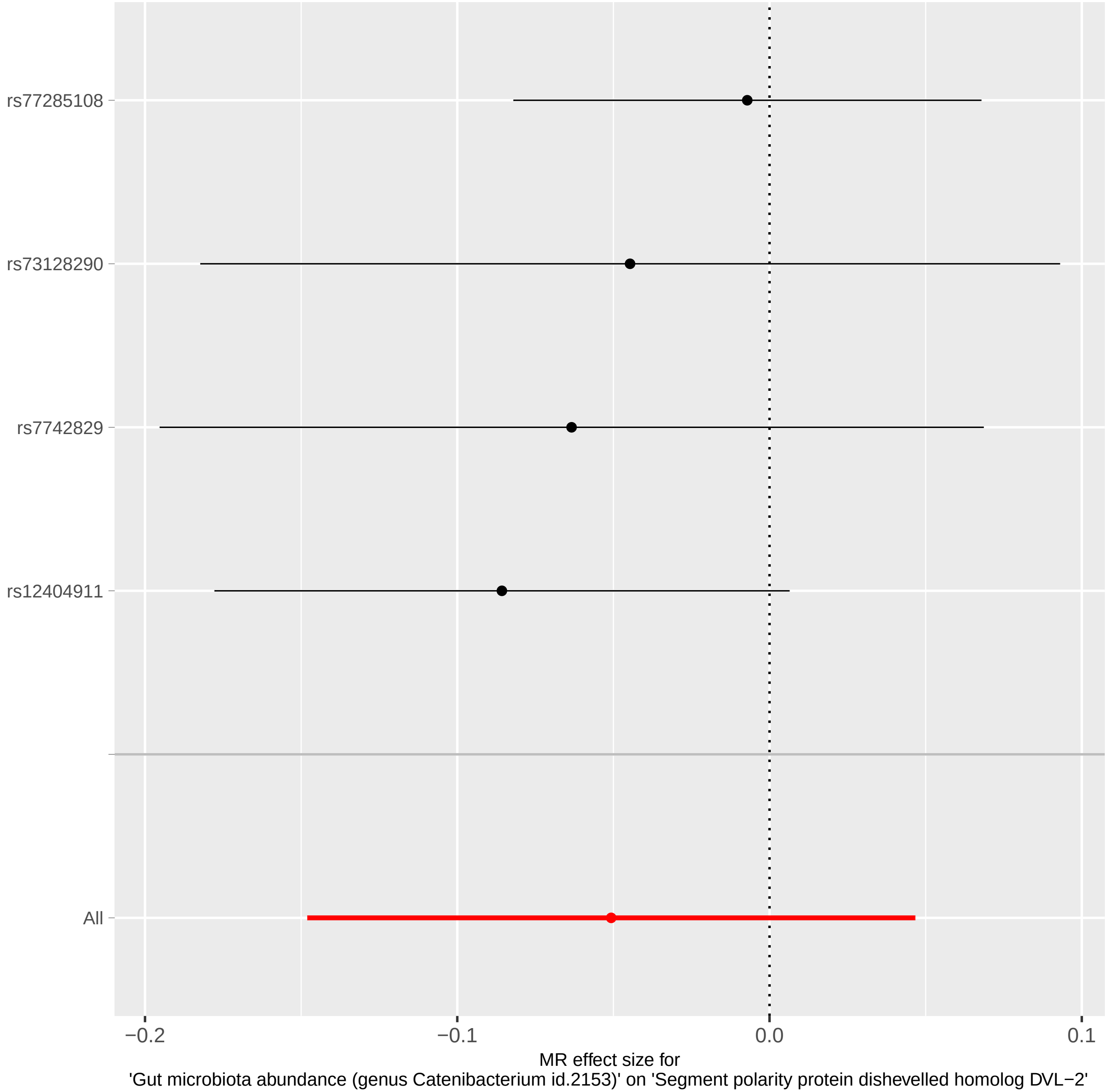

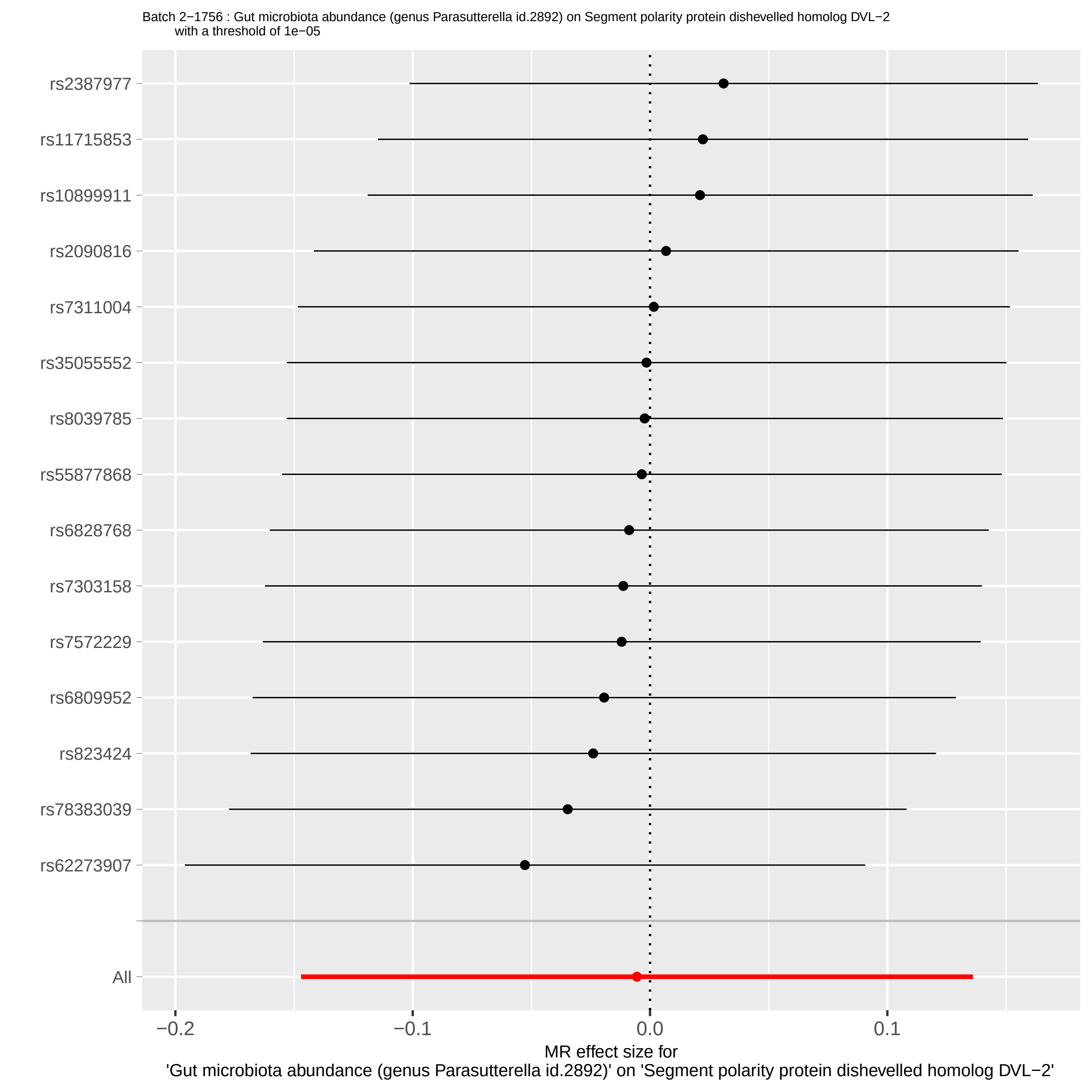
Batch 2−1756 : Gut microbiota abundance (genus Parasutterella id.2892) on Segment polarity protein dishevelled homolog DVL−2
with a threshold of 1e−05
rs2387977
rs11715853
rs10899911
rs2090816
rs7311004
rs35055552
rs8039785
rs55877868
rs6828768
rs7303158
rs7572229
rs6809952
rs823424
rs78383039
rs62273907
All
−0.2
−0.1
0.0
0.1
MR effect size for
'Gut microbiota abundance (genus Parasutterella id.2892)' on 'Segment polarity protein dishevelled homolog DVL−2'

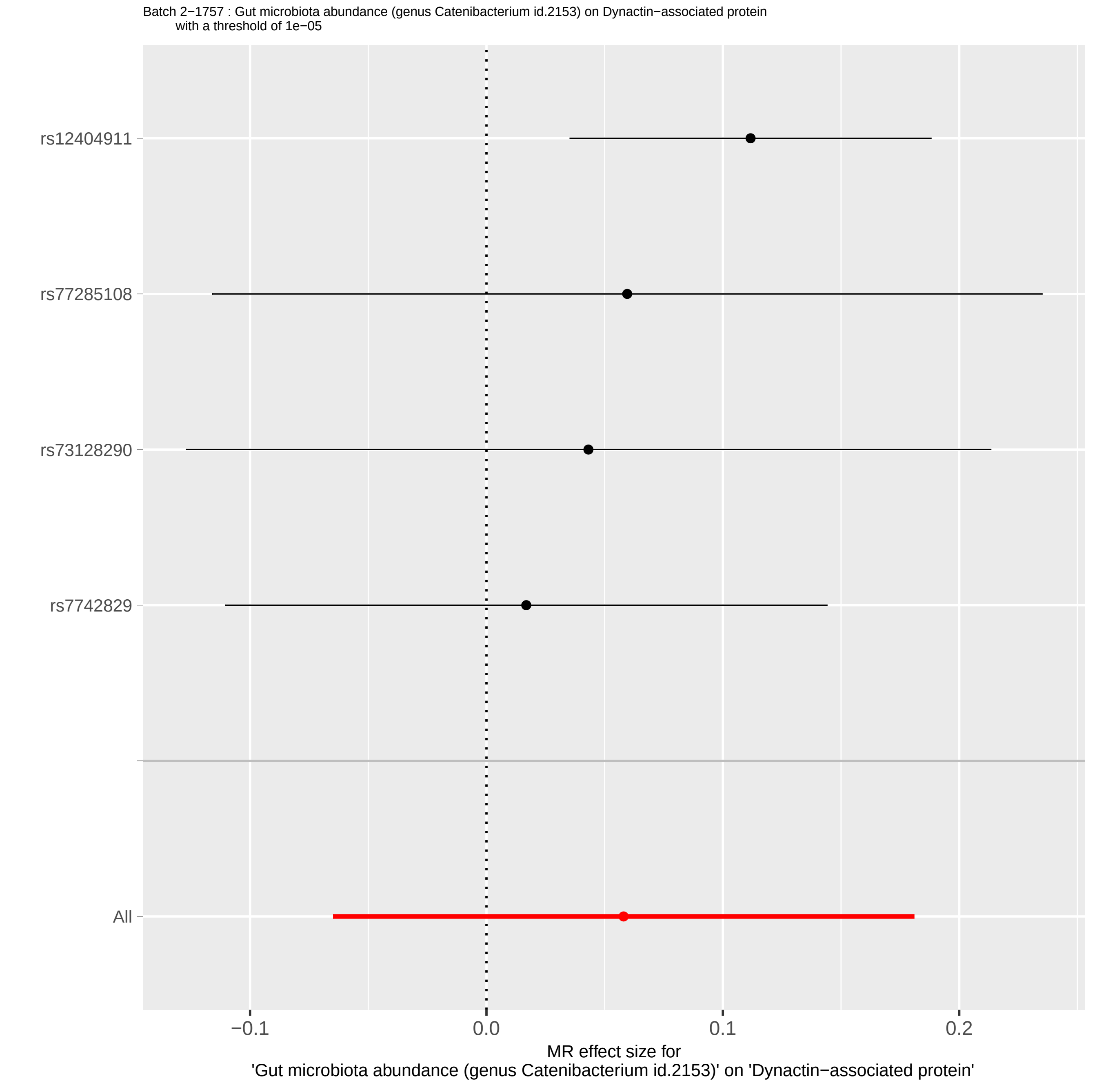

Batch 2−1757 : Gut microbiota abundance (genus Catenibacterium id.2153) on Dynactin−associated protein
with a threshold of 1e−05
rs12404911
rs77285108
rs73128290
rs7742829
All
−0.1
0.0
0.1
0.2
MR effect size for
'Gut microbiota abundance (genus Catenibacterium id.2153)' on 'Dynactin−associated protein'

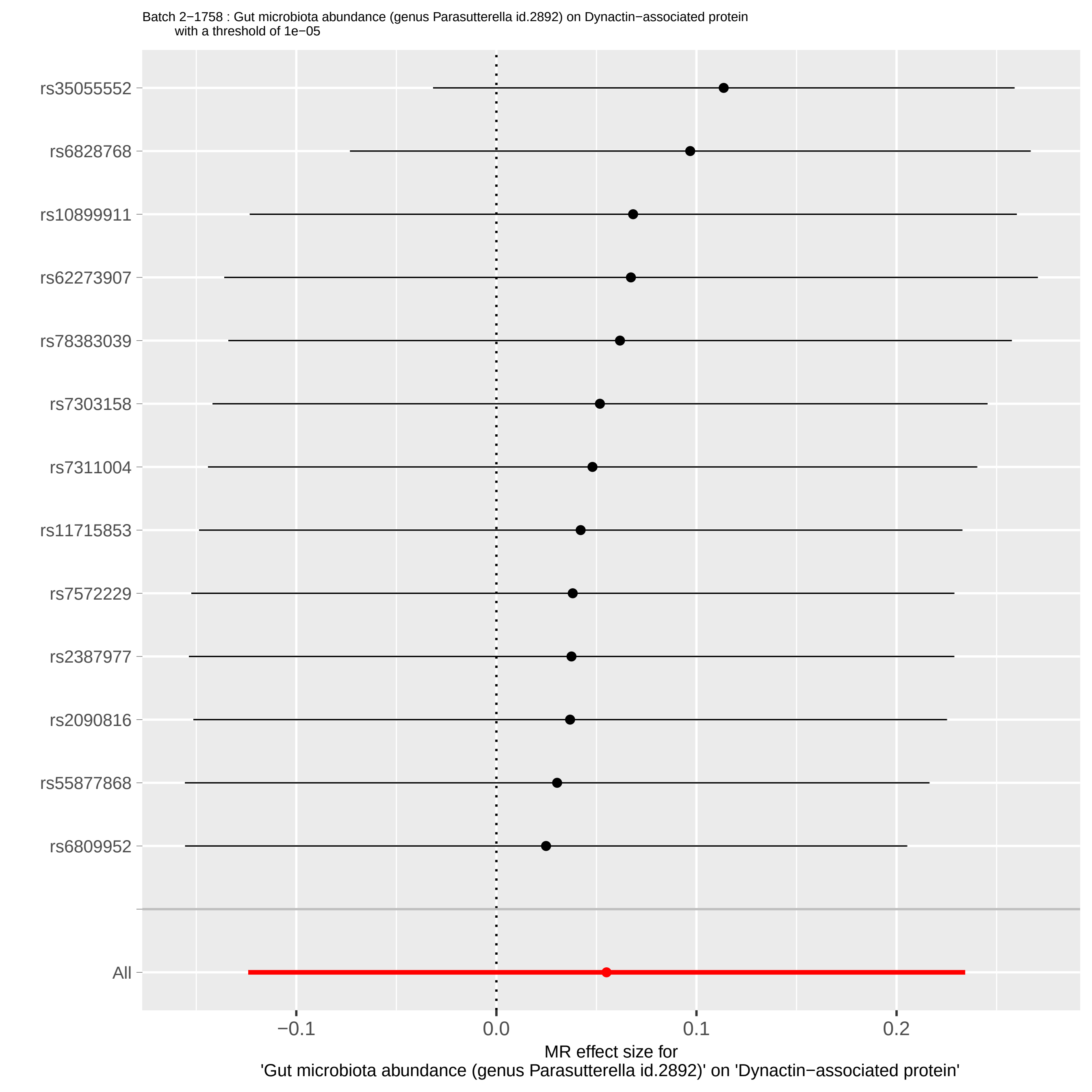
Batch 2−1758 : Gut microbiota abundance (genus Parasutterella id.2892) on Dynactin−associated protein
with a threshold of 1e−05
rs35055552
rs6828768
rs10899911
rs62273907
rs78383039
rs7303158
rs7311004
rs11715853
rs7572229
rs2387977
rs2090816
rs55877868
rs6809952
All
−0.1
0.0
0.1
0.2
MR effect size for
'Gut microbiota abundance (genus Parasutterella id.2892)' on 'Dynactin−associated protein'

Batch 2−1759 : Gut microbiota abundance (genus Catenibacterium id.2153) on Dynein light chain 1, cytoplasmic
with a threshold of 1e−05

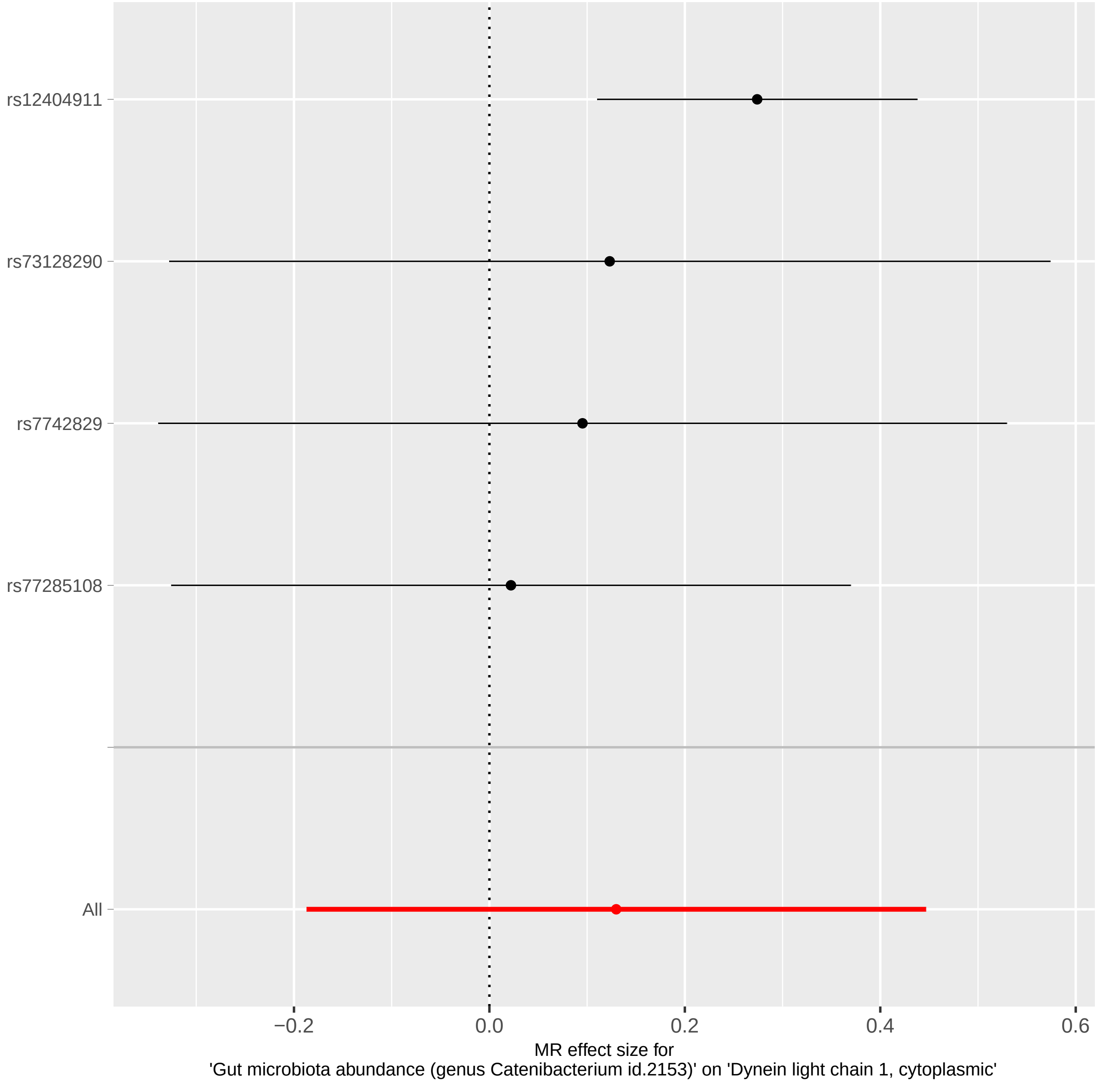

Batch 2−1760 : Gut microbiota abundance (genus Parasutterella id.2892) on Dynein light chain 1, cytoplasmic
with a threshold of 1e−05

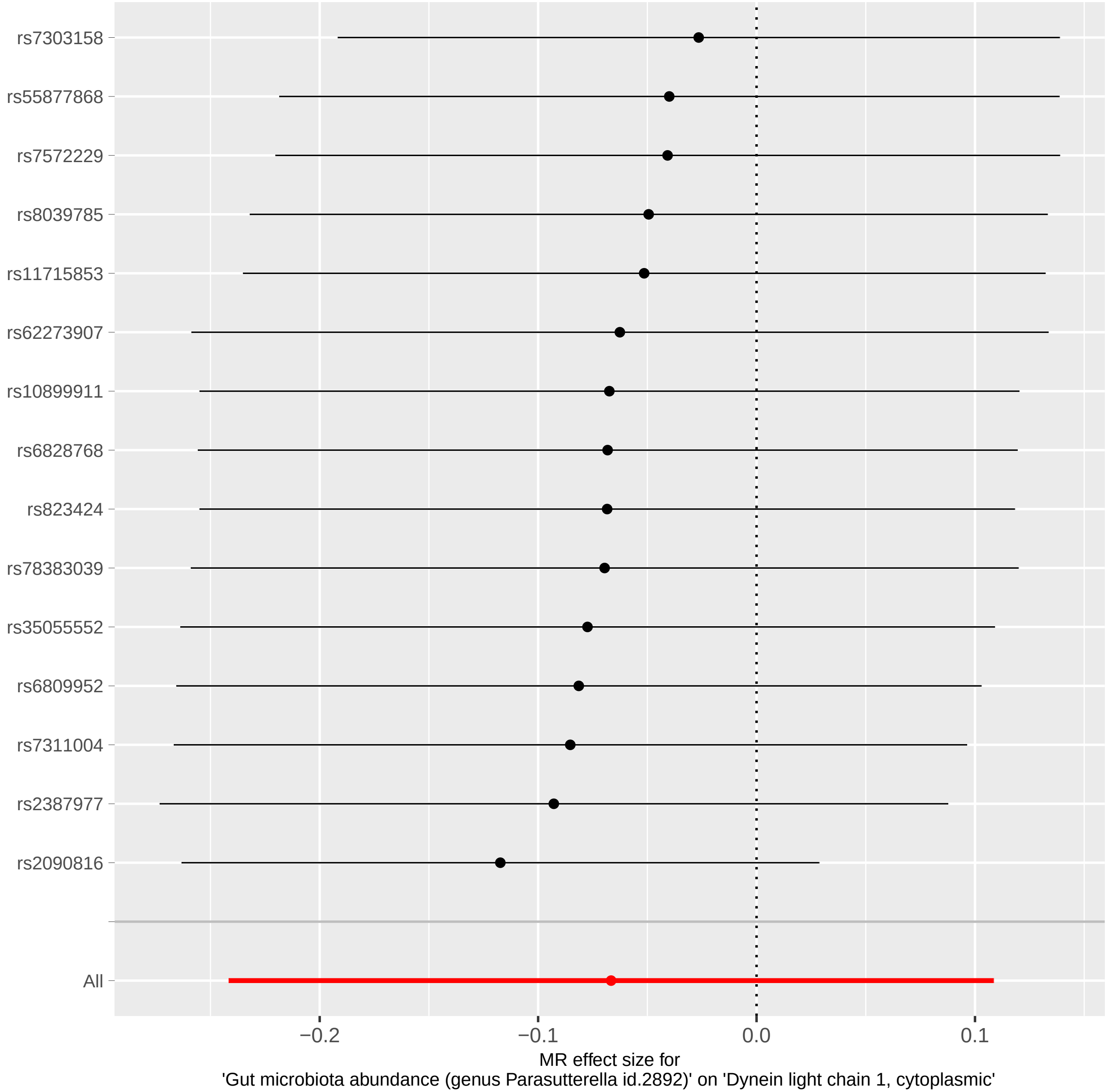

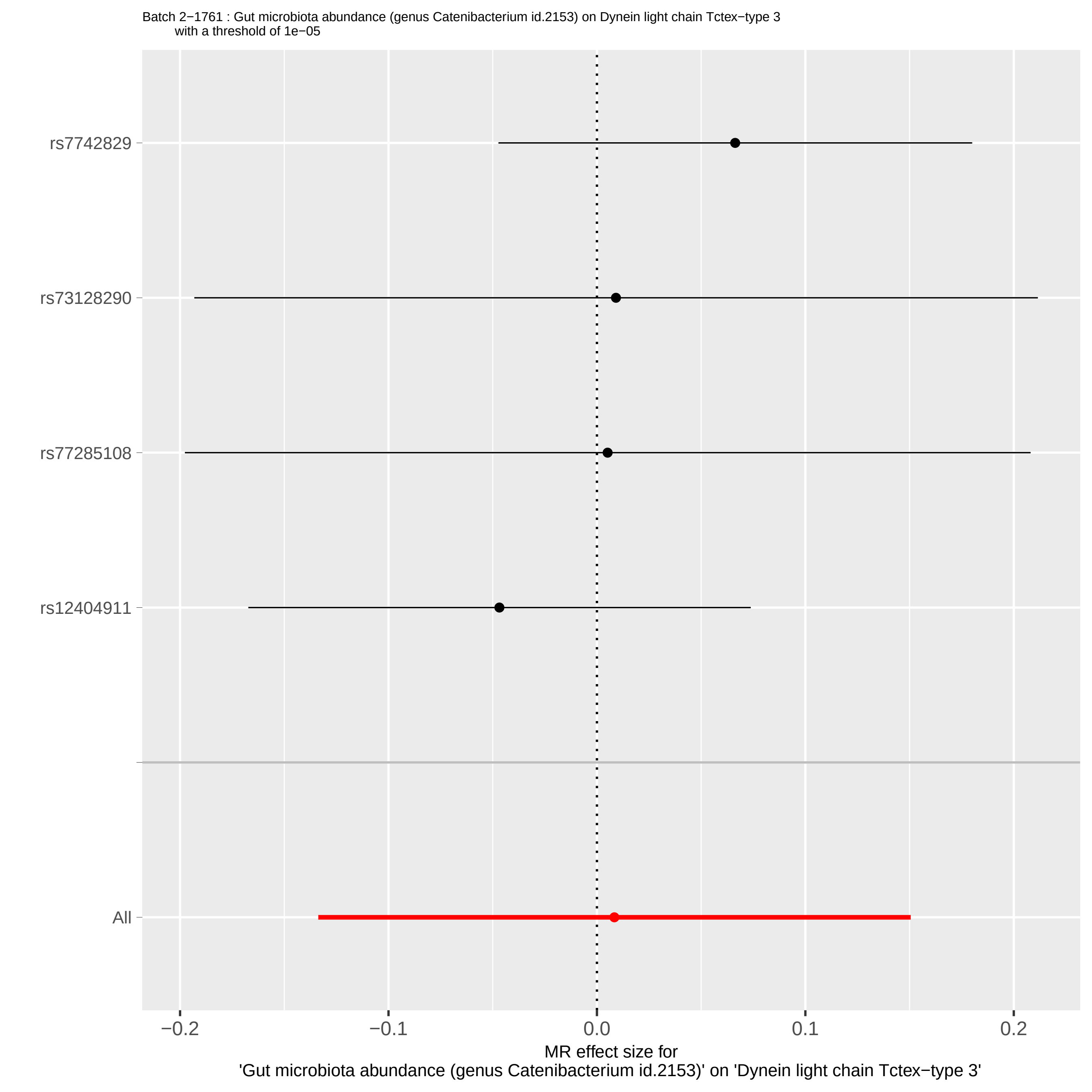

Batch 2−1761 : Gut microbiota abundance (genus Catenibacterium id.2153) on Dynein light chain Tctex−type 3
with a threshold of 1e−05
rs7742829
rs73128290
rs77285108
rs12404911
All
−0.2
−0.1
0.0
0.1
0.2
MR effect size for
'Gut microbiota abundance (genus Catenibacterium id.2153)' on 'Dynein light chain Tctex−type 3'

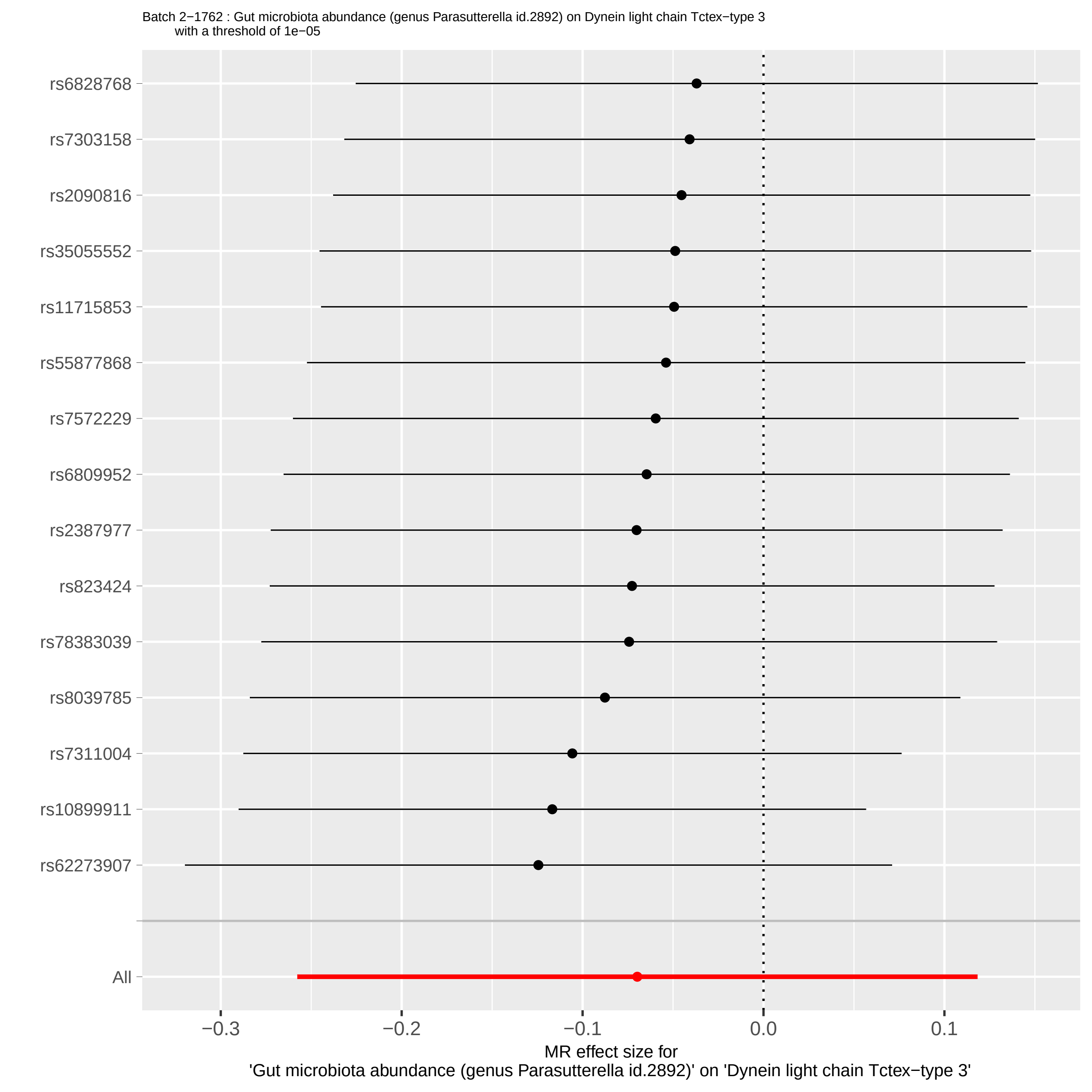

Batch 2−1762 : Gut microbiota abundance (genus Parasutterella id.2892) on Dynein light chain Tctex−type 3
with a threshold of 1e−05
rs6828768
rs7303158
rs2090816
rs35055552
rs11715853
rs55877868
rs7572229
rs6809952
rs2387977
rs823424
rs78383039
rs8039785
rs7311004
rs10899911
rs62273907
All
−0.3
−0.2
−0.1
0.0
0.1
MR effect size for
'Gut microbiota abundance (genus Parasutterella id.2892)' on 'Dynein light chain Tctex−type 3'

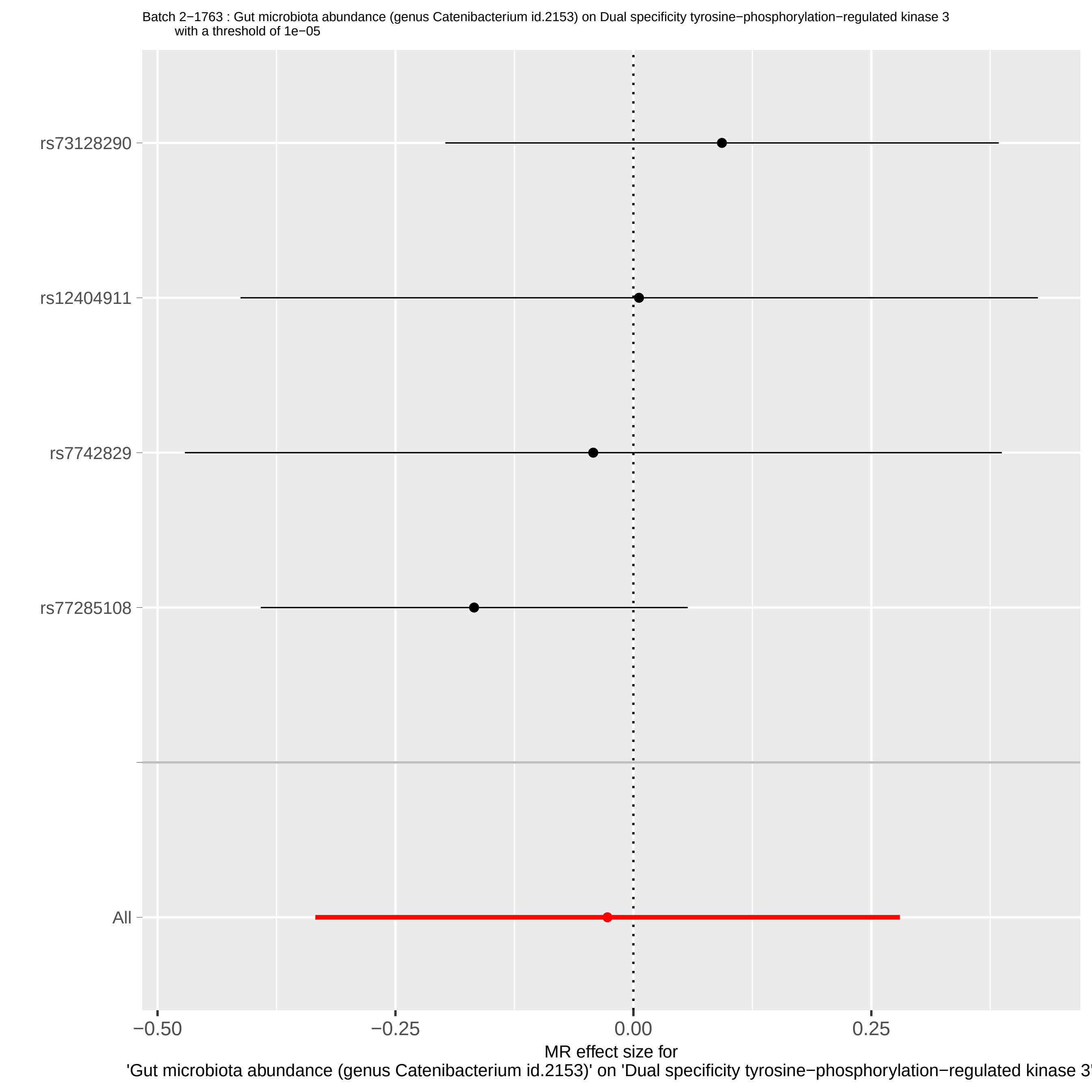
Batch 2−1763 : Gut microbiota abundance (genus Catenibacterium id.2153) on Dual specificity tyrosine−phosphorylation−regulated kinase 3
with a threshold of 1e−05
rs73128290
rs12404911
rs7742829
rs77285108
All
−0.50
−0.25
0.00
0.25
MR effect size for
'Gut microbiota abundance (genus Catenibacterium id.2153)' on 'Dual specificity tyrosine−phosphorylation−regulated kinase 3'

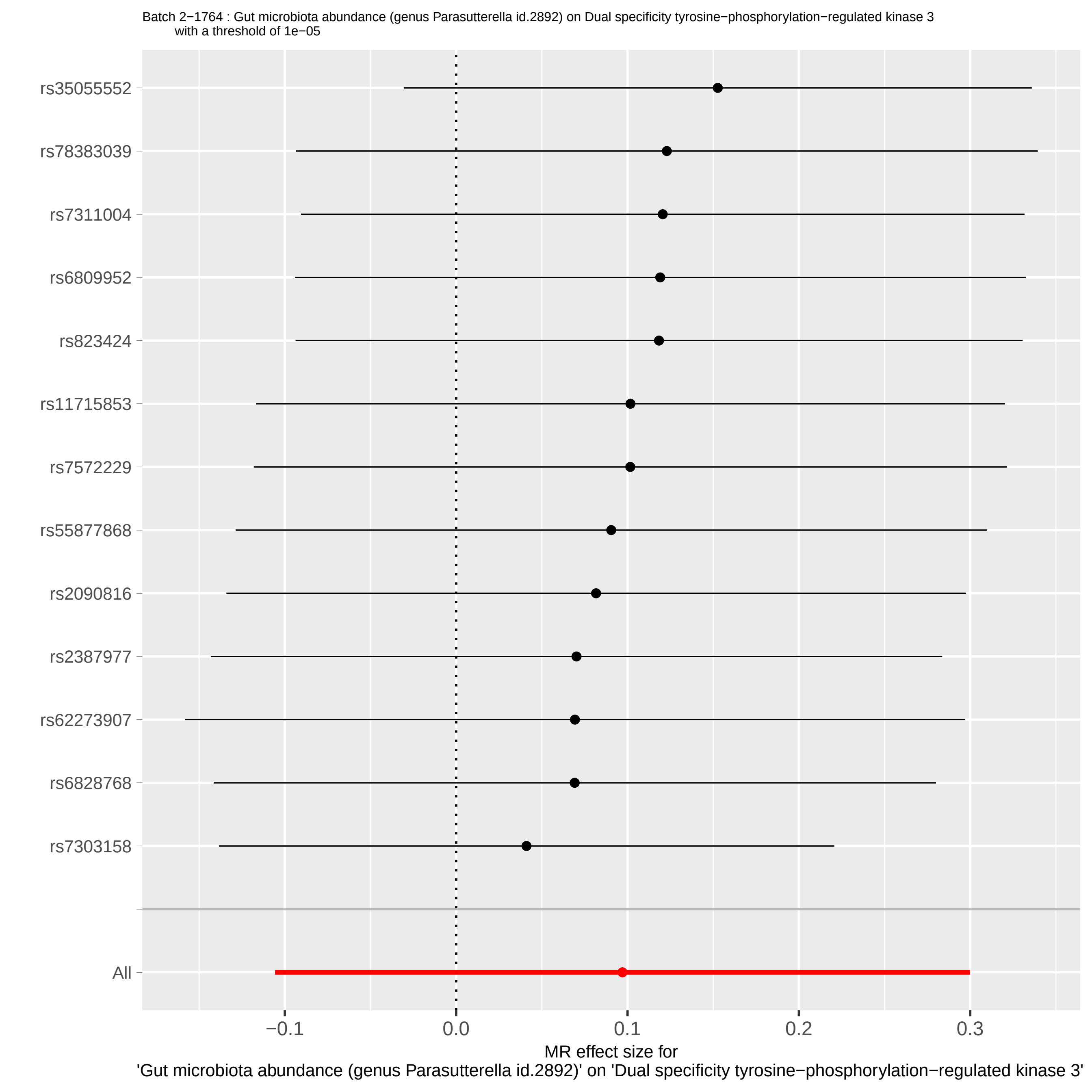

Batch 2−1764 : Gut microbiota abundance (genus Parasutterella id.2892) on Dual specificity tyrosine−phosphorylation−regulated kinase 3
with a threshold of 1e−05
rs35055552
rs78383039
rs7311004
rs6809952
rs823424
rs11715853
rs7572229
rs55877868
rs2090816
rs2387977
rs62273907
rs6828768
rs7303158
All
−0.1
0.0
0.1
0.2
0.3
MR effect size for
'Gut microbiota abundance (genus Parasutterella id.2892)' on 'Dual specificity tyrosine−phosphorylation−regulated kinase 3'

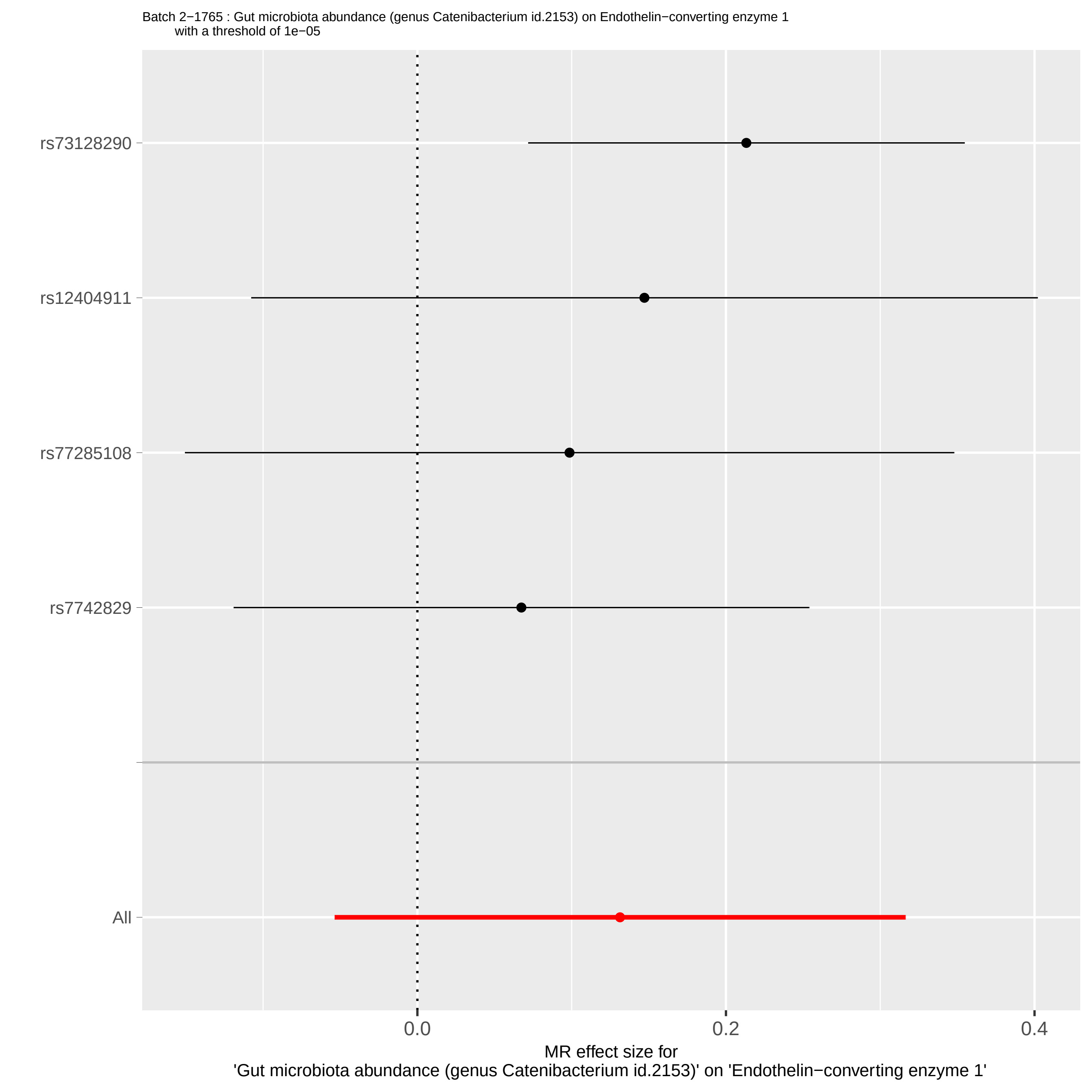
Batch 2−1765 : Gut microbiota abundance (genus Catenibacterium id.2153) on Endothelin−converting enzyme 1
with a threshold of 1e−05
rs73128290
rs12404911
rs77285108
rs7742829
All
0.0
0.2
0.4
MR effect size for
'Gut microbiota abundance (genus Catenibacterium id.2153)' on 'Endothelin−converting enzyme 1'

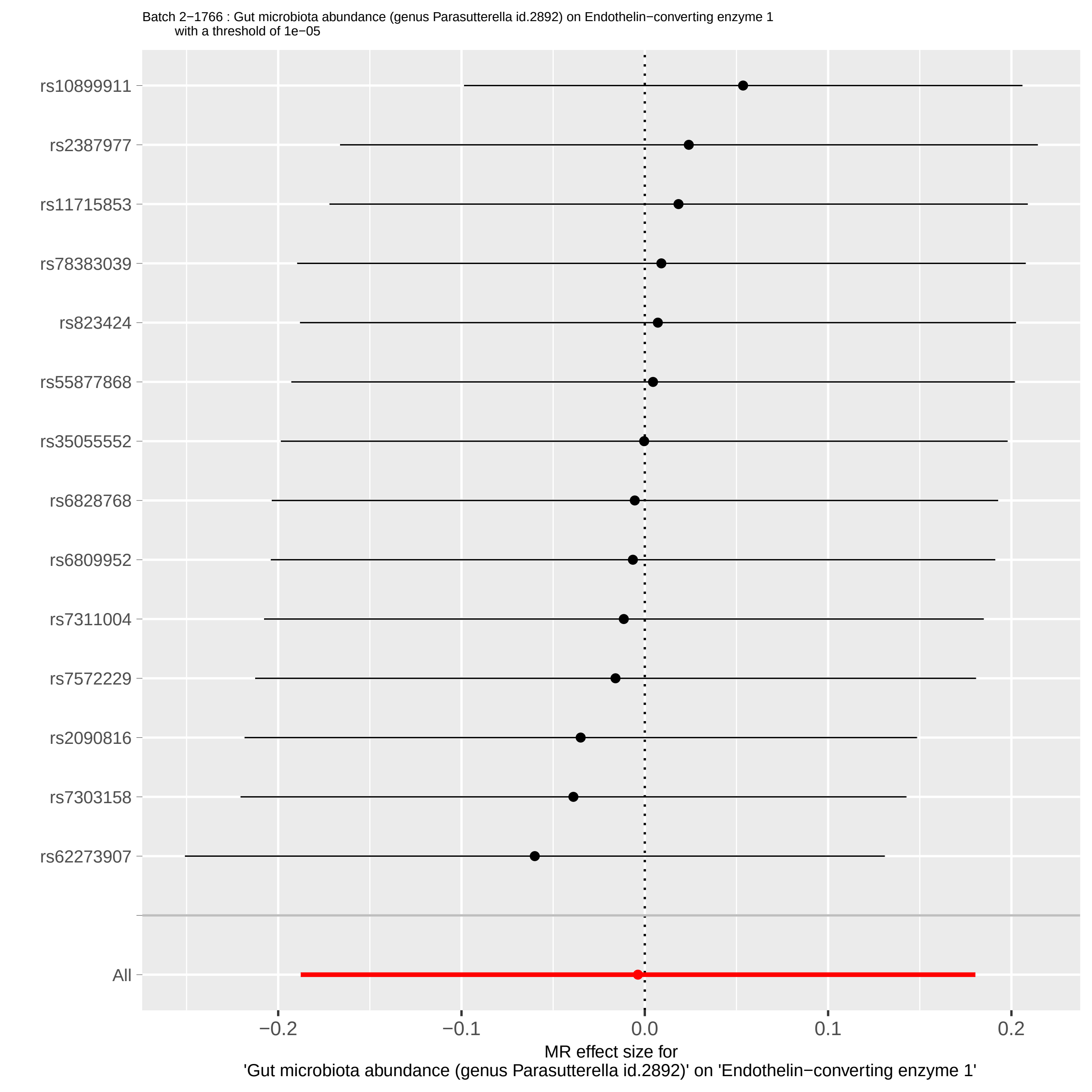
Batch 2−1766 : Gut microbiota abundance (genus Parasutterella id.2892) on Endothelin−converting enzyme 1
with a threshold of 1e−05
rs10899911
rs2387977
rs11715853
rs78383039
rs823424
rs55877868
rs35055552
rs6828768
rs6809952
rs7311004
rs7572229
rs2090816
rs7303158
rs62273907
All
−0.2
−0.1
0.0
0.1
0.2
MR effect size for
'Gut microbiota abundance (genus Parasutterella id.2892)' on 'Endothelin−converting enzyme 1'

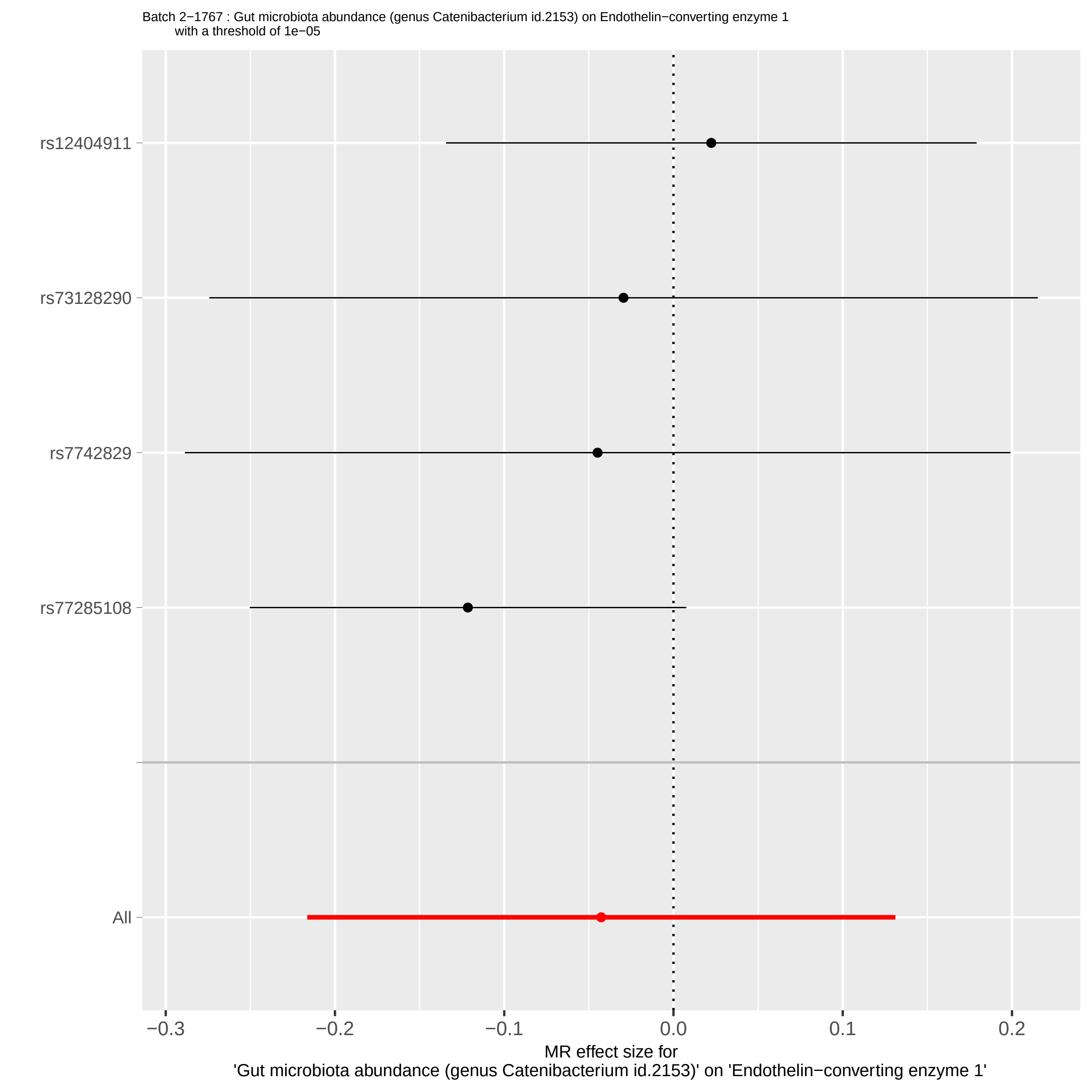
Batch 2−1767 : Gut microbiota abundance (genus Catenibacterium id.2153) on Endothelin−converting enzyme 1
with a threshold of 1e−05
rs12404911
rs73128290
rs7742829
rs77285108
All
−0.3
−0.2
−0.1
0.0
0.1
0.2
MR effect size for
'Gut microbiota abundance (genus Catenibacterium id.2153)' on 'Endothelin−converting enzyme 1'

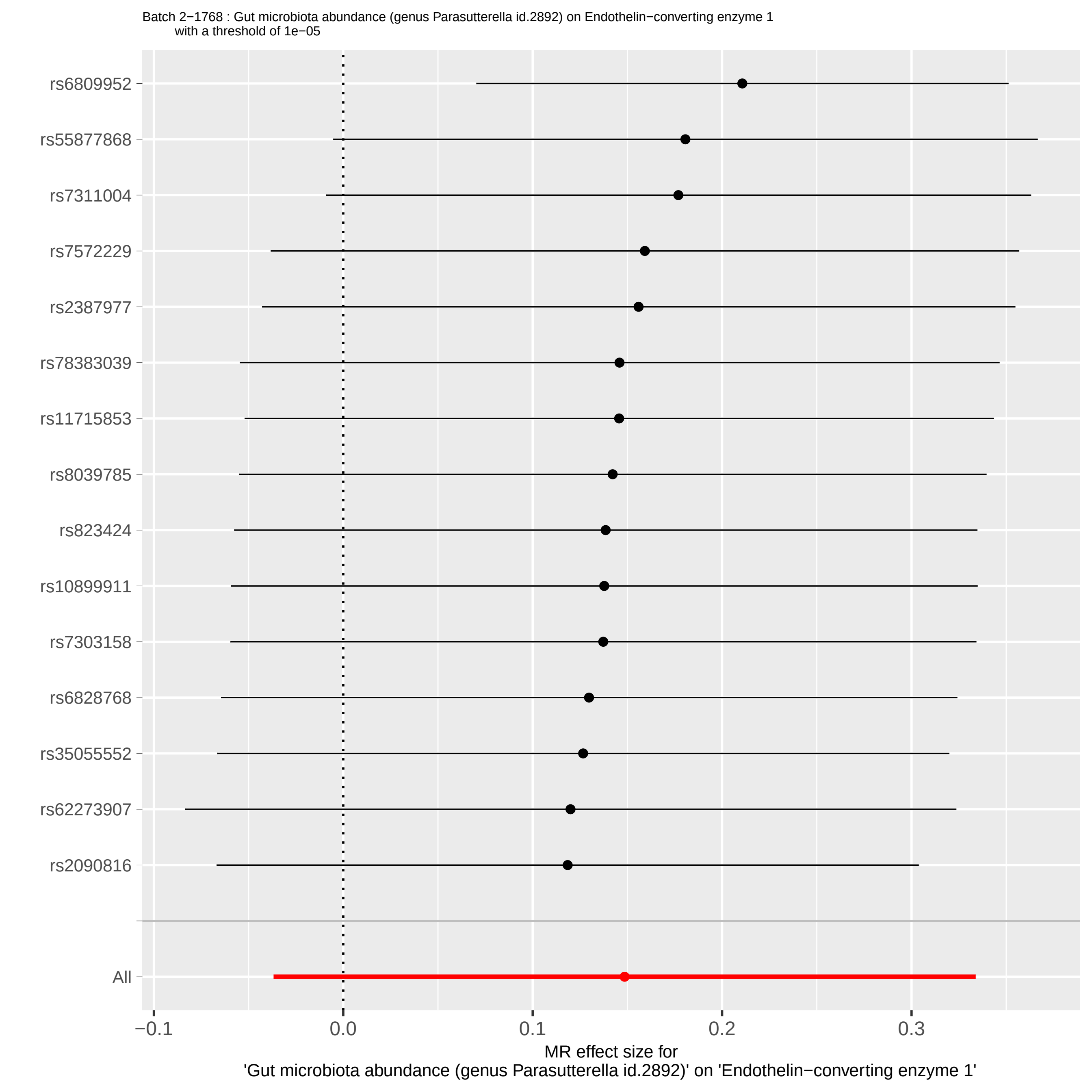
Batch 2−1768 : Gut microbiota abundance (genus Parasutterella id.2892) on Endothelin−converting enzyme 1
with a threshold of 1e−05
rs6809952
rs55877868
rs7311004
rs7572229
rs2387977
rs78383039
rs11715853
rs8039785
rs823424
rs10899911
rs7303158
rs6828768
rs35055552
rs62273907
rs2090816
All
−0.1
0.0
0.1
0.2
0.3
MR effect size for
'Gut microbiota abundance (genus Parasutterella id.2892)' on 'Endothelin−converting enzyme 1'

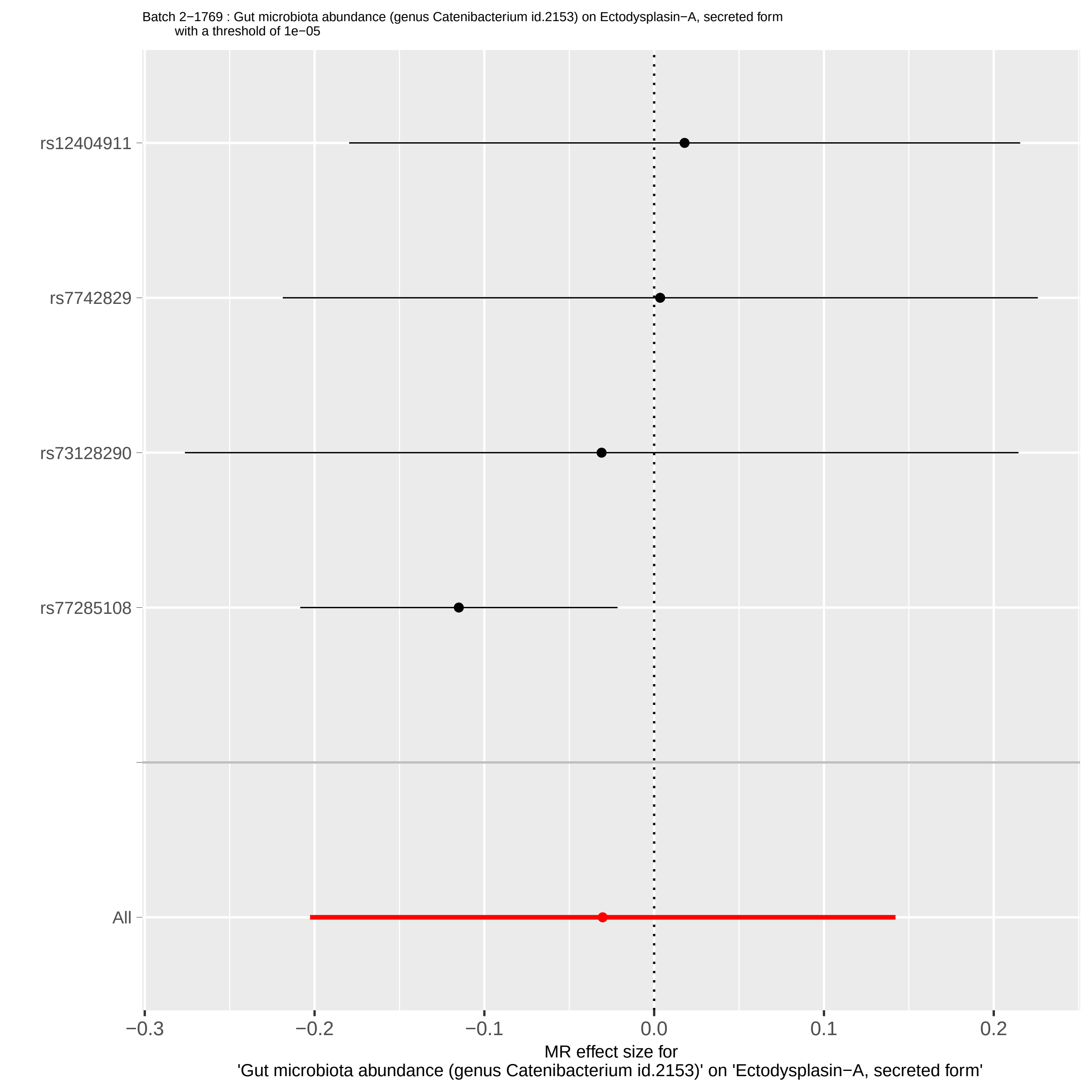
Batch 2−1769 : Gut microbiota abundance (genus Catenibacterium id.2153) on Ectodysplasin−A, secreted form
with a threshold of 1e−05
rs12404911
rs7742829
rs73128290
rs77285108
All
−0.3
−0.2
−0.1
0.0
0.1
0.2
MR effect size for
'Gut microbiota abundance (genus Catenibacterium id.2153)' on 'Ectodysplasin−A, secreted form'

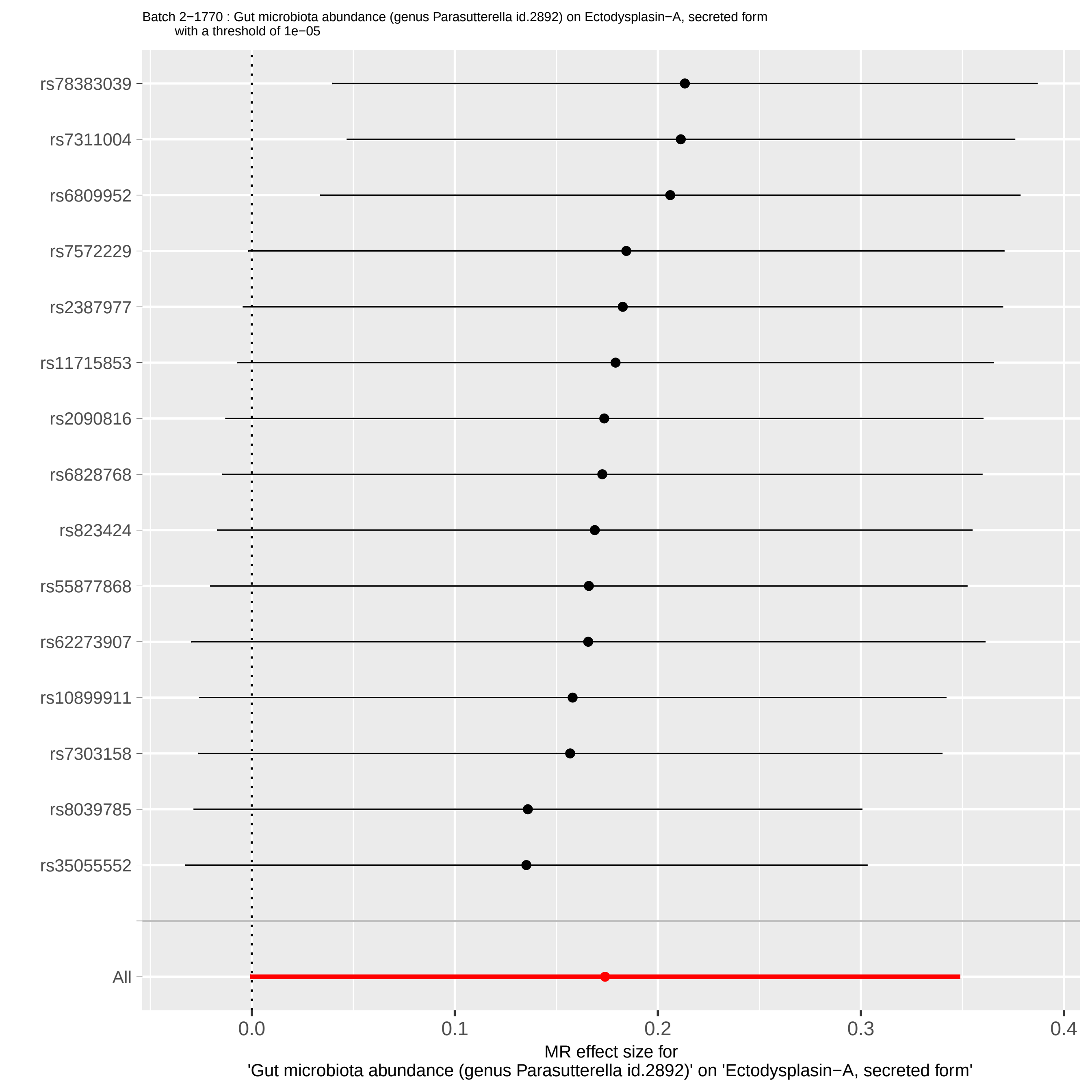

Batch 2−1770 : Gut microbiota abundance (genus Parasutterella id.2892) on Ectodysplasin−A, secreted form
with a threshold of 1e−05
rs78383039
rs7311004
rs6809952
rs7572229
rs2387977
rs11715853
rs2090816
rs6828768
rs823424
rs55877868
rs62273907
rs10899911
rs7303158
rs8039785
rs35055552
All
0.0
0.1
0.2
0.3
0.4
MR effect size for
'Gut microbiota abundance (genus Parasutterella id.2892)' on 'Ectodysplasin−A, secreted form'

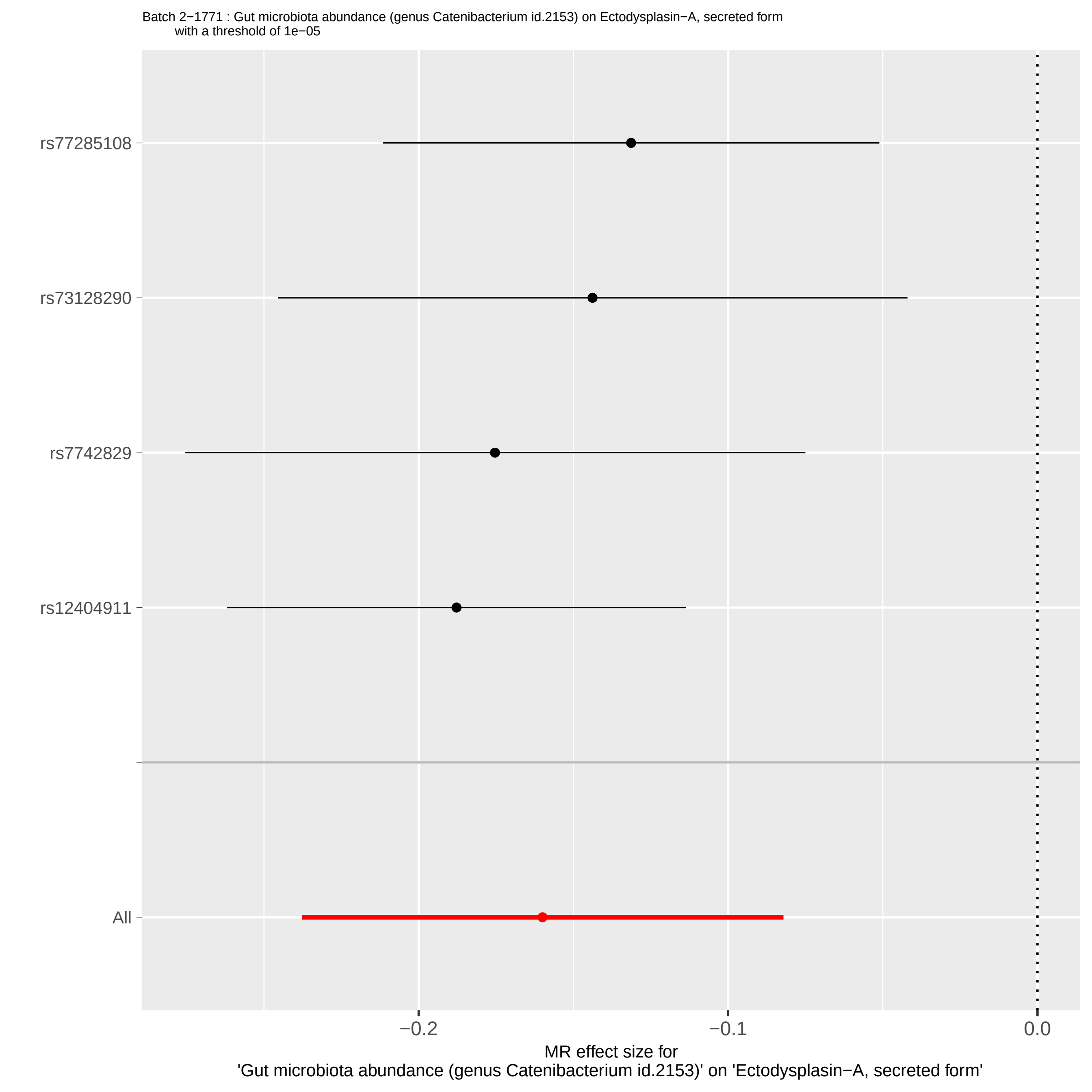
Batch 2−1771 : Gut microbiota abundance (genus Catenibacterium id.2153) on Ectodysplasin−A, secreted form
with a threshold of 1e−05
rs77285108
rs73128290
rs7742829
rs12404911
All
−0.2
−0.1
0.0
MR effect size for
'Gut microbiota abundance (genus Catenibacterium id.2153)' on 'Ectodysplasin−A, secreted form'

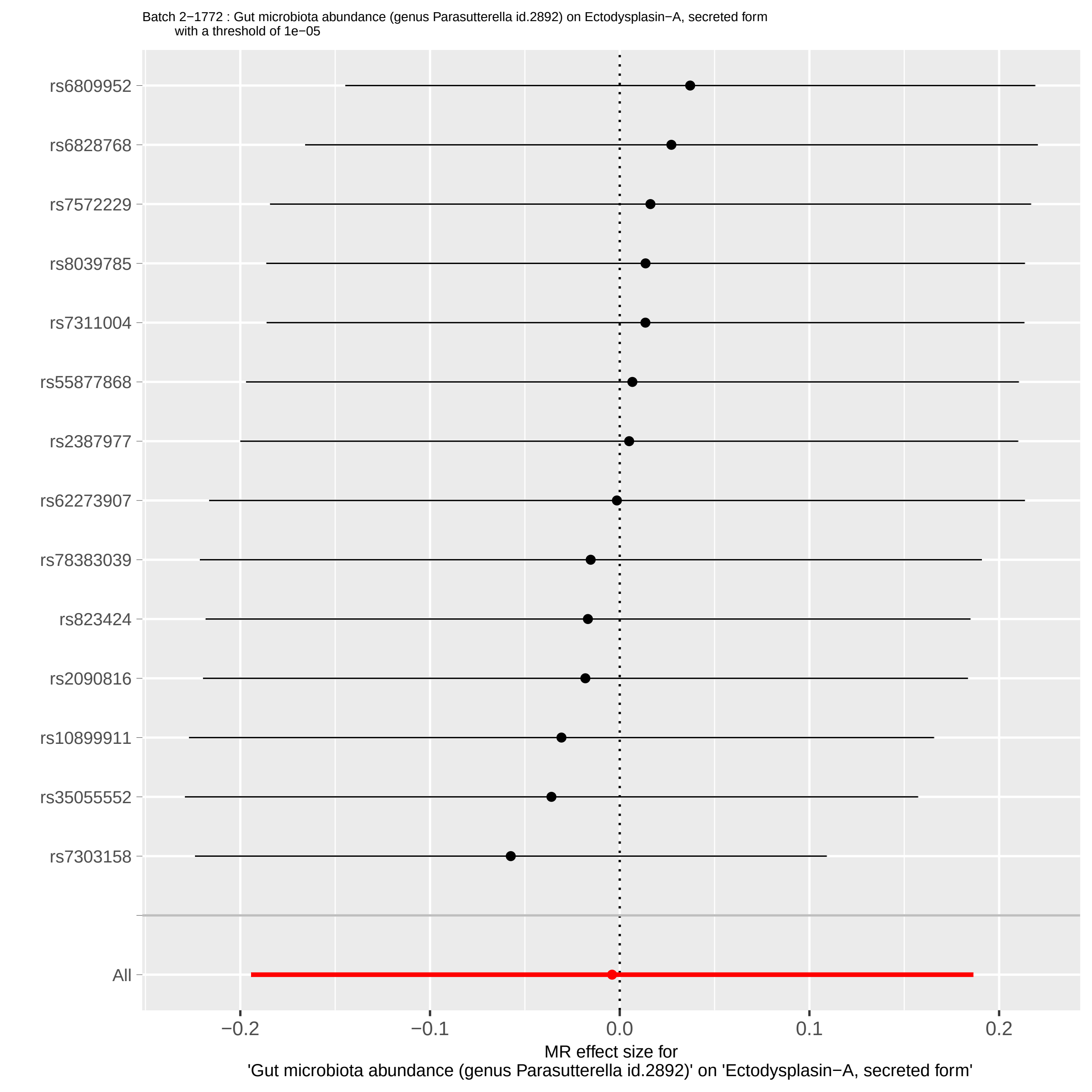

Batch 2−1772 : Gut microbiota abundance (genus Parasutterella id.2892) on Ectodysplasin−A, secreted form
with a threshold of 1e−05
rs6809952
rs6828768
rs7572229
rs8039785
rs7311004
rs55877868
rs2387977
rs62273907
rs78383039
rs823424
rs2090816
rs10899911
rs35055552
rs7303158
All
−0.2
−0.1
0.0
0.1
0.2
MR effect size for
'Gut microbiota abundance (genus Parasutterella id.2892)' on 'Ectodysplasin−A, secreted form'

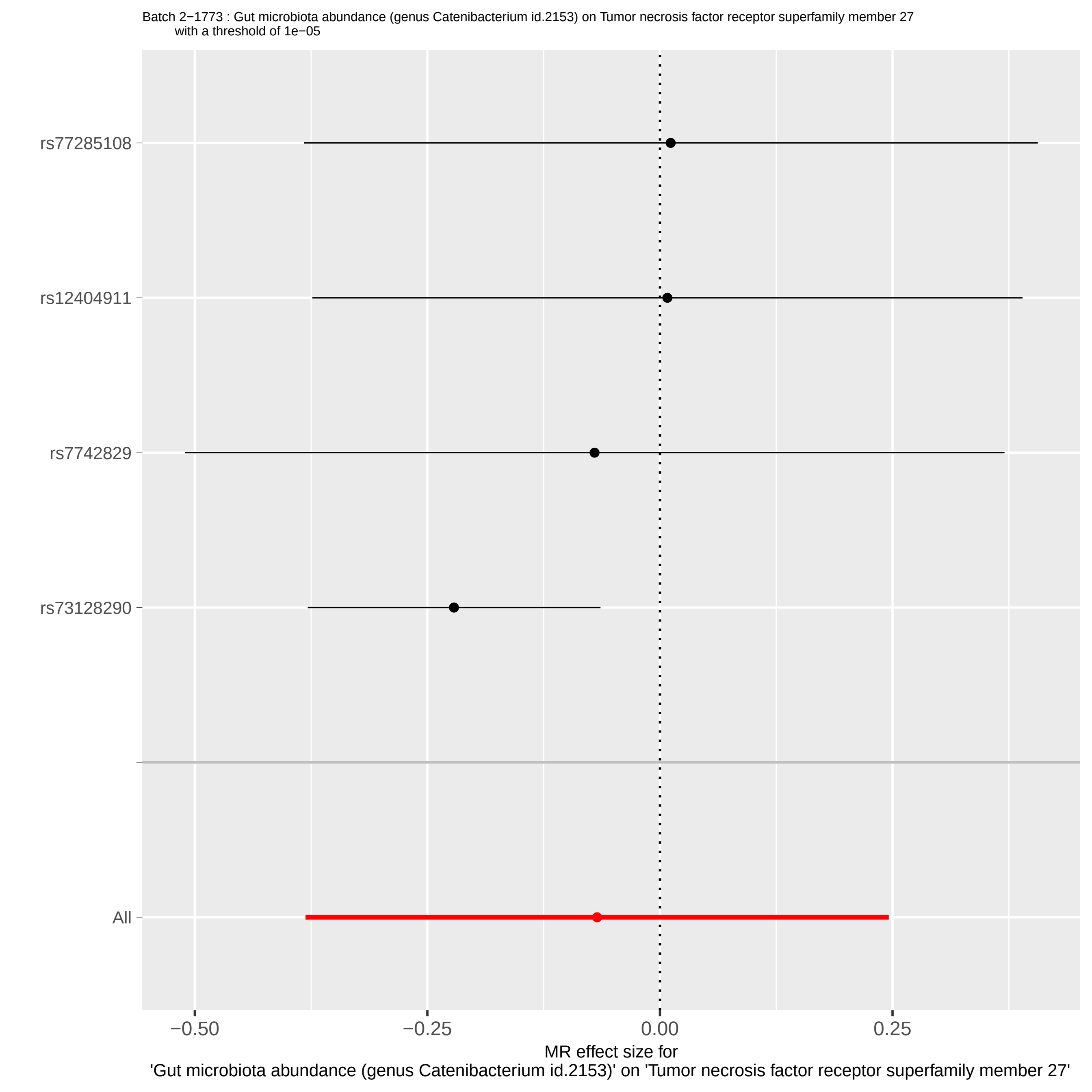

Batch 2−1773 : Gut microbiota abundance (genus Catenibacterium id.2153) on Tumor necrosis factor receptor superfamily member 27
with a threshold of 1e−05
rs77285108
rs12404911
rs7742829
rs73128290
All
−0.50
−0.25
0.00
0.25
MR effect size for
'Gut microbiota abundance (genus Catenibacterium id.2153)' on 'Tumor necrosis factor receptor superfamily member 27'

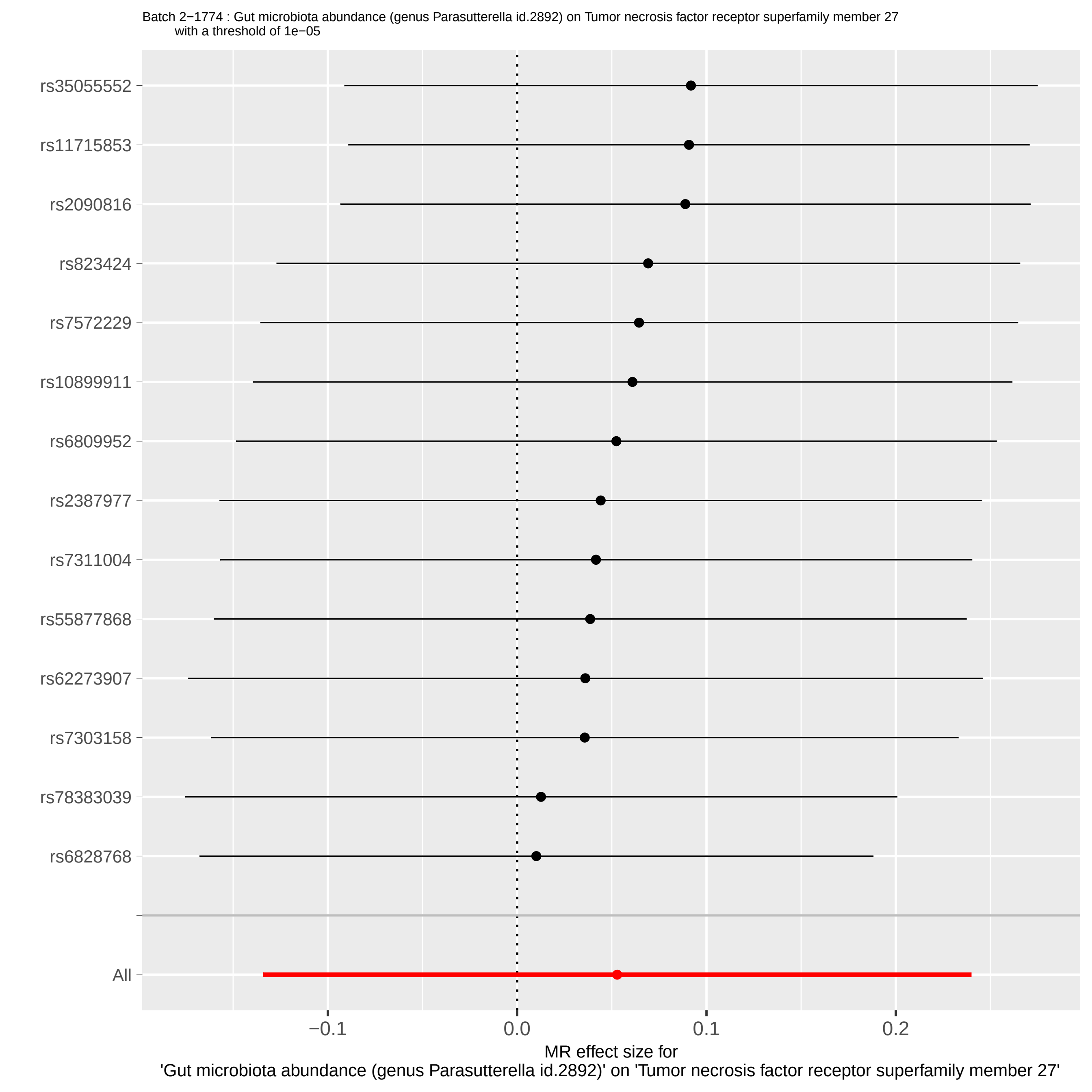

Batch 2−1774 : Gut microbiota abundance (genus Parasutterella id.2892) on Tumor necrosis factor receptor superfamily member 27
with a threshold of 1e−05
rs35055552
rs11715853
rs2090816
rs823424
rs7572229
rs10899911
rs6809952
rs2387977
rs7311004
rs55877868
rs62273907
rs7303158
rs78383039
rs6828768
All
−0.1
0.0
0.1
0.2
MR effect size for
'Gut microbiota abundance (genus Parasutterella id.2892)' on 'Tumor necrosis factor receptor superfamily member 27'

Batch 2−1775 : Gut microbiota abundance (genus Catenibacterium id.2153) on Tumor necrosis factor receptor superfamily member EDAR
with a threshold of 1e−05

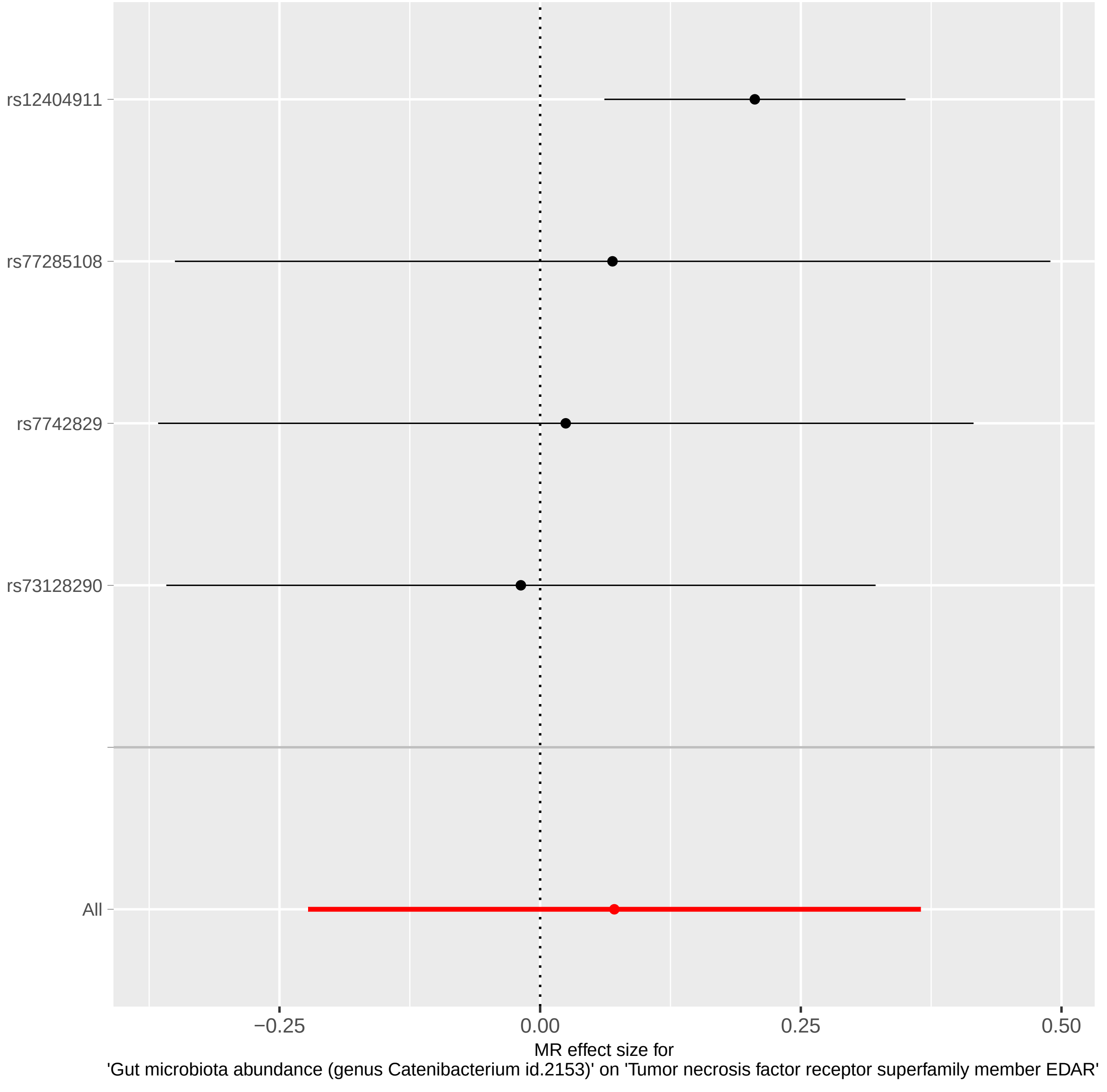

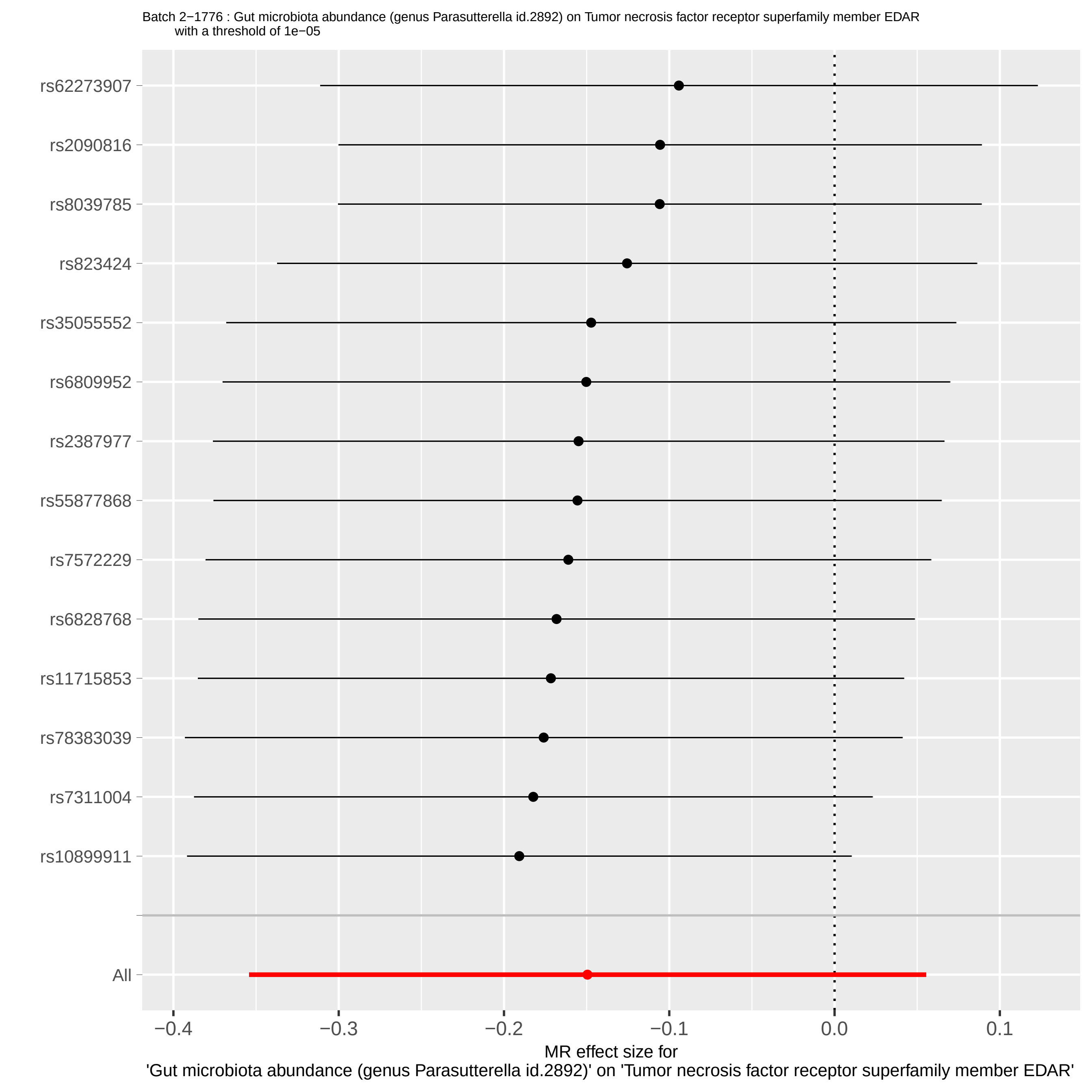

Batch 2−1776 : Gut microbiota abundance (genus Parasutterella id.2892) on Tumor necrosis factor receptor superfamily member EDAR
with a threshold of 1e−05
rs62273907
rs2090816
rs8039785
rs823424
rs35055552
rs6809952
rs2387977
rs55877868
rs7572229
rs6828768
rs11715853
rs78383039
rs7311004
rs10899911
All
−0.4
−0.3
−0.2
−0.1
0.0
0.1
MR effect size for
'Gut microbiota abundance (genus Parasutterella id.2892)' on 'Tumor necrosis factor receptor superfamily member EDAR'

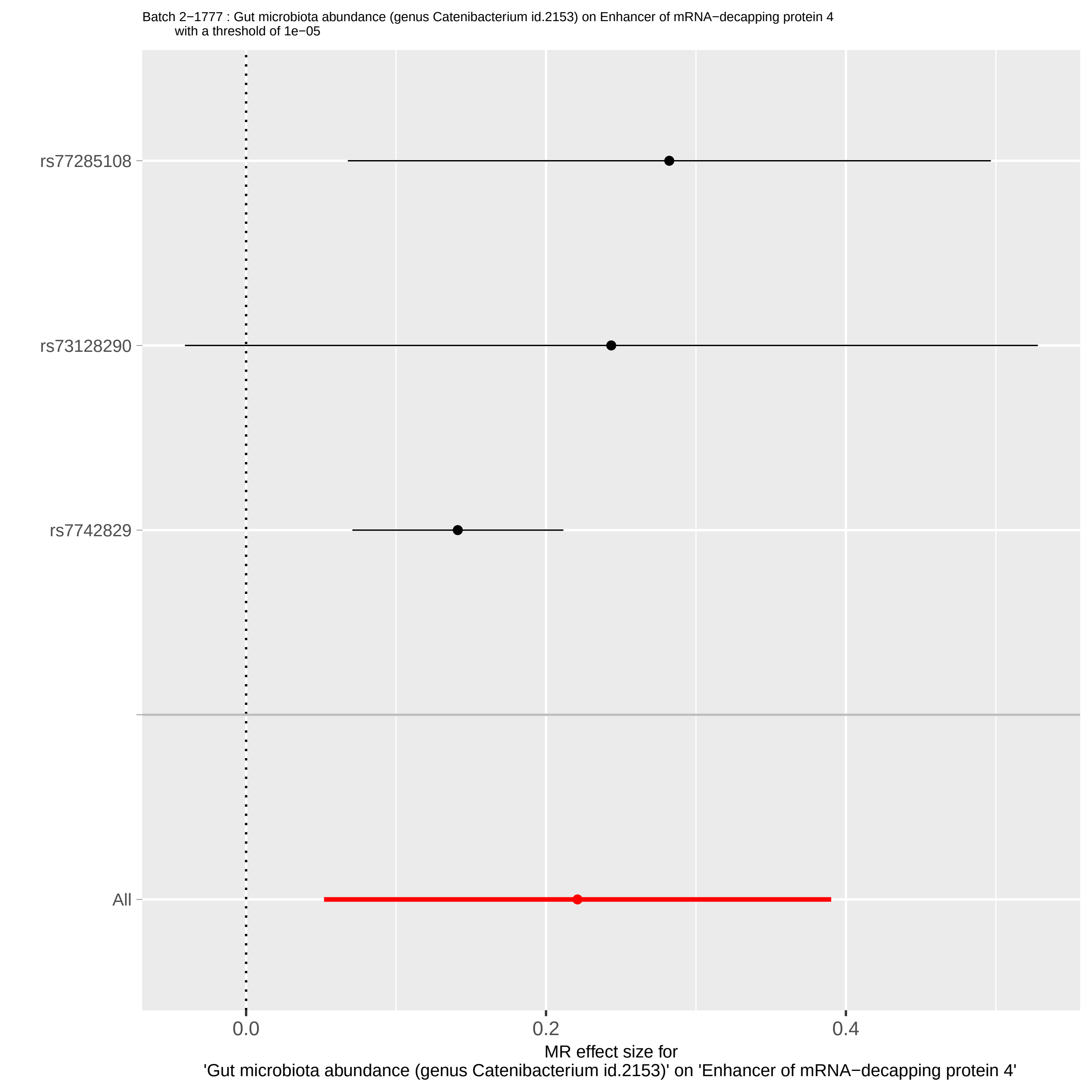

Batch 2−1777 : Gut microbiota abundance (genus Catenibacterium id.2153) on Enhancer of mRNA−decapping protein 4
with a threshold of 1e−05
rs77285108
rs73128290
rs7742829
All
0.0
0.2
0.4
MR effect size for
'Gut microbiota abundance (genus Catenibacterium id.2153)' on 'Enhancer of mRNA−decapping protein 4'

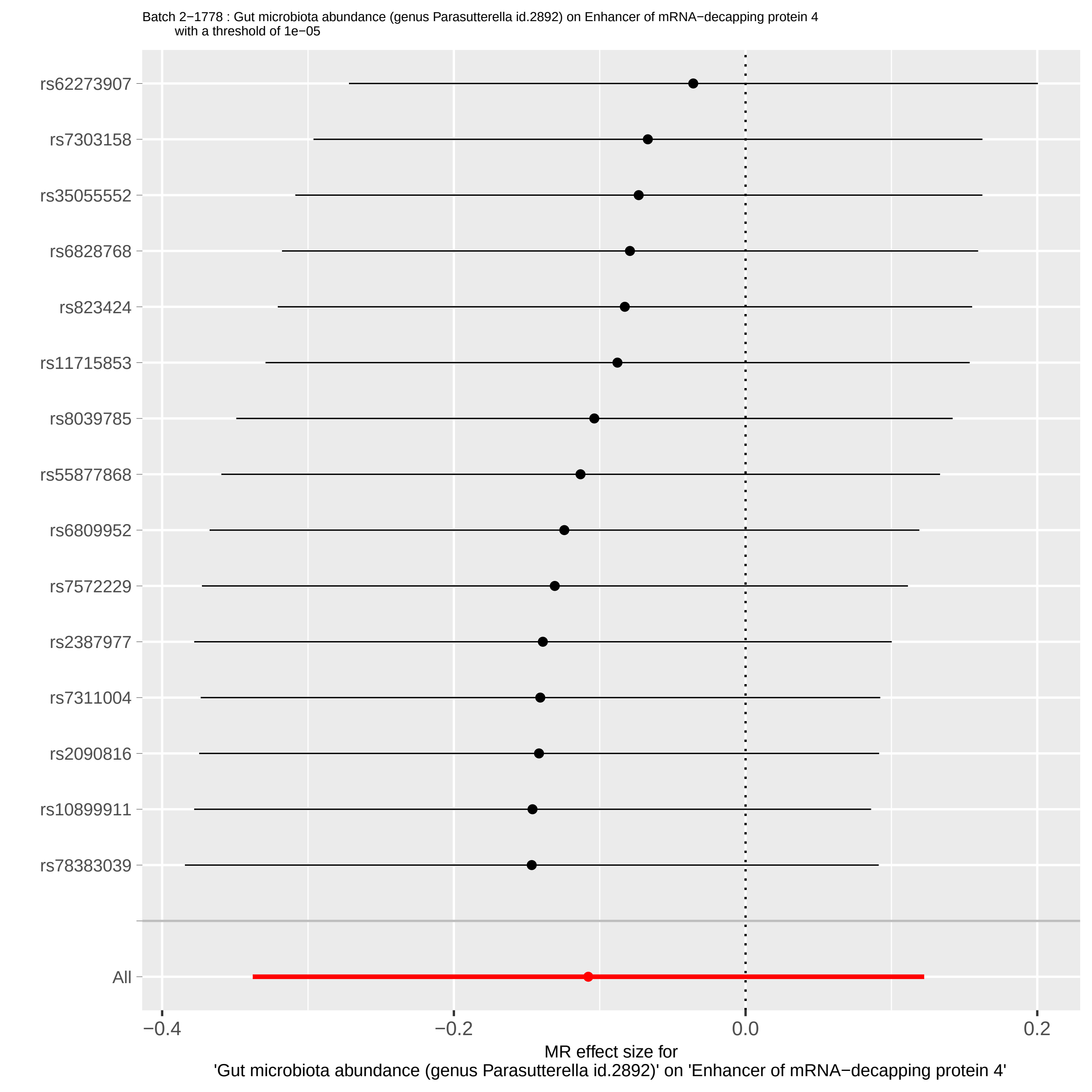

Batch 2−1778 : Gut microbiota abundance (genus Parasutterella id.2892) on Enhancer of mRNA−decapping protein 4
with a threshold of 1e−05
rs62273907
rs7303158
rs35055552
rs6828768
rs823424
rs11715853
rs8039785
rs55877868
rs6809952
rs7572229
rs2387977
rs7311004
rs2090816
rs10899911
rs78383039
All
−0.4
−0.2
0.0
0.2
MR effect size for
'Gut microbiota abundance (genus Parasutterella id.2892)' on 'Enhancer of mRNA−decapping protein 4'

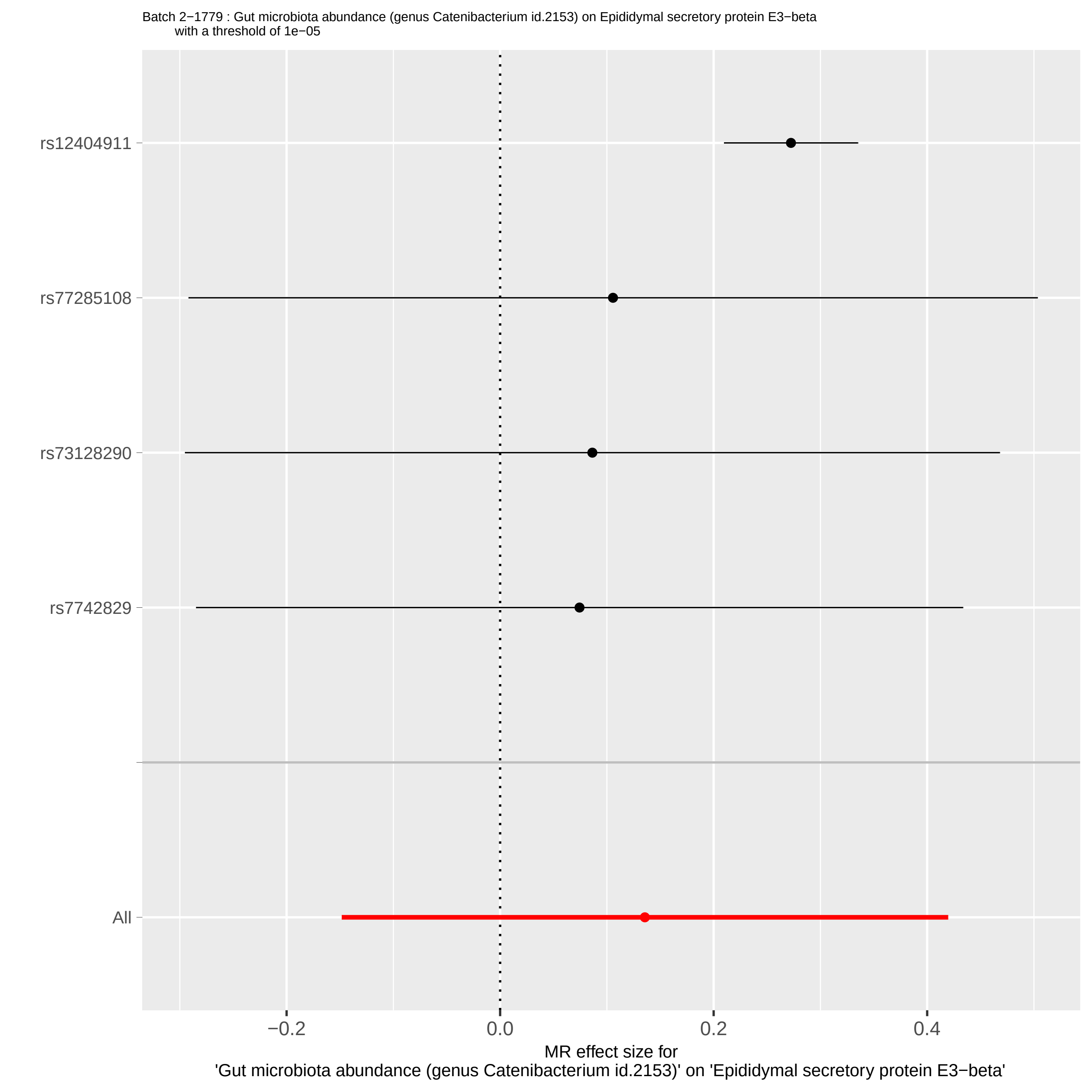
Batch 2−1779 : Gut microbiota abundance (genus Catenibacterium id.2153) on Epididymal secretory protein E3−beta
with a threshold of 1e−05
rs12404911
rs77285108
rs73128290
rs7742829
All
−0.2
0.0
0.2
0.4
MR effect size for
'Gut microbiota abundance (genus Catenibacterium id.2153)' on 'Epididymal secretory protein E3−beta'

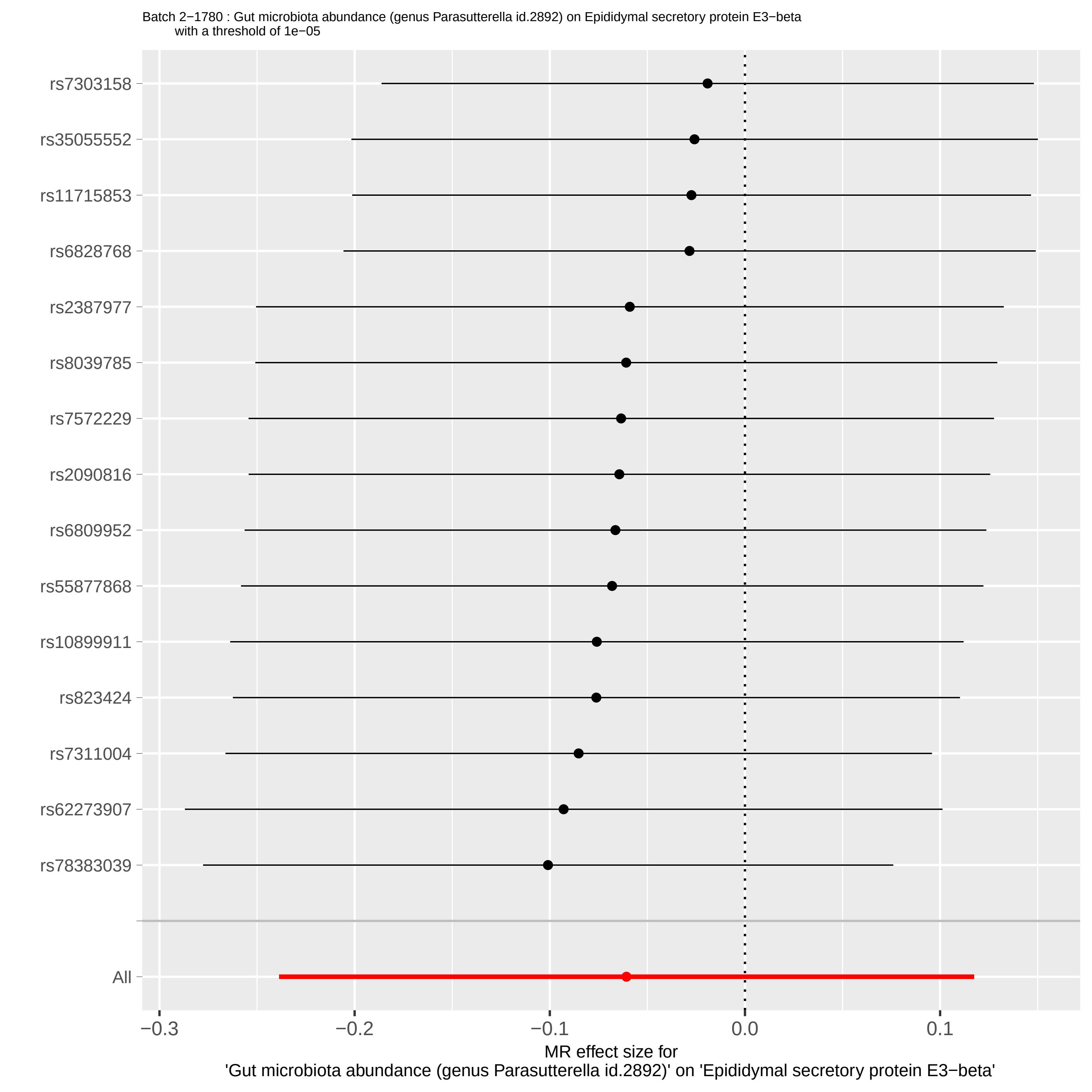

Batch 2−1780 : Gut microbiota abundance (genus Parasutterella id.2892) on Epididymal secretory protein E3−beta
with a threshold of 1e−05
rs7303158
rs35055552
rs11715853
rs6828768
rs2387977
rs8039785
rs7572229
rs2090816
rs6809952
rs55877868
rs10899911
rs823424
rs7311004
rs62273907
rs78383039
All
−0.3
−0.2
−0.1
0.0
0.1
MR effect size for
'Gut microbiota abundance (genus Parasutterella id.2892)' on 'Epididymal secretory protein E3−beta'

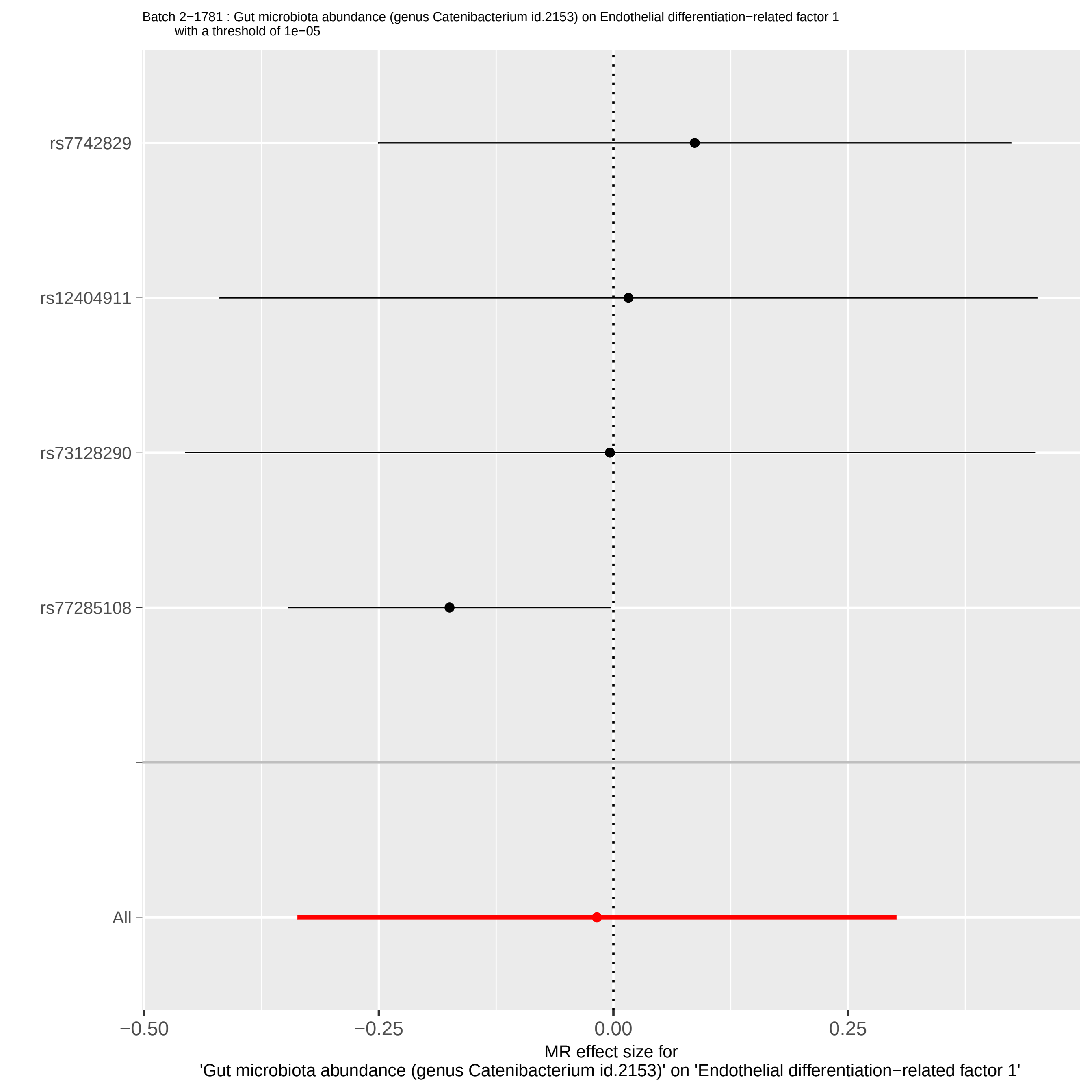
Batch 2−1781 : Gut microbiota abundance (genus Catenibacterium id.2153) on Endothelial differentiation−related factor 1
with a threshold of 1e−05
rs7742829
rs12404911
rs73128290
rs77285108
All
−0.50
−0.25
0.00
0.25
MR effect size for
'Gut microbiota abundance (genus Catenibacterium id.2153)' on 'Endothelial differentiation−related factor 1'

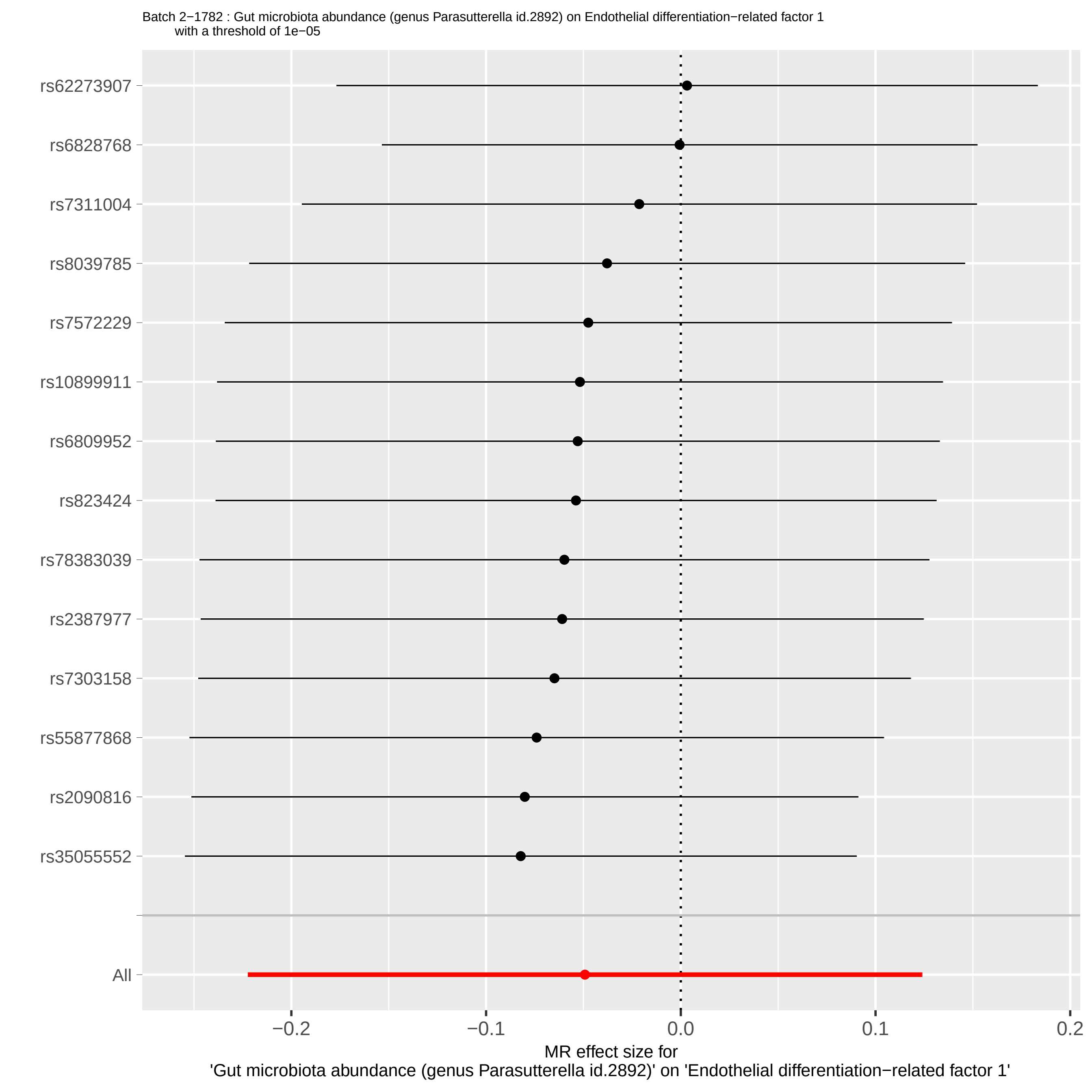
Batch 2−1782 : Gut microbiota abundance (genus Parasutterella id.2892) on Endothelial differentiation−related factor 1
with a threshold of 1e−05
rs62273907
rs6828768
rs7311004
rs8039785
rs7572229
rs10899911
rs6809952
rs823424
rs78383039
rs2387977
rs7303158
rs55877868
rs2090816
rs35055552
All
−0.2
−0.1
0.0
0.1
0.2
MR effect size for
'Gut microbiota abundance (genus Parasutterella id.2892)' on 'Endothelial differentiation−related factor 1'

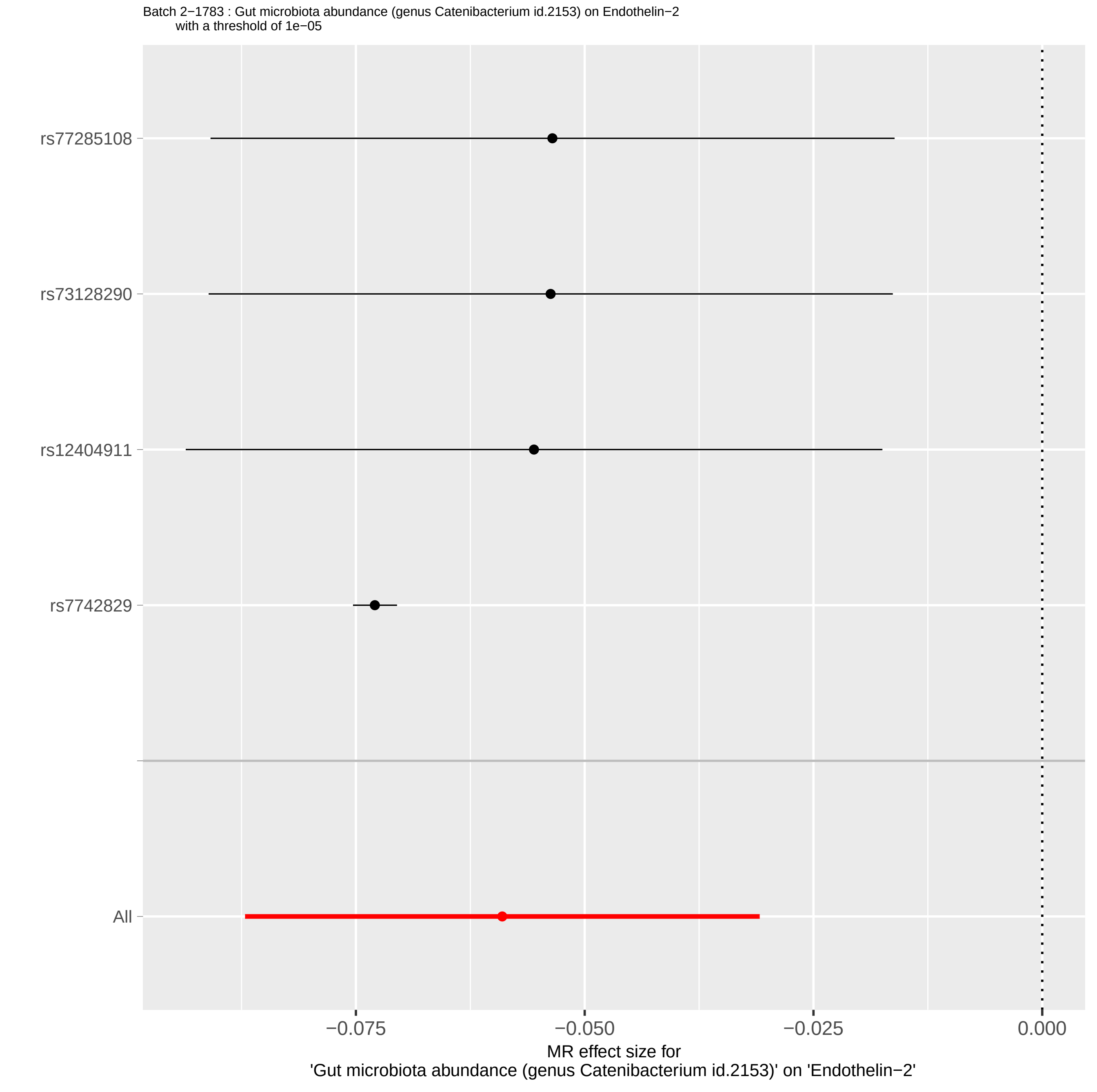
Batch 2−1783 : Gut microbiota abundance (genus Catenibacterium id.2153) on Endothelin−2
with a threshold of 1e−05
rs77285108
rs73128290
rs12404911
rs7742829
All
−0.075
−0.050
−0.025
0.000
MR effect size for
'Gut microbiota abundance (genus Catenibacterium id.2153)' on 'Endothelin−2'

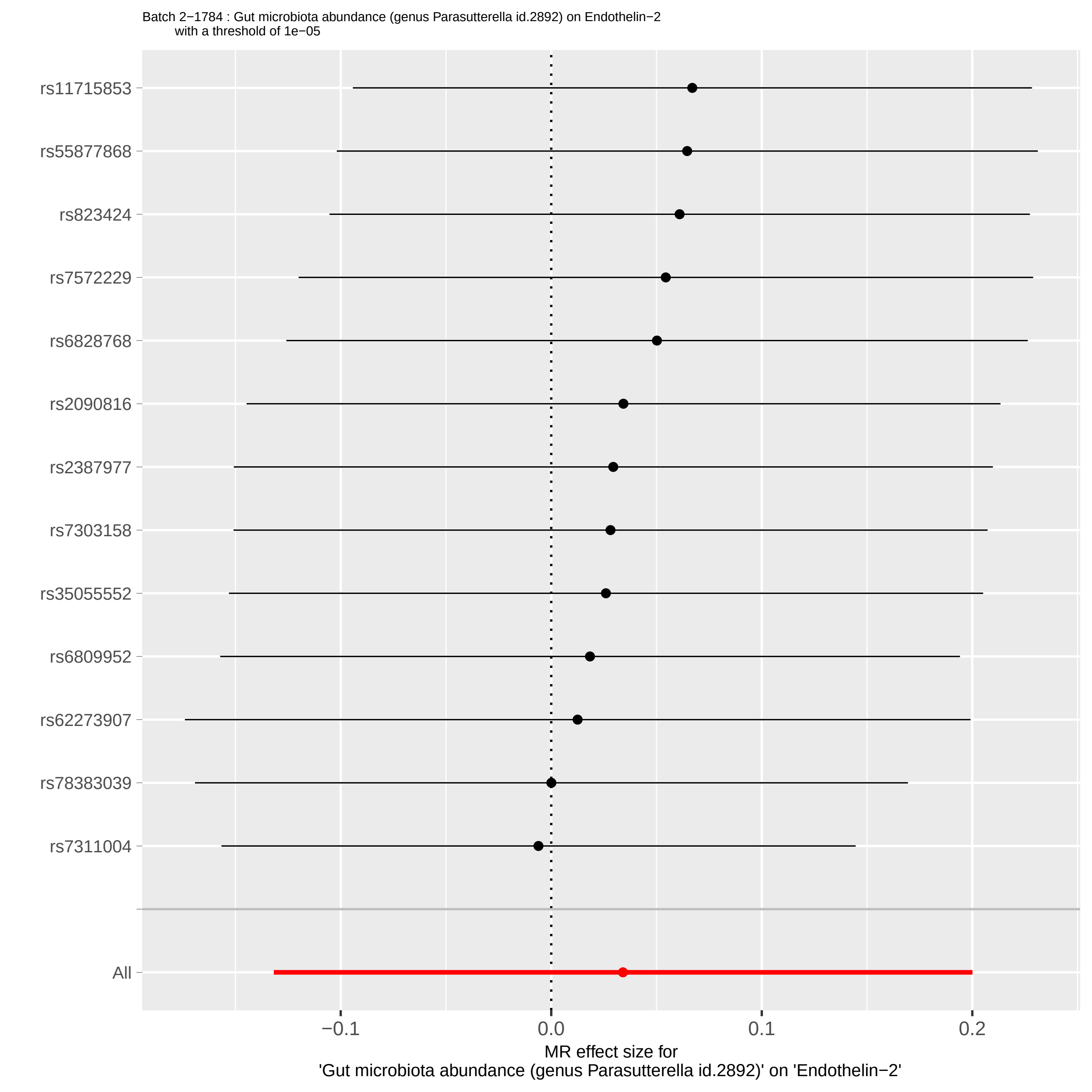

Batch 2−1784 : Gut microbiota abundance (genus Parasutterella id.2892) on Endothelin−2
with a threshold of 1e−05
rs11715853
rs55877868
rs823424
rs7572229
rs6828768
rs2090816
rs2387977
rs7303158
rs35055552
rs6809952
rs62273907
rs78383039
rs7311004
All
−0.1
0.0
0.1
0.2
MR effect size for
'Gut microbiota abundance (genus Parasutterella id.2892)' on 'Endothelin−2'

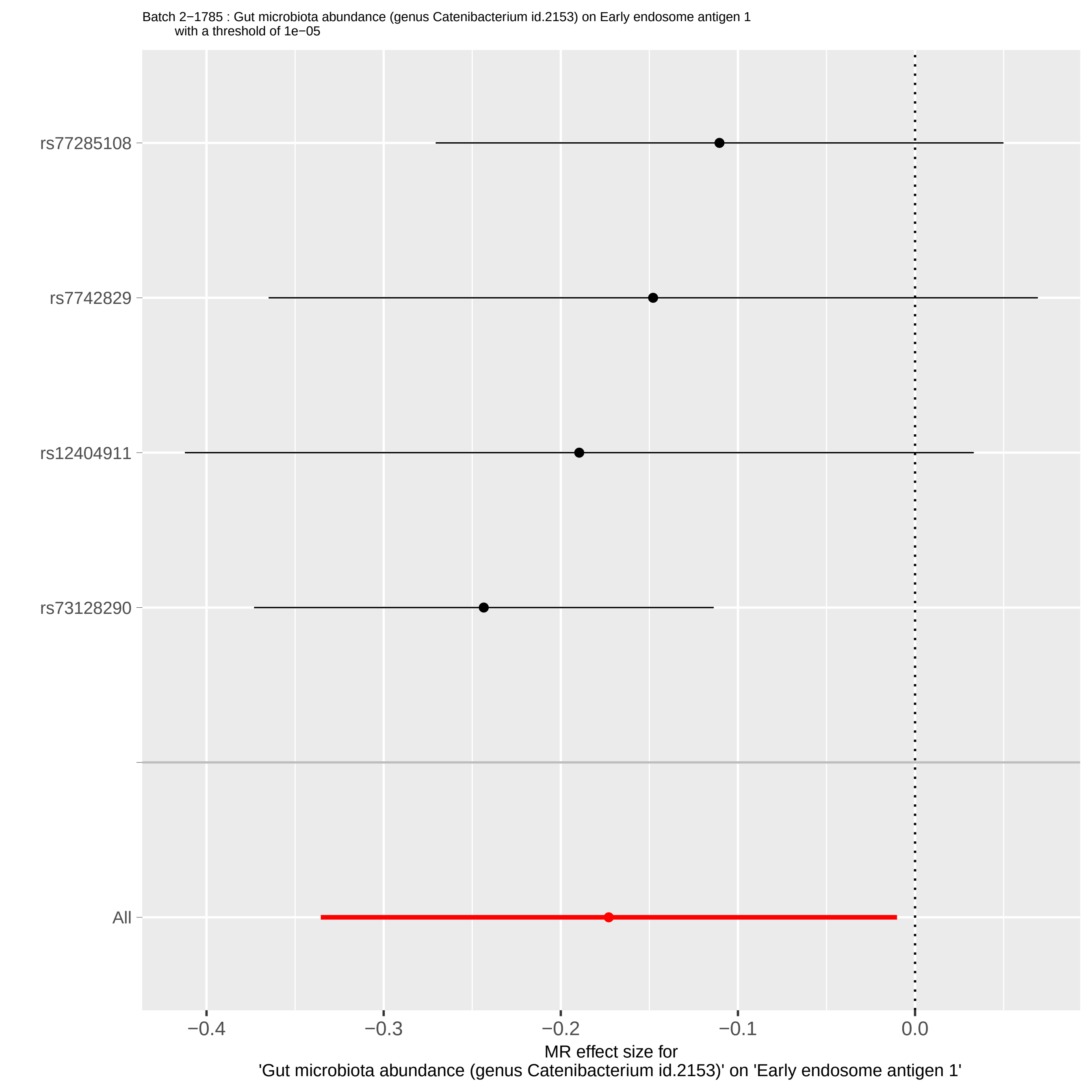
Batch 2−1785 : Gut microbiota abundance (genus Catenibacterium id.2153) on Early endosome antigen 1
with a threshold of 1e−05
rs77285108
rs7742829
rs12404911
rs73128290
All
−0.4
−0.3
−0.2
−0.1
0.0
MR effect size for
'Gut microbiota abundance (genus Catenibacterium id.2153)' on 'Early endosome antigen 1'

Batch 2−1786 : Gut microbiota abundance (genus Parasutterella id.2892) on Early endosome antigen 1
with a threshold of 1e−05

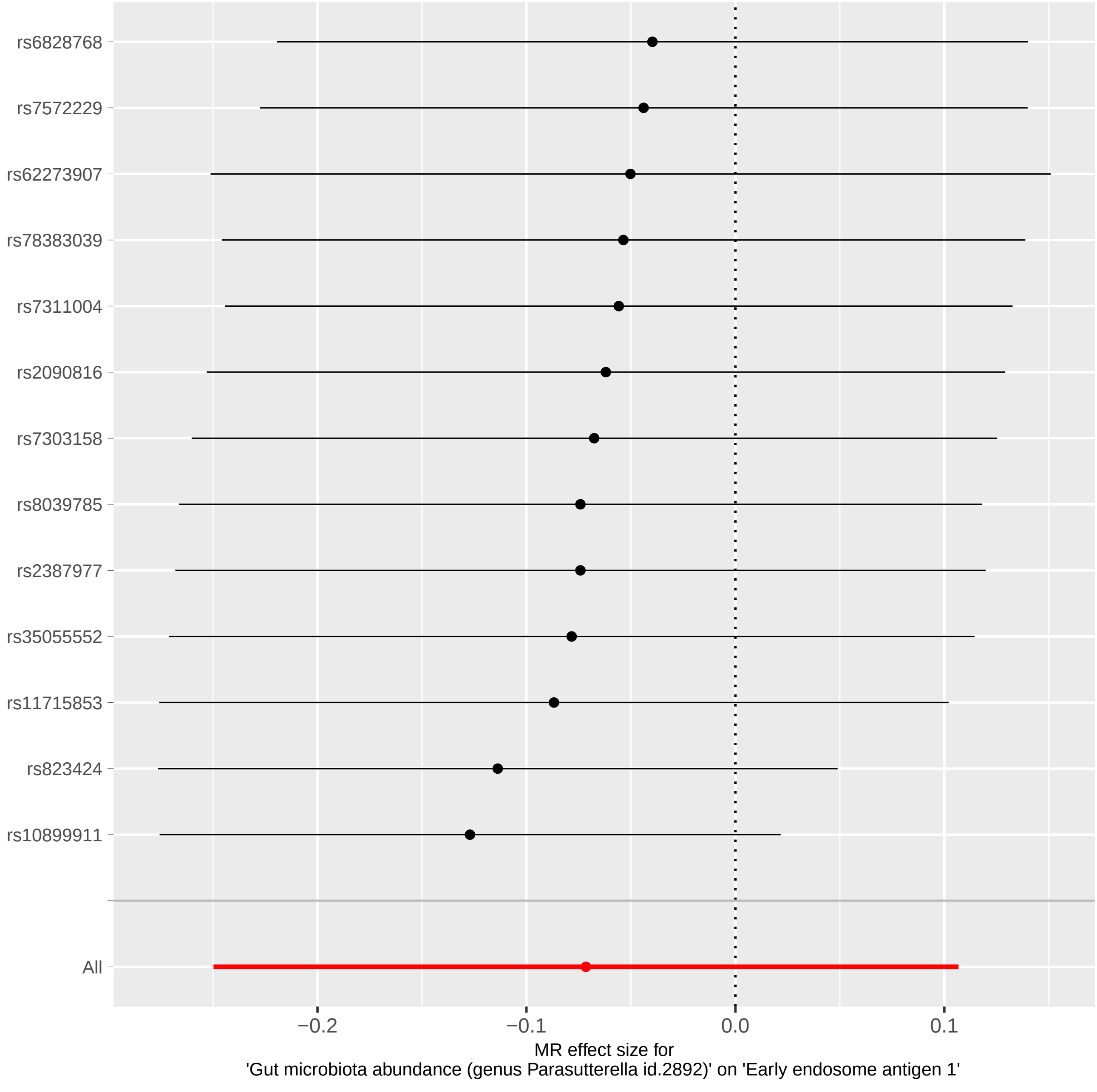

Batch 2−1787 : Gut microbiota abundance (genus Catenibacterium id.2153) on Elongation factor 1−beta
with a threshold of 1e−05

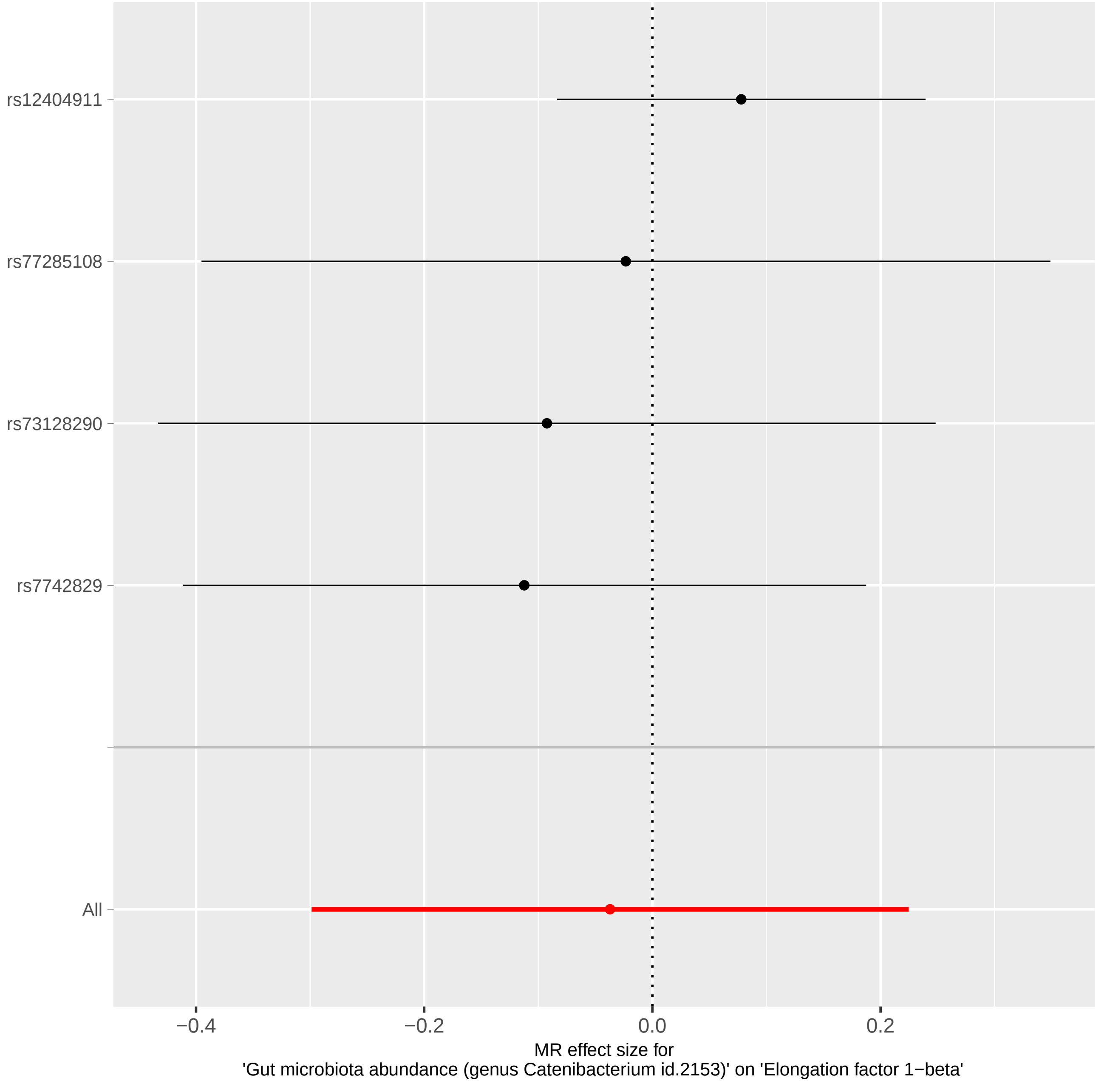

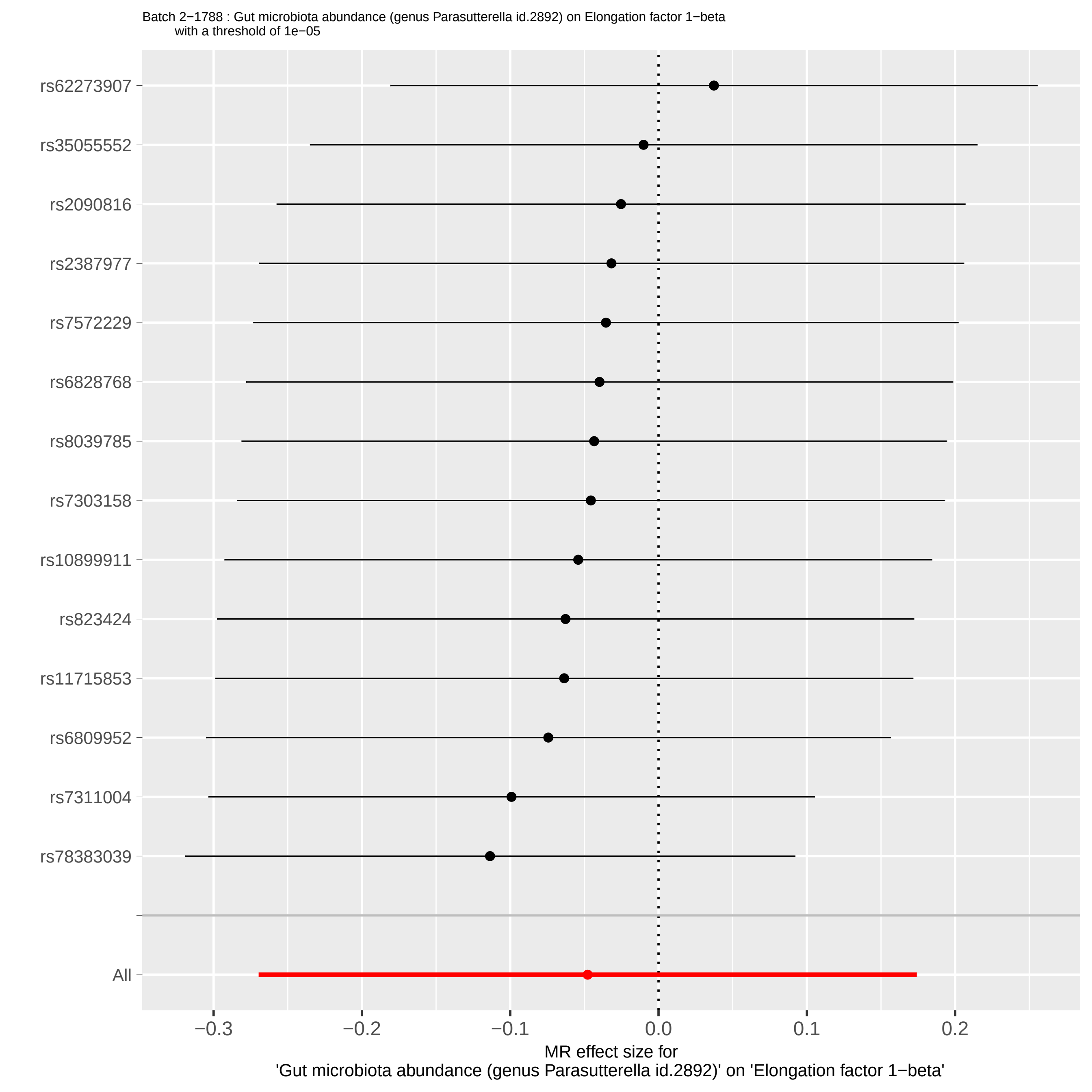

Batch 2−1788 : Gut microbiota abundance (genus Parasutterella id.2892) on Elongation factor 1−beta
with a threshold of 1e−05
rs62273907
rs35055552
rs2090816
rs2387977
rs7572229
rs6828768
rs8039785
rs7303158
rs10899911
rs823424
rs11715853
rs6809952
rs7311004
rs78383039
All
−0.3
−0.2
−0.1
0.0
0.1
0.2
MR effect size for
'Gut microbiota abundance (genus Parasutterella id.2892)' on 'Elongation factor 1−beta'

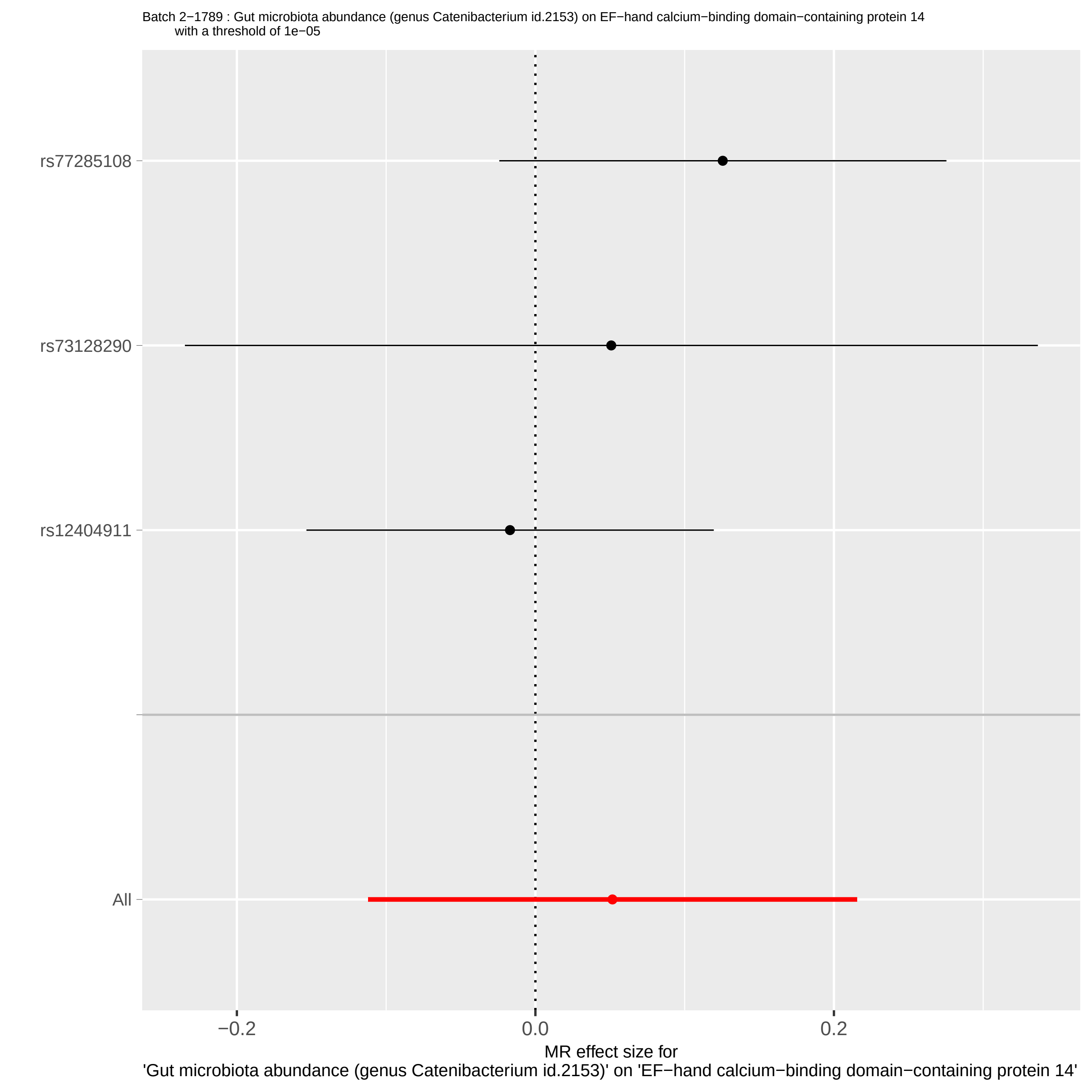
Batch 2−1789 : Gut microbiota abundance (genus Catenibacterium id.2153) on EF−hand calcium−binding domain−containing protein 14
with a threshold of 1e−05
rs77285108
rs73128290
rs12404911
All
−0.2
0.0
0.2
MR effect size for
'Gut microbiota abundance (genus Catenibacterium id.2153)' on 'EF−hand calcium−binding domain−containing protein 14'

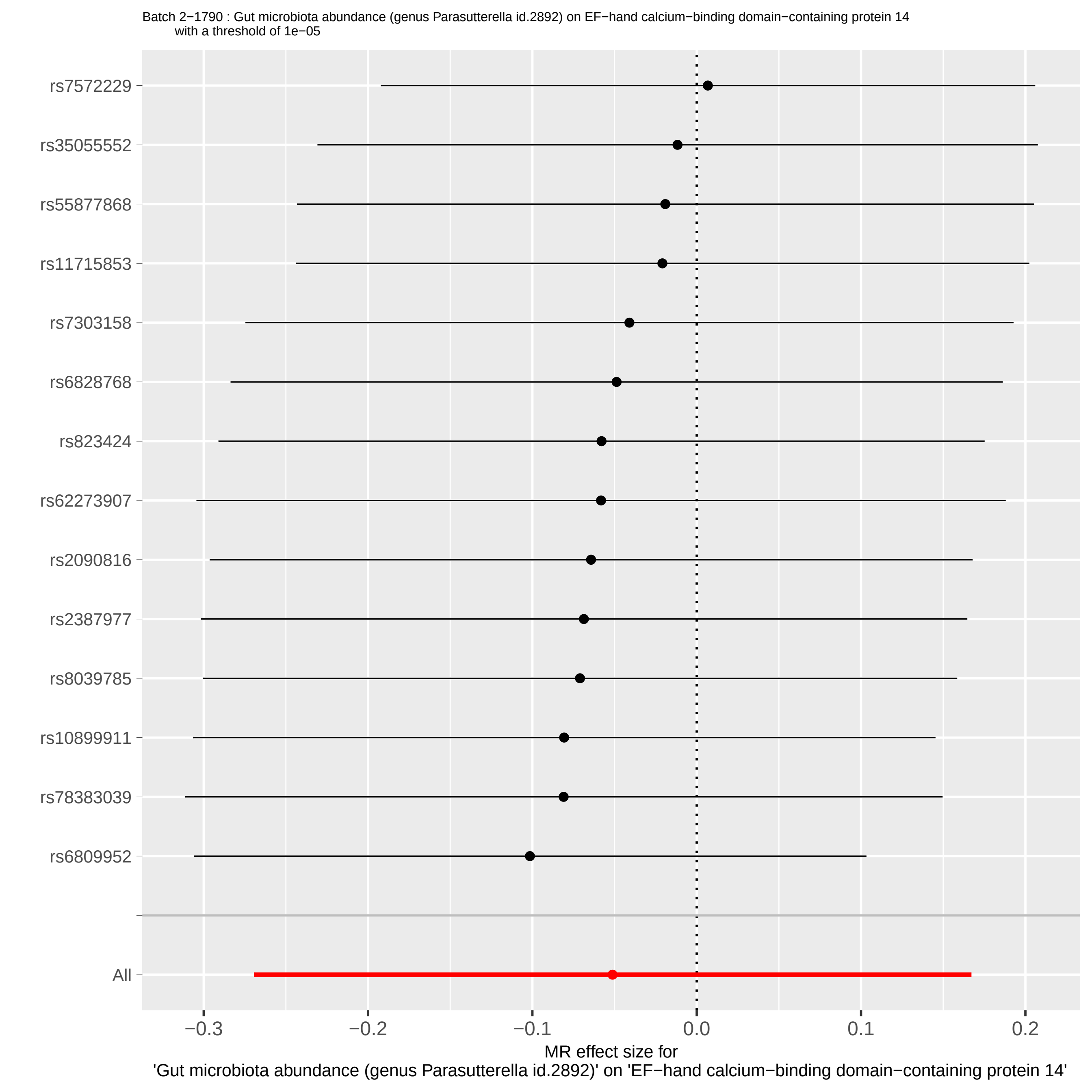
Batch 2−1790 : Gut microbiota abundance (genus Parasutterella id.2892) on EF−hand calcium−binding domain−containing protein 14
with a threshold of 1e−05
rs7572229
rs35055552
rs55877868
rs11715853
rs7303158
rs6828768
rs823424
rs62273907
rs2090816
rs2387977
rs8039785
rs10899911
rs78383039
rs6809952
All
−0.3
−0.2
−0.1
0.0
0.1
0.2
MR effect size for
'Gut microbiota abundance (genus Parasutterella id.2892)' on 'EF−hand calcium−binding domain−containing protein 14'

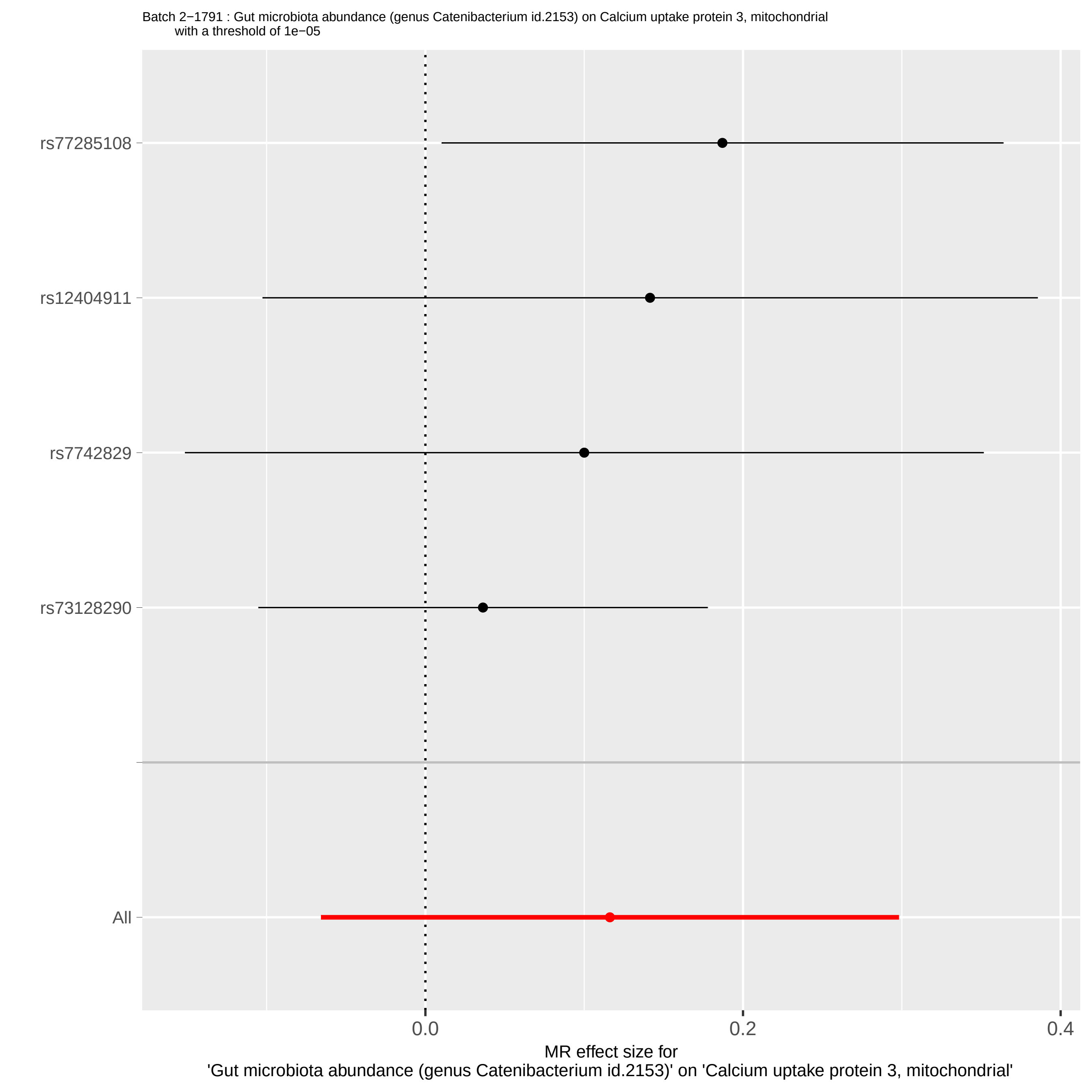

Batch 2−1791 : Gut microbiota abundance (genus Catenibacterium id.2153) on Calcium uptake protein 3, mitochondrial
with a threshold of 1e−05
rs77285108
rs12404911
rs7742829
rs73128290
All
0.0
0.2
0.4
MR effect size for
'Gut microbiota abundance (genus Catenibacterium id.2153)' on 'Calcium uptake protein 3, mitochondrial'

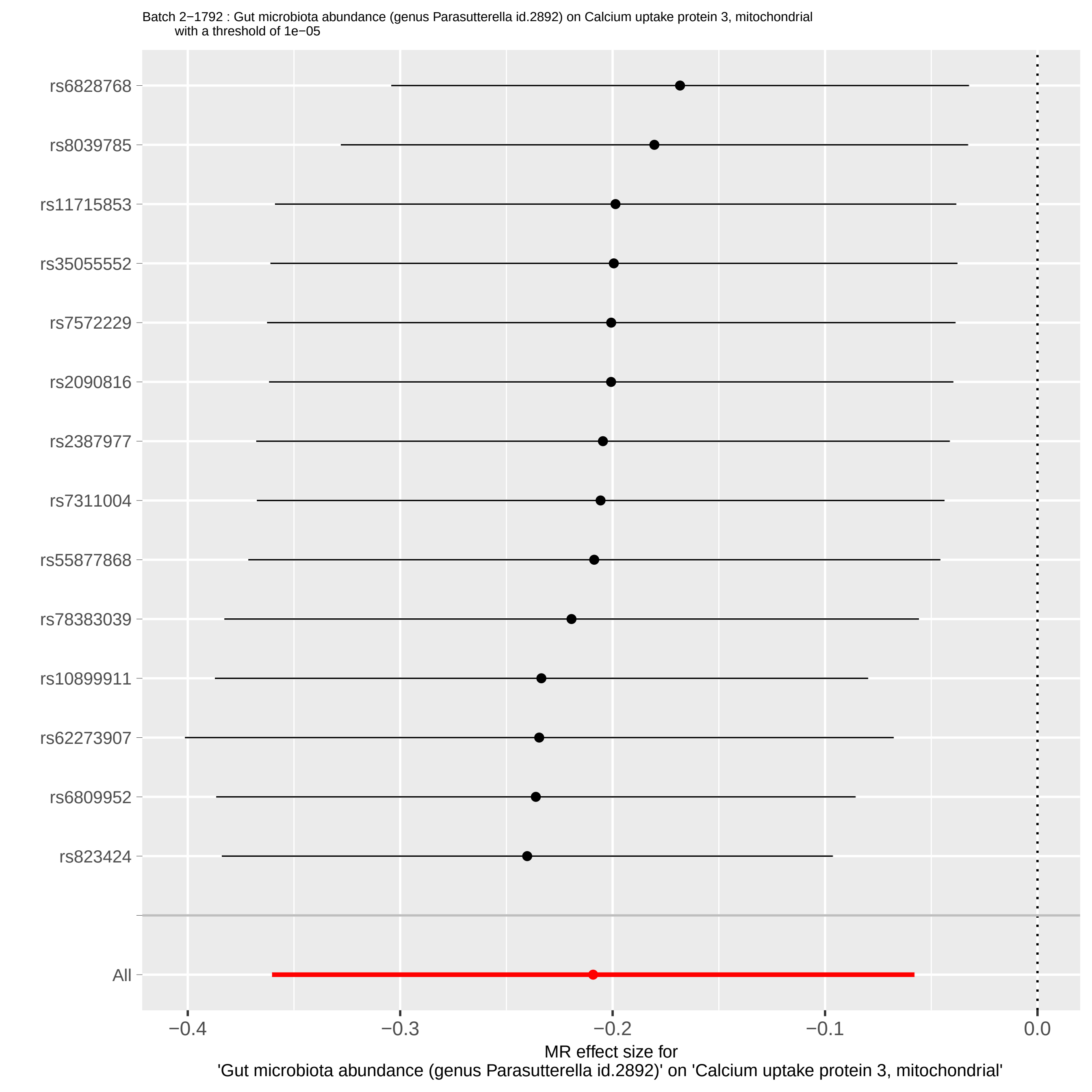

Batch 2−1792 : Gut microbiota abundance (genus Parasutterella id.2892) on Calcium uptake protein 3, mitochondrial with a threshold of 1e−05
rs6828768
rs8039785
rs11715853
rs35055552
rs7572229
rs2090816
rs2387977
rs7311004
rs55877868
rs78383039
rs10899911
rs62273907
rs6809952
rs823424
All
−0.4
−0.3
−0.2
−0.1
0.0
MR effect size for
'Gut microbiota abundance (genus Parasutterella id.2892)' on 'Calcium uptake protein 3, mitochondrial'

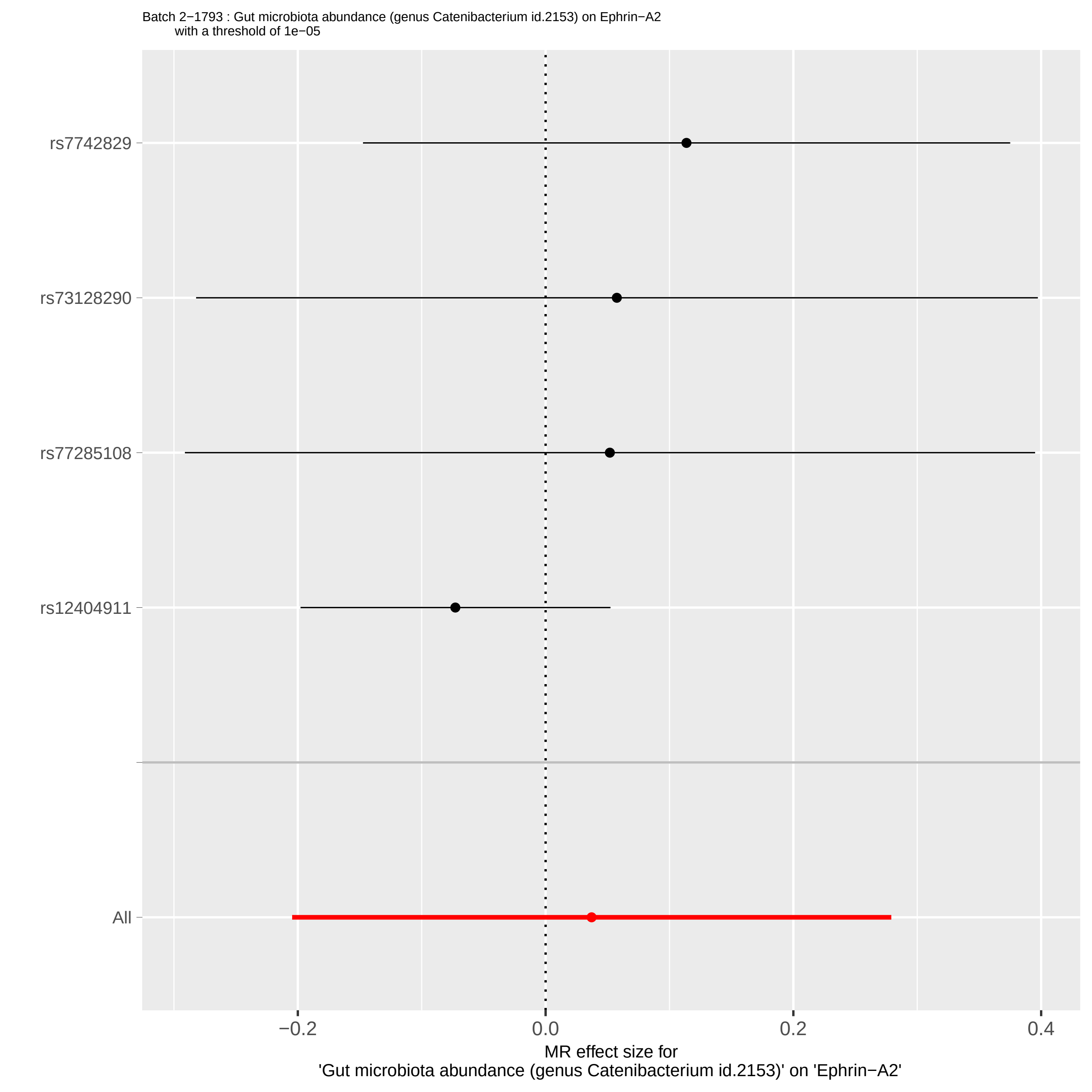
Batch 2−1793 : Gut microbiota abundance (genus Catenibacterium id.2153) on Ephrin−A2
with a threshold of 1e−05
rs7742829
rs73128290
rs77285108
rs12404911
All
−0.2
0.0
0.2
0.4
MR effect size for
'Gut microbiota abundance (genus Catenibacterium id.2153)' on 'Ephrin−A2'

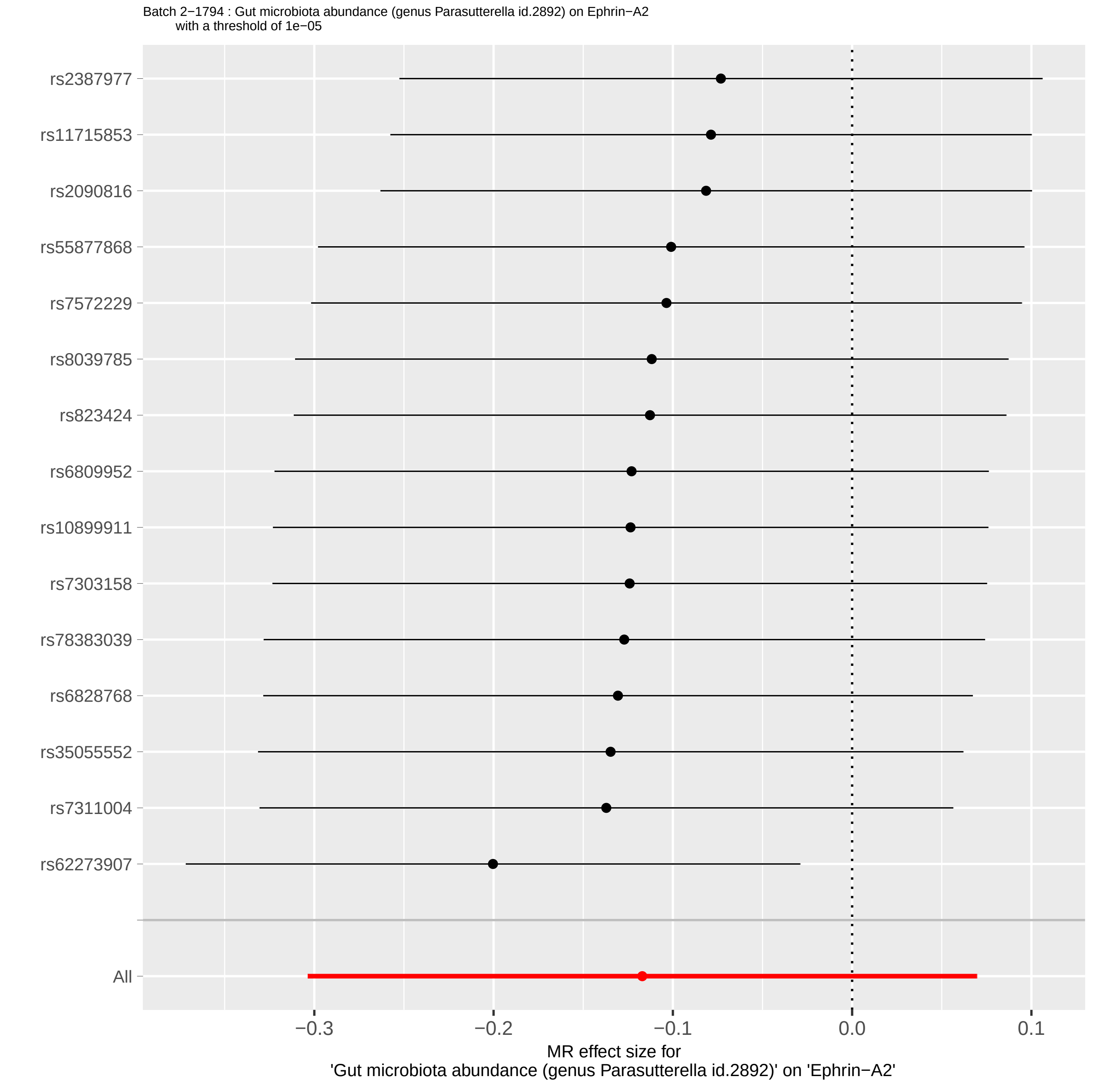

Batch 2−1794 : Gut microbiota abundance (genus Parasutterella id.2892) on Ephrin−A2
with a threshold of 1e−05
rs2387977
rs11715853
rs2090816
rs55877868
rs7572229
rs8039785
rs823424
rs6809952
rs10899911
rs7303158
rs78383039
rs6828768
rs35055552
rs7311004
rs62273907
All
−0.3
−0.2
−0.1
0.0
0.1
MR effect size for
'Gut microbiota abundance (genus Parasutterella id.2892)' on 'Ephrin−A2'

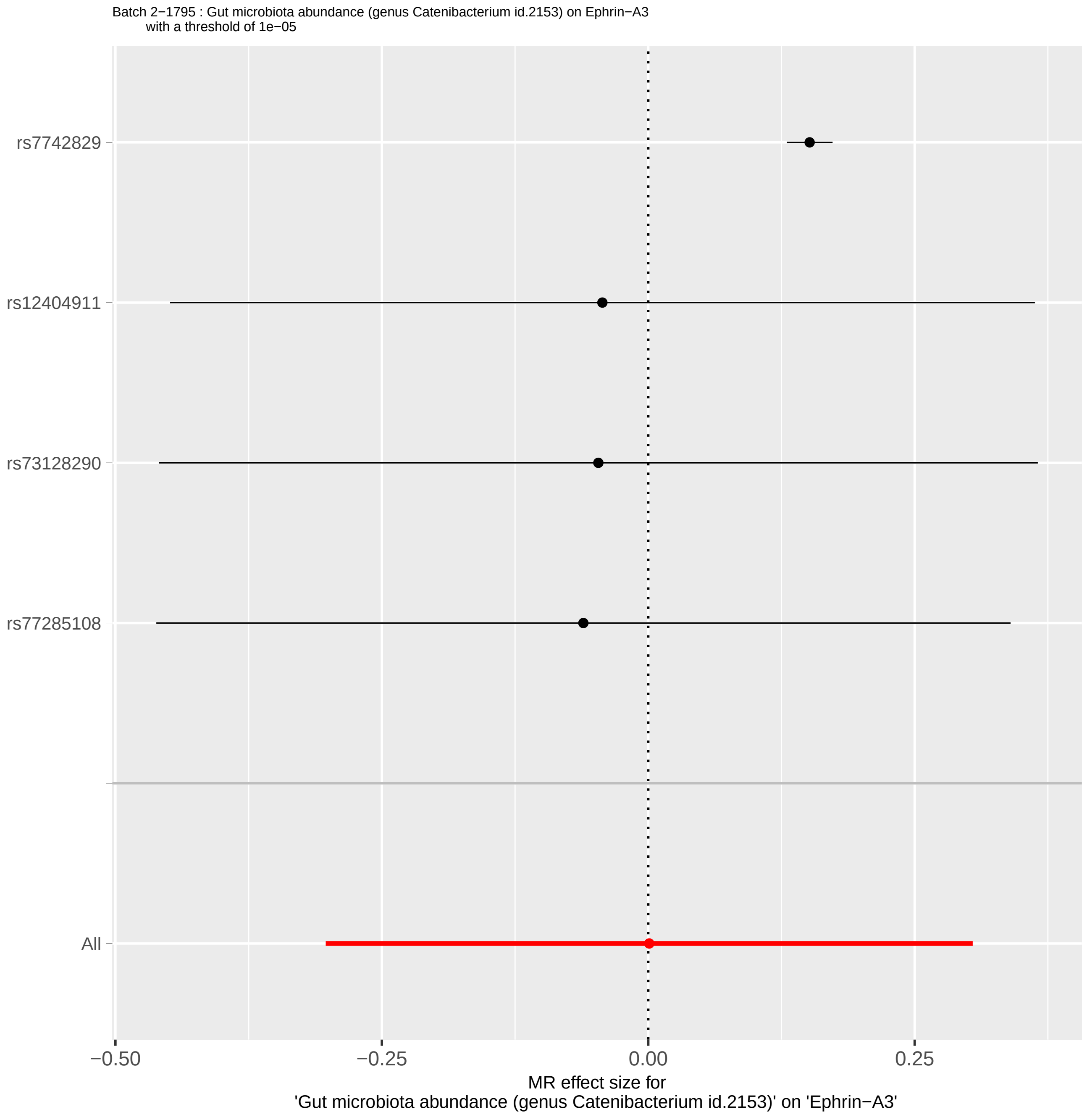

Batch 2−1795 : Gut microbiota abundance (genus Catenibacterium id.2153) on Ephrin−A3
with a threshold of 1e−05
rs7742829
rs12404911
rs73128290
rs77285108
All
−0.50
−0.25
0.00
0.25
MR effect size for
'Gut microbiota abundance (genus Catenibacterium id.2153)' on 'Ephrin−A3'

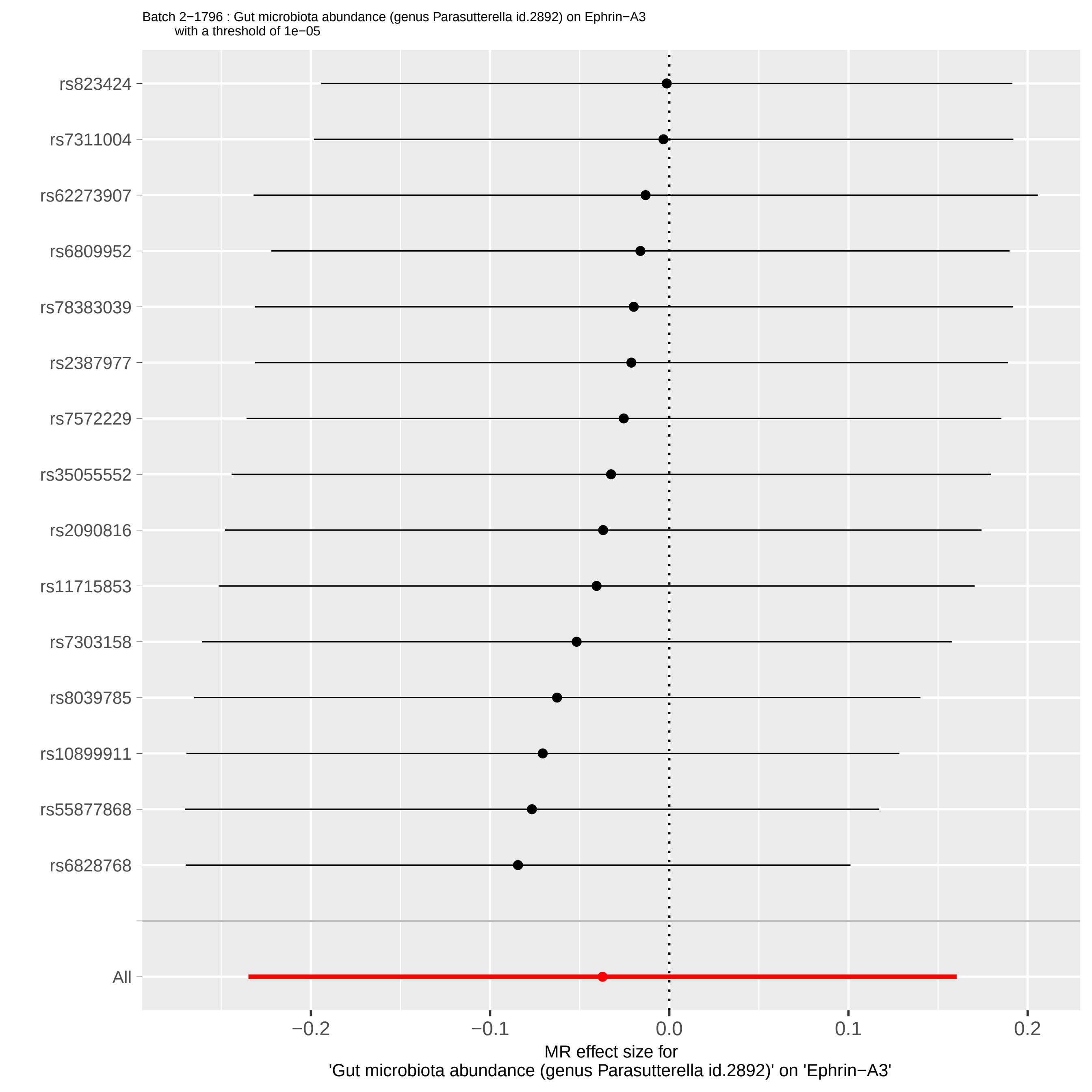
Batch 2−1796 : Gut microbiota abundance (genus Parasutterella id.2892) on Ephrin−A3
with a threshold of 1e−05
rs823424
rs7311004
rs62273907
rs6809952
rs78383039
rs2387977
rs7572229
rs35055552
rs2090816
rs11715853
rs7303158
rs8039785
rs10899911
rs55877868
rs6828768
All
−0.2
−0.1
0.0
0.1
0.2
MR effect size for
'Gut microbiota abundance (genus Parasutterella id.2892)' on 'Ephrin−A3'

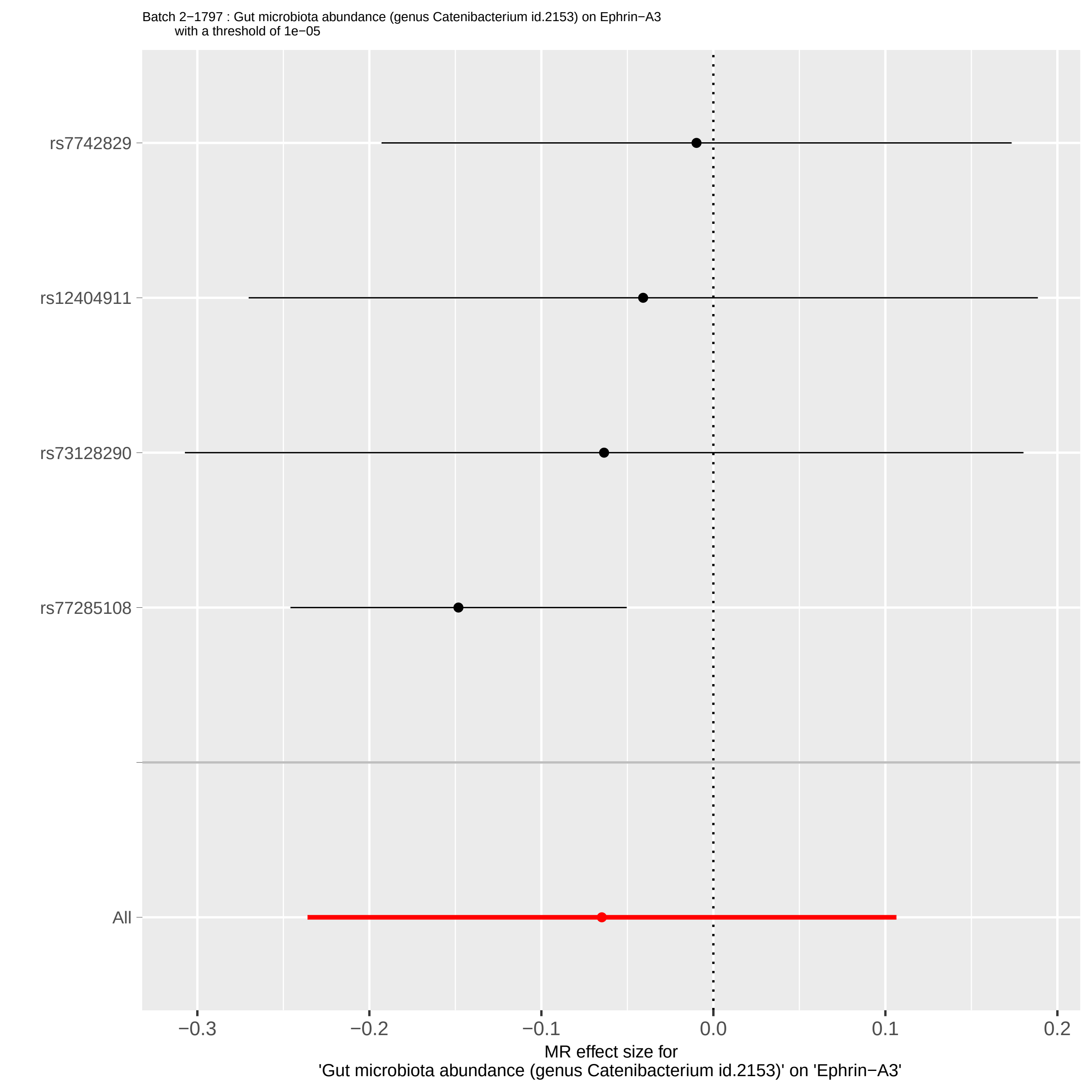
Batch 2−1797 : Gut microbiota abundance (genus Catenibacterium id.2153) on Ephrin−A3
with a threshold of 1e−05
rs7742829
rs12404911
rs73128290
rs77285108
All
−0.3
−0.2
−0.1
0.0
0.1
0.2
MR effect size for
'Gut microbiota abundance (genus Catenibacterium id.2153)' on 'Ephrin−A3'

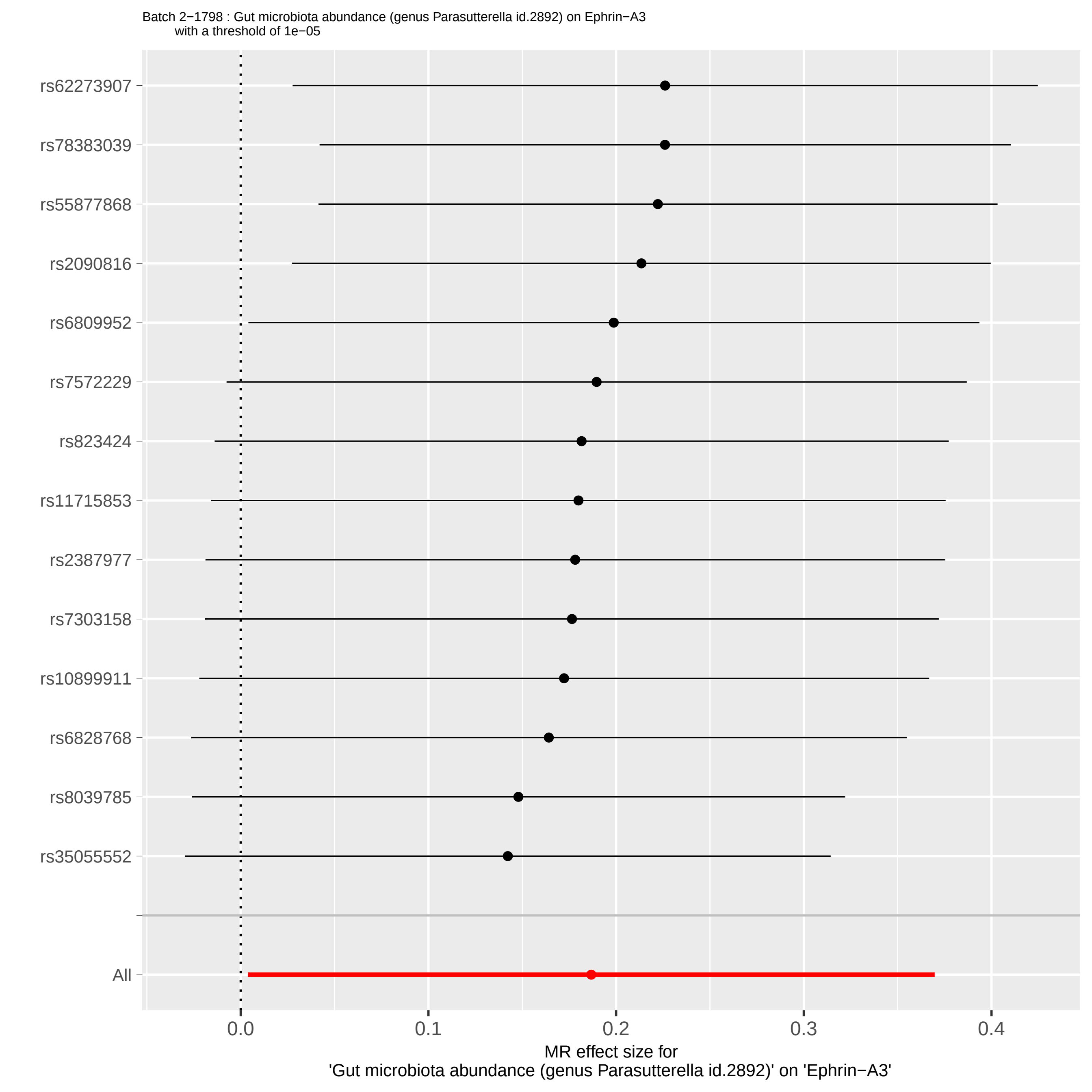

Batch 2−1798 : Gut microbiota abundance (genus Parasutterella id.2892) on Ephrin−A3
with a threshold of 1e−05
rs62273907
rs78383039
rs55877868
rs2090816
rs6809952
rs7572229
rs823424
rs11715853
rs2387977
rs7303158
rs10899911
rs6828768
rs8039785
rs35055552
All
0.0
0.1
0.2
0.3
0.4
MR effect size for
'Gut microbiota abundance (genus Parasutterella id.2892)' on 'Ephrin−A3'

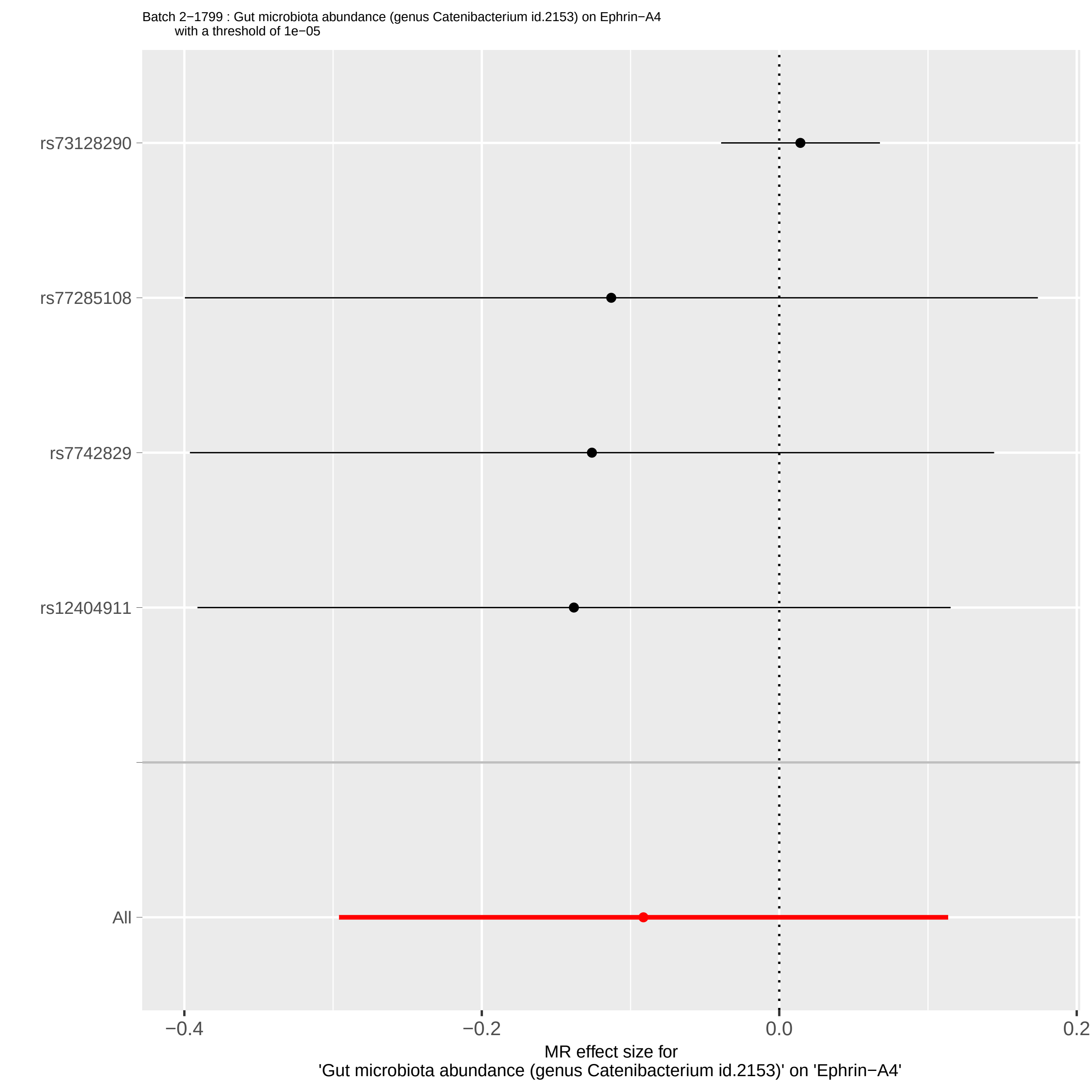
Batch 2−1799 : Gut microbiota abundance (genus Catenibacterium id.2153) on Ephrin−A4
with a threshold of 1e−05
rs73128290
rs77285108
rs7742829
rs12404911
All
−0.4
−0.2
0.0
0.2
MR effect size for
'Gut microbiota abundance (genus Catenibacterium id.2153)' on 'Ephrin−A4'

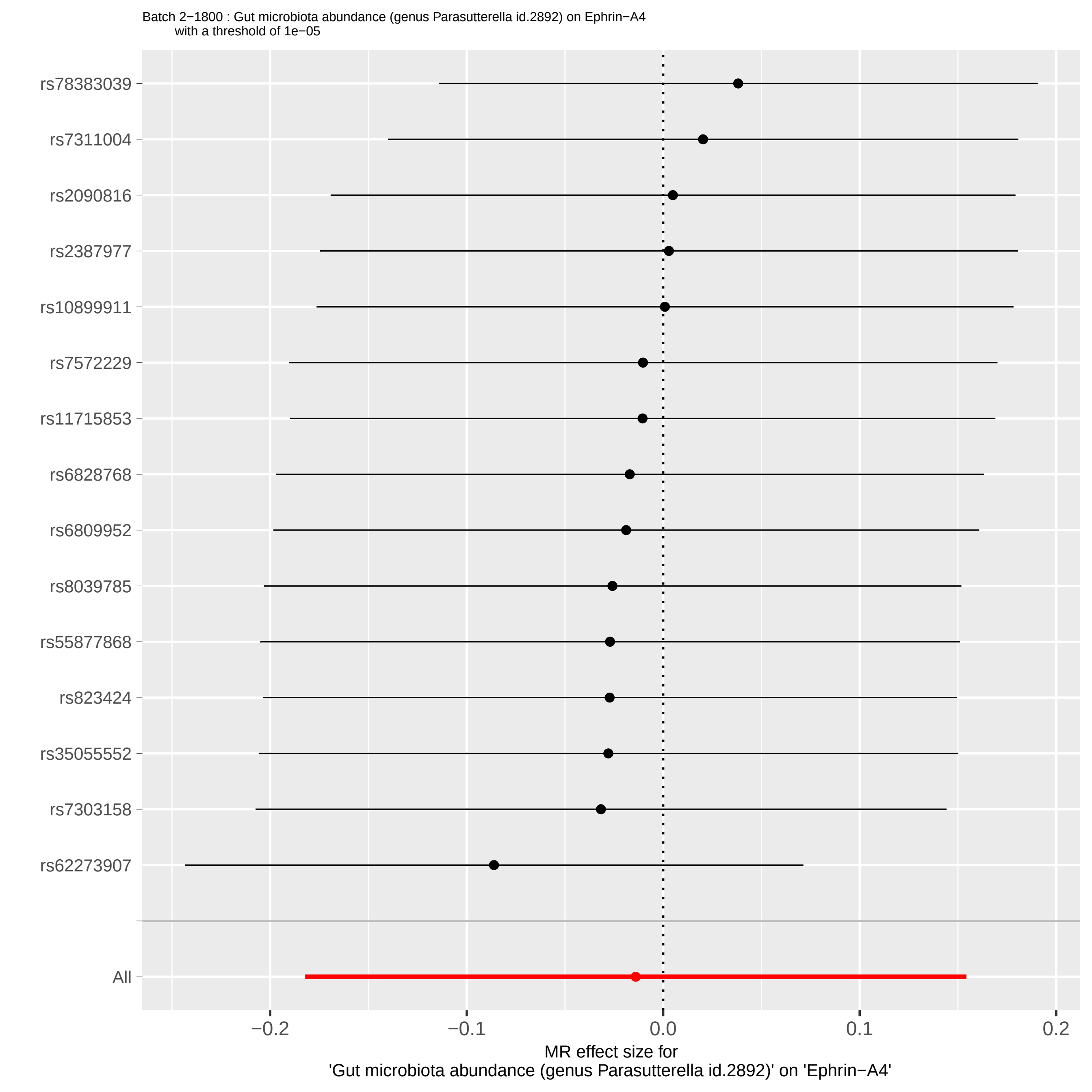

Batch 2−1800 : Gut microbiota abundance (genus Parasutterella id.2892) on Ephrin−A4
with a threshold of 1e−05
rs78383039
rs7311004
rs2090816
rs2387977
rs10899911
rs7572229
rs11715853
rs6828768
rs6809952
rs8039785
rs55877868
rs823424
rs35055552
rs7303158
rs62273907
All
−0.2
−0.1
0.0
0.1
0.2
MR effect size for
'Gut microbiota abundance (genus Parasutterella id.2892)' on 'Ephrin−A4'

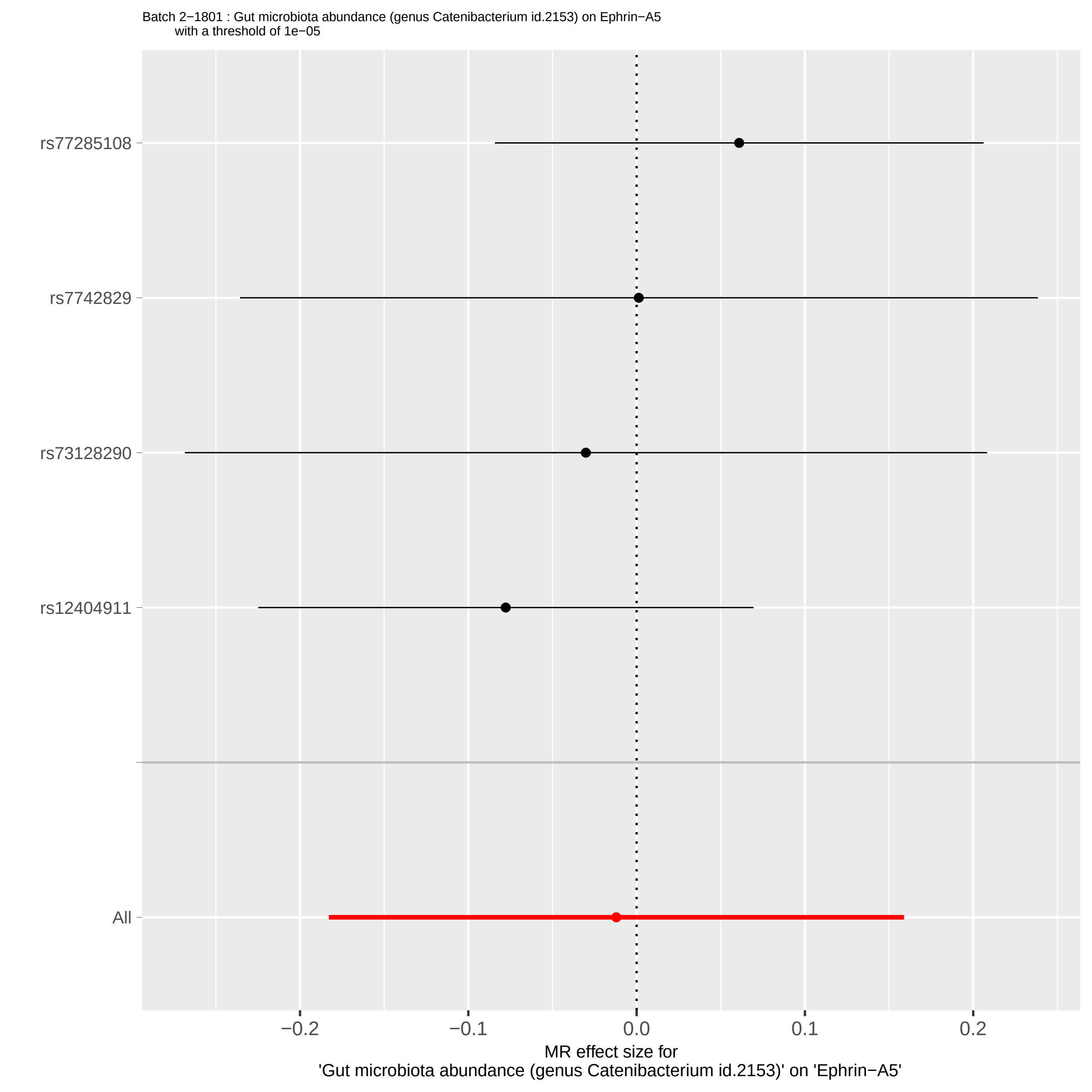
Batch 2−1801 : Gut microbiota abundance (genus Catenibacterium id.2153) on Ephrin−A5
with a threshold of 1e−05
rs77285108
rs7742829
rs73128290
rs12404911
All
−0.2
−0.1
0.0
0.1
0.2
MR effect size for
'Gut microbiota abundance (genus Catenibacterium id.2153)' on 'Ephrin−A5'

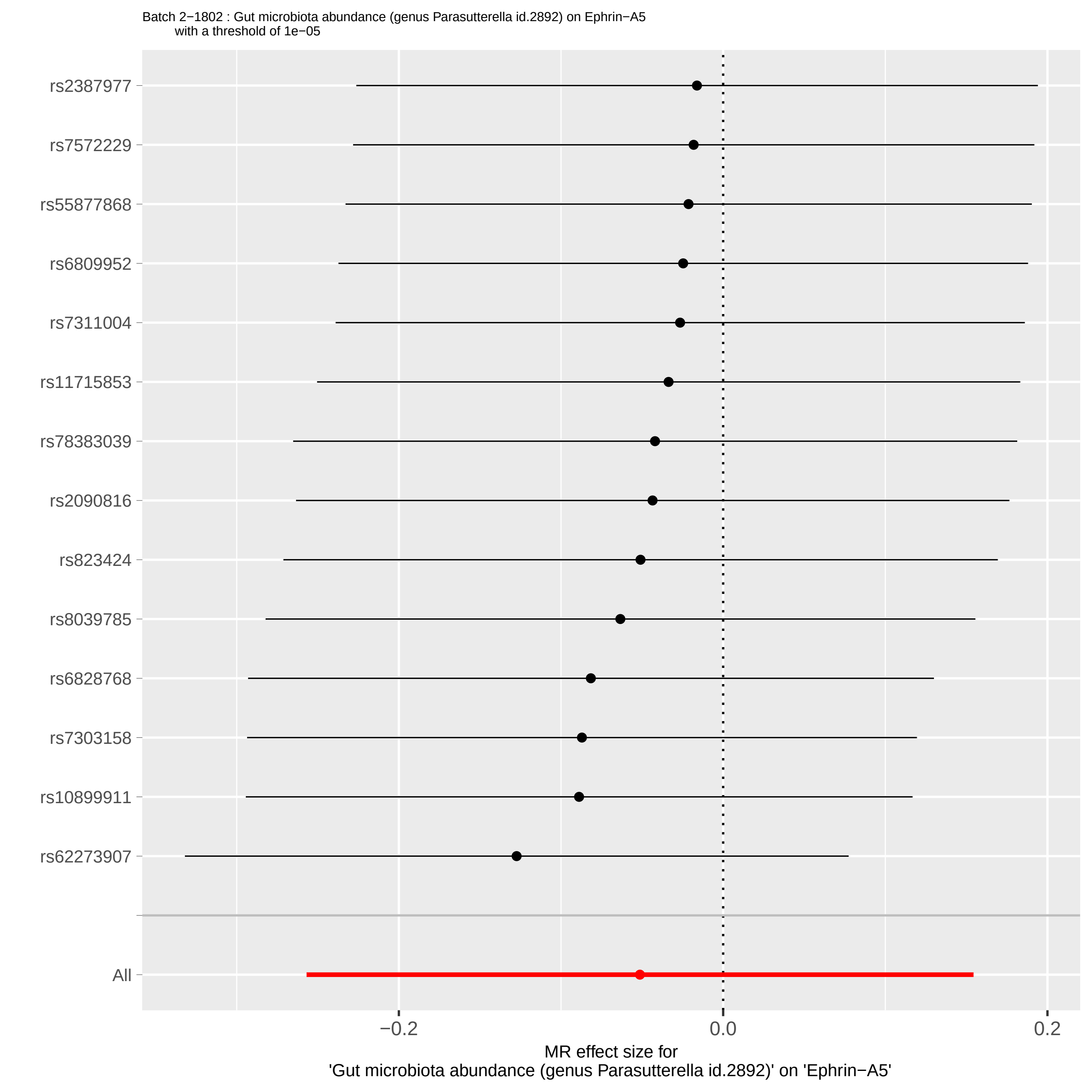

Batch 2−1802 : Gut microbiota abundance (genus Parasutterella id.2892) on Ephrin−A5
with a threshold of 1e−05
rs2387977
rs7572229
rs55877868
rs6809952
rs7311004
rs11715853
rs78383039
rs2090816
rs823424
rs8039785
rs6828768
rs7303158
rs10899911
rs62273907
All
−0.2
0.0
0.2
MR effect size for
'Gut microbiota abundance (genus Parasutterella id.2892)' on 'Ephrin−A5'

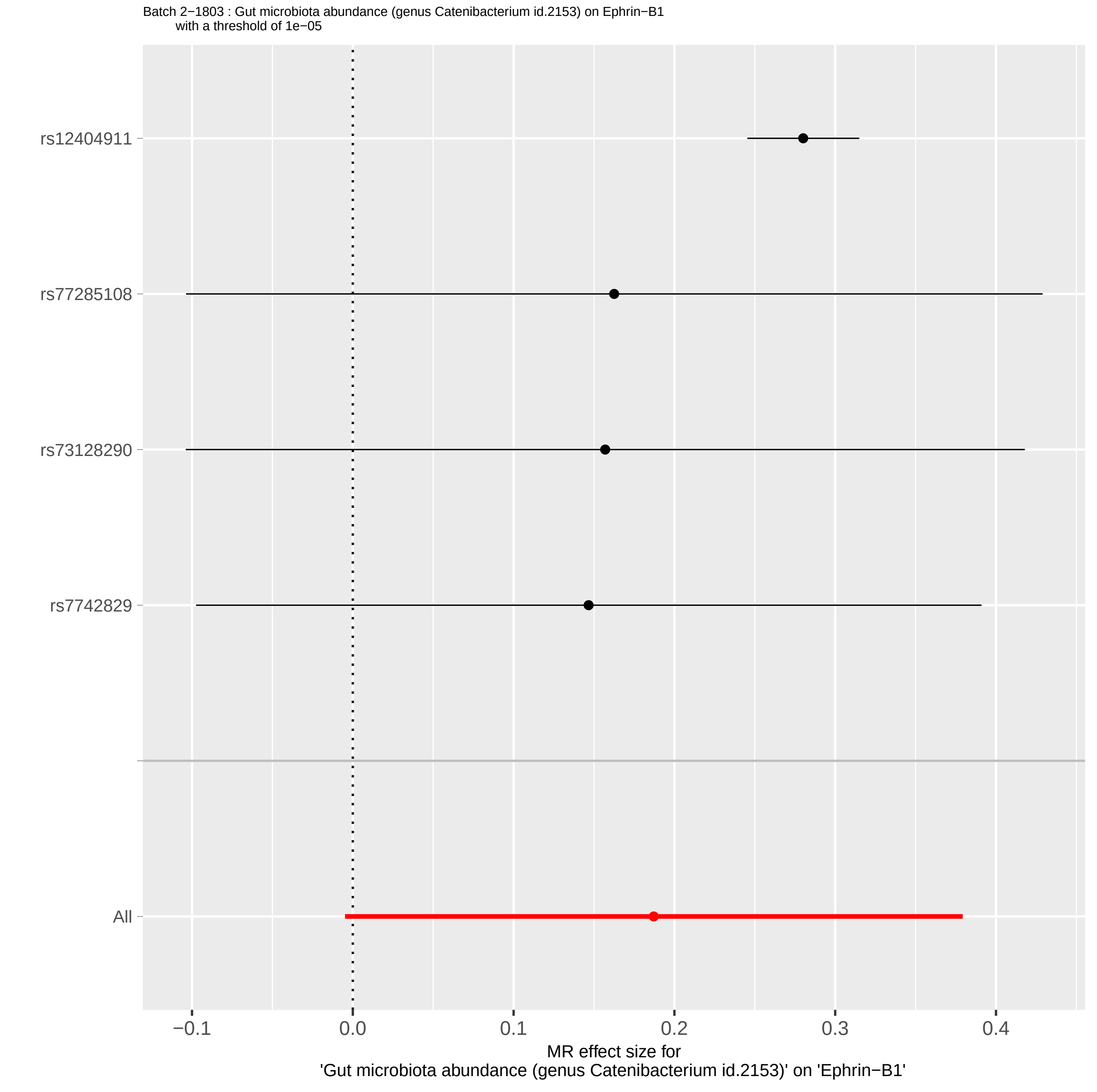

Batch 2−1803 : Gut microbiota abundance (genus Catenibacterium id.2153) on Ephrin−B1
with a threshold of 1e−05
rs12404911
rs77285108
rs73128290
rs7742829
All
−0.1
0.0
0.1
0.2
0.3
0.4
MR effect size for
'Gut microbiota abundance (genus Catenibacterium id.2153)' on 'Ephrin−B1'

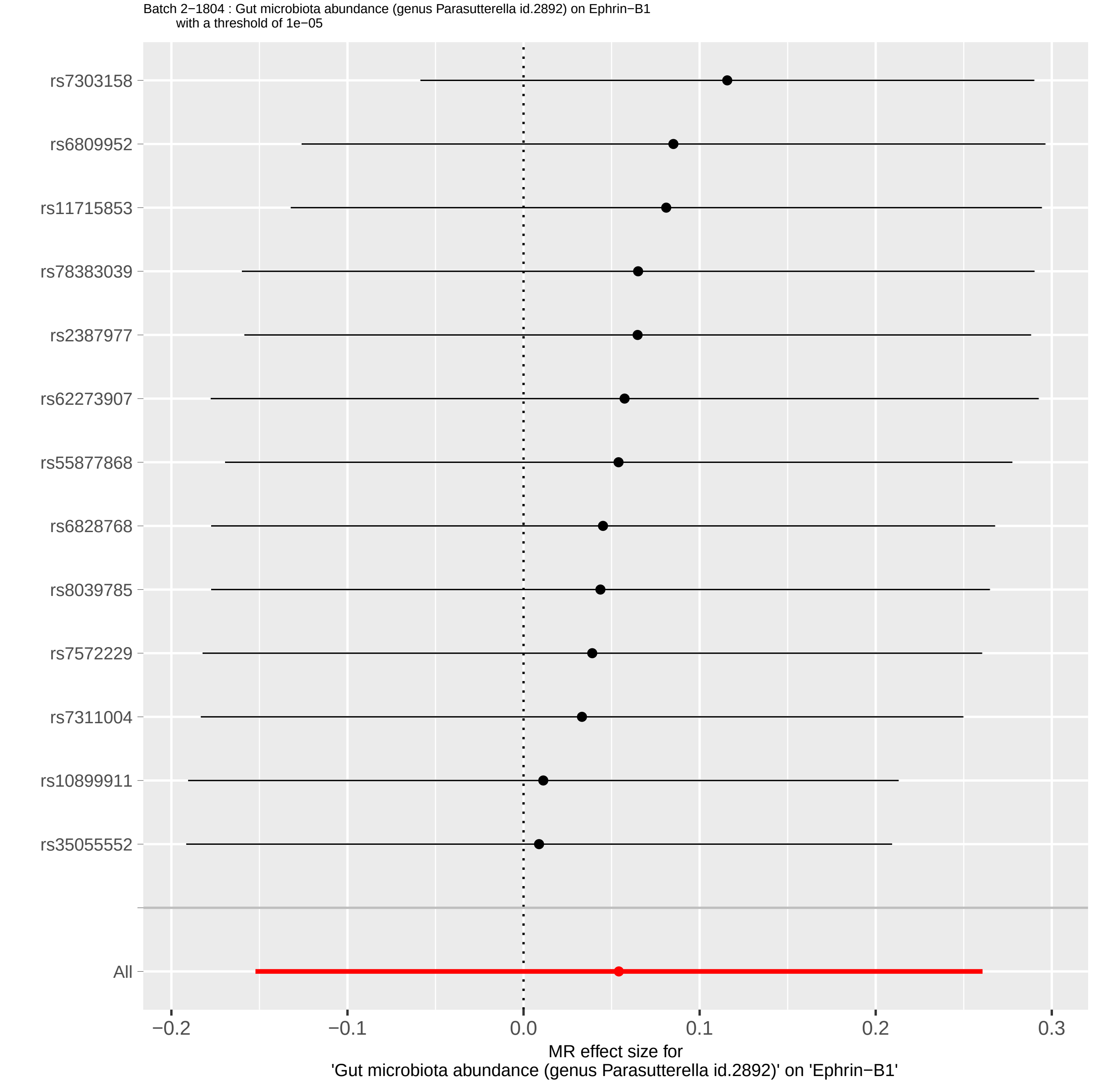
Batch 2−1804 : Gut microbiota abundance (genus Parasutterella id.2892) on Ephrin−B1
with a threshold of 1e−05
rs7303158
rs6809952
rs11715853
rs78383039
rs2387977
rs62273907
rs55877868
rs6828768
rs8039785
rs7572229
rs7311004
rs10899911
rs35055552
All
−0.2
−0.1
0.0
0.1
0.2
0.3
MR effect size for
'Gut microbiota abundance (genus Parasutterella id.2892)' on 'Ephrin−B1'

Batch 2−1805 : Gut microbiota abundance (genus Catenibacterium id.2153) on Ephrin−B1
with a threshold of 1e−05

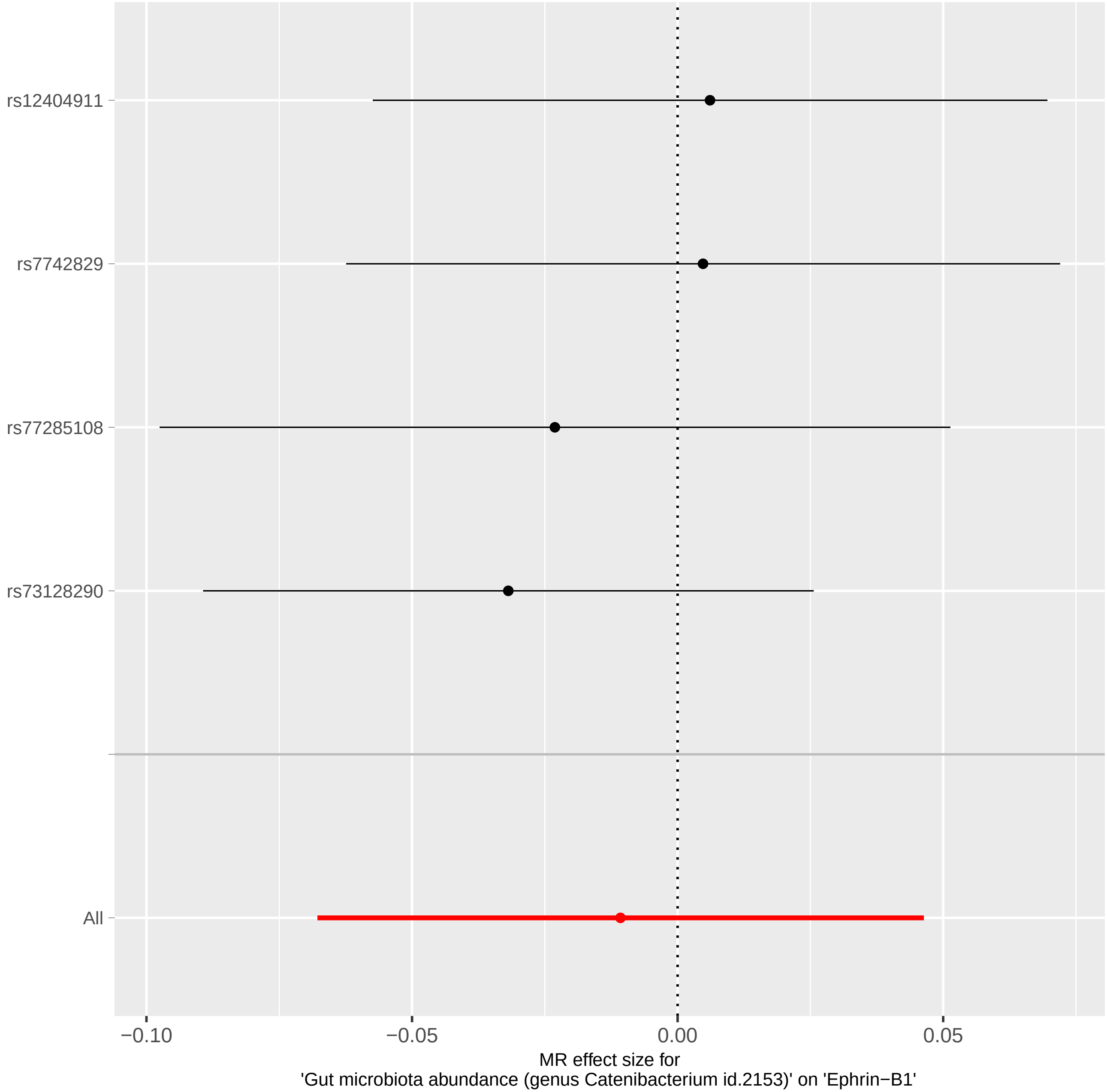

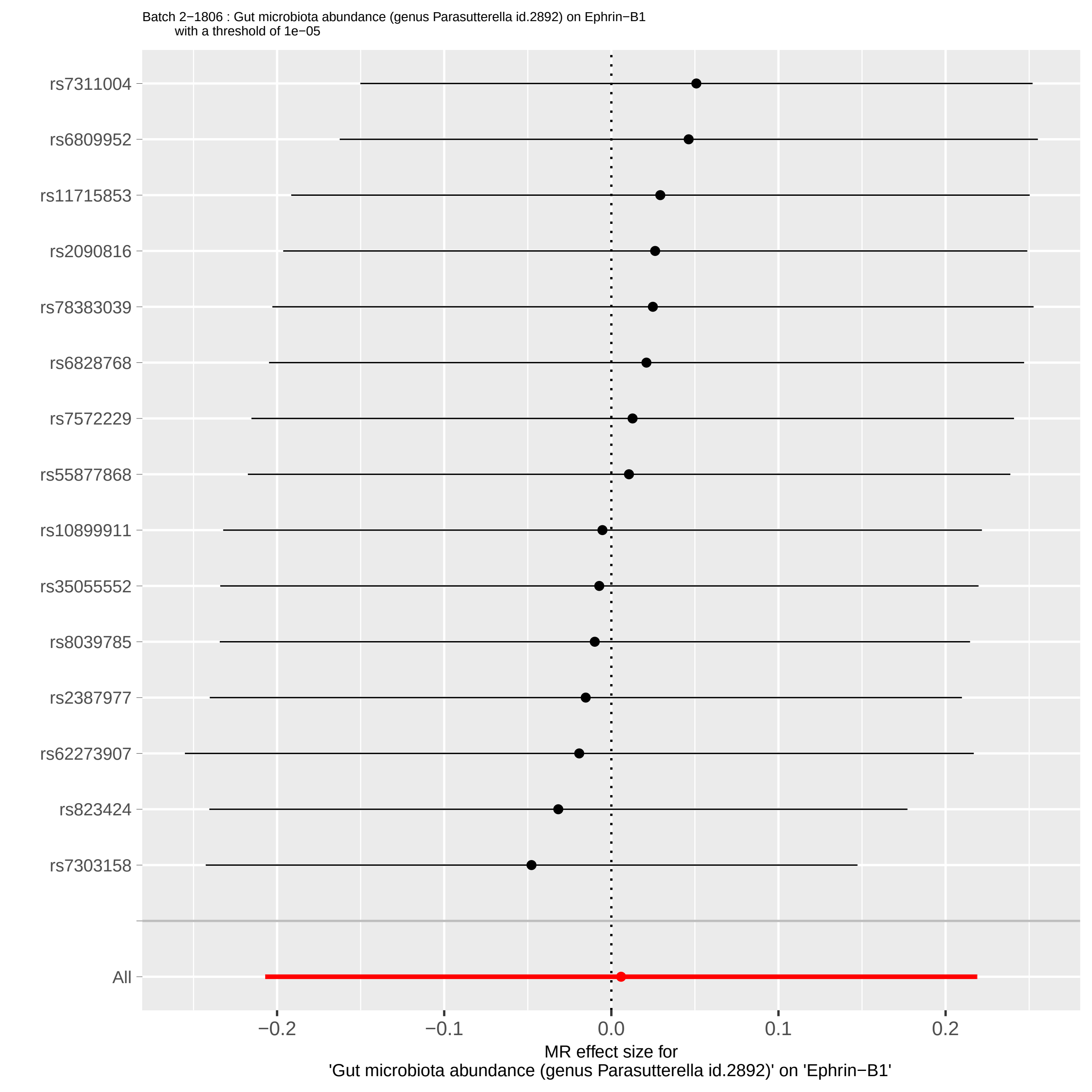
Batch 2−1806 : Gut microbiota abundance (genus Parasutterella id.2892) on Ephrin−B1
with a threshold of 1e−05
rs7311004
rs6809952
rs11715853
rs2090816
rs78383039
rs6828768
rs7572229
rs55877868
rs10899911
rs35055552
rs8039785
rs2387977
rs62273907
rs823424
rs7303158
All
−0.2
−0.1
0.0
0.1
0.2
MR effect size for
'Gut microbiota abundance (genus Parasutterella id.2892)' on 'Ephrin−B1'

Batch 2−1807 : Gut microbiota abundance (genus Catenibacterium id.2153) on Ephrin−B2
with a threshold of 1e−05

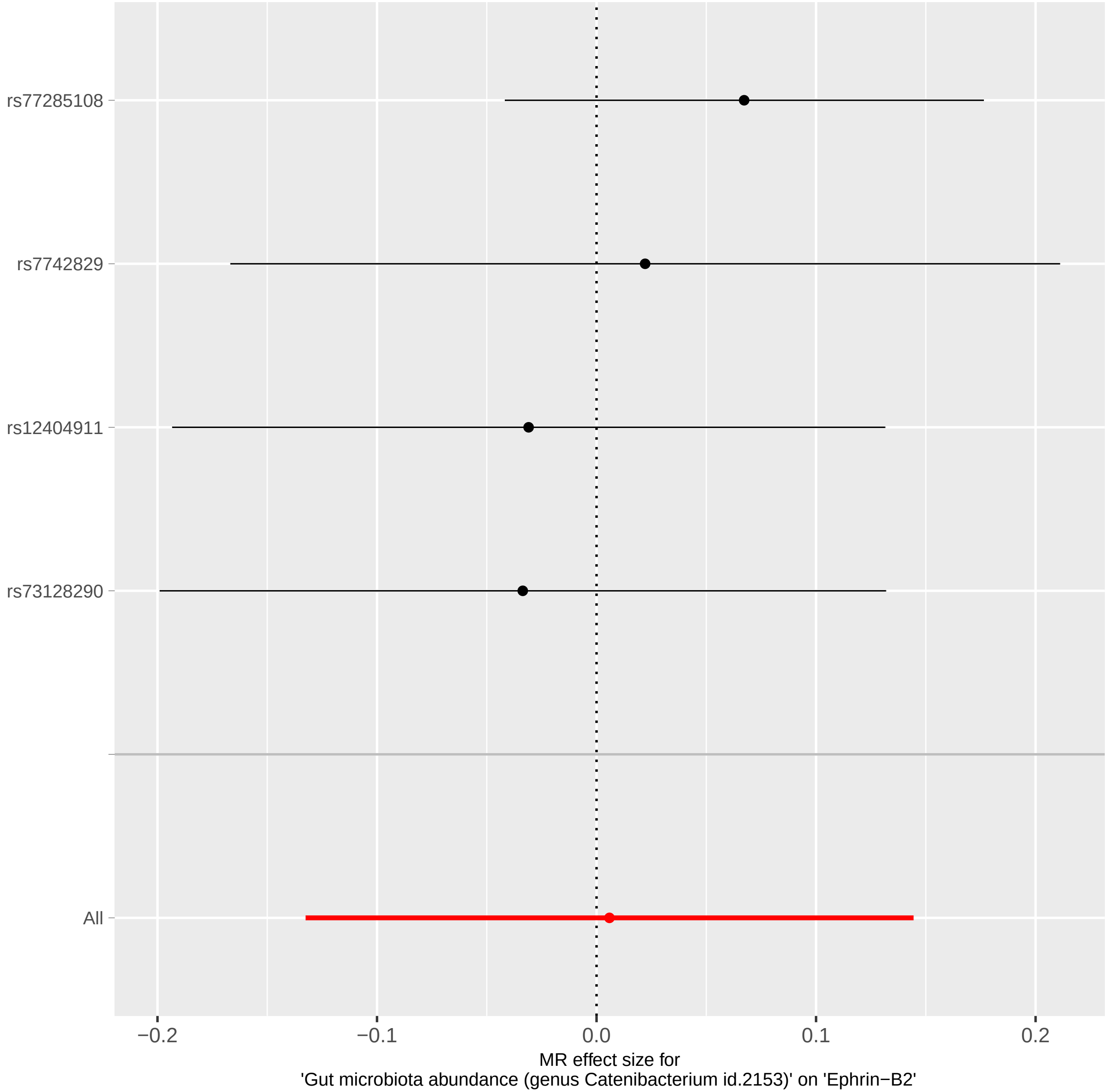

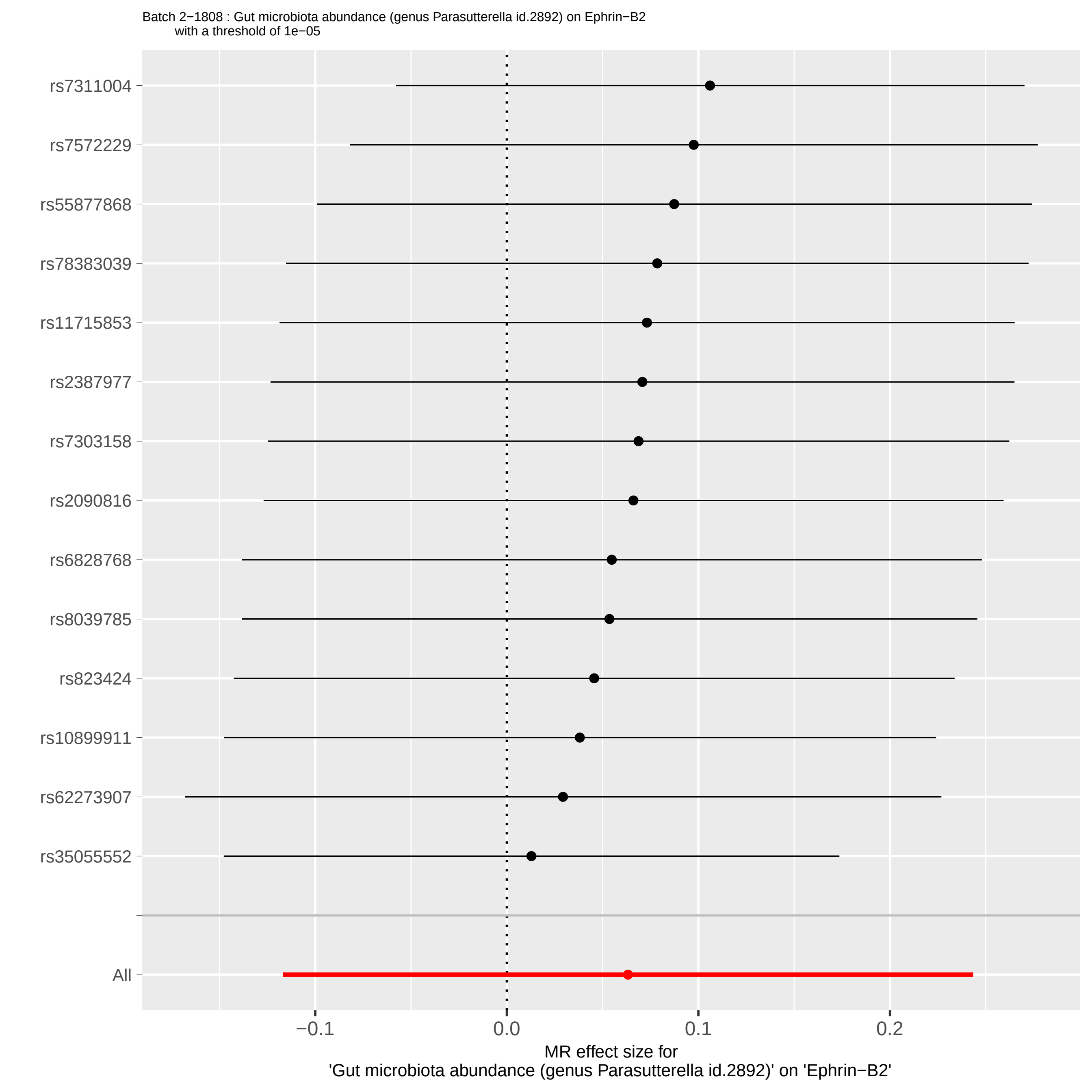

Batch 2−1808 : Gut microbiota abundance (genus Parasutterella id.2892) on Ephrin−B2
with a threshold of 1e−05
rs7311004
rs7572229
rs55877868
rs78383039
rs11715853
rs2387977
rs7303158
rs2090816
rs6828768
rs8039785
rs823424
rs10899911
rs62273907
rs35055552
All
−0.1
0.0
0.1
0.2
MR effect size for
'Gut microbiota abundance (genus Parasutterella id.2892)' on 'Ephrin−B2'

Batch 2−1809 : Gut microbiota abundance (genus Catenibacterium id.2153) on Ephrin−B2
with a threshold of 1e−05

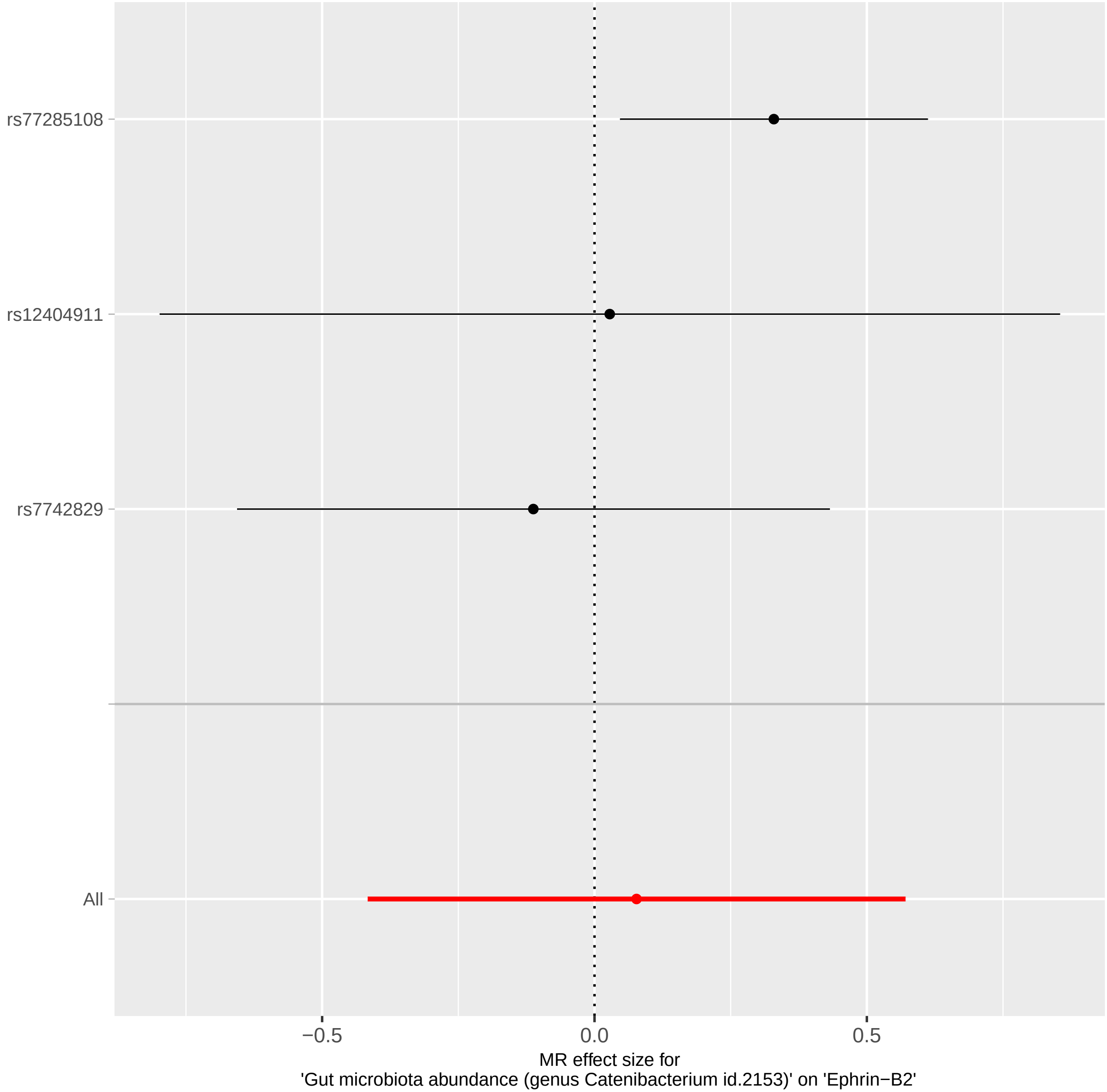

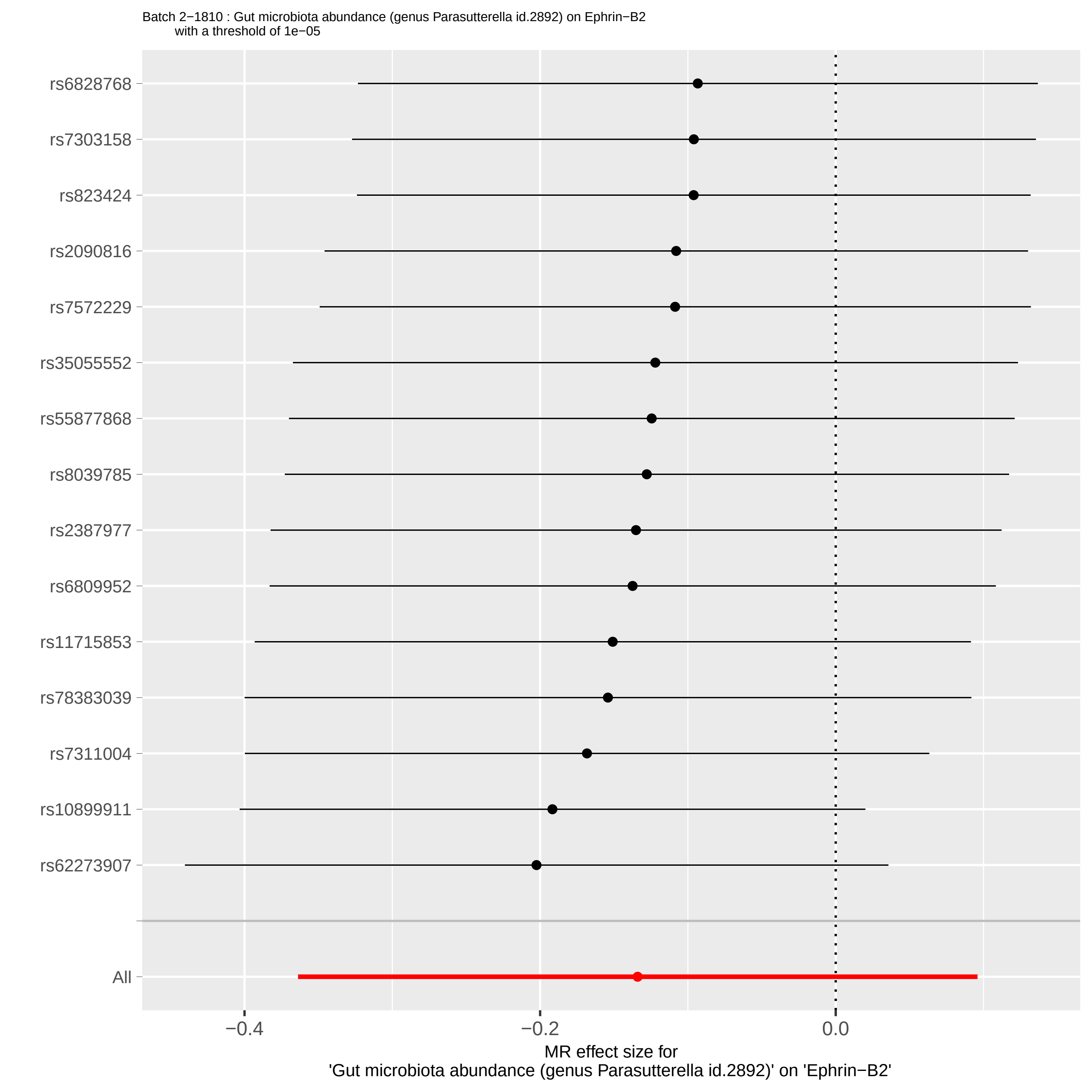
Batch 2−1810 : Gut microbiota abundance (genus Parasutterella id.2892) on Ephrin−B2
with a threshold of 1e−05
rs6828768
rs7303158
rs823424
rs2090816
rs7572229
rs35055552
rs55877868
rs8039785
rs2387977
rs6809952
rs11715853
rs78383039
rs7311004
rs10899911
rs62273907
All
−0.4
−0.2
0.0
MR effect size for
'Gut microbiota abundance (genus Parasutterella id.2892)' on 'Ephrin−B2'

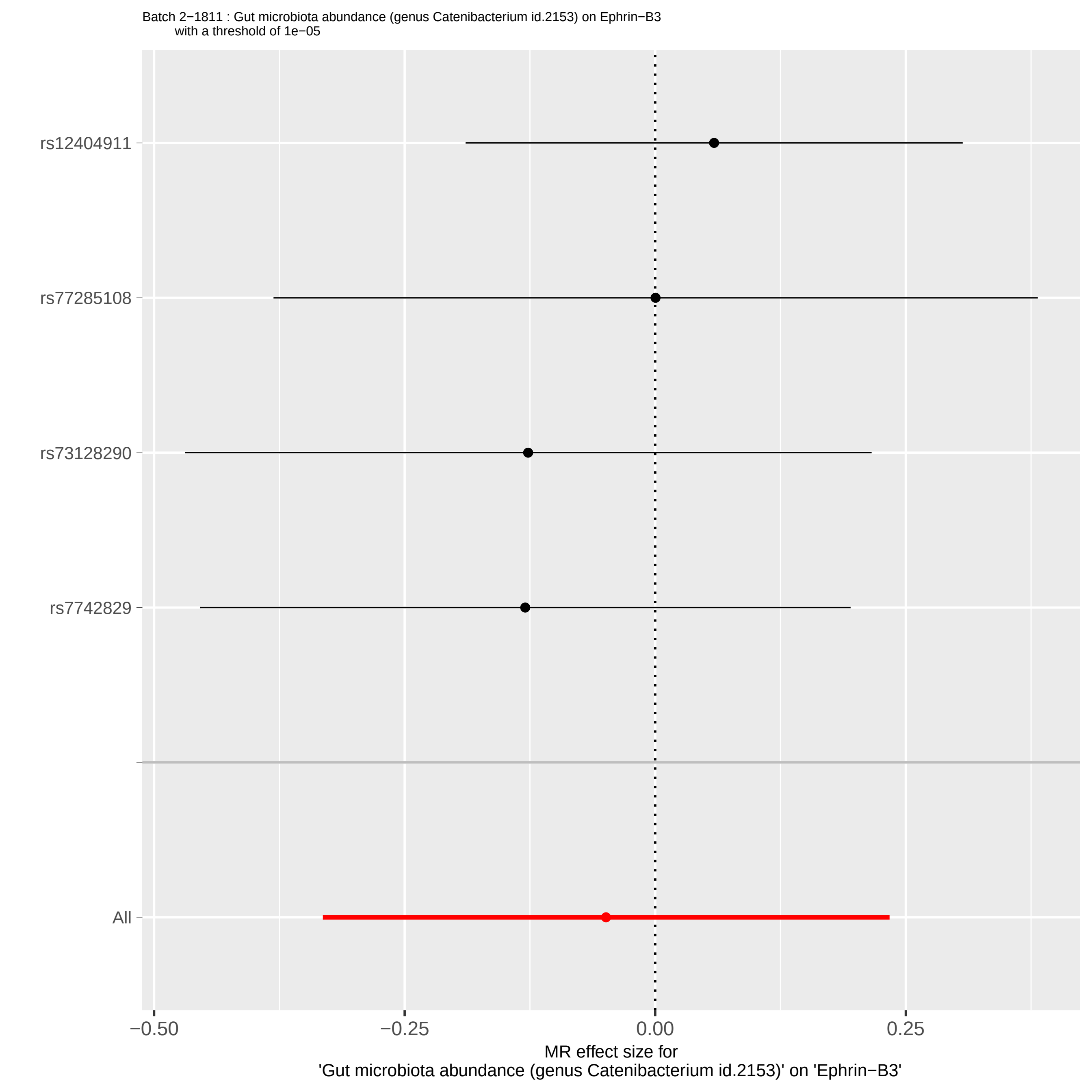

Batch 2−1811 : Gut microbiota abundance (genus Catenibacterium id.2153) on Ephrin−B3
with a threshold of 1e−05
rs12404911
rs77285108
rs73128290
rs7742829
All
−0.50
−0.25
0.00
0.25
MR effect size for
'Gut microbiota abundance (genus Catenibacterium id.2153)' on 'Ephrin−B3'

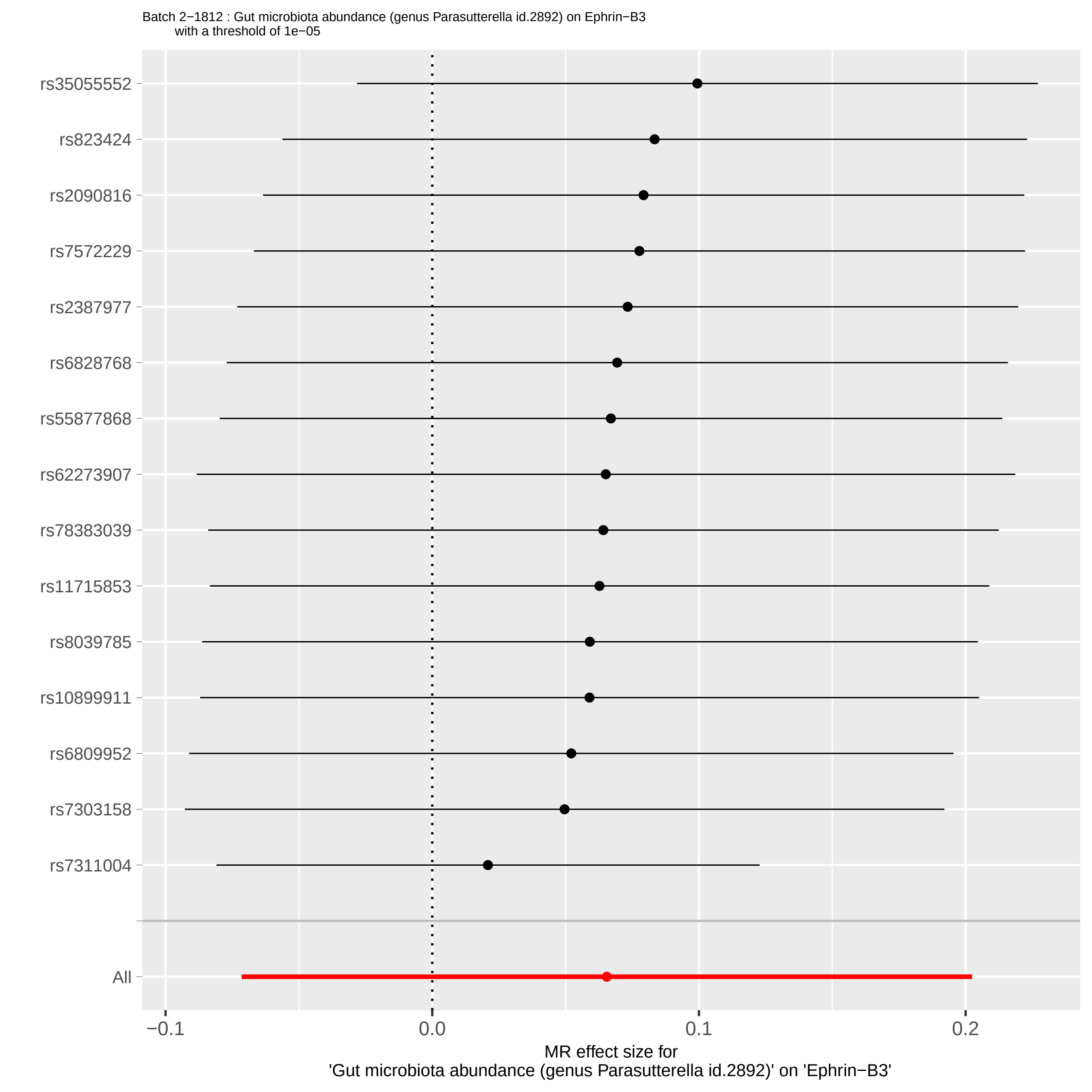

Batch 2−1812 : Gut microbiota abundance (genus Parasutterella id.2892) on Ephrin−B3
with a threshold of 1e−05
rs35055552
rs823424
rs2090816
rs7572229
rs2387977
rs6828768
rs55877868
rs62273907
rs78383039
rs11715853
rs8039785
rs10899911
rs6809952
rs7303158
rs7311004
All
−0.1
0.0
0.1
0.2
MR effect size for
'Gut microbiota abundance (genus Parasutterella id.2892)' on 'Ephrin−B3'

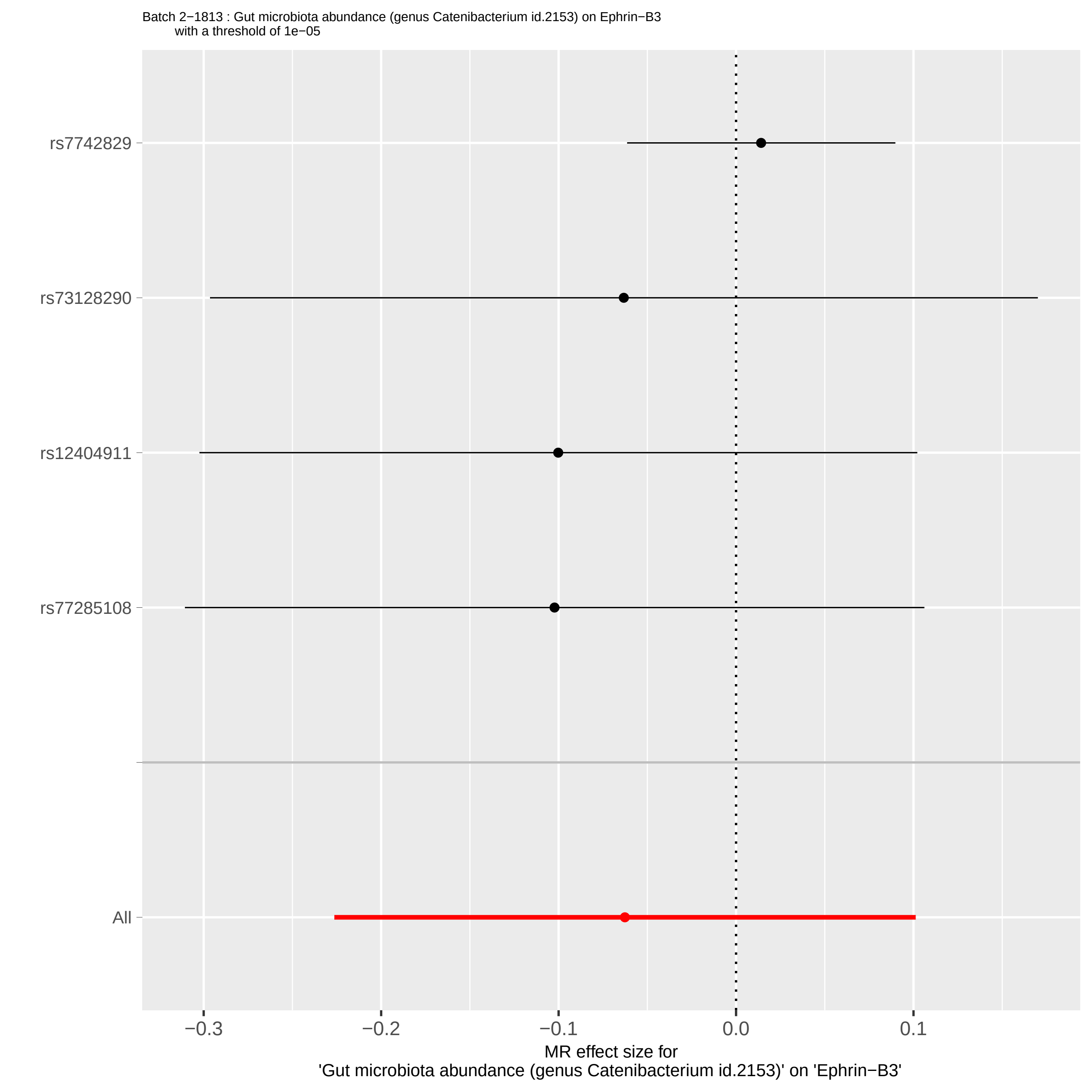
Batch 2−1813 : Gut microbiota abundance (genus Catenibacterium id.2153) on Ephrin−B3
with a threshold of 1e−05
rs7742829
rs73128290
rs12404911
rs77285108
All
−0.3
−0.2
−0.1
0.0
0.1
MR effect size for
'Gut microbiota abundance (genus Catenibacterium id.2153)' on 'Ephrin−B3'

Batch 2−1814 : Gut microbiota abundance (genus Parasutterella id.2892) on Ephrin−B3
with a threshold of 1e−05

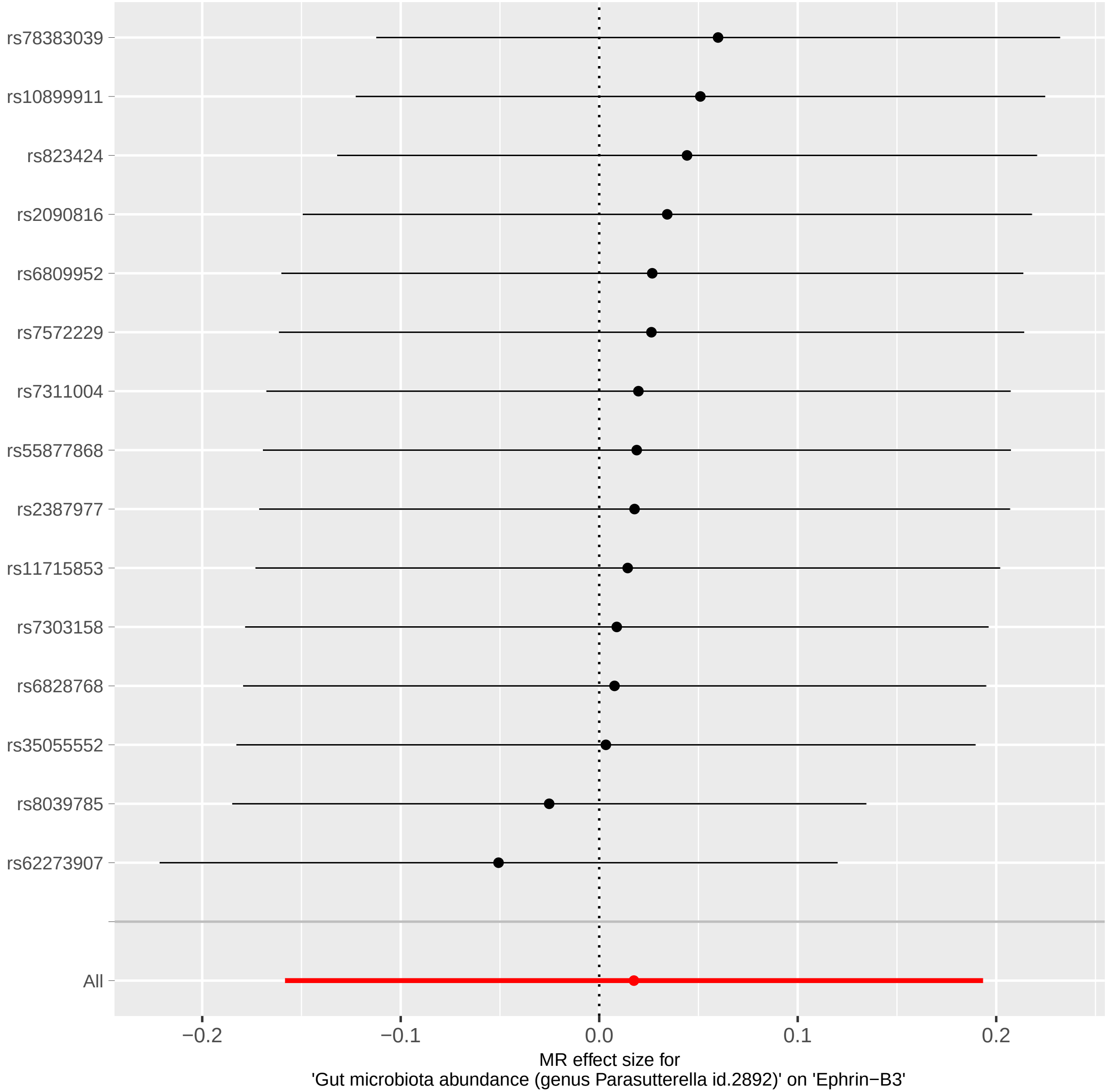

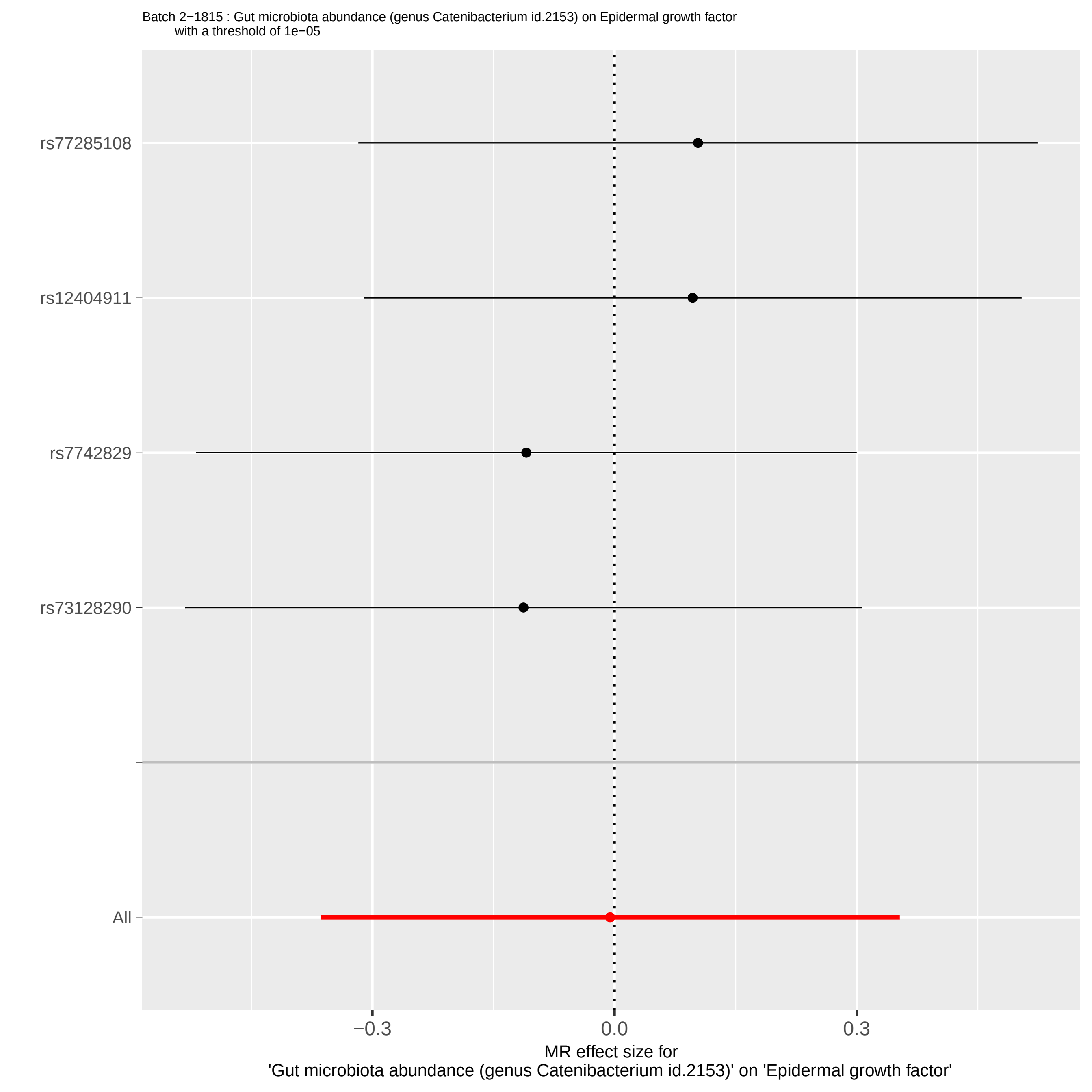
Batch 2−1815 : Gut microbiota abundance (genus Catenibacterium id.2153) on Epidermal growth factor
with a threshold of 1e−05
rs77285108
rs12404911
rs7742829
rs73128290
All
−0.3
0.0
0.3
MR effect size for
'Gut microbiota abundance (genus Catenibacterium id.2153)' on 'Epidermal growth factor'

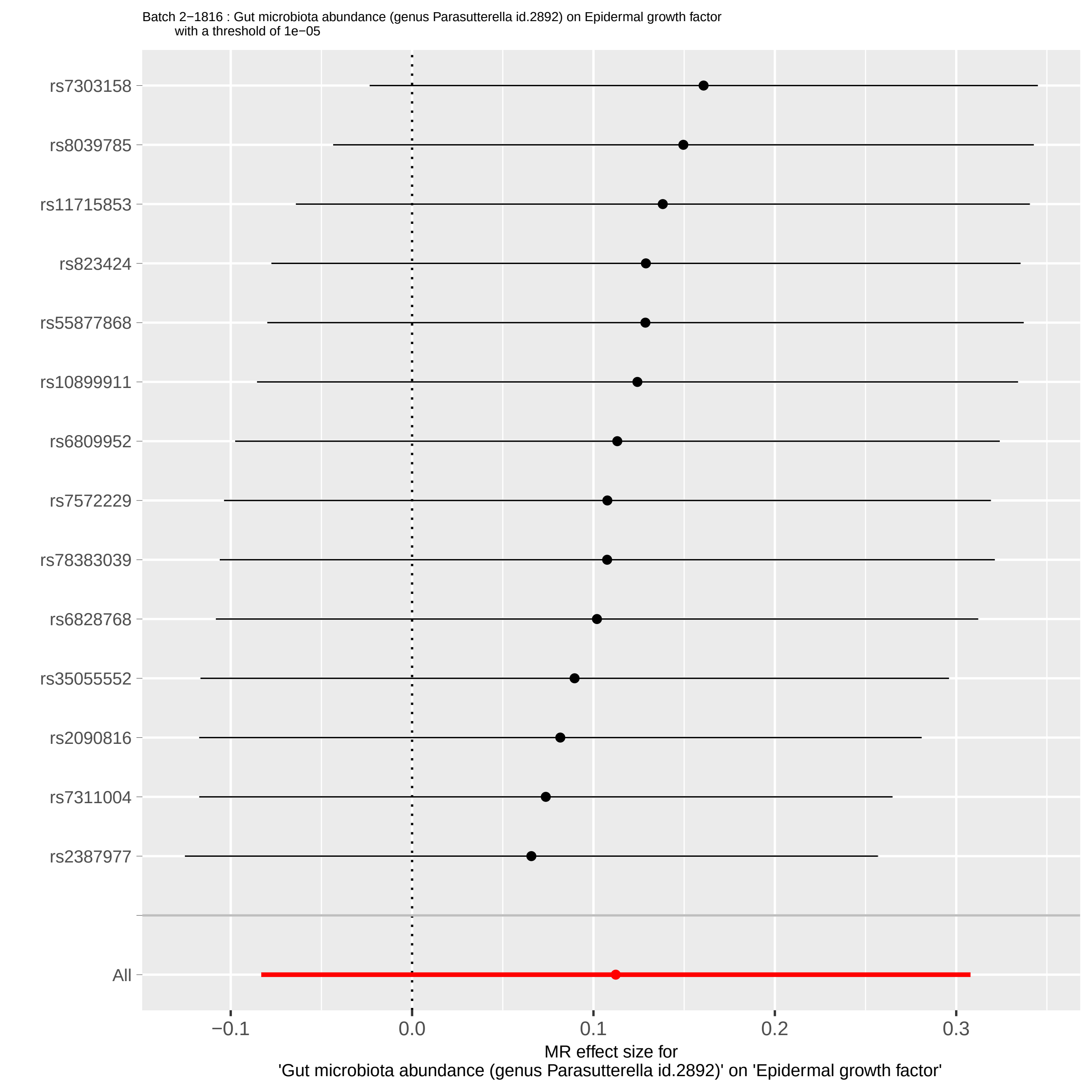

Batch 2−1816 : Gut microbiota abundance (genus Parasutterella id.2892) on Epidermal growth factor
with a threshold of 1e−05
rs7303158
rs8039785
rs11715853
rs823424
rs55877868
rs10899911
rs6809952
rs7572229
rs78383039
rs6828768
rs35055552
rs2090816
rs7311004
rs2387977
All
−0.1
0.0
0.1
0.2
0.3
MR effect size for
'Gut microbiota abundance (genus Parasutterella id.2892)' on 'Epidermal growth factor'

Batch 2−1817 : Gut microbiota abundance (genus Catenibacterium id.2153) on Epidermal growth factor receptor
with a threshold of 1e−05

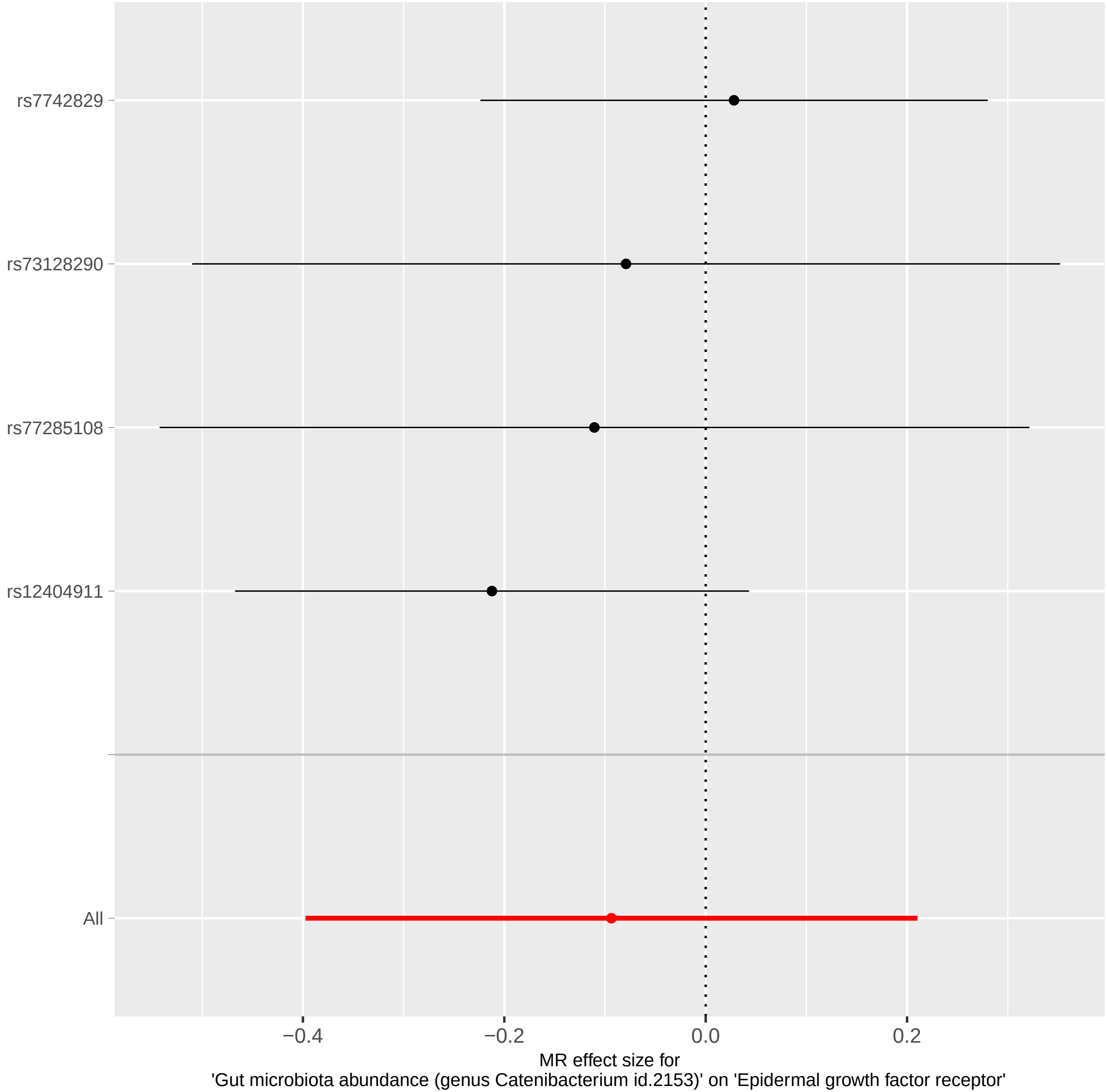

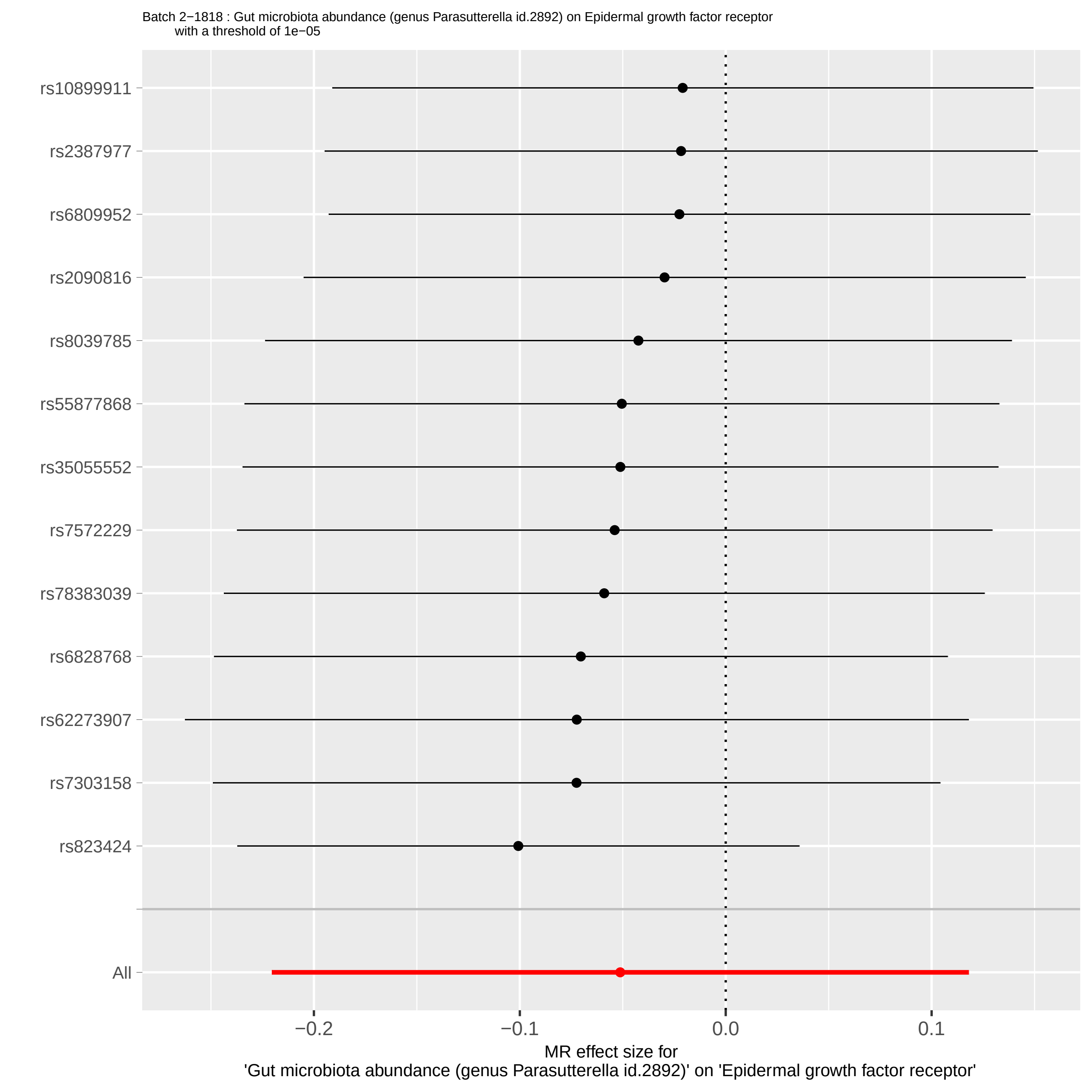

Batch 2−1818 : Gut microbiota abundance (genus Parasutterella id.2892) on Epidermal growth factor receptor
with a threshold of 1e−05
rs10899911
rs2387977
rs6809952
rs2090816
rs8039785
rs55877868
rs35055552
rs7572229
rs78383039
rs6828768
rs62273907
rs7303158
rs823424
All
−0.2
−0.1
0.0
0.1
MR effect size for
'Gut microbiota abundance (genus Parasutterella id.2892)' on 'Epidermal growth factor receptor'

Batch 2−1819 : Gut microbiota abundance (genus Catenibacterium id.2153) on Epidermal growth factor receptor variant III
with a threshold of 1e−05

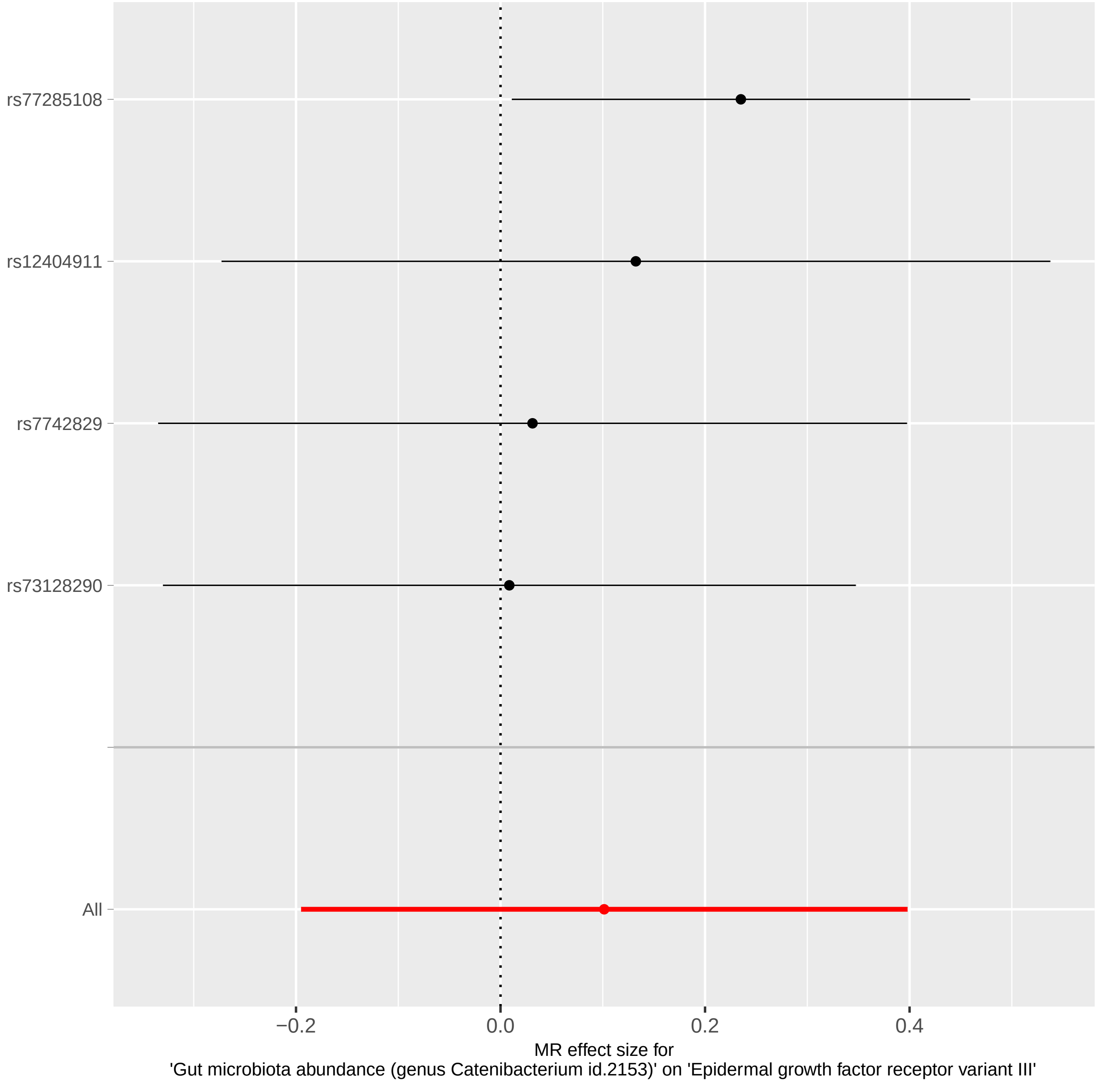

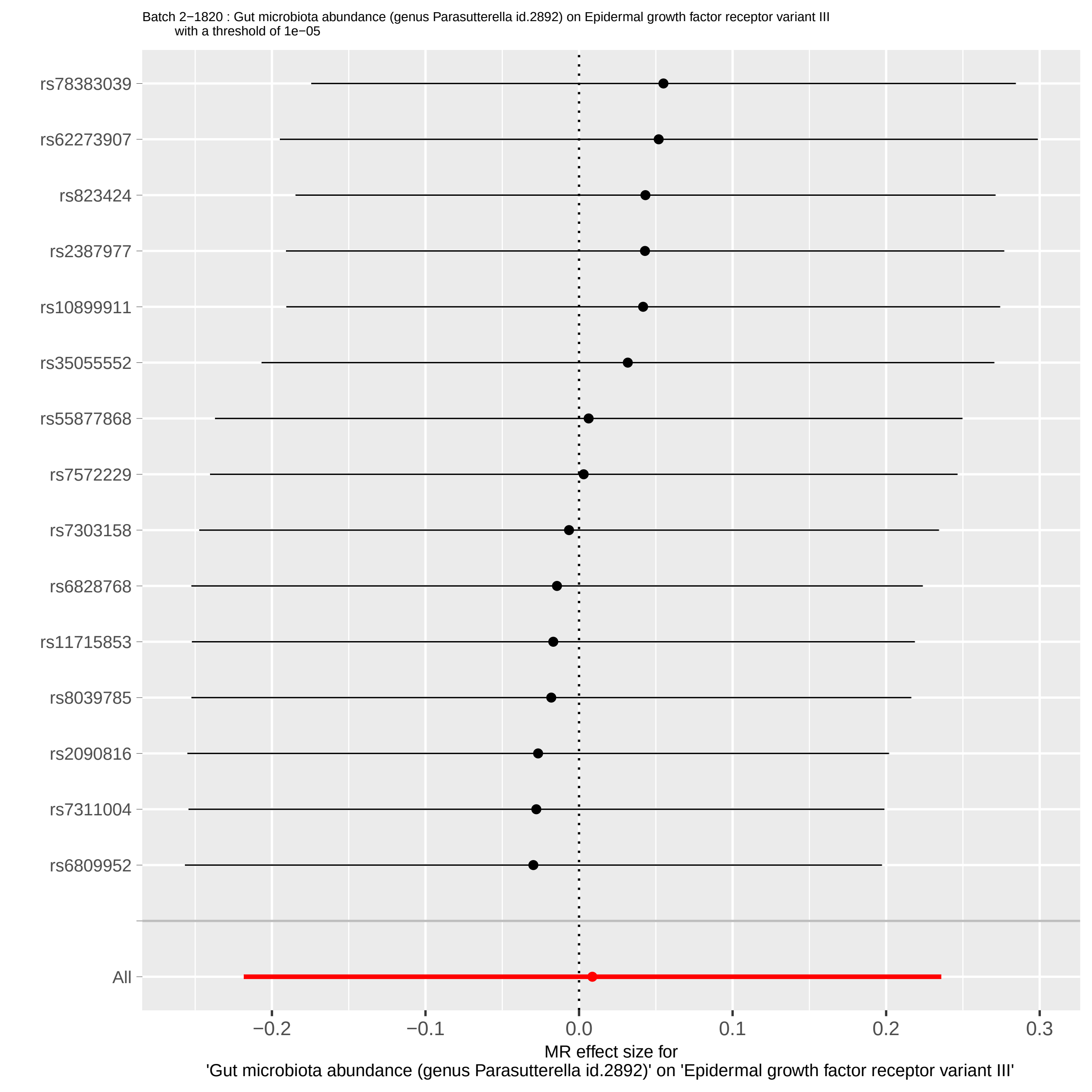
Batch 2−1820 : Gut microbiota abundance (genus Parasutterella id.2892) on Epidermal growth factor receptor variant III
with a threshold of 1e−05
rs78383039
rs62273907
rs823424
rs2387977
rs10899911
rs35055552
rs55877868
rs7572229
rs7303158
rs6828768
rs11715853
rs8039785
rs2090816
rs7311004
rs6809952
All
−0.2
−0.1
0.0
0.1
0.2
0.3
MR effect size for
'Gut microbiota abundance (genus Parasutterella id.2892)' on 'Epidermal growth factor receptor variant III'

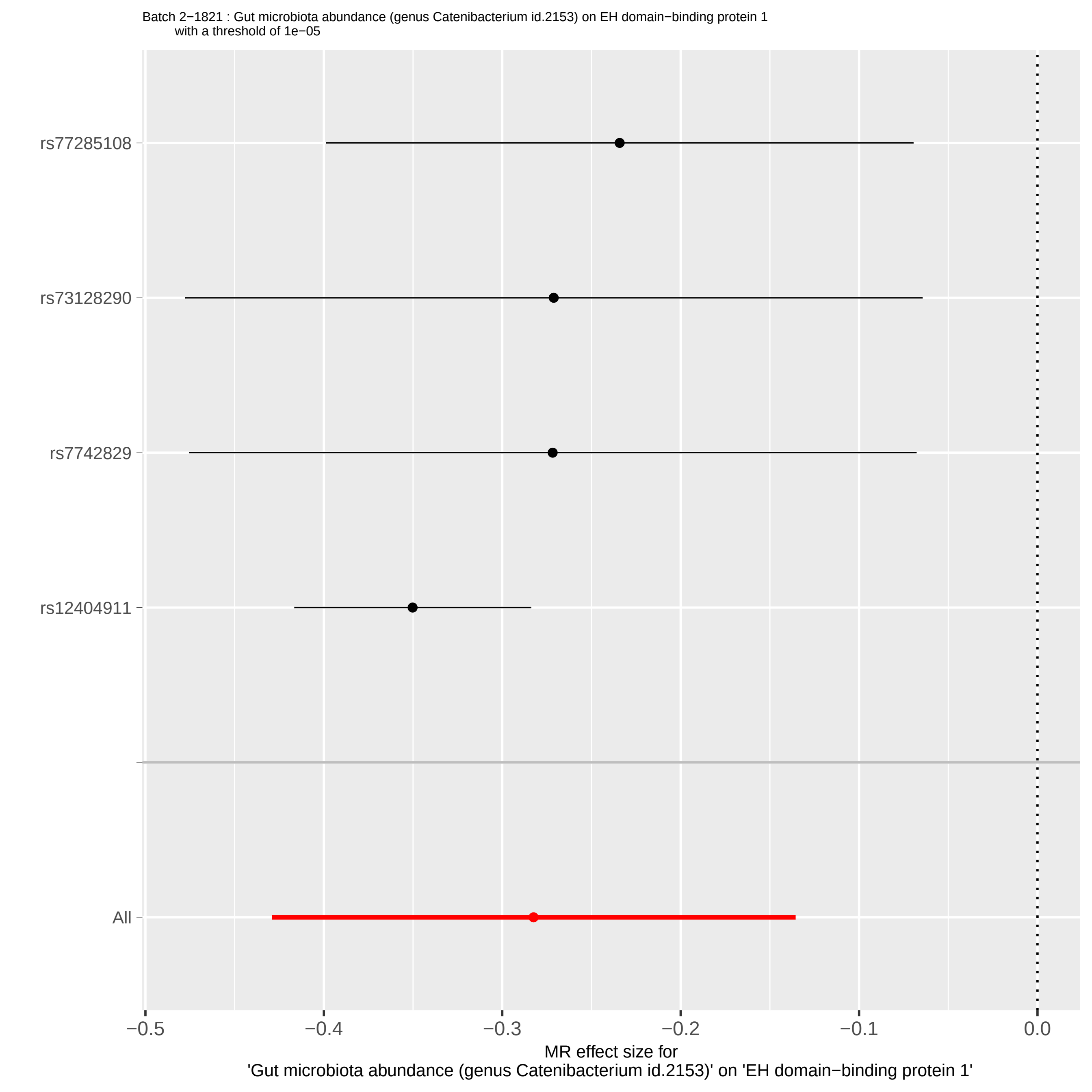
Batch 2−1821 : Gut microbiota abundance (genus Catenibacterium id.2153) on EH domain−binding protein 1
with a threshold of 1e−05
rs77285108
rs73128290
rs7742829
rs12404911
All
−0.5
−0.4
−0.3
−0.2
−0.1
0.0
MR effect size for
'Gut microbiota abundance (genus Catenibacterium id.2153)' on 'EH domain−binding protein 1'

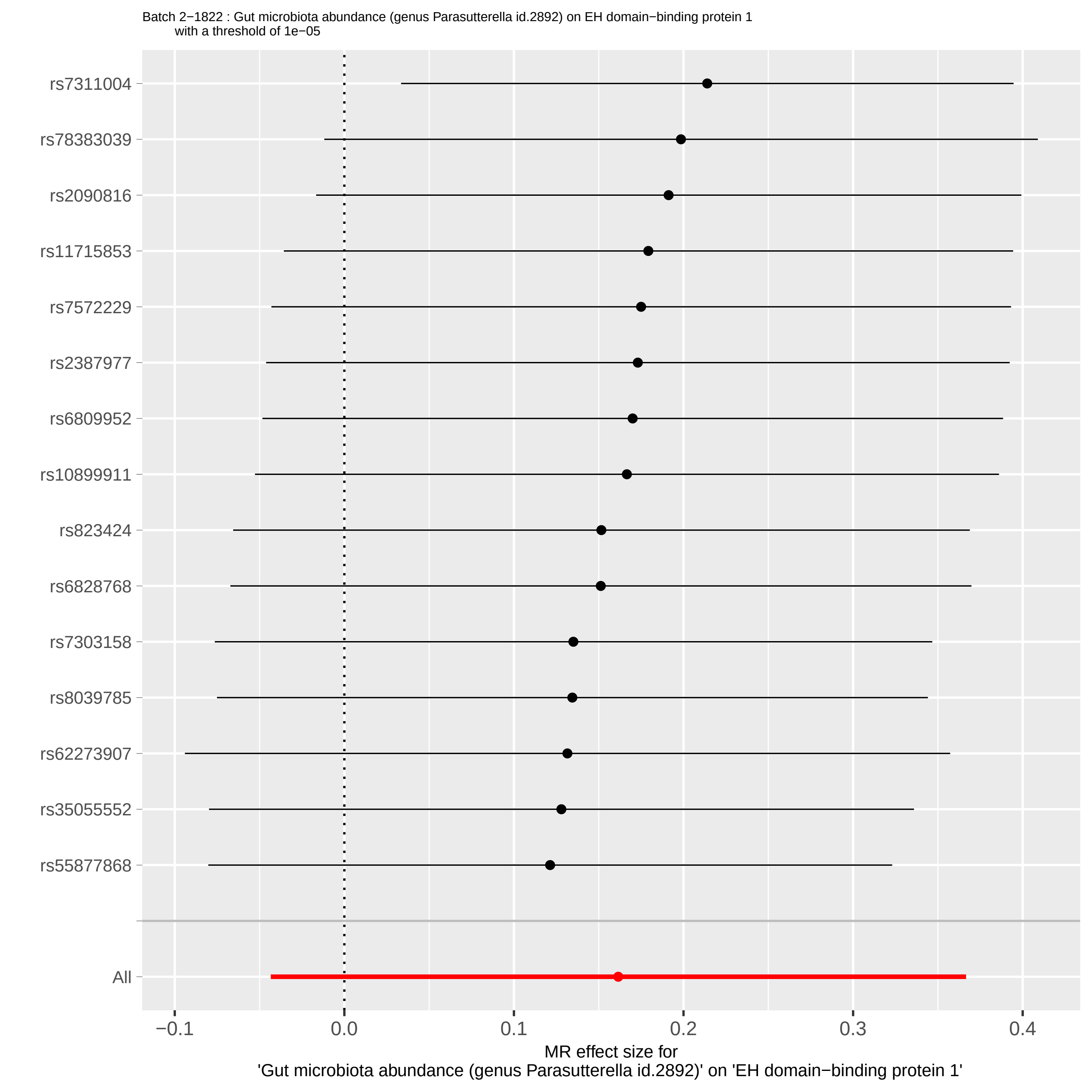

Batch 2−1822 : Gut microbiota abundance (genus Parasutterella id.2892) on EH domain−binding protein 1
with a threshold of 1e−05
rs7311004
rs78383039
rs2090816
rs11715853
rs7572229
rs2387977
rs6809952
rs10899911
rs823424
rs6828768
rs7303158
rs8039785
rs62273907
rs35055552
rs55877868
All
−0.1
0.0
0.1
0.2
0.3
0.4
MR effect size for
'Gut microbiota abundance (genus Parasutterella id.2892)' on 'EH domain−binding protein 1'

Batch 2−1823 : Gut microbiota abundance (genus Catenibacterium id.2153) on EH domain−containing protein 4
with a threshold of 1e−05

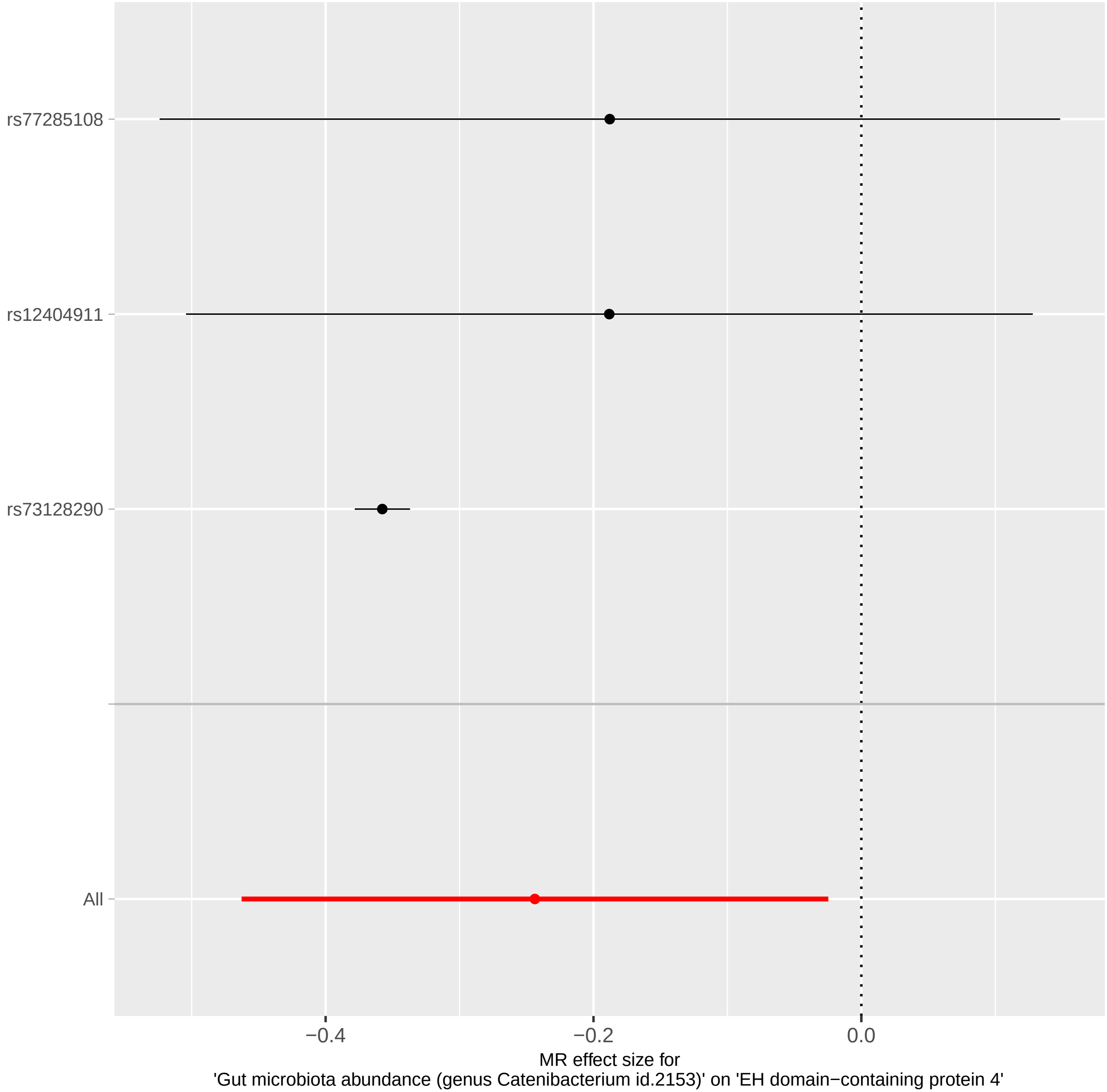

Batch 2−1824 : Gut microbiota abundance (genus Parasutterella id.2892) on EH domain−containing protein 4
with a threshold of 1e−05

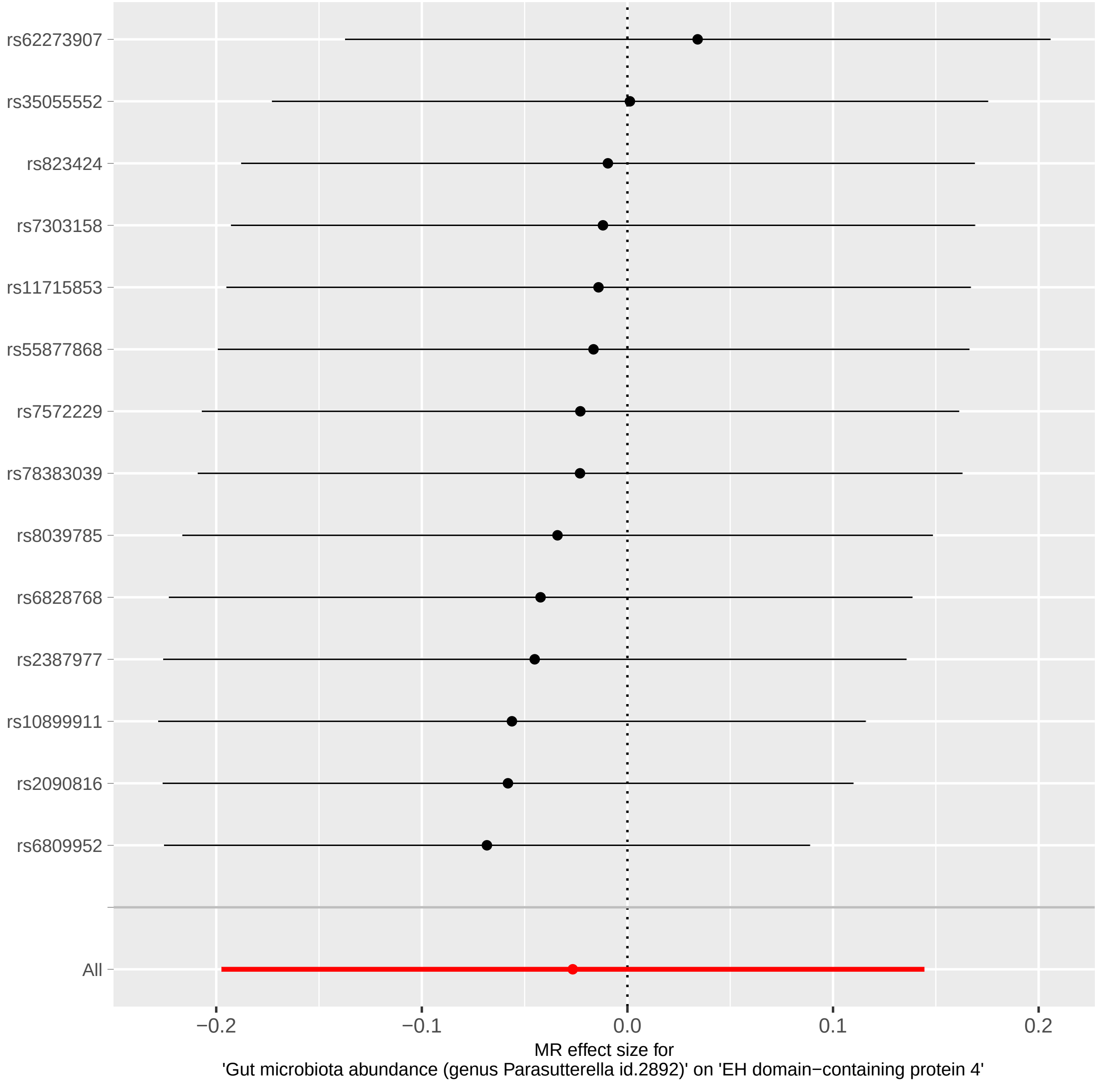

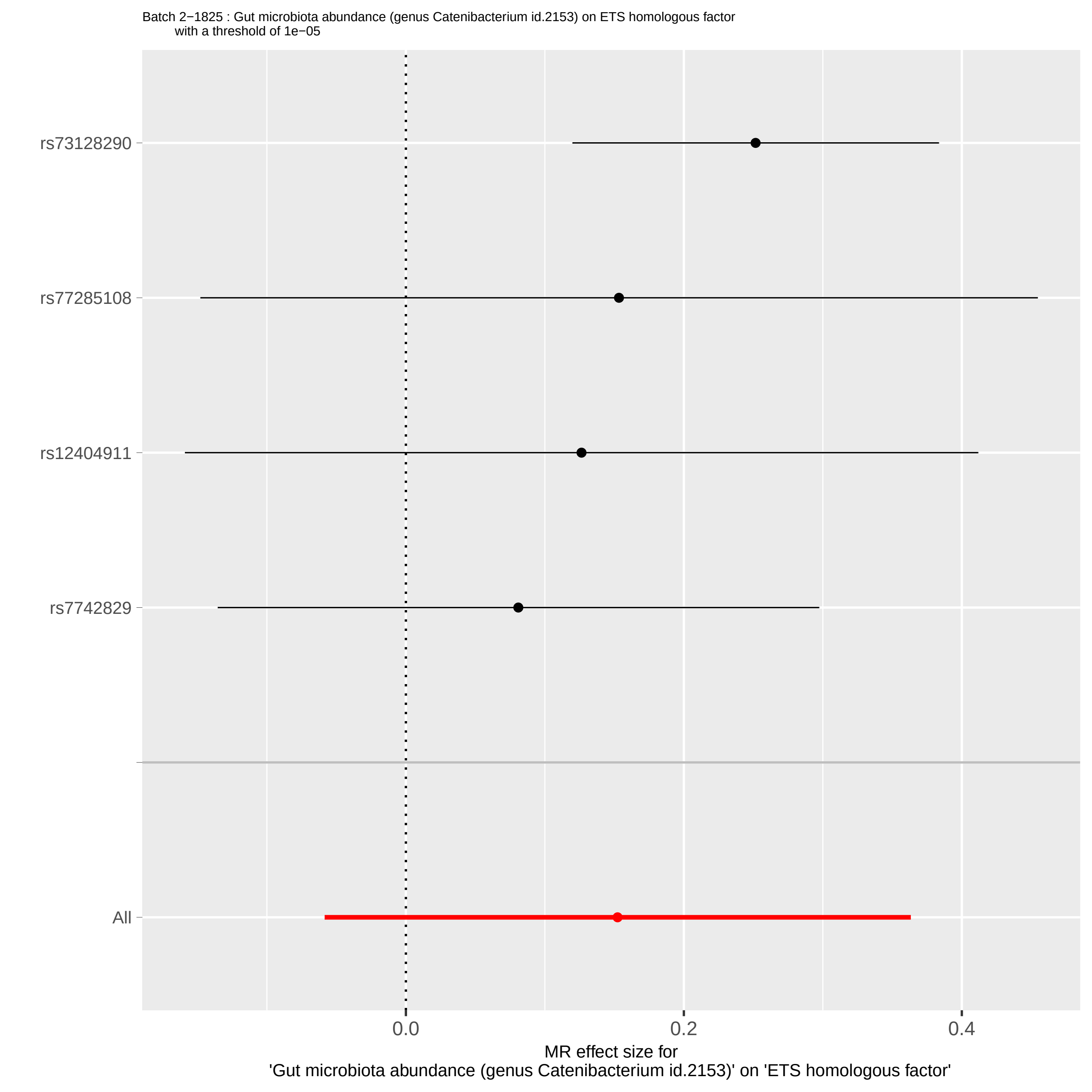
Batch 2−1825 : Gut microbiota abundance (genus Catenibacterium id.2153) on ETS homologous factor
with a threshold of 1e−05
rs73128290
rs77285108
rs12404911
rs7742829
All
0.0
0.2
0.4
MR effect size for
'Gut microbiota abundance (genus Catenibacterium id.2153)' on 'ETS homologous factor'

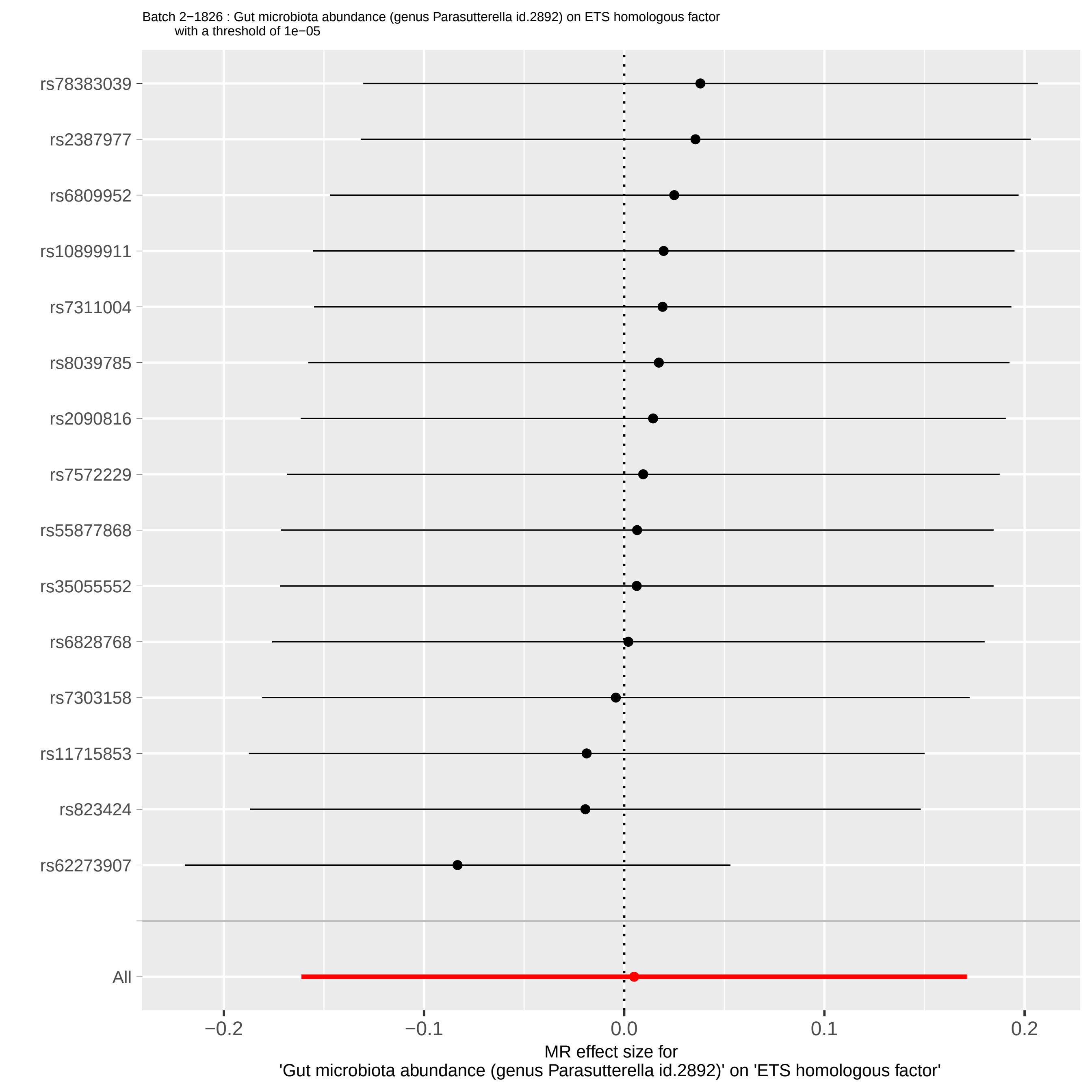
Batch 2−1826 : Gut microbiota abundance (genus Parasutterella id.2892) on ETS homologous factor
with a threshold of 1e−05
rs78383039
rs2387977
rs6809952
rs10899911
rs7311004
rs8039785
rs2090816
rs7572229
rs55877868
rs35055552
rs6828768
rs7303158
rs11715853
rs823424
rs62273907
All
−0.2
−0.1
0.0
0.1
0.2
MR effect size for
'Gut microbiota abundance (genus Parasutterella id.2892)' on 'ETS homologous factor'

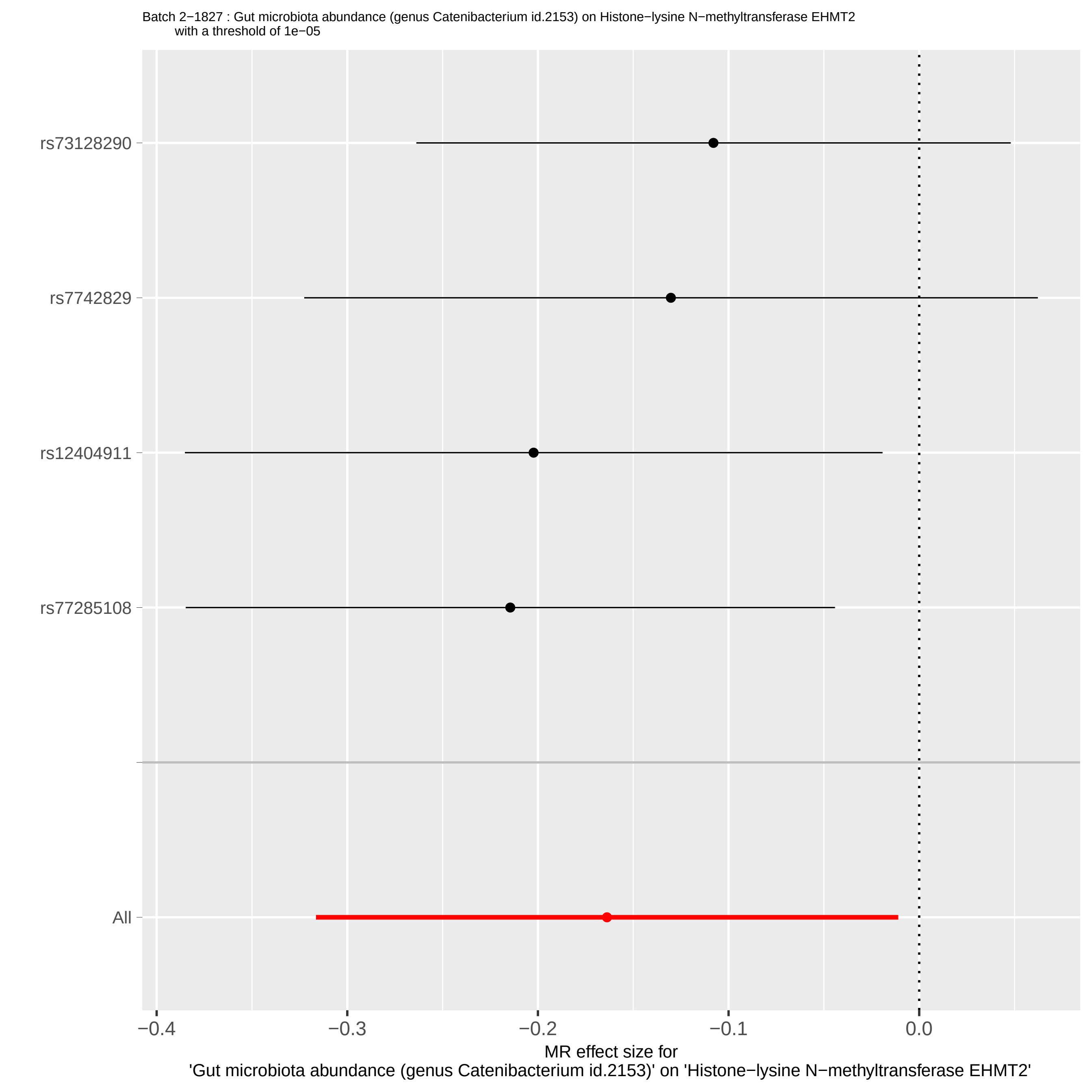

Batch 2−1827 : Gut microbiota abundance (genus Catenibacterium id.2153) on Histone−lysine N−methyltransferase EHMT2
with a threshold of 1e−05
rs73128290
rs7742829
rs12404911
rs77285108
All
−0.4
−0.3
−0.2
−0.1
0.0
MR effect size for
'Gut microbiota abundance (genus Catenibacterium id.2153)' on 'Histone−lysine N−methyltransferase EHMT2'

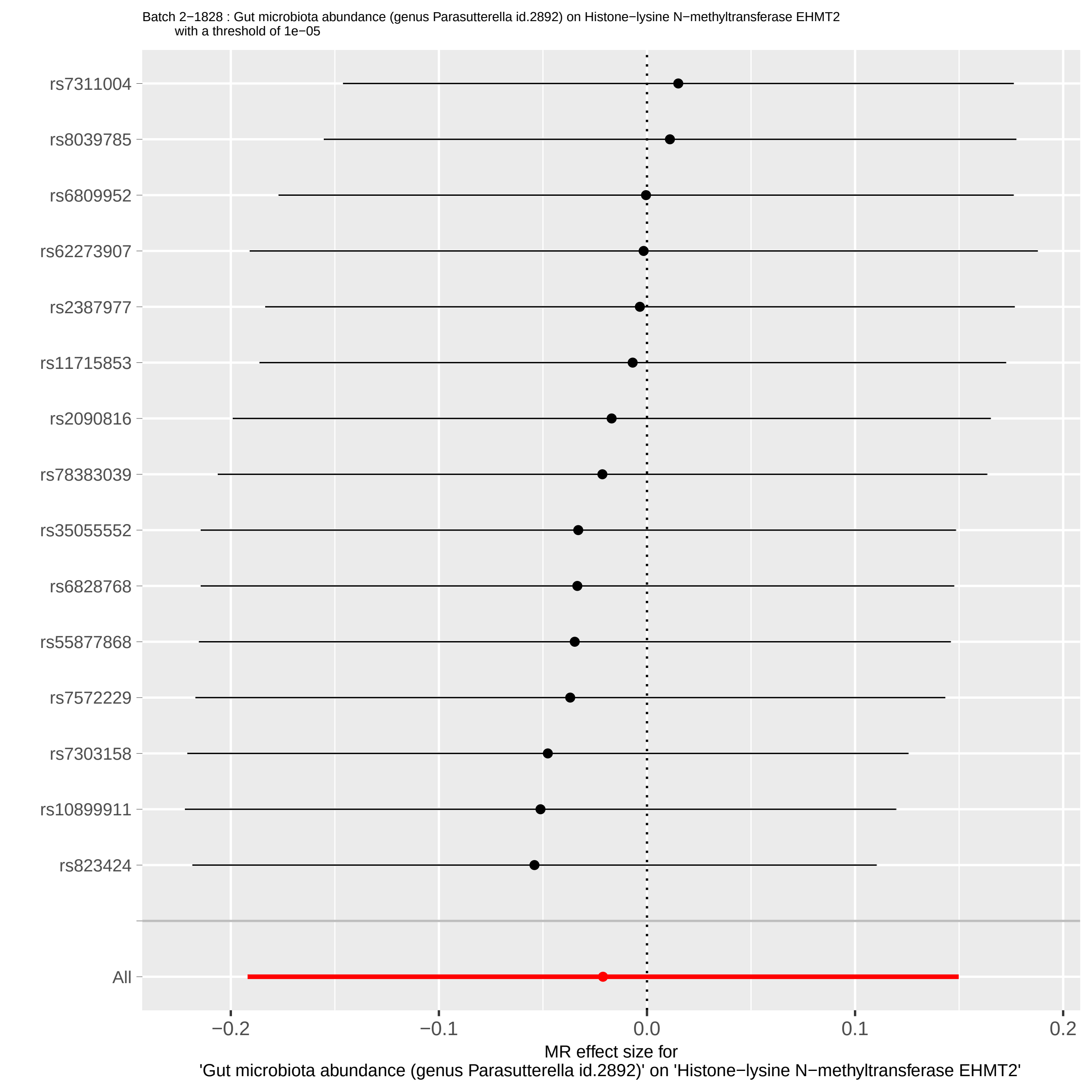

Batch 2−1828 : Gut microbiota abundance (genus Parasutterella id.2892) on Histone−lysine N−methyltransferase EHMT2
with a threshold of 1e−05
rs7311004
rs8039785
rs6809952
rs62273907
rs2387977
rs11715853
rs2090816
rs78383039
rs35055552
rs6828768
rs55877868
rs7572229
rs7303158
rs10899911
rs823424
All
−0.2
−0.1
0.0
0.1
0.2
MR effect size for
'Gut microbiota abundance (genus Parasutterella id.2892)' on 'Histone−lysine N−methyltransferase EHMT2'

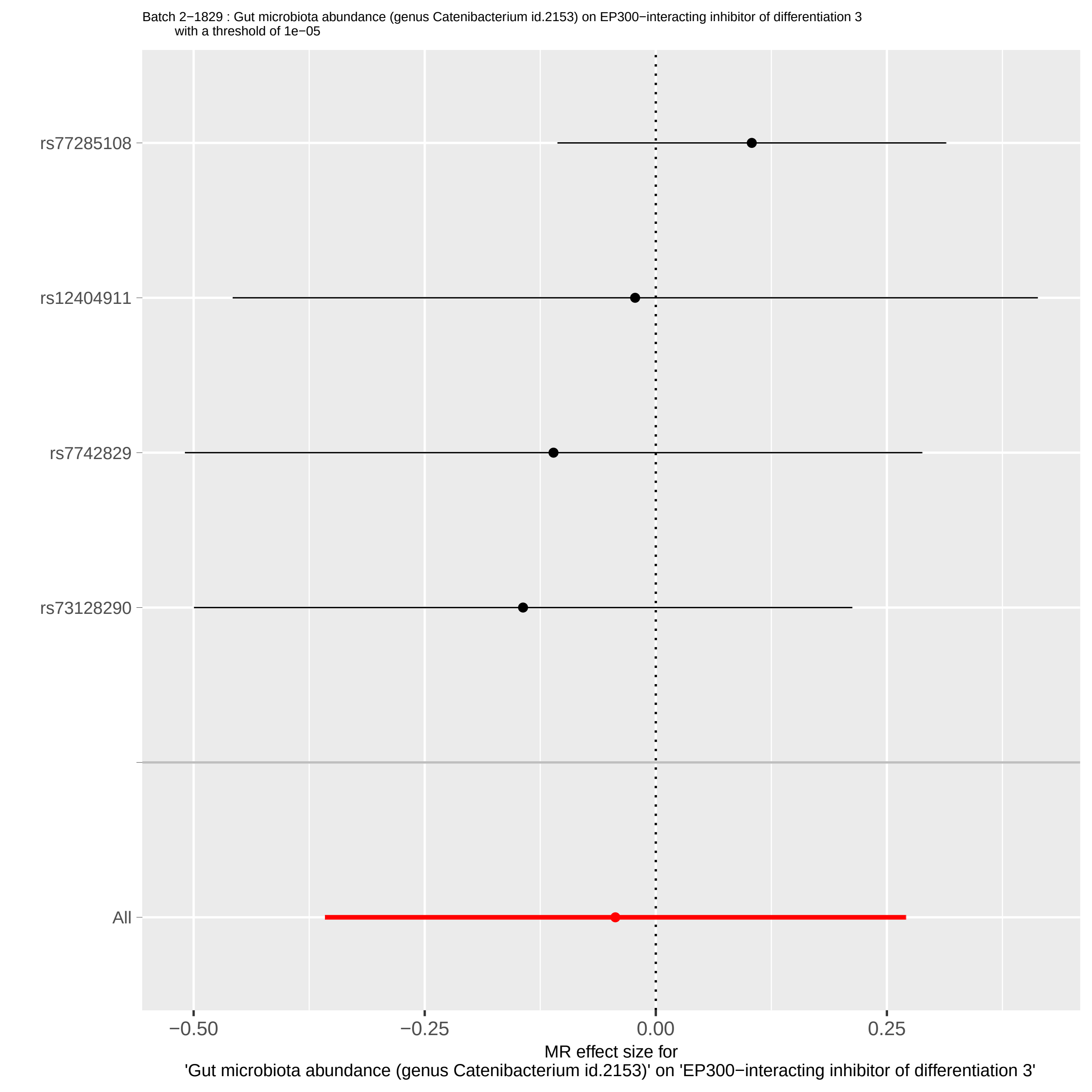
Batch 2−1829 : Gut microbiota abundance (genus Catenibacterium id.2153) on EP300−interacting inhibitor of differentiation 3
with a threshold of 1e−05
rs77285108
rs12404911
rs7742829
rs73128290
All
−0.50
−0.25
0.00
0.25
MR effect size for
'Gut microbiota abundance (genus Catenibacterium id.2153)' on 'EP300−interacting inhibitor of differentiation 3'

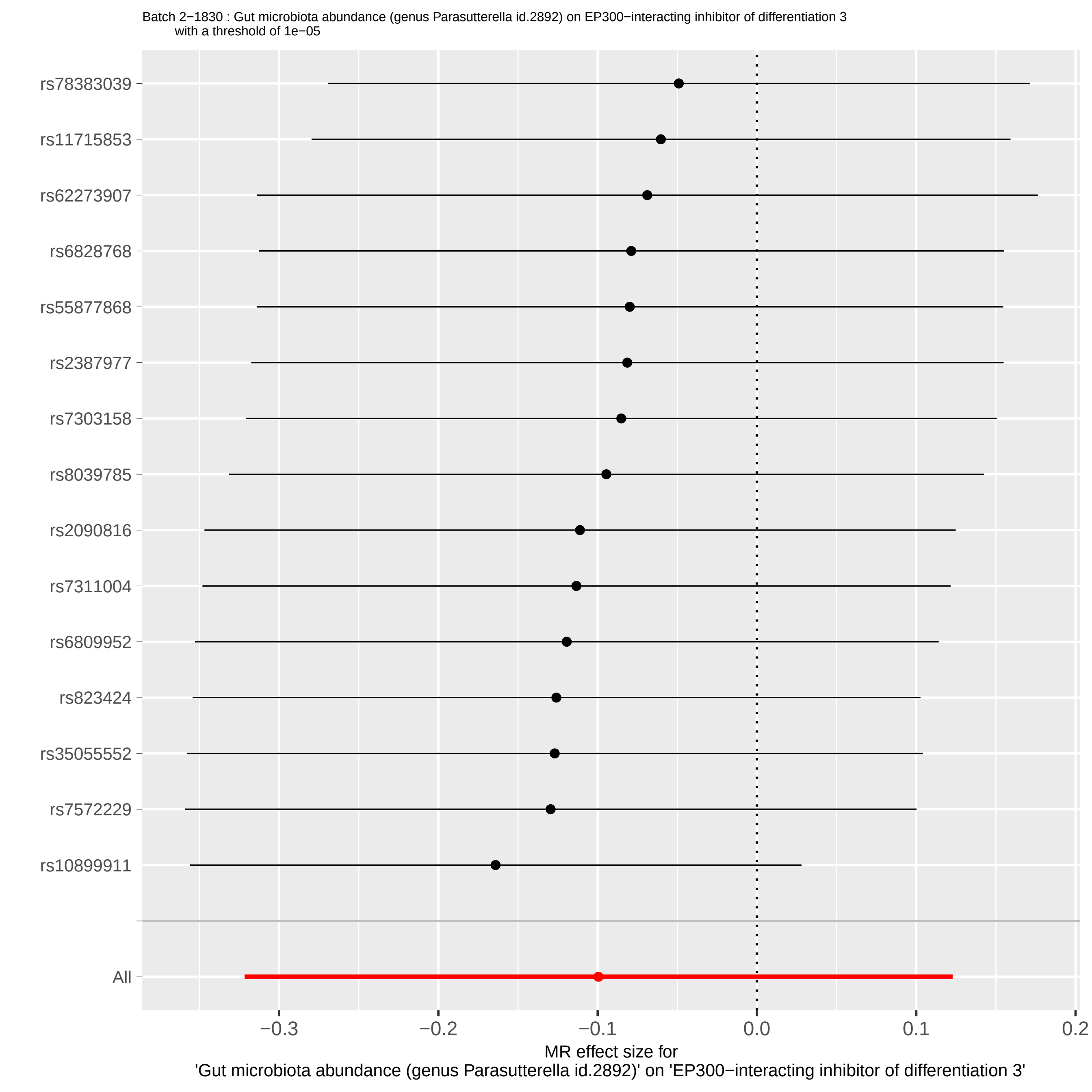
Batch 2−1830 : Gut microbiota abundance (genus Parasutterella id.2892) on EP300−interacting inhibitor of differentiation 3
with a threshold of 1e−05
rs78383039
rs11715853
rs62273907
rs6828768
rs55877868
rs2387977
rs7303158
rs8039785
rs2090816
rs7311004
rs6809952
rs823424
rs35055552
rs7572229
rs10899911
All
−0.3
−0.2
−0.1
0.0
0.1
0.2
MR effect size for
'Gut microbiota abundance (genus Parasutterella id.2892)' on 'EP300−interacting inhibitor of differentiation 3'

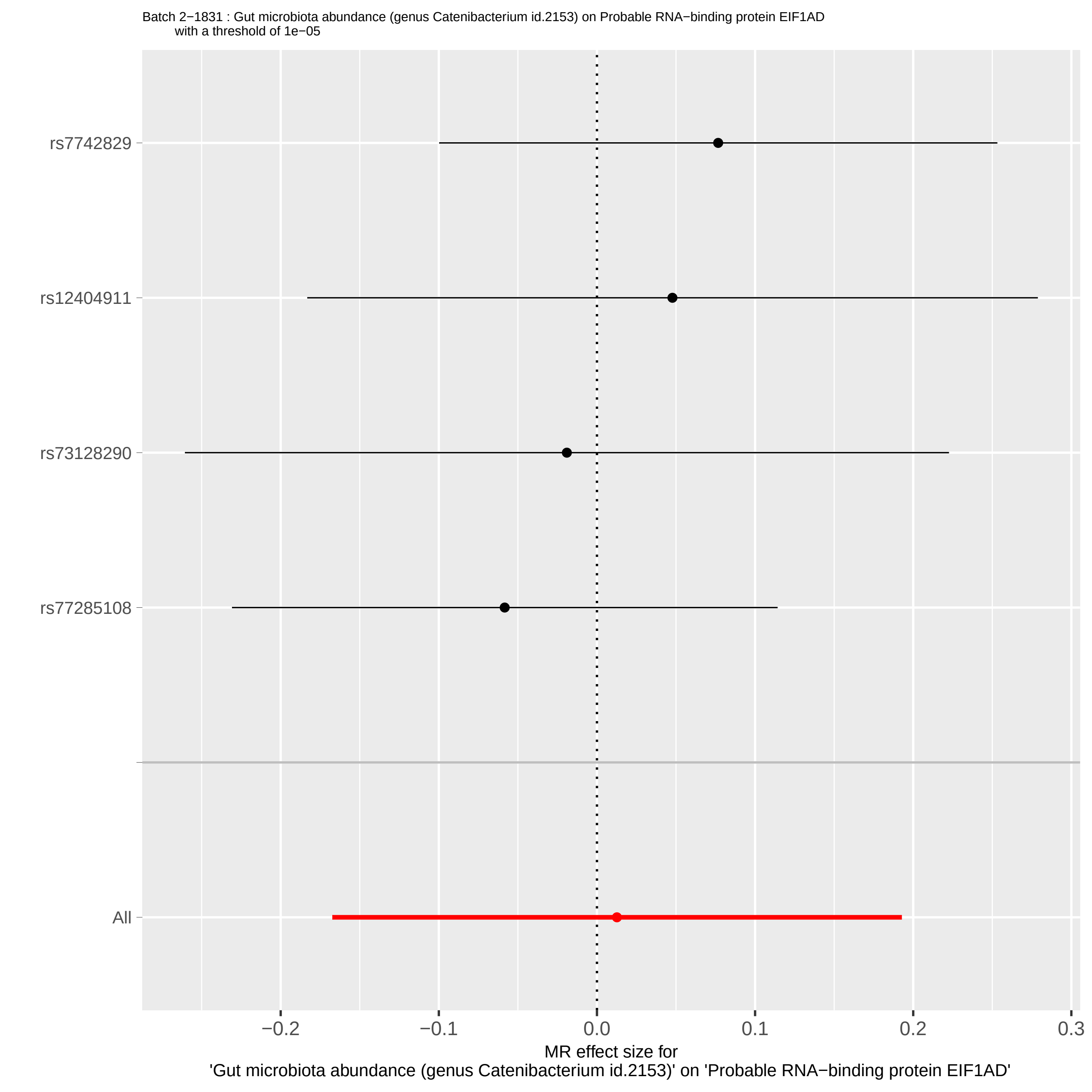

Batch 2−1831 : Gut microbiota abundance (genus Catenibacterium id.2153) on Probable RNA−binding protein EIF1AD
with a threshold of 1e−05
rs7742829
rs12404911
rs73128290
rs77285108
All
−0.2
−0.1
0.0
0.1
0.2
0.3
MR effect size for
'Gut microbiota abundance (genus Catenibacterium id.2153)' on 'Probable RNA−binding protein EIF1AD'

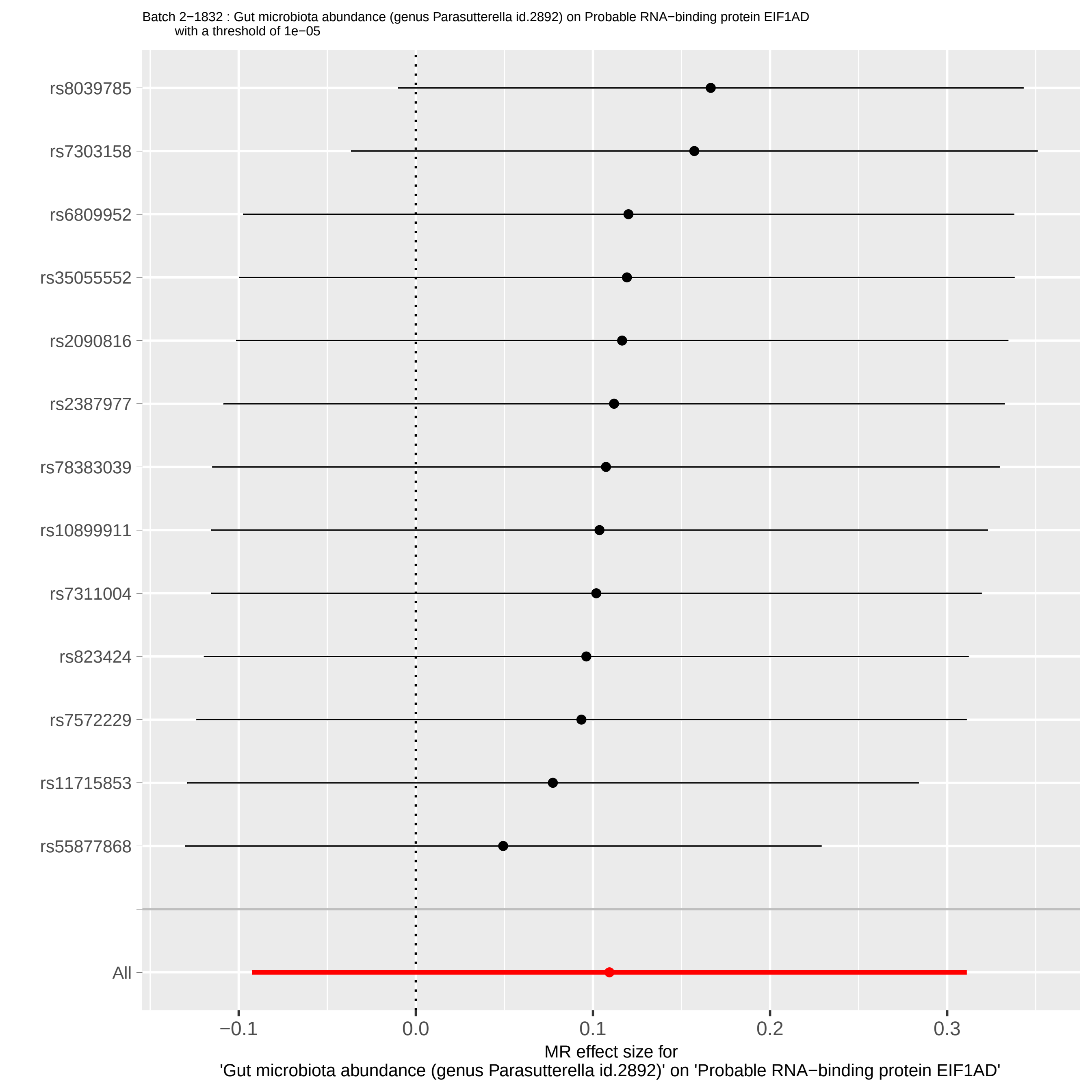
Batch 2−1832 : Gut microbiota abundance (genus Parasutterella id.2892) on Probable RNA−binding protein EIF1AD
with a threshold of 1e−05
rs8039785
rs7303158
rs6809952
rs35055552
rs2090816
rs2387977
rs78383039
rs10899911
rs7311004
rs823424
rs7572229
rs11715853
rs55877868
All
−0.1
0.0
0.1
0.2
0.3
MR effect size for
'Gut microbiota abundance (genus Parasutterella id.2892)' on 'Probable RNA−binding protein EIF1AD'

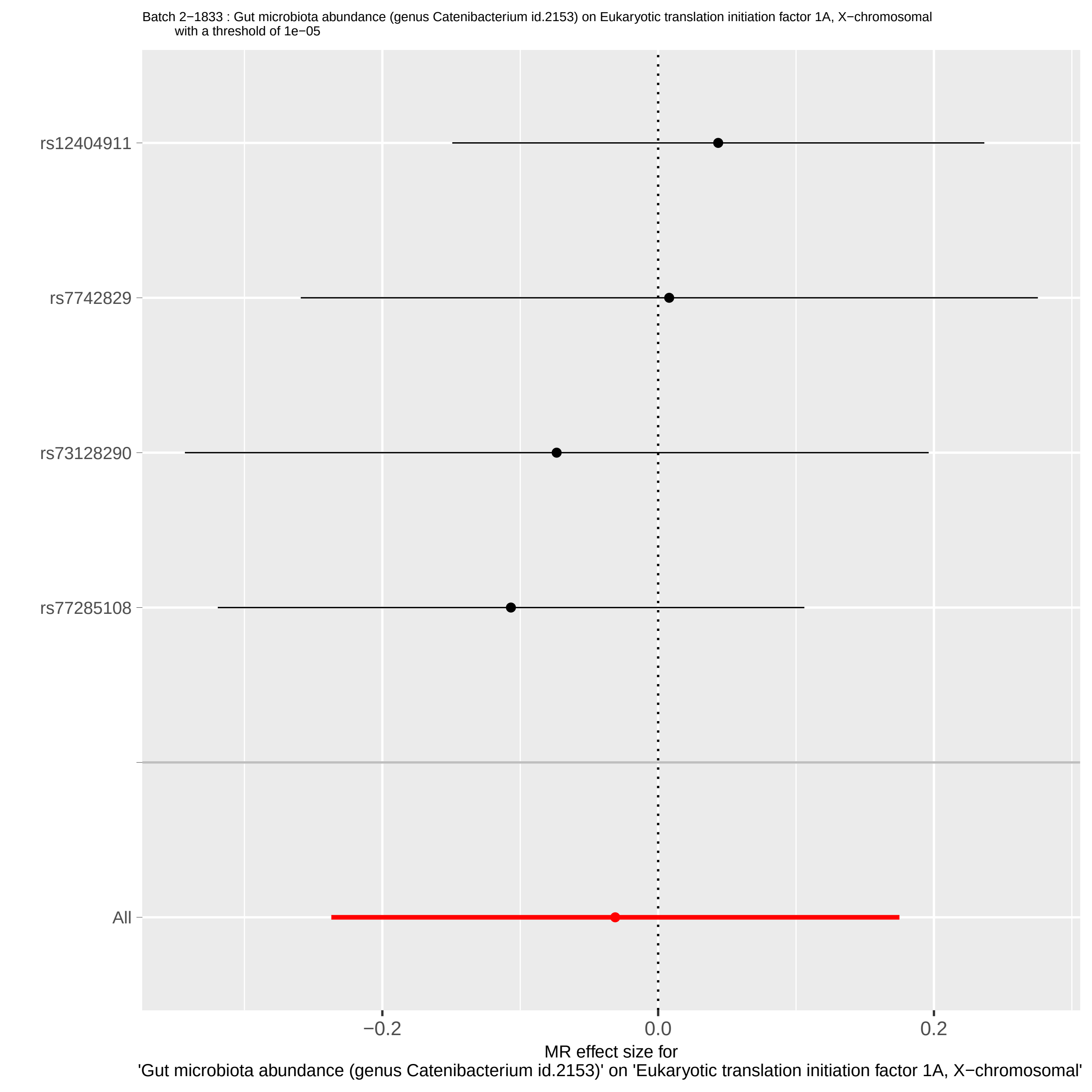

Batch 2−1833 : Gut microbiota abundance (genus Catenibacterium id.2153) on Eukaryotic translation initiation factor 1A, X−chromosomal
with a threshold of 1e−05
rs12404911
rs7742829
rs73128290
rs77285108
All
−0.2
0.0
0.2
MR effect size for
'Gut microbiota abundance (genus Catenibacterium id.2153)' on 'Eukaryotic translation initiation factor 1A, X−chromosomal'

Batch 2−1834 : Gut microbiota abundance (genus Parasutterella id.2892) on Eukaryotic translation initiation factor 1A, X−chromosomal
with a threshold of 1e−05

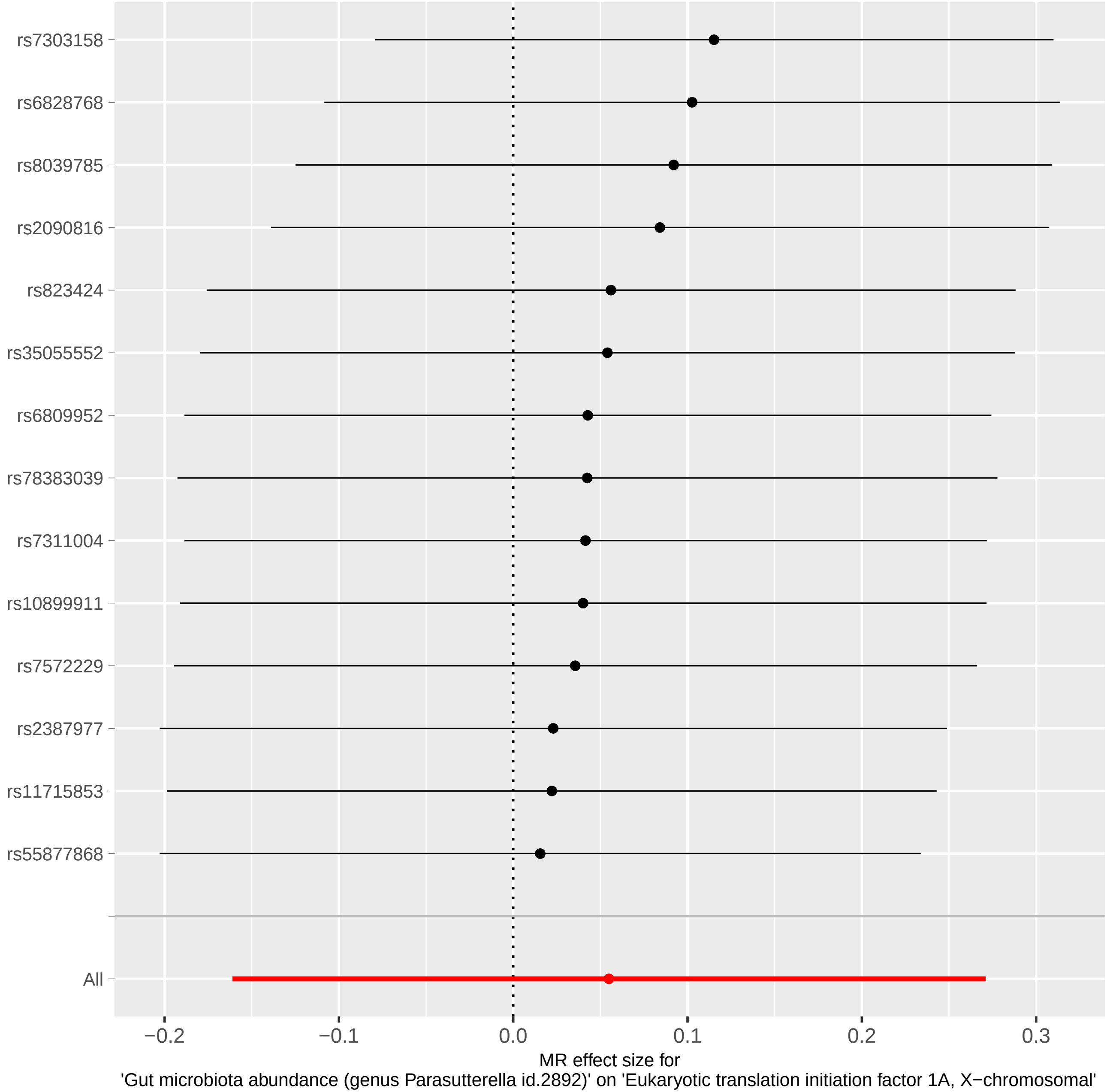

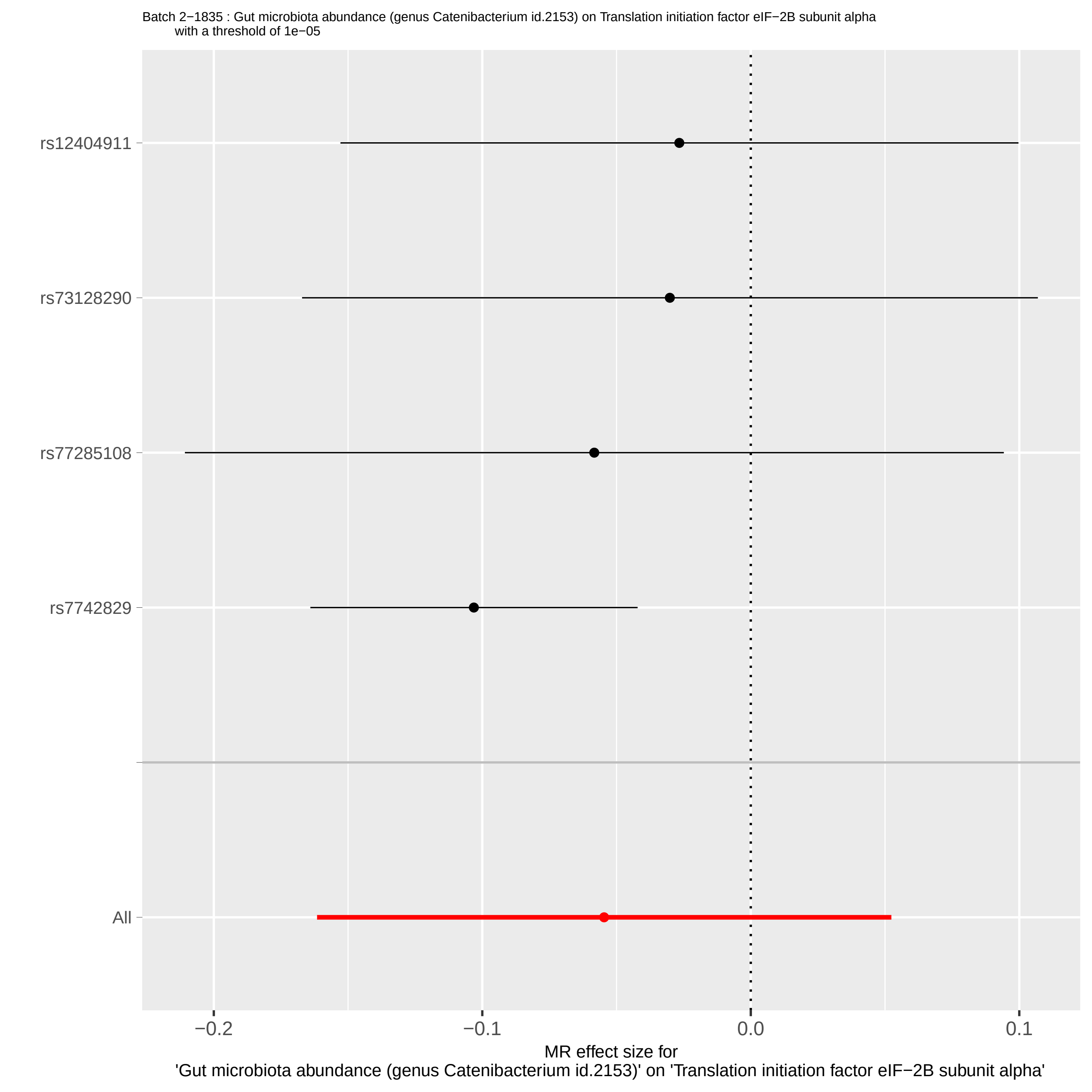

Batch 2−1835 : Gut microbiota abundance (genus Catenibacterium id.2153) on Translation initiation factor eIF−2B subunit alpha
with a threshold of 1e−05
rs12404911
rs73128290
rs77285108
rs7742829
All
−0.2
−0.1
0.0
0.1
MR effect size for
'Gut microbiota abundance (genus Catenibacterium id.2153)' on 'Translation initiation factor eIF−2B subunit alpha'

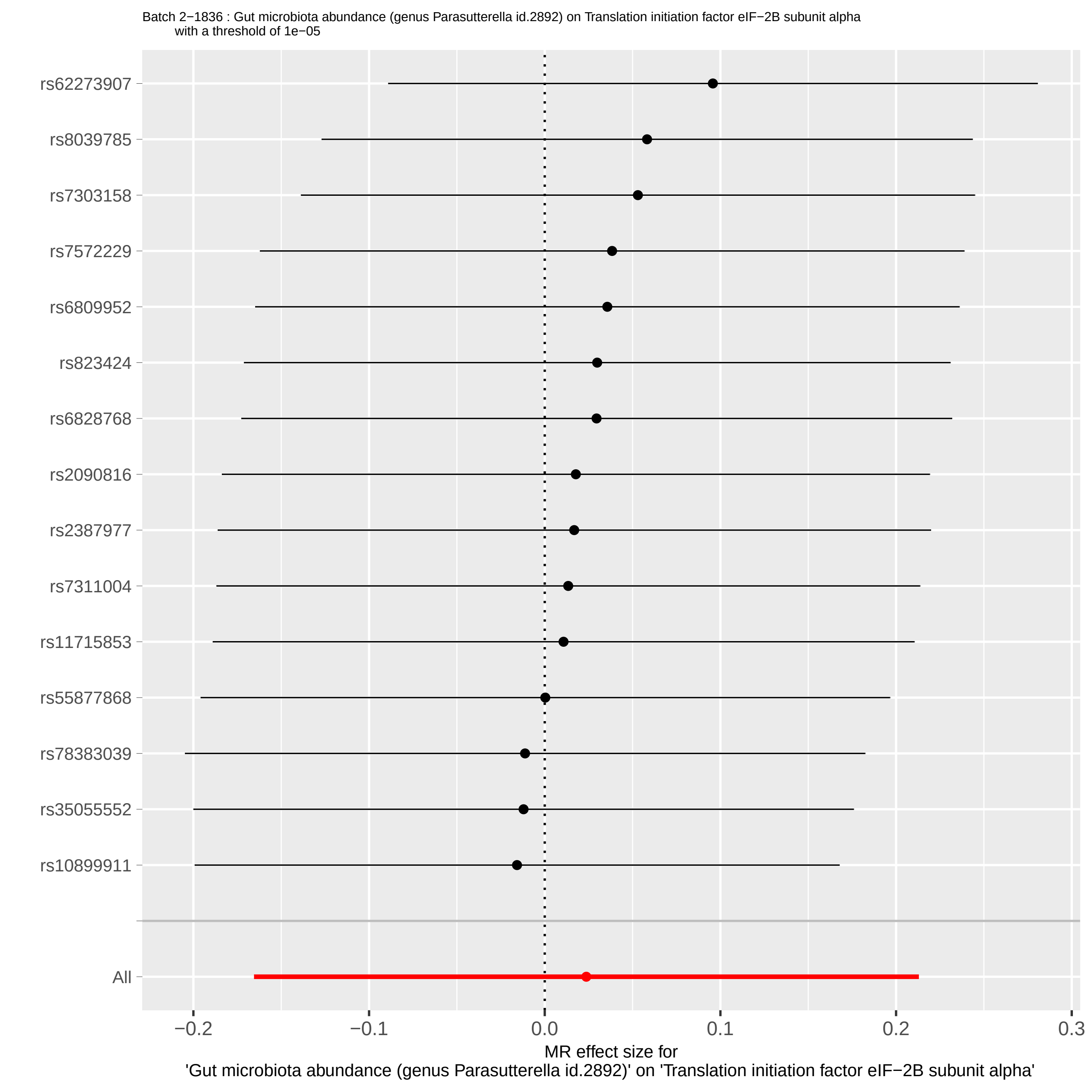

Batch 2−1836 : Gut microbiota abundance (genus Parasutterella id.2892) on Translation initiation factor eIF−2B subunit alpha
with a threshold of 1e−05
rs62273907
rs8039785
rs7303158
rs7572229
rs6809952
rs823424
rs6828768
rs2090816
rs2387977
rs7311004
rs11715853
rs55877868
rs78383039
rs35055552
rs10899911
All
−0.2
−0.1
0.0
0.1
0.2
0.3
MR effect size for
'Gut microbiota abundance (genus Parasutterella id.2892)' on 'Translation initiation factor eIF−2B subunit alpha'

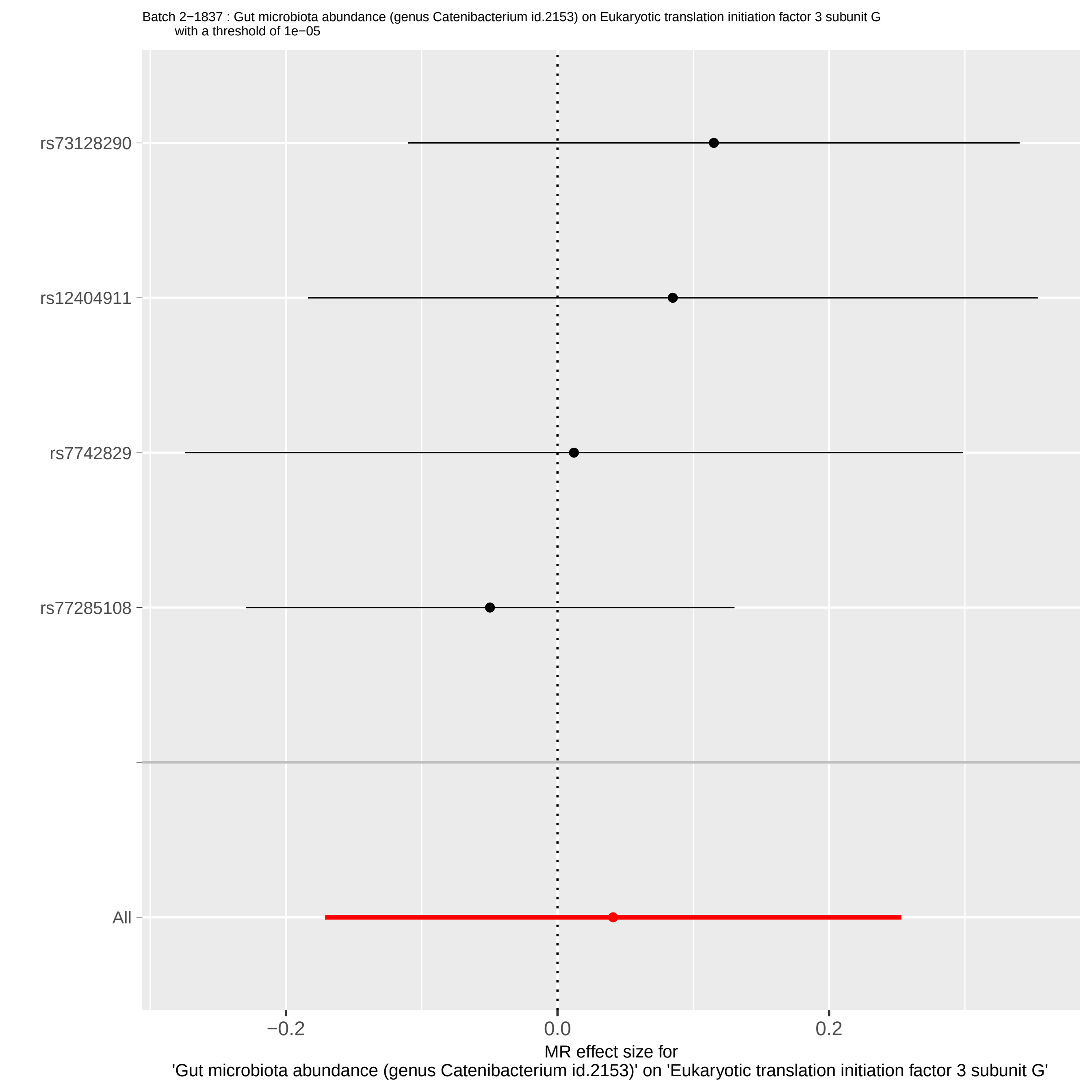
Batch 2−1837 : Gut microbiota abundance (genus Catenibacterium id.2153) on Eukaryotic translation initiation factor 3 subunit G
with a threshold of 1e−05
rs73128290
rs12404911
rs7742829
rs77285108
All
−0.2
0.0
0.2
MR effect size for
'Gut microbiota abundance (genus Catenibacterium id.2153)' on 'Eukaryotic translation initiation factor 3 subunit G'

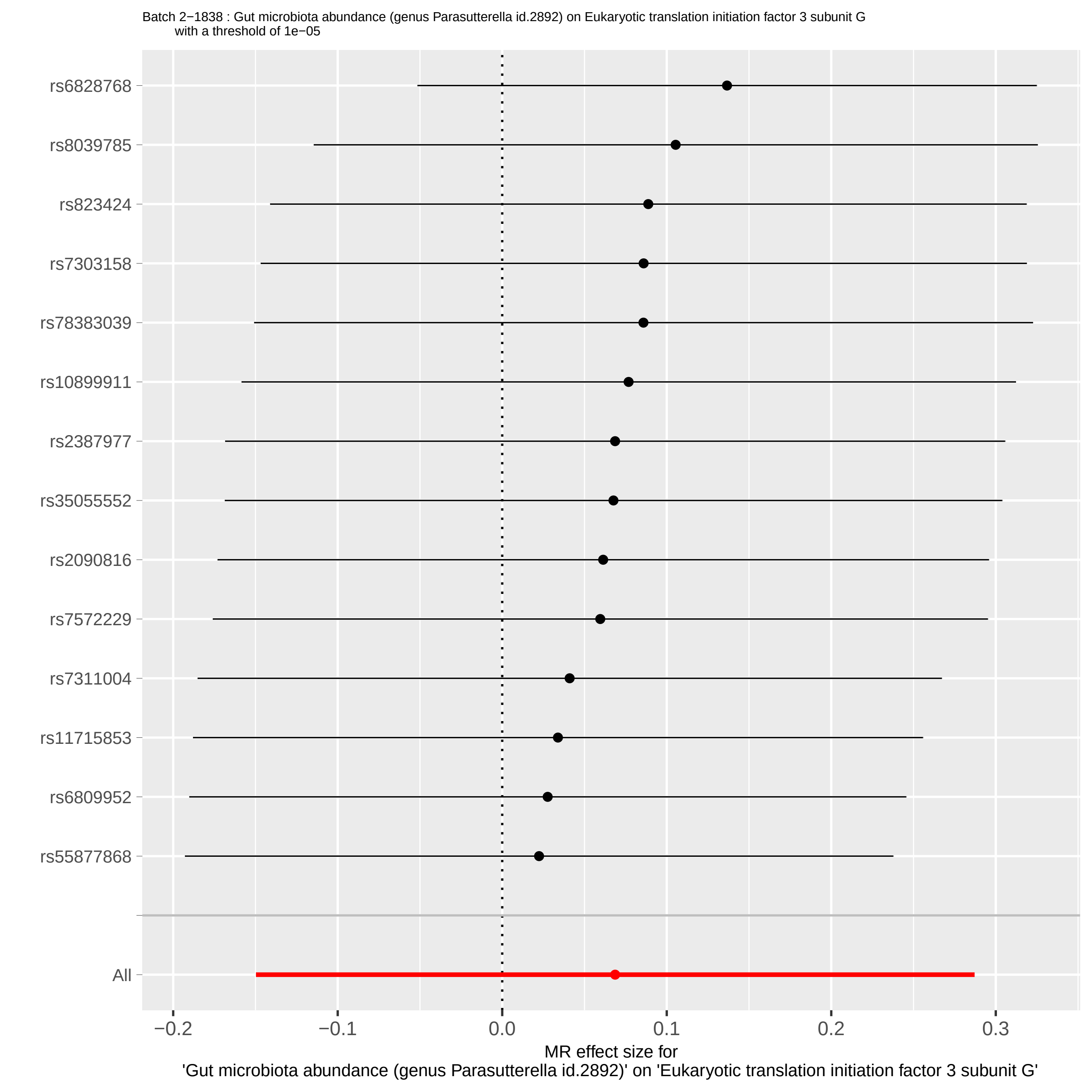
Batch 2−1838 : Gut microbiota abundance (genus Parasutterella id.2892) on Eukaryotic translation initiation factor 3 subunit G
with a threshold of 1e−05
rs6828768
rs8039785
rs823424
rs7303158
rs78383039
rs10899911
rs2387977
rs35055552
rs2090816
rs7572229
rs7311004
rs11715853
rs6809952
rs55877868
All
−0.2
−0.1
0.0
0.1
0.2
0.3
MR effect size for
'Gut microbiota abundance (genus Parasutterella id.2892)' on 'Eukaryotic translation initiation factor 3 subunit G'

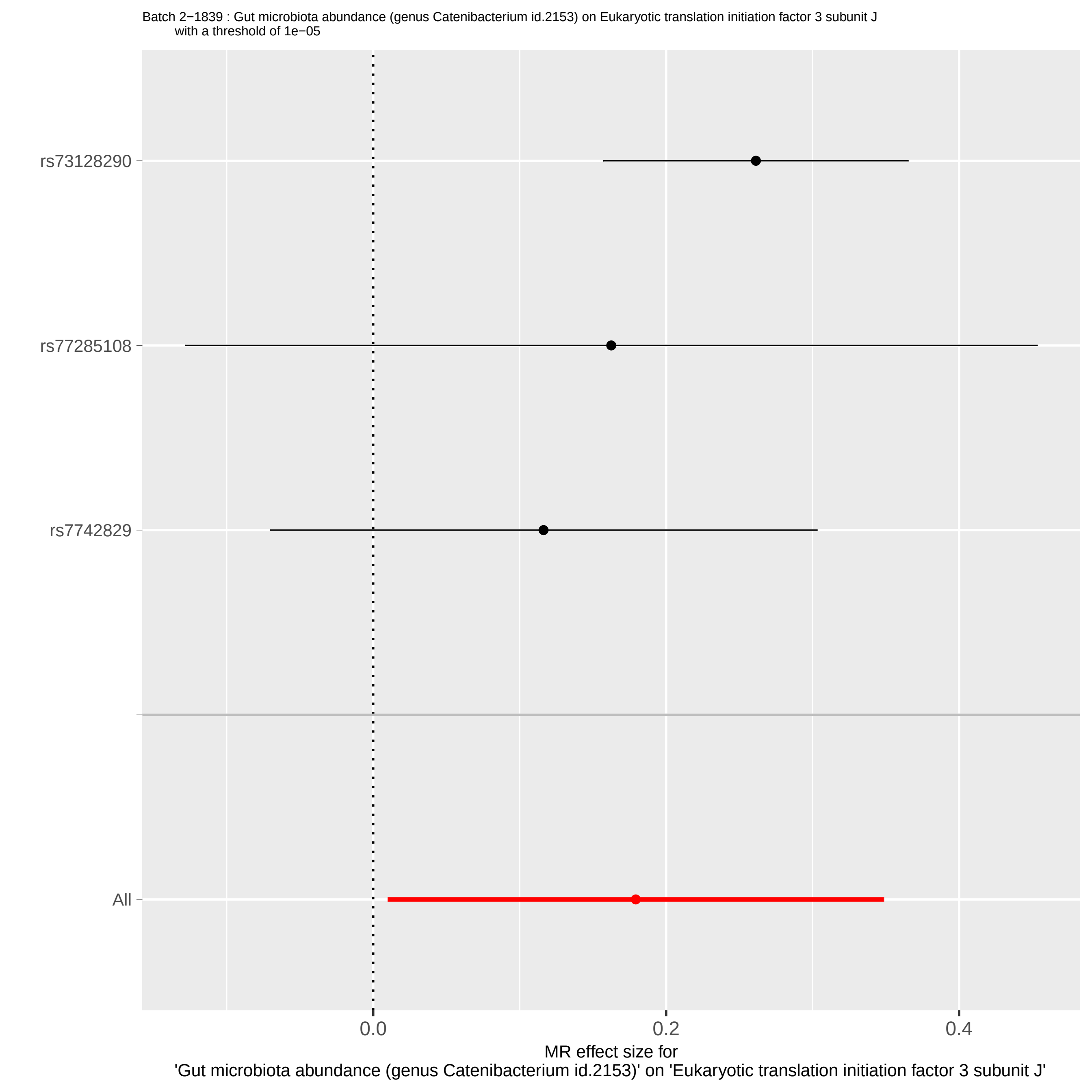
Batch 2−1839 : Gut microbiota abundance (genus Catenibacterium id.2153) on Eukaryotic translation initiation factor 3 subunit J
with a threshold of 1e−05
rs73128290
rs77285108
rs7742829
All
0.0
0.2
0.4
MR effect size for
'Gut microbiota abundance (genus Catenibacterium id.2153)' on 'Eukaryotic translation initiation factor 3 subunit J'

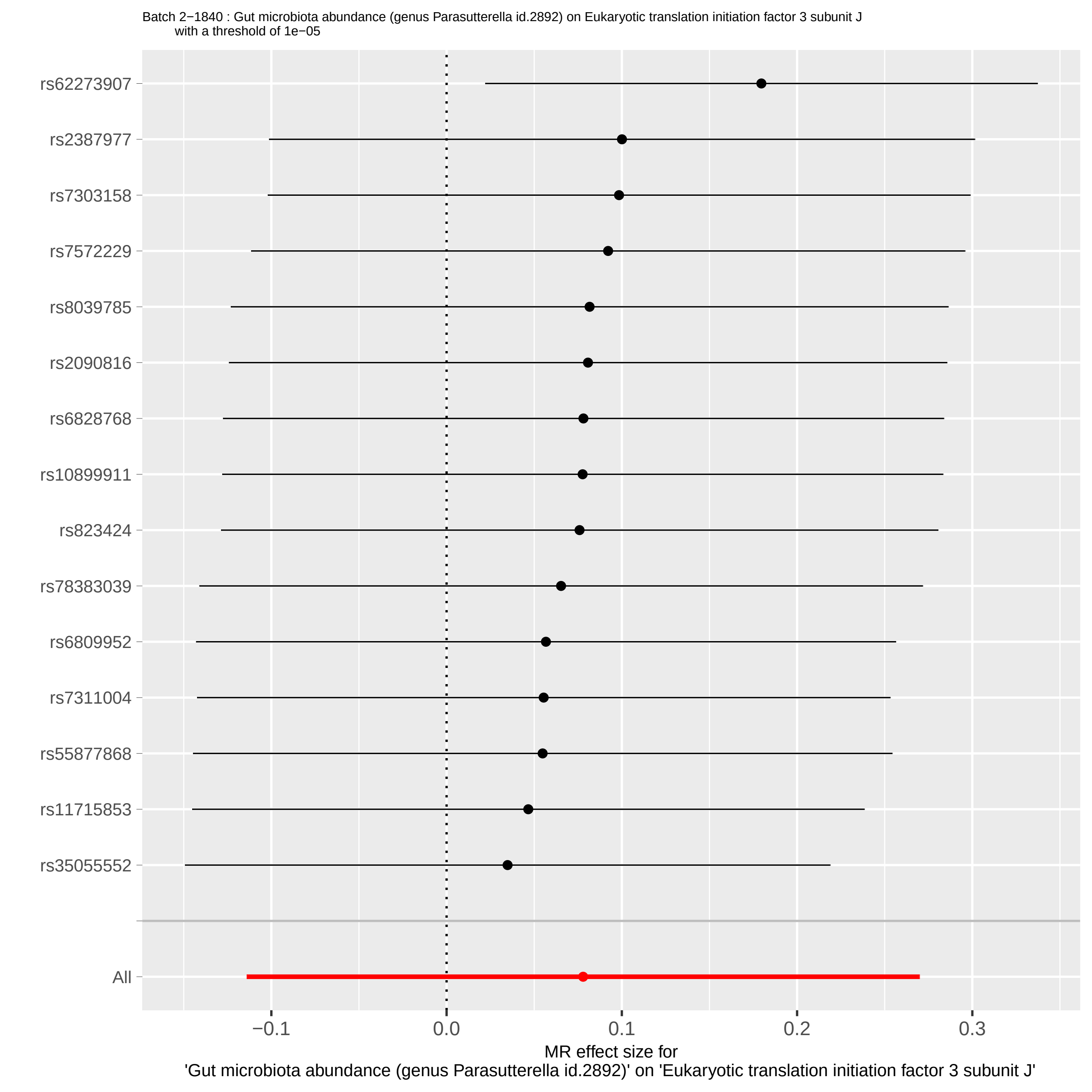
Batch 2−1840 : Gut microbiota abundance (genus Parasutterella id.2892) on Eukaryotic translation initiation factor 3 subunit J
with a threshold of 1e−05
rs62273907
rs2387977
rs7303158
rs7572229
rs8039785
rs2090816
rs6828768
rs10899911
rs823424
rs78383039
rs6809952
rs7311004
rs55877868
rs11715853
rs35055552
All
−0.1
0.0
0.1
0.2
0.3
MR effect size for
'Gut microbiota abundance (genus Parasutterella id.2892)' on 'Eukaryotic translation initiation factor 3 subunit J'

Batch 2−1841 : Gut microbiota abundance (genus Catenibacterium id.2153) on Eukaryotic initiation factor 4A−III
with a threshold of 1e−05

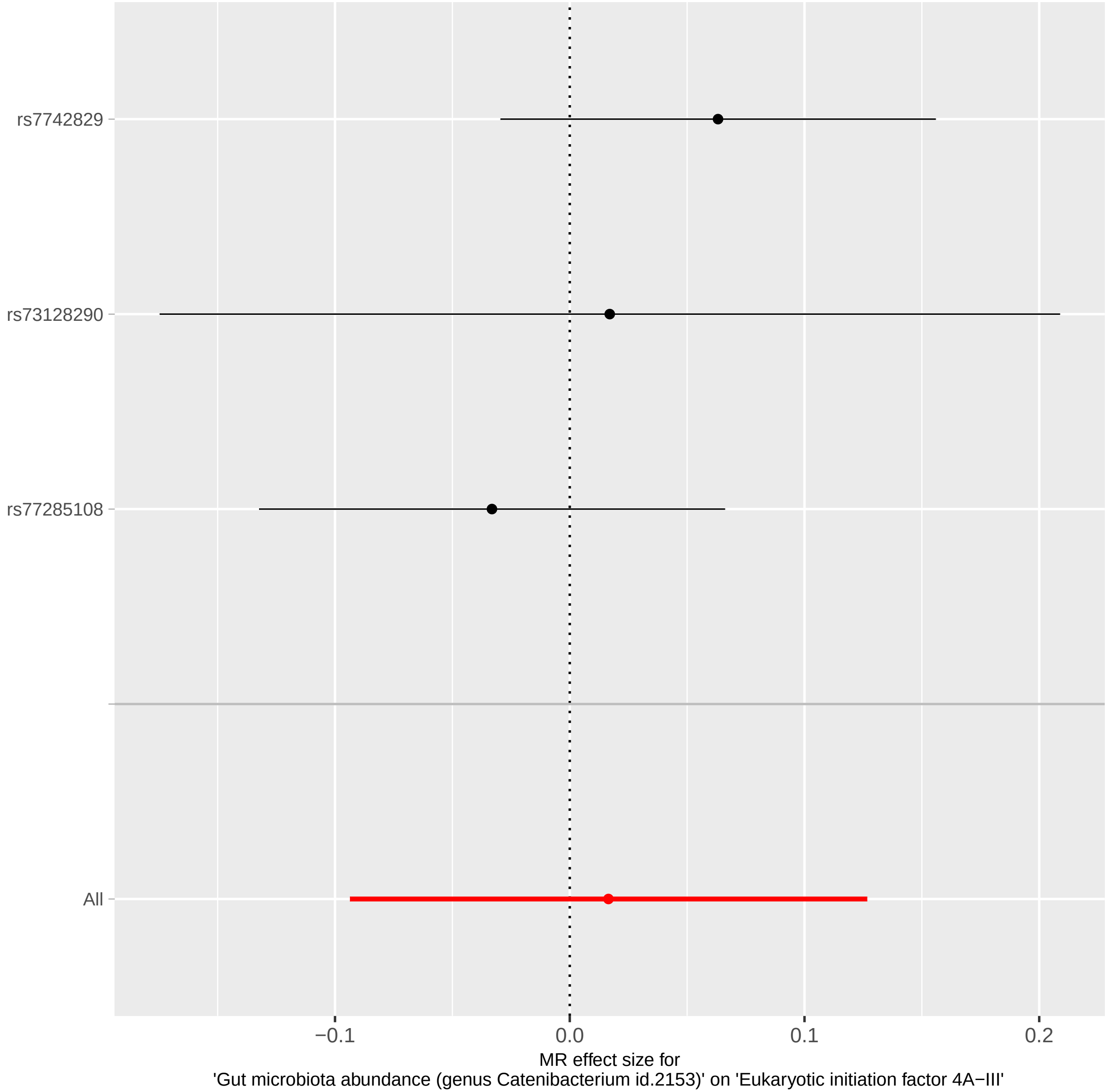

Batch 2−1842 : Gut microbiota abundance (genus Parasutterella id.2892) on Eukaryotic initiation factor 4A−III
with a threshold of 1e−05

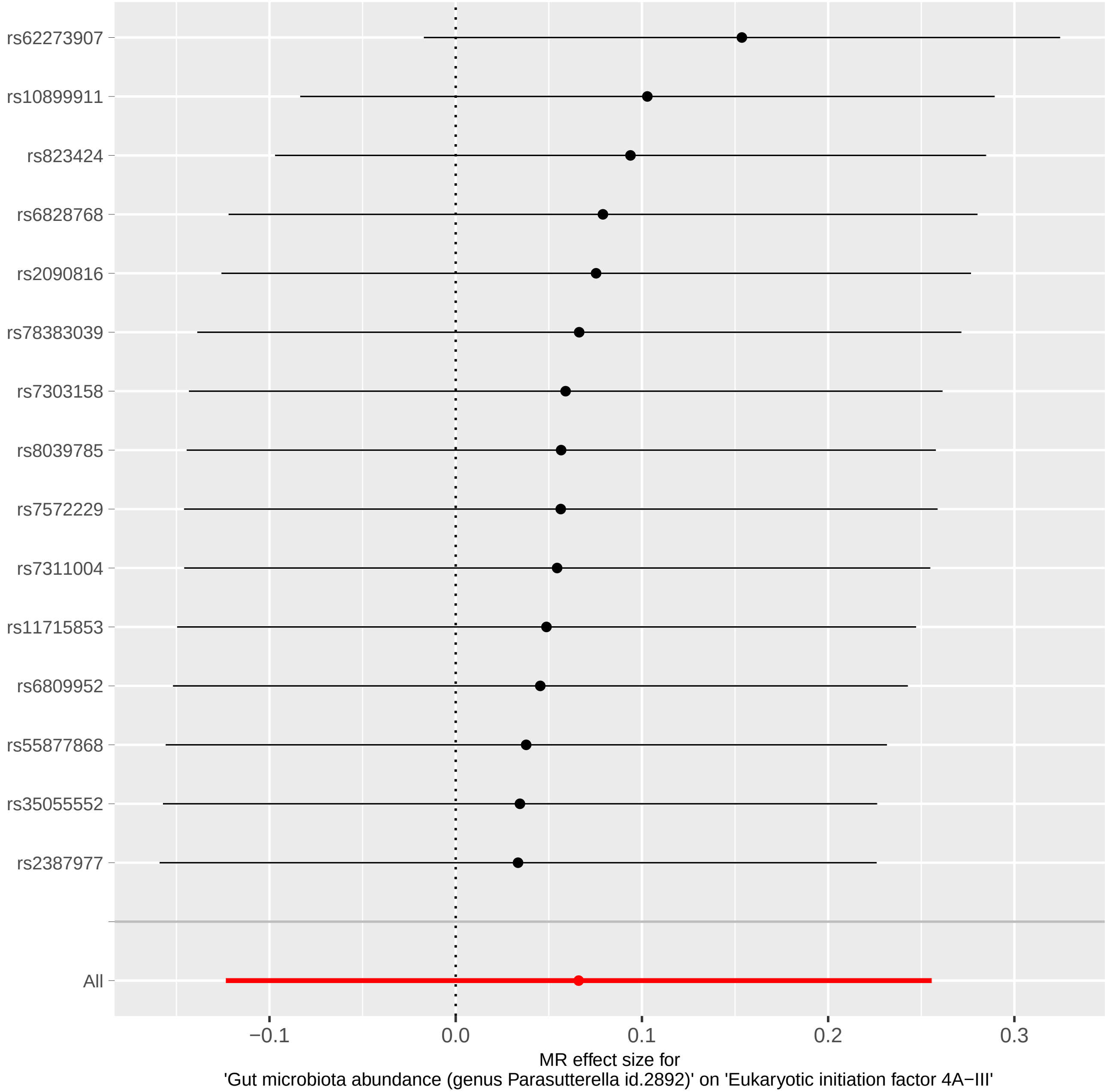

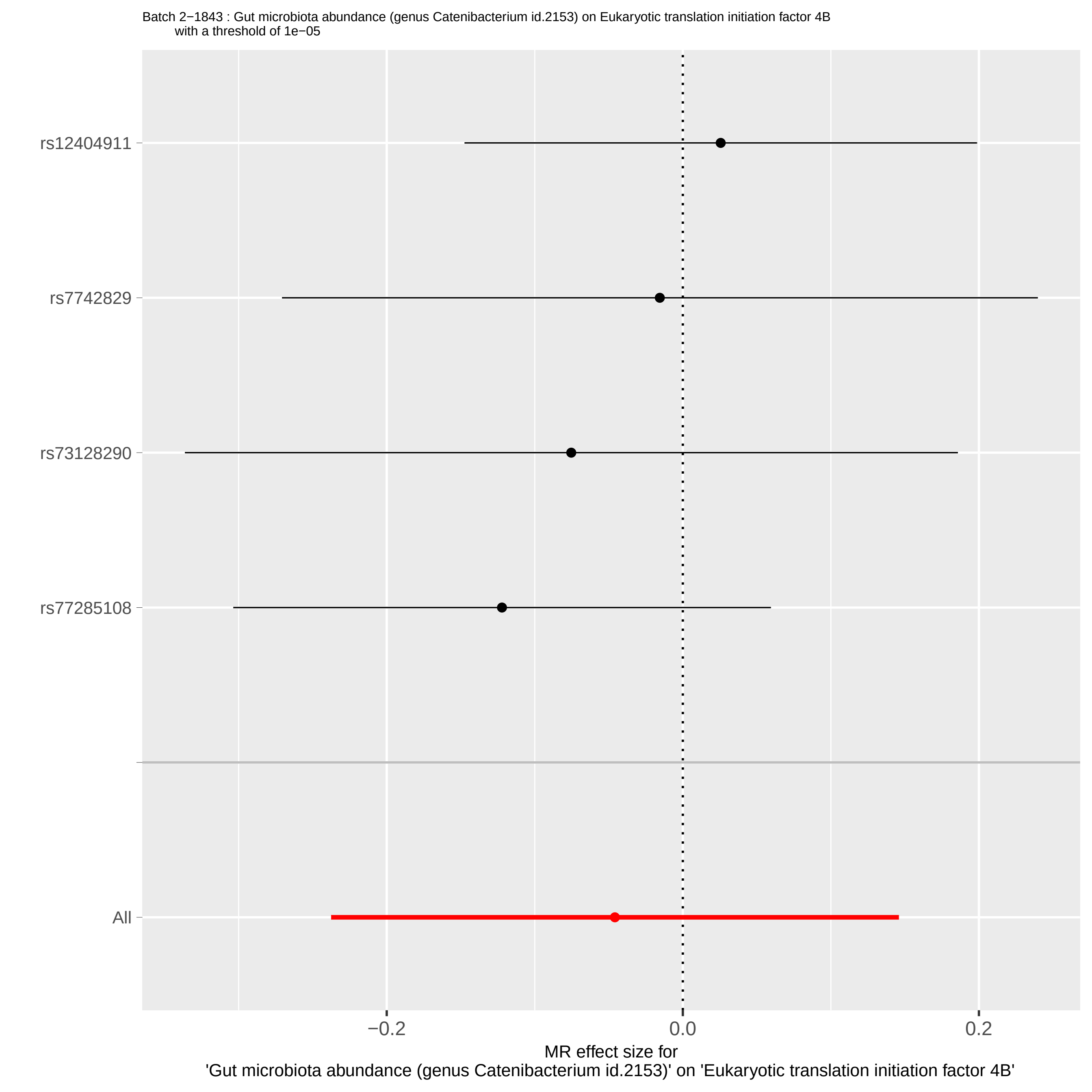
Batch 2−1843 : Gut microbiota abundance (genus Catenibacterium id.2153) on Eukaryotic translation initiation factor 4B
with a threshold of 1e−05
rs12404911
rs7742829
rs73128290
rs77285108
All
−0.2
0.0
0.2
MR effect size for
'Gut microbiota abundance (genus Catenibacterium id.2153)' on 'Eukaryotic translation initiation factor 4B'

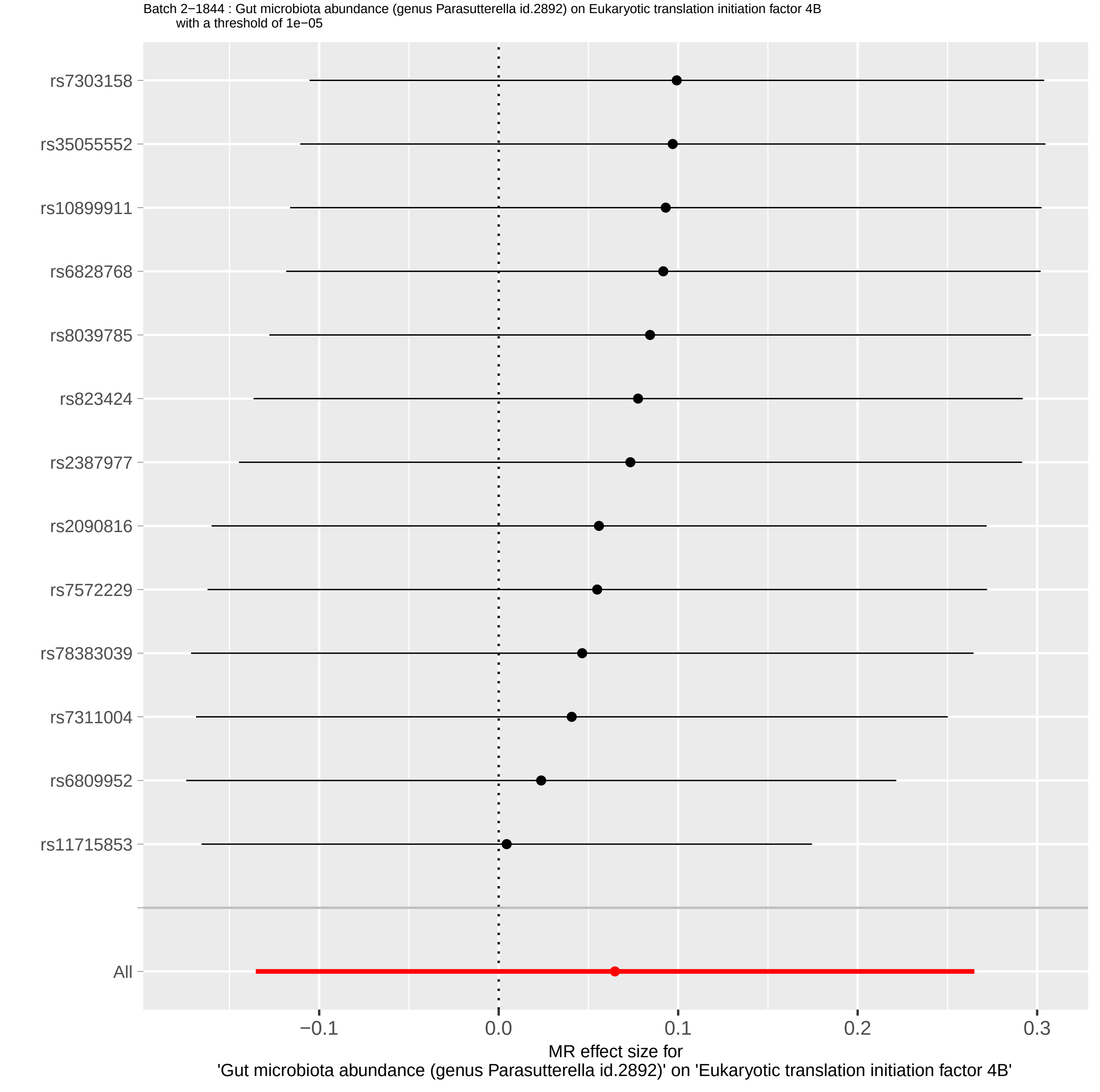

Batch 2−1844 : Gut microbiota abundance (genus Parasutterella id.2892) on Eukaryotic translation initiation factor 4B
with a threshold of 1e−05
rs7303158
rs35055552
rs10899911
rs6828768
rs8039785
rs823424
rs2387977
rs2090816
rs7572229
rs78383039
rs7311004
rs6809952
rs11715853
All
−0.1
0.0
0.1
0.2
0.3
MR effect size for
'Gut microbiota abundance (genus Parasutterella id.2892)' on 'Eukaryotic translation initiation factor 4B'

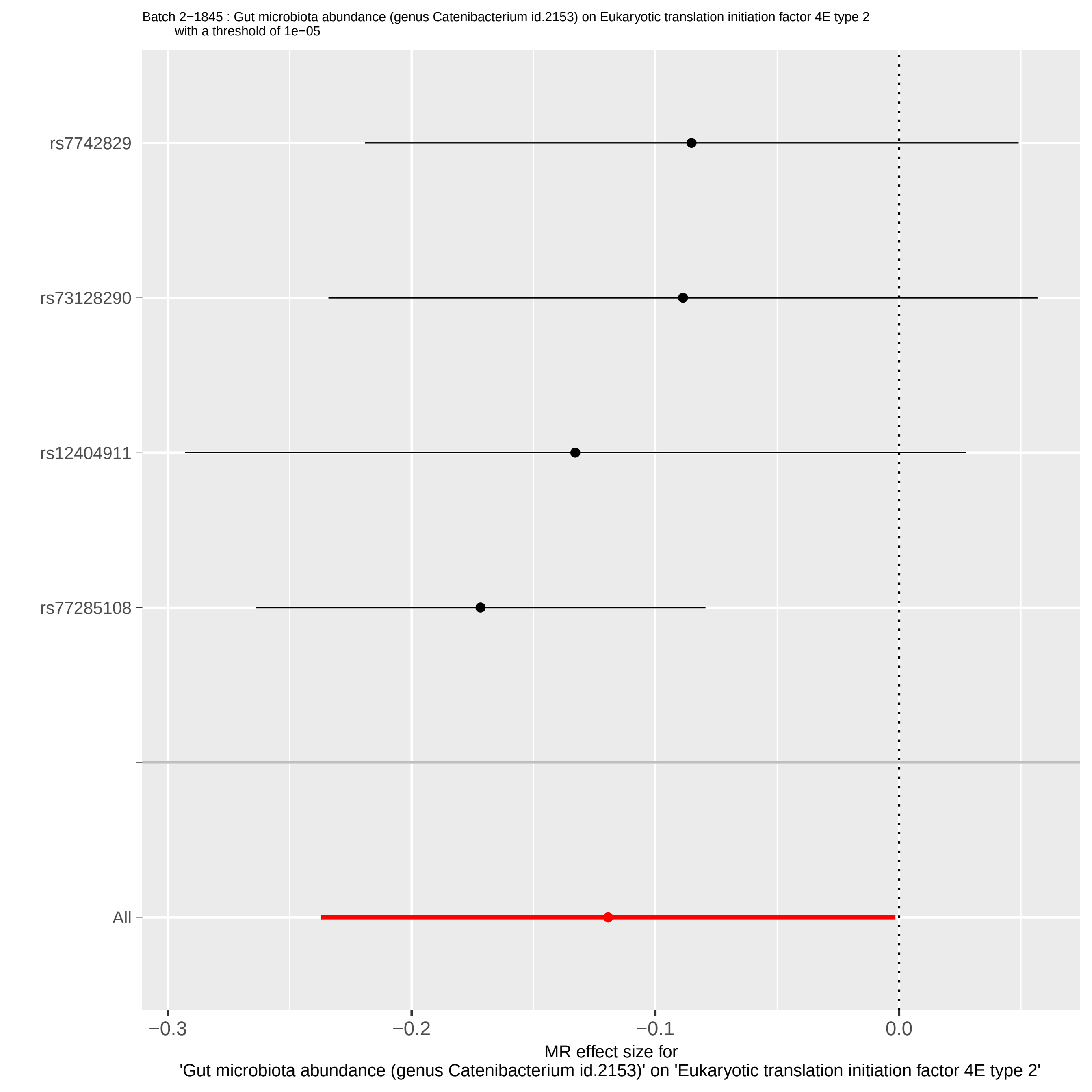
Batch 2−1845 : Gut microbiota abundance (genus Catenibacterium id.2153) on Eukaryotic translation initiation factor 4E type 2
with a threshold of 1e−05
rs7742829
rs73128290
rs12404911
rs77285108
All
−0.3
−0.2
−0.1
0.0
MR effect size for
'Gut microbiota abundance (genus Catenibacterium id.2153)' on 'Eukaryotic translation initiation factor 4E type 2'

Batch 2−1846 : Gut microbiota abundance (genus Parasutterella id.2892) on Eukaryotic translation initiation factor 4E type 2
with a threshold of 1e−05

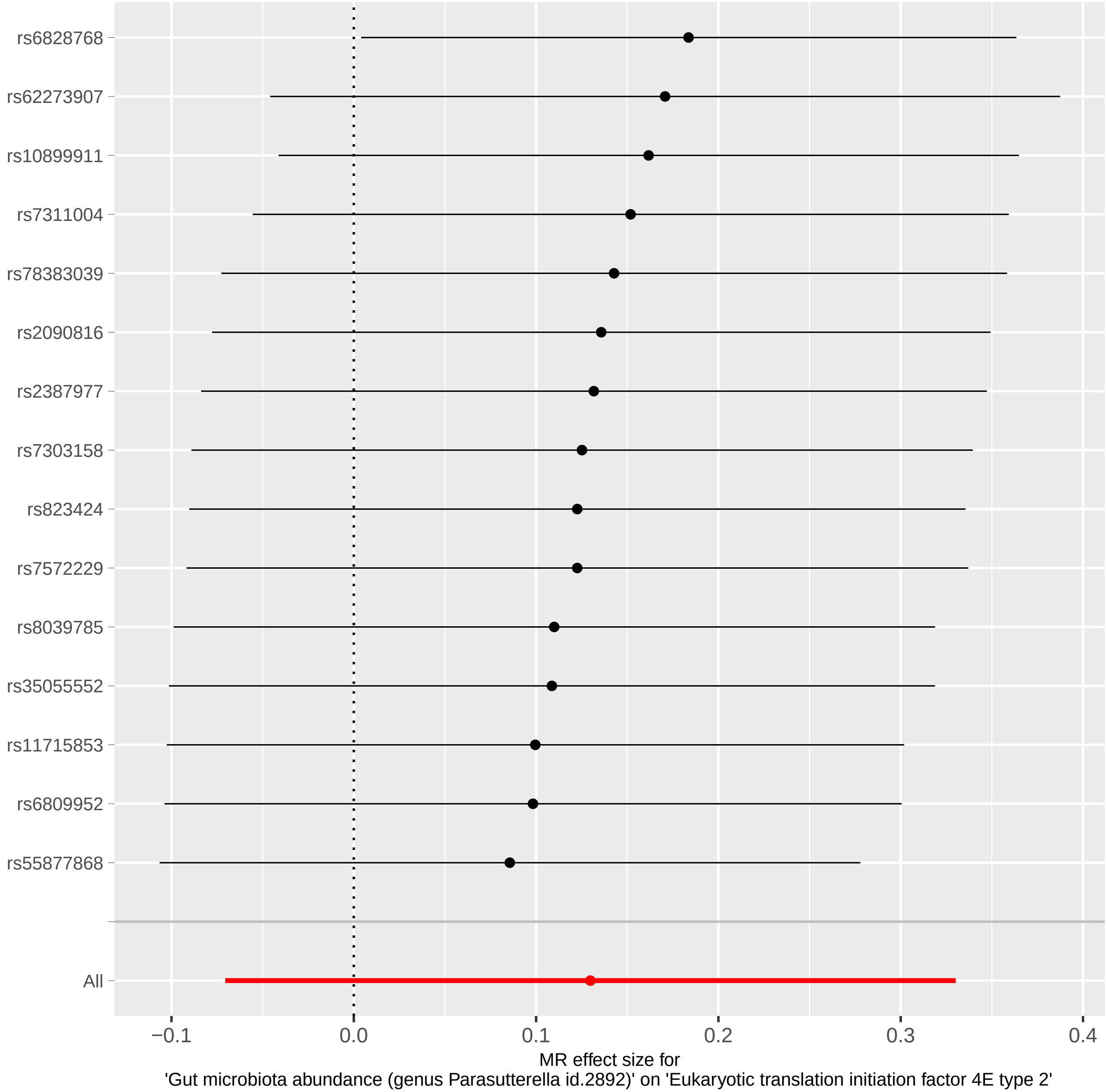

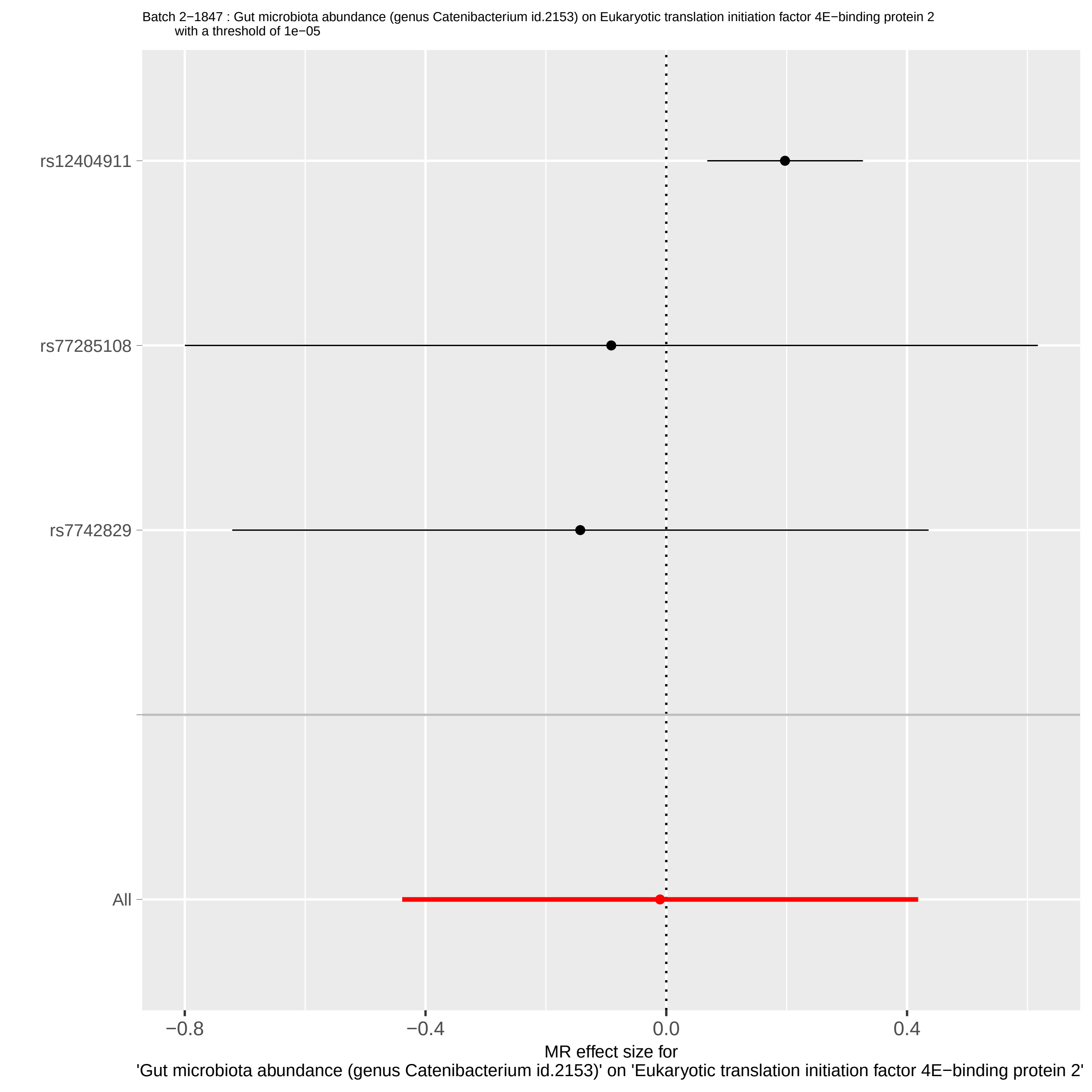
Batch 2−1847 : Gut microbiota abundance (genus Catenibacterium id.2153) on Eukaryotic translation initiation factor 4E−binding protein 2
with a threshold of 1e−05
rs12404911
rs77285108
rs7742829
All
−0.8
−0.4
0.0
0.4
MR effect size for
'Gut microbiota abundance (genus Catenibacterium id.2153)' on 'Eukaryotic translation initiation factor 4E−binding protein 2'

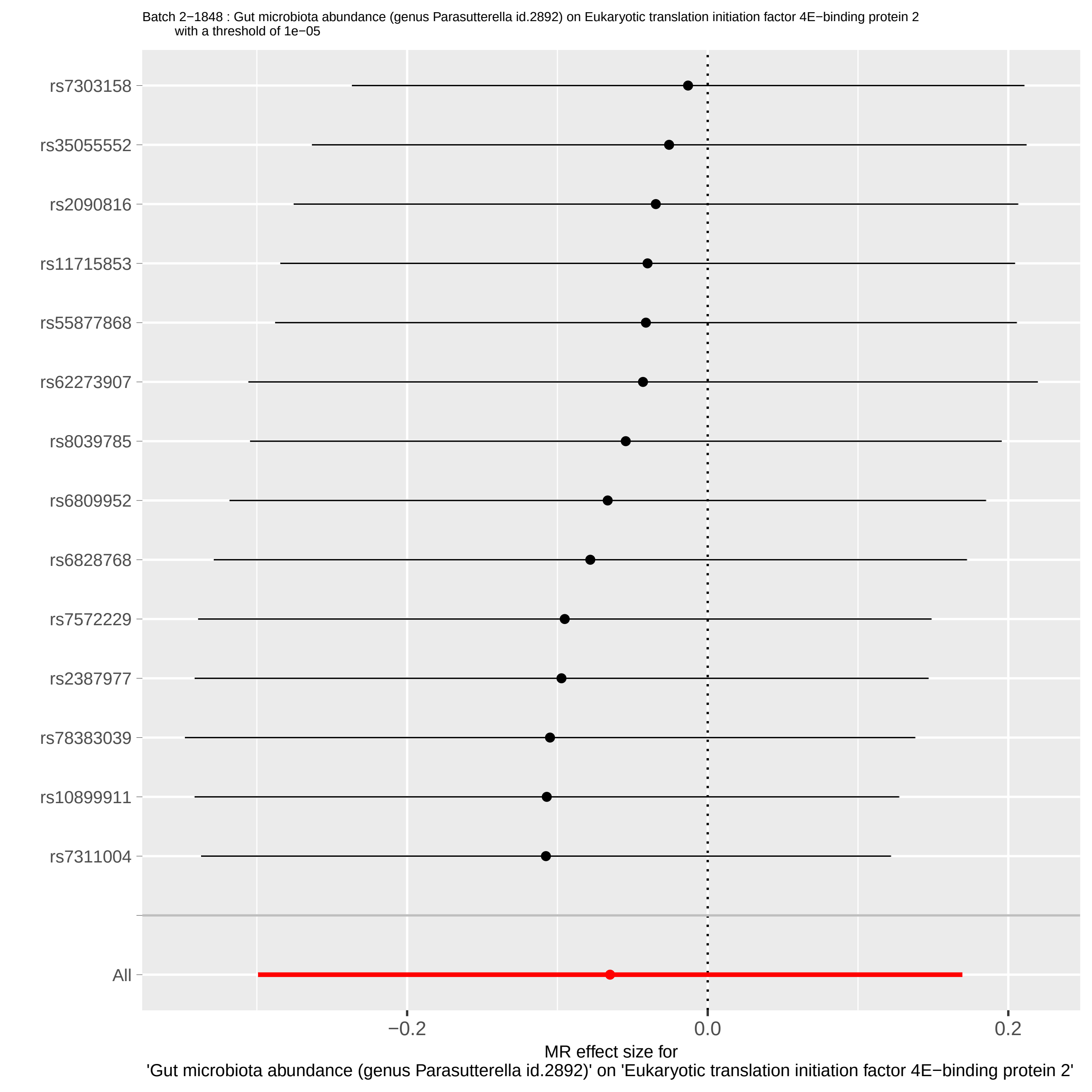
Batch 2−1848 : Gut microbiota abundance (genus Parasutterella id.2892) on Eukaryotic translation initiation factor 4E−binding protein 2
with a threshold of 1e−05
rs7303158
rs35055552
rs2090816
rs11715853
rs55877868
rs62273907
rs8039785
rs6809952
rs6828768
rs7572229
rs2387977
rs78383039
rs10899911
rs7311004
All
−0.2
0.0
0.2
MR effect size for
'Gut microbiota abundance (genus Parasutterella id.2892)' on 'Eukaryotic translation initiation factor 4E−binding protein 2'

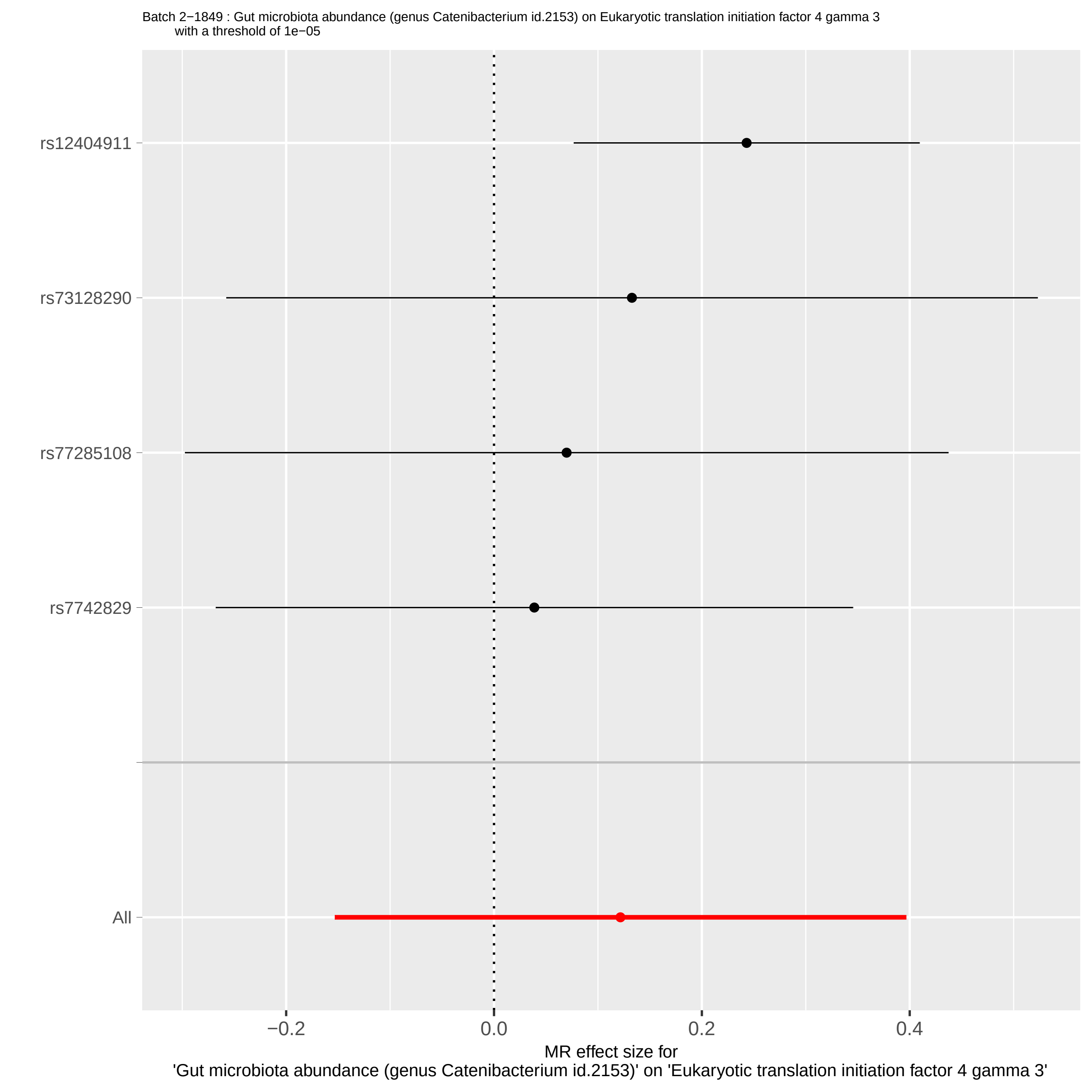
Batch 2−1849 : Gut microbiota abundance (genus Catenibacterium id.2153) on Eukaryotic translation initiation factor 4 gamma 3
with a threshold of 1e−05
rs12404911
rs73128290
rs77285108
rs7742829
All
−0.2
0.0
0.2
0.4
MR effect size for
'Gut microbiota abundance (genus Catenibacterium id.2153)' on 'Eukaryotic translation initiation factor 4 gamma 3'

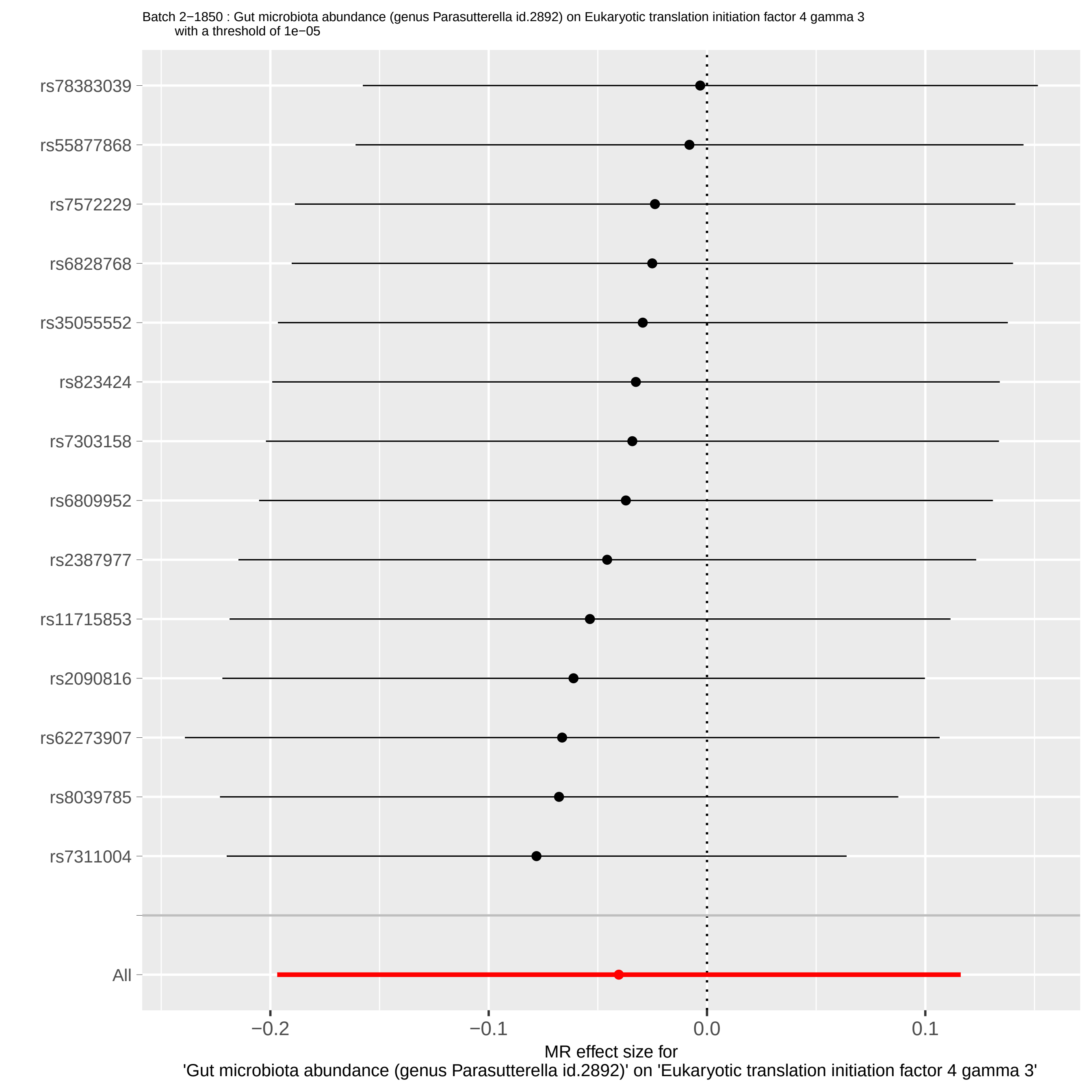
Batch 2−1850 : Gut microbiota abundance (genus Parasutterella id.2892) on Eukaryotic translation initiation factor 4 gamma 3
with a threshold of 1e−05
rs78383039
rs55877868
rs7572229
rs6828768
rs35055552
rs823424
rs7303158
rs6809952
rs2387977
rs11715853
rs2090816
rs62273907
rs8039785
rs7311004
All
−0.2
−0.1
0.0
0.1
MR effect size for
'Gut microbiota abundance (genus Parasutterella id.2892)' on 'Eukaryotic translation initiation factor 4 gamma 3'

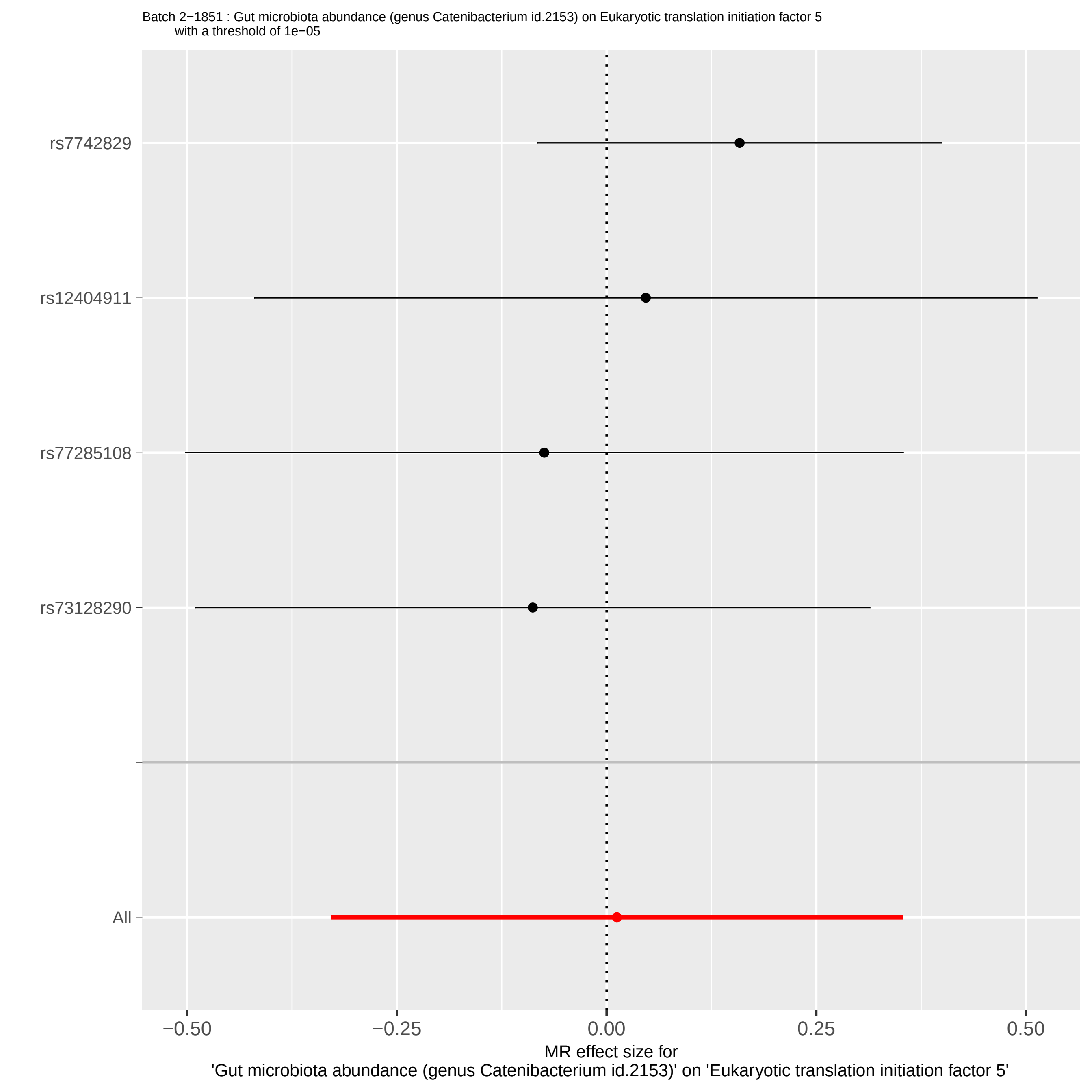

Batch 2−1851 : Gut microbiota abundance (genus Catenibacterium id.2153) on Eukaryotic translation initiation factor 5
with a threshold of 1e−05
rs7742829
rs12404911
rs77285108
rs73128290
All
−0.50
−0.25
0.00
0.25
0.50
MR effect size for
'Gut microbiota abundance (genus Catenibacterium id.2153)' on 'Eukaryotic translation initiation factor 5'

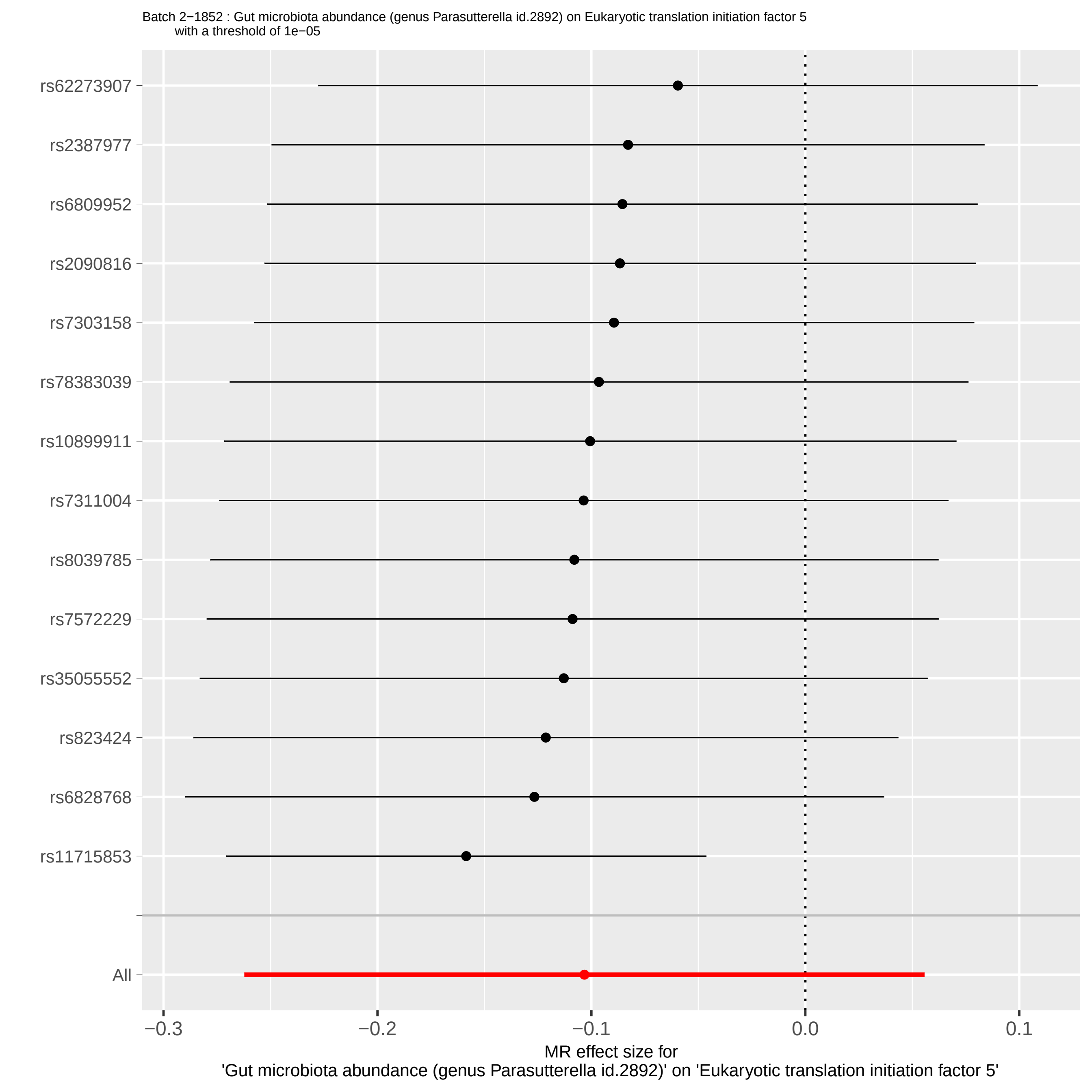

Batch 2−1852 : Gut microbiota abundance (genus Parasutterella id.2892) on Eukaryotic translation initiation factor 5
with a threshold of 1e−05
rs62273907
rs2387977
rs6809952
rs2090816
rs7303158
rs78383039
rs10899911
rs7311004
rs8039785
rs7572229
rs35055552
rs823424
rs6828768
rs11715853
All
−0.3
−0.2
−0.1
0.0
0.1
MR effect size for
'Gut microbiota abundance (genus Parasutterella id.2892)' on 'Eukaryotic translation initiation factor 5'

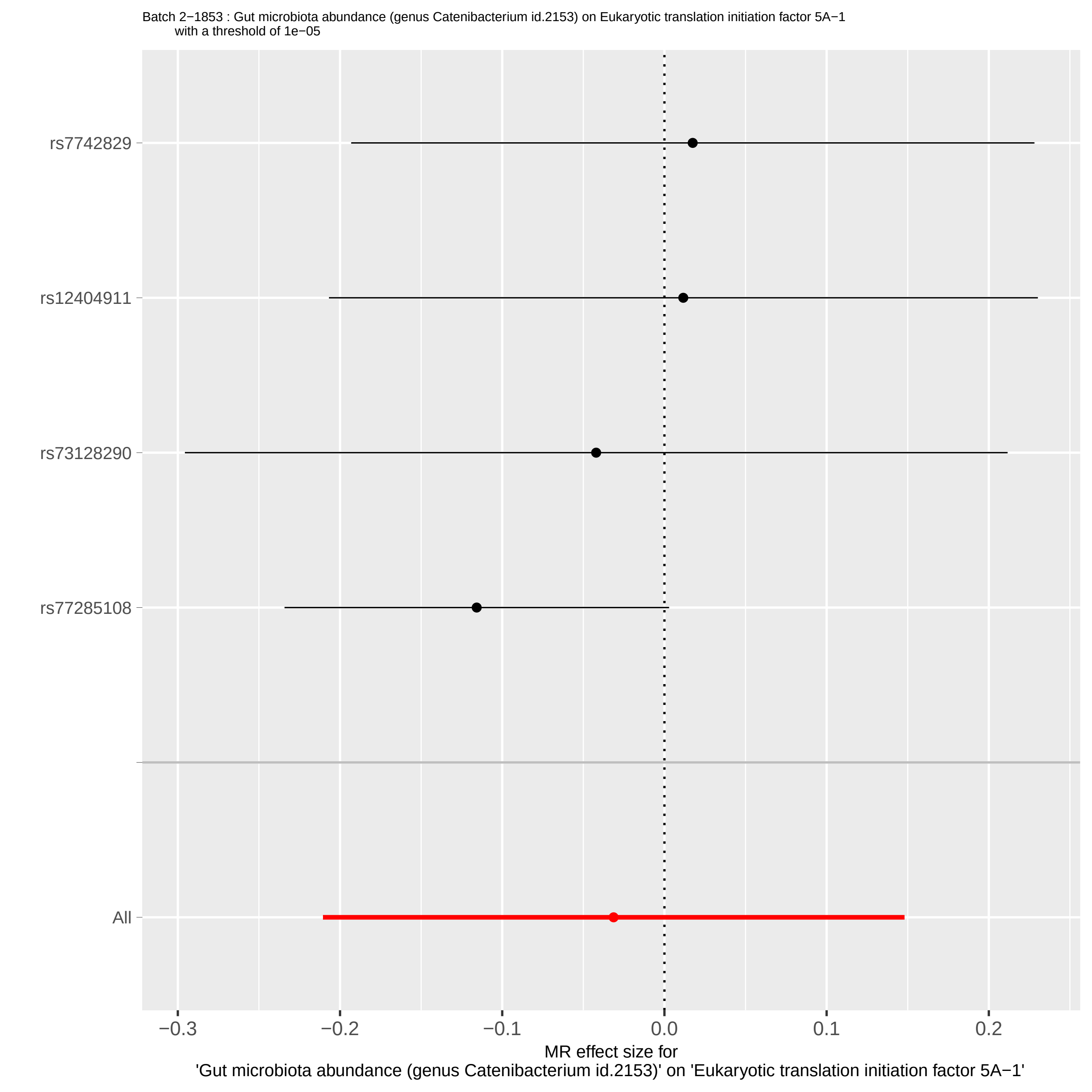
Batch 2−1853 : Gut microbiota abundance (genus Catenibacterium id.2153) on Eukaryotic translation initiation factor 5A−1
with a threshold of 1e−05
rs7742829
rs12404911
rs73128290
rs77285108
All
−0.3
−0.2
−0.1
0.0
0.1
0.2
MR effect size for
'Gut microbiota abundance (genus Catenibacterium id.2153)' on 'Eukaryotic translation initiation factor 5A−1'

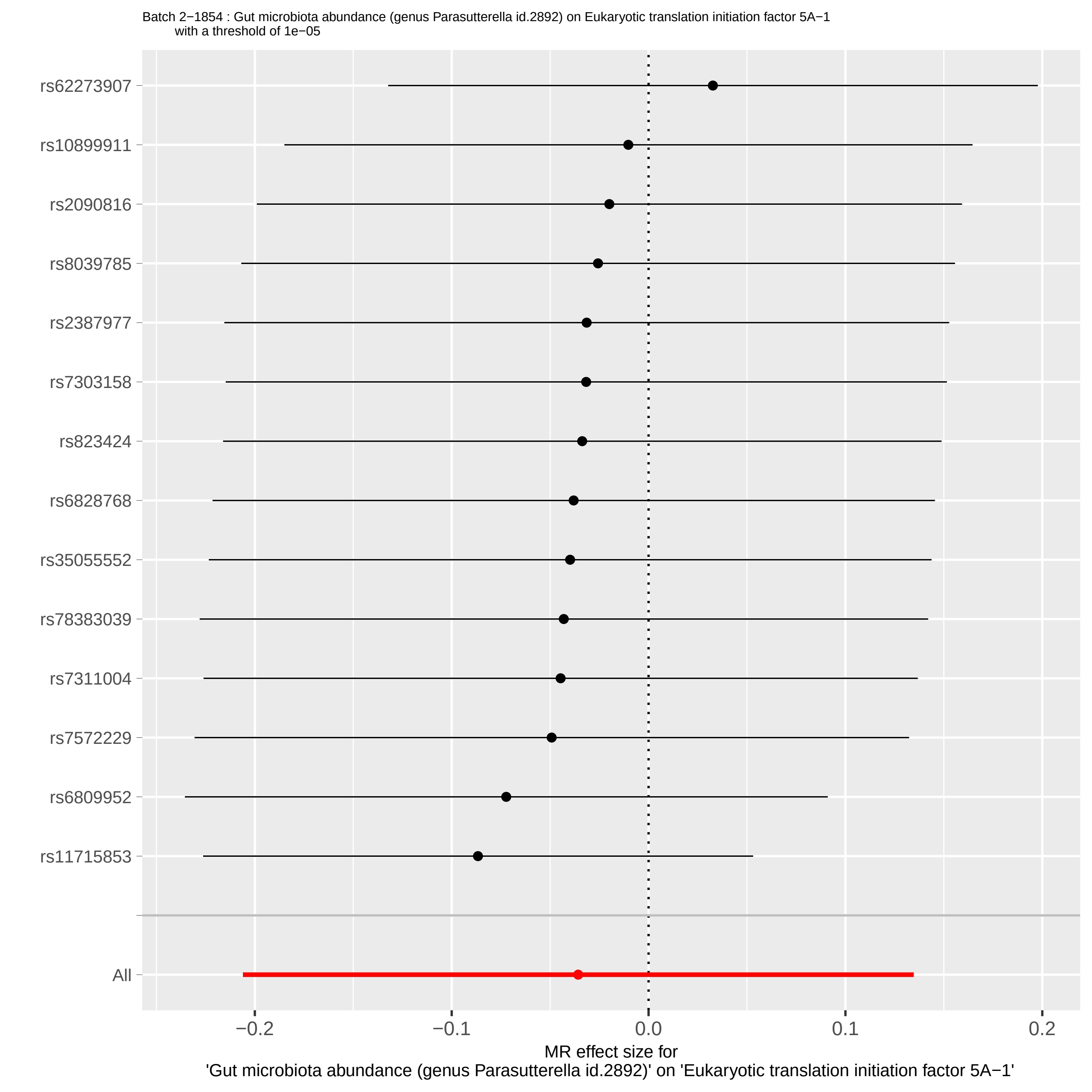

Batch 2−1854 : Gut microbiota abundance (genus Parasutterella id.2892) on Eukaryotic translation initiation factor 5A−1
with a threshold of 1e−05
rs62273907
rs10899911
rs2090816
rs8039785
rs2387977
rs7303158
rs823424
rs6828768
rs35055552
rs78383039
rs7311004
rs7572229
rs6809952
rs11715853
All
−0.2
−0.1
0.0
0.1
0.2
MR effect size for
'Gut microbiota abundance (genus Parasutterella id.2892)' on 'Eukaryotic translation initiation factor 5A−1'

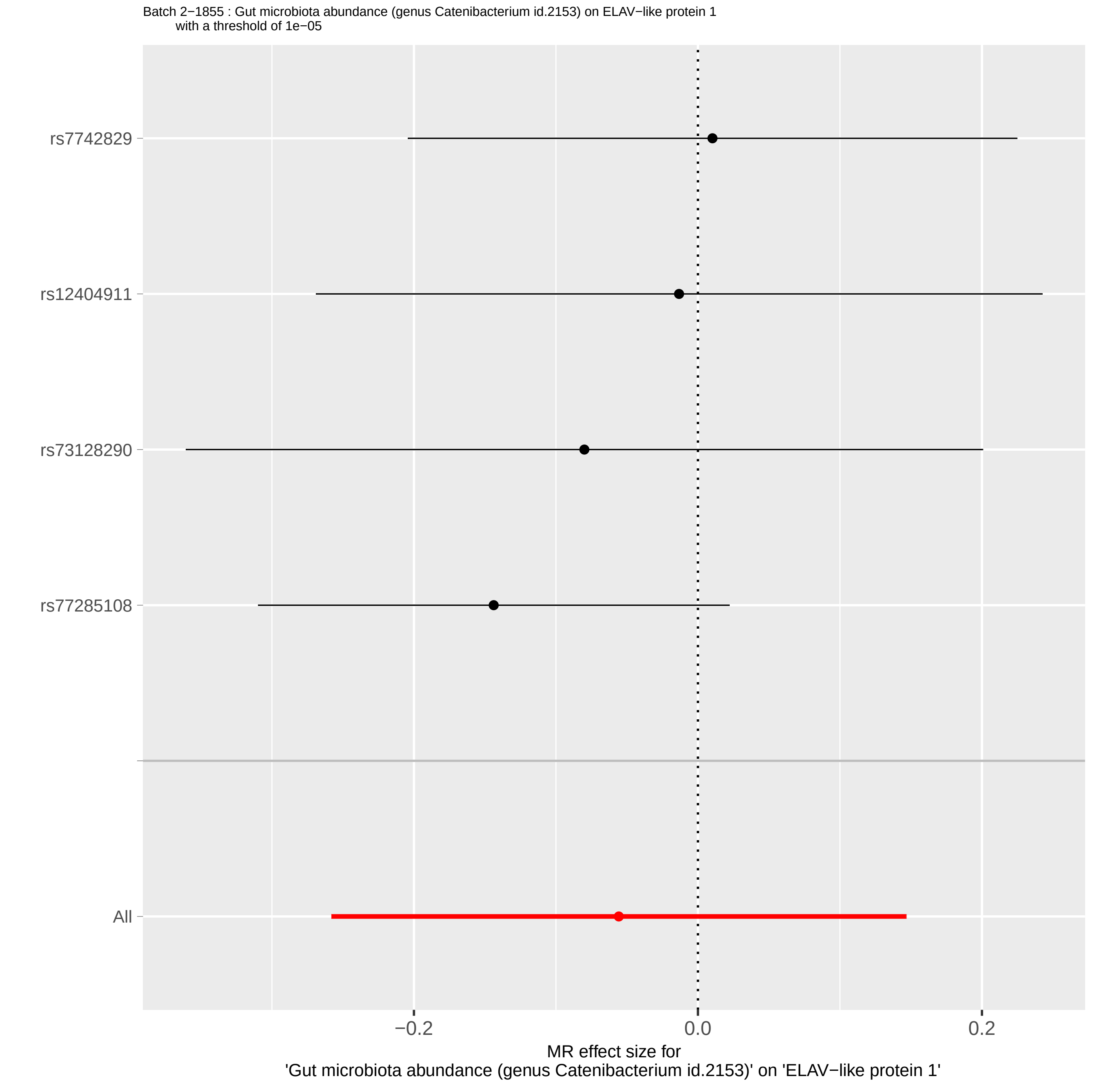
Batch 2−1855 : Gut microbiota abundance (genus Catenibacterium id.2153) on ELAV−like protein 1
with a threshold of 1e−05
rs7742829
rs12404911
rs73128290
rs77285108
All
−0.2
0.0
0.2
MR effect size for
'Gut microbiota abundance (genus Catenibacterium id.2153)' on 'ELAV−like protein 1'

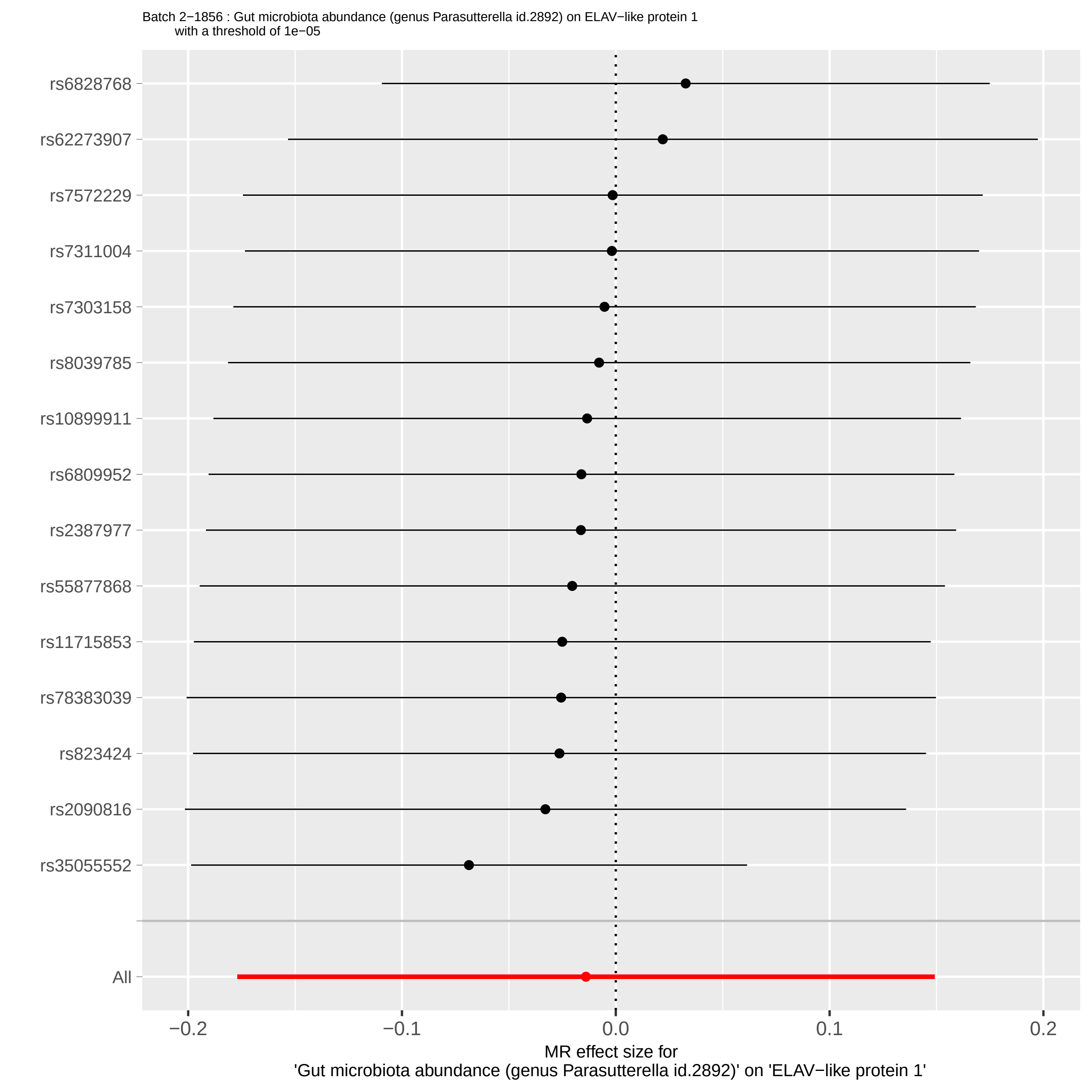
Batch 2−1856 : Gut microbiota abundance (genus Parasutterella id.2892) on ELAV−like protein 1
with a threshold of 1e−05
rs6828768
rs62273907
rs7572229
rs7311004
rs7303158
rs8039785
rs10899911
rs6809952
rs2387977
rs55877868
rs11715853
rs78383039
rs823424
rs2090816
rs35055552
All
−0.2
−0.1
0.0
0.1
0.2
MR effect size for
'Gut microbiota abundance (genus Parasutterella id.2892)' on 'ELAV−like protein 1'

Batch 2−1857 : Gut microbiota abundance (genus Catenibacterium id.2153) on ETS−related transcription factor Elf−5
with a threshold of 1e−05

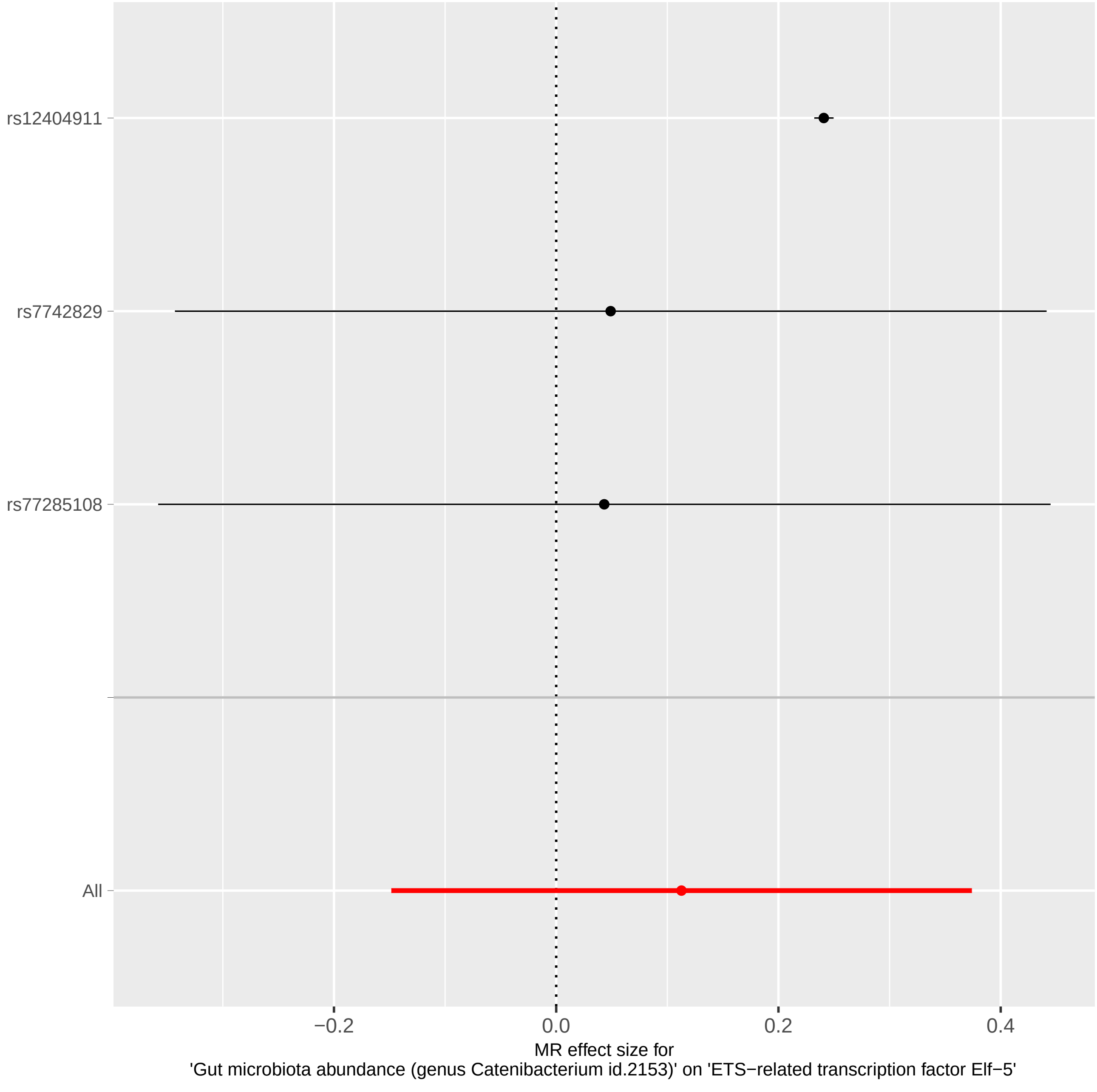

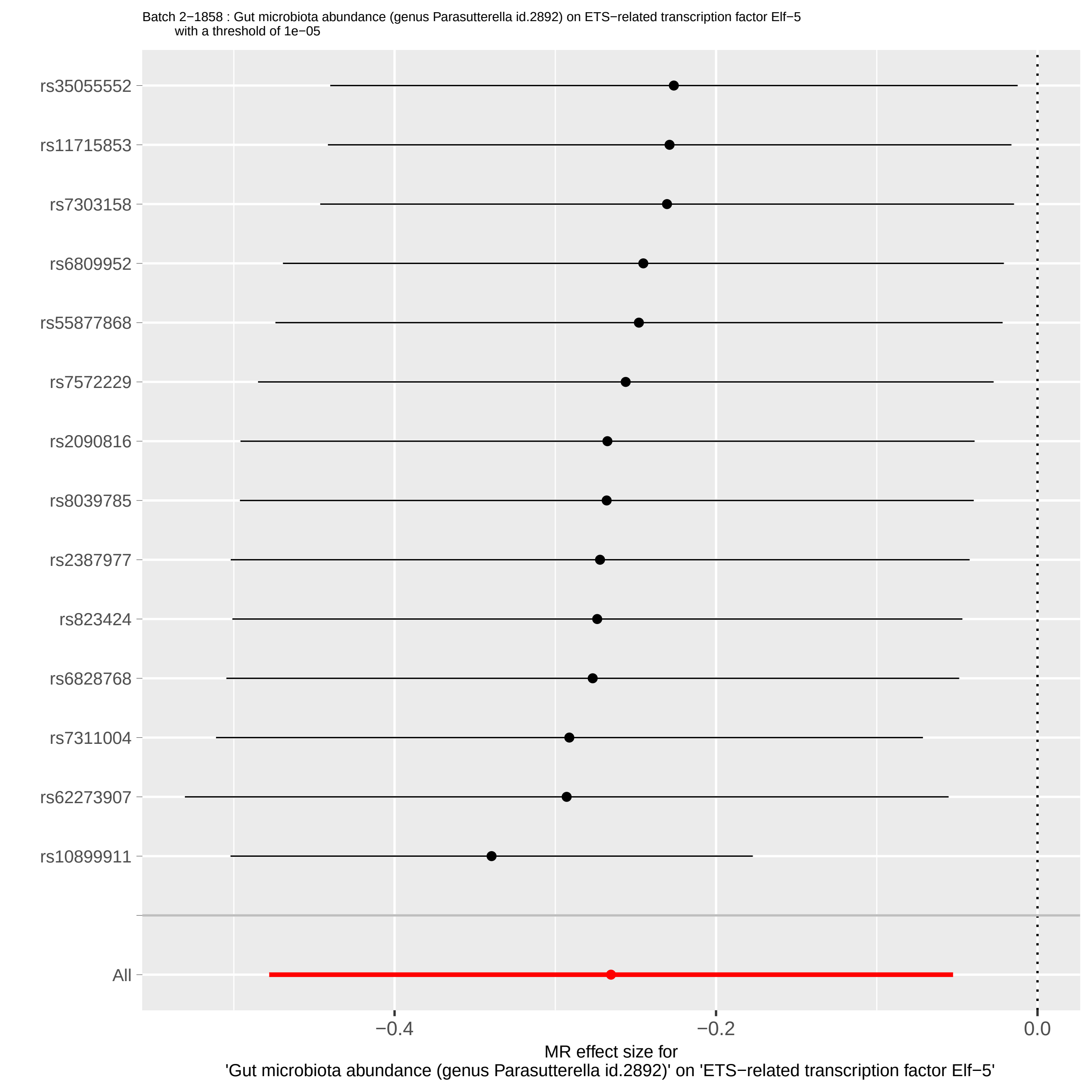

Batch 2−1858 : Gut microbiota abundance (genus Parasutterella id.2892) on ETS−related transcription factor Elf−5
with a threshold of 1e−05
rs35055552
rs11715853
rs7303158
rs6809952
rs55877868
rs7572229
rs2090816
rs8039785
rs2387977
rs823424
rs6828768
rs7311004
rs62273907
rs10899911
All
−0.4
−0.2
0.0
MR effect size for
'Gut microbiota abundance (genus Parasutterella id.2892)' on 'ETS−related transcription factor Elf−5'

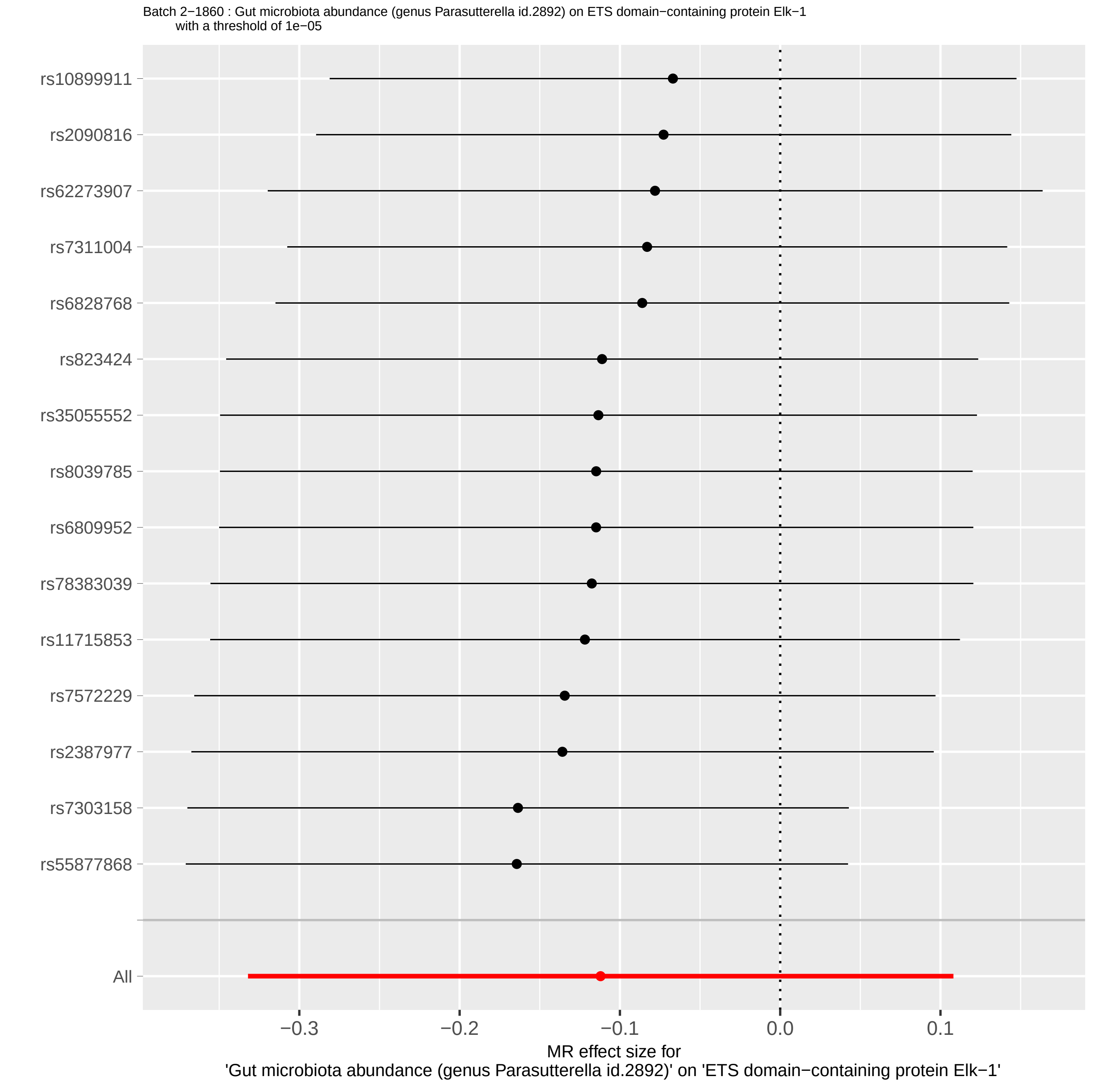

Batch 2−1860 : Gut microbiota abundance (genus Parasutterella id.2892) on ETS domain−containing protein Elk−1
with a threshold of 1e−05
rs10899911
rs2090816
rs62273907
rs7311004
rs6828768
rs823424
rs35055552
rs8039785
rs6809952
rs78383039
rs11715853
rs7572229
rs2387977
rs7303158
rs55877868
All
−0.3
−0.2
−0.1
0.0
0.1
MR effect size for
'Gut microbiota abundance (genus Parasutterella id.2892)' on 'ETS domain−containing protein Elk−1'

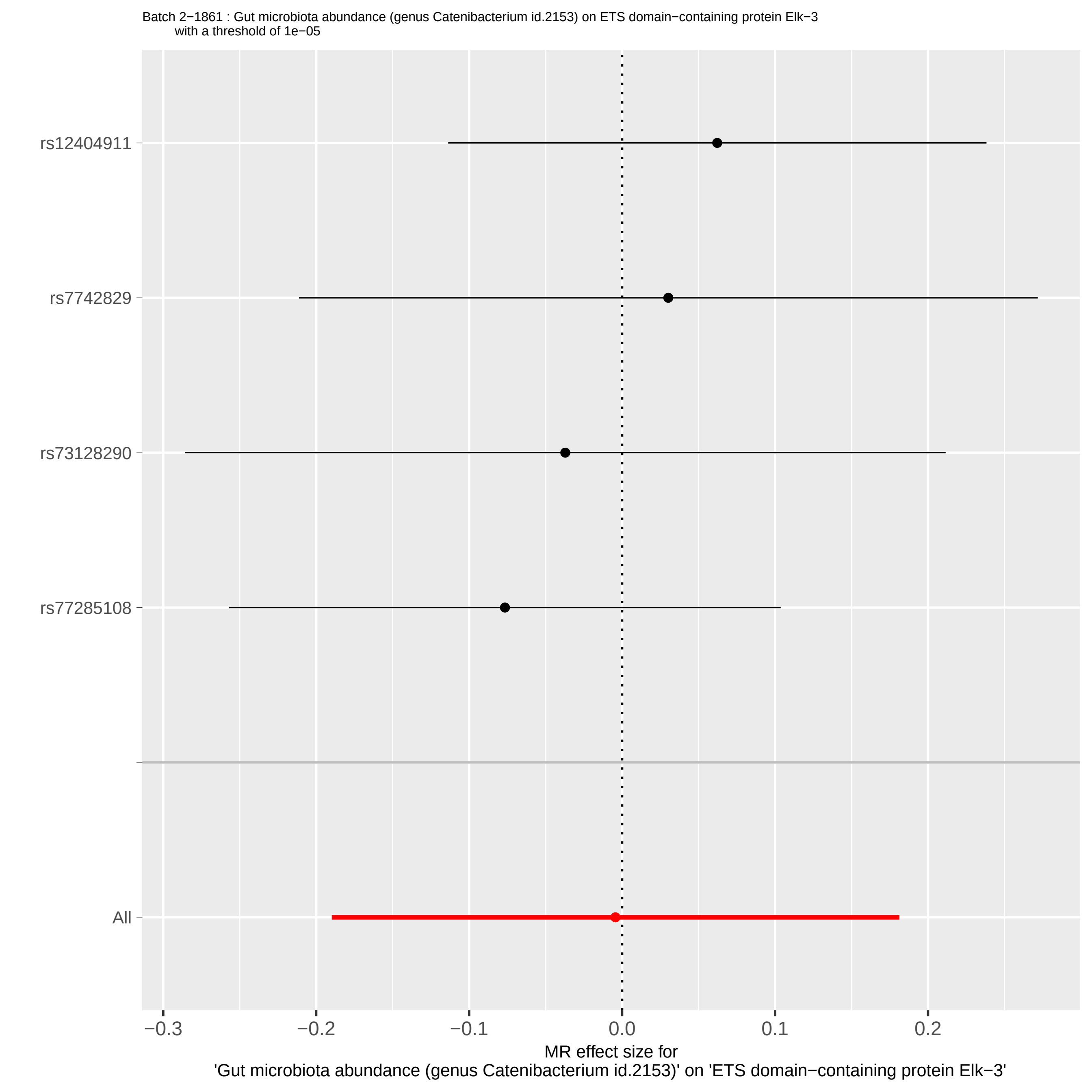

Batch 2−1861 : Gut microbiota abundance (genus Catenibacterium id.2153) on ETS domain−containing protein Elk−3
with a threshold of 1e−05
rs12404911
rs7742829
rs73128290
rs77285108
All
−0.3
−0.2
−0.1
0.0
0.1
0.2
MR effect size for
'Gut microbiota abundance (genus Catenibacterium id.2153)' on 'ETS domain−containing protein Elk−3'

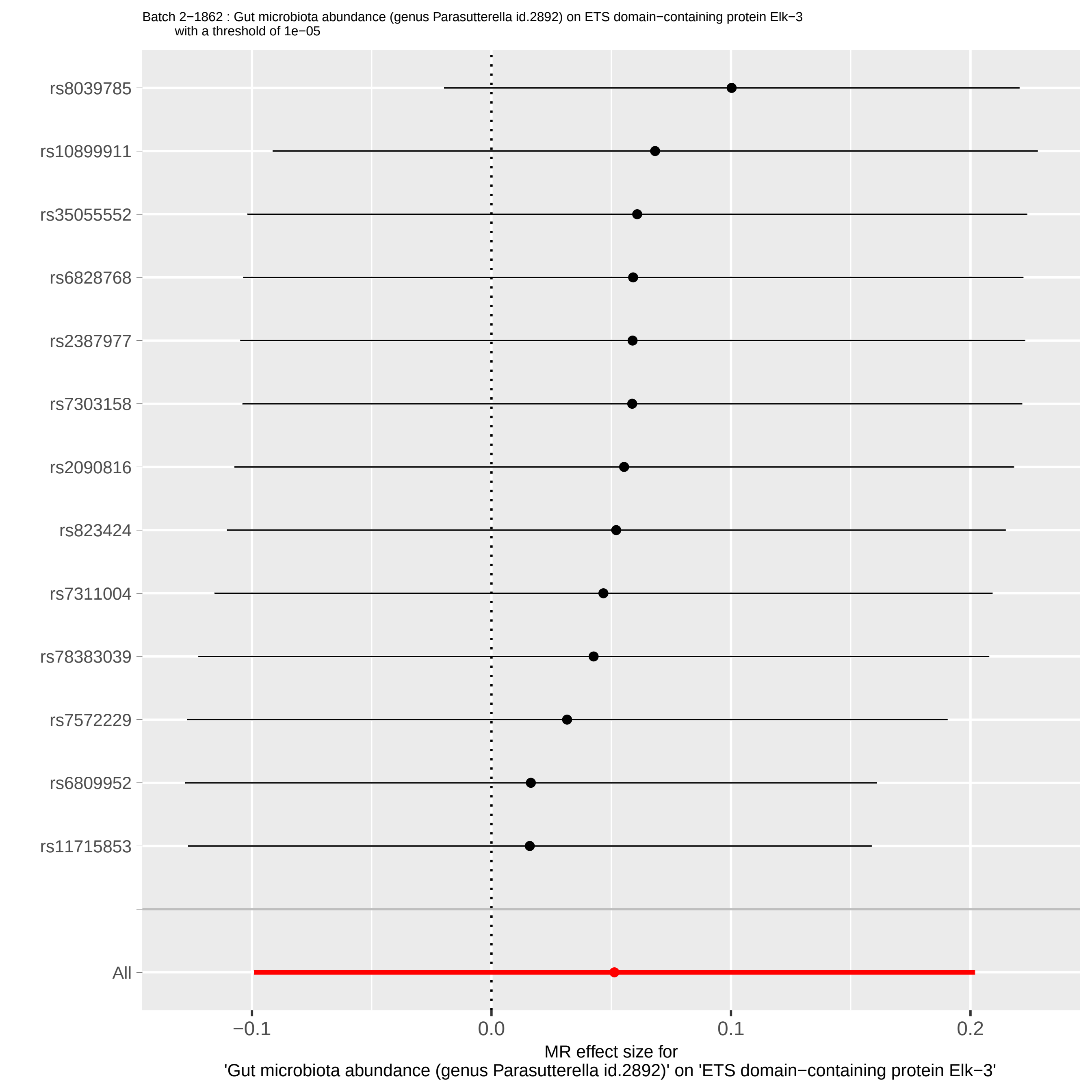

Batch 2−1862 : Gut microbiota abundance (genus Parasutterella id.2892) on ETS domain−containing protein Elk−3
with a threshold of 1e−05
rs8039785
rs10899911
rs35055552
rs6828768
rs2387977
rs7303158
rs2090816
rs823424
rs7311004
rs78383039
rs7572229
rs6809952
rs11715853
All
−0.1
0.0
0.1
0.2
MR effect size for
'Gut microbiota abundance (genus Parasutterella id.2892)' on 'ETS domain−containing protein Elk−3'

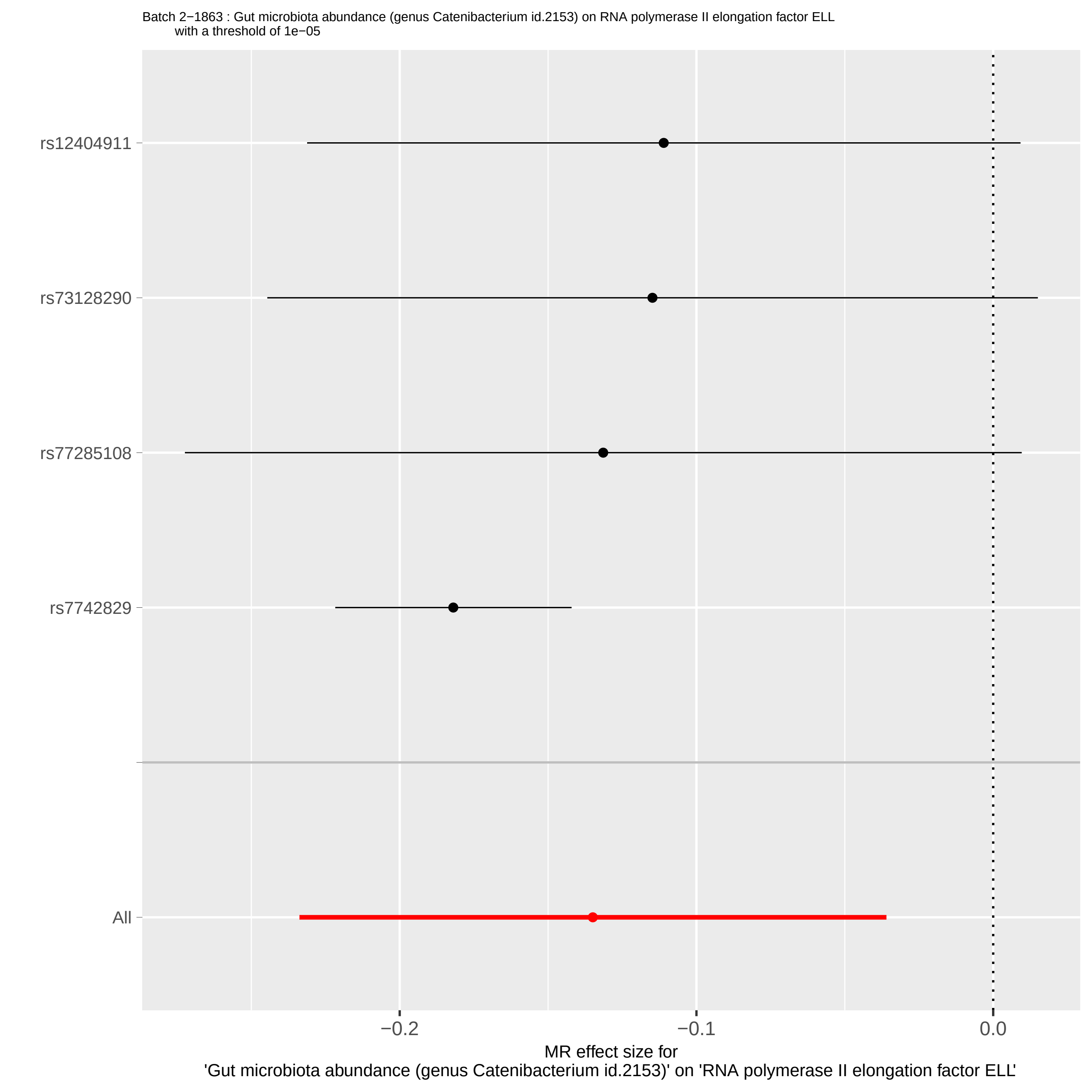

Batch 2−1863 : Gut microbiota abundance (genus Catenibacterium id.2153) on RNA polymerase II elongation factor ELL
with a threshold of 1e−05
rs12404911
rs73128290
rs77285108
rs7742829
All
−0.2
−0.1
0.0
MR effect size for
'Gut microbiota abundance (genus Catenibacterium id.2153)' on 'RNA polymerase II elongation factor ELL'

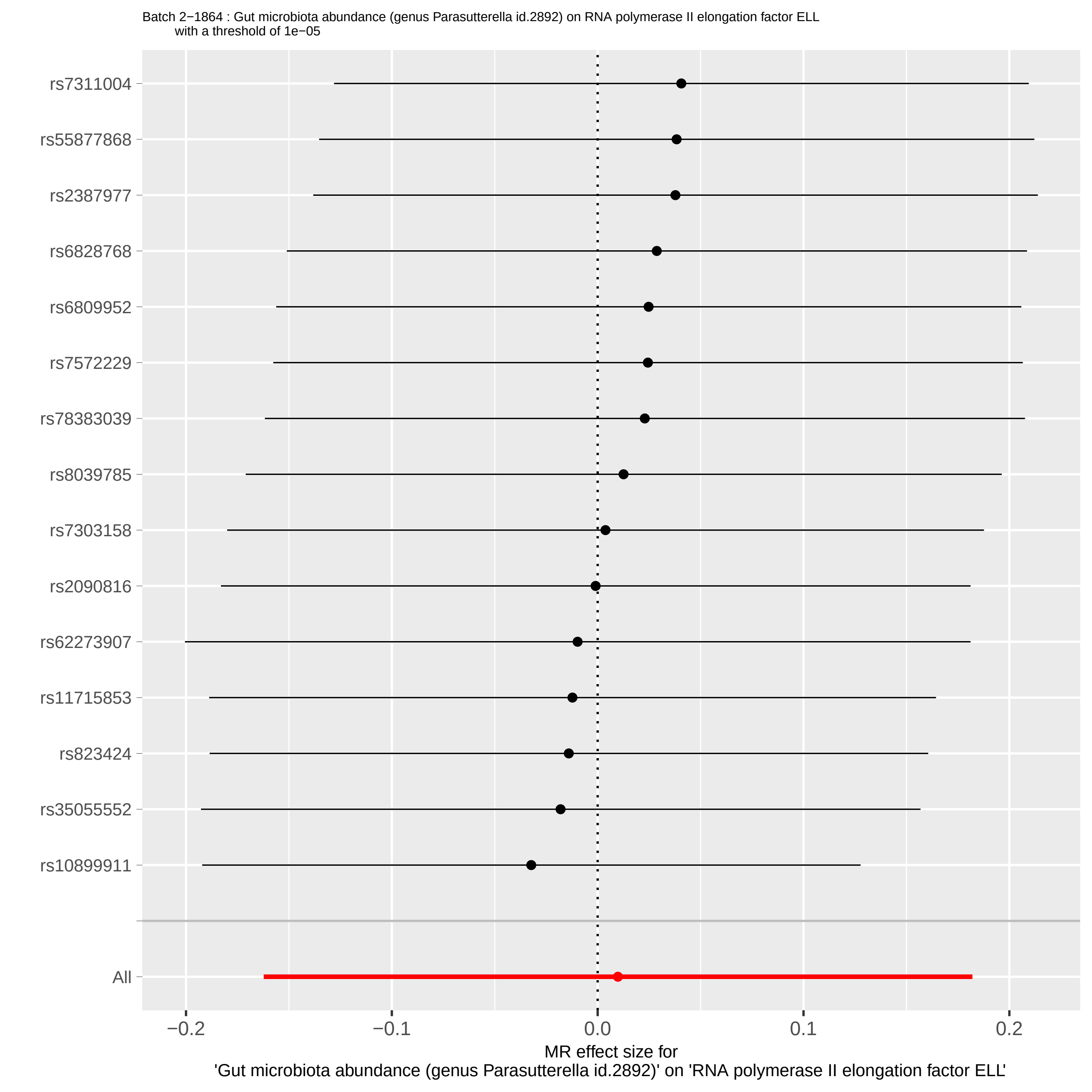
Batch 2−1864 : Gut microbiota abundance (genus Parasutterella id.2892) on RNA polymerase II elongation factor ELL
with a threshold of 1e−05
rs7311004
rs55877868
rs2387977
rs6828768
rs6809952
rs7572229
rs78383039
rs8039785
rs7303158
rs2090816
rs62273907
rs11715853
rs823424
rs35055552
rs10899911
All
−0.2
−0.1
0.0
0.1
0.2
MR effect size for
'Gut microbiota abundance (genus Parasutterella id.2892)' on 'RNA polymerase II elongation factor ELL'

Batch 2−1865 : Gut microbiota abundance (genus Catenibacterium id.2153) on RNA polymerase II elongation factor ELL2
with a threshold of 1e−05

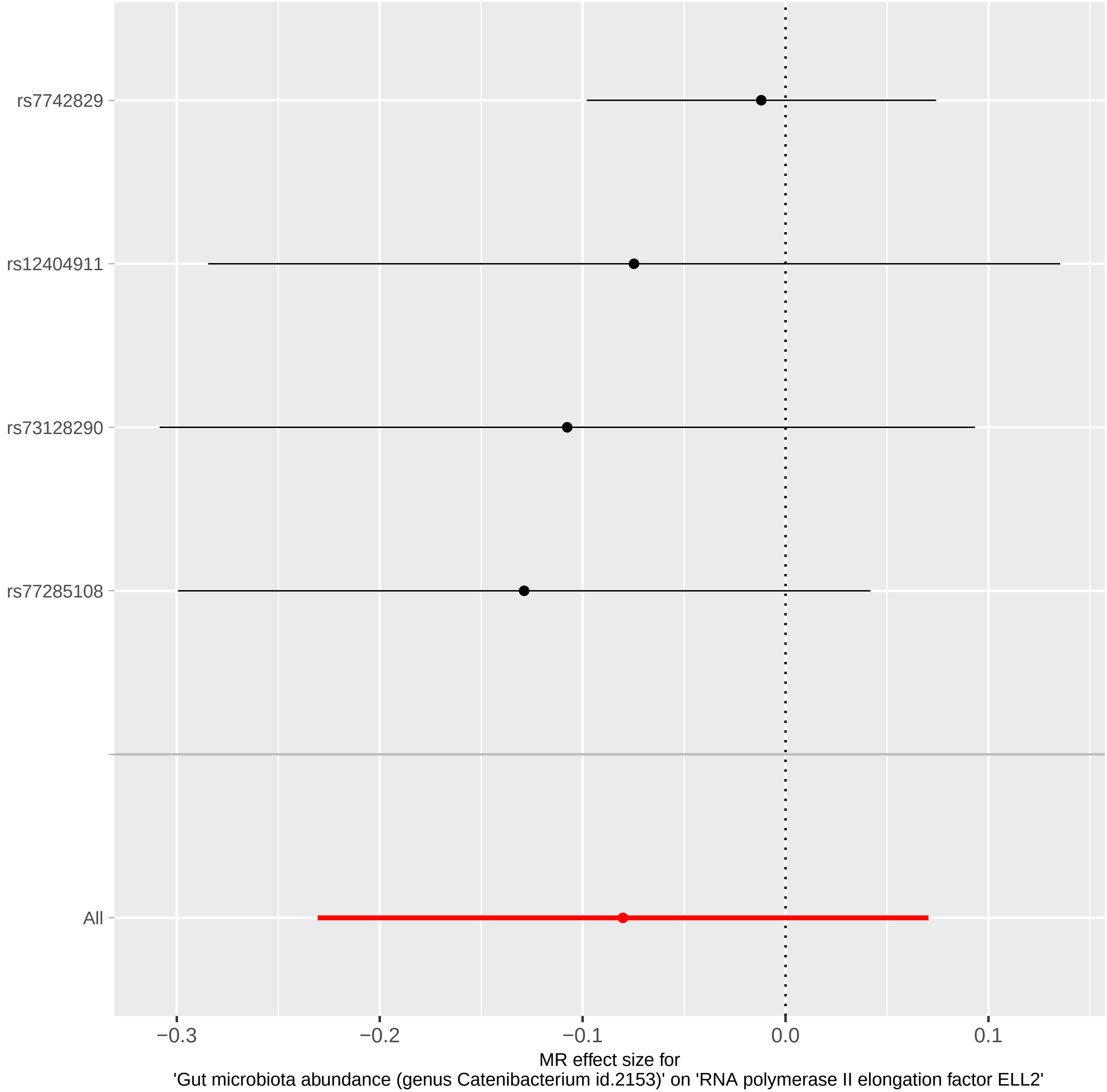

Batch 2−1866 : Gut microbiota abundance (genus Parasutterella id.2892) on RNA polymerase II elongation factor ELL2
with a threshold of 1e−05

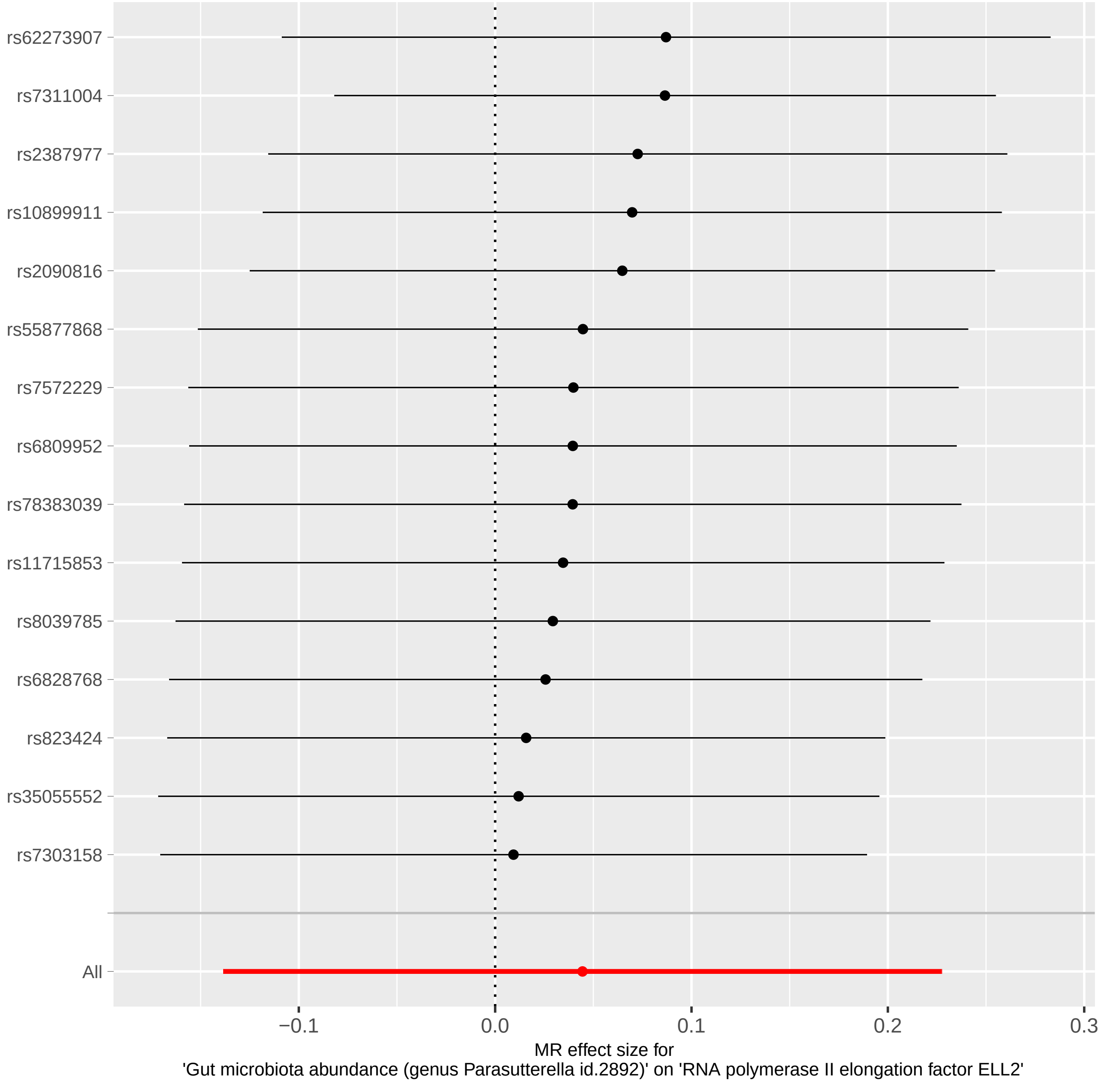

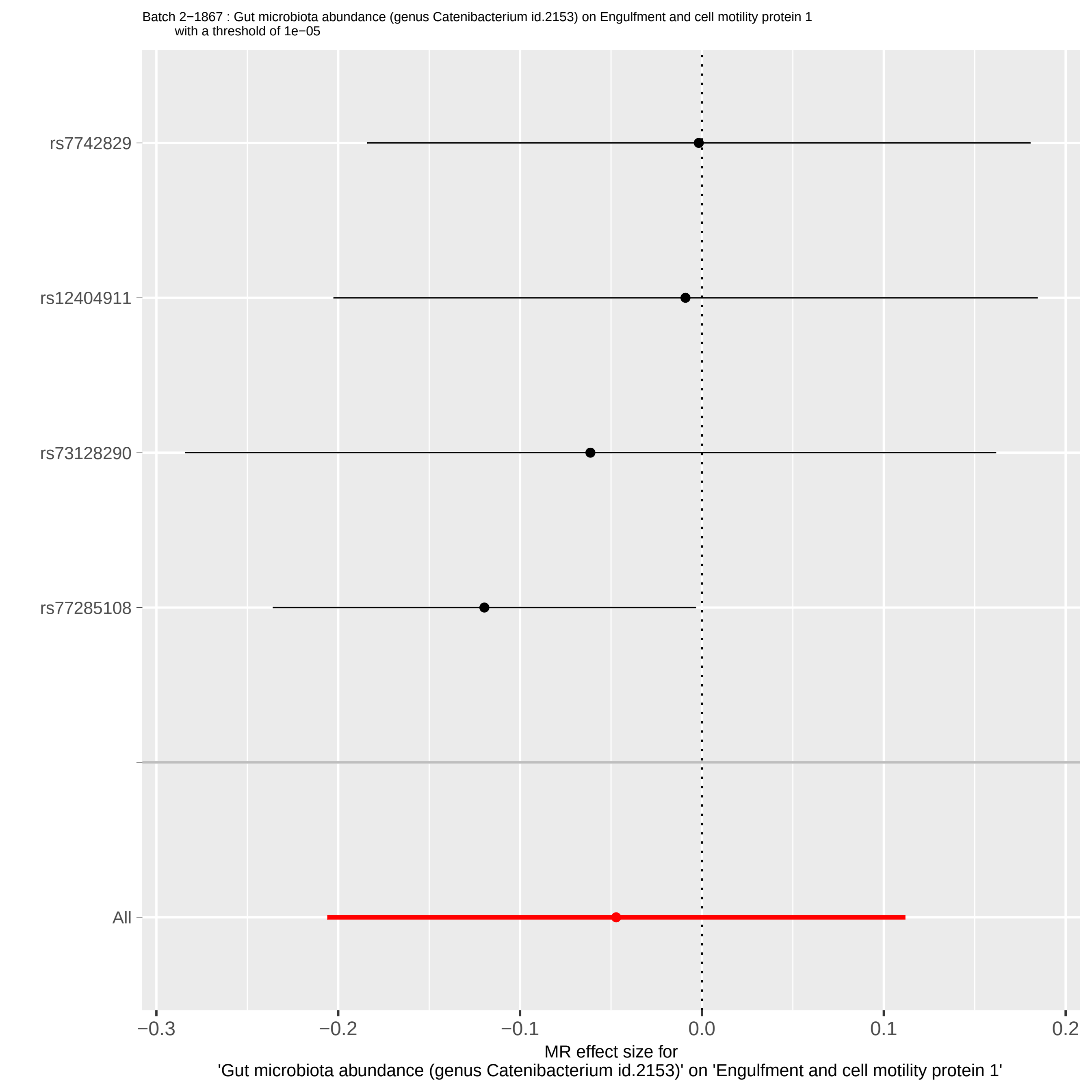

Batch 2−1867 : Gut microbiota abundance (genus Catenibacterium id.2153) on Engulfment and cell motility protein 1
with a threshold of 1e−05
rs7742829
rs12404911
rs73128290
rs77285108
All
−0.3
−0.2
−0.1
0.0
0.1
0.2
MR effect size for
'Gut microbiota abundance (genus Catenibacterium id.2153)' on 'Engulfment and cell motility protein 1'

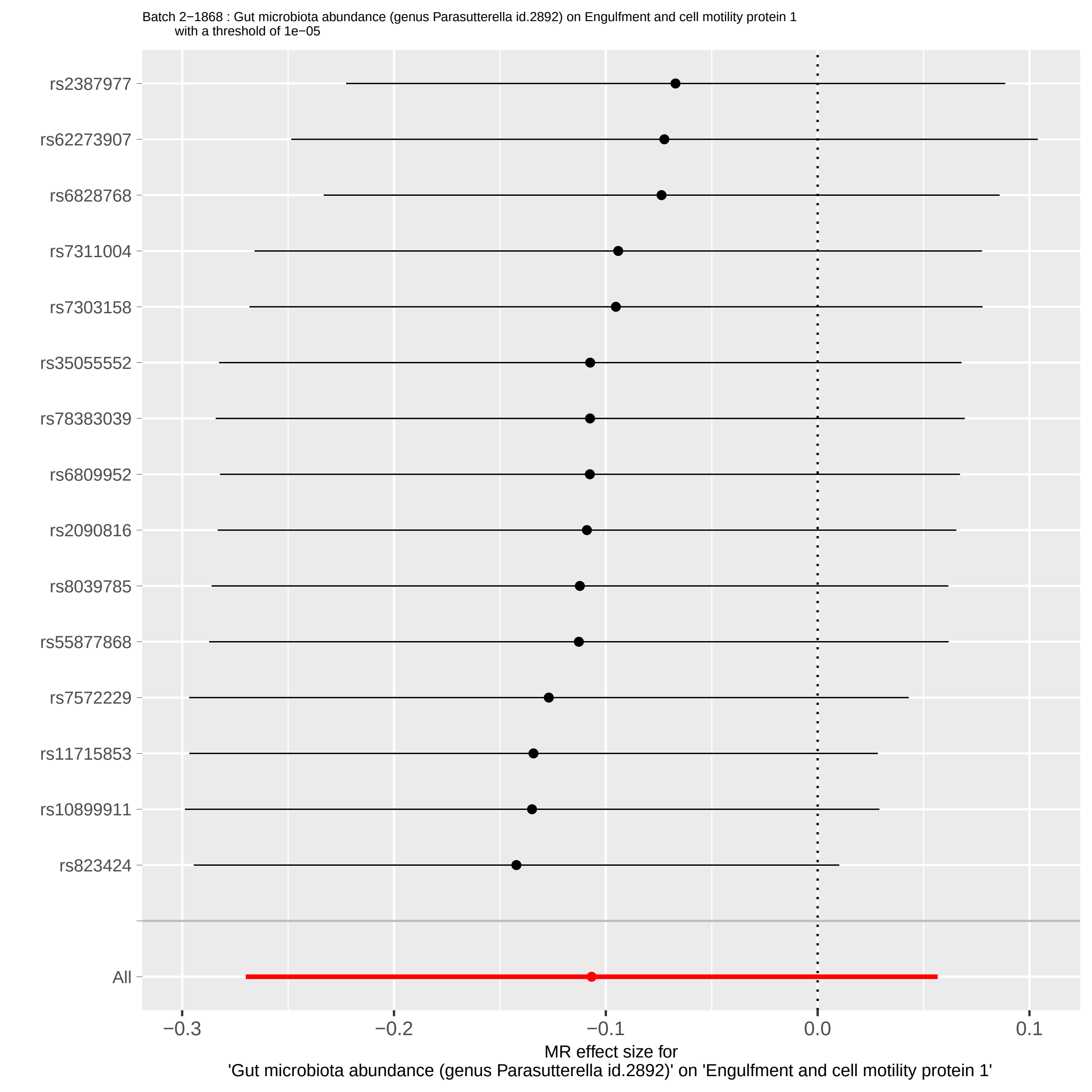

Batch 2−1868 : Gut microbiota abundance (genus Parasutterella id.2892) on Engulfment and cell motility protein 1
with a threshold of 1e−05
rs2387977
rs62273907
rs6828768
rs7311004
rs7303158
rs35055552
rs78383039
rs6809952
rs2090816
rs8039785
rs55877868
rs7572229
rs11715853
rs10899911
rs823424
All
−0.3
−0.2
−0.1
0.0
0.1
MR effect size for
'Gut microbiota abundance (genus Parasutterella id.2892)' on 'Engulfment and cell motility protein 1'

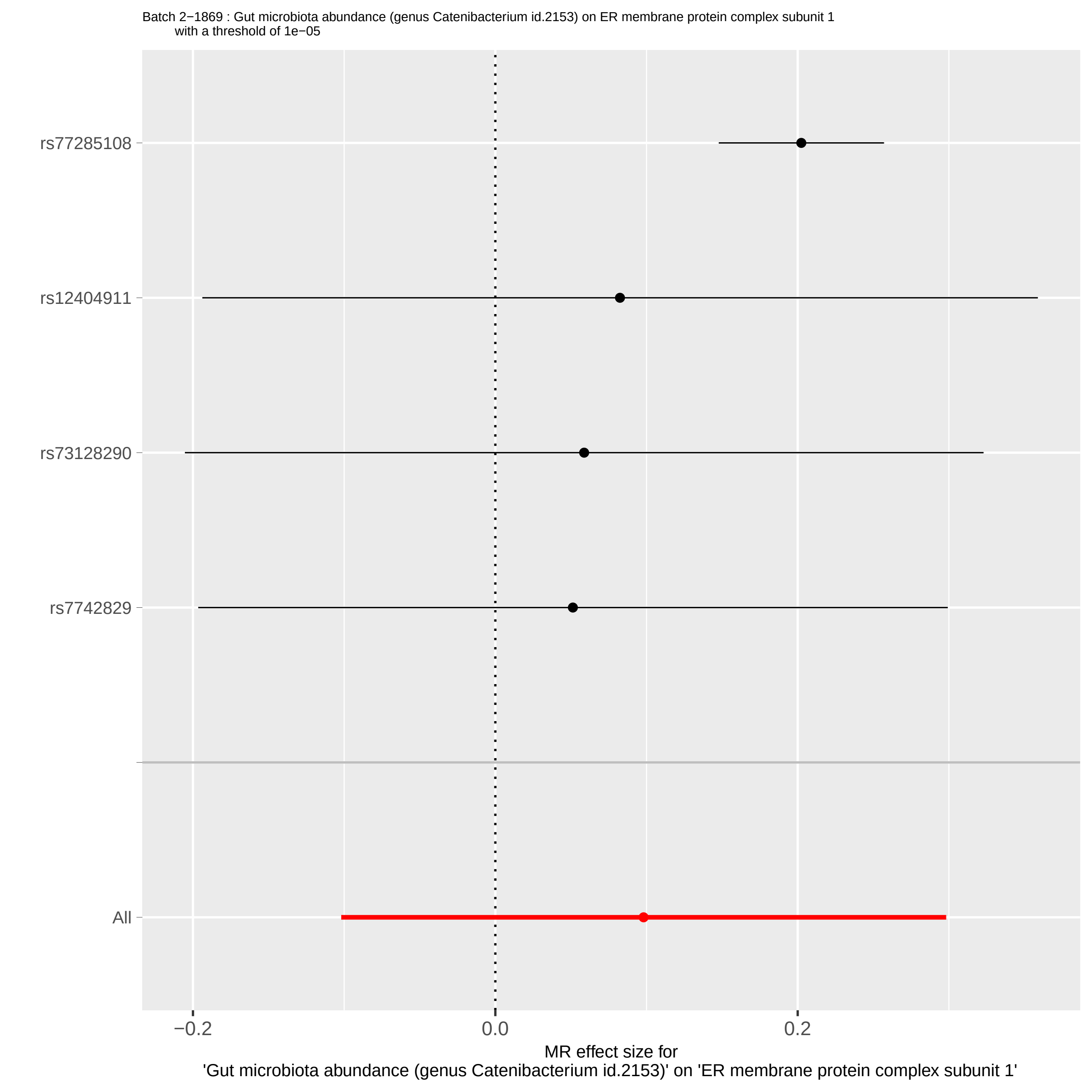

Batch 2−1869 : Gut microbiota abundance (genus Catenibacterium id.2153) on ER membrane protein complex subunit 1
with a threshold of 1e−05
rs77285108
rs12404911
rs73128290
rs7742829
All
−0.2
0.0
0.2
MR effect size for
'Gut microbiota abundance (genus Catenibacterium id.2153)' on 'ER membrane protein complex subunit 1'

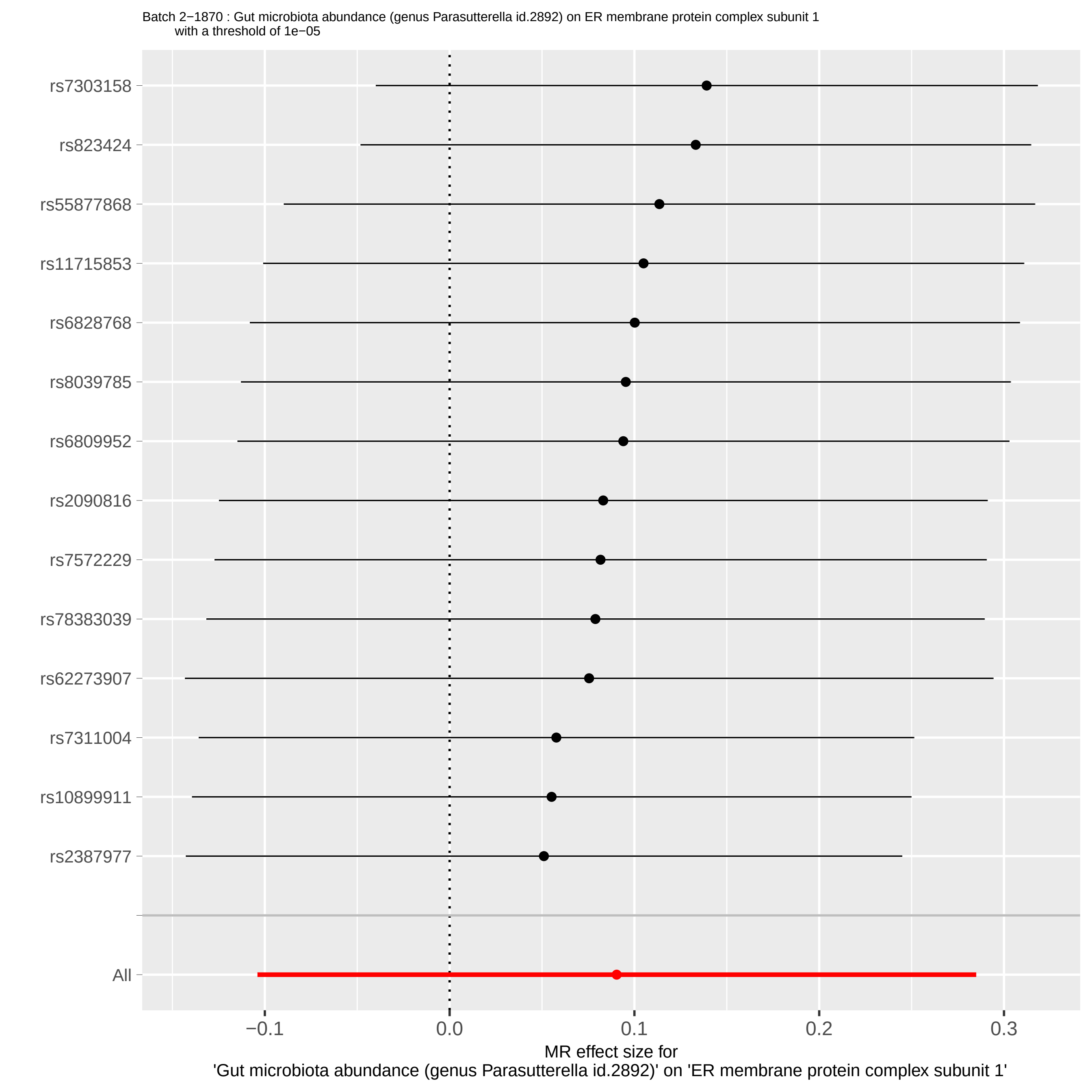

Batch 2−1870 : Gut microbiota abundance (genus Parasutterella id.2892) on ER membrane protein complex subunit 1
with a threshold of 1e−05
rs7303158
rs823424
rs55877868
rs11715853
rs6828768
rs8039785
rs6809952
rs2090816
rs7572229
rs78383039
rs62273907
rs7311004
rs10899911
rs2387977
All
−0.1
0.0
0.1
0.2
0.3
MR effect size for
'Gut microbiota abundance (genus Parasutterella id.2892)' on 'ER membrane protein complex subunit 1'

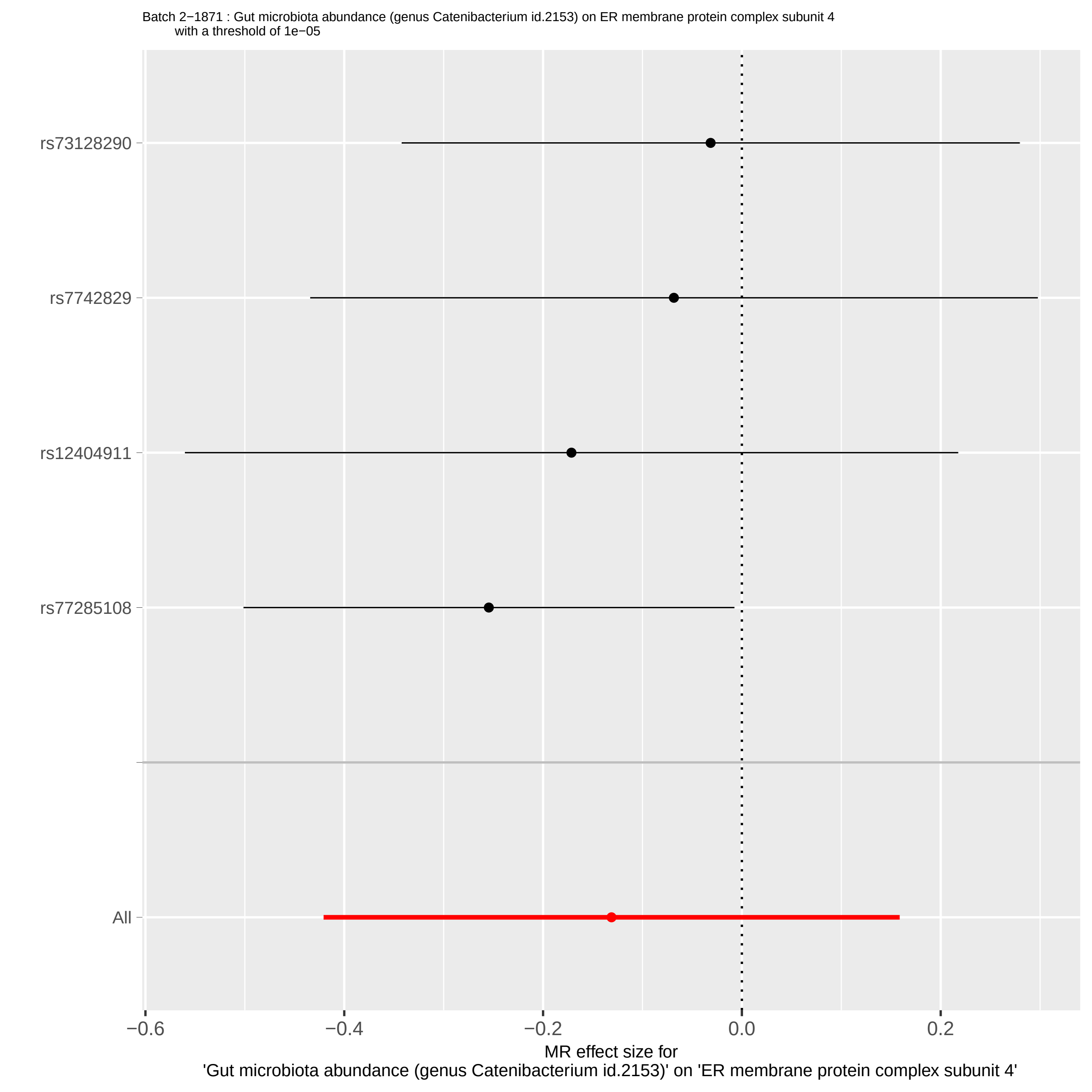
Batch 2−1871 : Gut microbiota abundance (genus Catenibacterium id.2153) on ER membrane protein complex subunit 4
with a threshold of 1e−05
rs73128290
rs7742829
rs12404911
rs77285108
All
−0.6
−0.4
−0.2
0.0
0.2
MR effect size for
'Gut microbiota abundance (genus Catenibacterium id.2153)' on 'ER membrane protein complex subunit 4'

Batch 2−1872 : Gut microbiota abundance (genus Parasutterella id.2892) on ER membrane protein complex subunit 4
with a threshold of 1e−05

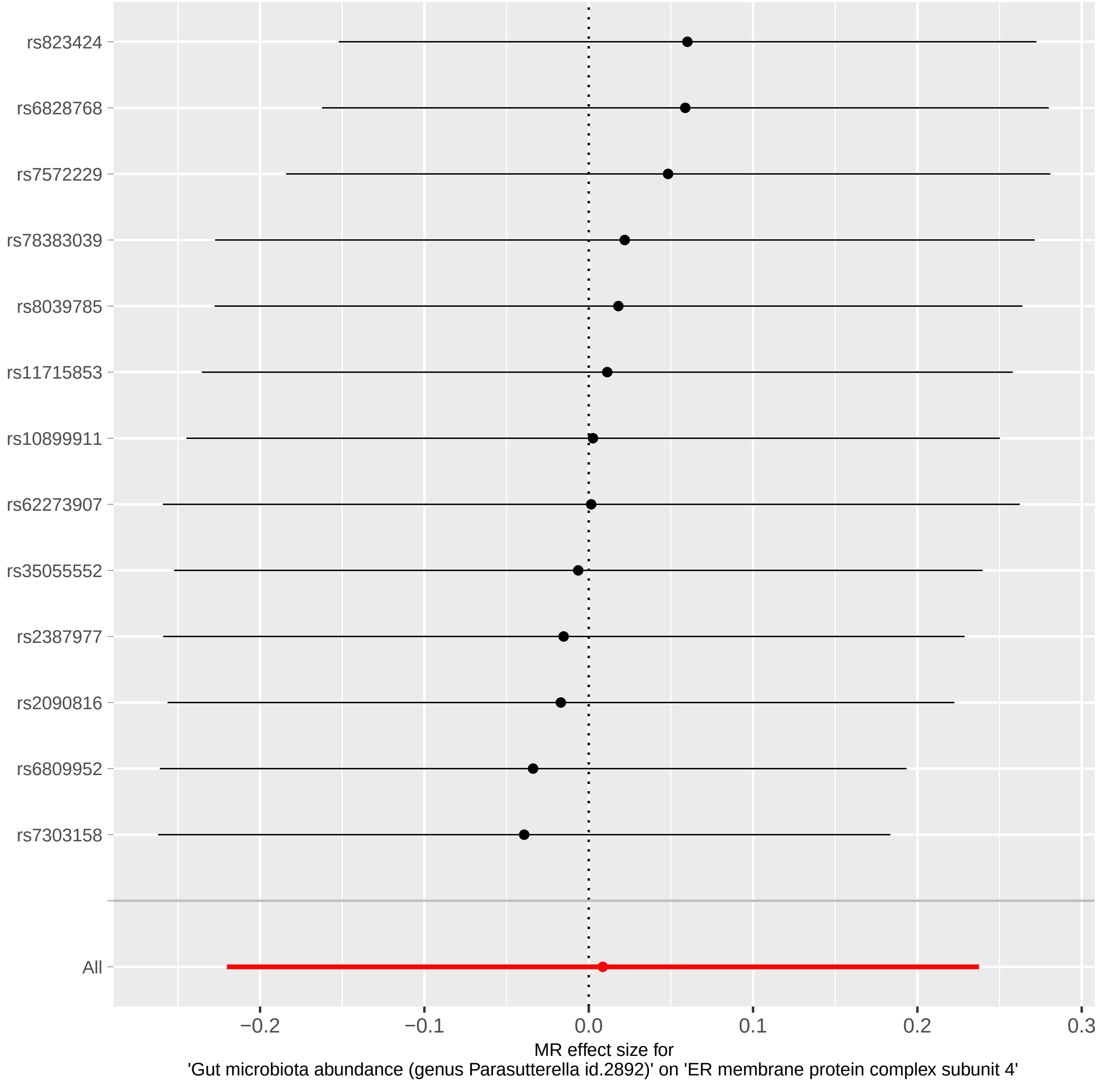

Batch 2−1873 : Gut microbiota abundance (genus Catenibacterium id.2153) on EMI domain−containing protein 1
with a threshold of 1e−05

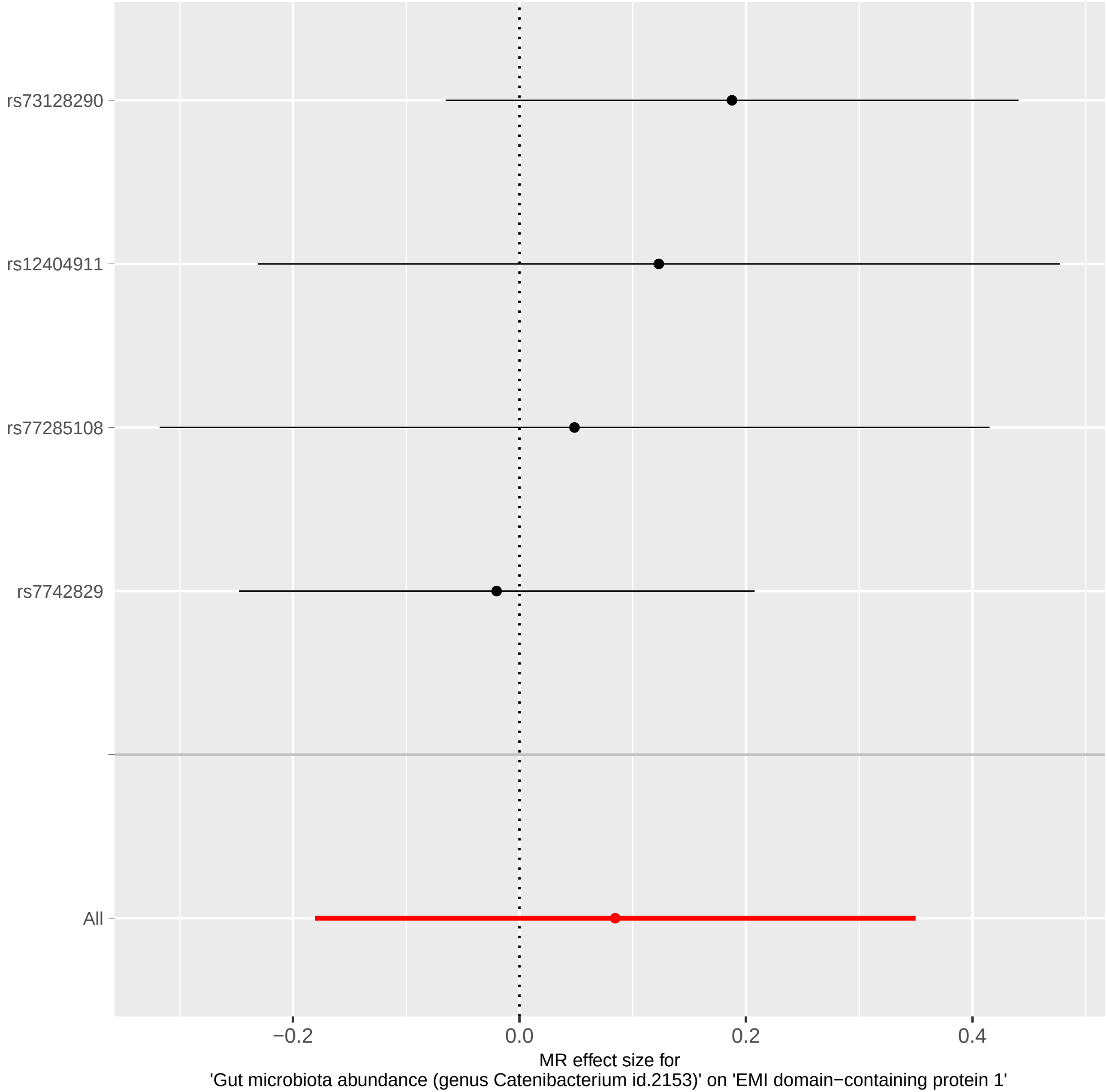

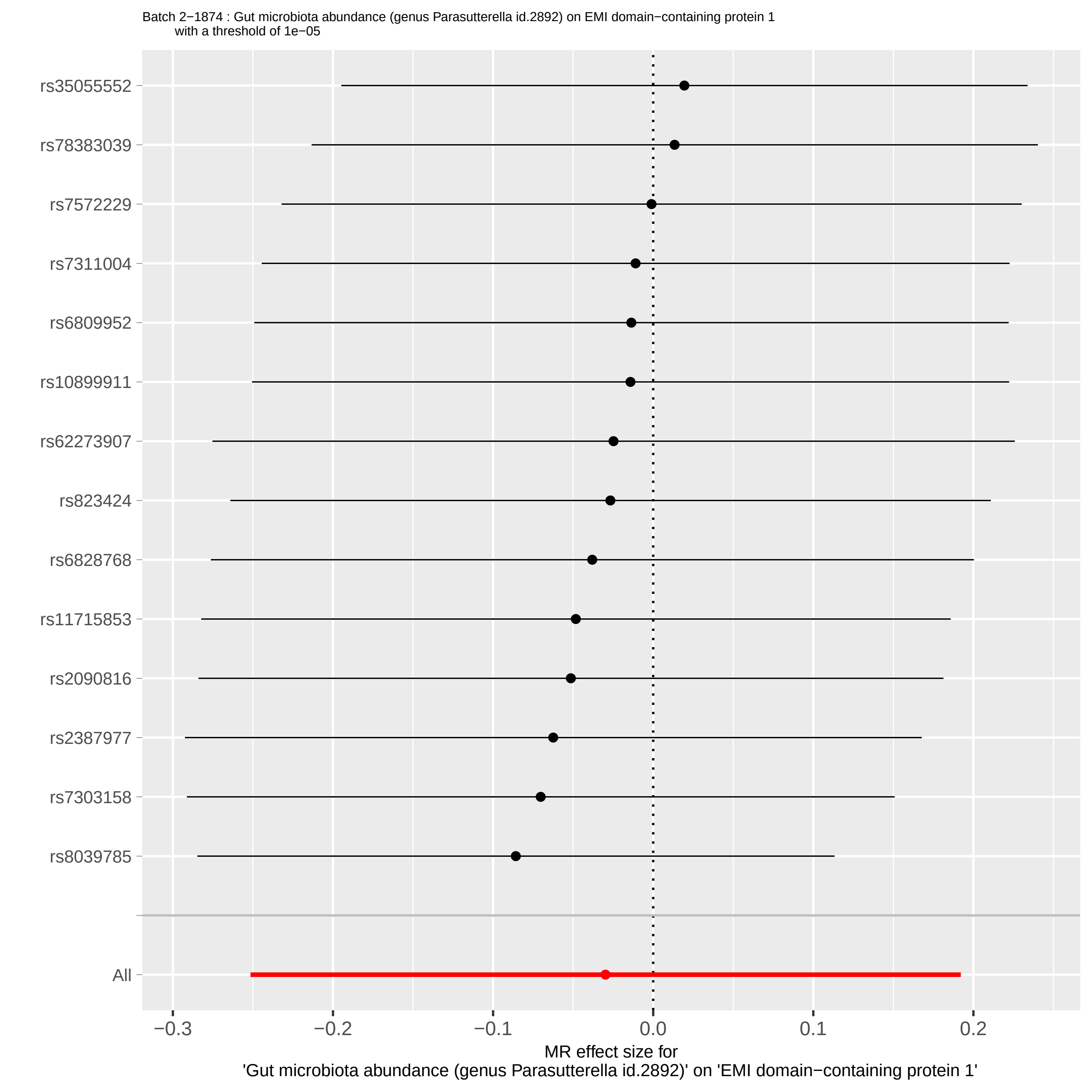

Batch 2−1874 : Gut microbiota abundance (genus Parasutterella id.2892) on EMI domain−containing protein 1
with a threshold of 1e−05
rs35055552
rs78383039
rs7572229
rs7311004
rs6809952
rs10899911
rs62273907
rs823424
rs6828768
rs11715853
rs2090816
rs2387977
rs7303158
rs8039785
All
−0.3
−0.2
−0.1
0.0
0.1
0.2
MR effect size for
'Gut microbiota abundance (genus Parasutterella id.2892)' on 'EMI domain−containing protein 1'

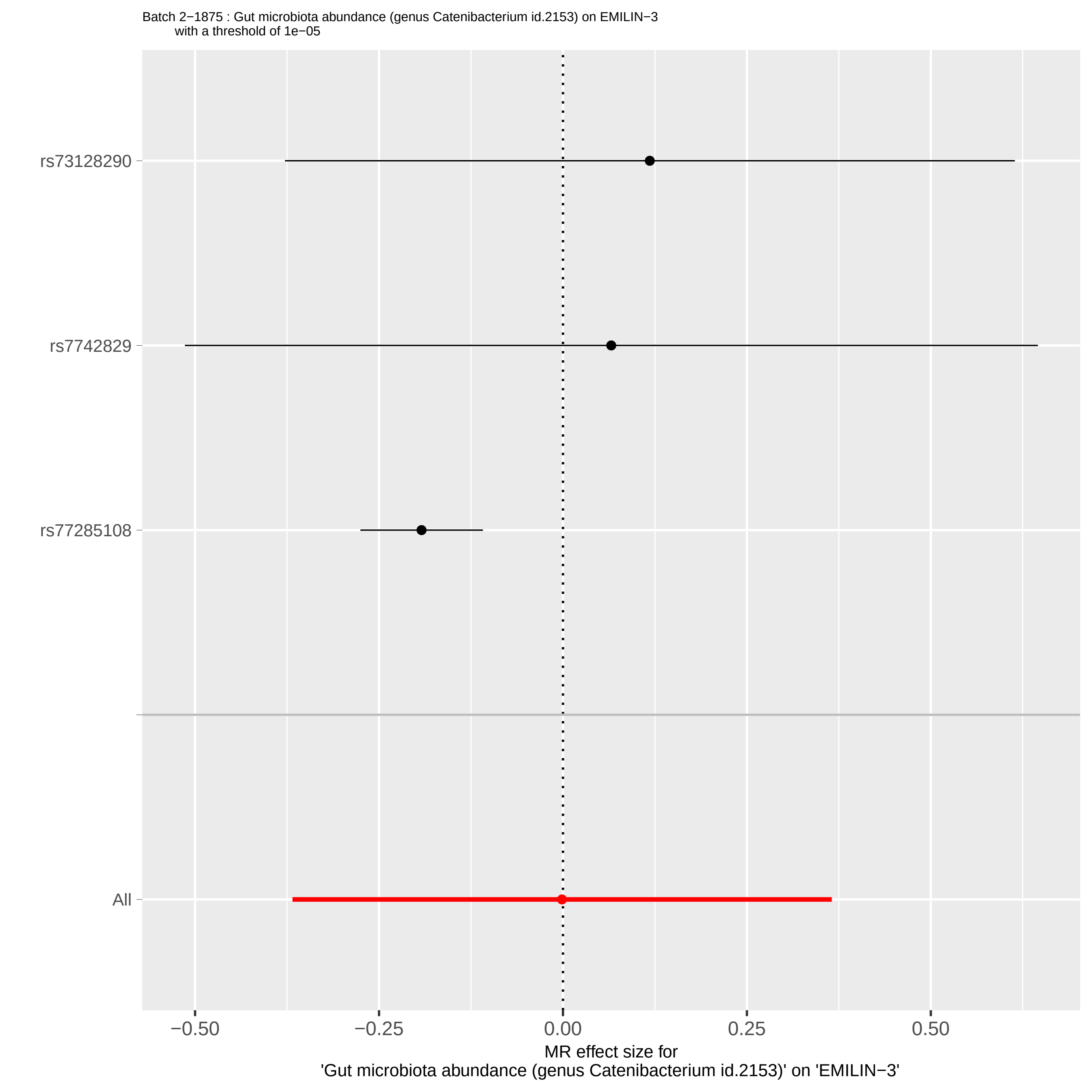
Batch 2−1875 : Gut microbiota abundance (genus Catenibacterium id.2153) on EMILIN−3
with a threshold of 1e−05
rs73128290
rs7742829
rs77285108
All
−0.50
−0.25
0.00
0.25
0.50
MR effect size for
'Gut microbiota abundance (genus Catenibacterium id.2153)' on 'EMILIN−3'

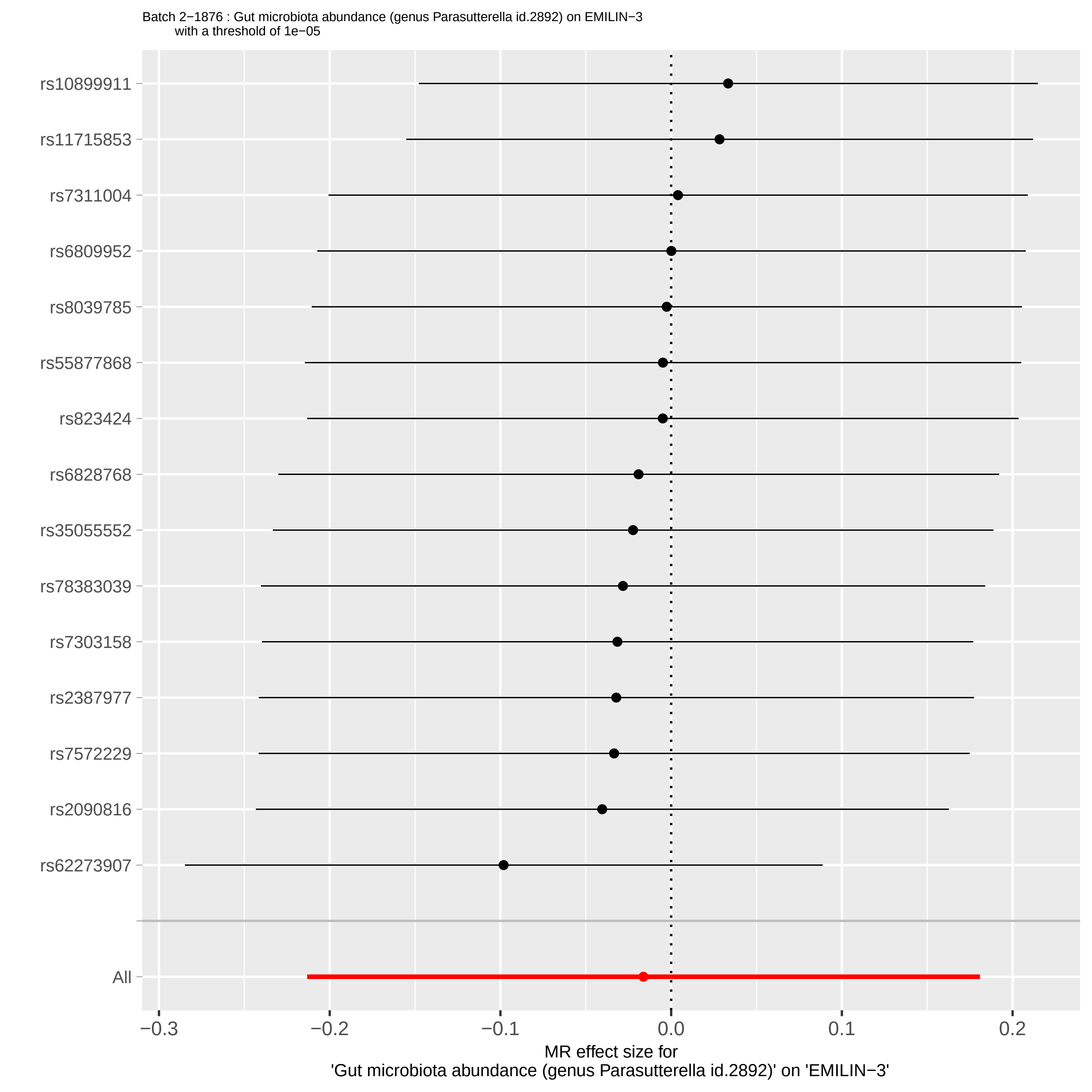
Batch 2−1876 : Gut microbiota abundance (genus Parasutterella id.2892) on EMILIN−3
with a threshold of 1e−05
rs10899911
rs11715853
rs7311004
rs6809952
rs8039785
rs55877868
rs823424
rs6828768
rs35055552
rs78383039
rs7303158
rs2387977
rs7572229
rs2090816
rs62273907
All
−0.3
−0.2
−0.1
0.0
0.1
0.2
MR effect size for
'Gut microbiota abundance (genus Parasutterella id.2892)' on 'EMILIN−3'

Batch 2−1877 : Gut microbiota abundance (genus Catenibacterium id.2153) on EMILIN−3
with a threshold of 1e−05

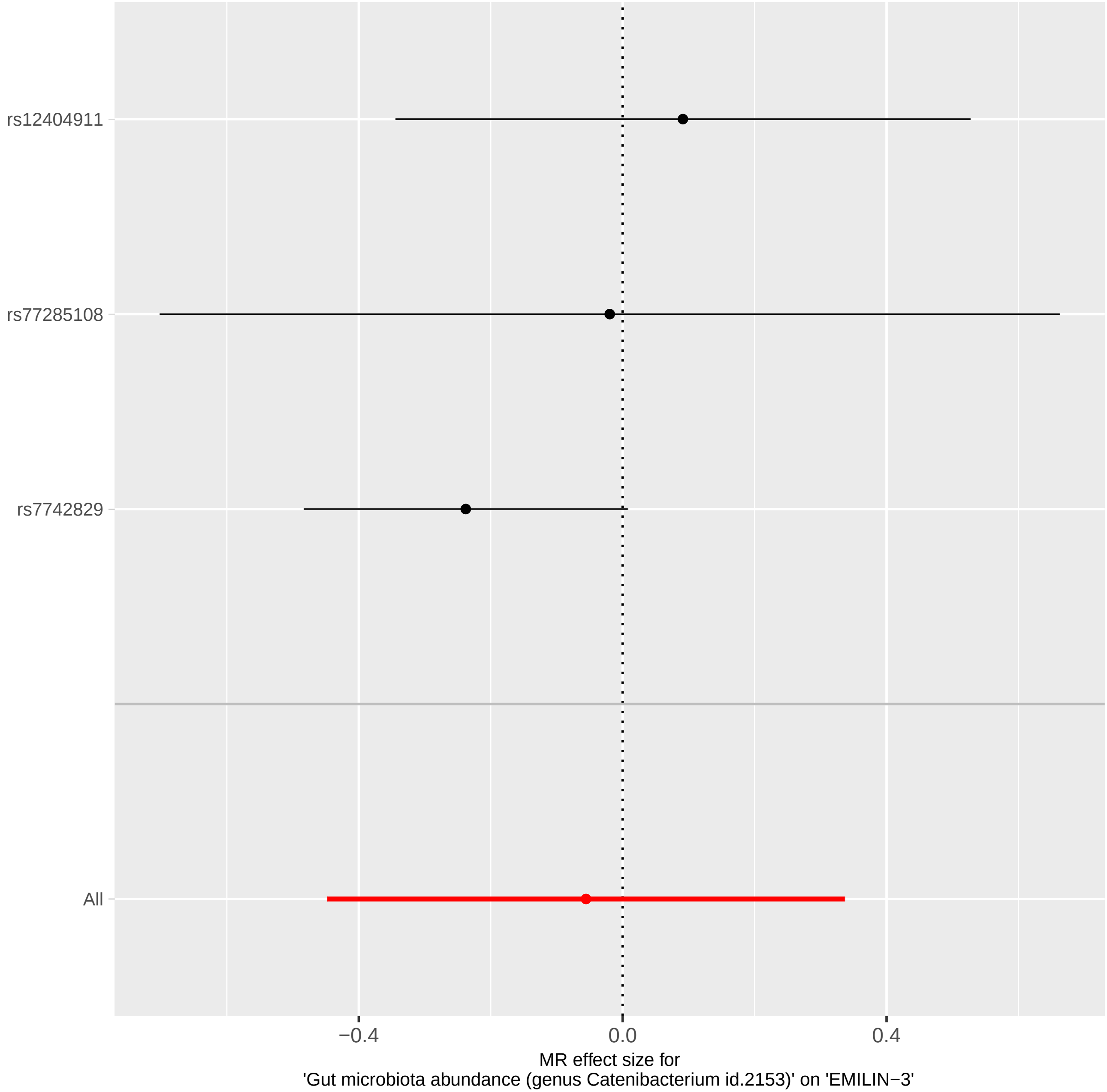

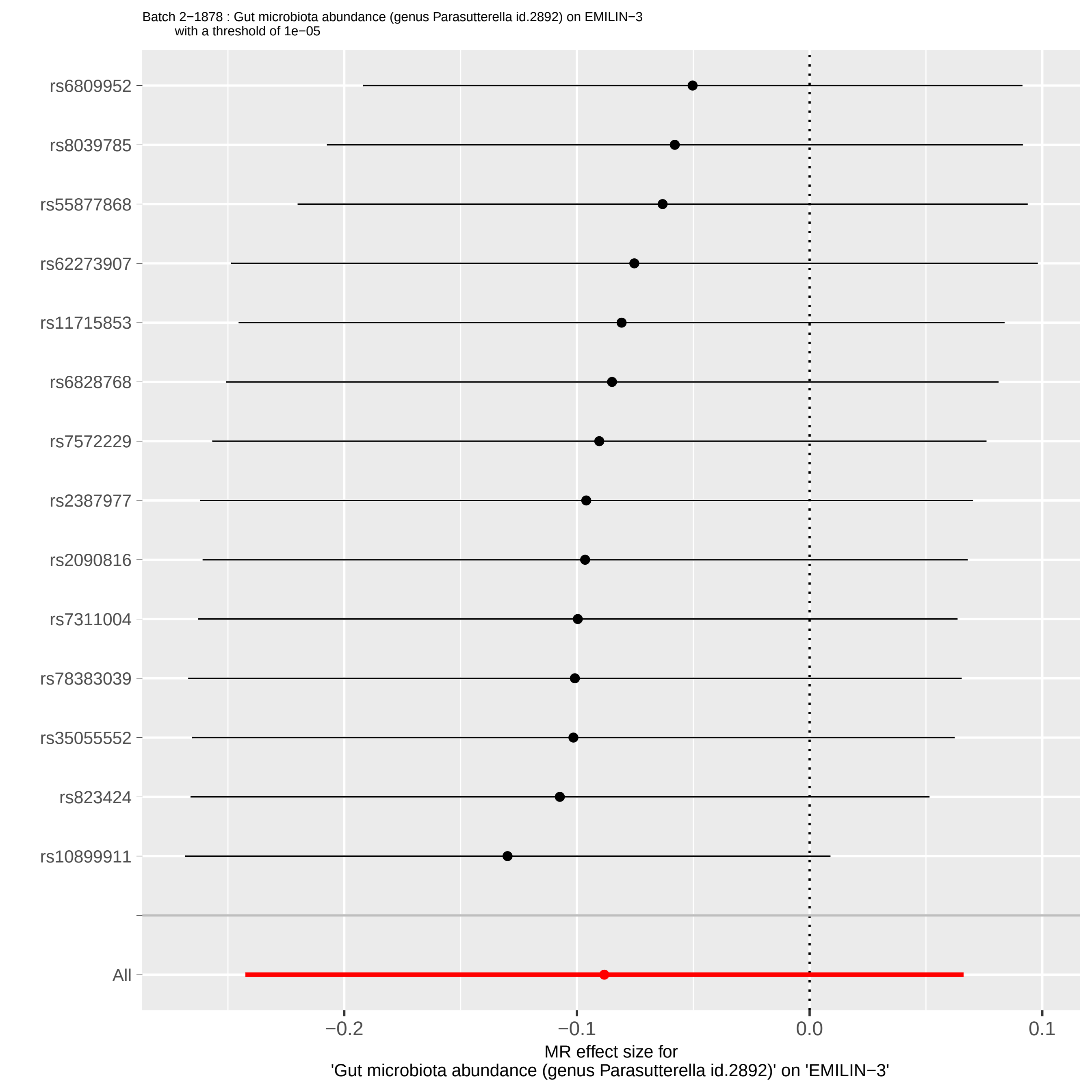

Batch 2−1878 : Gut microbiota abundance (genus Parasutterella id.2892) on EMILIN−3
with a threshold of 1e−05
rs6809952
rs8039785
rs55877868
rs62273907
rs11715853
rs6828768
rs7572229
rs2387977
rs2090816
rs7311004
rs78383039
rs35055552
rs823424
rs10899911
All
−0.2
−0.1
0.0
0.1
MR effect size for
'Gut microbiota abundance (genus Parasutterella id.2892)' on 'EMILIN−3'

Batch 2−1879 : Gut microbiota abundance (genus Catenibacterium id.2153) on EMILIN−3
with a threshold of 1e−05

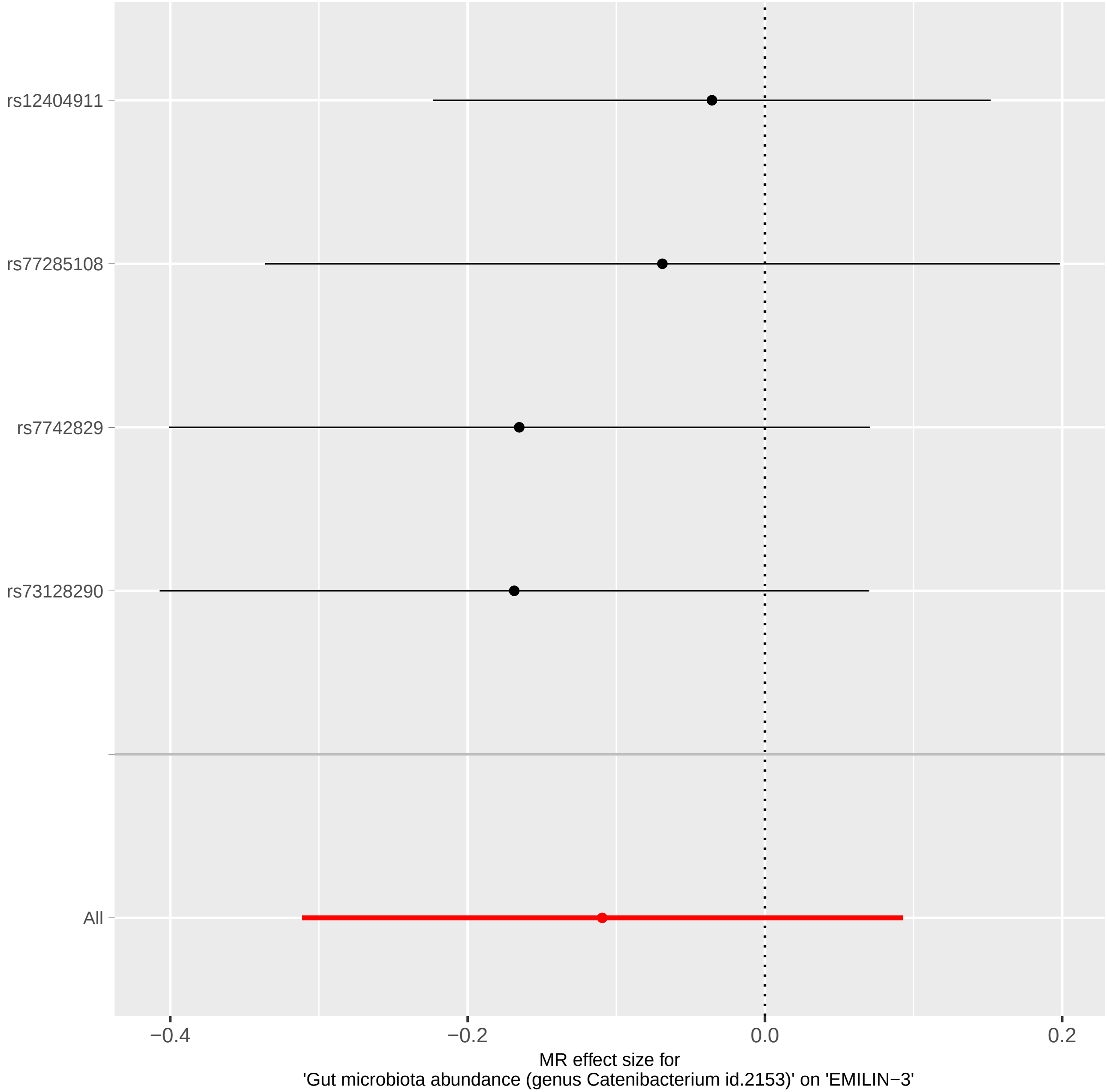

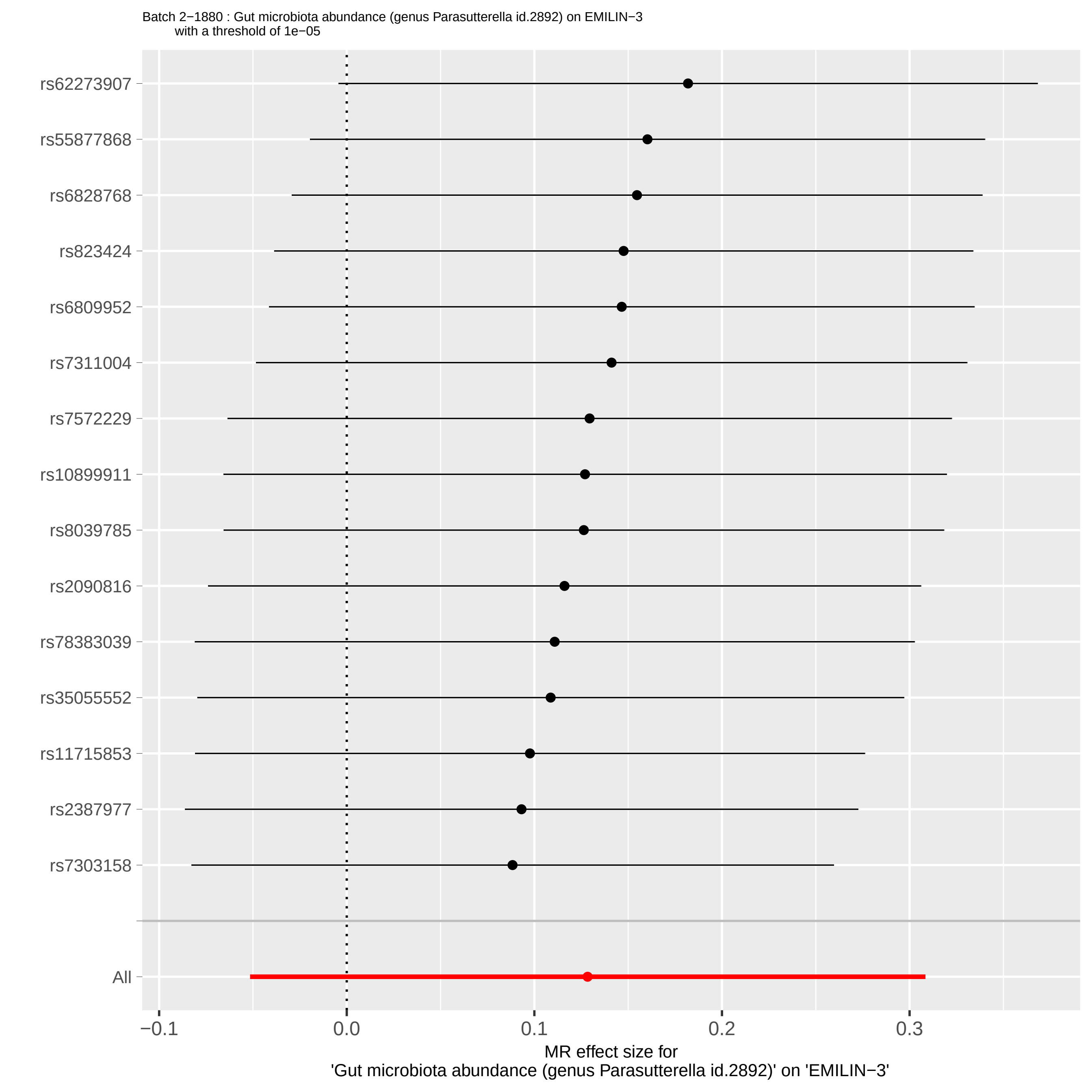

Batch 2−1880 : Gut microbiota abundance (genus Parasutterella id.2892) on EMILIN−3
with a threshold of 1e−05
rs62273907
rs55877868
rs6828768
rs823424
rs6809952
rs7311004
rs7572229
rs10899911
rs8039785
rs2090816
rs78383039
rs35055552
rs11715853
rs2387977
rs7303158
All
−0.1
0.0
0.1
0.2
0.3
MR effect size for
'Gut microbiota abundance (genus Parasutterella id.2892)' on 'EMILIN−3'

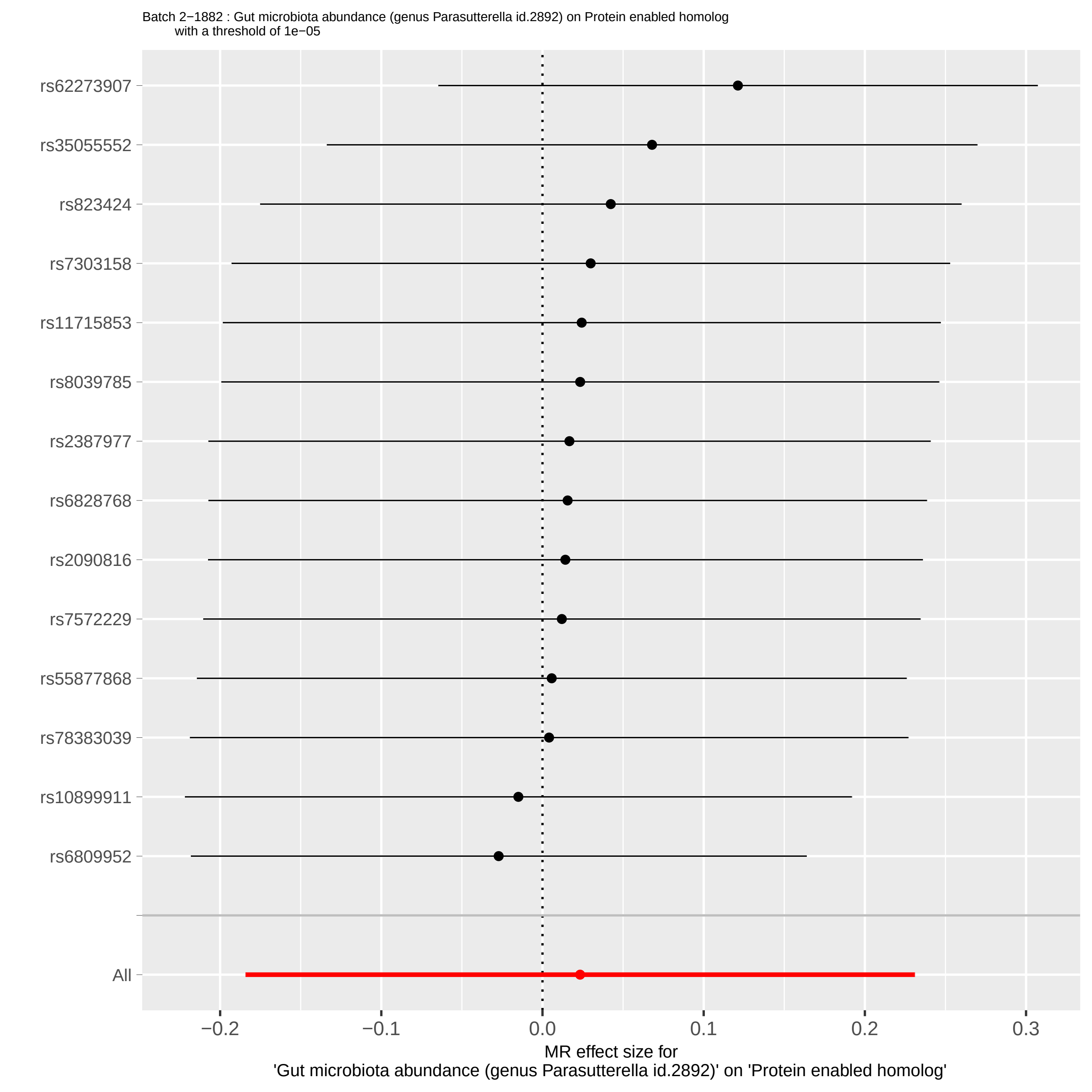
Batch 2−1882 : Gut microbiota abundance (genus Parasutterella id.2892) on Protein enabled homolog
with a threshold of 1e−05
rs62273907
rs35055552
rs823424
rs7303158
rs11715853
rs8039785
rs2387977
rs6828768
rs2090816
rs7572229
rs55877868
rs78383039
rs10899911
rs6809952
All
−0.2
−0.1
0.0
0.1
0.2
0.3
MR effect size for
'Gut microbiota abundance (genus Parasutterella id.2892)' on 'Protein enabled homolog'

Batch 2−1883 : Gut microbiota abundance (genus Catenibacterium id.2153) on Poly(U)−specific endoribonuclease
with a threshold of 1e−05

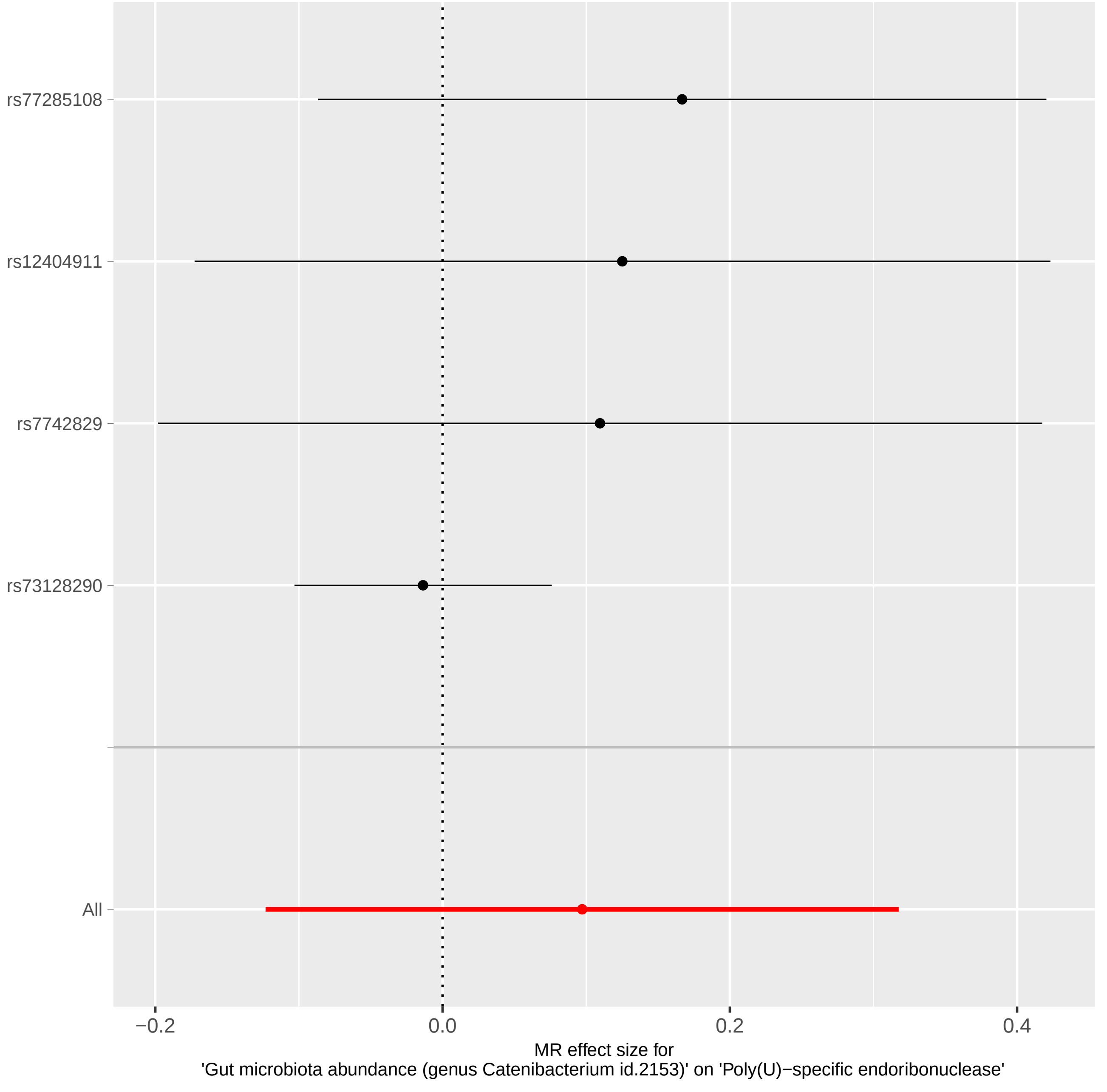

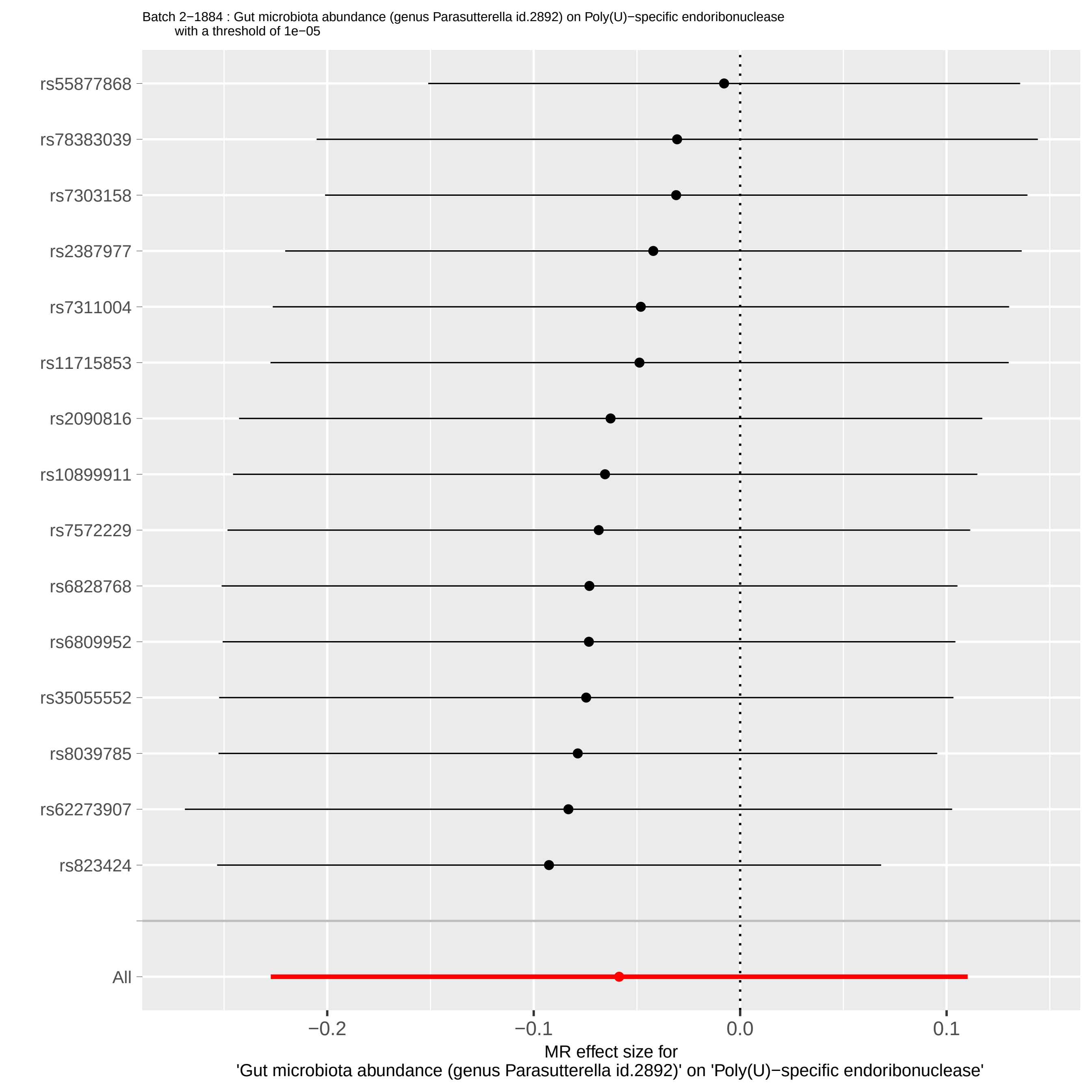

Batch 2−1884 : Gut microbiota abundance (genus Parasutterella id.2892) on Poly(U)−specific endoribonuclease
with a threshold of 1e−05
rs55877868
rs78383039
rs7303158
rs2387977
rs7311004
rs11715853
rs2090816
rs10899911
rs7572229
rs6828768
rs6809952
rs35055552
rs8039785
rs62273907
rs823424
All
−0.2
−0.1
0.0
0.1
MR effect size for
'Gut microbiota abundance (genus Parasutterella id.2892)' on 'Poly(U)−specific endoribonuclease'

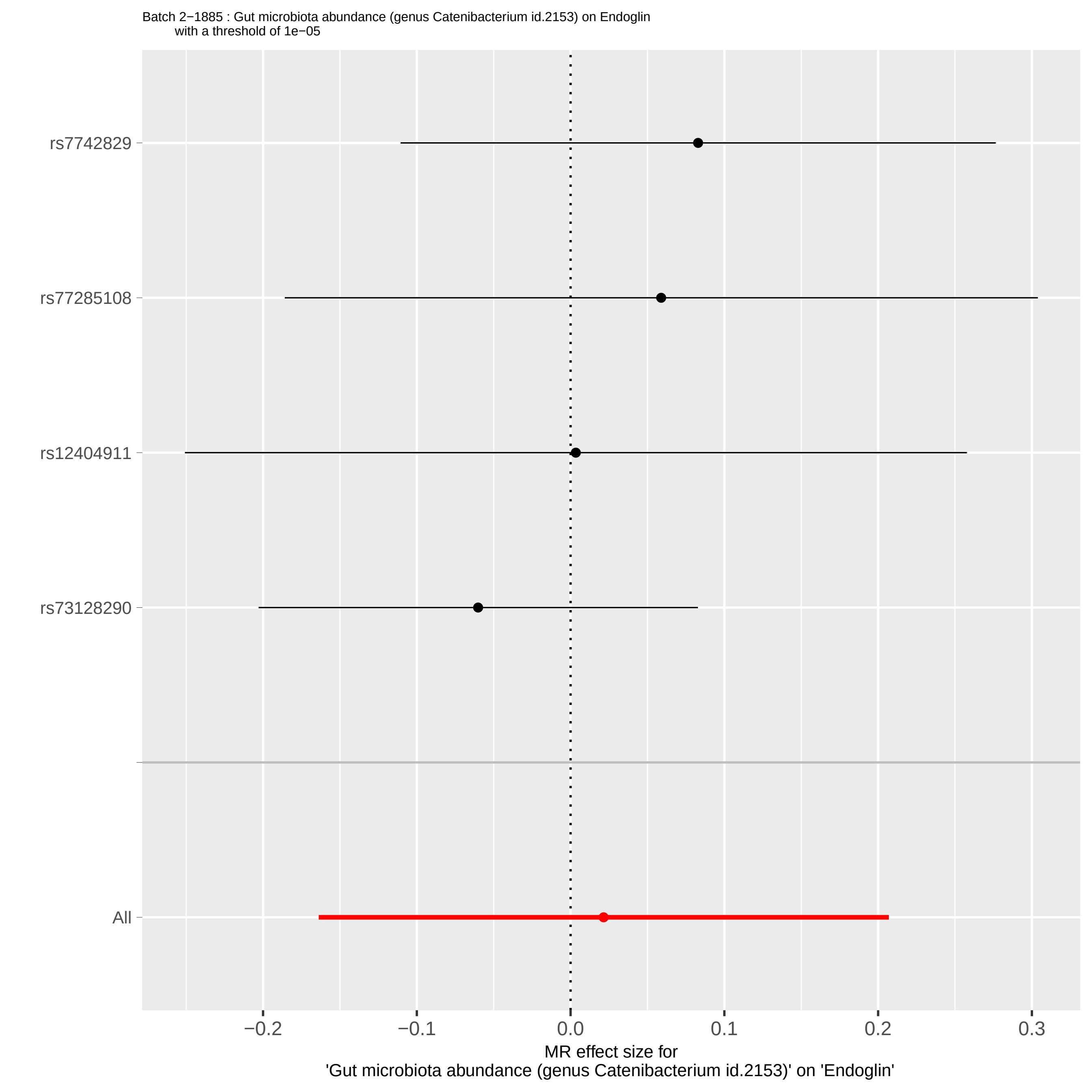

Batch 2−1885 : Gut microbiota abundance (genus Catenibacterium id.2153) on Endoglin
with a threshold of 1e−05
rs7742829
rs77285108
rs12404911
rs73128290
All
−0.2
−0.1
0.0
0.1
0.2
0.3
MR effect size for
'Gut microbiota abundance (genus Catenibacterium id.2153)' on 'Endoglin'

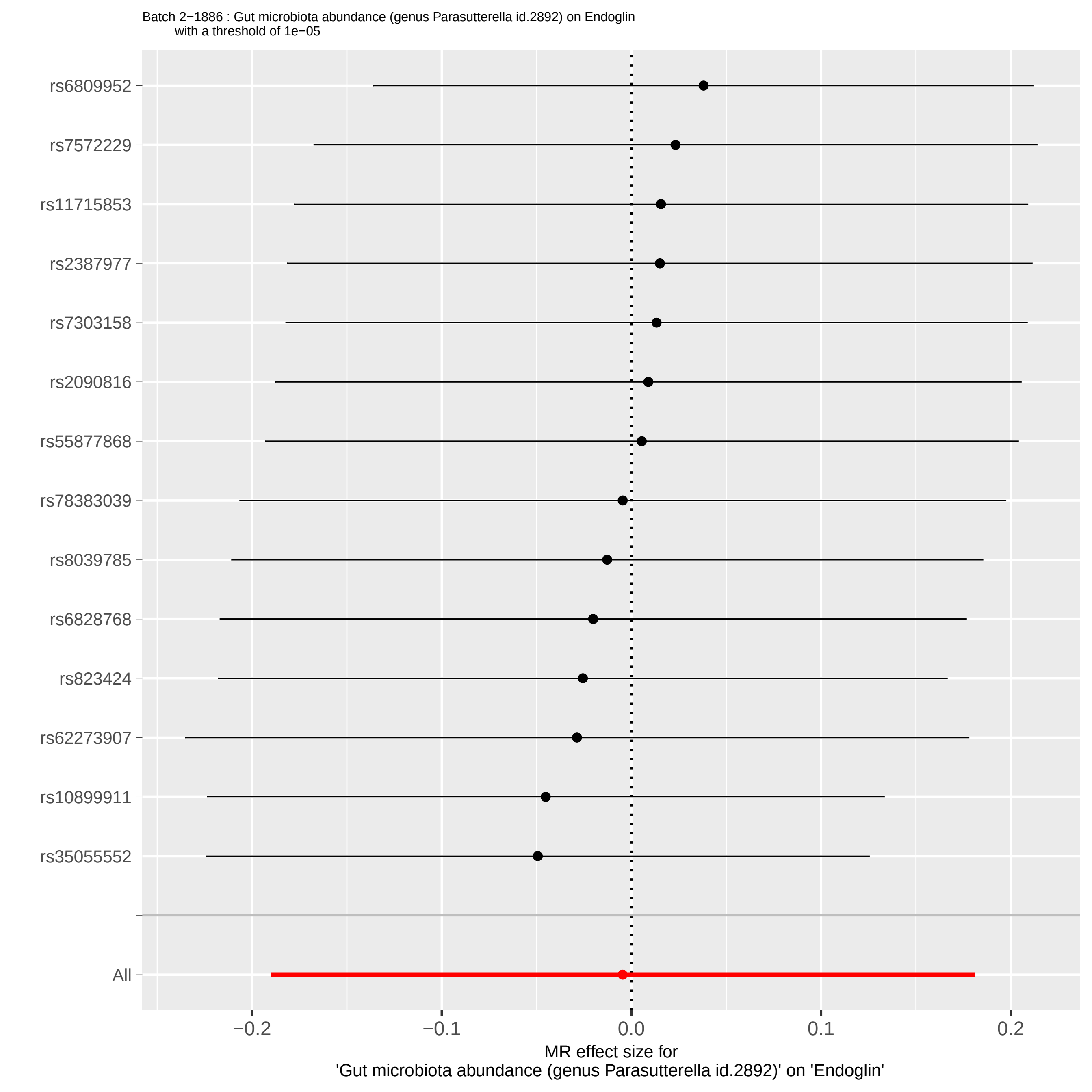
Batch 2−1886 : Gut microbiota abundance (genus Parasutterella id.2892) on Endoglin
with a threshold of 1e−05
rs6809952
rs7572229
rs11715853
rs2387977
rs7303158
rs2090816
rs55877868
rs78383039
rs8039785
rs6828768
rs823424
rs62273907
rs10899911
rs35055552
All
−0.2
−0.1
0.0
0.1
0.2
MR effect size for
'Gut microbiota abundance (genus Parasutterella id.2892)' on 'Endoglin'

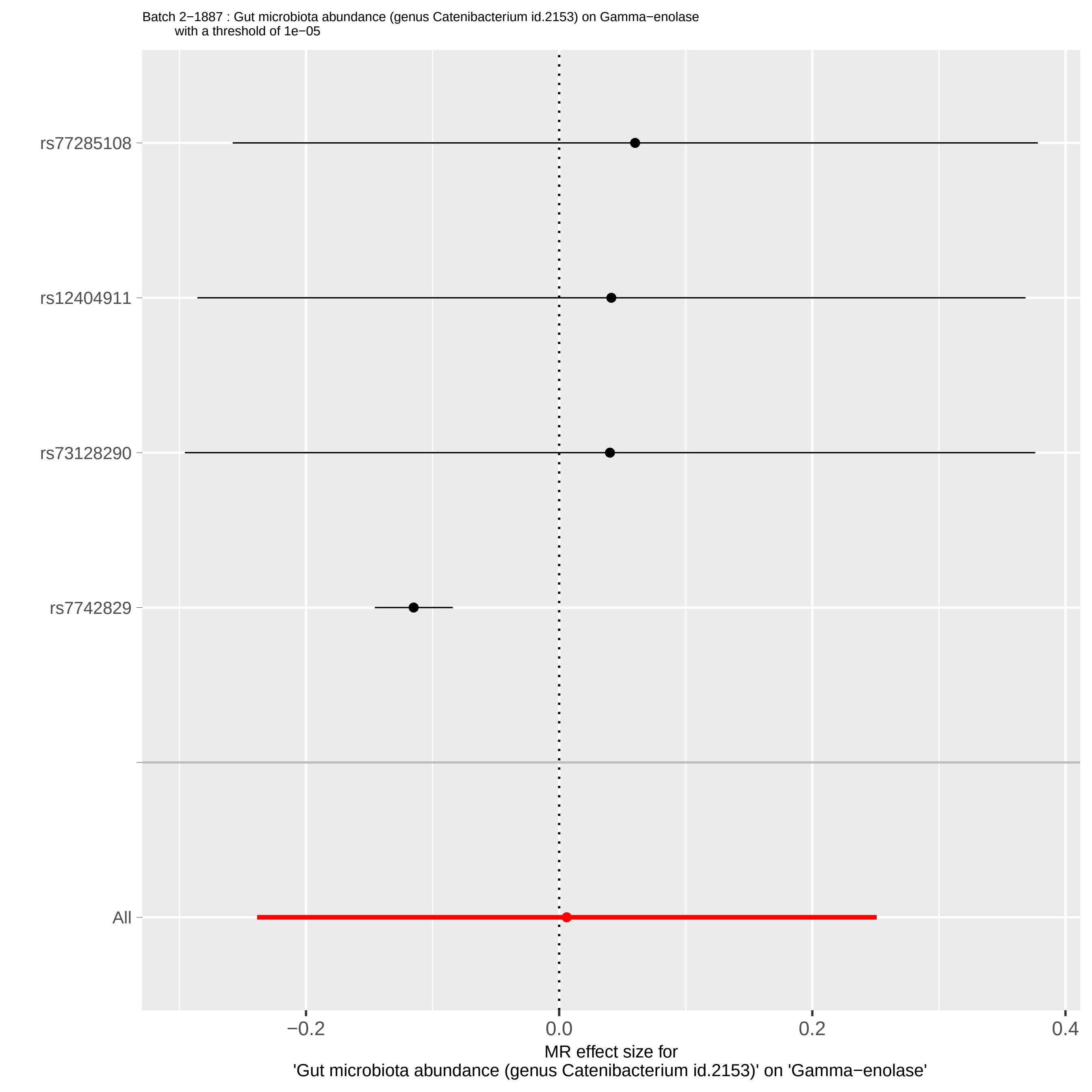
Batch 2−1887 : Gut microbiota abundance (genus Catenibacterium id.2153) on Gamma−enolase
with a threshold of 1e−05
rs77285108
rs12404911
rs73128290
rs7742829
All
−0.2
0.0
0.2
0.4
MR effect size for
'Gut microbiota abundance (genus Catenibacterium id.2153)' on 'Gamma−enolase'

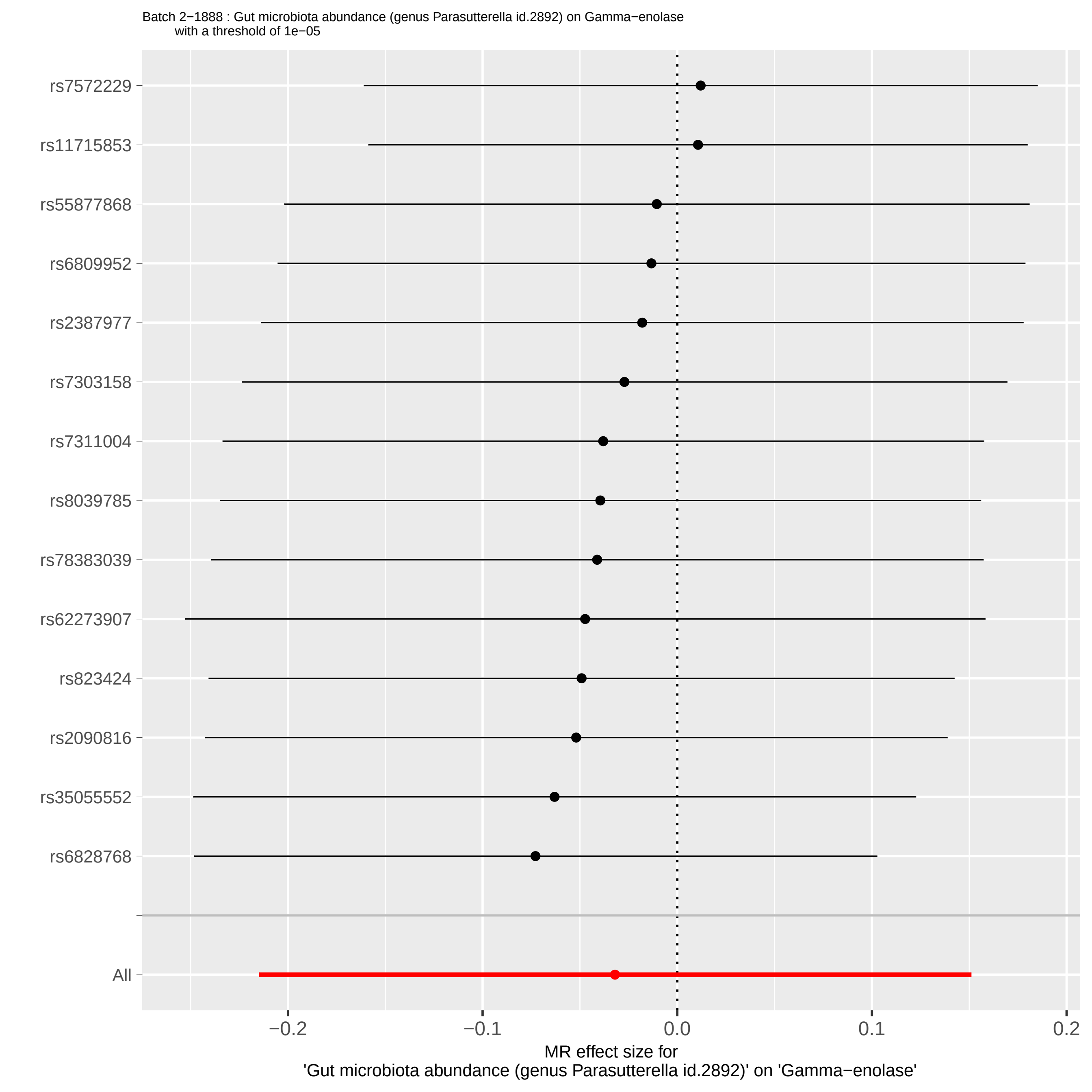

Batch 2−1888 : Gut microbiota abundance (genus Parasutterella id.2892) on Gamma−enolase
with a threshold of 1e−05
rs7572229
rs11715853
rs55877868
rs6809952
rs2387977
rs7303158
rs7311004
rs8039785
rs78383039
rs62273907
rs823424
rs2090816
rs35055552
rs6828768
All
−0.2
−0.1
0.0
0.1
0.2
MR effect size for
'Gut microbiota abundance (genus Parasutterella id.2892)' on 'Gamma−enolase'

Batch 2−1889 : Gut microbiota abundance (genus Catenibacterium id.2153) on Gamma−enolase
with a threshold of 1e−05

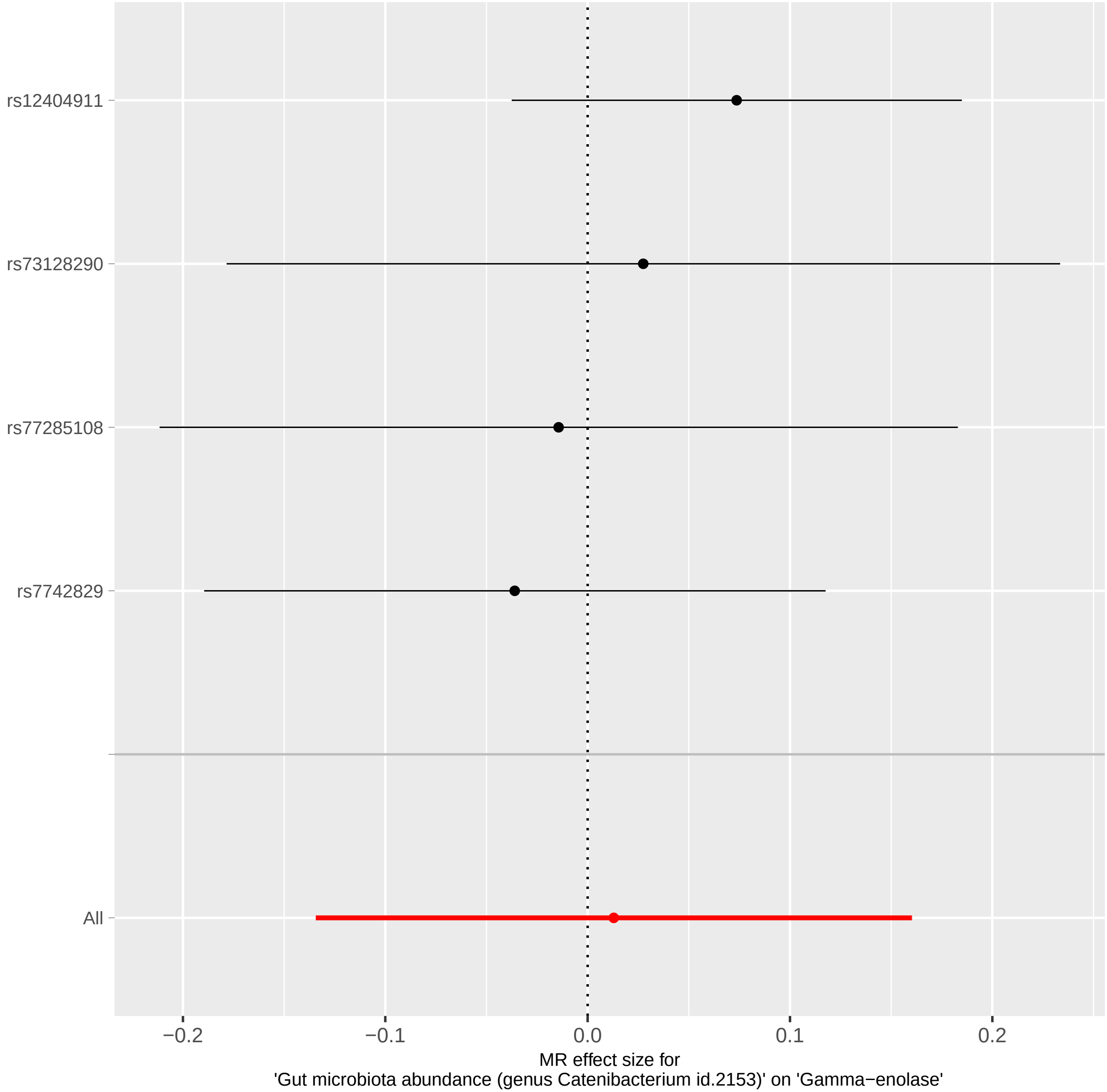

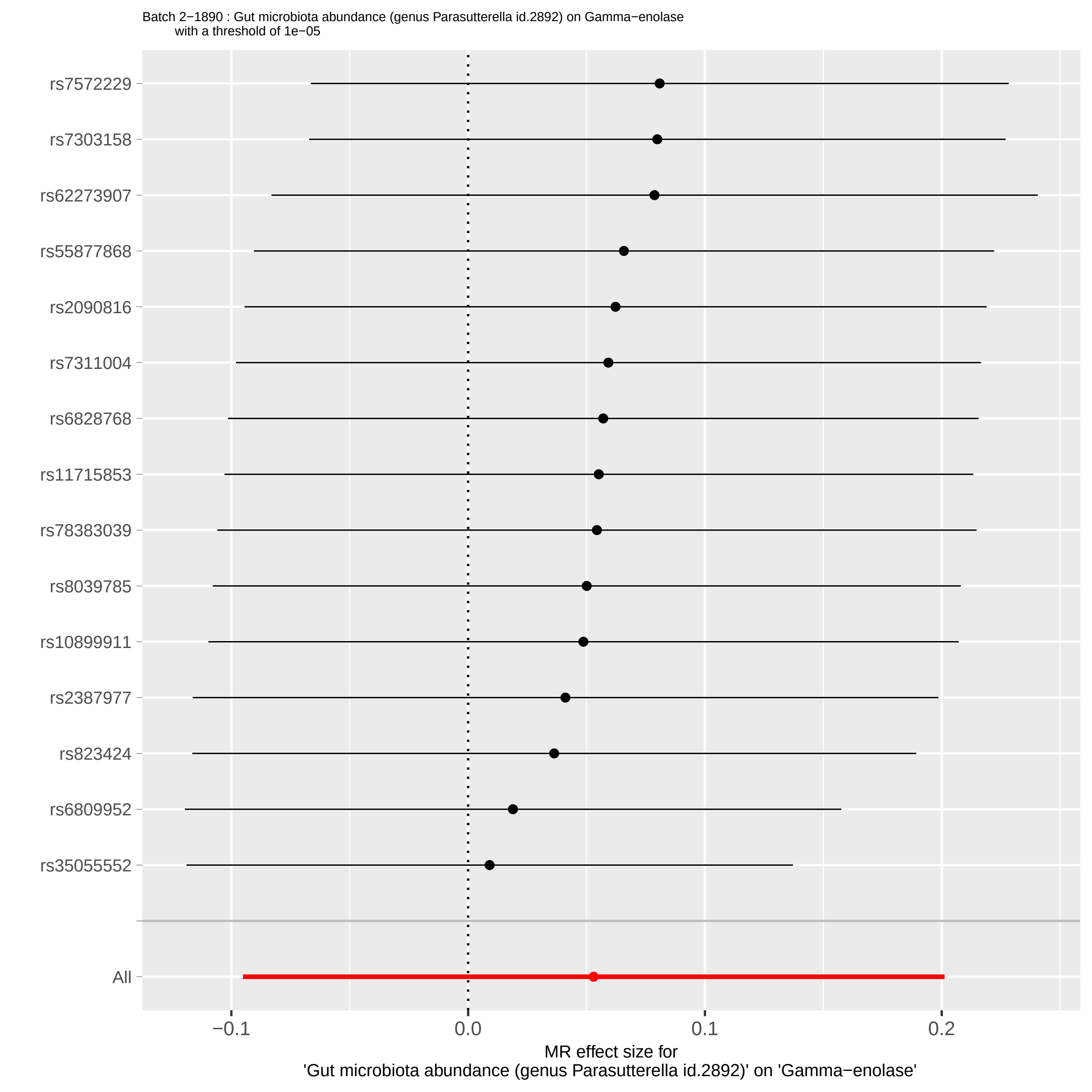
Batch 2−1890 : Gut microbiota abundance (genus Parasutterella id.2892) on Gamma−enolase
with a threshold of 1e−05
rs7572229
rs7303158
rs62273907
rs55877868
rs2090816
rs7311004
rs6828768
rs11715853
rs78383039
rs8039785
rs10899911
rs2387977
rs823424
rs6809952
rs35055552
All
−0.1
0.0
0.1
0.2
MR effect size for
'Gut microbiota abundance (genus Parasutterella id.2892)' on 'Gamma−enolase'

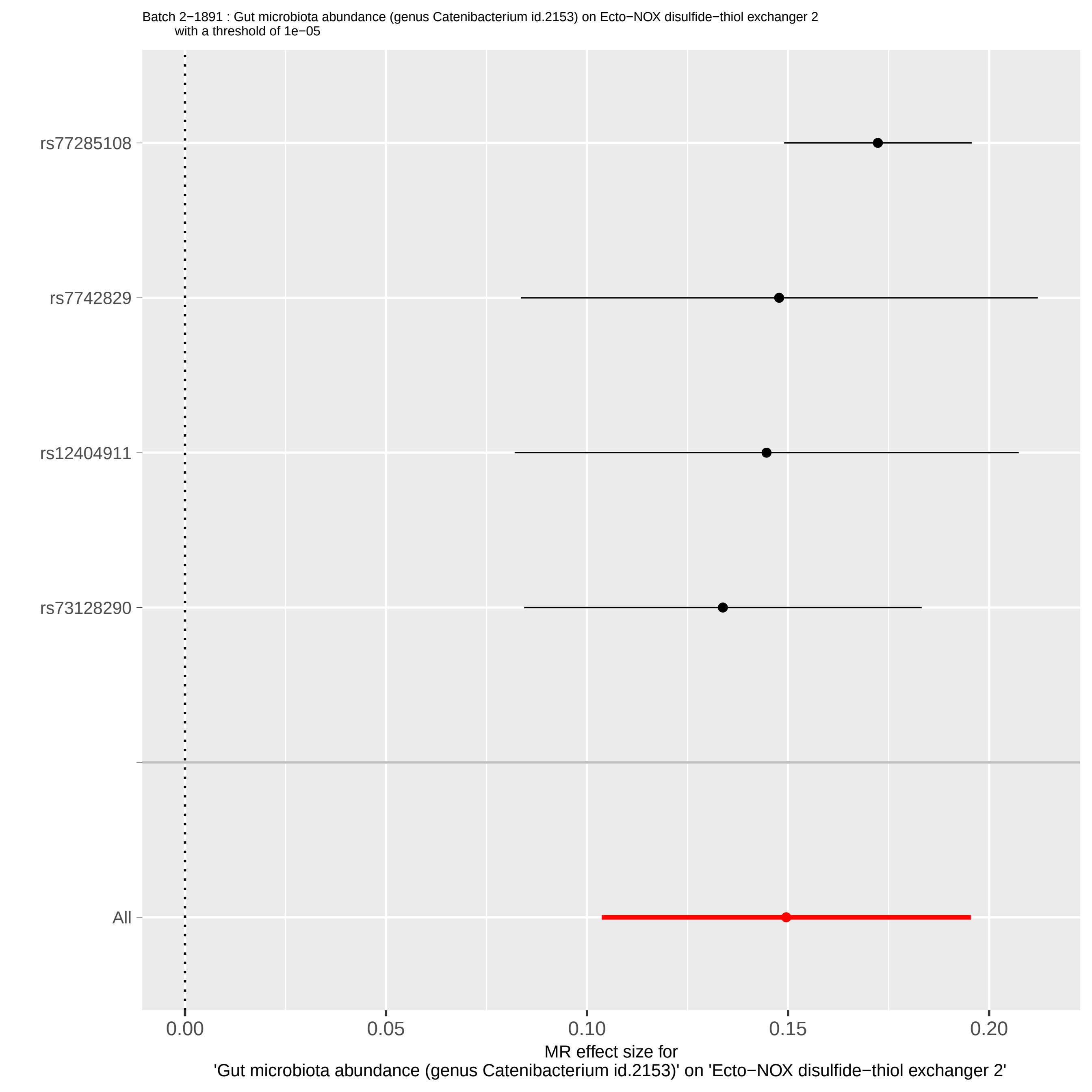
Batch 2−1891 : Gut microbiota abundance (genus Catenibacterium id.2153) on Ecto−NOX disulfide−thiol exchanger 2
with a threshold of 1e−05
rs77285108
rs7742829
rs12404911
rs73128290
All
0.00
0.05
0.10
0.15
0.20
MR effect size for
'Gut microbiota abundance (genus Catenibacterium id.2153)' on 'Ecto−NOX disulfide−thiol exchanger 2'

Batch 2−1892 : Gut microbiota abundance (genus Parasutterella id.2892) on Ecto−NOX disulfide−thiol exchanger 2
with a threshold of 1e−05

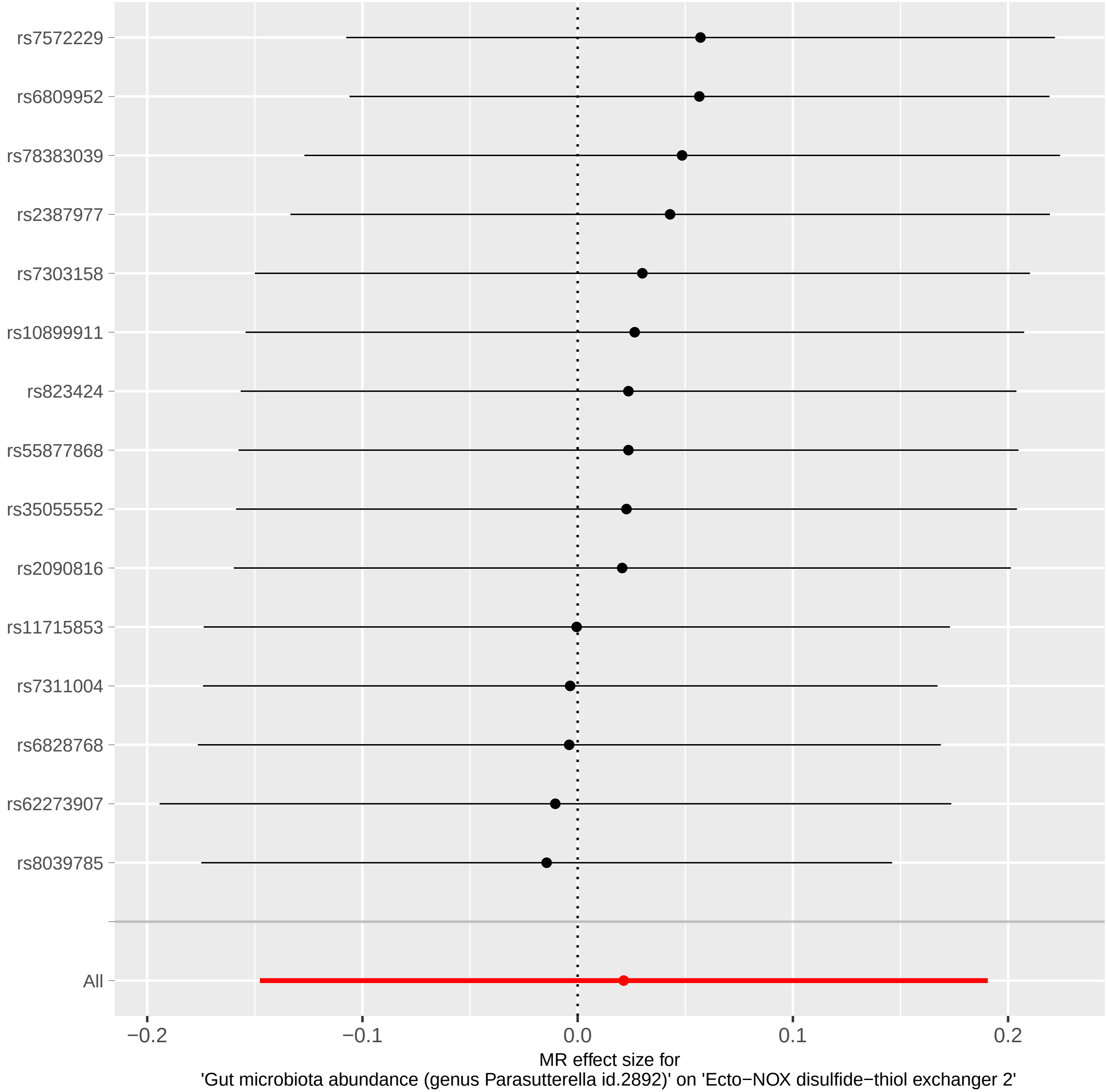

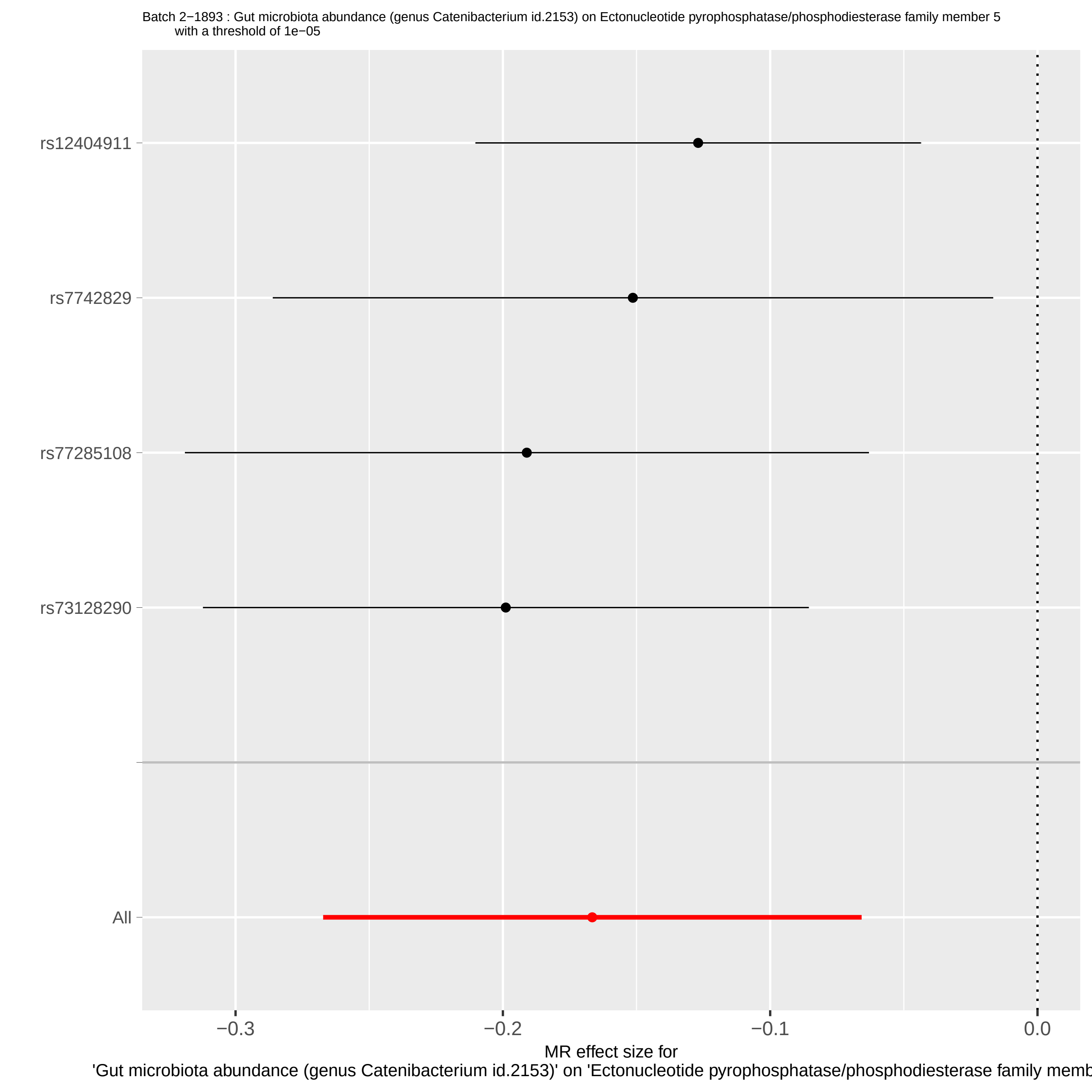
Batch 2−1893 : Gut microbiota abundance (genus Catenibacterium id.2153) on Ectonucleotide pyrophosphatase/phosphodiesterase family member 5
with a threshold of 1e−05
rs12404911
rs7742829
rs77285108
rs73128290
All
−0.3
−0.2
−0.1
0.0
MR effect size for
'Gut microbiota abundance (genus Catenibacterium id.2153)' on 'Ectonucleotide pyrophosphatase/phosphodiesterase family memb

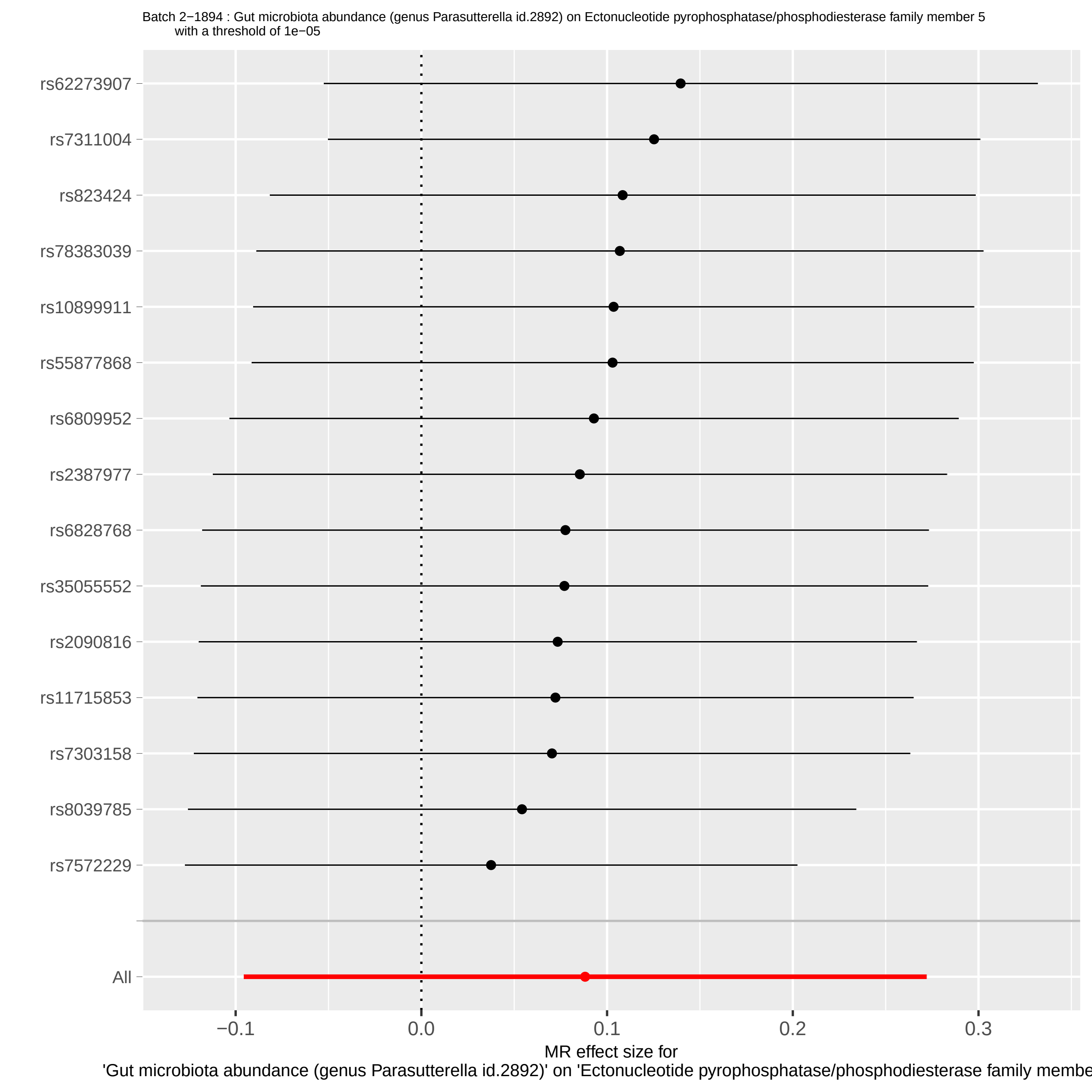
Batch 2−1894 : Gut microbiota abundance (genus Parasutterella id.2892) on Ectonucleotide pyrophosphatase/phosphodiesterase family member 5
with a threshold of 1e−05
rs62273907
rs7311004
rs823424
rs78383039
rs10899911
rs55877868
rs6809952
rs2387977
rs6828768
rs35055552
rs2090816
rs11715853
rs7303158
rs8039785
rs7572229
All
−0.1
0.0
0.1
0.2
0.3
MR effect size for
'Gut microbiota abundance (genus Parasutterella id.2892)' on 'Ectonucleotide pyrophosphatase/phosphodiesterase family membe

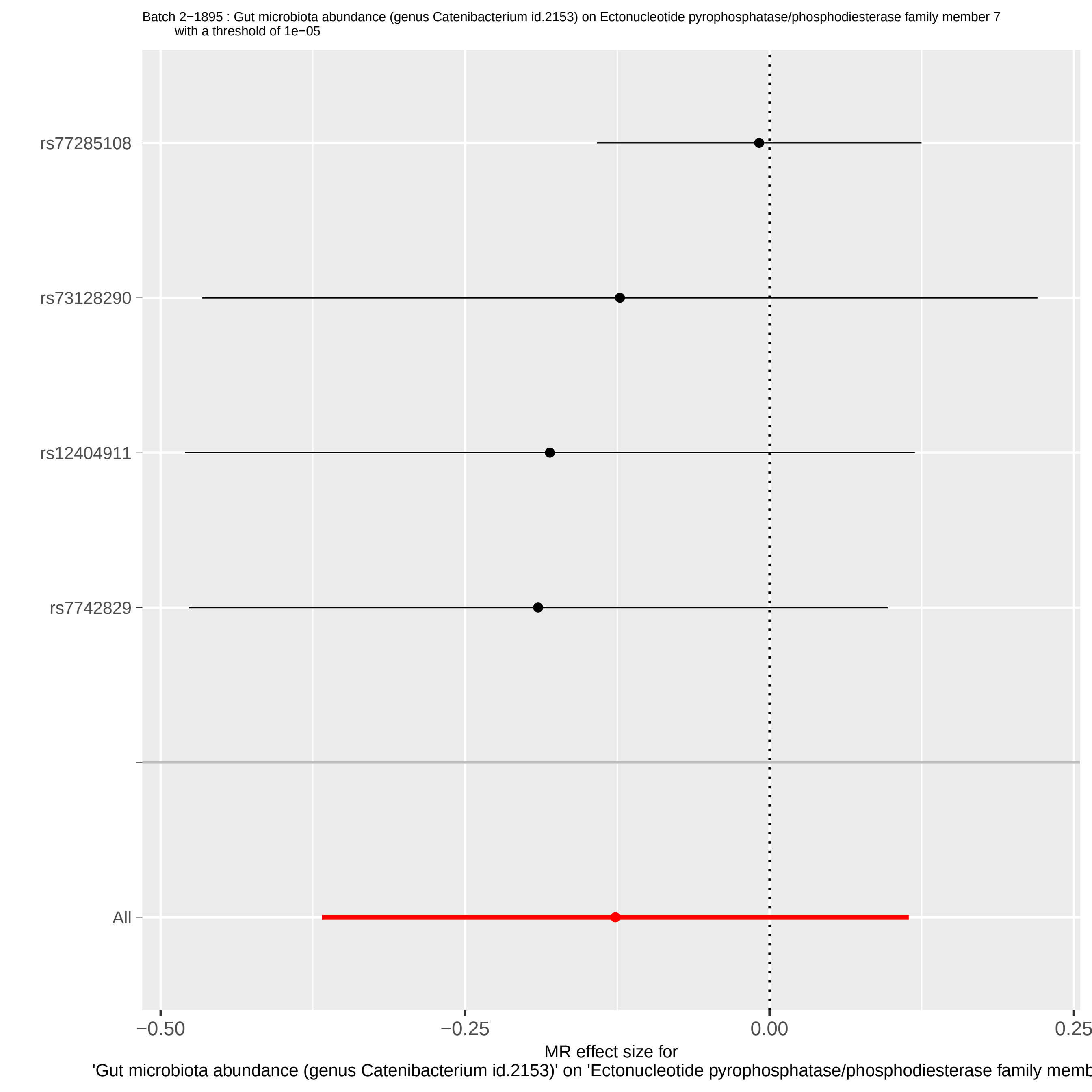
Batch 2−1895 : Gut microbiota abundance (genus Catenibacterium id.2153) on Ectonucleotide pyrophosphatase/phosphodiesterase family member 7
with a threshold of 1e−05
rs77285108
rs73128290
rs12404911
rs7742829
All
−0.50
−0.25
0.00
0.25
MR effect size for
'Gut microbiota abundance (genus Catenibacterium id.2153)' on 'Ectonucleotide pyrophosphatase/phosphodiesterase family memb

Batch 2−1896 : Gut microbiota abundance (genus Parasutterella id.2892) on Ectonucleotide pyrophosphatase/phosphodiesterase family member 7 with a threshold of 1e−05

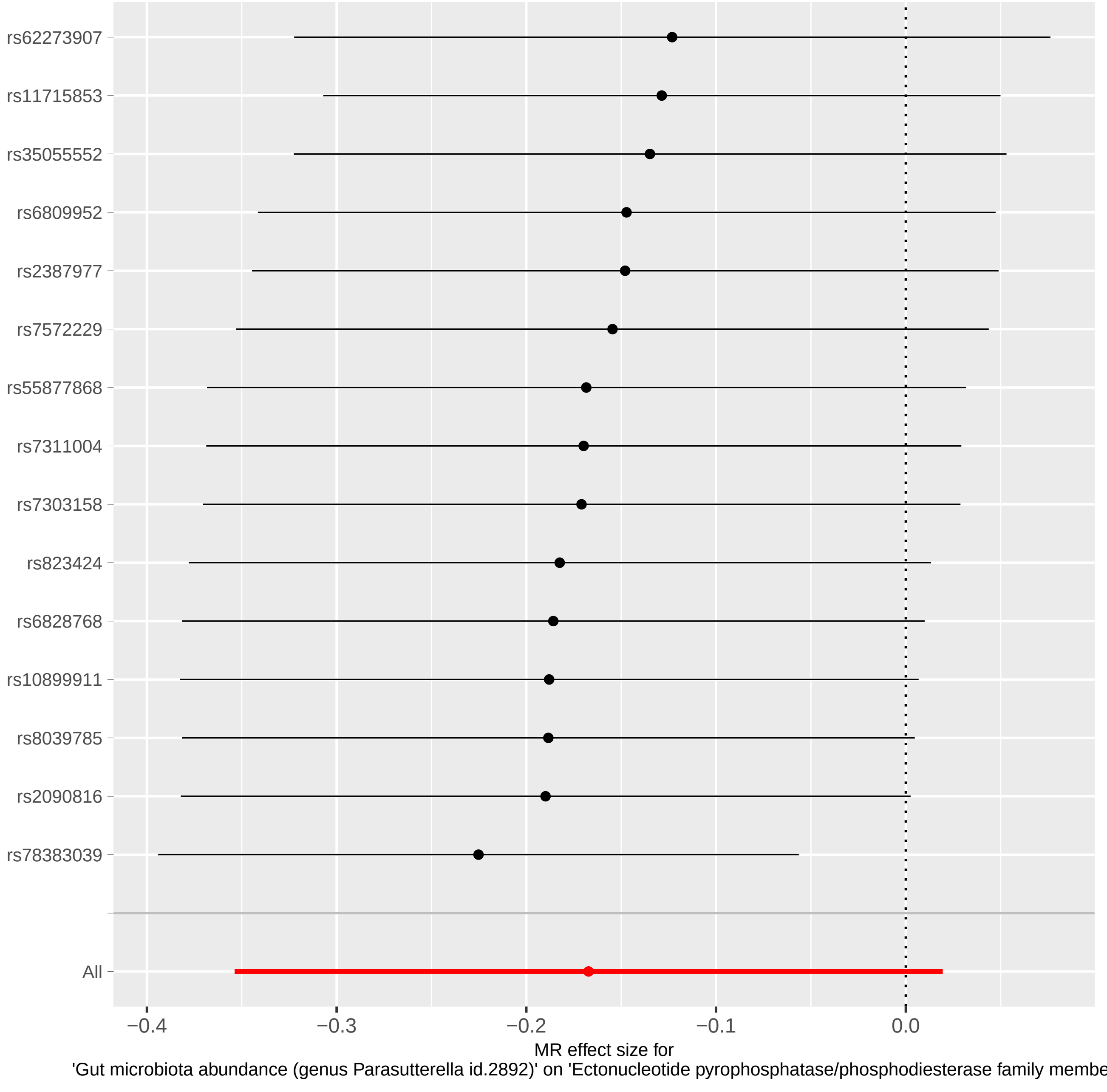

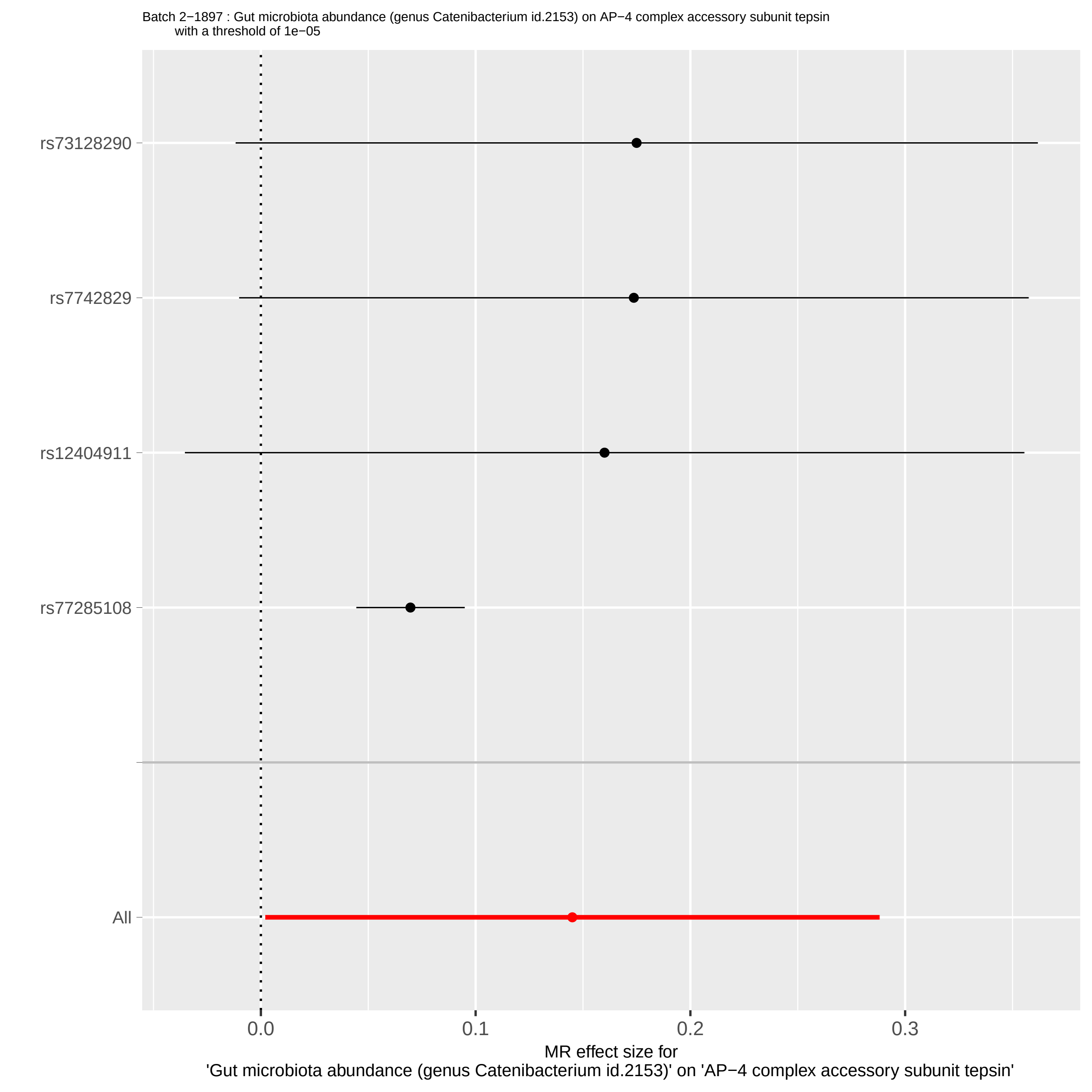
Batch 2−1897 : Gut microbiota abundance (genus Catenibacterium id.2153) on AP−4 complex accessory subunit tepsin
with a threshold of 1e−05
rs73128290
rs7742829
rs12404911
rs77285108
All
0.0
0.1
0.2
0.3
MR effect size for
'Gut microbiota abundance (genus Catenibacterium id.2153)' on 'AP−4 complex accessory subunit tepsin'

Batch 2−1898 : Gut microbiota abundance (genus Parasutterella id.2892) on AP−4 complex accessory subunit tepsin with a threshold of 1e−05

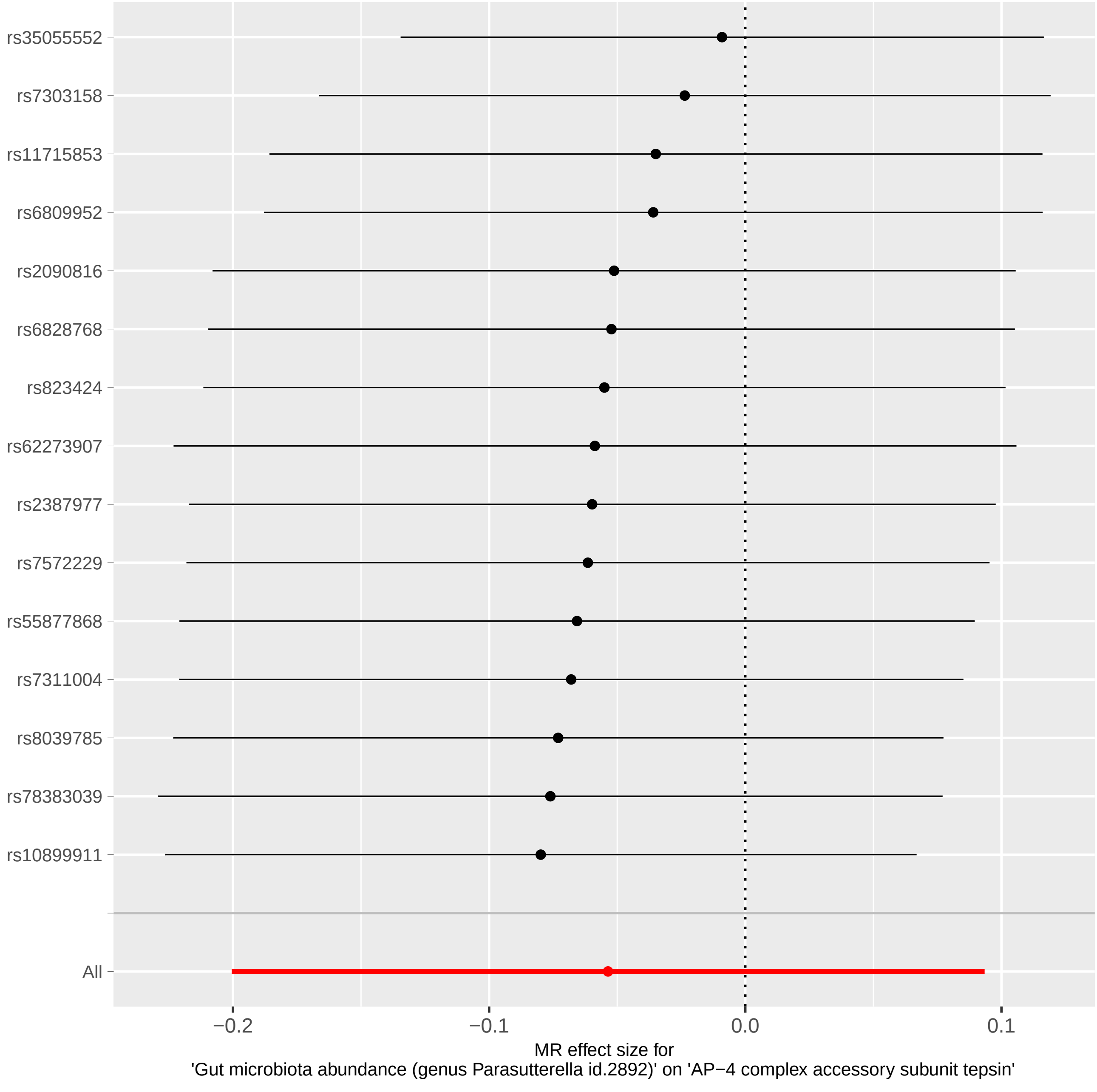

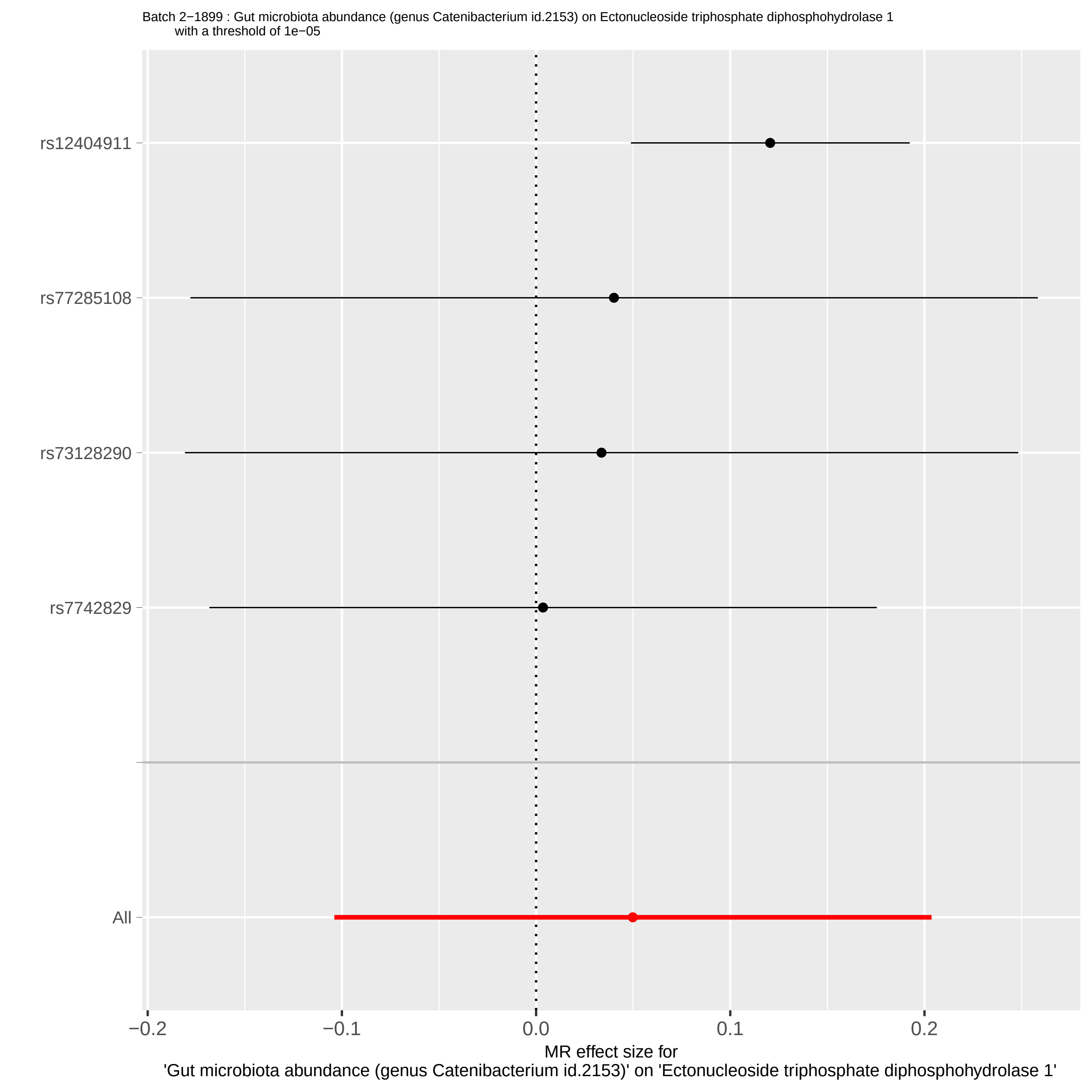

Batch 2−1899 : Gut microbiota abundance (genus Catenibacterium id.2153) on Ectonucleoside triphosphate diphosphohydrolase 1
with a threshold of 1e−05
rs12404911
rs77285108
rs73128290
rs7742829
All
−0.2
−0.1
0.0
0.1
0.2
MR effect size for
'Gut microbiota abundance (genus Catenibacterium id.2153)' on 'Ectonucleoside triphosphate diphosphohydrolase 1'

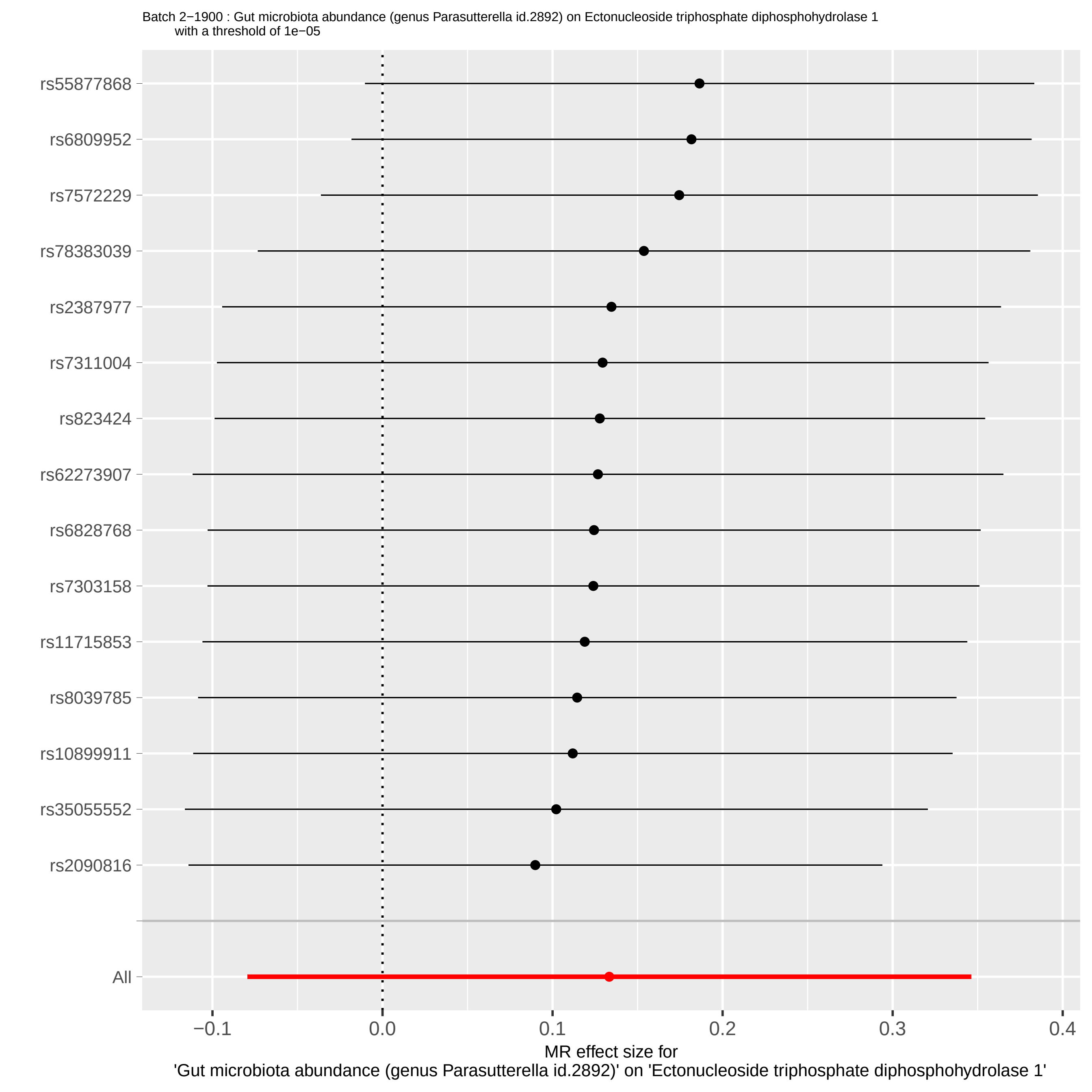

Batch 2−1900 : Gut microbiota abundance (genus Parasutterella id.2892) on Ectonucleoside triphosphate diphosphohydrolase 1
with a threshold of 1e−05
rs55877868
rs6809952
rs7572229
rs78383039
rs2387977
rs7311004
rs823424
rs62273907
rs6828768
rs7303158
rs11715853
rs8039785
rs10899911
rs35055552
rs2090816
All
−0.1
0.0
0.1
0.2
0.3
0.4
MR effect size for
'Gut microbiota abundance (genus Parasutterella id.2892)' on 'Ectonucleoside triphosphate diphosphohydrolase 1'

Batch 2−1901 : Gut microbiota abundance (genus Catenibacterium id.2153) on Ectonucleoside triphosphate diphosphohydrolase 1 with a threshold of 1e−05

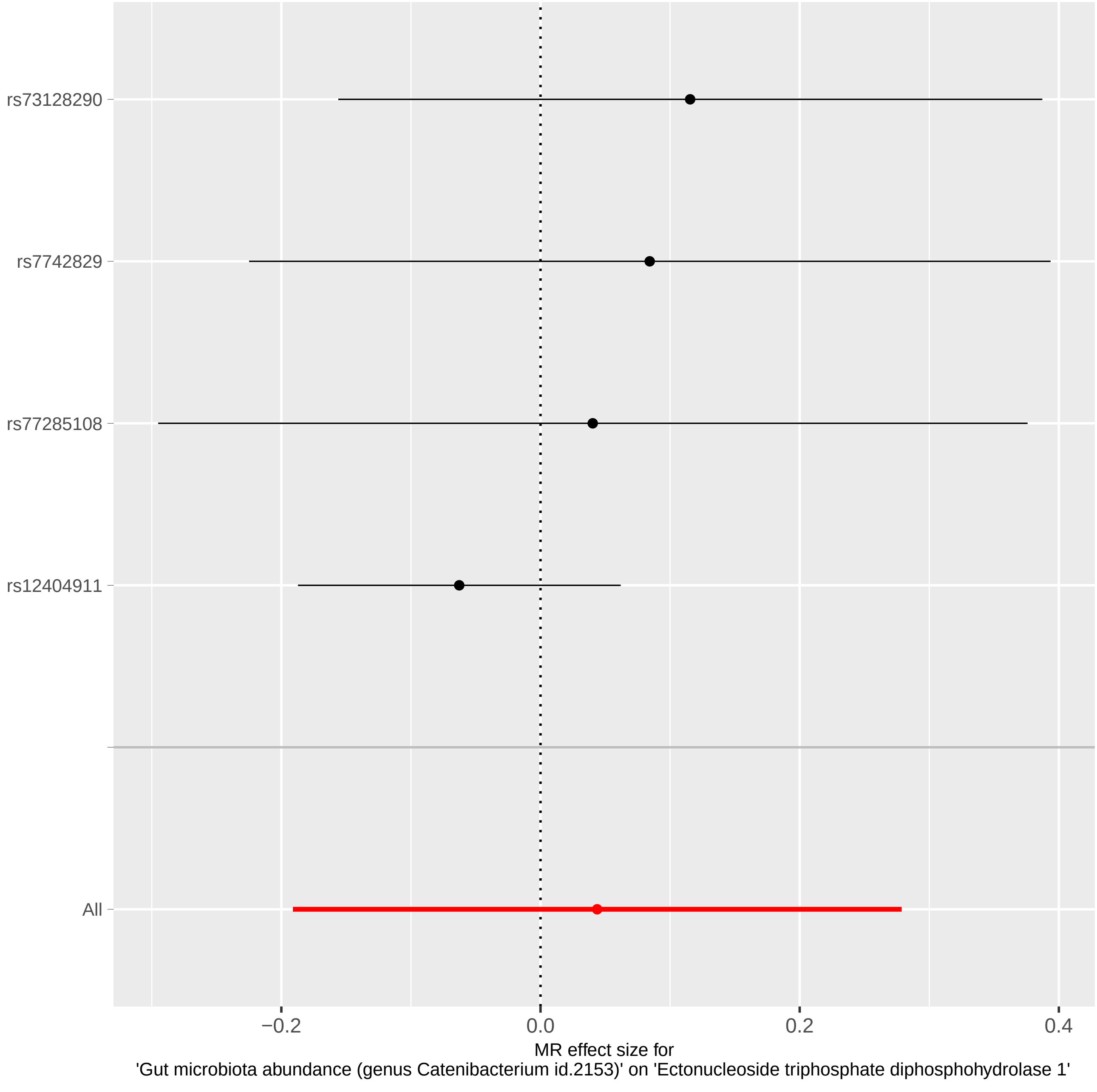

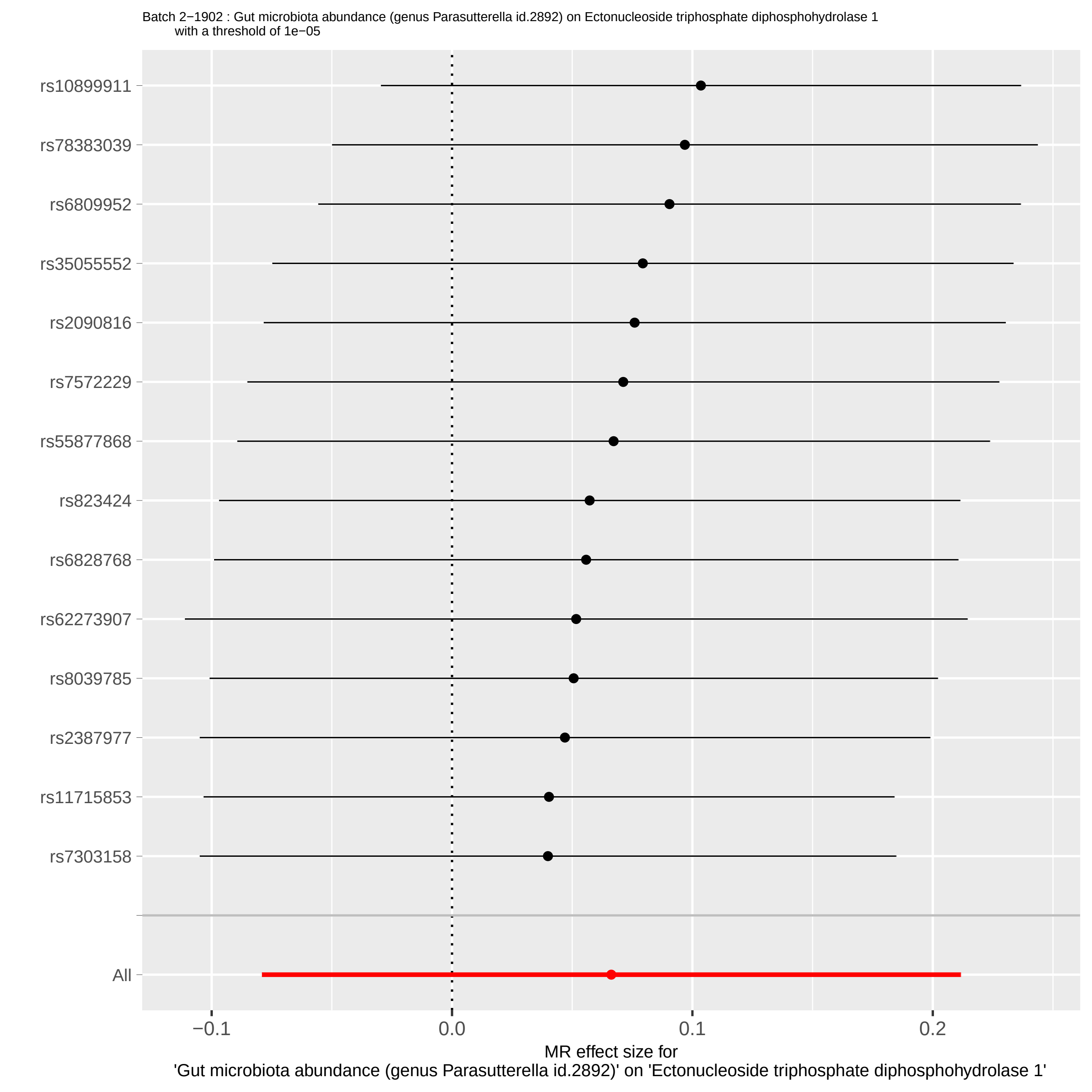
Batch 2−1902 : Gut microbiota abundance (genus Parasutterella id.2892) on Ectonucleoside triphosphate diphosphohydrolase 1
with a threshold of 1e−05
rs10899911
rs78383039
rs6809952
rs35055552
rs2090816
rs7572229
rs55877868
rs823424
rs6828768
rs62273907
rs8039785
rs2387977
rs11715853
rs7303158
All
−0.1
0.0
0.1
0.2
MR effect size for
'Gut microbiota abundance (genus Parasutterella id.2892)' on 'Ectonucleoside triphosphate diphosphohydrolase 1'

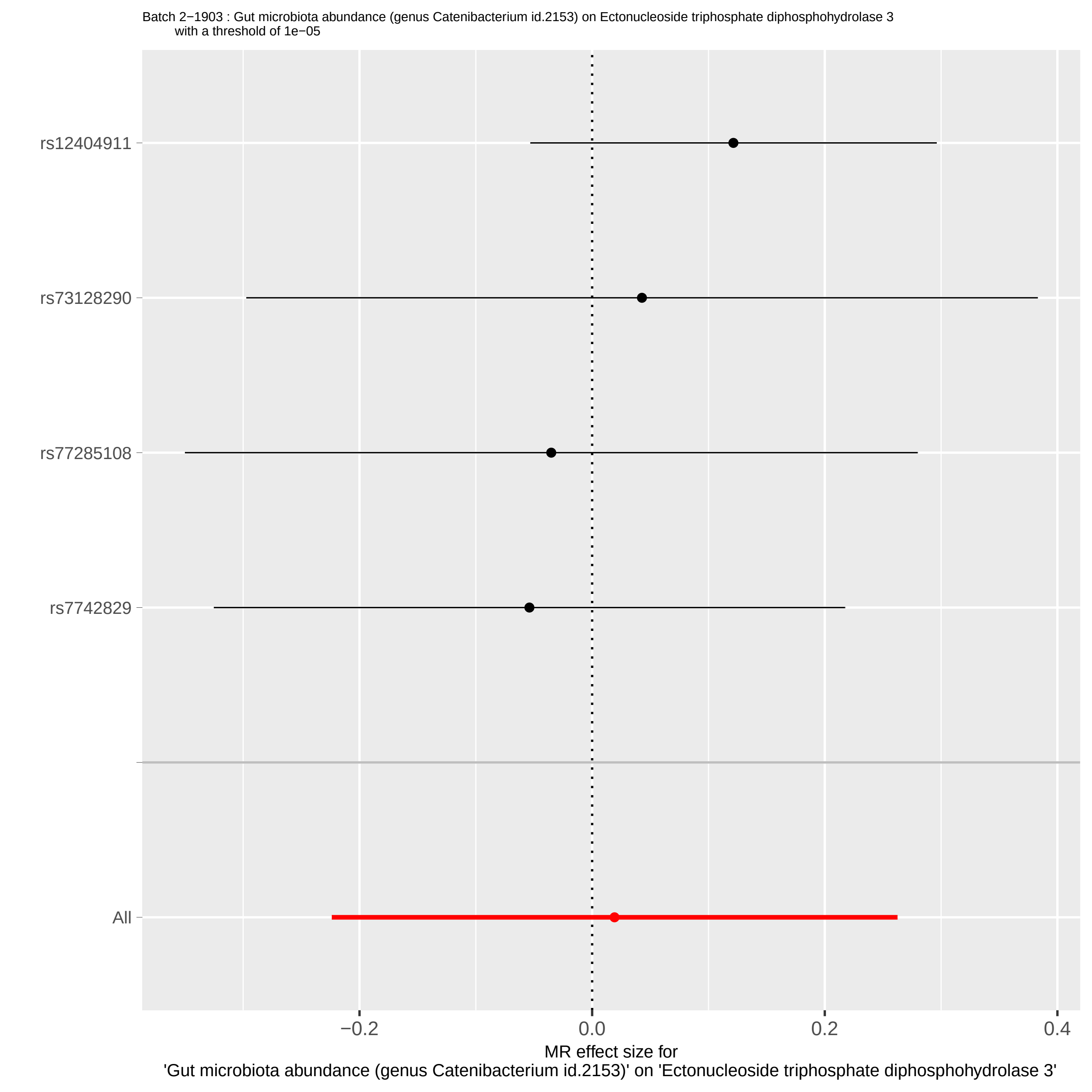
Batch 2−1903 : Gut microbiota abundance (genus Catenibacterium id.2153) on Ectonucleoside triphosphate diphosphohydrolase 3
with a threshold of 1e−05
rs12404911
rs73128290
rs77285108
rs7742829
All
−0.2
0.0
0.2
0.4
MR effect size for
'Gut microbiota abundance (genus Catenibacterium id.2153)' on 'Ectonucleoside triphosphate diphosphohydrolase 3'

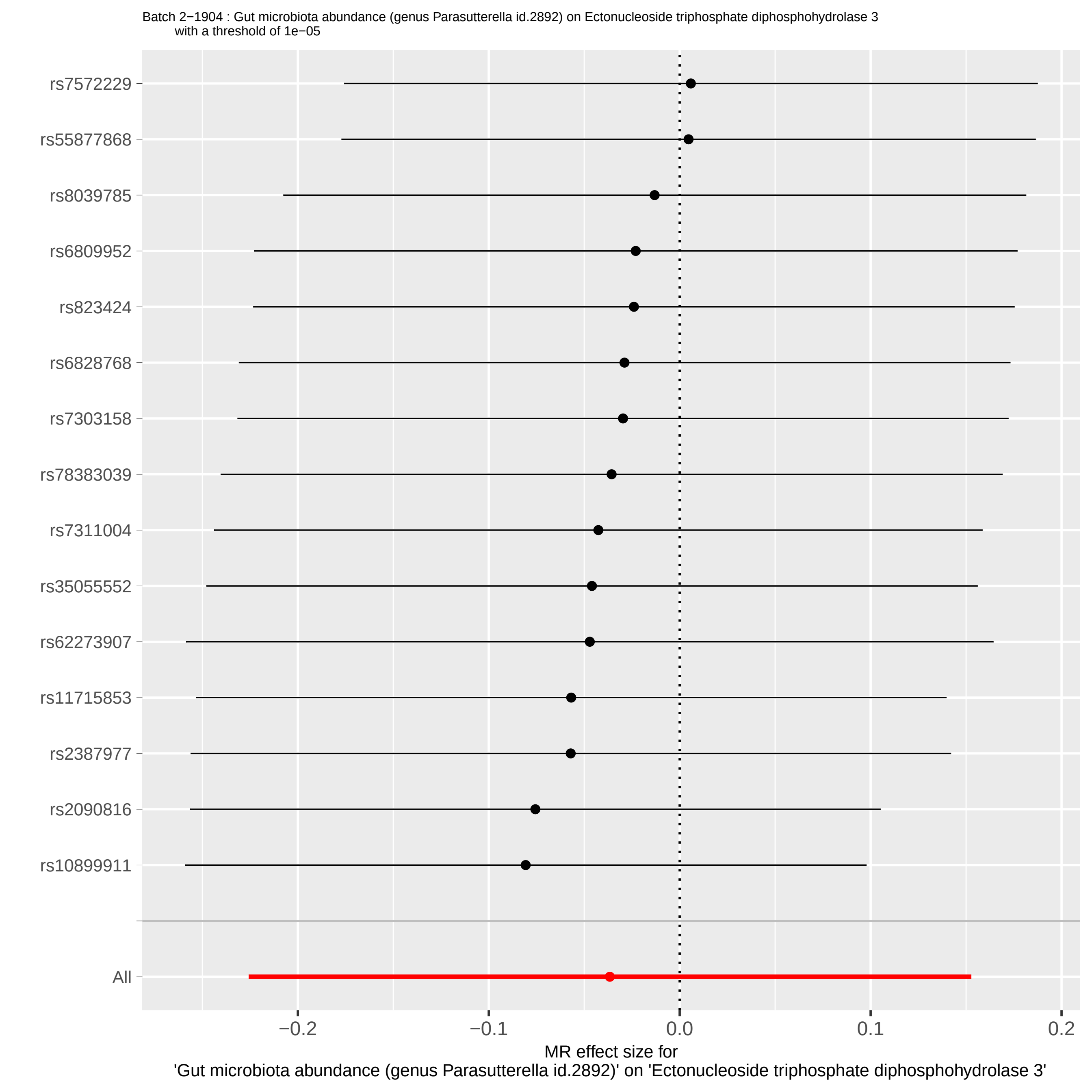

Batch 2−1904 : Gut microbiota abundance (genus Parasutterella id.2892) on Ectonucleoside triphosphate diphosphohydrolase 3
with a threshold of 1e−05
rs7572229
rs55877868
rs8039785
rs6809952
rs823424
rs6828768
rs7303158
rs78383039
rs7311004
rs35055552
rs62273907
rs11715853
rs2387977
rs2090816
rs10899911
All
−0.2
−0.1
0.0
0.1
0.2
MR effect size for
'Gut microbiota abundance (genus Parasutterella id.2892)' on 'Ectonucleoside triphosphate diphosphohydrolase 3'

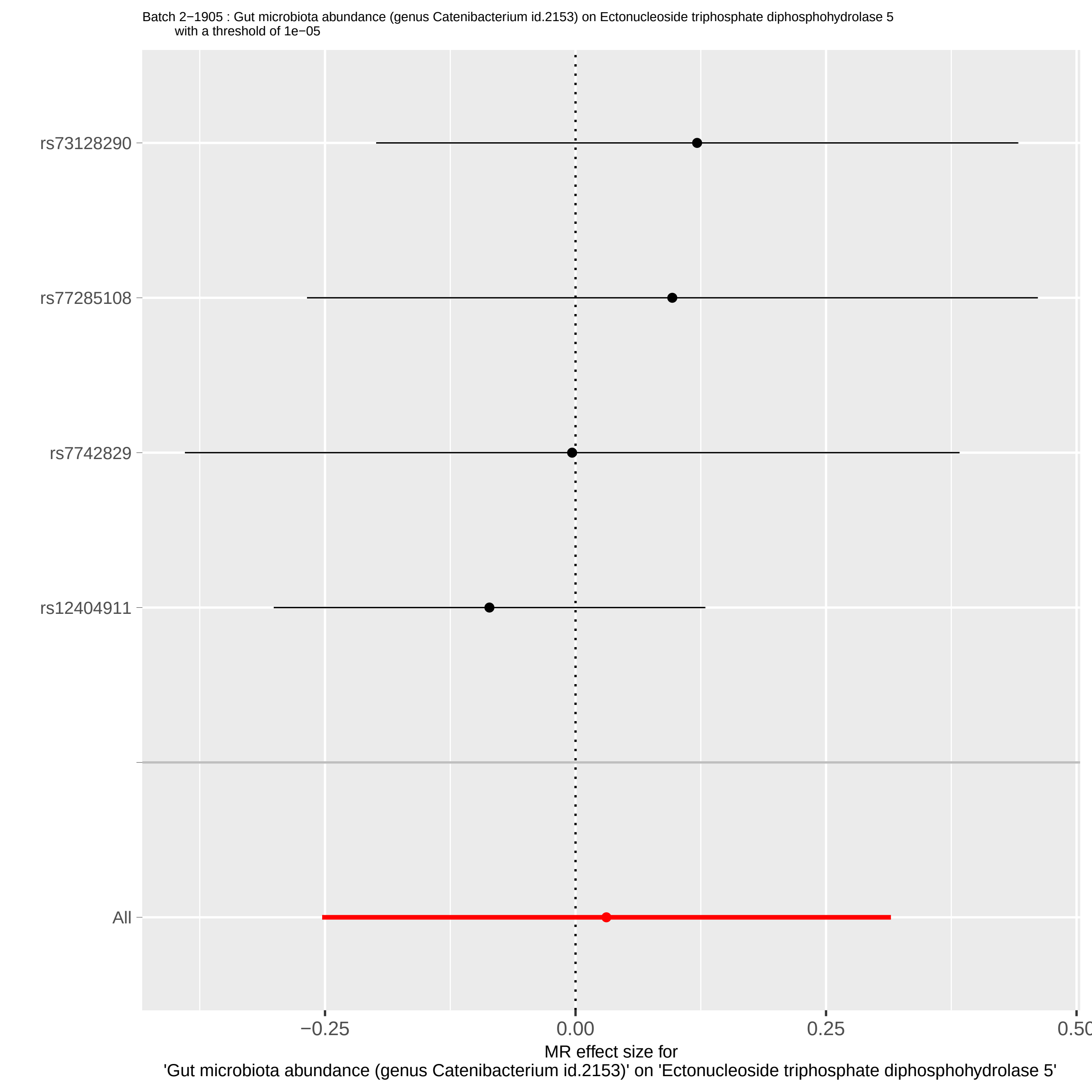

Batch 2−1905 : Gut microbiota abundance (genus Catenibacterium id.2153) on Ectonucleoside triphosphate diphosphohydrolase 5
with a threshold of 1e−05
rs73128290
rs77285108
rs7742829
rs12404911
All
−0.25
0.00
0.25
0.50
MR effect size for
'Gut microbiota abundance (genus Catenibacterium id.2153)' on 'Ectonucleoside triphosphate diphosphohydrolase 5'

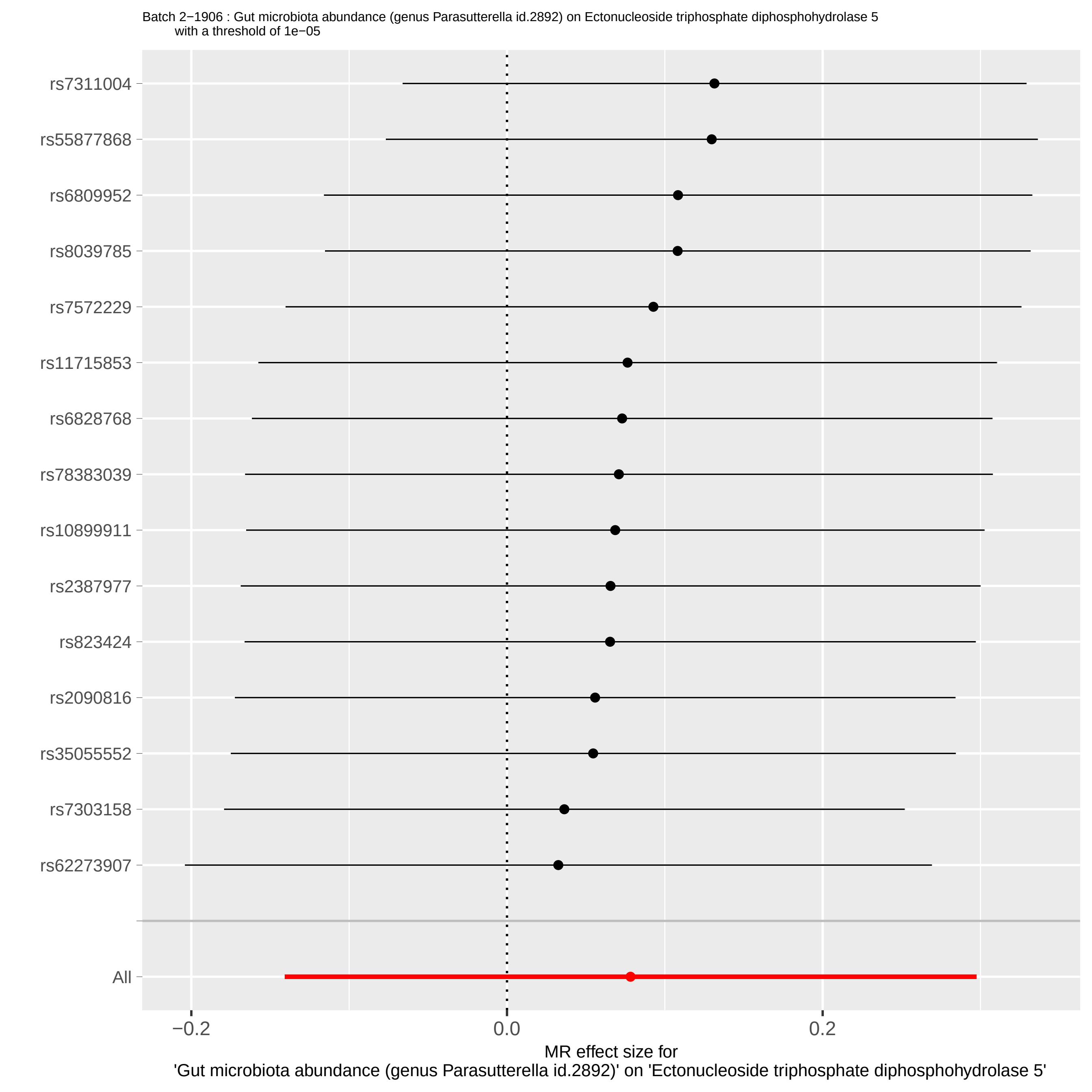

Batch 2−1906 : Gut microbiota abundance (genus Parasutterella id.2892) on Ectonucleoside triphosphate diphosphohydrolase 5
with a threshold of 1e−05
rs7311004
rs55877868
rs6809952
rs8039785
rs7572229
rs11715853
rs6828768
rs78383039
rs10899911
rs2387977
rs823424
rs2090816
rs35055552
rs7303158
rs62273907
All
−0.2
0.0
0.2
MR effect size for
'Gut microbiota abundance (genus Parasutterella id.2892)' on 'Ectonucleoside triphosphate diphosphohydrolase 5'

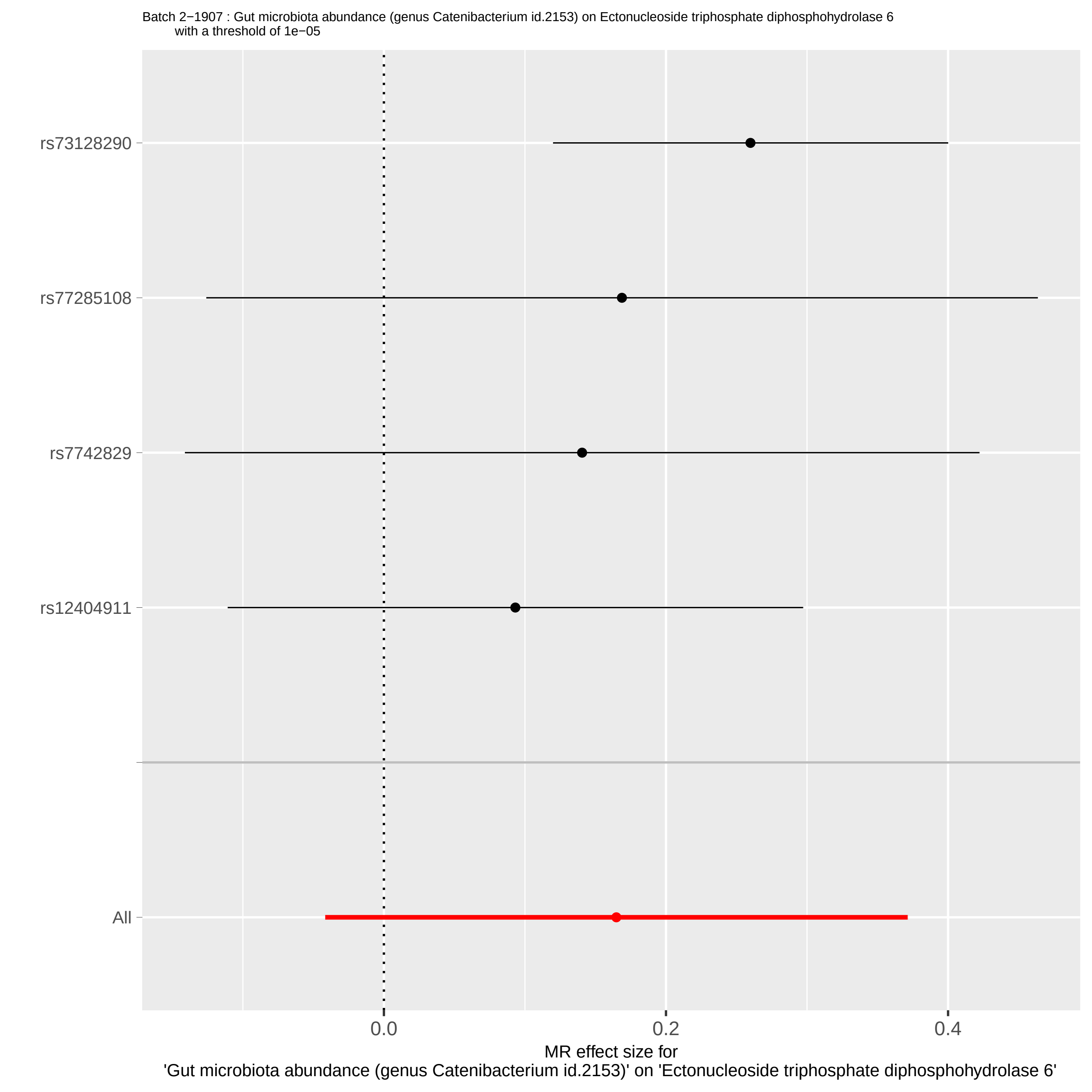
Batch 2−1907 : Gut microbiota abundance (genus Catenibacterium id.2153) on Ectonucleoside triphosphate diphosphohydrolase 6
with a threshold of 1e−05
rs73128290
rs77285108
rs7742829
rs12404911
All
0.0
0.2
0.4
MR effect size for
'Gut microbiota abundance (genus Catenibacterium id.2153)' on 'Ectonucleoside triphosphate diphosphohydrolase 6'

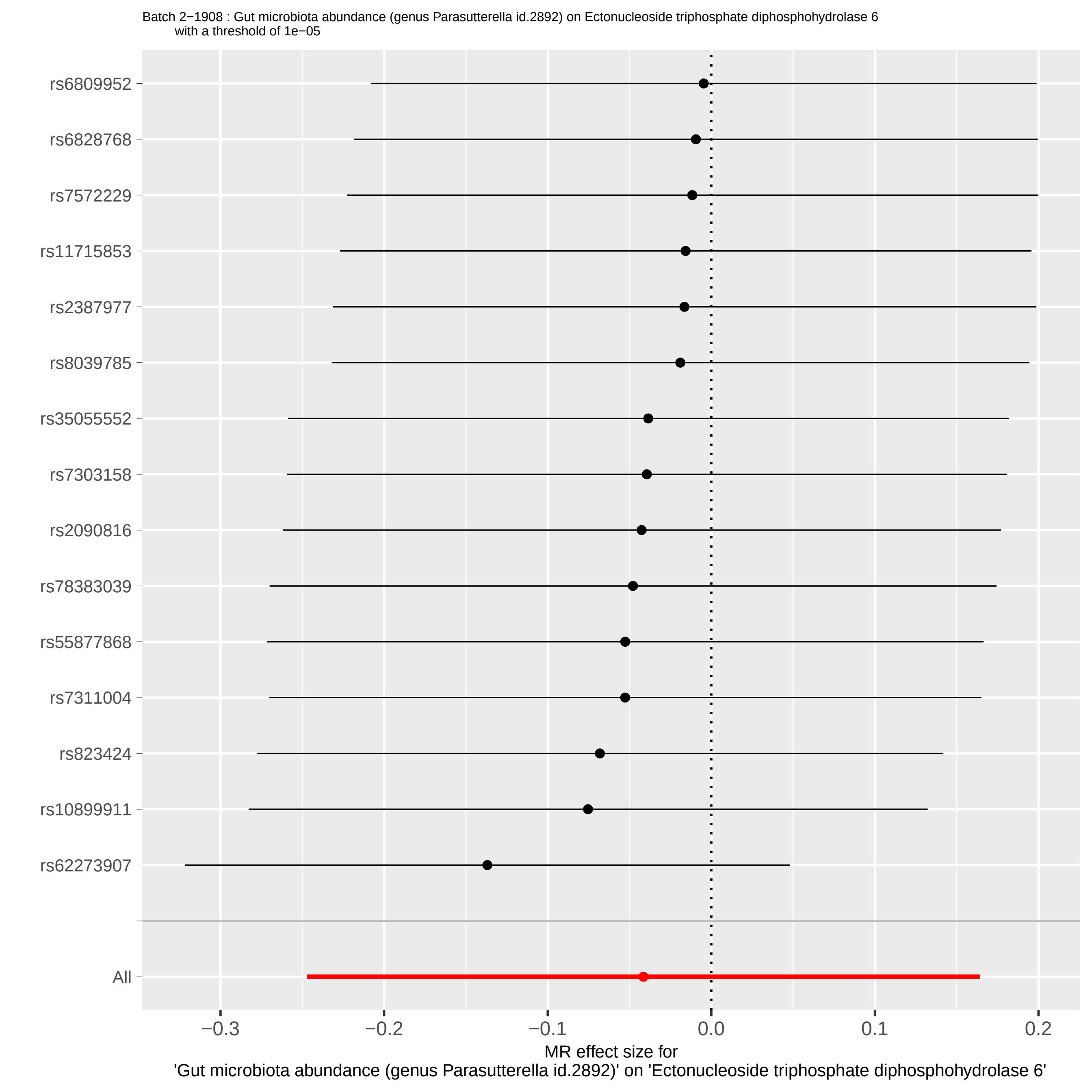
Batch 2−1908 : Gut microbiota abundance (genus Parasutterella id.2892) on Ectonucleoside triphosphate diphosphohydrolase 6
with a threshold of 1e−05
rs6809952
rs6828768
rs7572229
rs11715853
rs2387977
rs8039785
rs35055552
rs7303158
rs2090816
rs78383039
rs55877868
rs7311004
rs823424
rs10899911
rs62273907
All
−0.3
−0.2
−0.1
0.0
0.1
0.2
MR effect size for
'Gut microbiota abundance (genus Parasutterella id.2892)' on 'Ectonucleoside triphosphate diphosphohydrolase 6'

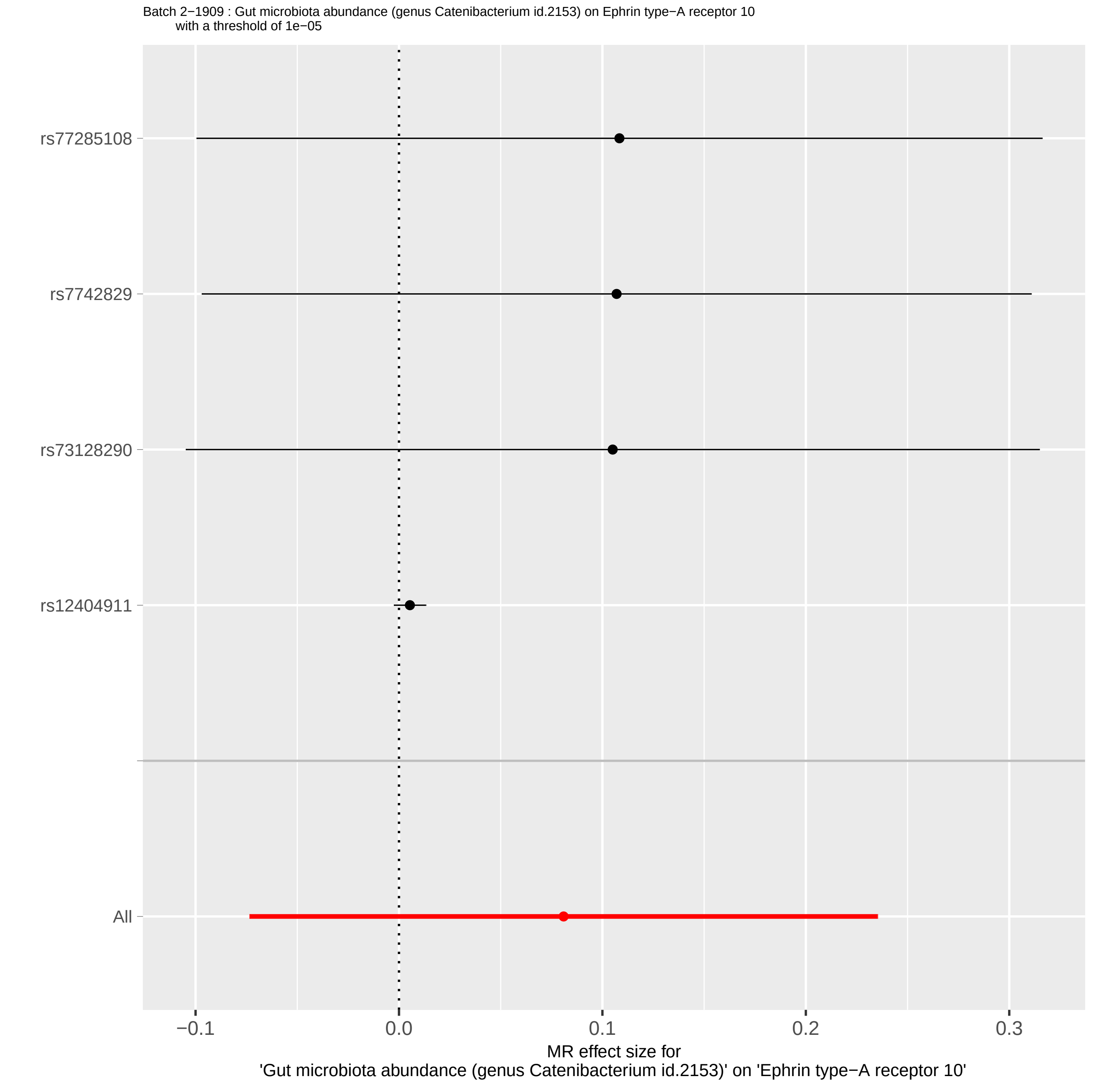

Batch 2−1909 : Gut microbiota abundance (genus Catenibacterium id.2153) on Ephrin type−A receptor 10
with a threshold of 1e−05
rs77285108
rs7742829
rs73128290
rs12404911
All
−0.1
0.0
0.1
0.2
0.3
MR effect size for
'Gut microbiota abundance (genus Catenibacterium id.2153)' on 'Ephrin type−A receptor 10'

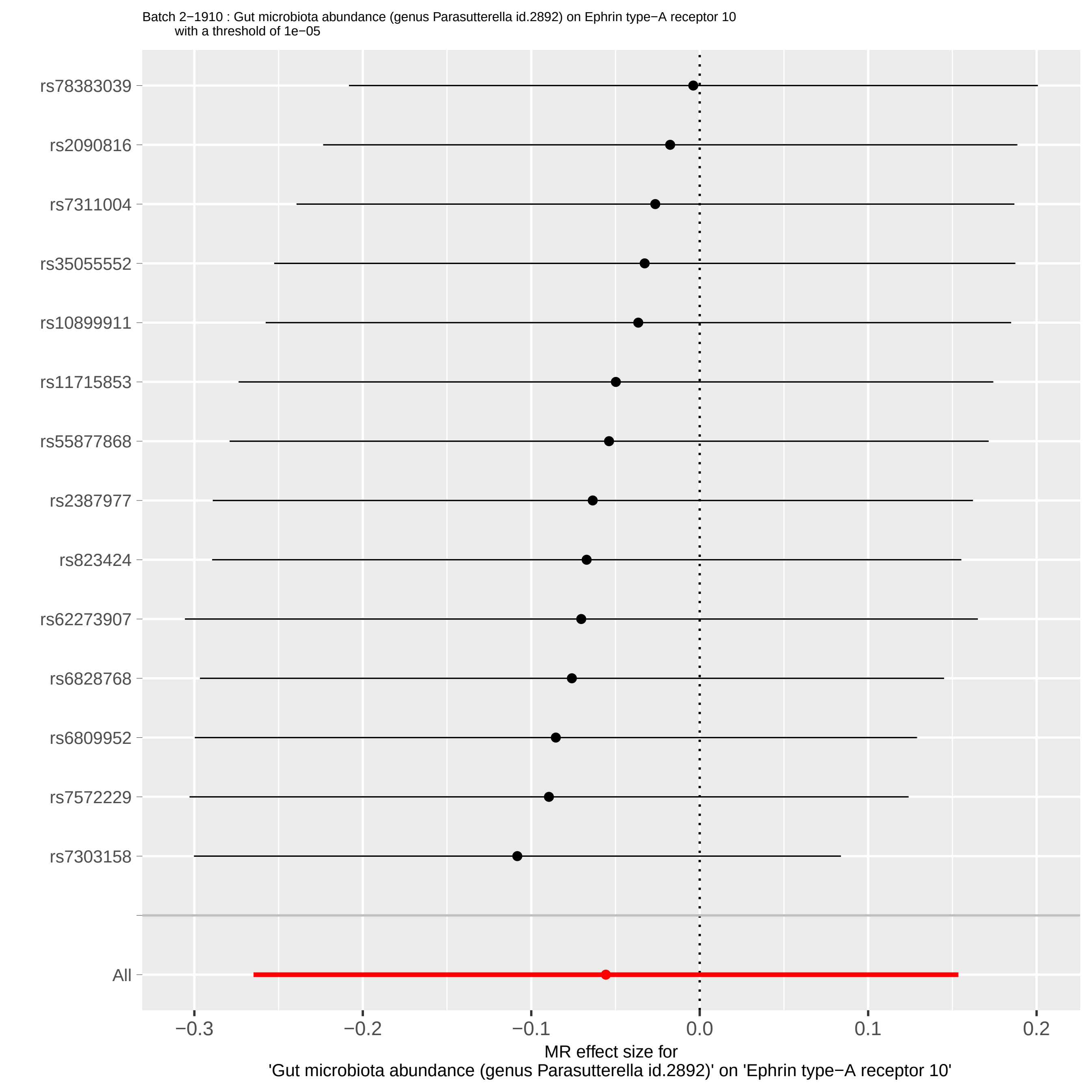
Batch 2−1910 : Gut microbiota abundance (genus Parasutterella id.2892) on Ephrin type−A receptor 10
with a threshold of 1e−05
rs78383039
rs2090816
rs7311004
rs35055552
rs10899911
rs11715853
rs55877868
rs2387977
rs823424
rs62273907
rs6828768
rs6809952
rs7572229
rs7303158
All
−0.3
−0.2
−0.1
0.0
0.1
0.2
MR effect size for
'Gut microbiota abundance (genus Parasutterella id.2892)' on 'Ephrin type−A receptor 10'

Batch 2−1911 : Gut microbiota abundance (genus Catenibacterium id.2153) on Ephrin type−A receptor 1
with a threshold of 1e−05

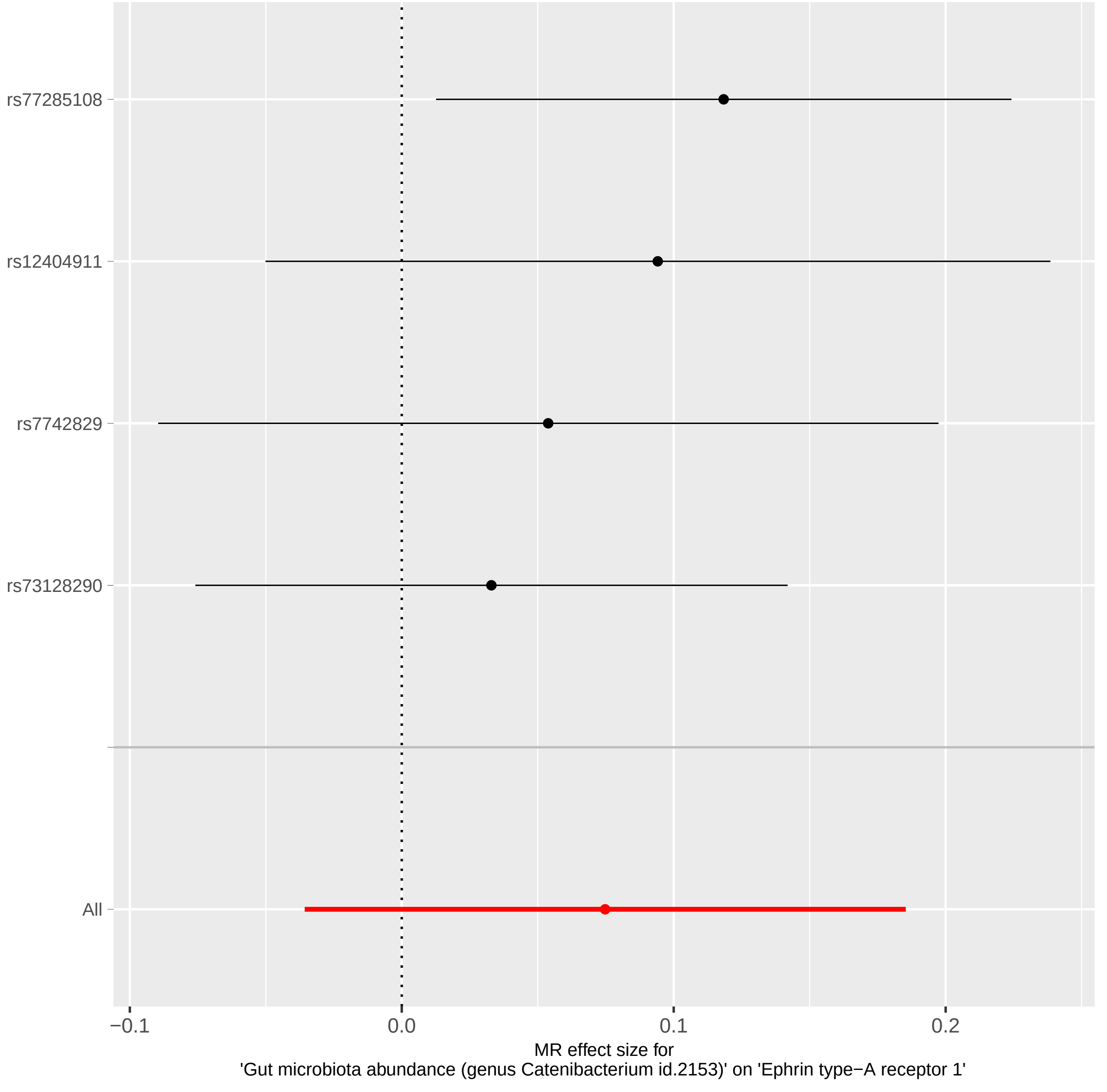

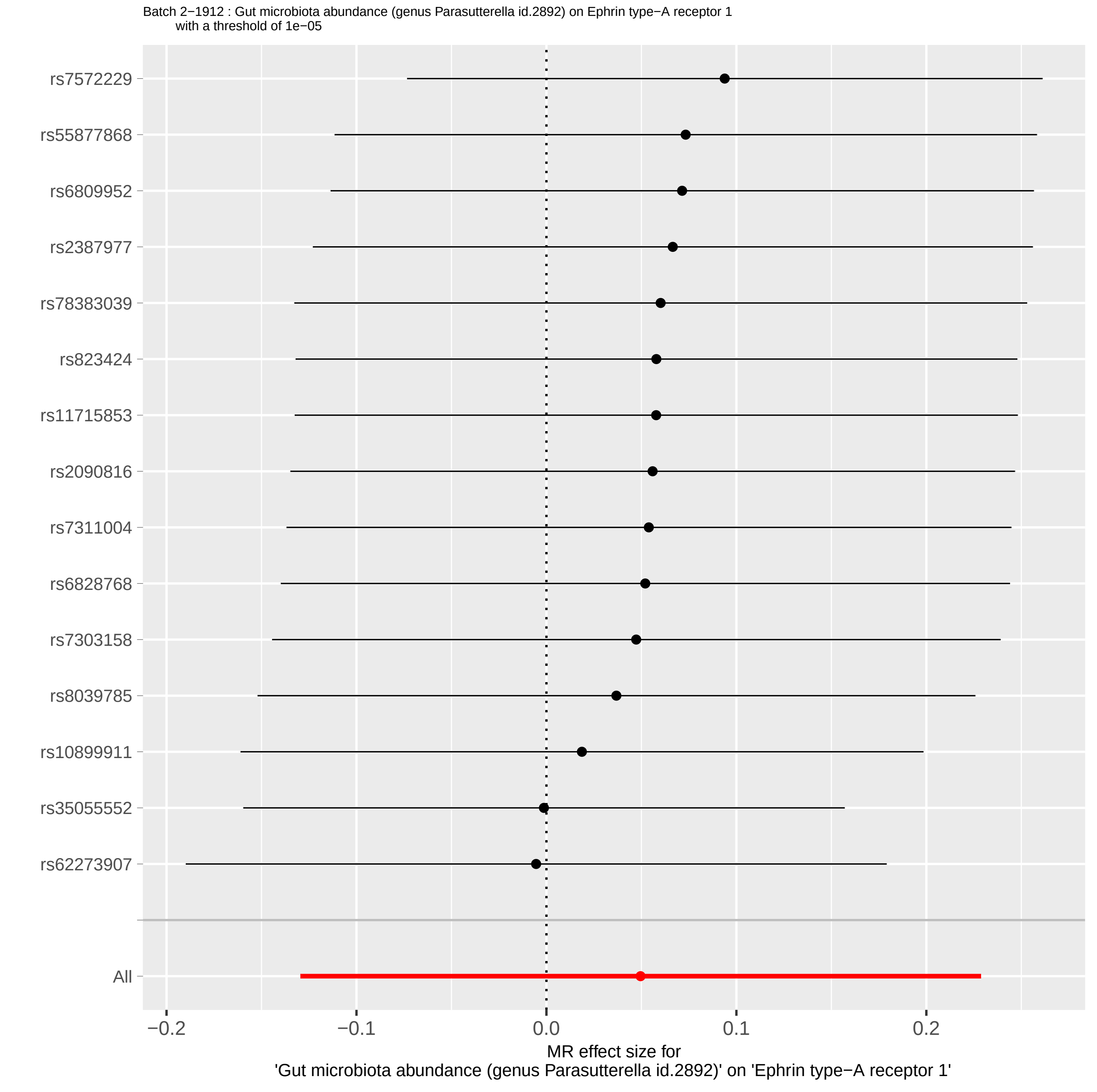

Batch 2−1912 : Gut microbiota abundance (genus Parasutterella id.2892) on Ephrin type−A receptor 1
with a threshold of 1e−05
rs7572229
rs55877868
rs6809952
rs2387977
rs78383039
rs823424
rs11715853
rs2090816
rs7311004
rs6828768
rs7303158
rs8039785
rs10899911
rs35055552
rs62273907
All
−0.2
−0.1
0.0
0.1
0.2
MR effect size for
'Gut microbiota abundance (genus Parasutterella id.2892)' on 'Ephrin type−A receptor 1'

Batch 2−1913 : Gut microbiota abundance (genus Catenibacterium id.2153) on Ephrin type−A receptor 2
with a threshold of 1e−05

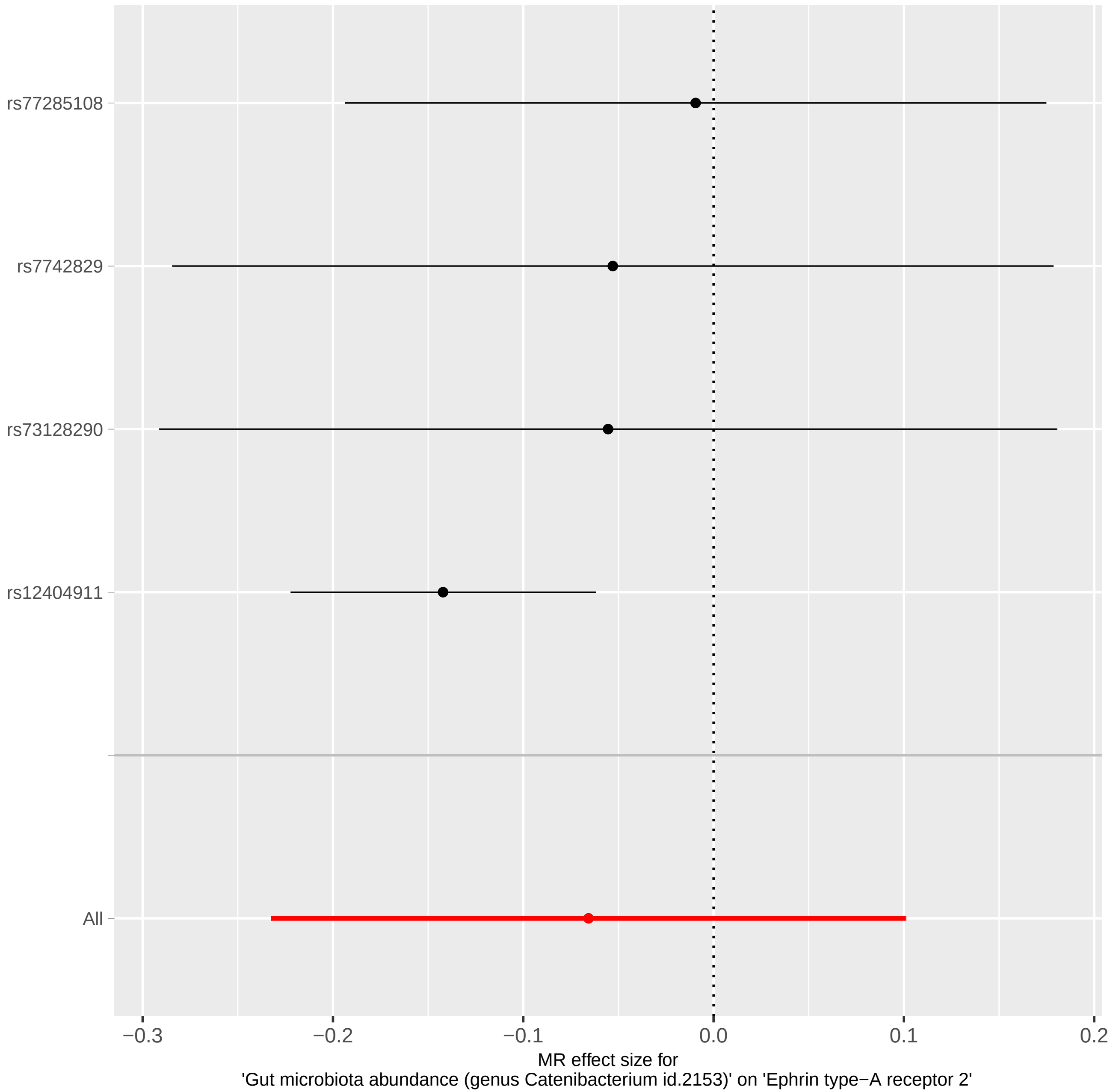

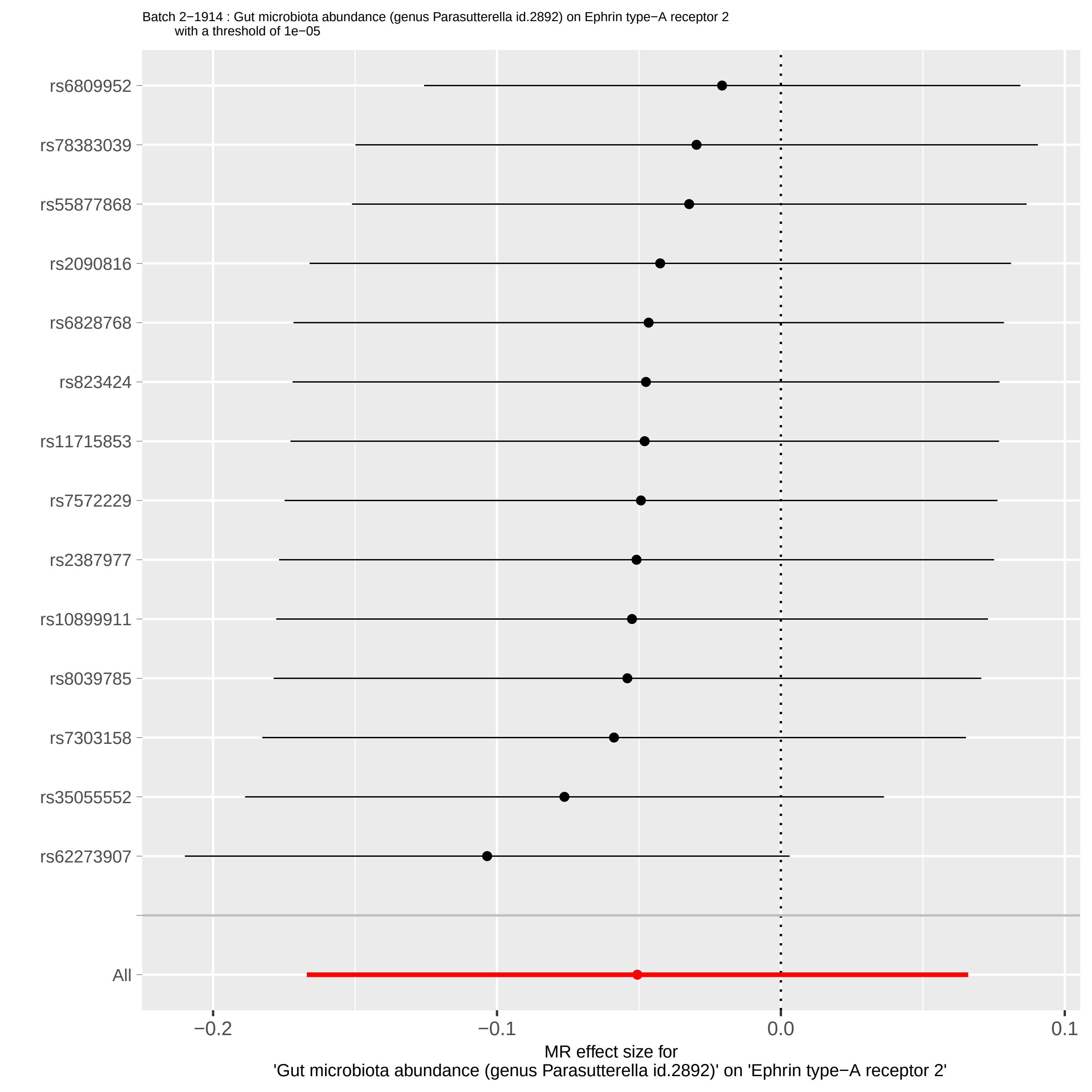
Batch 2−1914 : Gut microbiota abundance (genus Parasutterella id.2892) on Ephrin type−A receptor 2
with a threshold of 1e−05
rs6809952
rs78383039
rs55877868
rs2090816
rs6828768
rs823424
rs11715853
rs7572229
rs2387977
rs10899911
rs8039785
rs7303158
rs35055552
rs62273907
All
−0.2
−0.1
0.0
0.1
MR effect size for
'Gut microbiota abundance (genus Parasutterella id.2892)' on 'Ephrin type−A receptor 2'

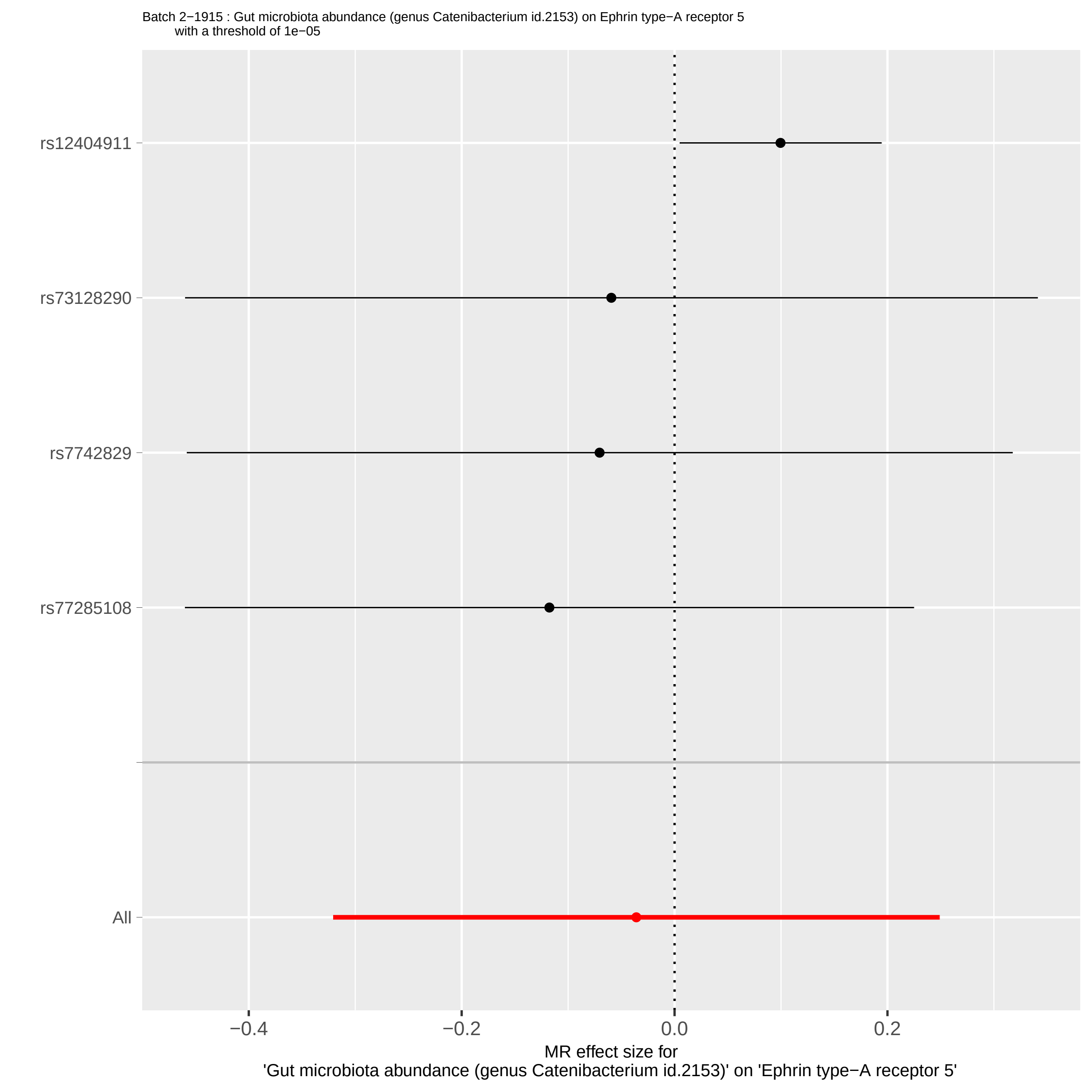

Batch 2−1915 : Gut microbiota abundance (genus Catenibacterium id.2153) on Ephrin type−A receptor 5
with a threshold of 1e−05
rs12404911
rs73128290
rs7742829
rs77285108
All
−0.4
−0.2
0.0
0.2
MR effect size for
'Gut microbiota abundance (genus Catenibacterium id.2153)' on 'Ephrin type−A receptor 5'

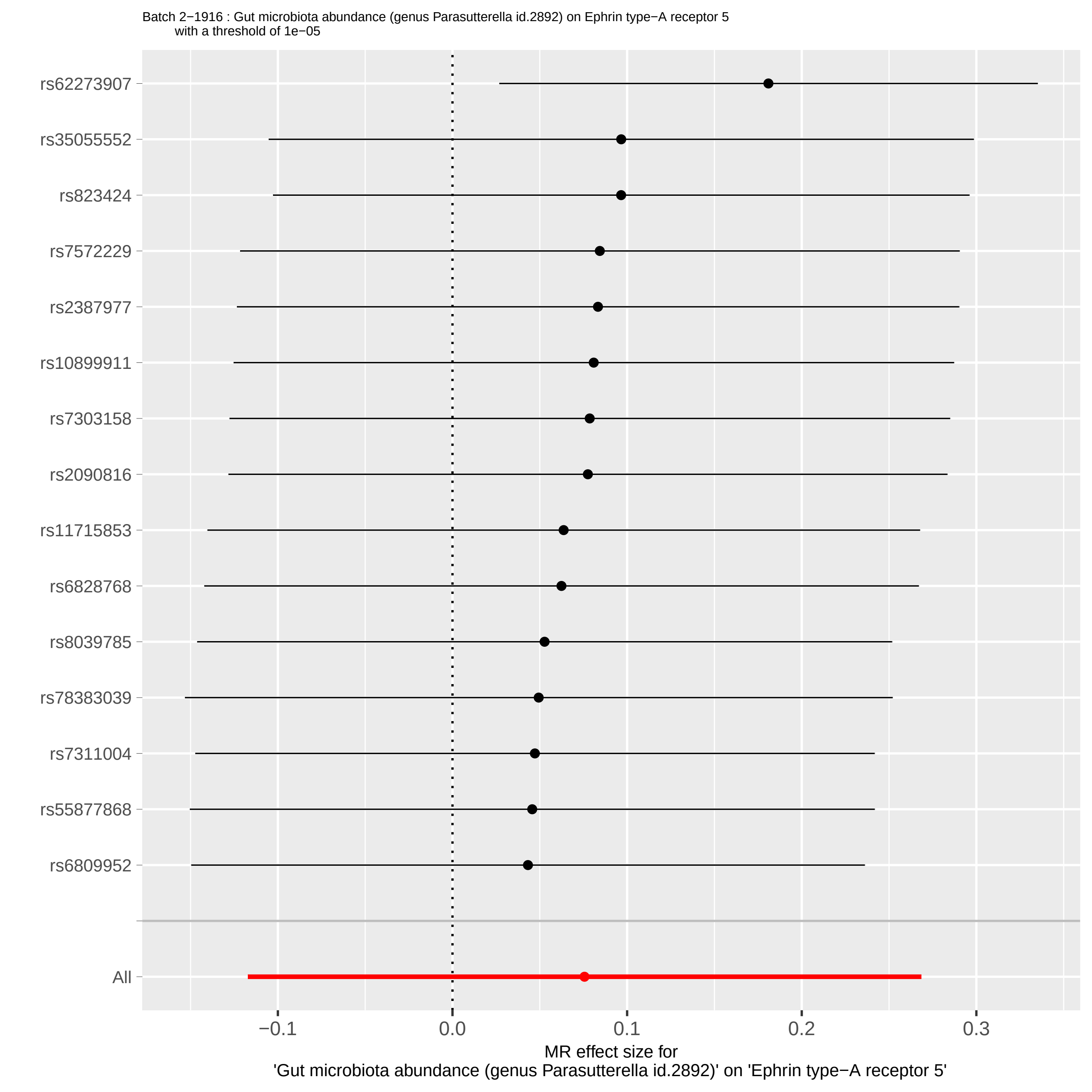
Batch 2−1916 : Gut microbiota abundance (genus Parasutterella id.2892) on Ephrin type−A receptor 5
with a threshold of 1e−05
rs62273907
rs35055552
rs823424
rs7572229
rs2387977
rs10899911
rs7303158
rs2090816
rs11715853
rs6828768
rs8039785
rs78383039
rs7311004
rs55877868
rs6809952
All
−0.1
0.0
0.1
0.2
0.3
MR effect size for
'Gut microbiota abundance (genus Parasutterella id.2892)' on 'Ephrin type−A receptor 5'

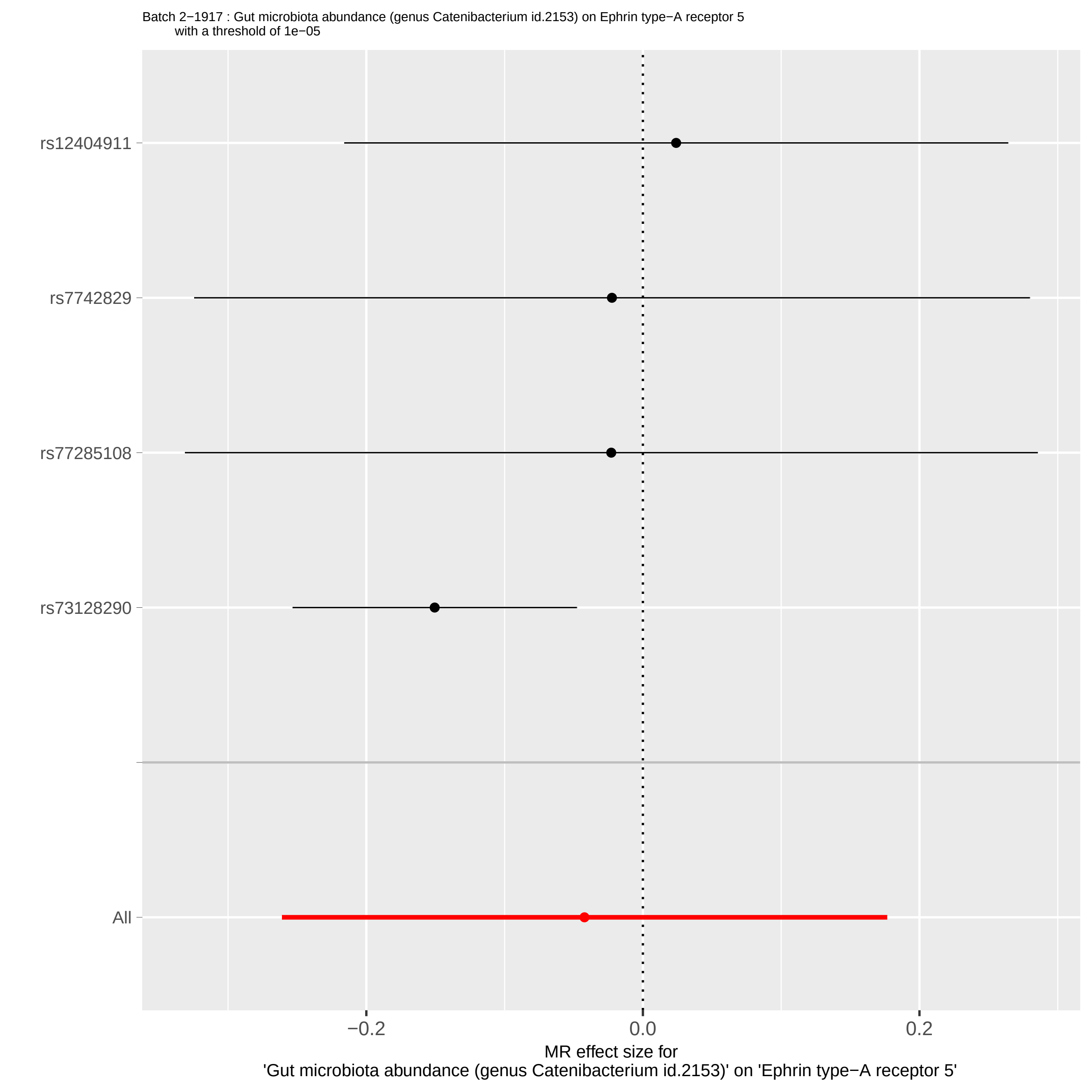
Batch 2−1917 : Gut microbiota abundance (genus Catenibacterium id.2153) on Ephrin type−A receptor 5
with a threshold of 1e−05
rs12404911
rs7742829
rs77285108
rs73128290
All
−0.2
0.0
0.2
MR effect size for
'Gut microbiota abundance (genus Catenibacterium id.2153)' on 'Ephrin type−A receptor 5'

Batch 2−1918 : Gut microbiota abundance (genus Parasutterella id.2892) on Ephrin type−A receptor 5
with a threshold of 1e−05

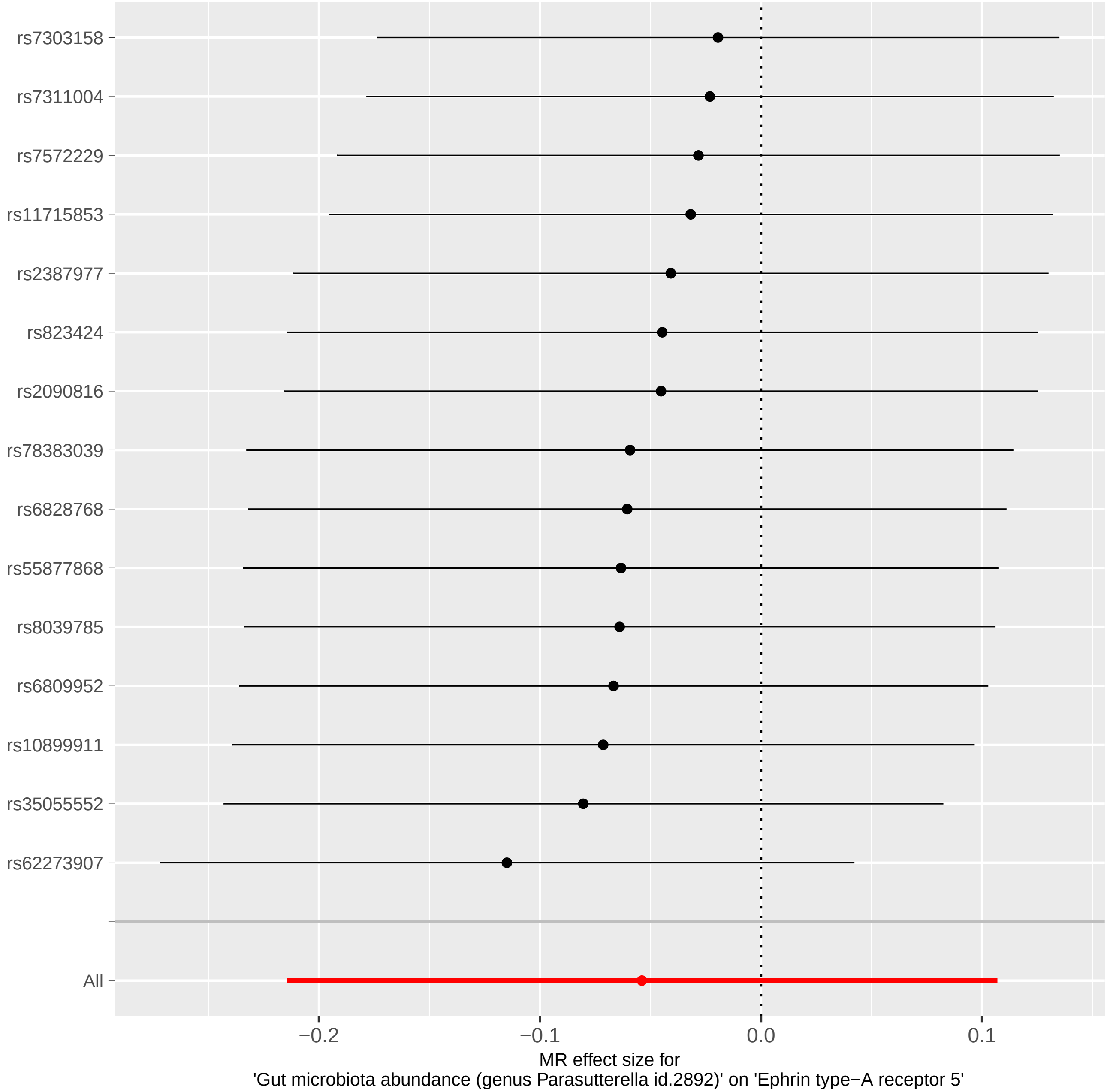

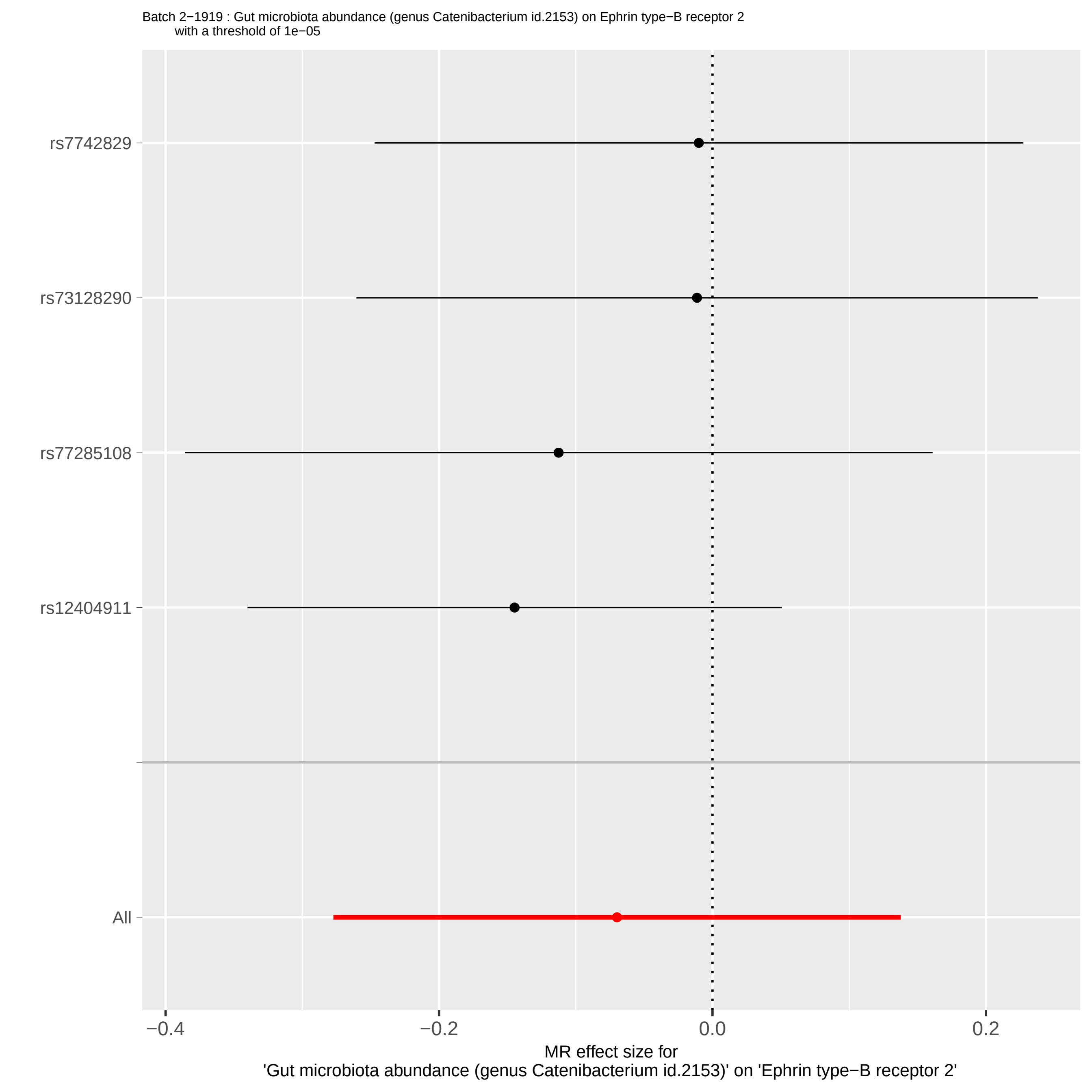

Batch 2−1919 : Gut microbiota abundance (genus Catenibacterium id.2153) on Ephrin type−B receptor 2
with a threshold of 1e−05
rs7742829
rs73128290
rs77285108
rs12404911
All
−0.4
−0.2
0.0
0.2
MR effect size for
'Gut microbiota abundance (genus Catenibacterium id.2153)' on 'Ephrin type−B receptor 2'

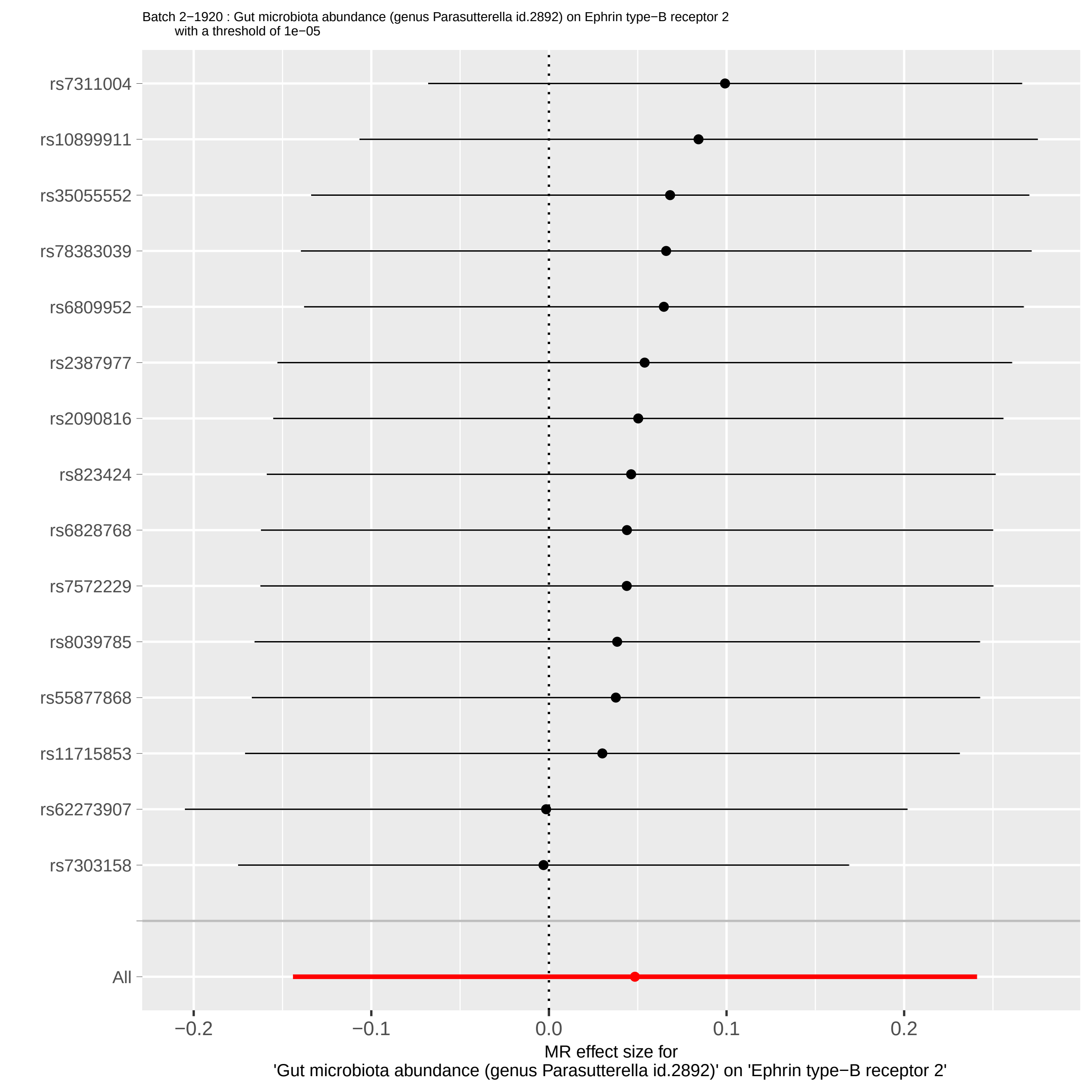
Batch 2−1920 : Gut microbiota abundance (genus Parasutterella id.2892) on Ephrin type−B receptor 2
with a threshold of 1e−05
rs7311004
rs10899911
rs35055552
rs78383039
rs6809952
rs2387977
rs2090816
rs823424
rs6828768
rs7572229
rs8039785
rs55877868
rs11715853
rs62273907
rs7303158
All
−0.2
−0.1
0.0
0.1
0.2
MR effect size for
'Gut microbiota abundance (genus Parasutterella id.2892)' on 'Ephrin type−B receptor 2'

Batch 2−1921 : Gut microbiota abundance (genus Catenibacterium id.2153) on Ephrin type−B receptor 2
with a threshold of 1e−05

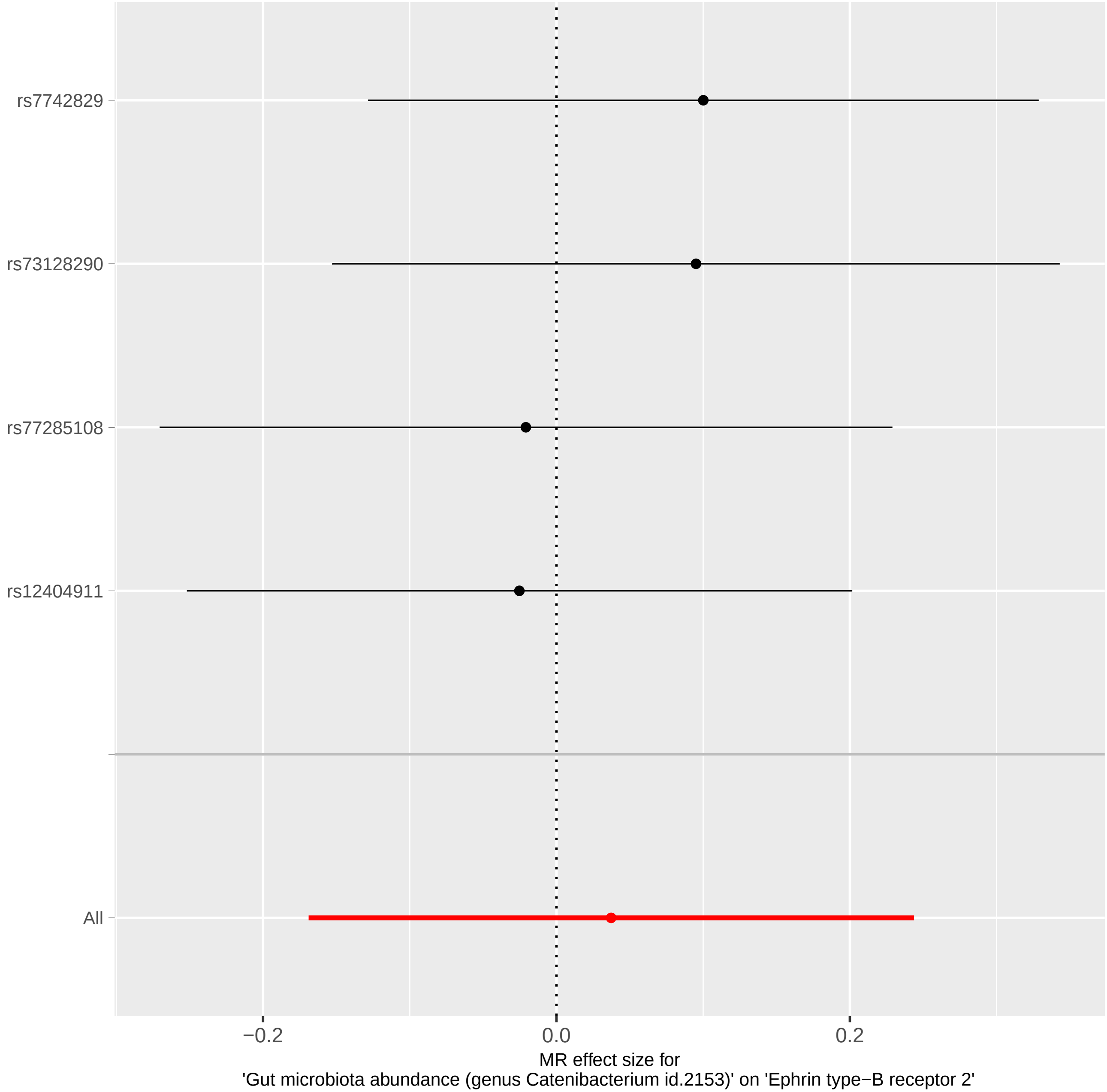

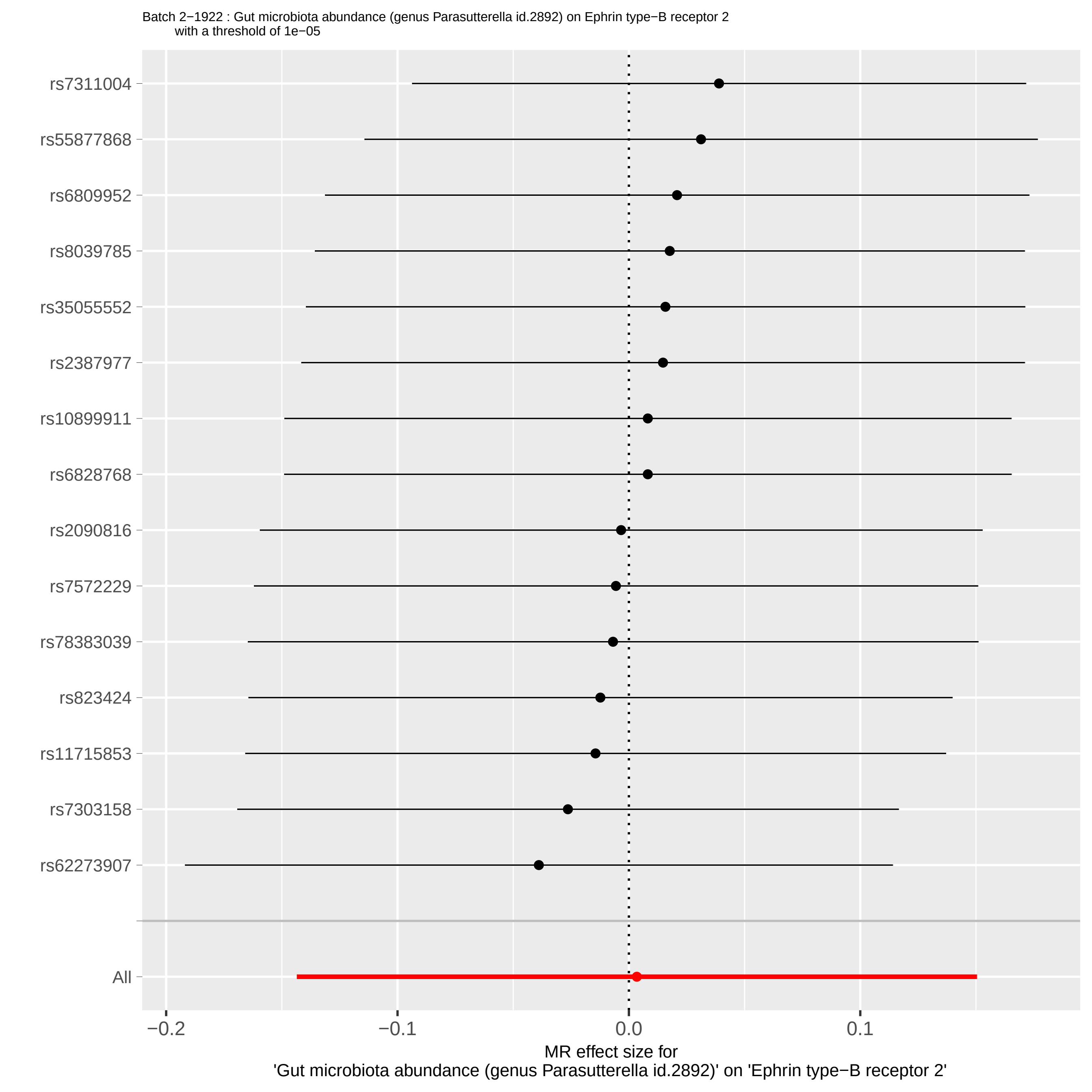

Batch 2−1922 : Gut microbiota abundance (genus Parasutterella id.2892) on Ephrin type−B receptor 2
with a threshold of 1e−05
rs7311004
rs55877868
rs6809952
rs8039785
rs35055552
rs2387977
rs10899911
rs6828768
rs2090816
rs7572229
rs78383039
rs823424
rs11715853
rs7303158
rs62273907
All
−0.2
−0.1
0.0
0.1
MR effect size for
'Gut microbiota abundance (genus Parasutterella id.2892)' on 'Ephrin type−B receptor 2'

Batch 2−1923 : Gut microbiota abundance (genus Catenibacterium id.2153) on Ephrin type−B receptor 2
with a threshold of 1e−05

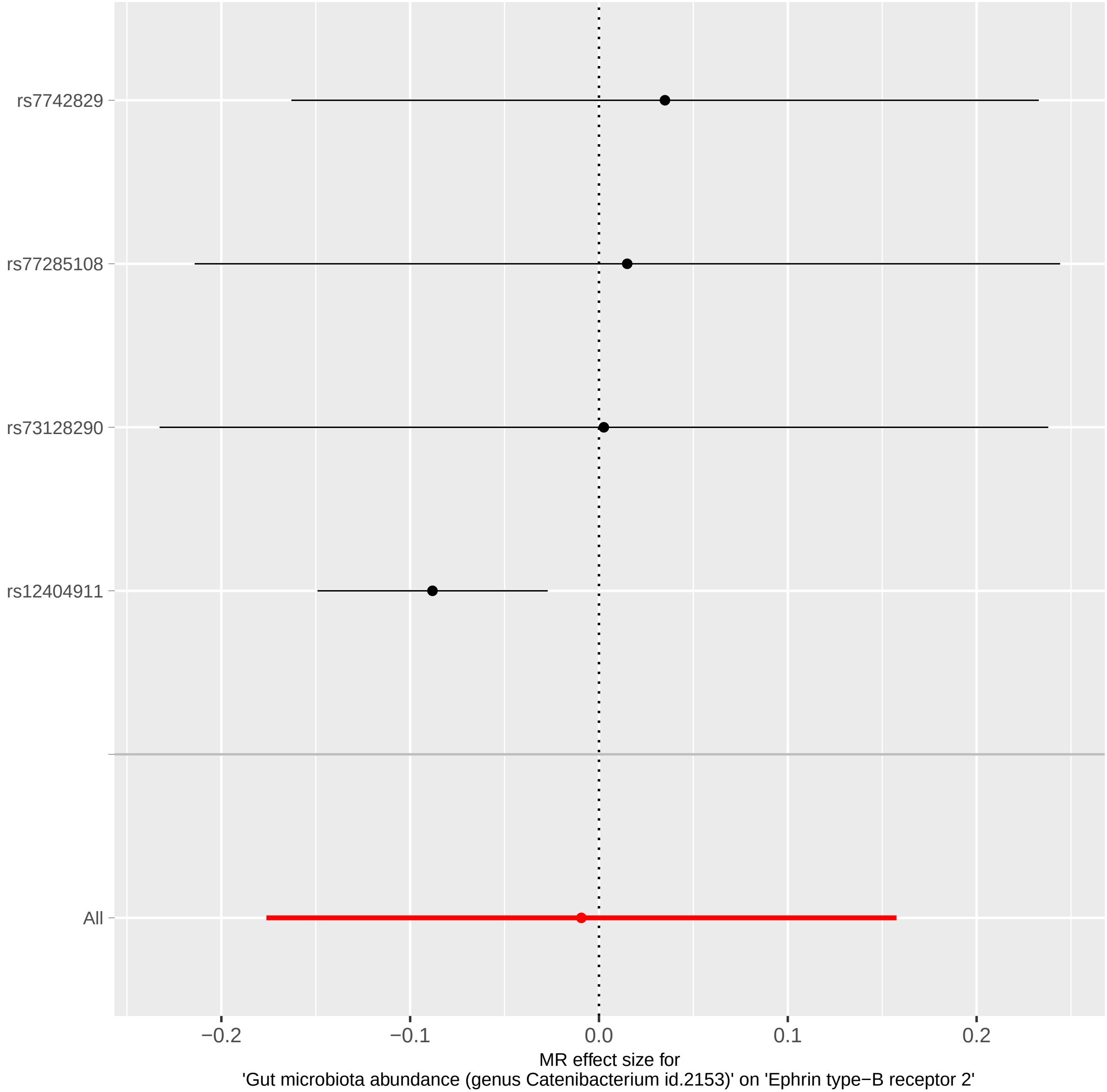

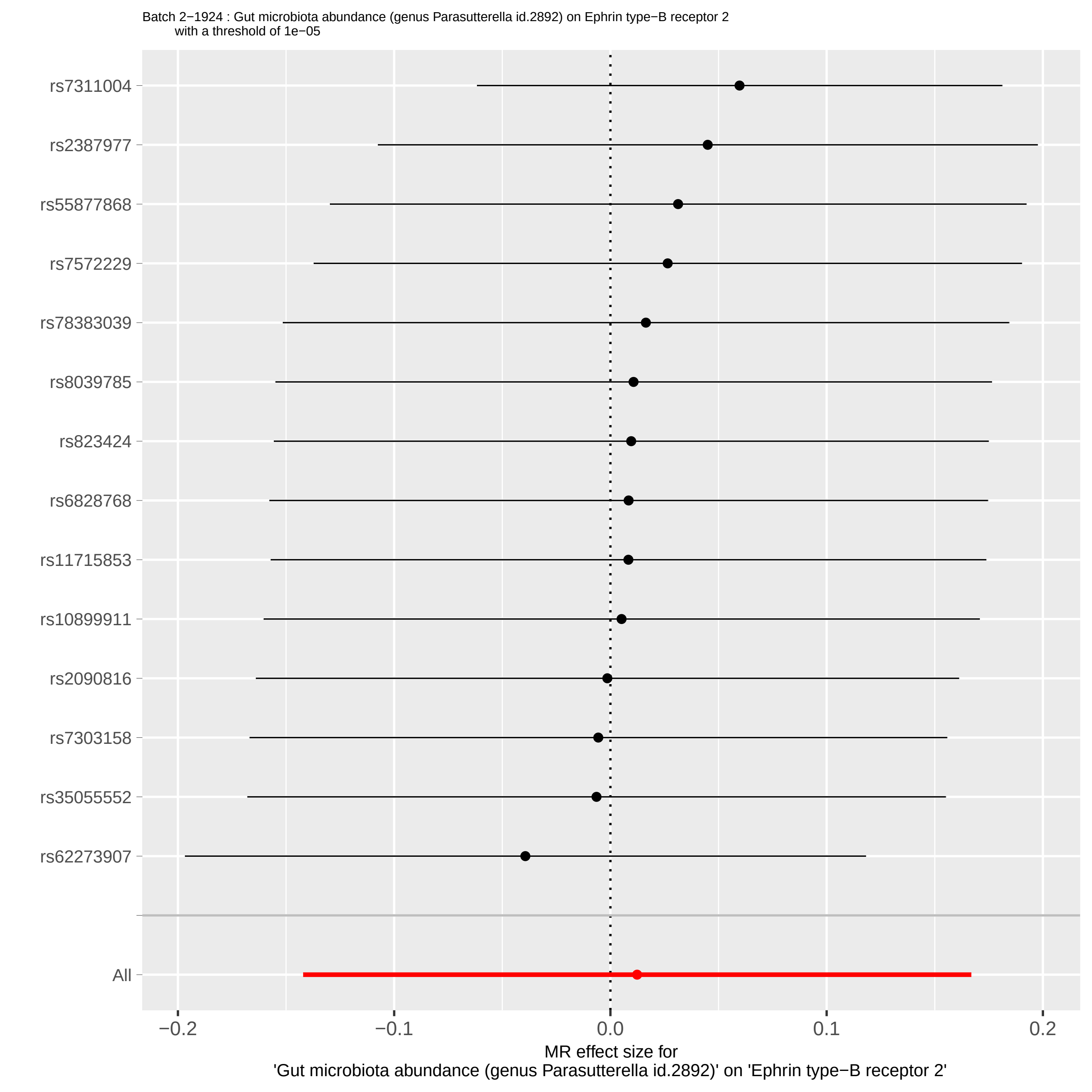
Batch 2−1924 : Gut microbiota abundance (genus Parasutterella id.2892) on Ephrin type−B receptor 2
with a threshold of 1e−05
rs7311004
rs2387977
rs55877868
rs7572229
rs78383039
rs8039785
rs823424
rs6828768
rs11715853
rs10899911
rs2090816
rs7303158
rs35055552
rs62273907
All
−0.2
−0.1
0.0
0.1
0.2
MR effect size for
'Gut microbiota abundance (genus Parasutterella id.2892)' on 'Ephrin type−B receptor 2'

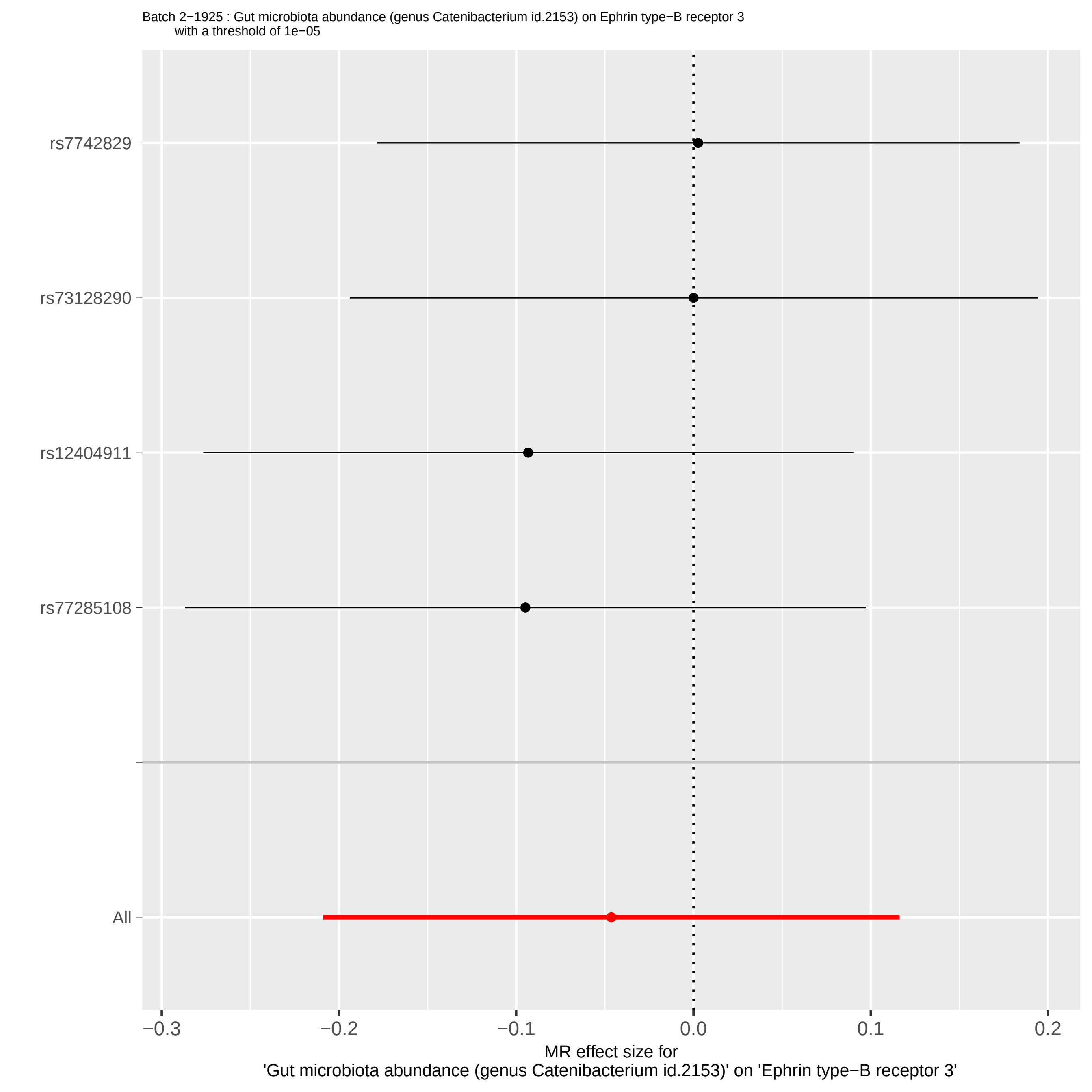

Batch 2−1925 : Gut microbiota abundance (genus Catenibacterium id.2153) on Ephrin type−B receptor 3
with a threshold of 1e−05
rs7742829
rs73128290
rs12404911
rs77285108
All
−0.3
−0.2
−0.1
0.0
0.1
0.2
MR effect size for
'Gut microbiota abundance (genus Catenibacterium id.2153)' on 'Ephrin type−B receptor 3'

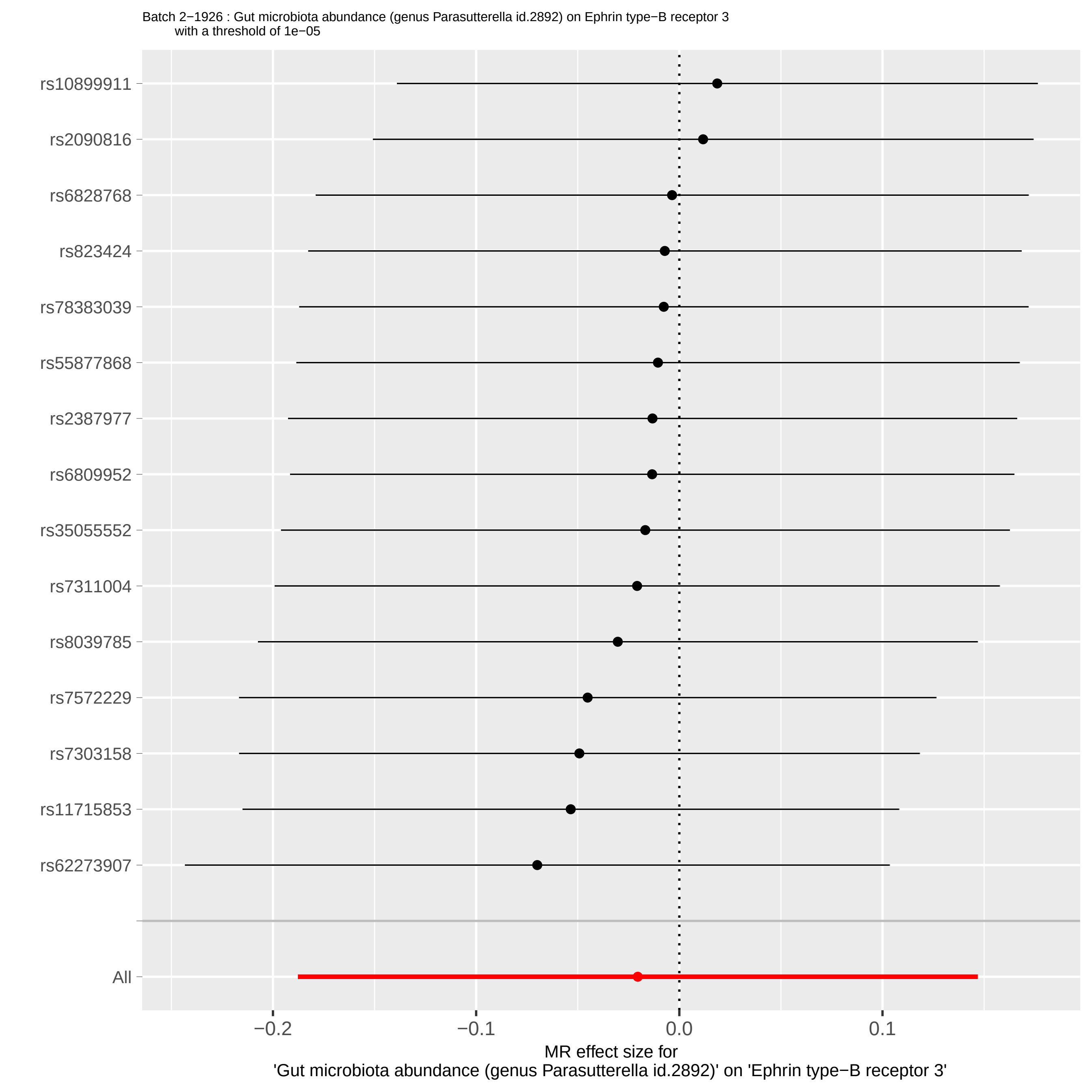

Batch 2−1926 : Gut microbiota abundance (genus Parasutterella id.2892) on Ephrin type−B receptor 3
with a threshold of 1e−05
rs10899911
rs2090816
rs6828768
rs823424
rs78383039
rs55877868
rs2387977
rs6809952
rs35055552
rs7311004
rs8039785
rs7572229
rs7303158
rs11715853
rs62273907
All
−0.2
−0.1
0.0
0.1
MR effect size for
'Gut microbiota abundance (genus Parasutterella id.2892)' on 'Ephrin type−B receptor 3'

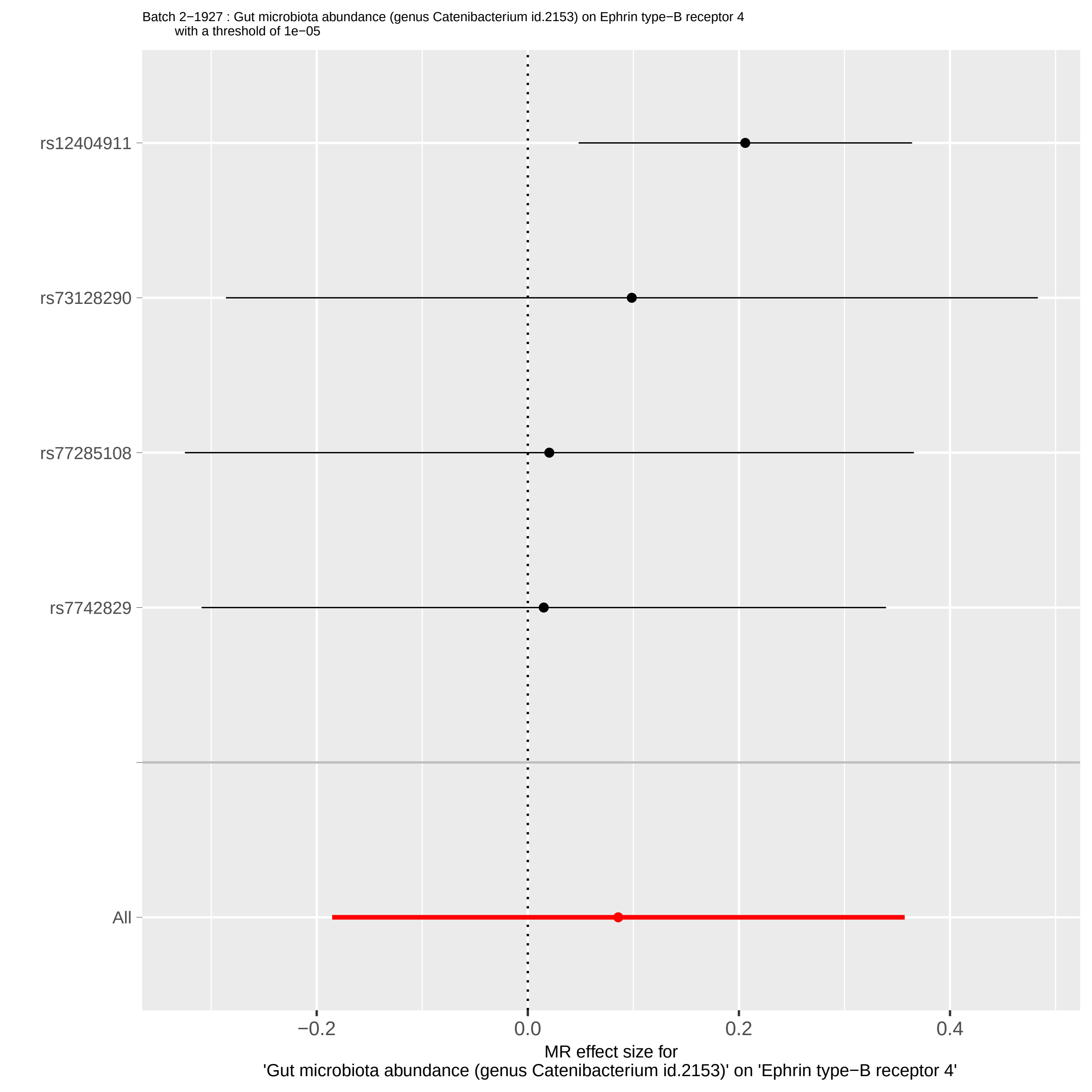
Batch 2−1927 : Gut microbiota abundance (genus Catenibacterium id.2153) on Ephrin type−B receptor 4
with a threshold of 1e−05
rs12404911
rs73128290
rs77285108
rs7742829
All
−0.2
0.0
0.2
0.4
MR effect size for
'Gut microbiota abundance (genus Catenibacterium id.2153)' on 'Ephrin type−B receptor 4'

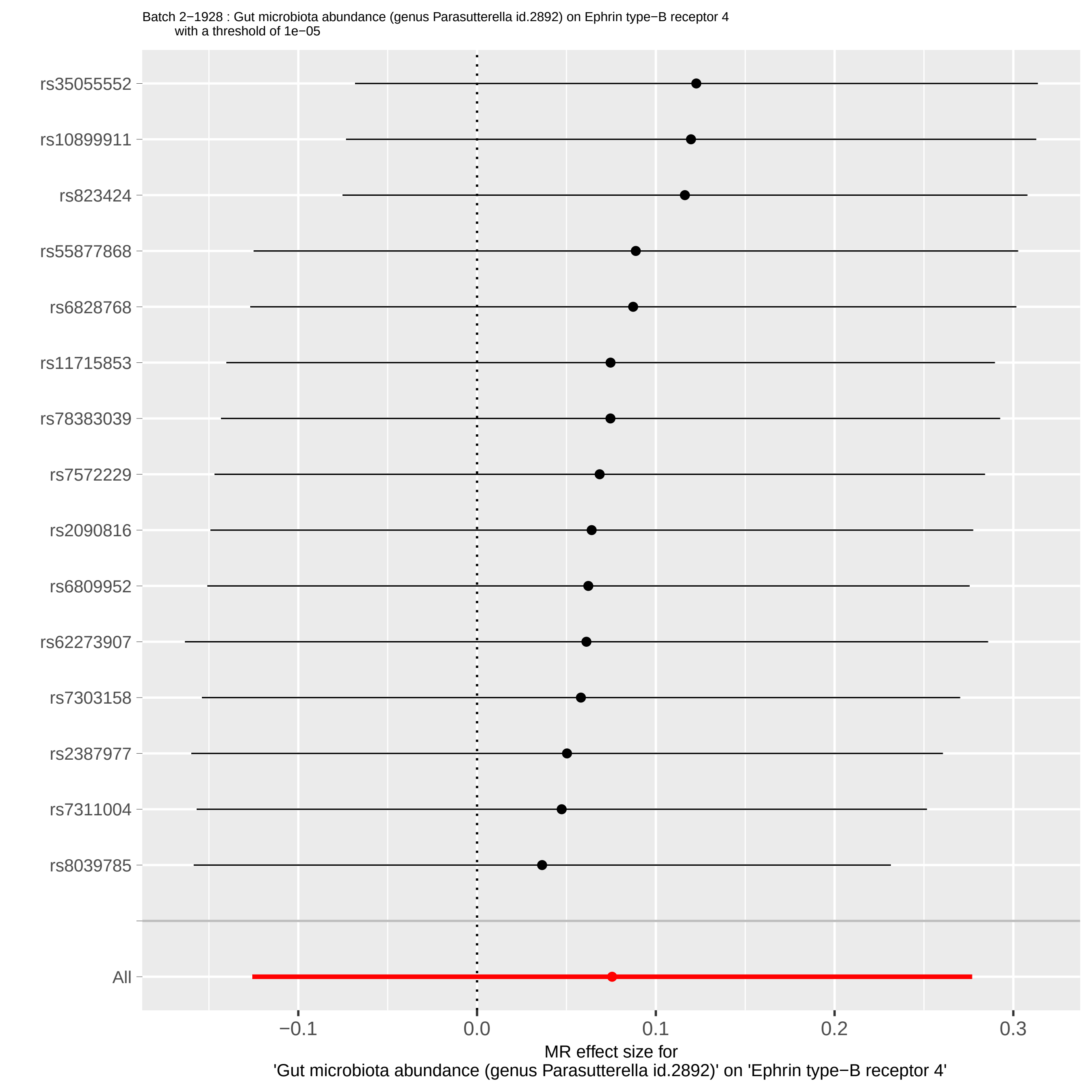
Batch 2−1928 : Gut microbiota abundance (genus Parasutterella id.2892) on Ephrin type−B receptor 4
with a threshold of 1e−05
rs35055552
rs10899911
rs823424
rs55877868
rs6828768
rs11715853
rs78383039
rs7572229
rs2090816
rs6809952
rs62273907
rs7303158
rs2387977
rs7311004
rs8039785
All
−0.1
0.0
0.1
0.2
0.3
MR effect size for
'Gut microbiota abundance (genus Parasutterella id.2892)' on 'Ephrin type−B receptor 4'

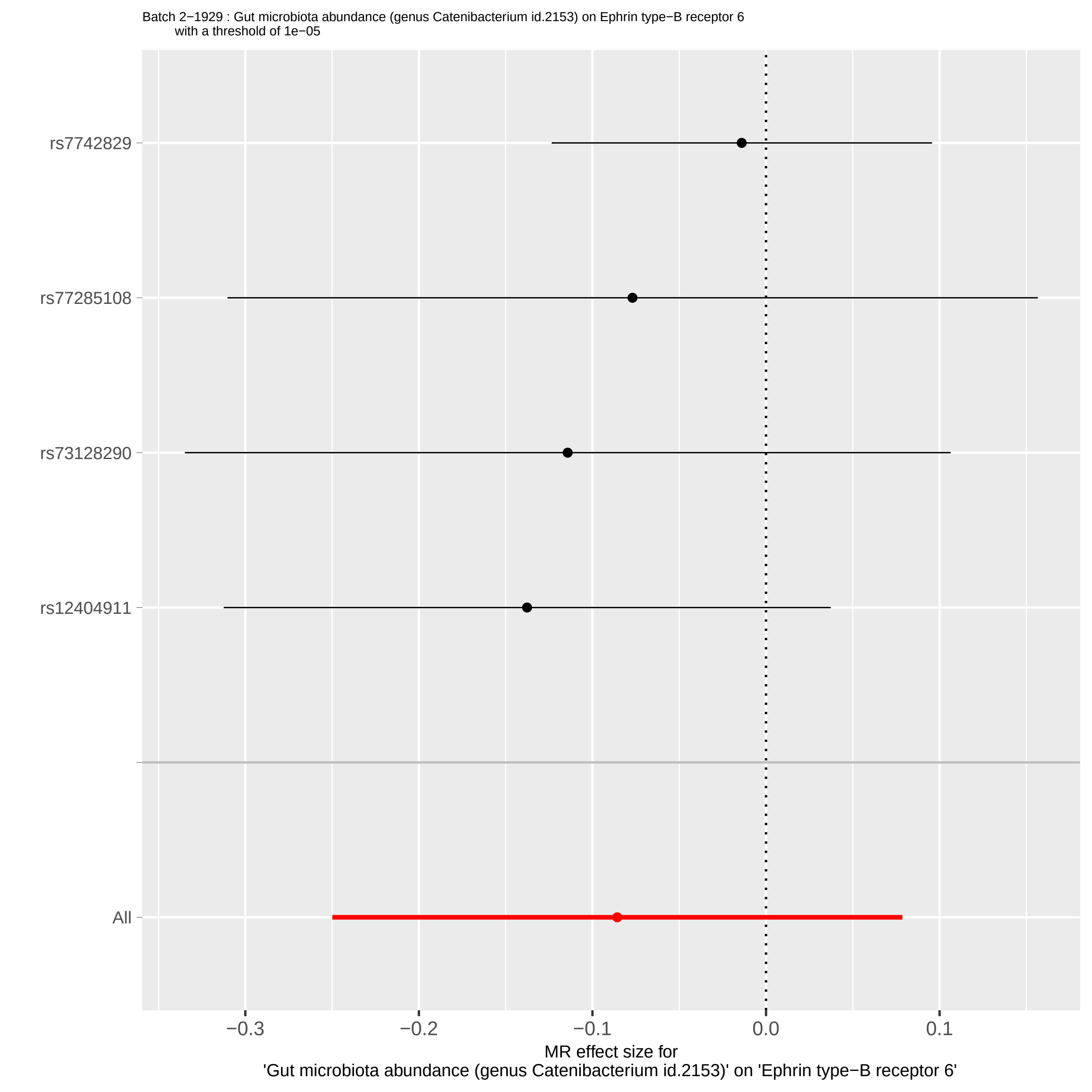

Batch 2−1929 : Gut microbiota abundance (genus Catenibacterium id.2153) on Ephrin type−B receptor 6
with a threshold of 1e−05
rs7742829
rs77285108
rs73128290
rs12404911
All
−0.3
−0.2
−0.1
0.0
0.1
MR effect size for
'Gut microbiota abundance (genus Catenibacterium id.2153)' on 'Ephrin type−B receptor 6'

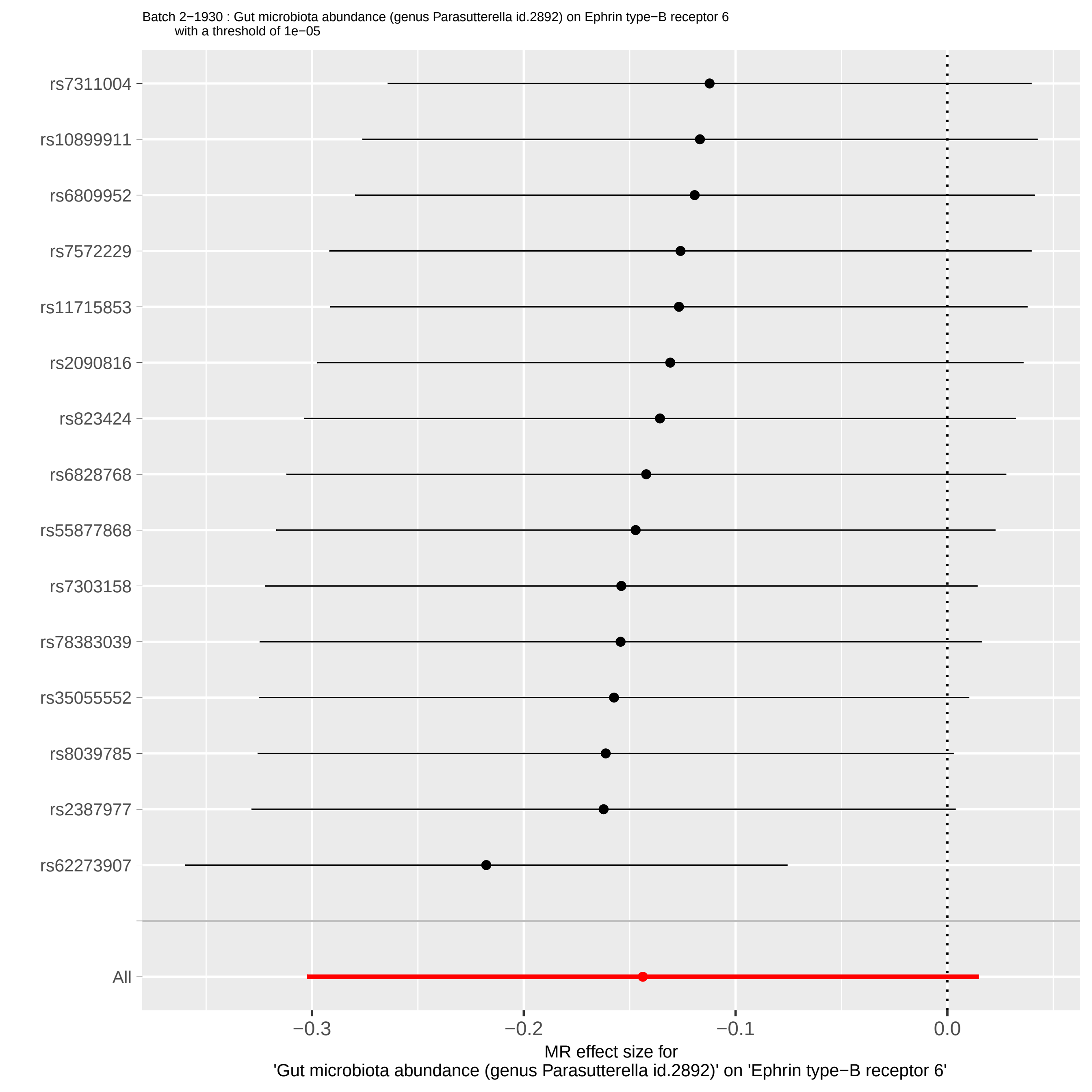

Batch 2−1930 : Gut microbiota abundance (genus Parasutterella id.2892) on Ephrin type−B receptor 6
with a threshold of 1e−05
rs7311004
rs10899911
rs6809952
rs7572229
rs11715853
rs2090816
rs823424
rs6828768
rs55877868
rs7303158
rs78383039
rs35055552
rs8039785
rs2387977
rs62273907
All
−0.3
−0.2
−0.1
0.0
MR effect size for
'Gut microbiota abundance (genus Parasutterella id.2892)' on 'Ephrin type−B receptor 6'

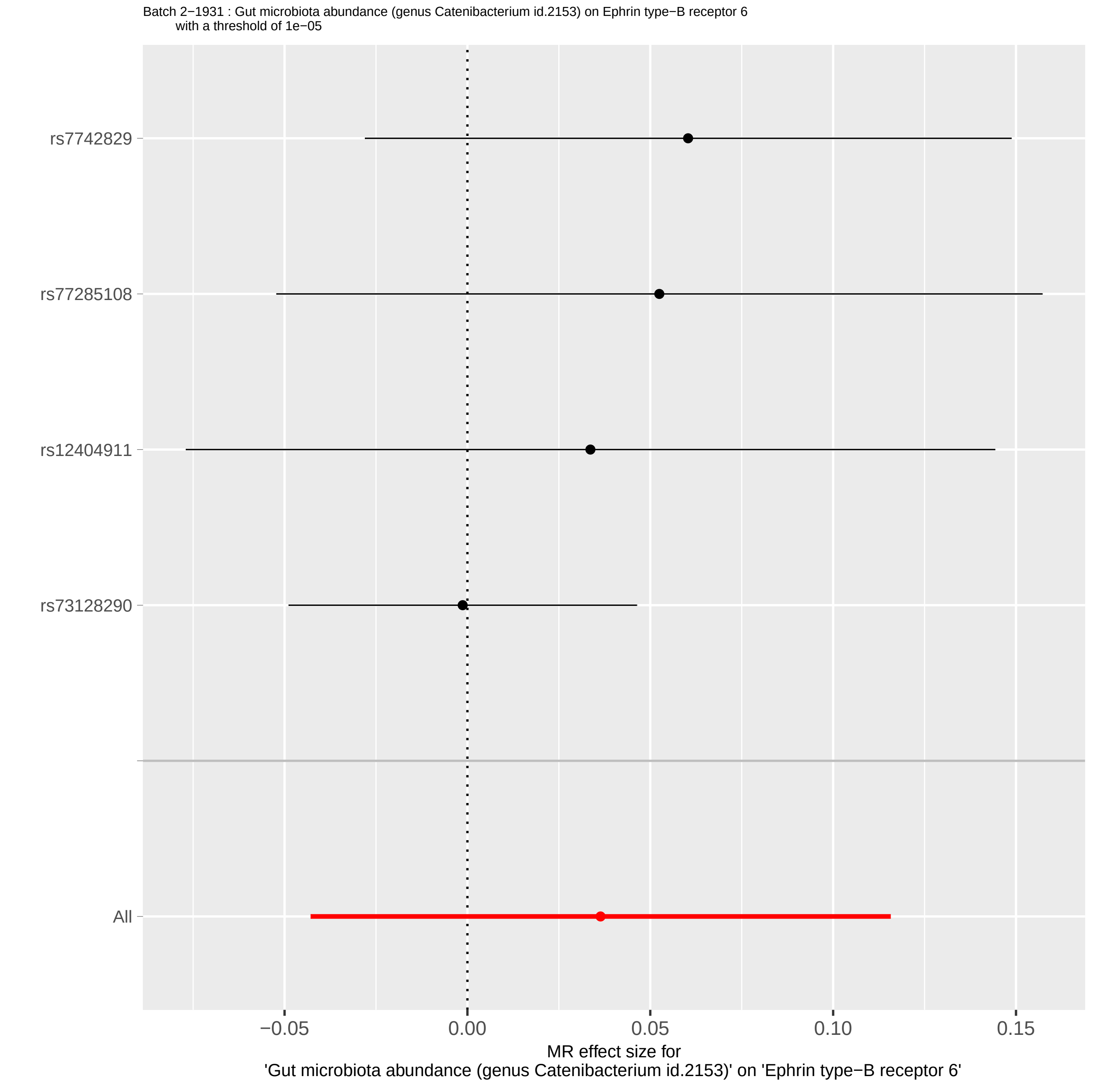
Batch 2−1931 : Gut microbiota abundance (genus Catenibacterium id.2153) on Ephrin type−B receptor 6
with a threshold of 1e−05
rs7742829
rs77285108
rs12404911
rs73128290
All
−0.05
0.00
0.05
0.10
0.15
MR effect size for
'Gut microbiota abundance (genus Catenibacterium id.2153)' on 'Ephrin type−B receptor 6'

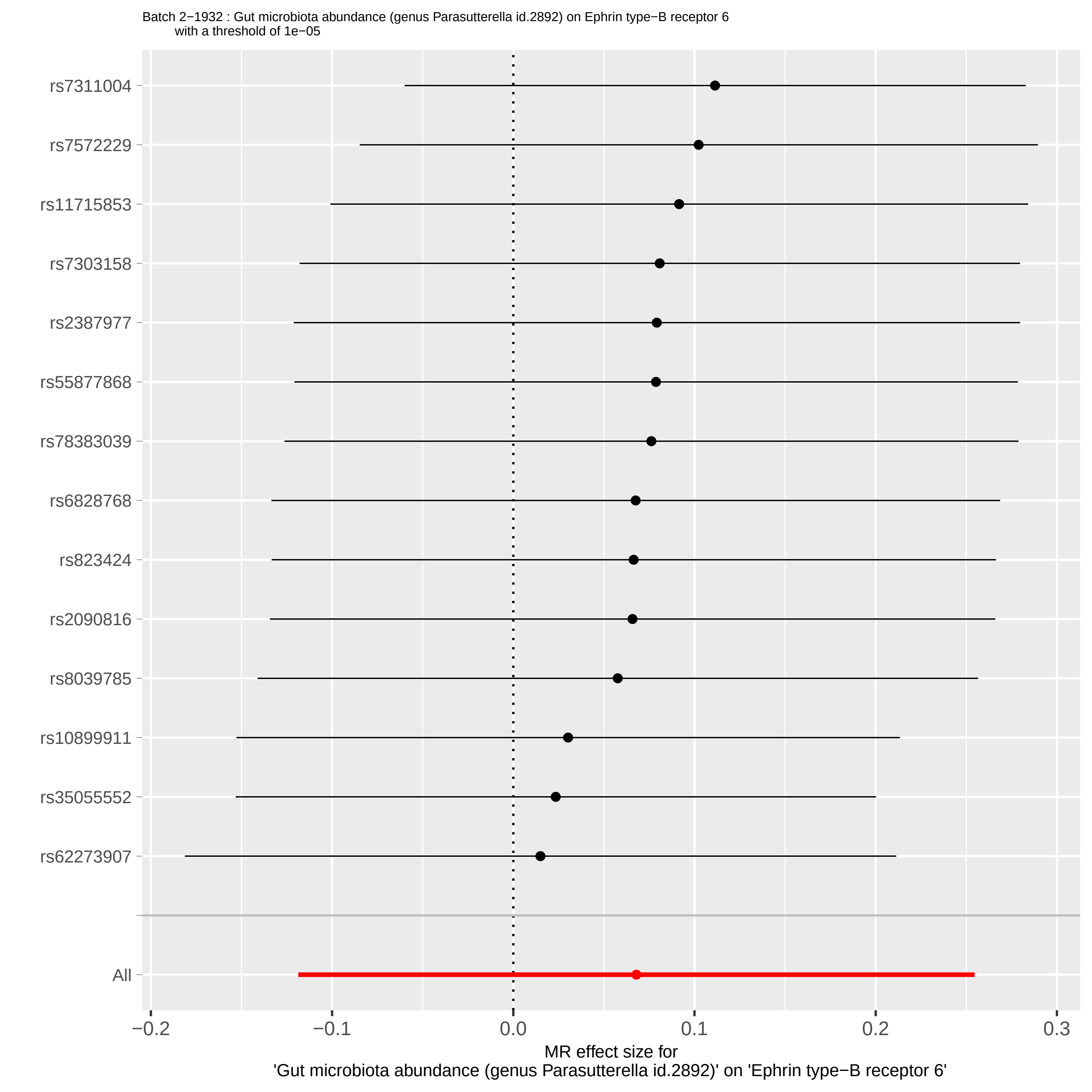
Batch 2−1932 : Gut microbiota abundance (genus Parasutterella id.2892) on Ephrin type−B receptor 6
with a threshold of 1e−05
rs7311004
rs7572229
rs11715853
rs7303158
rs2387977
rs55877868
rs78383039
rs6828768
rs823424
rs2090816
rs8039785
rs10899911
rs35055552
rs62273907
All
−0.2
−0.1
0.0
0.1
0.2
0.3
MR effect size for
'Gut microbiota abundance (genus Parasutterella id.2892)' on 'Ephrin type−B receptor 6'

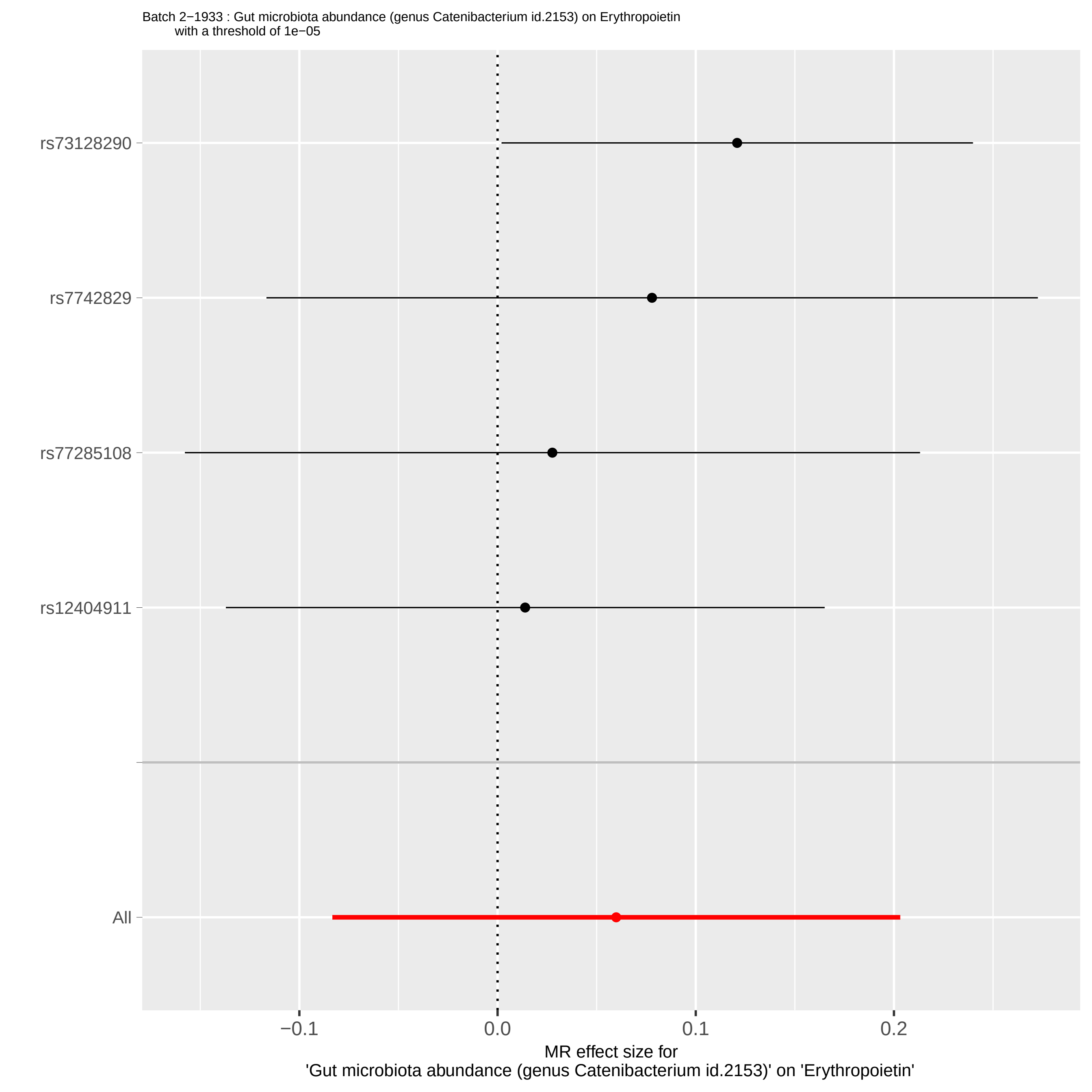
Batch 2−1933 : Gut microbiota abundance (genus Catenibacterium id.2153) on Erythropoietin
with a threshold of 1e−05
rs73128290
rs7742829
rs77285108
rs12404911
All
−0.1
0.0
0.1
0.2
MR effect size for
'Gut microbiota abundance (genus Catenibacterium id.2153)' on 'Erythropoietin'

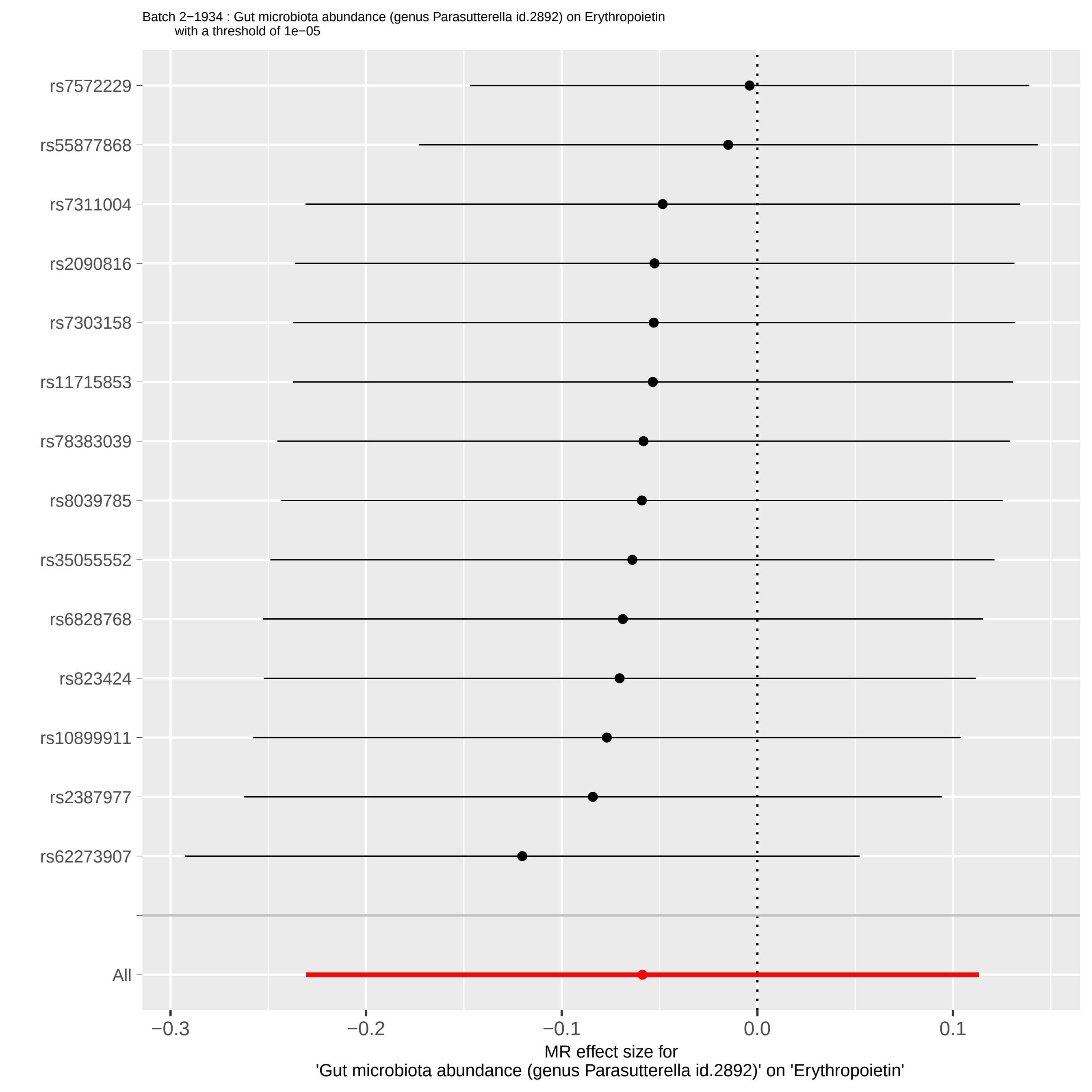
Batch 2−1934 : Gut microbiota abundance (genus Parasutterella id.2892) on Erythropoietin
with a threshold of 1e−05
rs7572229
rs55877868
rs7311004
rs2090816
rs7303158
rs11715853
rs78383039
rs8039785
rs35055552
rs6828768
rs823424
rs10899911
rs2387977
rs62273907
All
−0.3
−0.2
−0.1
0.0
0.1
MR effect size for
'Gut microbiota abundance (genus Parasutterella id.2892)' on 'Erythropoietin'

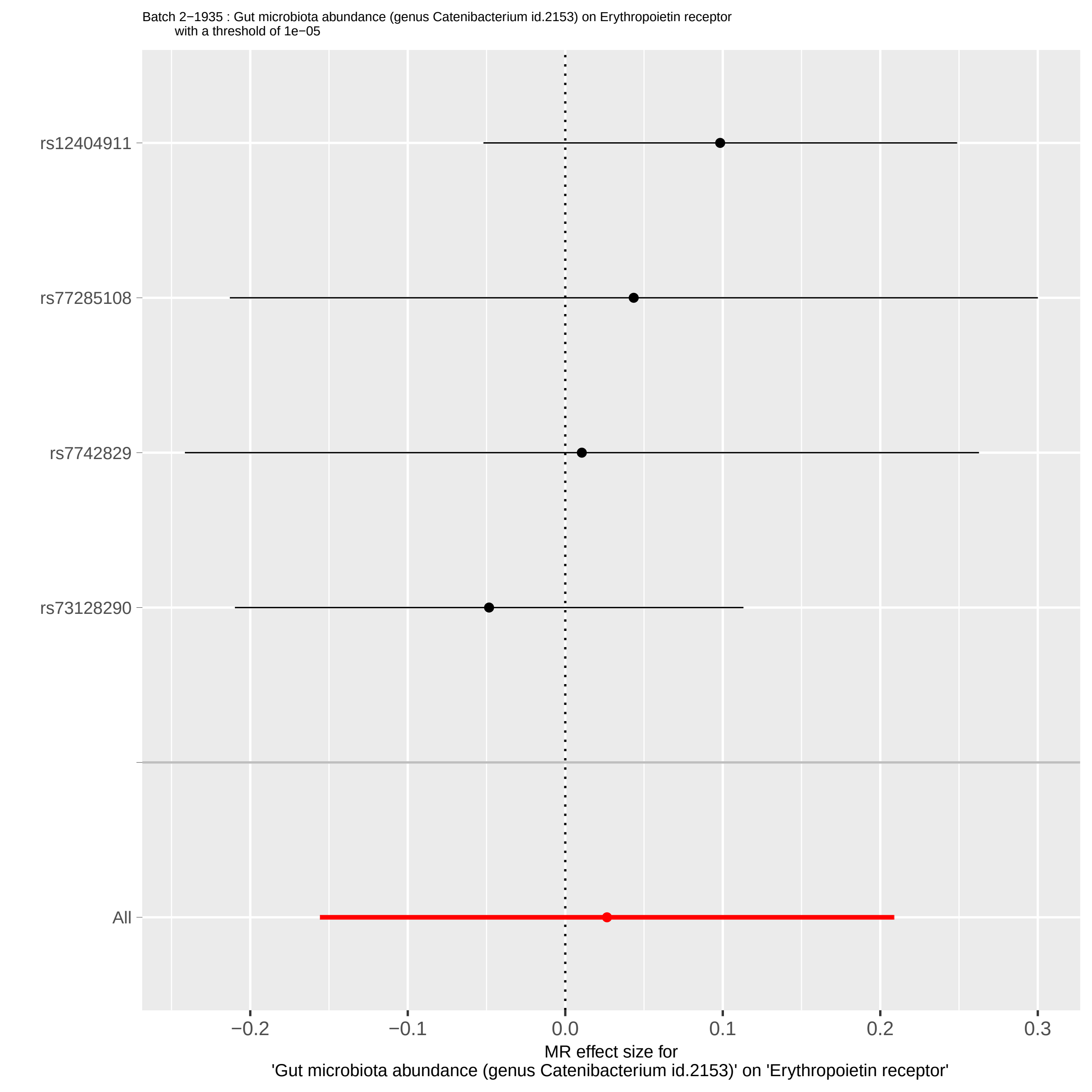

Batch 2−1935 : Gut microbiota abundance (genus Catenibacterium id.2153) on Erythropoietin receptor
with a threshold of 1e−05
rs12404911
rs77285108
rs7742829
rs73128290
All
−0.2
−0.1
0.0
0.1
0.2
0.3
MR effect size for
'Gut microbiota abundance (genus Catenibacterium id.2153)' on 'Erythropoietin receptor'

Batch 2−1936 : Gut microbiota abundance (genus Parasutterella id.2892) on Erythropoietin receptor
with a threshold of 1e−05

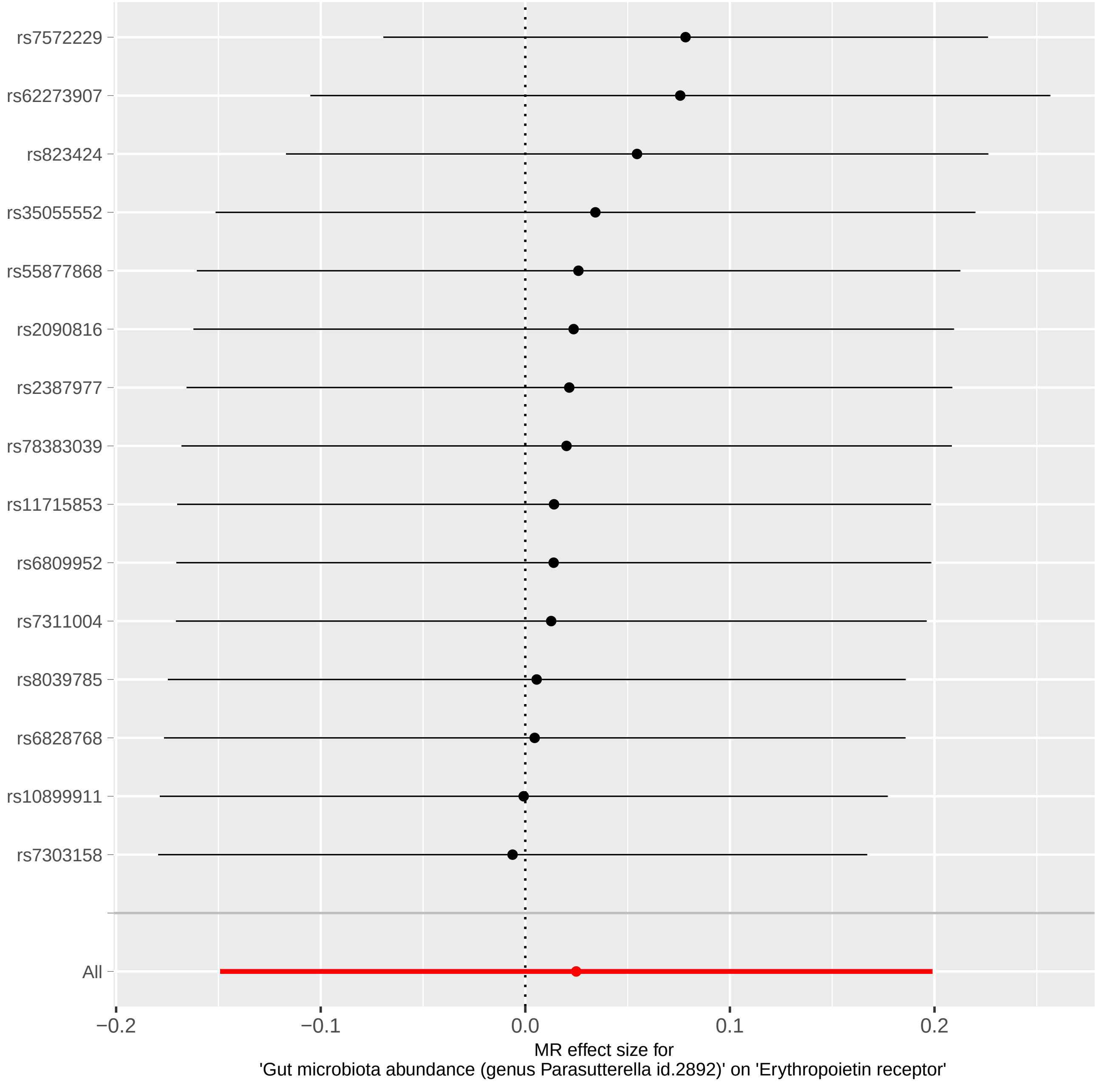

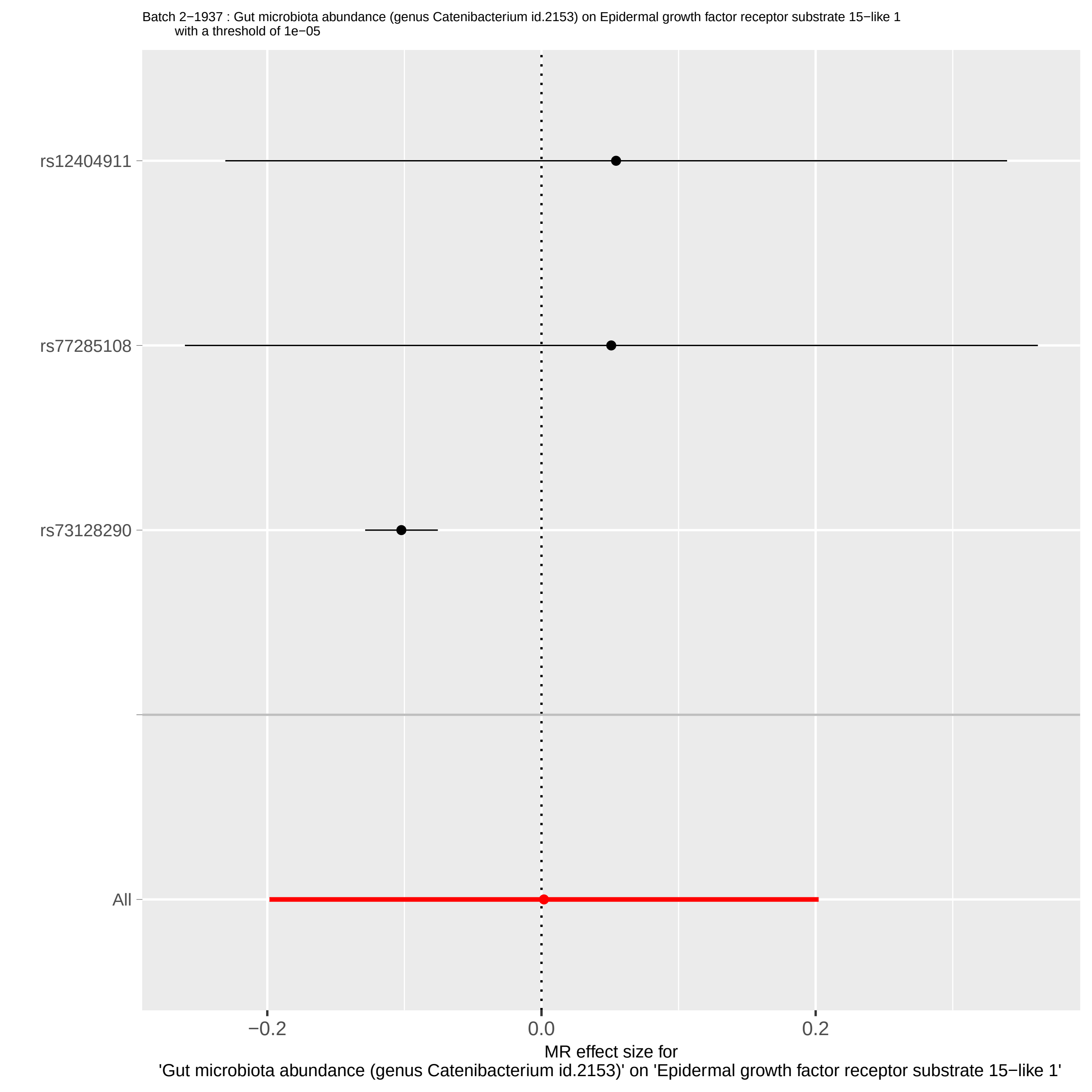

Batch 2−1937 : Gut microbiota abundance (genus Catenibacterium id.2153) on Epidermal growth factor receptor substrate 15−like 1
with a threshold of 1e−05
rs12404911
rs77285108
rs73128290
All
−0.2
0.0
0.2
MR effect size for
'Gut microbiota abundance (genus Catenibacterium id.2153)' on 'Epidermal growth factor receptor substrate 15−like 1'

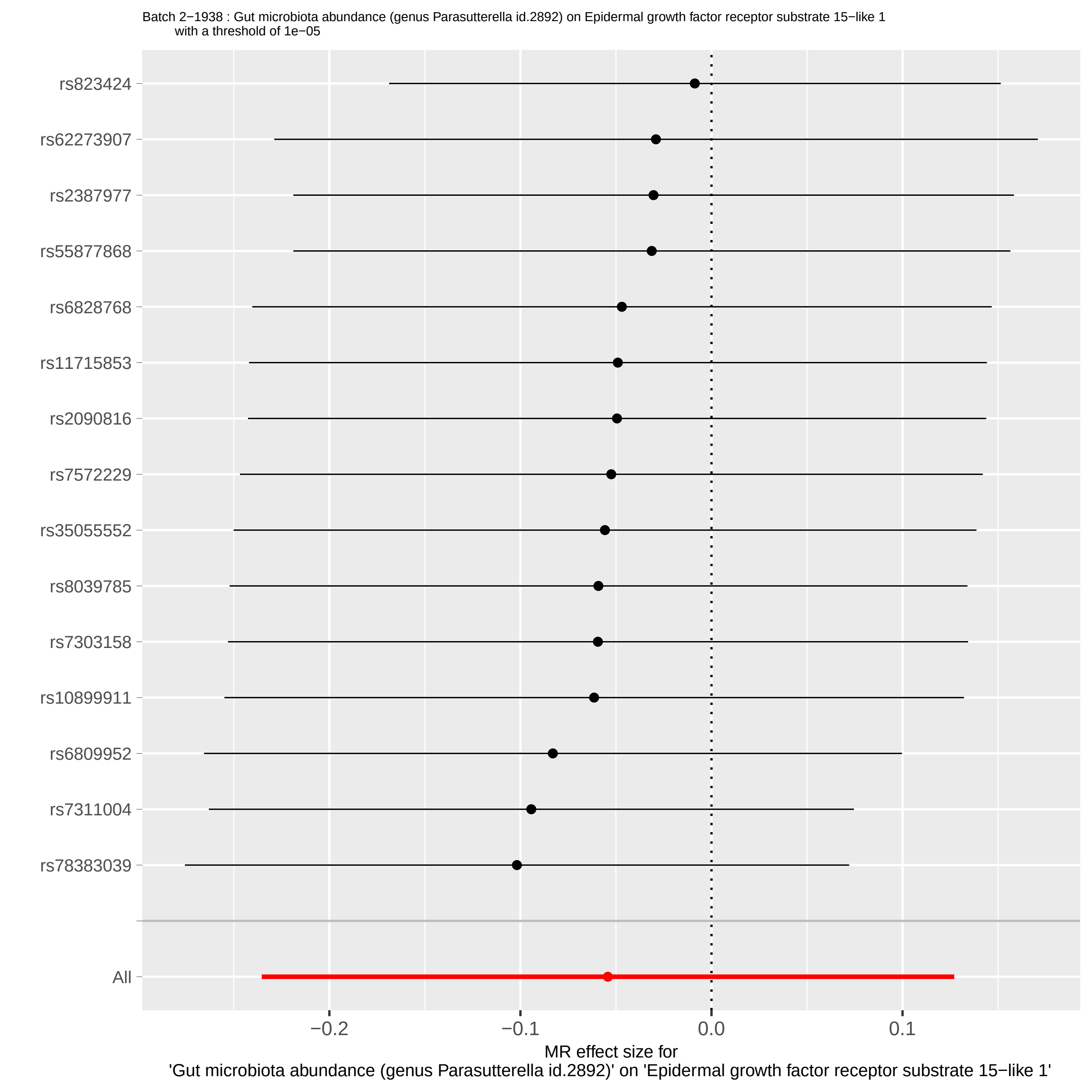
Batch 2−1938 : Gut microbiota abundance (genus Parasutterella id.2892) on Epidermal growth factor receptor substrate 15−like 1
with a threshold of 1e−05
rs823424
rs62273907
rs2387977
rs55877868
rs6828768
rs11715853
rs2090816
rs7572229
rs35055552
rs8039785
rs7303158
rs10899911
rs6809952
rs7311004
rs78383039
All
−0.2
−0.1
0.0
0.1
MR effect size for
'Gut microbiota abundance (genus Parasutterella id.2892)' on 'Epidermal growth factor receptor substrate 15−like 1'

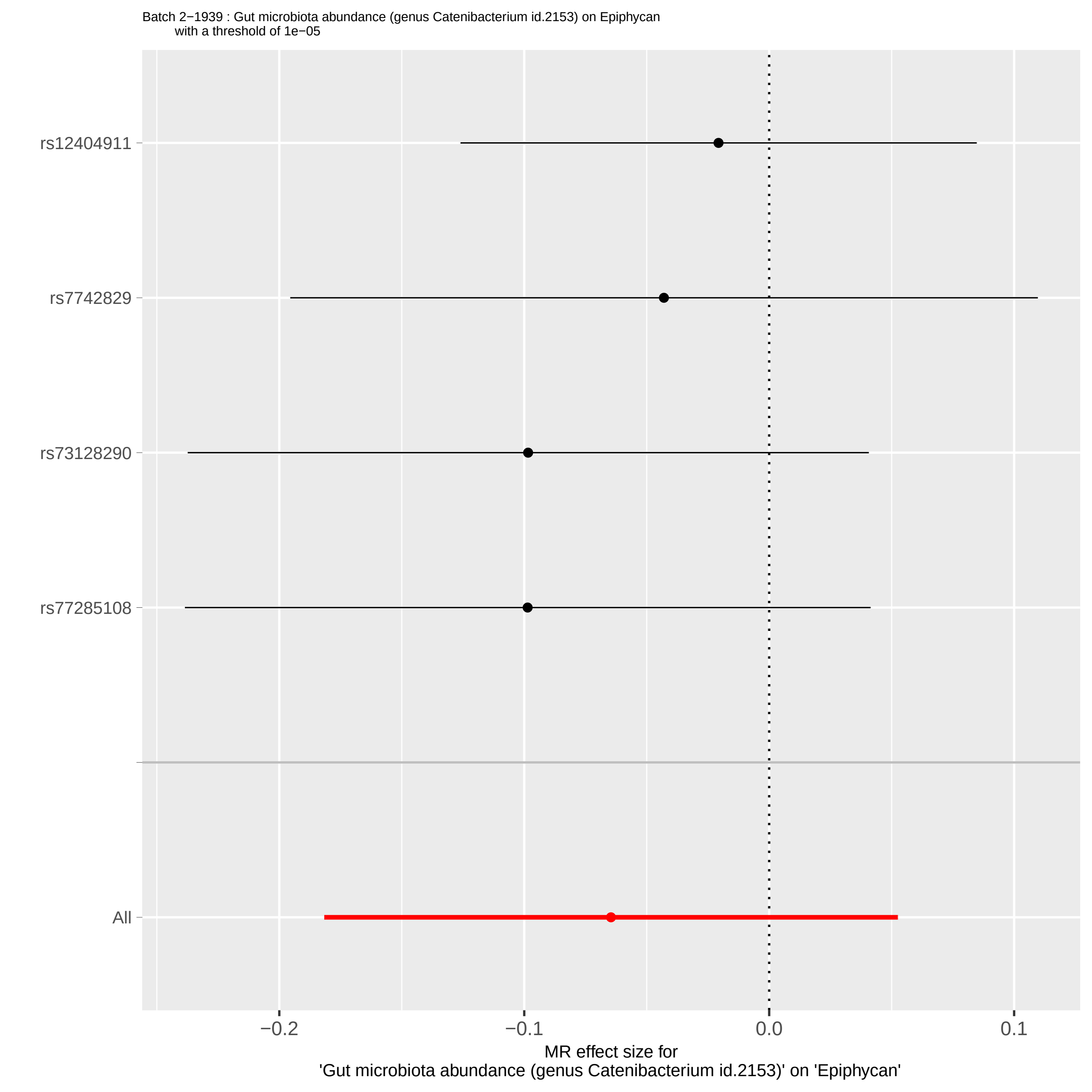
Batch 2−1939 : Gut microbiota abundance (genus Catenibacterium id.2153) on Epiphycan
with a threshold of 1e−05
rs12404911
rs7742829
rs73128290
rs77285108
All
−0.2
−0.1
0.0
0.1
MR effect size for
'Gut microbiota abundance (genus Catenibacterium id.2153)' on 'Epiphycan'

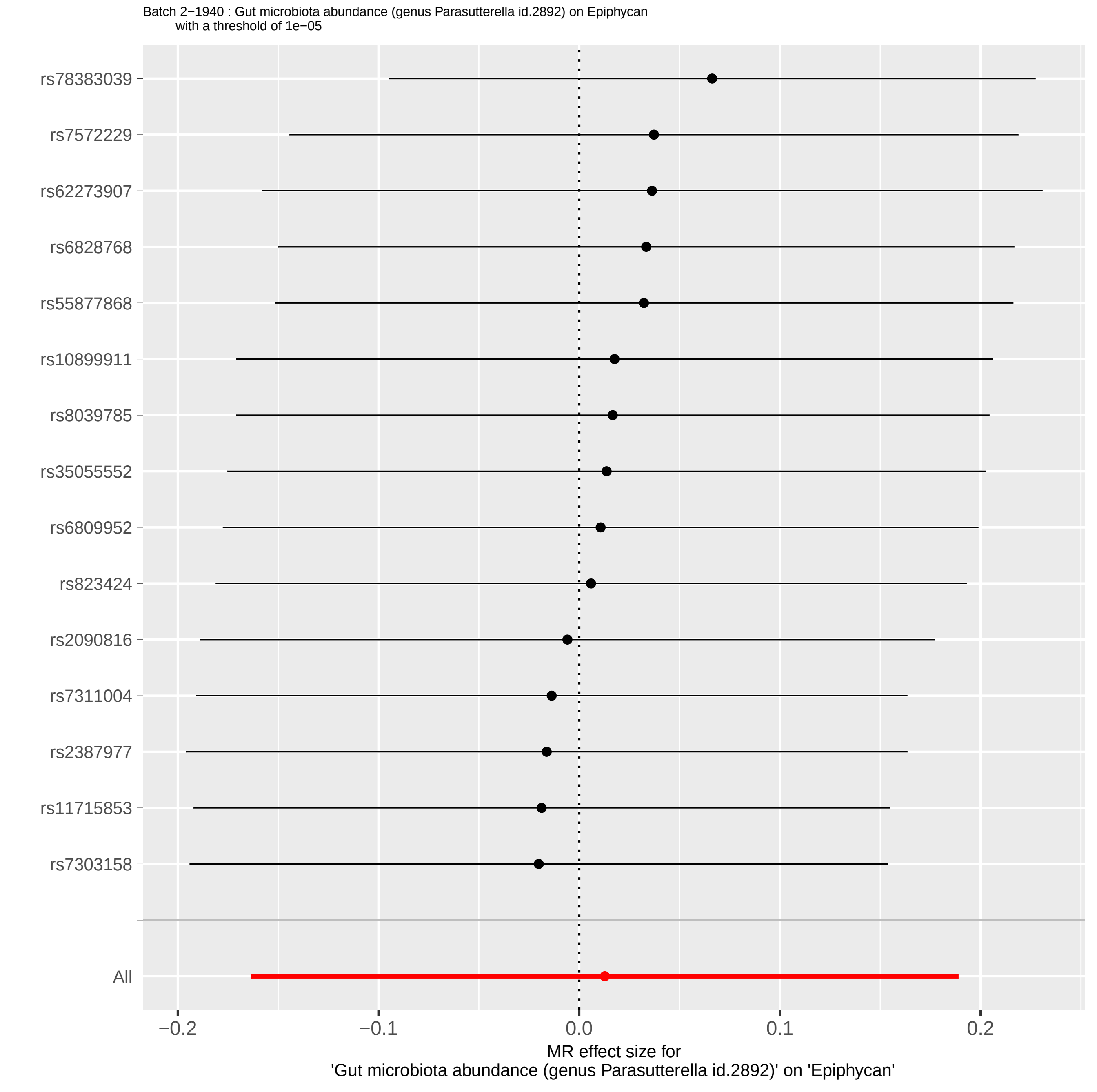
Batch 2−1940 : Gut microbiota abundance (genus Parasutterella id.2892) on Epiphycan
with a threshold of 1e−05
rs78383039
rs7572229
rs62273907
rs6828768
rs55877868
rs10899911
rs8039785
rs35055552
rs6809952
rs823424
rs2090816
rs7311004
rs2387977
rs11715853
rs7303158
All
−0.2
−0.1
0.0
0.1
0.2
MR effect size for
'Gut microbiota abundance (genus Parasutterella id.2892)' on 'Epiphycan'

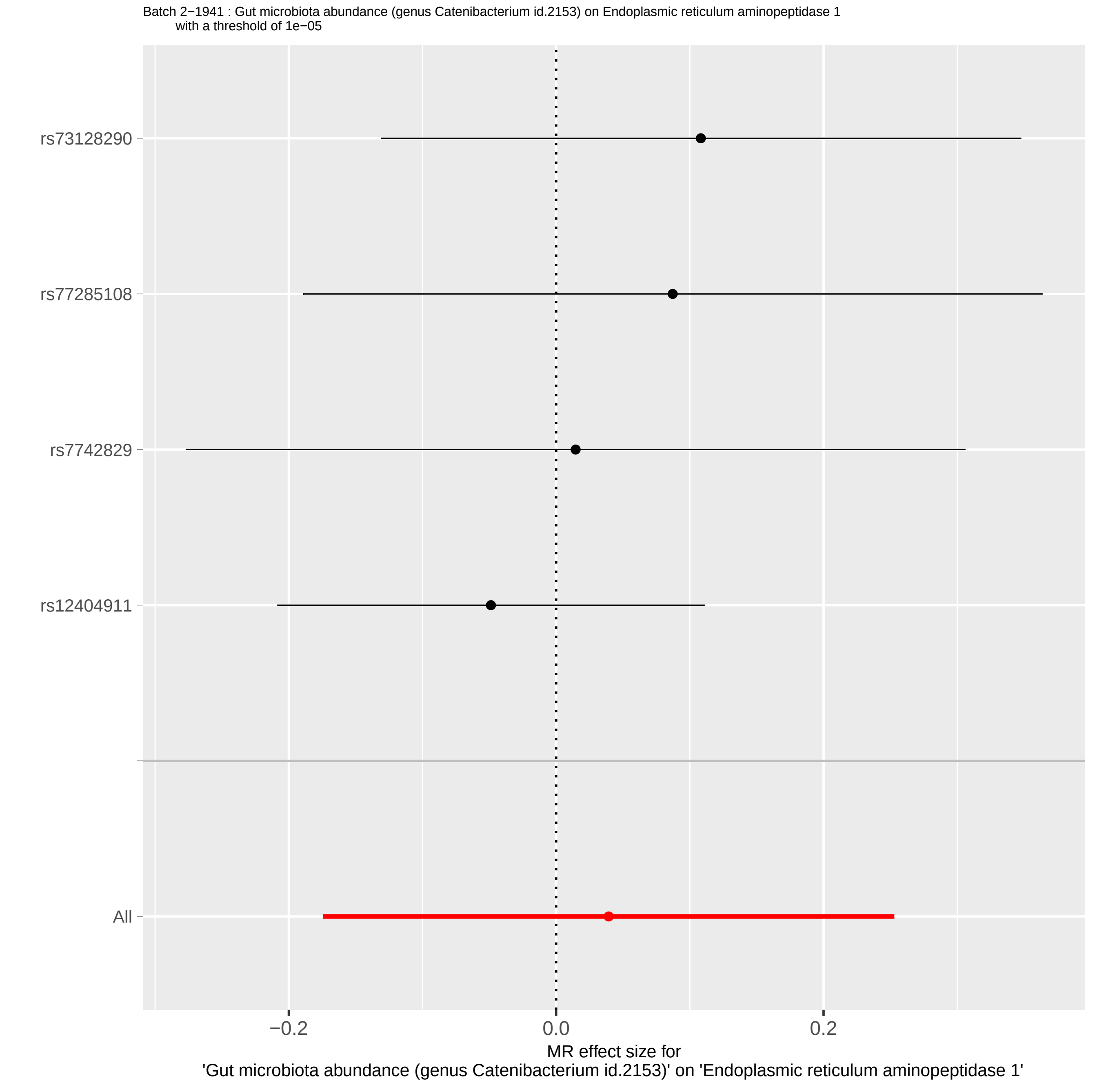

Batch 2−1941 : Gut microbiota abundance (genus Catenibacterium id.2153) on Endoplasmic reticulum aminopeptidase 1
with a threshold of 1e−05
rs73128290
rs77285108
rs7742829
rs12404911
All
−0.2
0.0
0.2
MR effect size for
'Gut microbiota abundance (genus Catenibacterium id.2153)' on 'Endoplasmic reticulum aminopeptidase 1'

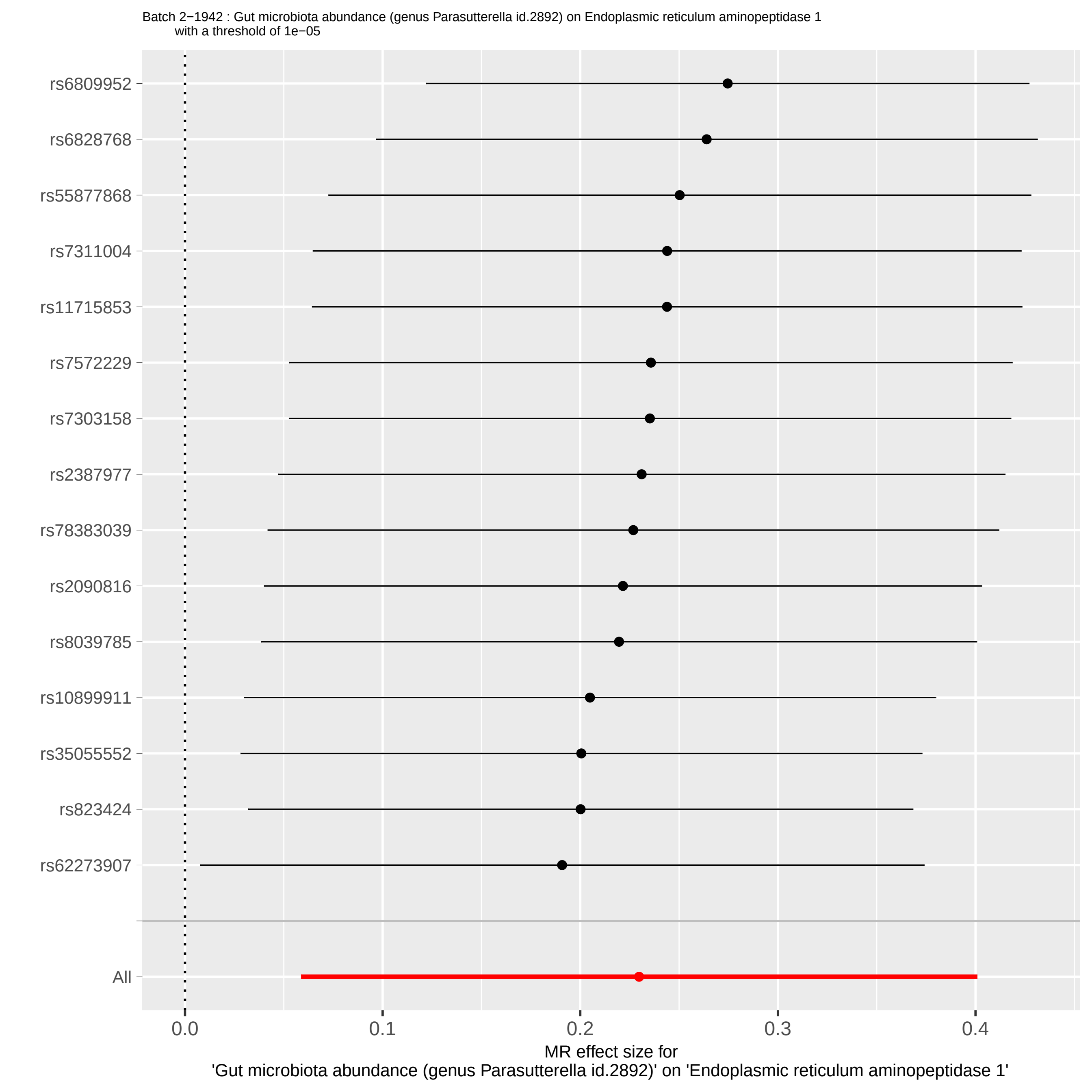

Batch 2−1942 : Gut microbiota abundance (genus Parasutterella id.2892) on Endoplasmic reticulum aminopeptidase 1
with a threshold of 1e−05
rs6809952
rs6828768
rs55877868
rs7311004
rs11715853
rs7572229
rs7303158
rs2387977
rs78383039
rs2090816
rs8039785
rs10899911
rs35055552
rs823424
rs62273907
All
0.0
0.1
0.2
0.3
0.4
MR effect size for
'Gut microbiota abundance (genus Parasutterella id.2892)' on 'Endoplasmic reticulum aminopeptidase 1'

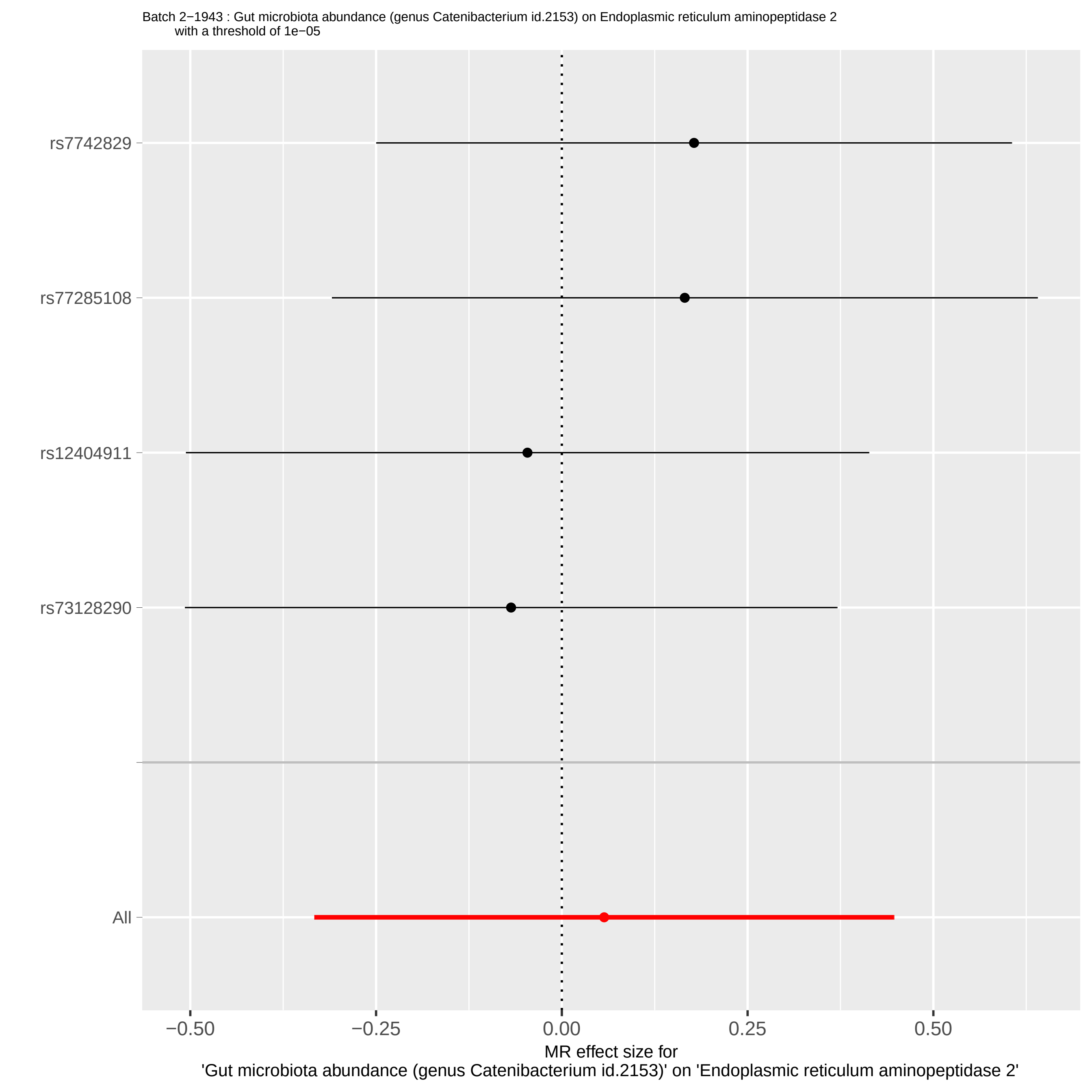

Batch 2−1943 : Gut microbiota abundance (genus Catenibacterium id.2153) on Endoplasmic reticulum aminopeptidase 2
with a threshold of 1e−05
rs7742829
rs77285108
rs12404911
rs73128290
All
−0.50
−0.25
0.00
0.25
0.50
MR effect size for
'Gut microbiota abundance (genus Catenibacterium id.2153)' on 'Endoplasmic reticulum aminopeptidase 2'

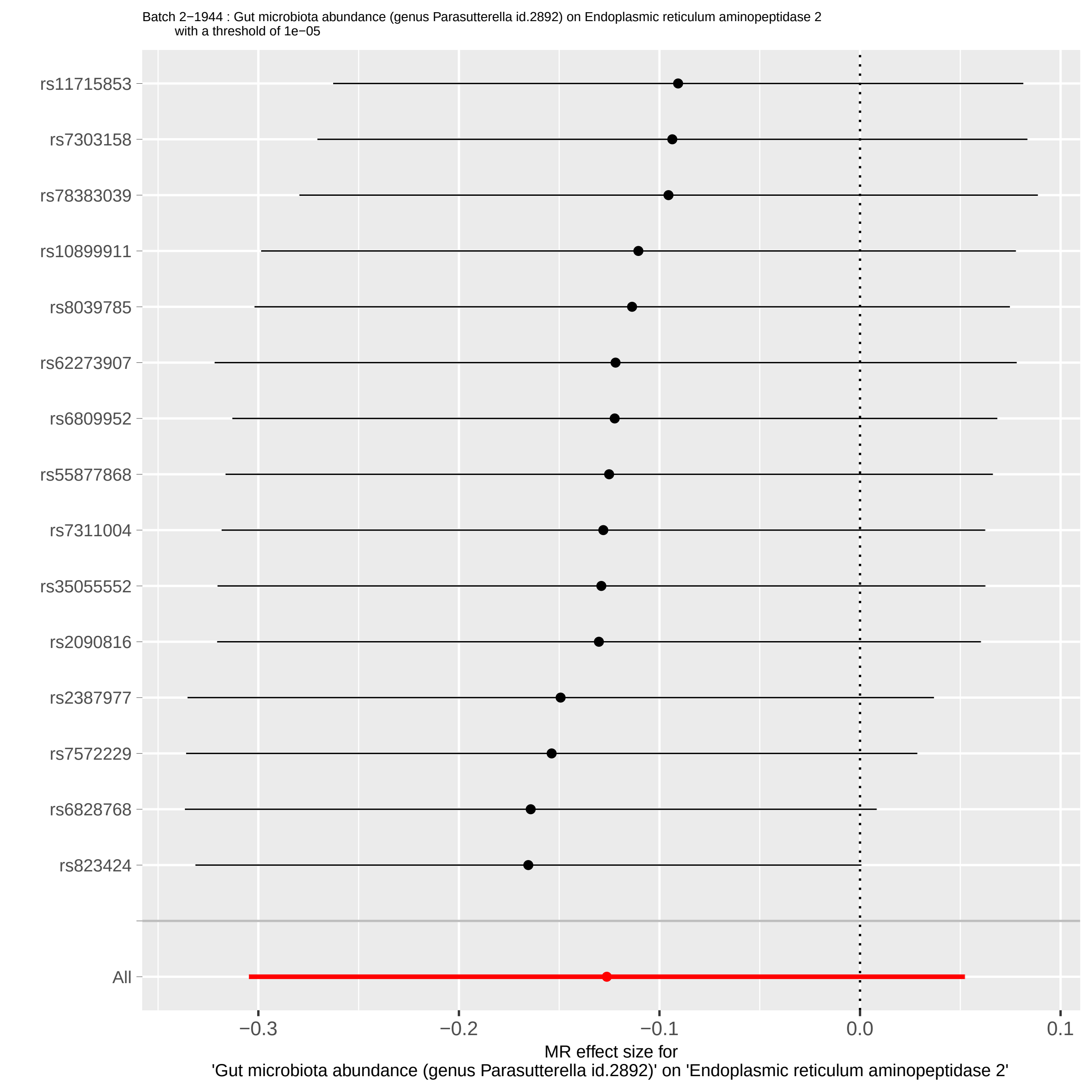

Batch 2−1944 : Gut microbiota abundance (genus Parasutterella id.2892) on Endoplasmic reticulum aminopeptidase 2
with a threshold of 1e−05
rs11715853
rs7303158
rs78383039
rs10899911
rs8039785
rs62273907
rs6809952
rs55877868
rs7311004
rs35055552
rs2090816
rs2387977
rs7572229
rs6828768
rs823424
All
−0.3
−0.2
−0.1
0.0
0.1
MR effect size for
'Gut microbiota abundance (genus Parasutterella id.2892)' on 'Endoplasmic reticulum aminopeptidase 2'

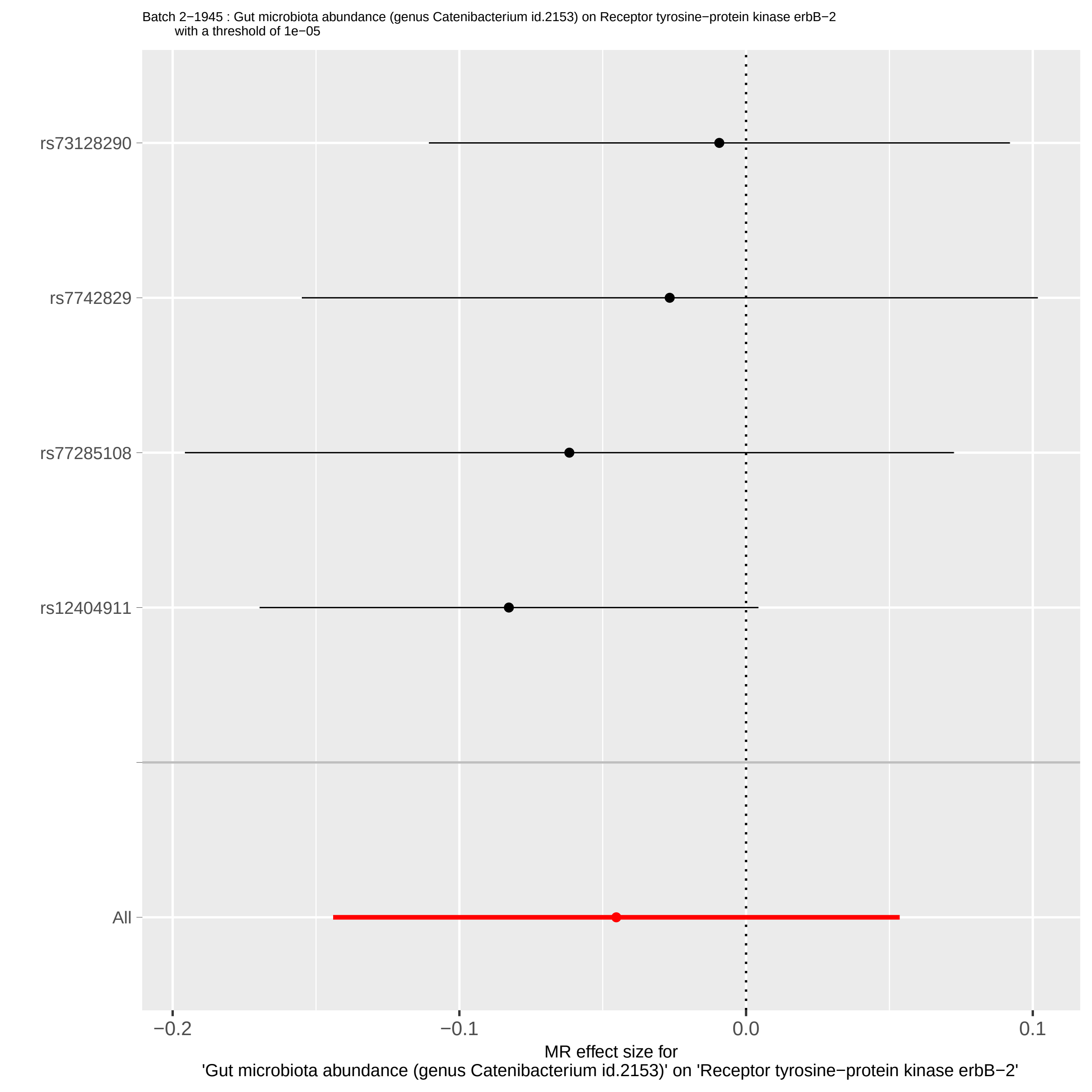
Batch 2−1945 : Gut microbiota abundance (genus Catenibacterium id.2153) on Receptor tyrosine−protein kinase erbB−2
with a threshold of 1e−05
rs73128290
rs7742829
rs77285108
rs12404911
All
−0.2
−0.1
0.0
0.1
MR effect size for
'Gut microbiota abundance (genus Catenibacterium id.2153)' on 'Receptor tyrosine−protein kinase erbB−2'

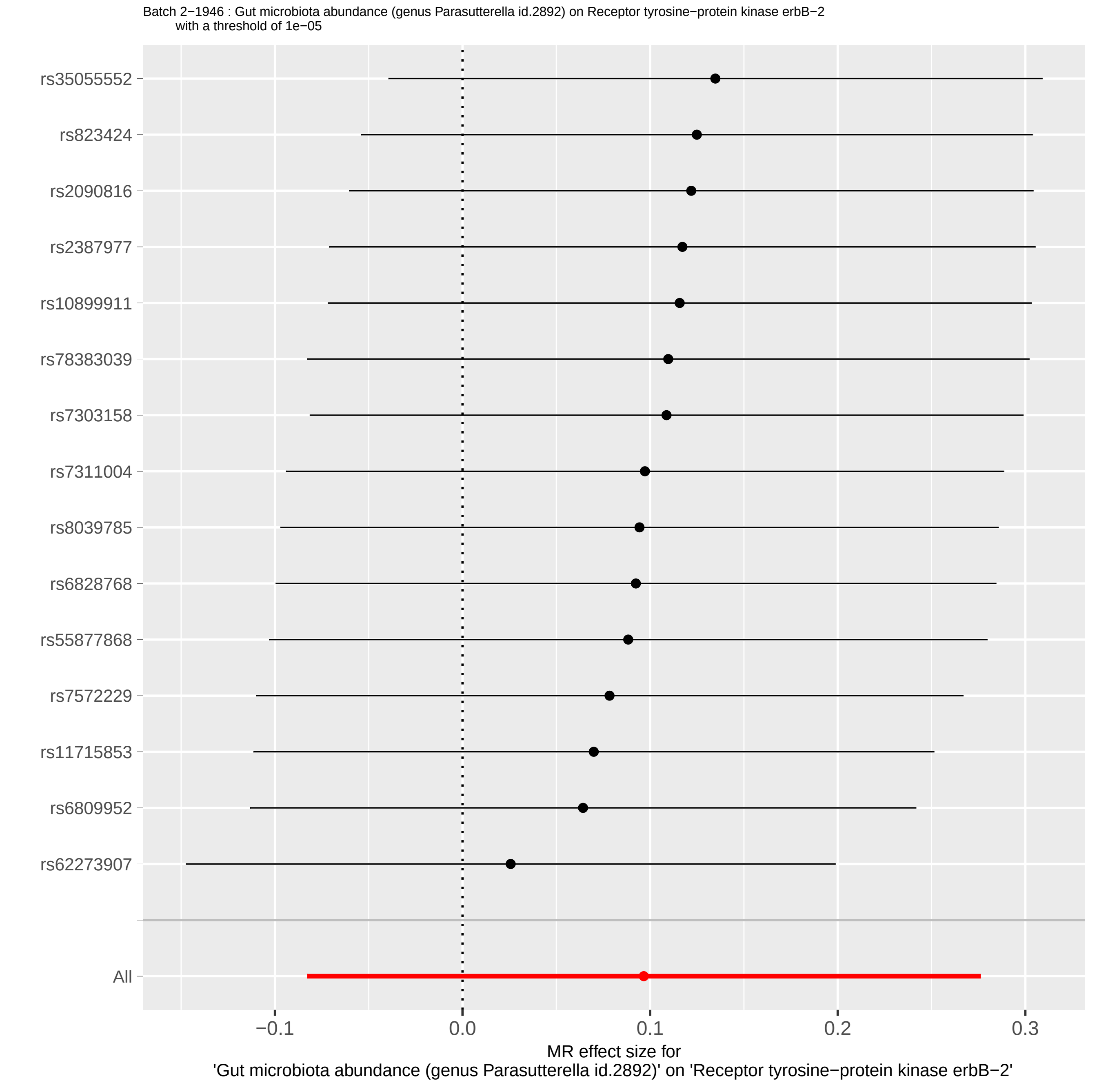
Batch 2−1946 : Gut microbiota abundance (genus Parasutterella id.2892) on Receptor tyrosine−protein kinase erbB−2
with a threshold of 1e−05
rs35055552
rs823424
rs2090816
rs2387977
rs10899911
rs78383039
rs7303158
rs7311004
rs8039785
rs6828768
rs55877868
rs7572229
rs11715853
rs6809952
rs62273907
All
−0.1
0.0
0.1
0.2
0.3
MR effect size for
'Gut microbiota abundance (genus Parasutterella id.2892)' on 'Receptor tyrosine−protein kinase erbB−2'

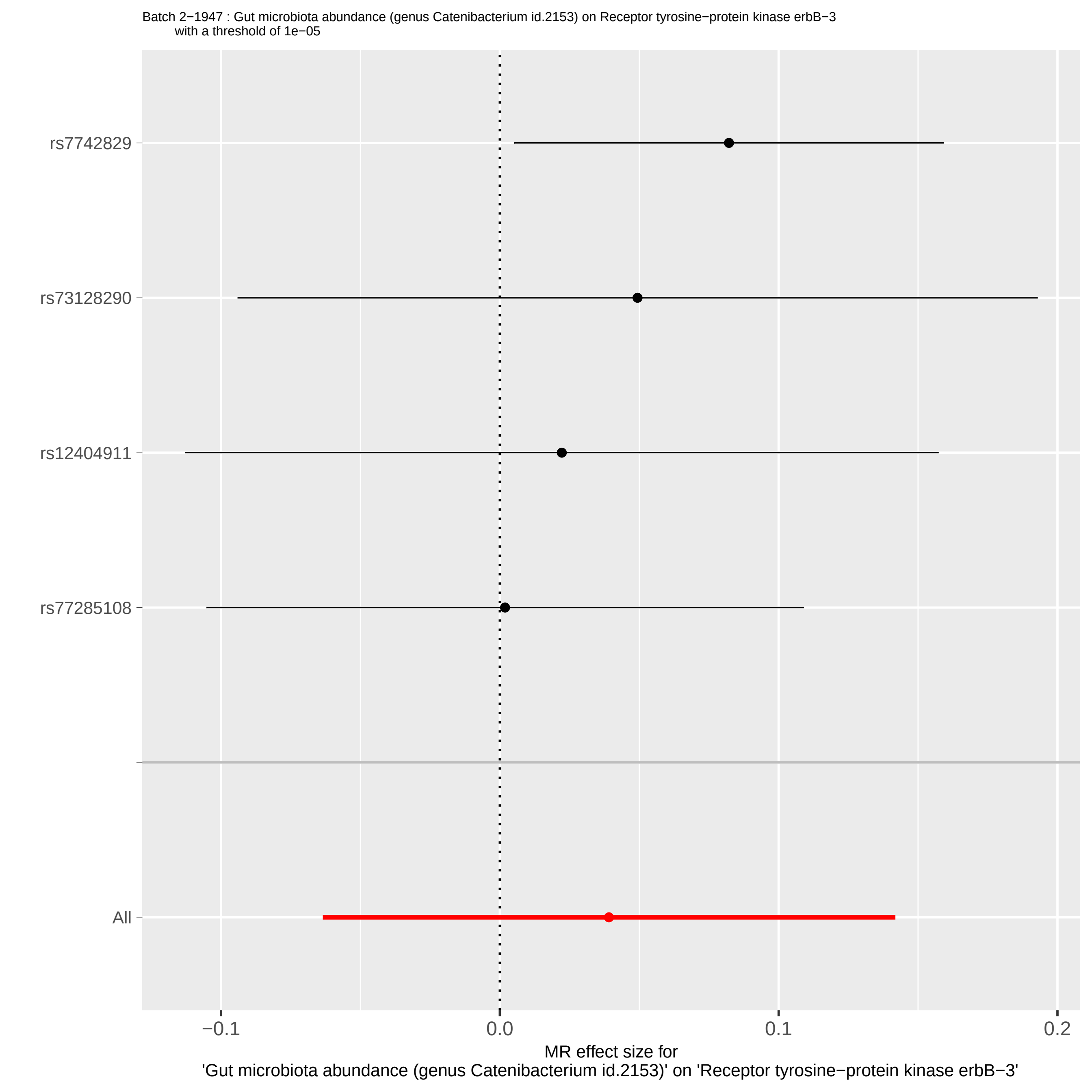
Batch 2−1947 : Gut microbiota abundance (genus Catenibacterium id.2153) on Receptor tyrosine−protein kinase erbB−3
with a threshold of 1e−05
rs7742829
rs73128290
rs12404911
rs77285108
All
−0.1
0.0
0.1
0.2
MR effect size for
'Gut microbiota abundance (genus Catenibacterium id.2153)' on 'Receptor tyrosine−protein kinase erbB−3'

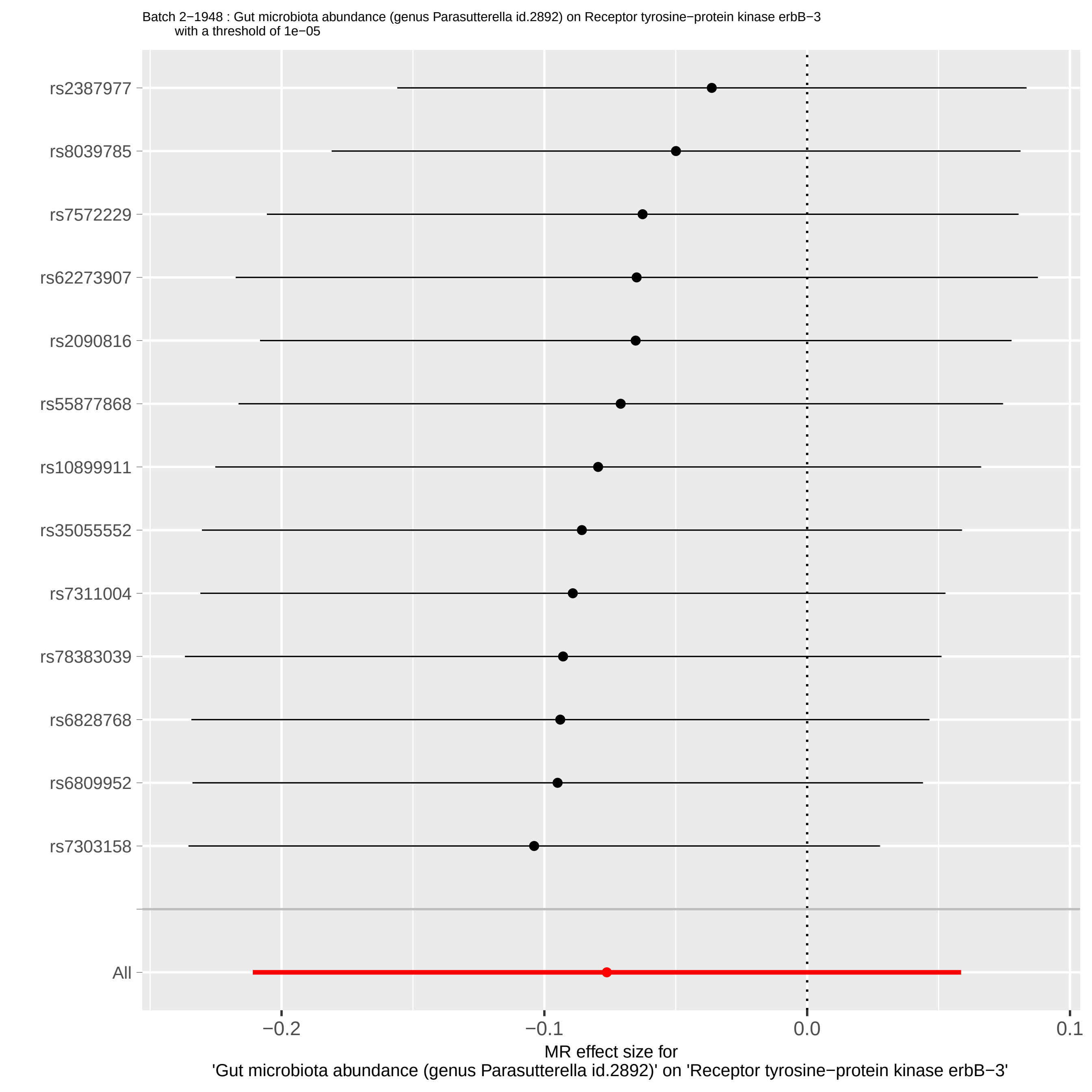

Batch 2−1948 : Gut microbiota abundance (genus Parasutterella id.2892) on Receptor tyrosine−protein kinase erbB−3
with a threshold of 1e−05
rs2387977
rs8039785
rs7572229
rs62273907
rs2090816
rs55877868
rs10899911
rs35055552
rs7311004
rs78383039
rs6828768
rs6809952
rs7303158
All
−0.2
−0.1
0.0
0.1
MR effect size for
'Gut microbiota abundance (genus Parasutterella id.2892)' on 'Receptor tyrosine−protein kinase erbB−3'

Batch 2−1949 : Gut microbiota abundance (genus Catenibacterium id.2153) on DNA excision repair protein ERCC−1 with a threshold of 1e−05

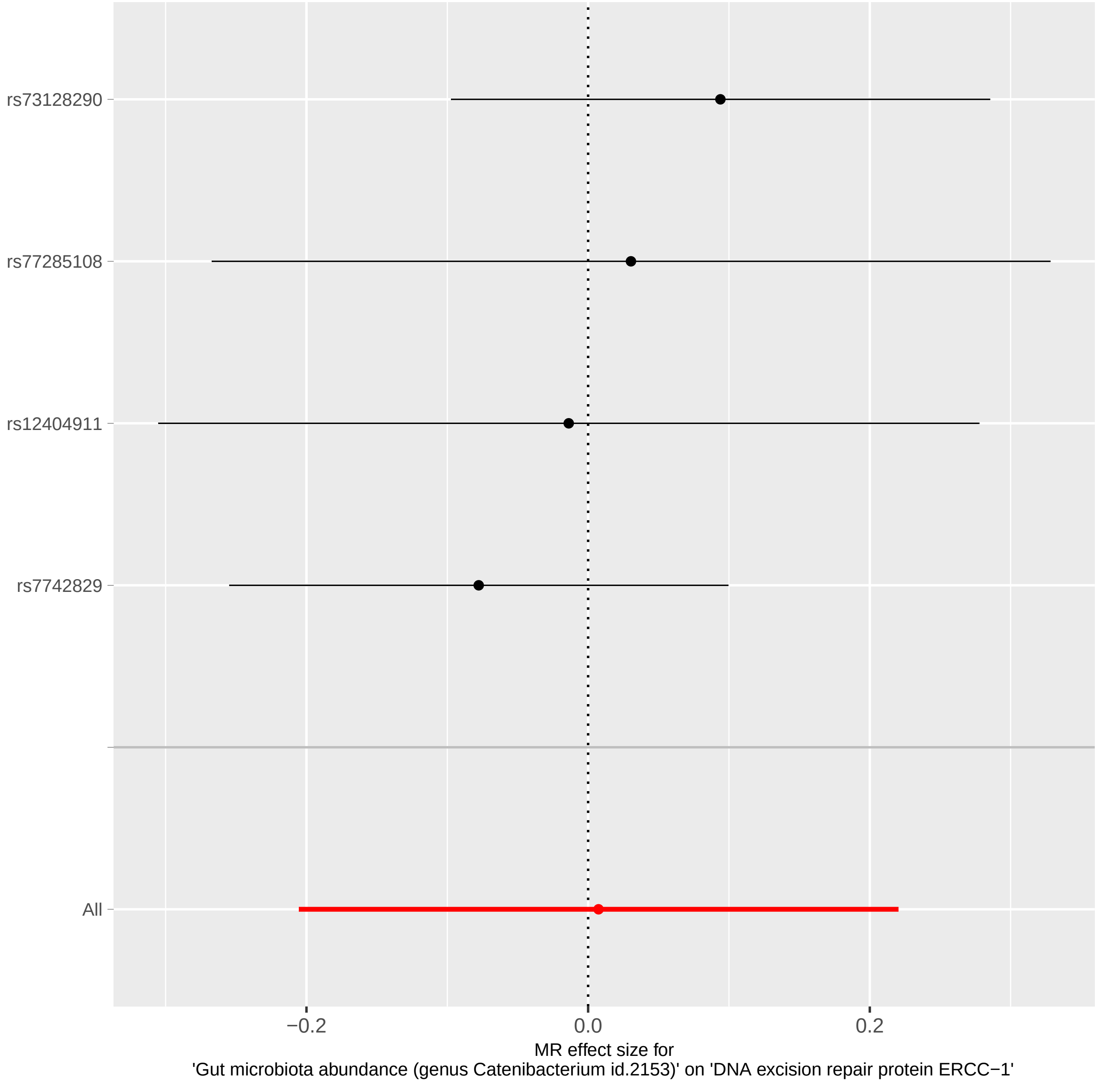

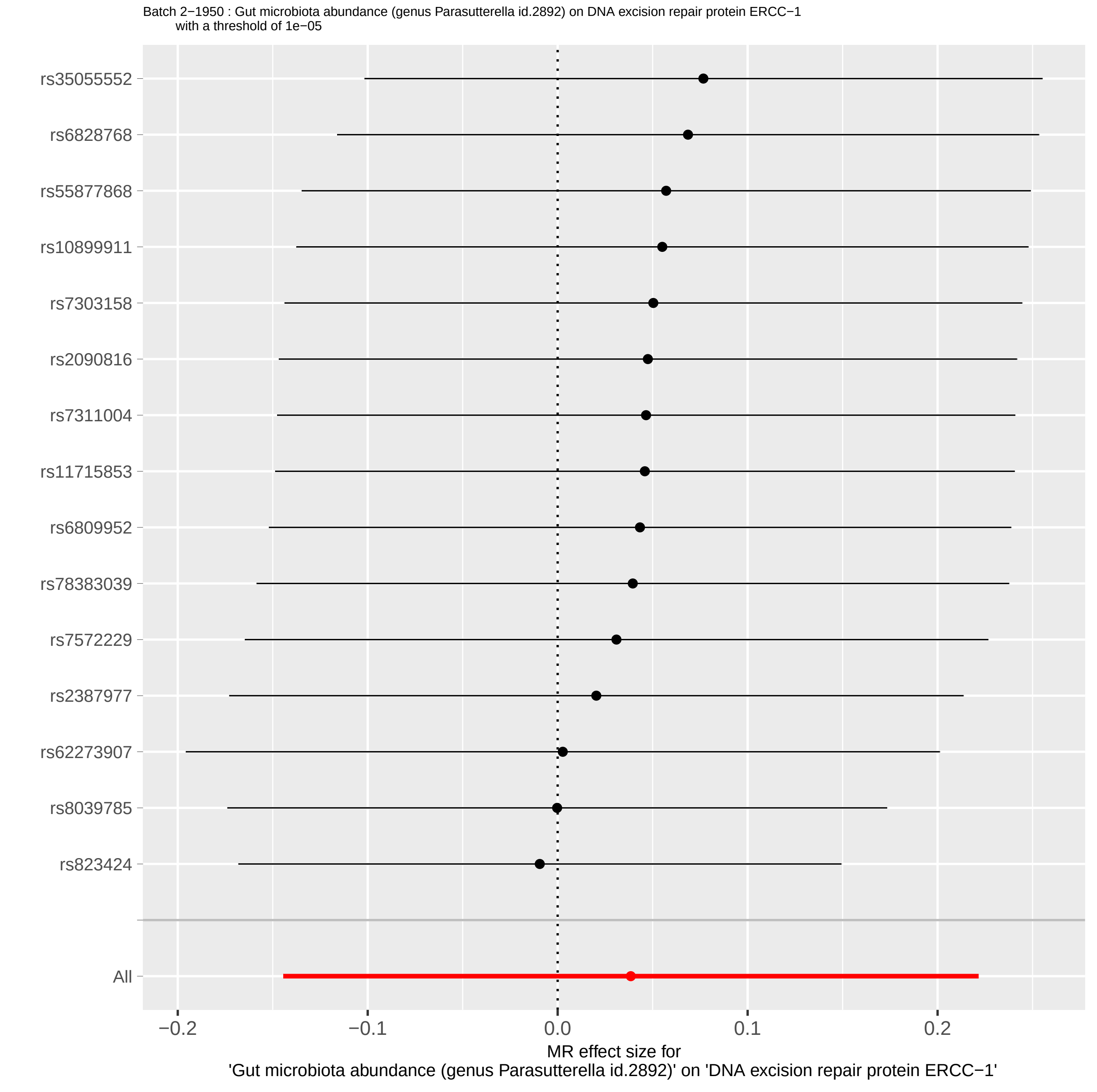

Batch 2−1950 : Gut microbiota abundance (genus Parasutterella id.2892) on DNA excision repair protein ERCC−1
with a threshold of 1e−05
rs35055552
rs6828768
rs55877868
rs10899911
rs7303158
rs2090816
rs7311004
rs11715853
rs6809952
rs78383039
rs7572229
rs2387977
rs62273907
rs8039785
rs823424
All
−0.2
−0.1
0.0
0.1
0.2
MR effect size for
'Gut microbiota abundance (genus Parasutterella id.2892)' on 'DNA excision repair protein ERCC−1'

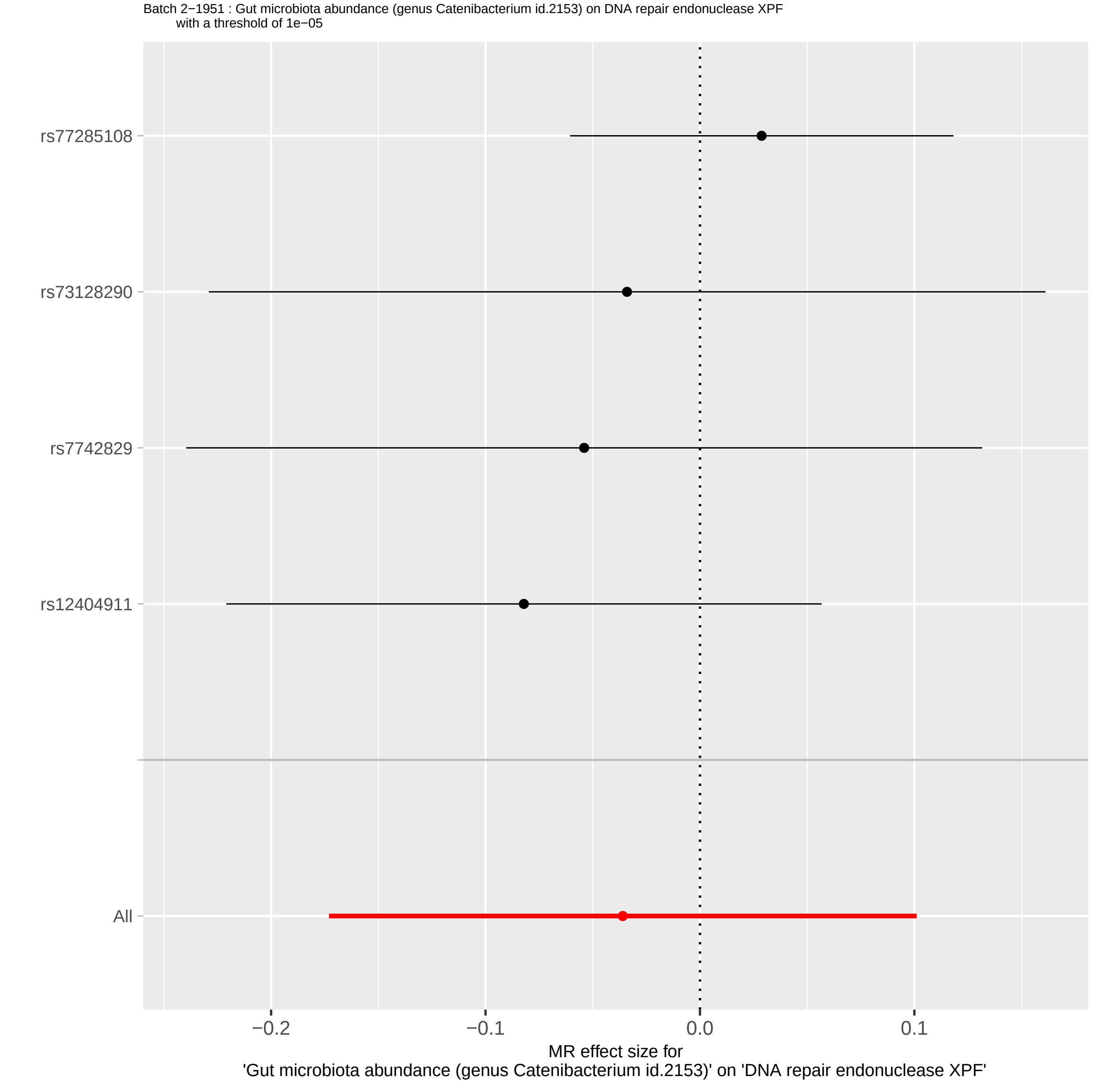

Batch 2−1951 : Gut microbiota abundance (genus Catenibacterium id.2153) on DNA repair endonuclease XPF
with a threshold of 1e−05
rs77285108
rs73128290
rs7742829
rs12404911
All
−0.2
−0.1
0.0
0.1
MR effect size for
'Gut microbiota abundance (genus Catenibacterium id.2153)' on 'DNA repair endonuclease XPF'

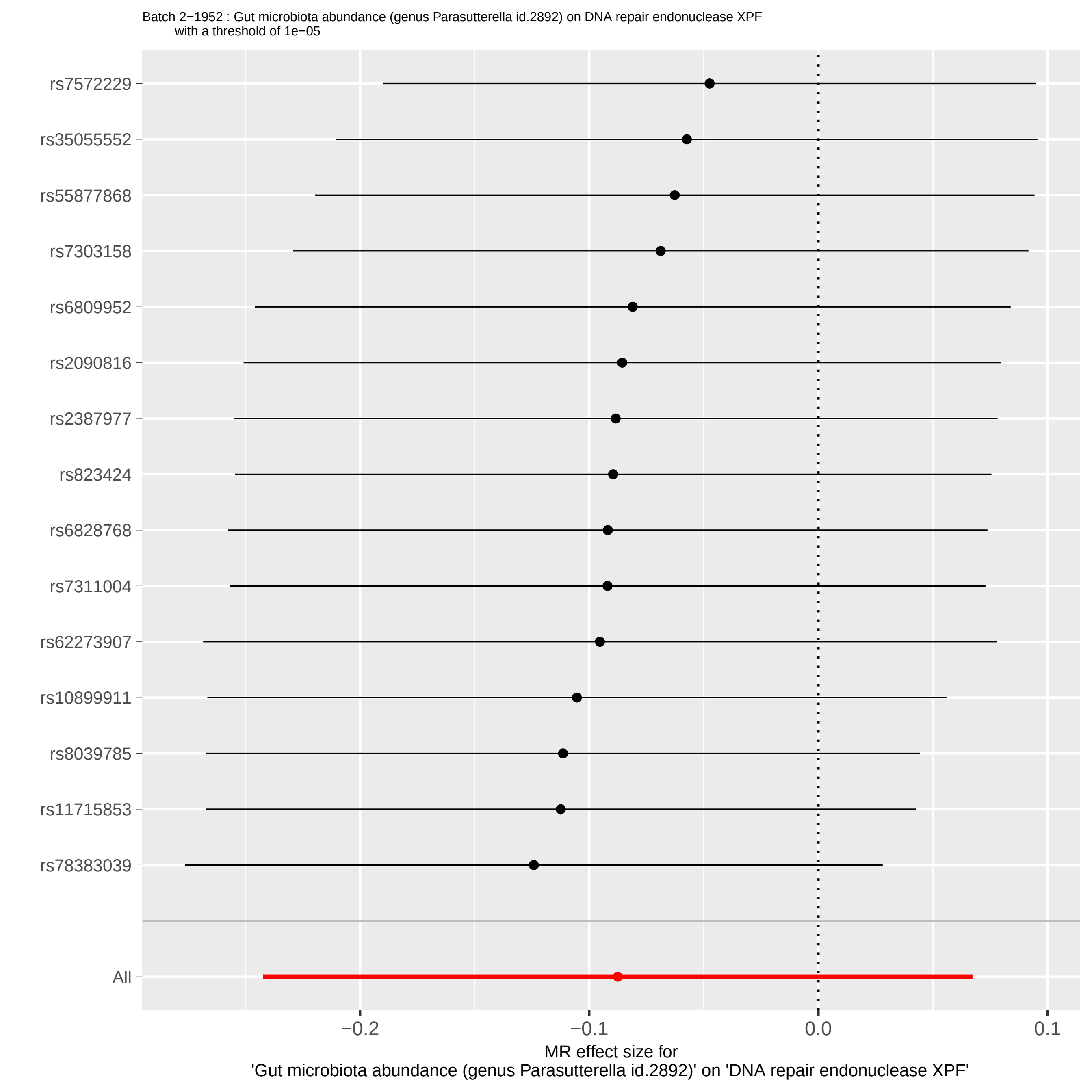

Batch 2−1952 : Gut microbiota abundance (genus Parasutterella id.2892) on DNA repair endonuclease XPF
with a threshold of 1e−05
rs7572229
rs35055552
rs55877868
rs7303158
rs6809952
rs2090816
rs2387977
rs823424
rs6828768
rs7311004
rs62273907
rs10899911
rs8039785
rs11715853
rs78383039
All
−0.2
−0.1
0.0
0.1
MR effect size for
'Gut microbiota abundance (genus Parasutterella id.2892)' on 'DNA repair endonuclease XPF'

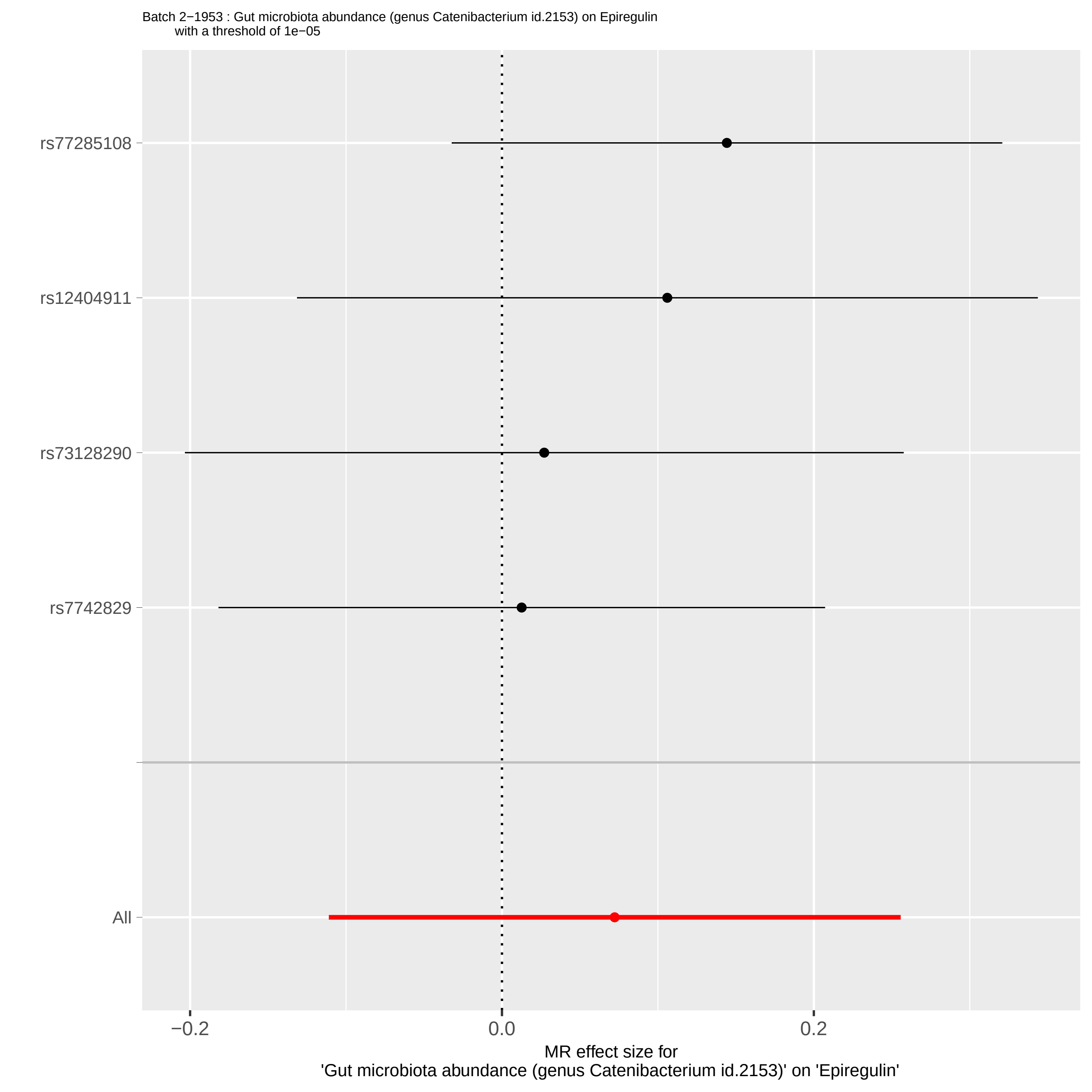
Batch 2−1953 : Gut microbiota abundance (genus Catenibacterium id.2153) on Epiregulin
with a threshold of 1e−05
rs77285108
rs12404911
rs73128290
rs7742829
All
−0.2
0.0
0.2
MR effect size for
'Gut microbiota abundance (genus Catenibacterium id.2153)' on 'Epiregulin'

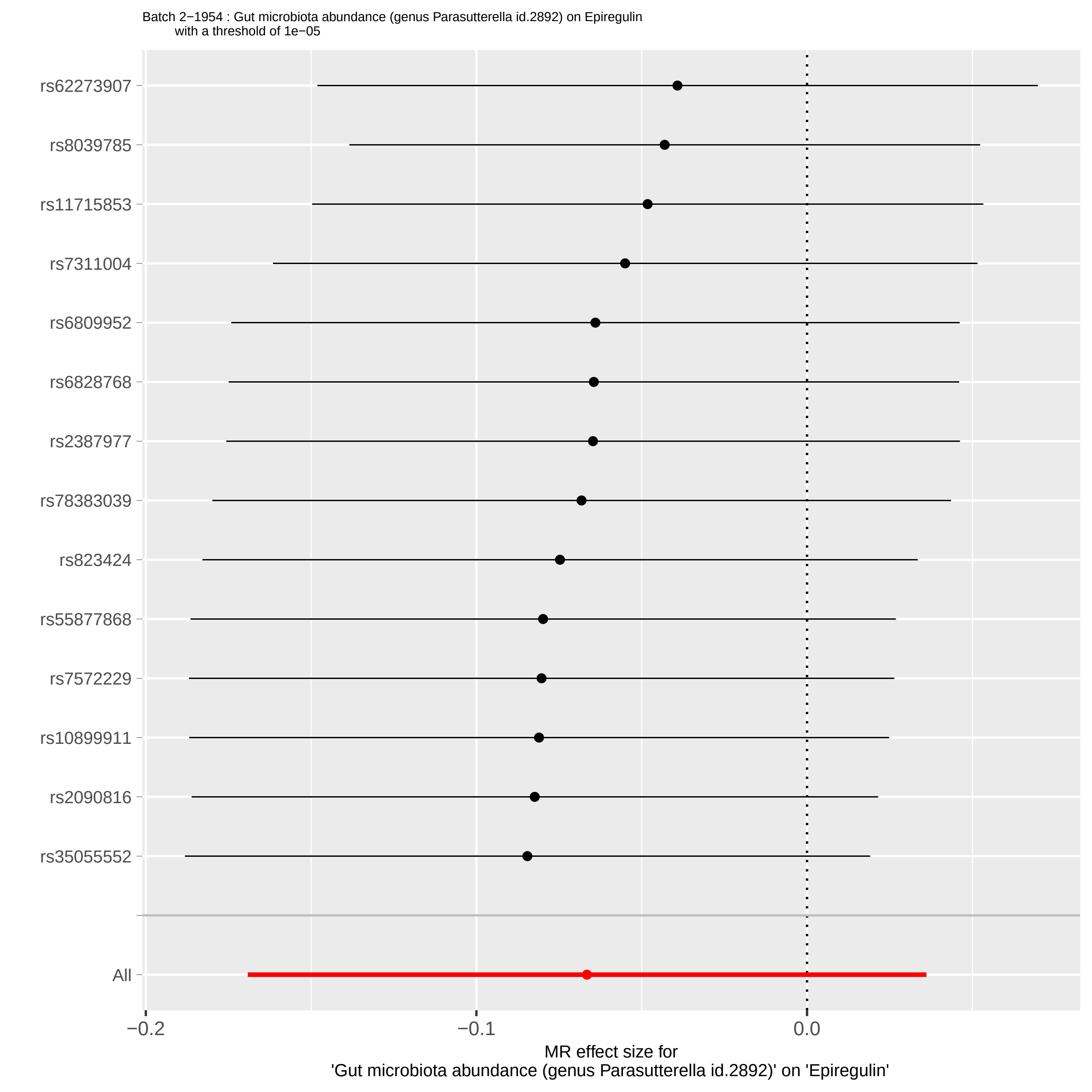

Batch 2−1954 : Gut microbiota abundance (genus Parasutterella id.2892) on Epiregulin
with a threshold of 1e−05
rs62273907
rs8039785
rs11715853
rs7311004
rs6809952
rs6828768
rs2387977
rs78383039
rs823424
rs55877868
rs7572229
rs10899911
rs2090816
rs35055552
All
−0.2
−0.1
0.0
MR effect size for
'Gut microbiota abundance (genus Parasutterella id.2892)' on 'Epiregulin'

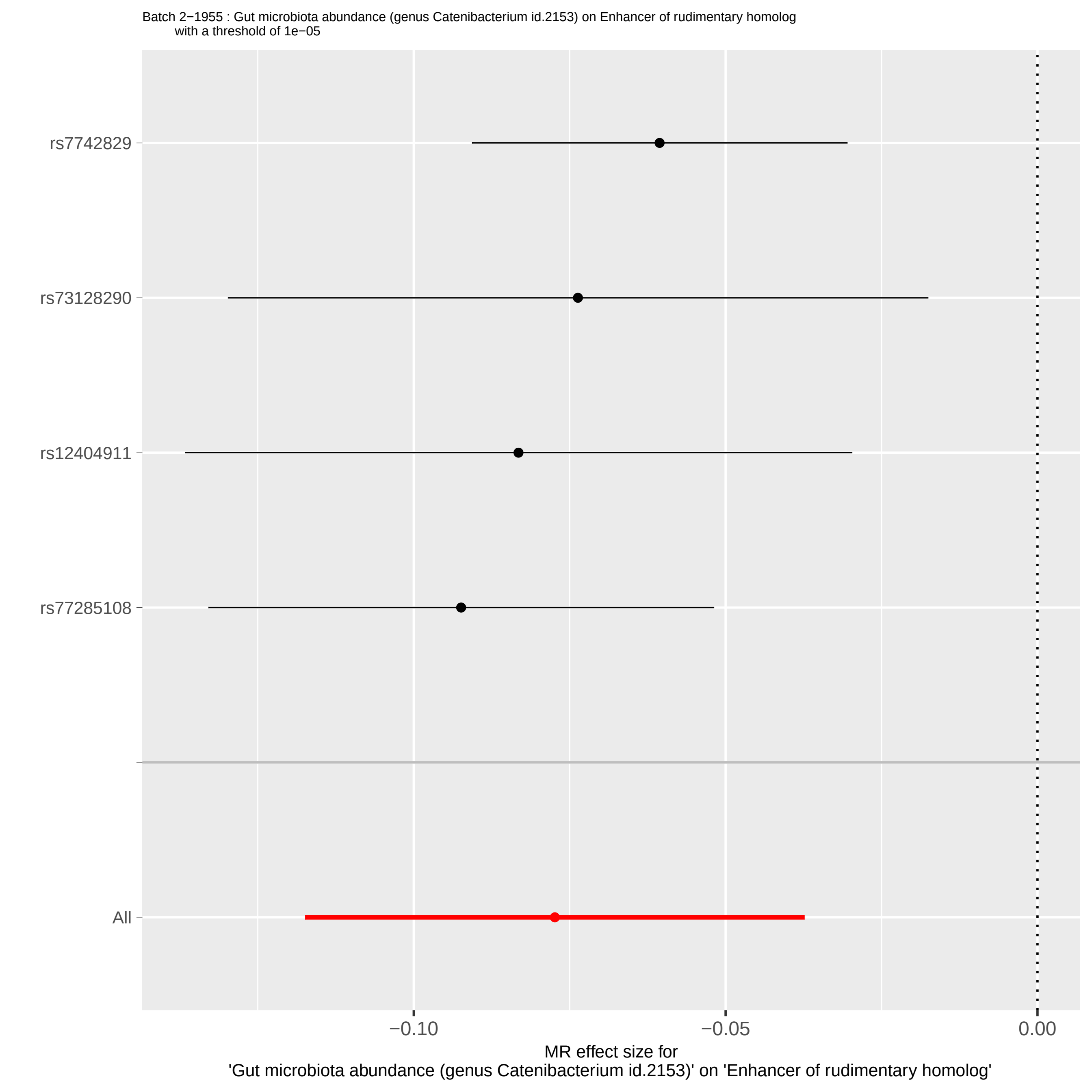
Batch 2−1955 : Gut microbiota abundance (genus Catenibacterium id.2153) on Enhancer of rudimentary homolog
with a threshold of 1e−05
rs7742829
rs73128290
rs12404911
rs77285108
All
−0.10
−0.05
0.00
MR effect size for
'Gut microbiota abundance (genus Catenibacterium id.2153)' on 'Enhancer of rudimentary homolog'

Batch 2−1956 : Gut microbiota abundance (genus Parasutterella id.2892) on Enhancer of rudimentary homolog
with a threshold of 1e−05

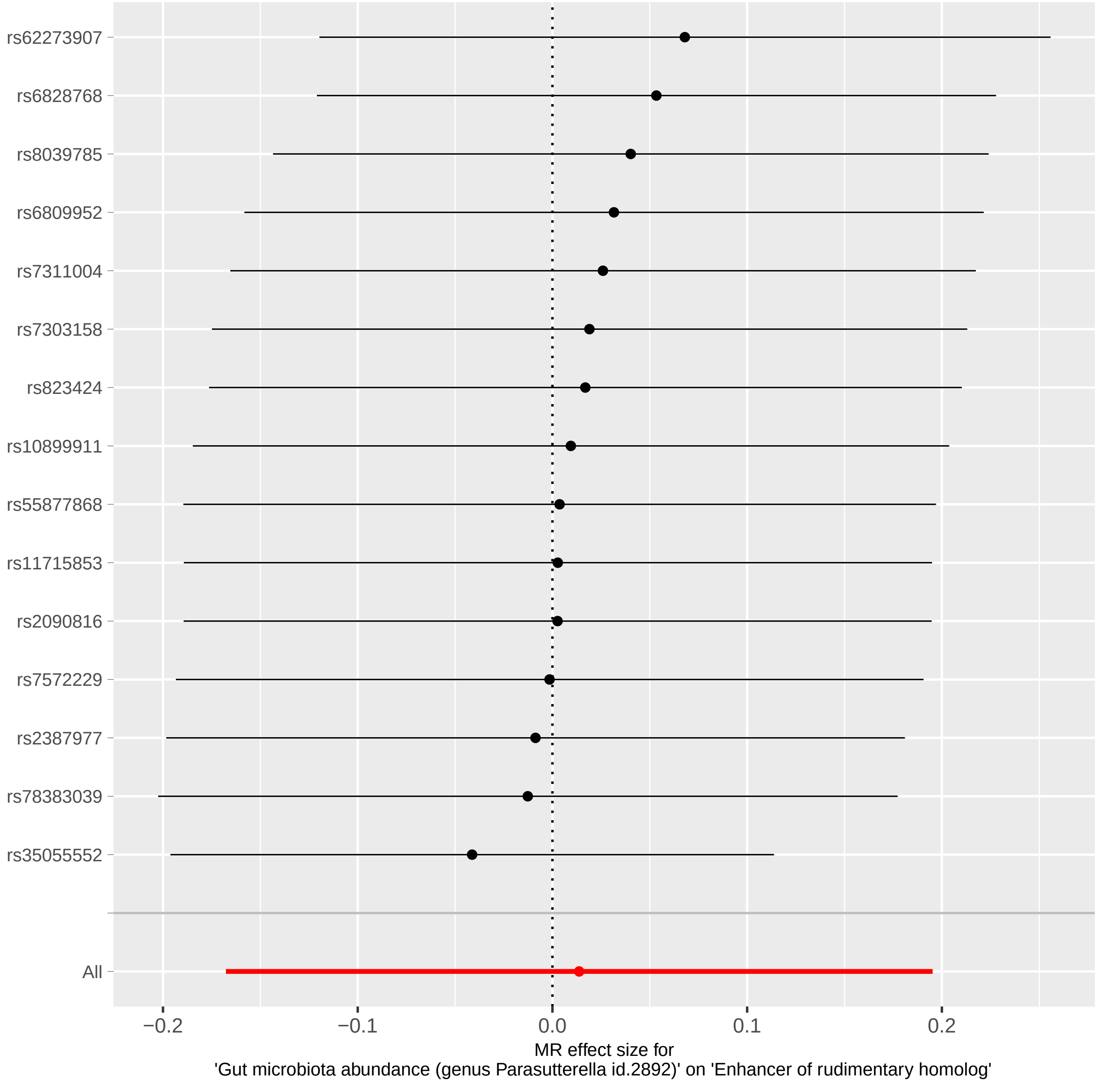

Batch 2−1957 : Gut microbiota abundance (genus Catenibacterium id.2153) on Endoplasmic reticulum lectin 1
with a threshold of 1e−05

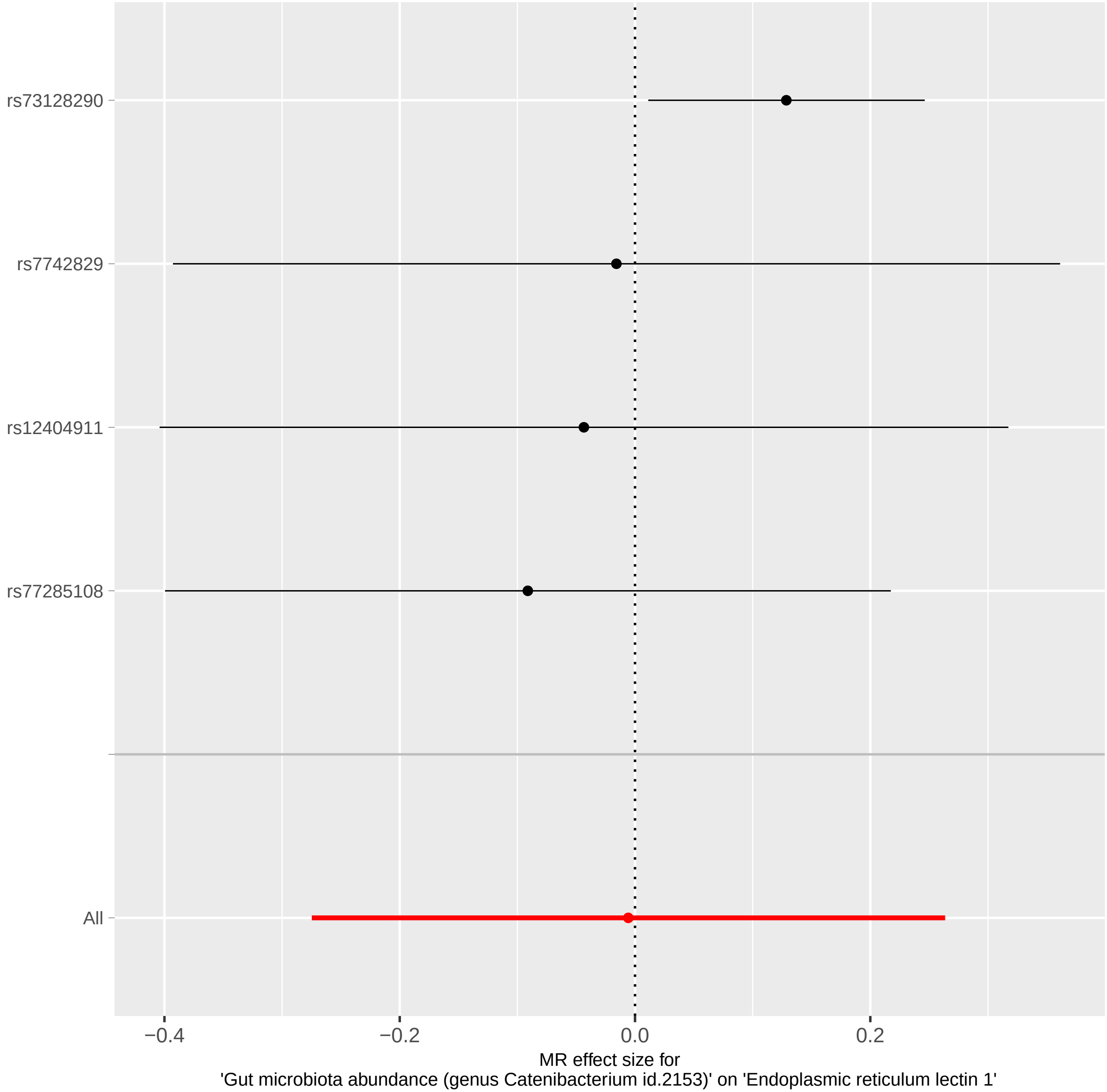

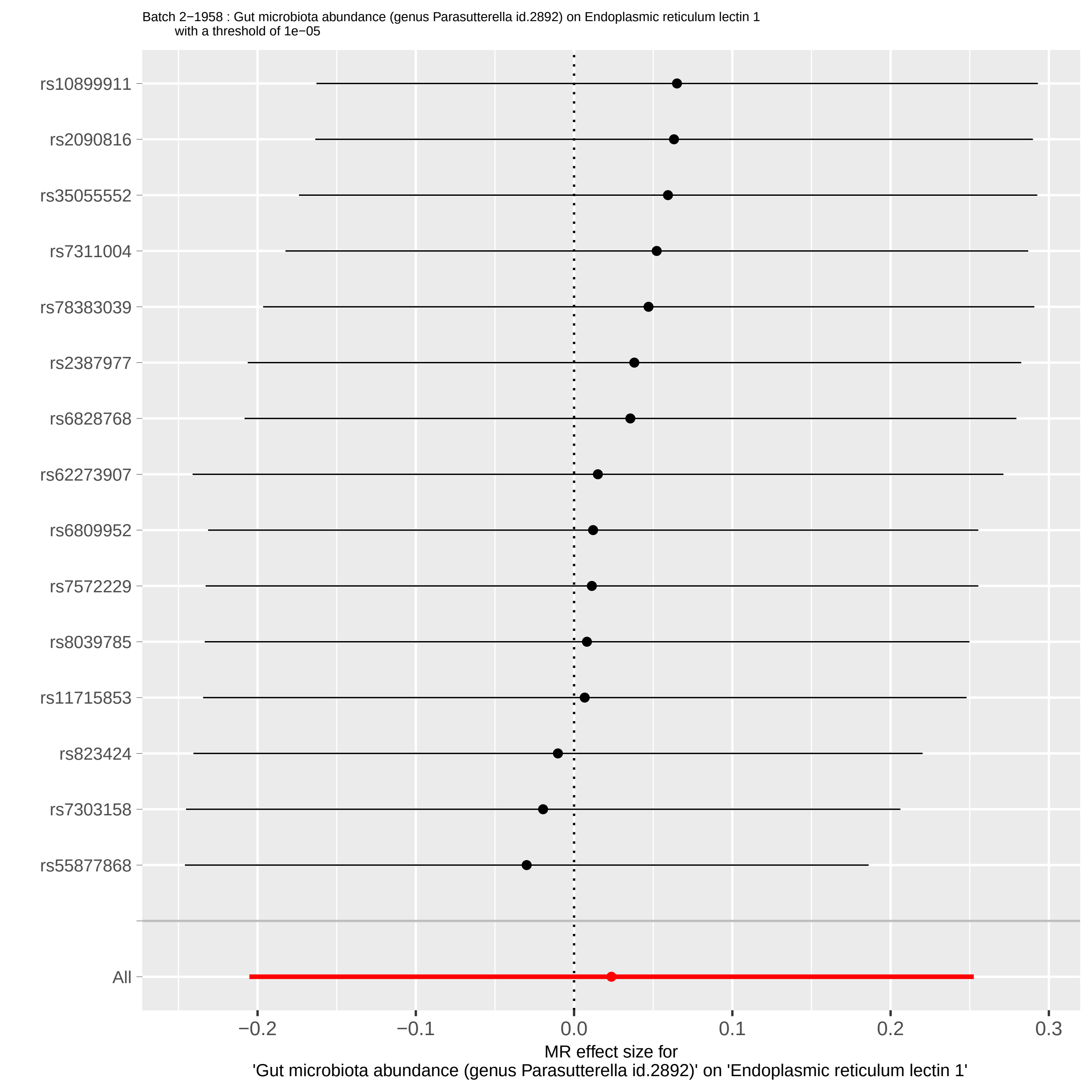

Batch 2−1958 : Gut microbiota abundance (genus Parasutterella id.2892) on Endoplasmic reticulum lectin 1
with a threshold of 1e−05
rs10899911
rs2090816
rs35055552
rs7311004
rs78383039
rs2387977
rs6828768
rs62273907
rs6809952
rs7572229
rs8039785
rs11715853
rs823424
rs7303158
rs55877868
All
−0.2
−0.1
0.0
0.1
0.2
0.3
MR effect size for
'Gut microbiota abundance (genus Parasutterella id.2892)' on 'Endoplasmic reticulum lectin 1'

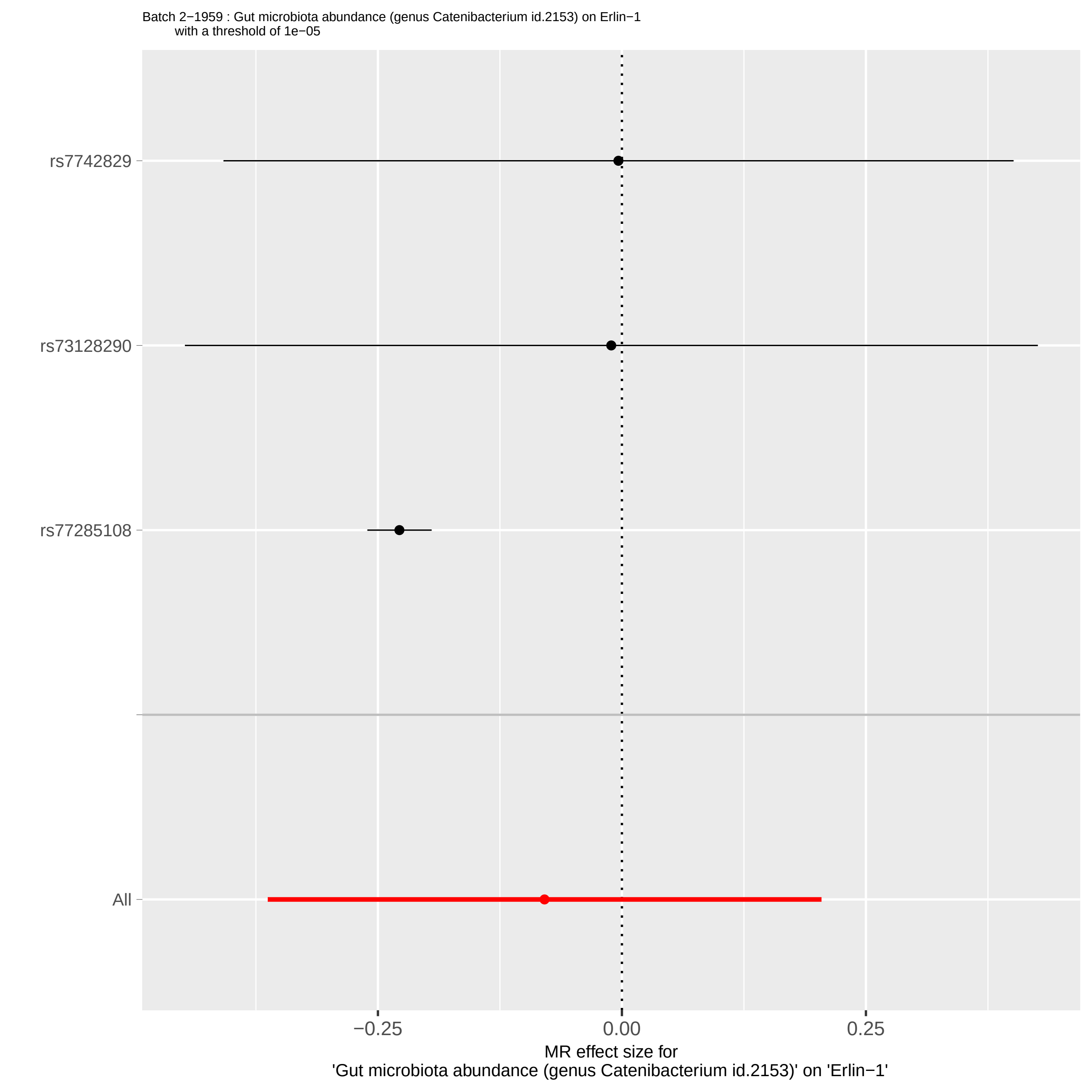
Batch 2−1959 : Gut microbiota abundance (genus Catenibacterium id.2153) on Erlin−1
with a threshold of 1e−05
rs7742829
rs73128290
rs77285108
All
−0.25
0.00
0.25
MR effect size for
'Gut microbiota abundance (genus Catenibacterium id.2153)' on 'Erlin−1'

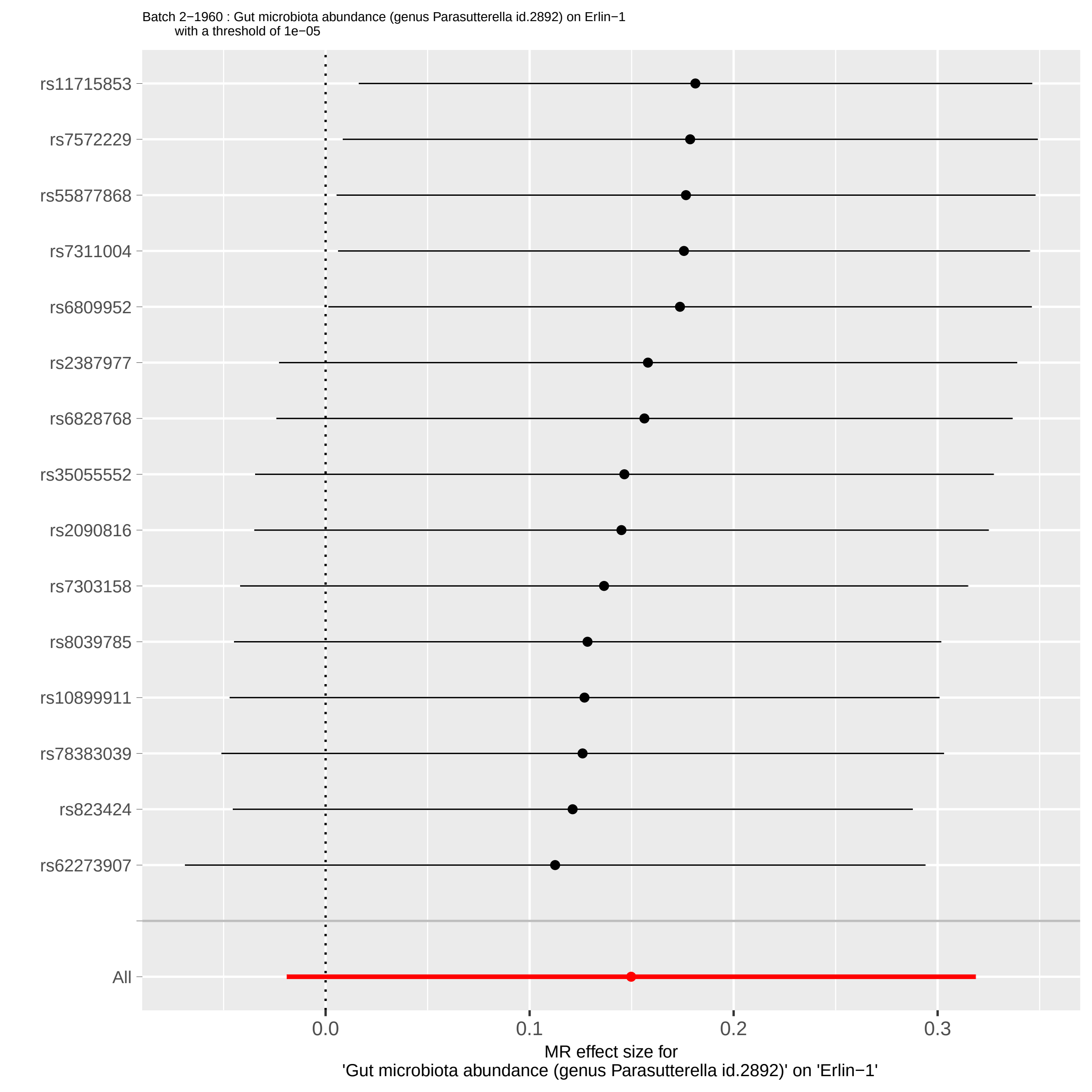

Batch 2−1960 : Gut microbiota abundance (genus Parasutterella id.2892) on Erlin−1
with a threshold of 1e−05
rs11715853
rs7572229
rs55877868
rs7311004
rs6809952
rs2387977
rs6828768
rs35055552
rs2090816
rs7303158
rs8039785
rs10899911
rs78383039
rs823424
rs62273907
All
0.0
0.1
0.2
0.3
MR effect size for
'Gut microbiota abundance (genus Parasutterella id.2892)' on 'Erlin−1'

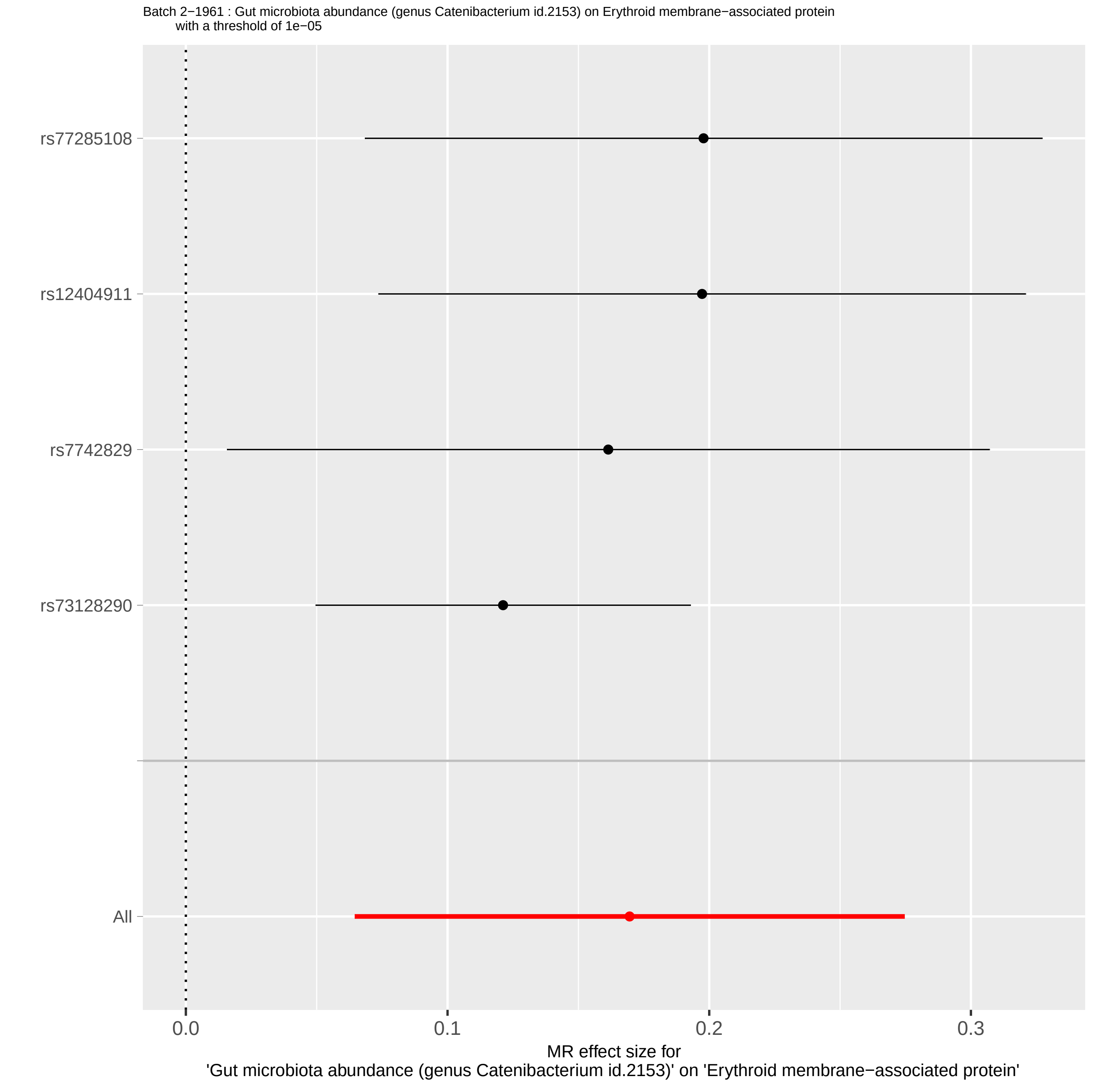
Batch 2−1961 : Gut microbiota abundance (genus Catenibacterium id.2153) on Erythroid membrane−associated protein
with a threshold of 1e−05
rs77285108
rs12404911
rs7742829
rs73128290
All
0.0
0.1
0.2
0.3
MR effect size for
'Gut microbiota abundance (genus Catenibacterium id.2153)' on 'Erythroid membrane−associated protein'

Batch 2−1962 : Gut microbiota abundance (genus Parasutterella id.2892) on Erythroid membrane−associated protein
with a threshold of 1e−05

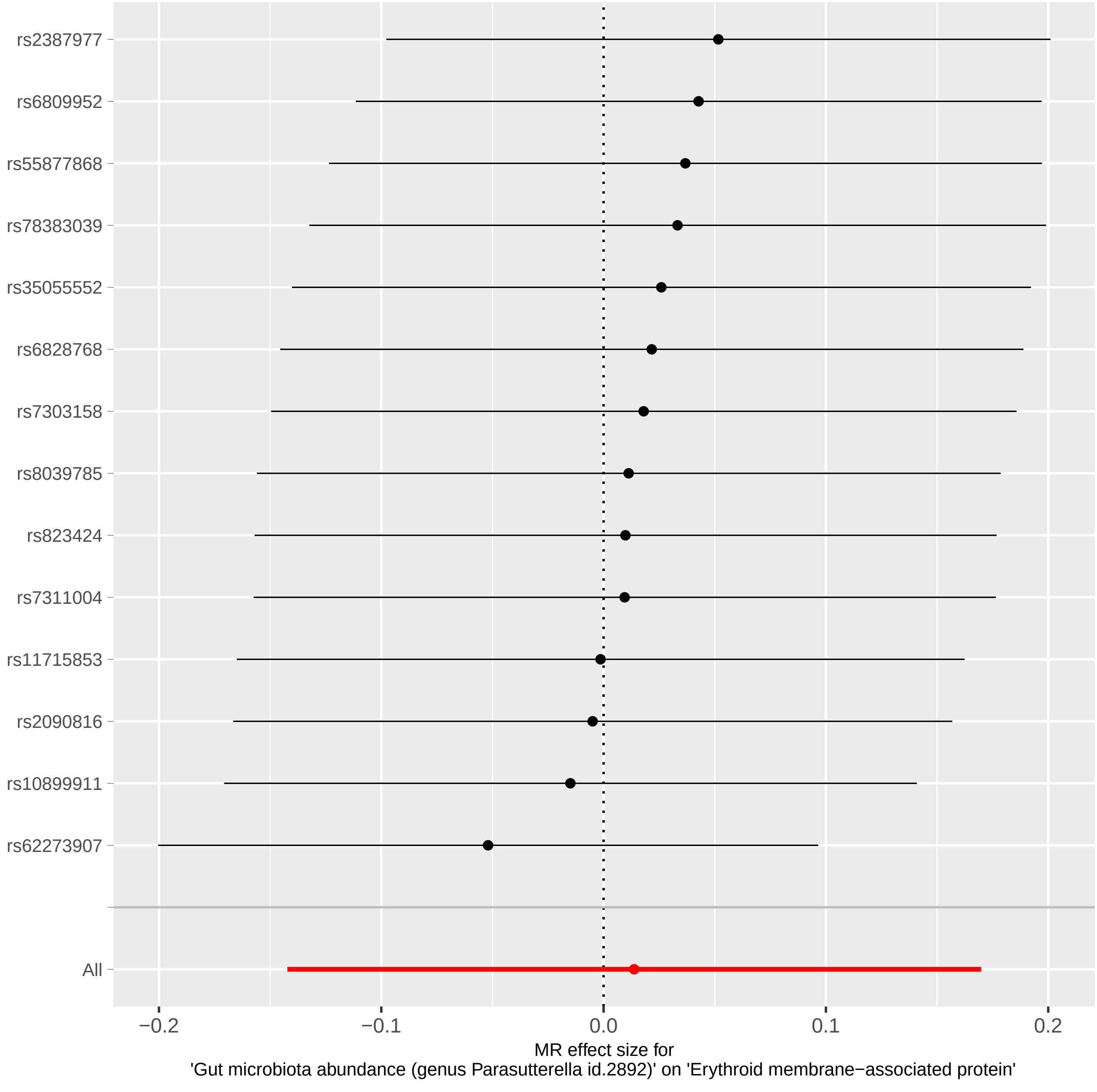

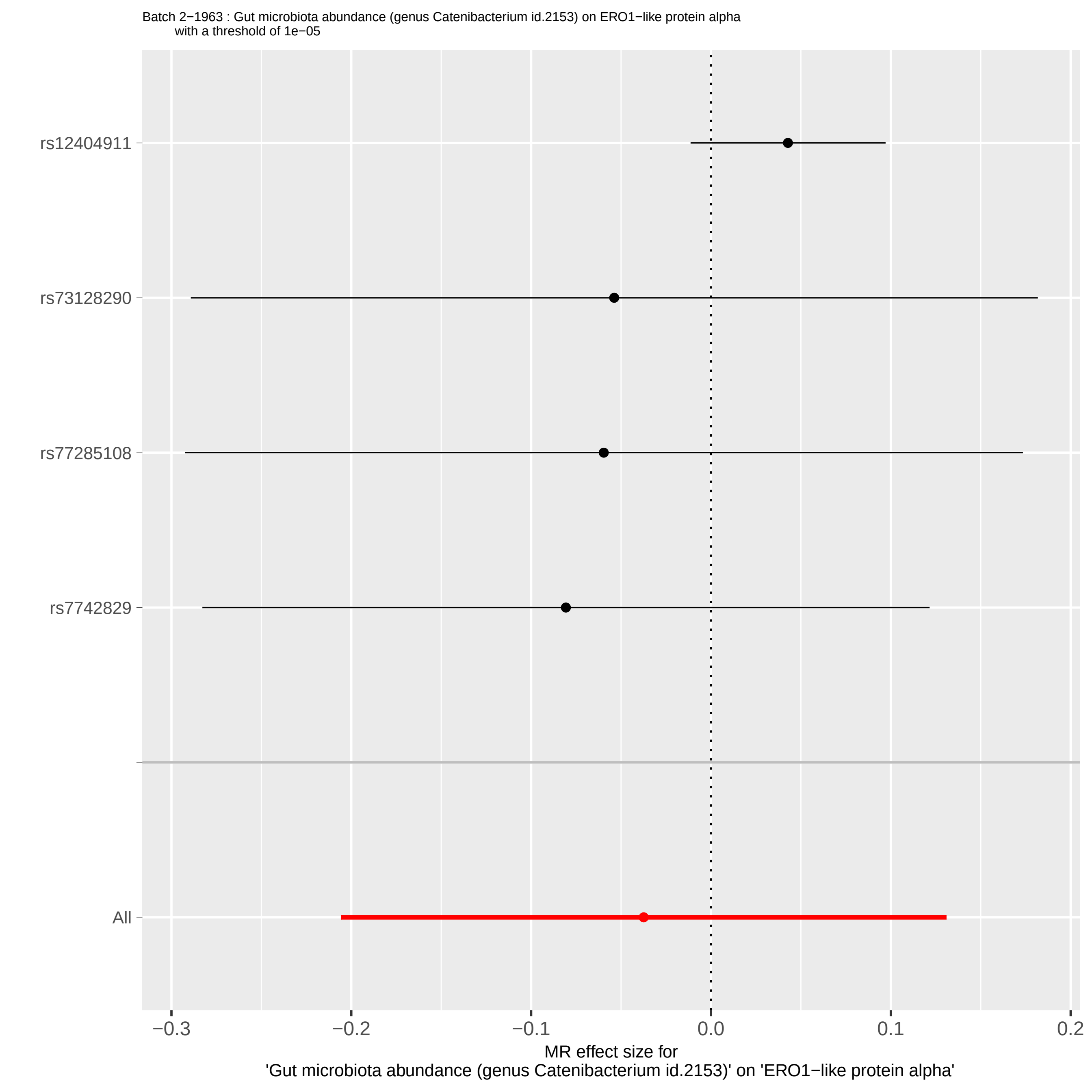
Batch 2−1963 : Gut microbiota abundance (genus Catenibacterium id.2153) on ERO1−like protein alpha
with a threshold of 1e−05
rs12404911
rs73128290
rs77285108
rs7742829
All
−0.3
−0.2
−0.1
0.0
0.1
0.2
MR effect size for
'Gut microbiota abundance (genus Catenibacterium id.2153)' on 'ERO1−like protein alpha'

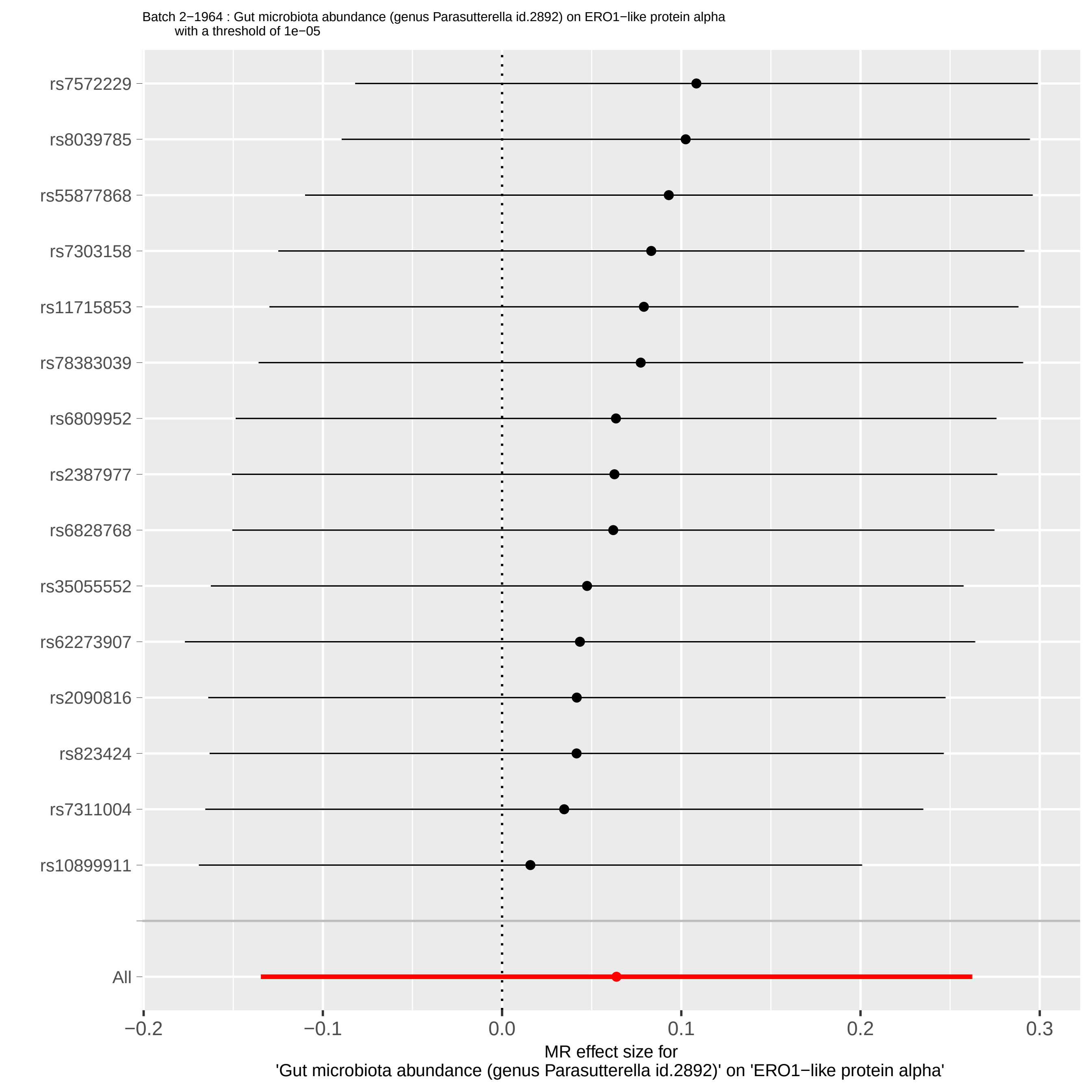

Batch 2−1964 : Gut microbiota abundance (genus Parasutterella id.2892) on ERO1−like protein alpha
with a threshold of 1e−05
rs7572229
rs8039785
rs55877868
rs7303158
rs11715853
rs78383039
rs6809952
rs2387977
rs6828768
rs35055552
rs62273907
rs2090816
rs823424
rs7311004
rs10899911
All
−0.2
−0.1
0.0
0.1
0.2
0.3
MR effect size for
'Gut microbiota abundance (genus Parasutterella id.2892)' on 'ERO1−like protein alpha'

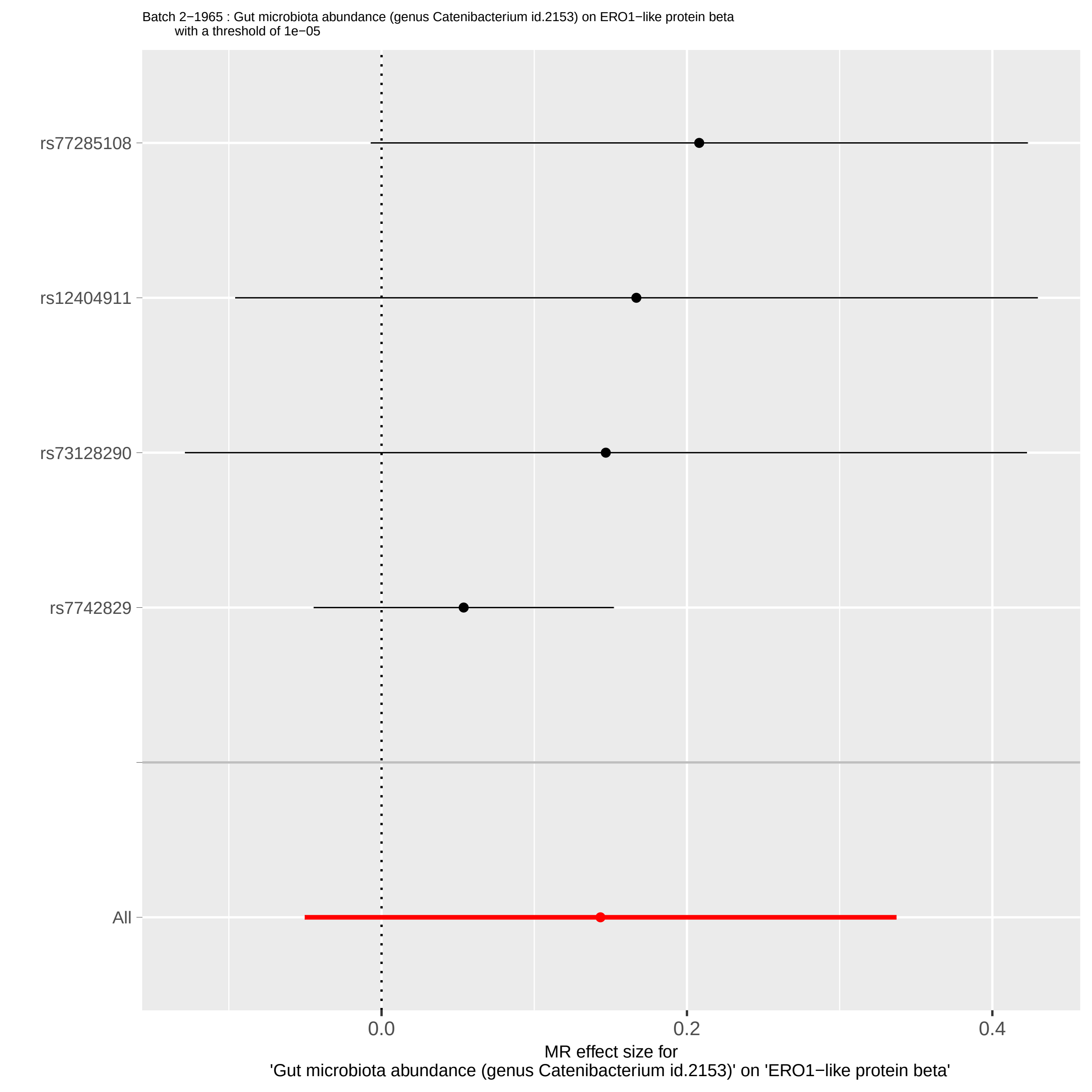
Batch 2−1965 : Gut microbiota abundance (genus Catenibacterium id.2153) on ERO1−like protein beta
with a threshold of 1e−05
rs77285108
rs12404911
rs73128290
rs7742829
All
0.0
0.2
0.4
MR effect size for
'Gut microbiota abundance (genus Catenibacterium id.2153)' on 'ERO1−like protein beta'

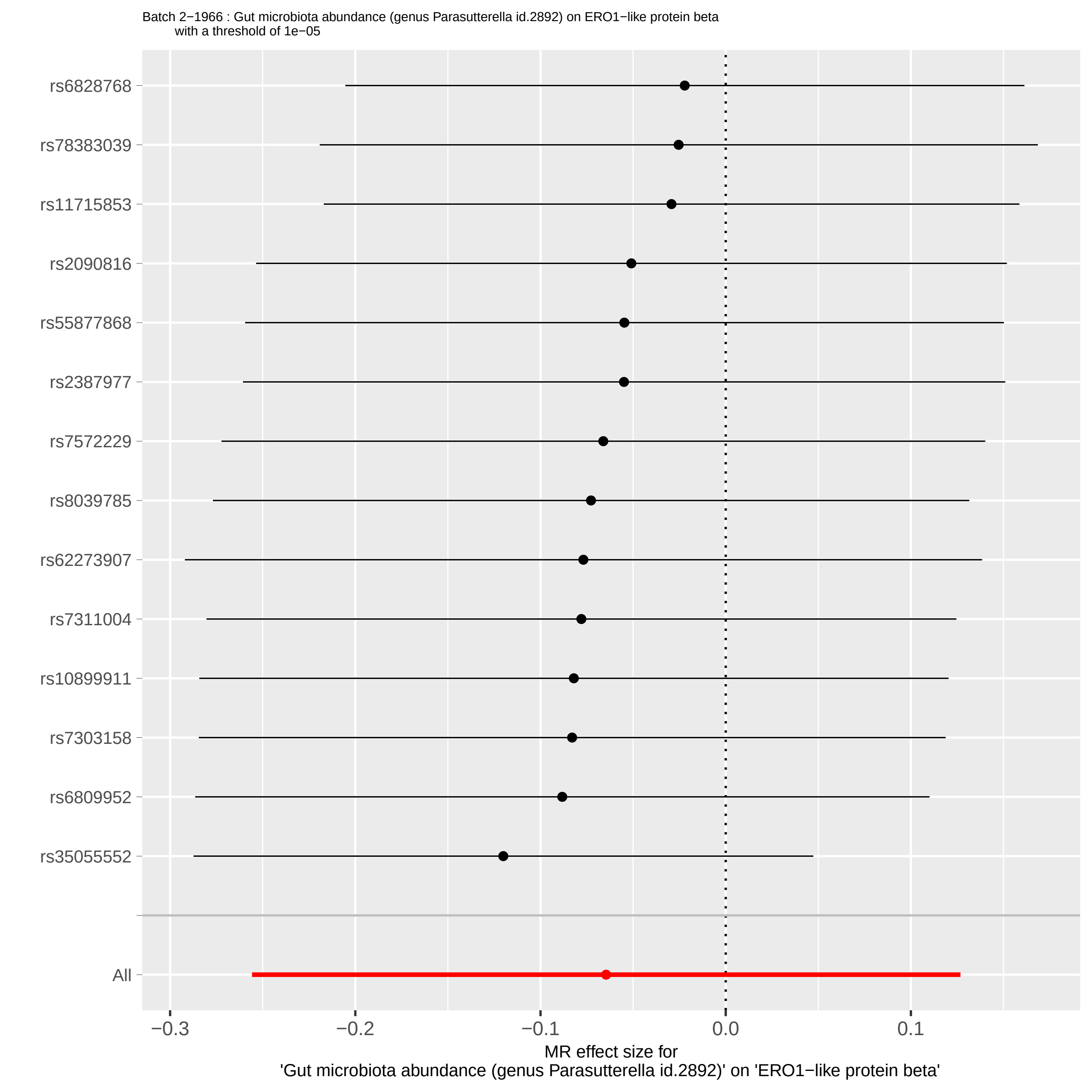

Batch 2−1966 : Gut microbiota abundance (genus Parasutterella id.2892) on ERO1−like protein beta
with a threshold of 1e−05
rs6828768
rs78383039
rs11715853
rs2090816
rs55877868
rs2387977
rs7572229
rs8039785
rs62273907
rs7311004
rs10899911
rs7303158
rs6809952
rs35055552
All
−0.3
−0.2
−0.1
0.0
0.1
MR effect size for
'Gut microbiota abundance (genus Parasutterella id.2892)' on 'ERO1−like protein beta'

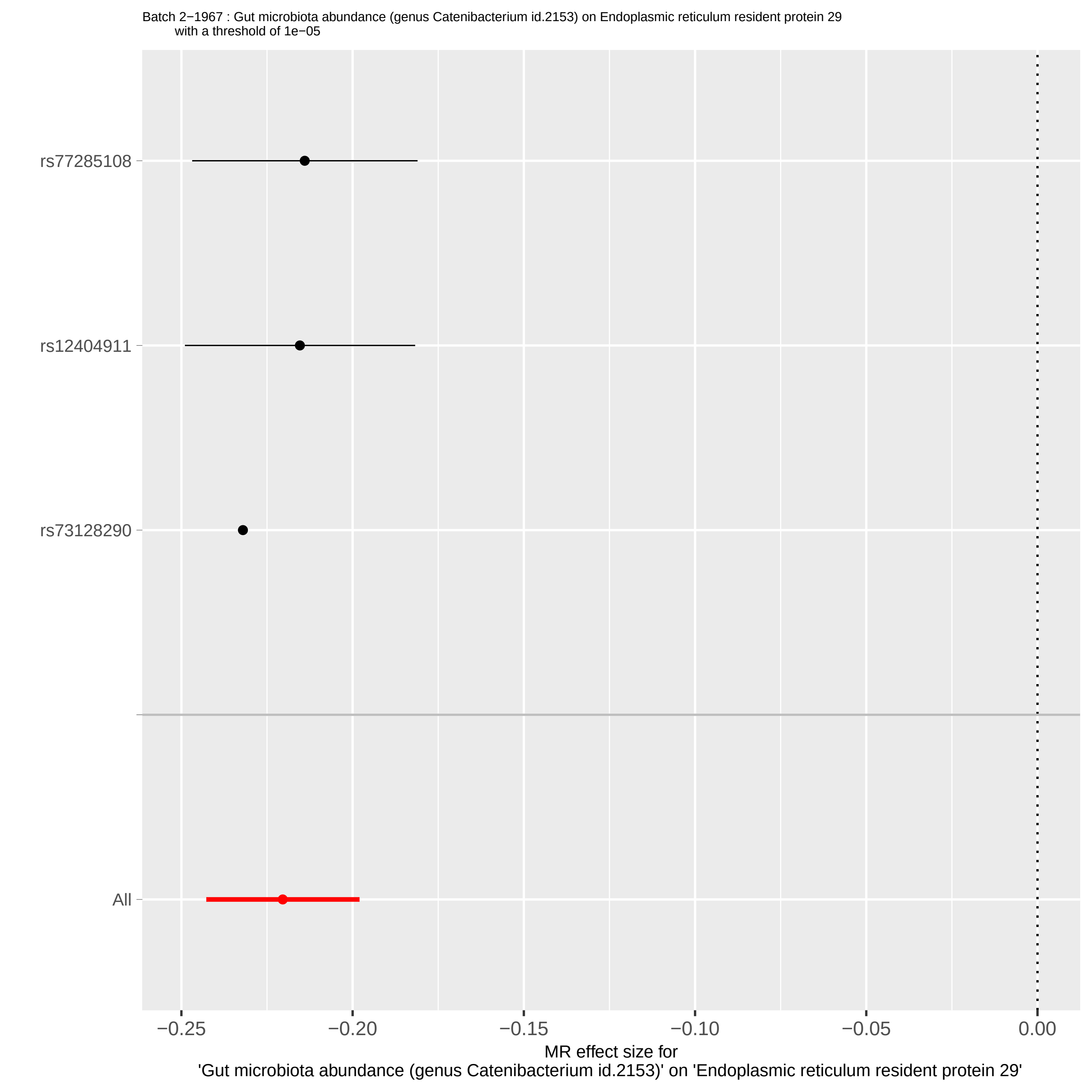
Batch 2−1967 : Gut microbiota abundance (genus Catenibacterium id.2153) on Endoplasmic reticulum resident protein 29
with a threshold of 1e−05
rs77285108
rs12404911
rs73128290
All
−0.25
−0.20
−0.15
−0.10
−0.05
0.00
MR effect size for
'Gut microbiota abundance (genus Catenibacterium id.2153)' on 'Endoplasmic reticulum resident protein 29'

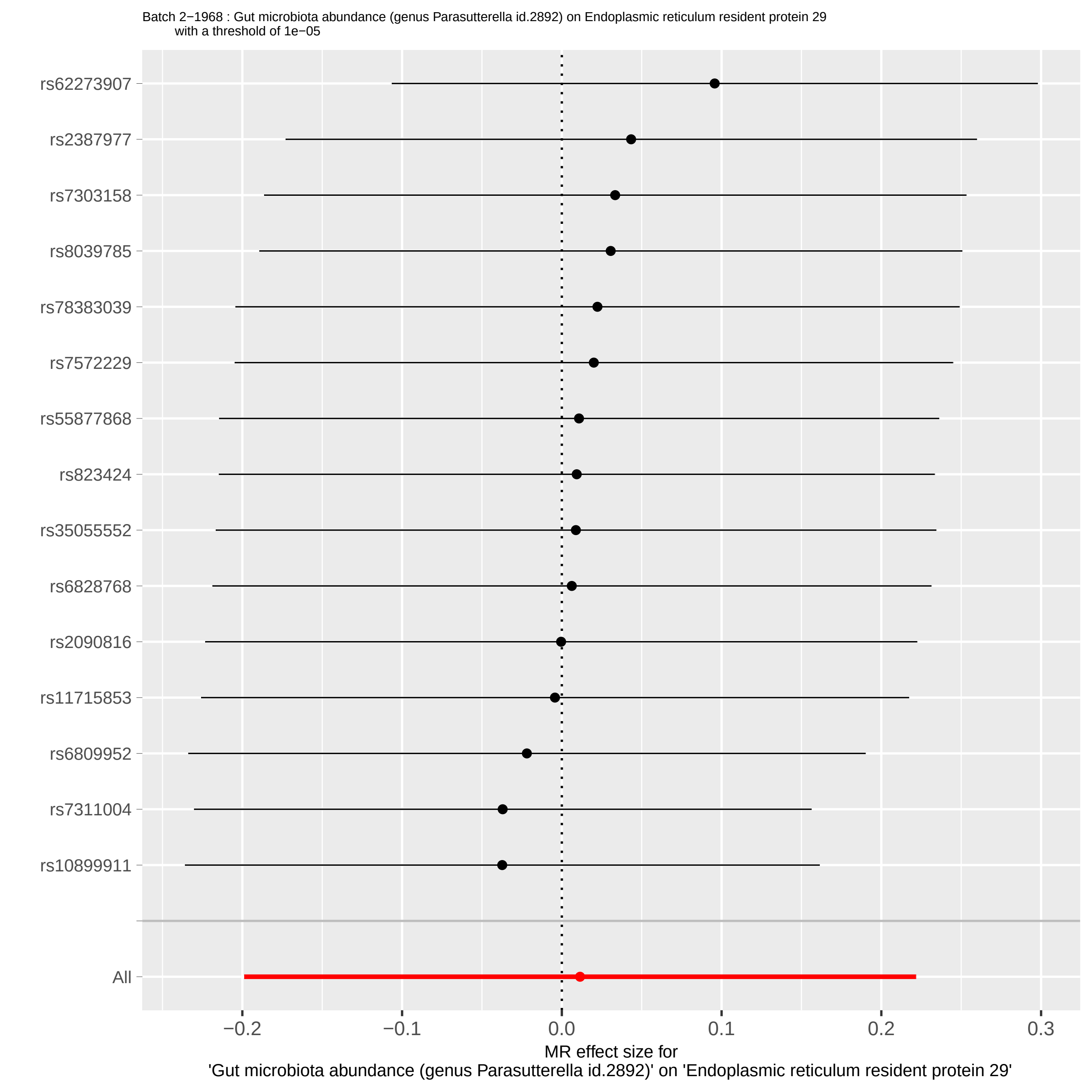

Batch 2−1968 : Gut microbiota abundance (genus Parasutterella id.2892) on Endoplasmic reticulum resident protein 29
with a threshold of 1e−05
rs62273907
rs2387977
rs7303158
rs8039785
rs78383039
rs7572229
rs55877868
rs823424
rs35055552
rs6828768
rs2090816
rs11715853
rs6809952
rs7311004
rs10899911
All
−0.2
−0.1
0.0
0.1
0.2
0.3
MR effect size for
'Gut microbiota abundance (genus Parasutterella id.2892)' on 'Endoplasmic reticulum resident protein 29'

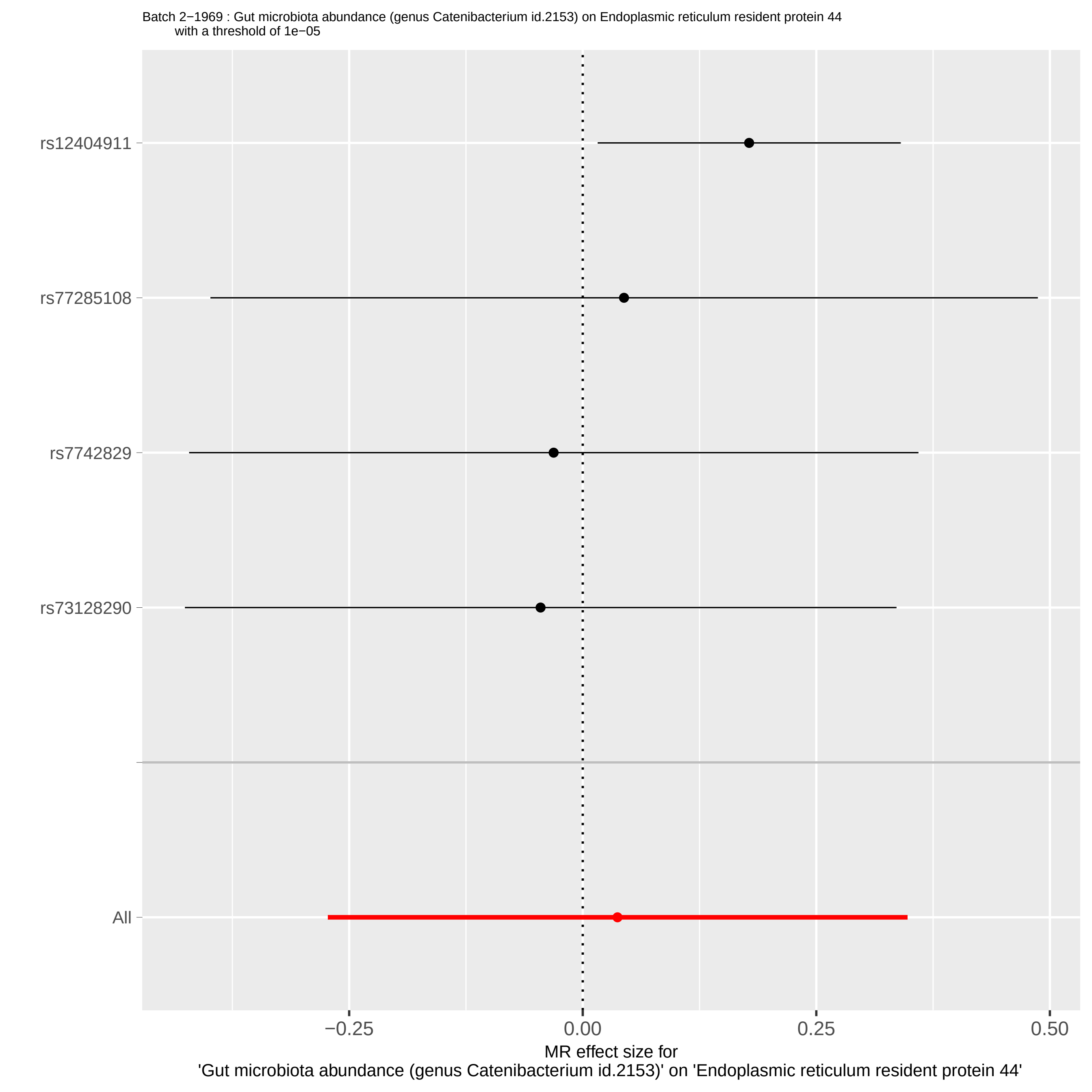
Batch 2−1969 : Gut microbiota abundance (genus Catenibacterium id.2153) on Endoplasmic reticulum resident protein 44
with a threshold of 1e−05
rs12404911
rs77285108
rs7742829
rs73128290
All
−0.25
0.00
0.25
0.50
MR effect size for
'Gut microbiota abundance (genus Catenibacterium id.2153)' on 'Endoplasmic reticulum resident protein 44'

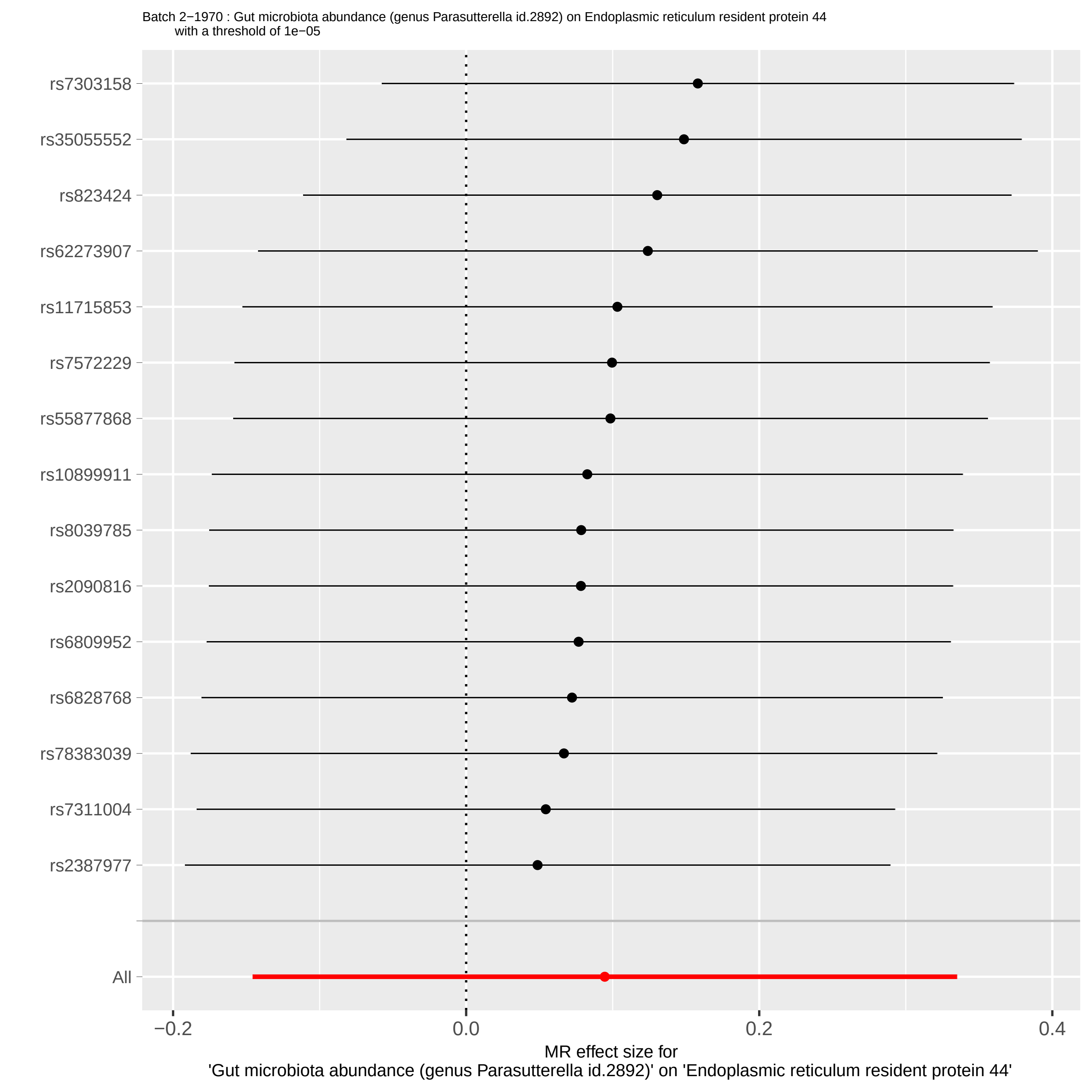
Batch 2−1970 : Gut microbiota abundance (genus Parasutterella id.2892) on Endoplasmic reticulum resident protein 44
with a threshold of 1e−05
rs7303158
rs35055552
rs823424
rs62273907
rs11715853
rs7572229
rs55877868
rs10899911
rs8039785
rs2090816
rs6809952
rs6828768
rs78383039
rs7311004
rs2387977
All
−0.2
0.0
0.2
0.4
MR effect size for
'Gut microbiota abundance (genus Parasutterella id.2892)' on 'Endoplasmic reticulum resident protein 44'

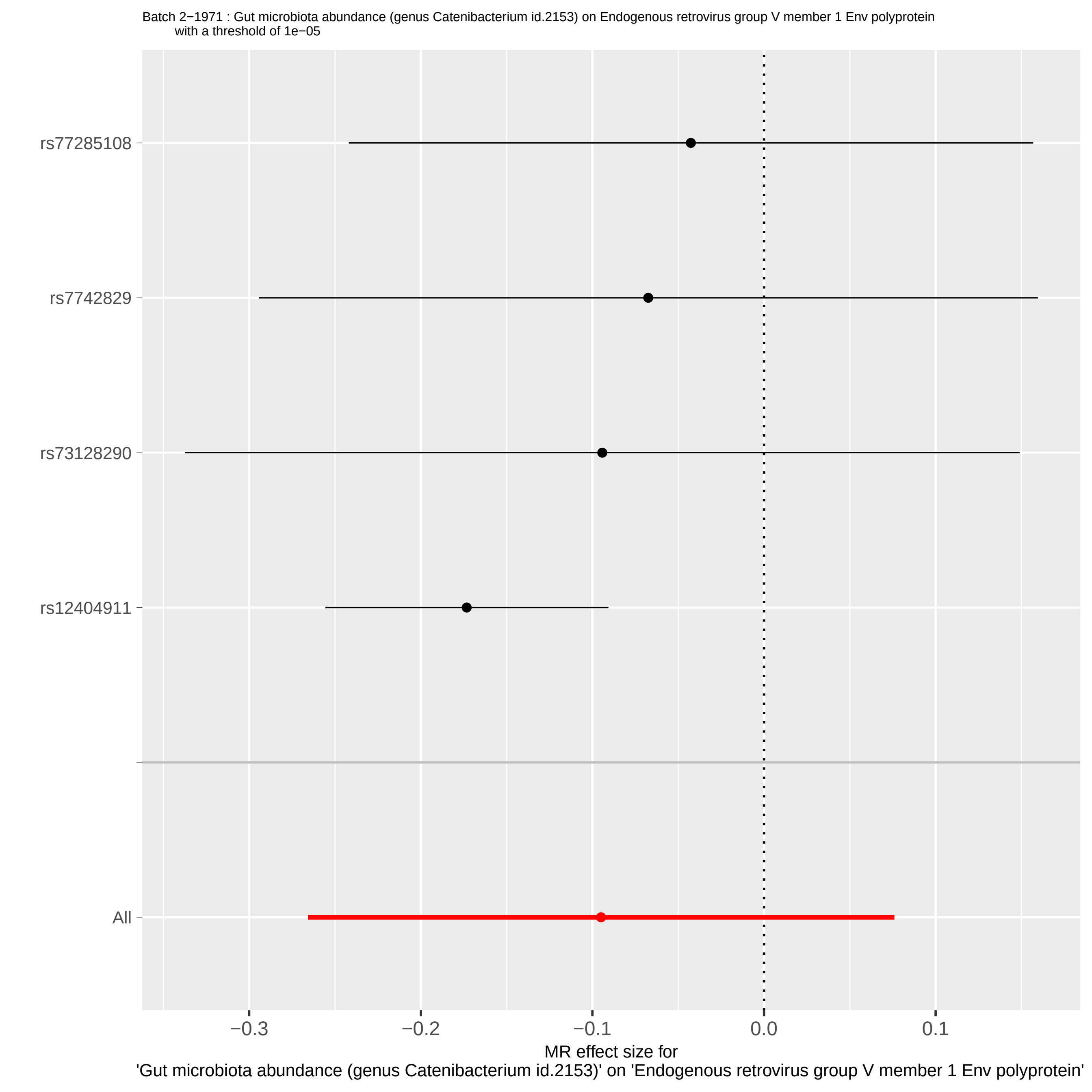

Batch 2−1971 : Gut microbiota abundance (genus Catenibacterium id.2153) on Endogenous retrovirus group V member 1 Env polyprotein
with a threshold of 1e−05
rs77285108
rs7742829
rs73128290
rs12404911
All
−0.3
−0.2
−0.1
0.0
0.1
MR effect size for
'Gut microbiota abundance (genus Catenibacterium id.2153)' on 'Endogenous retrovirus group V member 1 Env polyprotein'

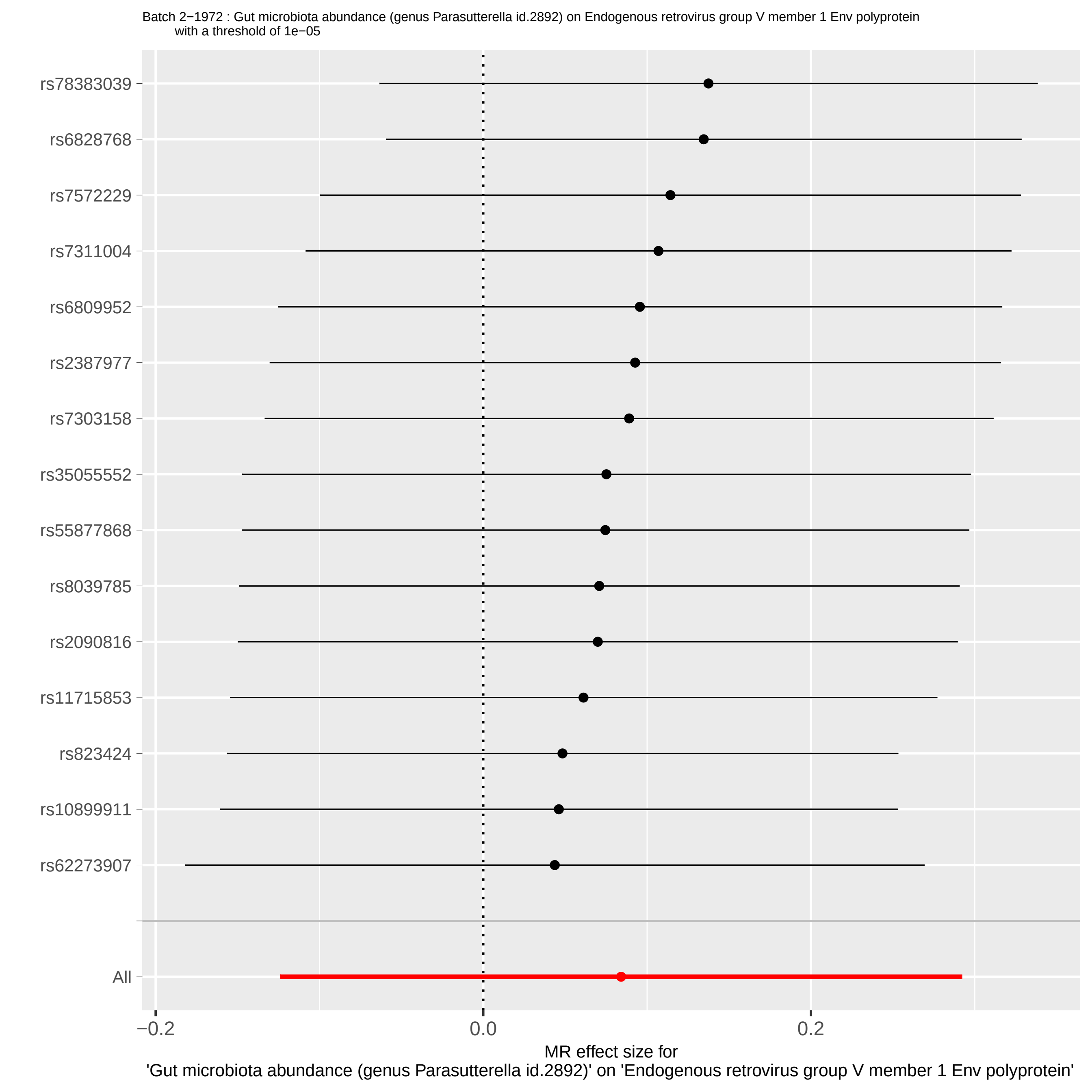

Batch 2−1972 : Gut microbiota abundance (genus Parasutterella id.2892) on Endogenous retrovirus group V member 1 Env polyprotein with a threshold of 1e−05
rs78383039
rs6828768
rs7572229
rs7311004
rs6809952
rs2387977
rs7303158
rs35055552
rs55877868
rs8039785
rs2090816
rs11715853
rs823424
rs10899911
rs62273907
All
−0.2
0.0
0.2
MR effect size for
'Gut microbiota abundance (genus Parasutterella id.2892)' on 'Endogenous retrovirus group V member 1 Env polyprotein'

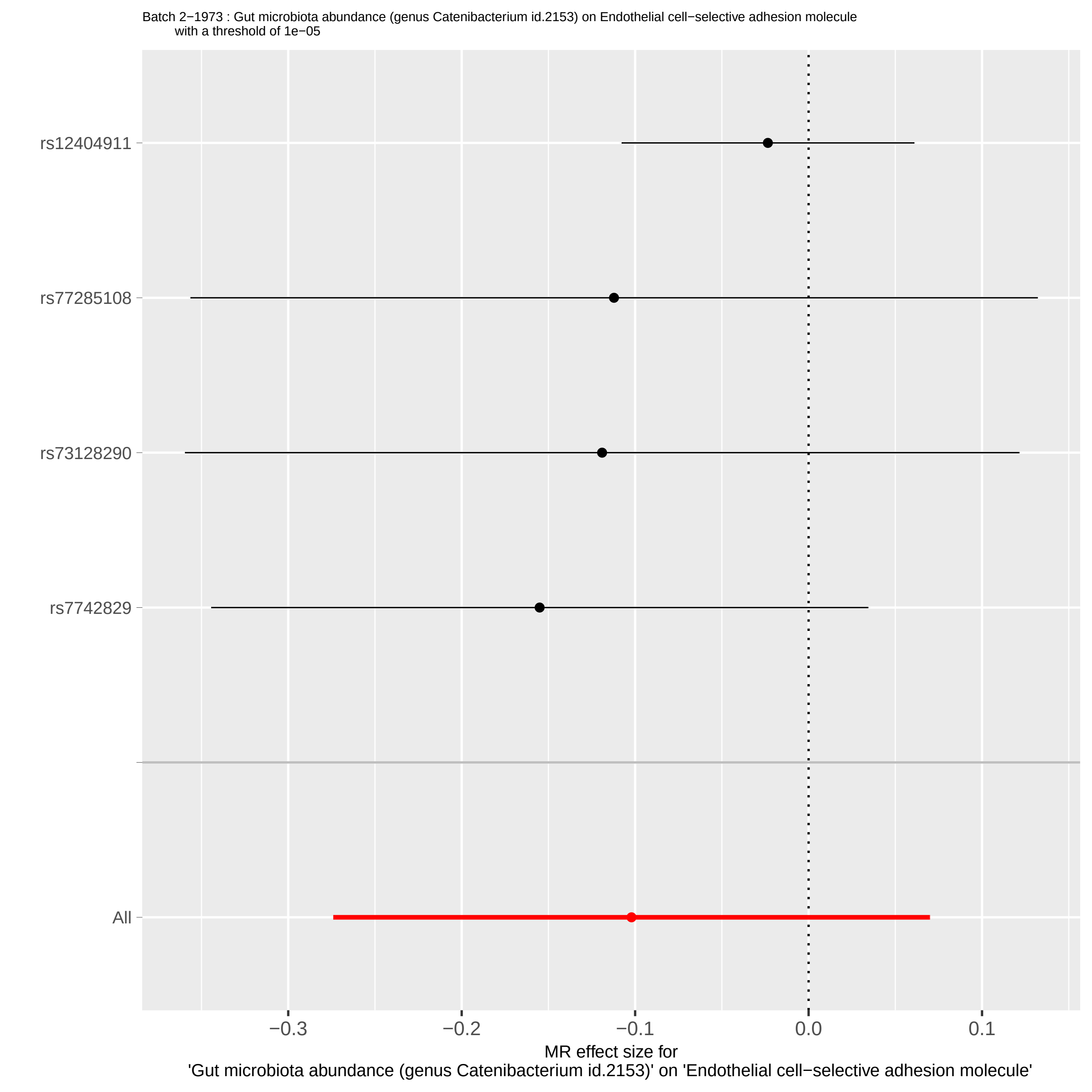
Batch 2−1973 : Gut microbiota abundance (genus Catenibacterium id.2153) on Endothelial cell−selective adhesion molecule
with a threshold of 1e−05
rs12404911
rs77285108
rs73128290
rs7742829
All
−0.3
−0.2
−0.1
0.0
0.1
MR effect size for
'Gut microbiota abundance (genus Catenibacterium id.2153)' on 'Endothelial cell−selective adhesion molecule'

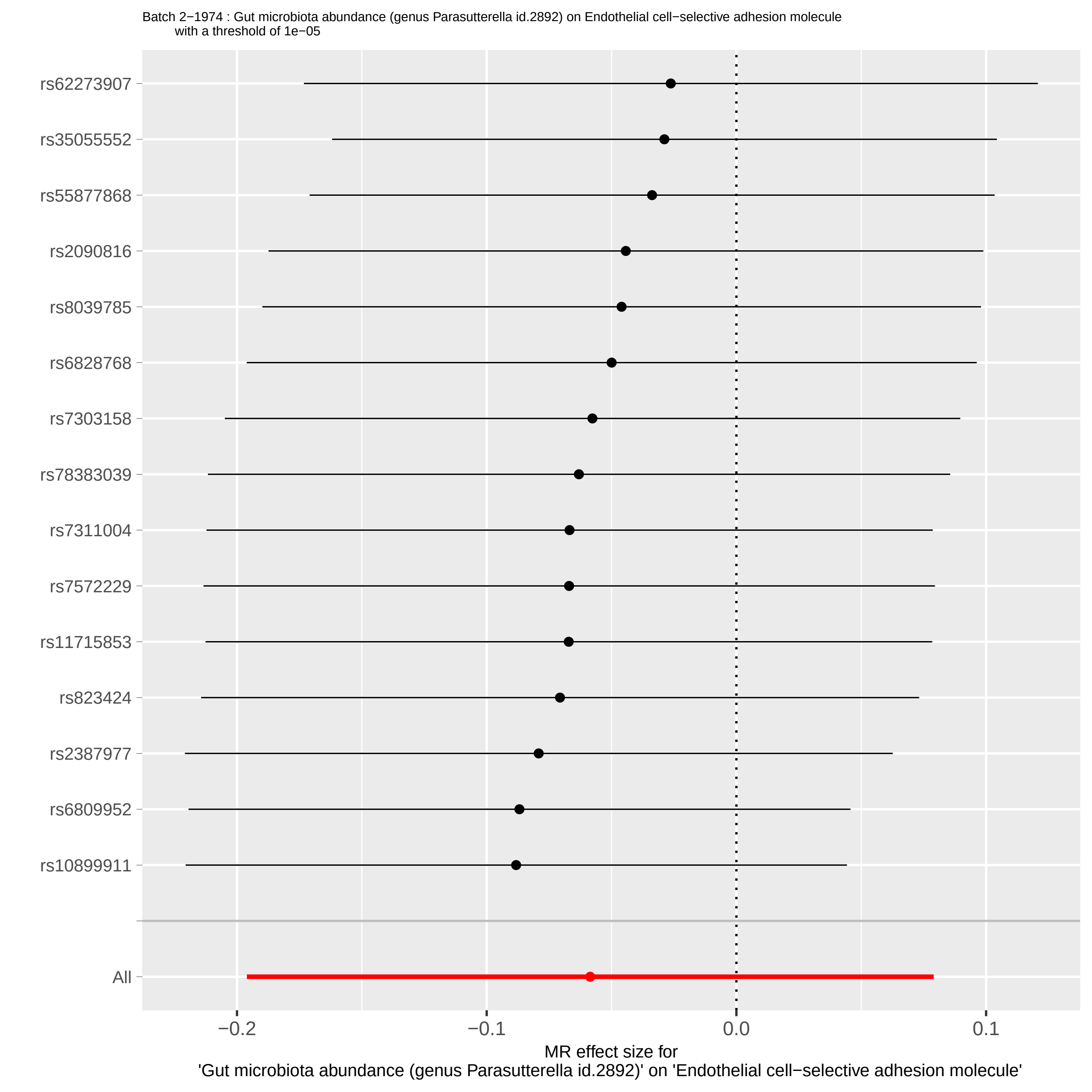

Batch 2−1974 : Gut microbiota abundance (genus Parasutterella id.2892) on Endothelial cell−selective adhesion molecule
with a threshold of 1e−05
rs62273907
rs35055552
rs55877868
rs2090816
rs8039785
rs6828768
rs7303158
rs78383039
rs7311004
rs7572229
rs11715853
rs823424
rs2387977
rs6809952
rs10899911
All
−0.2
−0.1
0.0
0.1
MR effect size for
'Gut microbiota abundance (genus Parasutterella id.2892)' on 'Endothelial cell−selective adhesion molecule'

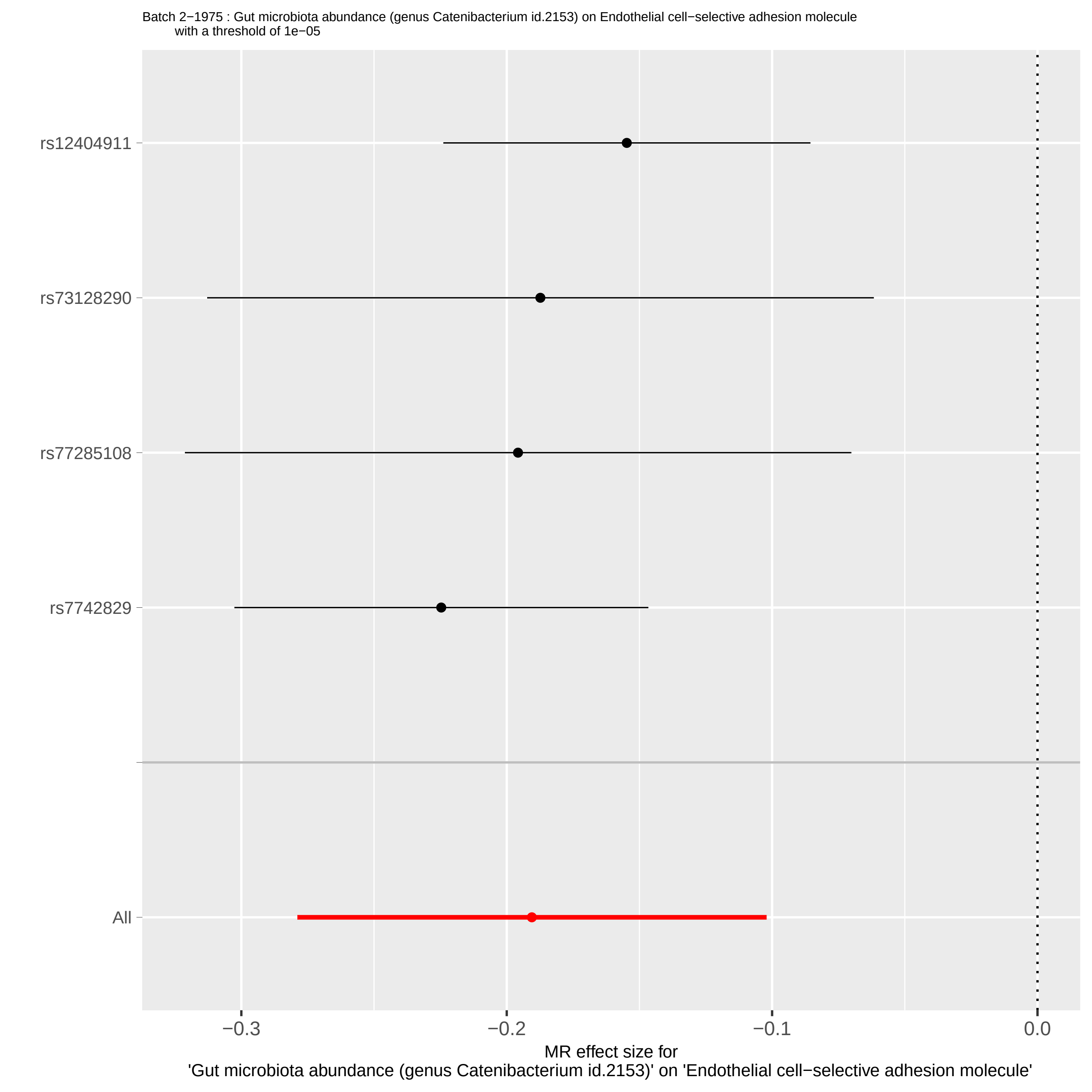
Batch 2−1975 : Gut microbiota abundance (genus Catenibacterium id.2153) on Endothelial cell−selective adhesion molecule
with a threshold of 1e−05
rs12404911
rs73128290
rs77285108
rs7742829
All
−0.3
−0.2
−0.1
0.0
MR effect size for
'Gut microbiota abundance (genus Catenibacterium id.2153)' on 'Endothelial cell−selective adhesion molecule'

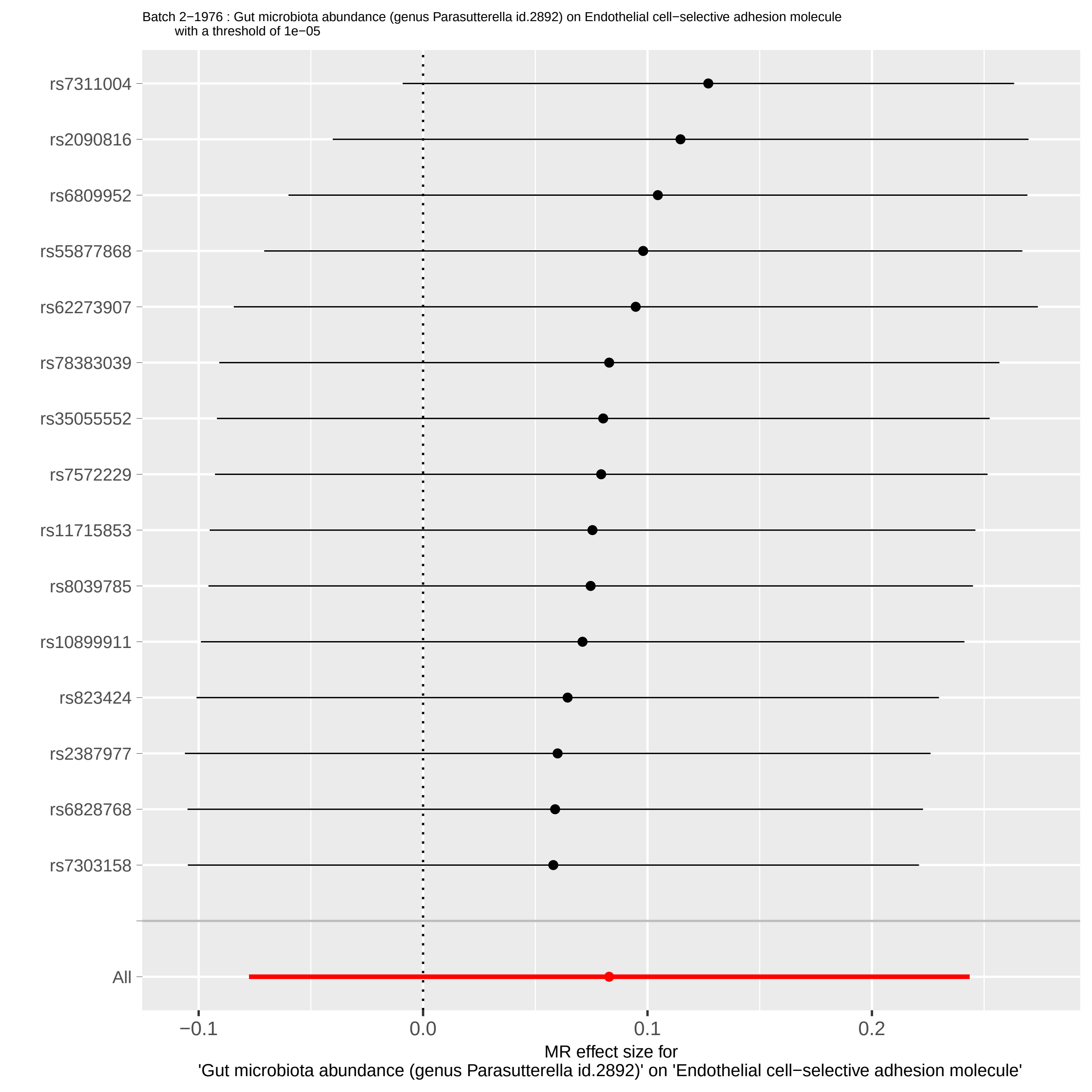

Batch 2−1976 : Gut microbiota abundance (genus Parasutterella id.2892) on Endothelial cell−selective adhesion molecule
with a threshold of 1e−05
rs7311004
rs2090816
rs6809952
rs55877868
rs62273907
rs78383039
rs35055552
rs7572229
rs11715853
rs8039785
rs10899911
rs823424
rs2387977
rs6828768
rs7303158
All
−0.1
0.0
0.1
0.2
MR effect size for
'Gut microbiota abundance (genus Parasutterella id.2892)' on 'Endothelial cell−selective adhesion molecule'

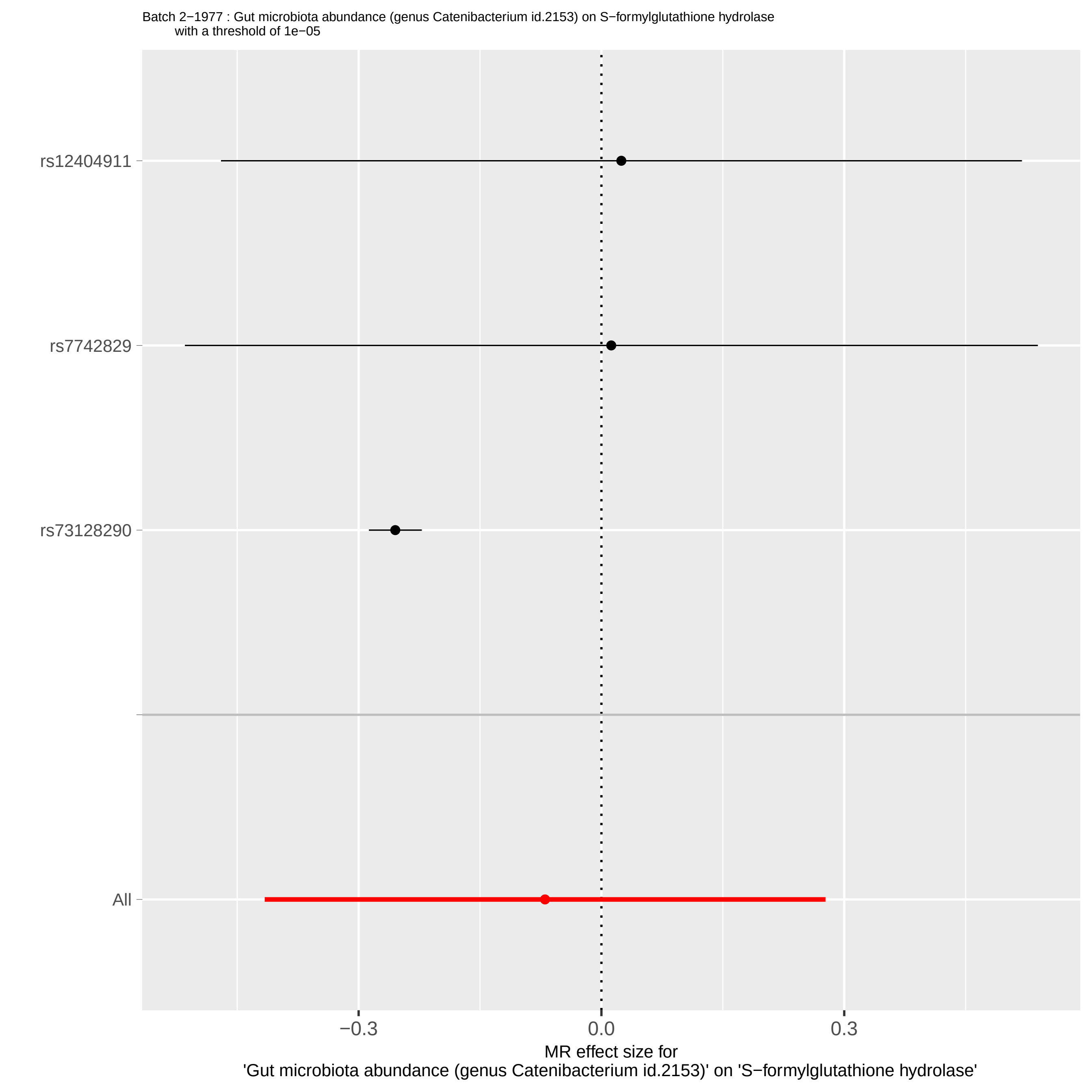
Batch 2−1977 : Gut microbiota abundance (genus Catenibacterium id.2153) on S−formylglutathione hydrolase
with a threshold of 1e−05
rs12404911
rs7742829
rs73128290
All
−0.3
0.0
0.3
MR effect size for
'Gut microbiota abundance (genus Catenibacterium id.2153)' on 'S−formylglutathione hydrolase'

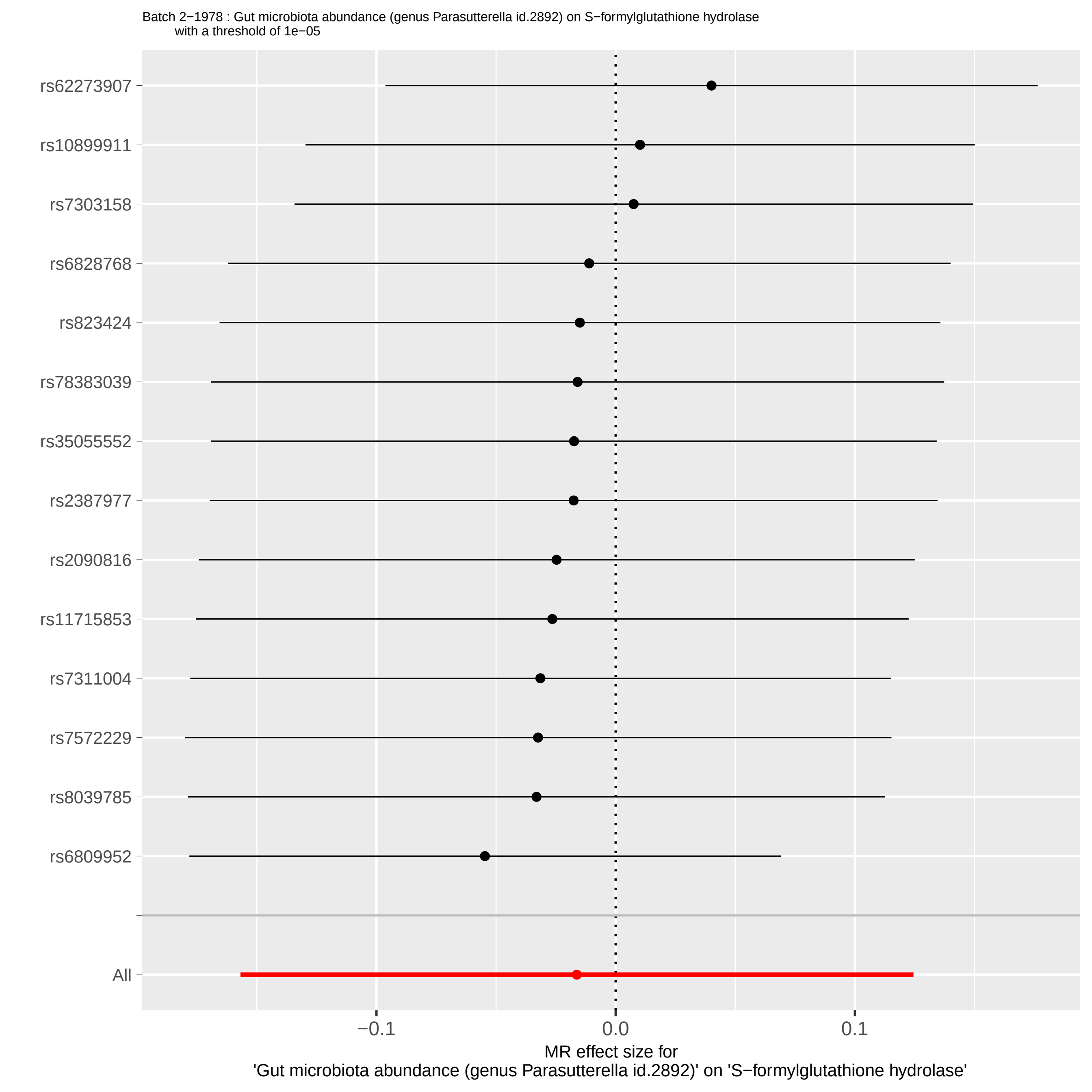
Batch 2−1978 : Gut microbiota abundance (genus Parasutterella id.2892) on S−formylglutathione hydrolase
with a threshold of 1e−05
rs62273907
rs10899911
rs7303158
rs6828768
rs823424
rs78383039
rs35055552
rs2387977
rs2090816
rs11715853
rs7311004
rs7572229
rs8039785
rs6809952
All
−0.1
0.0
0.1
MR effect size for
'Gut microbiota abundance (genus Parasutterella id.2892)' on 'S−formylglutathione hydrolase'

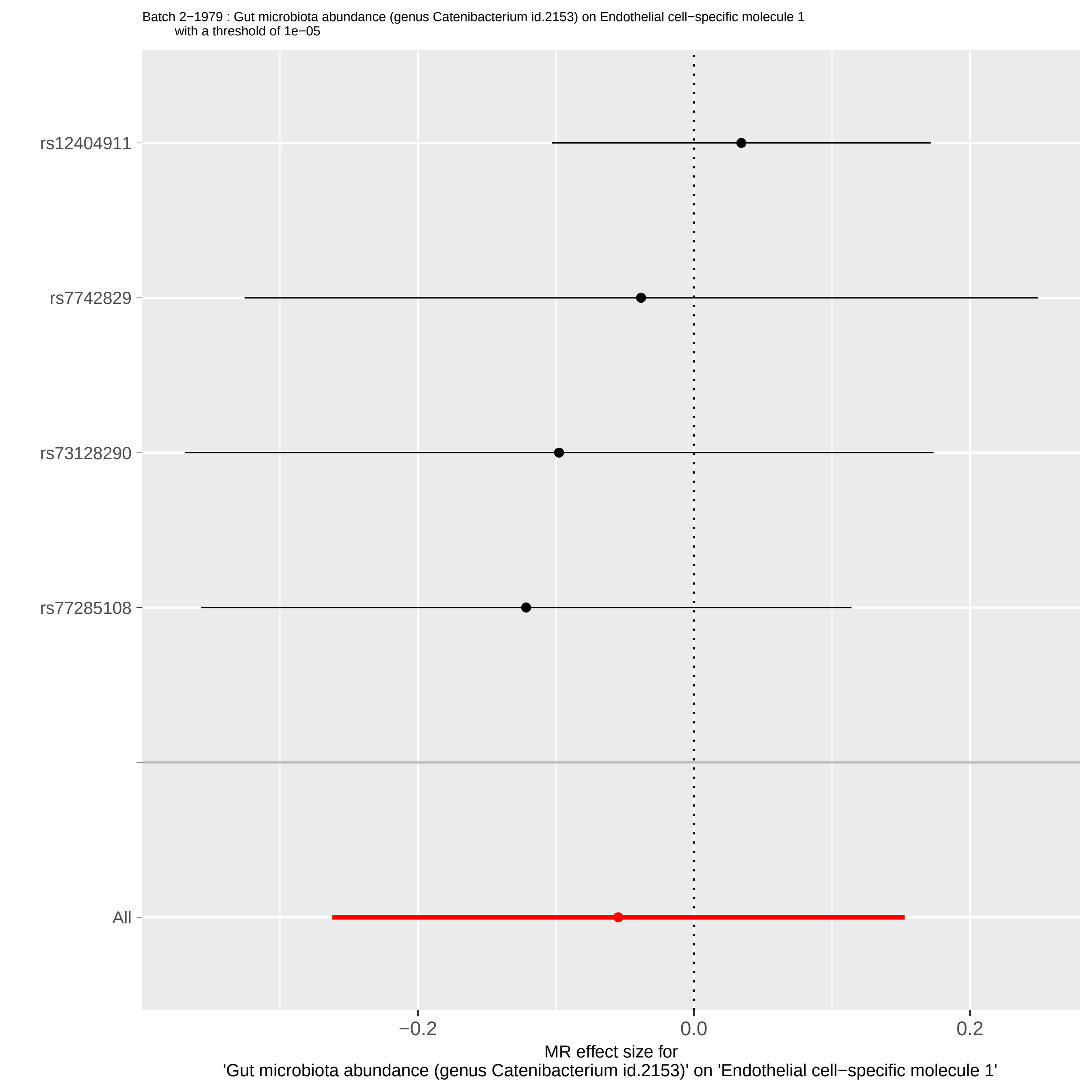
Batch 2−1979 : Gut microbiota abundance (genus Catenibacterium id.2153) on Endothelial cell−specific molecule 1
with a threshold of 1e−05
rs12404911
rs7742829
rs73128290
rs77285108
All
−0.2
0.0
0.2
MR effect size for
'Gut microbiota abundance (genus Catenibacterium id.2153)' on 'Endothelial cell−specific molecule 1'

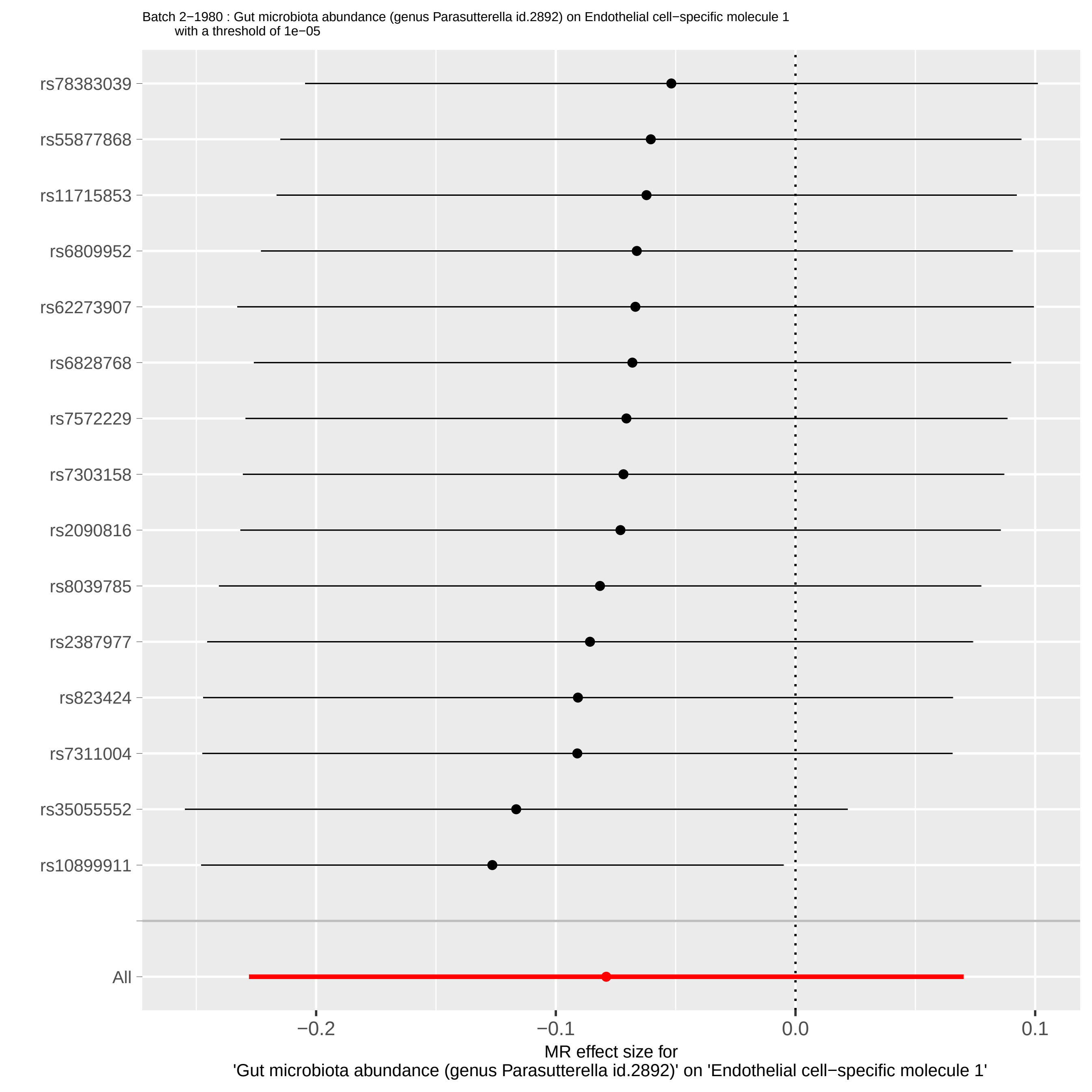

Batch 2−1980 : Gut microbiota abundance (genus Parasutterella id.2892) on Endothelial cell−specific molecule 1
with a threshold of 1e−05
rs78383039
rs55877868
rs11715853
rs6809952
rs62273907
rs6828768
rs7572229
rs7303158
rs2090816
rs8039785
rs2387977
rs823424
rs7311004
rs35055552
rs10899911
All
−0.2
−0.1
0.0
0.1
MR effect size for
'Gut microbiota abundance (genus Parasutterella id.2892)' on 'Endothelial cell−specific molecule 1'

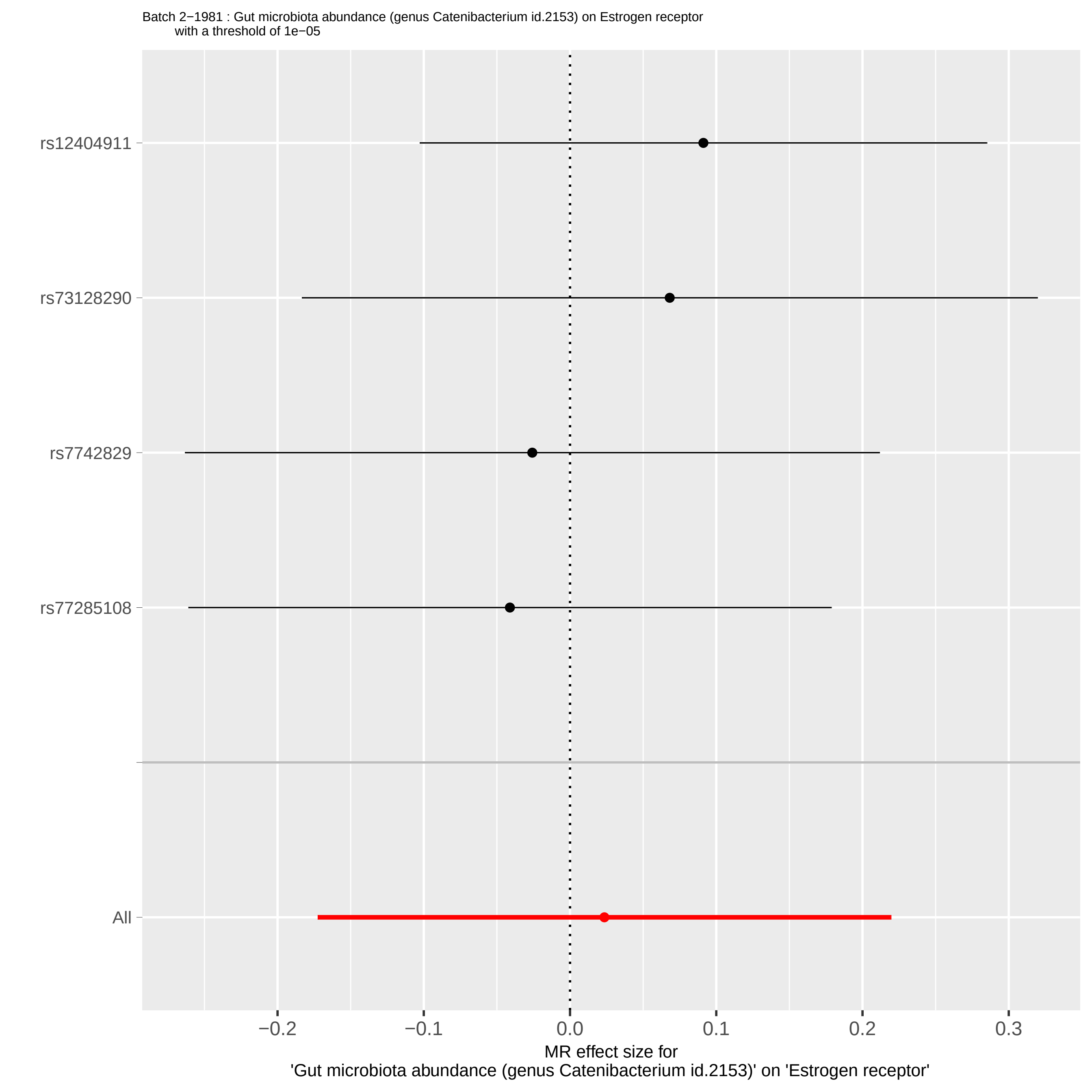

Batch 2−1981 : Gut microbiota abundance (genus Catenibacterium id.2153) on Estrogen receptor
with a threshold of 1e−05
rs12404911
rs73128290
rs7742829
rs77285108
All
−0.2
−0.1
0.0
0.1
0.2
0.3
MR effect size for
'Gut microbiota abundance (genus Catenibacterium id.2153)' on 'Estrogen receptor'

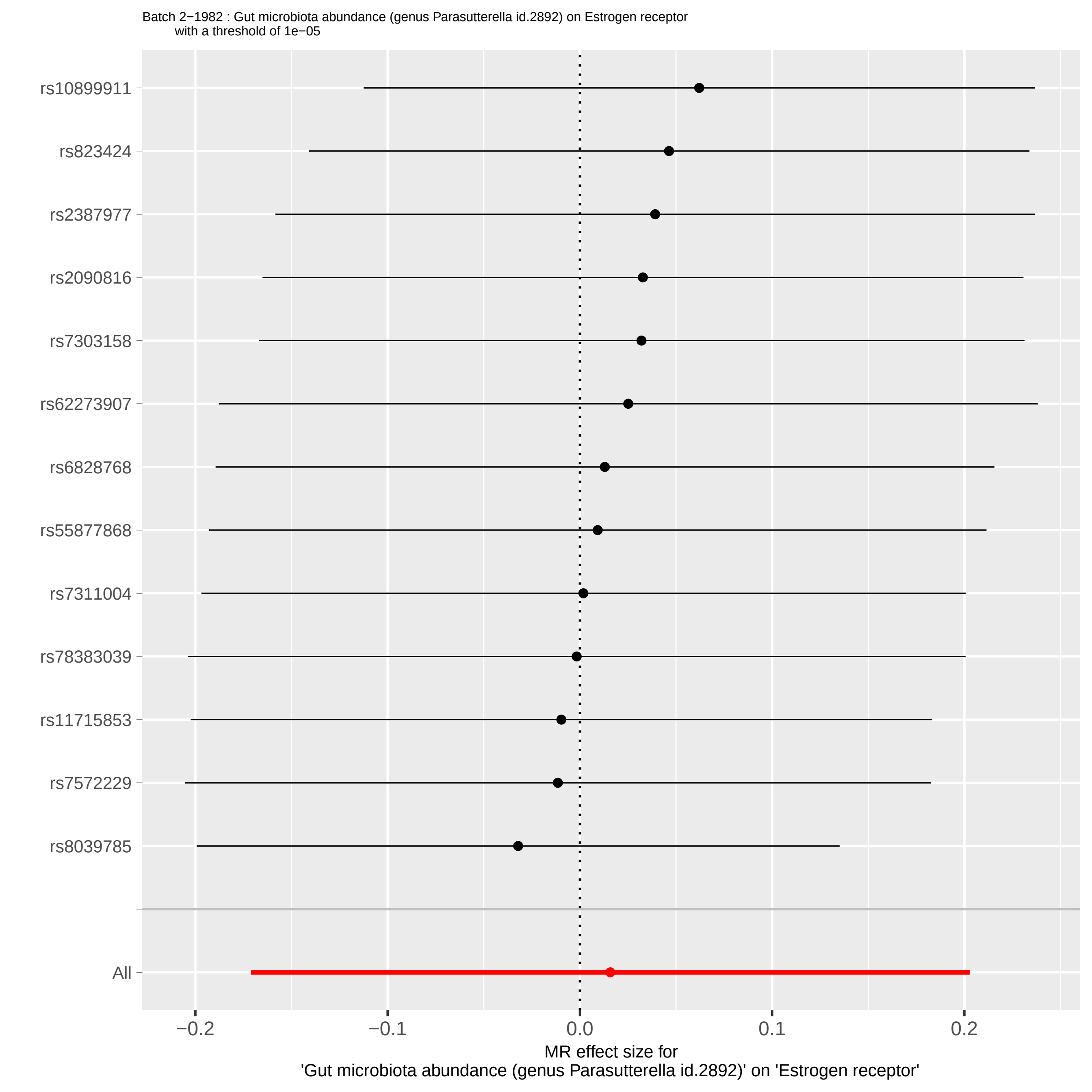
Batch 2−1982 : Gut microbiota abundance (genus Parasutterella id.2892) on Estrogen receptor
with a threshold of 1e−05
rs10899911
rs823424
rs2387977
rs2090816
rs7303158
rs62273907
rs6828768
rs55877868
rs7311004
rs78383039
rs11715853
rs7572229
rs8039785
All
−0.2
−0.1
0.0
0.1
0.2
MR effect size for
'Gut microbiota abundance (genus Parasutterella id.2892)' on 'Estrogen receptor'

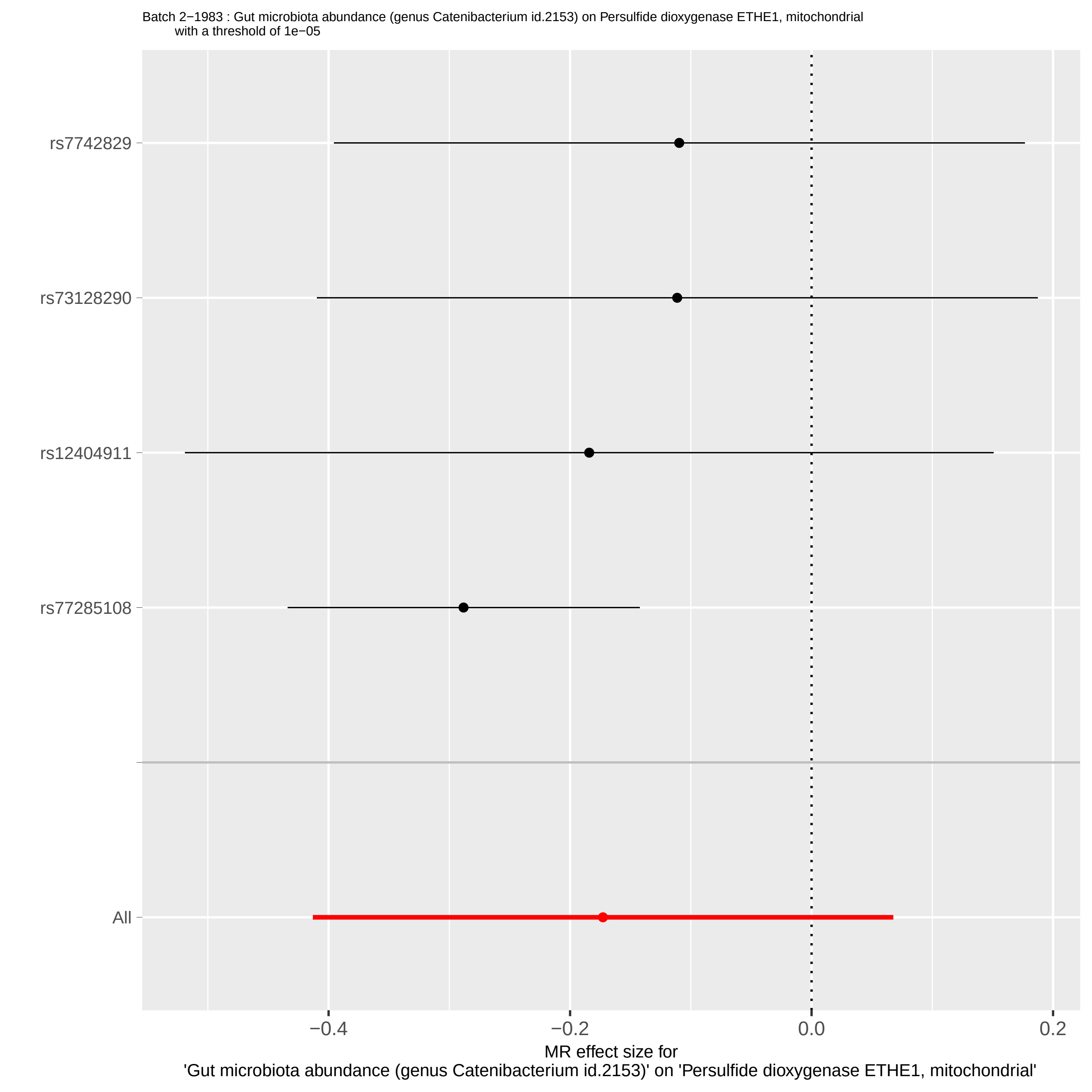
Batch 2−1983 : Gut microbiota abundance (genus Catenibacterium id.2153) on Persulfide dioxygenase ETHE1, mitochondrial
with a threshold of 1e−05
rs7742829
rs73128290
rs12404911
rs77285108
All
−0.4
−0.2
0.0
0.2
MR effect size for
'Gut microbiota abundance (genus Catenibacterium id.2153)' on 'Persulfide dioxygenase ETHE1, mitochondrial'

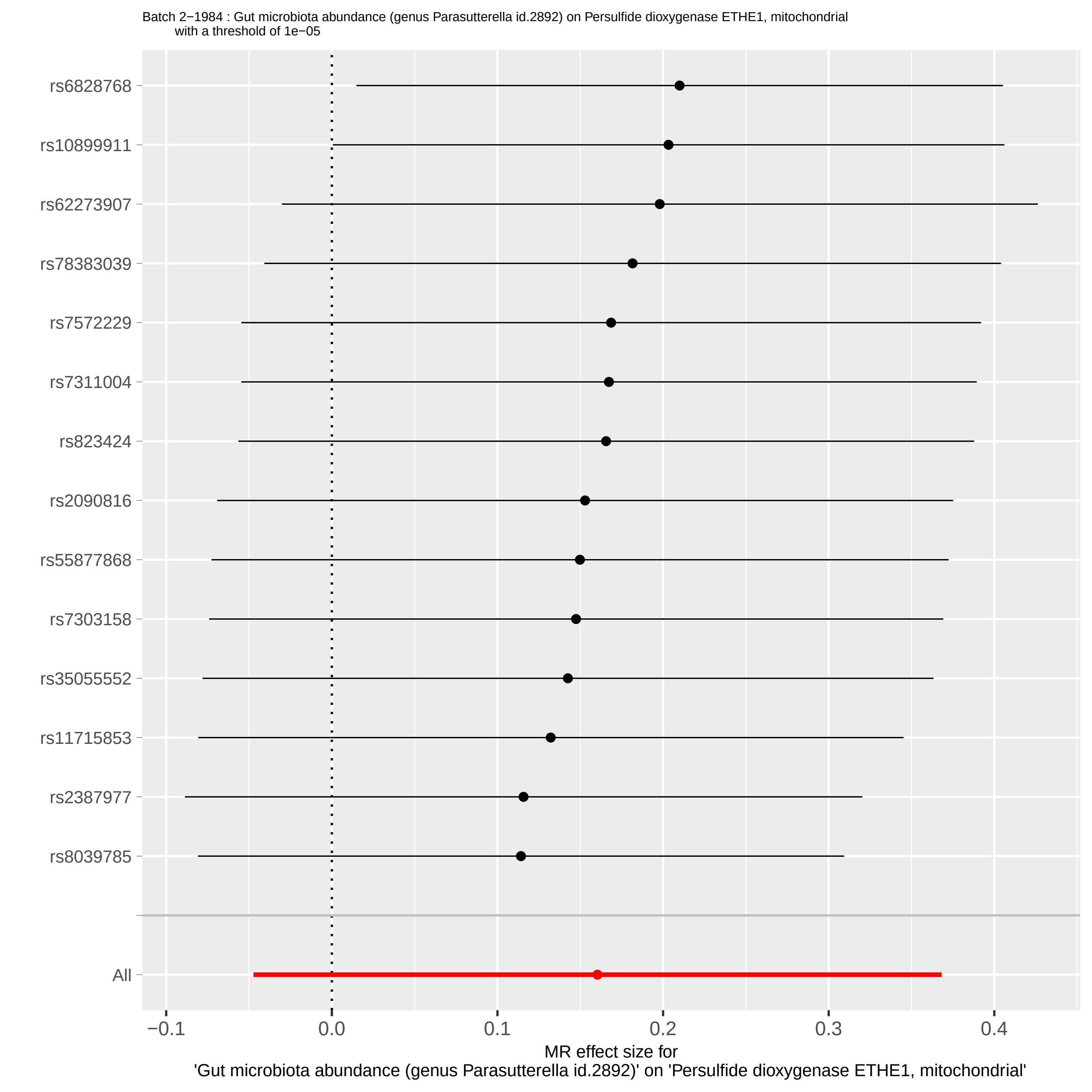

Batch 2−1984 : Gut microbiota abundance (genus Parasutterella id.2892) on Persulfide dioxygenase ETHE1, mitochondrial
with a threshold of 1e−05
rs6828768
rs10899911
rs62273907
rs78383039
rs7572229
rs7311004
rs823424
rs2090816
rs55877868
rs7303158
rs35055552
rs11715853
rs2387977
rs8039785
All
−0.1
0.0
0.1
0.2
0.3
0.4
MR effect size for
'Gut microbiota abundance (genus Parasutterella id.2892)' on 'Persulfide dioxygenase ETHE1, mitochondrial'

Batch 2−1985 : Gut microbiota abundance (genus Catenibacterium id.2153) on Ethanolamine kinase 1
with a threshold of 1e−05

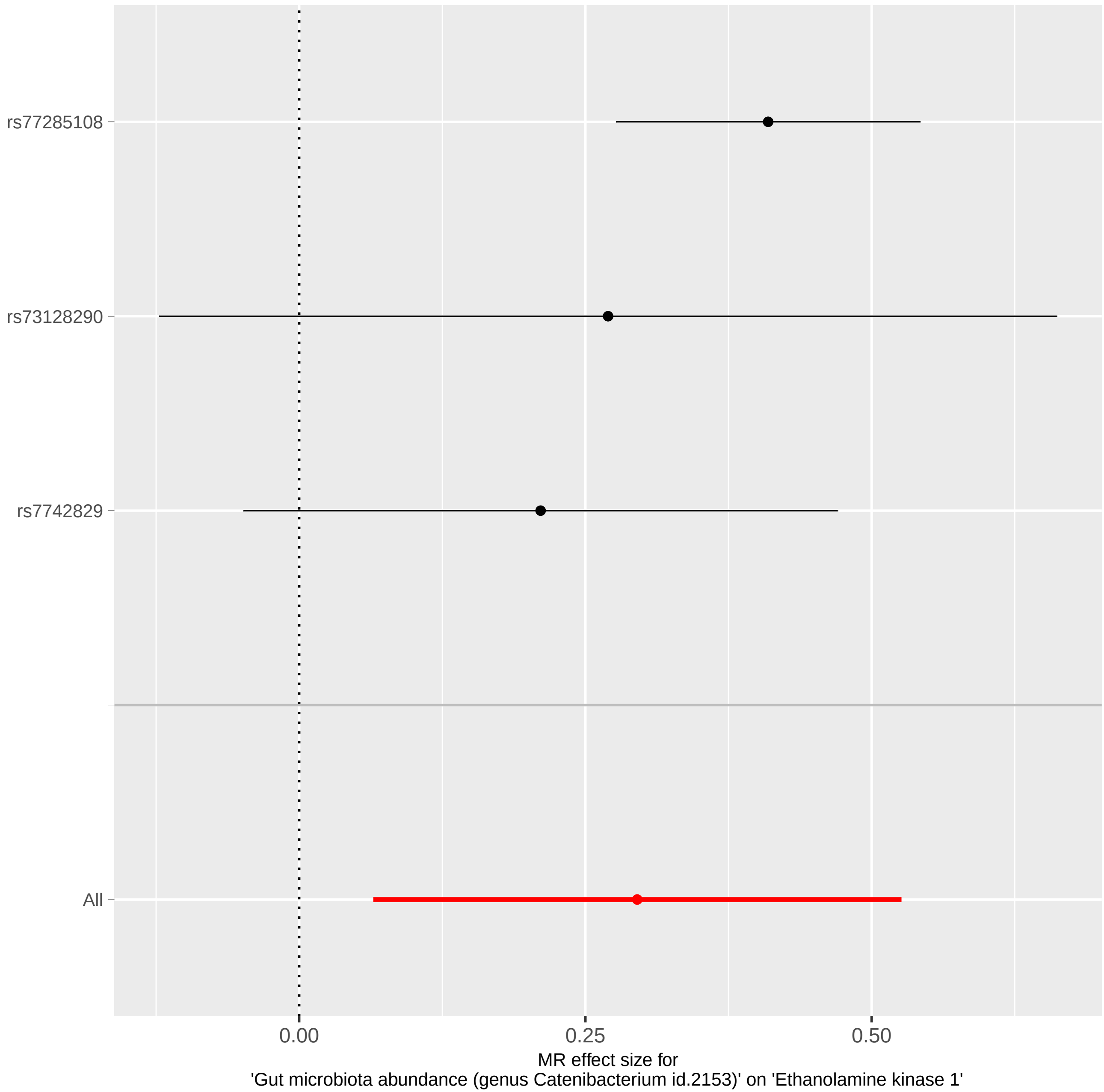

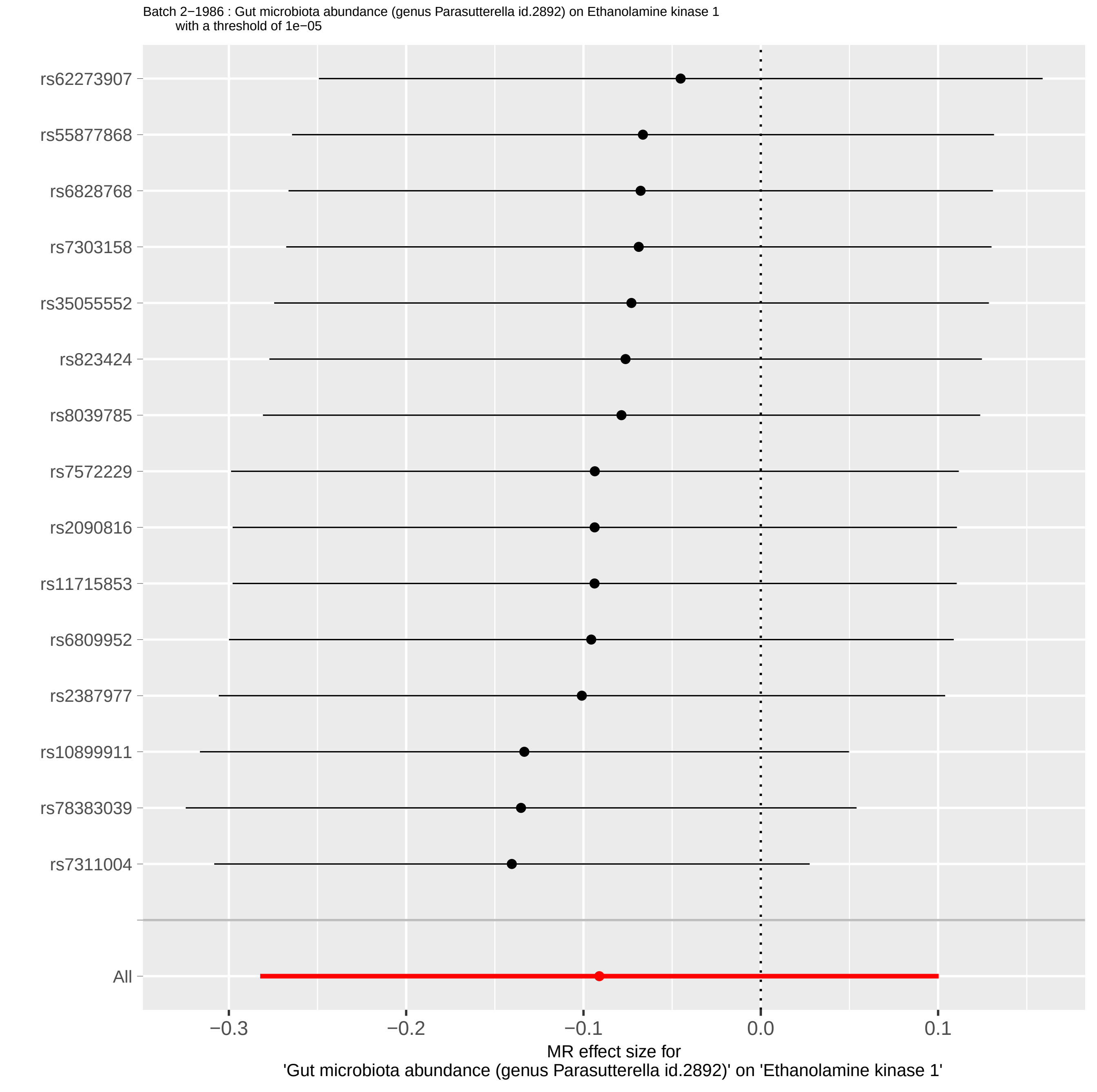

Batch 2−1986 : Gut microbiota abundance (genus Parasutterella id.2892) on Ethanolamine kinase 1
with a threshold of 1e−05
rs62273907
rs55877868
rs6828768
rs7303158
rs35055552
rs823424
rs8039785
rs7572229
rs2090816
rs11715853
rs6809952
rs2387977
rs10899911
rs78383039
rs7311004
All
−0.3
−0.2
−0.1
0.0
0.1
MR effect size for
'Gut microbiota abundance (genus Parasutterella id.2892)' on 'Ethanolamine kinase 1'

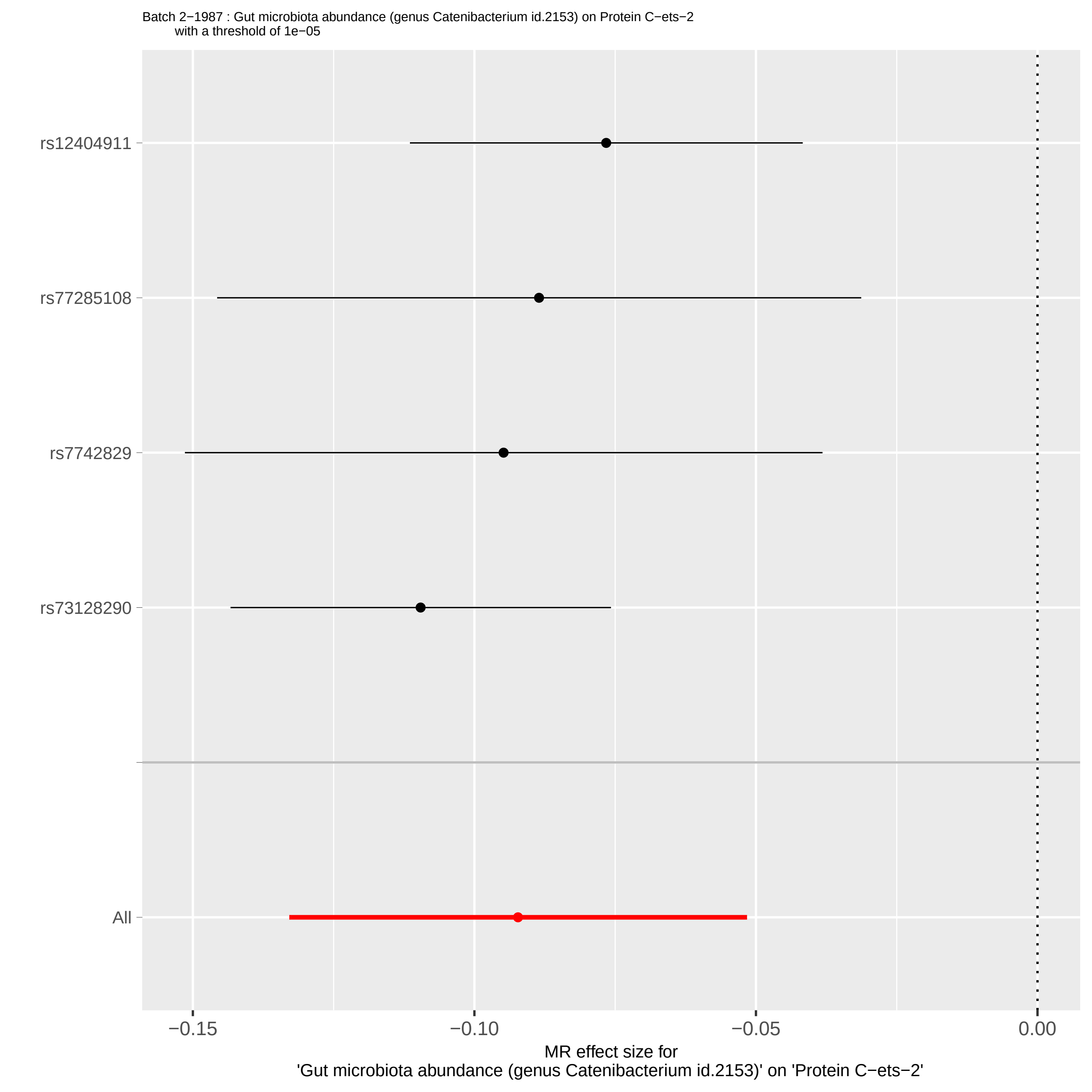
Batch 2−1987 : Gut microbiota abundance (genus Catenibacterium id.2153) on Protein C−ets−2
with a threshold of 1e−05
rs12404911
rs77285108
rs7742829
rs73128290
All
−0.15
−0.10
−0.05
0.00
MR effect size for
'Gut microbiota abundance (genus Catenibacterium id.2153)' on 'Protein C−ets−2'

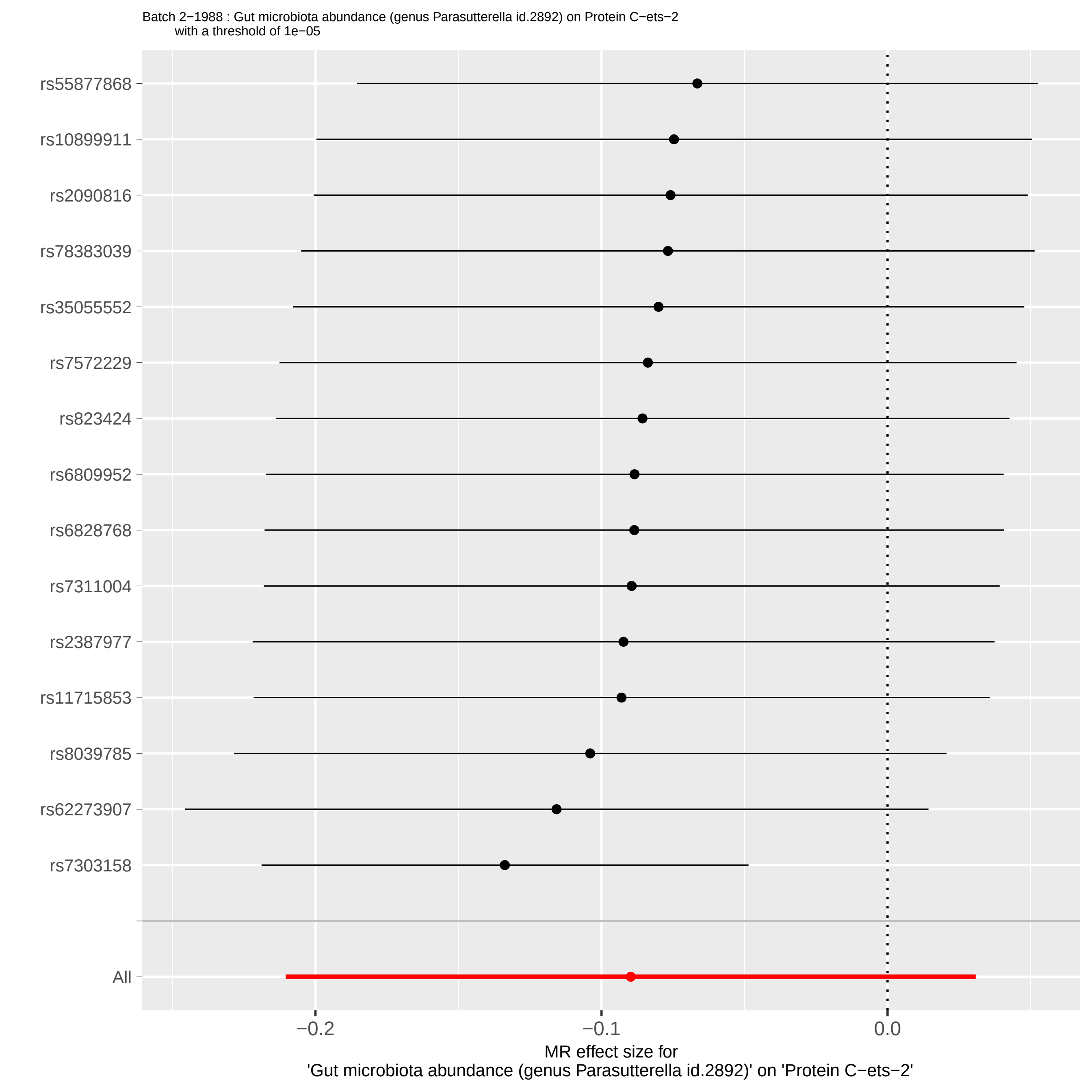

Batch 2−1988 : Gut microbiota abundance (genus Parasutterella id.2892) on Protein C−ets−2
with a threshold of 1e−05
rs55877868
rs10899911
rs2090816
rs78383039
rs35055552
rs7572229
rs823424
rs6809952
rs6828768
rs7311004
rs2387977
rs11715853
rs8039785
rs62273907
rs7303158
All
−0.2
−0.1
0.0
MR effect size for
'Gut microbiota abundance (genus Parasutterella id.2892)' on 'Protein C−ets−2'

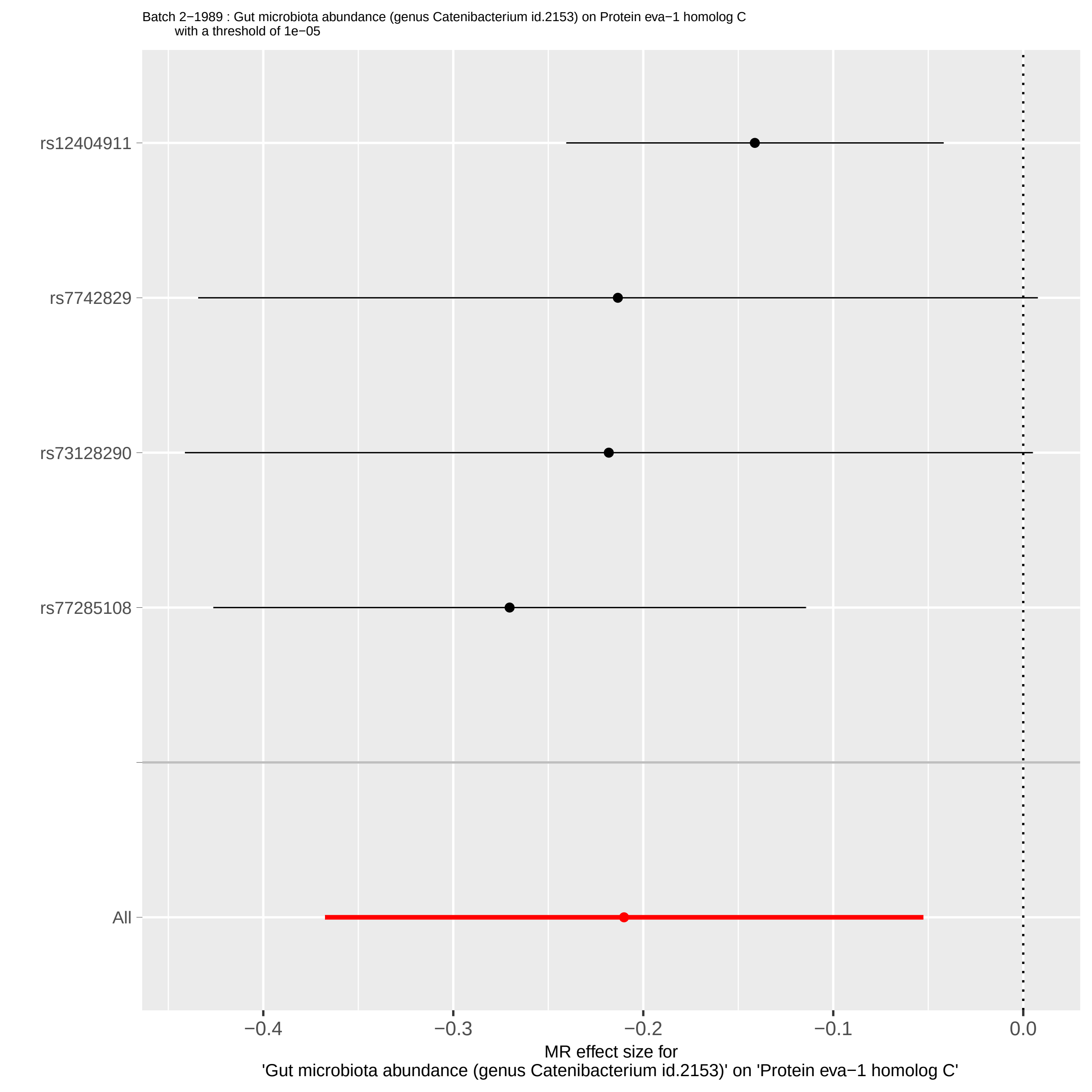
Batch 2−1989 : Gut microbiota abundance (genus Catenibacterium id.2153) on Protein eva−1 homolog C
with a threshold of 1e−05
rs12404911
rs7742829
rs73128290
rs77285108
All
−0.4
−0.3
−0.2
−0.1
0.0
MR effect size for
'Gut microbiota abundance (genus Catenibacterium id.2153)' on 'Protein eva−1 homolog C'

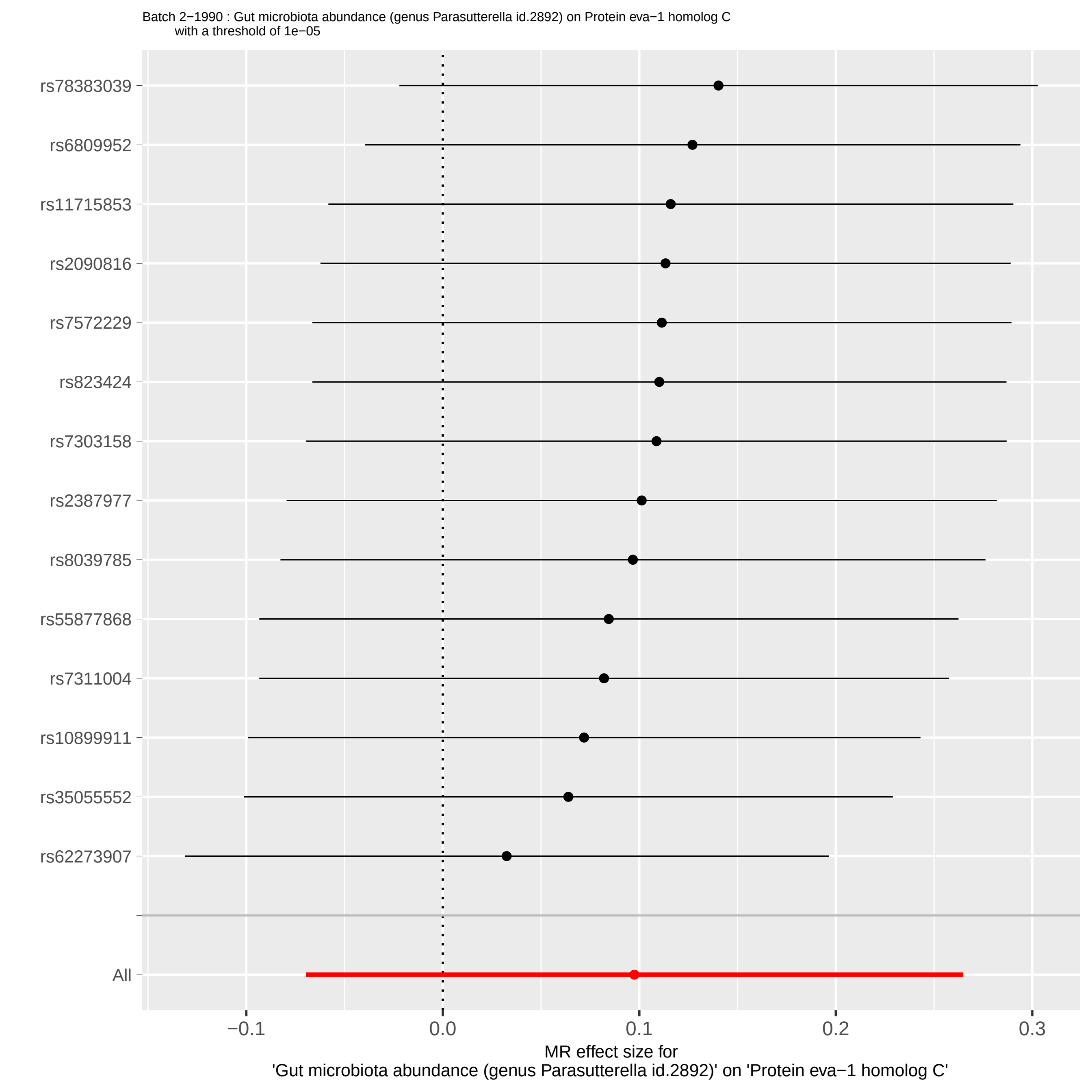
Batch 2−1990 : Gut microbiota abundance (genus Parasutterella id.2892) on Protein eva−1 homolog C
with a threshold of 1e−05
rs78383039
rs6809952
rs11715853
rs2090816
rs7572229
rs823424
rs7303158
rs2387977
rs8039785
rs55877868
rs7311004
rs10899911
rs35055552
rs62273907
All
−0.1
0.0
0.1
0.2
0.3
MR effect size for
'Gut microbiota abundance (genus Parasutterella id.2892)' on 'Protein eva−1 homolog C'

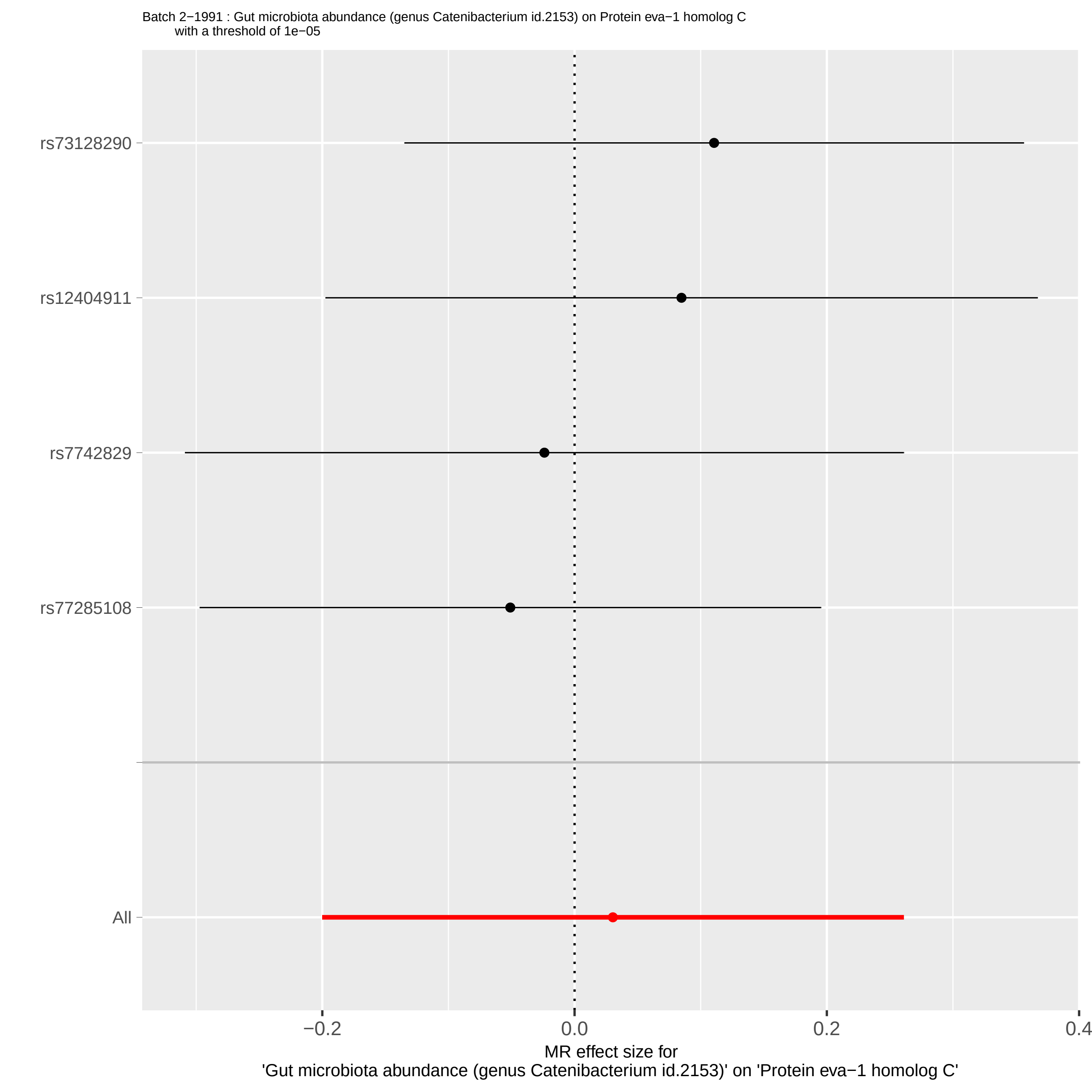

Batch 2−1991 : Gut microbiota abundance (genus Catenibacterium id.2153) on Protein eva−1 homolog C
with a threshold of 1e−05
rs73128290
rs12404911
rs7742829
rs77285108
All
−0.2
0.0
0.2
0.4
MR effect size for
'Gut microbiota abundance (genus Catenibacterium id.2153)' on 'Protein eva−1 homolog C'

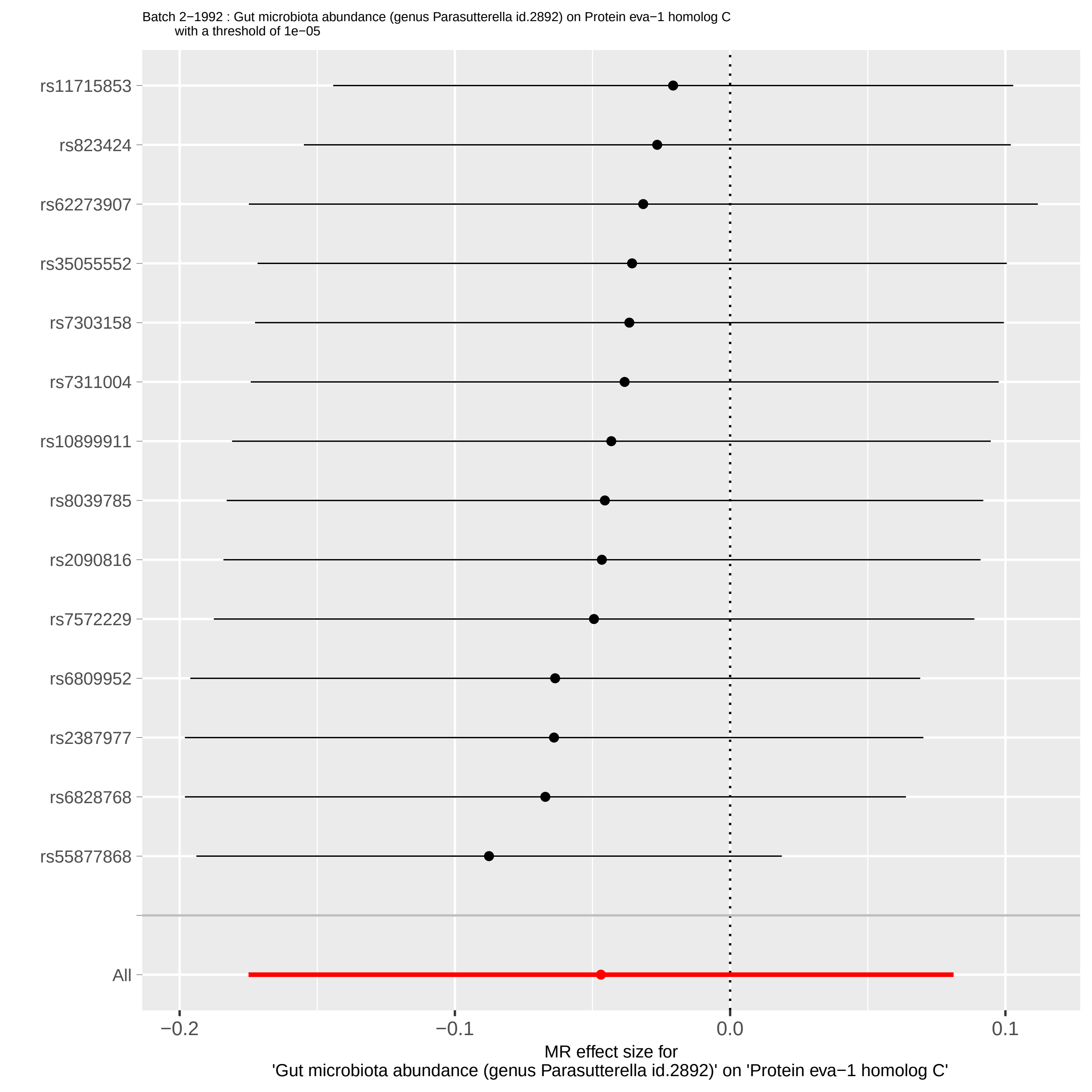
Batch 2−1992 : Gut microbiota abundance (genus Parasutterella id.2892) on Protein eva−1 homolog C
with a threshold of 1e−05
rs11715853
rs823424
rs62273907
rs35055552
rs7303158
rs7311004
rs10899911
rs8039785
rs2090816
rs7572229
rs6809952
rs2387977
rs6828768
rs55877868
All
−0.2
−0.1
0.0
0.1
MR effect size for
'Gut microbiota abundance (genus Parasutterella id.2892)' on 'Protein eva−1 homolog C'

Batch 2−1993 : Gut microbiota abundance (genus Catenibacterium id.2153) on Protein EVI2B
with a threshold of 1e−05

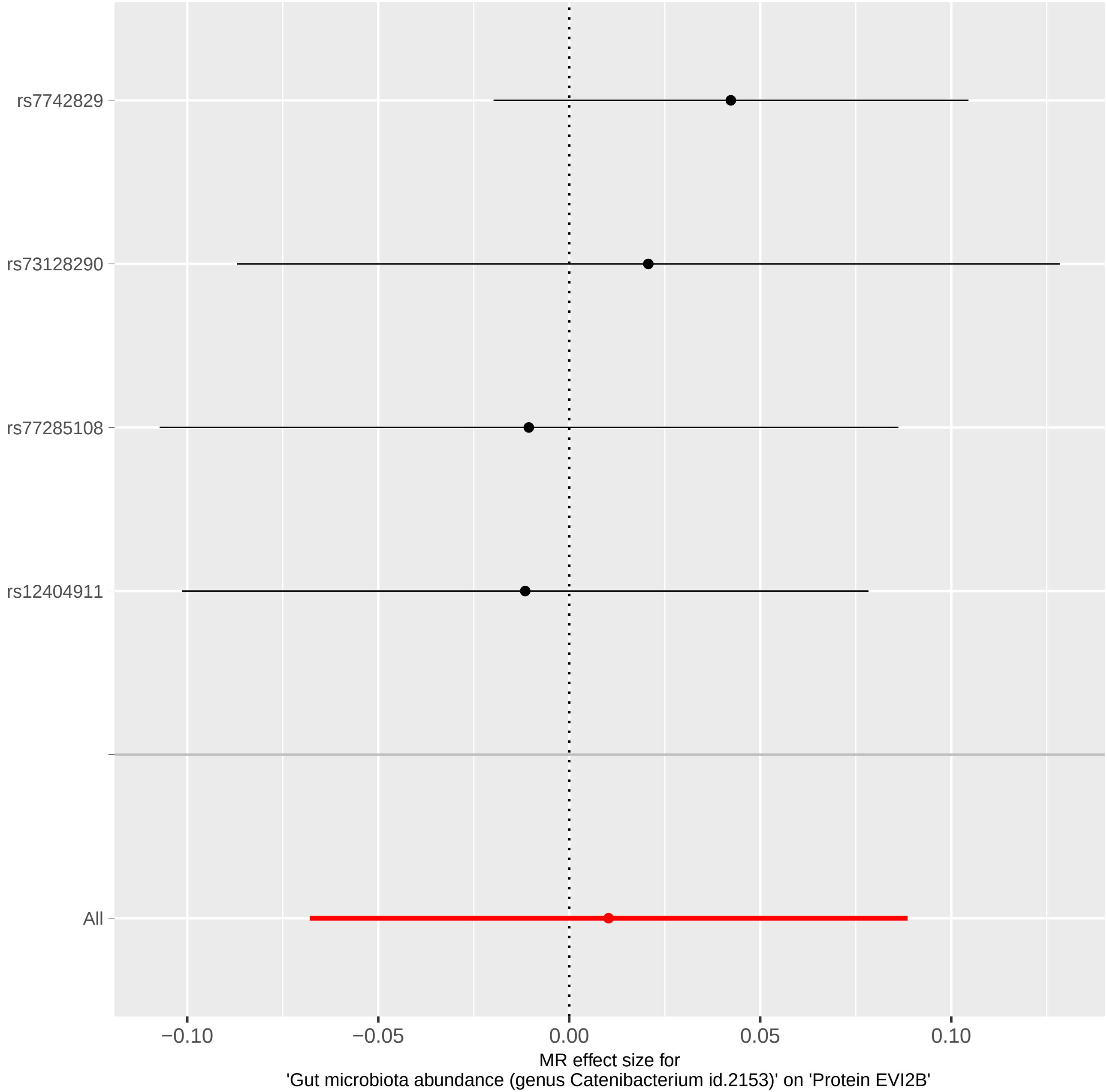

Batch 2−1994 : Gut microbiota abundance (genus Parasutterella id.2892) on Protein EVI2B
with a threshold of 1e−05

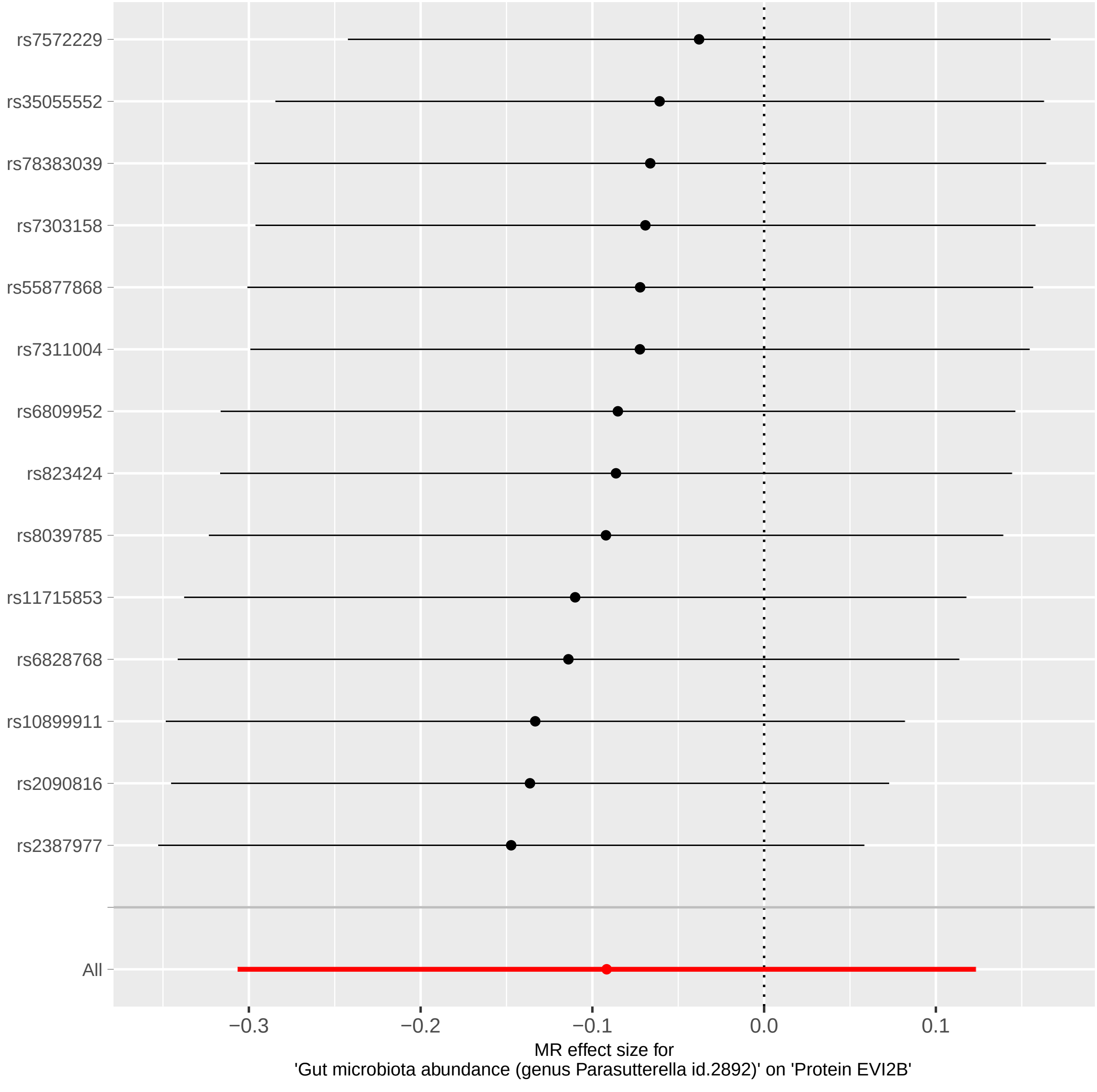

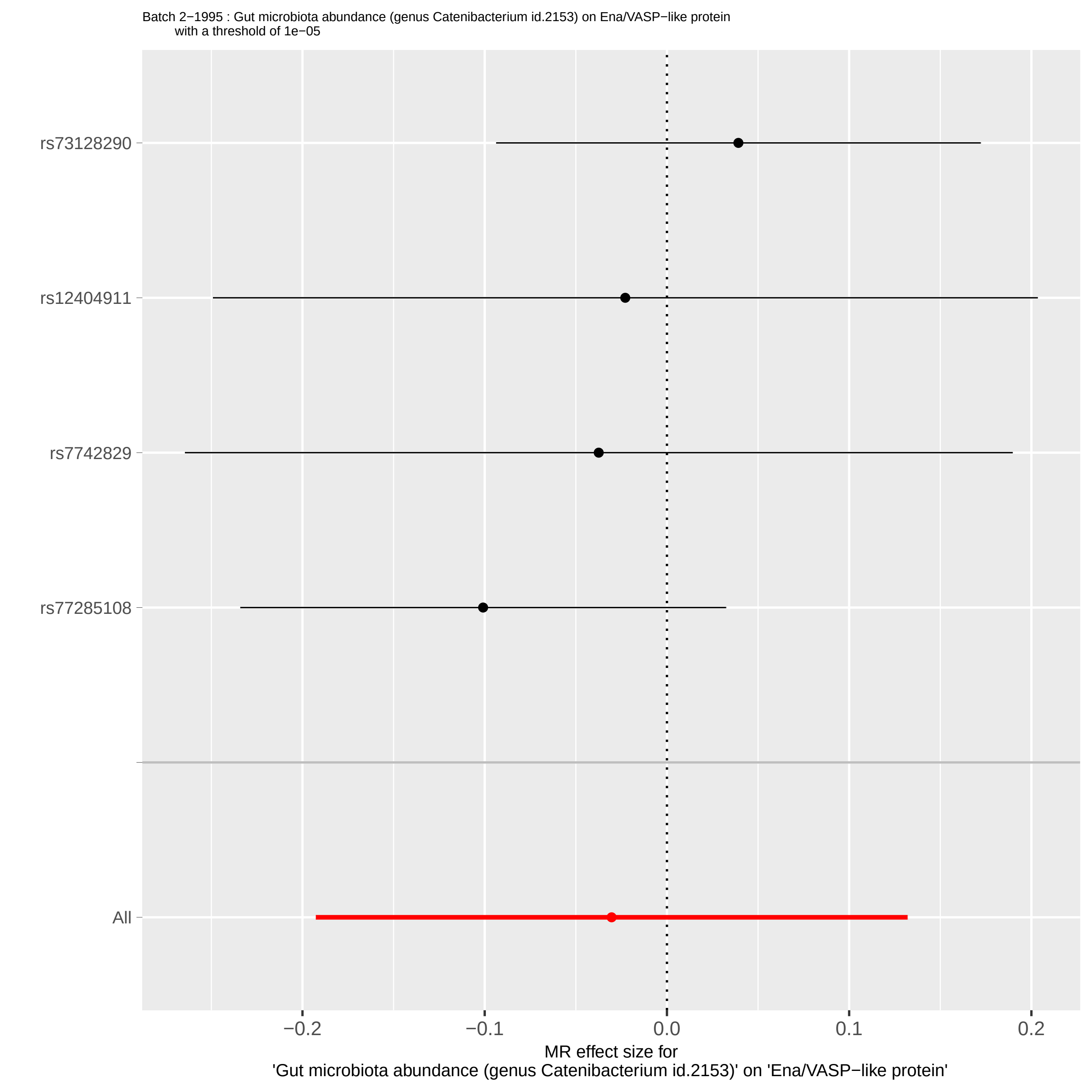
Batch 2−1995 : Gut microbiota abundance (genus Catenibacterium id.2153) on Ena/VASP−like protein
with a threshold of 1e−05
rs73128290
rs12404911
rs7742829
rs77285108
All
−0.2
−0.1
0.0
0.1
0.2
MR effect size for
'Gut microbiota abundance (genus Catenibacterium id.2153)' on 'Ena/VASP−like protein'

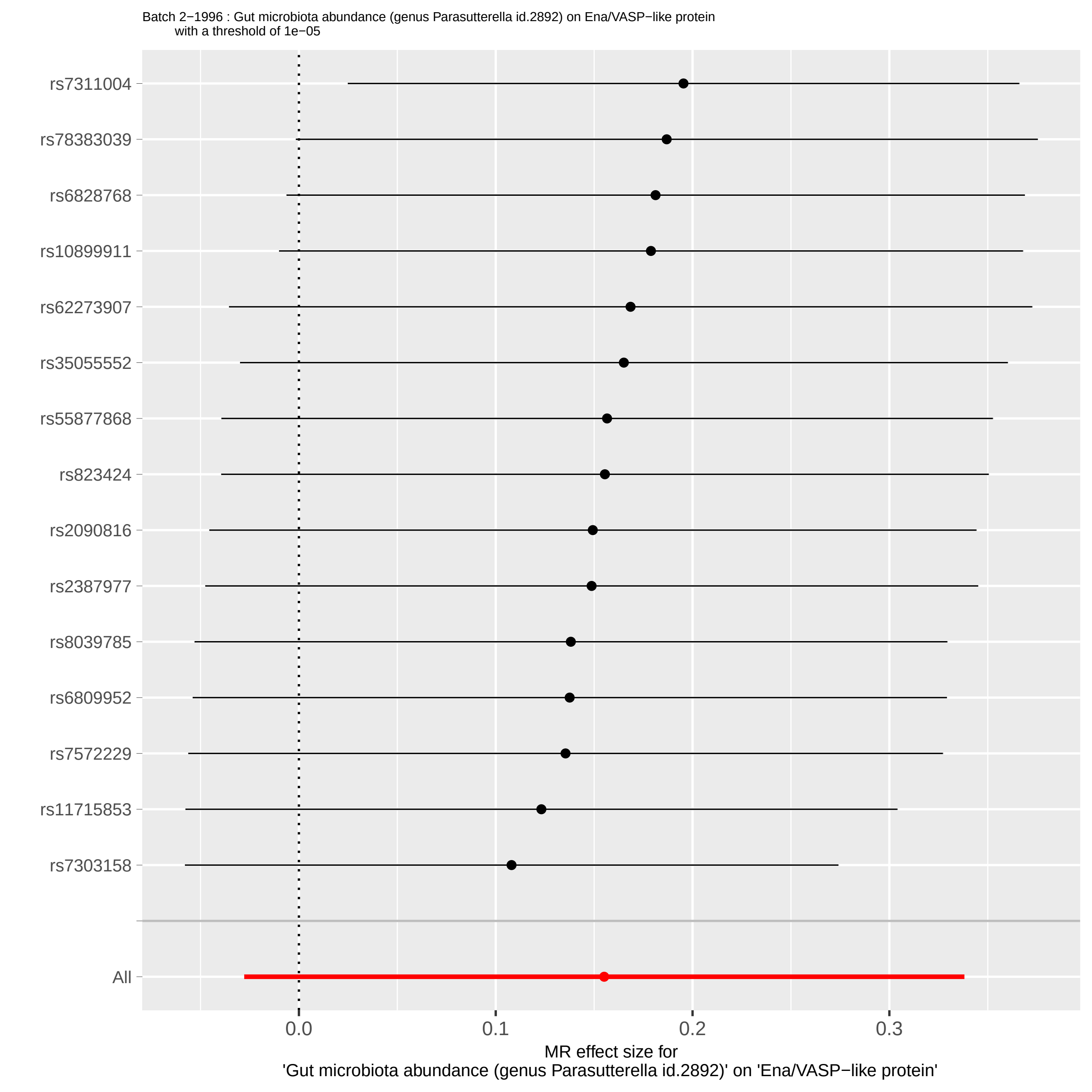
Batch 2−1996 : Gut microbiota abundance (genus Parasutterella id.2892) on Ena/VASP−like protein
with a threshold of 1e−05
rs7311004
rs78383039
rs6828768
rs10899911
rs62273907
rs35055552
rs55877868
rs823424
rs2090816
rs2387977
rs8039785
rs6809952
rs7572229
rs11715853
rs7303158
All
0.0
0.1
0.2
0.3
MR effect size for
'Gut microbiota abundance (genus Parasutterella id.2892)' on 'Ena/VASP−like protein'

Batch 2−1997 : Gut microbiota abundance (genus Catenibacterium id.2153) on Envoplakin
with a threshold of 1e−05

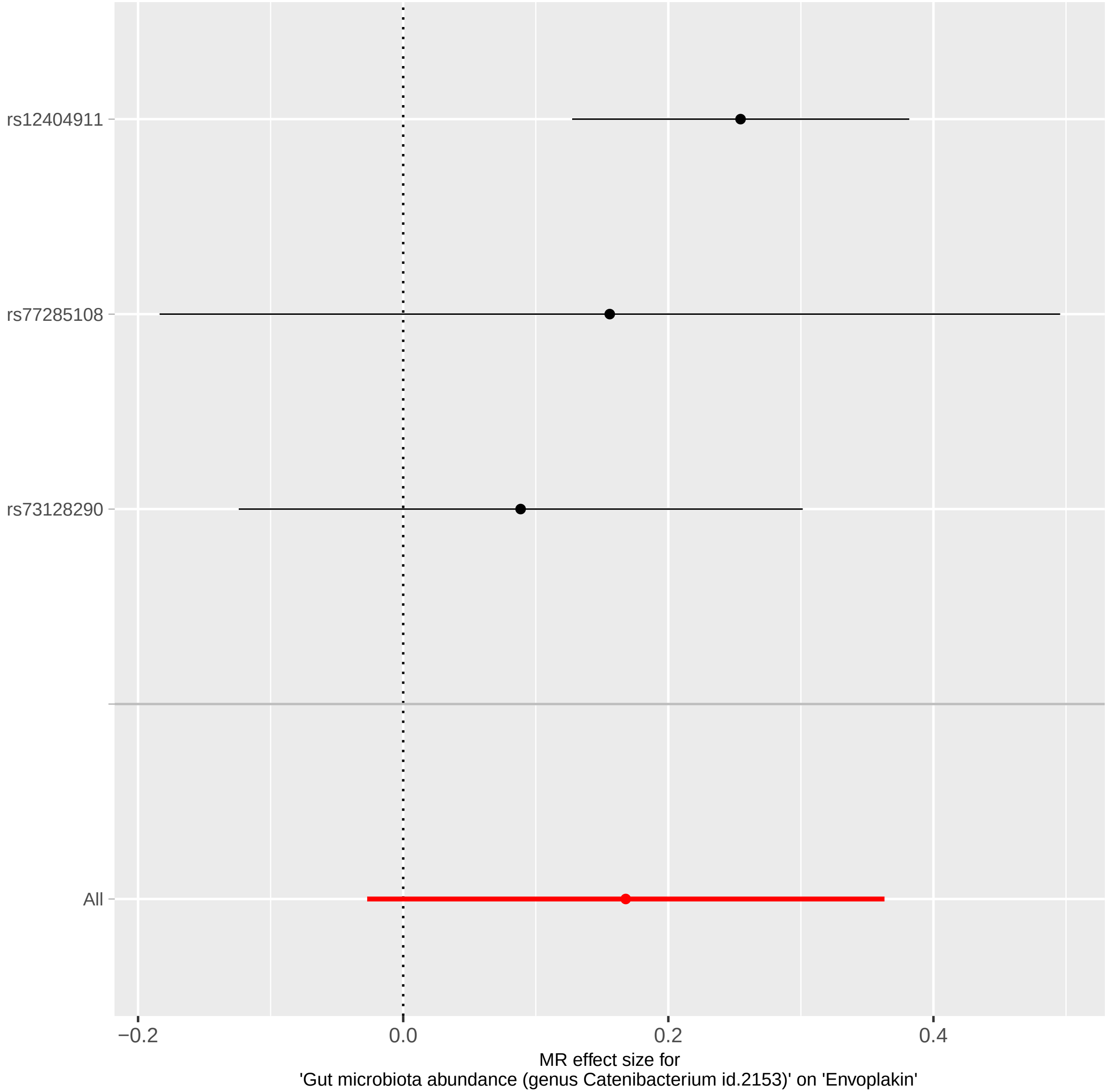

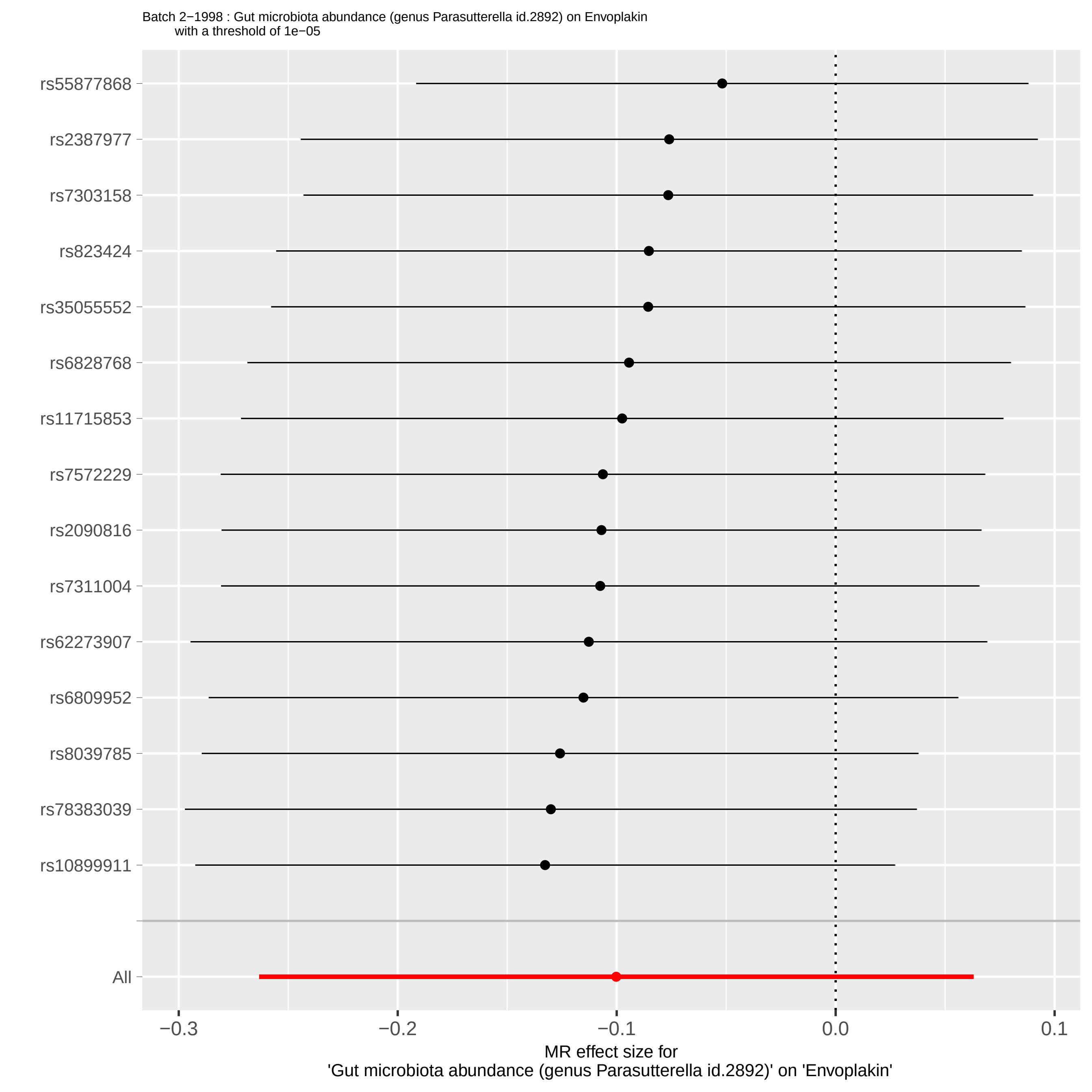
Batch 2−1998 : Gut microbiota abundance (genus Parasutterella id.2892) on Envoplakin
with a threshold of 1e−05
rs55877868
rs2387977
rs7303158
rs823424
rs35055552
rs6828768
rs11715853
rs7572229
rs2090816
rs7311004
rs62273907
rs6809952
rs8039785
rs78383039
rs10899911
All
−0.3
−0.2
−0.1
0.0
0.1
MR effect size for
'Gut microbiota abundance (genus Parasutterella id.2892)' on 'Envoplakin'

Batch 2−1999 : Gut microbiota abundance (genus Catenibacterium id.2153) on RNA−binding protein EWS
with a threshold of 1e−05

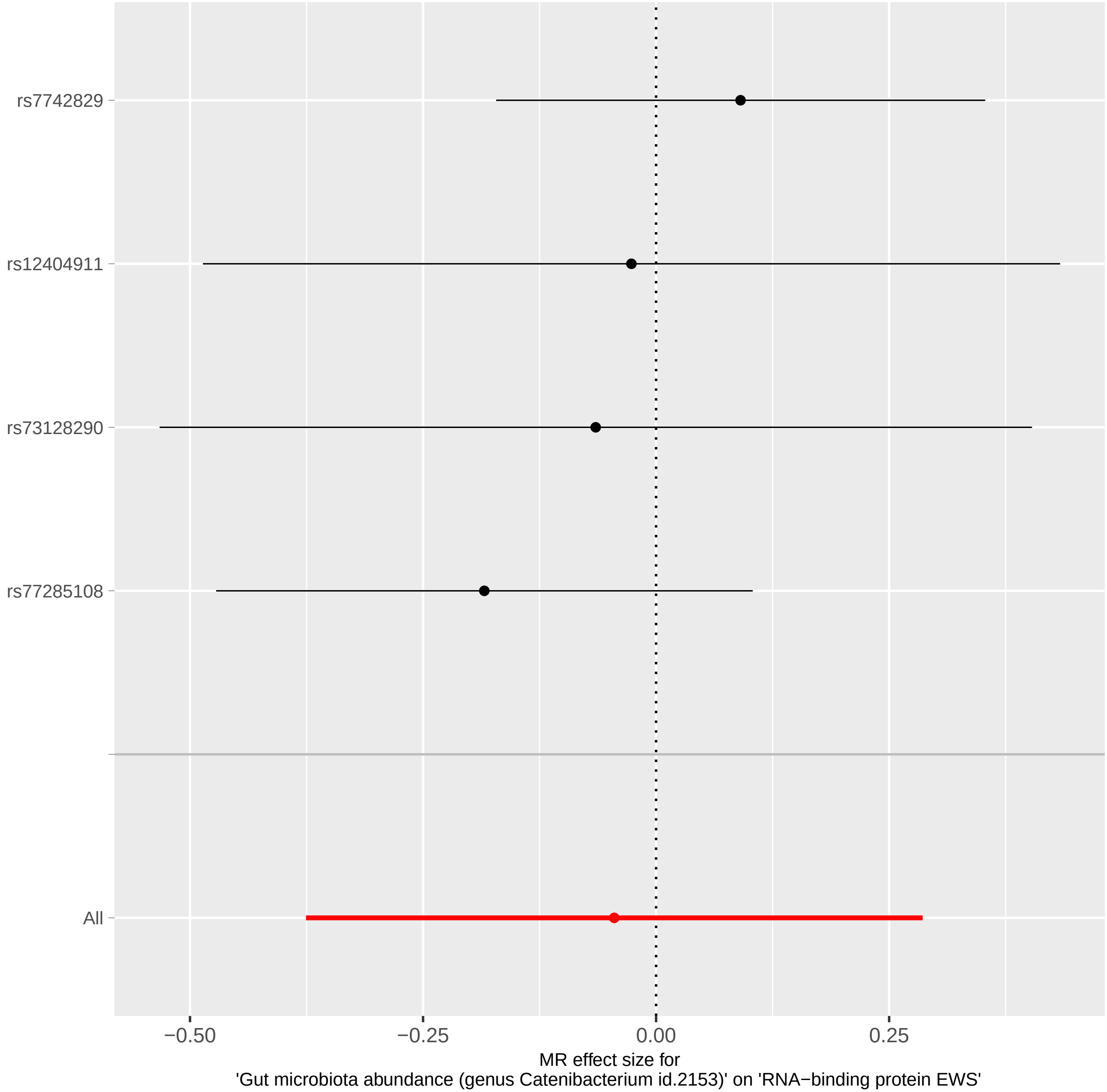

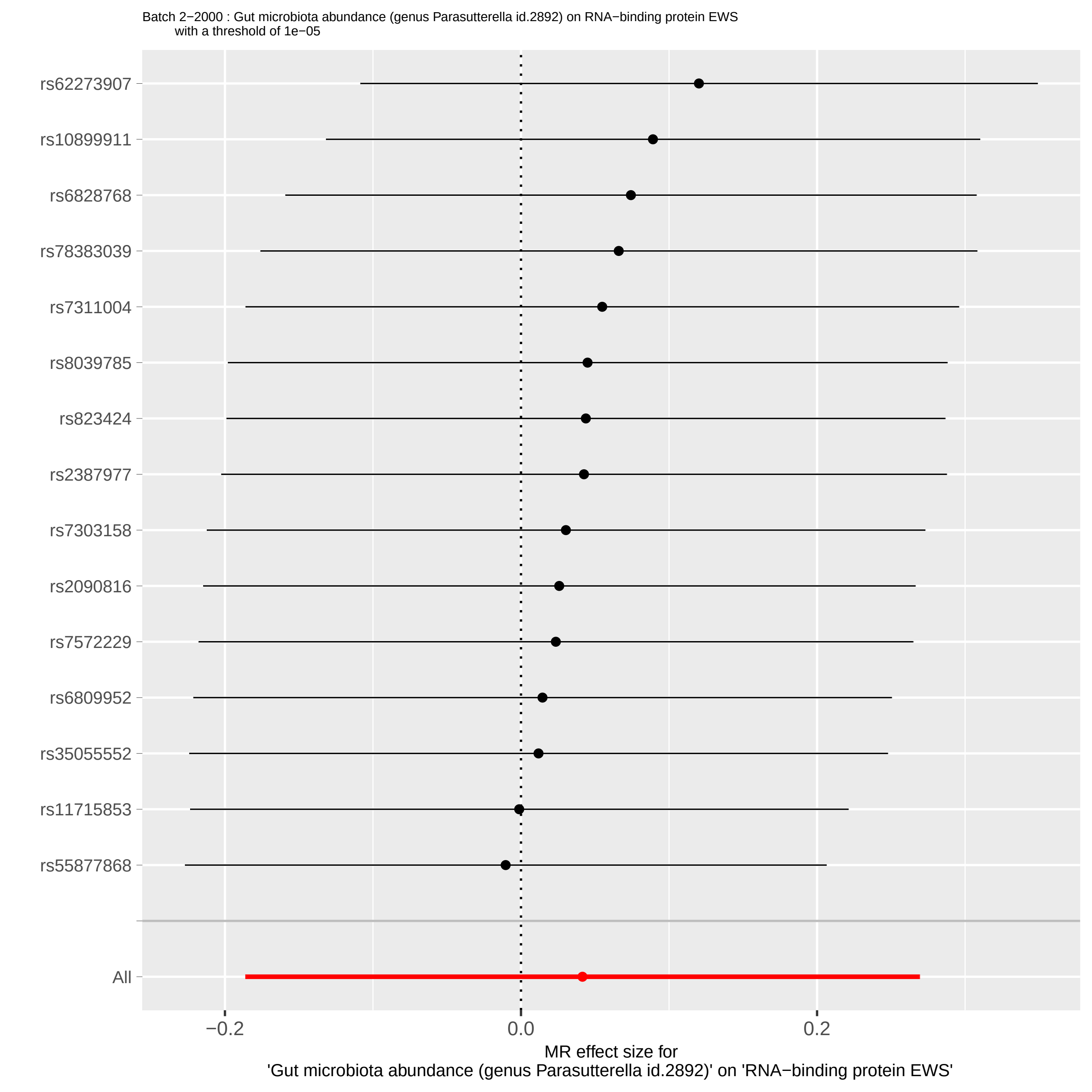
Batch 2−2000 : Gut microbiota abundance (genus Parasutterella id.2892) on RNA−binding protein EWS
with a threshold of 1e−05
rs62273907
rs10899911
rs6828768
rs78383039
rs7311004
rs8039785
rs823424
rs2387977
rs7303158
rs2090816
rs7572229
rs6809952
rs35055552
rs11715853
rs55877868
All
−0.2
0.0
0.2
MR effect size for
'Gut microbiota abundance (genus Parasutterella id.2892)' on 'RNA−binding protein EWS'

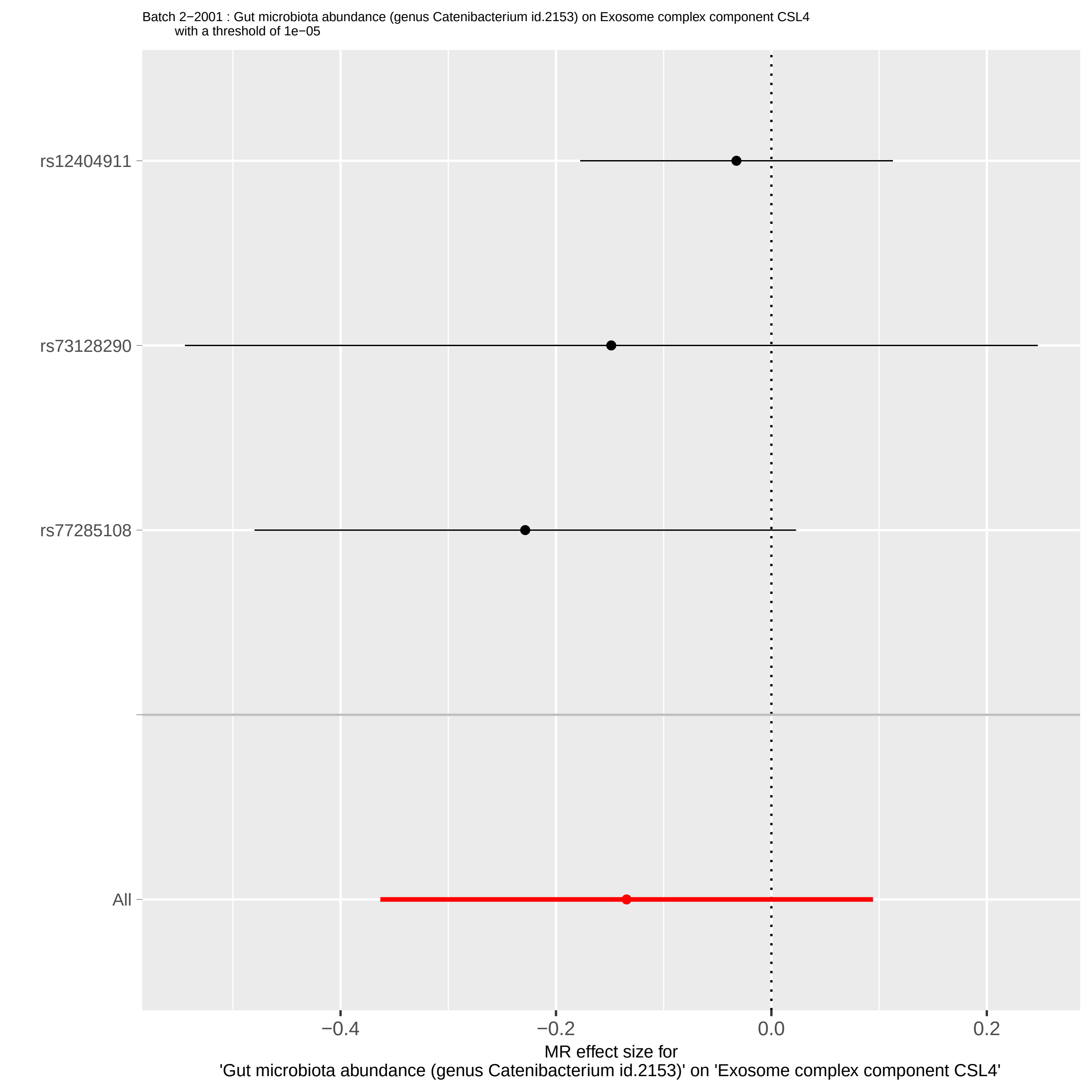
Batch 2−2001 : Gut microbiota abundance (genus Catenibacterium id.2153) on Exosome complex component CSL4
with a threshold of 1e−05
rs12404911
rs73128290
rs77285108
All
−0.4
−0.2
0.0
0.2
MR effect size for
'Gut microbiota abundance (genus Catenibacterium id.2153)' on 'Exosome complex component CSL4'

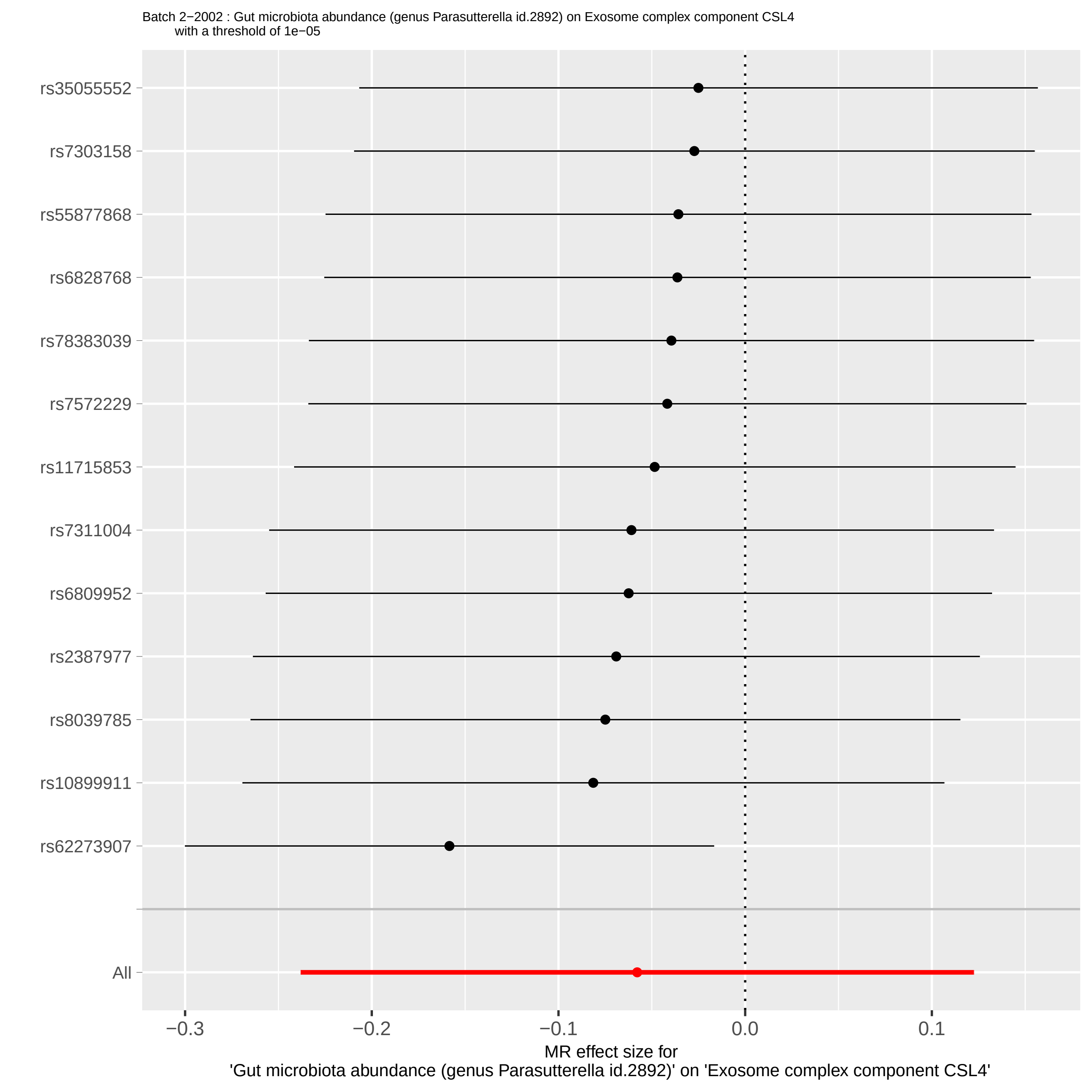

Batch 2−2002 : Gut microbiota abundance (genus Parasutterella id.2892) on Exosome complex component CSL4
with a threshold of 1e−05
rs35055552
rs7303158
rs55877868
rs6828768
rs78383039
rs7572229
rs11715853
rs7311004
rs6809952
rs2387977
rs8039785
rs10899911
rs62273907
All
−0.3
−0.2
−0.1
0.0
0.1
MR effect size for
'Gut microbiota abundance (genus Parasutterella id.2892)' on 'Exosome complex component CSL4'

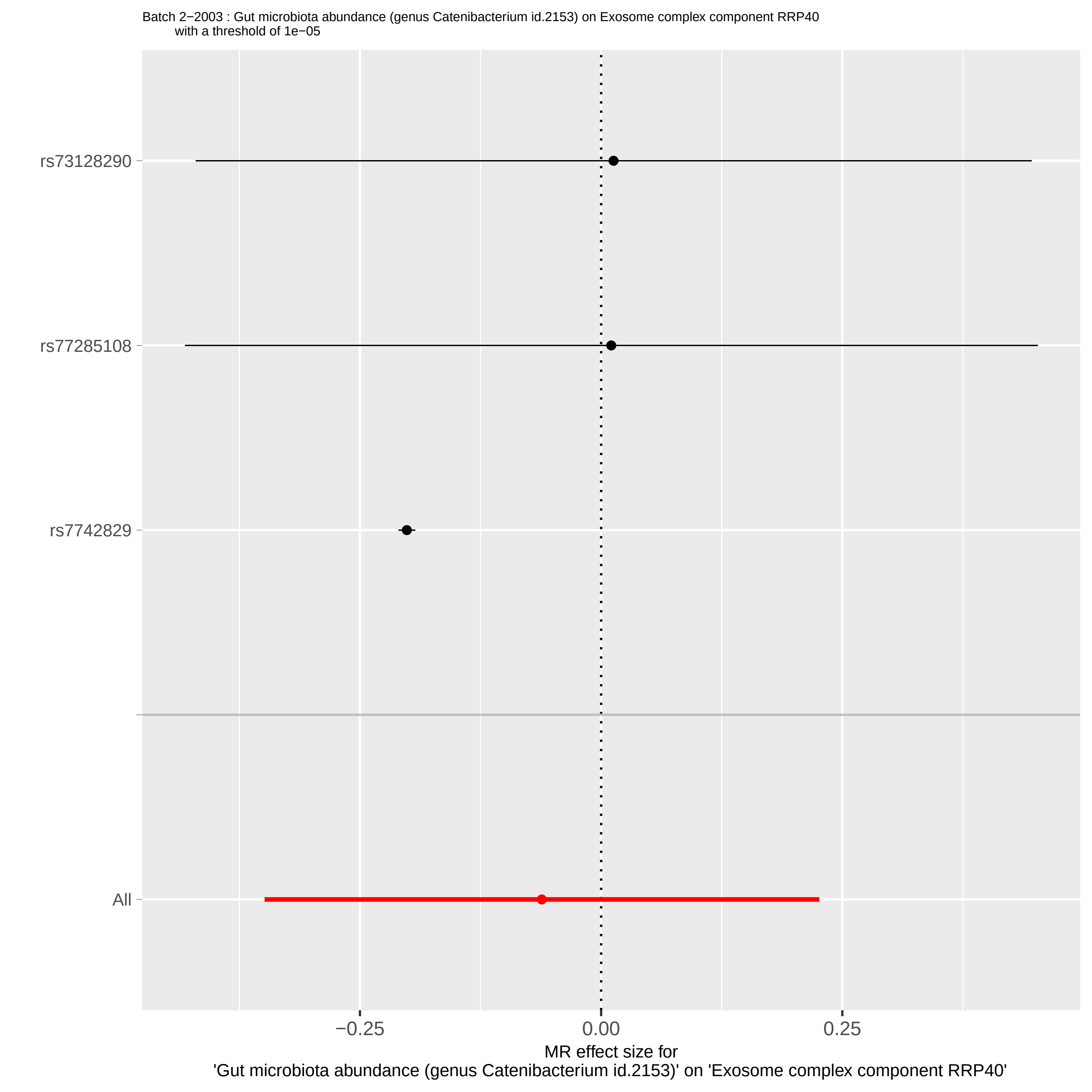

Batch 2−2003 : Gut microbiota abundance (genus Catenibacterium id.2153) on Exosome complex component RRP40
with a threshold of 1e−05
rs73128290
rs77285108
rs7742829
All
−0.25
0.00
0.25
MR effect size for
'Gut microbiota abundance (genus Catenibacterium id.2153)' on 'Exosome complex component RRP40'

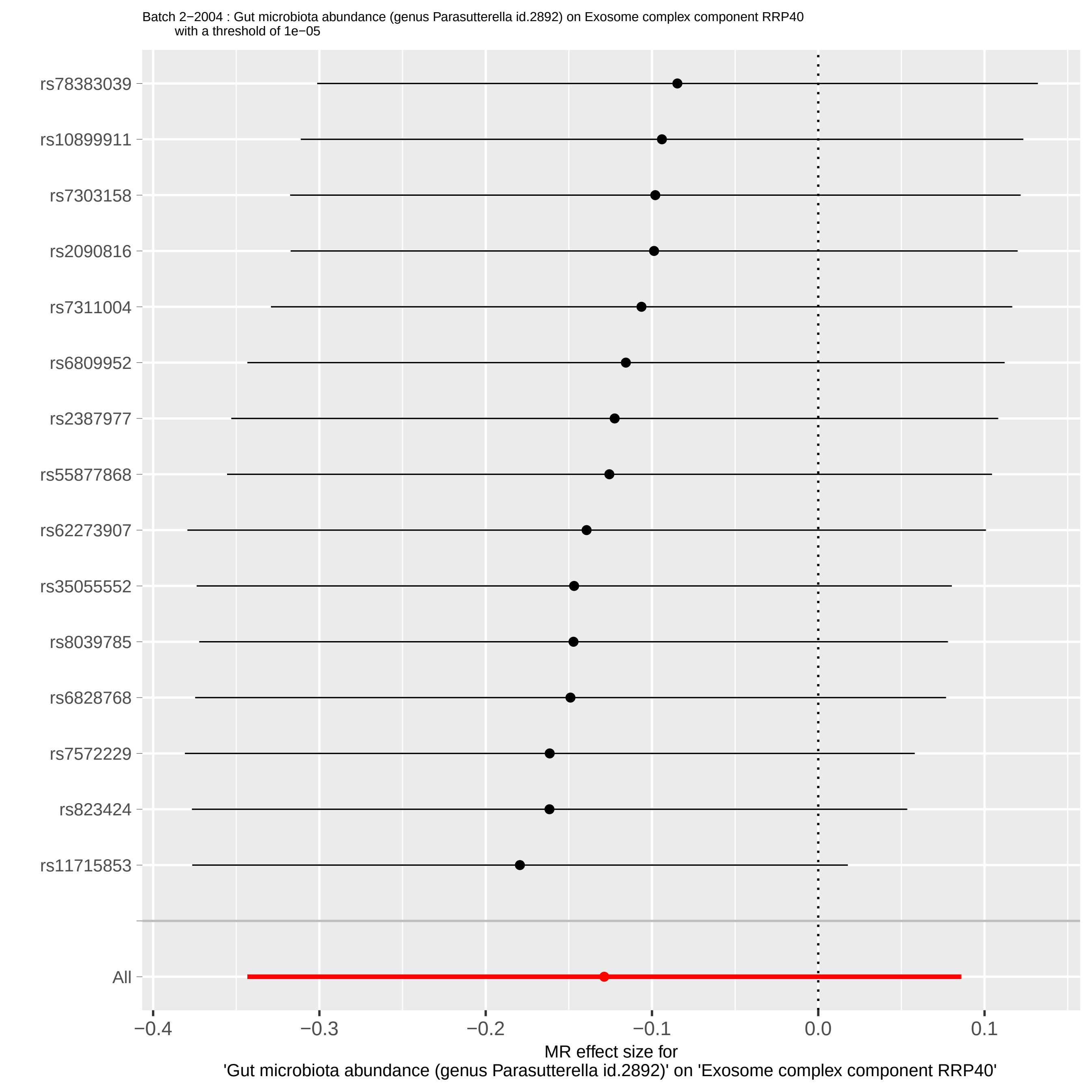
Batch 2−2004 : Gut microbiota abundance (genus Parasutterella id.2892) on Exosome complex component RRP40
with a threshold of 1e−05
rs78383039
rs10899911
rs7303158
rs2090816
rs7311004
rs6809952
rs2387977
rs55877868
rs62273907
rs35055552
rs8039785
rs6828768
rs7572229
rs823424
rs11715853
All
−0.4
−0.3
−0.2
−0.1
0.0
0.1
MR effect size for
'Gut microbiota abundance (genus Parasutterella id.2892)' on 'Exosome complex component RRP40'

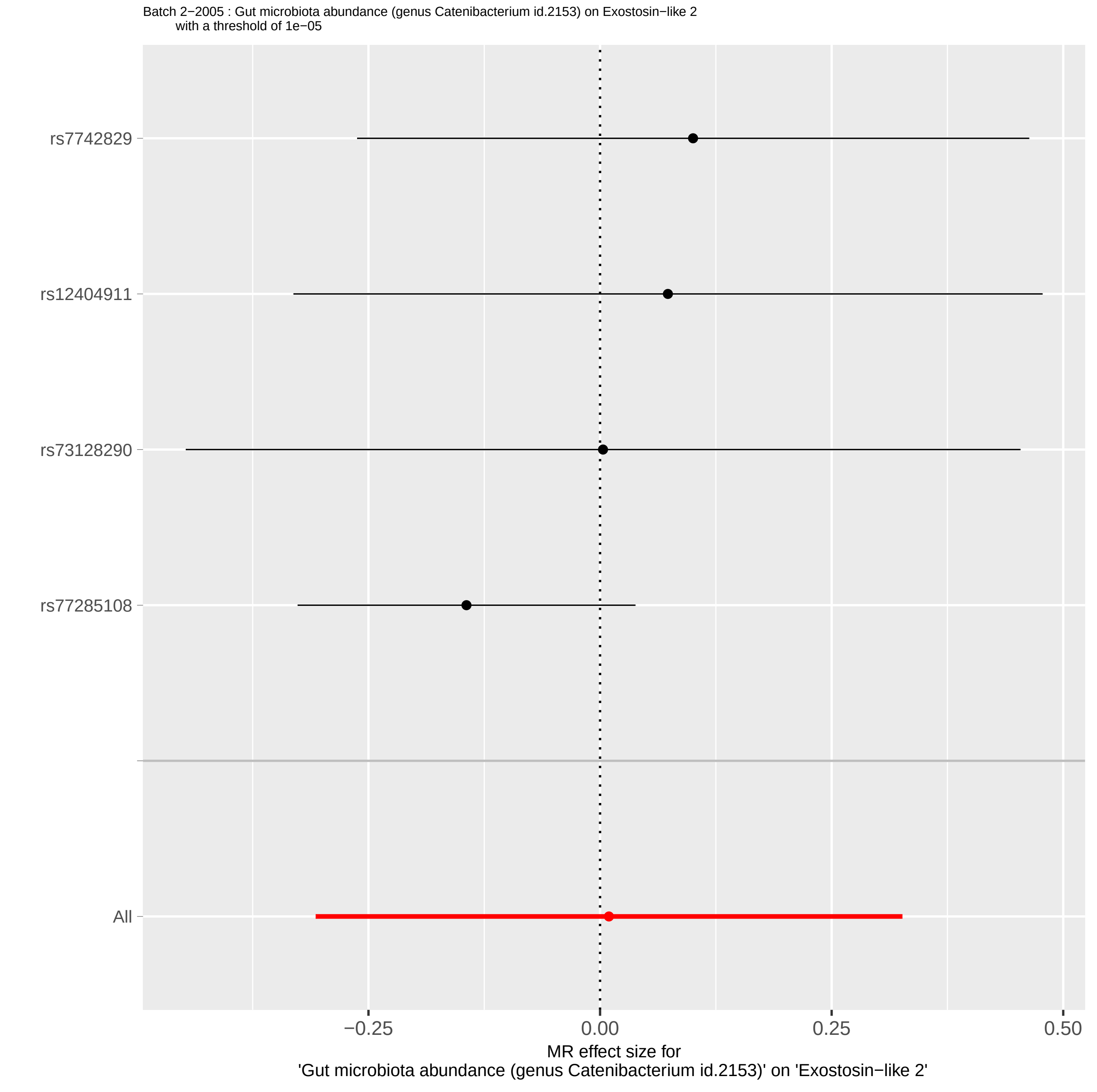

Batch 2−2005 : Gut microbiota abundance (genus Catenibacterium id.2153) on Exostosin−like 2
with a threshold of 1e−05
rs7742829
rs12404911
rs73128290
rs77285108
All
−0.25
0.00
0.25
0.50
MR effect size for
'Gut microbiota abundance (genus Catenibacterium id.2153)' on 'Exostosin−like 2'

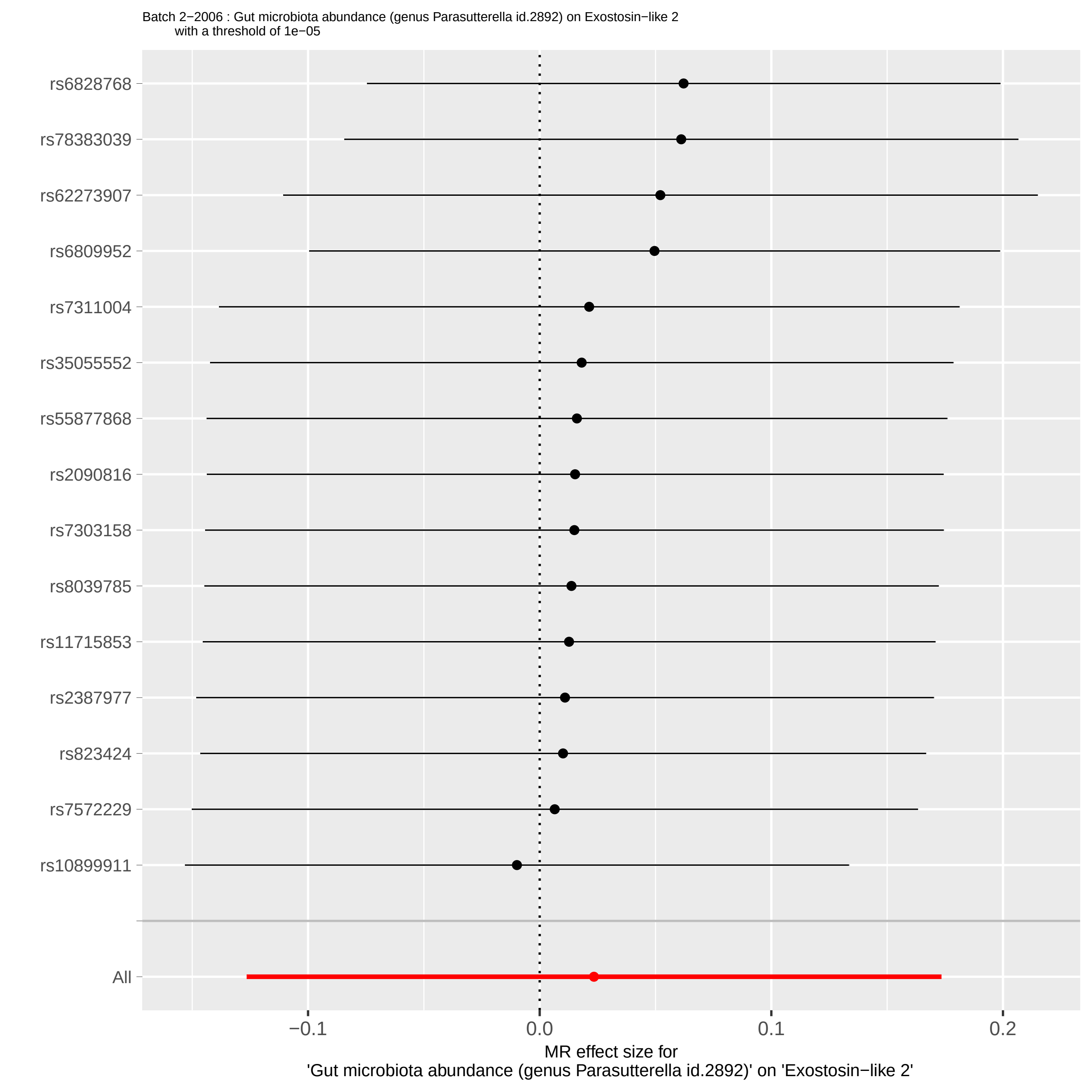

Batch 2−2006 : Gut microbiota abundance (genus Parasutterella id.2892) on Exostosin−like 2
with a threshold of 1e−05
rs6828768
rs78383039
rs62273907
rs6809952
rs7311004
rs35055552
rs55877868
rs2090816
rs7303158
rs8039785
rs11715853
rs2387977
rs823424
rs7572229
rs10899911
All
−0.1
0.0
0.1
0.2
MR effect size for
'Gut microbiota abundance (genus Parasutterella id.2892)' on 'Exostosin−like 2'

Batch 2−2007 : Gut microbiota abundance (genus Catenibacterium id.2153) on Ezrin
with a threshold of 1e−05

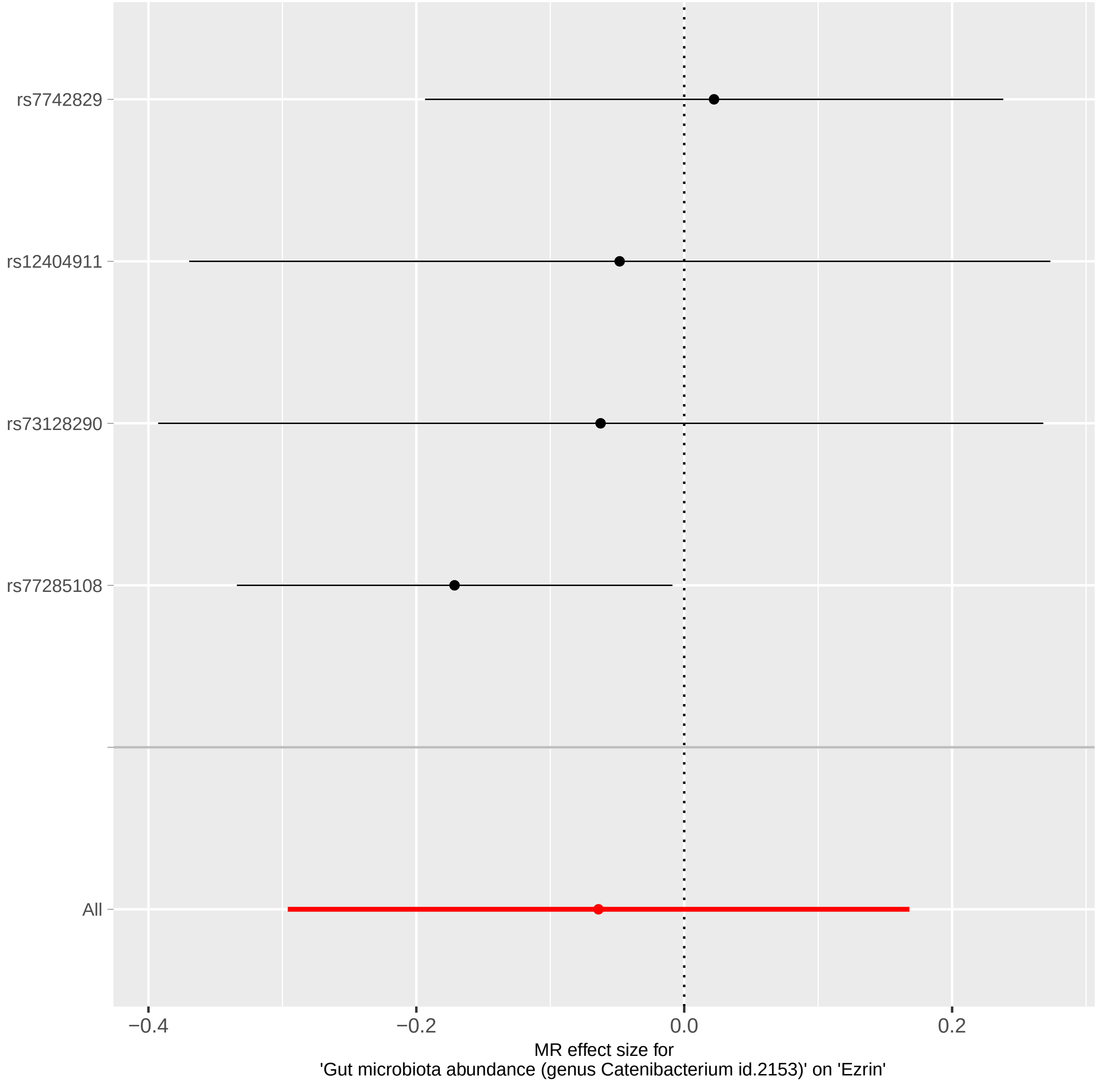

Batch 2−2008 : Gut microbiota abundance (genus Parasutterella id.2892) on Ezrin
with a threshold of 1e−05

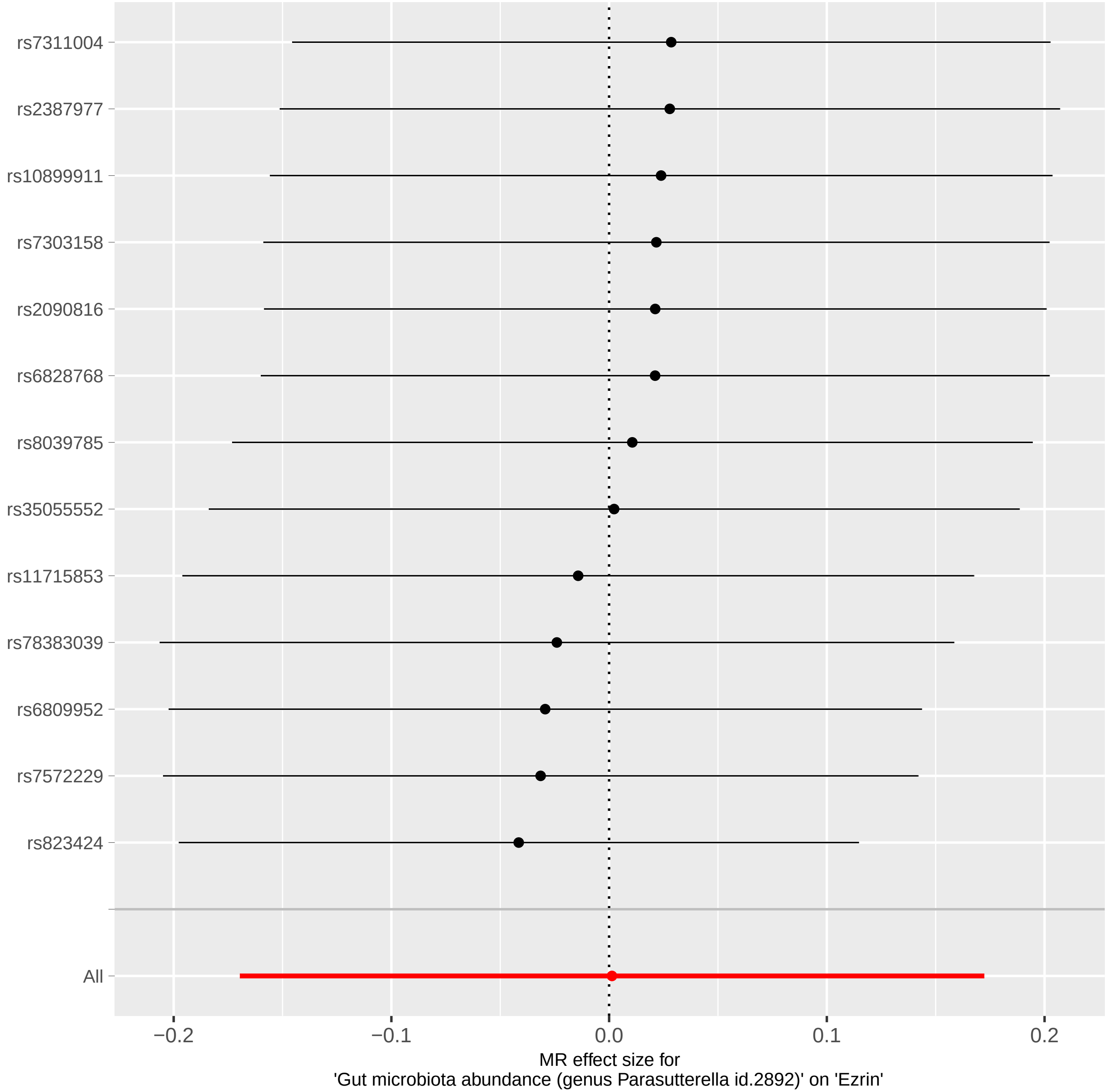

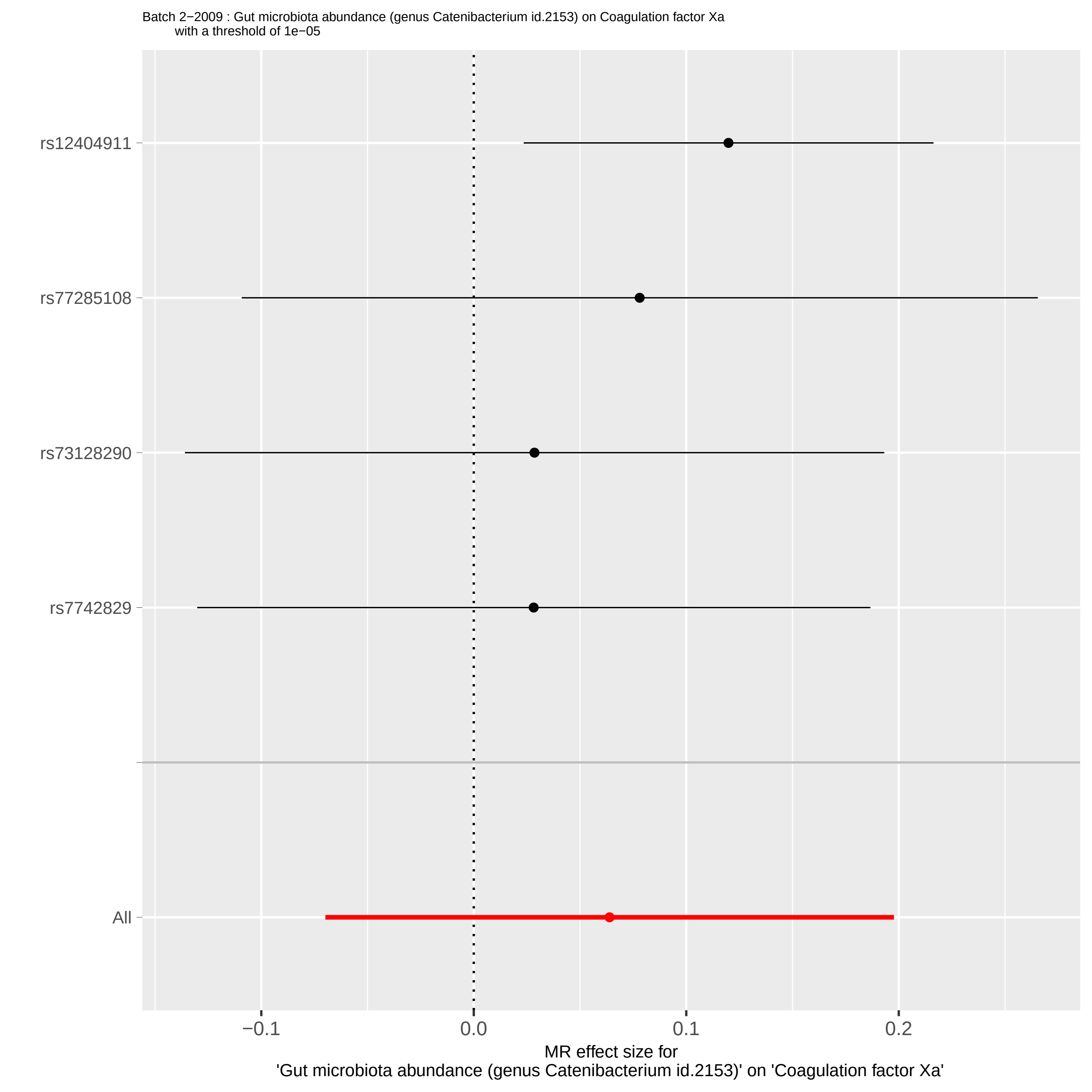
Batch 2−2009 : Gut microbiota abundance (genus Catenibacterium id.2153) on Coagulation factor Xa
with a threshold of 1e−05
rs12404911
rs77285108
rs73128290
rs7742829
All
−0.1
0.0
0.1
0.2
MR effect size for
'Gut microbiota abundance (genus Catenibacterium id.2153)' on 'Coagulation factor Xa'

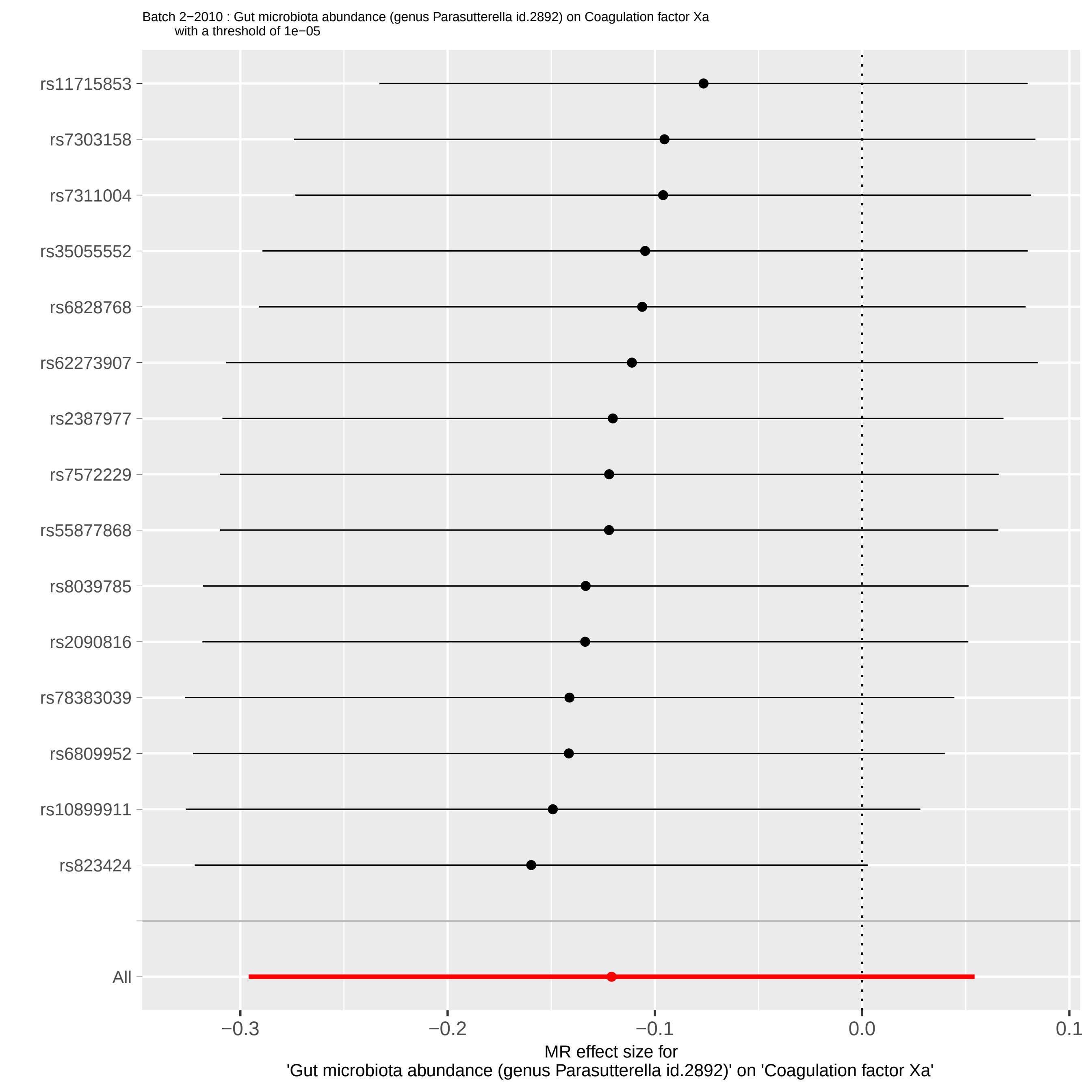

Batch 2−2010 : Gut microbiota abundance (genus Parasutterella id.2892) on Coagulation factor Xa
with a threshold of 1e−05
rs11715853
rs7303158
rs7311004
rs35055552
rs6828768
rs62273907
rs2387977
rs7572229
rs55877868
rs8039785
rs2090816
rs78383039
rs6809952
rs10899911
rs823424
All
−0.3
−0.2
−0.1
0.0
0.1
MR effect size for
'Gut microbiota abundance (genus Parasutterella id.2892)' on 'Coagulation factor Xa'

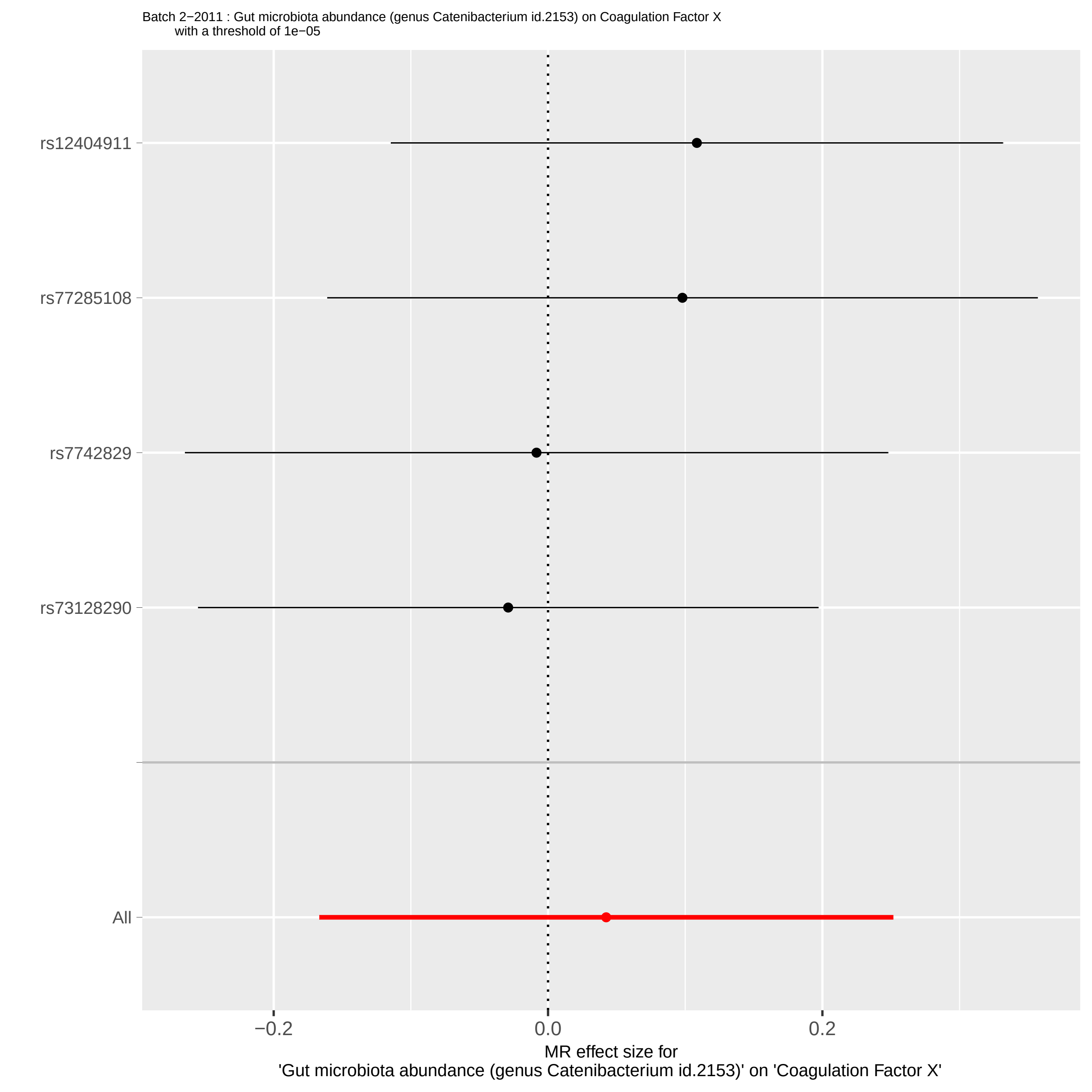
Batch 2−2011 : Gut microbiota abundance (genus Catenibacterium id.2153) on Coagulation Factor X
with a threshold of 1e−05
rs12404911
rs77285108
rs7742829
rs73128290
All
−0.2
0.0
0.2
MR effect size for
'Gut microbiota abundance (genus Catenibacterium id.2153)' on 'Coagulation Factor X'

Batch 2−2012 : Gut microbiota abundance (genus Parasutterella id.2892) on Coagulation Factor X
with a threshold of 1e−05

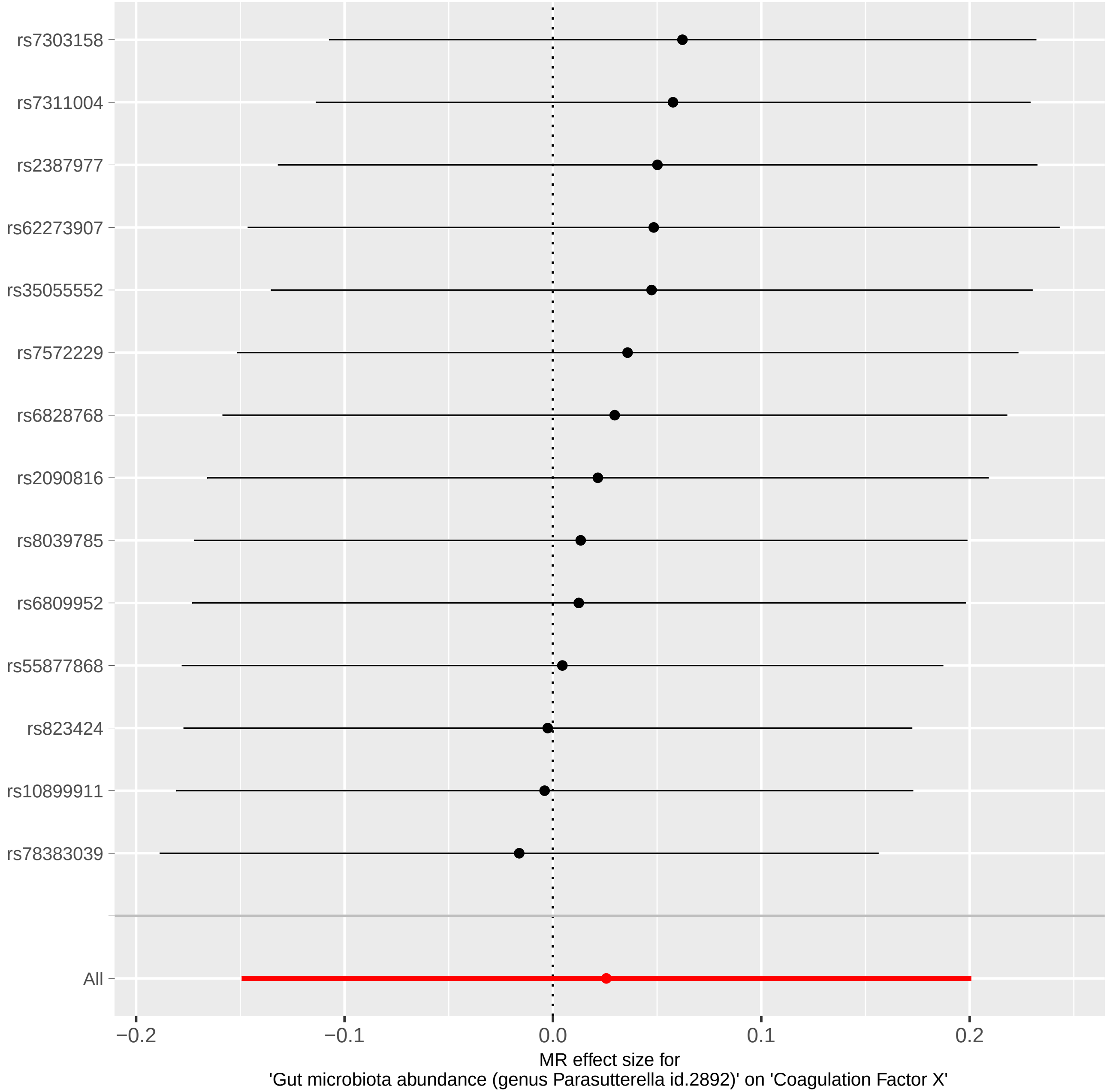

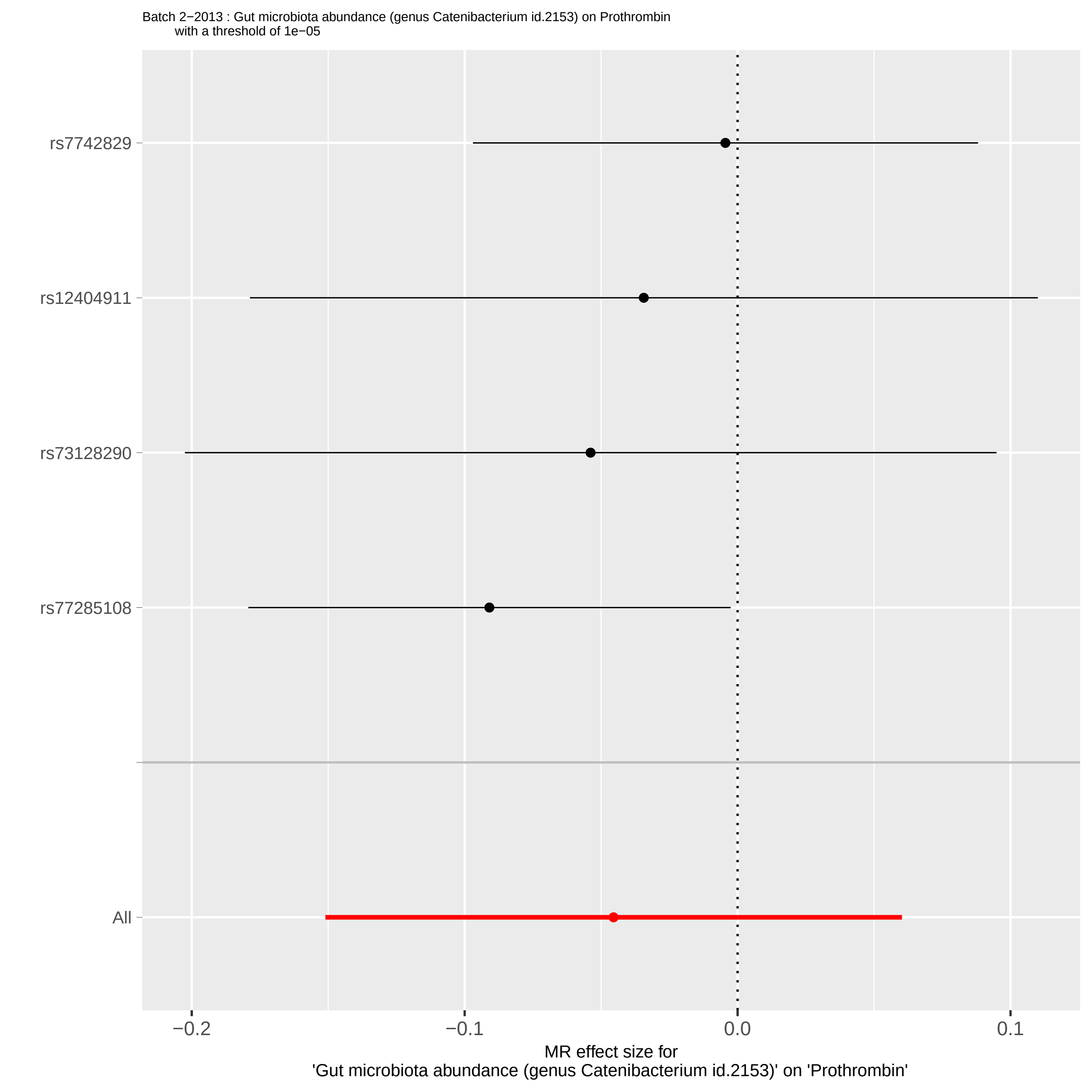
Batch 2−2013 : Gut microbiota abundance (genus Catenibacterium id.2153) on Prothrombin
with a threshold of 1e−05
rs7742829
rs12404911
rs73128290
rs77285108
All
−0.2
−0.1
0.0
0.1
MR effect size for
'Gut microbiota abundance (genus Catenibacterium id.2153)' on 'Prothrombin'

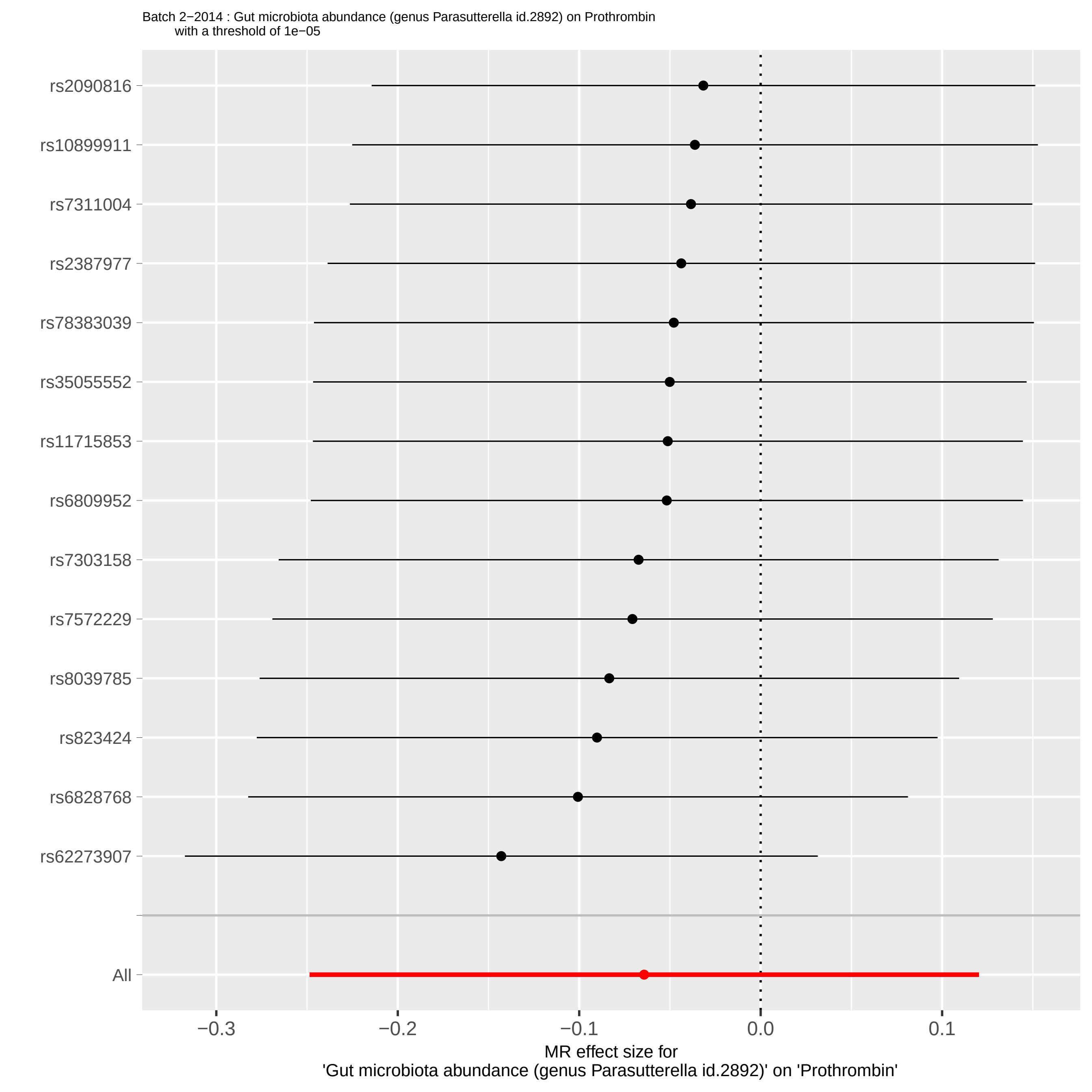
Batch 2−2014 : Gut microbiota abundance (genus Parasutterella id.2892) on Prothrombin
with a threshold of 1e−05
rs2090816
rs10899911
rs7311004
rs2387977
rs78383039
rs35055552
rs11715853
rs6809952
rs7303158
rs7572229
rs8039785
rs823424
rs6828768
rs62273907
All
−0.3
−0.2
−0.1
0.0
0.1
MR effect size for
'Gut microbiota abundance (genus Parasutterella id.2892)' on 'Prothrombin'

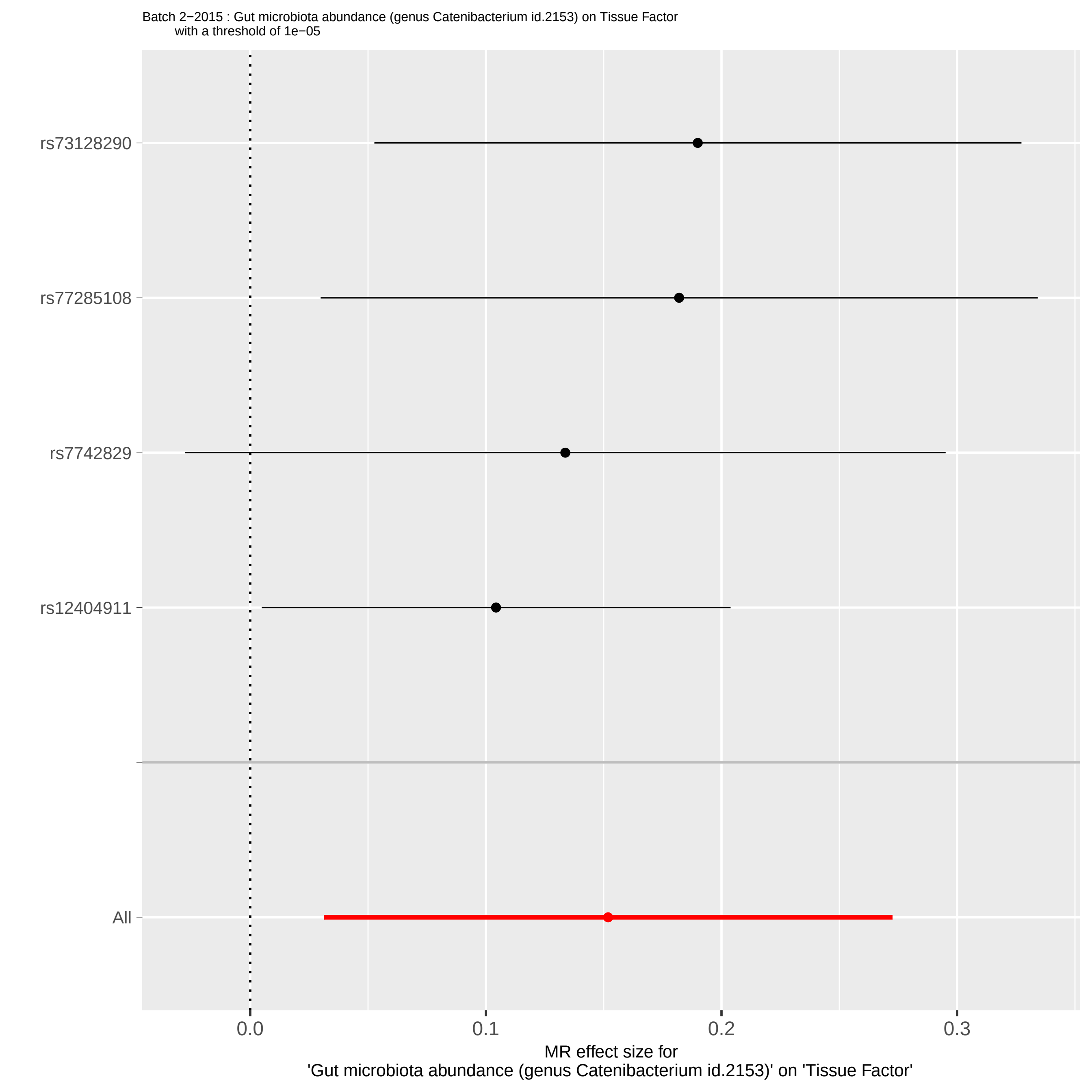
Batch 2−2015 : Gut microbiota abundance (genus Catenibacterium id.2153) on Tissue Factor
with a threshold of 1e−05
rs73128290
rs77285108
rs7742829
rs12404911
All
0.0
0.1
0.2
0.3
MR effect size for
'Gut microbiota abundance (genus Catenibacterium id.2153)' on 'Tissue Factor'

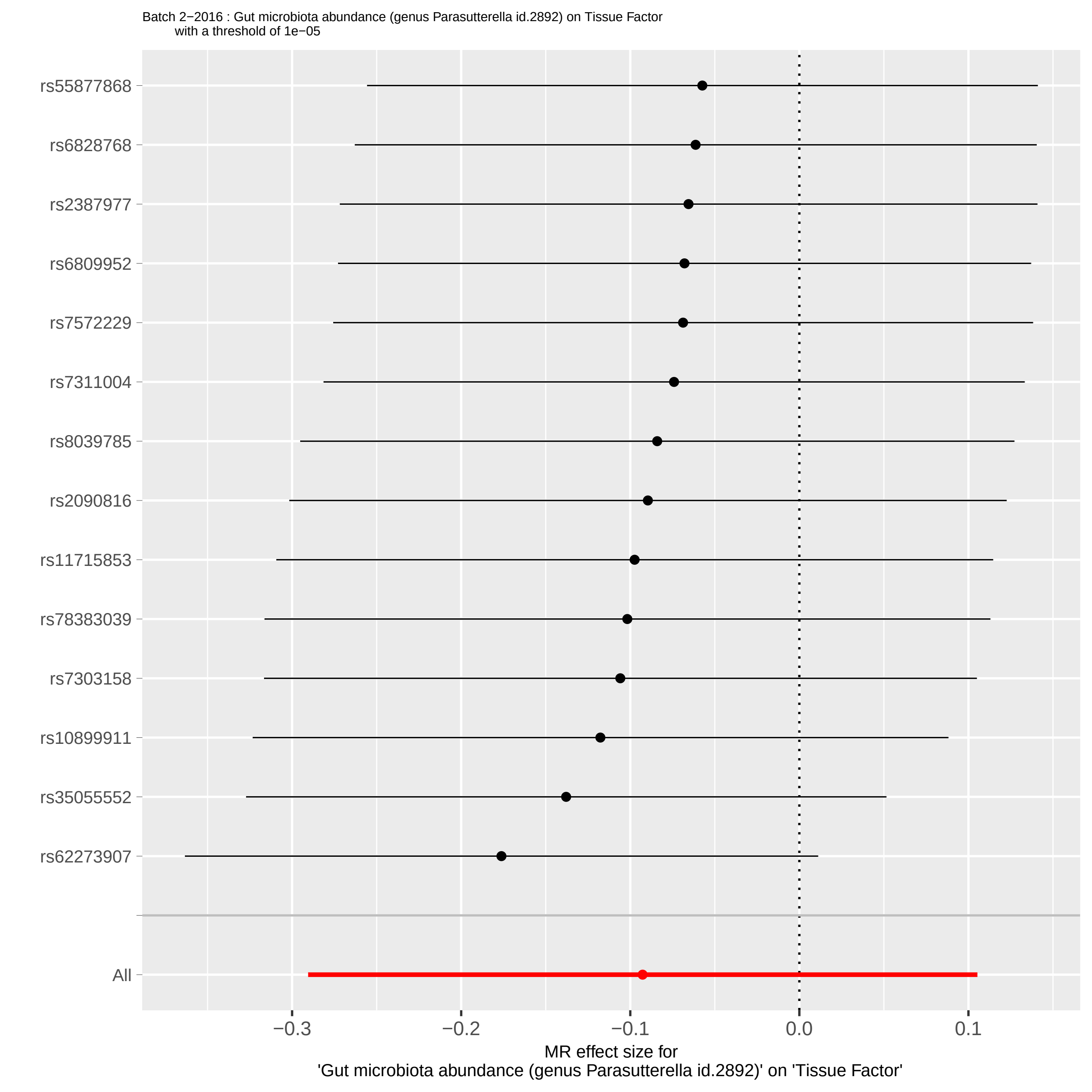
Batch 2−2016 : Gut microbiota abundance (genus Parasutterella id.2892) on Tissue Factor
with a threshold of 1e−05
rs55877868
rs6828768
rs2387977
rs6809952
rs7572229
rs7311004
rs8039785
rs2090816
rs11715853
rs78383039
rs7303158
rs10899911
rs35055552
rs62273907
All
−0.3
−0.2
−0.1
0.0
0.1
MR effect size for
'Gut microbiota abundance (genus Parasutterella id.2892)' on 'Tissue Factor'

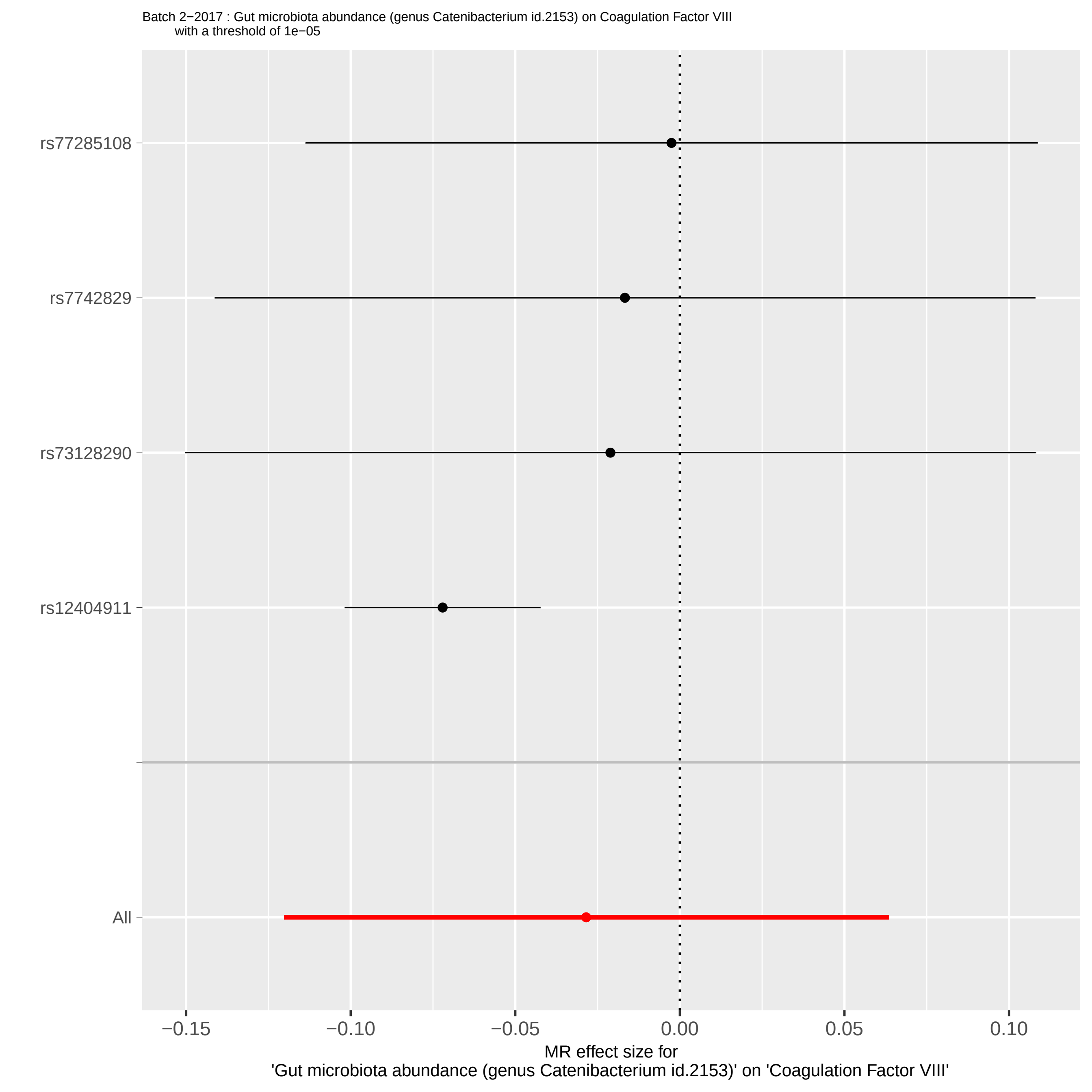

Batch 2−2017 : Gut microbiota abundance (genus Catenibacterium id.2153) on Coagulation Factor VIII
with a threshold of 1e−05
rs77285108
rs7742829
rs73128290
rs12404911
All
−0.15
−0.10
−0.05
0.00
0.05
0.10
MR effect size for
'Gut microbiota abundance (genus Catenibacterium id.2153)' on 'Coagulation Factor VIII'

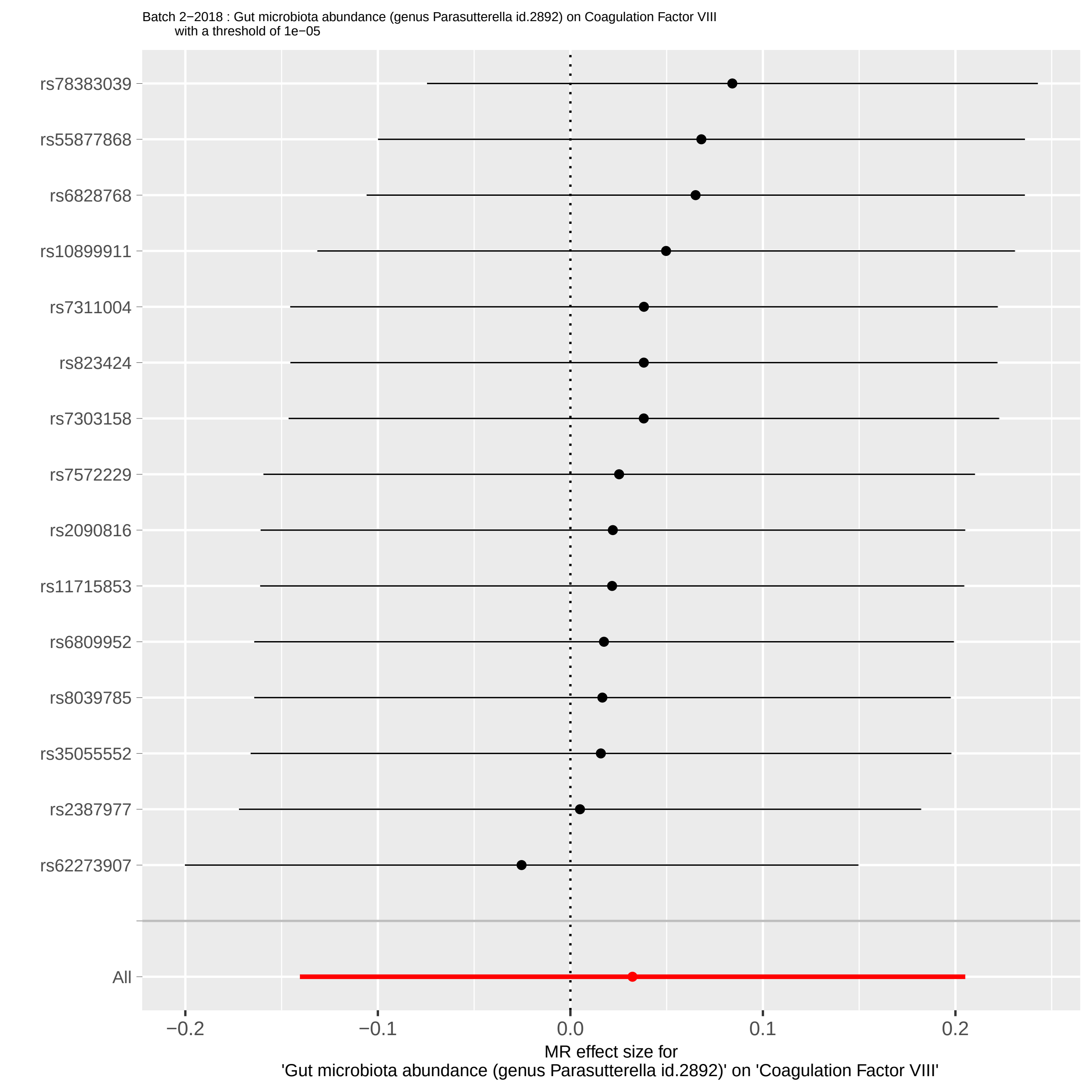

Batch 2−2018 : Gut microbiota abundance (genus Parasutterella id.2892) on Coagulation Factor VIII
with a threshold of 1e−05
rs78383039
rs55877868
rs6828768
rs10899911
rs7311004
rs823424
rs7303158
rs7572229
rs2090816
rs11715853
rs6809952
rs8039785
rs35055552
rs2387977
rs62273907
All
−0.2
−0.1
0.0
0.1
0.2
MR effect size for
'Gut microbiota abundance (genus Parasutterella id.2892)' on 'Coagulation Factor VIII'

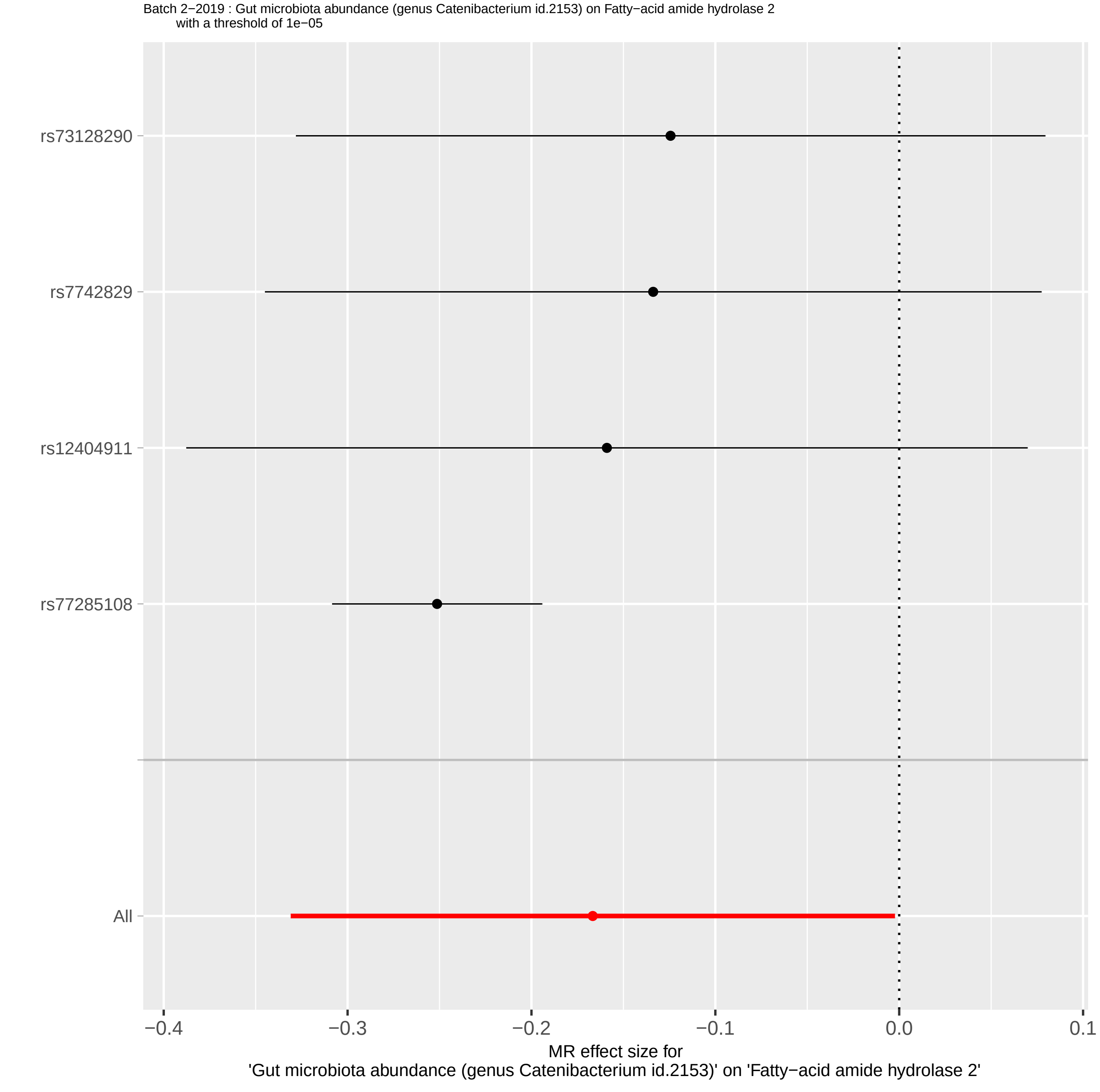
Batch 2−2019 : Gut microbiota abundance (genus Catenibacterium id.2153) on Fatty−acid amide hydrolase 2
with a threshold of 1e−05
rs73128290
rs7742829
rs12404911
rs77285108
All
−0.4
−0.3
−0.2
−0.1
0.0
0.1
MR effect size for
'Gut microbiota abundance (genus Catenibacterium id.2153)' on 'Fatty−acid amide hydrolase 2'

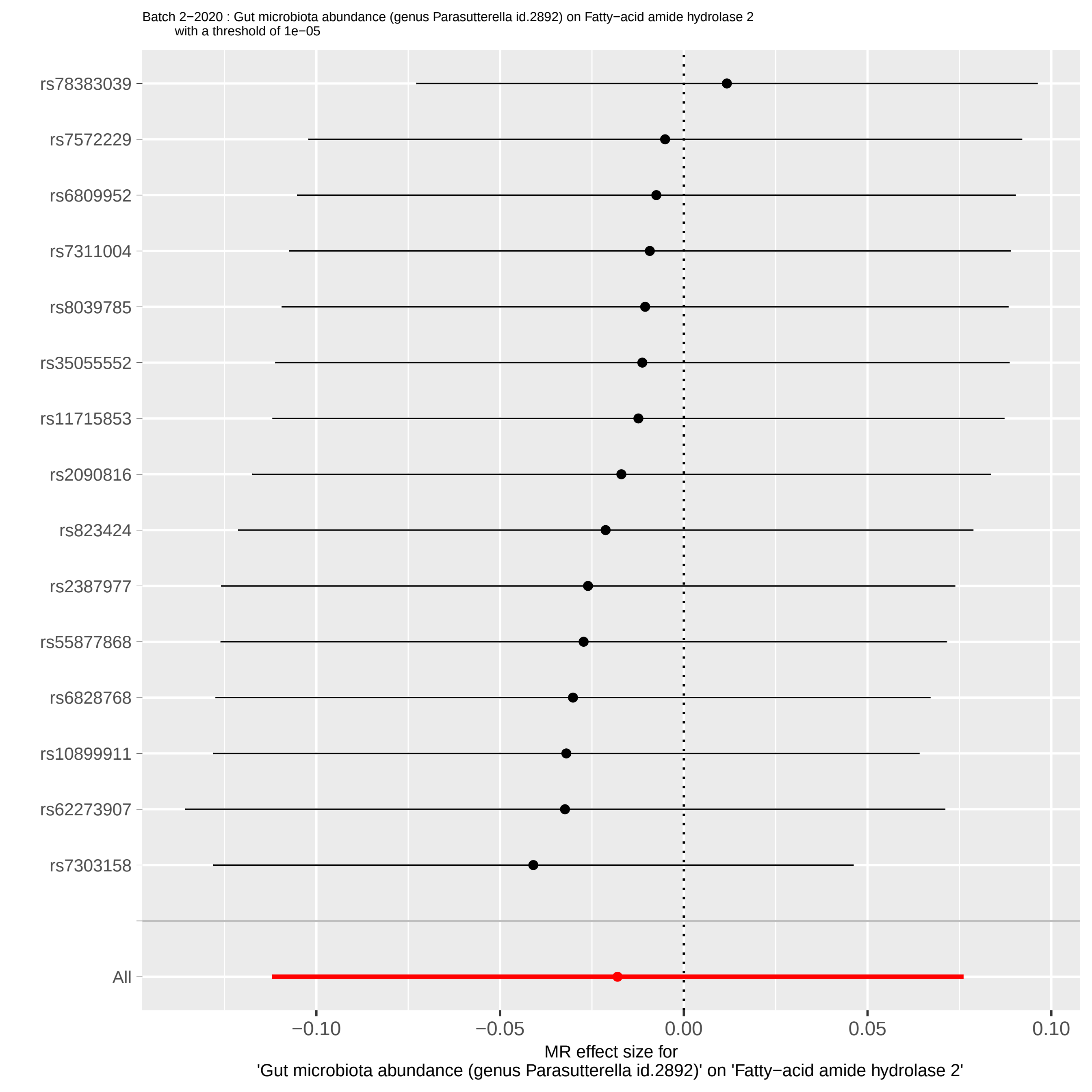
Batch 2−2020 : Gut microbiota abundance (genus Parasutterella id.2892) on Fatty−acid amide hydrolase 2
with a threshold of 1e−05
rs78383039
rs7572229
rs6809952
rs7311004
rs8039785
rs35055552
rs11715853
rs2090816
rs823424
rs2387977
rs55877868
rs6828768
rs10899911
rs62273907
rs7303158
All
−0.10
−0.05
0.00
0.05
0.10
MR effect size for
'Gut microbiota abundance (genus Parasutterella id.2892)' on 'Fatty−acid amide hydrolase 2'

Batch 2−2021 : Gut microbiota abundance (genus Catenibacterium id.2153) on Fatty acid−binding protein, liver
with a threshold of 1e−05

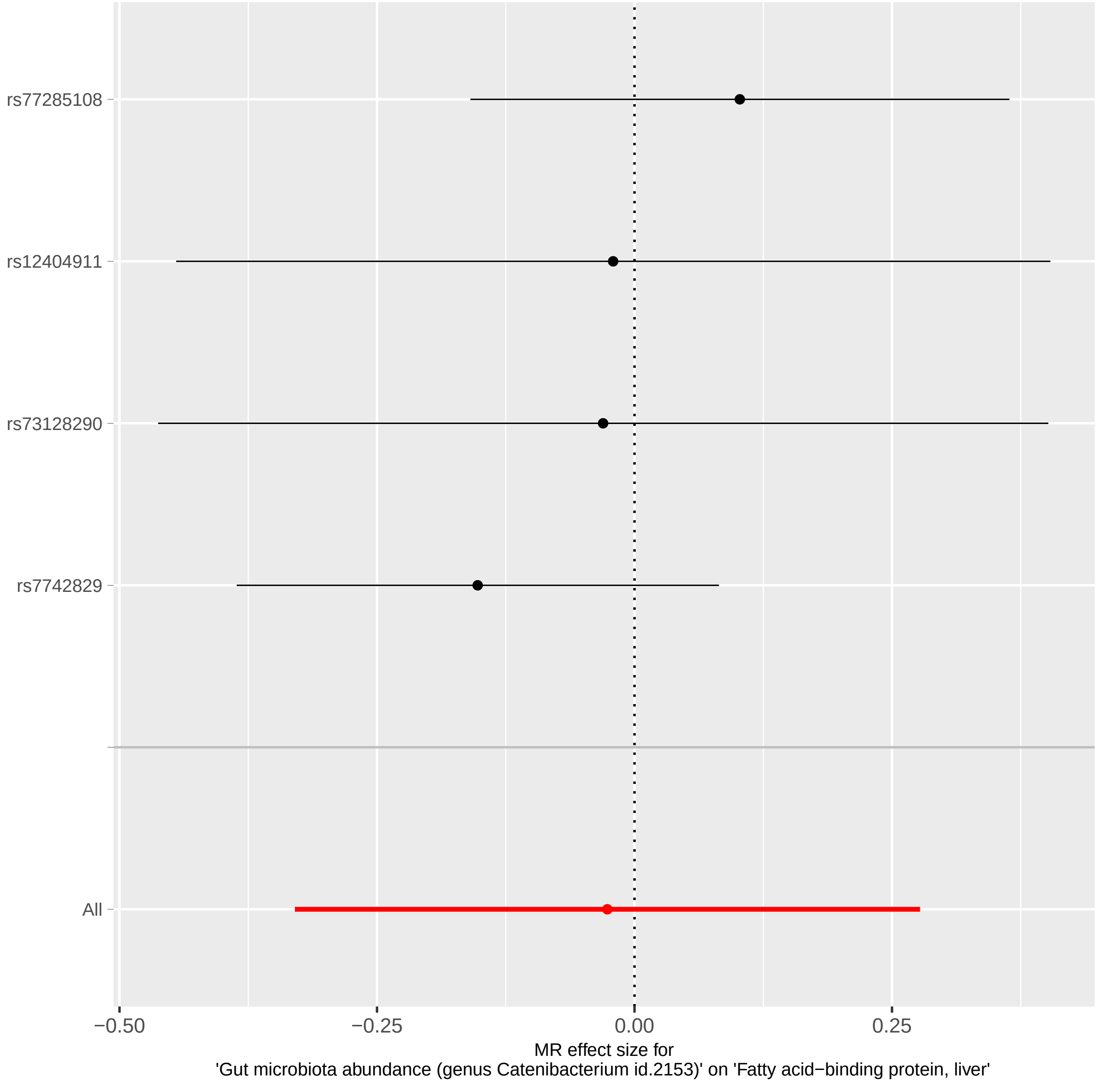

Batch 2−2022 : Gut microbiota abundance (genus Parasutterella id.2892) on Fatty acid−binding protein, liver
with a threshold of 1e−05

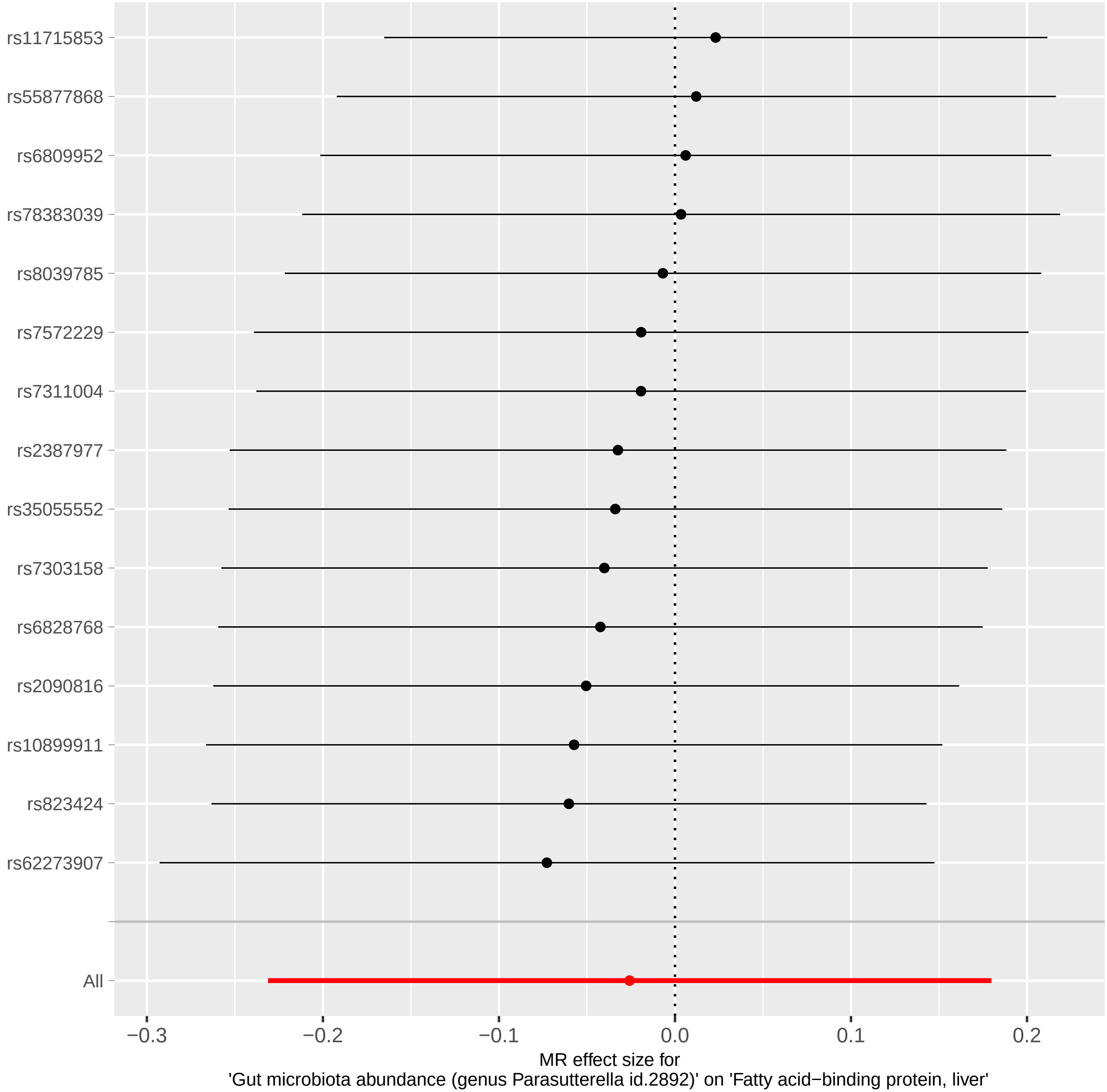

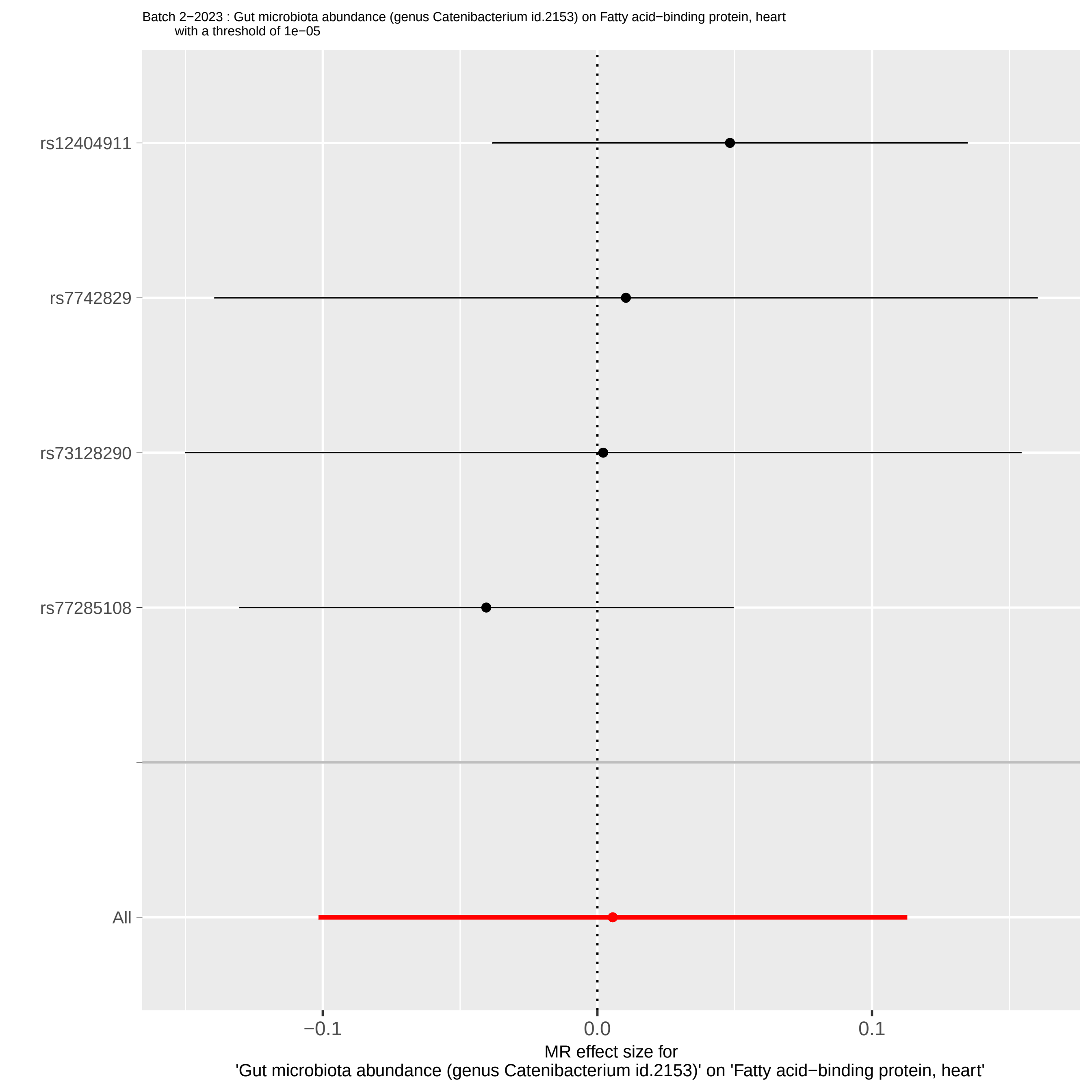

Batch 2−2023 : Gut microbiota abundance (genus Catenibacterium id.2153) on Fatty acid−binding protein, heart
with a threshold of 1e−05
rs12404911
rs7742829
rs73128290
rs77285108
All
−0.1
0.0
0.1
MR effect size for
'Gut microbiota abundance (genus Catenibacterium id.2153)' on 'Fatty acid−binding protein, heart'

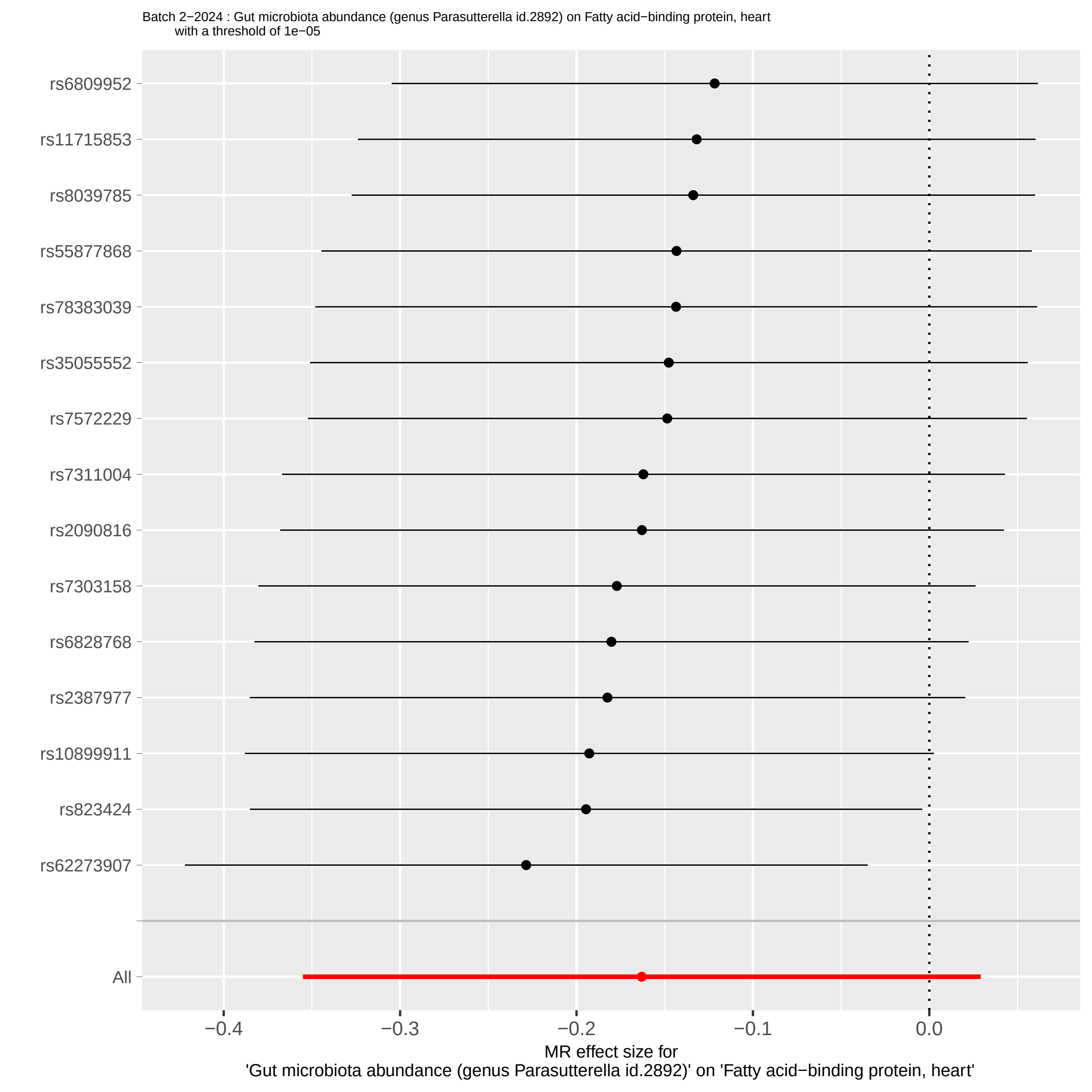

Batch 2−2024 : Gut microbiota abundance (genus Parasutterella id.2892) on Fatty acid−binding protein, heart
with a threshold of 1e−05
rs6809952
rs11715853
rs8039785
rs55877868
rs78383039
rs35055552
rs7572229
rs7311004
rs2090816
rs7303158
rs6828768
rs2387977
rs10899911
rs823424
rs62273907
All
−0.4
−0.3
−0.2
−0.1
0.0
MR effect size for
'Gut microbiota abundance (genus Parasutterella id.2892)' on 'Fatty acid−binding protein, heart'

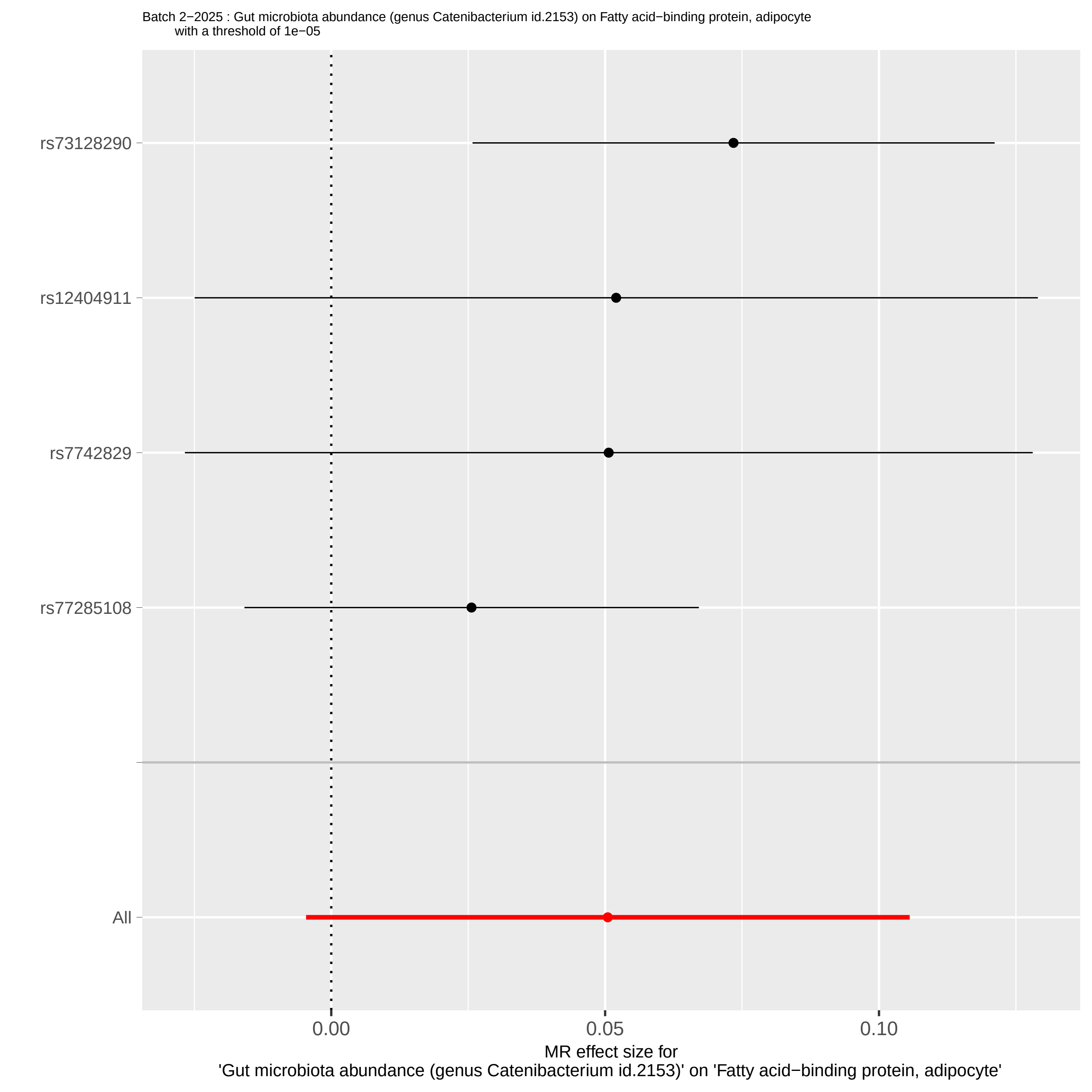
Batch 2−2025 : Gut microbiota abundance (genus Catenibacterium id.2153) on Fatty acid−binding protein, adipocyte
with a threshold of 1e−05
rs73128290
rs12404911
rs7742829
rs77285108
All
0.00
0.05
0.10
MR effect size for
'Gut microbiota abundance (genus Catenibacterium id.2153)' on 'Fatty acid−binding protein, adipocyte'

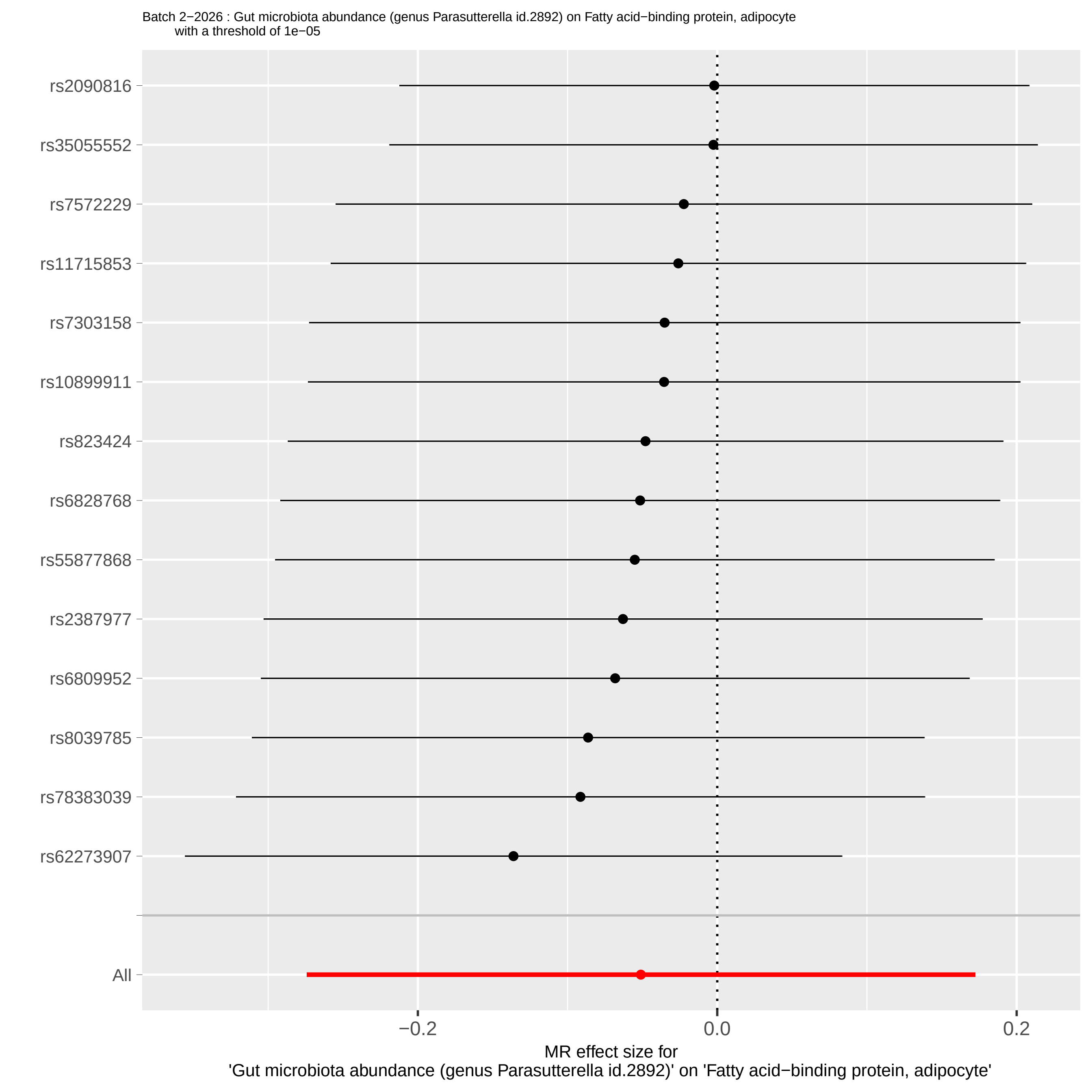
Batch 2−2026 : Gut microbiota abundance (genus Parasutterella id.2892) on Fatty acid−binding protein, adipocyte
with a threshold of 1e−05
rs2090816
rs35055552
rs7572229
rs11715853
rs7303158
rs10899911
rs823424
rs6828768
rs55877868
rs2387977
rs6809952
rs8039785
rs78383039
rs62273907
All
−0.2
0.0
0.2
MR effect size for
'Gut microbiota abundance (genus Parasutterella id.2892)' on 'Fatty acid−binding protein, adipocyte'

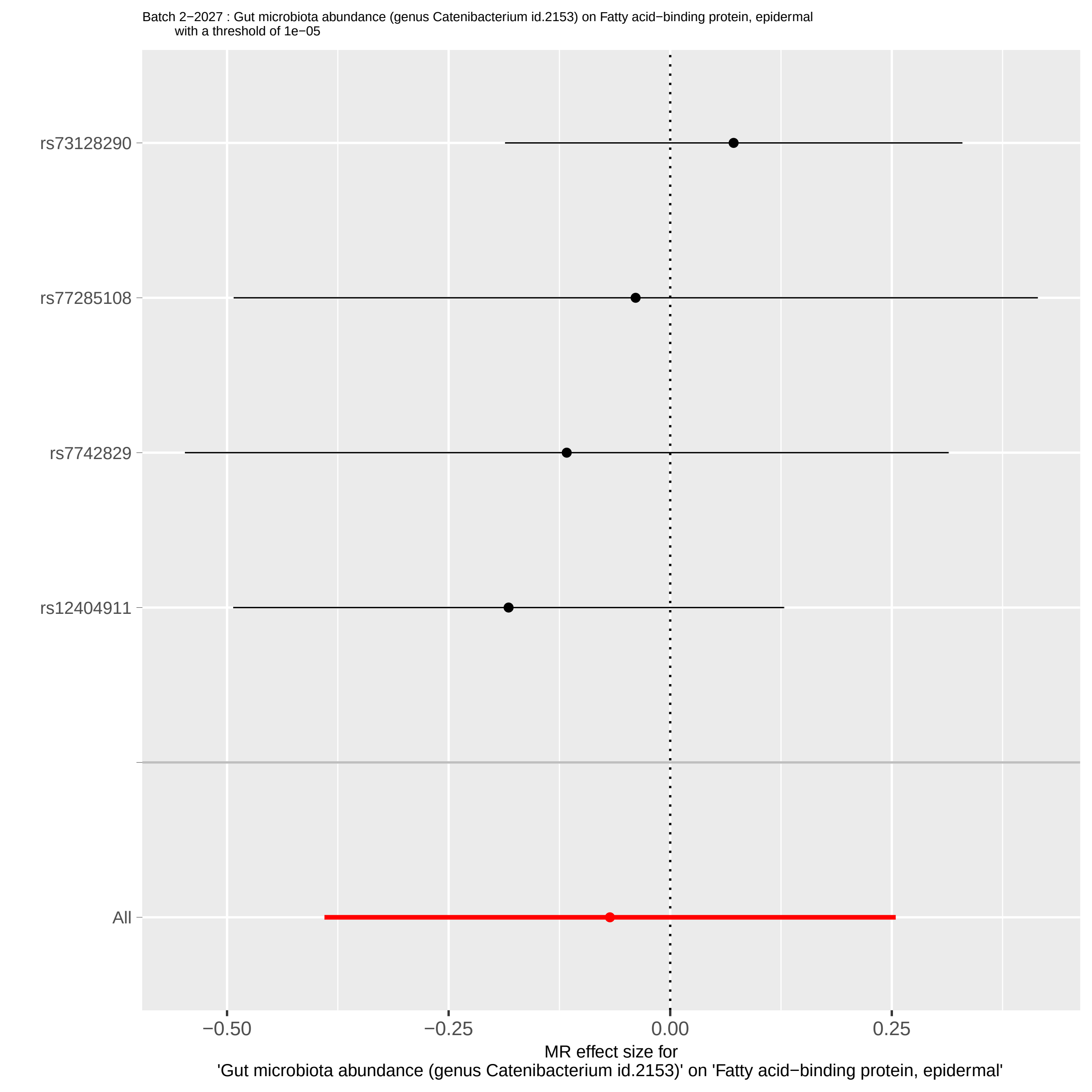

Batch 2−2027 : Gut microbiota abundance (genus Catenibacterium id.2153) on Fatty acid−binding protein, epidermal
with a threshold of 1e−05
rs73128290
rs77285108
rs7742829
rs12404911
All
−0.50
−0.25
0.00
0.25
MR effect size for
'Gut microbiota abundance (genus Catenibacterium id.2153)' on 'Fatty acid−binding protein, epidermal'

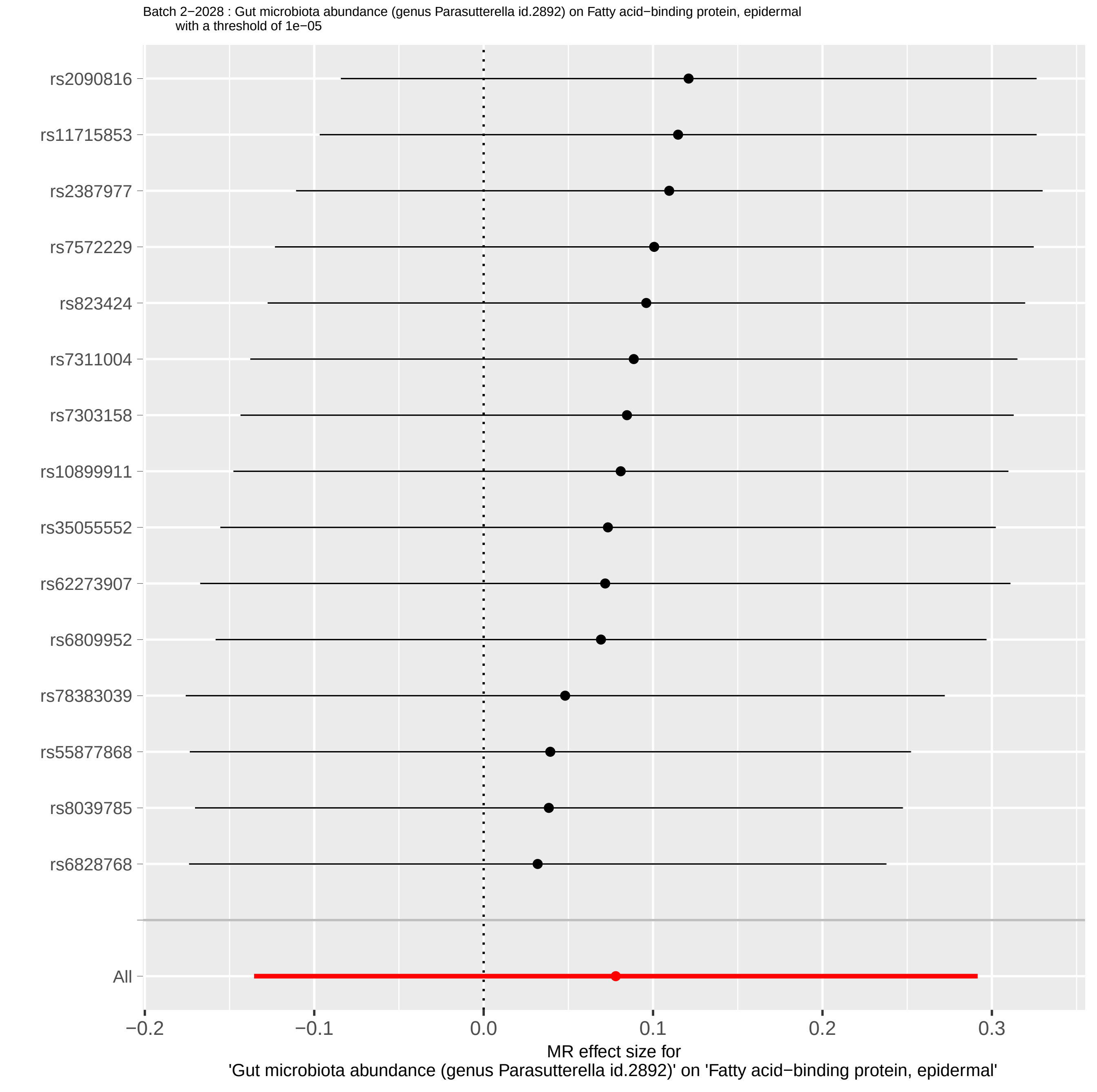

Batch 2−2028 : Gut microbiota abundance (genus Parasutterella id.2892) on Fatty acid−binding protein, epidermal
with a threshold of 1e−05
rs2090816
rs11715853
rs2387977
rs7572229
rs823424
rs7311004
rs7303158
rs10899911
rs35055552
rs62273907
rs6809952
rs78383039
rs55877868
rs8039785
rs6828768
All
−0.2
−0.1
0.0
0.1
0.2
0.3
MR effect size for
'Gut microbiota abundance (genus Parasutterella id.2892)' on 'Fatty acid−binding protein, epidermal'

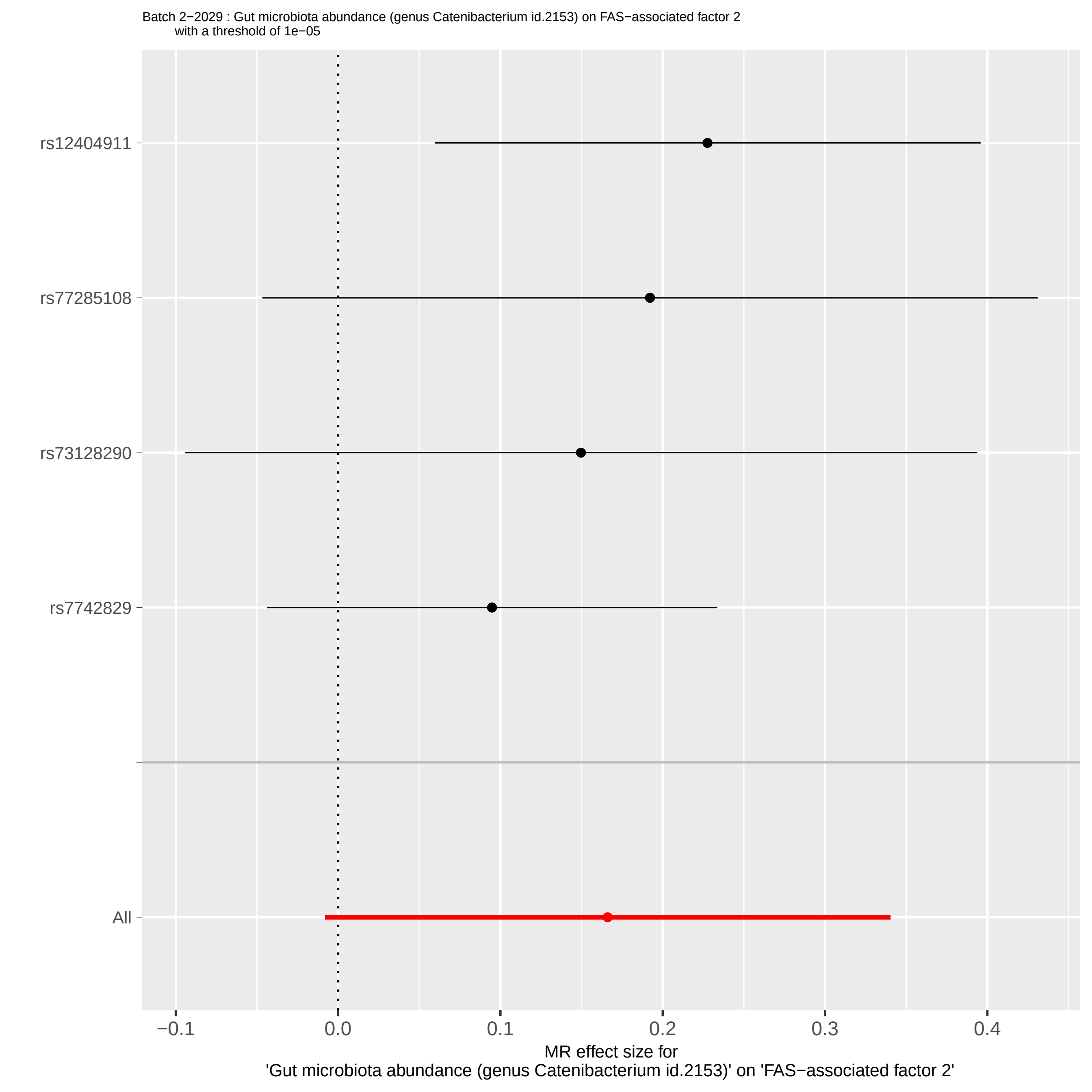
Batch 2−2029 : Gut microbiota abundance (genus Catenibacterium id.2153) on FAS−associated factor 2
with a threshold of 1e−05
rs12404911
rs77285108
rs73128290
rs7742829
All
−0.1
0.0
0.1
0.2
0.3
0.4
MR effect size for
'Gut microbiota abundance (genus Catenibacterium id.2153)' on 'FAS−associated factor 2'

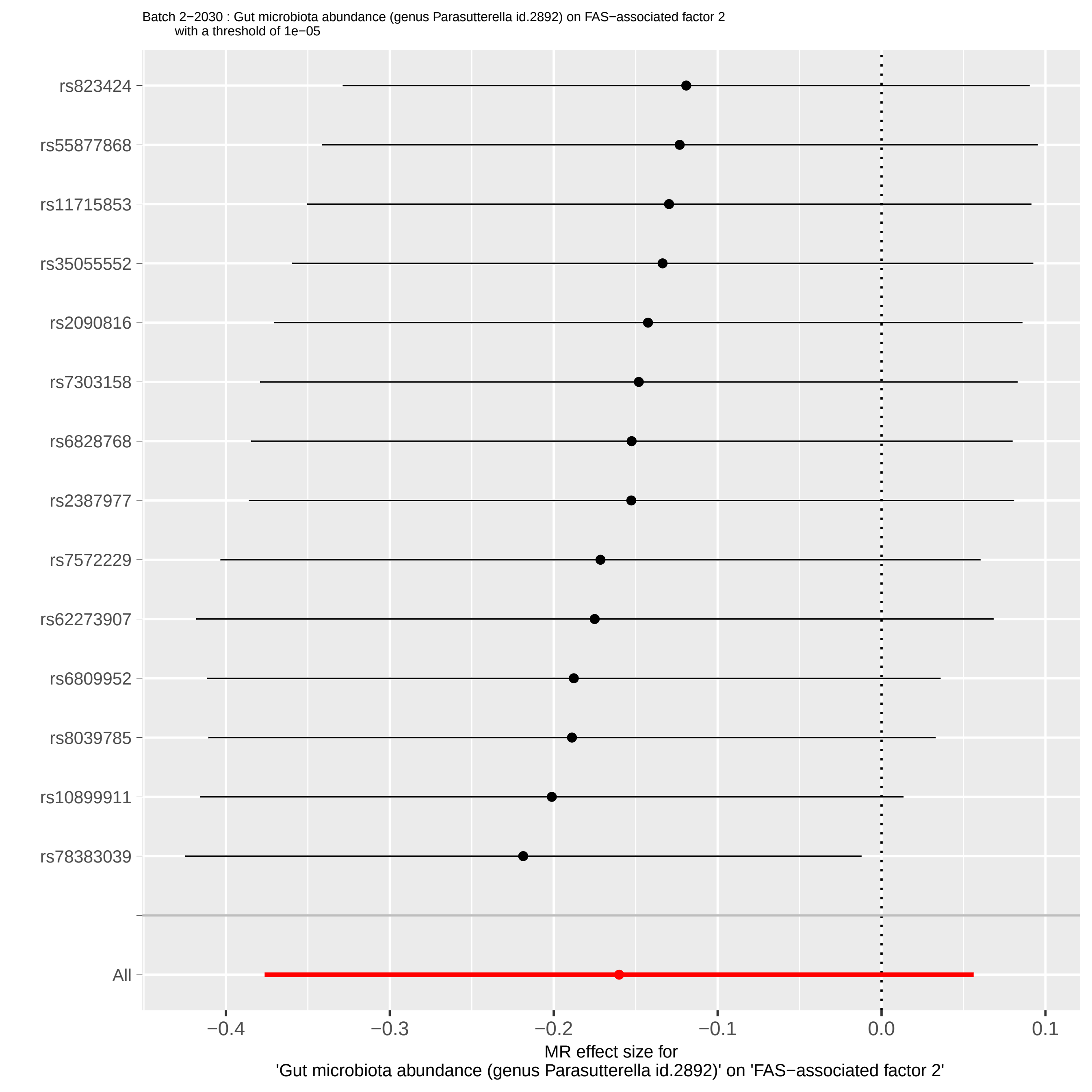

Batch 2−2030 : Gut microbiota abundance (genus Parasutterella id.2892) on FAS−associated factor 2
with a threshold of 1e−05
rs823424
rs55877868
rs11715853
rs35055552
rs2090816
rs7303158
rs6828768
rs2387977
rs7572229
rs62273907
rs6809952
rs8039785
rs10899911
rs78383039
All
−0.4
−0.3
−0.2
−0.1
0.0
0.1
MR effect size for
'Gut microbiota abundance (genus Parasutterella id.2892)' on 'FAS−associated factor 2'

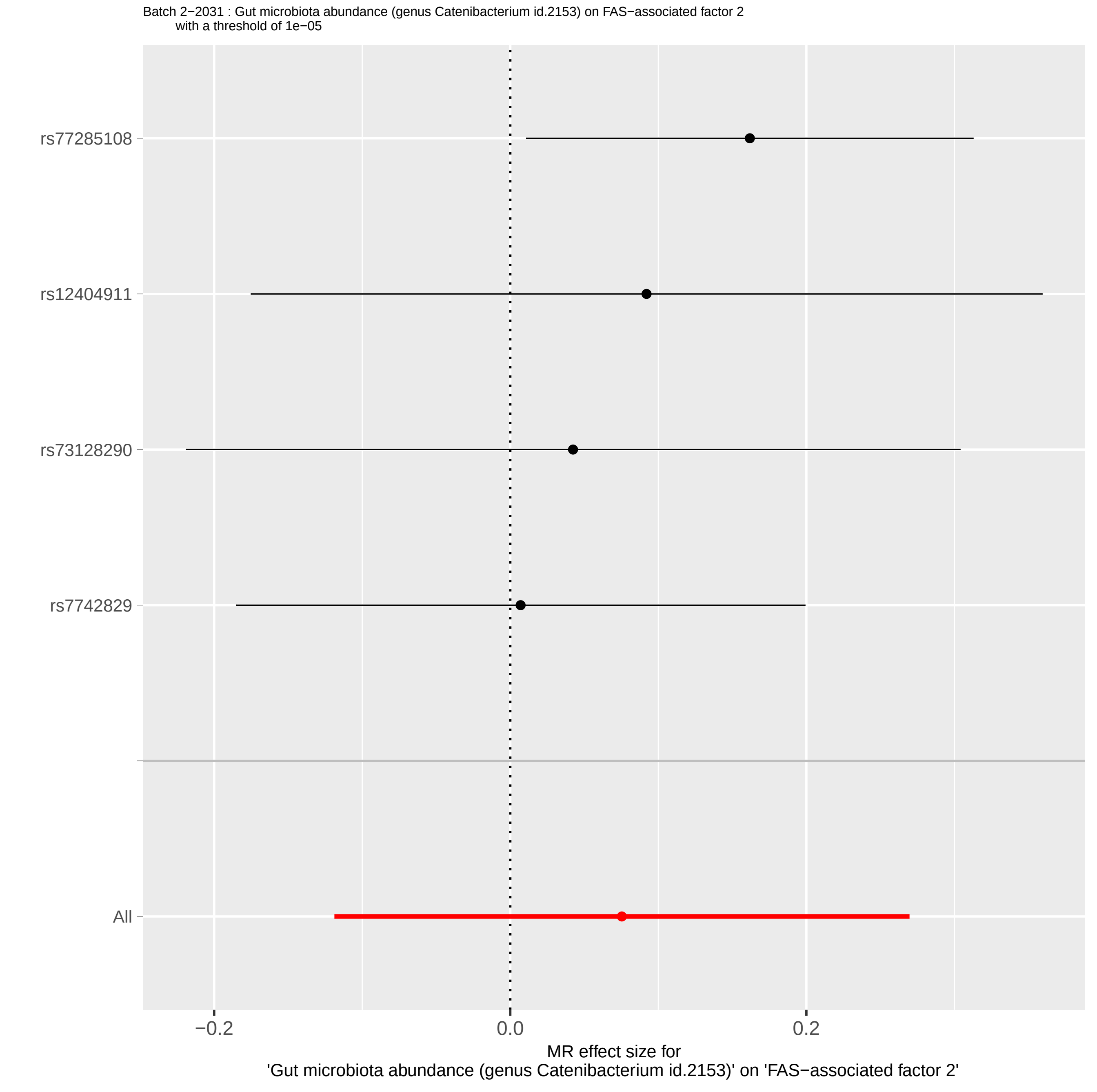
Batch 2−2031 : Gut microbiota abundance (genus Catenibacterium id.2153) on FAS−associated factor 2
with a threshold of 1e−05
rs77285108
rs12404911
rs73128290
rs7742829
All
−0.2
0.0
0.2
MR effect size for
'Gut microbiota abundance (genus Catenibacterium id.2153)' on 'FAS−associated factor 2'

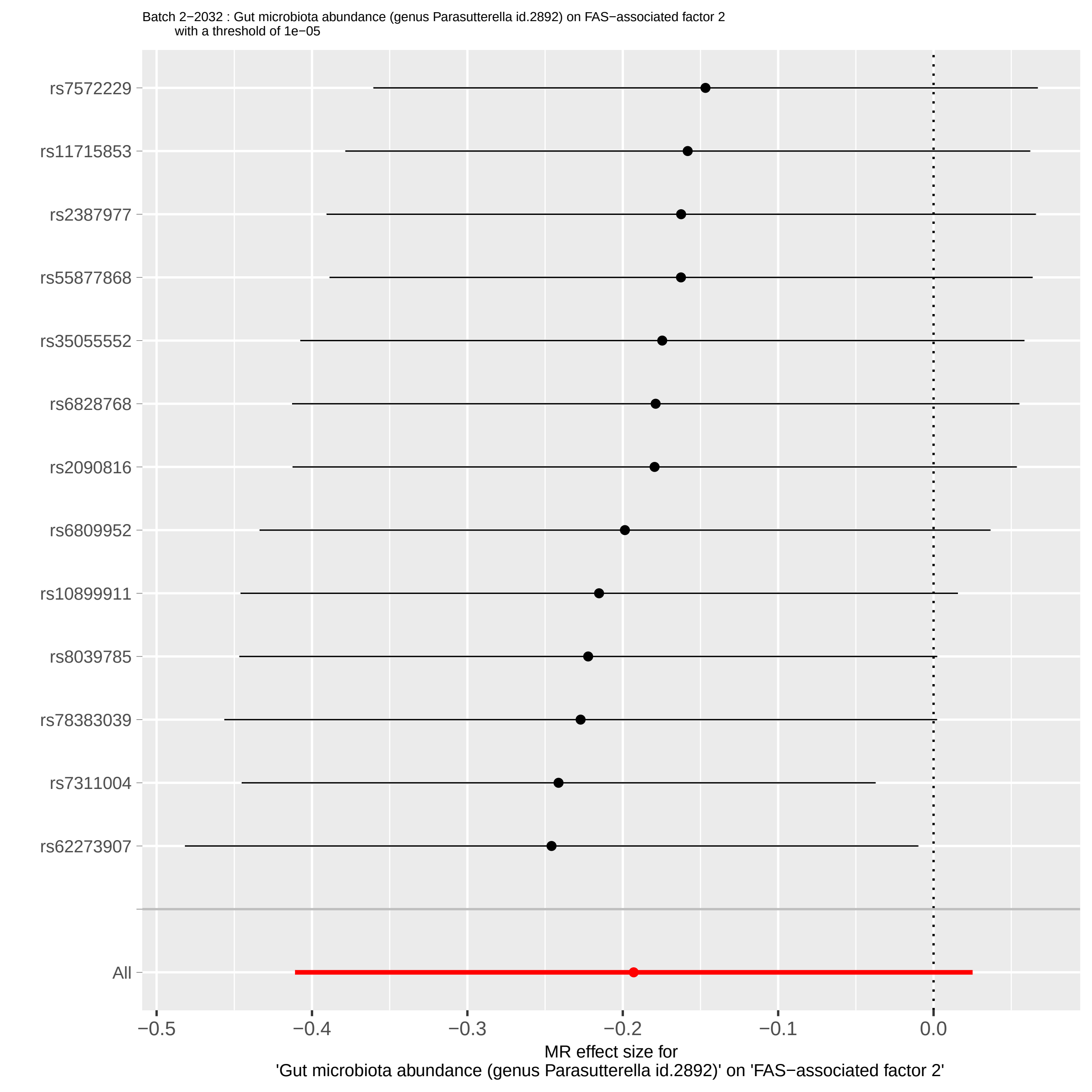

Batch 2−2032 : Gut microbiota abundance (genus Parasutterella id.2892) on FAS−associated factor 2
with a threshold of 1e−05
rs7572229
rs11715853
rs2387977
rs55877868
rs35055552
rs6828768
rs2090816
rs6809952
rs10899911
rs8039785
rs78383039
rs7311004
rs62273907
All
−0.5
−0.4
−0.3
−0.2
−0.1
0.0
MR effect size for
'Gut microbiota abundance (genus Parasutterella id.2892)' on 'FAS−associated factor 2'

Batch 2−2033 : Gut microbiota abundance (genus Catenibacterium id.2153) on FAS−associated factor 2
with a threshold of 1e−05

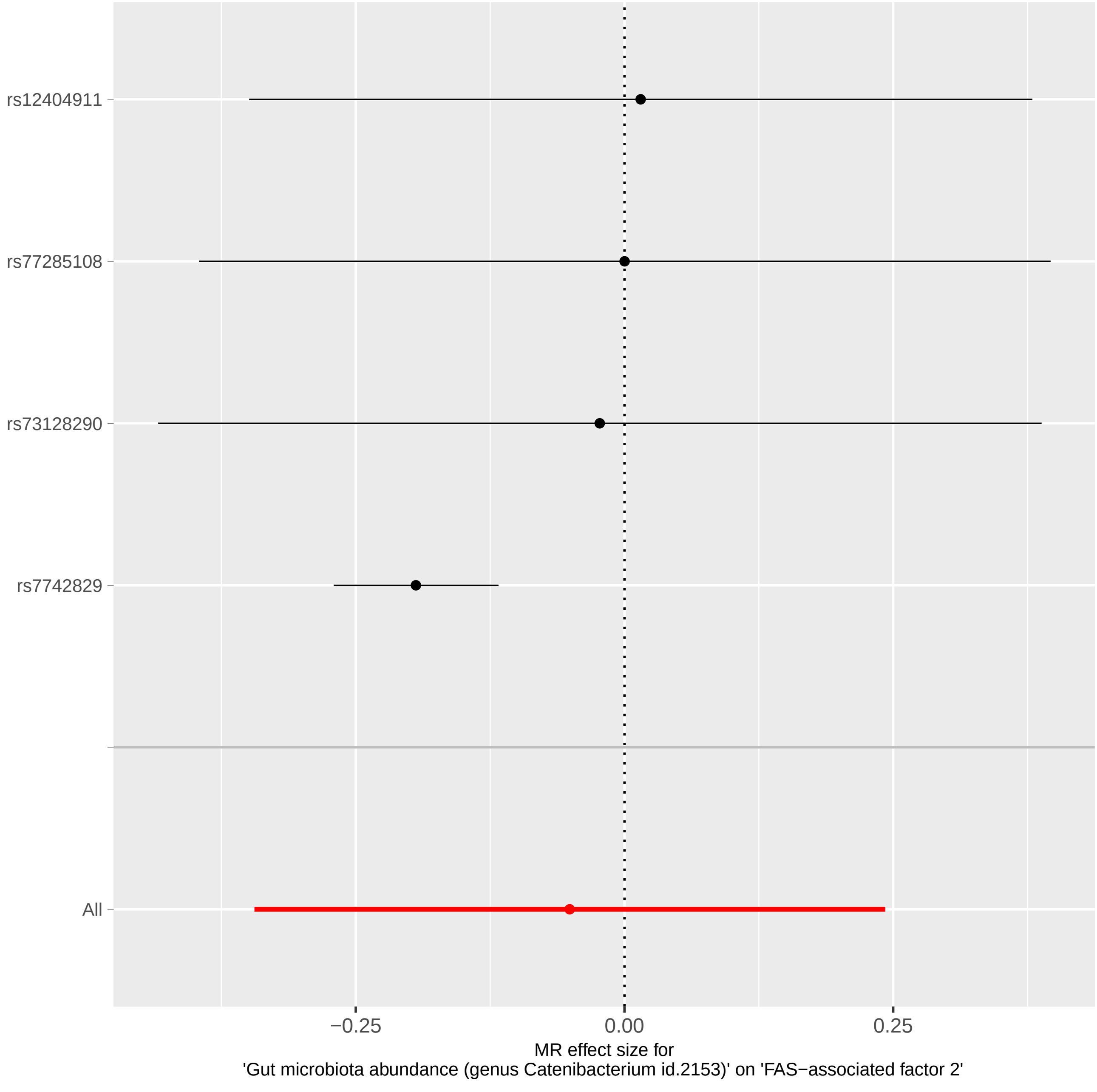

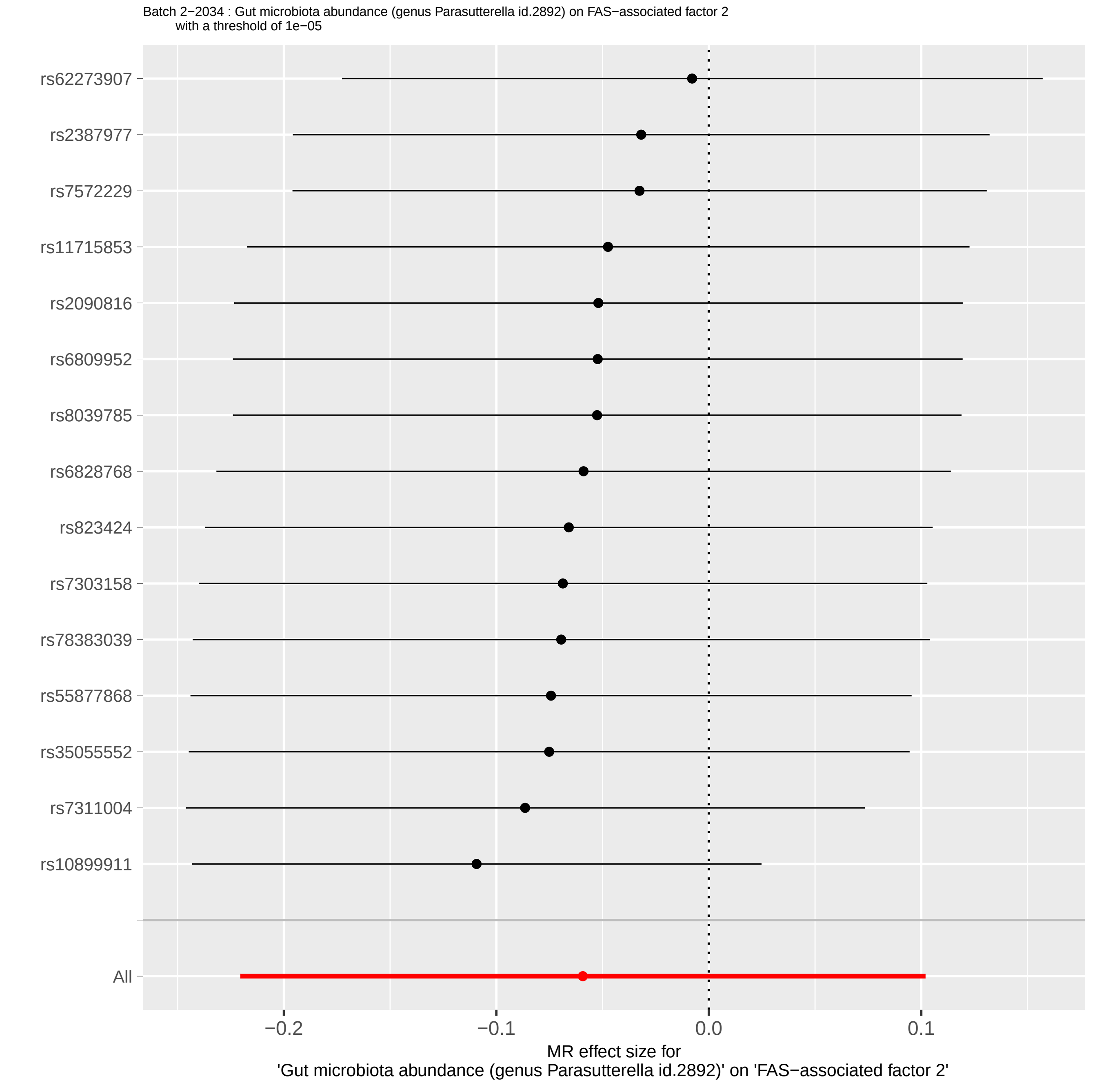
Batch 2−2034 : Gut microbiota abundance (genus Parasutterella id.2892) on FAS−associated factor 2
with a threshold of 1e−05
rs62273907
rs2387977
rs7572229
rs11715853
rs2090816
rs6809952
rs8039785
rs6828768
rs823424
rs7303158
rs78383039
rs55877868
rs35055552
rs7311004
rs10899911
All
−0.2
−0.1
0.0
0.1
MR effect size for
'Gut microbiota abundance (genus Parasutterella id.2892)' on 'FAS−associated factor 2'

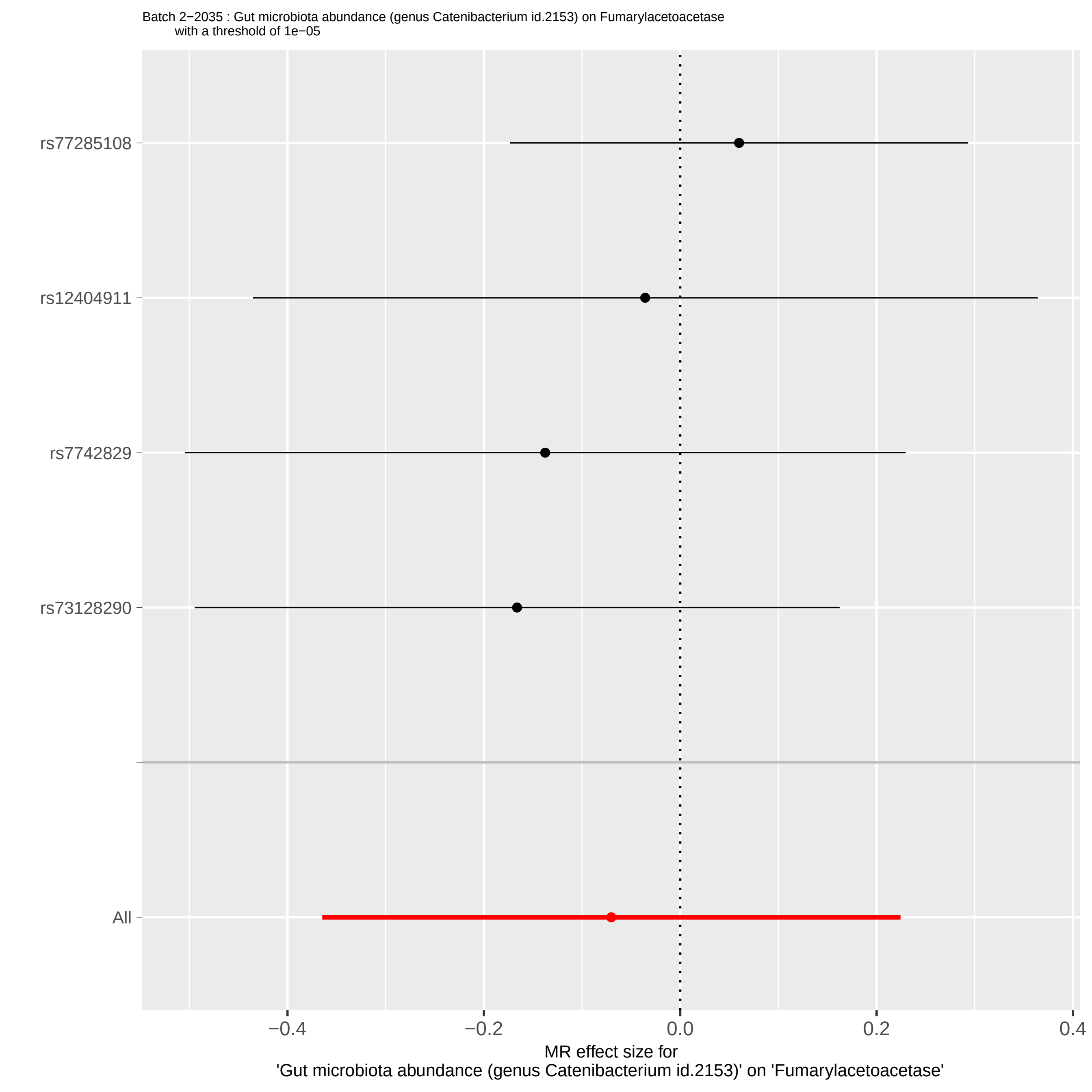
Batch 2−2035 : Gut microbiota abundance (genus Catenibacterium id.2153) on Fumarylacetoacetase
with a threshold of 1e−05
rs77285108
rs12404911
rs7742829
rs73128290
All
−0.4
−0.2
0.0
0.2
0.4
MR effect size for
'Gut microbiota abundance (genus Catenibacterium id.2153)' on 'Fumarylacetoacetase'

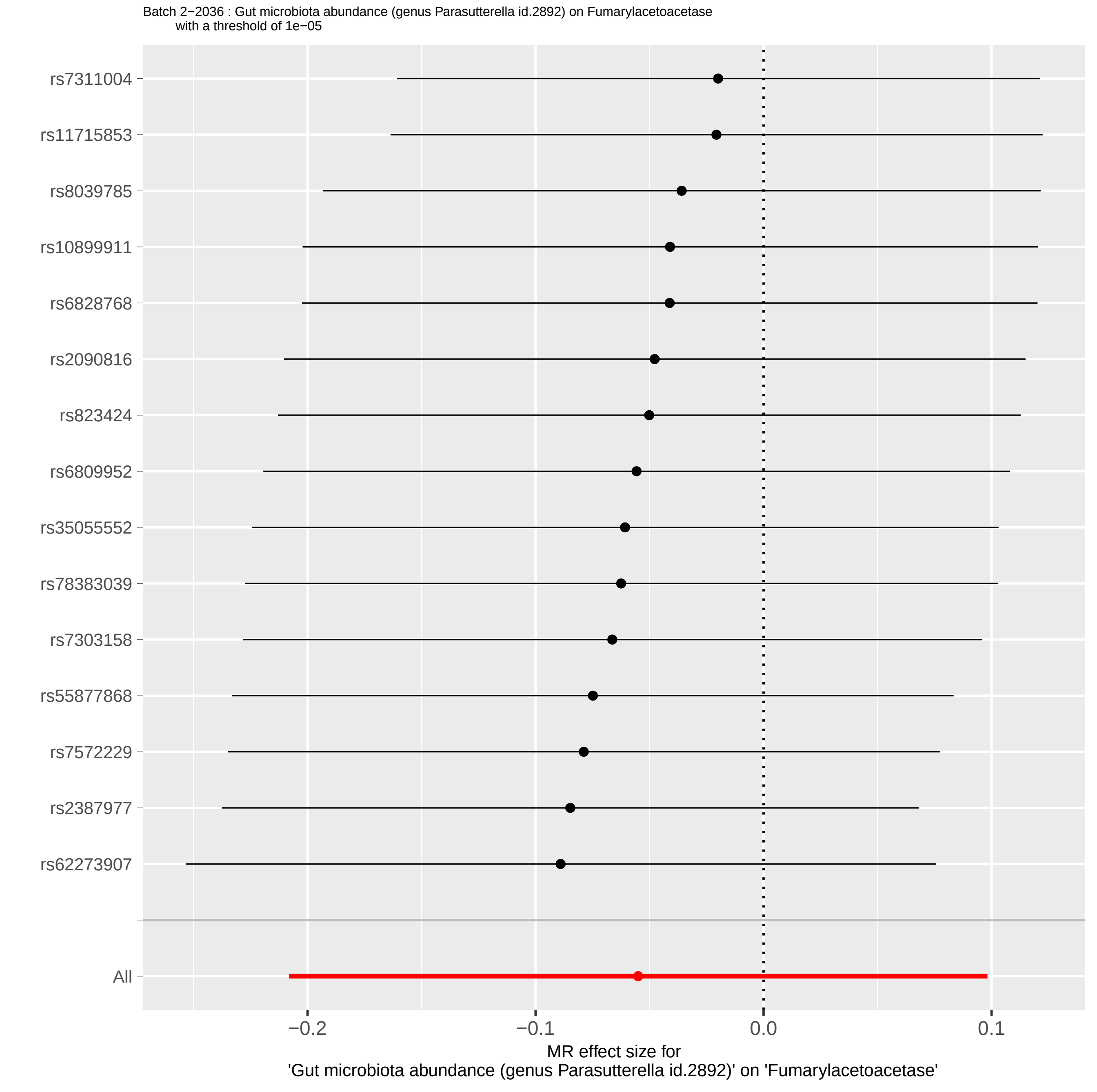

Batch 2−2036 : Gut microbiota abundance (genus Parasutterella id.2892) on Fumarylacetoacetase
with a threshold of 1e−05
rs7311004
rs11715853
rs8039785
rs10899911
rs6828768
rs2090816
rs823424
rs6809952
rs35055552
rs78383039
rs7303158
rs55877868
rs7572229
rs2387977
rs62273907
All
−0.2
−0.1
0.0
0.1
MR effect size for
'Gut microbiota abundance (genus Parasutterella id.2892)' on 'Fumarylacetoacetase'

Batch 2−2037 : Gut microbiota abundance (genus Catenibacterium id.2153) on Fas apoptotic inhibitory molecule 3
with a threshold of 1e−05

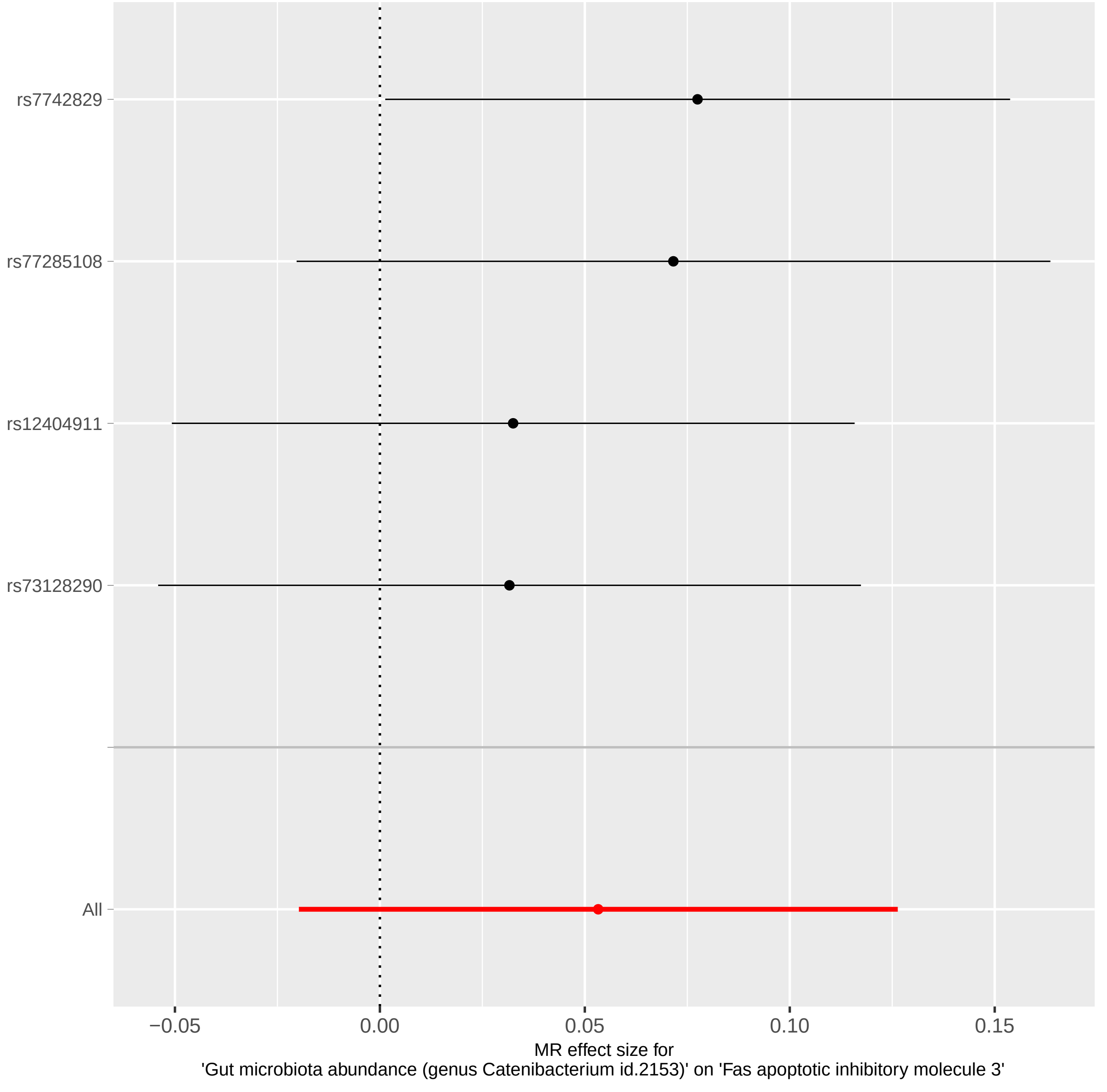

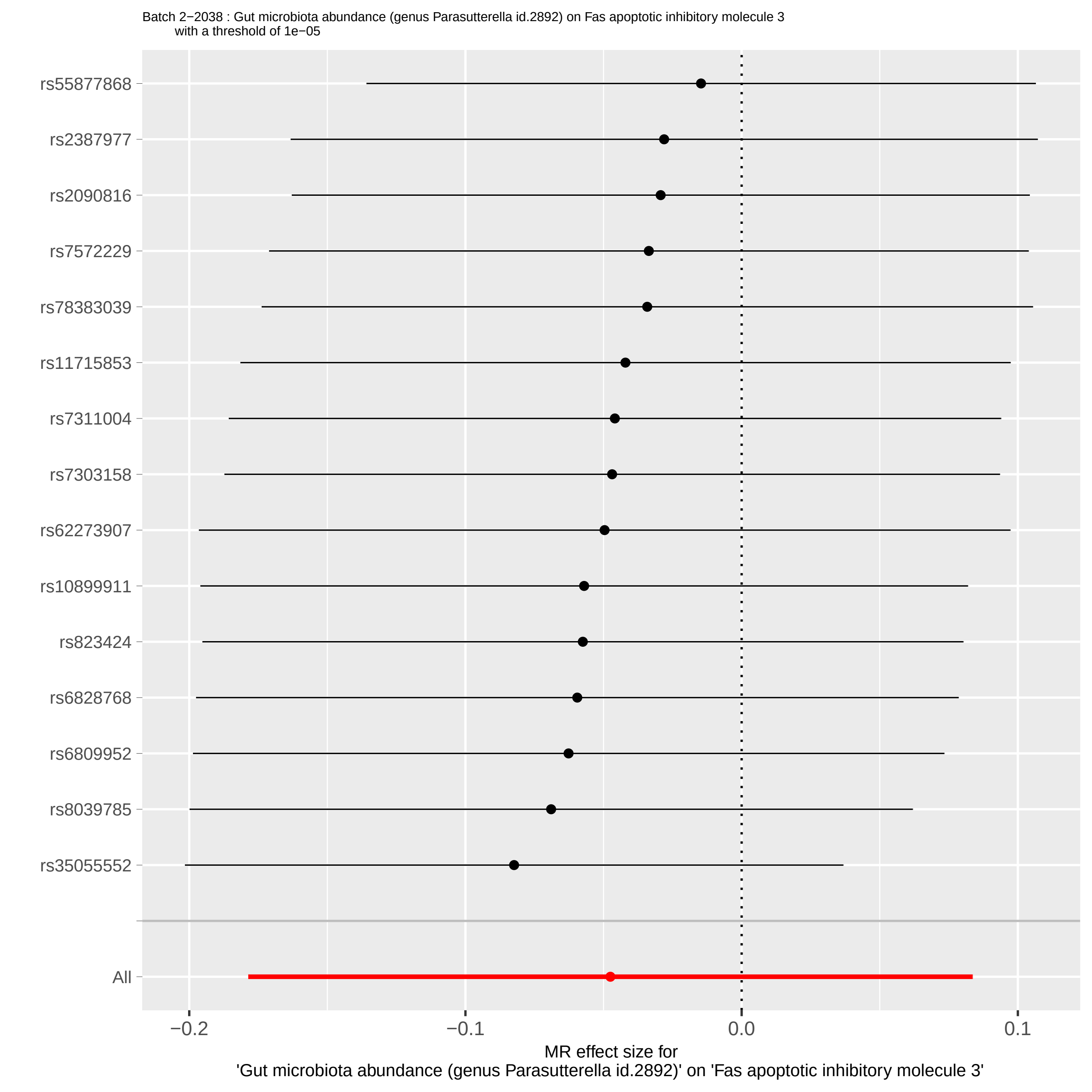
Batch 2−2038 : Gut microbiota abundance (genus Parasutterella id.2892) on Fas apoptotic inhibitory molecule 3
with a threshold of 1e−05
rs55877868
rs2387977
rs2090816
rs7572229
rs78383039
rs11715853
rs7311004
rs7303158
rs62273907
rs10899911
rs823424
rs6828768
rs6809952
rs8039785
rs35055552
All
−0.2
−0.1
0.0
0.1
MR effect size for
'Gut microbiota abundance (genus Parasutterella id.2892)' on 'Fas apoptotic inhibitory molecule 3'

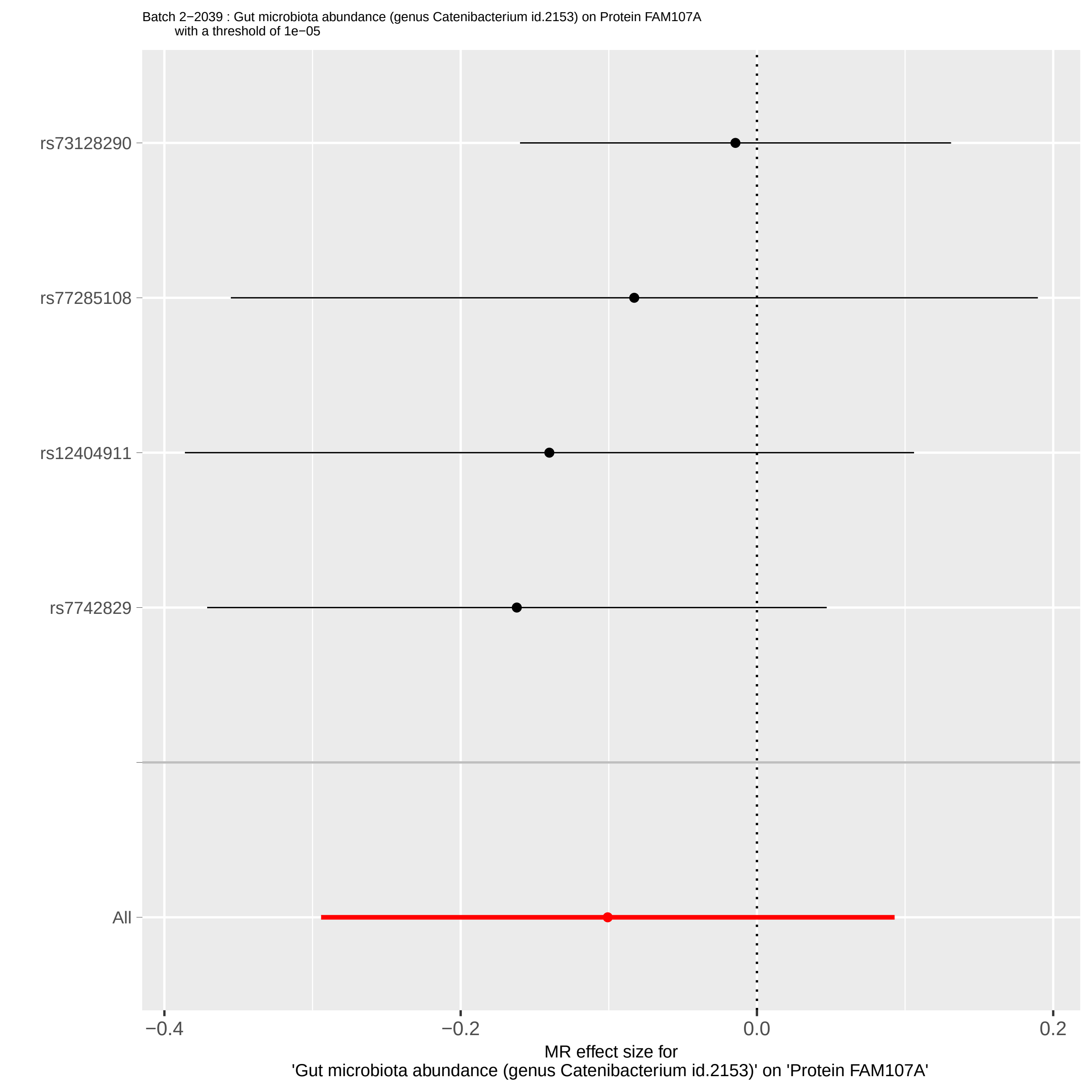
Batch 2−2039 : Gut microbiota abundance (genus Catenibacterium id.2153) on Protein FAM107A
with a threshold of 1e−05
rs73128290
rs77285108
rs12404911
rs7742829
All
−0.4
−0.2
0.0
0.2
MR effect size for
'Gut microbiota abundance (genus Catenibacterium id.2153)' on 'Protein FAM107A'

Batch 2−2040 : Gut microbiota abundance (genus Parasutterella id.2892) on Protein FAM107A
with a threshold of 1e−05

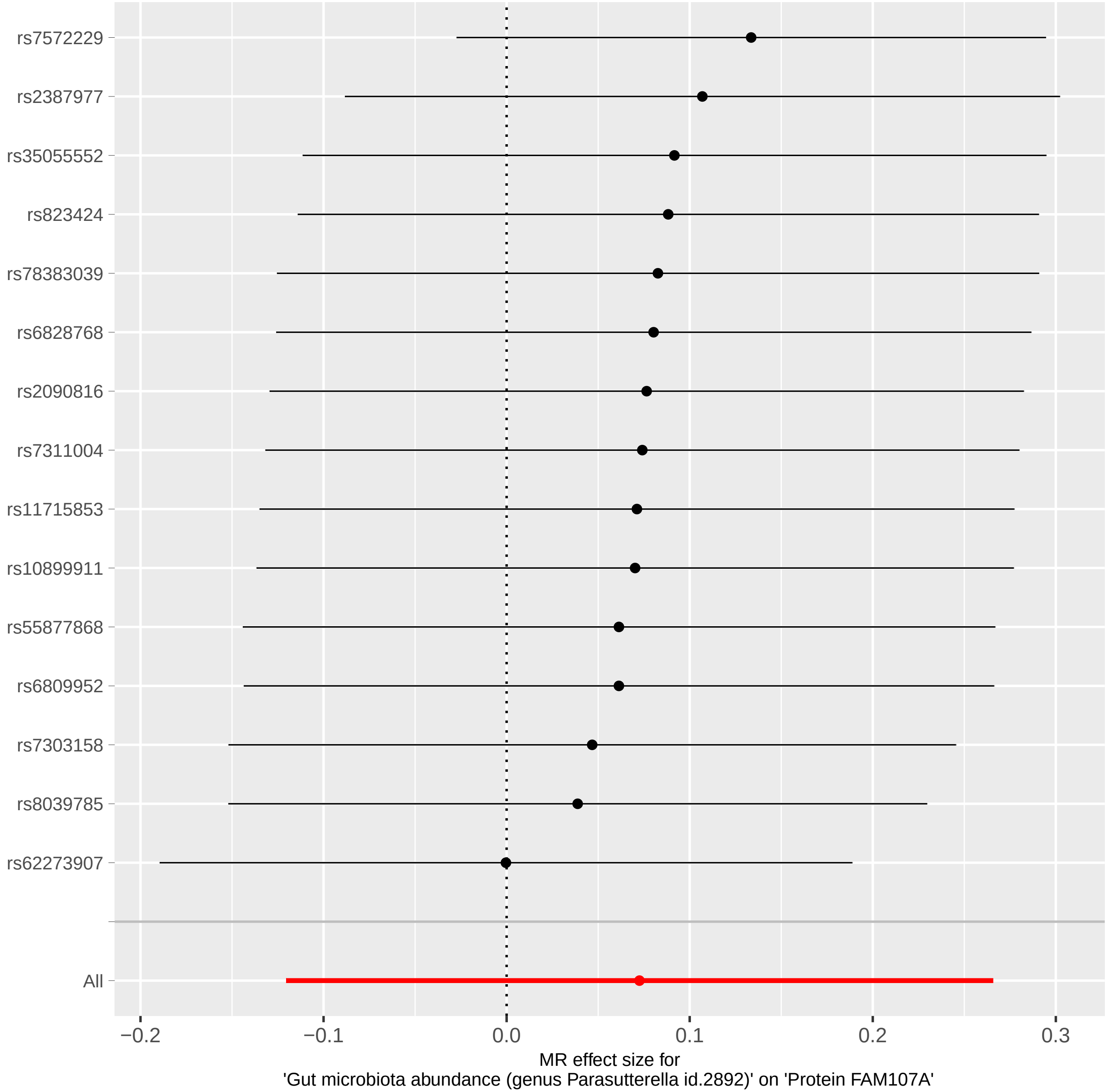

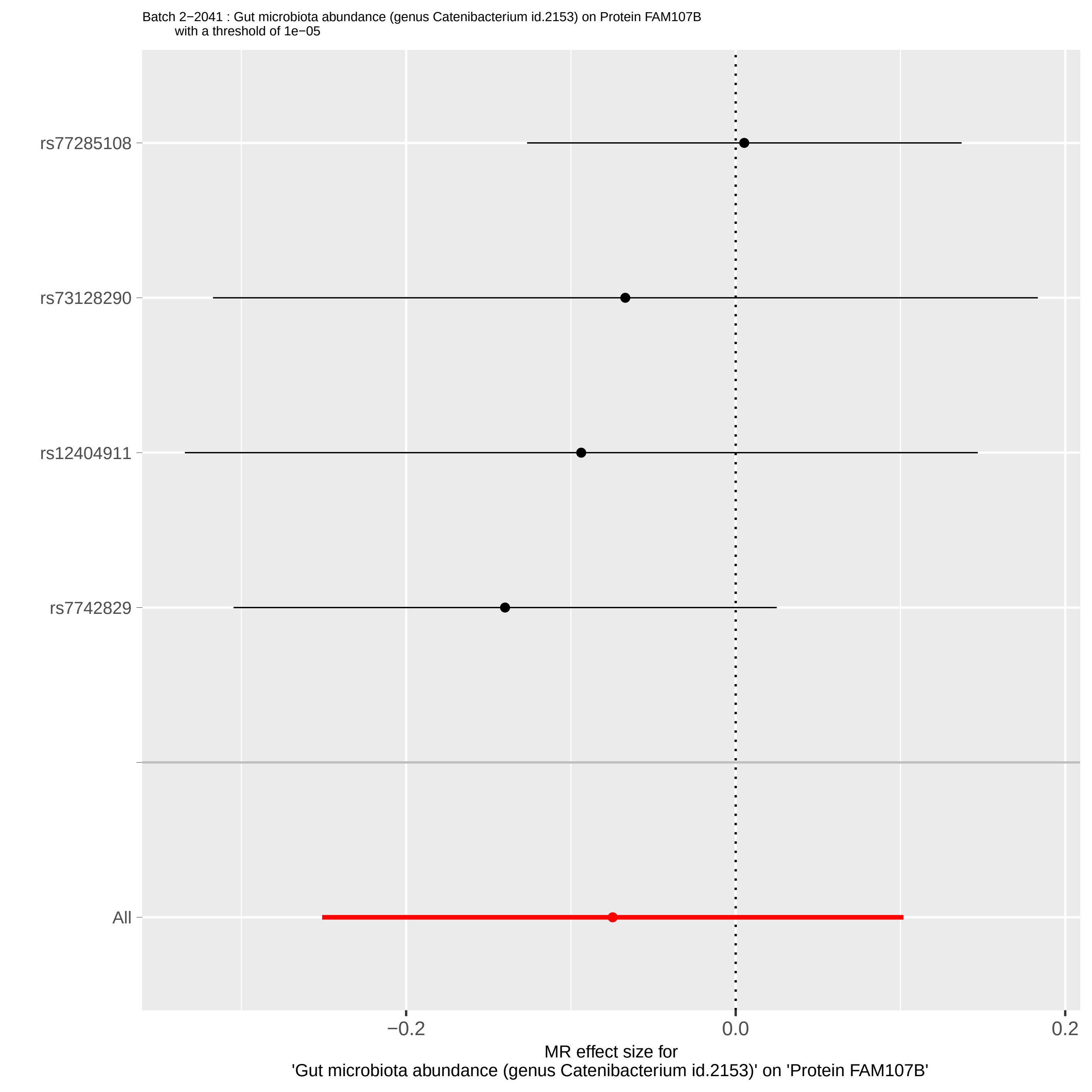
Batch 2−2041 : Gut microbiota abundance (genus Catenibacterium id.2153) on Protein FAM107B
with a threshold of 1e−05
rs77285108
rs73128290
rs12404911
rs7742829
All
−0.2
0.0
0.2
MR effect size for
'Gut microbiota abundance (genus Catenibacterium id.2153)' on 'Protein FAM107B'

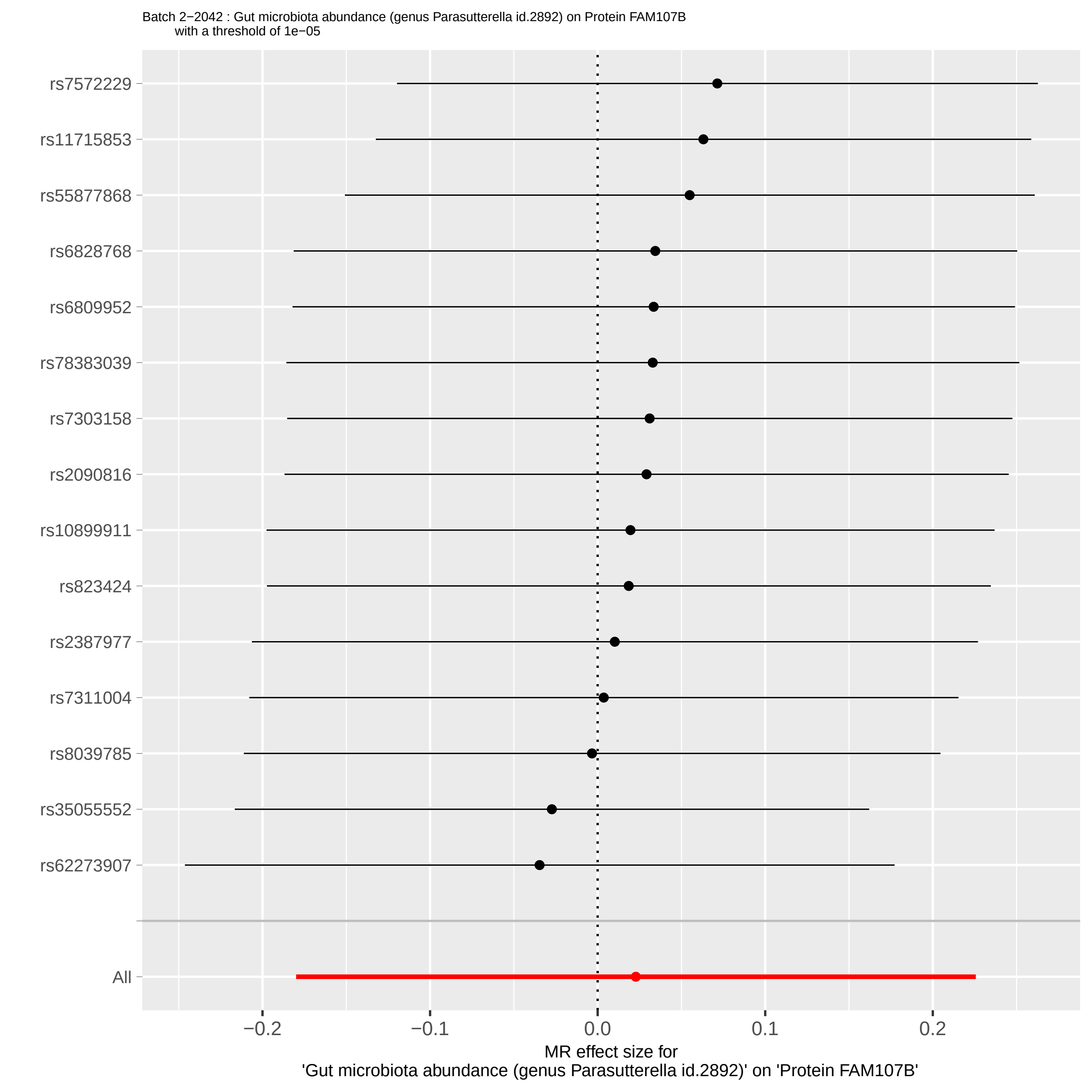

Batch 2−2042 : Gut microbiota abundance (genus Parasutterella id.2892) on Protein FAM107B
with a threshold of 1e−05
rs7572229
rs11715853
rs55877868
rs6828768
rs6809952
rs78383039
rs7303158
rs2090816
rs10899911
rs823424
rs2387977
rs7311004
rs8039785
rs35055552
rs62273907
All
−0.2
−0.1
0.0
0.1
0.2
MR effect size for
'Gut microbiota abundance (genus Parasutterella id.2892)' on 'Protein FAM107B'

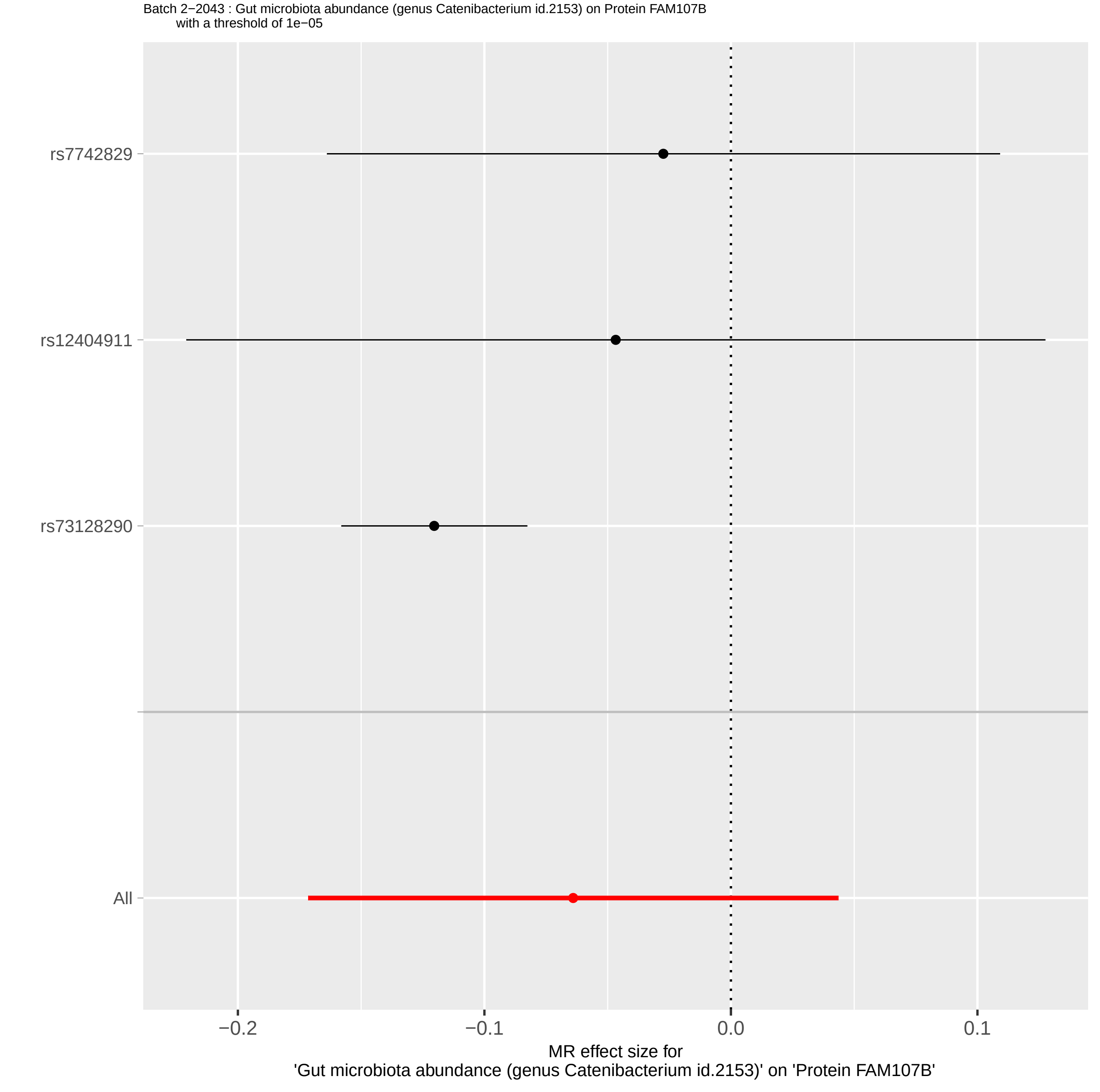
Batch 2−2043 : Gut microbiota abundance (genus Catenibacterium id.2153) on Protein FAM107B
with a threshold of 1e−05
rs7742829
rs12404911
rs73128290
All
−0.2
−0.1
0.0
0.1
MR effect size for
'Gut microbiota abundance (genus Catenibacterium id.2153)' on 'Protein FAM107B'

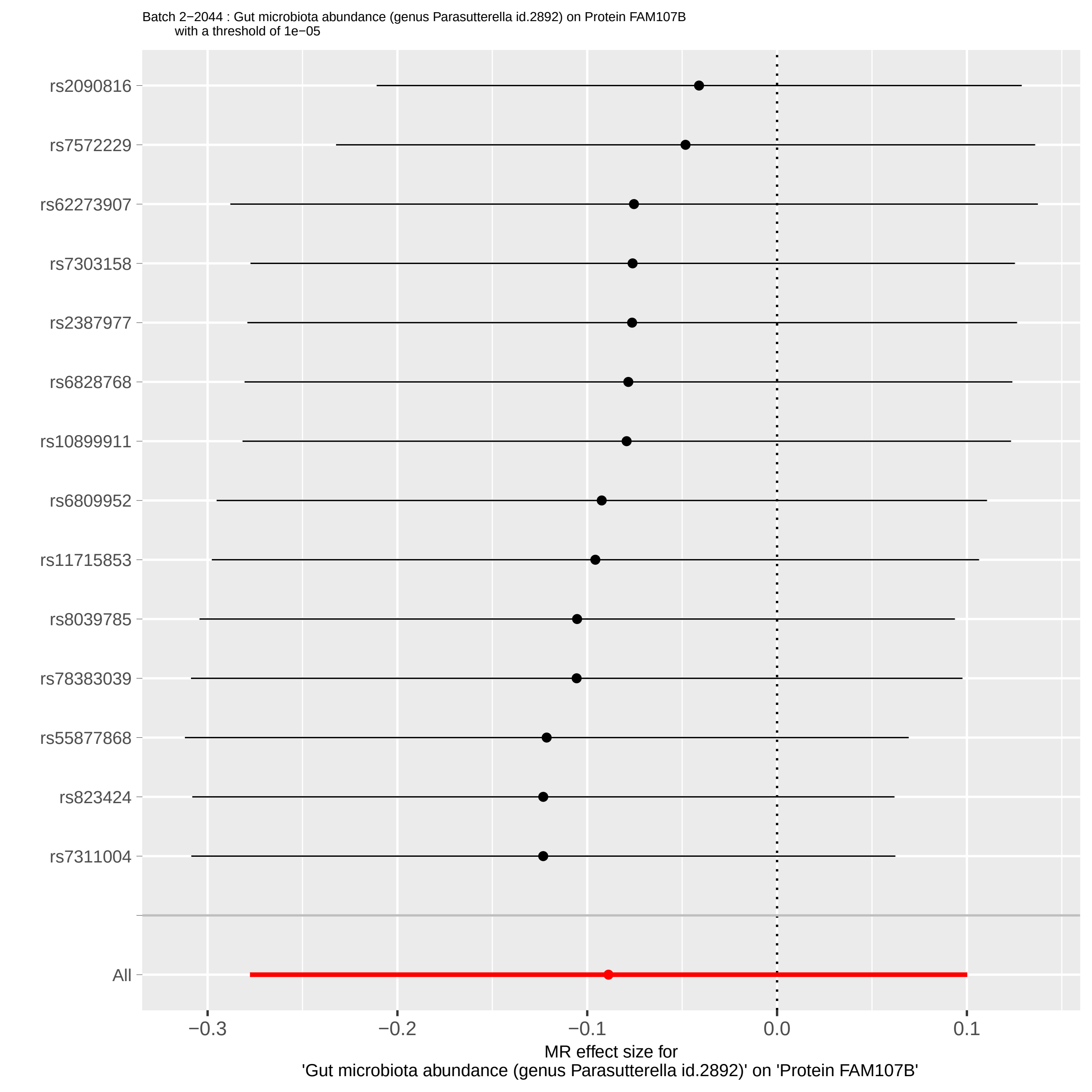

Batch 2−2044 : Gut microbiota abundance (genus Parasutterella id.2892) on Protein FAM107B
with a threshold of 1e−05
rs2090816
rs7572229
rs62273907
rs7303158
rs2387977
rs6828768
rs10899911
rs6809952
rs11715853
rs8039785
rs78383039
rs55877868
rs823424
rs7311004
All
−0.3
−0.2
−0.1
0.0
0.1
MR effect size for
'Gut microbiota abundance (genus Parasutterella id.2892)' on 'Protein FAM107B'

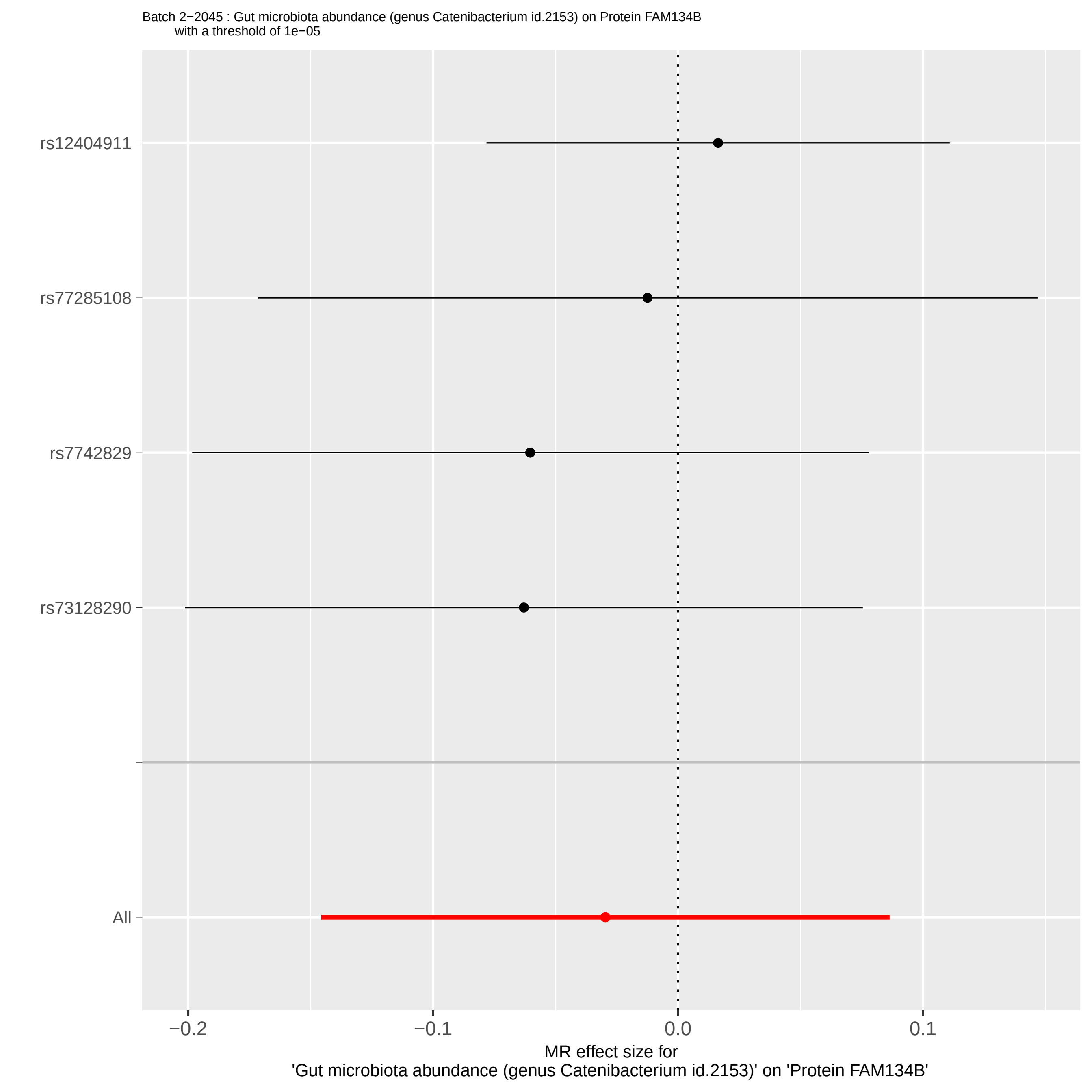
Batch 2−2045 : Gut microbiota abundance (genus Catenibacterium id.2153) on Protein FAM134B
with a threshold of 1e−05
rs12404911
rs77285108
rs7742829
rs73128290
All
−0.2
−0.1
0.0
0.1
MR effect size for
'Gut microbiota abundance (genus Catenibacterium id.2153)' on 'Protein FAM134B'

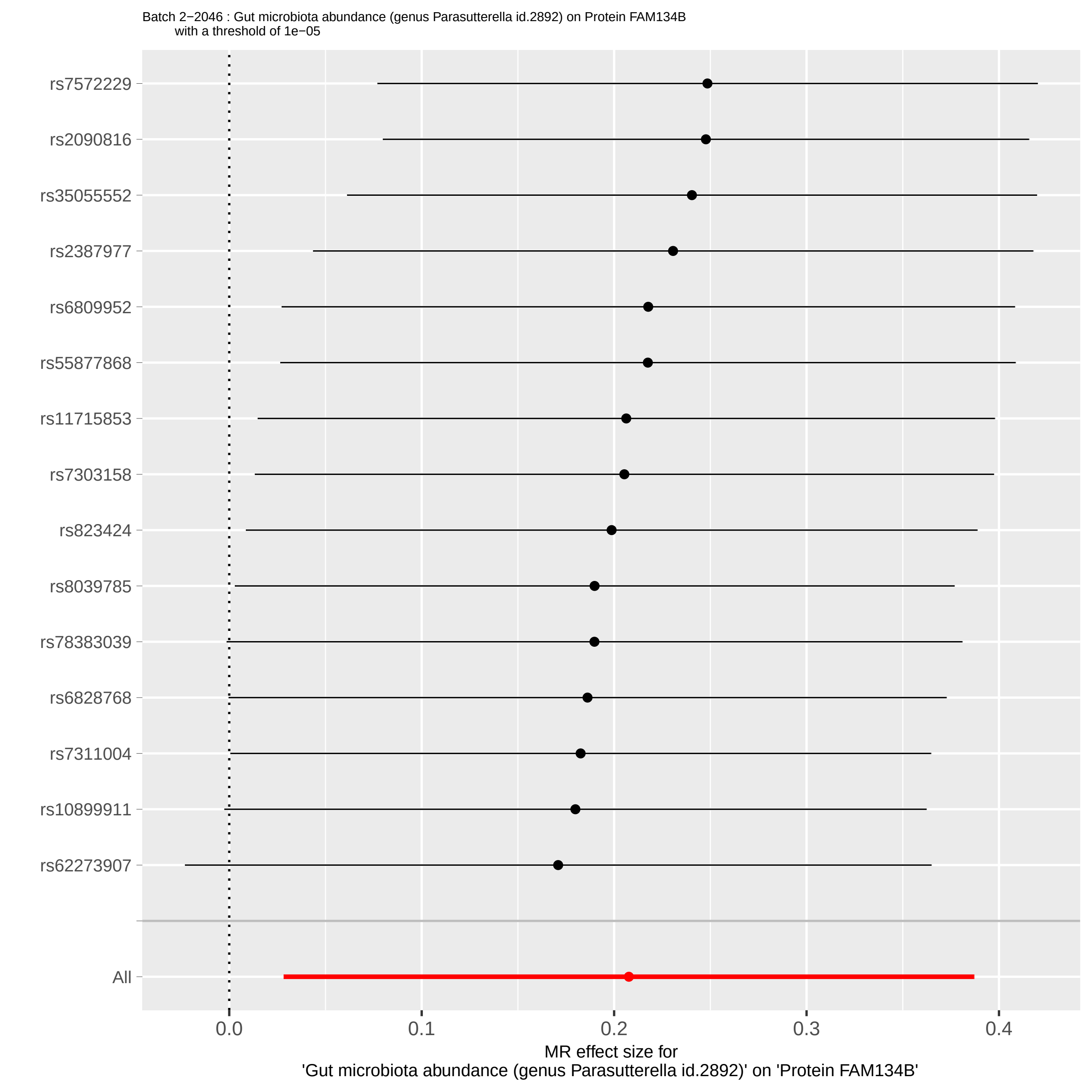

Batch 2−2046 : Gut microbiota abundance (genus Parasutterella id.2892) on Protein FAM134B
with a threshold of 1e−05
rs7572229
rs2090816
rs35055552
rs2387977
rs6809952
rs55877868
rs11715853
rs7303158
rs823424
rs8039785
rs78383039
rs6828768
rs7311004
rs10899911
rs62273907
All
0.0
0.1
0.2
0.3
0.4
MR effect size for
'Gut microbiota abundance (genus Parasutterella id.2892)' on 'Protein FAM134B'

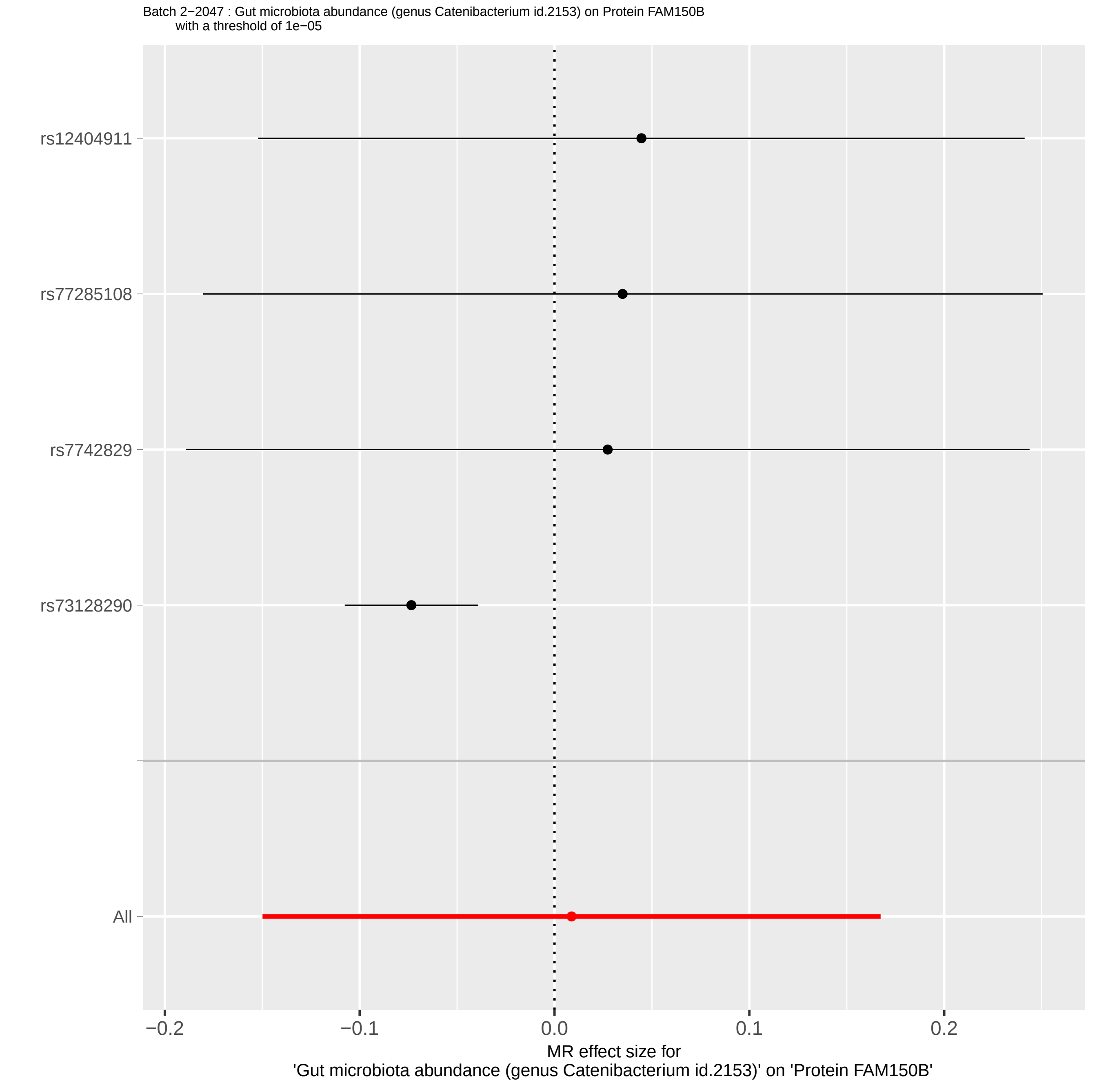

Batch 2−2047 : Gut microbiota abundance (genus Catenibacterium id.2153) on Protein FAM150B
with a threshold of 1e−05
rs12404911
rs77285108
rs7742829
rs73128290
All
−0.2
−0.1
0.0
0.1
0.2
MR effect size for
'Gut microbiota abundance (genus Catenibacterium id.2153)' on 'Protein FAM150B'

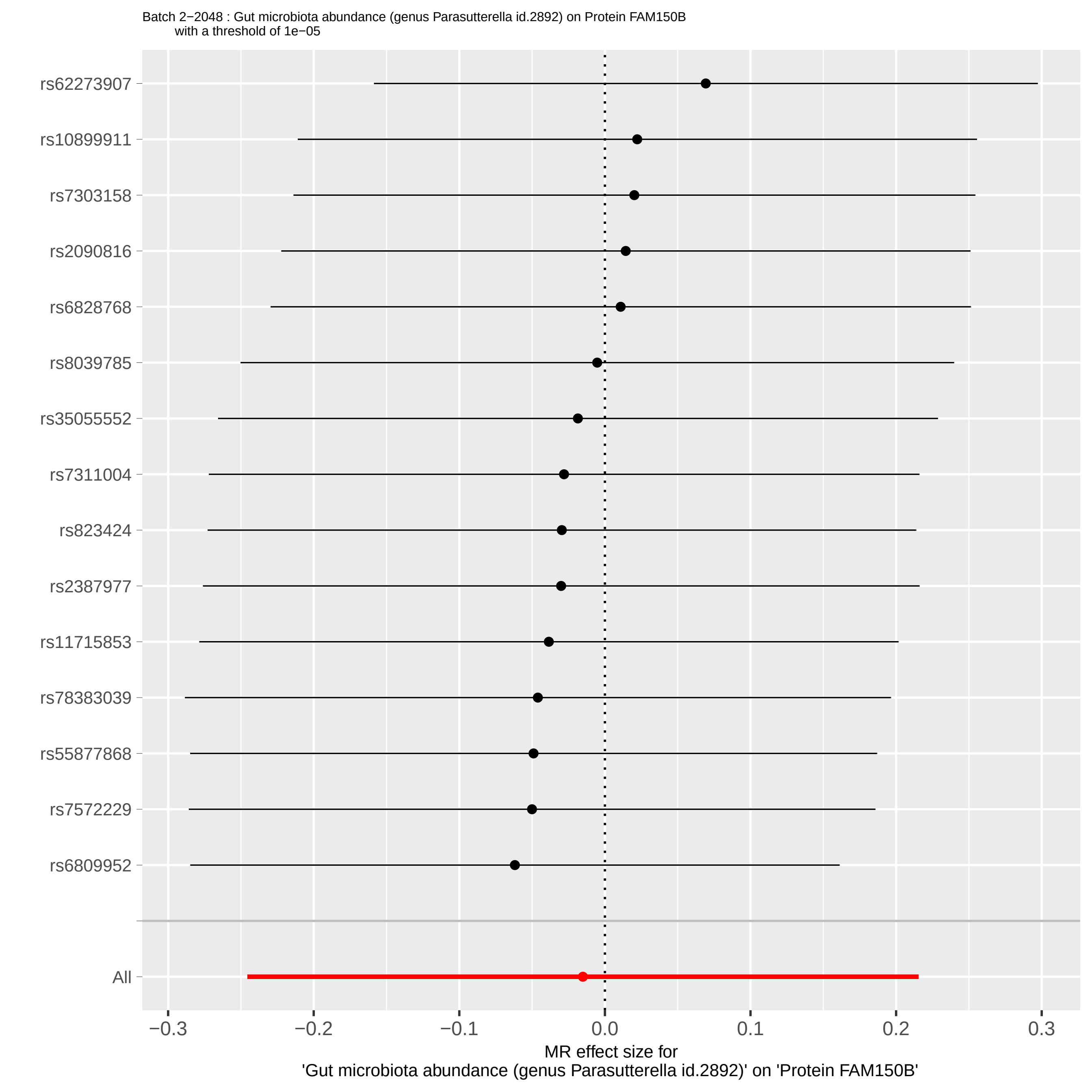

Batch 2−2048 : Gut microbiota abundance (genus Parasutterella id.2892) on Protein FAM150B
with a threshold of 1e−05
rs62273907
rs10899911
rs7303158
rs2090816
rs6828768
rs8039785
rs35055552
rs7311004
rs823424
rs2387977
rs11715853
rs78383039
rs55877868
rs7572229
rs6809952
All
−0.3
−0.2
−0.1
0.0
0.1
0.2
0.3
MR effect size for
'Gut microbiota abundance (genus Parasutterella id.2892)' on 'Protein FAM150B'

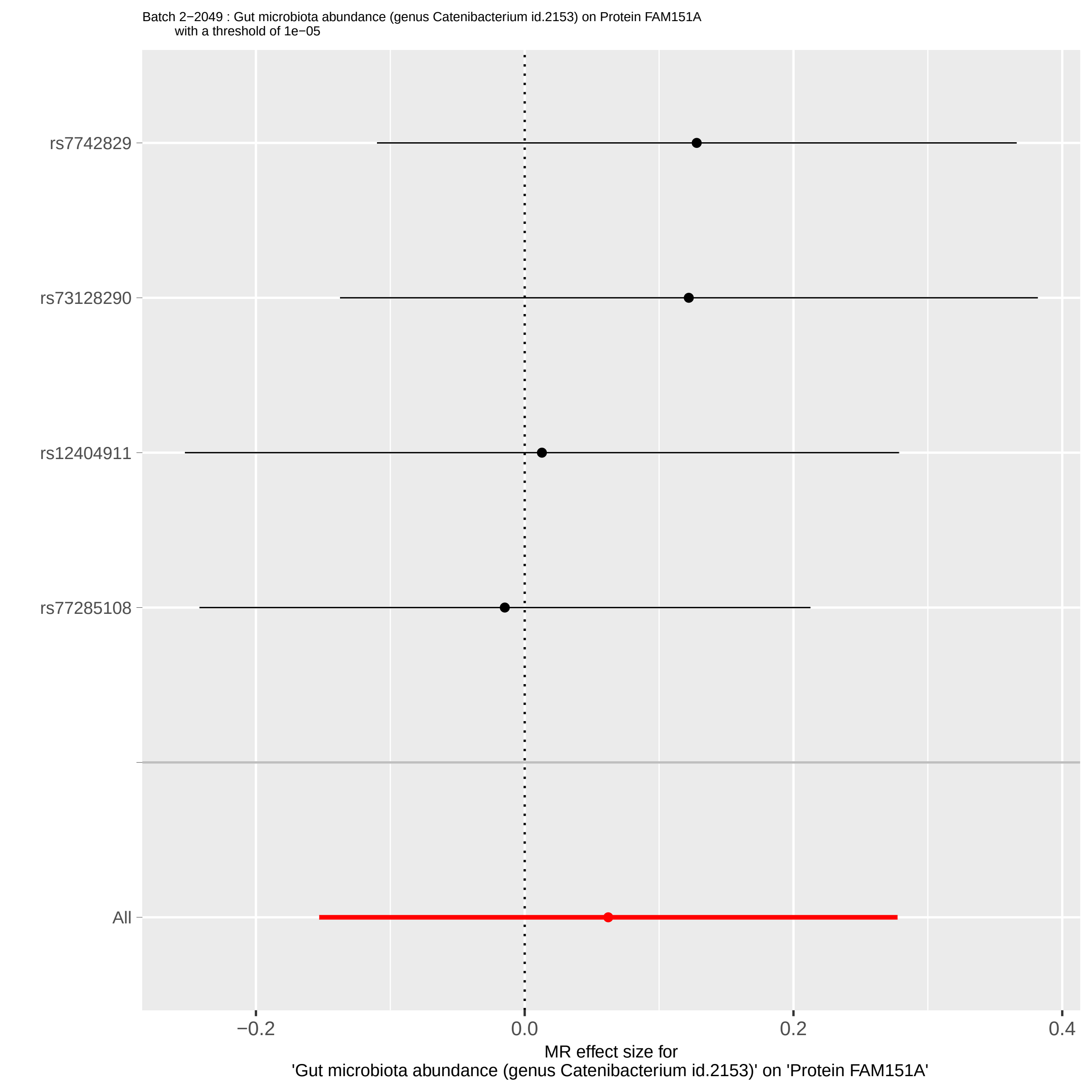

Batch 2−2049 : Gut microbiota abundance (genus Catenibacterium id.2153) on Protein FAM151A
with a threshold of 1e−05
rs7742829
rs73128290
rs12404911
rs77285108
All
−0.2
0.0
0.2
0.4
MR effect size for
'Gut microbiota abundance (genus Catenibacterium id.2153)' on 'Protein FAM151A'

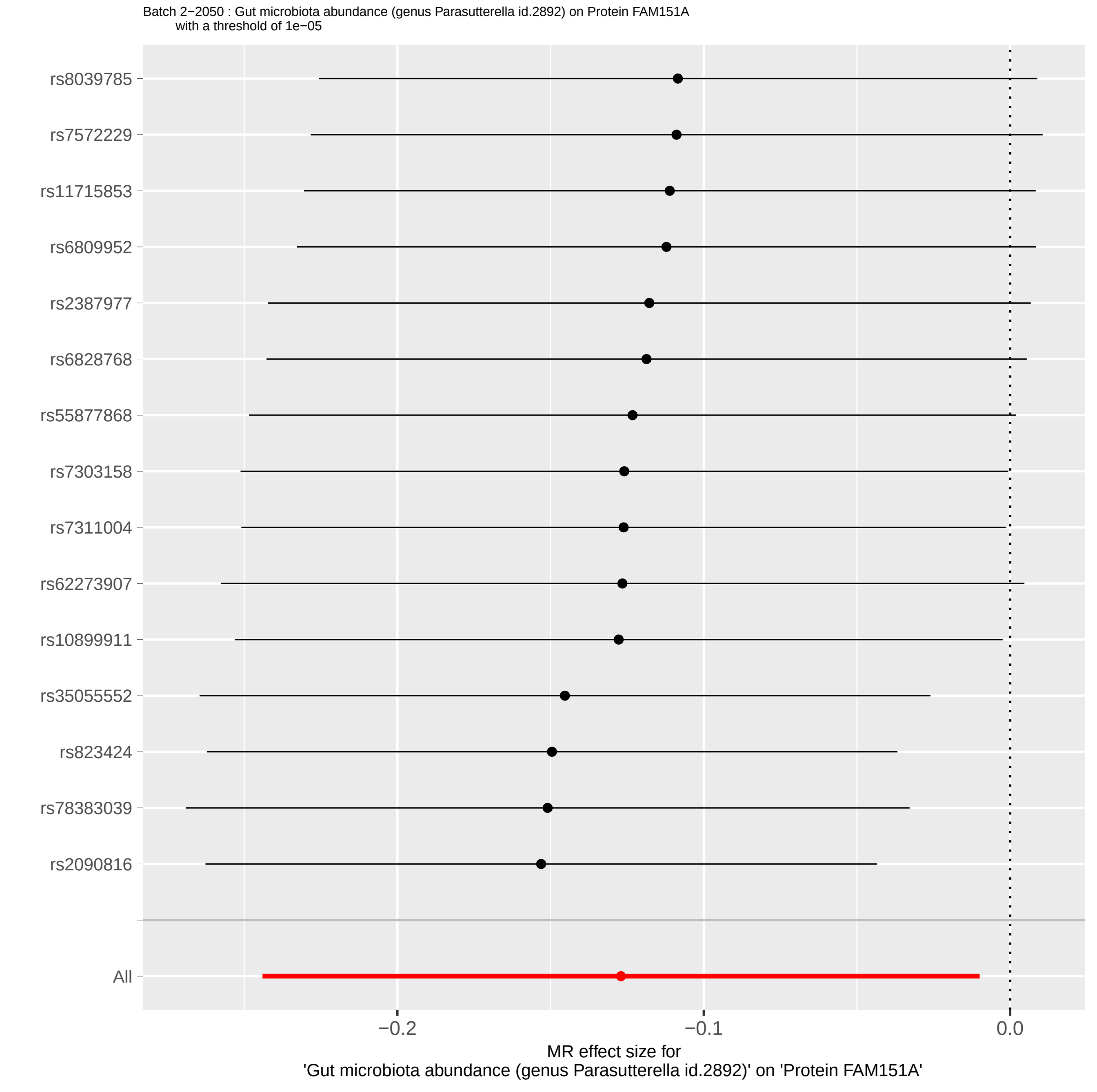

Batch 2−2050 : Gut microbiota abundance (genus Parasutterella id.2892) on Protein FAM151A
with a threshold of 1e−05
rs8039785
rs7572229
rs11715853
rs6809952
rs2387977
rs6828768
rs55877868
rs7303158
rs7311004
rs62273907
rs10899911
rs35055552
rs823424
rs78383039
rs2090816
All
−0.2
−0.1
0.0
MR effect size for
'Gut microbiota abundance (genus Parasutterella id.2892)' on 'Protein FAM151A'

Batch 2−2051 : Gut microbiota abundance (genus Catenibacterium id.2153) on Membrane protein FAM159B
with a threshold of 1e−05

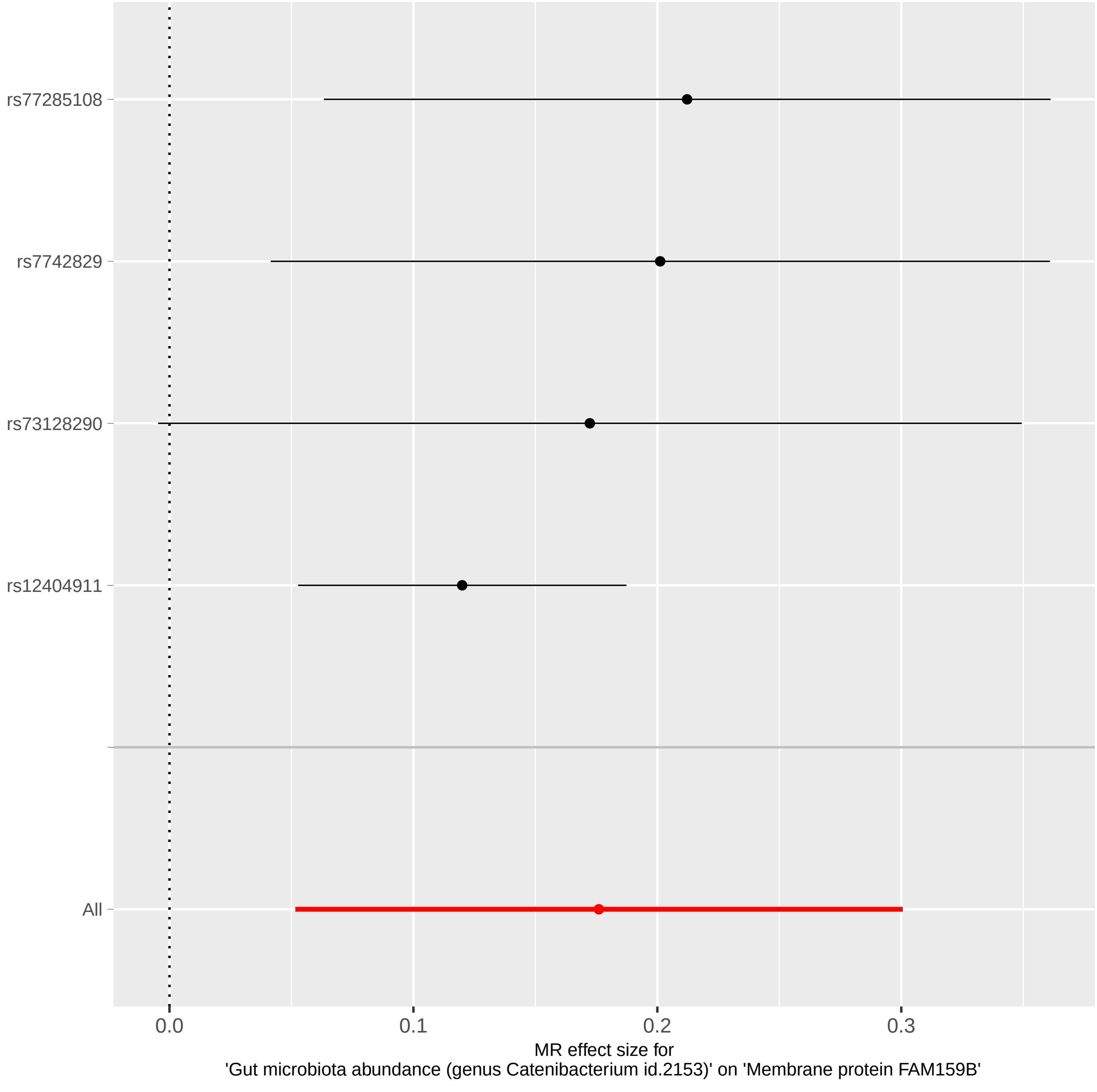

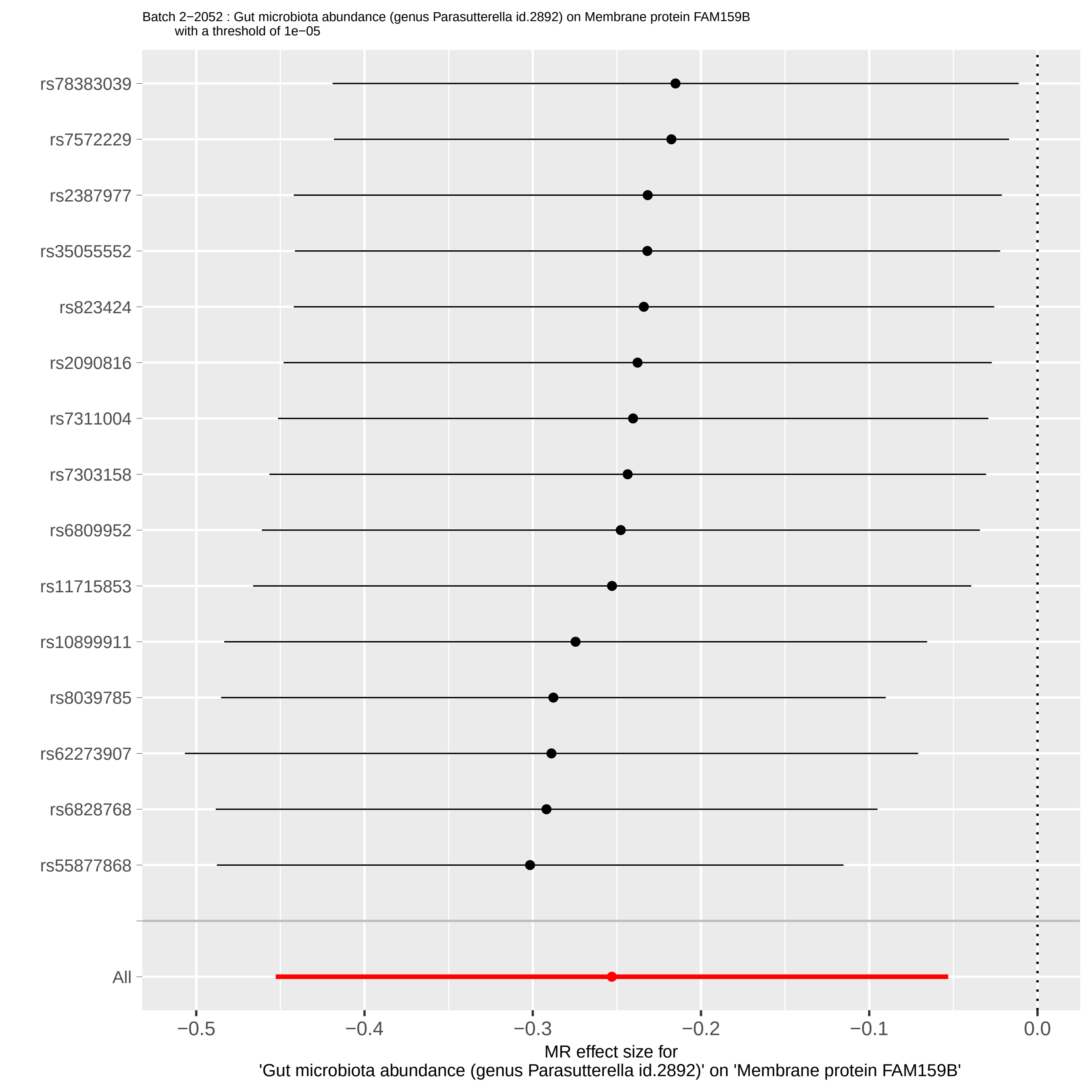

Batch 2−2052 : Gut microbiota abundance (genus Parasutterella id.2892) on Membrane protein FAM159B
with a threshold of 1e−05
rs78383039
rs7572229
rs2387977
rs35055552
rs823424
rs2090816
rs7311004
rs7303158
rs6809952
rs11715853
rs10899911
rs8039785
rs62273907
rs6828768
rs55877868
All
−0.5
−0.4
−0.3
−0.2
−0.1
0.0
MR effect size for
'Gut microbiota abundance (genus Parasutterella id.2892)' on 'Membrane protein FAM159B'

Batch 2−2053 : Gut microbiota abundance (genus Catenibacterium id.2153) on Protein FAM163A
with a threshold of 1e−05

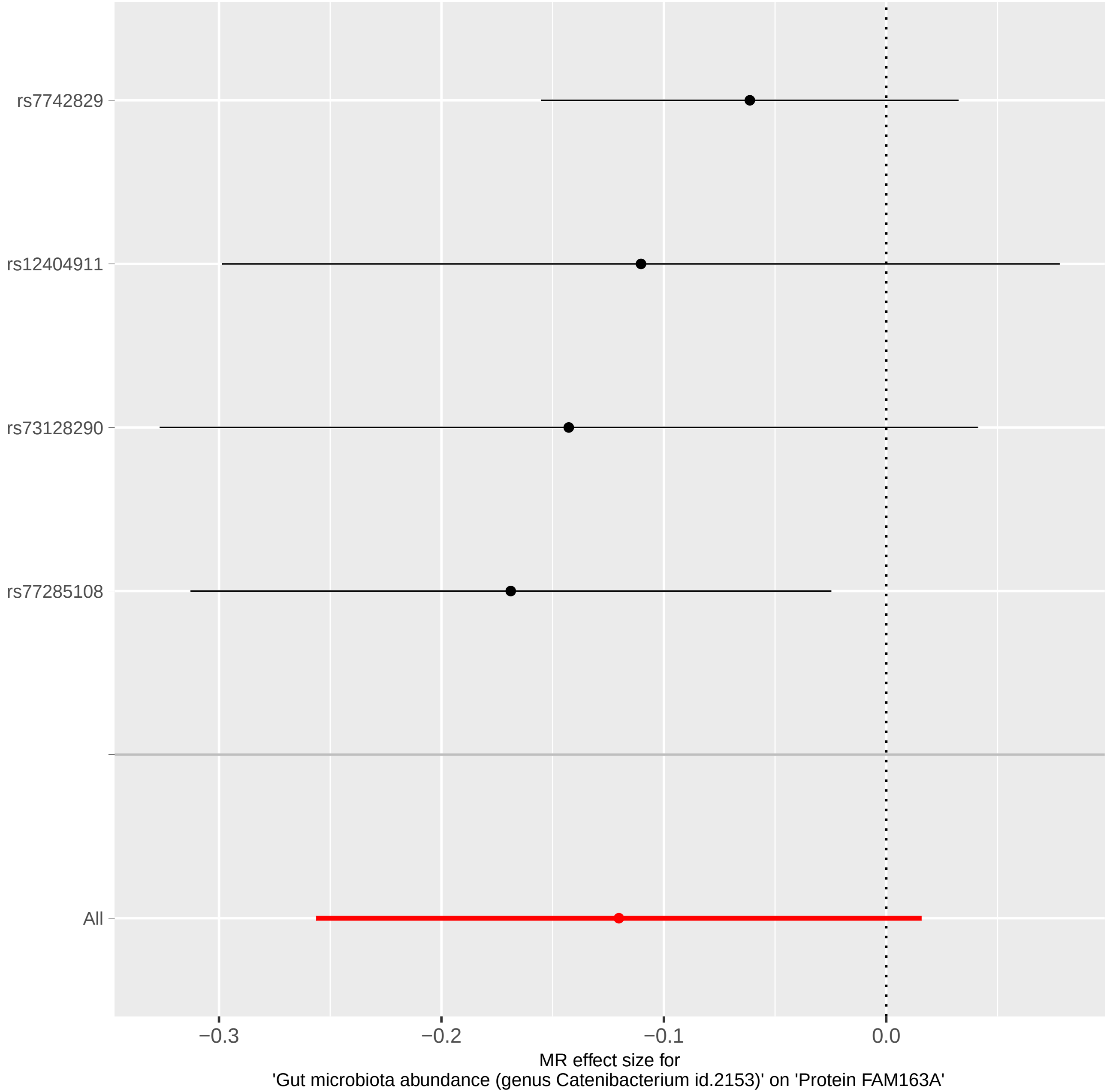

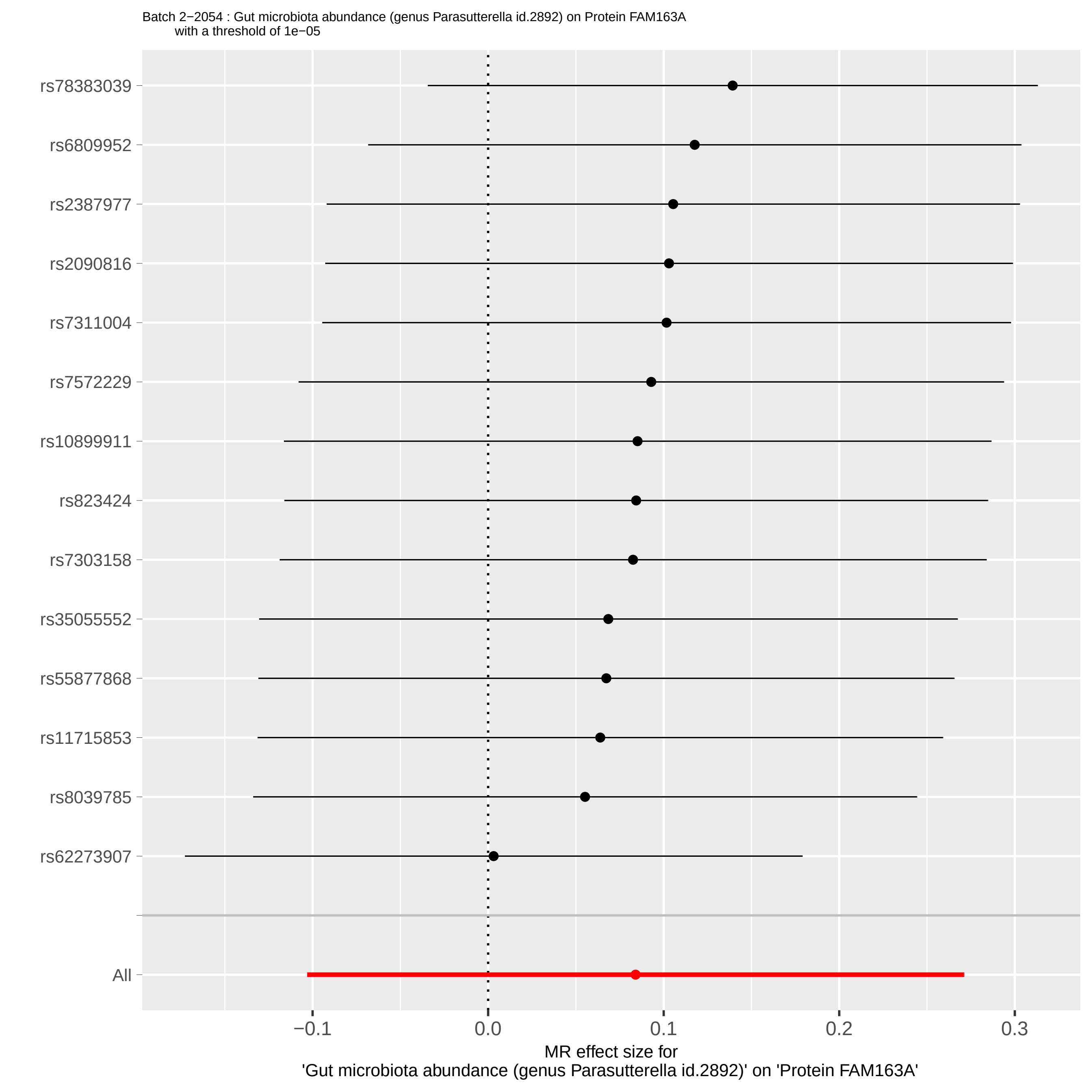

Batch 2−2054 : Gut microbiota abundance (genus Parasutterella id.2892) on Protein FAM163A
with a threshold of 1e−05
rs78383039
rs6809952
rs2387977
rs2090816
rs7311004
rs7572229
rs10899911
rs823424
rs7303158
rs35055552
rs55877868
rs11715853
rs8039785
rs62273907
All
−0.1
0.0
0.1
0.2
0.3
MR effect size for
'Gut microbiota abundance (genus Parasutterella id.2892)' on 'Protein FAM163A'

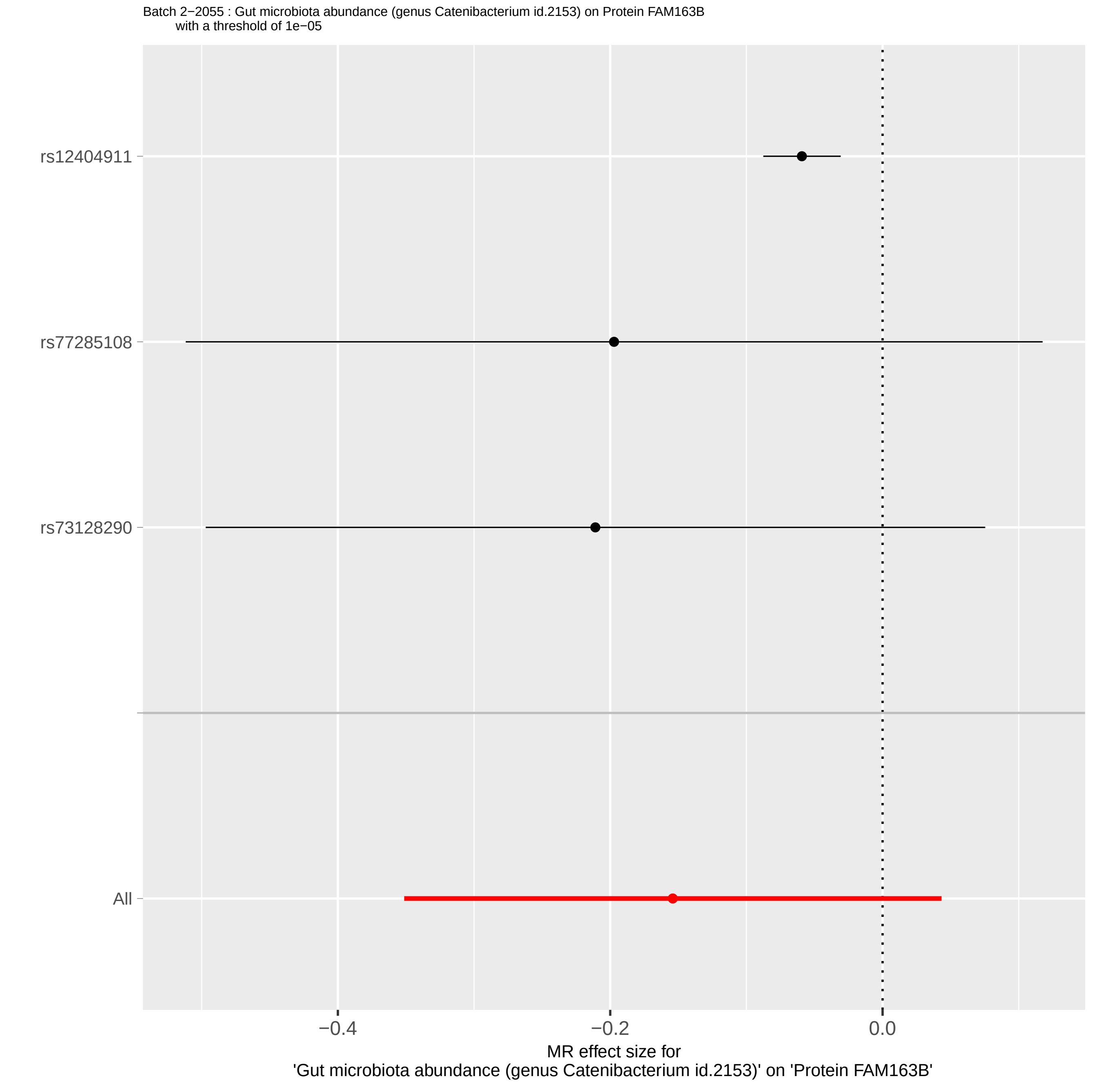
Batch 2−2055 : Gut microbiota abundance (genus Catenibacterium id.2153) on Protein FAM163B
with a threshold of 1e−05
rs12404911
rs77285108
rs73128290
All
−0.4
−0.2
0.0
MR effect size for
'Gut microbiota abundance (genus Catenibacterium id.2153)' on 'Protein FAM163B'

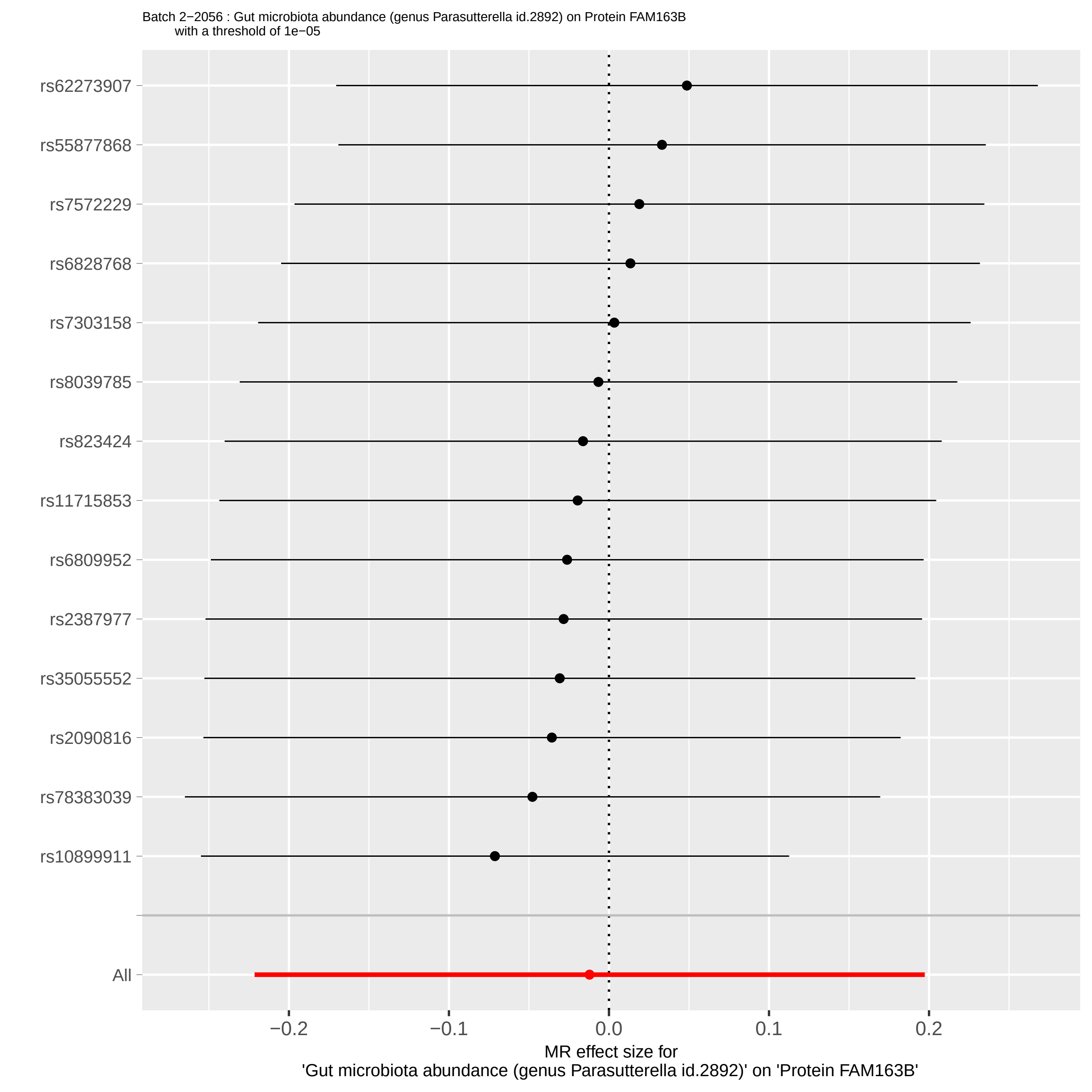

Batch 2−2056 : Gut microbiota abundance (genus Parasutterella id.2892) on Protein FAM163B
with a threshold of 1e−05
rs62273907
rs55877868
rs7572229
rs6828768
rs7303158
rs8039785
rs823424
rs11715853
rs6809952
rs2387977
rs35055552
rs2090816
rs78383039
rs10899911
All
−0.2
−0.1
0.0
0.1
0.2
MR effect size for
'Gut microbiota abundance (genus Parasutterella id.2892)' on 'Protein FAM163B'

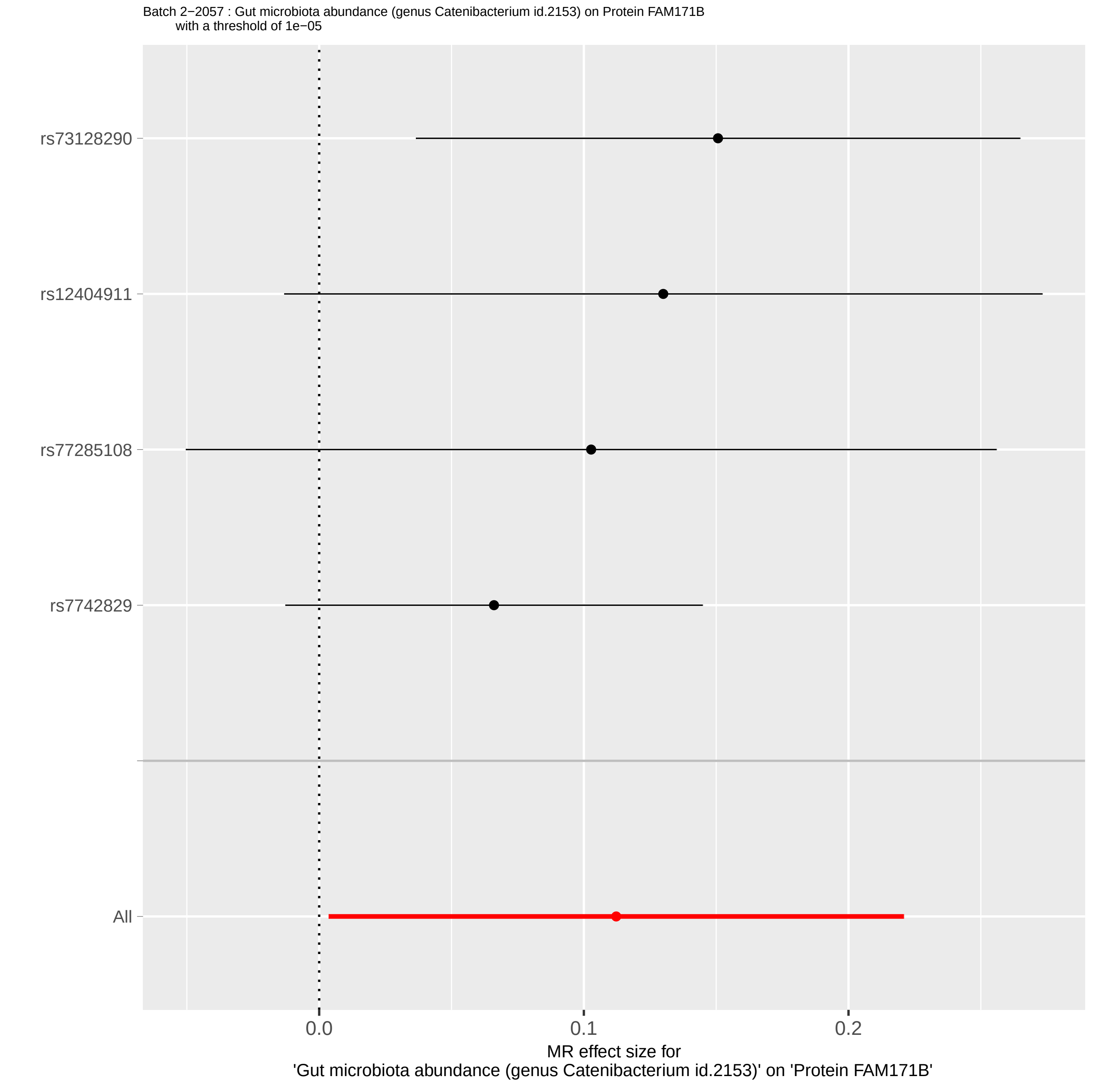
Batch 2−2057 : Gut microbiota abundance (genus Catenibacterium id.2153) on Protein FAM171B
with a threshold of 1e−05
rs73128290
rs12404911
rs77285108
rs7742829
All
0.0
0.1
0.2
MR effect size for
'Gut microbiota abundance (genus Catenibacterium id.2153)' on 'Protein FAM171B'

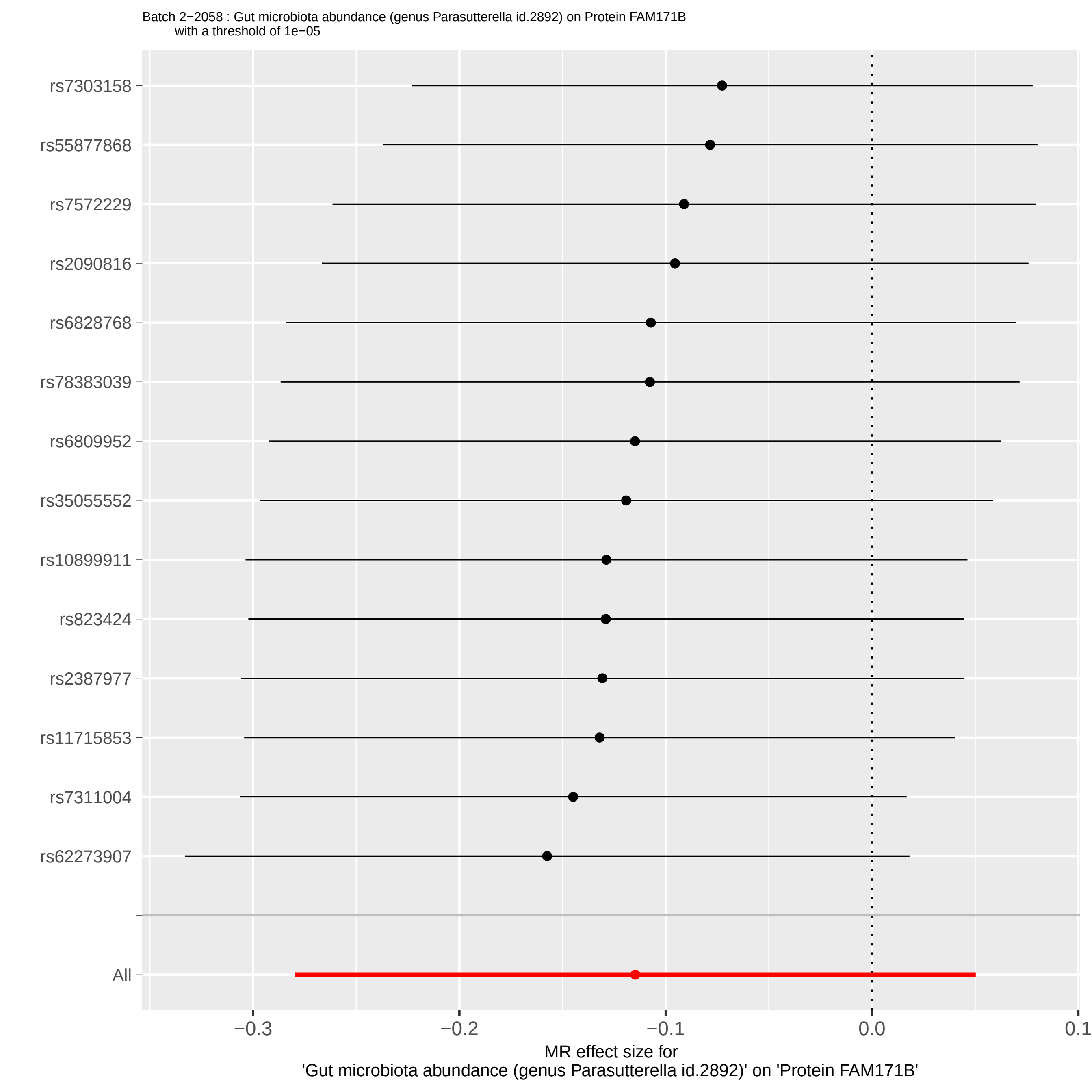

Batch 2−2058 : Gut microbiota abundance (genus Parasutterella id.2892) on Protein FAM171B
with a threshold of 1e−05
rs7303158
rs55877868
rs7572229
rs2090816
rs6828768
rs78383039
rs6809952
rs35055552
rs10899911
rs823424
rs2387977
rs11715853
rs7311004
rs62273907
All
−0.3
−0.2
−0.1
0.0
0.1
MR effect size for
'Gut microbiota abundance (genus Parasutterella id.2892)' on 'Protein FAM171B'

Batch 2−2059 : Gut microbiota abundance (genus Catenibacterium id.2153) on Protein FAM171B
with a threshold of 1e−05

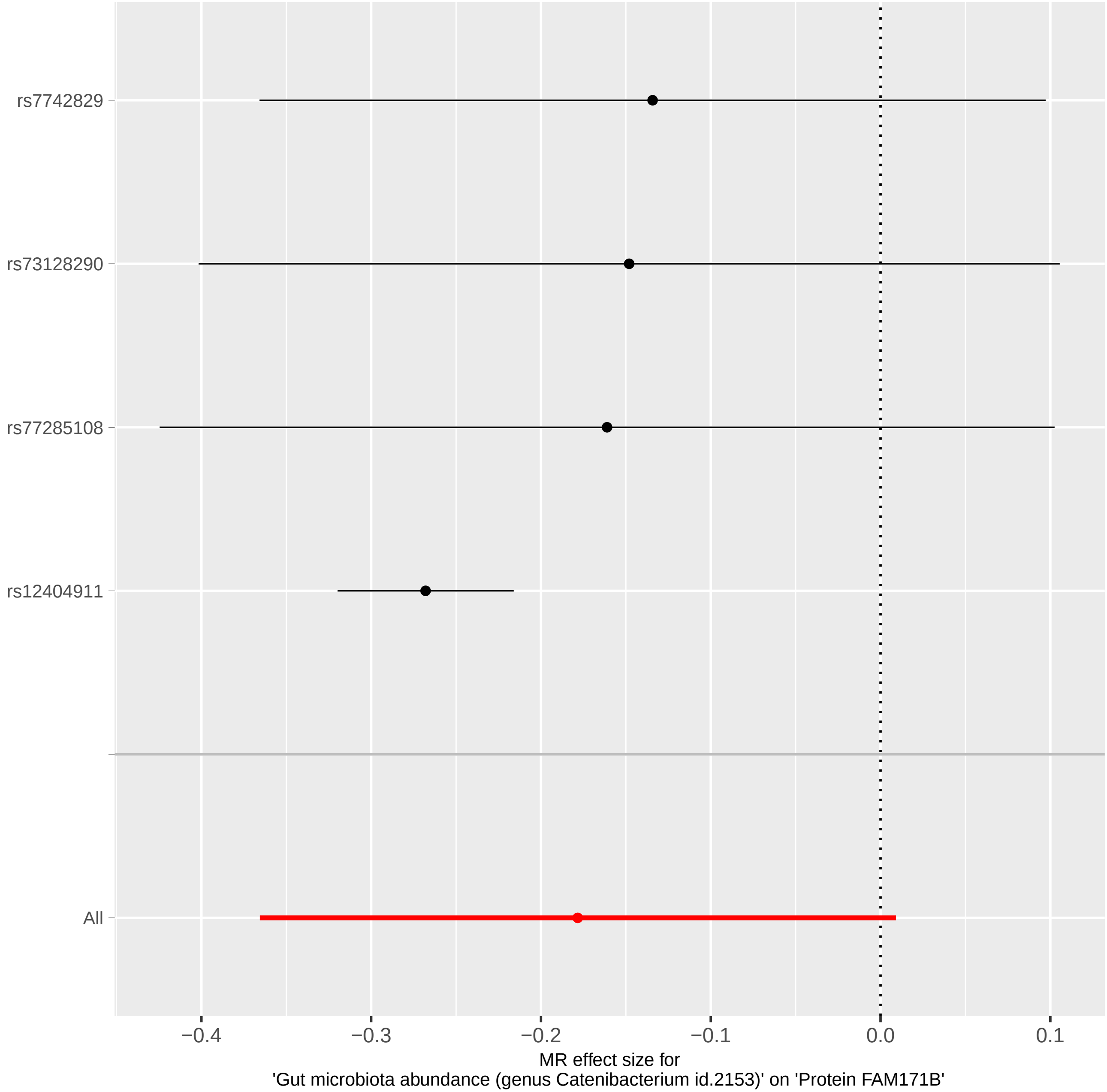

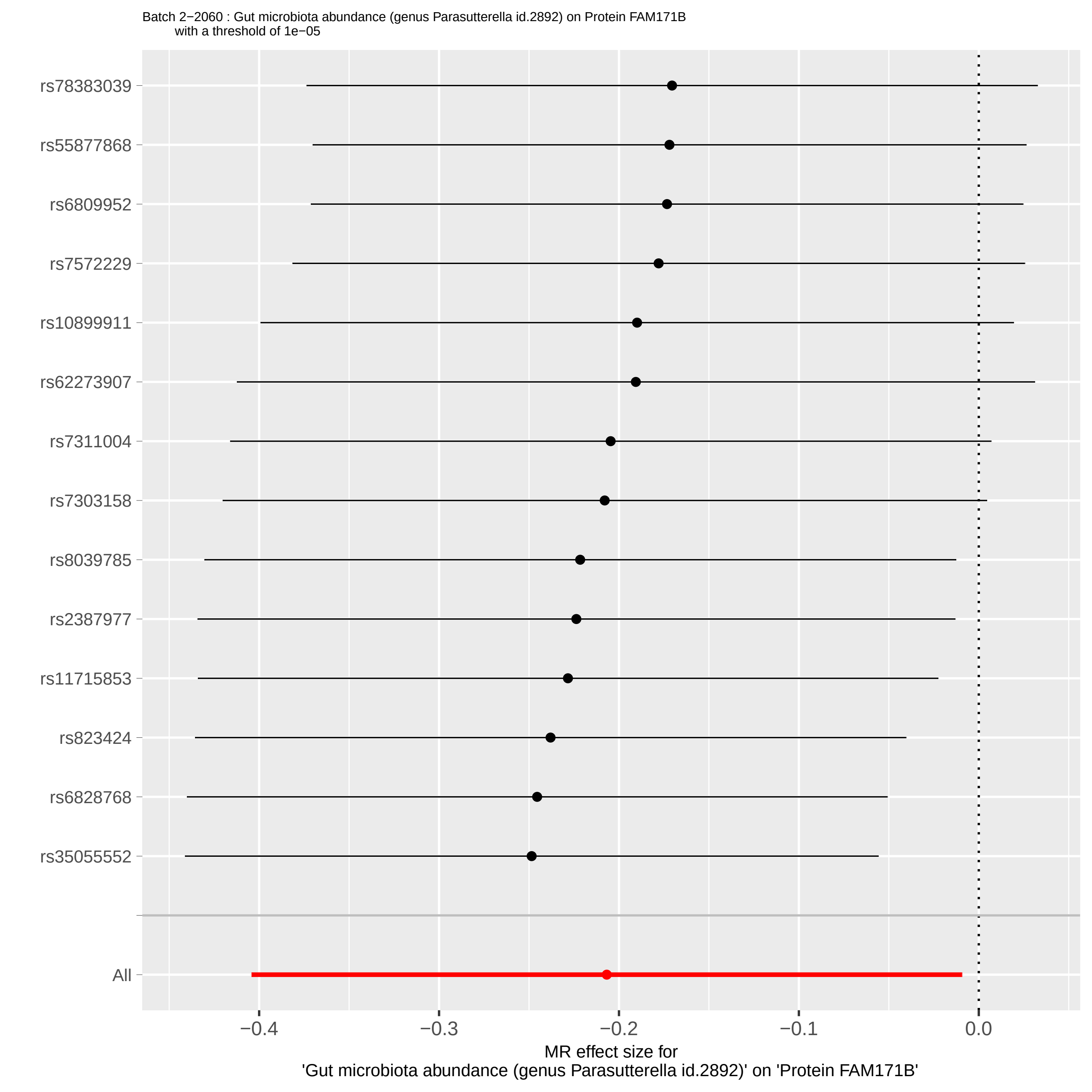
Batch 2−2060 : Gut microbiota abundance (genus Parasutterella id.2892) on Protein FAM171B
with a threshold of 1e−05
rs78383039
rs55877868
rs6809952
rs7572229
rs10899911
rs62273907
rs7311004
rs7303158
rs8039785
rs2387977
rs11715853
rs823424
rs6828768
rs35055552
All
−0.4
−0.3
−0.2
−0.1
0.0
MR effect size for
'Gut microbiota abundance (genus Parasutterella id.2892)' on 'Protein FAM171B'

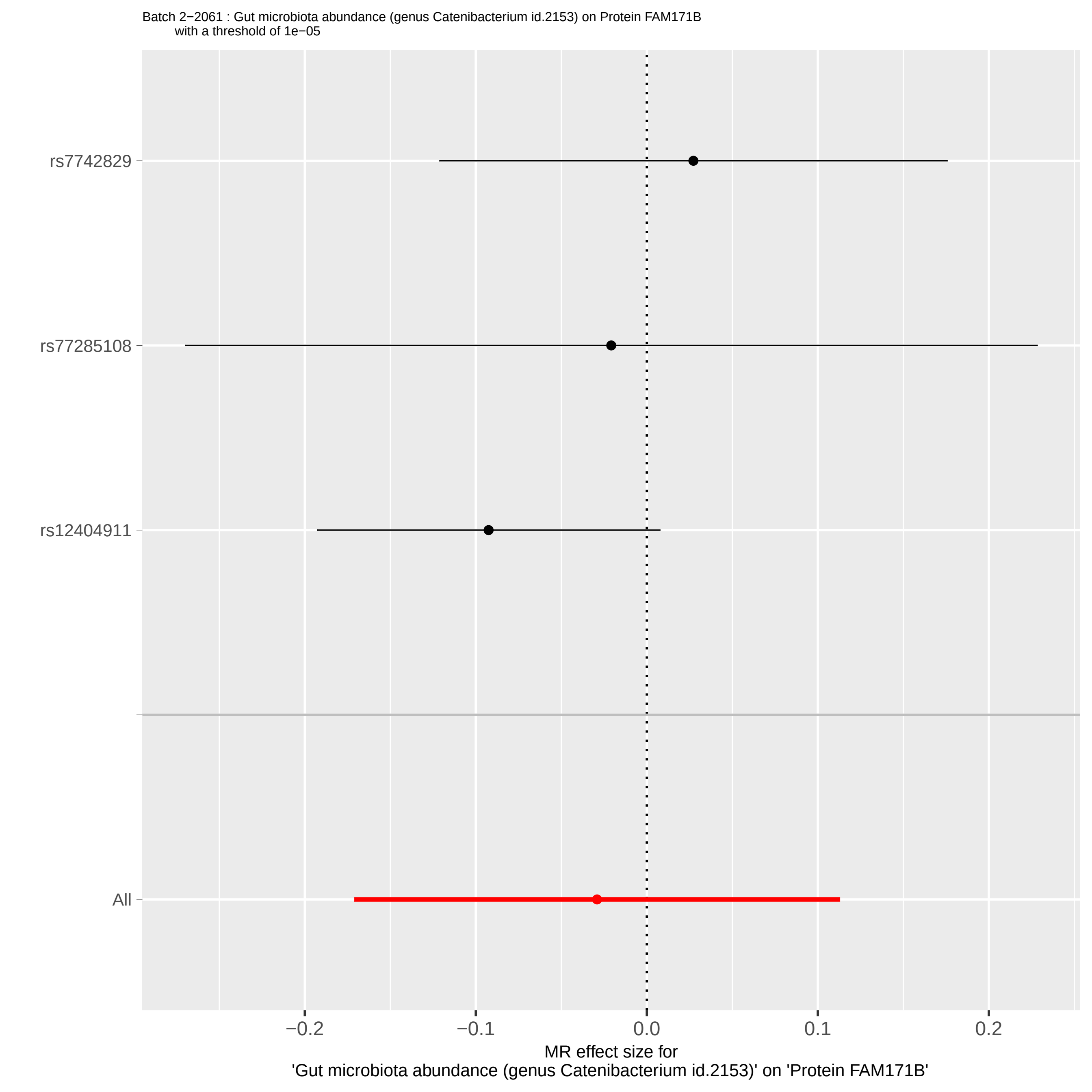

Batch 2−2061 : Gut microbiota abundance (genus Catenibacterium id.2153) on Protein FAM171B
with a threshold of 1e−05
rs7742829
rs77285108
rs12404911
All
−0.2
−0.1
0.0
0.1
0.2
MR effect size for
'Gut microbiota abundance (genus Catenibacterium id.2153)' on 'Protein FAM171B'

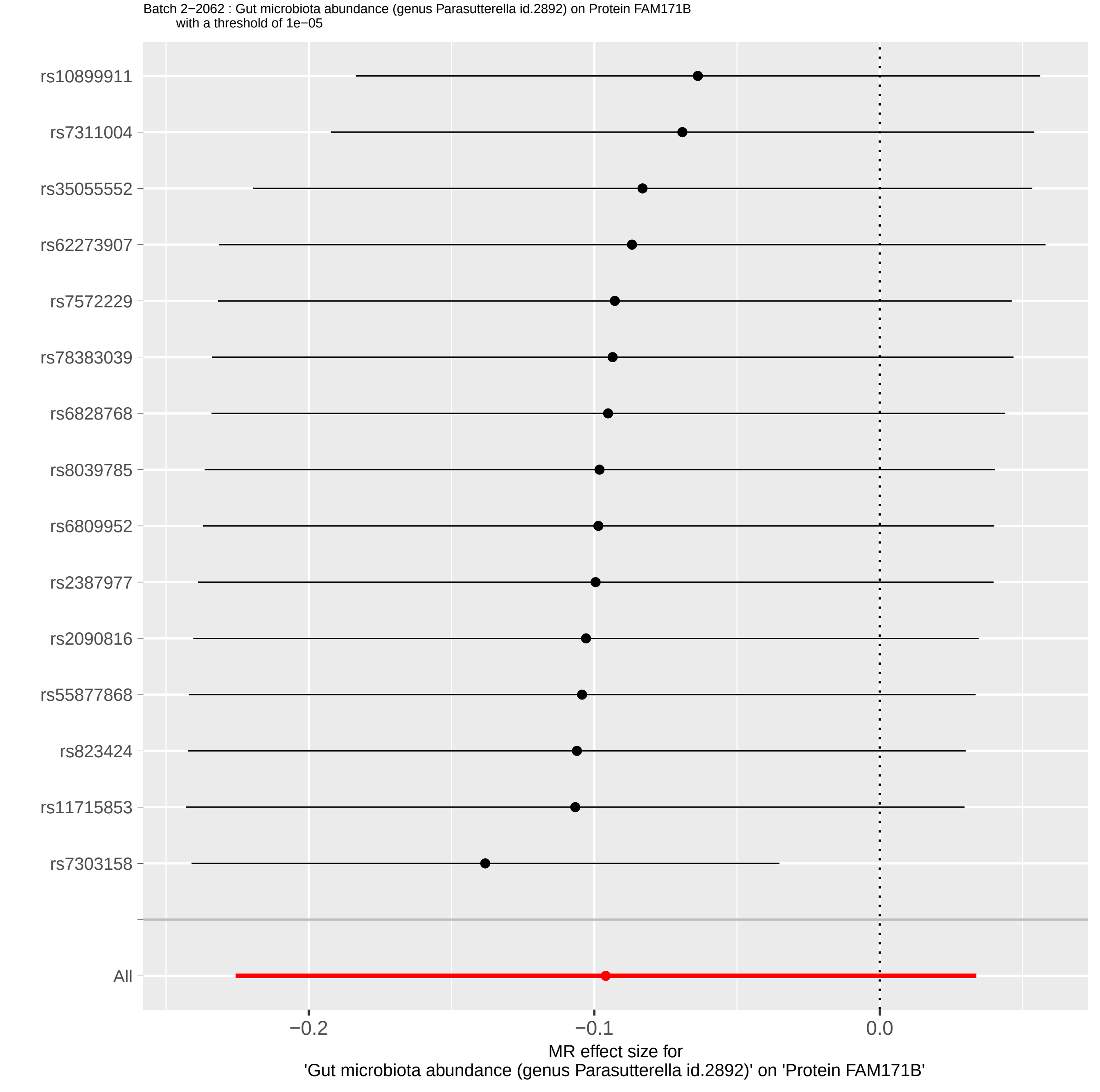
Batch 2−2062 : Gut microbiota abundance (genus Parasutterella id.2892) on Protein FAM171B
with a threshold of 1e−05
rs10899911
rs7311004
rs35055552
rs62273907
rs7572229
rs78383039
rs6828768
rs8039785
rs6809952
rs2387977
rs2090816
rs55877868
rs823424
rs11715853
rs7303158
All
−0.2
−0.1
0.0
MR effect size for
'Gut microbiota abundance (genus Parasutterella id.2892)' on 'Protein FAM171B'

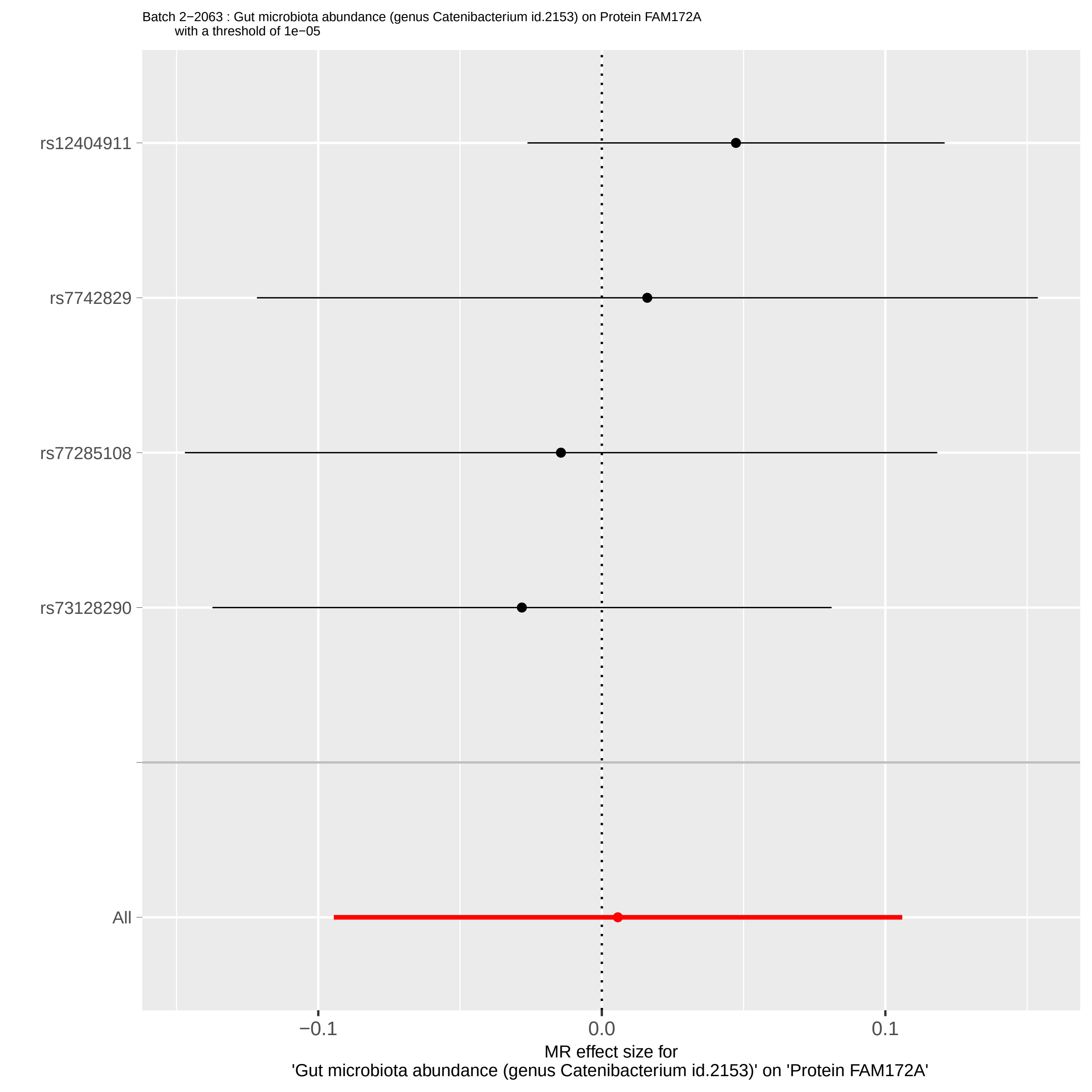
Batch 2−2063 : Gut microbiota abundance (genus Catenibacterium id.2153) on Protein FAM172A
with a threshold of 1e−05
rs12404911
rs7742829
rs77285108
rs73128290
All
−0.1
0.0
0.1
MR effect size for
'Gut microbiota abundance (genus Catenibacterium id.2153)' on 'Protein FAM172A'

Batch 2−2064 : Gut microbiota abundance (genus Parasutterella id.2892) on Protein FAM172A
with a threshold of 1e−05

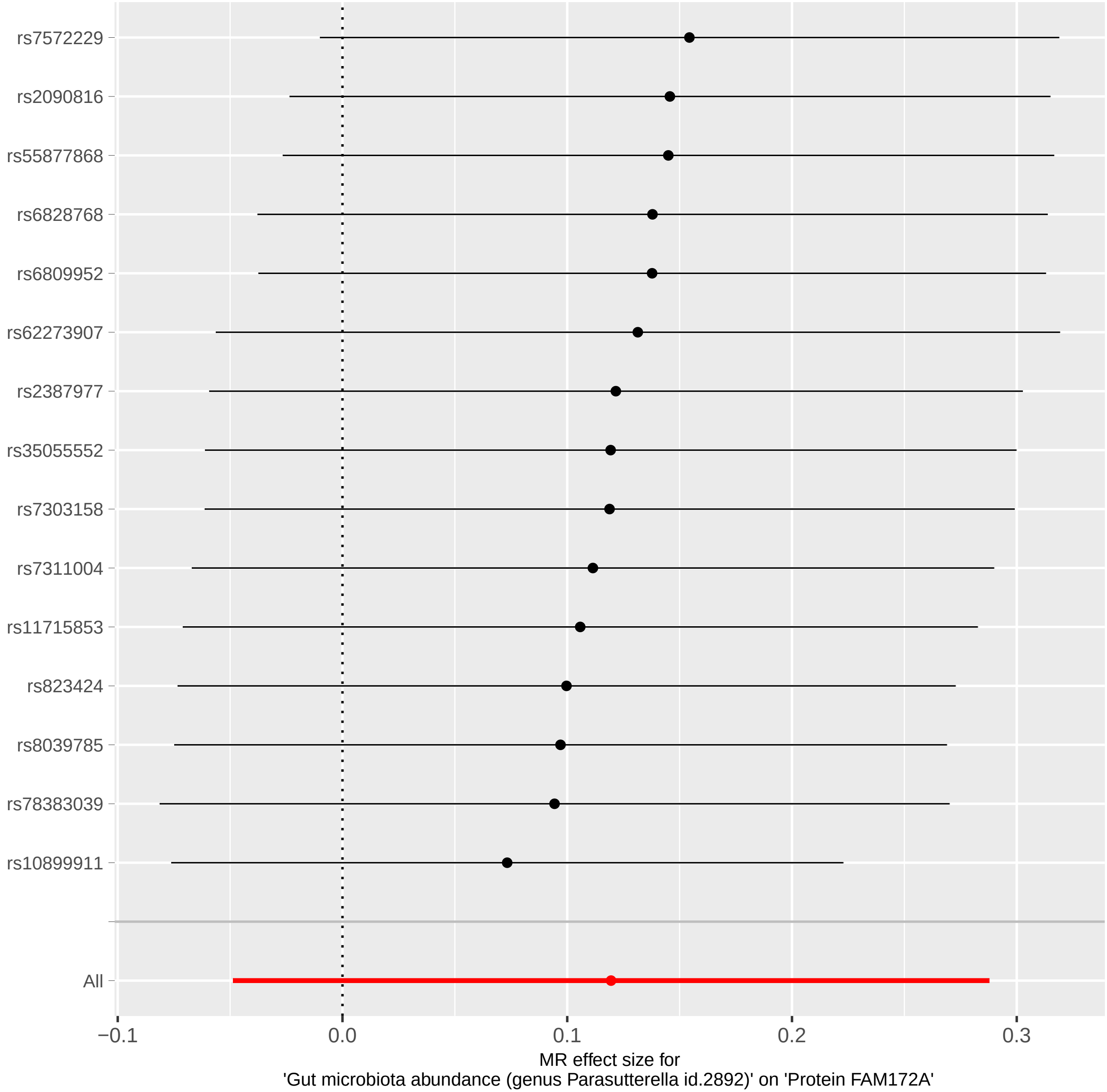

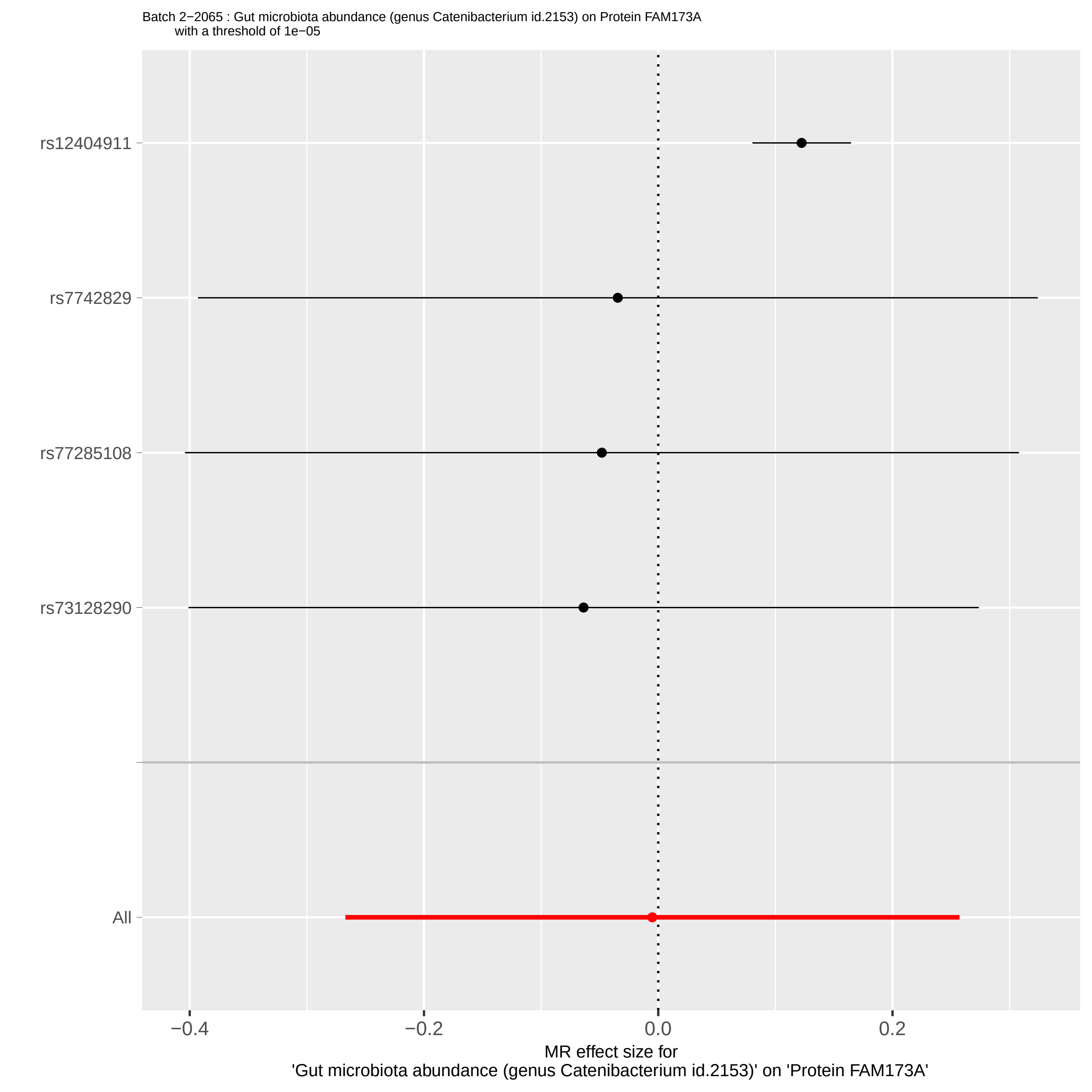
Batch 2−2065 : Gut microbiota abundance (genus Catenibacterium id.2153) on Protein FAM173A
with a threshold of 1e−05
rs12404911
rs7742829
rs77285108
rs73128290
All
−0.4
−0.2
0.0
0.2
MR effect size for
'Gut microbiota abundance (genus Catenibacterium id.2153)' on 'Protein FAM173A'

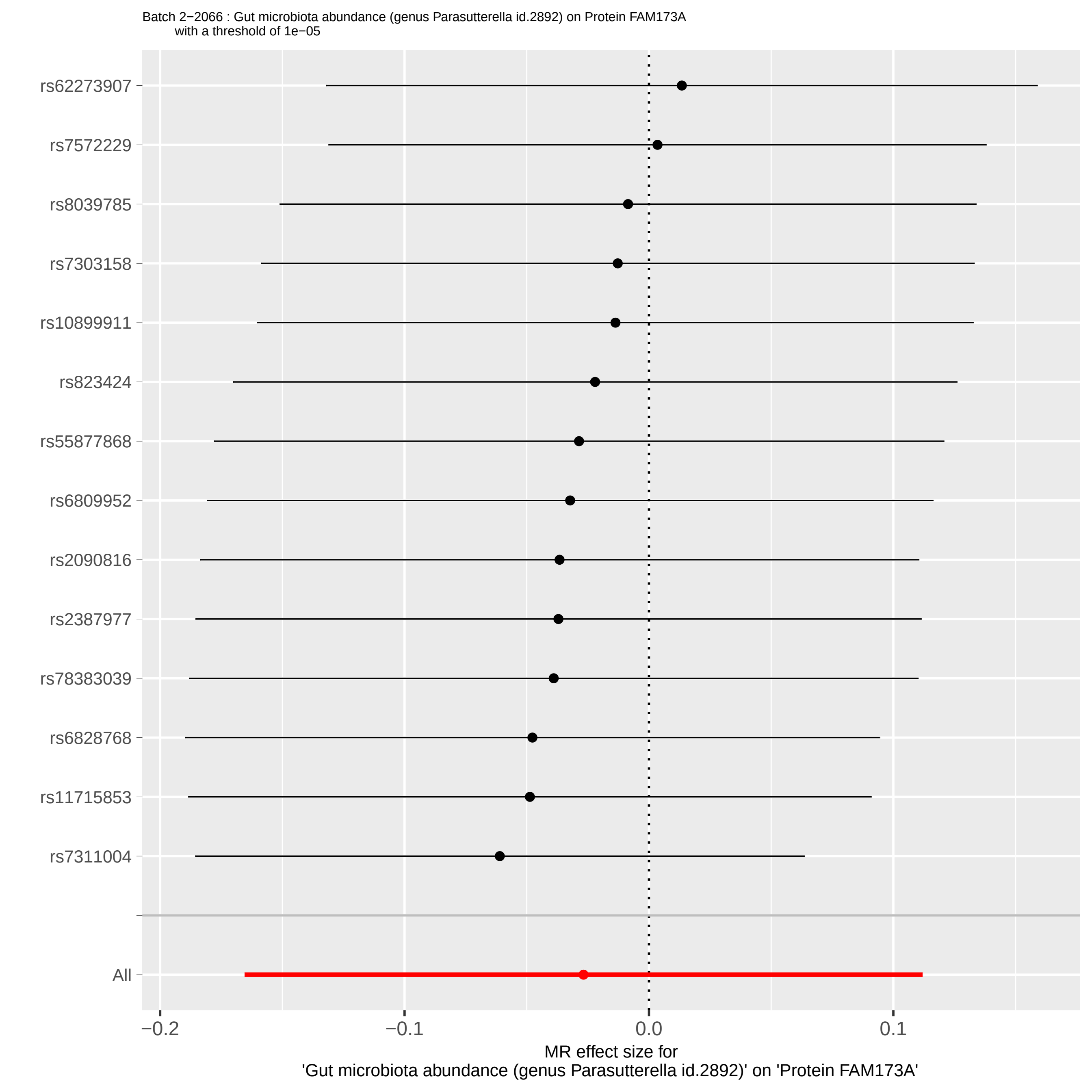
Batch 2−2066 : Gut microbiota abundance (genus Parasutterella id.2892) on Protein FAM173A
with a threshold of 1e−05
rs62273907
rs7572229
rs8039785
rs7303158
rs10899911
rs823424
rs55877868
rs6809952
rs2090816
rs2387977
rs78383039
rs6828768
rs11715853
rs7311004
All
−0.2
−0.1
0.0
0.1
MR effect size for
'Gut microbiota abundance (genus Parasutterella id.2892)' on 'Protein FAM173A'

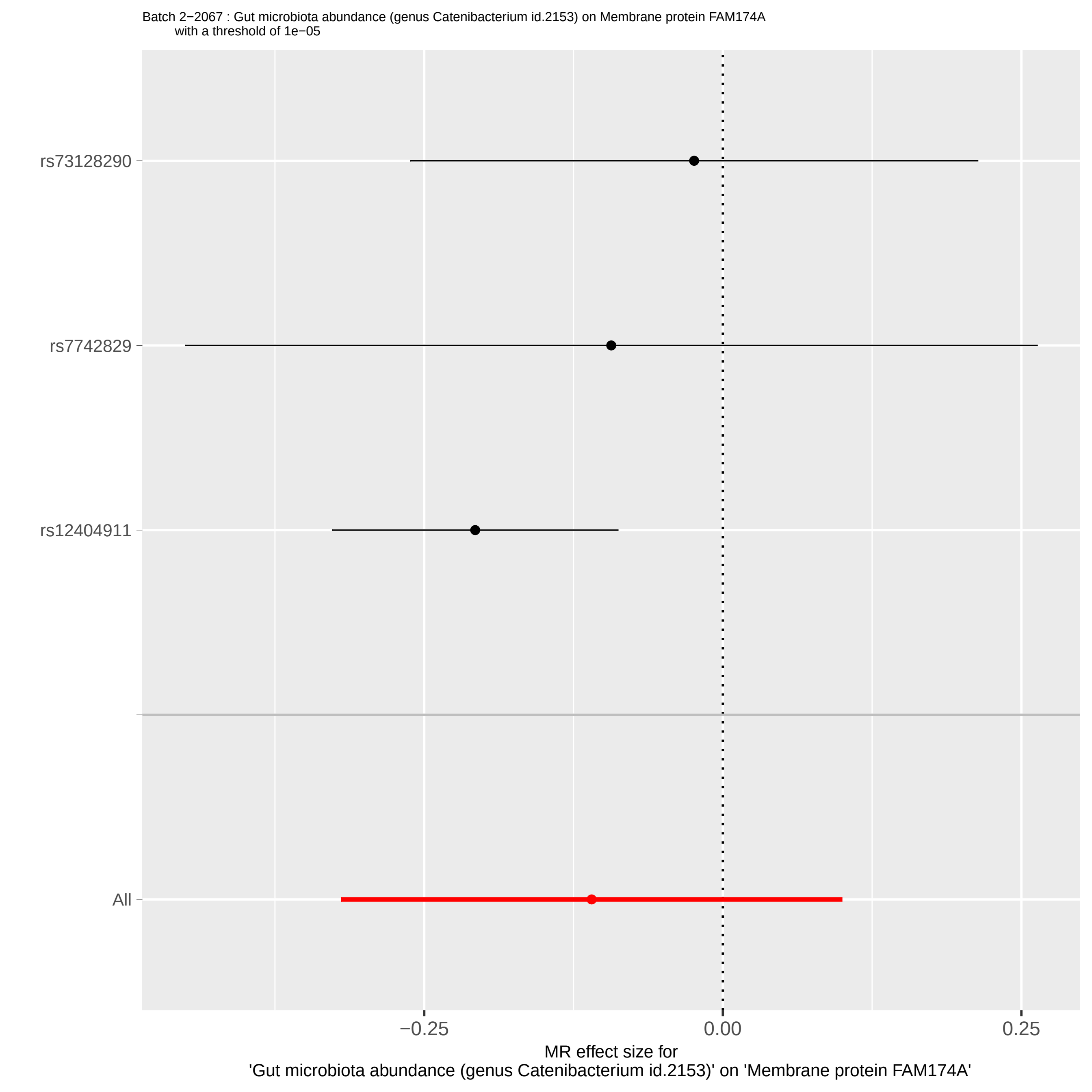

Batch 2−2067 : Gut microbiota abundance (genus Catenibacterium id.2153) on Membrane protein FAM174A
with a threshold of 1e−05
rs73128290
rs7742829
rs12404911
All
−0.25
0.00
0.25
MR effect size for
'Gut microbiota abundance (genus Catenibacterium id.2153)' on 'Membrane protein FAM174A'

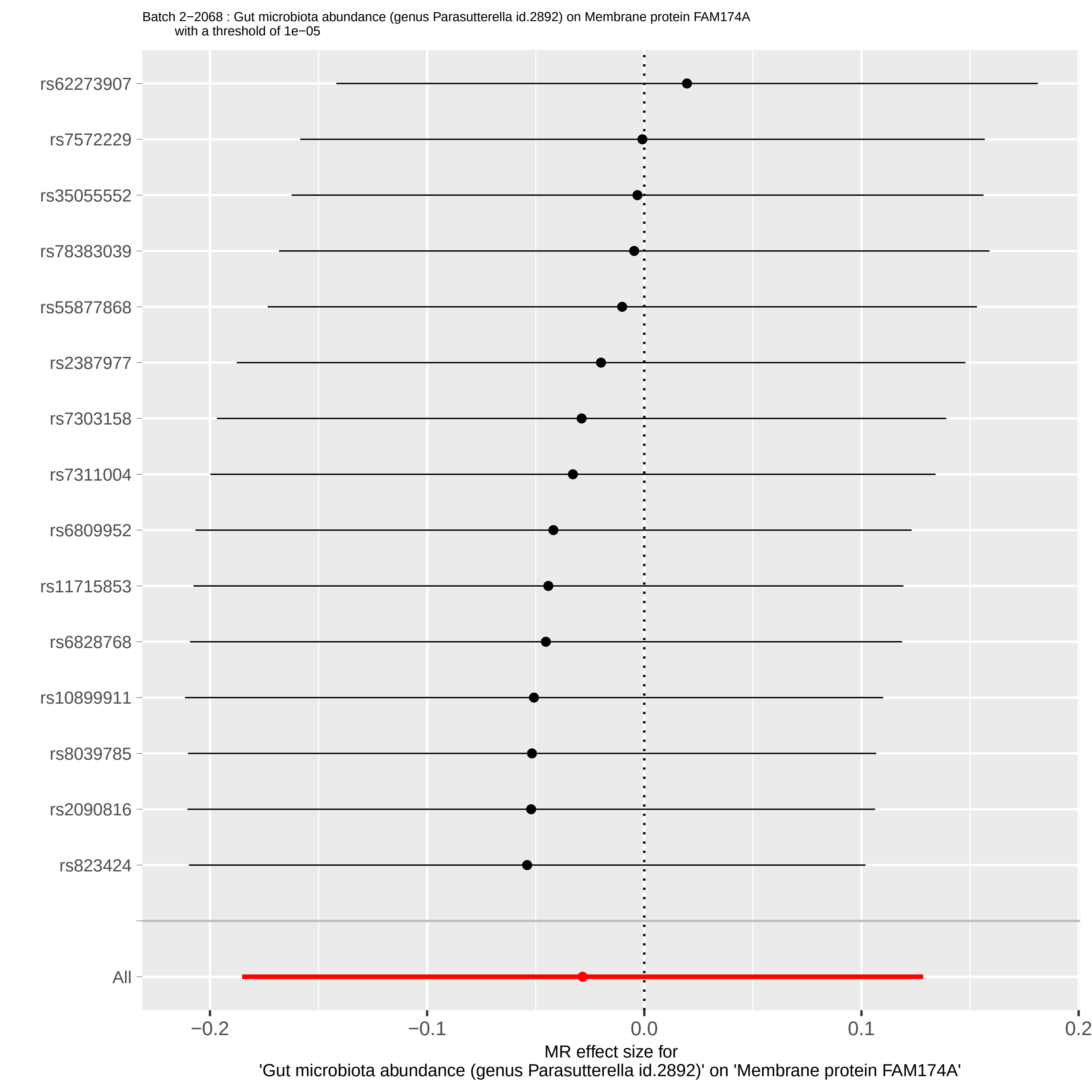
Batch 2−2068 : Gut microbiota abundance (genus Parasutterella id.2892) on Membrane protein FAM174A
with a threshold of 1e−05
rs62273907
rs7572229
rs35055552
rs78383039
rs55877868
rs2387977
rs7303158
rs7311004
rs6809952
rs11715853
rs6828768
rs10899911
rs8039785
rs2090816
rs823424
All
−0.2
−0.1
0.0
0.1
0.2
MR effect size for
'Gut microbiota abundance (genus Parasutterella id.2892)' on 'Membrane protein FAM174A'

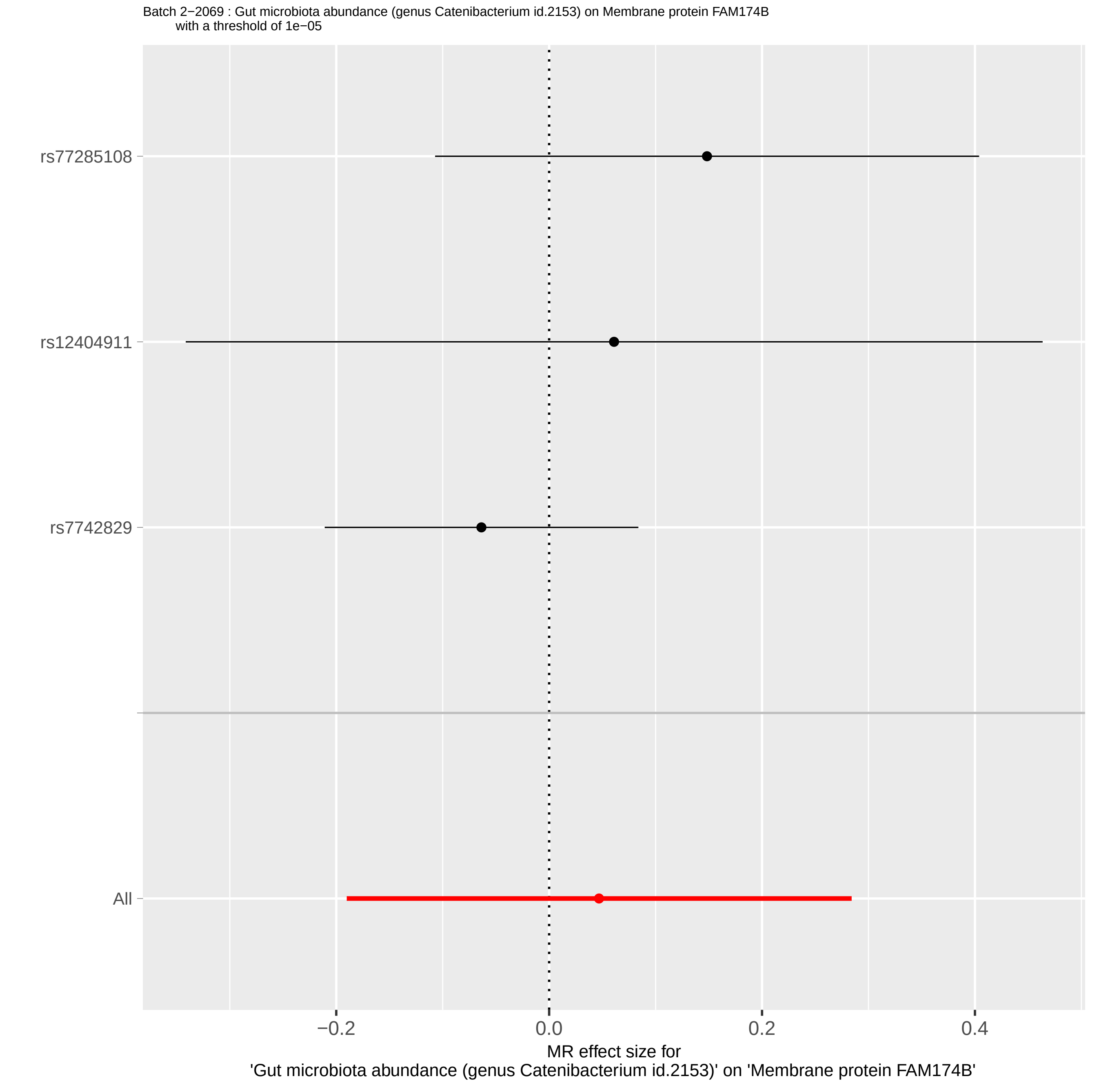

Batch 2−2069 : Gut microbiota abundance (genus Catenibacterium id.2153) on Membrane protein FAM174B
with a threshold of 1e−05
rs77285108
rs12404911
rs7742829
All
−0.2
0.0
0.2
0.4
MR effect size for
'Gut microbiota abundance (genus Catenibacterium id.2153)' on 'Membrane protein FAM174B'

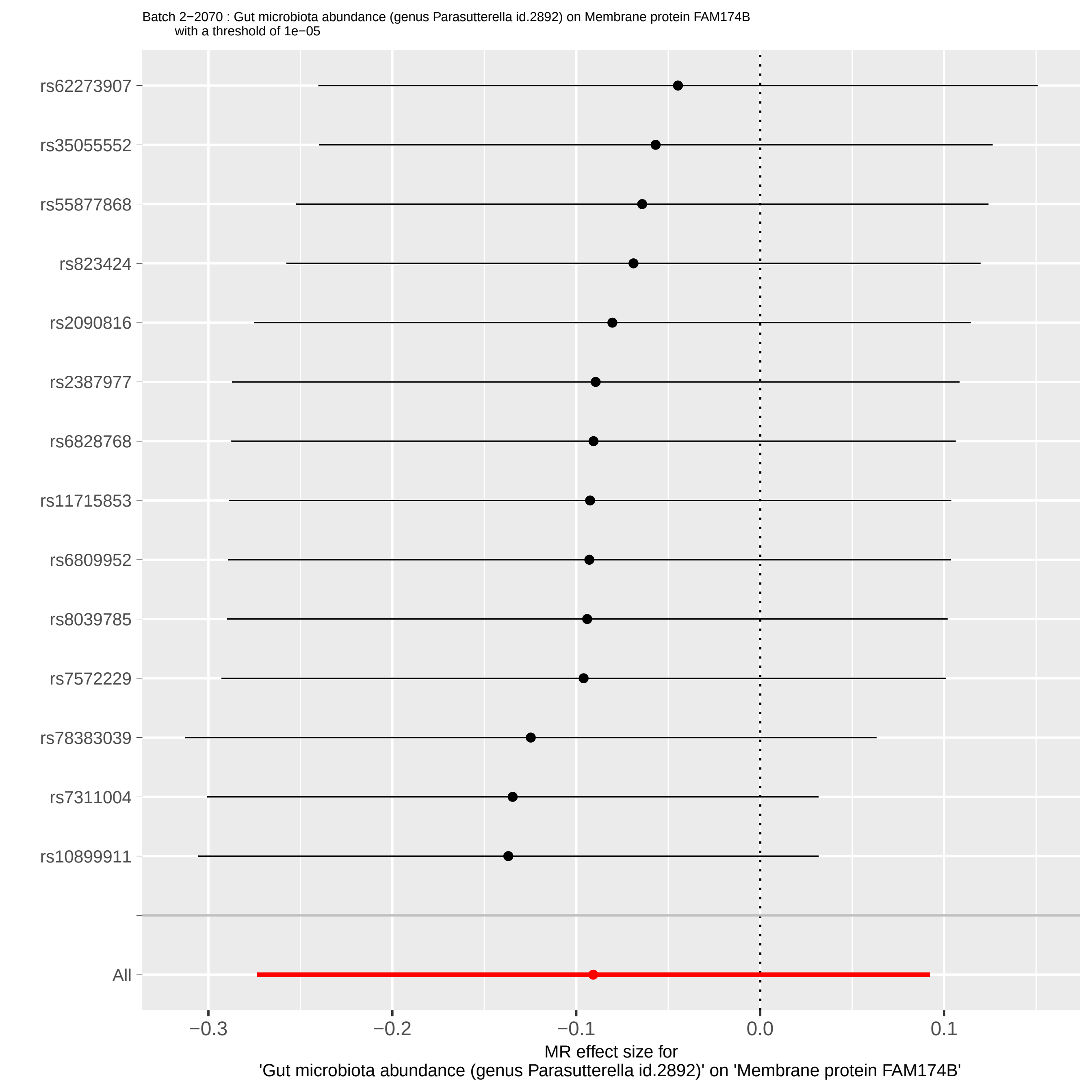

Batch 2−2070 : Gut microbiota abundance (genus Parasutterella id.2892) on Membrane protein FAM174B
with a threshold of 1e−05
rs62273907
rs35055552
rs55877868
rs823424
rs2090816
rs2387977
rs6828768
rs11715853
rs6809952
rs8039785
rs7572229
rs78383039
rs7311004
rs10899911
All
−0.3
−0.2
−0.1
0.0
0.1
MR effect size for
'Gut microbiota abundance (genus Parasutterella id.2892)' on 'Membrane protein FAM174B'

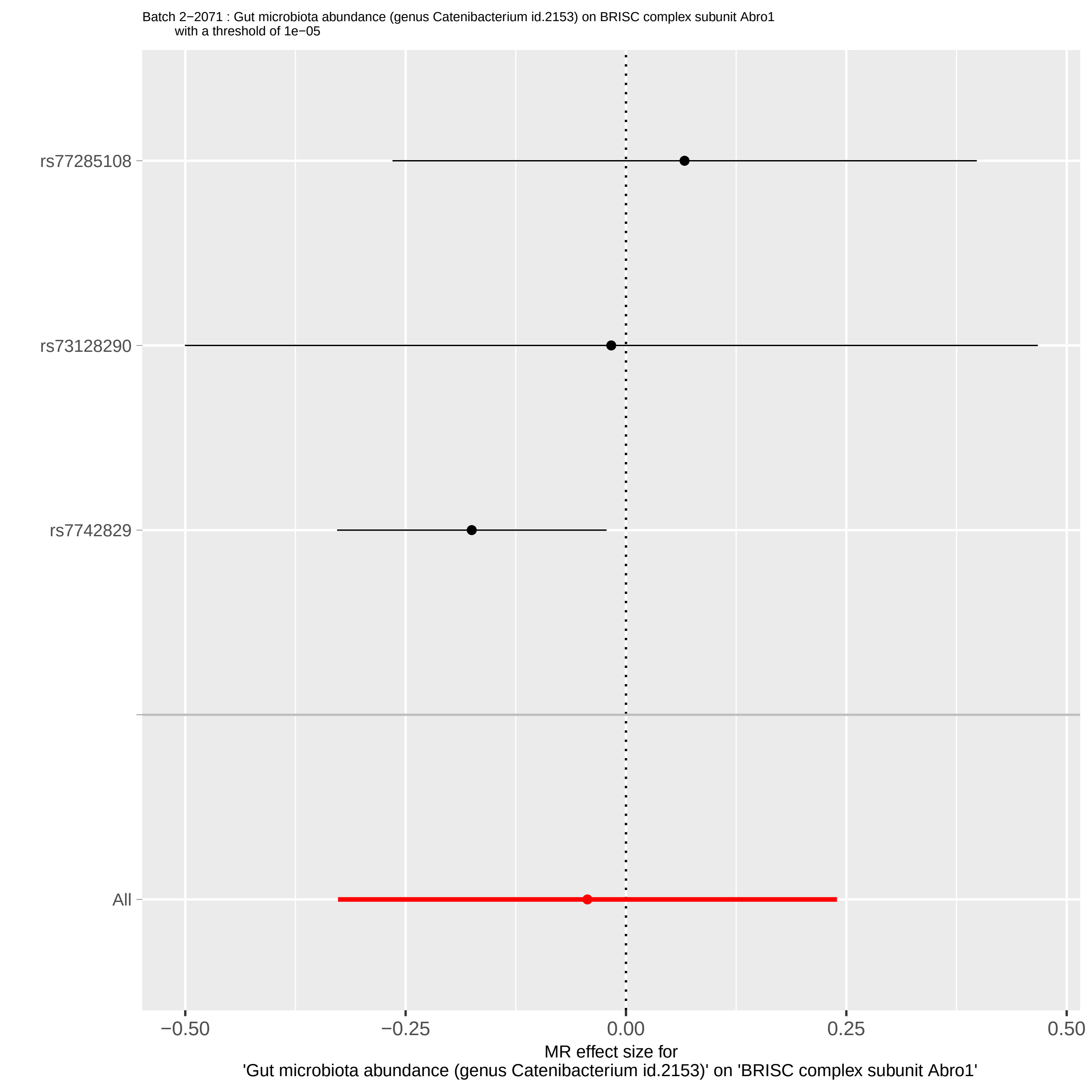
Batch 2−2071 : Gut microbiota abundance (genus Catenibacterium id.2153) on BRISC complex subunit Abro1
with a threshold of 1e−05
rs77285108
rs73128290
rs7742829
All
−0.50
−0.25
0.00
0.25
0.50
MR effect size for
'Gut microbiota abundance (genus Catenibacterium id.2153)' on 'BRISC complex subunit Abro1'

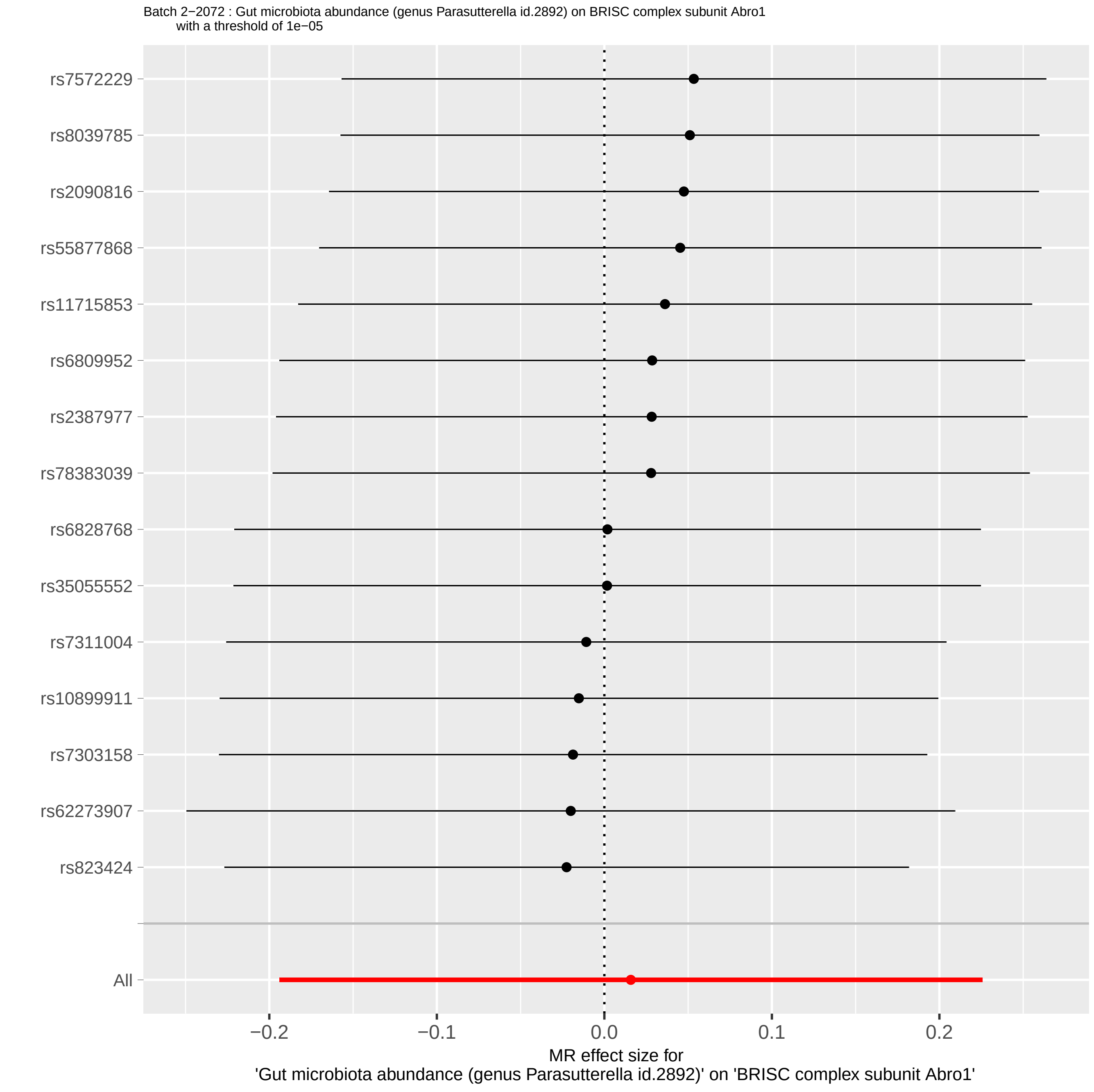

Batch 2−2072 : Gut microbiota abundance (genus Parasutterella id.2892) on BRISC complex subunit Abro1
with a threshold of 1e−05
rs7572229
rs8039785
rs2090816
rs55877868
rs11715853
rs6809952
rs2387977
rs78383039
rs6828768
rs35055552
rs7311004
rs10899911
rs7303158
rs62273907
rs823424
All
−0.2
−0.1
0.0
0.1
0.2
MR effect size for
'Gut microbiota abundance (genus Parasutterella id.2892)' on 'BRISC complex subunit Abro1'

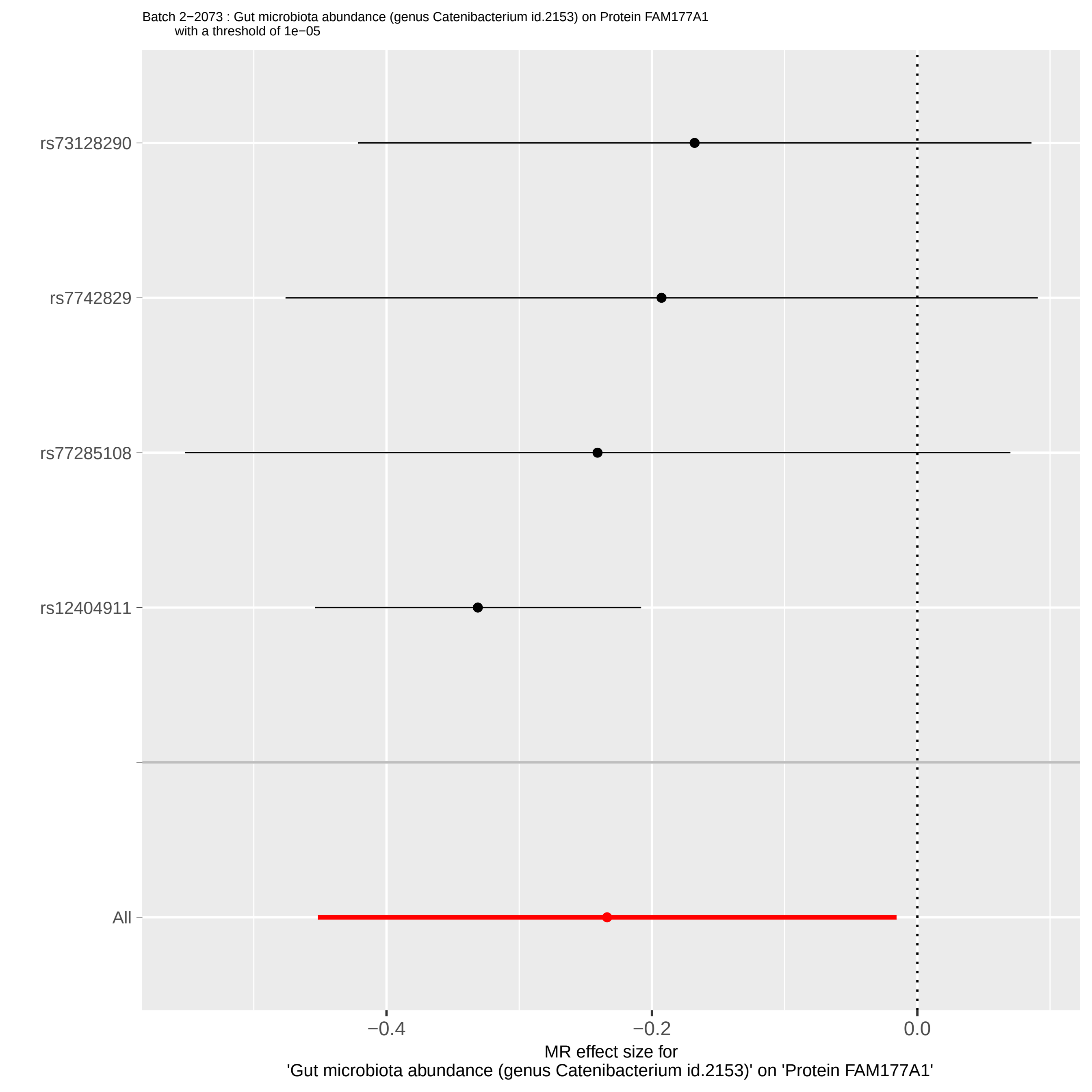
Batch 2−2073 : Gut microbiota abundance (genus Catenibacterium id.2153) on Protein FAM177A1
with a threshold of 1e−05
rs73128290
rs7742829
rs77285108
rs12404911
All
−0.4
−0.2
0.0
MR effect size for
'Gut microbiota abundance (genus Catenibacterium id.2153)' on 'Protein FAM177A1'

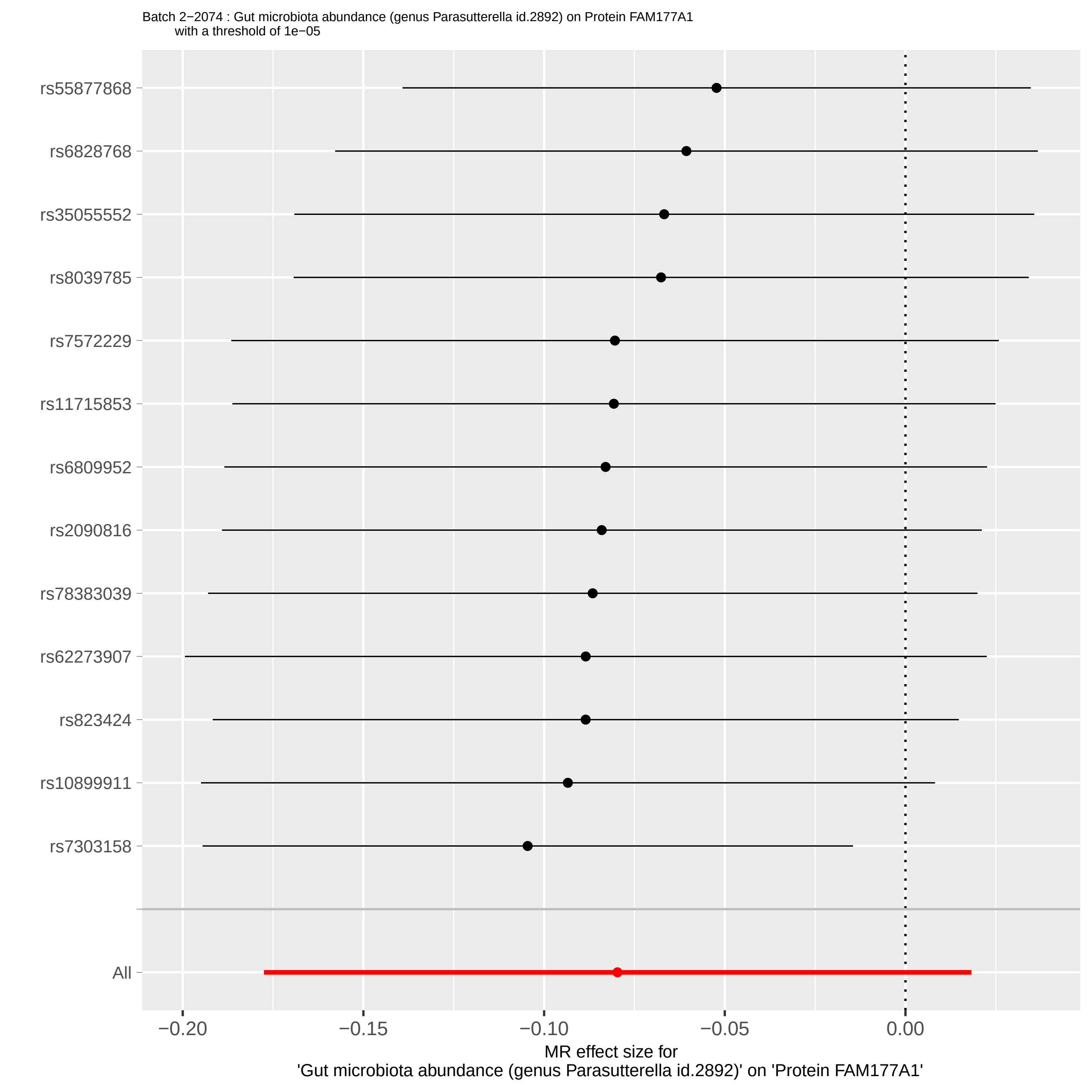
Batch 2−2074 : Gut microbiota abundance (genus Parasutterella id.2892) on Protein FAM177A1
with a threshold of 1e−05
rs55877868
rs6828768
rs35055552
rs8039785
rs7572229
rs11715853
rs6809952
rs2090816
rs78383039
rs62273907
rs823424
rs10899911
rs7303158
All
−0.20
−0.15
−0.10
−0.05
0.00
MR effect size for
'Gut microbiota abundance (genus Parasutterella id.2892)' on 'Protein FAM177A1'

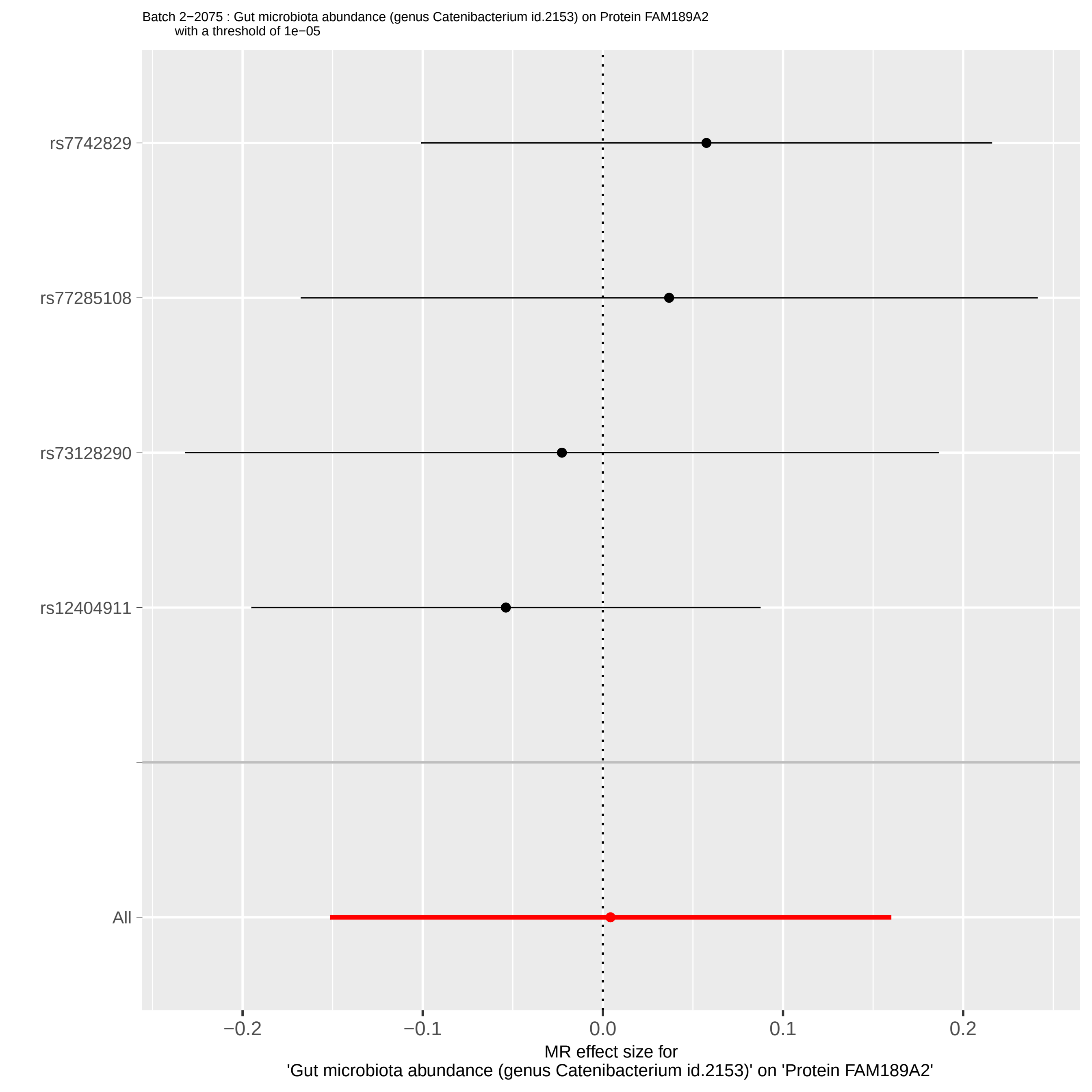
Batch 2−2075 : Gut microbiota abundance (genus Catenibacterium id.2153) on Protein FAM189A2
with a threshold of 1e−05
rs7742829
rs77285108
rs73128290
rs12404911
All
−0.2
−0.1
0.0
0.1
0.2
MR effect size for
'Gut microbiota abundance (genus Catenibacterium id.2153)' on 'Protein FAM189A2'

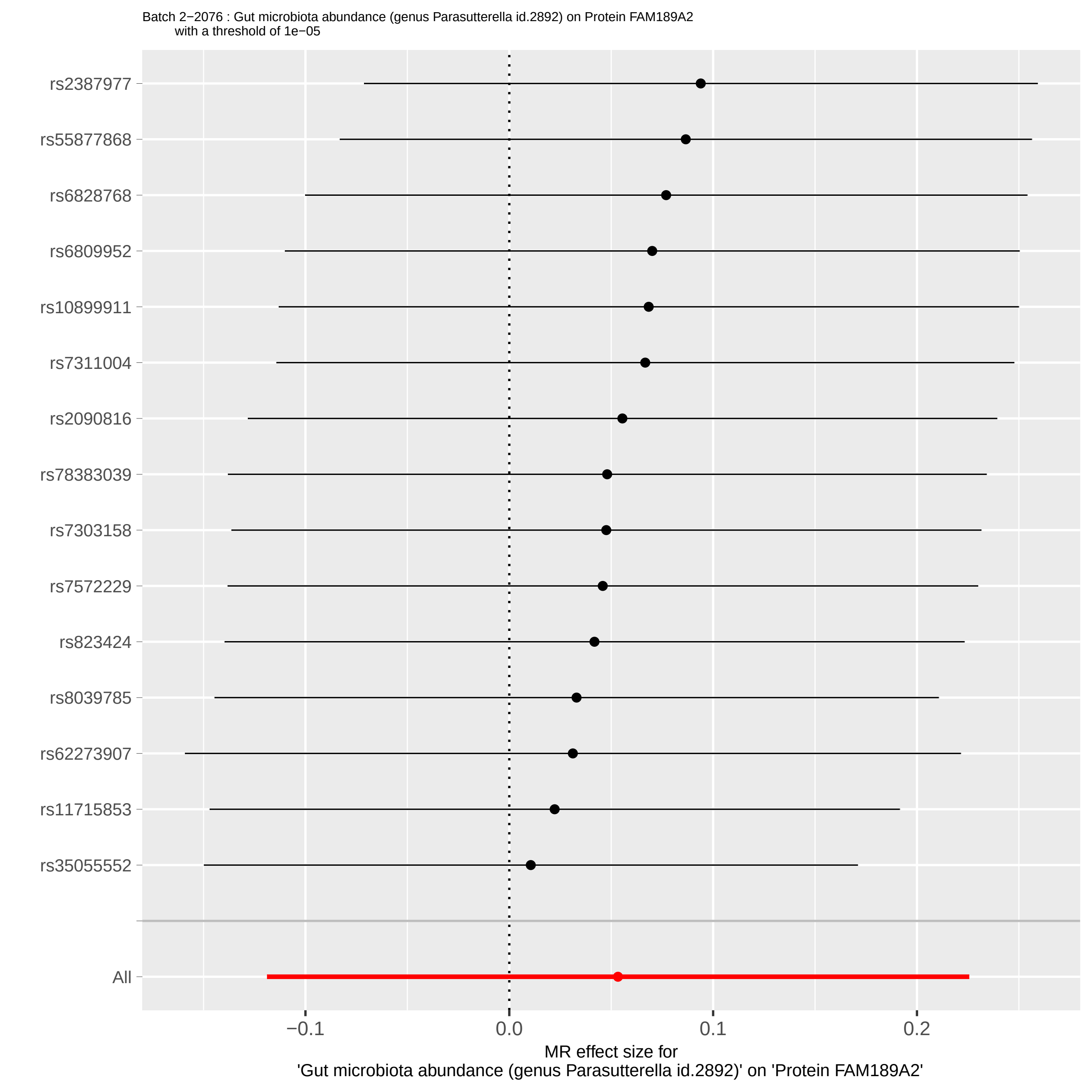
Batch 2−2076 : Gut microbiota abundance (genus Parasutterella id.2892) on Protein FAM189A2
with a threshold of 1e−05
rs2387977
rs55877868
rs6828768
rs6809952
rs10899911
rs7311004
rs2090816
rs78383039
rs7303158
rs7572229
rs823424
rs8039785
rs62273907
rs11715853
rs35055552
All
−0.1
0.0
0.1
0.2
MR effect size for
'Gut microbiota abundance (genus Parasutterella id.2892)' on 'Protein FAM189A2'

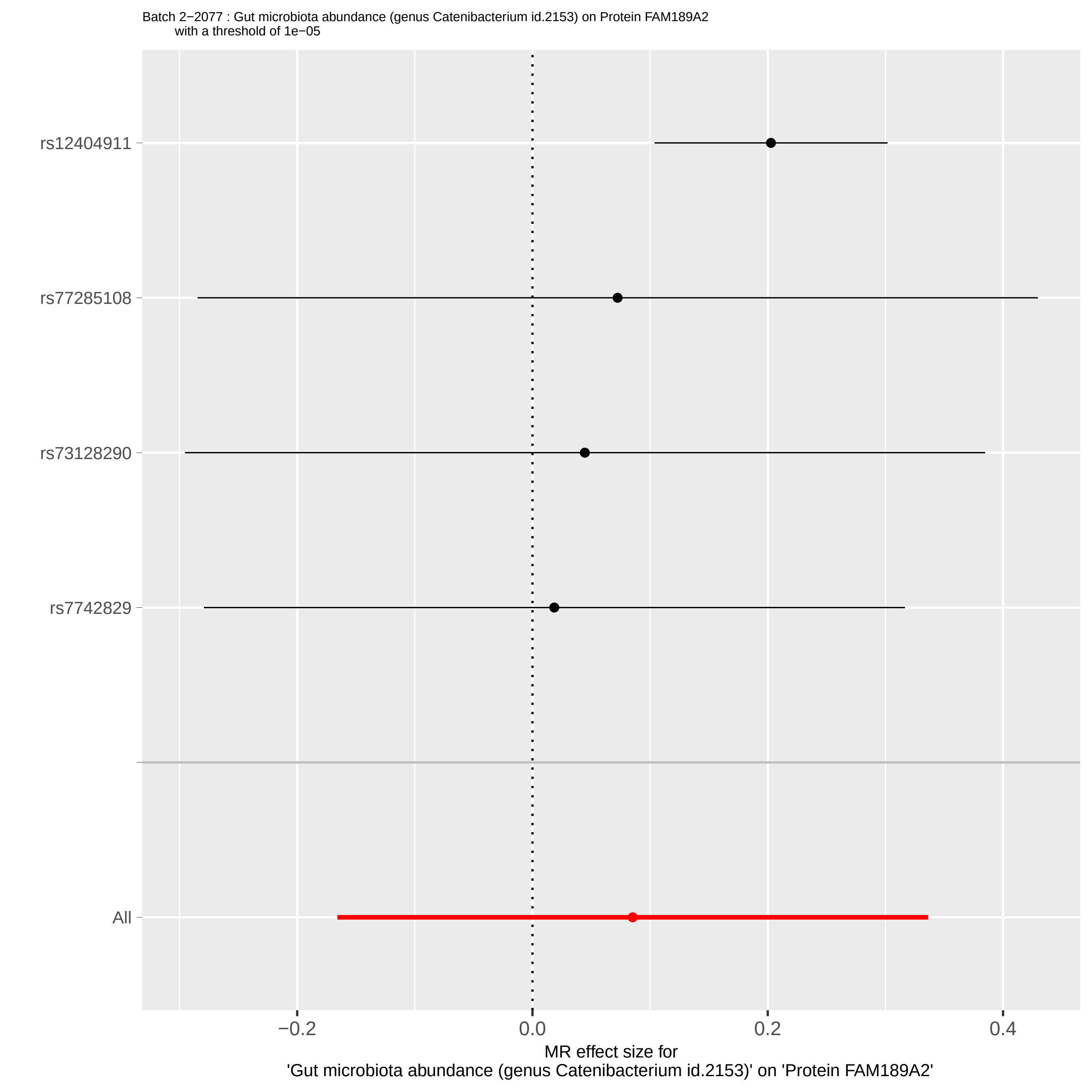

Batch 2−2077 : Gut microbiota abundance (genus Catenibacterium id.2153) on Protein FAM189A2
with a threshold of 1e−05
rs12404911
rs77285108
rs73128290
rs7742829
All
−0.2
0.0
0.2
0.4
MR effect size for
'Gut microbiota abundance (genus Catenibacterium id.2153)' on 'Protein FAM189A2'

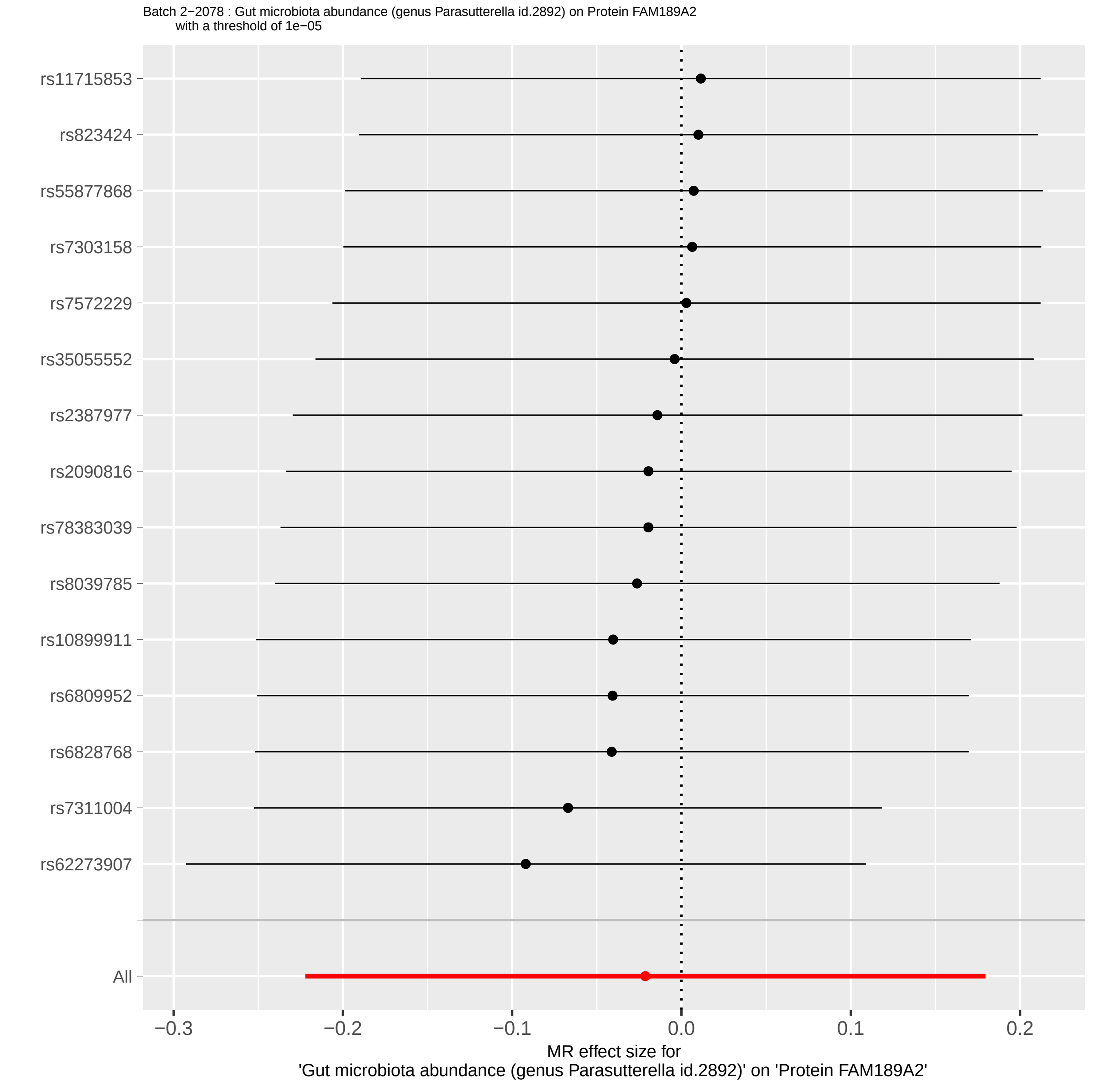
Batch 2−2078 : Gut microbiota abundance (genus Parasutterella id.2892) on Protein FAM189A2
with a threshold of 1e−05
rs11715853
rs823424
rs55877868
rs7303158
rs7572229
rs35055552
rs2387977
rs2090816
rs78383039
rs8039785
rs10899911
rs6809952
rs6828768
rs7311004
rs62273907
All
−0.3
−0.2
−0.1
0.0
0.1
0.2
MR effect size for
'Gut microbiota abundance (genus Parasutterella id.2892)' on 'Protein FAM189A2'

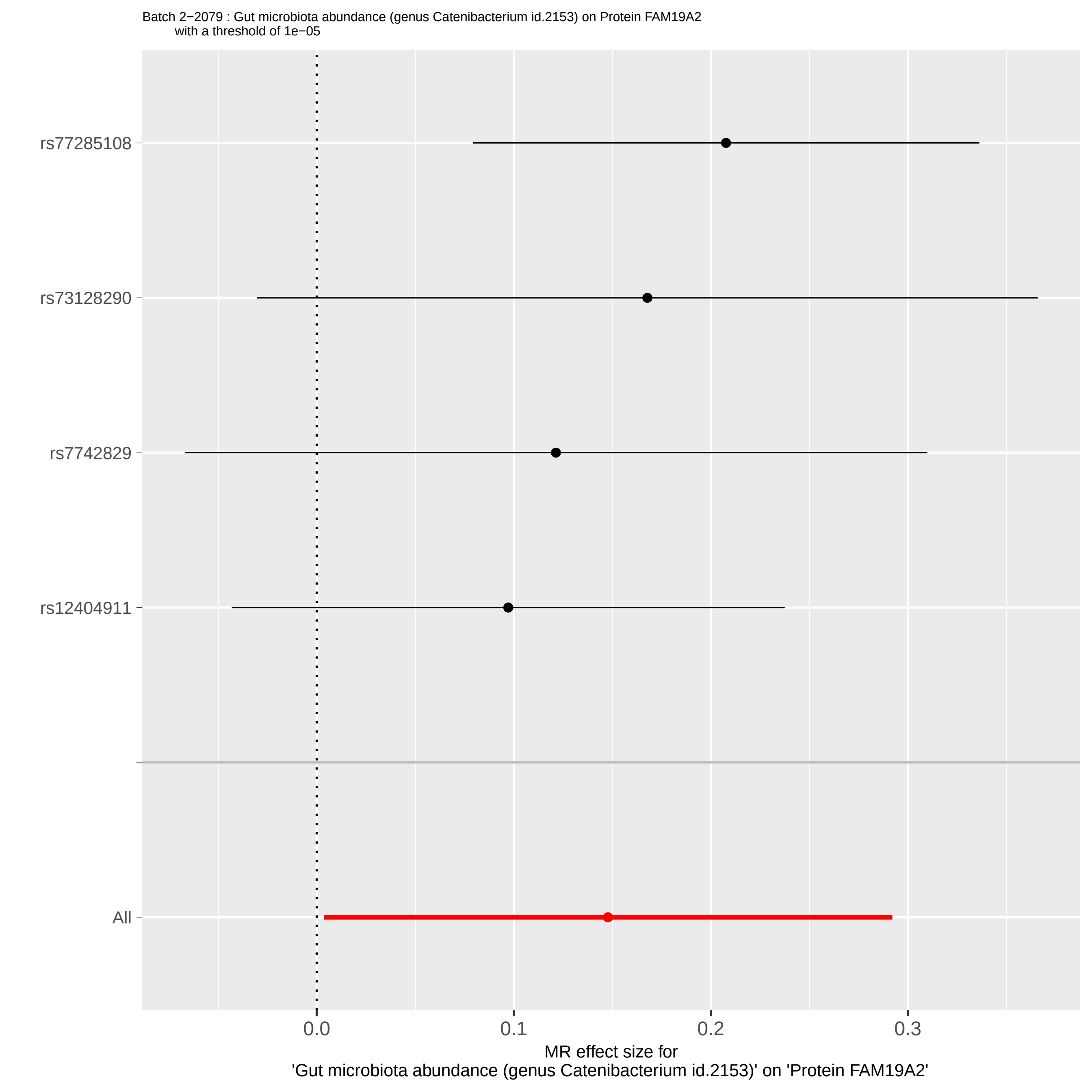

Batch 2−2079 : Gut microbiota abundance (genus Catenibacterium id.2153) on Protein FAM19A2
with a threshold of 1e−05
rs77285108
rs73128290
rs7742829
rs12404911
All
0.0
0.1
0.2
0.3
MR effect size for
'Gut microbiota abundance (genus Catenibacterium id.2153)' on 'Protein FAM19A2'

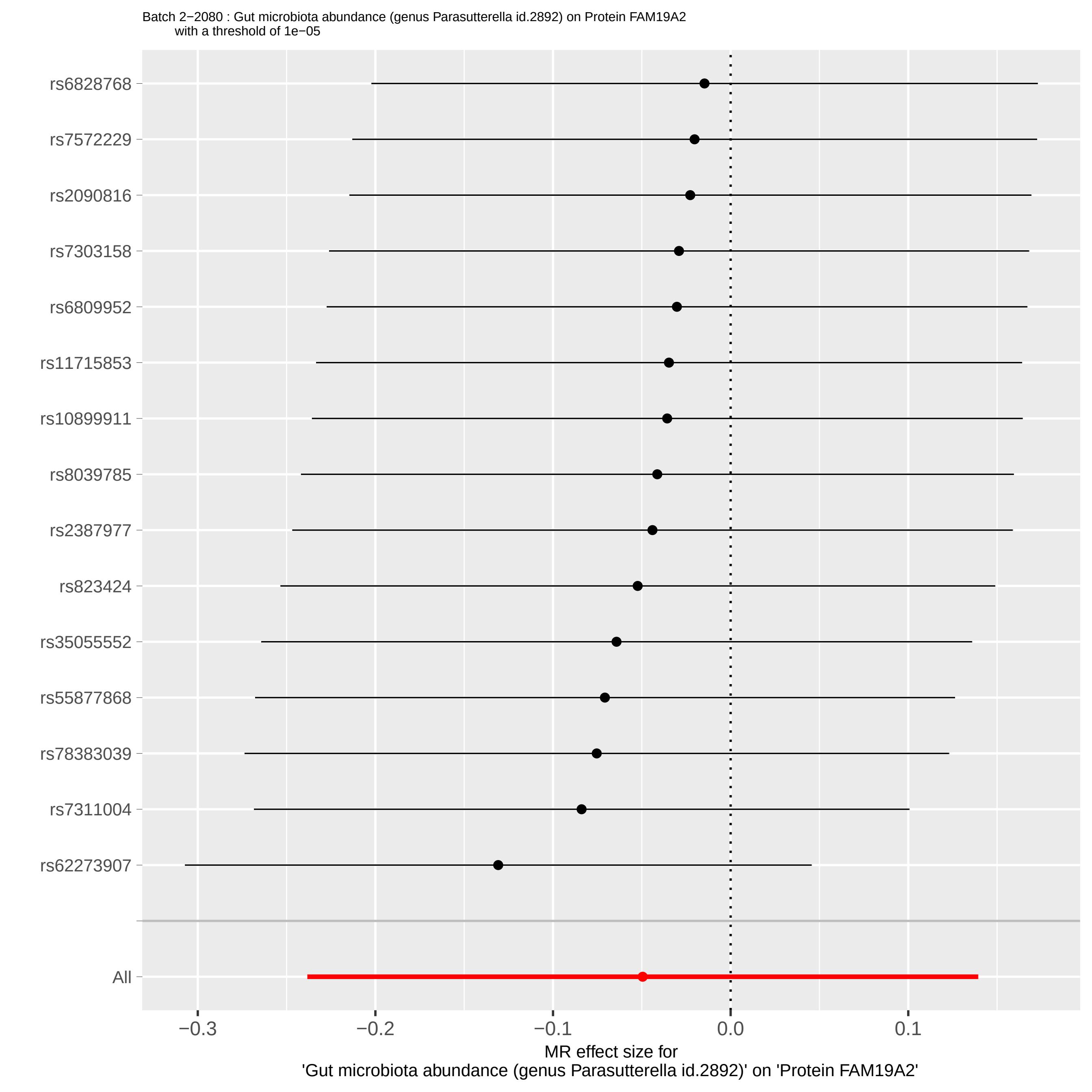
Batch 2−2080 : Gut microbiota abundance (genus Parasutterella id.2892) on Protein FAM19A2
with a threshold of 1e−05
rs6828768
rs7572229
rs2090816
rs7303158
rs6809952
rs11715853
rs10899911
rs8039785
rs2387977
rs823424
rs35055552
rs55877868
rs78383039
rs7311004
rs62273907
All
−0.3
−0.2
−0.1
0.0
0.1
MR effect size for
'Gut microbiota abundance (genus Parasutterella id.2892)' on 'Protein FAM19A2'

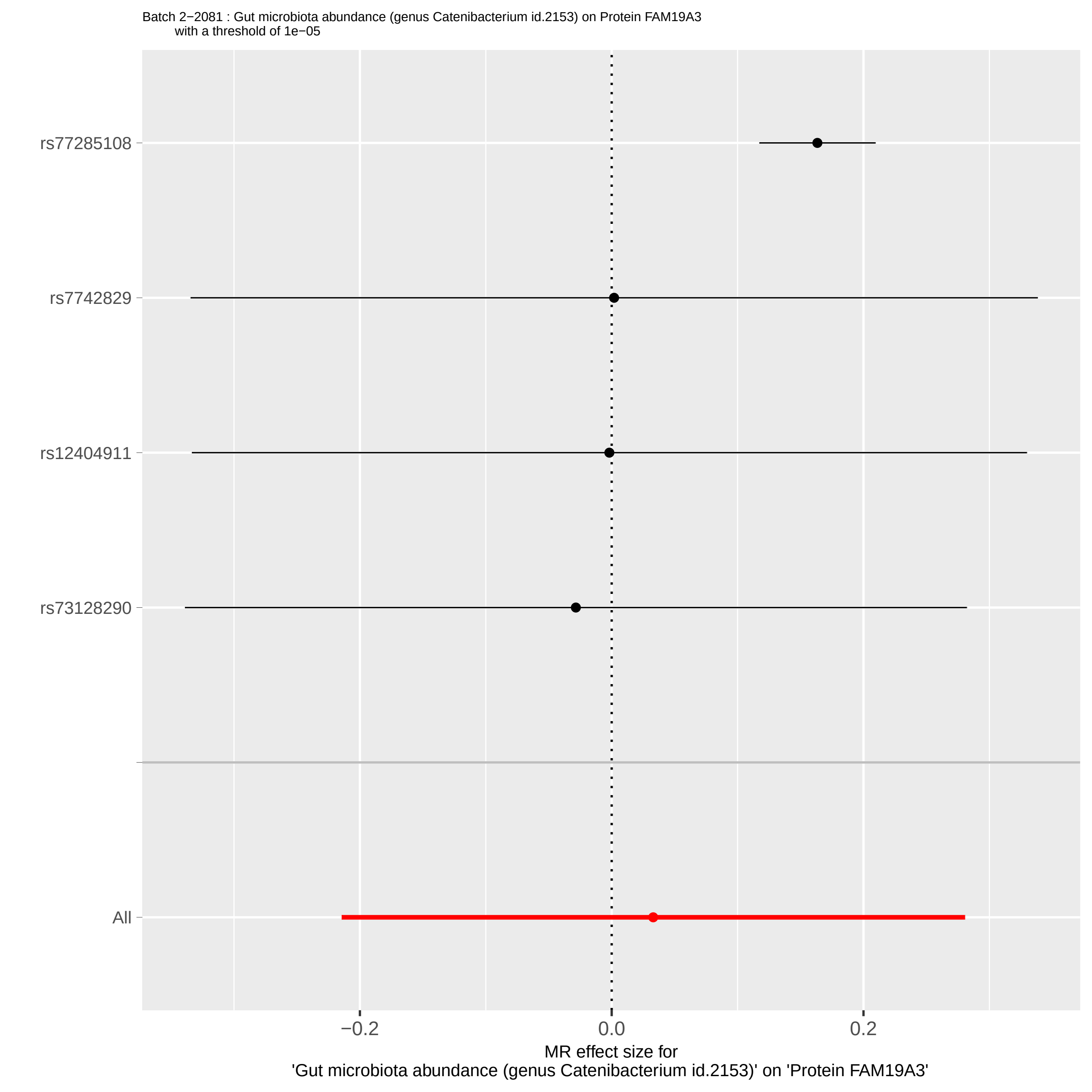
Batch 2−2081 : Gut microbiota abundance (genus Catenibacterium id.2153) on Protein FAM19A3
with a threshold of 1e−05
rs77285108
rs7742829
rs12404911
rs73128290
All
−0.2
0.0
0.2
MR effect size for
'Gut microbiota abundance (genus Catenibacterium id.2153)' on 'Protein FAM19A3'

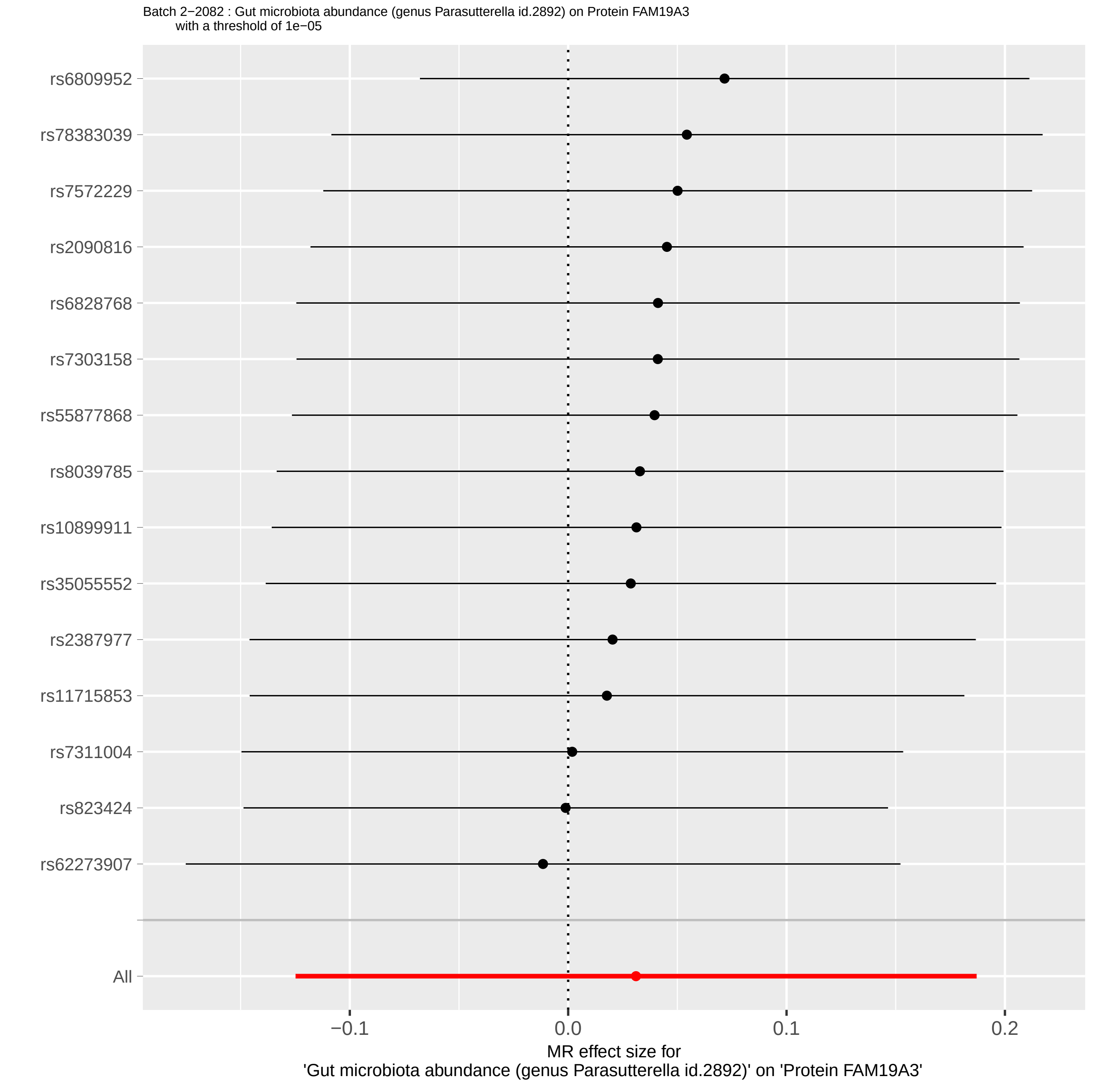
Batch 2−2082 : Gut microbiota abundance (genus Parasutterella id.2892) on Protein FAM19A3
with a threshold of 1e−05
rs6809952
rs78383039
rs7572229
rs2090816
rs6828768
rs7303158
rs55877868
rs8039785
rs10899911
rs35055552
rs2387977
rs11715853
rs7311004
rs823424
rs62273907
All
−0.1
0.0
0.1
0.2
MR effect size for
'Gut microbiota abundance (genus Parasutterella id.2892)' on 'Protein FAM19A3'

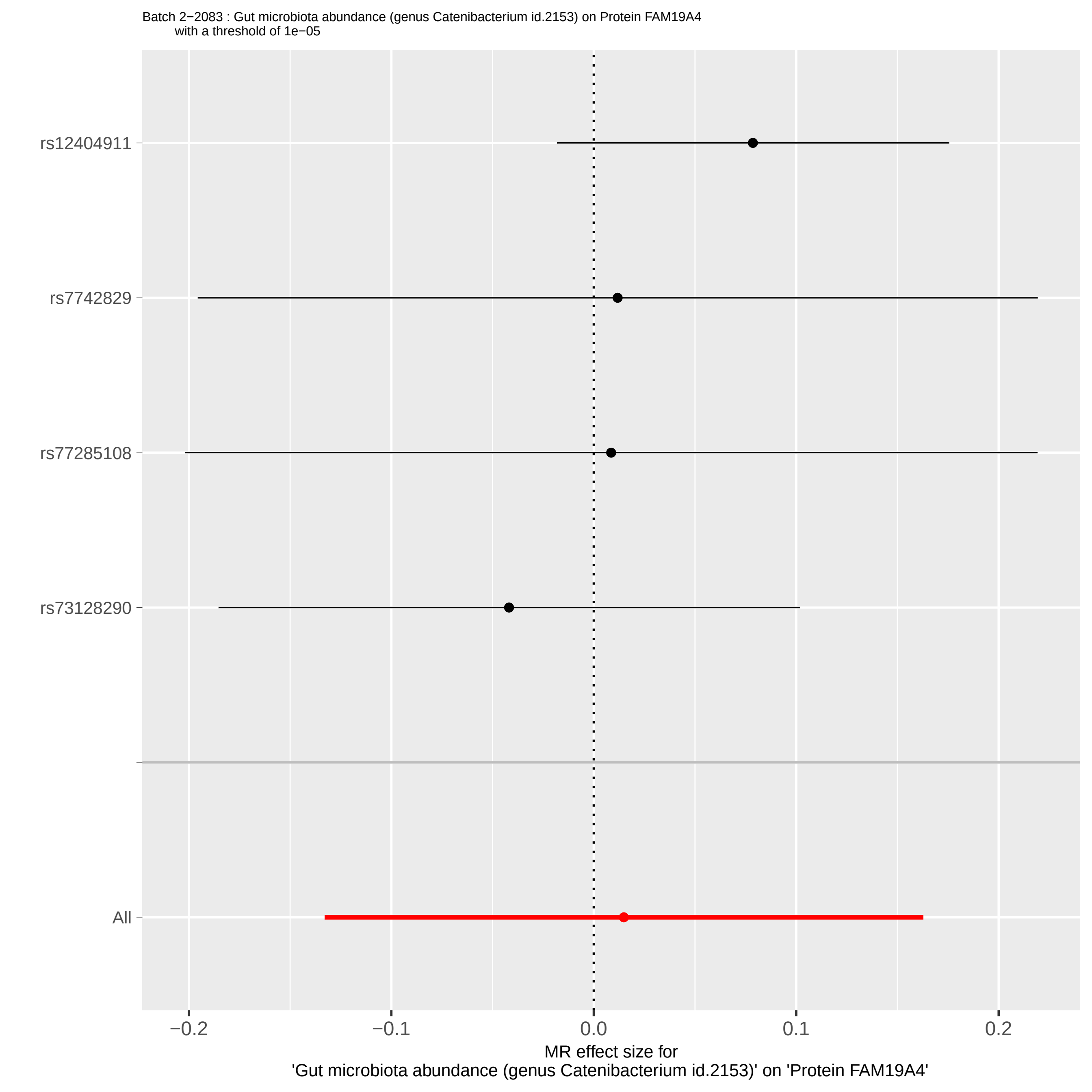

Batch 2−2083 : Gut microbiota abundance (genus Catenibacterium id.2153) on Protein FAM19A4
with a threshold of 1e−05
rs12404911
rs7742829
rs77285108
rs73128290
All
−0.2
−0.1
0.0
0.1
0.2
MR effect size for
'Gut microbiota abundance (genus Catenibacterium id.2153)' on 'Protein FAM19A4'

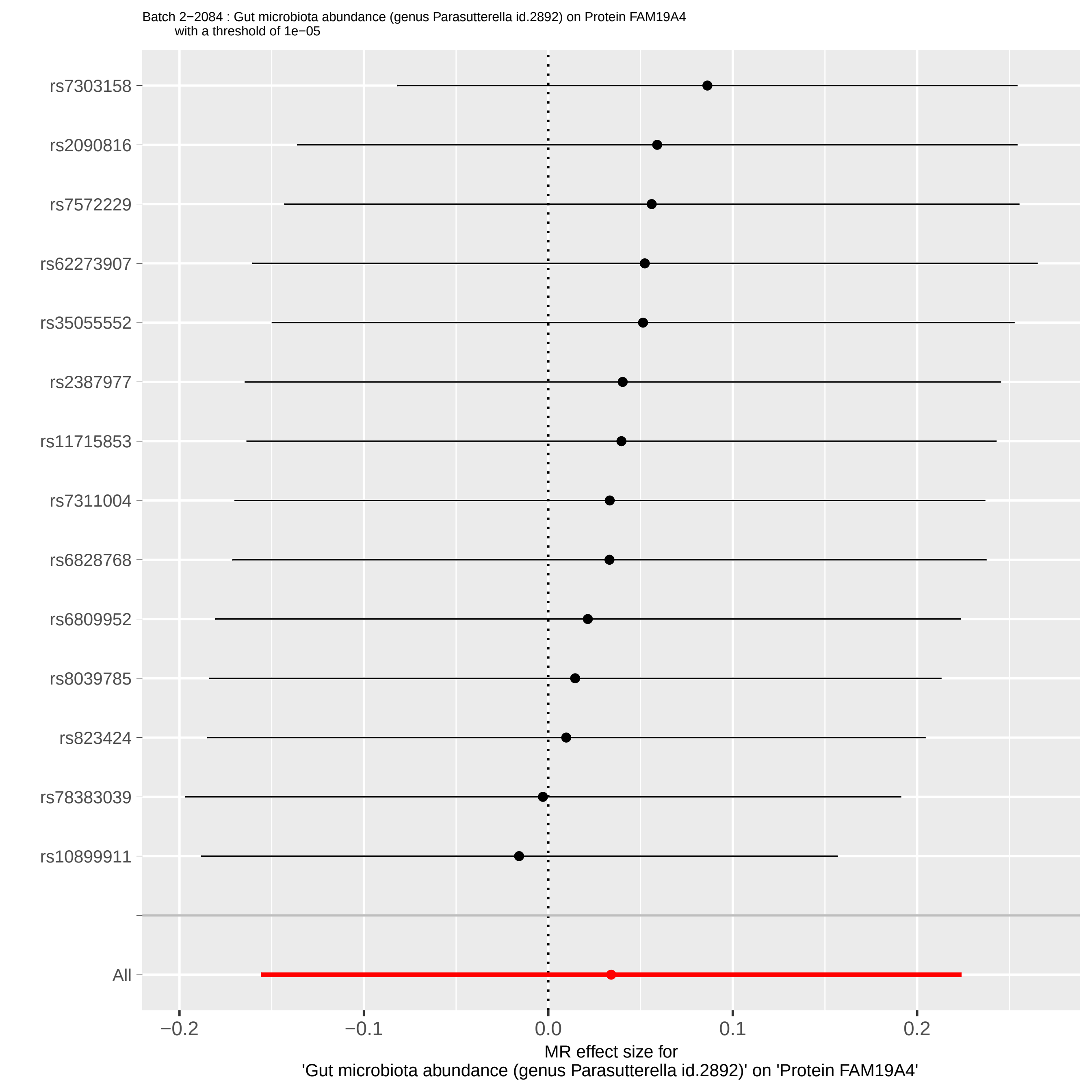

Batch 2−2084 : Gut microbiota abundance (genus Parasutterella id.2892) on Protein FAM19A4
with a threshold of 1e−05
rs7303158
rs2090816
rs7572229
rs62273907
rs35055552
rs2387977
rs11715853
rs7311004
rs6828768
rs6809952
rs8039785
rs823424
rs78383039
rs10899911
All
−0.2
−0.1
0.0
0.1
0.2
MR effect size for
'Gut microbiota abundance (genus Parasutterella id.2892)' on 'Protein FAM19A4'

Batch 2−2085 : Gut microbiota abundance (genus Catenibacterium id.2153) on Protein FAM19A5
with a threshold of 1e−05

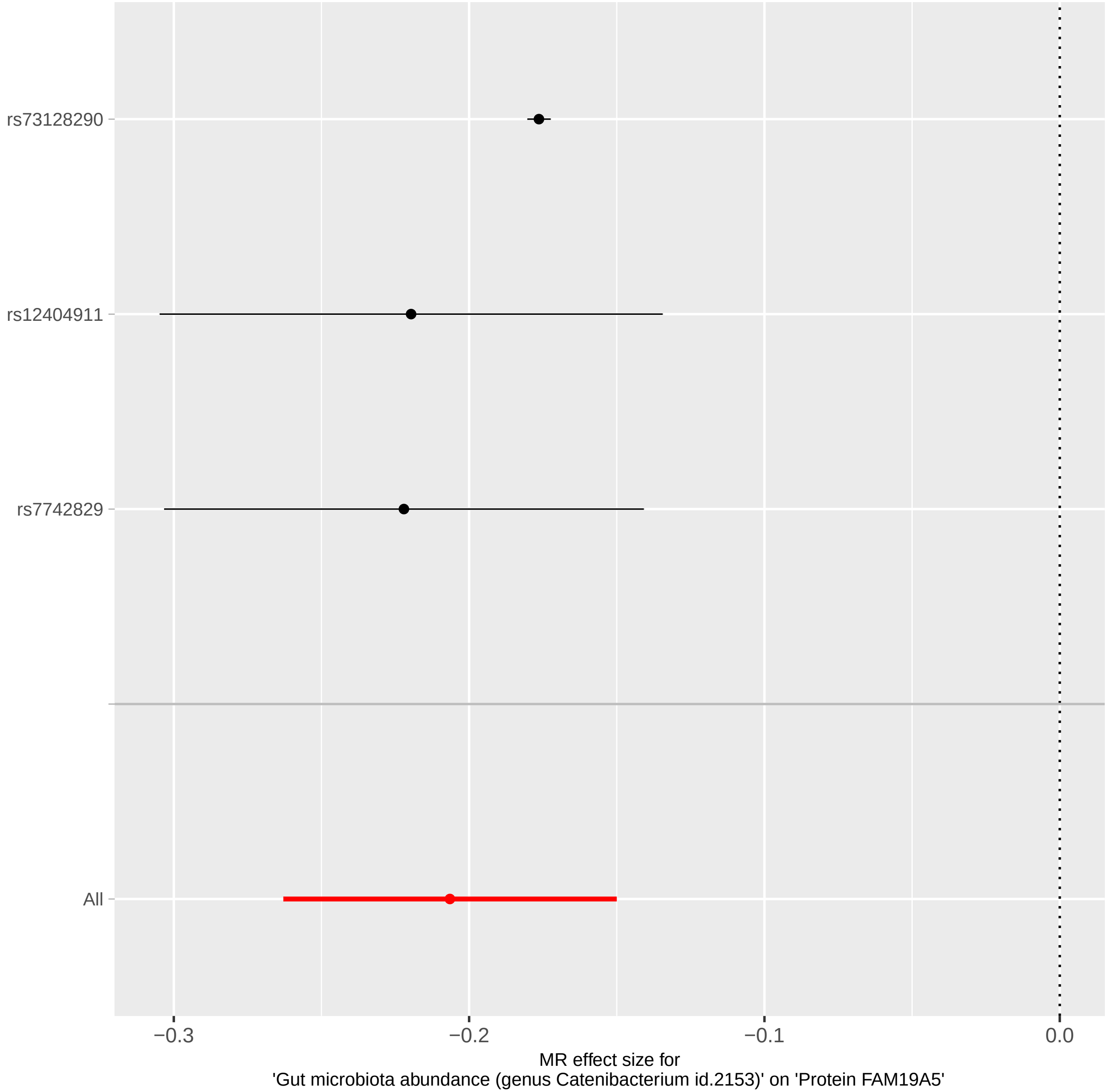

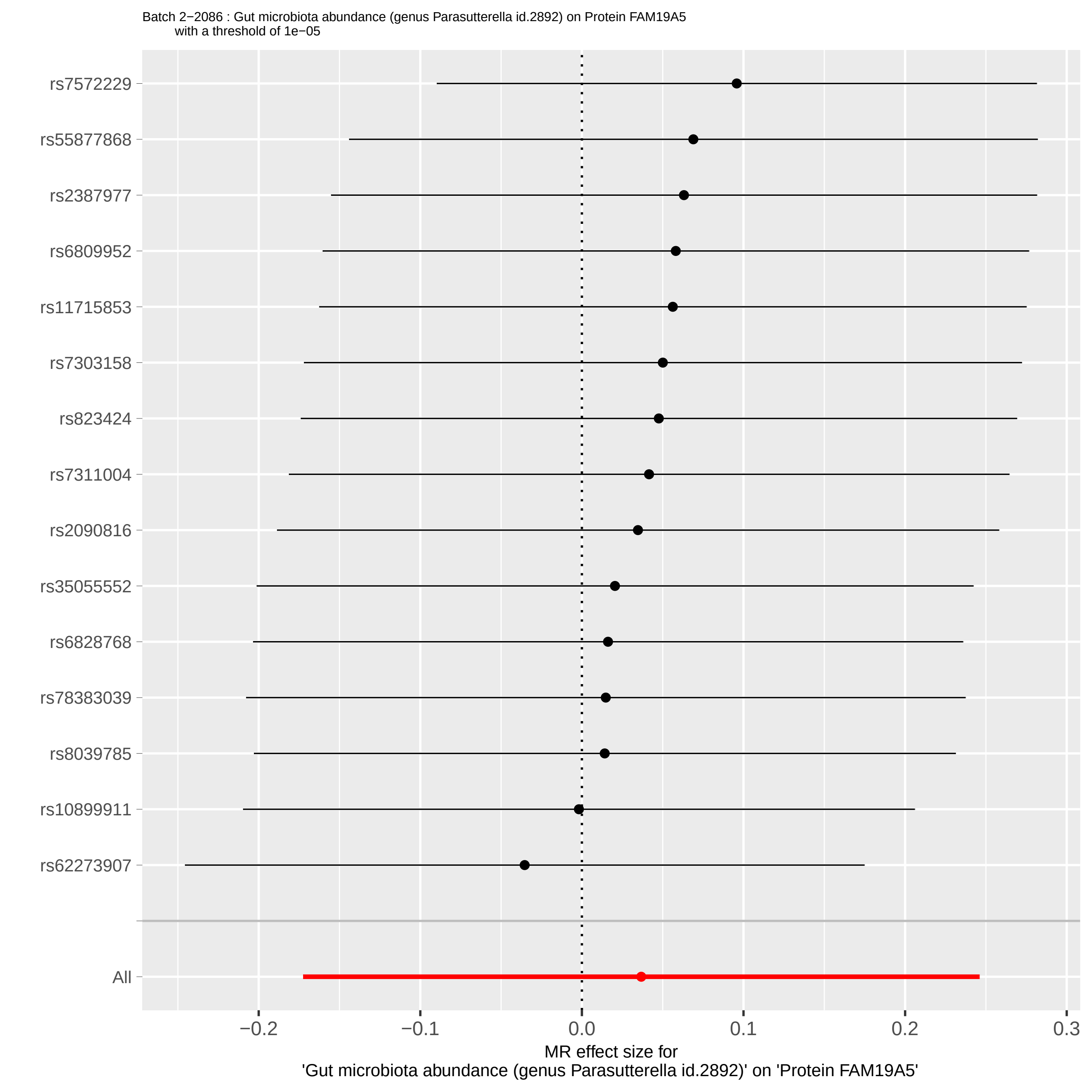

Batch 2−2086 : Gut microbiota abundance (genus Parasutterella id.2892) on Protein FAM19A5
with a threshold of 1e−05
rs7572229
rs55877868
rs2387977
rs6809952
rs11715853
rs7303158
rs823424
rs7311004
rs2090816
rs35055552
rs6828768
rs78383039
rs8039785
rs10899911
rs62273907
All
−0.2
−0.1
0.0
0.1
0.2
0.3
MR effect size for
'Gut microbiota abundance (genus Parasutterella id.2892)' on 'Protein FAM19A5'

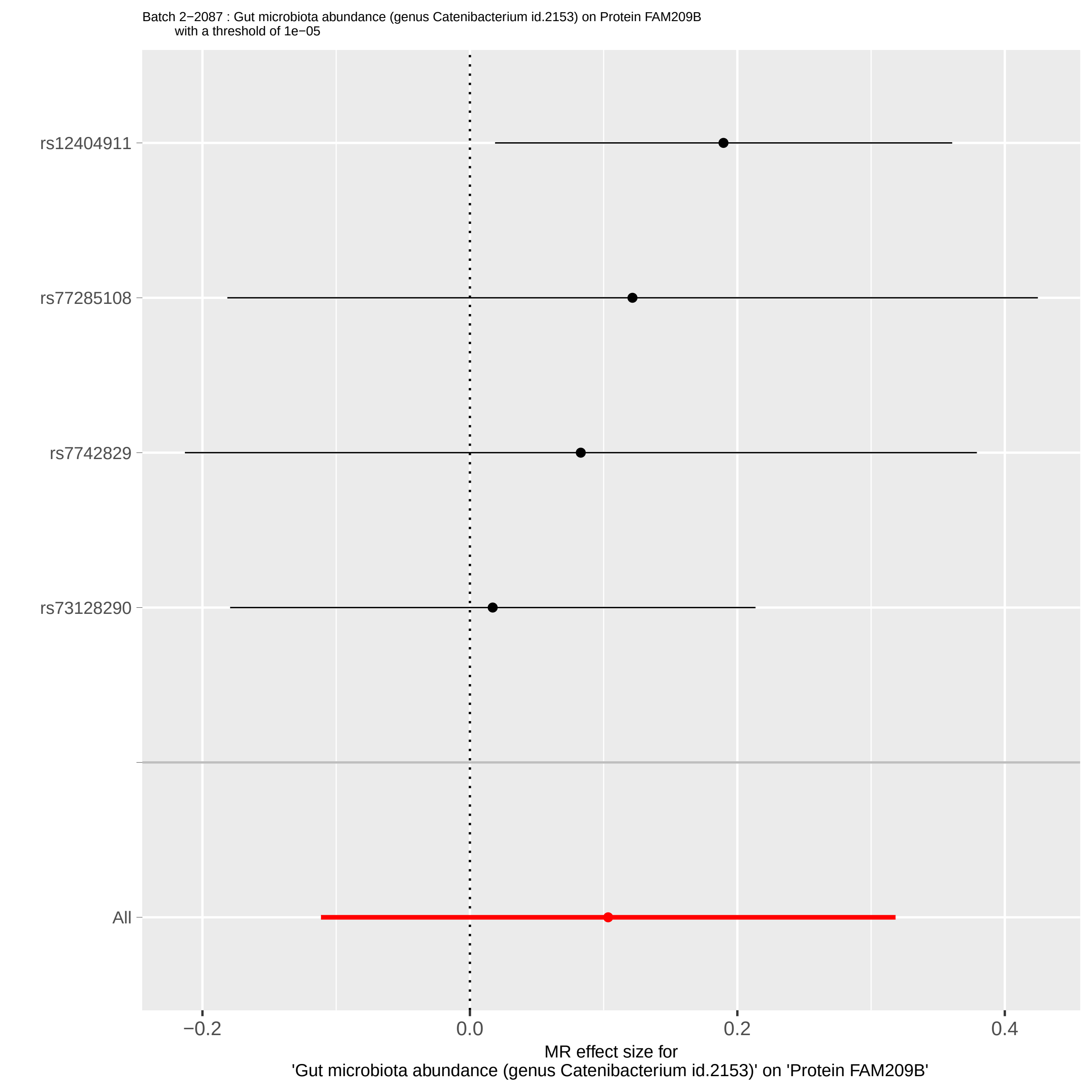
Batch 2−2087 : Gut microbiota abundance (genus Catenibacterium id.2153) on Protein FAM209B
with a threshold of 1e−05
rs12404911
rs77285108
rs7742829
rs73128290
All
−0.2
0.0
0.2
0.4
MR effect size for
'Gut microbiota abundance (genus Catenibacterium id.2153)' on 'Protein FAM209B'

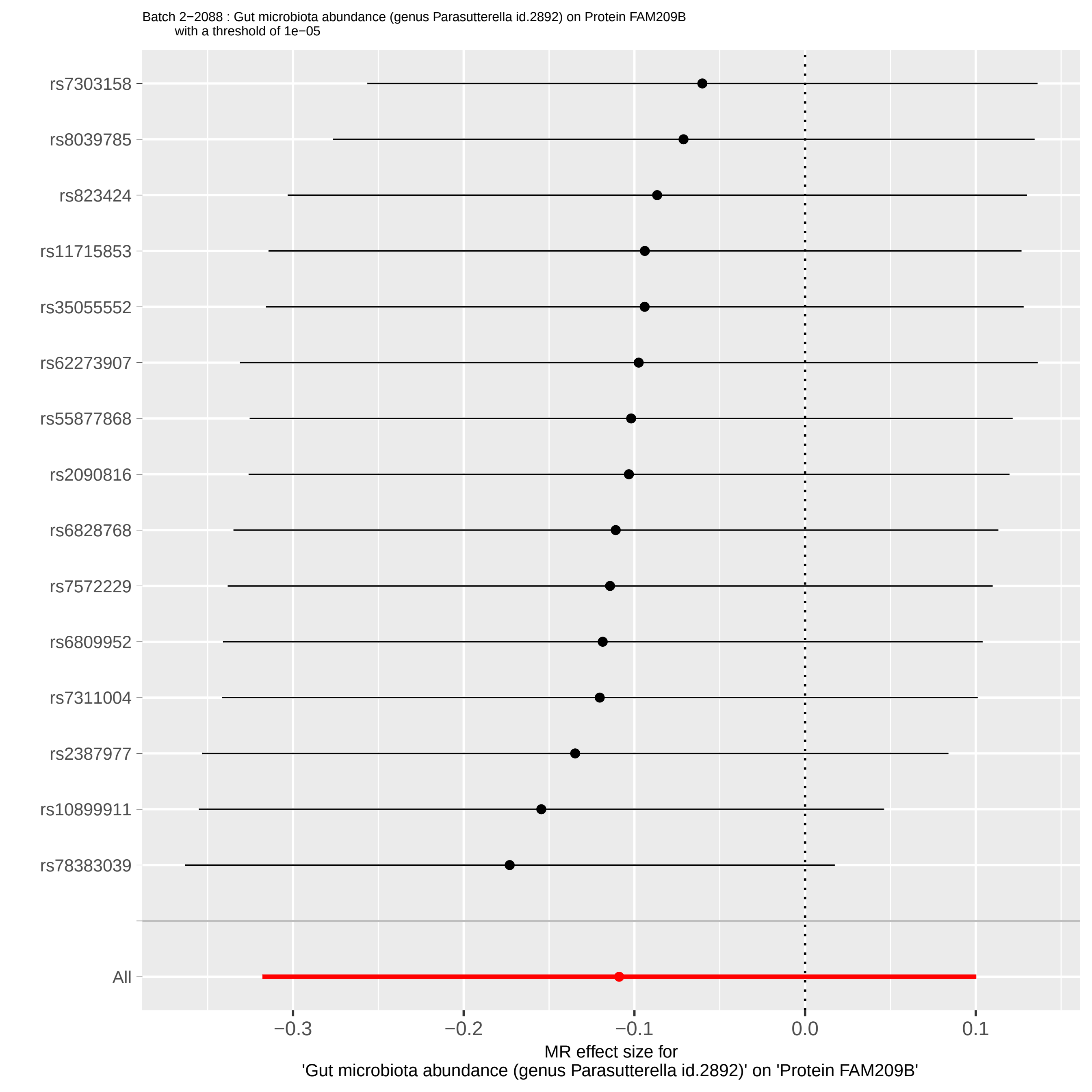

Batch 2−2088 : Gut microbiota abundance (genus Parasutterella id.2892) on Protein FAM209B
with a threshold of 1e−05
rs7303158
rs8039785
rs823424
rs11715853
rs35055552
rs62273907
rs55877868
rs2090816
rs6828768
rs7572229
rs6809952
rs7311004
rs2387977
rs10899911
rs78383039
All
−0.3
−0.2
−0.1
0.0
0.1
MR effect size for
'Gut microbiota abundance (genus Parasutterella id.2892)' on 'Protein FAM209B'

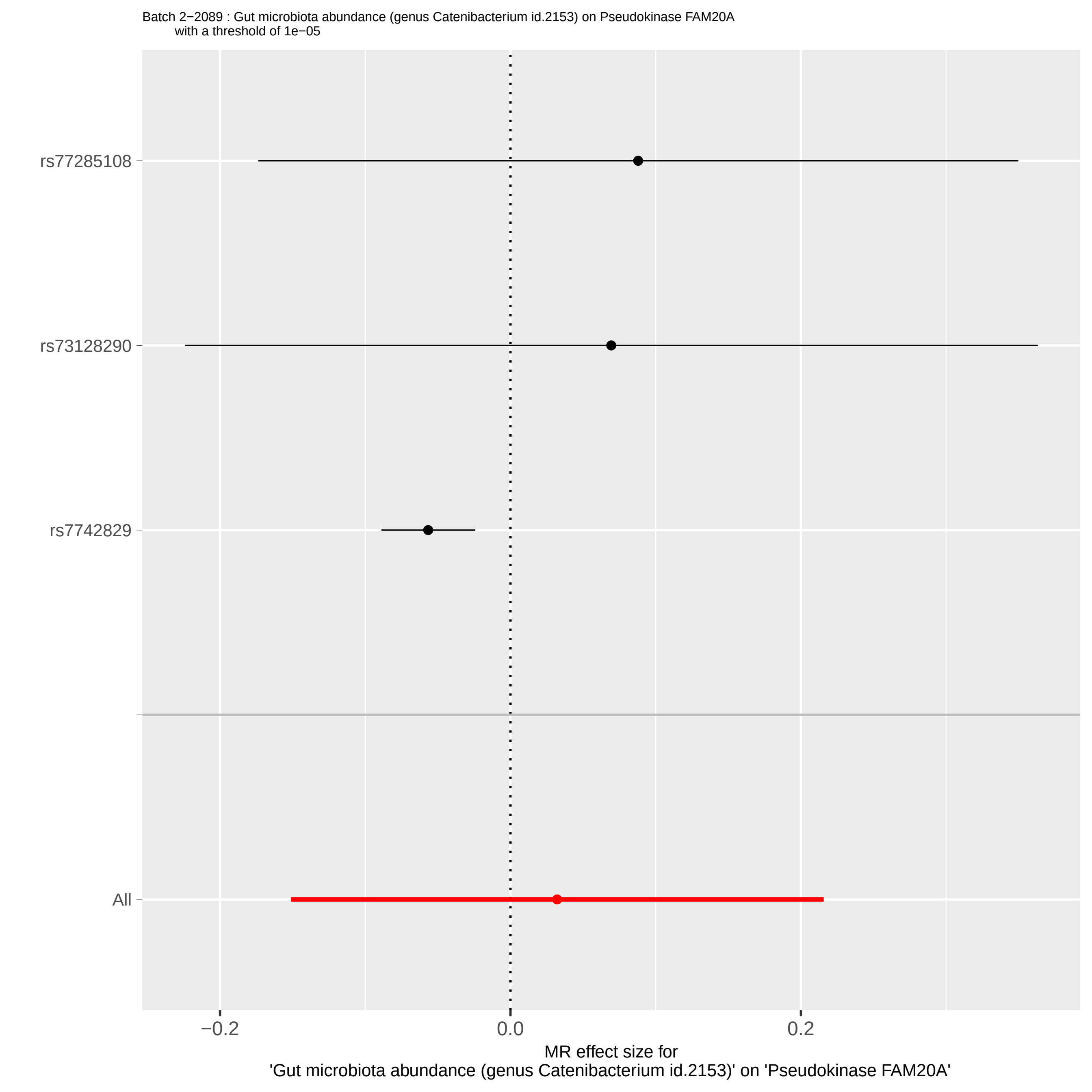
Batch 2−2089 : Gut microbiota abundance (genus Catenibacterium id.2153) on Pseudokinase FAM20A
with a threshold of 1e−05
rs77285108
rs73128290
rs7742829
All
−0.2
0.0
0.2
MR effect size for
'Gut microbiota abundance (genus Catenibacterium id.2153)' on 'Pseudokinase FAM20A'

Batch 2−2090 : Gut microbiota abundance (genus Parasutterella id.2892) on Pseudokinase FAM20A
with a threshold of 1e−05

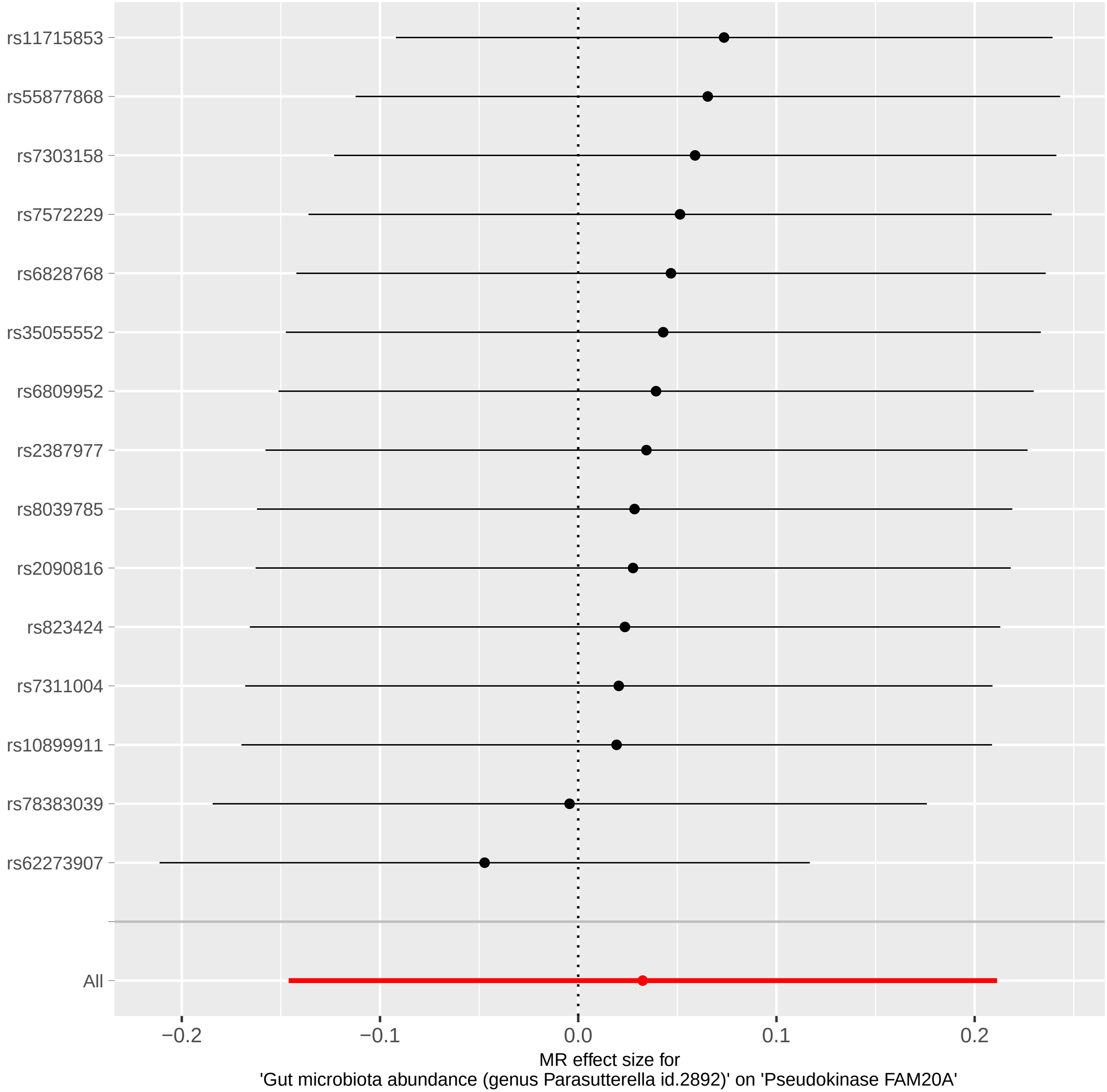

Batch 2−2091 : Gut microbiota abundance (genus Catenibacterium id.2153) on Glycosaminoglycan xylosylkinase
with a threshold of 1e−05

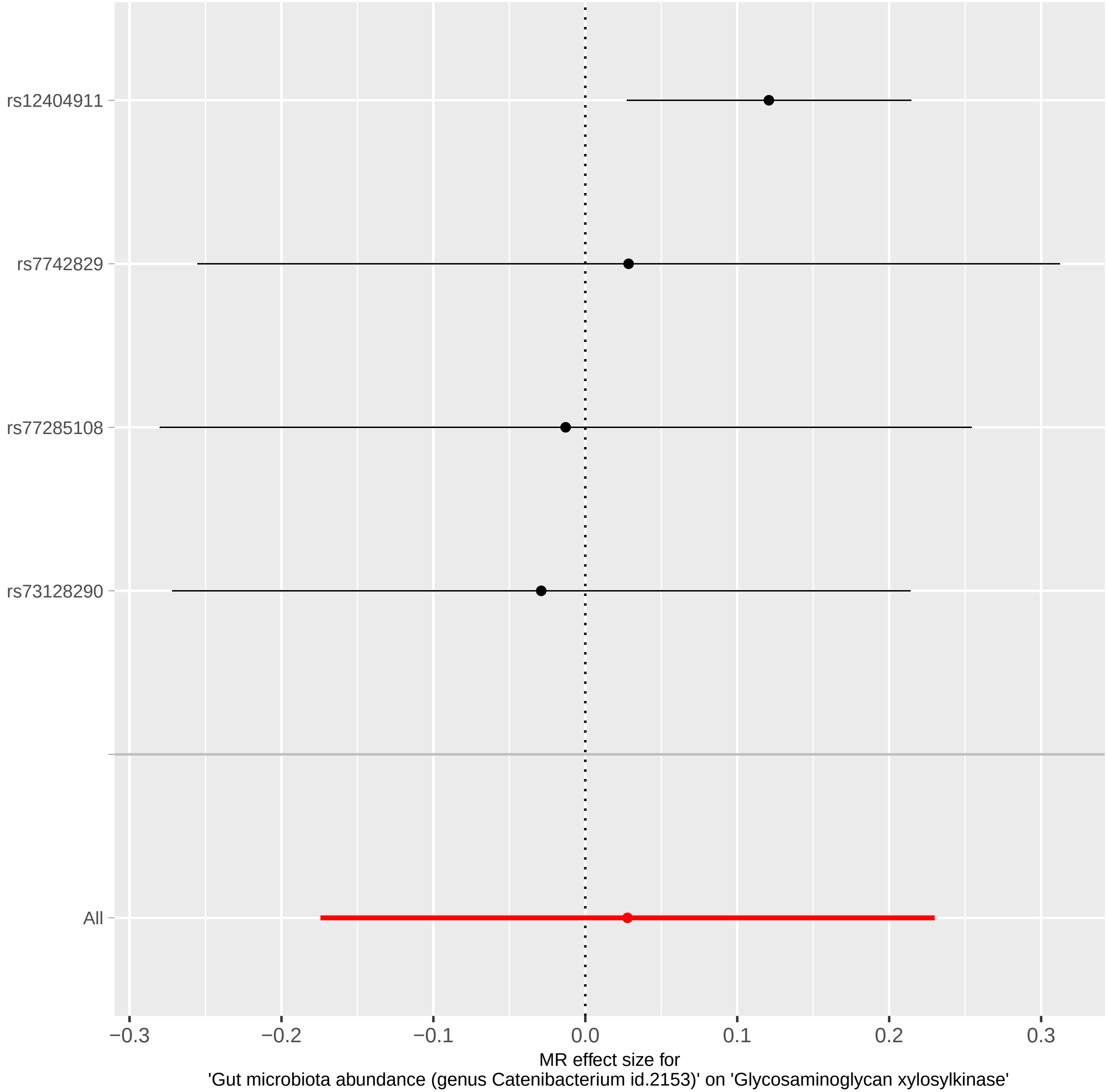

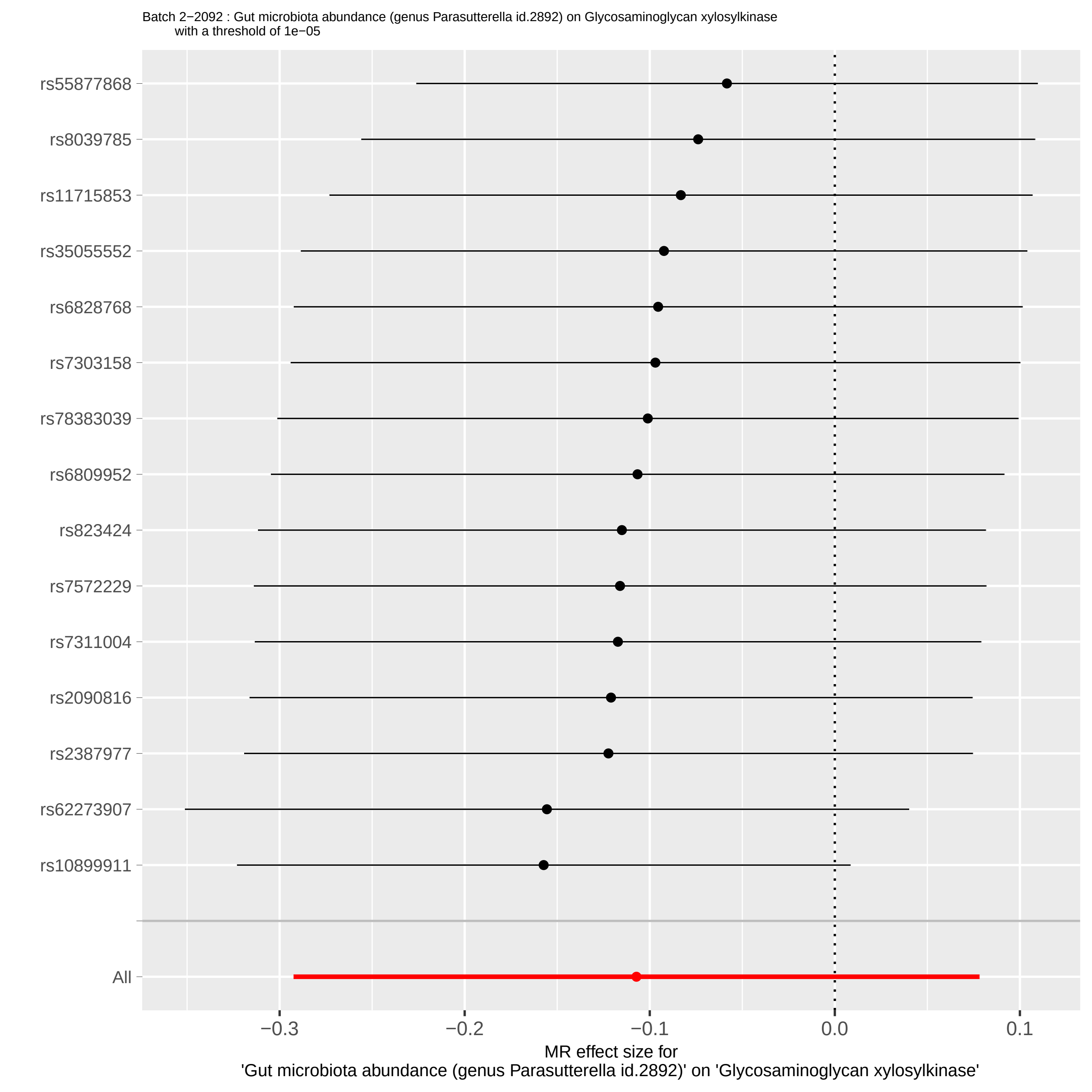

Batch 2−2092 : Gut microbiota abundance (genus Parasutterella id.2892) on Glycosaminoglycan xylosylkinase
with a threshold of 1e−05
rs55877868
rs8039785
rs11715853
rs35055552
rs6828768
rs7303158
rs78383039
rs6809952
rs823424
rs7572229
rs7311004
rs2090816
rs2387977
rs62273907
rs10899911
All
−0.3
−0.2
−0.1
0.0
0.1
MR effect size for
'Gut microbiota abundance (genus Parasutterella id.2892)' on 'Glycosaminoglycan xylosylkinase'

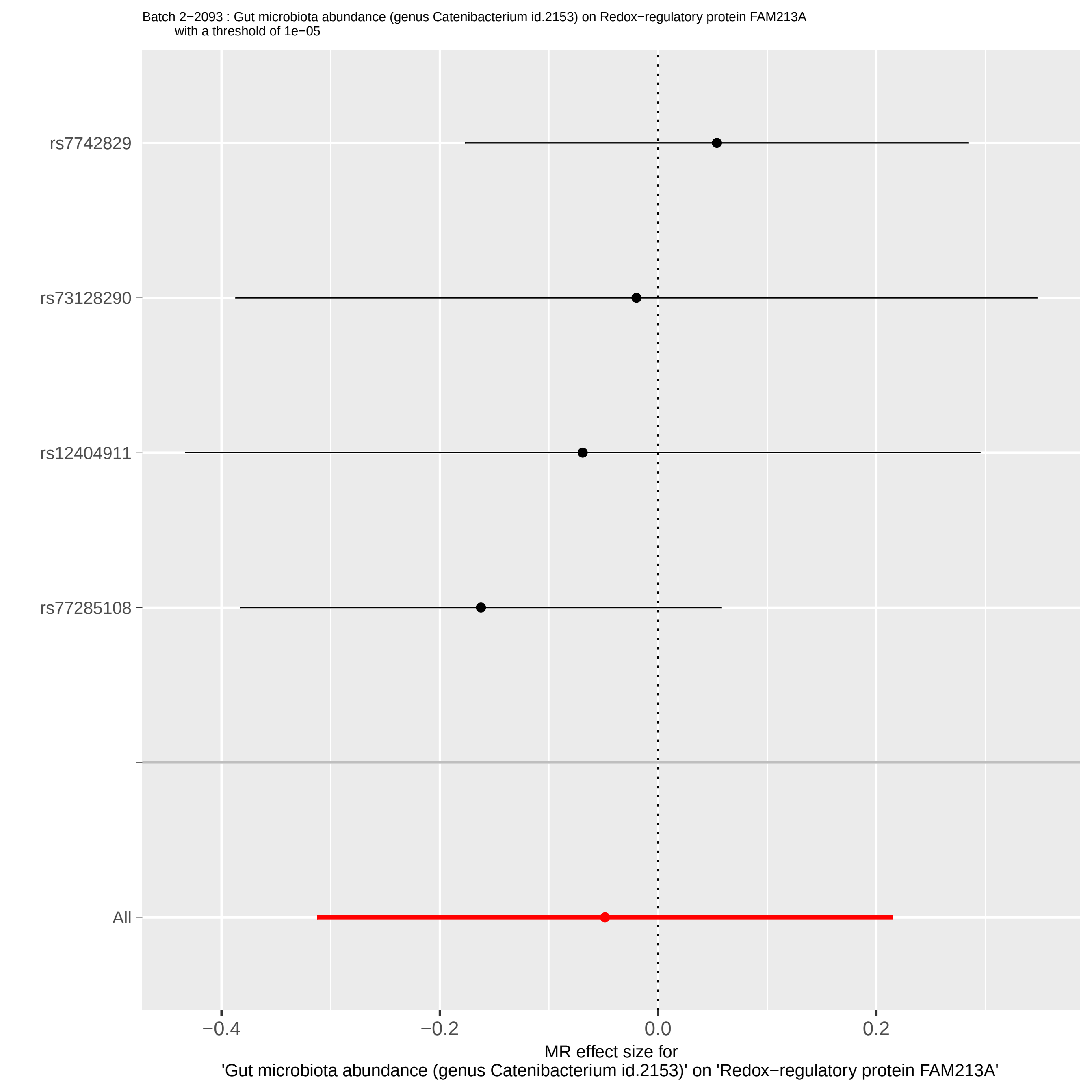

Batch 2−2093 : Gut microbiota abundance (genus Catenibacterium id.2153) on Redox−regulatory protein FAM213A
with a threshold of 1e−05
rs7742829
rs73128290
rs12404911
rs77285108
All
−0.4
−0.2
0.0
0.2
MR effect size for
'Gut microbiota abundance (genus Catenibacterium id.2153)' on 'Redox−regulatory protein FAM213A'

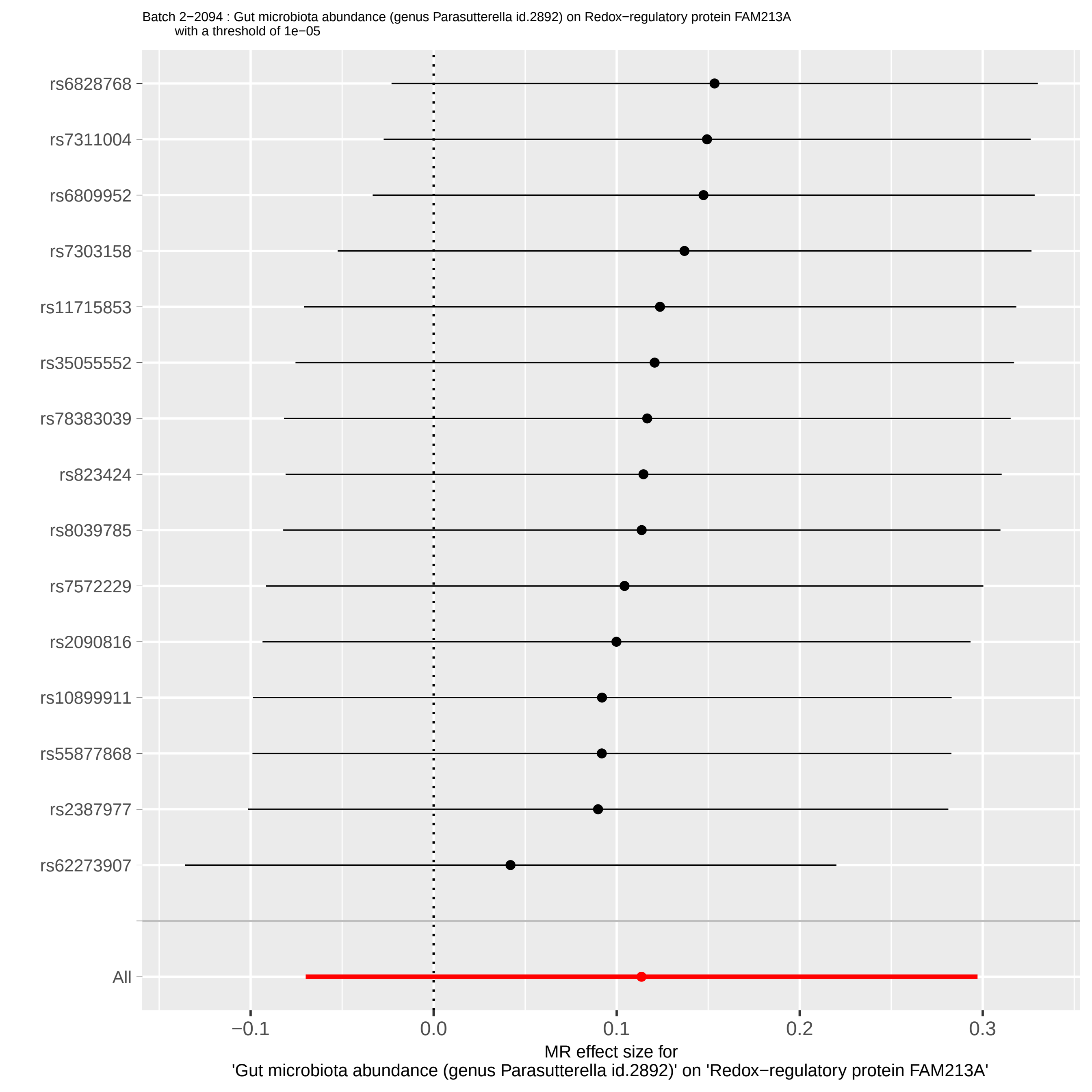
Batch 2−2094 : Gut microbiota abundance (genus Parasutterella id.2892) on Redox−regulatory protein FAM213A
with a threshold of 1e−05
rs6828768
rs7311004
rs6809952
rs7303158
rs11715853
rs35055552
rs78383039
rs823424
rs8039785
rs7572229
rs2090816
rs10899911
rs55877868
rs2387977
rs62273907
All
−0.1
0.0
0.1
0.2
0.3
MR effect size for
'Gut microbiota abundance (genus Parasutterella id.2892)' on 'Redox−regulatory protein FAM213A'

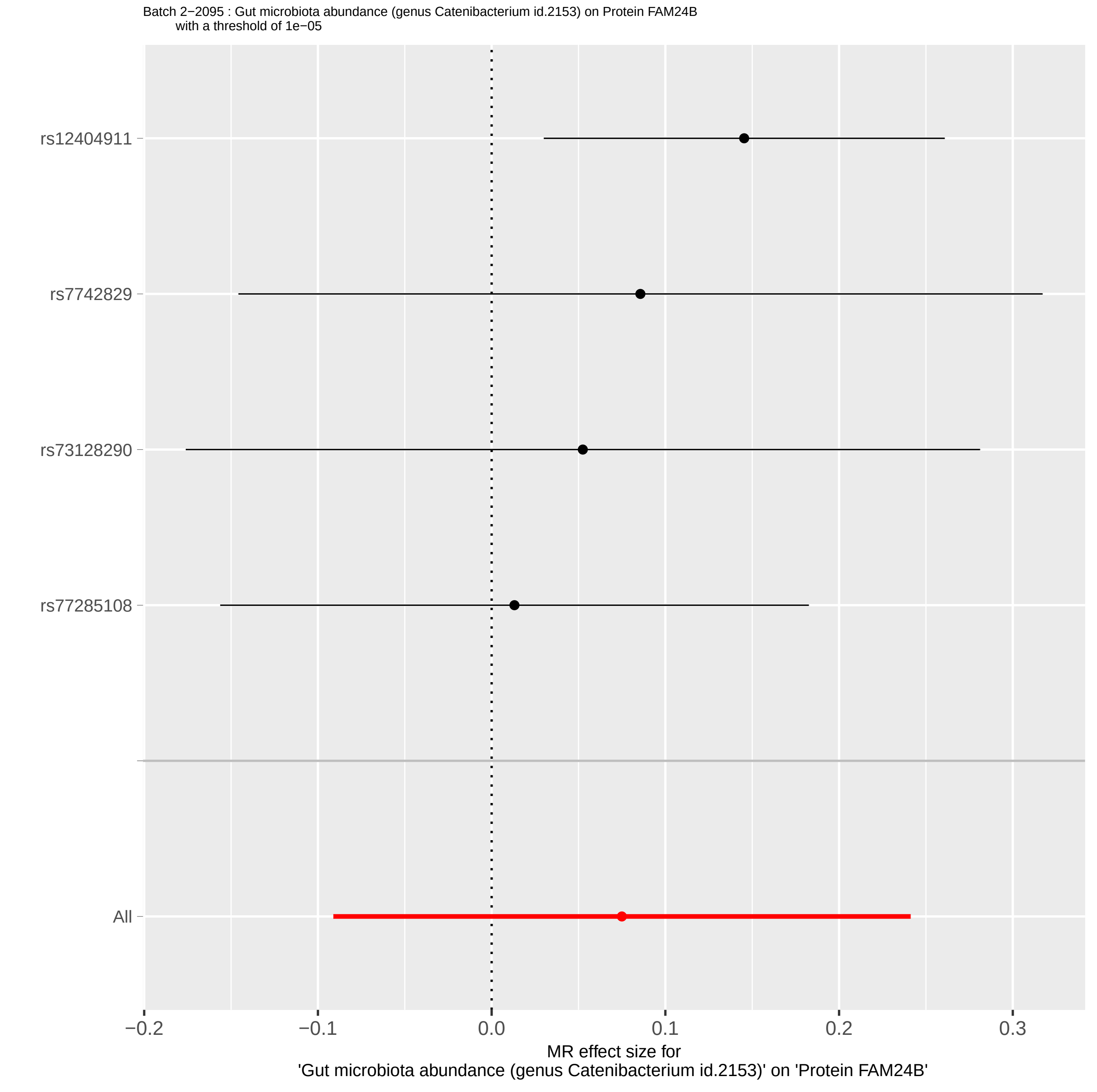

Batch 2−2095 : Gut microbiota abundance (genus Catenibacterium id.2153) on Protein FAM24B
with a threshold of 1e−05
rs12404911
rs7742829
rs73128290
rs77285108
All
−0.2
−0.1
0.0
0.1
0.2
0.3
MR effect size for
'Gut microbiota abundance (genus Catenibacterium id.2153)' on 'Protein FAM24B'

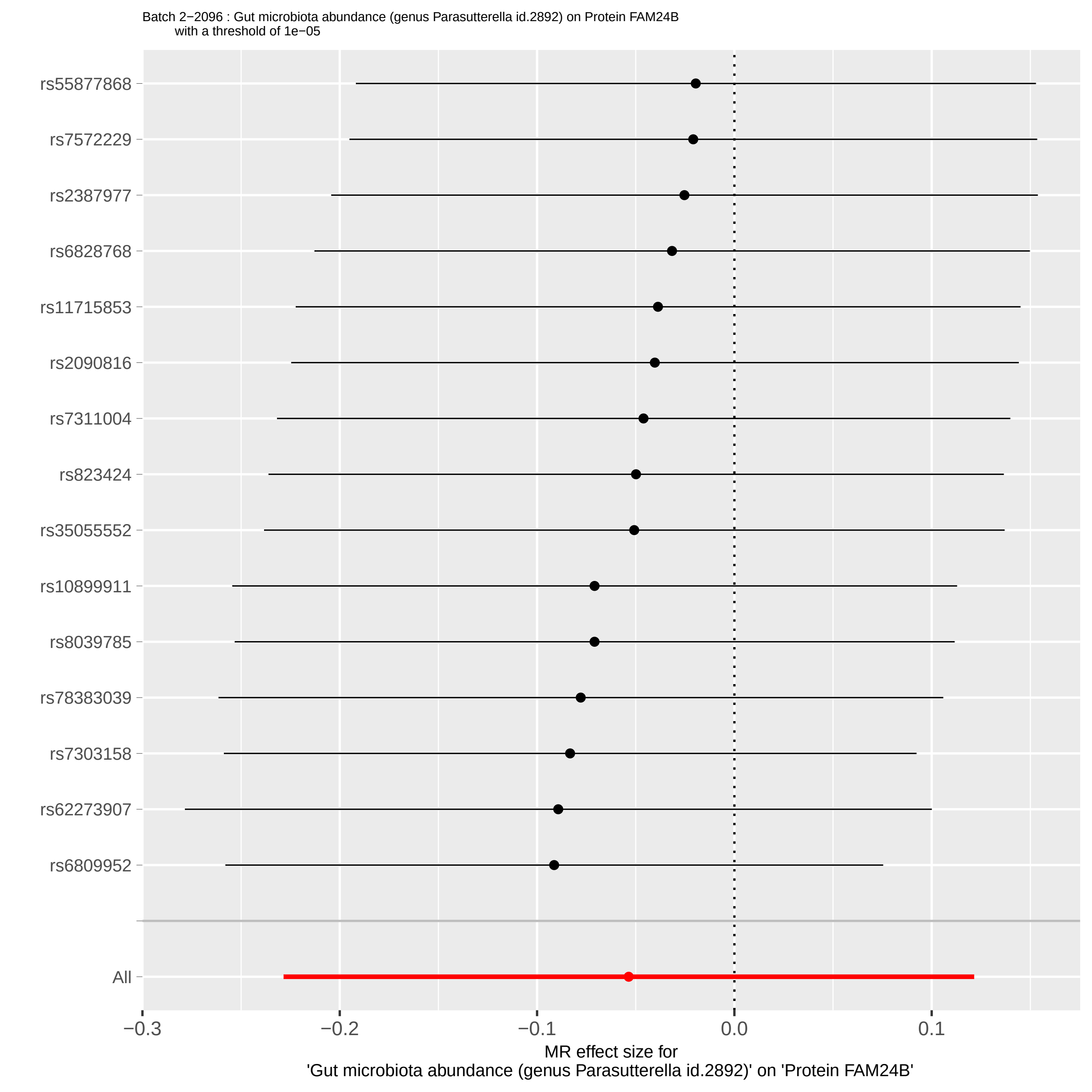
Batch 2−2096 : Gut microbiota abundance (genus Parasutterella id.2892) on Protein FAM24B
with a threshold of 1e−05
rs55877868
rs7572229
rs2387977
rs6828768
rs11715853
rs2090816
rs7311004
rs823424
rs35055552
rs10899911
rs8039785
rs78383039
rs7303158
rs62273907
rs6809952
All
−0.3
−0.2
−0.1
0.0
0.1
MR effect size for
'Gut microbiota abundance (genus Parasutterella id.2892)' on 'Protein FAM24B'

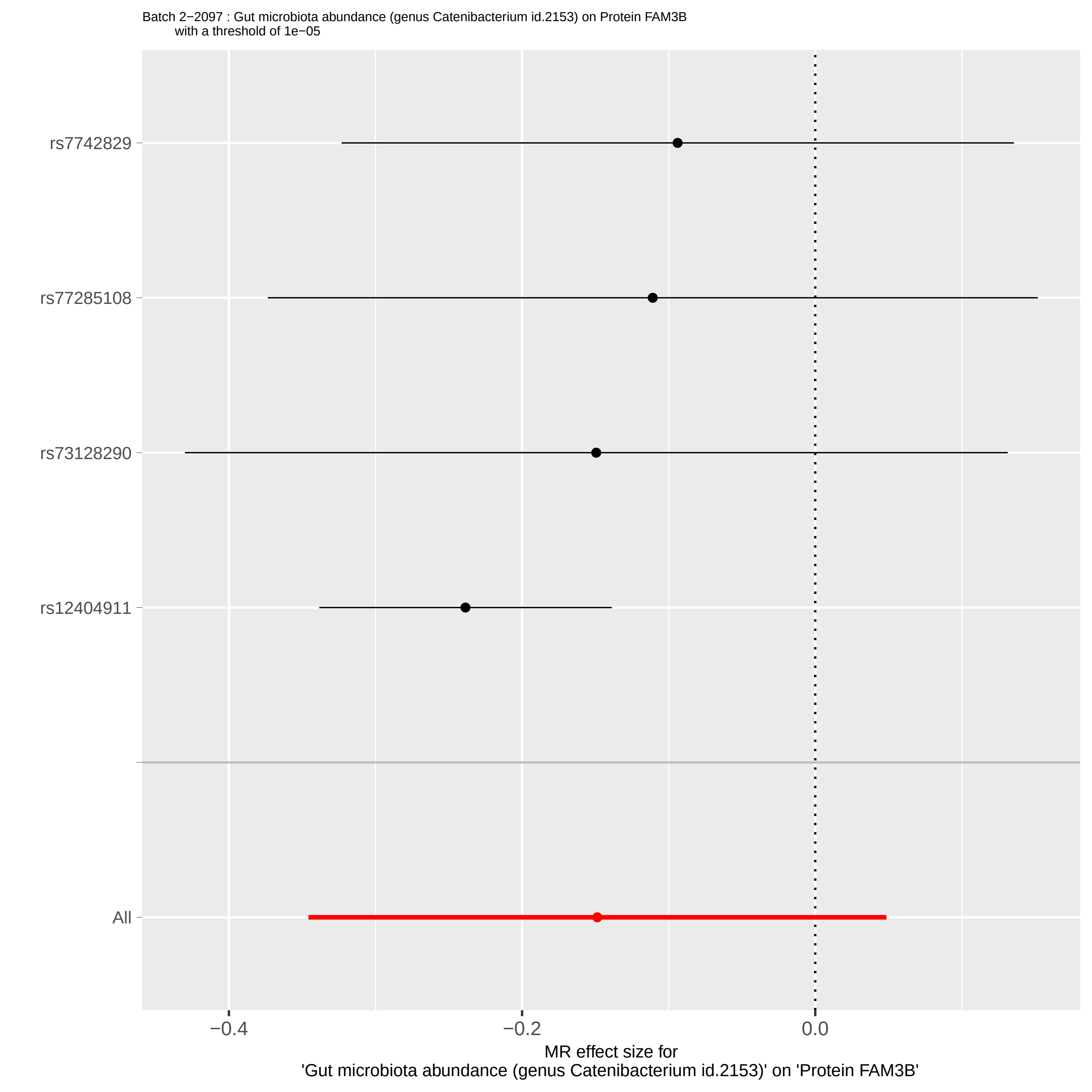
Batch 2−2097 : Gut microbiota abundance (genus Catenibacterium id.2153) on Protein FAM3B
with a threshold of 1e−05
rs7742829
rs77285108
rs73128290
rs12404911
All
−0.4
−0.2
0.0
MR effect size for
'Gut microbiota abundance (genus Catenibacterium id.2153)' on 'Protein FAM3B'

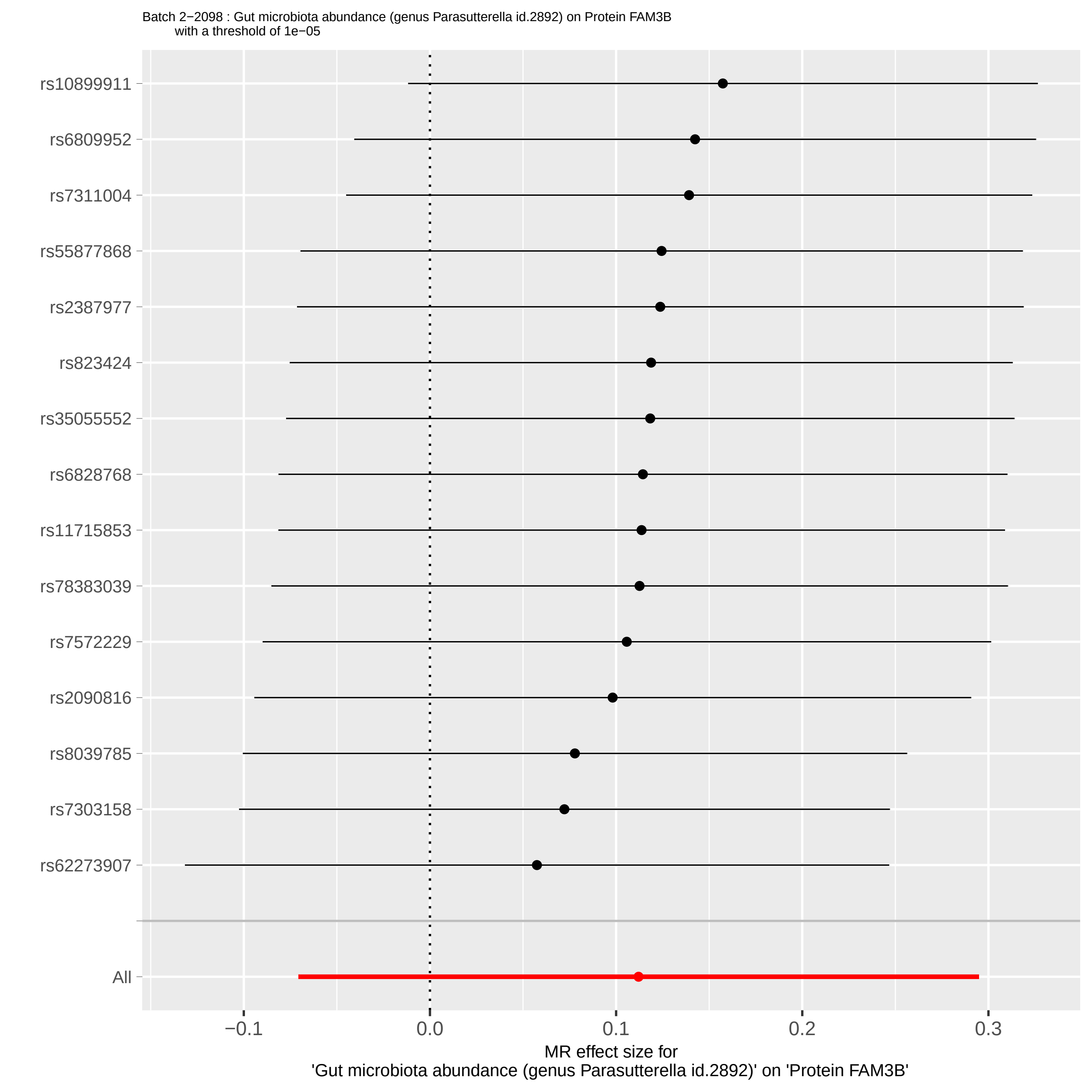
Batch 2−2098 : Gut microbiota abundance (genus Parasutterella id.2892) on Protein FAM3B
with a threshold of 1e−05
rs10899911
rs6809952
rs7311004
rs55877868
rs2387977
rs823424
rs35055552
rs6828768
rs11715853
rs78383039
rs7572229
rs2090816
rs8039785
rs7303158
rs62273907
All
−0.1
0.0
0.1
0.2
0.3
MR effect size for
'Gut microbiota abundance (genus Parasutterella id.2892)' on 'Protein FAM3B'

Batch 2−2099 : Gut microbiota abundance (genus Catenibacterium id.2153) on Protein FAM3B
with a threshold of 1e−05

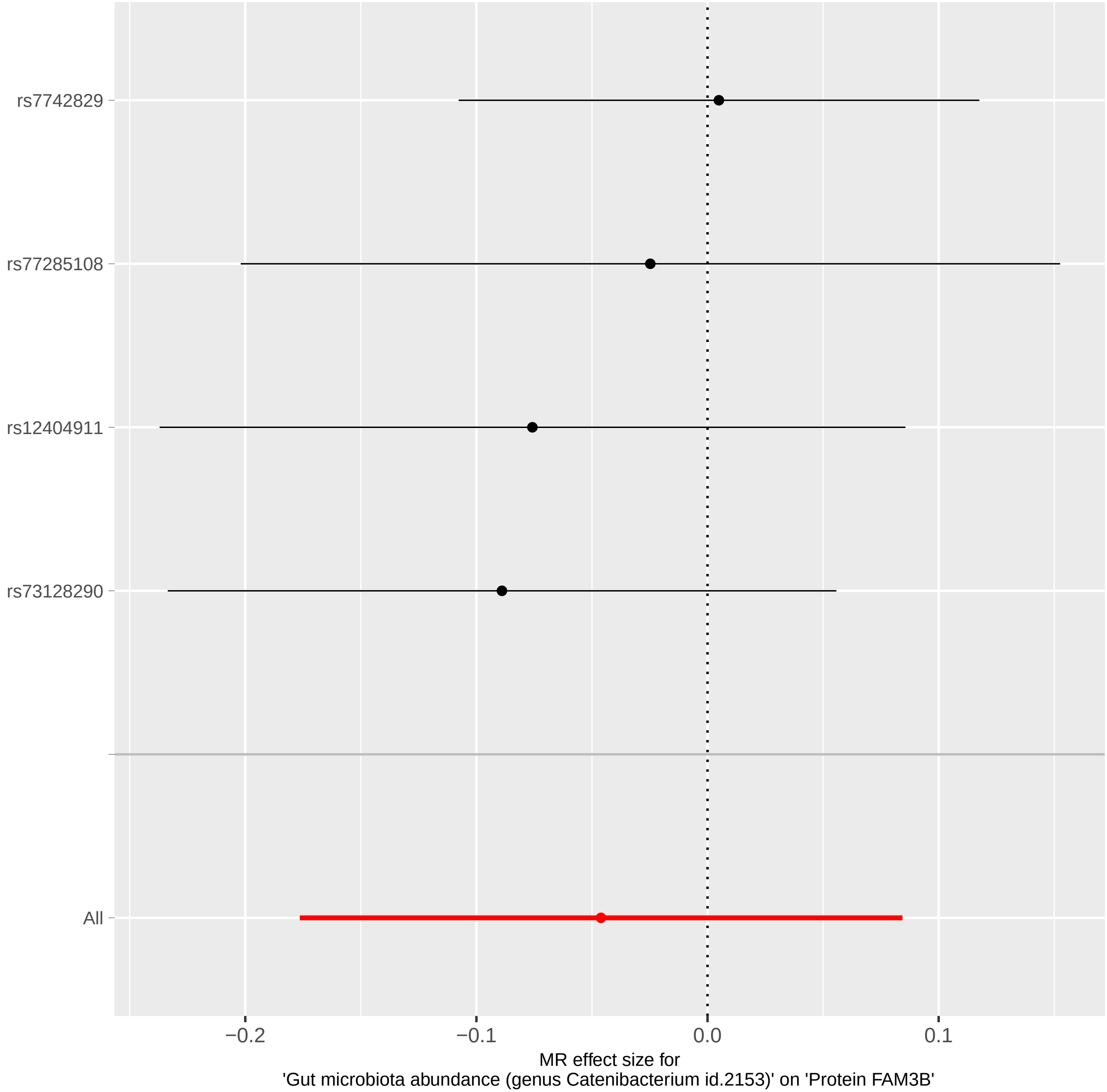

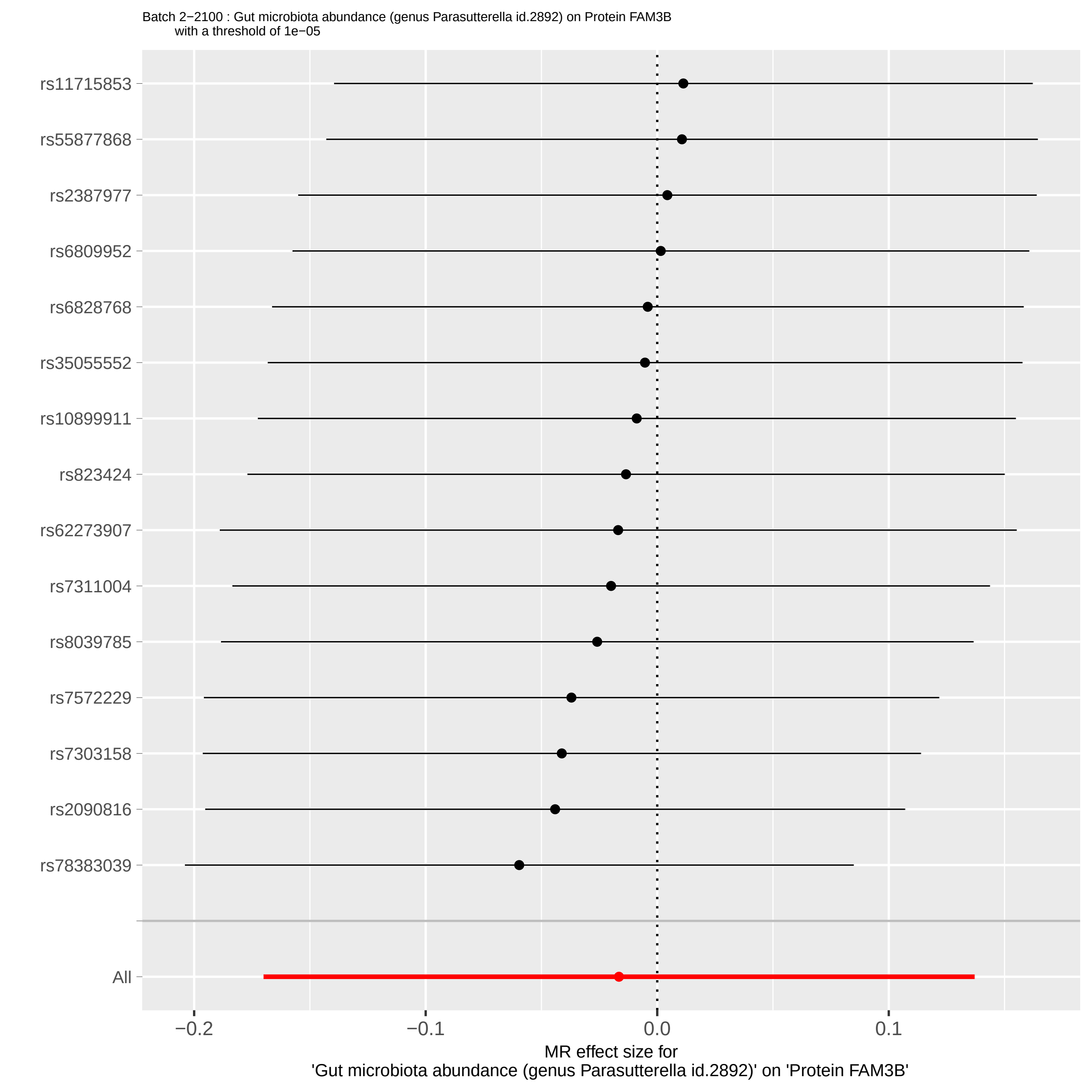

Batch 2−2100 : Gut microbiota abundance (genus Parasutterella id.2892) on Protein FAM3B
with a threshold of 1e−05
rs11715853
rs55877868
rs2387977
rs6809952
rs6828768
rs35055552
rs10899911
rs823424
rs62273907
rs7311004
rs8039785
rs7572229
rs7303158
rs2090816
rs78383039
All
−0.2
−0.1
0.0
0.1
MR effect size for
'Gut microbiota abundance (genus Parasutterella id.2892)' on 'Protein FAM3B'

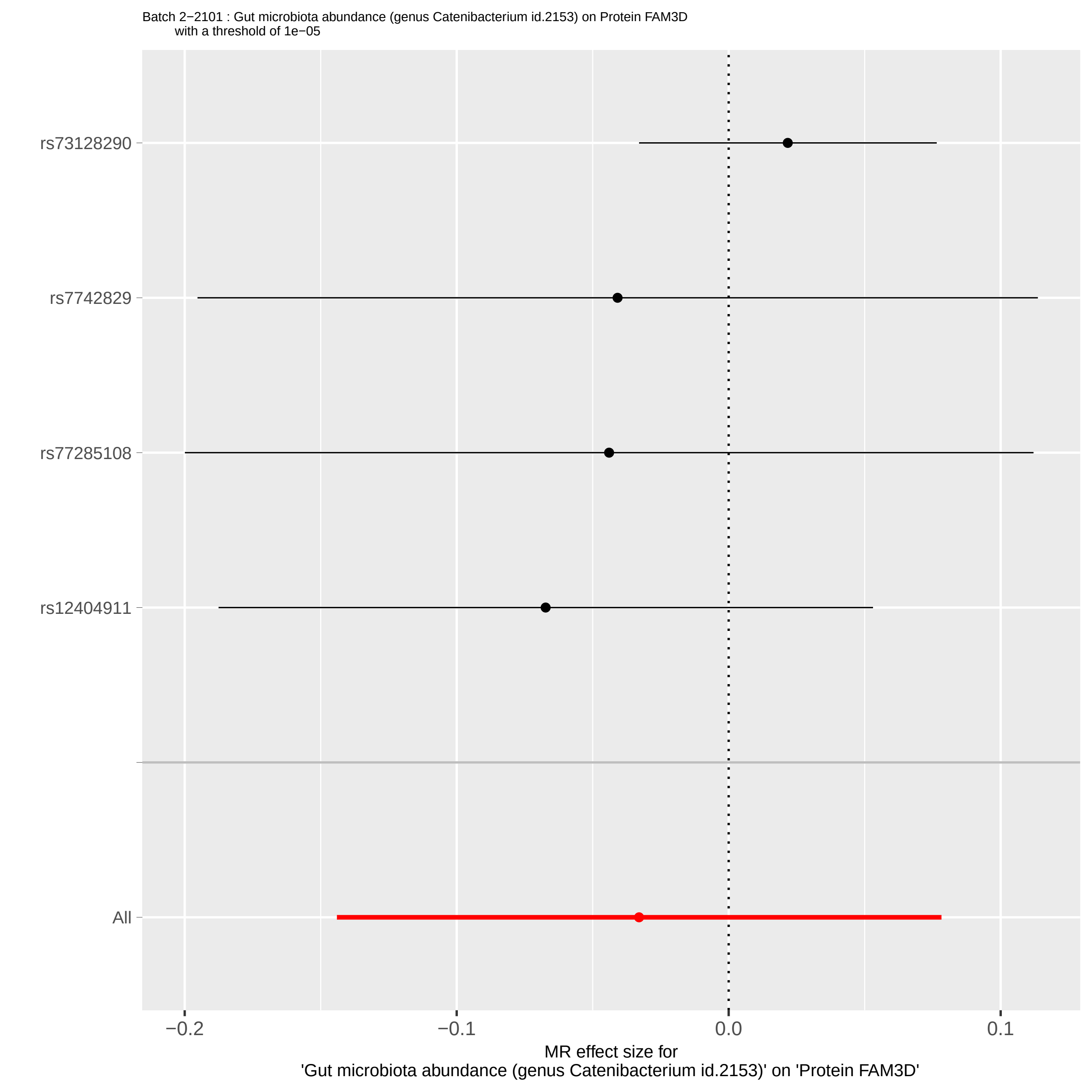

Batch 2−2101 : Gut microbiota abundance (genus Catenibacterium id.2153) on Protein FAM3D
with a threshold of 1e−05
rs73128290
rs7742829
rs77285108
rs12404911
All
−0.2
−0.1
0.0
0.1
MR effect size for
'Gut microbiota abundance (genus Catenibacterium id.2153)' on 'Protein FAM3D'

Batch 2−2102 : Gut microbiota abundance (genus Parasutterella id.2892) on Protein FAM3D
with a threshold of 1e−05

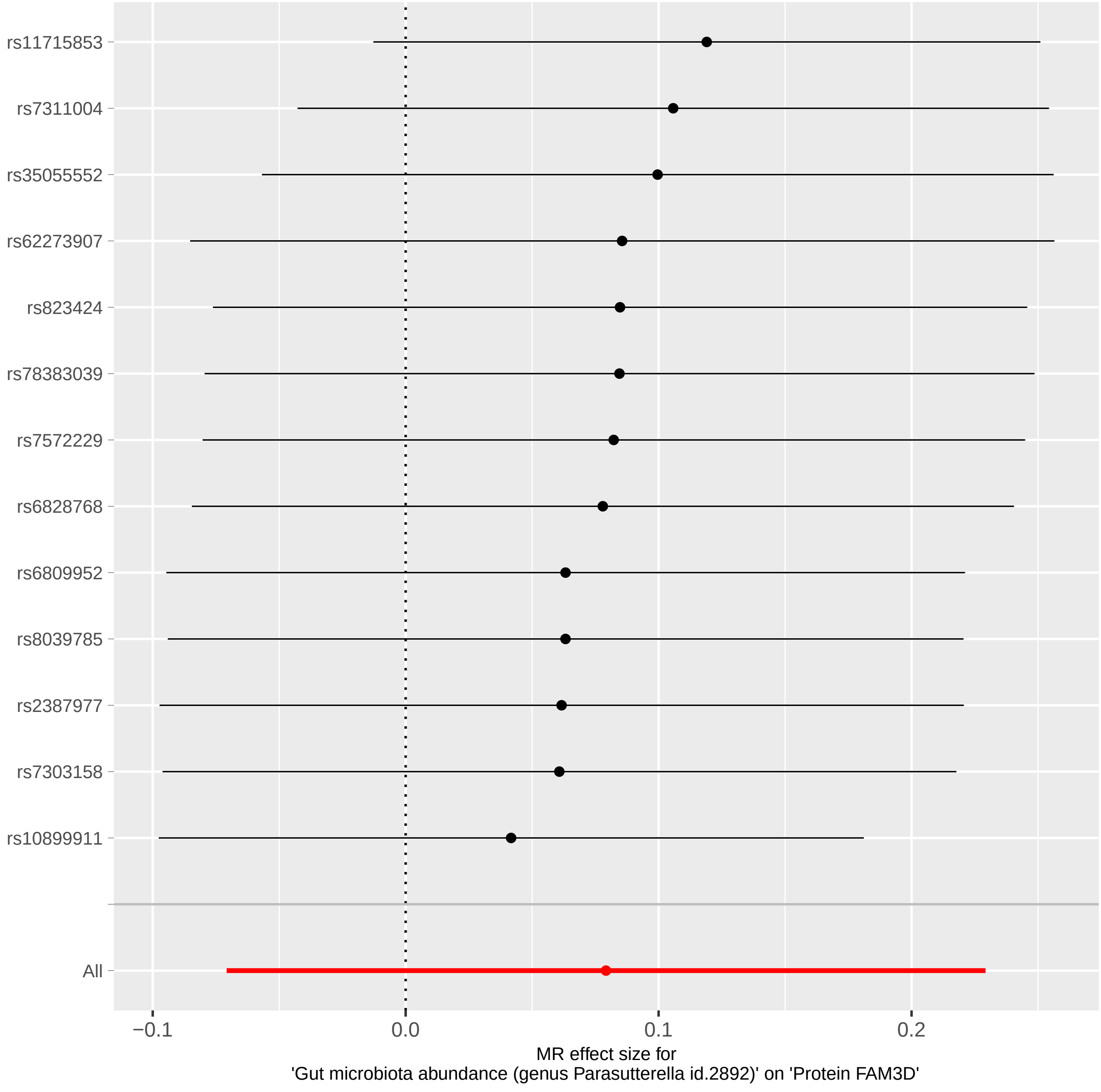

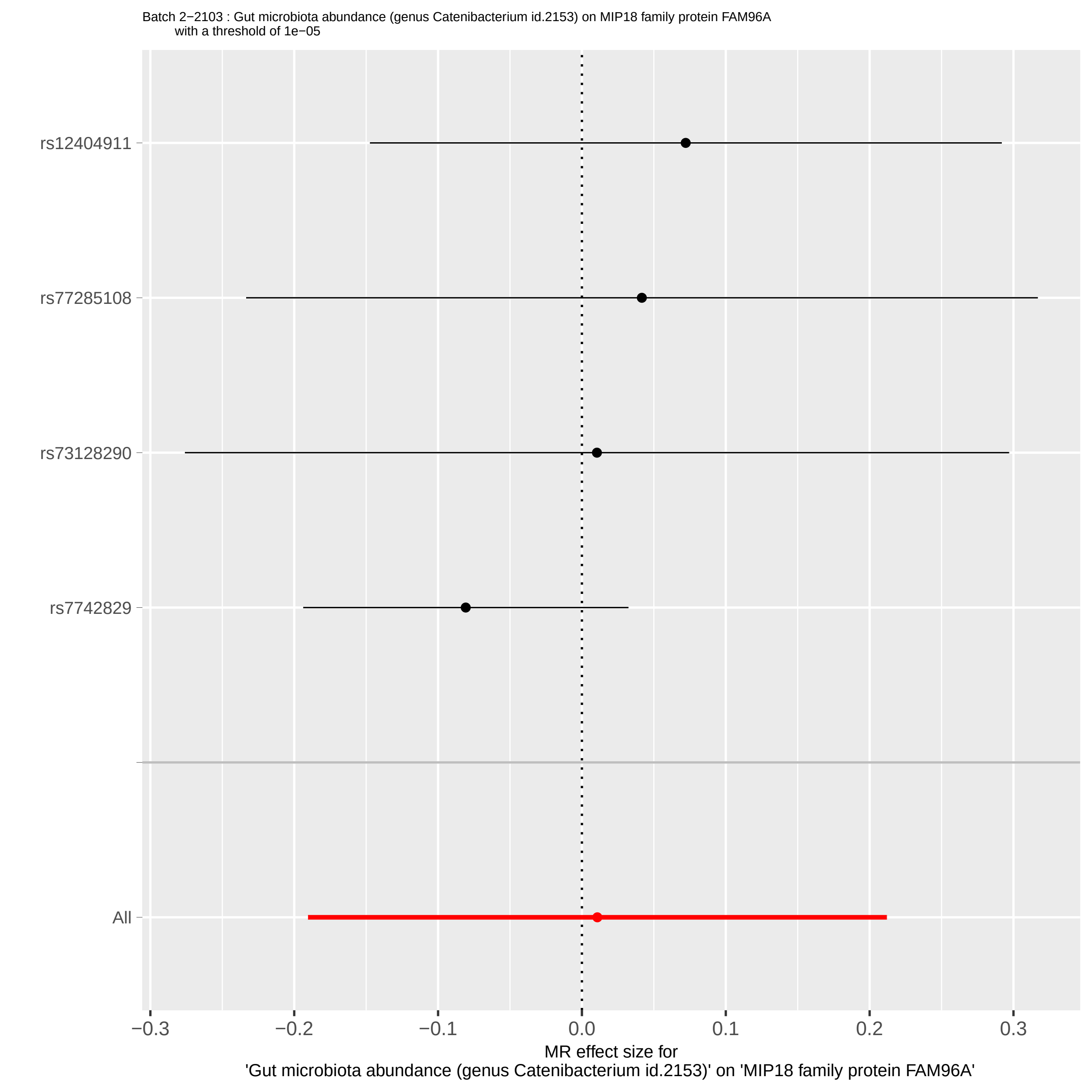

Batch 2−2103 : Gut microbiota abundance (genus Catenibacterium id.2153) on MIP18 family protein FAM96A
with a threshold of 1e−05
rs12404911
rs77285108
rs73128290
rs7742829
All
−0.3
−0.2
−0.1
0.0
0.1
0.2
0.3
MR effect size for
'Gut microbiota abundance (genus Catenibacterium id.2153)' on 'MIP18 family protein FAM96A'

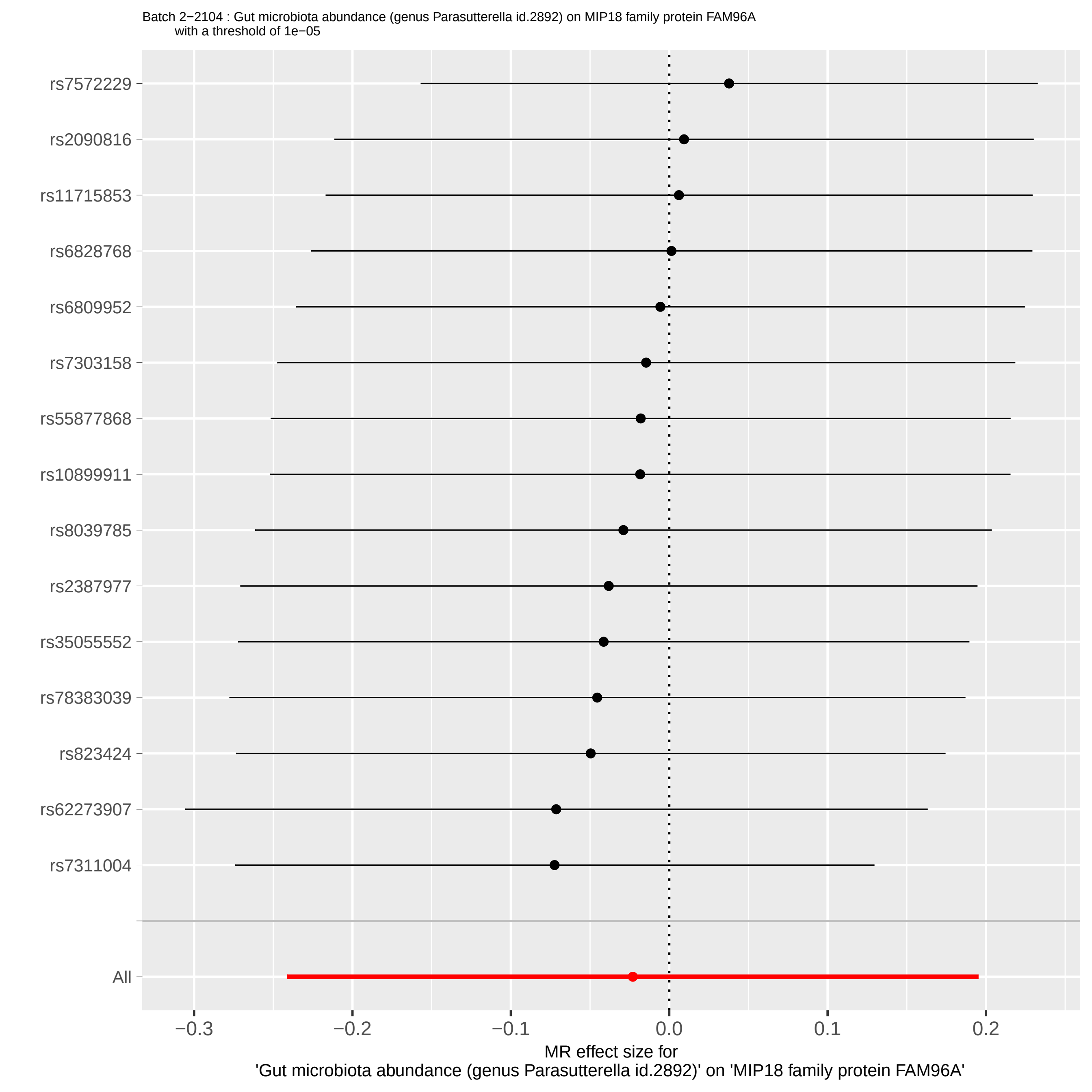

Batch 2−2104 : Gut microbiota abundance (genus Parasutterella id.2892) on MIP18 family protein FAM96A
with a threshold of 1e−05
rs7572229
rs2090816
rs11715853
rs6828768
rs6809952
rs7303158
rs55877868
rs10899911
rs8039785
rs2387977
rs35055552
rs78383039
rs823424
rs62273907
rs7311004
All
−0.3
−0.2
−0.1
0.0
0.1
0.2
MR effect size for
'Gut microbiota abundance (genus Parasutterella id.2892)' on 'MIP18 family protein FAM96A'

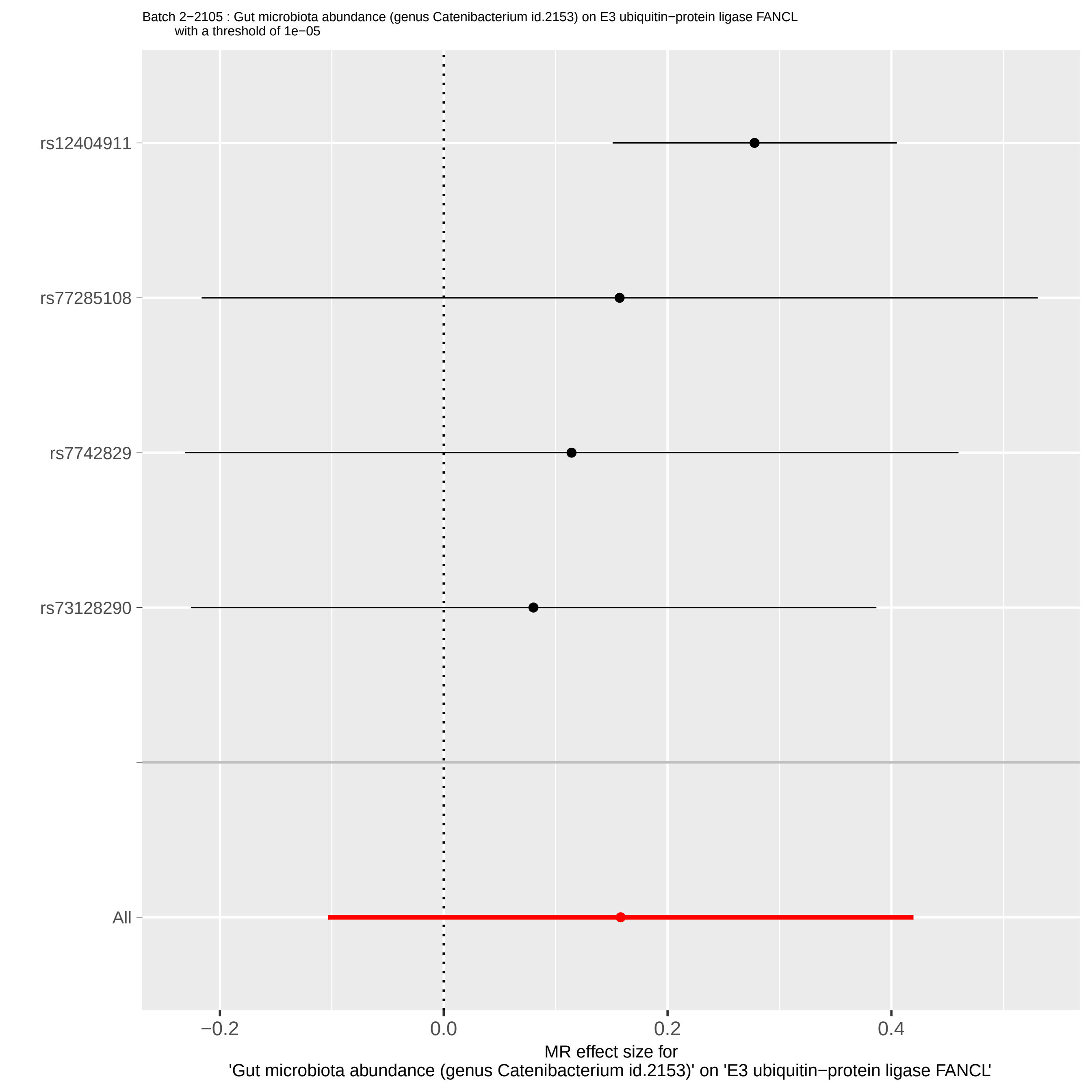
Batch 2−2105 : Gut microbiota abundance (genus Catenibacterium id.2153) on E3 ubiquitin−protein ligase FANCL
with a threshold of 1e−05
rs12404911
rs77285108
rs7742829
rs73128290
All
−0.2
0.0
0.2
0.4
MR effect size for
'Gut microbiota abundance (genus Catenibacterium id.2153)' on 'E3 ubiquitin−protein ligase FANCL'

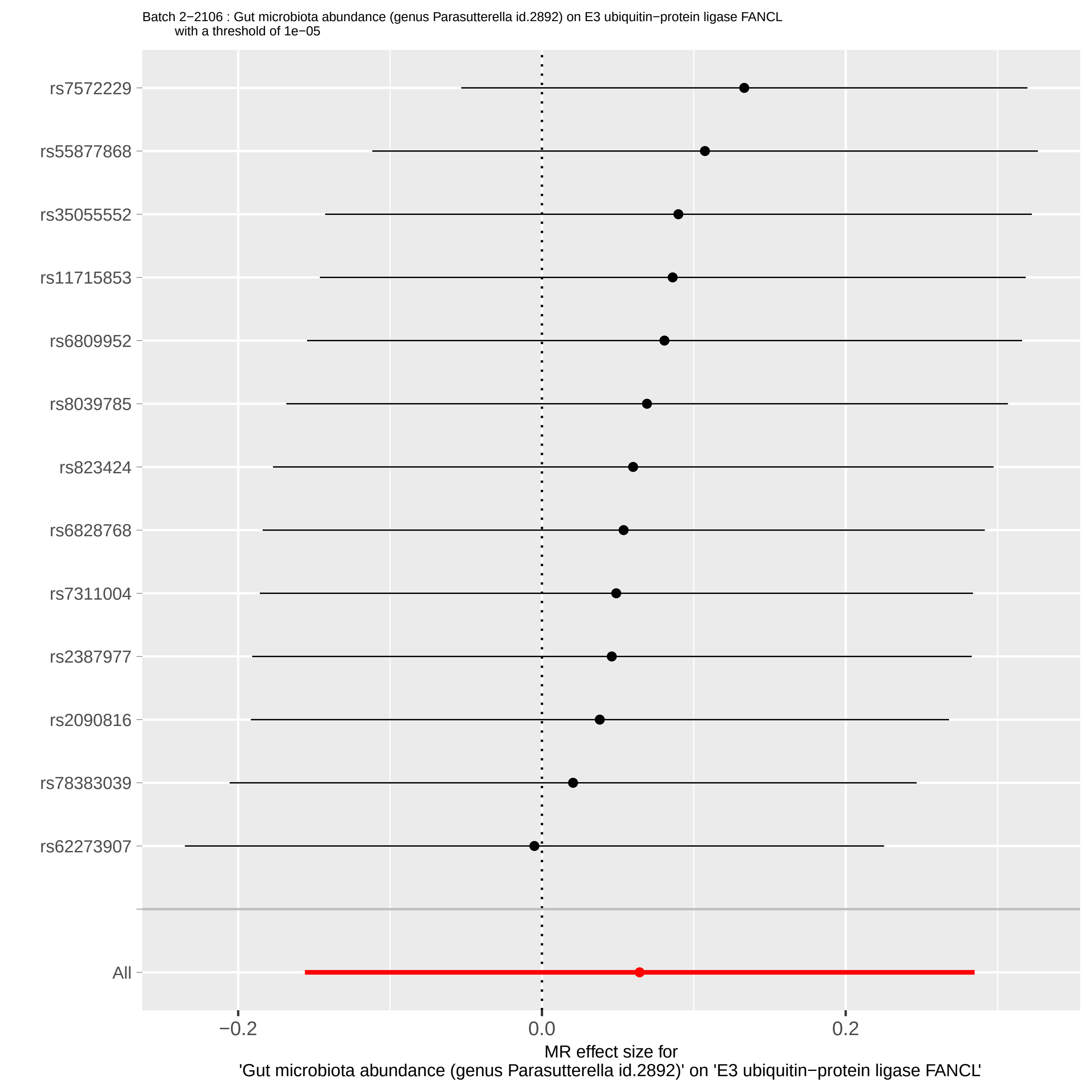
Batch 2−2106 : Gut microbiota abundance (genus Parasutterella id.2892) on E3 ubiquitin−protein ligase FANCL
with a threshold of 1e−05
rs7572229
rs55877868
rs35055552
rs11715853
rs6809952
rs8039785
rs823424
rs6828768
rs7311004
rs2387977
rs2090816
rs78383039
rs62273907
All
−0.2
0.0
0.2
MR effect size for
'Gut microbiota abundance (genus Parasutterella id.2892)' on 'E3 ubiquitin−protein ligase FANCL'

Batch 2−2107 : Gut microbiota abundance (genus Catenibacterium id.2153) on Prolyl endopeptidase FAP
with a threshold of 1e−05

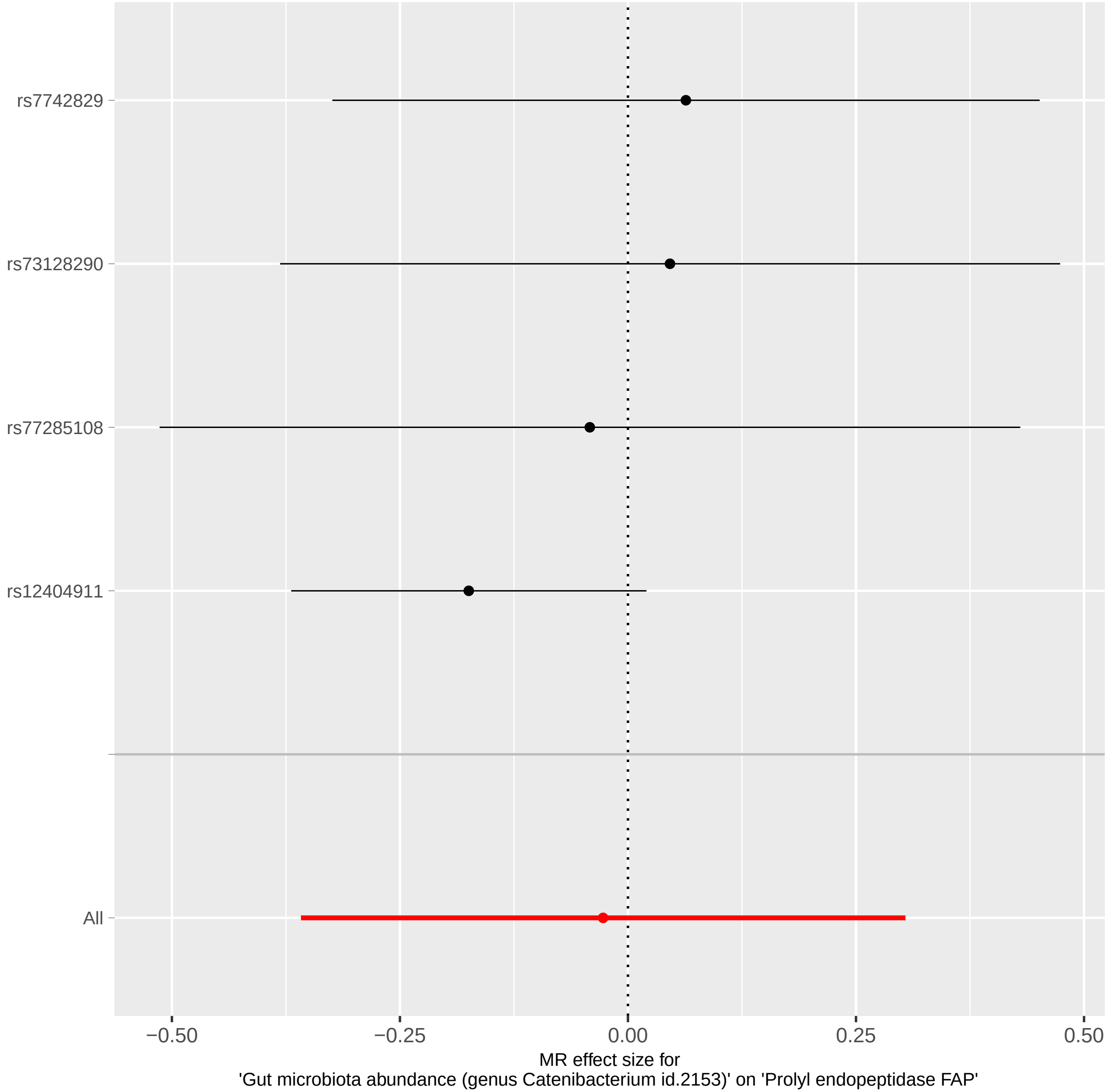

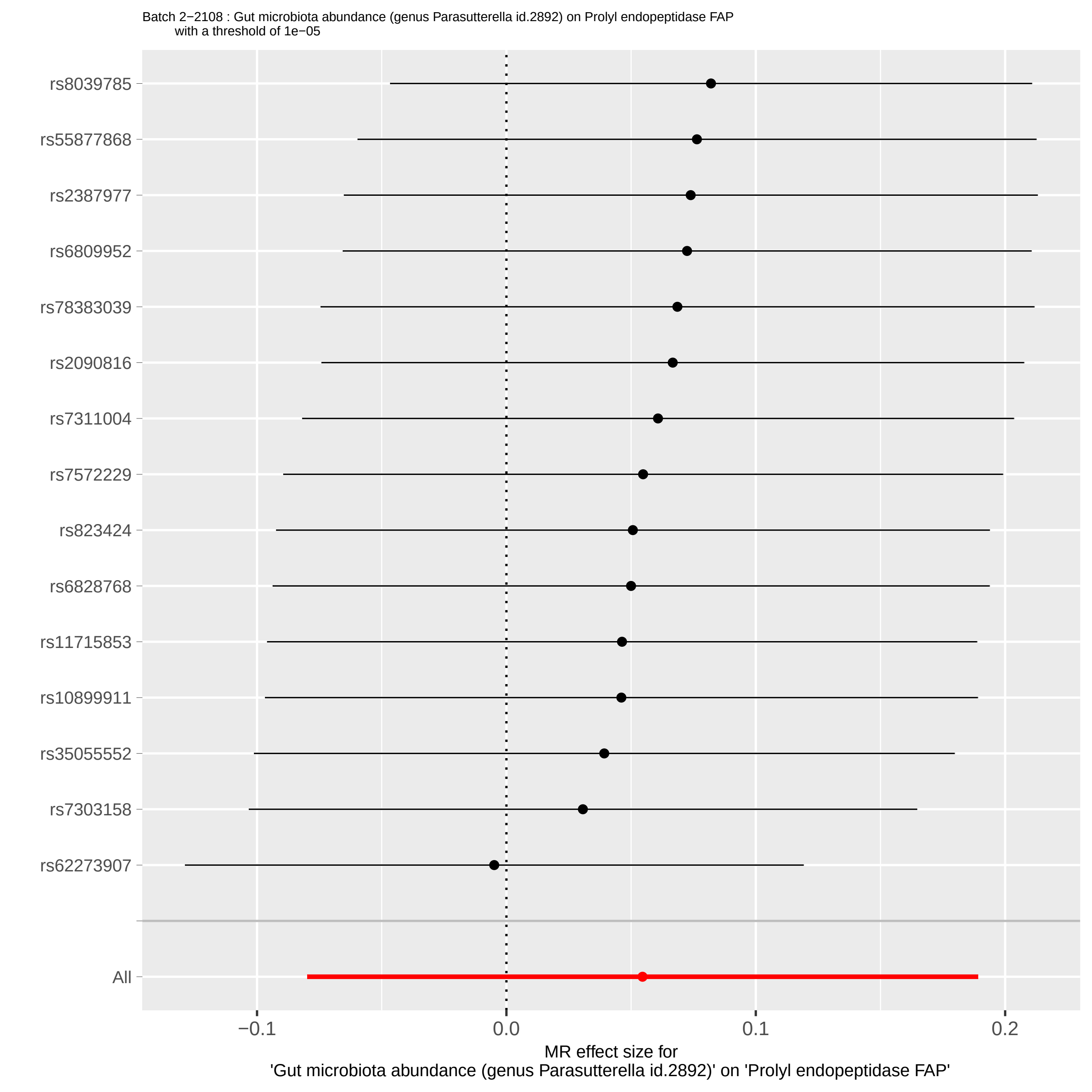

Batch 2−2108 : Gut microbiota abundance (genus Parasutterella id.2892) on Prolyl endopeptidase FAP
with a threshold of 1e−05
rs8039785
rs55877868
rs2387977
rs6809952
rs78383039
rs2090816
rs7311004
rs7572229
rs823424
rs6828768
rs11715853
rs10899911
rs35055552
rs7303158
rs62273907
All
−0.1
0.0
0.1
0.2
MR effect size for
'Gut microbiota abundance (genus Parasutterella id.2892)' on 'Prolyl endopeptidase FAP'

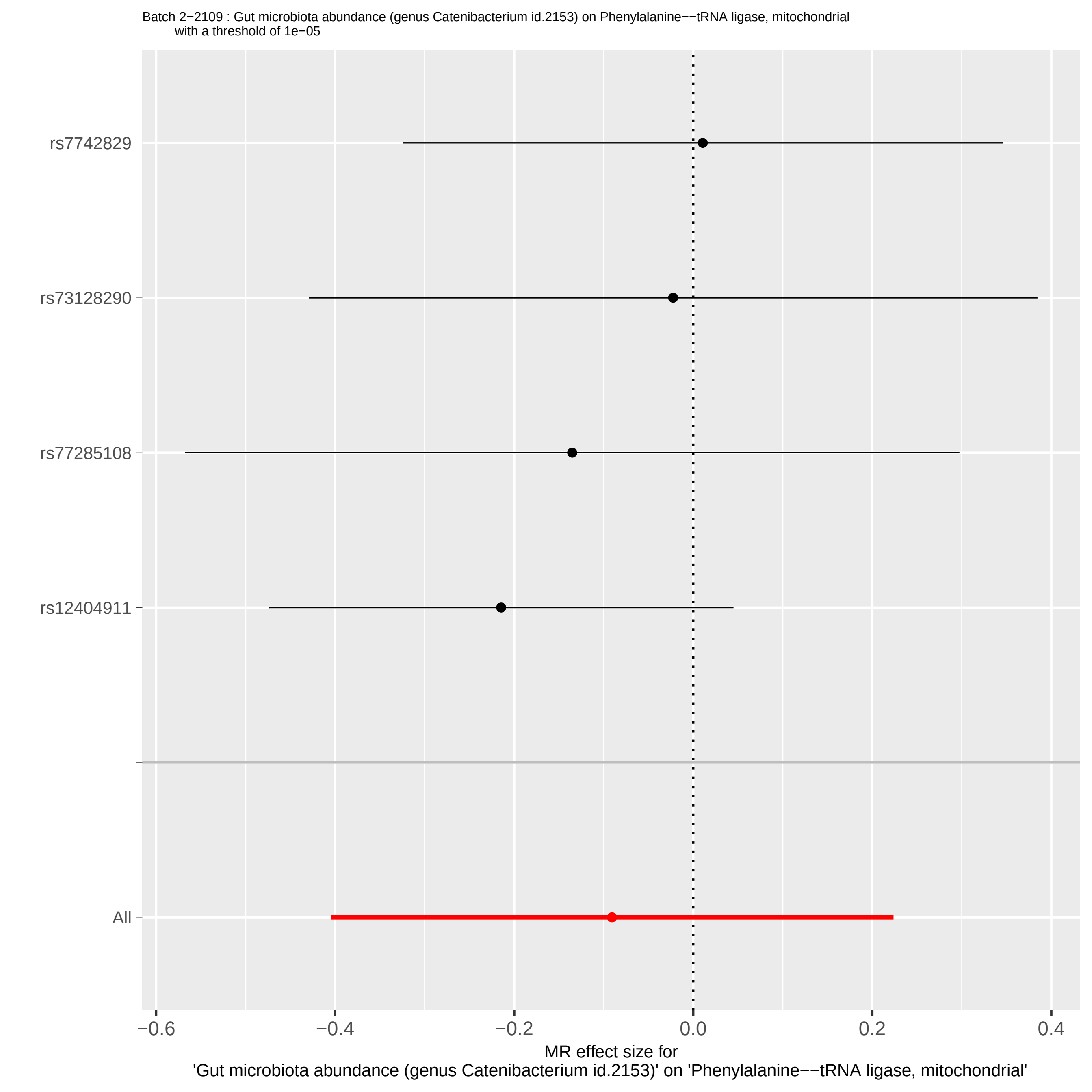

Batch 2−2109 : Gut microbiota abundance (genus Catenibacterium id.2153) on Phenylalanine−−tRNA ligase, mitochondrial
with a threshold of 1e−05
rs7742829
rs73128290
rs77285108
rs12404911
All
−0.6
−0.4
−0.2
0.0
0.2
0.4
MR effect size for
'Gut microbiota abundance (genus Catenibacterium id.2153)' on 'Phenylalanine−−tRNA ligase, mitochondrial'

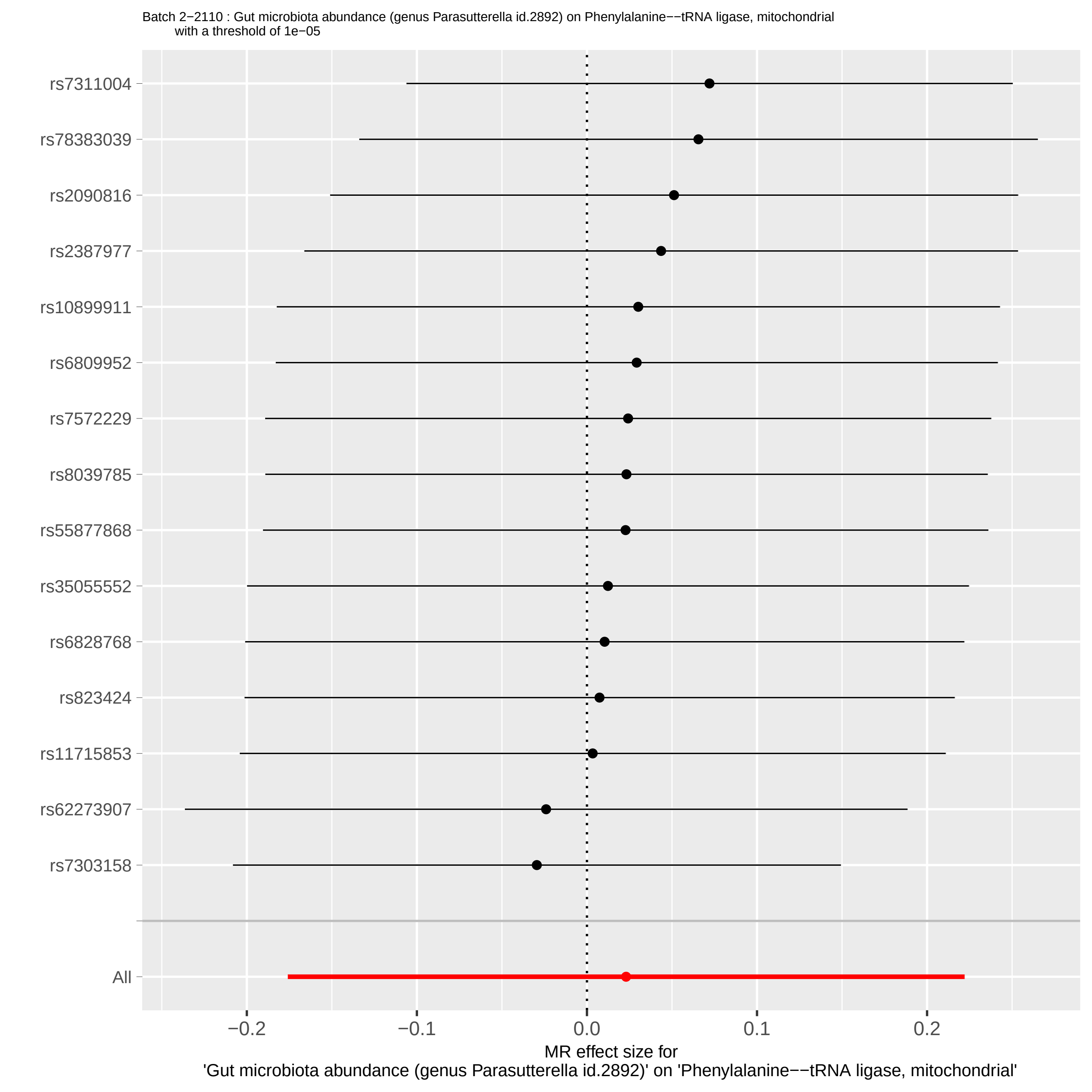

Batch 2−2110 : Gut microbiota abundance (genus Parasutterella id.2892) on Phenylalanine−−tRNA ligase, mitochondrial
with a threshold of 1e−05
rs7311004
rs78383039
rs2090816
rs2387977
rs10899911
rs6809952
rs7572229
rs8039785
rs55877868
rs35055552
rs6828768
rs823424
rs11715853
rs62273907
rs7303158
All
−0.2
−0.1
0.0
0.1
0.2
MR effect size for
'Gut microbiota abundance (genus Parasutterella id.2892)' on 'Phenylalanine−−tRNA ligase, mitochondrial'

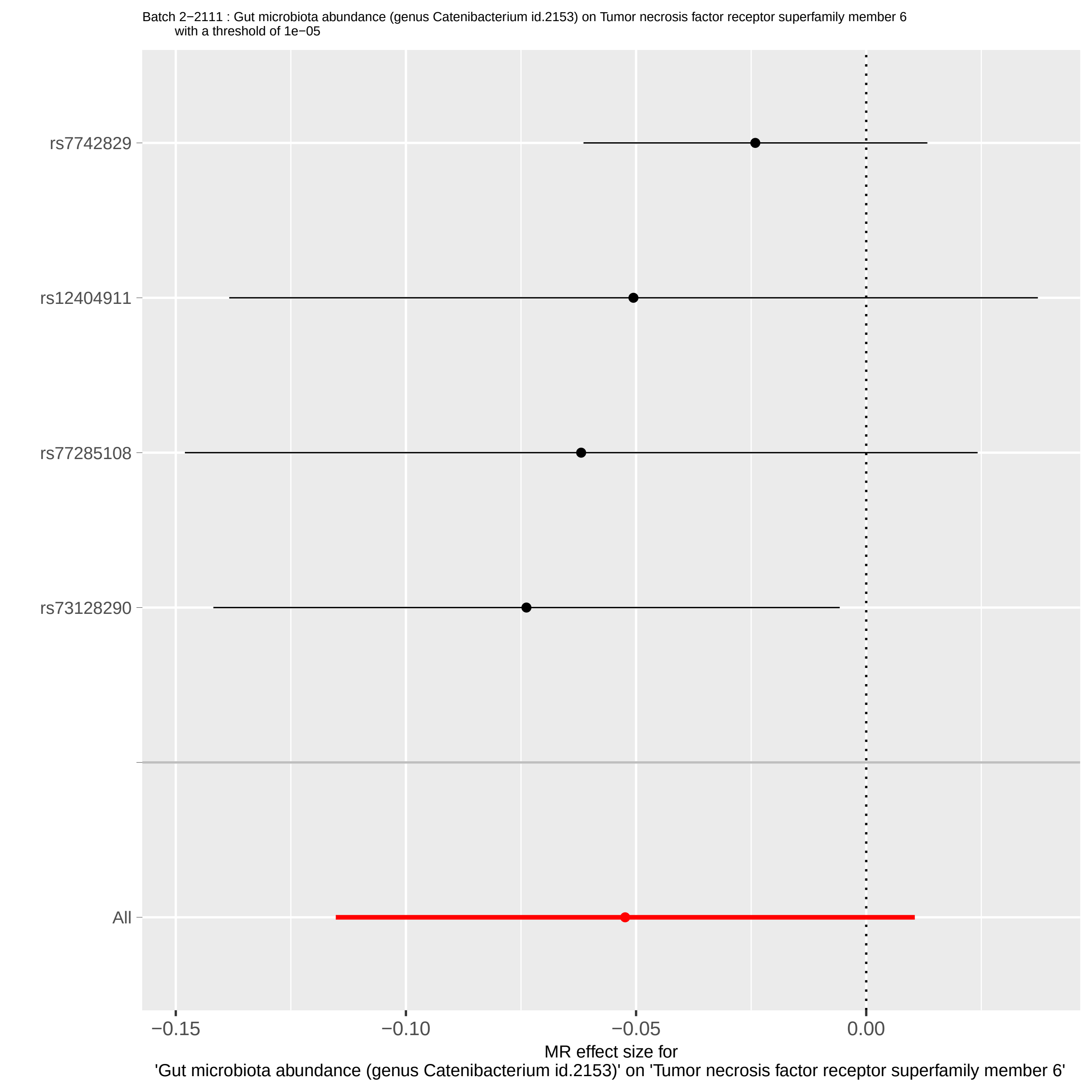
Batch 2−2111 : Gut microbiota abundance (genus Catenibacterium id.2153) on Tumor necrosis factor receptor superfamily member 6
with a threshold of 1e−05
rs7742829
rs12404911
rs77285108
rs73128290
All
−0.15
−0.10
−0.05
0.00
MR effect size for
'Gut microbiota abundance (genus Catenibacterium id.2153)' on 'Tumor necrosis factor receptor superfamily member 6'

Batch 2−2112 : Gut microbiota abundance (genus Parasutterella id.2892) on Tumor necrosis factor receptor superfamily member 6
with a threshold of 1e−05

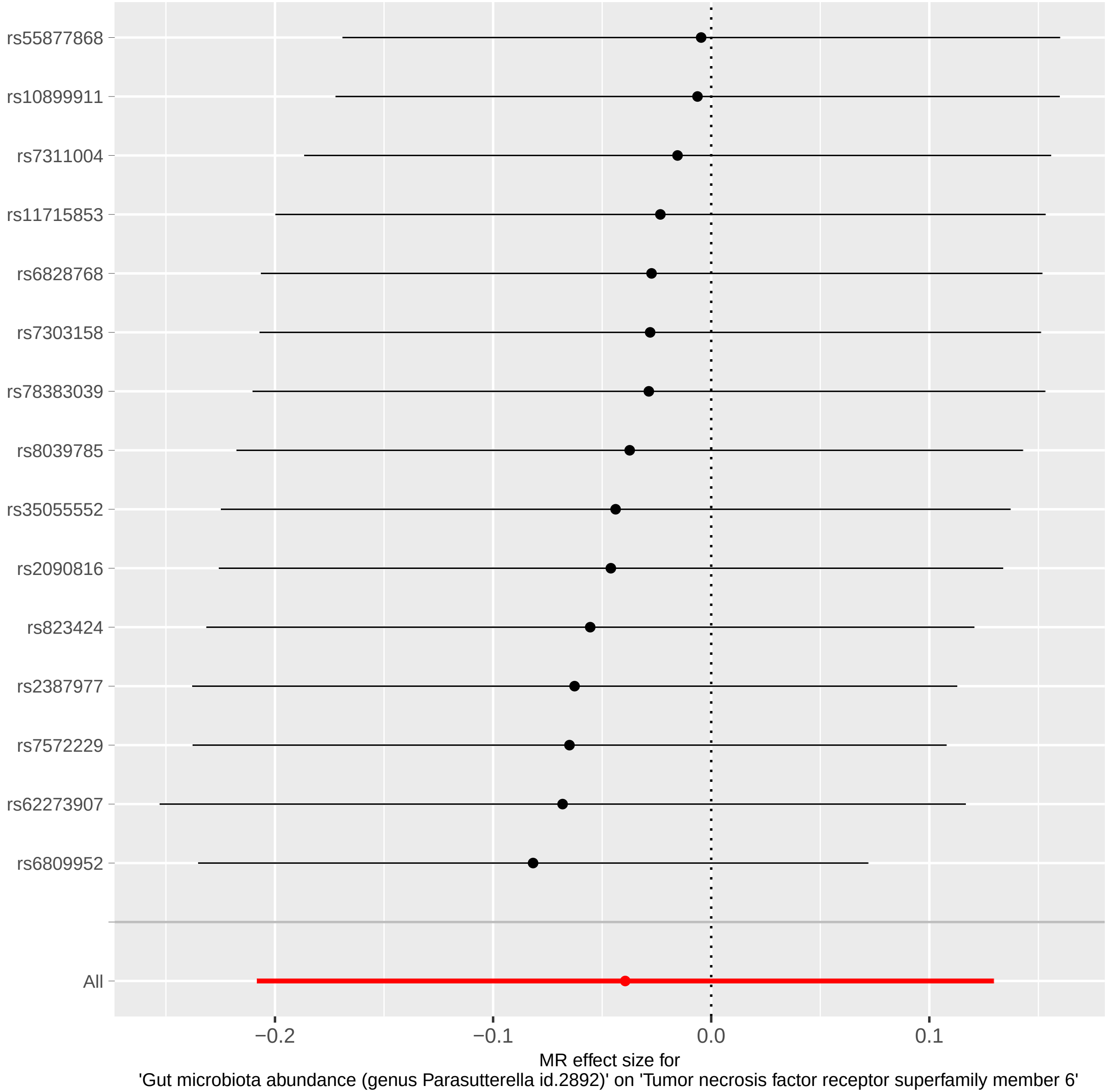

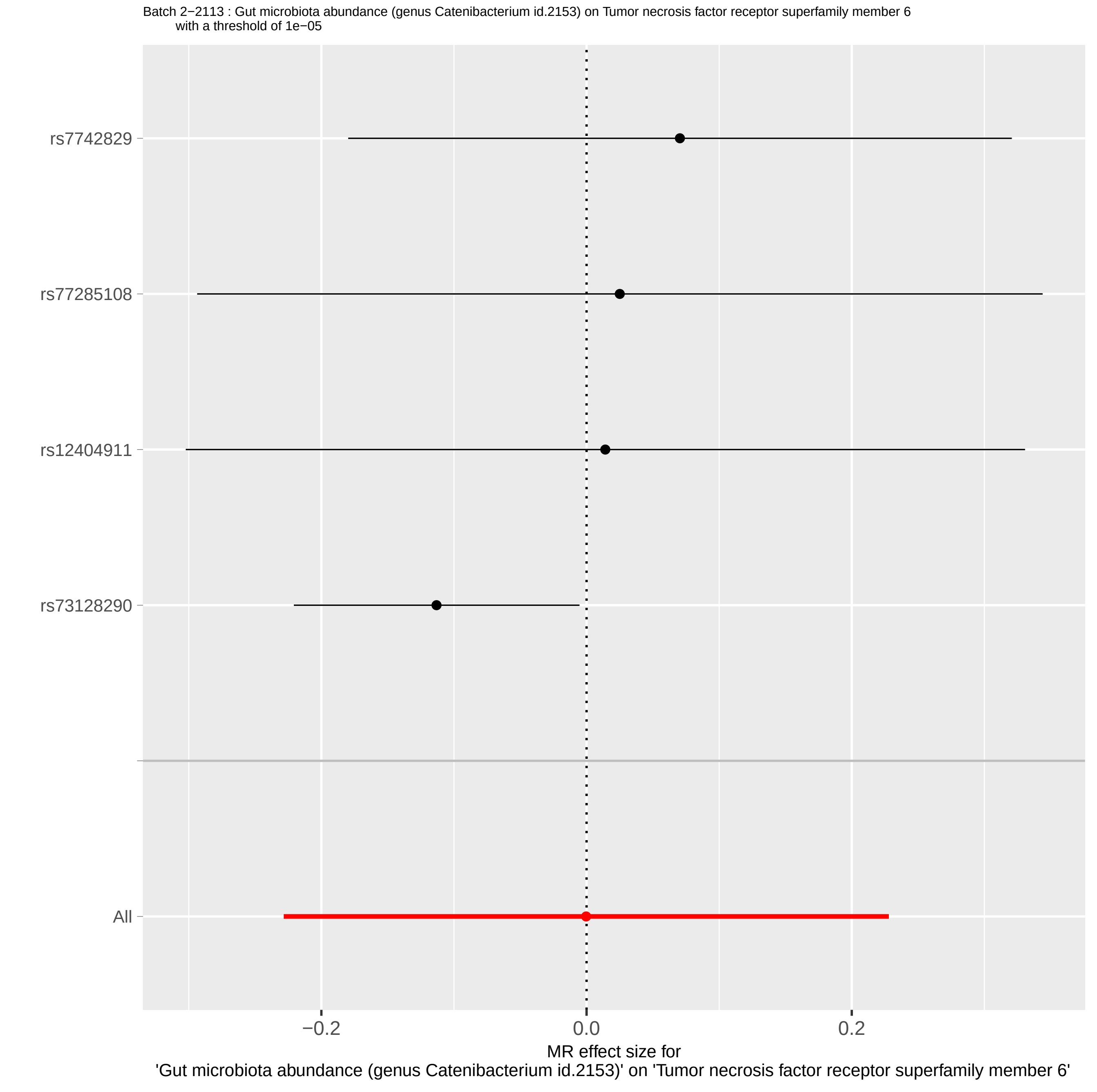

Batch 2−2113 : Gut microbiota abundance (genus Catenibacterium id.2153) on Tumor necrosis factor receptor superfamily member 6
with a threshold of 1e−05
rs7742829
rs77285108
rs12404911
rs73128290
All
−0.2
0.0
0.2
MR effect size for
'Gut microbiota abundance (genus Catenibacterium id.2153)' on 'Tumor necrosis factor receptor superfamily member 6'

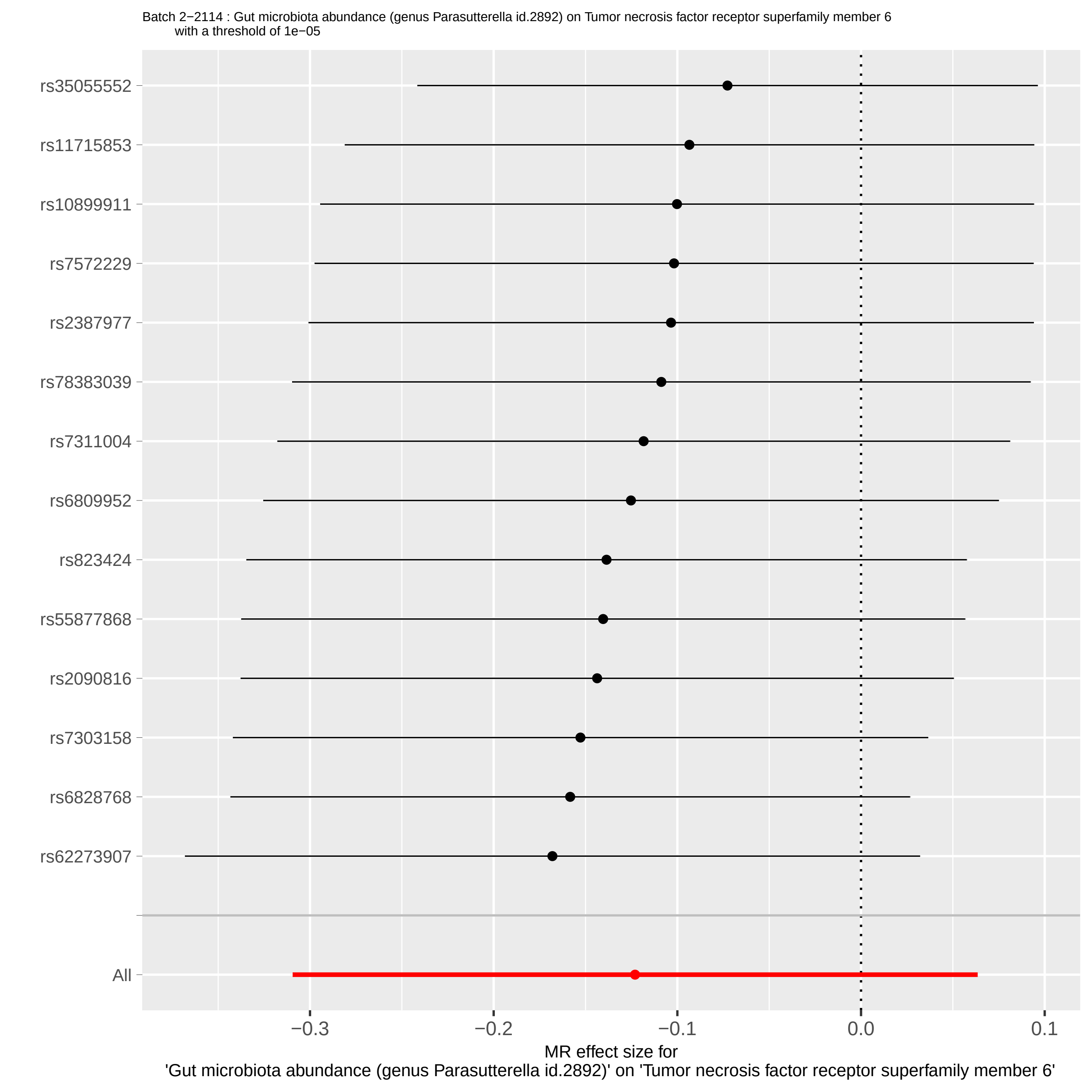
Batch 2−2114 : Gut microbiota abundance (genus Parasutterella id.2892) on Tumor necrosis factor receptor superfamily member 6
with a threshold of 1e−05
rs35055552
rs11715853
rs10899911
rs7572229
rs2387977
rs78383039
rs7311004
rs6809952
rs823424
rs55877868
rs2090816
rs7303158
rs6828768
rs62273907
All
−0.3
−0.2
−0.1
0.0
0.1
MR effect size for
'Gut microbiota abundance (genus Parasutterella id.2892)' on 'Tumor necrosis factor receptor superfamily member 6'

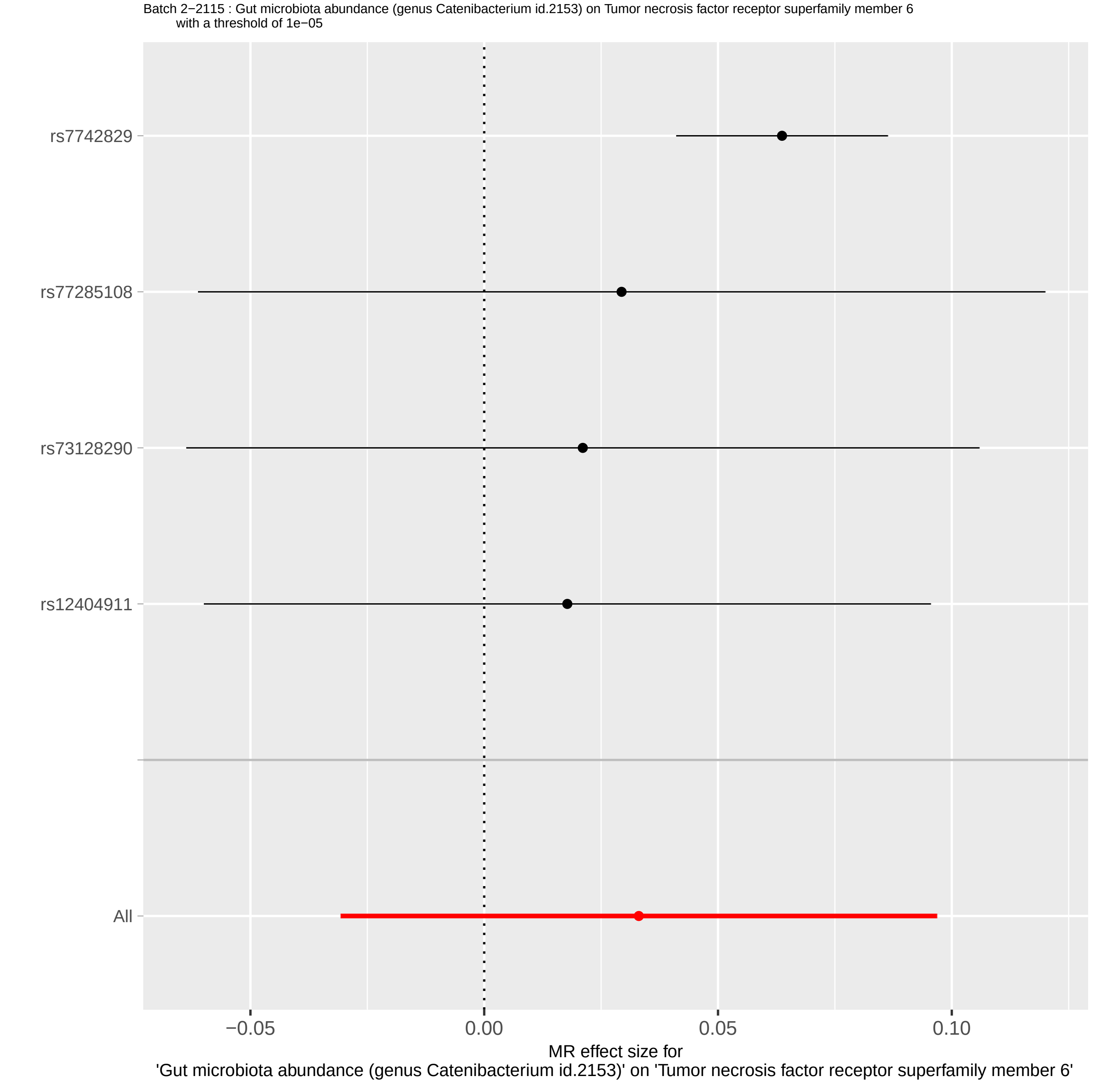

Batch 2−2115 : Gut microbiota abundance (genus Catenibacterium id.2153) on Tumor necrosis factor receptor superfamily member 6
with a threshold of 1e−05
rs7742829
rs77285108
rs73128290
rs12404911
All
−0.05
0.00
0.05
0.10
MR effect size for
'Gut microbiota abundance (genus Catenibacterium id.2153)' on 'Tumor necrosis factor receptor superfamily member 6'

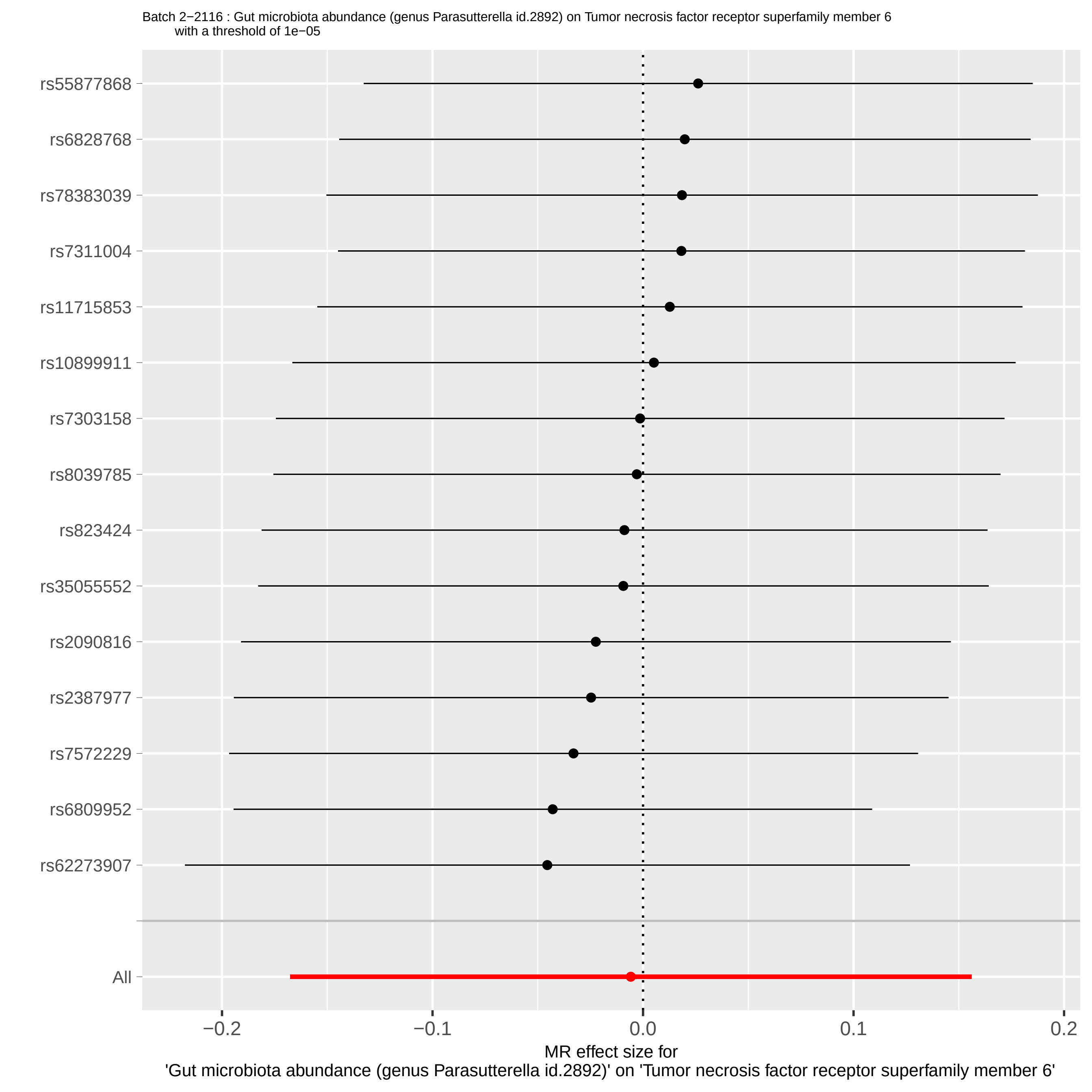
Batch 2−2116 : Gut microbiota abundance (genus Parasutterella id.2892) on Tumor necrosis factor receptor superfamily member 6
with a threshold of 1e−05
rs55877868
rs6828768
rs78383039
rs7311004
rs11715853
rs10899911
rs7303158
rs8039785
rs823424
rs35055552
rs2090816
rs2387977
rs7572229
rs6809952
rs62273907
All
−0.2
−0.1
0.0
0.1
0.2
MR effect size for
'Gut microbiota abundance (genus Parasutterella id.2892)' on 'Tumor necrosis factor receptor superfamily member 6'

Batch 2−2117 : Gut microbiota abundance (genus Catenibacterium id.2153) on Tumor necrosis factor ligand superfamily member 6, soluble form
with a threshold of 1e−05

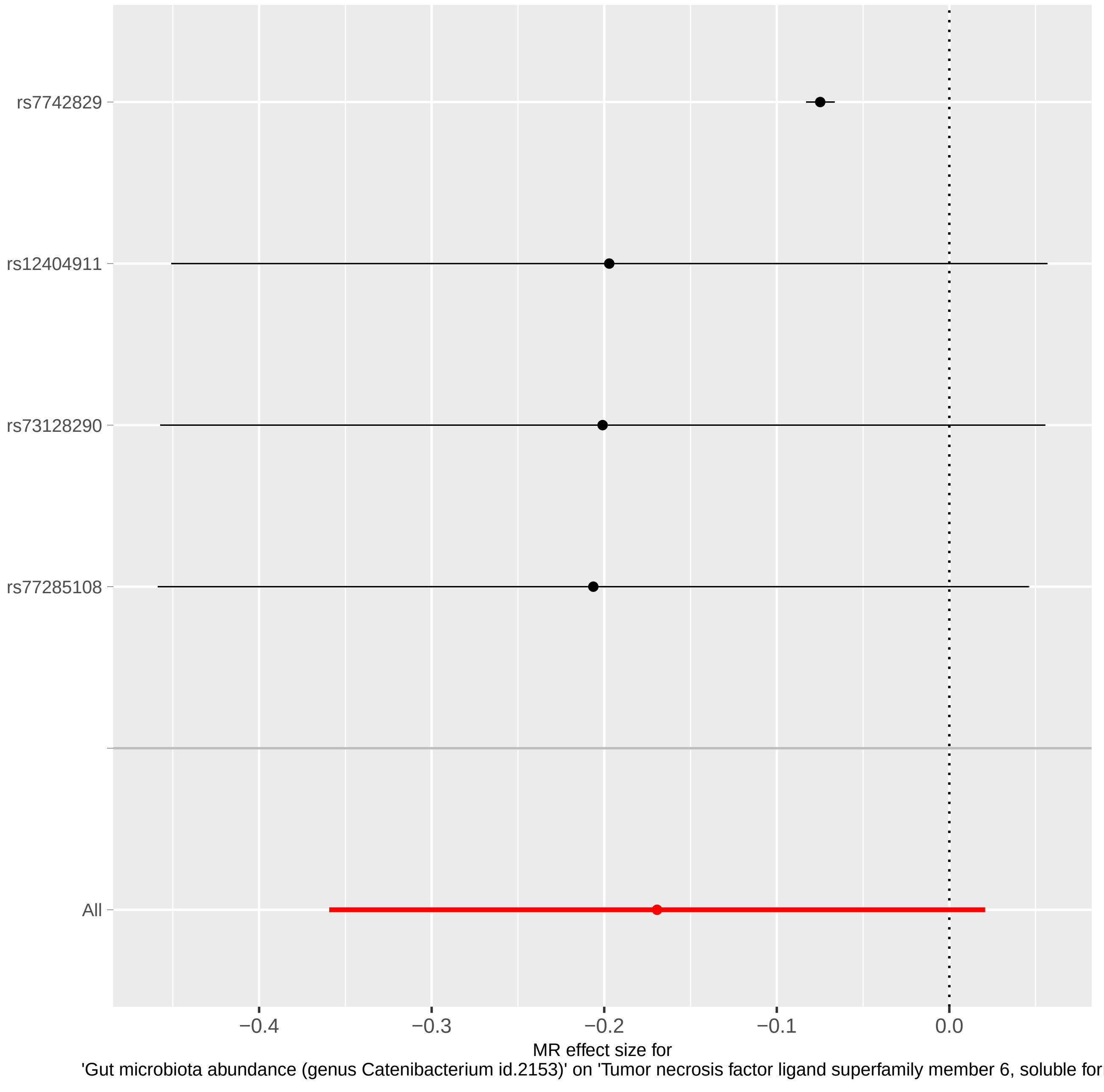

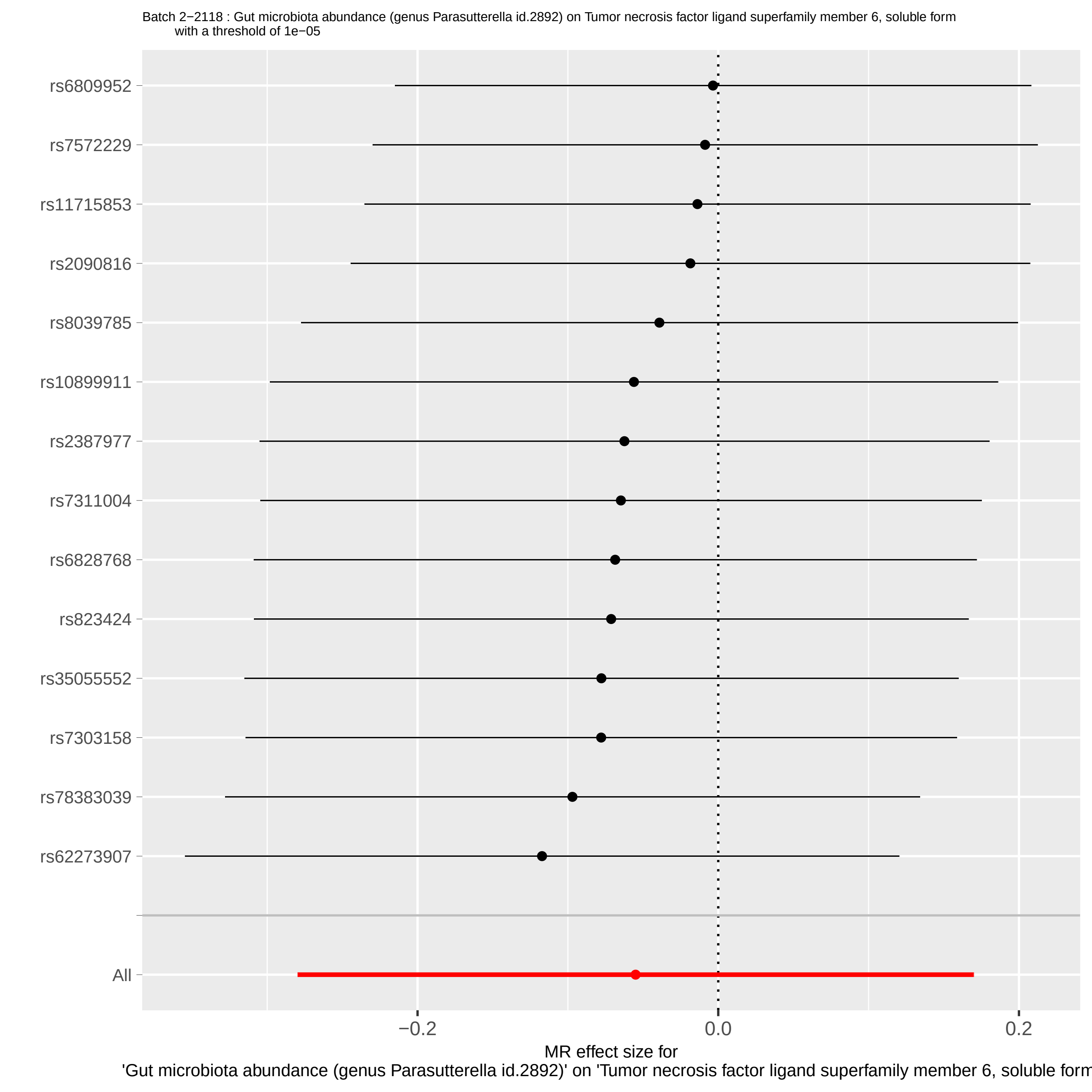
Batch 2−2118 : Gut microbiota abundance (genus Parasutterella id.2892) on Tumor necrosis factor ligand superfamily member 6, soluble form with a threshold of 1e−05
rs6809952
rs7572229
rs11715853
rs2090816
rs8039785
rs10899911
rs2387977
rs7311004
rs6828768
rs823424
rs35055552
rs7303158
rs78383039
rs62273907
All
−0.2
0.0
0.2
MR effect size for
'Gut microbiota abundance (genus Parasutterella id.2892)' on 'Tumor necrosis factor ligand superfamily member 6, soluble form

Batch 2−2119 : Gut microbiota abundance (genus Catenibacterium id.2153) on Fetal and adult testis−expressed transcript protein
with a threshold of 1e−05

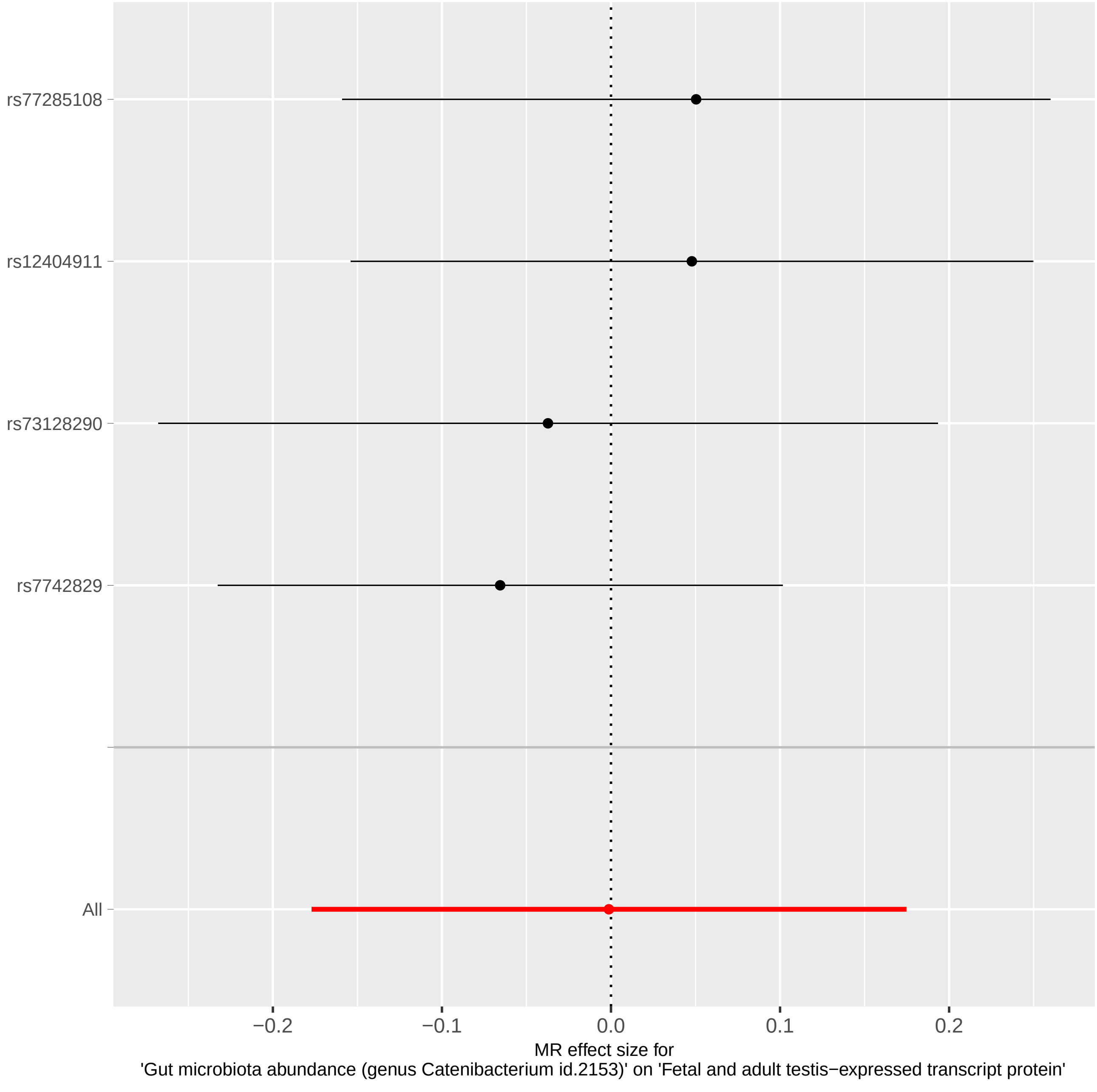

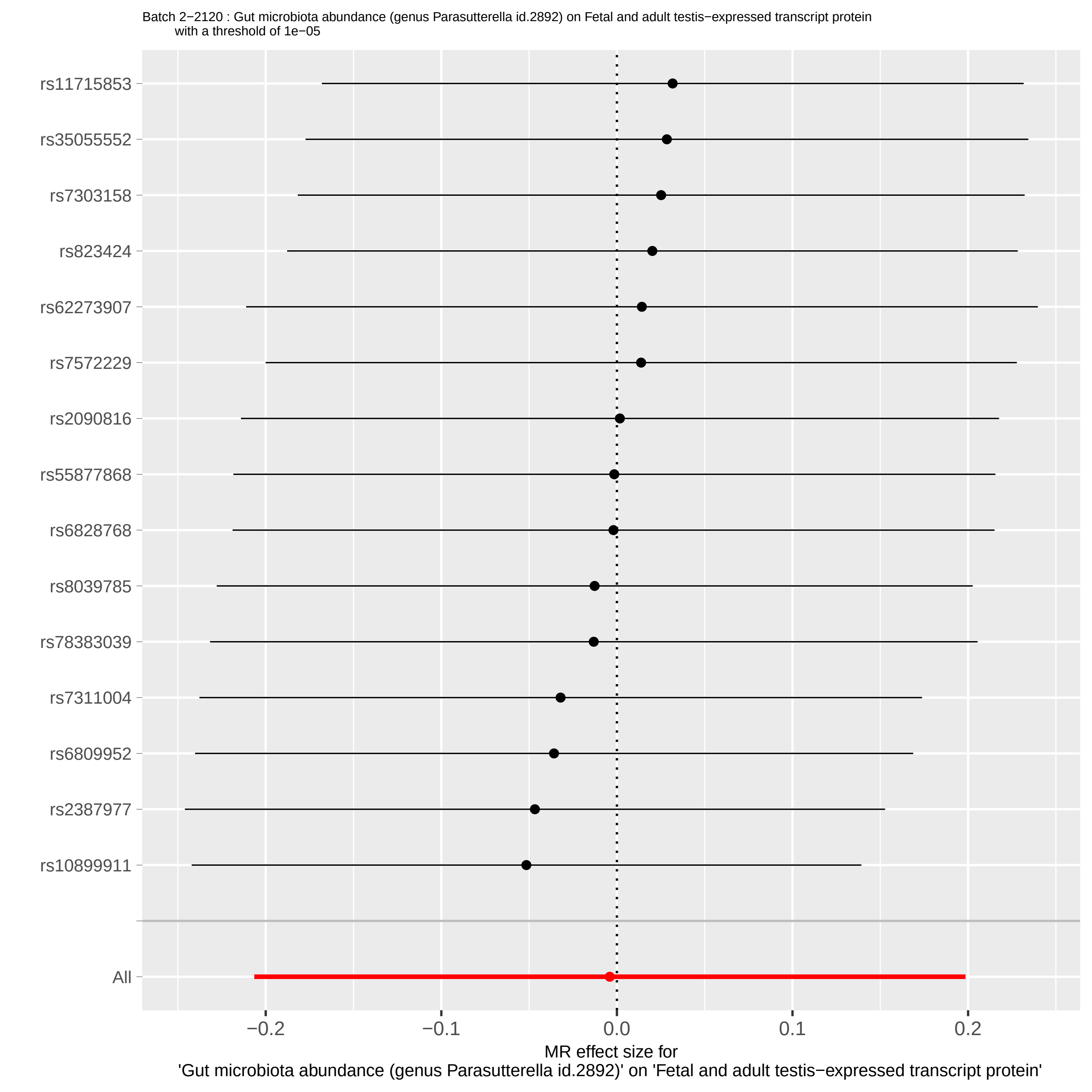
Batch 2−2120 : Gut microbiota abundance (genus Parasutterella id.2892) on Fetal and adult testis−expressed transcript protein
with a threshold of 1e−05
rs11715853
rs35055552
rs7303158
rs823424
rs62273907
rs7572229
rs2090816
rs55877868
rs6828768
rs8039785
rs78383039
rs7311004
rs6809952
rs2387977
rs10899911
All
−0.2
−0.1
0.0
0.1
0.2
MR effect size for
'Gut microbiota abundance (genus Parasutterella id.2892)' on 'Fetal and adult testis−expressed transcript protein'

Batch 2−2121 : Gut microbiota abundance (genus Catenibacterium id.2153) on Fatty acid hydroxylase domain−containing protein 2
with a threshold of 1e−05

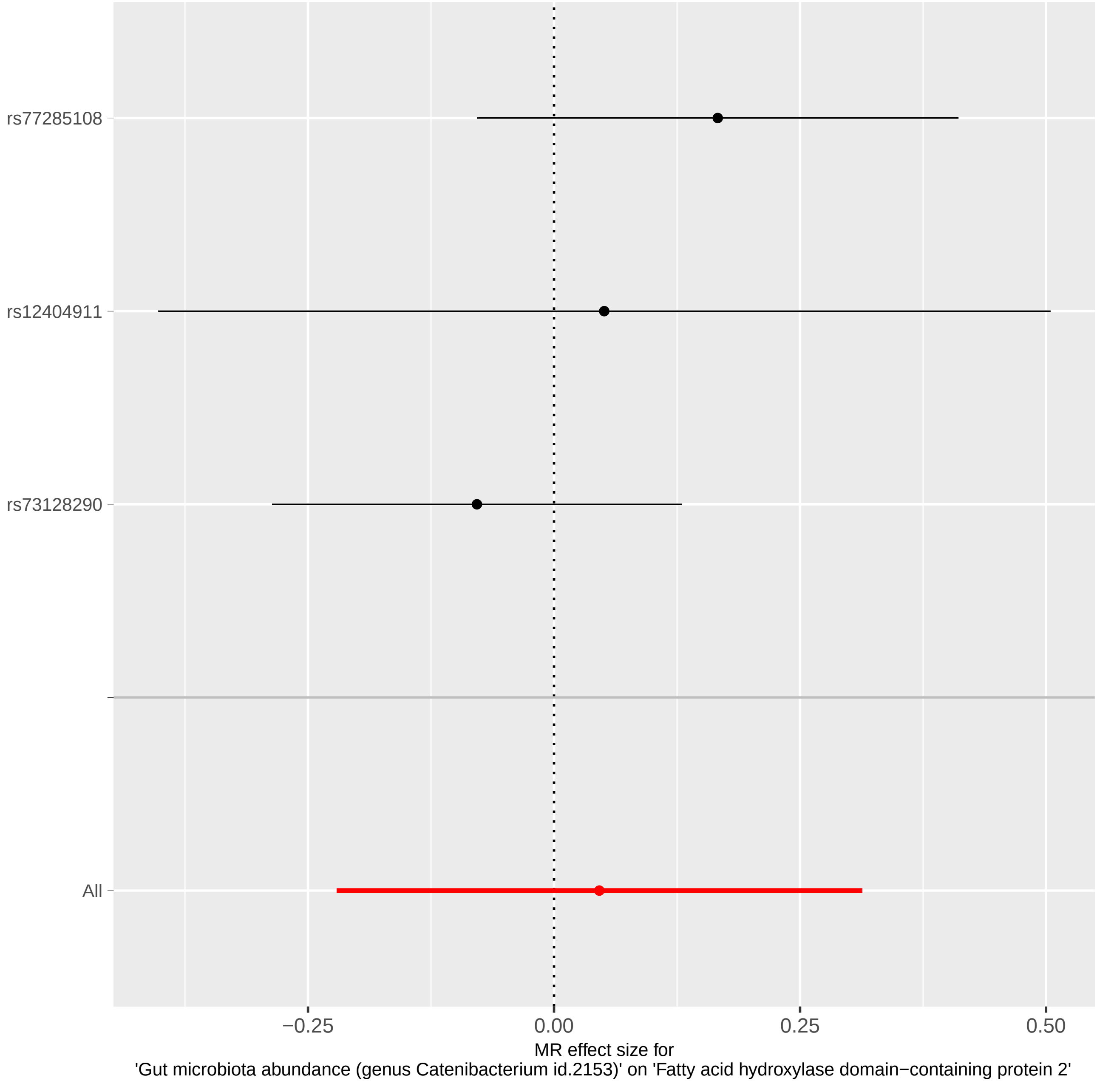

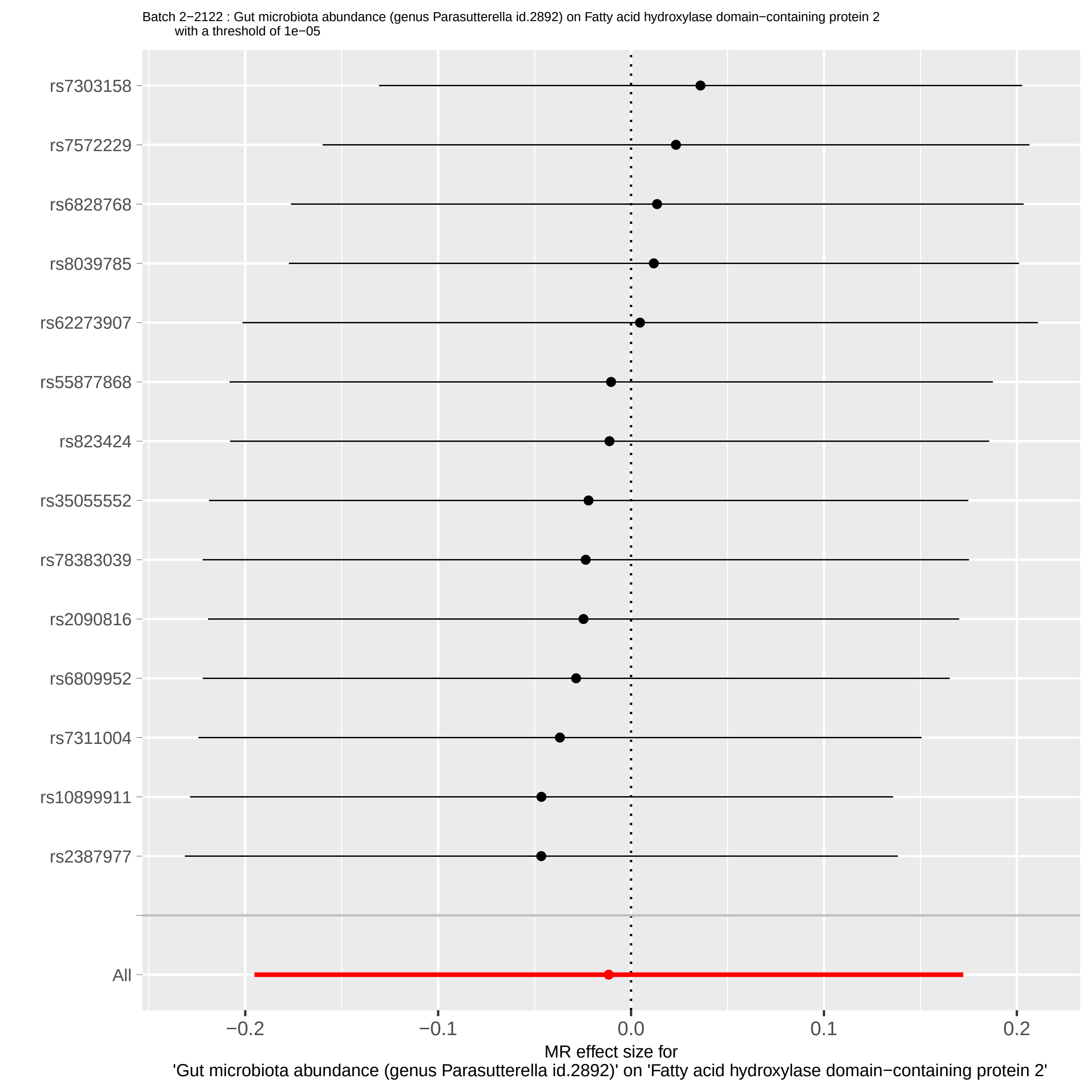

Batch 2−2122 : Gut microbiota abundance (genus Parasutterella id.2892) on Fatty acid hydroxylase domain−containing protein 2
with a threshold of 1e−05
rs7303158
rs7572229
rs6828768
rs8039785
rs62273907
rs55877868
rs823424
rs35055552
rs78383039
rs2090816
rs6809952
rs7311004
rs10899911
rs2387977
All
−0.2
−0.1
0.0
0.1
0.2
MR effect size for
'Gut microbiota abundance (genus Parasutterella id.2892)' on 'Fatty acid hydroxylase domain−containing protein 2'

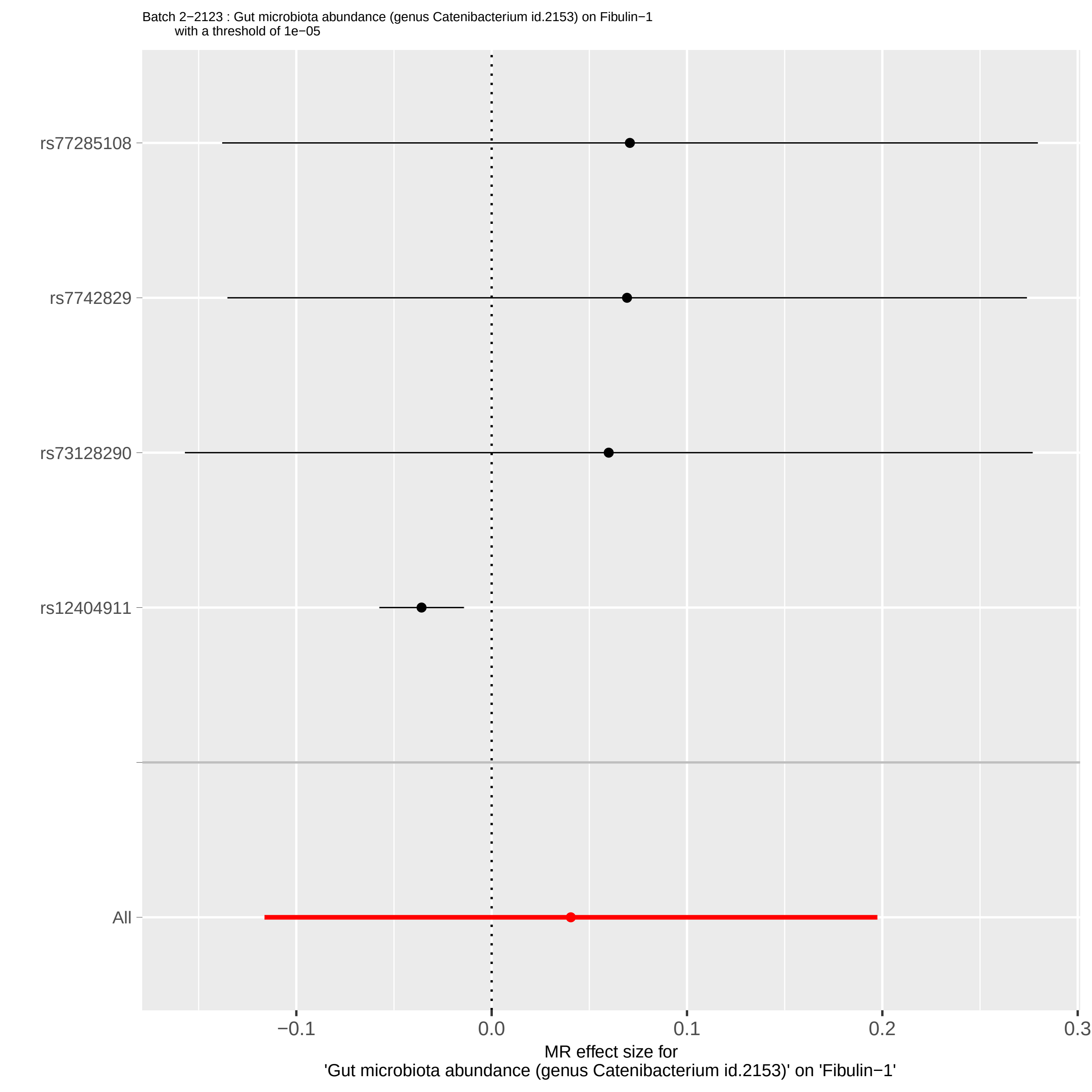
Batch 2−2123 : Gut microbiota abundance (genus Catenibacterium id.2153) on Fibulin−1
with a threshold of 1e−05
rs77285108
rs7742829
rs73128290
rs12404911
All
−0.1
0.0
0.1
0.2
0.3
MR effect size for
'Gut microbiota abundance (genus Catenibacterium id.2153)' on 'Fibulin−1'

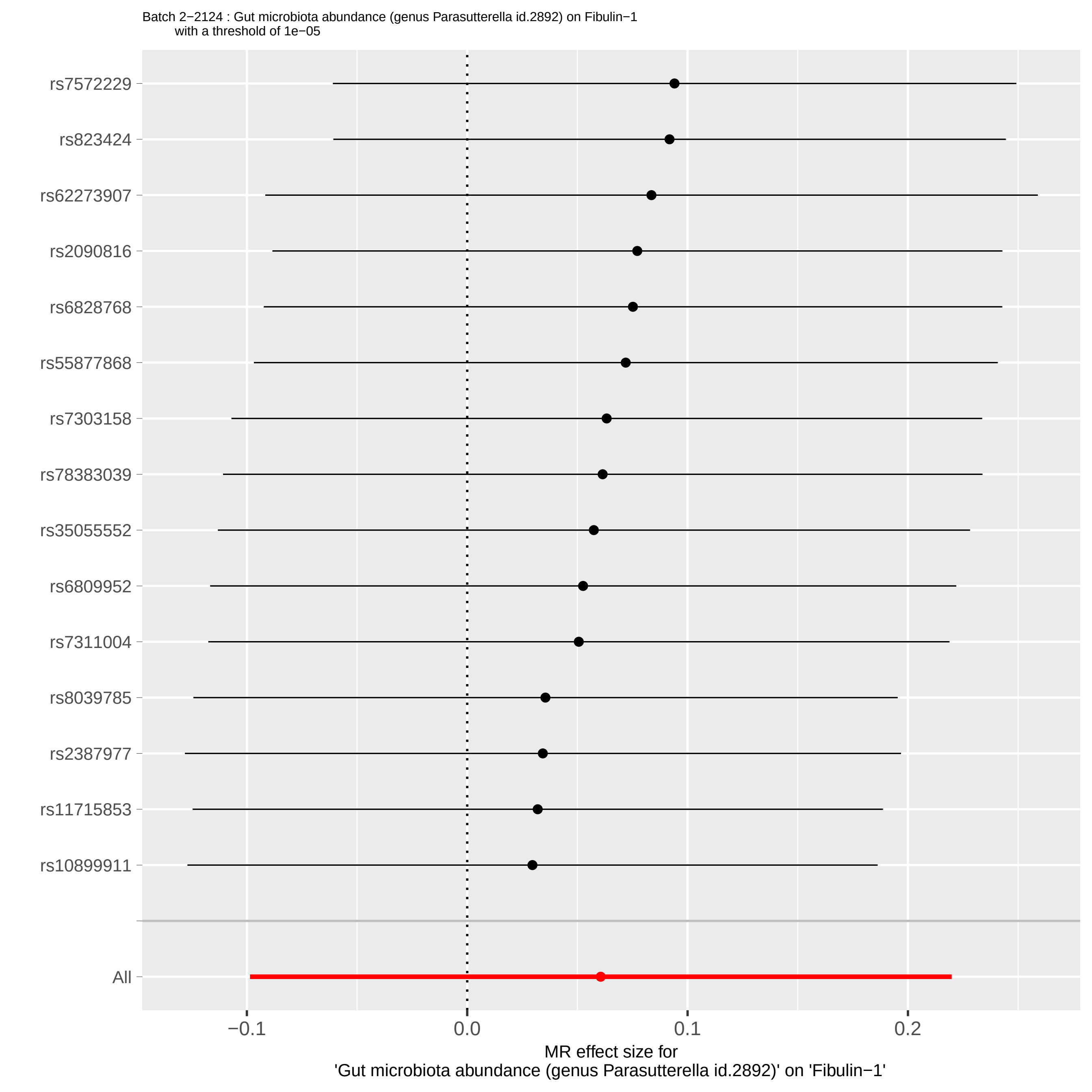
Batch 2−2124 : Gut microbiota abundance (genus Parasutterella id.2892) on Fibulin−1
with a threshold of 1e−05
rs7572229
rs823424
rs62273907
rs2090816
rs6828768
rs55877868
rs7303158
rs78383039
rs35055552
rs6809952
rs7311004
rs8039785
rs2387977
rs11715853
rs10899911
All
−0.1
0.0
0.1
0.2
MR effect size for
'Gut microbiota abundance (genus Parasutterella id.2892)' on 'Fibulin−1'

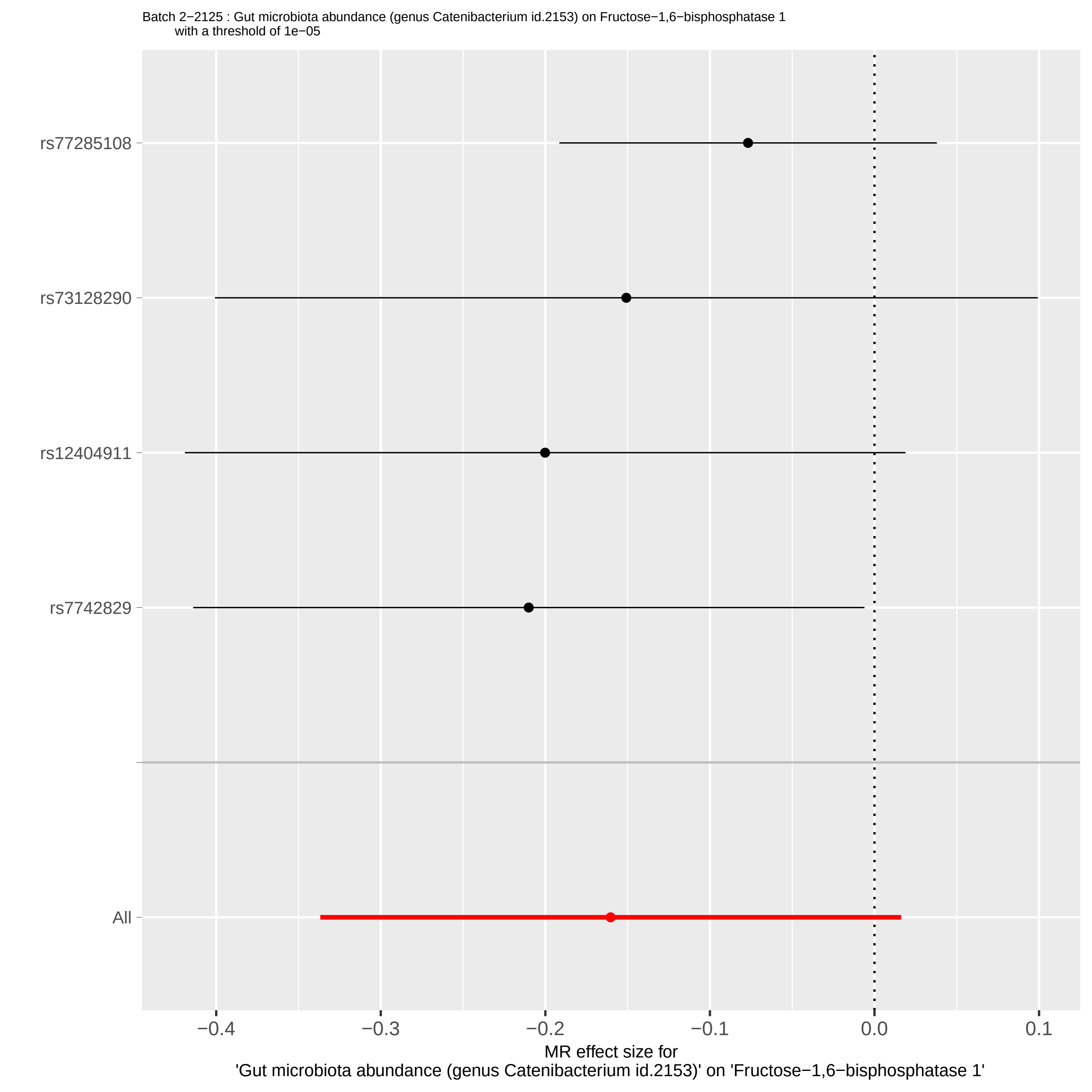
Batch 2−2125 : Gut microbiota abundance (genus Catenibacterium id.2153) on Fructose−1,6−bisphosphatase 1
with a threshold of 1e−05
rs77285108
rs73128290
rs12404911
rs7742829
All
−0.4
−0.3
−0.2
−0.1
0.0
0.1
MR effect size for
'Gut microbiota abundance (genus Catenibacterium id.2153)' on 'Fructose−1,6−bisphosphatase 1'

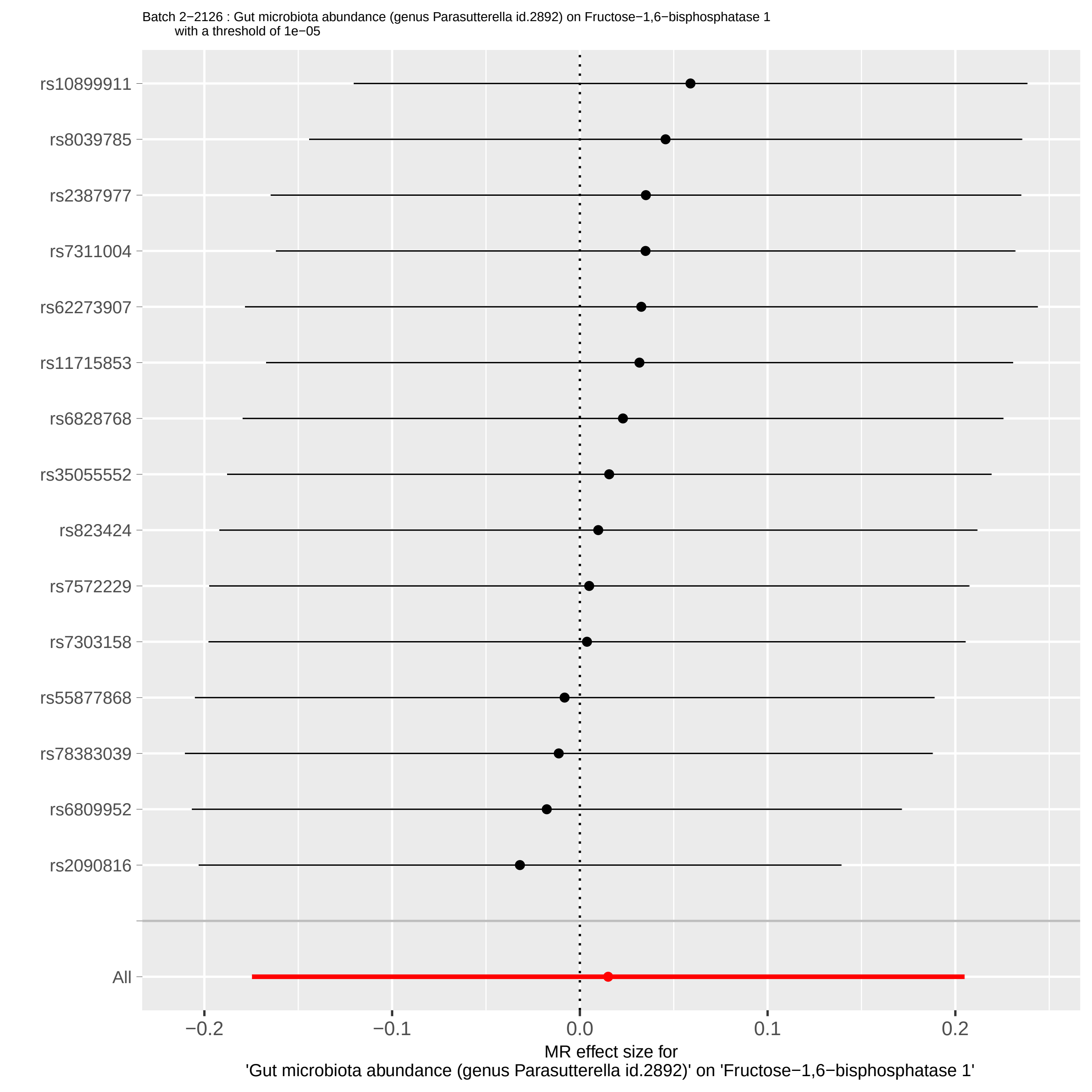

Batch 2−2126 : Gut microbiota abundance (genus Parasutterella id.2892) on Fructose−1,6−bisphosphatase 1
with a threshold of 1e−05
rs10899911
rs8039785
rs2387977
rs7311004
rs62273907
rs11715853
rs6828768
rs35055552
rs823424
rs7572229
rs7303158
rs55877868
rs78383039
rs6809952
rs2090816
All
−0.2
−0.1
0.0
0.1
0.2
MR effect size for
'Gut microbiota abundance (genus Parasutterella id.2892)' on 'Fructose−1,6−bisphosphatase 1'

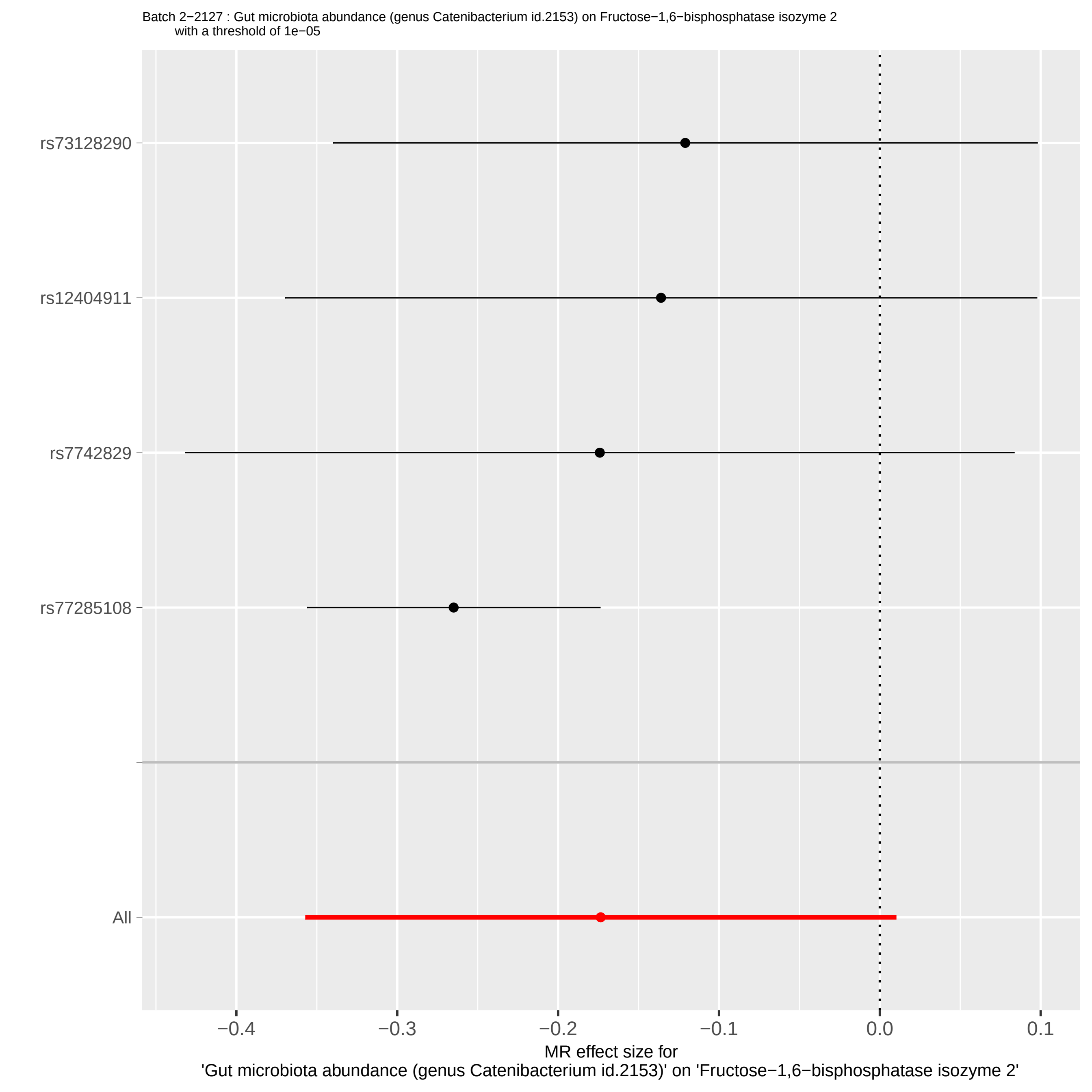

Batch 2−2127 : Gut microbiota abundance (genus Catenibacterium id.2153) on Fructose−1,6−bisphosphatase isozyme 2
with a threshold of 1e−05
rs73128290
rs12404911
rs7742829
rs77285108
All
−0.4
−0.3
−0.2
−0.1
0.0
0.1
MR effect size for
'Gut microbiota abundance (genus Catenibacterium id.2153)' on 'Fructose−1,6−bisphosphatase isozyme 2'

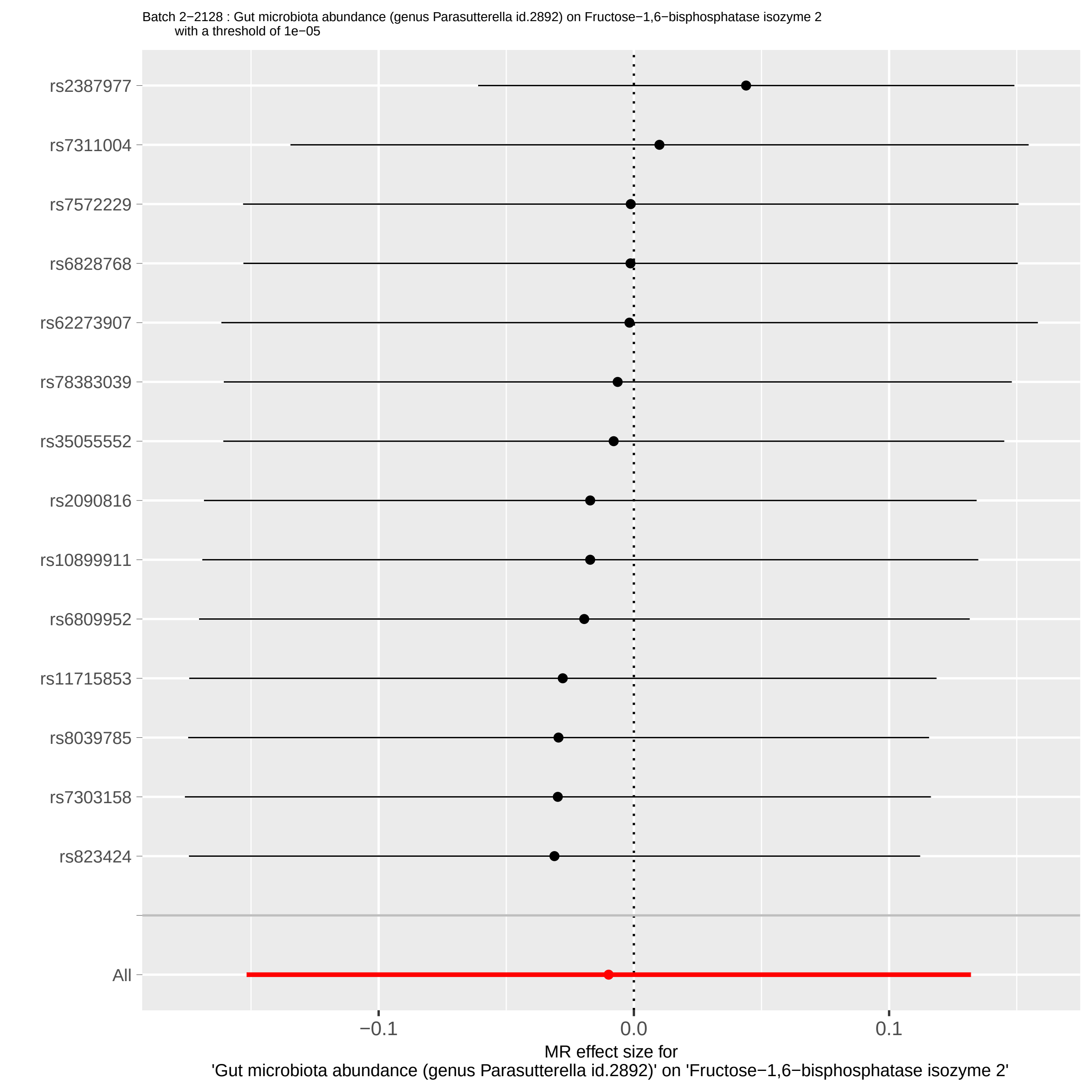

Batch 2−2128 : Gut microbiota abundance (genus Parasutterella id.2892) on Fructose−1,6−bisphosphatase isozyme 2
with a threshold of 1e−05
rs2387977
rs7311004
rs7572229
rs6828768
rs62273907
rs78383039
rs35055552
rs2090816
rs10899911
rs6809952
rs11715853
rs8039785
rs7303158
rs823424
All
−0.1
0.0
0.1
MR effect size for
'Gut microbiota abundance (genus Parasutterella id.2892)' on 'Fructose−1,6−bisphosphatase isozyme 2'

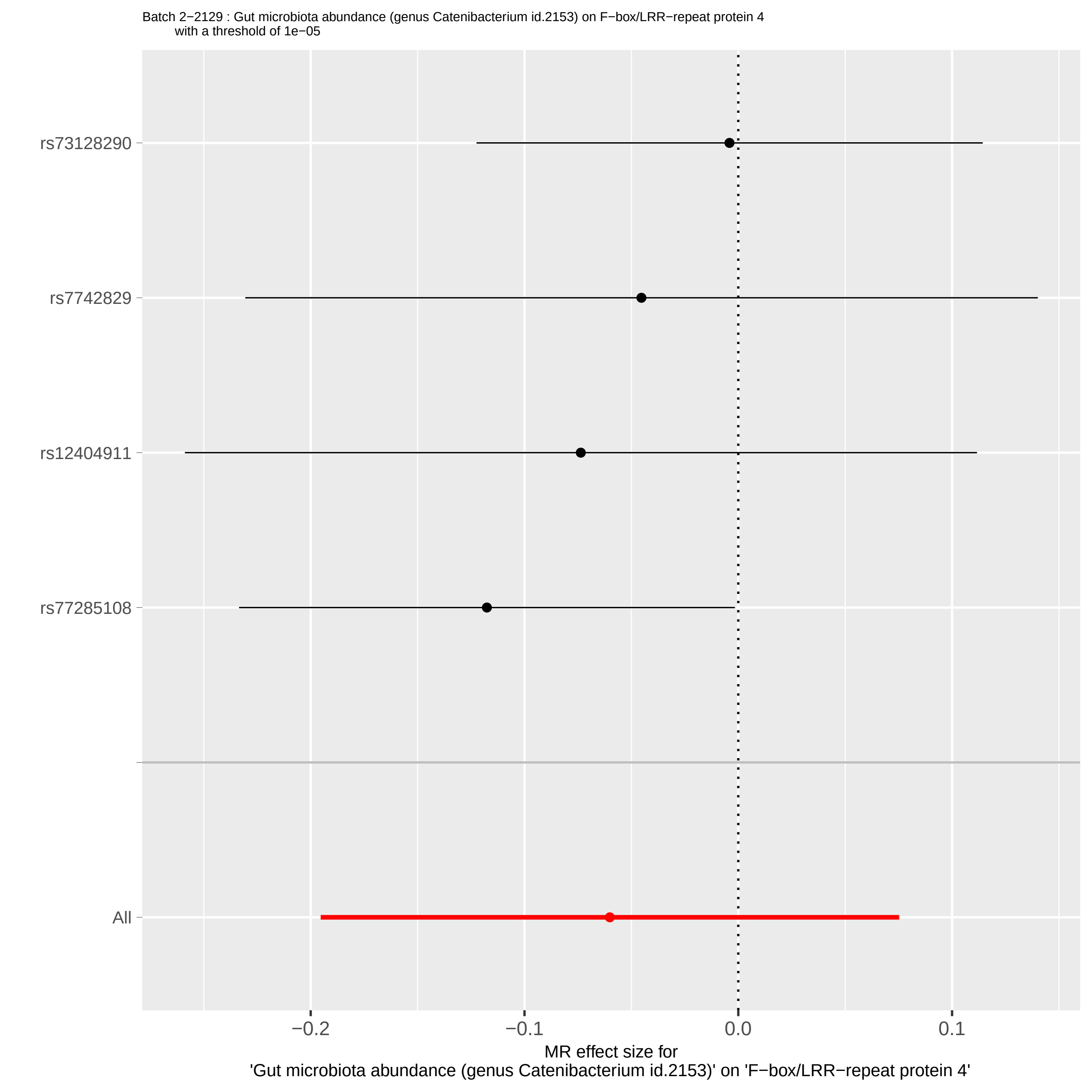
Batch 2−2129 : Gut microbiota abundance (genus Catenibacterium id.2153) on F−box/LRR−repeat protein 4
with a threshold of 1e−05
rs73128290
rs7742829
rs12404911
rs77285108
All
−0.2
−0.1
0.0
0.1
MR effect size for
'Gut microbiota abundance (genus Catenibacterium id.2153)' on 'F−box/LRR−repeat protein 4'

Batch 2−2130 : Gut microbiota abundance (genus Parasutterella id.2892) on F−box/LRR−repeat protein 4
with a threshold of 1e−05

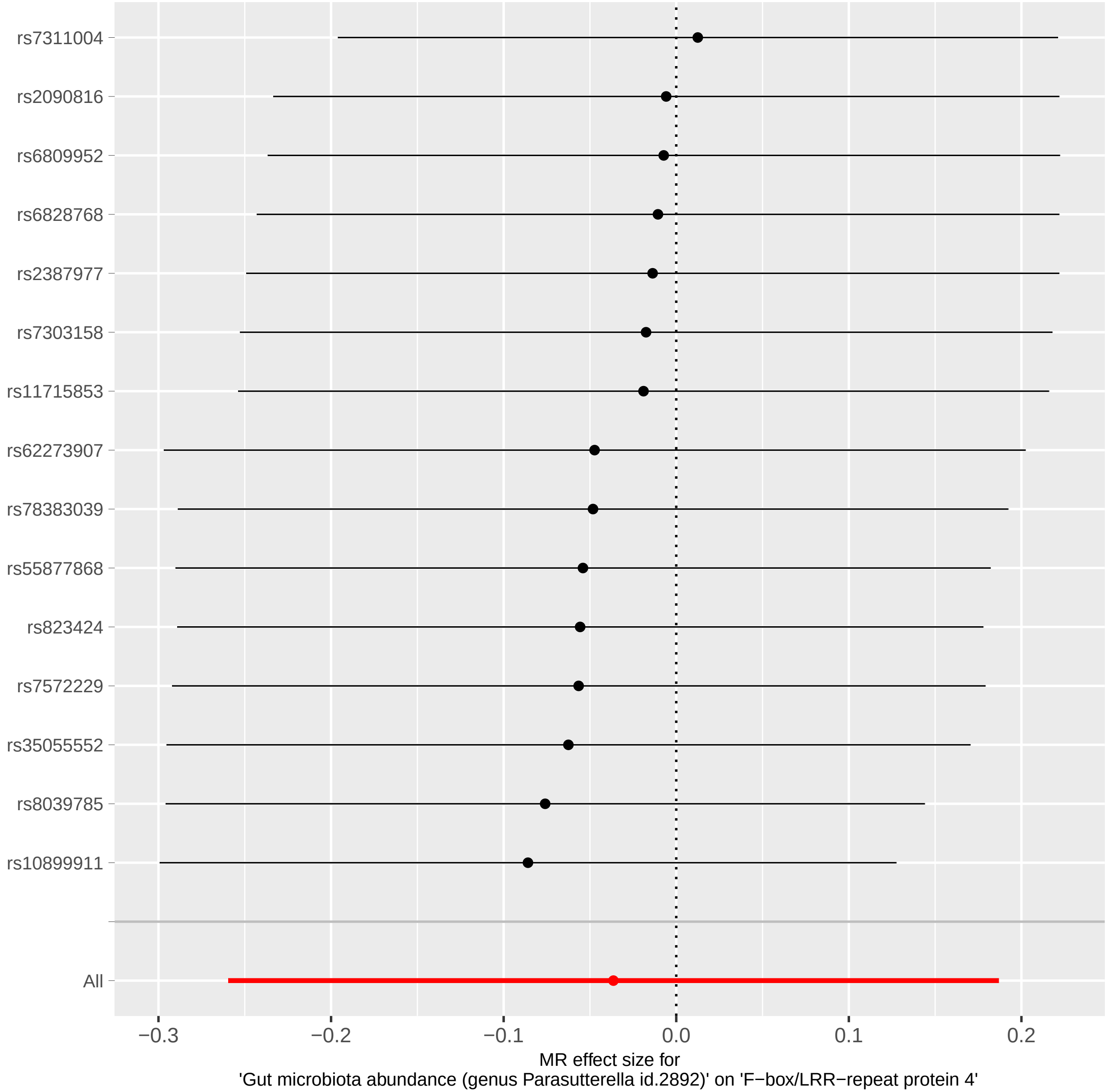

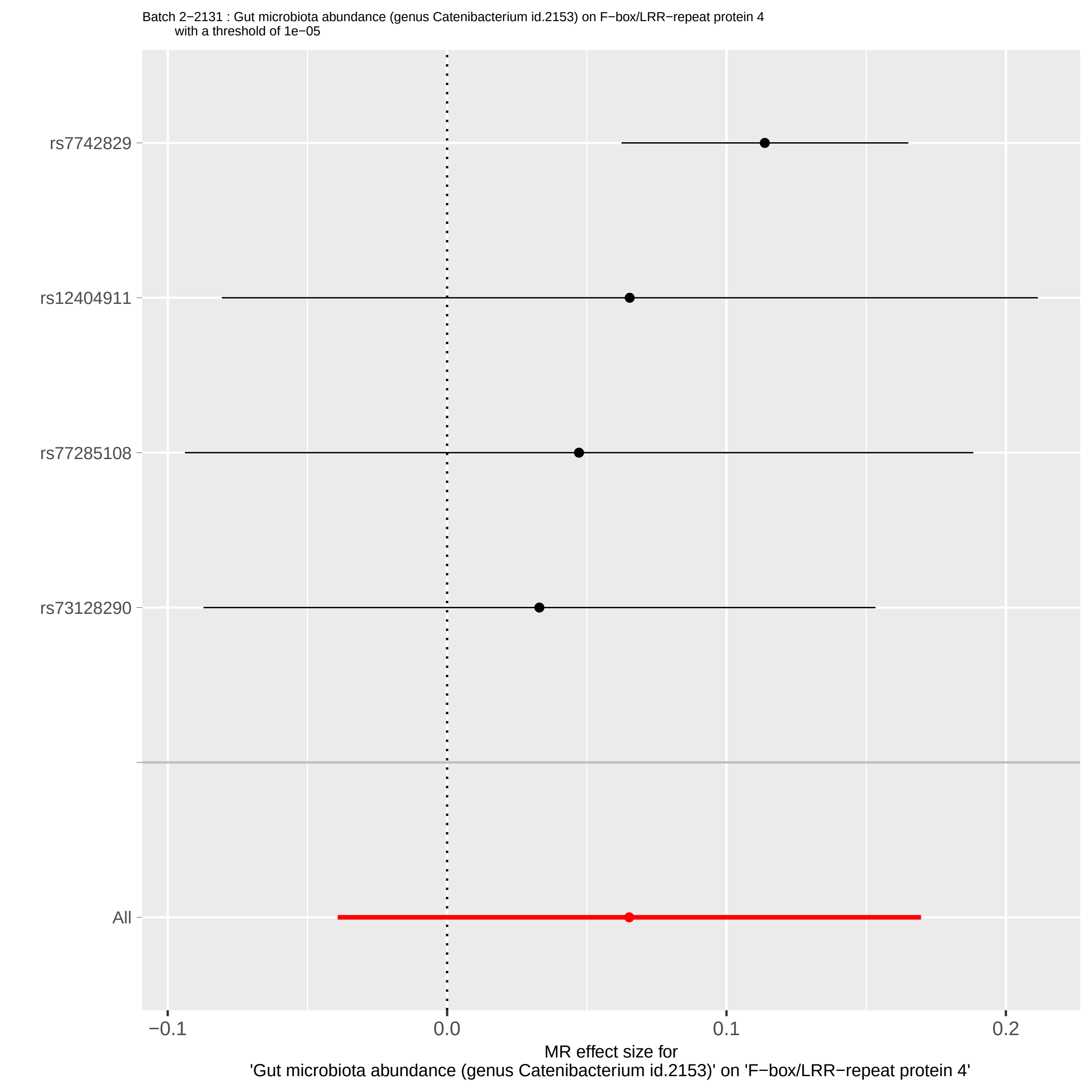

Batch 2−2131 : Gut microbiota abundance (genus Catenibacterium id.2153) on F−box/LRR−repeat protein 4
with a threshold of 1e−05
rs7742829
rs12404911
rs77285108
rs73128290
All
−0.1
0.0
0.1
0.2
MR effect size for
'Gut microbiota abundance (genus Catenibacterium id.2153)' on 'F−box/LRR−repeat protein 4'

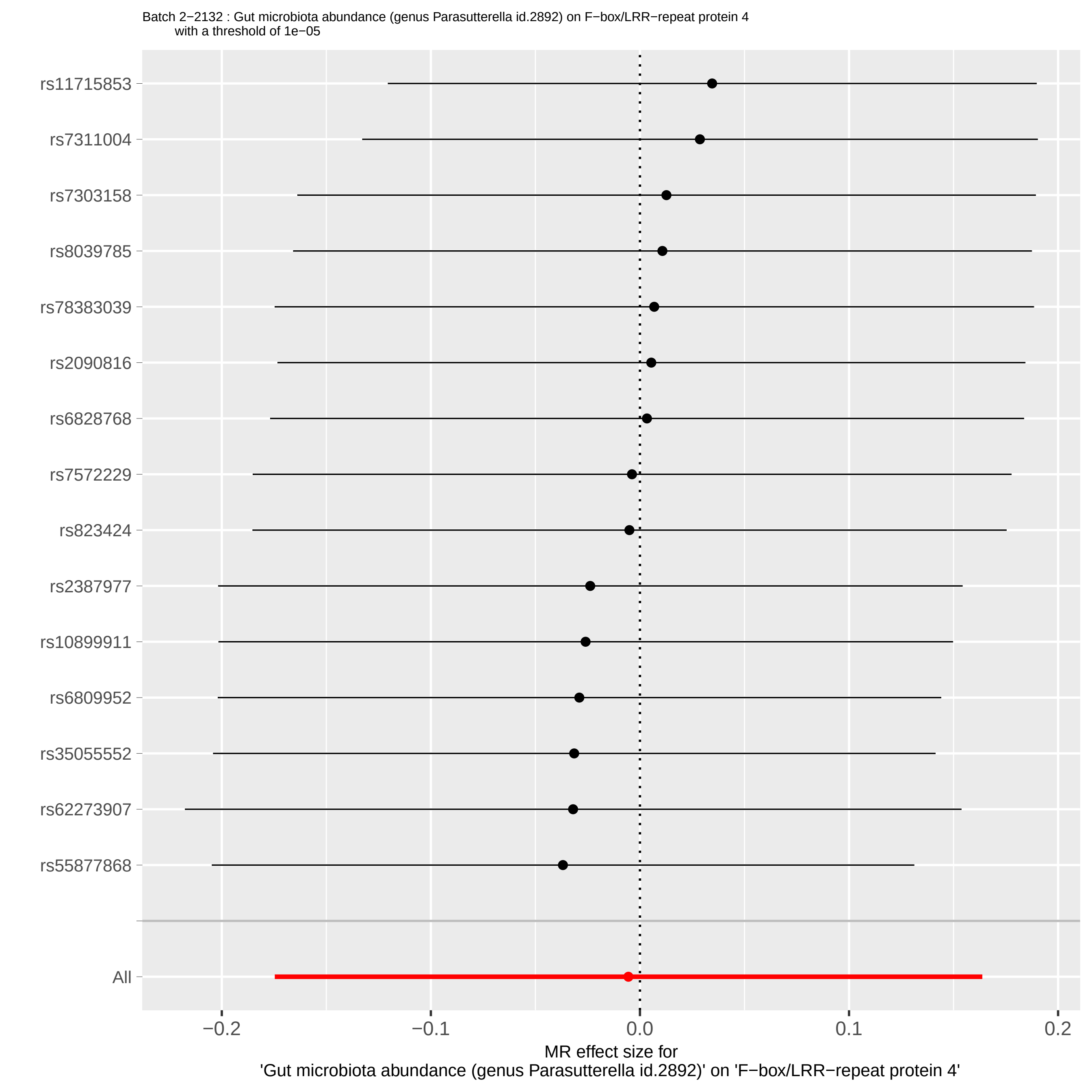
Batch 2−2132 : Gut microbiota abundance (genus Parasutterella id.2892) on F−box/LRR−repeat protein 4
with a threshold of 1e−05
rs11715853
rs7311004
rs7303158
rs8039785
rs78383039
rs2090816
rs6828768
rs7572229
rs823424
rs2387977
rs10899911
rs6809952
rs35055552
rs62273907
rs55877868
All
−0.2
−0.1
0.0
0.1
0.2
MR effect size for
'Gut microbiota abundance (genus Parasutterella id.2892)' on 'F−box/LRR−repeat protein 4'

Batch 2−2133 : Gut microbiota abundance (genus Catenibacterium id.2153) on F−box/LRR−repeat protein 4
with a threshold of 1e−05

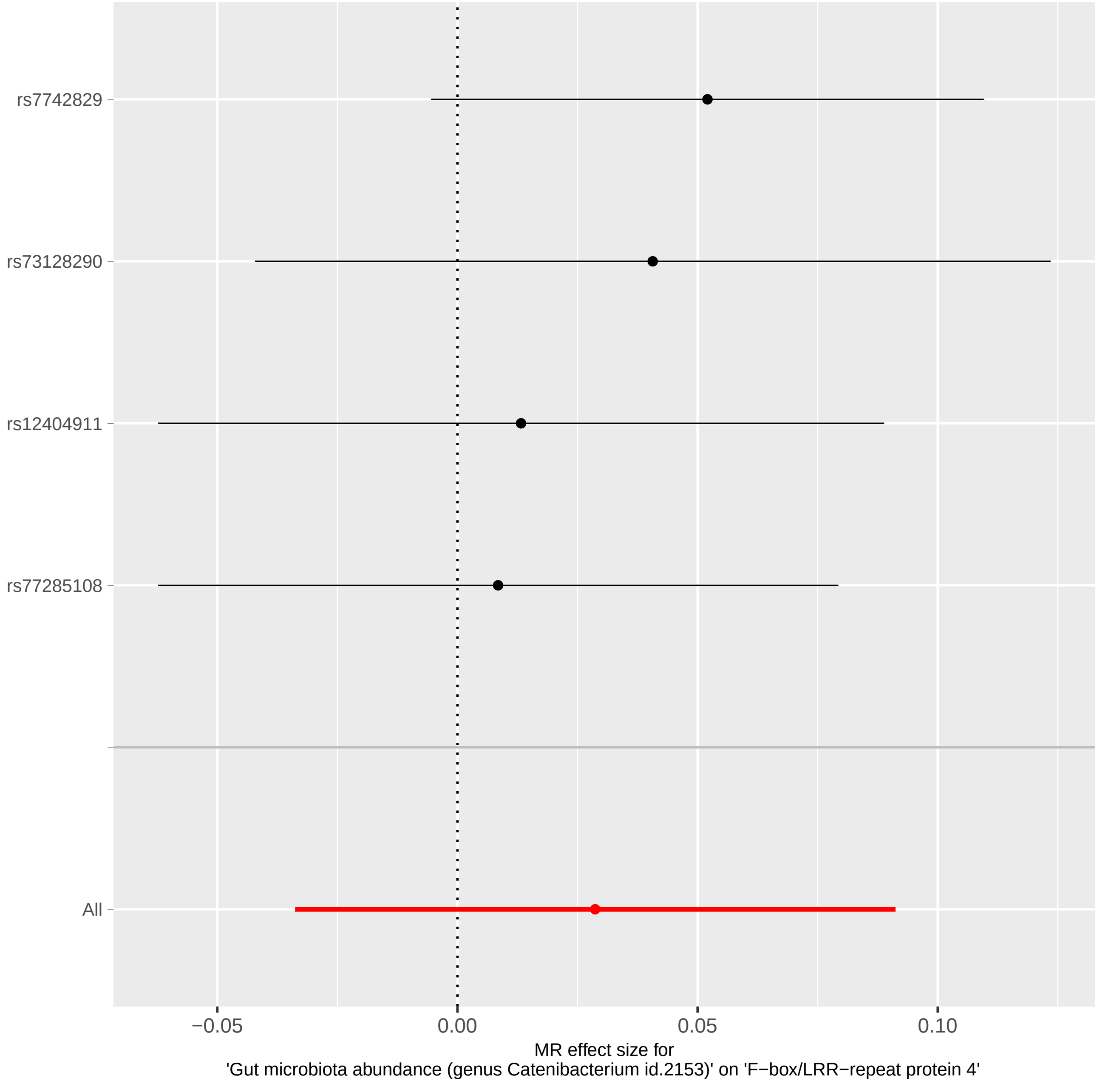

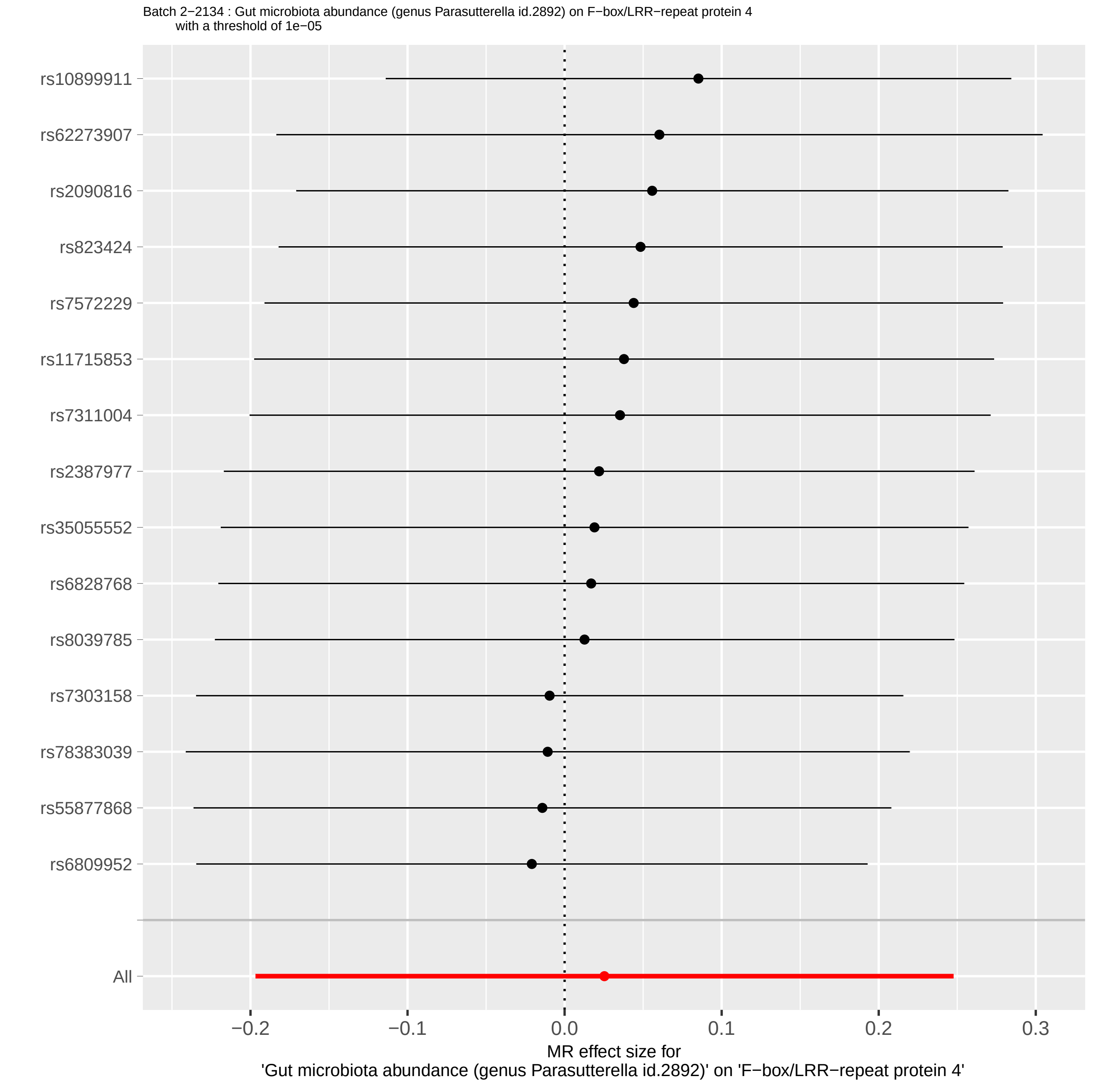

Batch 2−2134 : Gut microbiota abundance (genus Parasutterella id.2892) on F−box/LRR−repeat protein 4
with a threshold of 1e−05
rs10899911
rs62273907
rs2090816
rs823424
rs7572229
rs11715853
rs7311004
rs2387977
rs35055552
rs6828768
rs8039785
rs7303158
rs78383039
rs55877868
rs6809952
All
−0.2
−0.1
0.0
0.1
0.2
0.3
MR effect size for
'Gut microbiota abundance (genus Parasutterella id.2892)' on 'F−box/LRR−repeat protein 4'

Batch 2−2135 : Gut microbiota abundance (genus Catenibacterium id.2153) on F−box/LRR−repeat protein 5
with a threshold of 1e−05

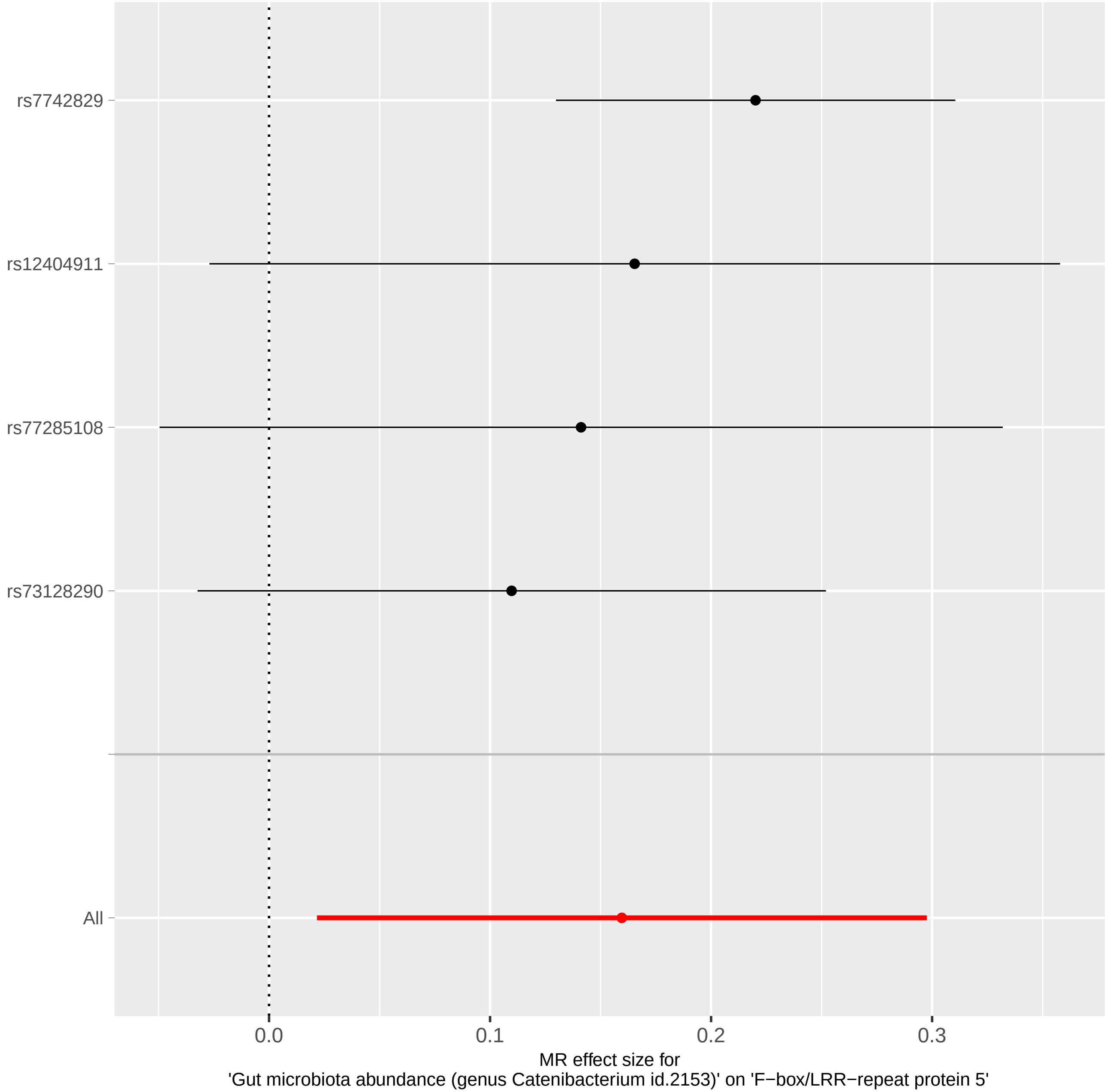

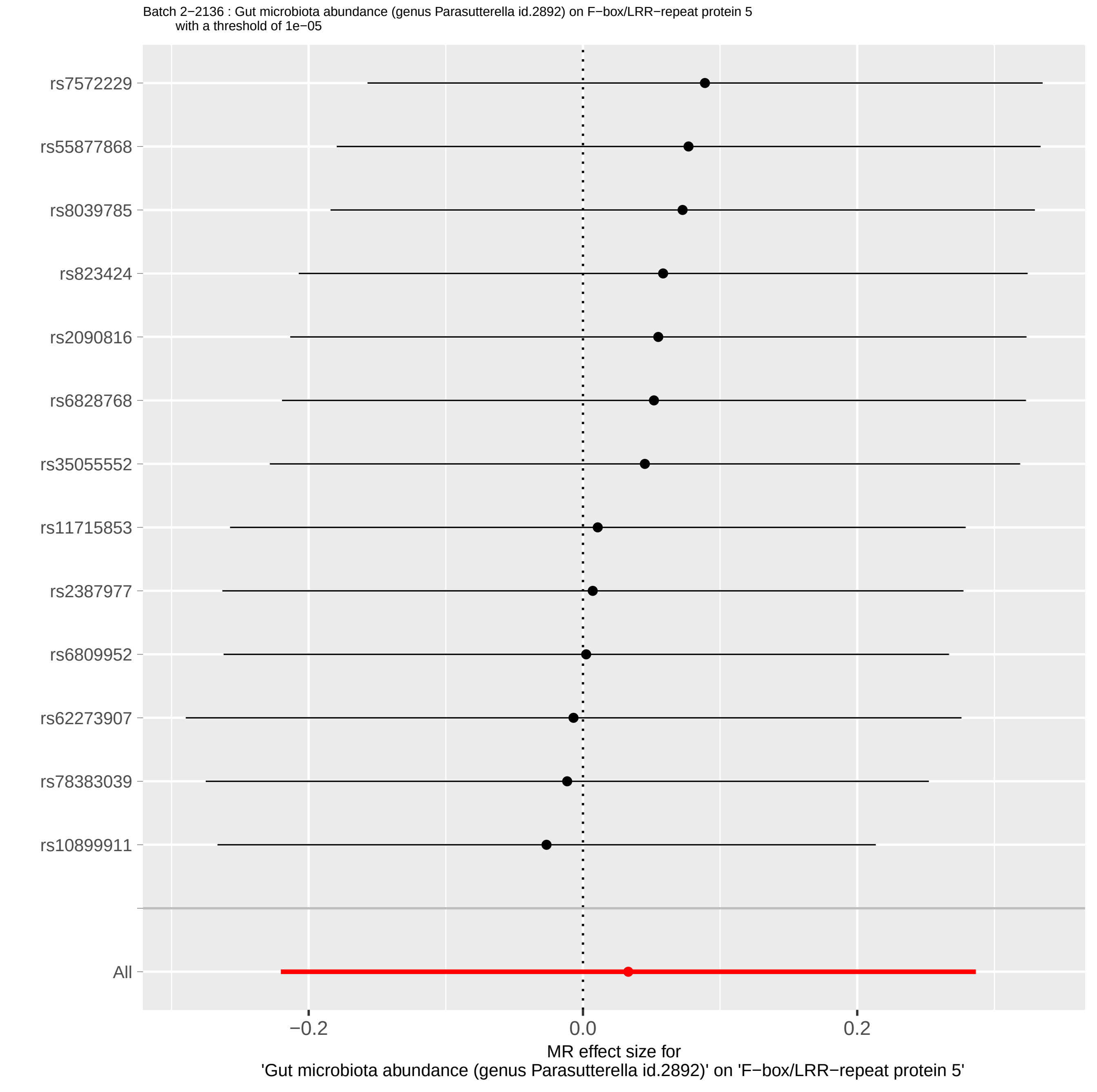
Batch 2−2136 : Gut microbiota abundance (genus Parasutterella id.2892) on F−box/LRR−repeat protein 5
with a threshold of 1e−05
rs7572229
rs55877868
rs8039785
rs823424
rs2090816
rs6828768
rs35055552
rs11715853
rs2387977
rs6809952
rs62273907
rs78383039
rs10899911
All
−0.2
0.0
0.2
MR effect size for
'Gut microbiota abundance (genus Parasutterella id.2892)' on 'F−box/LRR−repeat protein 5'

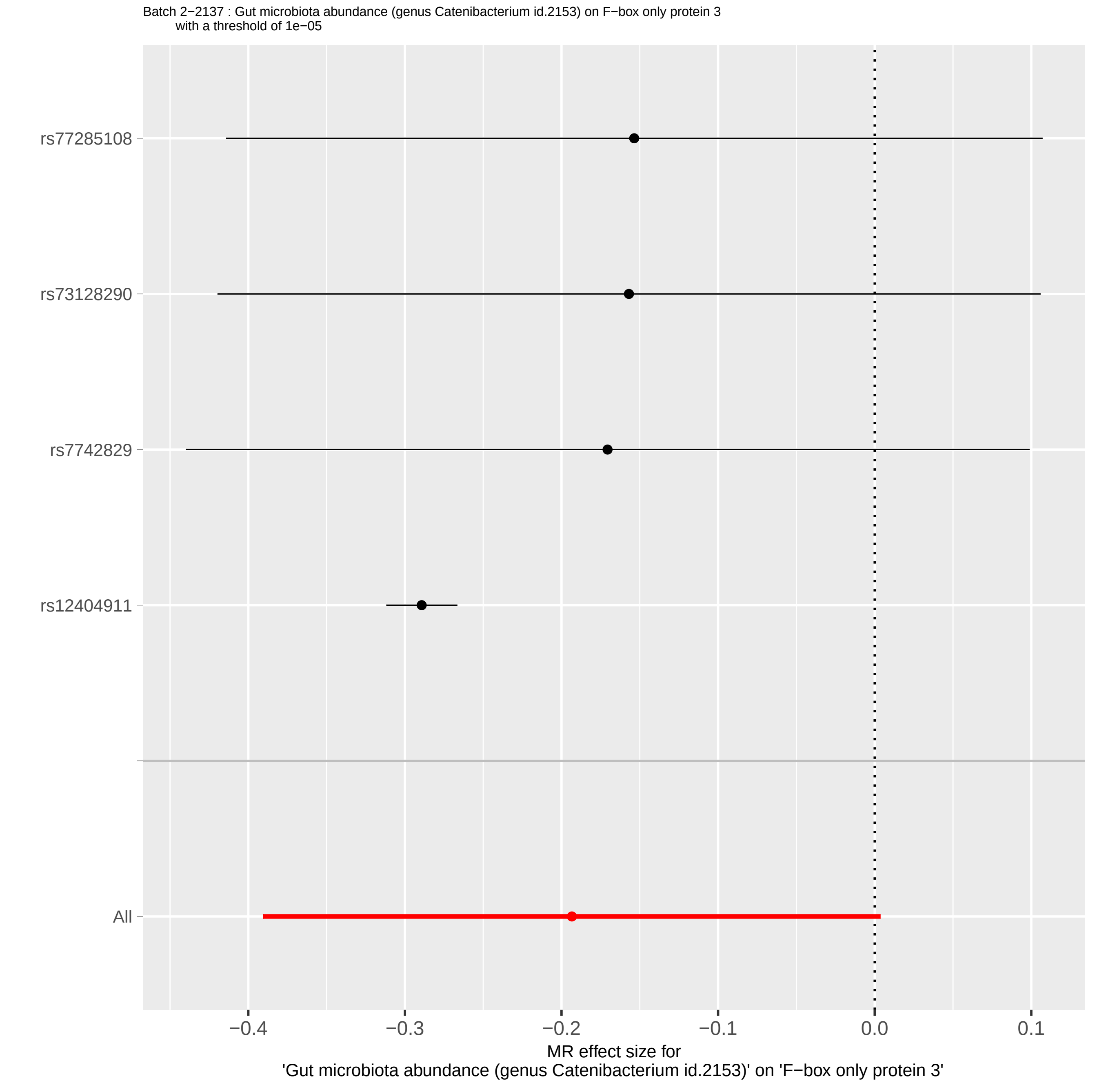
Batch 2−2137 : Gut microbiota abundance (genus Catenibacterium id.2153) on F−box only protein 3
with a threshold of 1e−05
rs77285108
rs73128290
rs7742829
rs12404911
All
−0.4
−0.3
−0.2
−0.1
0.0
0.1
MR effect size for
'Gut microbiota abundance (genus Catenibacterium id.2153)' on 'F−box only protein 3'

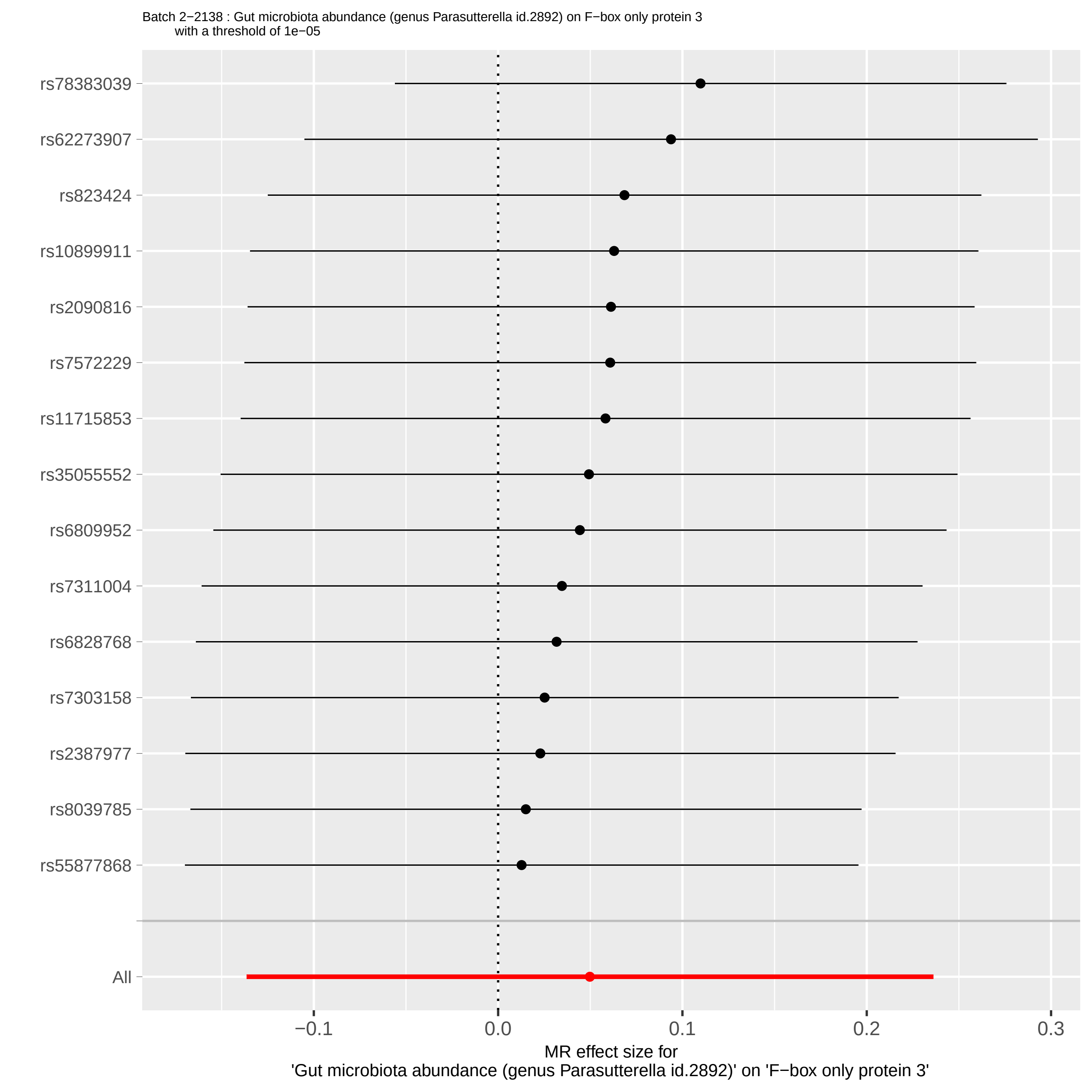

Batch 2−2138 : Gut microbiota abundance (genus Parasutterella id.2892) on F−box only protein 3
with a threshold of 1e−05
rs78383039
rs62273907
rs823424
rs10899911
rs2090816
rs7572229
rs11715853
rs35055552
rs6809952
rs7311004
rs6828768
rs7303158
rs2387977
rs8039785
rs55877868
All
−0.1
0.0
0.1
0.2
0.3
MR effect size for
'Gut microbiota abundance (genus Parasutterella id.2892)' on 'F−box only protein 3'

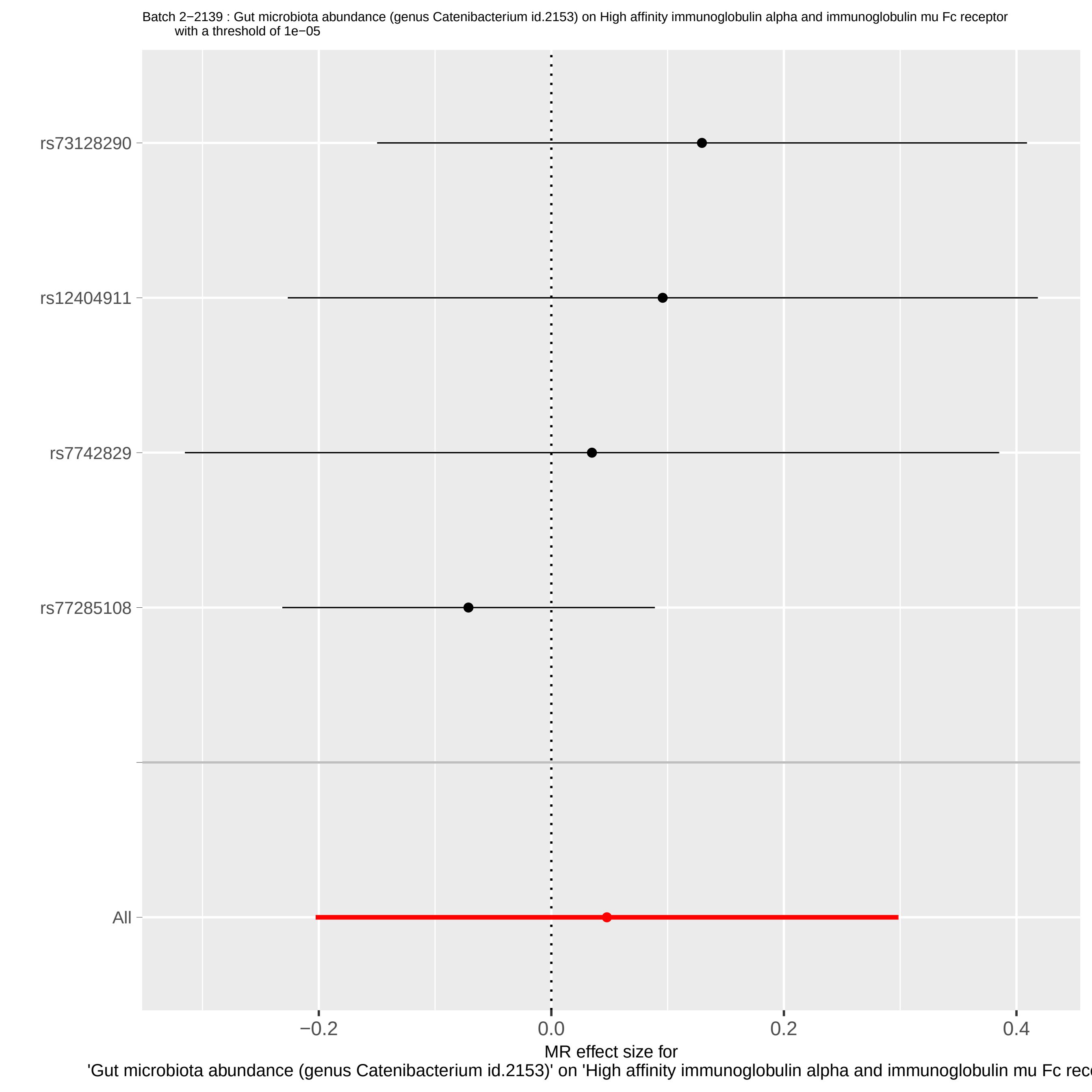
Batch 2−2139 : Gut microbiota abundance (genus Catenibacterium id.2153) on High affinity immunoglobulin alpha and immunoglobulin mu Fc receptor with a threshold of 1e−05
rs73128290
rs12404911
rs7742829
rs77285108
All
−0.2
0.0
0.2
0.4
MR effect size for
'Gut microbiota abundance (genus Catenibacterium id.2153)' on 'High affinity immunoglobulin alpha and immunoglobulin mu Fc rec

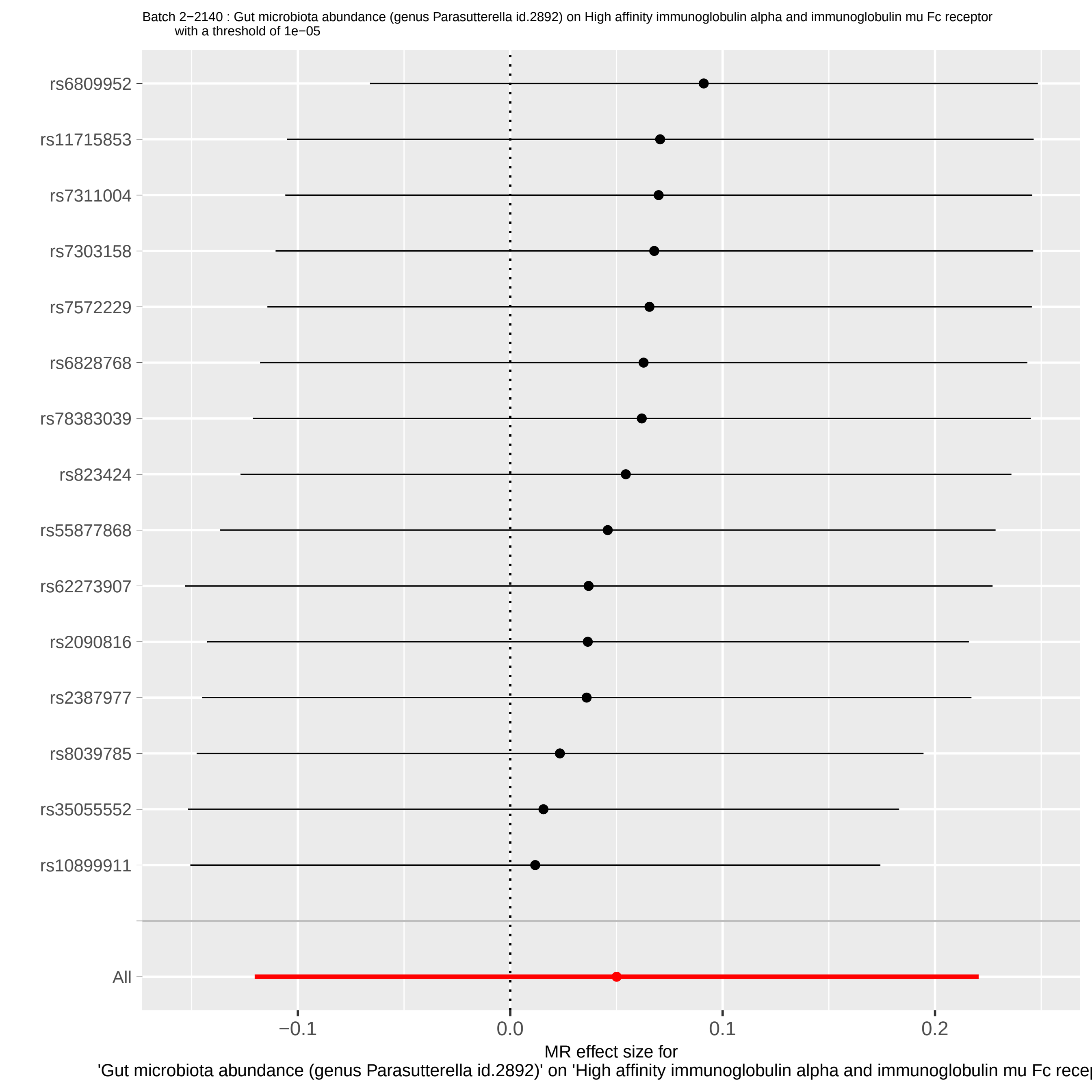

Batch 2−2140 : Gut microbiota abundance (genus Parasutterella id.2892) on High affinity immunoglobulin alpha and immunoglobulin mu Fc receptor with a threshold of 1e−05
rs6809952
rs11715853
rs7311004
rs7303158
rs7572229
rs6828768
rs78383039
rs823424
rs55877868
rs62273907
rs2090816
rs2387977
rs8039785
rs35055552
rs10899911
All
−0.1
0.0
0.1
0.2
MR effect size for
'Gut microbiota abundance (genus Parasutterella id.2892)' on 'High affinity immunoglobulin alpha and immunoglobulin mu Fc recep

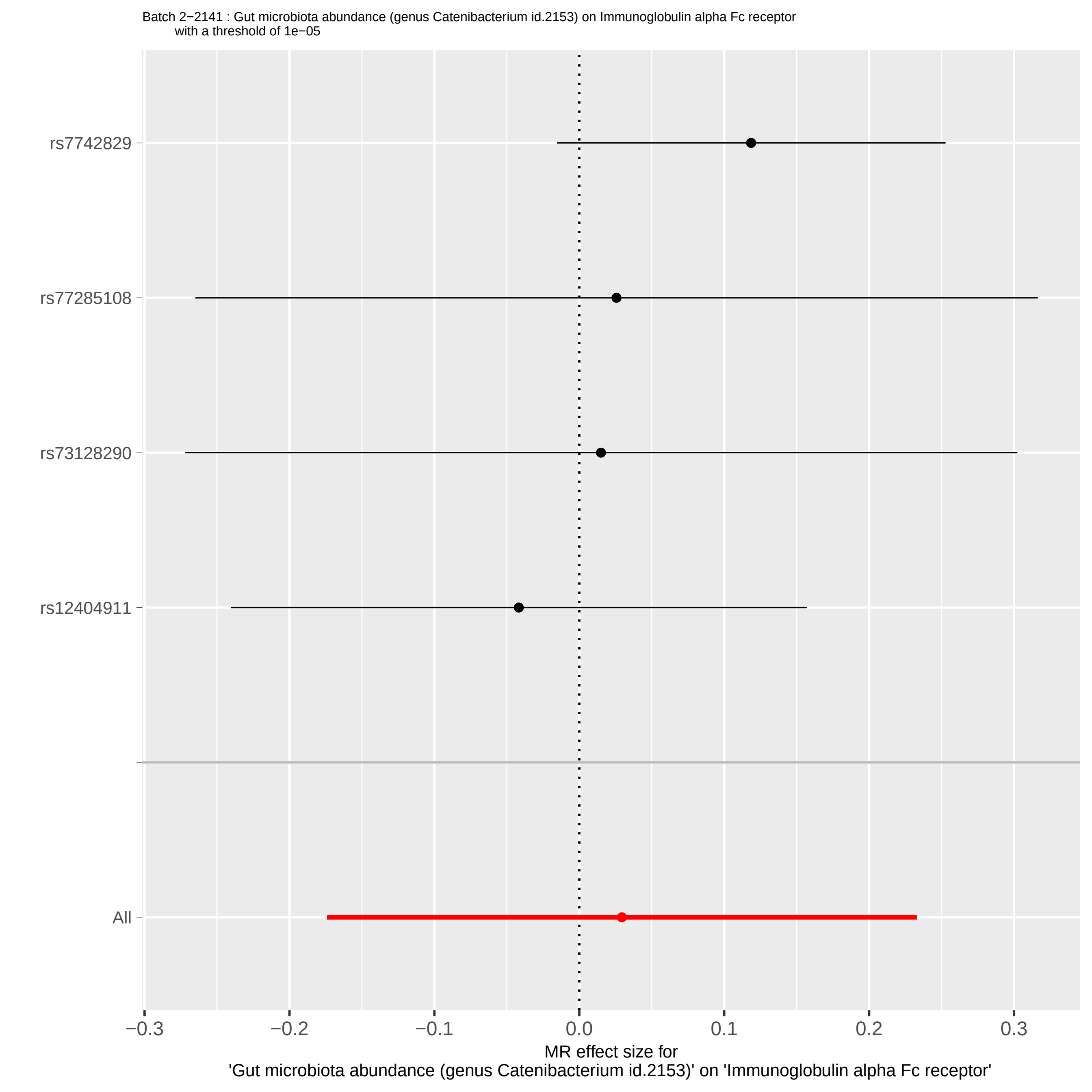
Batch 2−2141 : Gut microbiota abundance (genus Catenibacterium id.2153) on Immunoglobulin alpha Fc receptor
with a threshold of 1e−05
rs7742829
rs77285108
rs73128290
rs12404911
All
−0.3
−0.2
−0.1
0.0
0.1
0.2
0.3
MR effect size for
'Gut microbiota abundance (genus Catenibacterium id.2153)' on 'Immunoglobulin alpha Fc receptor'

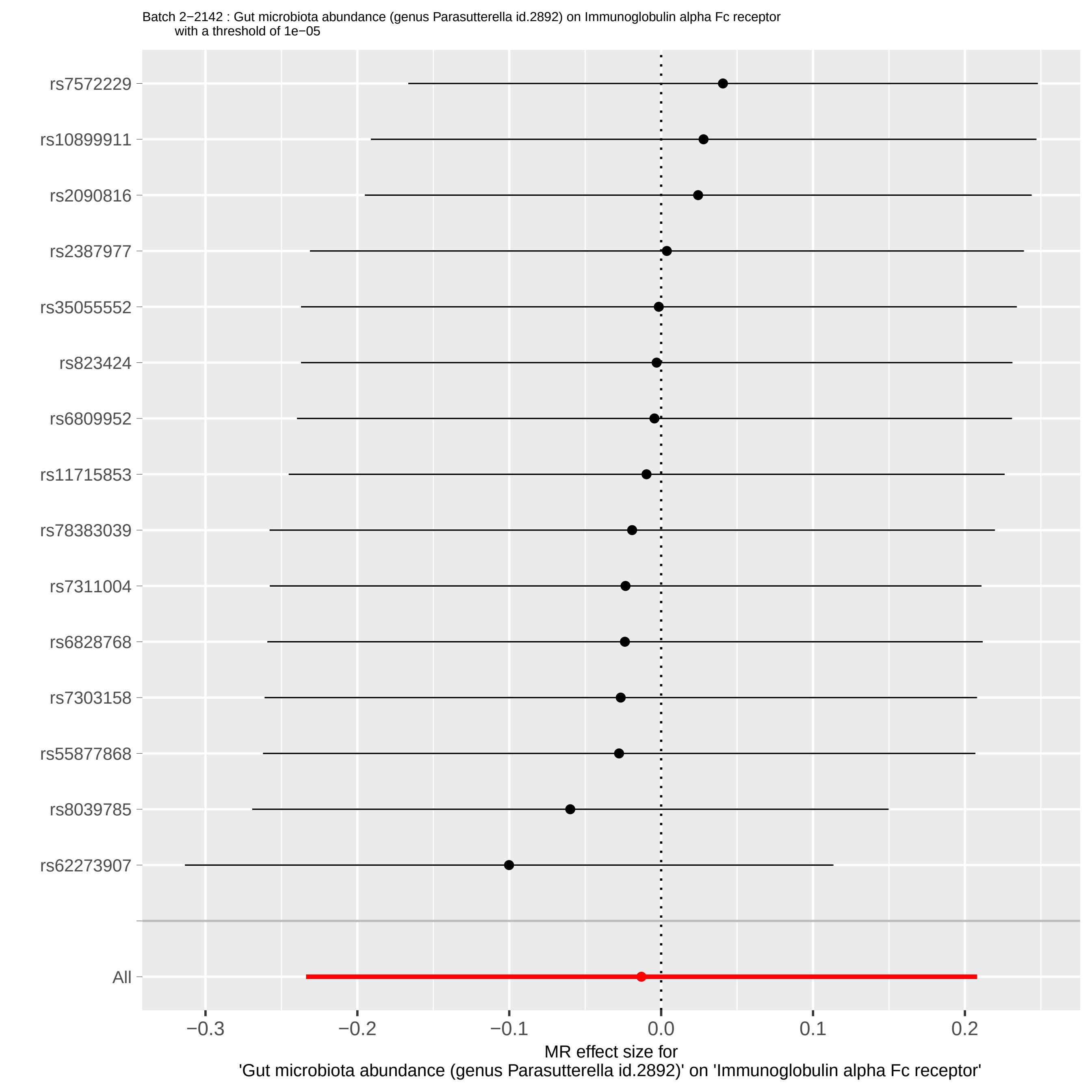
Batch 2−2142 : Gut microbiota abundance (genus Parasutterella id.2892) on Immunoglobulin alpha Fc receptor
with a threshold of 1e−05
rs7572229
rs10899911
rs2090816
rs2387977
rs35055552
rs823424
rs6809952
rs11715853
rs78383039
rs7311004
rs6828768
rs7303158
rs55877868
rs8039785
rs62273907
All
−0.3
−0.2
−0.1
0.0
0.1
0.2
MR effect size for
'Gut microbiota abundance (genus Parasutterella id.2892)' on 'Immunoglobulin alpha Fc receptor'

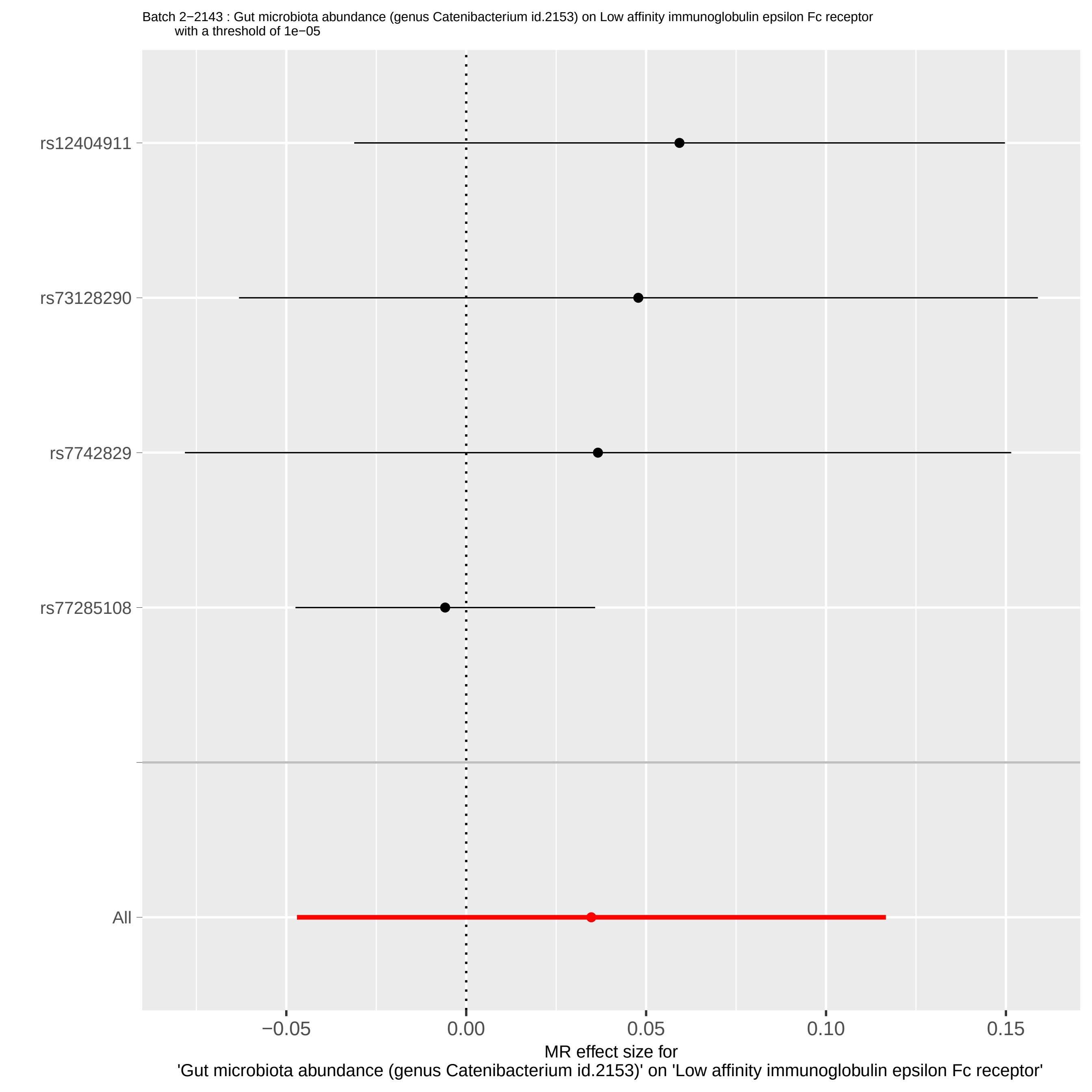
Batch 2−2143 : Gut microbiota abundance (genus Catenibacterium id.2153) on Low affinity immunoglobulin epsilon Fc receptor
with a threshold of 1e−05
rs12404911
rs73128290
rs7742829
rs77285108
All
−0.05
0.00
0.05
0.10
0.15
MR effect size for
'Gut microbiota abundance (genus Catenibacterium id.2153)' on 'Low affinity immunoglobulin epsilon Fc receptor'

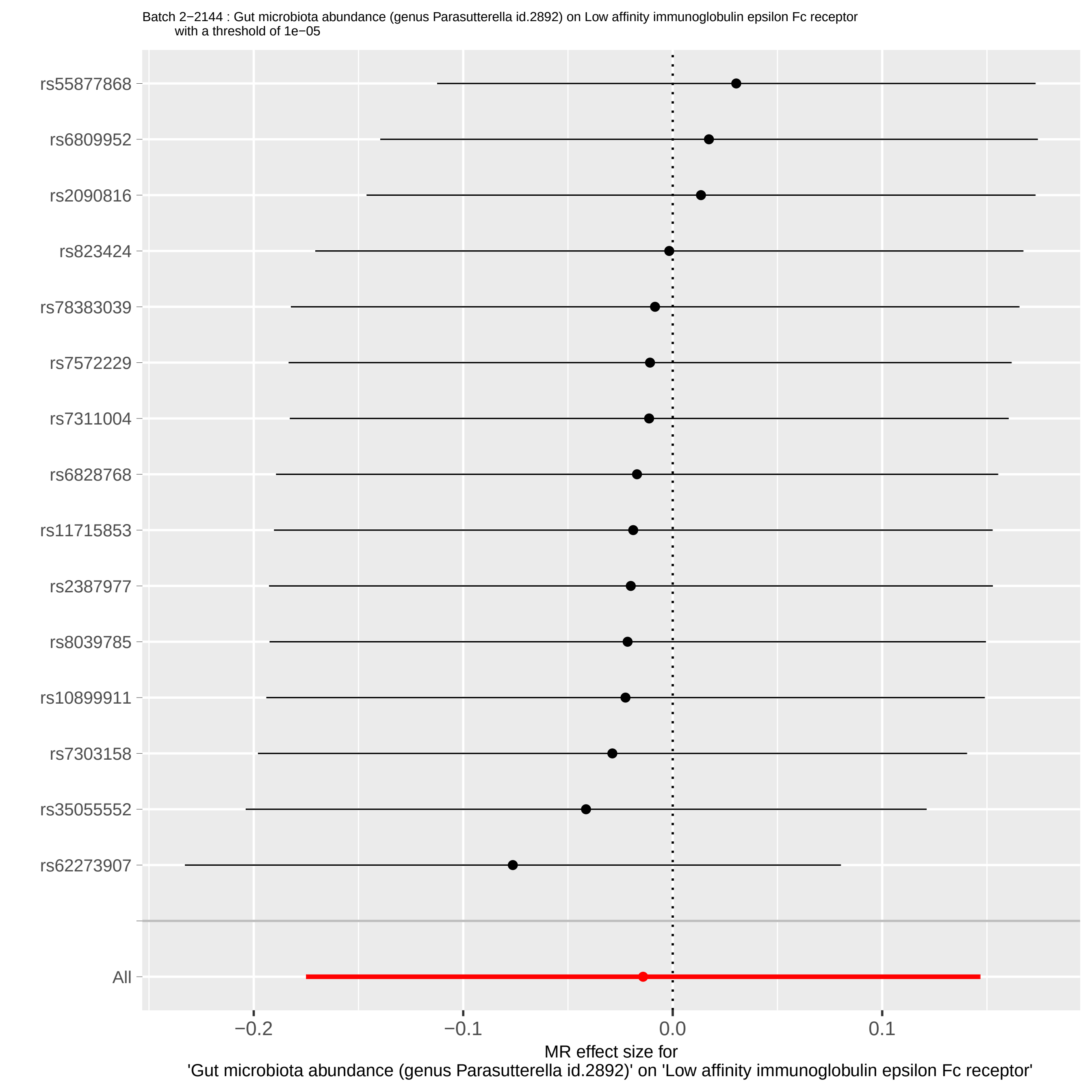

Batch 2−2144 : Gut microbiota abundance (genus Parasutterella id.2892) on Low affinity immunoglobulin epsilon Fc receptor
with a threshold of 1e−05
rs55877868
rs6809952
rs2090816
rs823424
rs78383039
rs7572229
rs7311004
rs6828768
rs11715853
rs2387977
rs8039785
rs10899911
rs7303158
rs35055552
rs62273907
All
−0.2
−0.1
0.0
0.1
MR effect size for
'Gut microbiota abundance (genus Parasutterella id.2892)' on 'Low affinity immunoglobulin epsilon Fc receptor'

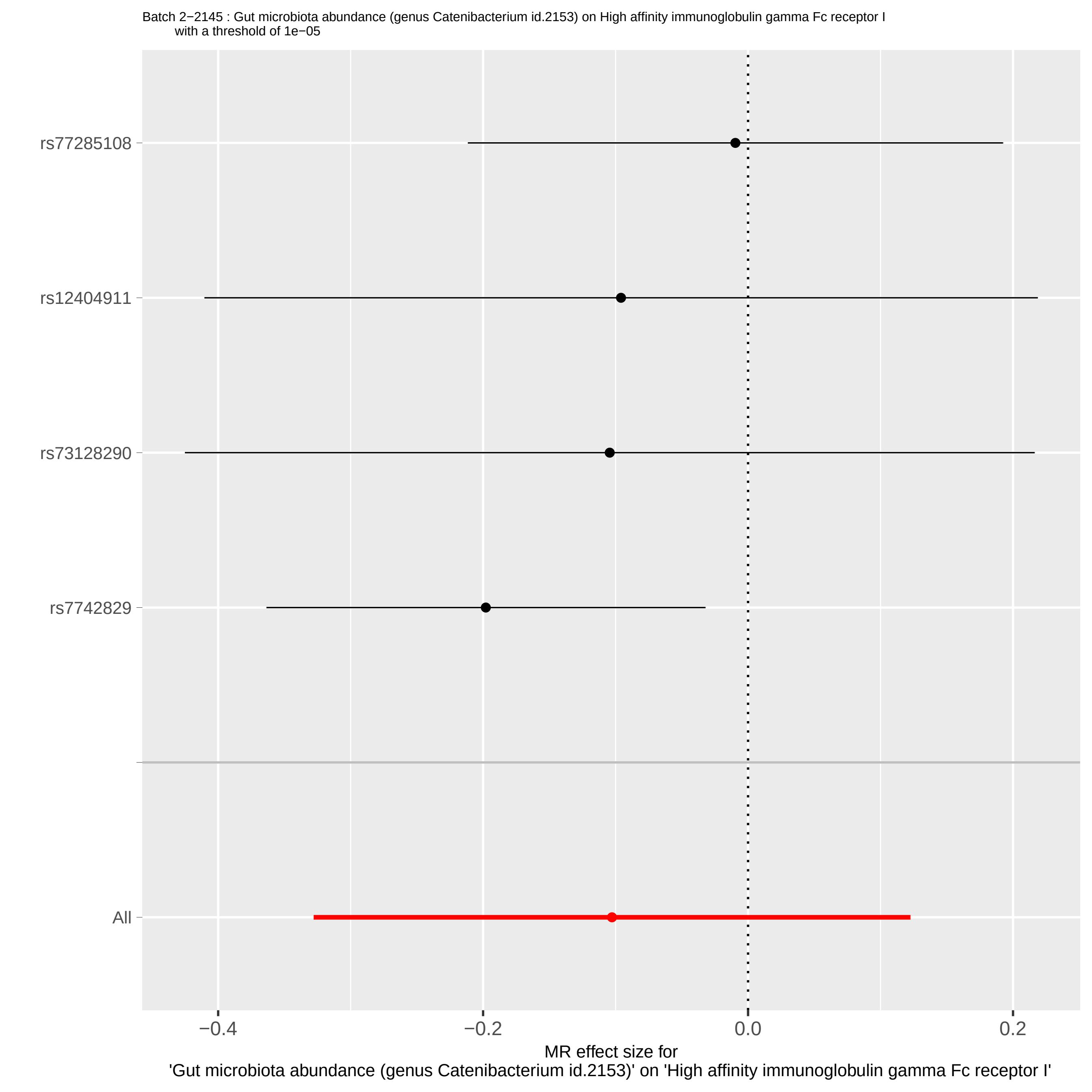
Batch 2−2145 : Gut microbiota abundance (genus Catenibacterium id.2153) on High affinity immunoglobulin gamma Fc receptor I
with a threshold of 1e−05
rs77285108
rs12404911
rs73128290
rs7742829
All
−0.4
−0.2
0.0
0.2
MR effect size for
'Gut microbiota abundance (genus Catenibacterium id.2153)' on 'High affinity immunoglobulin gamma Fc receptor I'

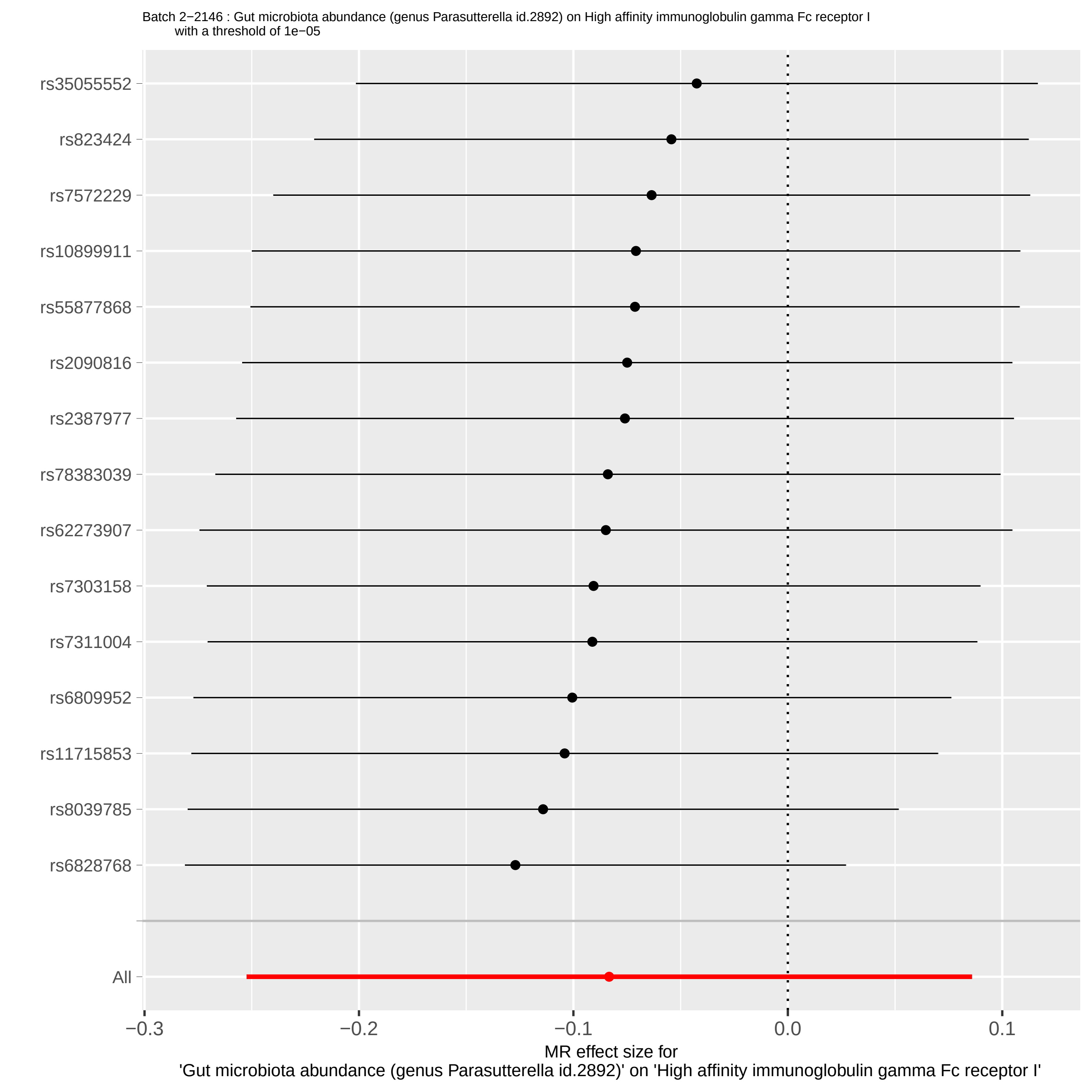
Batch 2−2146 : Gut microbiota abundance (genus Parasutterella id.2892) on High affinity immunoglobulin gamma Fc receptor I
with a threshold of 1e−05
rs35055552
rs823424
rs7572229
rs10899911
rs55877868
rs2090816
rs2387977
rs78383039
rs62273907
rs7303158
rs7311004
rs6809952
rs11715853
rs8039785
rs6828768
All
−0.3
−0.2
−0.1
0.0
0.1
MR effect size for
'Gut microbiota abundance (genus Parasutterella id.2892)' on 'High affinity immunoglobulin gamma Fc receptor I'

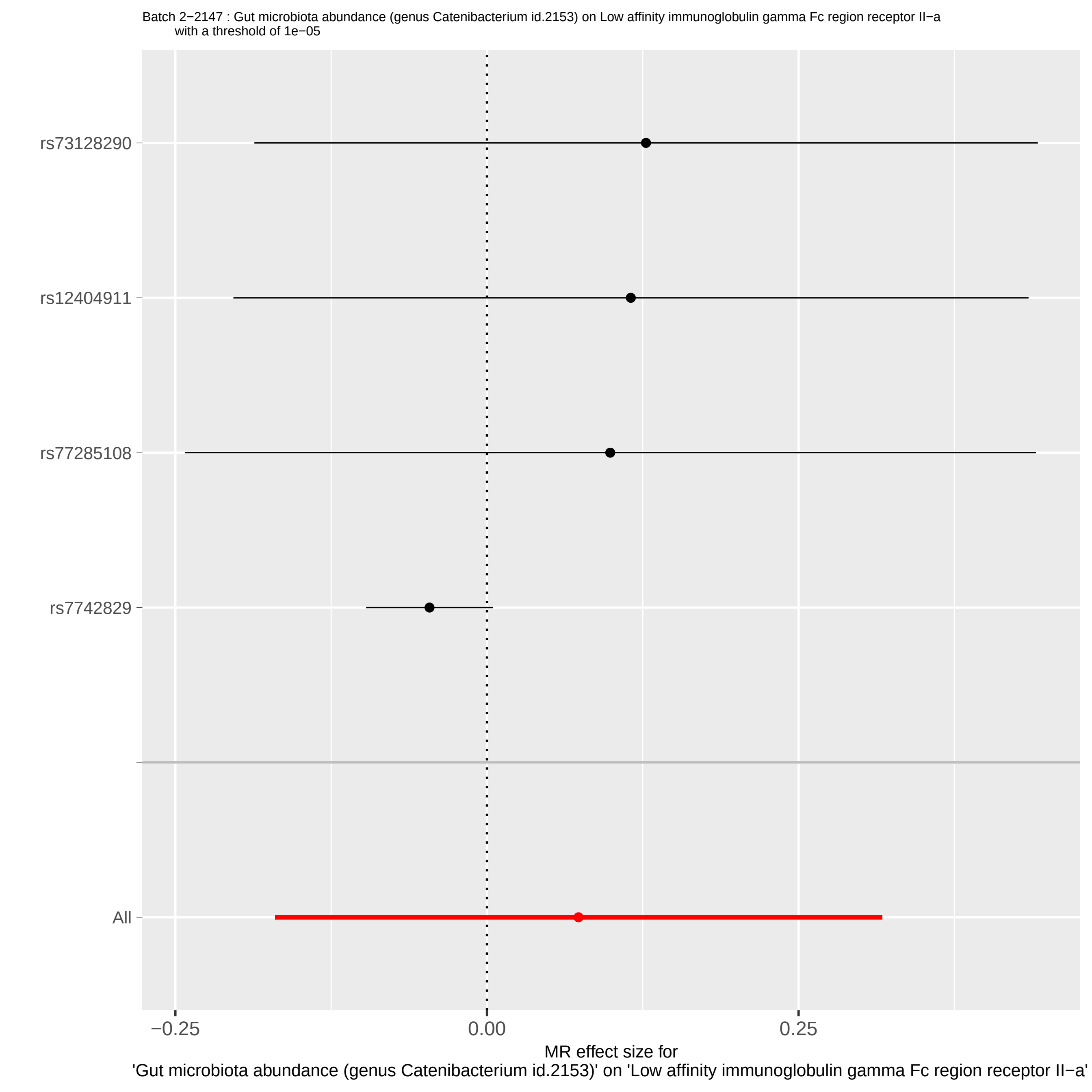
Batch 2−2147 : Gut microbiota abundance (genus Catenibacterium id.2153) on Low affinity immunoglobulin gamma Fc region receptor II−a
with a threshold of 1e−05
rs73128290
rs12404911
rs77285108
rs7742829
All
−0.25
0.00
0.25
MR effect size for
'Gut microbiota abundance (genus Catenibacterium id.2153)' on 'Low affinity immunoglobulin gamma Fc region receptor II−a'

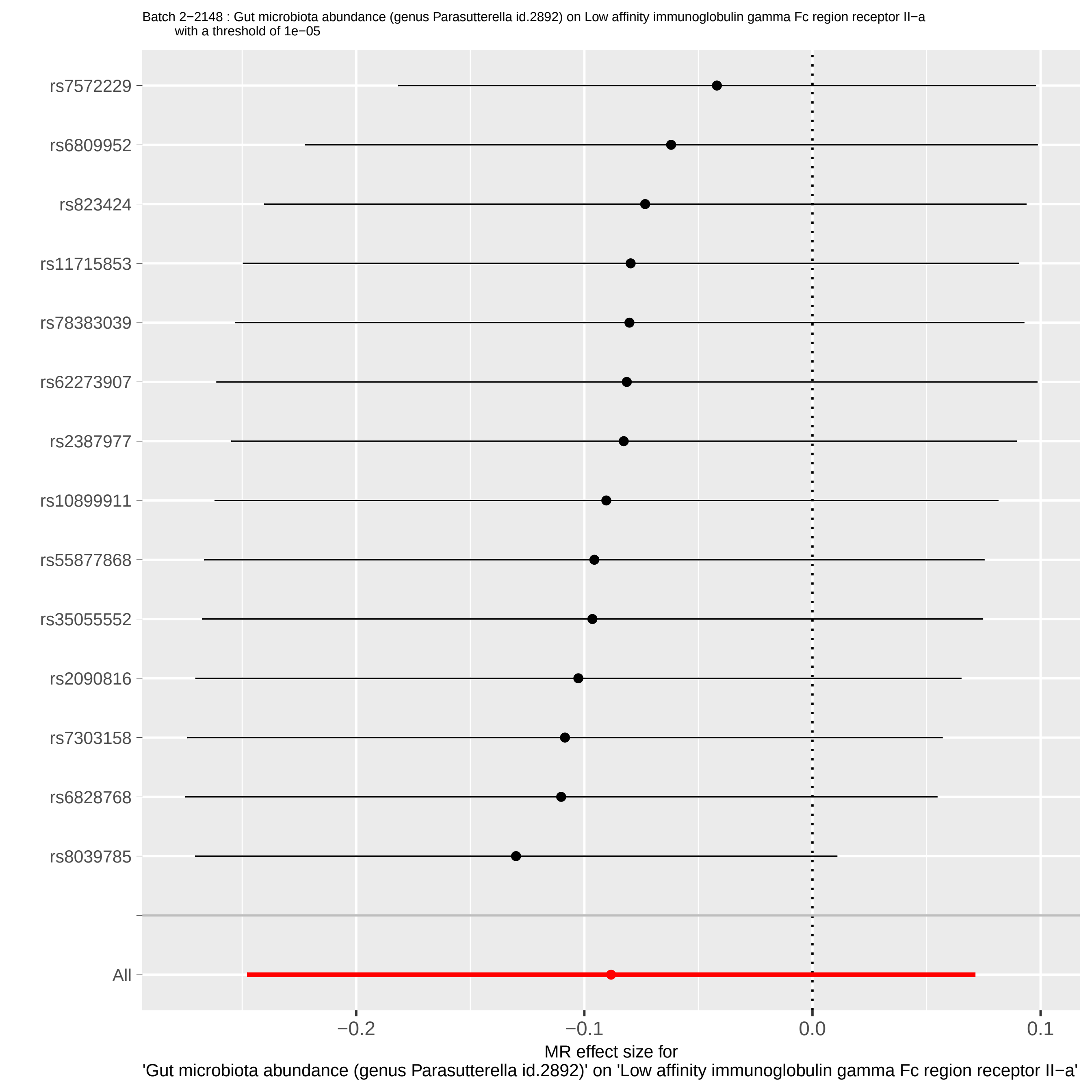

Batch 2−2148 : Gut microbiota abundance (genus Parasutterella id.2892) on Low affinity immunoglobulin gamma Fc region receptor II−a with a threshold of 1e−05
rs7572229
rs6809952
rs823424
rs11715853
rs78383039
rs62273907
rs2387977
rs10899911
rs55877868
rs35055552
rs2090816
rs7303158
rs6828768
rs8039785
All
−0.2
−0.1
0.0
0.1
MR effect size for
'Gut microbiota abundance (genus Parasutterella id.2892)' on 'Low affinity immunoglobulin gamma Fc region receptor II−a'

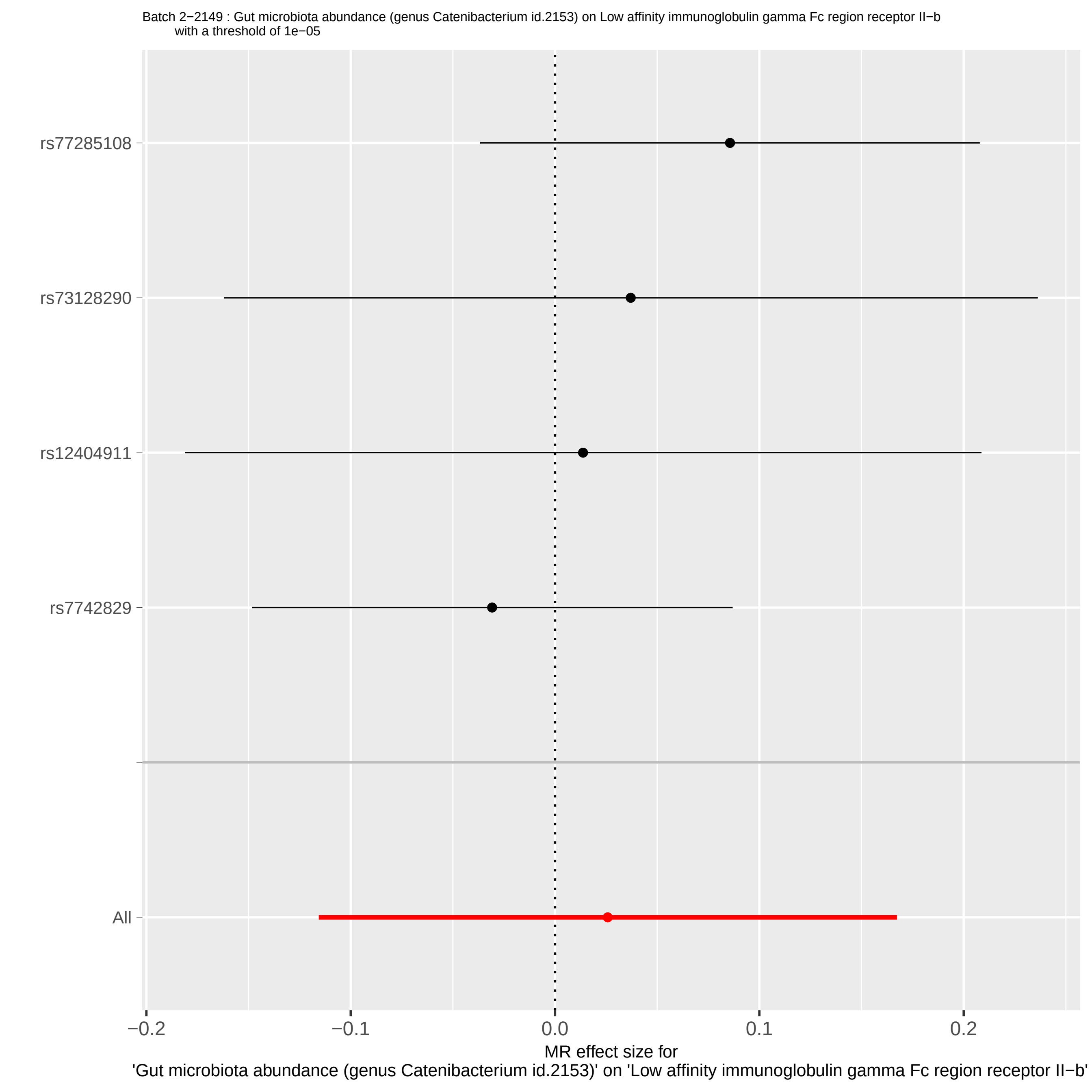
Batch 2−2149 : Gut microbiota abundance (genus Catenibacterium id.2153) on Low affinity immunoglobulin gamma Fc region receptor II−b
with a threshold of 1e−05
rs77285108
rs73128290
rs12404911
rs7742829
All
−0.2
−0.1
0.0
0.1
0.2
MR effect size for
'Gut microbiota abundance (genus Catenibacterium id.2153)' on 'Low affinity immunoglobulin gamma Fc region receptor II−b'

Batch 2−2150 : Gut microbiota abundance (genus Parasutterella id.2892) on Low affinity immunoglobulin gamma Fc region receptor II−b with a threshold of 1e−05

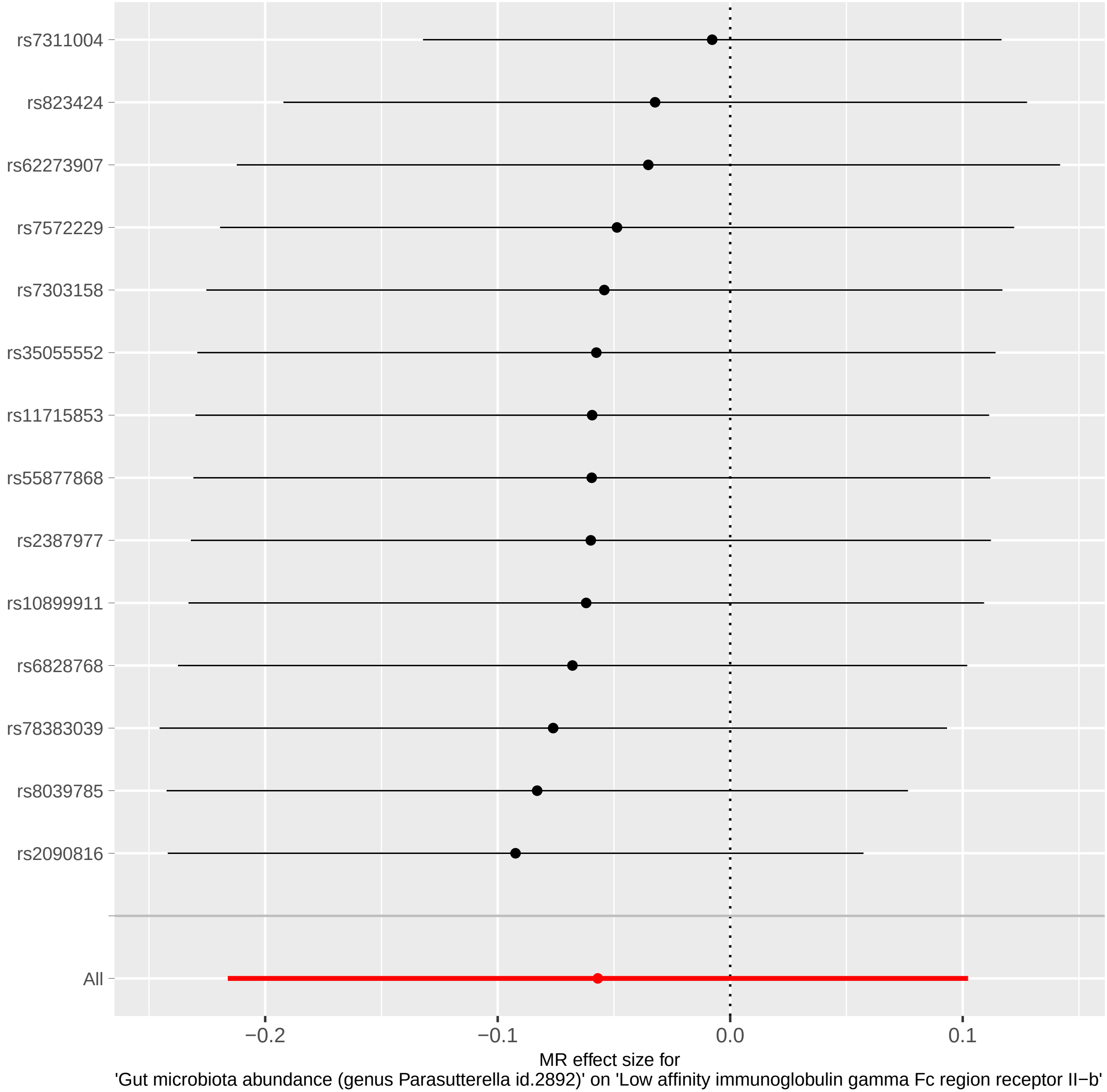

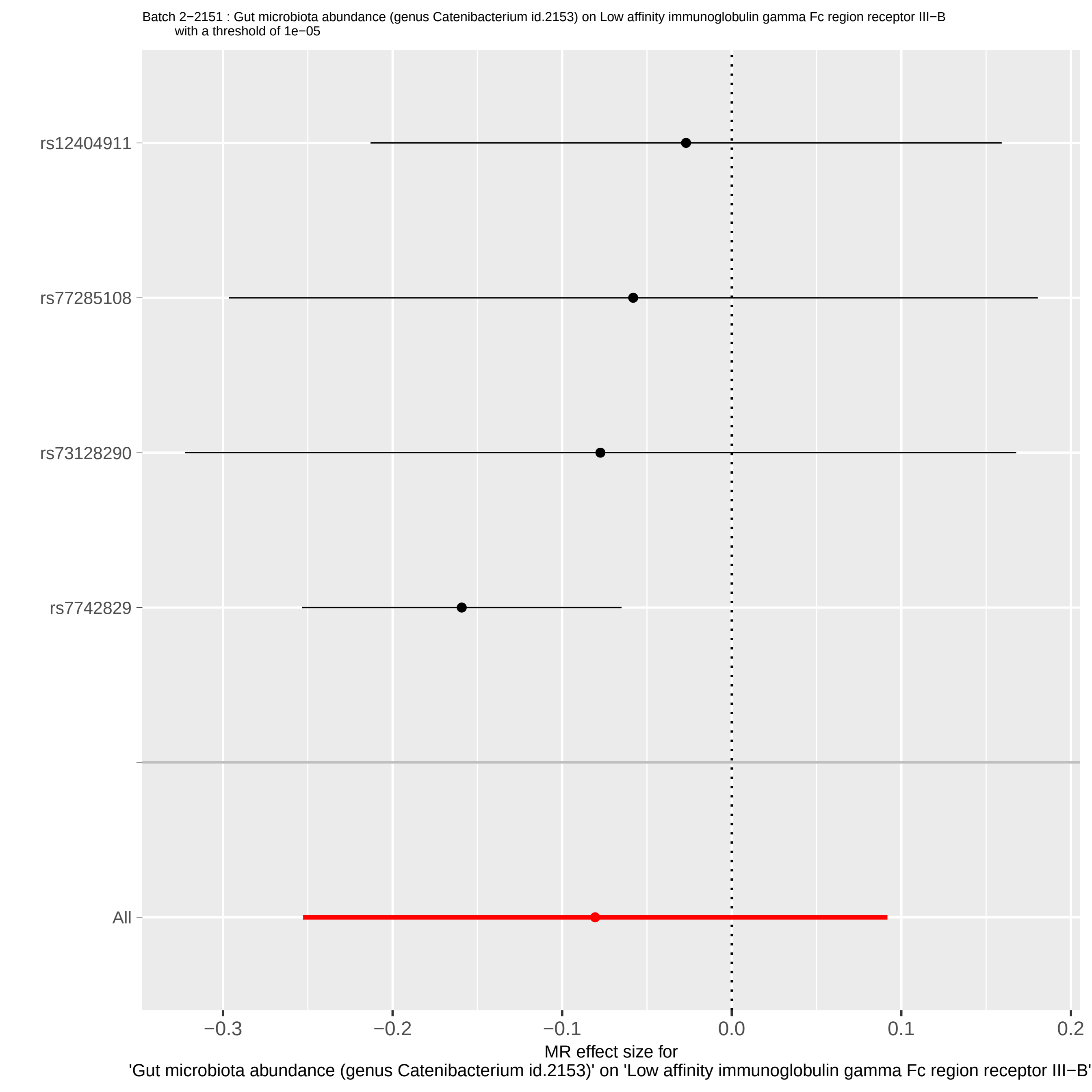

Batch 2−2151 : Gut microbiota abundance (genus Catenibacterium id.2153) on Low affinity immunoglobulin gamma Fc region receptor III−B
with a threshold of 1e−05
rs12404911
rs77285108
rs73128290
rs7742829
All
−0.3
−0.2
−0.1
0.0
0.1
0.2
MR effect size for
'Gut microbiota abundance (genus Catenibacterium id.2153)' on 'Low affinity immunoglobulin gamma Fc region receptor III−B'

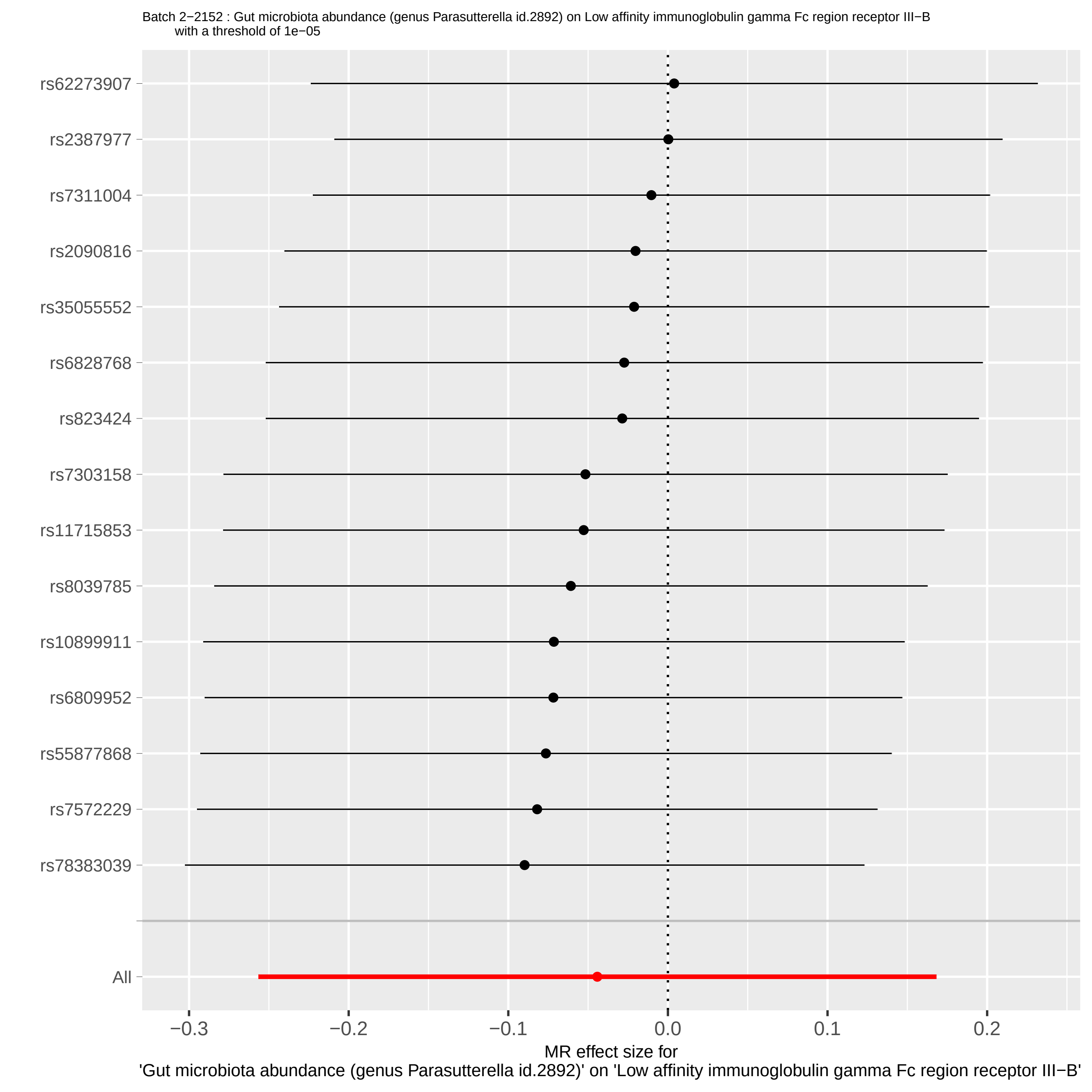

Batch 2−2152 : Gut microbiota abundance (genus Parasutterella id.2892) on Low affinity immunoglobulin gamma Fc region receptor III−B
with a threshold of 1e−05
rs62273907
rs2387977
rs7311004
rs2090816
rs35055552
rs6828768
rs823424
rs7303158
rs11715853
rs8039785
rs10899911
rs6809952
rs55877868
rs7572229
rs78383039
All
−0.3
−0.2
−0.1
0.0
0.1
0.2
MR effect size for
'Gut microbiota abundance (genus Parasutterella id.2892)' on 'Low affinity immunoglobulin gamma Fc region receptor III−B'

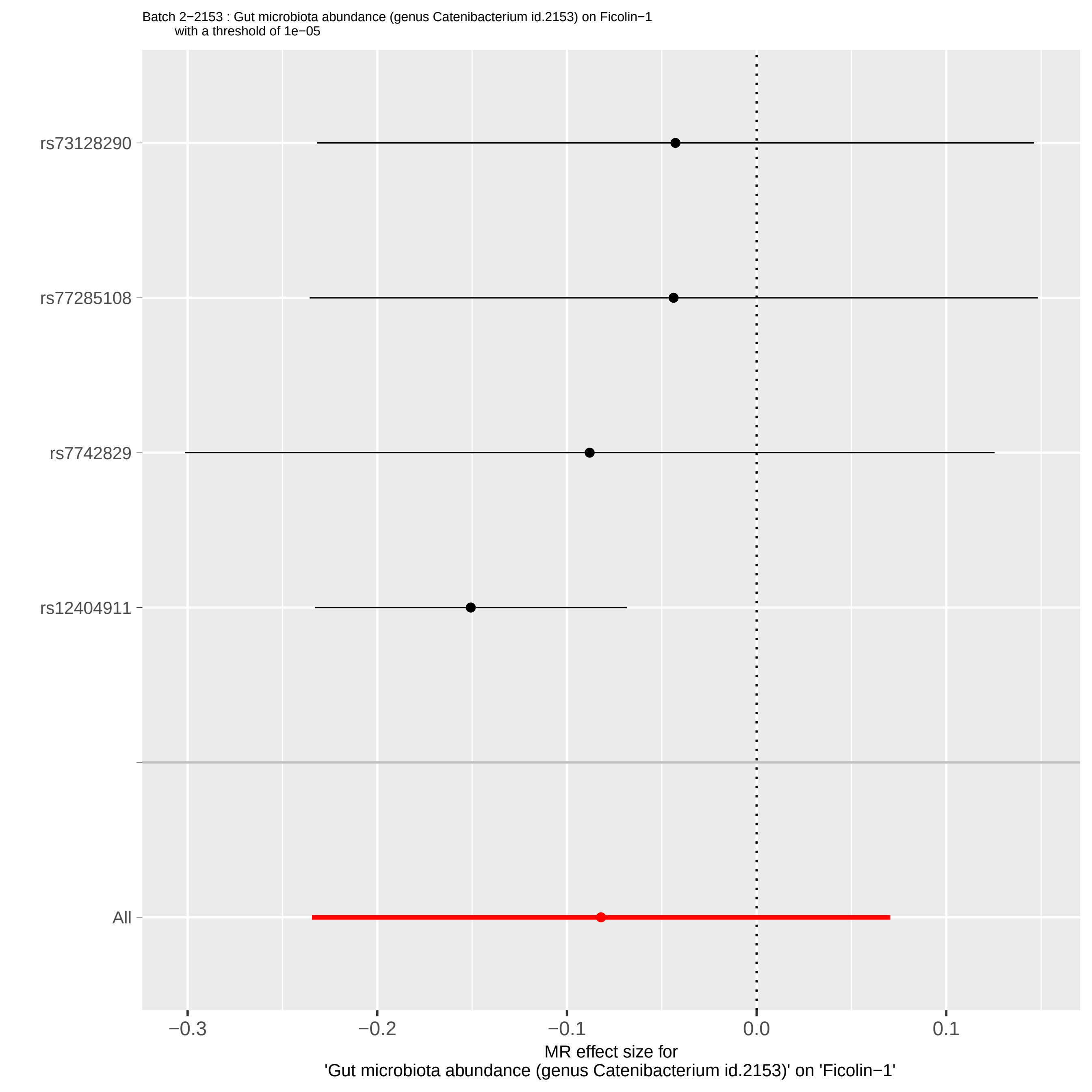

Batch 2−2153 : Gut microbiota abundance (genus Catenibacterium id.2153) on Ficolin−1
with a threshold of 1e−05
rs73128290
rs77285108
rs7742829
rs12404911
All
−0.3
−0.2
−0.1
0.0
0.1
MR effect size for
'Gut microbiota abundance (genus Catenibacterium id.2153)' on 'Ficolin−1'

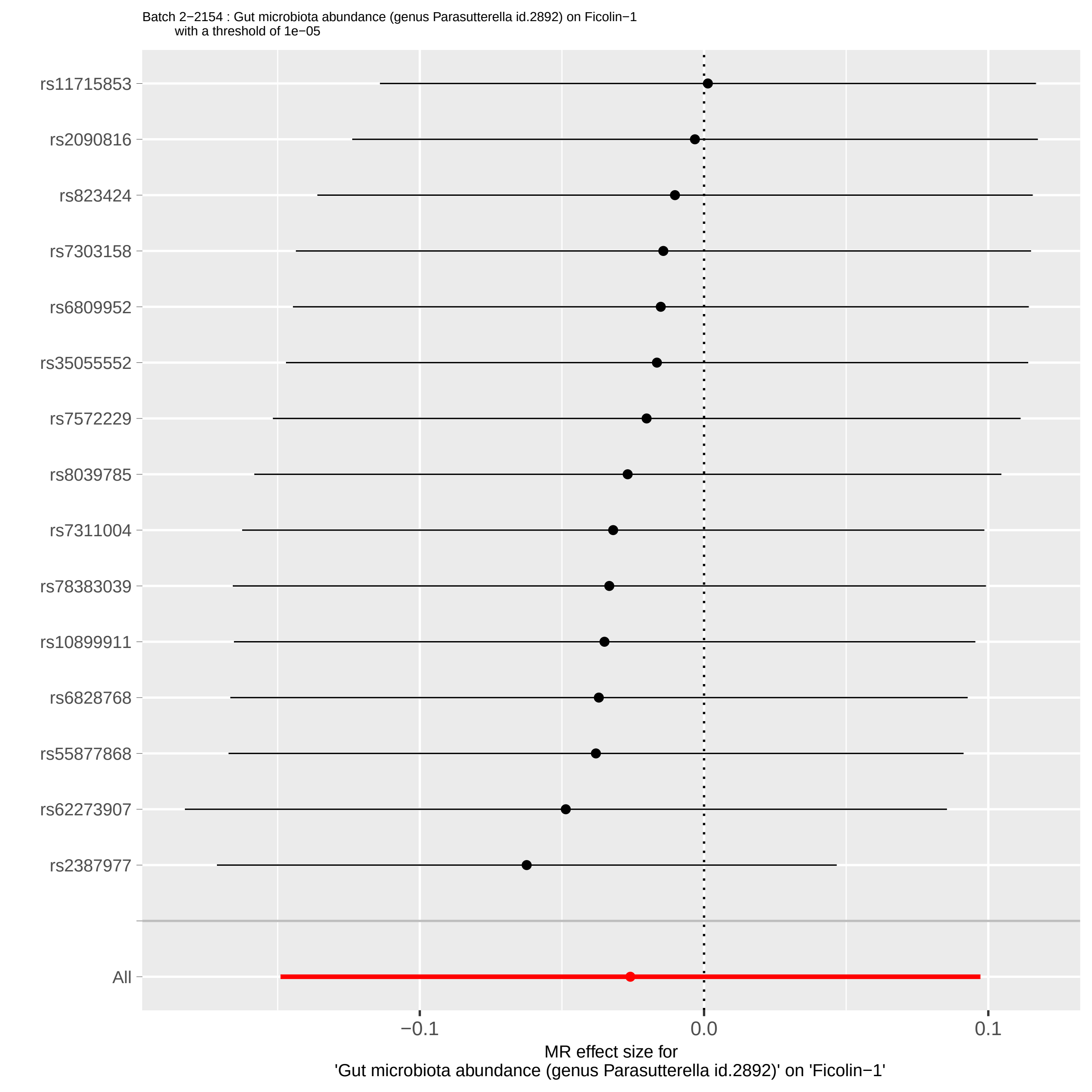
Batch 2−2154 : Gut microbiota abundance (genus Parasutterella id.2892) on Ficolin−1
with a threshold of 1e−05
rs11715853
rs2090816
rs823424
rs7303158
rs6809952
rs35055552
rs7572229
rs8039785
rs7311004
rs78383039
rs10899911
rs6828768
rs55877868
rs62273907
rs2387977
All
−0.1
0.0
0.1
MR effect size for
'Gut microbiota abundance (genus Parasutterella id.2892)' on 'Ficolin−1'

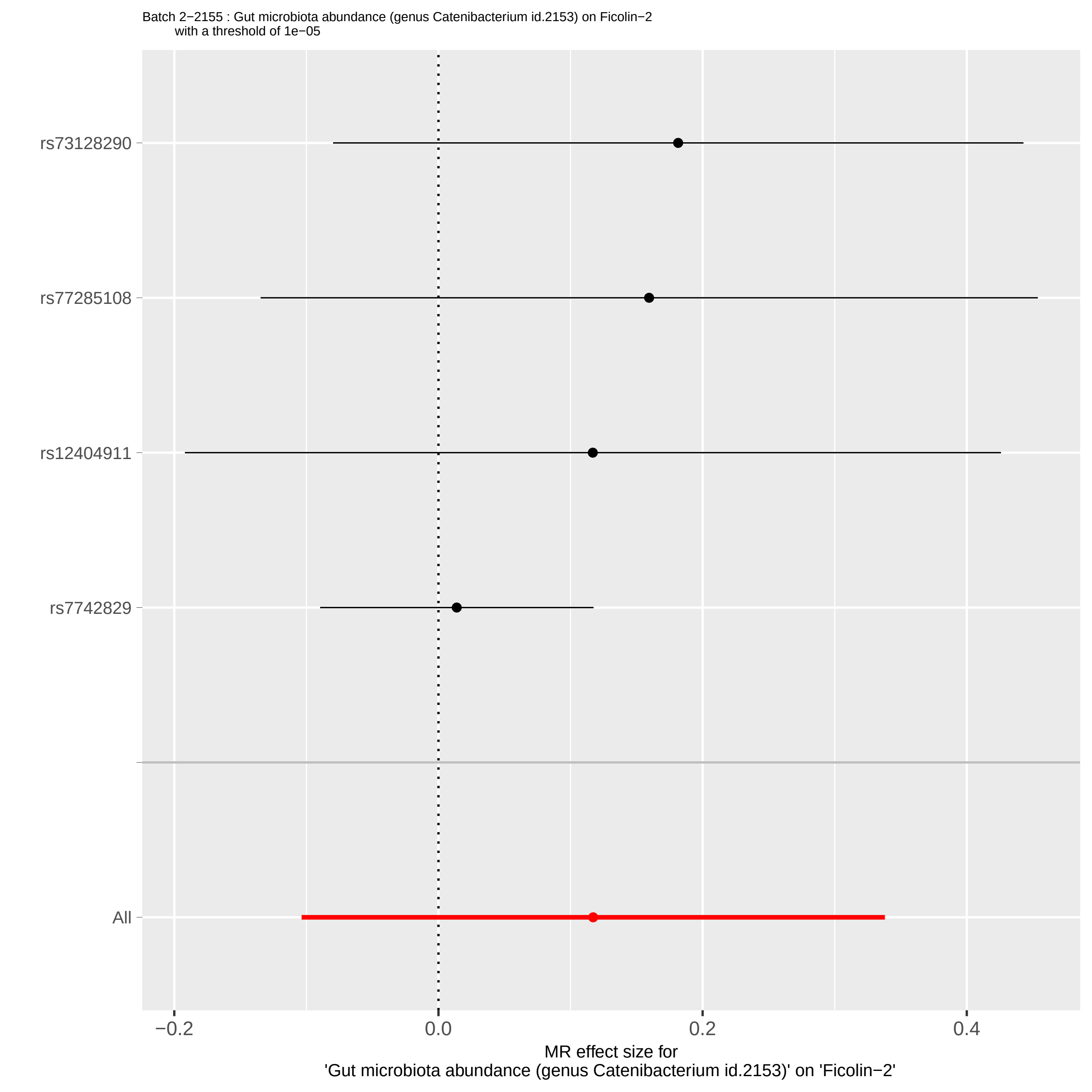
Batch 2−2155 : Gut microbiota abundance (genus Catenibacterium id.2153) on Ficolin−2
with a threshold of 1e−05
rs73128290
rs77285108
rs12404911
rs7742829
All
−0.2
0.0
0.2
0.4
MR effect size for
'Gut microbiota abundance (genus Catenibacterium id.2153)' on 'Ficolin−2'

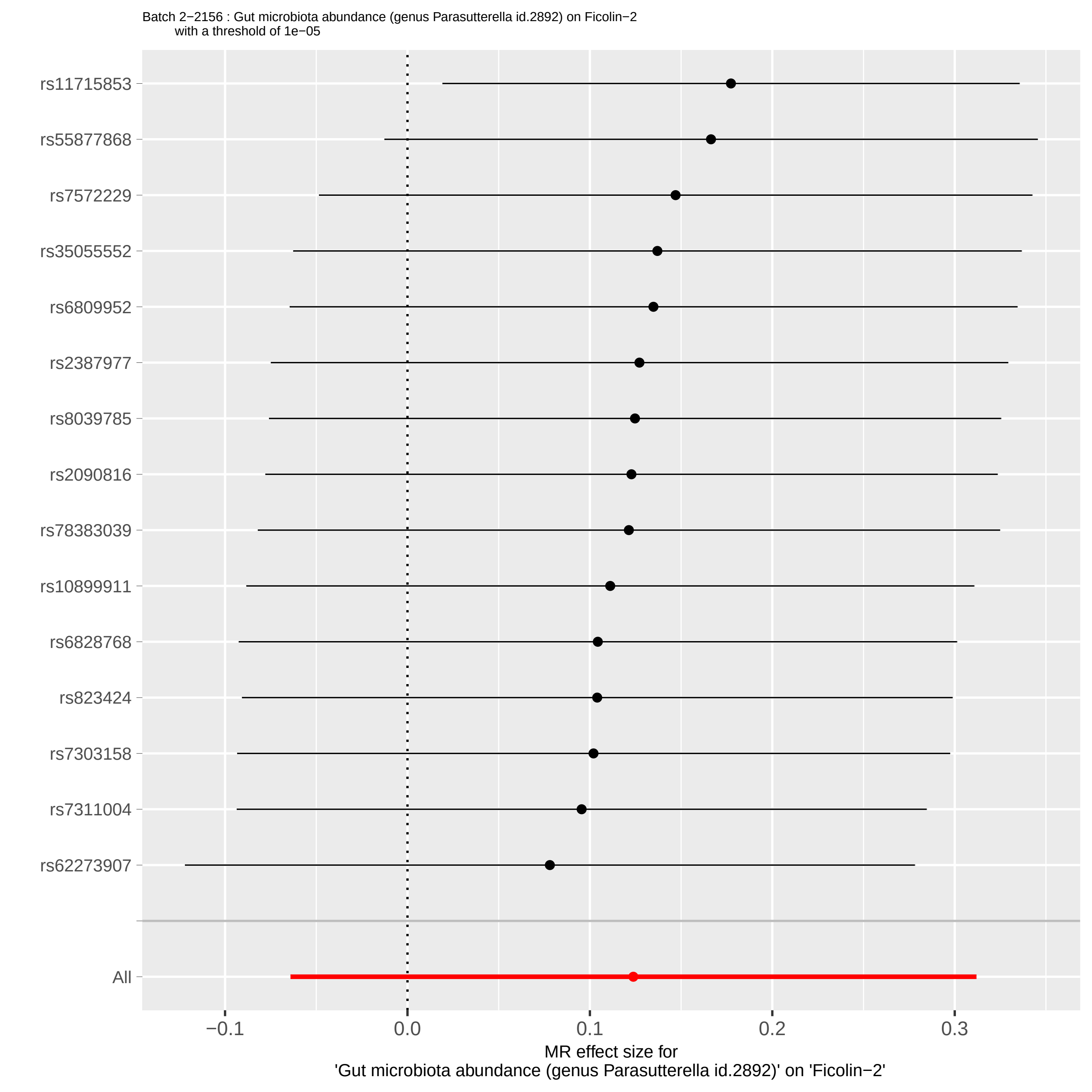
Batch 2−2156 : Gut microbiota abundance (genus Parasutterella id.2892) on Ficolin−2
with a threshold of 1e−05
rs11715853
rs55877868
rs7572229
rs35055552
rs6809952
rs2387977
rs8039785
rs2090816
rs78383039
rs10899911
rs6828768
rs823424
rs7303158
rs7311004
rs62273907
All
−0.1
0.0
0.1
0.2
0.3
MR effect size for
'Gut microbiota abundance (genus Parasutterella id.2892)' on 'Ficolin−2'

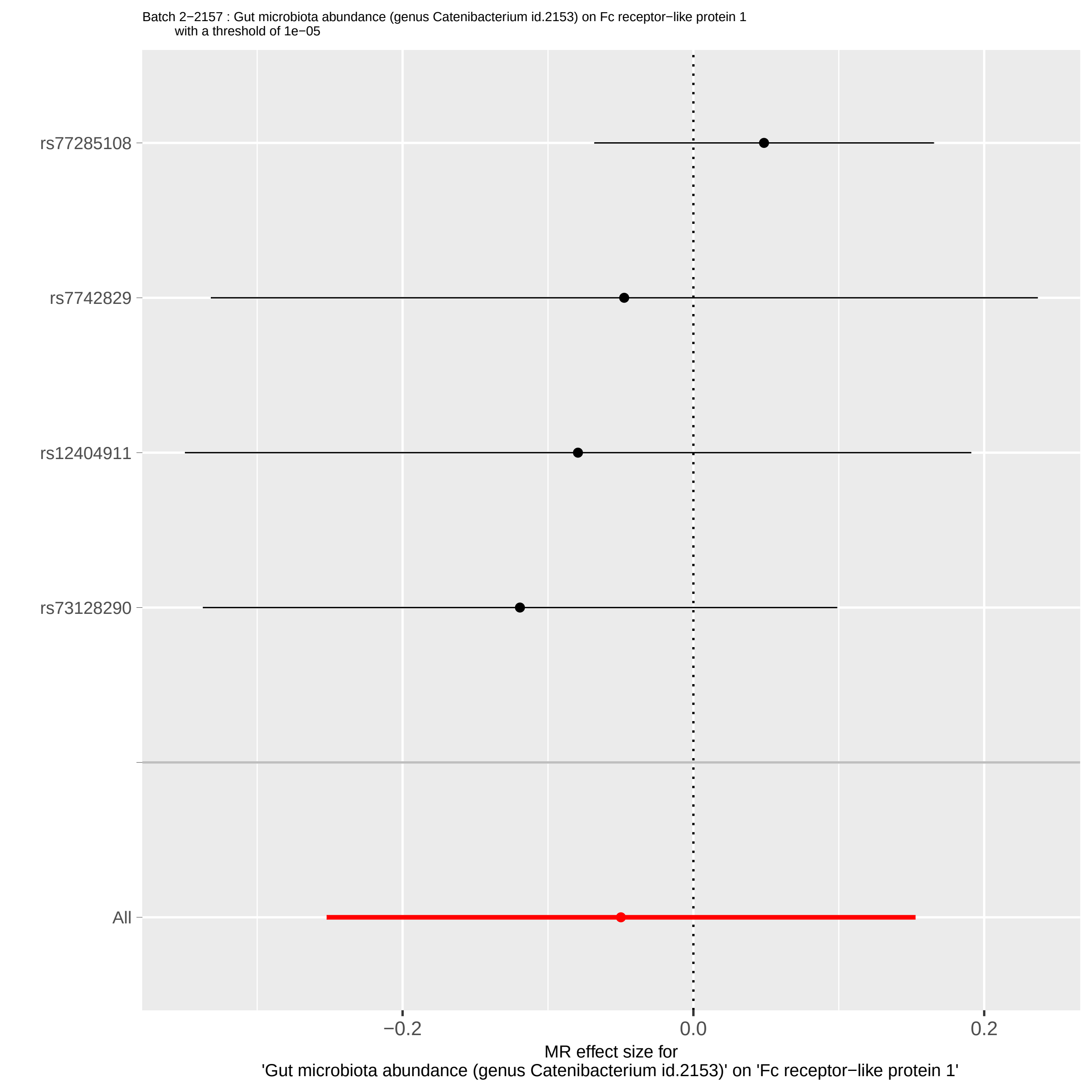
Batch 2−2157 : Gut microbiota abundance (genus Catenibacterium id.2153) on Fc receptor−like protein 1
with a threshold of 1e−05
rs77285108
rs7742829
rs12404911
rs73128290
All
−0.2
0.0
0.2
MR effect size for
'Gut microbiota abundance (genus Catenibacterium id.2153)' on 'Fc receptor−like protein 1'

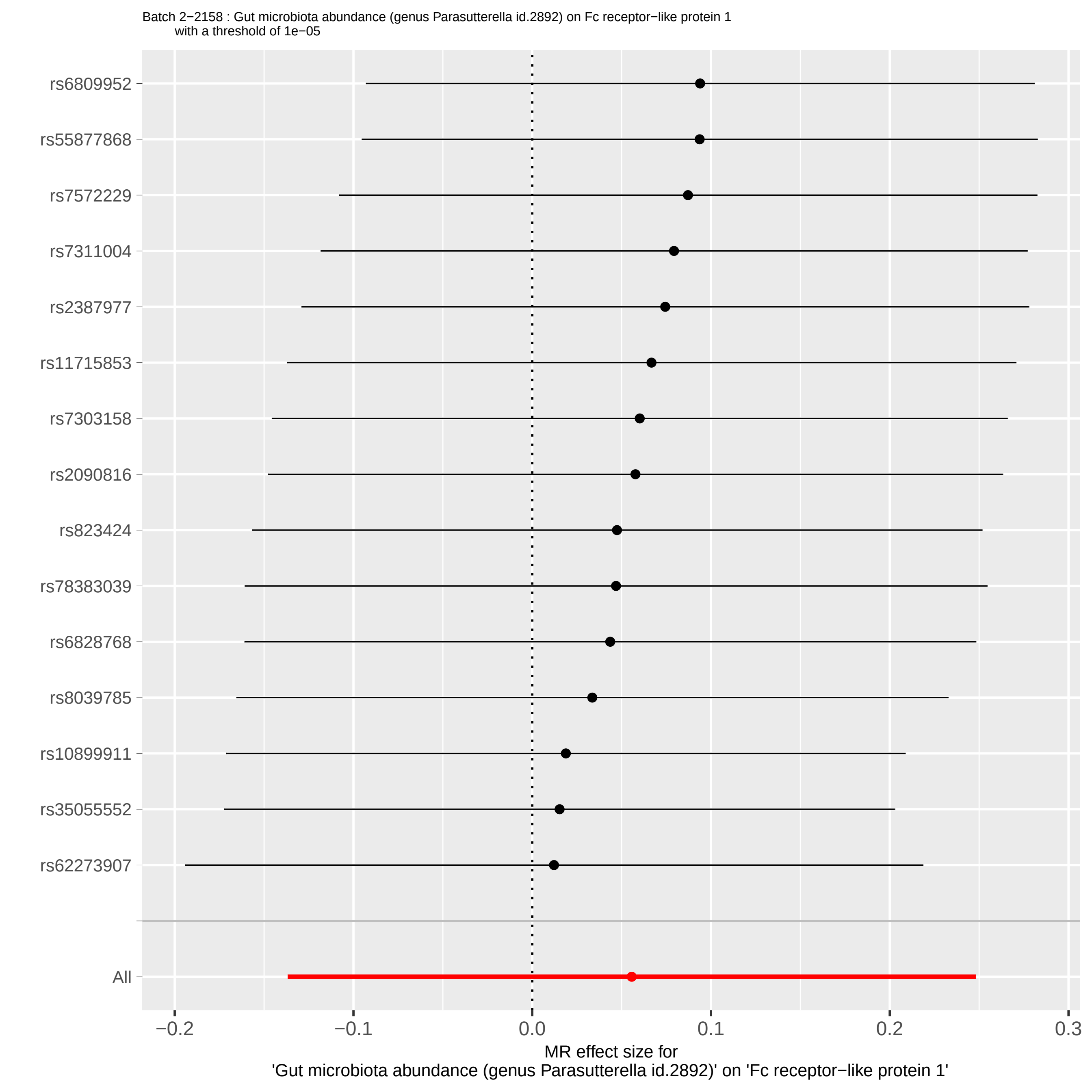

Batch 2−2158 : Gut microbiota abundance (genus Parasutterella id.2892) on Fc receptor−like protein 1
with a threshold of 1e−05
rs6809952
rs55877868
rs7572229
rs7311004
rs2387977
rs11715853
rs7303158
rs2090816
rs823424
rs78383039
rs6828768
rs8039785
rs10899911
rs35055552
rs62273907
All
−0.2
−0.1
0.0
0.1
0.2
0.3
MR effect size for
'Gut microbiota abundance (genus Parasutterella id.2892)' on 'Fc receptor−like protein 1'

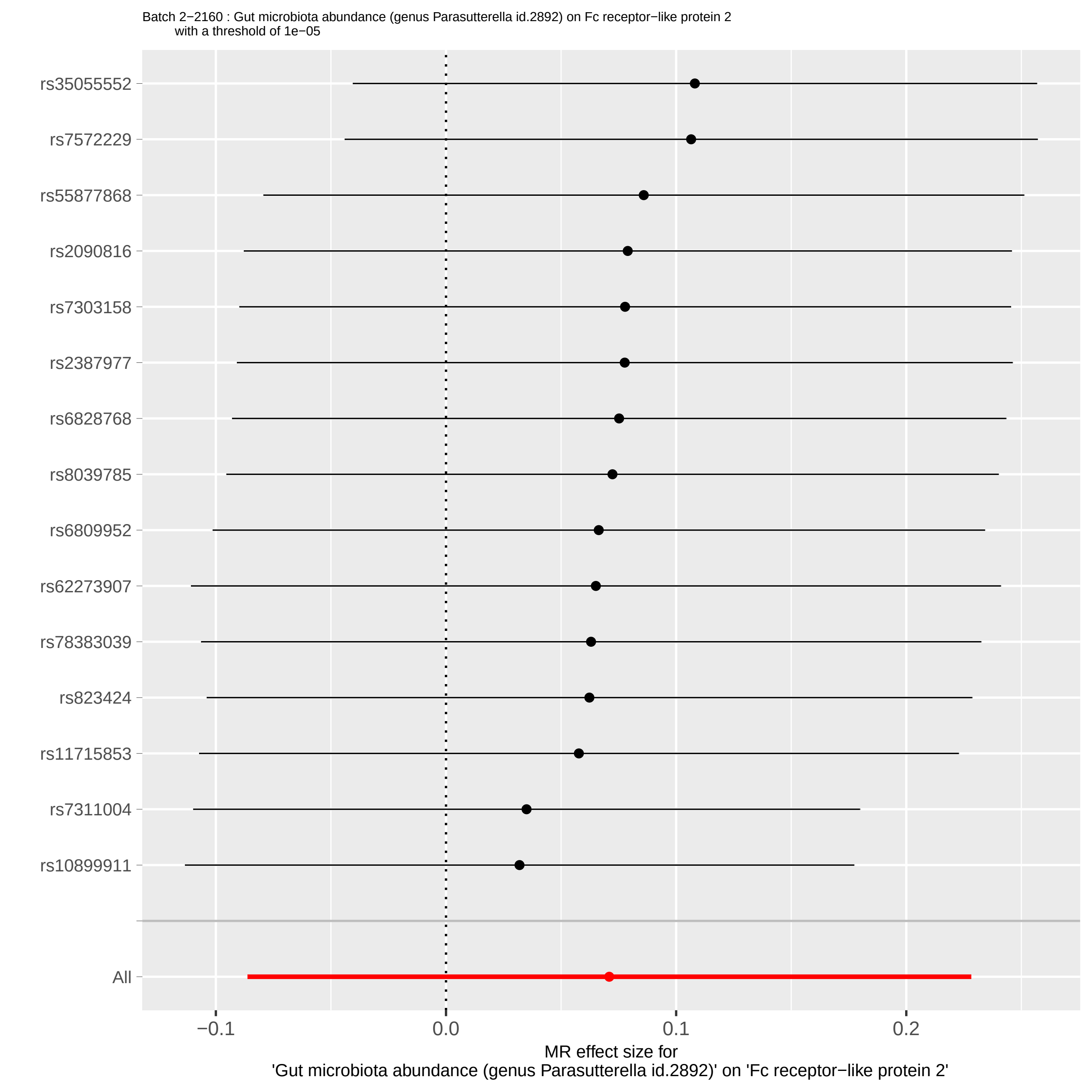

Batch 2−2160 : Gut microbiota abundance (genus Parasutterella id.2892) on Fc receptor−like protein 2
with a threshold of 1e−05
rs35055552
rs7572229
rs55877868
rs2090816
rs7303158
rs2387977
rs6828768
rs8039785
rs6809952
rs62273907
rs78383039
rs823424
rs11715853
rs7311004
rs10899911
All
−0.1
0.0
0.1
0.2
MR effect size for
'Gut microbiota abundance (genus Parasutterella id.2892)' on 'Fc receptor−like protein 2'

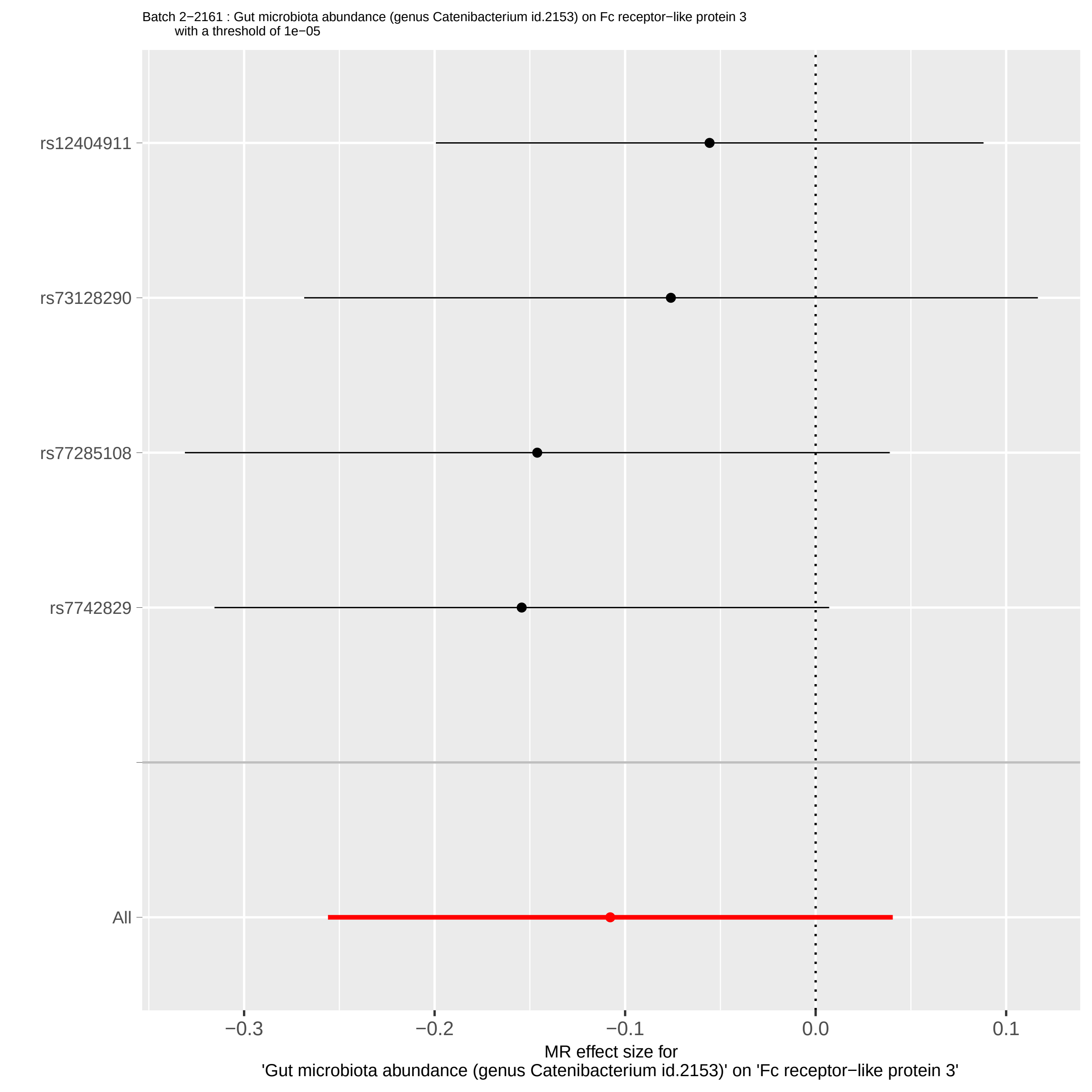
Batch 2−2161 : Gut microbiota abundance (genus Catenibacterium id.2153) on Fc receptor−like protein 3
with a threshold of 1e−05
rs12404911
rs73128290
rs77285108
rs7742829
All
−0.3
−0.2
−0.1
0.0
0.1
MR effect size for
'Gut microbiota abundance (genus Catenibacterium id.2153)' on 'Fc receptor−like protein 3'

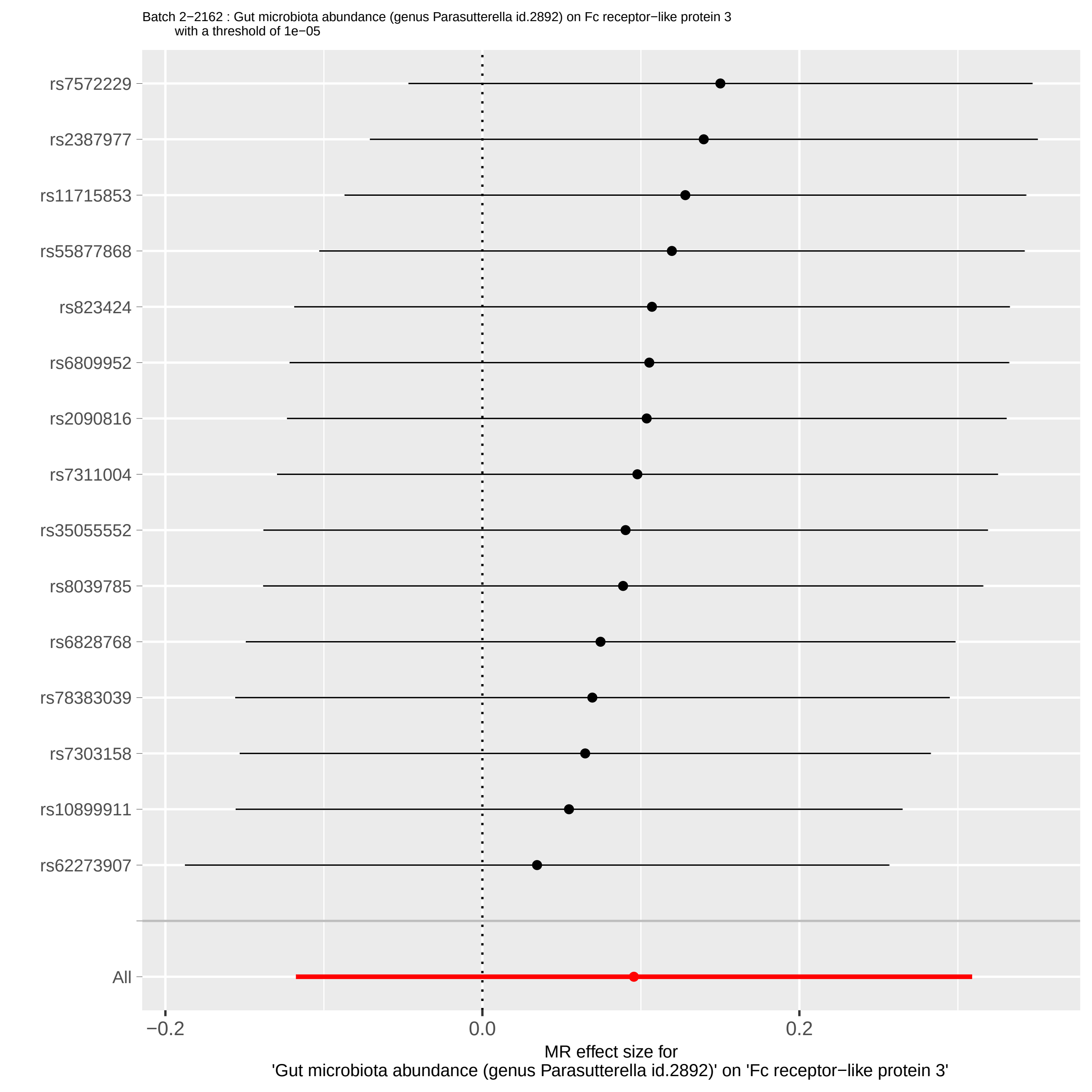
Batch 2−2162 : Gut microbiota abundance (genus Parasutterella id.2892) on Fc receptor−like protein 3
with a threshold of 1e−05
rs7572229
rs2387977
rs11715853
rs55877868
rs823424
rs6809952
rs2090816
rs7311004
rs35055552
rs8039785
rs6828768
rs78383039
rs7303158
rs10899911
rs62273907
All
−0.2
0.0
0.2
MR effect size for
'Gut microbiota abundance (genus Parasutterella id.2892)' on 'Fc receptor−like protein 3'

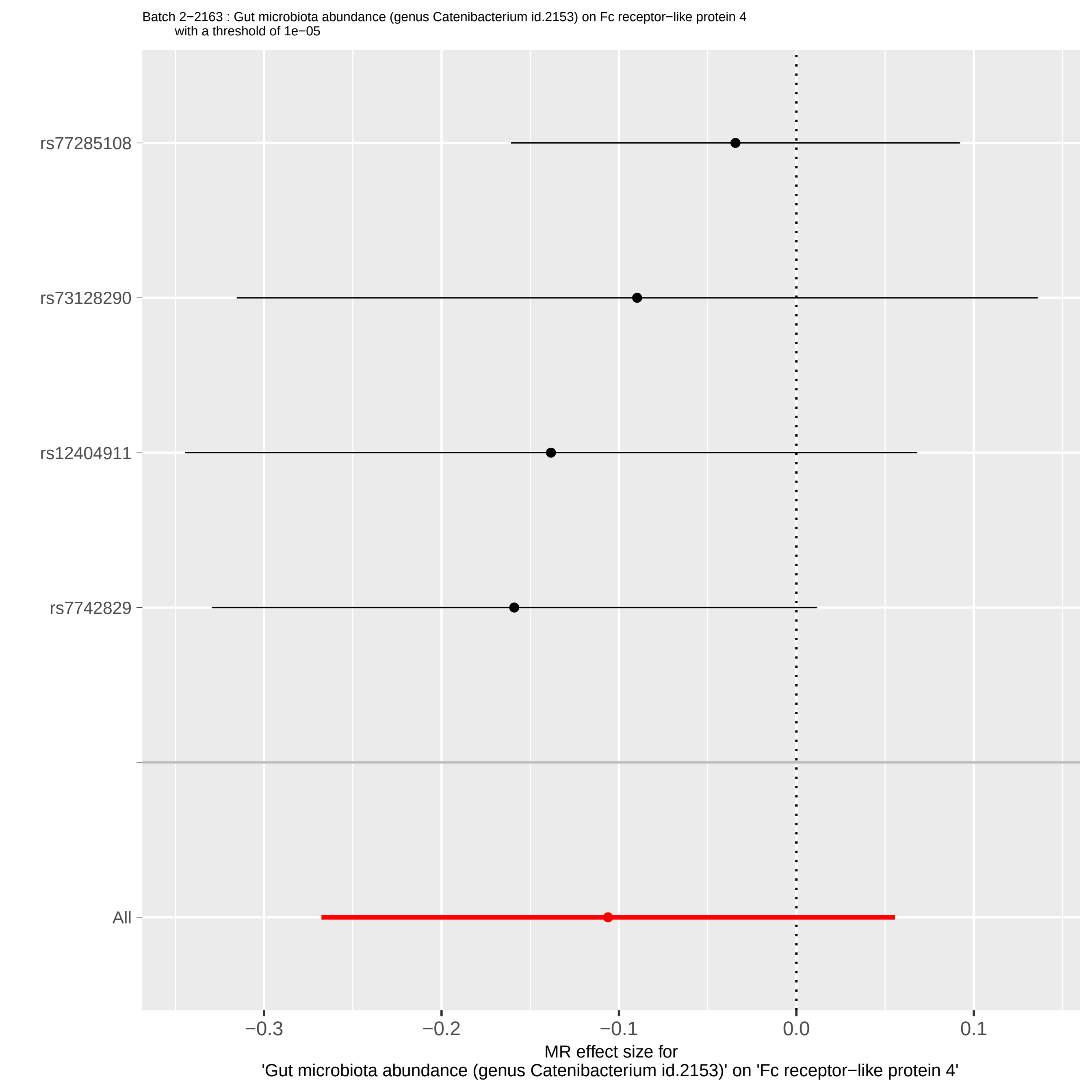
Batch 2−2163 : Gut microbiota abundance (genus Catenibacterium id.2153) on Fc receptor−like protein 4
with a threshold of 1e−05
rs77285108
rs73128290
rs12404911
rs7742829
All
−0.3
−0.2
−0.1
0.0
0.1
MR effect size for
'Gut microbiota abundance (genus Catenibacterium id.2153)' on 'Fc receptor−like protein 4'

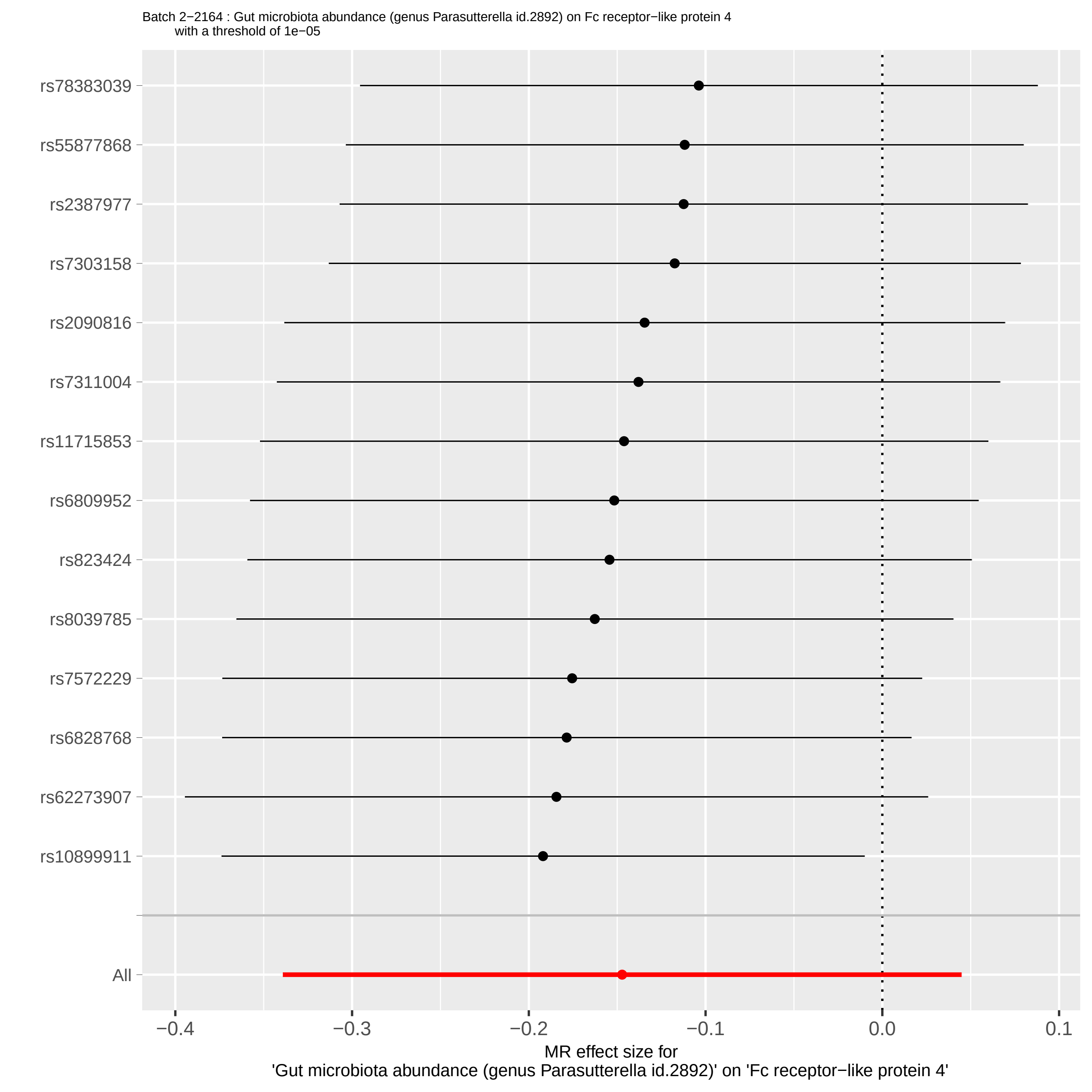
Batch 2−2164 : Gut microbiota abundance (genus Parasutterella id.2892) on Fc receptor−like protein 4
with a threshold of 1e−05
rs78383039
rs55877868
rs2387977
rs7303158
rs2090816
rs7311004
rs11715853
rs6809952
rs823424
rs8039785
rs7572229
rs6828768
rs62273907
rs10899911
All
−0.4
−0.3
−0.2
−0.1
0.0
0.1
MR effect size for
'Gut microbiota abundance (genus Parasutterella id.2892)' on 'Fc receptor−like protein 4'

Batch 2−2165 : Gut microbiota abundance (genus Catenibacterium id.2153) on Fc receptor−like protein 5
with a threshold of 1e−05

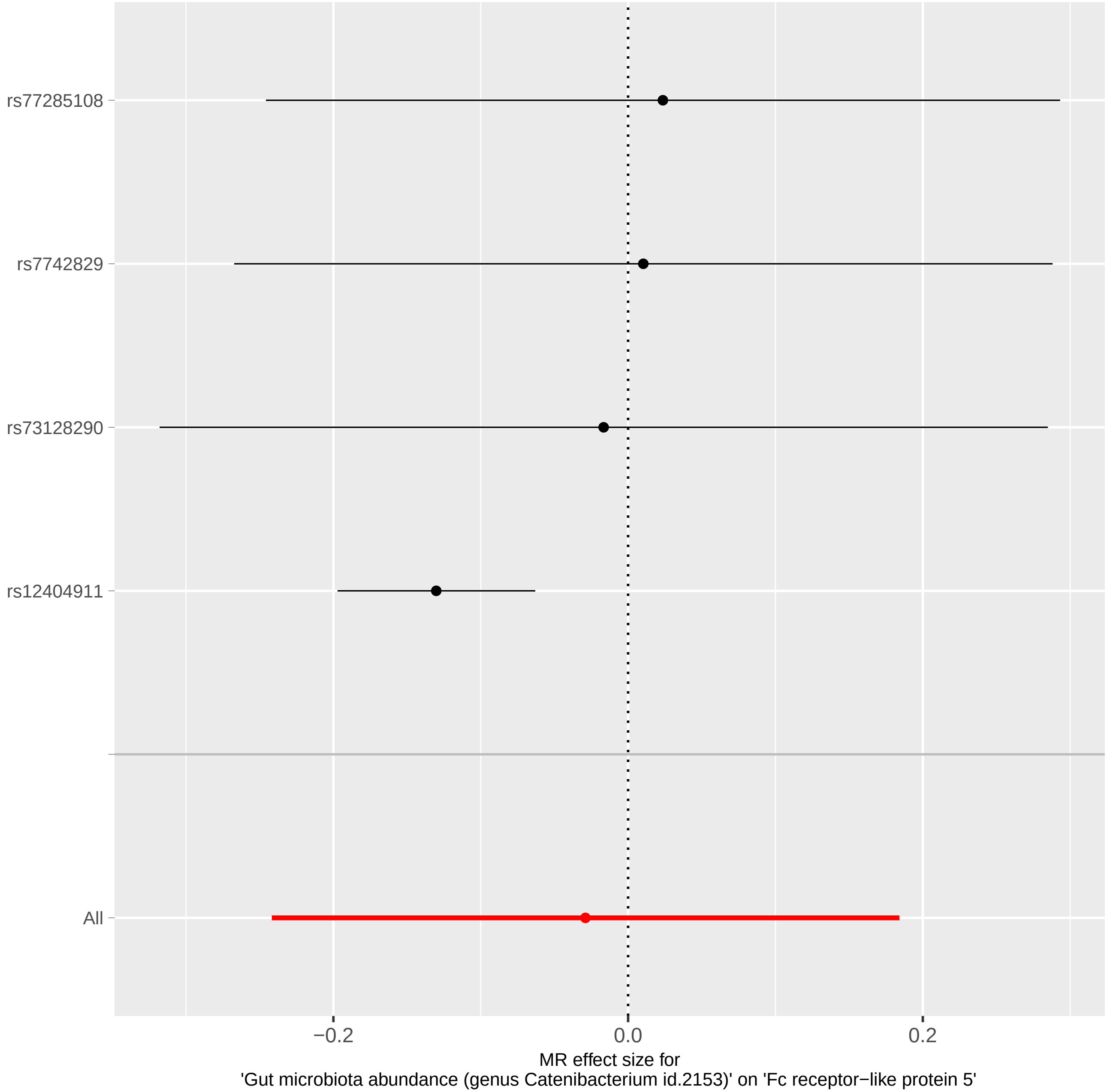

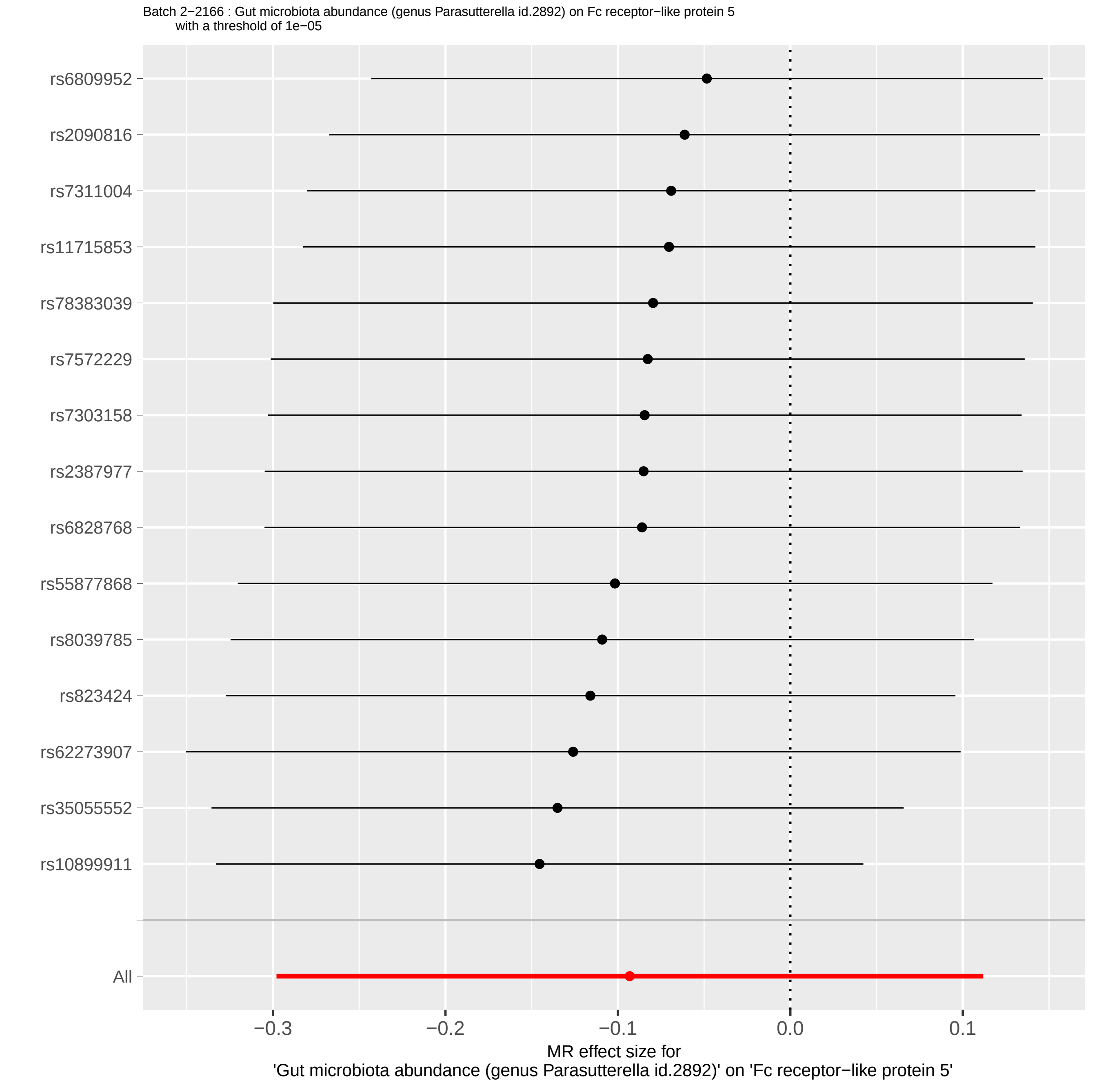

Batch 2−2166 : Gut microbiota abundance (genus Parasutterella id.2892) on Fc receptor−like protein 5
with a threshold of 1e−05
rs6809952
rs2090816
rs7311004
rs11715853
rs78383039
rs7572229
rs7303158
rs2387977
rs6828768
rs55877868
rs8039785
rs823424
rs62273907
rs35055552
rs10899911
All
−0.3
−0.2
−0.1
0.0
0.1
MR effect size for
'Gut microbiota abundance (genus Parasutterella id.2892)' on 'Fc receptor−like protein 5'

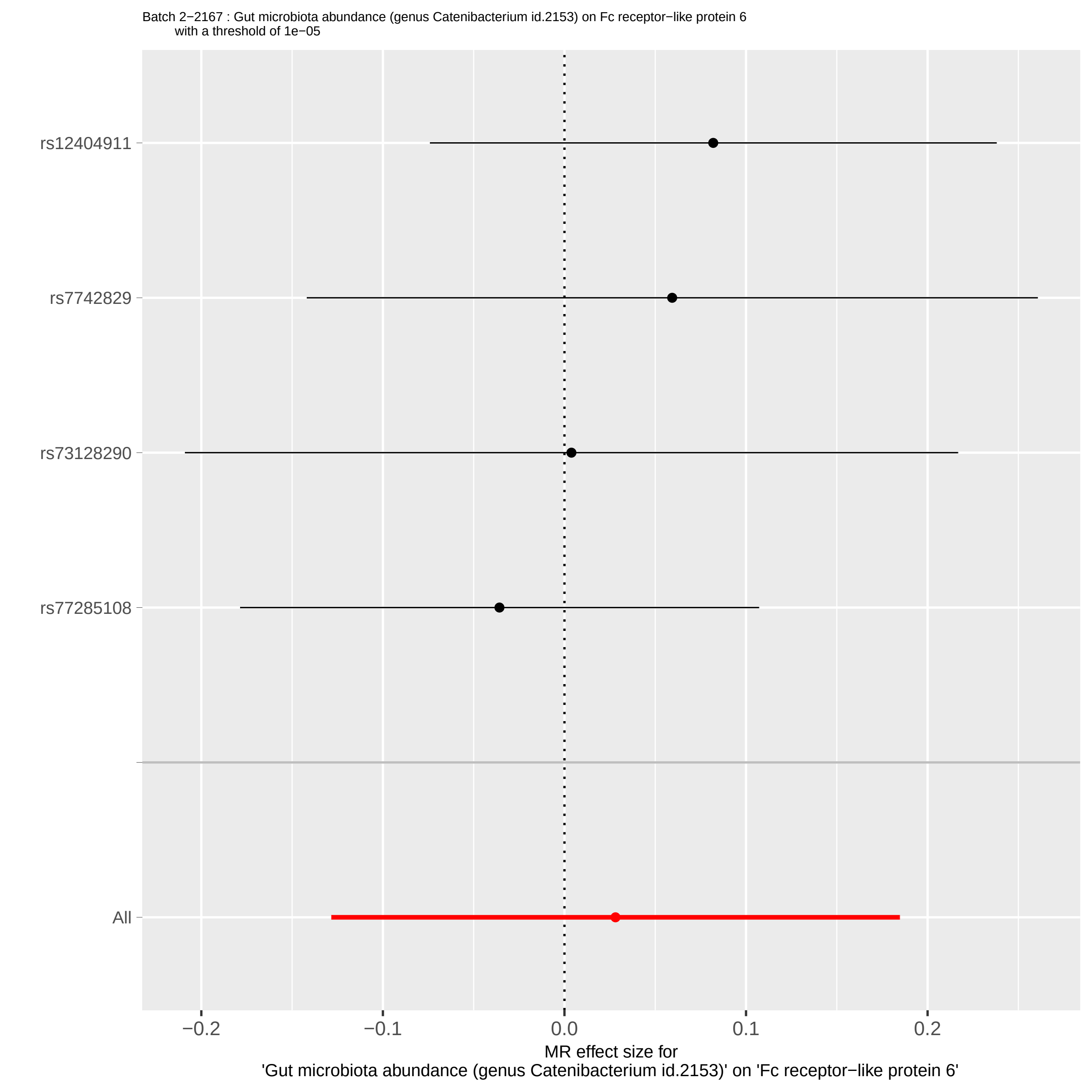
Batch 2−2167 : Gut microbiota abundance (genus Catenibacterium id.2153) on Fc receptor−like protein 6
with a threshold of 1e−05
rs12404911
rs7742829
rs73128290
rs77285108
All
−0.2
−0.1
0.0
0.1
0.2
MR effect size for
'Gut microbiota abundance (genus Catenibacterium id.2153)' on 'Fc receptor−like protein 6'

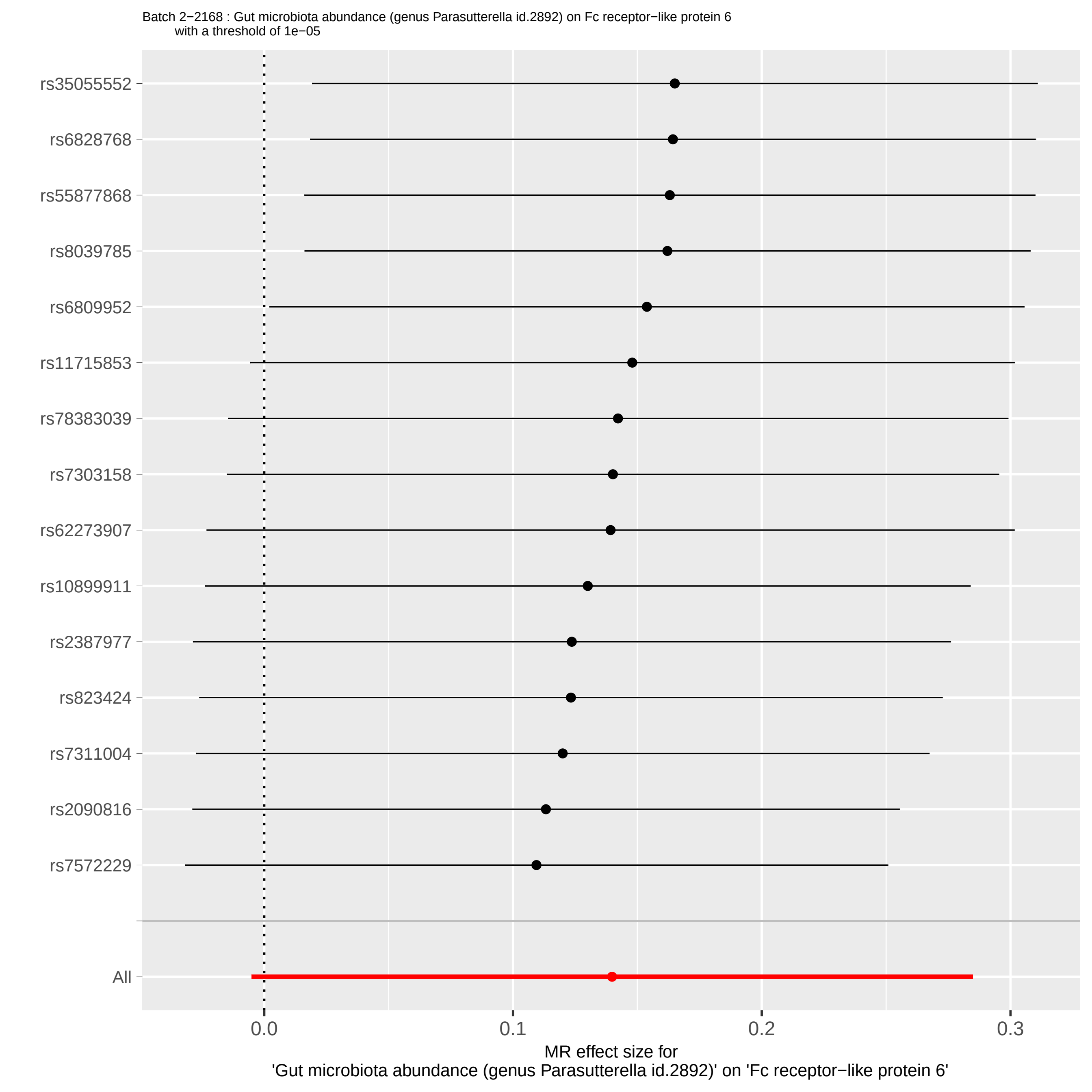

Batch 2−2168 : Gut microbiota abundance (genus Parasutterella id.2892) on Fc receptor−like protein 6
with a threshold of 1e−05
rs35055552
rs6828768
rs55877868
rs8039785
rs6809952
rs11715853
rs78383039
rs7303158
rs62273907
rs10899911
rs2387977
rs823424
rs7311004
rs2090816
rs7572229
All
0.0
0.1
0.2
0.3
MR effect size for
'Gut microbiota abundance (genus Parasutterella id.2892)' on 'Fc receptor−like protein 6'

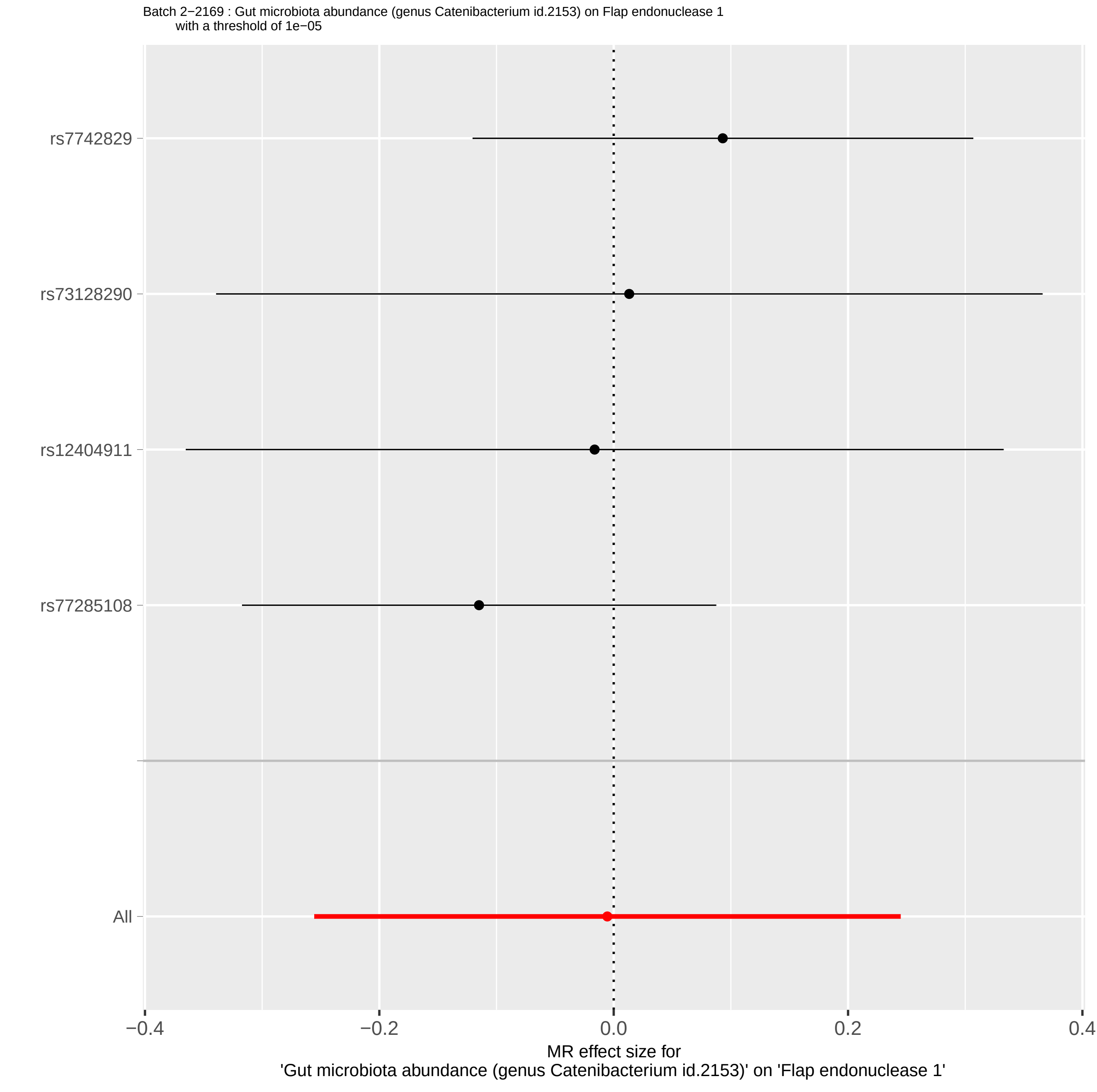
Batch 2−2169 : Gut microbiota abundance (genus Catenibacterium id.2153) on Flap endonuclease 1
with a threshold of 1e−05
rs7742829
rs73128290
rs12404911
rs77285108
All
−0.4
−0.2
0.0
0.2
0.4
MR effect size for
'Gut microbiota abundance (genus Catenibacterium id.2153)' on 'Flap endonuclease 1'

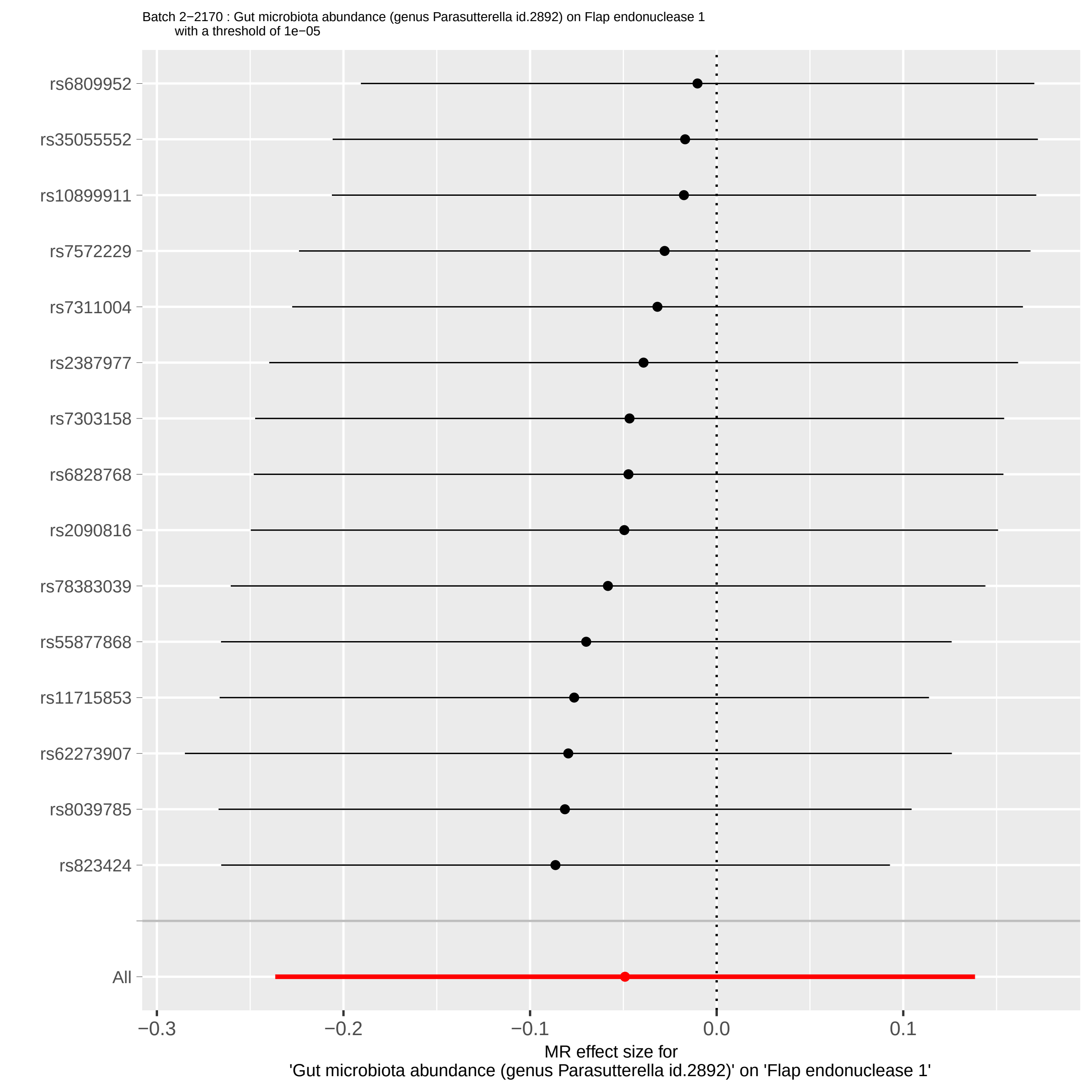

Batch 2−2170 : Gut microbiota abundance (genus Parasutterella id.2892) on Flap endonuclease 1
with a threshold of 1e−05
rs6809952
rs35055552
rs10899911
rs7572229
rs7311004
rs2387977
rs7303158
rs6828768
rs2090816
rs78383039
rs55877868
rs11715853
rs62273907
rs8039785
rs823424
All
−0.3
−0.2
−0.1
0.0
0.1
MR effect size for
'Gut microbiota abundance (genus Parasutterella id.2892)' on 'Flap endonuclease 1'

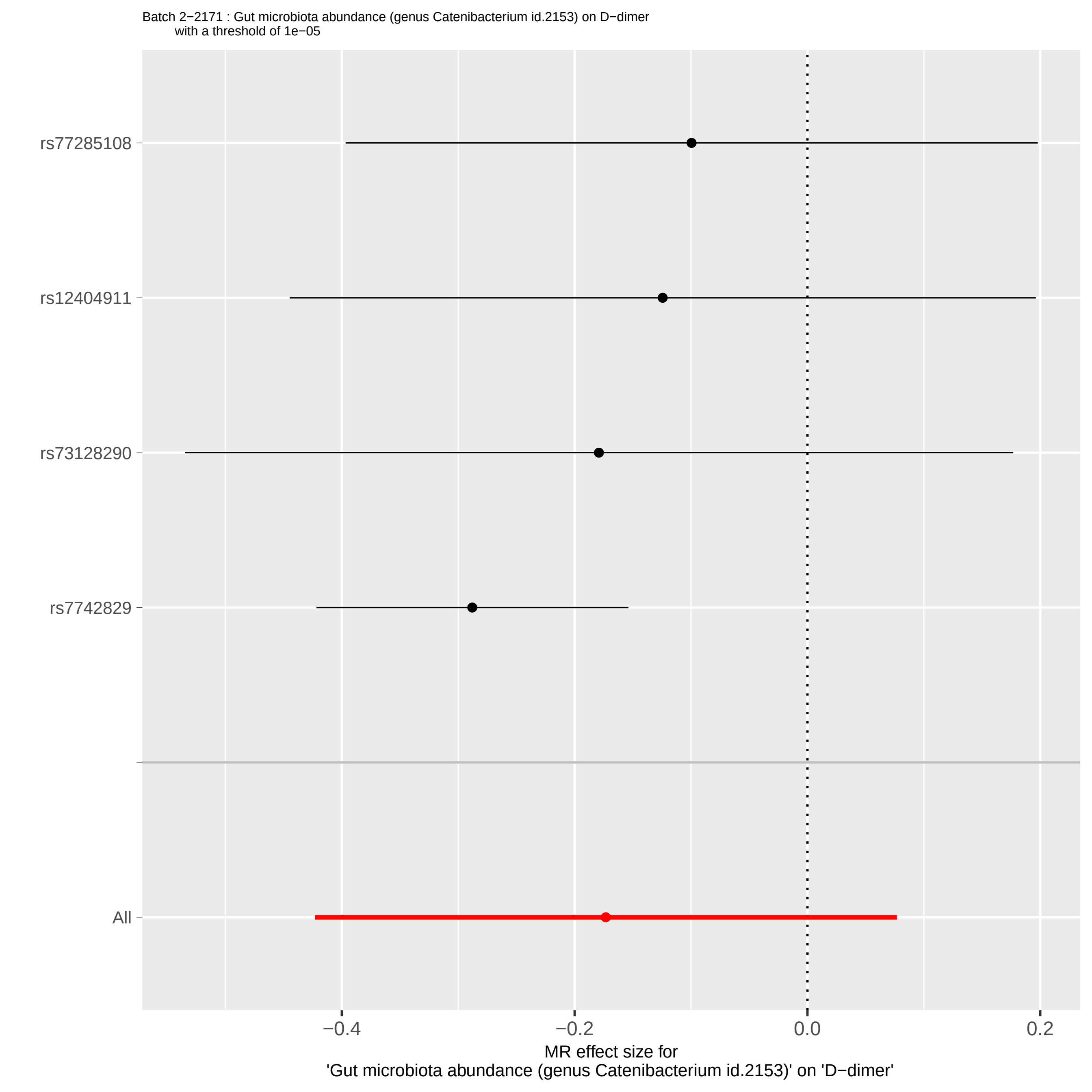
Batch 2−2171 : Gut microbiota abundance (genus Catenibacterium id.2153) on D−dimer
with a threshold of 1e−05
rs77285108
rs12404911
rs73128290
rs7742829
All
−0.4
−0.2
0.0
0.2
MR effect size for
'Gut microbiota abundance (genus Catenibacterium id.2153)' on 'D−dimer'

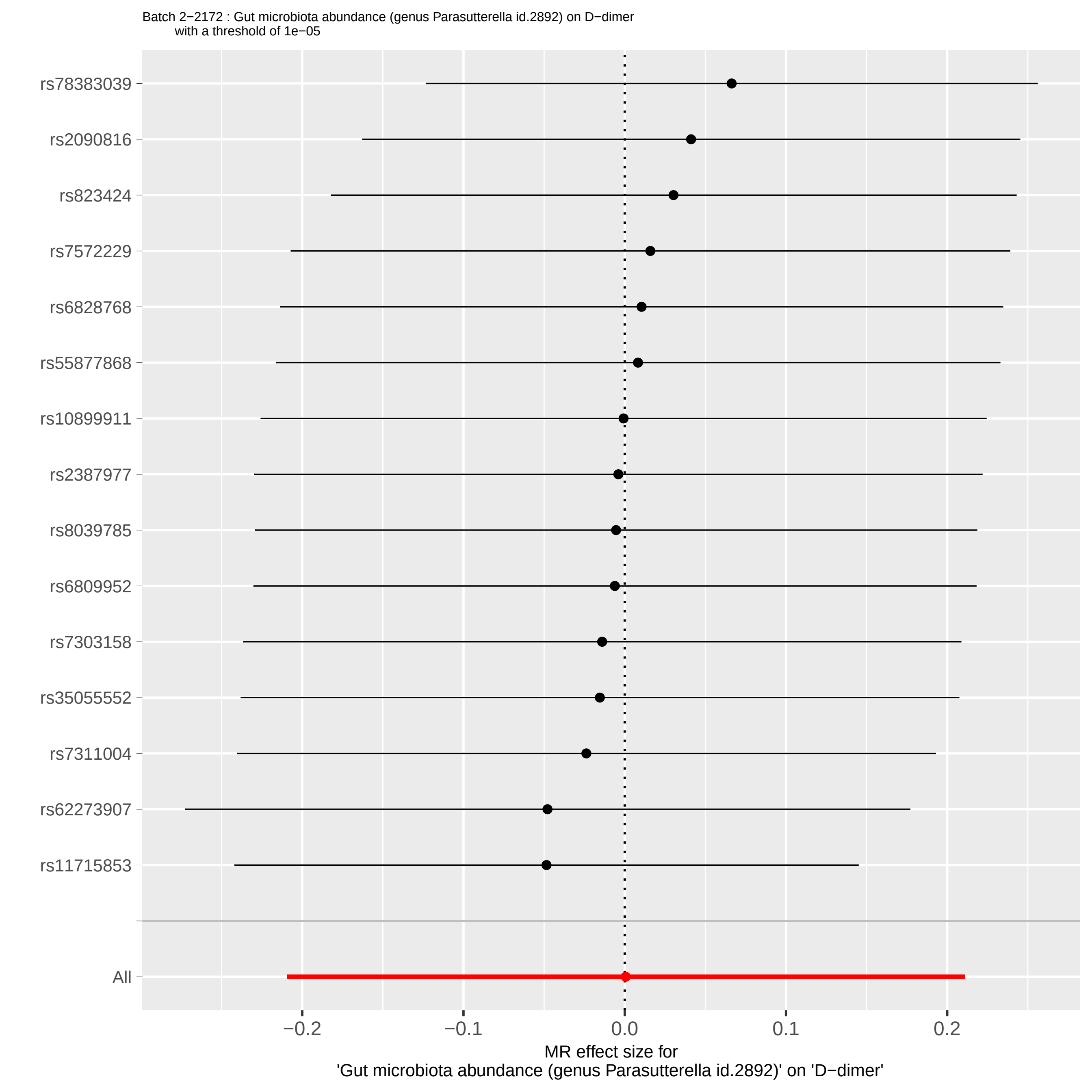
Batch 2−2172 : Gut microbiota abundance (genus Parasutterella id.2892) on D−dimer
with a threshold of 1e−05
rs78383039
rs2090816
rs823424
rs7572229
rs6828768
rs55877868
rs10899911
rs2387977
rs8039785
rs6809952
rs7303158
rs35055552
rs7311004
rs62273907
rs11715853
All
−0.2
−0.1
0.0
0.1
0.2
MR effect size for
'Gut microbiota abundance (genus Parasutterella id.2892)' on 'D−dimer'

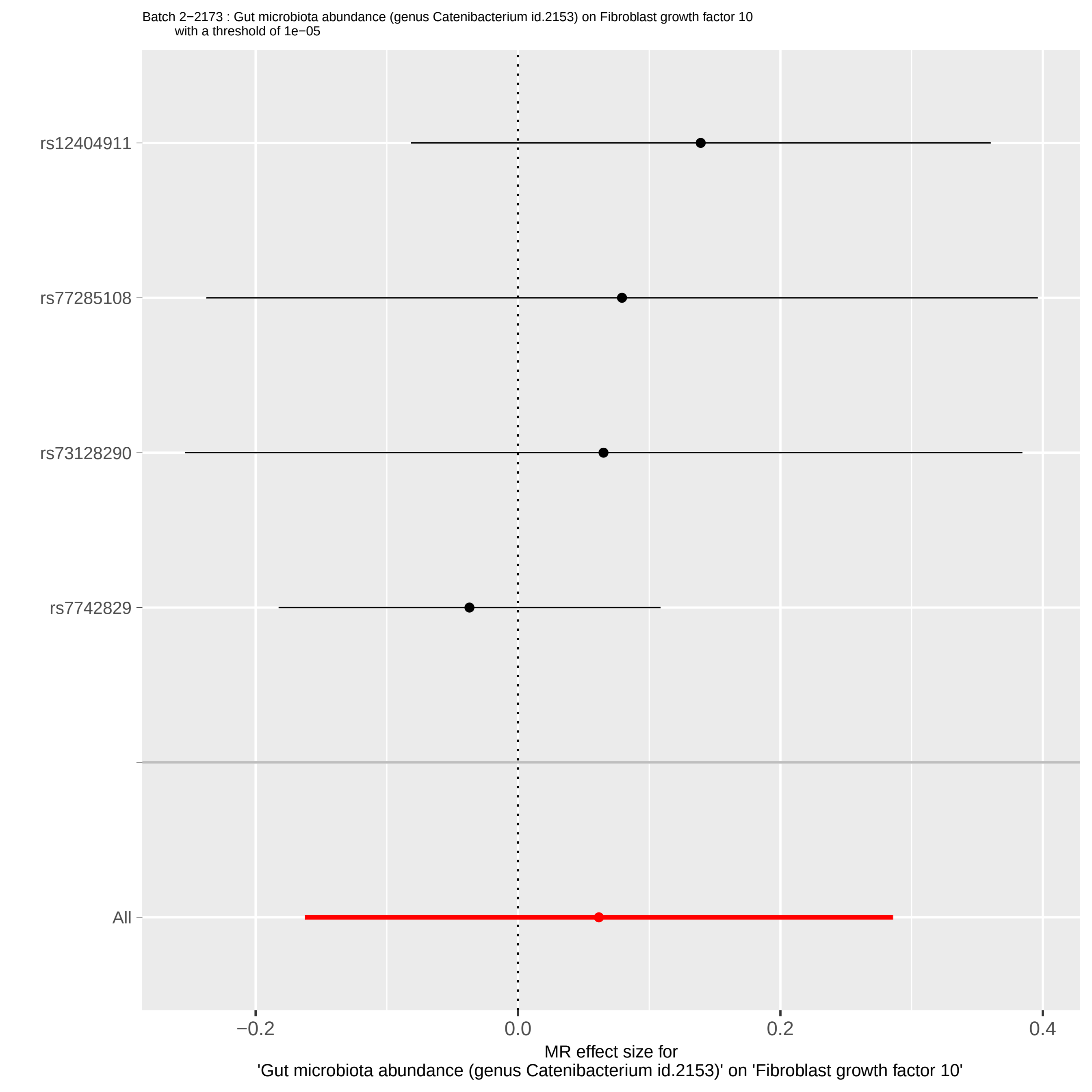
Batch 2−2173 : Gut microbiota abundance (genus Catenibacterium id.2153) on Fibroblast growth factor 10
with a threshold of 1e−05
rs12404911
rs77285108
rs73128290
rs7742829
All
−0.2
0.0
0.2
0.4
MR effect size for
'Gut microbiota abundance (genus Catenibacterium id.2153)' on 'Fibroblast growth factor 10'

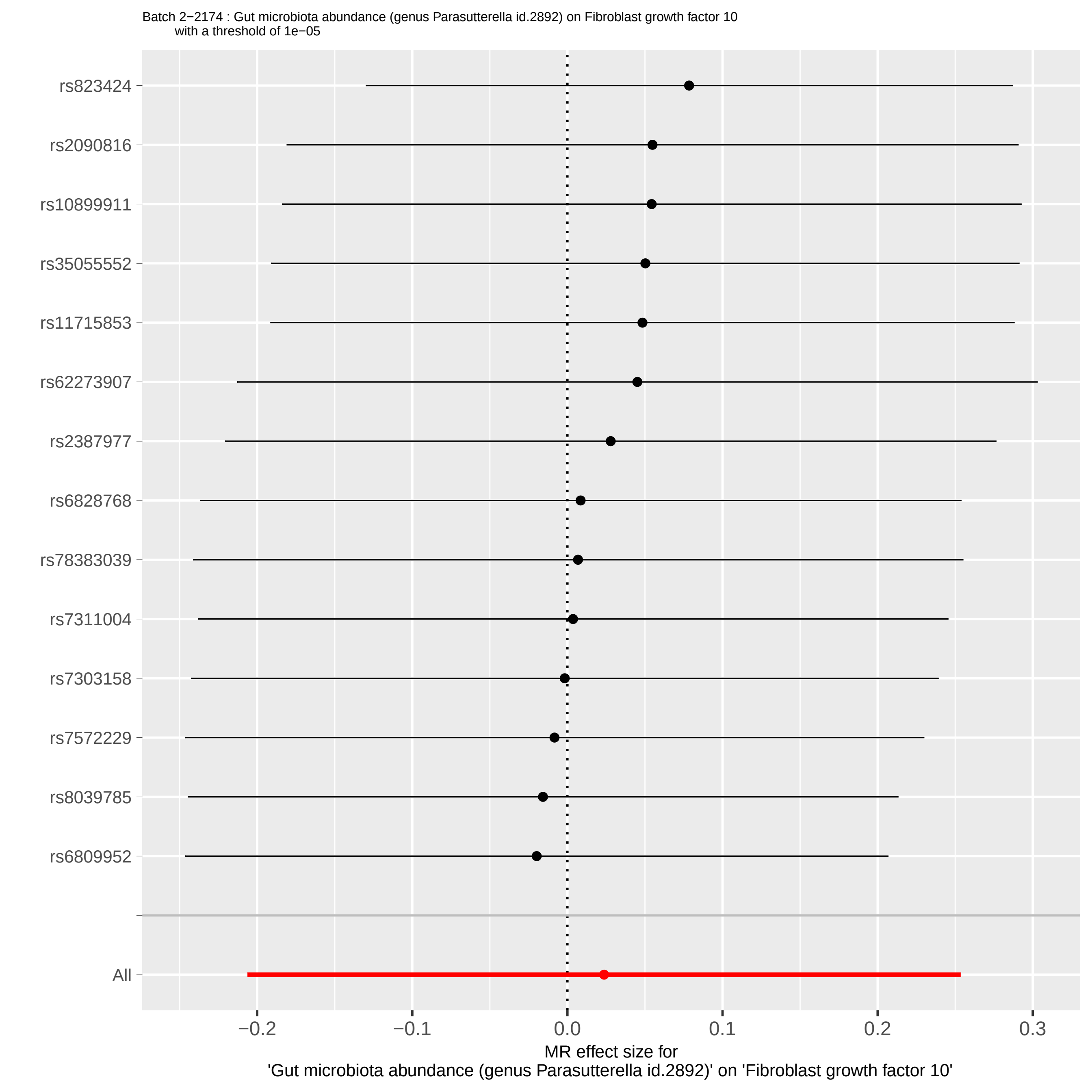
Batch 2−2174 : Gut microbiota abundance (genus Parasutterella id.2892) on Fibroblast growth factor 10
with a threshold of 1e−05
rs823424
rs2090816
rs10899911
rs35055552
rs11715853
rs62273907
rs2387977
rs6828768
rs78383039
rs7311004
rs7303158
rs7572229
rs8039785
rs6809952
All
−0.2
−0.1
0.0
0.1
0.2
0.3
MR effect size for
'Gut microbiota abundance (genus Parasutterella id.2892)' on 'Fibroblast growth factor 10'

Batch 2−2175 : Gut microbiota abundance (genus Catenibacterium id.2153) on Fibroblast growth factor 1
with a threshold of 1e−05

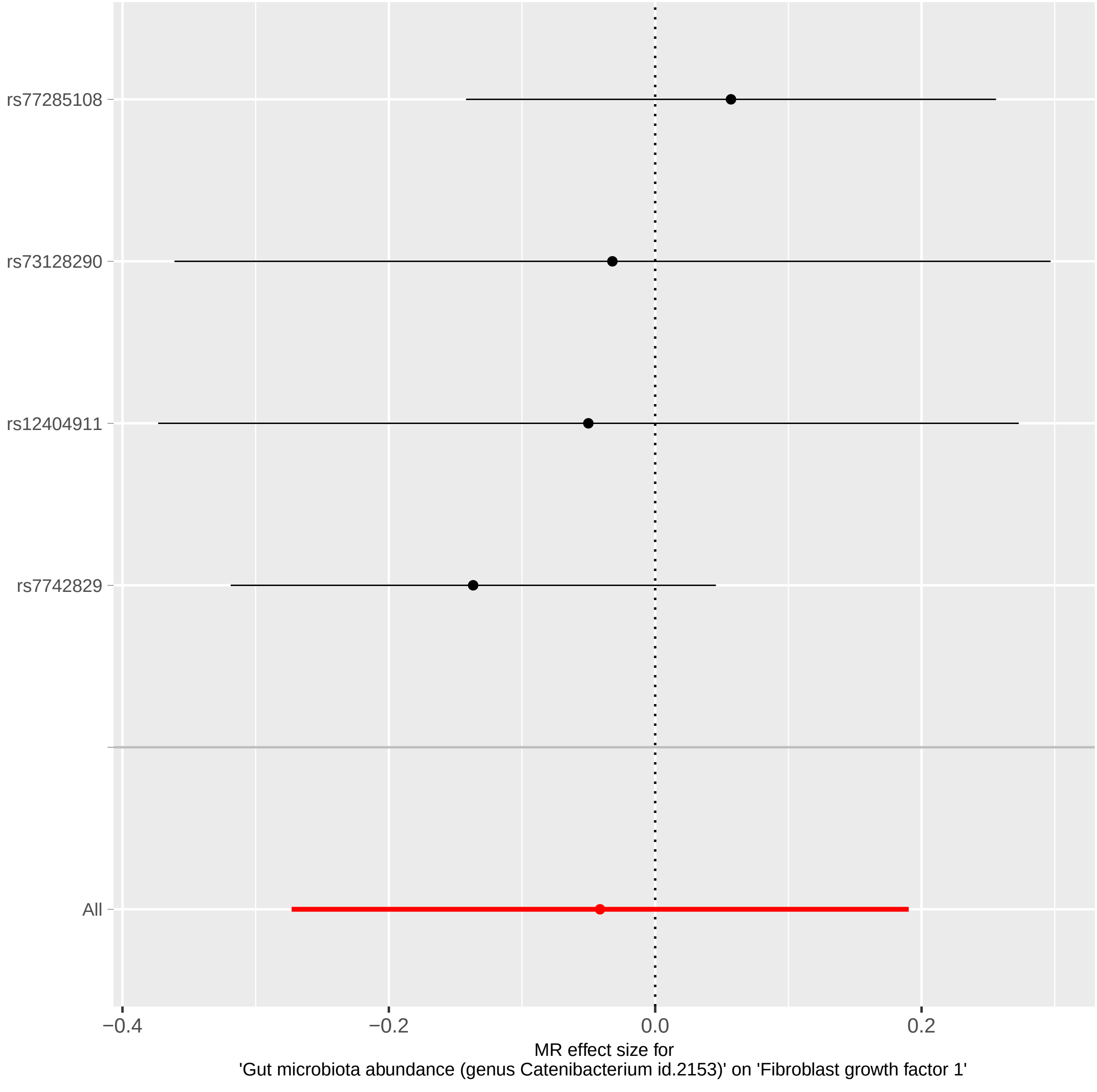

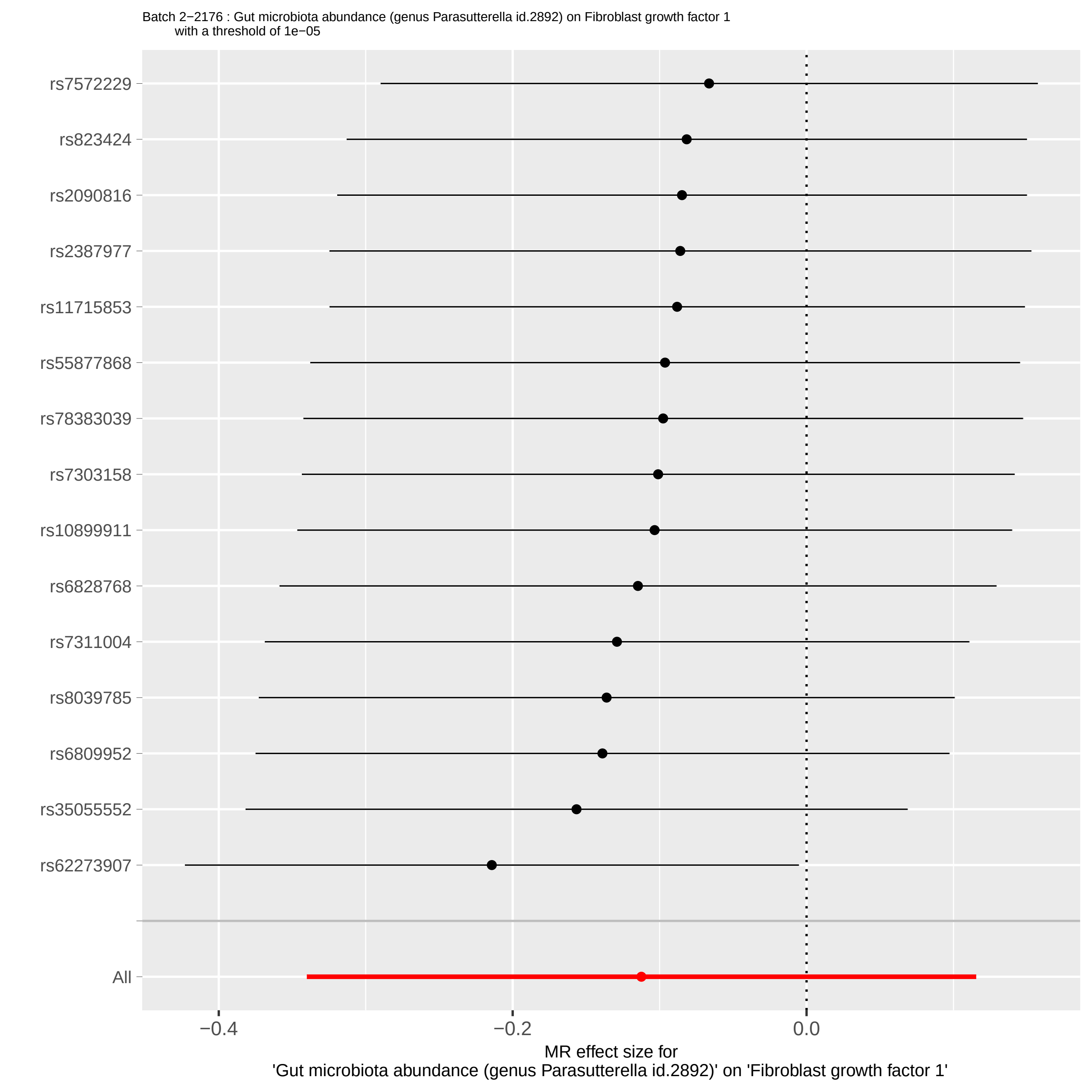

Batch 2−2176 : Gut microbiota abundance (genus Parasutterella id.2892) on Fibroblast growth factor 1
with a threshold of 1e−05
rs7572229
rs823424
rs2090816
rs2387977
rs11715853
rs55877868
rs78383039
rs7303158
rs10899911
rs6828768
rs7311004
rs8039785
rs6809952
rs35055552
rs62273907
All
−0.4
−0.2
0.0
MR effect size for
'Gut microbiota abundance (genus Parasutterella id.2892)' on 'Fibroblast growth factor 1'

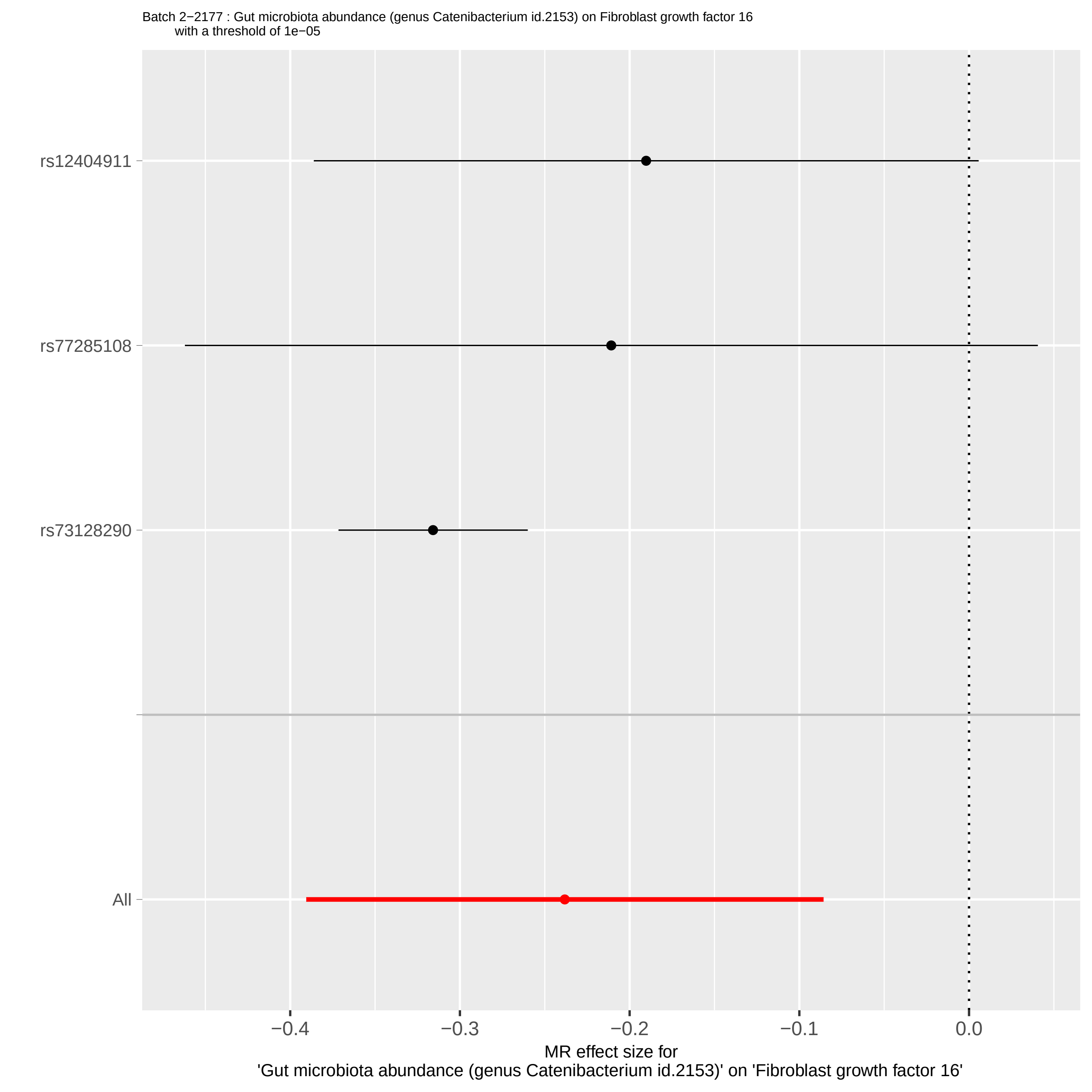

Batch 2−2177 : Gut microbiota abundance (genus Catenibacterium id.2153) on Fibroblast growth factor 16
with a threshold of 1e−05
rs12404911
rs77285108
rs73128290
All
−0.4
−0.3
−0.2
−0.1
0.0
MR effect size for
'Gut microbiota abundance (genus Catenibacterium id.2153)' on 'Fibroblast growth factor 16'

Batch 2−2178 : Gut microbiota abundance (genus Parasutterella id.2892) on Fibroblast growth factor 16
with a threshold of 1e−05

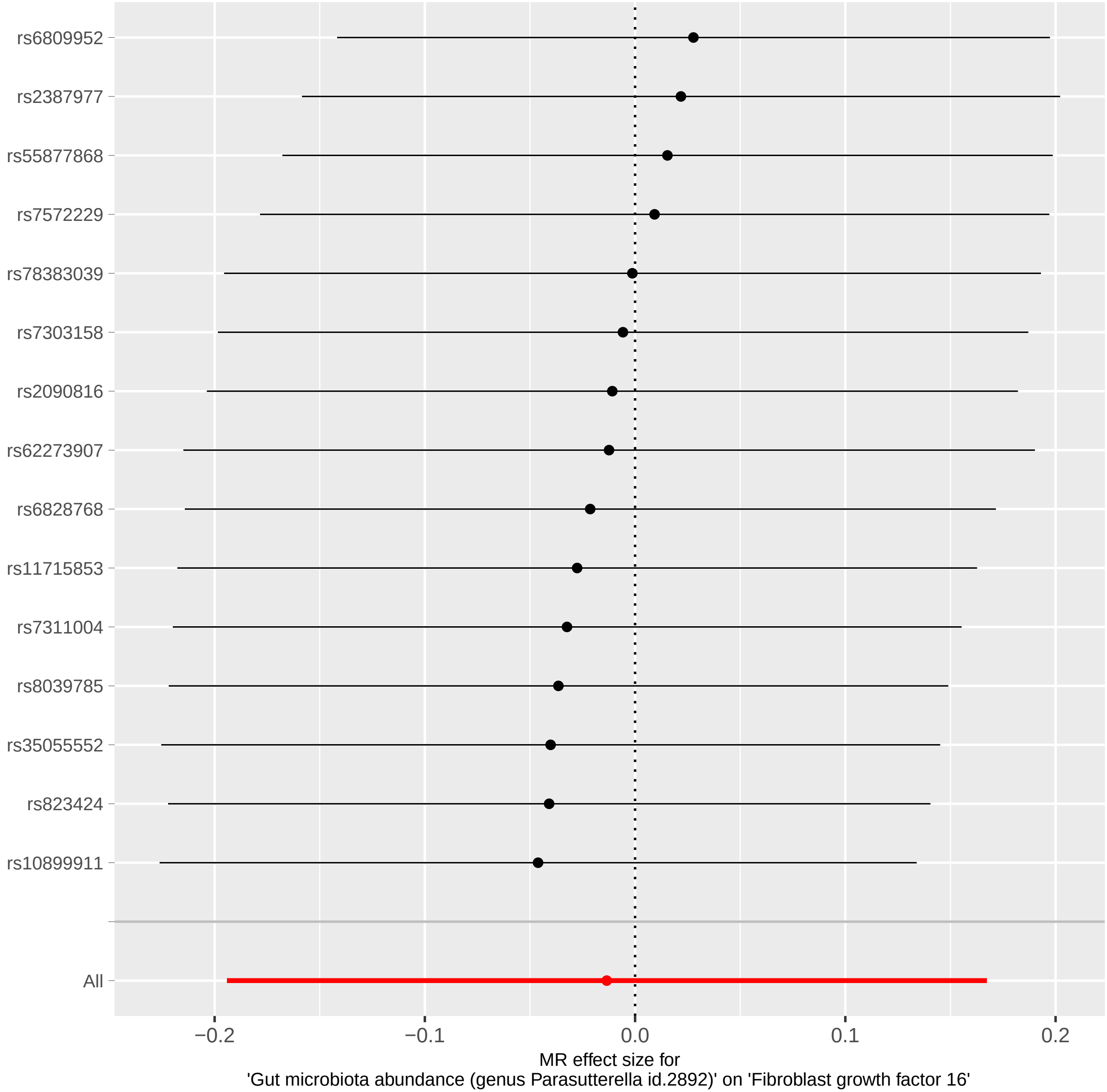

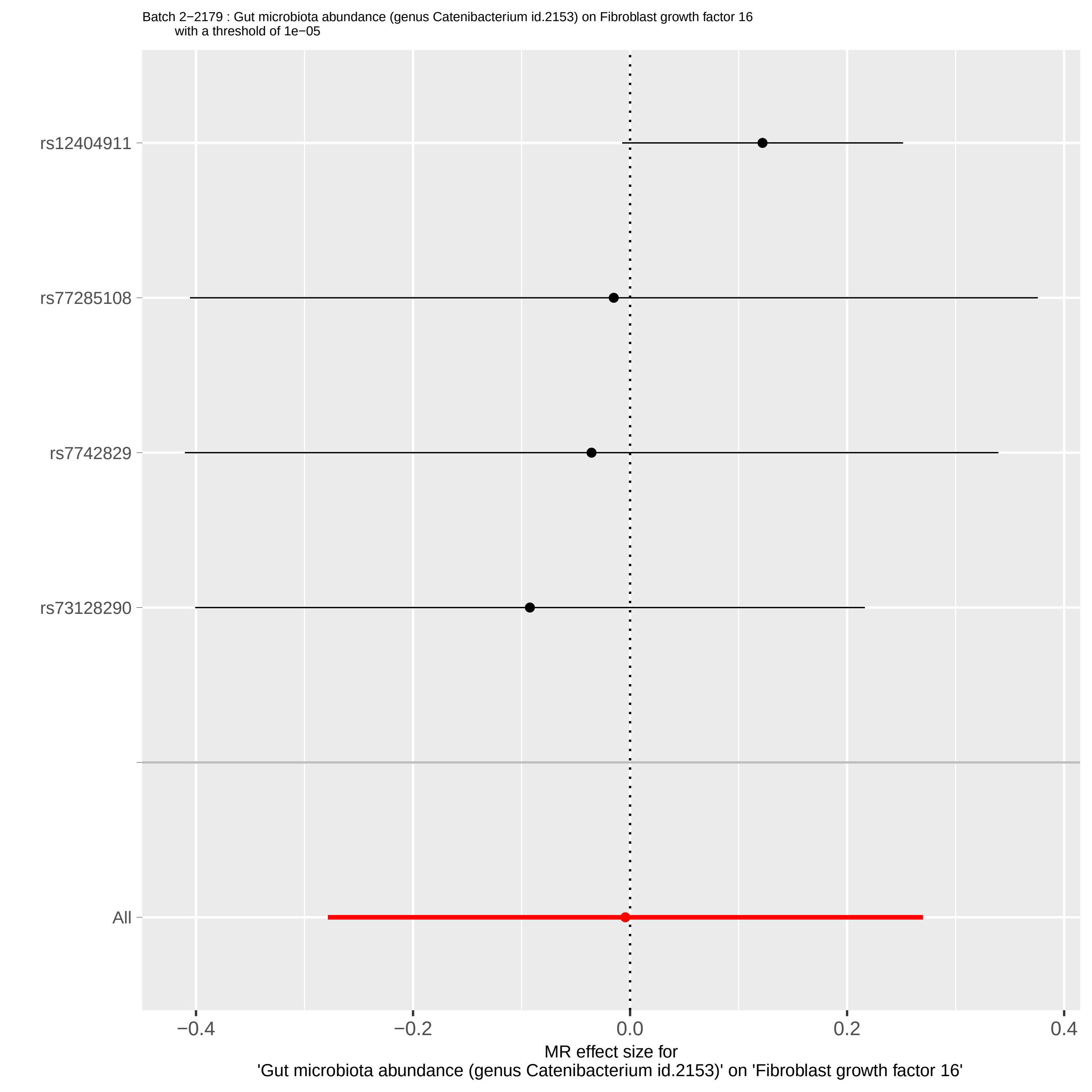
Batch 2−2179 : Gut microbiota abundance (genus Catenibacterium id.2153) on Fibroblast growth factor 16
with a threshold of 1e−05
rs12404911
rs77285108
rs7742829
rs73128290
All
−0.4
−0.2
0.0
0.2
0.4
MR effect size for
'Gut microbiota abundance (genus Catenibacterium id.2153)' on 'Fibroblast growth factor 16'

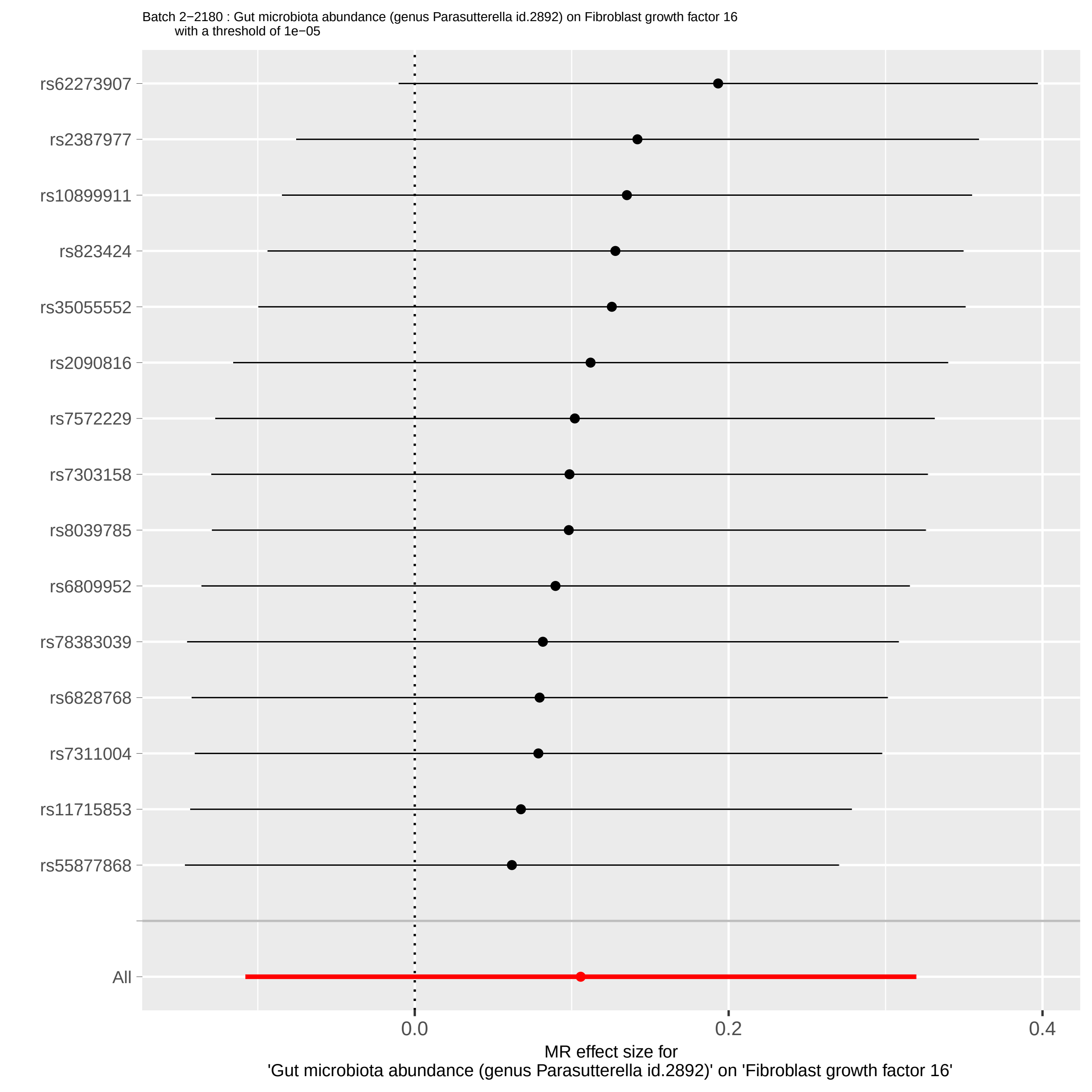
Batch 2−2180 : Gut microbiota abundance (genus Parasutterella id.2892) on Fibroblast growth factor 16
with a threshold of 1e−05
rs62273907
rs2387977
rs10899911
rs823424
rs35055552
rs2090816
rs7572229
rs7303158
rs8039785
rs6809952
rs78383039
rs6828768
rs7311004
rs11715853
rs55877868
All
0.0
0.2
0.4
MR effect size for
'Gut microbiota abundance (genus Parasutterella id.2892)' on 'Fibroblast growth factor 16'

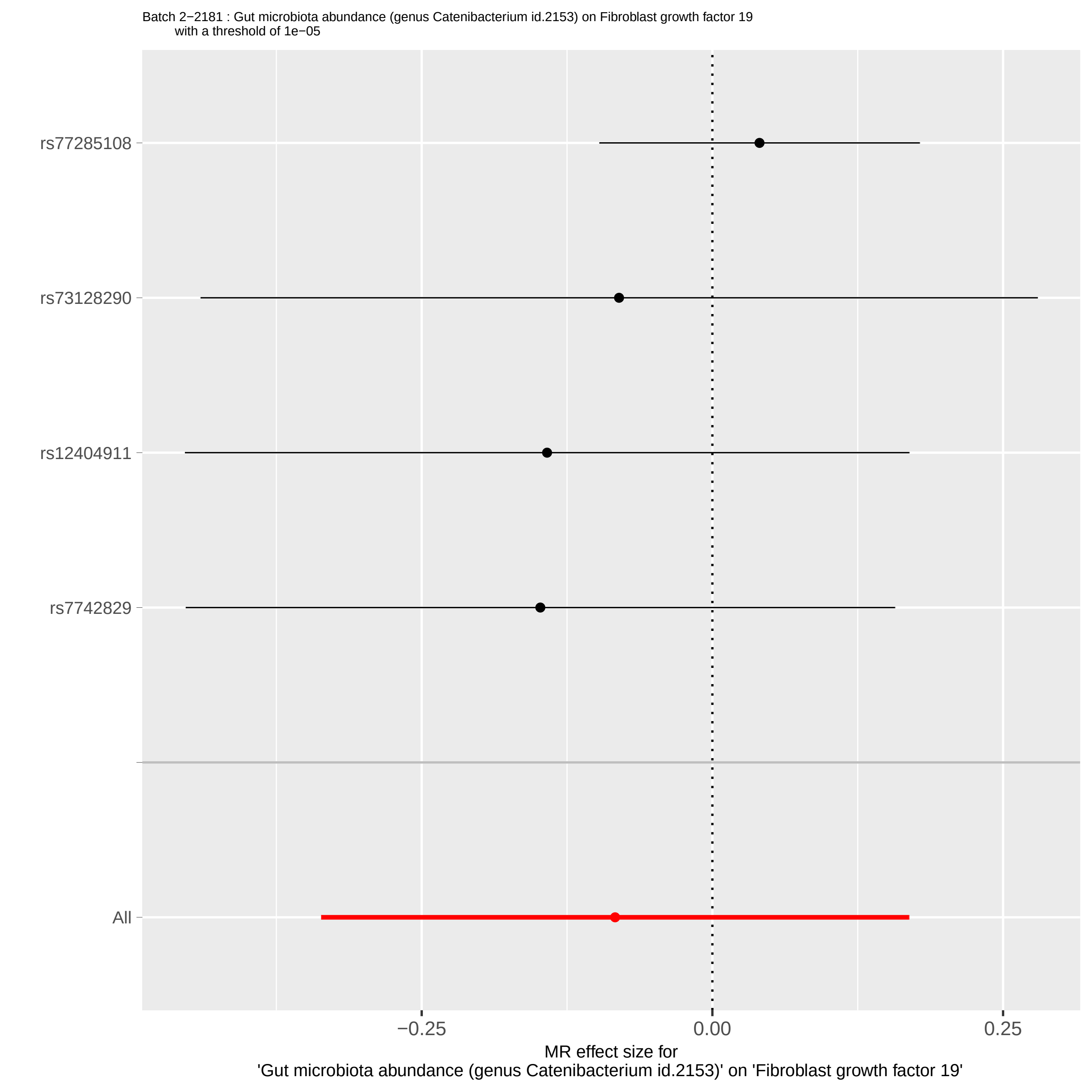
Batch 2−2181 : Gut microbiota abundance (genus Catenibacterium id.2153) on Fibroblast growth factor 19
with a threshold of 1e−05
rs77285108
rs73128290
rs12404911
rs7742829
All
−0.25
0.00
0.25
MR effect size for
'Gut microbiota abundance (genus Catenibacterium id.2153)' on 'Fibroblast growth factor 19'

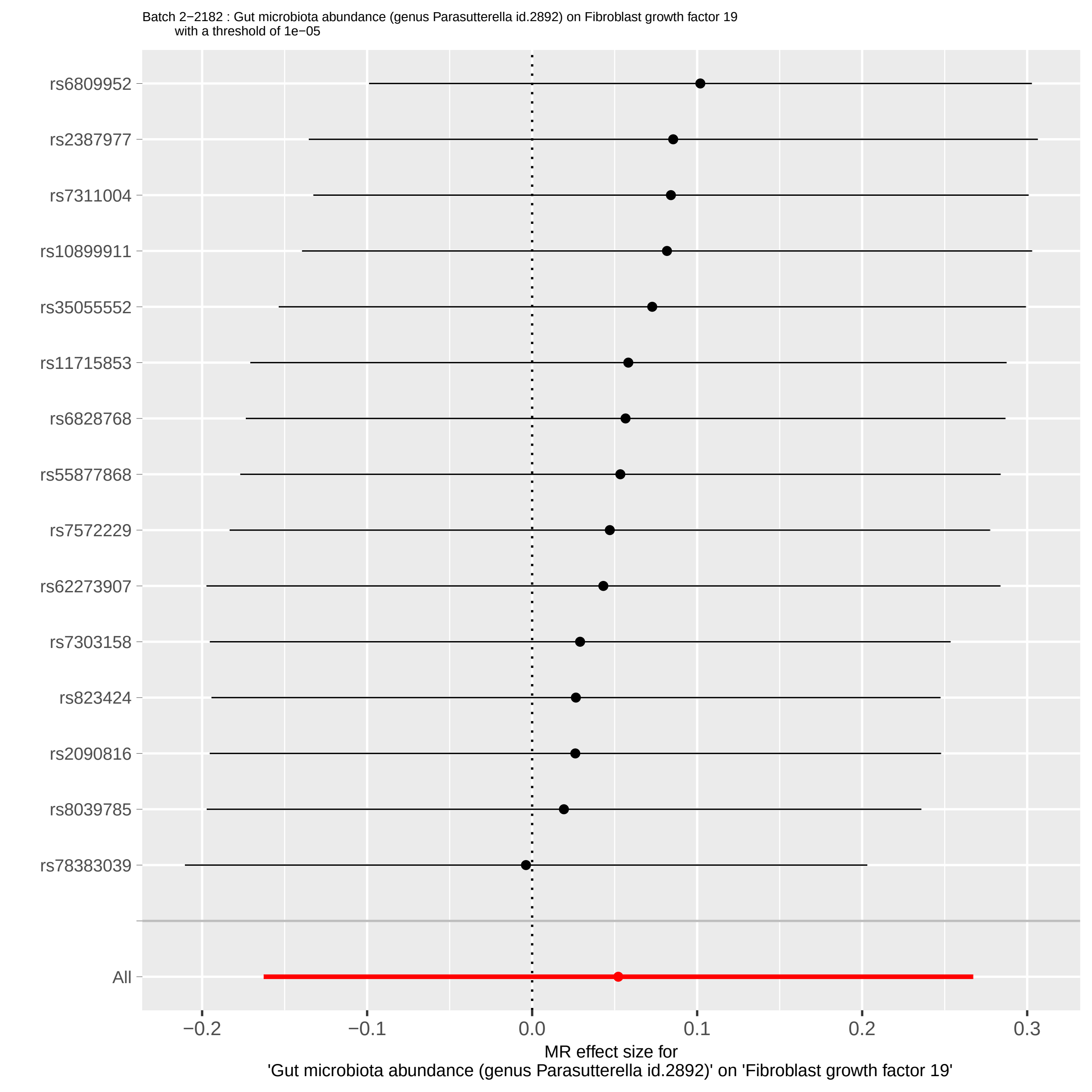

Batch 2−2182 : Gut microbiota abundance (genus Parasutterella id.2892) on Fibroblast growth factor 19
with a threshold of 1e−05
rs6809952
rs2387977
rs7311004
rs10899911
rs35055552
rs11715853
rs6828768
rs55877868
rs7572229
rs62273907
rs7303158
rs823424
rs2090816
rs8039785
rs78383039
All
−0.2
−0.1
0.0
0.1
0.2
0.3
MR effect size for
'Gut microbiota abundance (genus Parasutterella id.2892)' on 'Fibroblast growth factor 19'

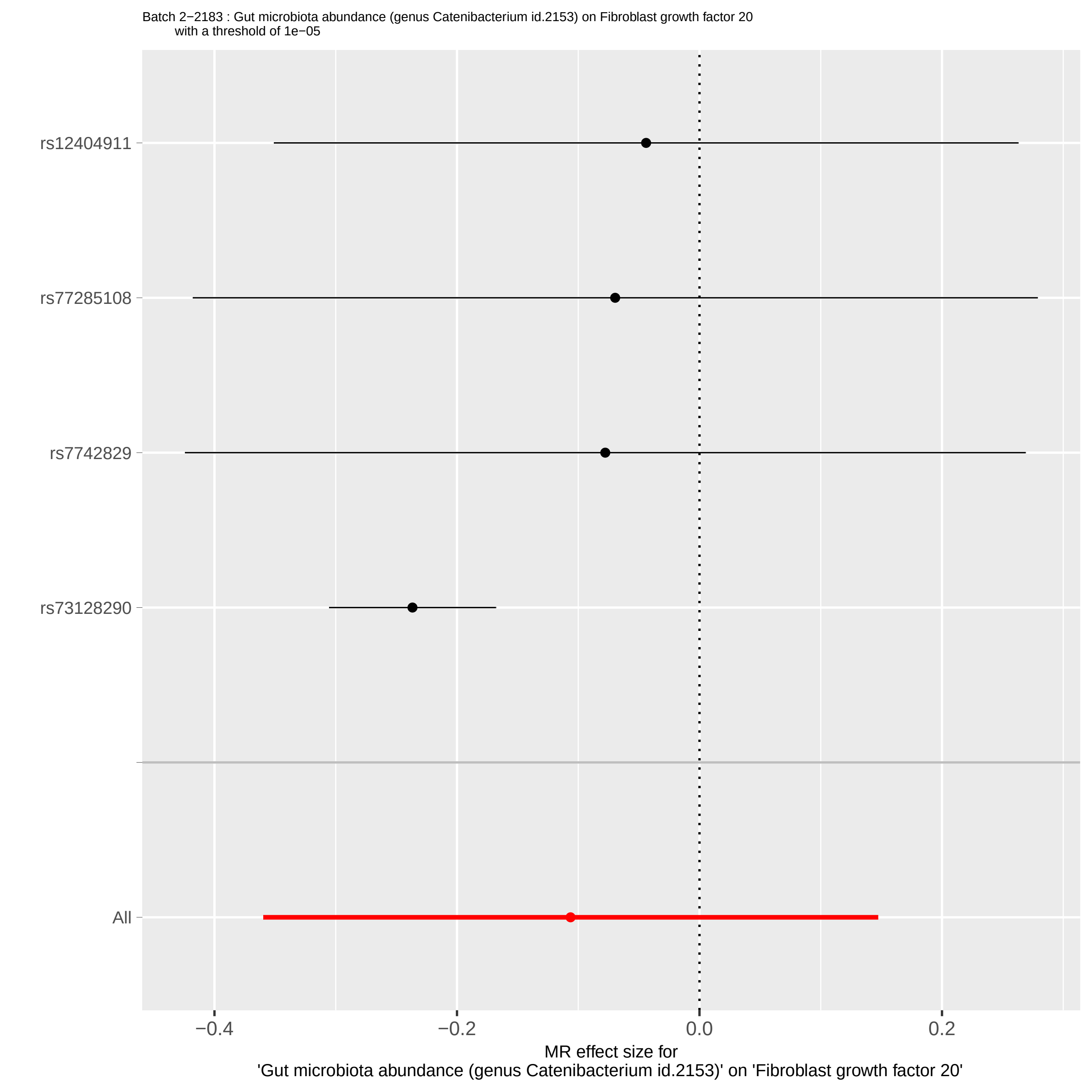
Batch 2−2183 : Gut microbiota abundance (genus Catenibacterium id.2153) on Fibroblast growth factor 20
with a threshold of 1e−05
rs12404911
rs77285108
rs7742829
rs73128290
All
−0.4
−0.2
0.0
0.2
MR effect size for
'Gut microbiota abundance (genus Catenibacterium id.2153)' on 'Fibroblast growth factor 20'

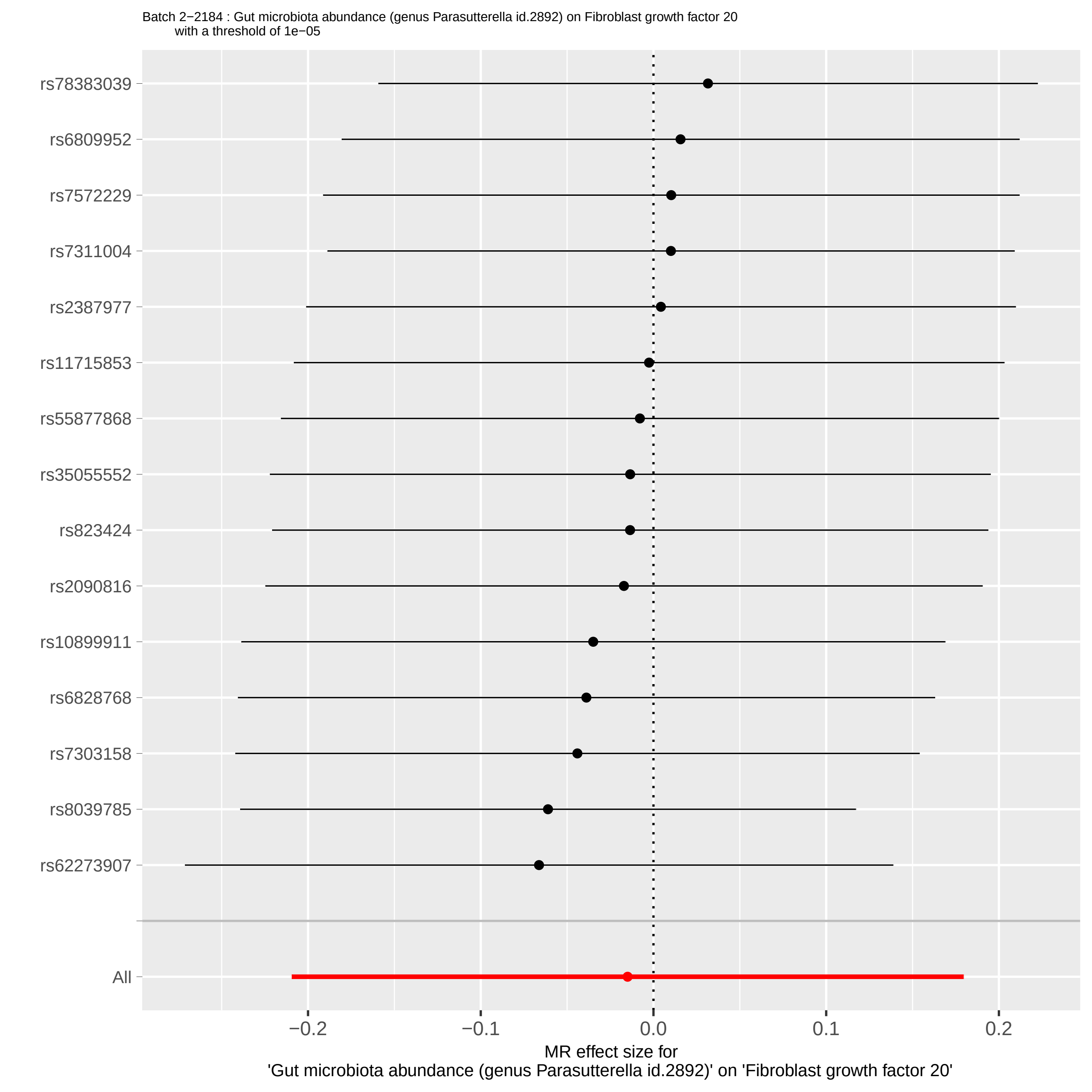
Batch 2−2184 : Gut microbiota abundance (genus Parasutterella id.2892) on Fibroblast growth factor 20
with a threshold of 1e−05
rs78383039
rs6809952
rs7572229
rs7311004
rs2387977
rs11715853
rs55877868
rs35055552
rs823424
rs2090816
rs10899911
rs6828768
rs7303158
rs8039785
rs62273907
All
−0.2
−0.1
0.0
0.1
0.2
MR effect size for
'Gut microbiota abundance (genus Parasutterella id.2892)' on 'Fibroblast growth factor 20'

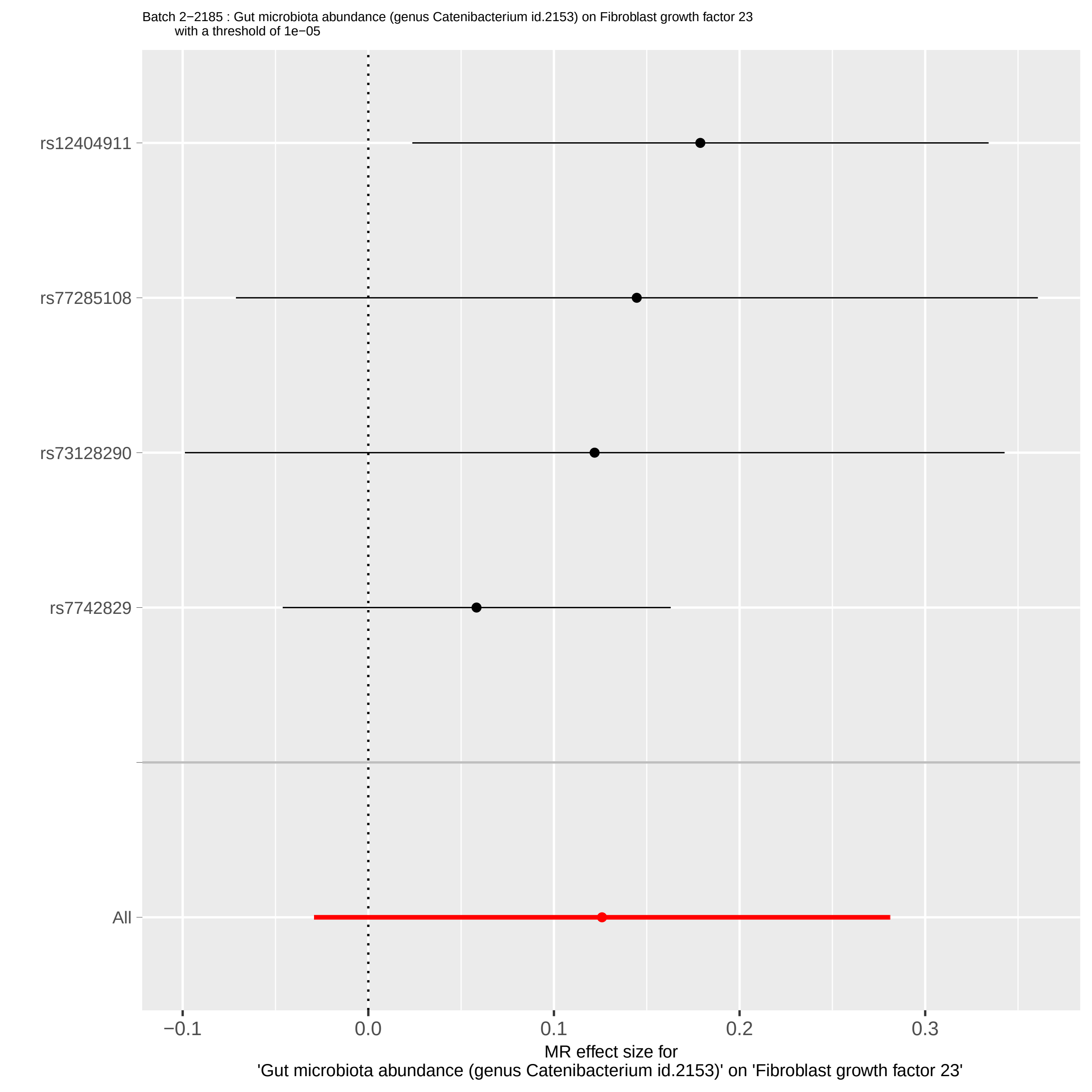
Batch 2−2185 : Gut microbiota abundance (genus Catenibacterium id.2153) on Fibroblast growth factor 23
with a threshold of 1e−05
rs12404911
rs77285108
rs73128290
rs7742829
All
−0.1
0.0
0.1
0.2
0.3
MR effect size for
'Gut microbiota abundance (genus Catenibacterium id.2153)' on 'Fibroblast growth factor 23'

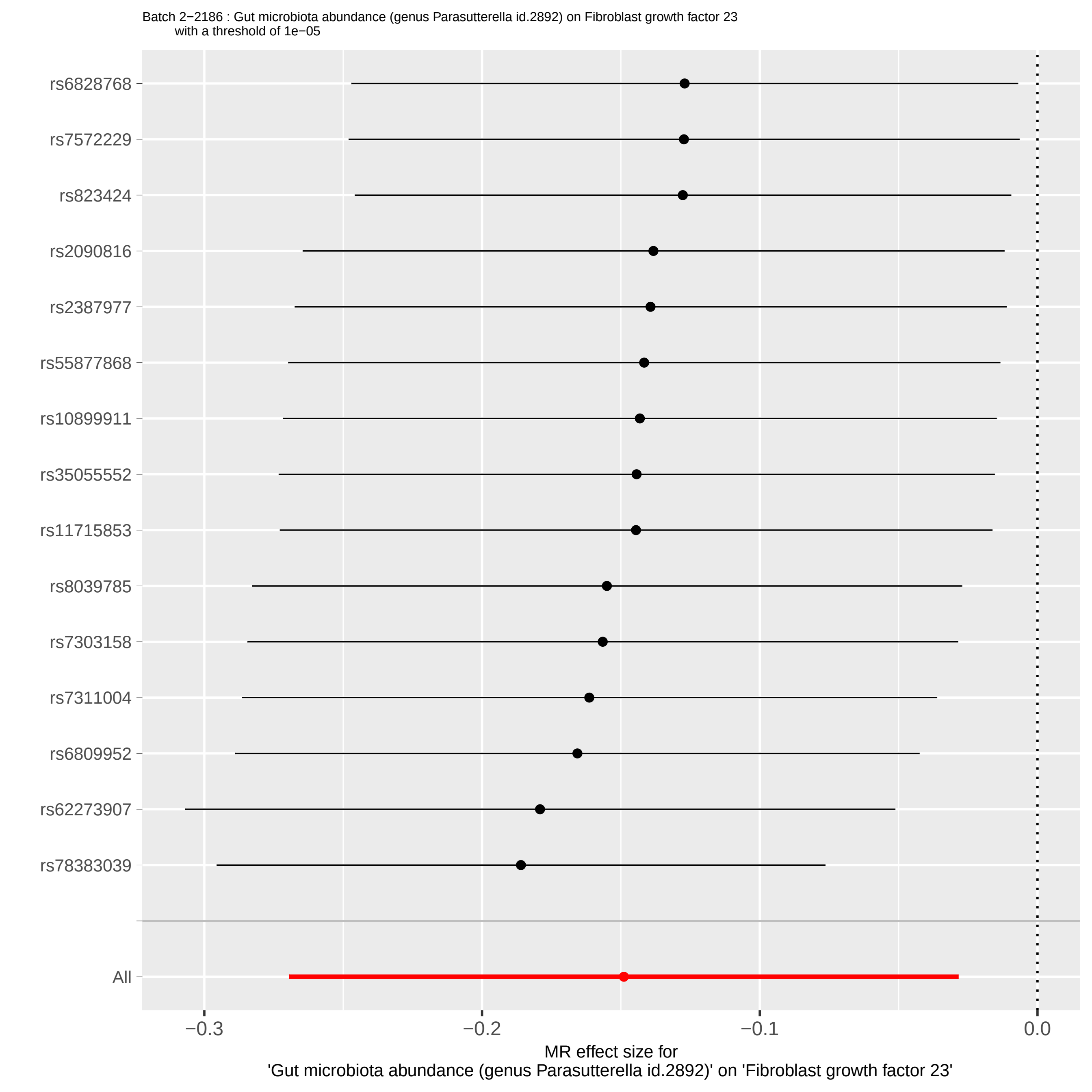
Batch 2−2186 : Gut microbiota abundance (genus Parasutterella id.2892) on Fibroblast growth factor 23
with a threshold of 1e−05
rs6828768
rs7572229
rs823424
rs2090816
rs2387977
rs55877868
rs10899911
rs35055552
rs11715853
rs8039785
rs7303158
rs7311004
rs6809952
rs62273907
rs78383039
All
−0.3
−0.2
−0.1
0.0
MR effect size for
'Gut microbiota abundance (genus Parasutterella id.2892)' on 'Fibroblast growth factor 23'

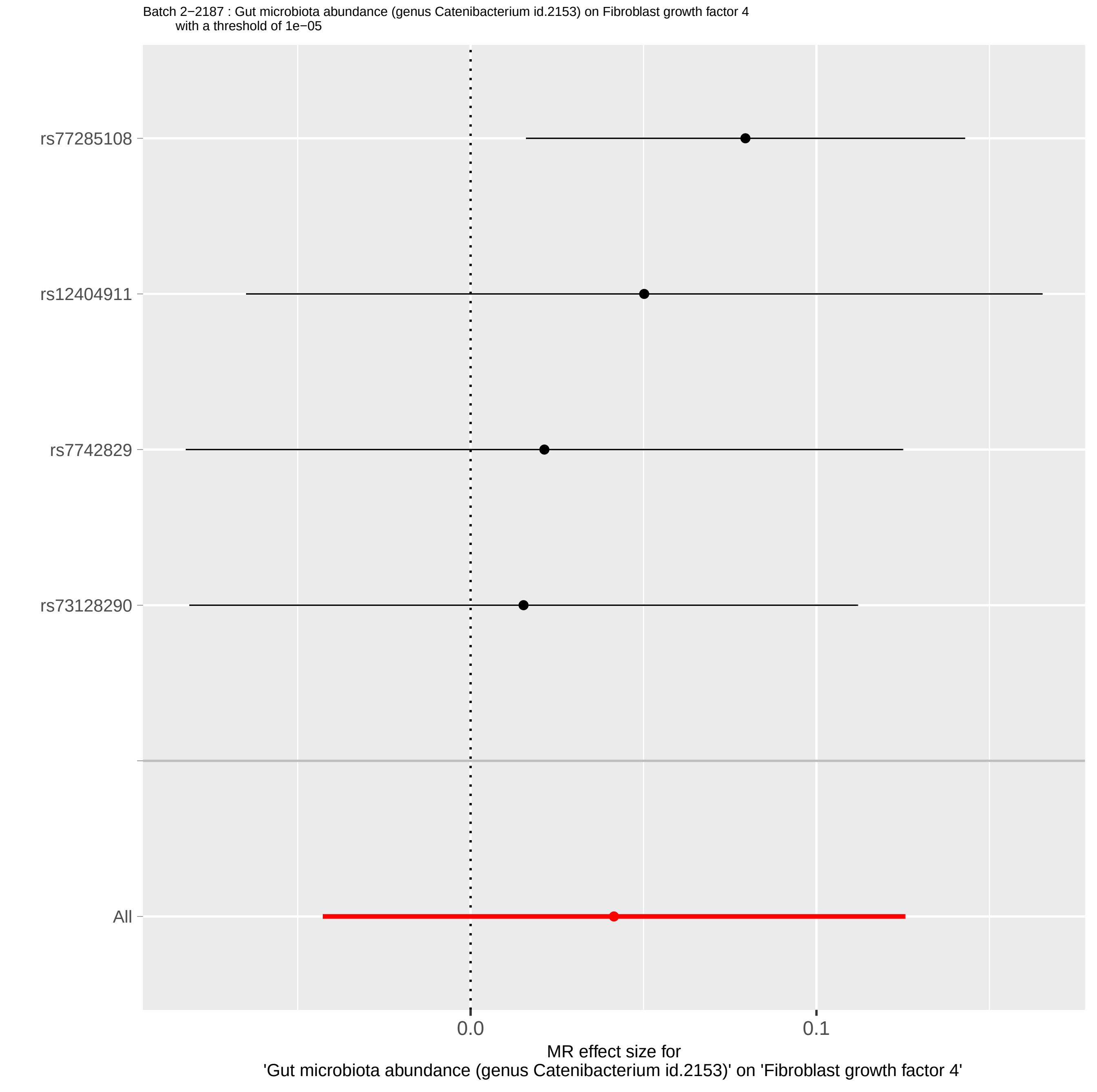

Batch 2−2187 : Gut microbiota abundance (genus Catenibacterium id.2153) on Fibroblast growth factor 4
with a threshold of 1e−05
rs77285108
rs12404911
rs7742829
rs73128290
All
0.0
0.1
MR effect size for
'Gut microbiota abundance (genus Catenibacterium id.2153)' on 'Fibroblast growth factor 4'

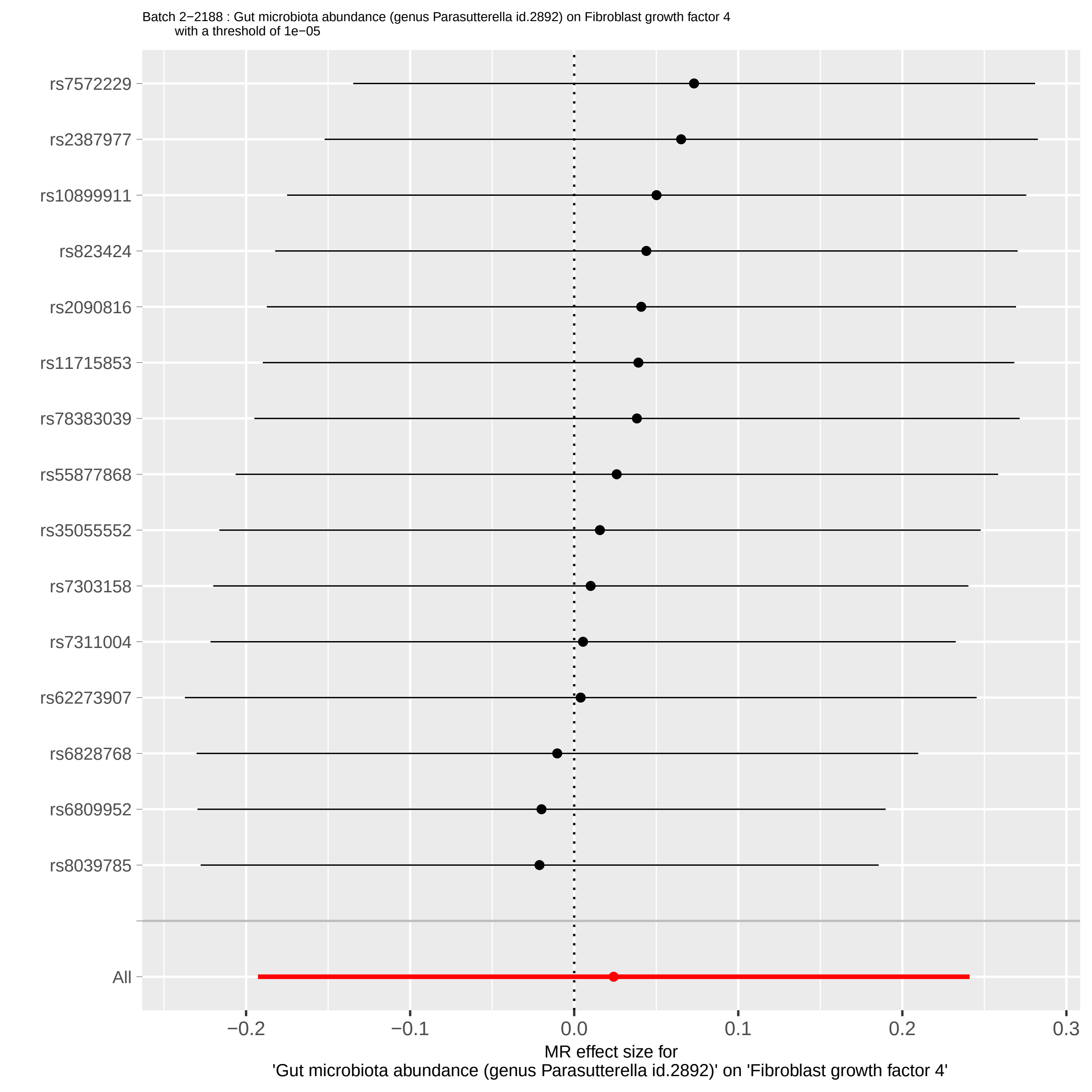
Batch 2−2188 : Gut microbiota abundance (genus Parasutterella id.2892) on Fibroblast growth factor 4
with a threshold of 1e−05
rs7572229
rs2387977
rs10899911
rs823424
rs2090816
rs11715853
rs78383039
rs55877868
rs35055552
rs7303158
rs7311004
rs62273907
rs6828768
rs6809952
rs8039785
All
−0.2
−0.1
0.0
0.1
0.2
0.3
MR effect size for
'Gut microbiota abundance (genus Parasutterella id.2892)' on 'Fibroblast growth factor 4'

Batch 2−2189 : Gut microbiota abundance (genus Catenibacterium id.2153) on Fibroblast growth factor 5
with a threshold of 1e−05

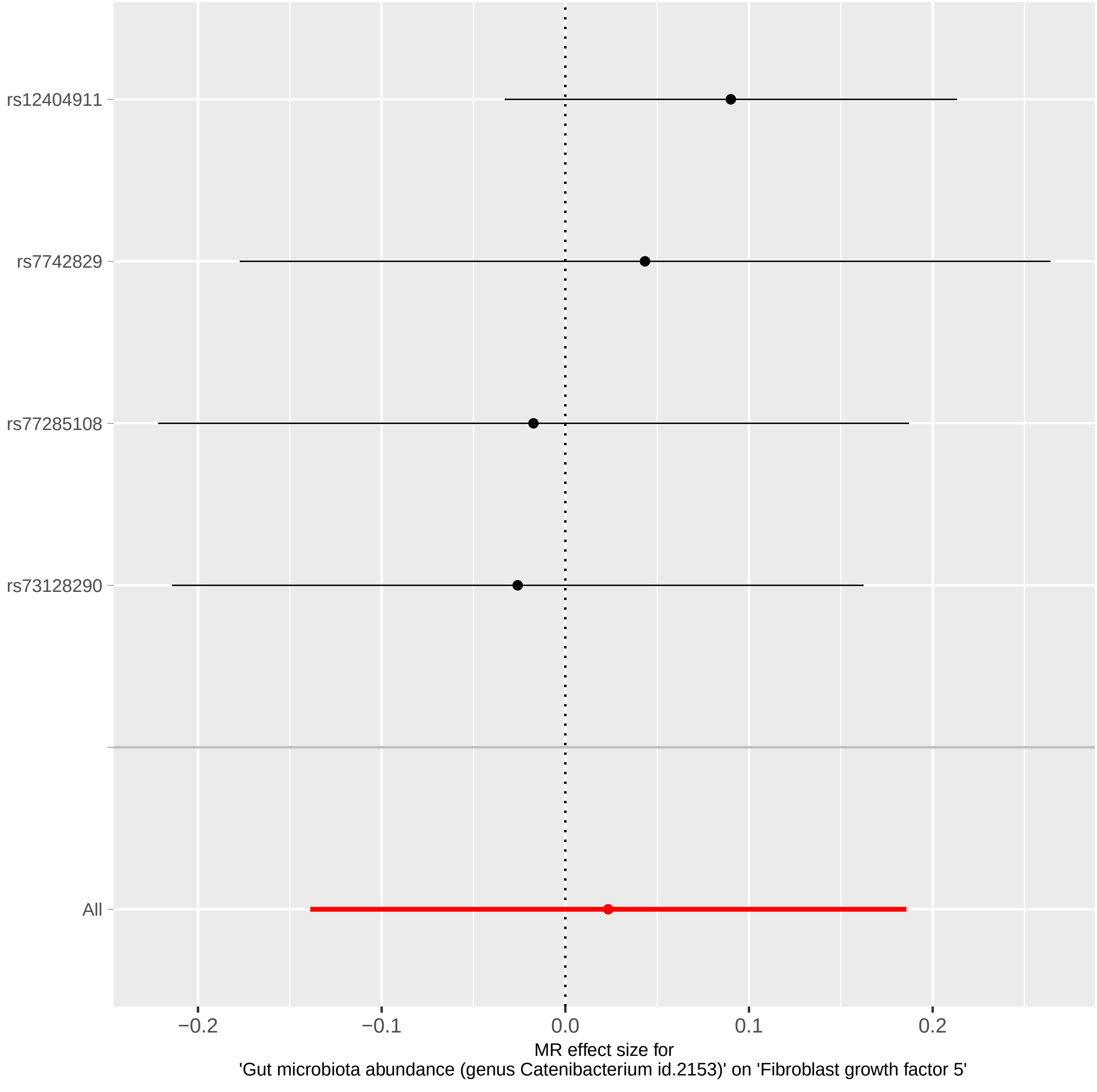

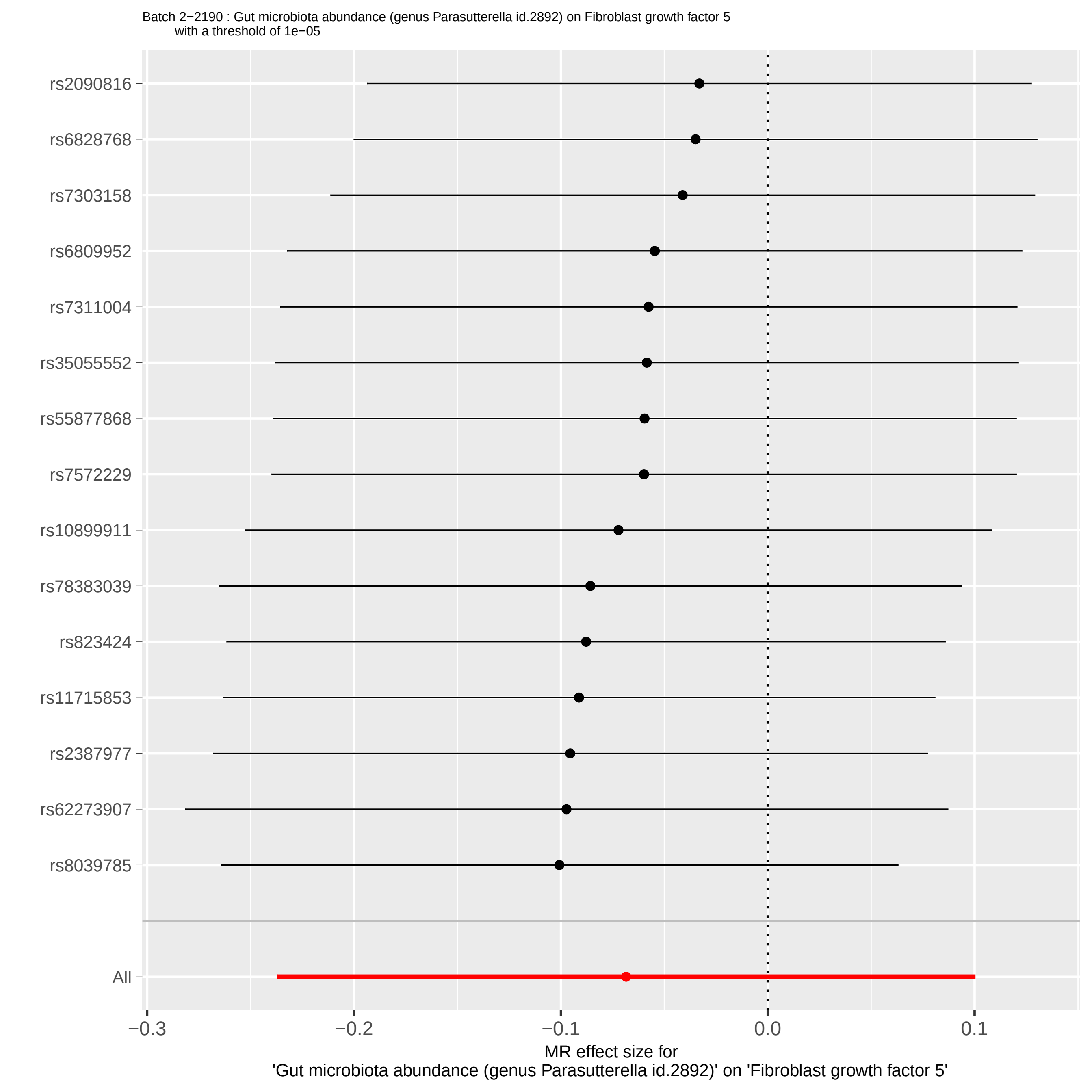
Batch 2−2190 : Gut microbiota abundance (genus Parasutterella id.2892) on Fibroblast growth factor 5
with a threshold of 1e−05
rs2090816
rs6828768
rs7303158
rs6809952
rs7311004
rs35055552
rs55877868
rs7572229
rs10899911
rs78383039
rs823424
rs11715853
rs2387977
rs62273907
rs8039785
All
−0.3
−0.2
−0.1
0.0
0.1
MR effect size for
'Gut microbiota abundance (genus Parasutterella id.2892)' on 'Fibroblast growth factor 5'

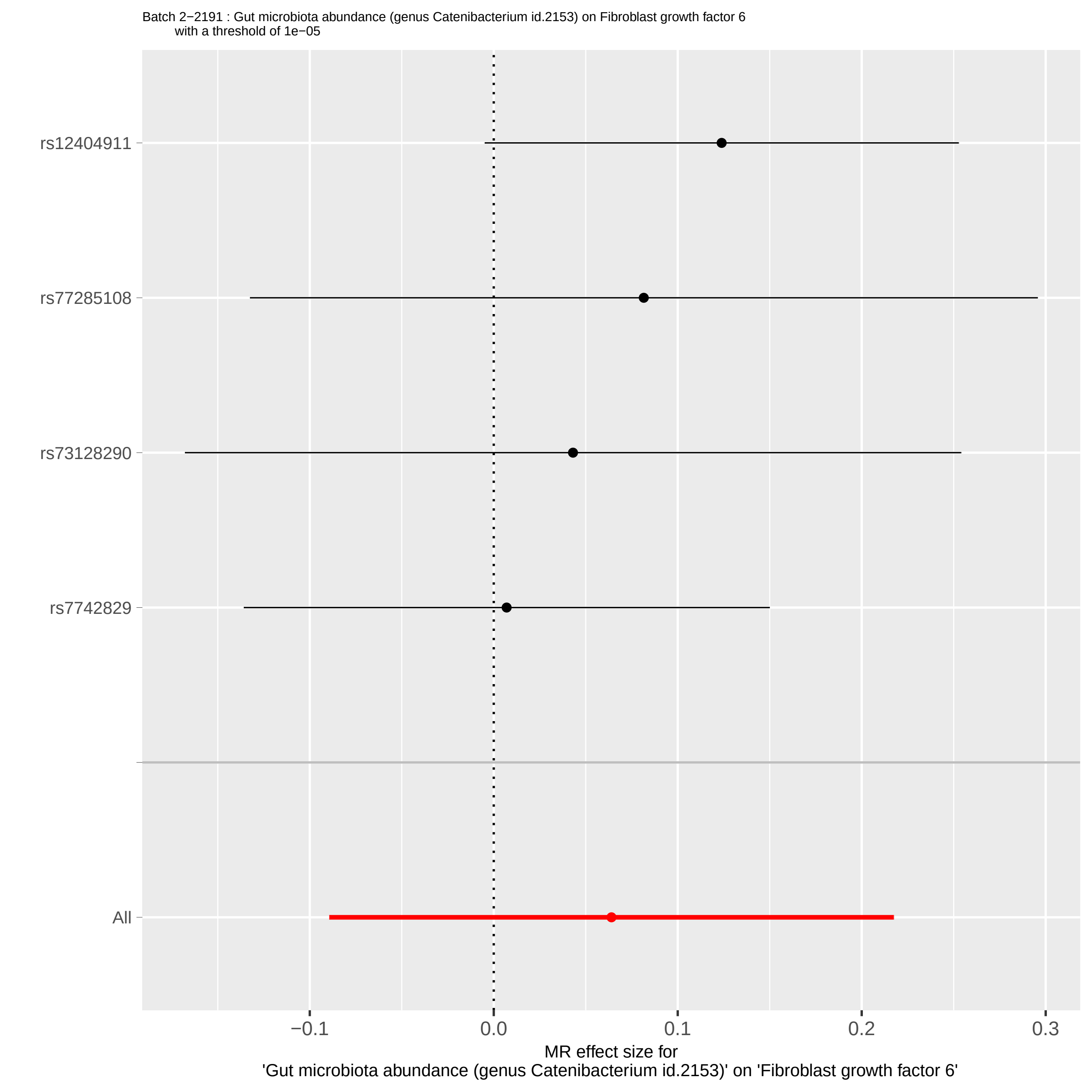
Batch 2−2191 : Gut microbiota abundance (genus Catenibacterium id.2153) on Fibroblast growth factor 6
with a threshold of 1e−05
rs12404911
rs77285108
rs73128290
rs7742829
All
−0.1
0.0
0.1
0.2
0.3
MR effect size for
'Gut microbiota abundance (genus Catenibacterium id.2153)' on 'Fibroblast growth factor 6'

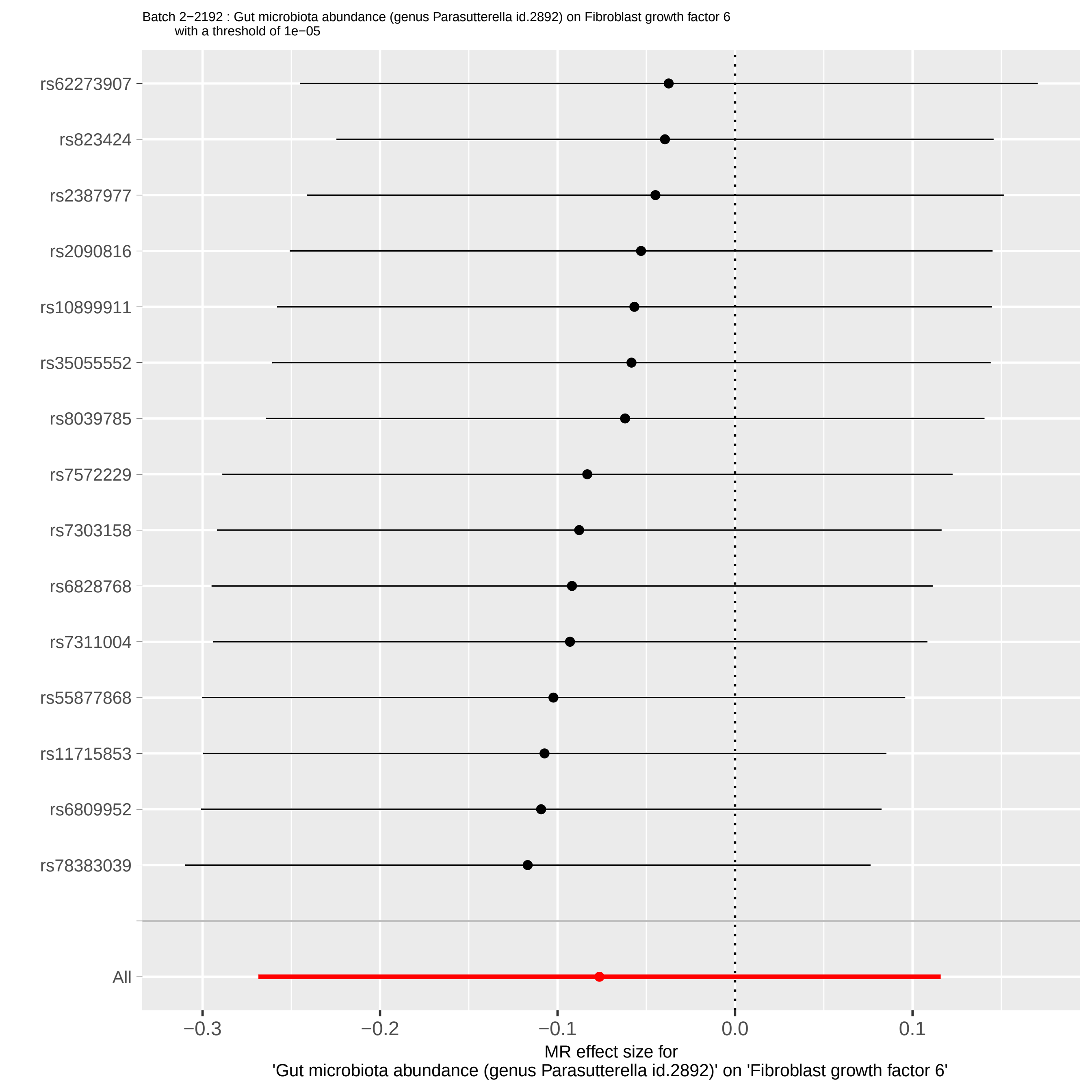
Batch 2−2192 : Gut microbiota abundance (genus Parasutterella id.2892) on Fibroblast growth factor 6
with a threshold of 1e−05
rs62273907
rs823424
rs2387977
rs2090816
rs10899911
rs35055552
rs8039785
rs7572229
rs7303158
rs6828768
rs7311004
rs55877868
rs11715853
rs6809952
rs78383039
All
−0.3
−0.2
−0.1
0.0
0.1
MR effect size for
'Gut microbiota abundance (genus Parasutterella id.2892)' on 'Fibroblast growth factor 6'

Batch 2−2193 : Gut microbiota abundance (genus Catenibacterium id.2153) on Fibroblast growth factor 7
with a threshold of 1e−05

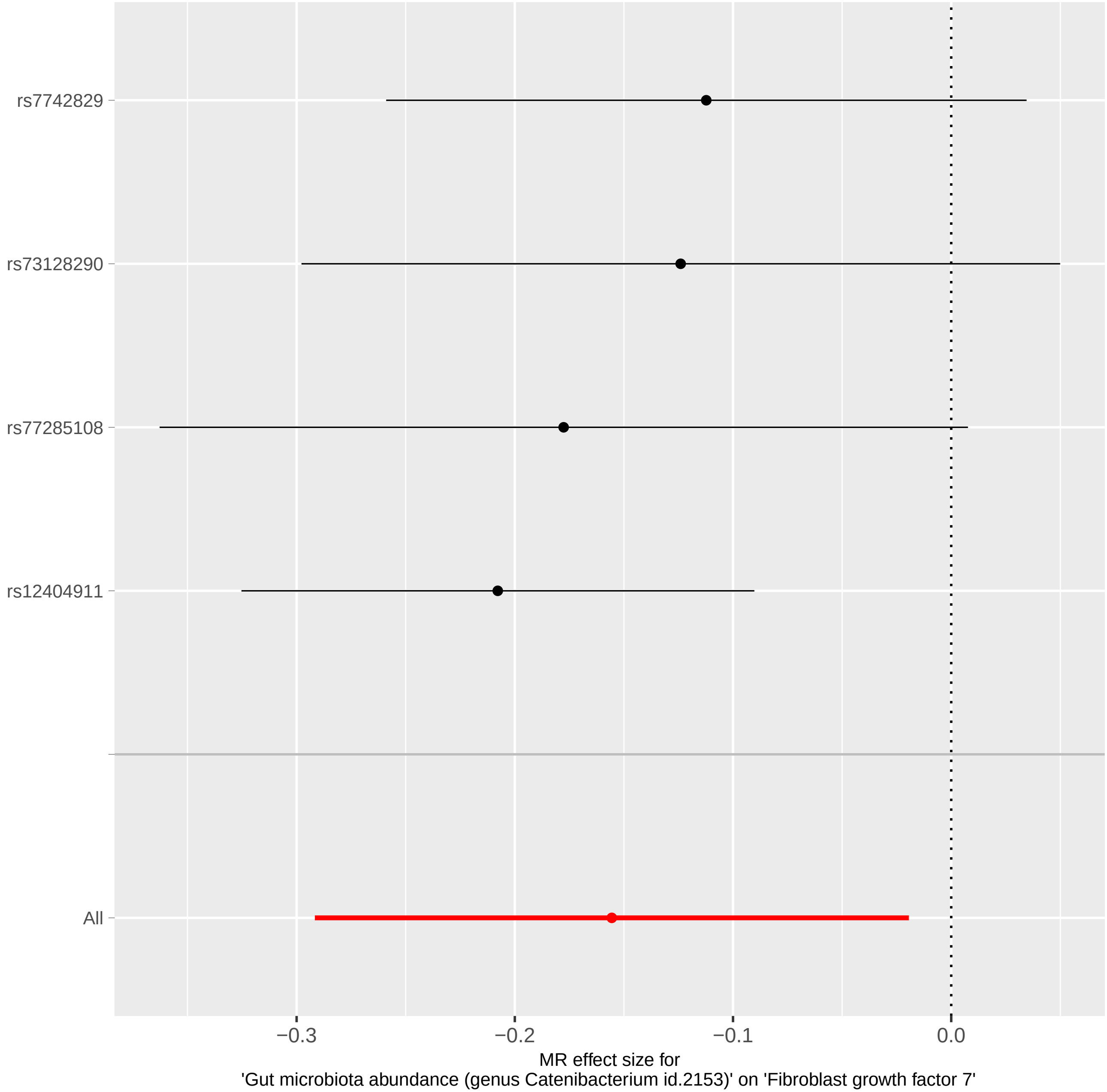

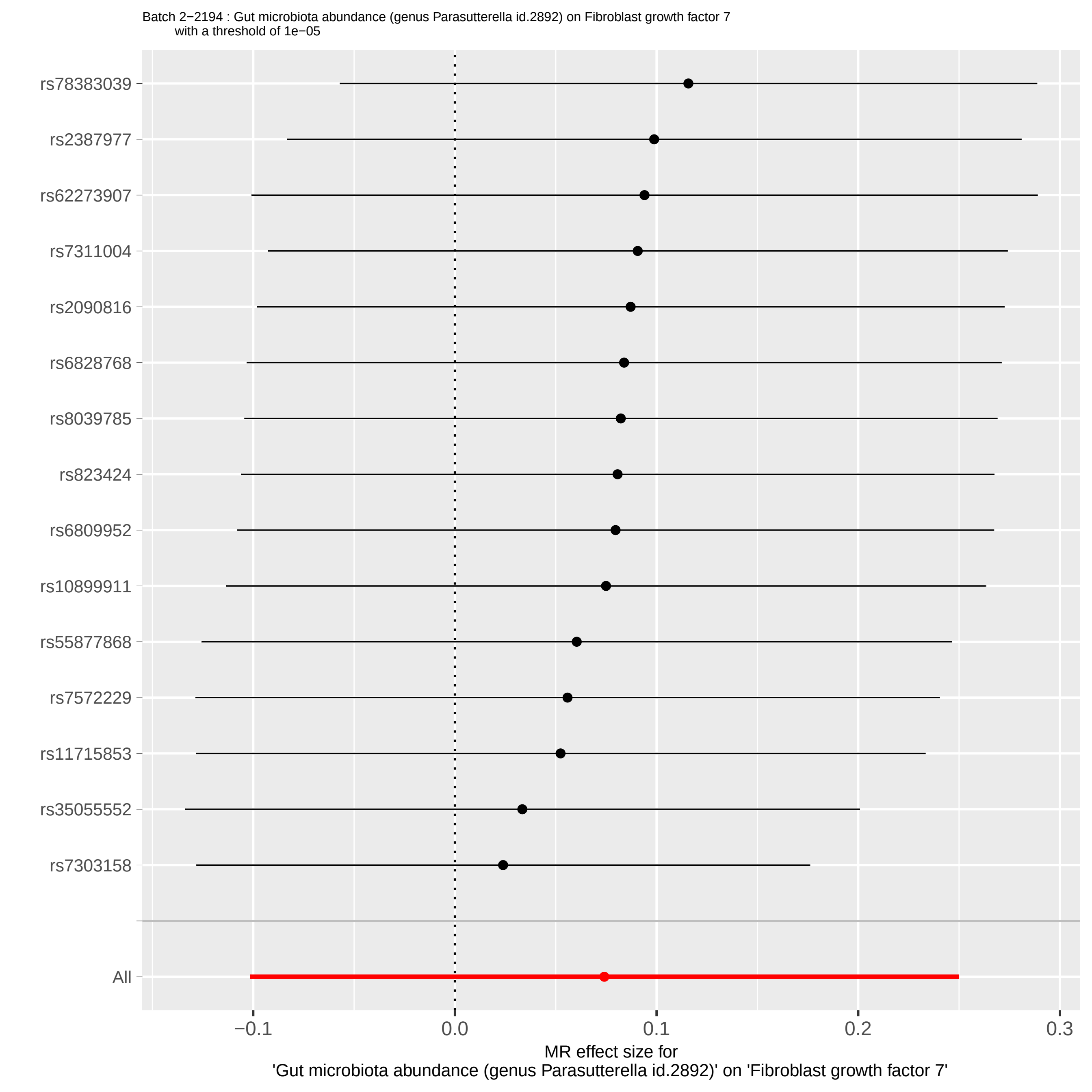

Batch 2−2194 : Gut microbiota abundance (genus Parasutterella id.2892) on Fibroblast growth factor 7
with a threshold of 1e−05
rs78383039
rs2387977
rs62273907
rs7311004
rs2090816
rs6828768
rs8039785
rs823424
rs6809952
rs10899911
rs55877868
rs7572229
rs11715853
rs35055552
rs7303158
All
−0.1
0.0
0.1
0.2
0.3
MR effect size for
'Gut microbiota abundance (genus Parasutterella id.2892)' on 'Fibroblast growth factor 7'

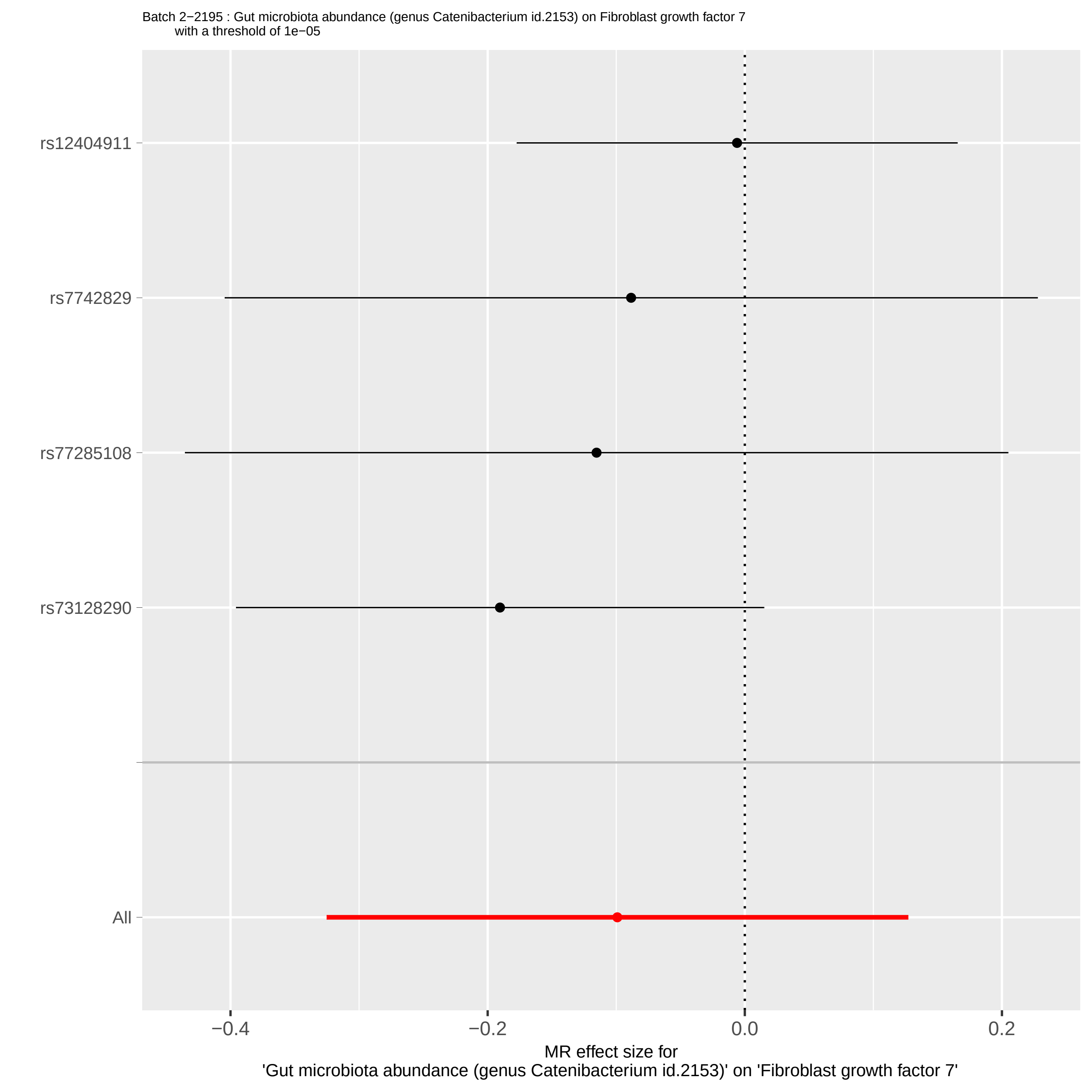

Batch 2−2195 : Gut microbiota abundance (genus Catenibacterium id.2153) on Fibroblast growth factor 7
with a threshold of 1e−05
rs12404911
rs7742829
rs77285108
rs73128290
All
−0.4
−0.2
0.0
0.2
MR effect size for
'Gut microbiota abundance (genus Catenibacterium id.2153)' on 'Fibroblast growth factor 7'

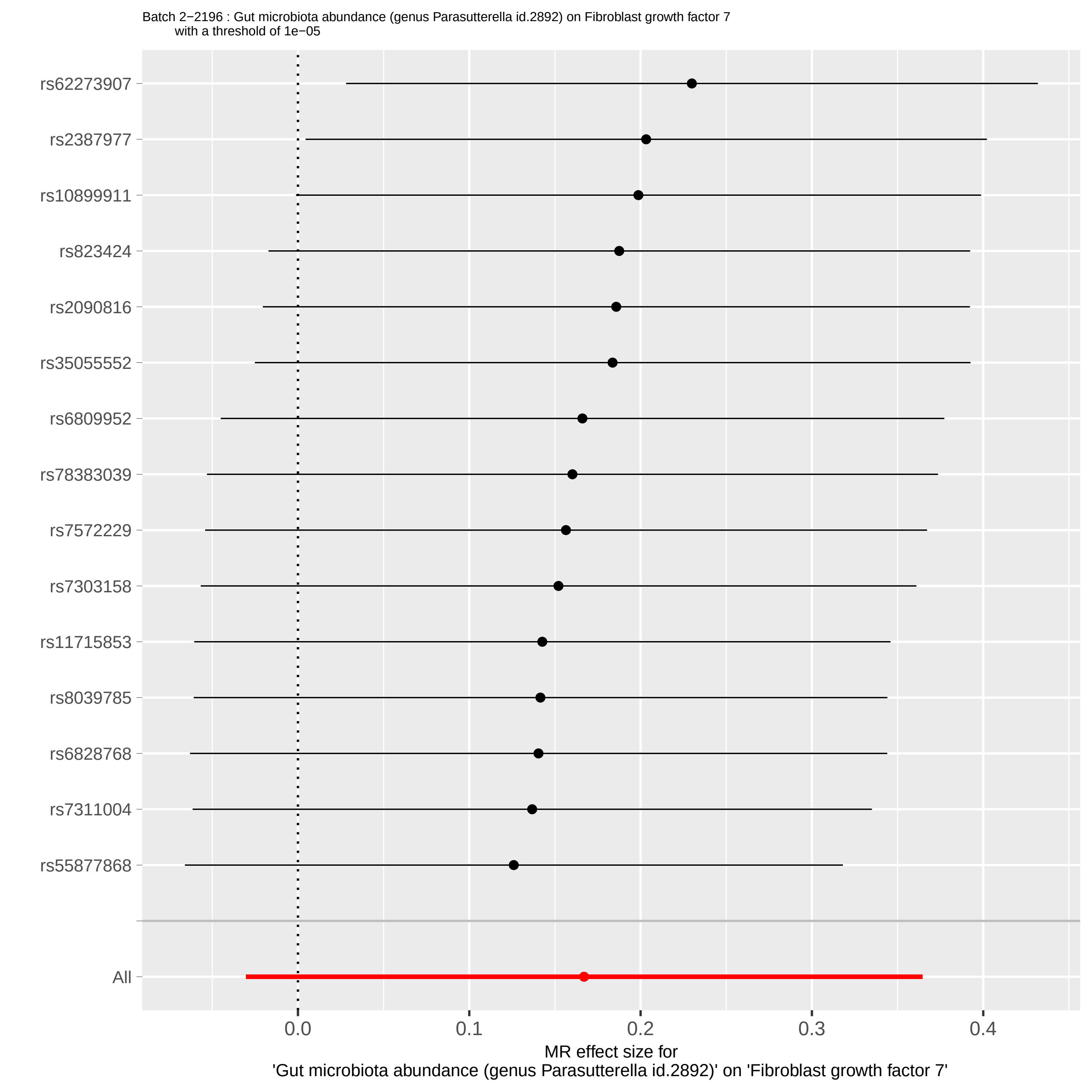

Batch 2−2196 : Gut microbiota abundance (genus Parasutterella id.2892) on Fibroblast growth factor 7
with a threshold of 1e−05
rs62273907
rs2387977
rs10899911
rs823424
rs2090816
rs35055552
rs6809952
rs78383039
rs7572229
rs7303158
rs11715853
rs8039785
rs6828768
rs7311004
rs55877868
All
0.0
0.1
0.2
0.3
0.4
MR effect size for
'Gut microbiota abundance (genus Parasutterella id.2892)' on 'Fibroblast growth factor 7'

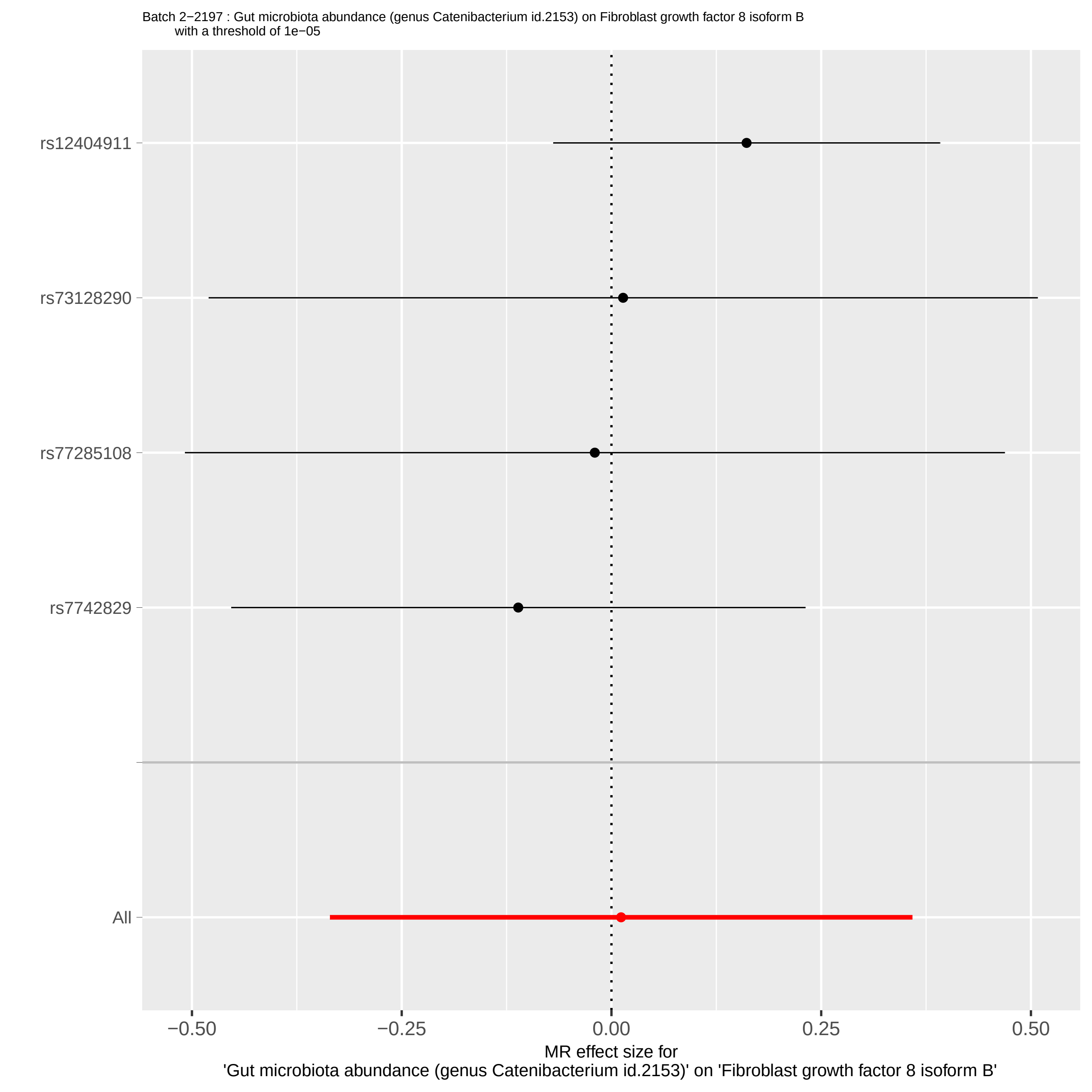
Batch 2−2197 : Gut microbiota abundance (genus Catenibacterium id.2153) on Fibroblast growth factor 8 isoform B
with a threshold of 1e−05
rs12404911
rs73128290
rs77285108
rs7742829
All
−0.50
−0.25
0.00
0.25
0.50
MR effect size for
'Gut microbiota abundance (genus Catenibacterium id.2153)' on 'Fibroblast growth factor 8 isoform B'

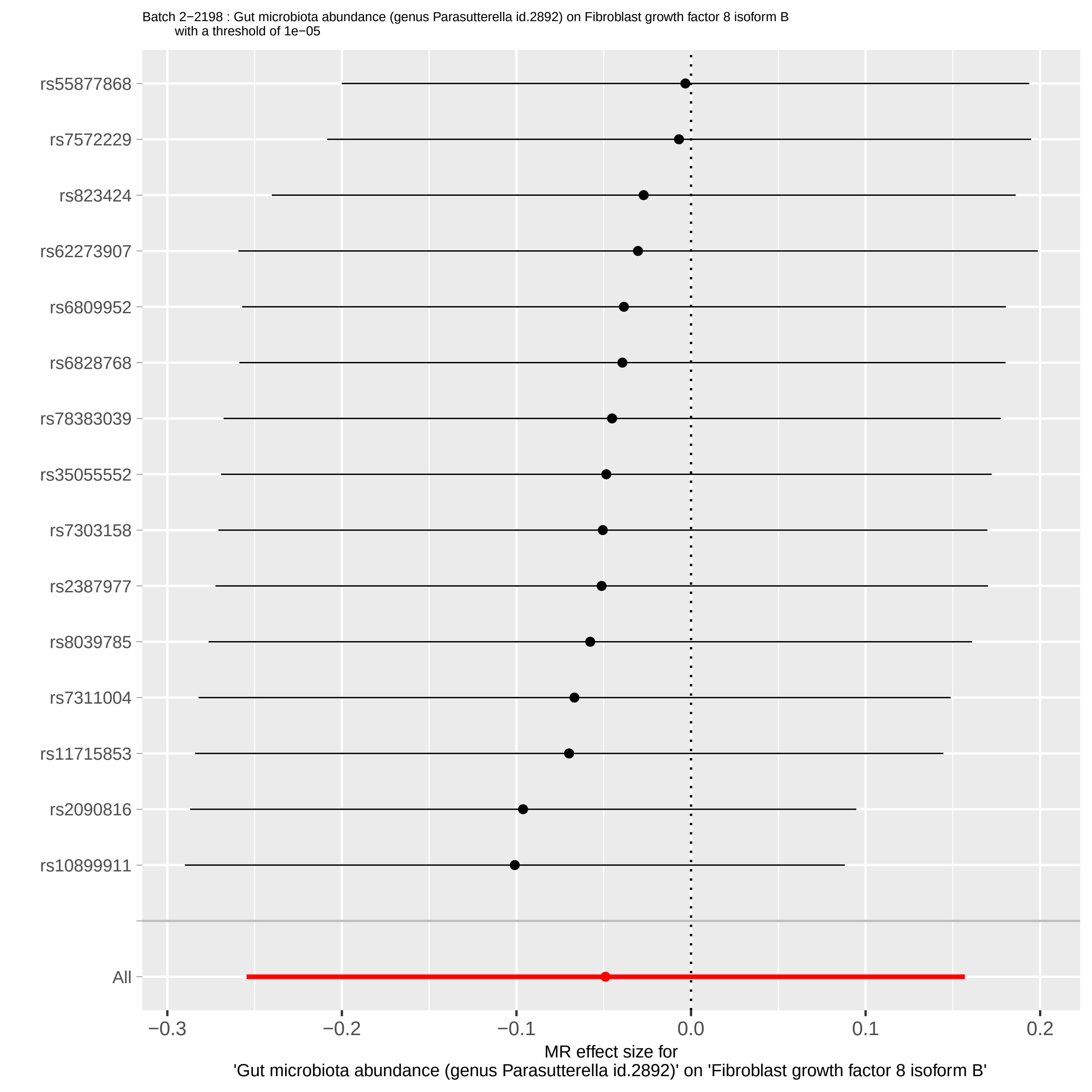

Batch 2−2198 : Gut microbiota abundance (genus Parasutterella id.2892) on Fibroblast growth factor 8 isoform B
with a threshold of 1e−05
rs55877868
rs7572229
rs823424
rs62273907
rs6809952
rs6828768
rs78383039
rs35055552
rs7303158
rs2387977
rs8039785
rs7311004
rs11715853
rs2090816
rs10899911
All
−0.3
−0.2
−0.1
0.0
0.1
0.2
MR effect size for
'Gut microbiota abundance (genus Parasutterella id.2892)' on 'Fibroblast growth factor 8 isoform B'

Batch 2−2199 : Gut microbiota abundance (genus Catenibacterium id.2153) on Fibroblast growth factor 8 isoform A
with a threshold of 1e−05

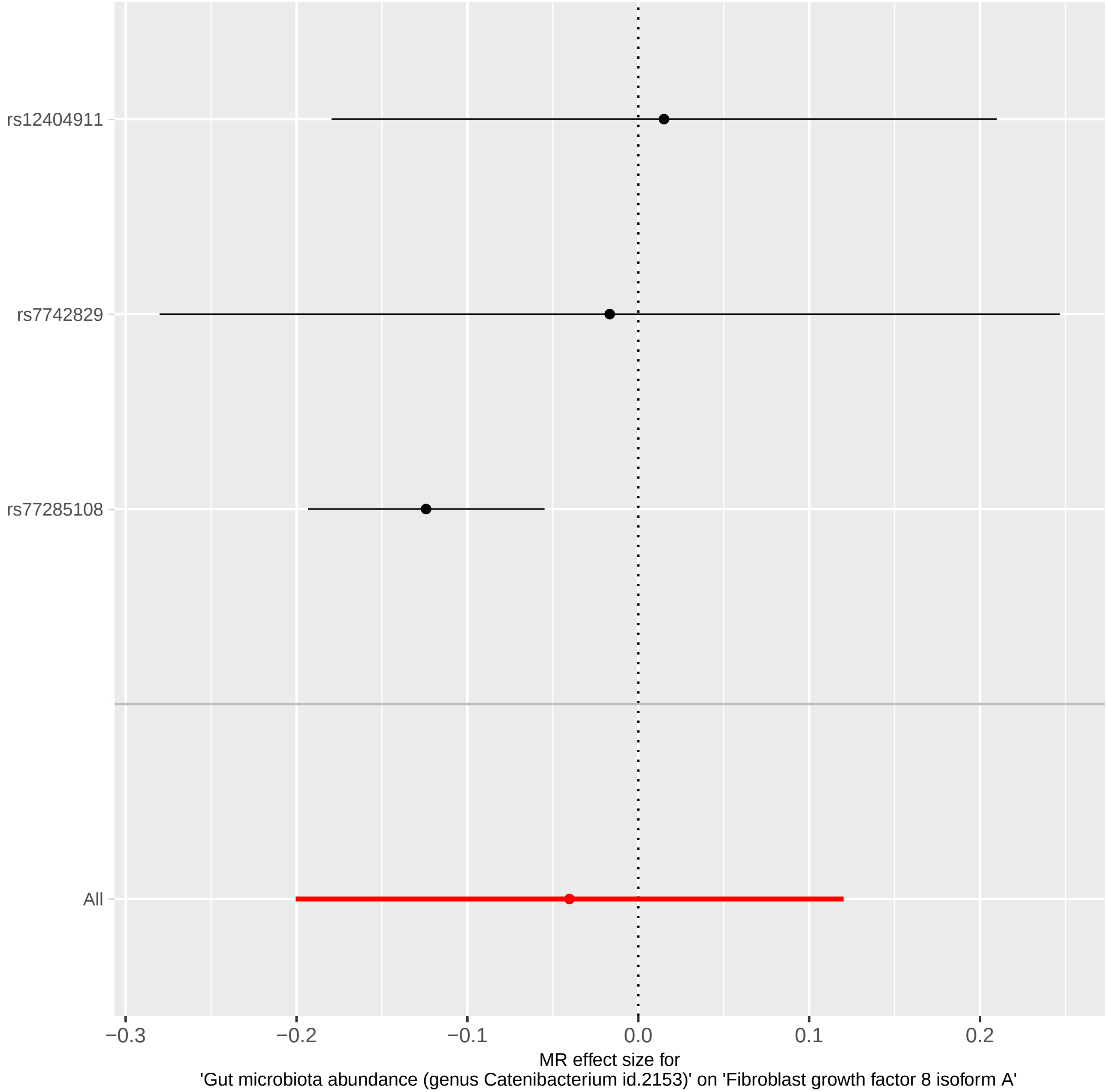

Batch 2−2200 : Gut microbiota abundance (genus Parasutterella id.2892) on Fibroblast growth factor 8 isoform A
with a threshold of 1e−05

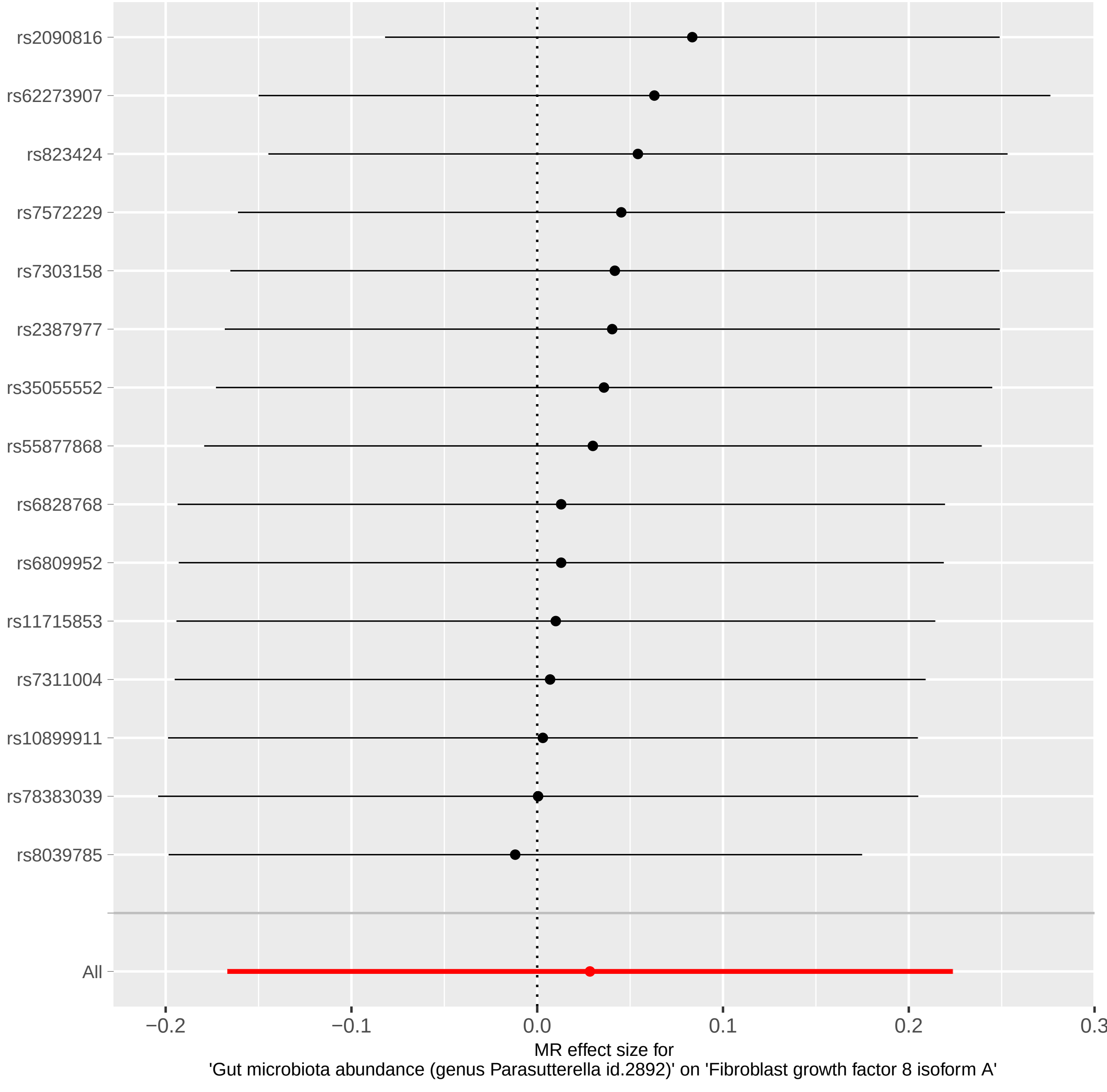

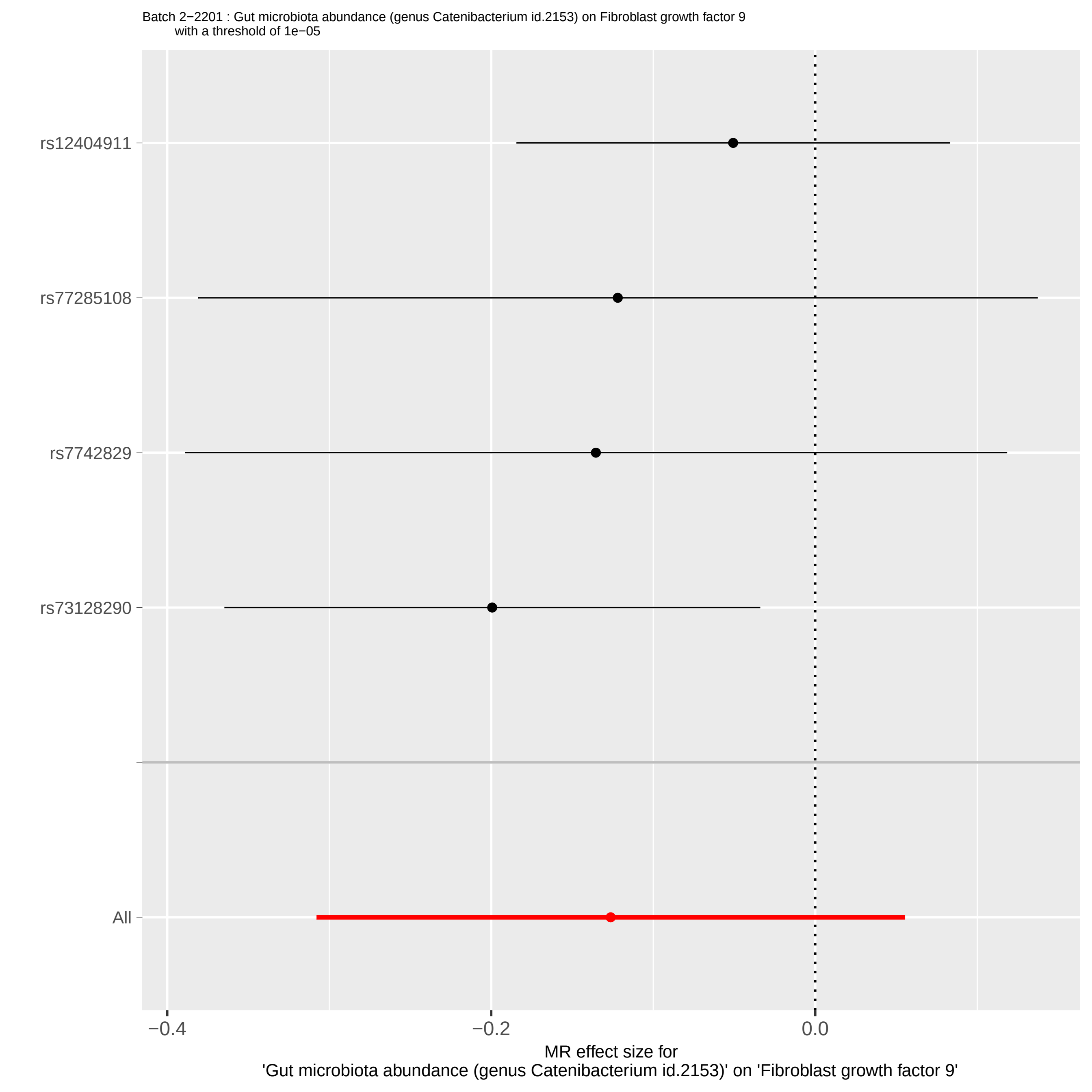

Batch 2−2201 : Gut microbiota abundance (genus Catenibacterium id.2153) on Fibroblast growth factor 9
with a threshold of 1e−05
rs12404911
rs77285108
rs7742829
rs73128290
All
−0.4
−0.2
0.0
MR effect size for
'Gut microbiota abundance (genus Catenibacterium id.2153)' on 'Fibroblast growth factor 9'

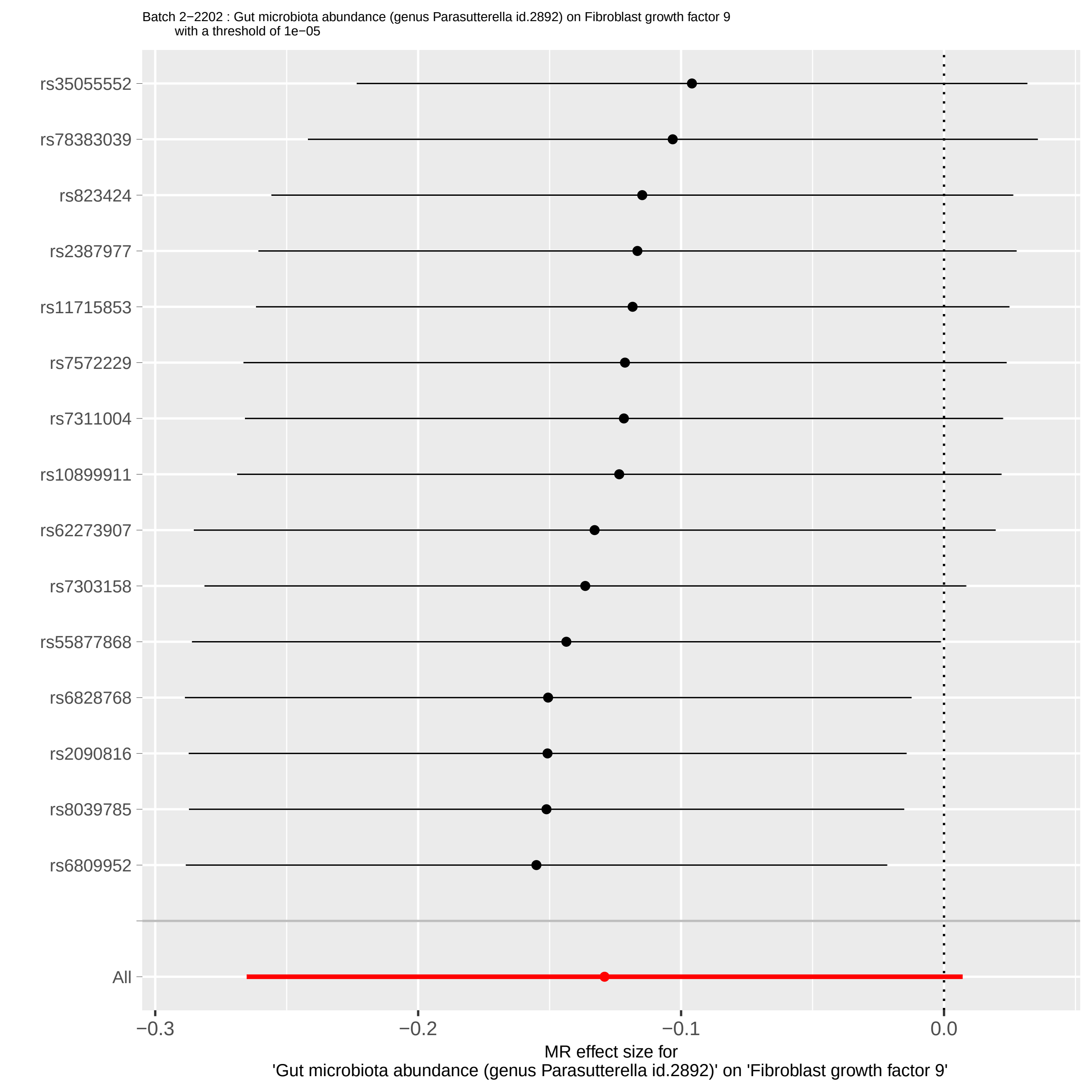
Batch 2−2202 : Gut microbiota abundance (genus Parasutterella id.2892) on Fibroblast growth factor 9
with a threshold of 1e−05
rs35055552
rs78383039
rs823424
rs2387977
rs11715853
rs7572229
rs7311004
rs10899911
rs62273907
rs7303158
rs55877868
rs6828768
rs2090816
rs8039785
rs6809952
All
−0.3
−0.2
−0.1
0.0
MR effect size for
'Gut microbiota abundance (genus Parasutterella id.2892)' on 'Fibroblast growth factor 9'

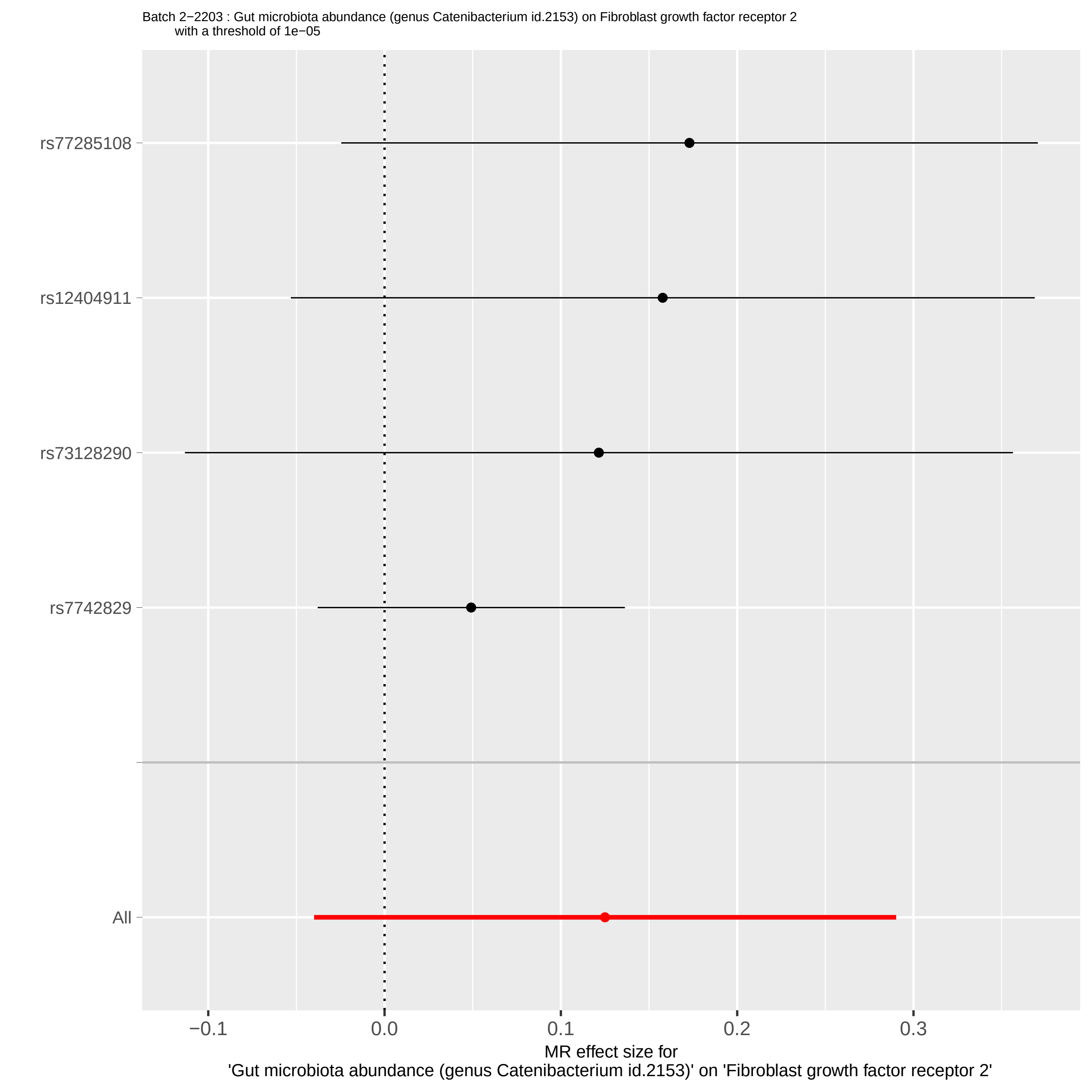
Batch 2−2203 : Gut microbiota abundance (genus Catenibacterium id.2153) on Fibroblast growth factor receptor 2
with a threshold of 1e−05
rs77285108
rs12404911
rs73128290
rs7742829
All
−0.1
0.0
0.1
0.2
0.3
MR effect size for
'Gut microbiota abundance (genus Catenibacterium id.2153)' on 'Fibroblast growth factor receptor 2'

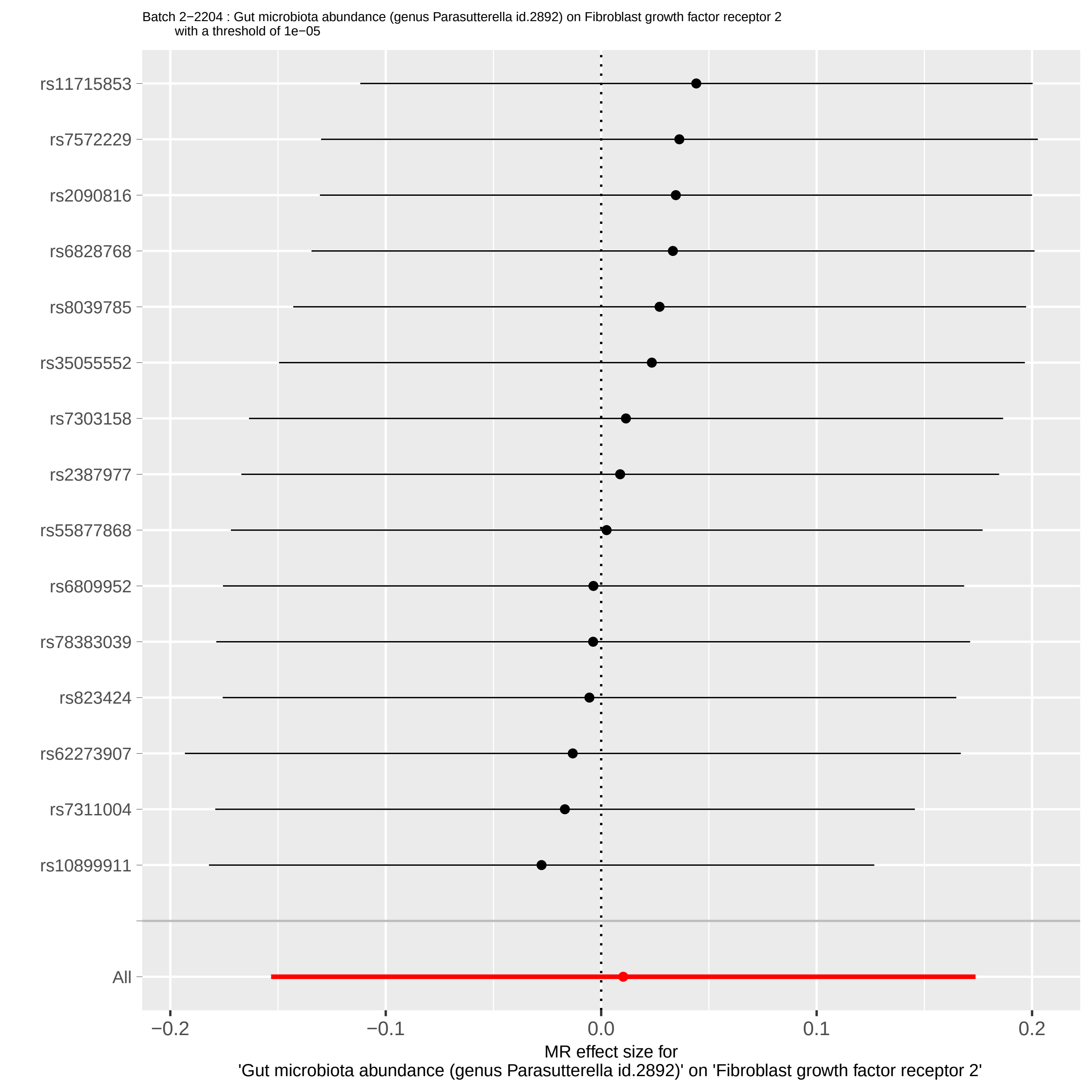
Batch 2−2204 : Gut microbiota abundance (genus Parasutterella id.2892) on Fibroblast growth factor receptor 2
with a threshold of 1e−05
rs11715853
rs7572229
rs2090816
rs6828768
rs8039785
rs35055552
rs7303158
rs2387977
rs55877868
rs6809952
rs78383039
rs823424
rs62273907
rs7311004
rs10899911
All
−0.2
−0.1
0.0
0.1
0.2
MR effect size for
'Gut microbiota abundance (genus Parasutterella id.2892)' on 'Fibroblast growth factor receptor 2'

Batch 2−2205 : Gut microbiota abundance (genus Catenibacterium id.2153) on Fibroblast growth factor receptor 3
with a threshold of 1e−05

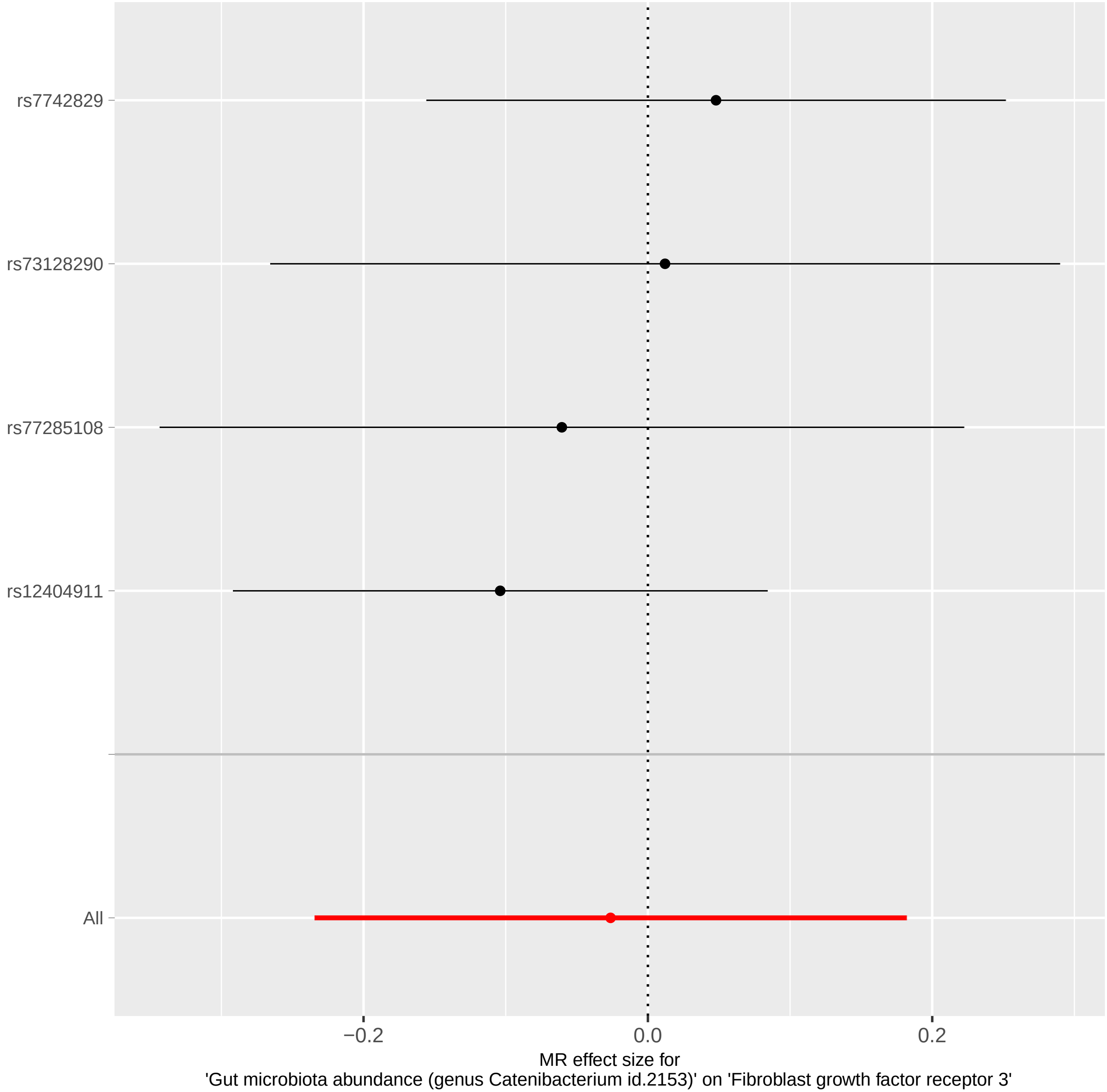

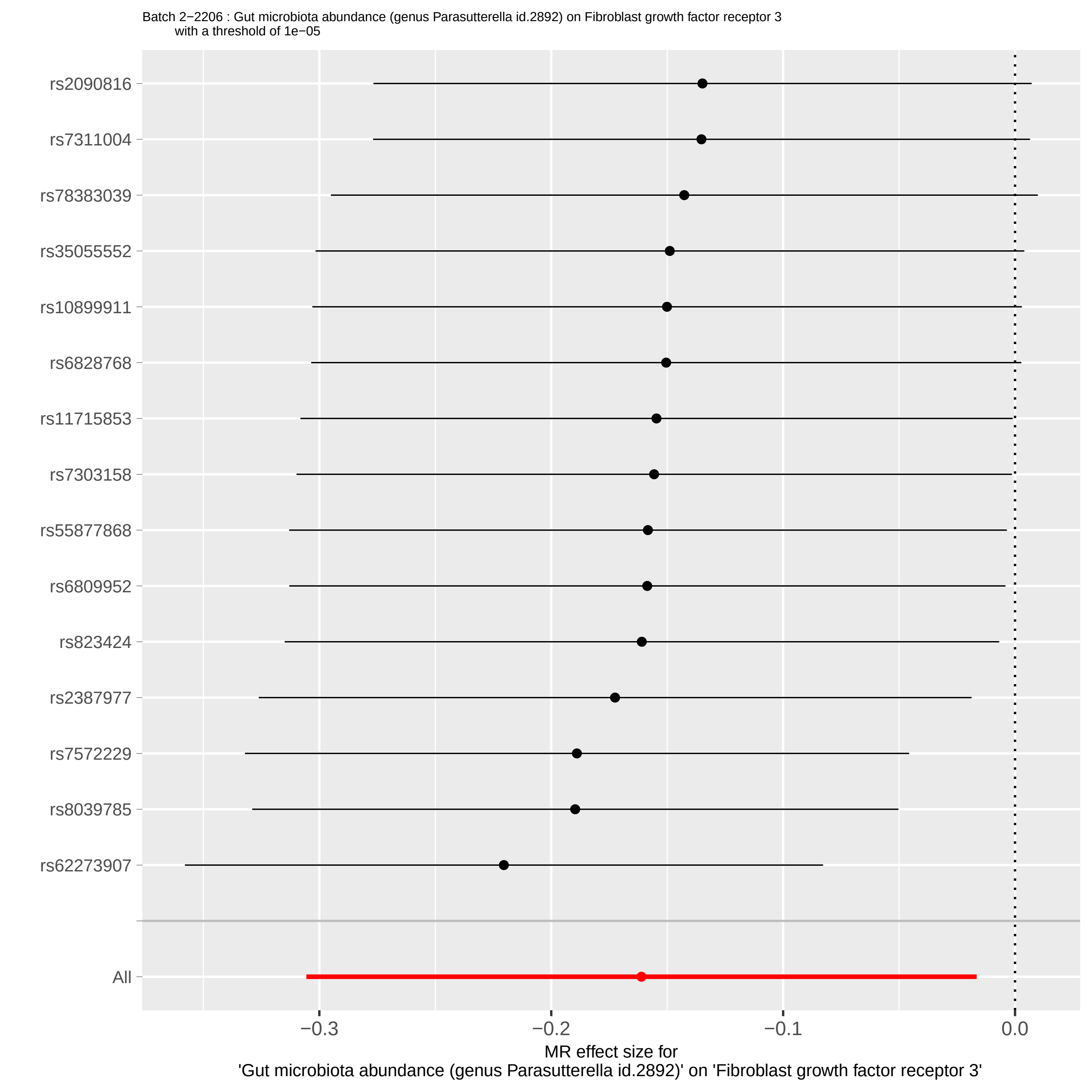
Batch 2−2206 : Gut microbiota abundance (genus Parasutterella id.2892) on Fibroblast growth factor receptor 3
with a threshold of 1e−05
rs2090816
rs7311004
rs78383039
rs35055552
rs10899911
rs6828768
rs11715853
rs7303158
rs55877868
rs6809952
rs823424
rs2387977
rs7572229
rs8039785
rs62273907
All
−0.3
−0.2
−0.1
0.0
MR effect size for
'Gut microbiota abundance (genus Parasutterella id.2892)' on 'Fibroblast growth factor receptor 3'

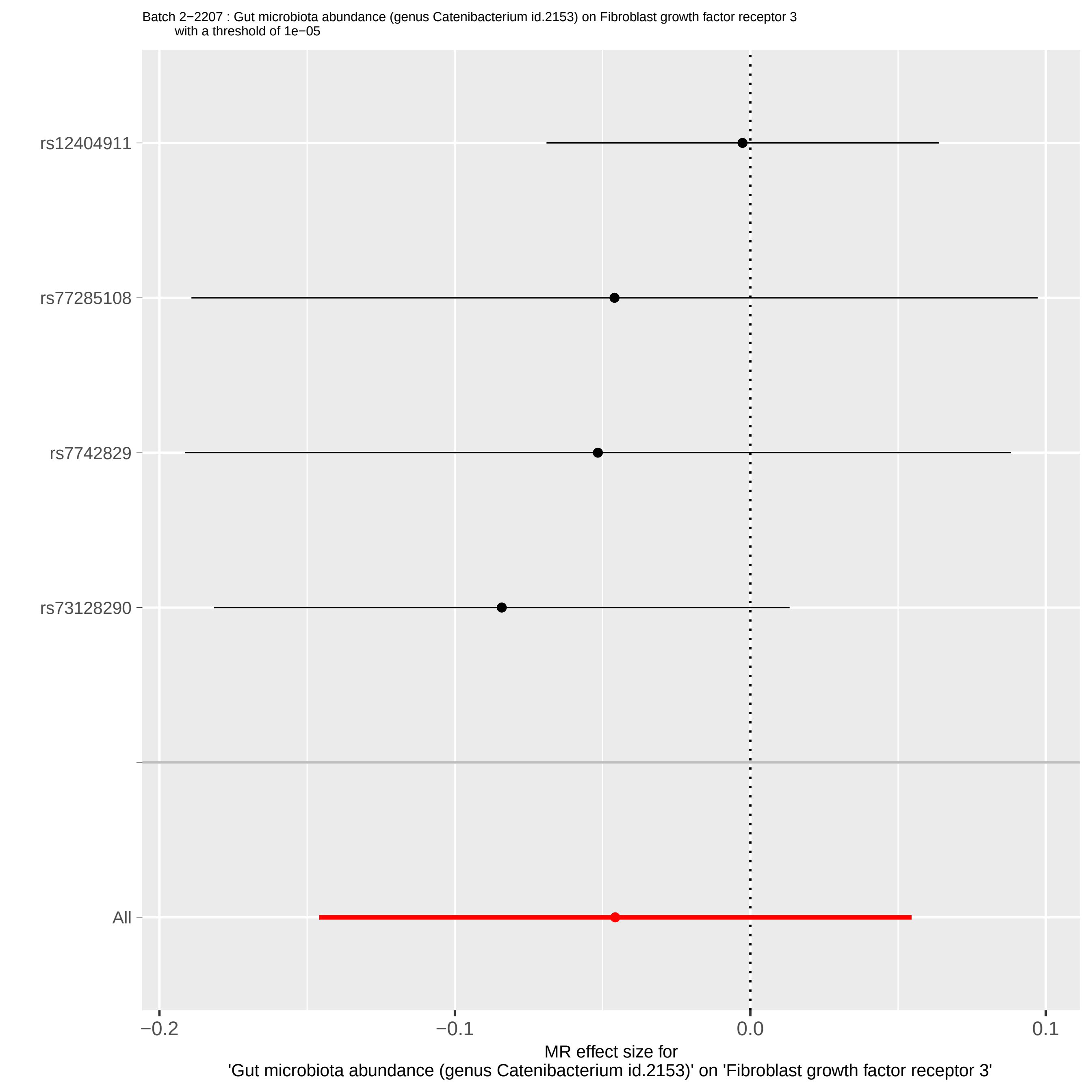
Batch 2−2207 : Gut microbiota abundance (genus Catenibacterium id.2153) on Fibroblast growth factor receptor 3
with a threshold of 1e−05
rs12404911
rs77285108
rs7742829
rs73128290
All
−0.2
−0.1
0.0
0.1
MR effect size for
'Gut microbiota abundance (genus Catenibacterium id.2153)' on 'Fibroblast growth factor receptor 3'

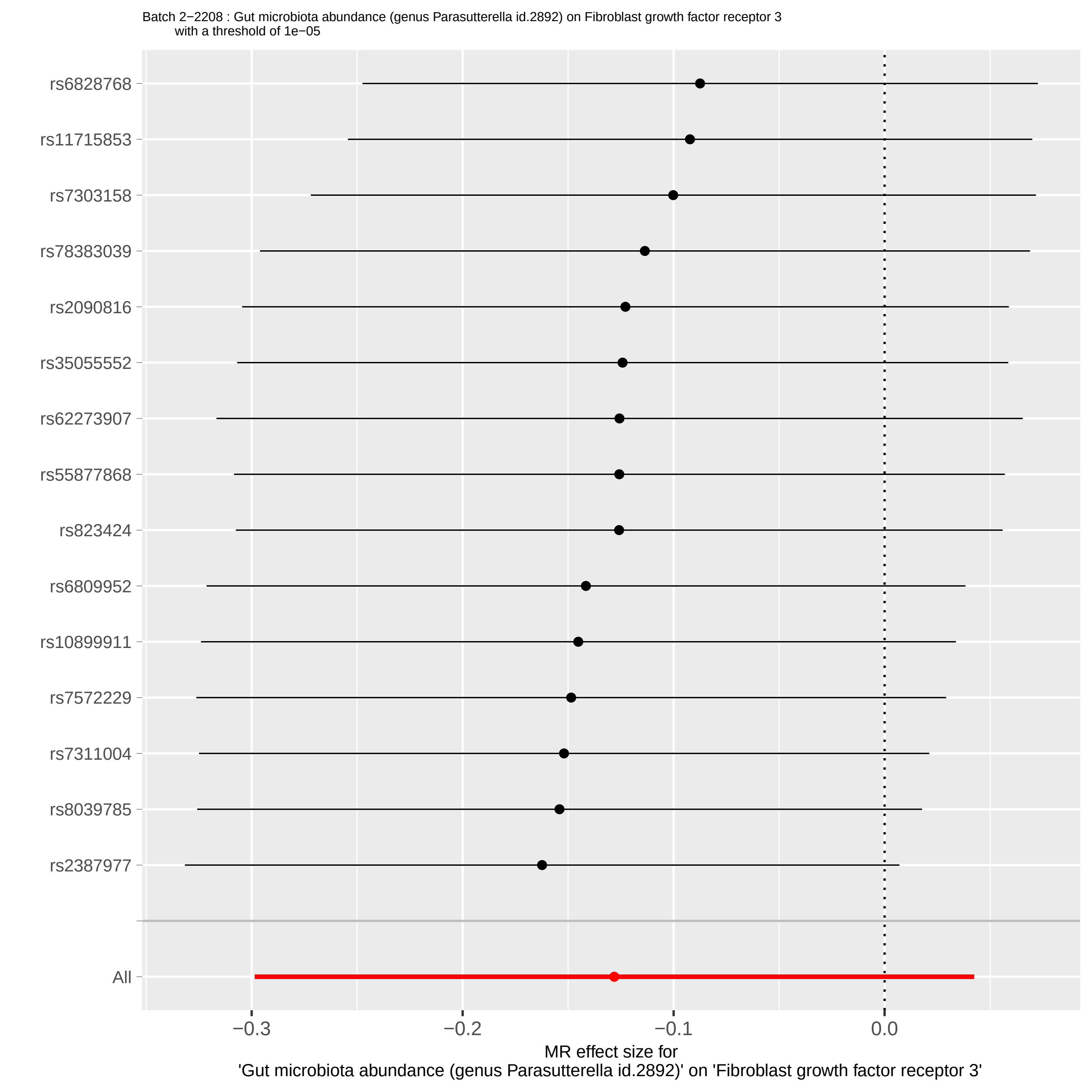
Batch 2−2208 : Gut microbiota abundance (genus Parasutterella id.2892) on Fibroblast growth factor receptor 3
with a threshold of 1e−05
rs6828768
rs11715853
rs7303158
rs78383039
rs2090816
rs35055552
rs62273907
rs55877868
rs823424
rs6809952
rs10899911
rs7572229
rs7311004
rs8039785
rs2387977
All
−0.3
−0.2
−0.1
0.0
MR effect size for
'Gut microbiota abundance (genus Parasutterella id.2892)' on 'Fibroblast growth factor receptor 3'

Batch 2−2209 : Gut microbiota abundance (genus Catenibacterium id.2153) on Fibroblast growth factor receptor 4
with a threshold of 1e−05

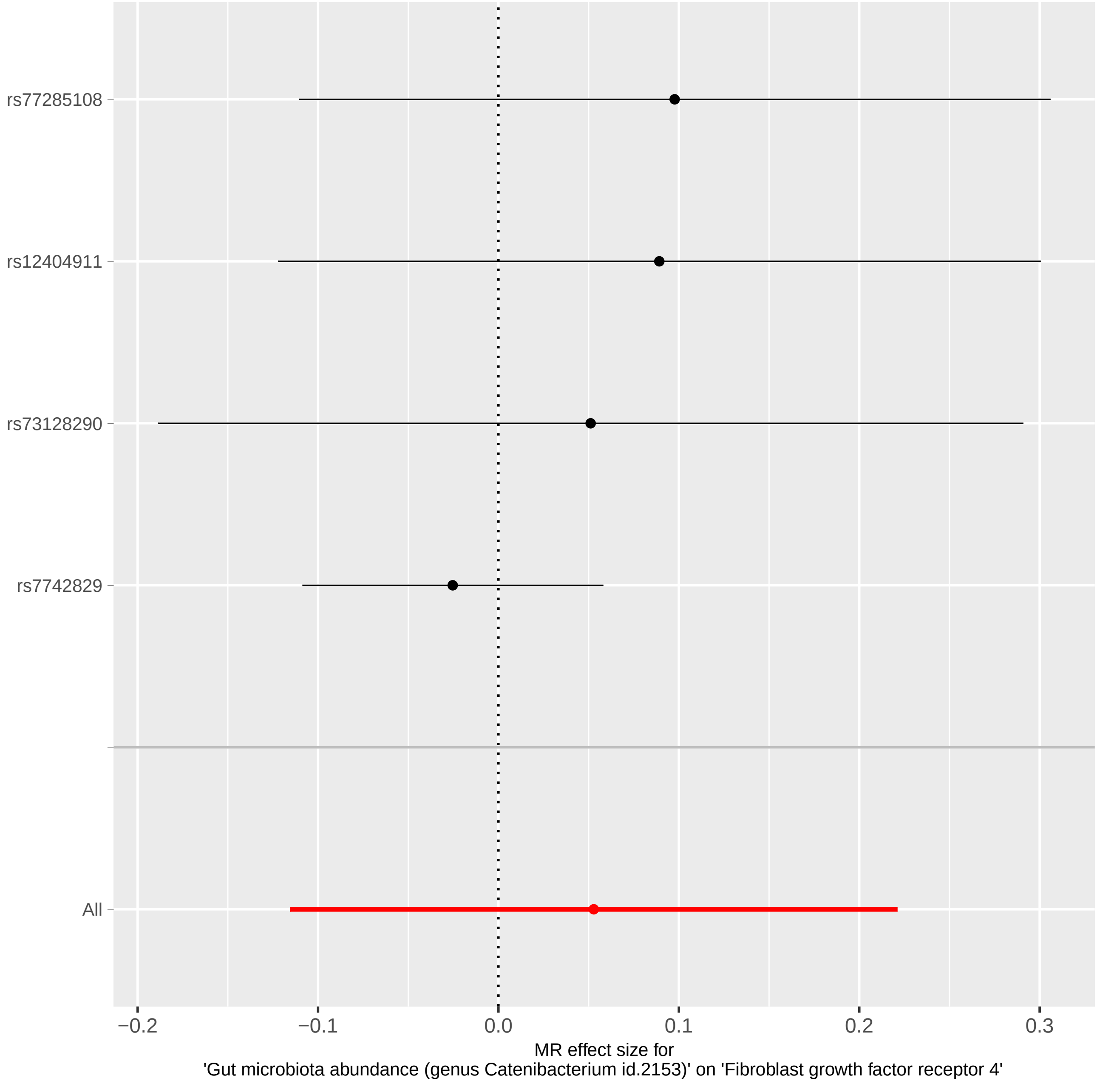

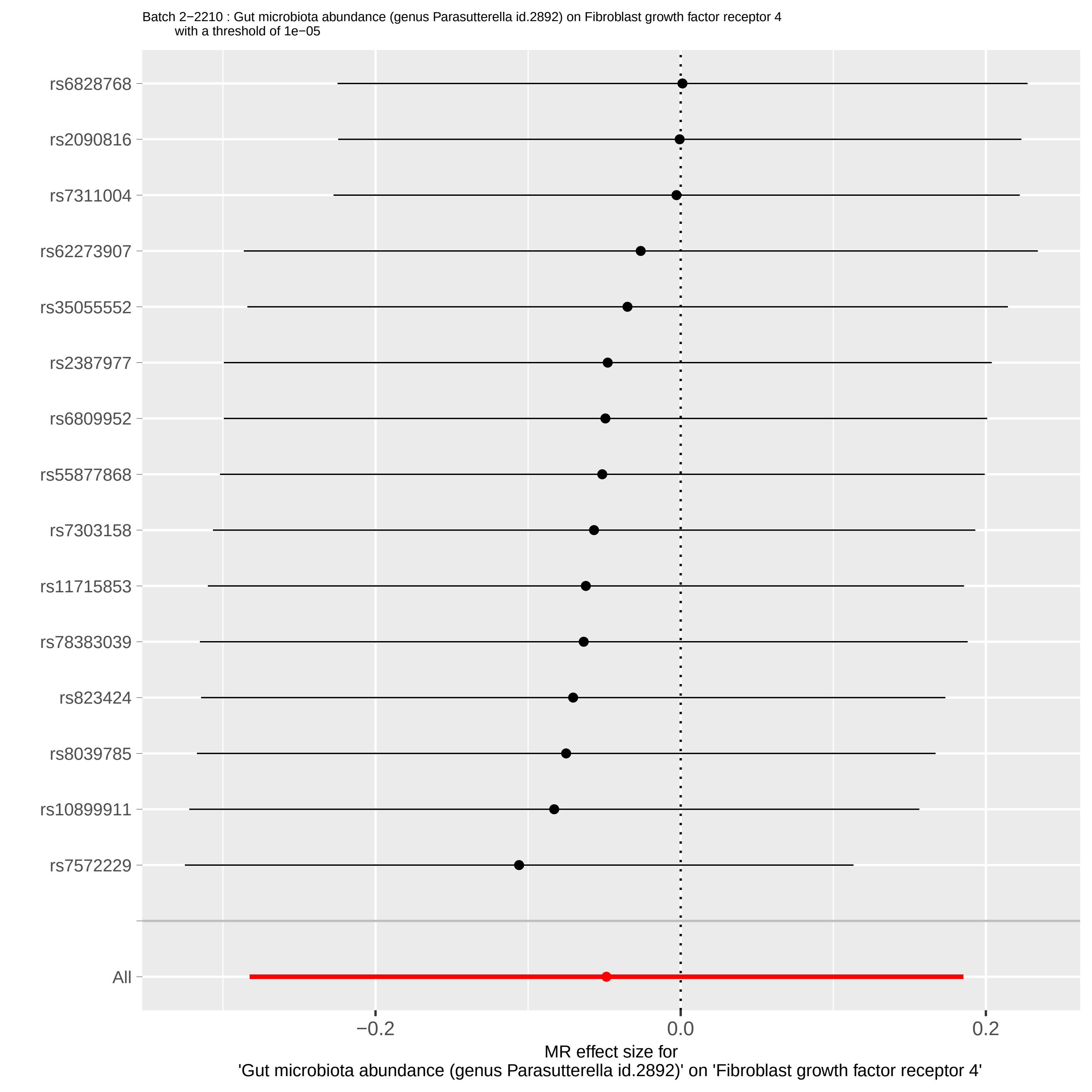

Batch 2−2210 : Gut microbiota abundance (genus Parasutterella id.2892) on Fibroblast growth factor receptor 4
with a threshold of 1e−05
rs6828768
rs2090816
rs7311004
rs62273907
rs35055552
rs2387977
rs6809952
rs55877868
rs7303158
rs11715853
rs78383039
rs823424
rs8039785
rs10899911
rs7572229
All
−0.2
0.0
0.2
MR effect size for
'Gut microbiota abundance (genus Parasutterella id.2892)' on 'Fibroblast growth factor receptor 4'

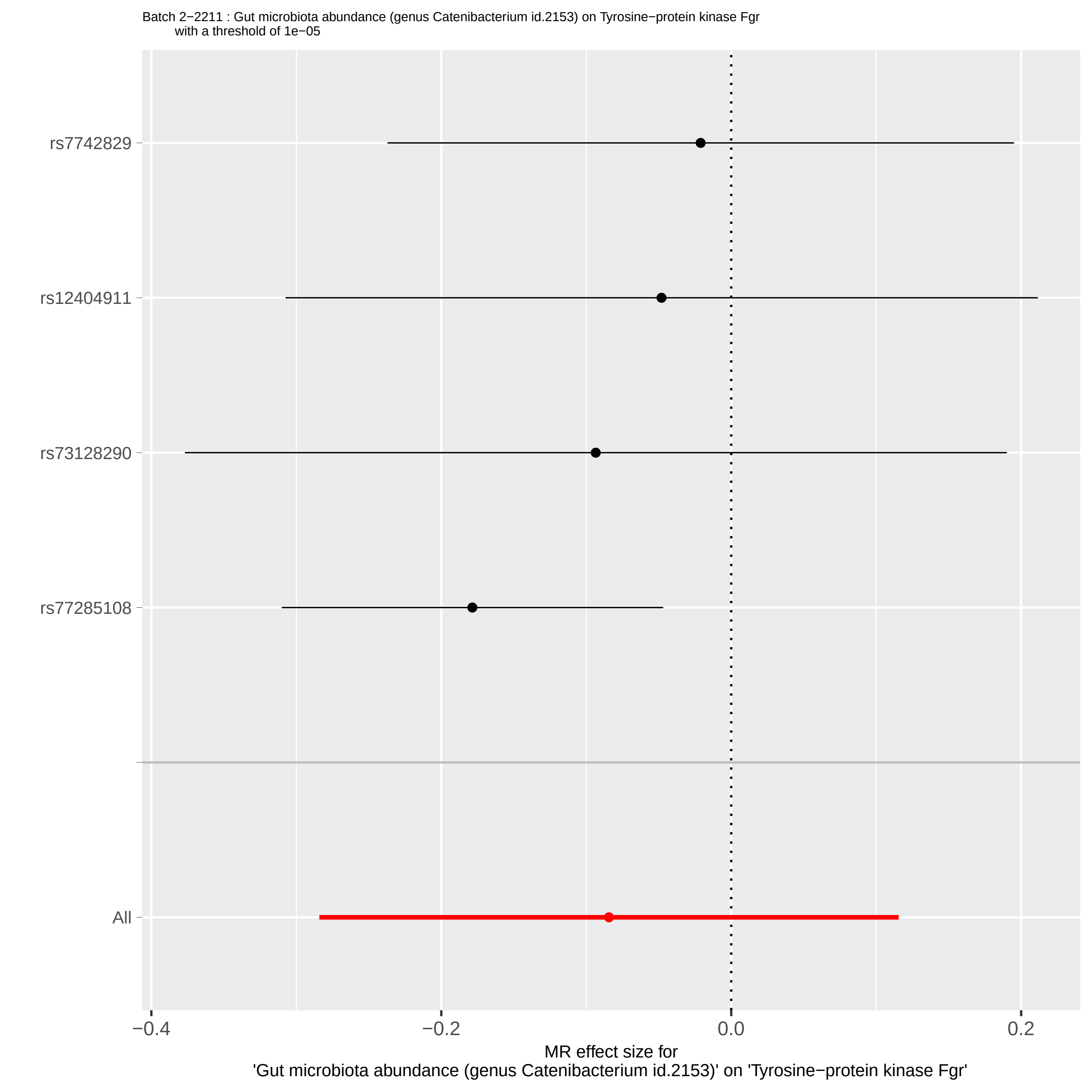
Batch 2−2211 : Gut microbiota abundance (genus Catenibacterium id.2153) on Tyrosine−protein kinase Fgr
with a threshold of 1e−05
rs7742829
rs12404911
rs73128290
rs77285108
All
−0.4
−0.2
0.0
0.2
MR effect size for
'Gut microbiota abundance (genus Catenibacterium id.2153)' on 'Tyrosine−protein kinase Fgr'

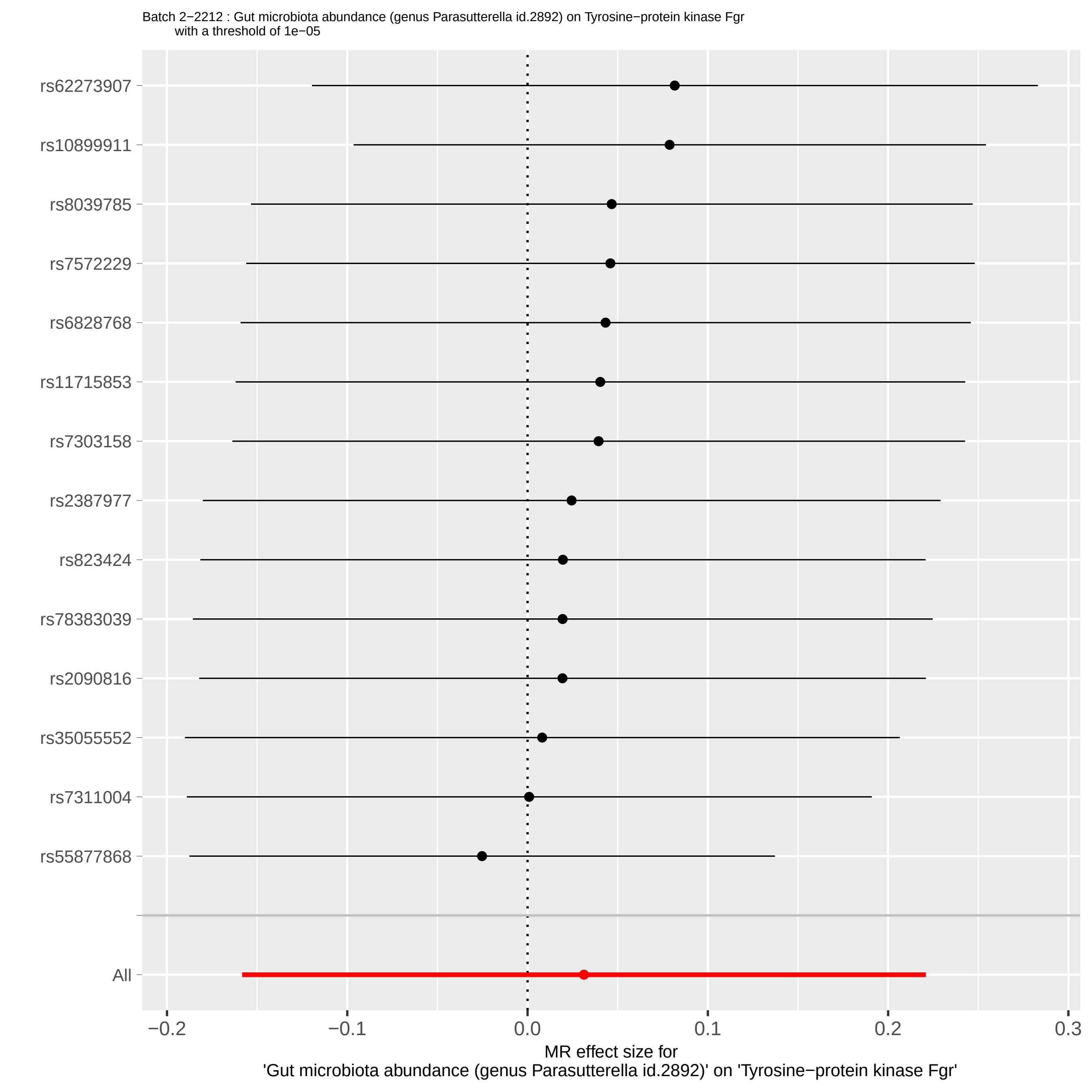
Batch 2−2212 : Gut microbiota abundance (genus Parasutterella id.2892) on Tyrosine−protein kinase Fgr
with a threshold of 1e−05
rs62273907
rs10899911
rs8039785
rs7572229
rs6828768
rs11715853
rs7303158
rs2387977
rs823424
rs78383039
rs2090816
rs35055552
rs7311004
rs55877868
All
−0.2
−0.1
0.0
0.1
0.2
0.3
MR effect size for
'Gut microbiota abundance (genus Parasutterella id.2892)' on 'Tyrosine−protein kinase Fgr'

Batch 2−2213 : Gut microbiota abundance (genus Catenibacterium id.2153) on Bis(5'−adenosyl)−triphosphatase
with a threshold of 1e−05

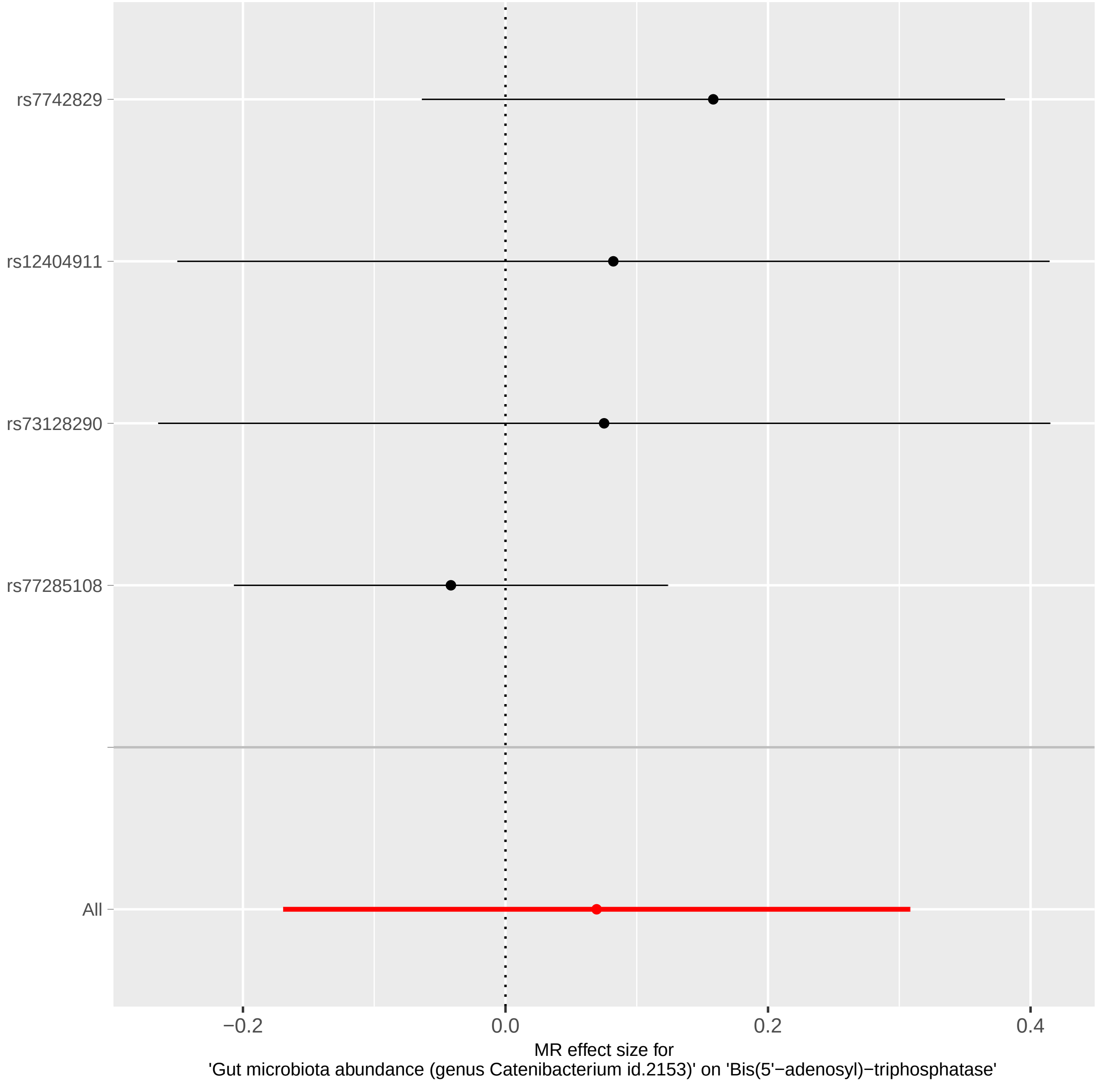

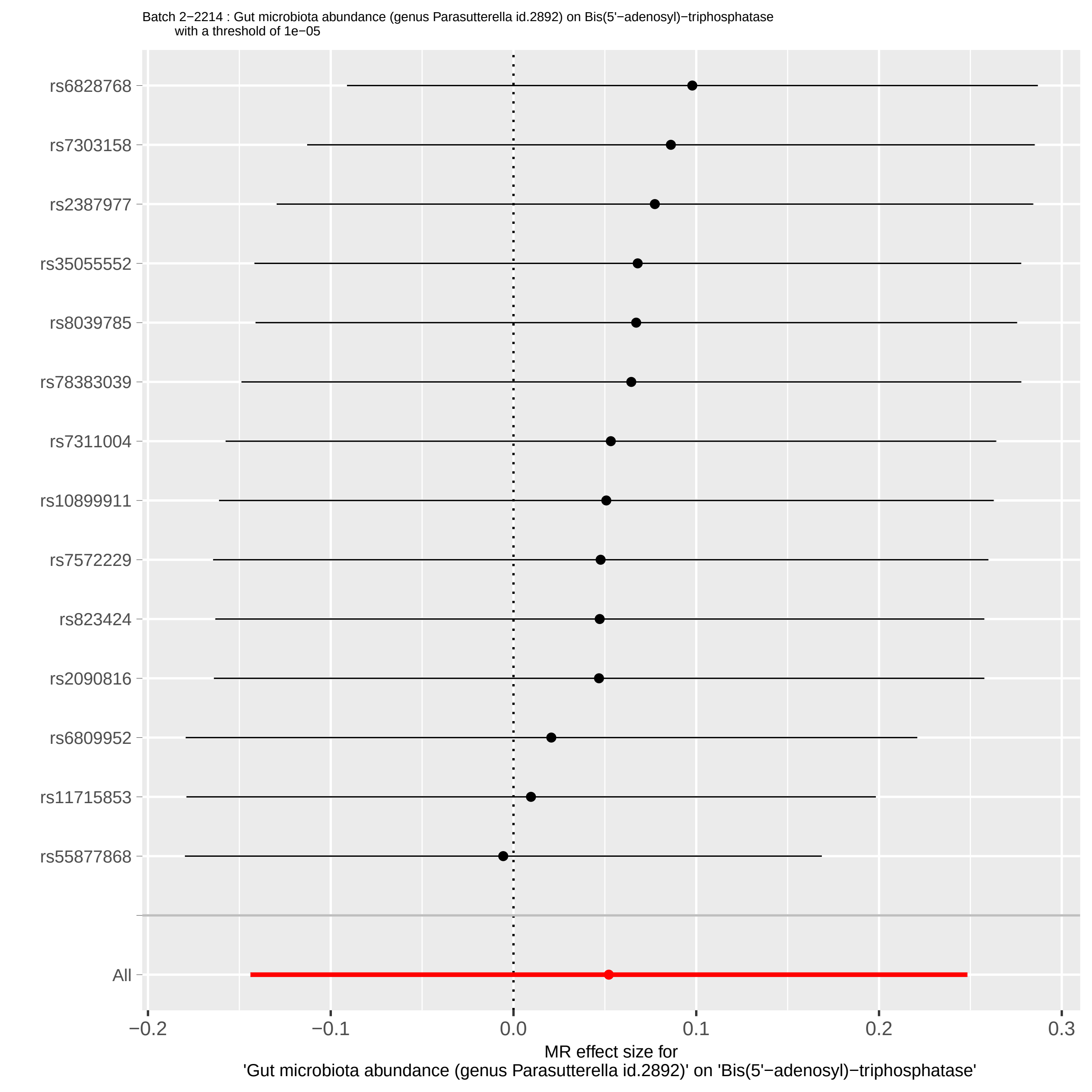

Batch 2−2214 : Gut microbiota abundance (genus Parasutterella id.2892) on Bis(5'−adenosyl)−triphosphatase
with a threshold of 1e−05
rs6828768
rs7303158
rs2387977
rs35055552
rs8039785
rs78383039
rs7311004
rs10899911
rs7572229
rs823424
rs2090816
rs6809952
rs11715853
rs55877868
All
−0.2
−0.1
0.0
0.1
0.2
0.3
MR effect size for
'Gut microbiota abundance (genus Parasutterella id.2892)' on 'Bis(5'−adenosyl)−triphosphatase'

Batch 2−2215 : Gut microbiota abundance (genus Catenibacterium id.2153) on Fibrinogen C domain−containing protein 1
with a threshold of 1e−05

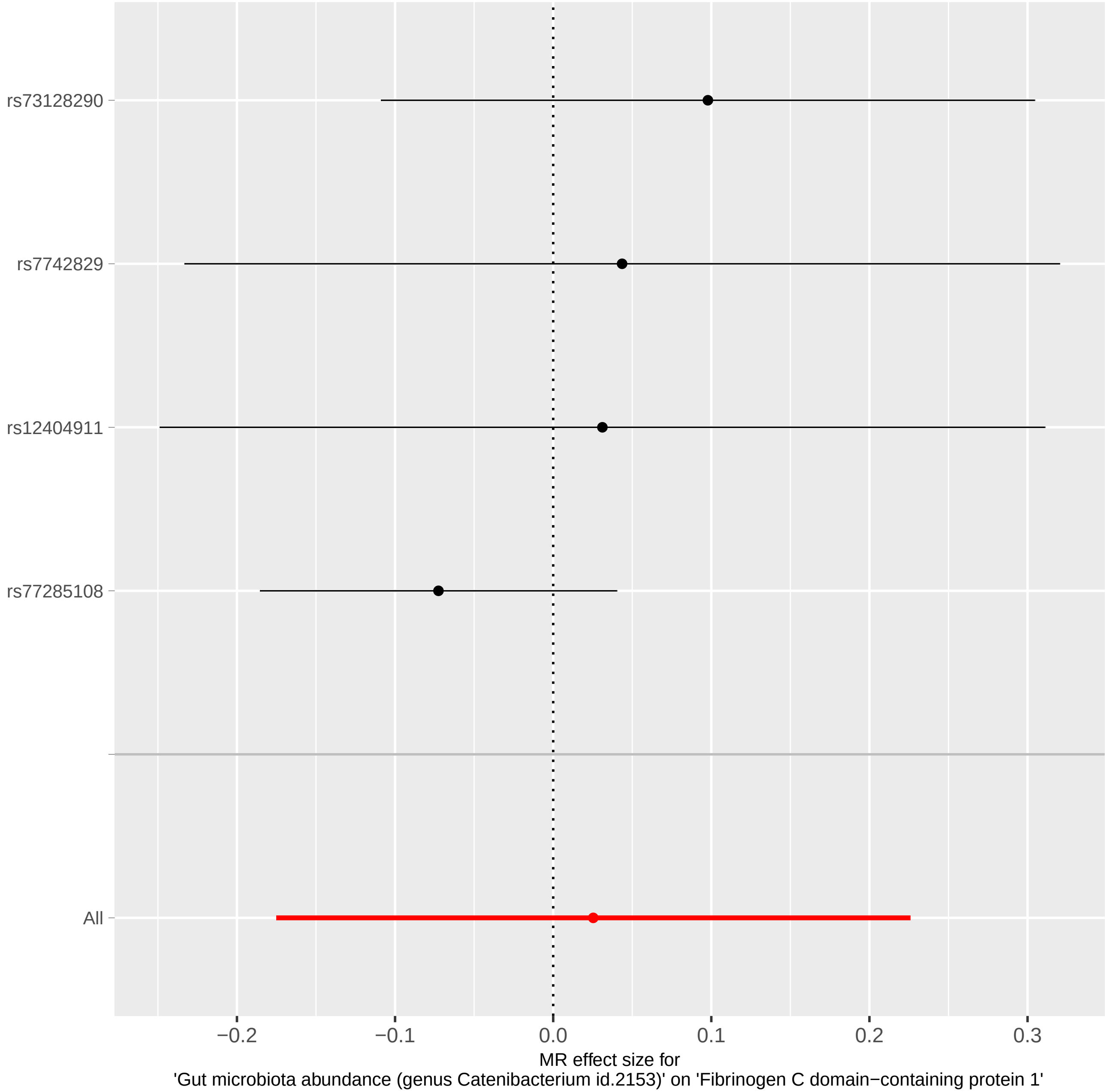

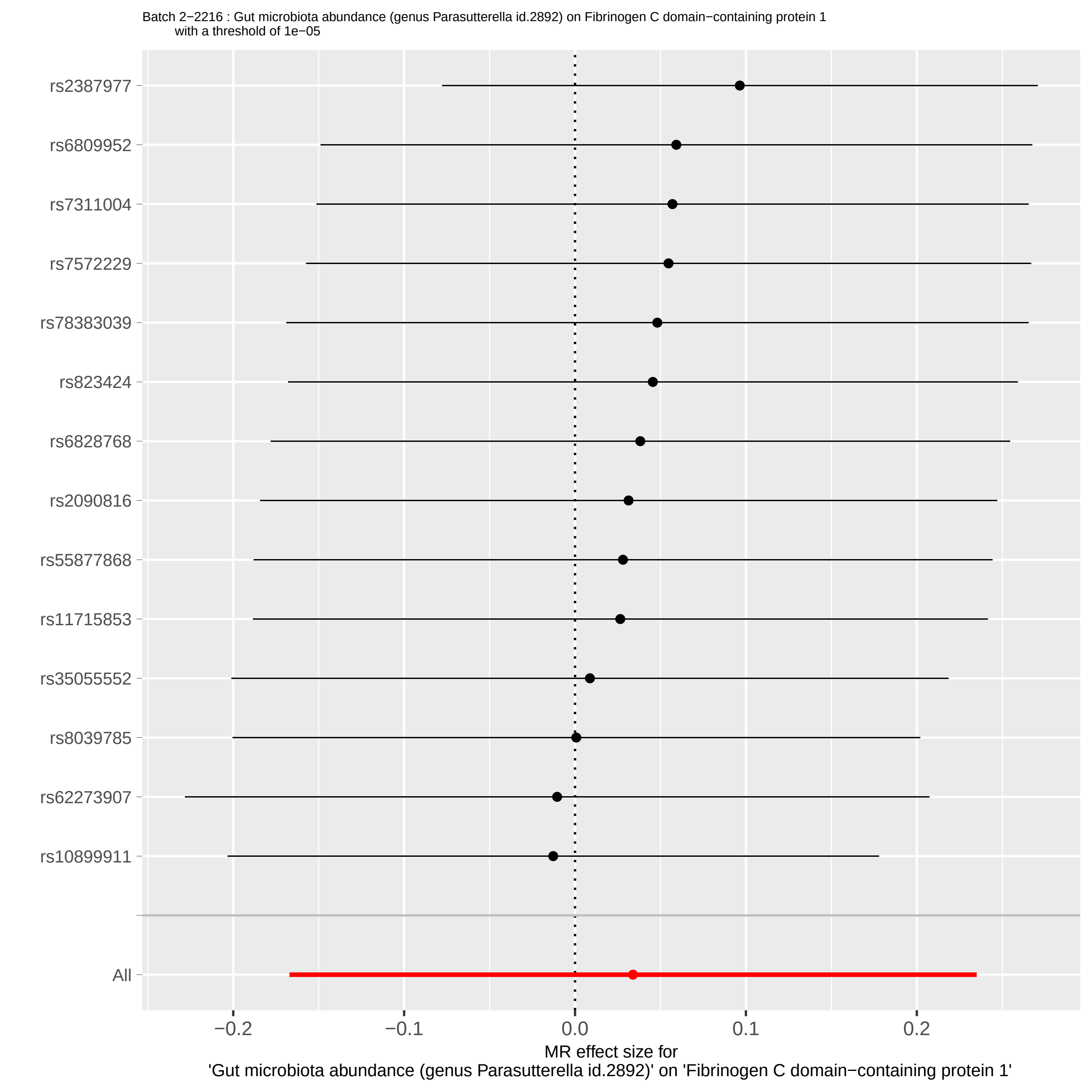
Batch 2−2216 : Gut microbiota abundance (genus Parasutterella id.2892) on Fibrinogen C domain−containing protein 1
with a threshold of 1e−05
rs2387977
rs6809952
rs7311004
rs7572229
rs78383039
rs823424
rs6828768
rs2090816
rs55877868
rs11715853
rs35055552
rs8039785
rs62273907
rs10899911
All
−0.2
−0.1
0.0
0.1
0.2
MR effect size for
'Gut microbiota abundance (genus Parasutterella id.2892)' on 'Fibrinogen C domain−containing protein 1'

Batch 2−2217 : Gut microbiota abundance (genus Catenibacterium id.2153) on Polyphosphoinositide phosphatase
with a threshold of 1e−05

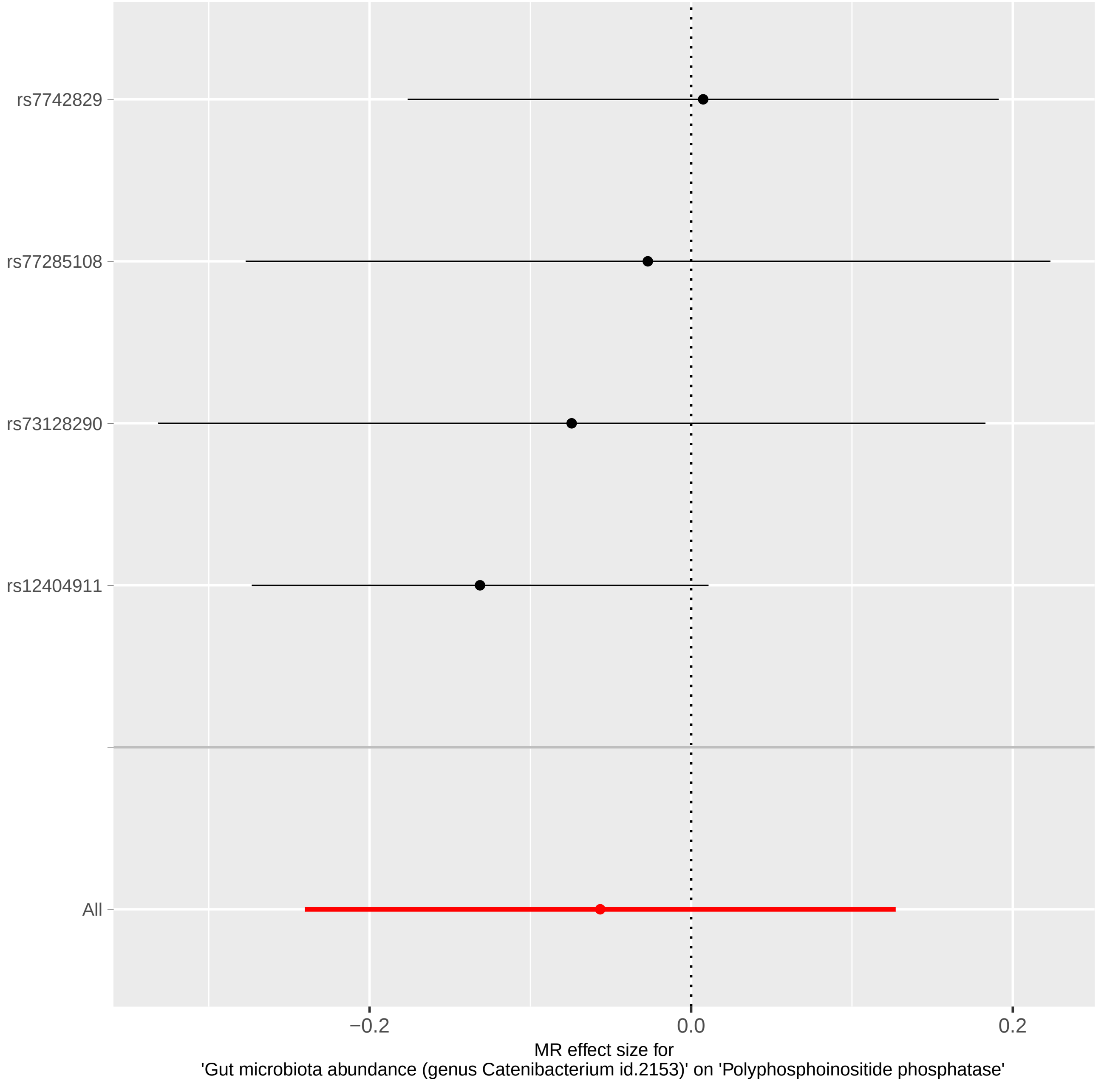

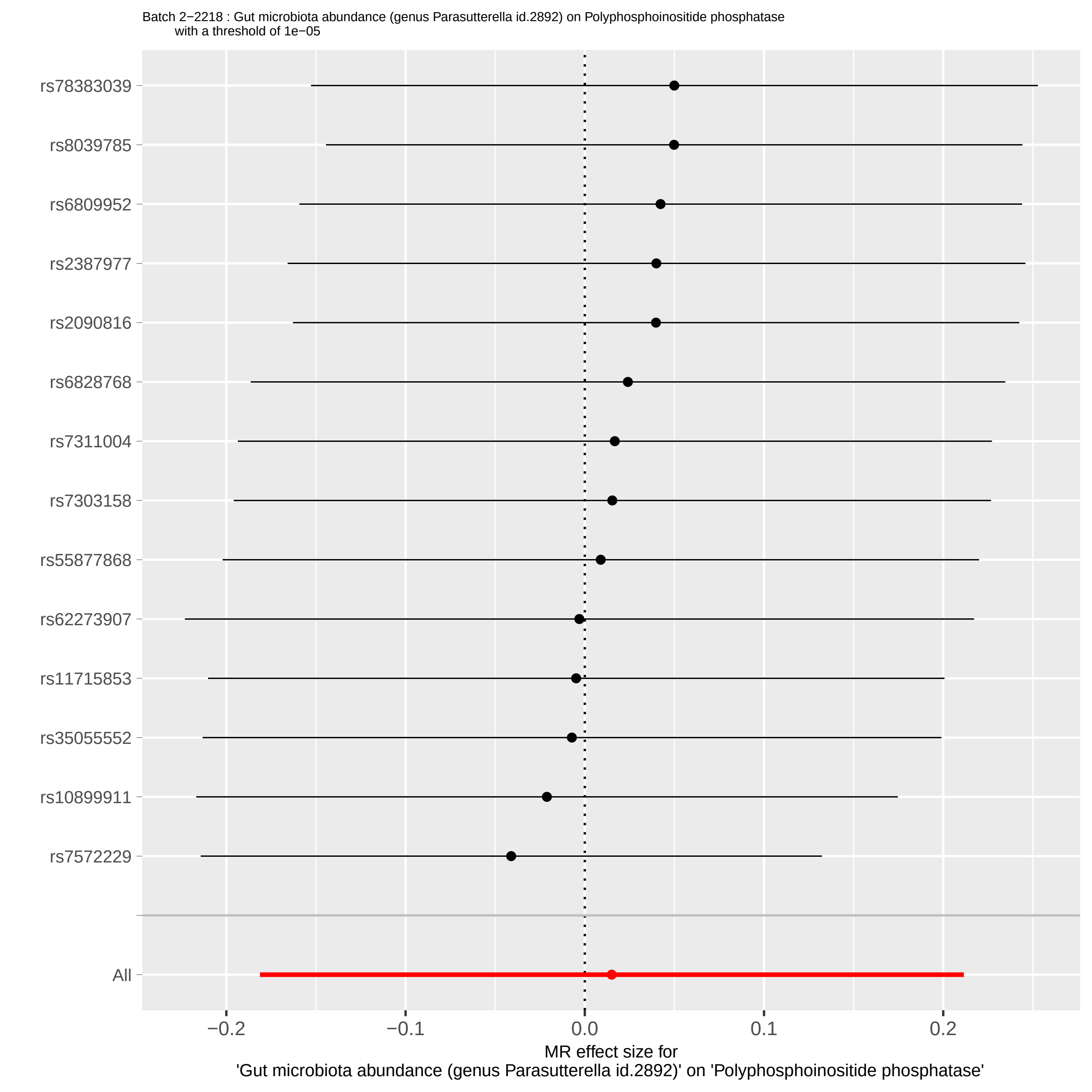

Batch 2−2218 : Gut microbiota abundance (genus Parasutterella id.2892) on Polyphosphoinositide phosphatase
with a threshold of 1e−05
rs78383039
rs8039785
rs6809952
rs2387977
rs2090816
rs6828768
rs7311004
rs7303158
rs55877868
rs62273907
rs11715853
rs35055552
rs10899911
rs7572229
All
−0.2
−0.1
0.0
0.1
0.2
MR effect size for
'Gut microbiota abundance (genus Parasutterella id.2892)' on 'Polyphosphoinositide phosphatase'

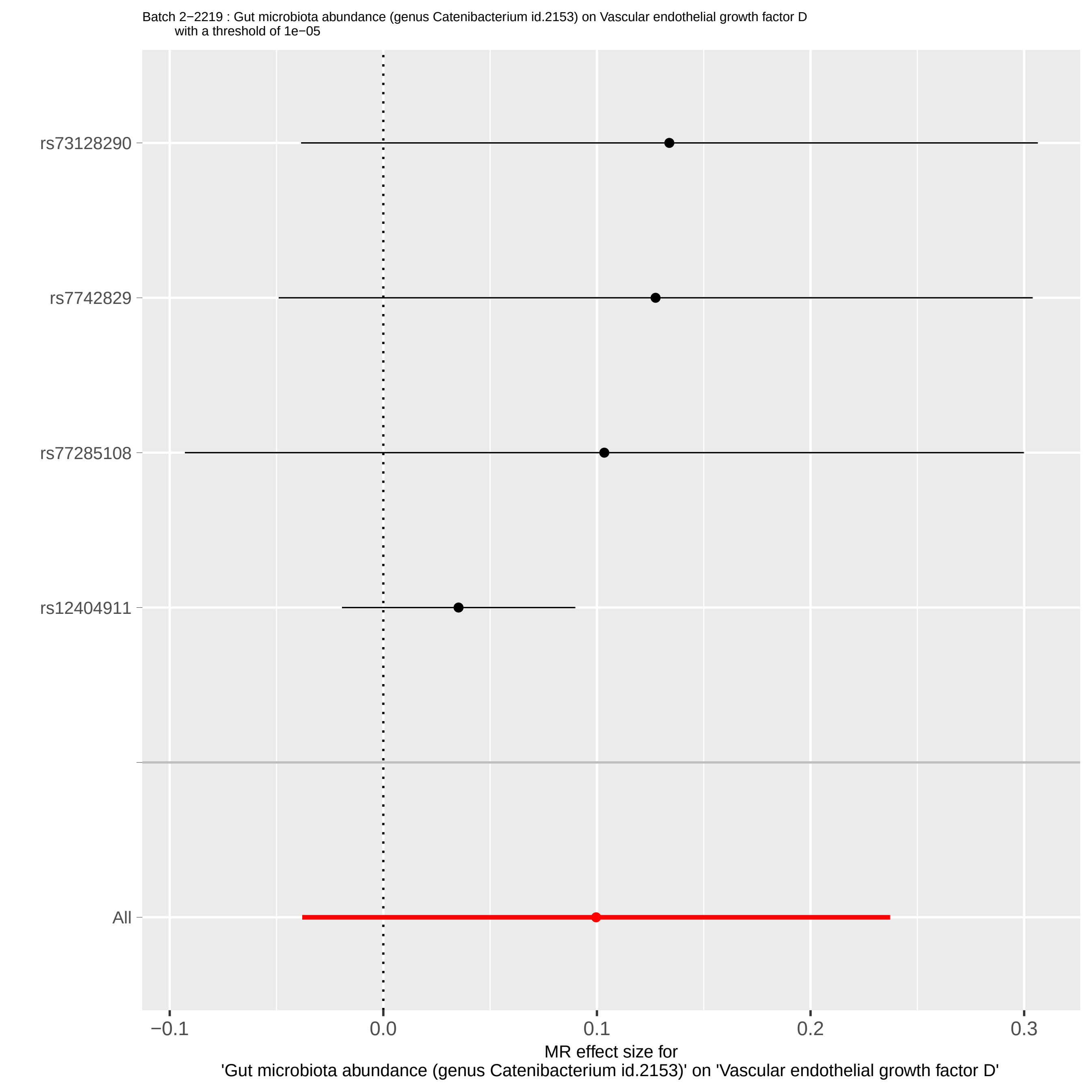
Batch 2−2219 : Gut microbiota abundance (genus Catenibacterium id.2153) on Vascular endothelial growth factor D
with a threshold of 1e−05
rs73128290
rs7742829
rs77285108
rs12404911
All
−0.1
0.0
0.1
0.2
0.3
MR effect size for
'Gut microbiota abundance (genus Catenibacterium id.2153)' on 'Vascular endothelial growth factor D'

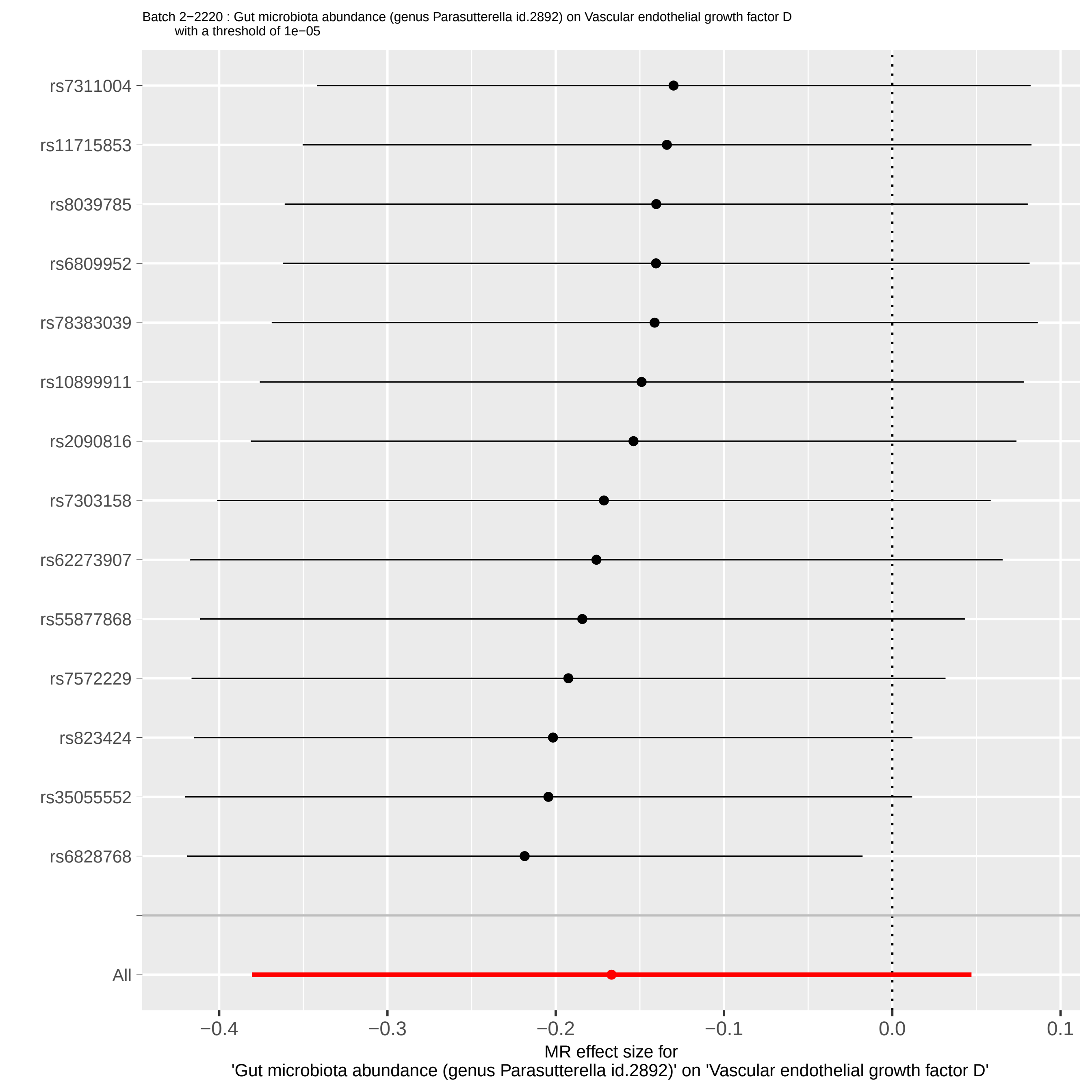

Batch 2−2220 : Gut microbiota abundance (genus Parasutterella id.2892) on Vascular endothelial growth factor D
with a threshold of 1e−05
rs7311004
rs11715853
rs8039785
rs6809952
rs78383039
rs10899911
rs2090816
rs7303158
rs62273907
rs55877868
rs7572229
rs823424
rs35055552
rs6828768
All
−0.4
−0.3
−0.2
−0.1
0.0
0.1
MR effect size for
'Gut microbiota abundance (genus Parasutterella id.2892)' on 'Vascular endothelial growth factor D'

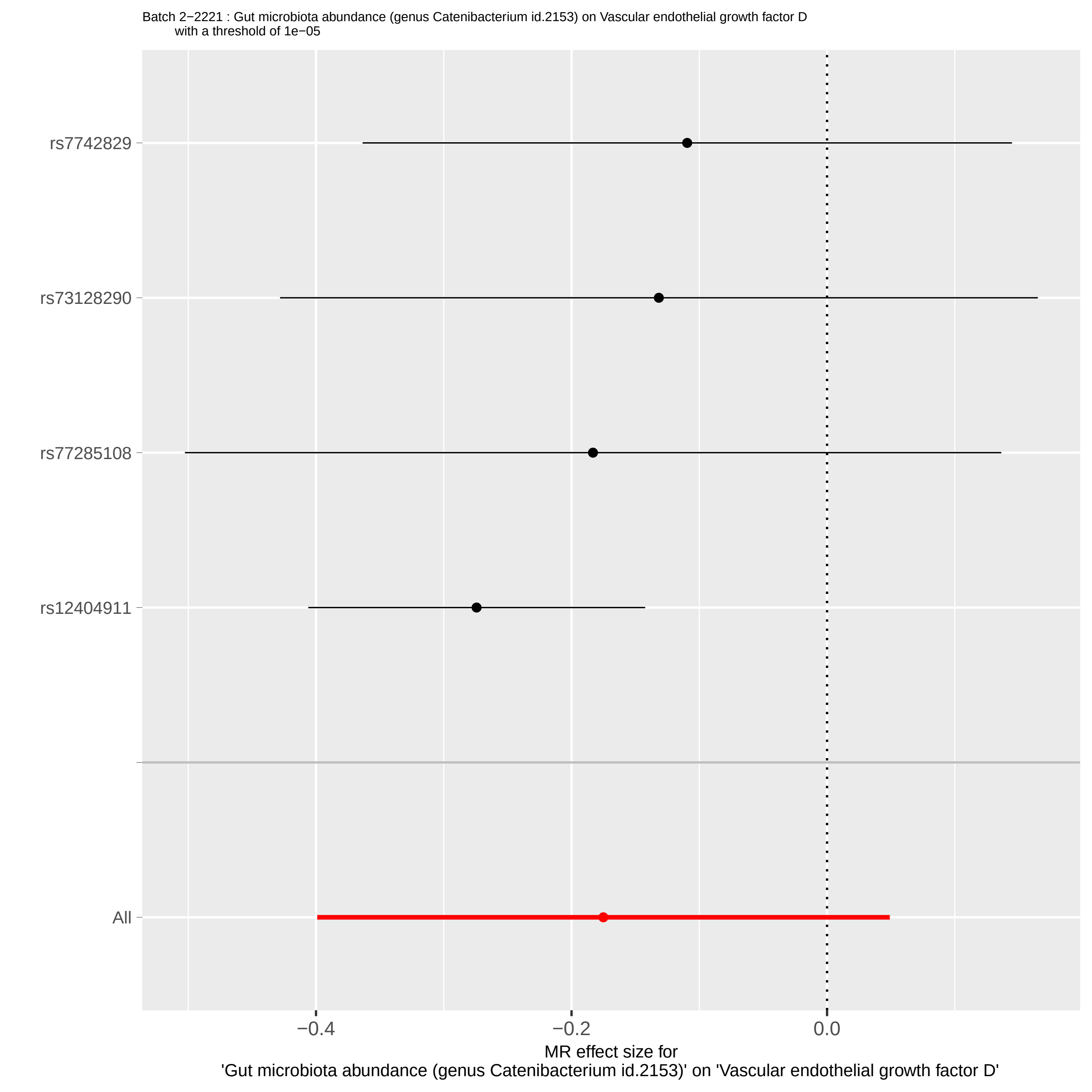
Batch 2−2221 : Gut microbiota abundance (genus Catenibacterium id.2153) on Vascular endothelial growth factor D
with a threshold of 1e−05
rs7742829
rs73128290
rs77285108
rs12404911
All
−0.4
−0.2
0.0
MR effect size for
'Gut microbiota abundance (genus Catenibacterium id.2153)' on 'Vascular endothelial growth factor D'

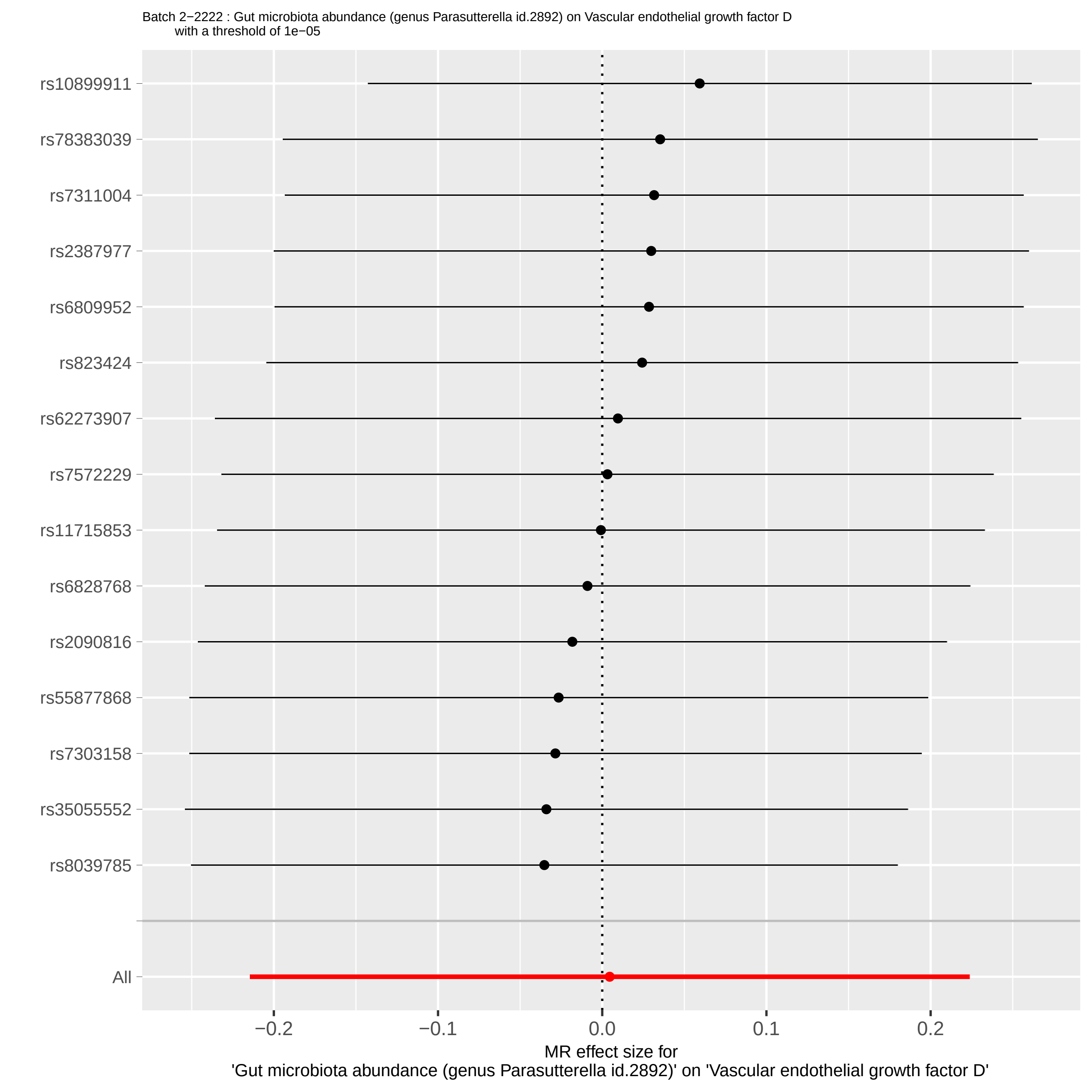

Batch 2−2222 : Gut microbiota abundance (genus Parasutterella id.2892) on Vascular endothelial growth factor D
with a threshold of 1e−05
rs10899911
rs78383039
rs7311004
rs2387977
rs6809952
rs823424
rs62273907
rs7572229
rs11715853
rs6828768
rs2090816
rs55877868
rs7303158
rs35055552
rs8039785
All
−0.2
−0.1
0.0
0.1
0.2
MR effect size for
'Gut microbiota abundance (genus Parasutterella id.2892)' on 'Vascular endothelial growth factor D'

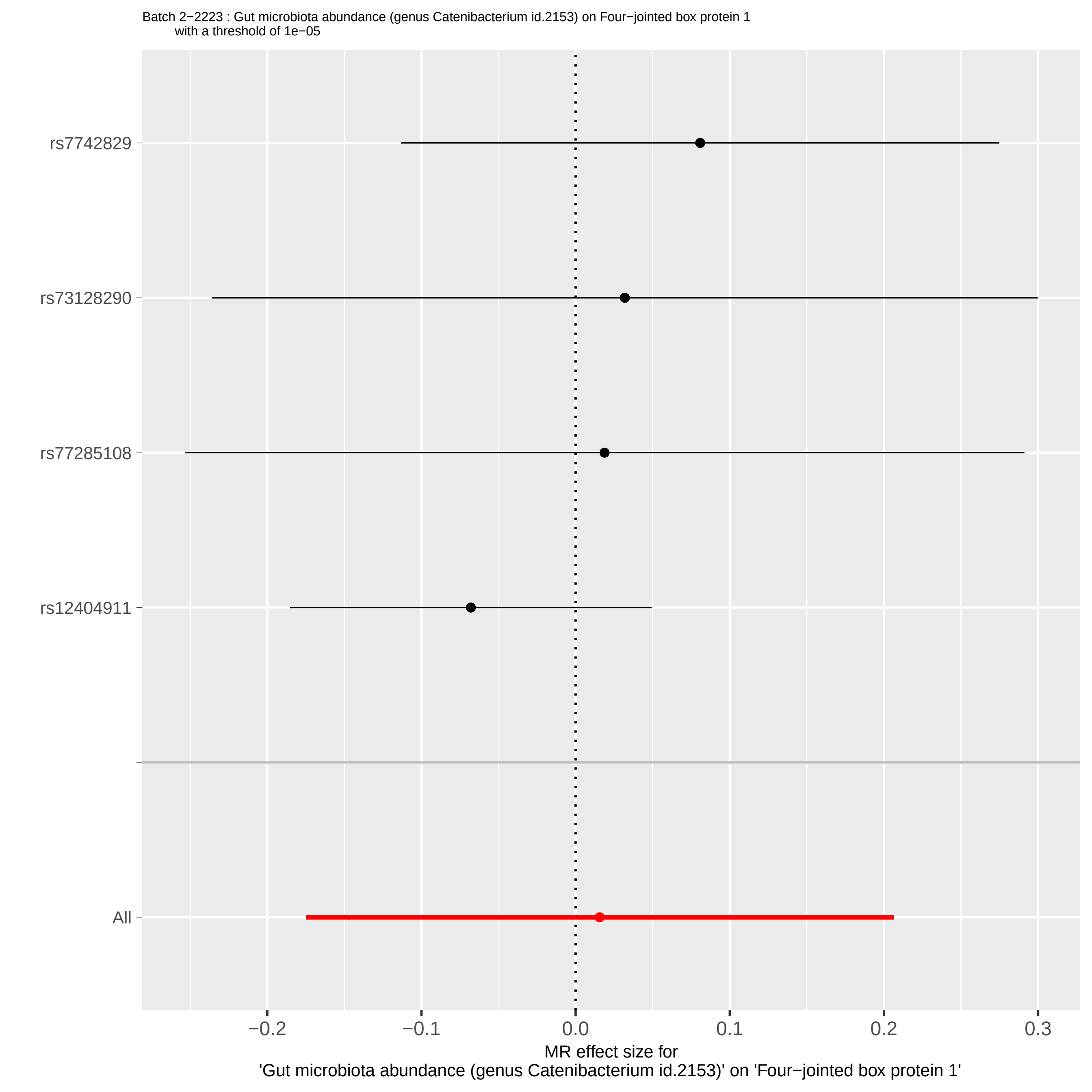

Batch 2−2223 : Gut microbiota abundance (genus Catenibacterium id.2153) on Four−jointed box protein 1
with a threshold of 1e−05
rs7742829
rs73128290
rs77285108
rs12404911
All
−0.2
−0.1
0.0
0.1
0.2
0.3
MR effect size for
'Gut microbiota abundance (genus Catenibacterium id.2153)' on 'Four−jointed box protein 1'

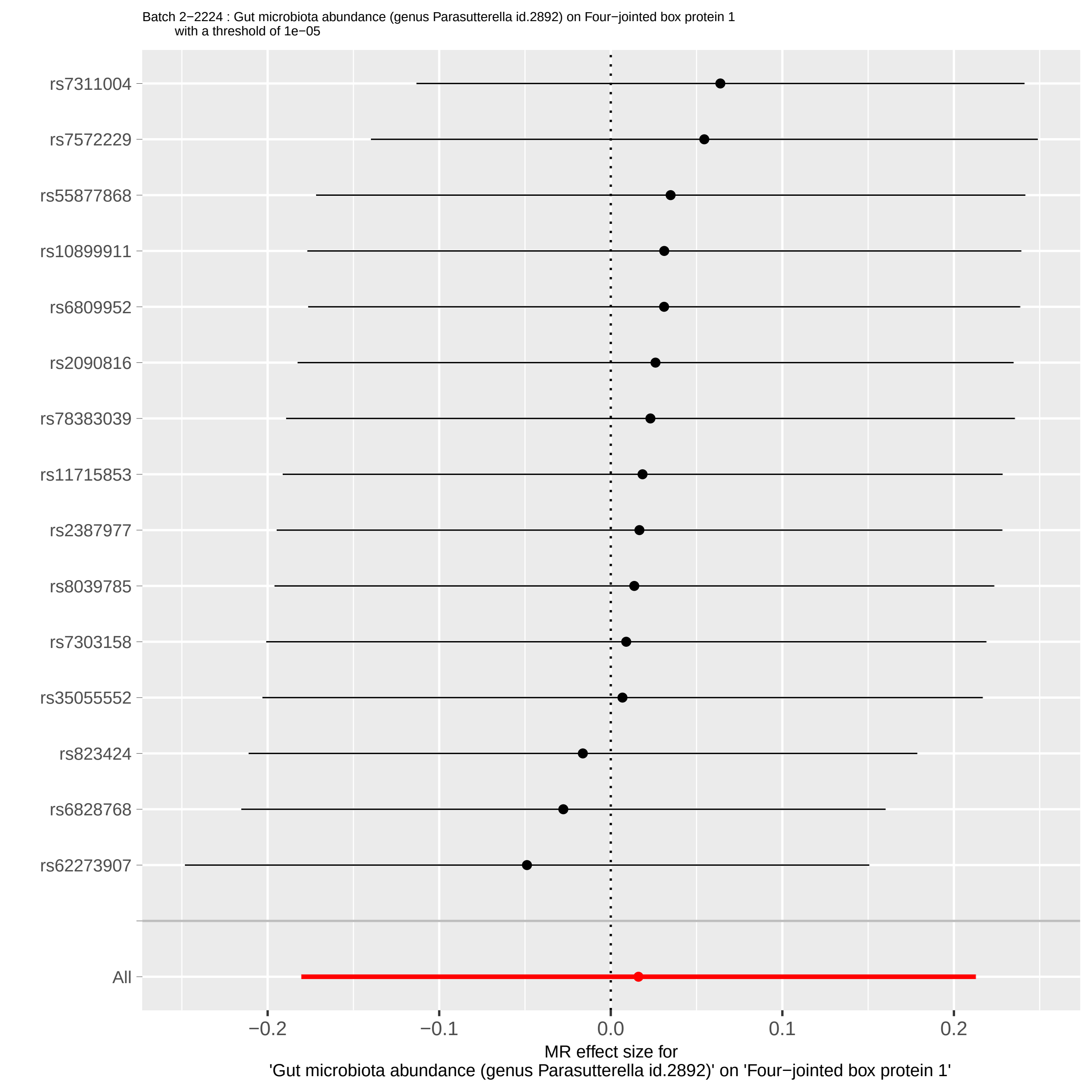

Batch 2−2224 : Gut microbiota abundance (genus Parasutterella id.2892) on Four−jointed box protein 1
with a threshold of 1e−05
rs7311004
rs7572229
rs55877868
rs10899911
rs6809952
rs2090816
rs78383039
rs11715853
rs2387977
rs8039785
rs7303158
rs35055552
rs823424
rs6828768
rs62273907
All
−0.2
−0.1
0.0
0.1
0.2
MR effect size for
'Gut microbiota abundance (genus Parasutterella id.2892)' on 'Four−jointed box protein 1'

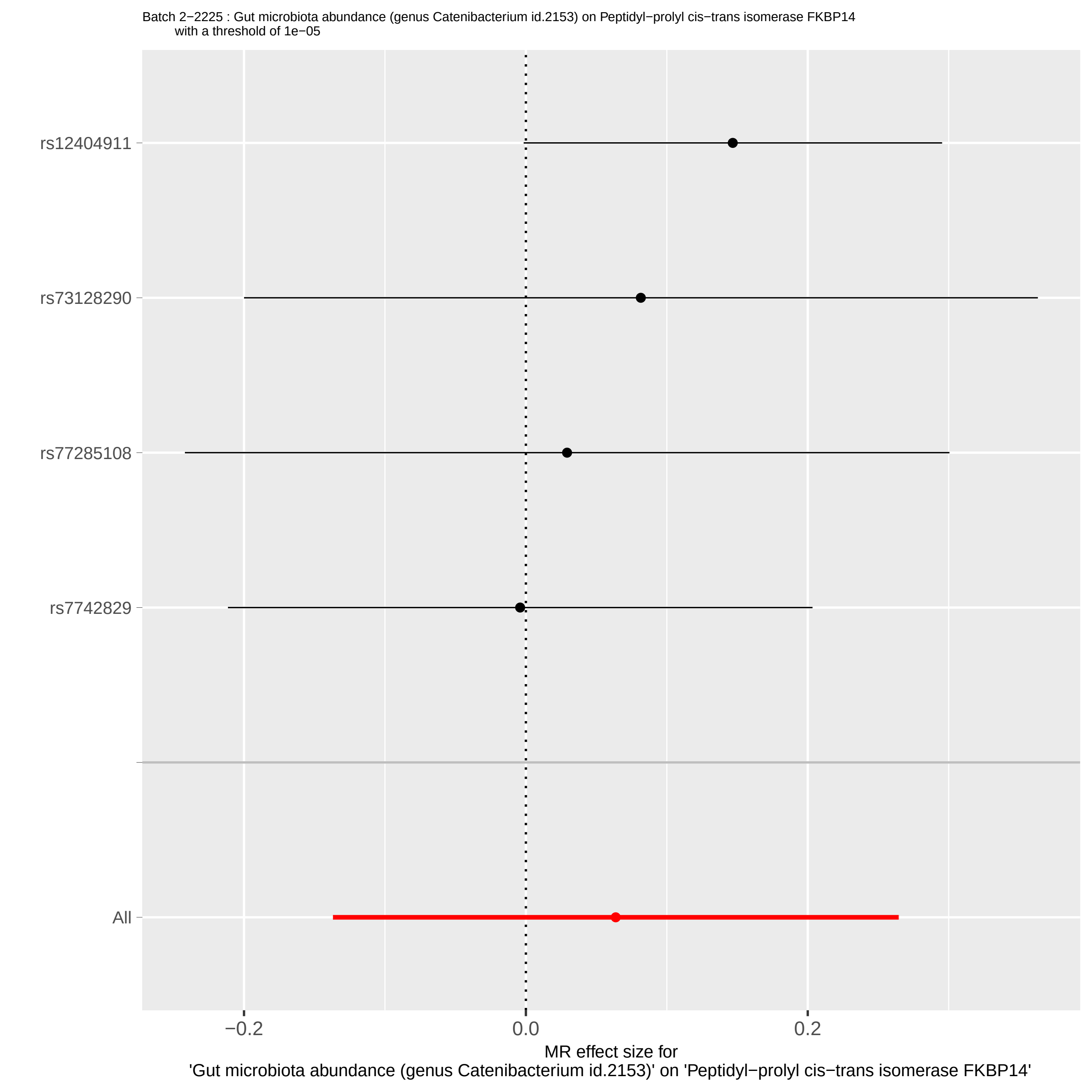

Batch 2−2225 : Gut microbiota abundance (genus Catenibacterium id.2153) on Peptidyl−prolyl cis−trans isomerase FKBP14
with a threshold of 1e−05
rs12404911
rs73128290
rs77285108
rs7742829
All
−0.2
0.0
0.2
MR effect size for
'Gut microbiota abundance (genus Catenibacterium id.2153)' on 'Peptidyl−prolyl cis−trans isomerase FKBP14'

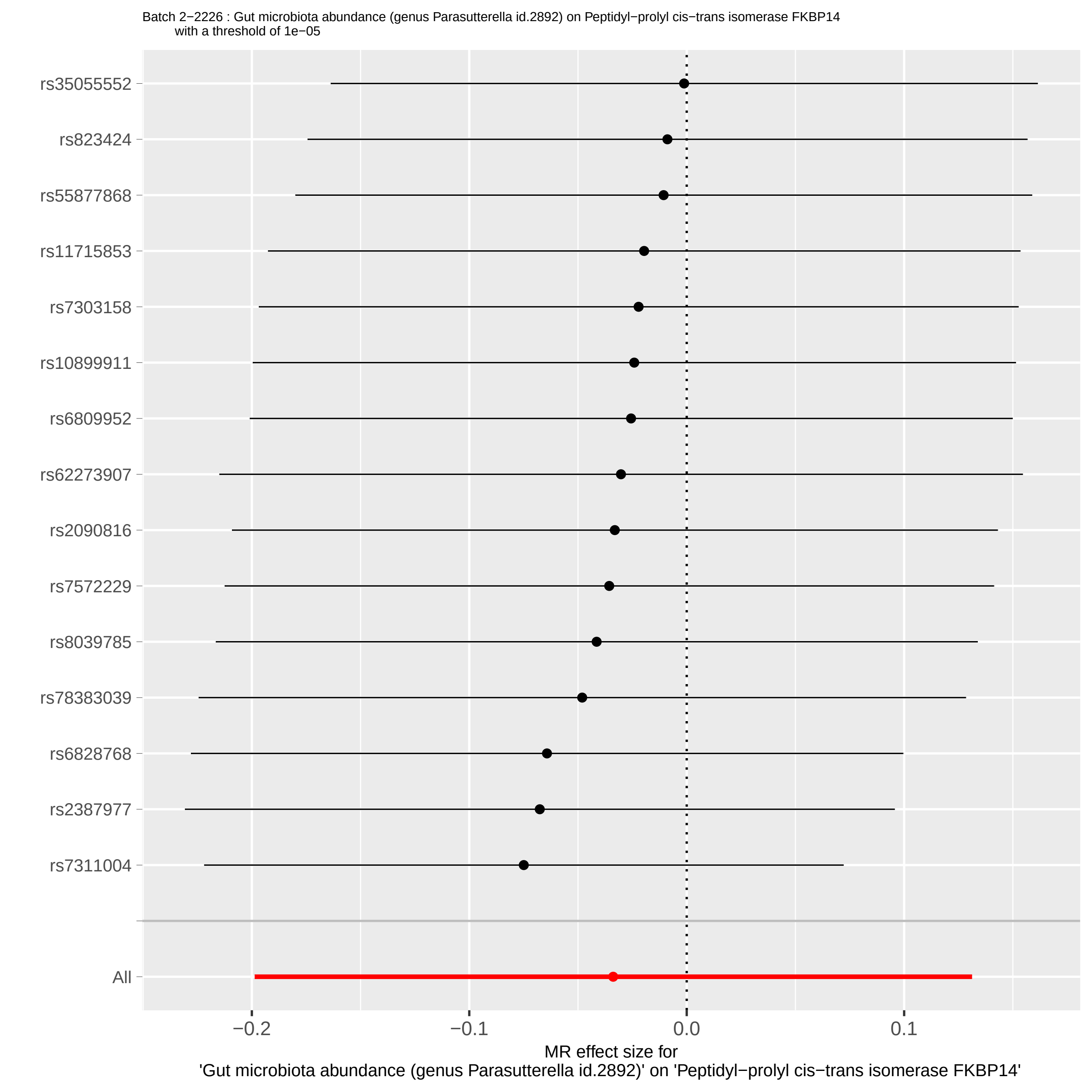

Batch 2−2226 : Gut microbiota abundance (genus Parasutterella id.2892) on Peptidyl−prolyl cis−trans isomerase FKBP14
with a threshold of 1e−05
rs35055552
rs823424
rs55877868
rs11715853
rs7303158
rs10899911
rs6809952
rs62273907
rs2090816
rs7572229
rs8039785
rs78383039
rs6828768
rs2387977
rs7311004
All
−0.2
−0.1
0.0
0.1
MR effect size for
'Gut microbiota abundance (genus Parasutterella id.2892)' on 'Peptidyl−prolyl cis−trans isomerase FKBP14'

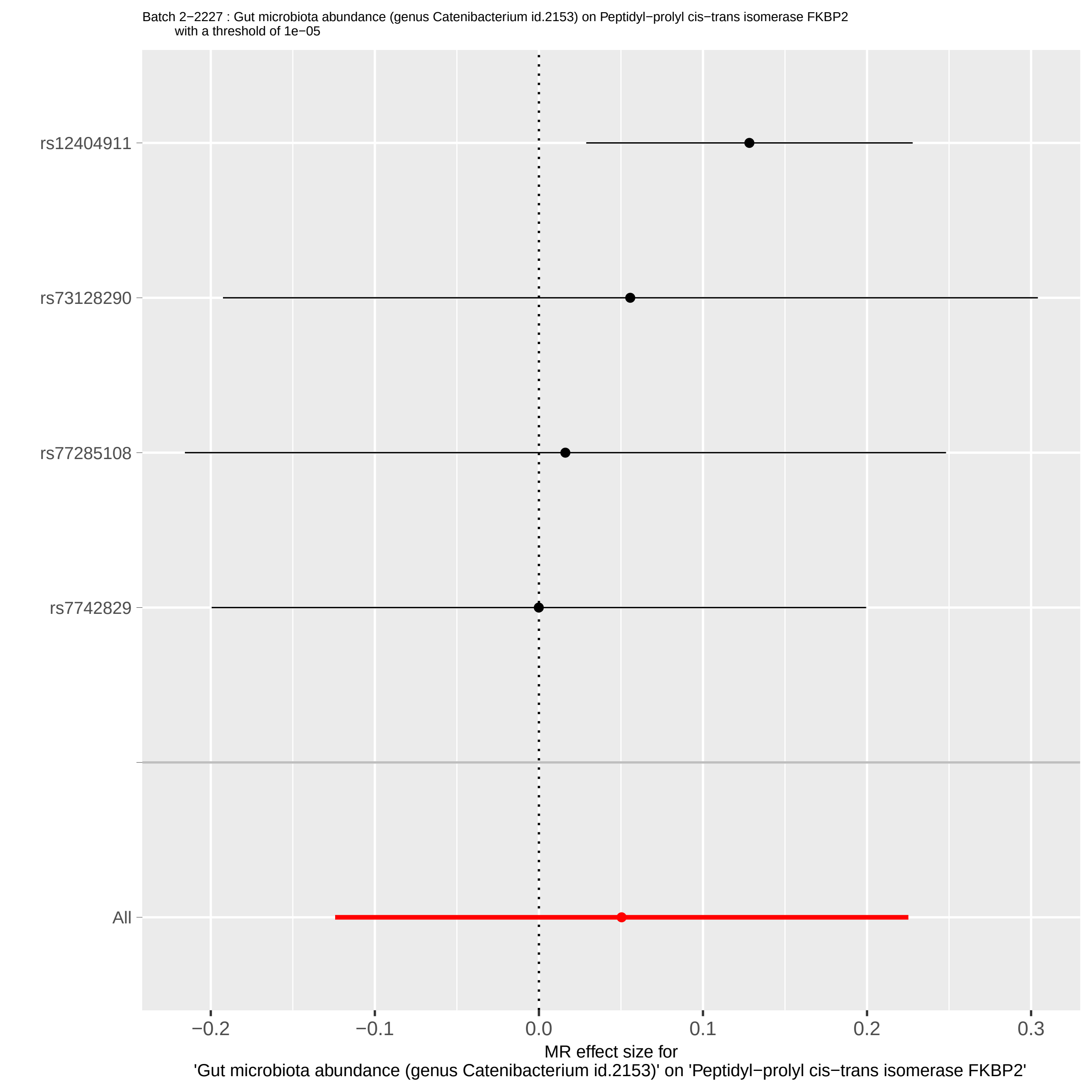
Batch 2−2227 : Gut microbiota abundance (genus Catenibacterium id.2153) on Peptidyl−prolyl cis−trans isomerase FKBP2
with a threshold of 1e−05
rs12404911
rs73128290
rs77285108
rs7742829
All
−0.2
−0.1
0.0
0.1
0.2
0.3
MR effect size for
'Gut microbiota abundance (genus Catenibacterium id.2153)' on 'Peptidyl−prolyl cis−trans isomerase FKBP2'

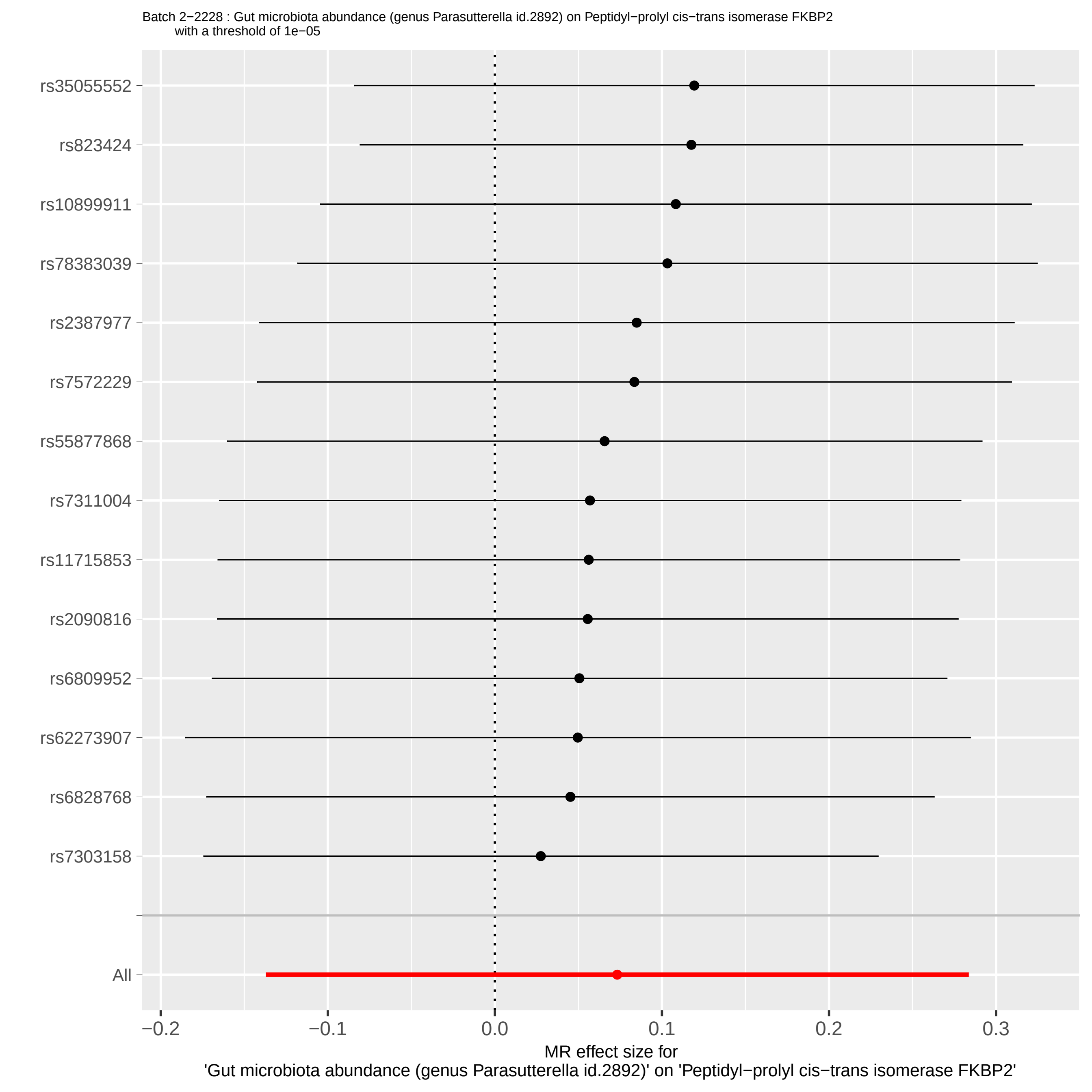

Batch 2−2228 : Gut microbiota abundance (genus Parasutterella id.2892) on Peptidyl−prolyl cis−trans isomerase FKBP2
with a threshold of 1e−05
rs35055552
rs823424
rs10899911
rs78383039
rs2387977
rs7572229
rs55877868
rs7311004
rs11715853
rs2090816
rs6809952
rs62273907
rs6828768
rs7303158
All
−0.2
−0.1
0.0
0.1
0.2
0.3
MR effect size for
'Gut microbiota abundance (genus Parasutterella id.2892)' on 'Peptidyl−prolyl cis−trans isomerase FKBP2'

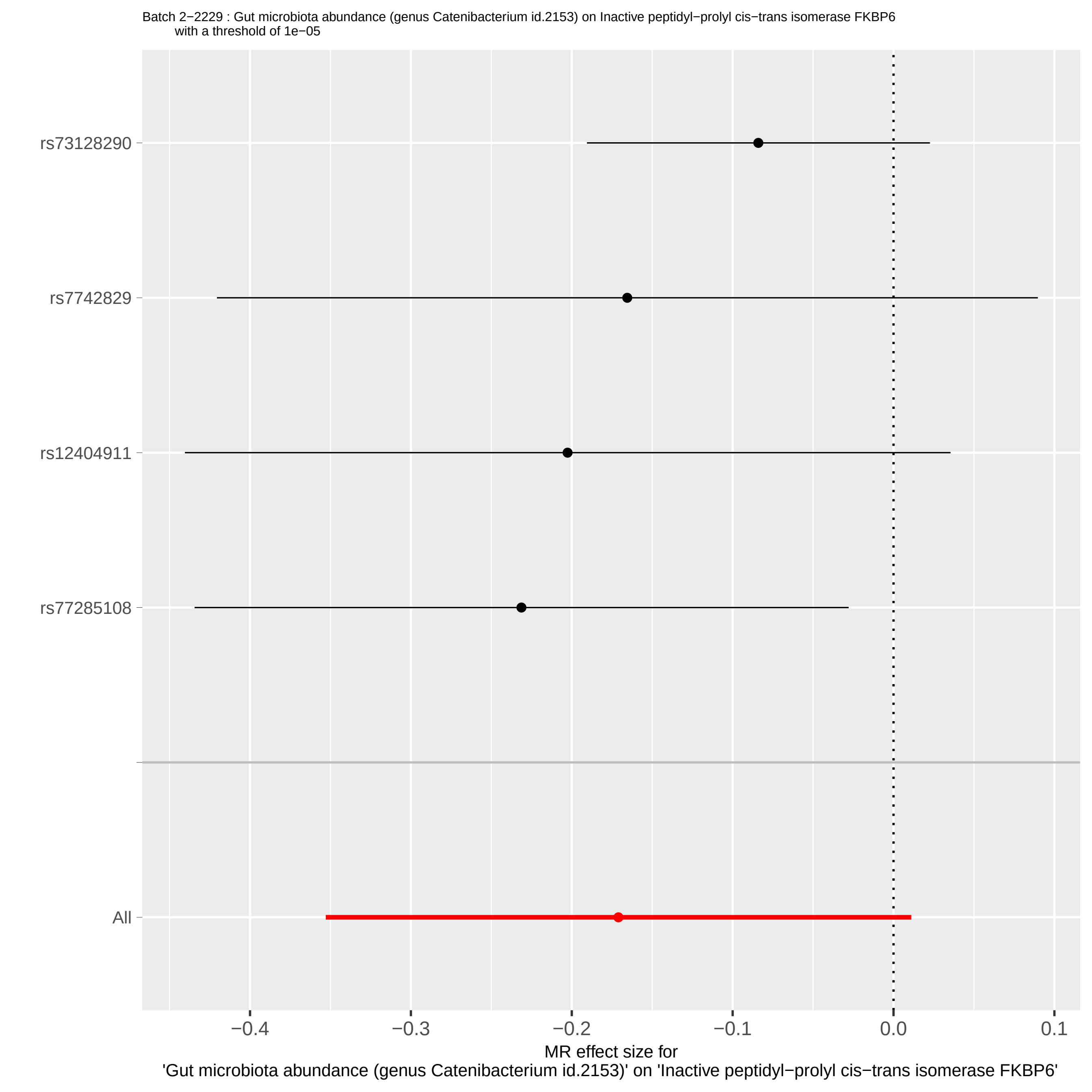
Batch 2−2229 : Gut microbiota abundance (genus Catenibacterium id.2153) on Inactive peptidyl−prolyl cis−trans isomerase FKBP6
with a threshold of 1e−05
rs73128290
rs7742829
rs12404911
rs77285108
All
−0.4
−0.3
−0.2
−0.1
0.0
0.1
MR effect size for
'Gut microbiota abundance (genus Catenibacterium id.2153)' on 'Inactive peptidyl−prolyl cis−trans isomerase FKBP6'

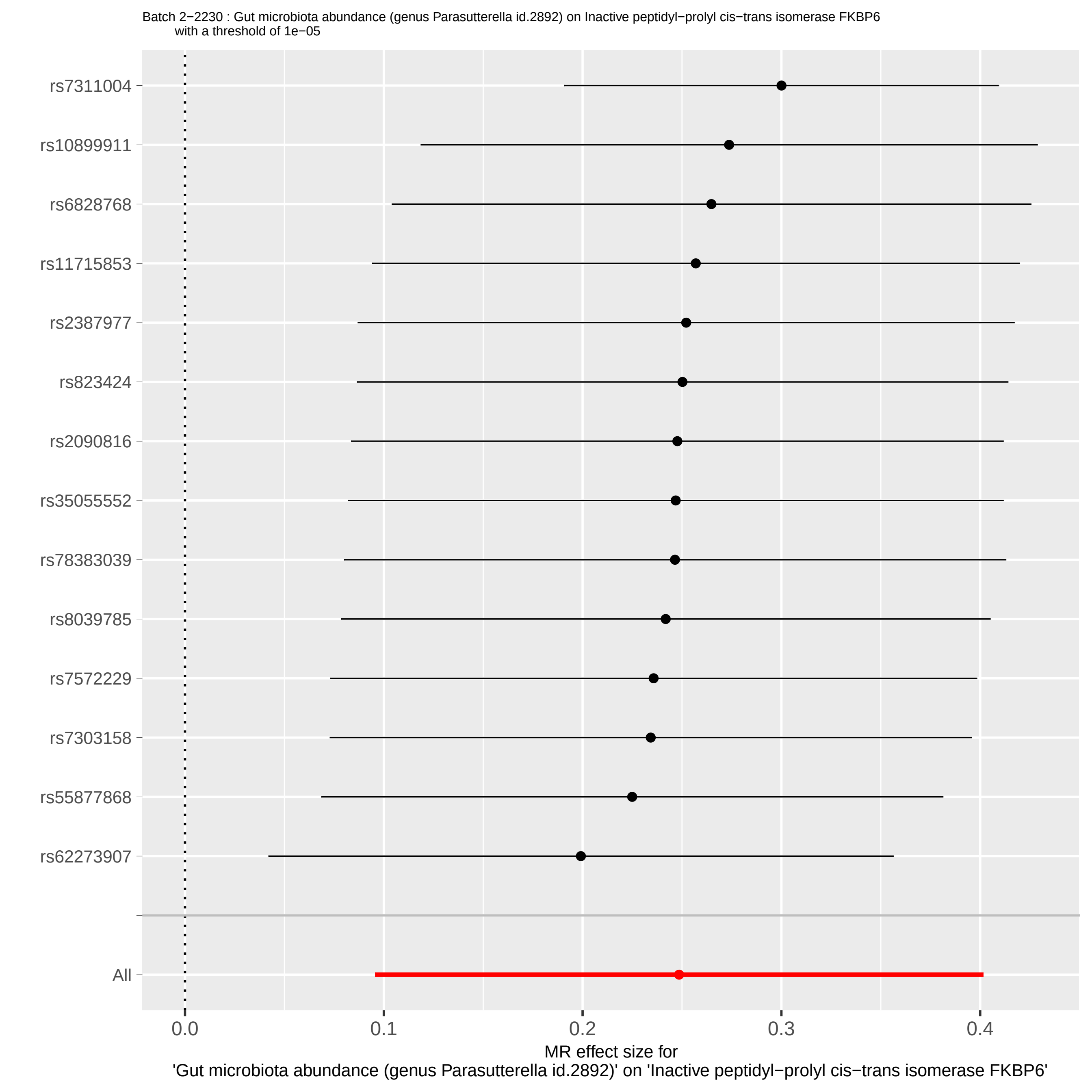

Batch 2−2230 : Gut microbiota abundance (genus Parasutterella id.2892) on Inactive peptidyl−prolyl cis−trans isomerase FKBP6
with a threshold of 1e−05
rs7311004
rs10899911
rs6828768
rs11715853
rs2387977
rs823424
rs2090816
rs35055552
rs78383039
rs8039785
rs7572229
rs7303158
rs55877868
rs62273907
All
0.0
0.1
0.2
0.3
0.4
MR effect size for
'Gut microbiota abundance (genus Parasutterella id.2892)' on 'Inactive peptidyl−prolyl cis−trans isomerase FKBP6'

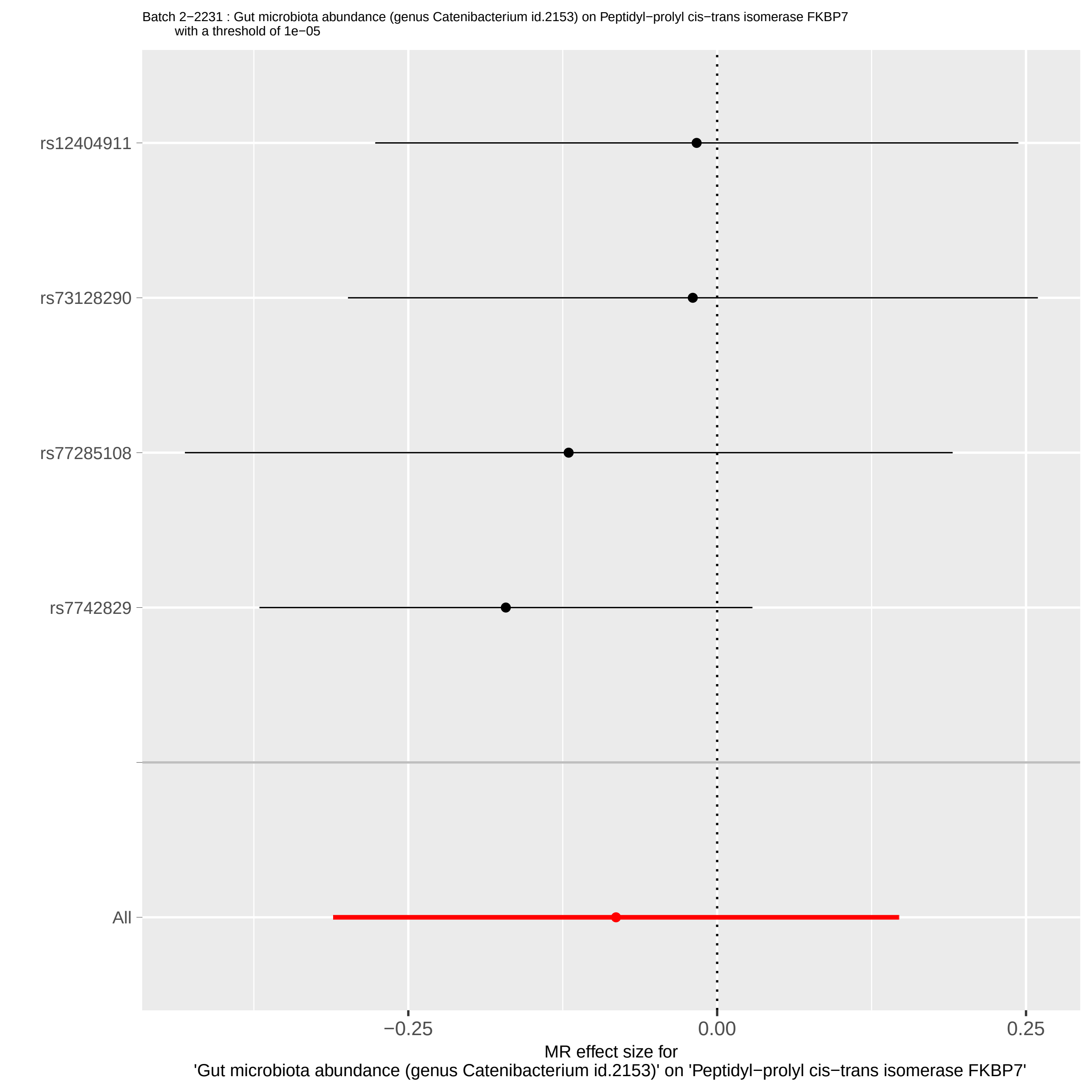
Batch 2−2231 : Gut microbiota abundance (genus Catenibacterium id.2153) on Peptidyl−prolyl cis−trans isomerase FKBP7
with a threshold of 1e−05
rs12404911
rs73128290
rs77285108
rs7742829
All
−0.25
0.00
0.25
MR effect size for
'Gut microbiota abundance (genus Catenibacterium id.2153)' on 'Peptidyl−prolyl cis−trans isomerase FKBP7'

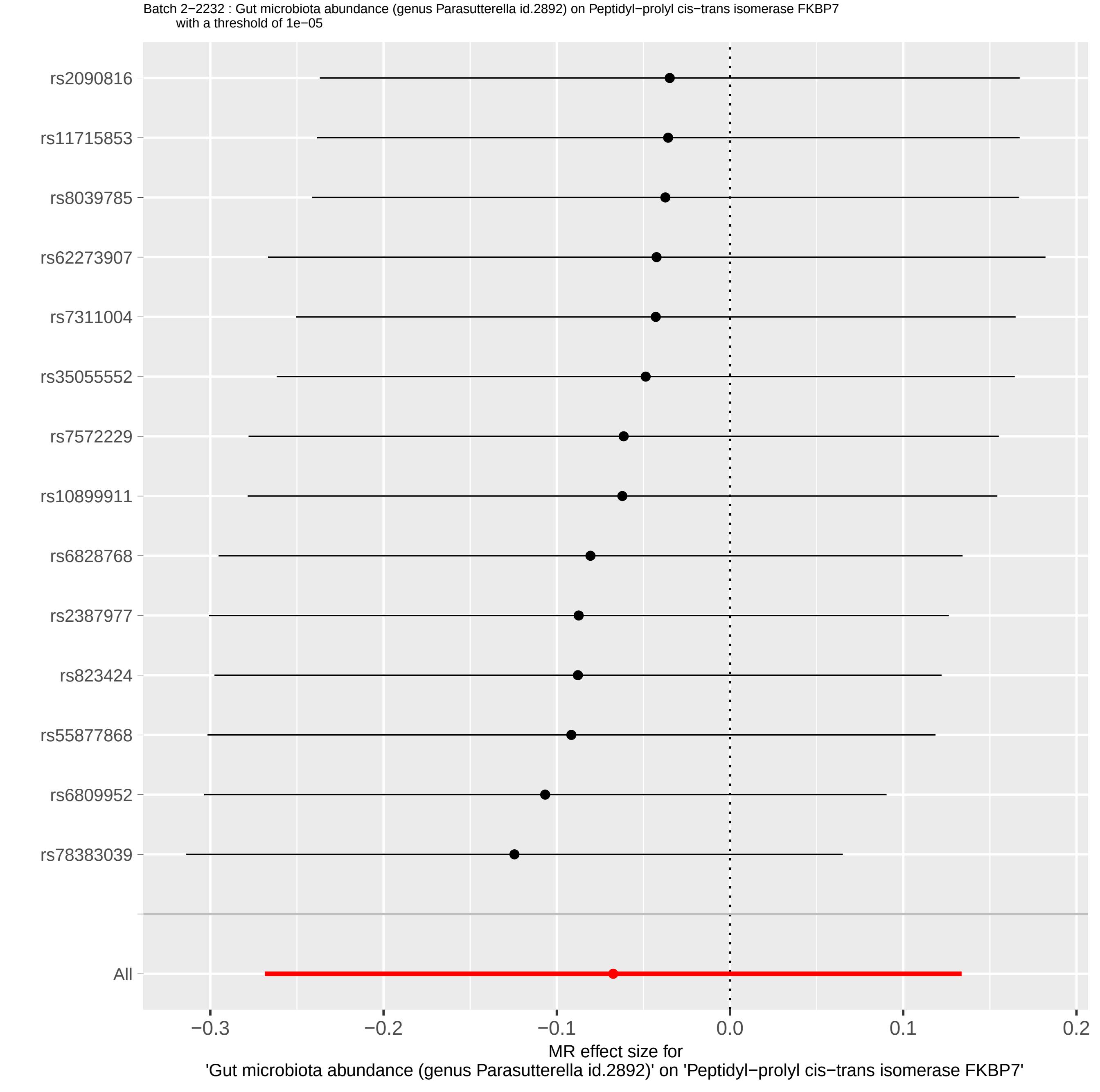
Batch 2−2232 : Gut microbiota abundance (genus Parasutterella id.2892) on Peptidyl−prolyl cis−trans isomerase FKBP7
with a threshold of 1e−05
rs2090816
rs11715853
rs8039785
rs62273907
rs7311004
rs35055552
rs7572229
rs10899911
rs6828768
rs2387977
rs823424
rs55877868
rs6809952
rs78383039
All
−0.3
−0.2
−0.1
0.0
0.1
0.2
MR effect size for
'Gut microbiota abundance (genus Parasutterella id.2892)' on 'Peptidyl−prolyl cis−trans isomerase FKBP7'

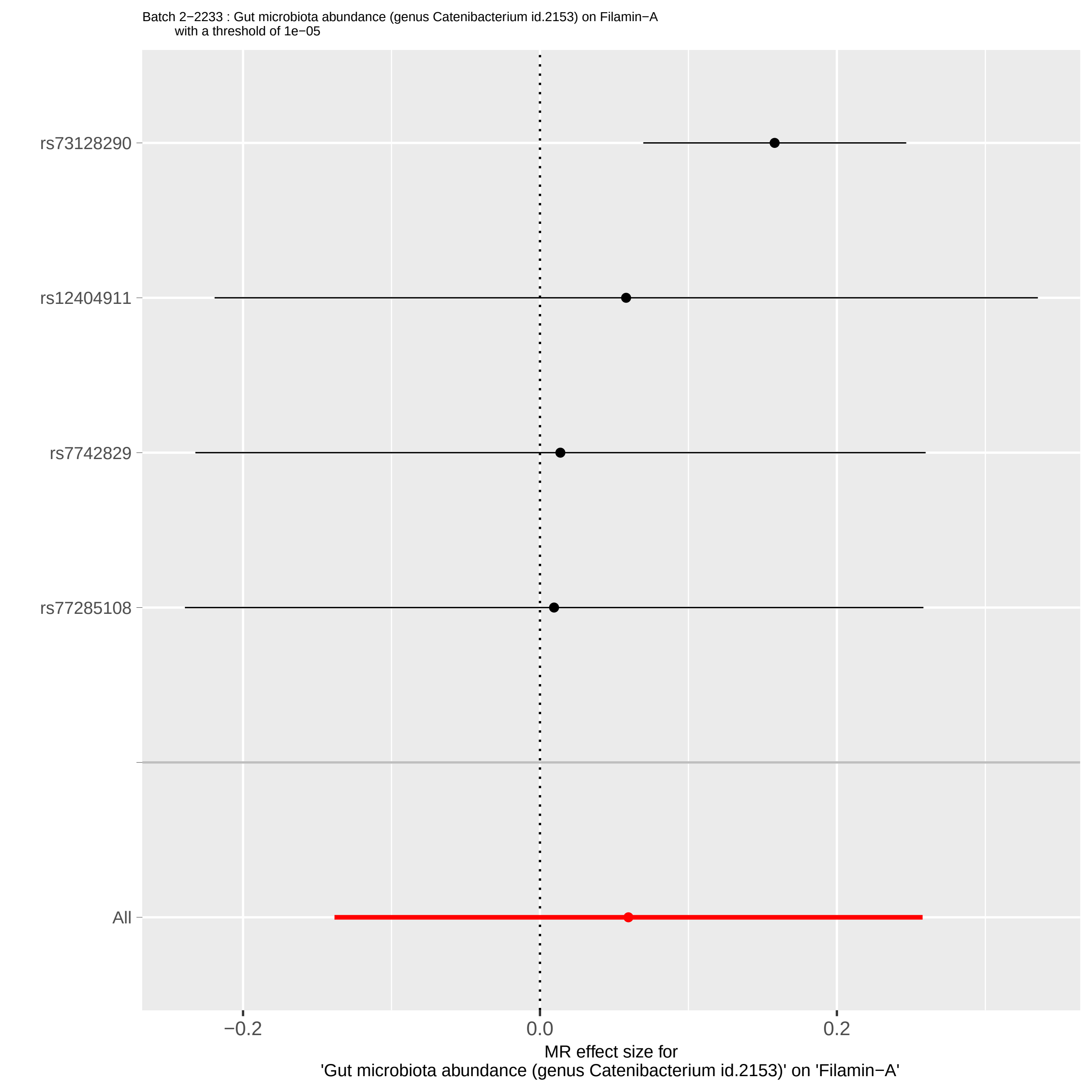
Batch 2−2233 : Gut microbiota abundance (genus Catenibacterium id.2153) on Filamin−A
with a threshold of 1e−05
rs73128290
rs12404911
rs7742829
rs77285108
All
−0.2
0.0
0.2
MR effect size for
'Gut microbiota abundance (genus Catenibacterium id.2153)' on 'Filamin−A'

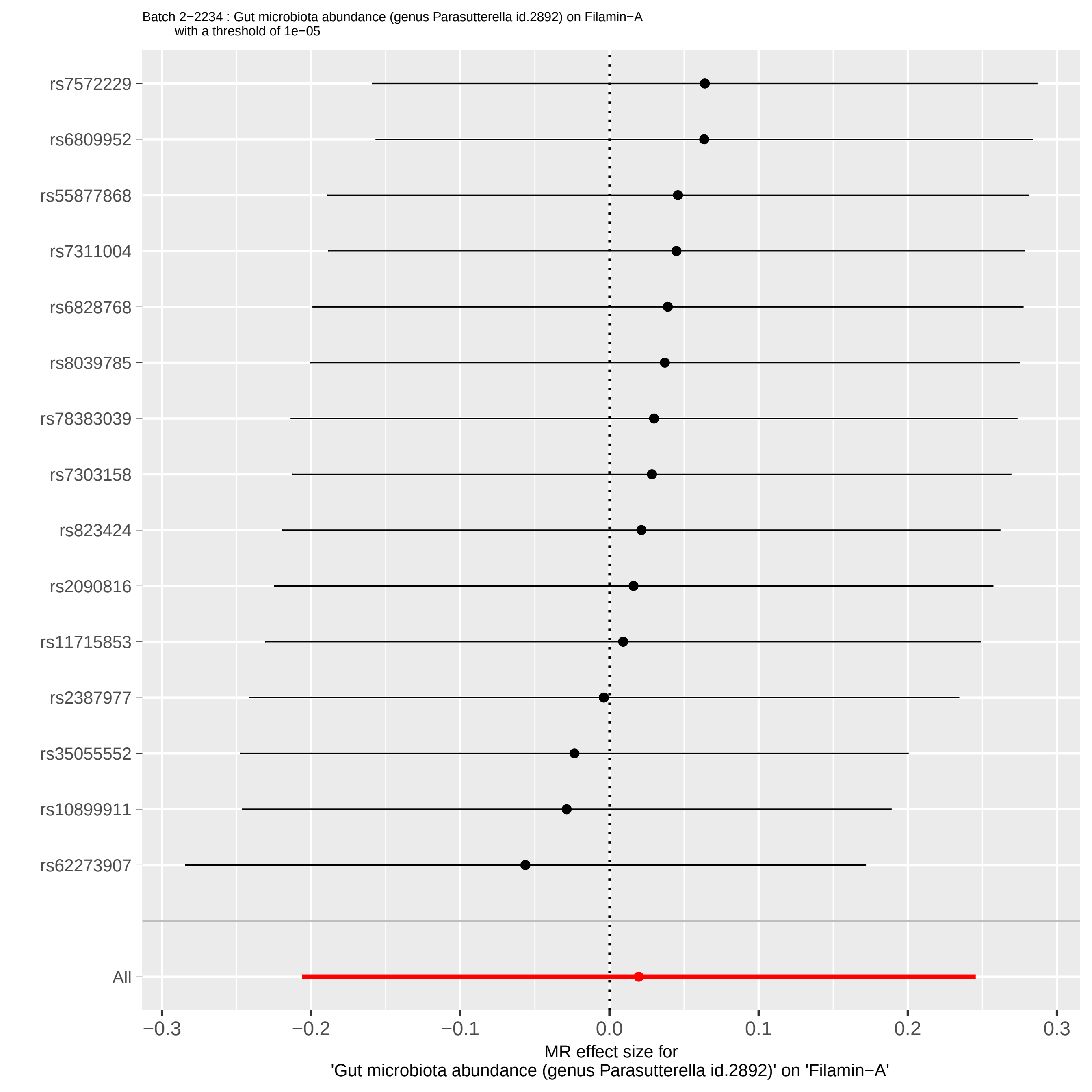
Batch 2−2234 : Gut microbiota abundance (genus Parasutterella id.2892) on Filamin−A
with a threshold of 1e−05
rs7572229
rs6809952
rs55877868
rs7311004
rs6828768
rs8039785
rs78383039
rs7303158
rs823424
rs2090816
rs11715853
rs2387977
rs35055552
rs10899911
rs62273907
All
−0.3
−0.2
−0.1
0.0
0.1
0.2
0.3
MR effect size for
'Gut microbiota abundance (genus Parasutterella id.2892)' on 'Filamin−A'

Batch 2−2235 : Gut microbiota abundance (genus Catenibacterium id.2153) on Filamin−A
with a threshold of 1e−05

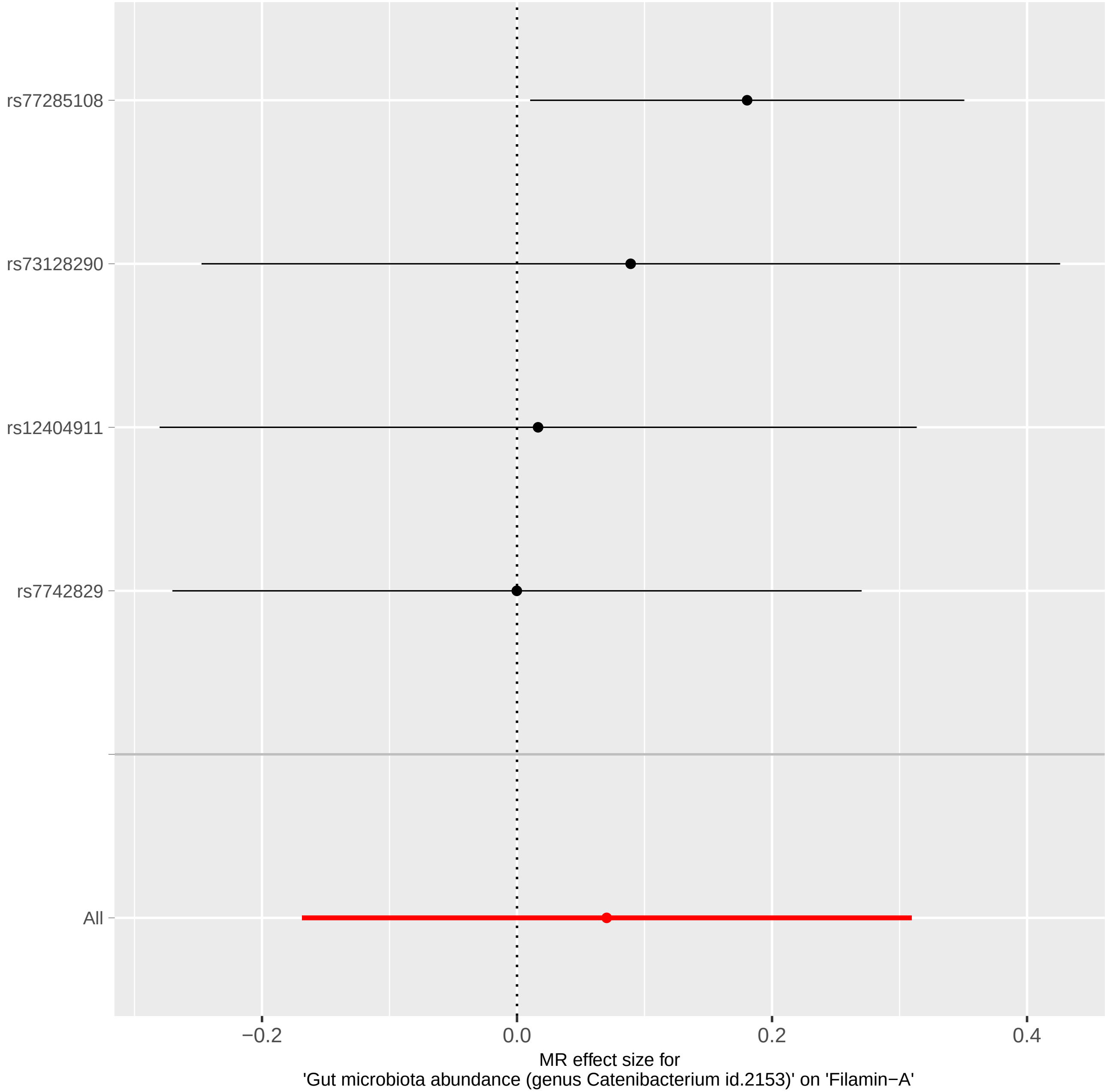

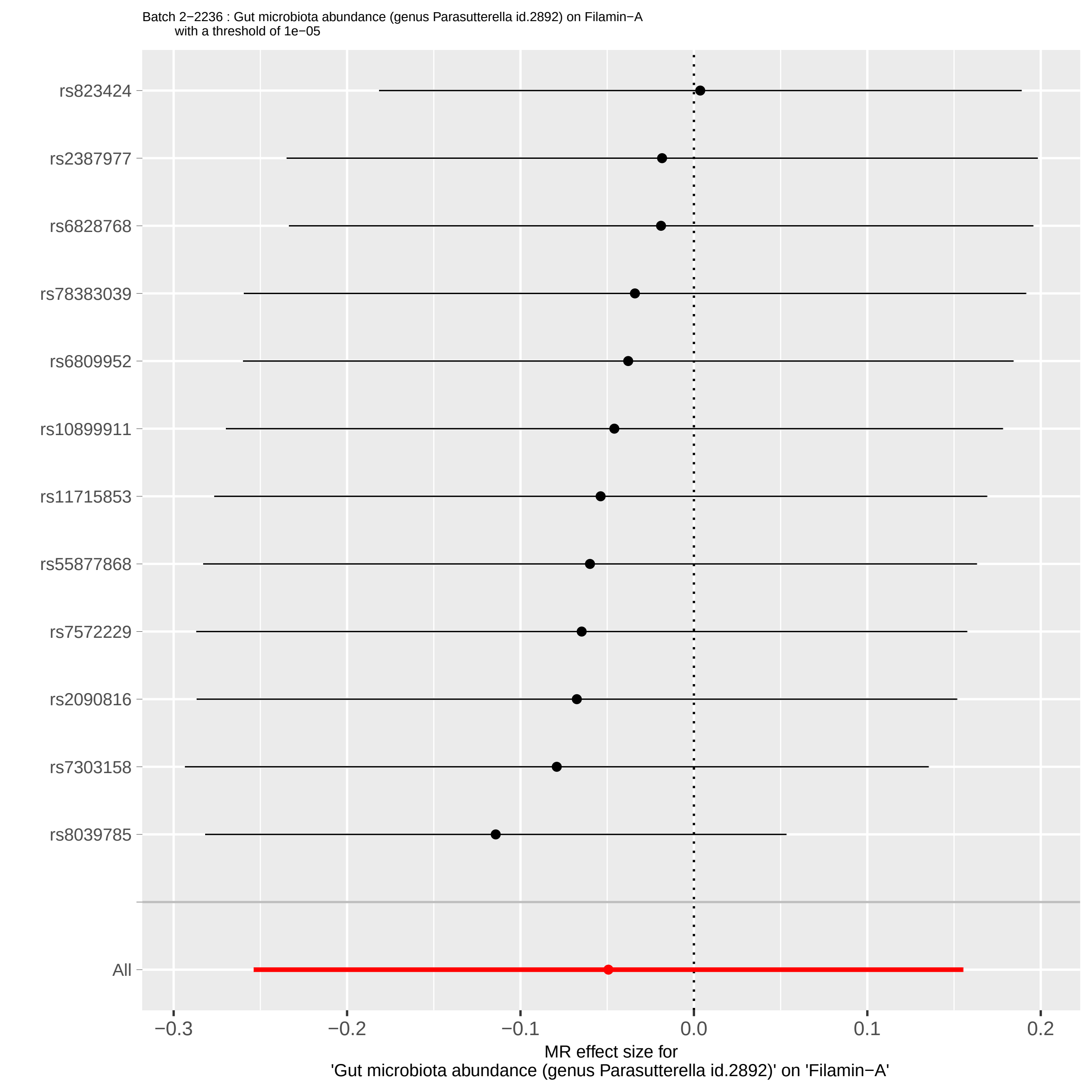
Batch 2−2236 : Gut microbiota abundance (genus Parasutterella id.2892) on Filamin−A
with a threshold of 1e−05
rs823424
rs2387977
rs6828768
rs78383039
rs6809952
rs10899911
rs11715853
rs55877868
rs7572229
rs2090816
rs7303158
rs8039785
All
−0.3
−0.2
−0.1
0.0
0.1
0.2
MR effect size for
'Gut microbiota abundance (genus Parasutterella id.2892)' on 'Filamin−A'

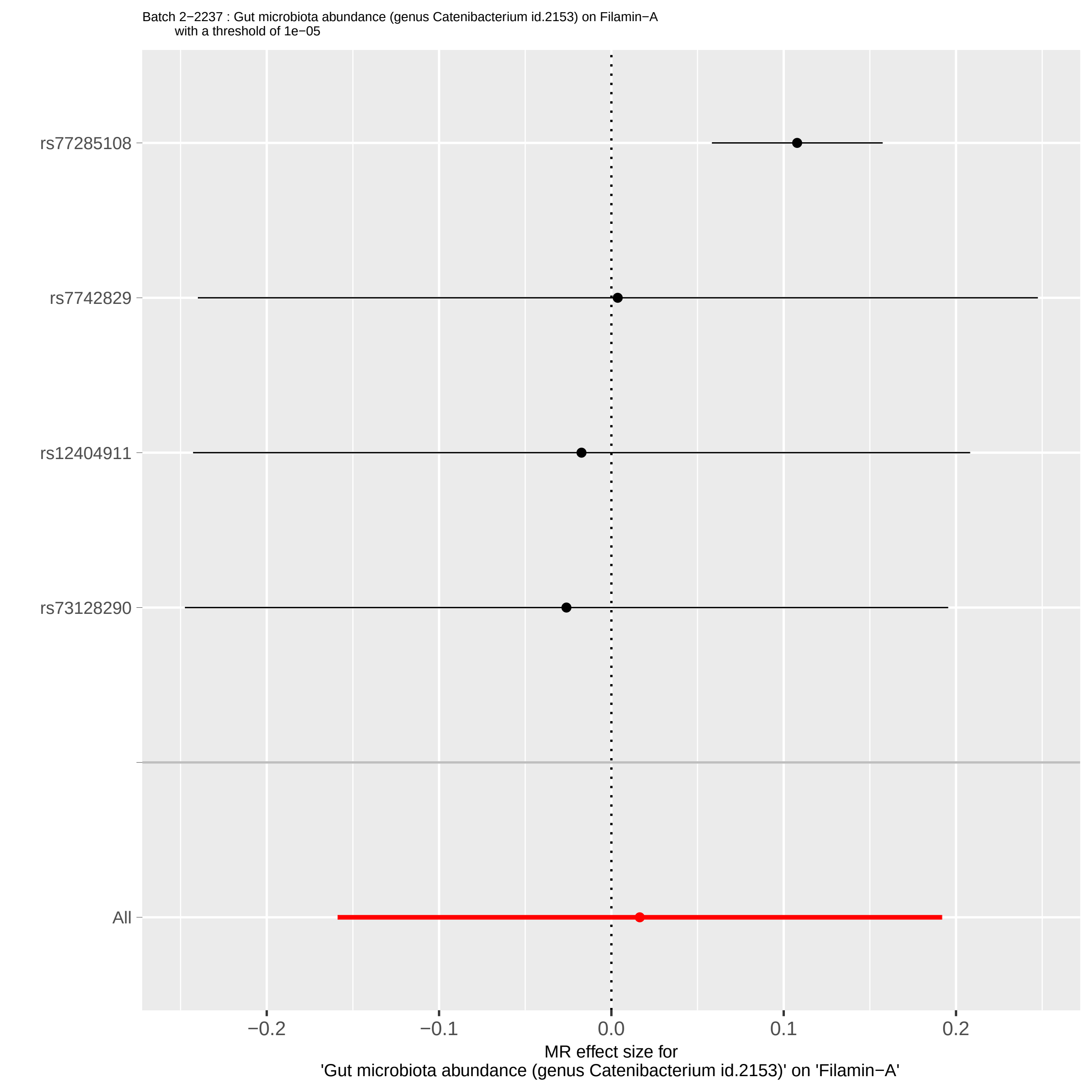

Batch 2−2237 : Gut microbiota abundance (genus Catenibacterium id.2153) on Filamin−A
with a threshold of 1e−05
rs77285108
rs7742829
rs12404911
rs73128290
All
−0.2
−0.1
0.0
0.1
0.2
MR effect size for
'Gut microbiota abundance (genus Catenibacterium id.2153)' on 'Filamin−A'

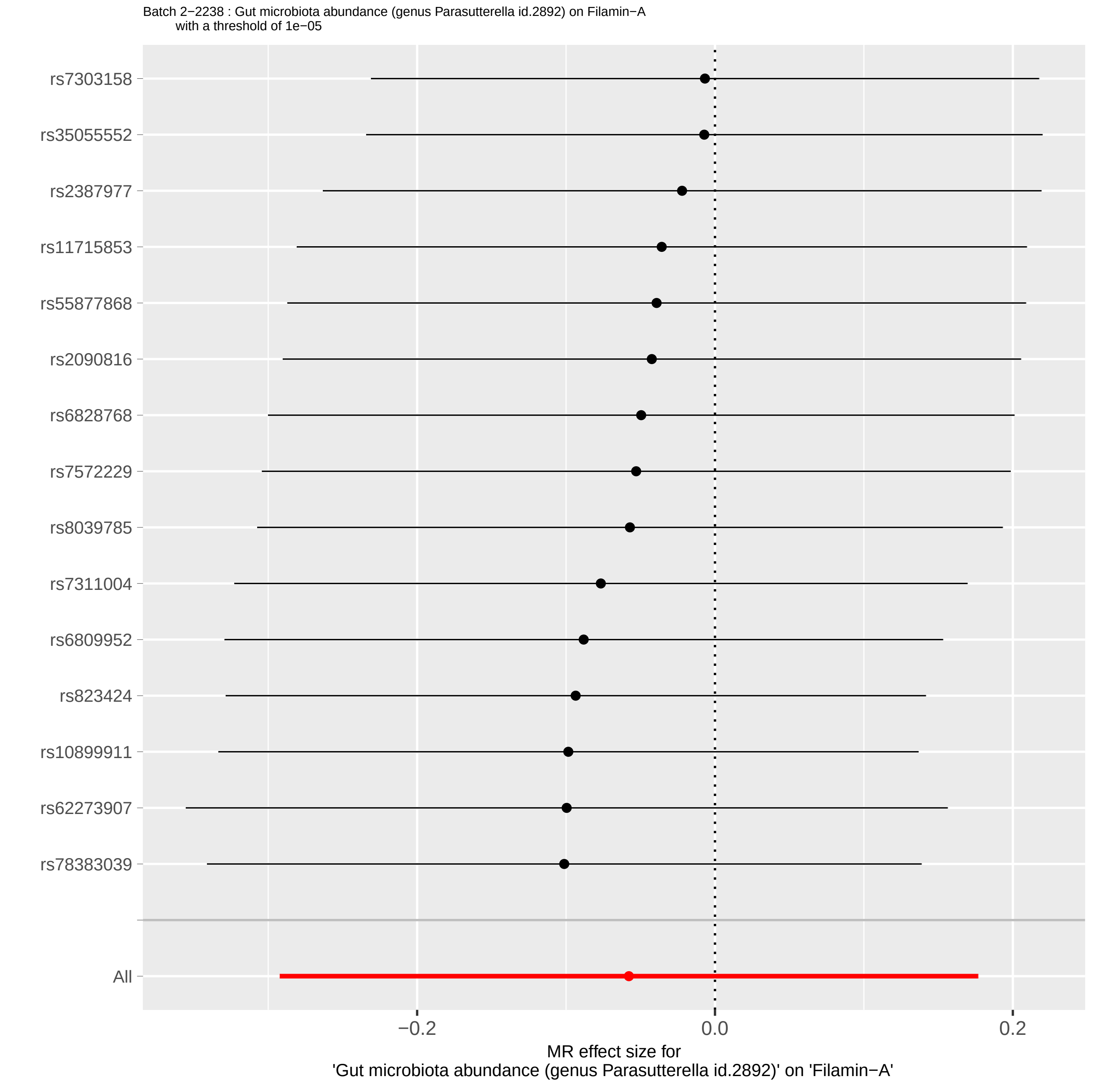
Batch 2−2238 : Gut microbiota abundance (genus Parasutterella id.2892) on Filamin−A
with a threshold of 1e−05
rs7303158
rs35055552
rs2387977
rs11715853
rs55877868
rs2090816
rs6828768
rs7572229
rs8039785
rs7311004
rs6809952
rs823424
rs10899911
rs62273907
rs78383039
All
−0.2
0.0
0.2
MR effect size for
'Gut microbiota abundance (genus Parasutterella id.2892)' on 'Filamin−A'

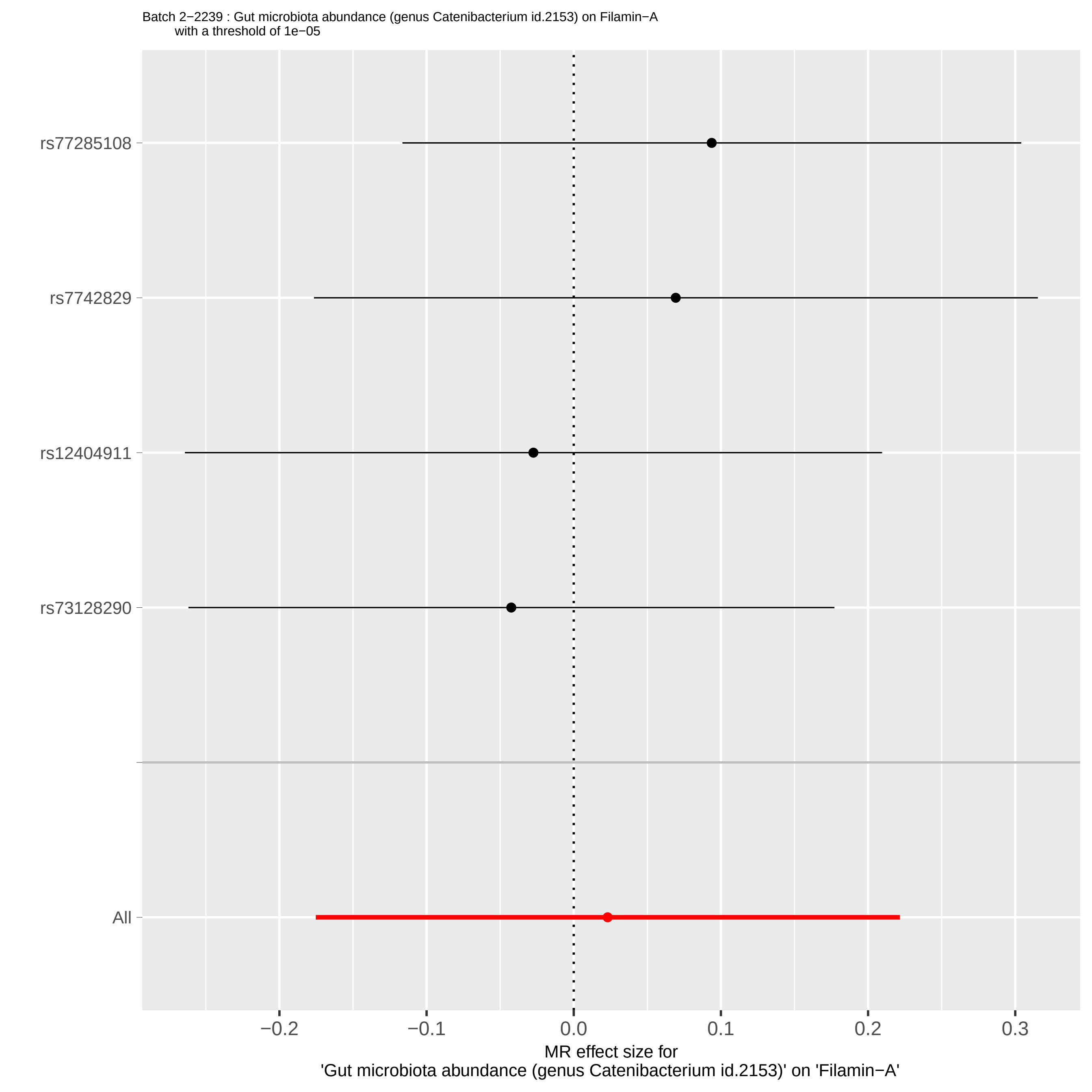
Batch 2−2239 : Gut microbiota abundance (genus Catenibacterium id.2153) on Filamin−A
with a threshold of 1e−05
rs77285108
rs7742829
rs12404911
rs73128290
All
−0.2
−0.1
0.0
0.1
0.2
0.3
MR effect size for
'Gut microbiota abundance (genus Catenibacterium id.2153)' on 'Filamin−A'

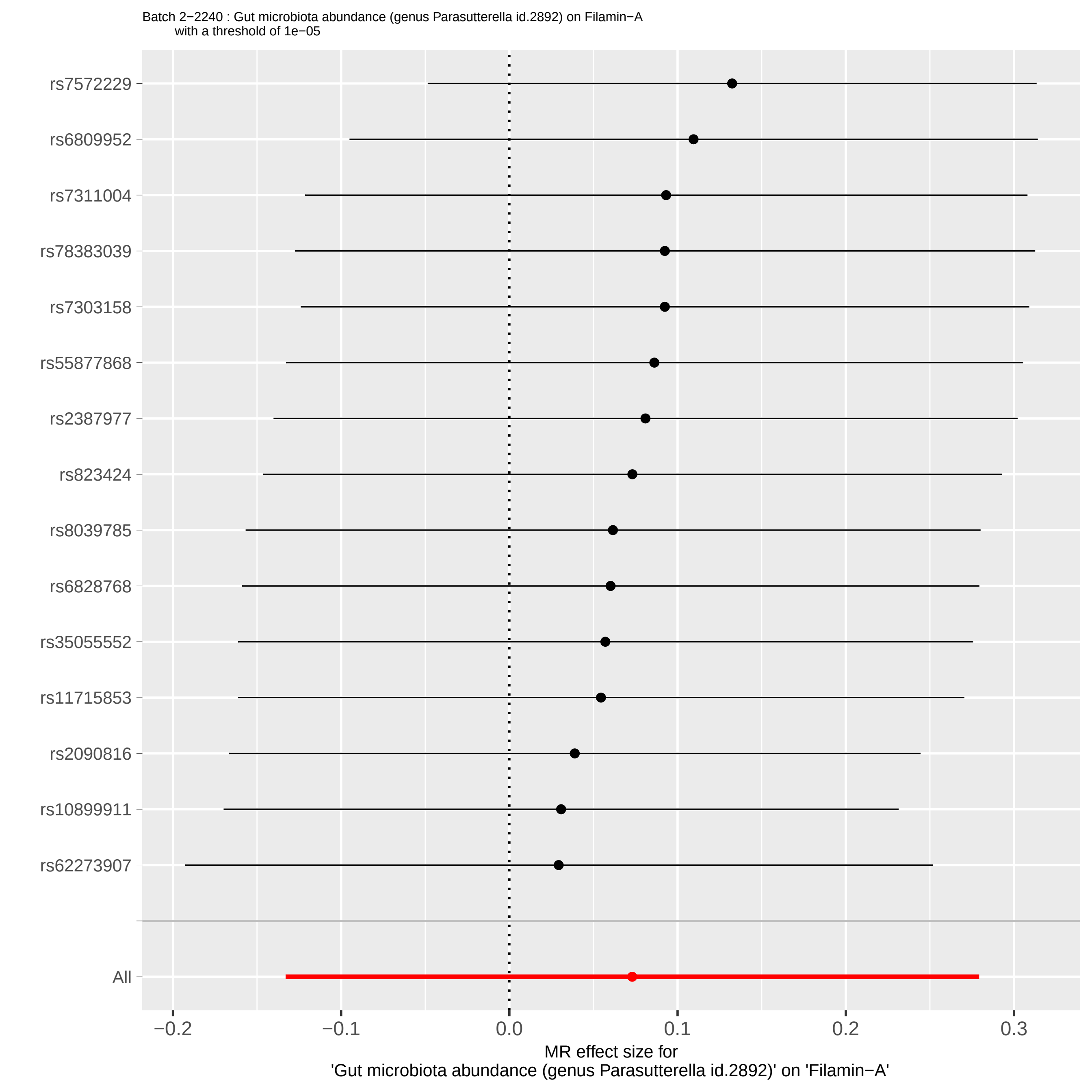

Batch 2−2240 : Gut microbiota abundance (genus Parasutterella id.2892) on Filamin−A
with a threshold of 1e−05
rs7572229
rs6809952
rs7311004
rs78383039
rs7303158
rs55877868
rs2387977
rs823424
rs8039785
rs6828768
rs35055552
rs11715853
rs2090816
rs10899911
rs62273907
All
−0.2
−0.1
0.0
0.1
0.2
0.3
MR effect size for
'Gut microbiota abundance (genus Parasutterella id.2892)' on 'Filamin−A'

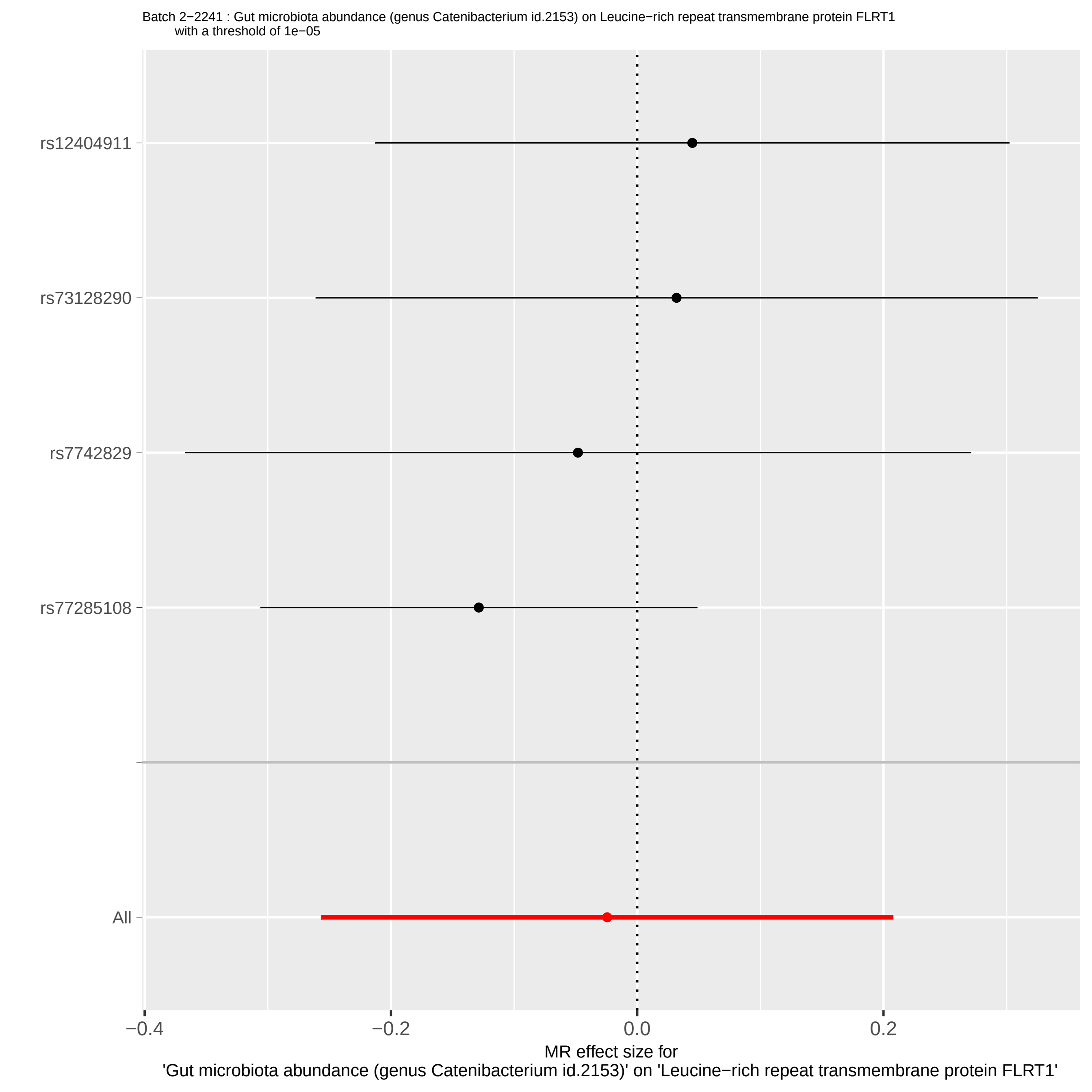

Batch 2−2241 : Gut microbiota abundance (genus Catenibacterium id.2153) on Leucine−rich repeat transmembrane protein FLRT1
with a threshold of 1e−05
rs12404911
rs73128290
rs7742829
rs77285108
All
−0.4
−0.2
0.0
0.2
MR effect size for
'Gut microbiota abundance (genus Catenibacterium id.2153)' on 'Leucine−rich repeat transmembrane protein FLRT1'

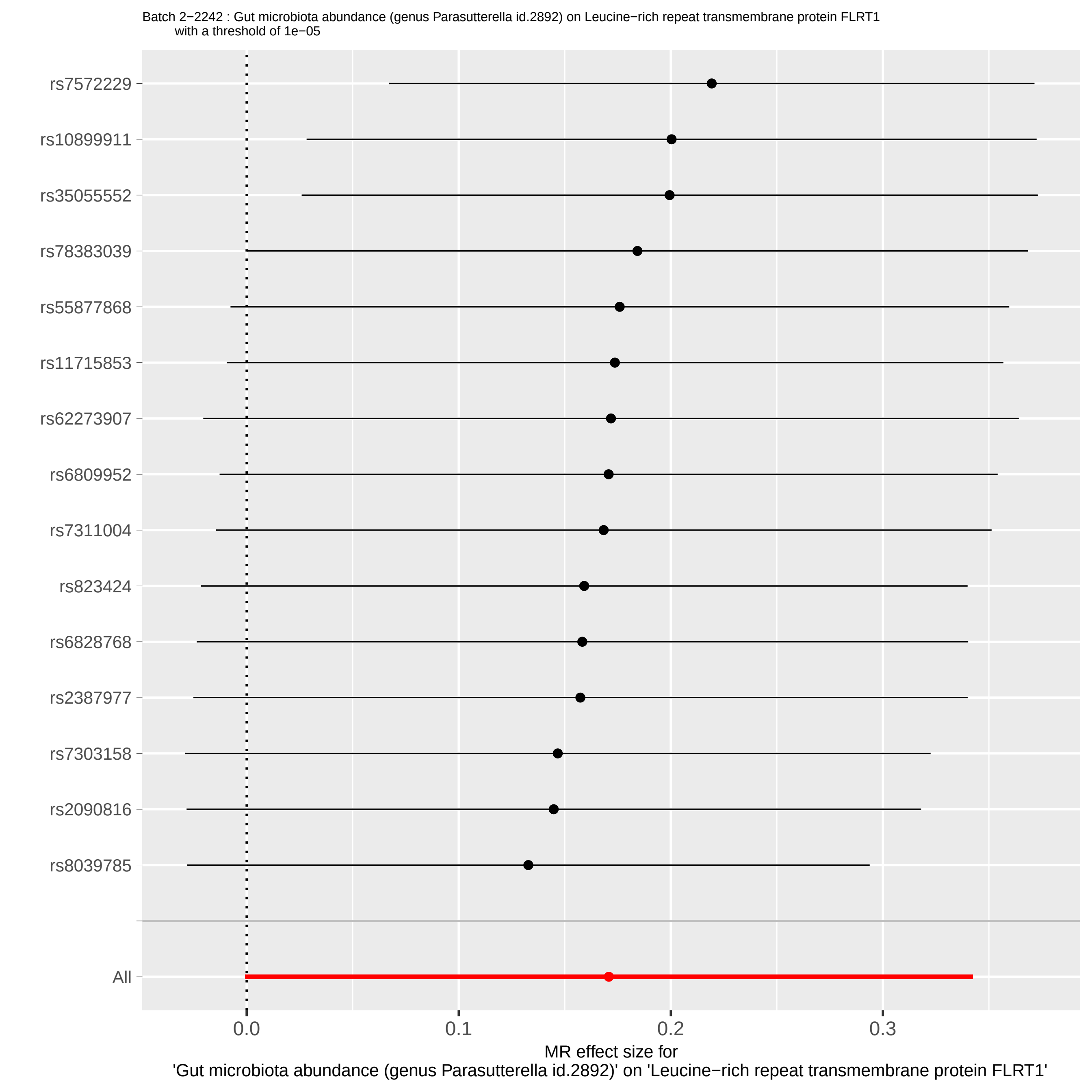

Batch 2−2242 : Gut microbiota abundance (genus Parasutterella id.2892) on Leucine−rich repeat transmembrane protein FLRT1
with a threshold of 1e−05
rs7572229
rs10899911
rs35055552
rs78383039
rs55877868
rs11715853
rs62273907
rs6809952
rs7311004
rs823424
rs6828768
rs2387977
rs7303158
rs2090816
rs8039785
All
0.0
0.1
0.2
0.3
MR effect size for
'Gut microbiota abundance (genus Parasutterella id.2892)' on 'Leucine−rich repeat transmembrane protein FLRT1'

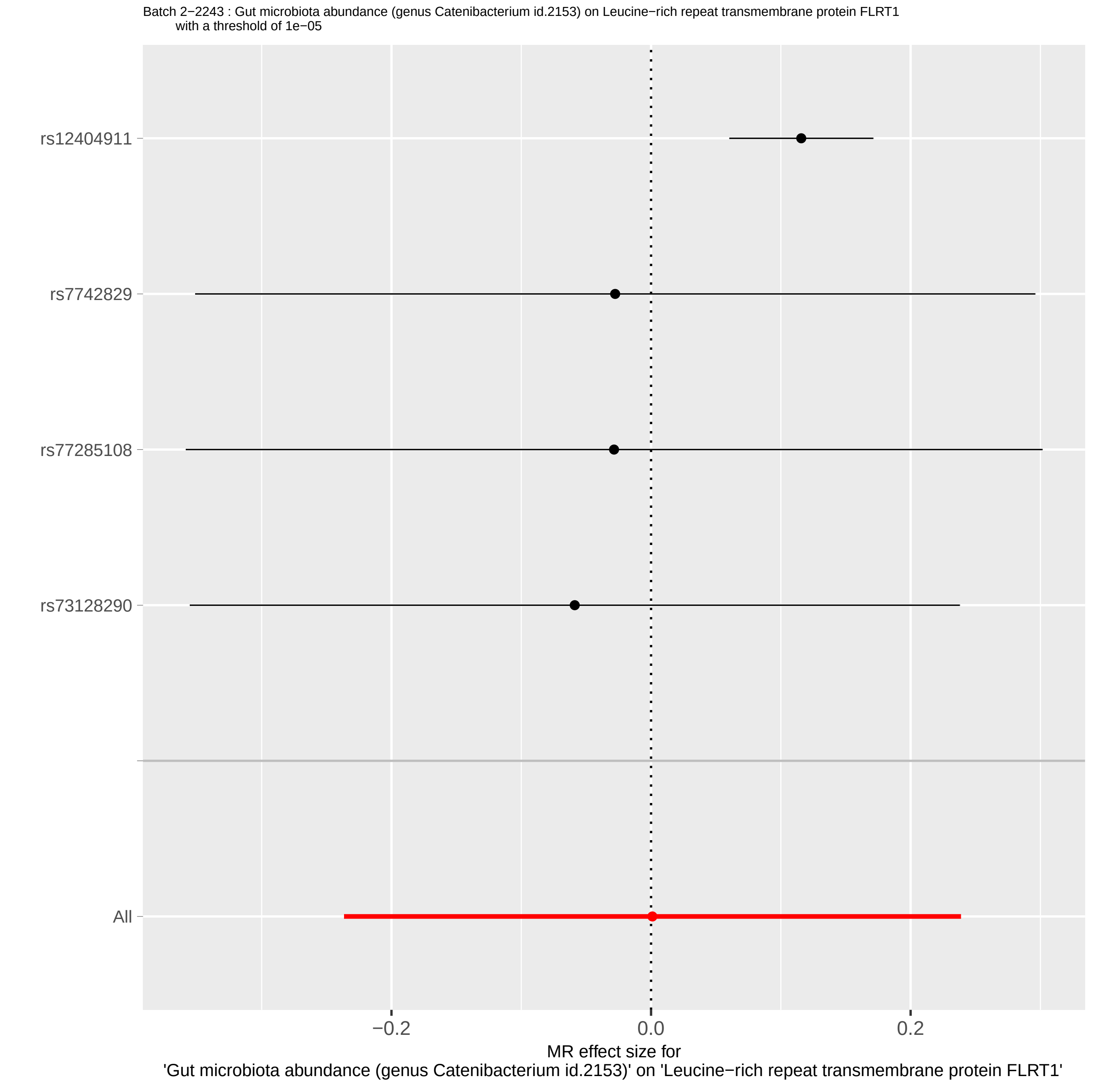

Batch 2−2243 : Gut microbiota abundance (genus Catenibacterium id.2153) on Leucine−rich repeat transmembrane protein FLRT1
with a threshold of 1e−05
rs12404911
rs7742829
rs77285108
rs73128290
All
−0.2
0.0
0.2
MR effect size for
'Gut microbiota abundance (genus Catenibacterium id.2153)' on 'Leucine−rich repeat transmembrane protein FLRT1'

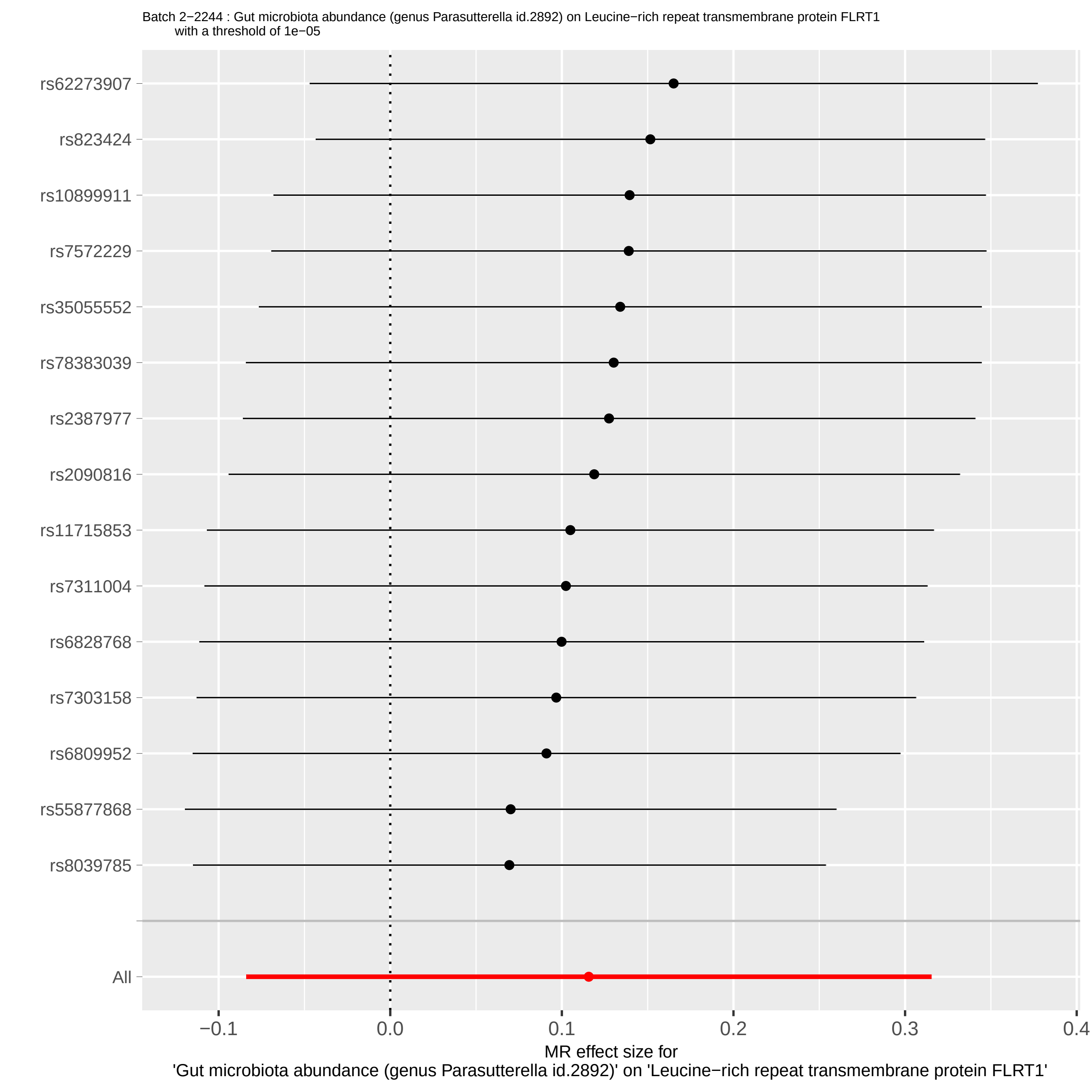

Batch 2−2244 : Gut microbiota abundance (genus Parasutterella id.2892) on Leucine−rich repeat transmembrane protein FLRT1
with a threshold of 1e−05
rs62273907
rs823424
rs10899911
rs7572229
rs35055552
rs78383039
rs2387977
rs2090816
rs11715853
rs7311004
rs6828768
rs7303158
rs6809952
rs55877868
rs8039785
All
−0.1
0.0
0.1
0.2
0.3
0.4
MR effect size for
'Gut microbiota abundance (genus Parasutterella id.2892)' on 'Leucine−rich repeat transmembrane protein FLRT1'

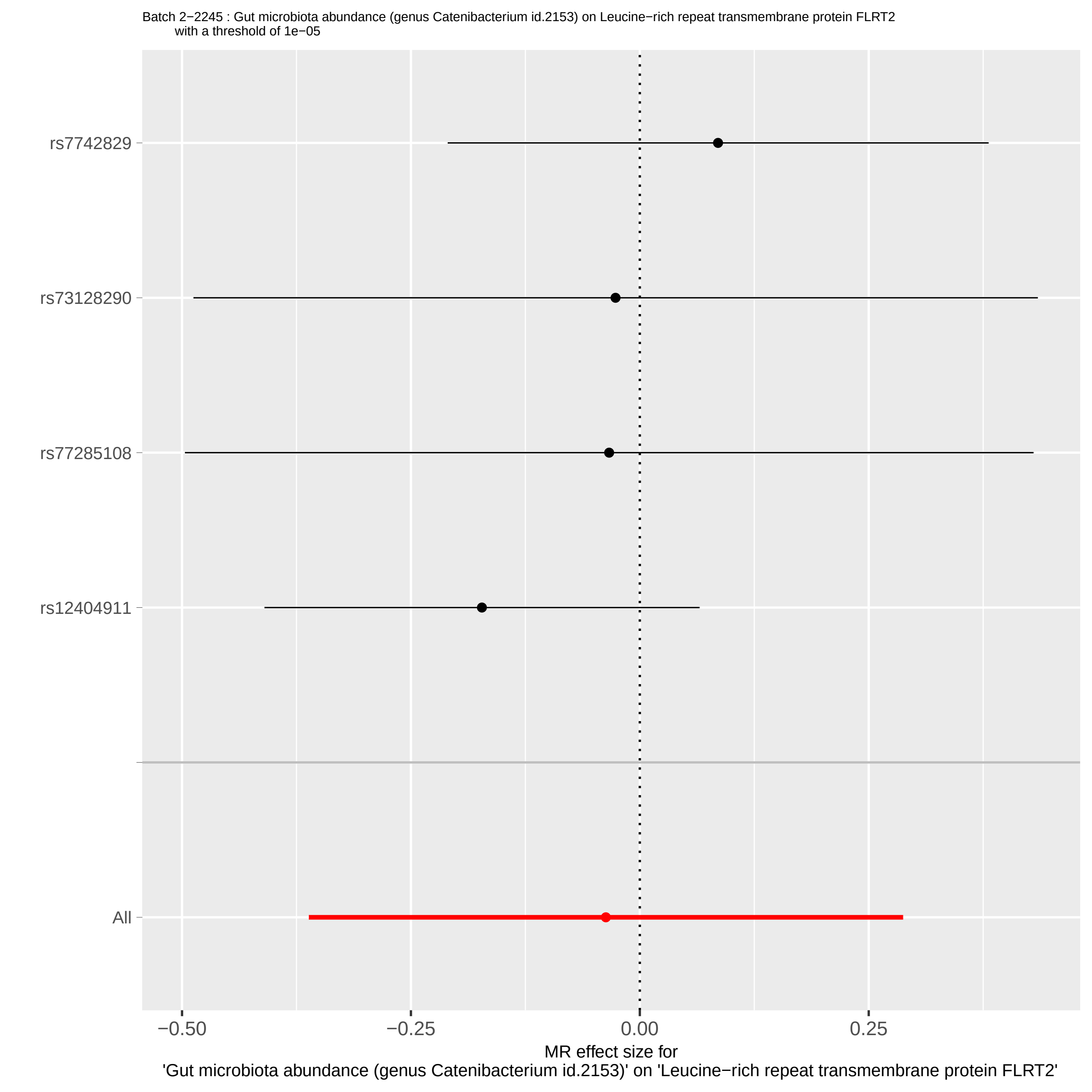

Batch 2−2245 : Gut microbiota abundance (genus Catenibacterium id.2153) on Leucine−rich repeat transmembrane protein FLRT2
with a threshold of 1e−05
rs7742829
rs73128290
rs77285108
rs12404911
All
−0.50
−0.25
0.00
0.25
MR effect size for
'Gut microbiota abundance (genus Catenibacterium id.2153)' on 'Leucine−rich repeat transmembrane protein FLRT2'

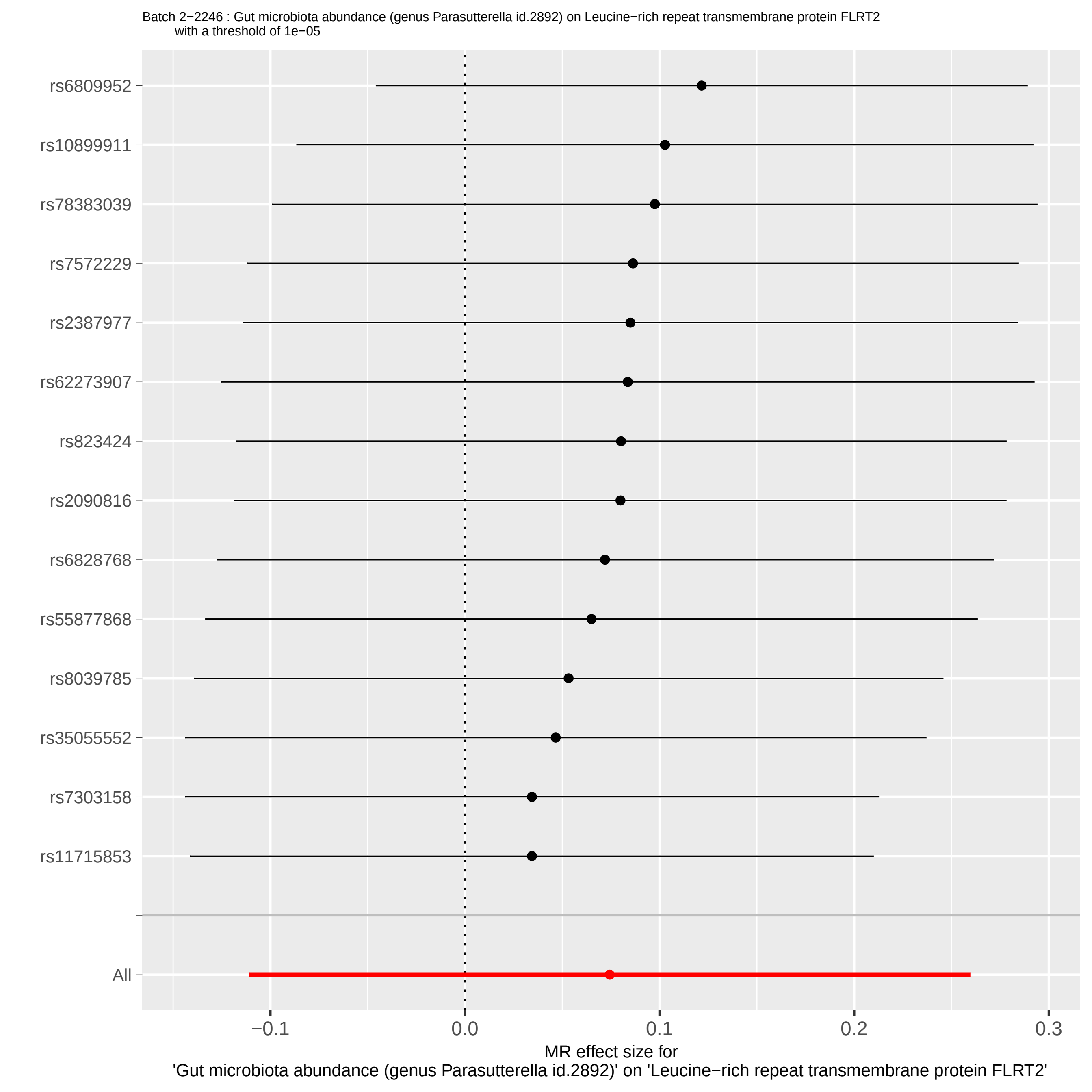

Batch 2−2246 : Gut microbiota abundance (genus Parasutterella id.2892) on Leucine−rich repeat transmembrane protein FLRT2
with a threshold of 1e−05
rs6809952
rs10899911
rs78383039
rs7572229
rs2387977
rs62273907
rs823424
rs2090816
rs6828768
rs55877868
rs8039785
rs35055552
rs7303158
rs11715853
All
−0.1
0.0
0.1
0.2
0.3
MR effect size for
'Gut microbiota abundance (genus Parasutterella id.2892)' on 'Leucine−rich repeat transmembrane protein FLRT2'

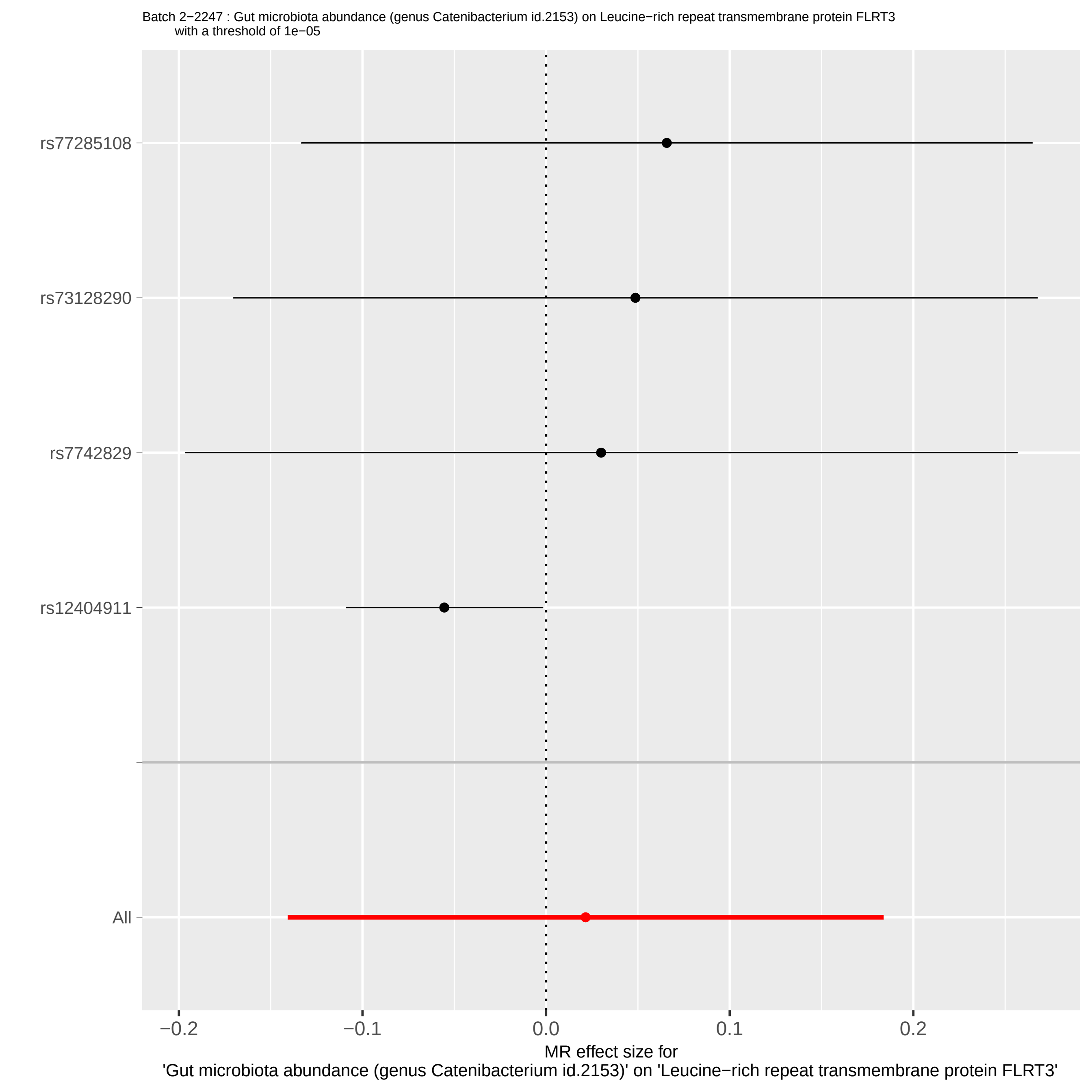
Batch 2−2247 : Gut microbiota abundance (genus Catenibacterium id.2153) on Leucine−rich repeat transmembrane protein FLRT3
with a threshold of 1e−05
rs77285108
rs73128290
rs7742829
rs12404911
All
−0.2
−0.1
0.0
0.1
0.2
MR effect size for
'Gut microbiota abundance (genus Catenibacterium id.2153)' on 'Leucine−rich repeat transmembrane protein FLRT3'

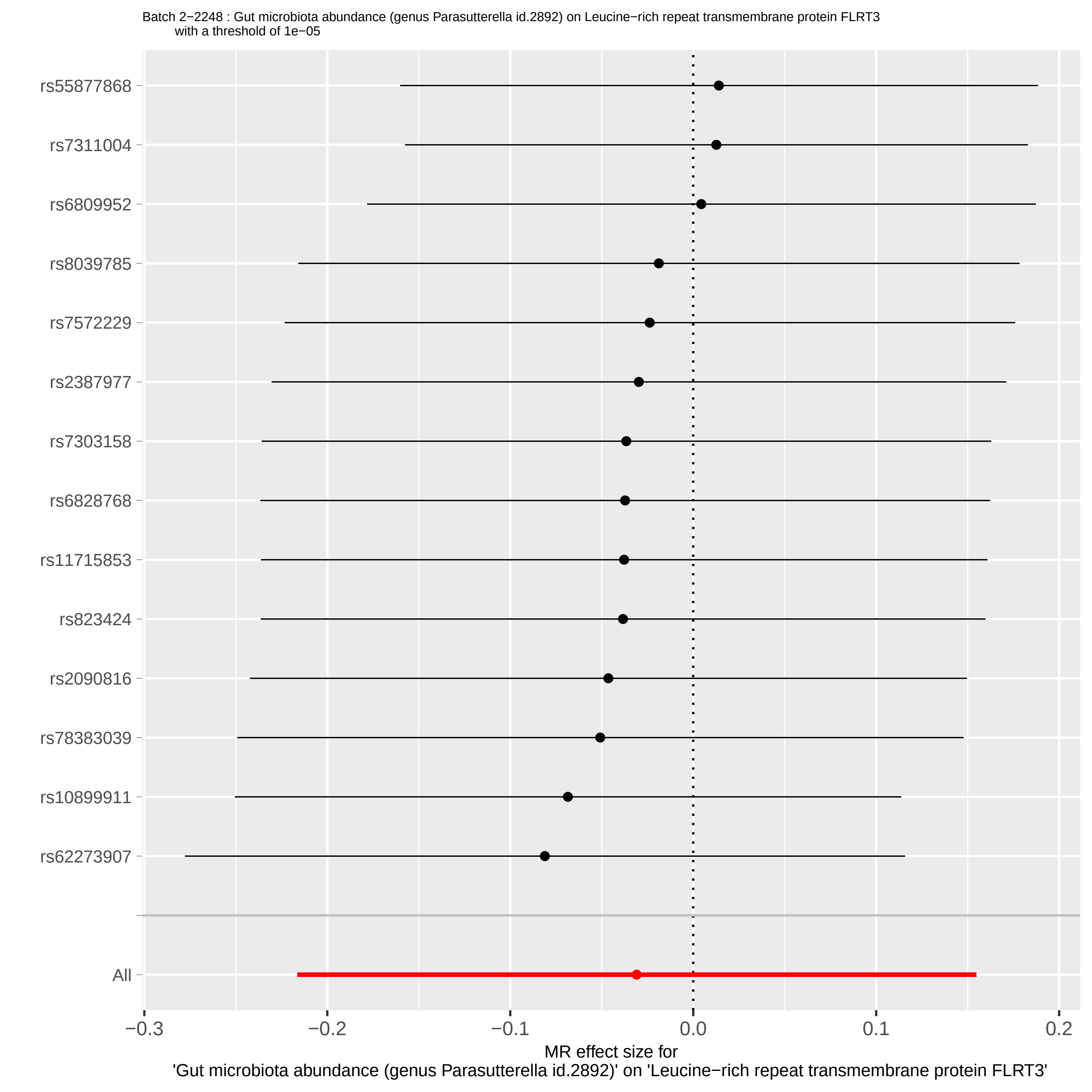
Batch 2−2248 : Gut microbiota abundance (genus Parasutterella id.2892) on Leucine−rich repeat transmembrane protein FLRT3
with a threshold of 1e−05
rs55877868
rs7311004
rs6809952
rs8039785
rs7572229
rs2387977
rs7303158
rs6828768
rs11715853
rs823424
rs2090816
rs78383039
rs10899911
rs62273907
All
−0.3
−0.2
−0.1
0.0
0.1
0.2
MR effect size for
'Gut microbiota abundance (genus Parasutterella id.2892)' on 'Leucine−rich repeat transmembrane protein FLRT3'

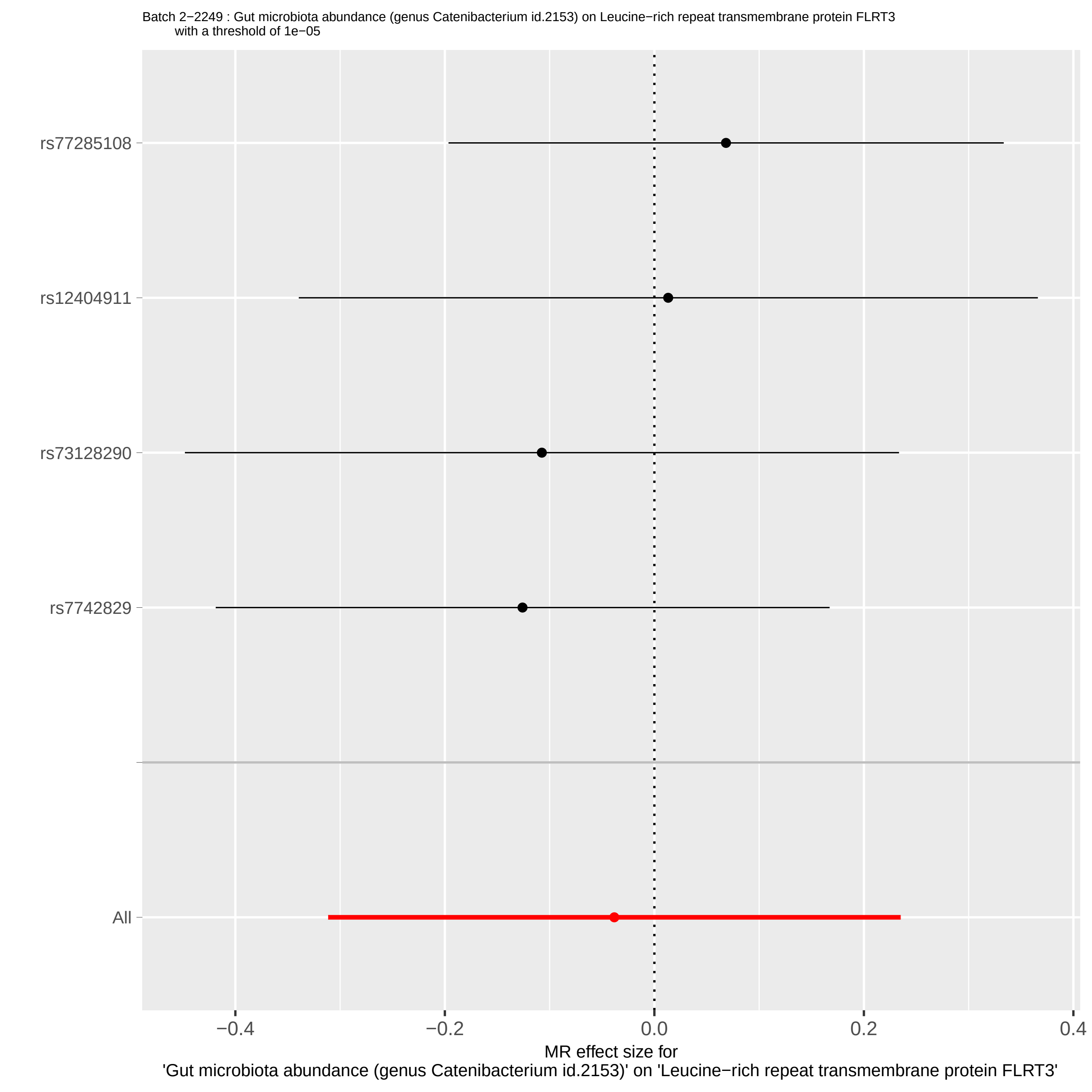
Batch 2−2249 : Gut microbiota abundance (genus Catenibacterium id.2153) on Leucine−rich repeat transmembrane protein FLRT3
with a threshold of 1e−05
rs77285108
rs12404911
rs73128290
rs7742829
All
−0.4
−0.2
0.0
0.2
0.4
MR effect size for
'Gut microbiota abundance (genus Catenibacterium id.2153)' on 'Leucine−rich repeat transmembrane protein FLRT3'

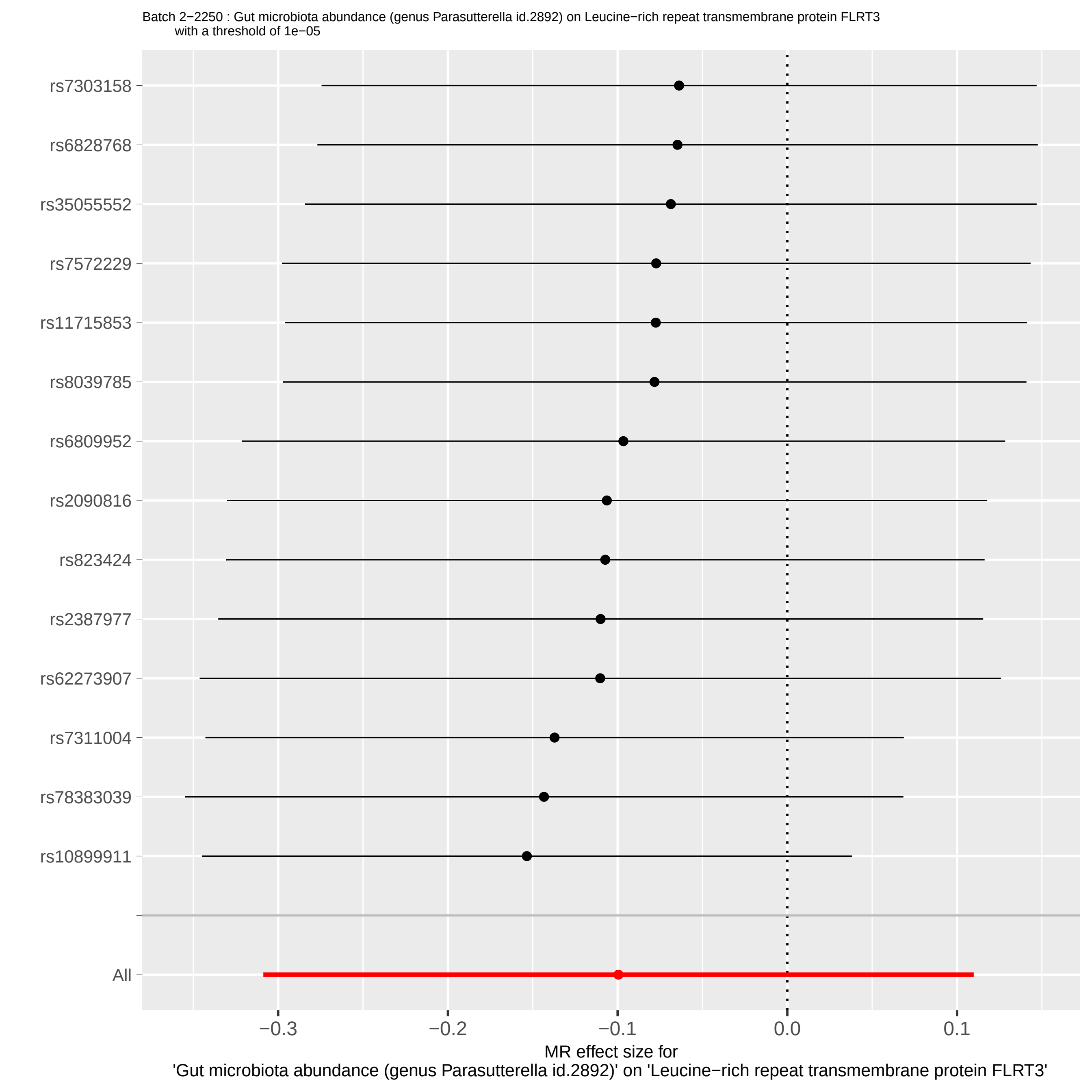
Batch 2−2250 : Gut microbiota abundance (genus Parasutterella id.2892) on Leucine−rich repeat transmembrane protein FLRT3
with a threshold of 1e−05
rs7303158
rs6828768
rs35055552
rs7572229
rs11715853
rs8039785
rs6809952
rs2090816
rs823424
rs2387977
rs62273907
rs7311004
rs78383039
rs10899911
All
−0.3
−0.2
−0.1
0.0
0.1
MR effect size for
'Gut microbiota abundance (genus Parasutterella id.2892)' on 'Leucine−rich repeat transmembrane protein FLRT3'

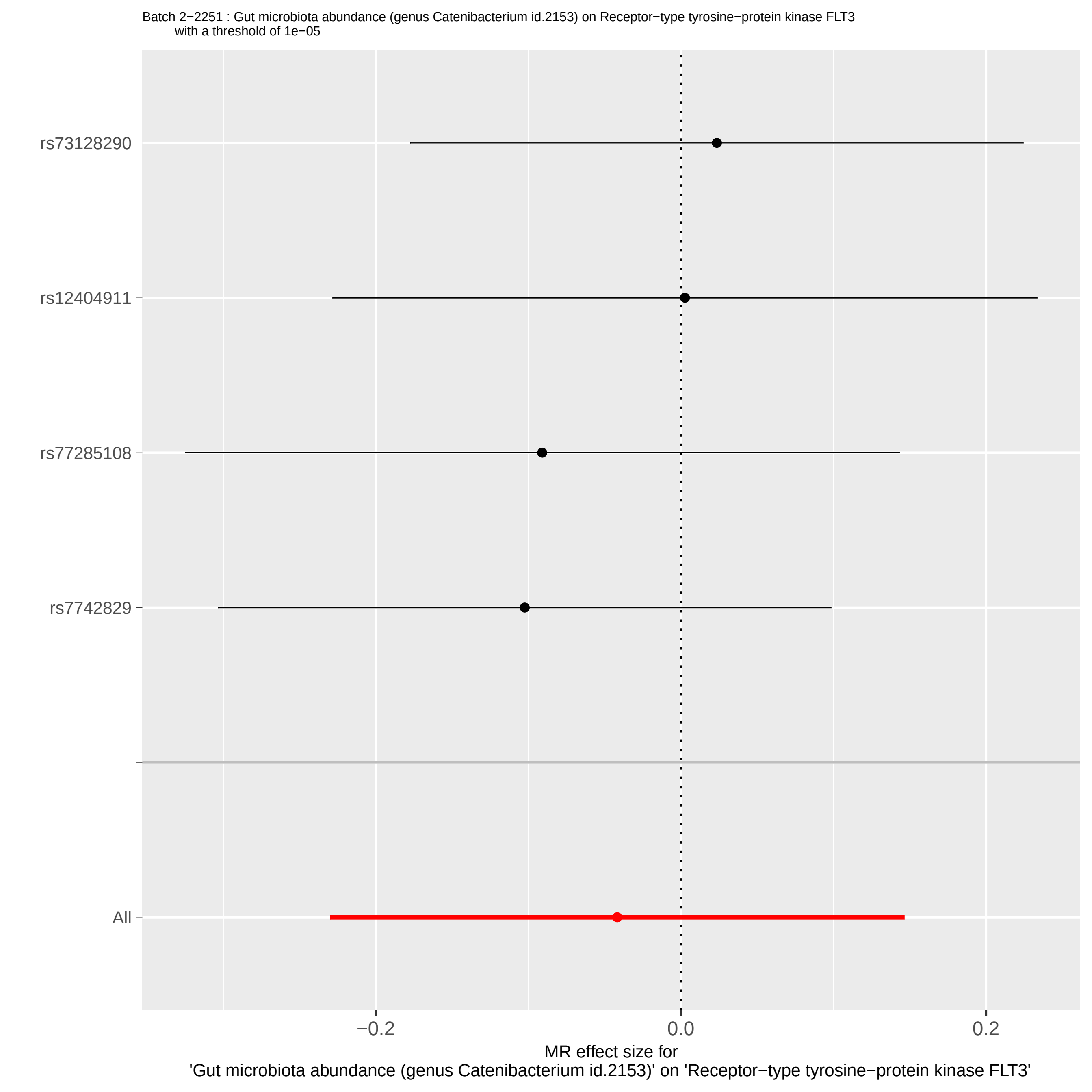
Batch 2−2251 : Gut microbiota abundance (genus Catenibacterium id.2153) on Receptor−type tyrosine−protein kinase FLT3
with a threshold of 1e−05
rs73128290
rs12404911
rs77285108
rs7742829
All
−0.2
0.0
0.2
MR effect size for
'Gut microbiota abundance (genus Catenibacterium id.2153)' on 'Receptor−type tyrosine−protein kinase FLT3'

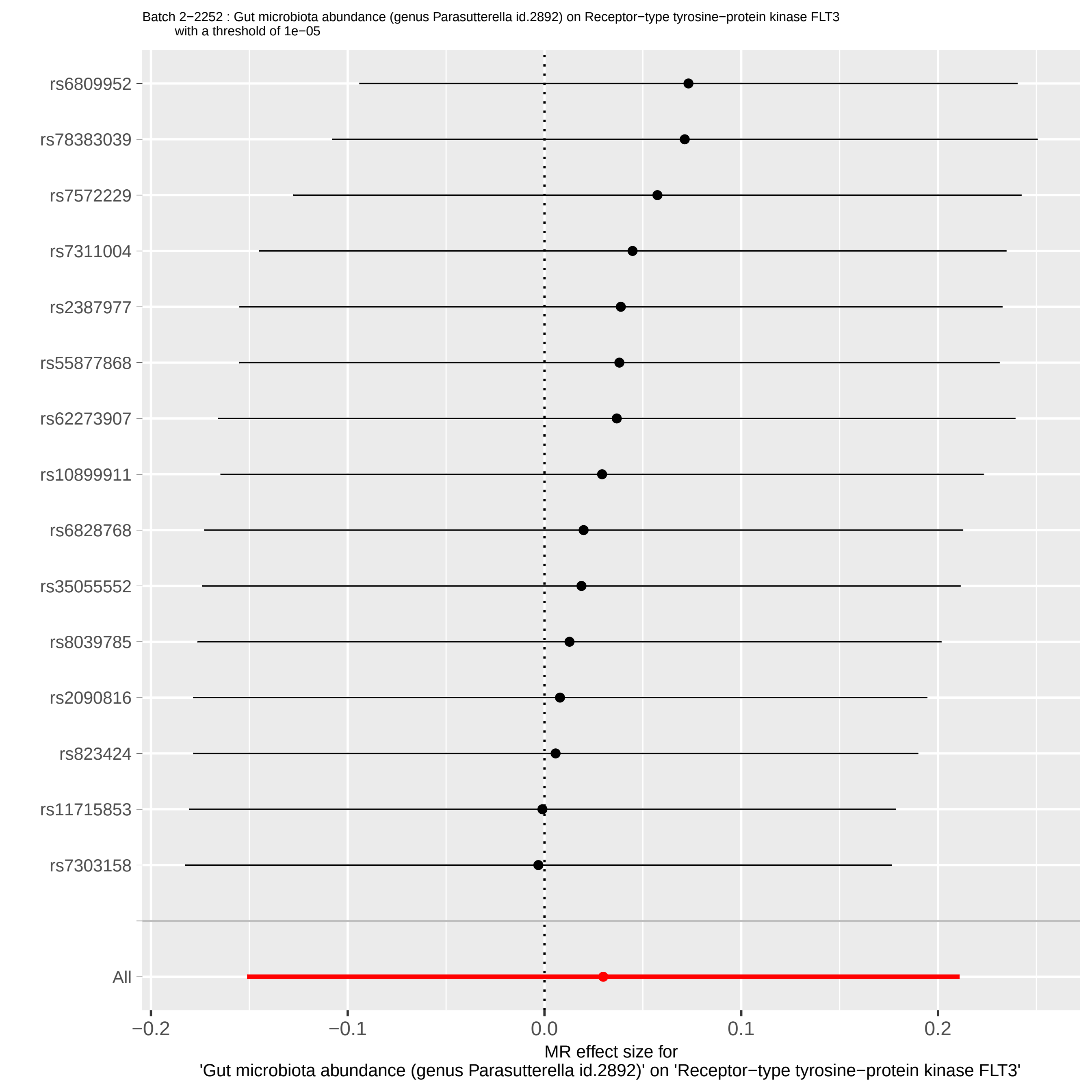

Batch 2−2252 : Gut microbiota abundance (genus Parasutterella id.2892) on Receptor−type tyrosine−protein kinase FLT3
with a threshold of 1e−05
rs6809952
rs78383039
rs7572229
rs7311004
rs2387977
rs55877868
rs62273907
rs10899911
rs6828768
rs35055552
rs8039785
rs2090816
rs823424
rs11715853
rs7303158
All
−0.2
−0.1
0.0
0.1
0.2
MR effect size for
'Gut microbiota abundance (genus Parasutterella id.2892)' on 'Receptor−type tyrosine−protein kinase FLT3'

Batch 2−2253 : Gut microbiota abundance (genus Catenibacterium id.2153) on Fms−related tyrosine kinase 3 ligand
with a threshold of 1e−05

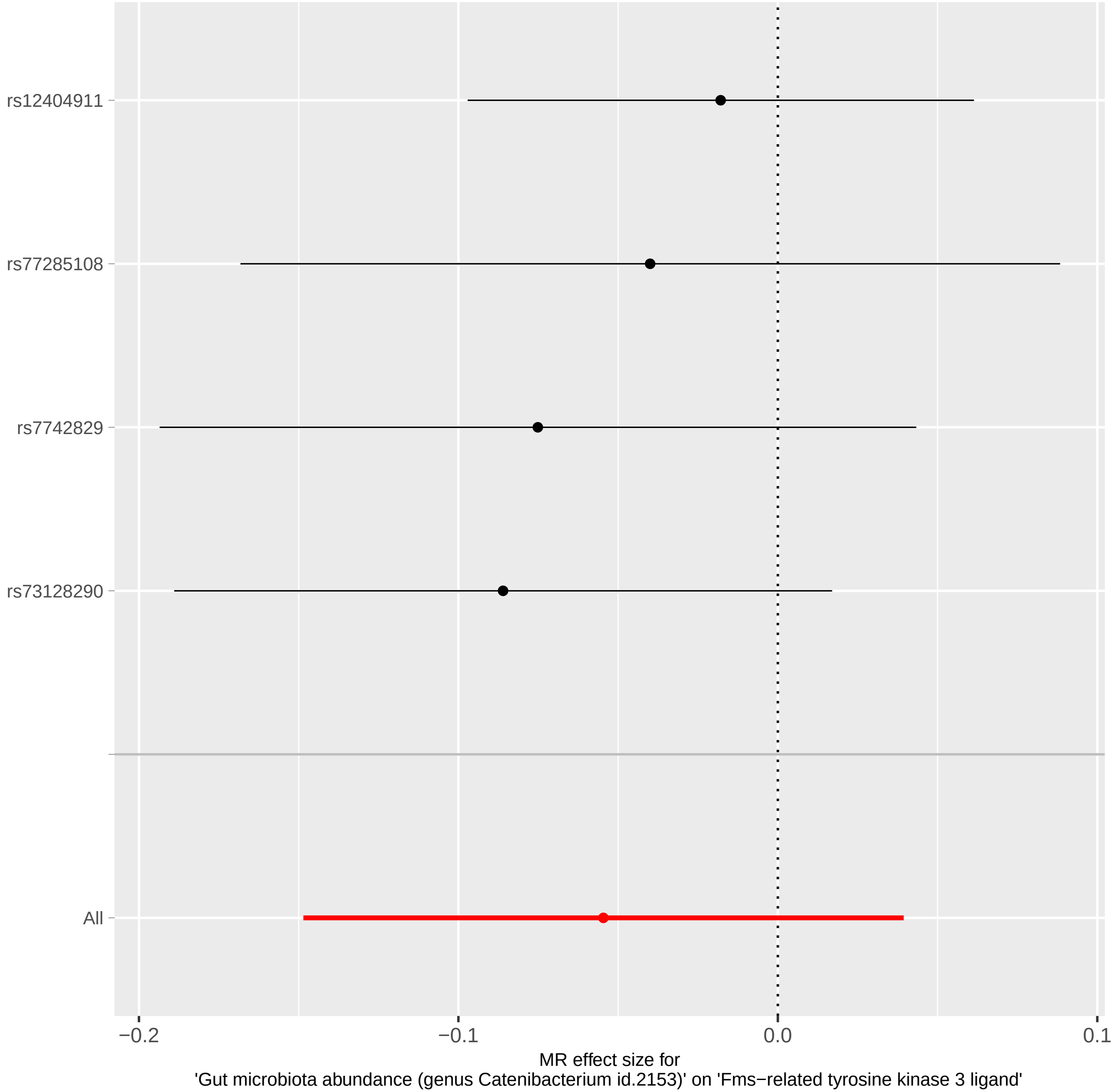

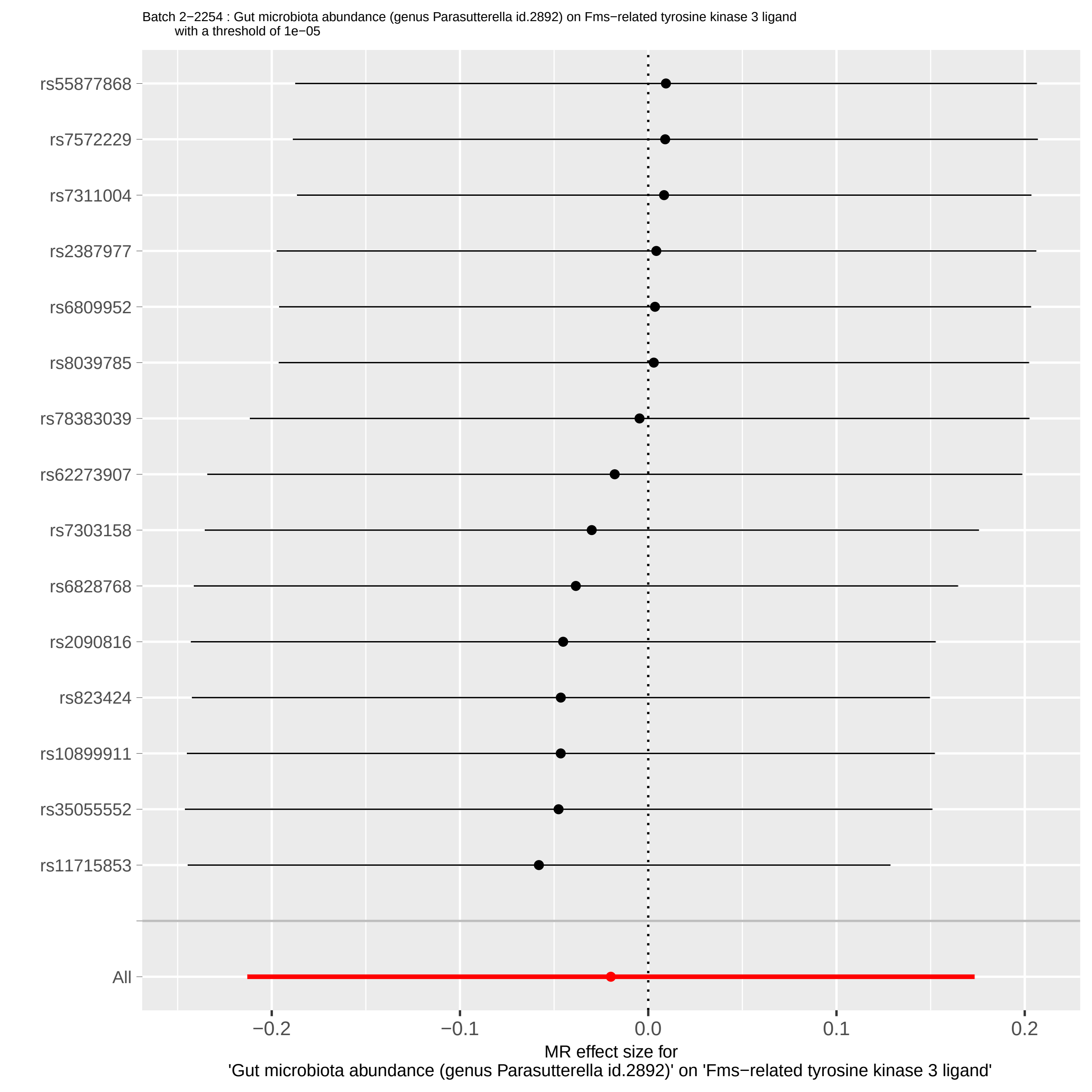
Batch 2−2254 : Gut microbiota abundance (genus Parasutterella id.2892) on Fms−related tyrosine kinase 3 ligand
with a threshold of 1e−05
rs55877868
rs7572229
rs7311004
rs2387977
rs6809952
rs8039785
rs78383039
rs62273907
rs7303158
rs6828768
rs2090816
rs823424
rs10899911
rs35055552
rs11715853
All
−0.2
−0.1
0.0
0.1
0.2
MR effect size for
'Gut microbiota abundance (genus Parasutterella id.2892)' on 'Fms−related tyrosine kinase 3 ligand'

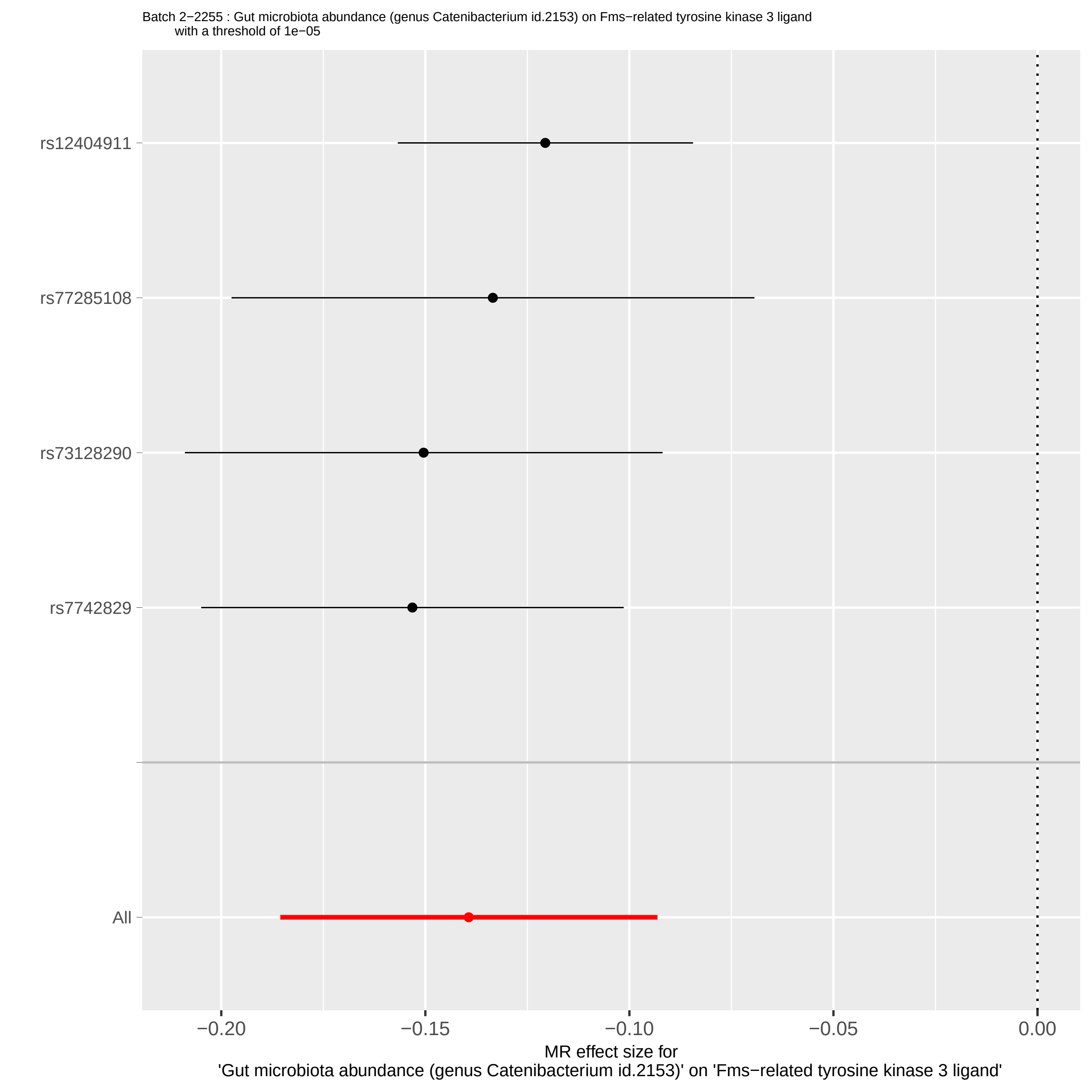
Batch 2−2255 : Gut microbiota abundance (genus Catenibacterium id.2153) on Fms−related tyrosine kinase 3 ligand
with a threshold of 1e−05
rs12404911
rs77285108
rs73128290
rs7742829
All
−0.20
−0.15
−0.10
−0.05
0.00
MR effect size for
'Gut microbiota abundance (genus Catenibacterium id.2153)' on 'Fms−related tyrosine kinase 3 ligand'

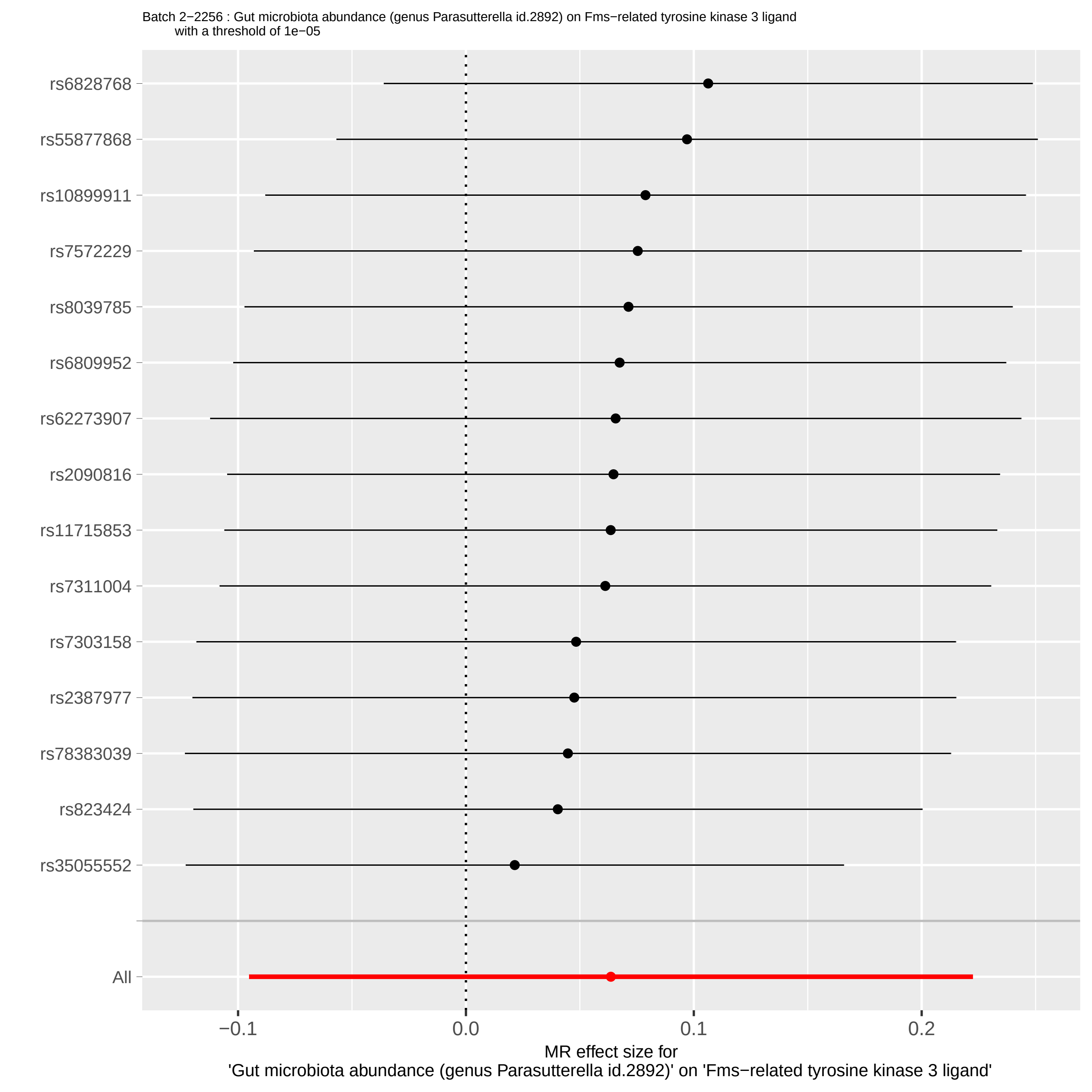
Batch 2−2256 : Gut microbiota abundance (genus Parasutterella id.2892) on Fms−related tyrosine kinase 3 ligand
with a threshold of 1e−05
rs6828768
rs55877868
rs10899911
rs7572229
rs8039785
rs6809952
rs62273907
rs2090816
rs11715853
rs7311004
rs7303158
rs2387977
rs78383039
rs823424
rs35055552
All
−0.1
0.0
0.1
0.2
MR effect size for
'Gut microbiota abundance (genus Parasutterella id.2892)' on 'Fms−related tyrosine kinase 3 ligand'

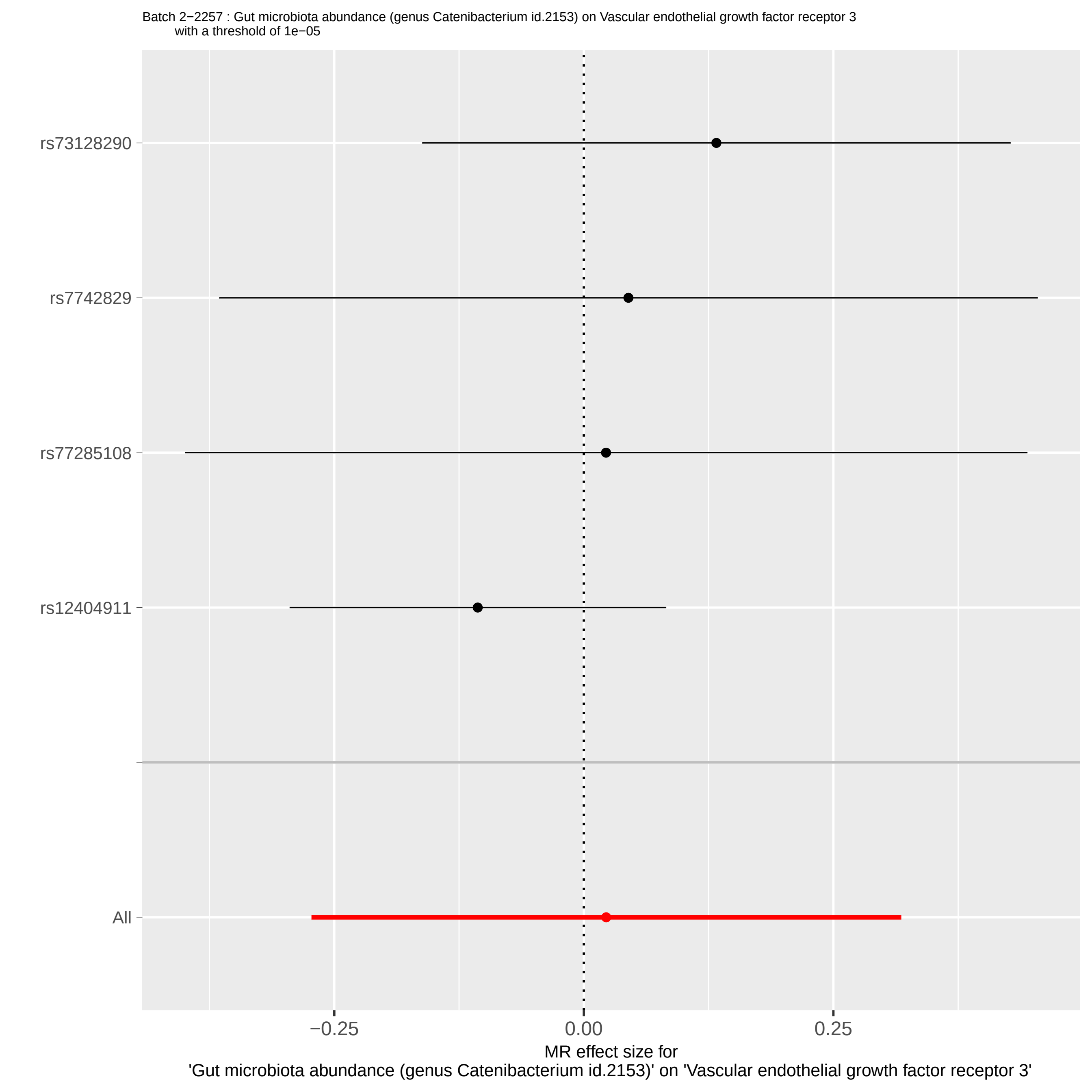

Batch 2−2257 : Gut microbiota abundance (genus Catenibacterium id.2153) on Vascular endothelial growth factor receptor 3
with a threshold of 1e−05
rs73128290
rs7742829
rs77285108
rs12404911
All
−0.25
0.00
0.25
MR effect size for
'Gut microbiota abundance (genus Catenibacterium id.2153)' on 'Vascular endothelial growth factor receptor 3'

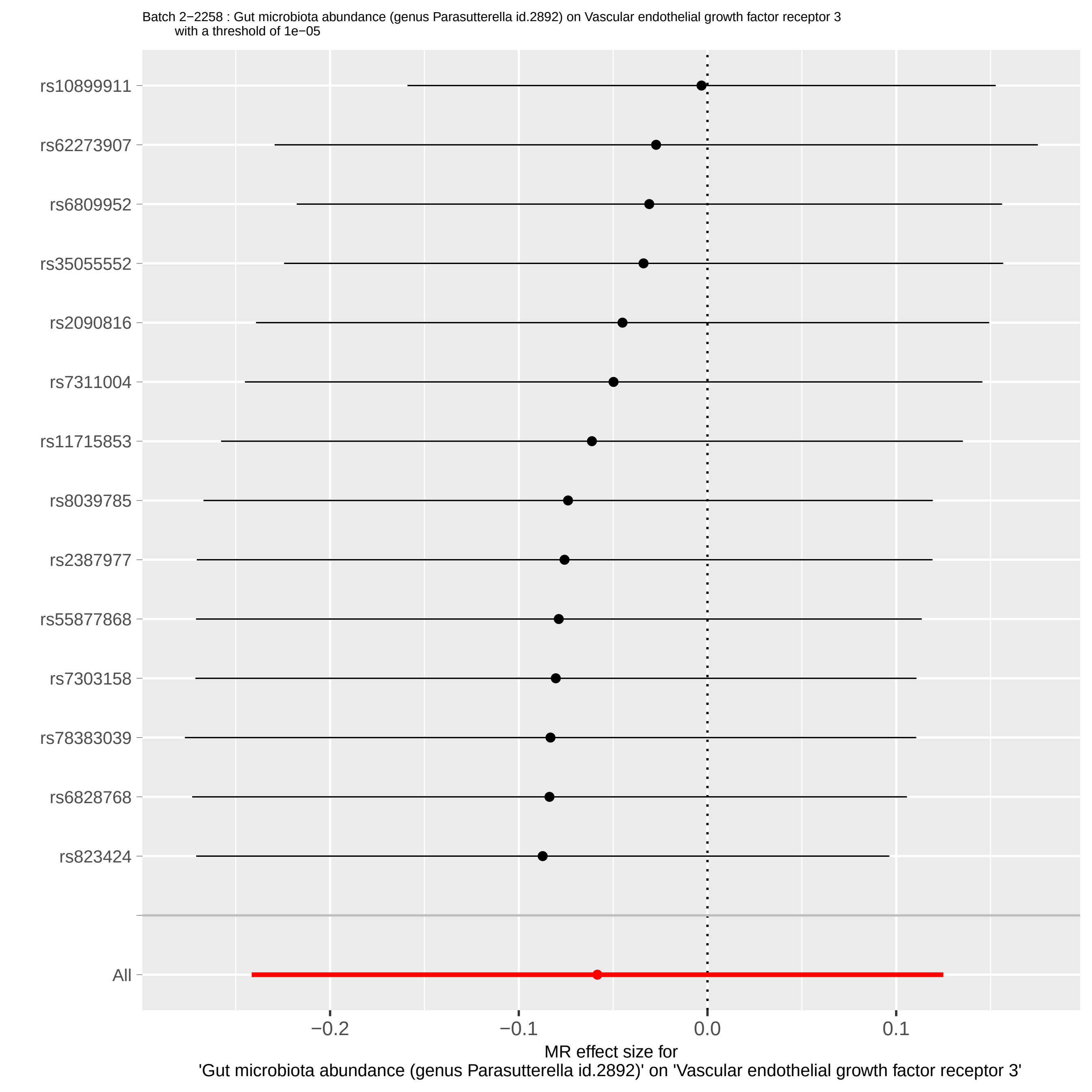
Batch 2−2258 : Gut microbiota abundance (genus Parasutterella id.2892) on Vascular endothelial growth factor receptor 3
with a threshold of 1e−05
rs10899911
rs62273907
rs6809952
rs35055552
rs2090816
rs7311004
rs11715853
rs8039785
rs2387977
rs55877868
rs7303158
rs78383039
rs6828768
rs823424
All
−0.2
−0.1
0.0
0.1
MR effect size for
'Gut microbiota abundance (genus Parasutterella id.2892)' on 'Vascular endothelial growth factor receptor 3'

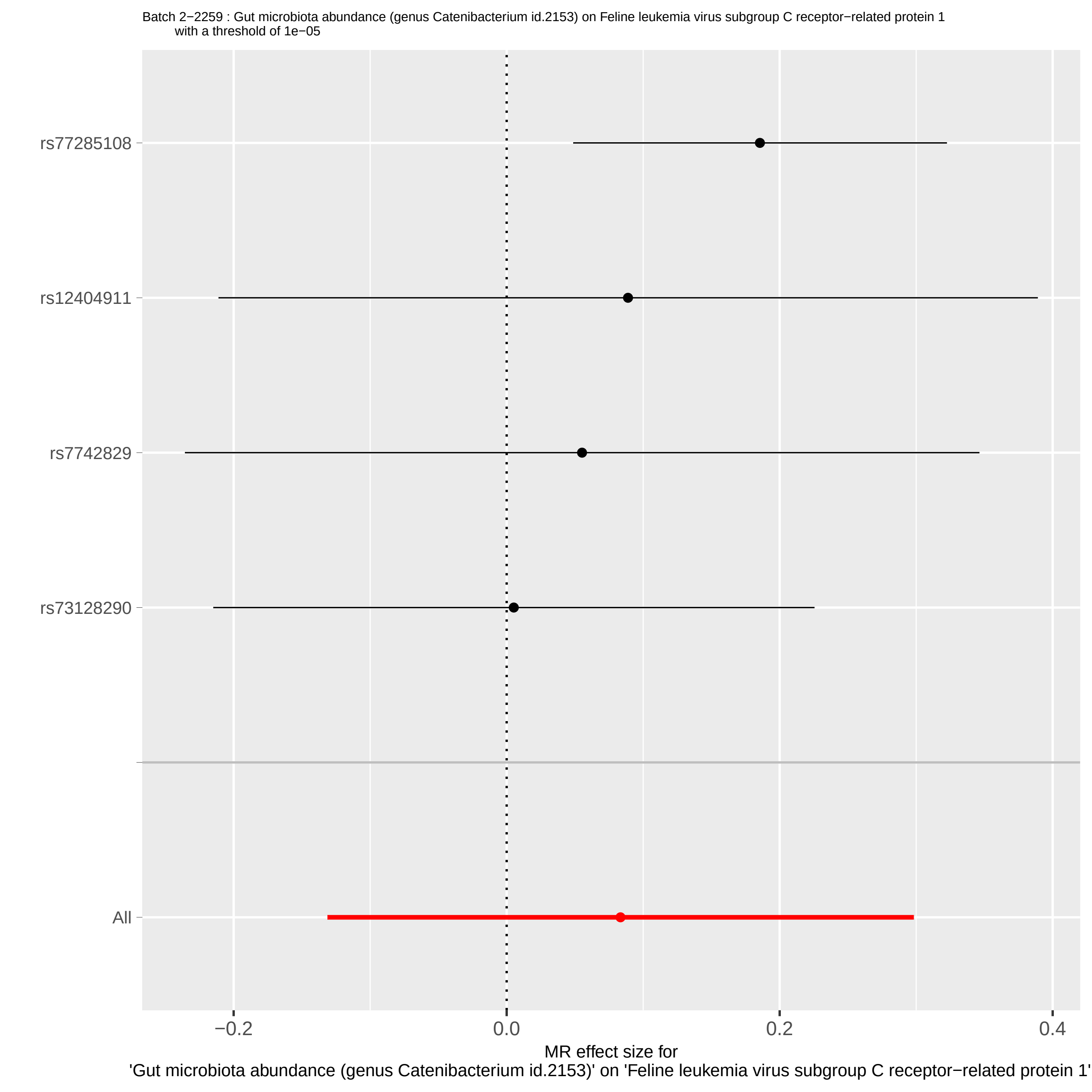
Batch 2−2259 : Gut microbiota abundance (genus Catenibacterium id.2153) on Feline leukemia virus subgroup C receptor−related protein 1
with a threshold of 1e−05
rs77285108
rs12404911
rs7742829
rs73128290
All
−0.2
0.0
0.2
0.4
MR effect size for
'Gut microbiota abundance (genus Catenibacterium id.2153)' on 'Feline leukemia virus subgroup C receptor−related protein 1'

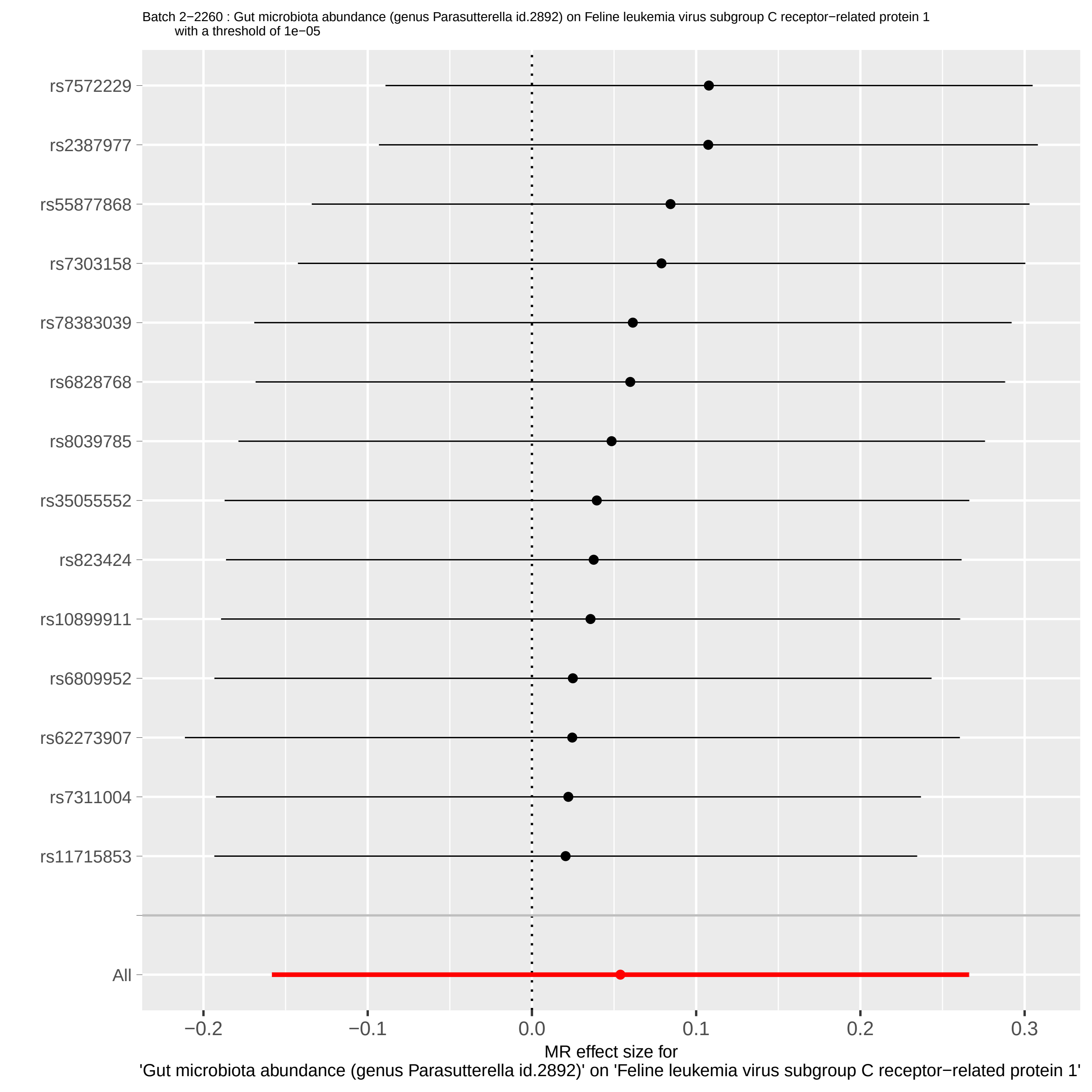
Batch 2−2260 : Gut microbiota abundance (genus Parasutterella id.2892) on Feline leukemia virus subgroup C receptor−related protein 1
with a threshold of 1e−05
rs7572229
rs2387977
rs55877868
rs7303158
rs78383039
rs6828768
rs8039785
rs35055552
rs823424
rs10899911
rs6809952
rs62273907
rs7311004
rs11715853
All
−0.2
−0.1
0.0
0.1
0.2
0.3
MR effect size for
'Gut microbiota abundance (genus Parasutterella id.2892)' on 'Feline leukemia virus subgroup C receptor−related protein 1'

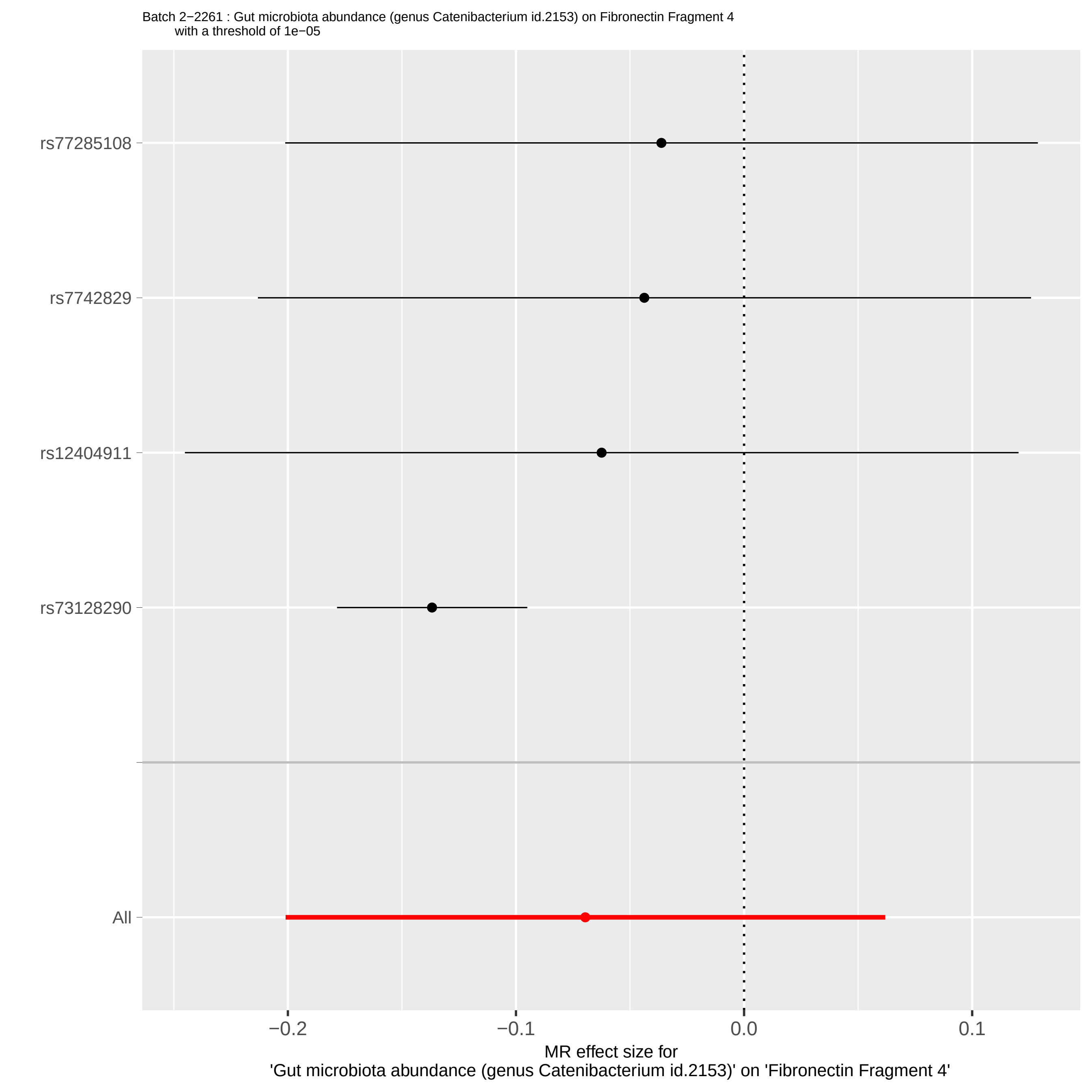
Batch 2−2261 : Gut microbiota abundance (genus Catenibacterium id.2153) on Fibronectin Fragment 4
with a threshold of 1e−05
rs77285108
rs7742829
rs12404911
rs73128290
All
−0.2
−0.1
0.0
0.1
MR effect size for
'Gut microbiota abundance (genus Catenibacterium id.2153)' on 'Fibronectin Fragment 4'

Batch 2−2262 : Gut microbiota abundance (genus Parasutterella id.2892) on Fibronectin Fragment 4
with a threshold of 1e−05

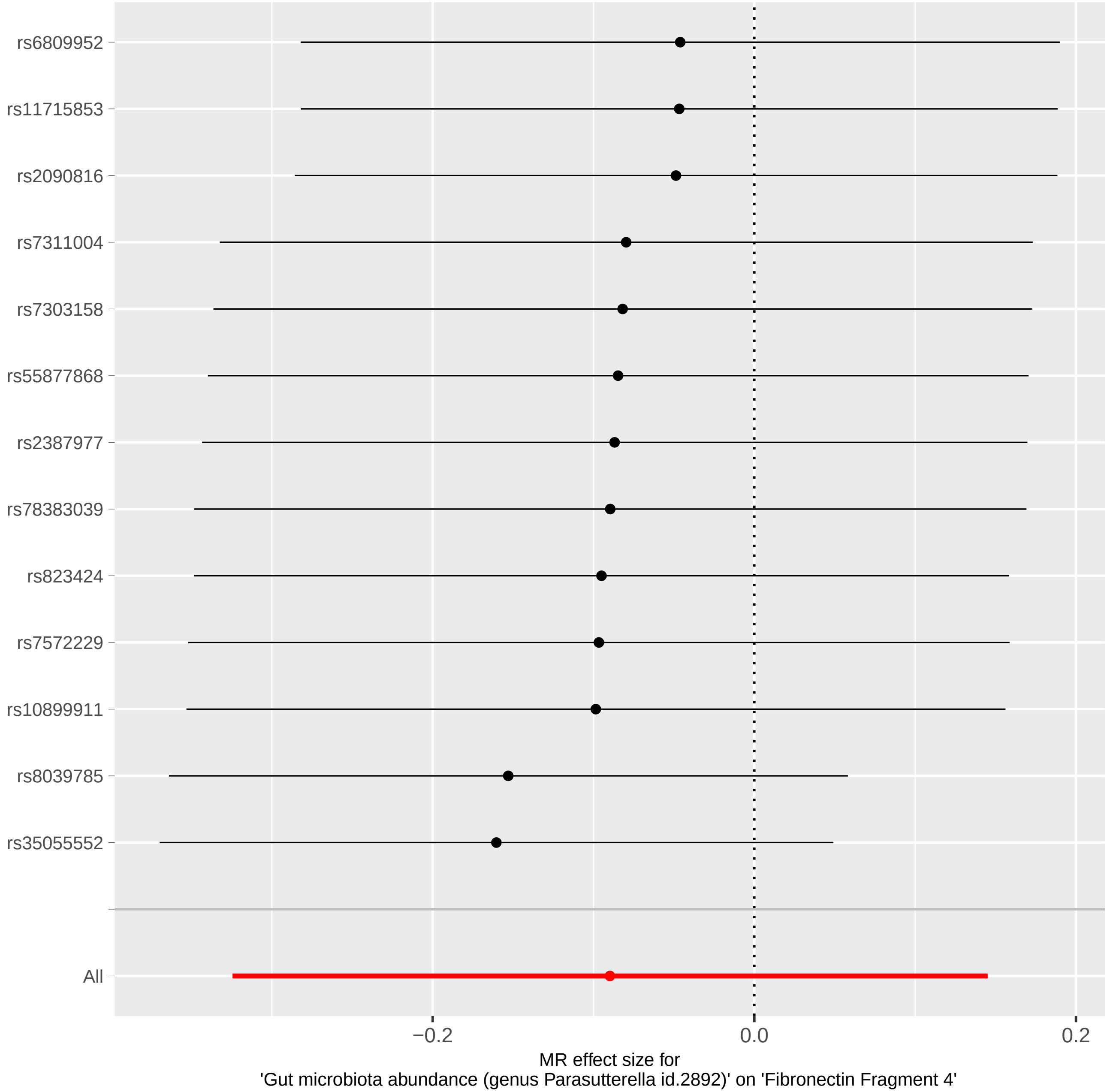

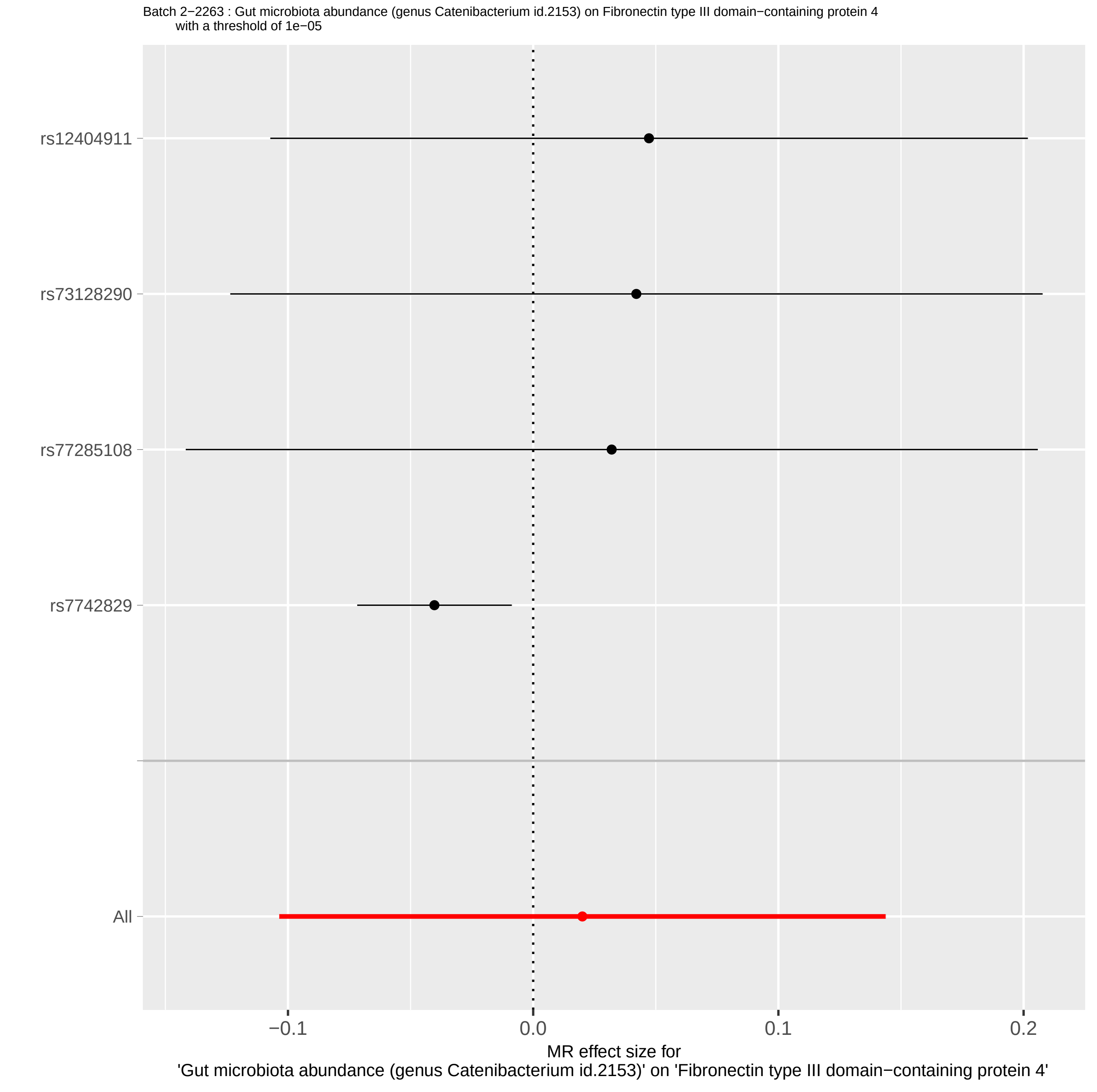
Batch 2−2263 : Gut microbiota abundance (genus Catenibacterium id.2153) on Fibronectin type III domain−containing protein 4
with a threshold of 1e−05
rs12404911
rs73128290
rs77285108
rs7742829
All
−0.1
0.0
0.1
0.2
MR effect size for
'Gut microbiota abundance (genus Catenibacterium id.2153)' on 'Fibronectin type III domain−containing protein 4'

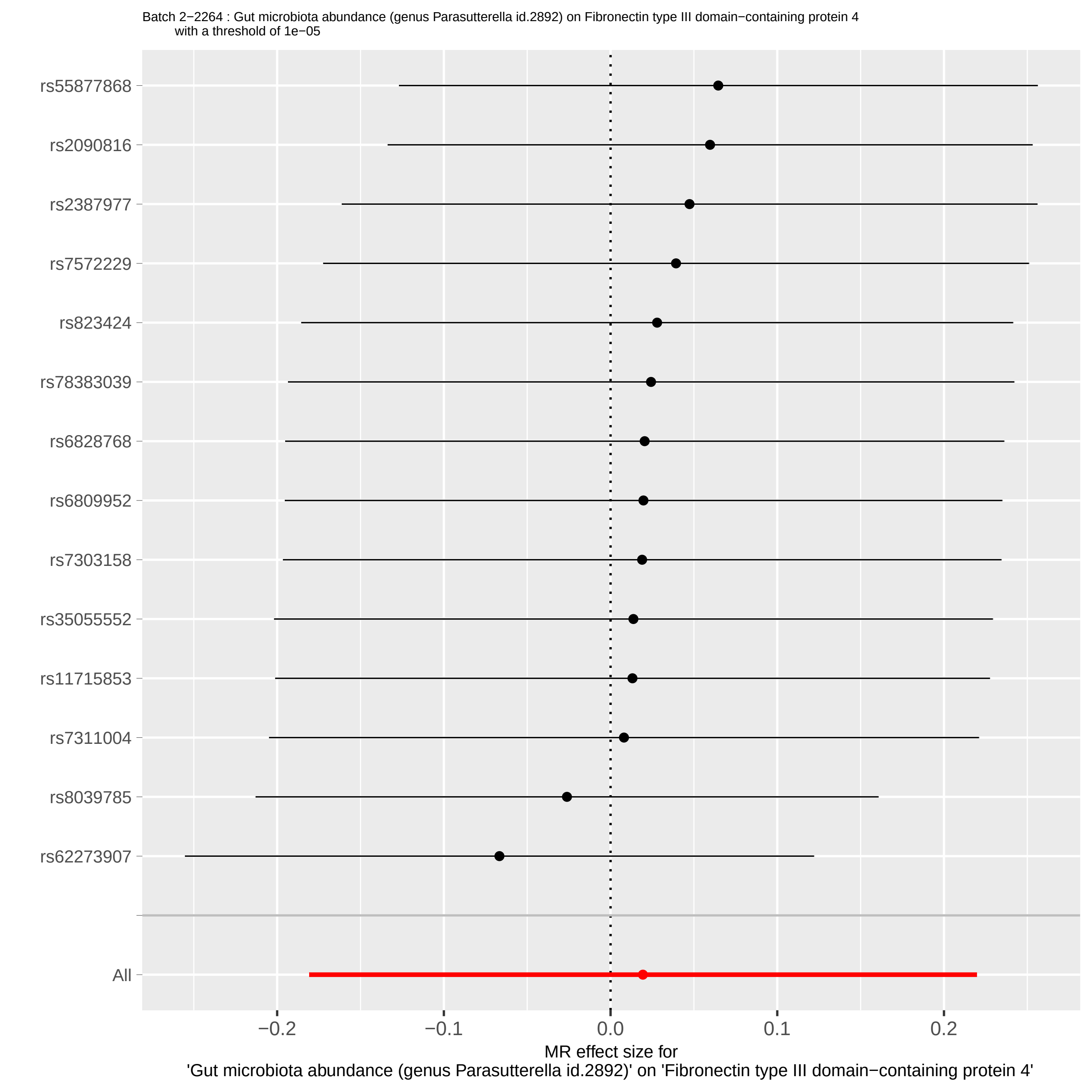

Batch 2−2264 : Gut microbiota abundance (genus Parasutterella id.2892) on Fibronectin type III domain−containing protein 4
with a threshold of 1e−05
rs55877868
rs2090816
rs2387977
rs7572229
rs823424
rs78383039
rs6828768
rs6809952
rs7303158
rs35055552
rs11715853
rs7311004
rs8039785
rs62273907
All
−0.2
−0.1
0.0
0.1
0.2
MR effect size for
'Gut microbiota abundance (genus Parasutterella id.2892)' on 'Fibronectin type III domain−containing protein 4'

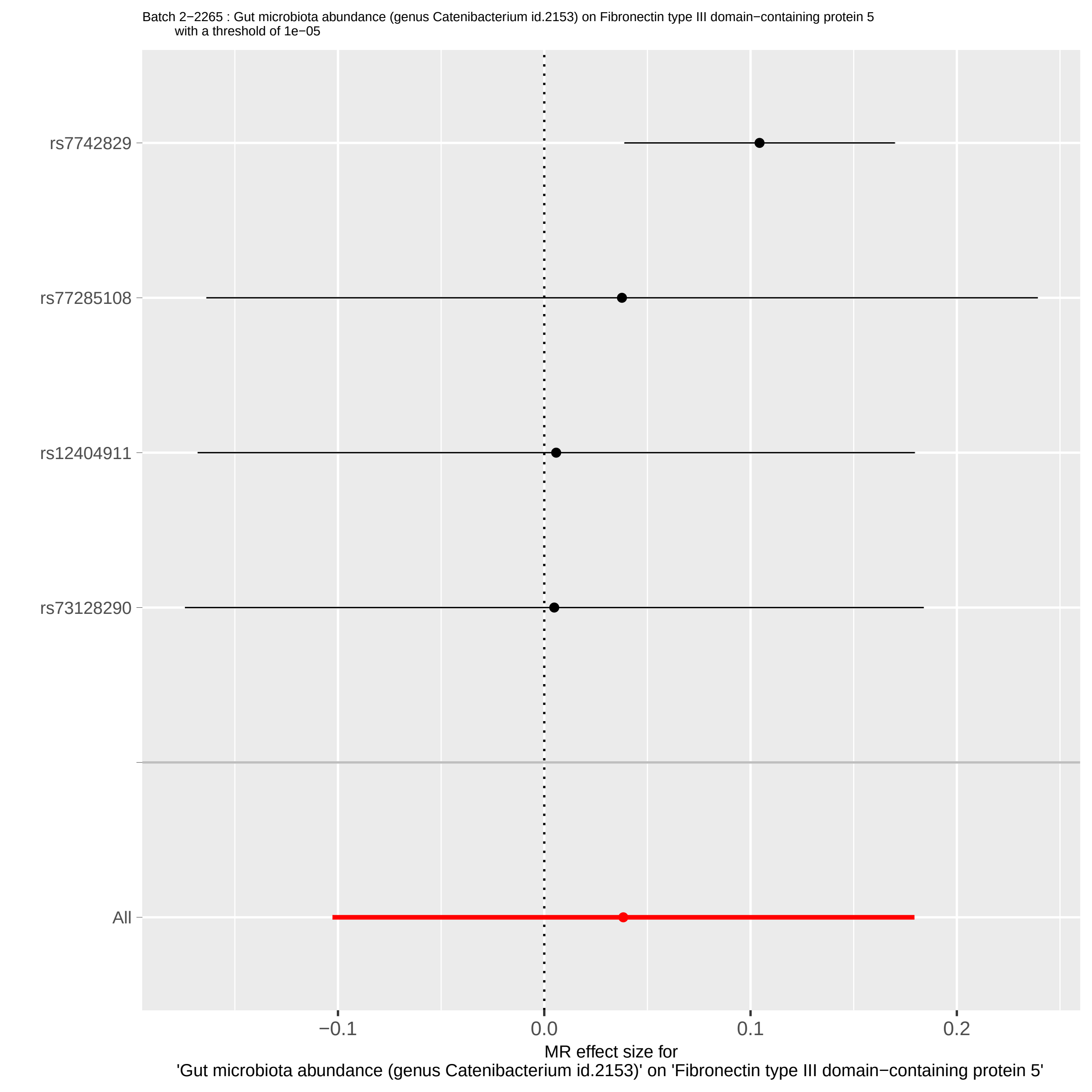
Batch 2−2265 : Gut microbiota abundance (genus Catenibacterium id.2153) on Fibronectin type III domain−containing protein 5
with a threshold of 1e−05
rs7742829
rs77285108
rs12404911
rs73128290
All
−0.1
0.0
0.1
0.2
MR effect size for
'Gut microbiota abundance (genus Catenibacterium id.2153)' on 'Fibronectin type III domain−containing protein 5'

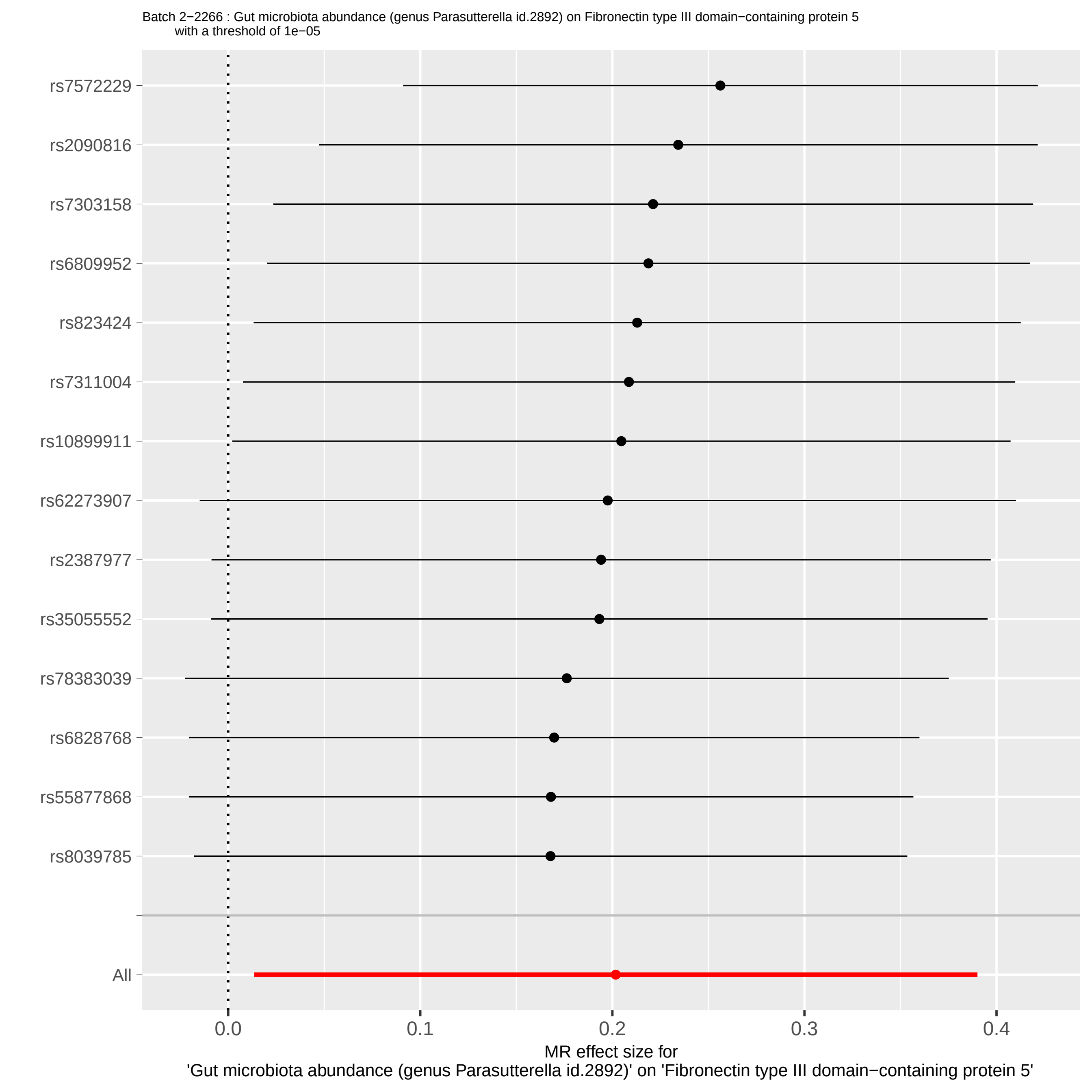

Batch 2−2266 : Gut microbiota abundance (genus Parasutterella id.2892) on Fibronectin type III domain−containing protein 5
with a threshold of 1e−05
rs7572229
rs2090816
rs7303158
rs6809952
rs823424
rs7311004
rs10899911
rs62273907
rs2387977
rs35055552
rs78383039
rs6828768
rs55877868
rs8039785
All
0.0
0.1
0.2
0.3
0.4
MR effect size for
'Gut microbiota abundance (genus Parasutterella id.2892)' on 'Fibronectin type III domain−containing protein 5'

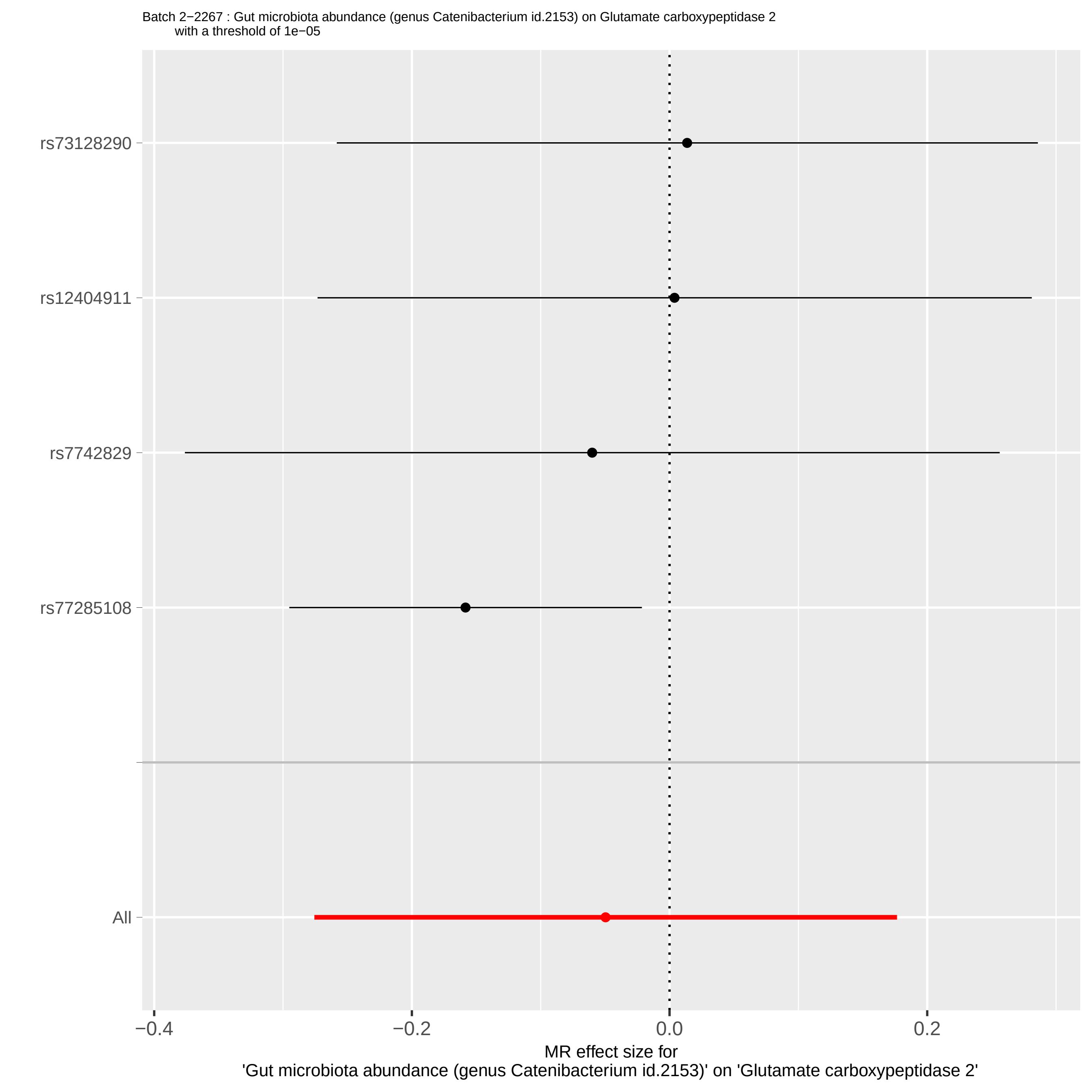
Batch 2−2267 : Gut microbiota abundance (genus Catenibacterium id.2153) on Glutamate carboxypeptidase 2
with a threshold of 1e−05
rs73128290
rs12404911
rs7742829
rs77285108
All
−0.4
−0.2
0.0
0.2
MR effect size for
'Gut microbiota abundance (genus Catenibacterium id.2153)' on 'Glutamate carboxypeptidase 2'

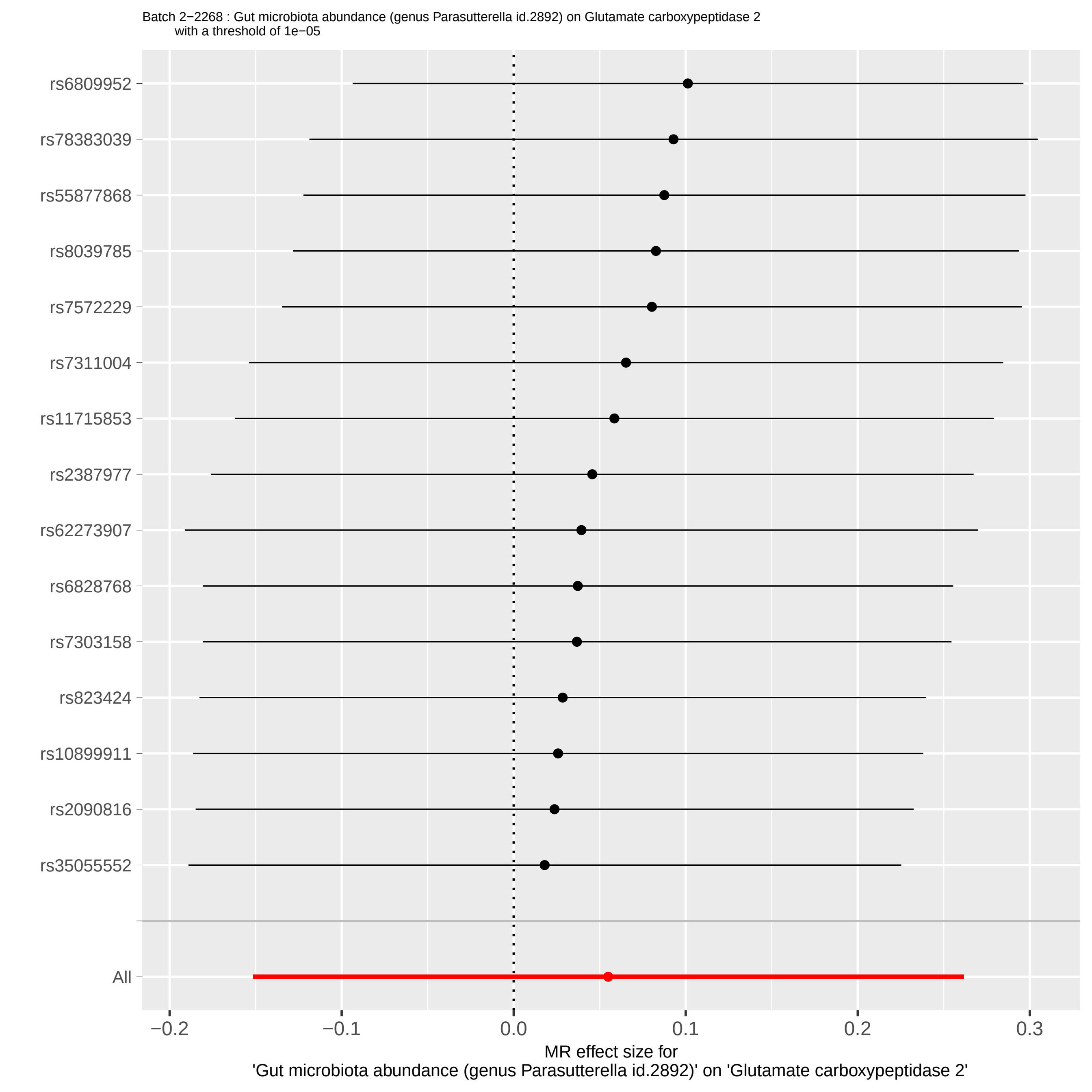

Batch 2−2268 : Gut microbiota abundance (genus Parasutterella id.2892) on Glutamate carboxypeptidase 2
with a threshold of 1e−05
rs6809952
rs78383039
rs55877868
rs8039785
rs7572229
rs7311004
rs11715853
rs2387977
rs62273907
rs6828768
rs7303158
rs823424
rs10899911
rs2090816
rs35055552
All
−0.2
−0.1
0.0
0.1
0.2
0.3
MR effect size for
'Gut microbiota abundance (genus Parasutterella id.2892)' on 'Glutamate carboxypeptidase 2'

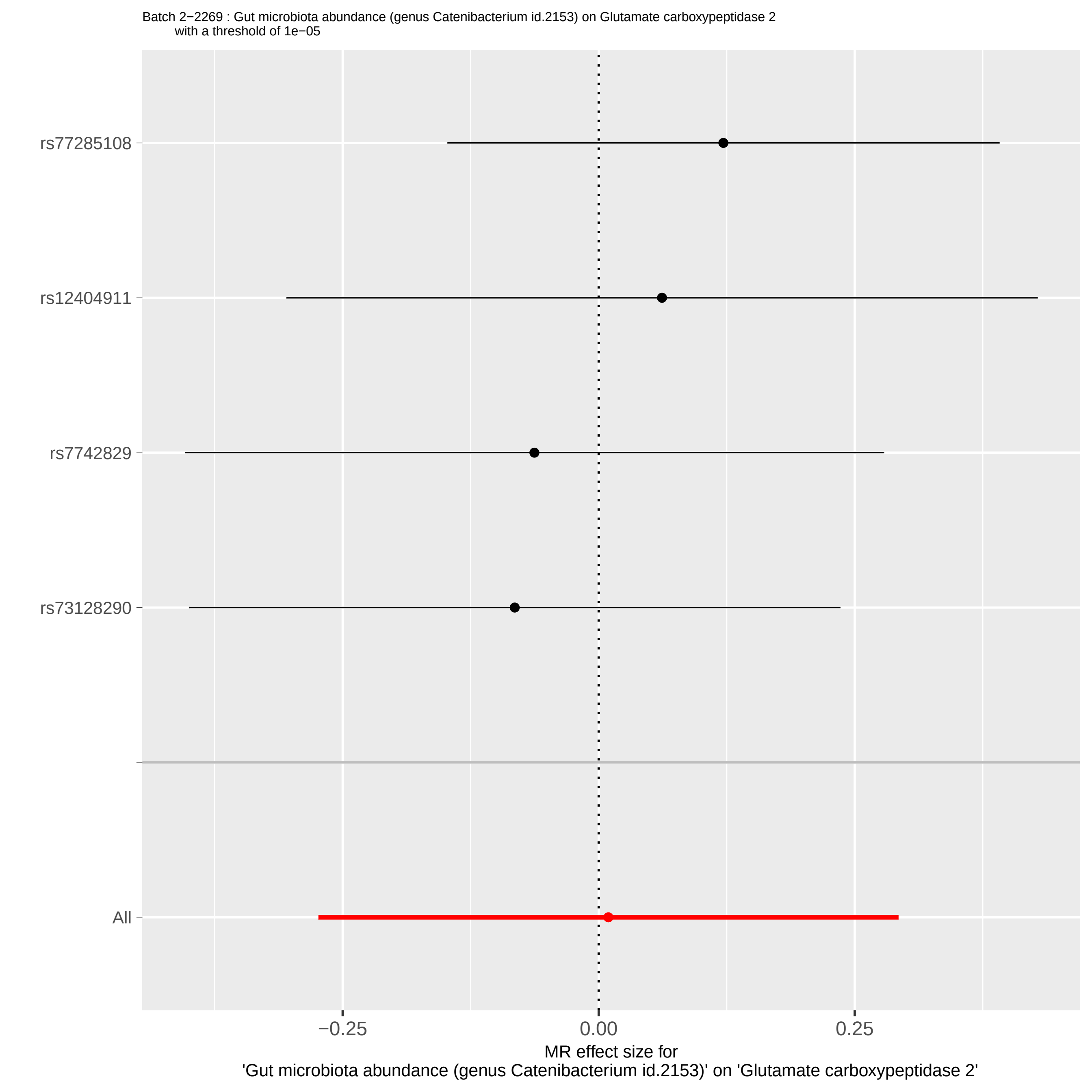

Batch 2−2269 : Gut microbiota abundance (genus Catenibacterium id.2153) on Glutamate carboxypeptidase 2
with a threshold of 1e−05
rs77285108
rs12404911
rs7742829
rs73128290
All
−0.25
0.00
0.25
MR effect size for
'Gut microbiota abundance (genus Catenibacterium id.2153)' on 'Glutamate carboxypeptidase 2'

Batch 2−2270 : Gut microbiota abundance (genus Parasutterella id.2892) on Glutamate carboxypeptidase 2
with a threshold of 1e−05

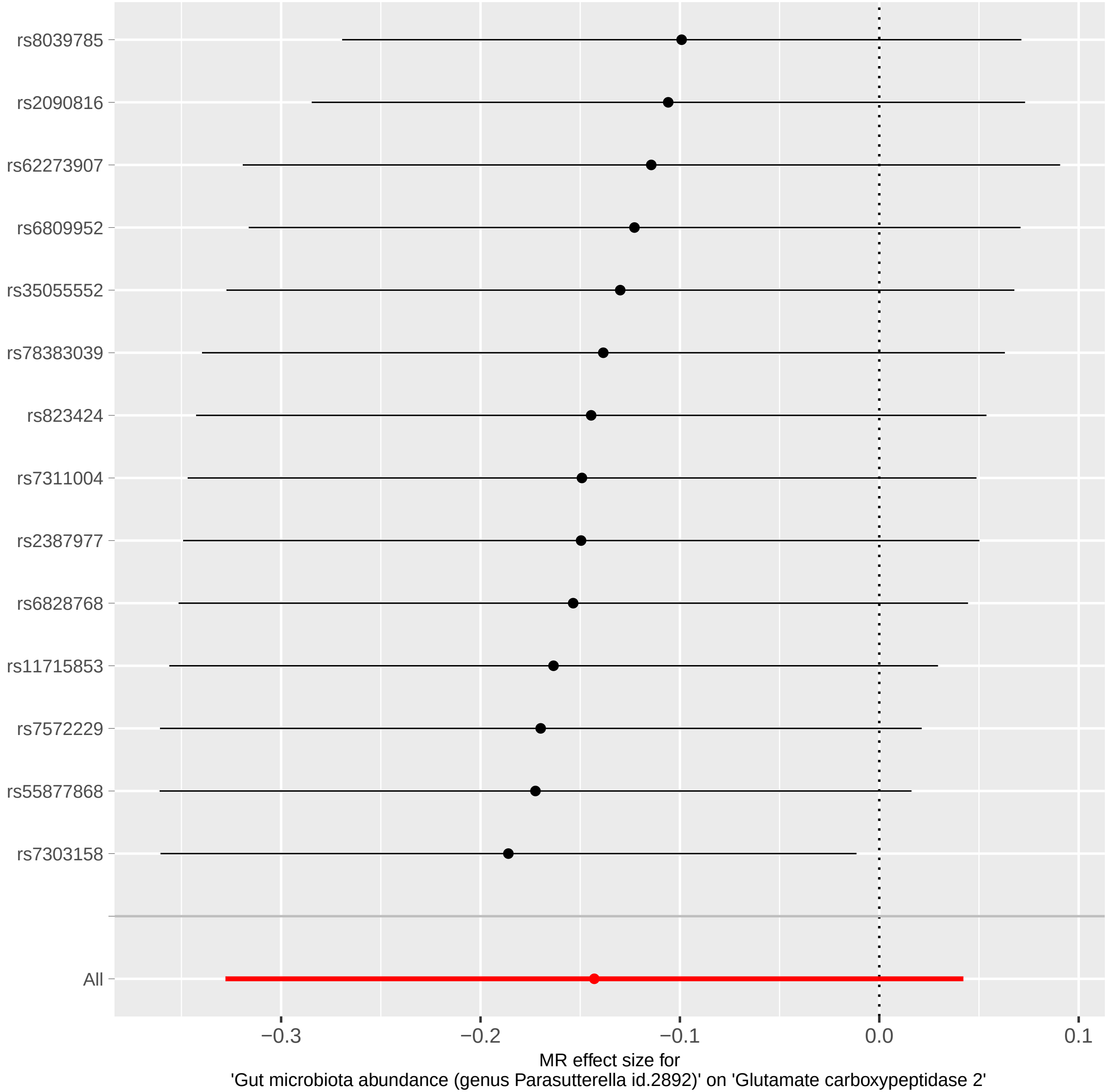

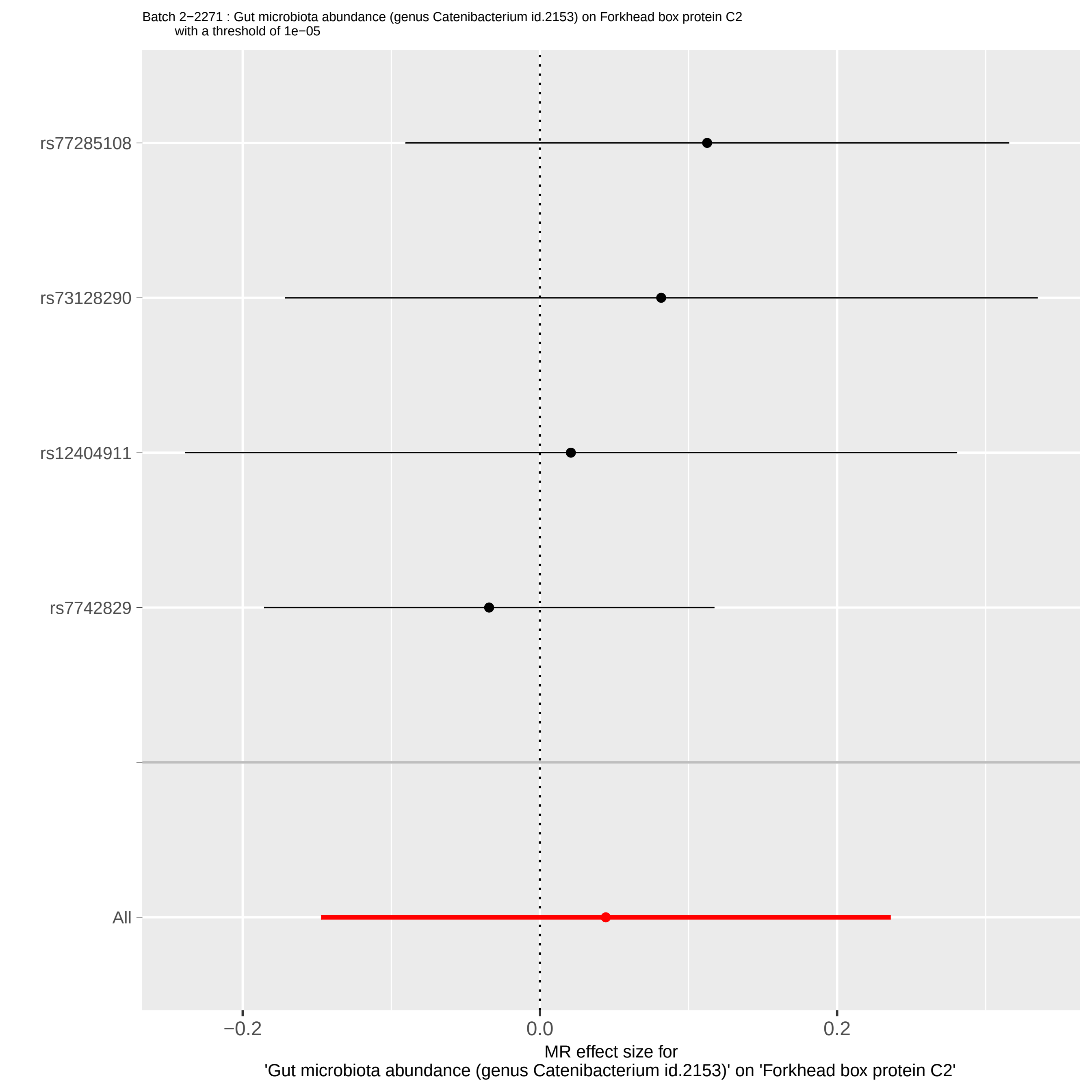
Batch 2−2271 : Gut microbiota abundance (genus Catenibacterium id.2153) on Forkhead box protein C2
with a threshold of 1e−05
rs77285108
rs73128290
rs12404911
rs7742829
All
−0.2
0.0
0.2
MR effect size for
'Gut microbiota abundance (genus Catenibacterium id.2153)' on 'Forkhead box protein C2'

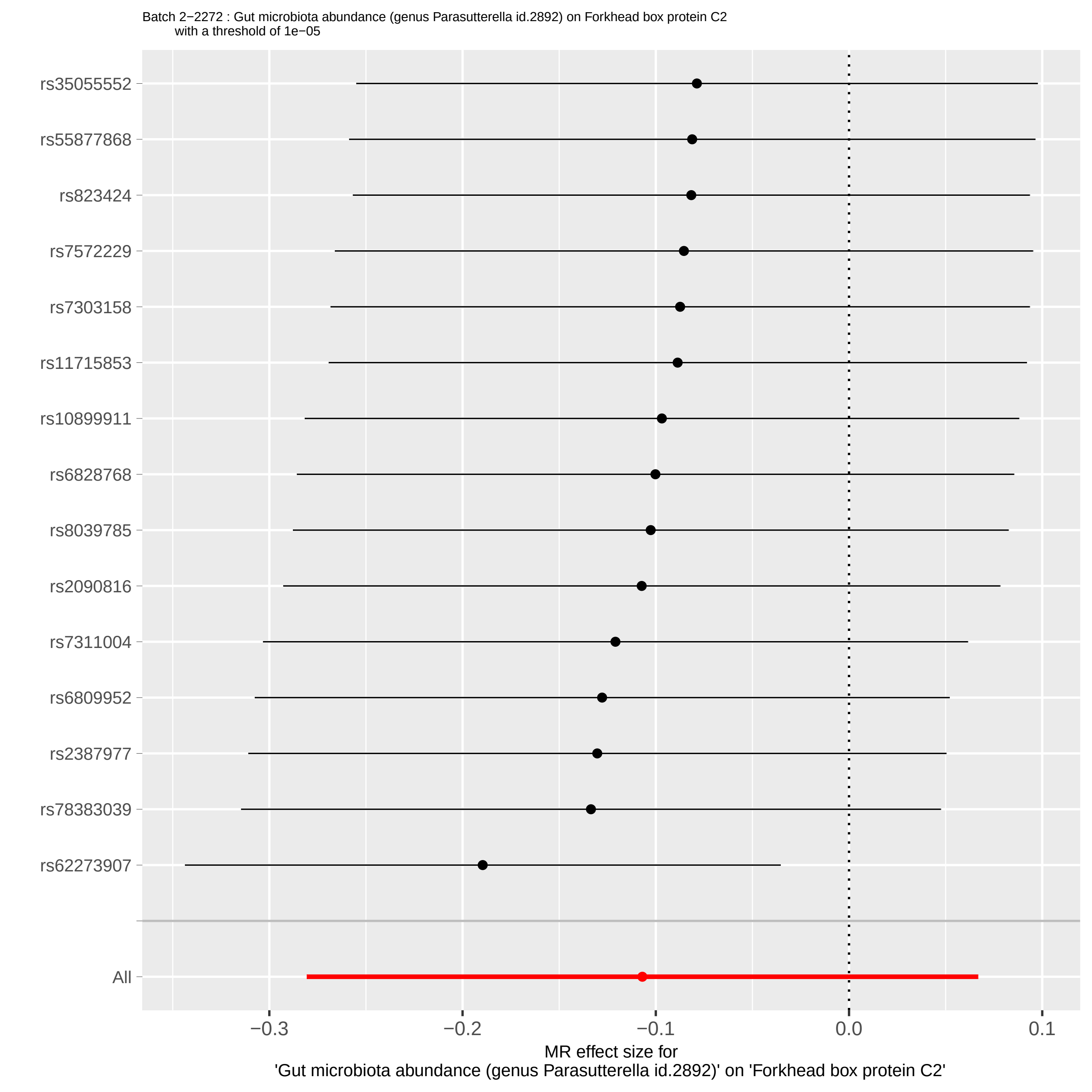

Batch 2−2272 : Gut microbiota abundance (genus Parasutterella id.2892) on Forkhead box protein C2
with a threshold of 1e−05
rs35055552
rs55877868
rs823424
rs7572229
rs7303158
rs11715853
rs10899911
rs6828768
rs8039785
rs2090816
rs7311004
rs6809952
rs2387977
rs78383039
rs62273907
All
−0.3
−0.2
−0.1
0.0
0.1
MR effect size for
'Gut microbiota abundance (genus Parasutterella id.2892)' on 'Forkhead box protein C2'

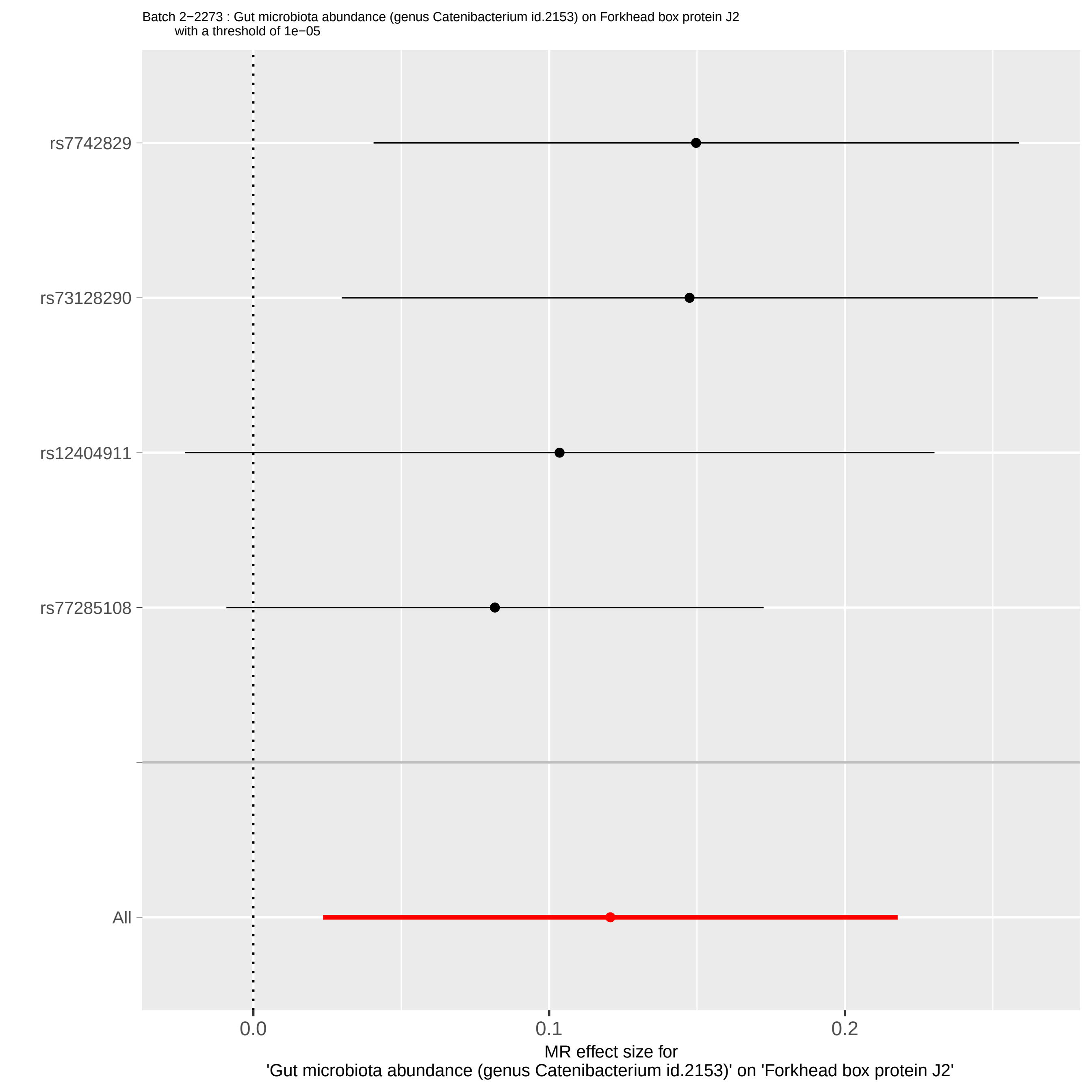

Batch 2−2273 : Gut microbiota abundance (genus Catenibacterium id.2153) on Forkhead box protein J2
with a threshold of 1e−05
rs7742829
rs73128290
rs12404911
rs77285108
All
0.0
0.1
0.2
MR effect size for
'Gut microbiota abundance (genus Catenibacterium id.2153)' on 'Forkhead box protein J2'

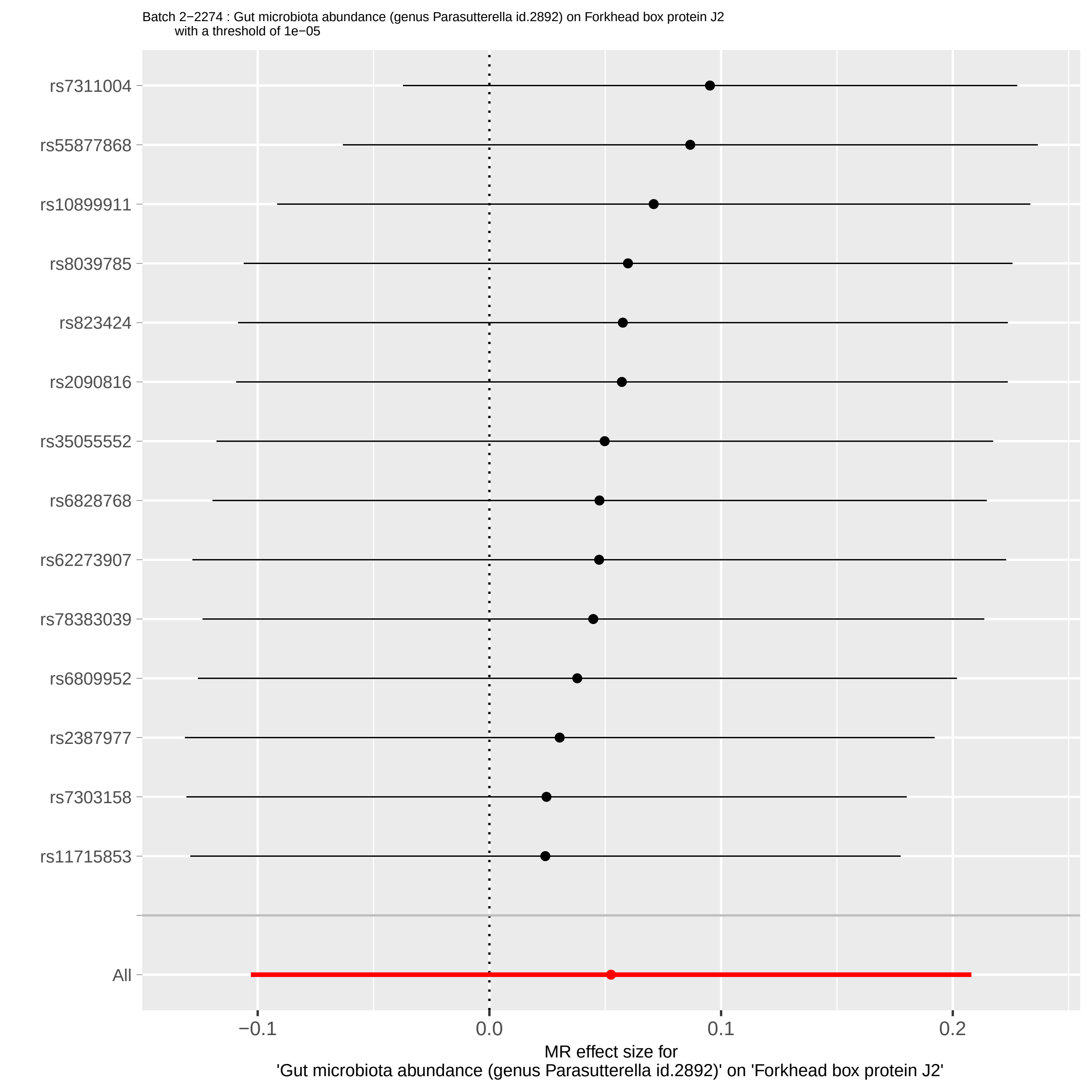

Batch 2−2274 : Gut microbiota abundance (genus Parasutterella id.2892) on Forkhead box protein J2
with a threshold of 1e−05
rs7311004
rs55877868
rs10899911
rs8039785
rs823424
rs2090816
rs35055552
rs6828768
rs62273907
rs78383039
rs6809952
rs2387977
rs7303158
rs11715853
All
−0.1
0.0
0.1
0.2
MR effect size for
'Gut microbiota abundance (genus Parasutterella id.2892)' on 'Forkhead box protein J2'

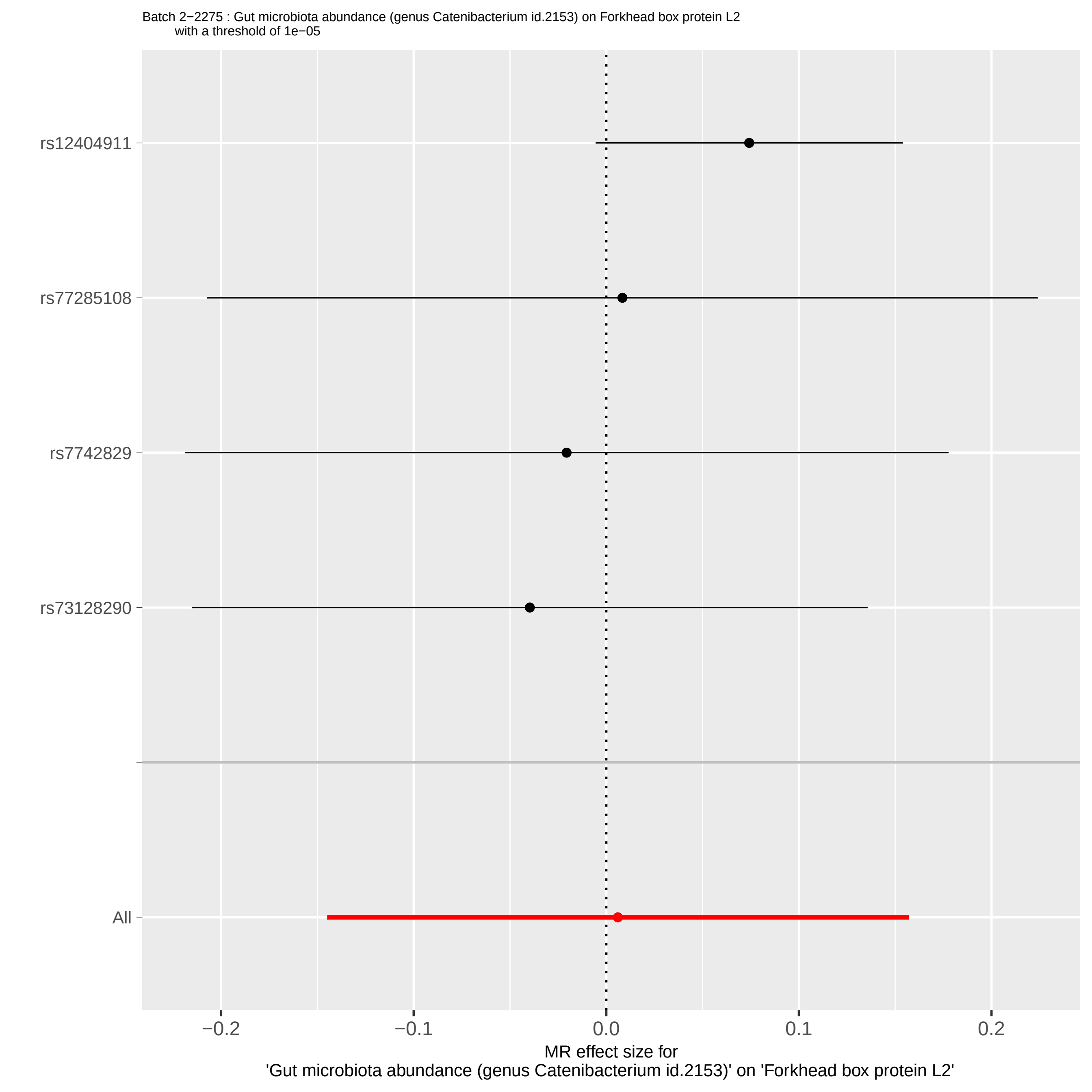
Batch 2−2275 : Gut microbiota abundance (genus Catenibacterium id.2153) on Forkhead box protein L2
with a threshold of 1e−05
rs12404911
rs77285108
rs7742829
rs73128290
All
−0.2
−0.1
0.0
0.1
0.2
MR effect size for
'Gut microbiota abundance (genus Catenibacterium id.2153)' on 'Forkhead box protein L2'

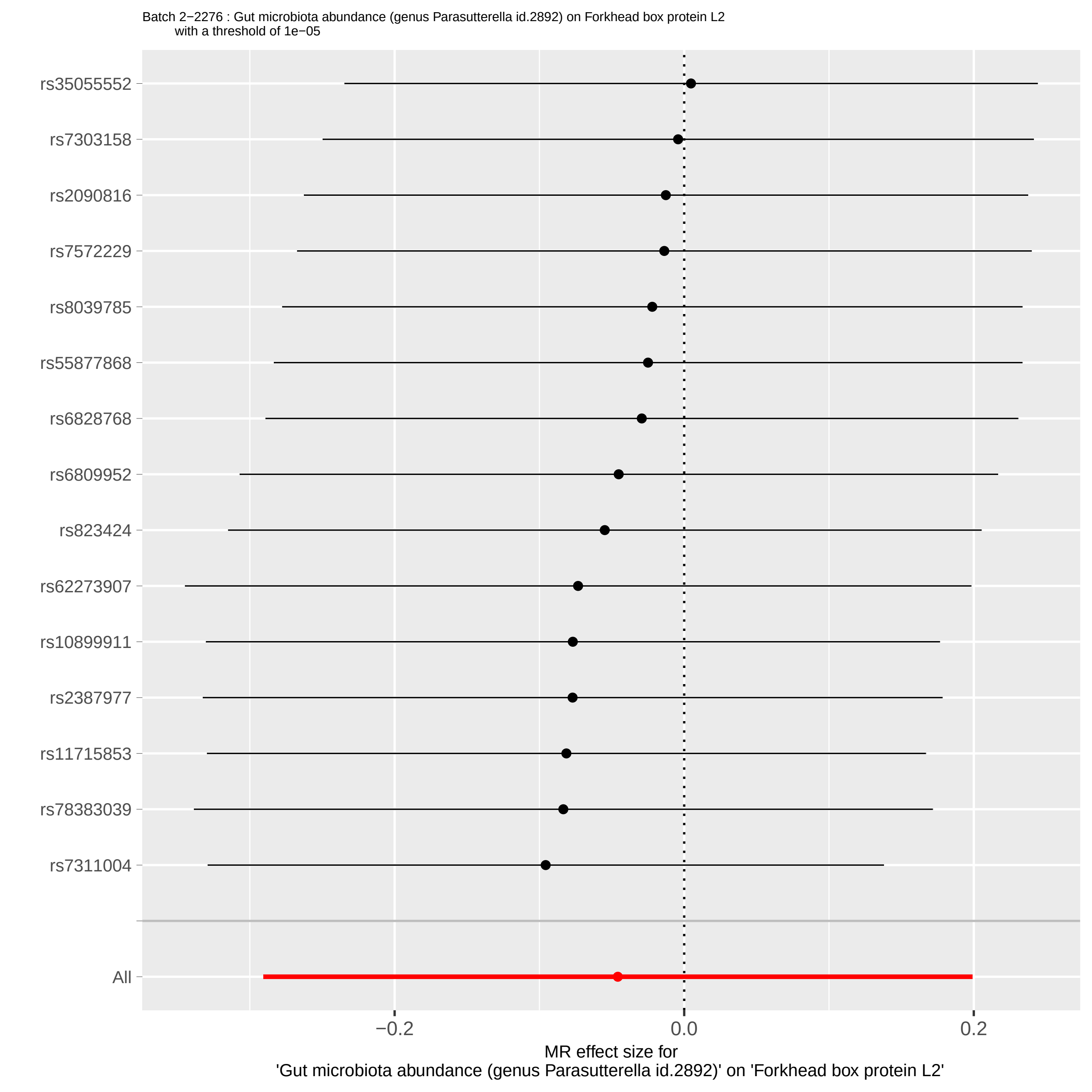
Batch 2−2276 : Gut microbiota abundance (genus Parasutterella id.2892) on Forkhead box protein L2
with a threshold of 1e−05
rs35055552
rs7303158
rs2090816
rs7572229
rs8039785
rs55877868
rs6828768
rs6809952
rs823424
rs62273907
rs10899911
rs2387977
rs11715853
rs78383039
rs7311004
All
−0.2
0.0
0.2
MR effect size for
'Gut microbiota abundance (genus Parasutterella id.2892)' on 'Forkhead box protein L2'

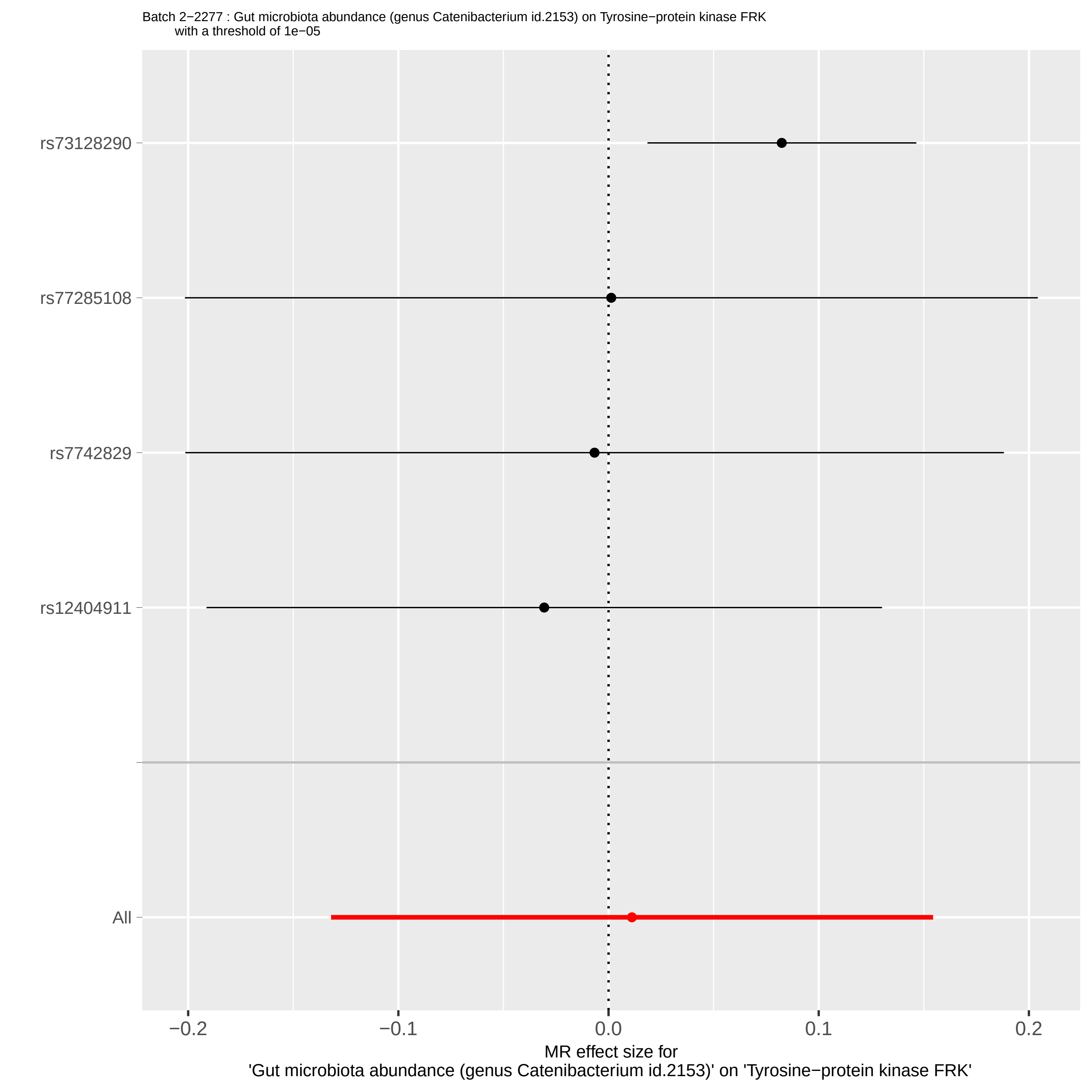
Batch 2−2277 : Gut microbiota abundance (genus Catenibacterium id.2153) on Tyrosine−protein kinase FRK
with a threshold of 1e−05
rs73128290
rs77285108
rs7742829
rs12404911
All
−0.2
−0.1
0.0
0.1
0.2
MR effect size for
'Gut microbiota abundance (genus Catenibacterium id.2153)' on 'Tyrosine−protein kinase FRK'

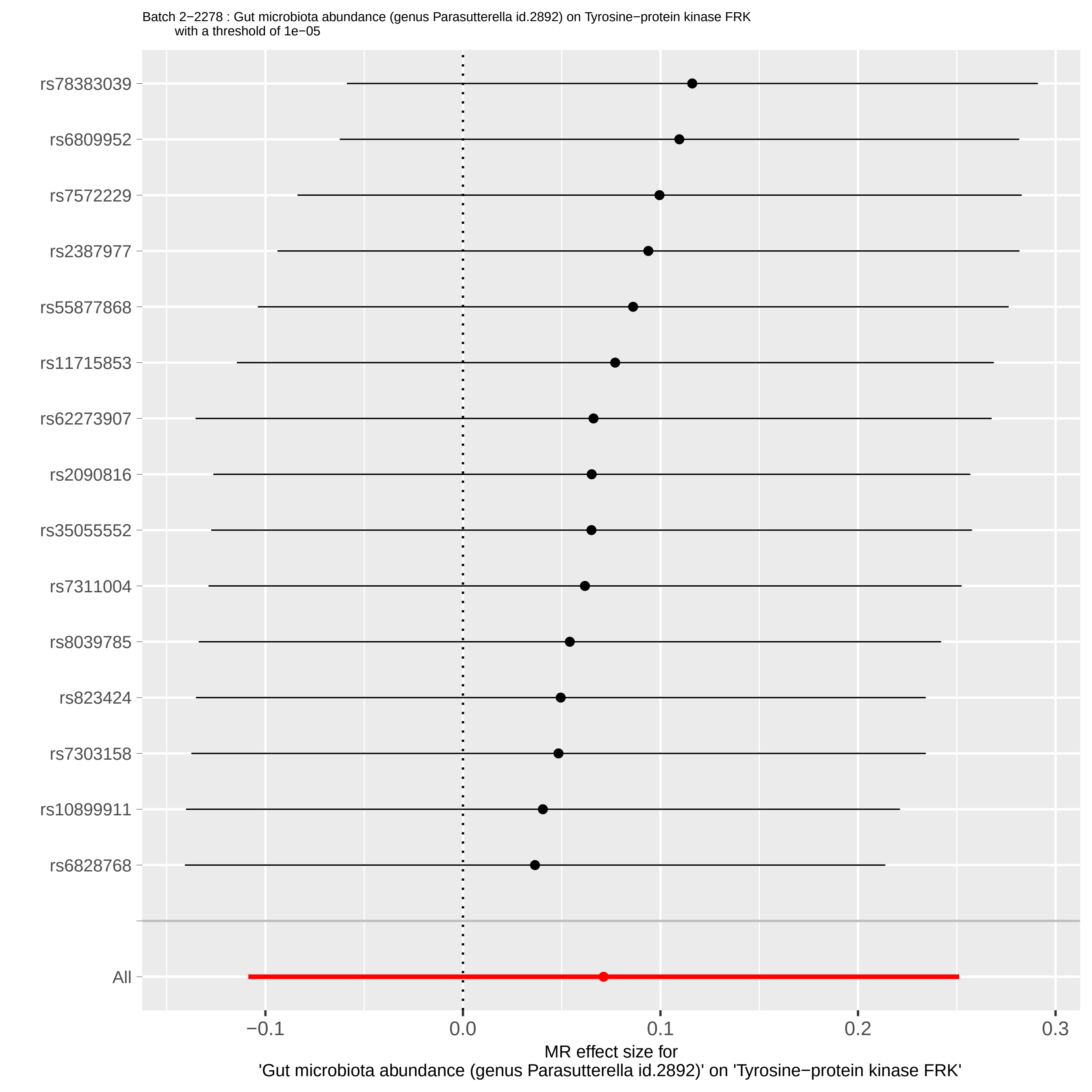
Batch 2−2278 : Gut microbiota abundance (genus Parasutterella id.2892) on Tyrosine−protein kinase FRK
with a threshold of 1e−05
rs78383039
rs6809952
rs7572229
rs2387977
rs55877868
rs11715853
rs62273907
rs2090816
rs35055552
rs7311004
rs8039785
rs823424
rs7303158
rs10899911
rs6828768
All
−0.1
0.0
0.1
0.2
0.3
MR effect size for
'Gut microbiota abundance (genus Parasutterella id.2892)' on 'Tyrosine−protein kinase FRK

Batch 2−2279 : Gut microbiota abundance (genus Catenibacterium id.2153) on Secreted frizzled−related protein 3
with a threshold of 1e−05

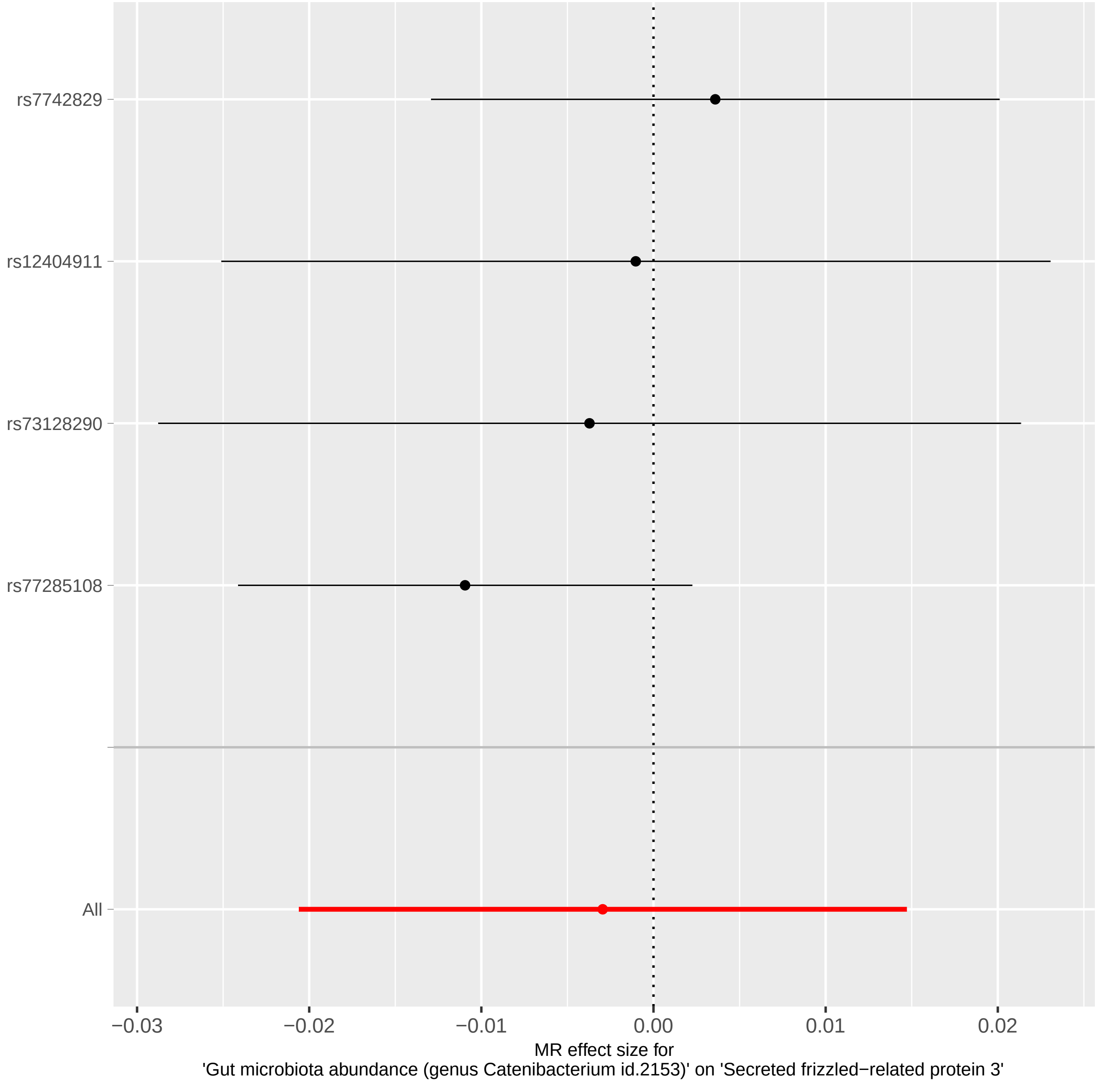

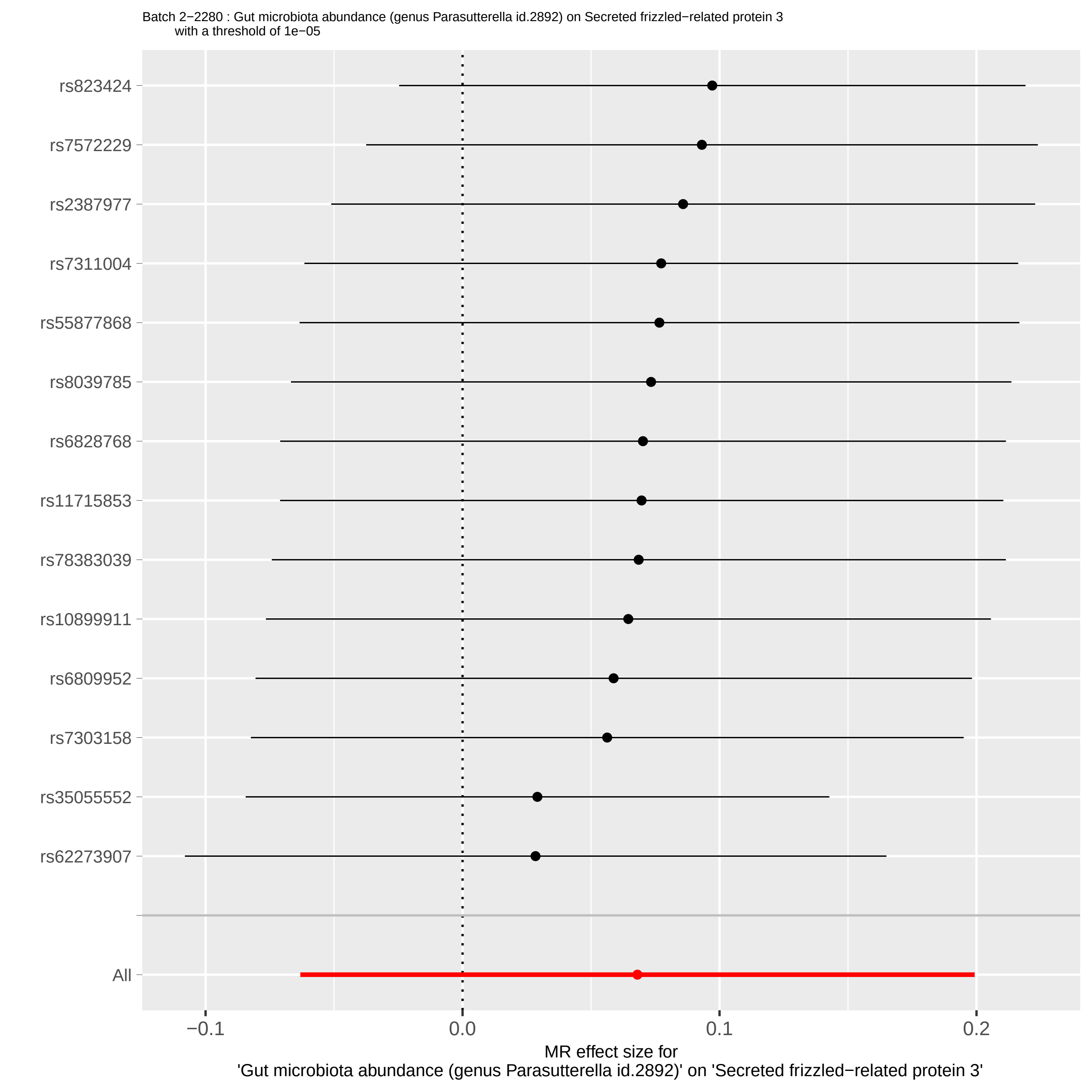

Batch 2−2280 : Gut microbiota abundance (genus Parasutterella id.2892) on Secreted frizzled−related protein 3
with a threshold of 1e−05
rs823424
rs7572229
rs2387977
rs7311004
rs55877868
rs8039785
rs6828768
rs11715853
rs78383039
rs10899911
rs6809952
rs7303158
rs35055552
rs62273907
All
−0.1
0.0
0.1
0.2
MR effect size for
'Gut microbiota abundance (genus Parasutterella id.2892)' on 'Secreted frizzled−related protein 3'

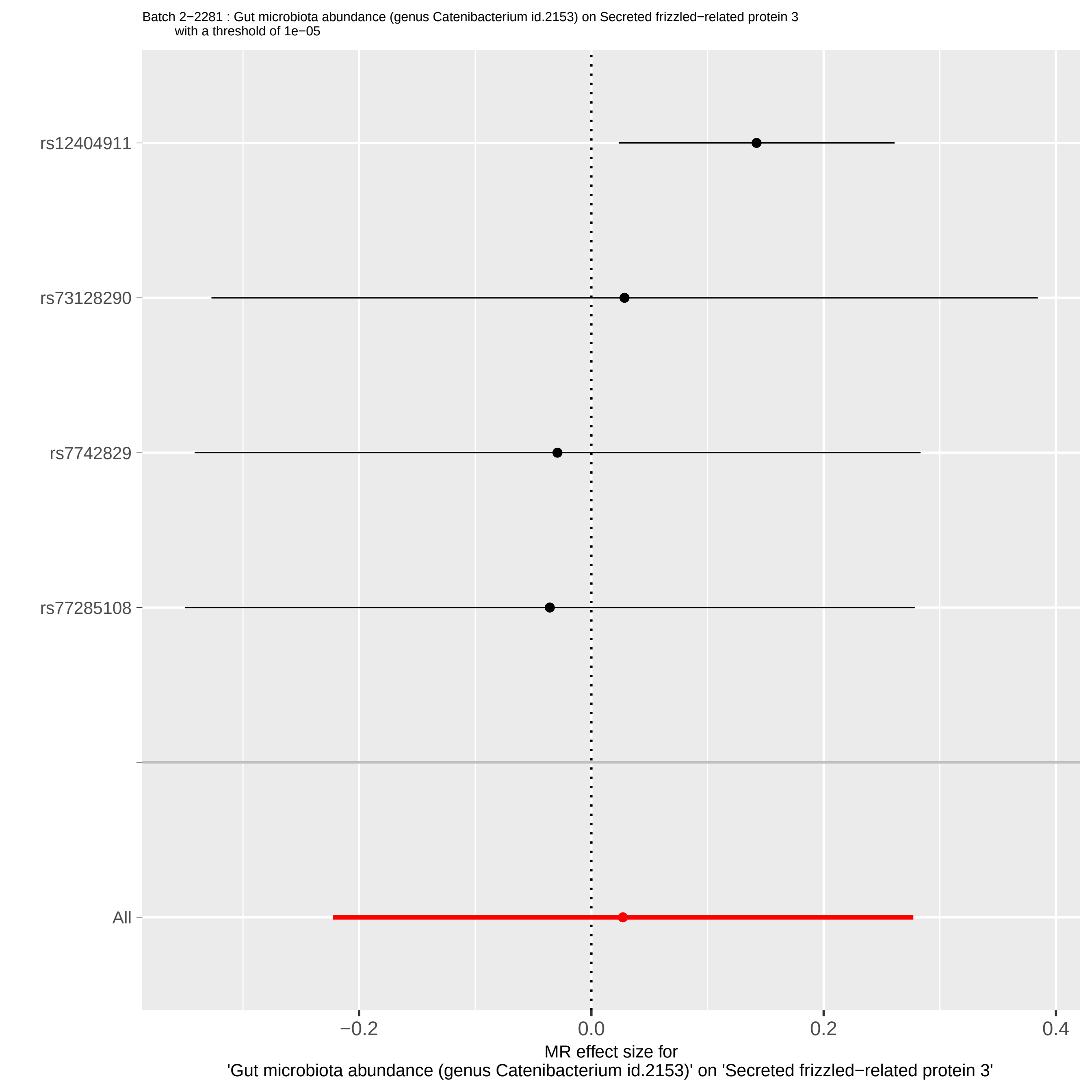
Batch 2−2281 : Gut microbiota abundance (genus Catenibacterium id.2153) on Secreted frizzled−related protein 3
with a threshold of 1e−05
rs12404911
rs73128290
rs7742829
rs77285108
All
−0.2
0.0
0.2
0.4
MR effect size for
'Gut microbiota abundance (genus Catenibacterium id.2153)' on 'Secreted frizzled−related protein 3'

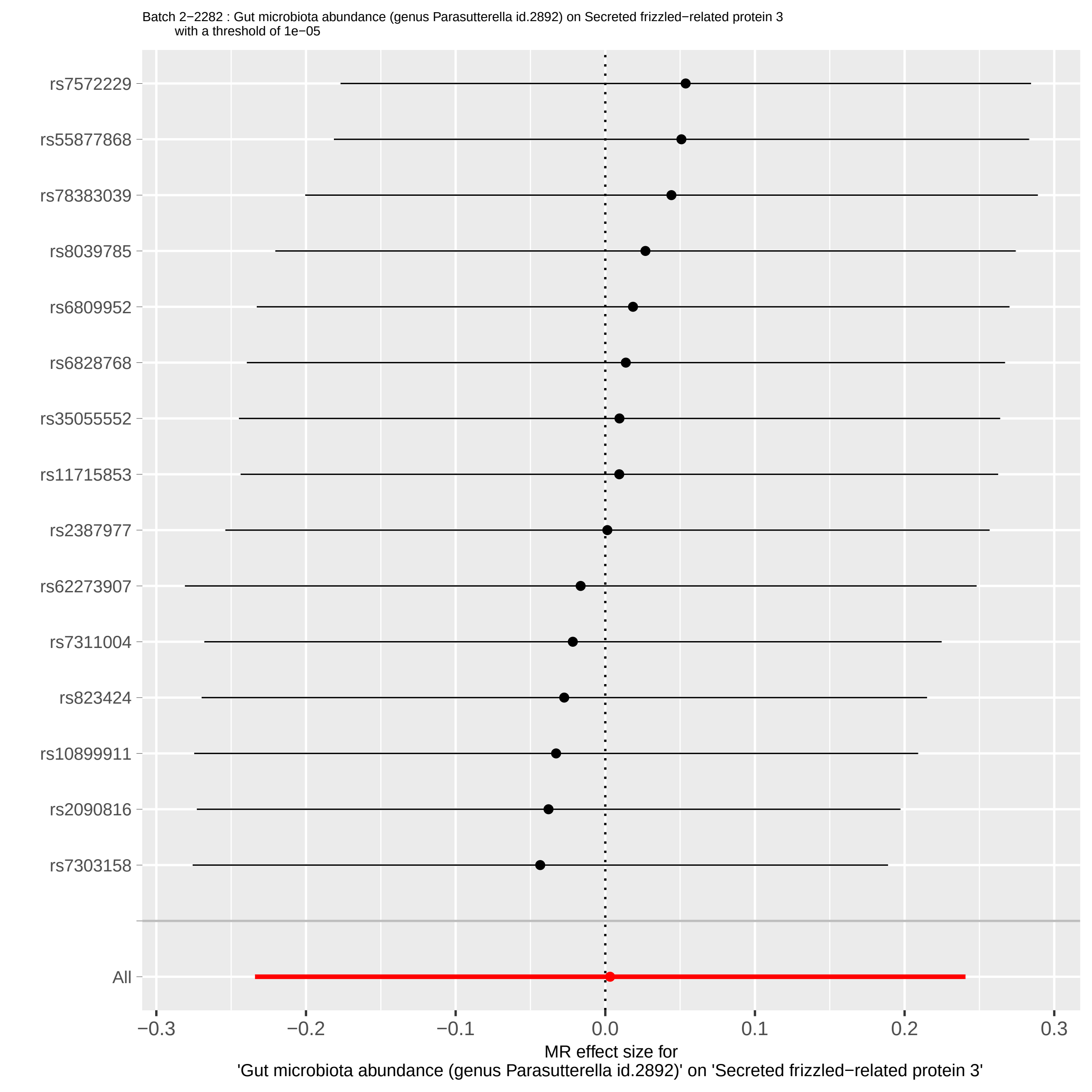
Batch 2−2282 : Gut microbiota abundance (genus Parasutterella id.2892) on Secreted frizzled−related protein 3
with a threshold of 1e−05
rs7572229
rs55877868
rs78383039
rs8039785
rs6809952
rs6828768
rs35055552
rs11715853
rs2387977
rs62273907
rs7311004
rs823424
rs10899911
rs2090816
rs7303158
All
−0.3
−0.2
−0.1
0.0
0.1
0.2
0.3
MR effect size for
'Gut microbiota abundance (genus Parasutterella id.2892)' on 'Secreted frizzled−related protein 3'

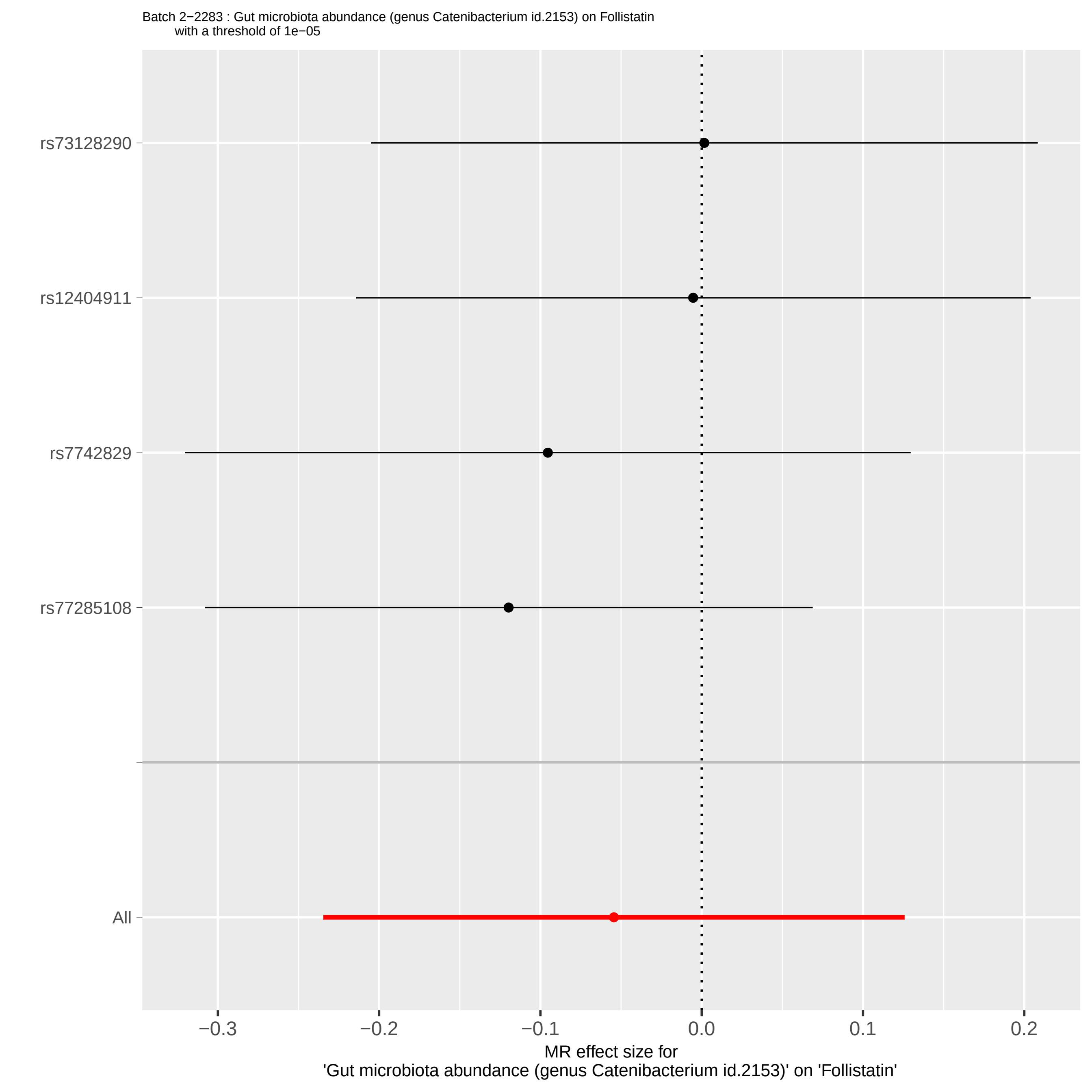
Batch 2−2283 : Gut microbiota abundance (genus Catenibacterium id.2153) on Follistatin
with a threshold of 1e−05
rs73128290
rs12404911
rs7742829
rs77285108
All
−0.3
−0.2
−0.1
0.0
0.1
0.2
MR effect size for
'Gut microbiota abundance (genus Catenibacterium id.2153)' on 'Follistatin'

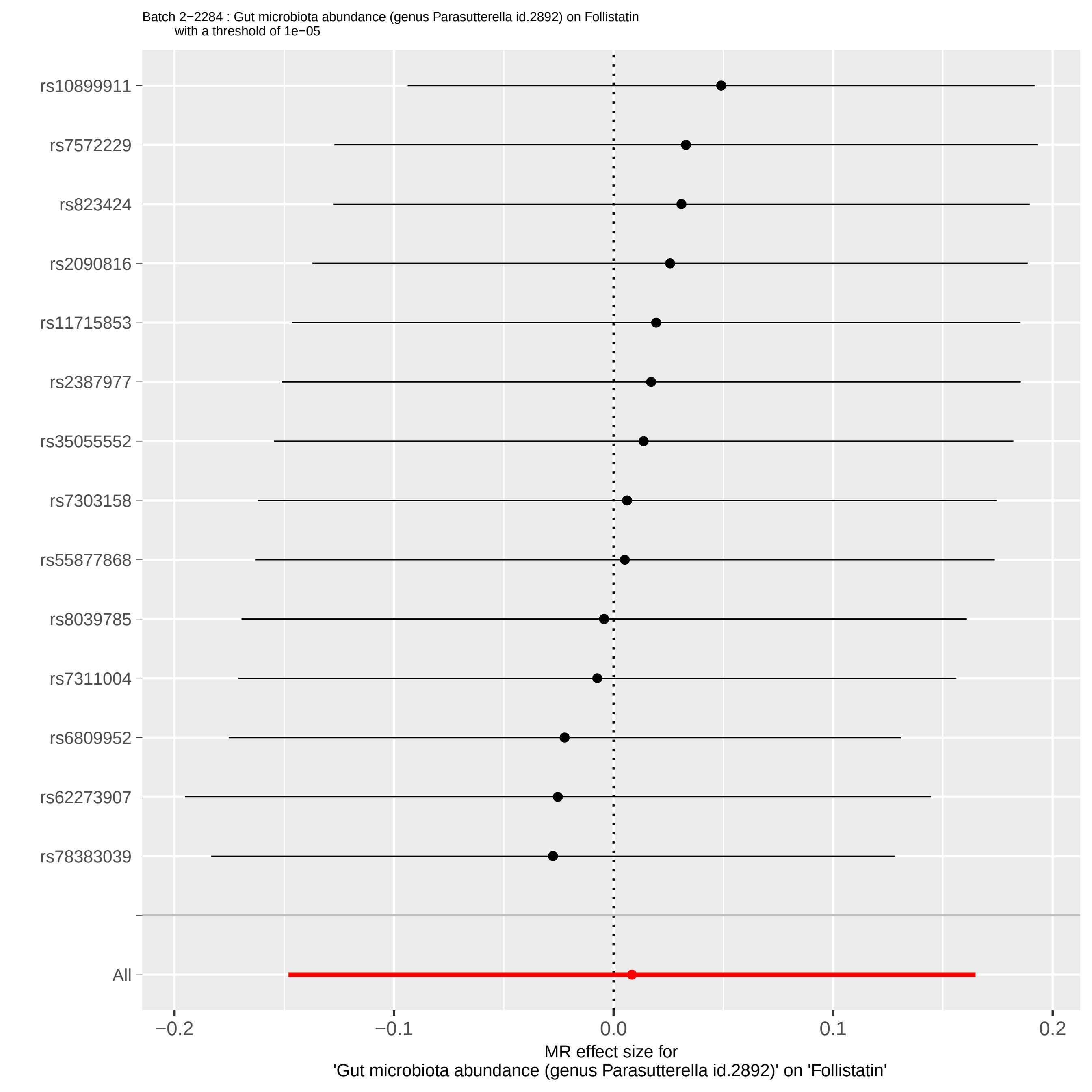
Batch 2−2284 : Gut microbiota abundance (genus Parasutterella id.2892) on Follistatin
with a threshold of 1e−05
rs10899911
rs7572229
rs823424
rs2090816
rs11715853
rs2387977
rs35055552
rs7303158
rs55877868
rs8039785
rs7311004
rs6809952
rs62273907
rs78383039
All
−0.2
−0.1
0.0
0.1
0.2
MR effect size for
'Gut microbiota abundance (genus Parasutterella id.2892)' on 'Follistatin'

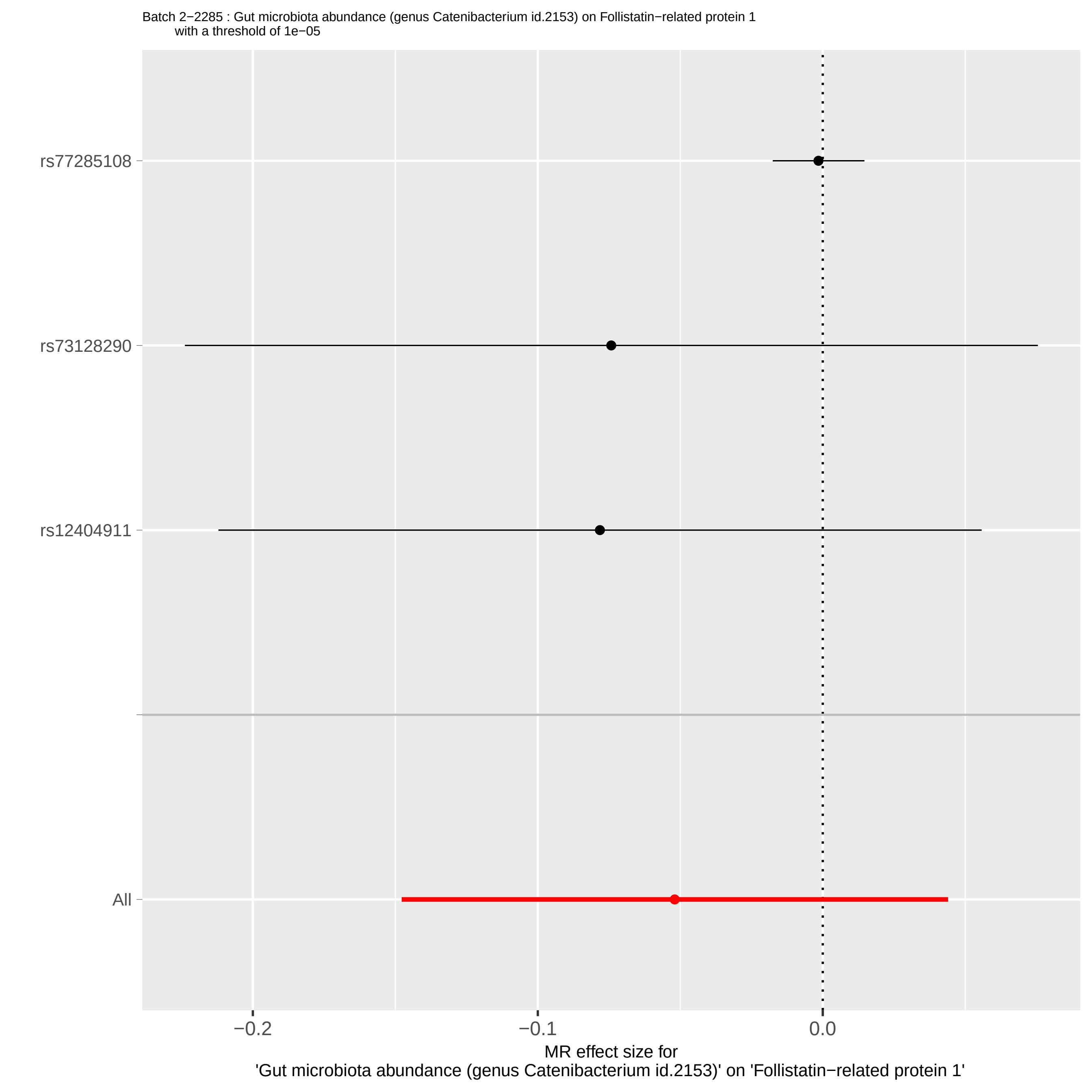

Batch 2−2285 : Gut microbiota abundance (genus Catenibacterium id.2153) on Follistatin−related protein 1
with a threshold of 1e−05
rs77285108
rs73128290
rs12404911
All
−0.2
−0.1
0.0
MR effect size for
'Gut microbiota abundance (genus Catenibacterium id.2153)' on 'Follistatin−related protein 1'

Batch 2−2286 : Gut microbiota abundance (genus Parasutterella id.2892) on Follistatin−related protein 1
with a threshold of 1e−05

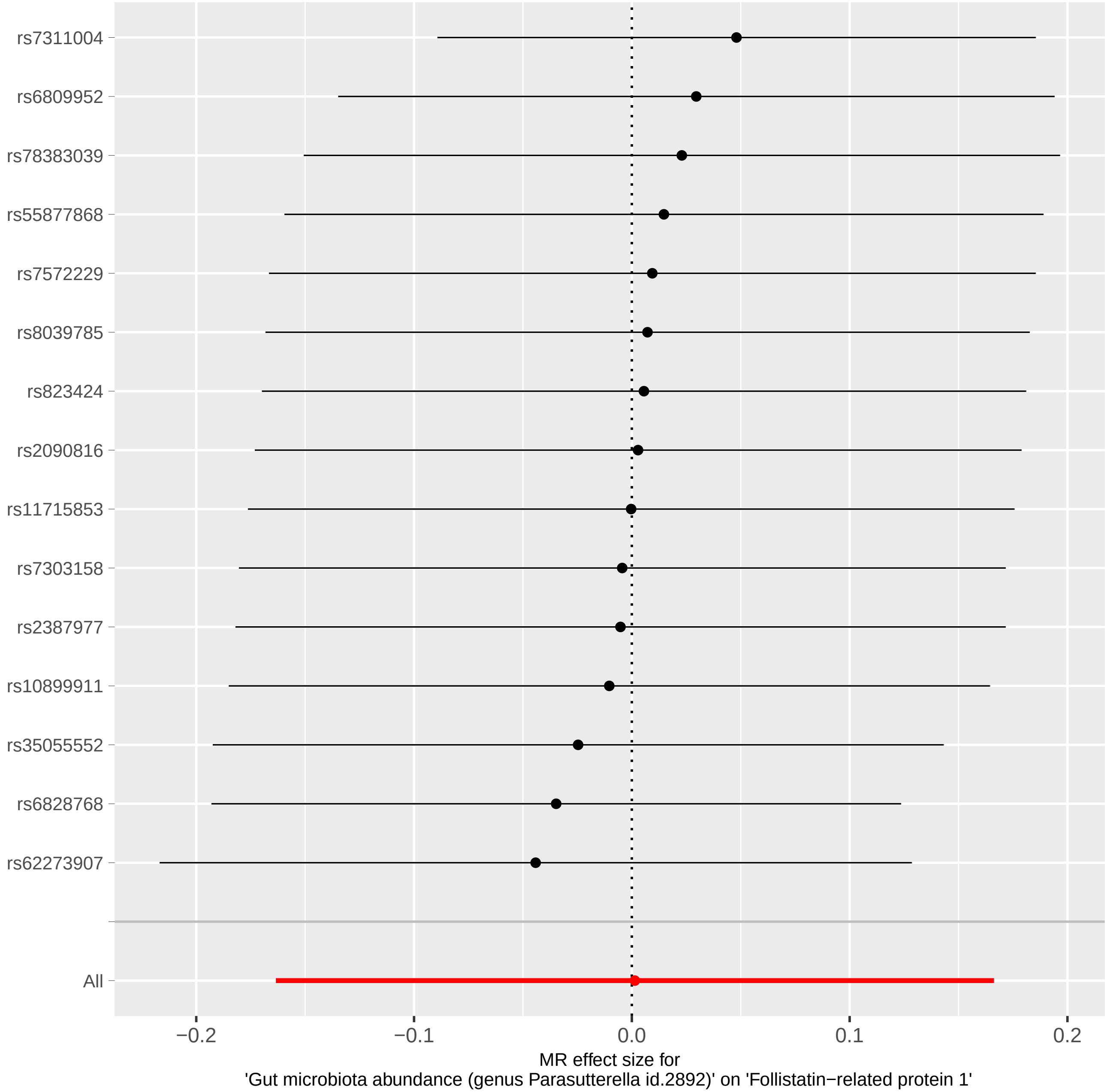

Batch 2−2287 : Gut microbiota abundance (genus Catenibacterium id.2153) on Follistatin−related protein 1
with a threshold of 1e−05

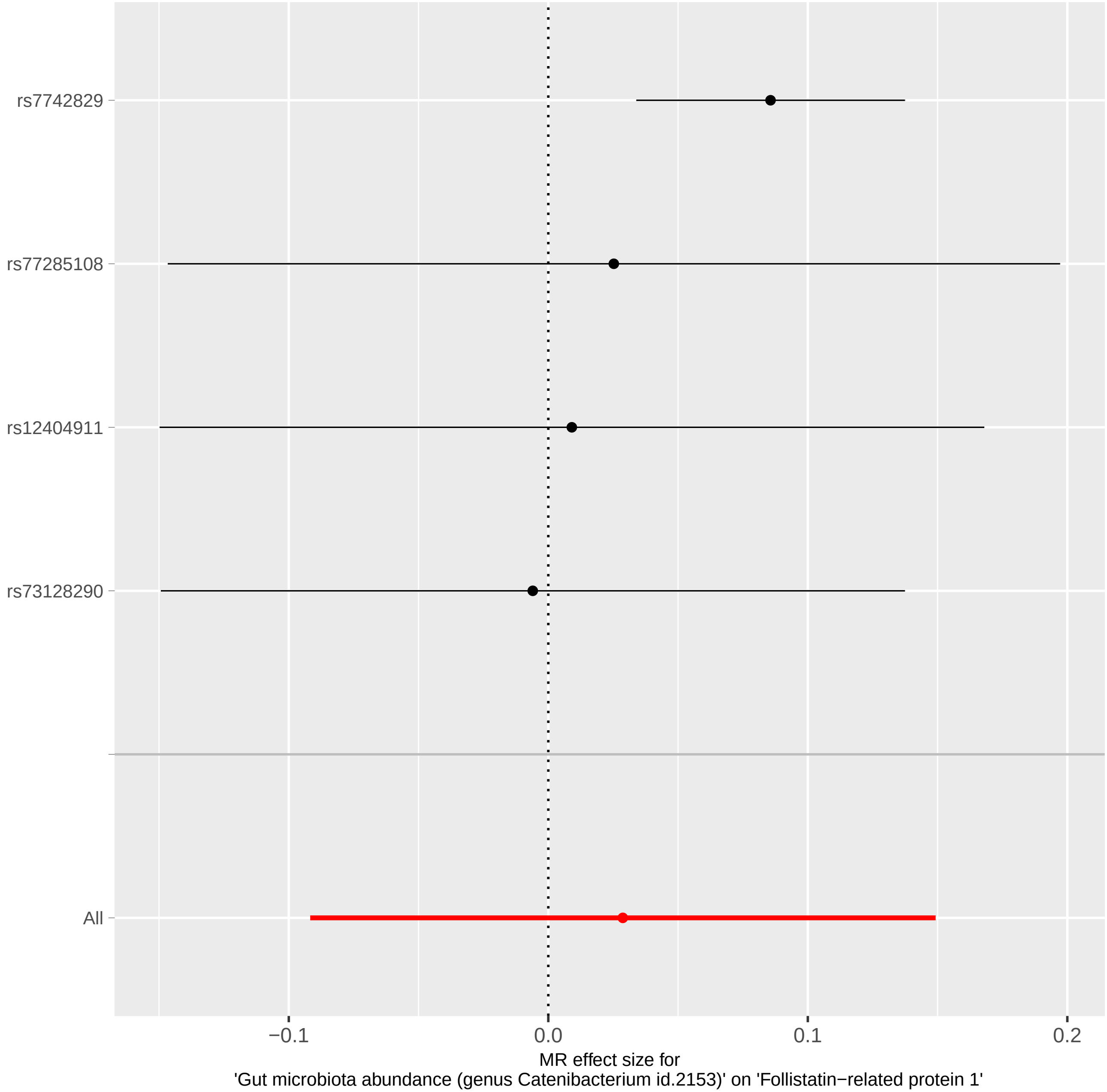

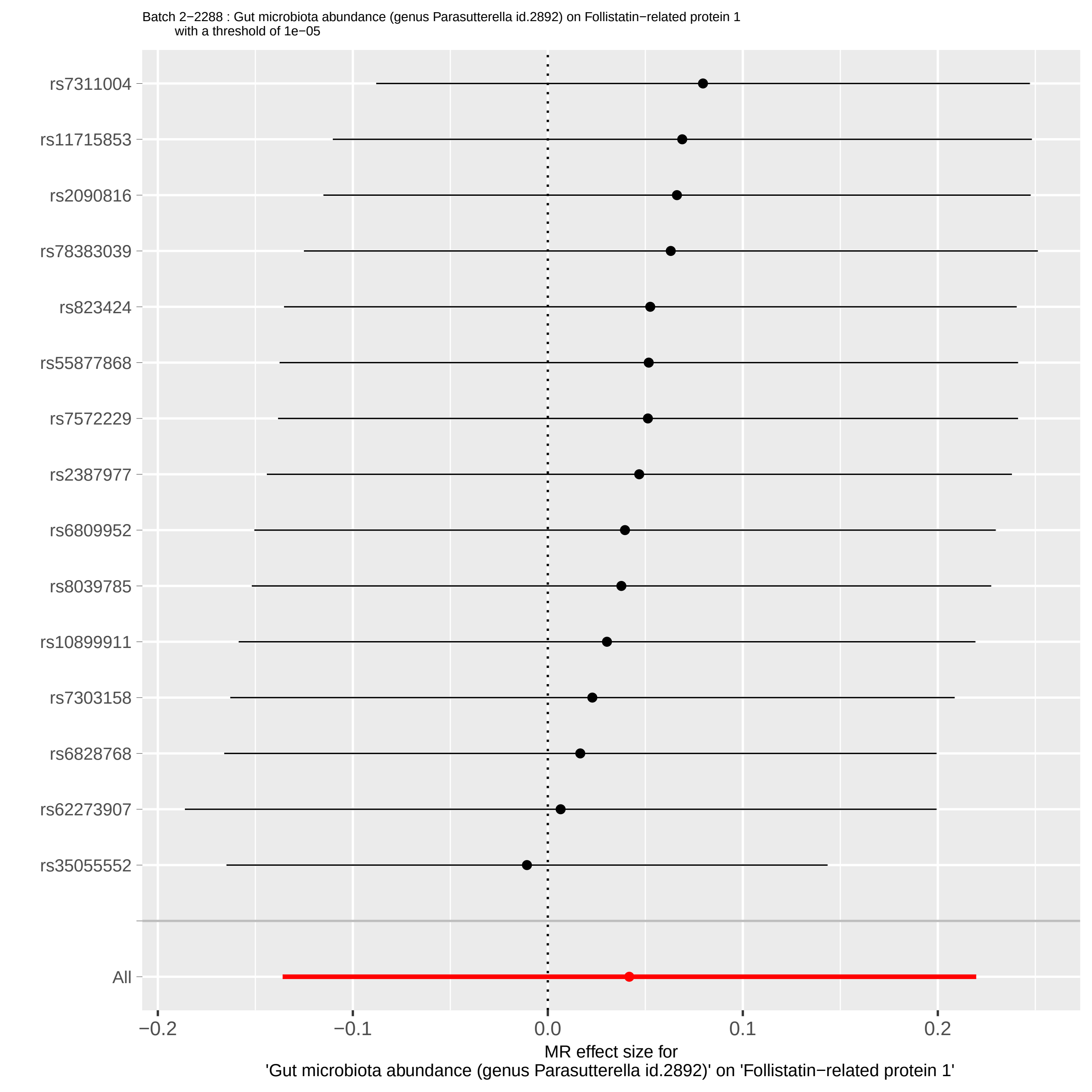
Batch 2−2288 : Gut microbiota abundance (genus Parasutterella id.2892) on Follistatin−related protein 1
with a threshold of 1e−05
rs7311004
rs11715853
rs2090816
rs78383039
rs823424
rs55877868
rs7572229
rs2387977
rs6809952
rs8039785
rs10899911
rs7303158
rs6828768
rs62273907
rs35055552
All
−0.2
−0.1
0.0
0.1
0.2
MR effect size for
'Gut microbiota abundance (genus Parasutterella id.2892)' on 'Follistatin−related protein 1'

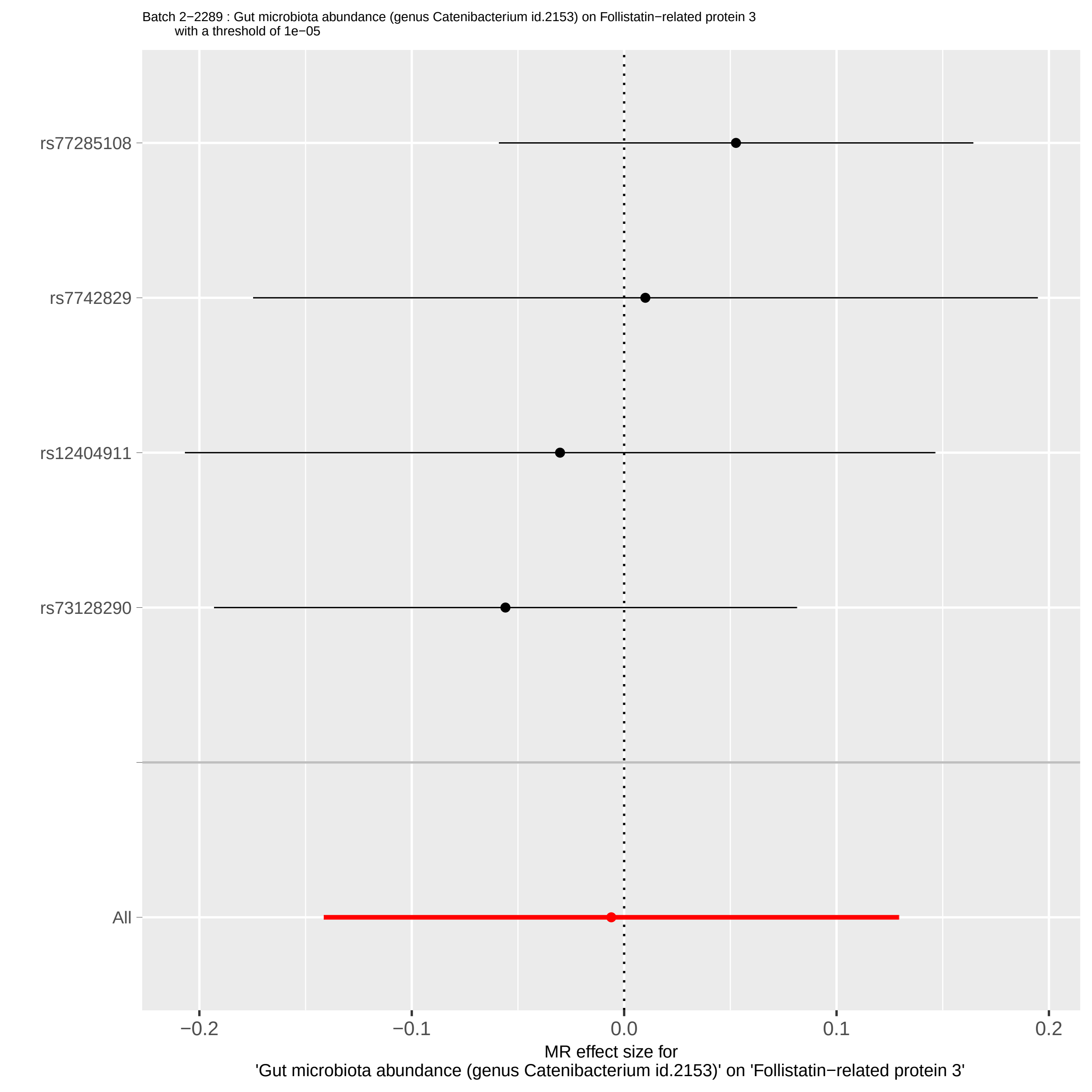
Batch 2−2289 : Gut microbiota abundance (genus Catenibacterium id.2153) on Follistatin−related protein 3
with a threshold of 1e−05
rs77285108
rs7742829
rs12404911
rs73128290
All
−0.2
−0.1
0.0
0.1
0.2
MR effect size for
'Gut microbiota abundance (genus Catenibacterium id.2153)' on 'Follistatin−related protein 3'

Batch 2−2290 : Gut microbiota abundance (genus Parasutterella id.2892) on Follistatin−related protein 3
with a threshold of 1e−05

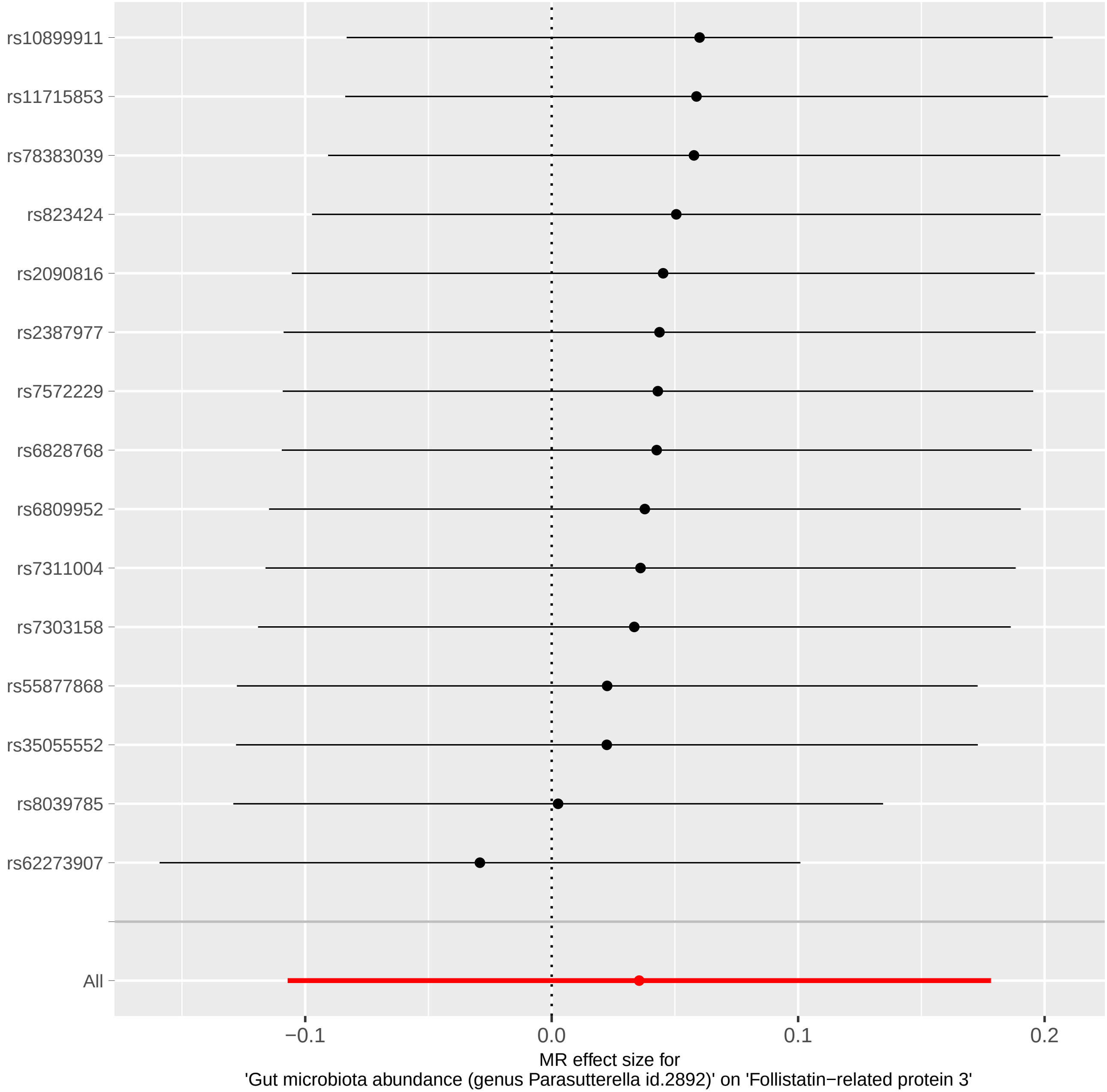

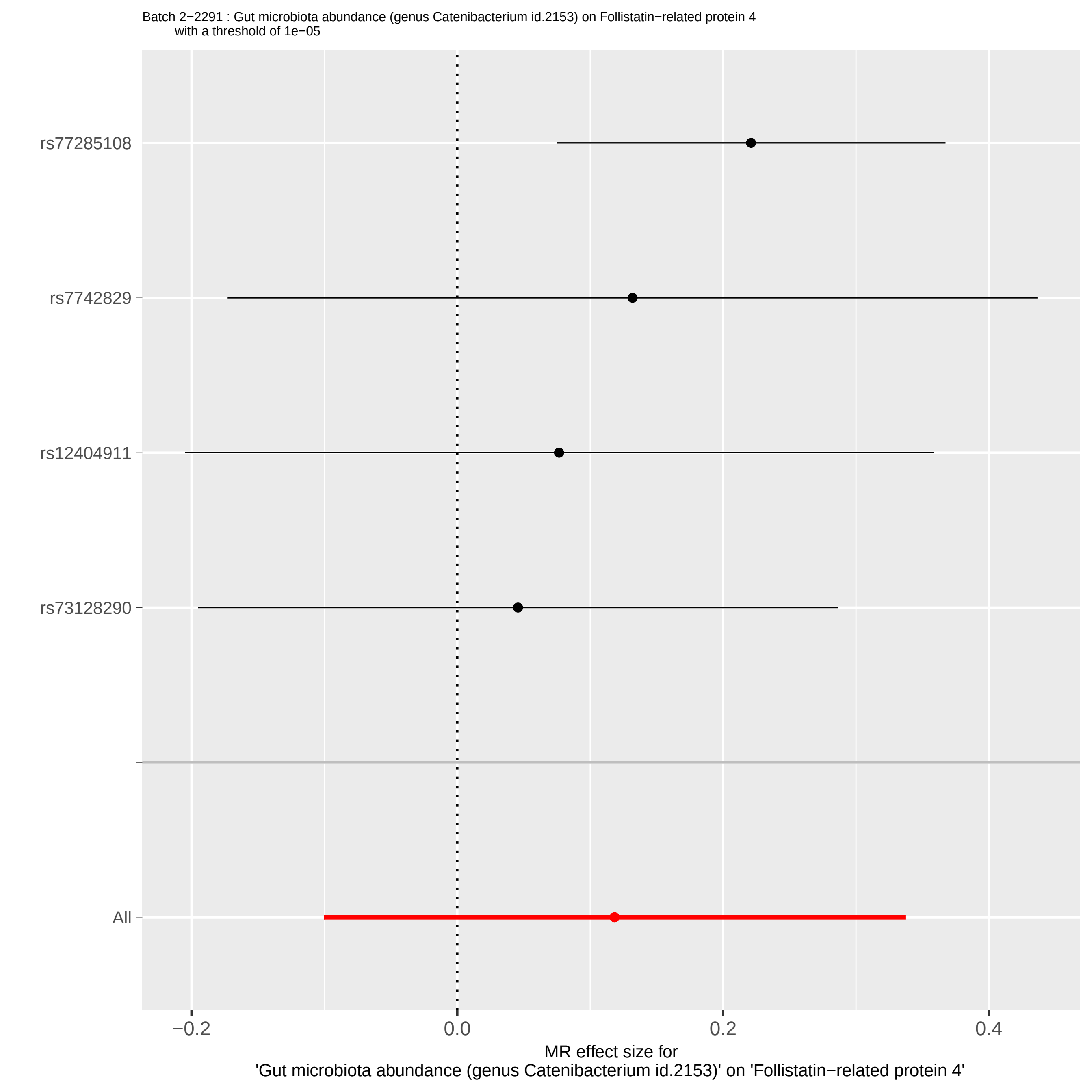

Batch 2−2291 : Gut microbiota abundance (genus Catenibacterium id.2153) on Follistatin−related protein 4
with a threshold of 1e−05
rs77285108
rs7742829
rs12404911
rs73128290
All
−0.2
0.0
0.2
0.4
MR effect size for
'Gut microbiota abundance (genus Catenibacterium id.2153)' on 'Follistatin−related protein 4'

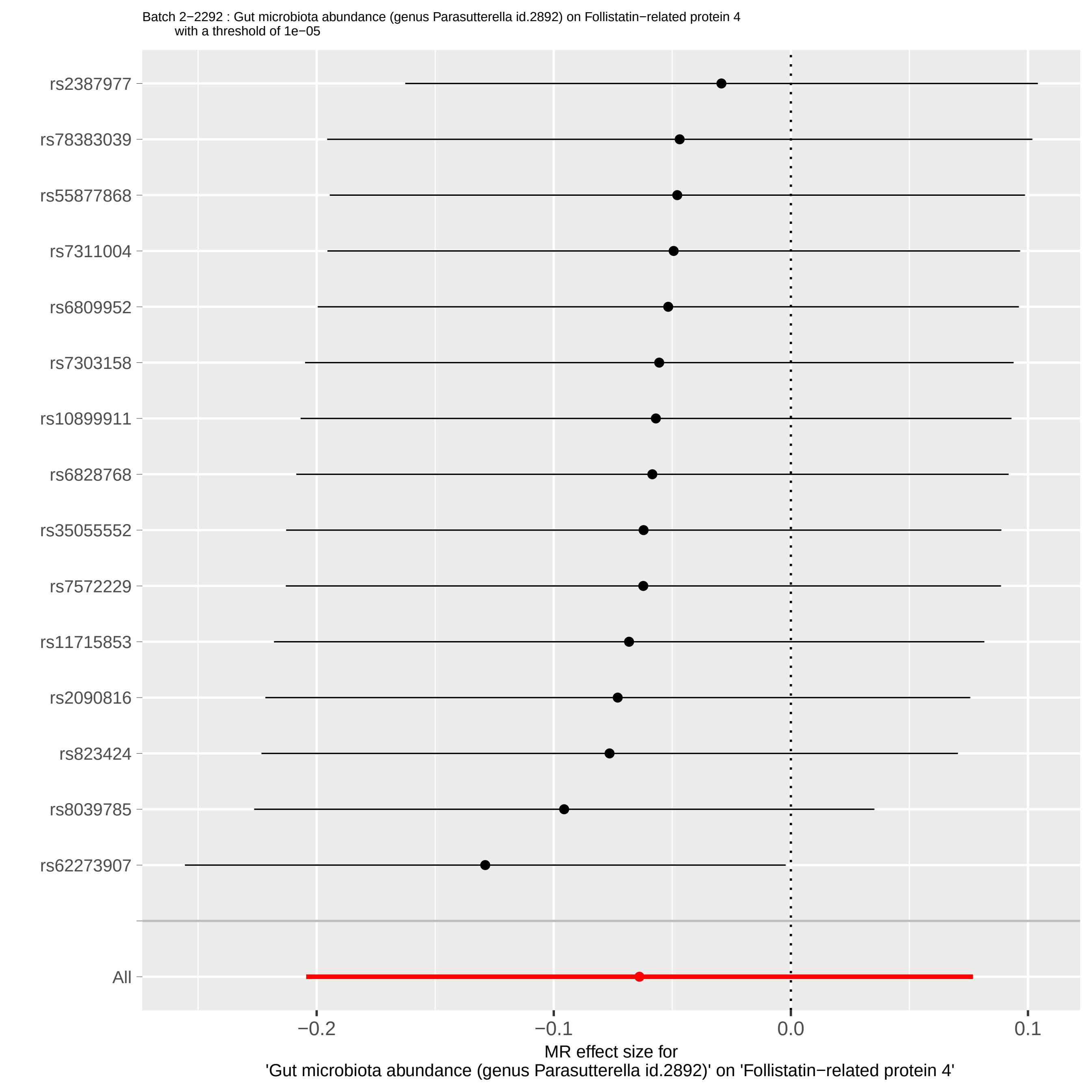
Batch 2−2292 : Gut microbiota abundance (genus Parasutterella id.2892) on Follistatin−related protein 4
with a threshold of 1e−05
rs2387977
rs78383039
rs55877868
rs7311004
rs6809952
rs7303158
rs10899911
rs6828768
rs35055552
rs7572229
rs11715853
rs2090816
rs823424
rs8039785
rs62273907
All
−0.2
−0.1
0.0
0.1
MR effect size for
'Gut microbiota abundance (genus Parasutterella id.2892)' on 'Follistatin−related protein 4'

Batch 2−2293 : Gut microbiota abundance (genus Catenibacterium id.2153) on Follistatin−related protein 5
with a threshold of 1e−05

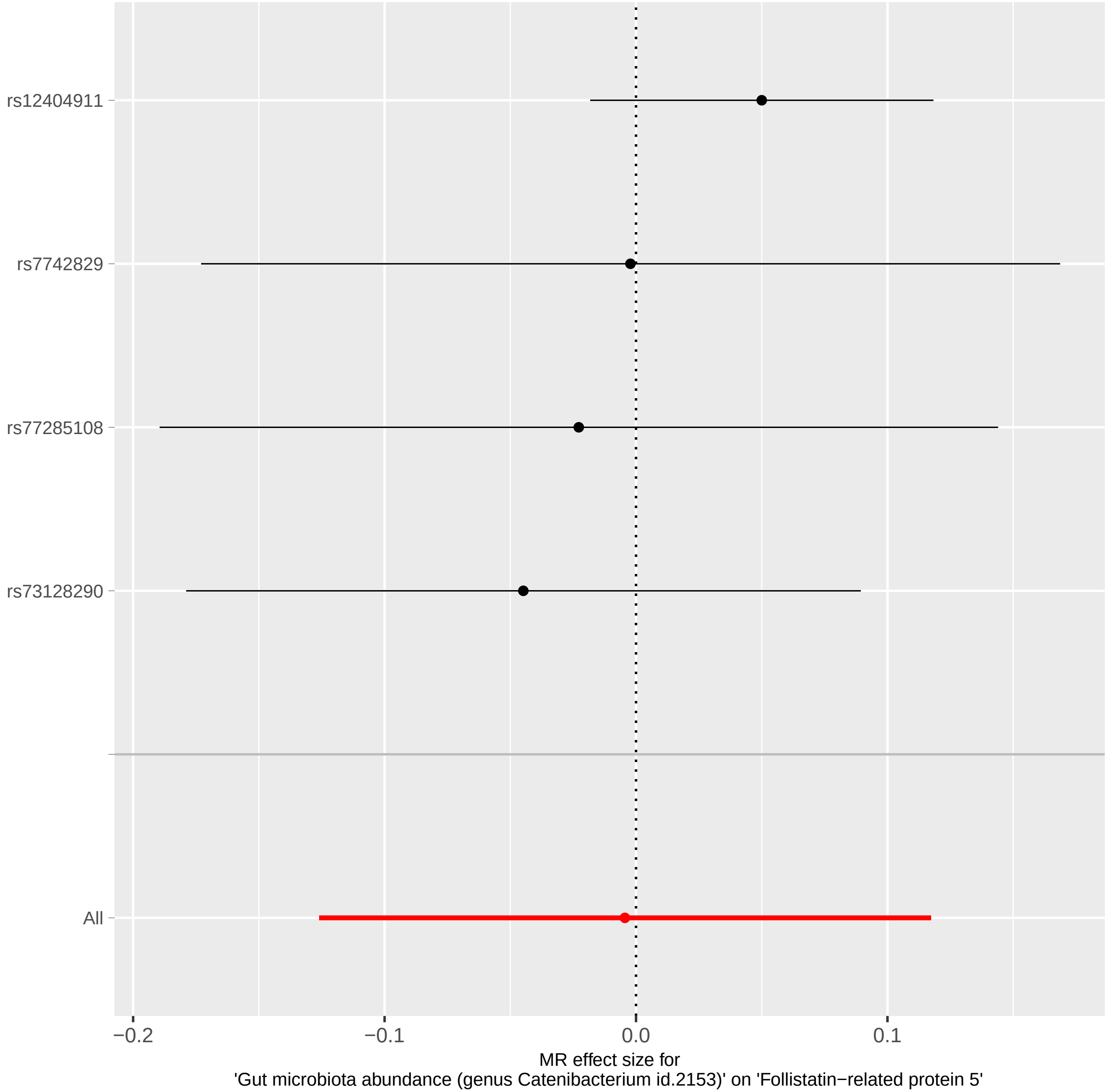

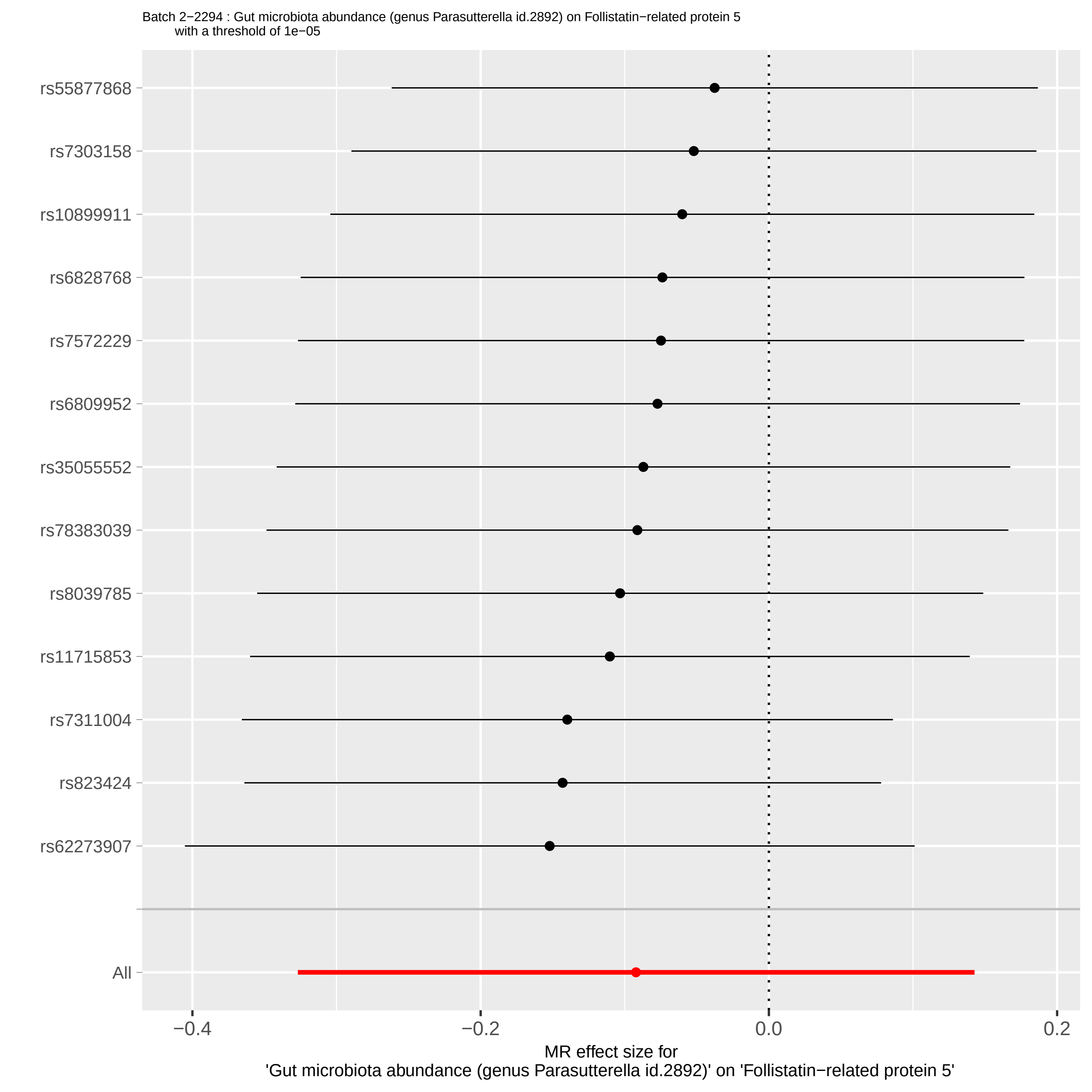
Batch 2−2294 : Gut microbiota abundance (genus Parasutterella id.2892) on Follistatin−related protein 5
with a threshold of 1e−05
rs55877868
rs7303158
rs10899911
rs6828768
rs7572229
rs6809952
rs35055552
rs78383039
rs8039785
rs11715853
rs7311004
rs823424
rs62273907
All
−0.4
−0.2
0.0
0.2
MR effect size for
'Gut microbiota abundance (genus Parasutterella id.2892)' on 'Follistatin−related protein 5'

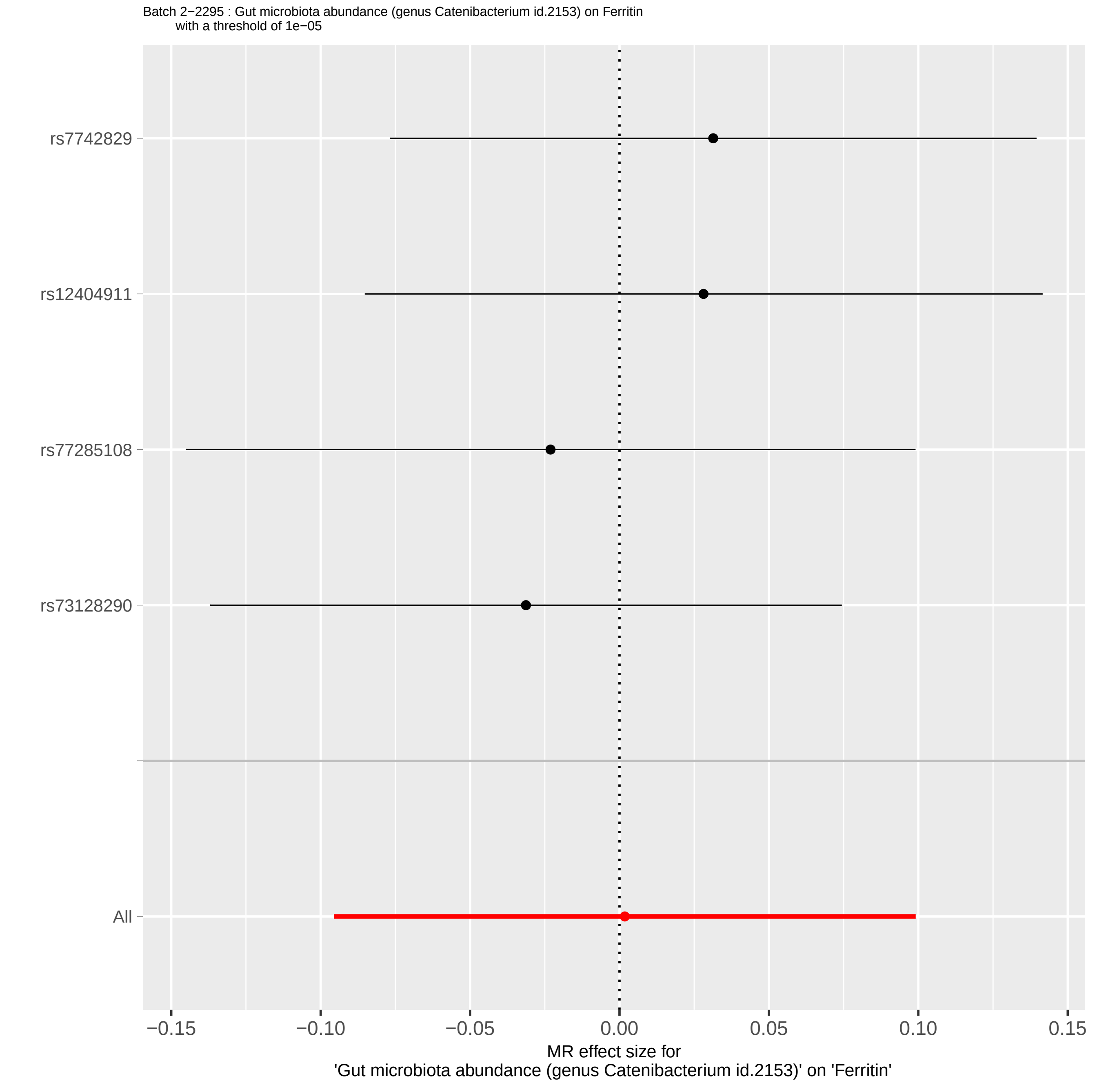

Batch 2−2295 : Gut microbiota abundance (genus Catenibacterium id.2153) on Ferritin
with a threshold of 1e−05
rs7742829
rs12404911
rs77285108
rs73128290
All
−0.15
−0.10
−0.05
0.00
0.05
0.10
0.15
MR effect size for
'Gut microbiota abundance (genus Catenibacterium id.2153)' on 'Ferritin'

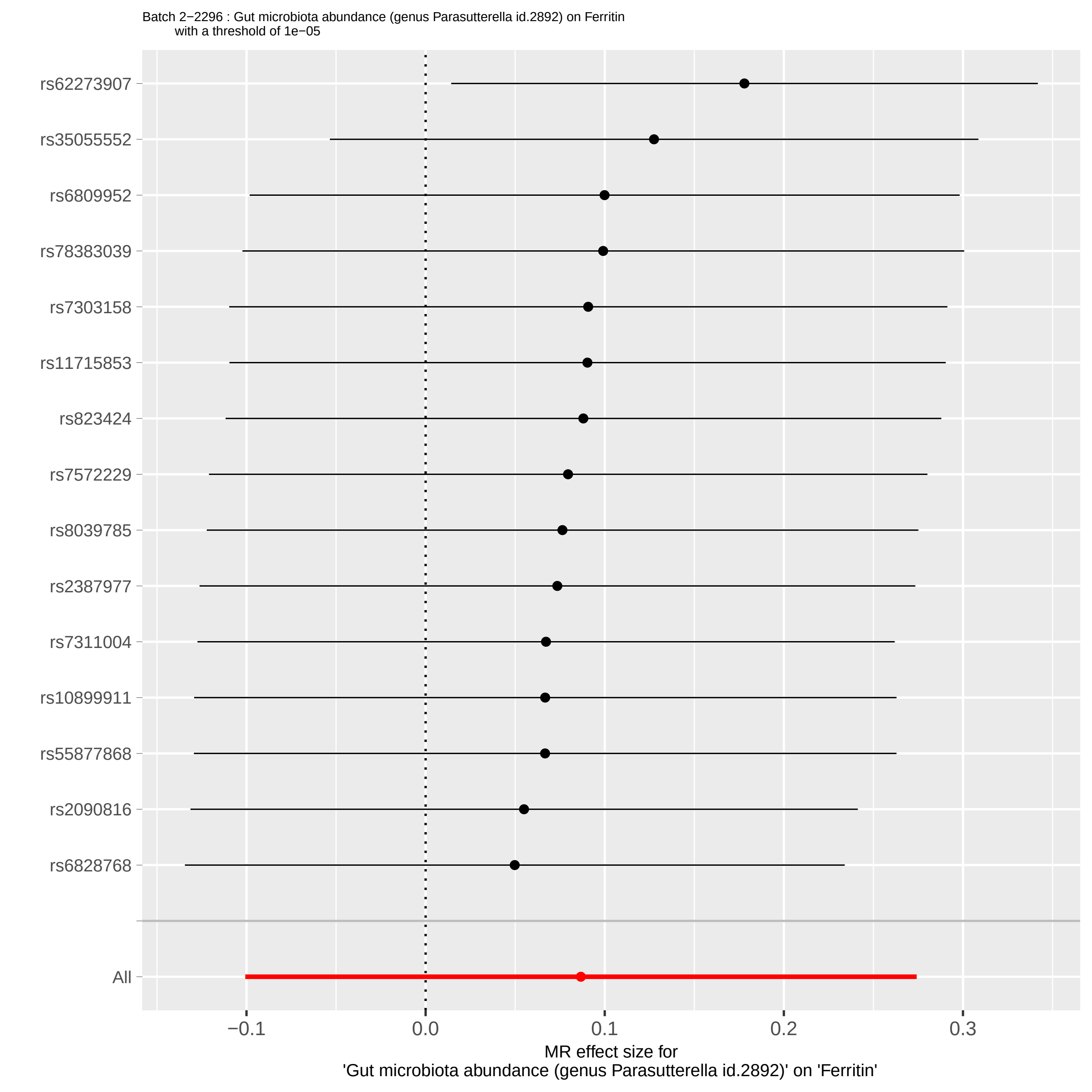
Batch 2−2296 : Gut microbiota abundance (genus Parasutterella id.2892) on Ferritin
with a threshold of 1e−05
rs62273907
rs35055552
rs6809952
rs78383039
rs7303158
rs11715853
rs823424
rs7572229
rs8039785
rs2387977
rs7311004
rs10899911
rs55877868
rs2090816
rs6828768
All
−0.1
0.0
0.1
0.2
0.3
MR effect size for
'Gut microbiota abundance (genus Parasutterella id.2892)' on 'Ferritin'

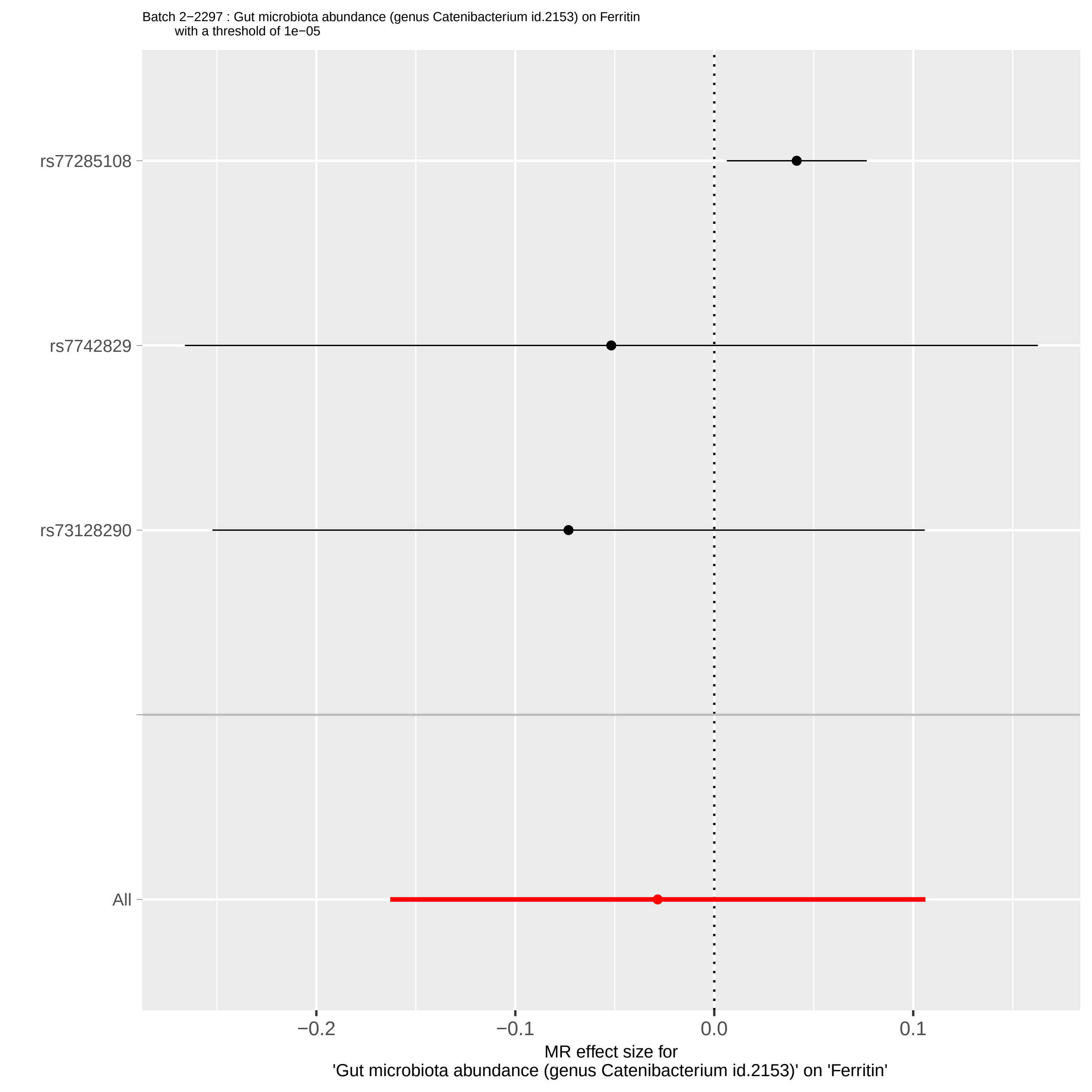
Batch 2−2297 : Gut microbiota abundance (genus Catenibacterium id.2153) on Ferritin
with a threshold of 1e−05
rs77285108
rs7742829
rs73128290
All
−0.2
−0.1
0.0
0.1
MR effect size for
'Gut microbiota abundance (genus Catenibacterium id.2153)' on 'Ferritin'

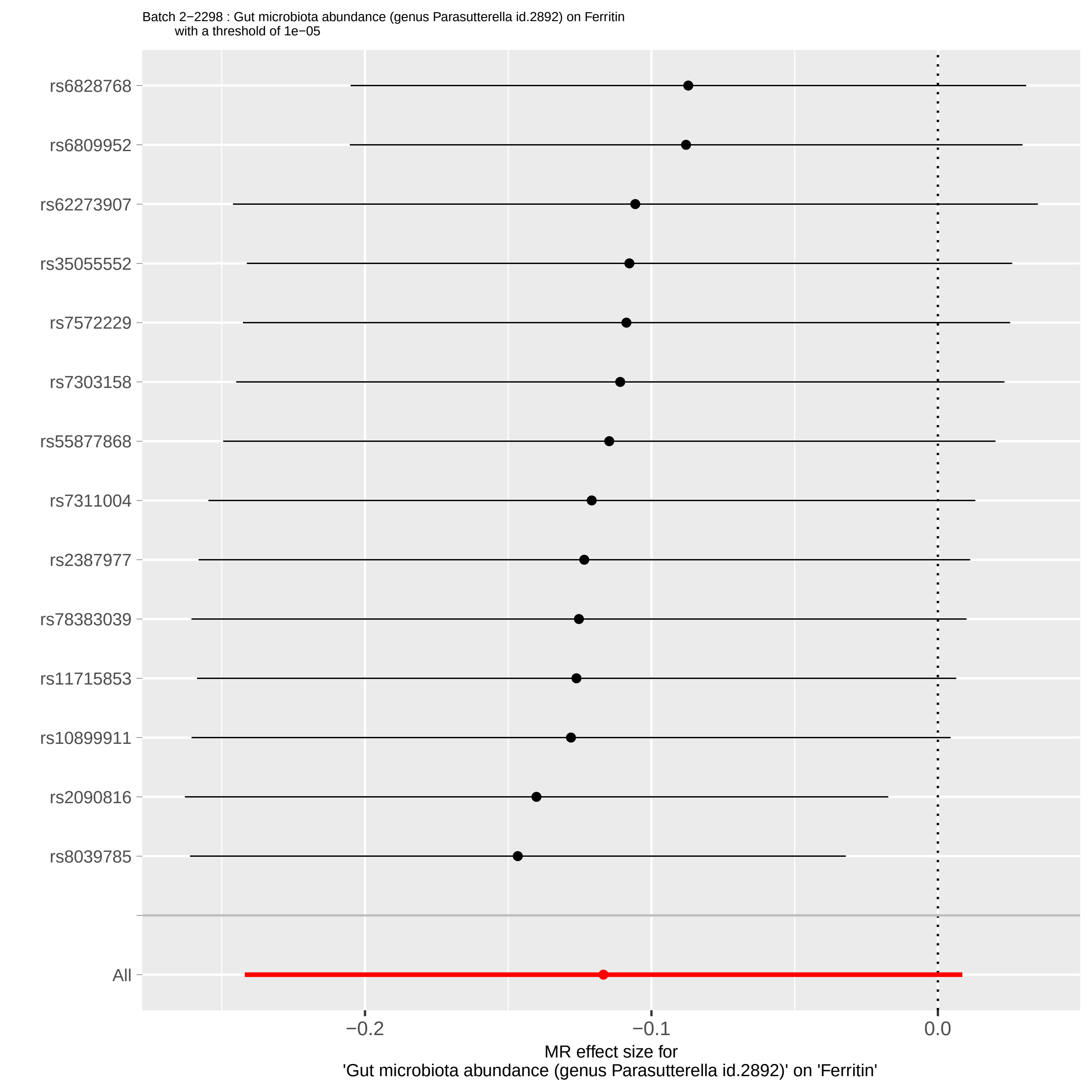
Batch 2−2298 : Gut microbiota abundance (genus Parasutterella id.2892) on Ferritin
with a threshold of 1e−05
rs6828768
rs6809952
rs62273907
rs35055552
rs7572229
rs7303158
rs55877868
rs7311004
rs2387977
rs78383039
rs11715853
rs10899911
rs2090816
rs8039785
All
−0.2
−0.1
0.0
MR effect size for
'Gut microbiota abundance (genus Parasutterella id.2892)' on 'Ferritin'

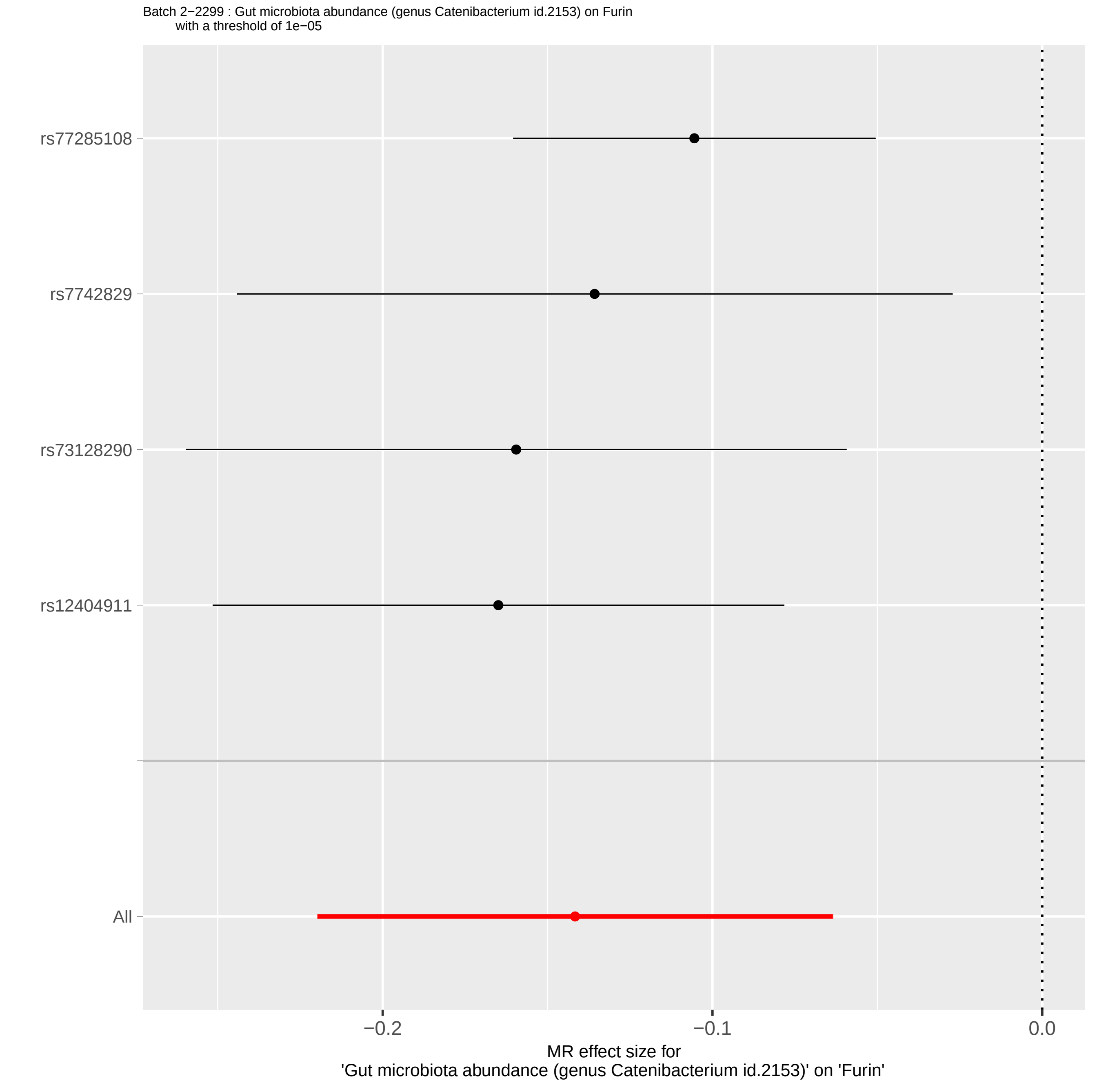

Batch 2−2299 : Gut microbiota abundance (genus Catenibacterium id.2153) on Furin
with a threshold of 1e−05
rs77285108
rs7742829
rs73128290
rs12404911
All
−0.2
−0.1
0.0
MR effect size for
'Gut microbiota abundance (genus Catenibacterium id.2153)' on 'Furin'

Batch 2−2300 : Gut microbiota abundance (genus Parasutterella id.2892) on Furin
with a threshold of 1e−05

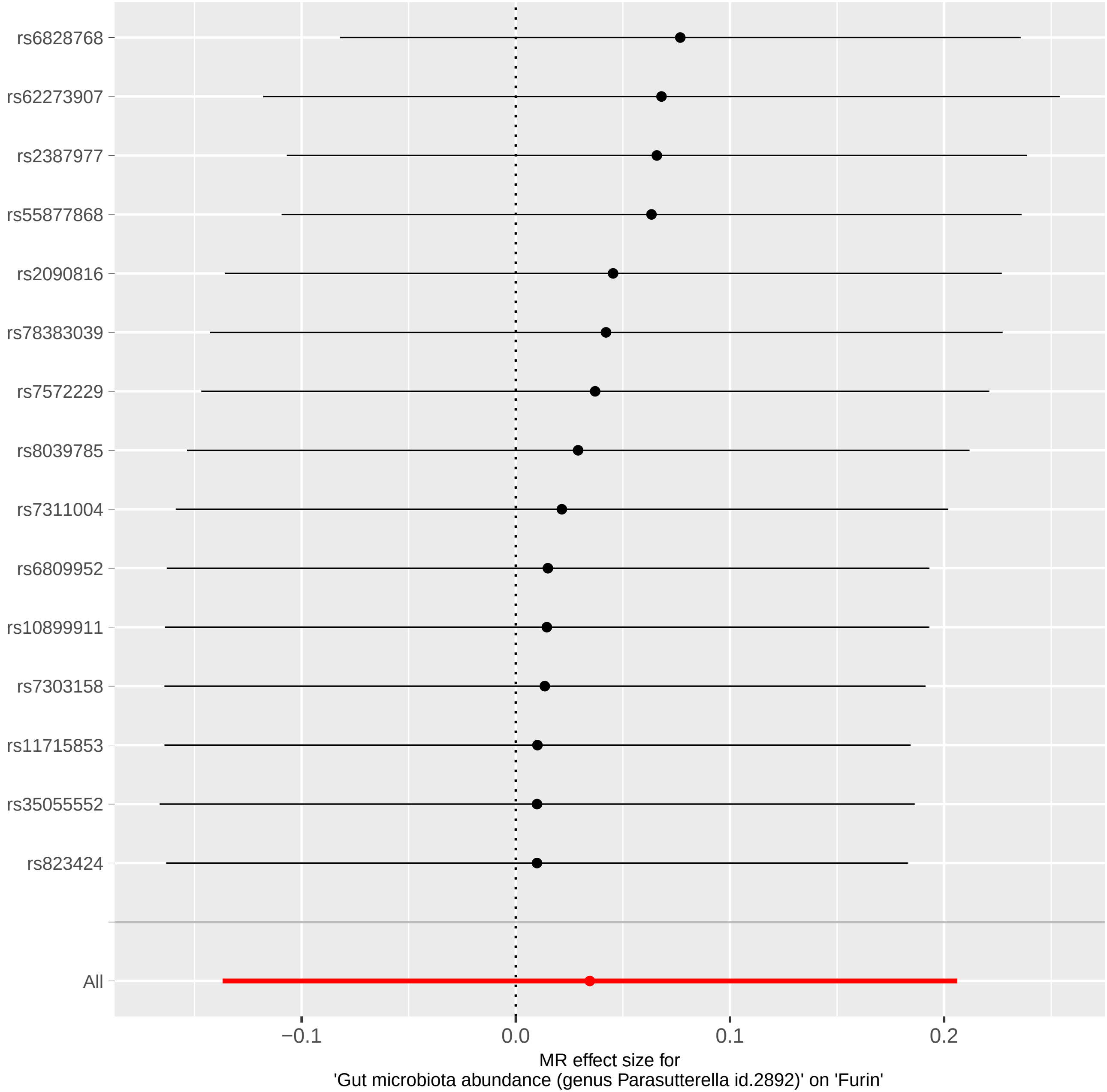

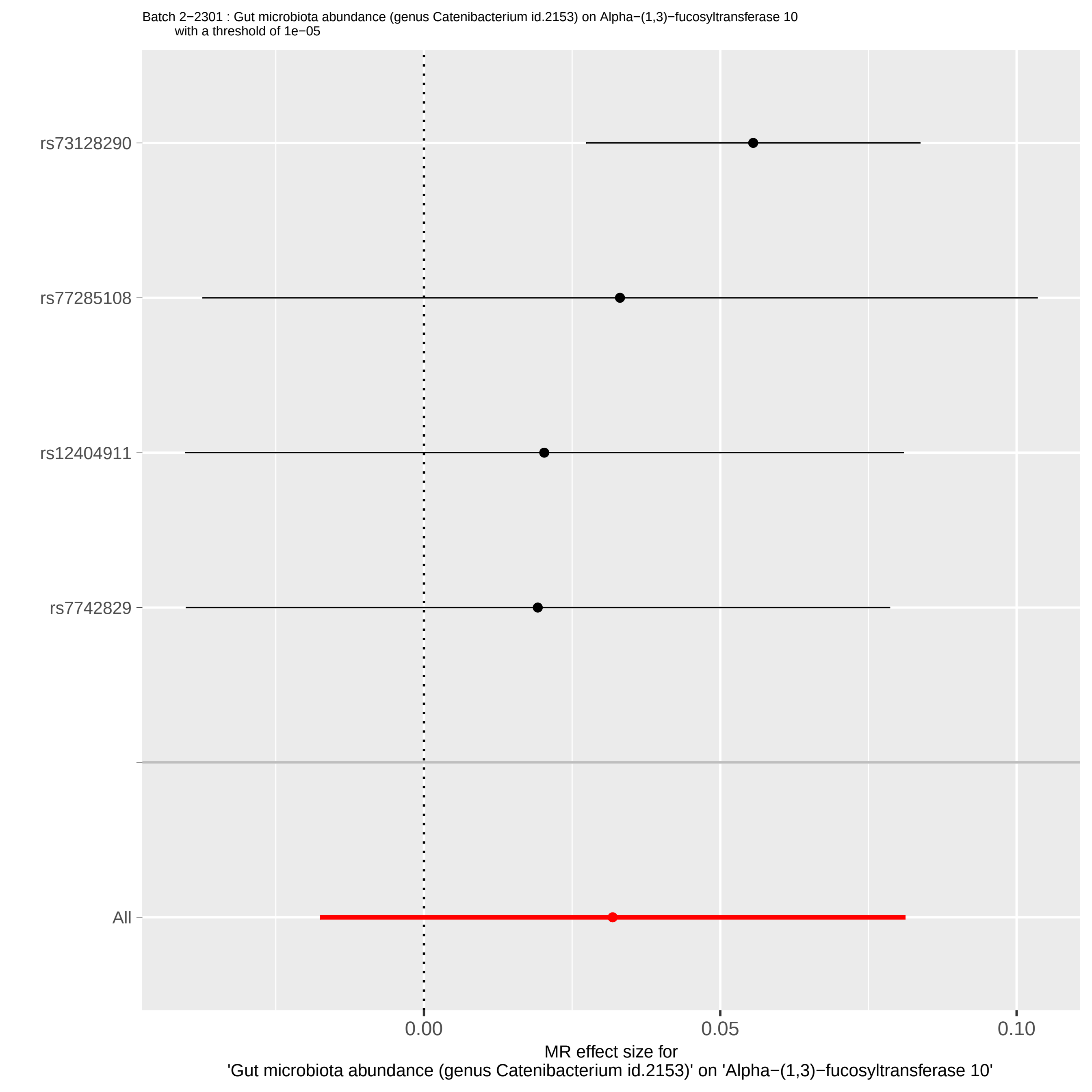

Batch 2−2301 : Gut microbiota abundance (genus Catenibacterium id.2153) on Alpha−(1,3)−fucosyltransferase 10
with a threshold of 1e−05
rs73128290
rs77285108
rs12404911
rs7742829
All
0.00
0.05
0.10
MR effect size for
'Gut microbiota abundance (genus Catenibacterium id.2153)' on 'Alpha−(1,3)−fucosyltransferase 10'

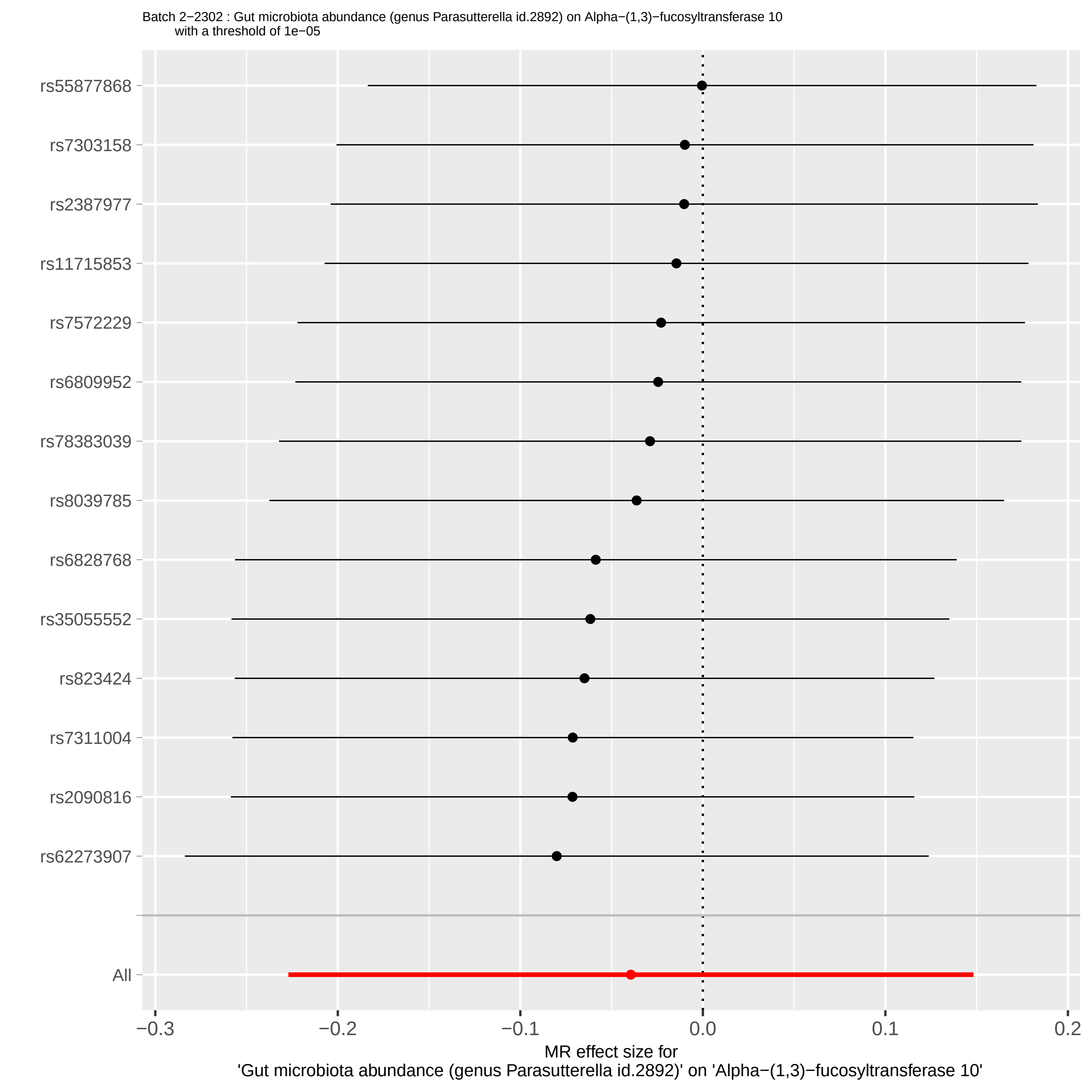
Batch 2−2302 : Gut microbiota abundance (genus Parasutterella id.2892) on Alpha−(1,3)−fucosyltransferase 10
with a threshold of 1e−05
rs55877868
rs7303158
rs2387977
rs11715853
rs7572229
rs6809952
rs78383039
rs8039785
rs6828768
rs35055552
rs823424
rs7311004
rs2090816
rs62273907
All
−0.3
−0.2
−0.1
0.0
0.1
0.2
MR effect size for
'Gut microbiota abundance (genus Parasutterella id.2892)' on 'Alpha−(1,3)−fucosyltransferase 10'

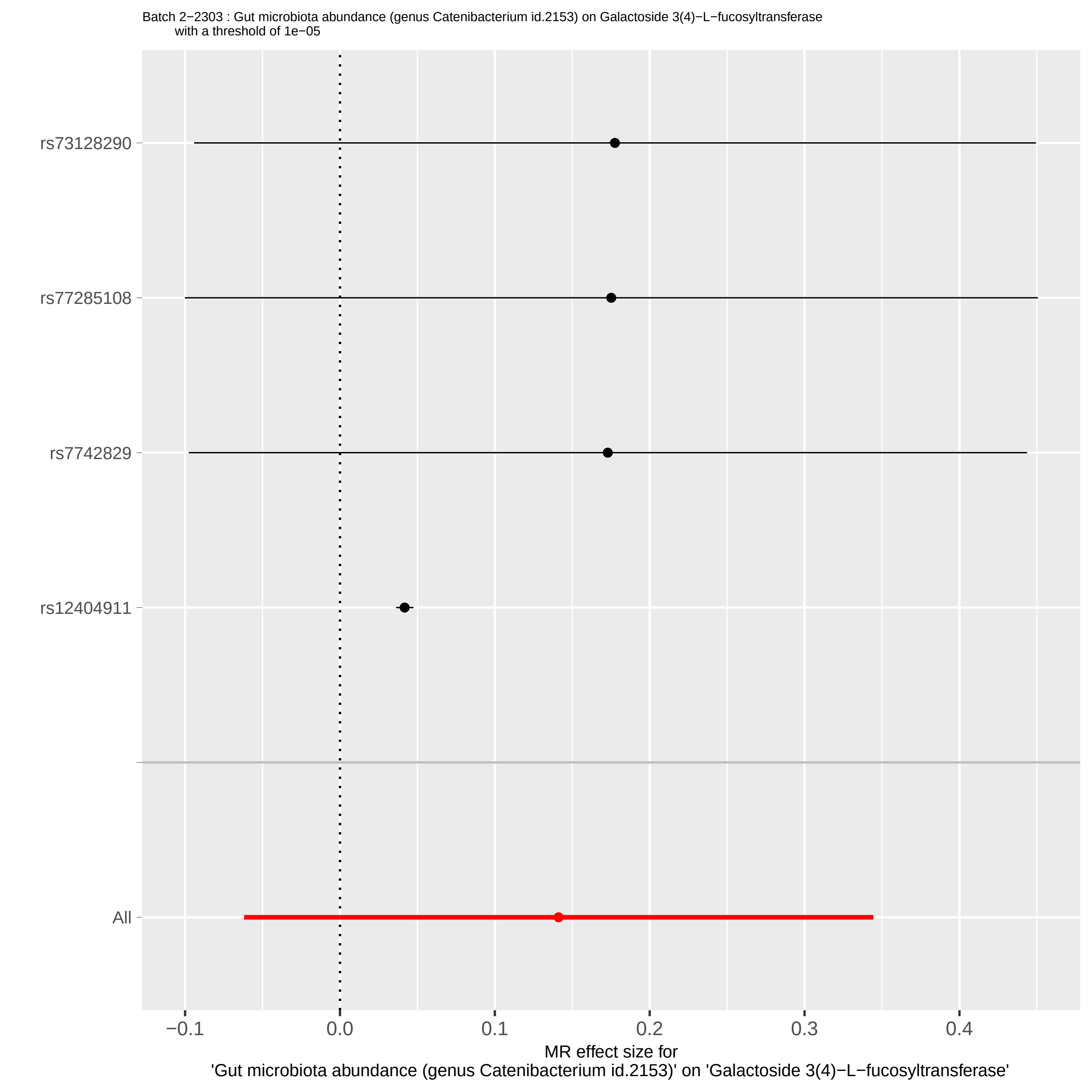

Batch 2−2303 : Gut microbiota abundance (genus Catenibacterium id.2153) on Galactoside 3(4)−L−fucosyltransferase
with a threshold of 1e−05
rs73128290
rs77285108
rs7742829
rs12404911
All
−0.1
0.0
0.1
0.2
0.3
0.4
MR effect size for
'Gut microbiota abundance (genus Catenibacterium id.2153)' on 'Galactoside 3(4)−L−fucosyltransferase'

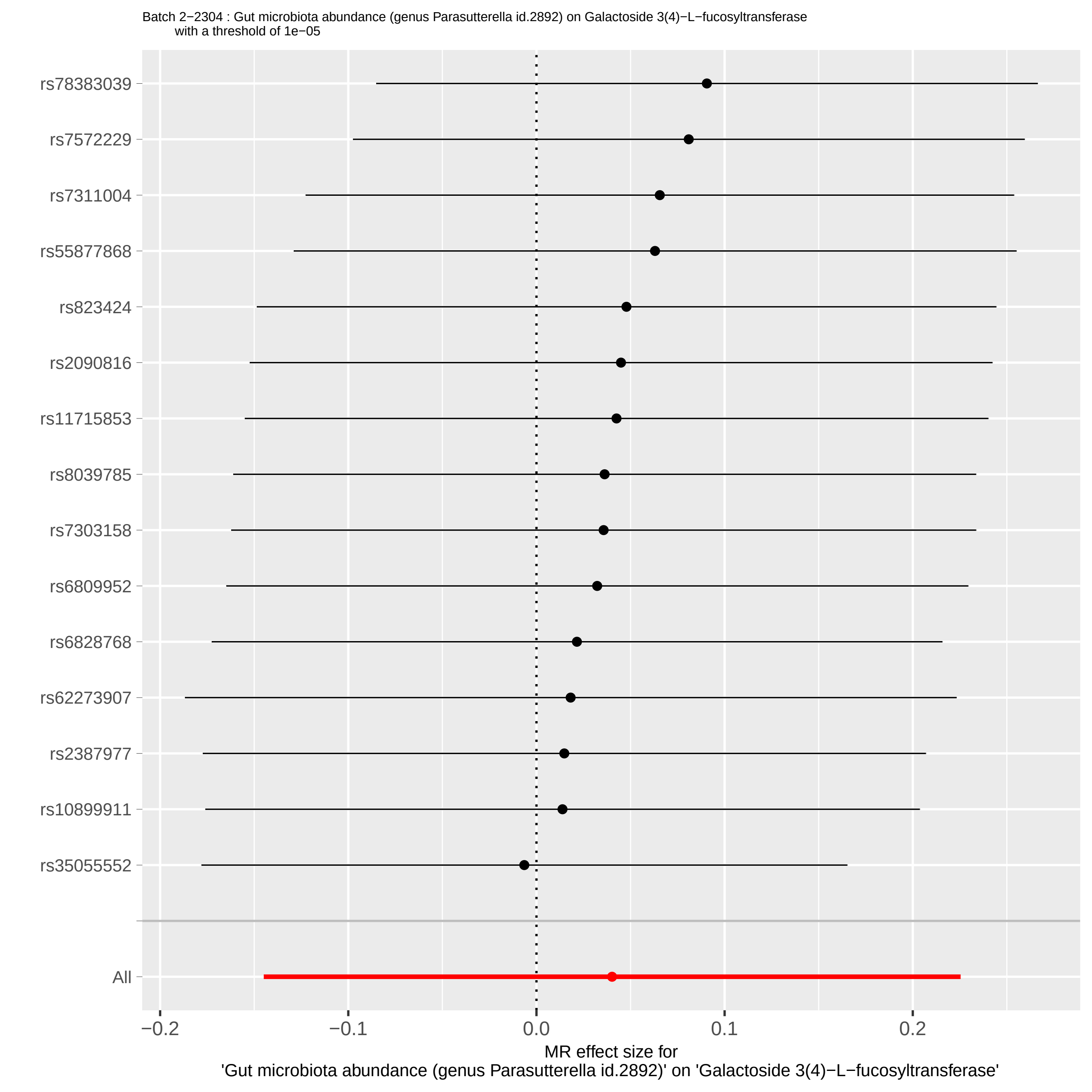
Batch 2−2304 : Gut microbiota abundance (genus Parasutterella id.2892) on Galactoside 3(4)−L−fucosyltransferase
with a threshold of 1e−05
rs78383039
rs7572229
rs7311004
rs55877868
rs823424
rs2090816
rs11715853
rs8039785
rs7303158
rs6809952
rs6828768
rs62273907
rs2387977
rs10899911
rs35055552
All
−0.2
−0.1
0.0
0.1
0.2
MR effect size for
'Gut microbiota abundance (genus Parasutterella id.2892)' on 'Galactoside 3(4)−L−fucosyltransferase'

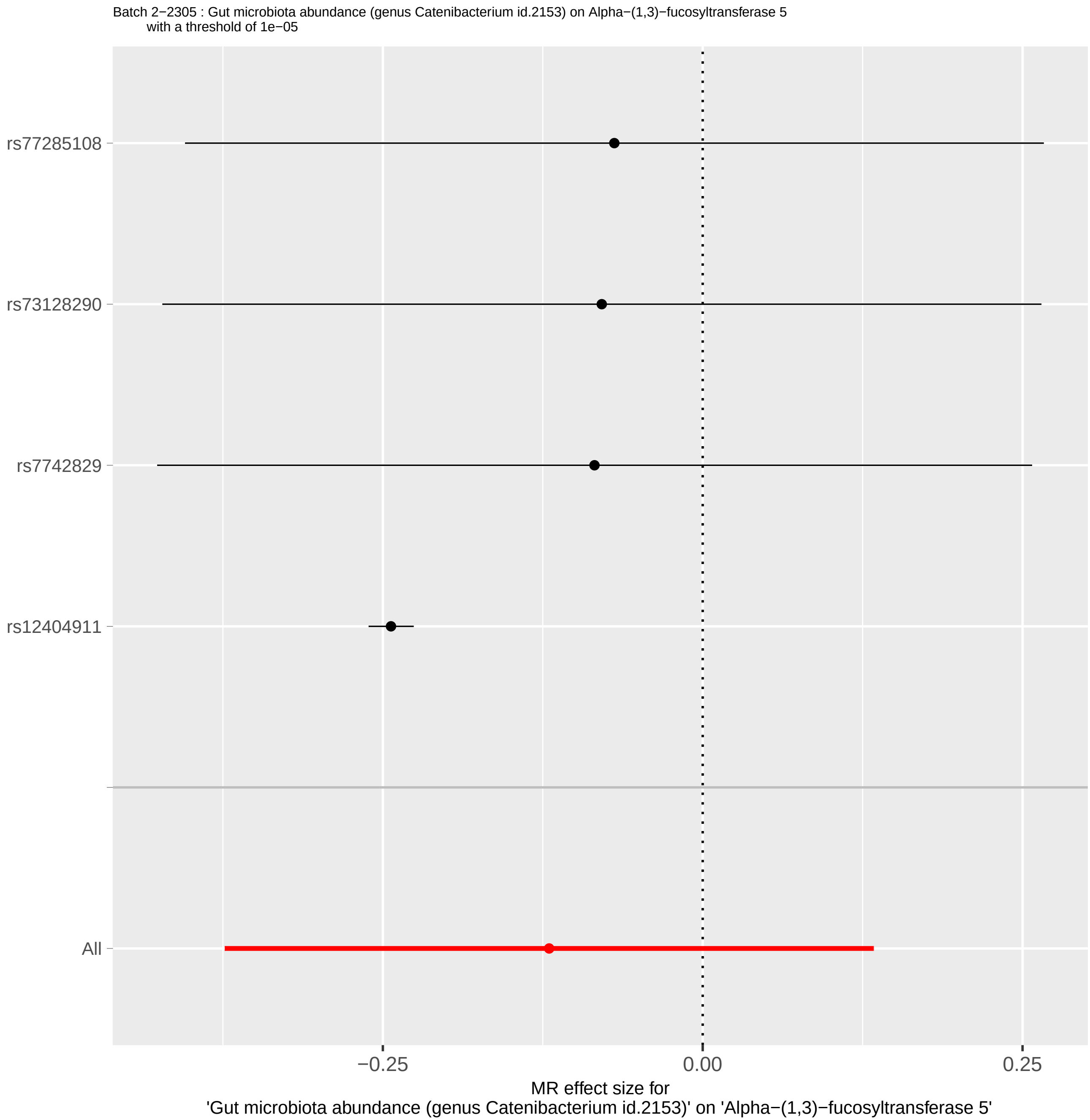

Batch 2−2305 : Gut microbiota abundance (genus Catenibacterium id.2153) on Alpha−(1,3)−fucosyltransferase 5
with a threshold of 1e−05
rs77285108
rs73128290
rs7742829
rs12404911
All
−0.25
0.00
0.25
MR effect size for
'Gut microbiota abundance (genus Catenibacterium id.2153)' on 'Alpha−(1,3)−fucosyltransferase 5'

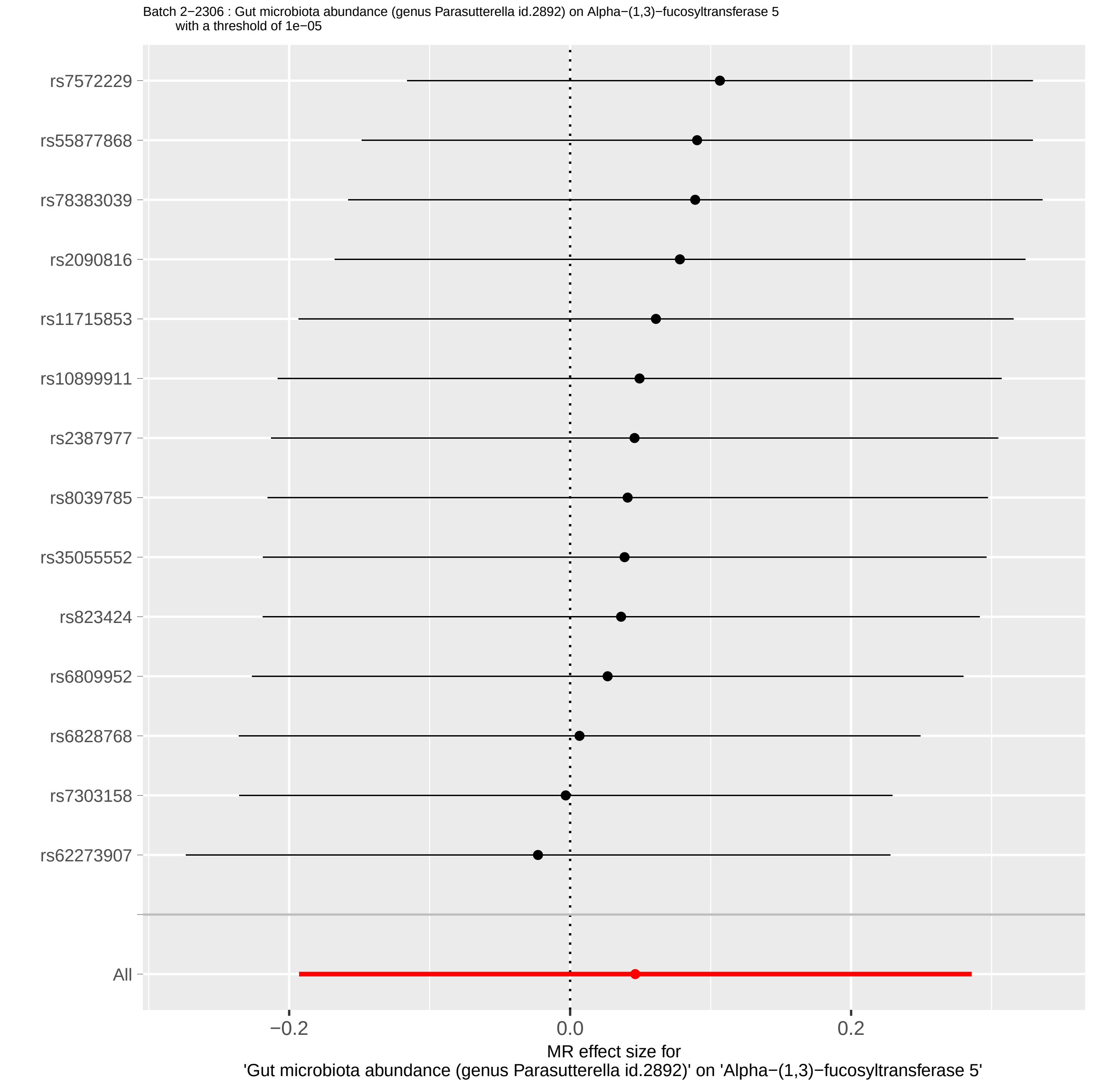

Batch 2−2306 : Gut microbiota abundance (genus Parasutterella id.2892) on Alpha−(1,3)−fucosyltransferase 5
with a threshold of 1e−05
rs7572229
rs55877868
rs78383039
rs2090816
rs11715853
rs10899911
rs2387977
rs8039785
rs35055552
rs823424
rs6809952
rs6828768
rs7303158
rs62273907
All
−0.2
0.0
0.2
MR effect size for
'Gut microbiota abundance (genus Parasutterella id.2892)' on 'Alpha−(1,3)−fucosyltransferase 5'

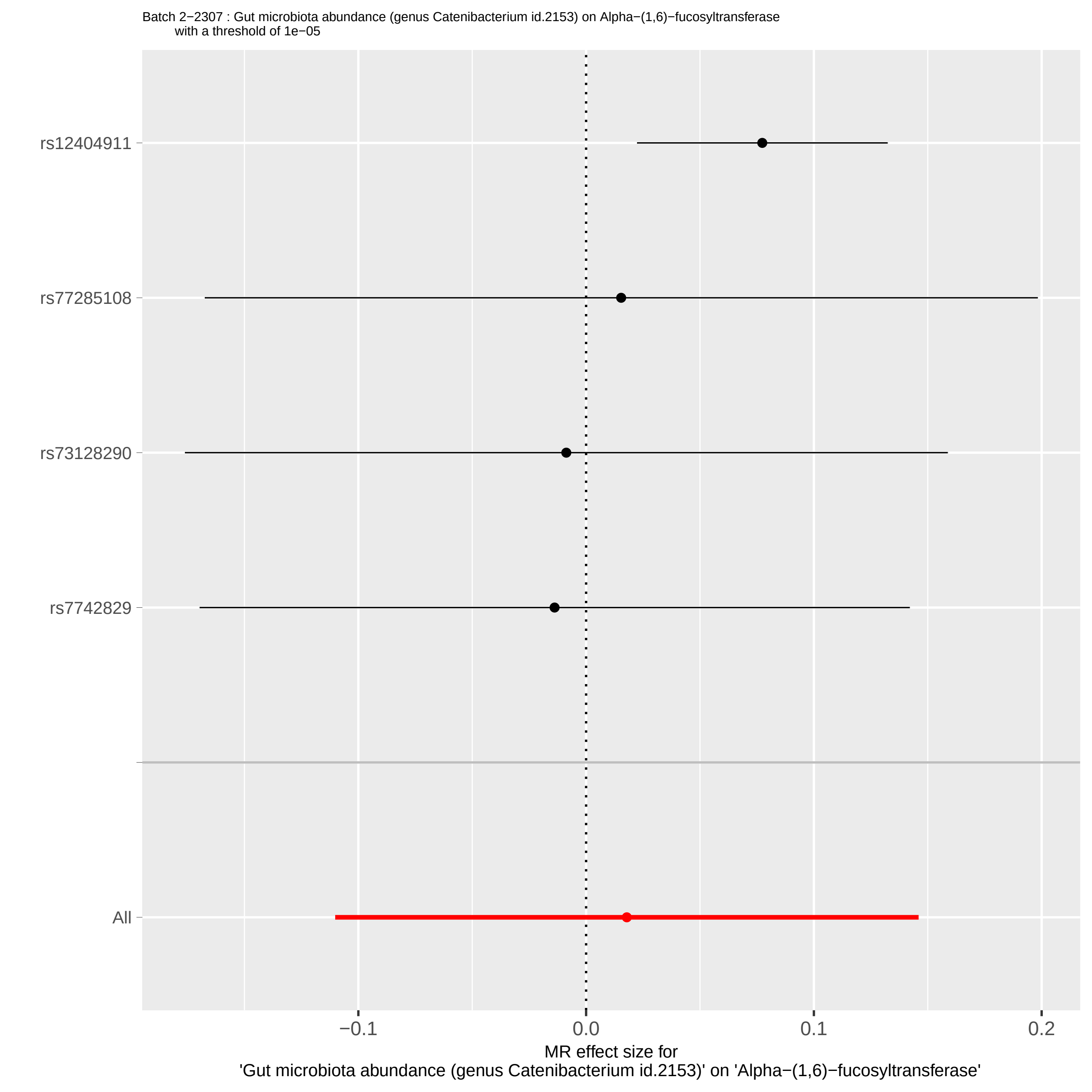
Batch 2−2307 : Gut microbiota abundance (genus Catenibacterium id.2153) on Alpha−(1,6)−fucosyltransferase
with a threshold of 1e−05
rs12404911
rs77285108
rs73128290
rs7742829
All
−0.1
0.0
0.1
0.2
MR effect size for
'Gut microbiota abundance (genus Catenibacterium id.2153)' on 'Alpha−(1,6)−fucosyltransferase'

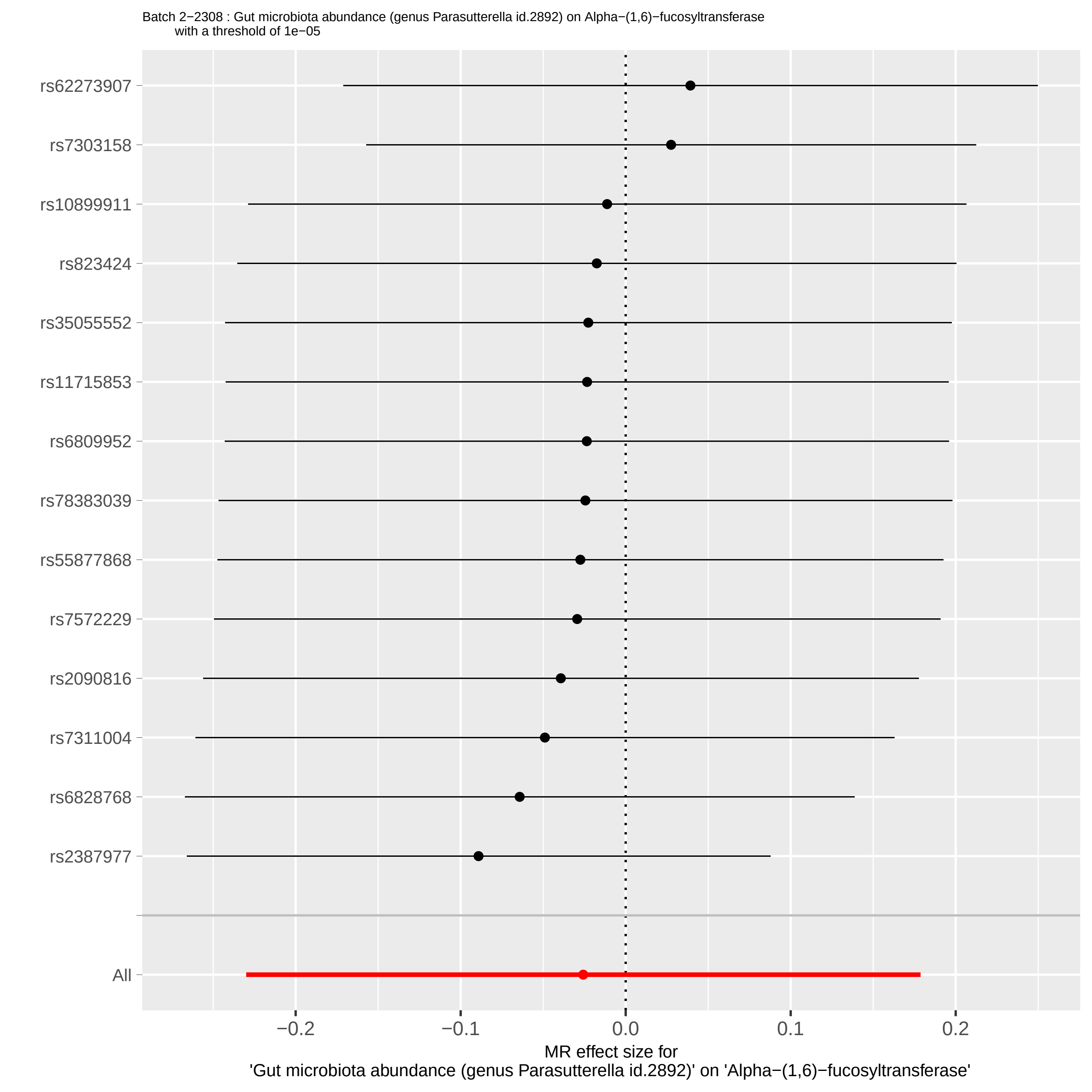
Batch 2−2308 : Gut microbiota abundance (genus Parasutterella id.2892) on Alpha−(1,6)−fucosyltransferase
with a threshold of 1e−05
rs62273907
rs7303158
rs10899911
rs823424
rs35055552
rs11715853
rs6809952
rs78383039
rs55877868
rs7572229
rs2090816
rs7311004
rs6828768
rs2387977
All
−0.2
−0.1
0.0
0.1
0.2
MR effect size for
'Gut microbiota abundance (genus Parasutterella id.2892)' on 'Alpha−(1,6)−fucosyltransferase'

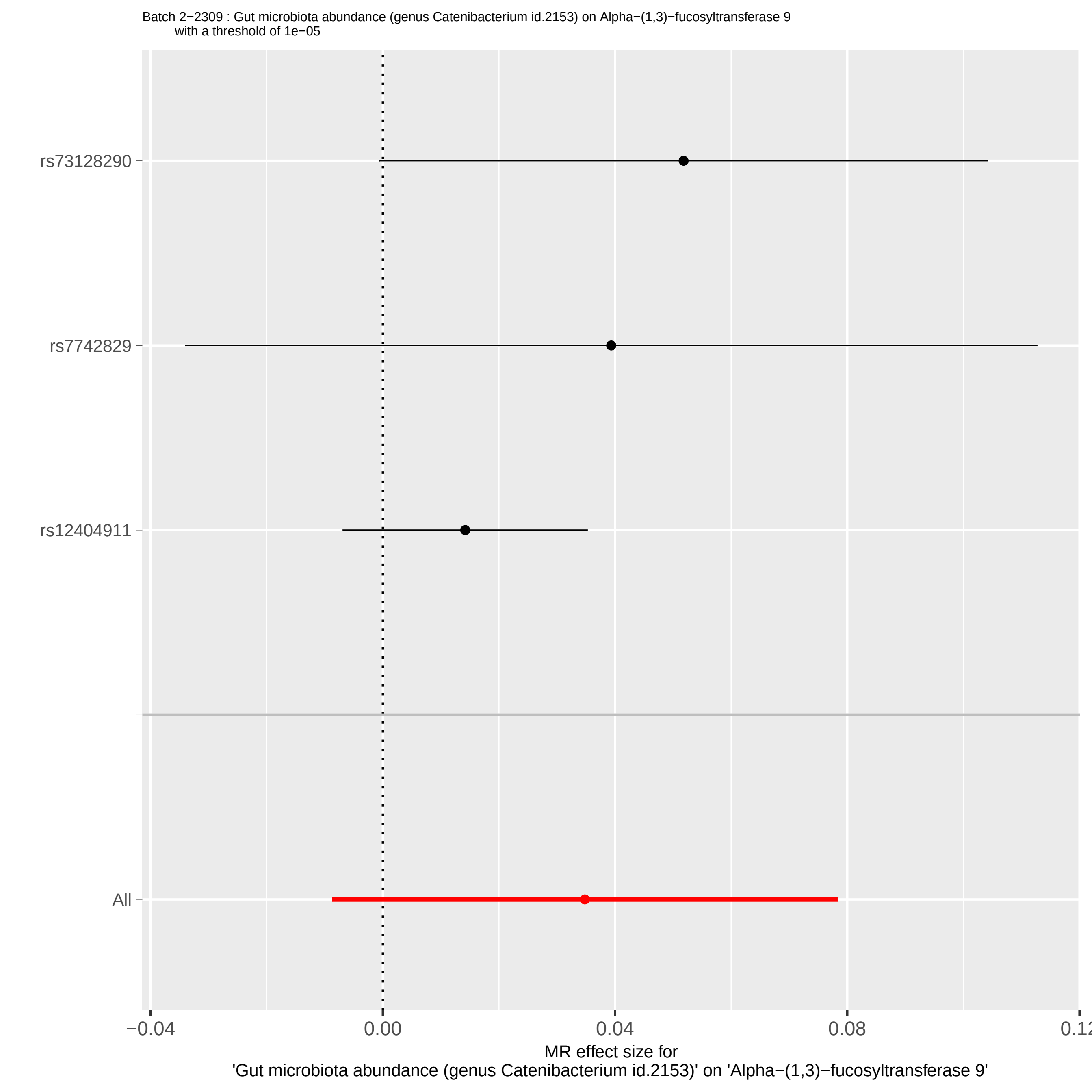

Batch 2−2309 : Gut microbiota abundance (genus Catenibacterium id.2153) on Alpha−(1,3)−fucosyltransferase 9
with a threshold of 1e−05
rs73128290
rs7742829
rs12404911
All
−0.04
0.00
0.04
0.08
0.12
MR effect size for
'Gut microbiota abundance (genus Catenibacterium id.2153)' on 'Alpha−(1,3)−fucosyltransferase 9'

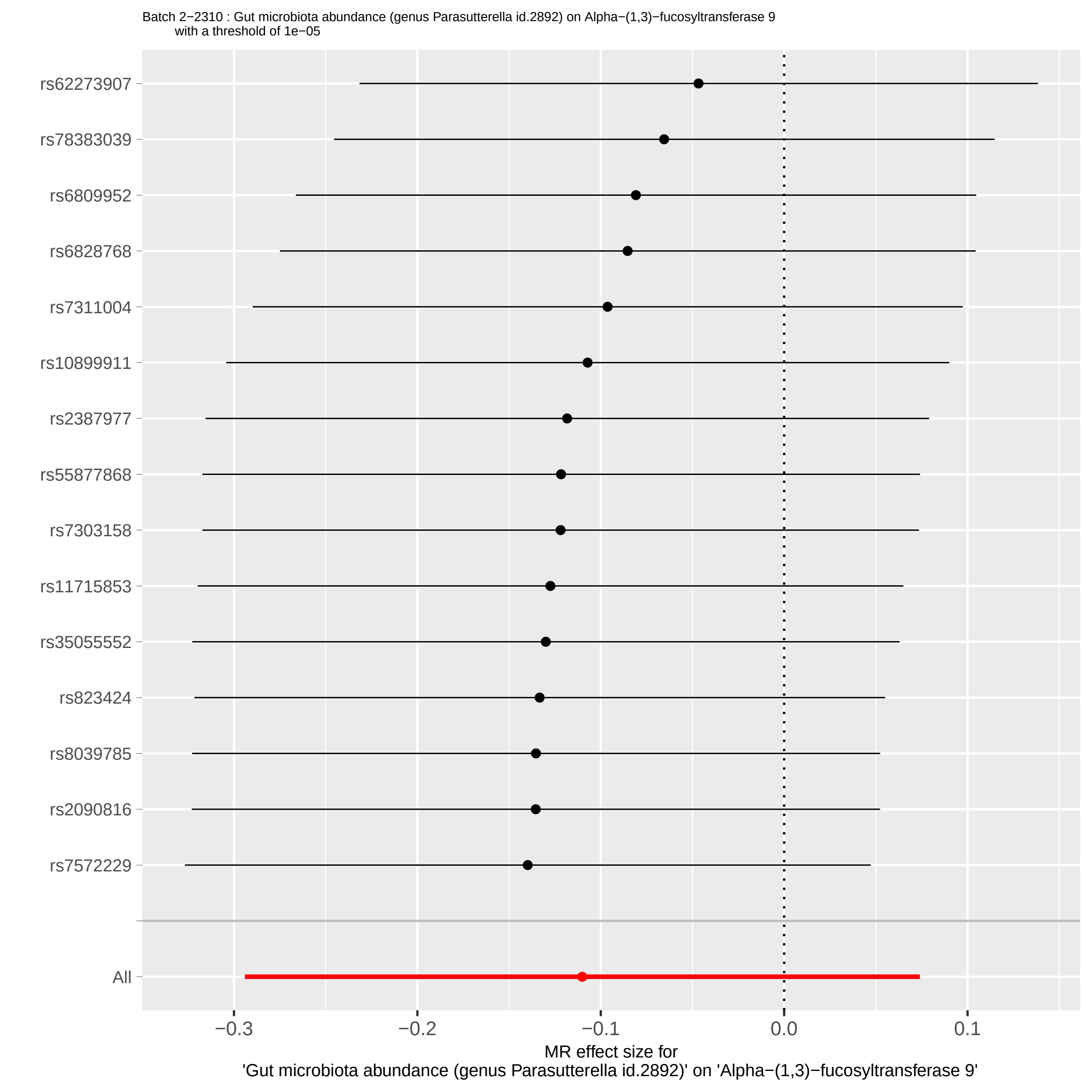

Batch 2−2310 : Gut microbiota abundance (genus Parasutterella id.2892) on Alpha−(1,3)−fucosyltransferase 9
with a threshold of 1e−05
rs62273907
rs78383039
rs6809952
rs6828768
rs7311004
rs10899911
rs2387977
rs55877868
rs7303158
rs11715853
rs35055552
rs823424
rs8039785
rs2090816
rs7572229
All
−0.3
−0.2
−0.1
0.0
0.1
MR effect size for
'Gut microbiota abundance (genus Parasutterella id.2892)' on 'Alpha−(1,3)−fucosyltransferase 9'

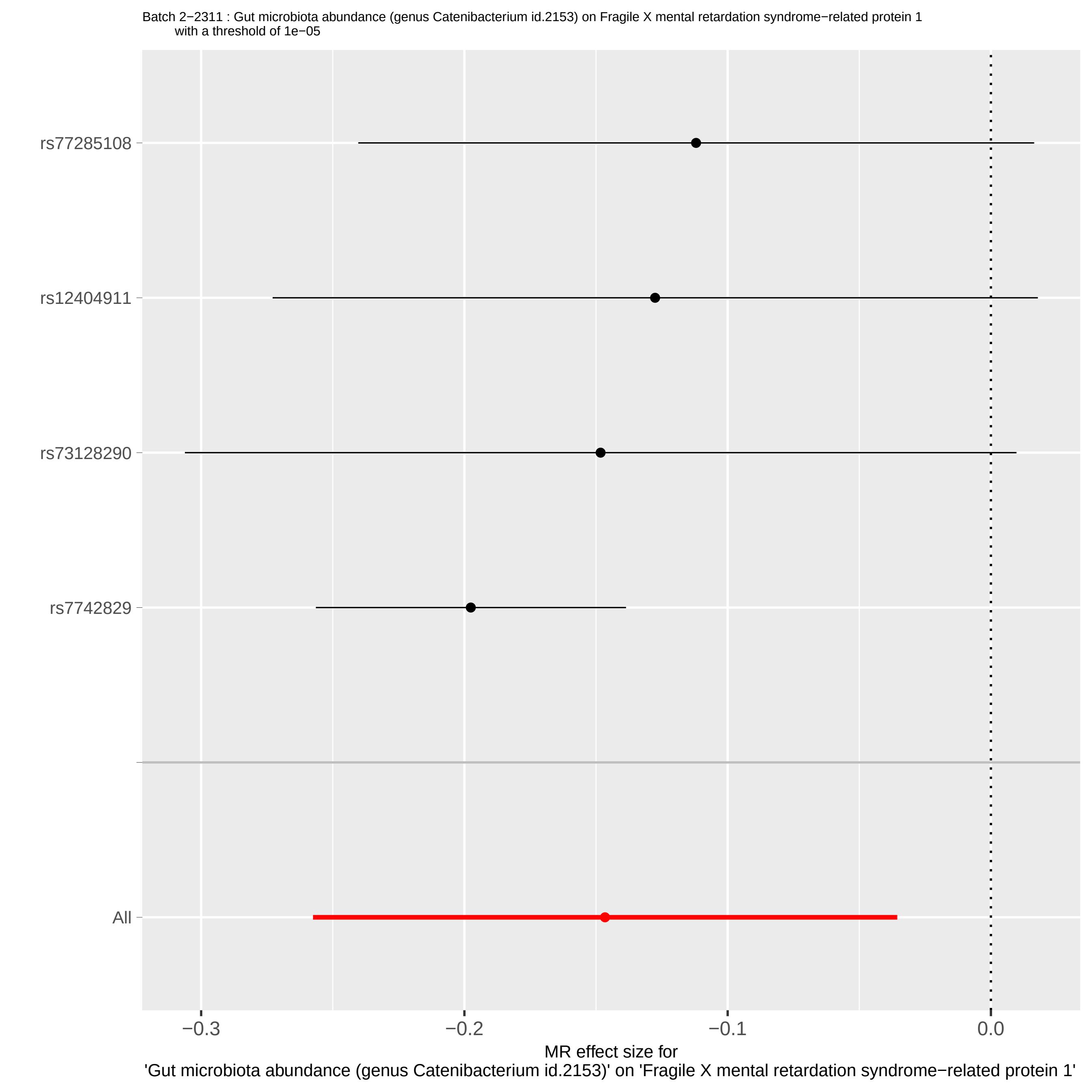
Batch 2−2311 : Gut microbiota abundance (genus Catenibacterium id.2153) on Fragile X mental retardation syndrome−related protein 1
with a threshold of 1e−05
rs77285108
rs12404911
rs73128290
rs7742829
All
−0.3
−0.2
−0.1
0.0
MR effect size for
'Gut microbiota abundance (genus Catenibacterium id.2153)' on 'Fragile X mental retardation syndrome−related protein 1'

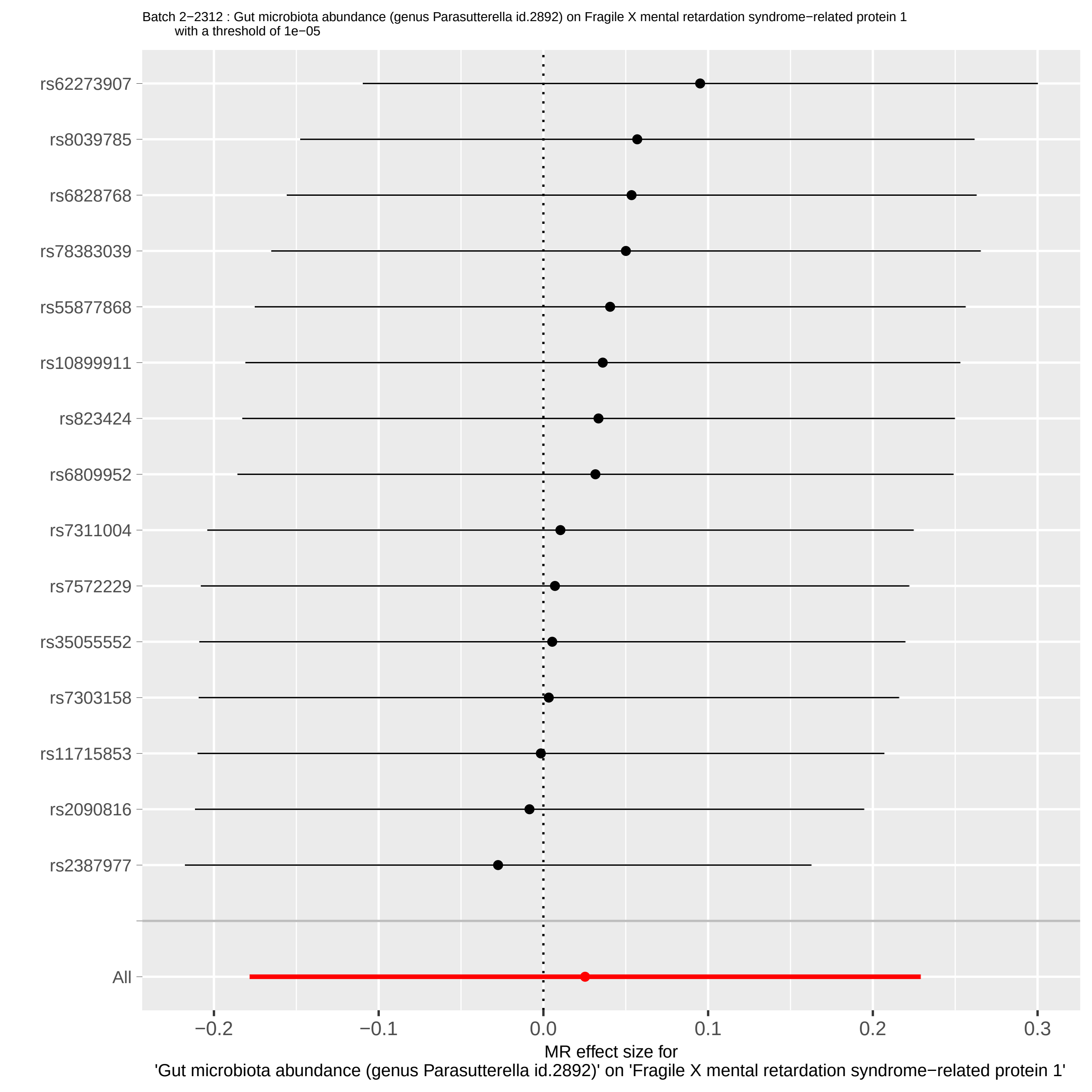
Batch 2−2312 : Gut microbiota abundance (genus Parasutterella id.2892) on Fragile X mental retardation syndrome−related protein 1
with a threshold of 1e−05
rs62273907
rs8039785
rs6828768
rs78383039
rs55877868
rs10899911
rs823424
rs6809952
rs7311004
rs7572229
rs35055552
rs7303158
rs11715853
rs2090816
rs2387977
All
−0.2
−0.1
0.0
0.1
0.2
0.3
MR effect size for
'Gut microbiota abundance (genus Parasutterella id.2892)' on 'Fragile X mental retardation syndrome−related protein 1'

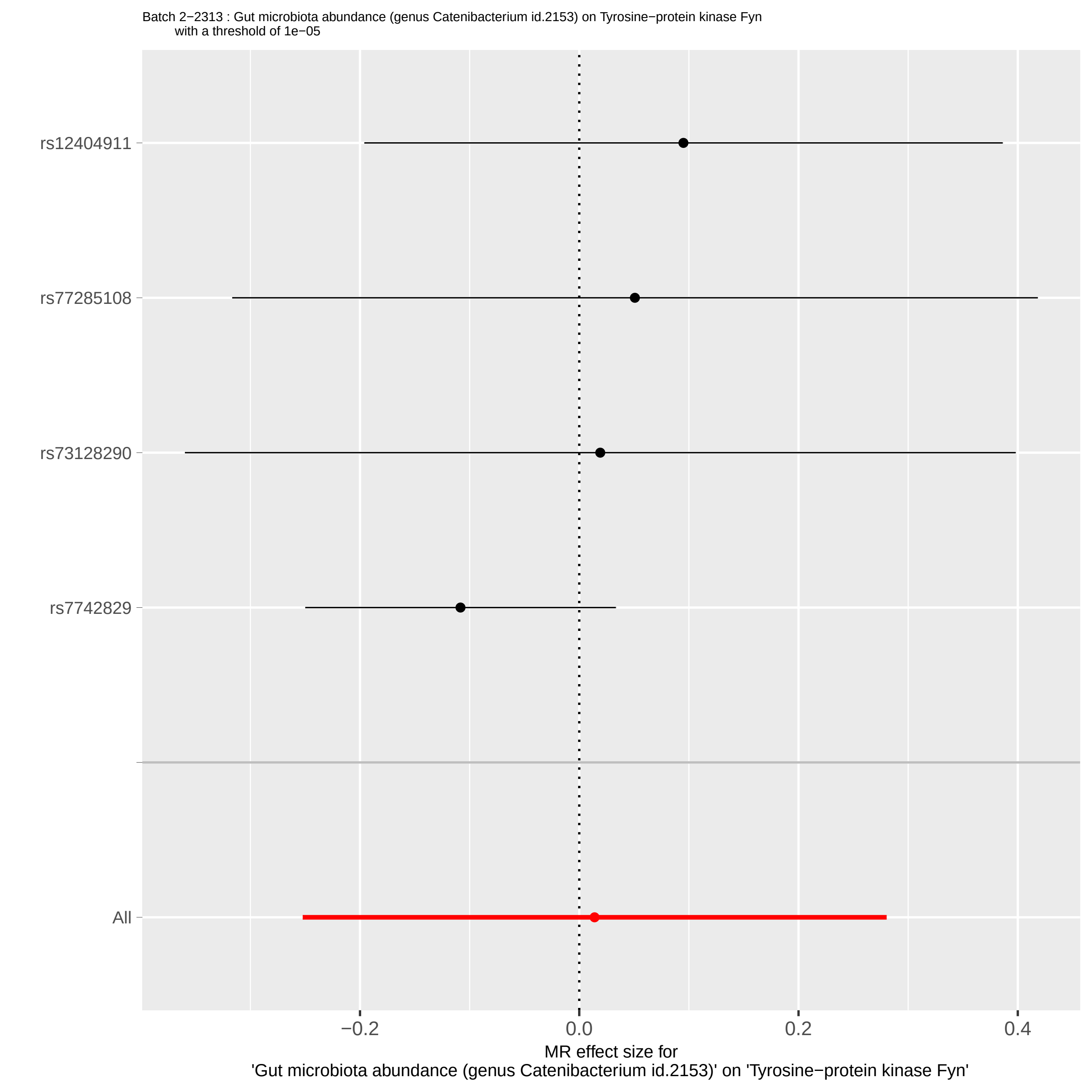
Batch 2−2313 : Gut microbiota abundance (genus Catenibacterium id.2153) on Tyrosine−protein kinase Fyn
with a threshold of 1e−05
rs12404911
rs77285108
rs73128290
rs7742829
All
−0.2
0.0
0.2
0.4
MR effect size for
'Gut microbiota abundance (genus Catenibacterium id.2153)' on 'Tyrosine−protein kinase Fyn'

Batch 2−2314 : Gut microbiota abundance (genus Parasutterella id.2892) on Tyrosine−protein kinase Fyn
with a threshold of 1e−05

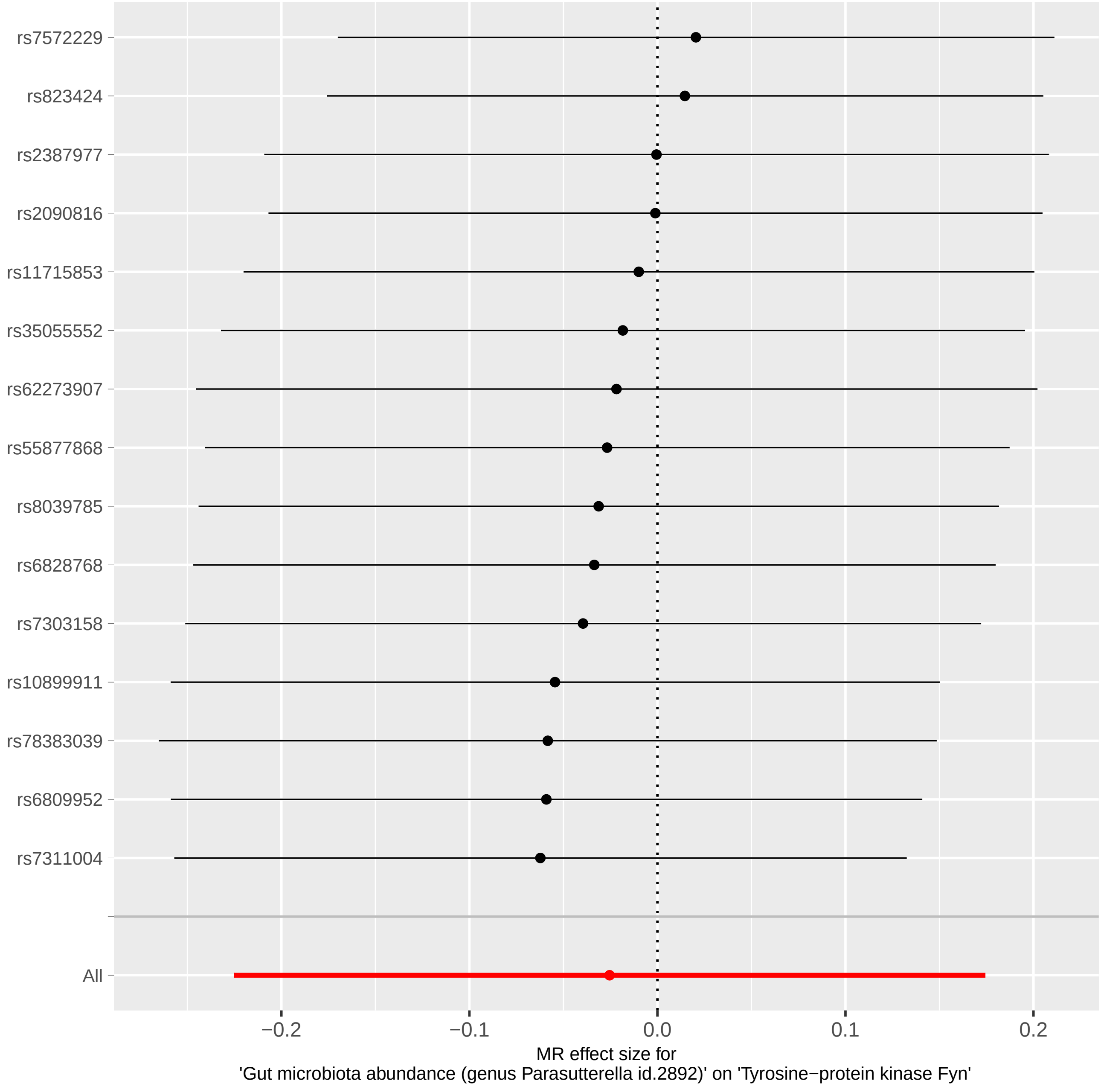

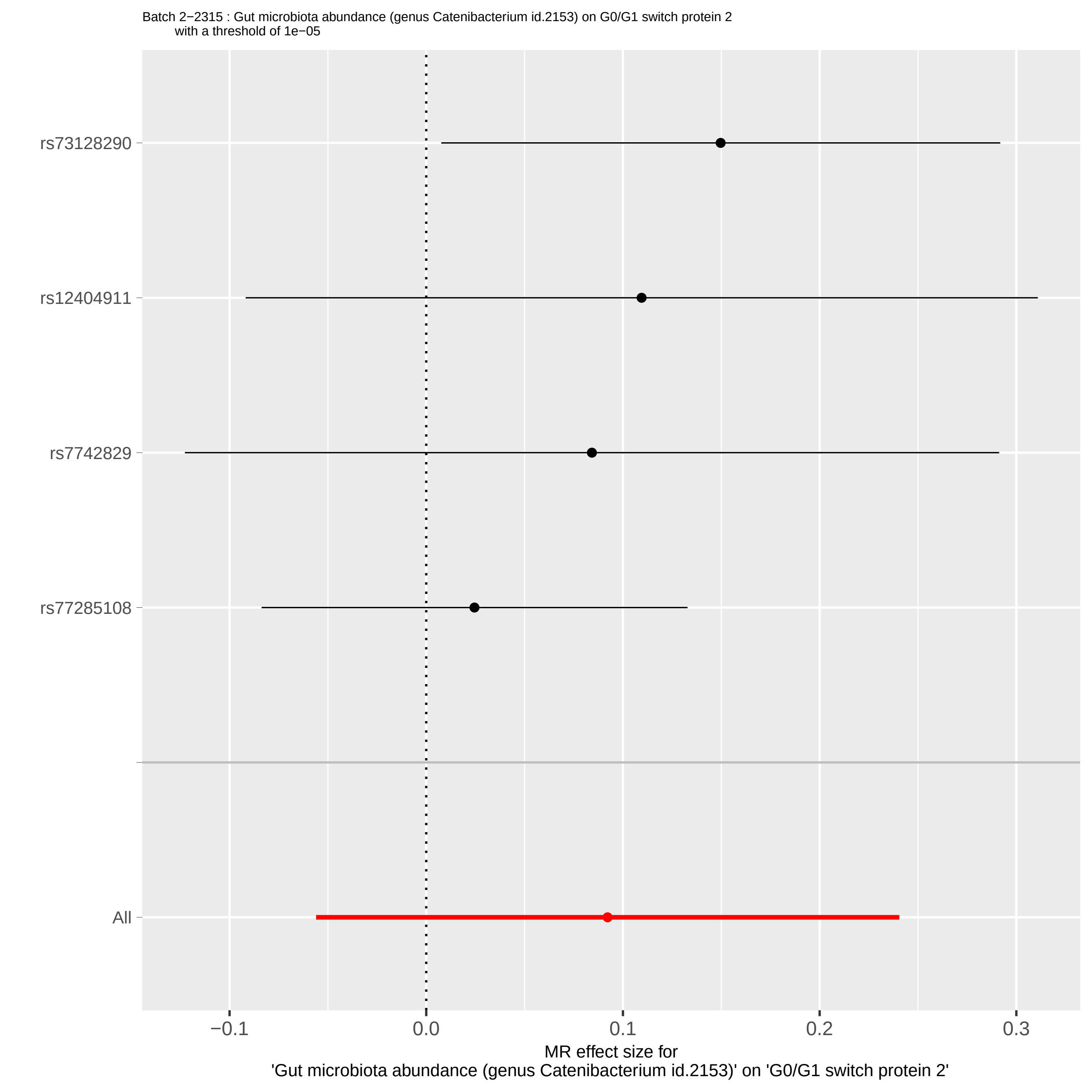

Batch 2−2315 : Gut microbiota abundance (genus Catenibacterium id.2153) on G0/G1 switch protein 2
with a threshold of 1e−05
rs73128290
rs12404911
rs7742829
rs77285108
All
−0.1
0.0
0.1
0.2
0.3
MR effect size for
'Gut microbiota abundance (genus Catenibacterium id.2153)' on 'G0/G1 switch protein 2'

Batch 2−2316 : Gut microbiota abundance (genus Parasutterella id.2892) on G0/G1 switch protein 2
with a threshold of 1e−05

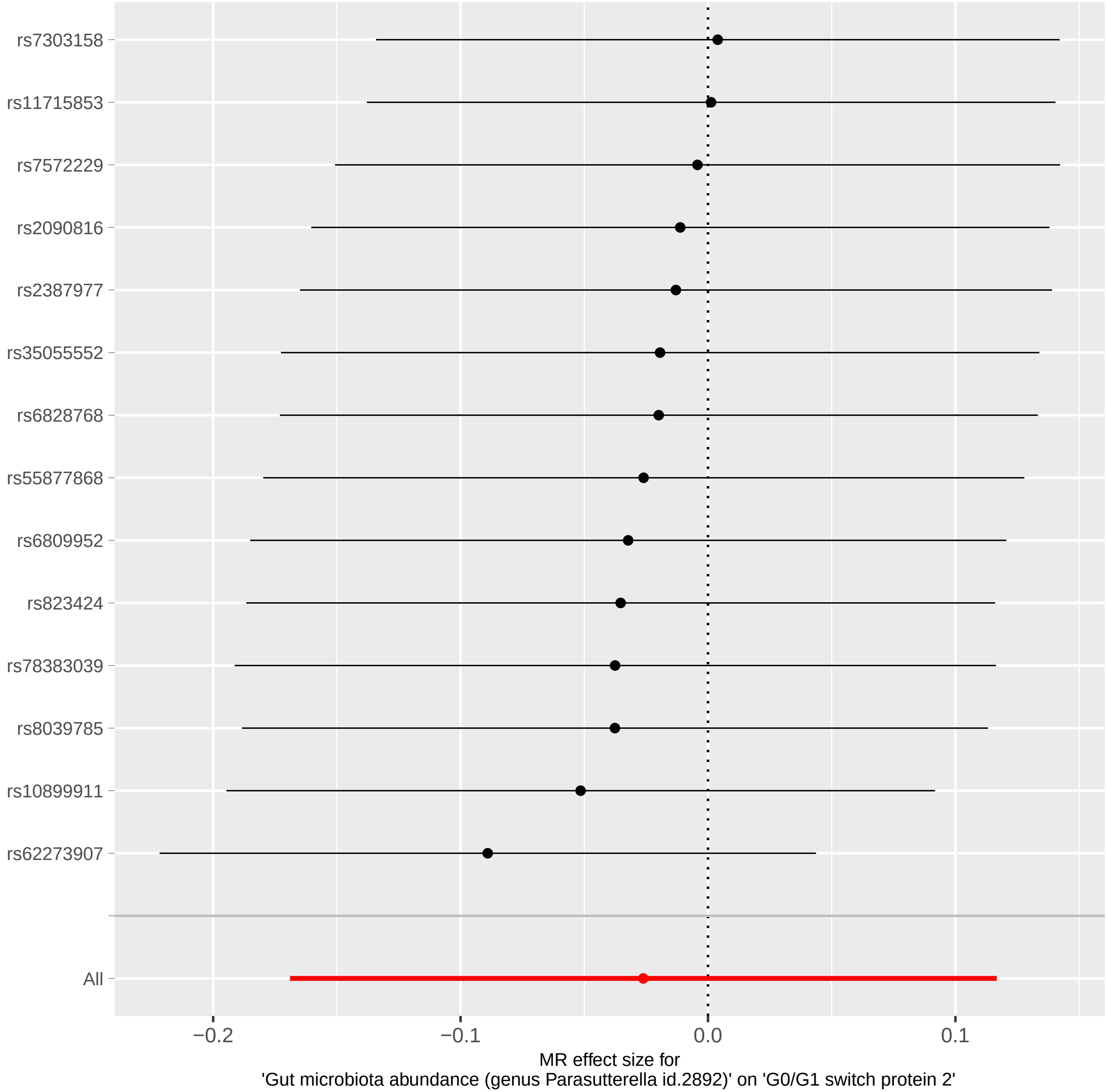

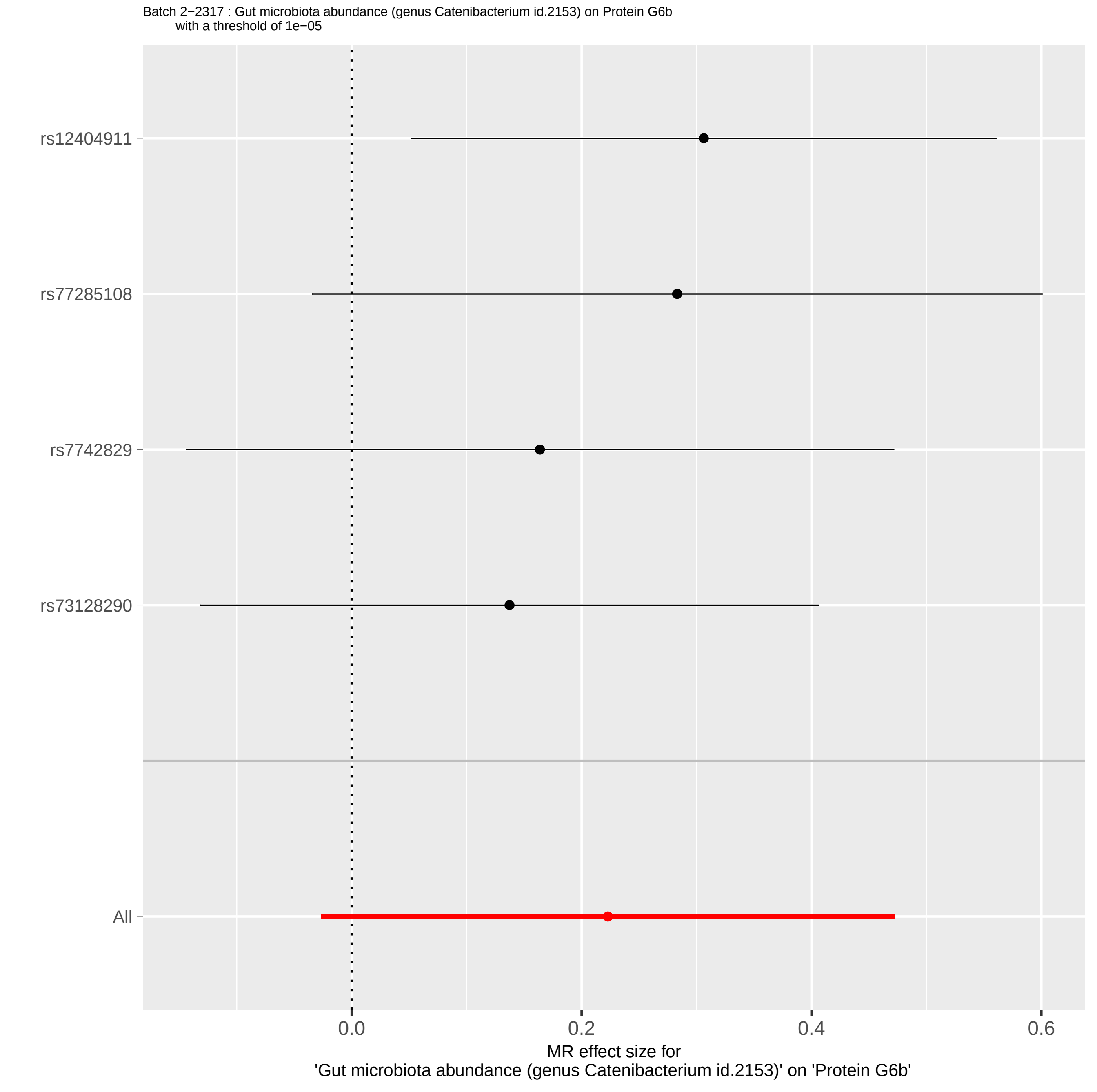
Batch 2−2317 : Gut microbiota abundance (genus Catenibacterium id.2153) on Protein G6b
with a threshold of 1e−05
rs12404911
rs77285108
rs7742829
rs73128290
All
0.0
0.2
0.4
0.6
MR effect size for
'Gut microbiota abundance (genus Catenibacterium id.2153)' on 'Protein G6b'

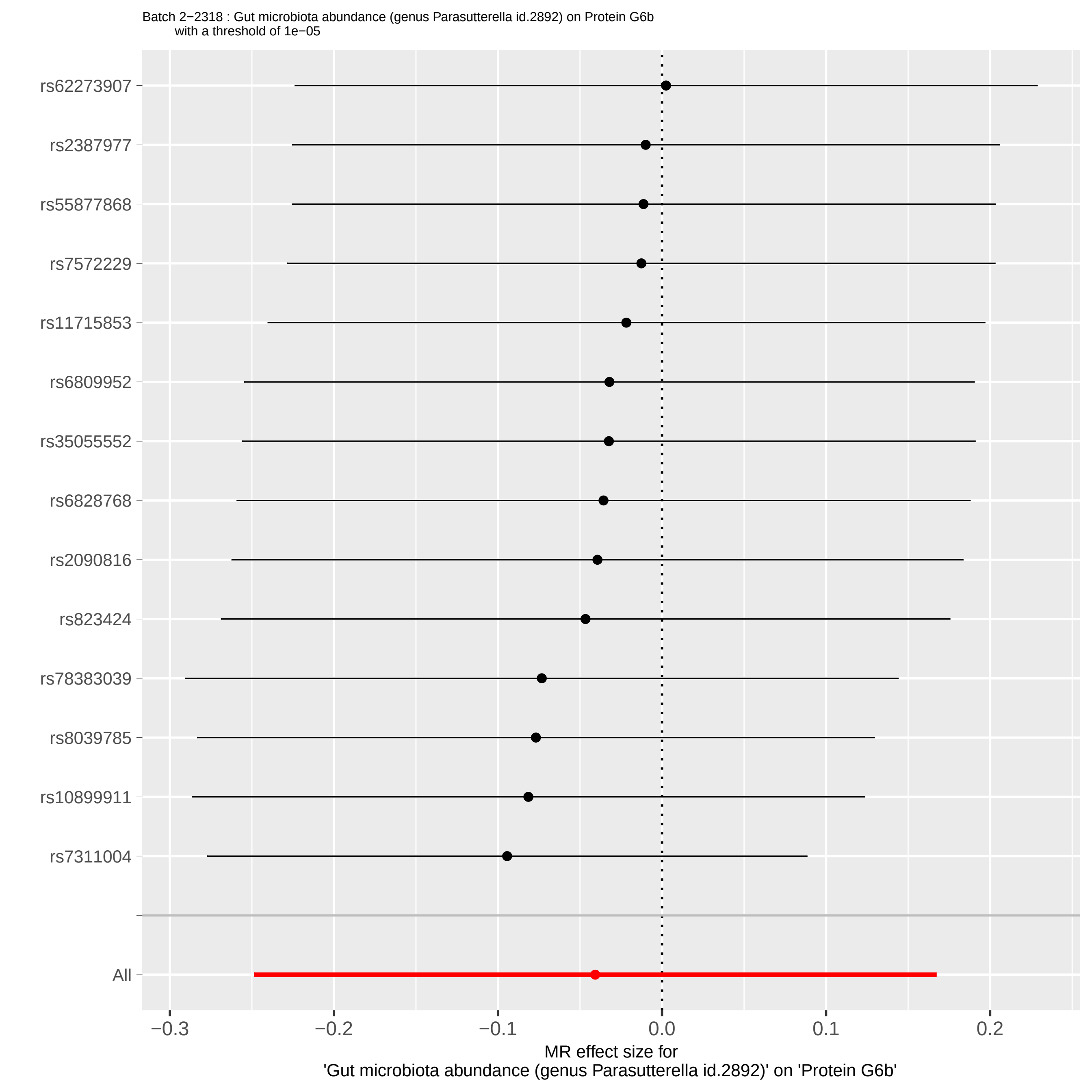
Batch 2−2318 : Gut microbiota abundance (genus Parasutterella id.2892) on Protein G6b
with a threshold of 1e−05
rs62273907
rs2387977
rs55877868
rs7572229
rs11715853
rs6809952
rs35055552
rs6828768
rs2090816
rs823424
rs78383039
rs8039785
rs10899911
rs7311004
All
−0.3
−0.2
−0.1
0.0
0.1
0.2
MR effect size for
'Gut microbiota abundance (genus Parasutterella id.2892)' on 'Protein G6b'

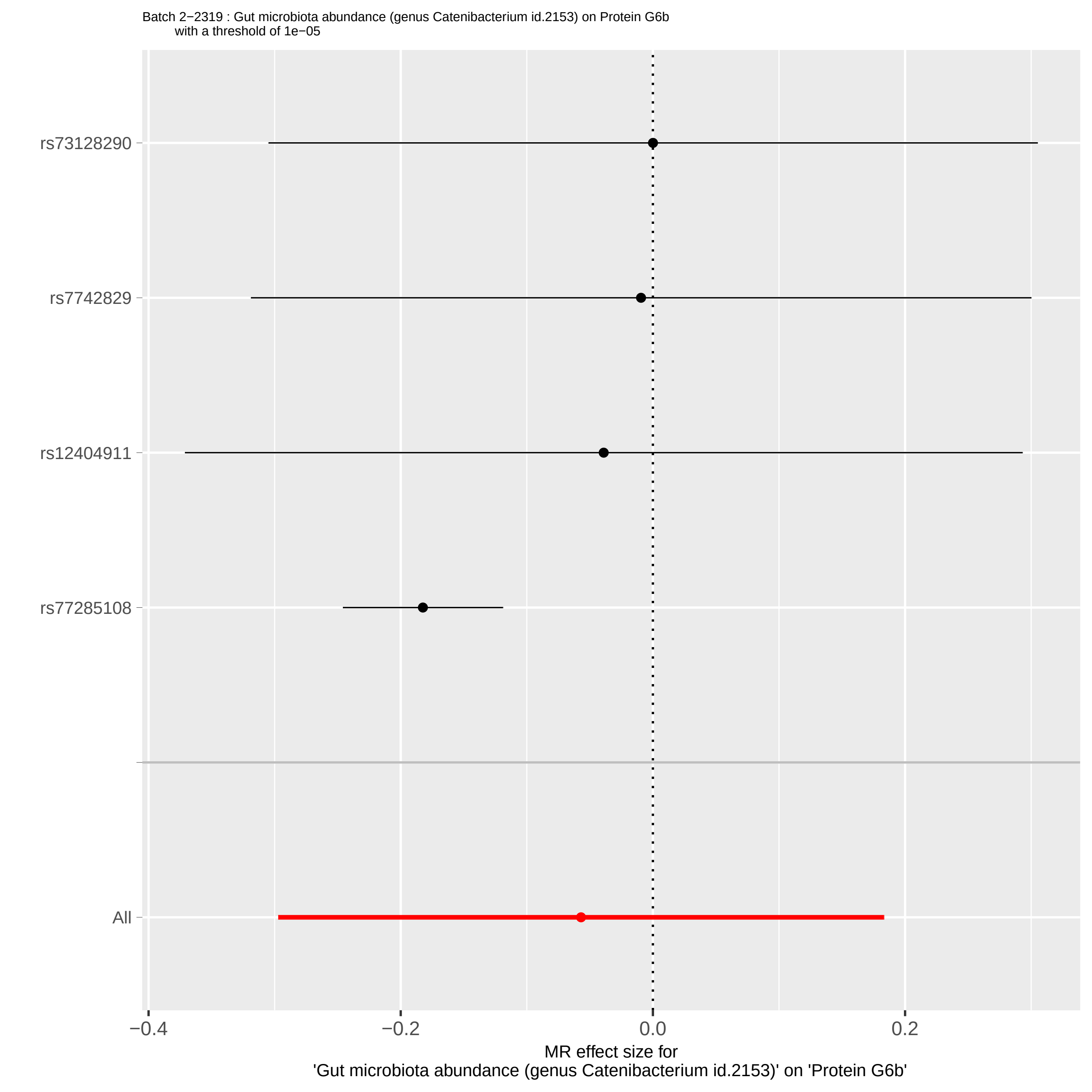

Batch 2−2319 : Gut microbiota abundance (genus Catenibacterium id.2153) on Protein G6b
with a threshold of 1e−05
rs73128290
rs7742829
rs12404911
rs77285108
All
−0.4
−0.2
0.0
0.2
MR effect size for
'Gut microbiota abundance (genus Catenibacterium id.2153)' on 'Protein G6b'

Batch 2−2320 : Gut microbiota abundance (genus Parasutterella id.2892) on Protein G6b
with a threshold of 1e−05

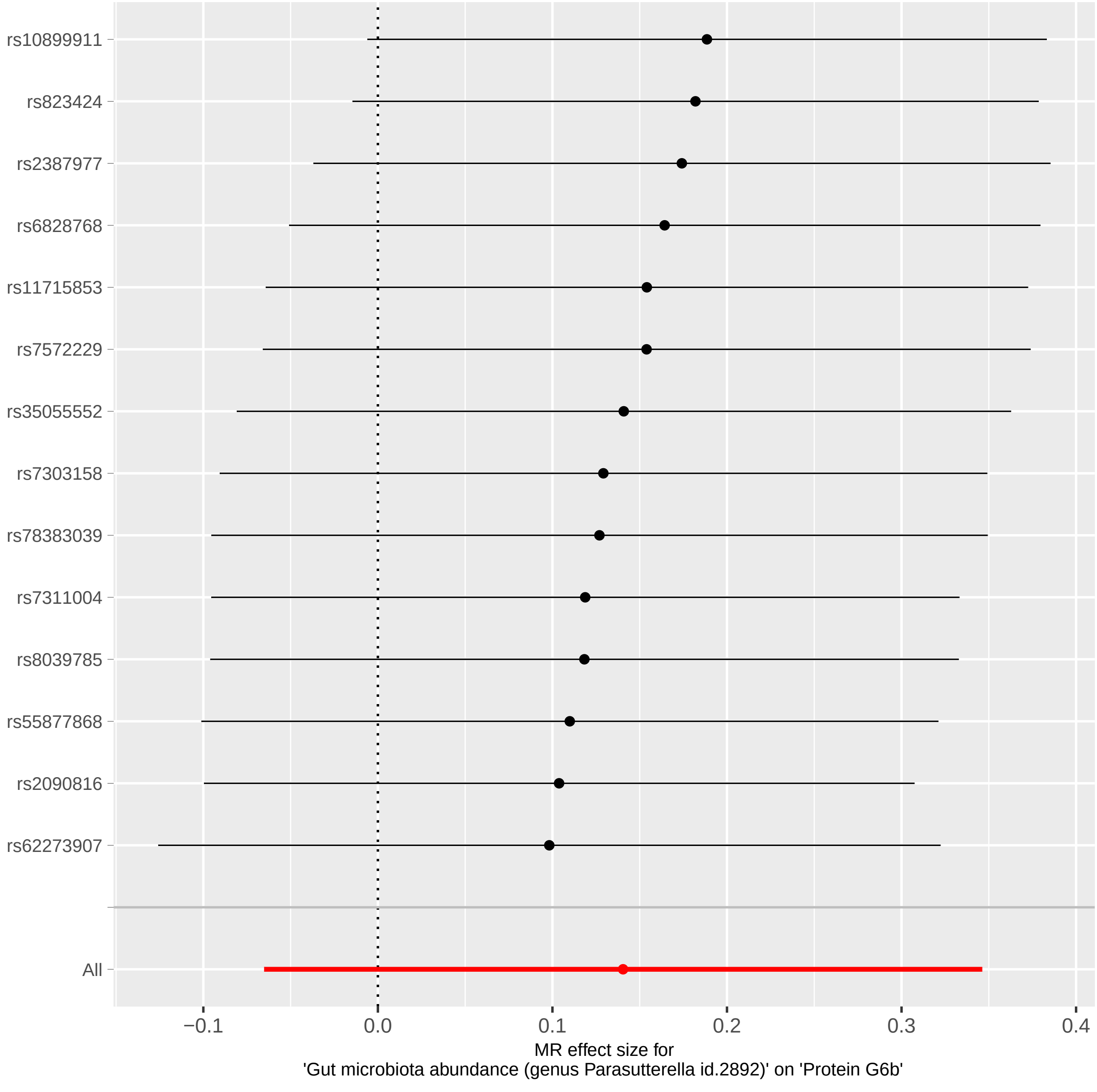

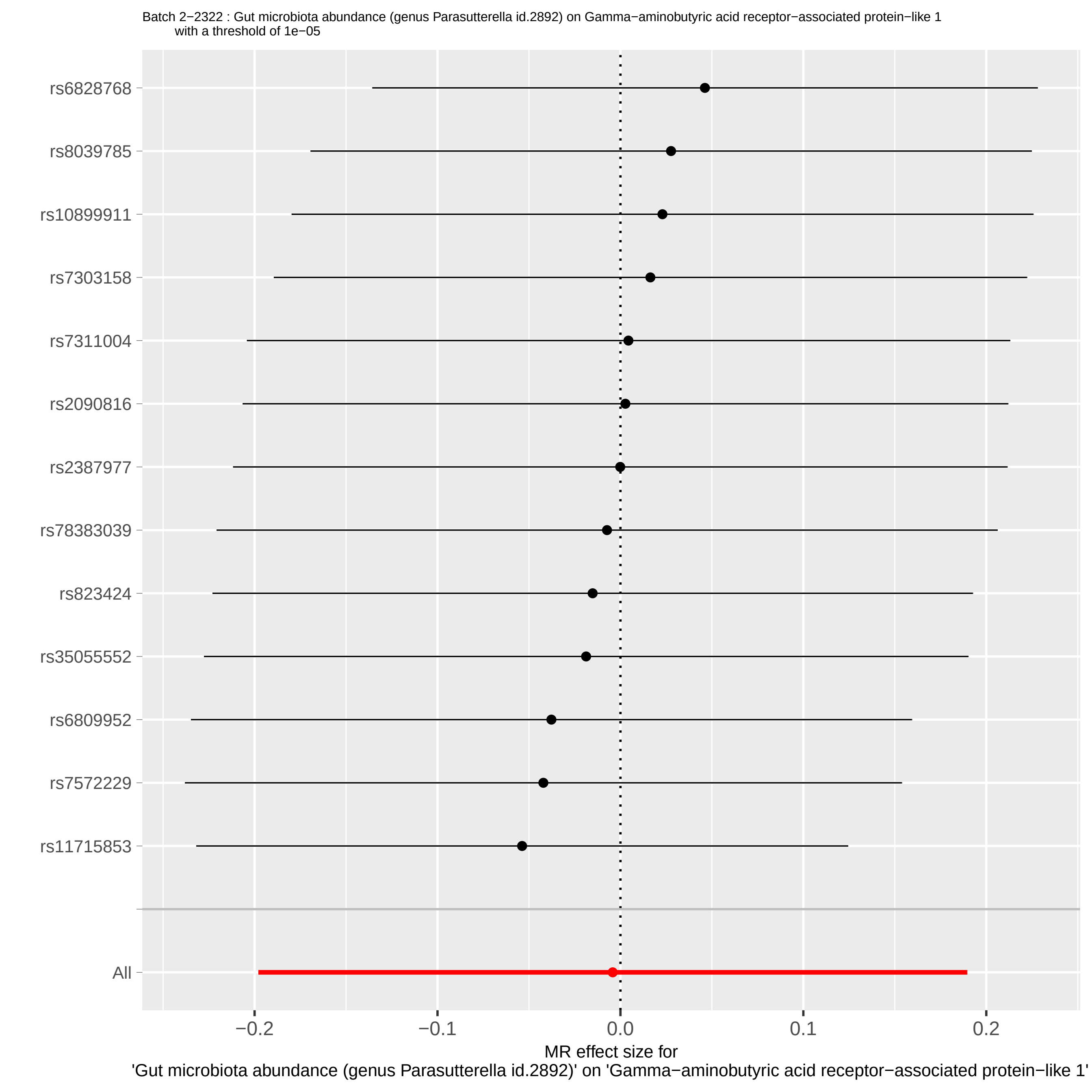

Batch 2−2322 : Gut microbiota abundance (genus Parasutterella id.2892) on Gamma−aminobutyric acid receptor−associated protein−like 1
with a threshold of 1e−05
rs6828768
rs8039785
rs10899911
rs7303158
rs7311004
rs2090816
rs2387977
rs78383039
rs823424
rs35055552
rs6809952
rs7572229
rs11715853
All
−0.2
−0.1
0.0
0.1
0.2
MR effect size for
'Gut microbiota abundance (genus Parasutterella id.2892)' on 'Gamma−aminobutyric acid receptor−associated protein−like 1'

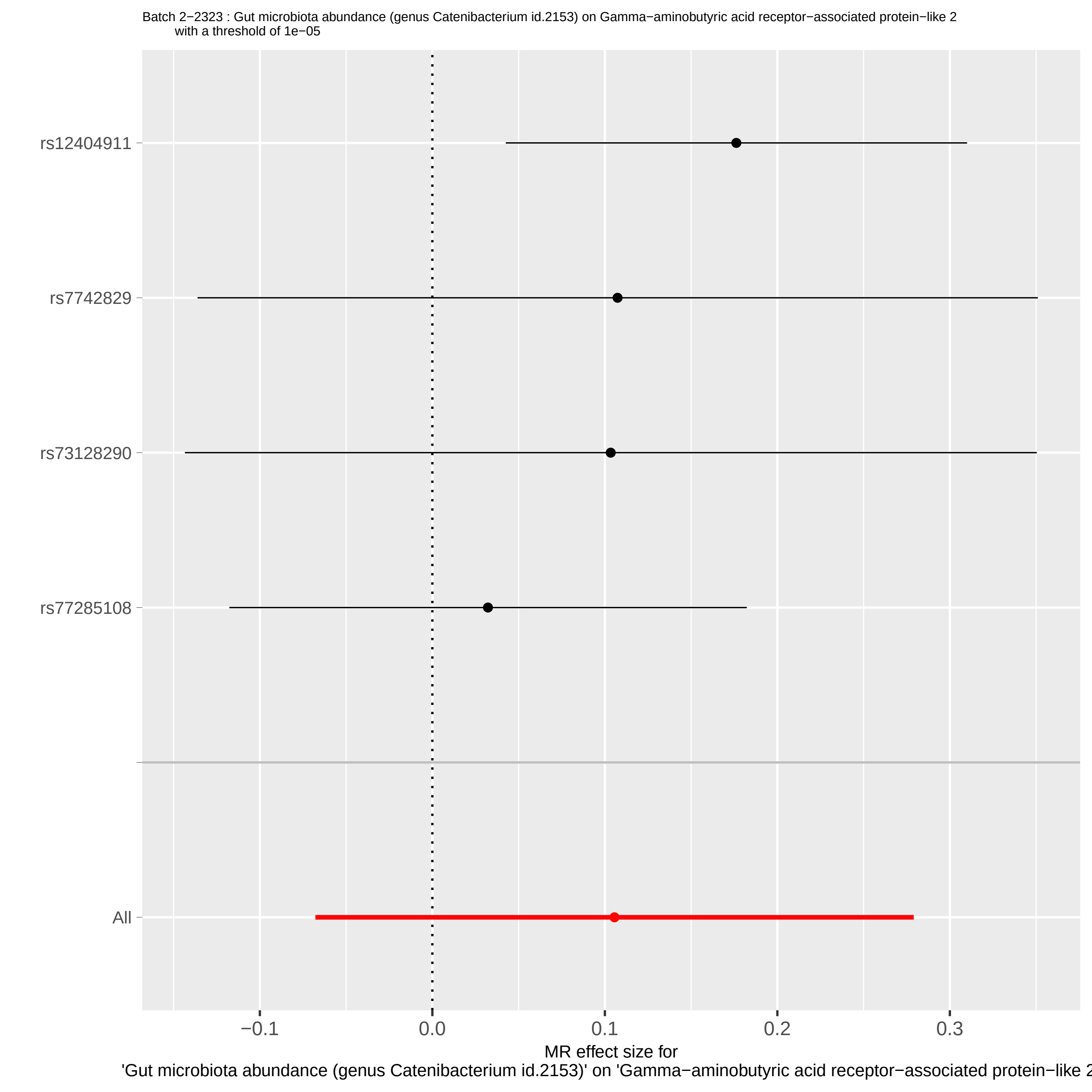

Batch 2−2323 : Gut microbiota abundance (genus Catenibacterium id.2153) on Gamma−aminobutyric acid receptor−associated protein−like 2
with a threshold of 1e−05
rs12404911
rs7742829
rs73128290
rs77285108
All
−0.1
0.0
0.1
0.2
0.3
MR effect size for
'Gut microbiota abundance (genus Catenibacterium id.2153)' on 'Gamma−aminobutyric acid receptor−associated protein−like 2

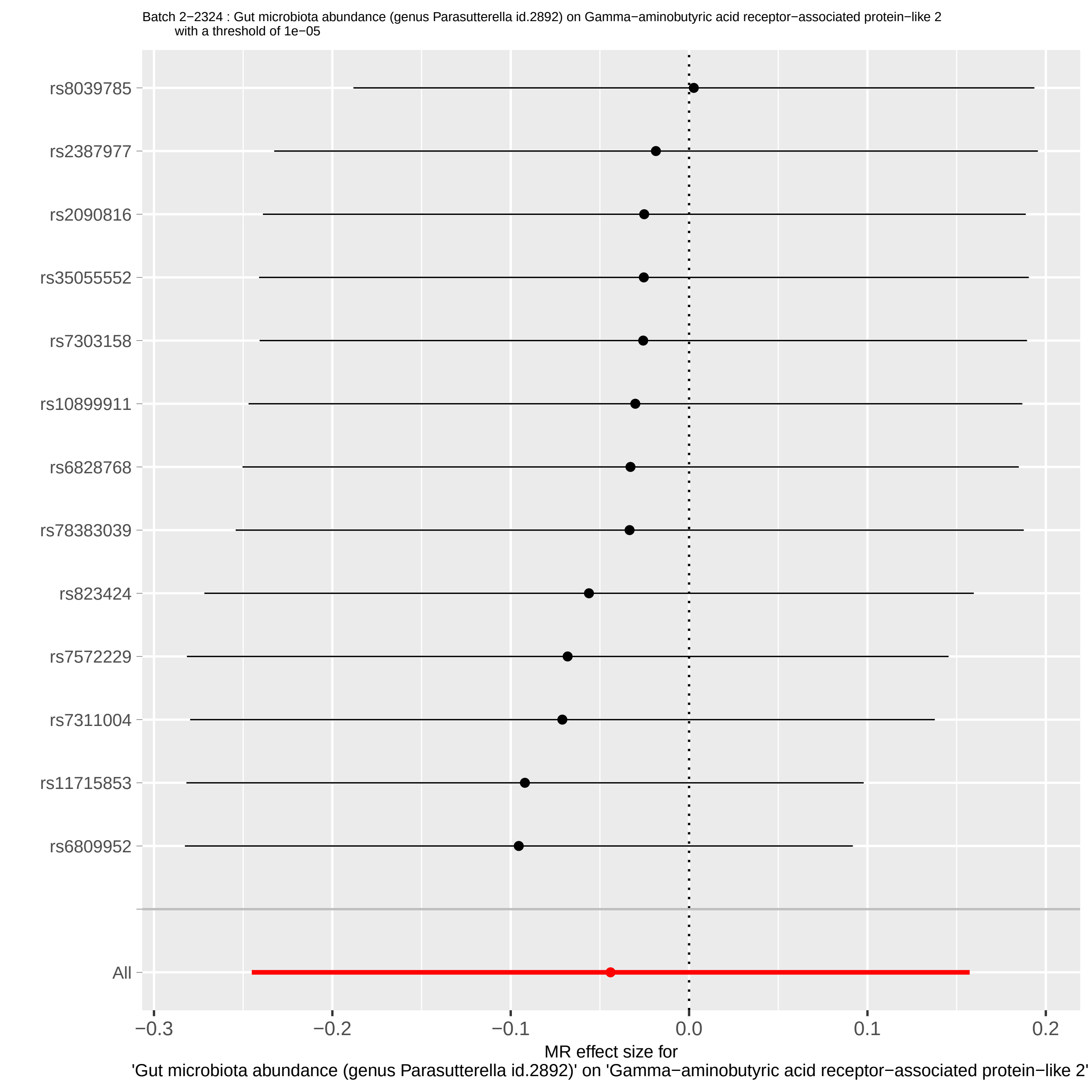

Batch 2−2324 : Gut microbiota abundance (genus Parasutterella id.2892) on Gamma−aminobutyric acid receptor−associated protein−like 2 with a threshold of 1e−05
rs8039785
rs2387977
rs2090816
rs35055552
rs7303158
rs10899911
rs6828768
rs78383039
rs823424
rs7572229
rs7311004
rs11715853
rs6809952
All
−0.3
−0.2
−0.1
0.0
0.1
0.2
MR effect size for
'Gut microbiota abundance (genus Parasutterella id.2892)' on 'Gamma−aminobutyric acid receptor−associated protein−like 2'

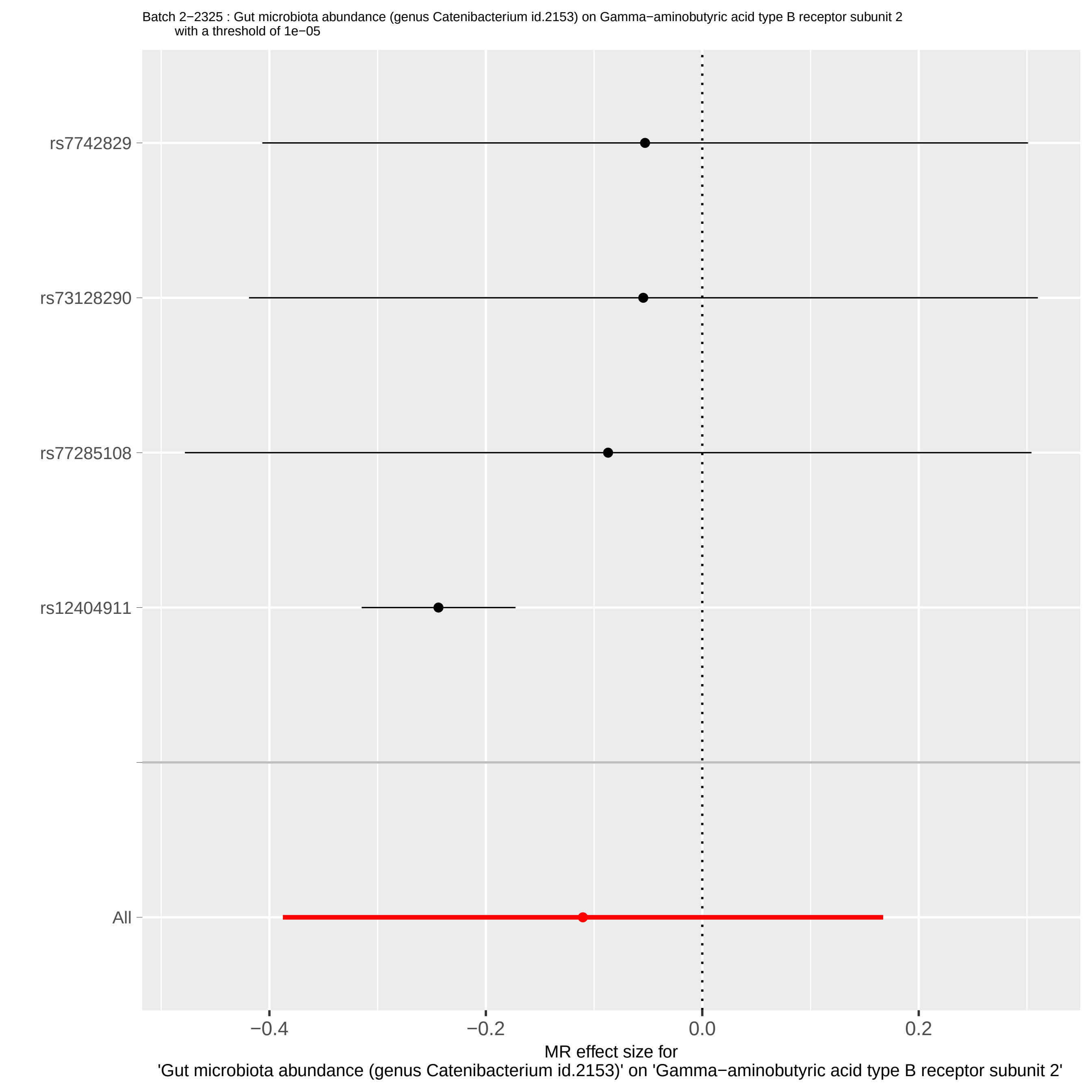

Batch 2−2325 : Gut microbiota abundance (genus Catenibacterium id.2153) on Gamma−aminobutyric acid type B receptor subunit 2
with a threshold of 1e−05
rs7742829
rs73128290
rs77285108
rs12404911
All
−0.4
−0.2
0.0
0.2
MR effect size for
'Gut microbiota abundance (genus Catenibacterium id.2153)' on 'Gamma−aminobutyric acid type B receptor subunit 2'

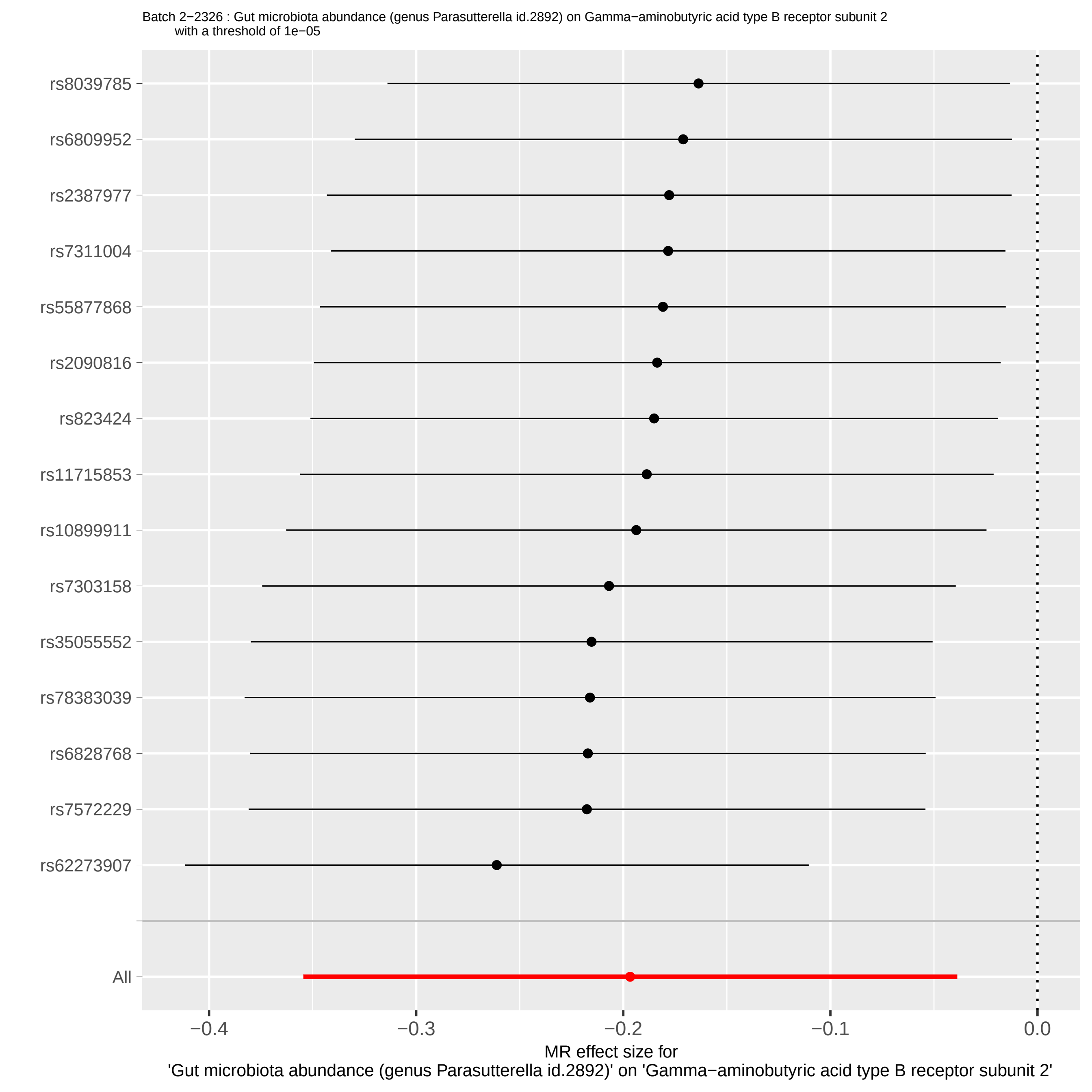

Batch 2−2326 : Gut microbiota abundance (genus Parasutterella id.2892) on Gamma−aminobutyric acid type B receptor subunit 2
with a threshold of 1e−05
rs8039785
rs6809952
rs2387977
rs7311004
rs55877868
rs2090816
rs823424
rs11715853
rs10899911
rs7303158
rs35055552
rs78383039
rs6828768
rs7572229
rs62273907
All
−0.4
−0.3
−0.2
−0.1
0.0
MR effect size for
'Gut microbiota abundance (genus Parasutterella id.2892)' on 'Gamma−aminobutyric acid type B receptor subunit 2'

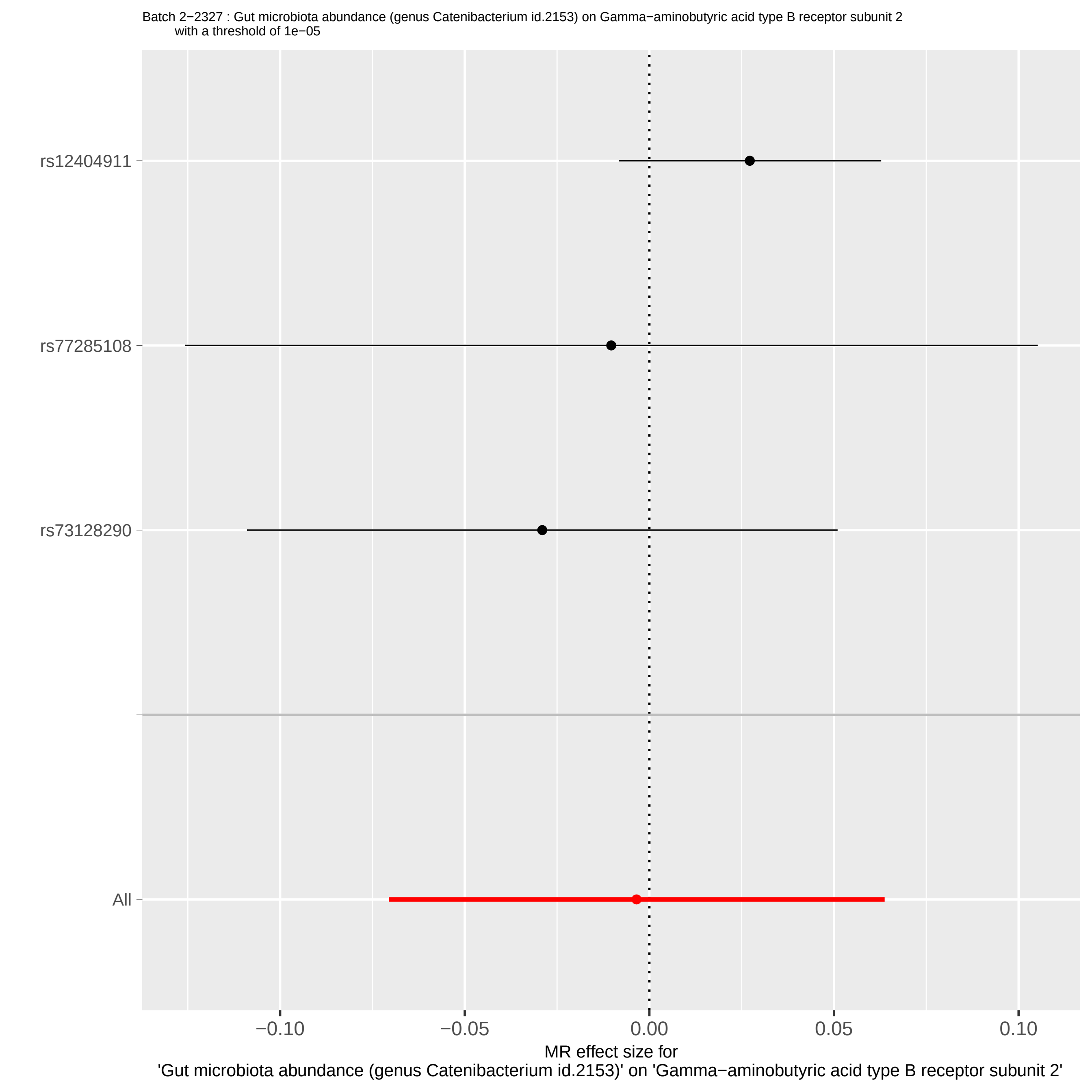
Batch 2−2327 : Gut microbiota abundance (genus Catenibacterium id.2153) on Gamma−aminobutyric acid type B receptor subunit 2
with a threshold of 1e−05
rs12404911
rs77285108
rs73128290
All
−0.10
−0.05
0.00
0.05
0.10
MR effect size for
'Gut microbiota abundance (genus Catenibacterium id.2153)' on 'Gamma−aminobutyric acid type B receptor subunit 2'

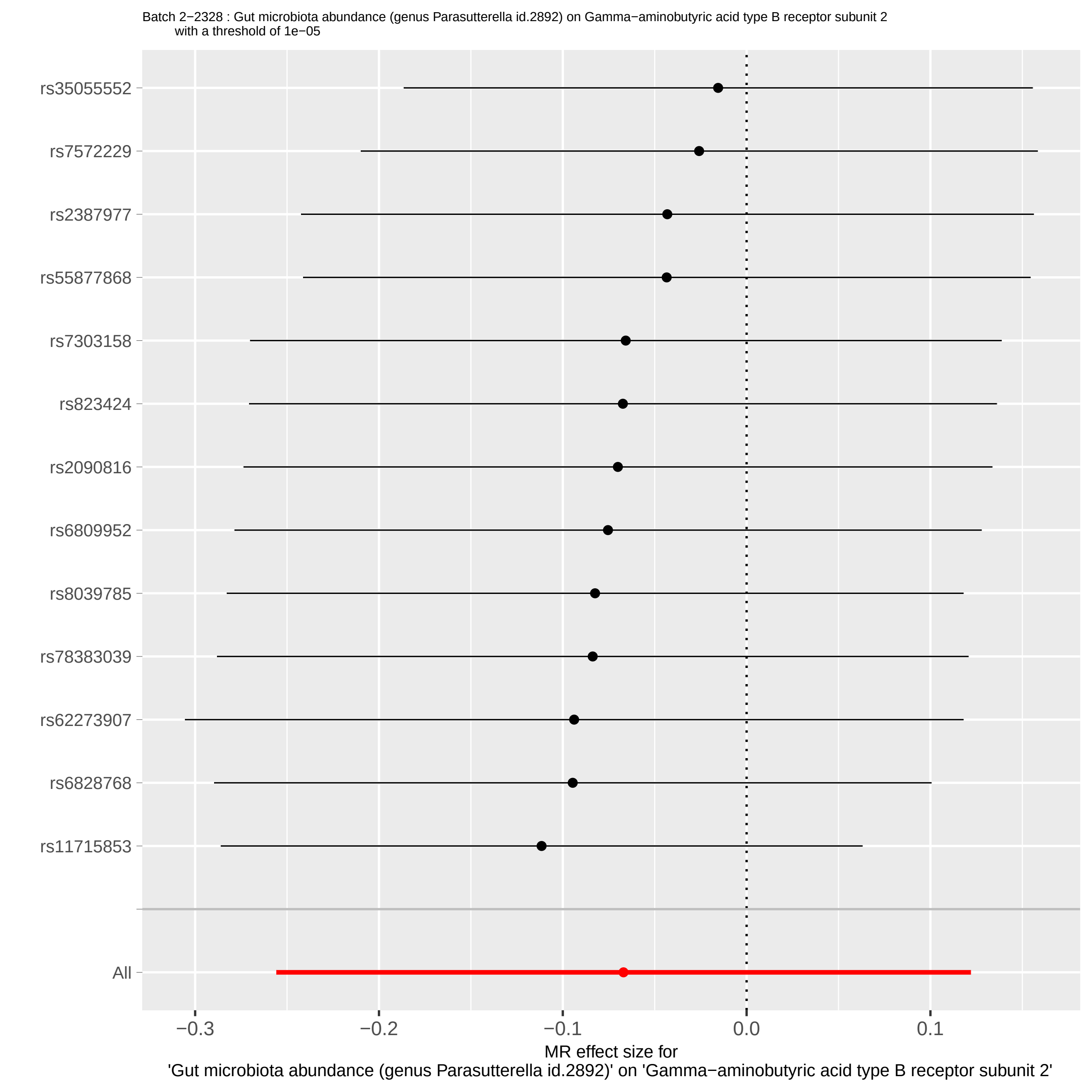

Batch 2−2328 : Gut microbiota abundance (genus Parasutterella id.2892) on Gamma−aminobutyric acid type B receptor subunit 2
with a threshold of 1e−05
rs35055552
rs7572229
rs2387977
rs55877868
rs7303158
rs823424
rs2090816
rs6809952
rs8039785
rs78383039
rs62273907
rs6828768
rs11715853
All
−0.3
−0.2
−0.1
0.0
0.1
MR effect size for
'Gut microbiota abundance (genus Parasutterella id.2892)' on 'Gamma−aminobutyric acid type B receptor subunit 2'

Batch 2−2329 : Gut microbiota abundance (genus Catenibacterium id.2153) on Growth arrest and DNA damage−inducible proteins−interacting protein 1
with a threshold of 1e−05

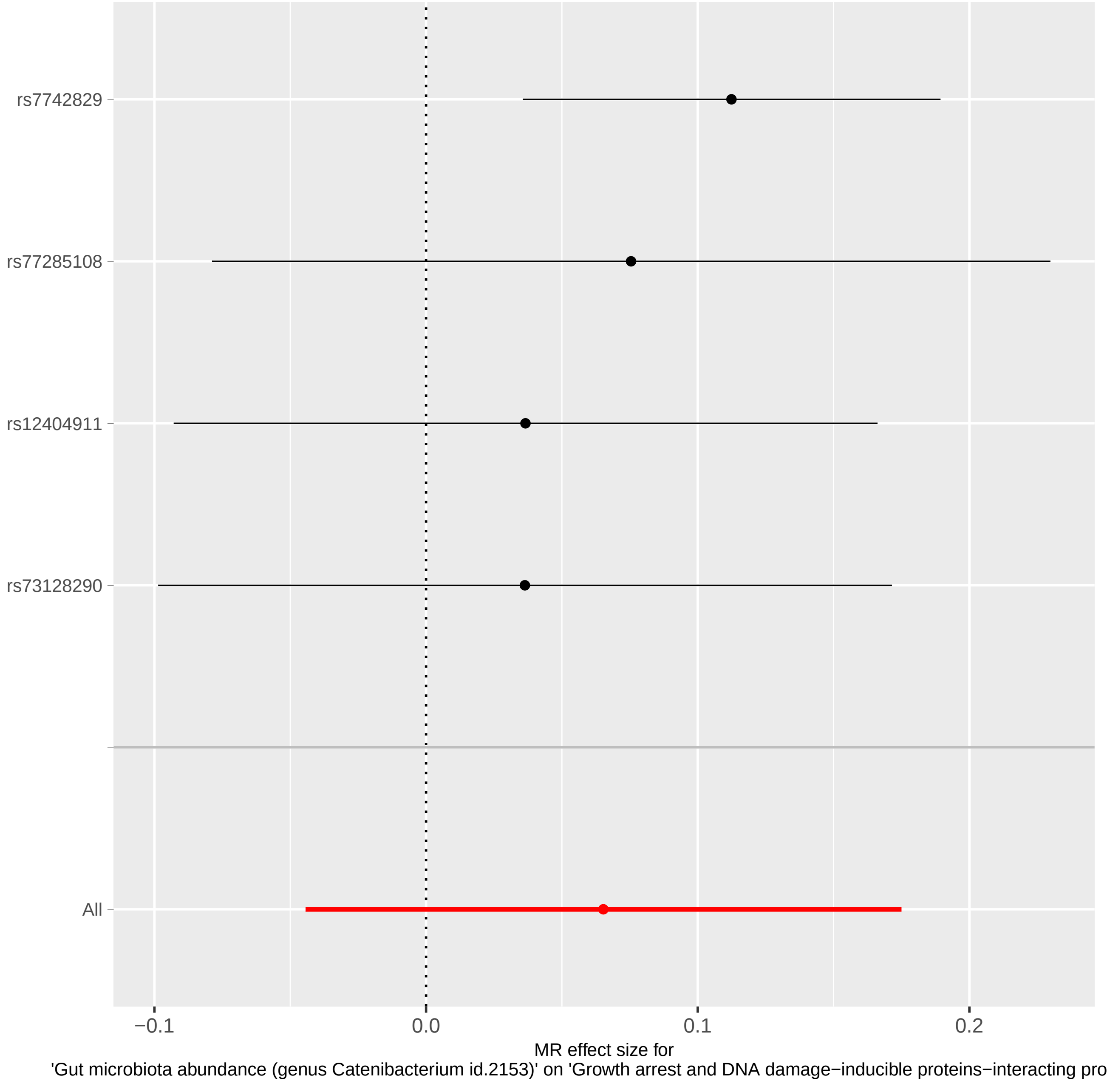

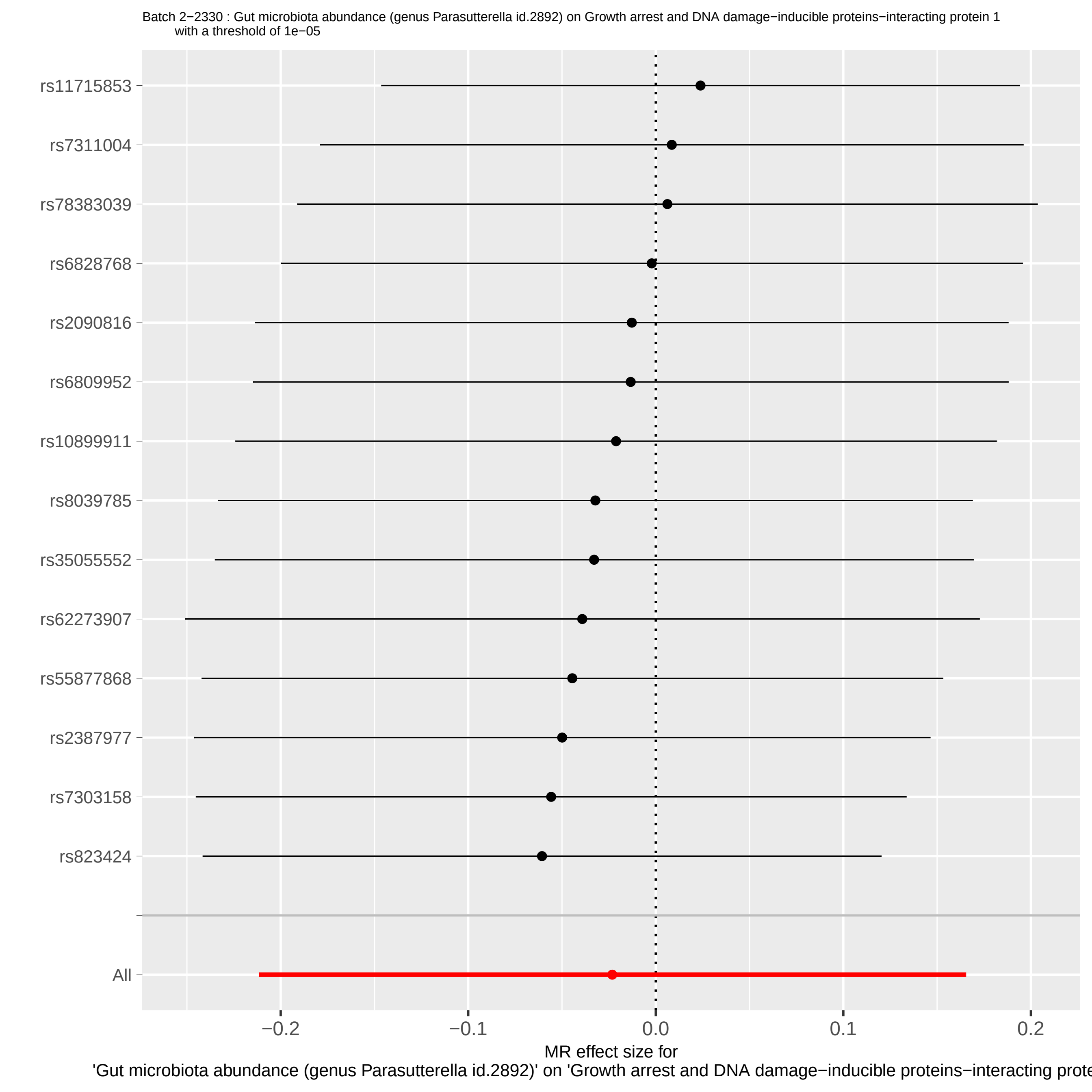
Batch 2−2330 : Gut microbiota abundance (genus Parasutterella id.2892) on Growth arrest and DNA damage−inducible proteins−interacting protein 1
with a threshold of 1e−05
rs11715853
rs7311004
rs78383039
rs6828768
rs2090816
rs6809952
rs10899911
rs8039785
rs35055552
rs62273907
rs55877868
rs2387977
rs7303158
rs823424
All
−0.2
−0.1
0.0
0.1
0.2
MR effect size for
'Gut microbiota abundance (genus Parasutterella id.2892)' on 'Growth arrest and DNA damage−inducible proteins−interacting prote

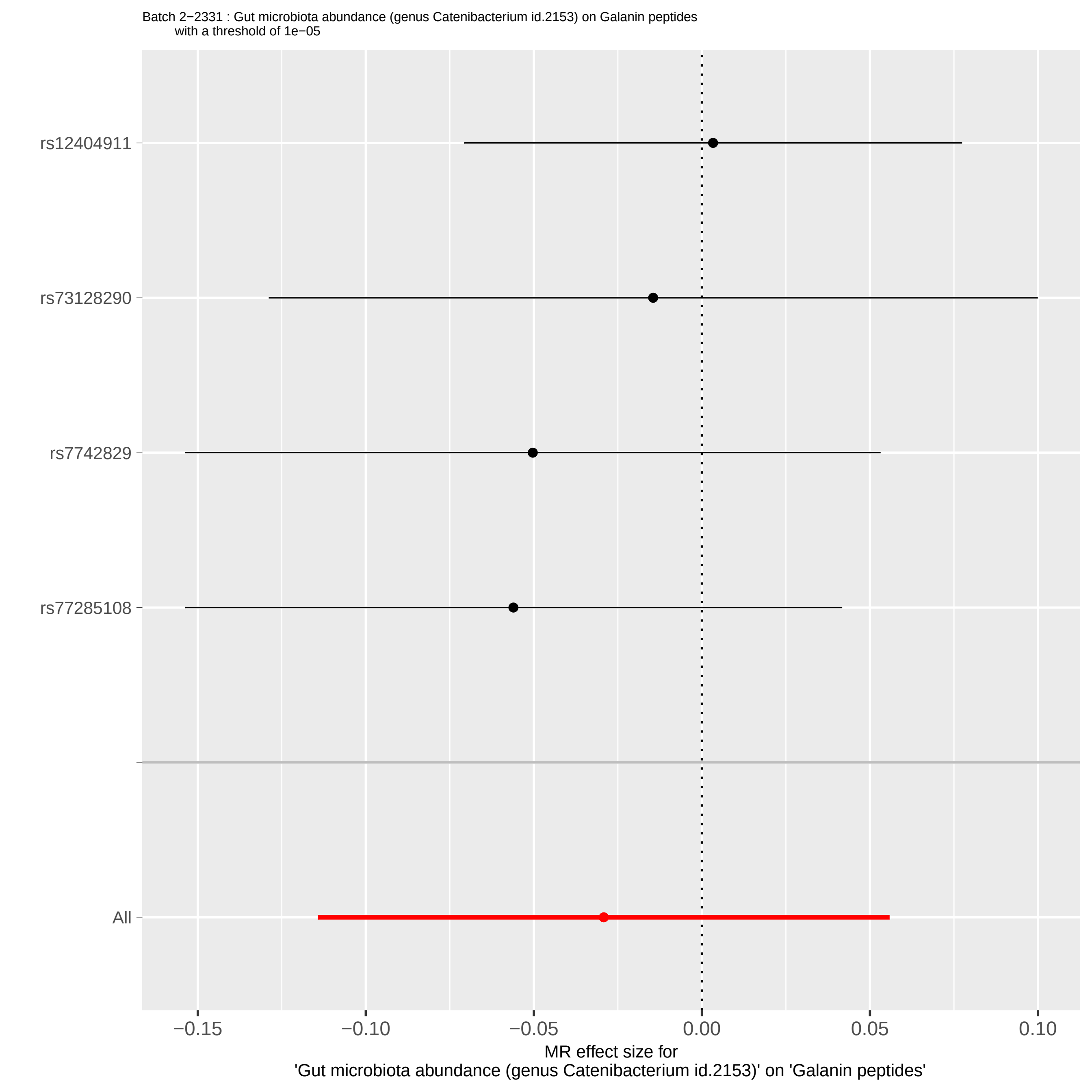

Batch 2−2331 : Gut microbiota abundance (genus Catenibacterium id.2153) on Galanin peptides
with a threshold of 1e−05
rs12404911
rs73128290
rs7742829
rs77285108
All
−0.15
−0.10
−0.05
0.00
0.05
0.10
MR effect size for
'Gut microbiota abundance (genus Catenibacterium id.2153)' on 'Galanin peptides'

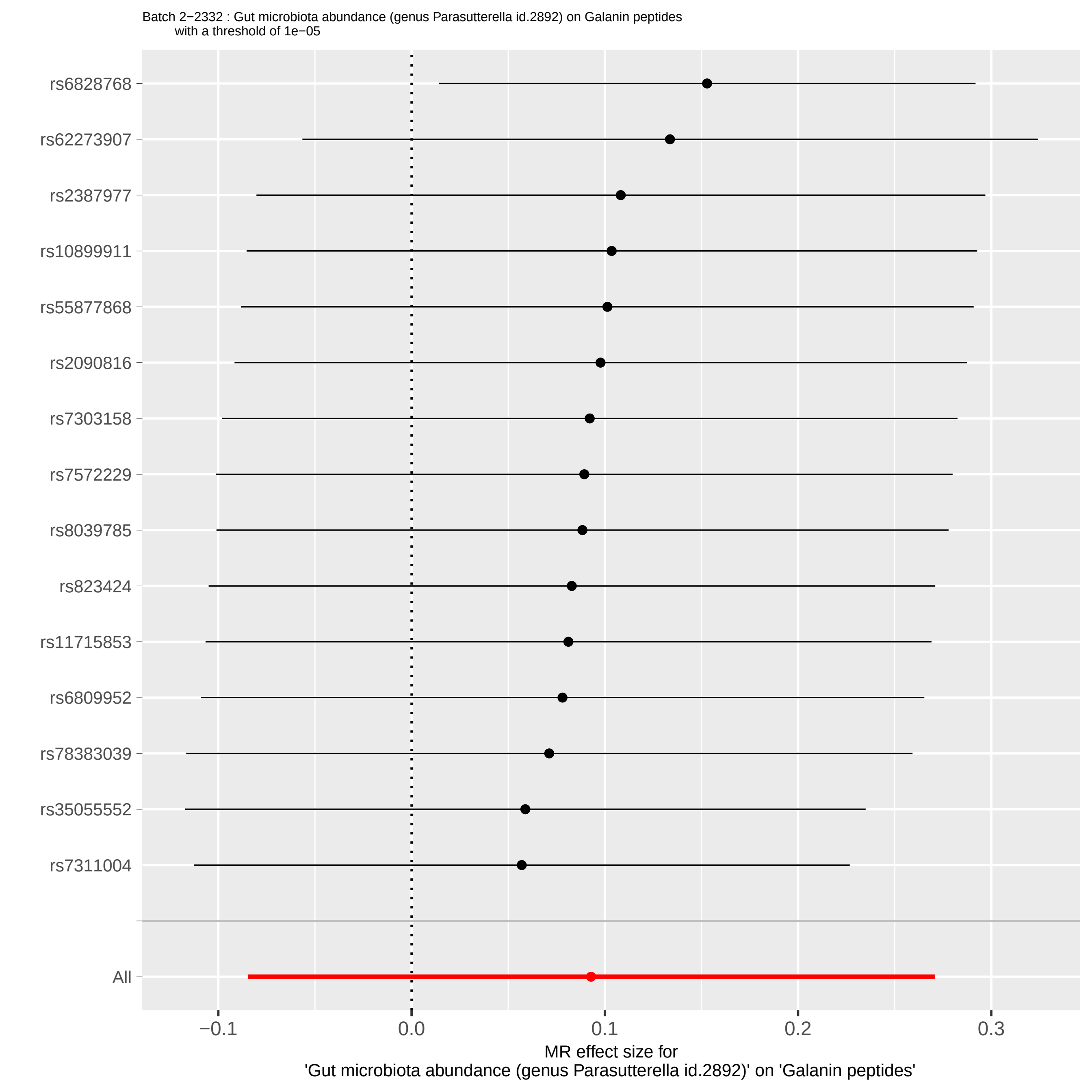

Batch 2−2332 : Gut microbiota abundance (genus Parasutterella id.2892) on Galanin peptides
with a threshold of 1e−05
rs6828768
rs62273907
rs2387977
rs10899911
rs55877868
rs2090816
rs7303158
rs7572229
rs8039785
rs823424
rs11715853
rs6809952
rs78383039
rs35055552
rs7311004
All
−0.1
0.0
0.1
0.2
0.3
MR effect size for
'Gut microbiota abundance (genus Parasutterella id.2892)' on 'Galanin peptides'

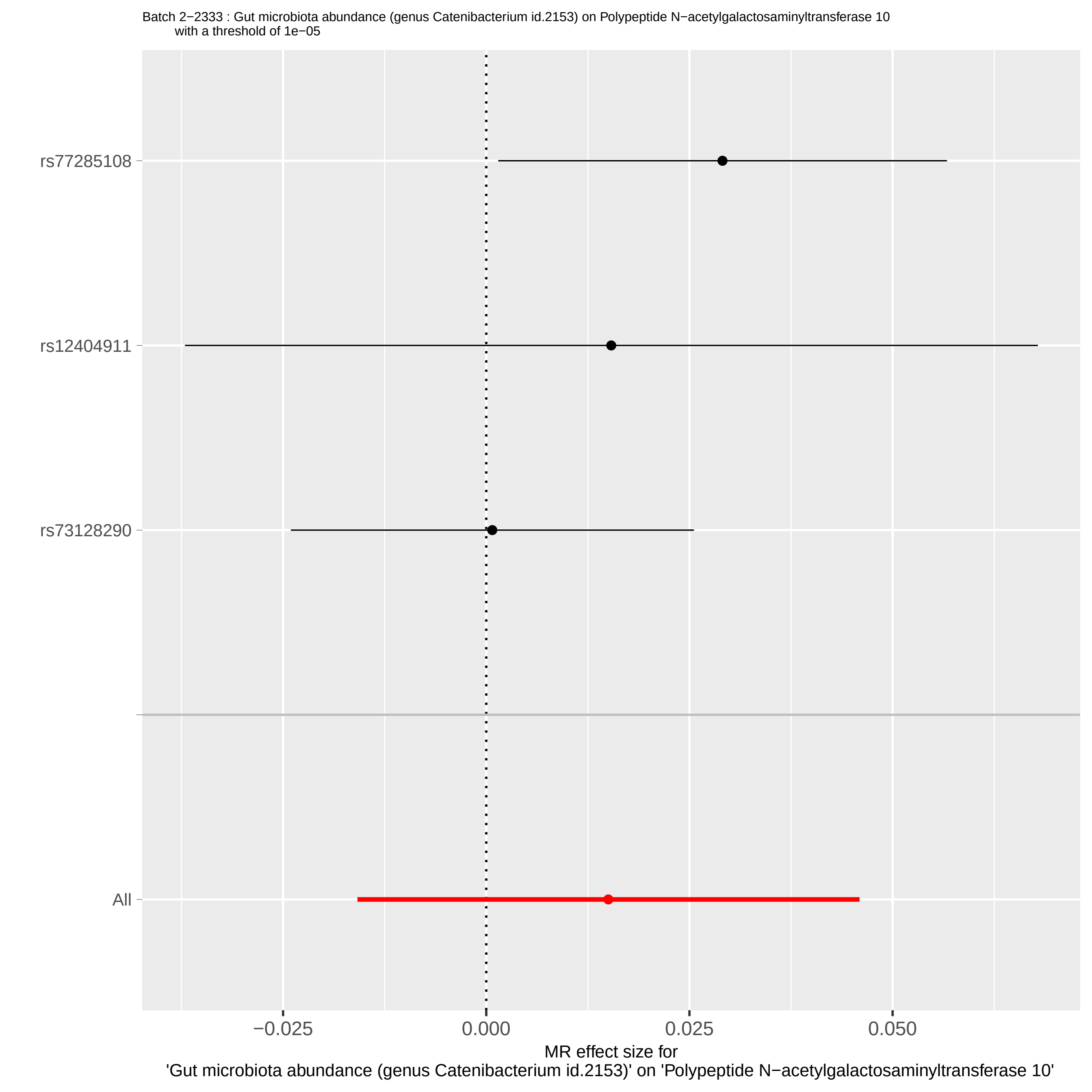
Batch 2−2333 : Gut microbiota abundance (genus Catenibacterium id.2153) on Polypeptide N−acetylgalactosaminyltransferase 10
with a threshold of 1e−05
rs77285108
rs12404911
rs73128290
All
−0.025
0.000
0.025
0.050
MR effect size for
'Gut microbiota abundance (genus Catenibacterium id.2153)' on 'Polypeptide N−acetylgalactosaminyltransferase 10'

Batch 2−2334 : Gut microbiota abundance (genus Parasutterella id.2892) on Polypeptide N−acetylgalactosaminyltransferase 10
with a threshold of 1e−05

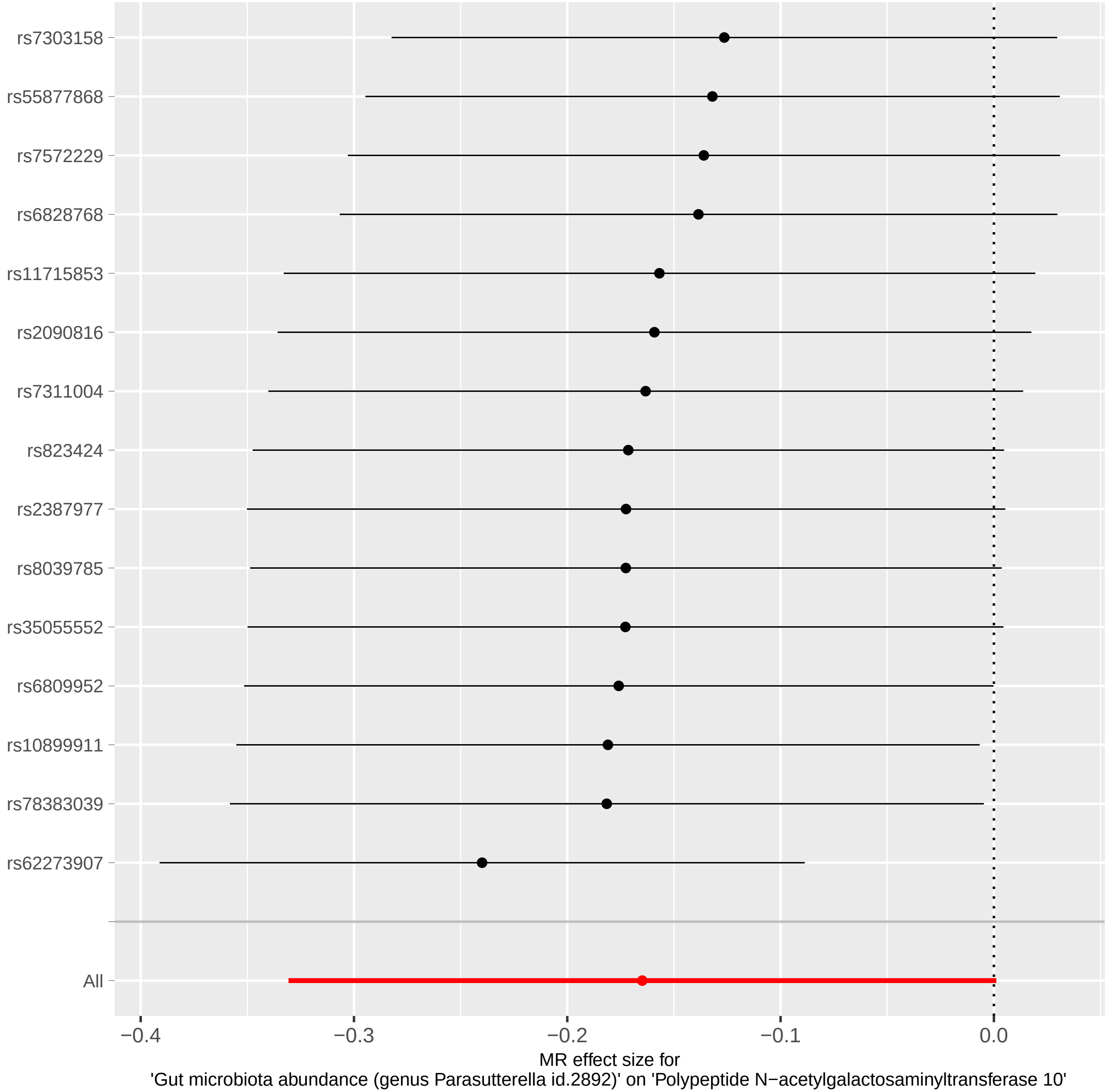

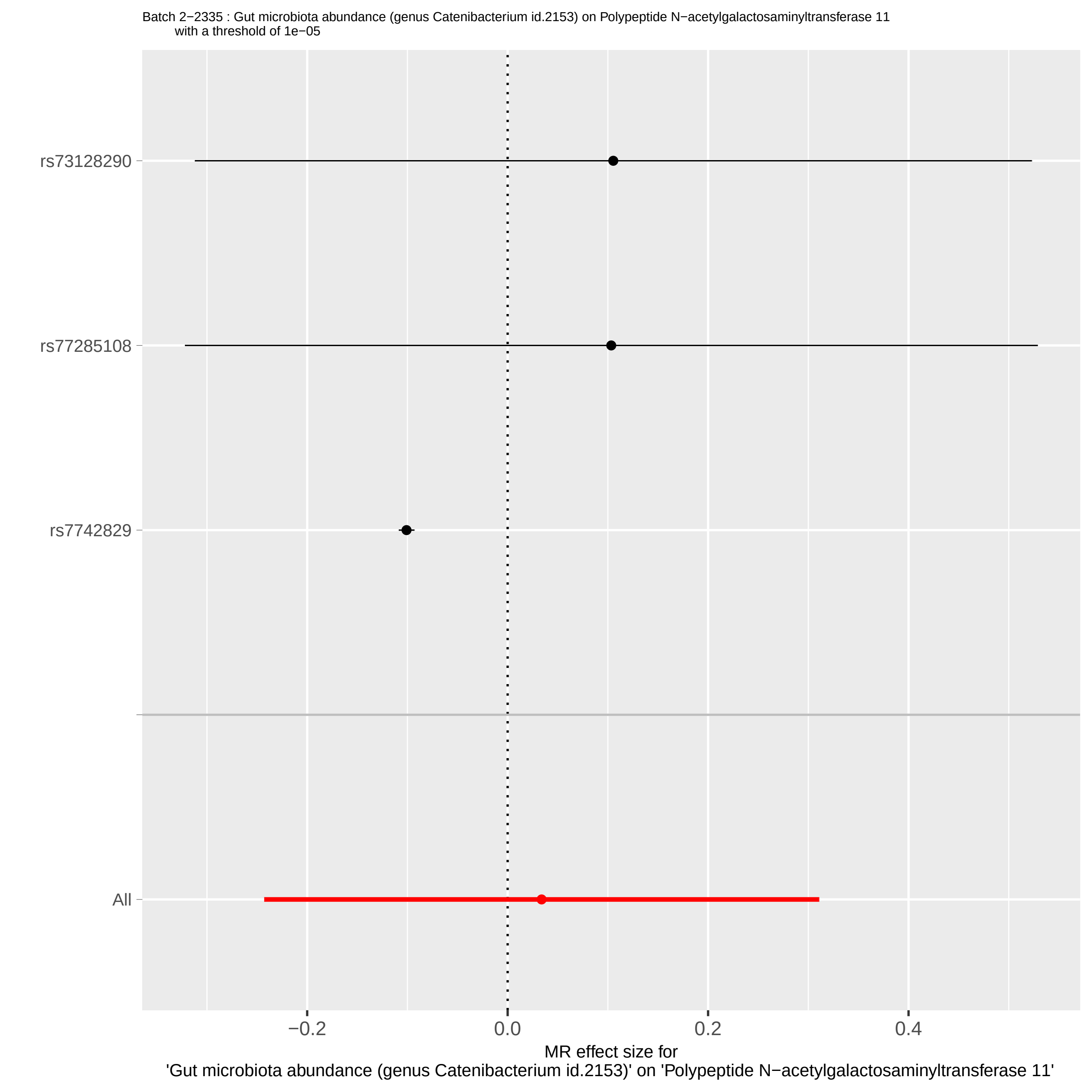

Batch 2−2335 : Gut microbiota abundance (genus Catenibacterium id.2153) on Polypeptide N−acetylgalactosaminyltransferase 11
with a threshold of 1e−05
rs73128290
rs77285108
rs7742829
All
−0.2
0.0
0.2
0.4
MR effect size for
'Gut microbiota abundance (genus Catenibacterium id.2153)' on 'Polypeptide N−acetylgalactosaminyltransferase 11'

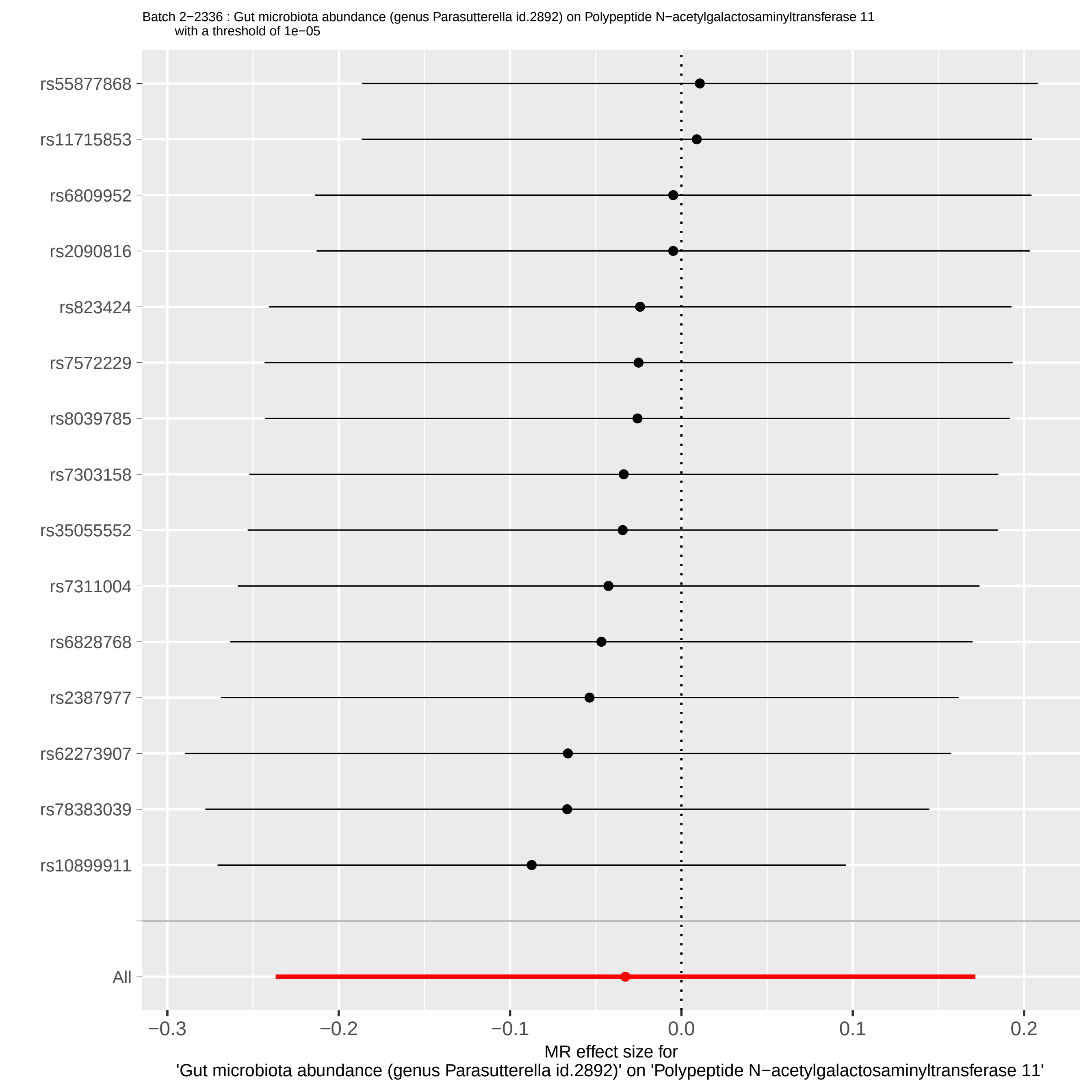

Batch 2−2336 : Gut microbiota abundance (genus Parasutterella id.2892) on Polypeptide N−acetylgalactosaminyltransferase 11 with a threshold of 1e−05
rs55877868
rs11715853
rs6809952
rs2090816
rs823424
rs7572229
rs8039785
rs7303158
rs35055552
rs7311004
rs6828768
rs2387977
rs62273907
rs78383039
rs10899911
All
−0.3
−0.2
−0.1
0.0
0.1
0.2
MR effect size for
'Gut microbiota abundance (genus Parasutterella id.2892)' on 'Polypeptide N−acetylgalactosaminyltransferase 11'

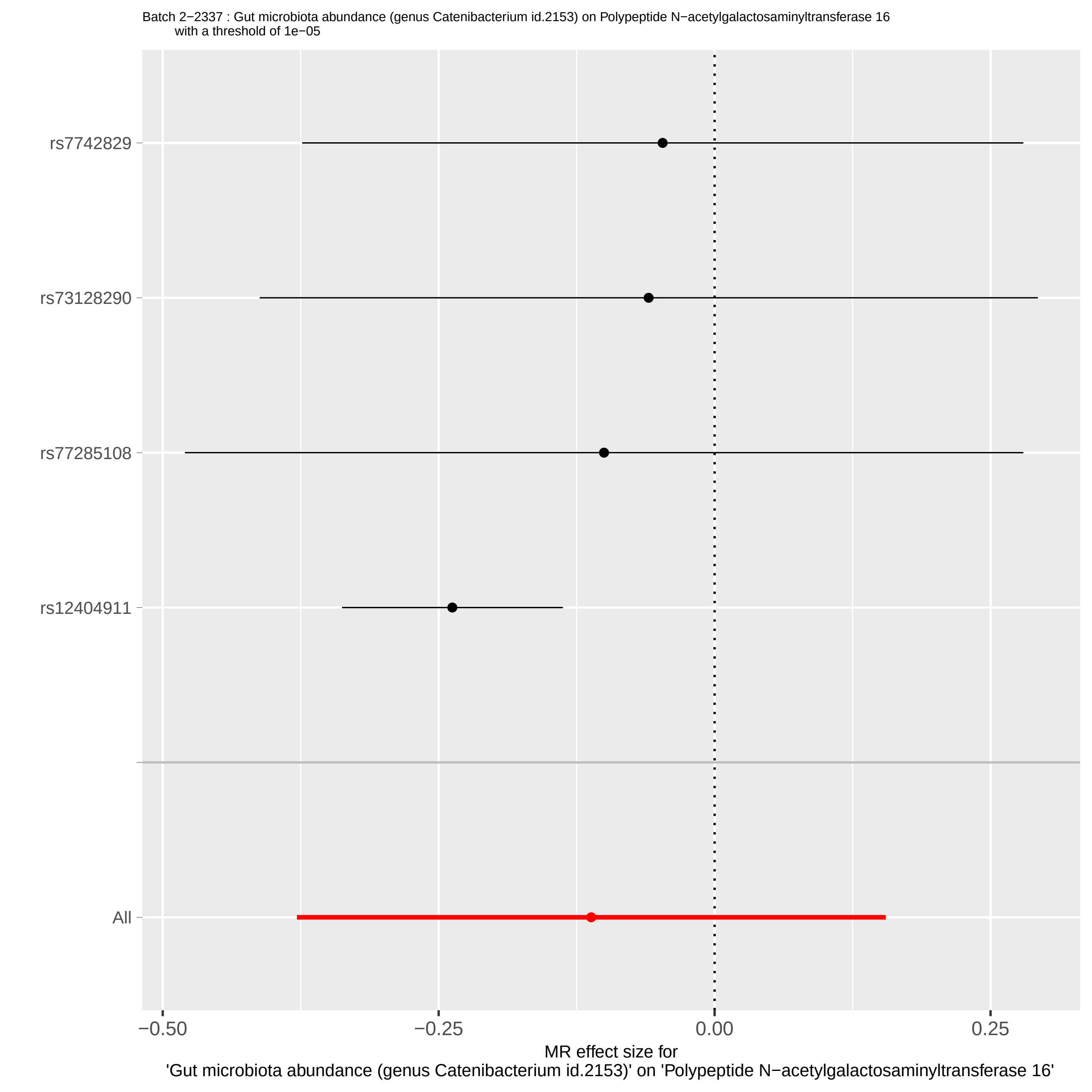
Batch 2−2337 : Gut microbiota abundance (genus Catenibacterium id.2153) on Polypeptide N−acetylgalactosaminyltransferase 16
with a threshold of 1e−05
rs7742829
rs73128290
rs77285108
rs12404911
All
−0.50
−0.25
0.00
0.25
MR effect size for
'Gut microbiota abundance (genus Catenibacterium id.2153)' on 'Polypeptide N−acetylgalactosaminyltransferase 16'

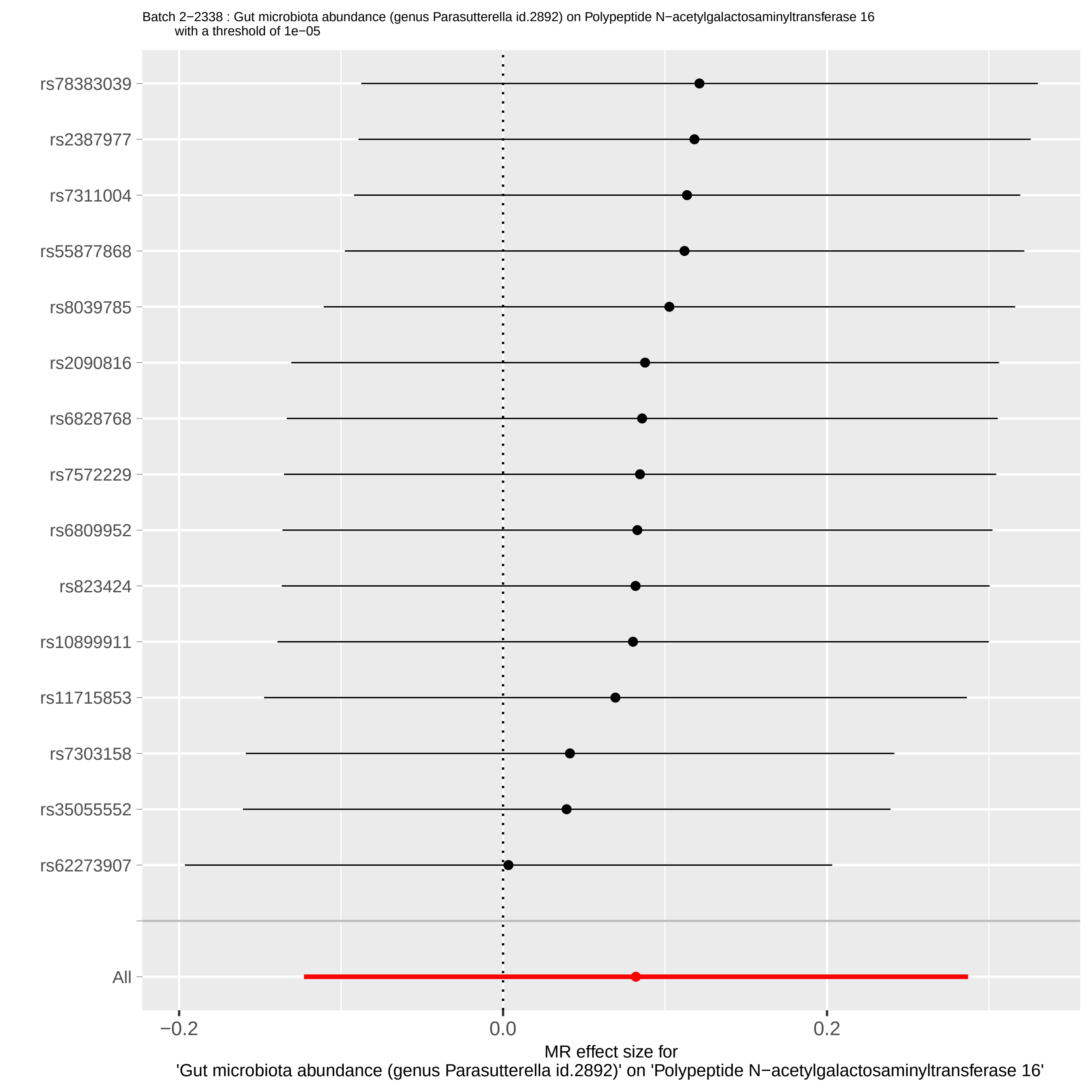

Batch 2−2338 : Gut microbiota abundance (genus Parasutterella id.2892) on Polypeptide N−acetylgalactosaminyltransferase 16
with a threshold of 1e−05
rs78383039
rs2387977
rs7311004
rs55877868
rs8039785
rs2090816
rs6828768
rs7572229
rs6809952
rs823424
rs10899911
rs11715853
rs7303158
rs35055552
rs62273907
All
−0.2
0.0
0.2
MR effect size for
'Gut microbiota abundance (genus Parasutterella id.2892)' on 'Polypeptide N−acetylgalactosaminyltransferase 16'

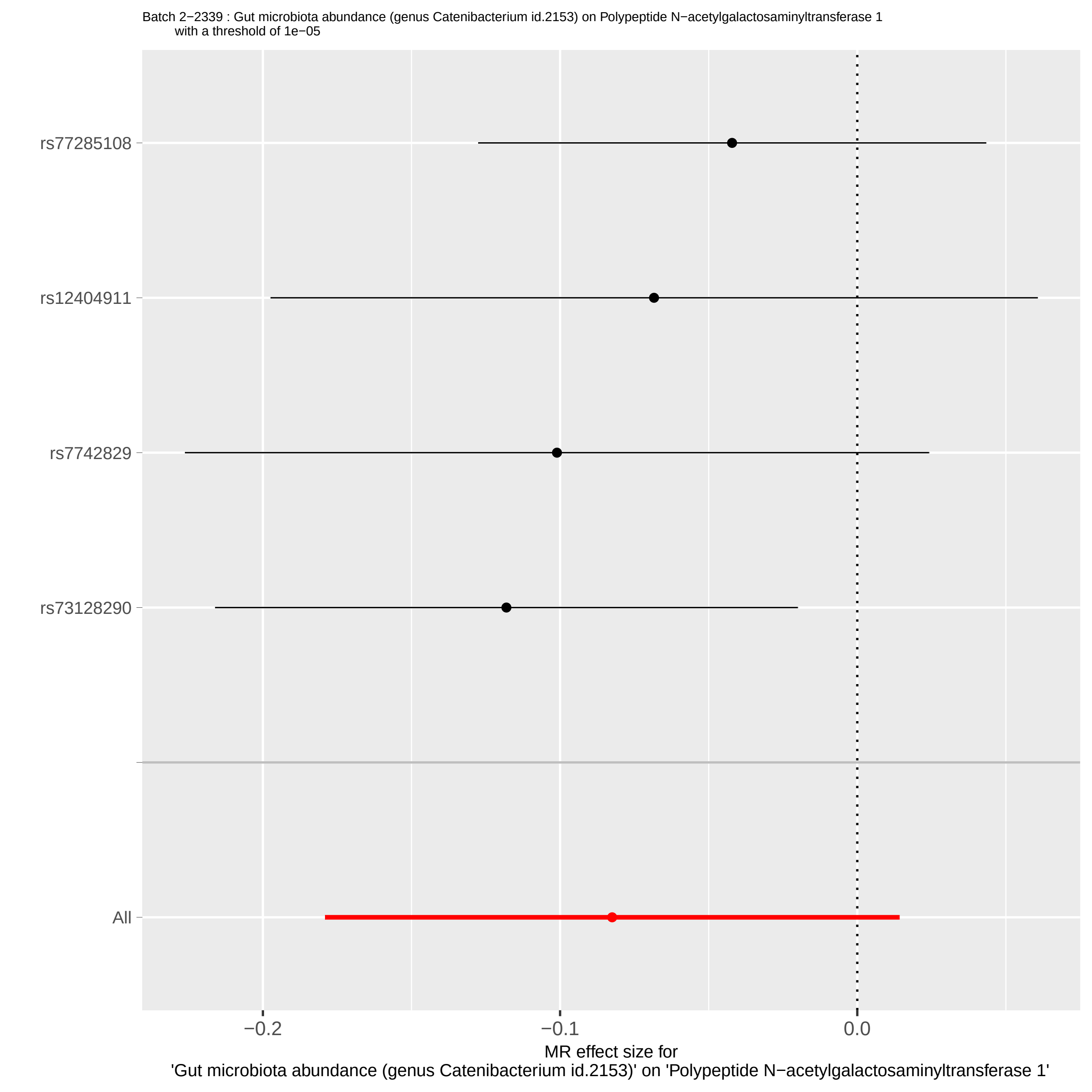

Batch 2−2339 : Gut microbiota abundance (genus Catenibacterium id.2153) on Polypeptide N−acetylgalactosaminyltransferase 1
with a threshold of 1e−05
rs77285108
rs12404911
rs7742829
rs73128290
All
−0.2
−0.1
0.0
MR effect size for
'Gut microbiota abundance (genus Catenibacterium id.2153)' on 'Polypeptide N−acetylgalactosaminyltransferase 1'

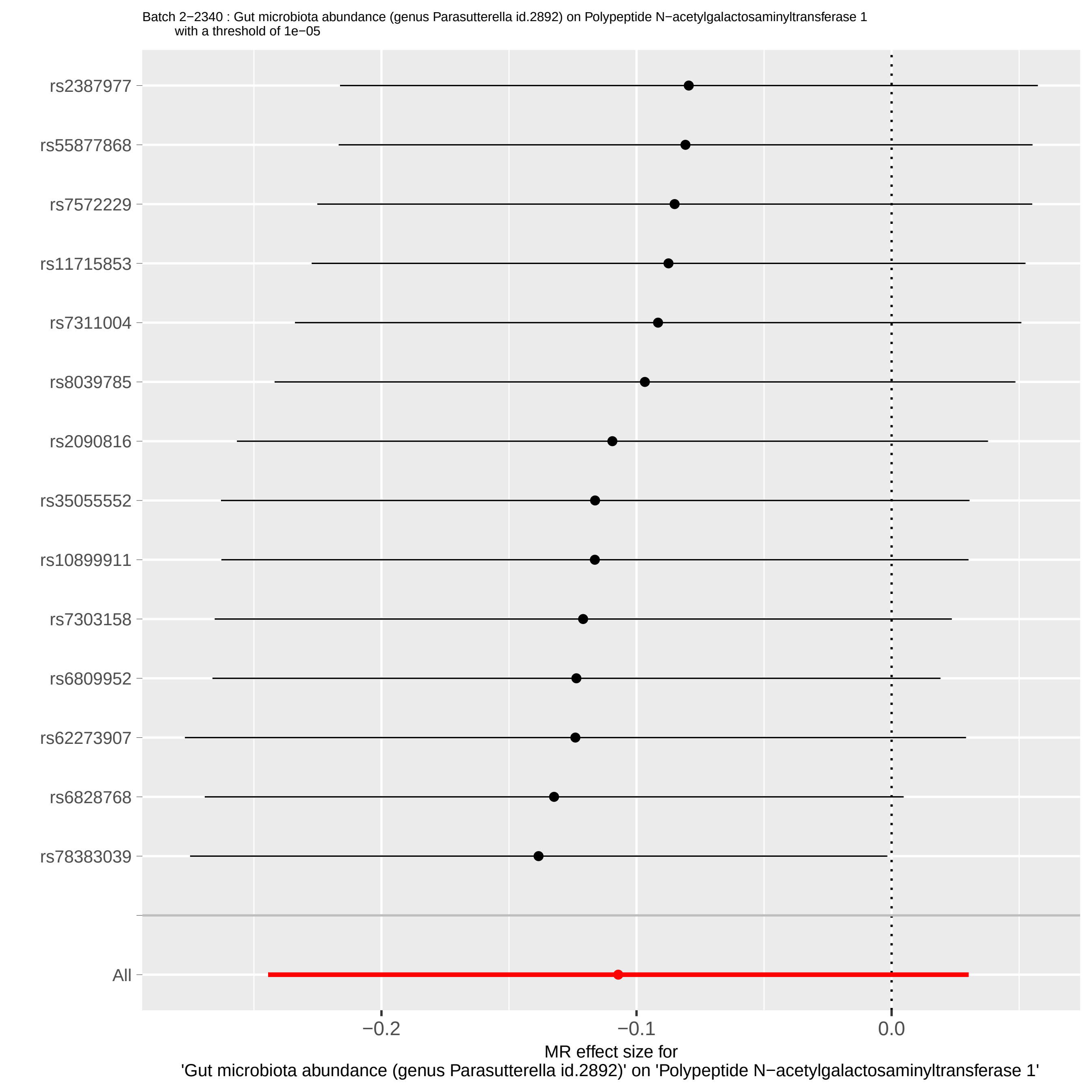

Batch 2−2340 : Gut microbiota abundance (genus Parasutterella id.2892) on Polypeptide N−acetylgalactosaminyltransferase 1
with a threshold of 1e−05
rs2387977
rs55877868
rs7572229
rs11715853
rs7311004
rs8039785
rs2090816
rs35055552
rs10899911
rs7303158
rs6809952
rs62273907
rs6828768
rs78383039
All
−0.2
−0.1
0.0
MR effect size for
'Gut microbiota abundance (genus Parasutterella id.2892)' on 'Polypeptide N−acetylgalactosaminyltransferase 1'

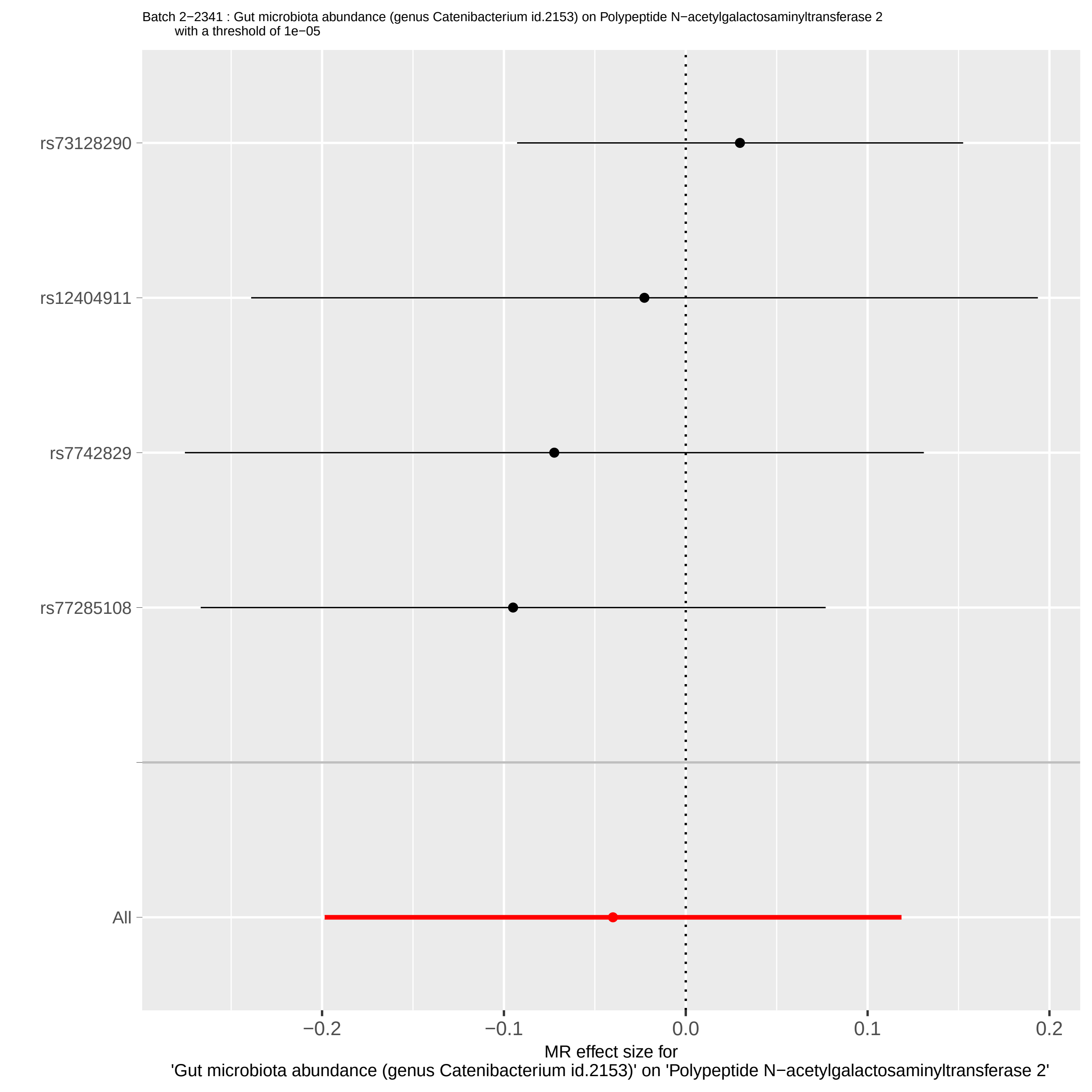
Batch 2−2341 : Gut microbiota abundance (genus Catenibacterium id.2153) on Polypeptide N−acetylgalactosaminyltransferase 2
with a threshold of 1e−05
rs73128290
rs12404911
rs7742829
rs77285108
All
−0.2
−0.1
0.0
0.1
0.2
MR effect size for
'Gut microbiota abundance (genus Catenibacterium id.2153)' on 'Polypeptide N−acetylgalactosaminyltransferase 2'

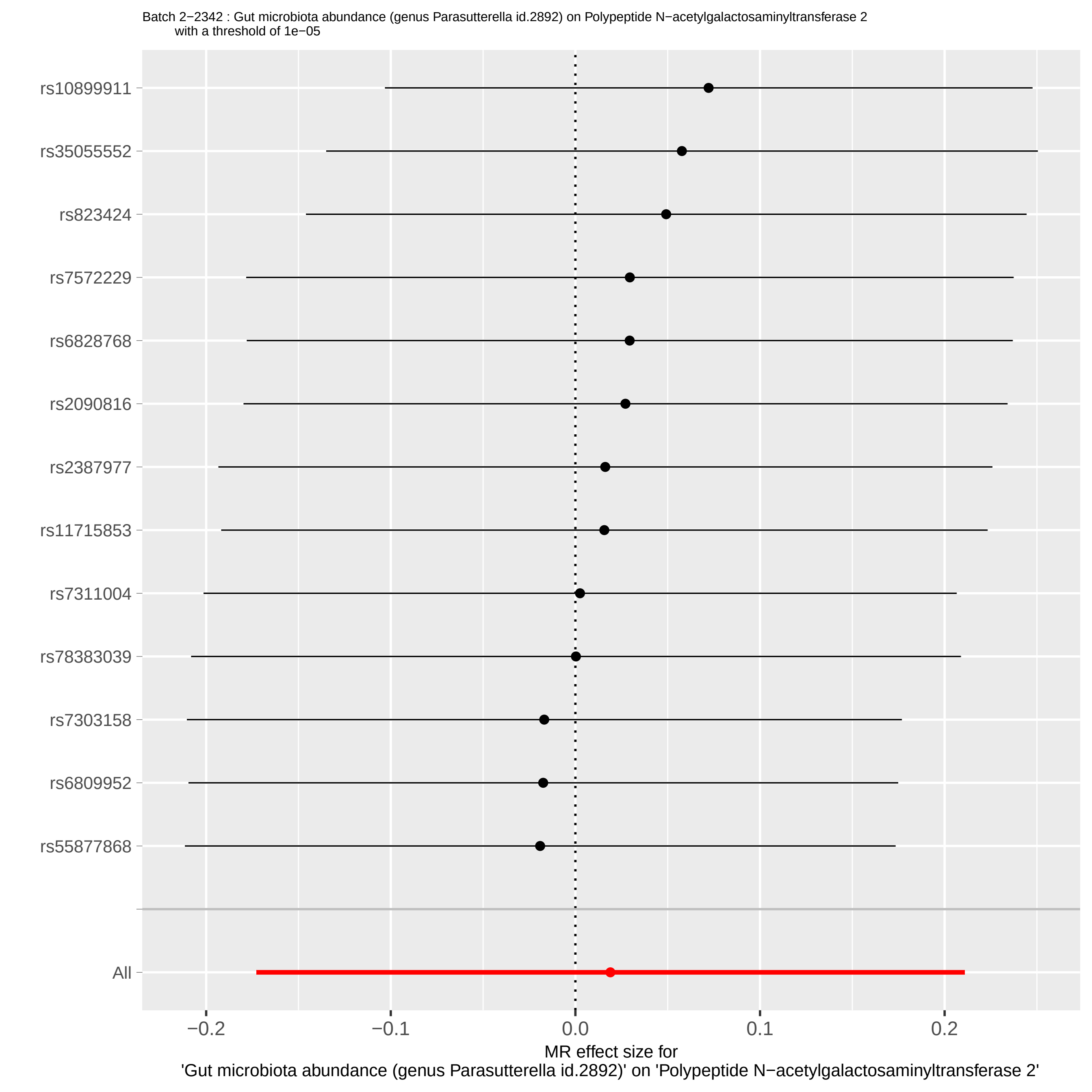
Batch 2−2342 : Gut microbiota abundance (genus Parasutterella id.2892) on Polypeptide N−acetylgalactosaminyltransferase 2
with a threshold of 1e−05
rs10899911
rs35055552
rs823424
rs7572229
rs6828768
rs2090816
rs2387977
rs11715853
rs7311004
rs78383039
rs7303158
rs6809952
rs55877868
All
−0.2
−0.1
0.0
0.1
0.2
MR effect size for
'Gut microbiota abundance (genus Parasutterella id.2892)' on 'Polypeptide N−acetylgalactosaminyltransferase 2'

Batch 2−2343 : Gut microbiota abundance (genus Catenibacterium id.2153) on Polypeptide N−acetylgalactosaminyltransferase 3
with a threshold of 1e−05

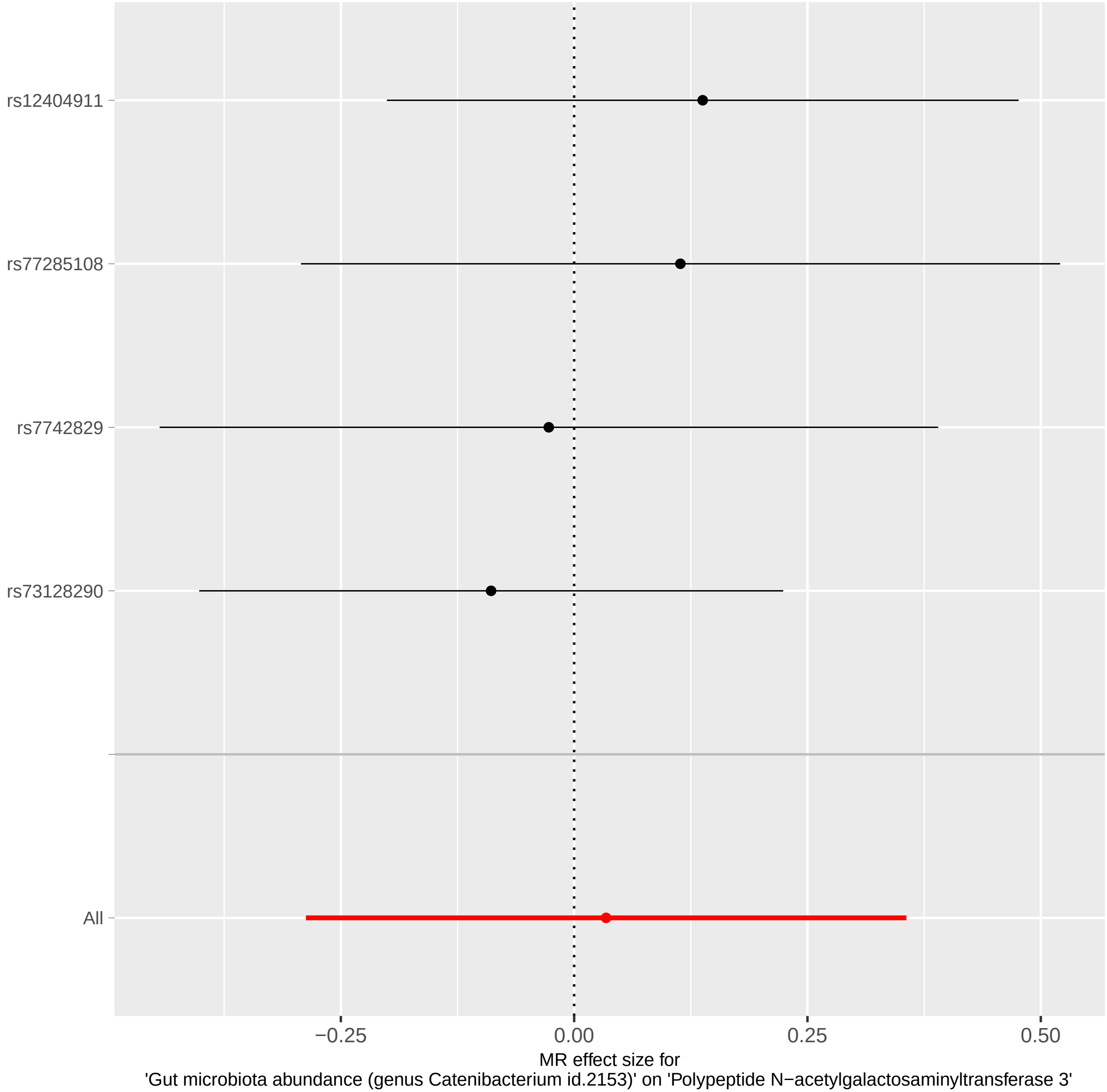

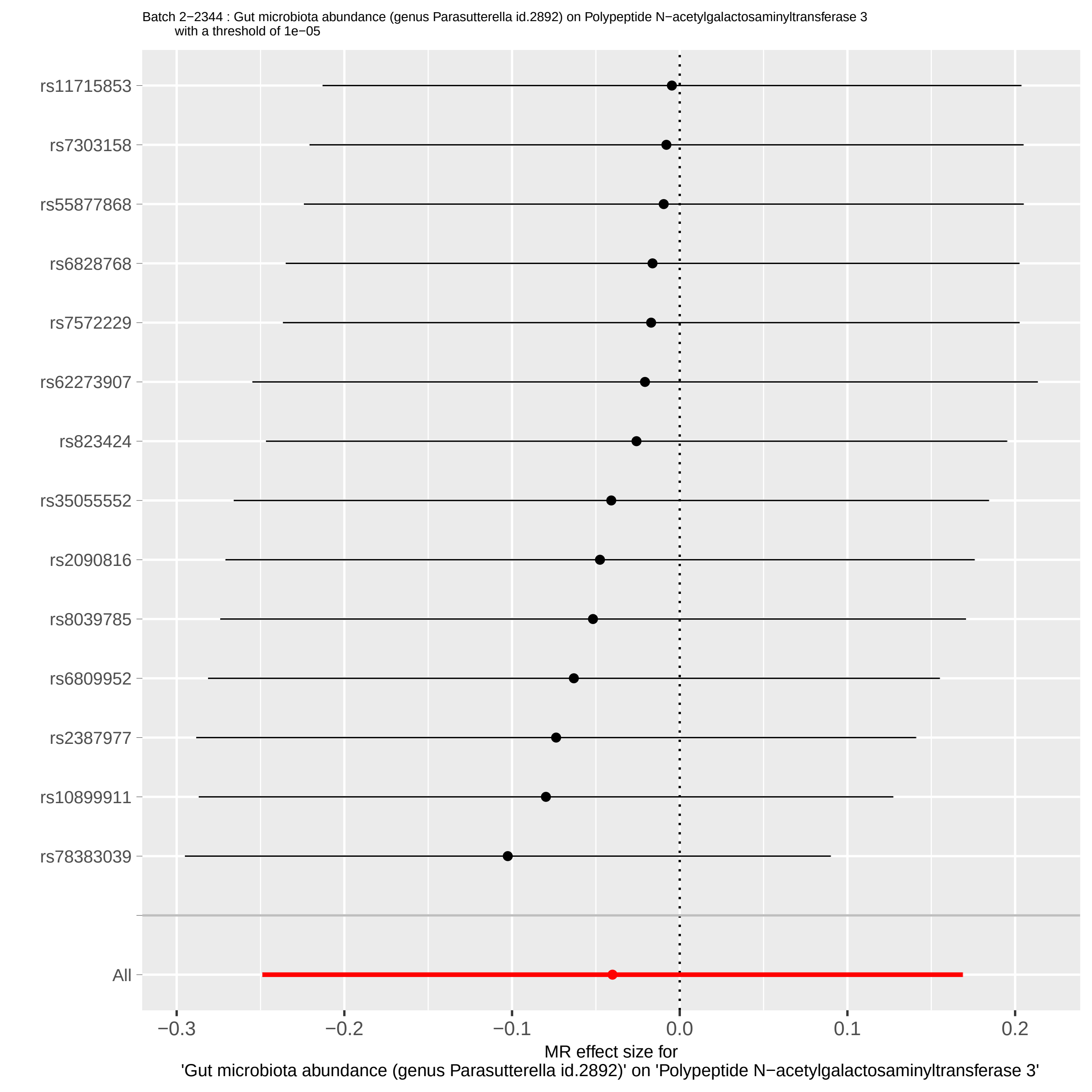
Batch 2−2344 : Gut microbiota abundance (genus Parasutterella id.2892) on Polypeptide N−acetylgalactosaminyltransferase 3
with a threshold of 1e−05
rs11715853
rs7303158
rs55877868
rs6828768
rs7572229
rs62273907
rs823424
rs35055552
rs2090816
rs8039785
rs6809952
rs2387977
rs10899911
rs78383039
All
−0.3
−0.2
−0.1
0.0
0.1
0.2
MR effect size for
'Gut microbiota abundance (genus Parasutterella id.2892)' on 'Polypeptide N−acetylgalactosaminyltransferase 3'

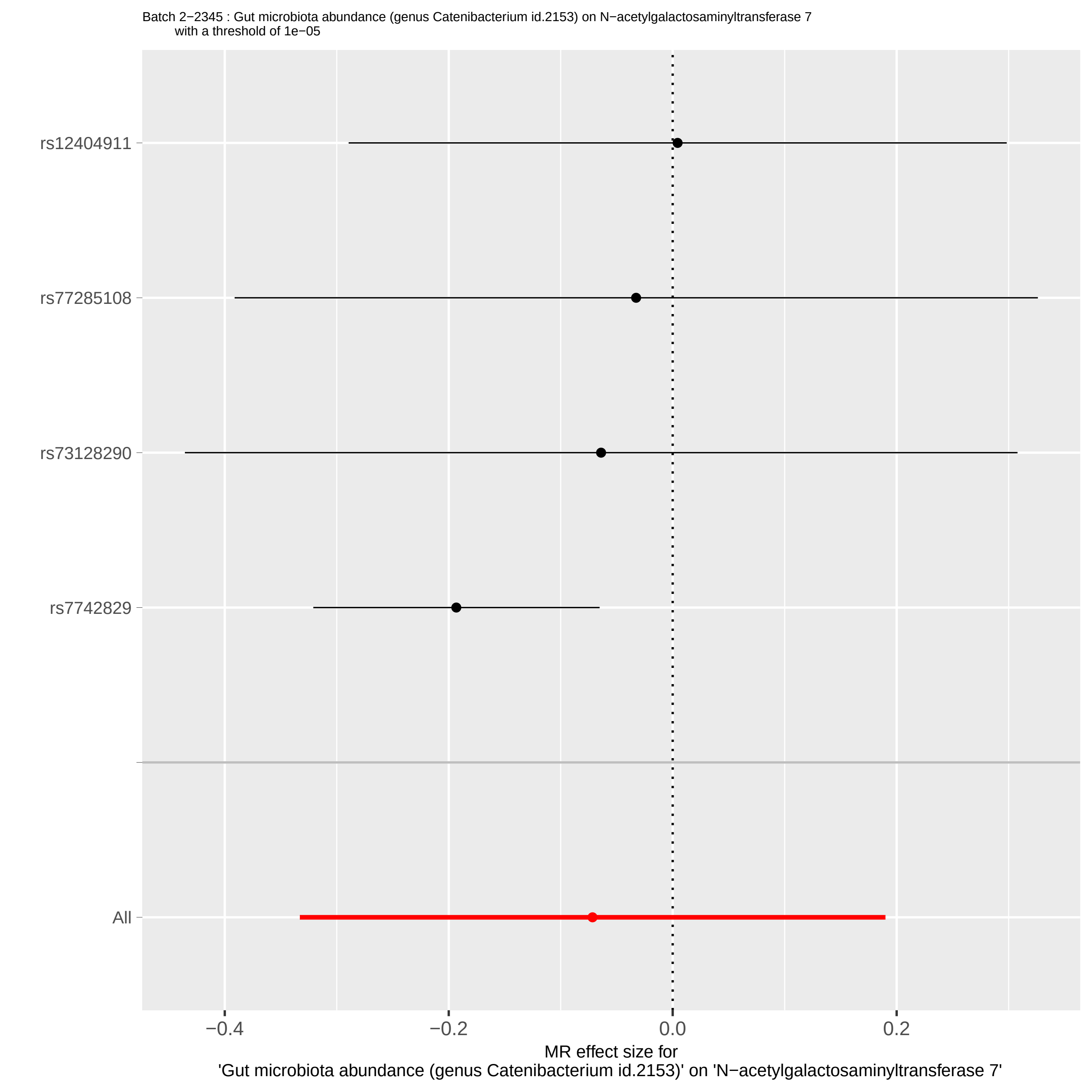
Batch 2−2345 : Gut microbiota abundance (genus Catenibacterium id.2153) on N−acetylgalactosaminyltransferase 7
with a threshold of 1e−05
rs12404911
rs77285108
rs73128290
rs7742829
All
−0.4
−0.2
0.0
0.2
MR effect size for
'Gut microbiota abundance (genus Catenibacterium id.2153)' on 'N−acetylgalactosaminyltransferase 7'

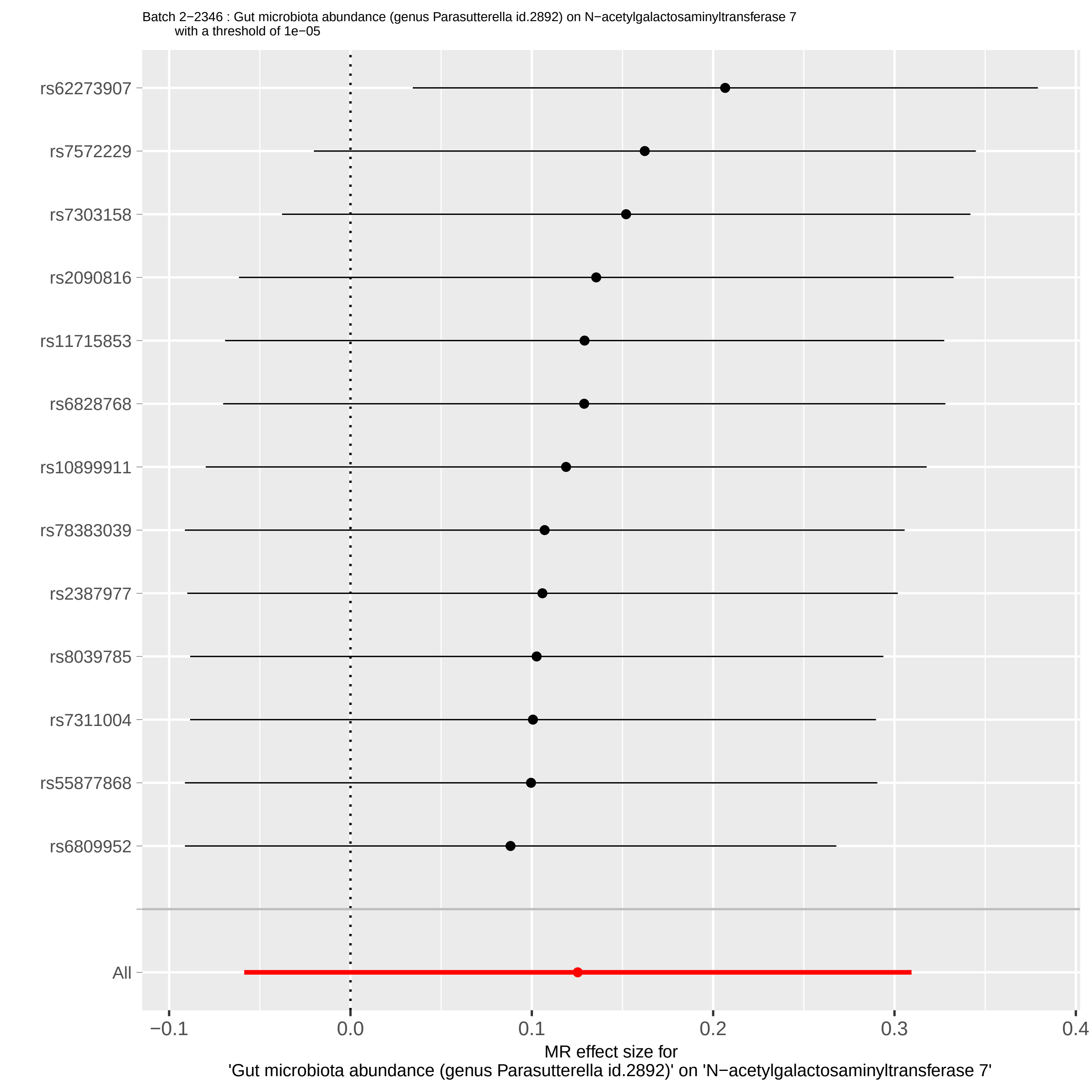

Batch 2−2346 : Gut microbiota abundance (genus Parasutterella id.2892) on N−acetylgalactosaminyltransferase 7
with a threshold of 1e−05
rs62273907
rs7572229
rs7303158
rs2090816
rs11715853
rs6828768
rs10899911
rs78383039
rs2387977
rs8039785
rs7311004
rs55877868
rs6809952
All
−0.1
0.0
0.1
0.2
0.3
0.4
MR effect size for
'Gut microbiota abundance (genus Parasutterella id.2892)' on 'N−acetylgalactosaminyltransferase 7'

Batch 2−2347 : Gut microbiota abundance (genus Catenibacterium id.2153) on Galanin−like peptide
with a threshold of 1e−05

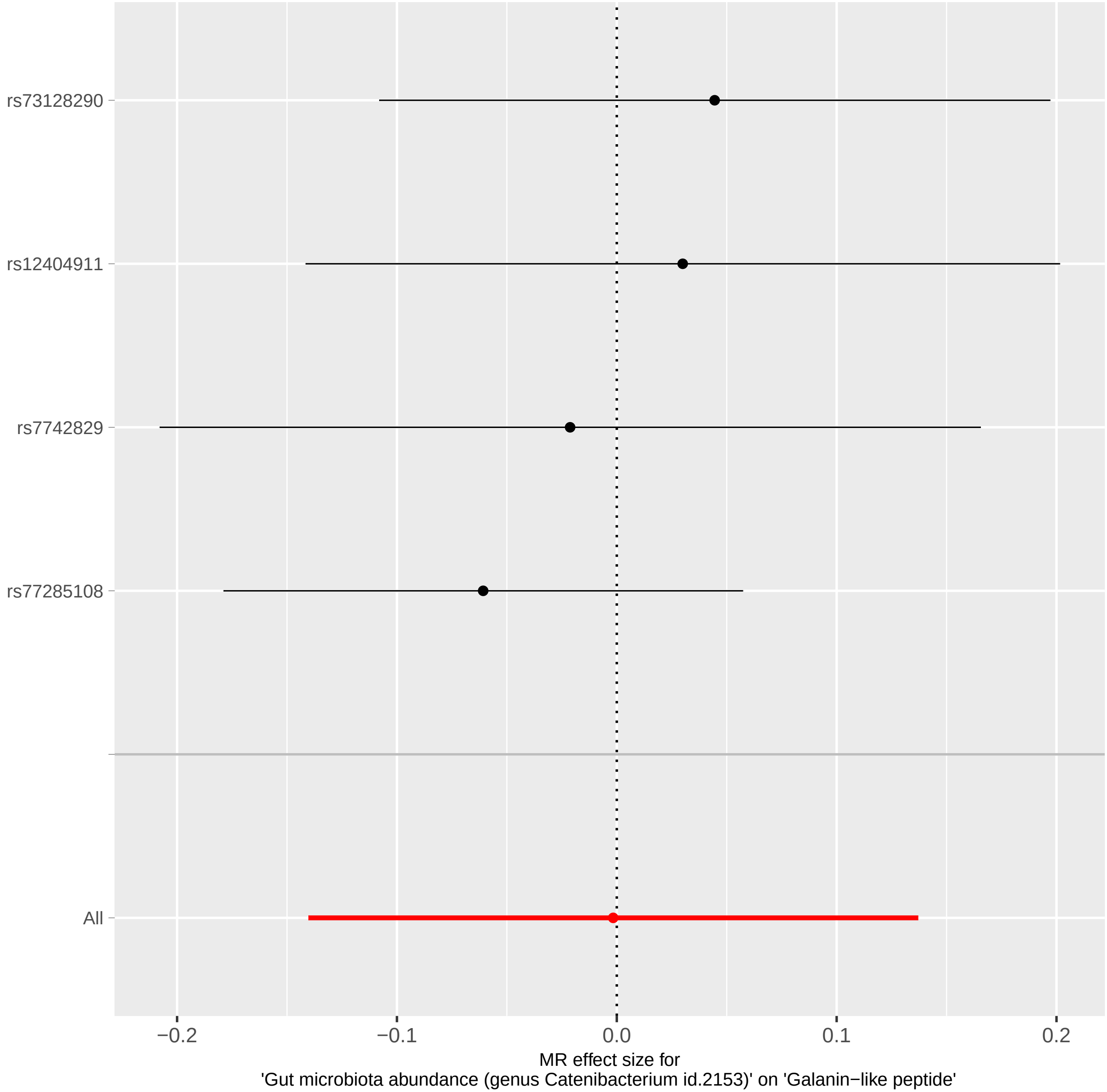

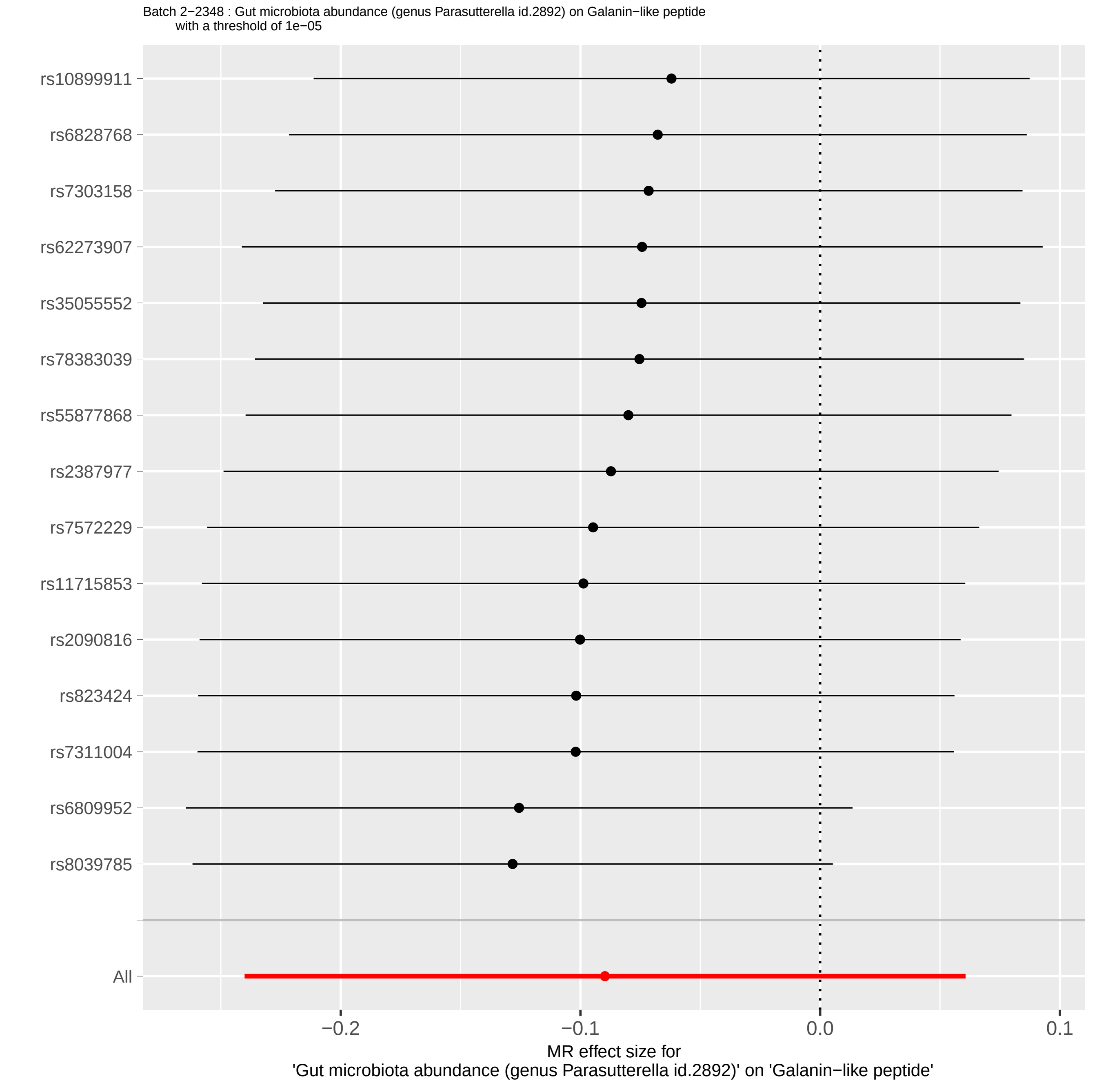

Batch 2−2348 : Gut microbiota abundance (genus Parasutterella id.2892) on Galanin−like peptide
with a threshold of 1e−05
rs10899911
rs6828768
rs7303158
rs62273907
rs35055552
rs78383039
rs55877868
rs2387977
rs7572229
rs11715853
rs2090816
rs823424
rs7311004
rs6809952
rs8039785
All
−0.2
−0.1
0.0
0.1
MR effect size for
'Gut microbiota abundance (genus Parasutterella id.2892)' on 'Galanin−like peptide'

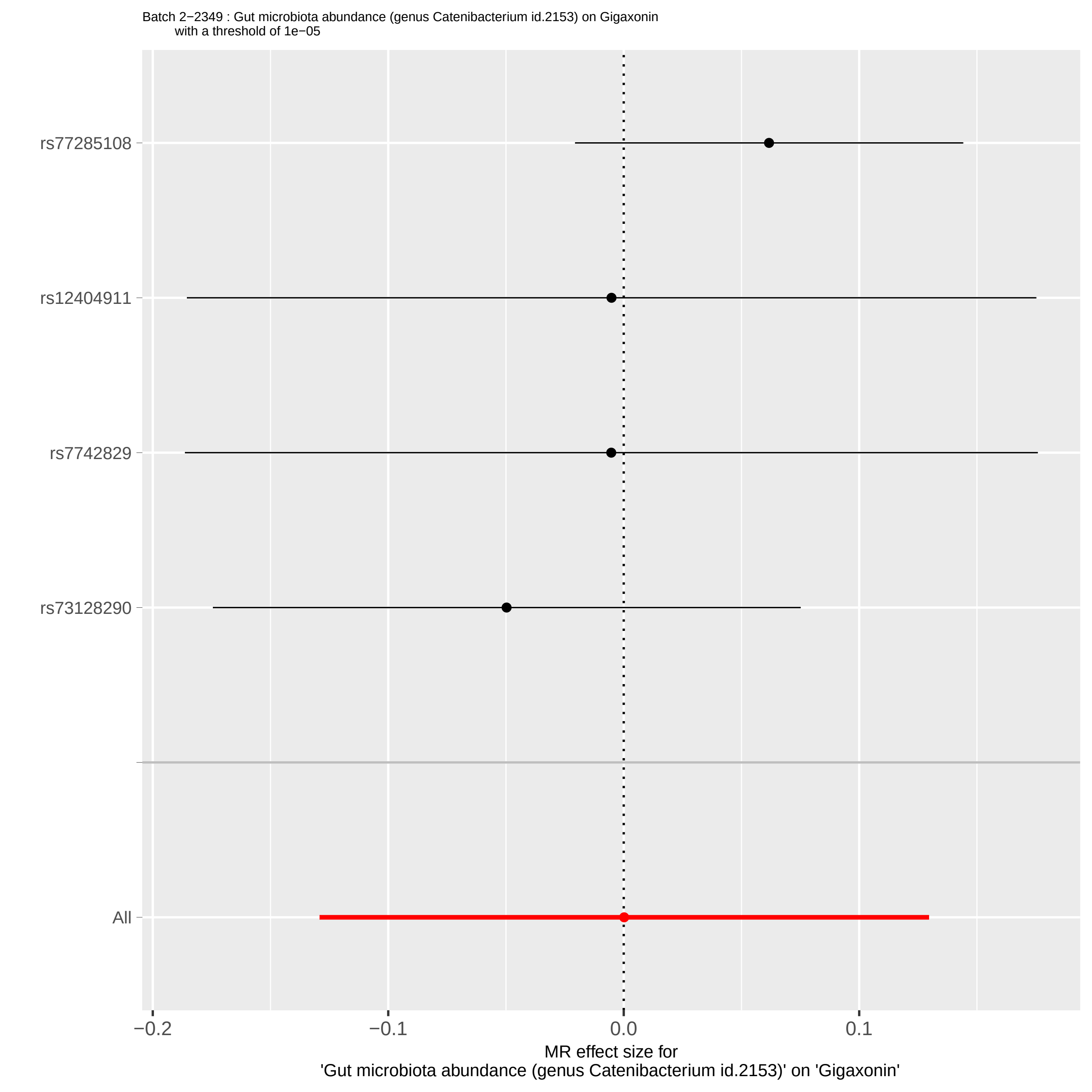
Batch 2−2349 : Gut microbiota abundance (genus Catenibacterium id.2153) on Gigaxonin
with a threshold of 1e−05
rs77285108
rs12404911
rs7742829
rs73128290
All
−0.2
−0.1
0.0
0.1
MR effect size for
'Gut microbiota abundance (genus Catenibacterium id.2153)' on 'Gigaxonin'

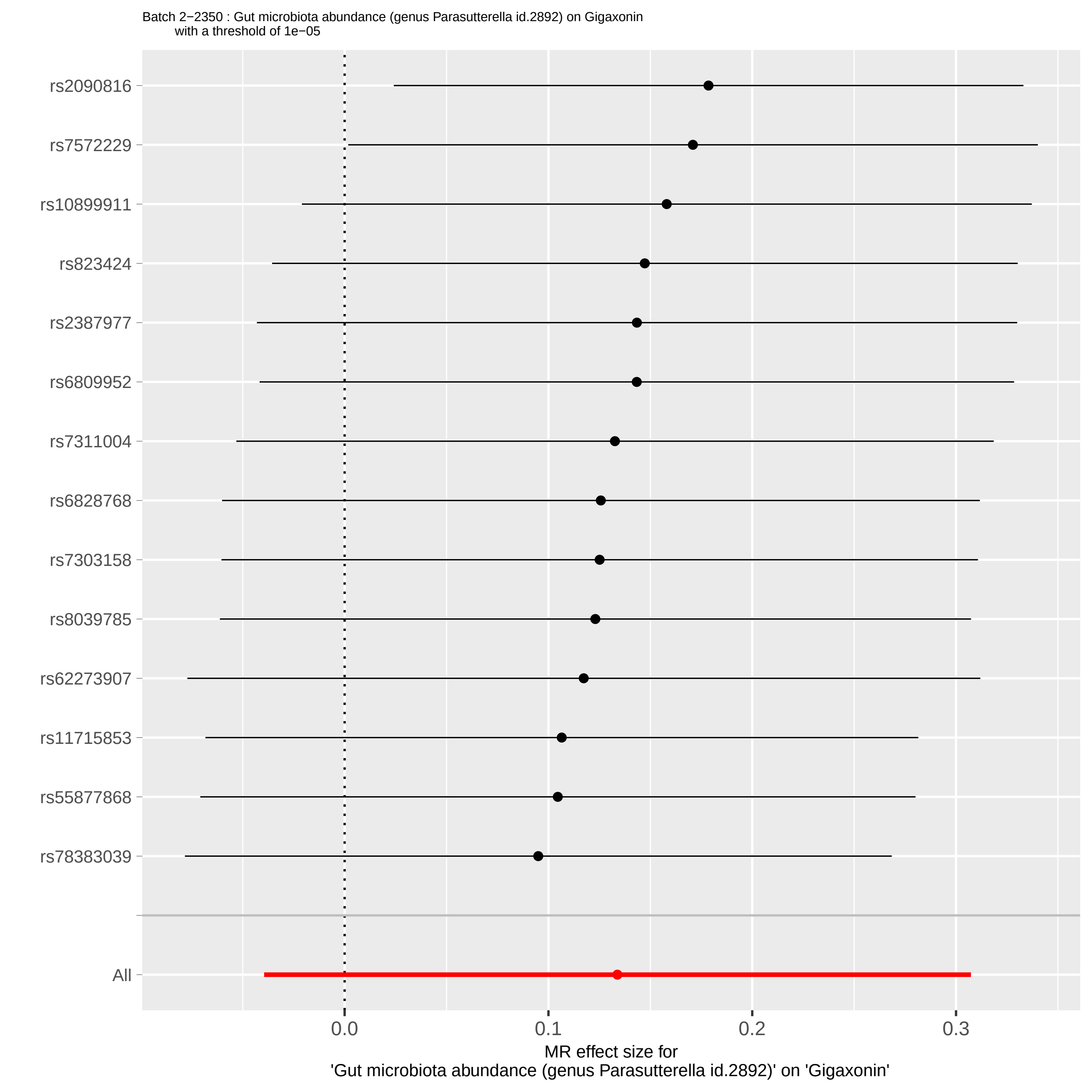
Batch 2−2350 : Gut microbiota abundance (genus Parasutterella id.2892) on Gigaxonin
with a threshold of 1e−05
rs2090816
rs7572229
rs10899911
rs823424
rs2387977
rs6809952
rs7311004
rs6828768
rs7303158
rs8039785
rs62273907
rs11715853
rs55877868
rs78383039
All
0.0
0.1
0.2
0.3
MR effect size for
'Gut microbiota abundance (genus Parasutterella id.2892)' on 'Gigaxonin'

Batch 2−2351 : Gut microbiota abundance (genus Catenibacterium id.2153) on Glyceraldehyde−3−phosphate dehydrogenase, testis−specific
with a threshold of 1e−05

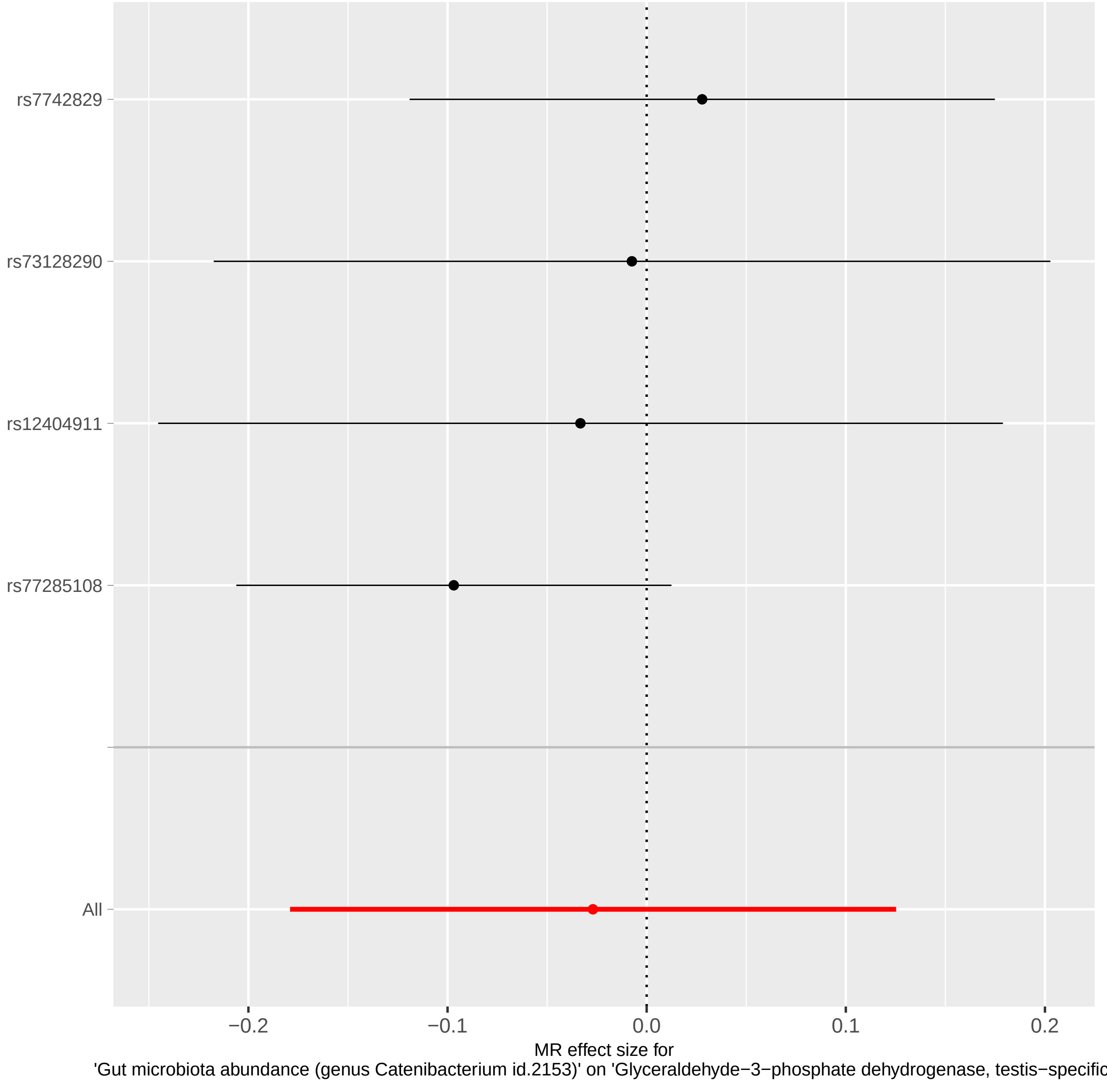

Batch 2−2352 : Gut microbiota abundance (genus Parasutterella id.2892) on Glyceraldehyde−3−phosphate dehydrogenase, testis−specific
with a threshold of 1e−05

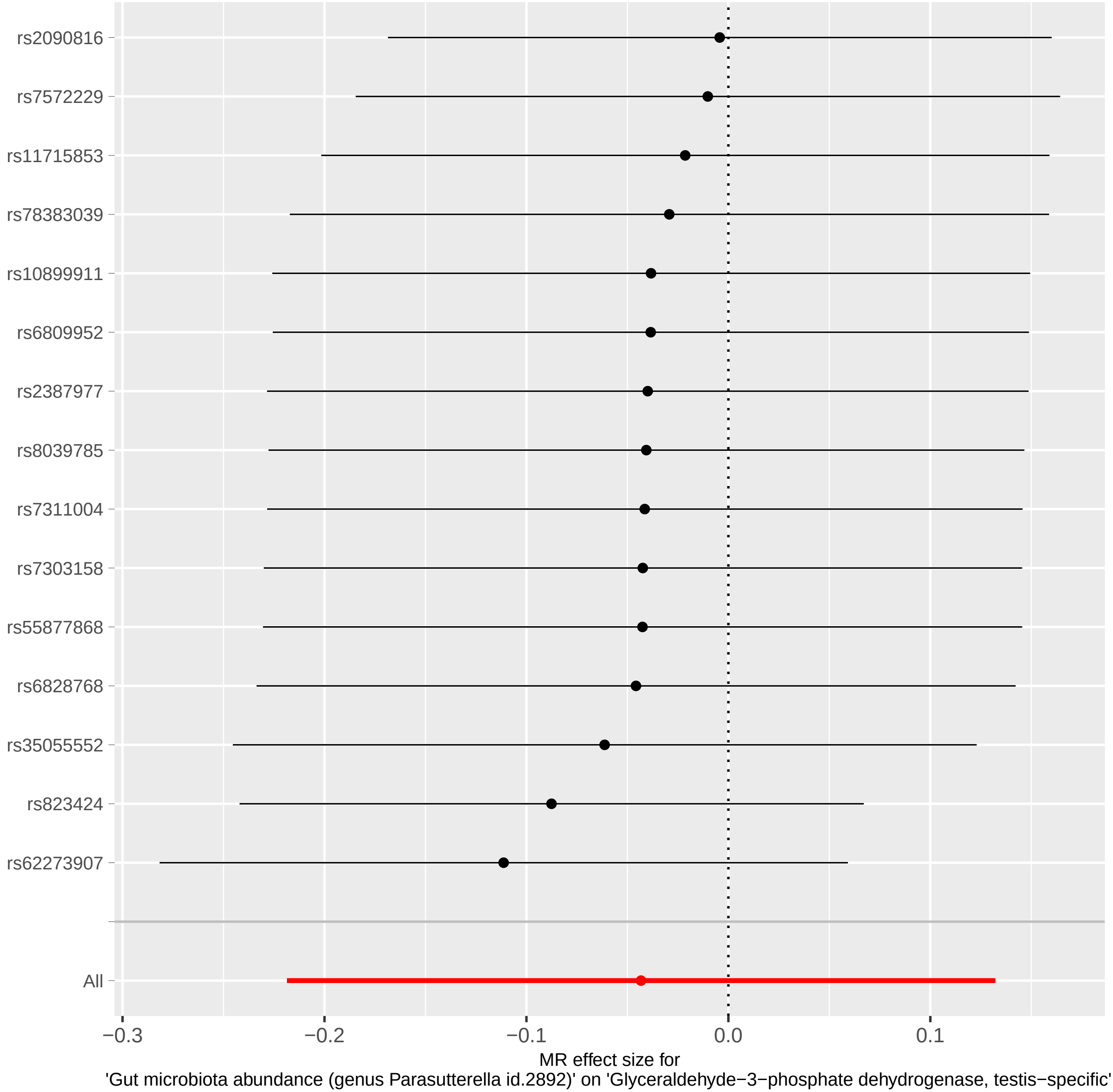

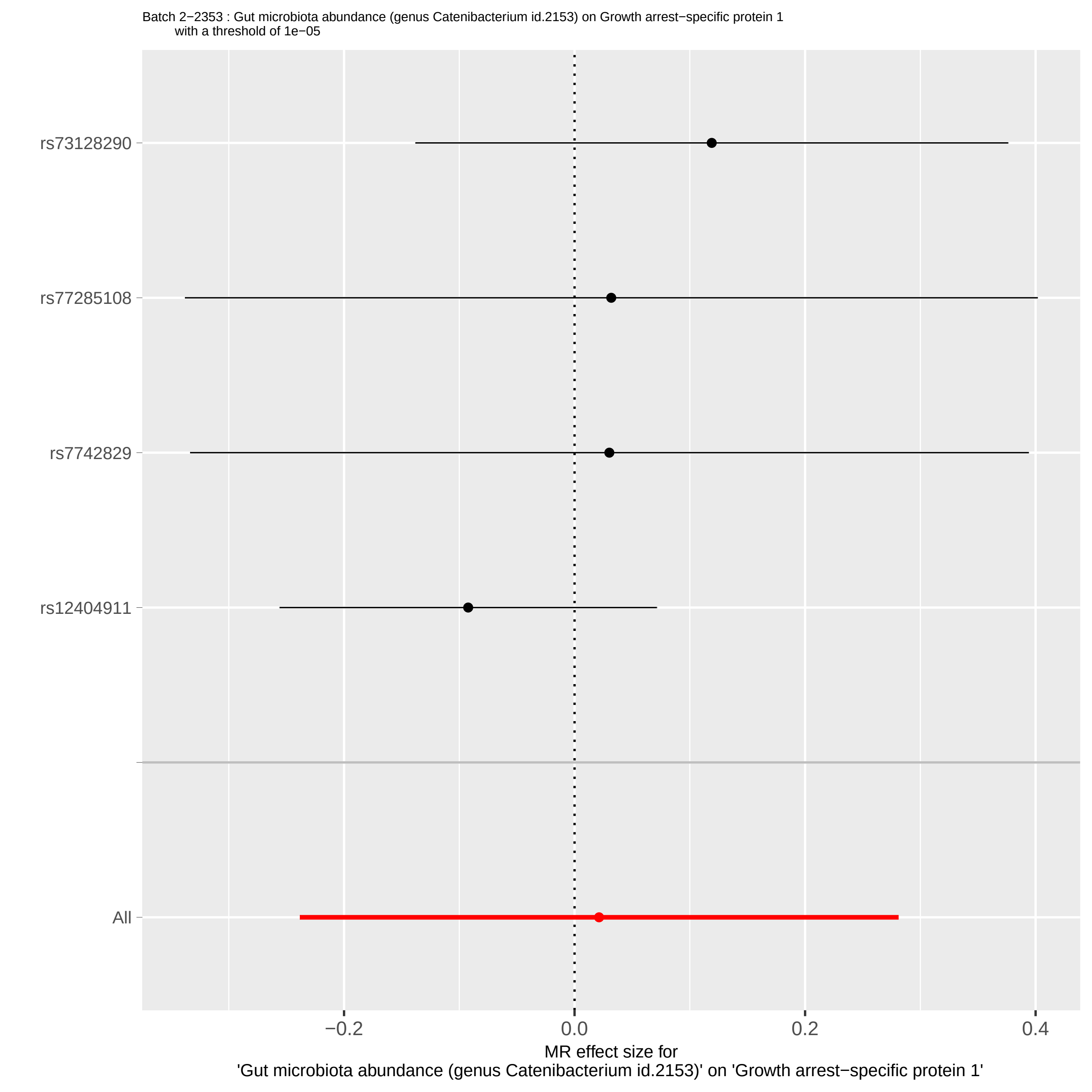
Batch 2−2353 : Gut microbiota abundance (genus Catenibacterium id.2153) on Growth arrest−specific protein 1
with a threshold of 1e−05
rs73128290
rs77285108
rs7742829
rs12404911
All
−0.2
0.0
0.2
0.4
MR effect size for
'Gut microbiota abundance (genus Catenibacterium id.2153)' on 'Growth arrest−specific protein 1'

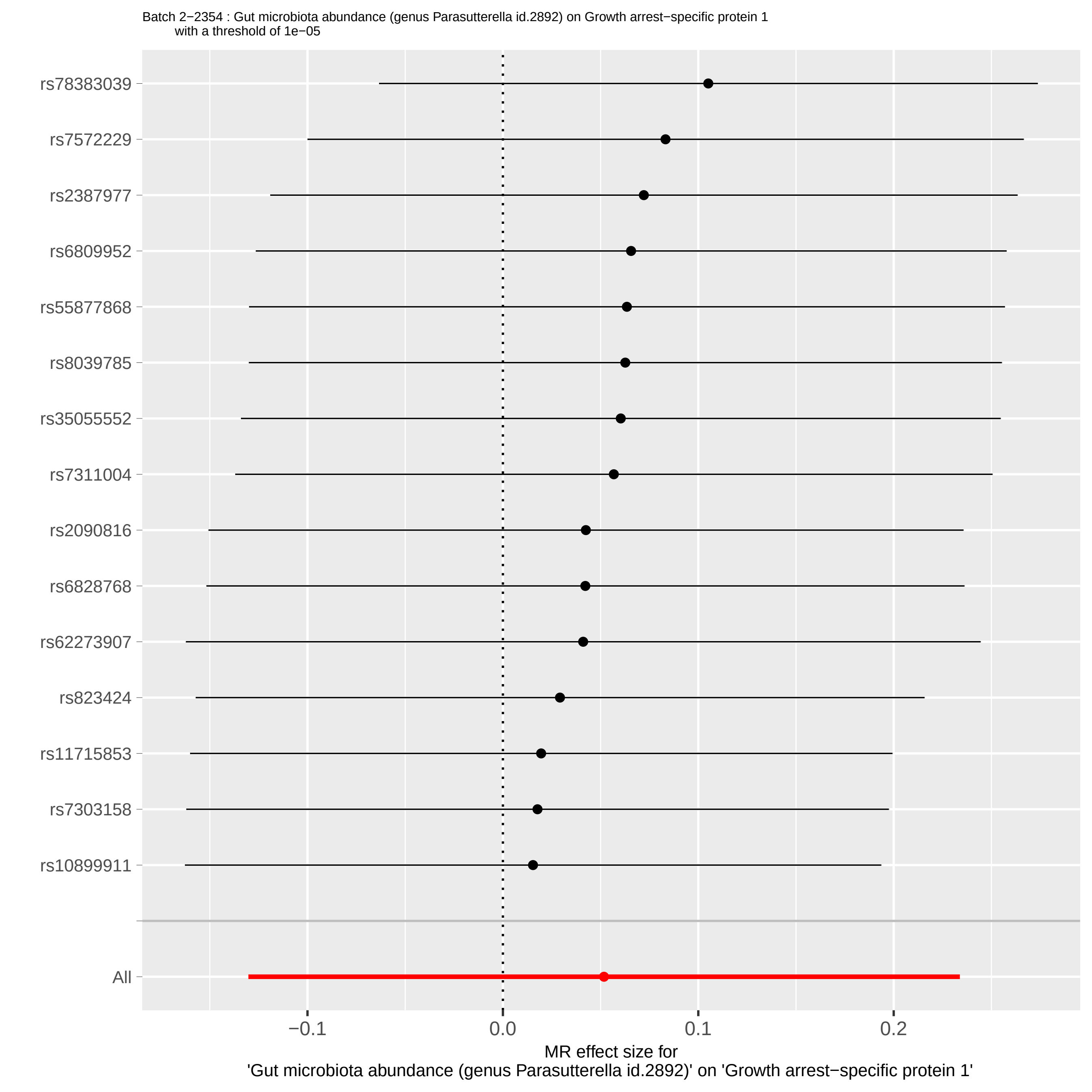

Batch 2−2354 : Gut microbiota abundance (genus Parasutterella id.2892) on Growth arrest−specific protein 1
with a threshold of 1e−05
rs78383039
rs7572229
rs2387977
rs6809952
rs55877868
rs8039785
rs35055552
rs7311004
rs2090816
rs6828768
rs62273907
rs823424
rs11715853
rs7303158
rs10899911
All
−0.1
0.0
0.1
0.2
MR effect size for
'Gut microbiota abundance (genus Parasutterella id.2892)' on 'Growth arrest−specific protein 1'

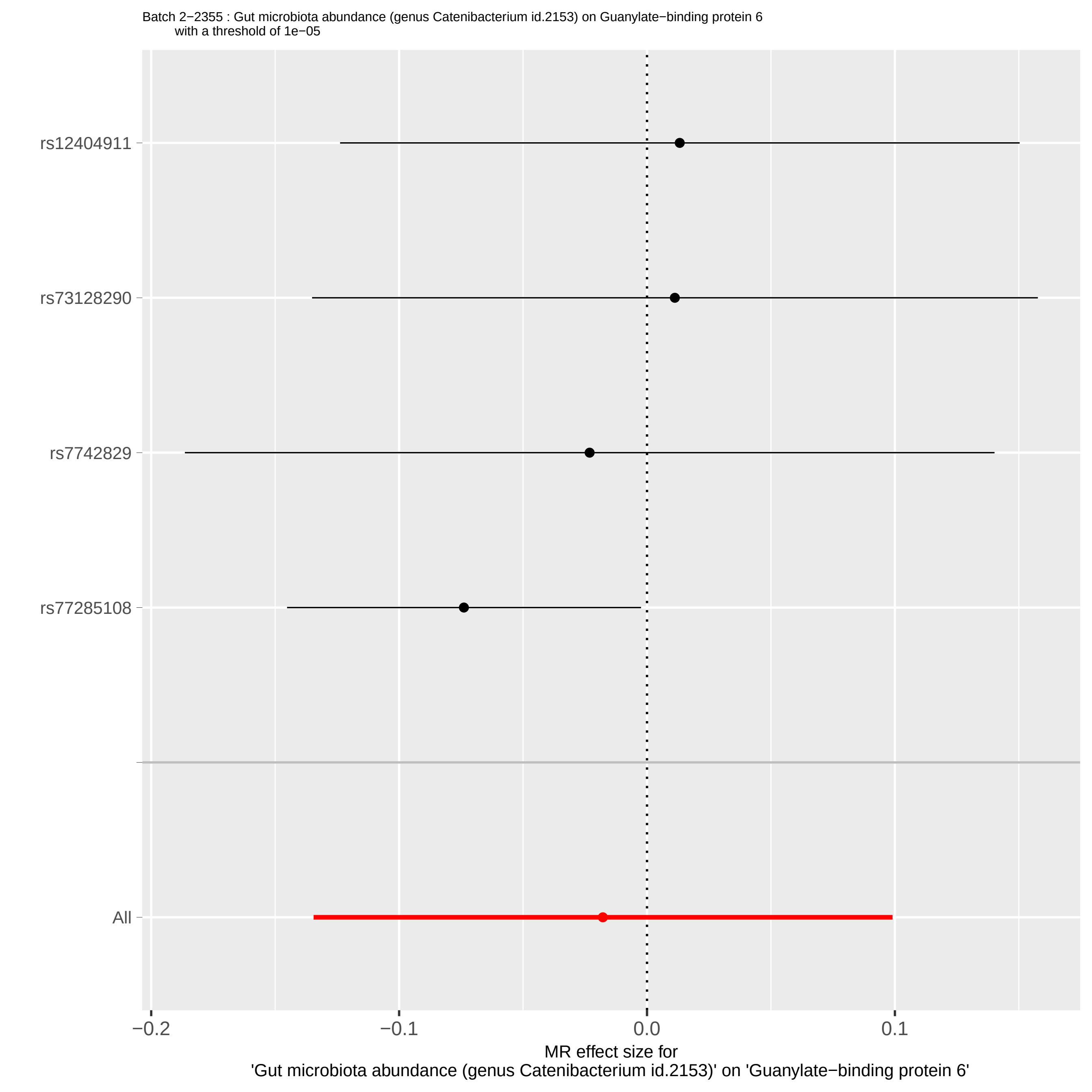
Batch 2−2355 : Gut microbiota abundance (genus Catenibacterium id.2153) on Guanylate−binding protein 6
with a threshold of 1e−05
rs12404911
rs73128290
rs7742829
rs77285108
All
−0.2
−0.1
0.0
0.1
MR effect size for
'Gut microbiota abundance (genus Catenibacterium id.2153)' on 'Guanylate−binding protein 6'

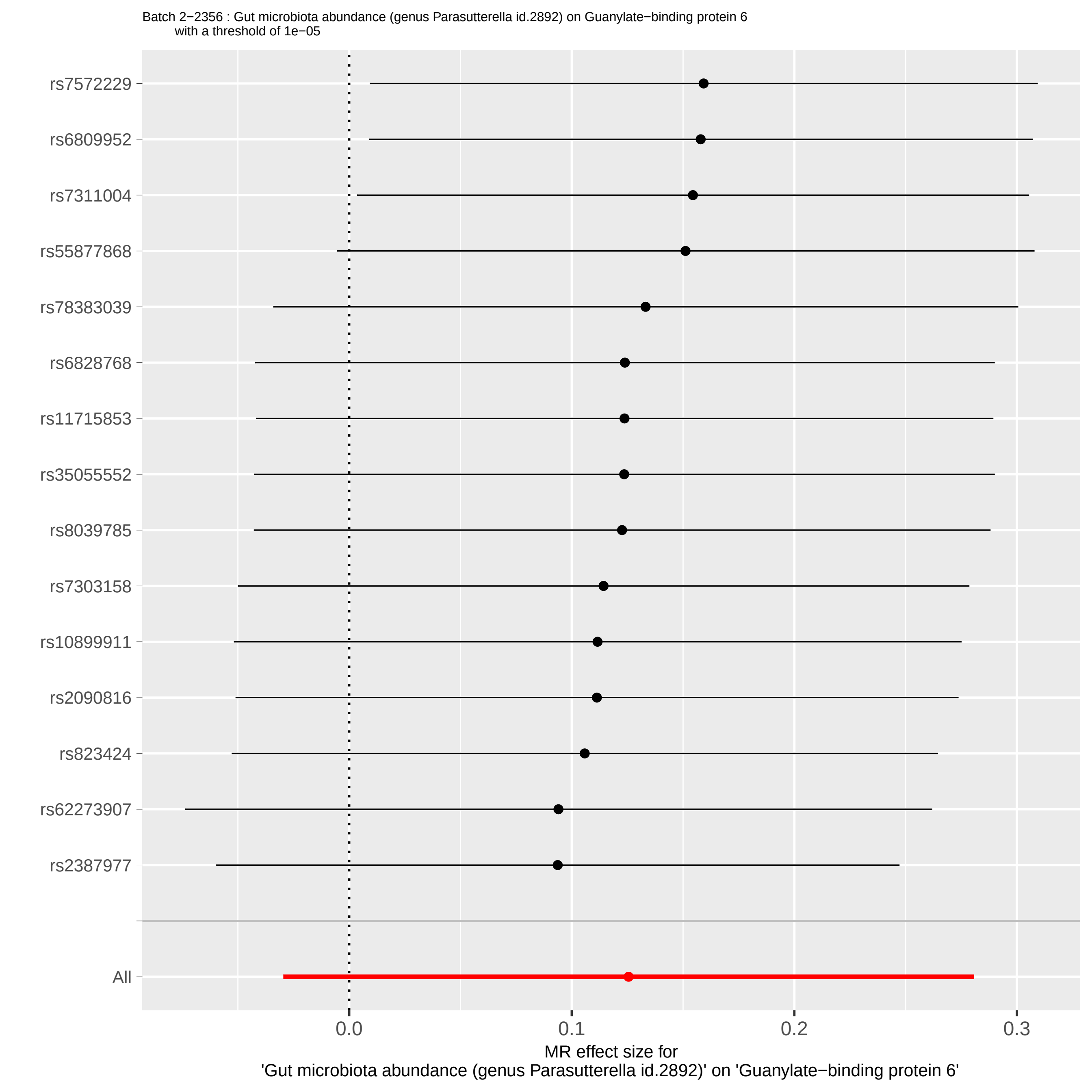

Batch 2−2356 : Gut microbiota abundance (genus Parasutterella id.2892) on Guanylate−binding protein 6
with a threshold of 1e−05
rs7572229
rs6809952
rs7311004
rs55877868
rs78383039
rs6828768
rs11715853
rs35055552
rs8039785
rs7303158
rs10899911
rs2090816
rs823424
rs62273907
rs2387977
All
0.0
0.1
0.2
0.3
MR effect size for
'Gut microbiota abundance (genus Parasutterella id.2892)' on 'Guanylate−binding protein 6'

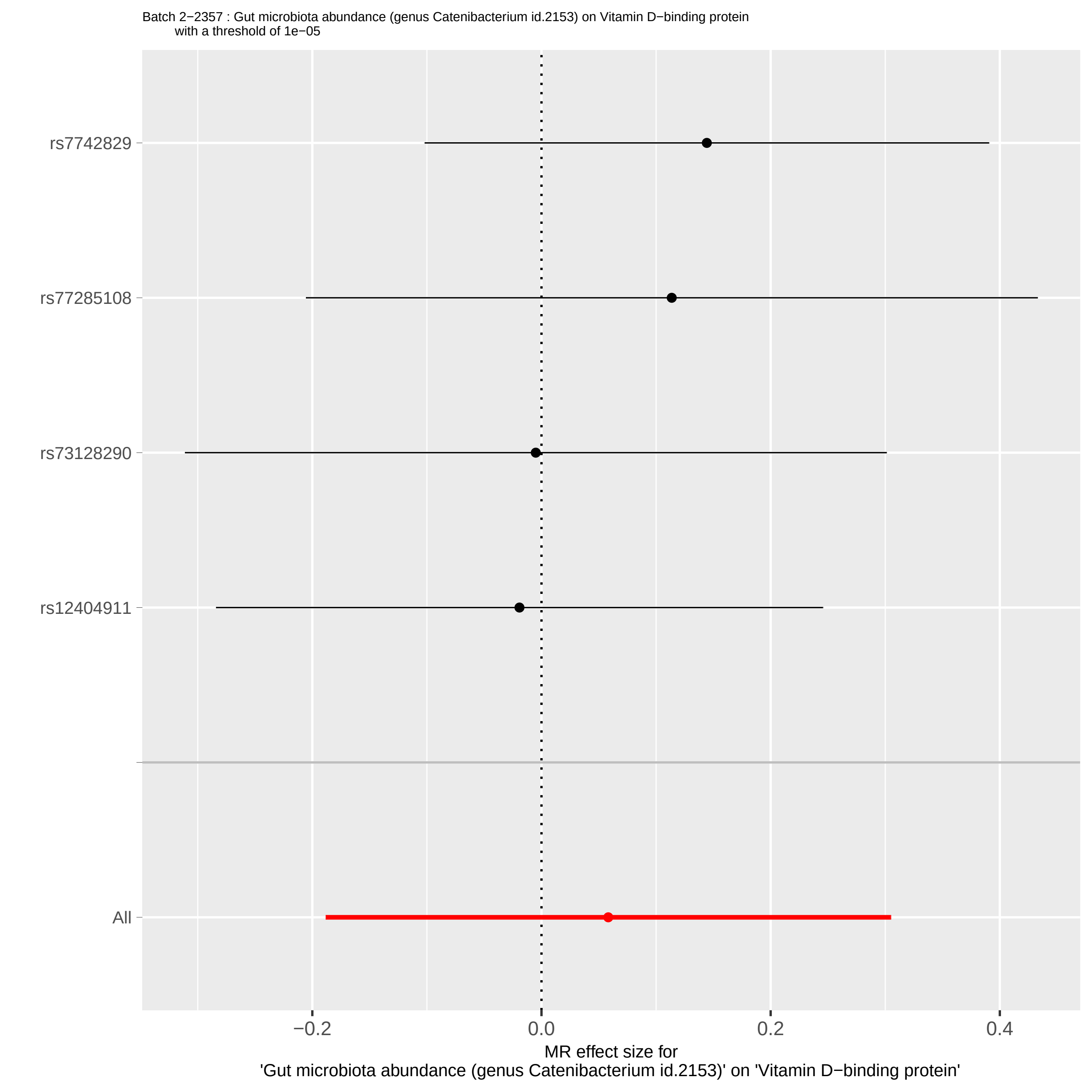
Batch 2−2357 : Gut microbiota abundance (genus Catenibacterium id.2153) on Vitamin D−binding protein
with a threshold of 1e−05
rs7742829
rs77285108
rs73128290
rs12404911
All
−0.2
0.0
0.2
0.4
MR effect size for
'Gut microbiota abundance (genus Catenibacterium id.2153)' on 'Vitamin D−binding protein'

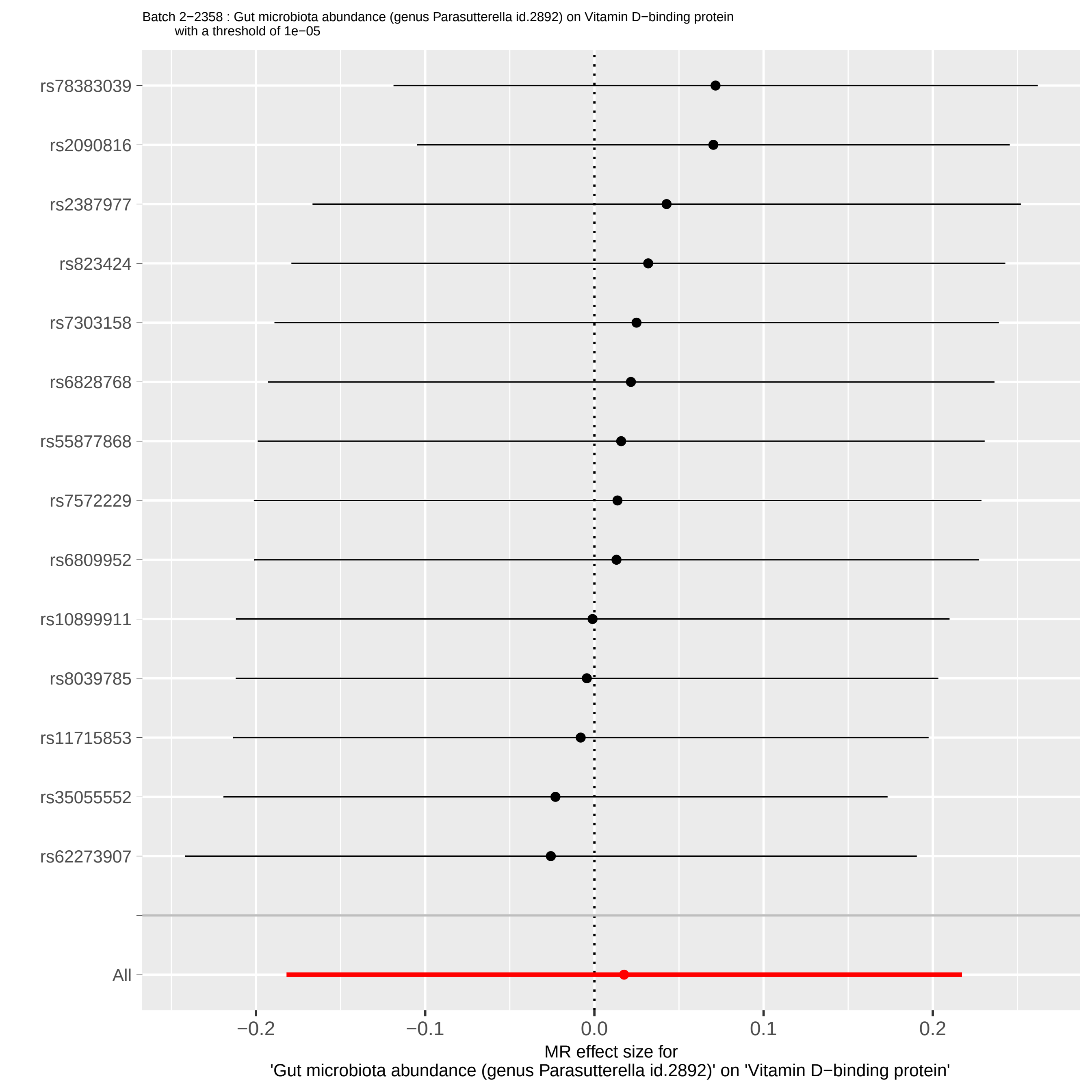

Batch 2−2358 : Gut microbiota abundance (genus Parasutterella id.2892) on Vitamin D−binding protein
with a threshold of 1e−05
rs78383039
rs2090816
rs2387977
rs823424
rs7303158
rs6828768
rs55877868
rs7572229
rs6809952
rs10899911
rs8039785
rs11715853
rs35055552
rs62273907
All
−0.2
−0.1
0.0
0.1
0.2
MR effect size for
'Gut microbiota abundance (genus Parasutterella id.2892)' on 'Vitamin D−binding protein'

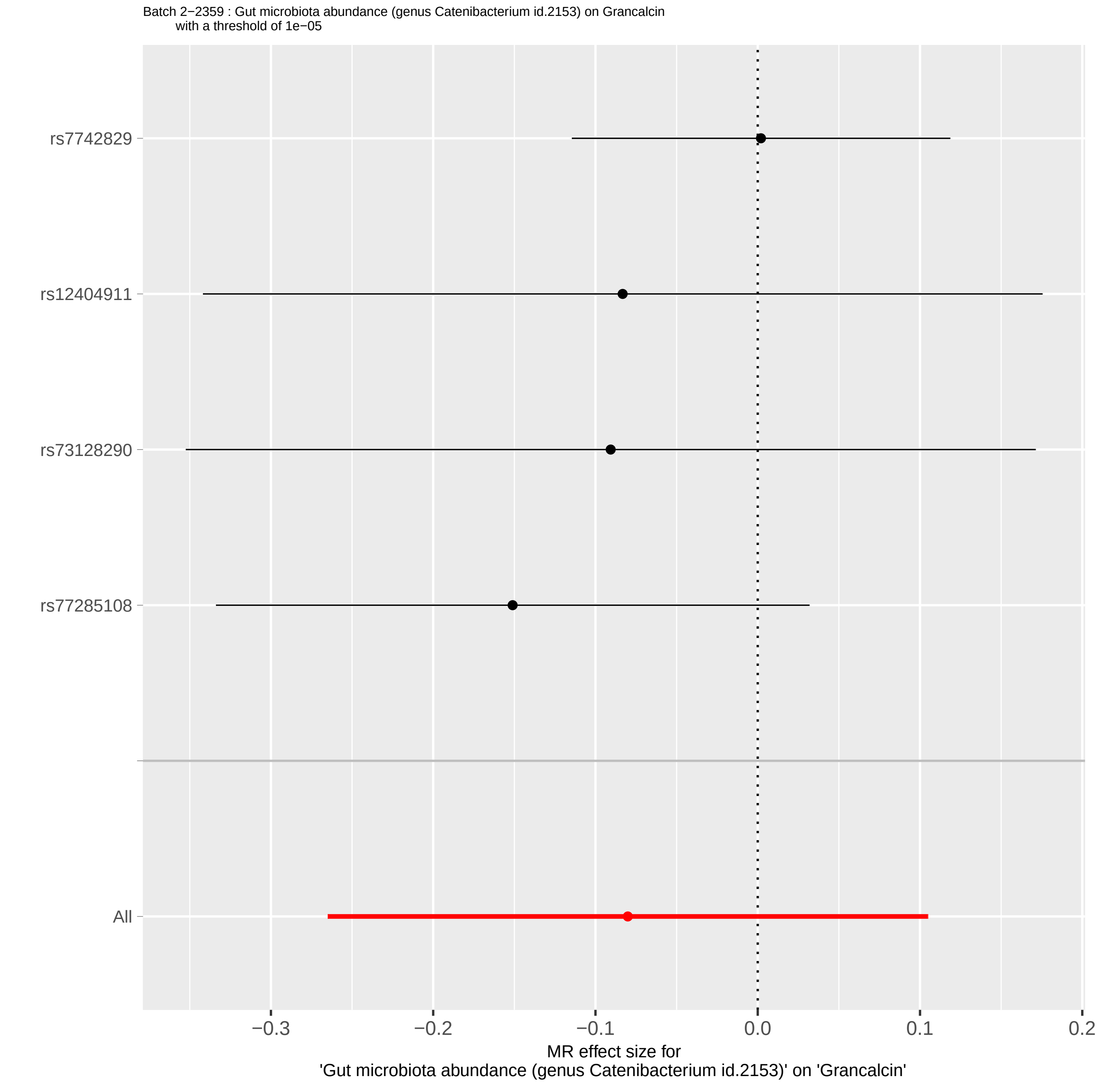
Batch 2−2359 : Gut microbiota abundance (genus Catenibacterium id.2153) on Grancalcin
with a threshold of 1e−05
rs7742829
rs12404911
rs73128290
rs77285108
All
−0.3
−0.2
−0.1
0.0
0.1
0.2
MR effect size for
'Gut microbiota abundance (genus Catenibacterium id.2153)' on 'Grancalcin'

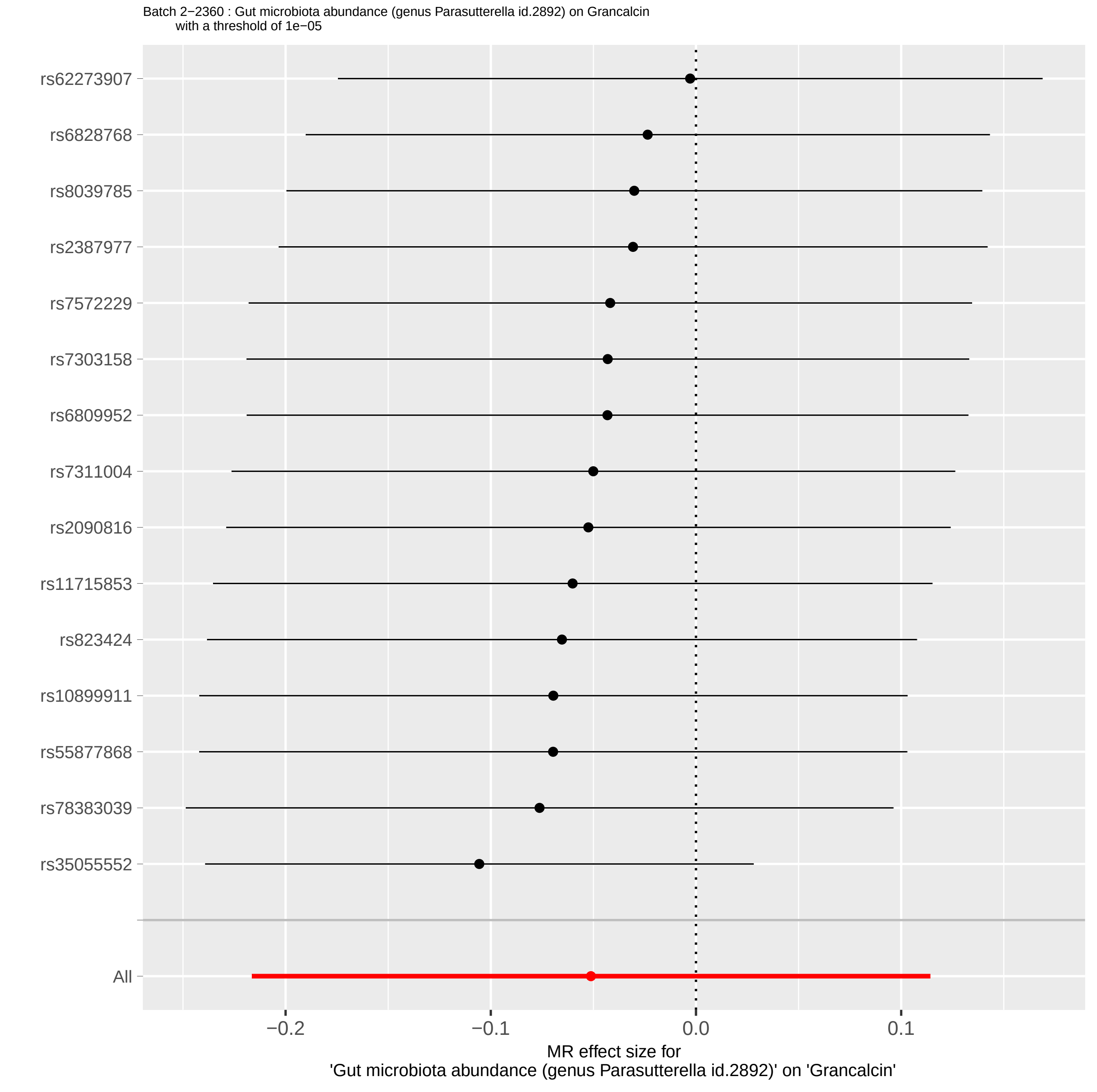

Batch 2−2360 : Gut microbiota abundance (genus Parasutterella id.2892) on Grancalcin
with a threshold of 1e−05
rs62273907
rs6828768
rs8039785
rs2387977
rs7572229
rs7303158
rs6809952
rs7311004
rs2090816
rs11715853
rs823424
rs10899911
rs55877868
rs78383039
rs35055552
All
−0.2
−0.1
0.0
0.1
MR effect size for
'Gut microbiota abundance (genus Parasutterella id.2892)' on 'Grancalcin'

Batch 2−2361 : Gut microbiota abundance (genus Catenibacterium id.2153) on Glucagon
with a threshold of 1e−05

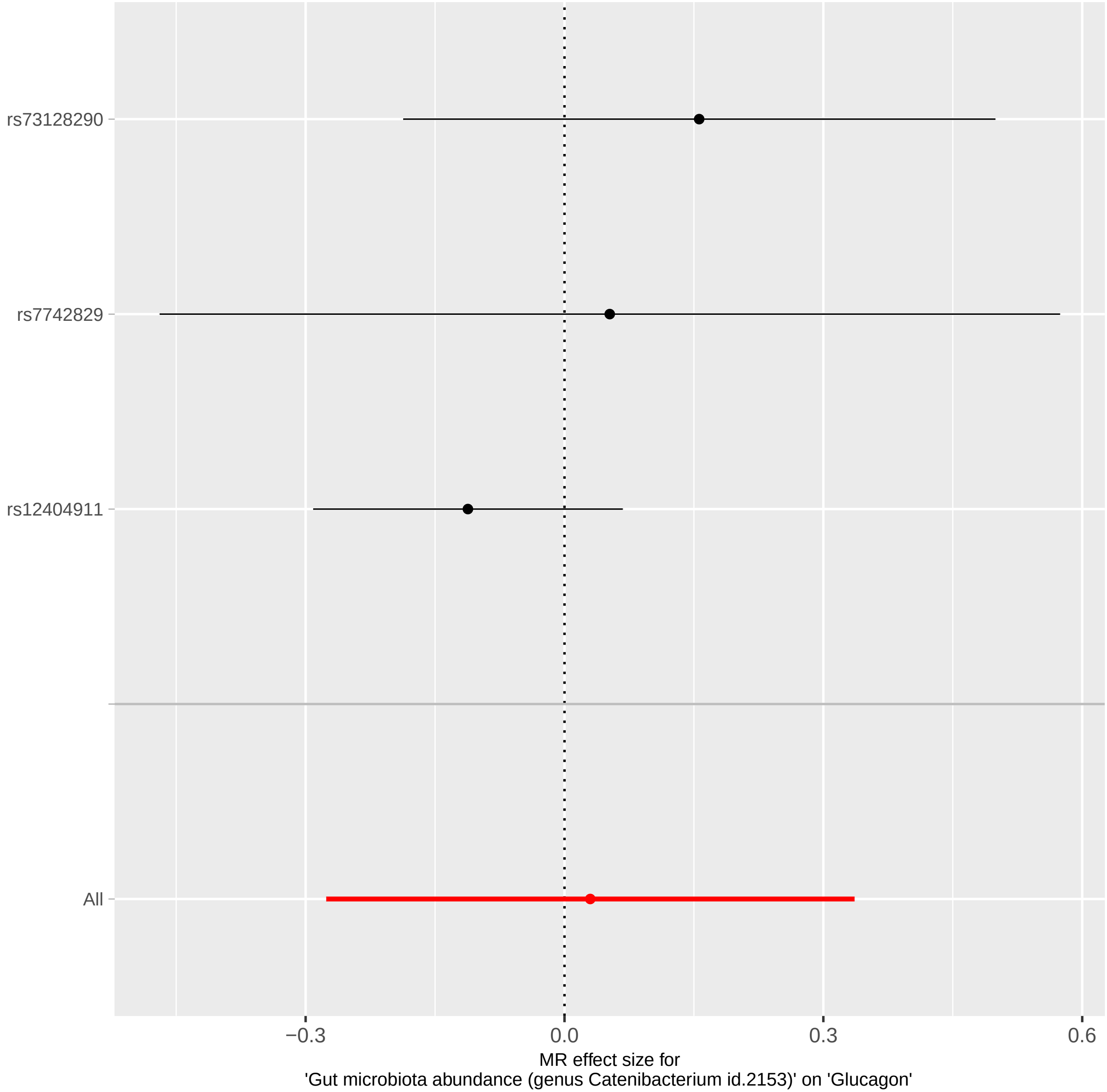

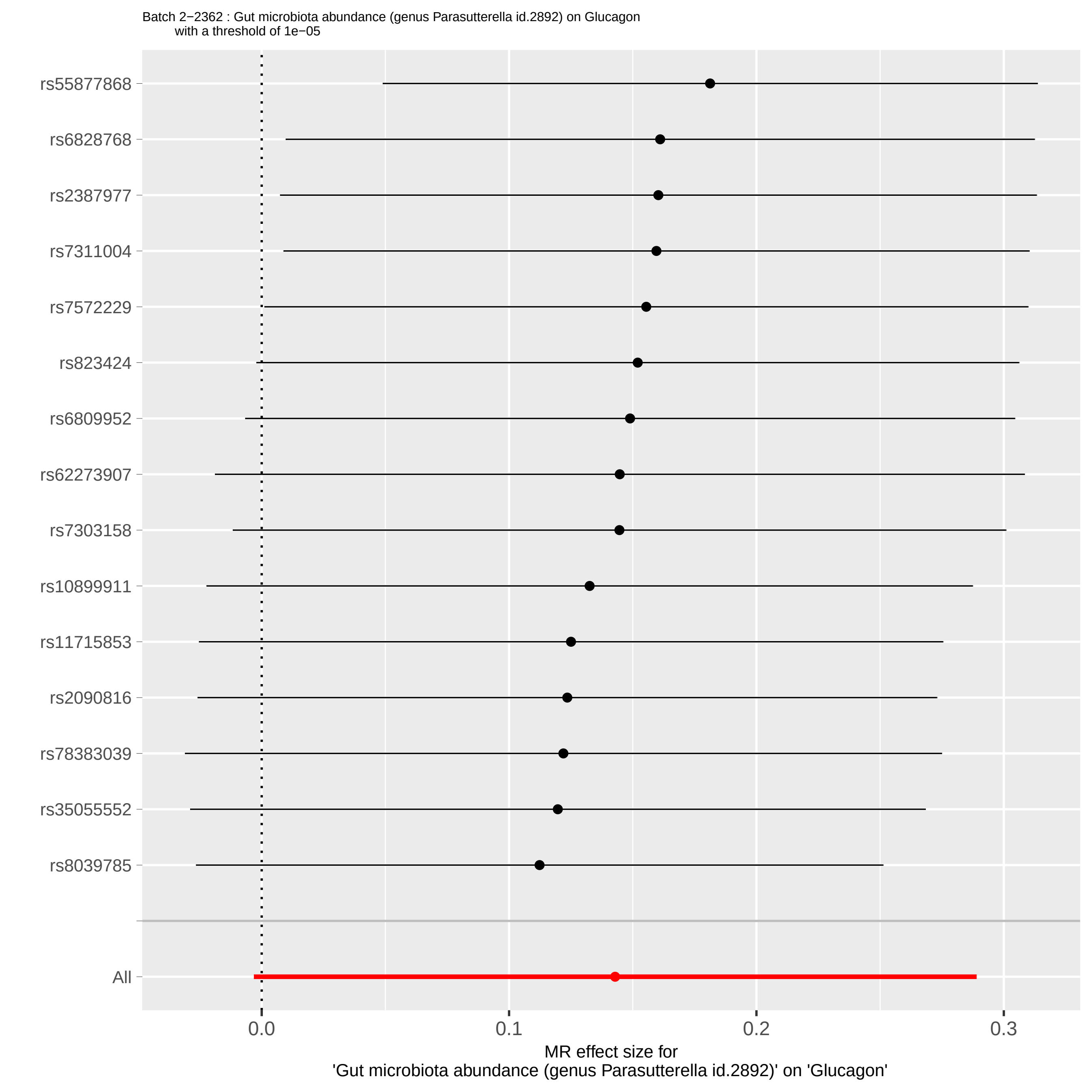

Batch 2−2362 : Gut microbiota abundance (genus Parasutterella id.2892) on Glucagon
with a threshold of 1e−05
rs55877868
rs6828768
rs2387977
rs7311004
rs7572229
rs823424
rs6809952
rs62273907
rs7303158
rs10899911
rs11715853
rs2090816
rs78383039
rs35055552
rs8039785
All
0.0
0.1
0.2
0.3
MR effect size for
'Gut microbiota abundance (genus Parasutterella id.2892)' on 'Glucagon'

Batch 2−2363 : Gut microbiota abundance (genus Catenibacterium id.2153) on GTP cyclohydrolase 1
with a threshold of 1e−05

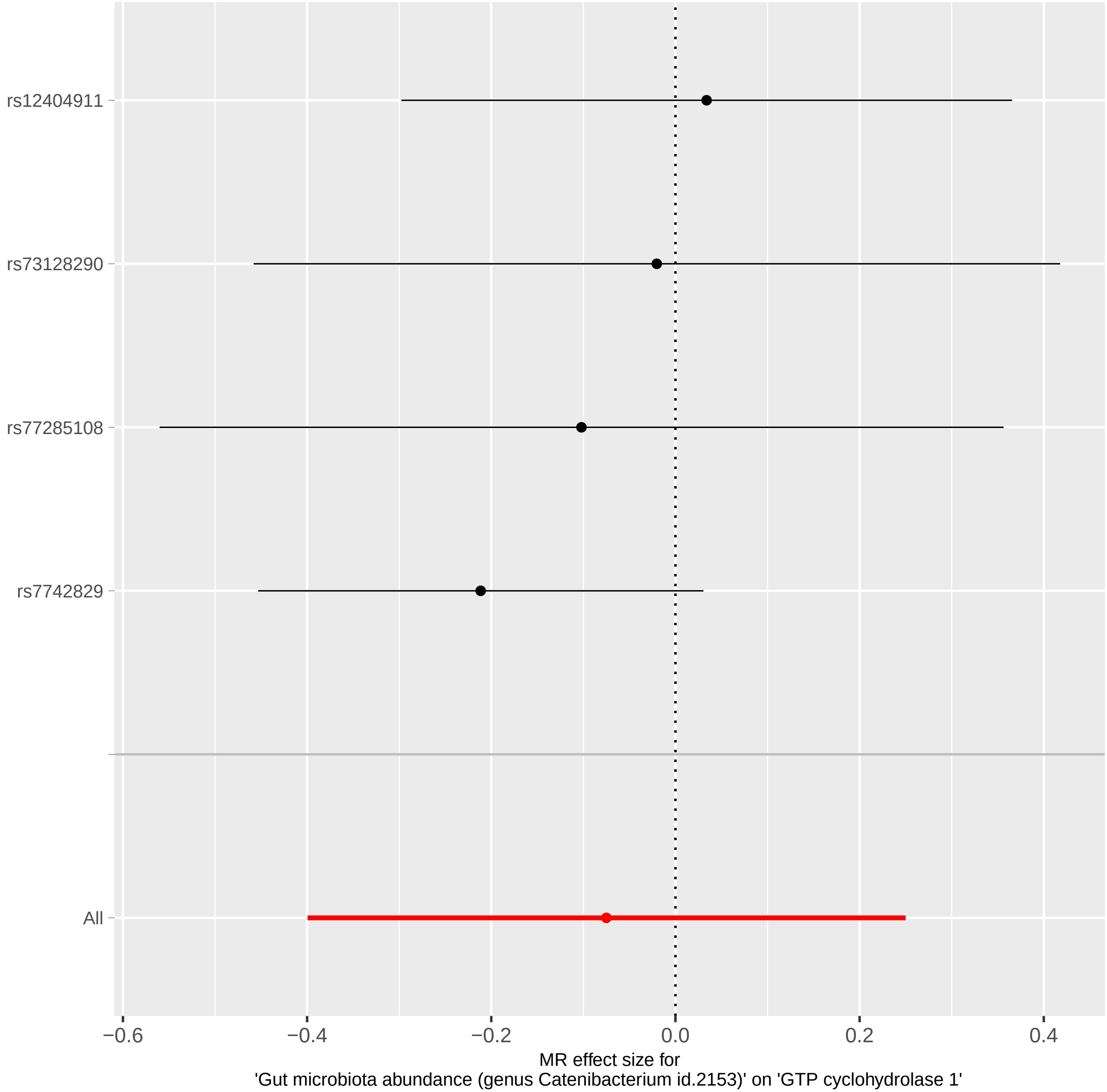

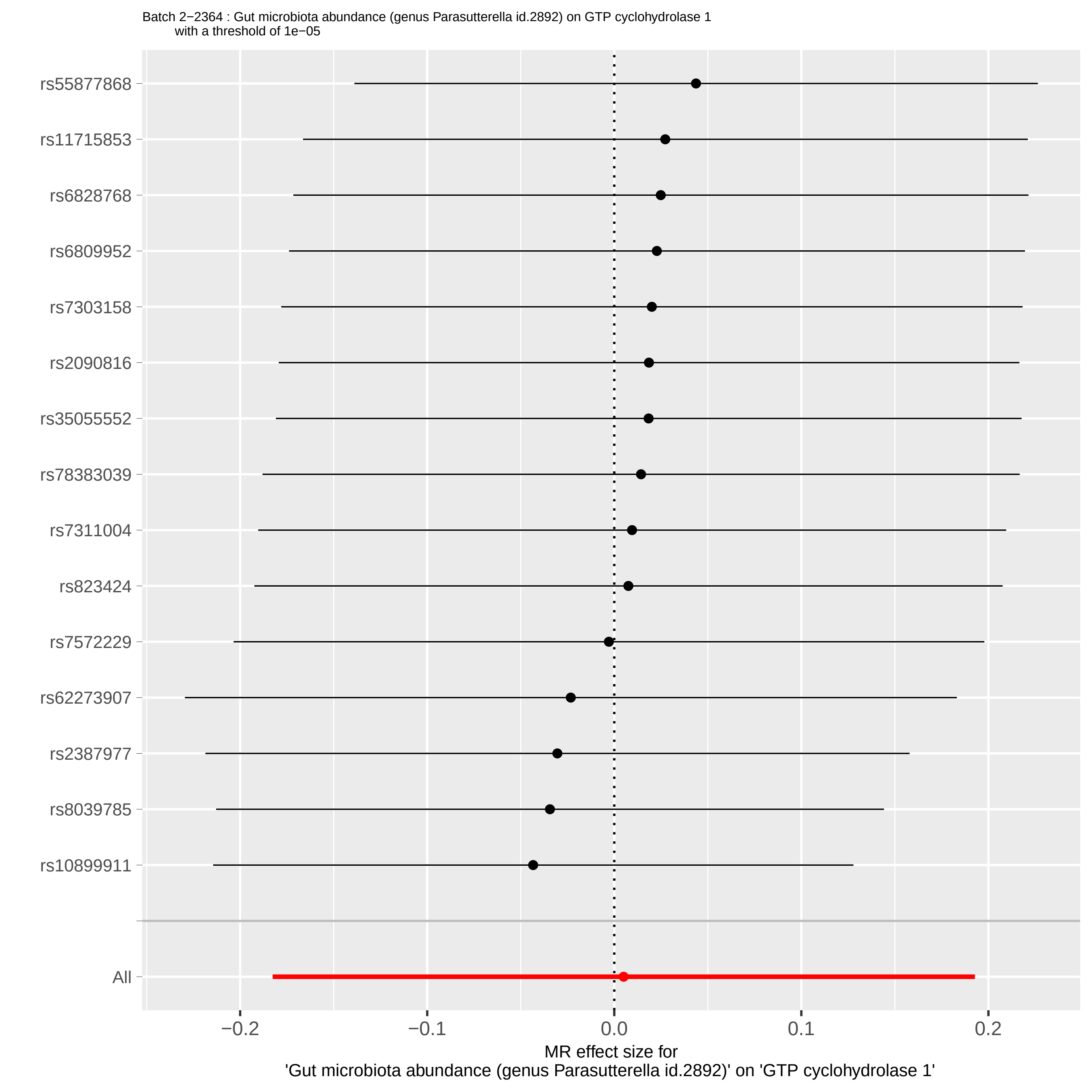

Batch 2−2364 : Gut microbiota abundance (genus Parasutterella id.2892) on GTP cyclohydrolase 1
with a threshold of 1e−05
rs55877868
rs11715853
rs6828768
rs6809952
rs7303158
rs2090816
rs35055552
rs78383039
rs7311004
rs823424
rs7572229
rs62273907
rs2387977
rs8039785
rs10899911
All
−0.2
−0.1
0.0
0.1
0.2
MR effect size for
'Gut microbiota abundance (genus Parasutterella id.2892)' on 'GTP cyclohydrolase 1'

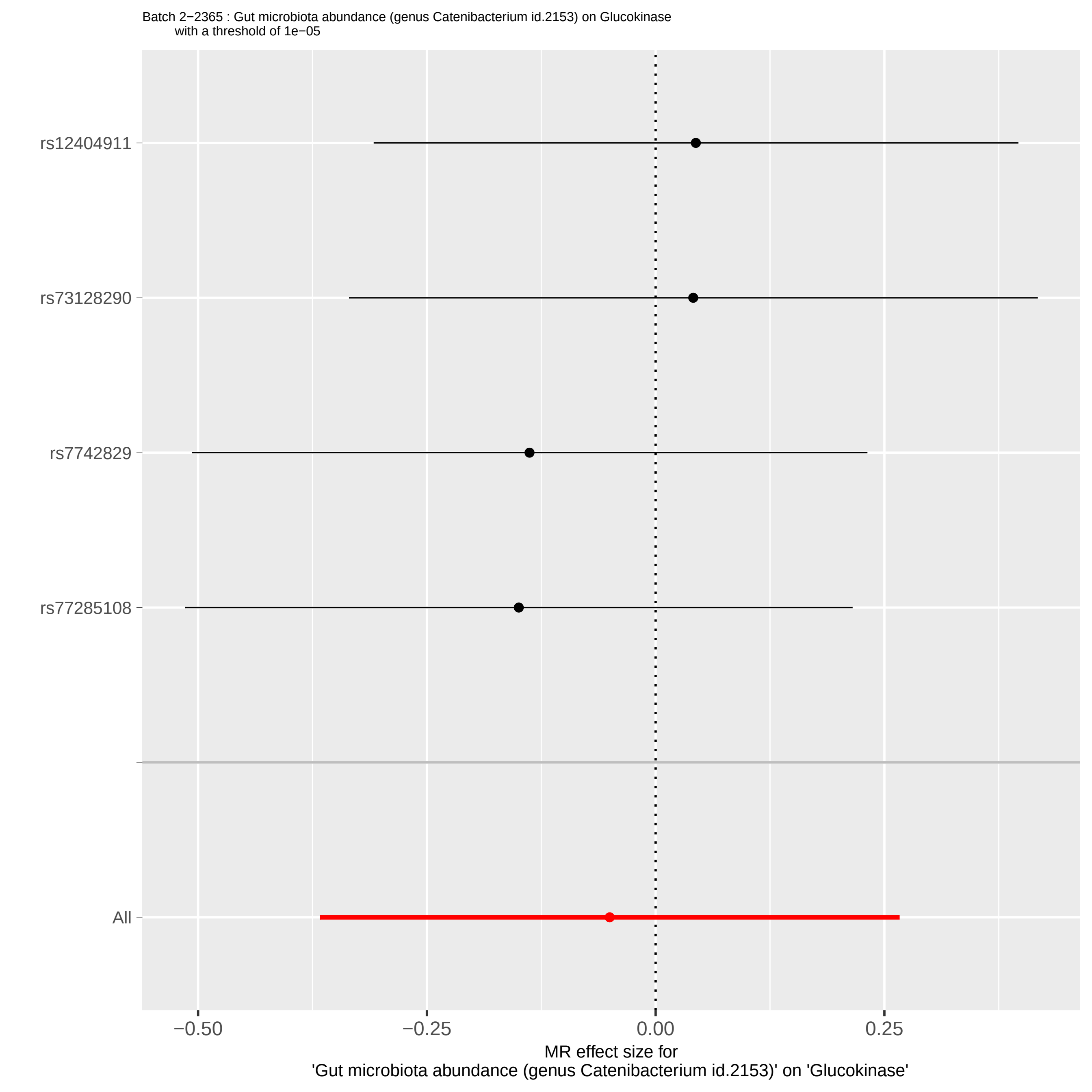

Batch 2−2365 : Gut microbiota abundance (genus Catenibacterium id.2153) on Glucokinase
with a threshold of 1e−05
rs12404911
rs73128290
rs7742829
rs77285108
All
−0.50
−0.25
0.00
0.25
MR effect size for
'Gut microbiota abundance (genus Catenibacterium id.2153)' on 'Glucokinase'

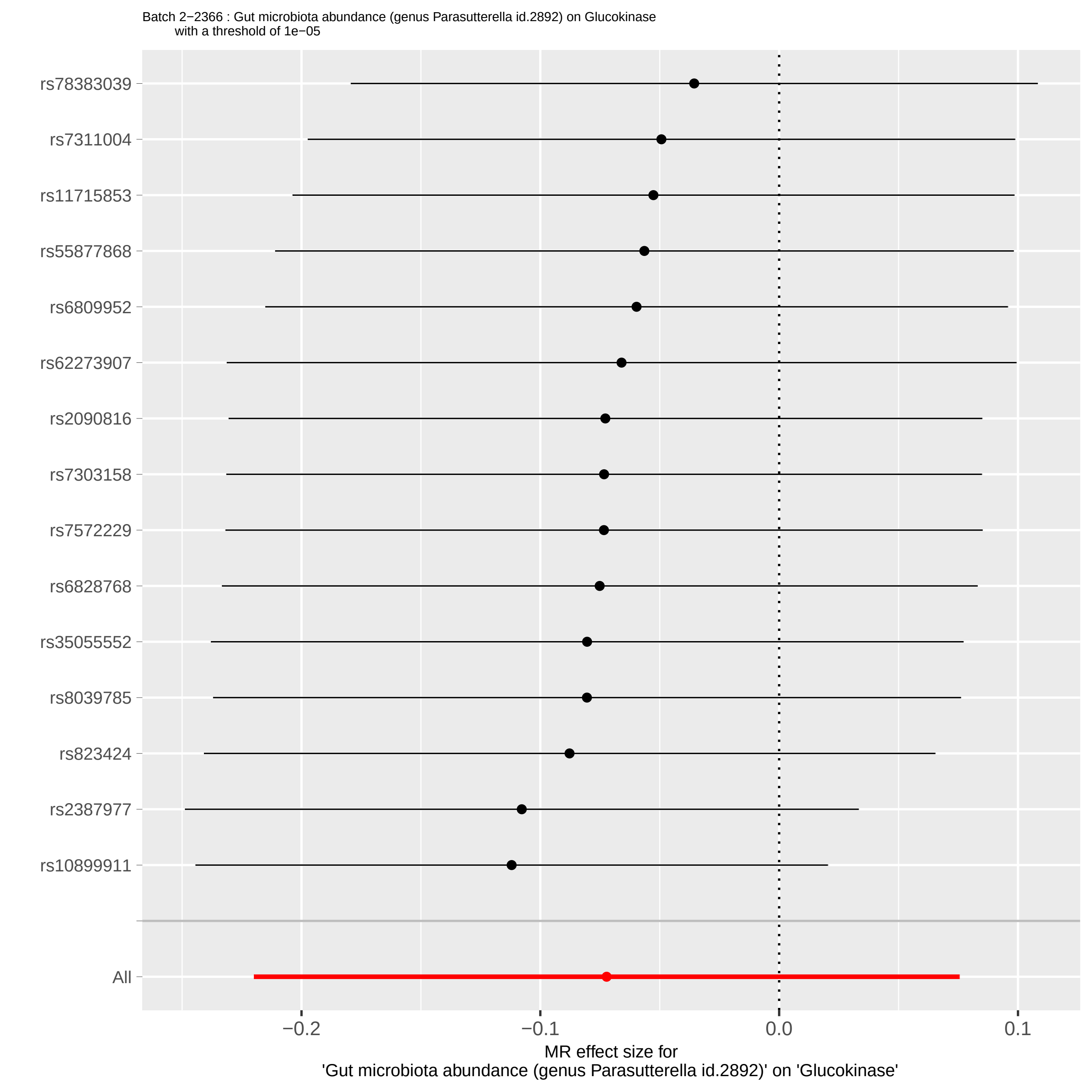

Batch 2−2366 : Gut microbiota abundance (genus Parasutterella id.2892) on Glucokinase
with a threshold of 1e−05
rs78383039
rs7311004
rs11715853
rs55877868
rs6809952
rs62273907
rs2090816
rs7303158
rs7572229
rs6828768
rs35055552
rs8039785
rs823424
rs2387977
rs10899911
All
−0.2
−0.1
0.0
0.1
MR effect size for
'Gut microbiota abundance (genus Parasutterella id.2892)' on 'Glucokinase'

Batch 2−2367 : Gut microbiota abundance (genus Catenibacterium id.2153) on Glucokinase regulatory protein
with a threshold of 1e−05

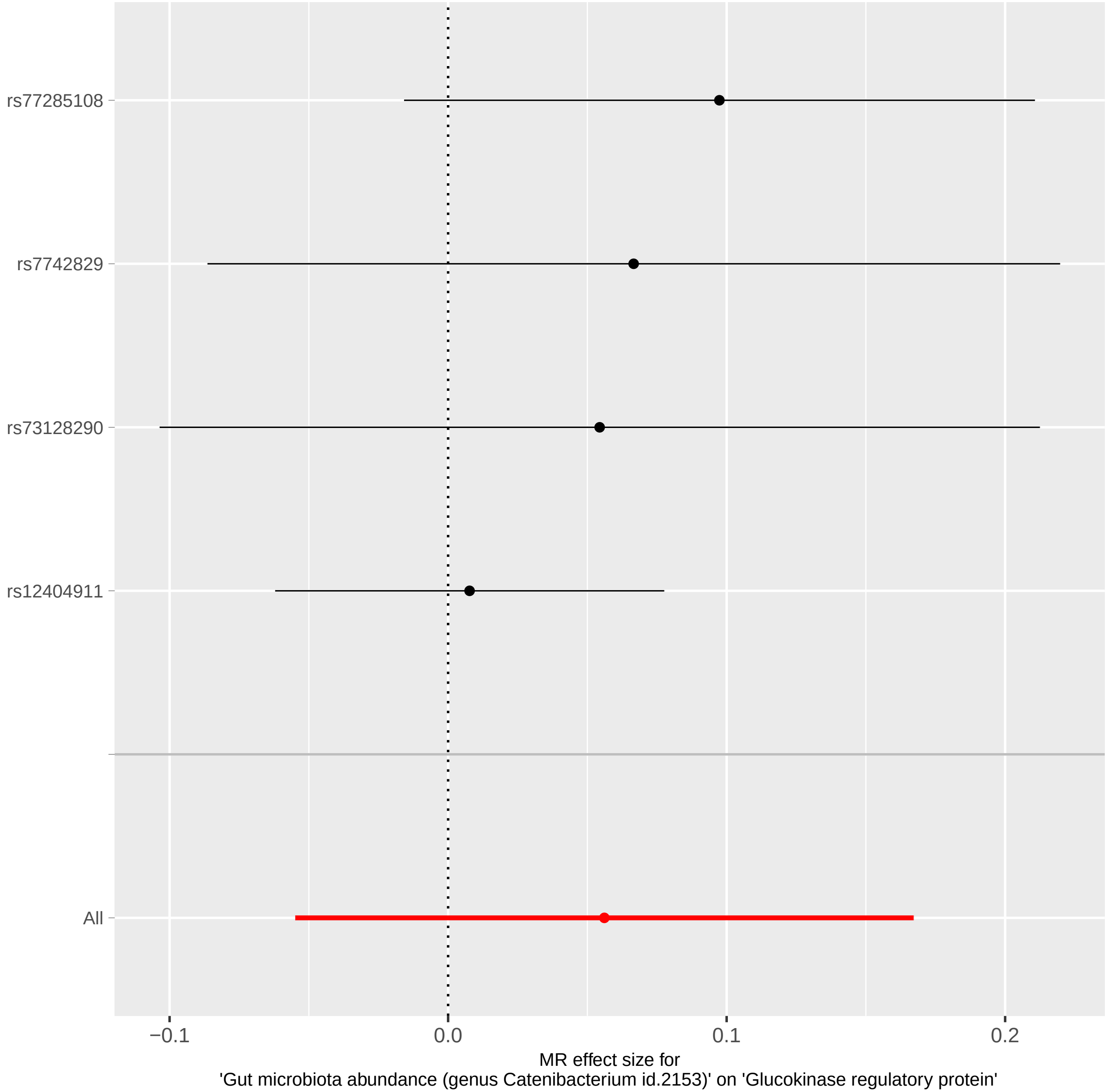

Batch 2−2368 : Gut microbiota abundance (genus Parasutterella id.2892) on Glucokinase regulatory protein
with a threshold of 1e−05

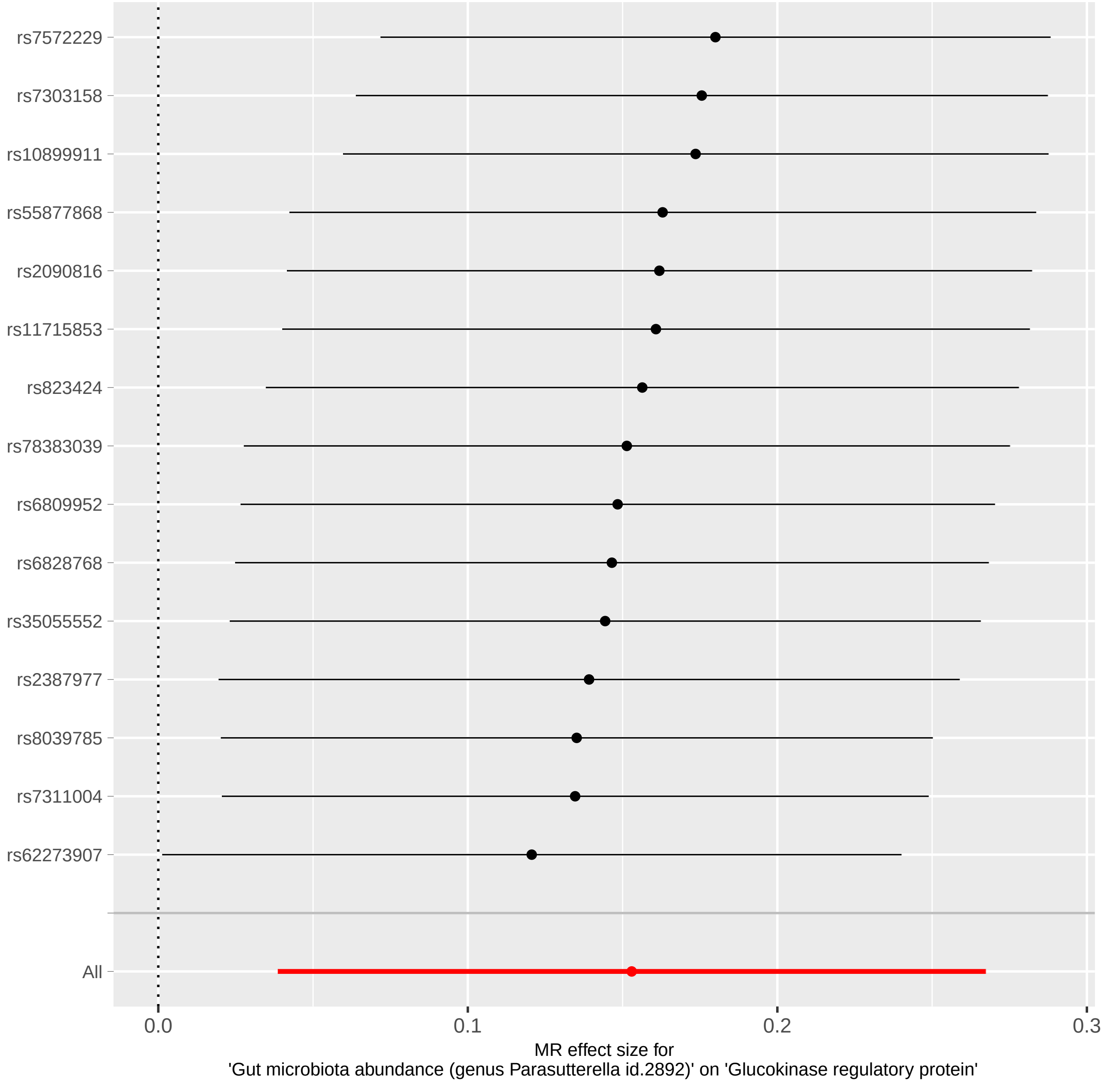

Batch 2−2369 : Gut microbiota abundance (genus Catenibacterium id.2153) on Beta−1,3−galactosyl−O−glycosyl−glycoprotein beta−1,6−N−acetylglucosaminyltransfe
with a threshold of 1e−05

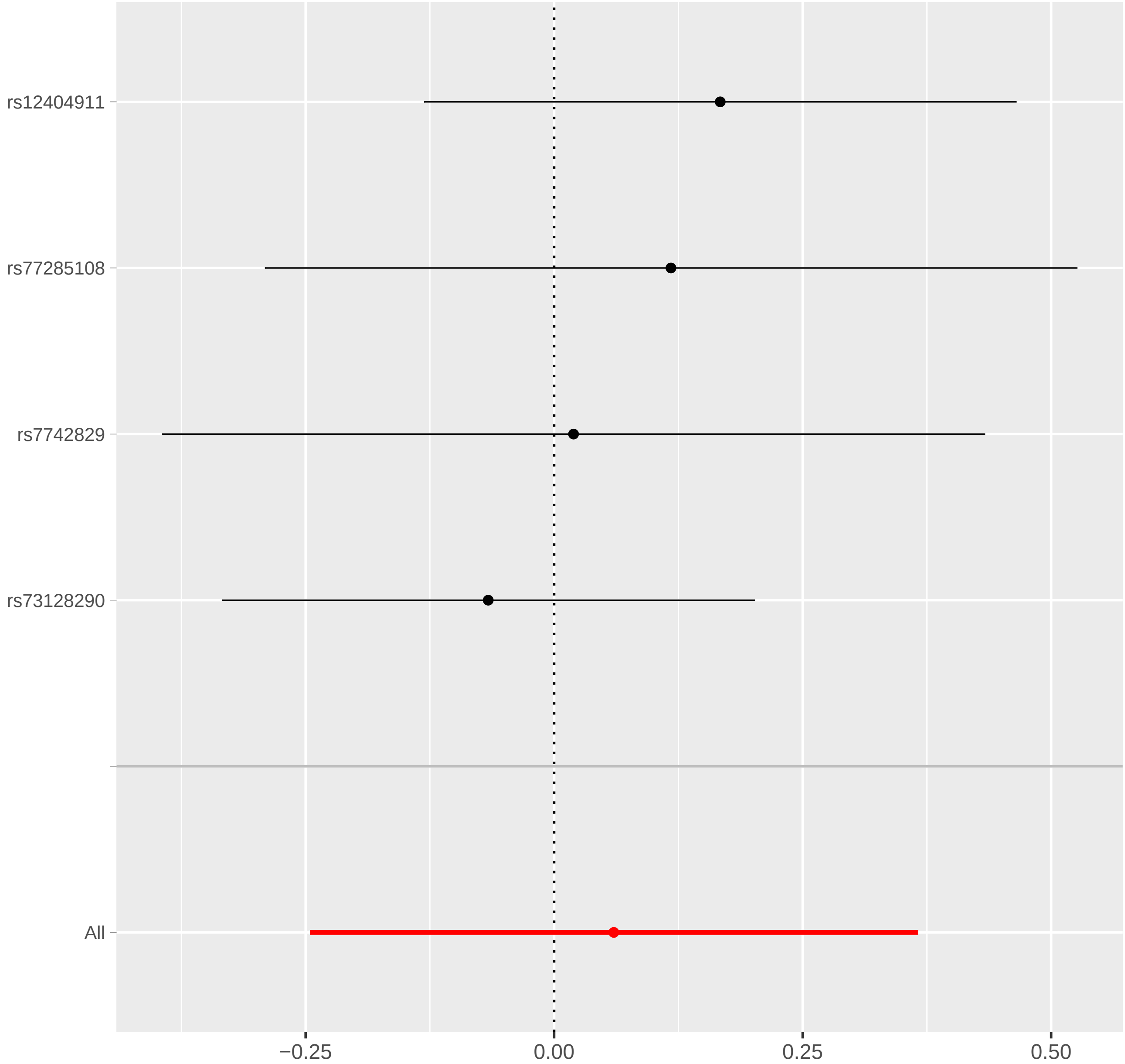

MR effect size for
'Gut microbiota abundance (genus Catenibacterium id.2153)' on 'Beta−1,3−galactosyl−O−glycosyl−glycoprotein beta−1,6−N−acetylglucosam

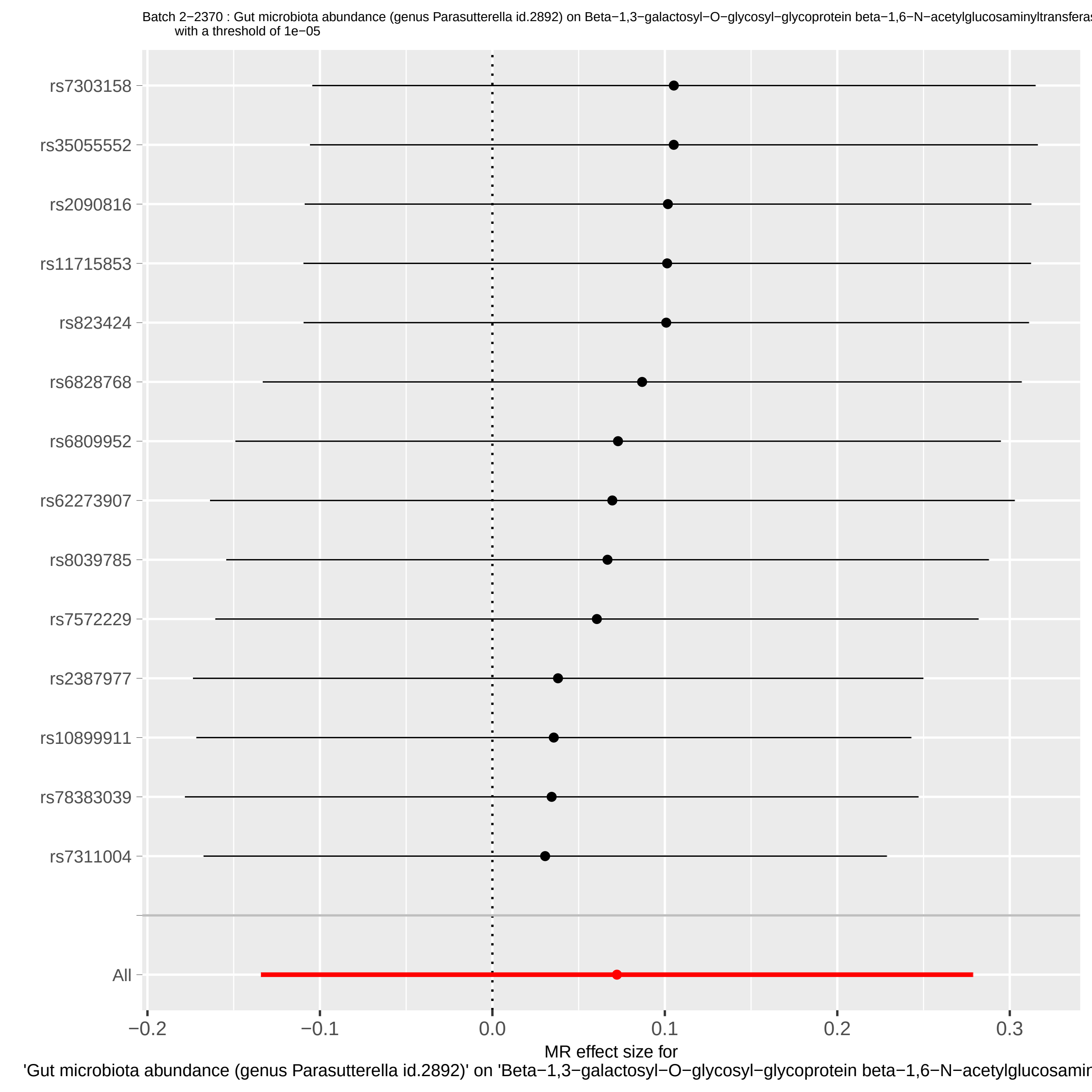

Batch 2−2370 : Gut microbiota abundance (genus Parasutterella id.2892) on Beta−1,3−galactosyl−O−glycosyl−glycoprotein beta−1,6−N−acetylglucosaminyltransfera
with a threshold of 1e−05
rs7303158
rs35055552
rs2090816
rs11715853
rs823424
rs6828768
rs6809952
rs62273907
rs8039785
rs7572229
rs2387977
rs10899911
rs78383039
rs7311004
All
−0.2
−0.1
0.0
0.1
0.2
0.3
MR effect size for
'Gut microbiota abundance (genus Parasutterella id.2892)' on 'Beta−1,3−galactosyl−O−glycosyl−glycoprotein beta−1,6−N−acetylglucosamin

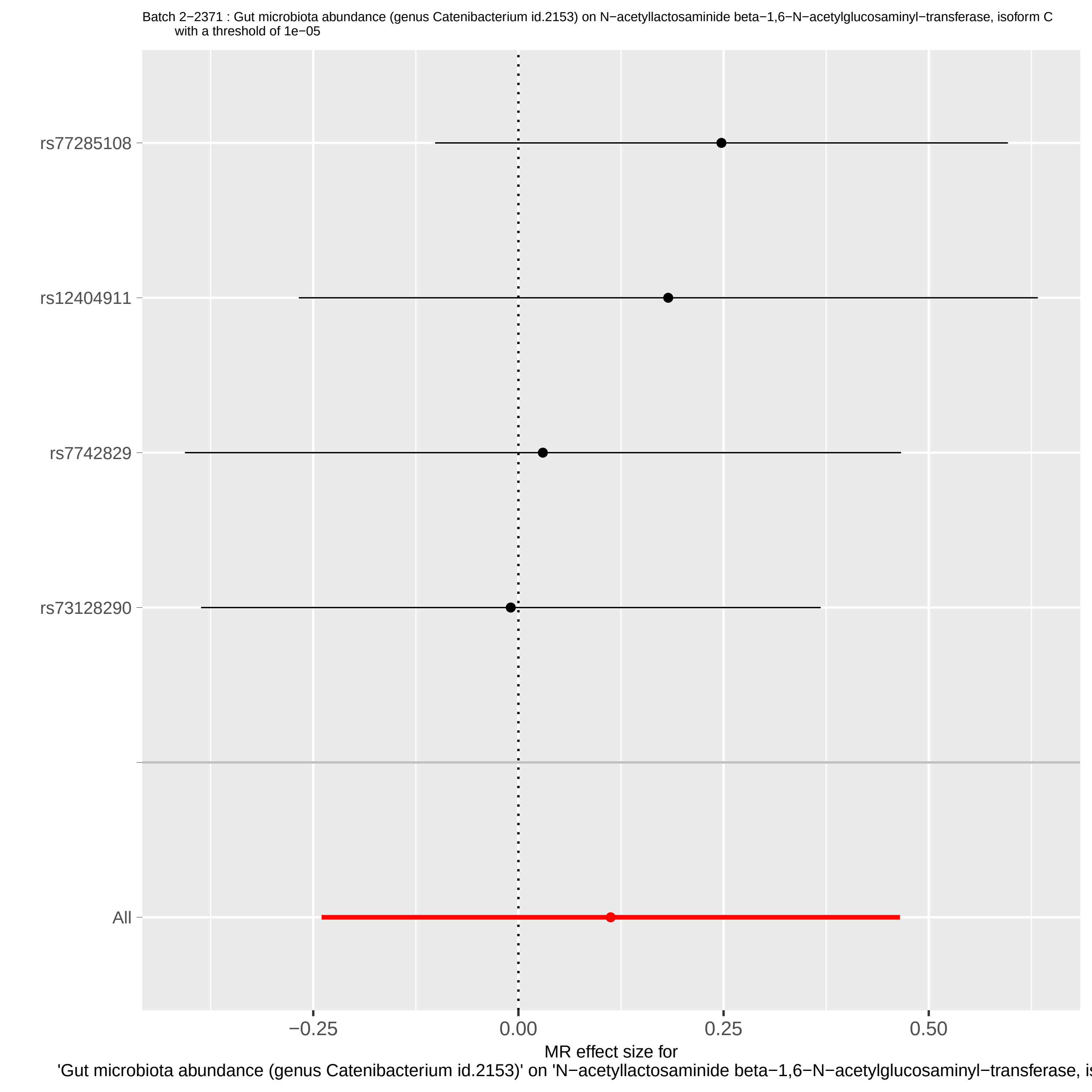
Batch 2−2371 : Gut microbiota abundance (genus Catenibacterium id.2153) on N−acetyllactosaminide beta−1,6−N−acetylglucosaminyl−transferase, isoform C
with a threshold of 1e−05
rs77285108
rs12404911
rs7742829
rs73128290
All
−0.25
0.00
0.25
0.50
MR effect size for
'Gut microbiota abundance (genus Catenibacterium id.2153)' on 'N−acetyllactosaminide beta−1,6−N−acetylglucosaminyl−transferase, is

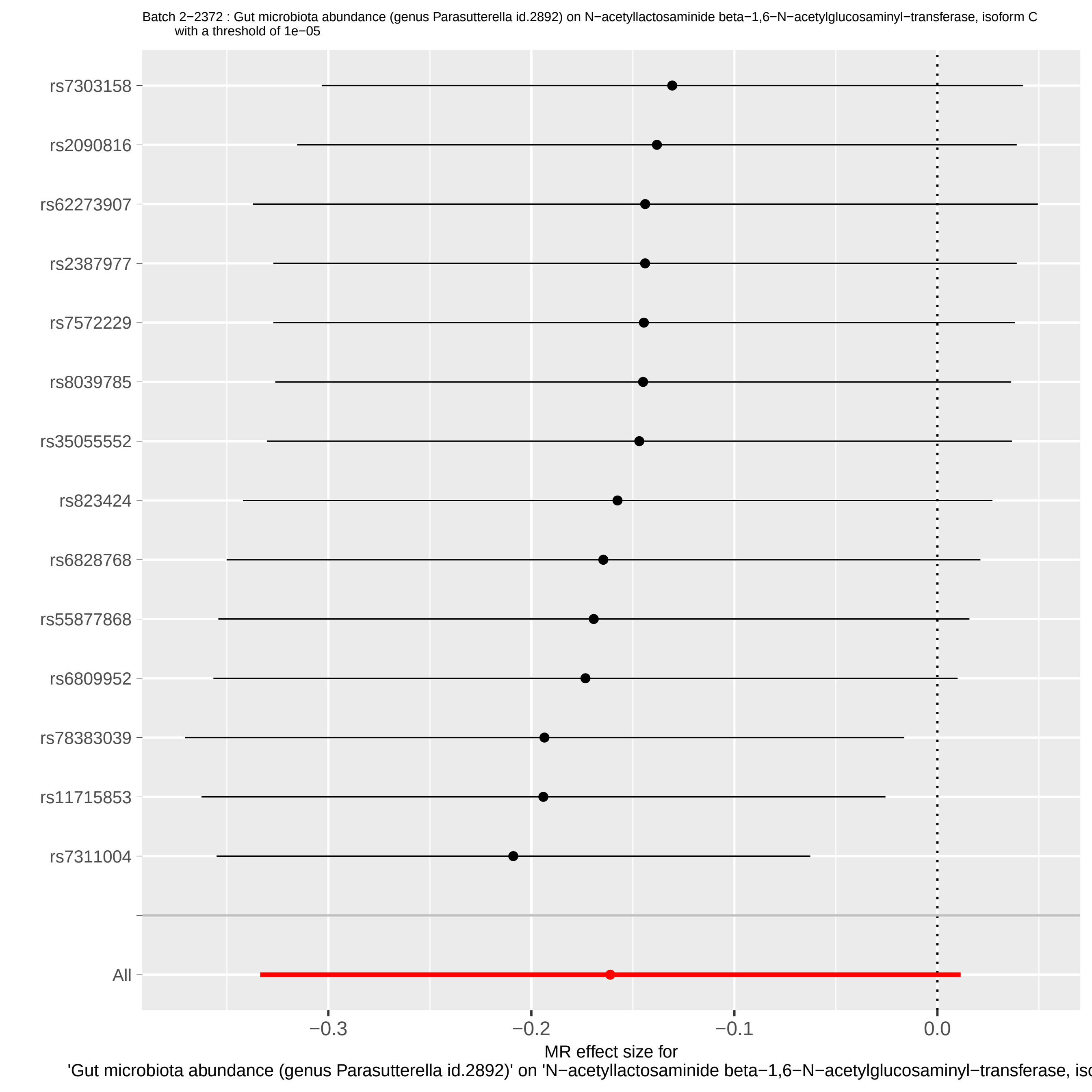
Batch 2−2372 : Gut microbiota abundance (genus Parasutterella id.2892) on N−acetyllactosaminide beta−1,6−N−acetylglucosaminyl−transferase, isoform C with a threshold of 1e−05
rs7303158
rs2090816
rs62273907
rs2387977
rs7572229
rs8039785
rs35055552
rs823424
rs6828768
rs55877868
rs6809952
rs78383039
rs11715853
rs7311004
All
−0.3
−0.2
−0.1
0.0
MR effect size for
'Gut microbiota abundance (genus Parasutterella id.2892)' on 'N−acetyllactosaminide beta−1,6−N−acetylglucosaminyl−transferase, isc

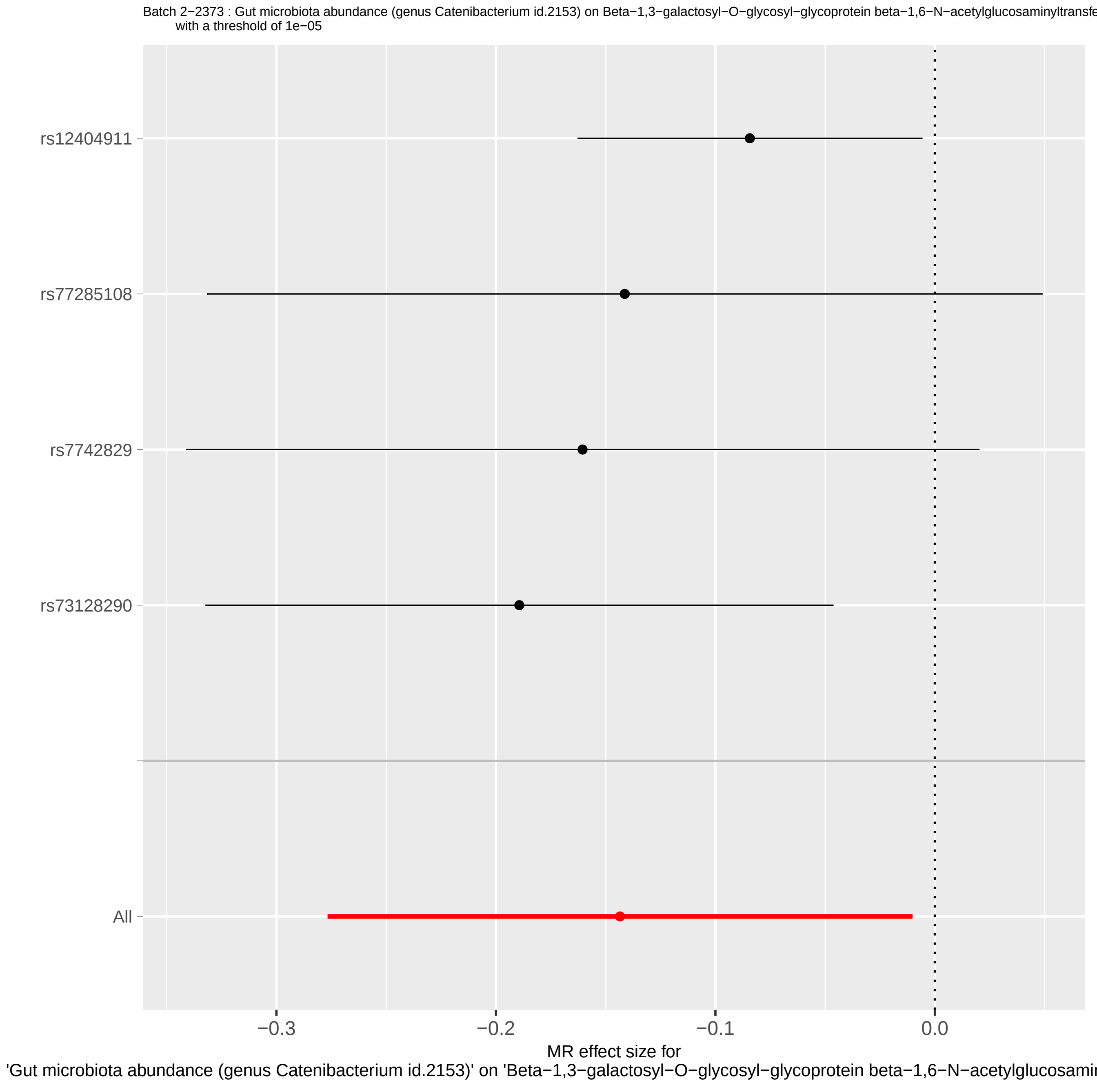

Batch 2−2373 : Gut microbiota abundance (genus Catenibacterium id.2153) on Beta−1,3−galactosyl−O−glycosyl−glycoprotein beta−1,6−N−acetylglucosaminyltransfe
with a threshold of 1e−05
rs12404911
rs77285108
rs7742829
rs73128290
All
−0.3
−0.2
−0.1
0.0
MR effect size for
'Gut microbiota abundance (genus Catenibacterium id.2153)' on 'Beta−1,3−galactosyl−O−glycosyl−glycoprotein beta−1,6−N−acetylglucosami

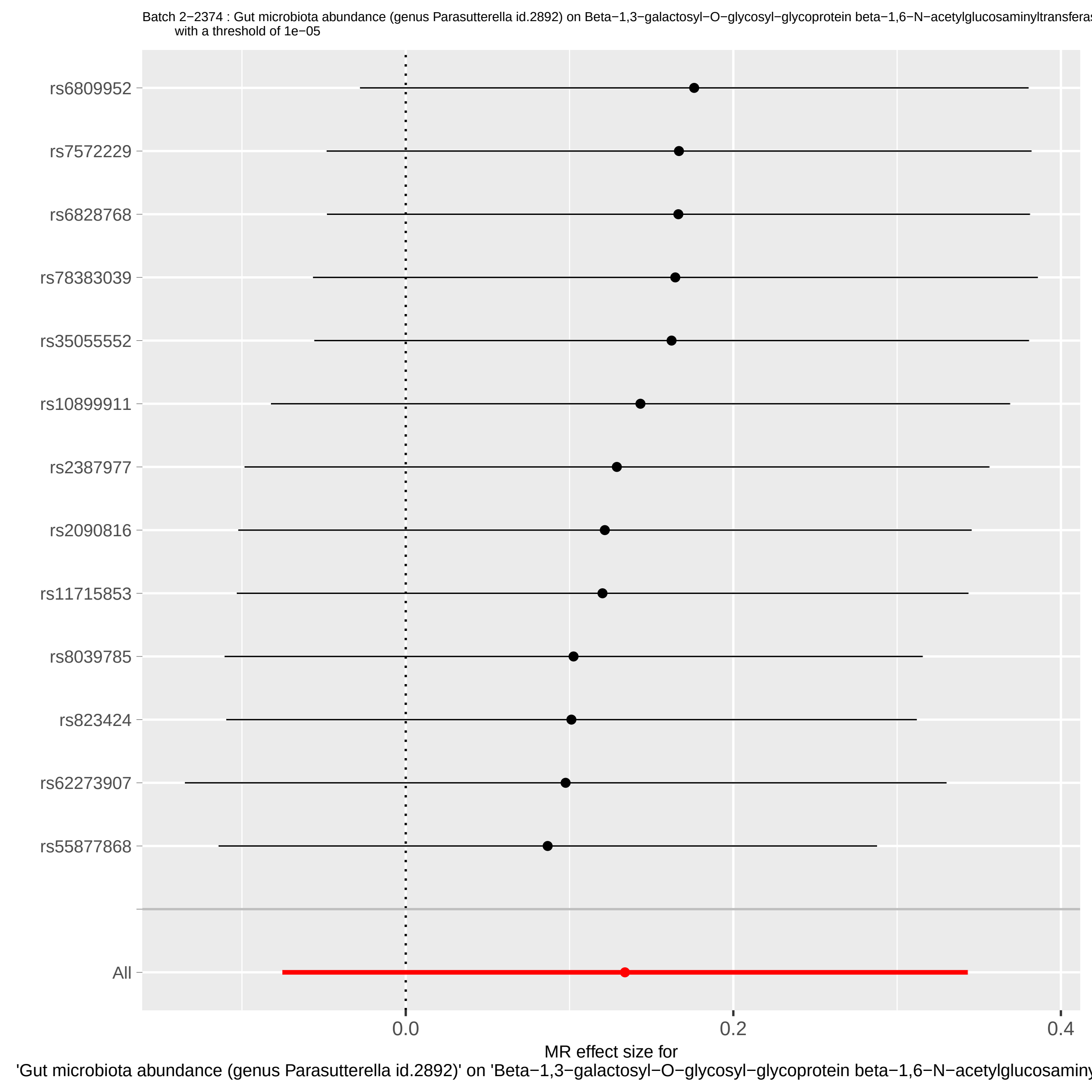
Batch 2−2374 : Gut microbiota abundance (genus Parasutterella id.2892) on Beta−1,3−galactosyl−O−glycosyl−glycoprotein beta−1,6−N−acetylglucosaminyltransfera
with a threshold of 1e−05
rs6809952
rs7572229
rs6828768
rs78383039
rs35055552
rs10899911
rs2387977
rs2090816
rs11715853
rs8039785
rs823424
rs62273907
rs55877868
All
0.0
0.2
0.4
MR effect size for
'Gut microbiota abundance (genus Parasutterella id.2892)' on 'Beta−1,3−galactosyl−O−glycosyl−glycoprotein beta−1,6−N−acetylglucosaminy

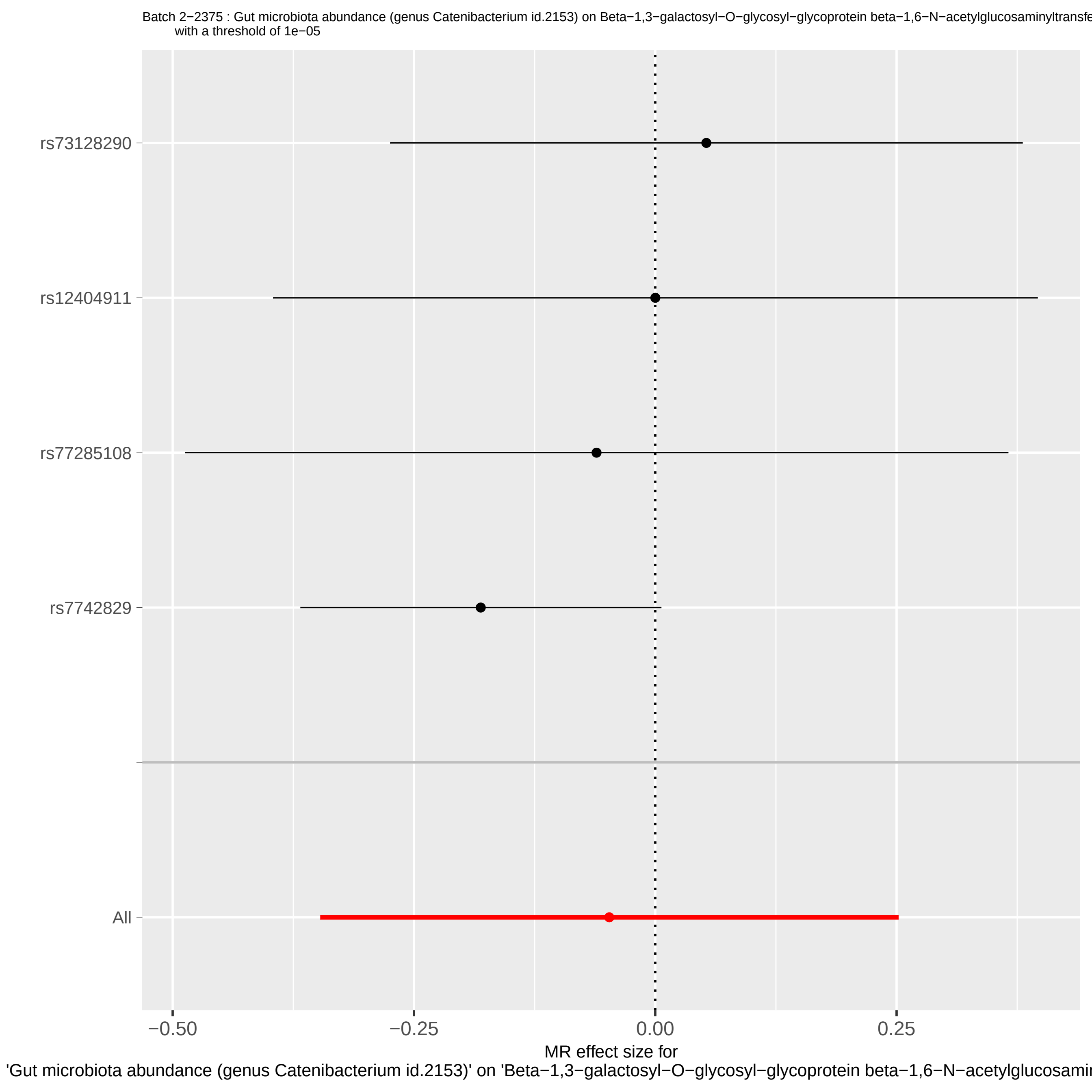

Batch 2−2375 : Gut microbiota abundance (genus Catenibacterium id.2153) on Beta−1,3−galactosyl−O−glycosyl−glycoprotein beta−1,6−N−acetylglucosaminyltransfe
with a threshold of 1e−05
rs73128290
rs12404911
rs77285108
rs7742829
All
−0.50
−0.25
0.00
0.25
MR effect size for
'Gut microbiota abundance (genus Catenibacterium id.2153)' on 'Beta−1,3−galactosyl−O−glycosyl−glycoprotein beta−1,6−N−acetylglucosami

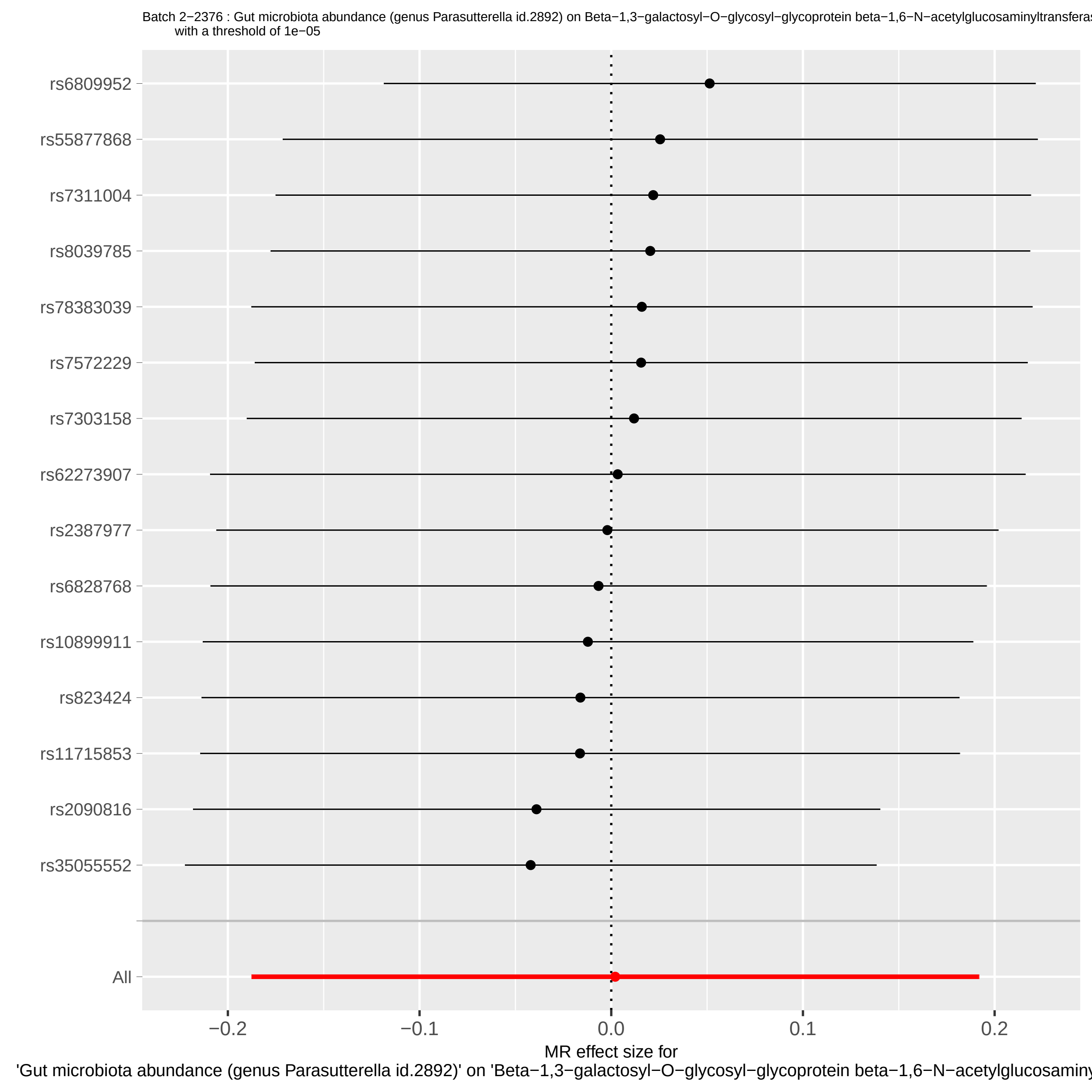

Batch 2−2376 : Gut microbiota abundance (genus Parasutterella id.2892) on Beta−1,3−galactosyl−O−glycosyl−glycoprotein beta−1,6−N−acetylglucosaminyltransferas
with a threshold of 1e−05
rs6809952
rs55877868
rs7311004
rs8039785
rs78383039
rs7572229
rs7303158
rs62273907
rs2387977
rs6828768
rs10899911
rs823424
rs11715853
rs2090816
rs35055552
All
−0.2
−0.1
0.0
0.1
0.2
MR effect size for
'Gut microbiota abundance (genus Parasutterella id.2892)' on 'Beta−1,3−galactosyl−O−glycosyl−glycoprotein beta−1,6−N−acetylglucosaminy

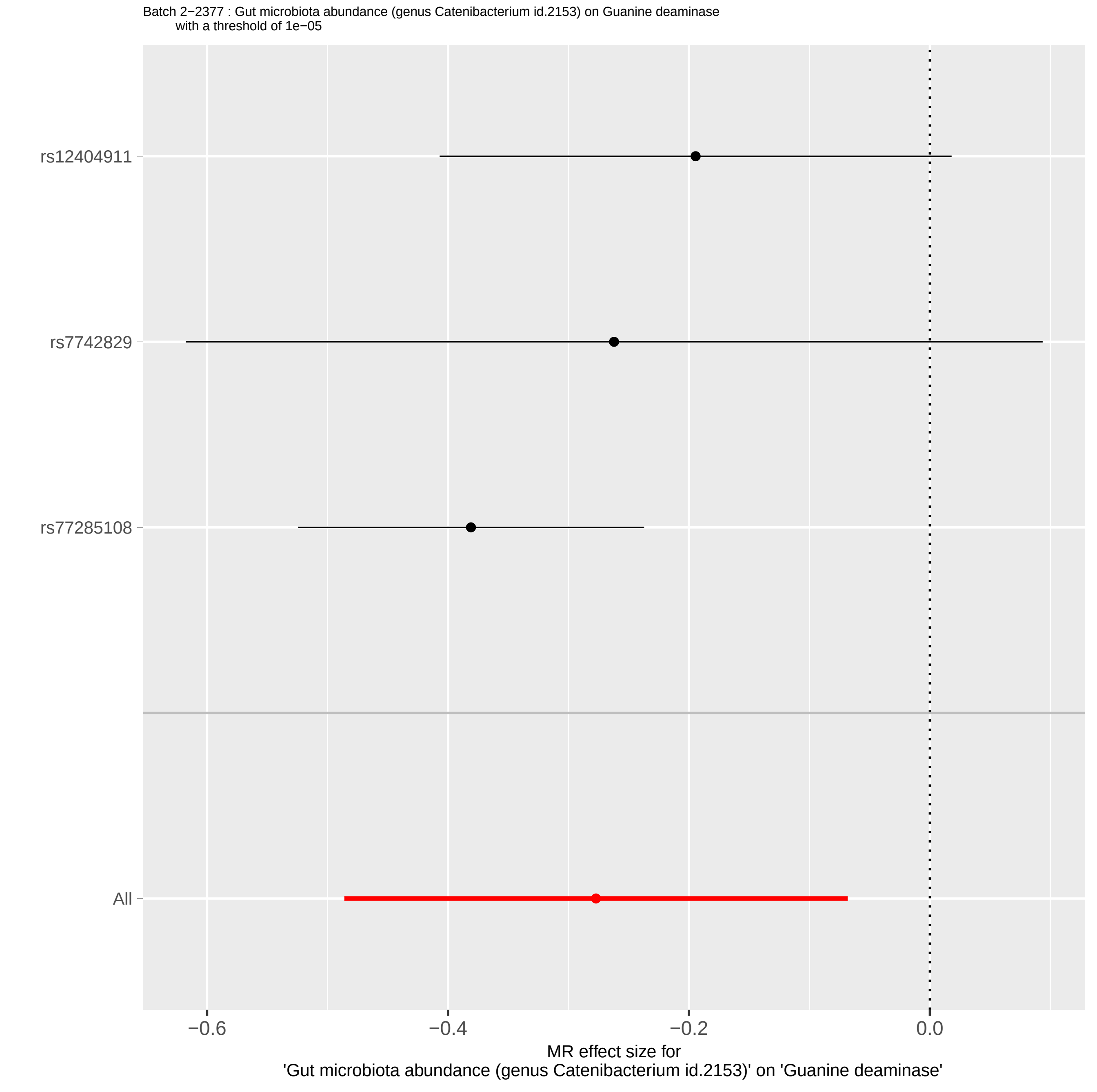

Batch 2−2377 : Gut microbiota abundance (genus Catenibacterium id.2153) on Guanine deaminase
with a threshold of 1e−05
rs12404911
rs7742829
rs77285108
All
−0.6
−0.4
−0.2
0.0
MR effect size for
'Gut microbiota abundance (genus Catenibacterium id.2153)' on 'Guanine deaminase'

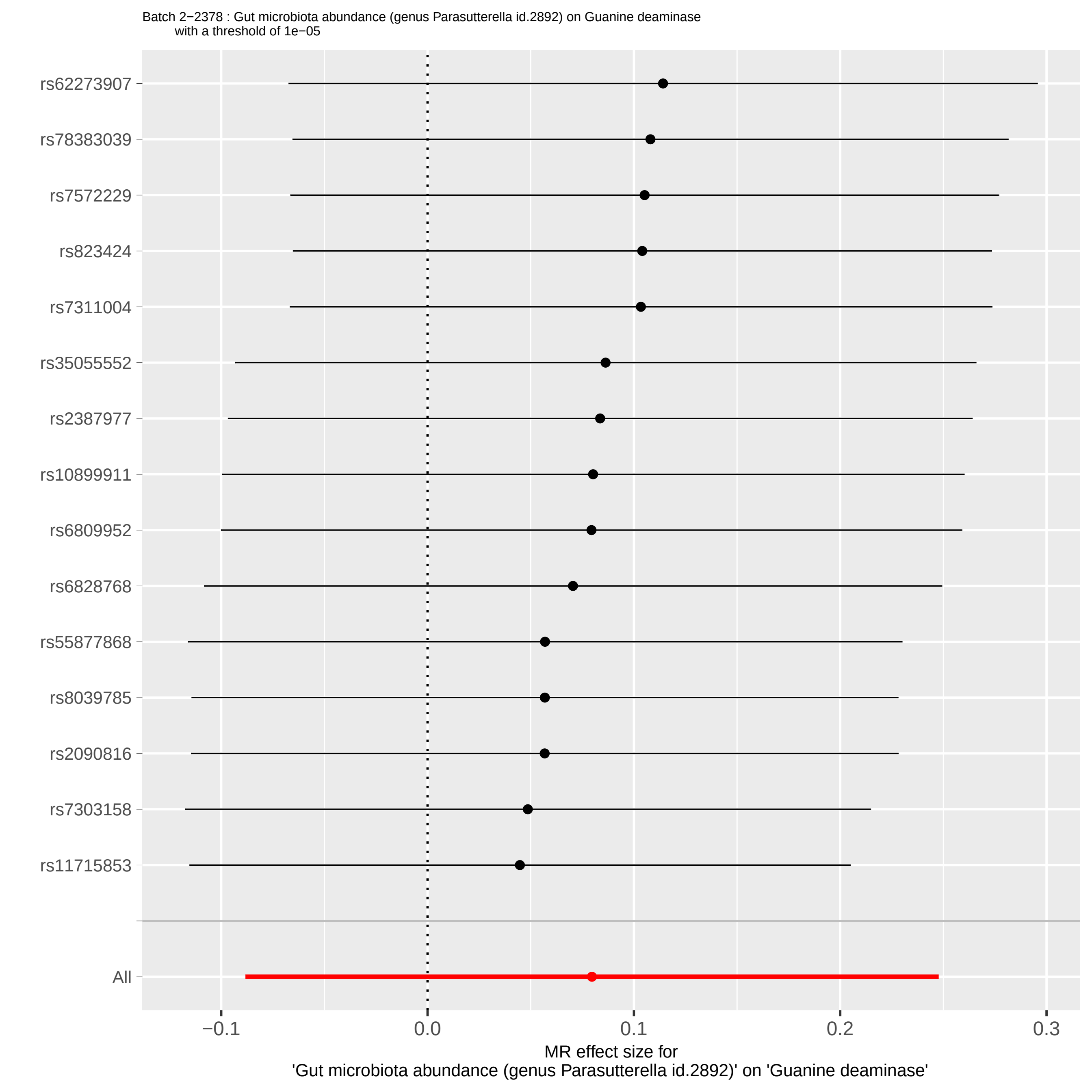

Batch 2−2378 : Gut microbiota abundance (genus Parasutterella id.2892) on Guanine deaminase
with a threshold of 1e−05
rs62273907
rs78383039
rs7572229
rs823424
rs7311004
rs35055552
rs2387977
rs10899911
rs6809952
rs6828768
rs55877868
rs8039785
rs2090816
rs7303158
rs11715853
All
−0.1
0.0
0.1
0.2
0.3
MR effect size for
'Gut microbiota abundance (genus Parasutterella id.2892)' on 'Guanine deaminase'

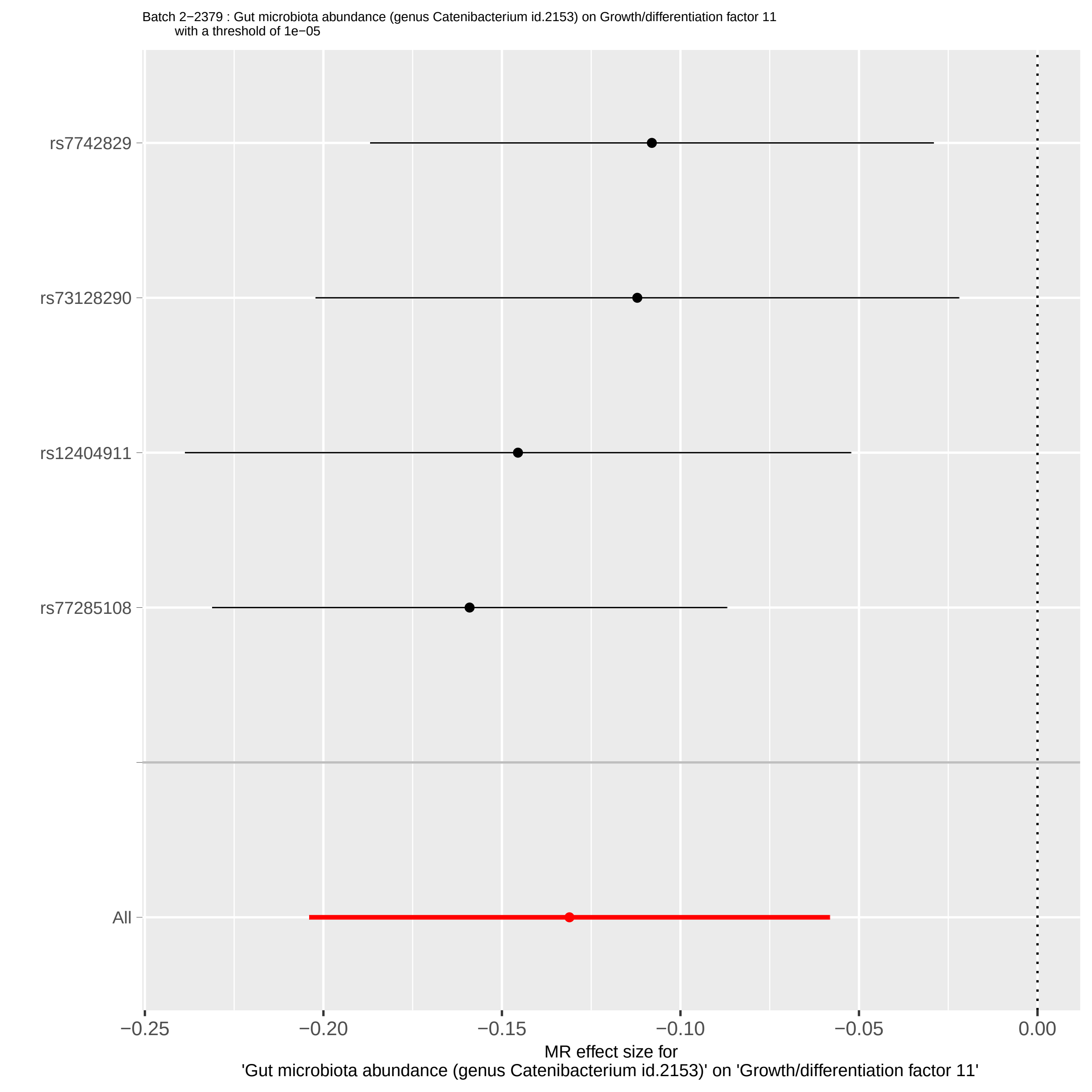
Batch 2−2379 : Gut microbiota abundance (genus Catenibacterium id.2153) on Growth/differentiation factor 11
with a threshold of 1e−05
rs7742829
rs73128290
rs12404911
rs77285108
All
−0.25
−0.20
−0.15
−0.10
−0.05
0.00
MR effect size for
'Gut microbiota abundance (genus Catenibacterium id.2153)' on 'Growth/differentiation factor 11'

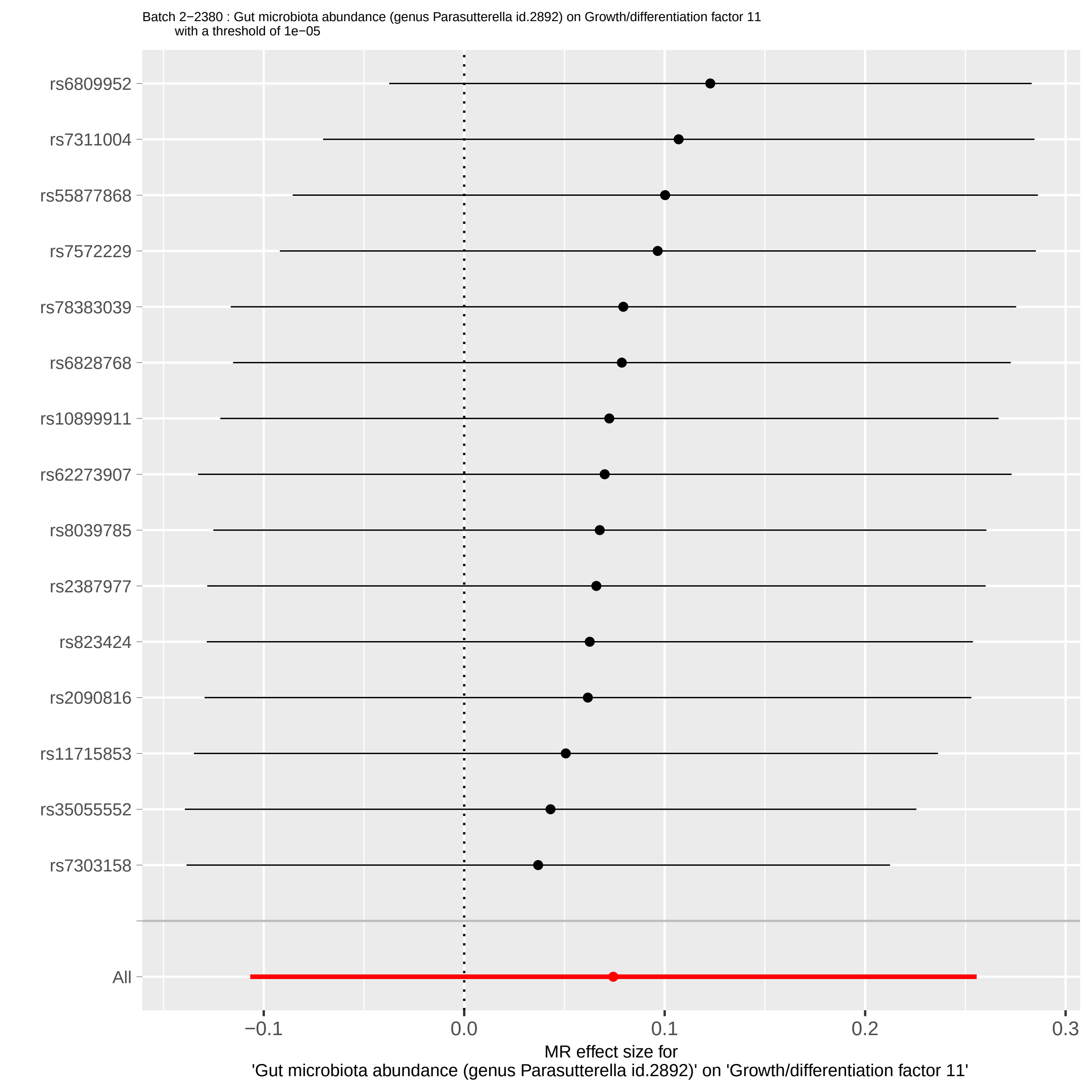
Batch 2−2380 : Gut microbiota abundance (genus Parasutterella id.2892) on Growth/differentiation factor 11
with a threshold of 1e−05
rs6809952
rs7311004
rs55877868
rs7572229
rs78383039
rs6828768
rs10899911
rs62273907
rs8039785
rs2387977
rs823424
rs2090816
rs11715853
rs35055552
rs7303158
All
−0.1
0.0
0.1
0.2
0.3
MR effect size for
'Gut microbiota abundance (genus Parasutterella id.2892)' on 'Growth/differentiation factor 11'

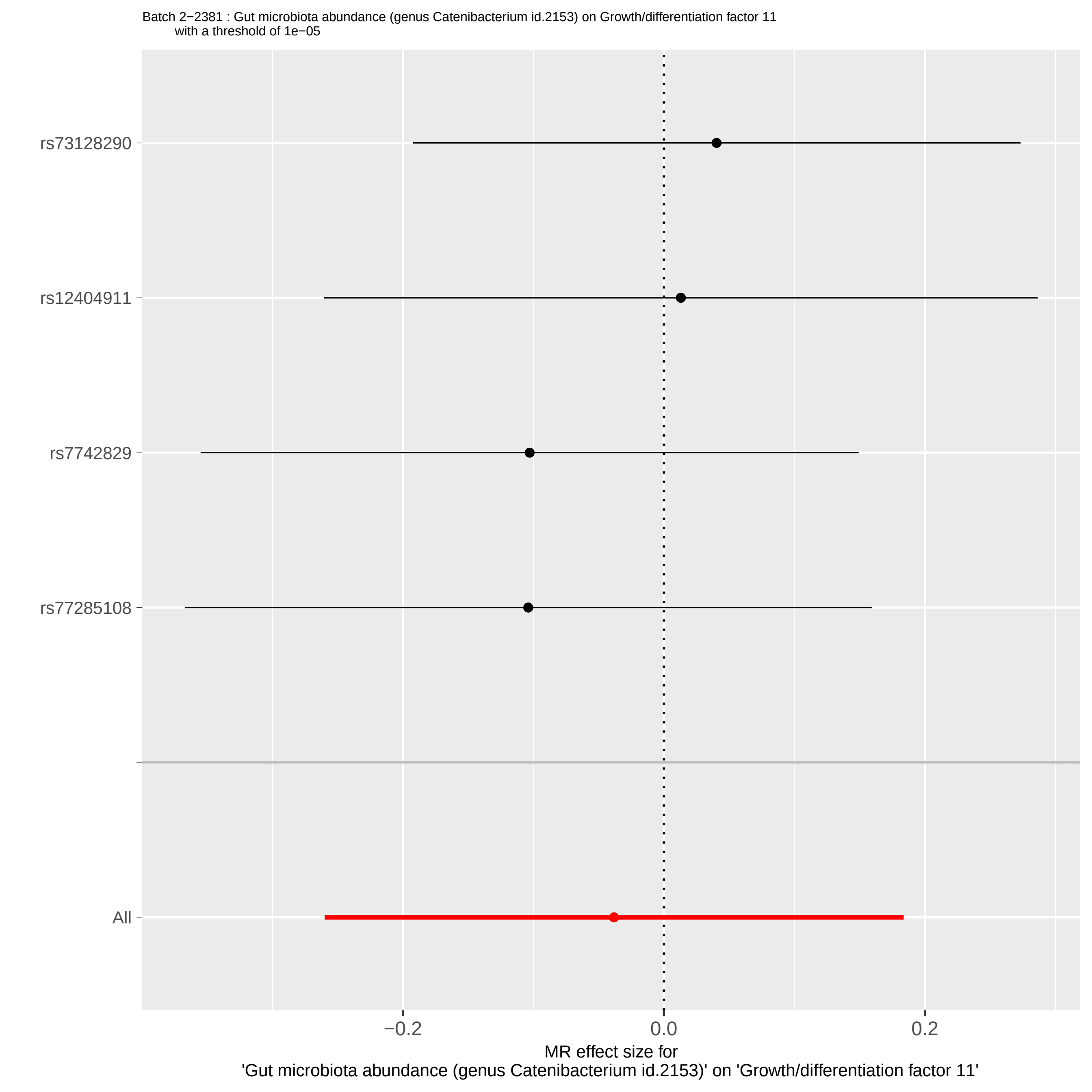
Batch 2−2381 : Gut microbiota abundance (genus Catenibacterium id.2153) on Growth/differentiation factor 11
with a threshold of 1e−05
rs73128290
rs12404911
rs7742829
rs77285108
All
−0.2
0.0
0.2
MR effect size for
'Gut microbiota abundance (genus Catenibacterium id.2153)' on 'Growth/differentiation factor 11'

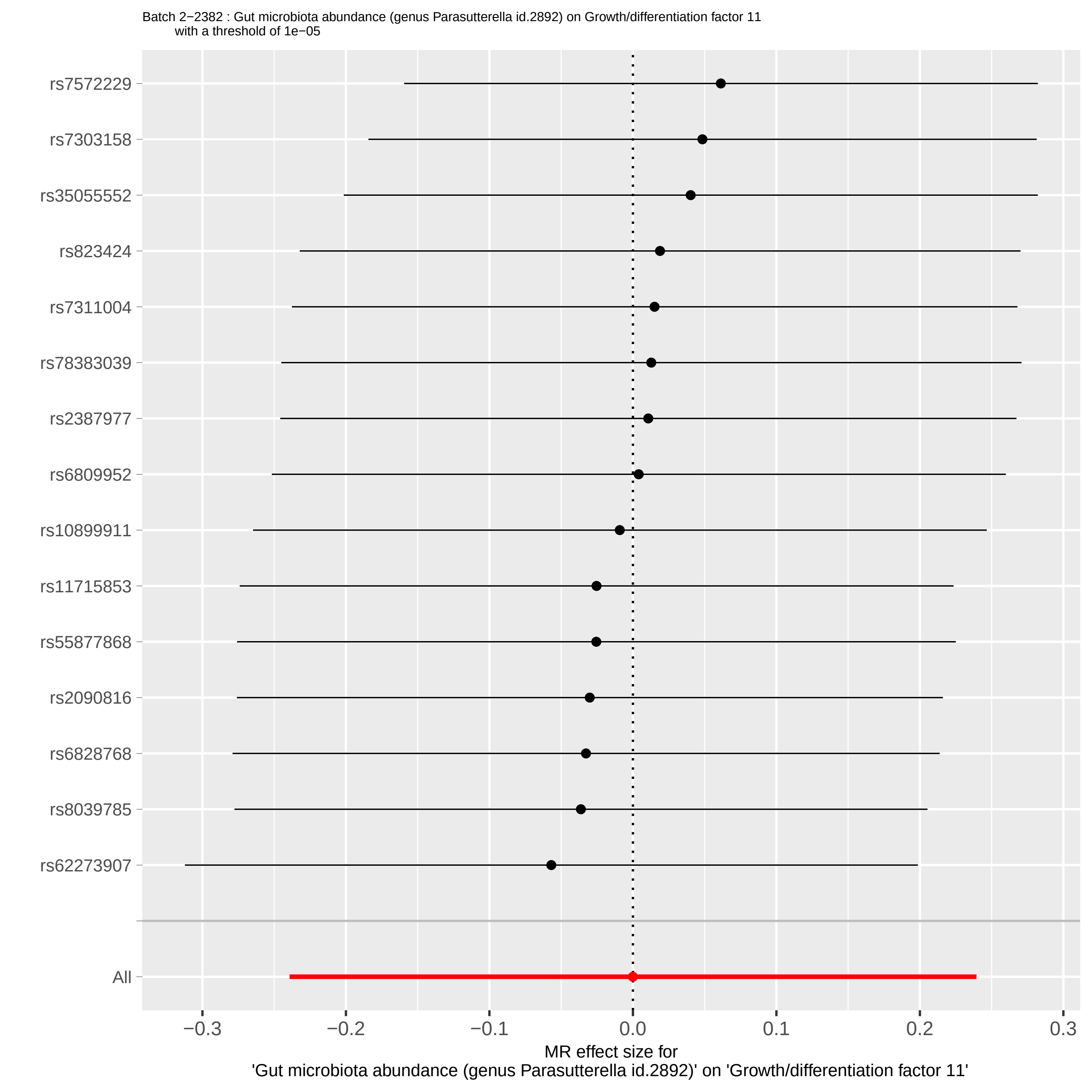
Batch 2−2382 : Gut microbiota abundance (genus Parasutterella id.2892) on Growth/differentiation factor 11
with a threshold of 1e−05
rs7572229
rs7303158
rs35055552
rs823424
rs7311004
rs78383039
rs2387977
rs6809952
rs10899911
rs11715853
rs55877868
rs2090816
rs6828768
rs8039785
rs62273907
All
−0.3
−0.2
−0.1
0.0
0.1
0.2
0.3
MR effect size for
'Gut microbiota abundance (genus Parasutterella id.2892)' on 'Growth/differentiation factor 11'

Batch 2−2383 : Gut microbiota abundance (genus Catenibacterium id.2153) on Growth/differentiation factor 11
with a threshold of 1e−05

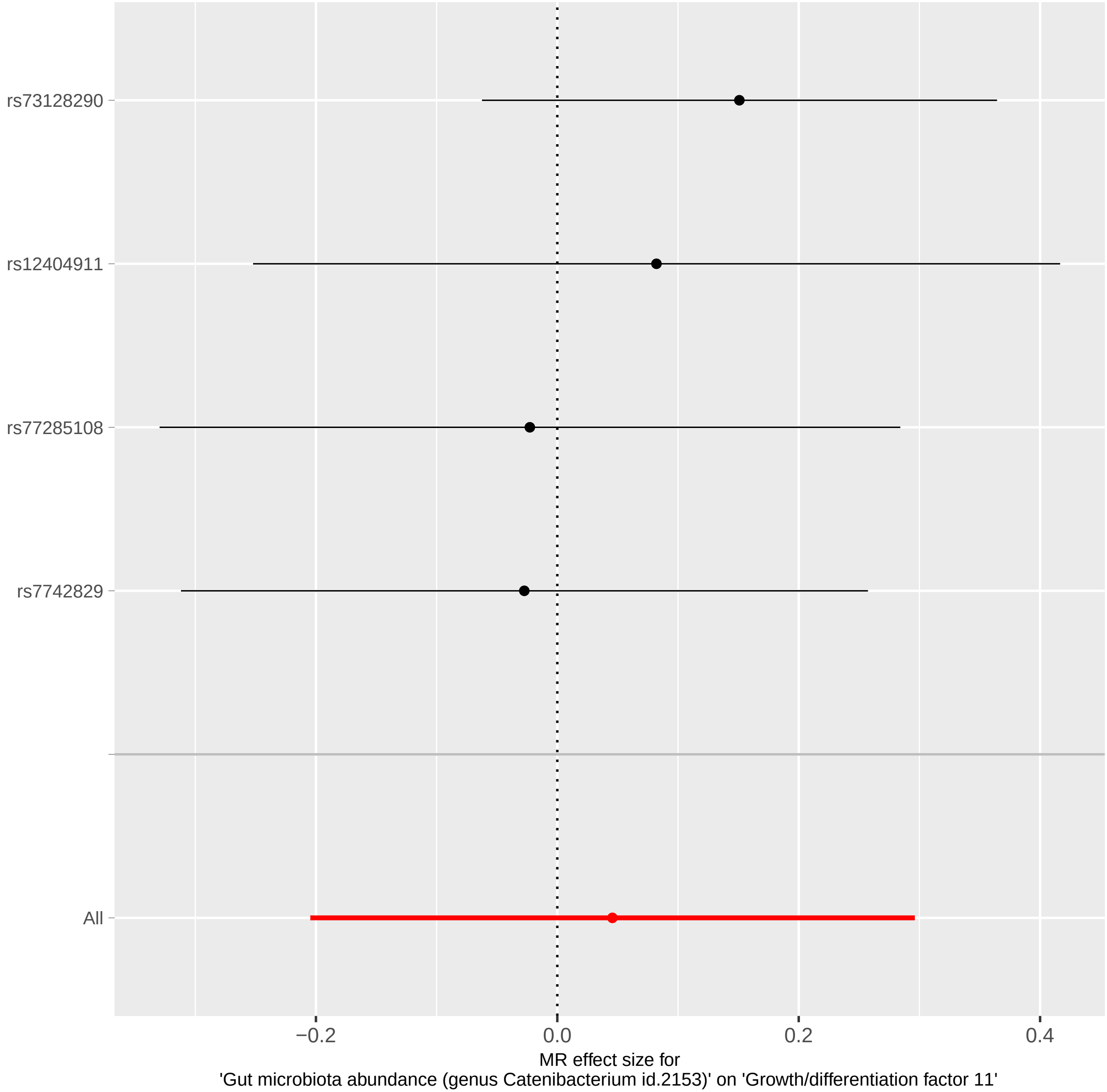

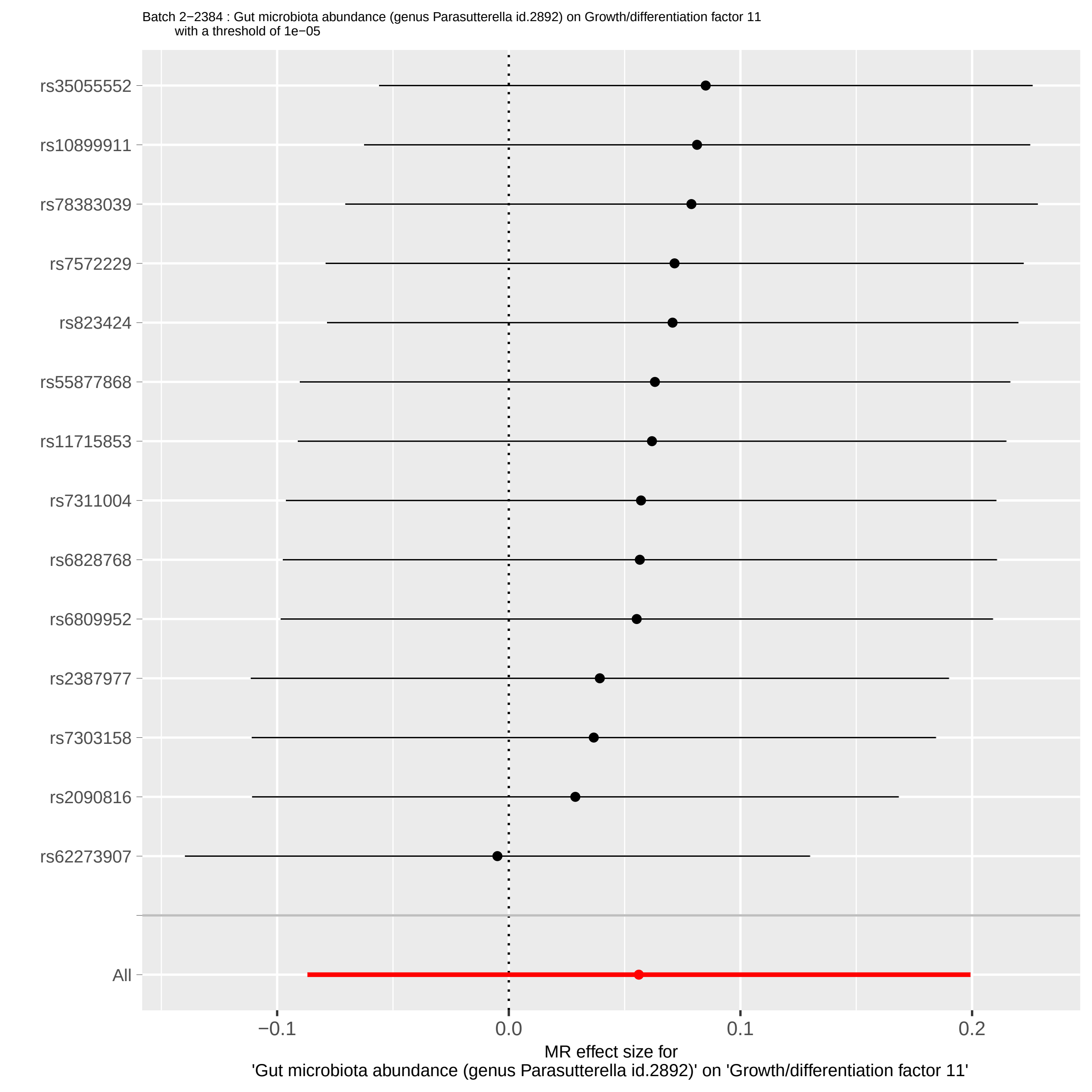

Batch 2−2384 : Gut microbiota abundance (genus Parasutterella id.2892) on Growth/differentiation factor 11
with a threshold of 1e−05
rs35055552
rs10899911
rs78383039
rs7572229
rs823424
rs55877868
rs11715853
rs7311004
rs6828768
rs6809952
rs2387977
rs7303158
rs2090816
rs62273907
All
−0.1
0.0
0.1
0.2
MR effect size for
'Gut microbiota abundance (genus Parasutterella id.2892)' on 'Growth/differentiation factor 11'

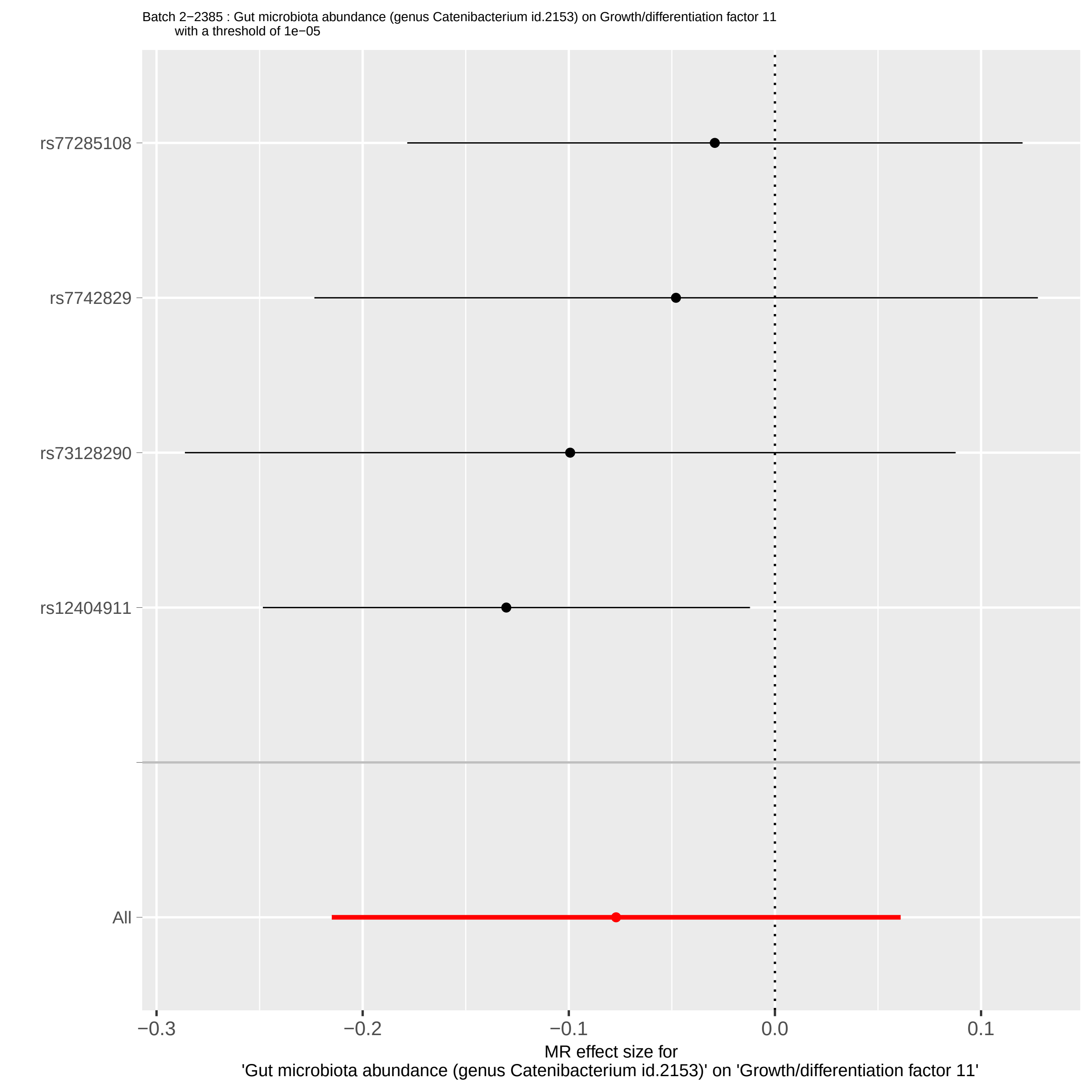
Batch 2−2385 : Gut microbiota abundance (genus Catenibacterium id.2153) on Growth/differentiation factor 11
with a threshold of 1e−05
rs77285108
rs7742829
rs73128290
rs12404911
All
−0.3
−0.2
−0.1
0.0
0.1
MR effect size for
'Gut microbiota abundance (genus Catenibacterium id.2153)' on 'Growth/differentiation factor 11'

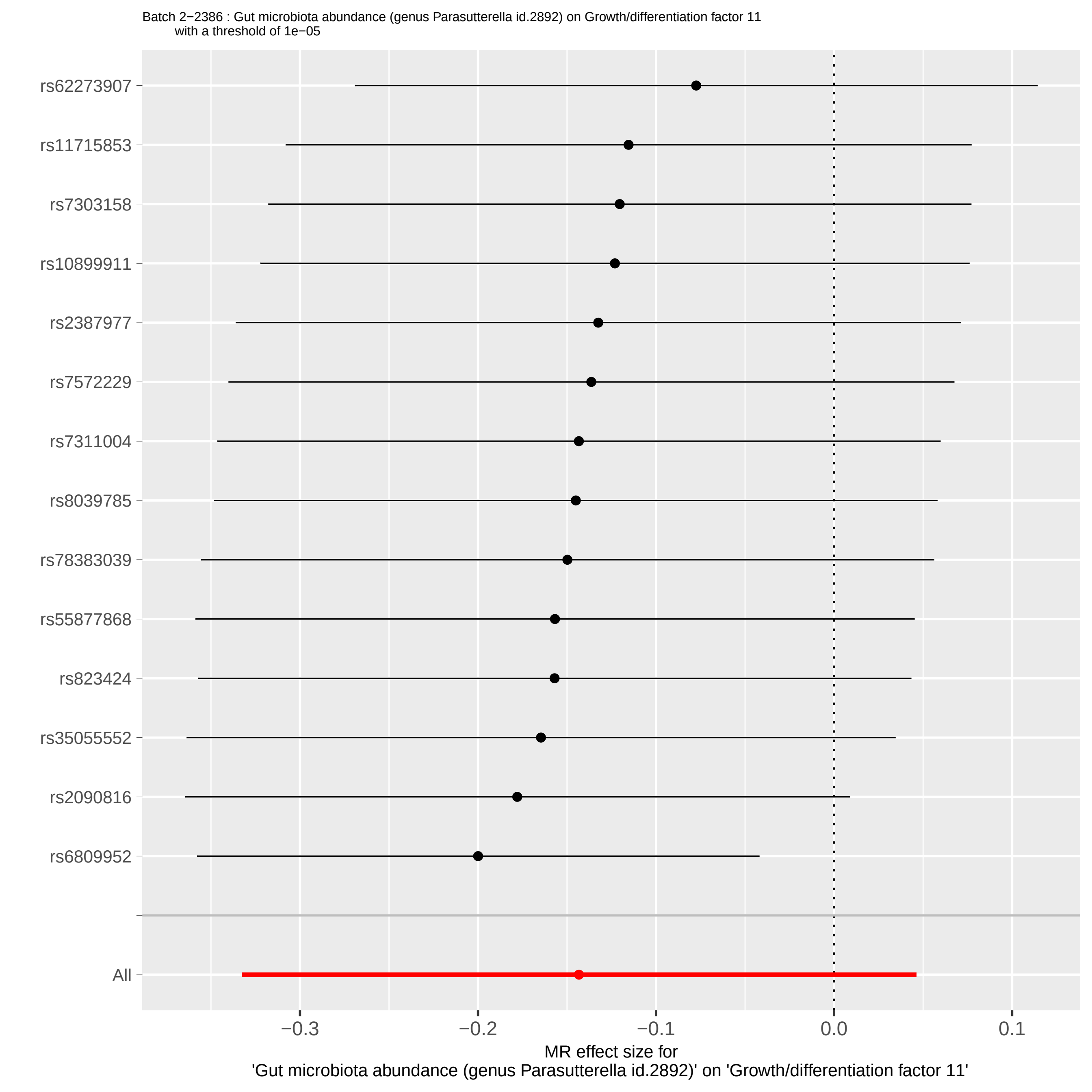

Batch 2−2386 : Gut microbiota abundance (genus Parasutterella id.2892) on Growth/differentiation factor 11
with a threshold of 1e−05
rs62273907
rs11715853
rs7303158
rs10899911
rs2387977
rs7572229
rs7311004
rs8039785
rs78383039
rs55877868
rs823424
rs35055552
rs2090816
rs6809952
All
−0.3
−0.2
−0.1
0.0
0.1
MR effect size for
'Gut microbiota abundance (genus Parasutterella id.2892)' on 'Growth/differentiation factor 11'

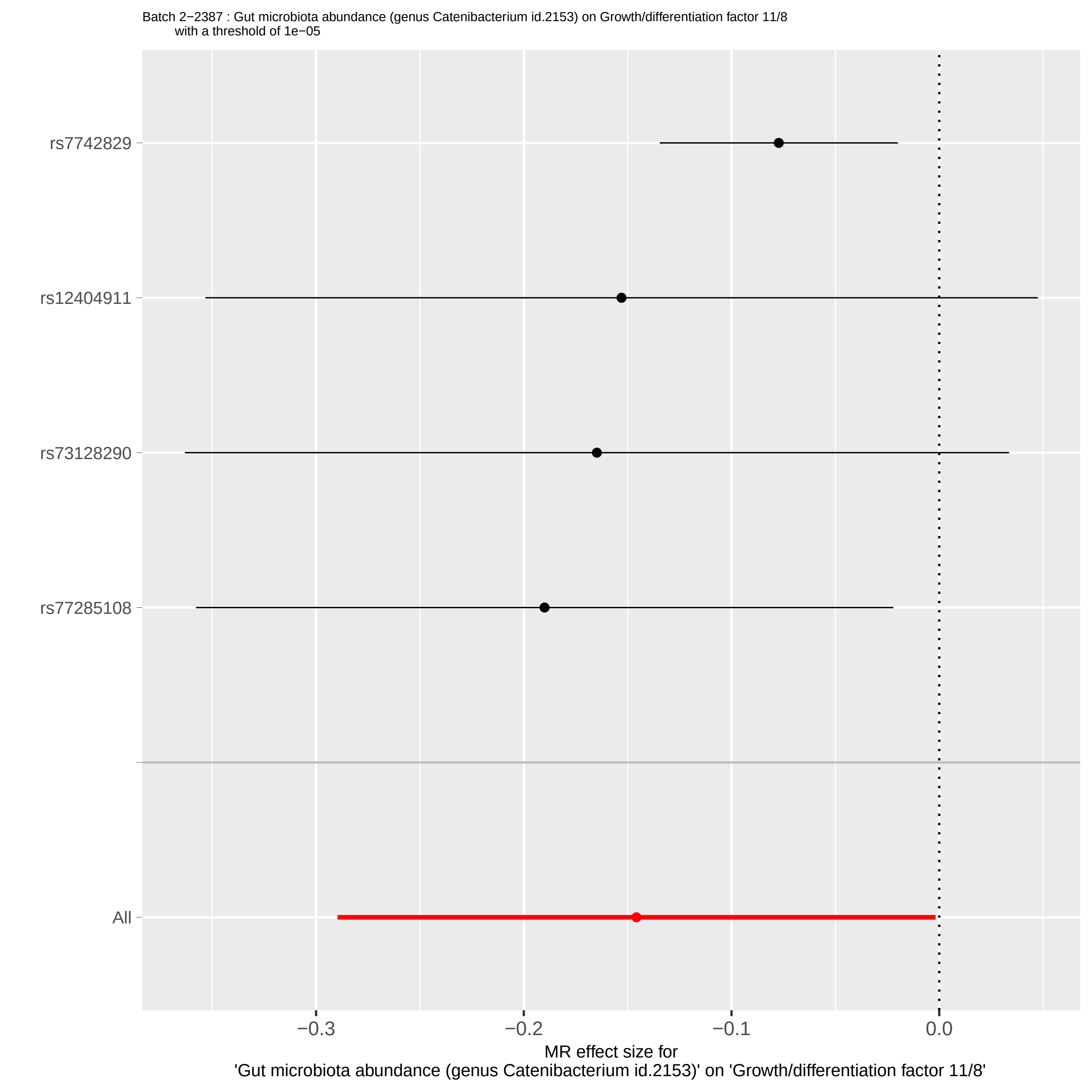

Batch 2−2387 : Gut microbiota abundance (genus Catenibacterium id.2153) on Growth/differentiation factor 11/8
with a threshold of 1e−05
rs7742829
rs12404911
rs73128290
rs77285108
All
−0.3
−0.2
−0.1
0.0
MR effect size for
'Gut microbiota abundance (genus Catenibacterium id.2153)' on 'Growth/differentiation factor 11/8'

Batch 2−2388 : Gut microbiota abundance (genus Parasutterella id.2892) on Growth/differentiation factor 11/8
with a threshold of 1e−05

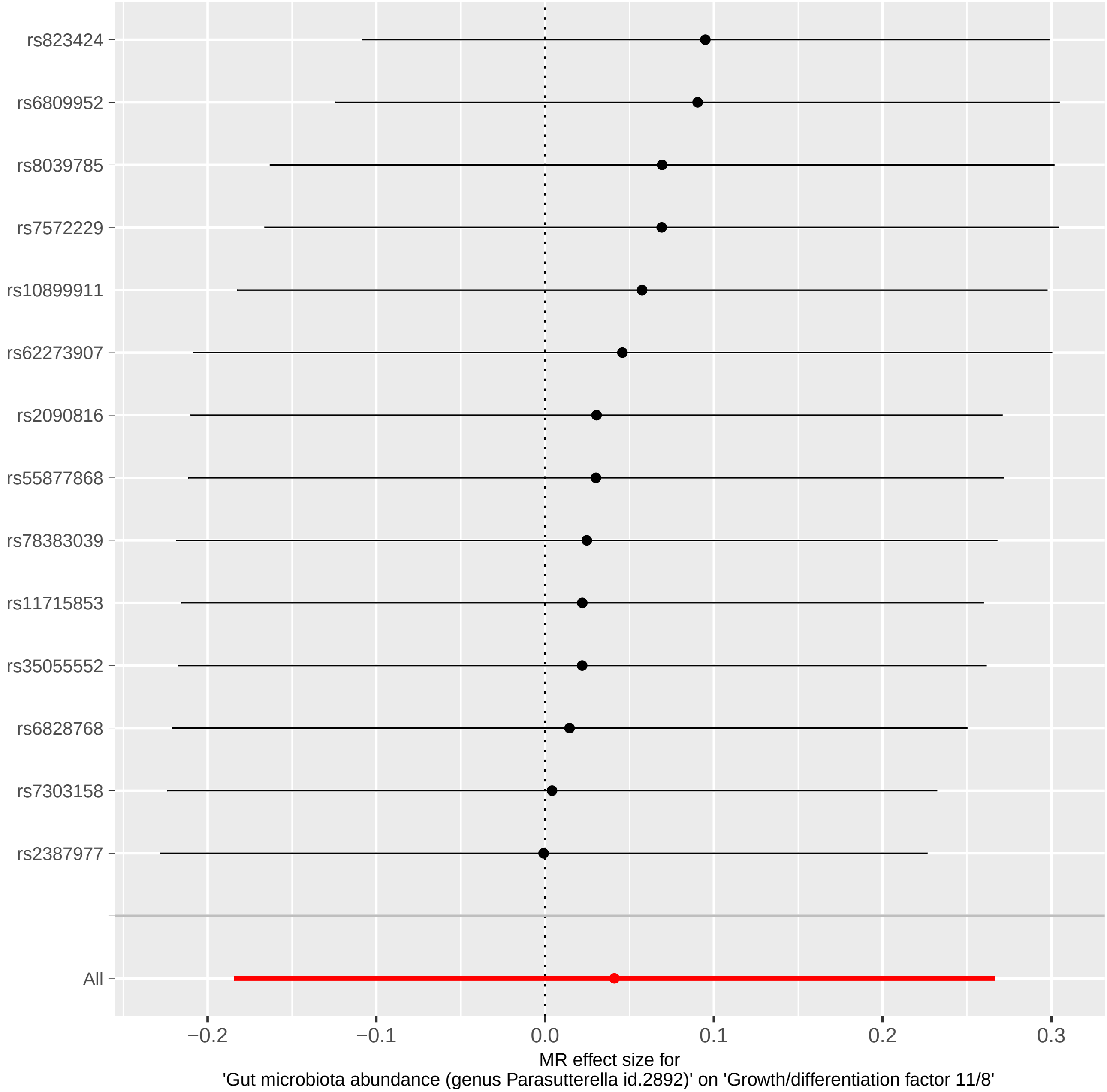

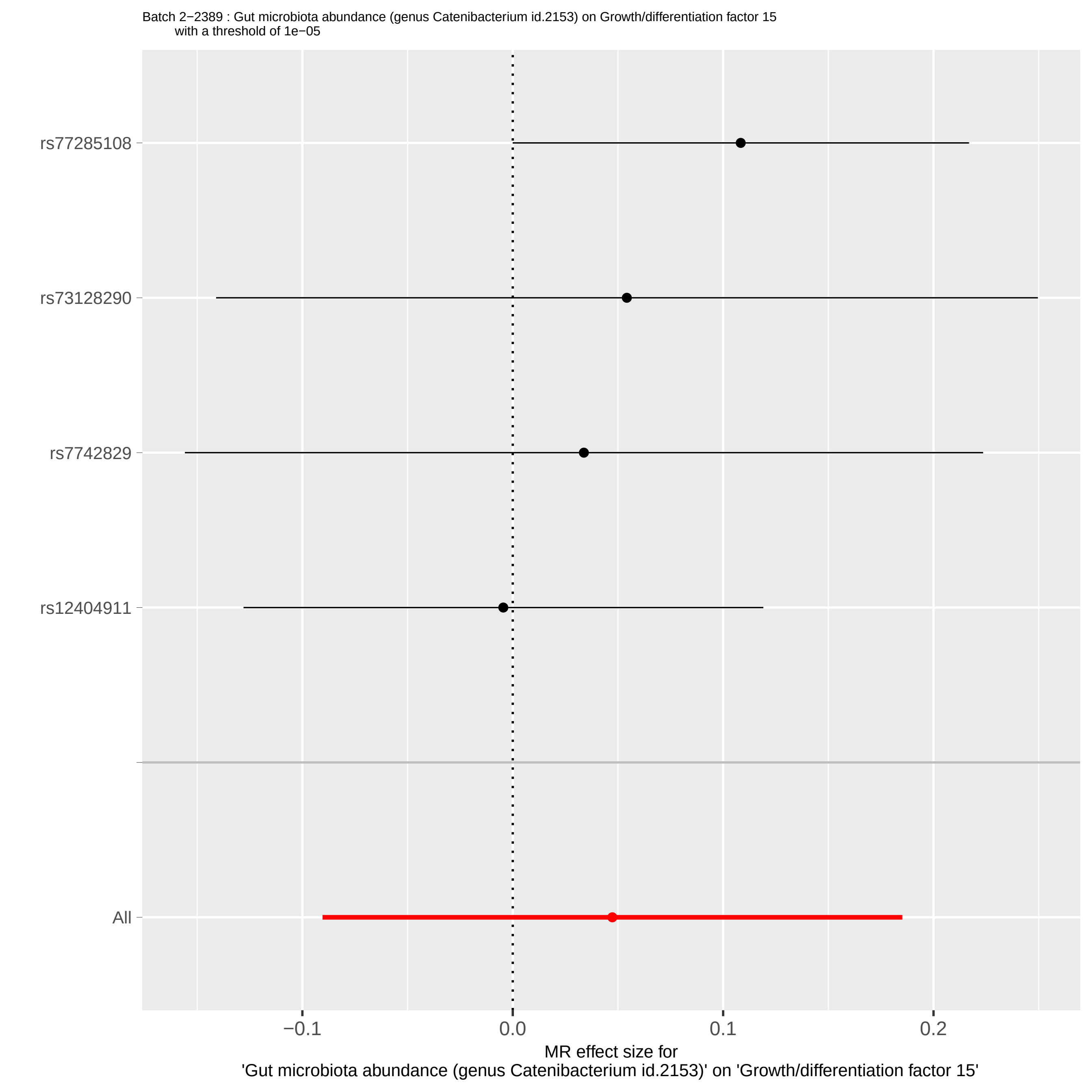
Batch 2−2389 : Gut microbiota abundance (genus Catenibacterium id.2153) on Growth/differentiation factor 15
with a threshold of 1e−05
rs77285108
rs73128290
rs7742829
rs12404911
All
−0.1
0.0
0.1
0.2
MR effect size for
'Gut microbiota abundance (genus Catenibacterium id.2153)' on 'Growth/differentiation factor 15'

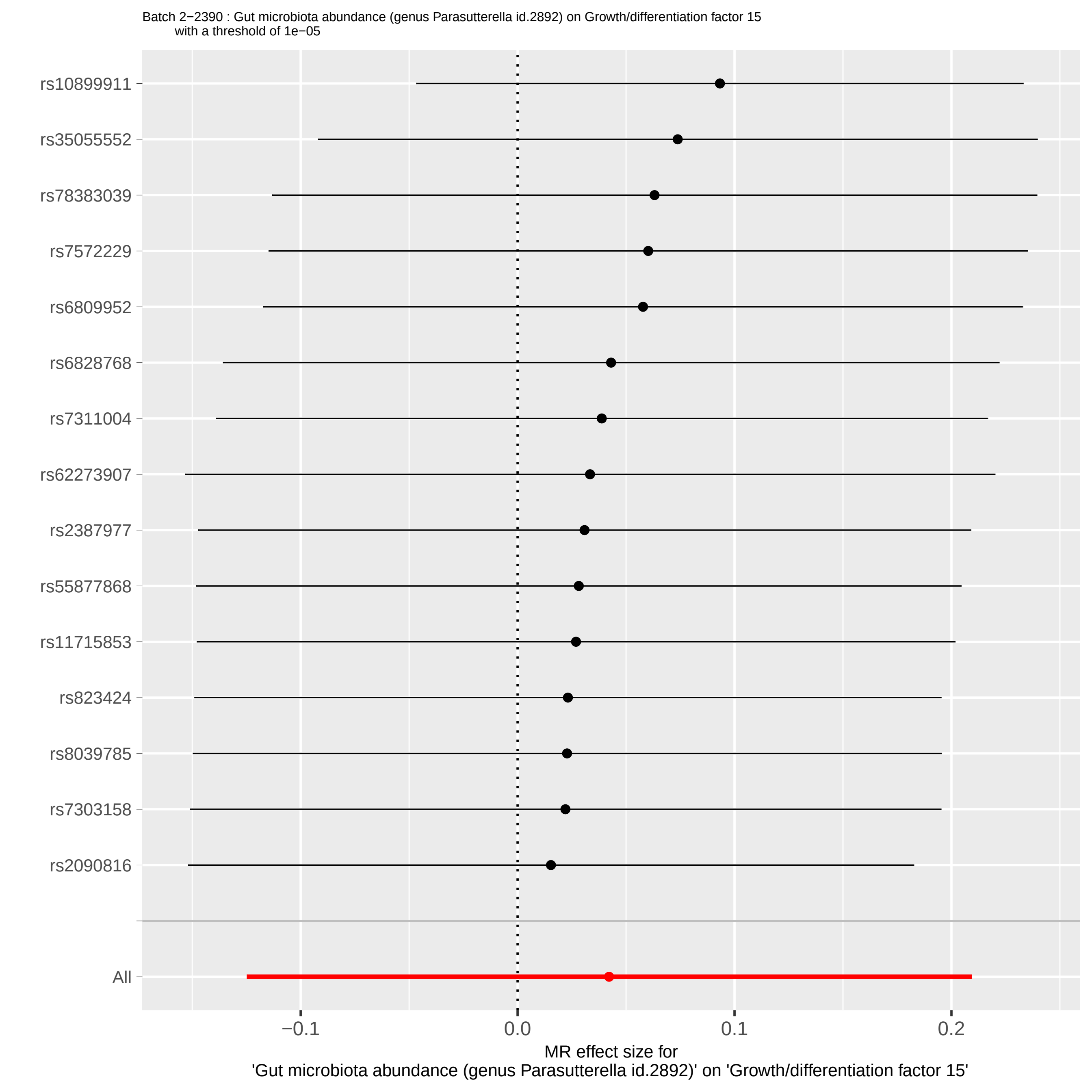

Batch 2−2390 : Gut microbiota abundance (genus Parasutterella id.2892) on Growth/differentiation factor 15
with a threshold of 1e−05
rs10899911
rs35055552
rs78383039
rs7572229
rs6809952
rs6828768
rs7311004
rs62273907
rs2387977
rs55877868
rs11715853
rs823424
rs8039785
rs7303158
rs2090816
All
−0.1
0.0
0.1
0.2
MR effect size for
'Gut microbiota abundance (genus Parasutterella id.2892)' on 'Growth/differentiation factor 15'

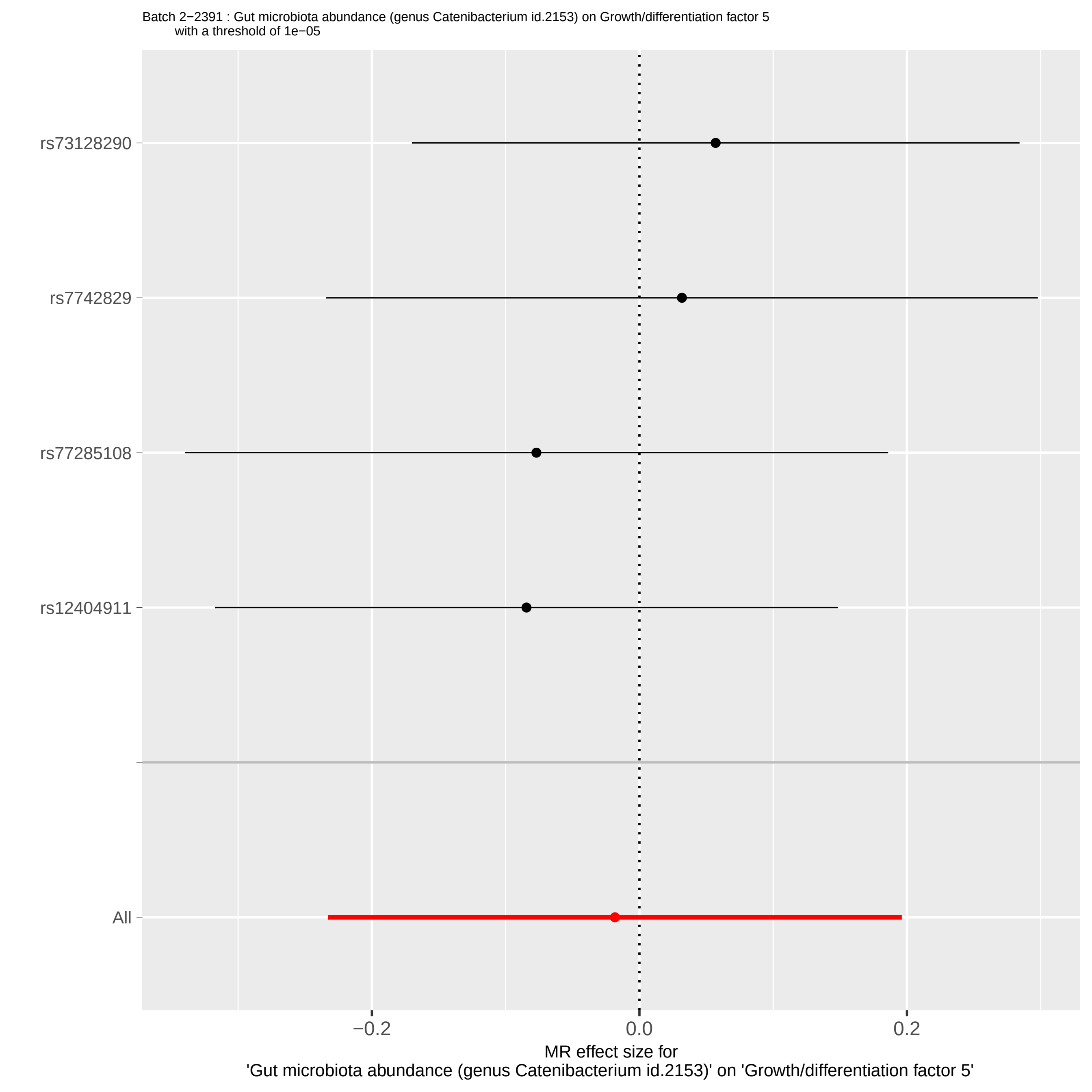
Batch 2−2391 : Gut microbiota abundance (genus Catenibacterium id.2153) on Growth/differentiation factor 5
with a threshold of 1e−05
rs73128290
rs7742829
rs77285108
rs12404911
All
−0.2
0.0
0.2
MR effect size for
'Gut microbiota abundance (genus Catenibacterium id.2153)' on 'Growth/differentiation factor 5'

Batch 2−2392 : Gut microbiota abundance (genus Parasutterella id.2892) on Growth/differentiation factor 5
with a threshold of 1e−05

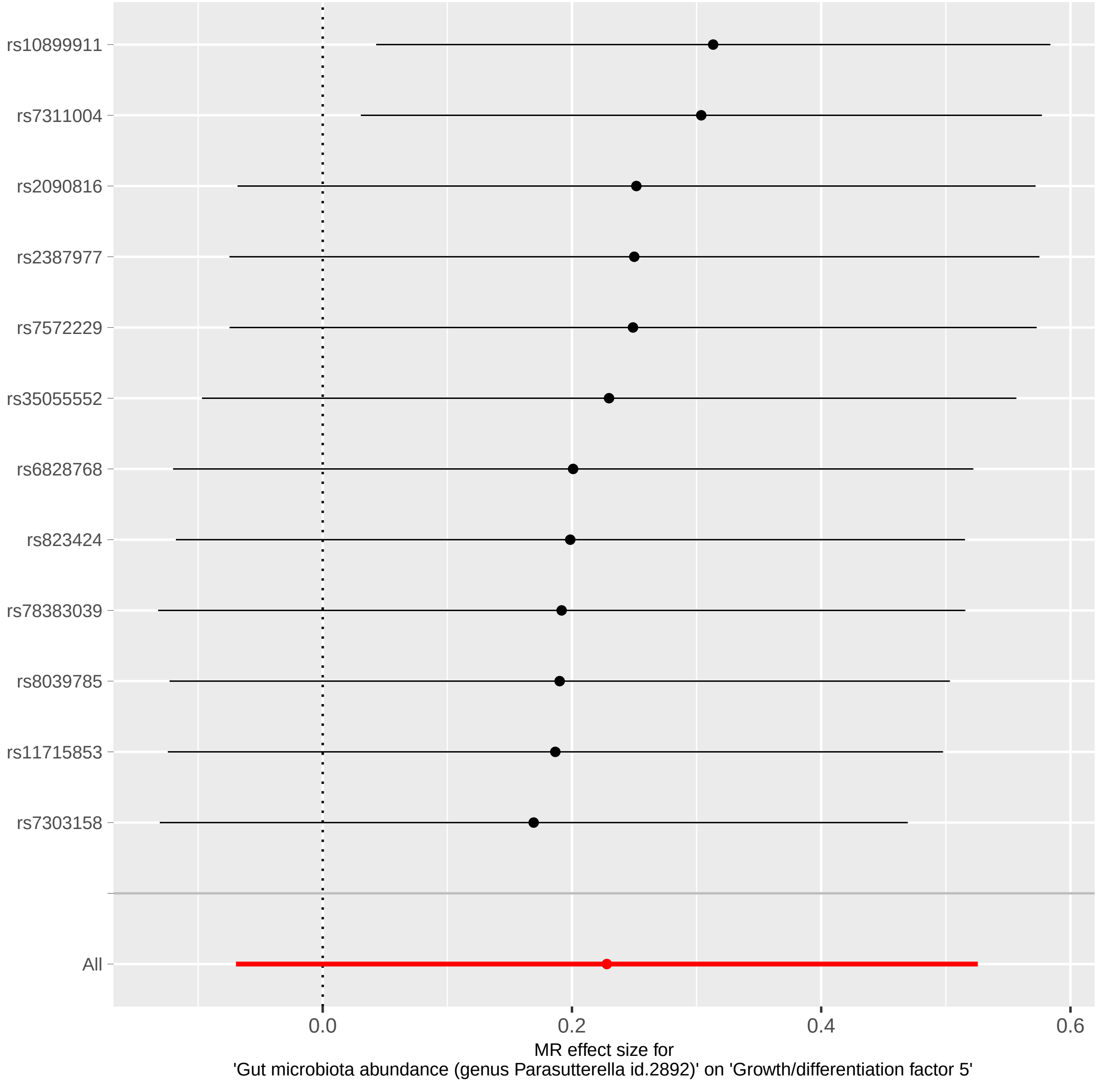

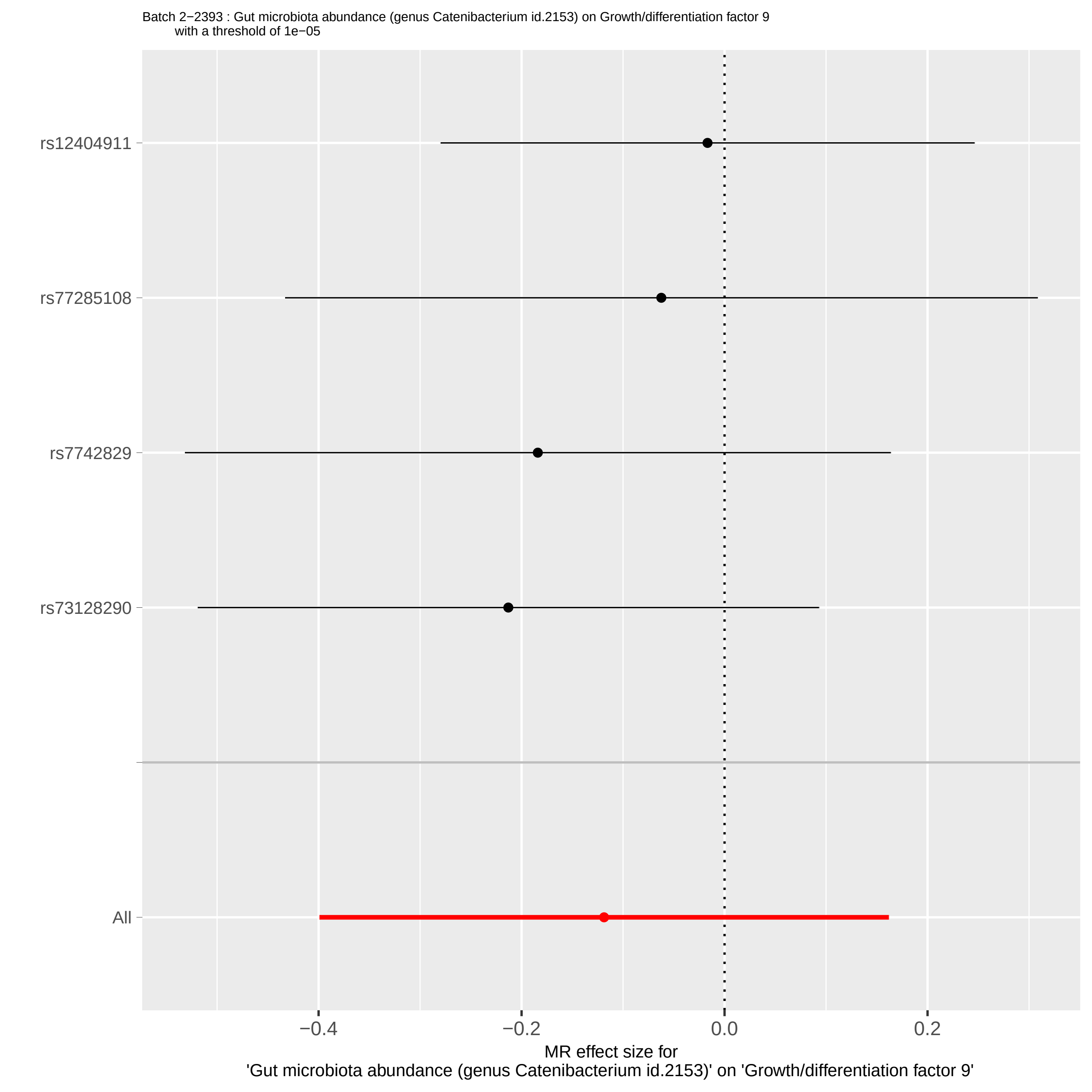
Batch 2−2393 : Gut microbiota abundance (genus Catenibacterium id.2153) on Growth/differentiation factor 9
with a threshold of 1e−05
rs12404911
rs77285108
rs7742829
rs73128290
All
−0.4
−0.2
0.0
0.2
MR effect size for
'Gut microbiota abundance (genus Catenibacterium id.2153)' on 'Growth/differentiation factor 9'

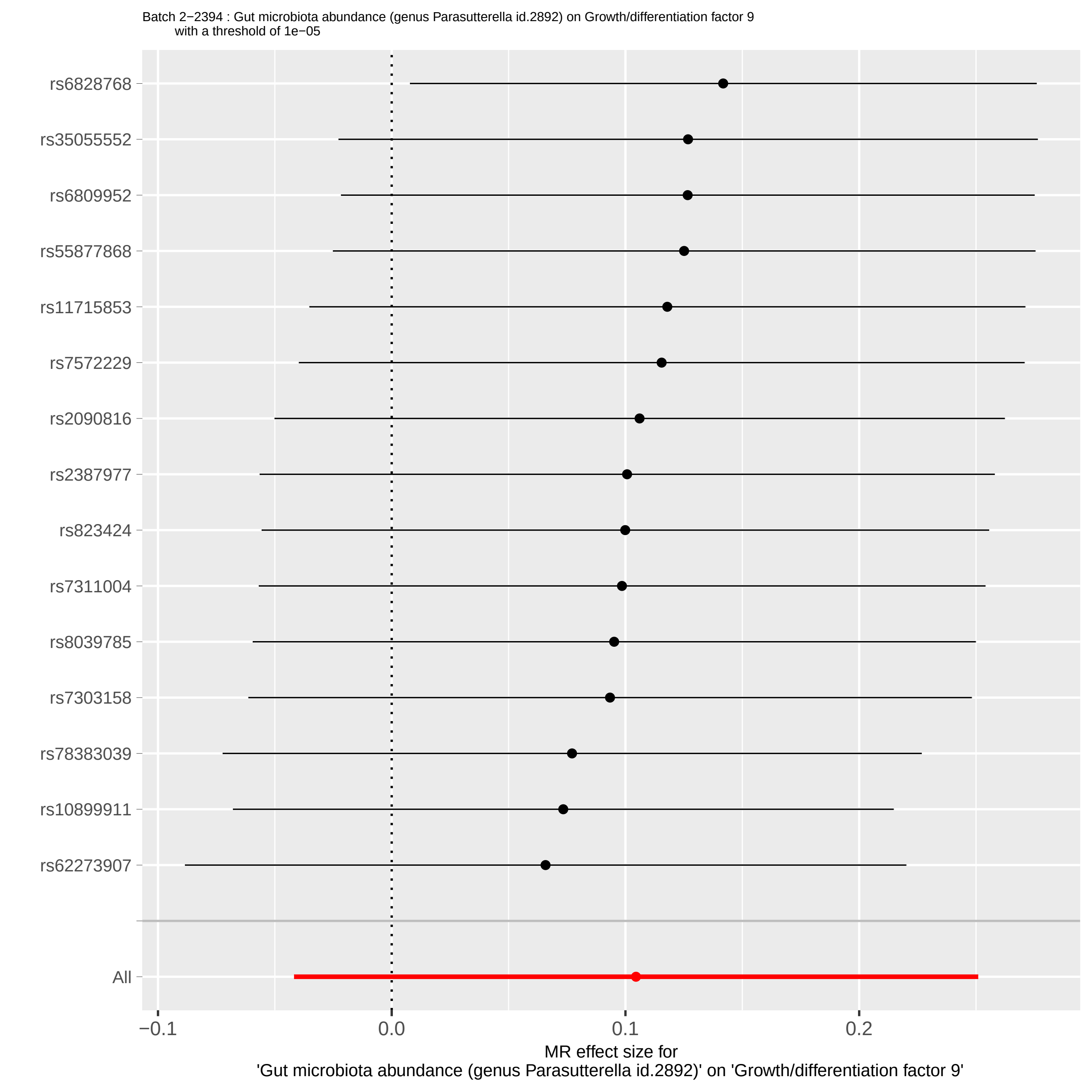
Batch 2−2394 : Gut microbiota abundance (genus Parasutterella id.2892) on Growth/differentiation factor 9
with a threshold of 1e−05
rs6828768
rs35055552
rs6809952
rs55877868
rs11715853
rs7572229
rs2090816
rs2387977
rs823424
rs7311004
rs8039785
rs7303158
rs78383039
rs10899911
rs62273907
All
−0.1
0.0
0.1
0.2
MR effect size for
'Gut microbiota abundance (genus Parasutterella id.2892)' on 'Growth/differentiation factor 9'

Batch 2−2395 : Gut microbiota abundance (genus Catenibacterium id.2153) on Glial cell line−derived neurotrophic factor
with a threshold of 1e−05

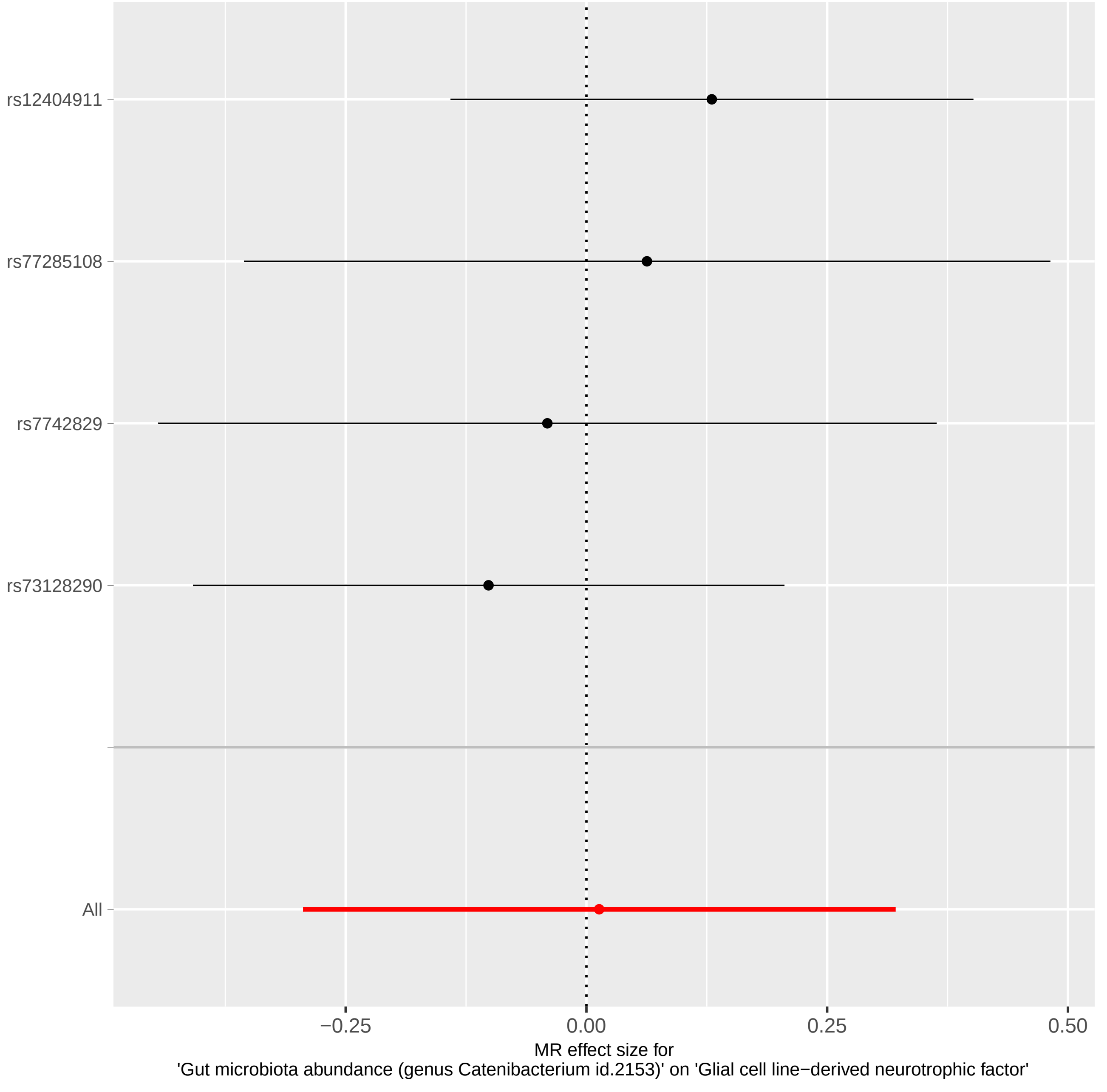

Batch 2−2396 : Gut microbiota abundance (genus Parasutterella id.2892) on Glial cell line−derived neurotrophic factor
with a threshold of 1e−05

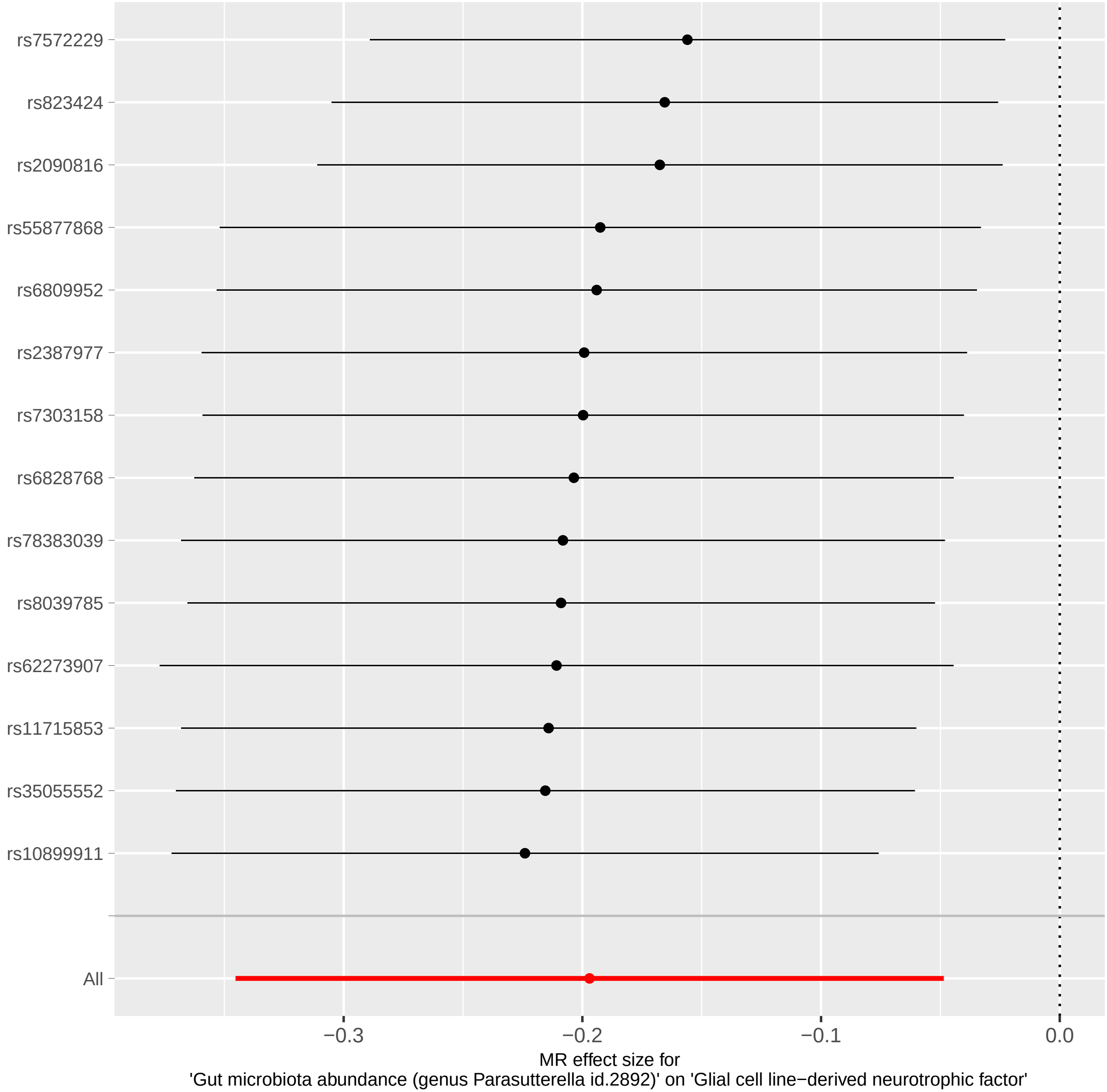

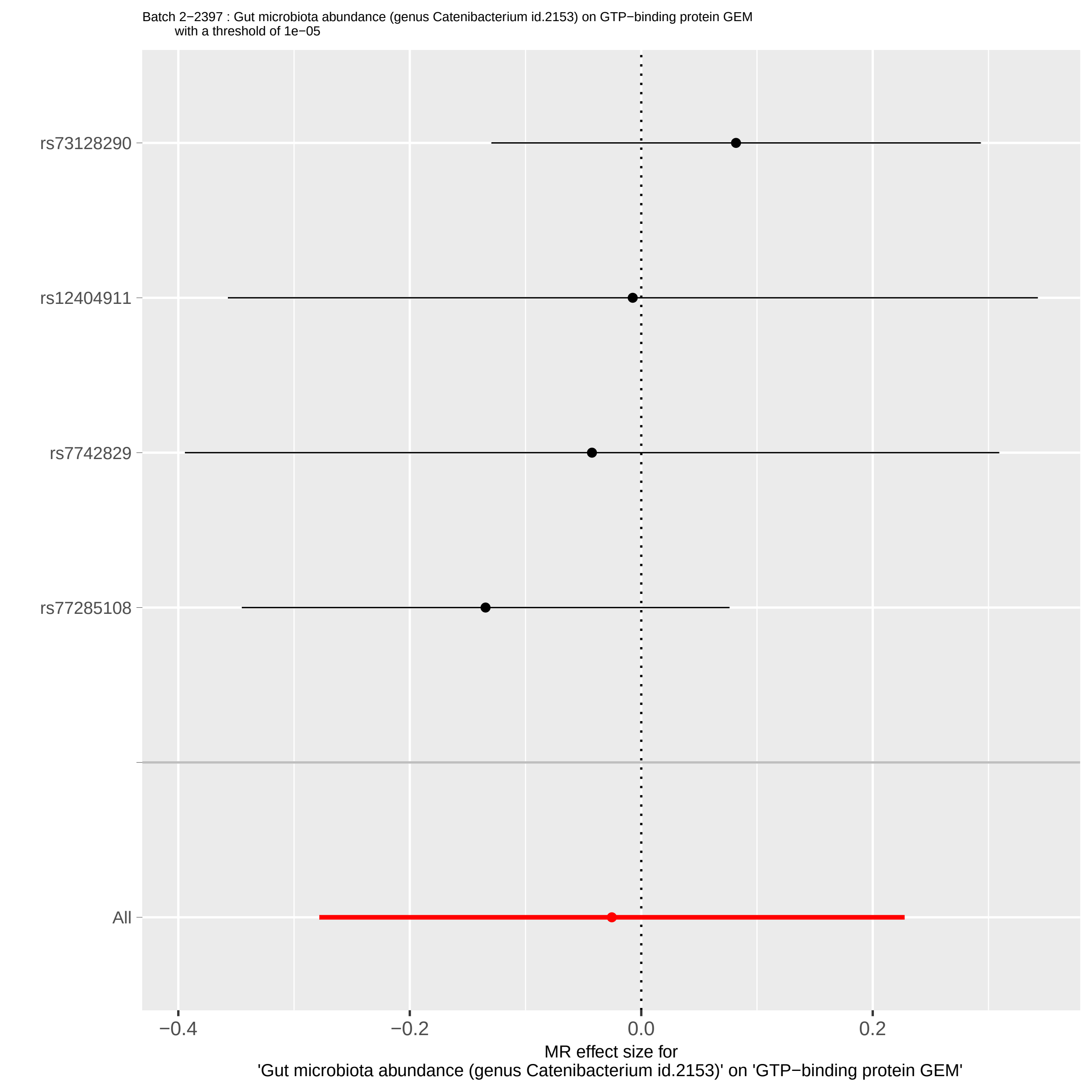
Batch 2−2397 : Gut microbiota abundance (genus Catenibacterium id.2153) on GTP−binding protein GEM
with a threshold of 1e−05
rs73128290
rs12404911
rs7742829
rs77285108
All
−0.4
−0.2
0.0
0.2
MR effect size for
'Gut microbiota abundance (genus Catenibacterium id.2153)' on 'GTP−binding protein GEM'

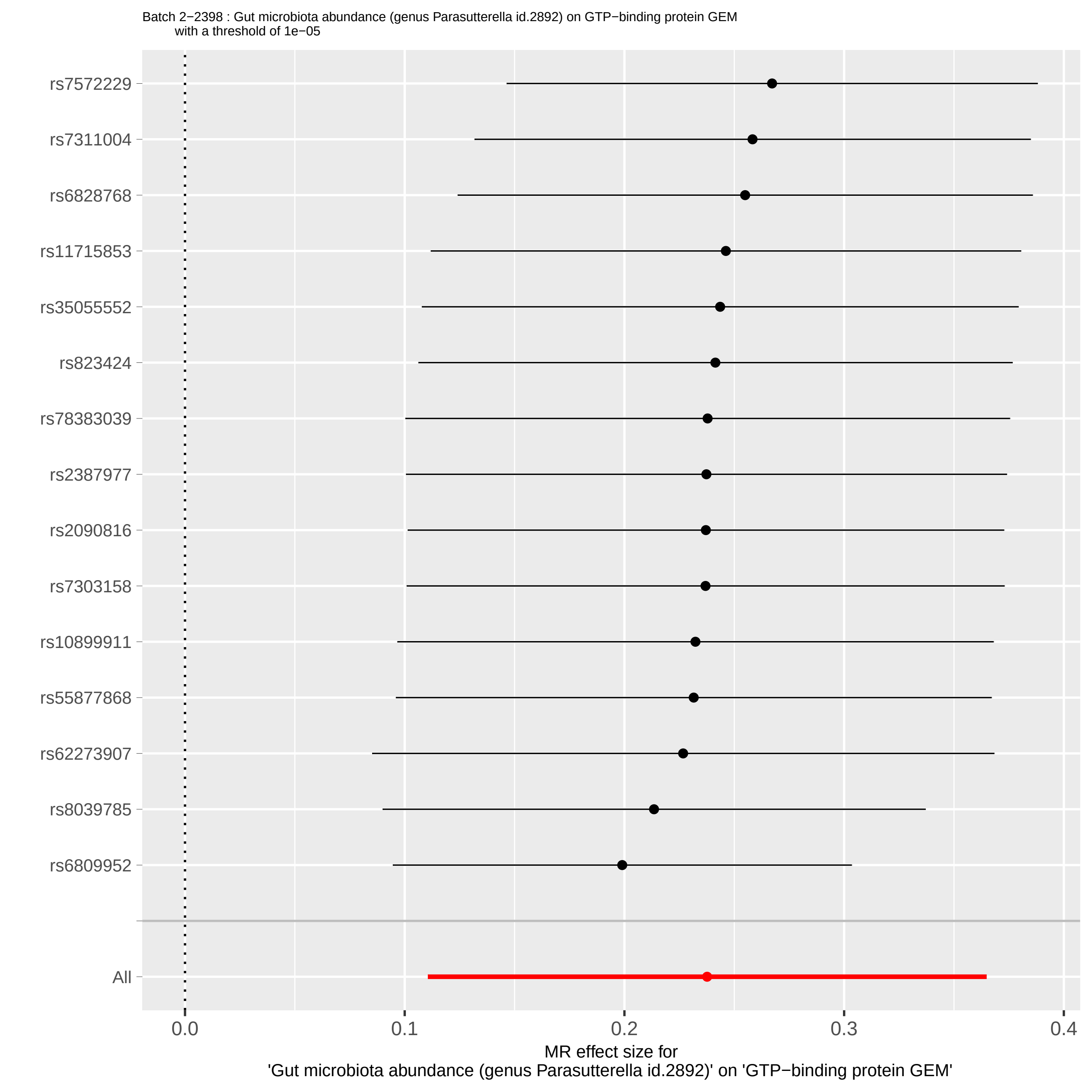

Batch 2−2398 : Gut microbiota abundance (genus Parasutterella id.2892) on GTP−binding protein GEM
with a threshold of 1e−05
rs7572229
rs7311004
rs6828768
rs11715853
rs35055552
rs823424
rs78383039
rs2387977
rs2090816
rs7303158
rs10899911
rs55877868
rs62273907
rs8039785
rs6809952
All
0.0
0.1
0.2
0.3
0.4
MR effect size for
'Gut microbiota abundance (genus Parasutterella id.2892)' on 'GTP−binding protein GEM'

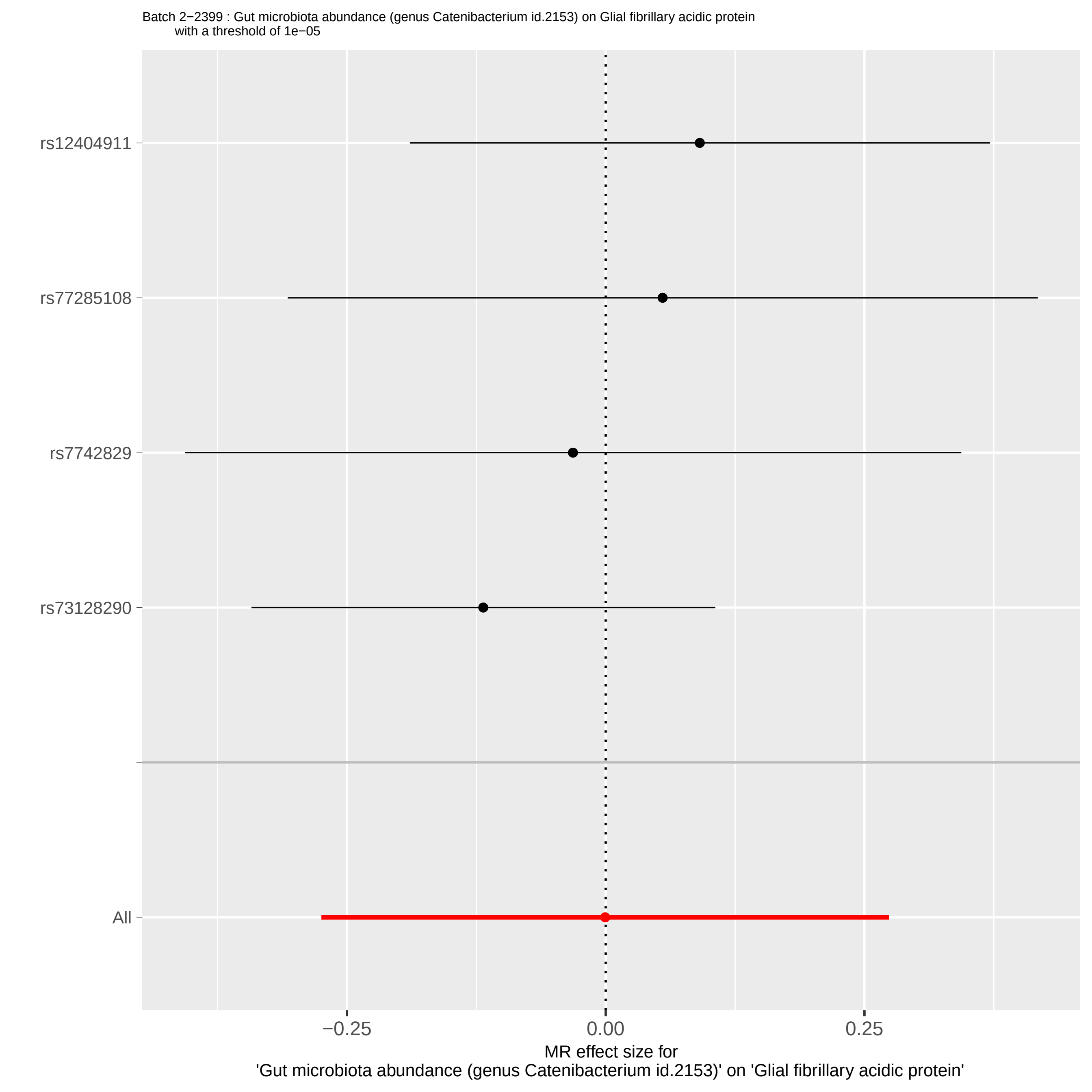
Batch 2−2399 : Gut microbiota abundance (genus Catenibacterium id.2153) on Glial fibrillary acidic protein
with a threshold of 1e−05
rs12404911
rs77285108
rs7742829
rs73128290
All
−0.25
0.00
0.25
MR effect size for
'Gut microbiota abundance (genus Catenibacterium id.2153)' on 'Glial fibrillary acidic protein'

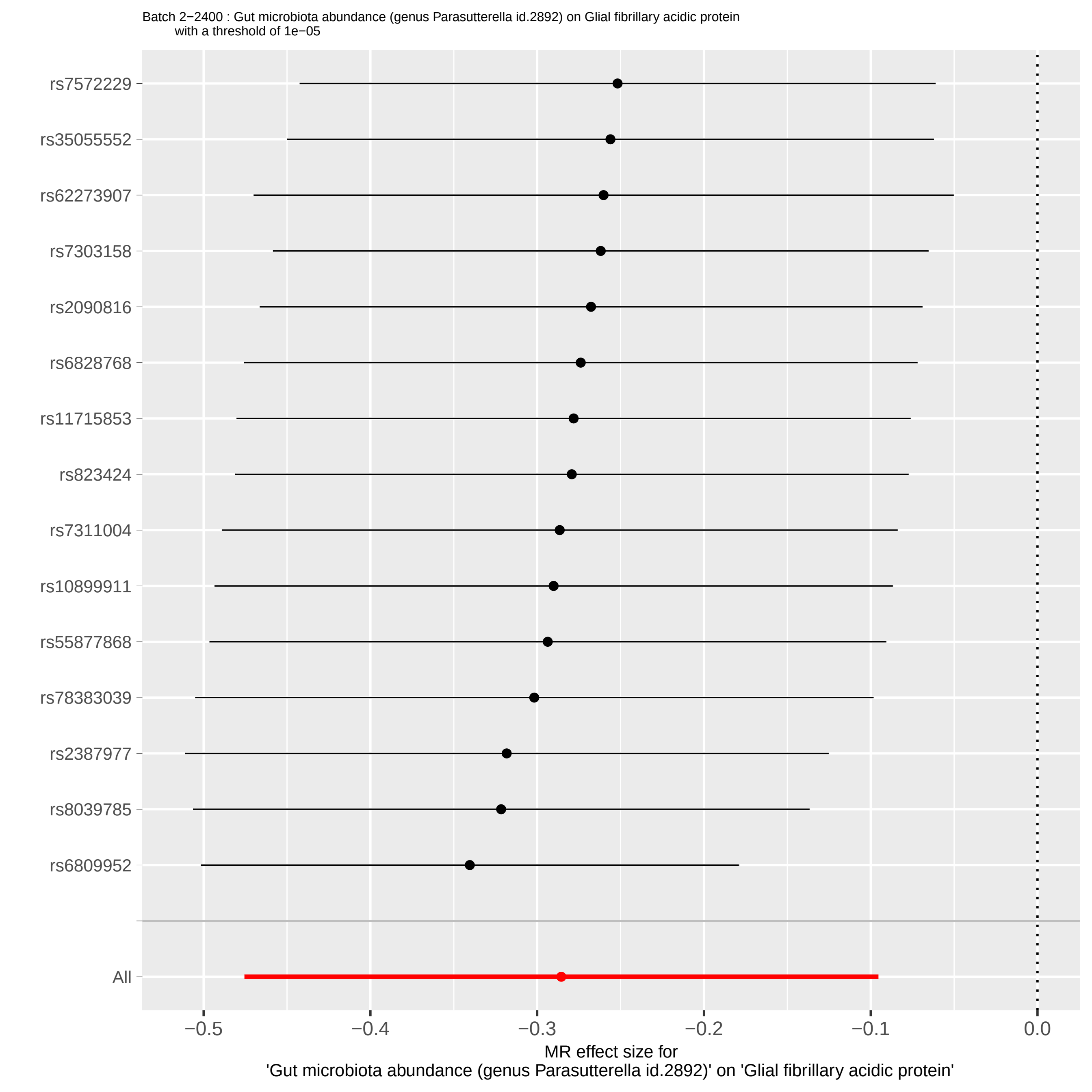

Batch 2−2400 : Gut microbiota abundance (genus Parasutterella id.2892) on Glial fibrillary acidic protein
with a threshold of 1e−05
rs7572229
rs35055552
rs62273907
rs7303158
rs2090816
rs6828768
rs11715853
rs823424
rs7311004
rs10899911
rs55877868
rs78383039
rs2387977
rs8039785
rs6809952
All
−0.5
−0.4
−0.3
−0.2
−0.1
0.0
MR effect size for
'Gut microbiota abundance (genus Parasutterella id.2892)' on 'Glial fibrillary acidic protein'

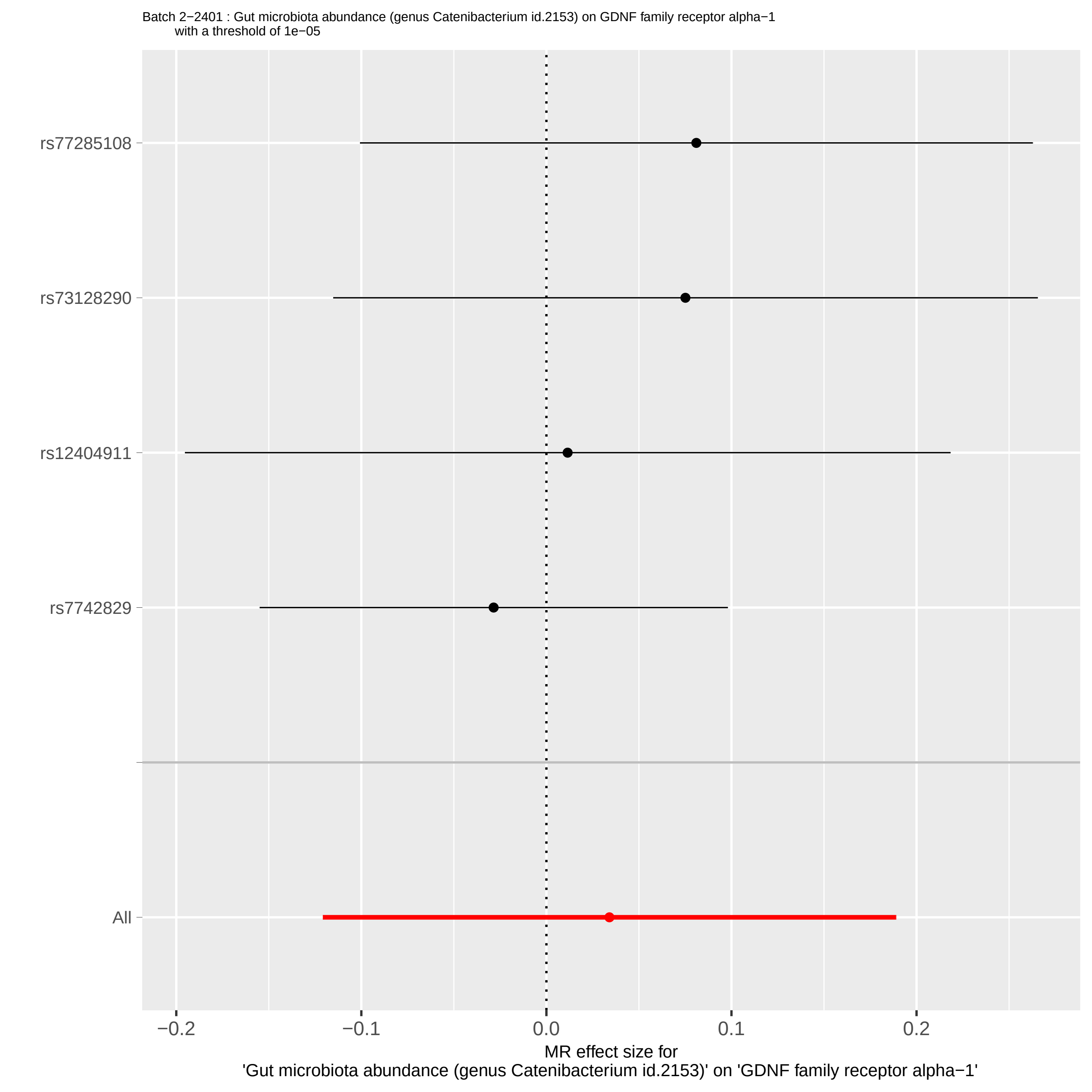
Batch 2−2401 : Gut microbiota abundance (genus Catenibacterium id.2153) on GDNF family receptor alpha−1
with a threshold of 1e−05
rs77285108
rs73128290
rs12404911
rs7742829
All
−0.2
−0.1
0.0
0.1
0.2
MR effect size for
'Gut microbiota abundance (genus Catenibacterium id.2153)' on 'GDNF family receptor alpha−1'

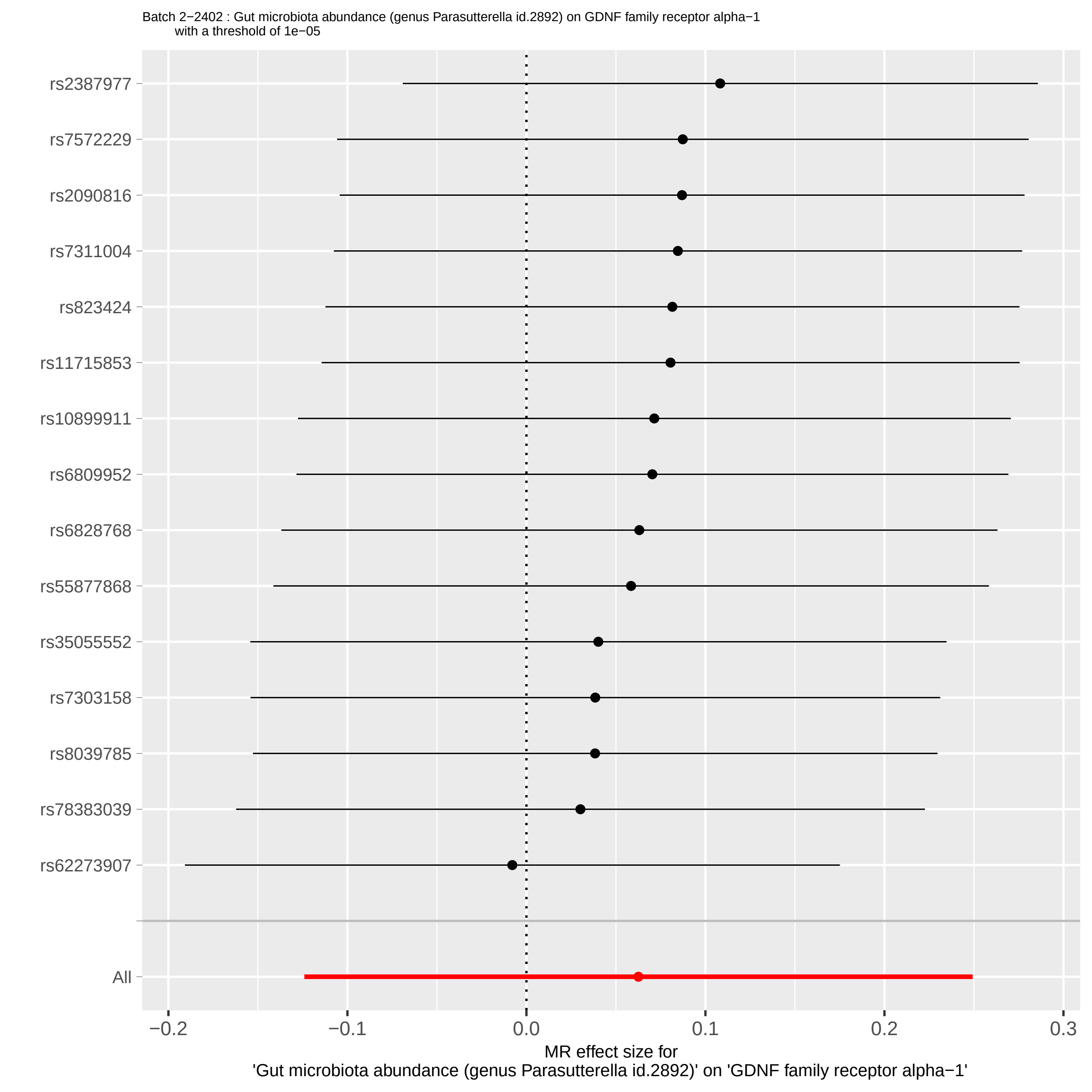

Batch 2−2402 : Gut microbiota abundance (genus Parasutterella id.2892) on GDNF family receptor alpha−1
with a threshold of 1e−05
rs2387977
rs7572229
rs2090816
rs7311004
rs823424
rs11715853
rs10899911
rs6809952
rs6828768
rs55877868
rs35055552
rs7303158
rs8039785
rs78383039
rs62273907
All
−0.2
−0.1
0.0
0.1
0.2
0.3
MR effect size for
'Gut microbiota abundance (genus Parasutterella id.2892)' on 'GDNF family receptor alpha−1'

Batch 2−2403 : Gut microbiota abundance (genus Catenibacterium id.2153) on GDNF family receptor alpha−2
with a threshold of 1e−05

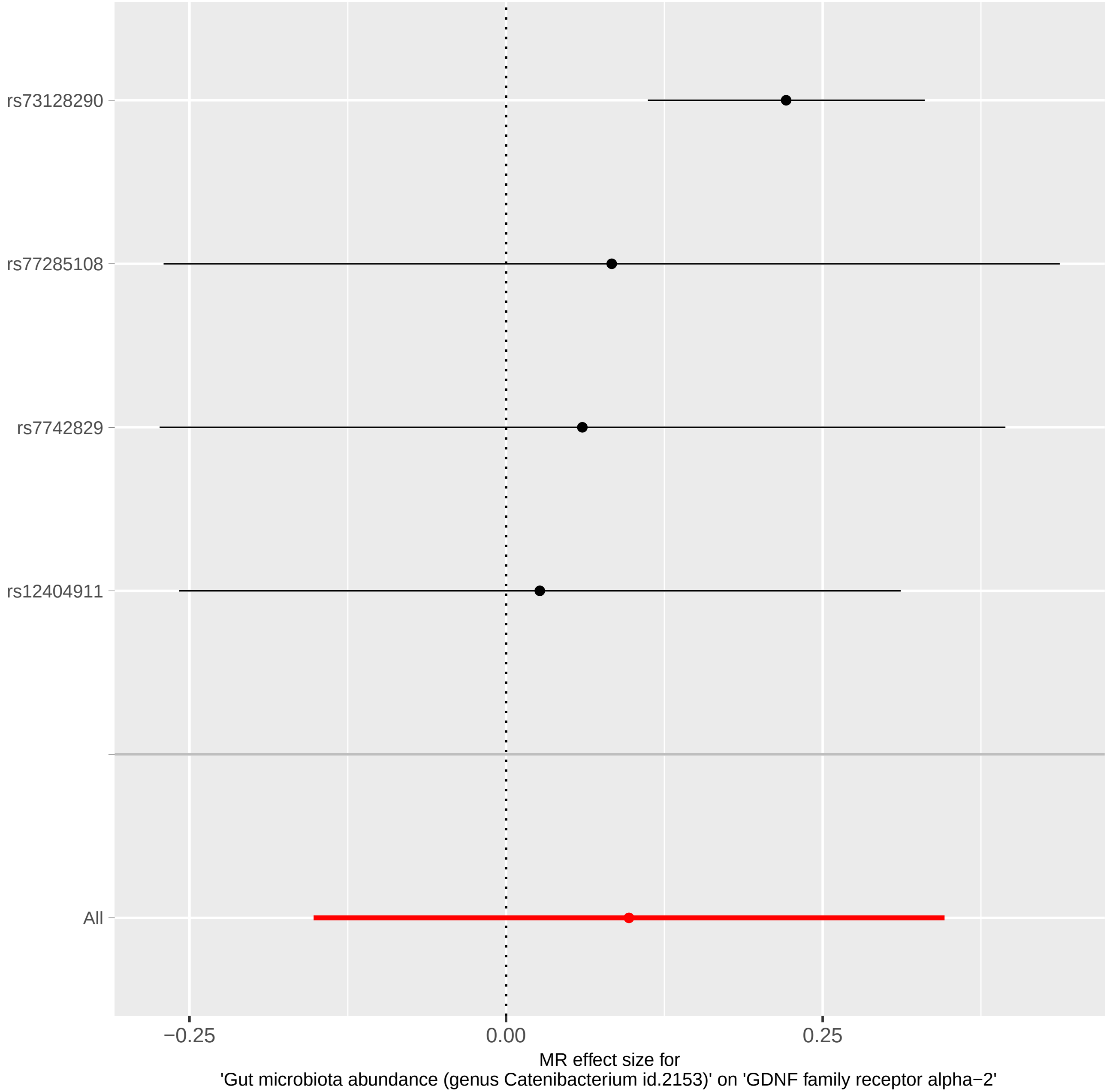

Batch 2−2404 : Gut microbiota abundance (genus Parasutterella id.2892) on GDNF family receptor alpha−2
with a threshold of 1e−05

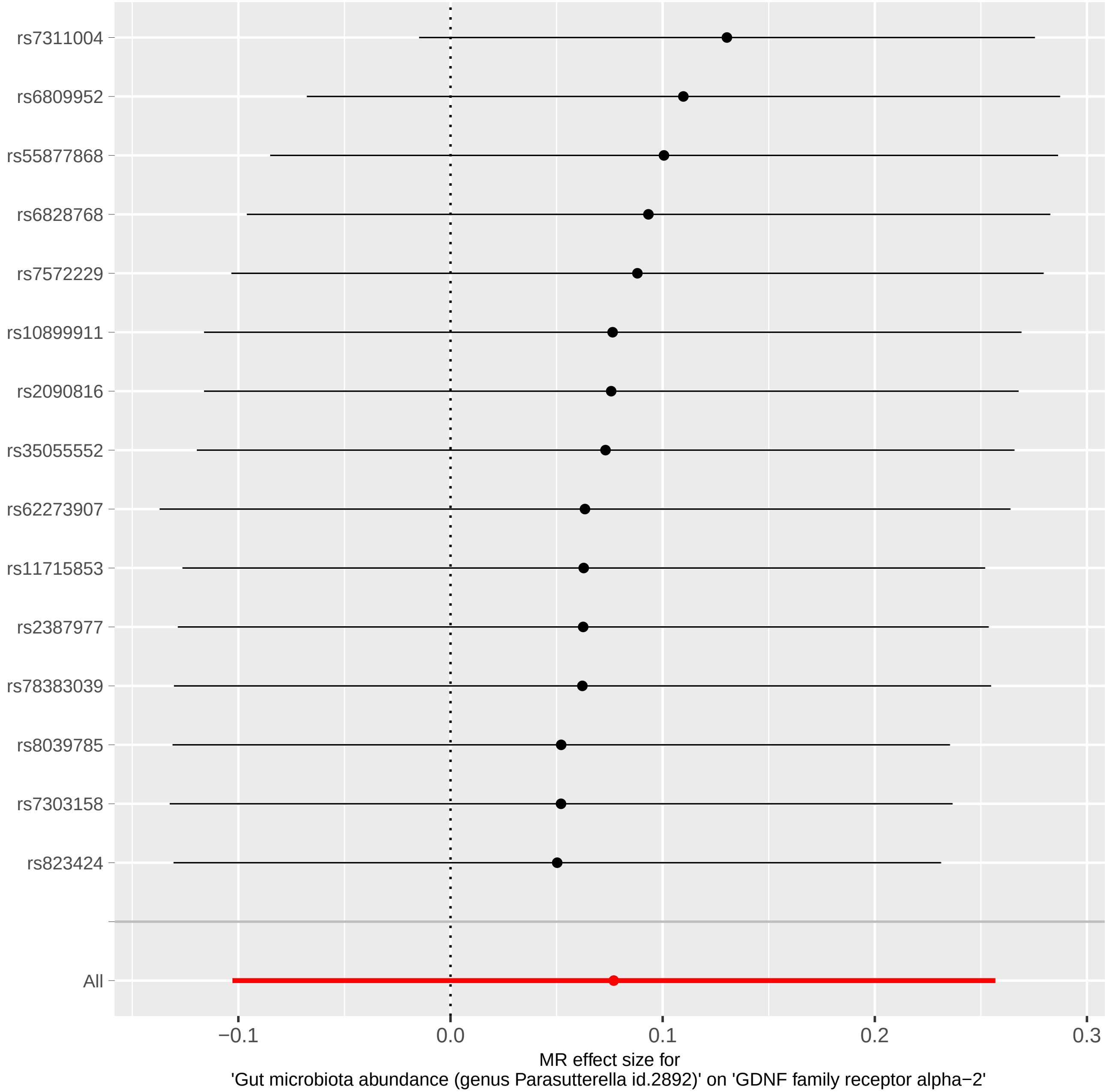

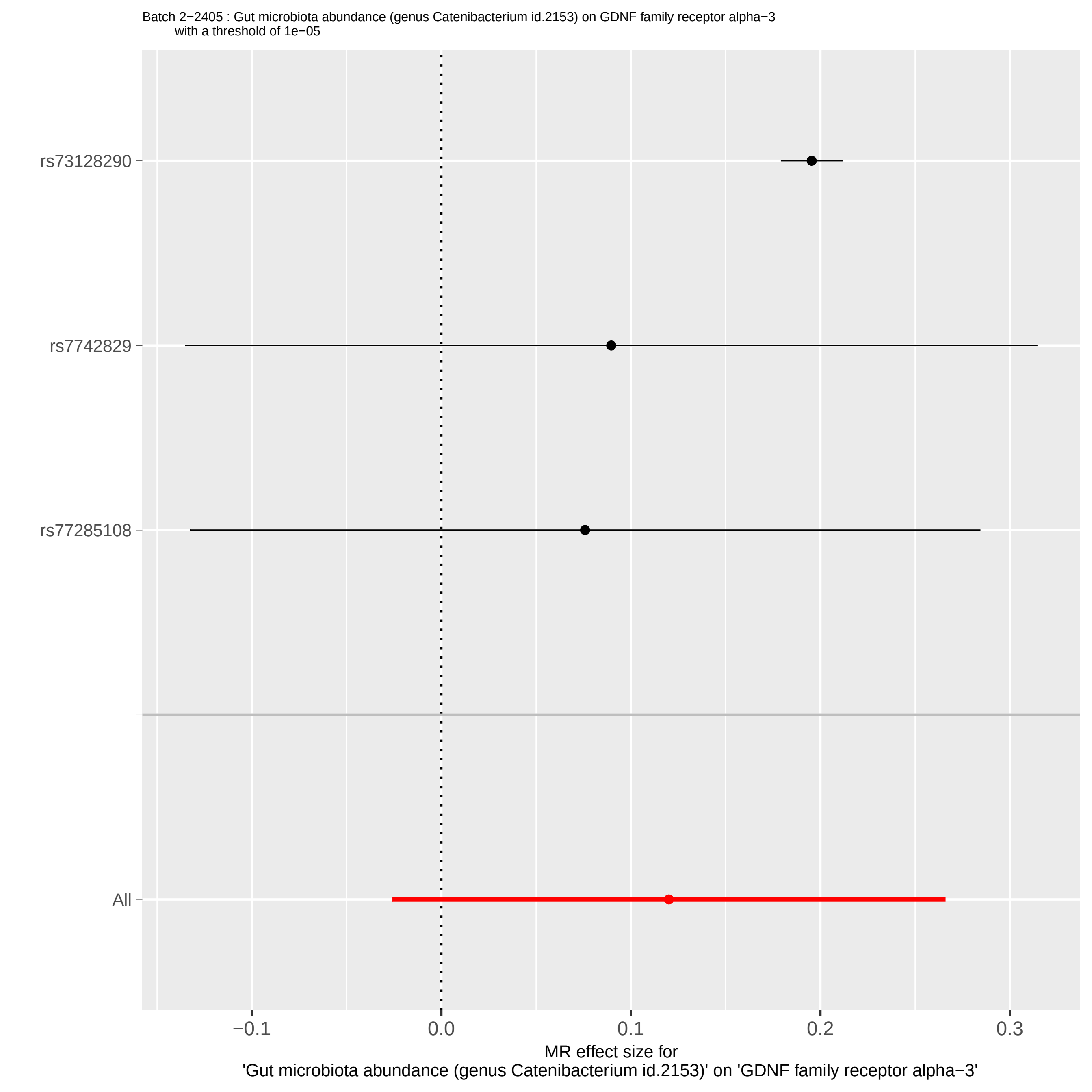
Batch 2−2405 : Gut microbiota abundance (genus Catenibacterium id.2153) on GDNF family receptor alpha−3
with a threshold of 1e−05
rs73128290
rs7742829
rs77285108
All
−0.1
0.0
0.1
0.2
0.3
MR effect size for
'Gut microbiota abundance (genus Catenibacterium id.2153)' on 'GDNF family receptor alpha−3'

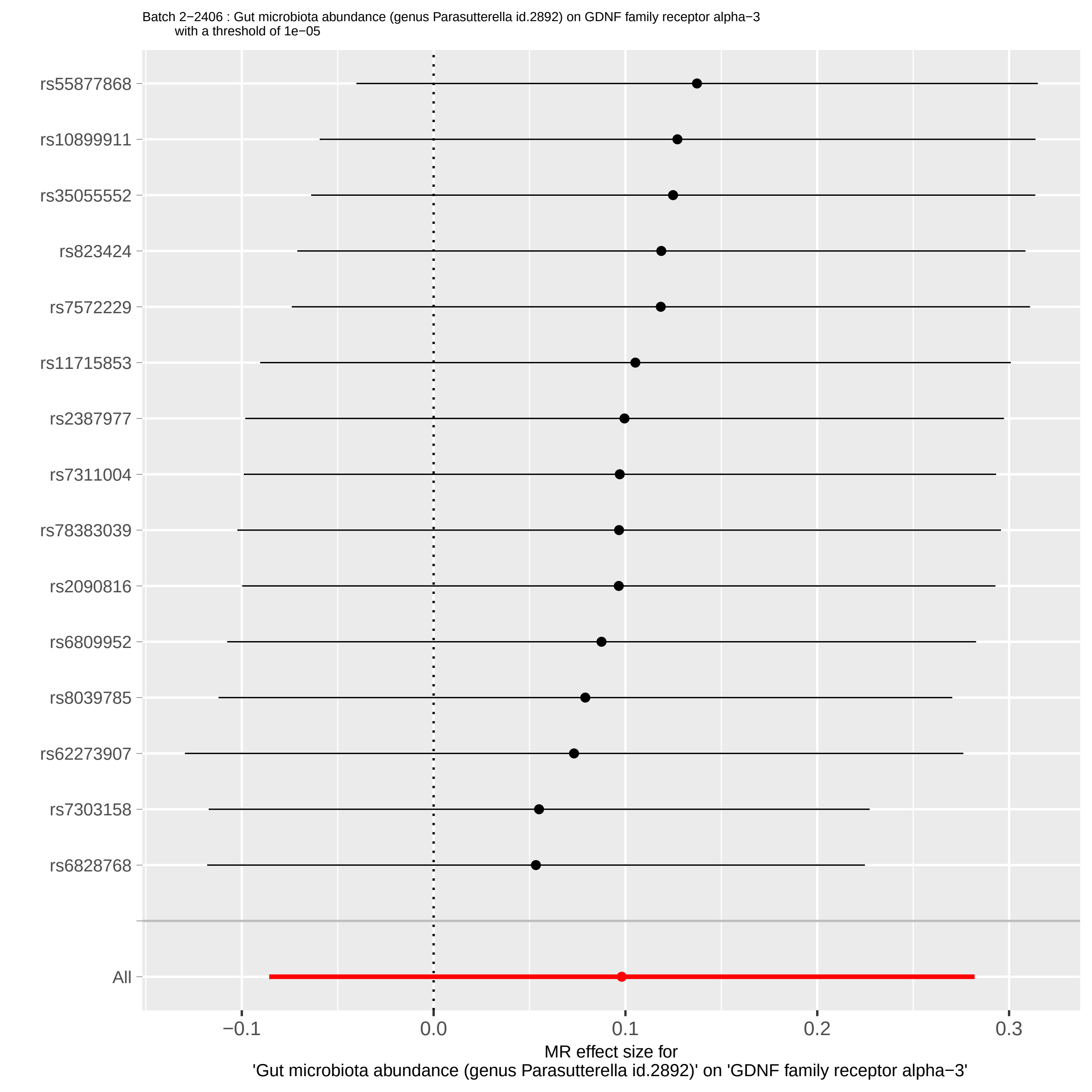
Batch 2−2406 : Gut microbiota abundance (genus Parasutterella id.2892) on GDNF family receptor alpha−3
with a threshold of 1e−05
rs55877868
rs10899911
rs35055552
rs823424
rs7572229
rs11715853
rs2387977
rs7311004
rs78383039
rs2090816
rs6809952
rs8039785
rs62273907
rs7303158
rs6828768
All
−0.1
0.0
0.1
0.2
0.3
MR effect size for
'Gut microbiota abundance (genus Parasutterella id.2892)' on 'GDNF family receptor alpha−3'

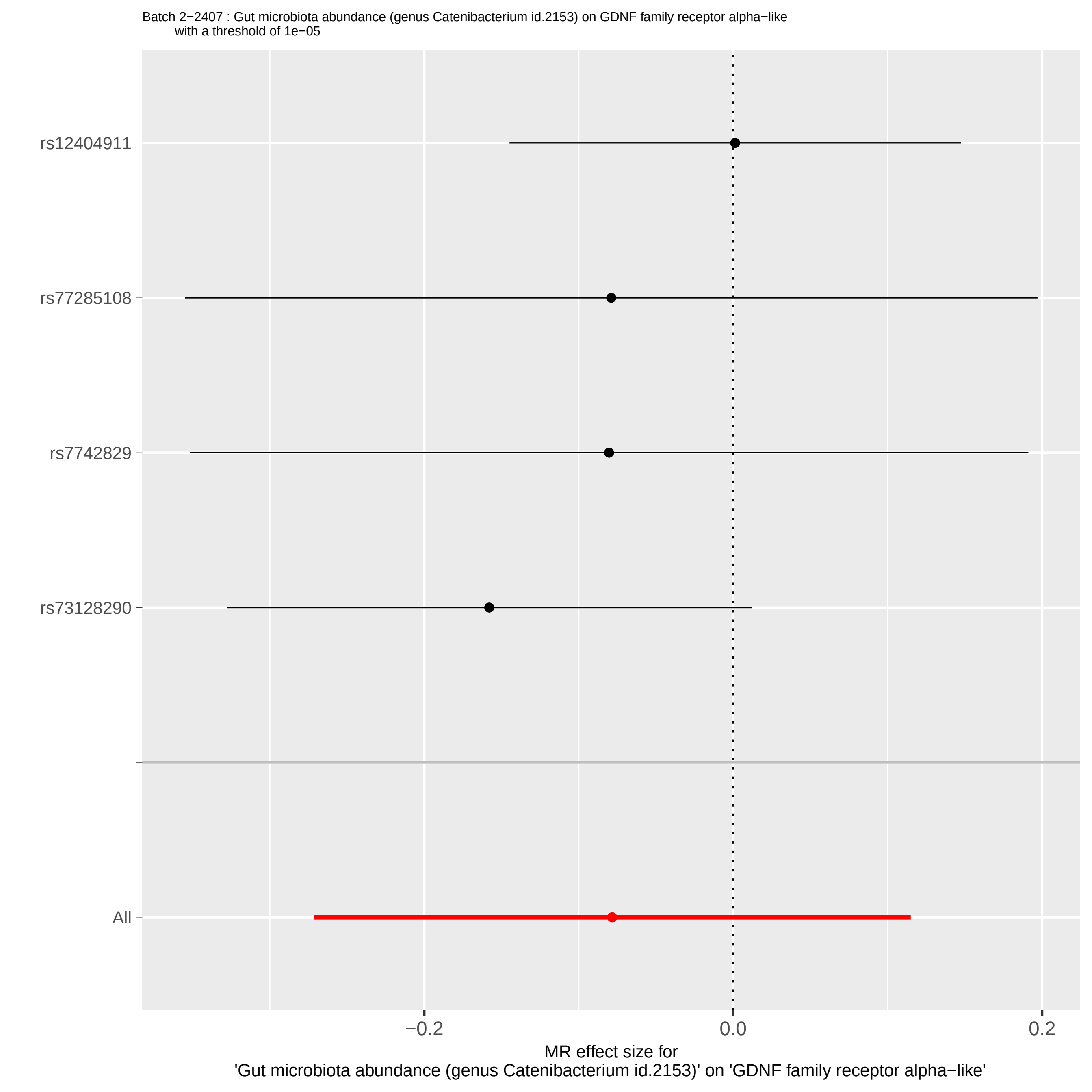
Batch 2−2407 : Gut microbiota abundance (genus Catenibacterium id.2153) on GDNF family receptor alpha−like
with a threshold of 1e−05
rs12404911
rs77285108
rs7742829
rs73128290
All
−0.2
0.0
0.2
MR effect size for
'Gut microbiota abundance (genus Catenibacterium id.2153)' on 'GDNF family receptor alpha−like'

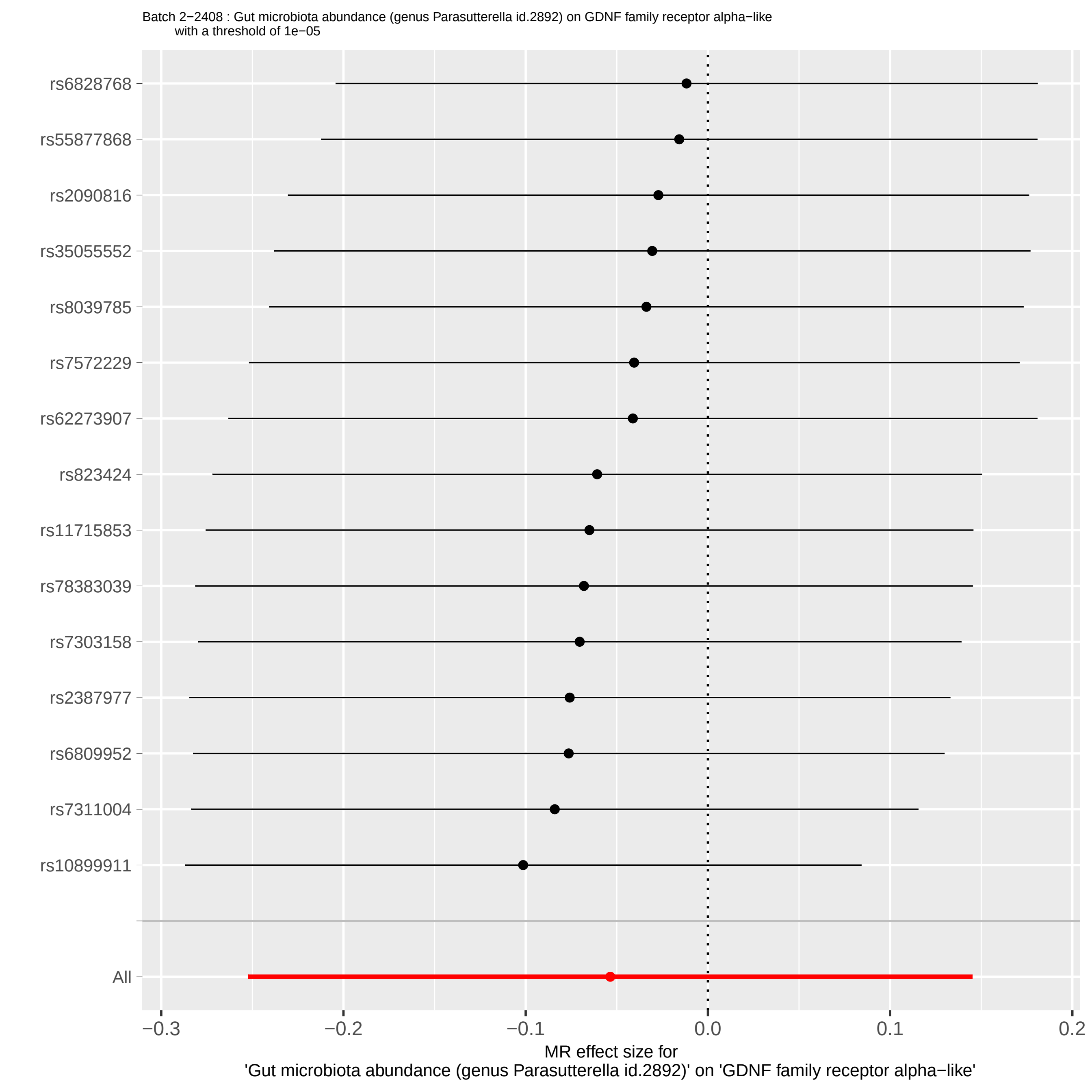
Batch 2−2408 : Gut microbiota abundance (genus Parasutterella id.2892) on GDNF family receptor alpha−like
with a threshold of 1e−05
rs6828768
rs55877868
rs2090816
rs35055552
rs8039785
rs7572229
rs62273907
rs823424
rs11715853
rs78383039
rs7303158
rs2387977
rs6809952
rs7311004
rs10899911
All
−0.3
−0.2
−0.1
0.0
0.1
0.2
MR effect size for
'Gut microbiota abundance (genus Parasutterella id.2892)' on 'GDNF family receptor alpha−like'

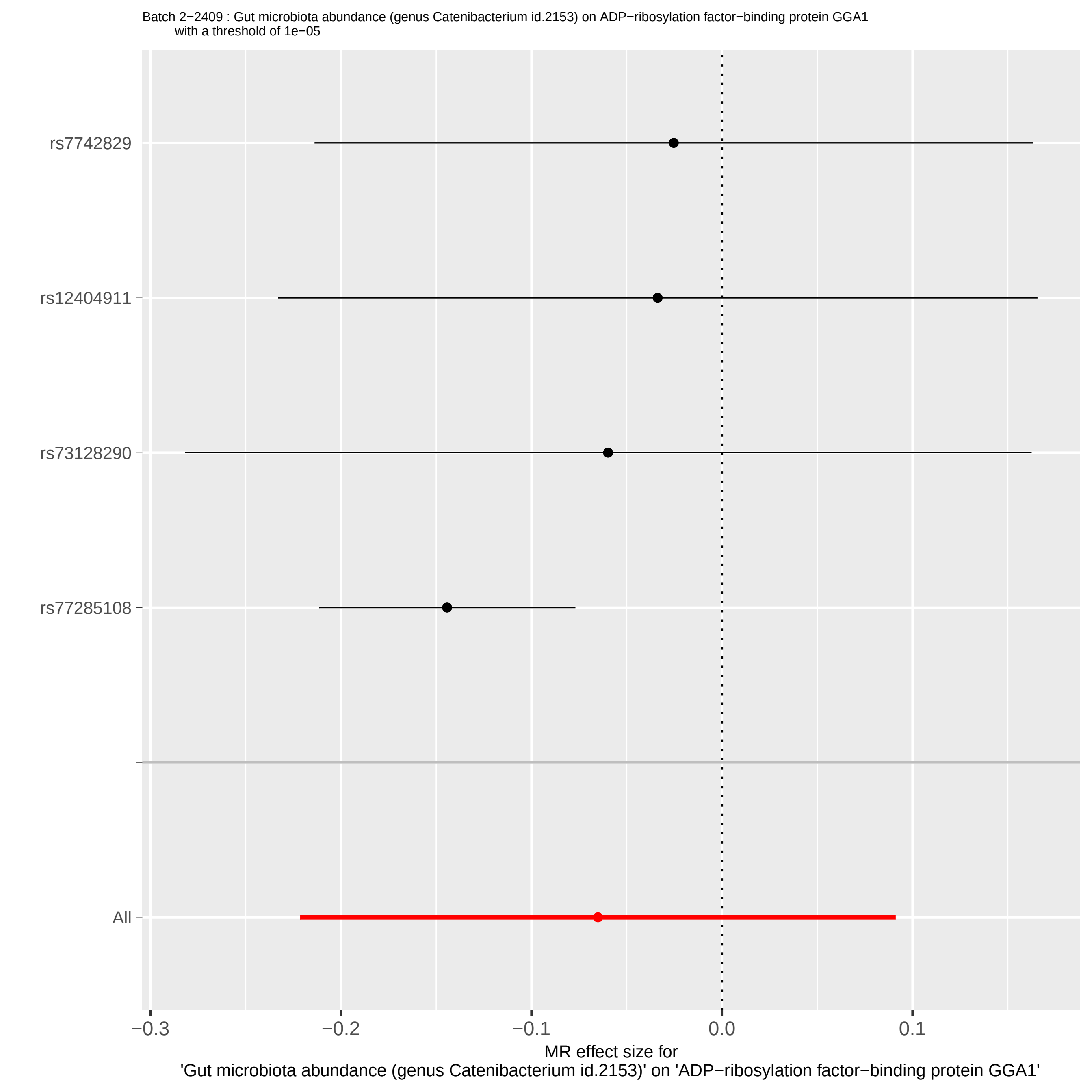

Batch 2−2409 : Gut microbiota abundance (genus Catenibacterium id.2153) on ADP−ribosylation factor−binding protein GGA1
with a threshold of 1e−05
rs7742829
rs12404911
rs73128290
rs77285108
All
−0.3
−0.2
−0.1
0.0
0.1
MR effect size for
'Gut microbiota abundance (genus Catenibacterium id.2153)' on 'ADP−ribosylation factor−binding protein GGA1'

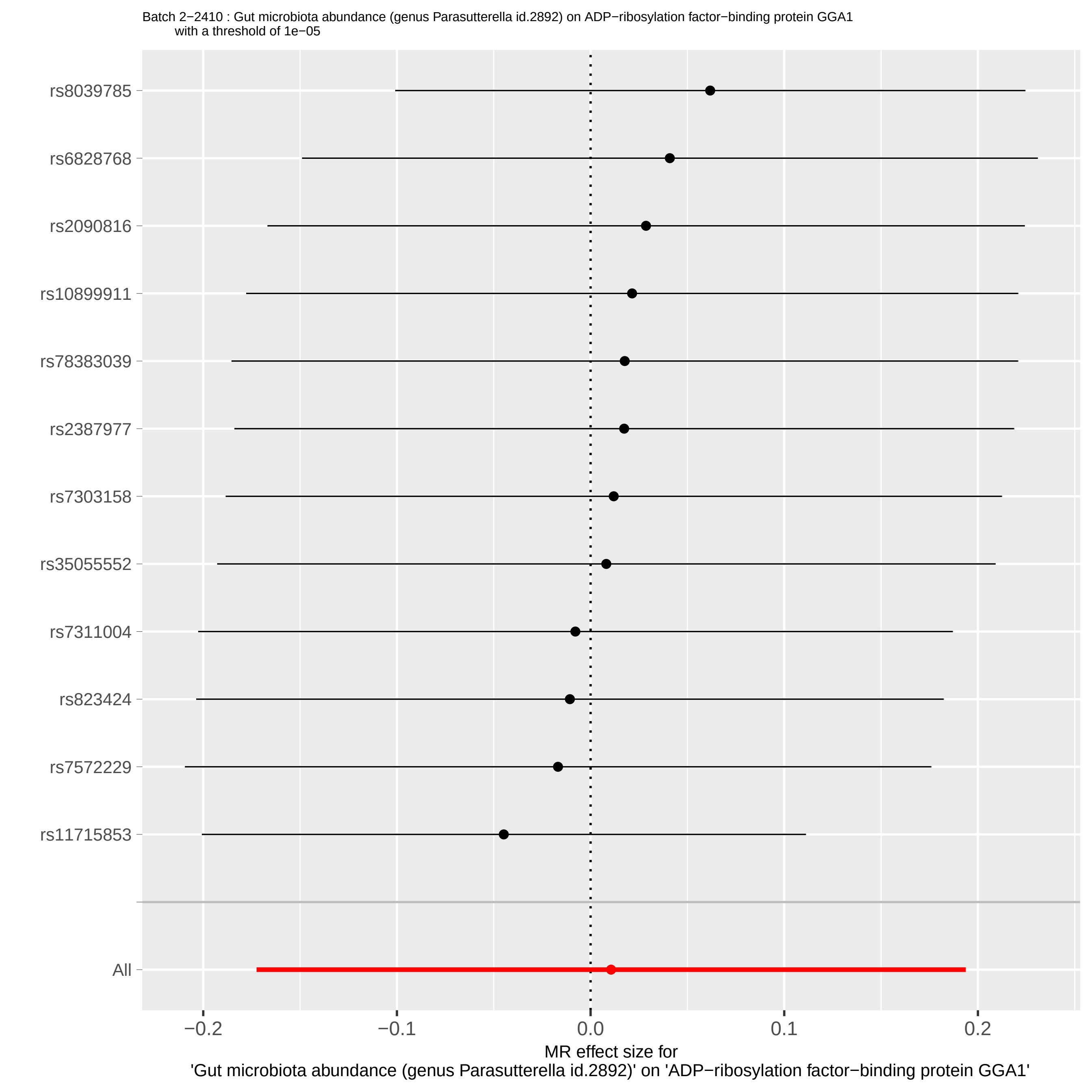
Batch 2−2410 : Gut microbiota abundance (genus Parasutterella id.2892) on ADP−ribosylation factor−binding protein GGA1
with a threshold of 1e−05
rs8039785
rs6828768
rs2090816
rs10899911
rs78383039
rs2387977
rs7303158
rs35055552
rs7311004
rs823424
rs7572229
rs11715853
All
−0.2
−0.1
0.0
0.1
0.2
MR effect size for
'Gut microbiota abundance (genus Parasutterella id.2892)' on 'ADP−ribosylation factor−binding protein GGA1'

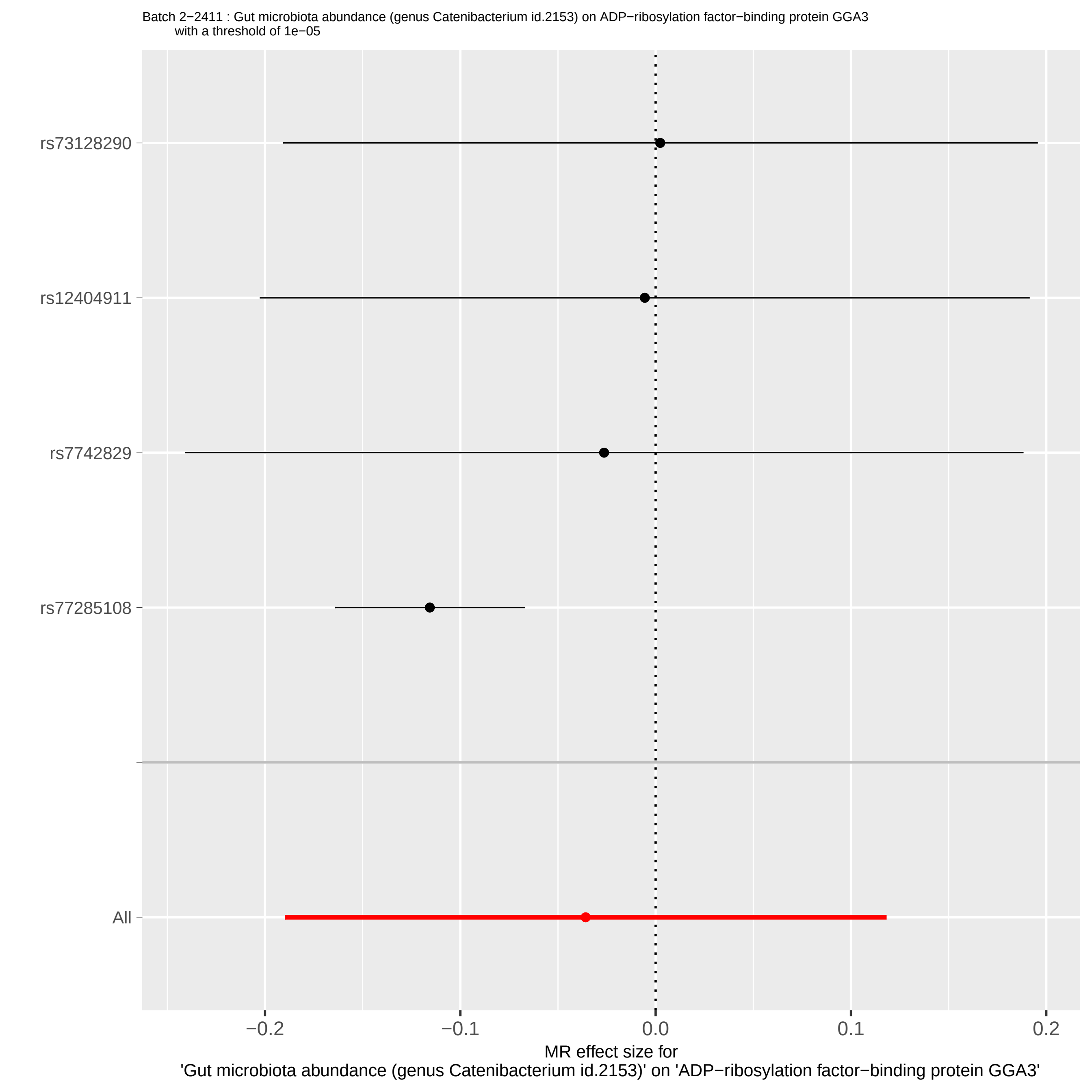

Batch 2−2411 : Gut microbiota abundance (genus Catenibacterium id.2153) on ADP−ribosylation factor−binding protein GGA3
with a threshold of 1e−05
rs73128290
rs12404911
rs7742829
rs77285108
All
−0.2
−0.1
0.0
0.1
0.2
MR effect size for
'Gut microbiota abundance (genus Catenibacterium id.2153)' on 'ADP−ribosylation factor−binding protein GGA3'

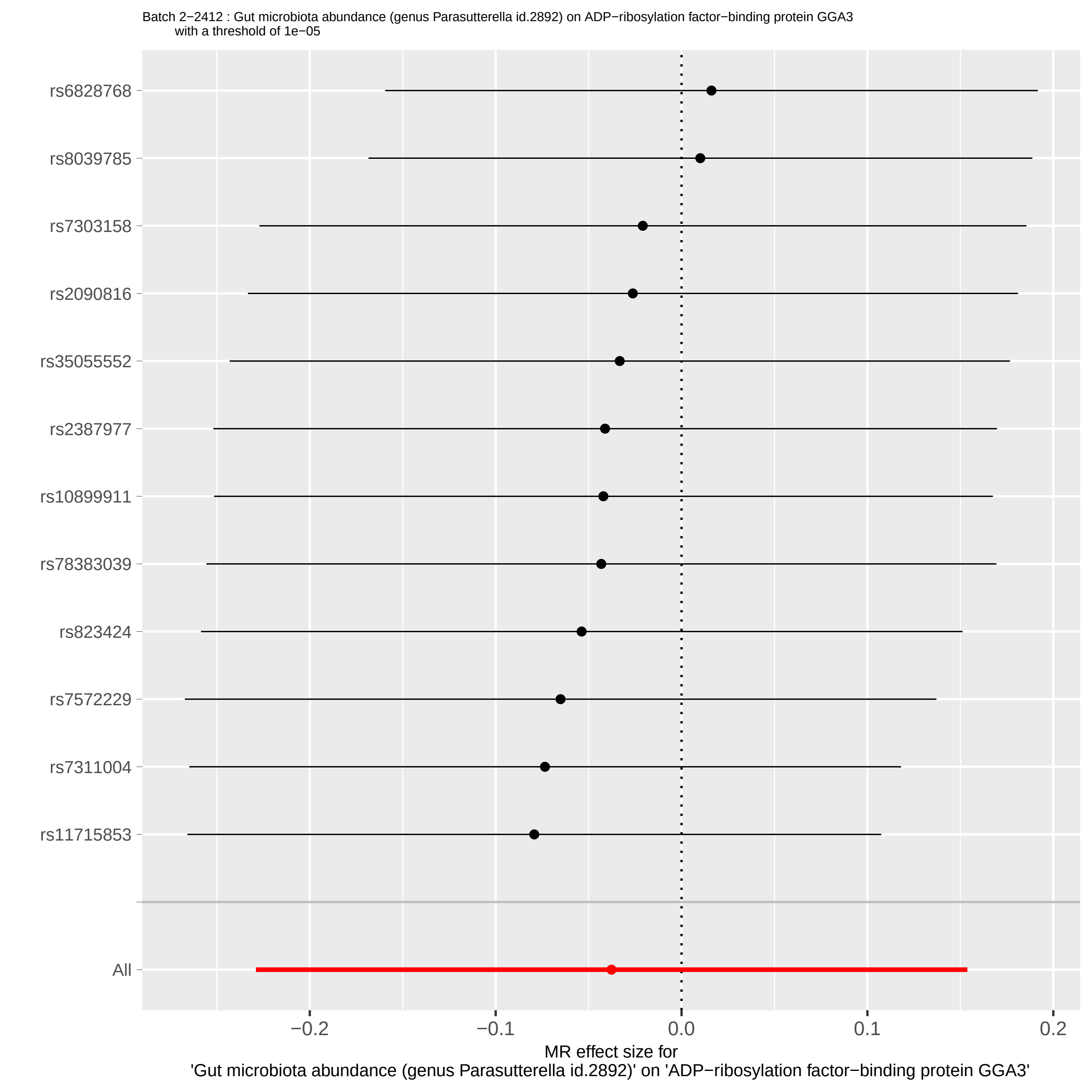
Batch 2−2412 : Gut microbiota abundance (genus Parasutterella id.2892) on ADP−ribosylation factor−binding protein GGA3
with a threshold of 1e−05
rs6828768
rs8039785
rs7303158
rs2090816
rs35055552
rs2387977
rs10899911
rs78383039
rs823424
rs7572229
rs7311004
rs11715853
All
−0.2
−0.1
0.0
0.1
0.2
MR effect size for
'Gut microbiota abundance (genus Parasutterella id.2892)' on 'ADP−ribosylation factor−binding protein GGA3'

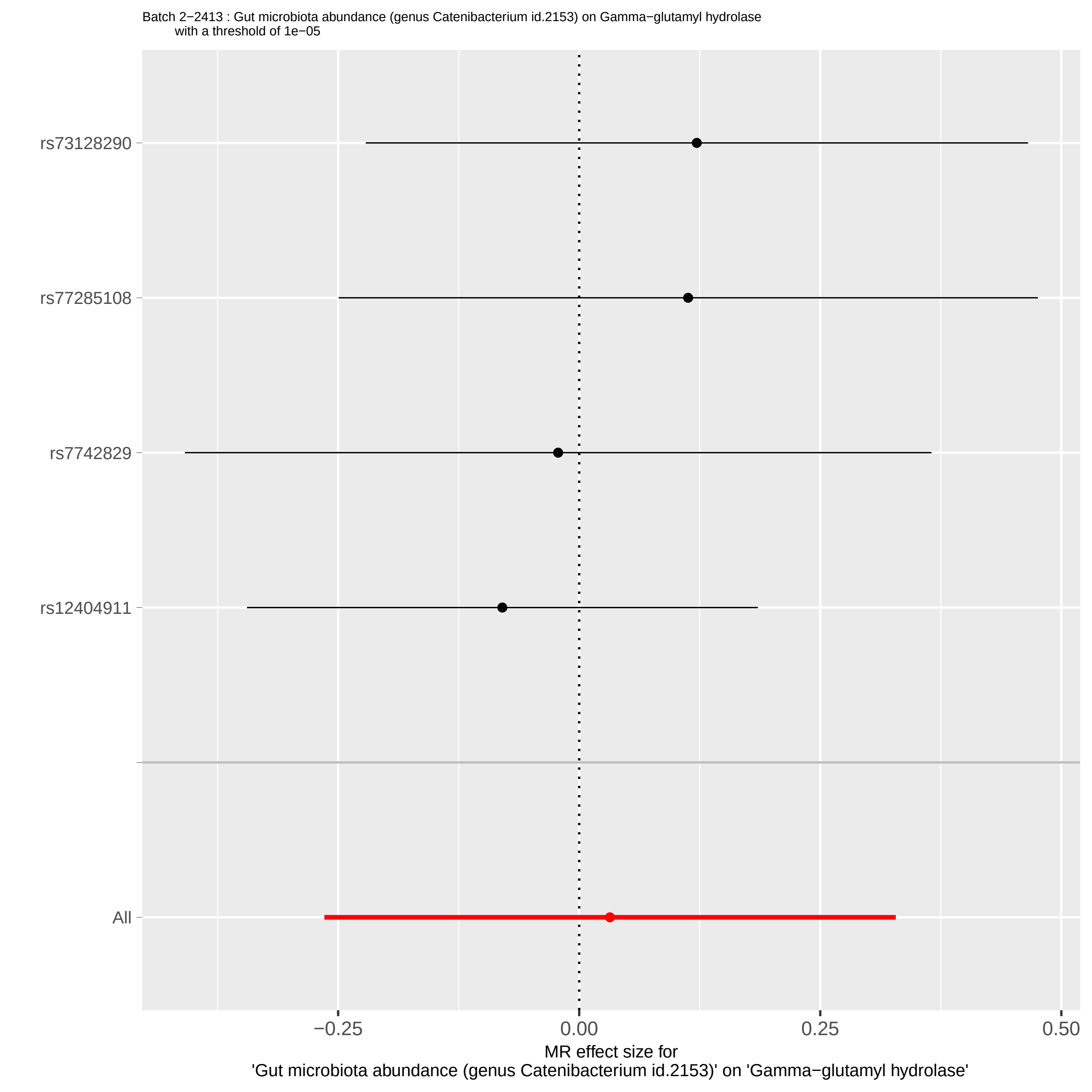

Batch 2−2413 : Gut microbiota abundance (genus Catenibacterium id.2153) on Gamma−glutamyl hydrolase
with a threshold of 1e−05
rs73128290
rs77285108
rs7742829
rs12404911
All
−0.25
0.00
0.25
0.50
MR effect size for
'Gut microbiota abundance (genus Catenibacterium id.2153)' on 'Gamma−glutamyl hydrolase'

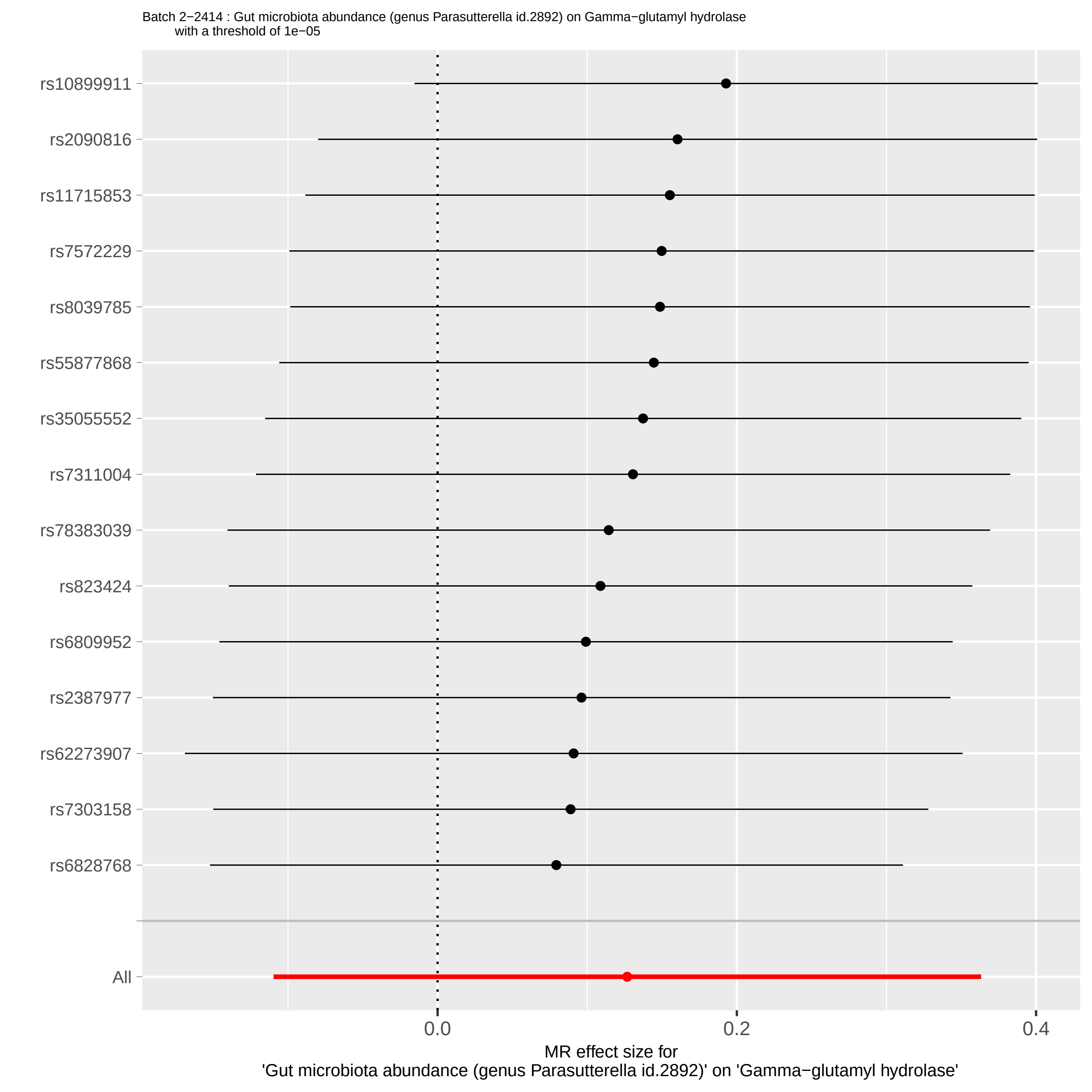
Batch 2−2414 : Gut microbiota abundance (genus Parasutterella id.2892) on Gamma−glutamyl hydrolase
with a threshold of 1e−05
rs10899911
rs2090816
rs11715853
rs7572229
rs8039785
rs55877868
rs35055552
rs7311004
rs78383039
rs823424
rs6809952
rs2387977
rs62273907
rs7303158
rs6828768
All
0.0
0.2
0.4
MR effect size for
'Gut microbiota abundance (genus Parasutterella id.2892)' on 'Gamma−glutamyl hydrolase'

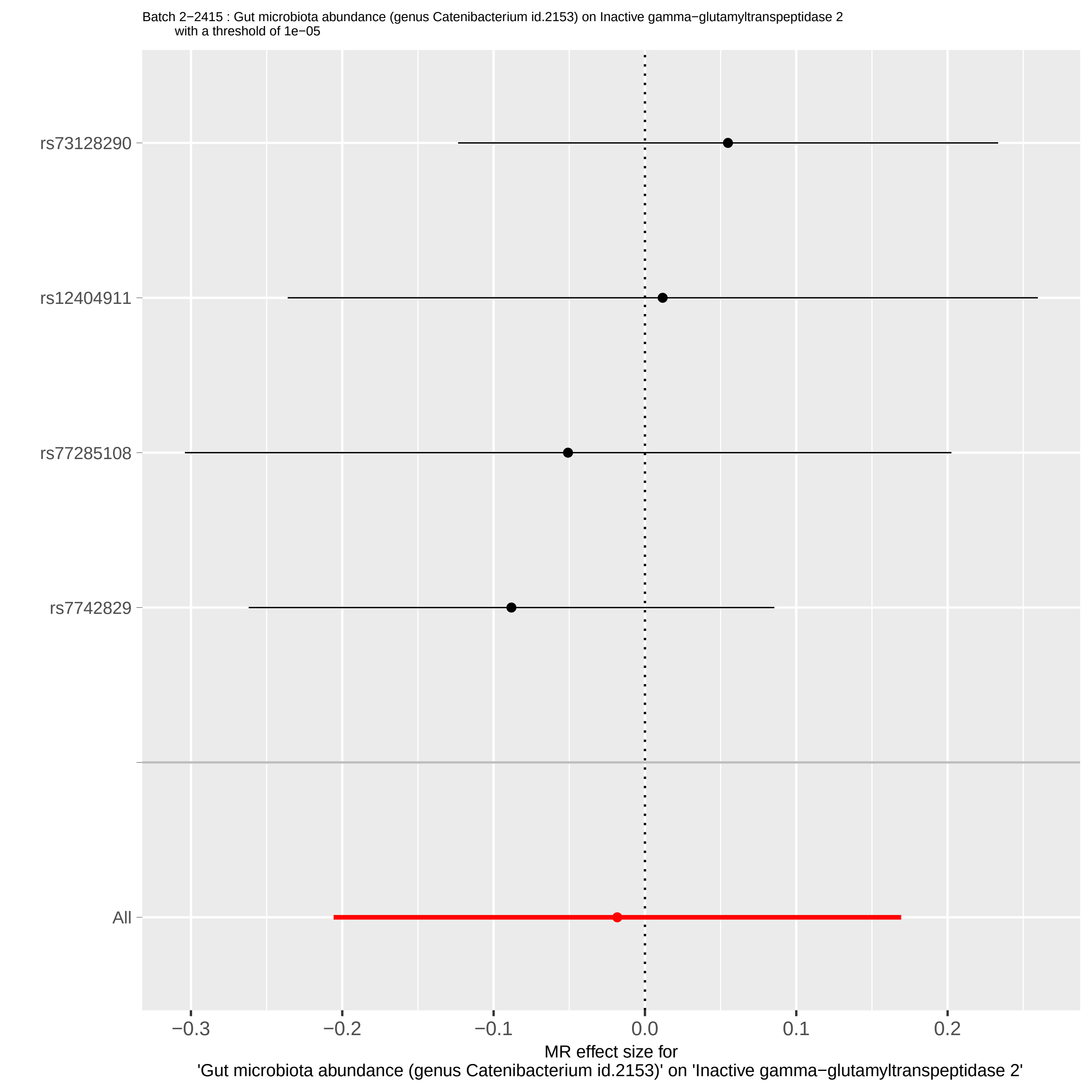

Batch 2−2415 : Gut microbiota abundance (genus Catenibacterium id.2153) on Inactive gamma−glutamyltranspeptidase 2
with a threshold of 1e−05
rs73128290
rs12404911
rs77285108
rs7742829
All
−0.3
−0.2
−0.1
0.0
0.1
0.2
MR effect size for
'Gut microbiota abundance (genus Catenibacterium id.2153)' on 'Inactive gamma−glutamyltranspeptidase 2'

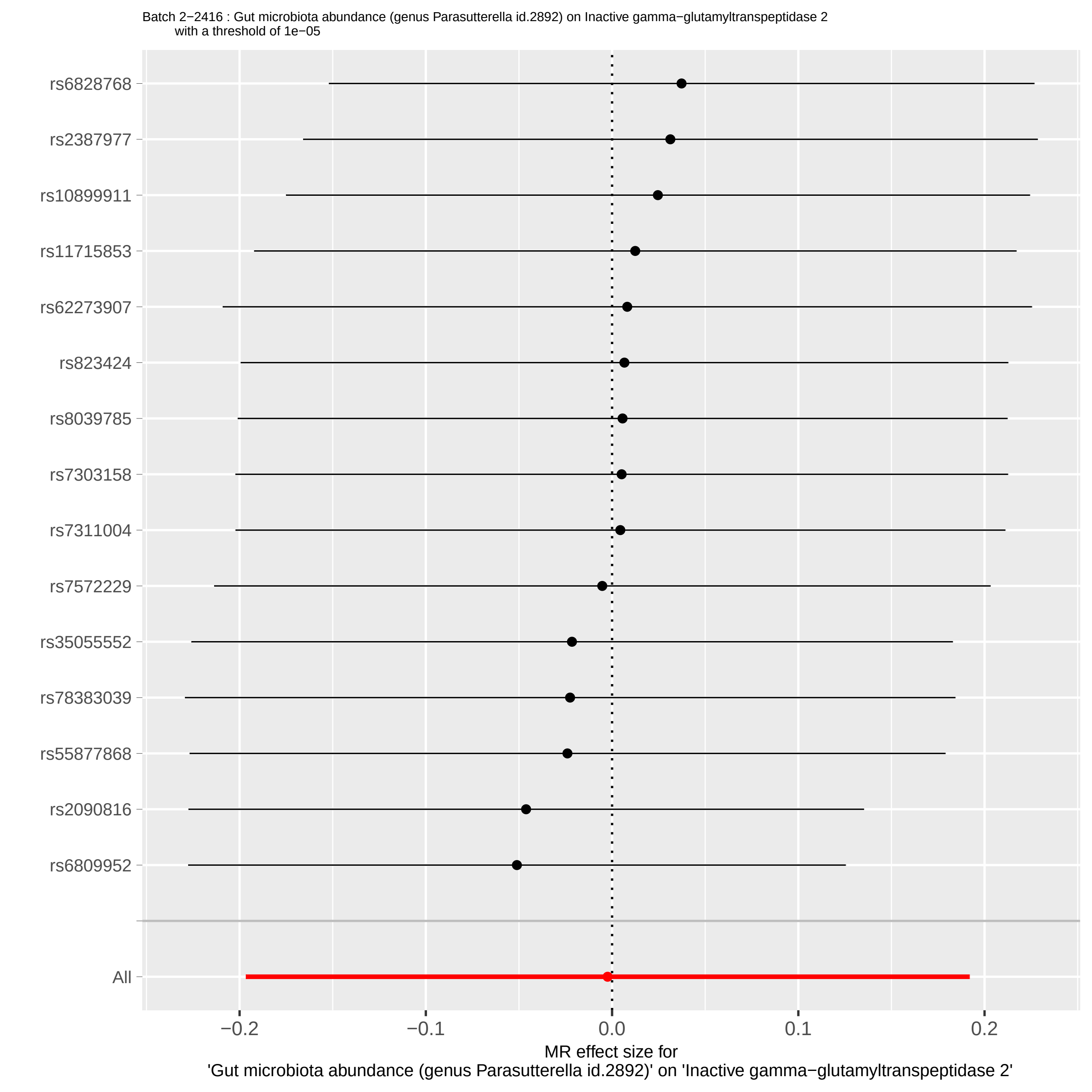

Batch 2−2416 : Gut microbiota abundance (genus Parasutterella id.2892) on Inactive gamma−glutamyltranspeptidase 2
with a threshold of 1e−05
rs6828768
rs2387977
rs10899911
rs11715853
rs62273907
rs823424
rs8039785
rs7303158
rs7311004
rs7572229
rs35055552
rs78383039
rs55877868
rs2090816
rs6809952
All
−0.2
−0.1
0.0
0.1
0.2
MR effect size for
'Gut microbiota abundance (genus Parasutterella id.2892)' on 'Inactive gamma−glutamyltranspeptidase 2'

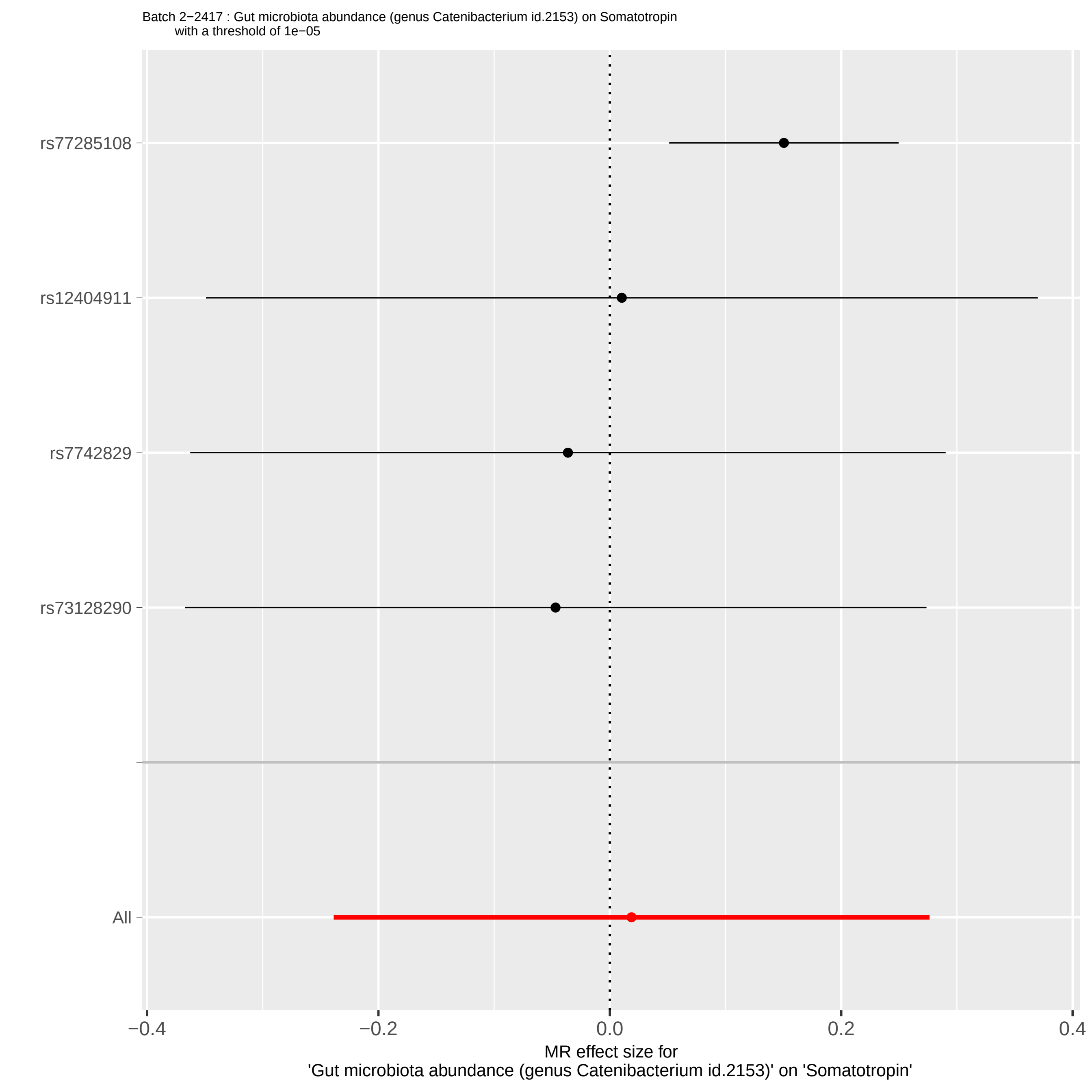
Batch 2−2417 : Gut microbiota abundance (genus Catenibacterium id.2153) on Somatotropin
with a threshold of 1e−05
rs77285108
rs12404911
rs7742829
rs73128290
All
−0.4
−0.2
0.0
0.2
0.4
MR effect size for
'Gut microbiota abundance (genus Catenibacterium id.2153)' on 'Somatotropin'

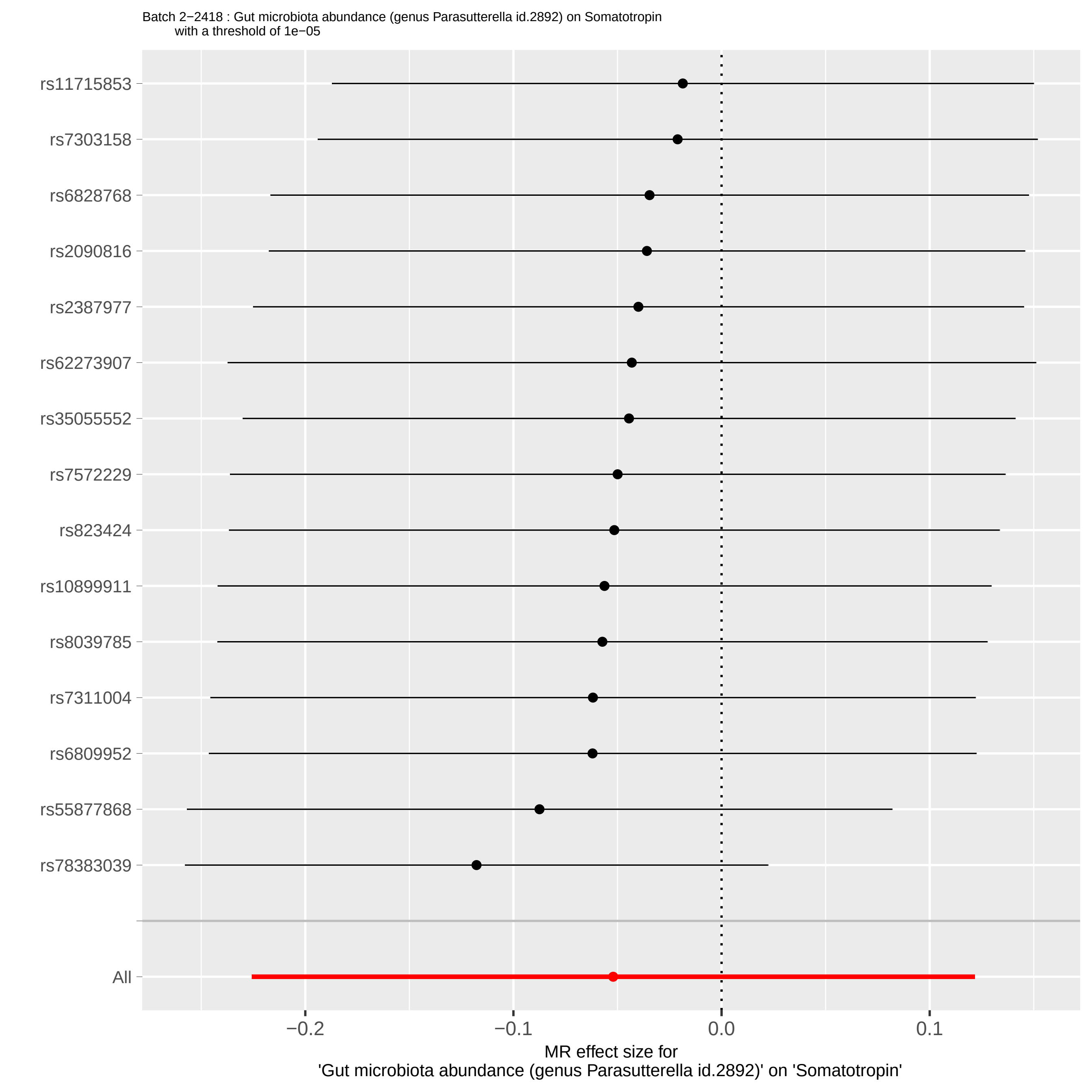

Batch 2−2418 : Gut microbiota abundance (genus Parasutterella id.2892) on Somatotropin
with a threshold of 1e−05
rs11715853
rs7303158
rs6828768
rs2090816
rs2387977
rs62273907
rs35055552
rs7572229
rs823424
rs10899911
rs8039785
rs7311004
rs6809952
rs55877868
rs78383039
All
−0.2
−0.1
0.0
0.1
MR effect size for
'Gut microbiota abundance (genus Parasutterella id.2892)' on 'Somatotropin'

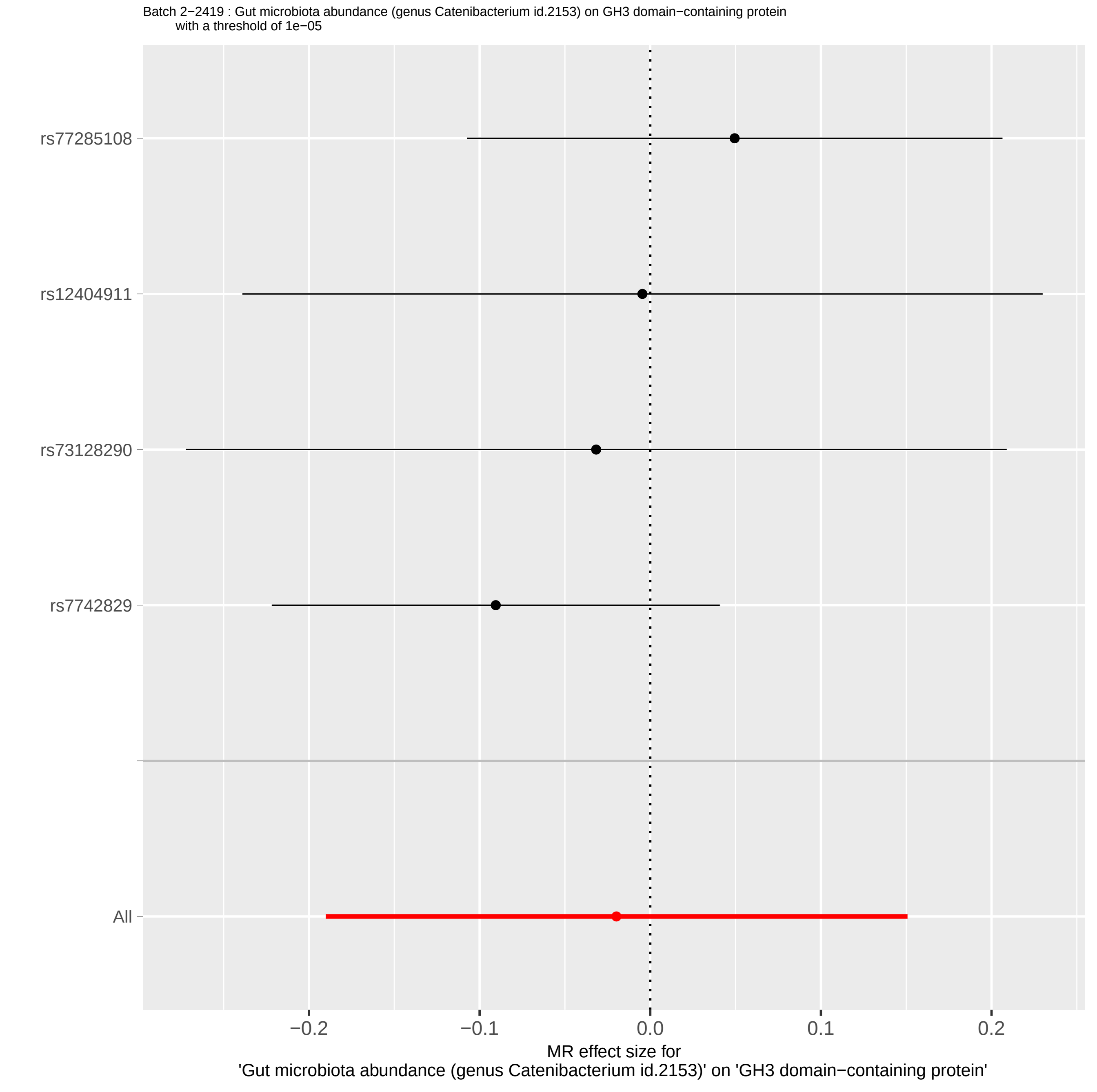
Batch 2−2419 : Gut microbiota abundance (genus Catenibacterium id.2153) on GH3 domain−containing protein
with a threshold of 1e−05
rs77285108
rs12404911
rs73128290
rs7742829
All
−0.2
−0.1
0.0
0.1
0.2
MR effect size for
'Gut microbiota abundance (genus Catenibacterium id.2153)' on 'GH3 domain−containing protein'

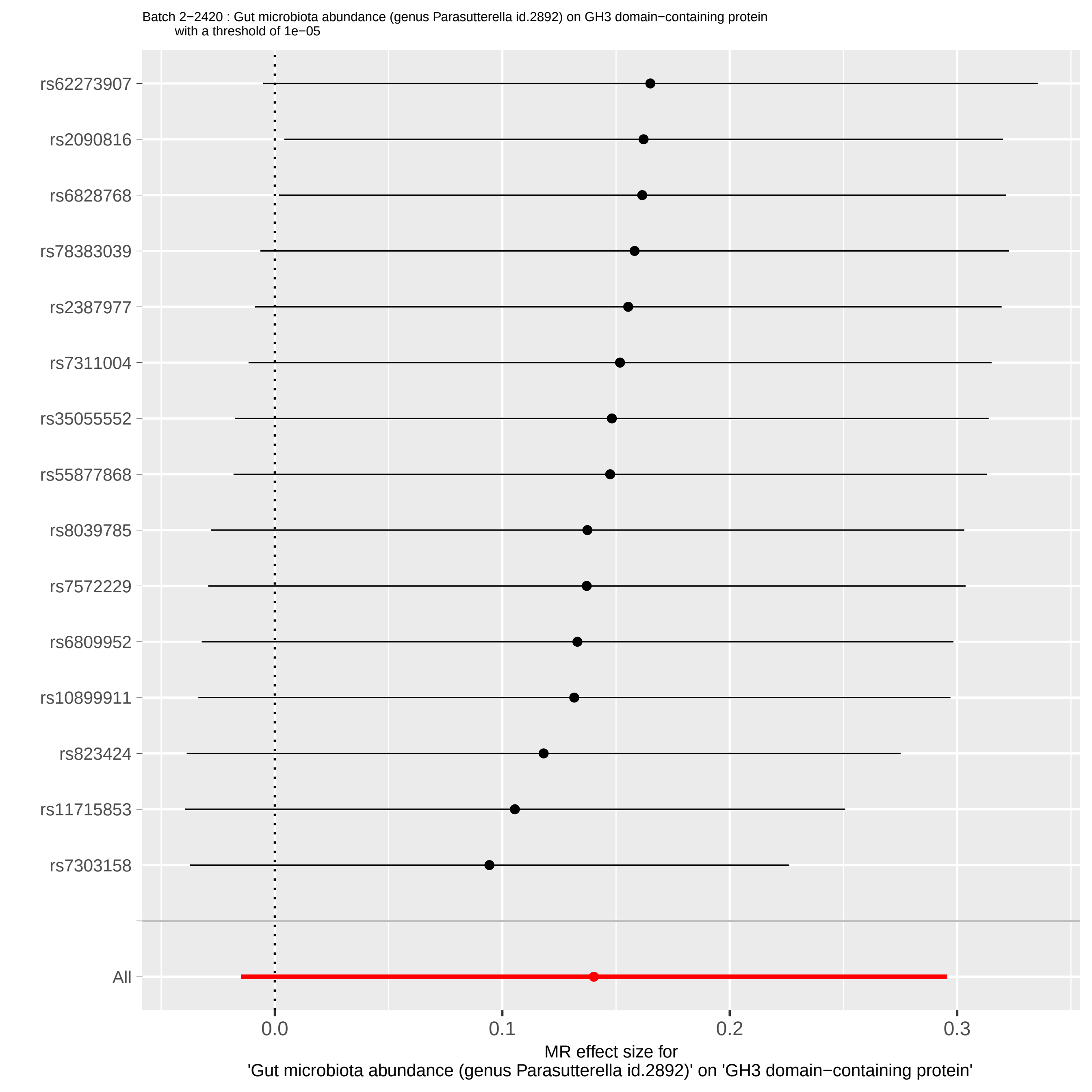

Batch 2−2420 : Gut microbiota abundance (genus Parasutterella id.2892) on GH3 domain−containing protein
with a threshold of 1e−05
rs62273907
rs2090816
rs6828768
rs78383039
rs2387977
rs7311004
rs35055552
rs55877868
rs8039785
rs7572229
rs6809952
rs10899911
rs823424
rs11715853
rs7303158
All
0.0
0.1
0.2
0.3
MR effect size for
'Gut microbiota abundance (genus Parasutterella id.2892)' on 'GH3 domain−containing protein'

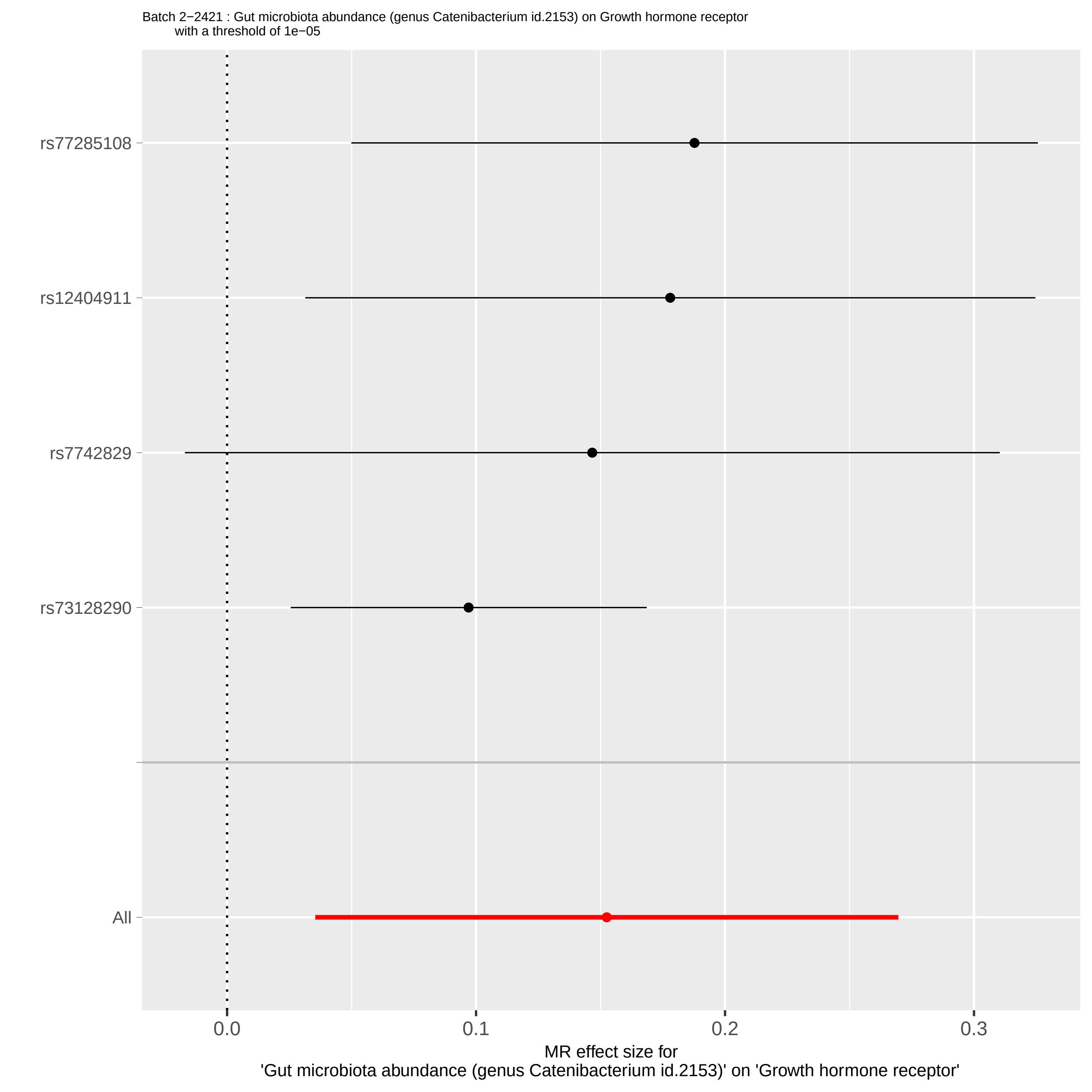

Batch 2−2421 : Gut microbiota abundance (genus Catenibacterium id.2153) on Growth hormone receptor
with a threshold of 1e−05
rs77285108
rs12404911
rs7742829
rs73128290
All
0.0
0.1
0.2
0.3
MR effect size for
'Gut microbiota abundance (genus Catenibacterium id.2153)' on 'Growth hormone receptor'

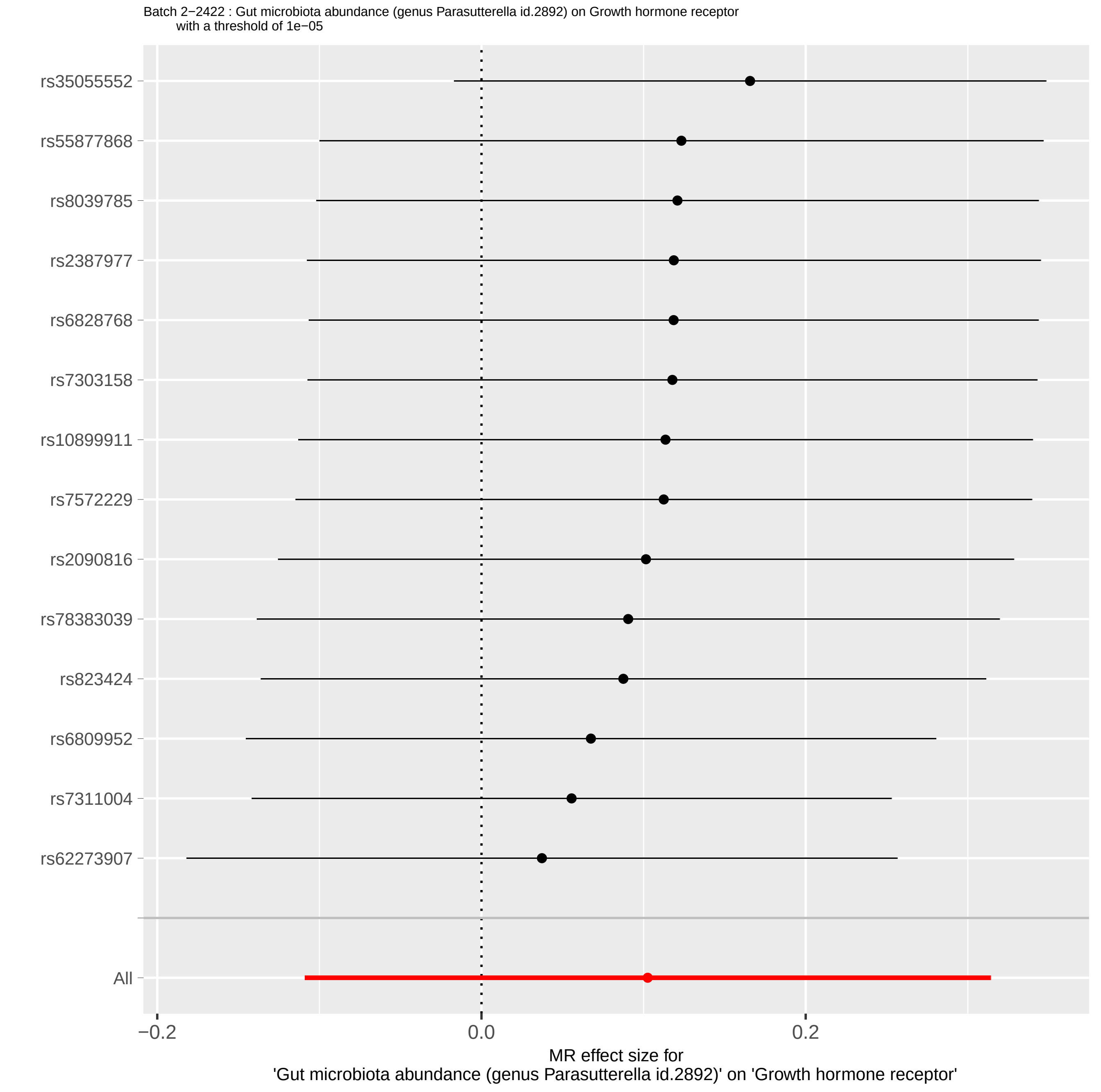
Batch 2−2422 : Gut microbiota abundance (genus Parasutterella id.2892) on Growth hormone receptor
with a threshold of 1e−05
rs35055552
rs55877868
rs8039785
rs2387977
rs6828768
rs7303158
rs10899911
rs7572229
rs2090816
rs78383039
rs823424
rs6809952
rs7311004
rs62273907
All
−0.2
0.0
0.2
MR effect size for
'Gut microbiota abundance (genus Parasutterella id.2892)' on 'Growth hormone receptor'

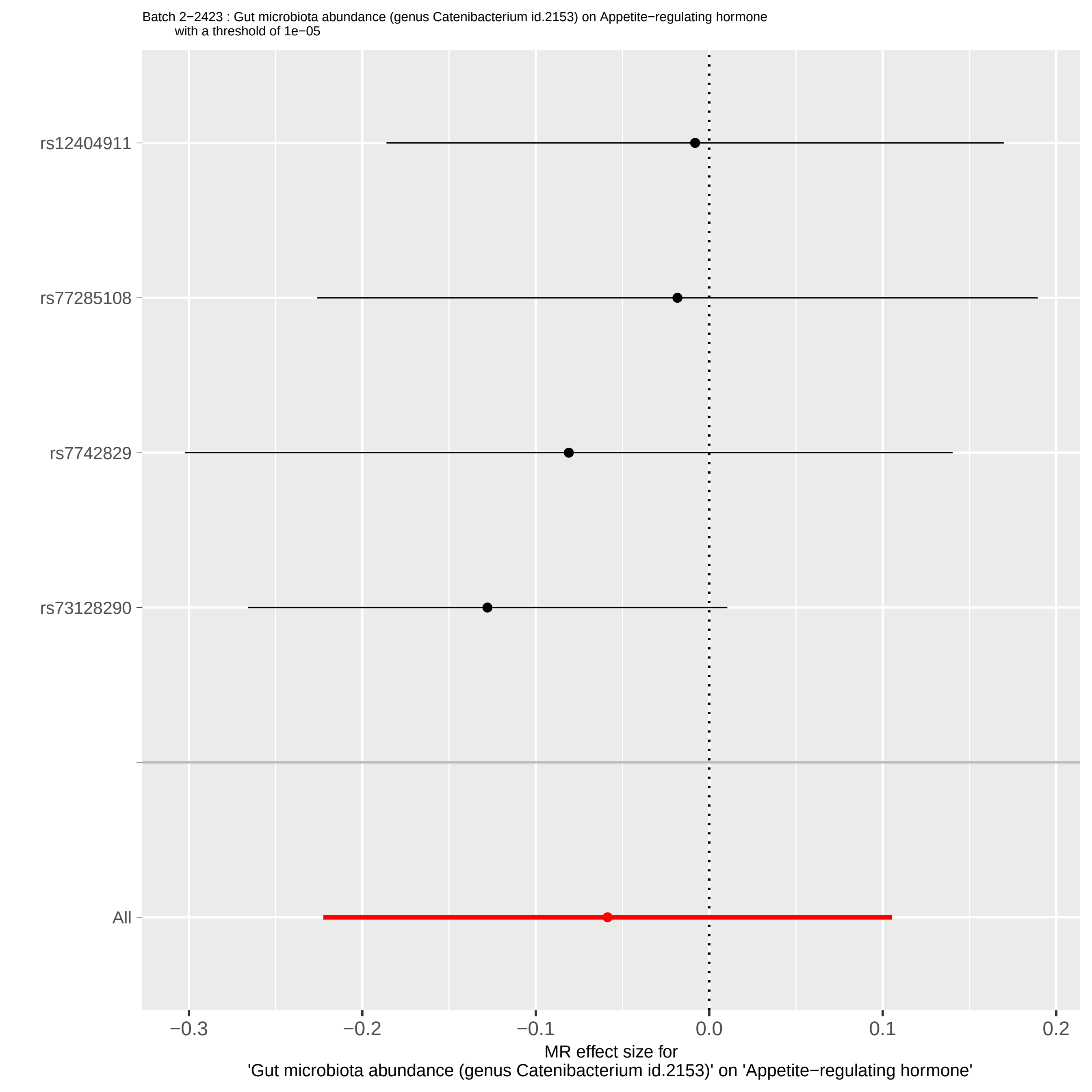
Batch 2−2423 : Gut microbiota abundance (genus Catenibacterium id.2153) on Appetite−regulating hormone
with a threshold of 1e−05
rs12404911
rs77285108
rs7742829
rs73128290
All
−0.3
−0.2
−0.1
0.0
0.1
0.2
MR effect size for
'Gut microbiota abundance (genus Catenibacterium id.2153)' on 'Appetite−regulating hormone'

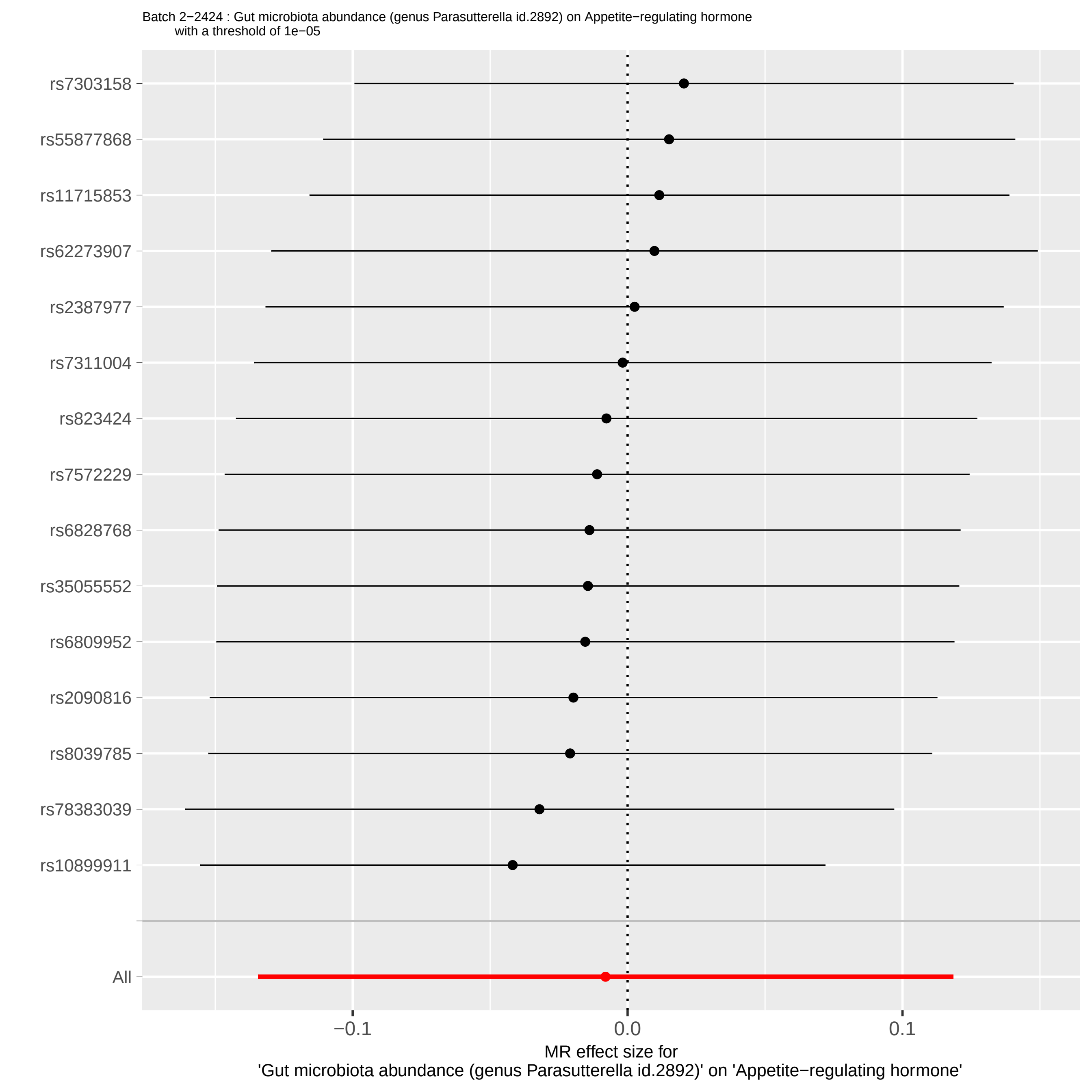
Batch 2−2424 : Gut microbiota abundance (genus Parasutterella id.2892) on Appetite−regulating hormone
with a threshold of 1e−05
rs7303158
rs55877868
rs11715853
rs62273907
rs2387977
rs7311004
rs823424
rs7572229
rs6828768
rs35055552
rs6809952
rs2090816
rs8039785
rs78383039
rs10899911
All
−0.1
0.0
0.1
MR effect size for
'Gut microbiota abundance (genus Parasutterella id.2892)' on 'Appetite−regulating hormone'

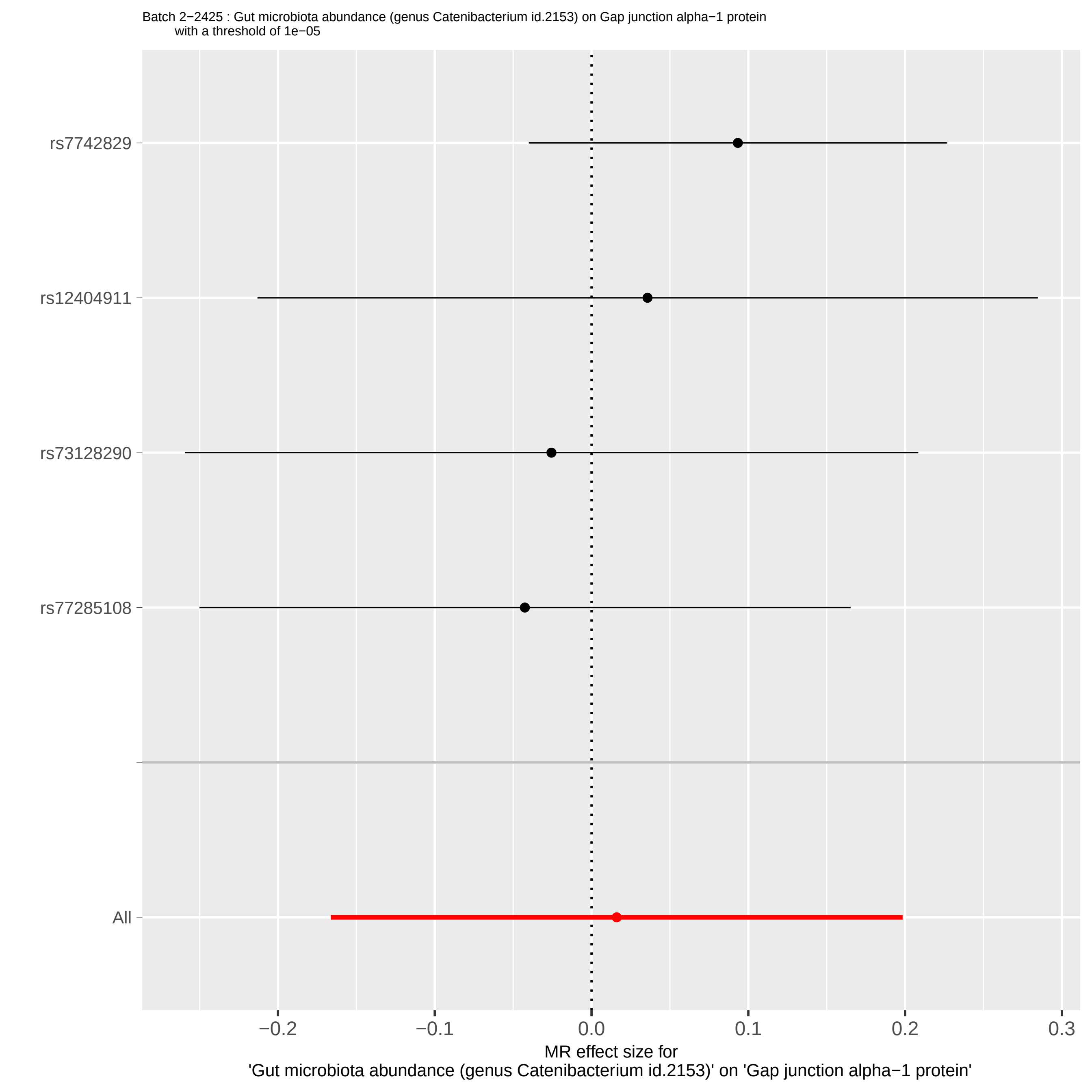
Batch 2−2425 : Gut microbiota abundance (genus Catenibacterium id.2153) on Gap junction alpha−1 protein
with a threshold of 1e−05
rs7742829
rs12404911
rs73128290
rs77285108
All
−0.2
−0.1
0.0
0.1
0.2
0.3
MR effect size for
'Gut microbiota abundance (genus Catenibacterium id.2153)' on 'Gap junction alpha−1 protein'

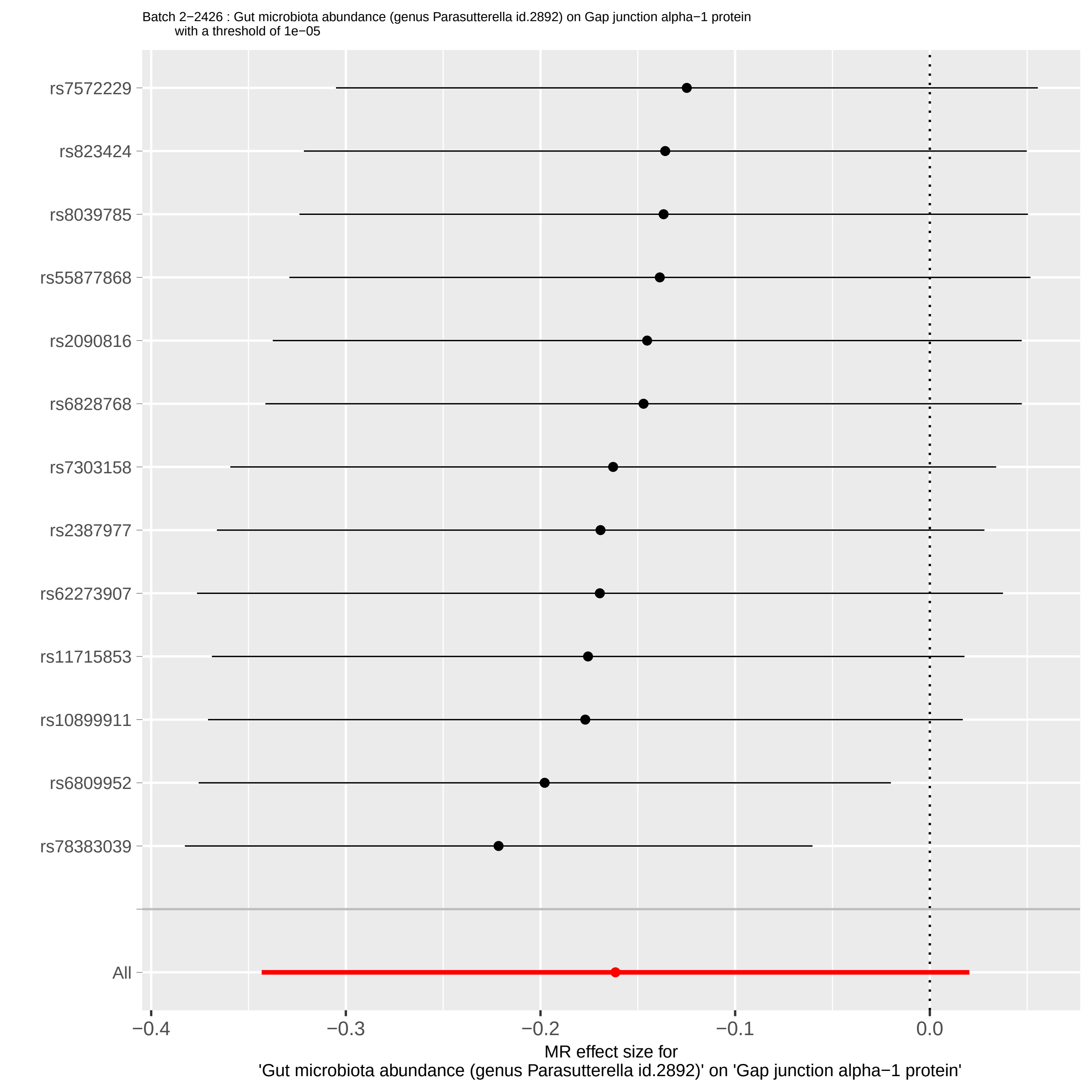

Batch 2−2426 : Gut microbiota abundance (genus Parasutterella id.2892) on Gap junction alpha−1 protein
with a threshold of 1e−05
rs7572229
rs823424
rs8039785
rs55877868
rs2090816
rs6828768
rs7303158
rs2387977
rs62273907
rs11715853
rs10899911
rs6809952
rs78383039
All
−0.4
−0.3
−0.2
−0.1
0.0
MR effect size for
'Gut microbiota abundance (genus Parasutterella id.2892)' on 'Gap junction alpha−1 protein'

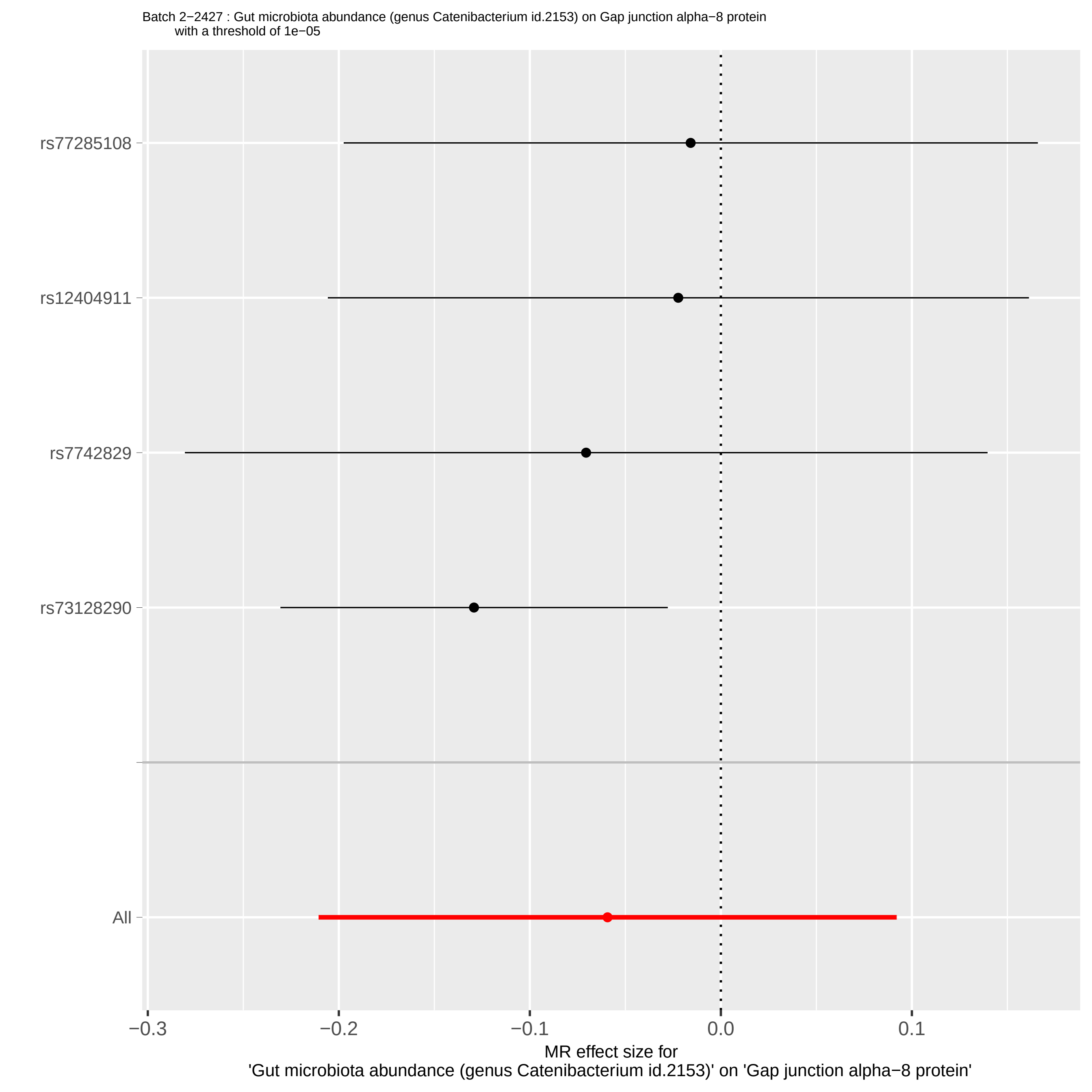
Batch 2−2427 : Gut microbiota abundance (genus Catenibacterium id.2153) on Gap junction alpha−8 protein
with a threshold of 1e−05
rs77285108
rs12404911
rs7742829
rs73128290
All
−0.3
−0.2
−0.1
0.0
0.1
MR effect size for
'Gut microbiota abundance (genus Catenibacterium id.2153)' on 'Gap junction alpha−8 protein'

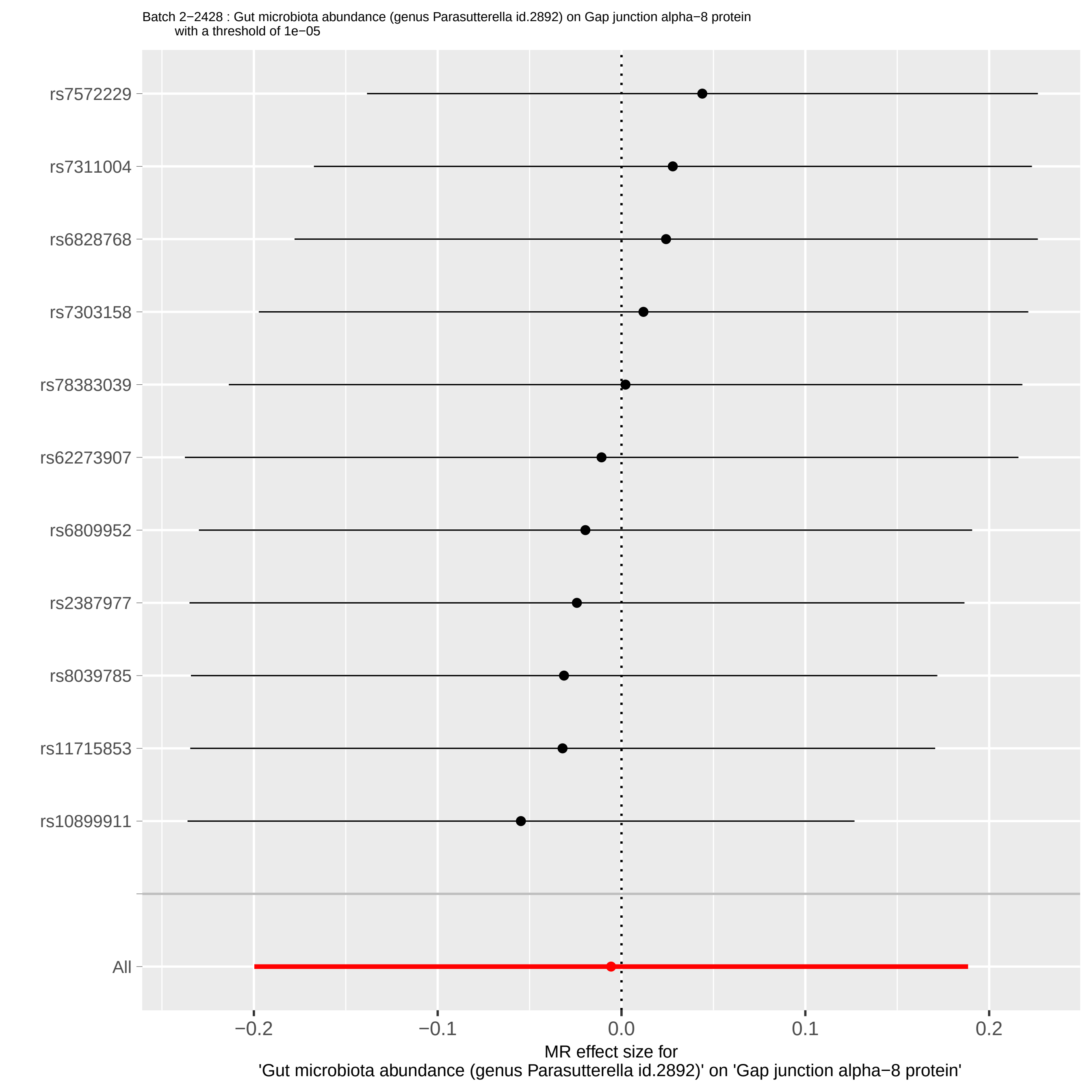

Batch 2−2428 : Gut microbiota abundance (genus Parasutterella id.2892) on Gap junction alpha−8 protein
with a threshold of 1e−05
rs7572229
rs7311004
rs6828768
rs7303158
rs78383039
rs62273907
rs6809952
rs2387977
rs8039785
rs11715853
rs10899911
All
−0.2
−0.1
0.0
0.1
0.2
MR effect size for
'Gut microbiota abundance (genus Parasutterella id.2892)' on 'Gap junction alpha−8 protein'

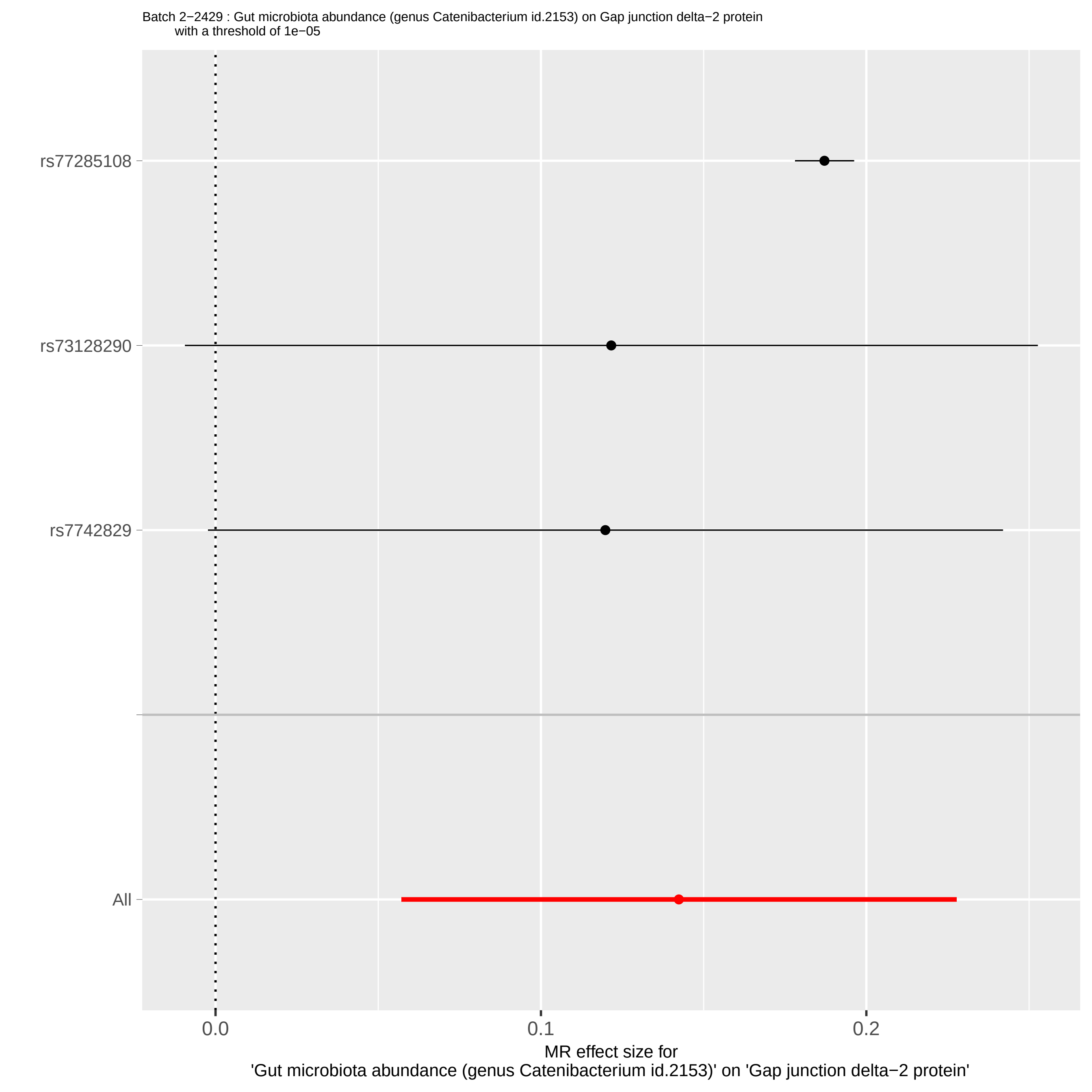
Batch 2−2429 : Gut microbiota abundance (genus Catenibacterium id.2153) on Gap junction delta−2 protein
with a threshold of 1e−05
rs77285108
rs73128290
rs7742829
All
0.0
0.1
0.2
MR effect size for
'Gut microbiota abundance (genus Catenibacterium id.2153)' on 'Gap junction delta−2 protein'

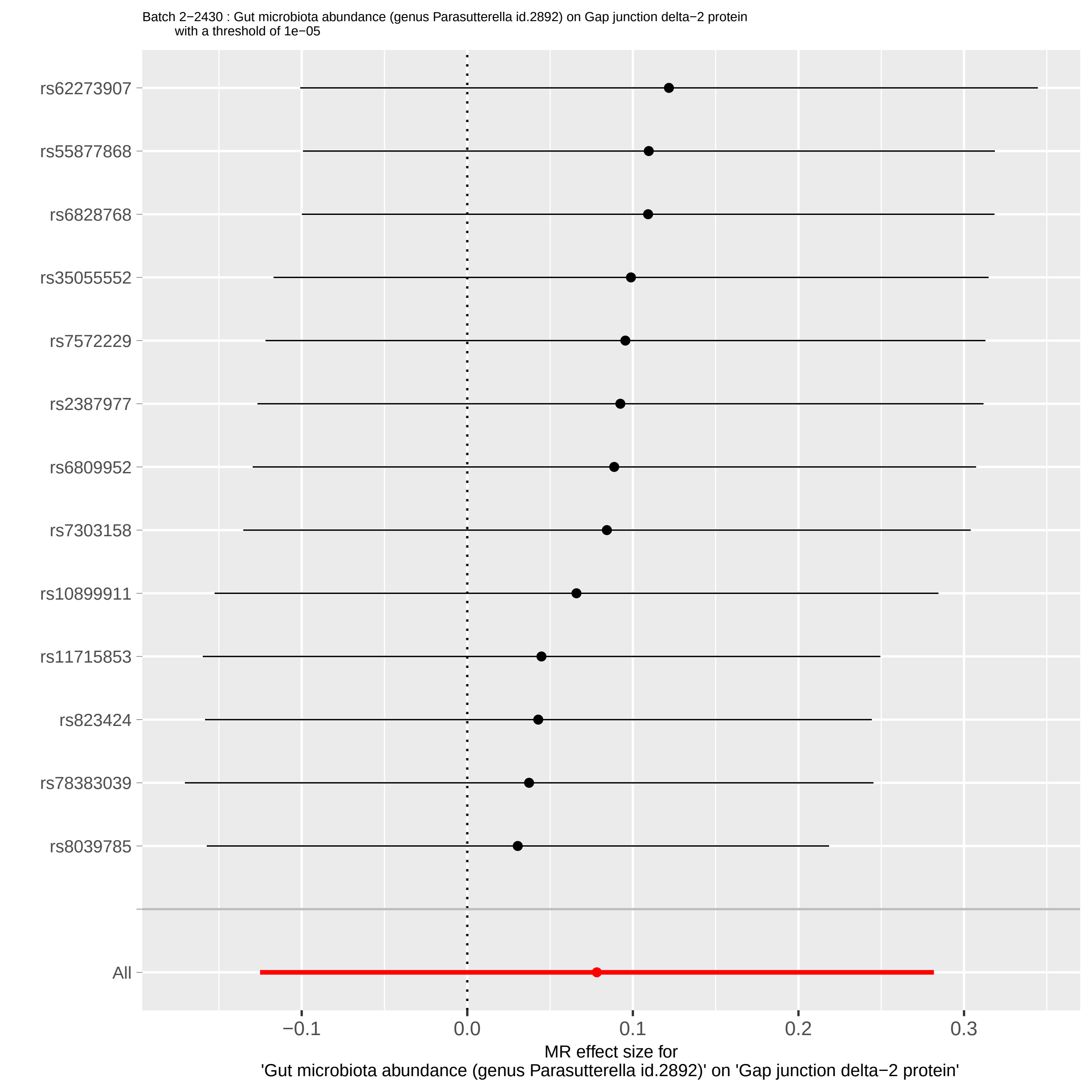

Batch 2−2430 : Gut microbiota abundance (genus Parasutterella id.2892) on Gap junction delta−2 protein
with a threshold of 1e−05
rs62273907
rs55877868
rs6828768
rs35055552
rs7572229
rs2387977
rs6809952
rs7303158
rs10899911
rs11715853
rs823424
rs78383039
rs8039785
All
−0.1
0.0
0.1
0.2
0.3
MR effect size for
'Gut microbiota abundance (genus Parasutterella id.2892)' on 'Gap junction delta−2 protein'

Batch 2−2431 : Gut microbiota abundance (genus Catenibacterium id.2153) on D−glucuronyl C5−epimerase
with a threshold of 1e−05

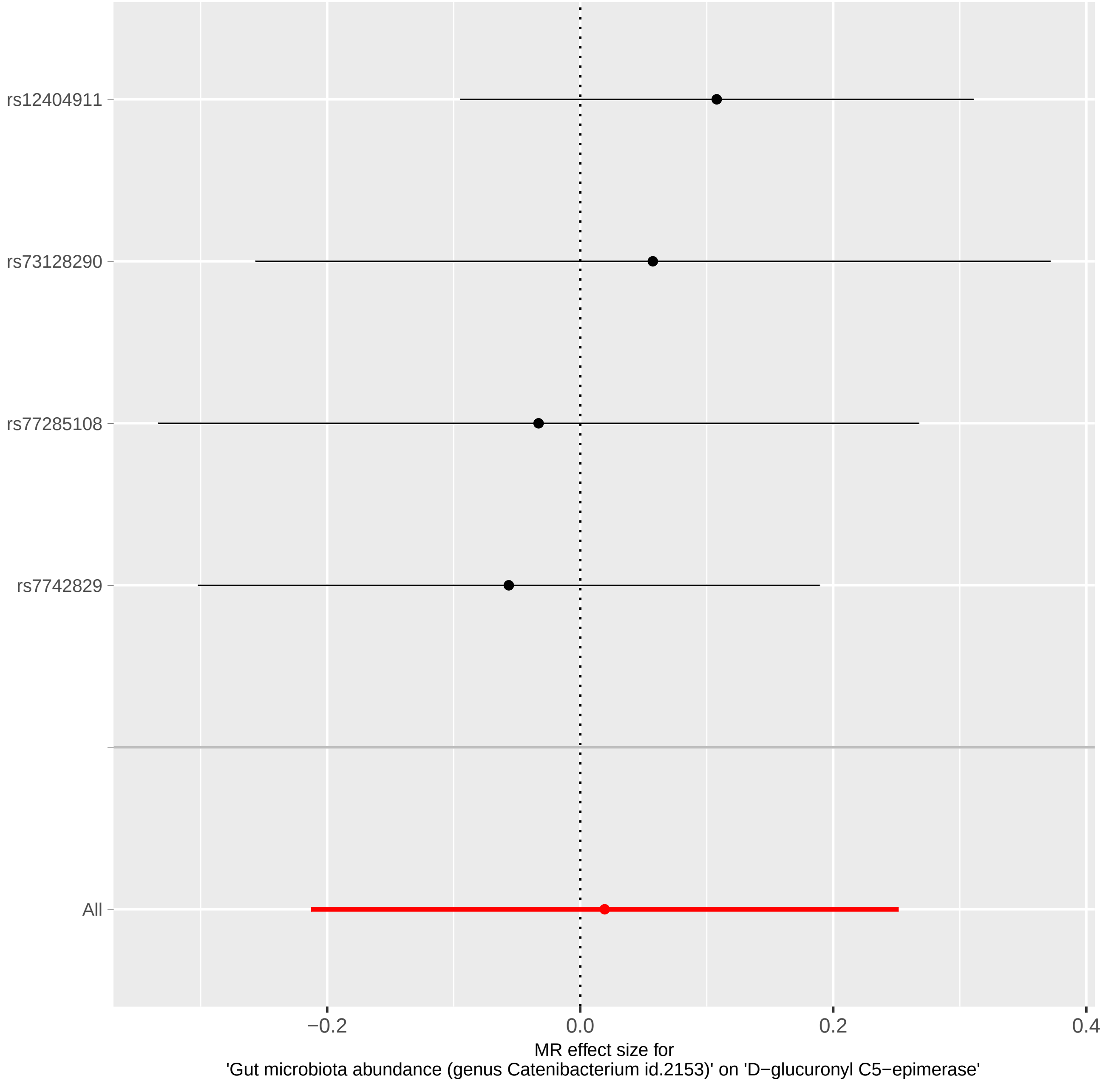

Batch 2−2432 : Gut microbiota abundance (genus Parasutterella id.2892) on D−glucuronyl C5−epimerase
with a threshold of 1e−05

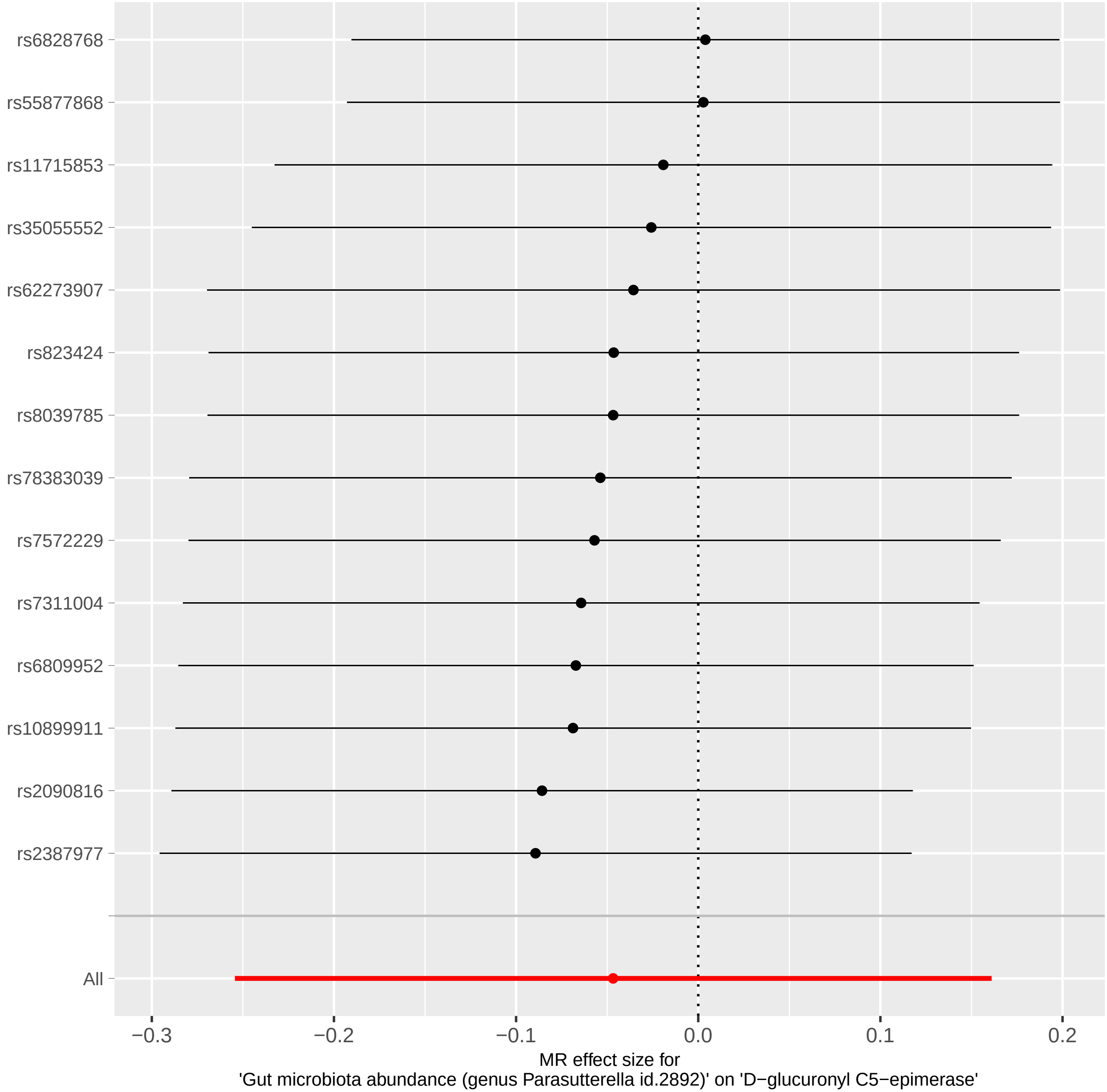

Batch 2−2433 : Gut microbiota abundance (genus Catenibacterium id.2153) on Glioma pathogenesis−related protein 1
with a threshold of 1e−05

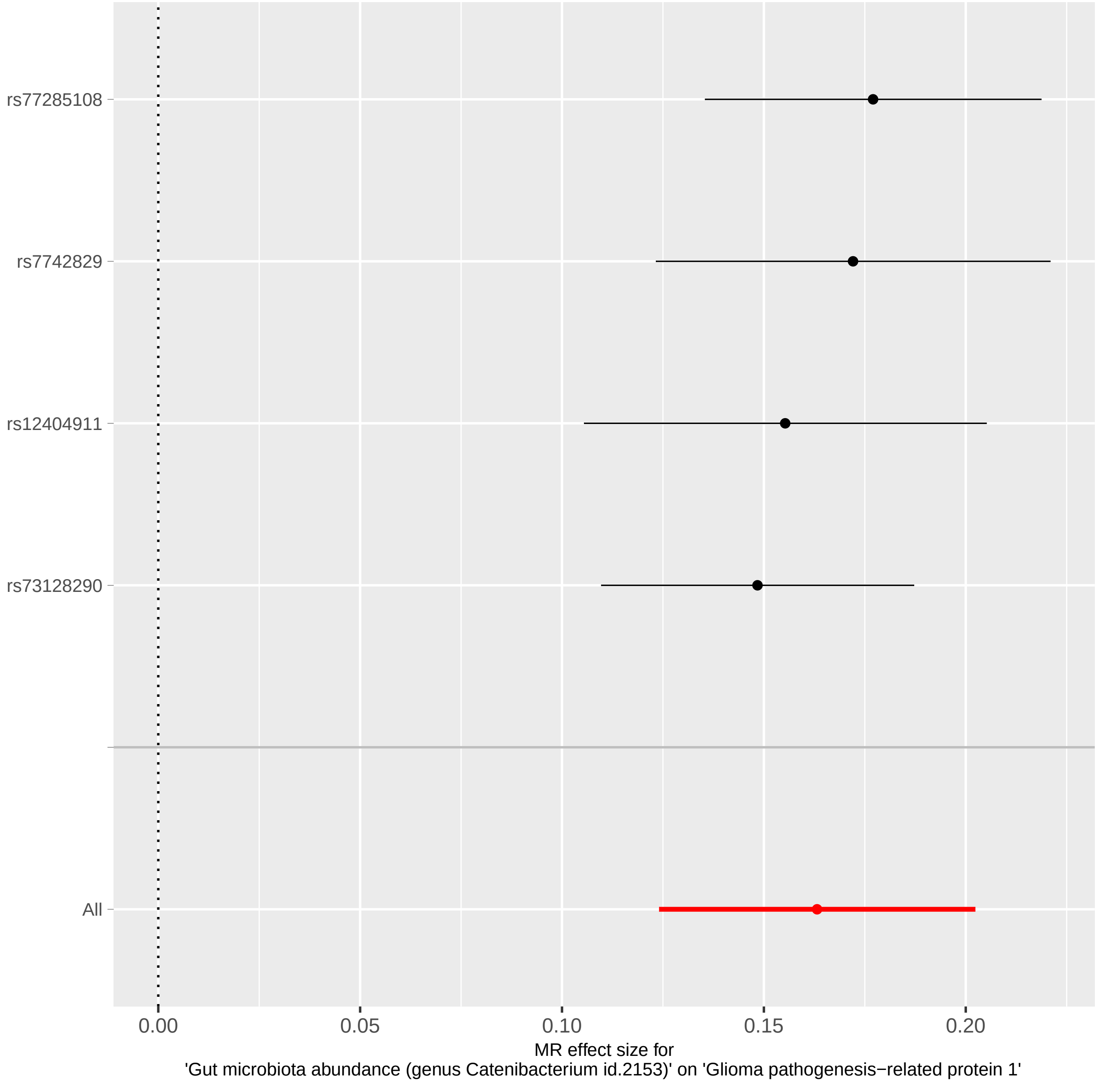

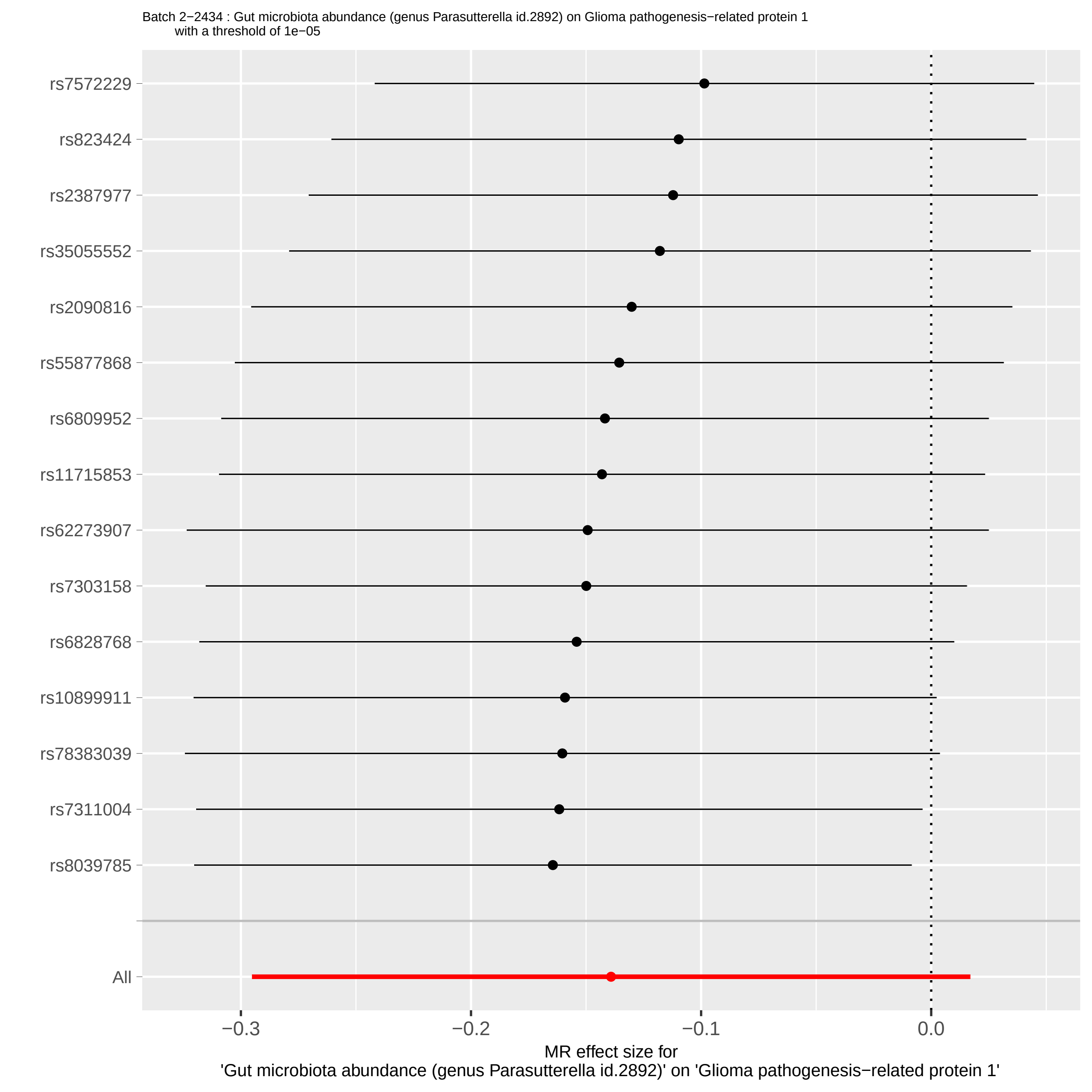

Batch 2−2434 : Gut microbiota abundance (genus Parasutterella id.2892) on Glioma pathogenesis−related protein 1
with a threshold of 1e−05
rs7572229
rs823424
rs2387977
rs35055552
rs2090816
rs55877868
rs6809952
rs11715853
rs62273907
rs7303158
rs6828768
rs10899911
rs78383039
rs7311004
rs8039785
All
−0.3
−0.2
−0.1
0.0
MR effect size for
'Gut microbiota abundance (genus Parasutterella id.2892)' on 'Glioma pathogenesis−related protein 1'

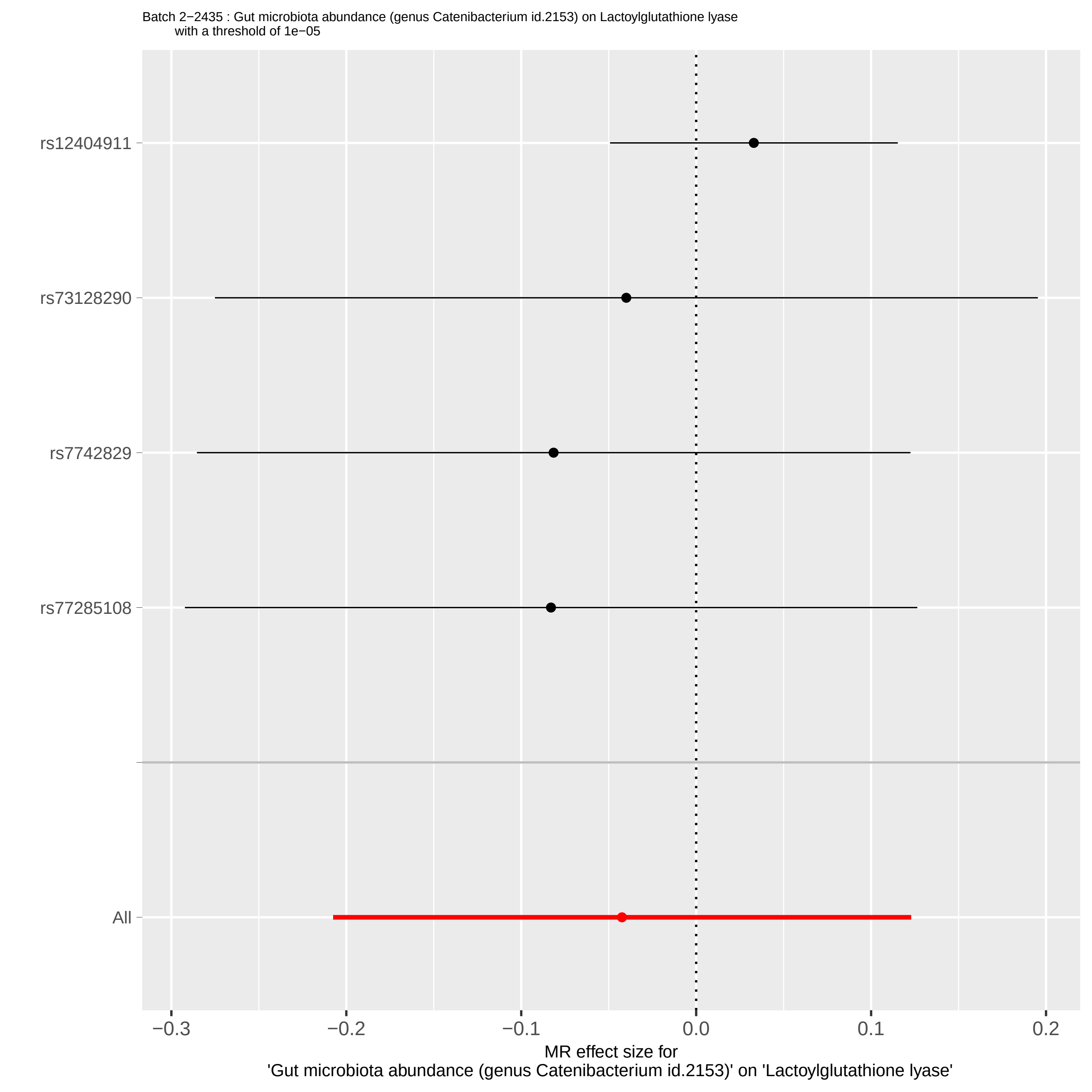
Batch 2−2435 : Gut microbiota abundance (genus Catenibacterium id.2153) on Lactoylglutathione lyase
with a threshold of 1e−05
rs12404911
rs73128290
rs7742829
rs77285108
All
−0.3
−0.2
−0.1
0.0
0.1
0.2
MR effect size for
'Gut microbiota abundance (genus Catenibacterium id.2153)' on 'Lactoylglutathione lyase'

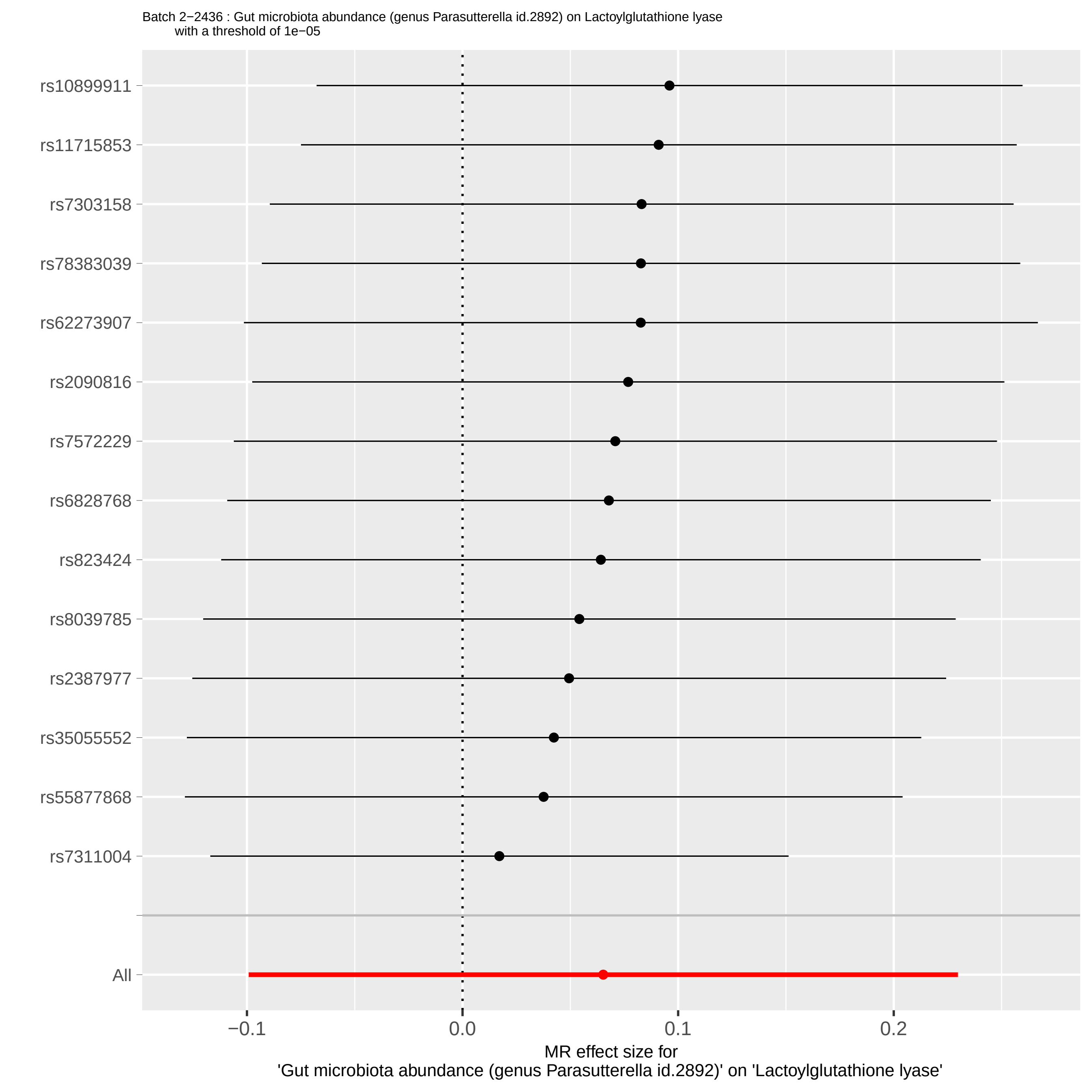

Batch 2−2436 : Gut microbiota abundance (genus Parasutterella id.2892) on Lactoylglutathione lyase
with a threshold of 1e−05
rs10899911
rs11715853
rs7303158
rs78383039
rs62273907
rs2090816
rs7572229
rs6828768
rs823424
rs8039785
rs2387977
rs35055552
rs55877868
rs7311004
All
−0.1
0.0
0.1
0.2
MR effect size for
'Gut microbiota abundance (genus Parasutterella id.2892)' on 'Lactoylglutathione lyase'

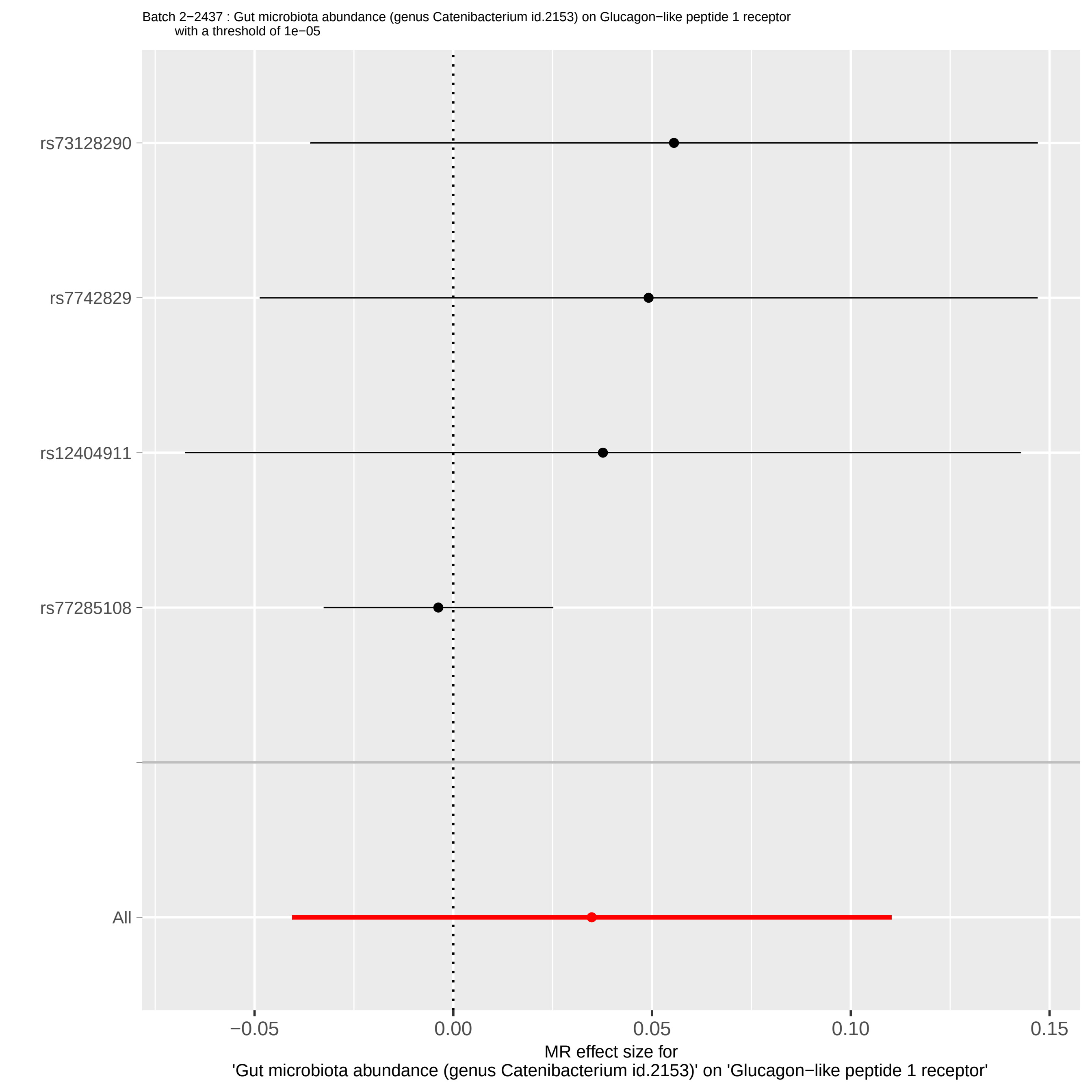
Batch 2−2437 : Gut microbiota abundance (genus Catenibacterium id.2153) on Glucagon−like peptide 1 receptor
with a threshold of 1e−05
rs73128290
rs7742829
rs12404911
rs77285108
All
−0.05
0.00
0.05
0.10
0.15
MR effect size for
'Gut microbiota abundance (genus Catenibacterium id.2153)' on 'Glucagon−like peptide 1 receptor'

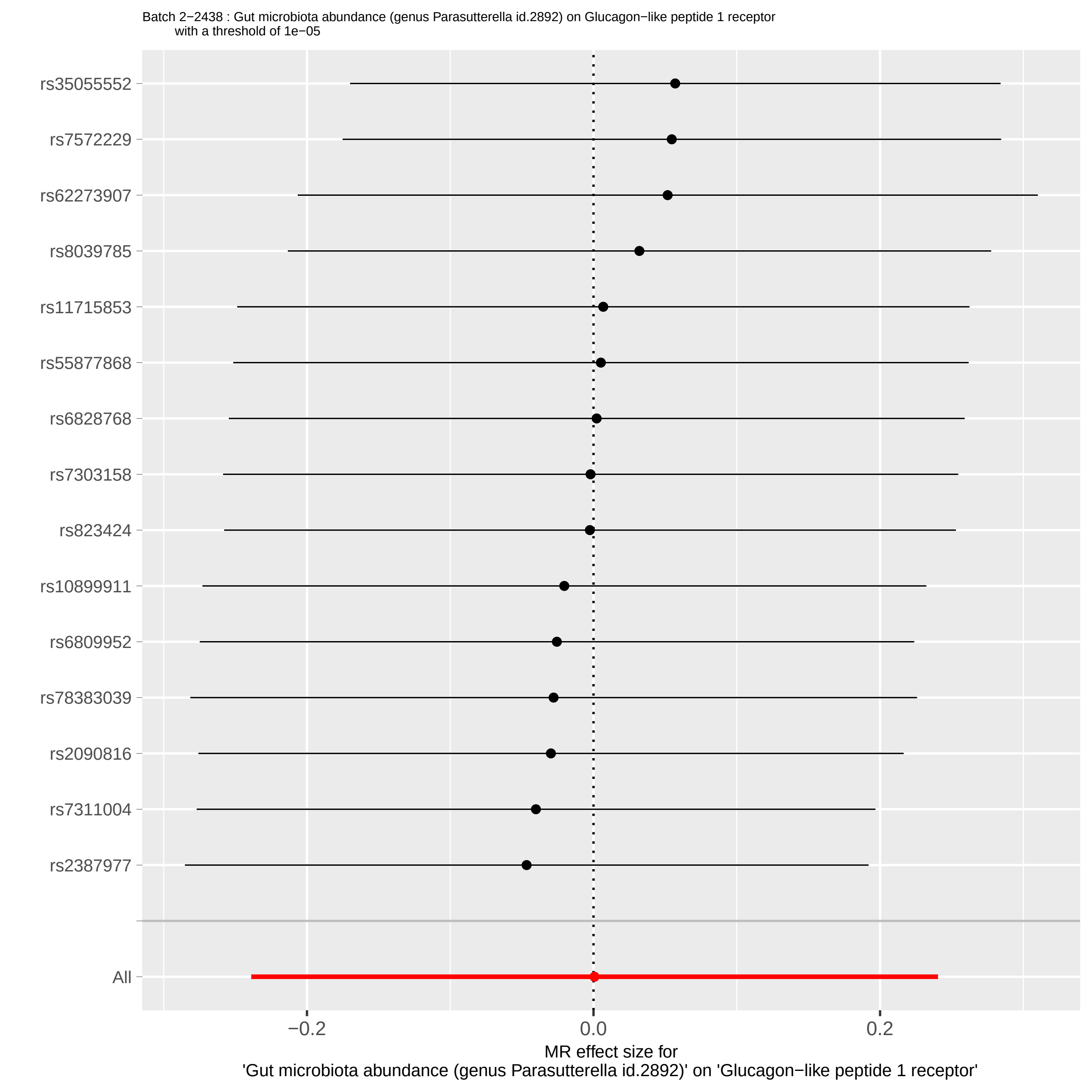
Batch 2−2438 : Gut microbiota abundance (genus Parasutterella id.2892) on Glucagon−like peptide 1 receptor
with a threshold of 1e−05
rs35055552
rs7572229
rs62273907
rs8039785
rs11715853
rs55877868
rs6828768
rs7303158
rs823424
rs10899911
rs6809952
rs78383039
rs2090816
rs7311004
rs2387977
All
−0.2
0.0
0.2
MR effect size for
'Gut microbiota abundance (genus Parasutterella id.2892)' on 'Glucagon−like peptide 1 receptor'

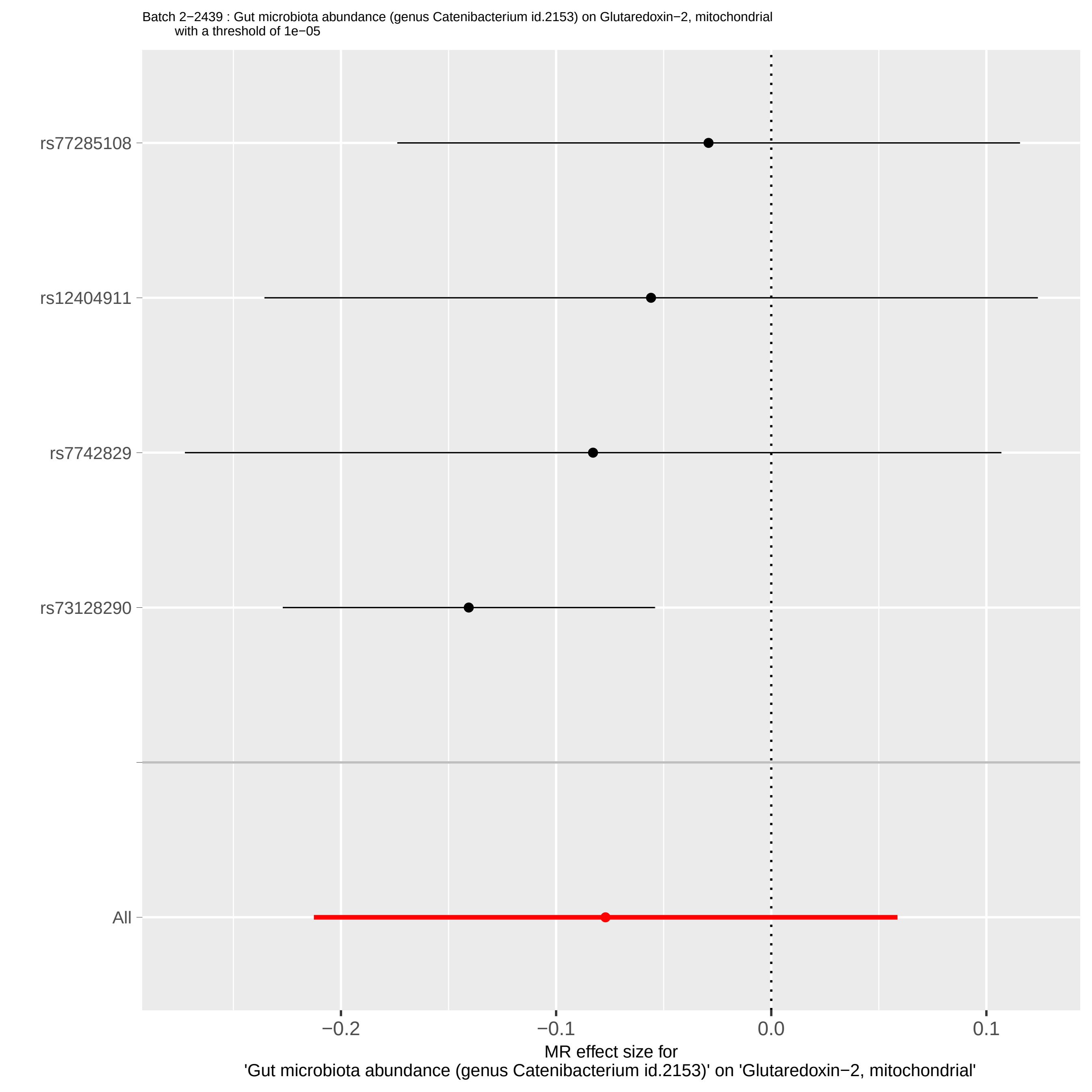
Batch 2−2439 : Gut microbiota abundance (genus Catenibacterium id.2153) on Glutaredoxin−2, mitochondrial
with a threshold of 1e−05
rs77285108
rs12404911
rs7742829
rs73128290
All
−0.2
−0.1
0.0
0.1
MR effect size for
'Gut microbiota abundance (genus Catenibacterium id.2153)' on 'Glutaredoxin−2, mitochondrial'

Batch 2−2440 : Gut microbiota abundance (genus Parasutterella id.2892) on Glutaredoxin−2, mitochondrial
with a threshold of 1e−05

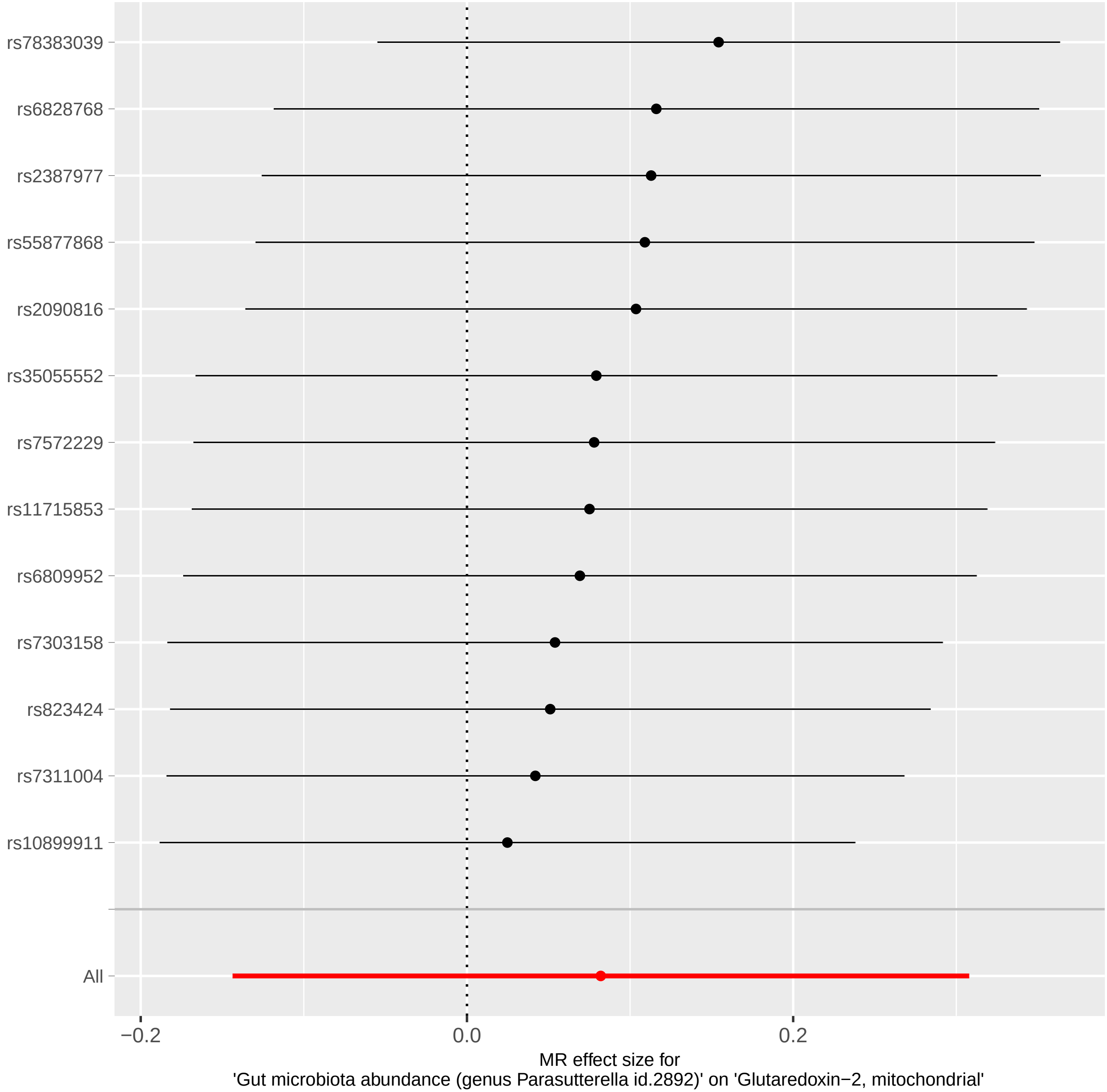

Batch 2−2441 : Gut microbiota abundance (genus Catenibacterium id.2153) on Glycosyltransferase 8 domain−containing protein 1
with a threshold of 1e−05

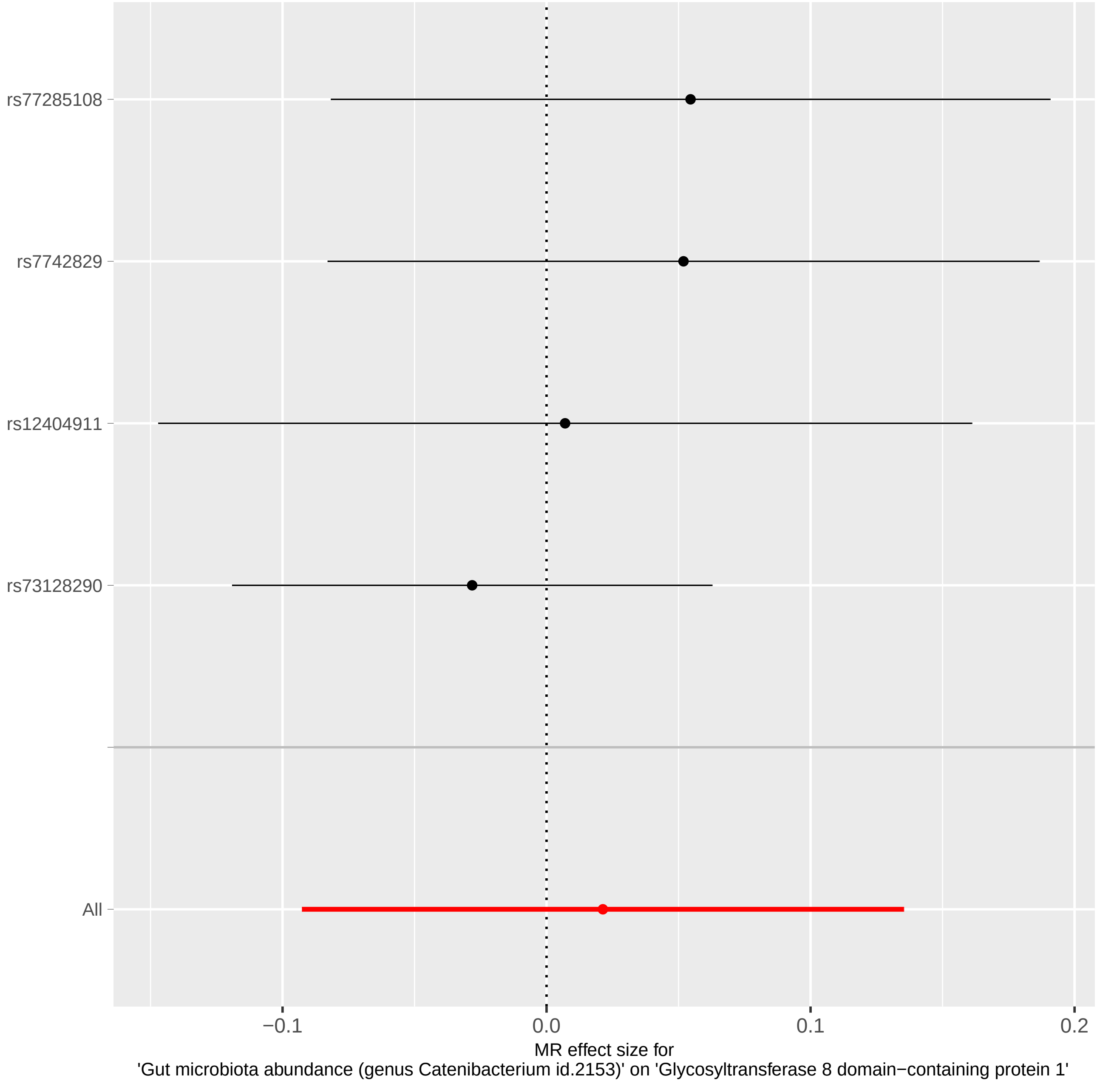

Batch 2−2442 : Gut microbiota abundance (genus Parasutterella id.2892) on Glycosyltransferase 8 domain−containing protein 1
with a threshold of 1e−05

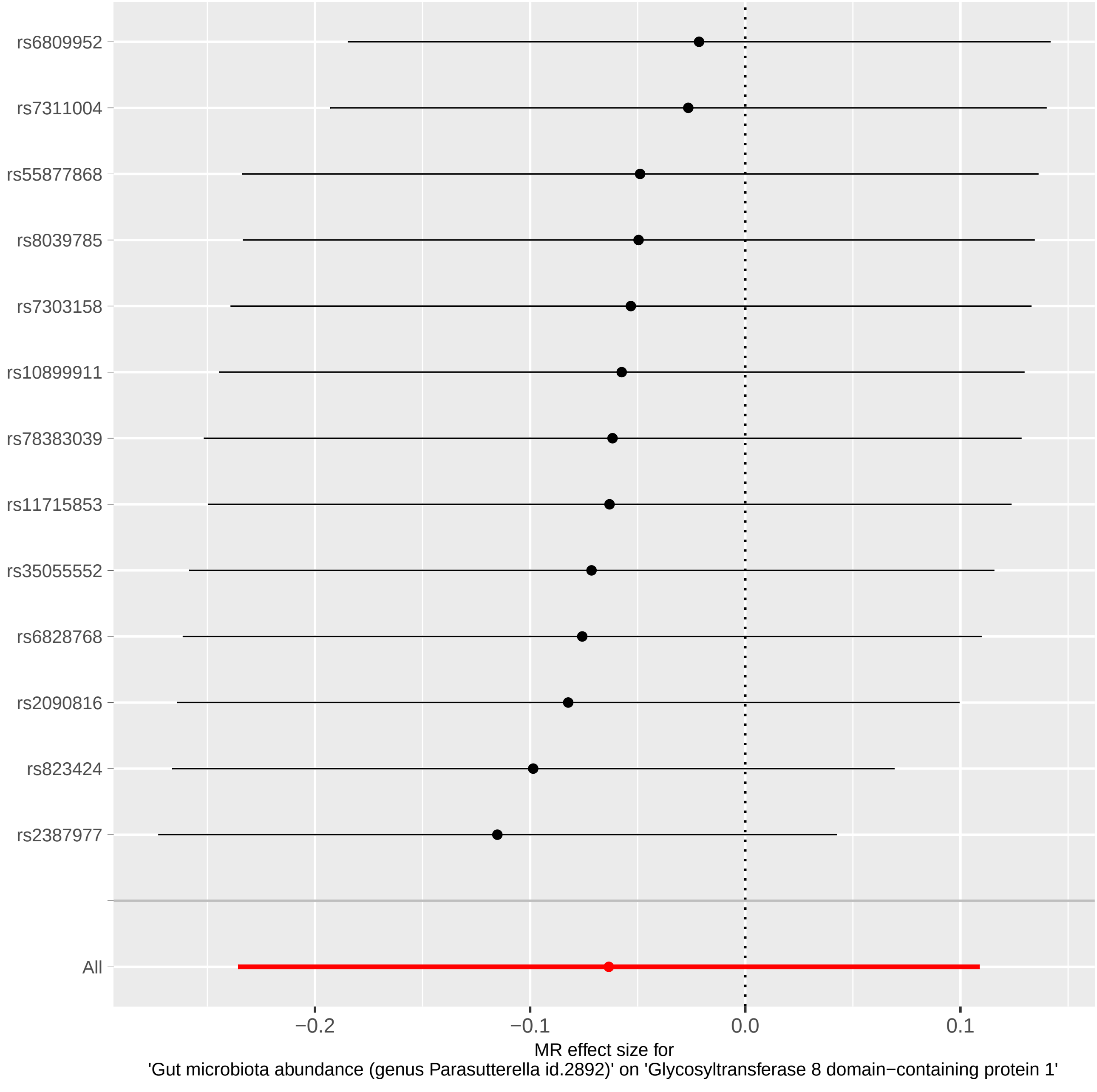

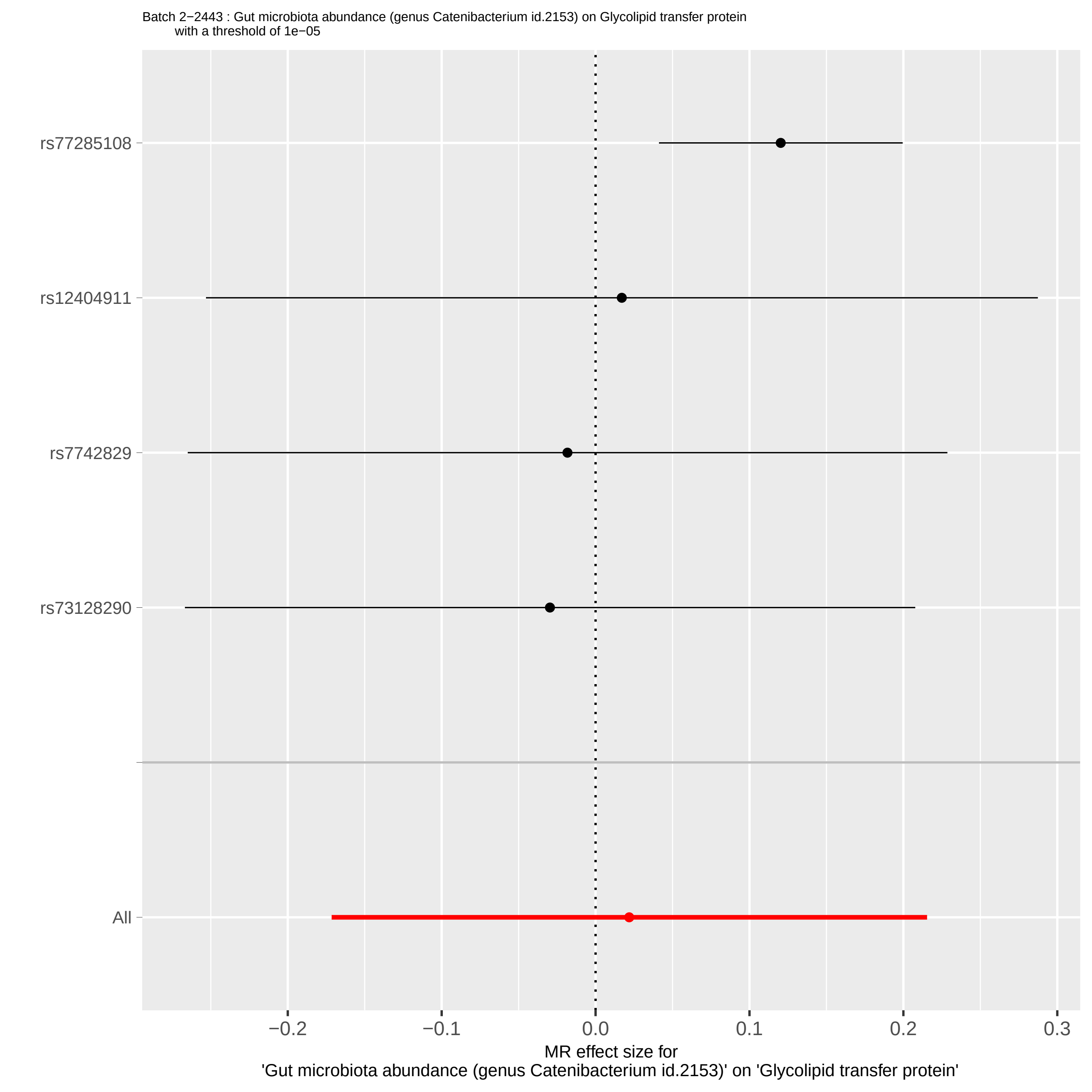
Batch 2−2443 : Gut microbiota abundance (genus Catenibacterium id.2153) on Glycolipid transfer protein
with a threshold of 1e−05
rs77285108
rs12404911
rs7742829
rs73128290
All
−0.2
−0.1
0.0
0.1
0.2
0.3
MR effect size for
'Gut microbiota abundance (genus Catenibacterium id.2153)' on 'Glycolipid transfer protein'

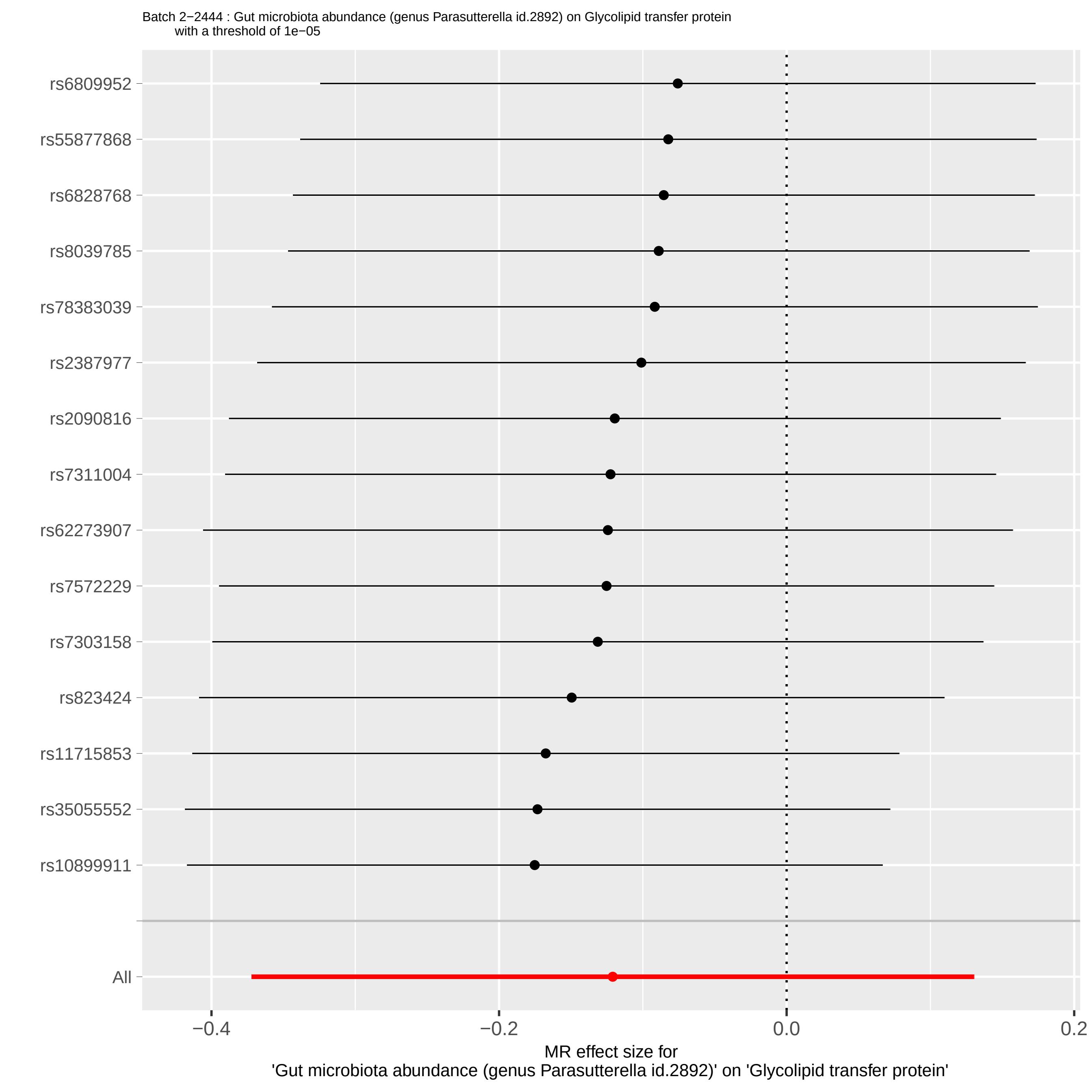
Batch 2−2444 : Gut microbiota abundance (genus Parasutterella id.2892) on Glycolipid transfer protein
with a threshold of 1e−05
rs6809952
rs55877868
rs6828768
rs8039785
rs78383039
rs2387977
rs2090816
rs7311004
rs62273907
rs7572229
rs7303158
rs823424
rs11715853
rs35055552
rs10899911
All
−0.4
−0.2
0.0
0.2
MR effect size for
'Gut microbiota abundance (genus Parasutterella id.2892)' on 'Glycolipid transfer protein'

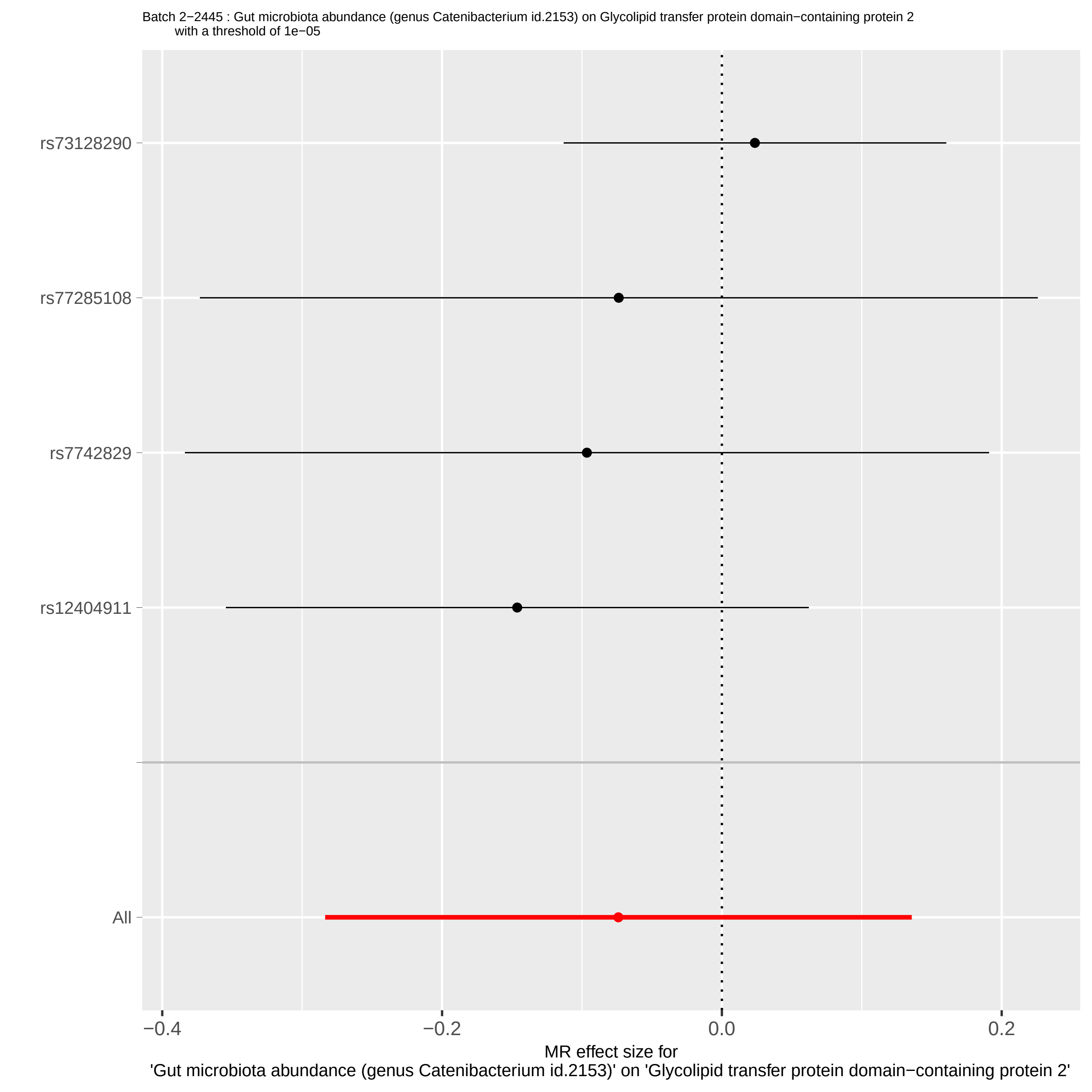
Batch 2−2445 : Gut microbiota abundance (genus Catenibacterium id.2153) on Glycolipid transfer protein domain−containing protein 2
with a threshold of 1e−05
rs73128290
rs77285108
rs7742829
rs12404911
All
−0.4
−0.2
0.0
0.2
MR effect size for
'Gut microbiota abundance (genus Catenibacterium id.2153)' on 'Glycolipid transfer protein domain−containing protein 2'

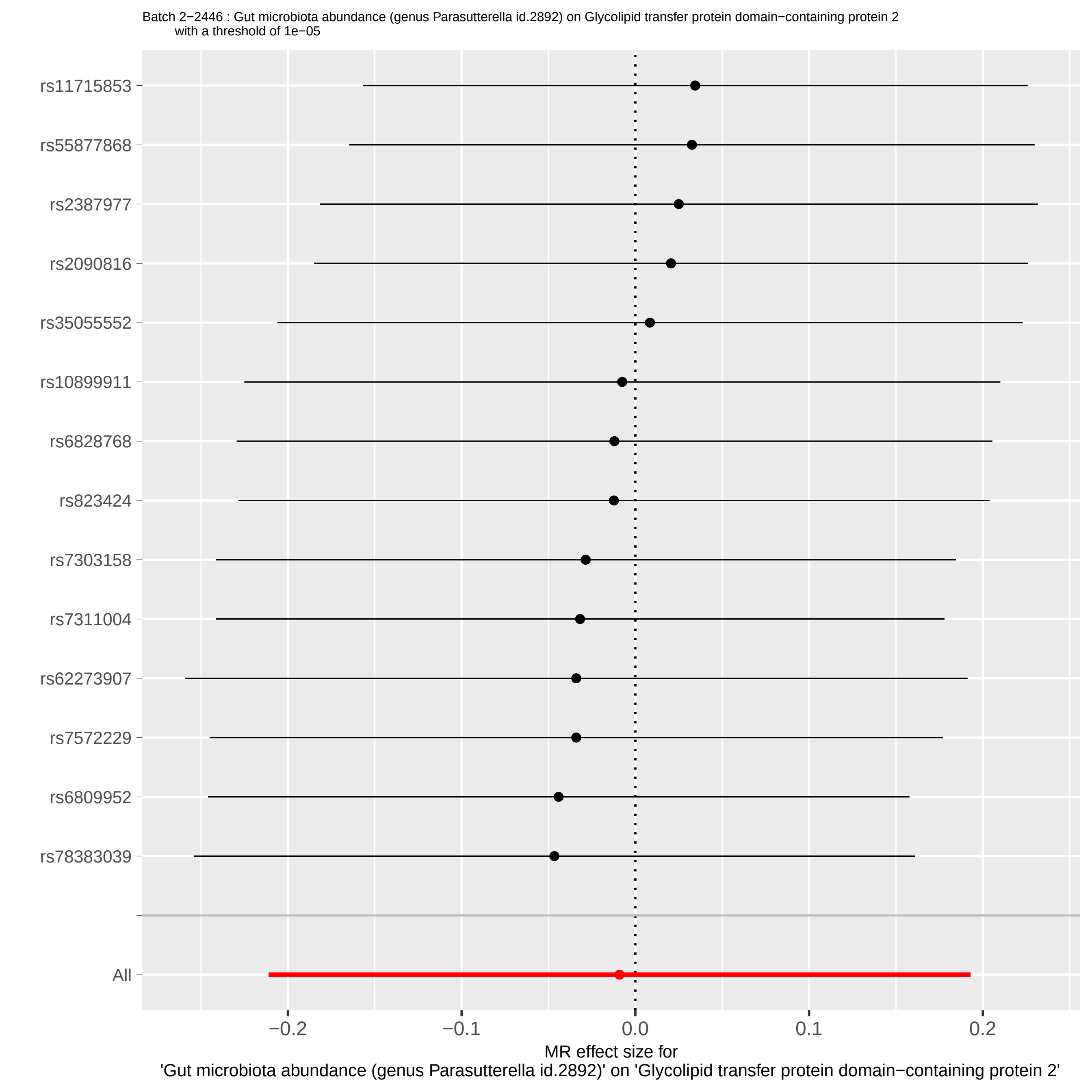
Batch 2−2446 : Gut microbiota abundance (genus Parasutterella id.2892) on Glycolipid transfer protein domain−containing protein 2
with a threshold of 1e−05
rs11715853
rs55877868
rs2387977
rs2090816
rs35055552
rs10899911
rs6828768
rs823424
rs7303158
rs7311004
rs62273907
rs7572229
rs6809952
rs78383039
All
−0.2
−0.1
0.0
0.1
0.2
MR effect size for
'Gut microbiota abundance (genus Parasutterella id.2892)' on 'Glycolipid transfer protein domain−containing protein 2'

Batch 2−2447 : Gut microbiota abundance (genus Catenibacterium id.2153) on Glucocorticoid modulatory element−binding protein 2
with a threshold of 1e−05

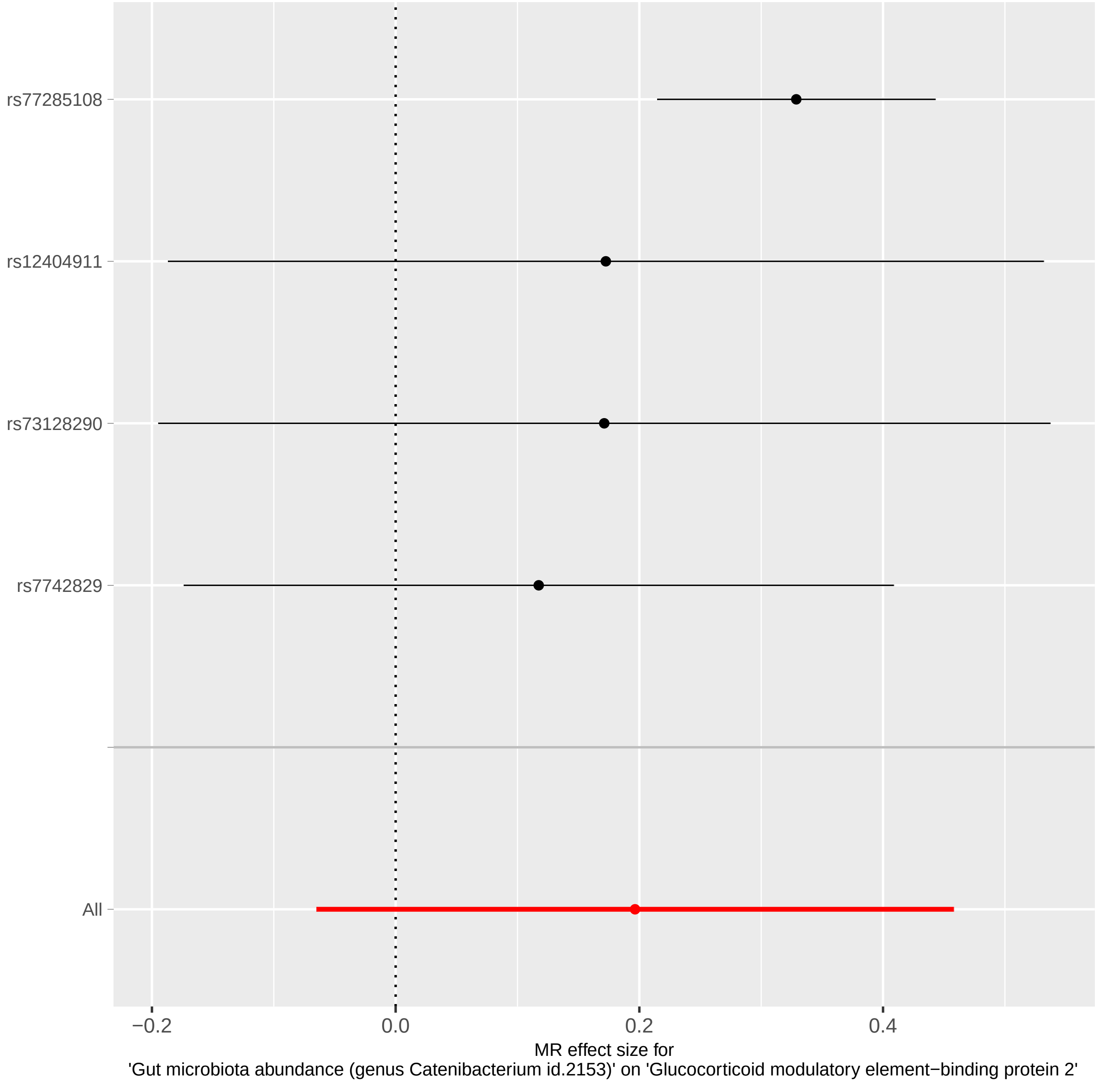

Batch 2−2448 : Gut microbiota abundance (genus Parasutterella id.2892) on Glucocorticoid modulatory element−binding protein 2
with a threshold of 1e−05

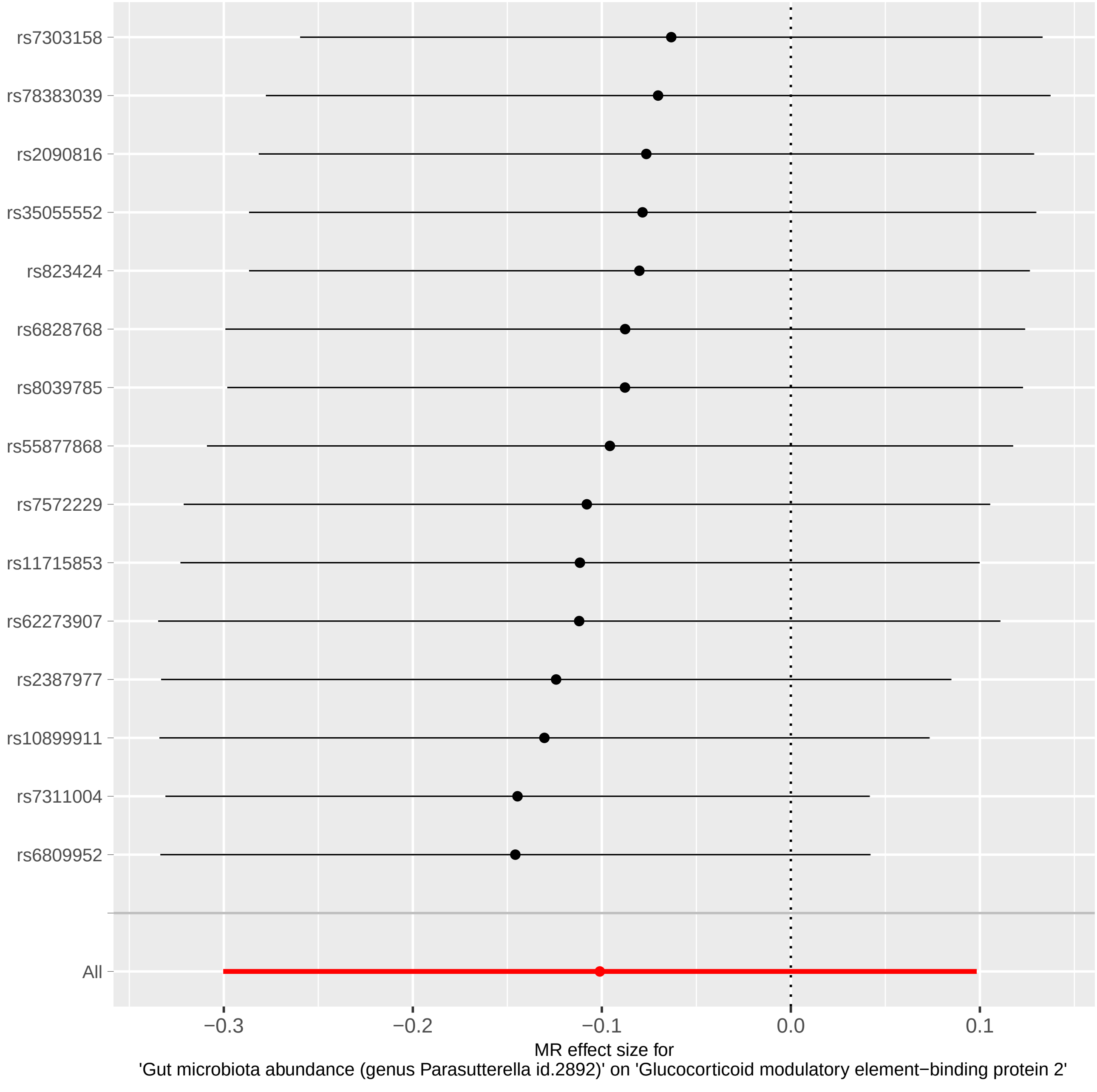

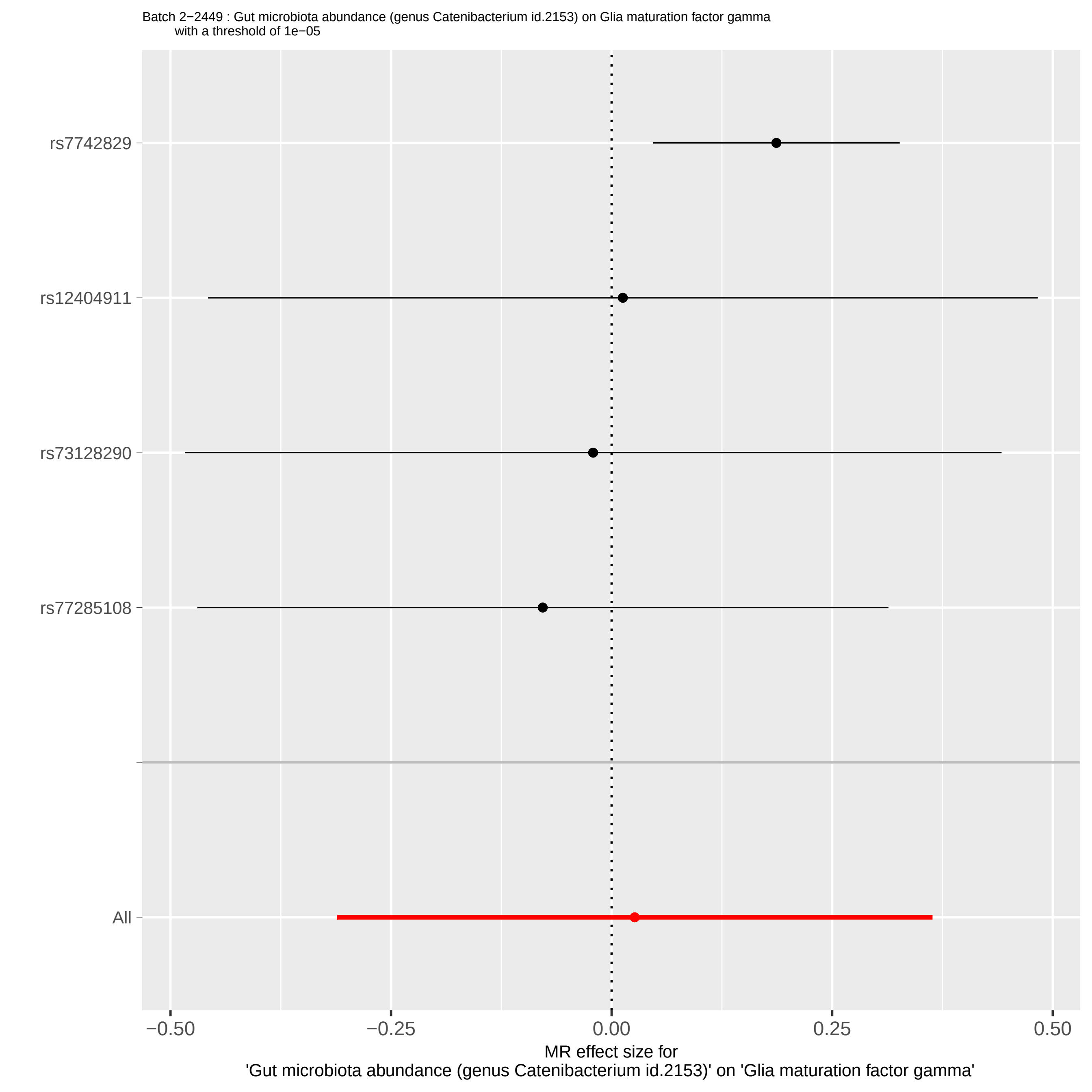

Batch 2−2449 : Gut microbiota abundance (genus Catenibacterium id.2153) on Glia maturation factor gamma
with a threshold of 1e−05
rs7742829
rs12404911
rs73128290
rs77285108
All
−0.50
−0.25
0.00
0.25
0.50
MR effect size for
'Gut microbiota abundance (genus Catenibacterium id.2153)' on 'Glia maturation factor gamma'

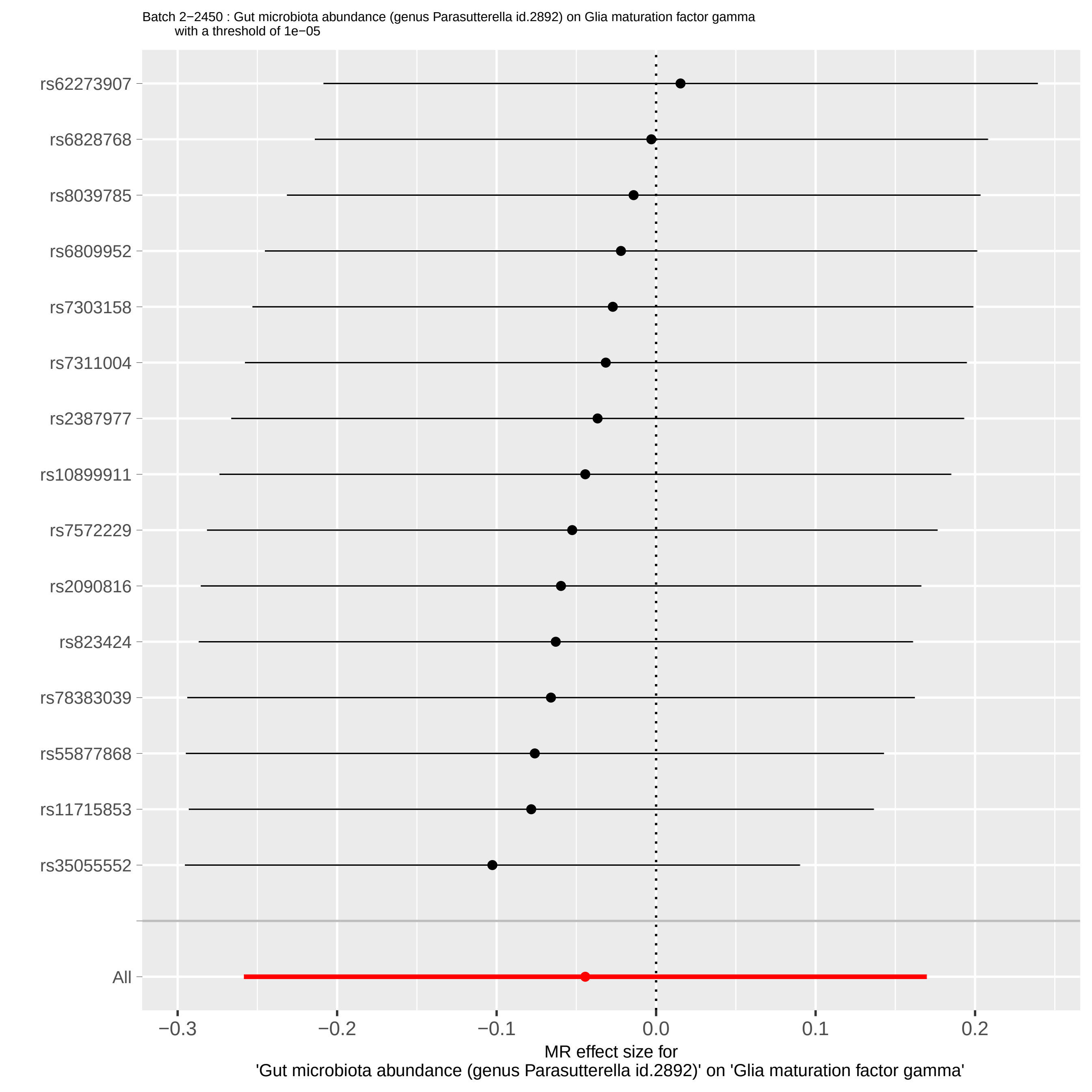
Batch 2−2450 : Gut microbiota abundance (genus Parasutterella id.2892) on Glia maturation factor gamma
with a threshold of 1e−05
rs62273907
rs6828768
rs8039785
rs6809952
rs7303158
rs7311004
rs2387977
rs10899911
rs7572229
rs2090816
rs823424
rs78383039
rs55877868
rs11715853
rs35055552
All
−0.3
−0.2
−0.1
0.0
0.1
0.2
MR effect size for
'Gut microbiota abundance (genus Parasutterella id.2892)' on 'Glia maturation factor gamma'

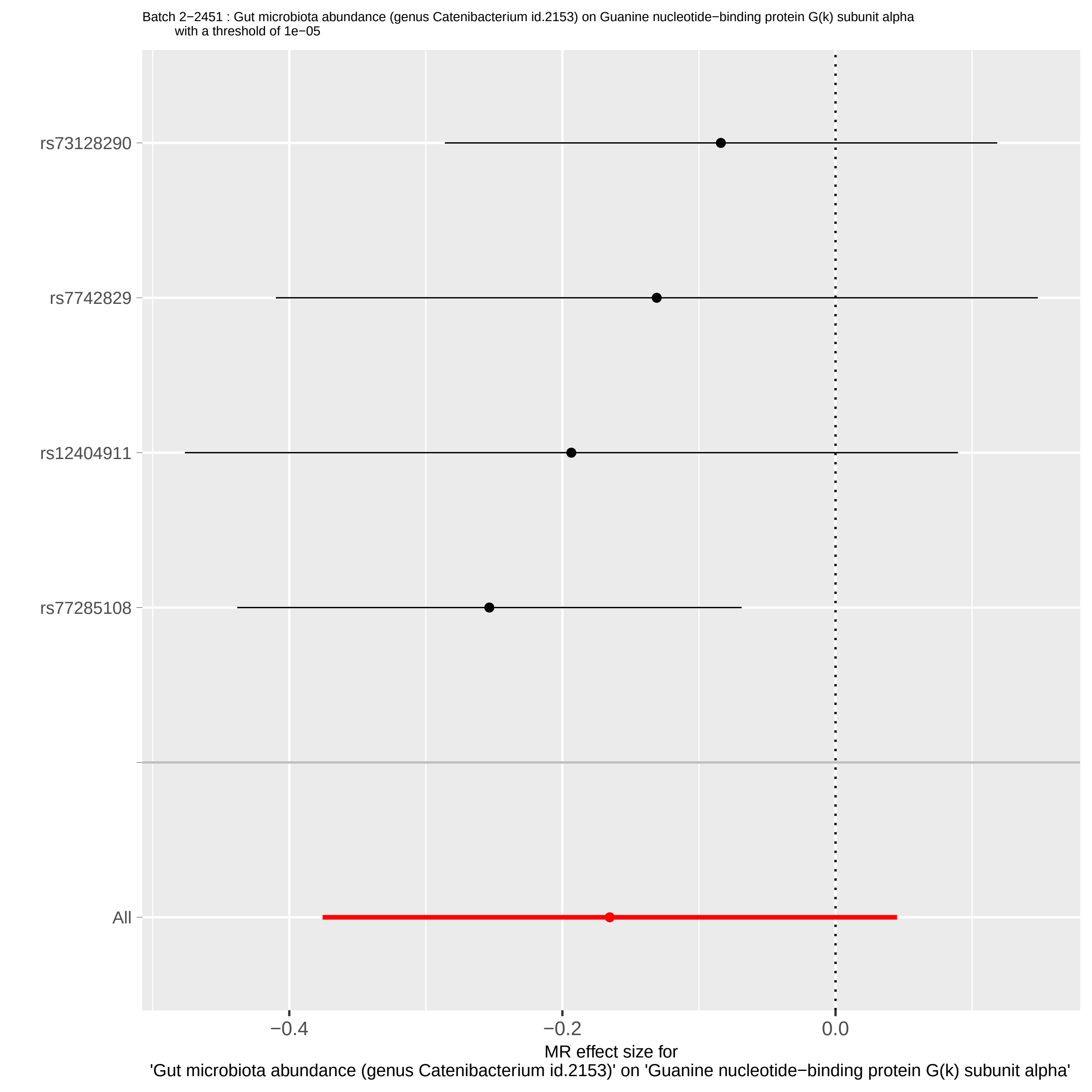

Batch 2−2451 : Gut microbiota abundance (genus Catenibacterium id.2153) on Guanine nucleotide−binding protein G(k) subunit alpha
with a threshold of 1e−05
rs73128290
rs7742829
rs12404911
rs77285108
All
−0.4
−0.2
0.0
MR effect size for
'Gut microbiota abundance (genus Catenibacterium id.2153)' on 'Guanine nucleotide−binding protein G(k) subunit alpha'

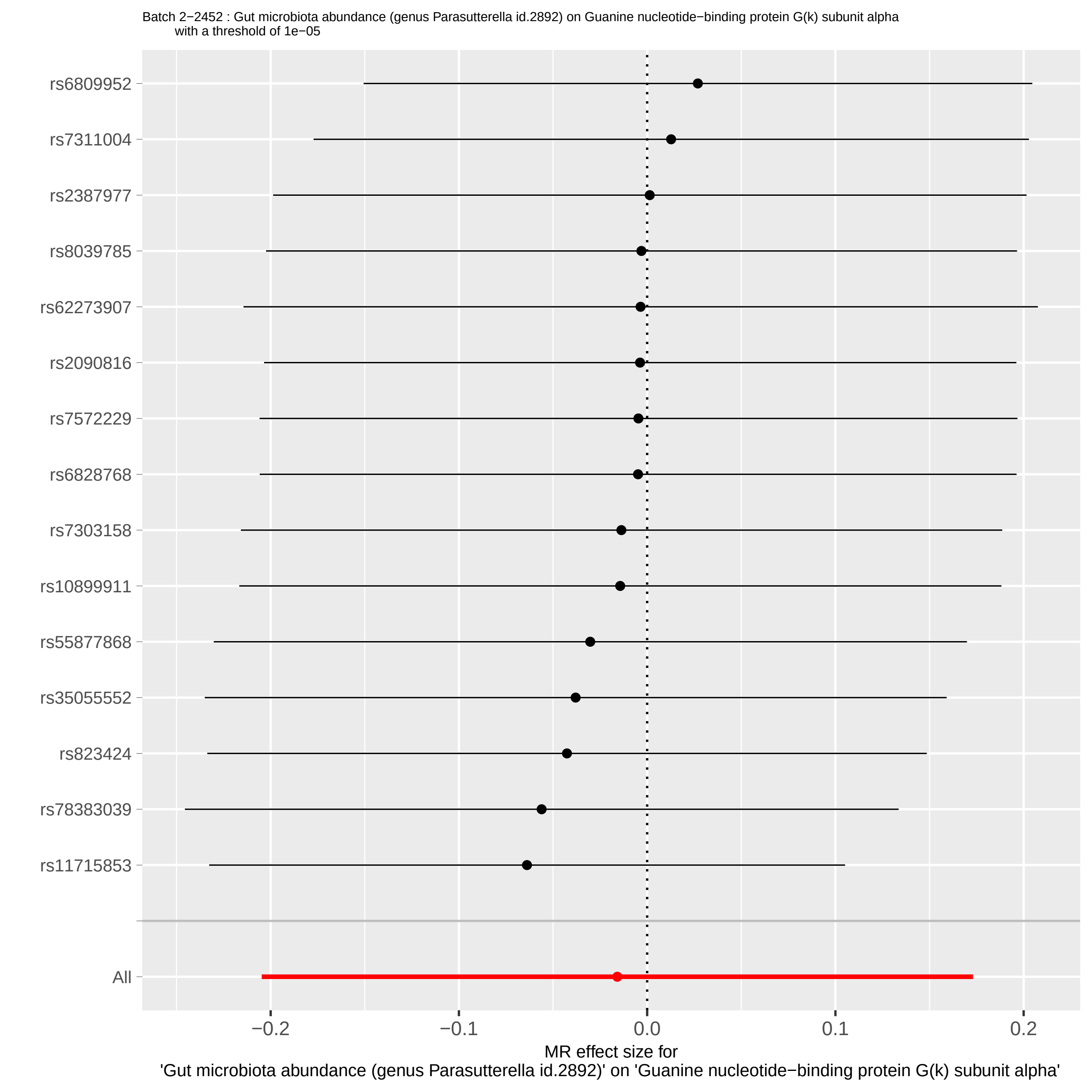
Batch 2−2452 : Gut microbiota abundance (genus Parasutterella id.2892) on Guanine nucleotide−binding protein G(k) subunit alpha
with a threshold of 1e−05
rs6809952
rs7311004
rs2387977
rs8039785
rs62273907
rs2090816
rs7572229
rs6828768
rs7303158
rs10899911
rs55877868
rs35055552
rs823424
rs78383039
rs11715853
All
−0.2
−0.1
0.0
0.1
0.2
MR effect size for
'Gut microbiota abundance (genus Parasutterella id.2892)' on 'Guanine nucleotide−binding protein G(k) subunit alpha'

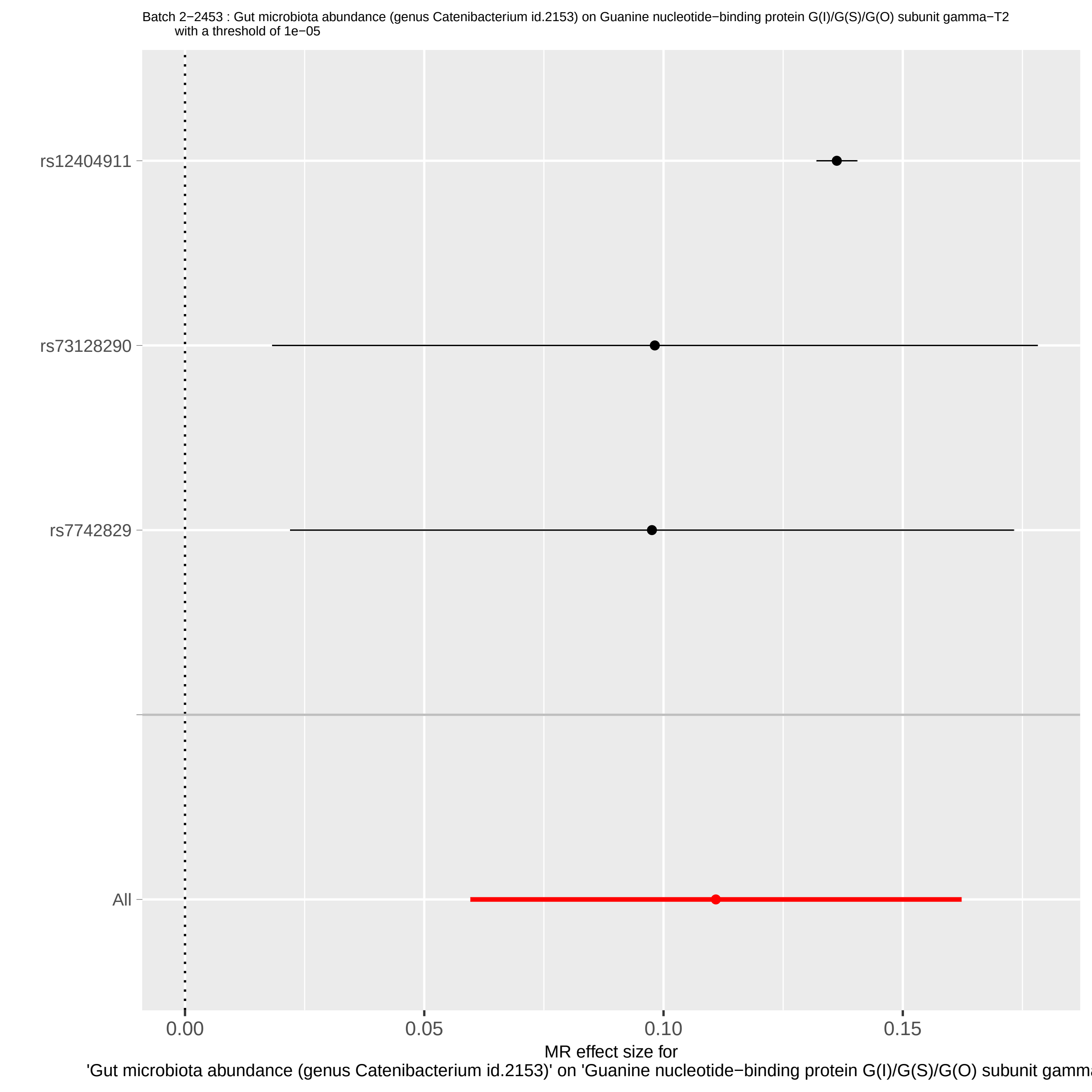

Batch 2−2453 : Gut microbiota abundance (genus Catenibacterium id.2153) on Guanine nucleotide−binding protein G(I)/G(S)/G(O) subunit gamma−T2
with a threshold of 1e−05
rs12404911
rs73128290
rs7742829
All
0.00
0.05
0.10
0.15
MR effect size for
'Gut microbiota abundance (genus Catenibacterium id.2153)' on 'Guanine nucleotide−binding protein G(I)/G(S)/G(O) subunit gamm

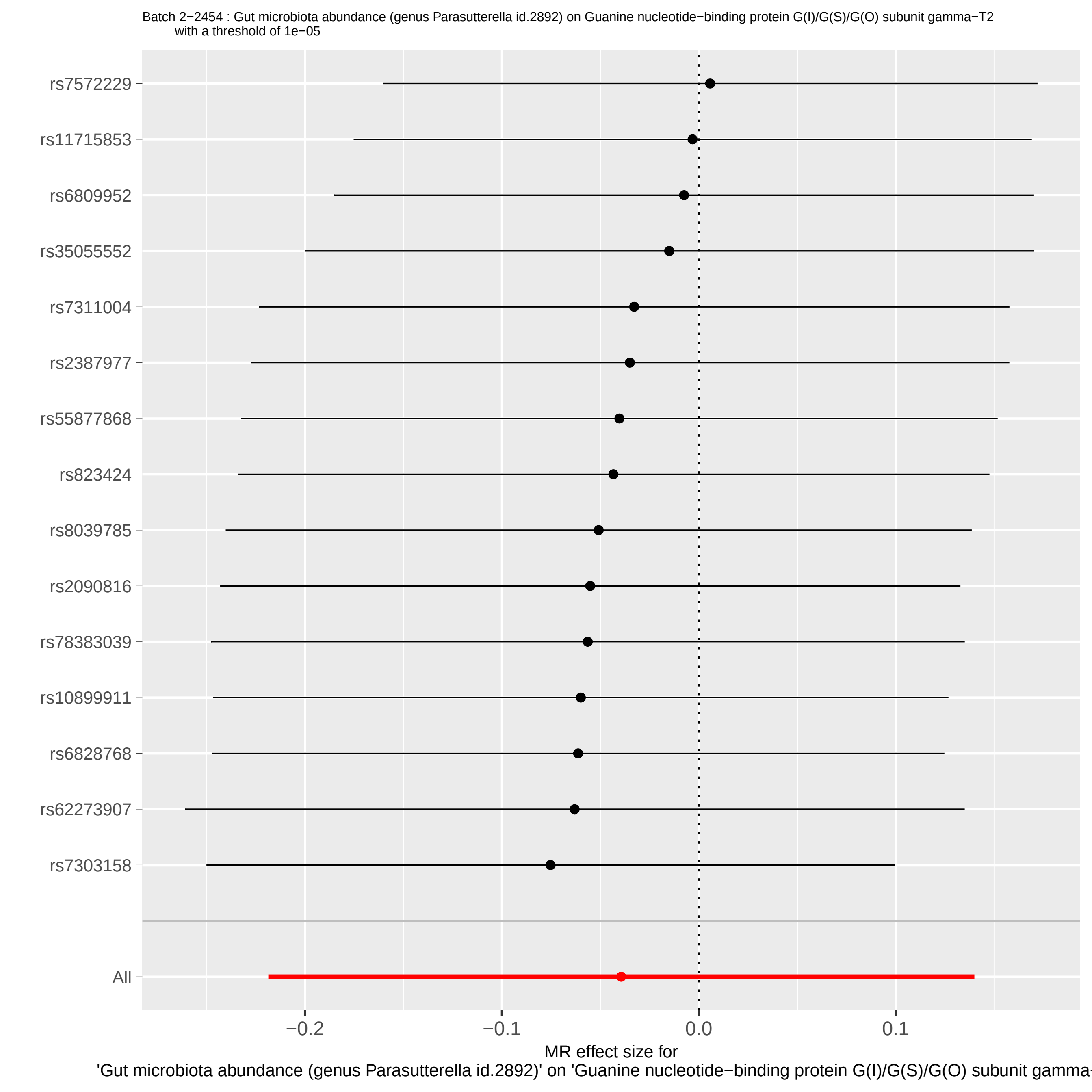

Batch 2−2454 : Gut microbiota abundance (genus Parasutterella id.2892) on Guanine nucleotide−binding protein G(I)/G(S)/G(O) subunit gamma−T2
with a threshold of 1e−05
rs7572229
rs11715853
rs6809952
rs35055552
rs7311004
rs2387977
rs55877868
rs823424
rs8039785
rs2090816
rs78383039
rs10899911
rs6828768
rs62273907
rs7303158
All
−0.2
−0.1
0.0
0.1
MR effect size for
'Gut microbiota abundance (genus Parasutterella id.2892)' on 'Guanine nucleotide−binding protein G(I)/G(S)/G(O) subunit gamma

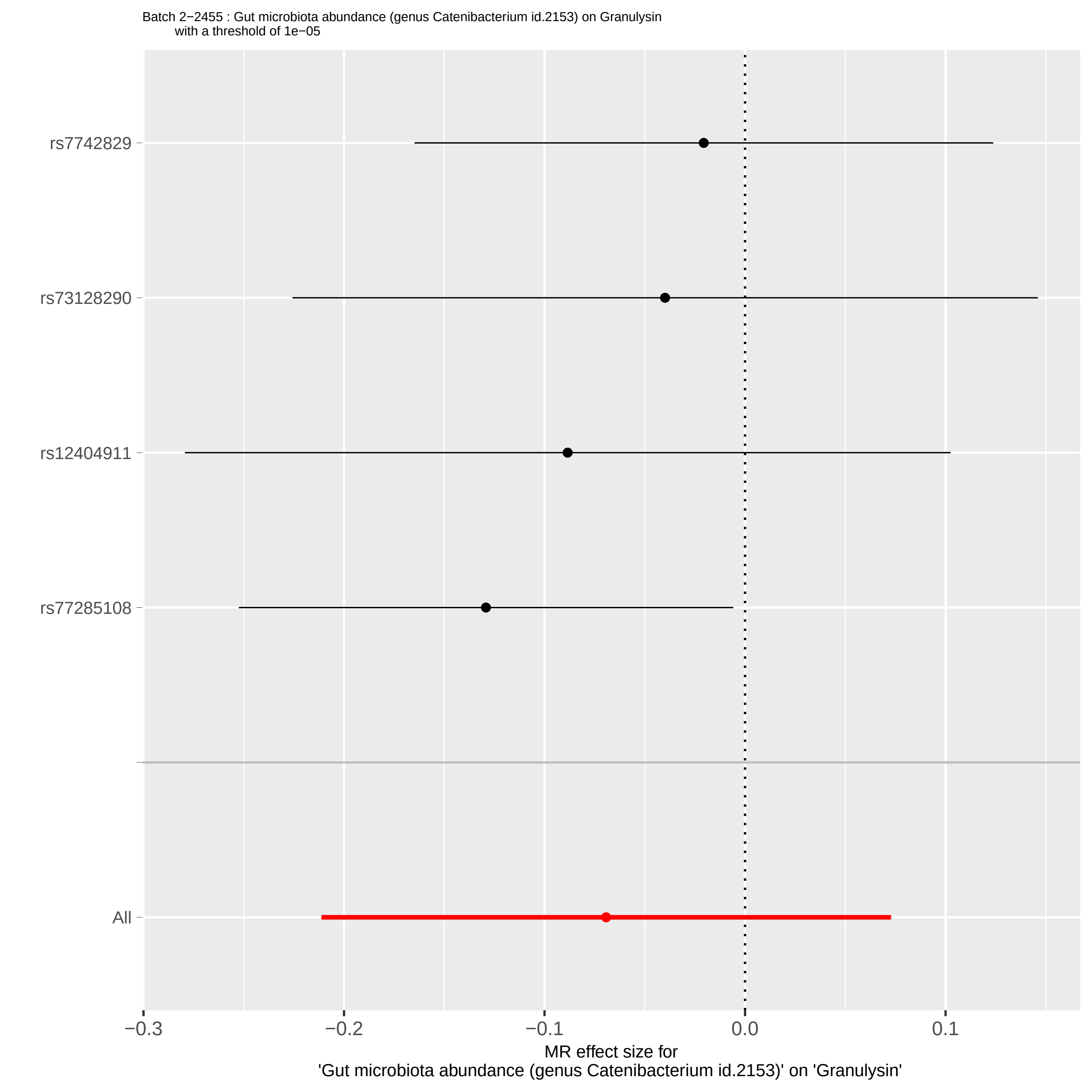
Batch 2−2455 : Gut microbiota abundance (genus Catenibacterium id.2153) on Granulysin
with a threshold of 1e−05
rs7742829
rs73128290
rs12404911
rs77285108
All
−0.3
−0.2
−0.1
0.0
0.1
MR effect size for
'Gut microbiota abundance (genus Catenibacterium id.2153)' on 'Granulysin'

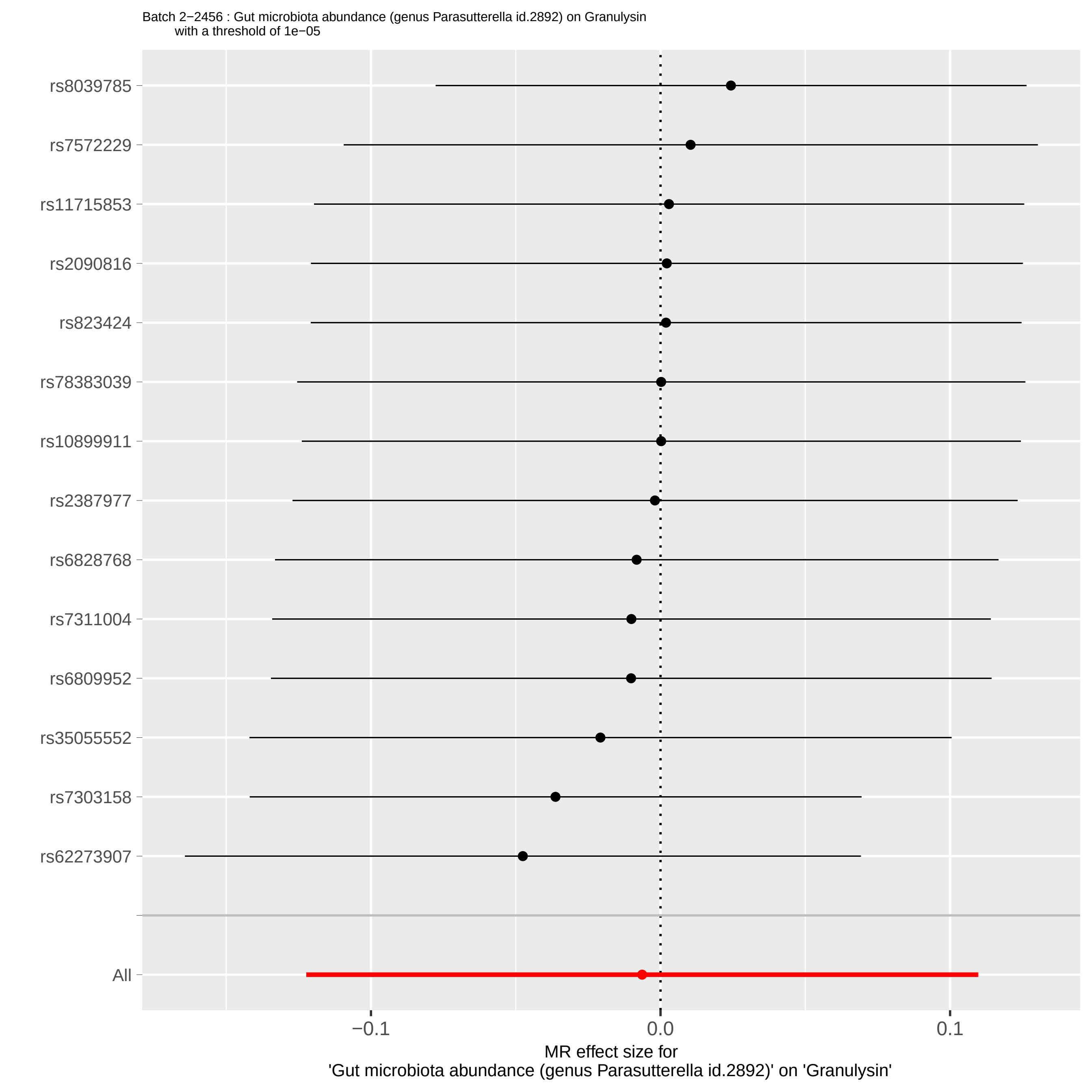

Batch 2−2456 : Gut microbiota abundance (genus Parasutterella id.2892) on Granulysin
with a threshold of 1e−05
rs8039785
rs7572229
rs11715853
rs2090816
rs823424
rs78383039
rs10899911
rs2387977
rs6828768
rs7311004
rs6809952
rs35055552
rs7303158
rs62273907
All
−0.1
0.0
0.1
MR effect size for
'Gut microbiota abundance (genus Parasutterella id.2892)' on 'Granulysin'

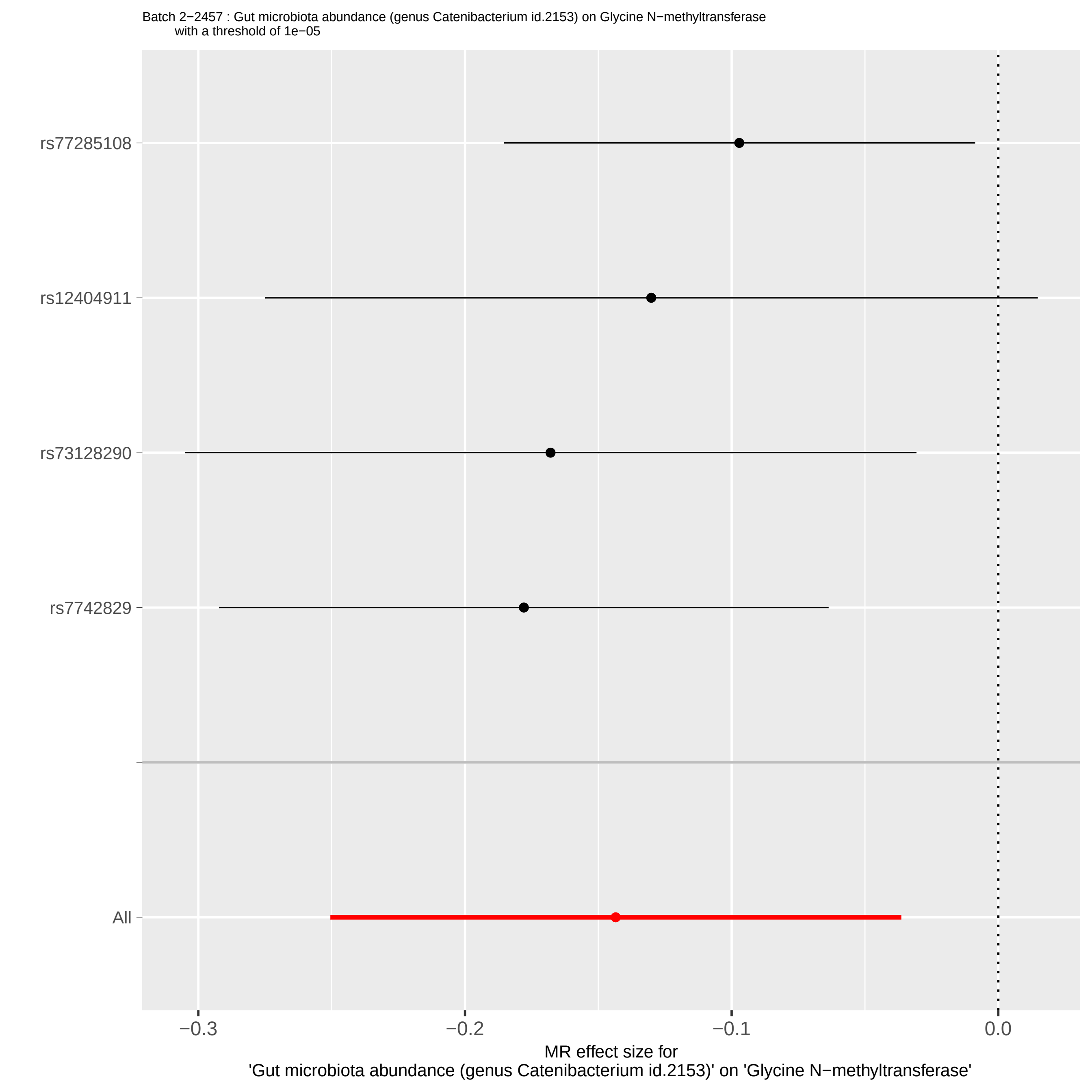
Batch 2−2457 : Gut microbiota abundance (genus Catenibacterium id.2153) on Glycine N−methyltransferase
with a threshold of 1e−05
rs77285108
rs12404911
rs73128290
rs7742829
All
−0.3
−0.2
−0.1
0.0
MR effect size for
'Gut microbiota abundance (genus Catenibacterium id.2153)' on 'Glycine N−methyltransferase'

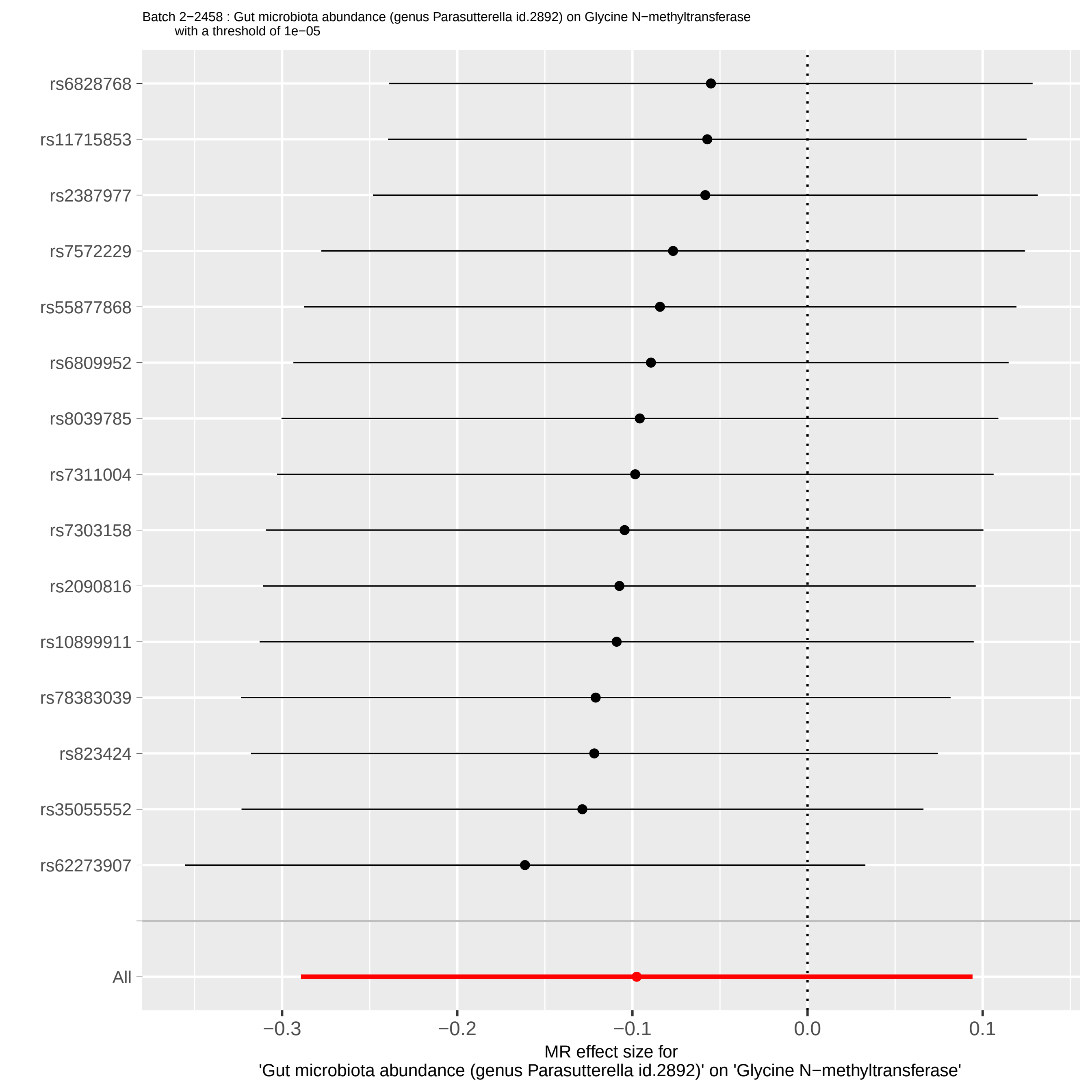

Batch 2−2458 : Gut microbiota abundance (genus Parasutterella id.2892) on Glycine N−methyltransferase
with a threshold of 1e−05
rs6828768
rs11715853
rs2387977
rs7572229
rs55877868
rs6809952
rs8039785
rs7311004
rs7303158
rs2090816
rs10899911
rs78383039
rs823424
rs35055552
rs62273907
All
−0.3
−0.2
−0.1
0.0
0.1
MR effect size for
'Gut microbiota abundance (genus Parasutterella id.2892)' on 'Glycine N−methyltransferase'

Batch 2−2459 : Gut microbiota abundance (genus Catenibacterium id.2153) on Glucosamine−6−phosphate isomerase 1
with a threshold of 1e−05

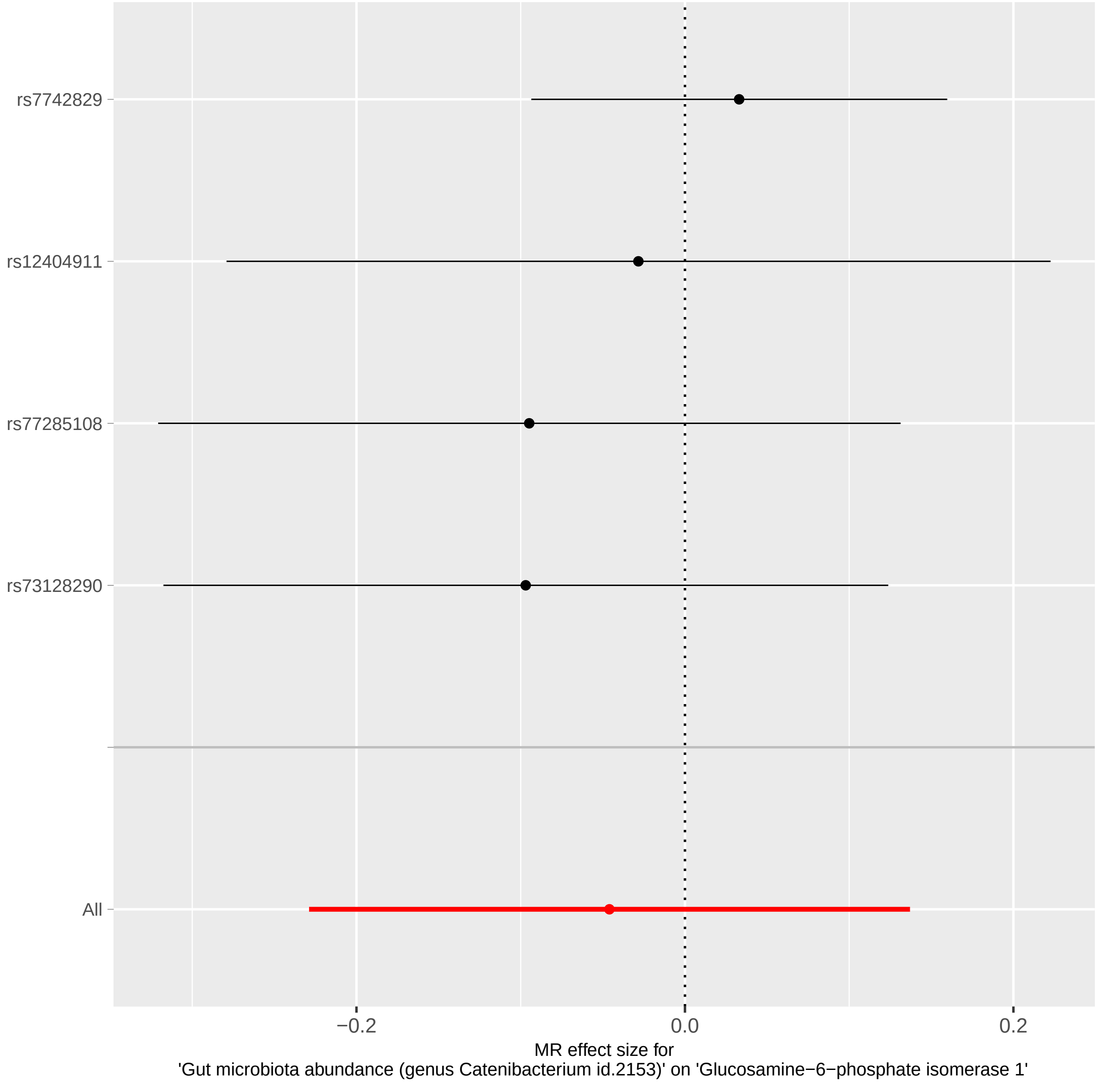

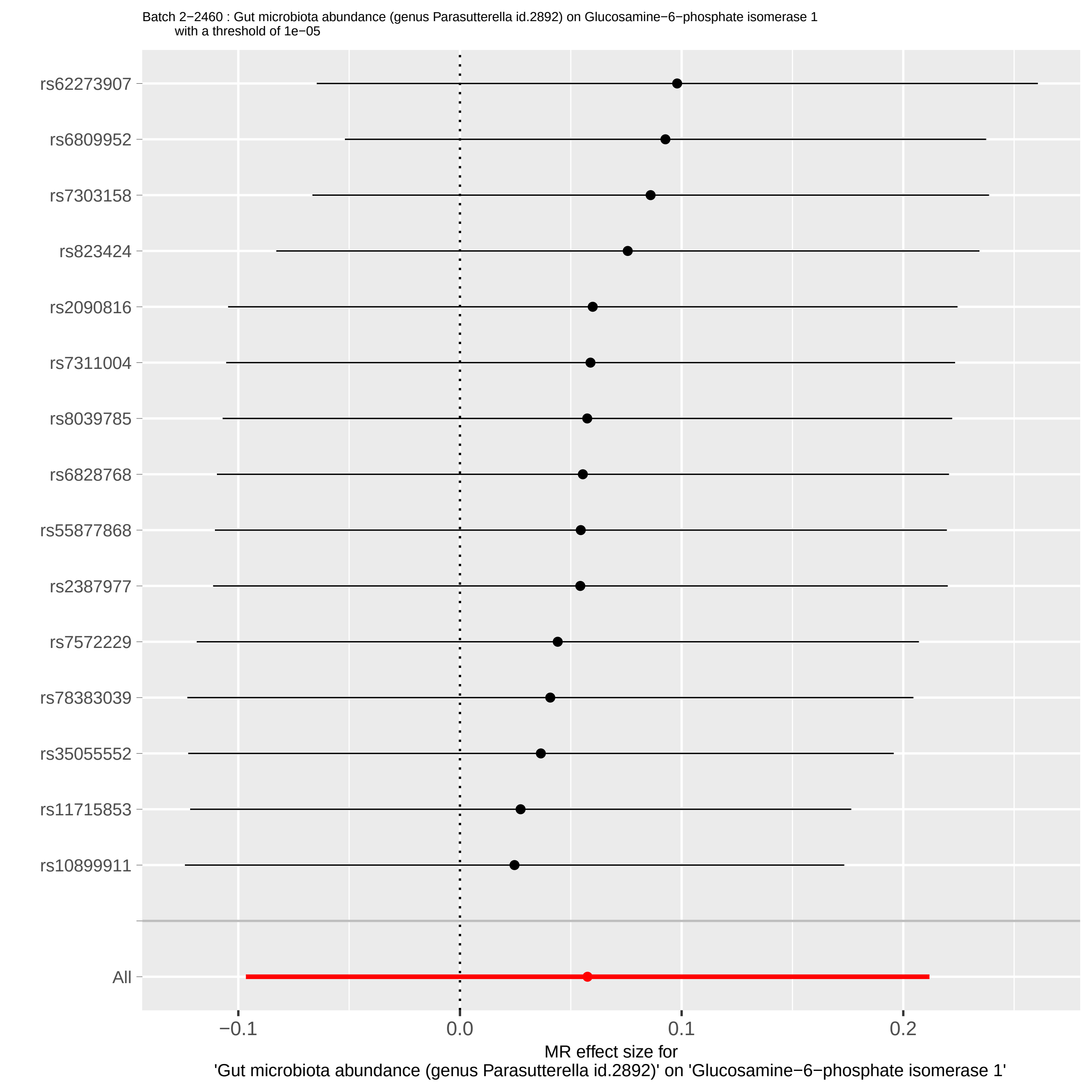
Batch 2−2460 : Gut microbiota abundance (genus Parasutterella id.2892) on Glucosamine−6−phosphate isomerase 1
with a threshold of 1e−05
rs62273907
rs6809952
rs7303158
rs823424
rs2090816
rs7311004
rs8039785
rs6828768
rs55877868
rs2387977
rs7572229
rs78383039
rs35055552
rs11715853
rs10899911
All
−0.1
0.0
0.1
0.2
MR effect size for
'Gut microbiota abundance (genus Parasutterella id.2892)' on 'Glucosamine−6−phosphate isomerase 1'

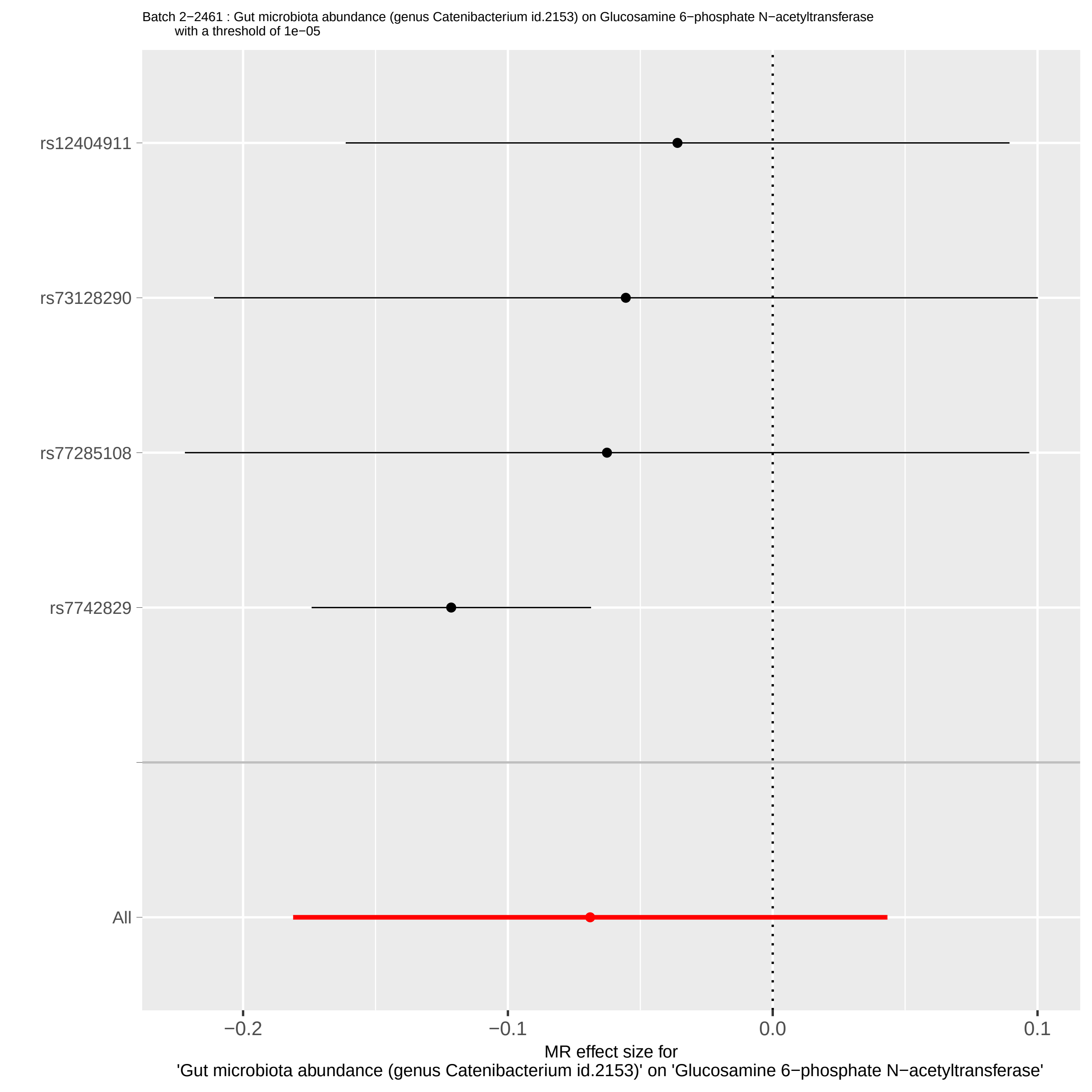
Batch 2−2461 : Gut microbiota abundance (genus Catenibacterium id.2153) on Glucosamine 6−phosphate N−acetyltransferase
with a threshold of 1e−05
rs12404911
rs73128290
rs77285108
rs7742829
All
−0.2
−0.1
0.0
0.1
MR effect size for
'Gut microbiota abundance (genus Catenibacterium id.2153)' on 'Glucosamine 6−phosphate N−acetyltransferase'

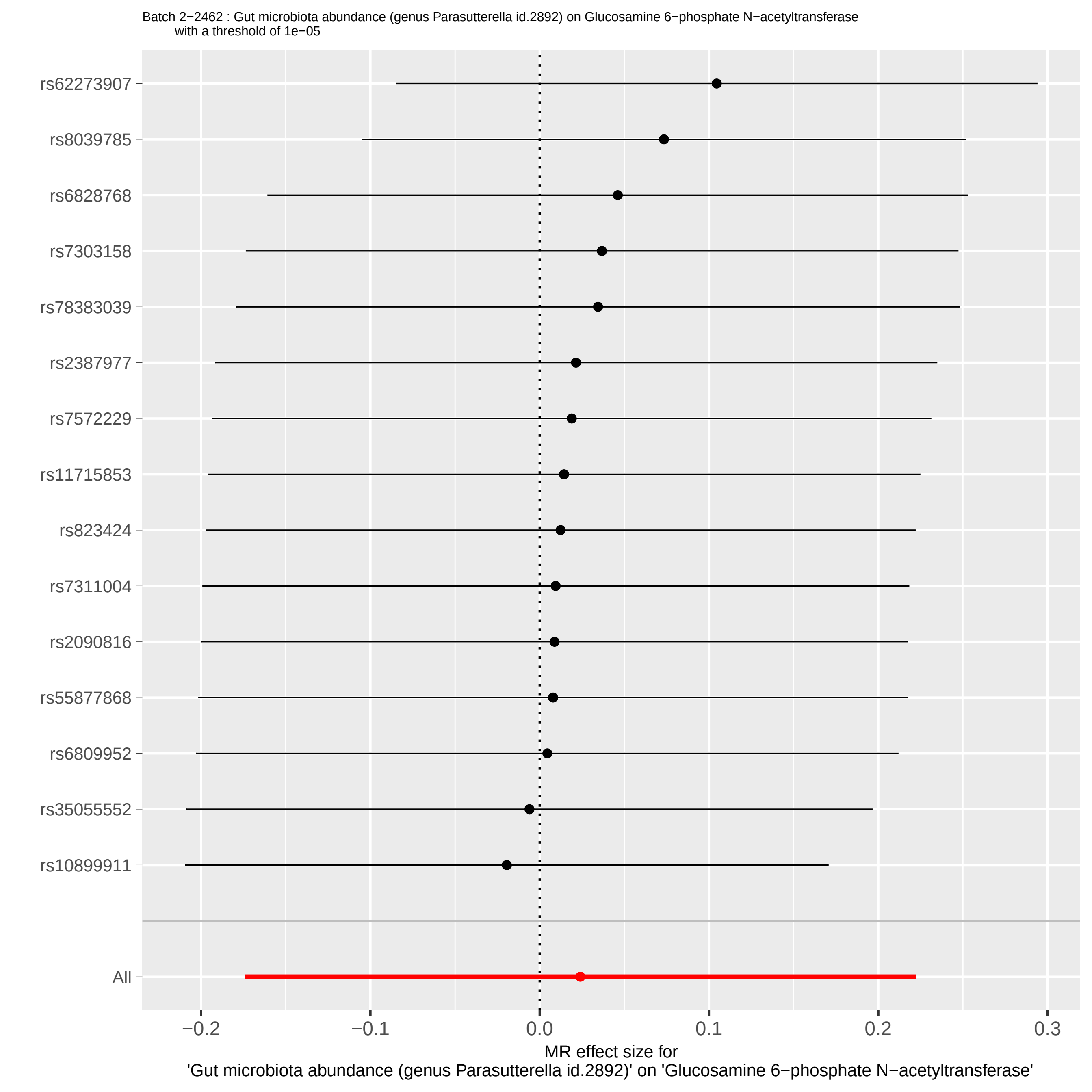

Batch 2−2462 : Gut microbiota abundance (genus Parasutterella id.2892) on Glucosamine 6−phosphate N−acetyltransferase
with a threshold of 1e−05
rs62273907
rs8039785
rs6828768
rs7303158
rs78383039
rs2387977
rs7572229
rs11715853
rs823424
rs7311004
rs2090816
rs55877868
rs6809952
rs35055552
rs10899911
All
−0.2
−0.1
0.0
0.1
0.2
0.3
MR effect size for
'Gut microbiota abundance (genus Parasutterella id.2892)' on 'Glucosamine 6−phosphate N−acetyltransferase'

Batch 2−2463 : Gut microbiota abundance (genus Catenibacterium id.2153) on N−acetylglucosamine−1−phosphotransferase subunit gamma
with a threshold of 1e−05

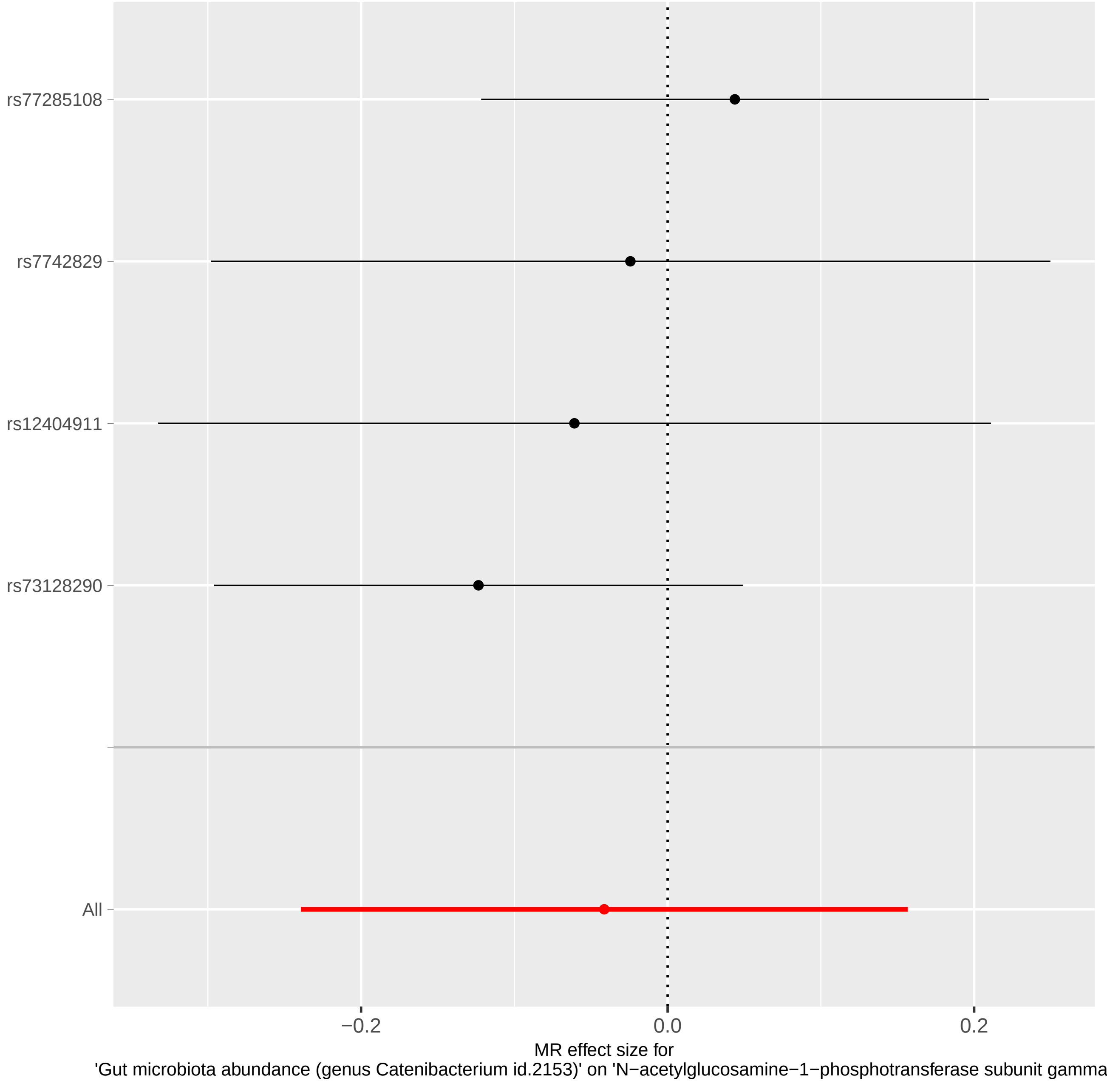

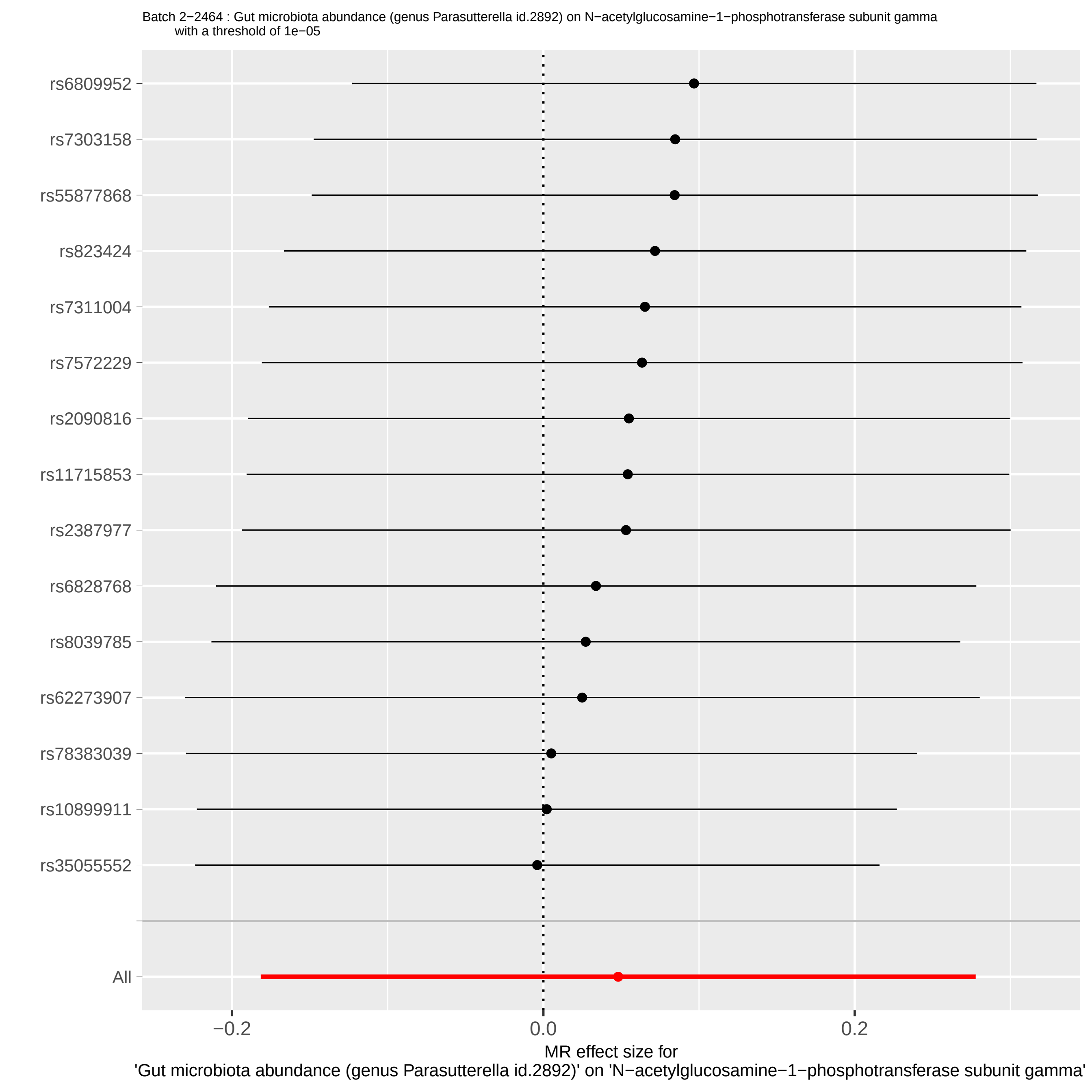
Batch 2−2464 : Gut microbiota abundance (genus Parasutterella id.2892) on N−acetylglucosamine−1−phosphotransferase subunit gamma
with a threshold of 1e−05
rs6809952
rs7303158
rs55877868
rs823424
rs7311004
rs7572229
rs2090816
rs11715853
rs2387977
rs6828768
rs8039785
rs62273907
rs78383039
rs10899911
rs35055552
All
−0.2
0.0
0.2
MR effect size for
'Gut microbiota abundance (genus Parasutterella id.2892)' on 'N−acetylglucosamine−1−phosphotransferase subunit gamma'

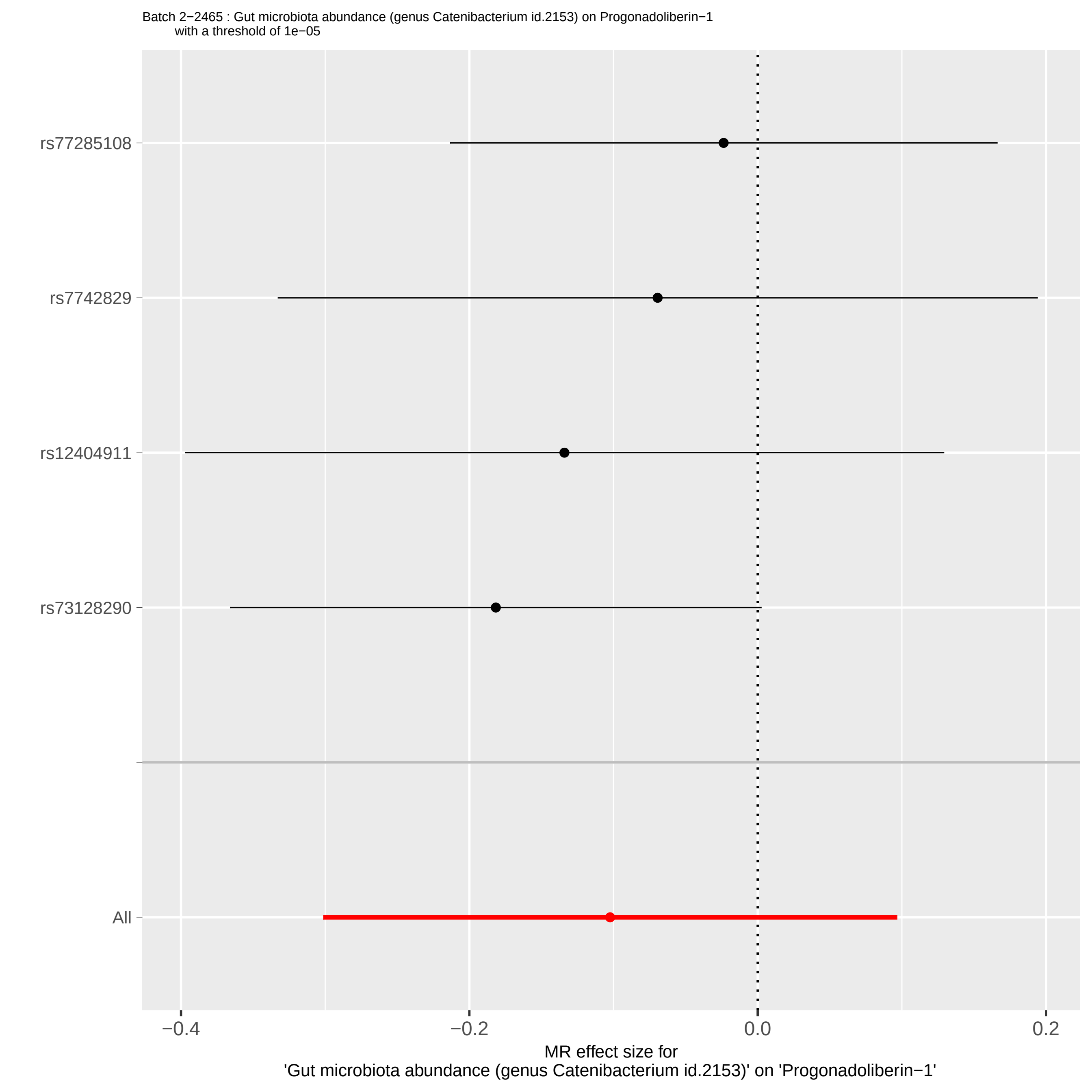
Batch 2−2465 : Gut microbiota abundance (genus Catenibacterium id.2153) on Progonadoliberin−1
with a threshold of 1e−05
rs77285108
rs7742829
rs12404911
rs73128290
All
−0.4
−0.2
0.0
0.2
MR effect size for
'Gut microbiota abundance (genus Catenibacterium id.2153)' on 'Progonadoliberin−1'

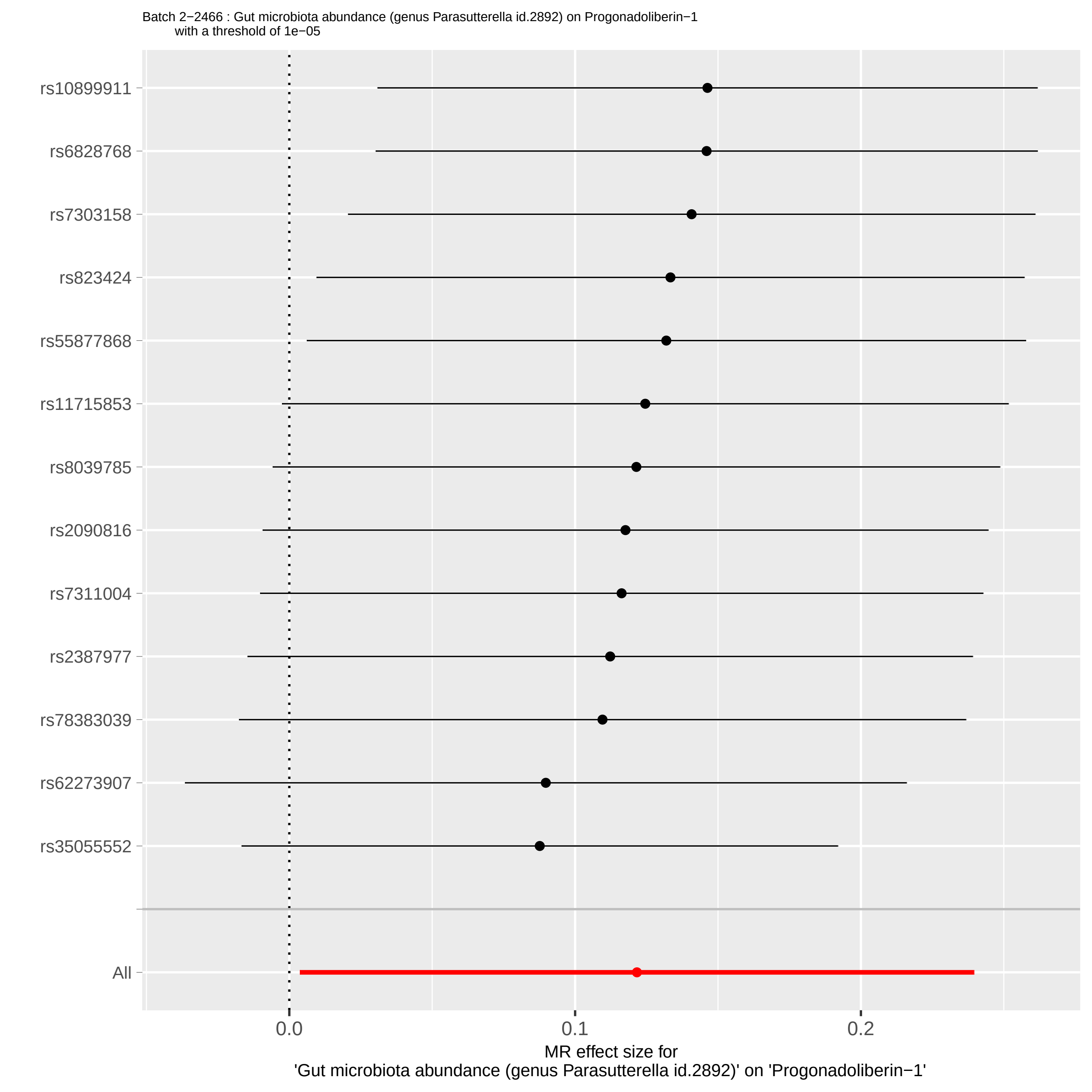

Batch 2−2466 : Gut microbiota abundance (genus Parasutterella id.2892) on Progonadoliberin−1
with a threshold of 1e−05
rs10899911
rs6828768
rs7303158
rs823424
rs55877868
rs11715853
rs8039785
rs2090816
rs7311004
rs2387977
rs78383039
rs62273907
rs35055552
All
0.0
0.1
0.2
MR effect size for
'Gut microbiota abundance (genus Parasutterella id.2892)' on 'Progonadoliberin−1'

Batch 2−2467 : Gut microbiota abundance (genus Catenibacterium id.2153) on Progonadoliberin−2
with a threshold of 1e−05

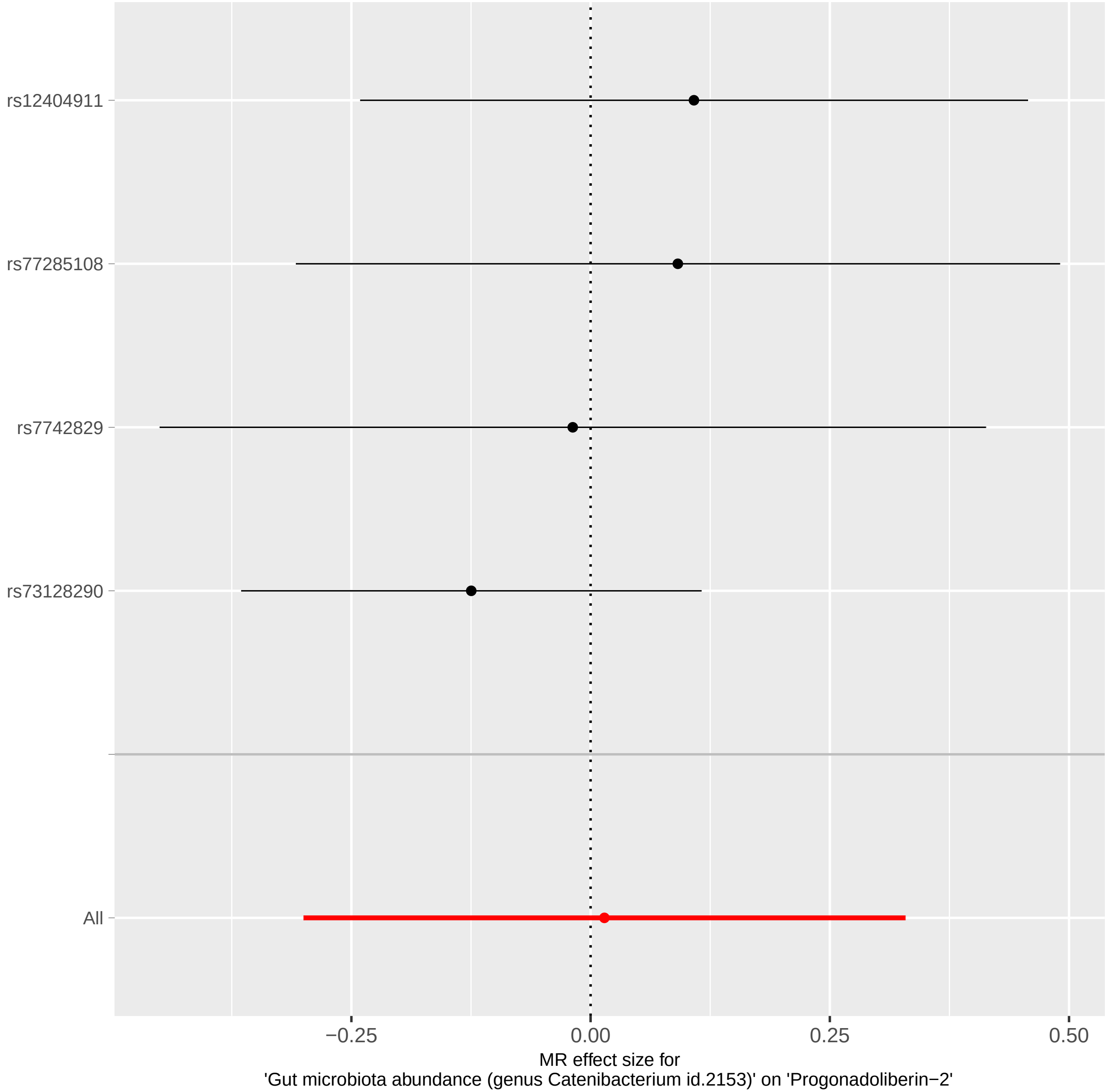

Batch 2−2468 : Gut microbiota abundance (genus Parasutterella id.2892) on Progonadoliberin−2
with a threshold of 1e−05

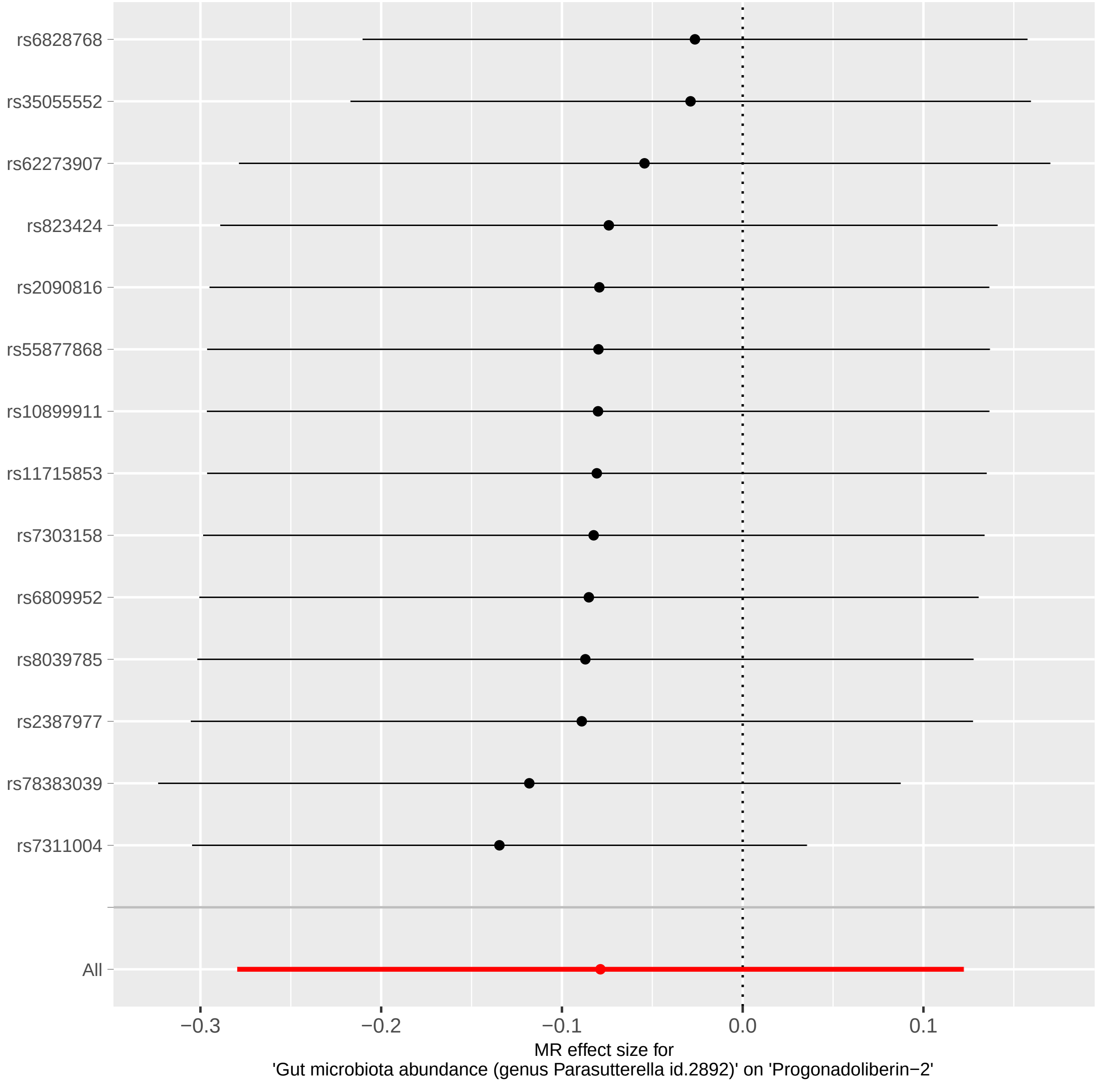

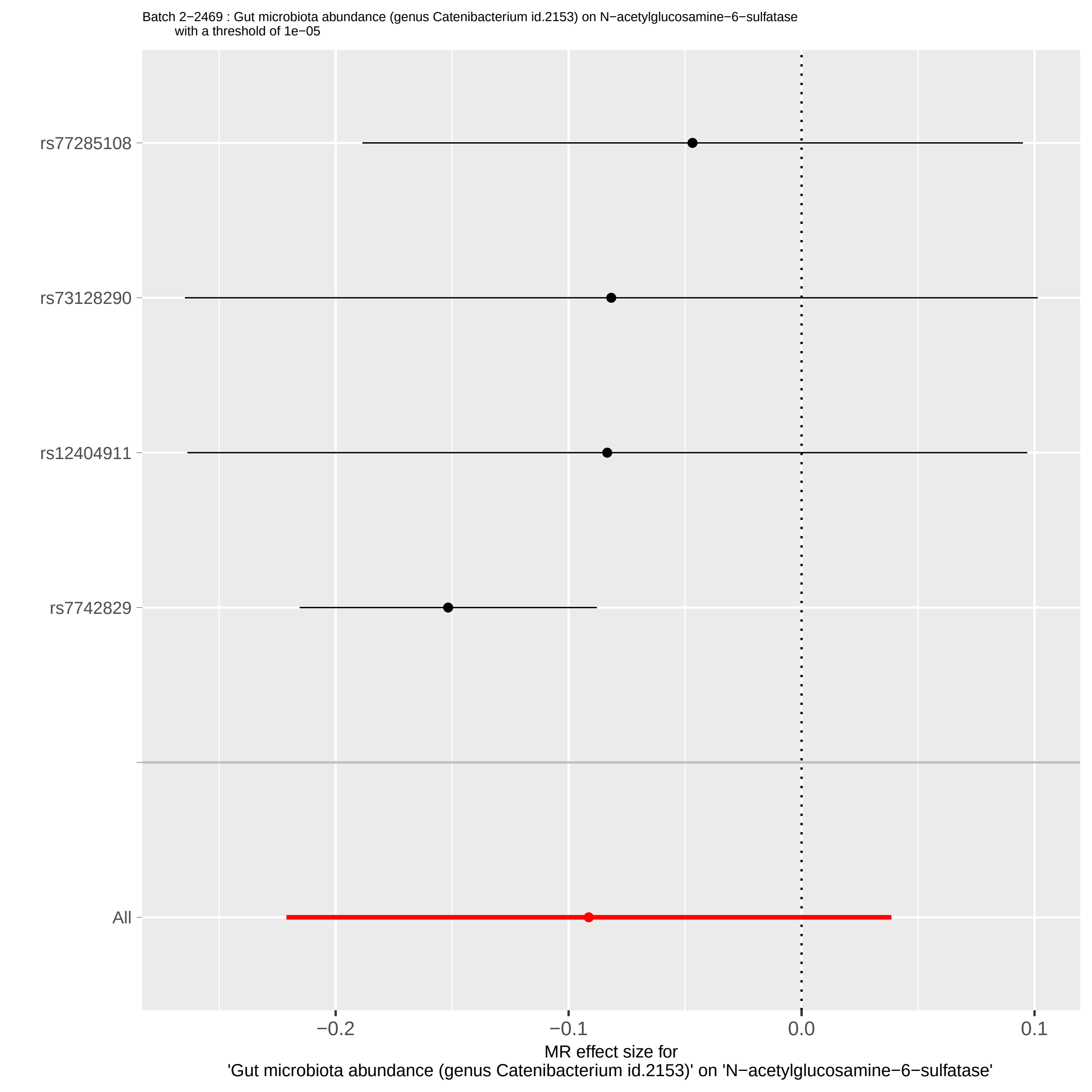
Batch 2−2469 : Gut microbiota abundance (genus Catenibacterium id.2153) on N−acetylglucosamine−6−sulfatase
with a threshold of 1e−05
rs77285108
rs73128290
rs12404911
rs7742829
All
−0.2
−0.1
0.0
0.1
MR effect size for
'Gut microbiota abundance (genus Catenibacterium id.2153)' on 'N−acetylglucosamine−6−sulfatase'

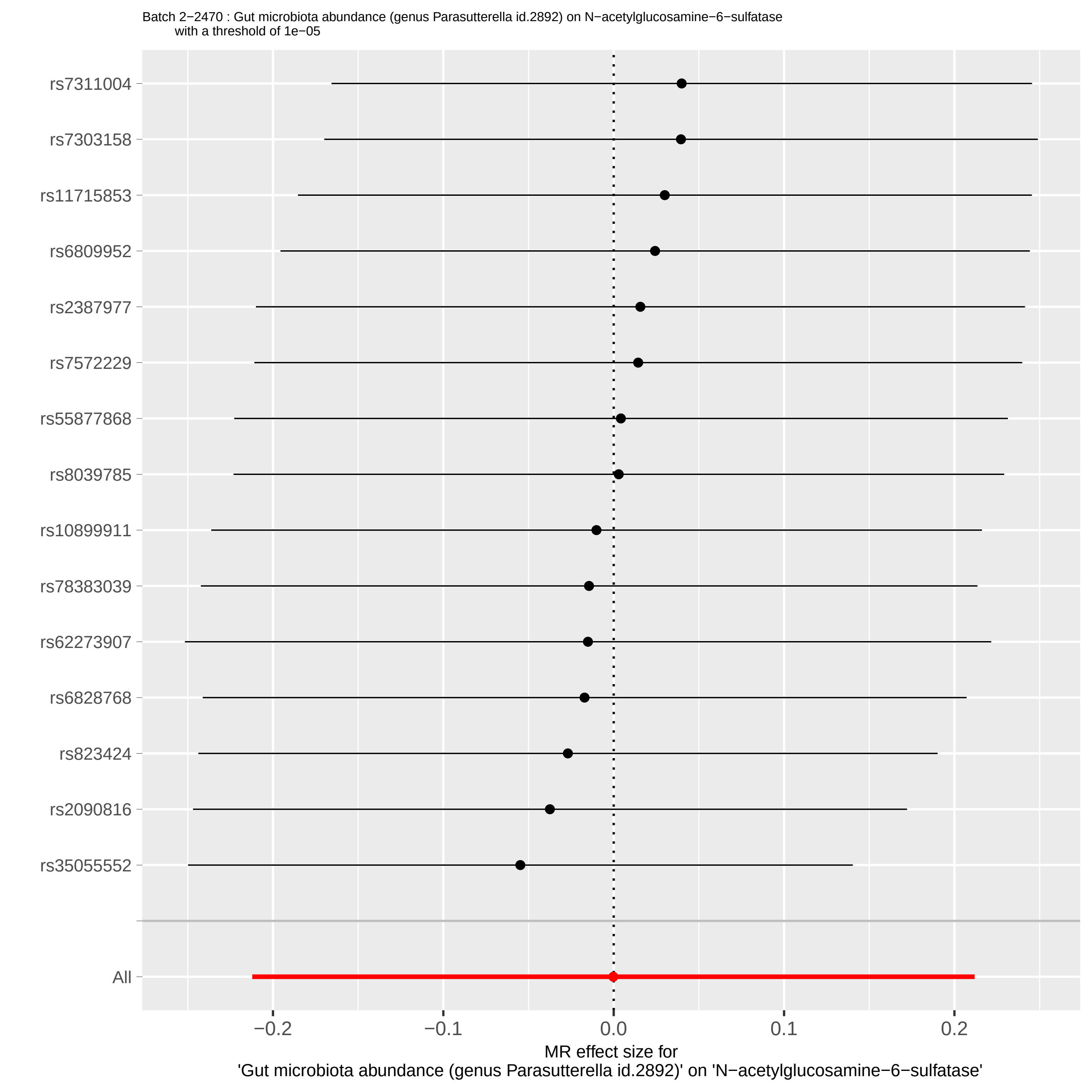
Batch 2−2470 : Gut microbiota abundance (genus Parasutterella id.2892) on N−acetylglucosamine−6−sulfatase
with a threshold of 1e−05
rs7311004
rs7303158
rs11715853
rs6809952
rs2387977
rs7572229
rs55877868
rs8039785
rs10899911
rs78383039
rs62273907
rs6828768
rs823424
rs2090816
rs35055552
All
−0.2
−0.1
0.0
0.1
0.2
MR effect size for
'Gut microbiota abundance (genus Parasutterella id.2892)' on 'N−acetylglucosamine−6−sulfatase'

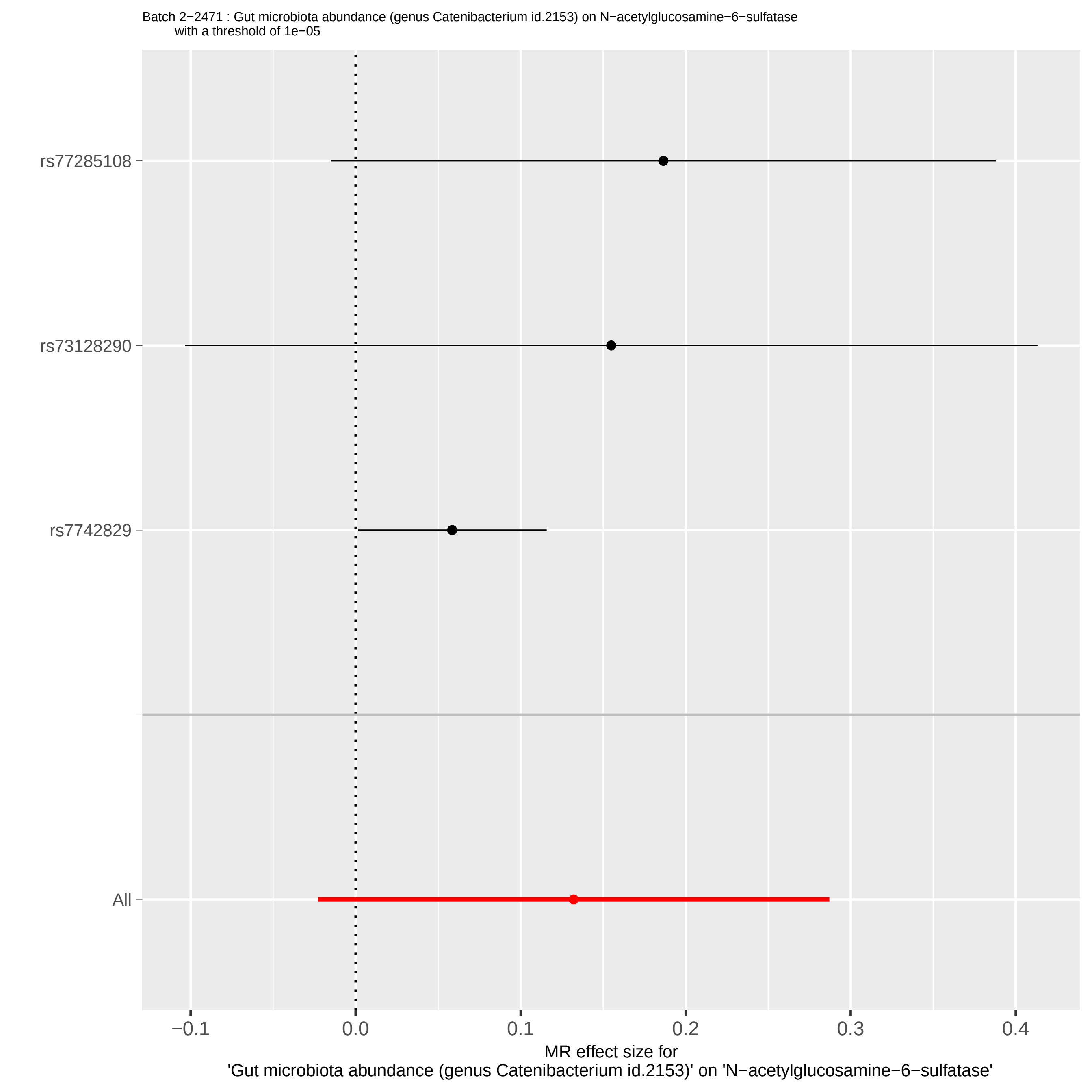

Batch 2−2471 : Gut microbiota abundance (genus Catenibacterium id.2153) on N−acetylglucosamine−6−sulfatase
with a threshold of 1e−05
rs77285108
rs73128290
rs7742829
All
−0.1
0.0
0.1
0.2
0.3
0.4
MR effect size for
'Gut microbiota abundance (genus Catenibacterium id.2153)' on 'N−acetylglucosamine−6−sulfatase'

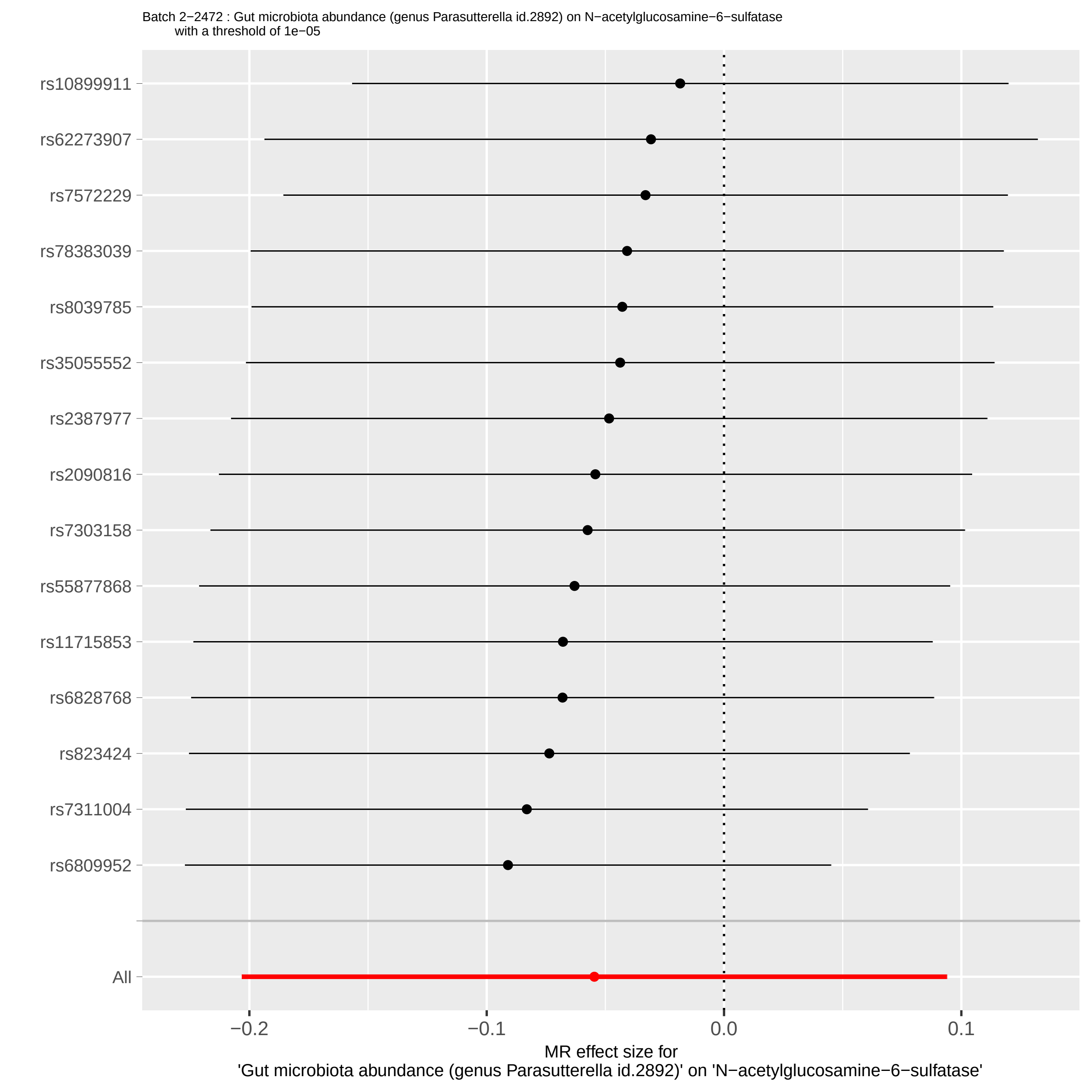

Batch 2−2472 : Gut microbiota abundance (genus Parasutterella id.2892) on N−acetylglucosamine−6−sulfatase
with a threshold of 1e−05
rs10899911
rs62273907
rs7572229
rs78383039
rs8039785
rs35055552
rs2387977
rs2090816
rs7303158
rs55877868
rs11715853
rs6828768
rs823424
rs7311004
rs6809952
All
−0.2
−0.1
0.0
0.1
MR effect size for
'Gut microbiota abundance (genus Parasutterella id.2892)' on 'N−acetylglucosamine−6−sulfatase'

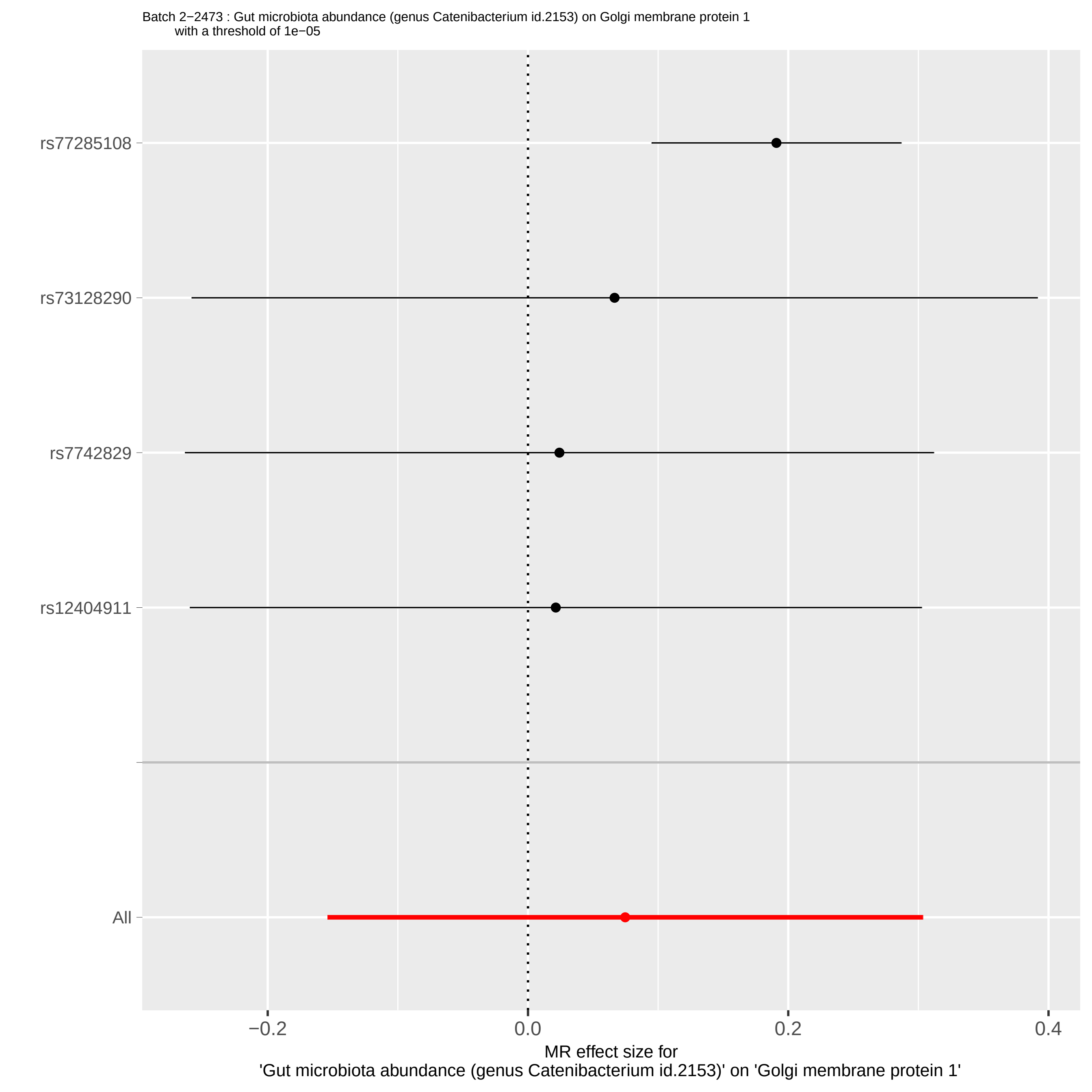

Batch 2−2473 : Gut microbiota abundance (genus Catenibacterium id.2153) on Golgi membrane protein 1
with a threshold of 1e−05
rs77285108
rs73128290
rs7742829
rs12404911
All
−0.2
0.0
0.2
0.4
MR effect size for
'Gut microbiota abundance (genus Catenibacterium id.2153)' on 'Golgi membrane protein 1'

Batch 2−2474 : Gut microbiota abundance (genus Parasutterella id.2892) on Golgi membrane protein 1
with a threshold of 1e−05

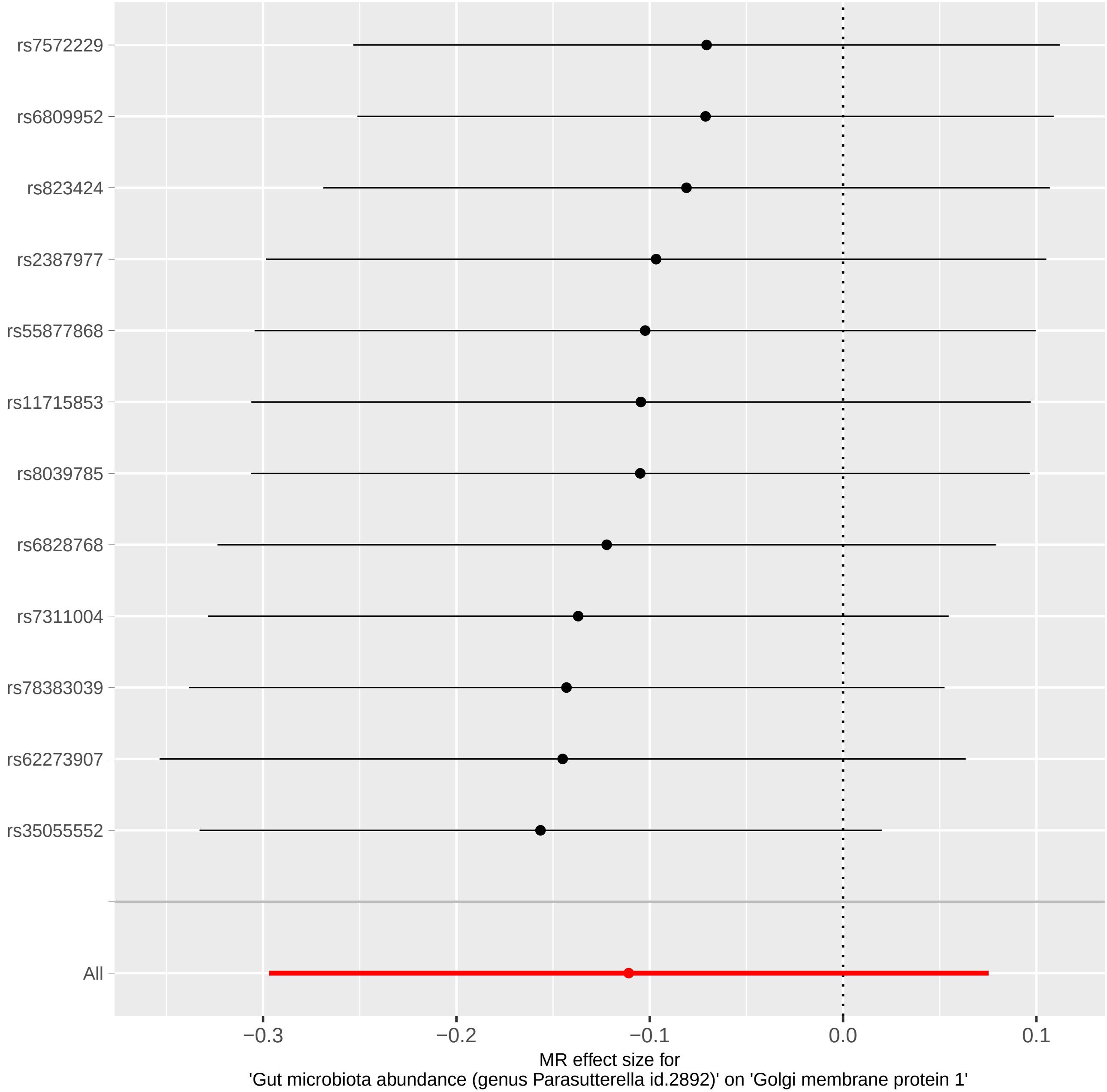

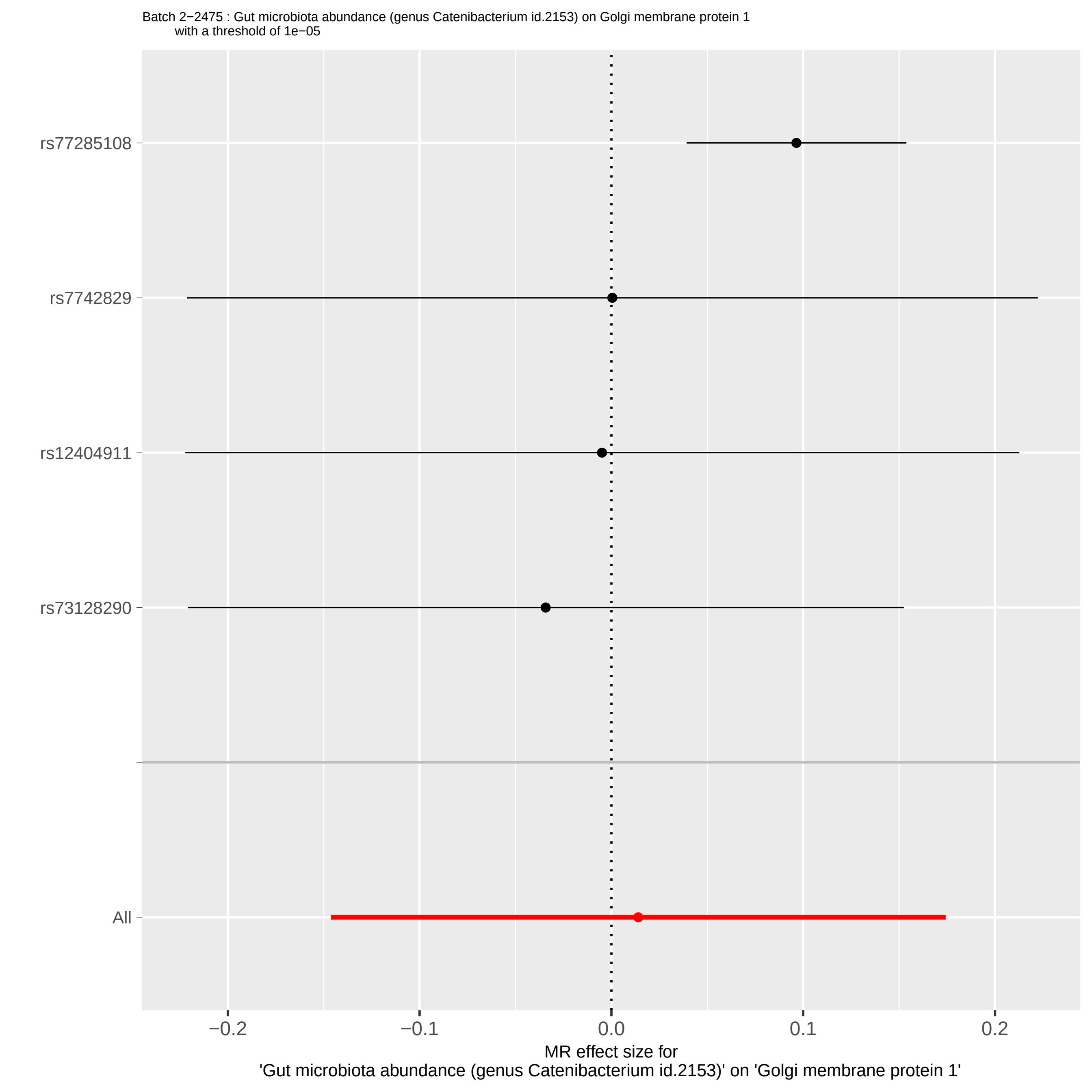
Batch 2−2475 : Gut microbiota abundance (genus Catenibacterium id.2153) on Golgi membrane protein 1
with a threshold of 1e−05
rs77285108
rs7742829
rs12404911
rs73128290
All
−0.2
−0.1
0.0
0.1
0.2
MR effect size for
'Gut microbiota abundance (genus Catenibacterium id.2153)' on 'Golgi membrane protein 1'

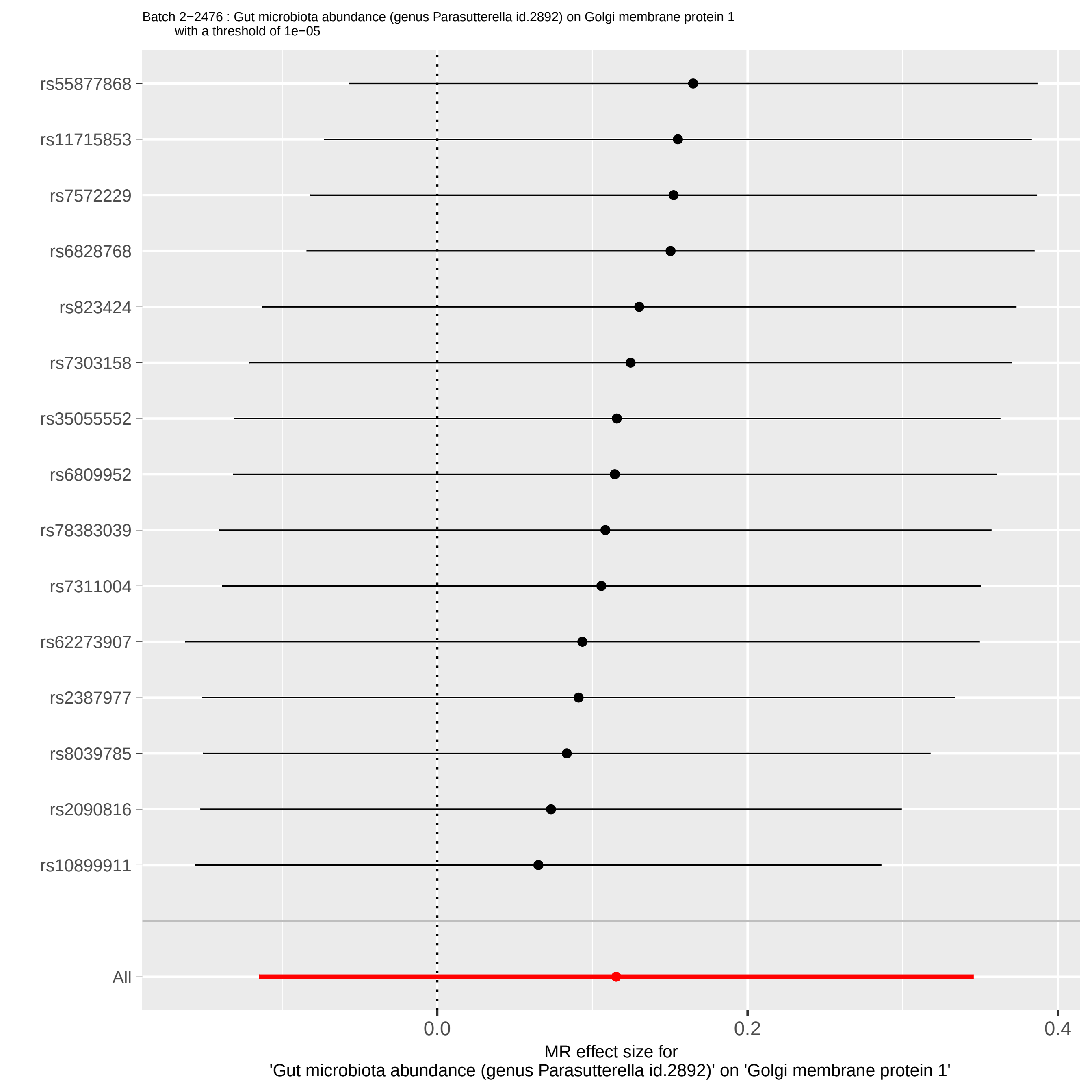

Batch 2−2476 : Gut microbiota abundance (genus Parasutterella id.2892) on Golgi membrane protein 1
with a threshold of 1e−05
rs55877868
rs11715853
rs7572229
rs6828768
rs823424
rs7303158
rs35055552
rs6809952
rs78383039
rs7311004
rs62273907
rs2387977
rs8039785
rs2090816
rs10899911
All
0.0
0.2
0.4
MR effect size for
'Gut microbiota abundance (genus Parasutterella id.2892)' on 'Golgi membrane protein 1'

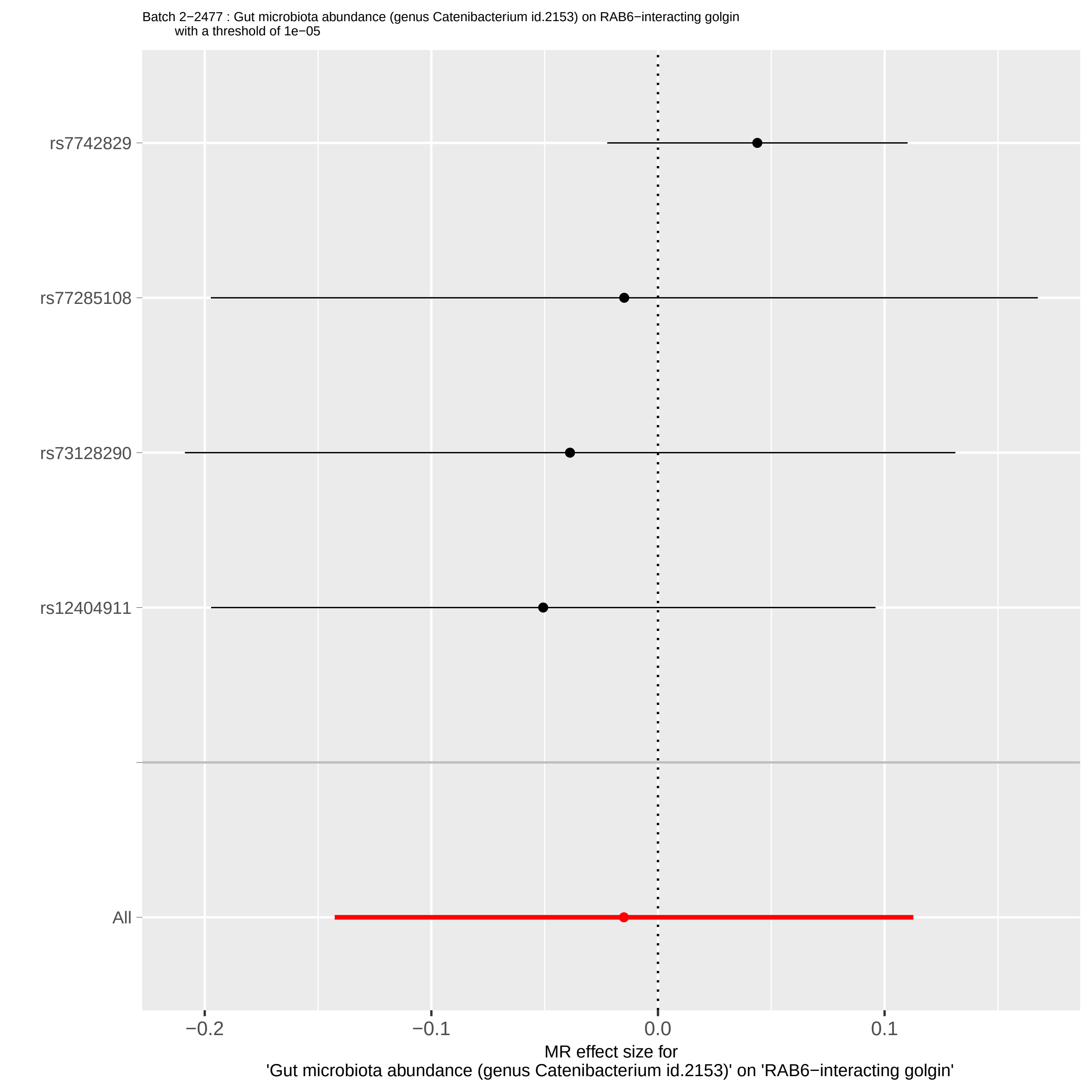
Batch 2−2477 : Gut microbiota abundance (genus Catenibacterium id.2153) on RAB6−interacting golgin
with a threshold of 1e−05
rs7742829
rs77285108
rs73128290
rs12404911
All
−0.2
−0.1
0.0
0.1
MR effect size for
'Gut microbiota abundance (genus Catenibacterium id.2153)' on 'RAB6−interacting golgin'

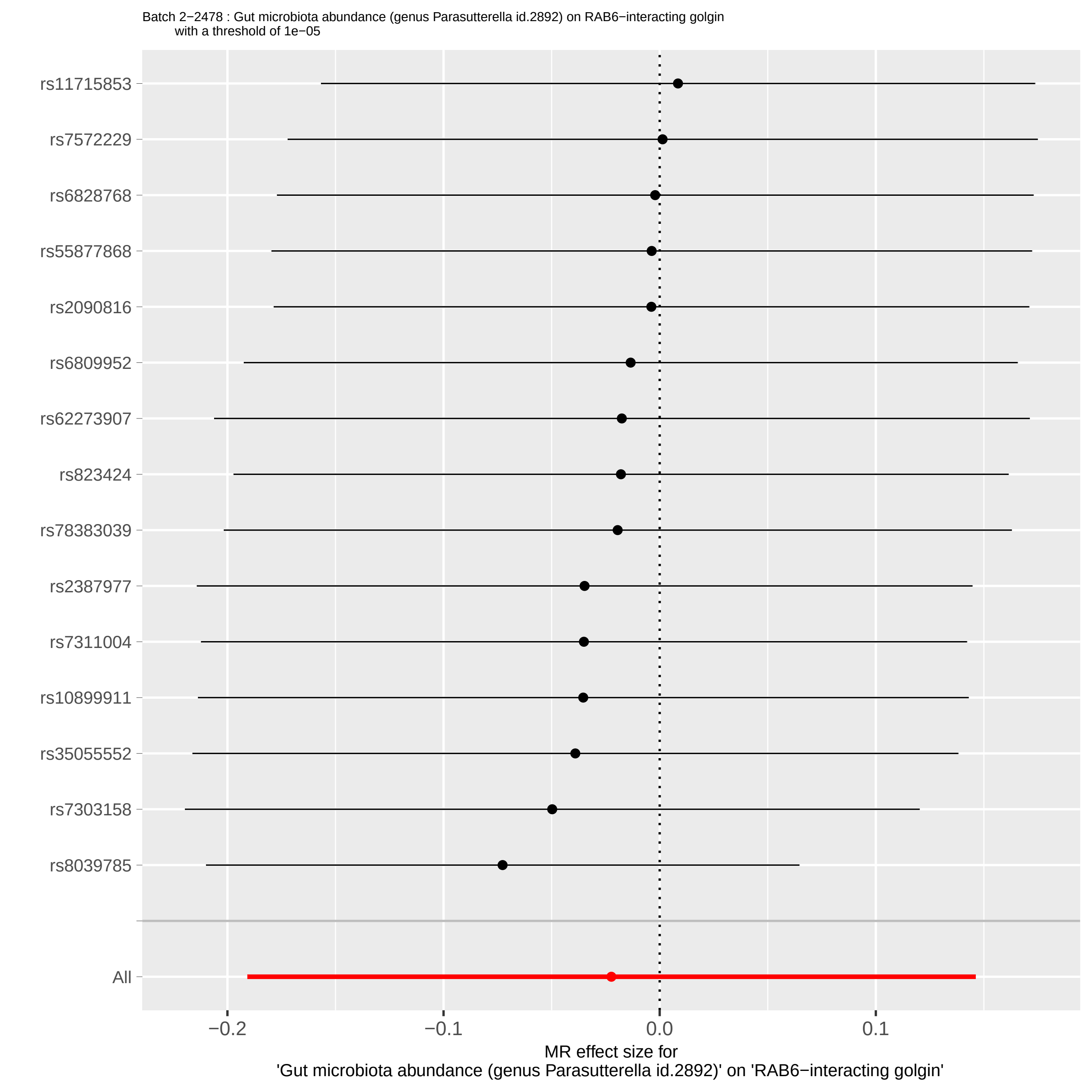
Batch 2−2478 : Gut microbiota abundance (genus Parasutterella id.2892) on RAB6−interacting golgin
with a threshold of 1e−05
rs11715853
rs7572229
rs6828768
rs55877868
rs2090816
rs6809952
rs62273907
rs823424
rs78383039
rs2387977
rs7311004
rs10899911
rs35055552
rs7303158
rs8039785
All
−0.2
−0.1
0.0
0.1
MR effect size for
'Gut microbiota abundance (genus Parasutterella id.2892)' on 'RAB6−interacting golgin'

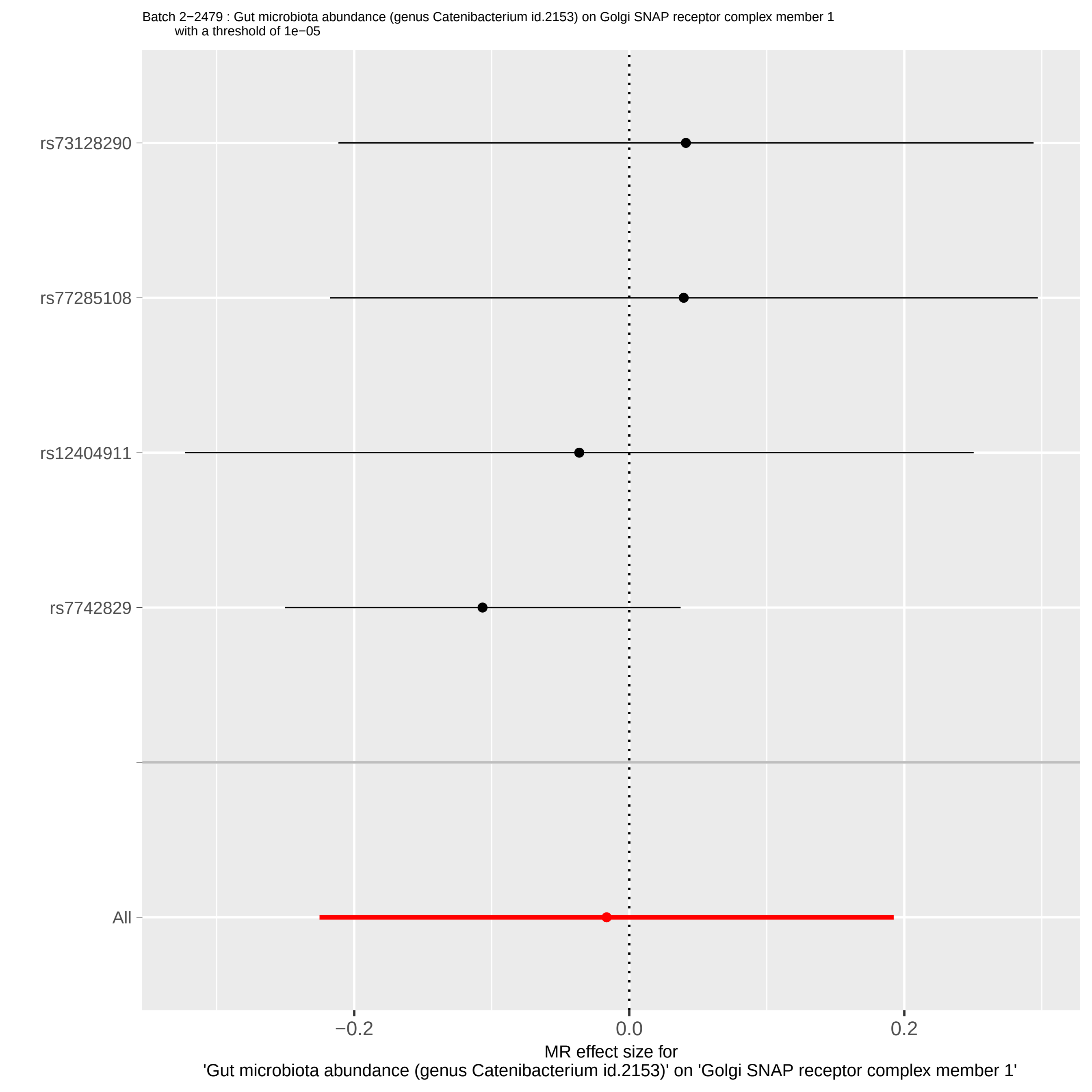
Batch 2−2479 : Gut microbiota abundance (genus Catenibacterium id.2153) on Golgi SNAP receptor complex member 1
with a threshold of 1e−05
rs73128290
rs77285108
rs12404911
rs7742829
All
−0.2
0.0
0.2
MR effect size for
'Gut microbiota abundance (genus Catenibacterium id.2153)' on 'Golgi SNAP receptor complex member 1'

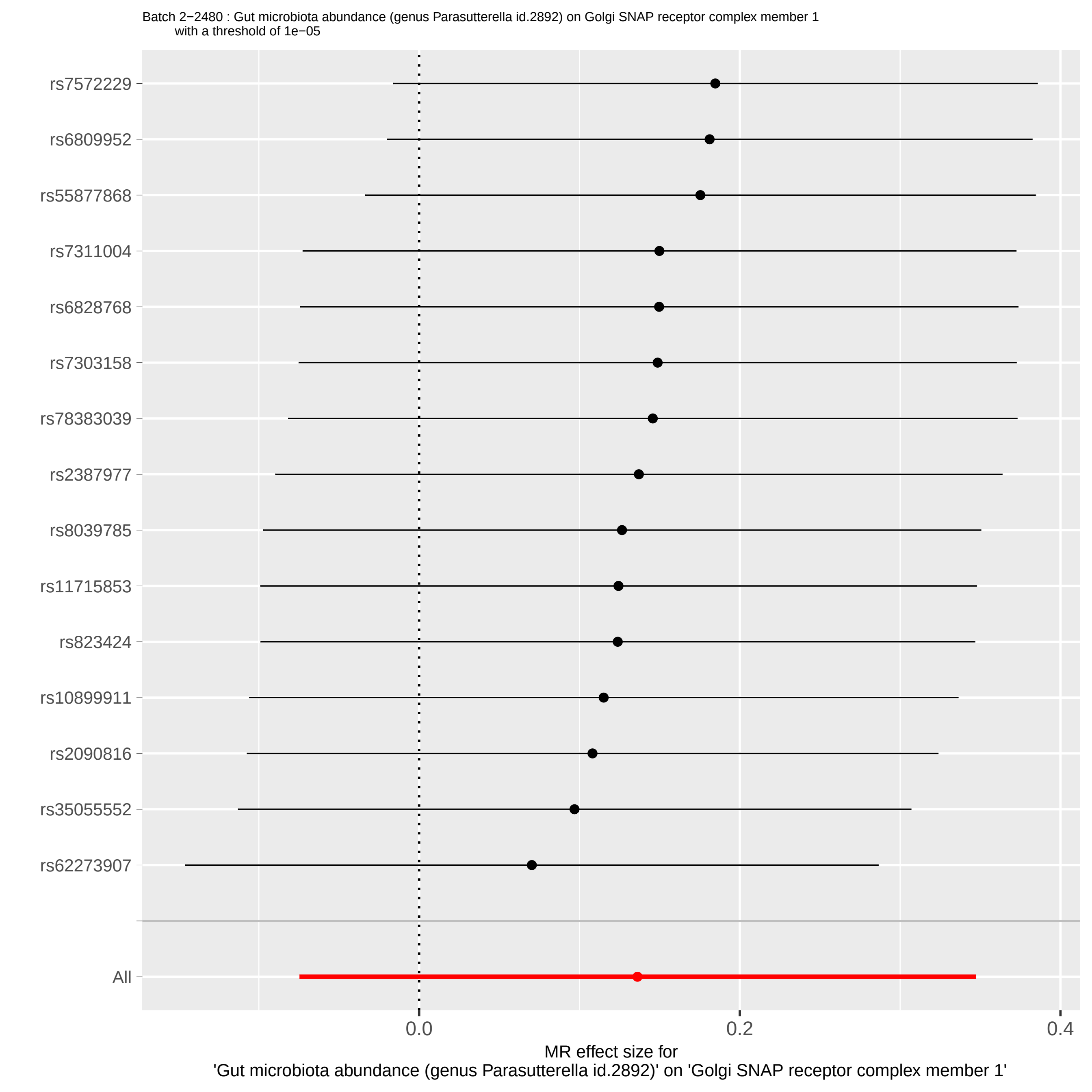

Batch 2−2480 : Gut microbiota abundance (genus Parasutterella id.2892) on Golgi SNAP receptor complex member 1
with a threshold of 1e−05
rs7572229
rs6809952
rs55877868
rs7311004
rs6828768
rs7303158
rs78383039
rs2387977
rs8039785
rs11715853
rs823424
rs10899911
rs2090816
rs35055552
rs62273907
All
0.0
0.2
0.4
MR effect size for
'Gut microbiota abundance (genus Parasutterella id.2892)' on 'Golgi SNAP receptor complex member 1'

Batch 2−2481 : Gut microbiota abundance (genus Catenibacterium id.2153) on Aspartate aminotransferase, cytoplasmic
with a threshold of 1e−05

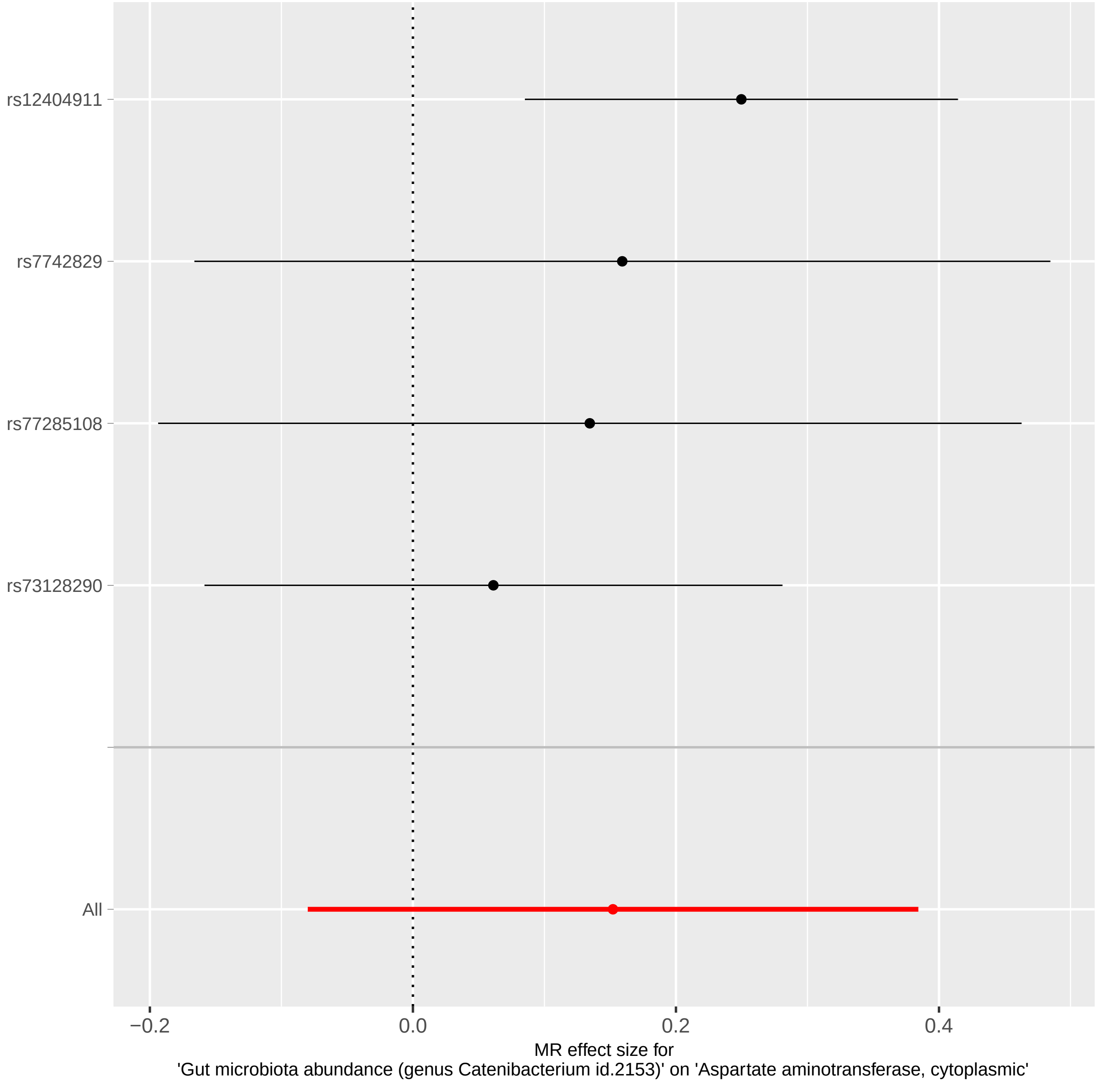

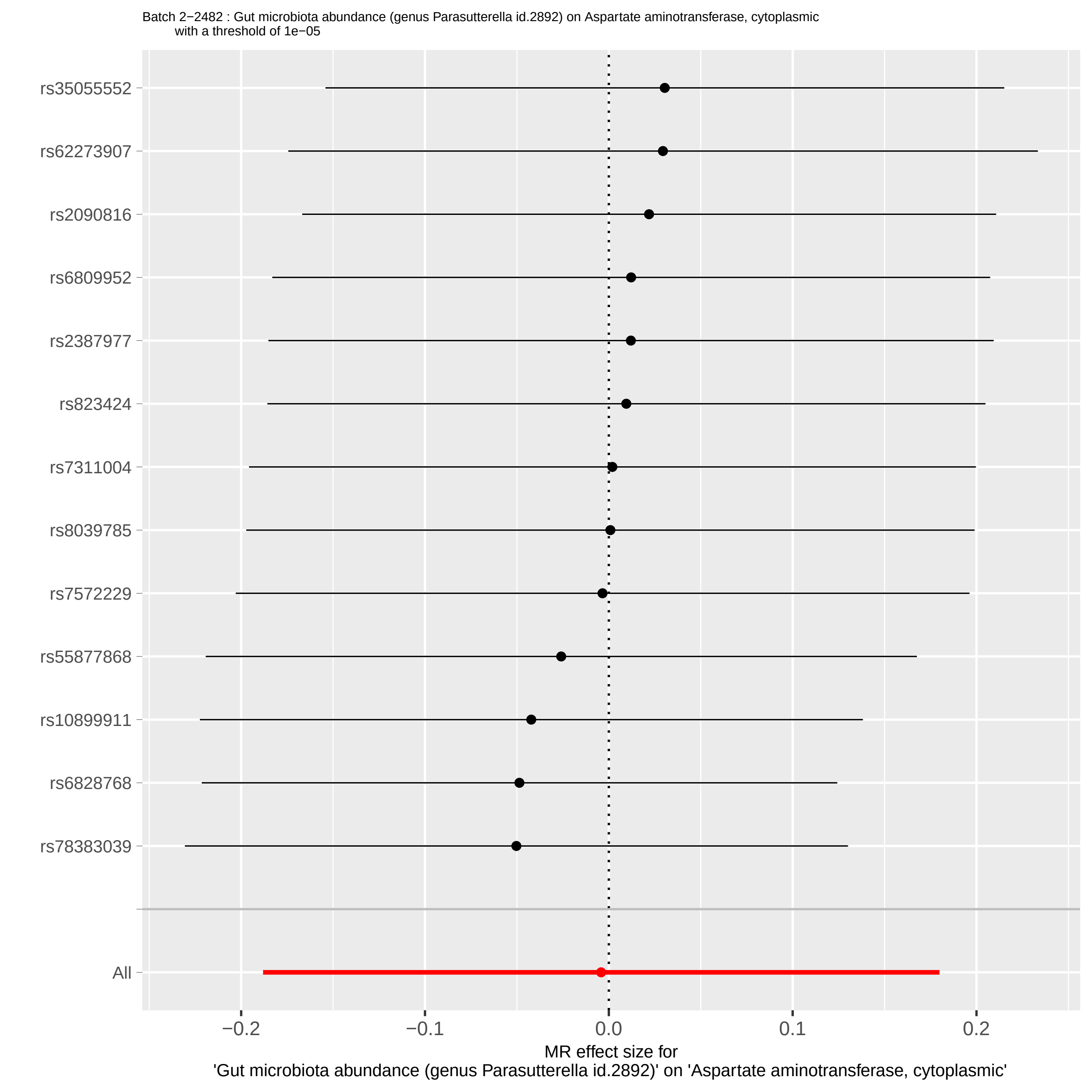
Batch 2−2482 : Gut microbiota abundance (genus Parasutterella id.2892) on Aspartate aminotransferase, cytoplasmic
with a threshold of 1e−05
rs35055552
rs62273907
rs2090816
rs6809952
rs2387977
rs823424
rs7311004
rs8039785
rs7572229
rs55877868
rs10899911
rs6828768
rs78383039
All
−0.2
−0.1
0.0
0.1
0.2
MR effect size for
'Gut microbiota abundance (genus Parasutterella id.2892)' on 'Aspartate aminotransferase, cytoplasmic'

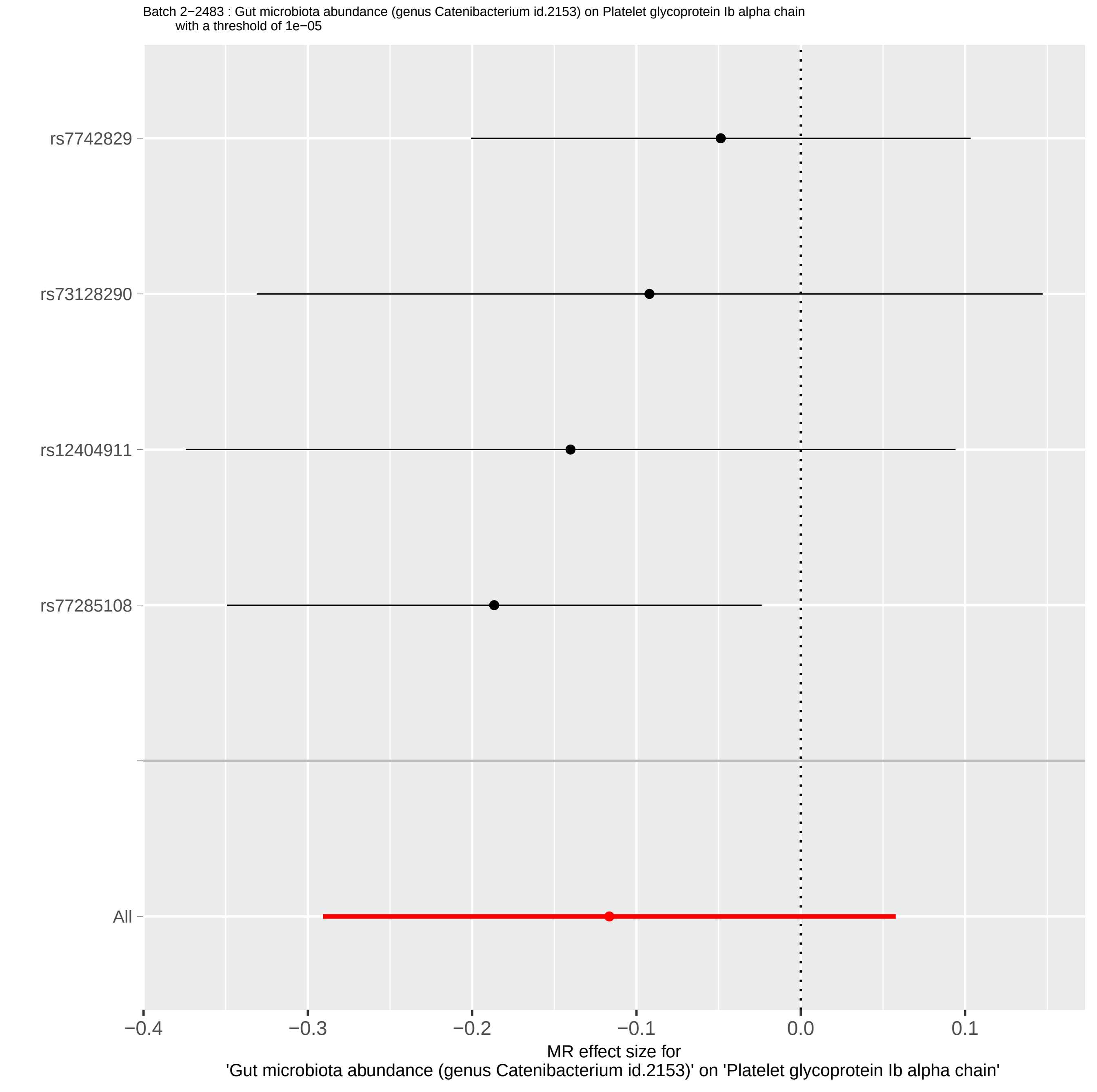

Batch 2−2483 : Gut microbiota abundance (genus Catenibacterium id.2153) on Platelet glycoprotein Ib alpha chain
with a threshold of 1e−05
rs7742829
rs73128290
rs12404911
rs77285108
All
−0.4
−0.3
−0.2
−0.1
0.0
0.1
MR effect size for
'Gut microbiota abundance (genus Catenibacterium id.2153)' on 'Platelet glycoprotein Ib alpha chain'

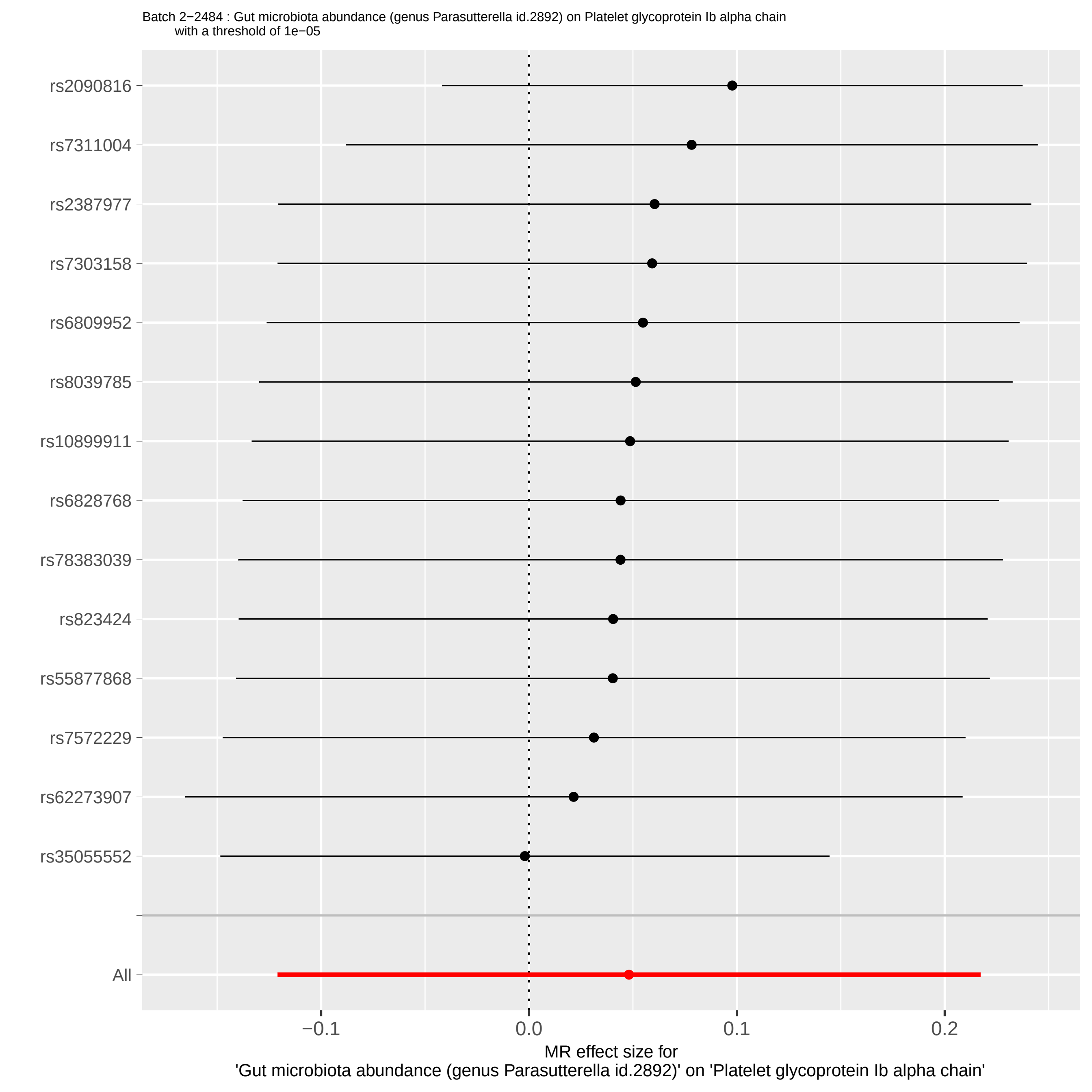
Batch 2−2484 : Gut microbiota abundance (genus Parasutterella id.2892) on Platelet glycoprotein Ib alpha chain
with a threshold of 1e−05
rs2090816
rs7311004
rs2387977
rs7303158
rs6809952
rs8039785
rs10899911
rs6828768
rs78383039
rs823424
rs55877868
rs7572229
rs62273907
rs35055552
All
−0.1
0.0
0.1
0.2
MR effect size for
'Gut microbiota abundance (genus Parasutterella id.2892)' on 'Platelet glycoprotein Ib alpha chain'

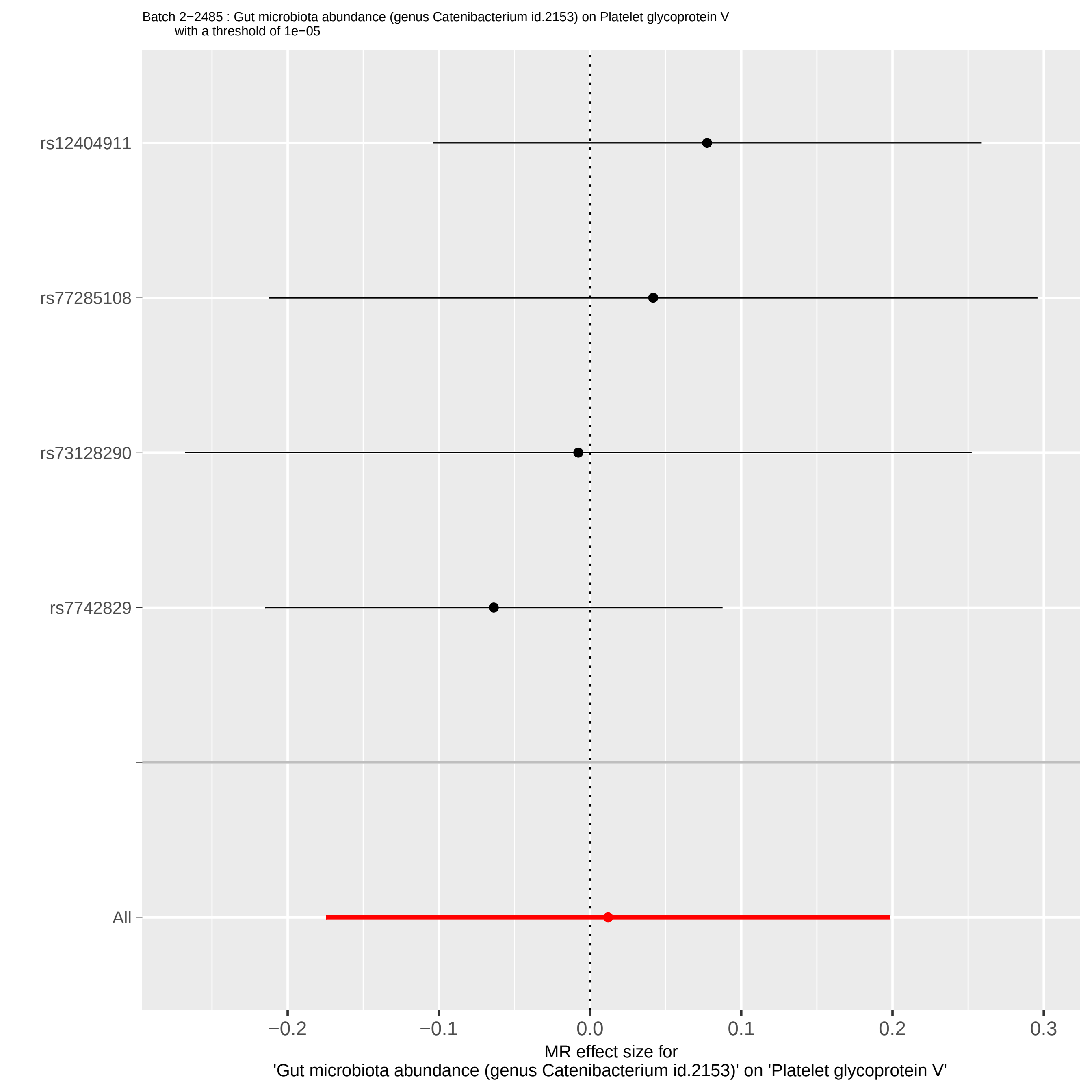
Batch 2−2485 : Gut microbiota abundance (genus Catenibacterium id.2153) on Platelet glycoprotein V
with a threshold of 1e−05
rs12404911
rs77285108
rs73128290
rs7742829
All
−0.2
−0.1
0.0
0.1
0.2
0.3
MR effect size for
'Gut microbiota abundance (genus Catenibacterium id.2153)' on 'Platelet glycoprotein V'

Batch 2−2486 : Gut microbiota abundance (genus Parasutterella id.2892) on Platelet glycoprotein V
with a threshold of 1e−05

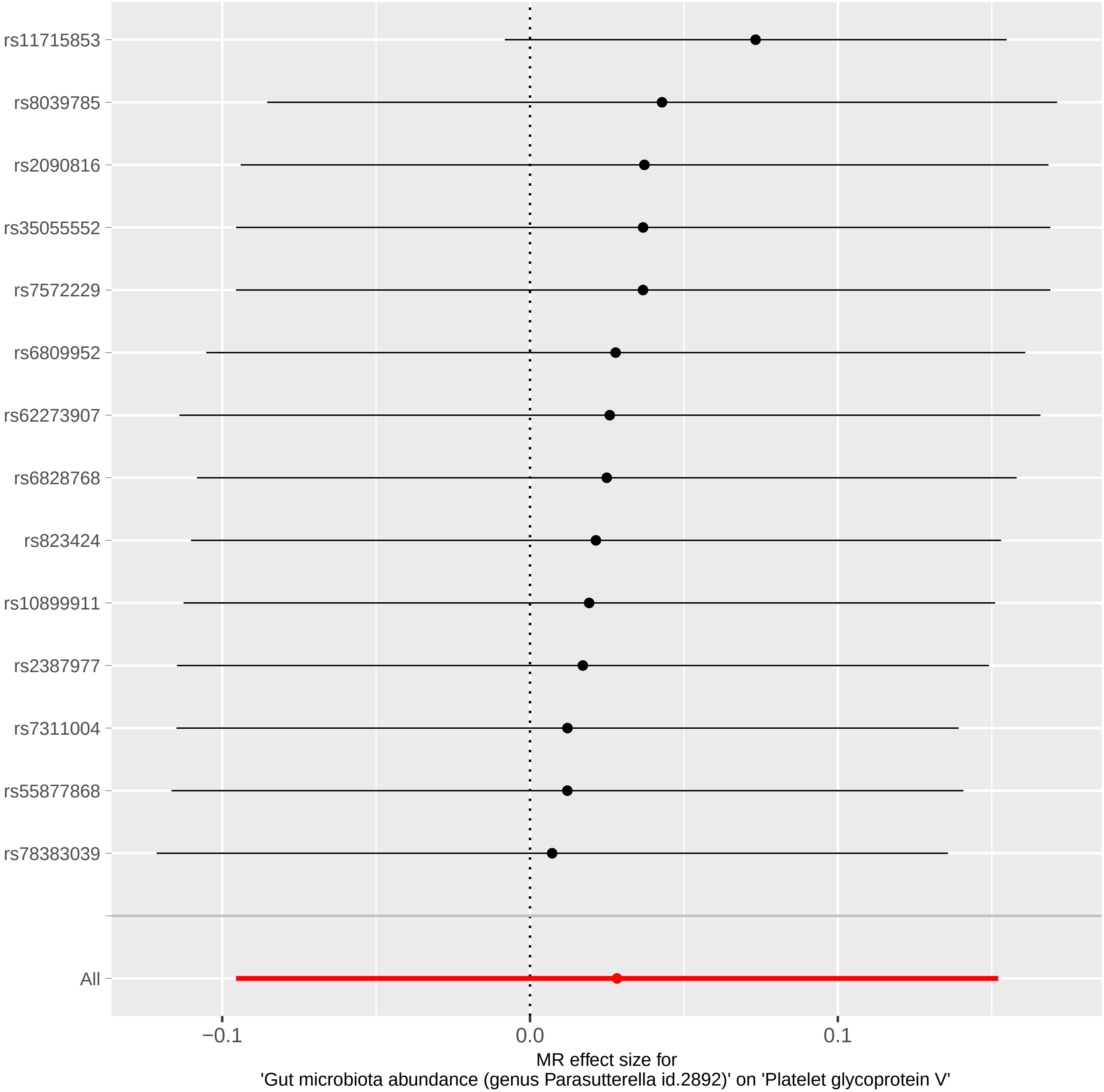

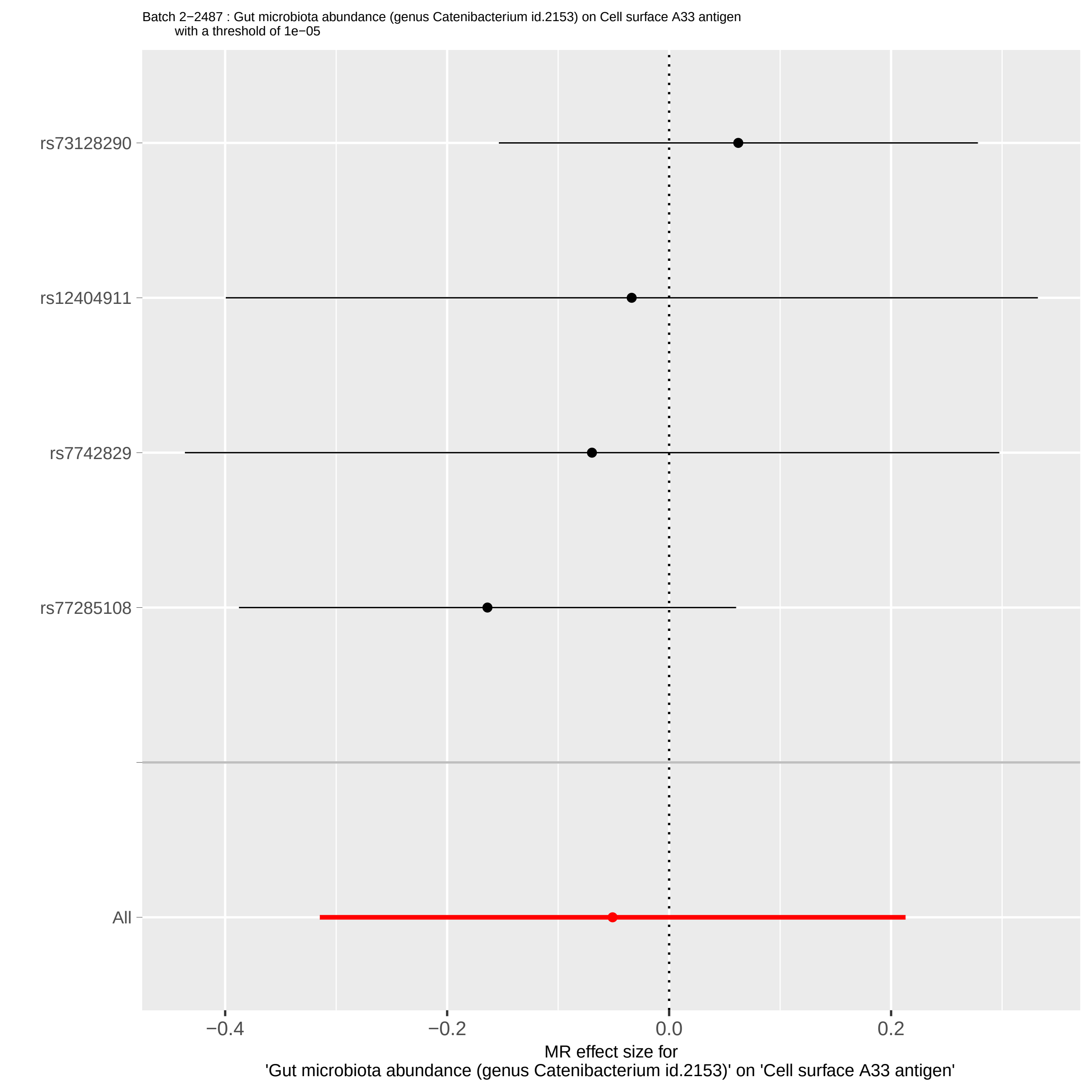
Batch 2−2487 : Gut microbiota abundance (genus Catenibacterium id.2153) on Cell surface A33 antigen
with a threshold of 1e−05
rs73128290
rs12404911
rs7742829
rs77285108
All
−0.4
−0.2
0.0
0.2
MR effect size for
'Gut microbiota abundance (genus Catenibacterium id.2153)' on 'Cell surface A33 antigen'

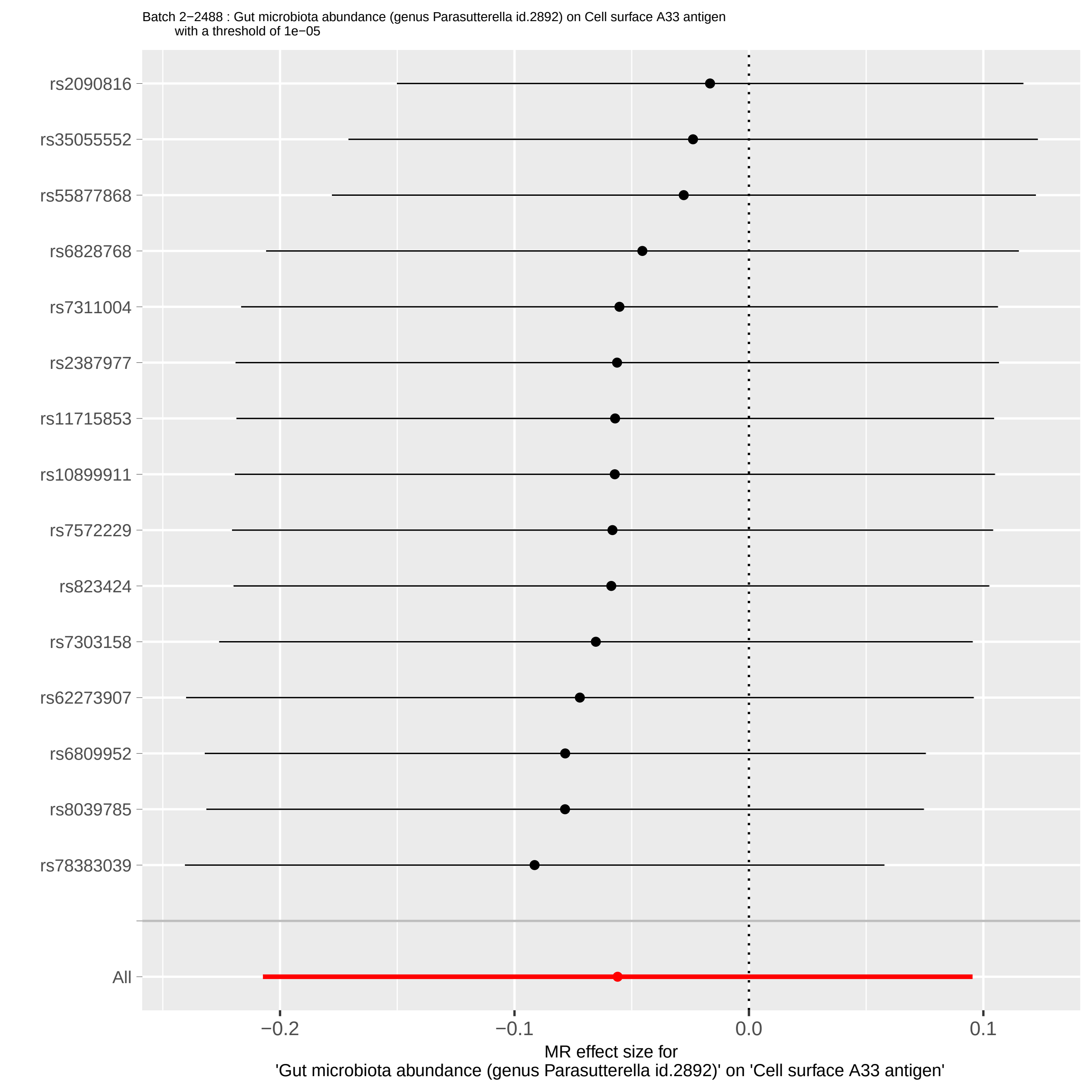

Batch 2−2488 : Gut microbiota abundance (genus Parasutterella id.2892) on Cell surface A33 antigen
with a threshold of 1e−05
rs2090816
rs35055552
rs55877868
rs6828768
rs7311004
rs2387977
rs11715853
rs10899911
rs7572229
rs823424
rs7303158
rs62273907
rs6809952
rs8039785
rs78383039
All
−0.2
−0.1
0.0
0.1
MR effect size for
'Gut microbiota abundance (genus Parasutterella id.2892)' on 'Cell surface A33 antigen'

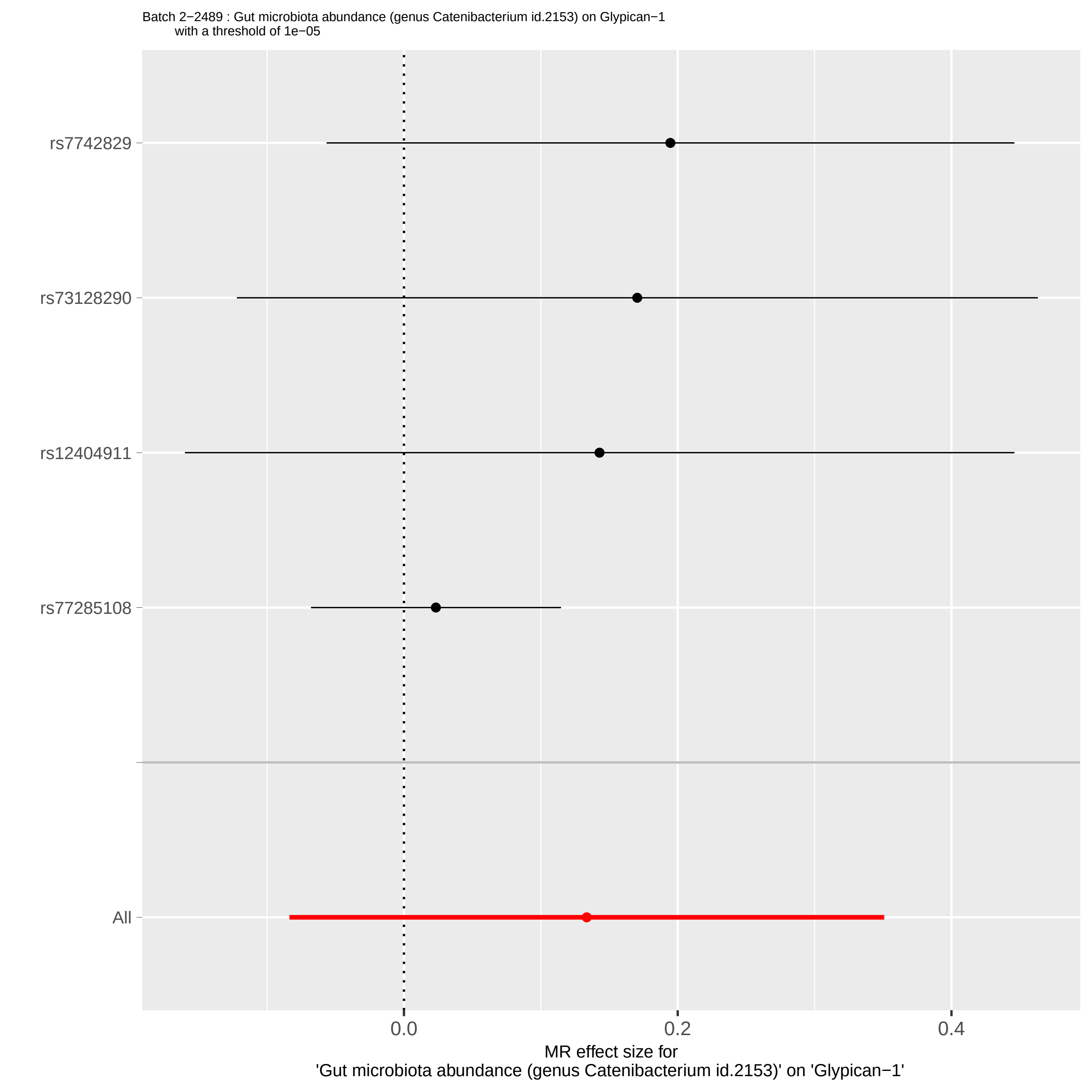
Batch 2−2489 : Gut microbiota abundance (genus Catenibacterium id.2153) on Glypican−1
with a threshold of 1e−05
rs7742829
rs73128290
rs12404911
rs77285108
All
0.0
0.2
0.4
MR effect size for
'Gut microbiota abundance (genus Catenibacterium id.2153)' on 'Glypican−1'

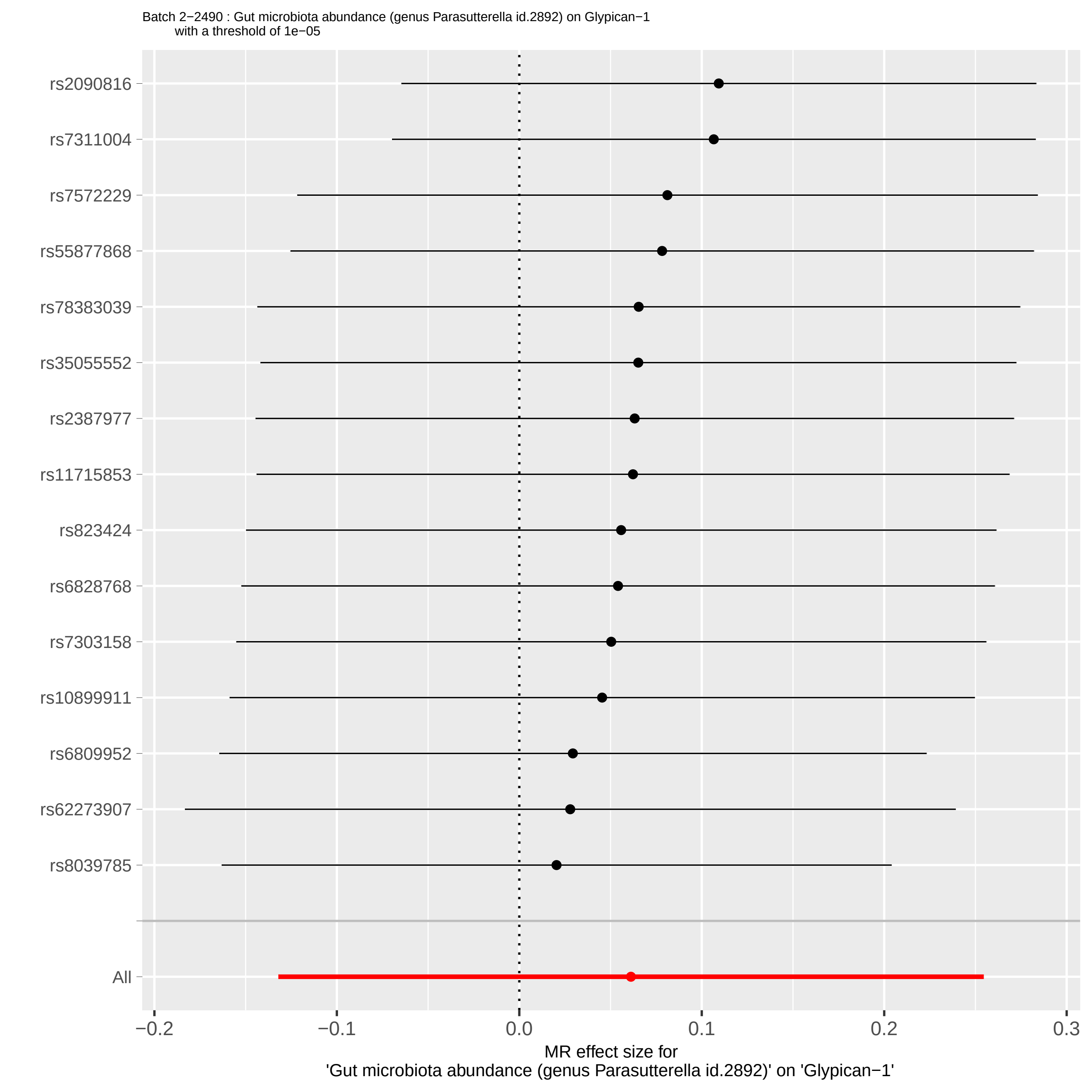
Batch 2−2490 : Gut microbiota abundance (genus Parasutterella id.2892) on Glypican−1
with a threshold of 1e−05
rs2090816
rs7311004
rs7572229
rs55877868
rs78383039
rs35055552
rs2387977
rs11715853
rs823424
rs6828768
rs7303158
rs10899911
rs6809952
rs62273907
rs8039785
All
−0.2
−0.1
0.0
0.1
0.2
0.3
MR effect size for
'Gut microbiota abundance (genus Parasutterella id.2892)' on 'Glypican−1'

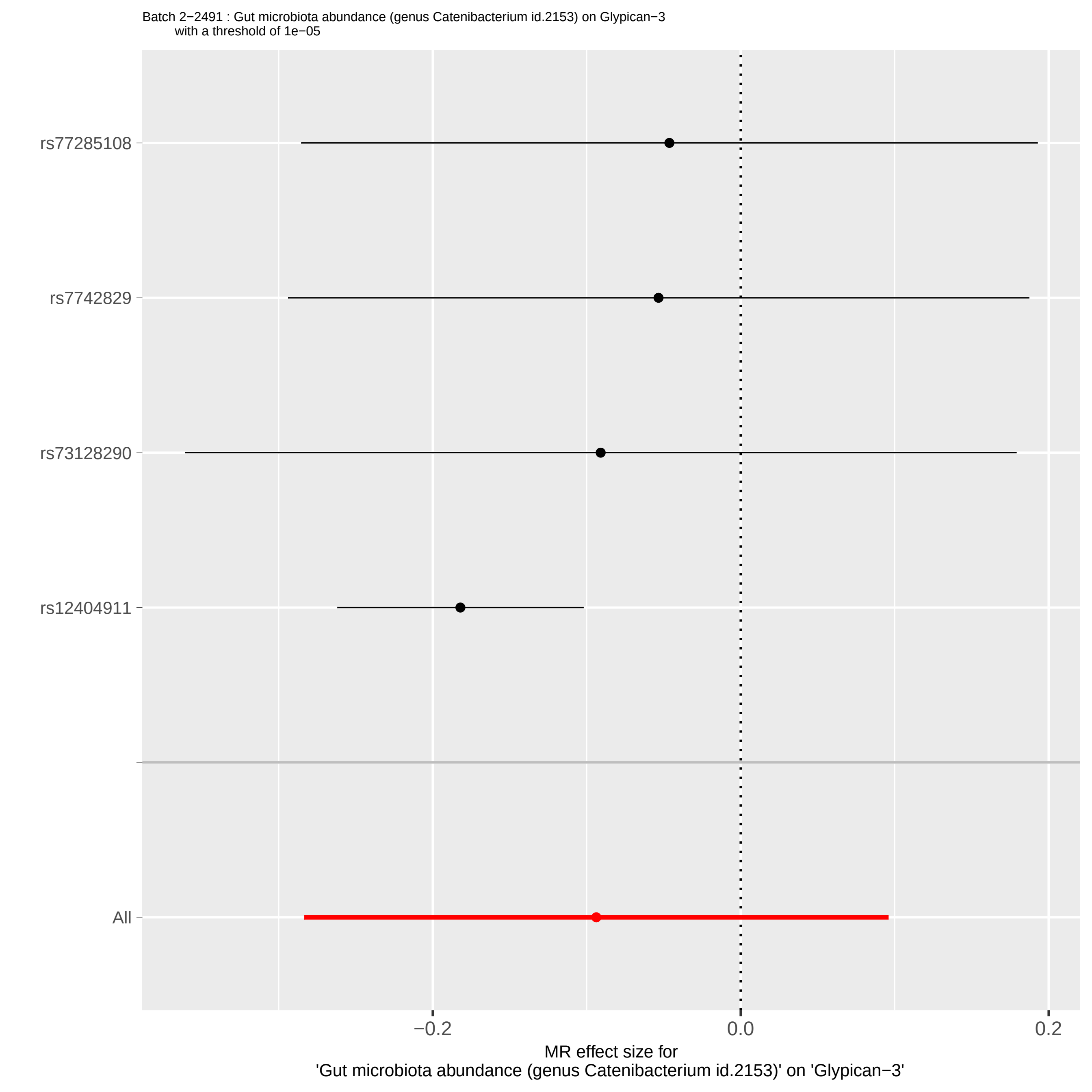

Batch 2−2491 : Gut microbiota abundance (genus Catenibacterium id.2153) on Glypican−3
with a threshold of 1e−05
rs77285108
rs7742829
rs73128290
rs12404911
All
−0.2
0.0
0.2
MR effect size for
'Gut microbiota abundance (genus Catenibacterium id.2153)' on 'Glypican−3'

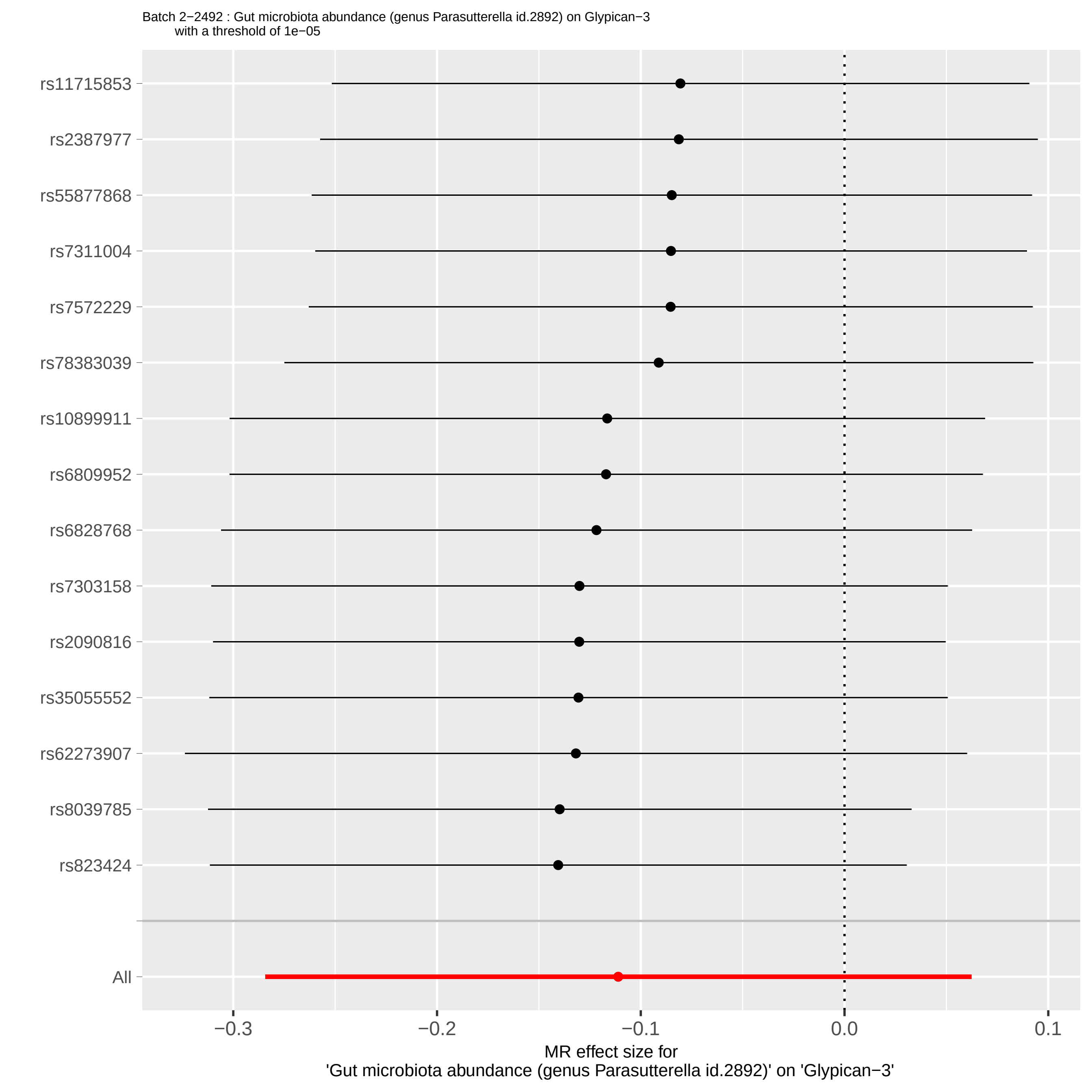

Batch 2−2492 : Gut microbiota abundance (genus Parasutterella id.2892) on Glypican−3
with a threshold of 1e−05
rs11715853
rs2387977
rs55877868
rs7311004
rs7572229
rs78383039
rs10899911
rs6809952
rs6828768
rs7303158
rs2090816
rs35055552
rs62273907
rs8039785
rs823424
All
−0.3
−0.2
−0.1
0.0
0.1
MR effect size for
'Gut microbiota abundance (genus Parasutterella id.2892)' on 'Glypican−3'

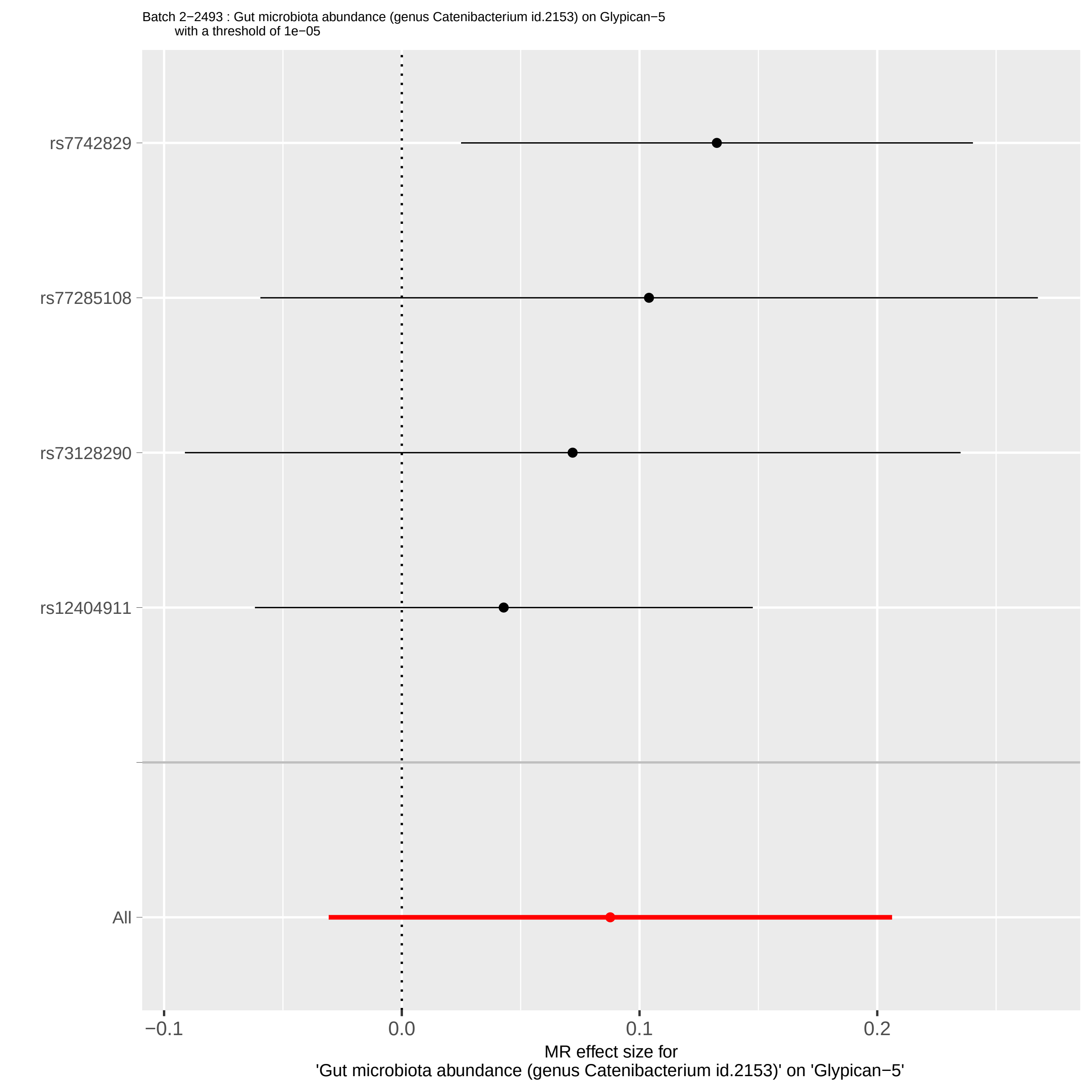
Batch 2−2493 : Gut microbiota abundance (genus Catenibacterium id.2153) on Glypican−5
with a threshold of 1e−05
rs7742829
rs77285108
rs73128290
rs12404911
All
−0.1
0.0
0.1
0.2
MR effect size for
'Gut microbiota abundance (genus Catenibacterium id.2153)' on 'Glypican−5'

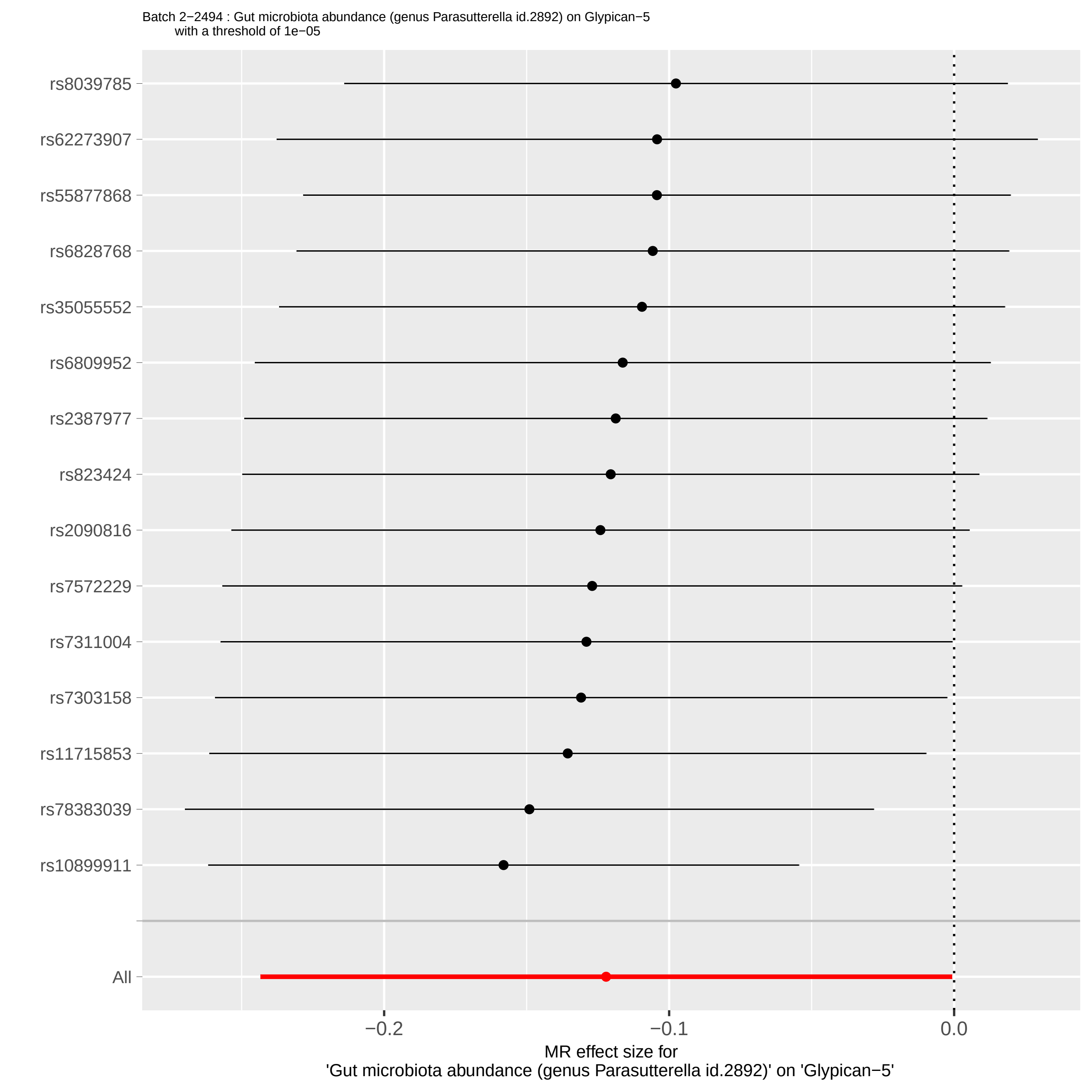

Batch 2−2494 : Gut microbiota abundance (genus Parasutterella id.2892) on Glypican−5
with a threshold of 1e−05
rs8039785
rs62273907
rs55877868
rs6828768
rs35055552
rs6809952
rs2387977
rs823424
rs2090816
rs7572229
rs7311004
rs7303158
rs11715853
rs78383039
rs10899911
All
−0.2
−0.1
0.0
MR effect size for
'Gut microbiota abundance (genus Parasutterella id.2892)' on 'Glypican−5'

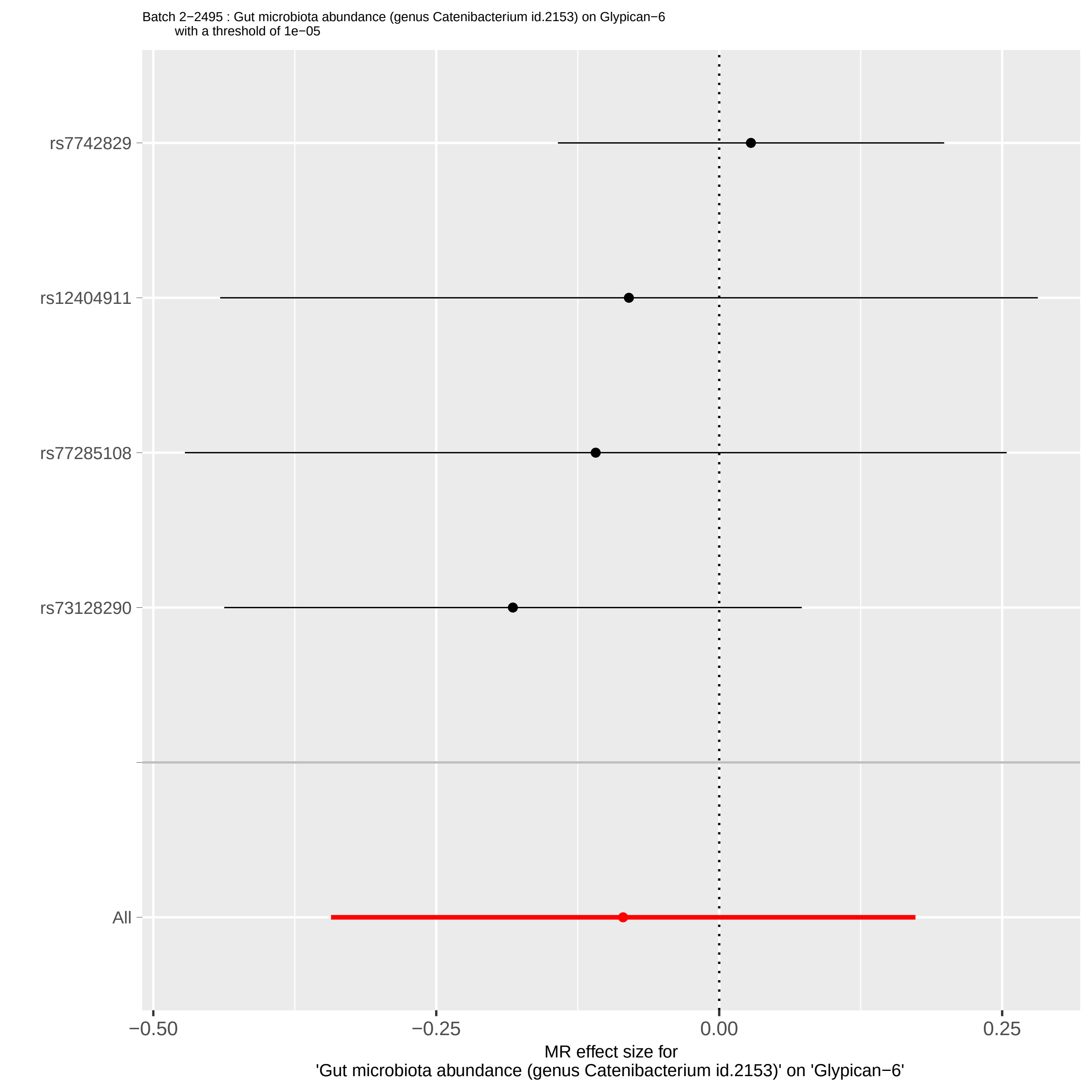

Batch 2−2495 : Gut microbiota abundance (genus Catenibacterium id.2153) on Glypican−6
with a threshold of 1e−05
rs7742829
rs12404911
rs77285108
rs73128290
All
−0.50
−0.25
0.00
0.25
MR effect size for
'Gut microbiota abundance (genus Catenibacterium id.2153)' on 'Glypican−6'

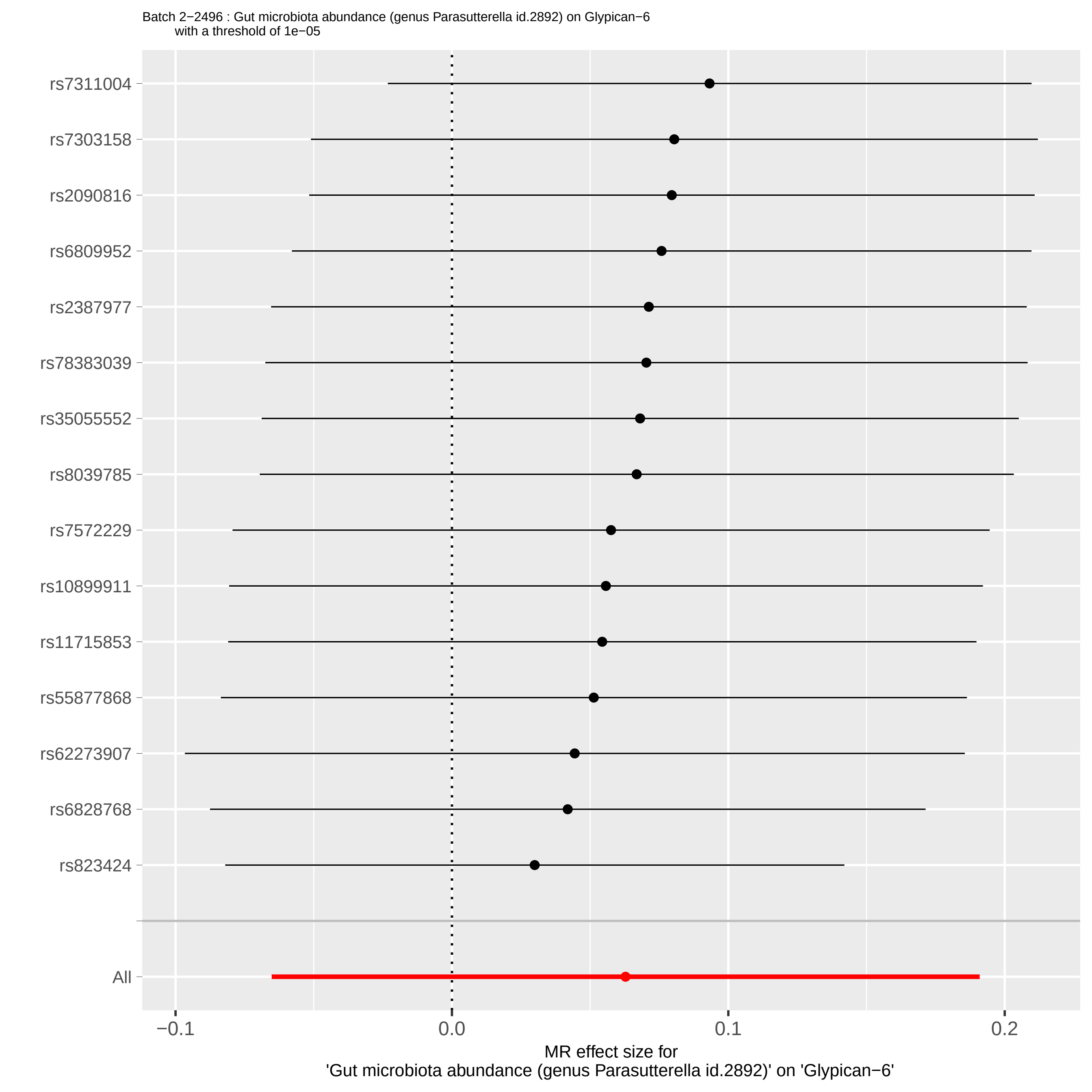

Batch 2−2496 : Gut microbiota abundance (genus Parasutterella id.2892) on Glypican−6
with a threshold of 1e−05
rs7311004
rs7303158
rs2090816
rs6809952
rs2387977
rs78383039
rs35055552
rs8039785
rs7572229
rs10899911
rs11715853
rs55877868
rs62273907
rs6828768
rs823424
All
−0.1
0.0
0.1
0.2
MR effect size for
'Gut microbiota abundance (genus Parasutterella id.2892)' on 'Glypican−6'

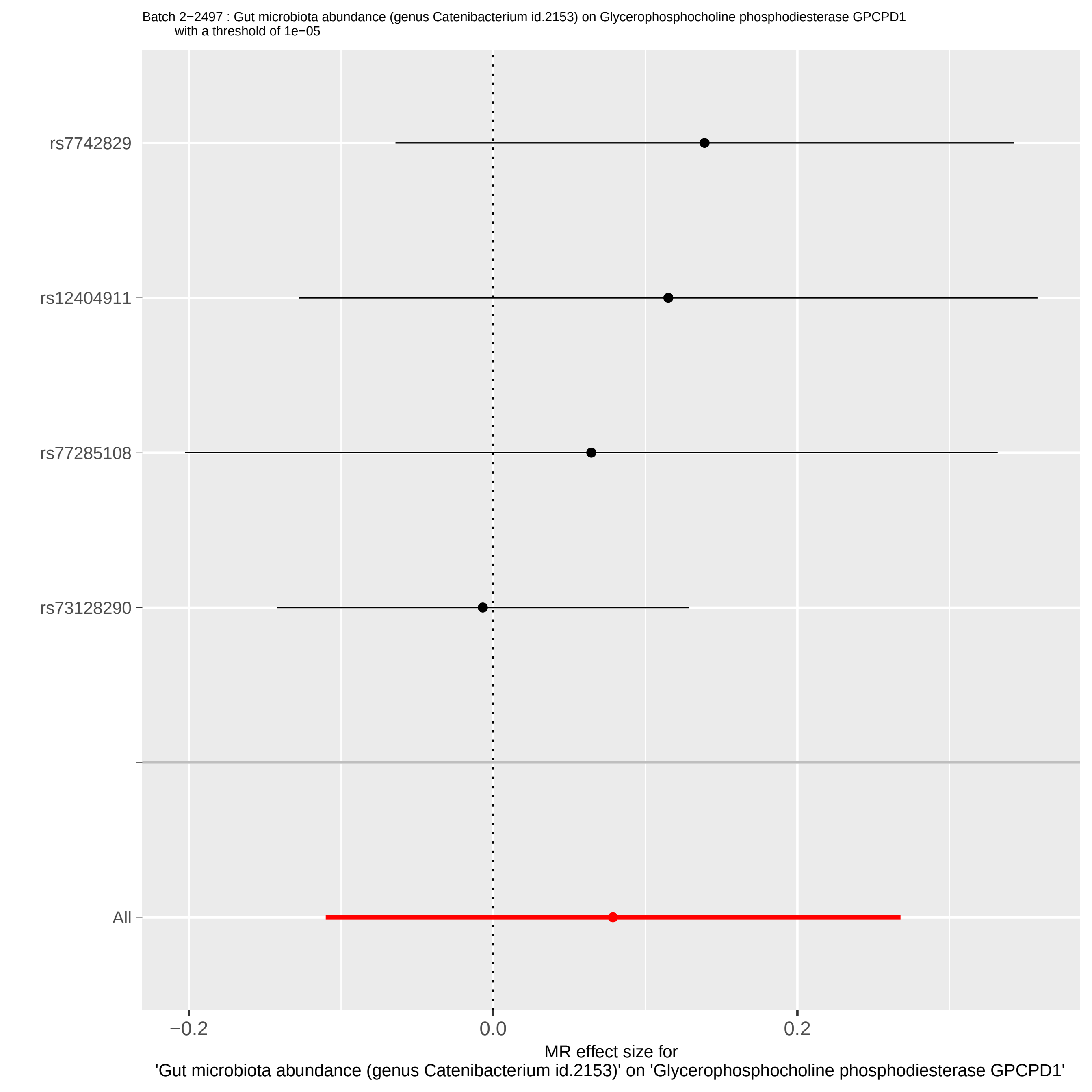
Batch 2−2497 : Gut microbiota abundance (genus Catenibacterium id.2153) on Glycerophosphocholine phosphodiesterase GPCPD1
with a threshold of 1e−05
rs7742829
rs12404911
rs77285108
rs73128290
All
−0.2
0.0
0.2
MR effect size for
'Gut microbiota abundance (genus Catenibacterium id.2153)' on 'Glycerophosphocholine phosphodiesterase GPCPD1'

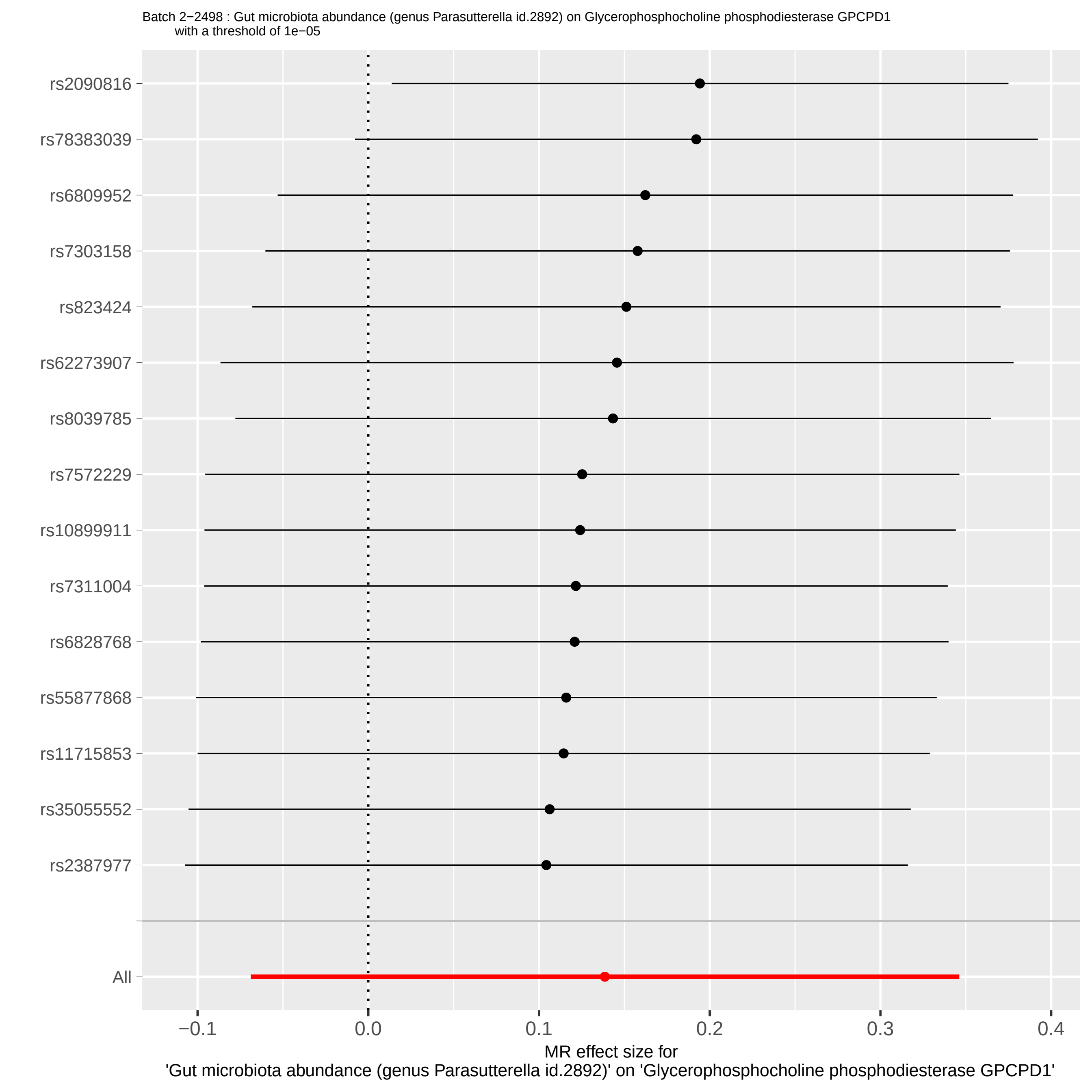

Batch 2−2498 : Gut microbiota abundance (genus Parasutterella id.2892) on Glycerophosphocholine phosphodiesterase GPCPD1
with a threshold of 1e−05
rs2090816
rs78383039
rs6809952
rs7303158
rs823424
rs62273907
rs8039785
rs7572229
rs10899911
rs7311004
rs6828768
rs55877868
rs11715853
rs35055552
rs2387977
All
−0.1
0.0
0.1
0.2
0.3
0.4
MR effect size for
'Gut microbiota abundance (genus Parasutterella id.2892)' on 'Glycerophosphocholine phosphodiesterase GPCPD1'

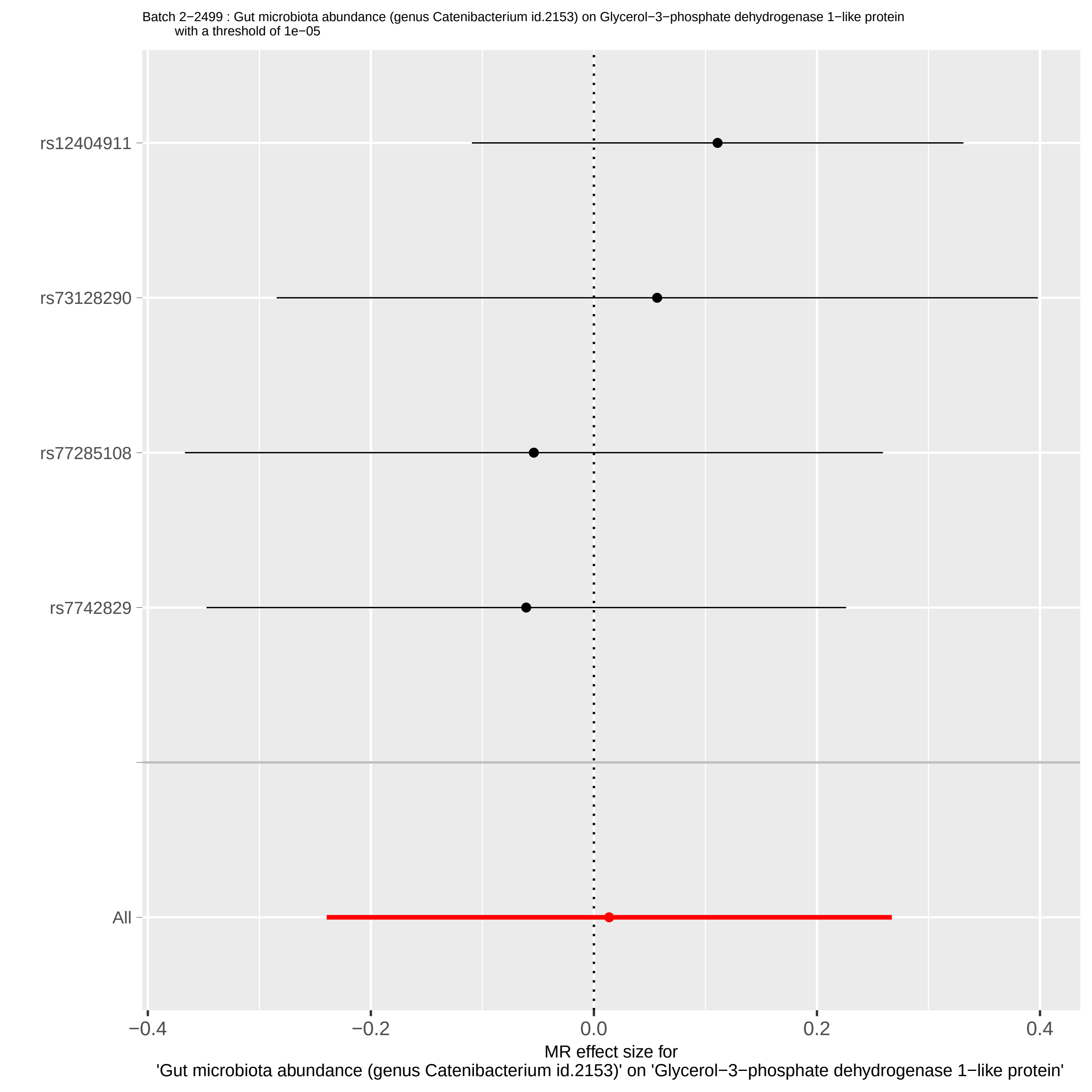

Batch 2−2499 : Gut microbiota abundance (genus Catenibacterium id.2153) on Glycerol−3−phosphate dehydrogenase 1−like protein
with a threshold of 1e−05
rs12404911
rs73128290
rs77285108
rs7742829
All
−0.4
−0.2
0.0
0.2
0.4
MR effect size for
'Gut microbiota abundance (genus Catenibacterium id.2153)' on 'Glycerol−3−phosphate dehydrogenase 1−like protein'

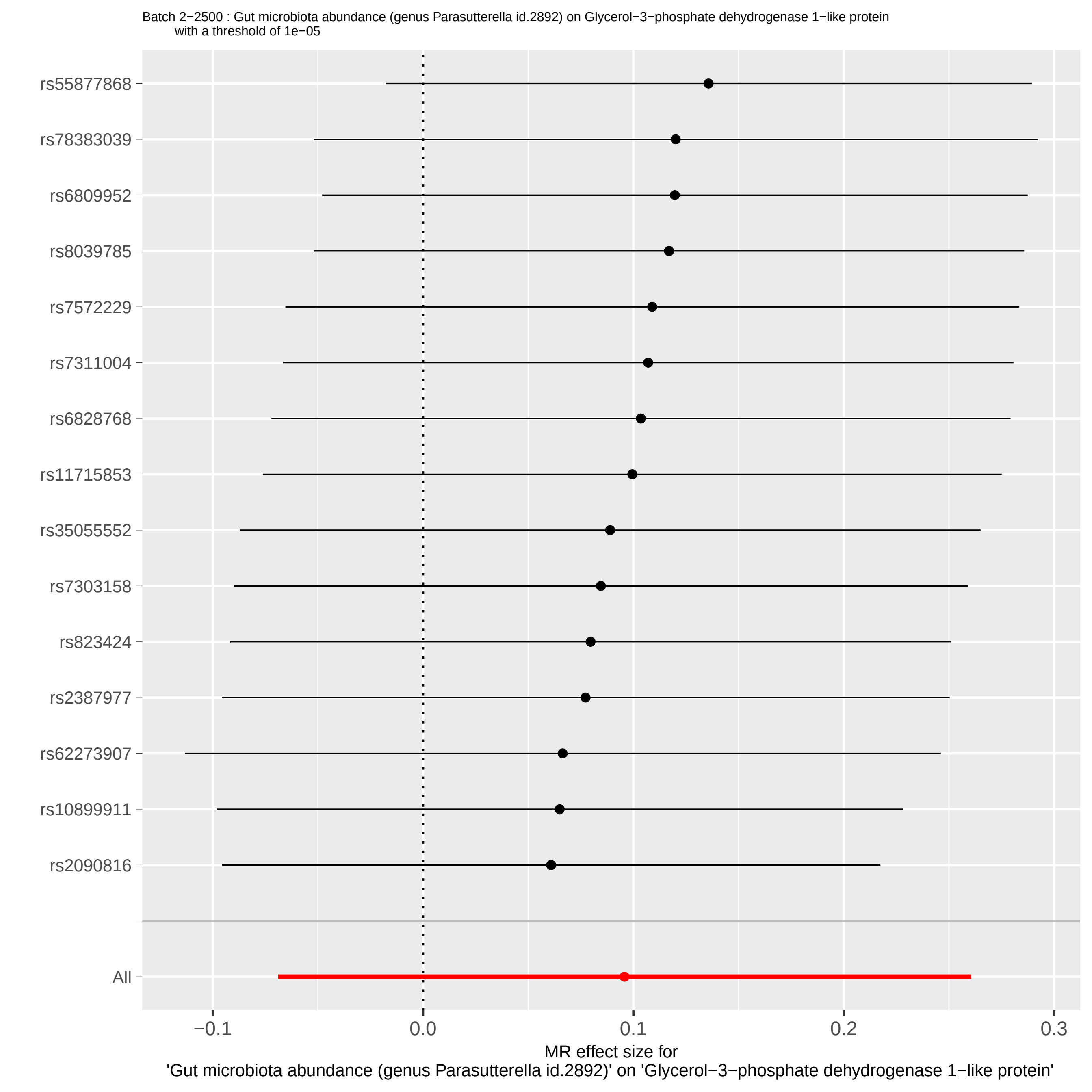

Batch 2−2500 : Gut microbiota abundance (genus Parasutterella id.2892) on Glycerol−3−phosphate dehydrogenase 1−like protein
with a threshold of 1e−05
rs55877868
rs78383039
rs6809952
rs8039785
rs7572229
rs7311004
rs6828768
rs11715853
rs35055552
rs7303158
rs823424
rs2387977
rs62273907
rs10899911
rs2090816
All
−0.1
0.0
0.1
0.2
0.3
MR effect size for
'Gut microbiota abundance (genus Parasutterella id.2892)' on 'Glycerol−3−phosphate dehydrogenase 1−like protein'

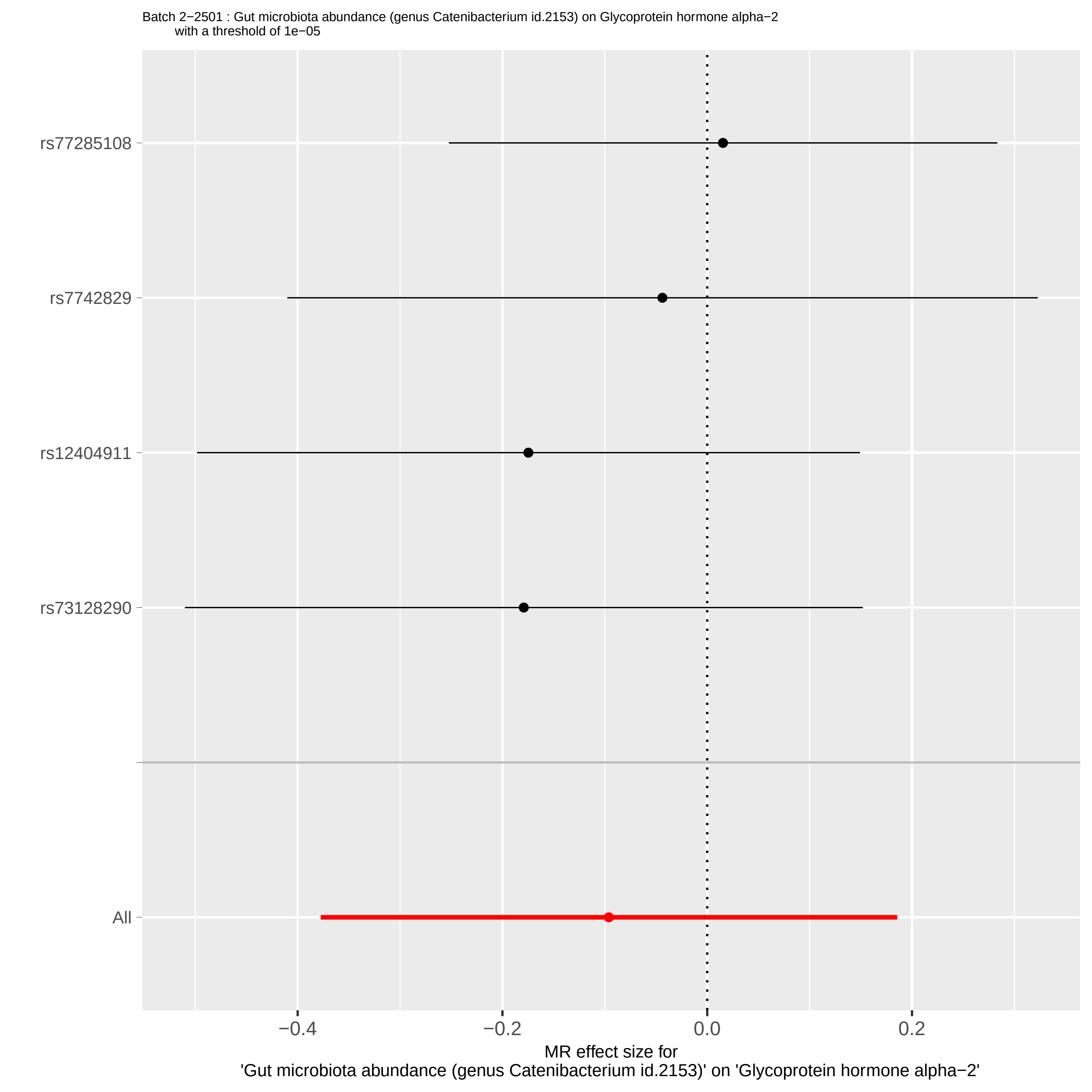

Batch 2−2501 : Gut microbiota abundance (genus Catenibacterium id.2153) on Glycoprotein hormone alpha−2
with a threshold of 1e−05
rs77285108
rs7742829
rs12404911
rs73128290
All
−0.4
−0.2
0.0
0.2
MR effect size for
'Gut microbiota abundance (genus Catenibacterium id.2153)' on 'Glycoprotein hormone alpha−2'

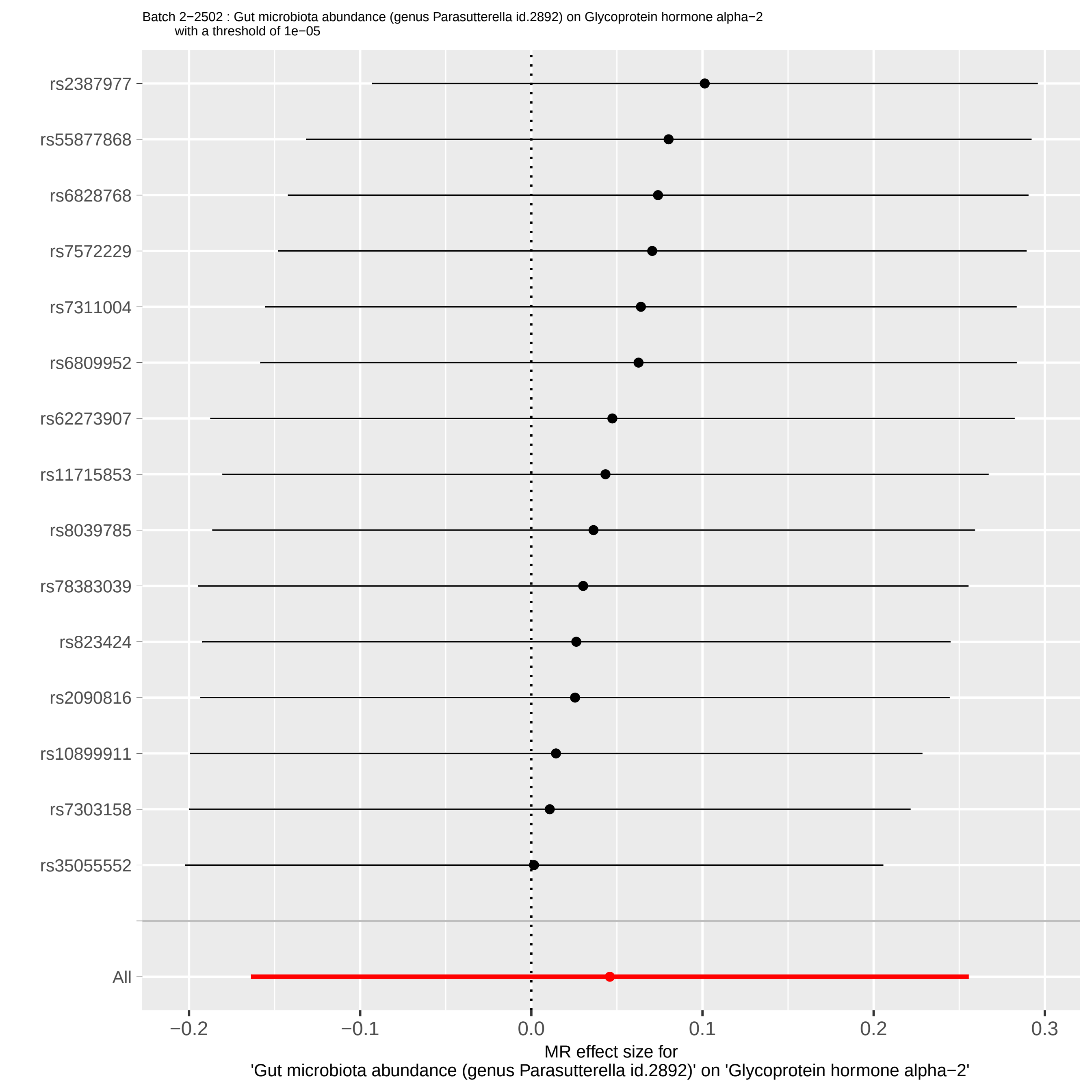
Batch 2−2502 : Gut microbiota abundance (genus Parasutterella id.2892) on Glycoprotein hormone alpha−2
with a threshold of 1e−05
rs2387977
rs55877868
rs6828768
rs7572229
rs7311004
rs6809952
rs62273907
rs11715853
rs8039785
rs78383039
rs823424
rs2090816
rs10899911
rs7303158
rs35055552
All
−0.2
−0.1
0.0
0.1
0.2
0.3
MR effect size for
'Gut microbiota abundance (genus Parasutterella id.2892)' on 'Glycoprotein hormone alpha−2'

Batch 2−2503 : Gut microbiota abundance (genus Catenibacterium id.2153) on Glucose−6−phosphate isomerase
with a threshold of 1e−05

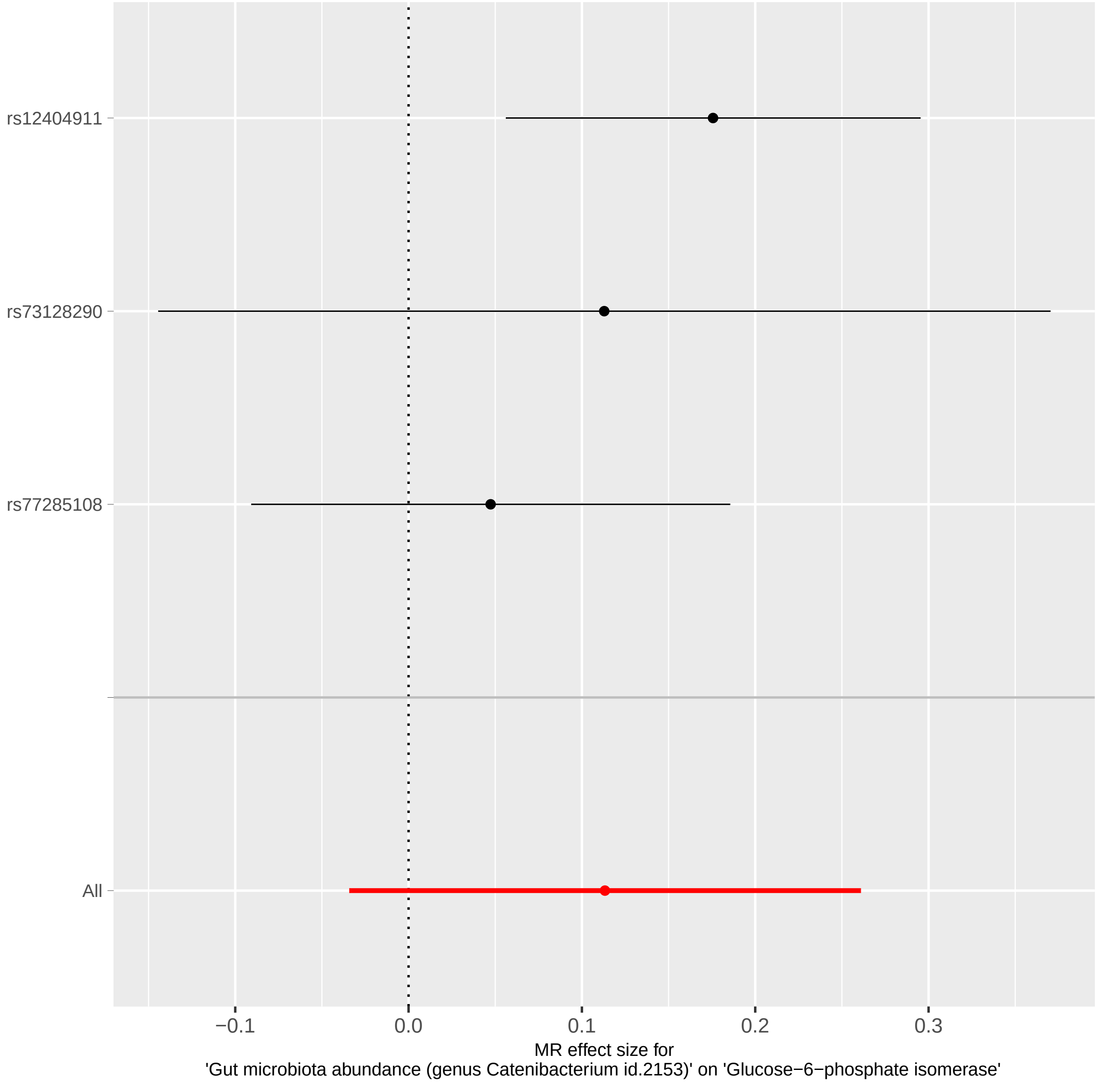

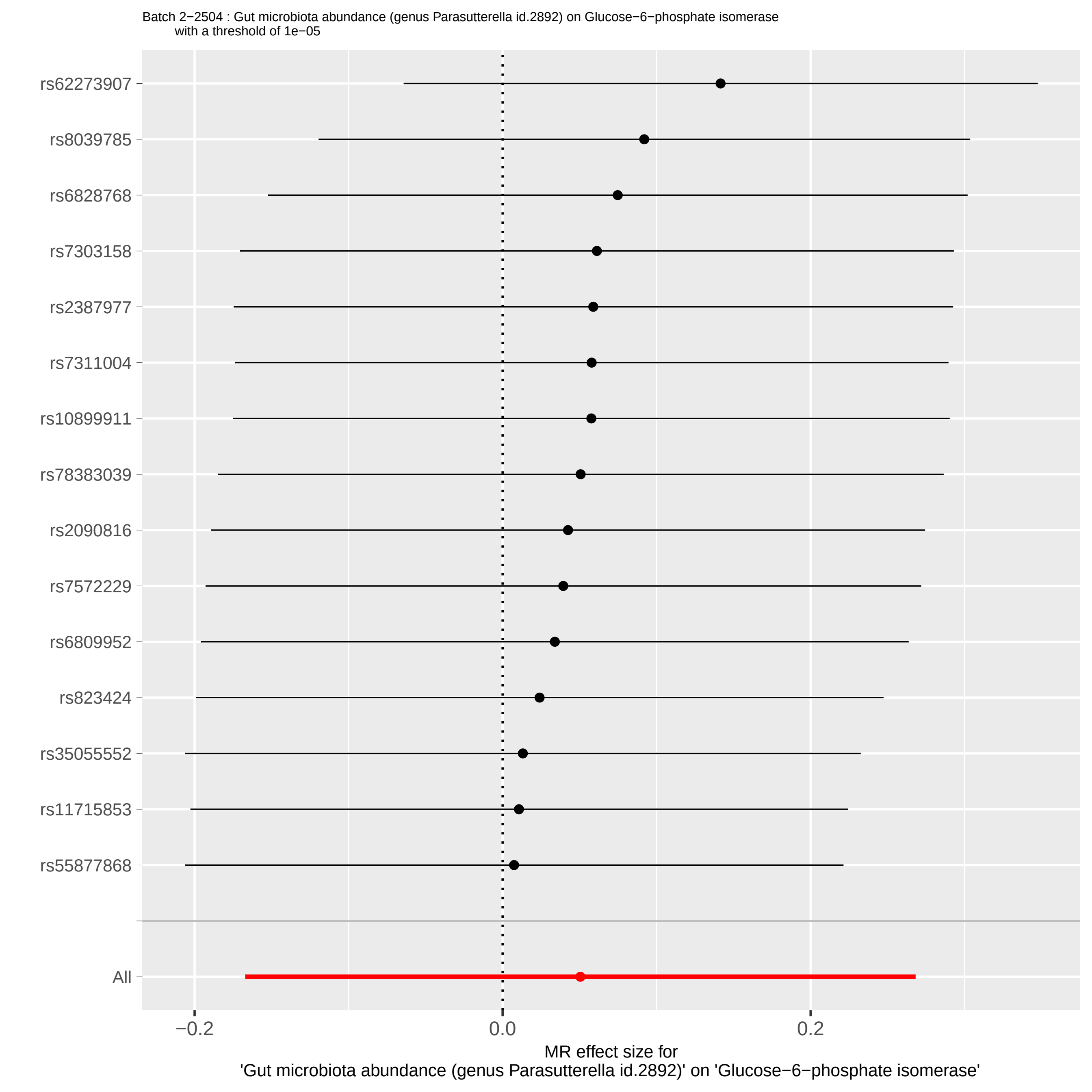

Batch 2−2504 : Gut microbiota abundance (genus Parasutterella id.2892) on Glucose−6−phosphate isomerase
with a threshold of 1e−05
rs62273907
rs8039785
rs6828768
rs7303158
rs2387977
rs7311004
rs10899911
rs78383039
rs2090816
rs7572229
rs6809952
rs823424
rs35055552
rs11715853
rs55877868
All
−0.2
0.0
0.2
MR effect size for
'Gut microbiota abundance (genus Parasutterella id.2892)' on 'Glucose−6−phosphate isomerase'

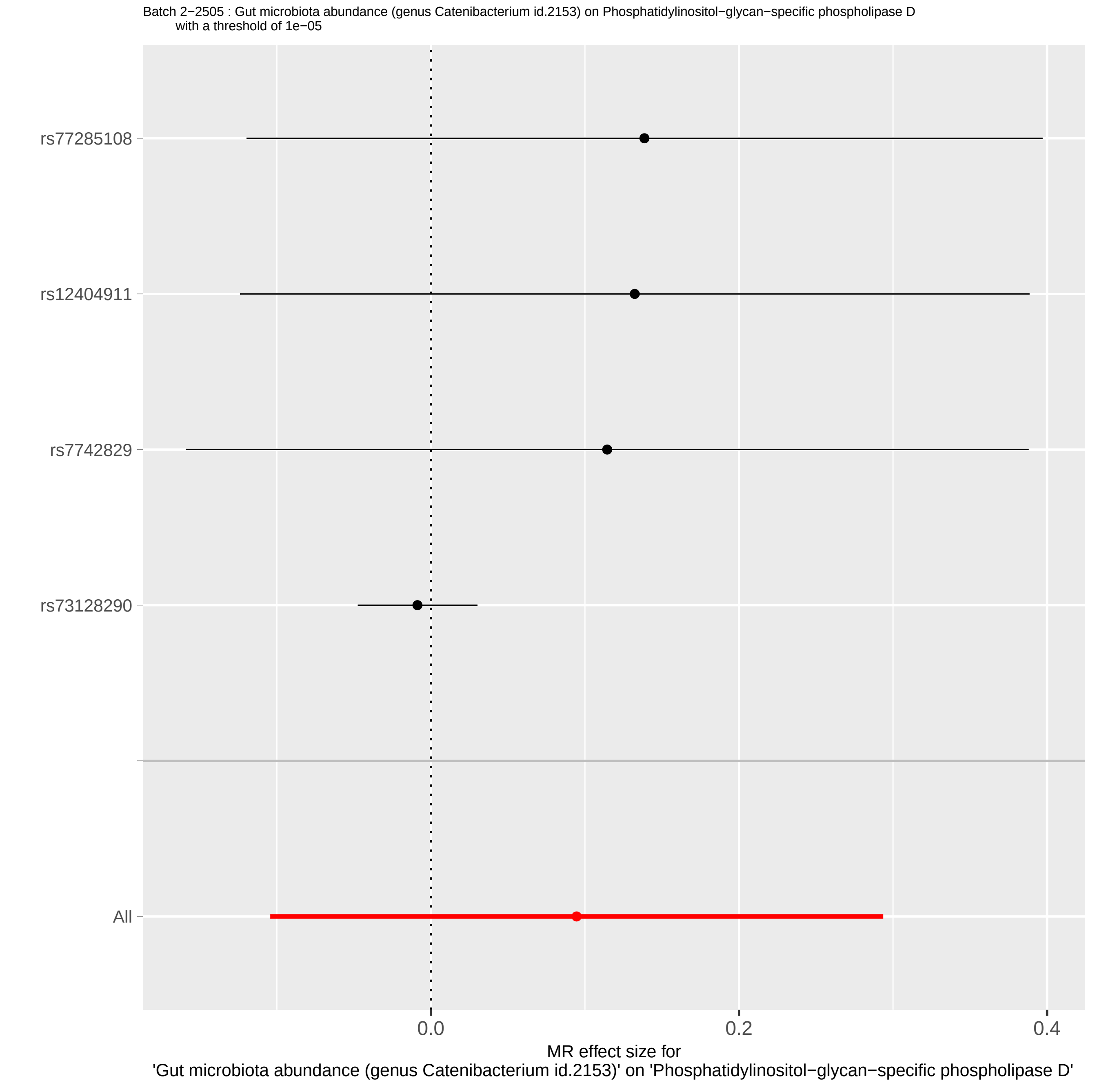
Batch 2−2505 : Gut microbiota abundance (genus Catenibacterium id.2153) on Phosphatidylinositol−glycan−specific phospholipase D
with a threshold of 1e−05
rs77285108
rs12404911
rs7742829
rs73128290
All
0.0
0.2
0.4
MR effect size for
'Gut microbiota abundance (genus Catenibacterium id.2153)' on 'Phosphatidylinositol−glycan−specific phospholipase D'

Batch 2−2506 : Gut microbiota abundance (genus Parasutterella id.2892) on Phosphatidylinositol−glycan−specific phospholipase D with a threshold of 1e−05

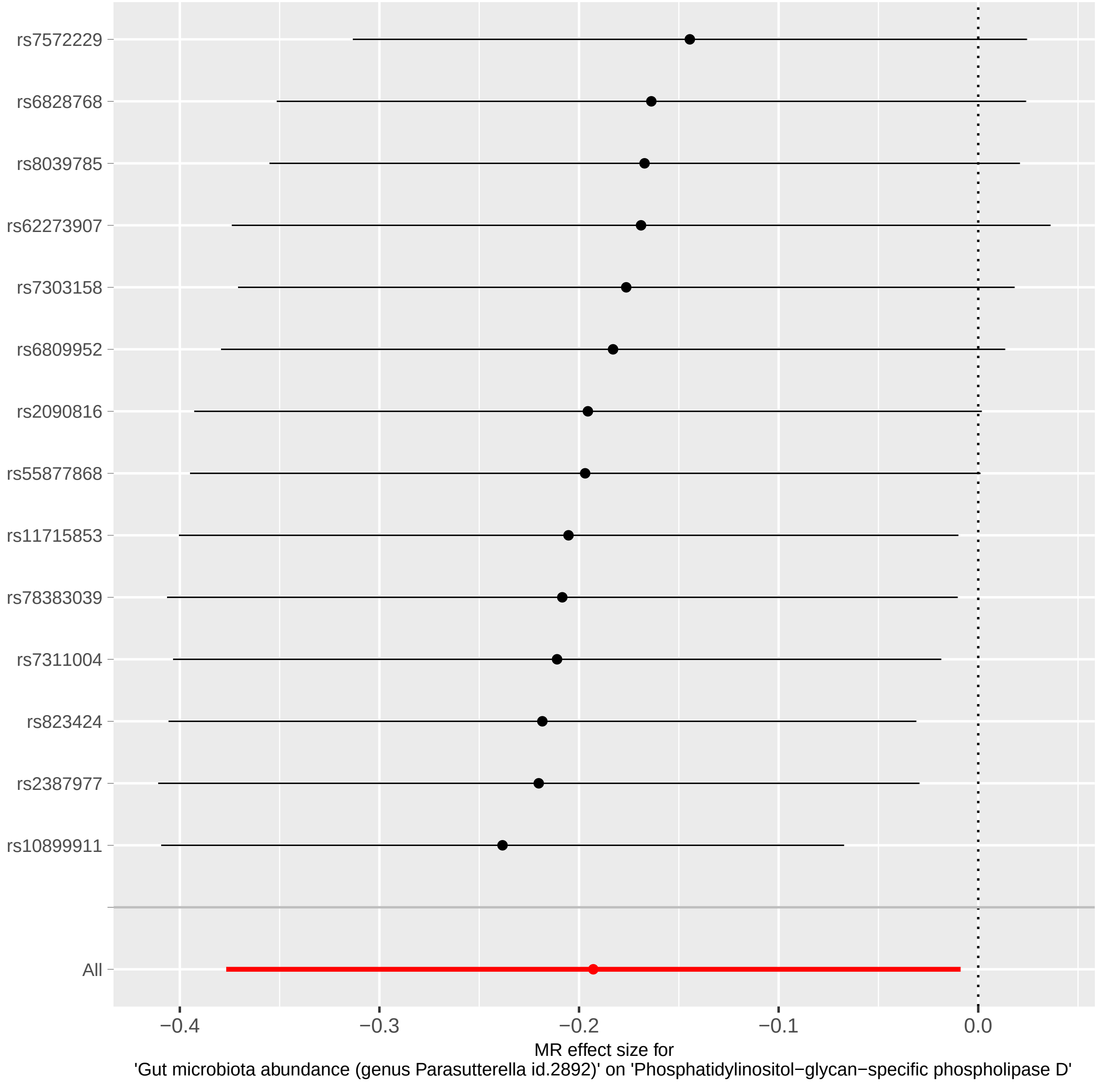

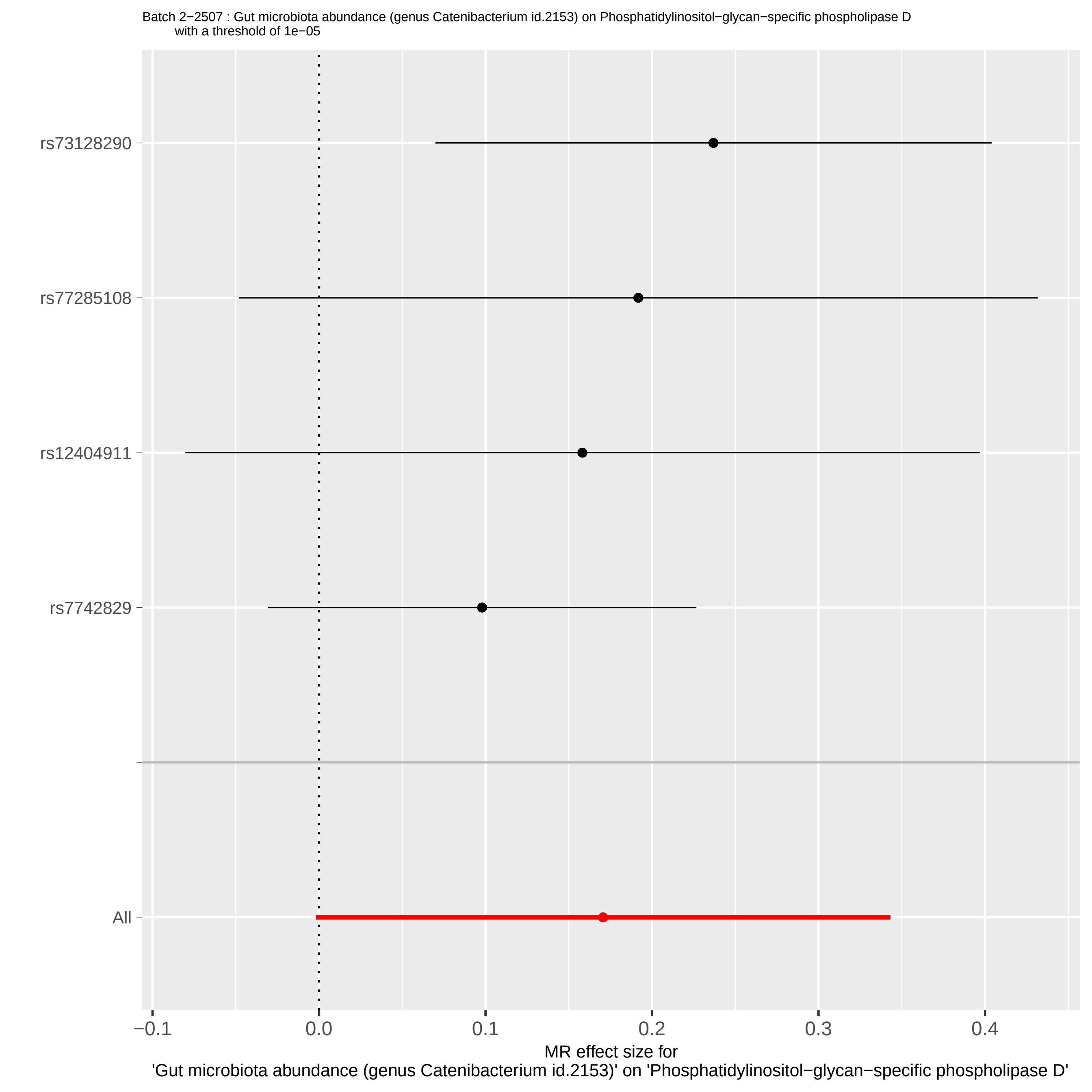

Batch 2−2507 : Gut microbiota abundance (genus Catenibacterium id.2153) on Phosphatidylinositol−glycan−specific phospholipase D
with a threshold of 1e−05
rs73128290
rs77285108
rs12404911
rs7742829
All
−0.1
0.0
0.1
0.2
0.3
0.4
MR effect size for
'Gut microbiota abundance (genus Catenibacterium id.2153)' on 'Phosphatidylinositol−glycan−specific phospholipase D'

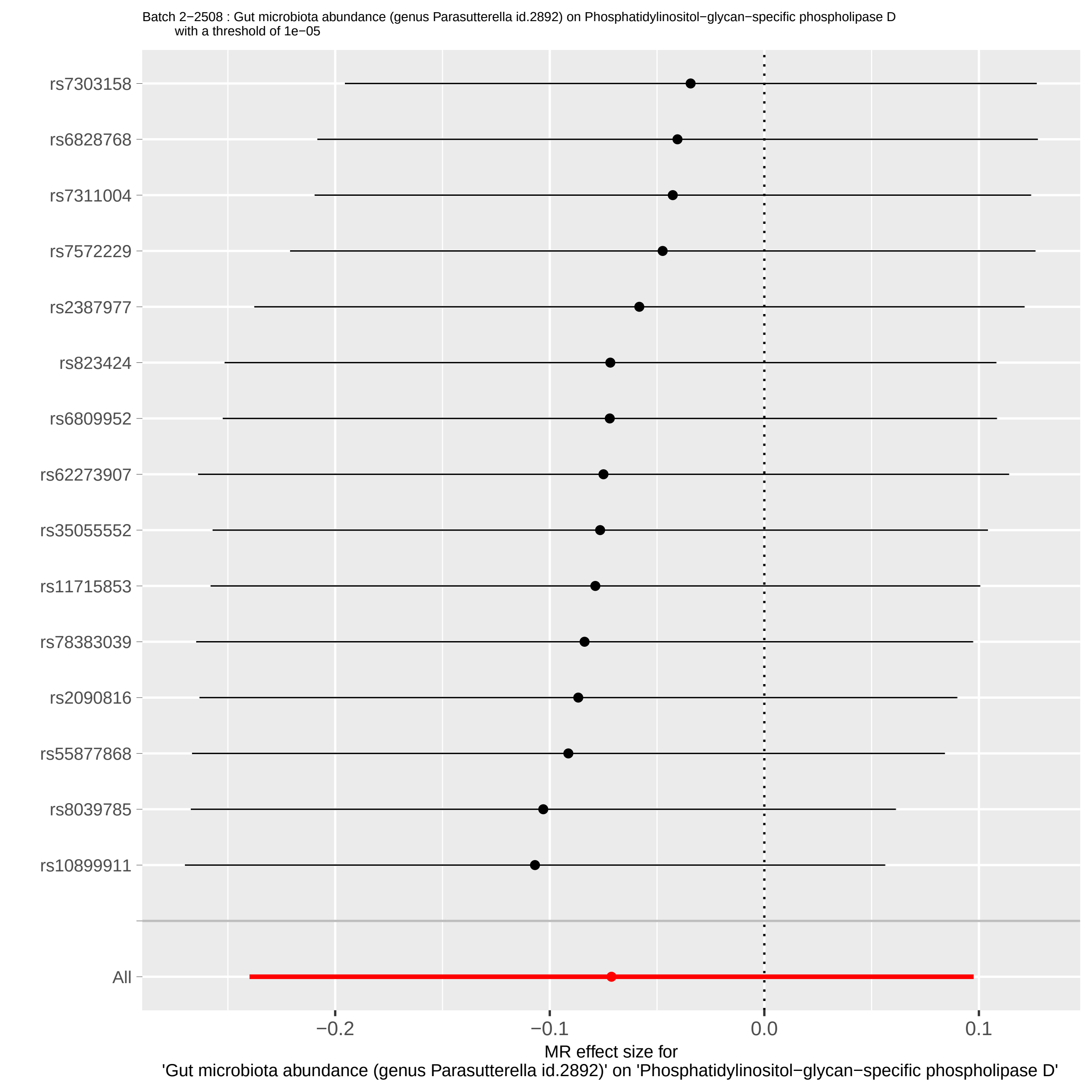
Batch 2−2508 : Gut microbiota abundance (genus Parasutterella id.2892) on Phosphatidylinositol−glycan−specific phospholipase D
with a threshold of 1e−05
rs7303158
rs6828768
rs7311004
rs7572229
rs2387977
rs823424
rs6809952
rs62273907
rs35055552
rs11715853
rs78383039
rs2090816
rs55877868
rs8039785
rs10899911
All
−0.2
−0.1
0.0
0.1
MR effect size for
'Gut microbiota abundance (genus Parasutterella id.2892)' on 'Phosphatidylinositol−glycan−specific phospholipase D'

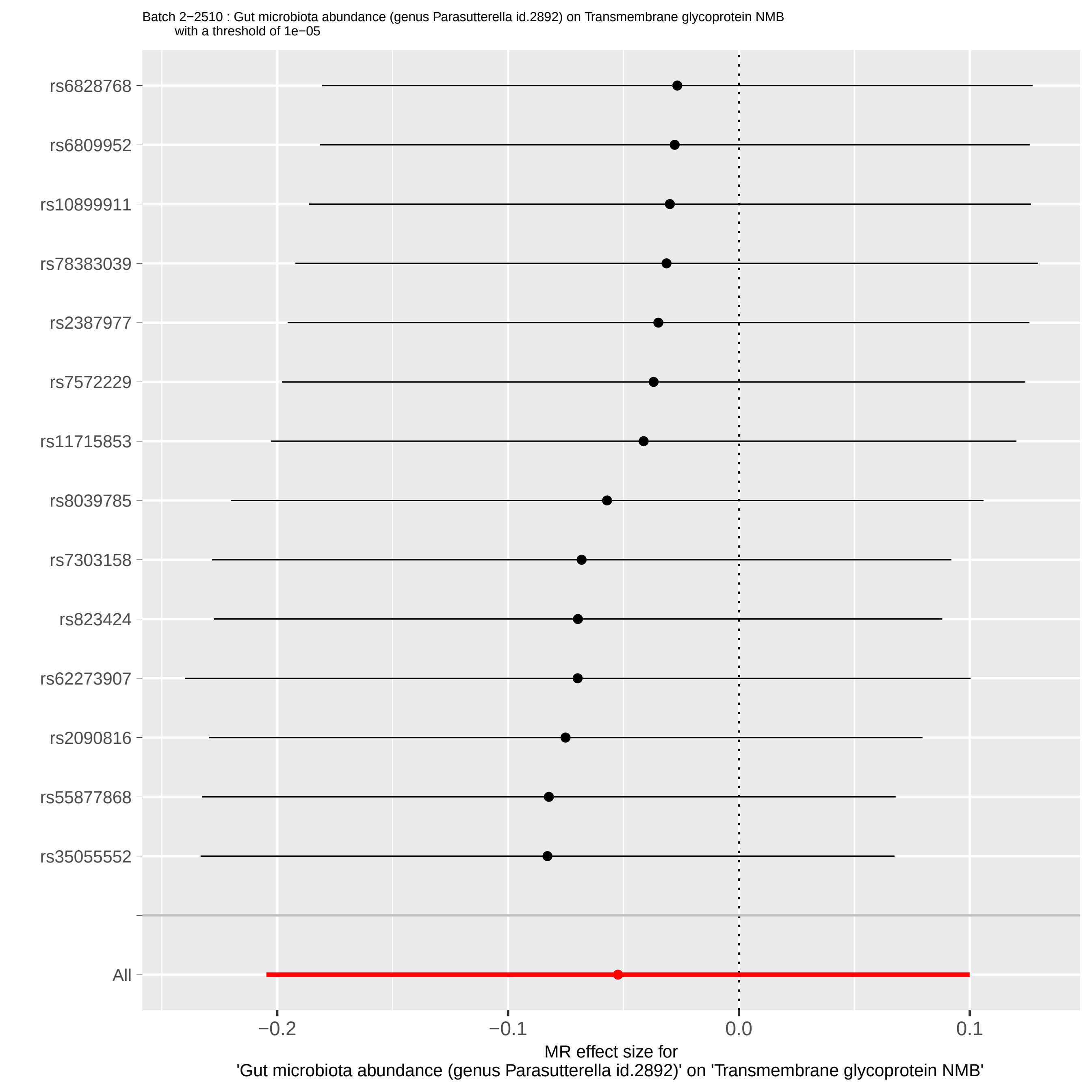

Batch 2−2510 : Gut microbiota abundance (genus Parasutterella id.2892) on Transmembrane glycoprotein NMB
with a threshold of 1e−05
rs6828768
rs6809952
rs10899911
rs78383039
rs2387977
rs7572229
rs11715853
rs8039785
rs7303158
rs823424
rs62273907
rs2090816
rs55877868
rs35055552
All
−0.2
−0.1
0.0
0.1
MR effect size for
'Gut microbiota abundance (genus Parasutterella id.2892)' on 'Transmembrane glycoprotein NMB'

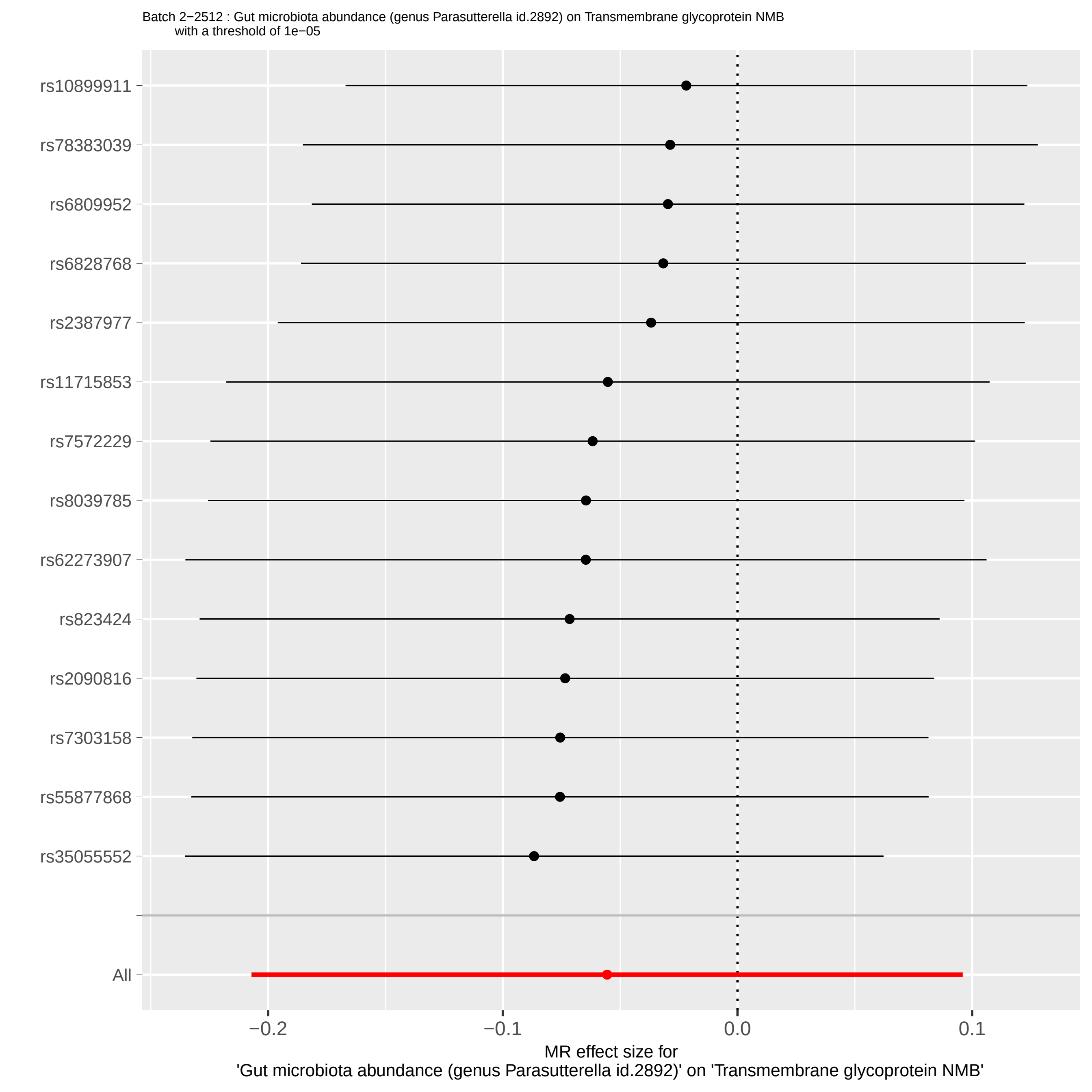
Batch 2−2512 : Gut microbiota abundance (genus Parasutterella id.2892) on Transmembrane glycoprotein NMB
with a threshold of 1e−05
rs10899911
rs78383039
rs6809952
rs6828768
rs2387977
rs11715853
rs7572229
rs8039785
rs62273907
rs823424
rs2090816
rs7303158
rs55877868
rs35055552
All
−0.2
−0.1
0.0
0.1
MR effect size for
'Gut microbiota abundance (genus Parasutterella id.2892)' on 'Transmembrane glycoprotein NMB'

Batch 2−2513 : Gut microbiota abundance (genus Catenibacterium id.2153) on Transmembrane glycoprotein NMB
with a threshold of 1e−05

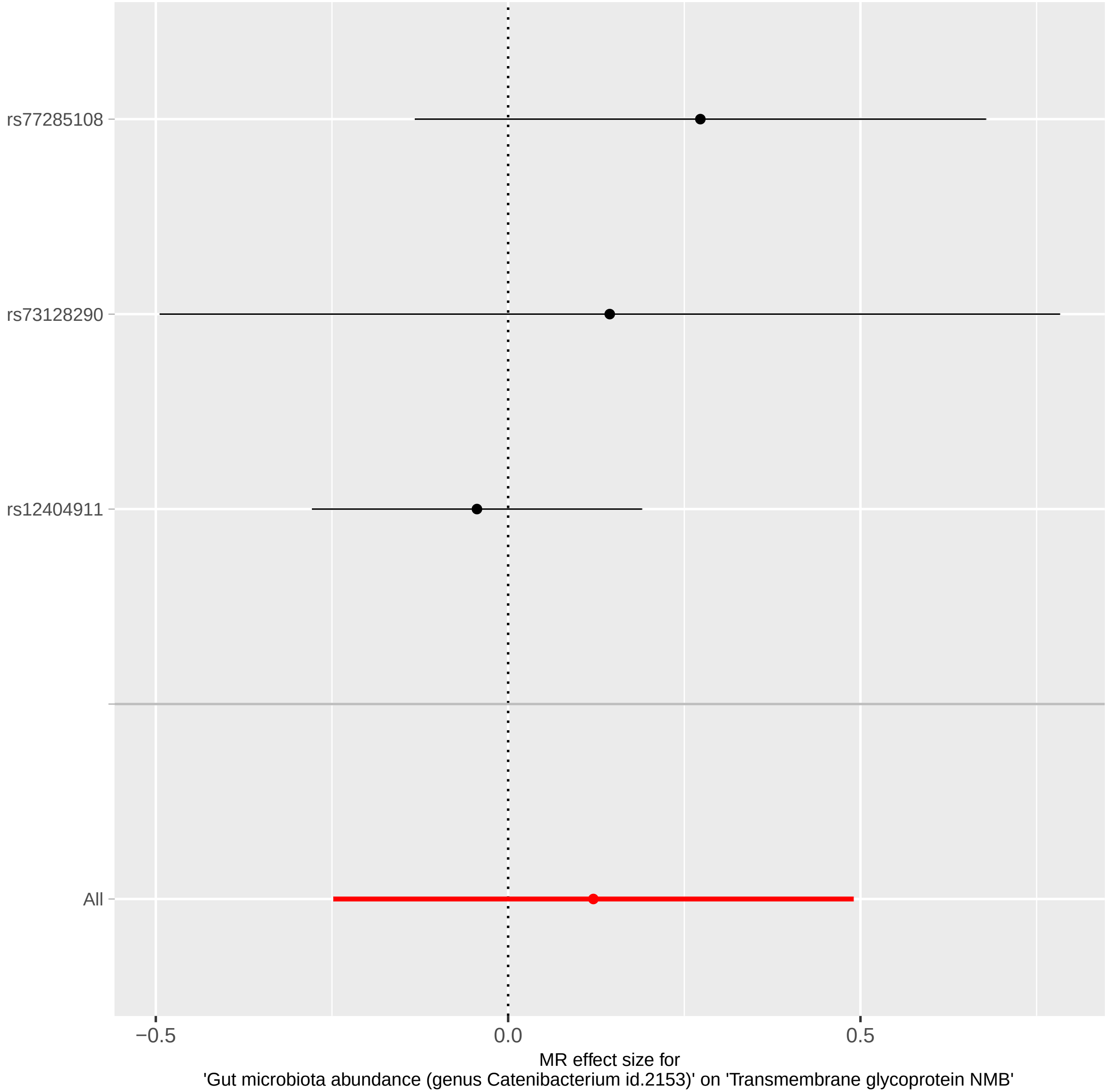

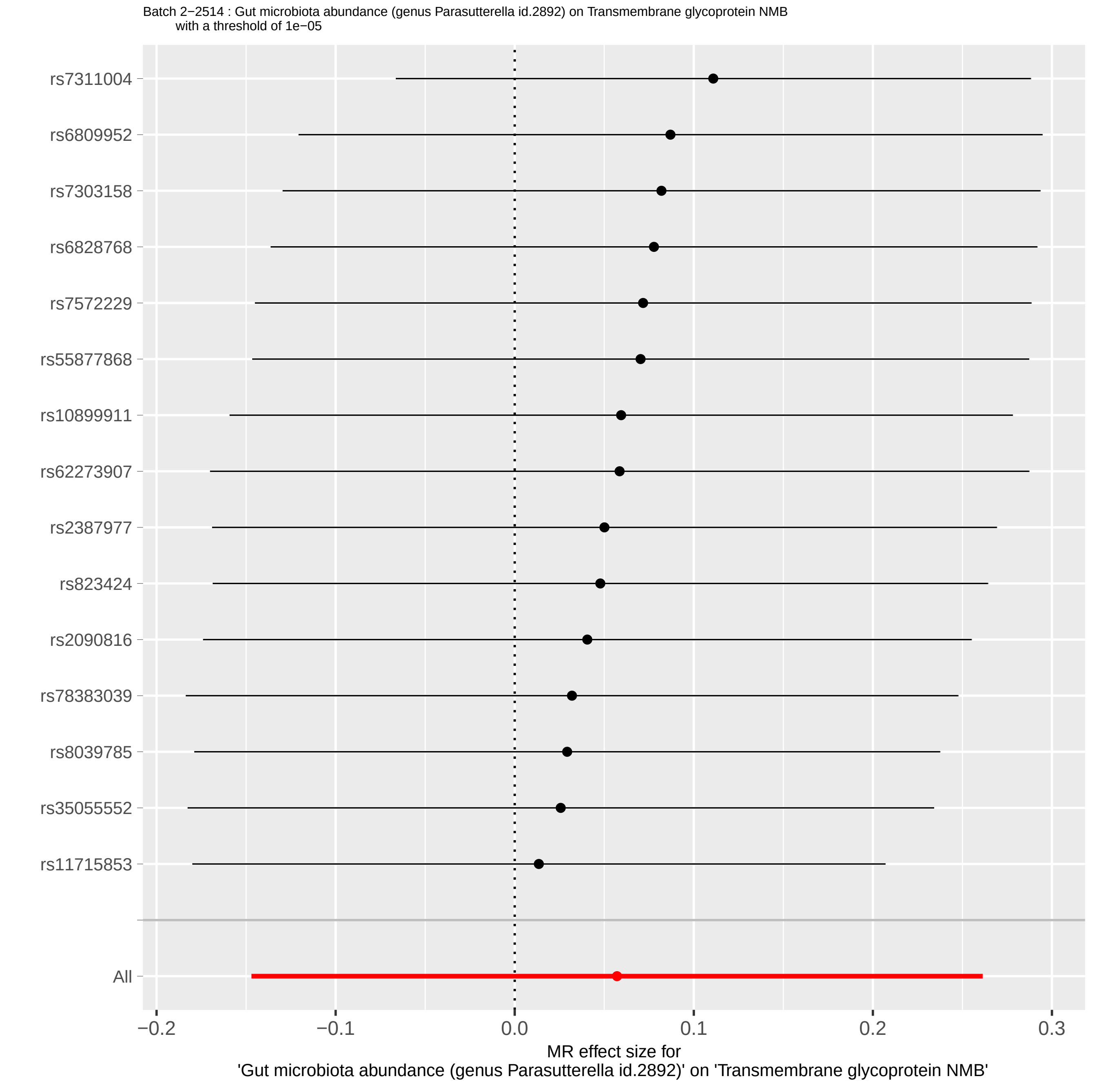

Batch 2−2514 : Gut microbiota abundance (genus Parasutterella id.2892) on Transmembrane glycoprotein NMB
with a threshold of 1e−05
rs7311004
rs6809952
rs7303158
rs6828768
rs7572229
rs55877868
rs10899911
rs62273907
rs2387977
rs823424
rs2090816
rs78383039
rs8039785
rs35055552
rs11715853
All
−0.2
−0.1
0.0
0.1
0.2
0.3
MR effect size for
'Gut microbiota abundance (genus Parasutterella id.2892)' on 'Transmembrane glycoprotein NMB'

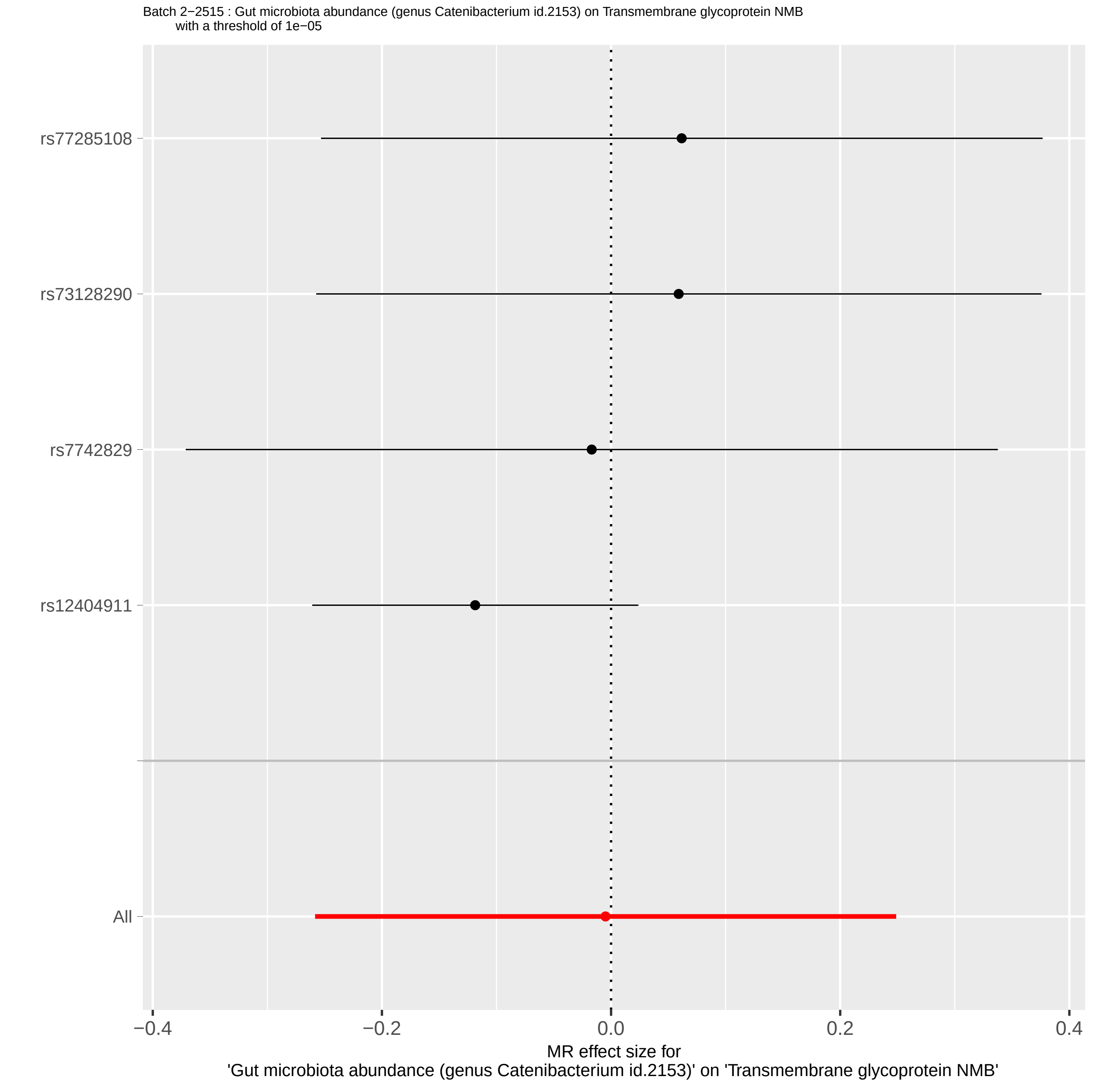
Batch 2−2515 : Gut microbiota abundance (genus Catenibacterium id.2153) on Transmembrane glycoprotein NMB
with a threshold of 1e−05
rs77285108
rs73128290
rs7742829
rs12404911
All
−0.4
−0.2
0.0
0.2
0.4
MR effect size for
'Gut microbiota abundance (genus Catenibacterium id.2153)' on 'Transmembrane glycoprotein NMB'

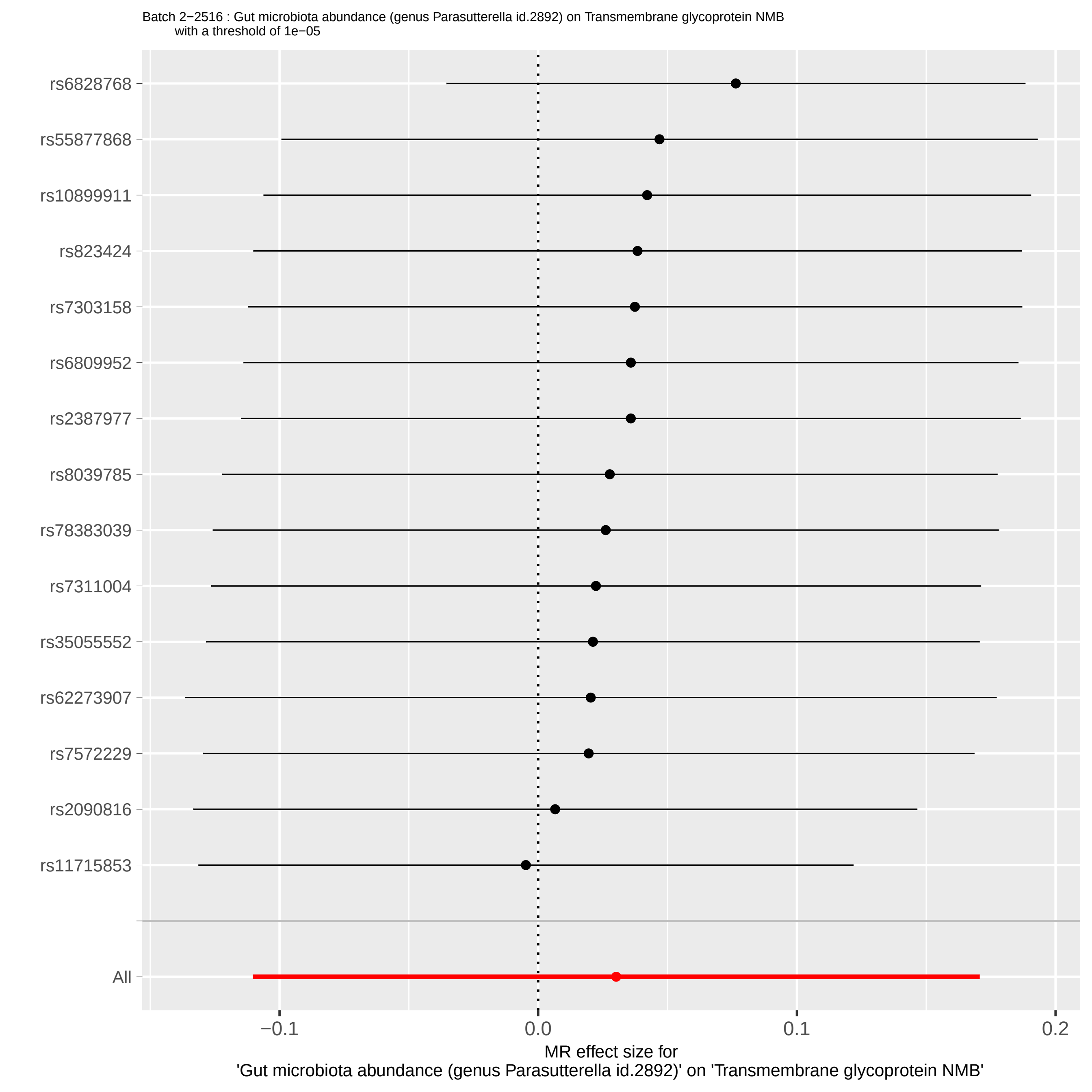
Batch 2−2516 : Gut microbiota abundance (genus Parasutterella id.2892) on Transmembrane glycoprotein NMB
with a threshold of 1e−05
rs6828768
rs55877868
rs10899911
rs823424
rs7303158
rs6809952
rs2387977
rs8039785
rs78383039
rs7311004
rs35055552
rs62273907
rs7572229
rs2090816
rs11715853
All
−0.1
0.0
0.1
0.2
MR effect size for
'Gut microbiota abundance (genus Parasutterella id.2892)' on 'Transmembrane glycoprotein NMB'

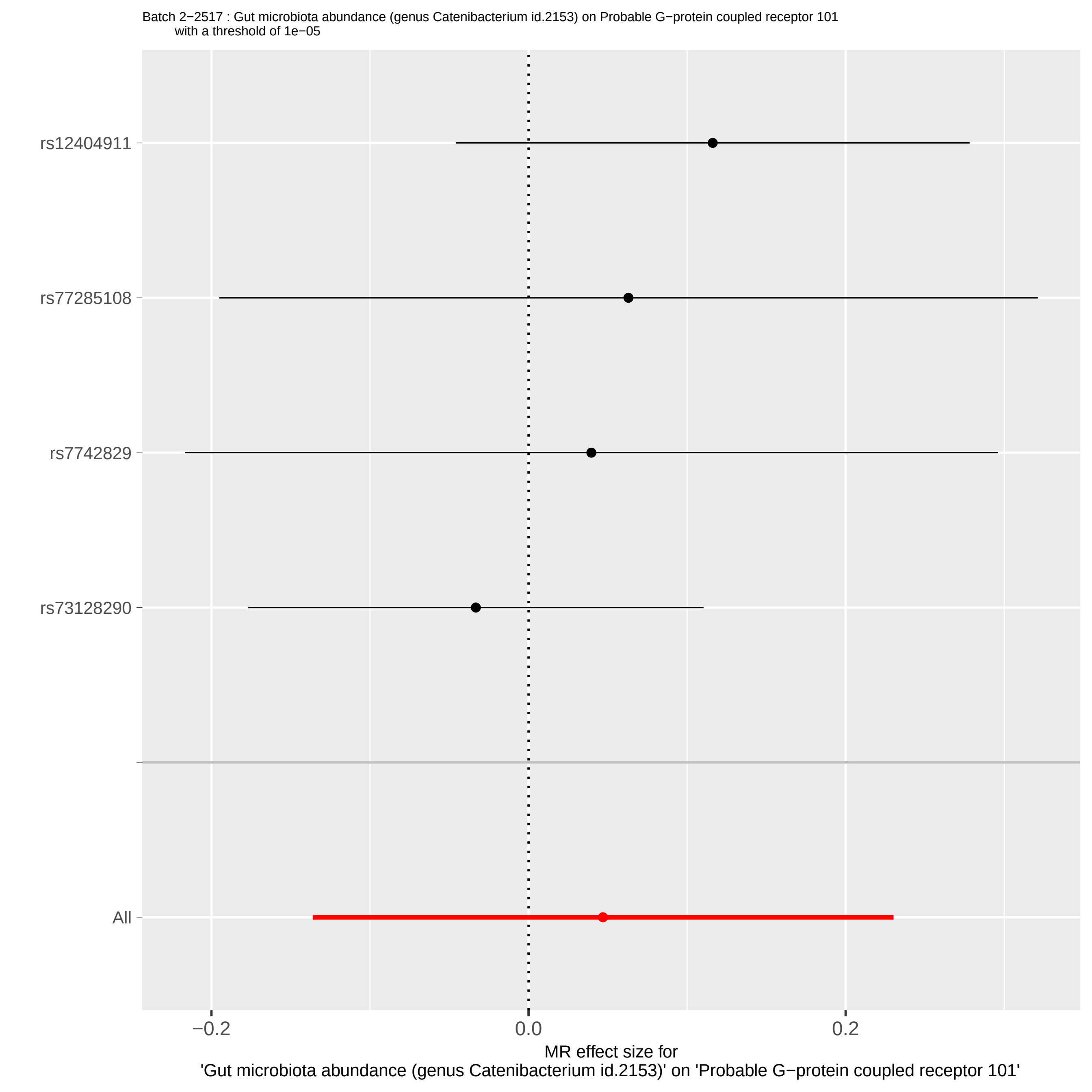

Batch 2−2517 : Gut microbiota abundance (genus Catenibacterium id.2153) on Probable G−protein coupled receptor 101
with a threshold of 1e−05
rs12404911
rs77285108
rs7742829
rs73128290
All
−0.2
0.0
0.2
MR effect size for
'Gut microbiota abundance (genus Catenibacterium id.2153)' on 'Probable G−protein coupled receptor 101'

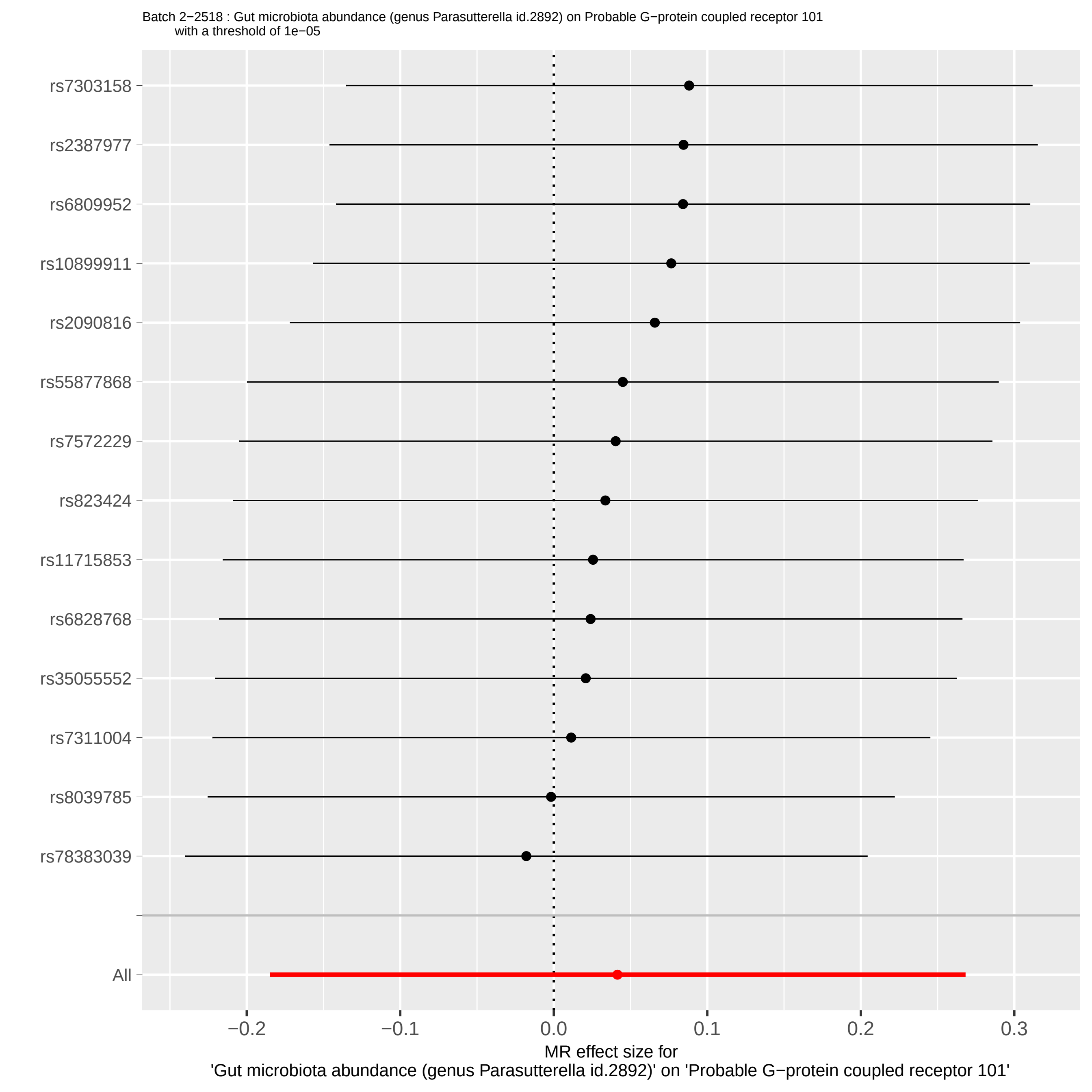
Batch 2−2518 : Gut microbiota abundance (genus Parasutterella id.2892) on Probable G−protein coupled receptor 101
with a threshold of 1e−05
rs7303158
rs2387977
rs6809952
rs10899911
rs2090816
rs55877868
rs7572229
rs823424
rs11715853
rs6828768
rs35055552
rs7311004
rs8039785
rs78383039
All
−0.2
−0.1
0.0
0.1
0.2
0.3
MR effect size for
'Gut microbiota abundance (genus Parasutterella id.2892)' on 'Probable G−protein coupled receptor 101'

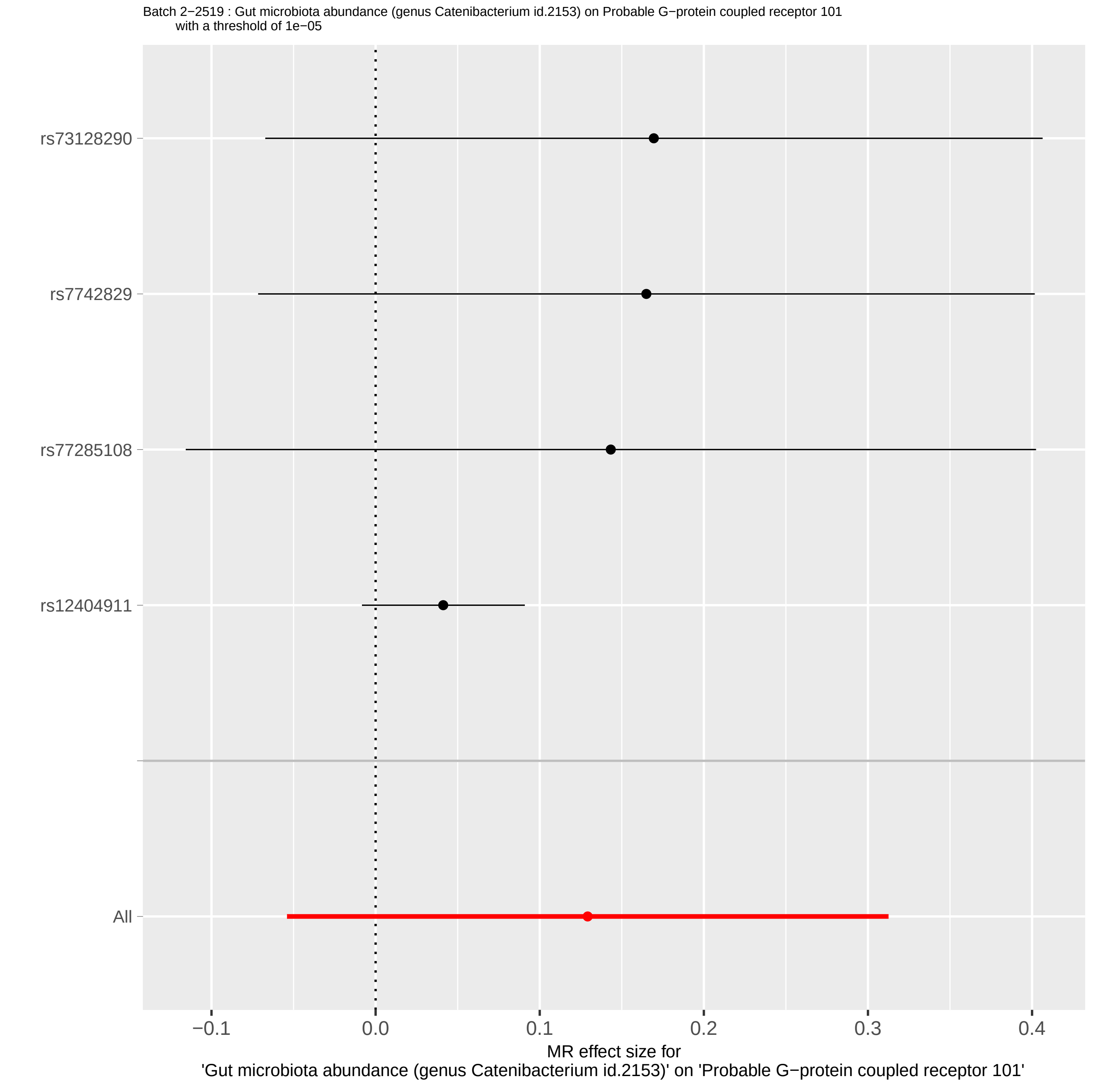
Batch 2−2519 : Gut microbiota abundance (genus Catenibacterium id.2153) on Probable G−protein coupled receptor 101
with a threshold of 1e−05
rs73128290
rs7742829
rs77285108
rs12404911
All
−0.1
0.0
0.1
0.2
0.3
0.4
MR effect size for
'Gut microbiota abundance (genus Catenibacterium id.2153)' on 'Probable G−protein coupled receptor 101'

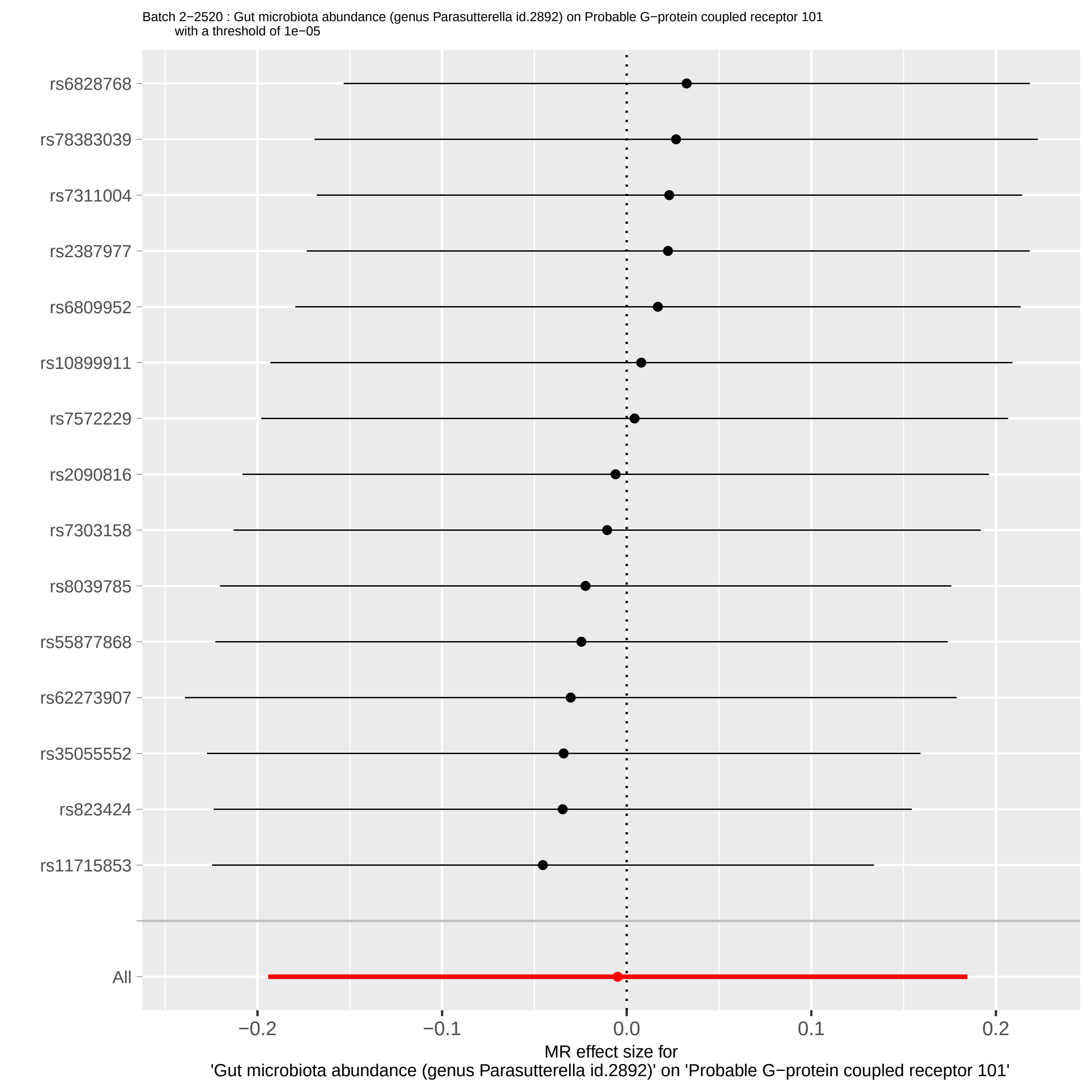
Batch 2−2520 : Gut microbiota abundance (genus Parasutterella id.2892) on Probable G−protein coupled receptor 101
with a threshold of 1e−05
rs6828768
rs78383039
rs7311004
rs2387977
rs6809952
rs10899911
rs7572229
rs2090816
rs7303158
rs8039785
rs55877868
rs62273907
rs35055552
rs823424
rs11715853
All
−0.2
−0.1
0.0
0.1
0.2
MR effect size for
'Gut microbiota abundance (genus Parasutterella id.2892)' on 'Probable G−protein coupled receptor 101'

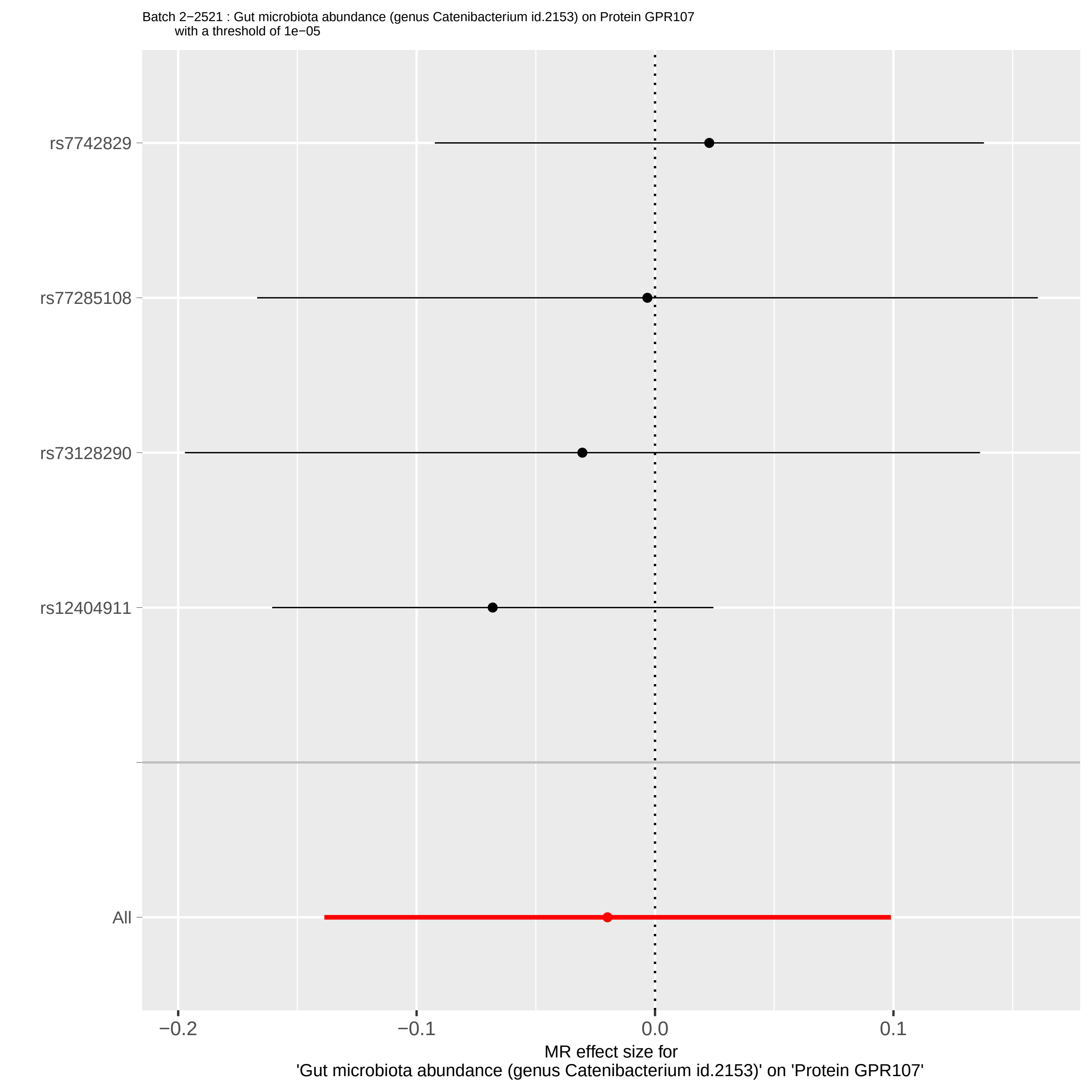
Batch 2−2521 : Gut microbiota abundance (genus Catenibacterium id.2153) on Protein GPR107
with a threshold of 1e−05
rs7742829
rs77285108
rs73128290
rs12404911
All
−0.2
−0.1
0.0
0.1
MR effect size for
'Gut microbiota abundance (genus Catenibacterium id.2153)' on 'Protein GPR107'

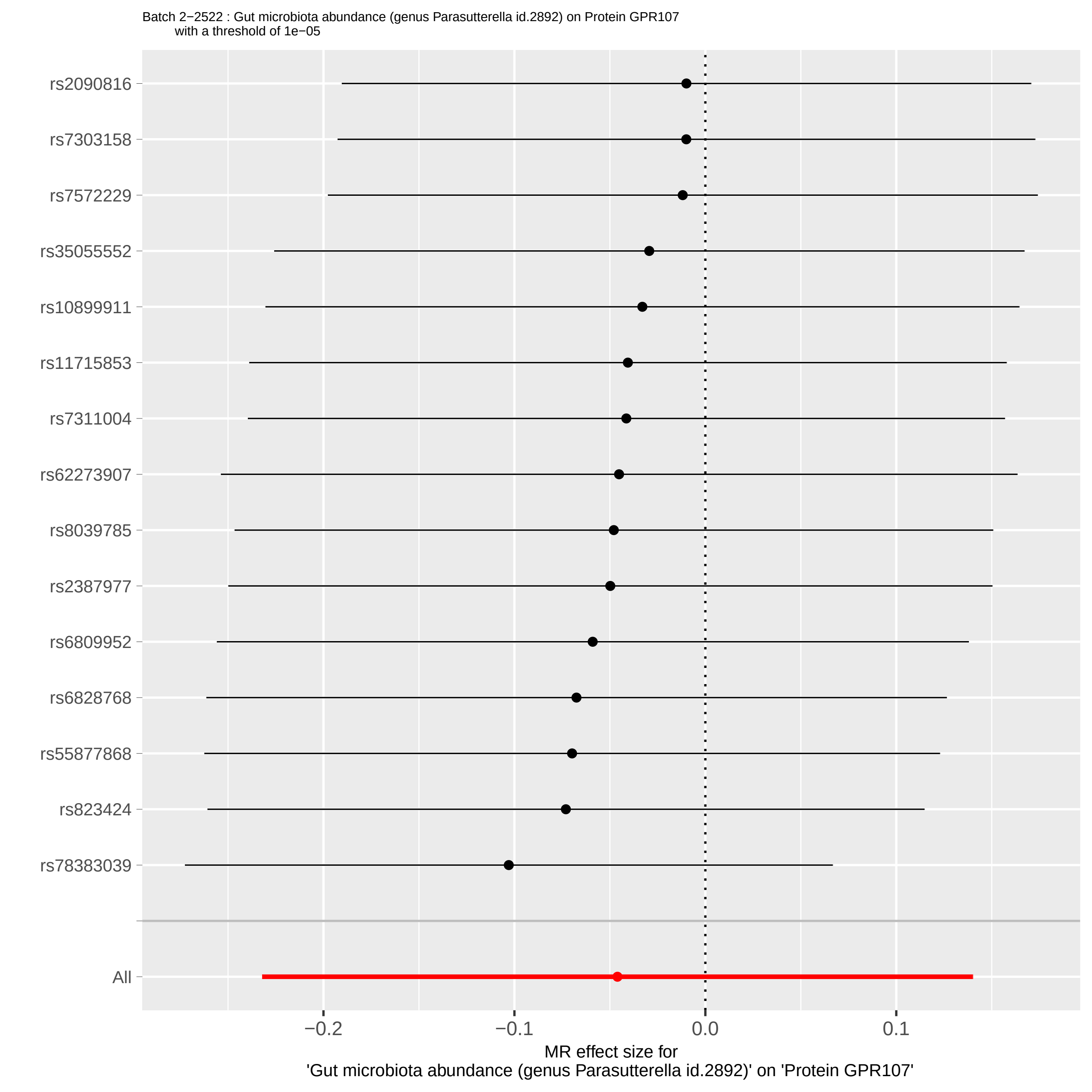

Batch 2−2522 : Gut microbiota abundance (genus Parasutterella id.2892) on Protein GPR107
with a threshold of 1e−05
rs2090816
rs7303158
rs7572229
rs35055552
rs10899911
rs11715853
rs7311004
rs62273907
rs8039785
rs2387977
rs6809952
rs6828768
rs55877868
rs823424
rs78383039
All
−0.2
−0.1
0.0
0.1
MR effect size for
'Gut microbiota abundance (genus Parasutterella id.2892)' on 'Protein GPR107'

Batch 2−2523 : Gut microbiota abundance (genus Catenibacterium id.2153) on Probable G−protein coupled receptor 135
with a threshold of 1e−05

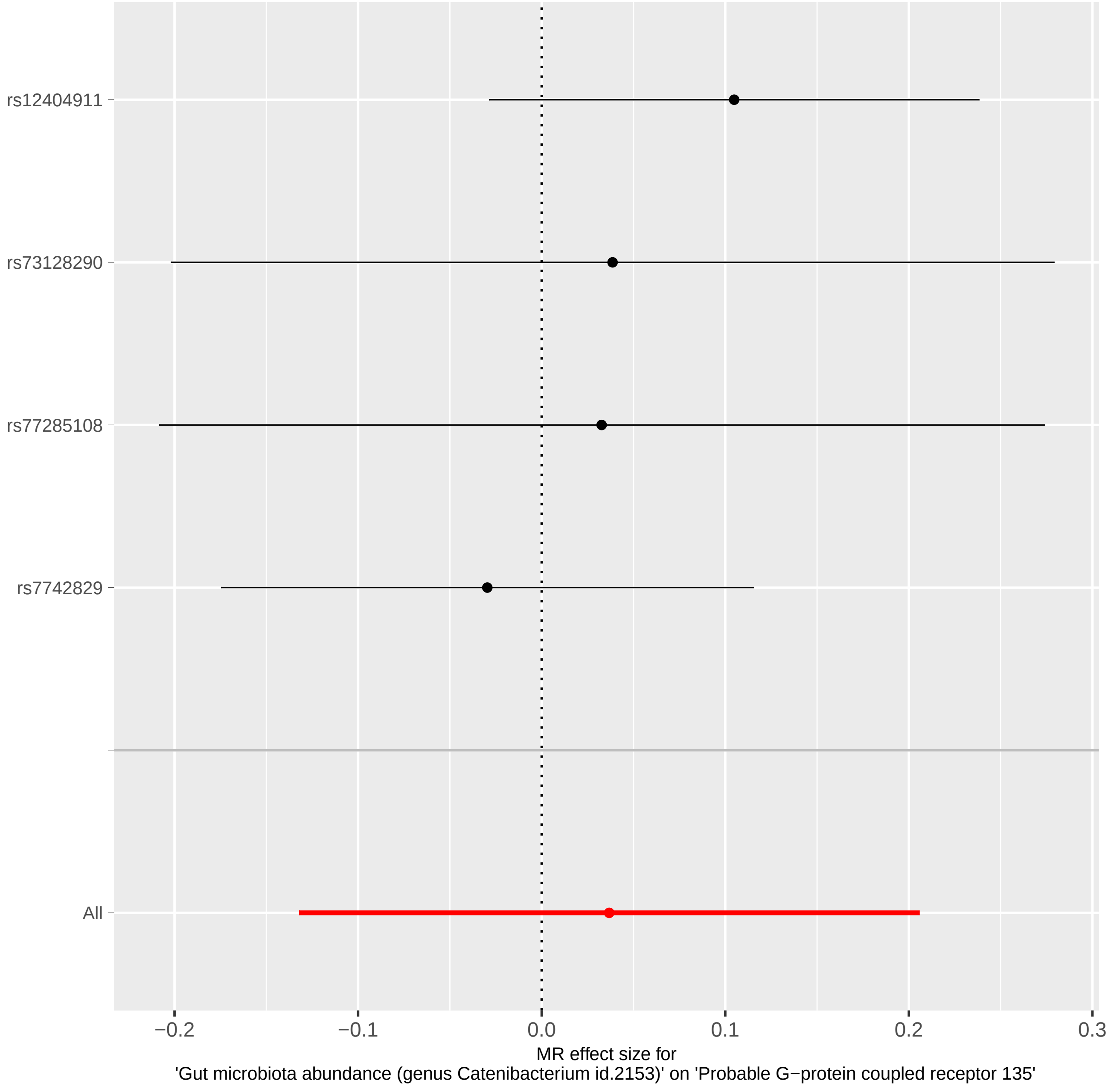

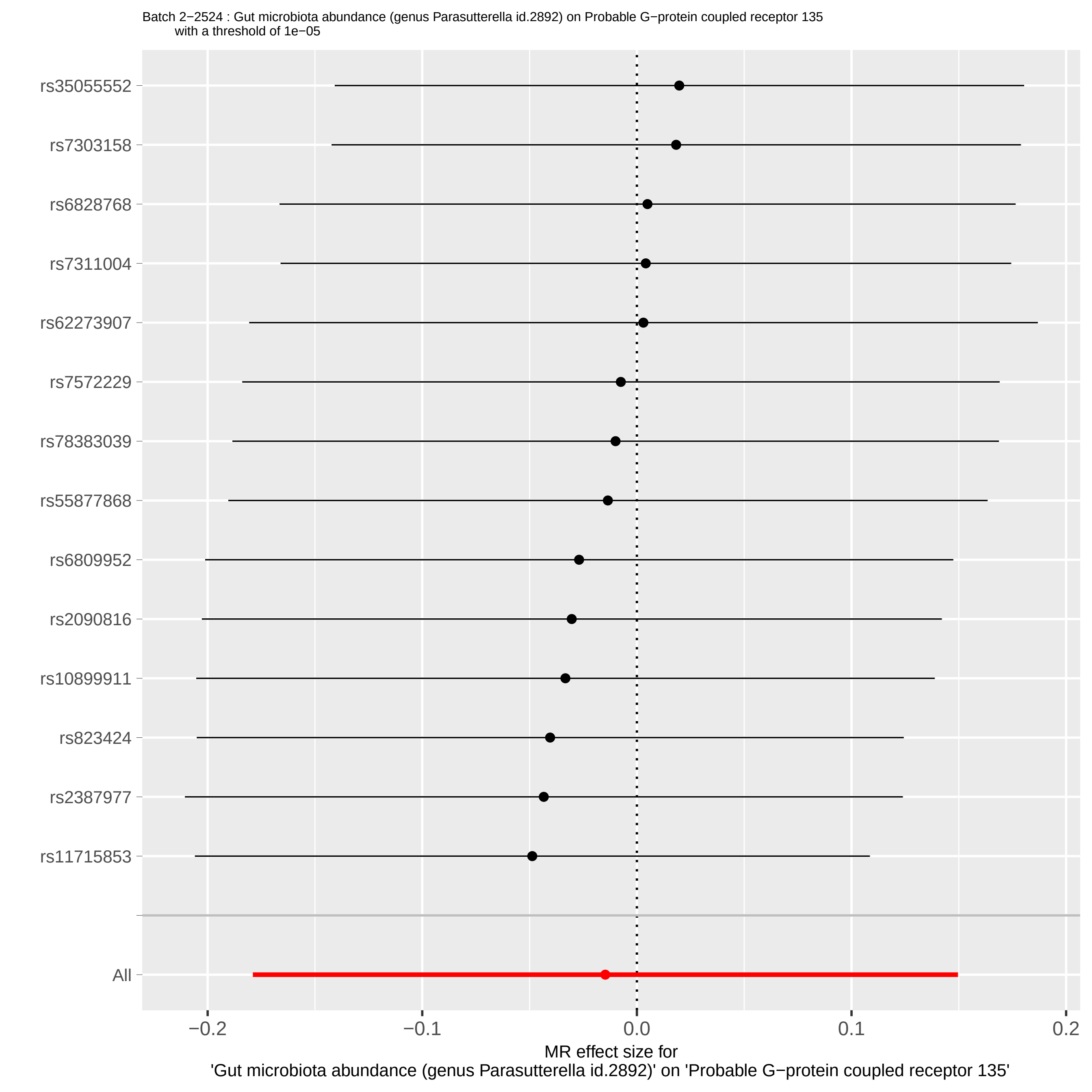

Batch 2−2524 : Gut microbiota abundance (genus Parasutterella id.2892) on Probable G−protein coupled receptor 135
with a threshold of 1e−05
rs35055552
rs7303158
rs6828768
rs7311004
rs62273907
rs7572229
rs78383039
rs55877868
rs6809952
rs2090816
rs10899911
rs823424
rs2387977
rs11715853
All
−0.2
−0.1
0.0
0.1
0.2
MR effect size for
'Gut microbiota abundance (genus Parasutterella id.2892)' on 'Probable G−protein coupled receptor 135'

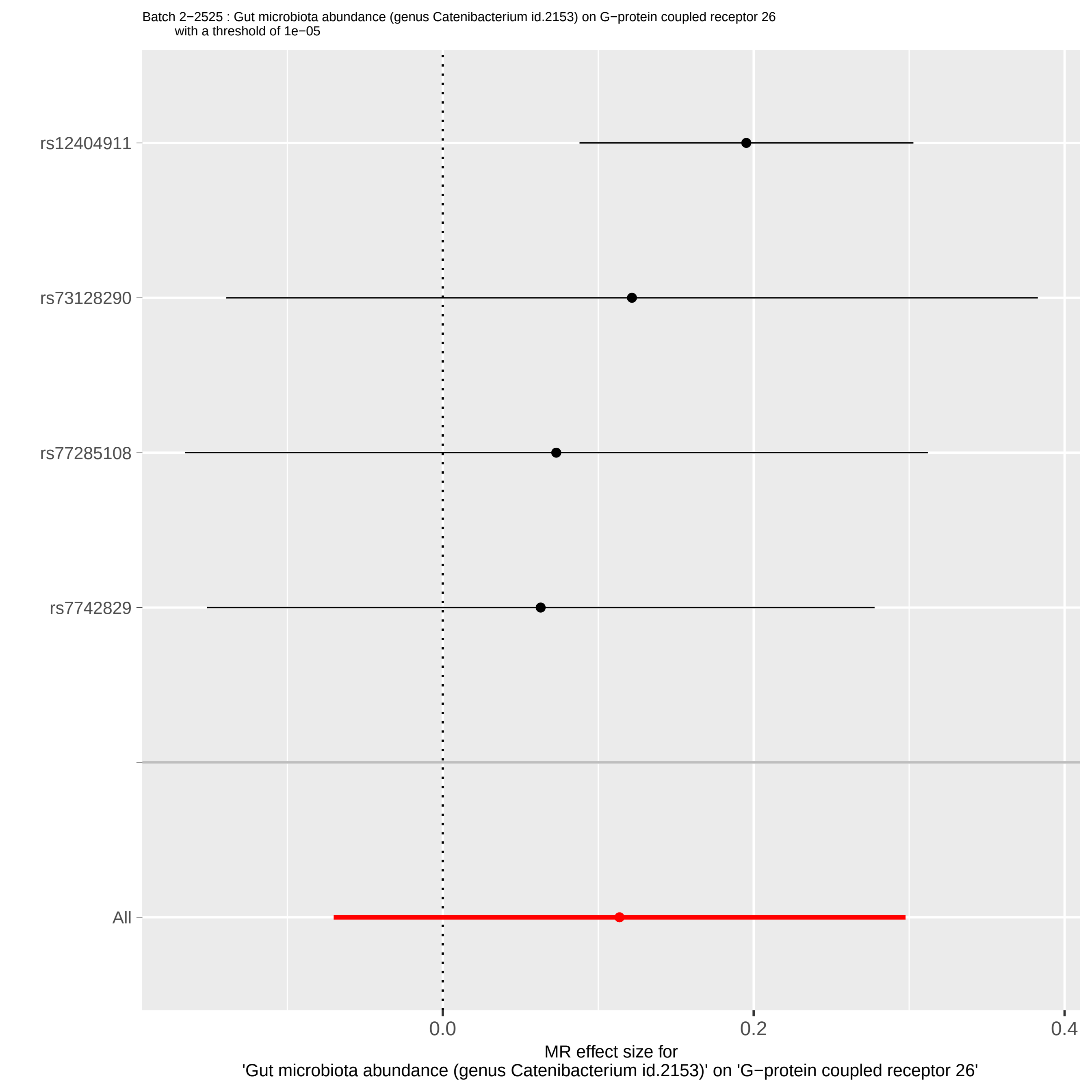
Batch 2−2525 : Gut microbiota abundance (genus Catenibacterium id.2153) on G−protein coupled receptor 26
with a threshold of 1e−05
rs12404911
rs73128290
rs77285108
rs7742829
All
0.0
0.2
0.4
MR effect size for
'Gut microbiota abundance (genus Catenibacterium id.2153)' on 'G−protein coupled receptor 26'

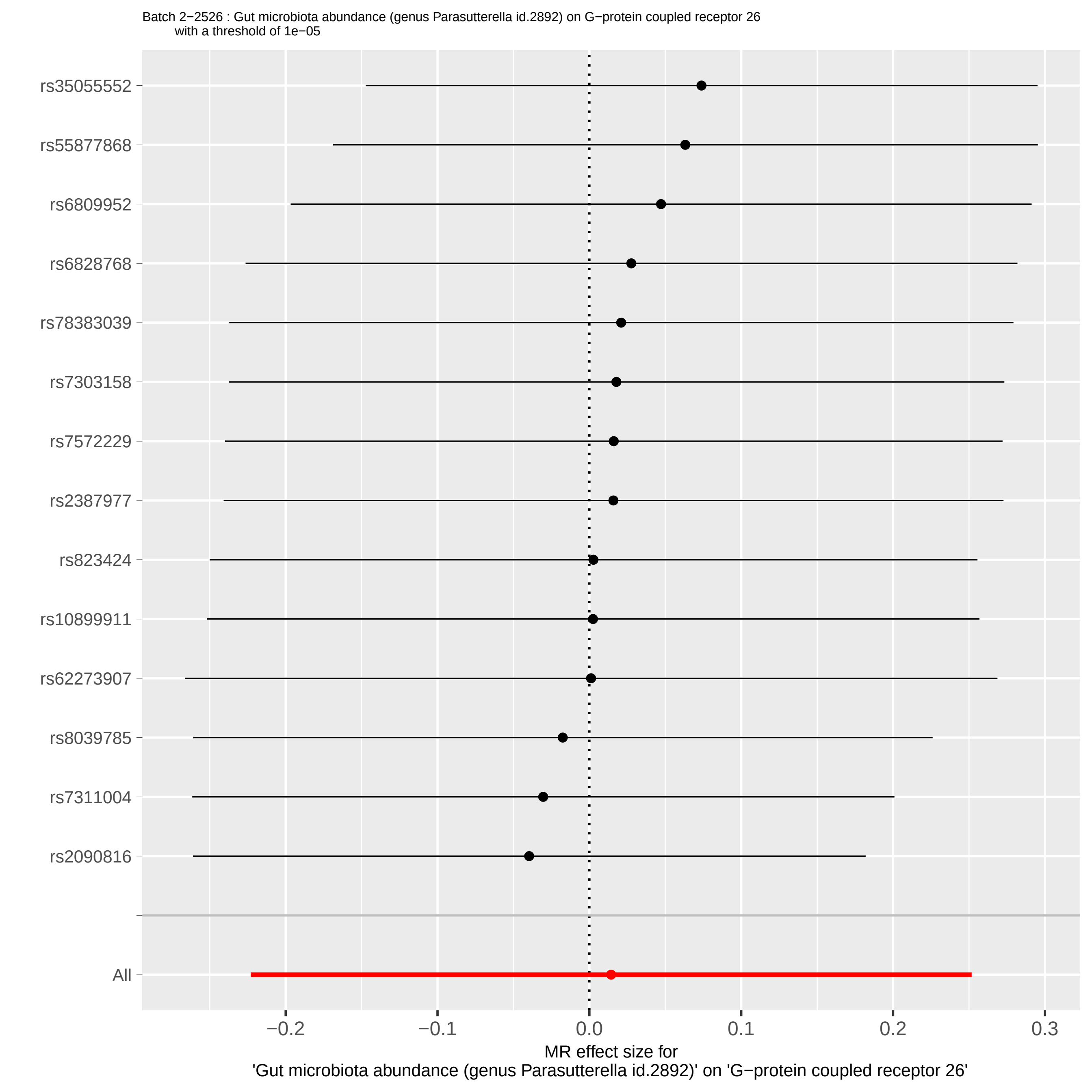
Batch 2−2526 : Gut microbiota abundance (genus Parasutterella id.2892) on G−protein coupled receptor 26
with a threshold of 1e−05
rs35055552
rs55877868
rs6809952
rs6828768
rs78383039
rs7303158
rs7572229
rs2387977
rs823424
rs10899911
rs62273907
rs8039785
rs7311004
rs2090816
All
−0.2
−0.1
0.0
0.1
0.2
0.3
MR effect size for
'Gut microbiota abundance (genus Parasutterella id.2892)' on 'G−protein coupled receptor 26'

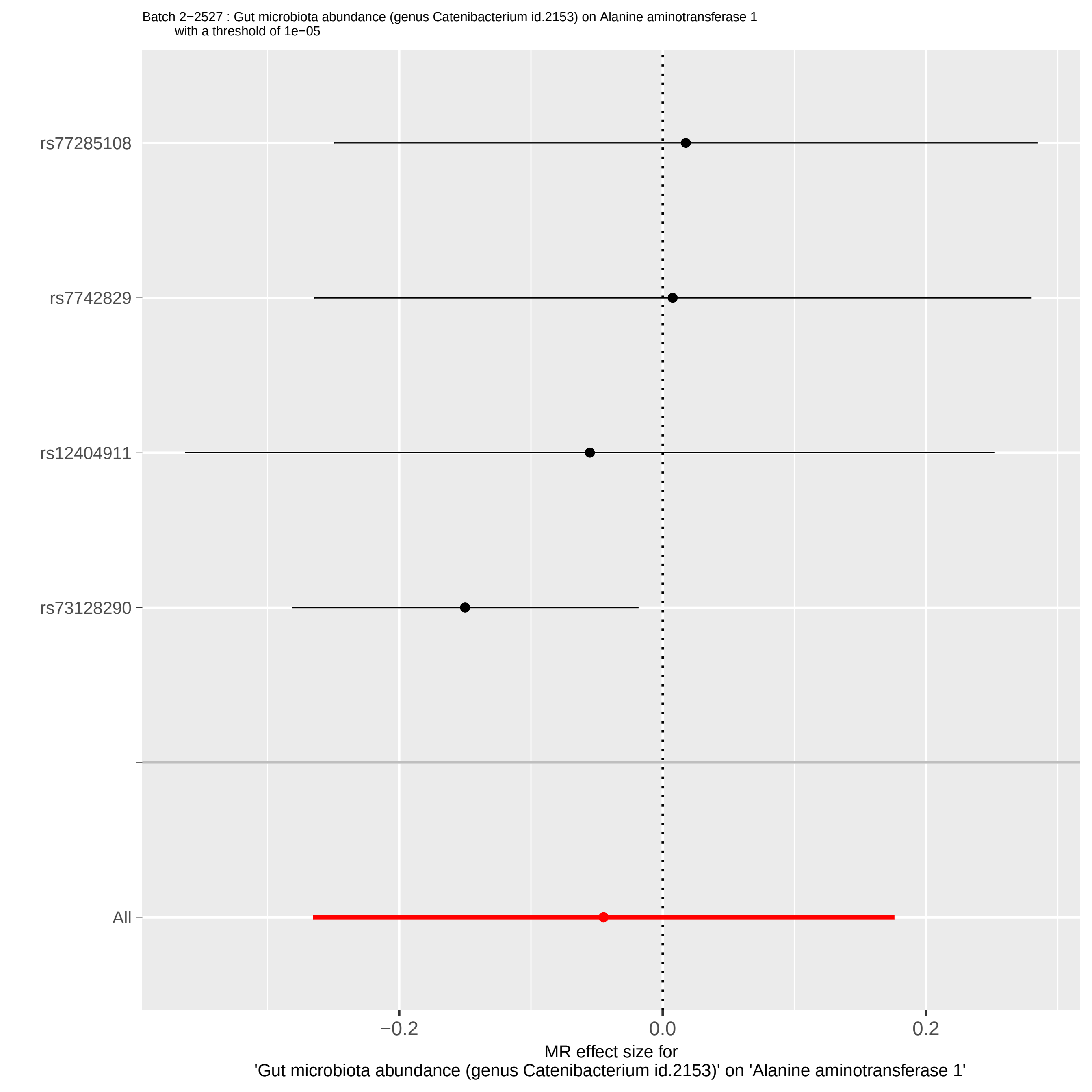

Batch 2−2527 : Gut microbiota abundance (genus Catenibacterium id.2153) on Alanine aminotransferase 1
with a threshold of 1e−05
rs77285108
rs7742829
rs12404911
rs73128290
All
−0.2
0.0
0.2
MR effect size for
'Gut microbiota abundance (genus Catenibacterium id.2153)' on 'Alanine aminotransferase 1'

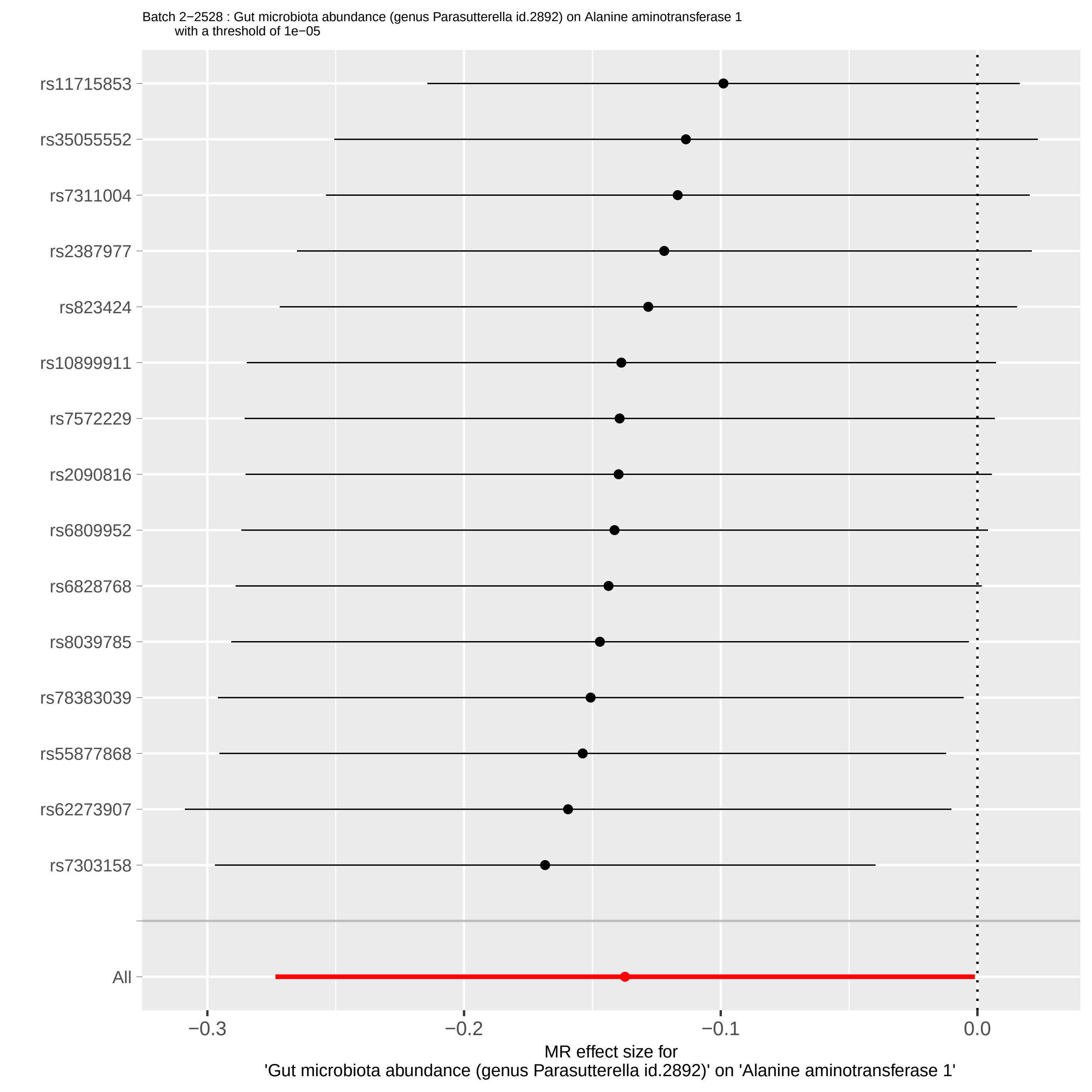

Batch 2−2528 : Gut microbiota abundance (genus Parasutterella id.2892) on Alanine aminotransferase 1
with a threshold of 1e−05
rs11715853
rs35055552
rs7311004
rs2387977
rs823424
rs10899911
rs7572229
rs2090816
rs6809952
rs6828768
rs8039785
rs78383039
rs55877868
rs62273907
rs7303158
All
−0.3
−0.2
−0.1
0.0
MR effect size for
'Gut microbiota abundance (genus Parasutterella id.2892)' on 'Alanine aminotransferase 1'

Batch 2−2529 : Gut microbiota abundance (genus Catenibacterium id.2153) on Glutathione peroxidase 7
with a threshold of 1e−05

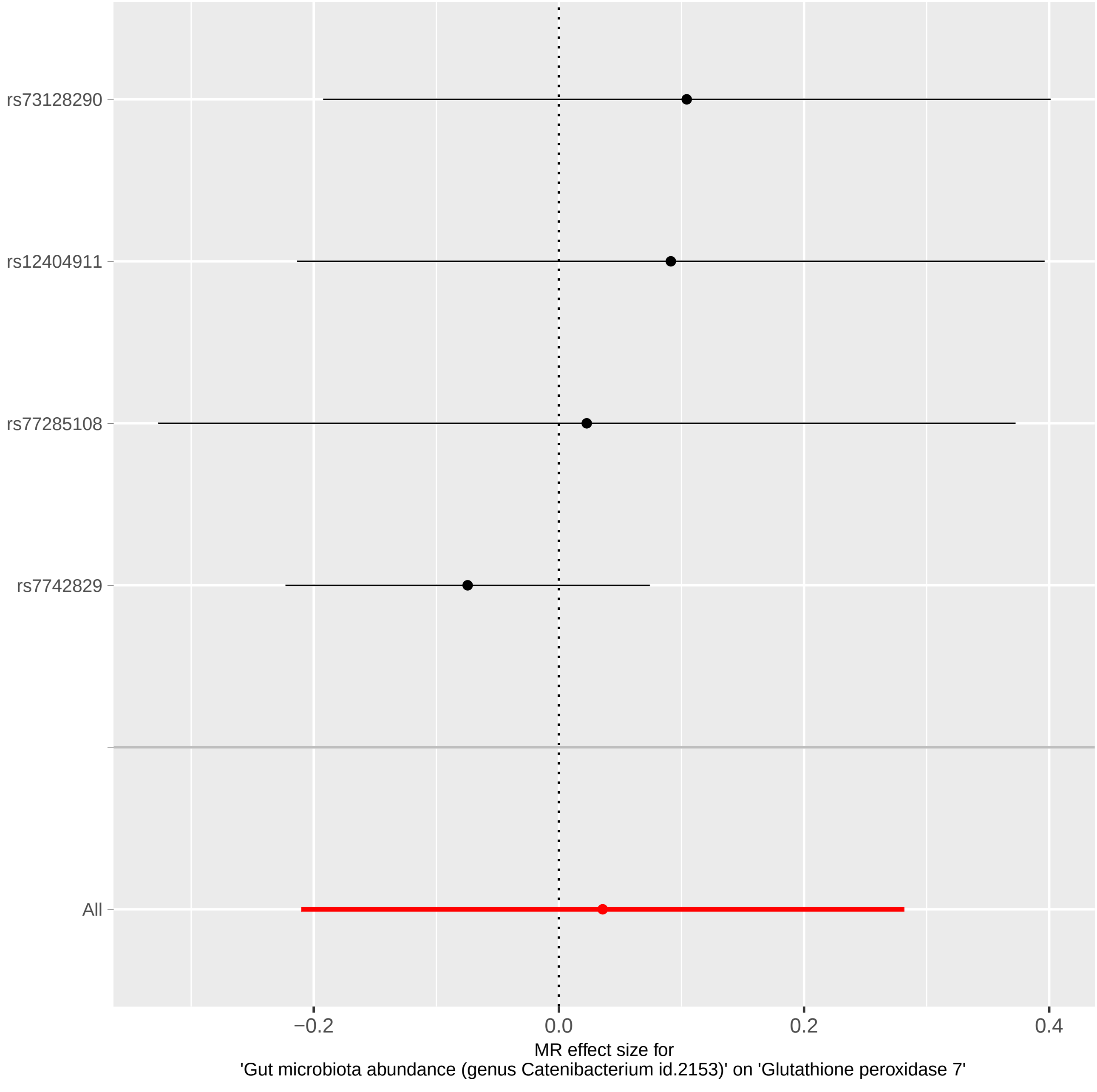

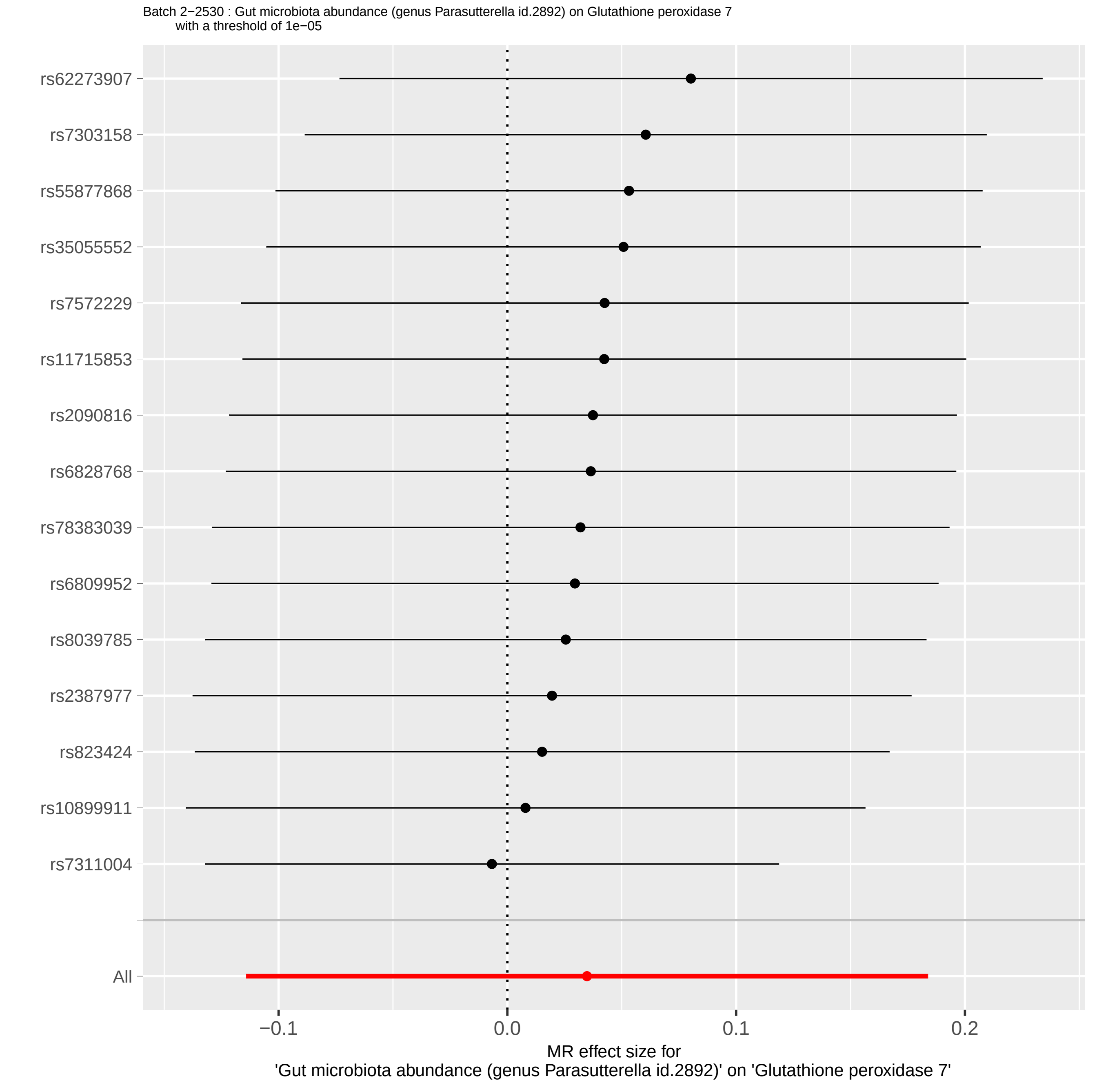
Batch 2−2530 : Gut microbiota abundance (genus Parasutterella id.2892) on Glutathione peroxidase 7
with a threshold of 1e−05
rs62273907
rs7303158
rs55877868
rs35055552
rs7572229
rs11715853
rs2090816
rs6828768
rs78383039
rs6809952
rs8039785
rs2387977
rs823424
rs10899911
rs7311004
All
−0.1
0.0
0.1
0.2
MR effect size for
'Gut microbiota abundance (genus Parasutterella id.2892)' on 'Glutathione peroxidase 7'

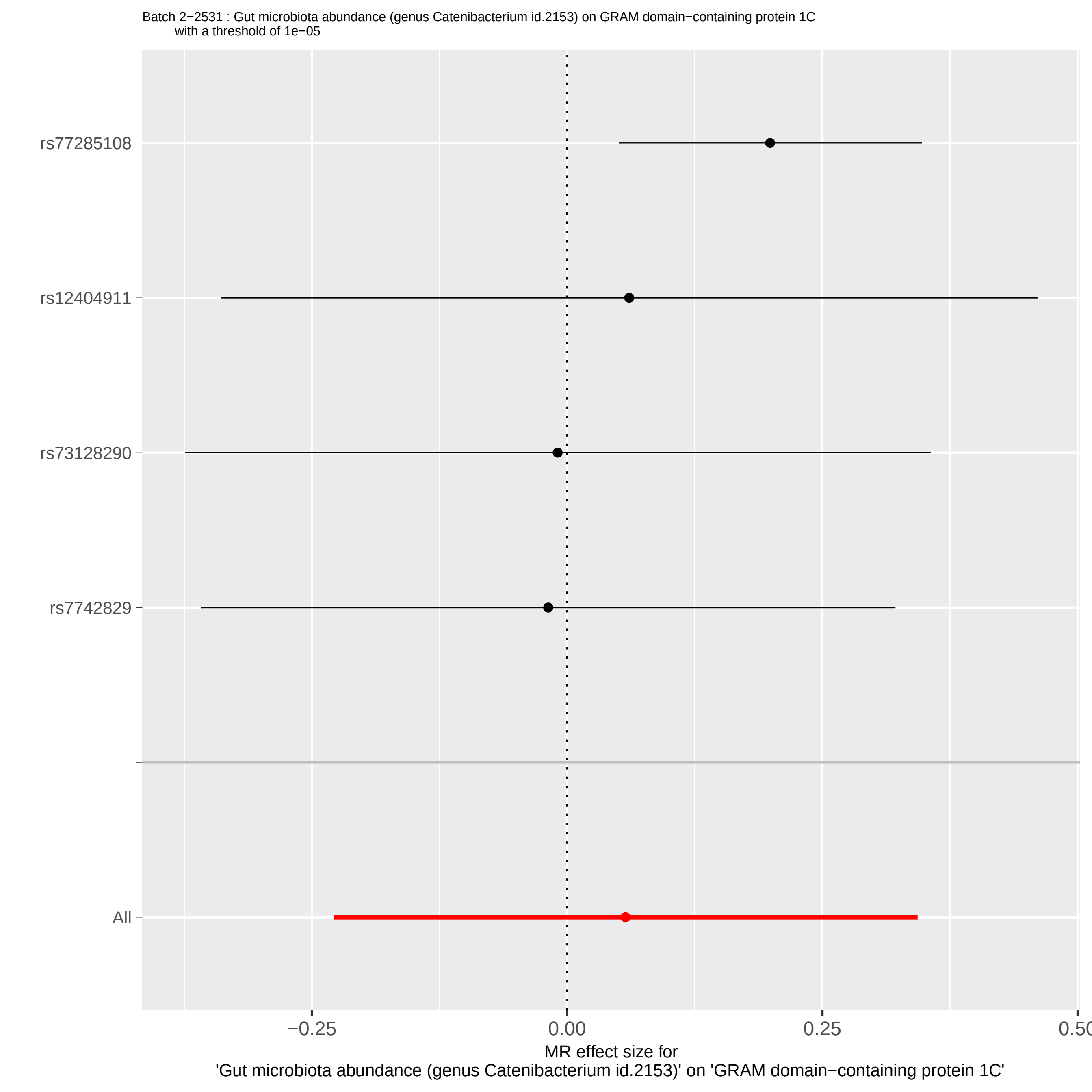
Batch 2−2531 : Gut microbiota abundance (genus Catenibacterium id.2153) on GRAM domain−containing protein 1C
with a threshold of 1e−05
rs77285108
rs12404911
rs73128290
rs7742829
All
−0.25
0.00
0.25
0.50
MR effect size for
'Gut microbiota abundance (genus Catenibacterium id.2153)' on 'GRAM domain−containing protein 1C'

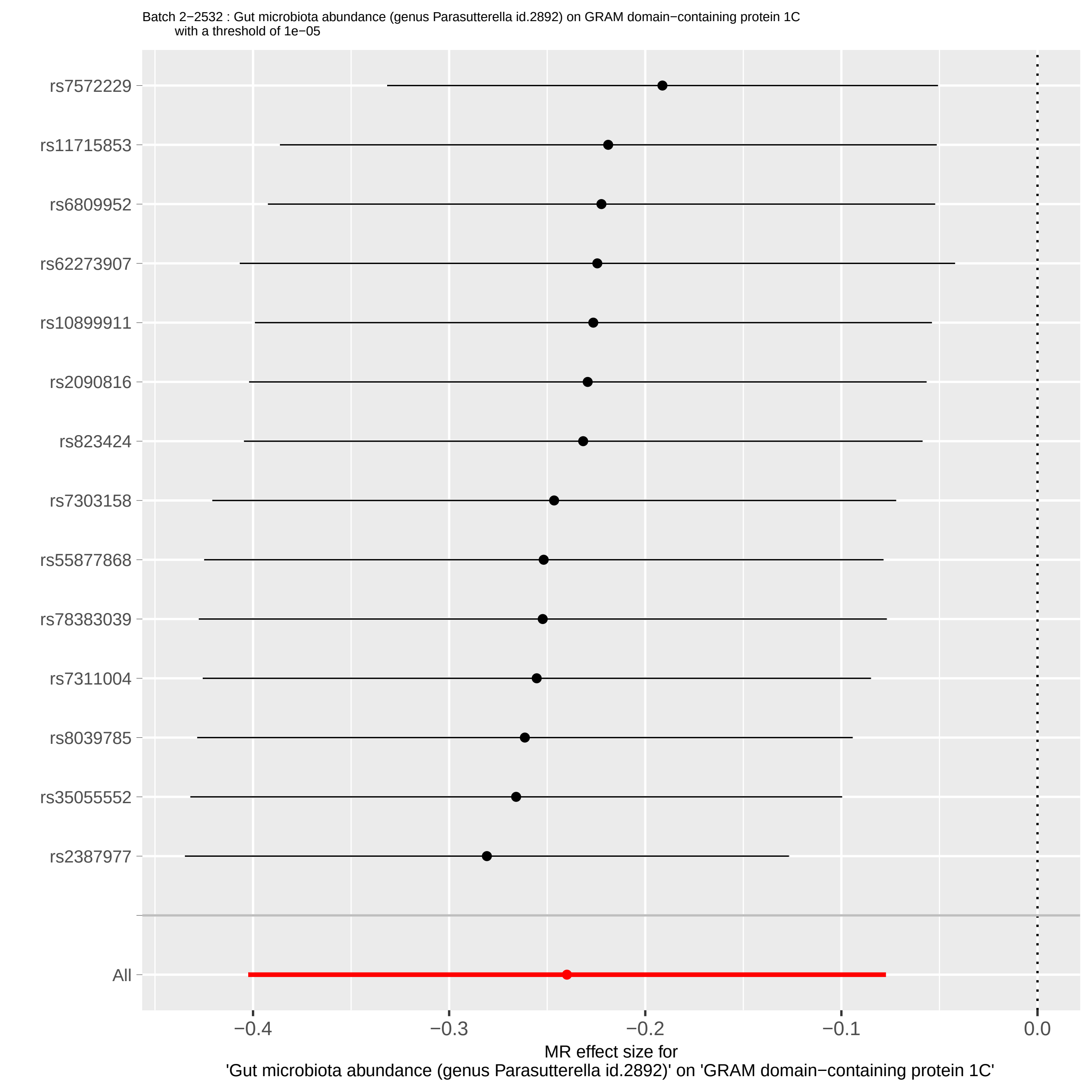

Batch 2−2532 : Gut microbiota abundance (genus Parasutterella id.2892) on GRAM domain−containing protein 1C
with a threshold of 1e−05
rs7572229
rs11715853
rs6809952
rs62273907
rs10899911
rs2090816
rs823424
rs7303158
rs55877868
rs78383039
rs7311004
rs8039785
rs35055552
rs2387977
All
−0.4
−0.3
−0.2
−0.1
0.0
MR effect size for
'Gut microbiota abundance (genus Parasutterella id.2892)' on 'GRAM domain−containing protein 1C'

Batch 2−2533 : Gut microbiota abundance (genus Catenibacterium id.2153) on GRAM domain−containing protein 1C
with a threshold of 1e−05

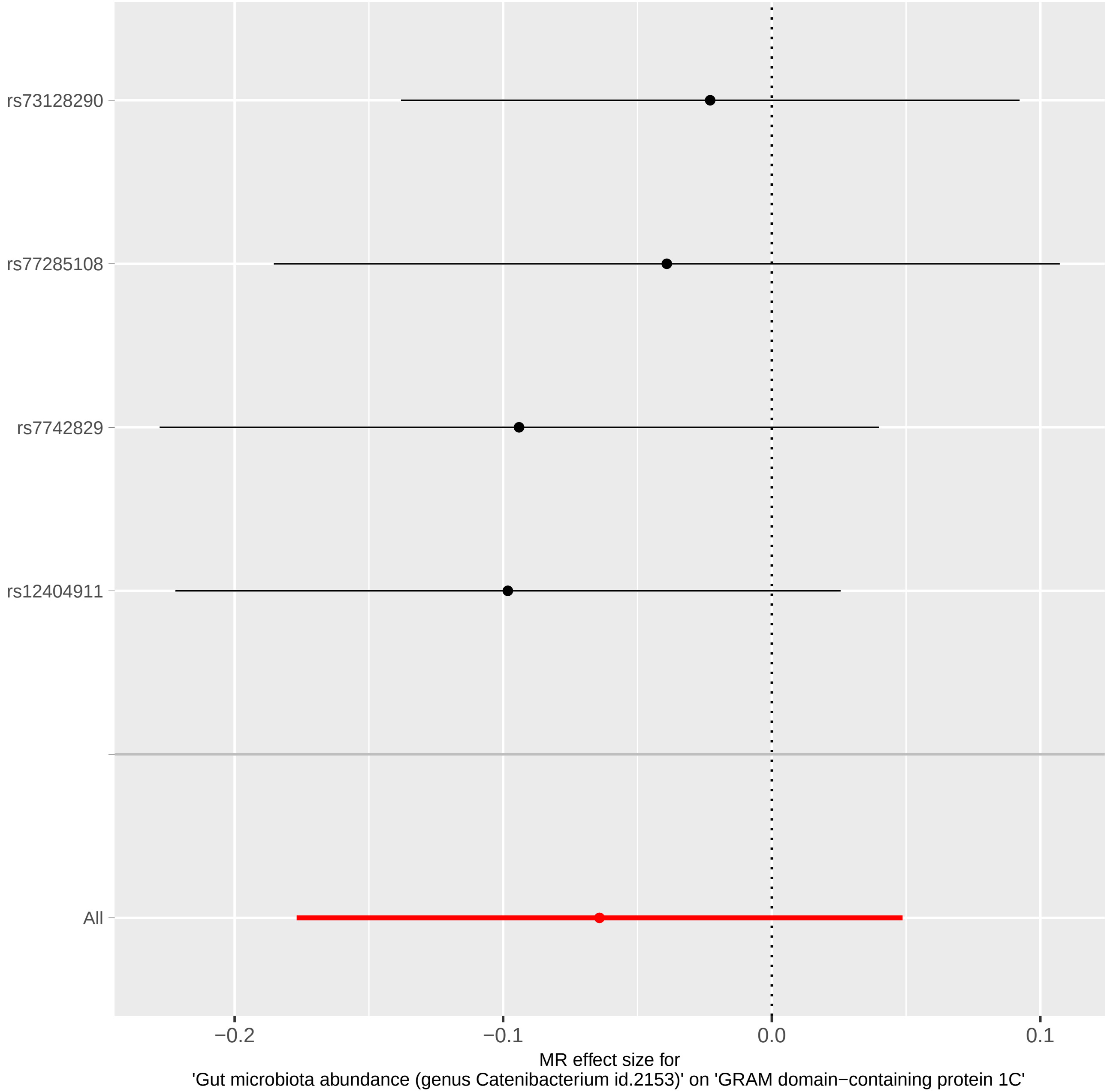

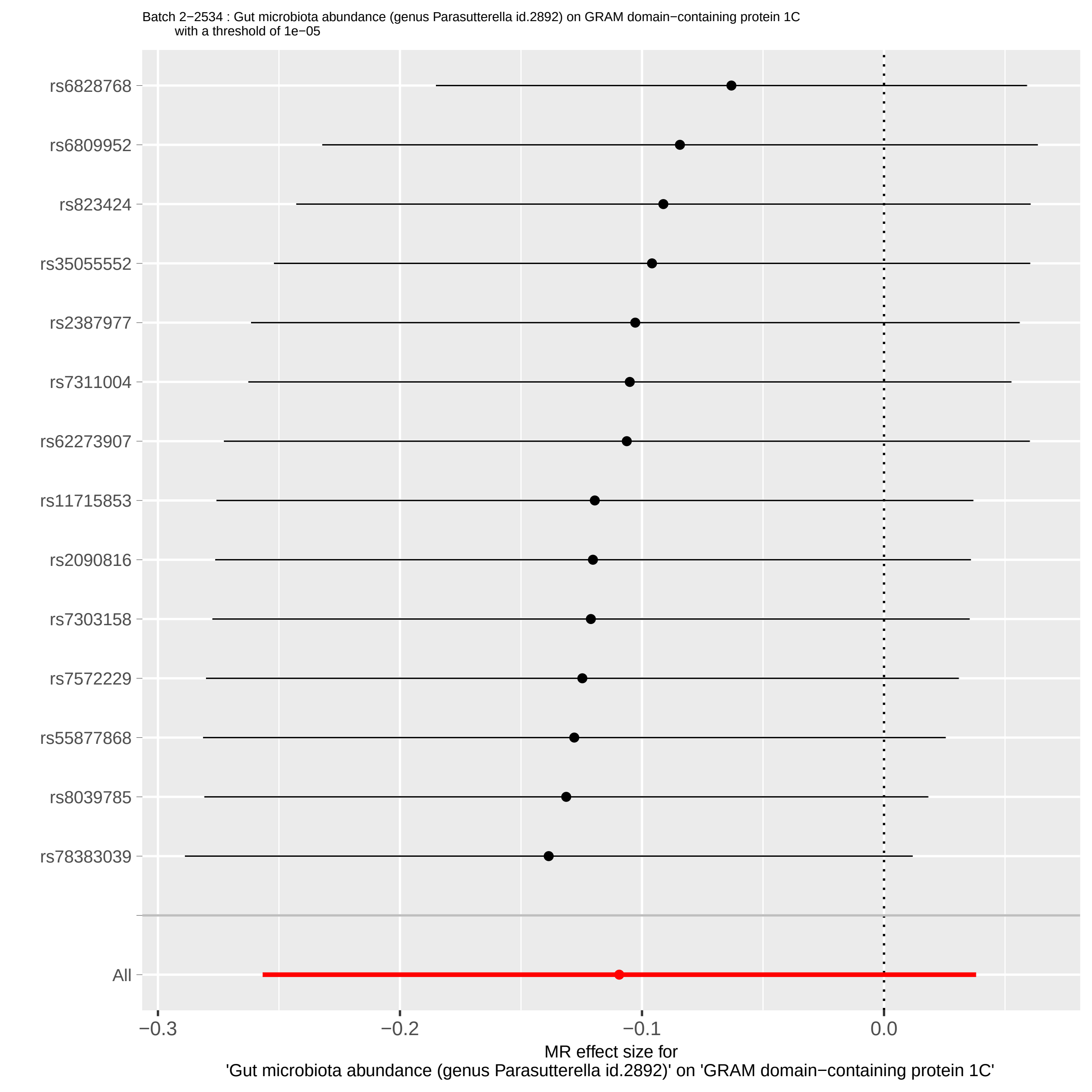
Batch 2−2534 : Gut microbiota abundance (genus Parasutterella id.2892) on GRAM domain−containing protein 1C
with a threshold of 1e−05
rs6828768
rs6809952
rs823424
rs35055552
rs2387977
rs7311004
rs62273907
rs11715853
rs2090816
rs7303158
rs7572229
rs55877868
rs8039785
rs78383039
All
−0.3
−0.2
−0.1
0.0
MR effect size for
'Gut microbiota abundance (genus Parasutterella id.2892)' on 'GRAM domain−containing protein 1C'

Batch 2−2535 : Gut microbiota abundance (genus Catenibacterium id.2153) on GRB2−related adapter protein
with a threshold of 1e−05

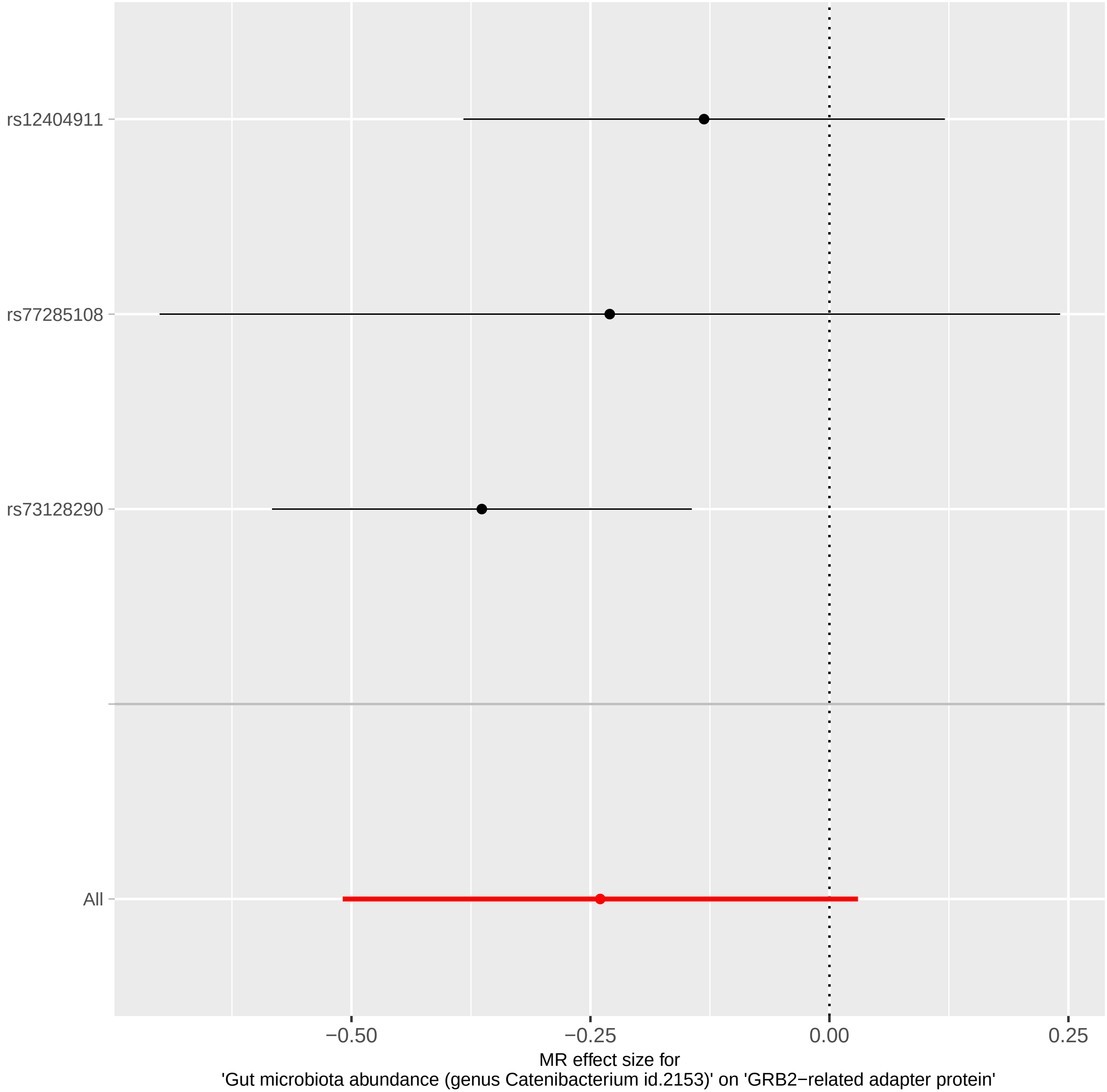

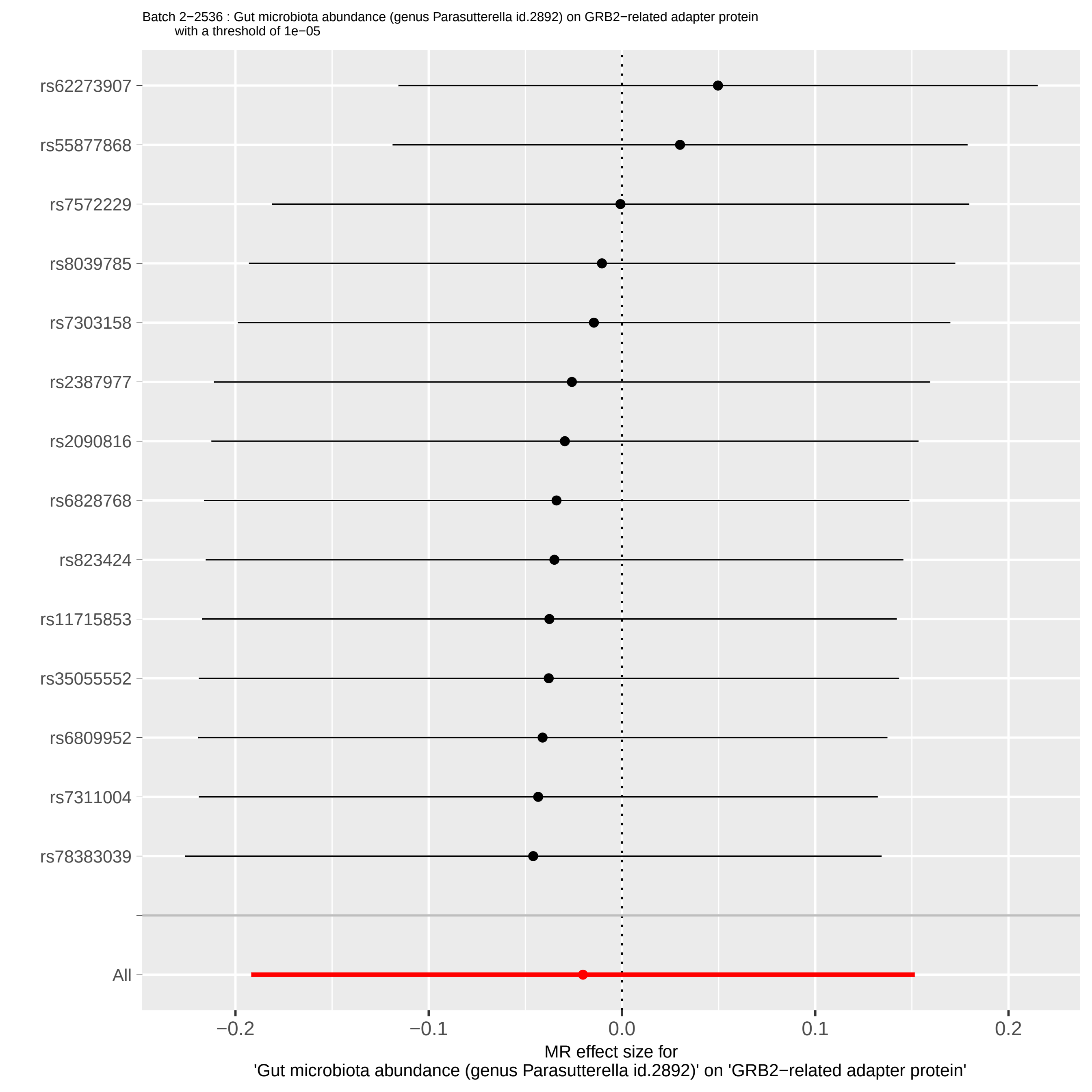

Batch 2−2536 : Gut microbiota abundance (genus Parasutterella id.2892) on GRB2−related adapter protein
with a threshold of 1e−05
rs62273907
rs55877868
rs7572229
rs8039785
rs7303158
rs2387977
rs2090816
rs6828768
rs823424
rs11715853
rs35055552
rs6809952
rs7311004
rs78383039
All
−0.2
−0.1
0.0
0.1
0.2
MR effect size for
'Gut microbiota abundance (genus Parasutterella id.2892)' on 'GRB2−related adapter protein'

Batch 2−2537 : Gut microbiota abundance (genus Catenibacterium id.2153) on GRB2−related adapter protein 2
with a threshold of 1e−05

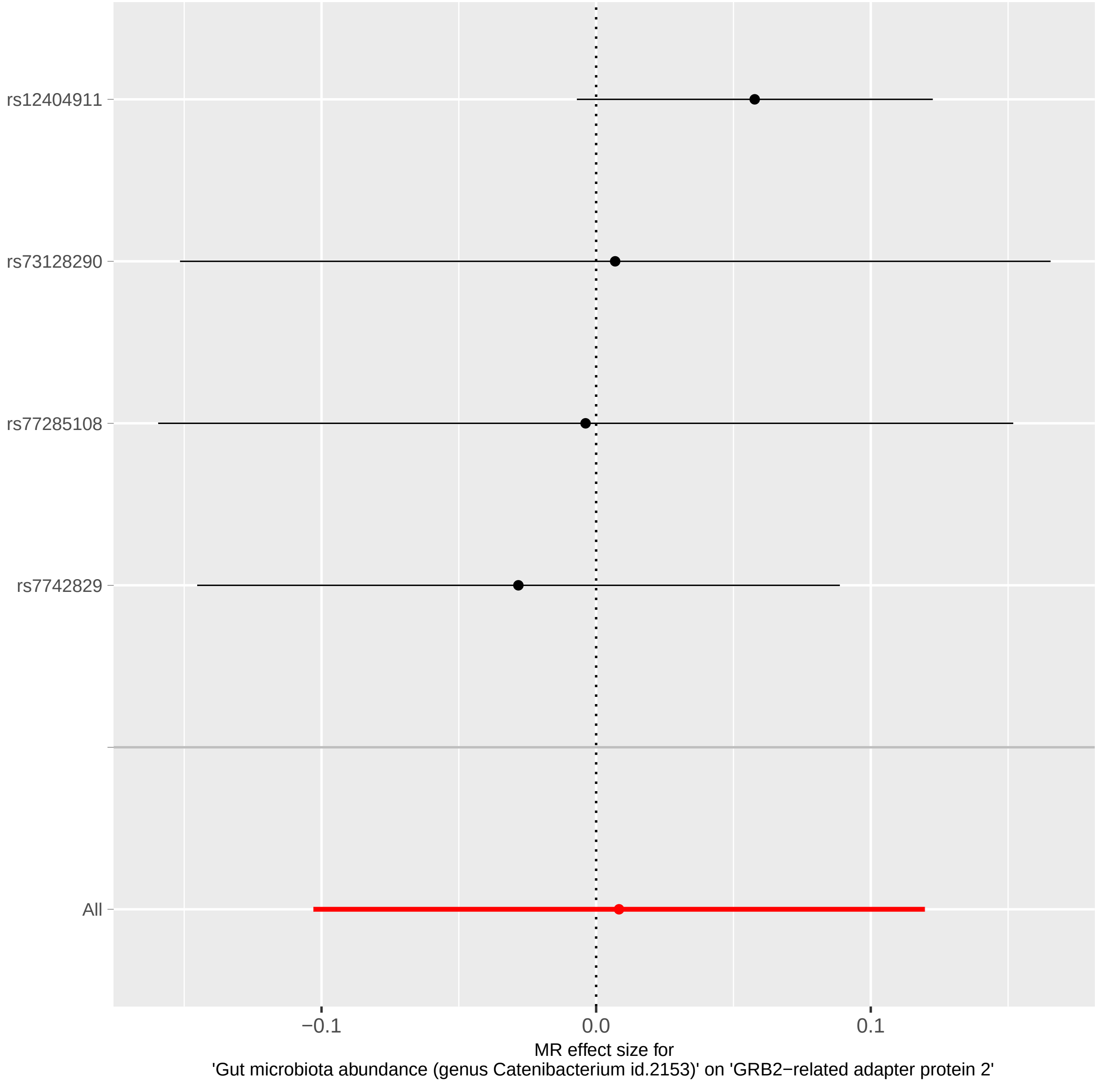

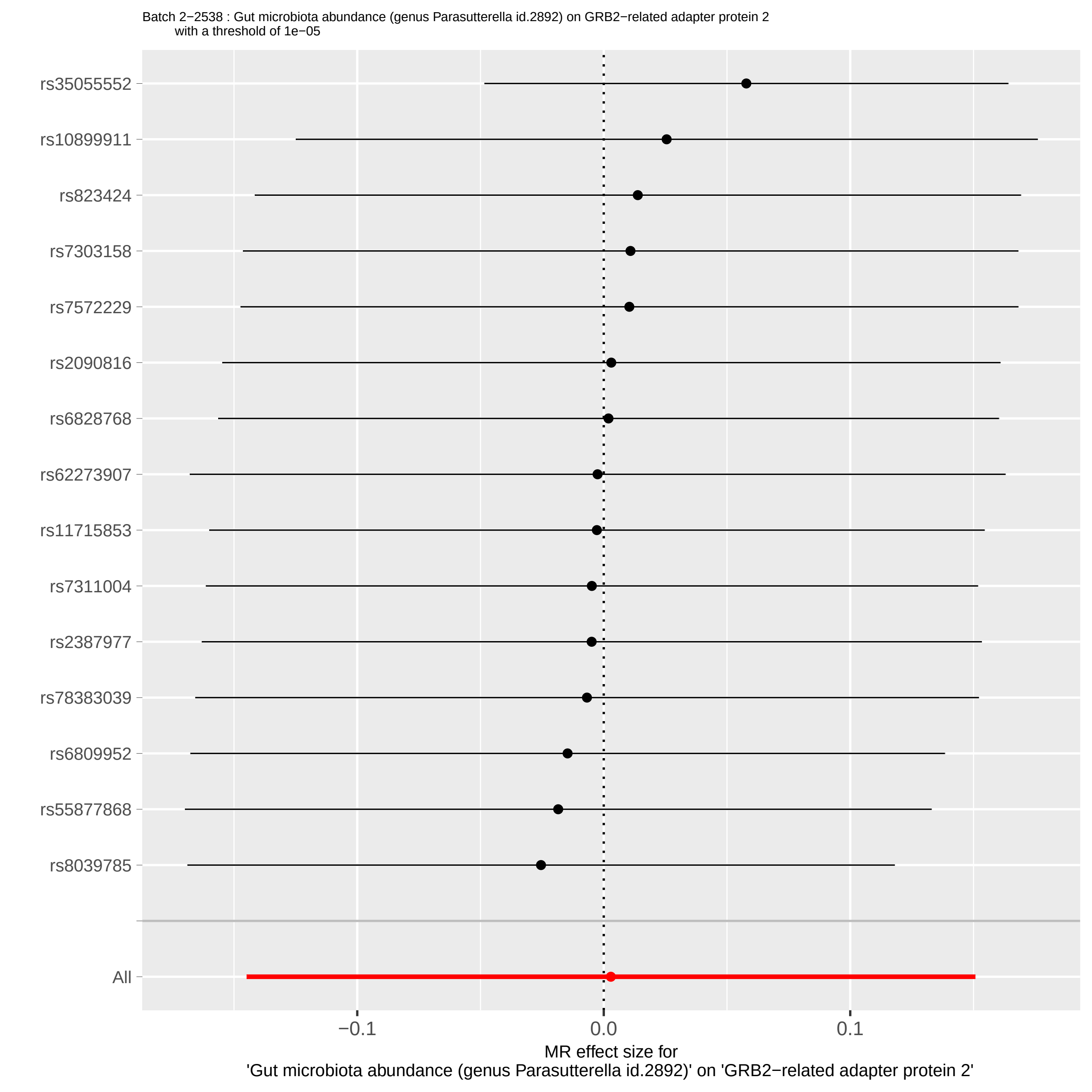
Batch 2−2538 : Gut microbiota abundance (genus Parasutterella id.2892) on GRB2−related adapter protein 2
with a threshold of 1e−05
rs35055552
rs10899911
rs823424
rs7303158
rs7572229
rs2090816
rs6828768
rs62273907
rs11715853
rs7311004
rs2387977
rs78383039
rs6809952
rs55877868
rs8039785
All
−0.1
0.0
0.1
MR effect size for
'Gut microbiota abundance (genus Parasutterella id.2892)' on 'GRB2−related adapter protein 2'

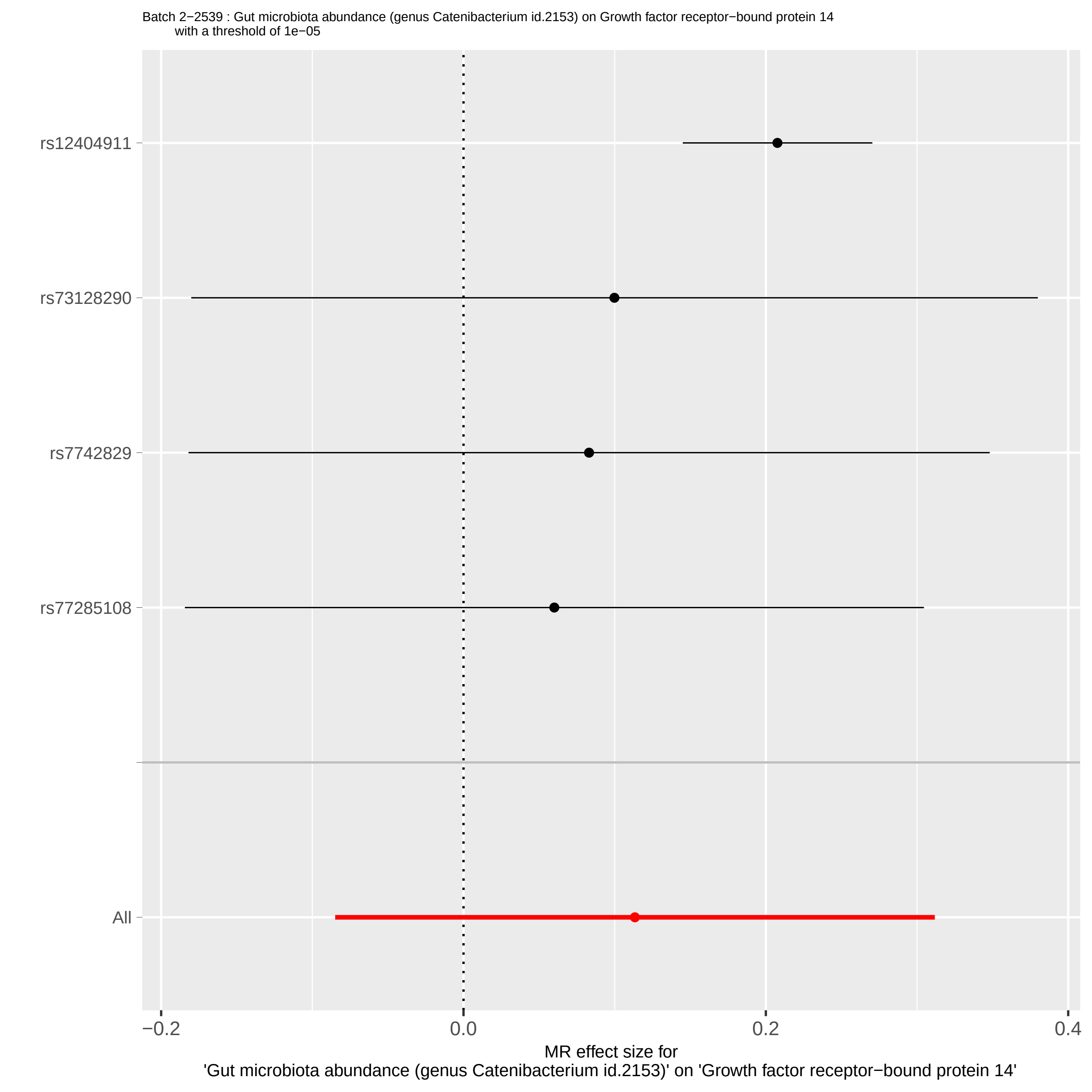

Batch 2−2539 : Gut microbiota abundance (genus Catenibacterium id.2153) on Growth factor receptor−bound protein 14
with a threshold of 1e−05
rs12404911
rs73128290
rs7742829
rs77285108
All
−0.2
0.0
0.2
0.4
MR effect size for
'Gut microbiota abundance (genus Catenibacterium id.2153)' on 'Growth factor receptor−bound protein 14'

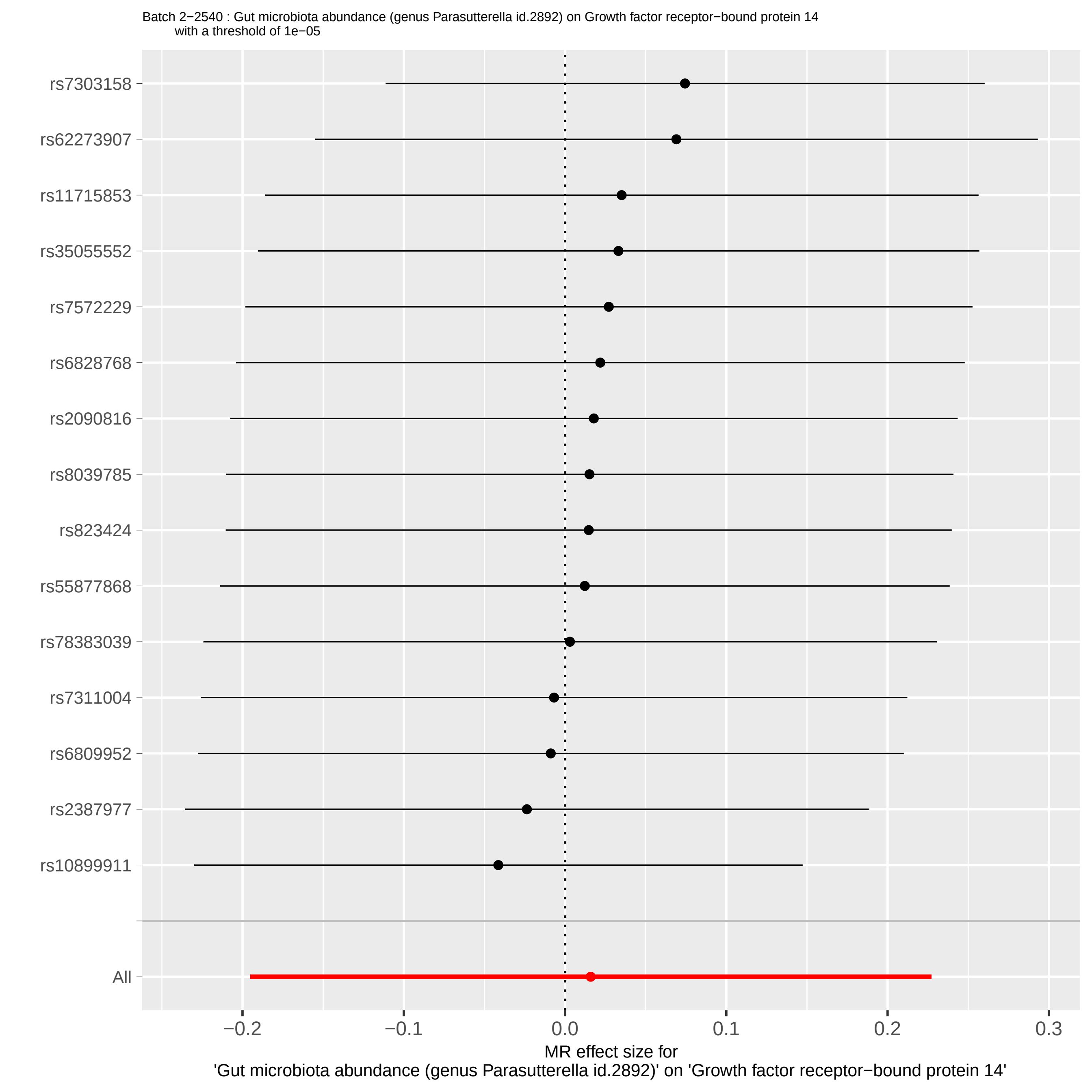

Batch 2−2540 : Gut microbiota abundance (genus Parasutterella id.2892) on Growth factor receptor−bound protein 14
with a threshold of 1e−05
rs7303158
rs62273907
rs11715853
rs35055552
rs7572229
rs6828768
rs2090816
rs8039785
rs823424
rs55877868
rs78383039
rs7311004
rs6809952
rs2387977
rs10899911
All
−0.2
−0.1
0.0
0.1
0.2
0.3
MR effect size for
'Gut microbiota abundance (genus Parasutterella id.2892)' on 'Growth factor receptor−bound protein 14'

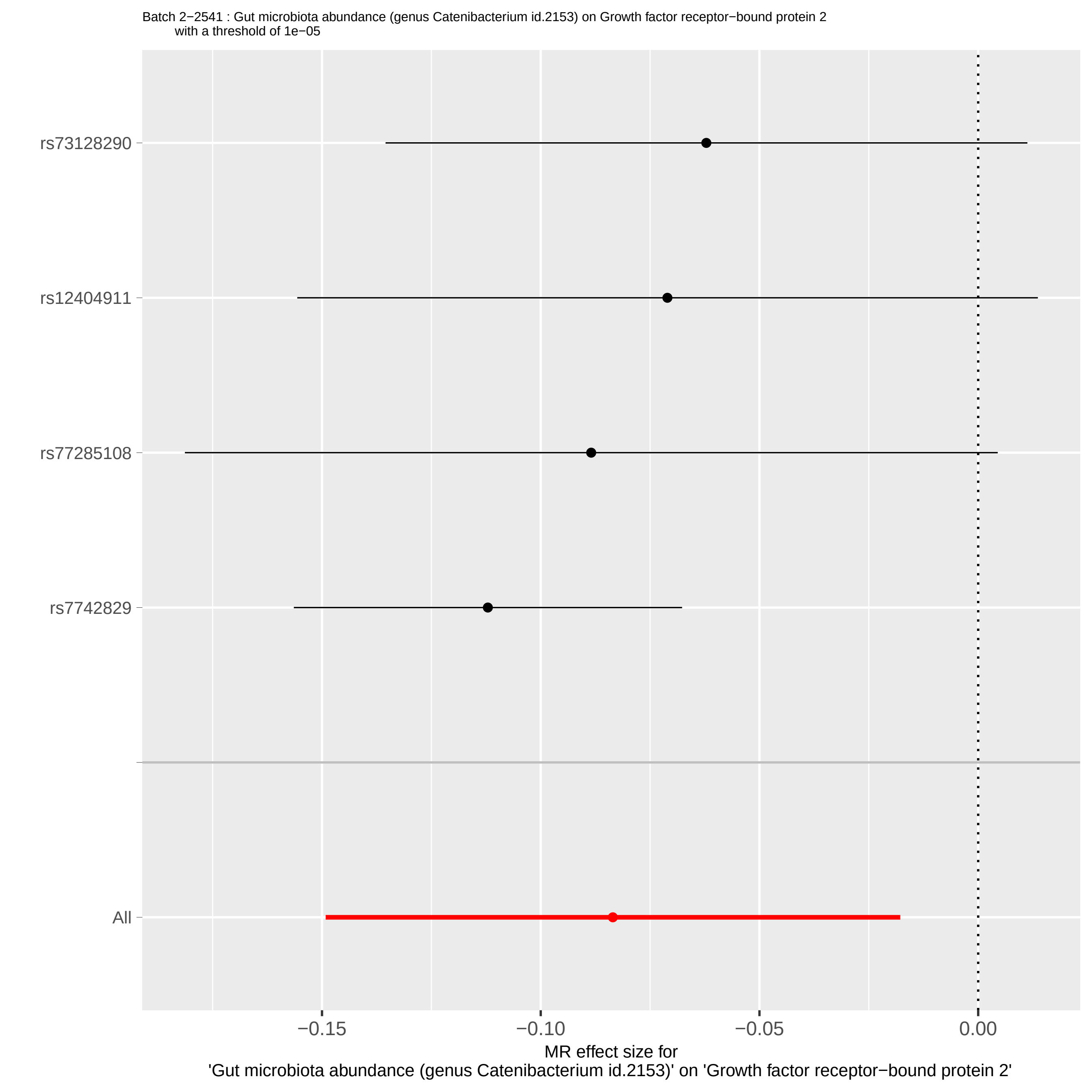
Batch 2−2541 : Gut microbiota abundance (genus Catenibacterium id.2153) on Growth factor receptor−bound protein 2
with a threshold of 1e−05
rs73128290
rs12404911
rs77285108
rs7742829
All
−0.15
−0.10
−0.05
0.00
MR effect size for
'Gut microbiota abundance (genus Catenibacterium id.2153)' on 'Growth factor receptor−bound protein 2'

Batch 2−2542 : Gut microbiota abundance (genus Parasutterella id.2892) on Growth factor receptor−bound protein 2
with a threshold of 1e−05

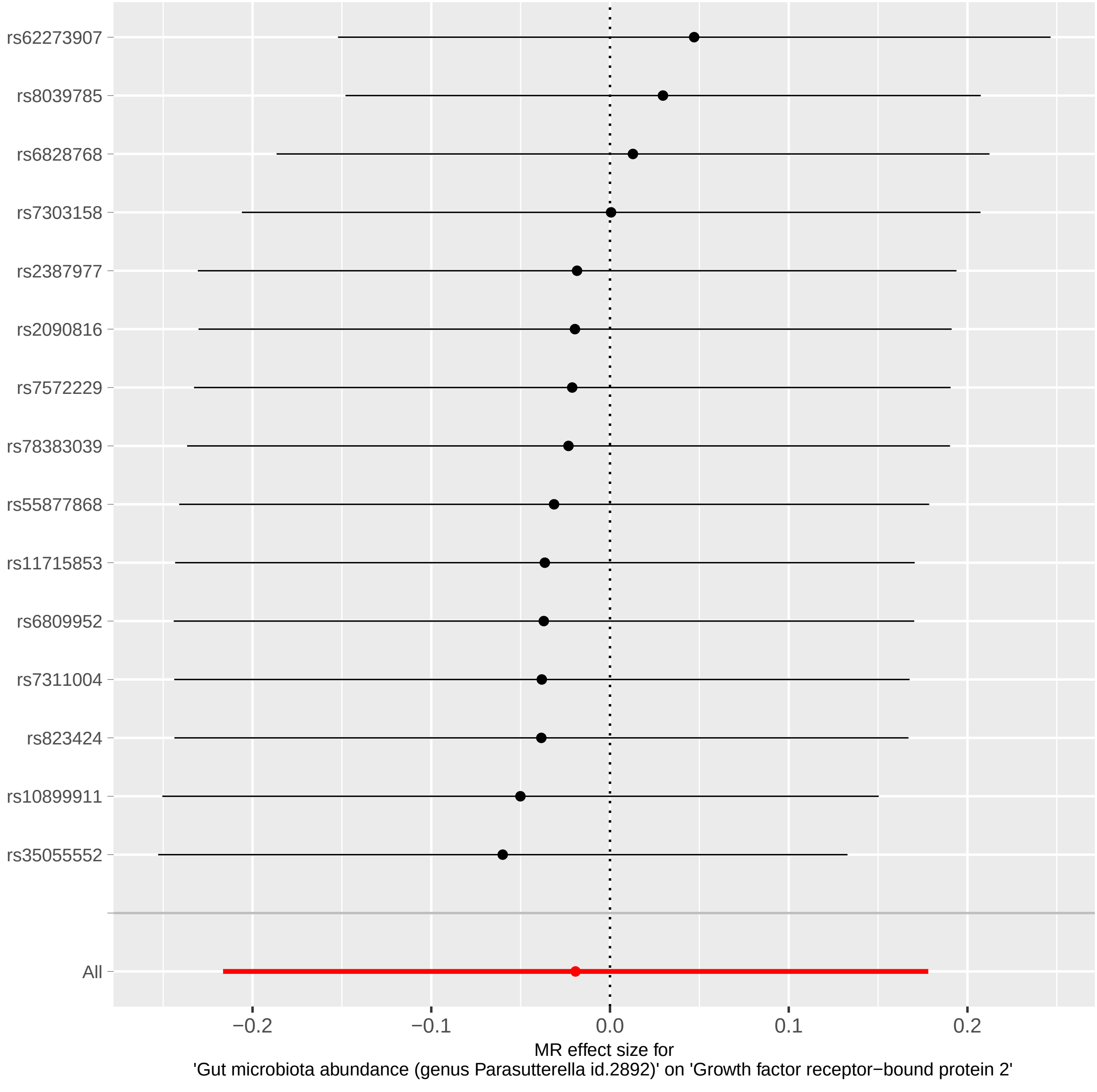

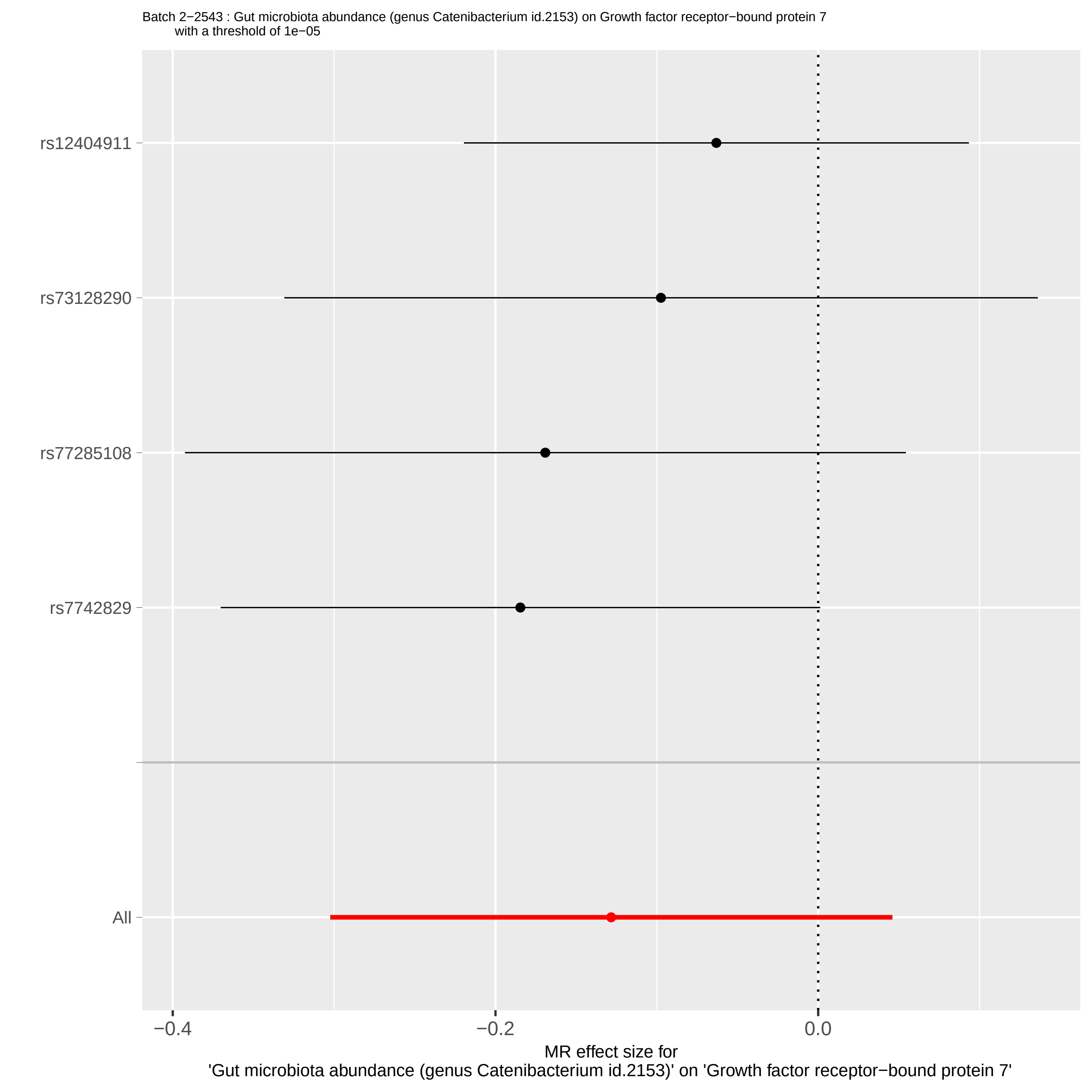

Batch 2−2543 : Gut microbiota abundance (genus Catenibacterium id.2153) on Growth factor receptor−bound protein 7
with a threshold of 1e−05
rs12404911
rs73128290
rs77285108
rs7742829
All
−0.4
−0.2
0.0
MR effect size for
'Gut microbiota abundance (genus Catenibacterium id.2153)' on 'Growth factor receptor−bound protein 7'

Batch 2−2544 : Gut microbiota abundance (genus Parasutterella id.2892) on Growth factor receptor−bound protein 7
with a threshold of 1e−05

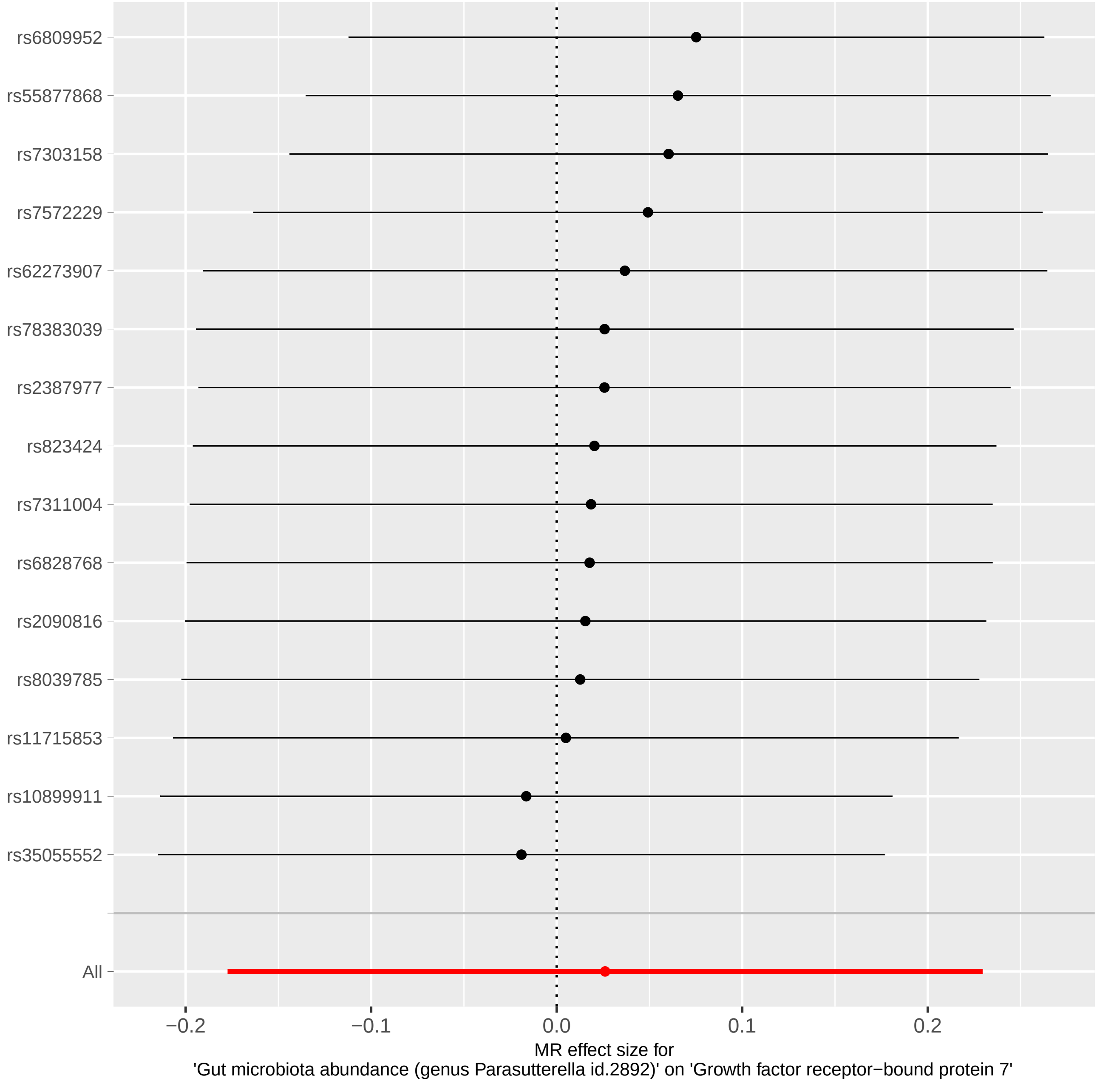

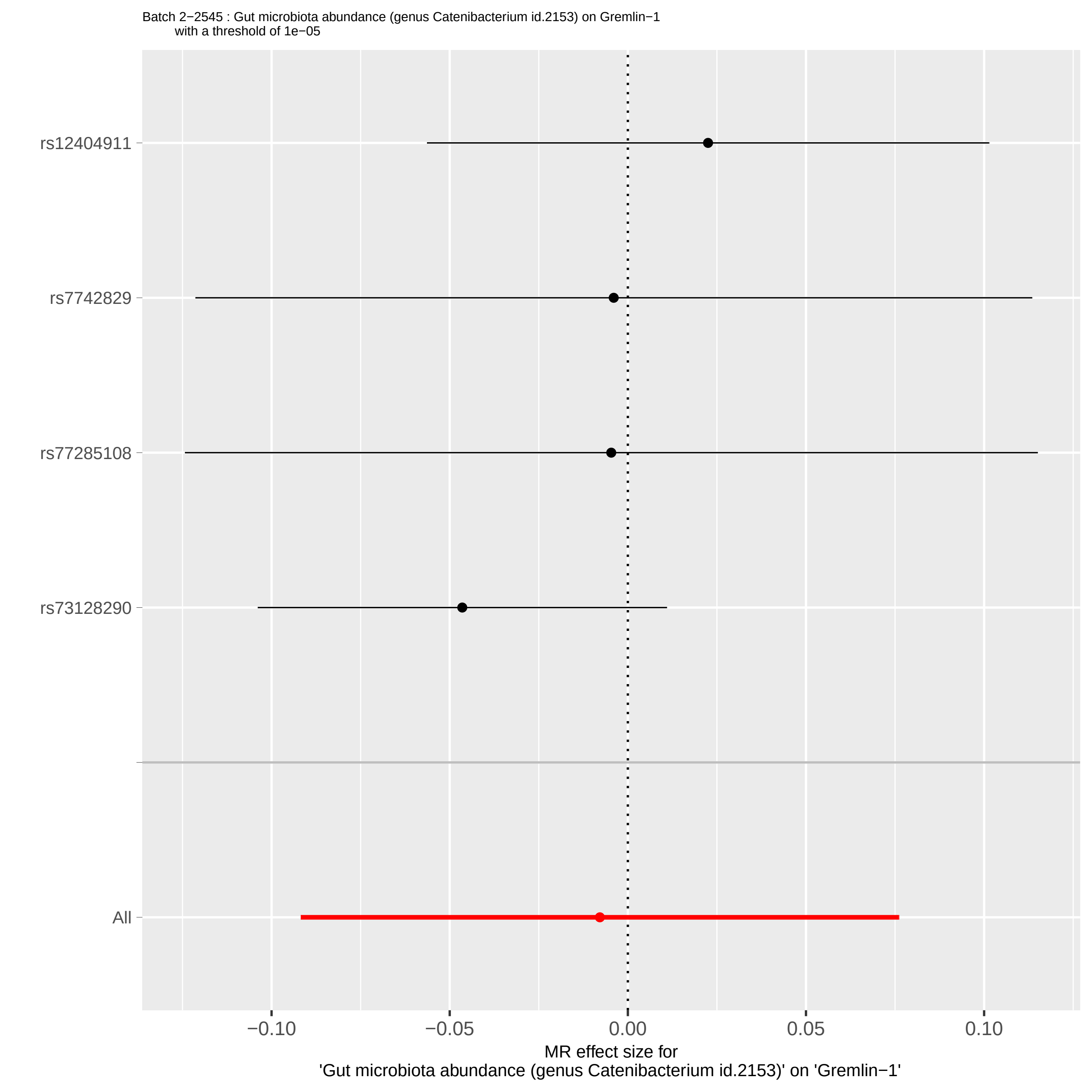
Batch 2−2545 : Gut microbiota abundance (genus Catenibacterium id.2153) on Gremlin−1
with a threshold of 1e−05
rs12404911
rs7742829
rs77285108
rs73128290
All
−0.10
−0.05
0.00
0.05
0.10
MR effect size for
'Gut microbiota abundance (genus Catenibacterium id.2153)' on 'Gremlin−1'

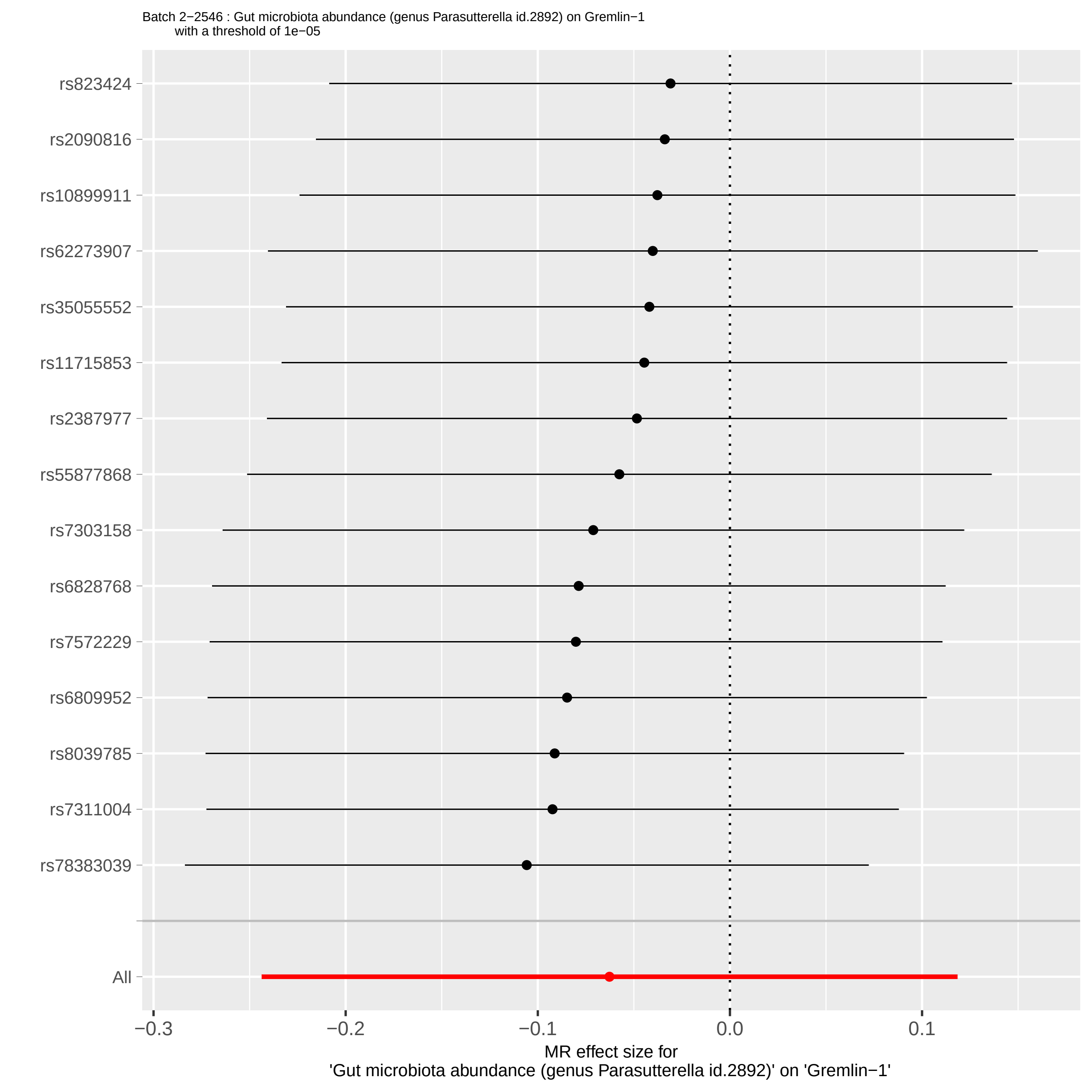
Batch 2−2546 : Gut microbiota abundance (genus Parasutterella id.2892) on Gremlin−1
with a threshold of 1e−05
rs823424
rs2090816
rs10899911
rs62273907
rs35055552
rs11715853
rs2387977
rs55877868
rs7303158
rs6828768
rs7572229
rs6809952
rs8039785
rs7311004
rs78383039
All
−0.3
−0.2
−0.1
0.0
0.1
MR effect size for
'Gut microbiota abundance (genus Parasutterella id.2892)' on 'Gremlin−1'

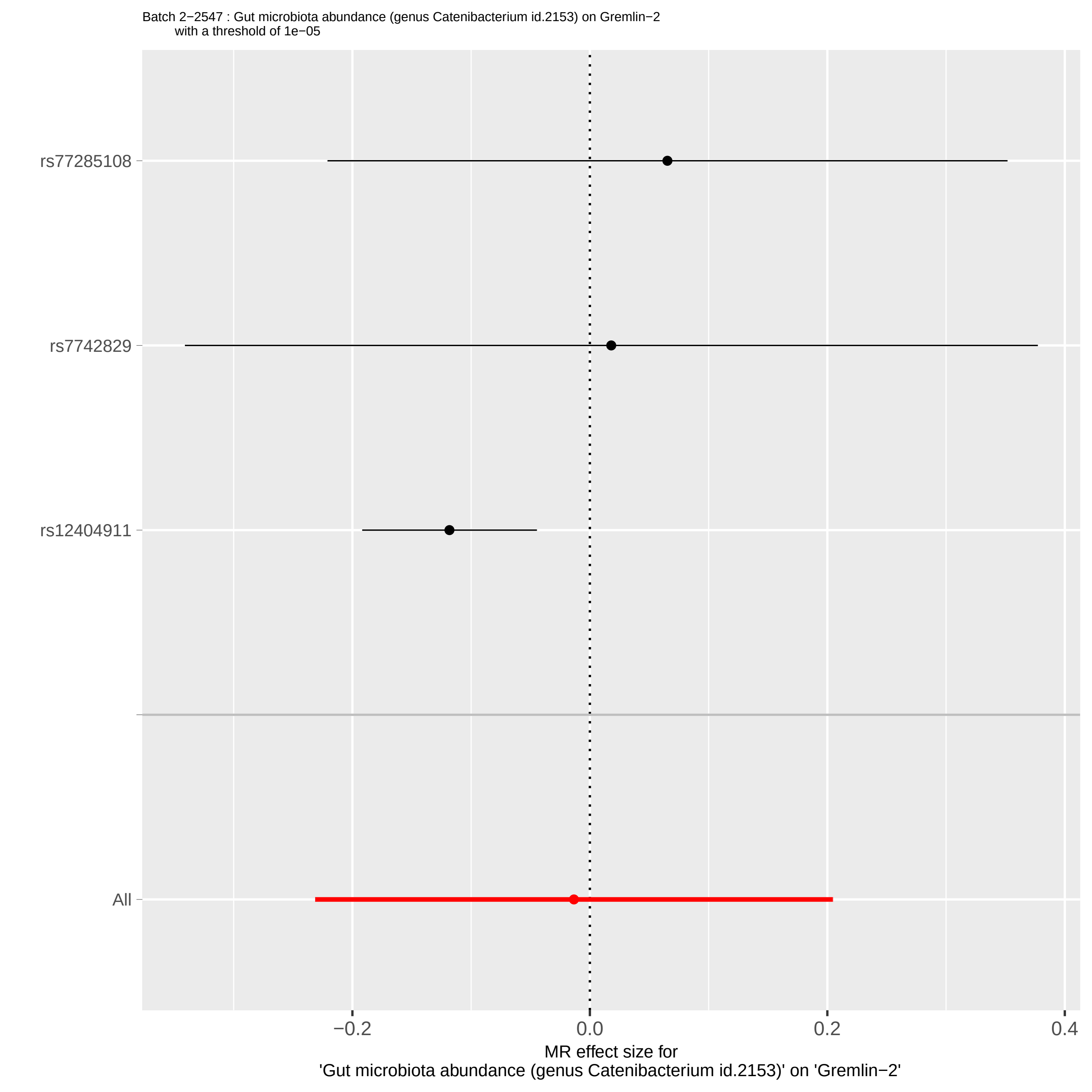

Batch 2−2547 : Gut microbiota abundance (genus Catenibacterium id.2153) on Gremlin−2
with a threshold of 1e−05
rs77285108
rs7742829
rs12404911
All
−0.2
0.0
0.2
0.4
MR effect size for
'Gut microbiota abundance (genus Catenibacterium id.2153)' on 'Gremlin−2'

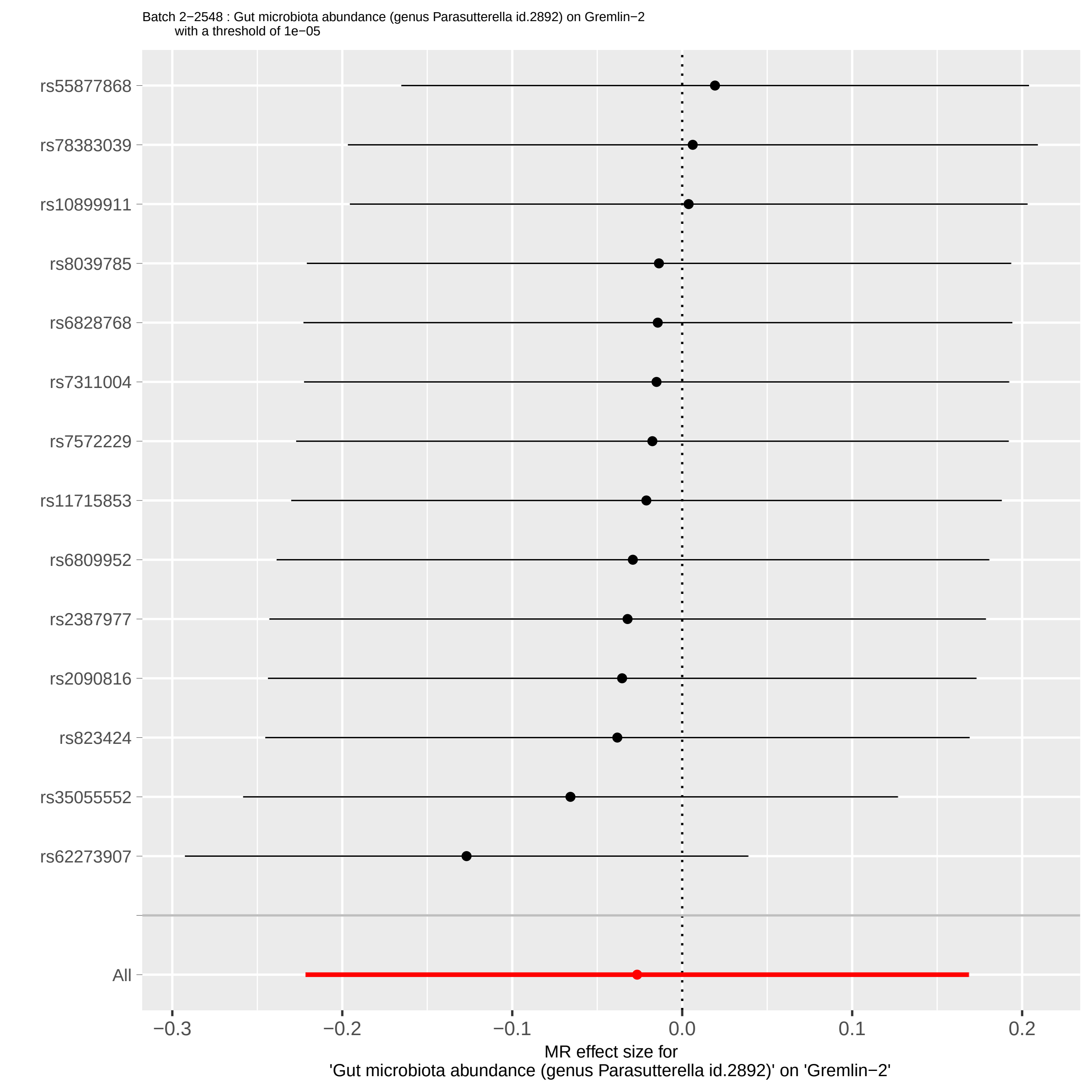

Batch 2−2548 : Gut microbiota abundance (genus Parasutterella id.2892) on Gremlin−2
with a threshold of 1e−05
rs55877868
rs78383039
rs10899911
rs8039785
rs6828768
rs7311004
rs7572229
rs11715853
rs6809952
rs2387977
rs2090816
rs823424
rs35055552
rs62273907
All
−0.3
−0.2
−0.1
0.0
0.1
0.2
MR effect size for
'Gut microbiota abundance (genus Parasutterella id.2892)' on 'Gremlin−2'

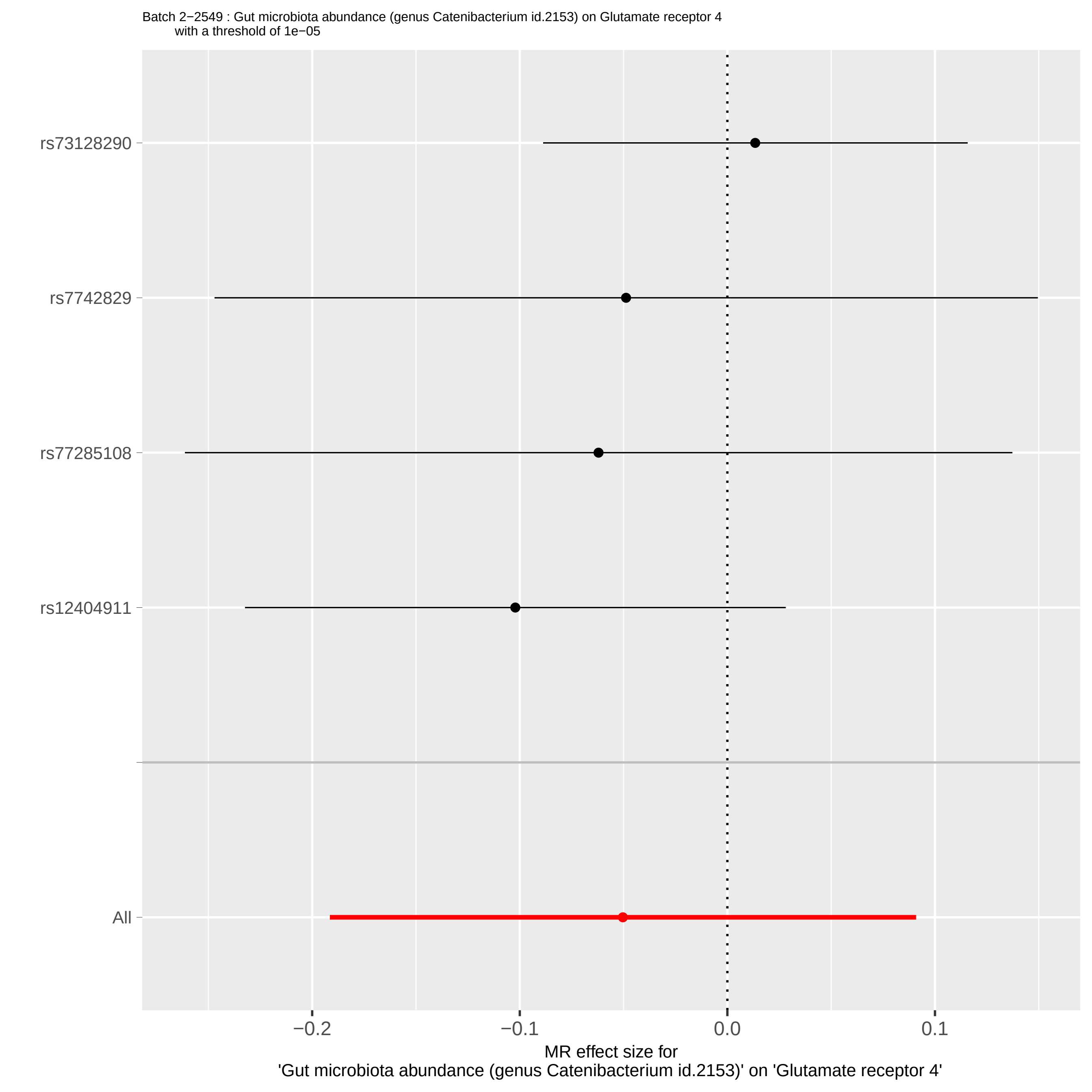
Batch 2−2549 : Gut microbiota abundance (genus Catenibacterium id.2153) on Glutamate receptor 4
with a threshold of 1e−05
rs73128290
rs7742829
rs77285108
rs12404911
All
−0.2
−0.1
0.0
0.1
MR effect size for
'Gut microbiota abundance (genus Catenibacterium id.2153)' on 'Glutamate receptor 4'

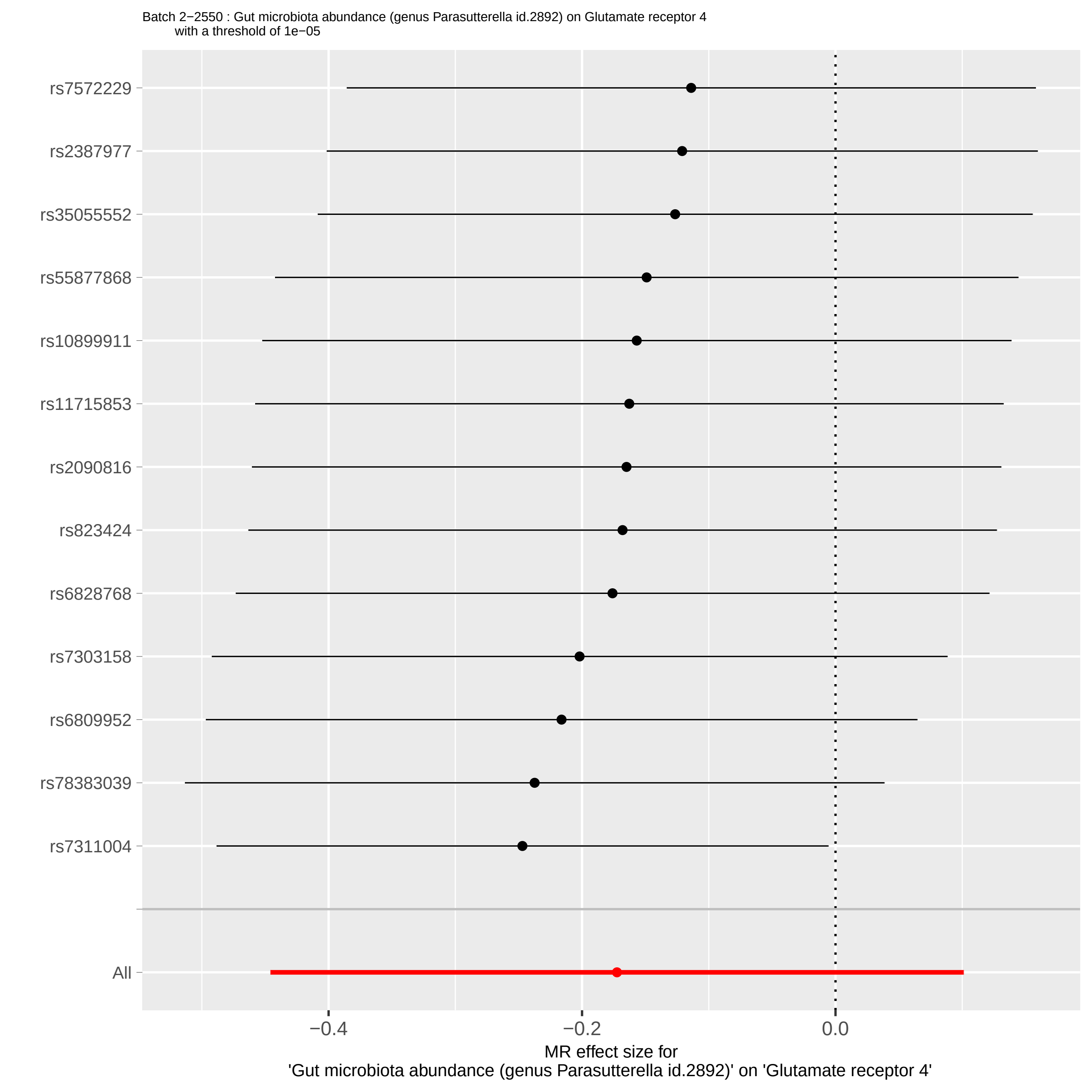
Batch 2−2550 : Gut microbiota abundance (genus Parasutterella id.2892) on Glutamate receptor 4
with a threshold of 1e−05
rs7572229
rs2387977
rs35055552
rs55877868
rs10899911
rs11715853
rs2090816
rs823424
rs6828768
rs7303158
rs6809952
rs78383039
rs7311004
All
−0.4
−0.2
0.0
MR effect size for
'Gut microbiota abundance (genus Parasutterella id.2892)' on 'Glutamate receptor 4'

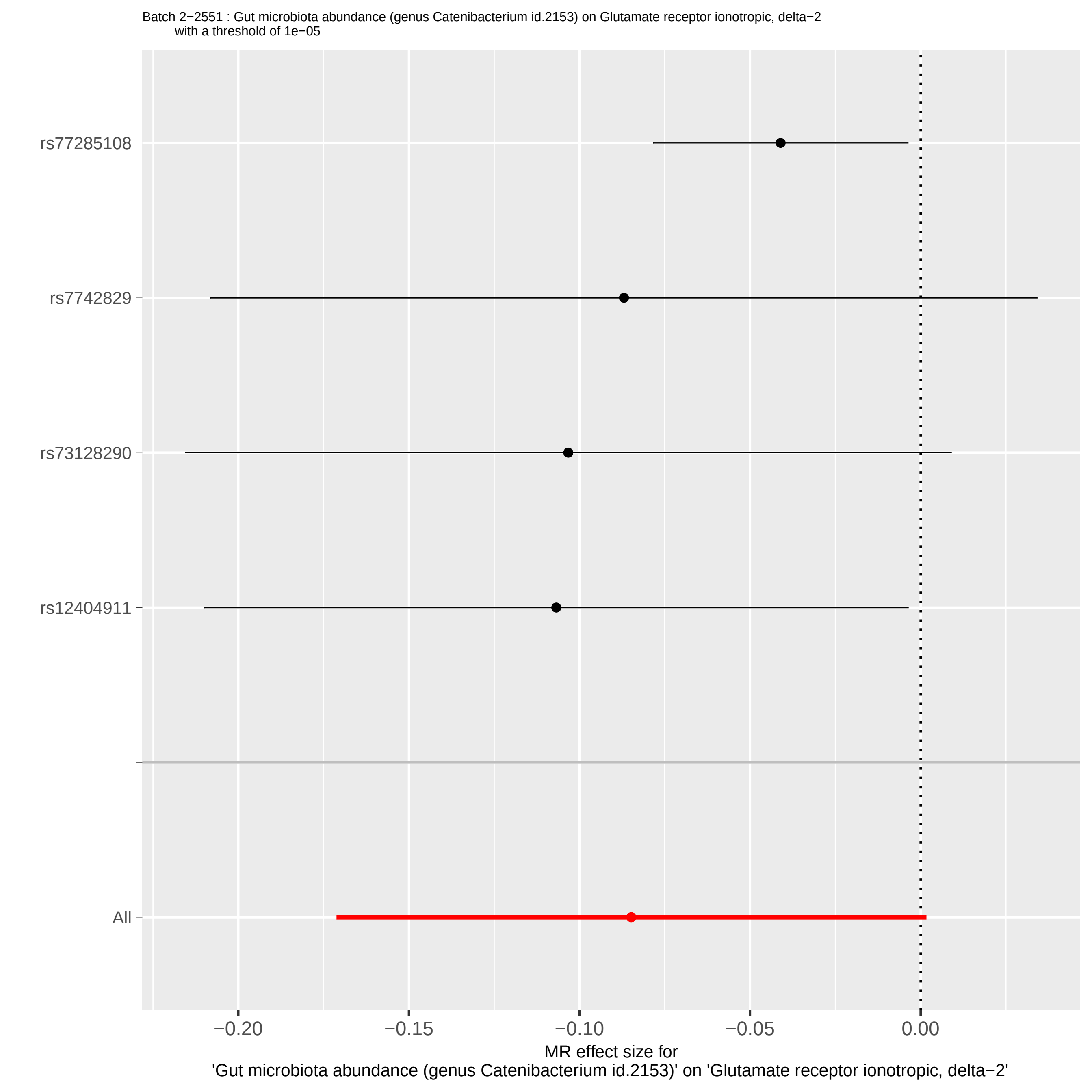
Batch 2−2551 : Gut microbiota abundance (genus Catenibacterium id.2153) on Glutamate receptor ionotropic, delta−2
with a threshold of 1e−05
rs77285108
rs7742829
rs73128290
rs12404911
All
−0.20
−0.15
−0.10
−0.05
0.00
MR effect size for
'Gut microbiota abundance (genus Catenibacterium id.2153)' on 'Glutamate receptor ionotropic, delta−2'

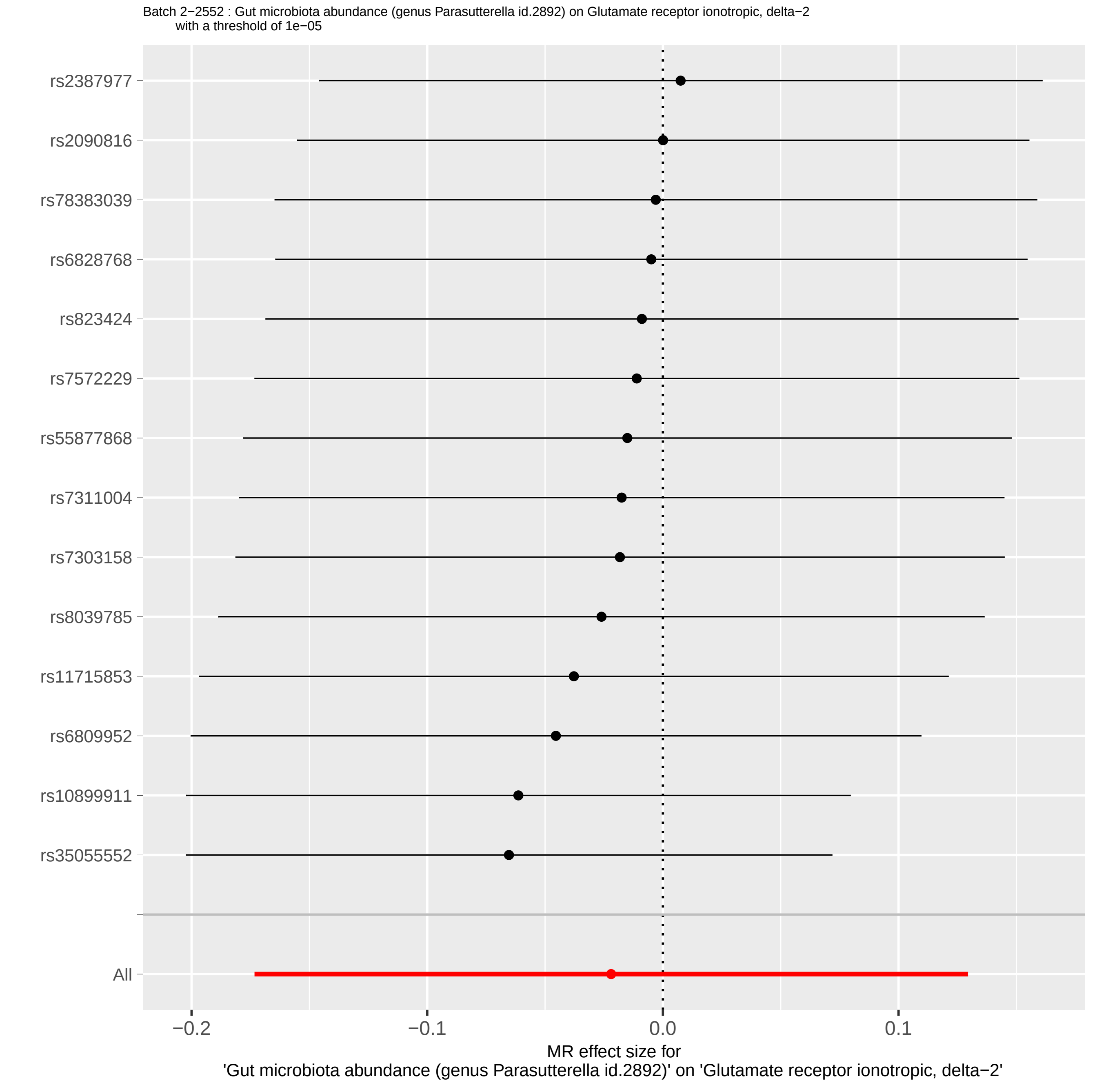
Batch 2−2552 : Gut microbiota abundance (genus Parasutterella id.2892) on Glutamate receptor ionotropic, delta−2
with a threshold of 1e−05
rs2387977
rs2090816
rs78383039
rs6828768
rs823424
rs7572229
rs55877868
rs7311004
rs7303158
rs8039785
rs11715853
rs6809952
rs10899911
rs35055552
All
−0.2
−0.1
0.0
0.1
MR effect size for
'Gut microbiota abundance (genus Parasutterella id.2892)' on 'Glutamate receptor ionotropic, delta−2'

Batch 2−2553 : Gut microbiota abundance (genus Catenibacterium id.2153) on Granulins
with a threshold of 1e−05

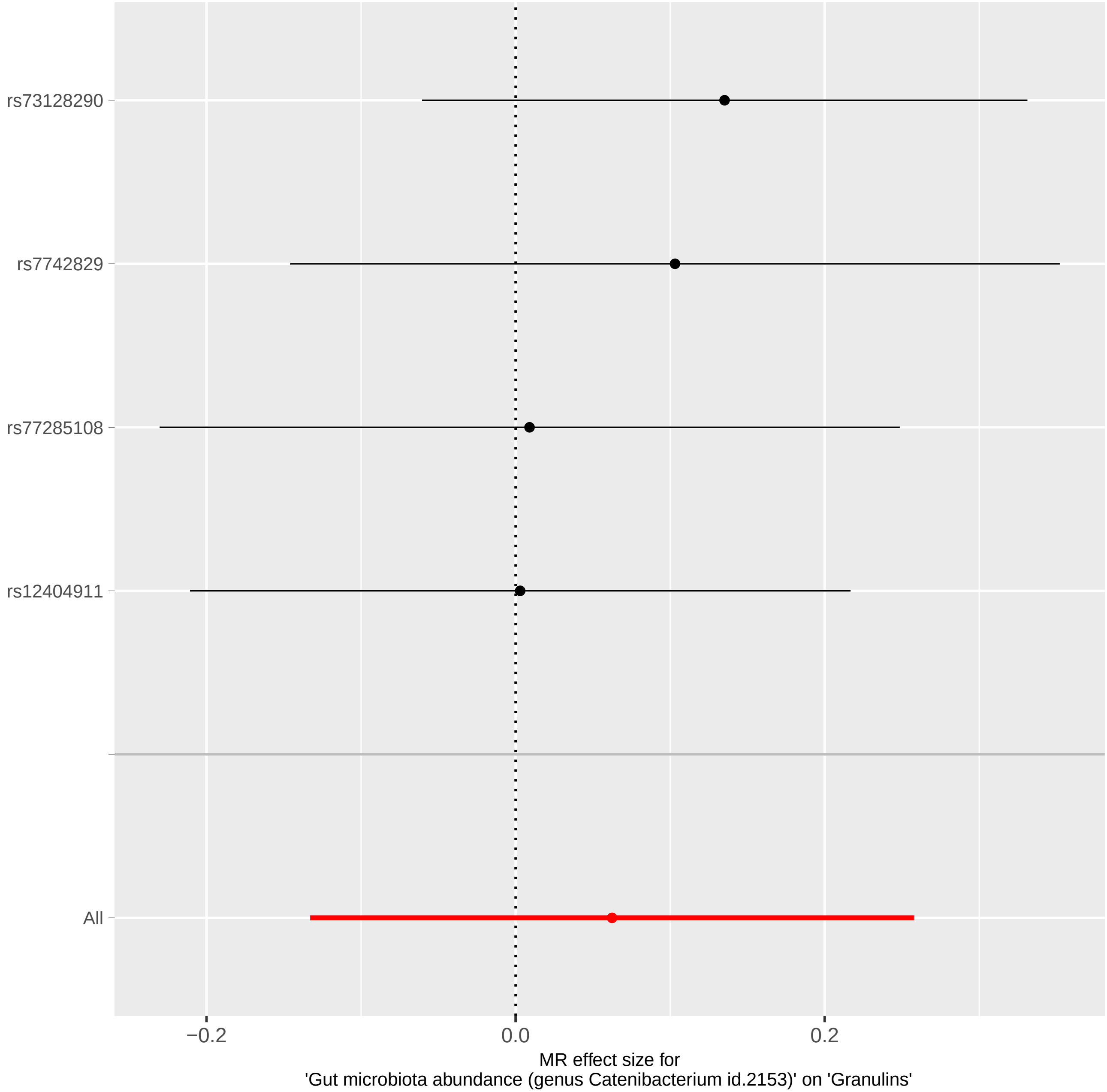

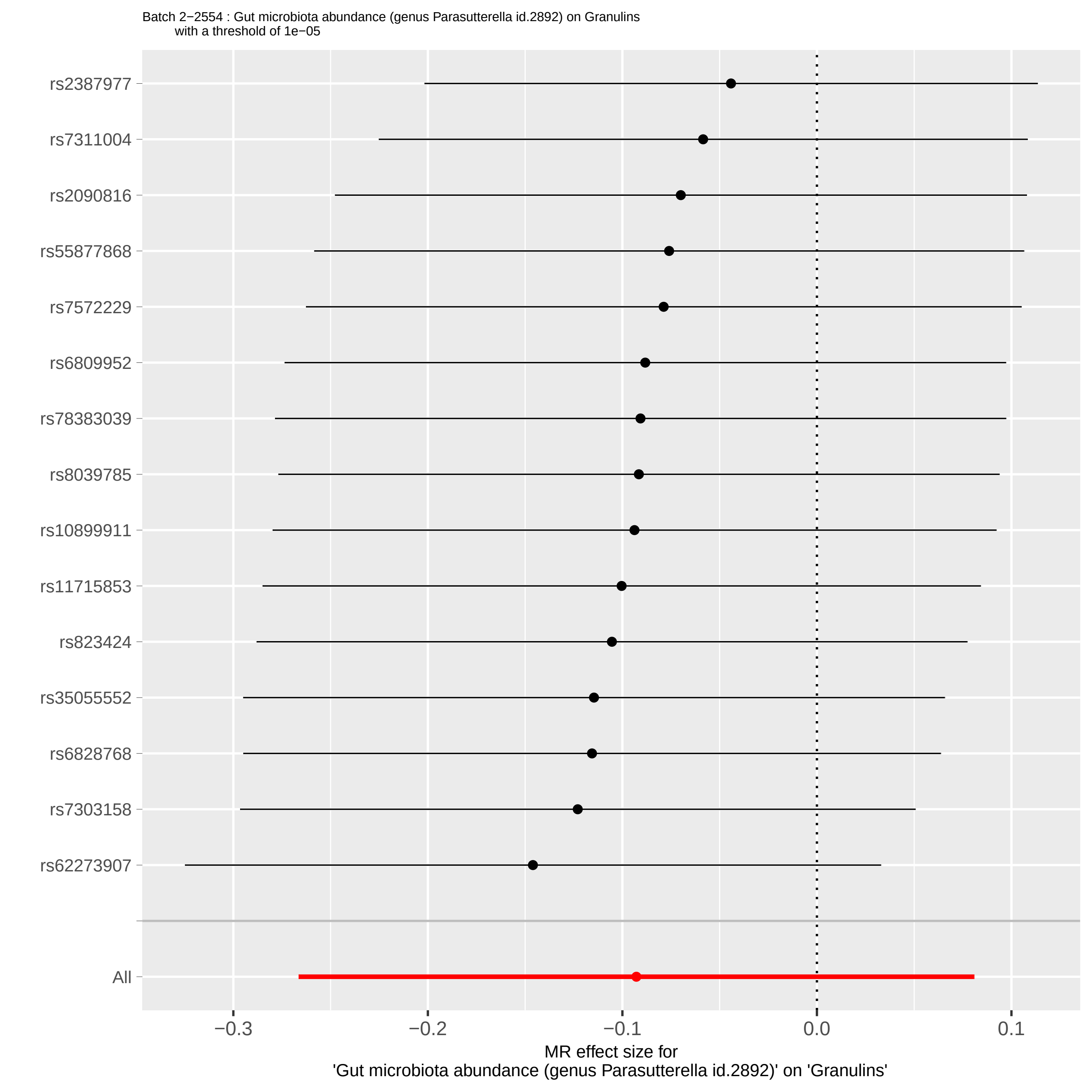

Batch 2−2554 : Gut microbiota abundance (genus Parasutterella id.2892) on Granulins
with a threshold of 1e−05
rs2387977
rs7311004
rs2090816
rs55877868
rs7572229
rs6809952
rs78383039
rs8039785
rs10899911
rs11715853
rs823424
rs35055552
rs6828768
rs7303158
rs62273907
All
−0.3
−0.2
−0.1
0.0
0.1
MR effect size for
'Gut microbiota abundance (genus Parasutterella id.2892)' on 'Granulins'

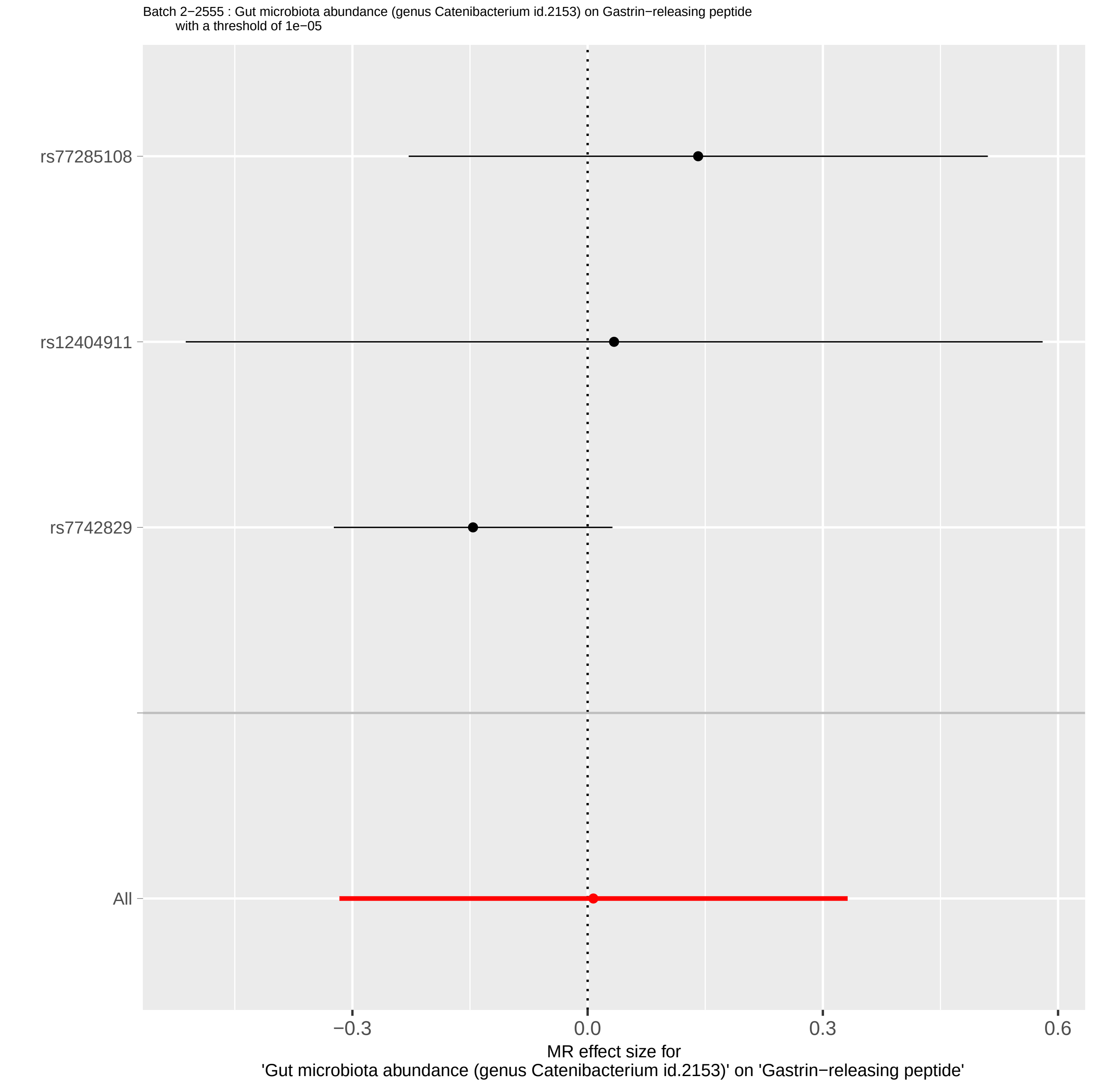

Batch 2−2555 : Gut microbiota abundance (genus Catenibacterium id.2153) on Gastrin−releasing peptide
with a threshold of 1e−05
rs77285108
rs12404911
rs7742829
All
−0.3
0.0
0.3
0.6
MR effect size for
'Gut microbiota abundance (genus Catenibacterium id.2153)' on 'Gastrin−releasing peptide'

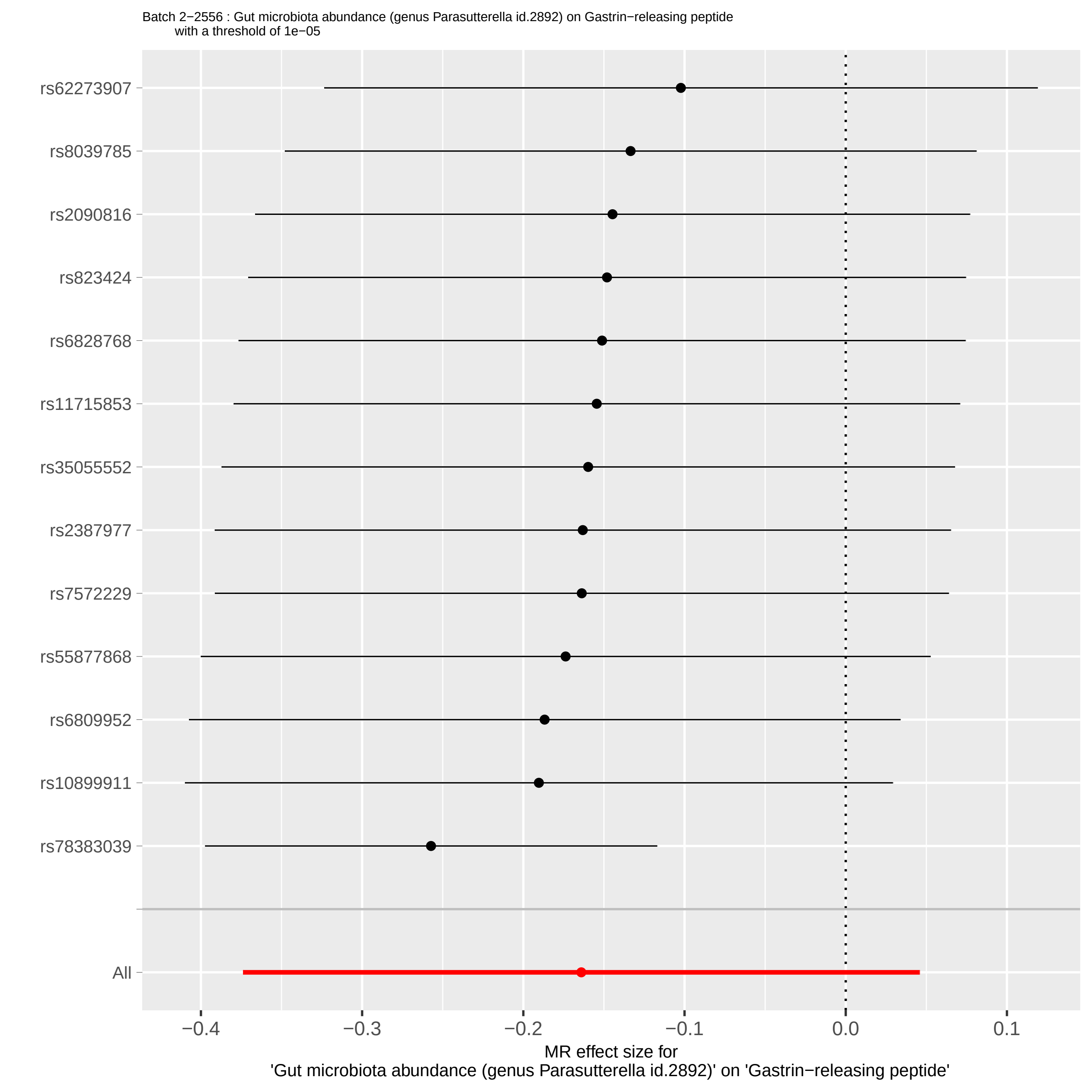

Batch 2−2556 : Gut microbiota abundance (genus Parasutterella id.2892) on Gastrin−releasing peptide
with a threshold of 1e−05
rs62273907
rs8039785
rs2090816
rs823424
rs6828768
rs11715853
rs35055552
rs2387977
rs7572229
rs55877868
rs6809952
rs10899911
rs78383039
All
−0.4
−0.3
−0.2
−0.1
0.0
0.1
MR effect size for
'Gut microbiota abundance (genus Parasutterella id.2892)' on 'Gastrin−releasing peptide'

Batch 2−2557 : Gut microbiota abundance (genus Catenibacterium id.2153) on Gastrin−releasing peptide
with a threshold of 1e−05

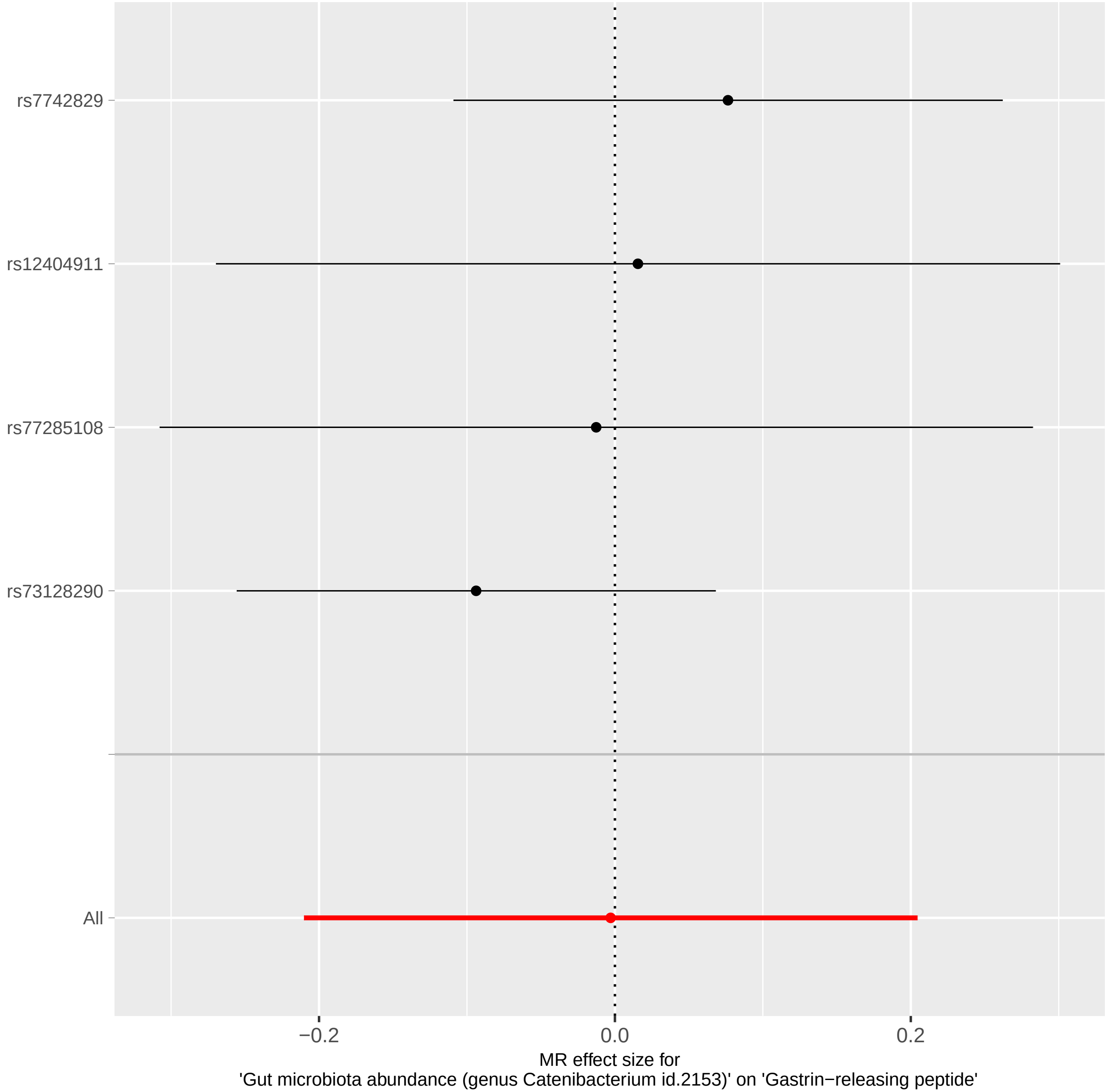

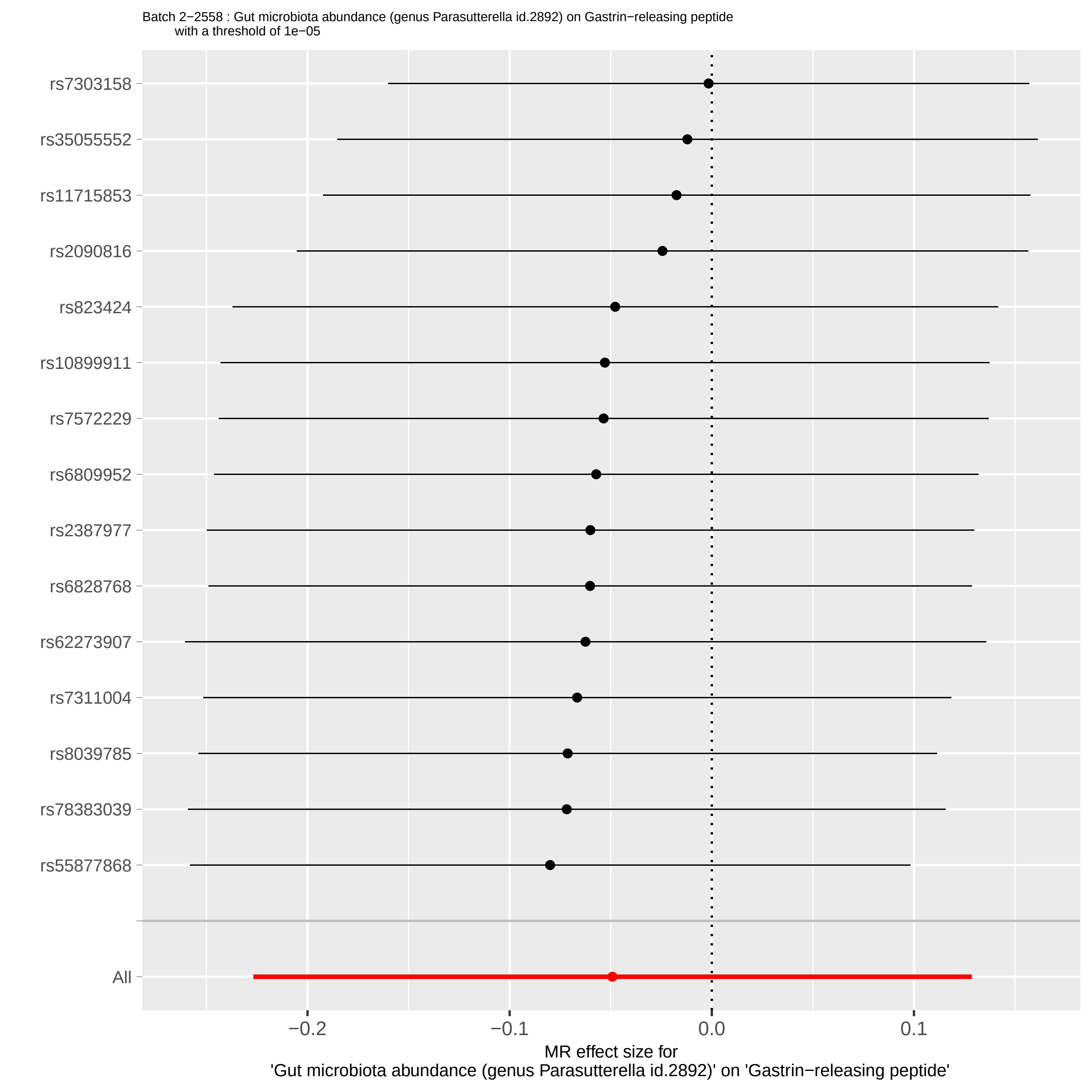
Batch 2−2558 : Gut microbiota abundance (genus Parasutterella id.2892) on Gastrin−releasing peptide
with a threshold of 1e−05
rs7303158
rs35055552
rs11715853
rs2090816
rs823424
rs10899911
rs7572229
rs6809952
rs2387977
rs6828768
rs62273907
rs7311004
rs8039785
rs78383039
rs55877868
All
−0.2
−0.1
0.0
0.1
MR effect size for
'Gut microbiota abundance (genus Parasutterella id.2892)' on 'Gastrin−releasing peptide'

Batch 2−2559 : Gut microbiota abundance (genus Catenibacterium id.2153) on Gastrin−releasing peptide
with a threshold of 1e−05

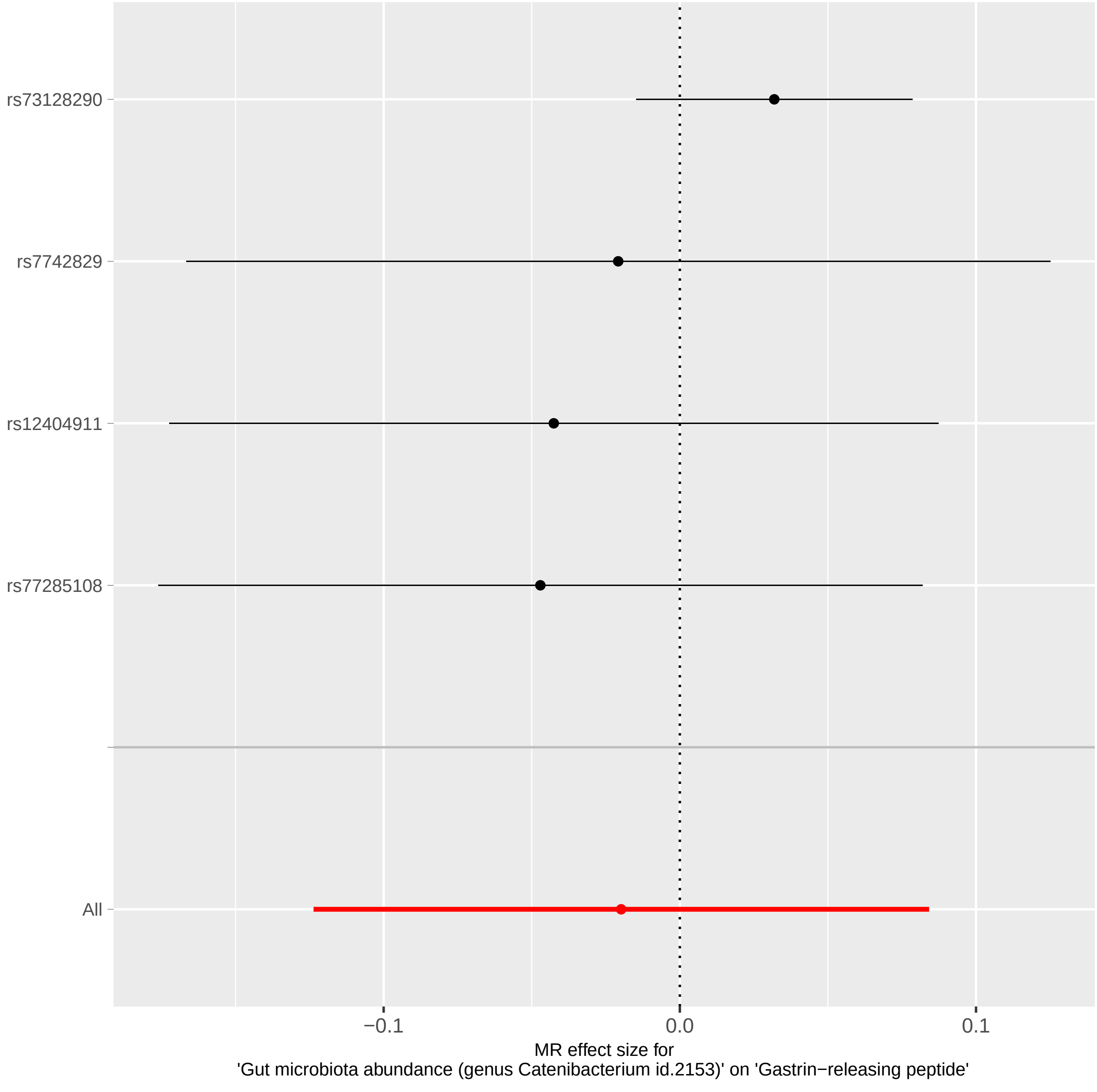

Batch 2−2560 : Gut microbiota abundance (genus Parasutterella id.2892) on Gastrin−releasing peptide
with a threshold of 1e−05

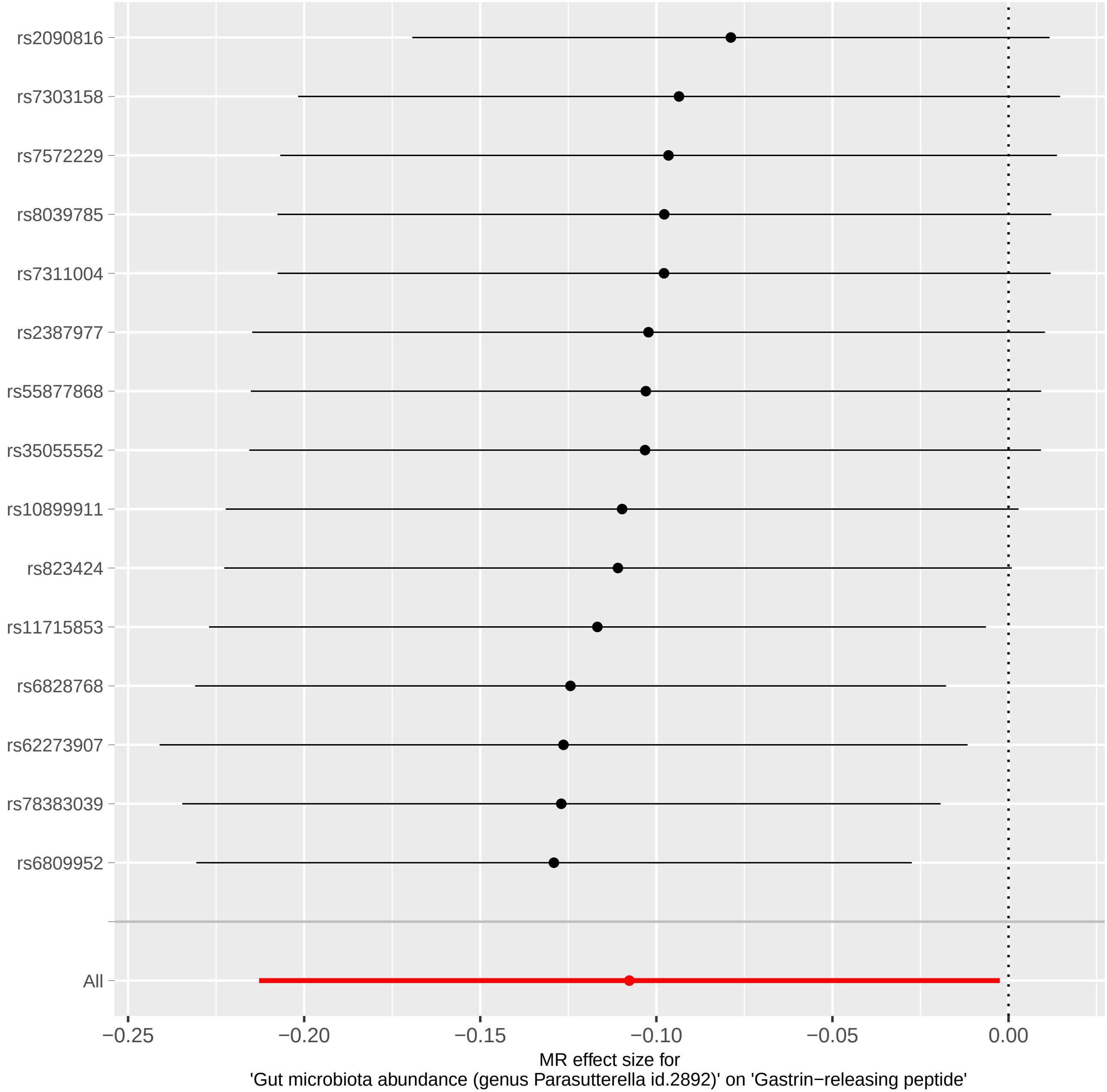

Batch 2−2562 : Gut microbiota abundance (genus Parasutterella id.2892) on GrpE protein homolog 1, mitochondrial
with a threshold of 1e−05

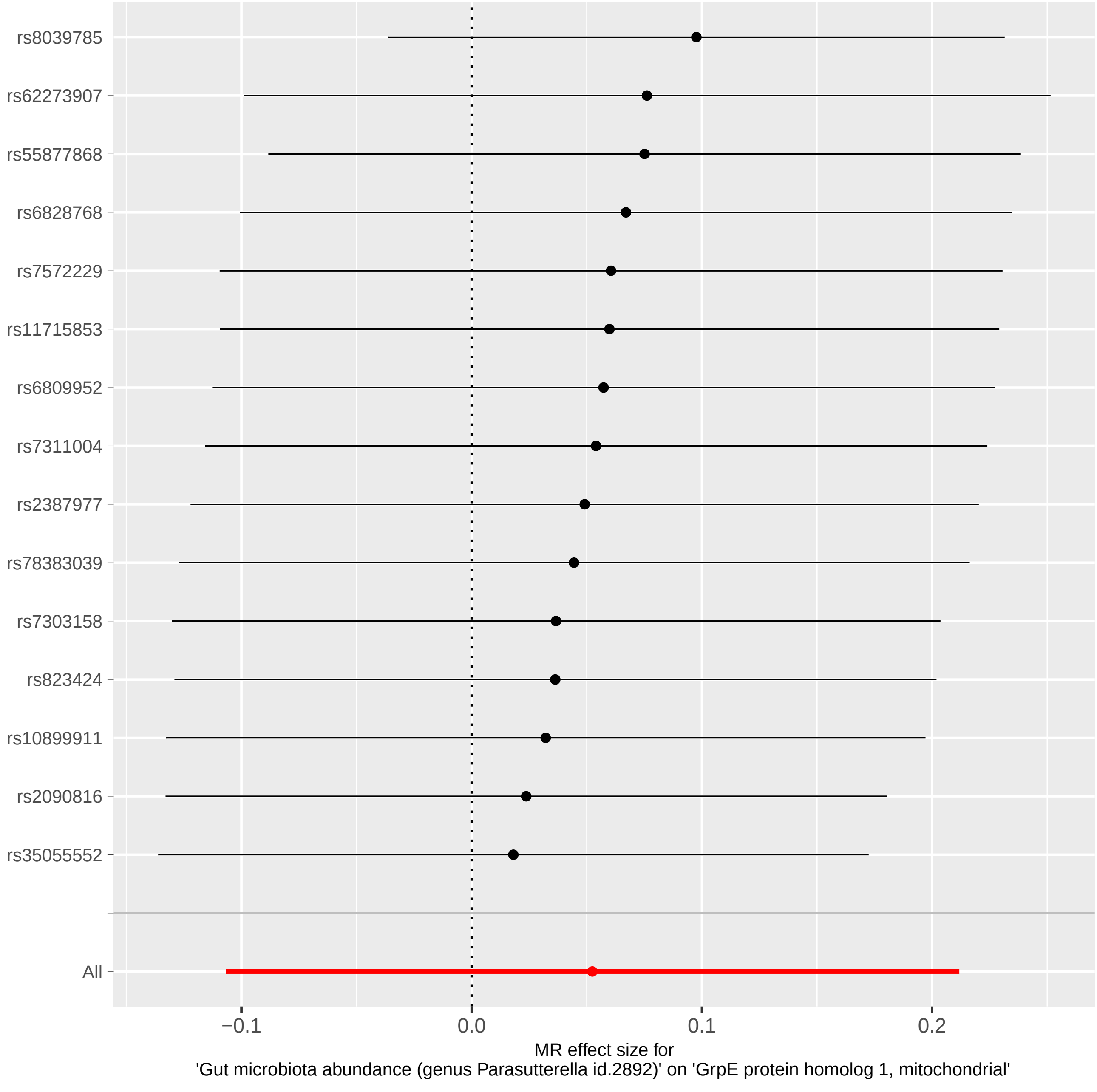

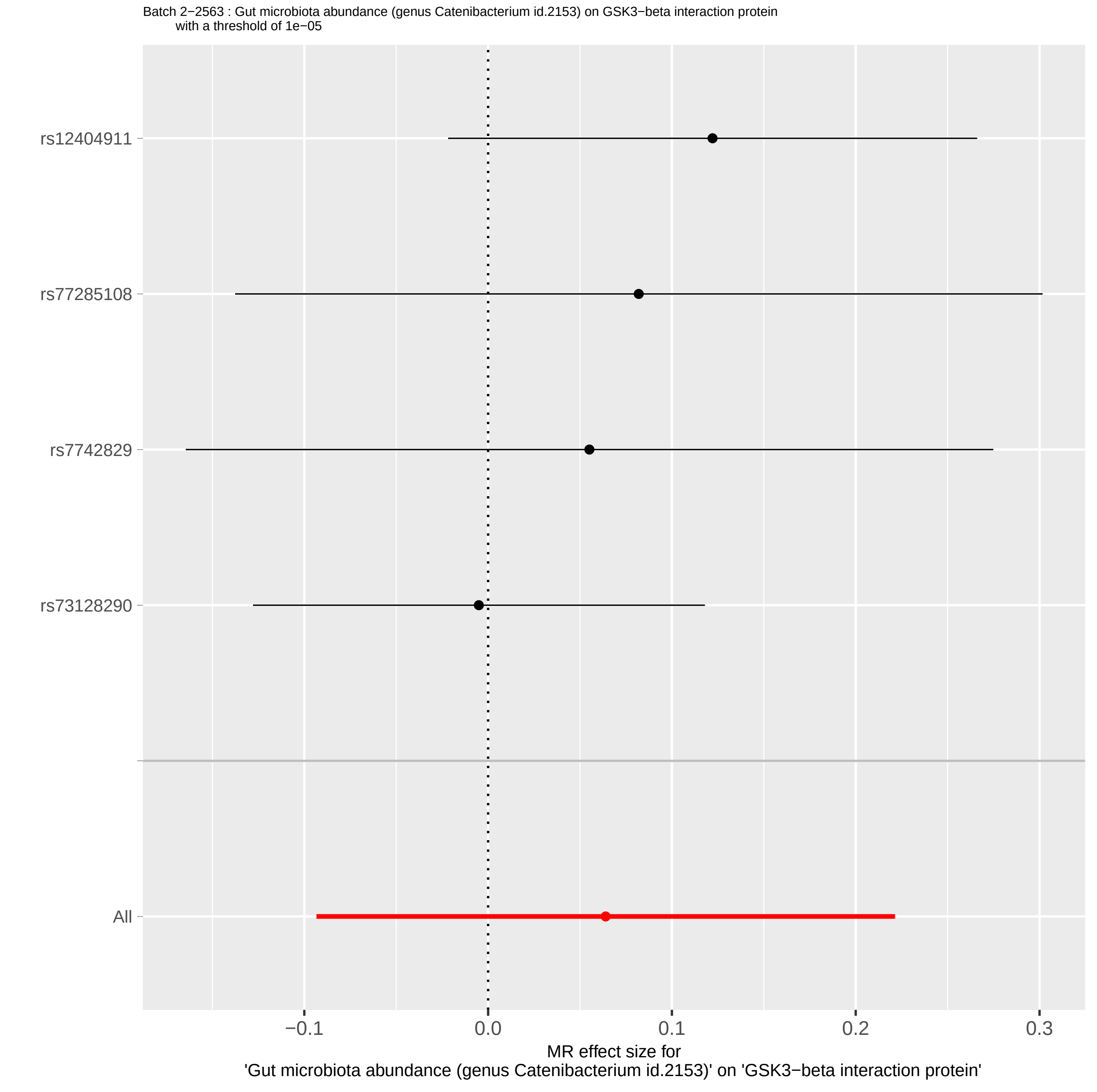

Batch 2−2563 : Gut microbiota abundance (genus Catenibacterium id.2153) on GSK3−beta interaction protein
with a threshold of 1e−05
rs12404911
rs77285108
rs7742829
rs73128290
All
−0.1
0.0
0.1
0.2
0.3
MR effect size for
'Gut microbiota abundance (genus Catenibacterium id.2153)' on 'GSK3−beta interaction protein'

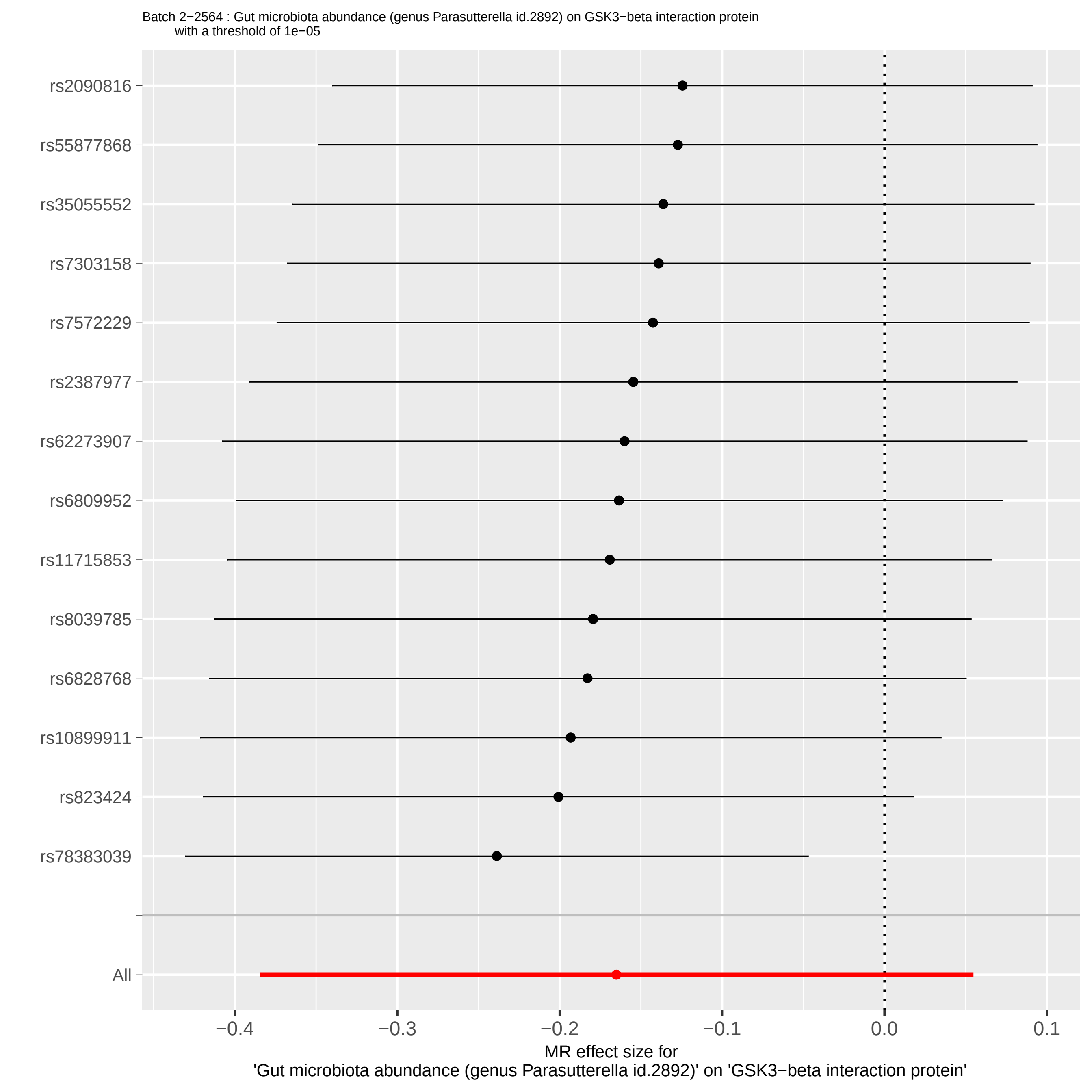
Batch 2−2564 : Gut microbiota abundance (genus Parasutterella id.2892) on GSK3−beta interaction protein
with a threshold of 1e−05
rs2090816
rs55877868
rs35055552
rs7303158
rs7572229
rs2387977
rs62273907
rs6809952
rs11715853
rs8039785
rs6828768
rs10899911
rs823424
rs78383039
All
−0.4
−0.3
−0.2
−0.1
0.0
0.1
MR effect size for
'Gut microbiota abundance (genus Parasutterella id.2892)' on 'GSK3−beta interaction protein'

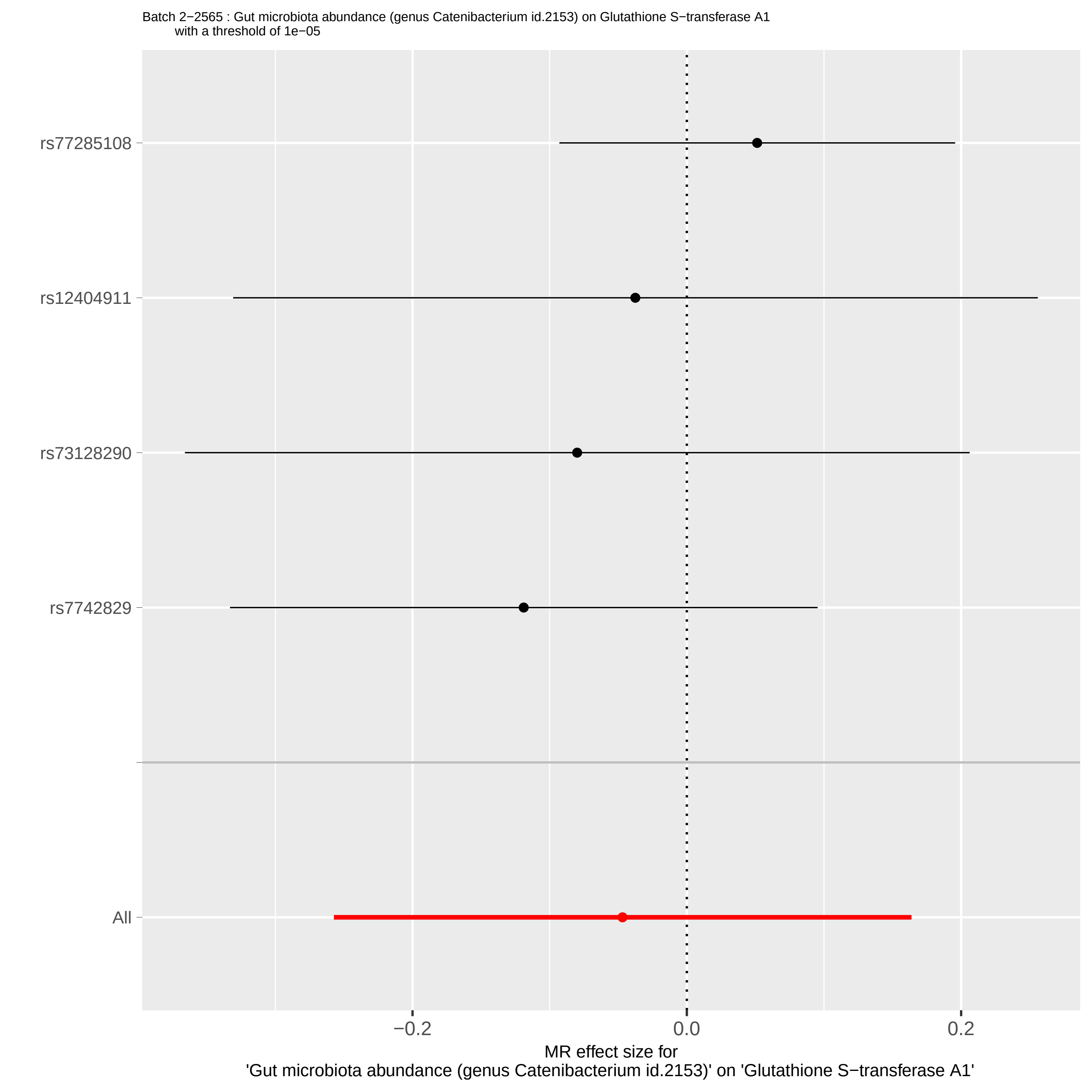
Batch 2−2565 : Gut microbiota abundance (genus Catenibacterium id.2153) on Glutathione S−transferase A1
with a threshold of 1e−05
rs77285108
rs12404911
rs73128290
rs7742829
All
−0.2
0.0
0.2
MR effect size for
'Gut microbiota abundance (genus Catenibacterium id.2153)' on 'Glutathione S−transferase A1'

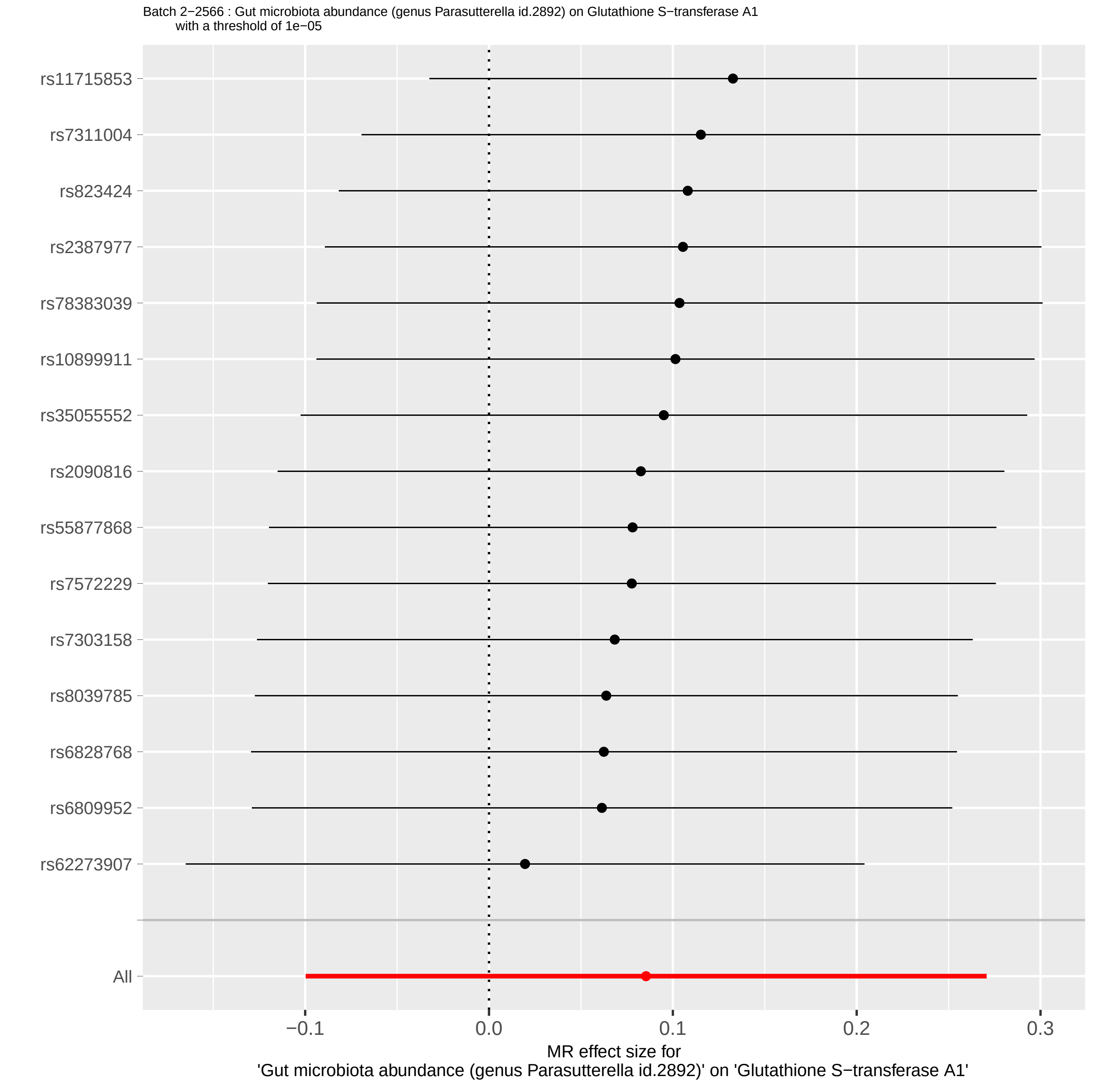

Batch 2−2566 : Gut microbiota abundance (genus Parasutterella id.2892) on Glutathione S−transferase A1
with a threshold of 1e−05
rs11715853
rs7311004
rs823424
rs2387977
rs78383039
rs10899911
rs35055552
rs2090816
rs55877868
rs7572229
rs7303158
rs8039785
rs6828768
rs6809952
rs62273907
All
−0.1
0.0
0.1
0.2
0.3
MR effect size for
'Gut microbiota abundance (genus Parasutterella id.2892)' on 'Glutathione S−transferase A1'

Batch 2−2567 : Gut microbiota abundance (genus Catenibacterium id.2153) on Glutathione S−transferase A3
with a threshold of 1e−05

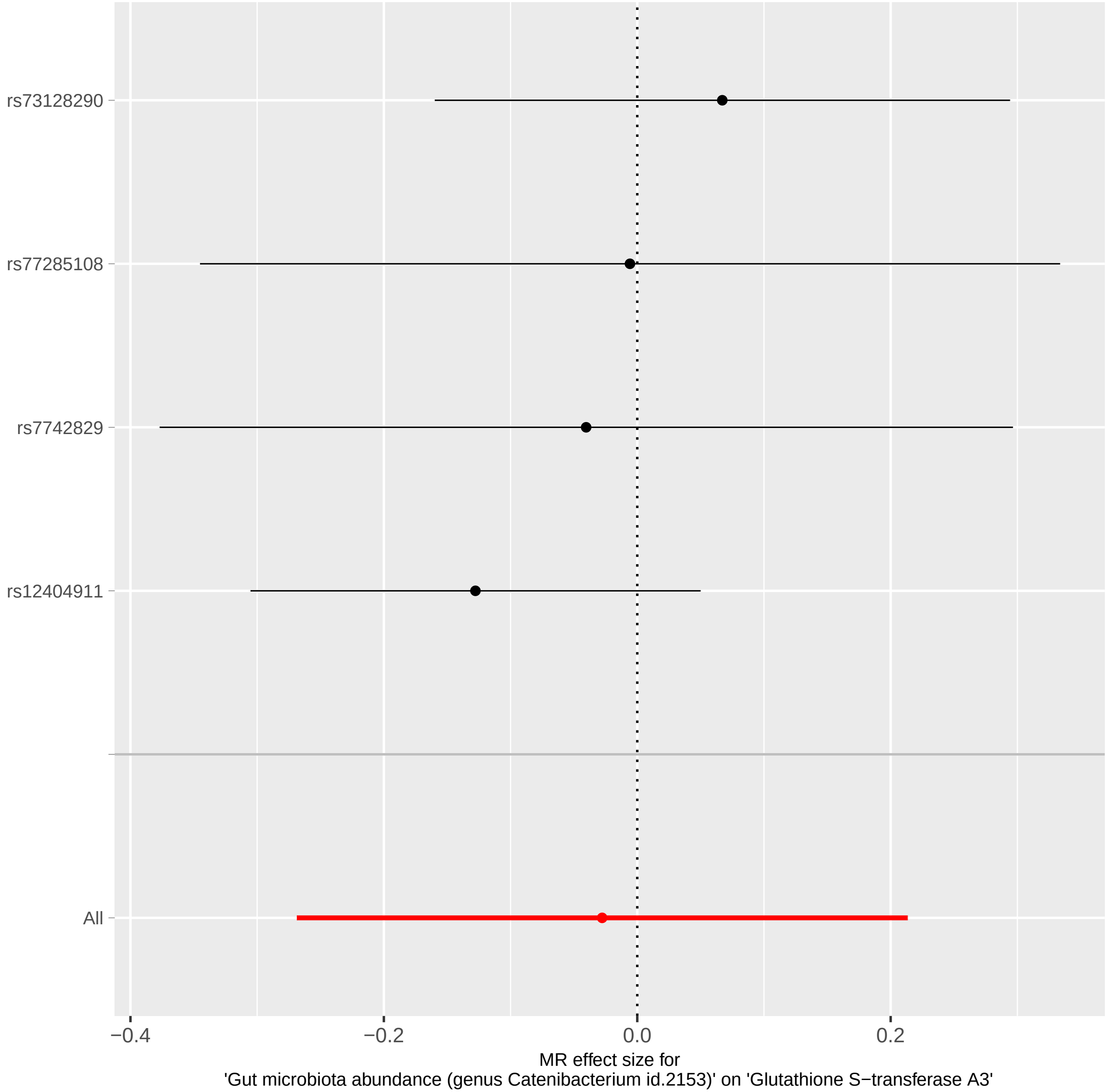

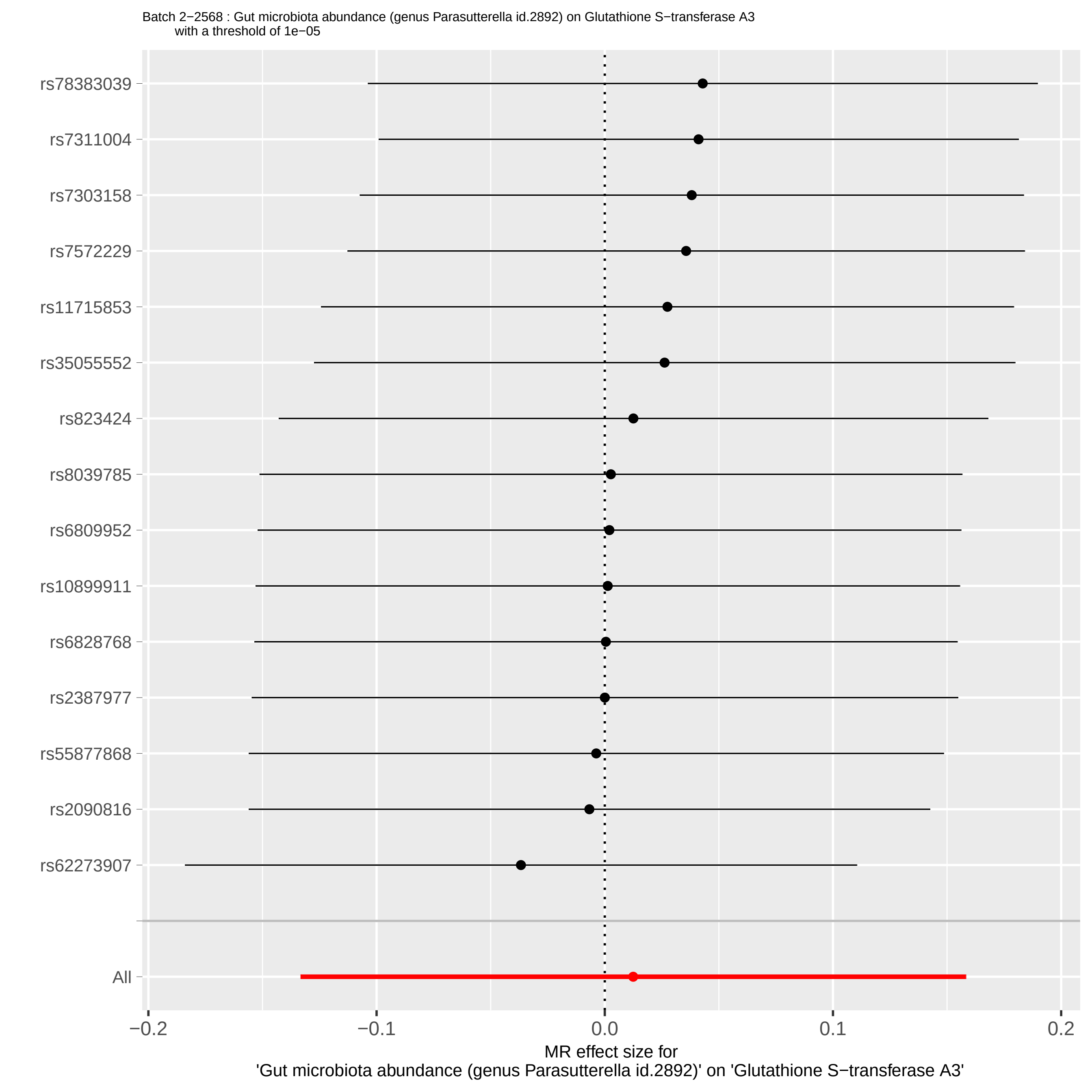
Batch 2−2568 : Gut microbiota abundance (genus Parasutterella id.2892) on Glutathione S−transferase A3
with a threshold of 1e−05
rs78383039
rs7311004
rs7303158
rs7572229
rs11715853
rs35055552
rs823424
rs8039785
rs6809952
rs10899911
rs6828768
rs2387977
rs55877868
rs2090816
rs62273907
All
−0.2
−0.1
0.0
0.1
0.2
MR effect size for
'Gut microbiota abundance (genus Parasutterella id.2892)' on 'Glutathione S−transferase A3'

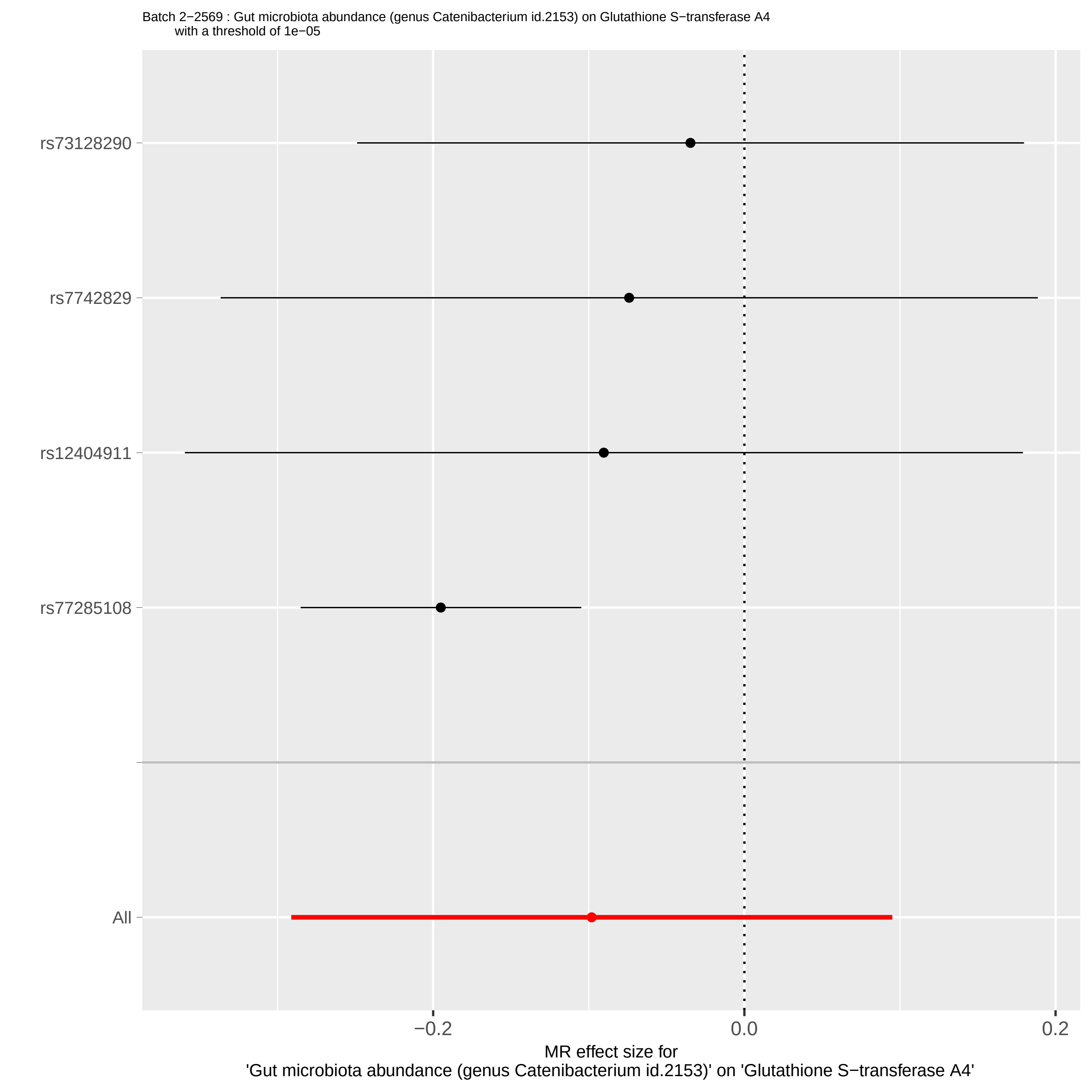
Batch 2−2569 : Gut microbiota abundance (genus Catenibacterium id.2153) on Glutathione S−transferase A4
with a threshold of 1e−05
rs73128290
rs7742829
rs12404911
rs77285108
All
−0.2
0.0
0.2
MR effect size for
'Gut microbiota abundance (genus Catenibacterium id.2153)' on 'Glutathione S−transferase A4'

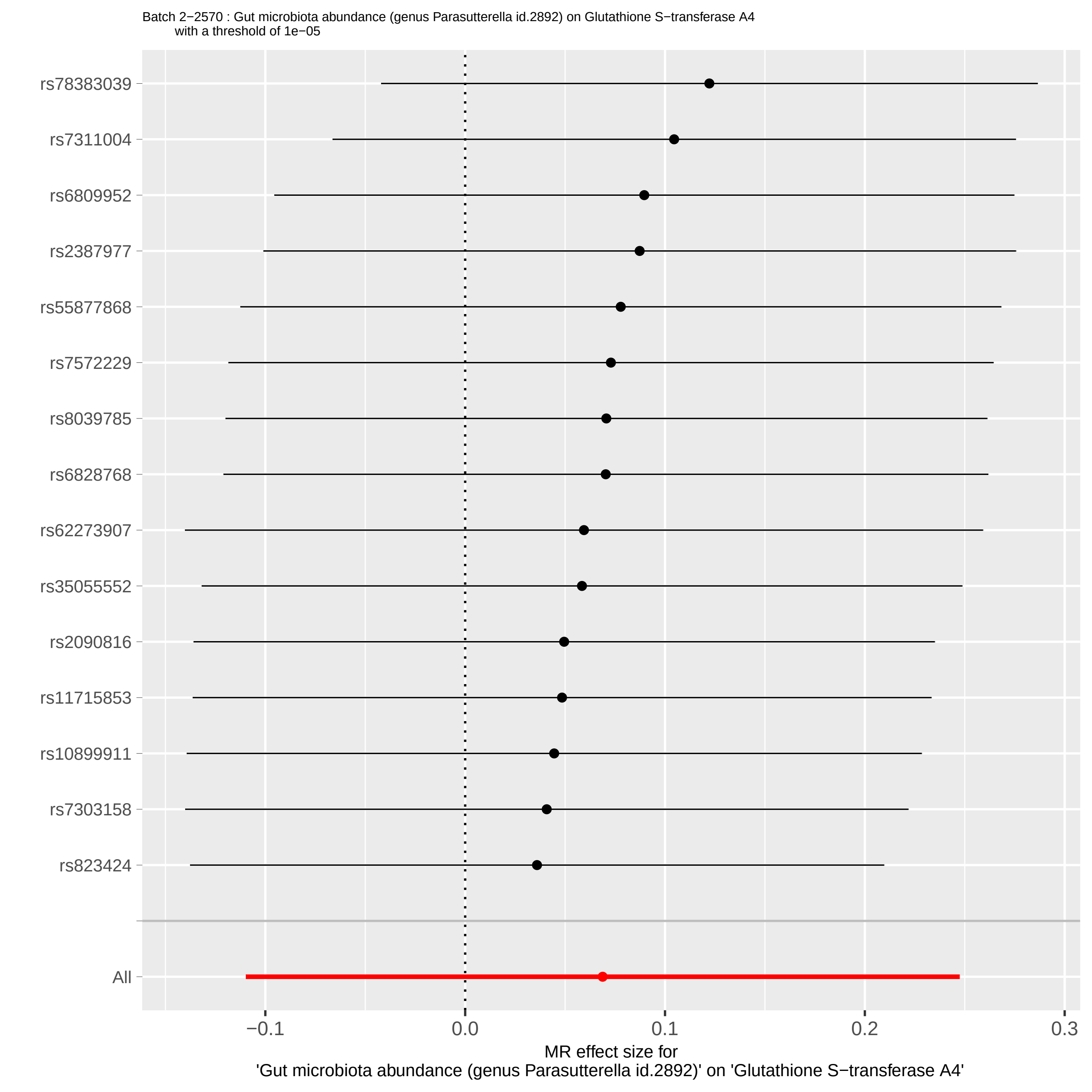
Batch 2−2570 : Gut microbiota abundance (genus Parasutterella id.2892) on Glutathione S−transferase A4
with a threshold of 1e−05
rs78383039
rs7311004
rs6809952
rs2387977
rs55877868
rs7572229
rs8039785
rs6828768
rs62273907
rs35055552
rs2090816
rs11715853
rs10899911
rs7303158
rs823424
All
−0.1
0.0
0.1
0.2
0.3
MR effect size for
'Gut microbiota abundance (genus Parasutterella id.2892)' on 'Glutathione S−transferase A4'

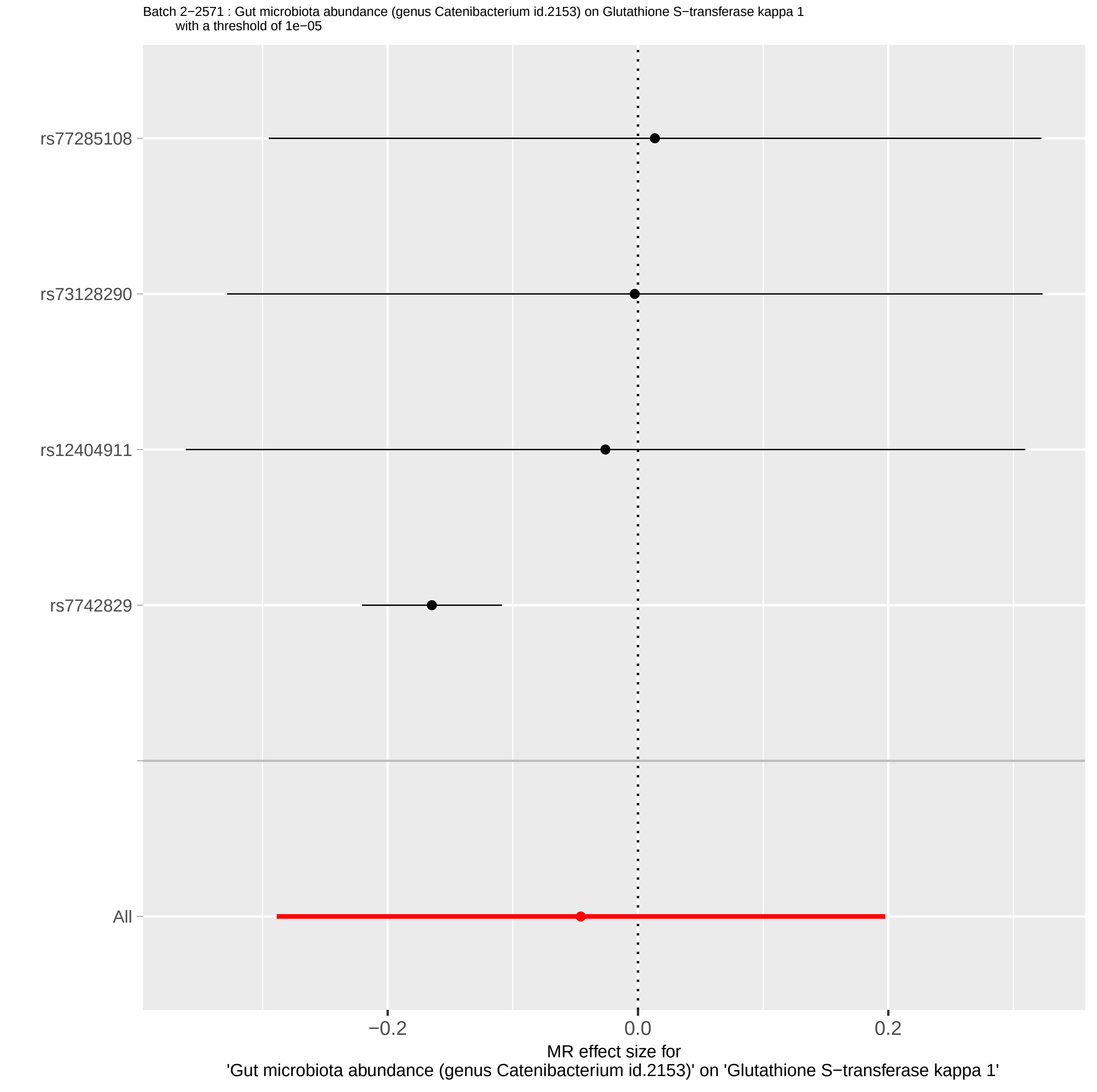

Batch 2−2571 : Gut microbiota abundance (genus Catenibacterium id.2153) on Glutathione S−transferase kappa 1
with a threshold of 1e−05
rs77285108
rs73128290
rs12404911
rs7742829
All
−0.2
0.0
0.2
MR effect size for
'Gut microbiota abundance (genus Catenibacterium id.2153)' on 'Glutathione S−transferase kappa 1'

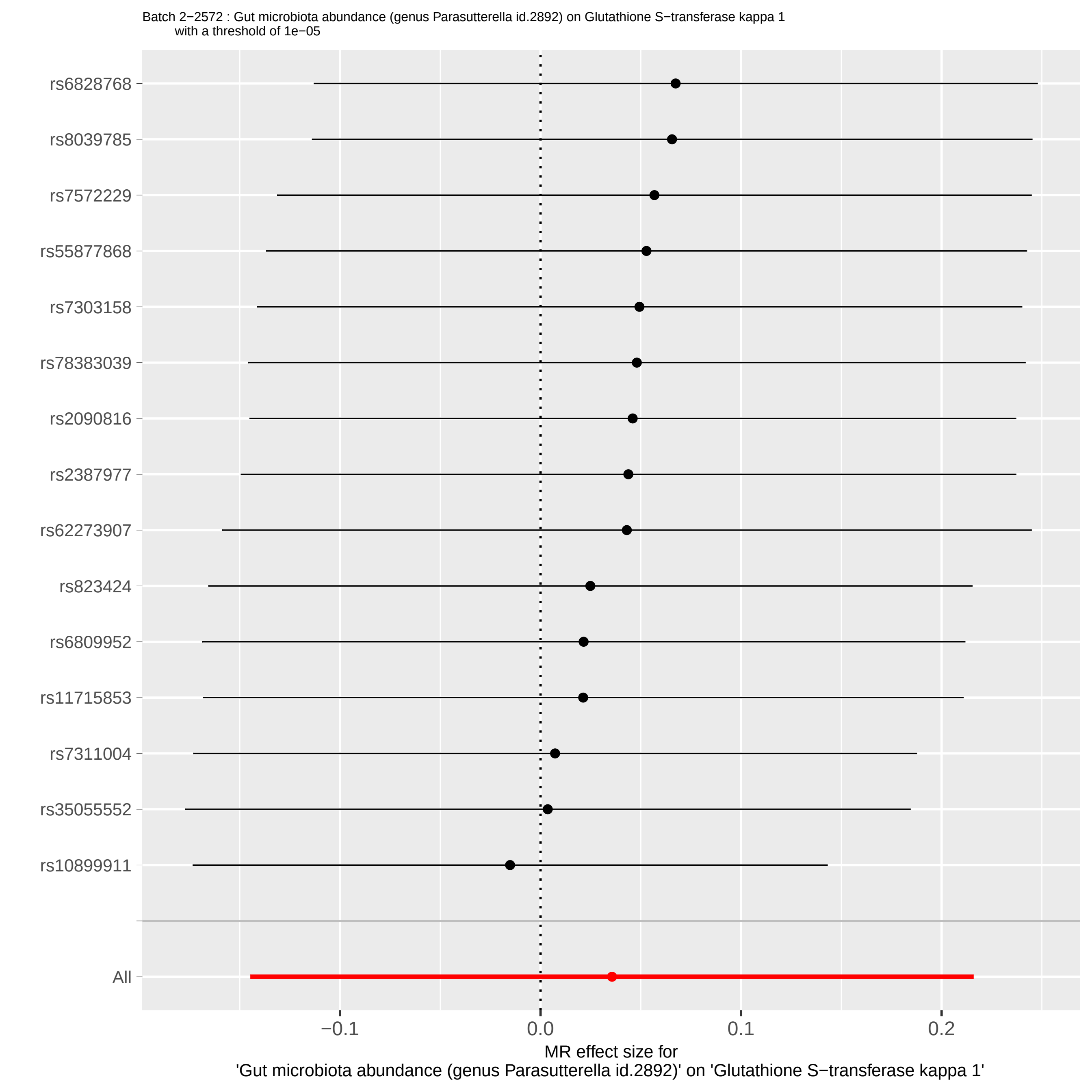
Batch 2−2572 : Gut microbiota abundance (genus Parasutterella id.2892) on Glutathione S−transferase kappa 1
with a threshold of 1e−05
rs6828768
rs8039785
rs7572229
rs55877868
rs7303158
rs78383039
rs2090816
rs2387977
rs62273907
rs823424
rs6809952
rs11715853
rs7311004
rs35055552
rs10899911
All
−0.1
0.0
0.1
0.2
MR effect size for
'Gut microbiota abundance (genus Parasutterella id.2892)' on 'Glutathione S−transferase kappa 1'

Batch 2−2573 : Gut microbiota abundance (genus Catenibacterium id.2153) on Glutathione S−transferase Mu 1
with a threshold of 1e−05

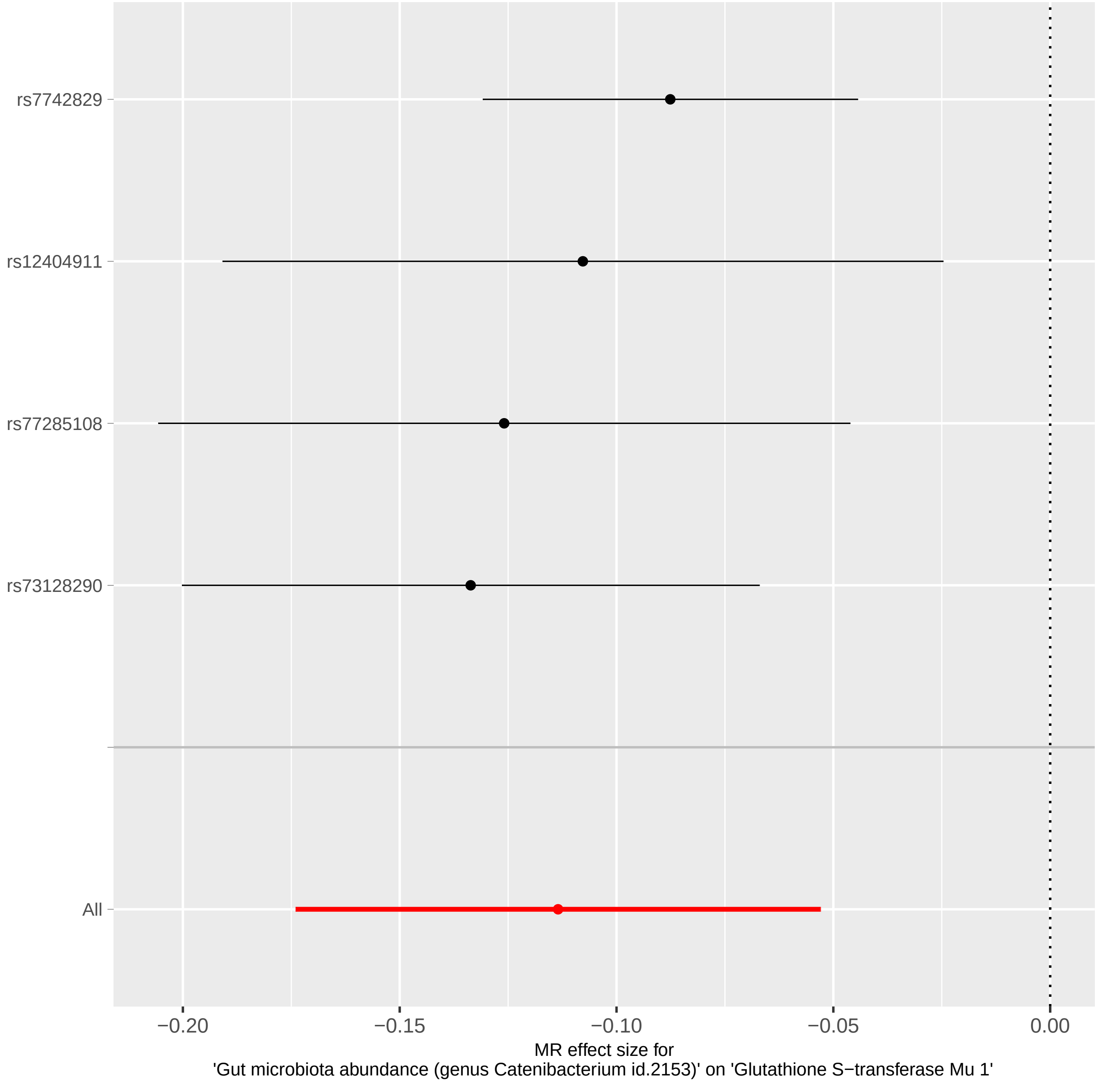

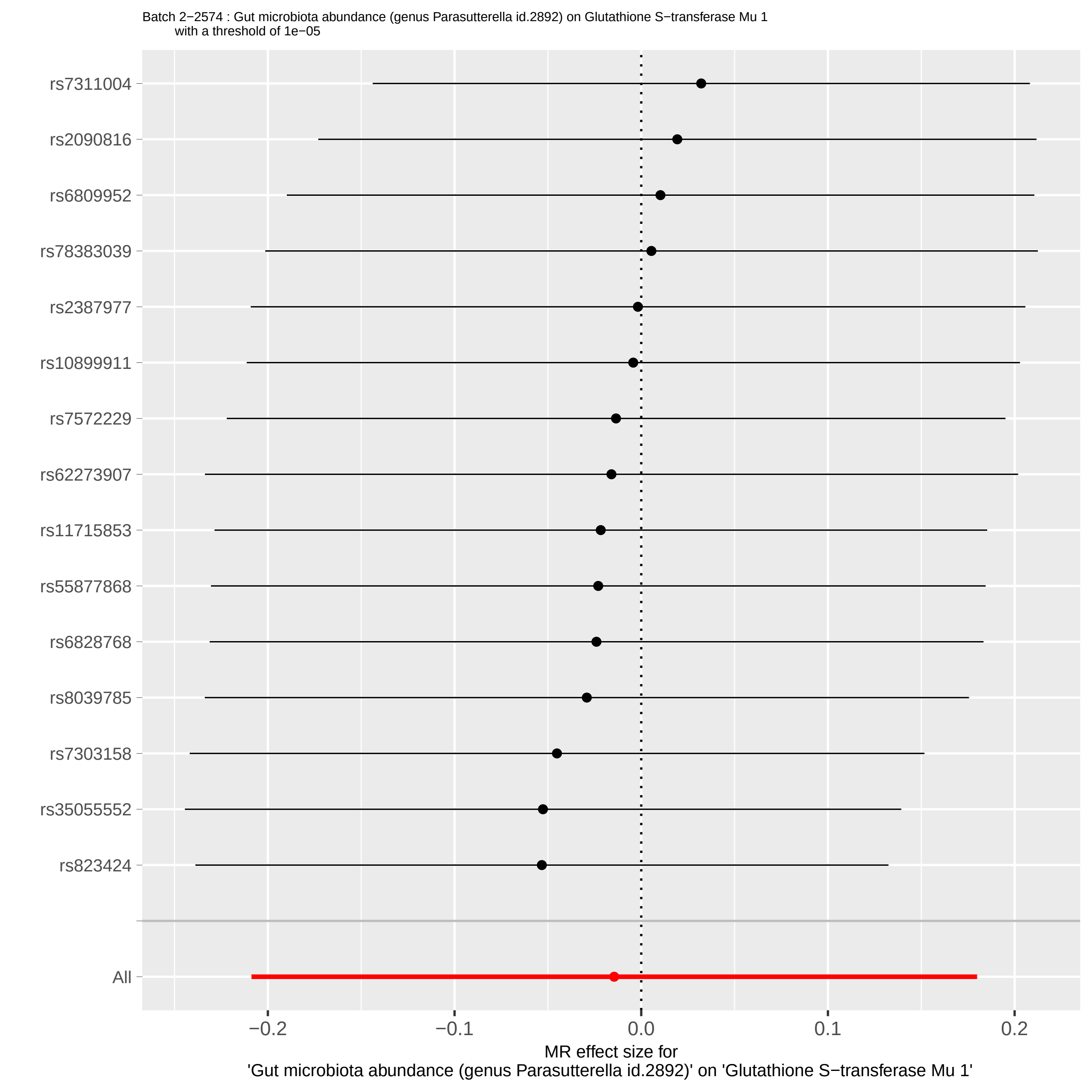

Batch 2−2574 : Gut microbiota abundance (genus Parasutterella id.2892) on Glutathione S−transferase Mu 1
with a threshold of 1e−05
rs7311004
rs2090816
rs6809952
rs78383039
rs2387977
rs10899911
rs7572229
rs62273907
rs11715853
rs55877868
rs6828768
rs8039785
rs7303158
rs35055552
rs823424
All
−0.2
−0.1
0.0
0.1
0.2
MR effect size for
'Gut microbiota abundance (genus Parasutterella id.2892)' on 'Glutathione S−transferase Mu 1'

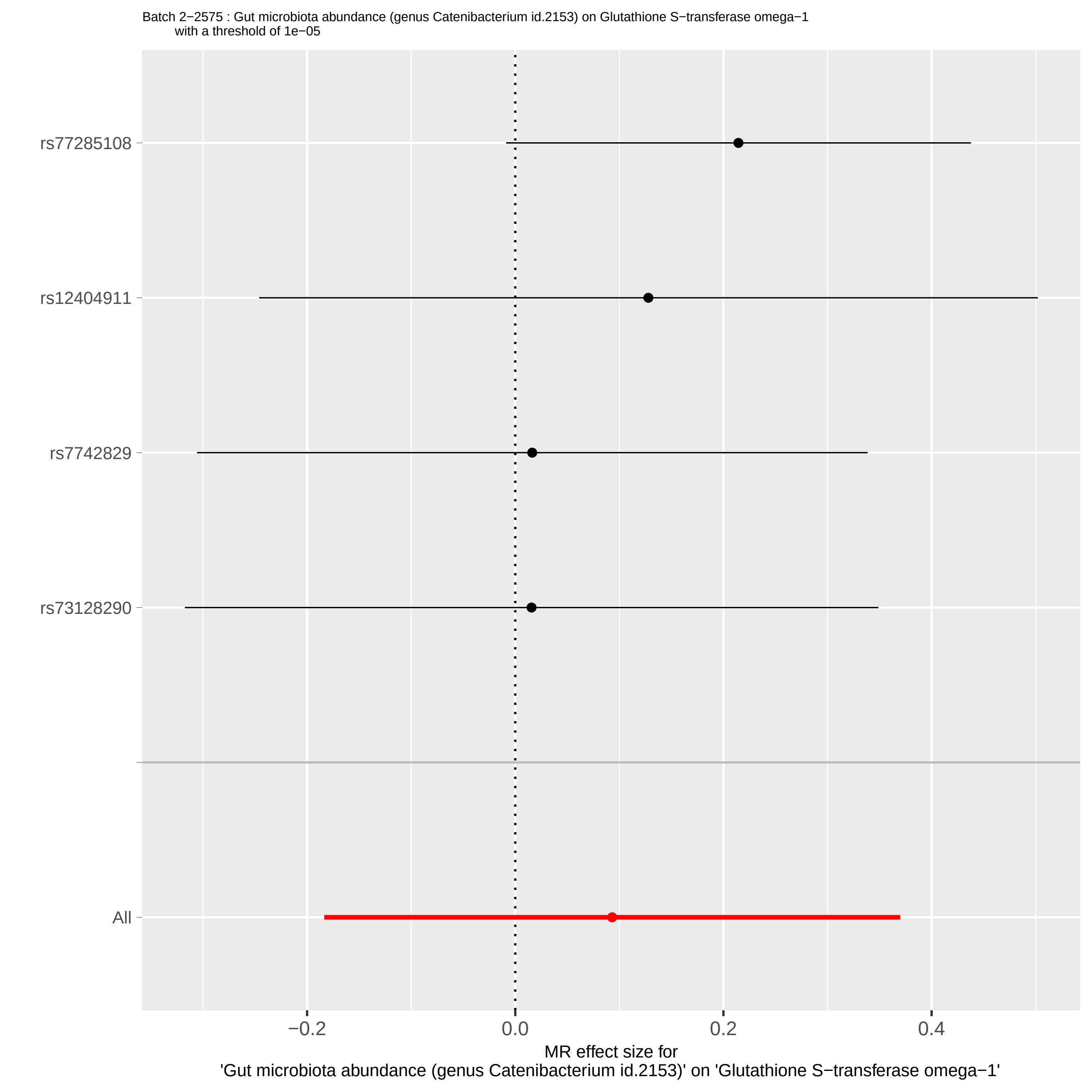

Batch 2−2575 : Gut microbiota abundance (genus Catenibacterium id.2153) on Glutathione S−transferase omega−1
with a threshold of 1e−05
rs77285108
rs12404911
rs7742829
rs73128290
All
−0.2
0.0
0.2
0.4
MR effect size for
'Gut microbiota abundance (genus Catenibacterium id.2153)' on 'Glutathione S−transferase omega−1'

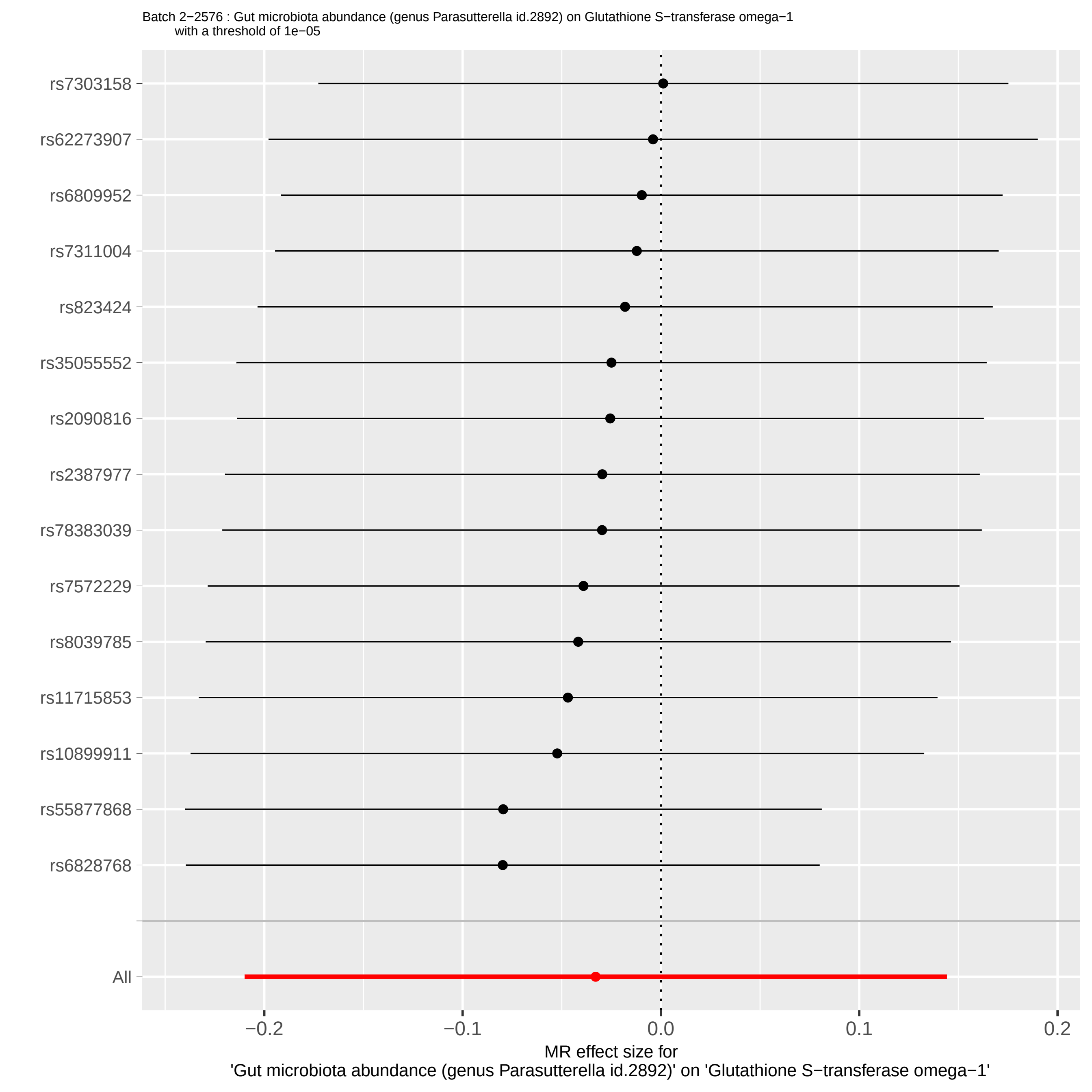

Batch 2−2576 : Gut microbiota abundance (genus Parasutterella id.2892) on Glutathione S−transferase omega−1
with a threshold of 1e−05
rs7303158
rs62273907
rs6809952
rs7311004
rs823424
rs35055552
rs2090816
rs2387977
rs78383039
rs7572229
rs8039785
rs11715853
rs10899911
rs55877868
rs6828768
All
−0.2
−0.1
0.0
0.1
0.2
MR effect size for
'Gut microbiota abundance (genus Parasutterella id.2892)' on 'Glutathione S−transferase omega−1'

Batch 2−2577 : Gut microbiota abundance (genus Catenibacterium id.2153) on Glutathione S−transferase P
with a threshold of 1e−05

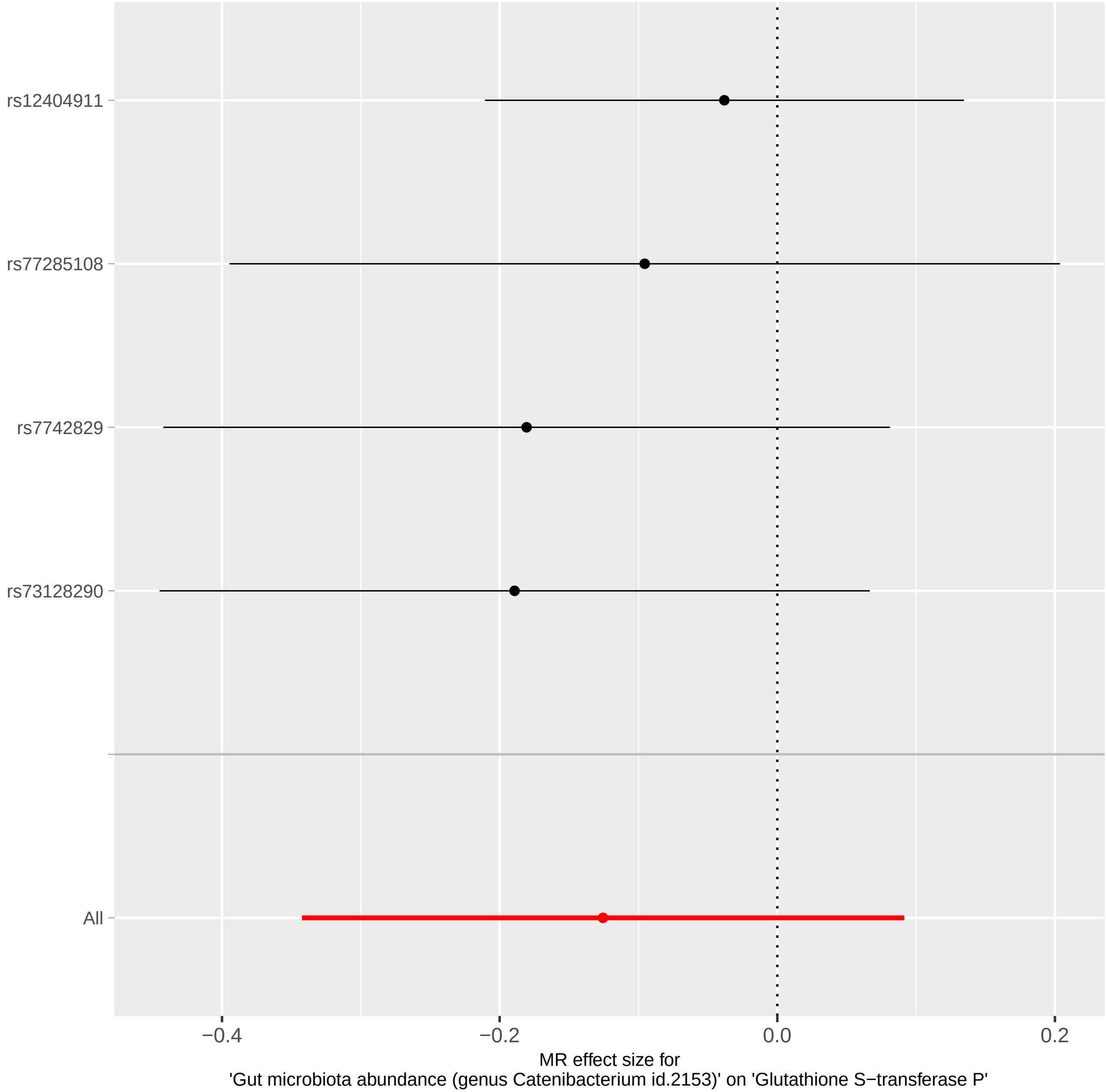

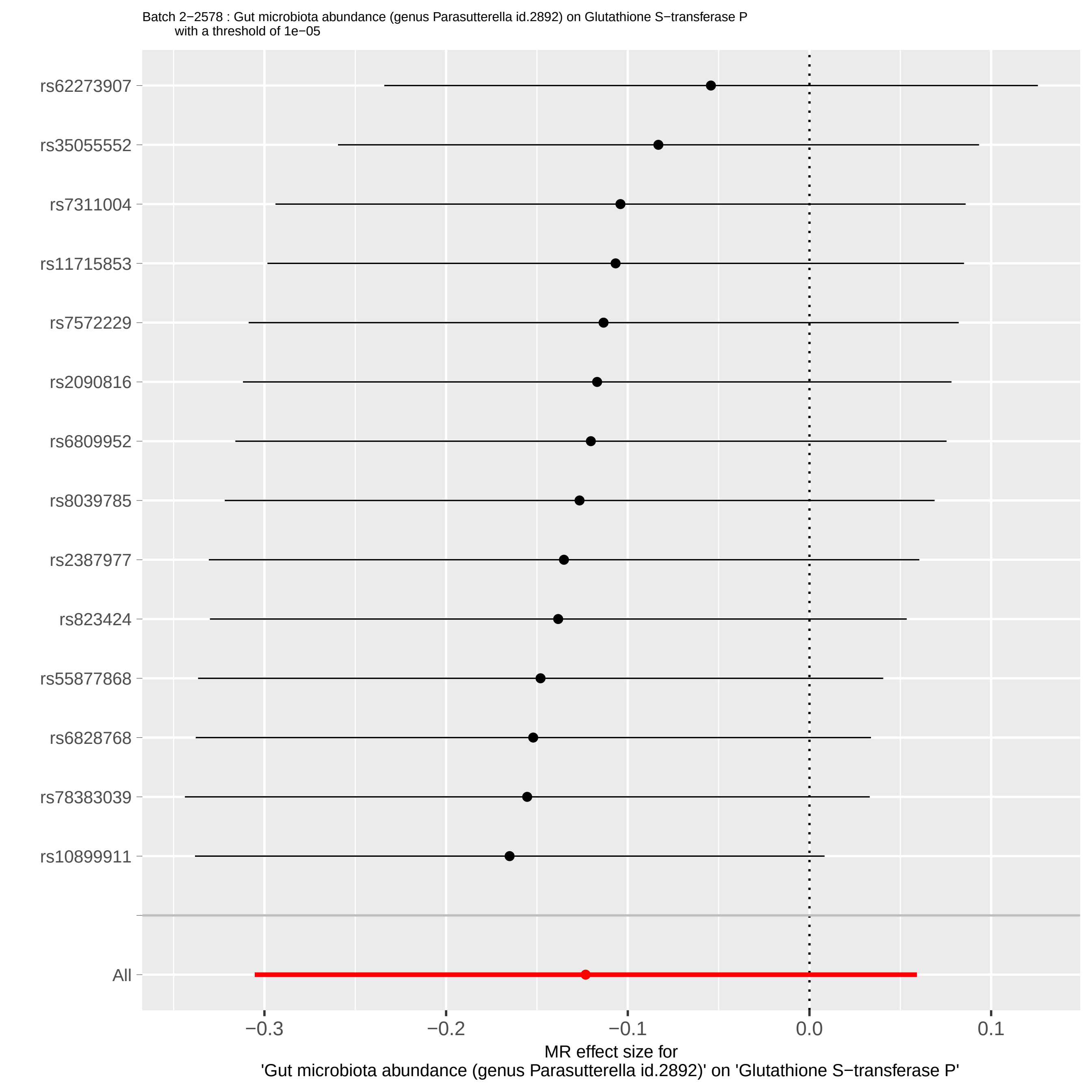

Batch 2−2578 : Gut microbiota abundance (genus Parasutterella id.2892) on Glutathione S−transferase P
with a threshold of 1e−05
rs62273907
rs35055552
rs7311004
rs11715853
rs7572229
rs2090816
rs6809952
rs8039785
rs2387977
rs823424
rs55877868
rs6828768
rs78383039
rs10899911
All
−0.3
−0.2
−0.1
0.0
0.1
MR effect size for
'Gut microbiota abundance (genus Parasutterella id.2892)' on 'Glutathione S−transferase P'

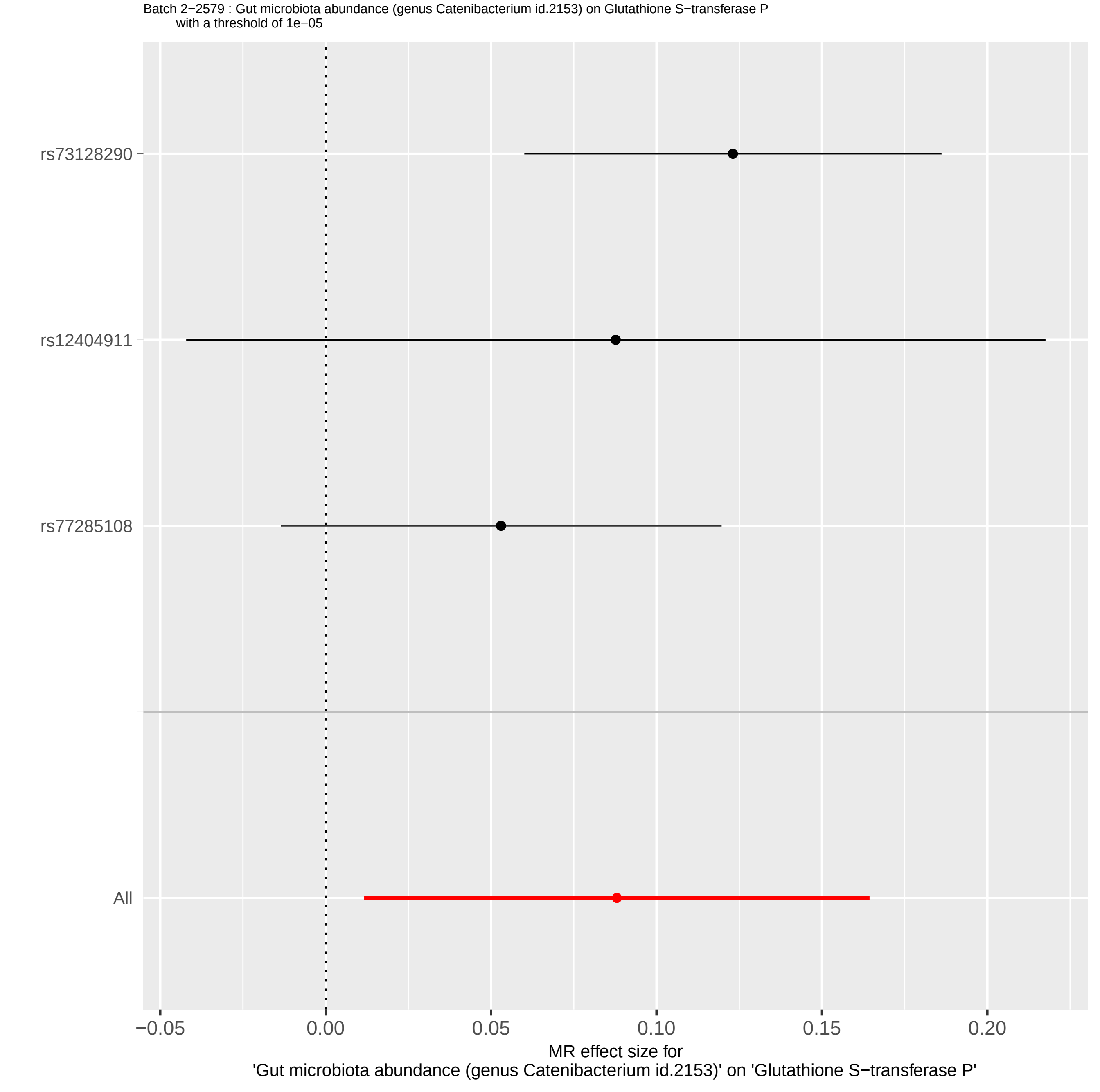
Batch 2−2579 : Gut microbiota abundance (genus Catenibacterium id.2153) on Glutathione S−transferase P
with a threshold of 1e−05
rs73128290
rs12404911
rs77285108
All
−0.05
0.00
0.05
0.10
0.15
0.20
MR effect size for
'Gut microbiota abundance (genus Catenibacterium id.2153)' on 'Glutathione S−transferase P'

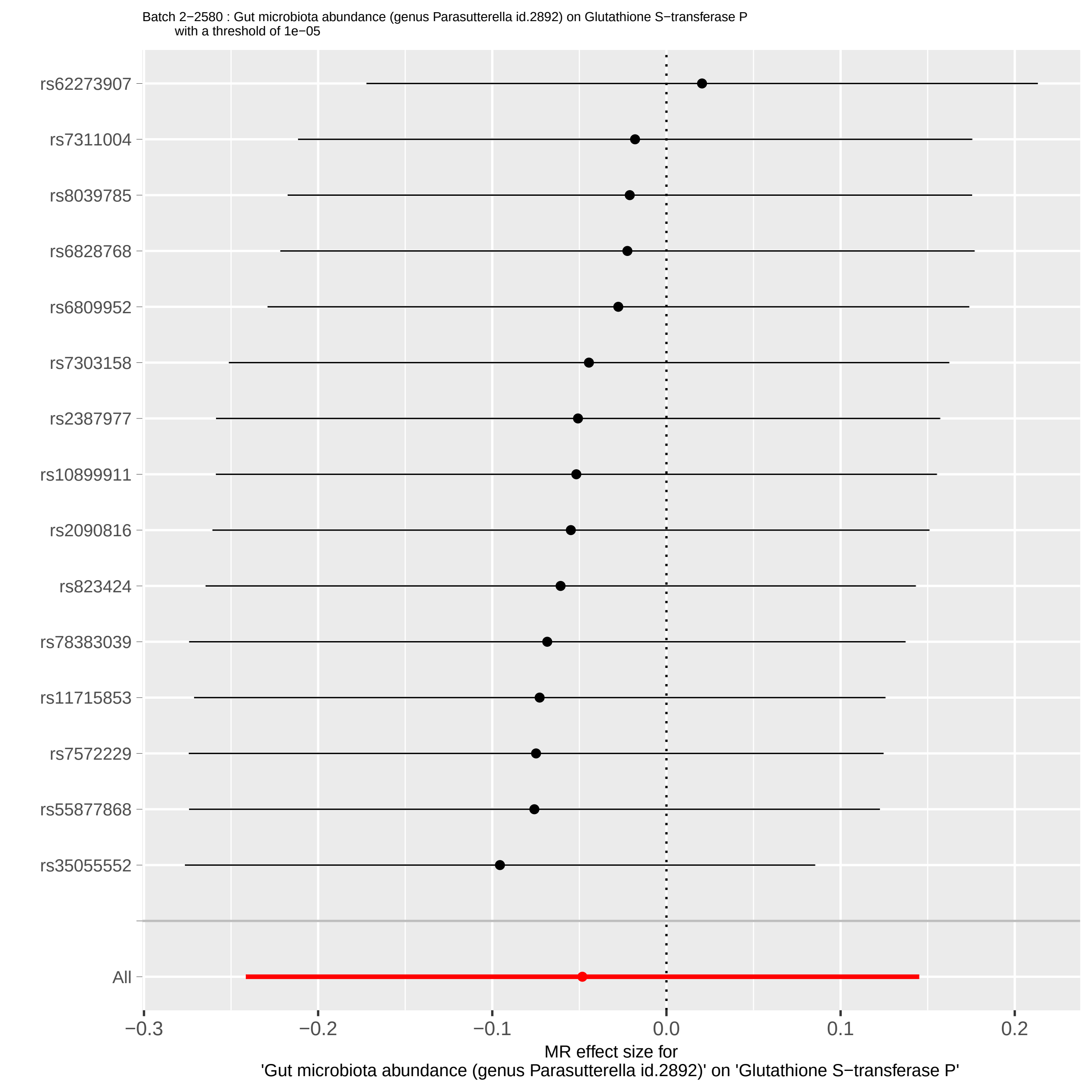

Batch 2−2580 : Gut microbiota abundance (genus Parasutterella id.2892) on Glutathione S−transferase P
with a threshold of 1e−05
rs62273907
rs7311004
rs8039785
rs6828768
rs6809952
rs7303158
rs2387977
rs10899911
rs2090816
rs823424
rs78383039
rs11715853
rs7572229
rs55877868
rs35055552
All
−0.3
−0.2
−0.1
0.0
0.1
0.2
MR effect size for
'Gut microbiota abundance (genus Parasutterella id.2892)' on 'Glutathione S−transferase P'

Batch 2−2581 : Gut microbiota abundance (genus Catenibacterium id.2153) on Glutathione S−transferase theta−2B
with a threshold of 1e−05

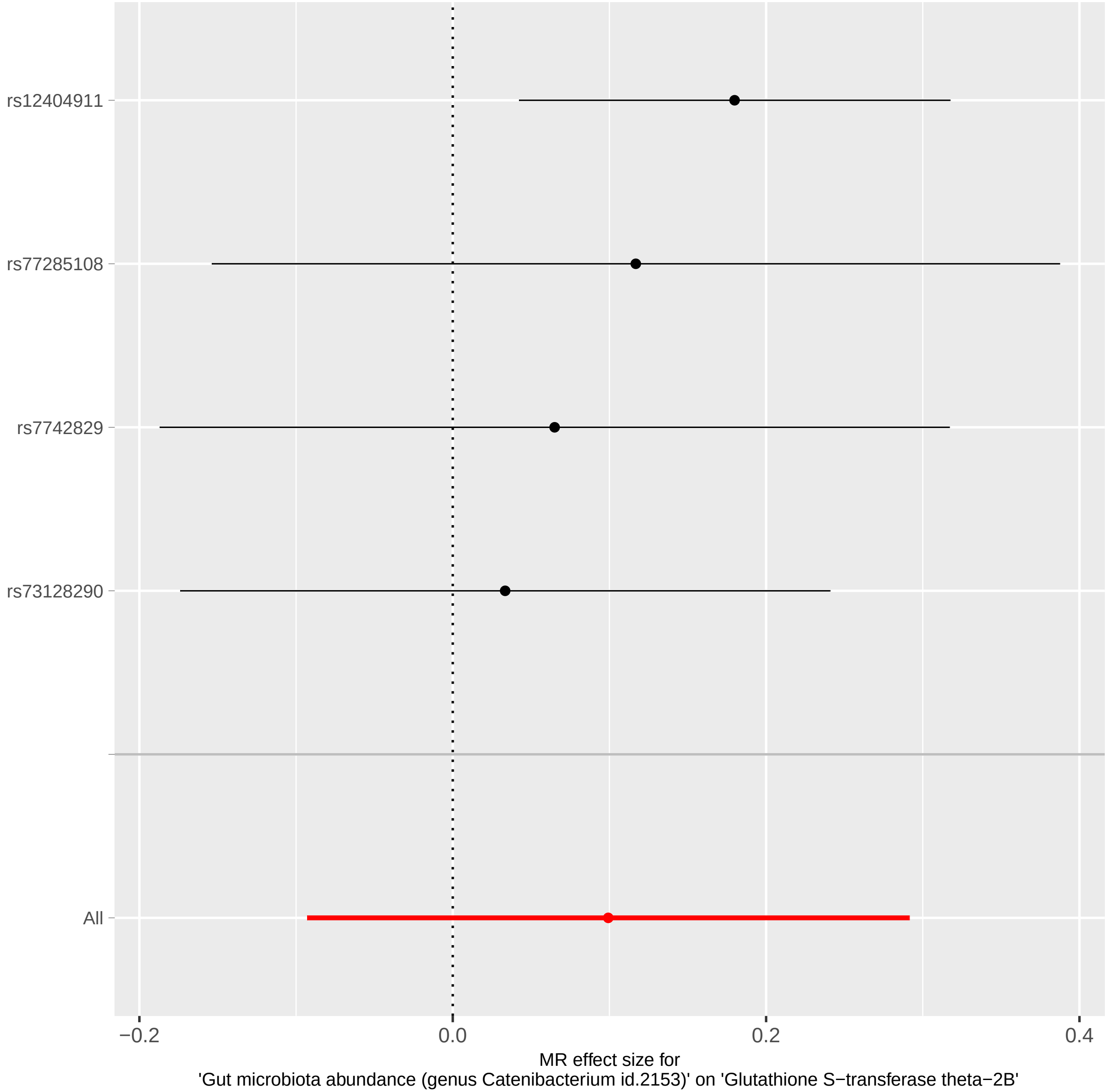

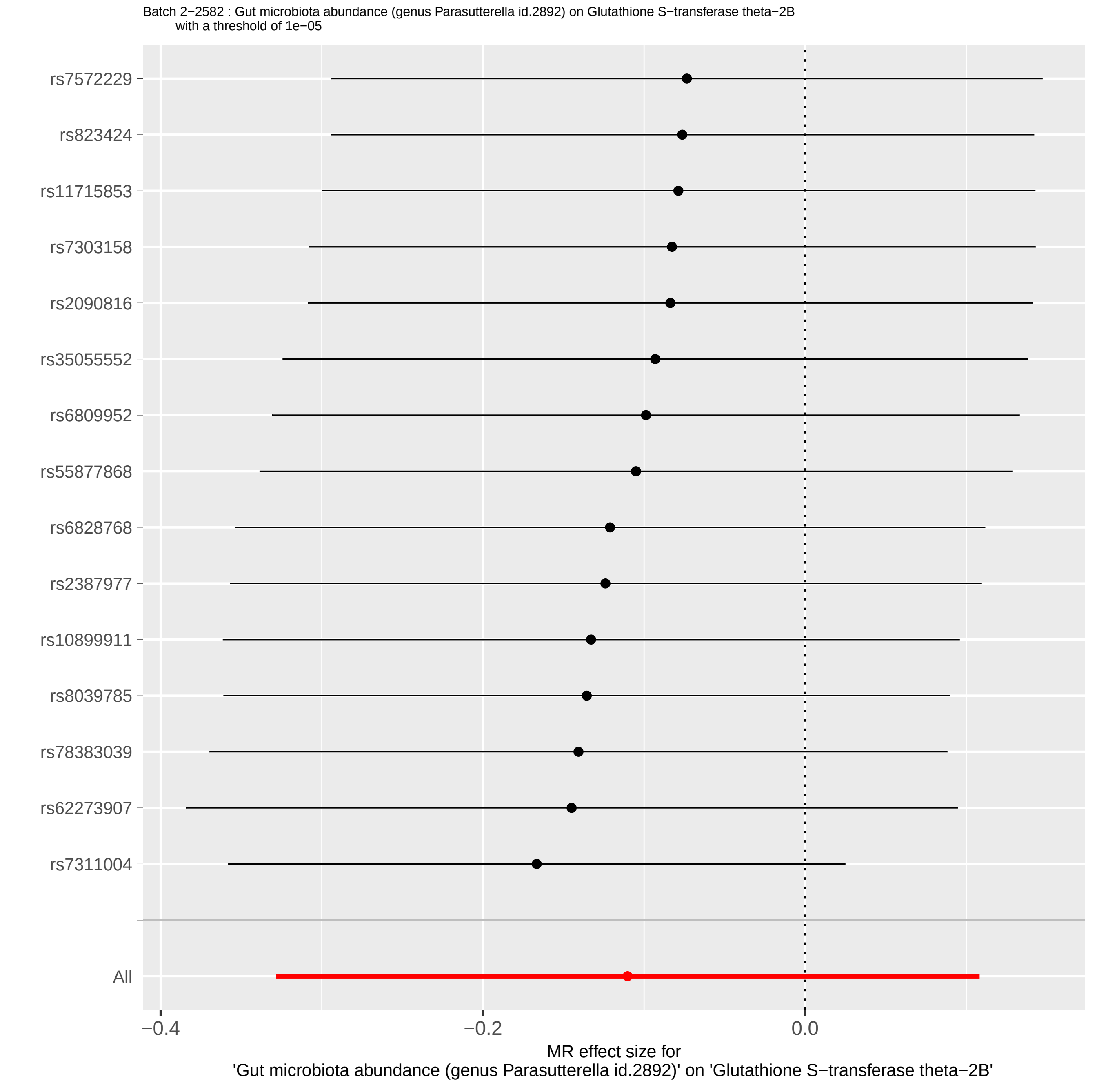

Batch 2−2582 : Gut microbiota abundance (genus Parasutterella id.2892) on Glutathione S−transferase theta−2B
with a threshold of 1e−05
rs7572229
rs823424
rs11715853
rs7303158
rs2090816
rs35055552
rs6809952
rs55877868
rs6828768
rs2387977
rs10899911
rs8039785
rs78383039
rs62273907
rs7311004
All
−0.4
−0.2
0.0
MR effect size for
'Gut microbiota abundance (genus Parasutterella id.2892)' on 'Glutathione S−transferase theta−2B'

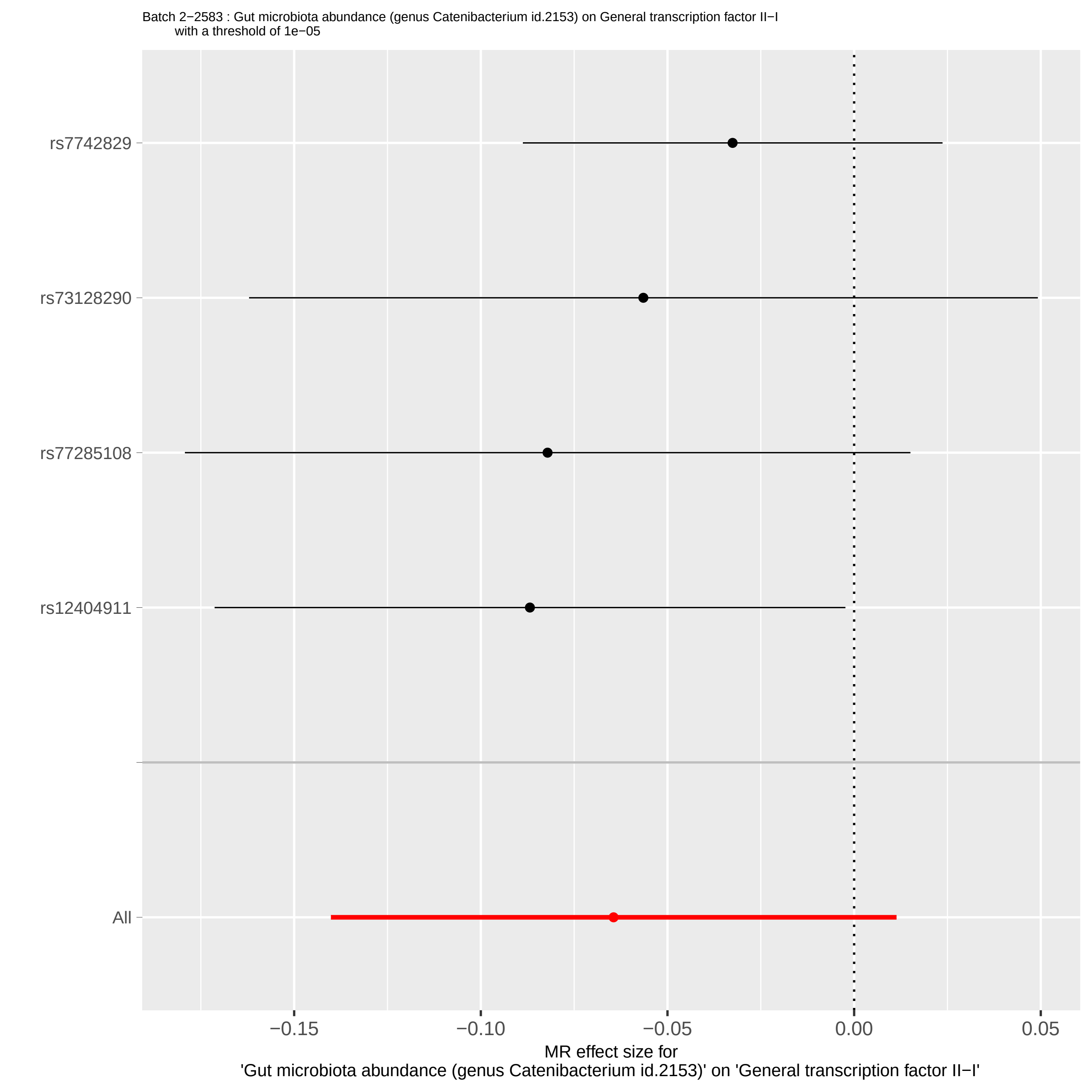
Batch 2−2583 : Gut microbiota abundance (genus Catenibacterium id.2153) on General transcription factor II−I
with a threshold of 1e−05
rs7742829
rs73128290
rs77285108
rs12404911
All
−0.15
−0.10
−0.05
0.00
0.05
MR effect size for
'Gut microbiota abundance (genus Catenibacterium id.2153)' on 'General transcription factor II−I'

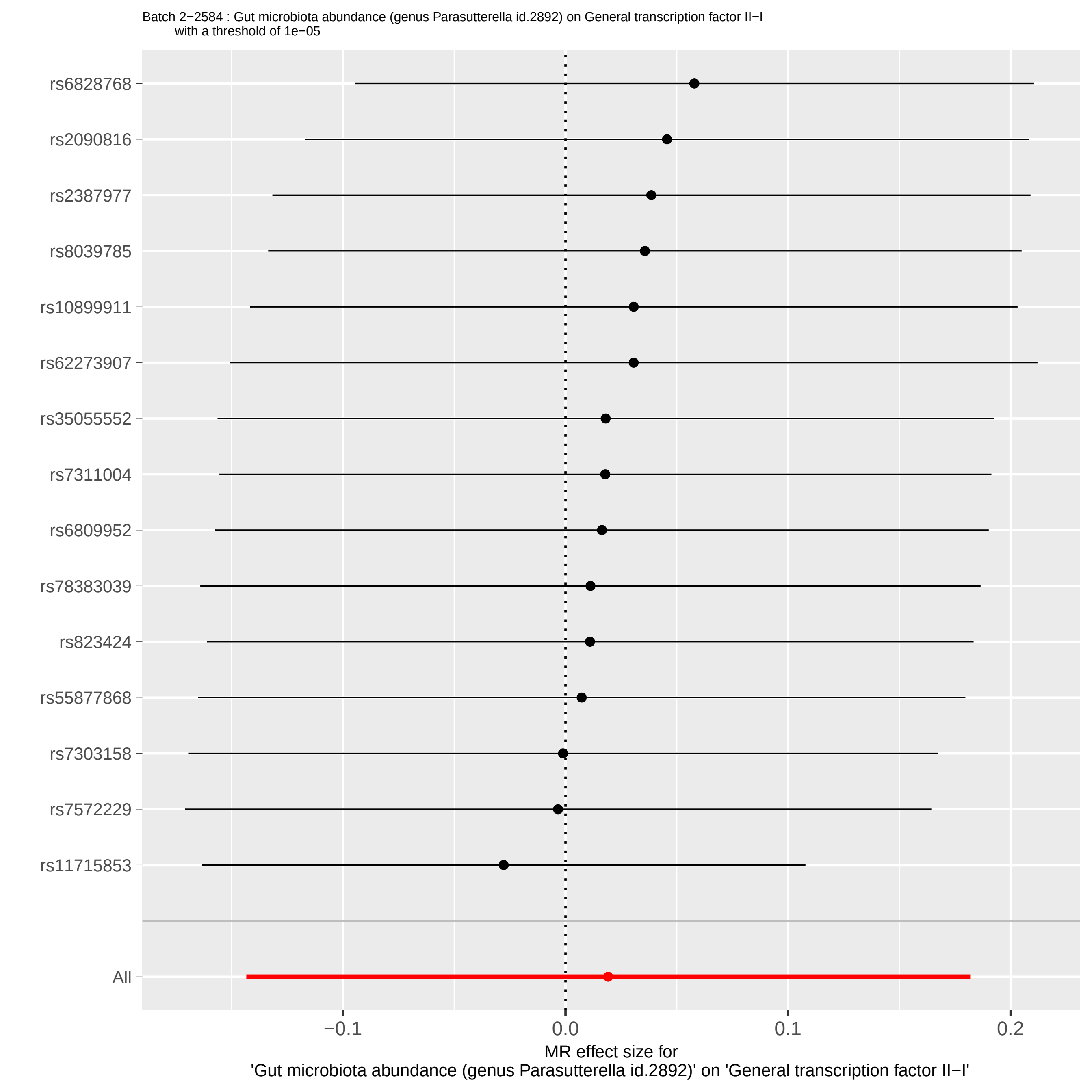

Batch 2−2584 : Gut microbiota abundance (genus Parasutterella id.2892) on General transcription factor II−I
with a threshold of 1e−05
rs6828768
rs2090816
rs2387977
rs8039785
rs10899911
rs62273907
rs35055552
rs7311004
rs6809952
rs78383039
rs823424
rs55877868
rs7303158
rs7572229
rs11715853
All
−0.1
0.0
0.1
0.2
MR effect size for
'Gut microbiota abundance (genus Parasutterella id.2892)' on 'General transcription factor II−I'

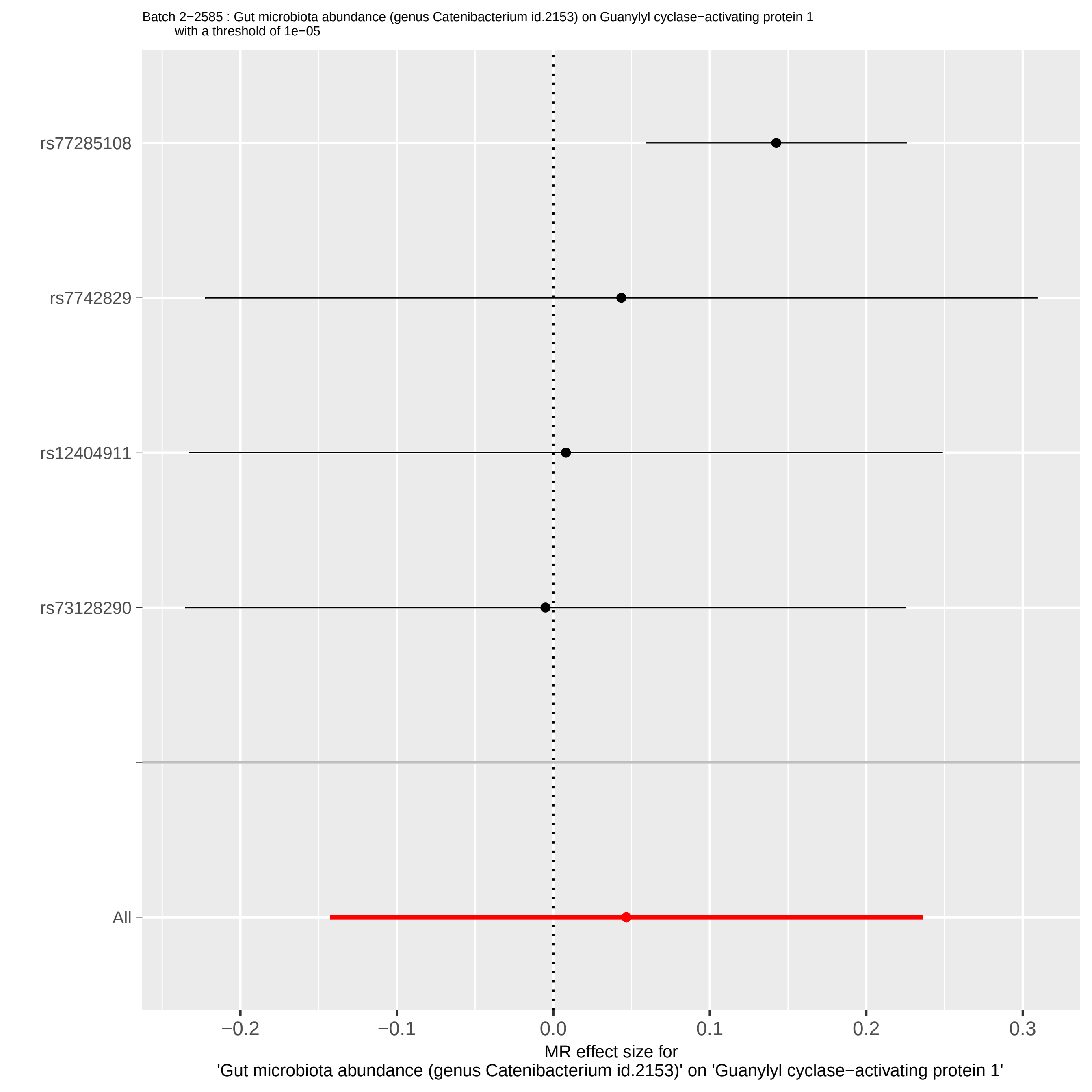
Batch 2−2585 : Gut microbiota abundance (genus Catenibacterium id.2153) on Guanylyl cyclase−activating protein 1
with a threshold of 1e−05
rs77285108
rs7742829
rs12404911
rs73128290
All
−0.2
−0.1
0.0
0.1
0.2
0.3
MR effect size for
'Gut microbiota abundance (genus Catenibacterium id.2153)' on 'Guanylyl cyclase−activating protein 1'

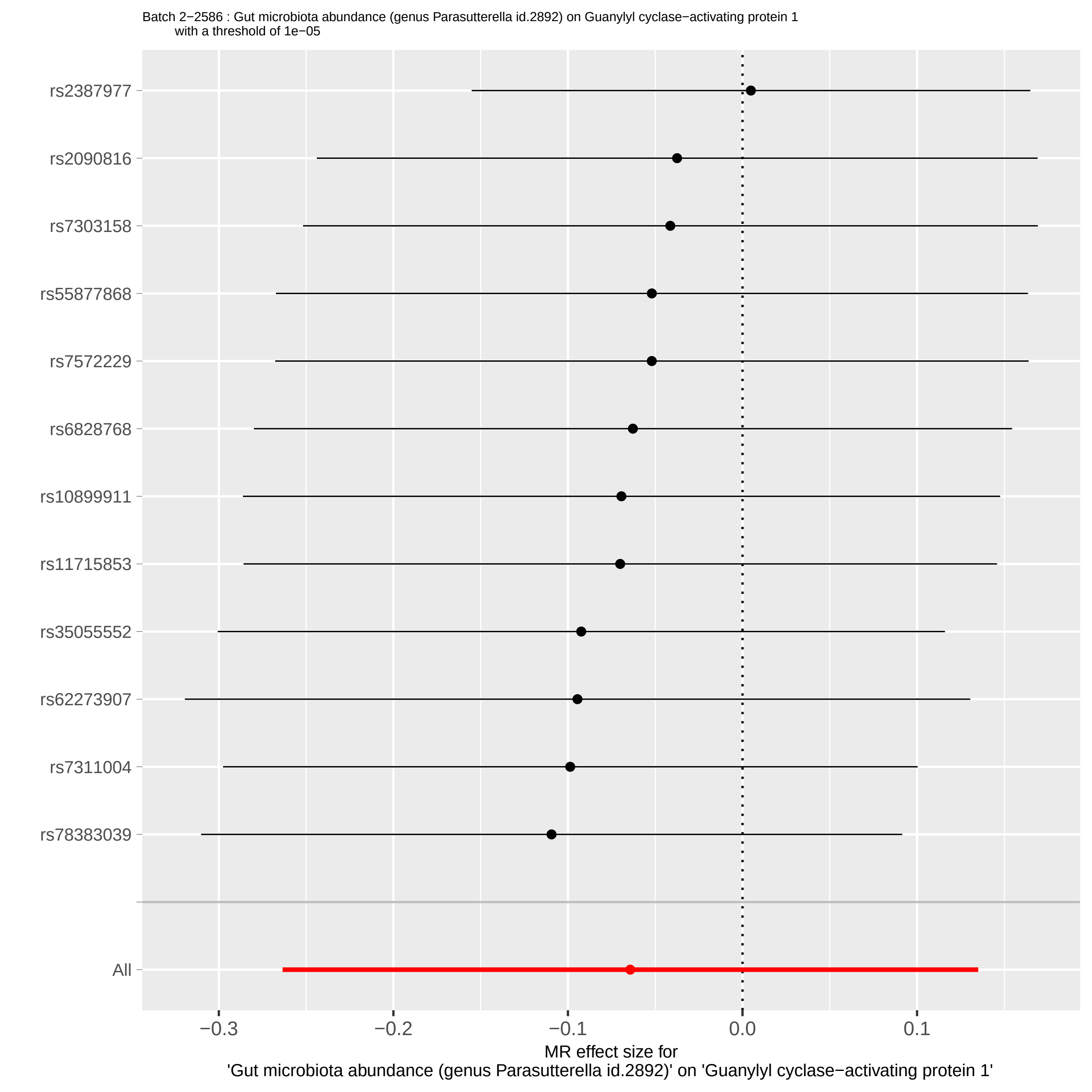
Batch 2−2586 : Gut microbiota abundance (genus Parasutterella id.2892) on Guanylyl cyclase−activating protein 1
with a threshold of 1e−05
rs2387977
rs2090816
rs7303158
rs55877868
rs7572229
rs6828768
rs10899911
rs11715853
rs35055552
rs62273907
rs7311004
rs78383039
All
−0.3
−0.2
−0.1
0.0
0.1
MR effect size for
'Gut microbiota abundance (genus Parasutterella id.2892)' on 'Guanylyl cyclase−activating protein 1'

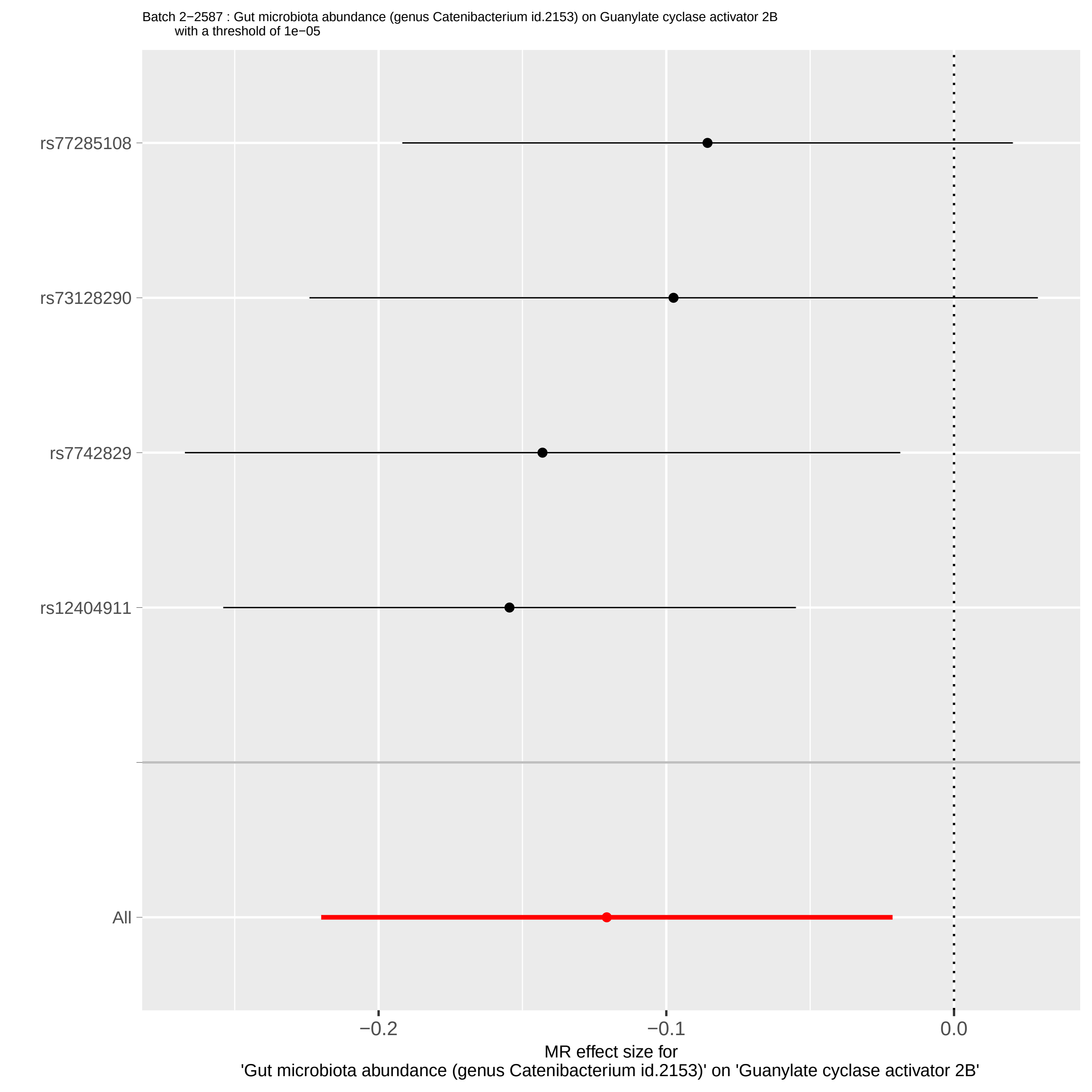

Batch 2−2587 : Gut microbiota abundance (genus Catenibacterium id.2153) on Guanylate cyclase activator 2B
with a threshold of 1e−05
rs77285108
rs73128290
rs7742829
rs12404911
All
−0.2
−0.1
0.0
MR effect size for
'Gut microbiota abundance (genus Catenibacterium id.2153)' on 'Guanylate cyclase activator 2B'

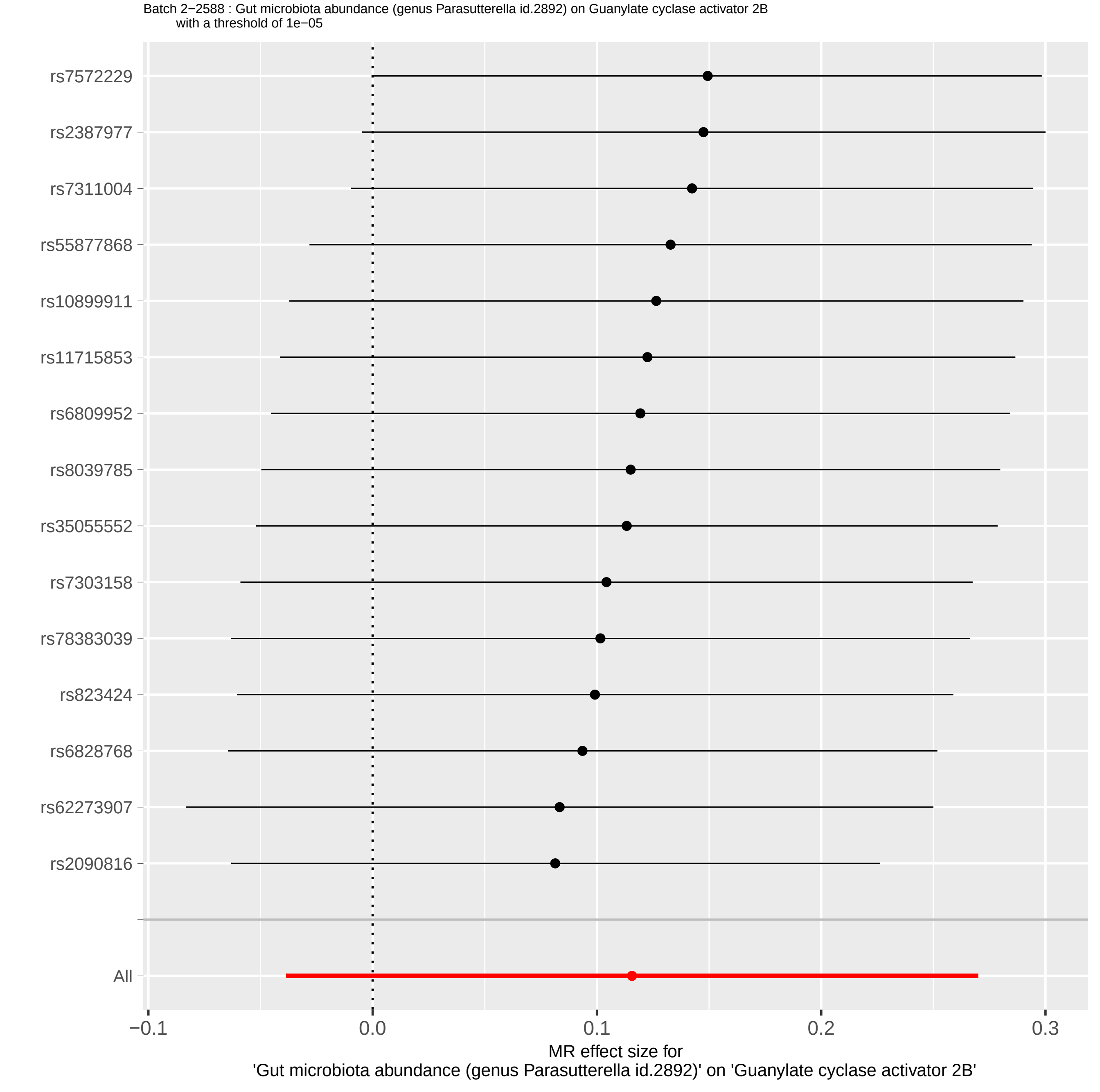

Batch 2−2588 : Gut microbiota abundance (genus Parasutterella id.2892) on Guanylate cyclase activator 2B
with a threshold of 1e−05
rs7572229
rs2387977
rs7311004
rs55877868
rs10899911
rs11715853
rs6809952
rs8039785
rs35055552
rs7303158
rs78383039
rs823424
rs6828768
rs62273907
rs2090816
All
−0.1
0.0
0.1
0.2
0.3
MR effect size for
'Gut microbiota abundance (genus Parasutterella id.2892)' on 'Guanylate cyclase activator 2B'

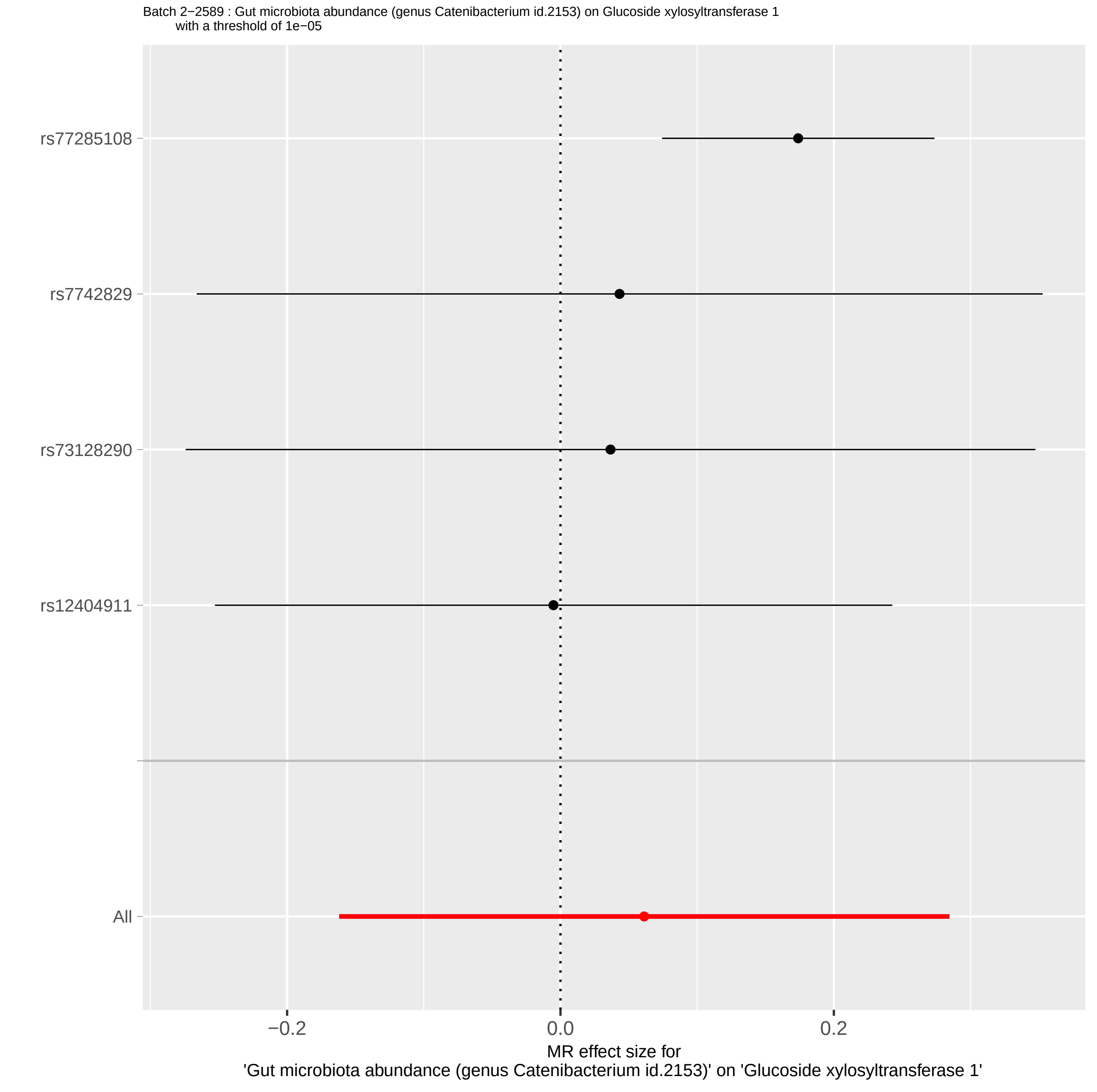

Batch 2−2589 : Gut microbiota abundance (genus Catenibacterium id.2153) on Glucoside xylosyltransferase 1
with a threshold of 1e−05
rs77285108
rs7742829
rs73128290
rs12404911
All
−0.2
0.0
0.2
MR effect size for
'Gut microbiota abundance (genus Catenibacterium id.2153)' on 'Glucoside xylosyltransferase 1'

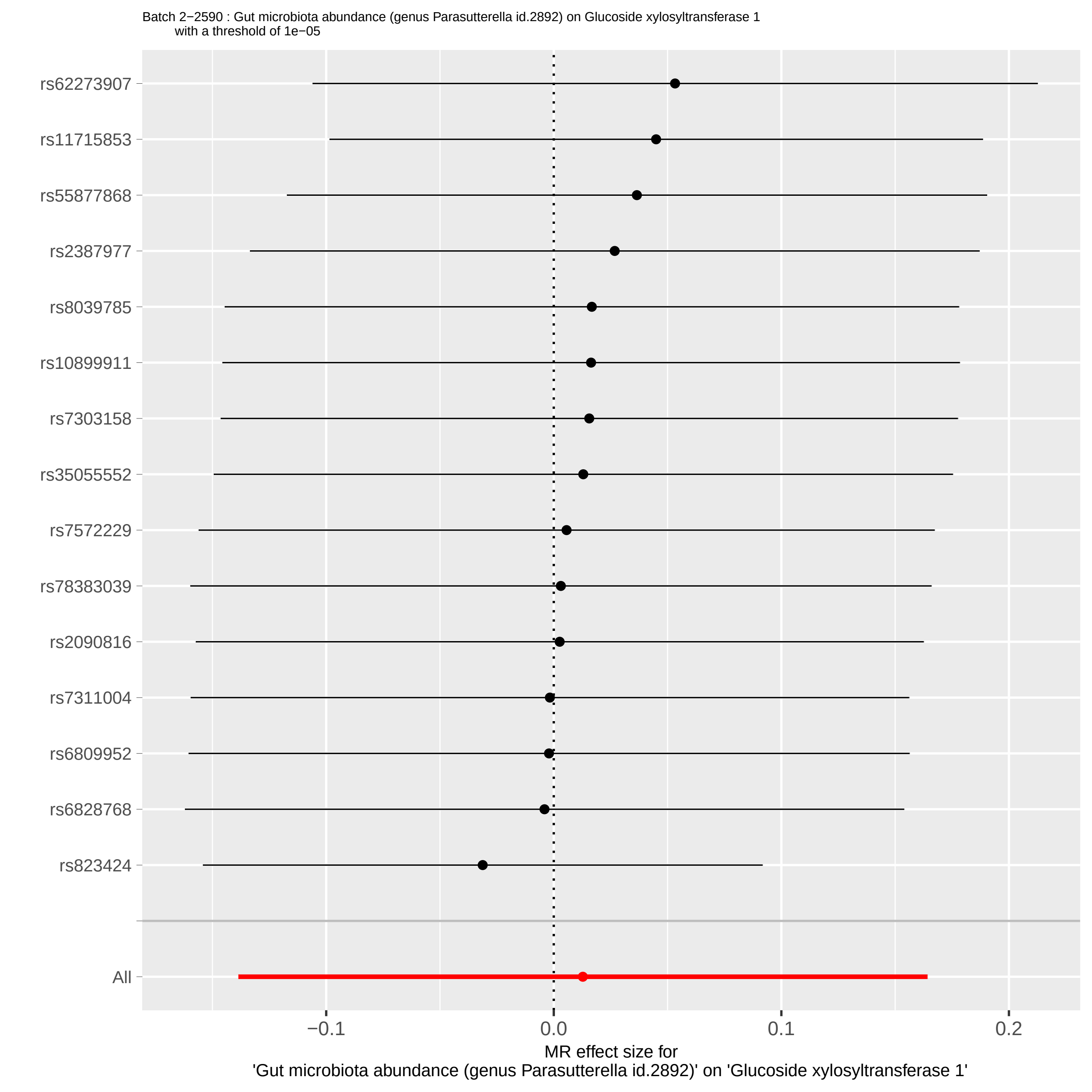
Batch 2−2590 : Gut microbiota abundance (genus Parasutterella id.2892) on Glucoside xylosyltransferase 1
with a threshold of 1e−05
rs62273907
rs11715853
rs55877868
rs2387977
rs8039785
rs10899911
rs7303158
rs35055552
rs7572229
rs78383039
rs2090816
rs7311004
rs6809952
rs6828768
rs823424
All
−0.1
0.0
0.1
0.2
MR effect size for
'Gut microbiota abundance (genus Parasutterella id.2892)' on 'Glucoside xylosyltransferase 1'

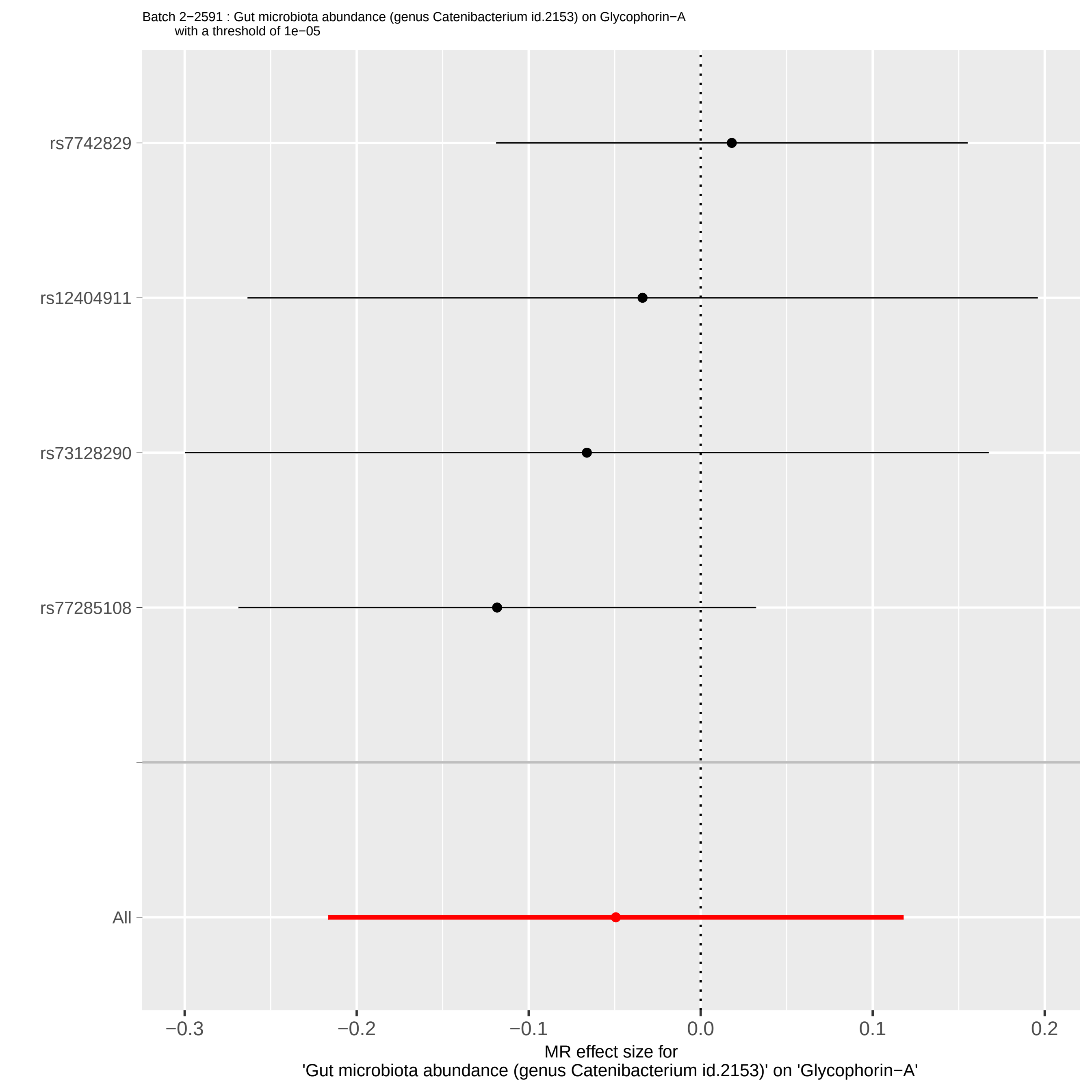
Batch 2−2591 : Gut microbiota abundance (genus Catenibacterium id.2153) on Glycophorin−A
with a threshold of 1e−05
rs7742829
rs12404911
rs73128290
rs77285108
All
−0.3
−0.2
−0.1
0.0
0.1
0.2
MR effect size for
'Gut microbiota abundance (genus Catenibacterium id.2153)' on 'Glycophorin−A'

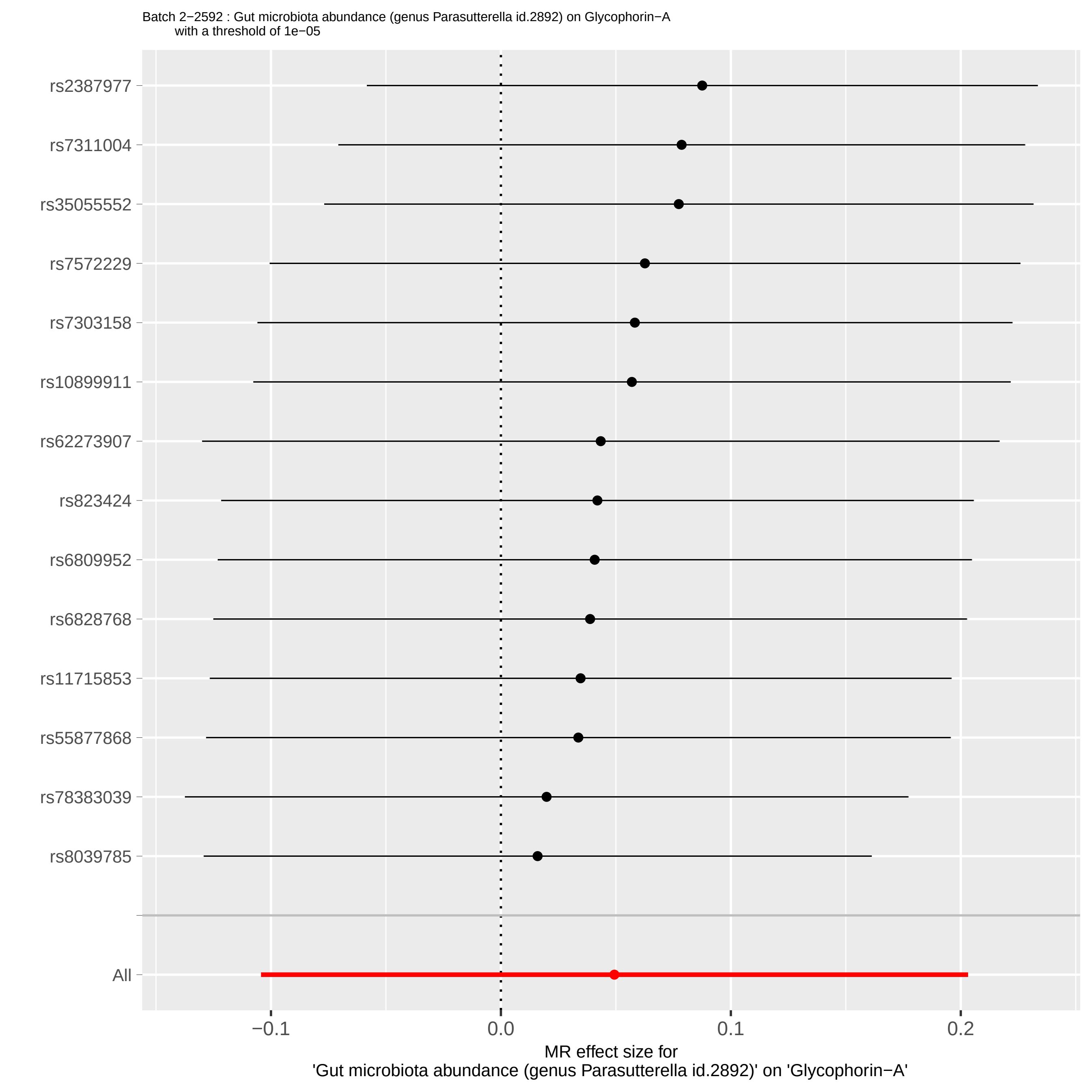

Batch 2−2592 : Gut microbiota abundance (genus Parasutterella id.2892) on Glycophorin−A
with a threshold of 1e−05
rs2387977
rs7311004
rs35055552
rs7572229
rs7303158
rs10899911
rs62273907
rs823424
rs6809952
rs6828768
rs11715853
rs55877868
rs78383039
rs8039785
All
−0.1
0.0
0.1
0.2
MR effect size for
'Gut microbiota abundance (genus Parasutterella id.2892)' on 'Glycophorin−A'

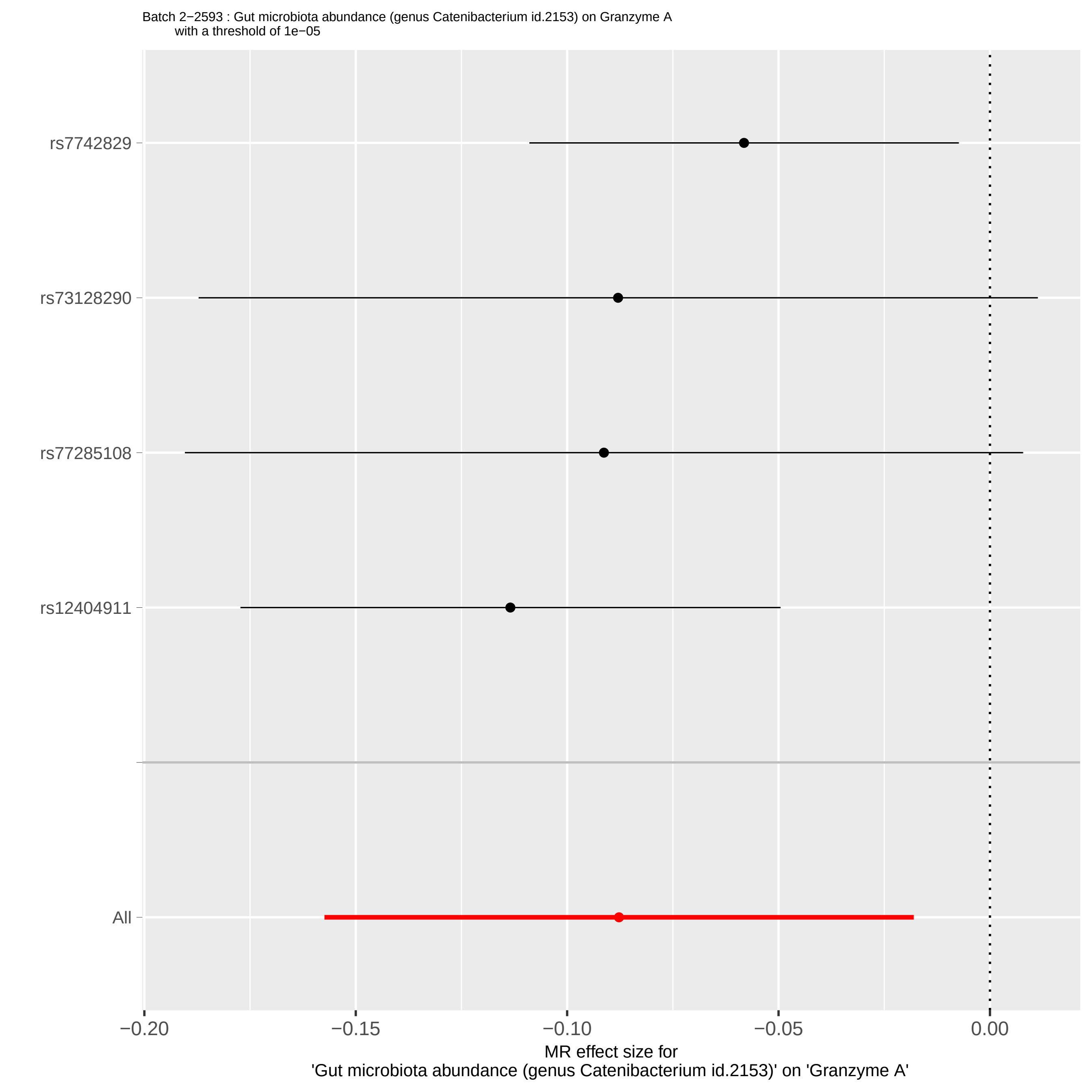
Batch 2−2593 : Gut microbiota abundance (genus Catenibacterium id.2153) on Granzyme A
with a threshold of 1e−05
rs7742829
rs73128290
rs77285108
rs12404911
All
−0.20
−0.15
−0.10
−0.05
0.00
MR effect size for
'Gut microbiota abundance (genus Catenibacterium id.2153)' on 'Granzyme A'

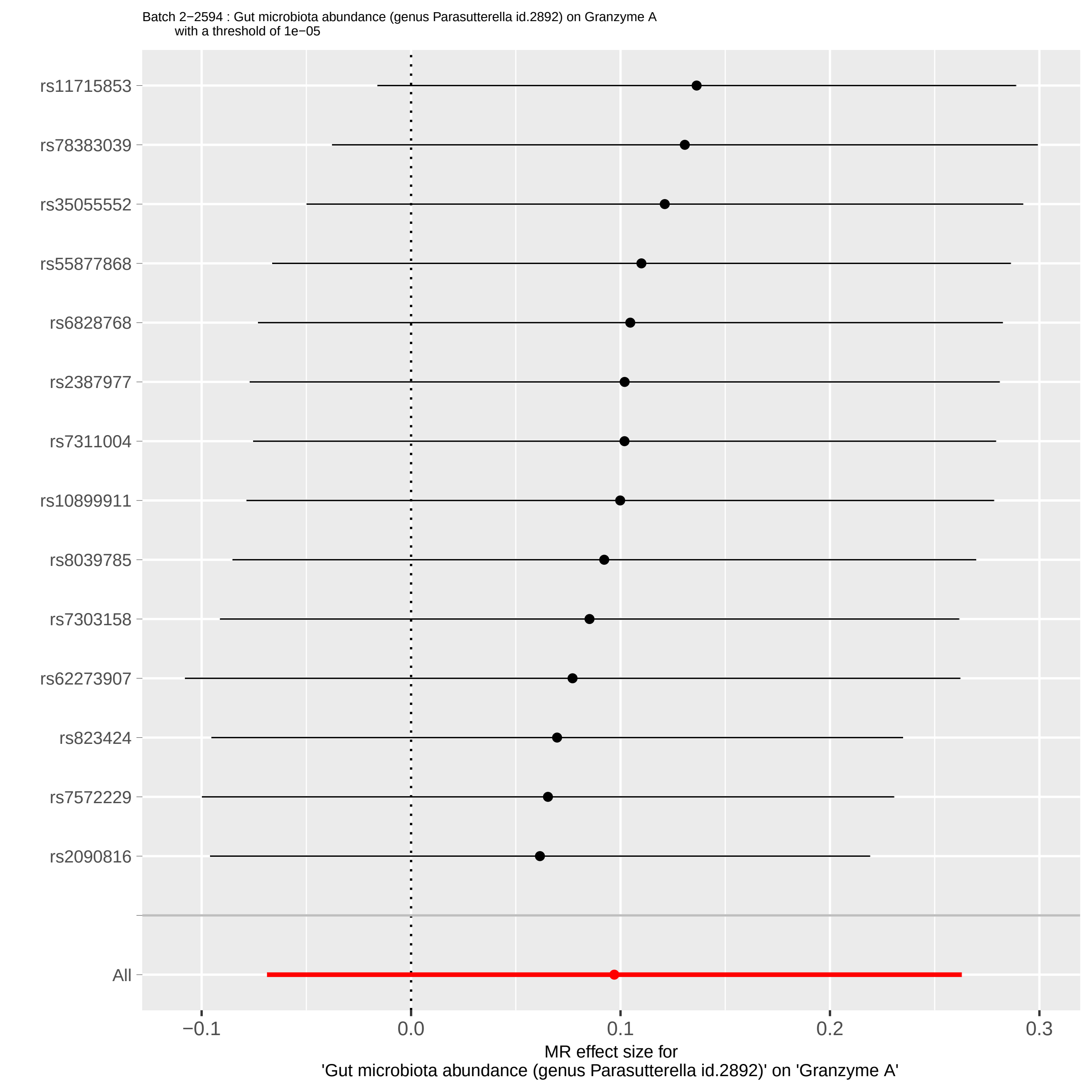
Batch 2−2594 : Gut microbiota abundance (genus Parasutterella id.2892) on Granzyme A
with a threshold of 1e−05
rs11715853
rs78383039
rs35055552
rs55877868
rs6828768
rs2387977
rs7311004
rs10899911
rs8039785
rs7303158
rs62273907
rs823424
rs7572229
rs2090816
All
−0.1
0.0
0.1
0.2
0.3
MR effect size for
'Gut microbiota abundance (genus Parasutterella id.2892)' on 'Granzyme A'

Batch 2−2595 : Gut microbiota abundance (genus Catenibacterium id.2153) on Granzyme B
with a threshold of 1e−05

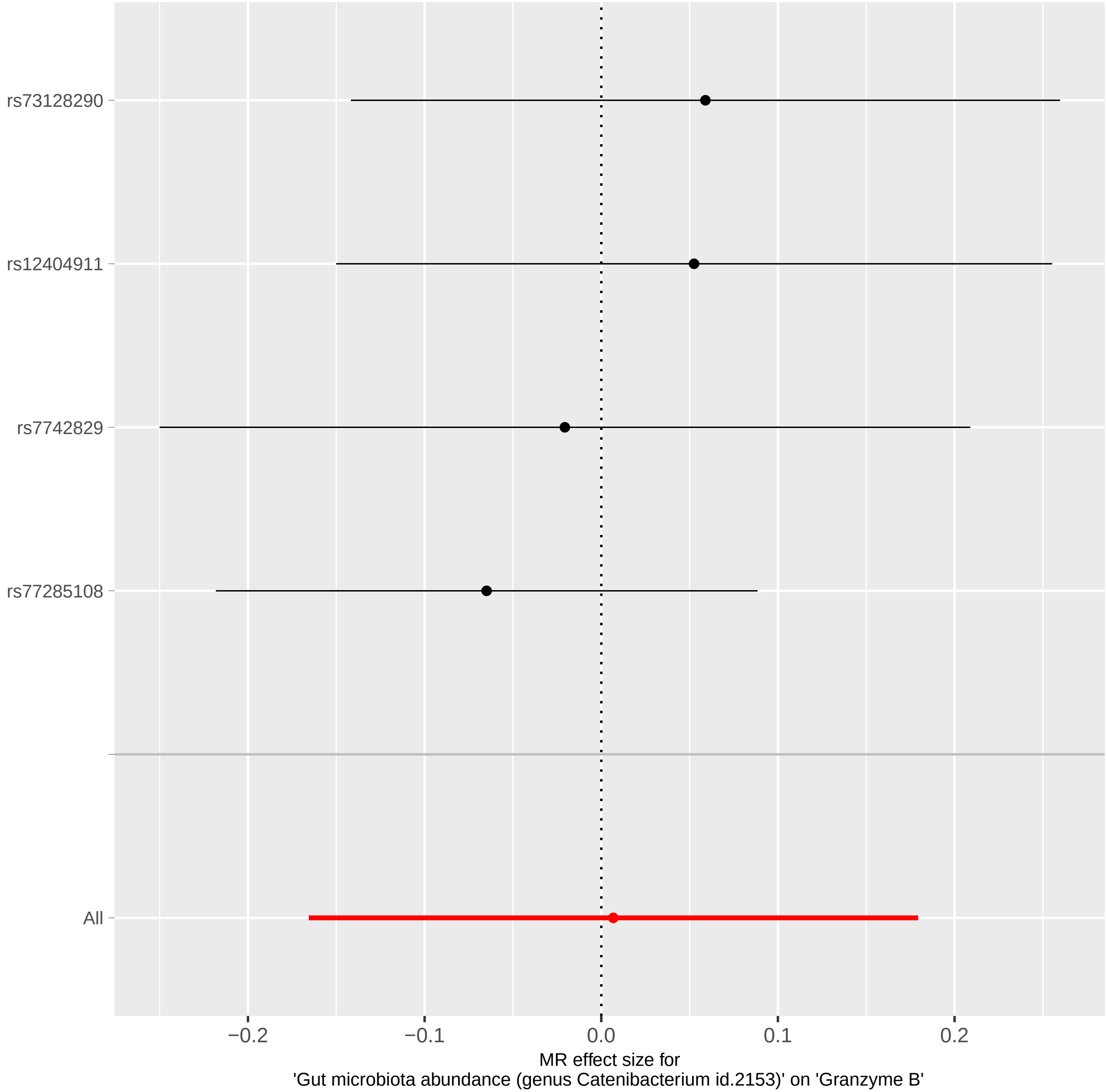

Batch 2−2596 : Gut microbiota abundance (genus Parasutterella id.2892) on Granzyme B
with a threshold of 1e−05

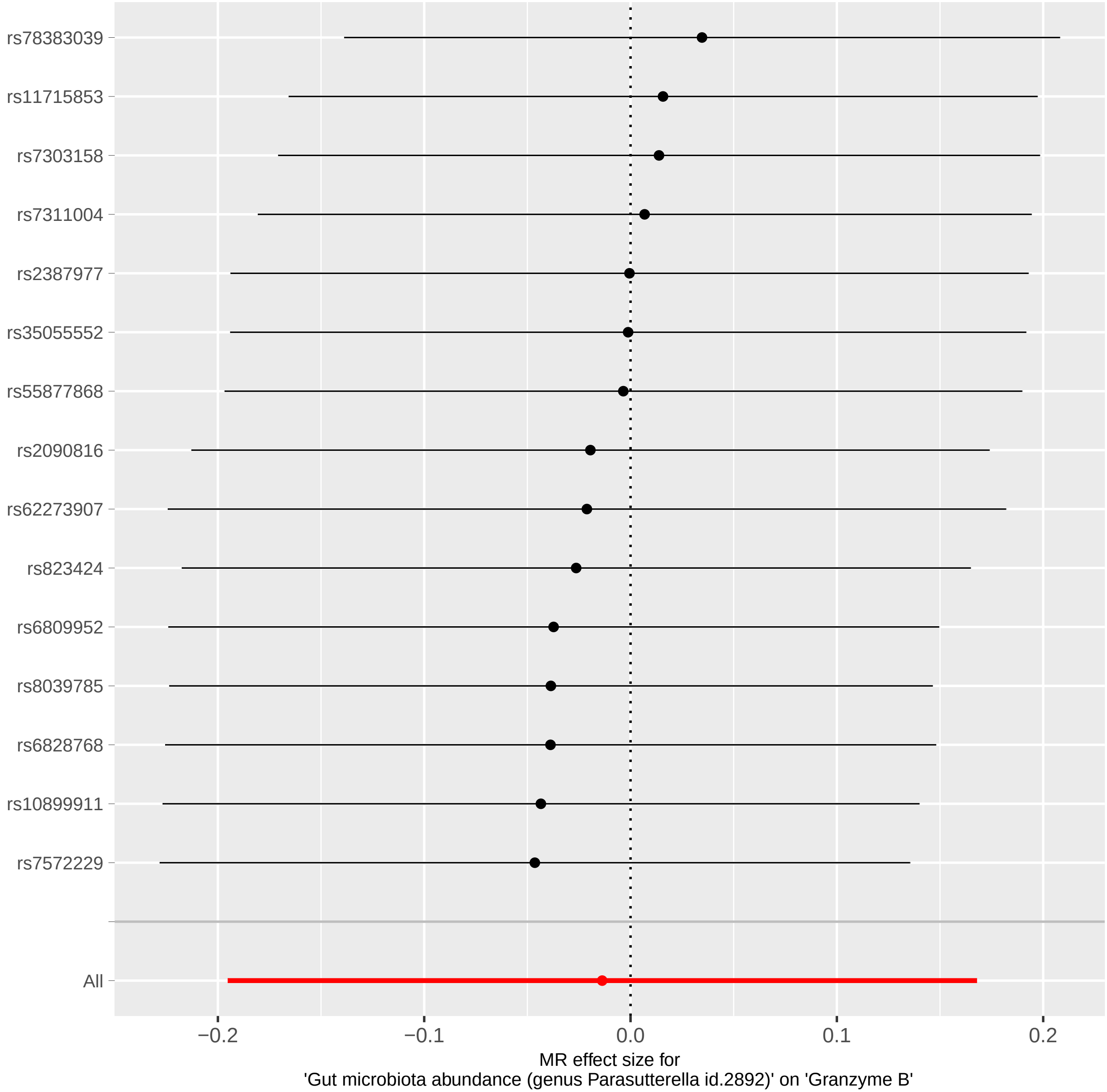

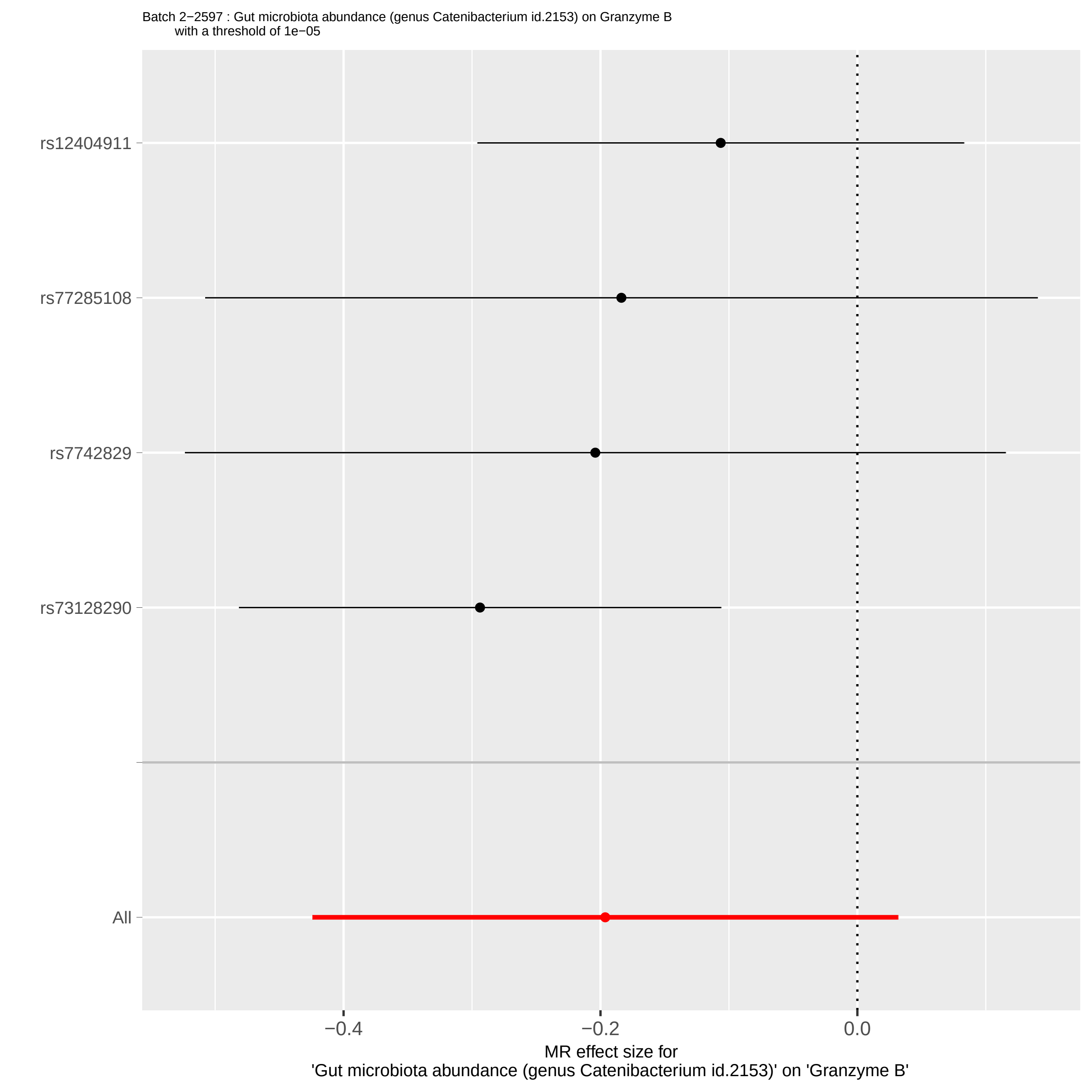

Batch 2−2597 : Gut microbiota abundance (genus Catenibacterium id.2153) on Granzyme B
with a threshold of 1e−05
rs12404911
rs77285108
rs7742829
rs73128290
All
−0.4
−0.2
0.0
MR effect size for
'Gut microbiota abundance (genus Catenibacterium id.2153)' on 'Granzyme B'

Batch 2−2598 : Gut microbiota abundance (genus Parasutterella id.2892) on Granzyme B
with a threshold of 1e−05

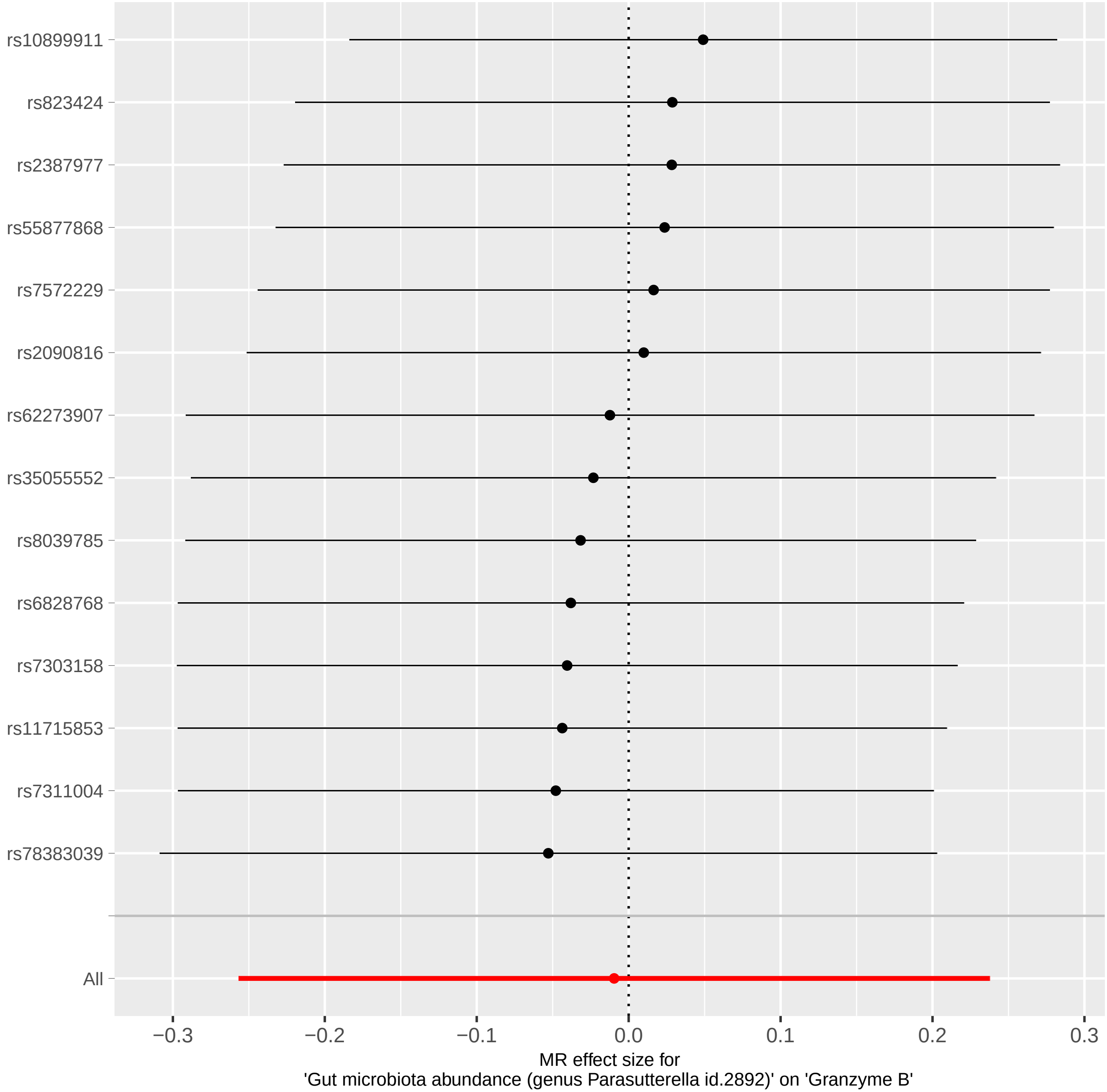

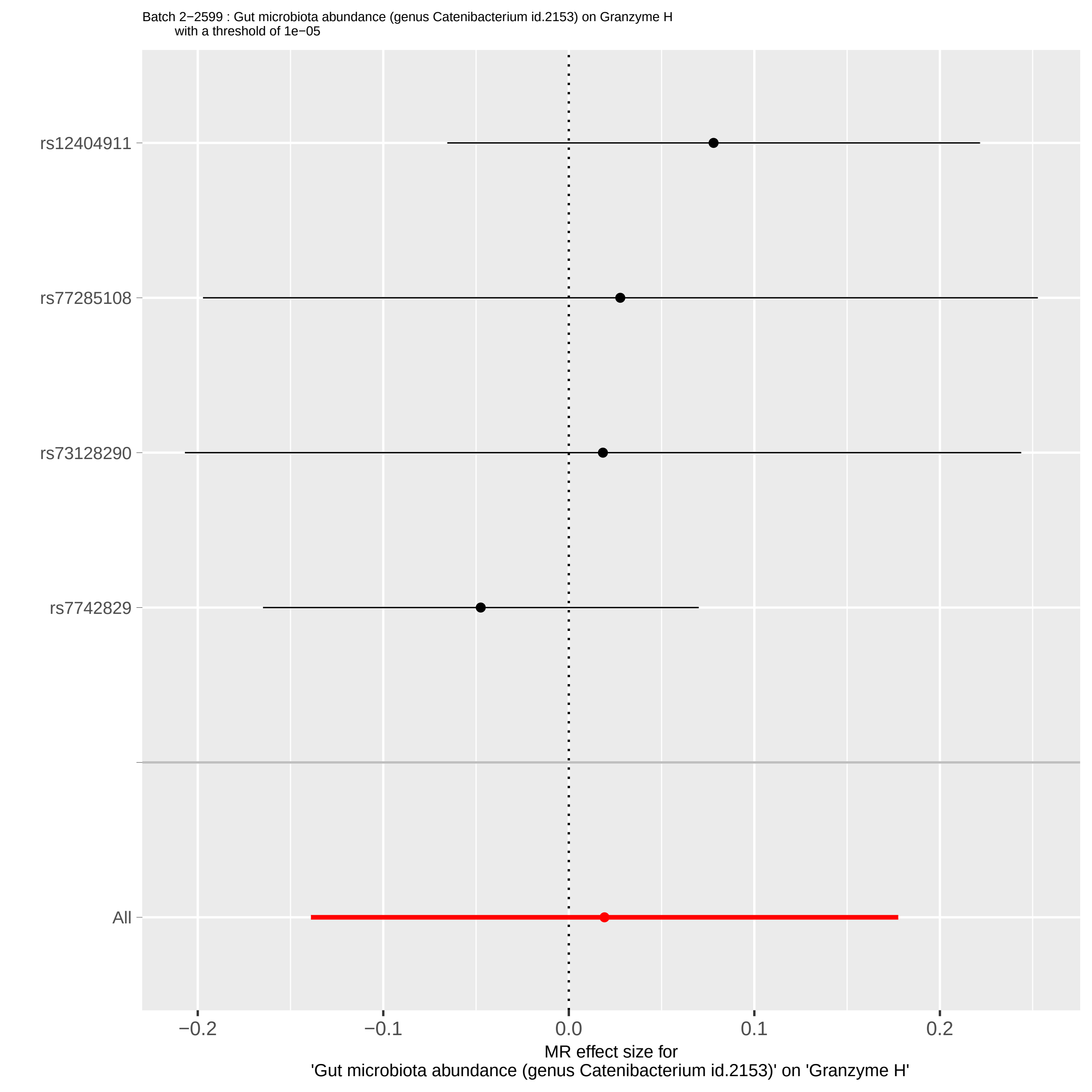
Batch 2−2599 : Gut microbiota abundance (genus Catenibacterium id.2153) on Granzyme H
with a threshold of 1e−05
rs12404911
rs77285108
rs73128290
rs7742829
All
−0.2
−0.1
0.0
0.1
0.2
MR effect size for
'Gut microbiota abundance (genus Catenibacterium id.2153)' on 'Granzyme H'

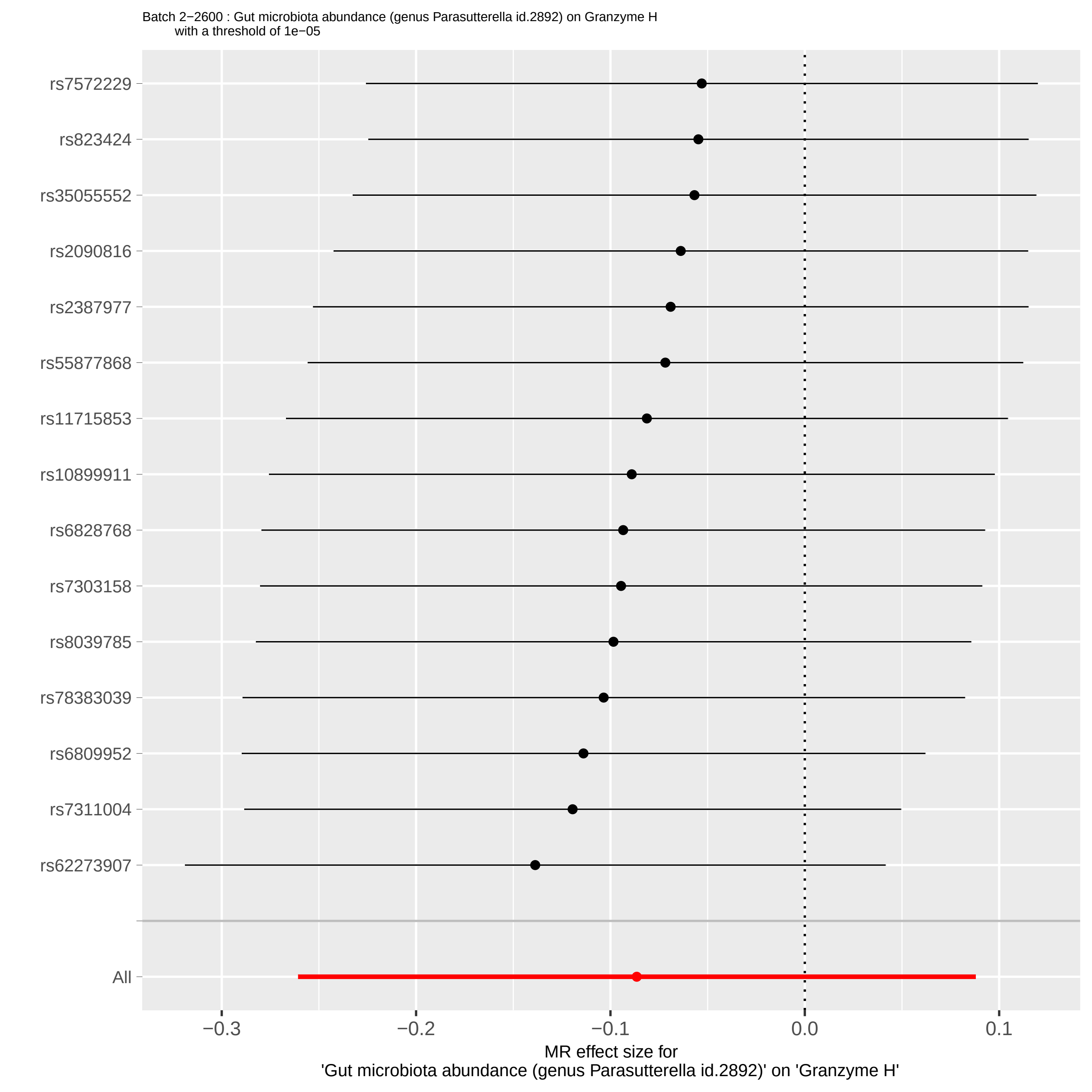
Batch 2−2600 : Gut microbiota abundance (genus Parasutterella id.2892) on Granzyme H
with a threshold of 1e−05
rs7572229
rs823424
rs35055552
rs2090816
rs2387977
rs55877868
rs11715853
rs10899911
rs6828768
rs7303158
rs8039785
rs78383039
rs6809952
rs7311004
rs62273907
All
−0.3
−0.2
−0.1
0.0
0.1
MR effect size for
'Gut microbiota abundance (genus Parasutterella id.2892)' on 'Granzyme H'

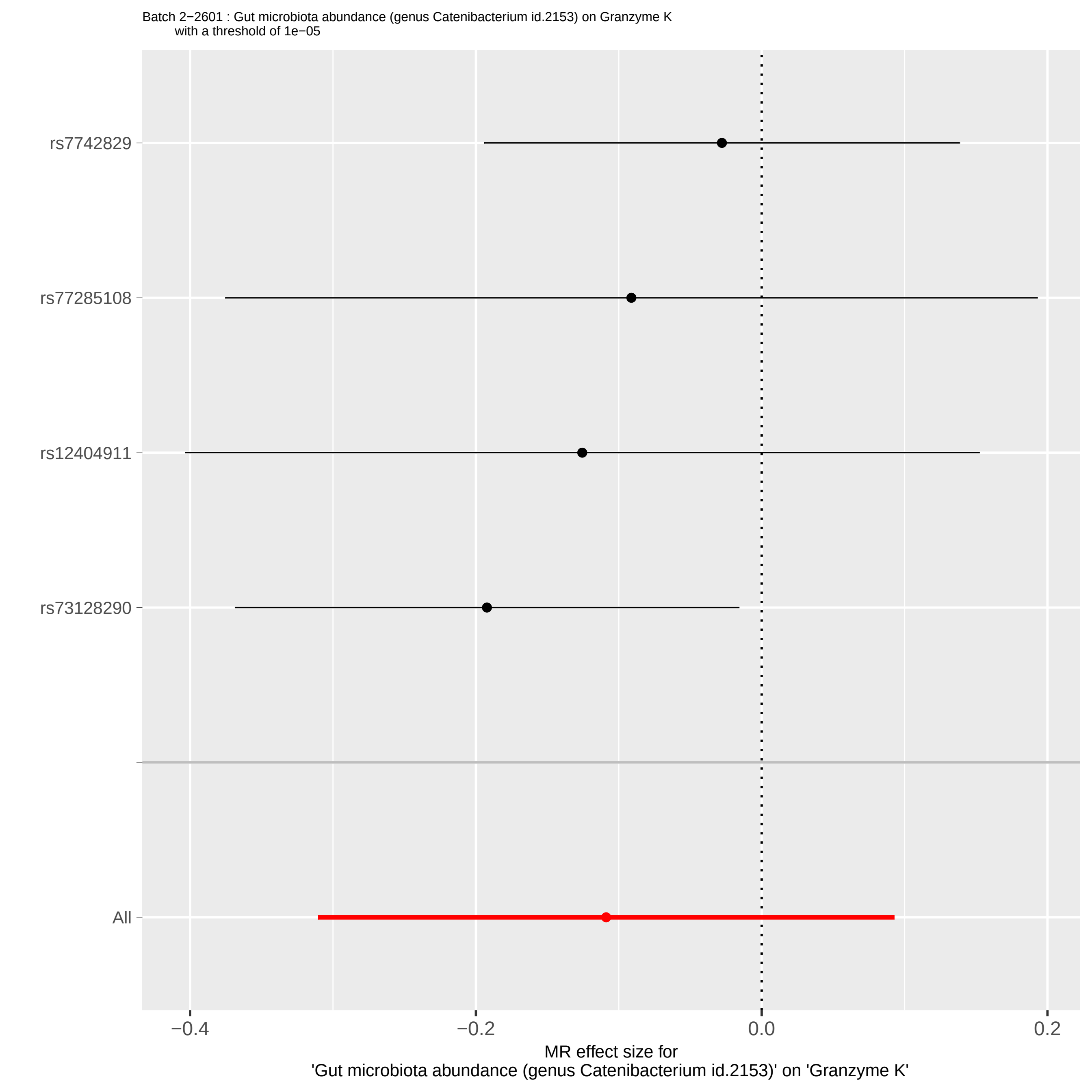

Batch 2−2601 : Gut microbiota abundance (genus Catenibacterium id.2153) on Granzyme K
with a threshold of 1e−05
rs7742829
rs77285108
rs12404911
rs73128290
All
−0.4
−0.2
0.0
0.2
MR effect size for
'Gut microbiota abundance (genus Catenibacterium id.2153)' on 'Granzyme K'

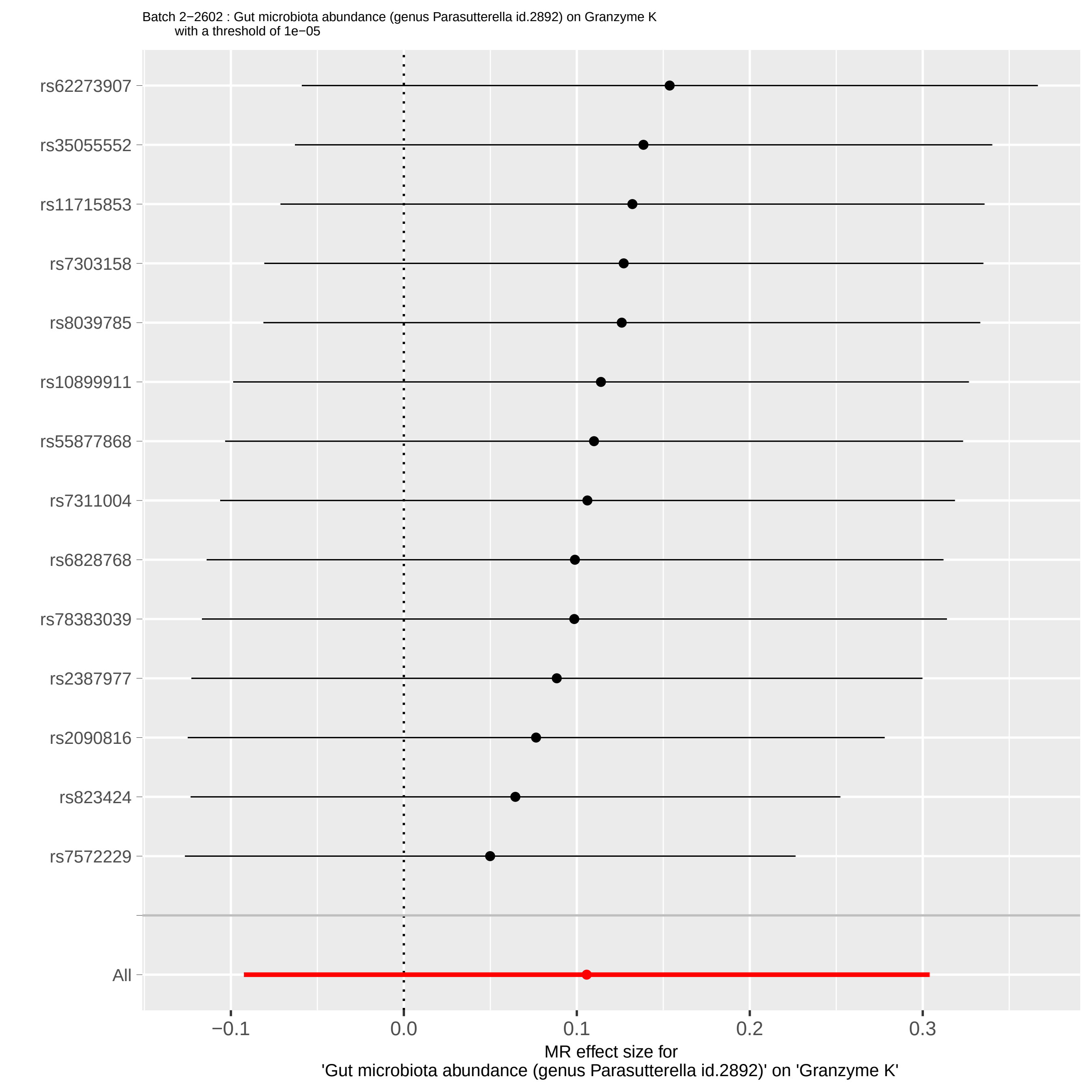

Batch 2−2602 : Gut microbiota abundance (genus Parasutterella id.2892) on Granzyme K
with a threshold of 1e−05
rs62273907
rs35055552
rs11715853
rs7303158
rs8039785
rs10899911
rs55877868
rs7311004
rs6828768
rs78383039
rs2387977
rs2090816
rs823424
rs7572229
All
−0.1
0.0
0.1
0.2
0.3
MR effect size for
'Gut microbiota abundance (genus Parasutterella id.2892)' on 'Granzyme K'

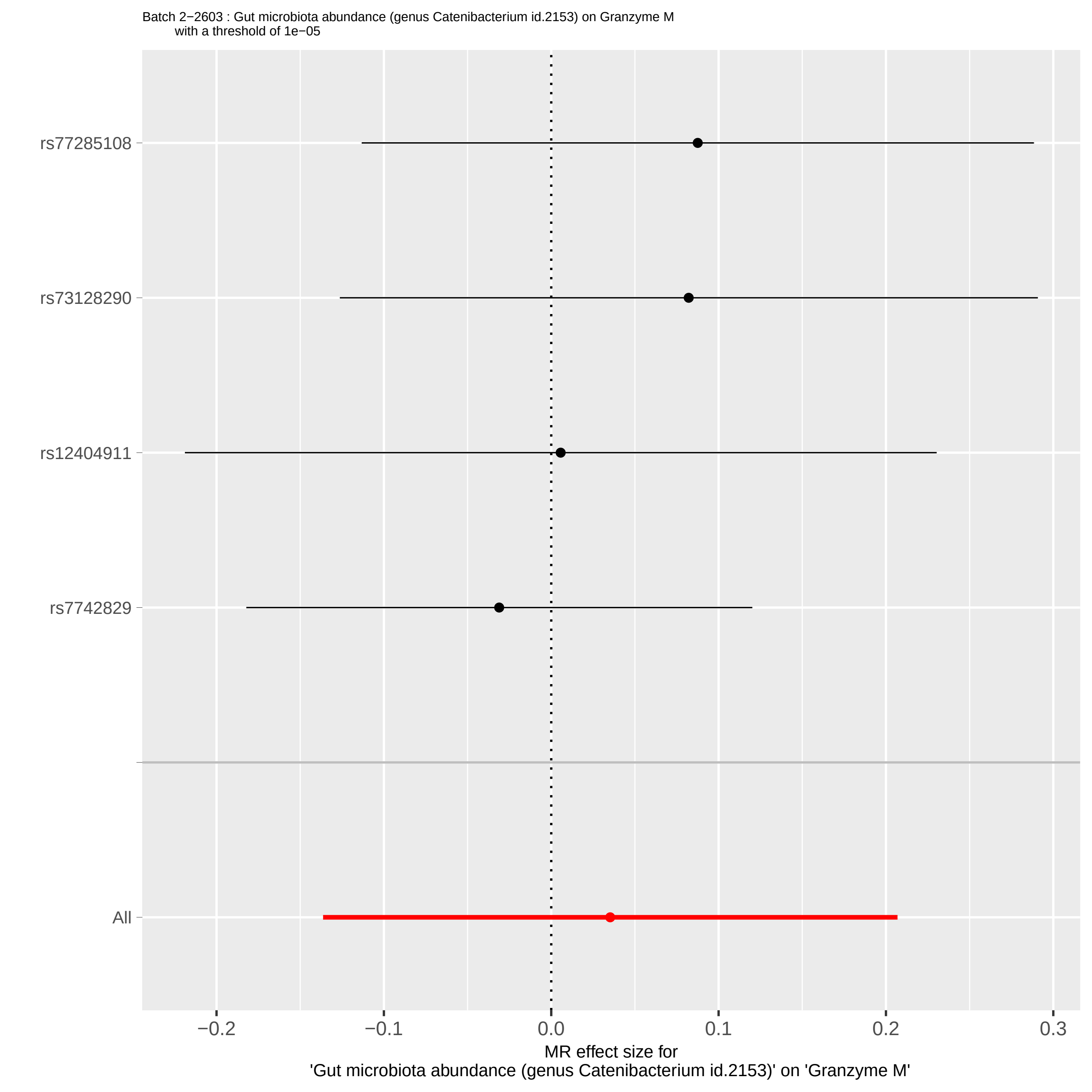
Batch 2−2603 : Gut microbiota abundance (genus Catenibacterium id.2153) on Granzyme M
with a threshold of 1e−05
rs77285108
rs73128290
rs12404911
rs7742829
All
−0.2
−0.1
0.0
0.1
0.2
0.3
MR effect size for
'Gut microbiota abundance (genus Catenibacterium id.2153)' on 'Granzyme M'

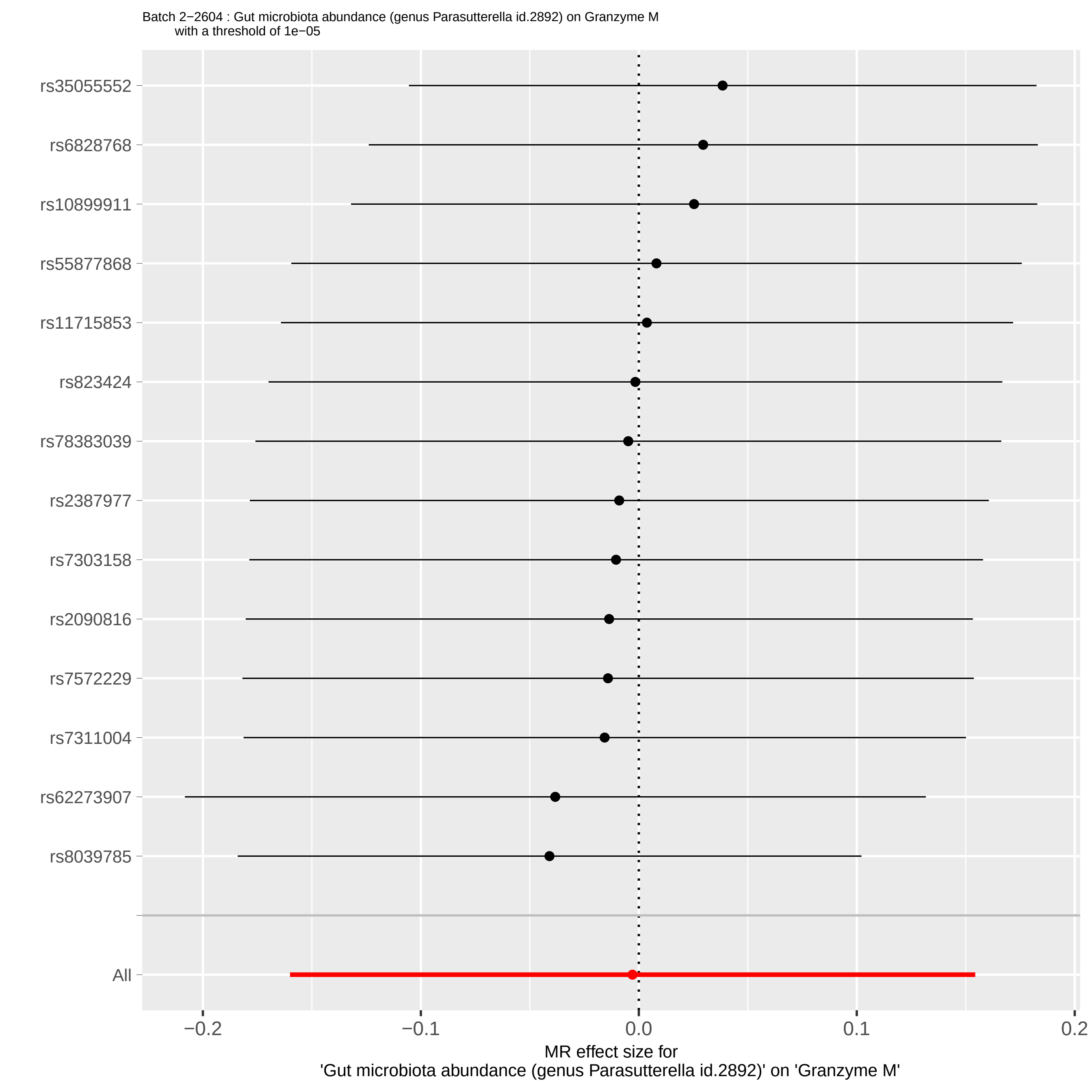
Batch 2−2604 : Gut microbiota abundance (genus Parasutterella id.2892) on Granzyme M
with a threshold of 1e−05
rs35055552
rs6828768
rs10899911
rs55877868
rs11715853
rs823424
rs78383039
rs2387977
rs7303158
rs2090816
rs7572229
rs7311004
rs62273907
rs8039785
All
−0.2
−0.1
0.0
0.1
0.2
MR effect size for
'Gut microbiota abundance (genus Parasutterella id.2892)' on 'Granzyme M'

Batch 2−2605 : Gut microbiota abundance (genus Catenibacterium id.2153) on Histone H1x
with a threshold of 1e−05

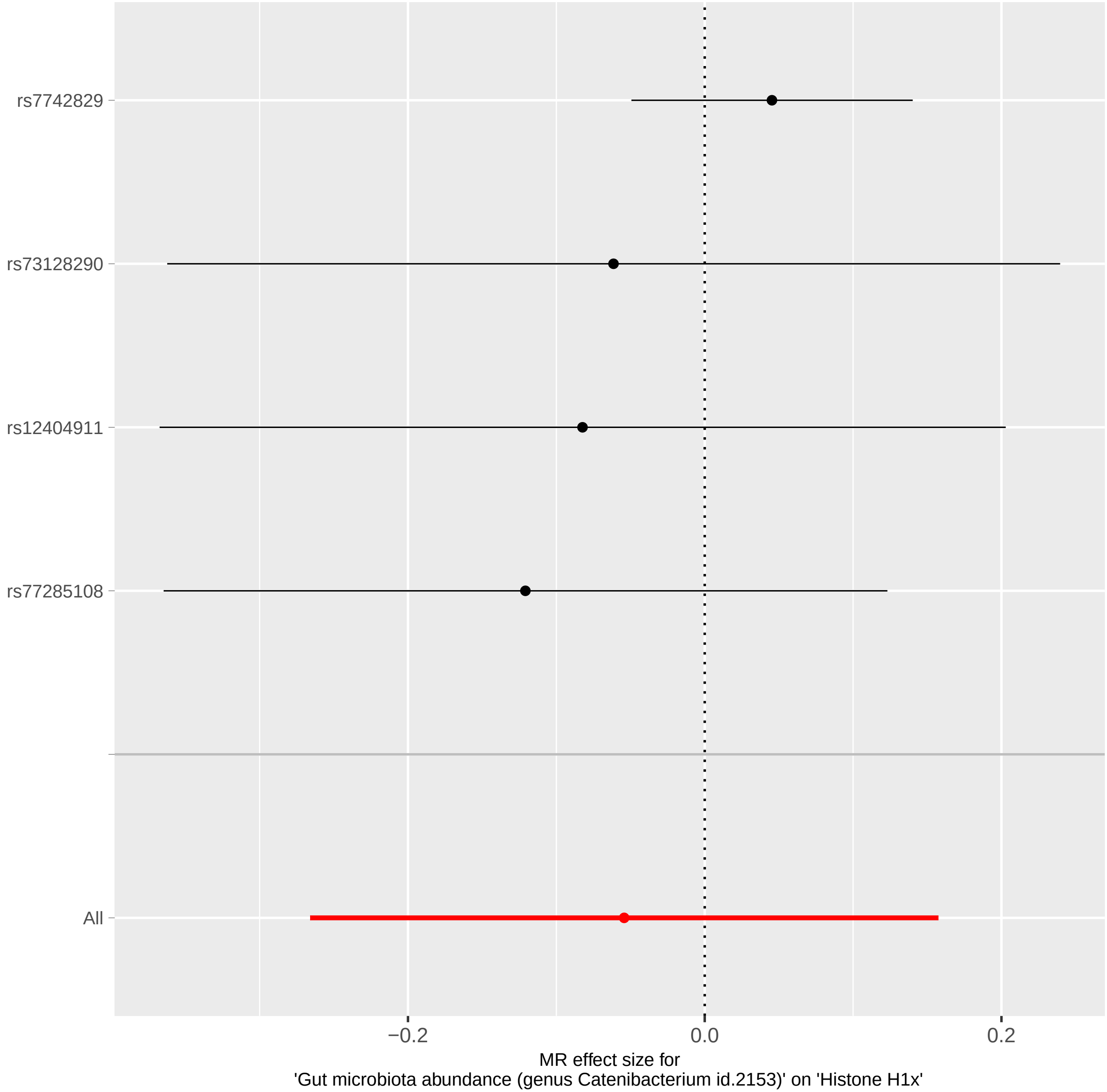

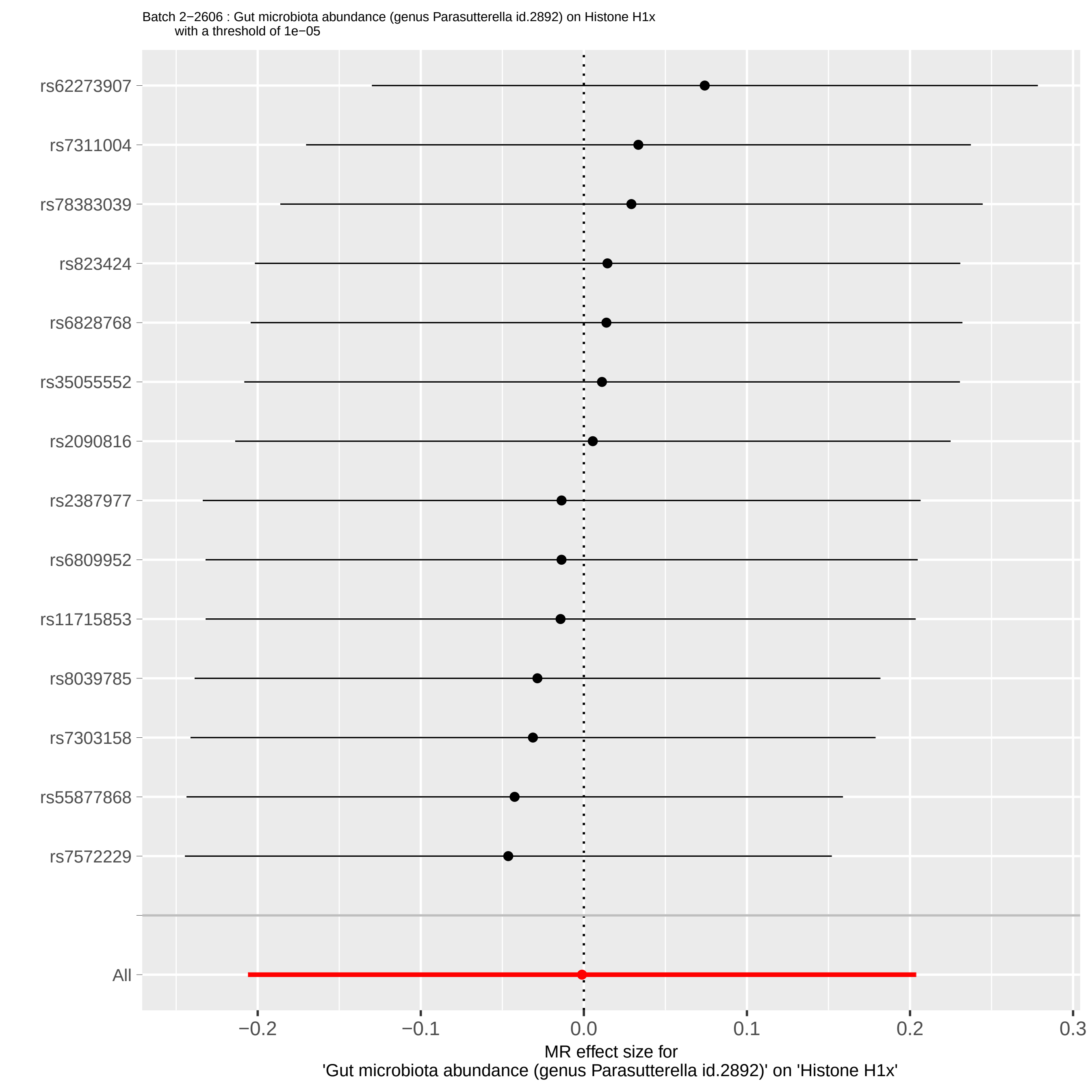
Batch 2−2606 : Gut microbiota abundance (genus Parasutterella id.2892) on Histone H1x
with a threshold of 1e−05
rs62273907
rs7311004
rs78383039
rs823424
rs6828768
rs35055552
rs2090816
rs2387977
rs6809952
rs11715853
rs8039785
rs7303158
rs55877868
rs7572229
All
−0.2
−0.1
0.0
0.1
0.2
0.3
MR effect size for
'Gut microbiota abundance (genus Parasutterella id.2892)' on 'Histone H1x'

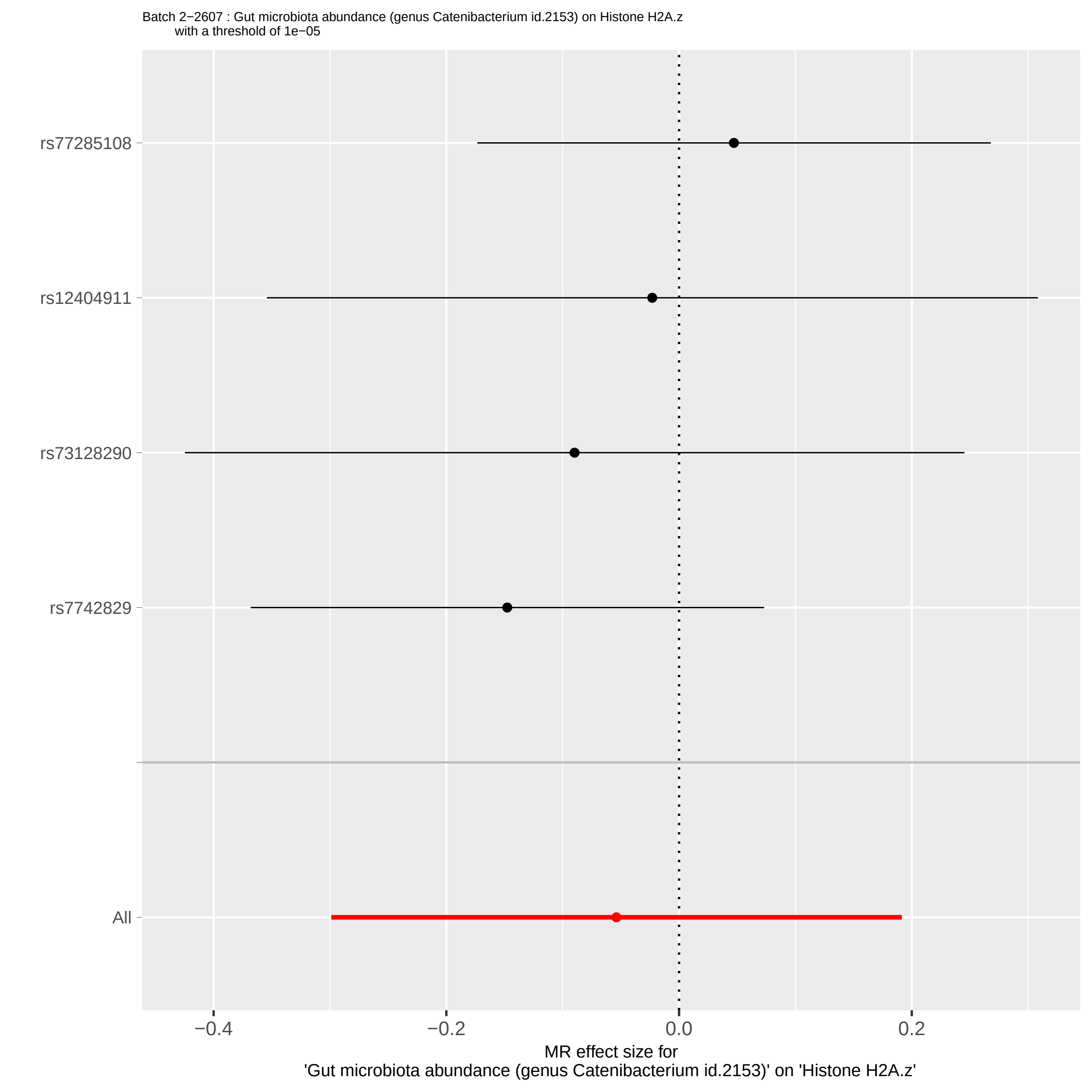

Batch 2−2607 : Gut microbiota abundance (genus Catenibacterium id.2153) on Histone H2A.z
with a threshold of 1e−05
rs77285108
rs12404911
rs73128290
rs7742829
All
−0.4
−0.2
0.0
0.2
MR effect size for
'Gut microbiota abundance (genus Catenibacterium id.2153)' on 'Histone H2A.z'

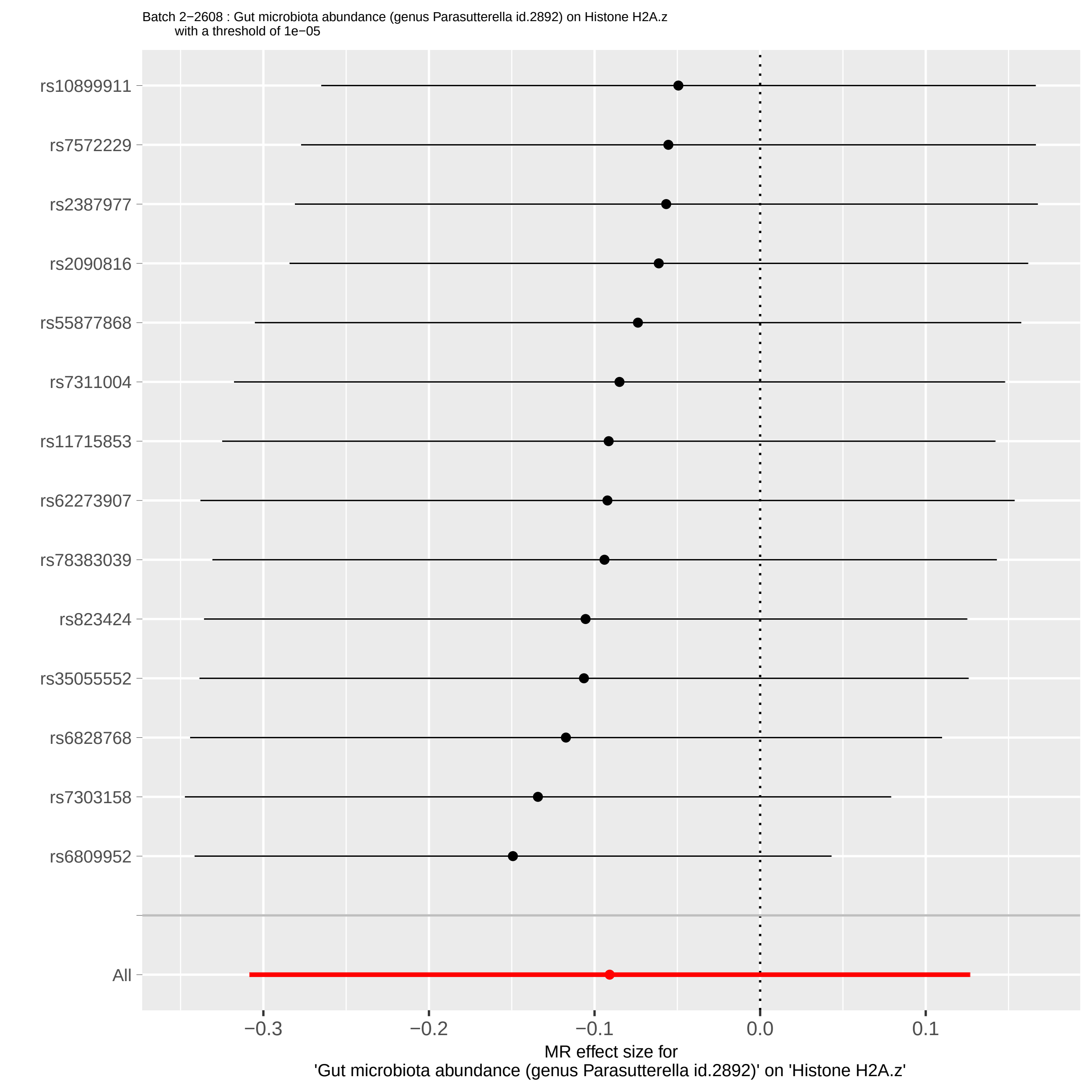

Batch 2−2608 : Gut microbiota abundance (genus Parasutterella id.2892) on Histone H2A.z
with a threshold of 1e−05
rs10899911
rs7572229
rs2387977
rs2090816
rs55877868
rs7311004
rs11715853
rs62273907
rs78383039
rs823424
rs35055552
rs6828768
rs7303158
rs6809952
All
−0.3
−0.2
−0.1
0.0
0.1
MR effect size for
'Gut microbiota abundance (genus Parasutterella id.2892)' on 'Histone H2A.z'

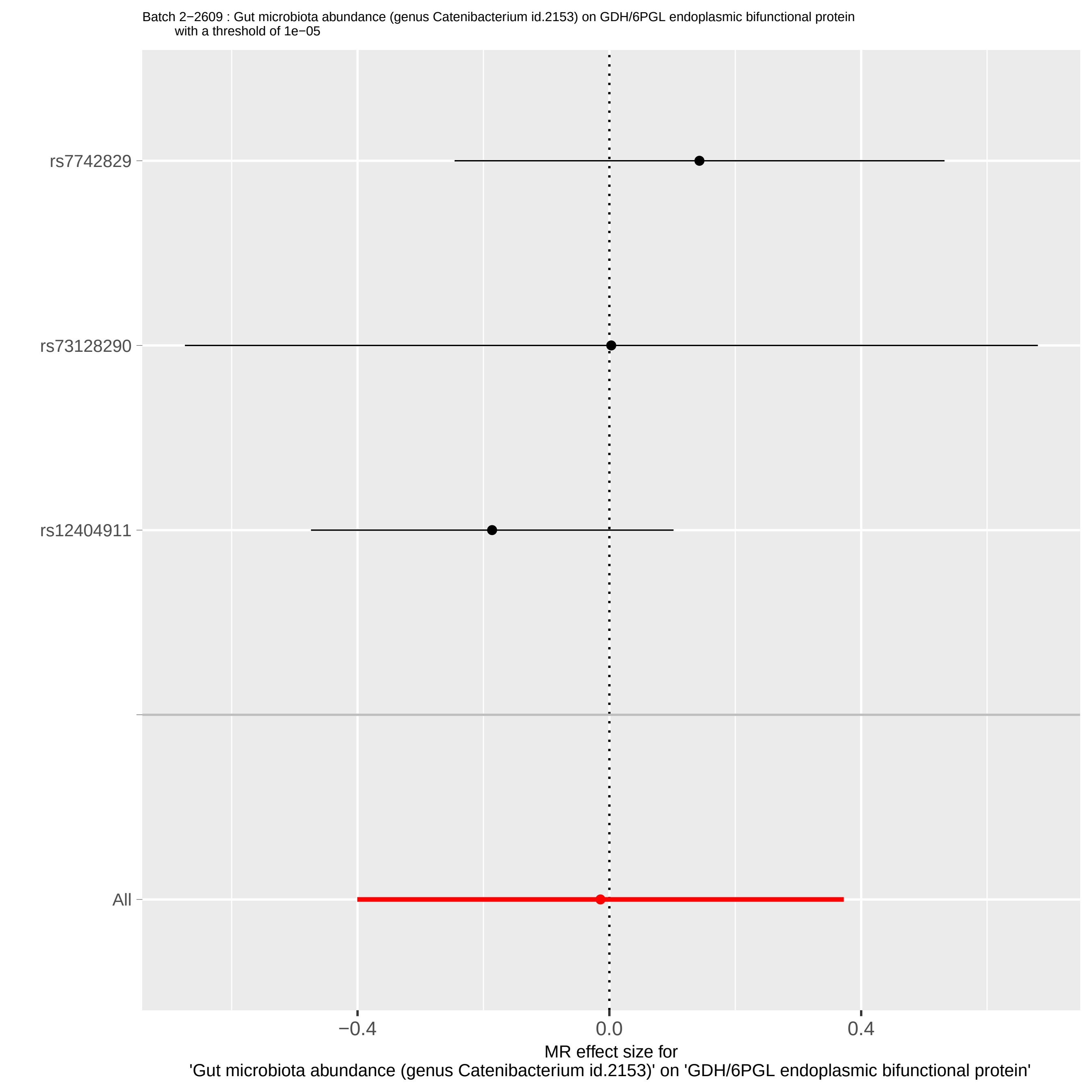
Batch 2−2609 : Gut microbiota abundance (genus Catenibacterium id.2153) on GDH/6PGL endoplasmic bifunctional protein
with a threshold of 1e−05
rs7742829
rs73128290
rs12404911
All
−0.4
0.0
0.4
MR effect size for
'Gut microbiota abundance (genus Catenibacterium id.2153)' on 'GDH/6PGL endoplasmic bifunctional protein'

Batch 2−2610 : Gut microbiota abundance (genus Parasutterella id.2892) on GDH/6PGL endoplasmic bifunctional protein with a threshold of 1e−05

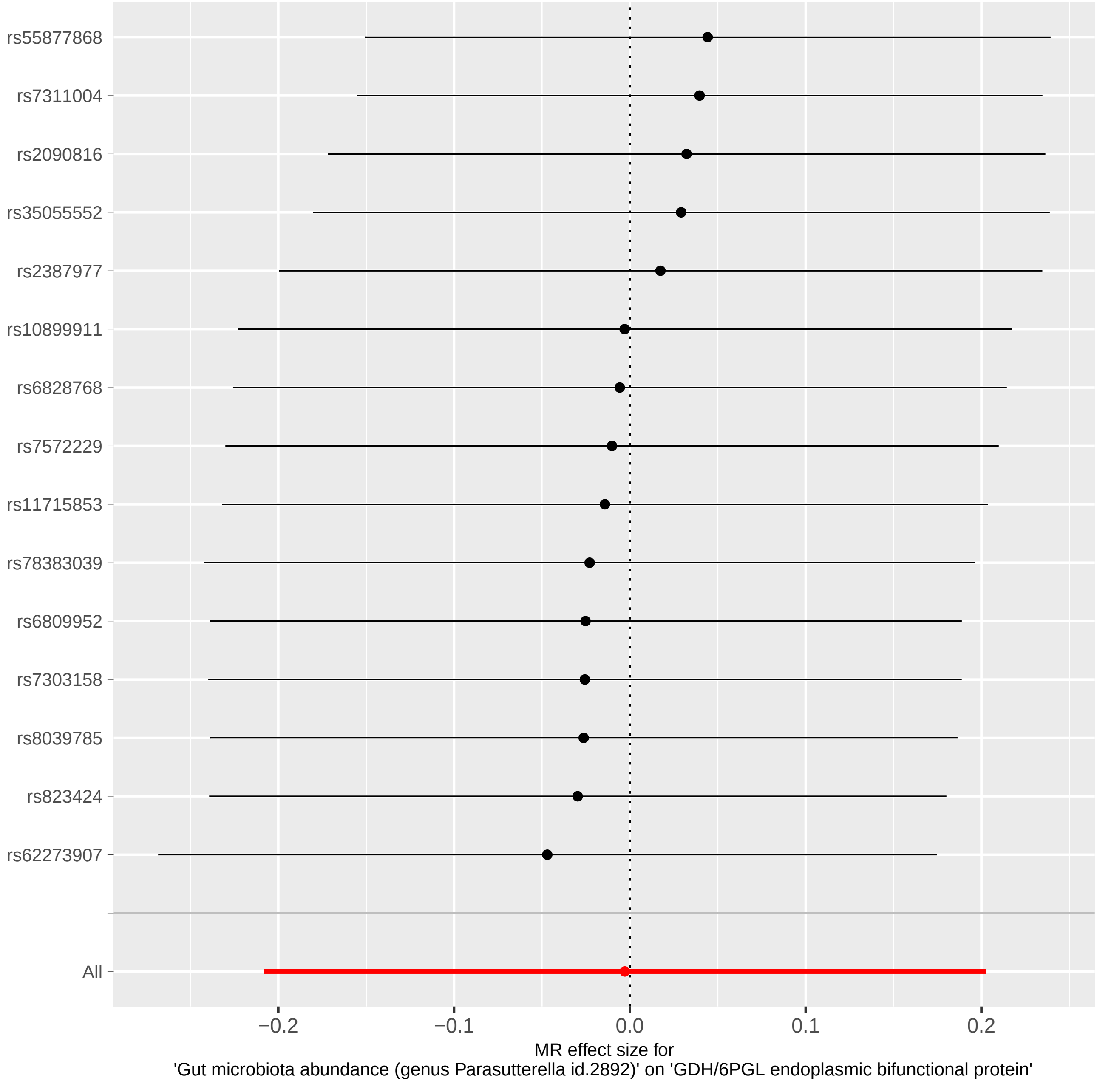

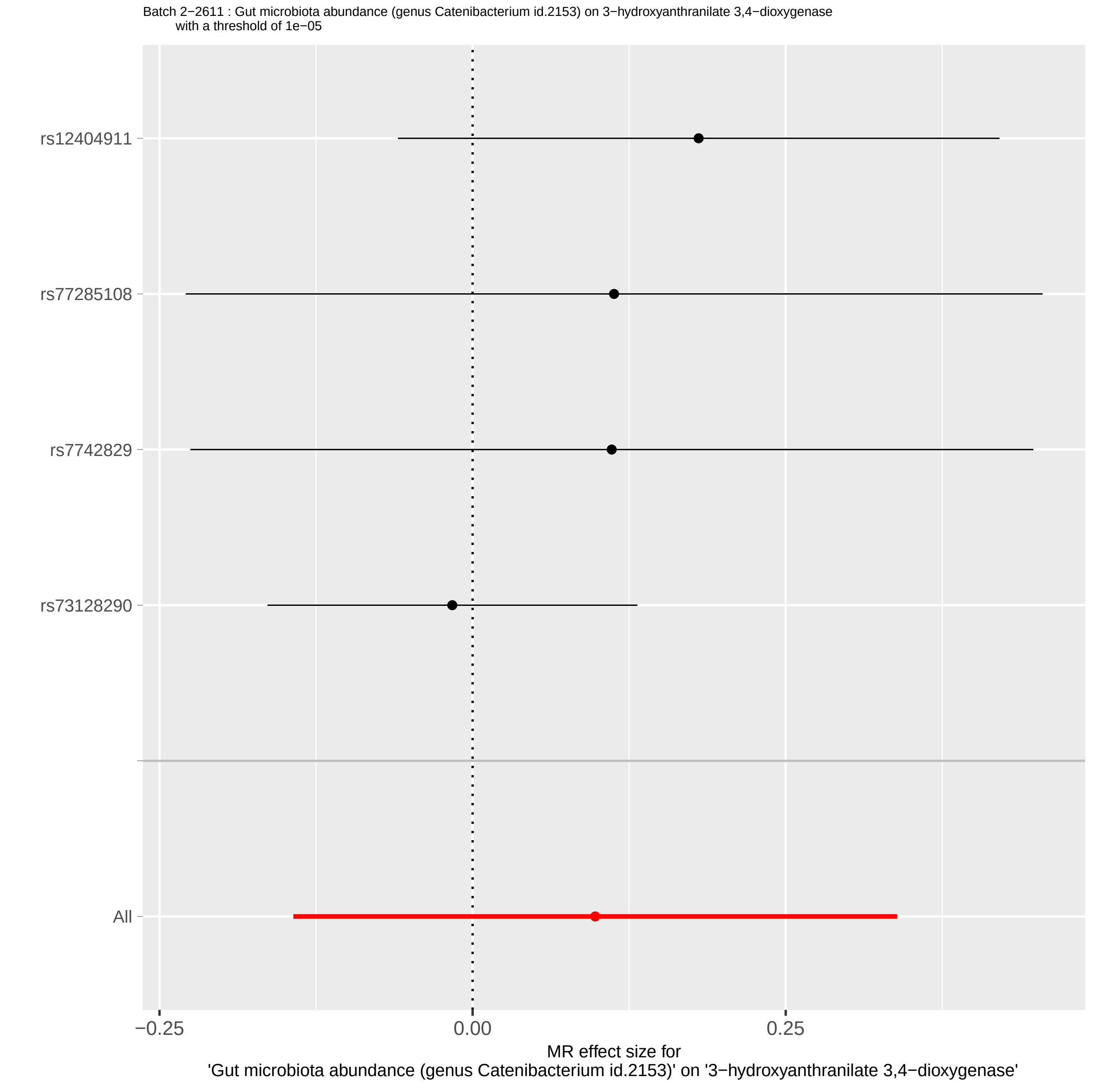

Batch 2−2611 : Gut microbiota abundance (genus Catenibacterium id.2153) on 3−hydroxyanthranilate 3,4−dioxygenase
with a threshold of 1e−05
rs12404911
rs77285108
rs7742829
rs73128290
All
−0.25
0.00
0.25
MR effect size for
'Gut microbiota abundance (genus Catenibacterium id.2153)' on '3−hydroxyanthranilate 3,4−dioxygenase'

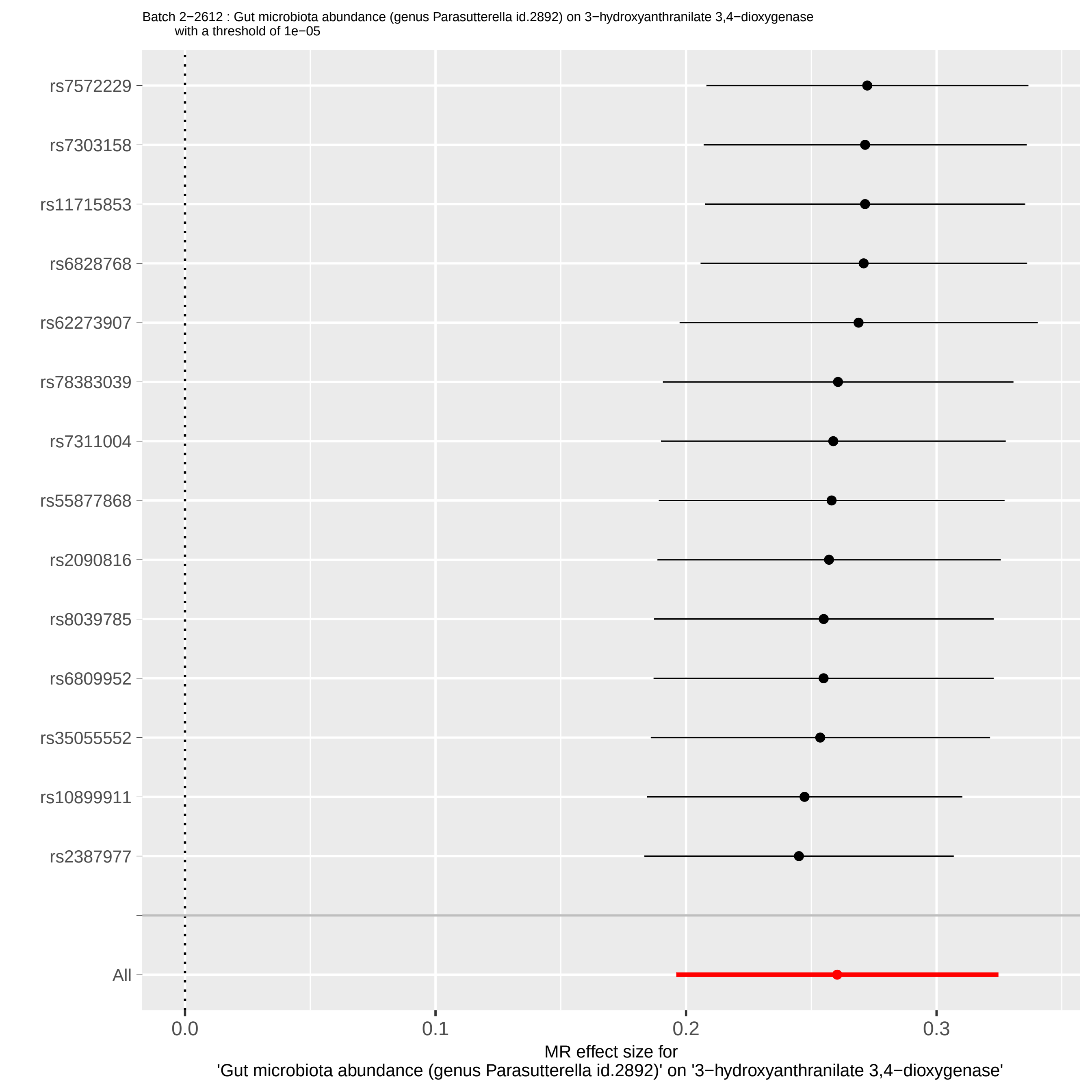
Batch 2−2612 : Gut microbiota abundance (genus Parasutterella id.2892) on 3−hydroxyanthranilate 3,4−dioxygenase
with a threshold of 1e−05
rs7572229
rs7303158
rs11715853
rs6828768
rs62273907
rs78383039
rs7311004
rs55877868
rs2090816
rs8039785
rs6809952
rs35055552
rs10899911
rs2387977
All
0.0
0.1
0.2
0.3
MR effect size for
'Gut microbiota abundance (genus Parasutterella id.2892)' on '3−hydroxyanthranilate 3,4−dioxygenase'

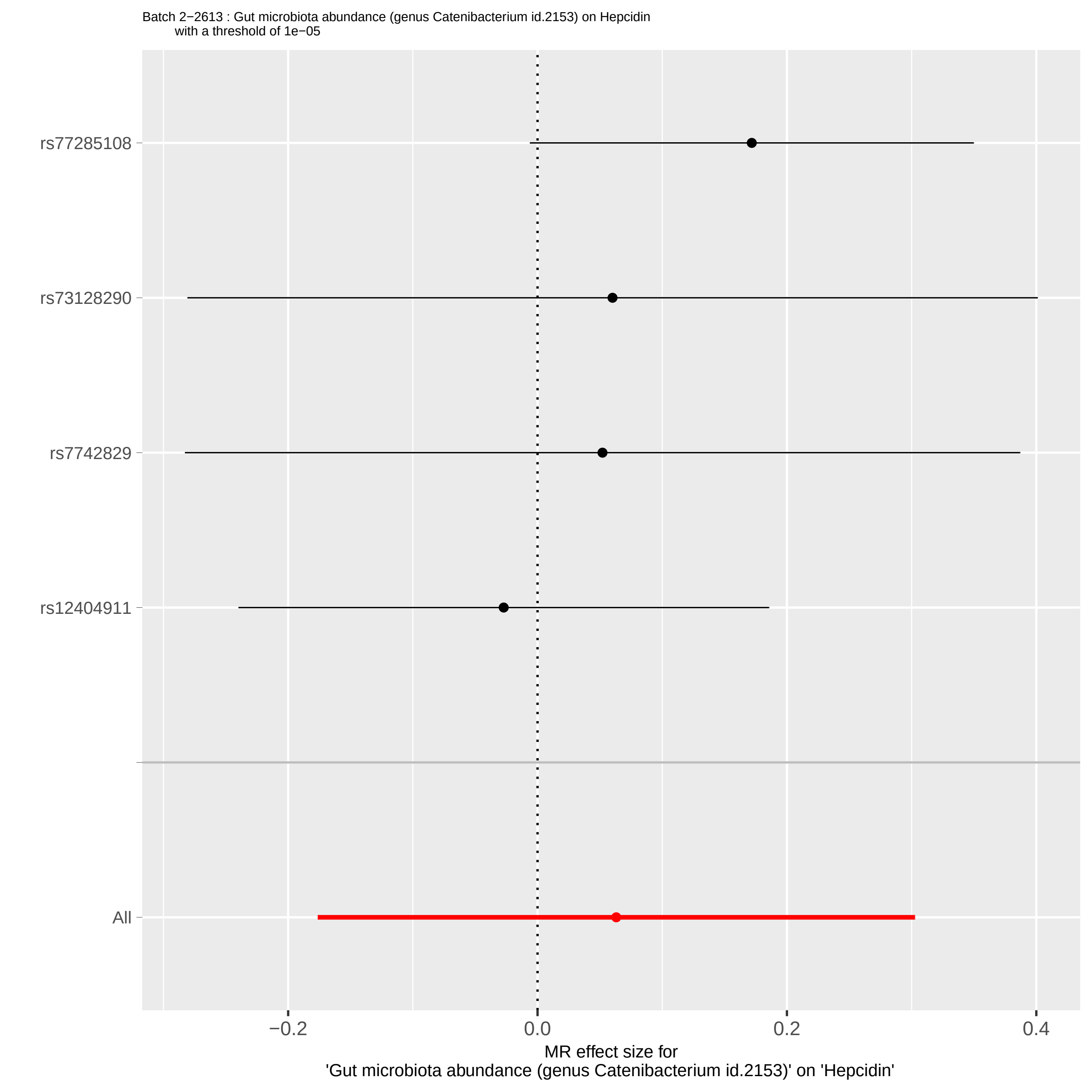

Batch 2−2613 : Gut microbiota abundance (genus Catenibacterium id.2153) on Hepcidin
with a threshold of 1e−05
rs77285108
rs73128290
rs7742829
rs12404911
All
−0.2
0.0
0.2
0.4
MR effect size for
'Gut microbiota abundance (genus Catenibacterium id.2153)' on 'Hepcidin'

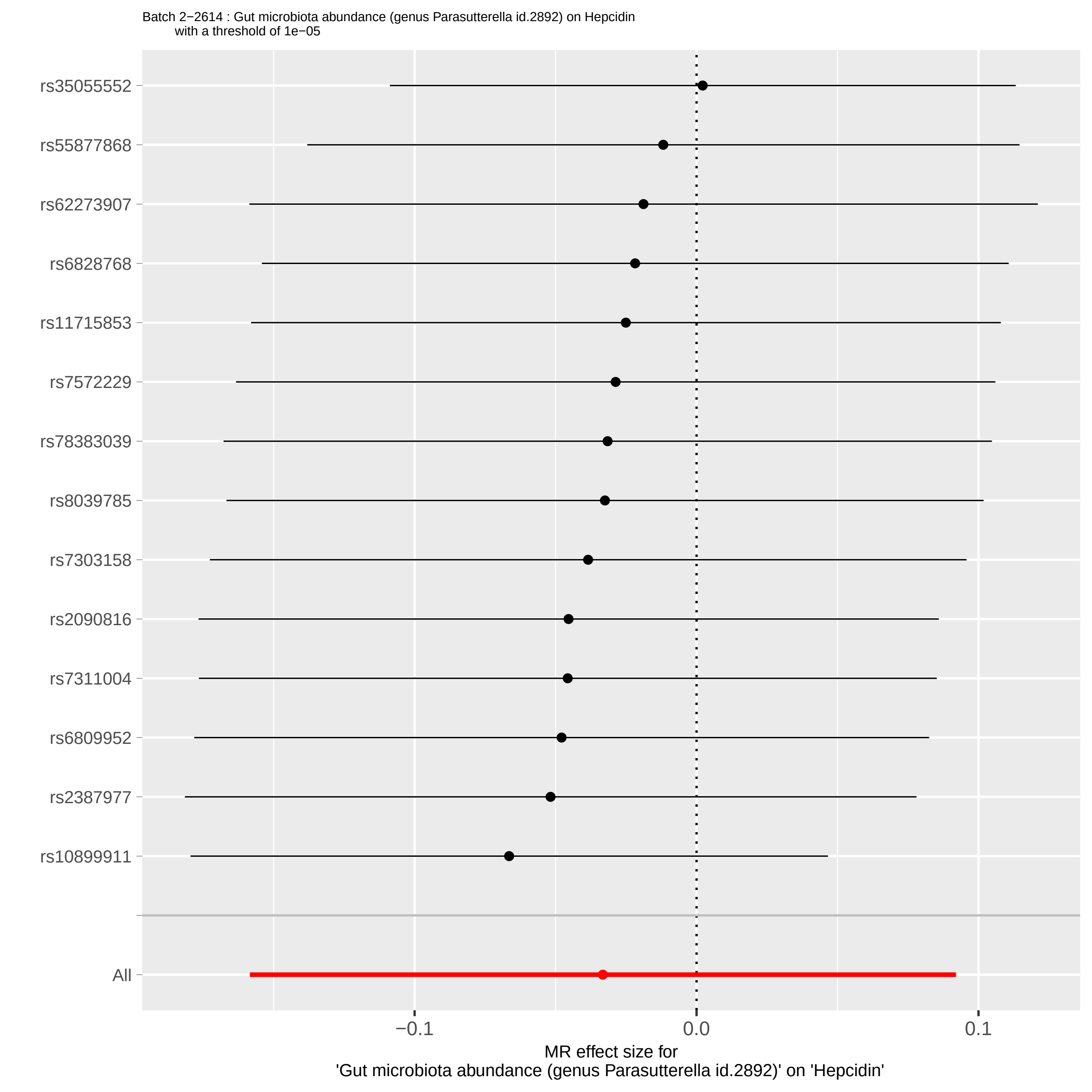
Batch 2−2614 : Gut microbiota abundance (genus Parasutterella id.2892) on Hepcidin
with a threshold of 1e−05
rs35055552
rs55877868
rs62273907
rs6828768
rs11715853
rs7572229
rs78383039
rs8039785
rs7303158
rs2090816
rs7311004
rs6809952
rs2387977
rs10899911
All
−0.1
0.0
0.1
MR effect size for
'Gut microbiota abundance (genus Parasutterella id.2892)' on 'Hepcidin'

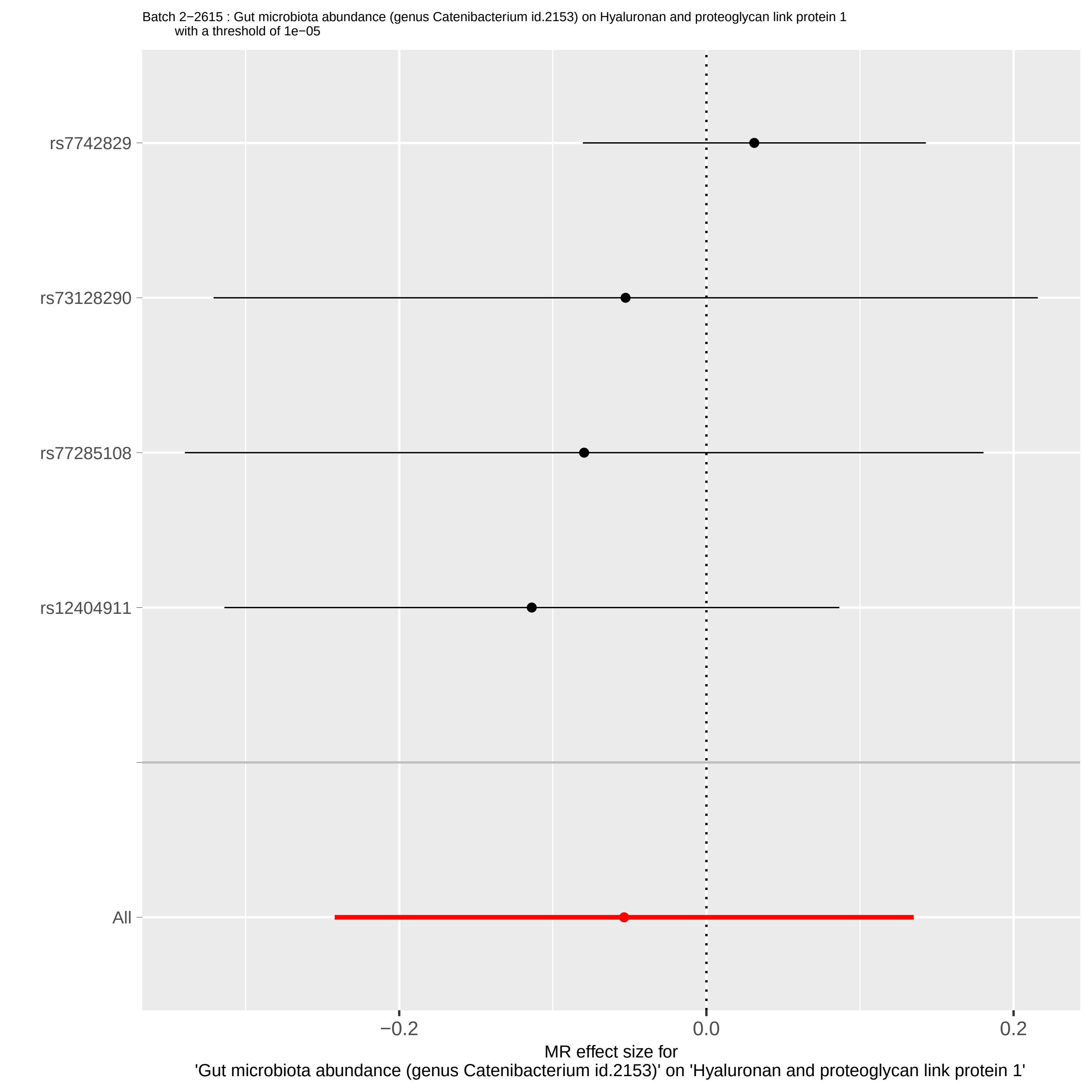
Batch 2−2615 : Gut microbiota abundance (genus Catenibacterium id.2153) on Hyaluronan and proteoglycan link protein 1
with a threshold of 1e−05
rs7742829
rs73128290
rs77285108
rs12404911
All
−0.2
0.0
0.2
MR effect size for
'Gut microbiota abundance (genus Catenibacterium id.2153)' on 'Hyaluronan and proteoglycan link protein 1'

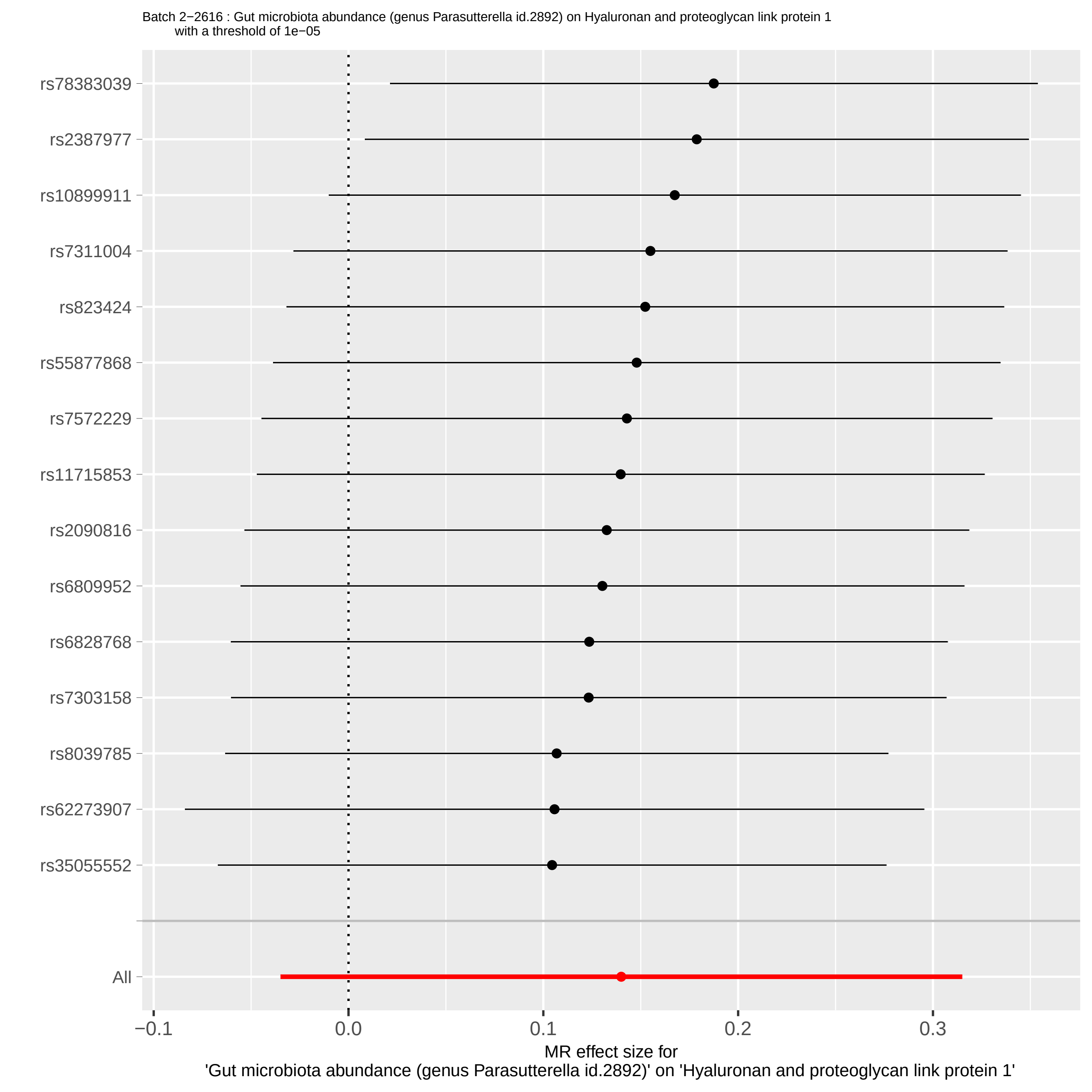

Batch 2−2616 : Gut microbiota abundance (genus Parasutterella id.2892) on Hyaluronan and proteoglycan link protein 1
with a threshold of 1e−05
rs78383039
rs2387977
rs10899911
rs7311004
rs823424
rs55877868
rs7572229
rs11715853
rs2090816
rs6809952
rs6828768
rs7303158
rs8039785
rs62273907
rs35055552
All
−0.1
0.0
0.1
0.2
0.3
MR effect size for
'Gut microbiota abundance (genus Parasutterella id.2892)' on 'Hyaluronan and proteoglycan link protein 1'

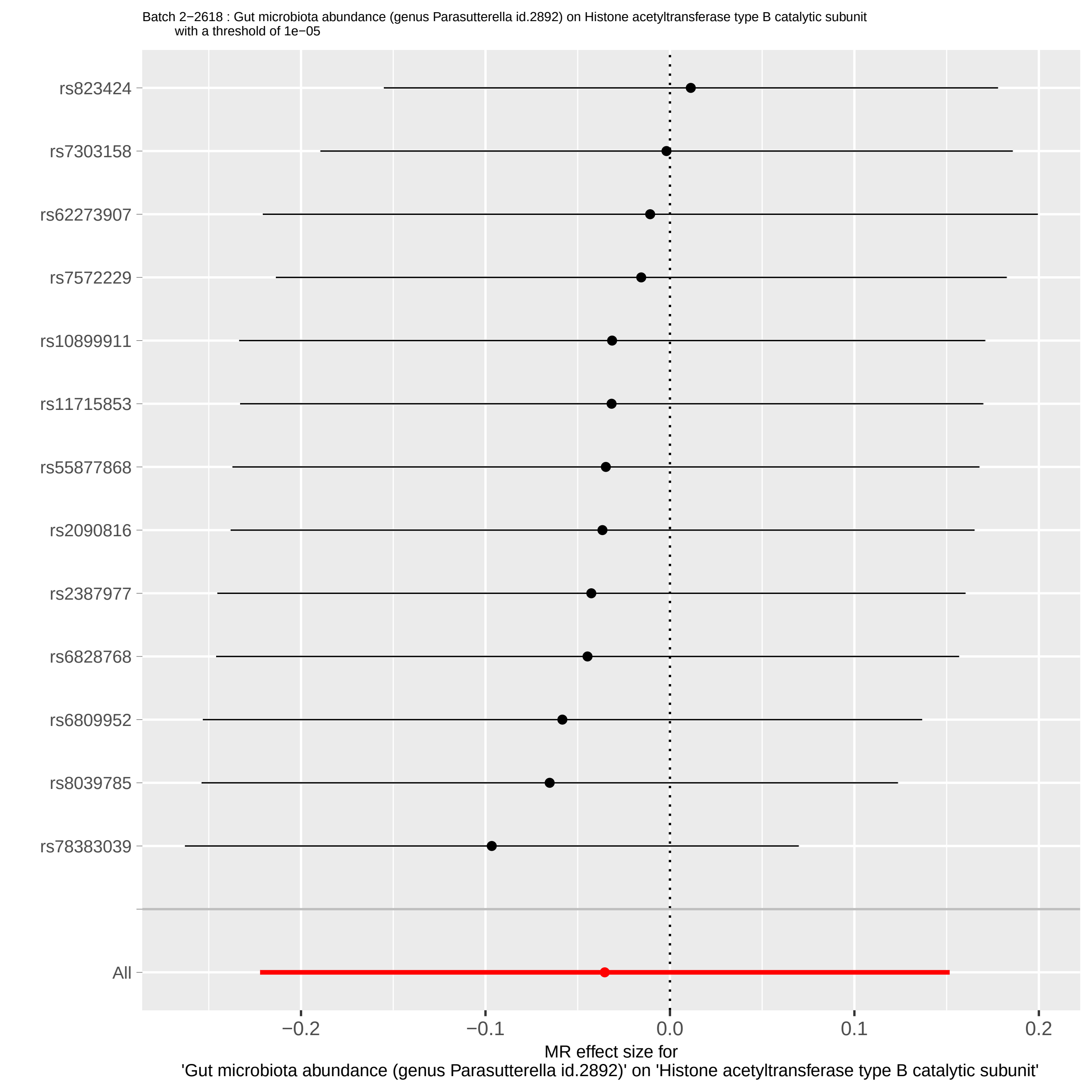
Batch 2−2618 : Gut microbiota abundance (genus Parasutterella id.2892) on Histone acetyltransferase type B catalytic subunit
with a threshold of 1e−05
rs823424
rs7303158
rs62273907
rs7572229
rs10899911
rs11715853
rs55877868
rs2090816
rs2387977
rs6828768
rs6809952
rs8039785
rs78383039
All
−0.2
−0.1
0.0
0.1
0.2
MR effect size for
'Gut microbiota abundance (genus Parasutterella id.2892)' on 'Histone acetyltransferase type B catalytic subunit'

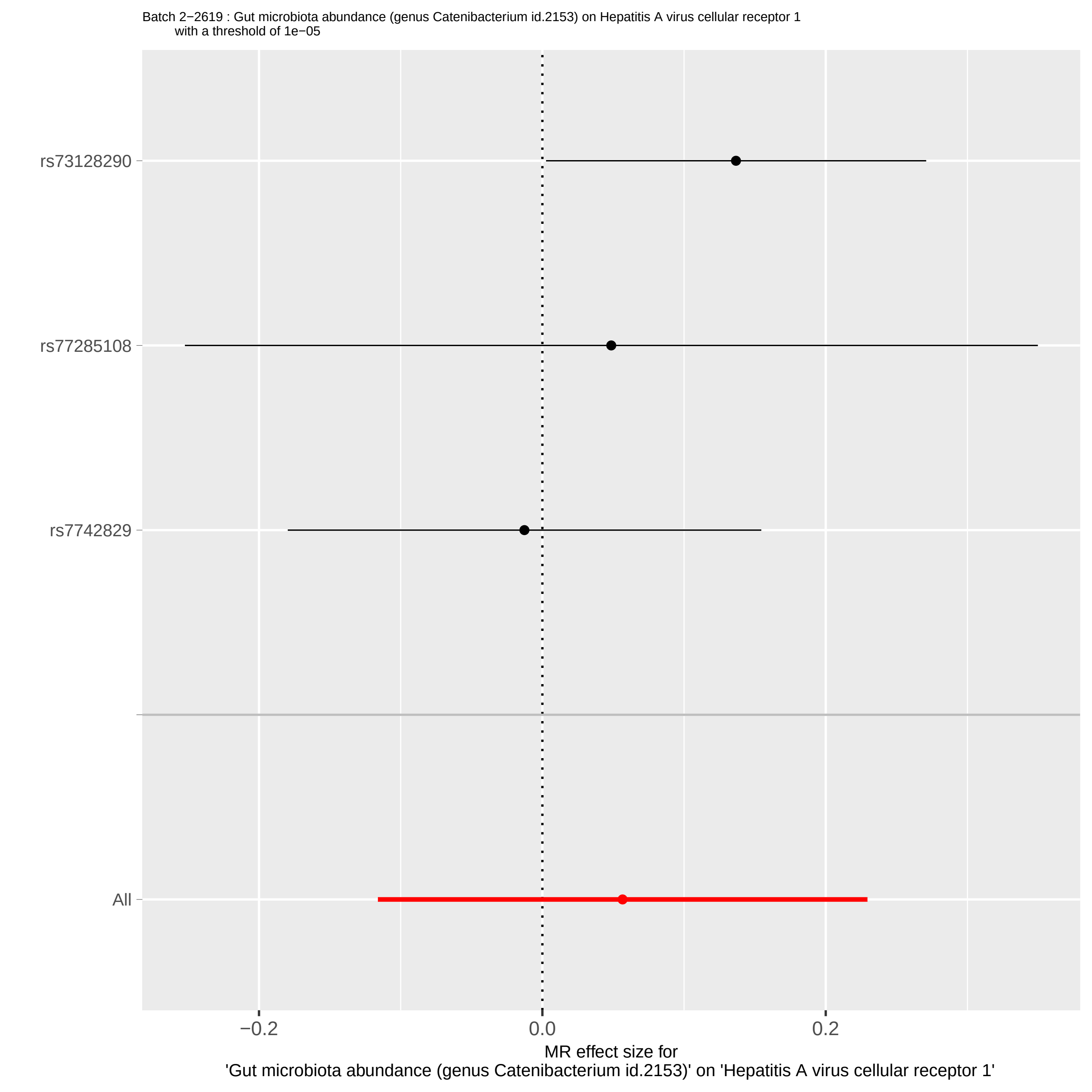
Batch 2−2619 : Gut microbiota abundance (genus Catenibacterium id.2153) on Hepatitis A virus cellular receptor 1
with a threshold of 1e−05
rs73128290
rs77285108
rs7742829
All
−0.2
0.0
0.2
MR effect size for
'Gut microbiota abundance (genus Catenibacterium id.2153)' on 'Hepatitis A virus cellular receptor 1'

Batch 2−2620 : Gut microbiota abundance (genus Parasutterella id.2892) on Hepatitis A virus cellular receptor 1
with a threshold of 1e−05

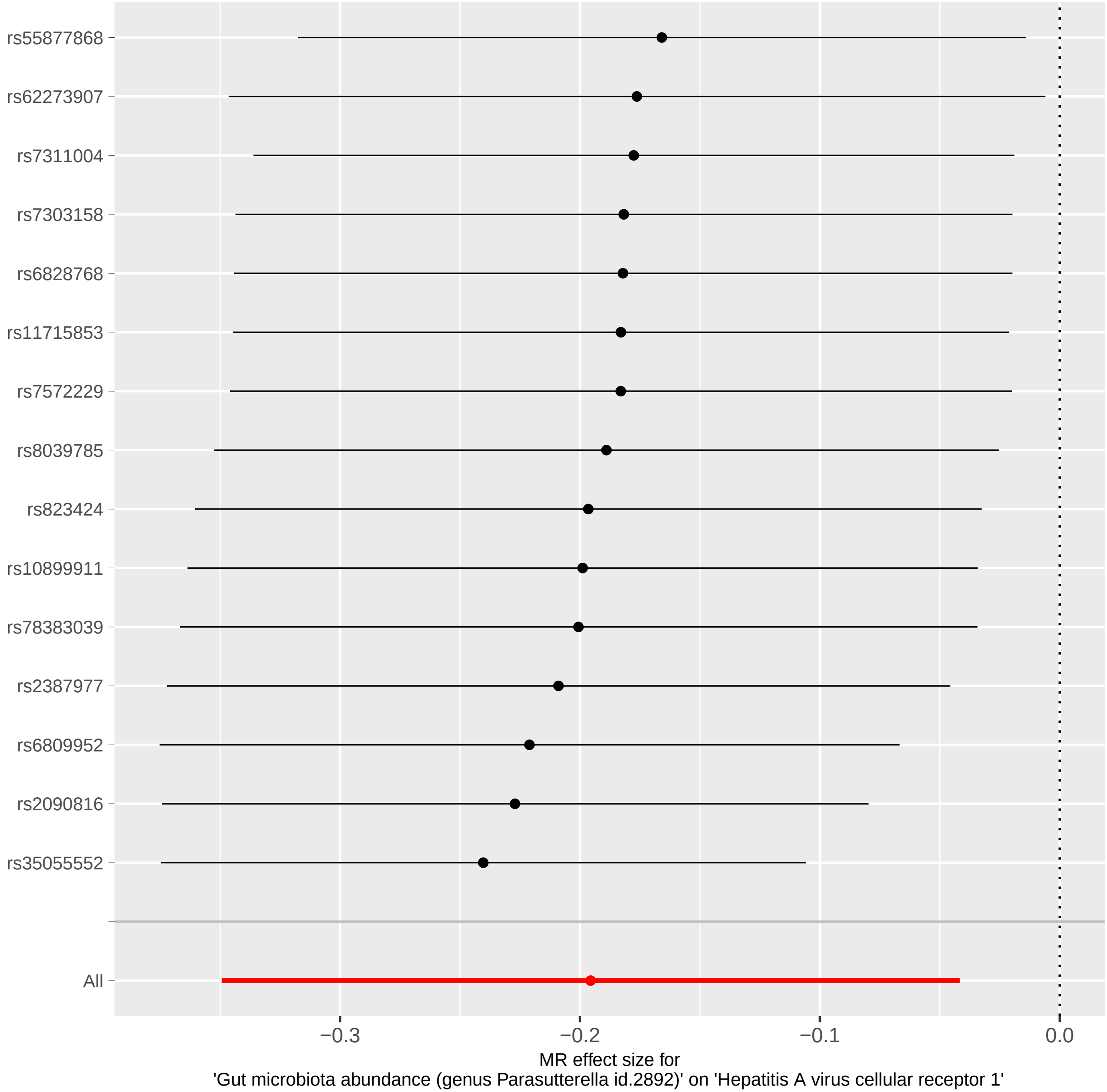

Batch 2−2621 : Gut microbiota abundance (genus Catenibacterium id.2153) on Hepatitis A virus cellular receptor 2
with a threshold of 1e−05

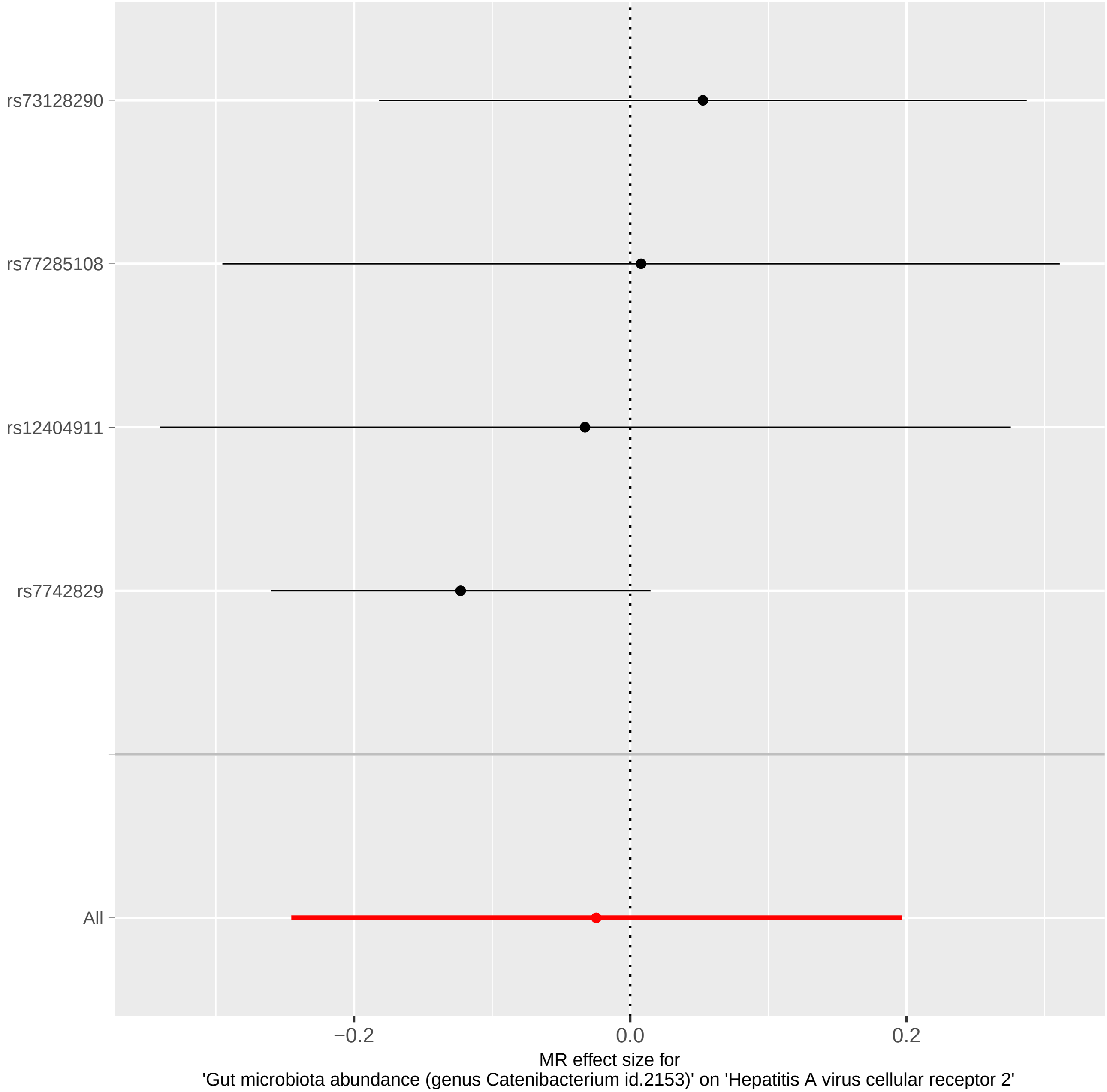

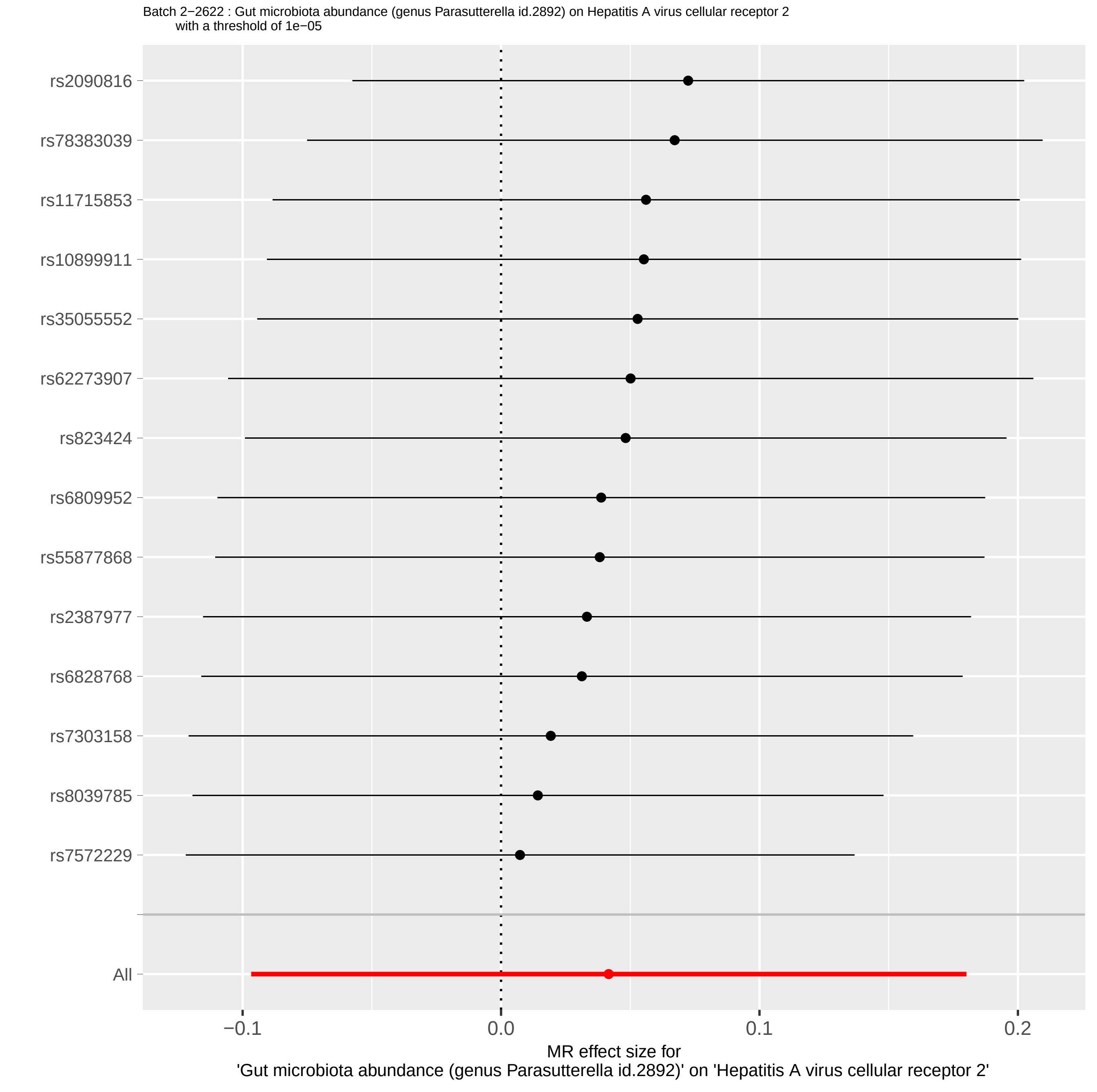

Batch 2−2622 : Gut microbiota abundance (genus Parasutterella id.2892) on Hepatitis A virus cellular receptor 2
with a threshold of 1e−05
rs2090816
rs78383039
rs11715853
rs10899911
rs35055552
rs62273907
rs823424
rs6809952
rs55877868
rs2387977
rs6828768
rs7303158
rs8039785
rs7572229
All
−0.1
0.0
0.1
0.2
MR effect size for
'Gut microbiota abundance (genus Parasutterella id.2892)' on 'Hepatitis A virus cellular receptor 2'

Batch 2−2623 : Gut microbiota abundance (genus Catenibacterium id.2153) on Hepatitis A virus cellular receptor 2
with a threshold of 1e−05

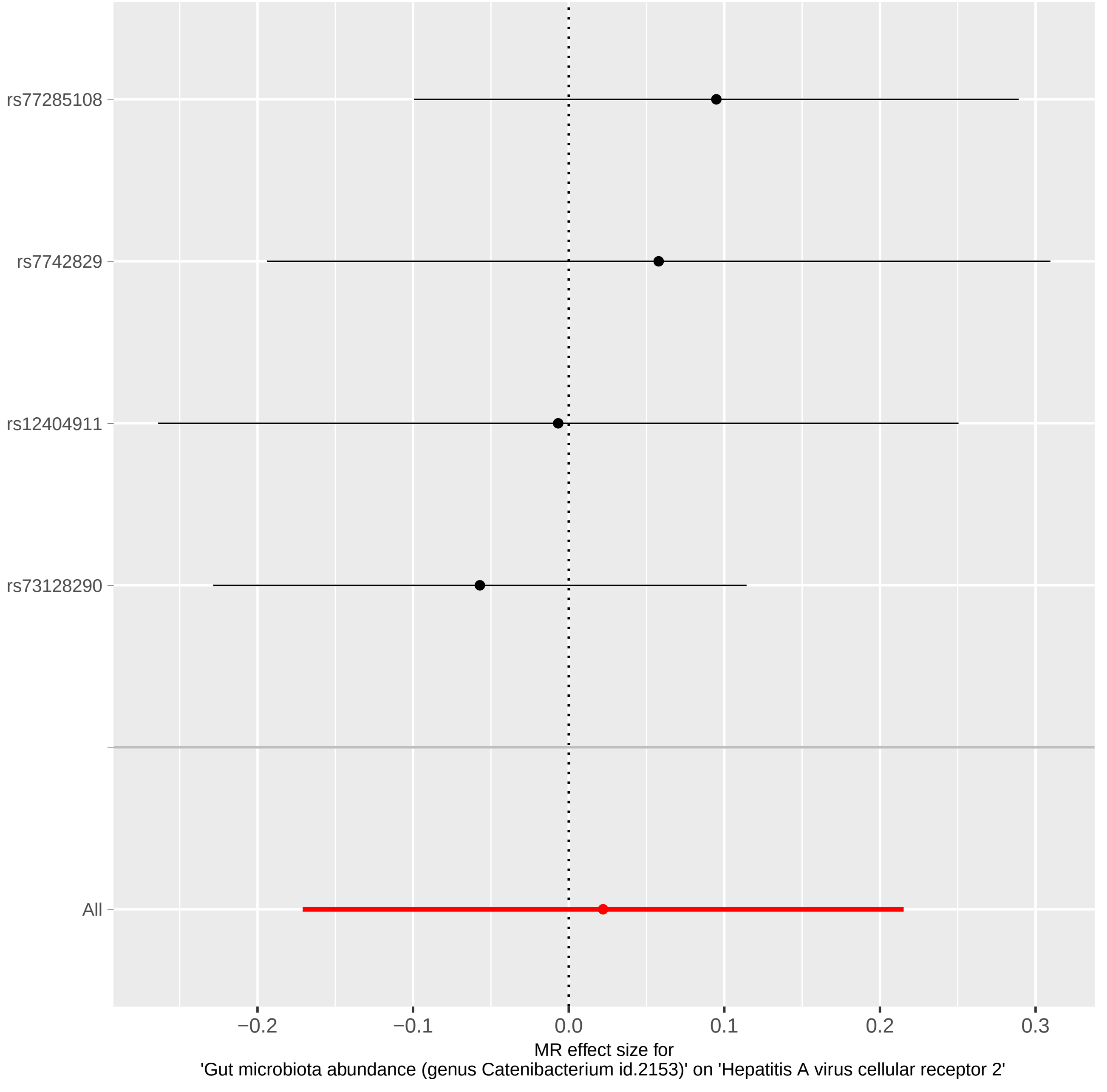

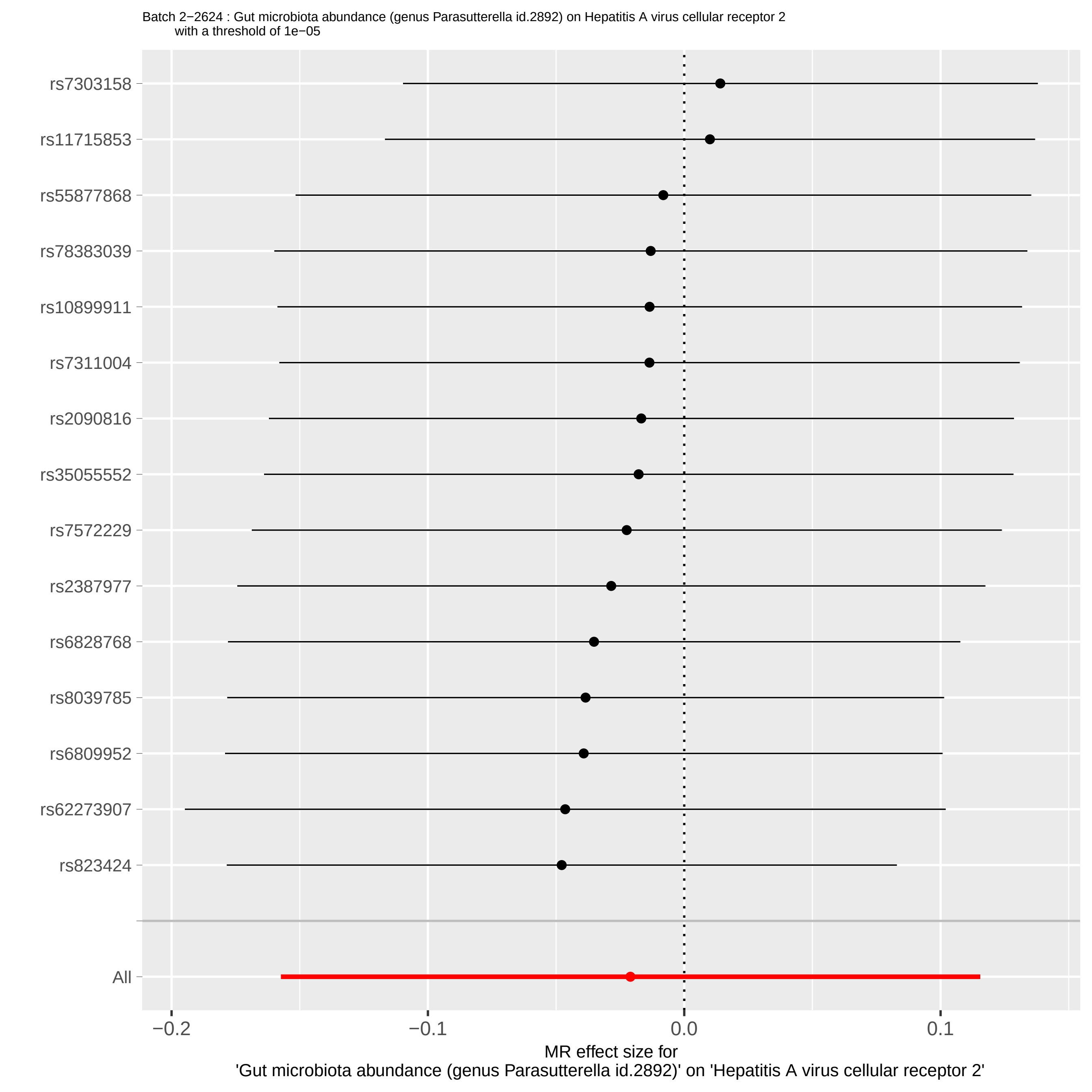

Batch 2−2624 : Gut microbiota abundance (genus Parasutterella id.2892) on Hepatitis A virus cellular receptor 2
with a threshold of 1e−05
rs7303158
rs11715853
rs55877868
rs78383039
rs10899911
rs7311004
rs2090816
rs35055552
rs7572229
rs2387977
rs6828768
rs8039785
rs6809952
rs62273907
rs823424
All
−0.2
−0.1
0.0
0.1
MR effect size for
'Gut microbiota abundance (genus Parasutterella id.2892)' on 'Hepatitis A virus cellular receptor 2'

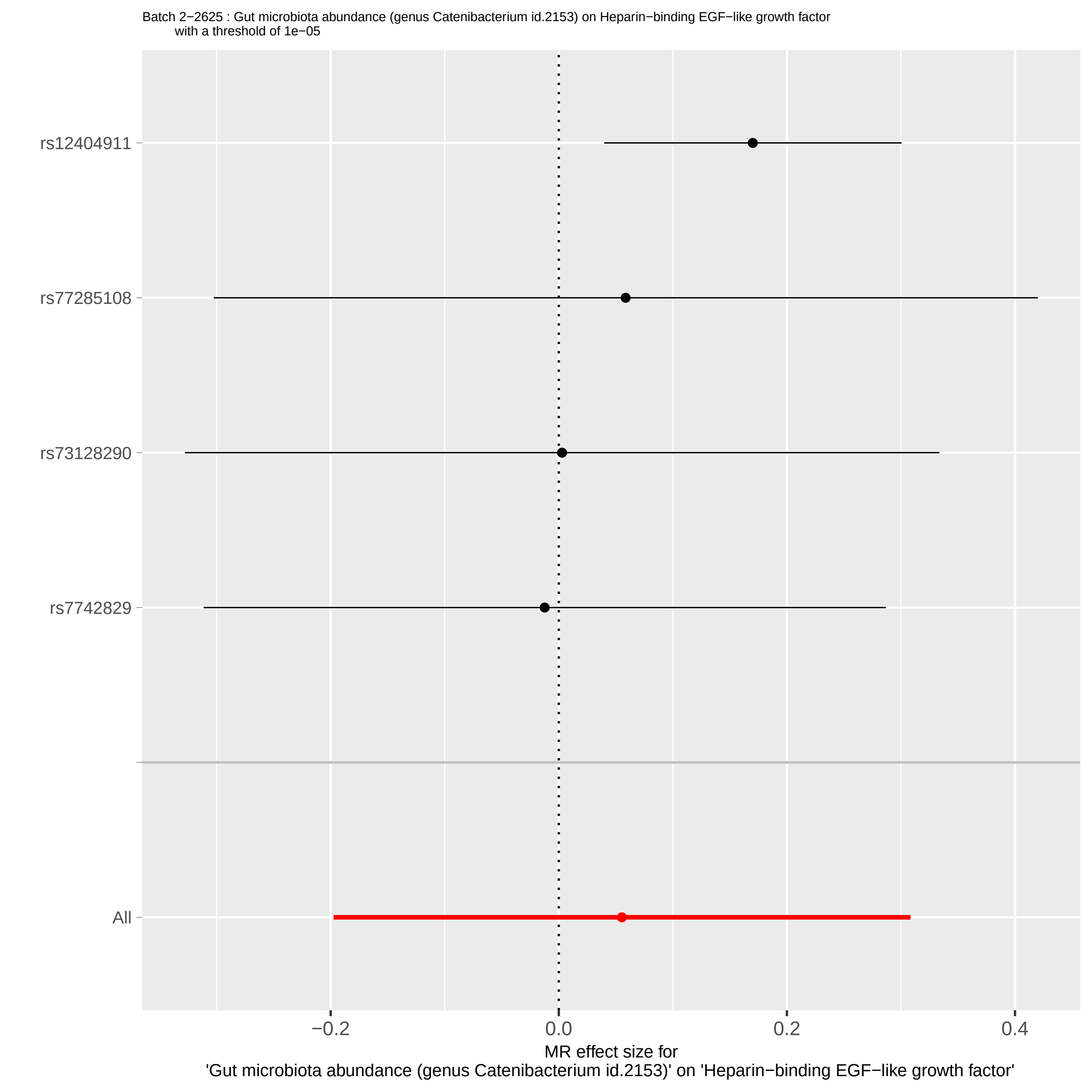
Batch 2−2625 : Gut microbiota abundance (genus Catenibacterium id.2153) on Heparin−binding EGF−like growth factor
with a threshold of 1e−05
rs12404911
rs77285108
rs73128290
rs7742829
All
−0.2
0.0
0.2
0.4
MR effect size for
'Gut microbiota abundance (genus Catenibacterium id.2153)' on 'Heparin−binding EGF−like growth factor'

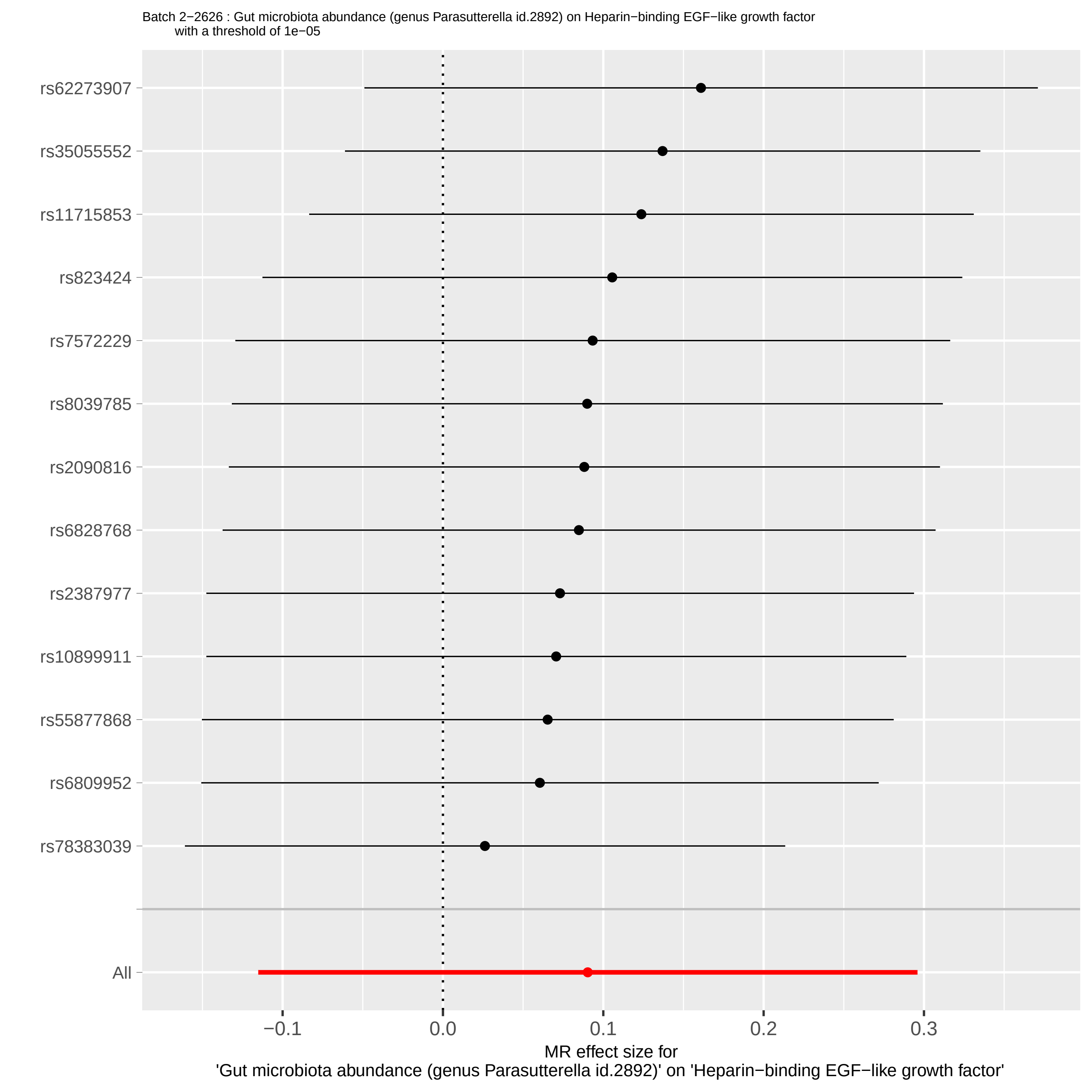

Batch 2−2626 : Gut microbiota abundance (genus Parasutterella id.2892) on Heparin−binding EGF−like growth factor
with a threshold of 1e−05
rs62273907
rs35055552
rs11715853
rs823424
rs7572229
rs8039785
rs2090816
rs6828768
rs2387977
rs10899911
rs55877868
rs6809952
rs78383039
All
−0.1
0.0
0.1
0.2
0.3
MR effect size for
'Gut microbiota abundance (genus Parasutterella id.2892)' on 'Heparin−binding EGF−like growth factor'

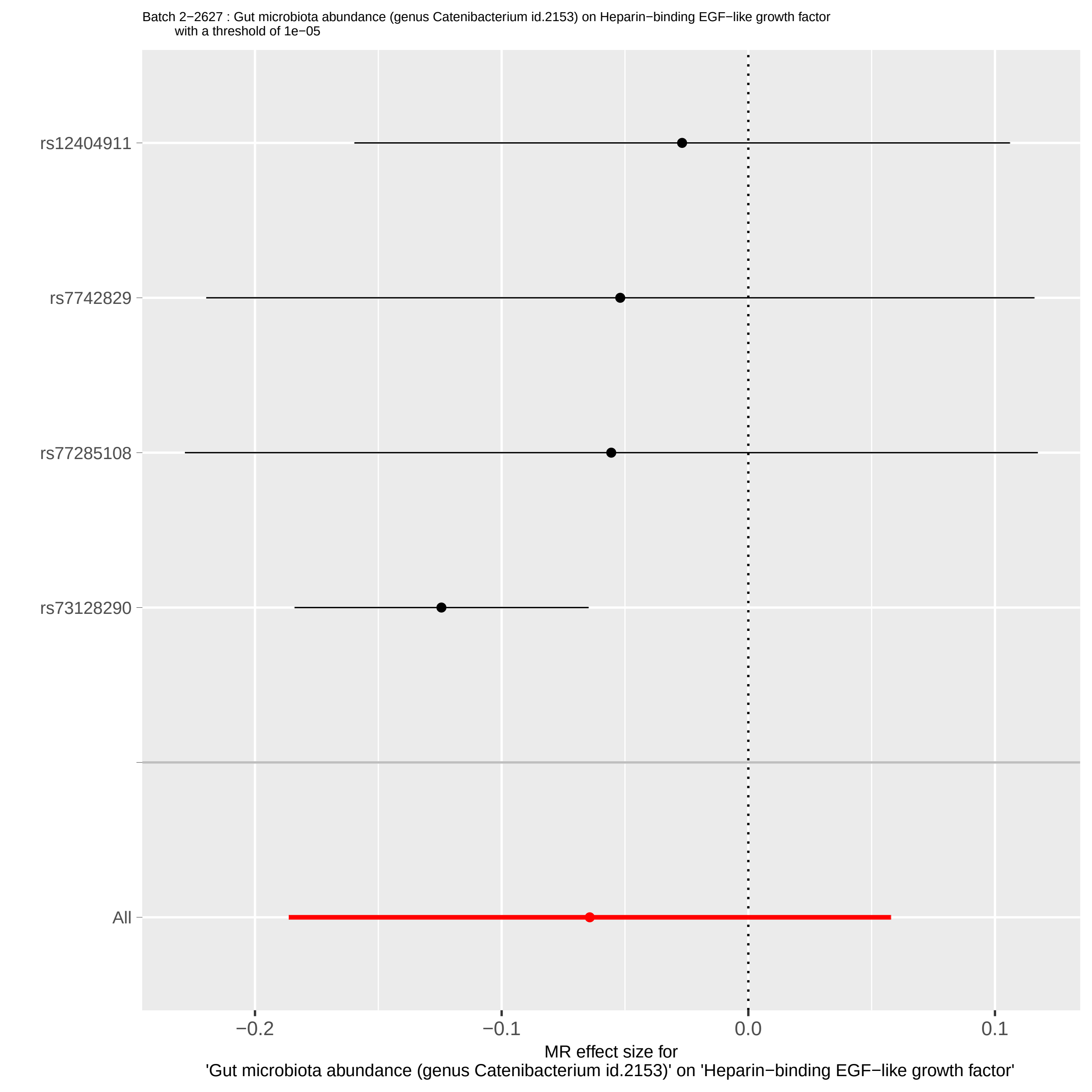
Batch 2−2627 : Gut microbiota abundance (genus Catenibacterium id.2153) on Heparin−binding EGF−like growth factor
with a threshold of 1e−05
rs12404911
rs7742829
rs77285108
rs73128290
All
−0.2
−0.1
0.0
0.1
MR effect size for
'Gut microbiota abundance (genus Catenibacterium id.2153)' on 'Heparin−binding EGF−like growth factor'

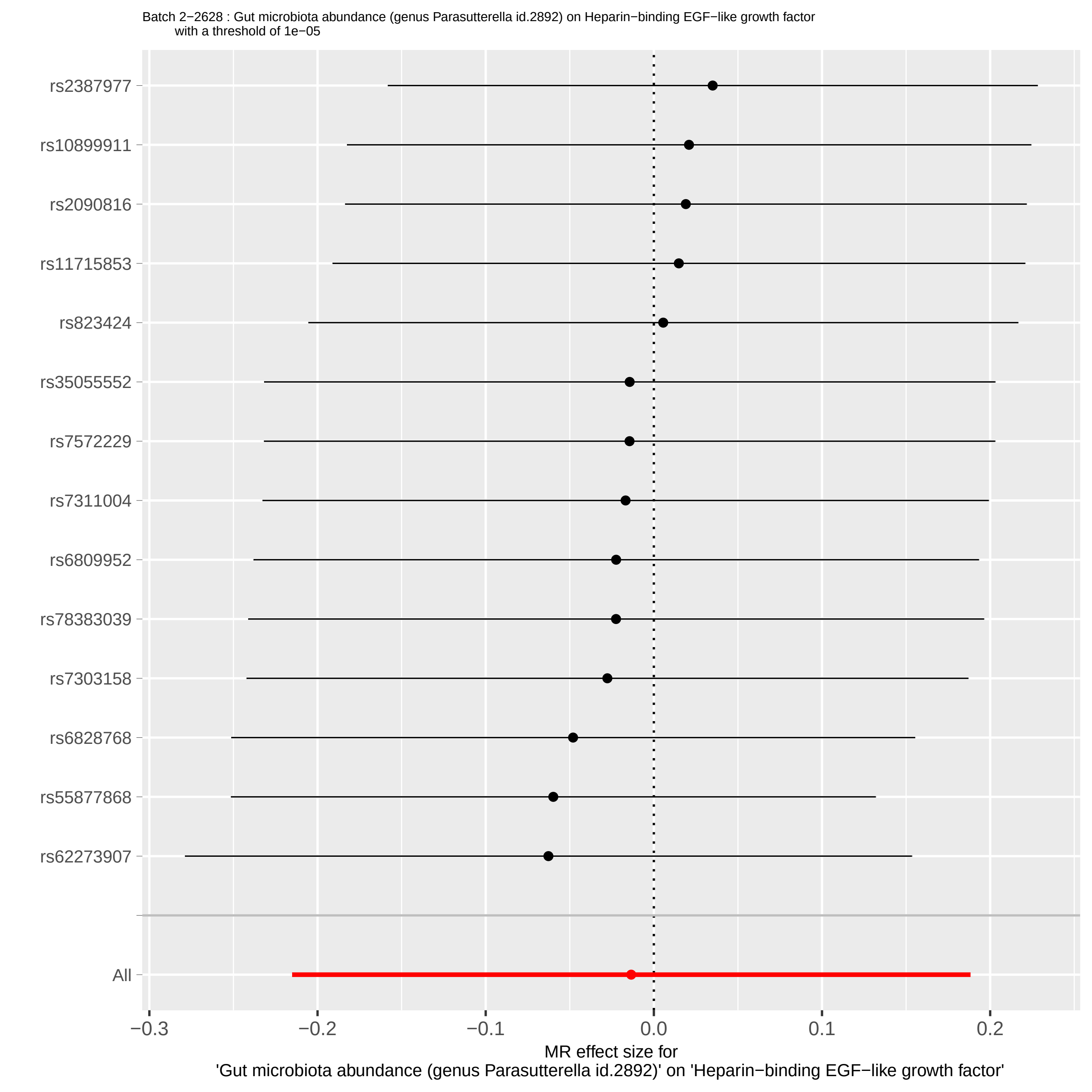
Batch 2−2628 : Gut microbiota abundance (genus Parasutterella id.2892) on Heparin−binding EGF−like growth factor
with a threshold of 1e−05
rs2387977
rs10899911
rs2090816
rs11715853
rs823424
rs35055552
rs7572229
rs7311004
rs6809952
rs78383039
rs7303158
rs6828768
rs55877868
rs62273907
All
−0.3
−0.2
−0.1
0.0
0.1
0.2
MR effect size for
'Gut microbiota abundance (genus Parasutterella id.2892)' on 'Heparin−binding EGF−like growth factor'

Batch 2−2629 : Gut microbiota abundance (genus Catenibacterium id.2153) on Hemoglobin subunit gamma−1
with a threshold of 1e−05

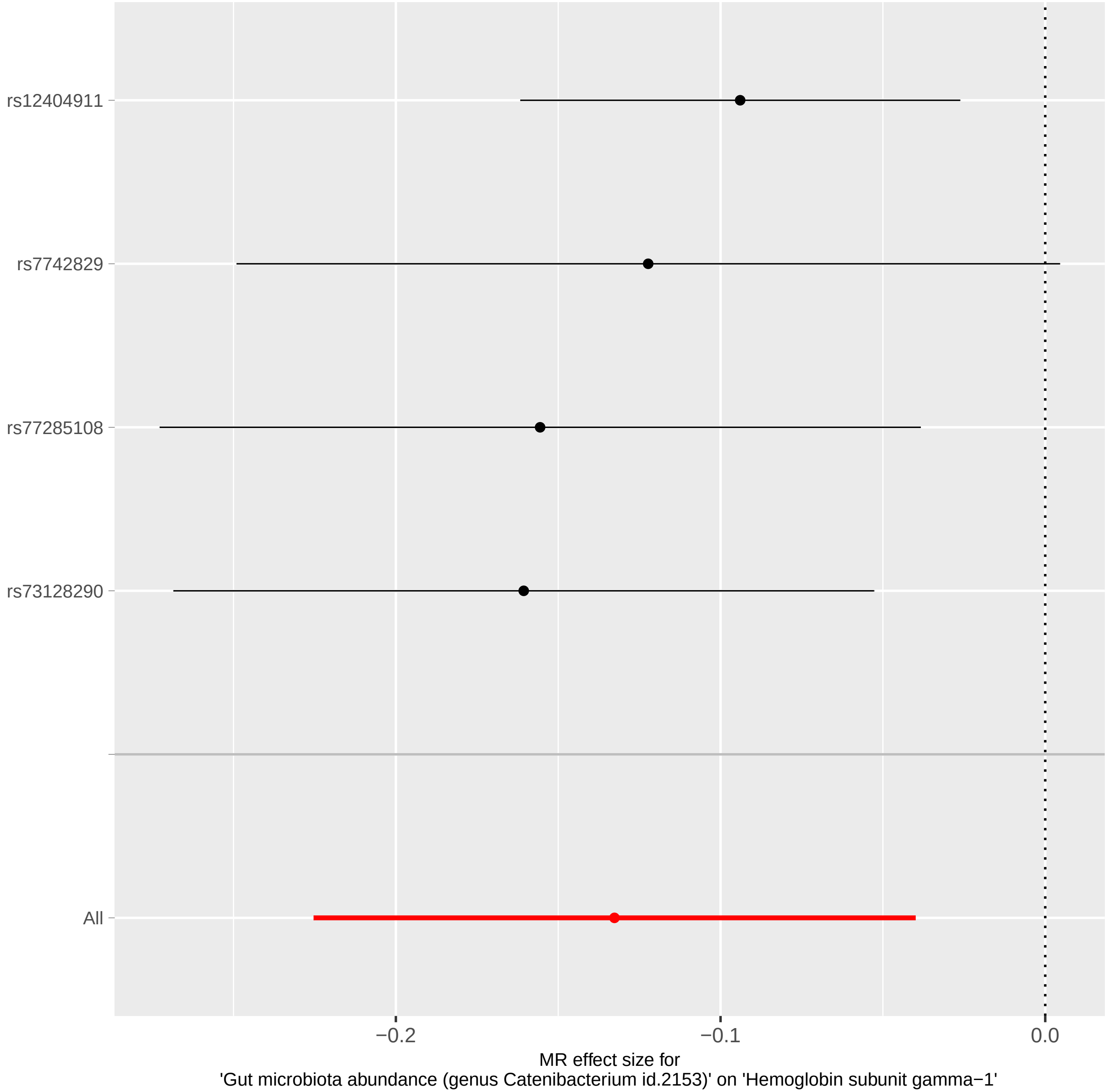

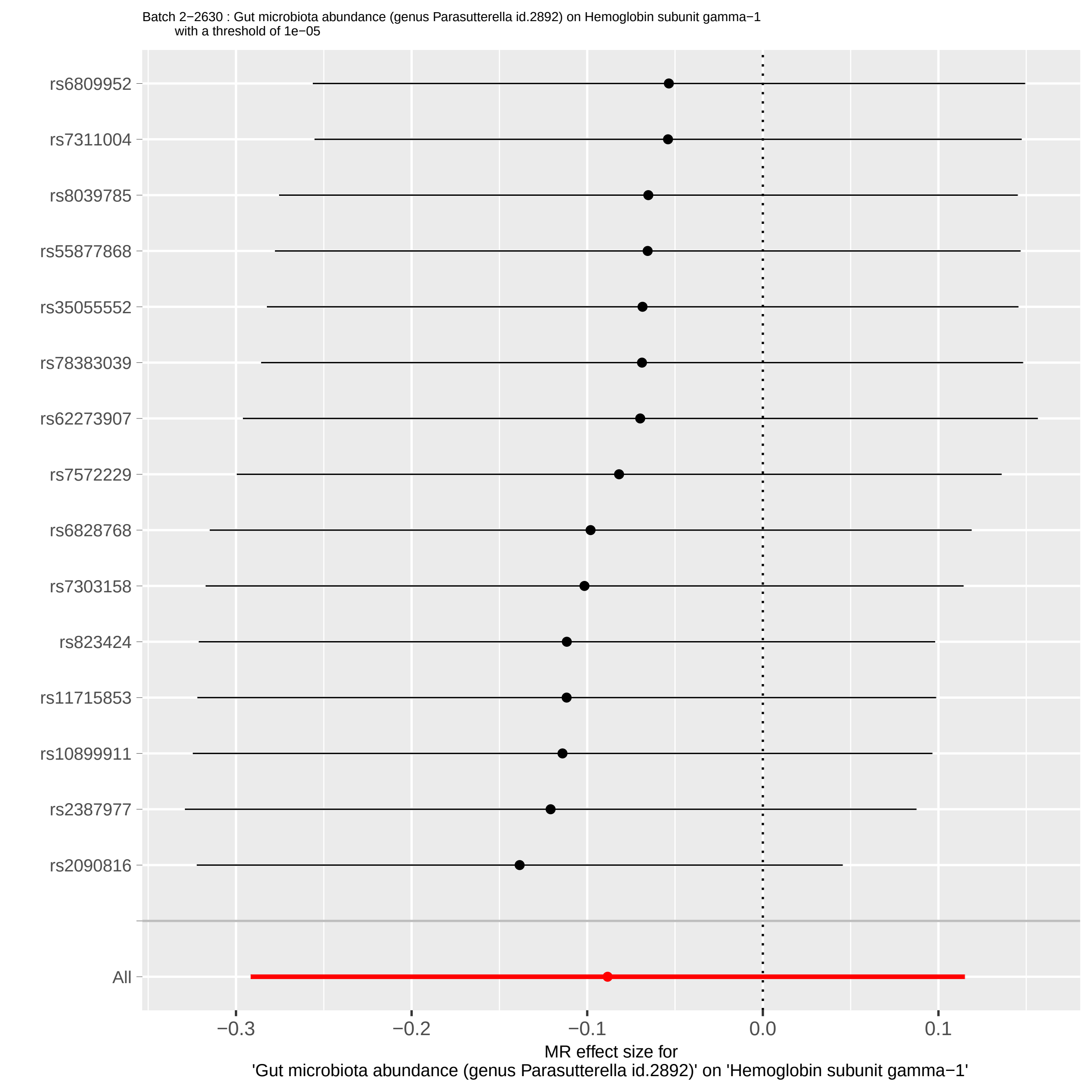

Batch 2−2630 : Gut microbiota abundance (genus Parasutterella id.2892) on Hemoglobin subunit gamma−1
with a threshold of 1e−05
rs6809952
rs7311004
rs8039785
rs55877868
rs35055552
rs78383039
rs62273907
rs7572229
rs6828768
rs7303158
rs823424
rs11715853
rs10899911
rs2387977
rs2090816
All
−0.3
−0.2
−0.1
0.0
0.1
MR effect size for
'Gut microbiota abundance (genus Parasutterella id.2892)' on 'Hemoglobin subunit gamma−1'

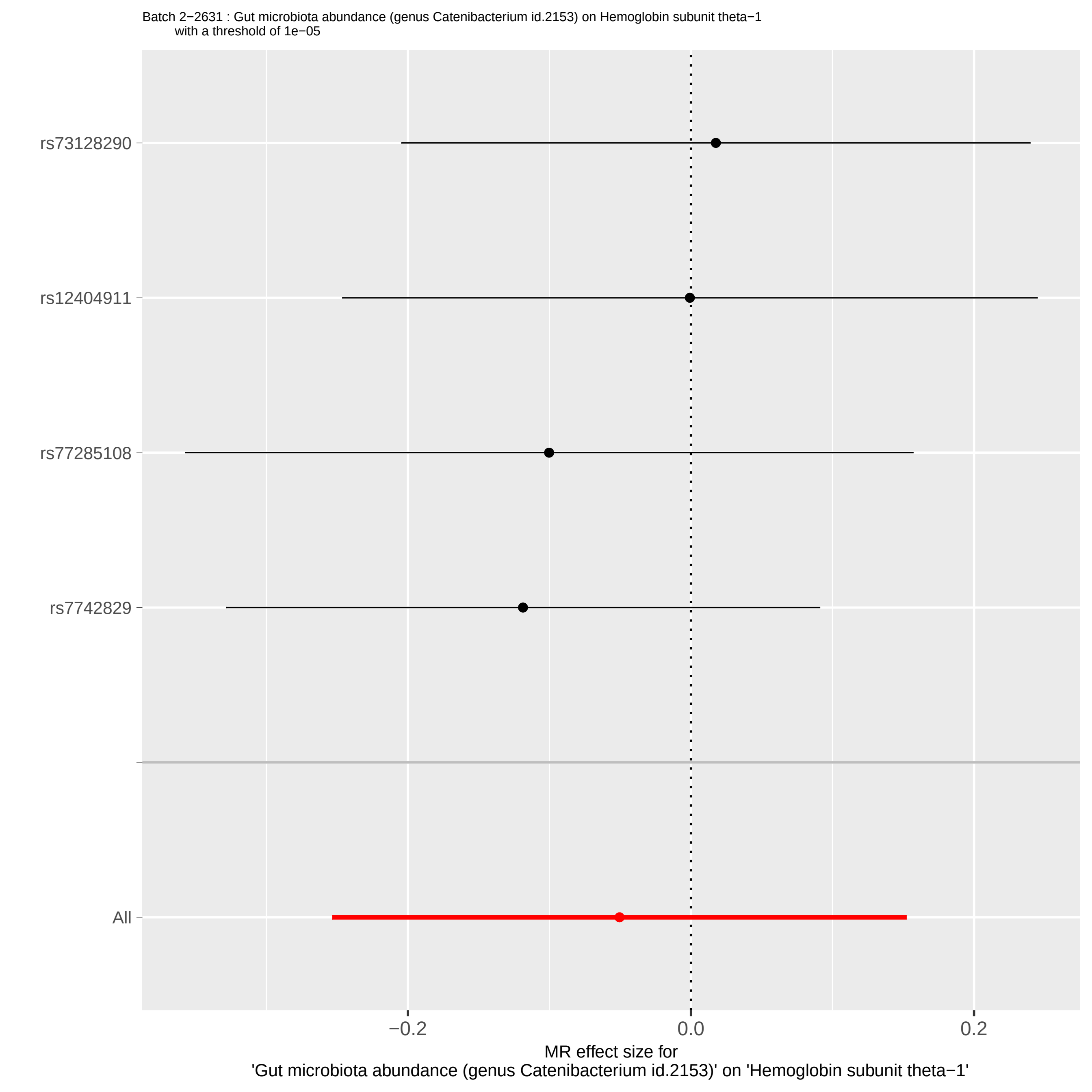

Batch 2−2631 : Gut microbiota abundance (genus Catenibacterium id.2153) on Hemoglobin subunit theta−1
with a threshold of 1e−05
rs73128290
rs12404911
rs77285108
rs7742829
All
−0.2
0.0
0.2
MR effect size for
'Gut microbiota abundance (genus Catenibacterium id.2153)' on 'Hemoglobin subunit theta−1'

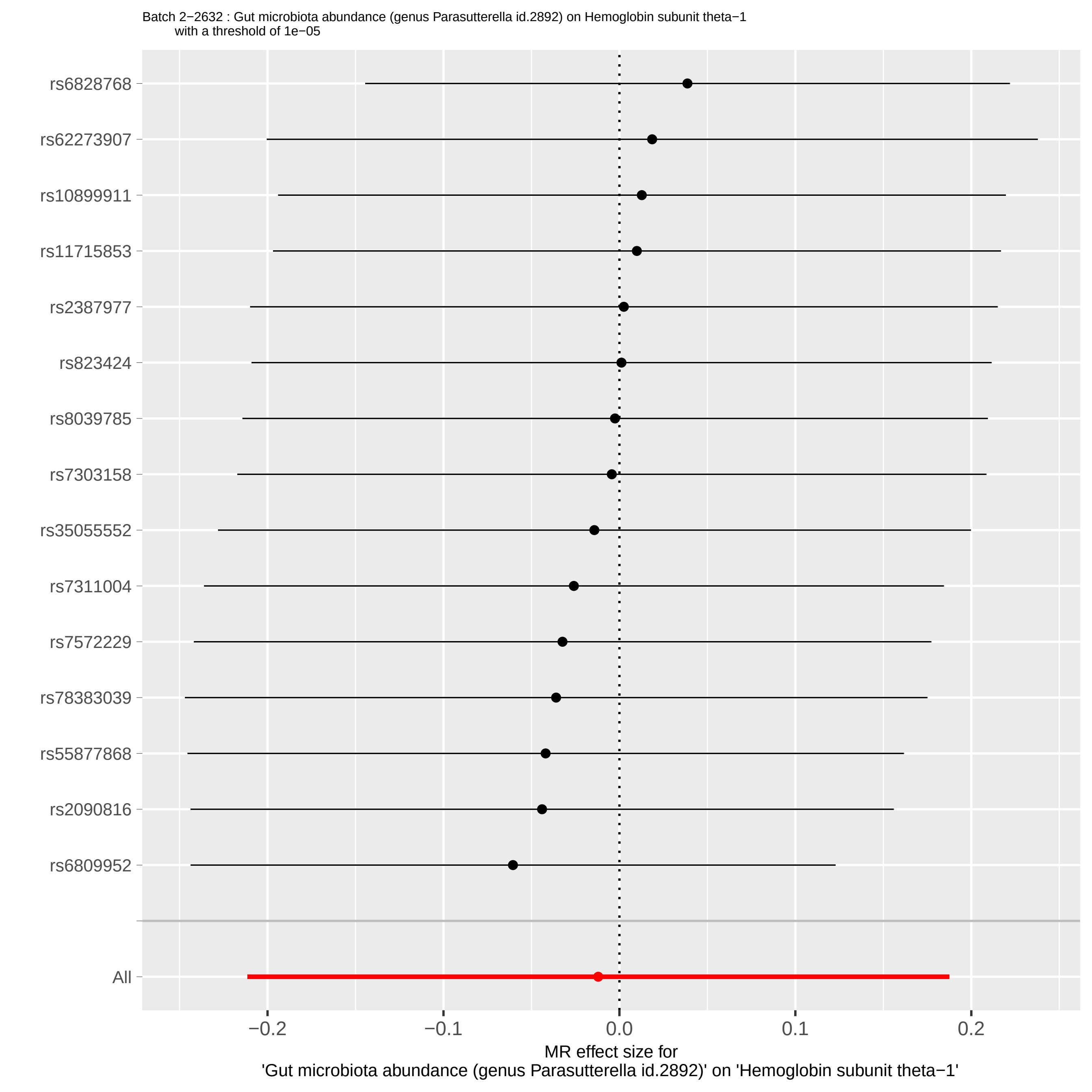

Batch 2−2632 : Gut microbiota abundance (genus Parasutterella id.2892) on Hemoglobin subunit theta−1
with a threshold of 1e−05
rs6828768
rs62273907
rs10899911
rs11715853
rs2387977
rs823424
rs8039785
rs7303158
rs35055552
rs7311004
rs7572229
rs78383039
rs55877868
rs2090816
rs6809952
All
−0.2
−0.1
0.0
0.1
0.2
MR effect size for
'Gut microbiota abundance (genus Parasutterella id.2892)' on 'Hemoglobin subunit theta−1'

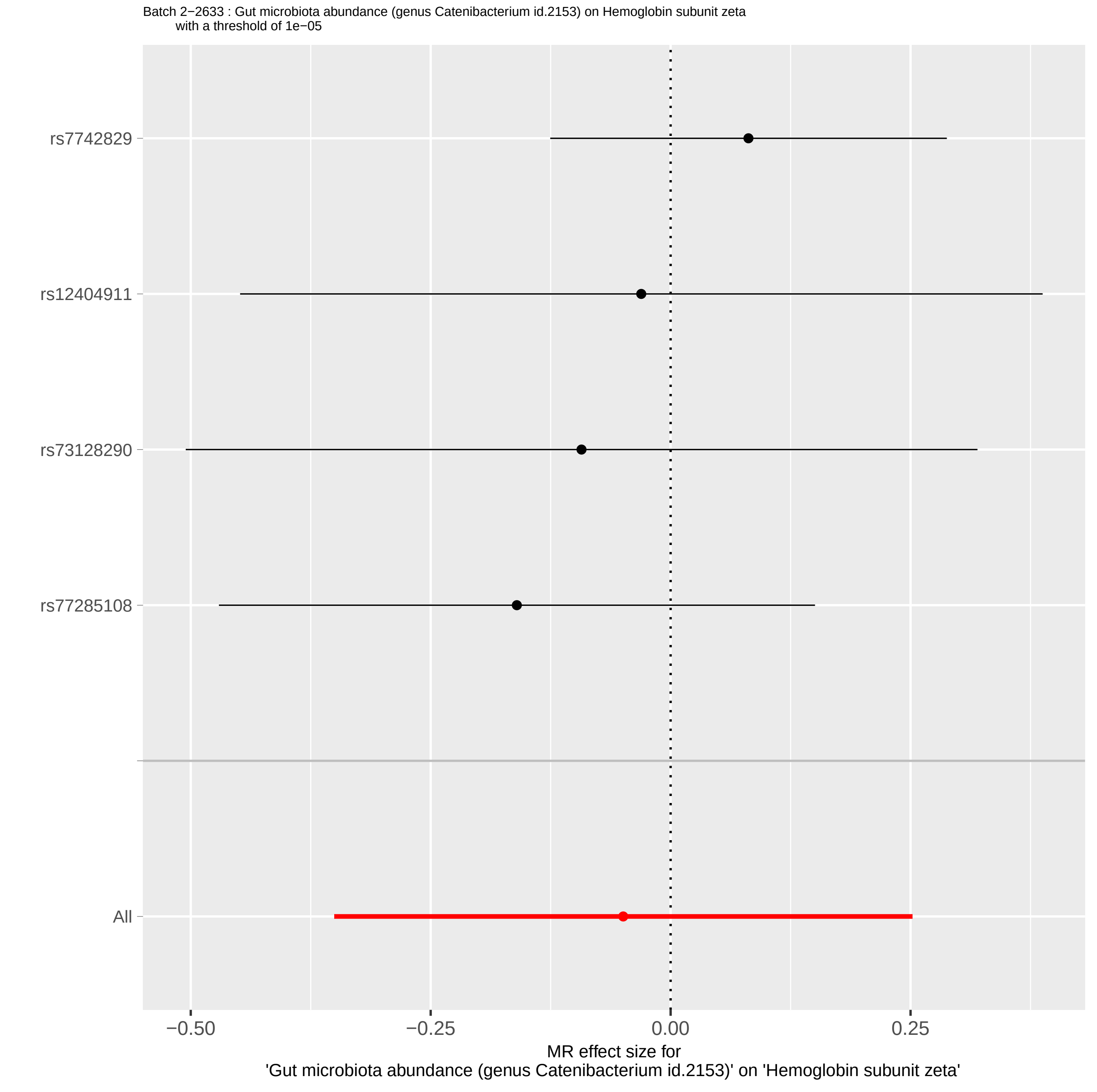

Batch 2−2633 : Gut microbiota abundance (genus Catenibacterium id.2153) on Hemoglobin subunit zeta
with a threshold of 1e−05
rs7742829
rs12404911
rs73128290
rs77285108
All
−0.50
−0.25
0.00
0.25
MR effect size for
'Gut microbiota abundance (genus Catenibacterium id.2153)' on 'Hemoglobin subunit zeta'

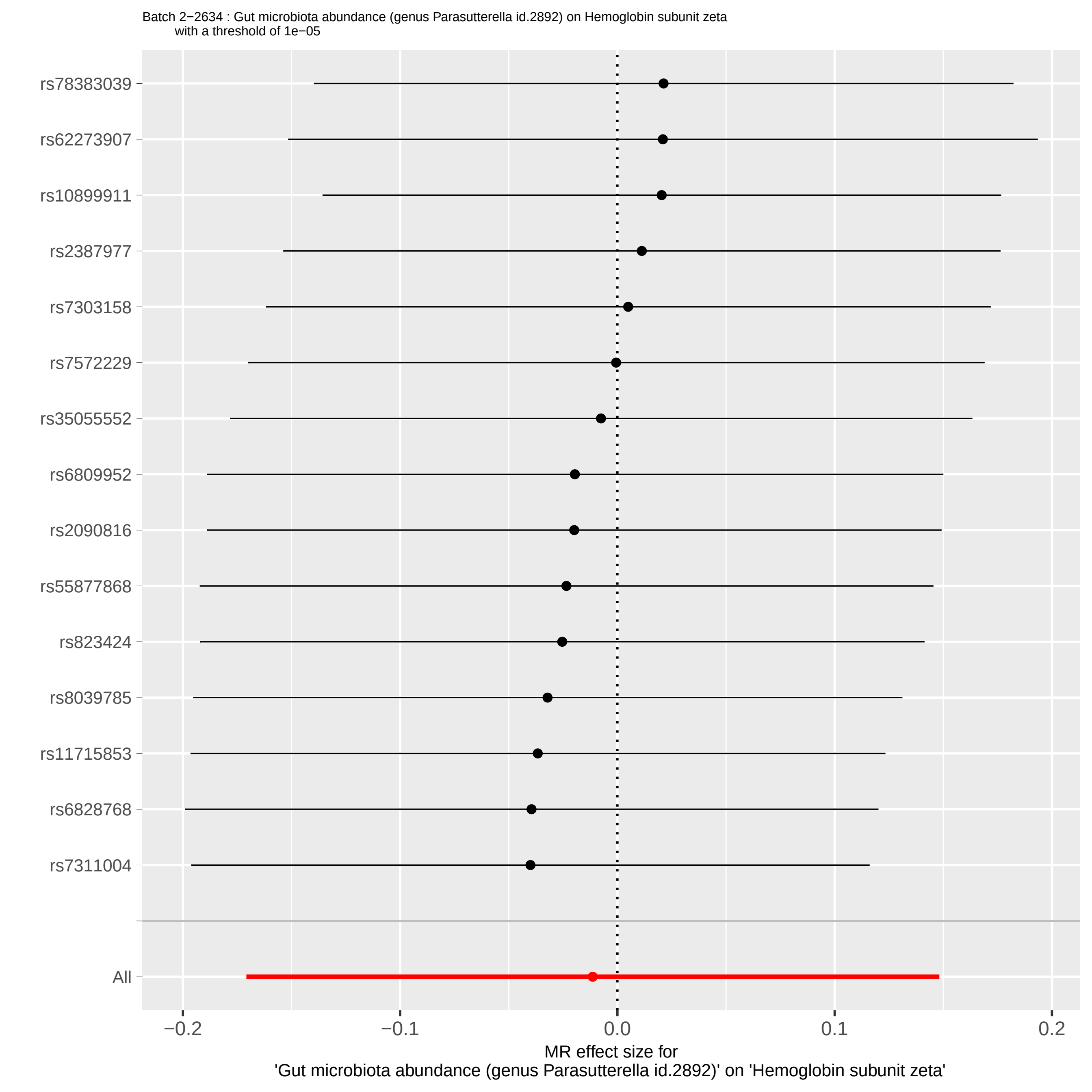
Batch 2−2634 : Gut microbiota abundance (genus Parasutterella id.2892) on Hemoglobin subunit zeta
with a threshold of 1e−05
rs78383039
rs62273907
rs10899911
rs2387977
rs7303158
rs7572229
rs35055552
rs6809952
rs2090816
rs55877868
rs823424
rs8039785
rs11715853
rs6828768
rs7311004
All
−0.2
−0.1
0.0
0.1
0.2
MR effect size for
'Gut microbiota abundance (genus Parasutterella id.2892)' on 'Hemoglobin subunit zeta'

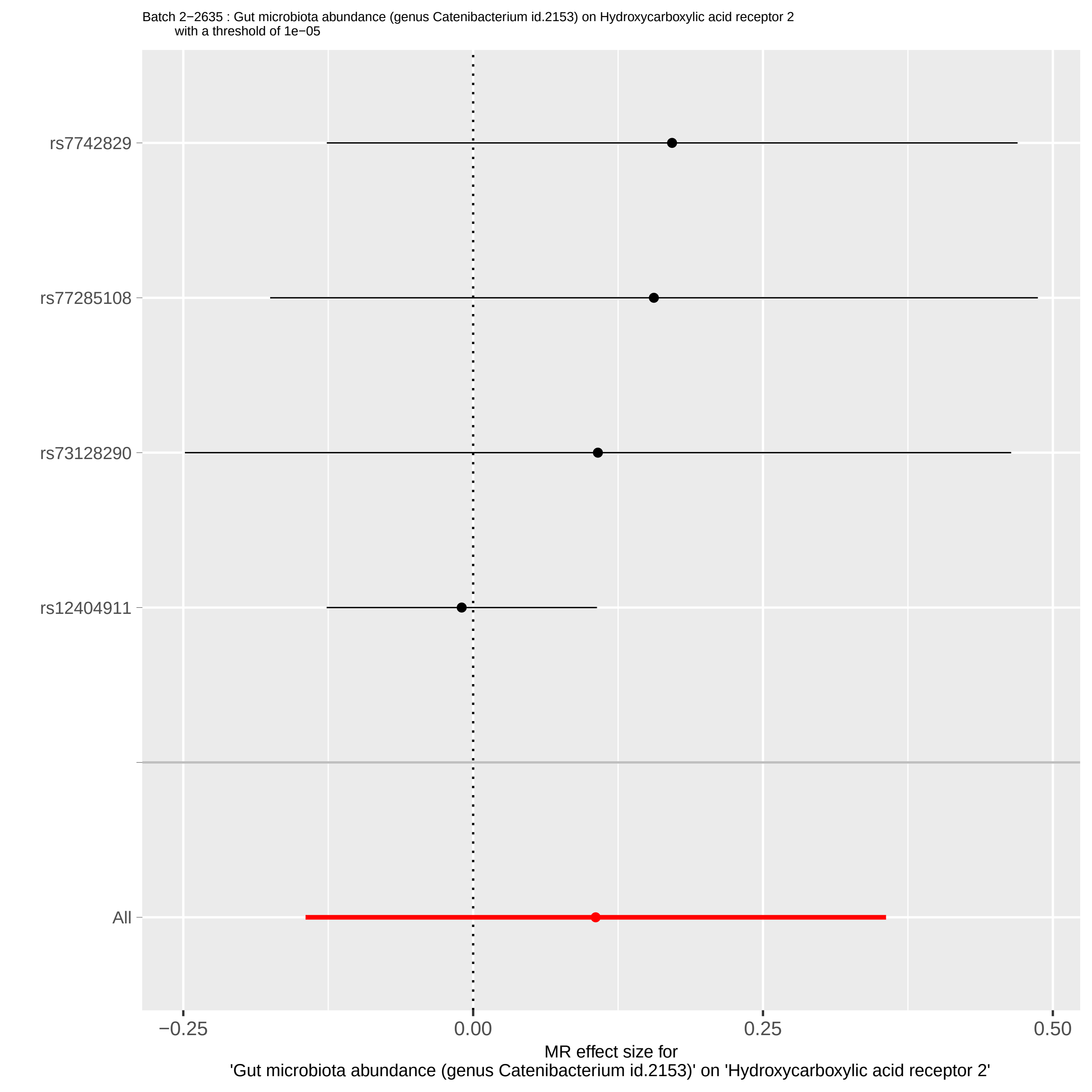
Batch 2−2635 : Gut microbiota abundance (genus Catenibacterium id.2153) on Hydroxycarboxylic acid receptor 2
with a threshold of 1e−05
rs7742829
rs77285108
rs73128290
rs12404911
All
−0.25
0.00
0.25
0.50
MR effect size for
'Gut microbiota abundance (genus Catenibacterium id.2153)' on 'Hydroxycarboxylic acid receptor 2'

Batch 2−2636 : Gut microbiota abundance (genus Parasutterella id.2892) on Hydroxycarboxylic acid receptor 2
with a threshold of 1e−05

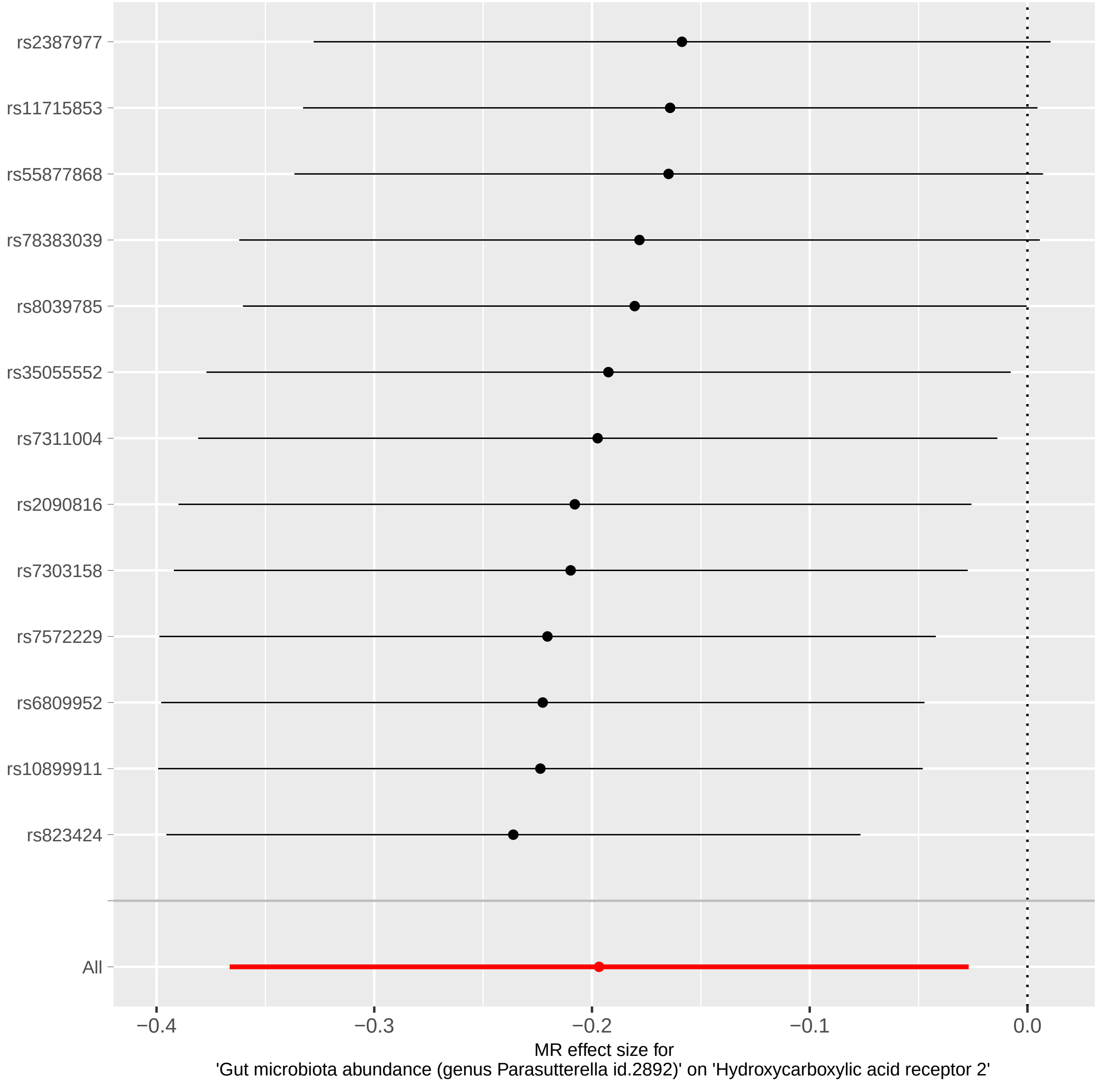

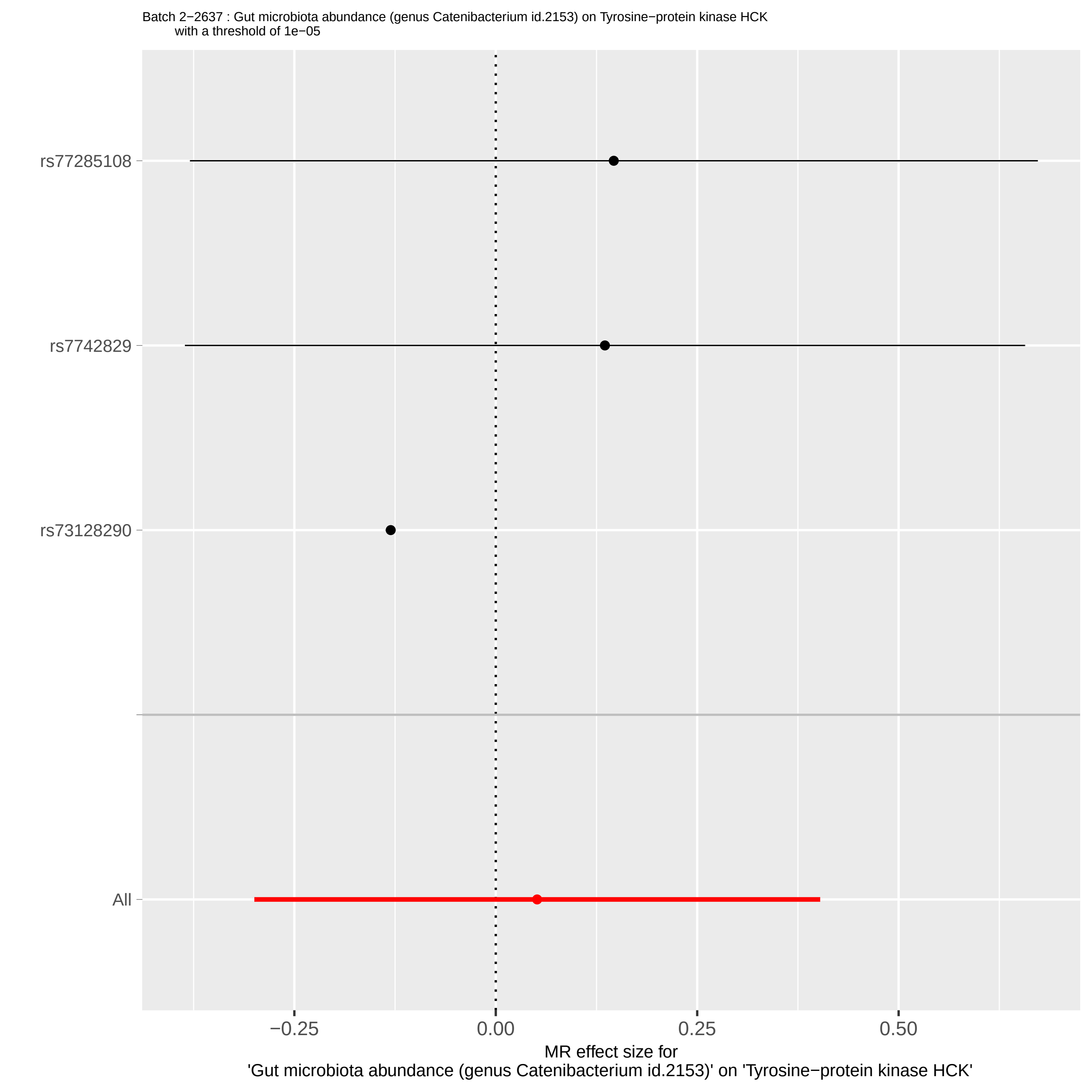
Batch 2−2637 : Gut microbiota abundance (genus Catenibacterium id.2153) on Tyrosine−protein kinase HCK
with a threshold of 1e−05
rs77285108
rs7742829
rs73128290
All
−0.25
0.00
0.25
0.50
MR effect size for
'Gut microbiota abundance (genus Catenibacterium id.2153)' on 'Tyrosine−protein kinase HCK'

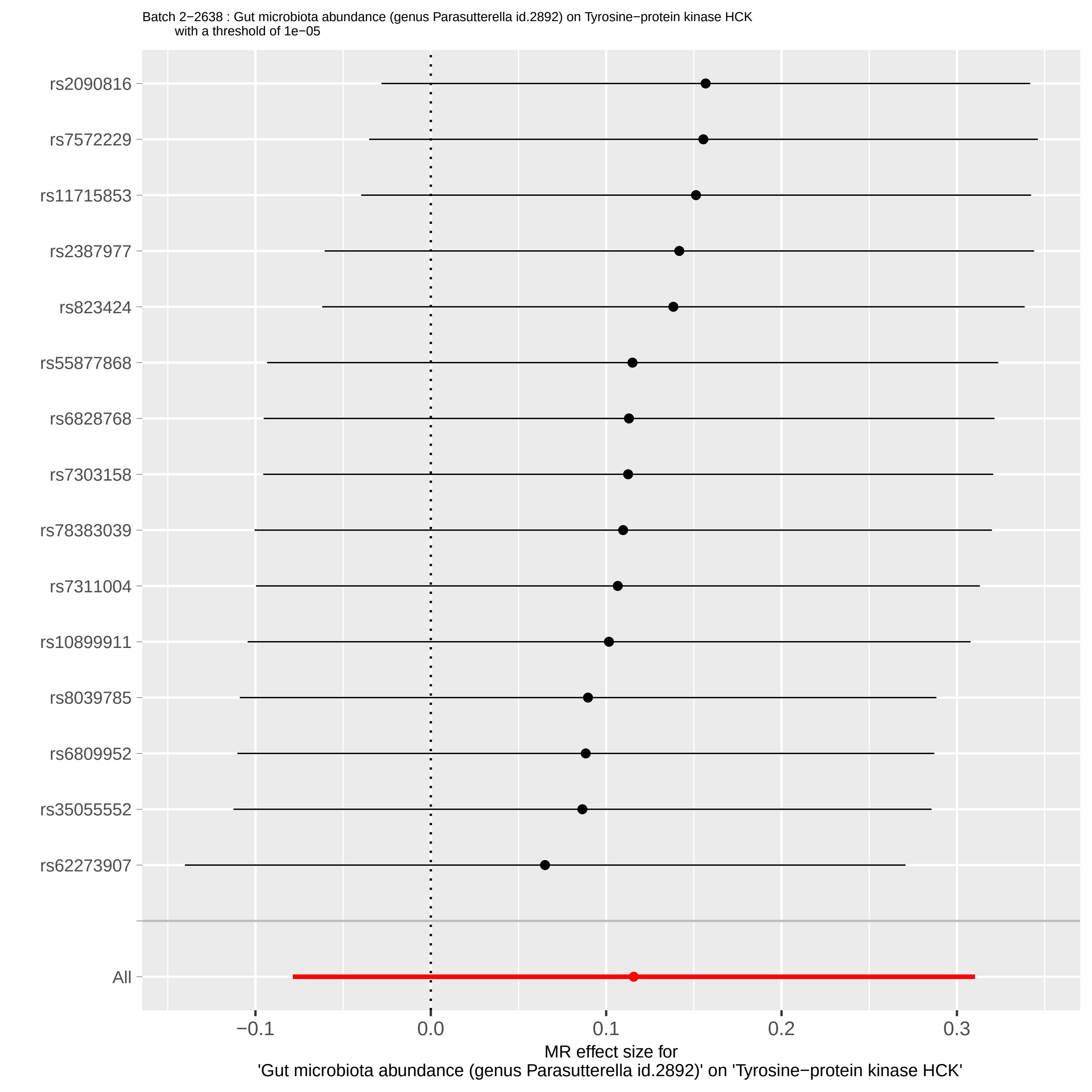

Batch 2−2638 : Gut microbiota abundance (genus Parasutterella id.2892) on Tyrosine−protein kinase HCK
with a threshold of 1e−05
rs2090816
rs7572229
rs11715853
rs2387977
rs823424
rs55877868
rs6828768
rs7303158
rs78383039
rs7311004
rs10899911
rs8039785
rs6809952
rs35055552
rs62273907
All
−0.1
0.0
0.1
0.2
0.3
MR effect size for
'Gut microbiota abundance (genus Parasutterella id.2892)' on 'Tyrosine−protein kinase HCK'

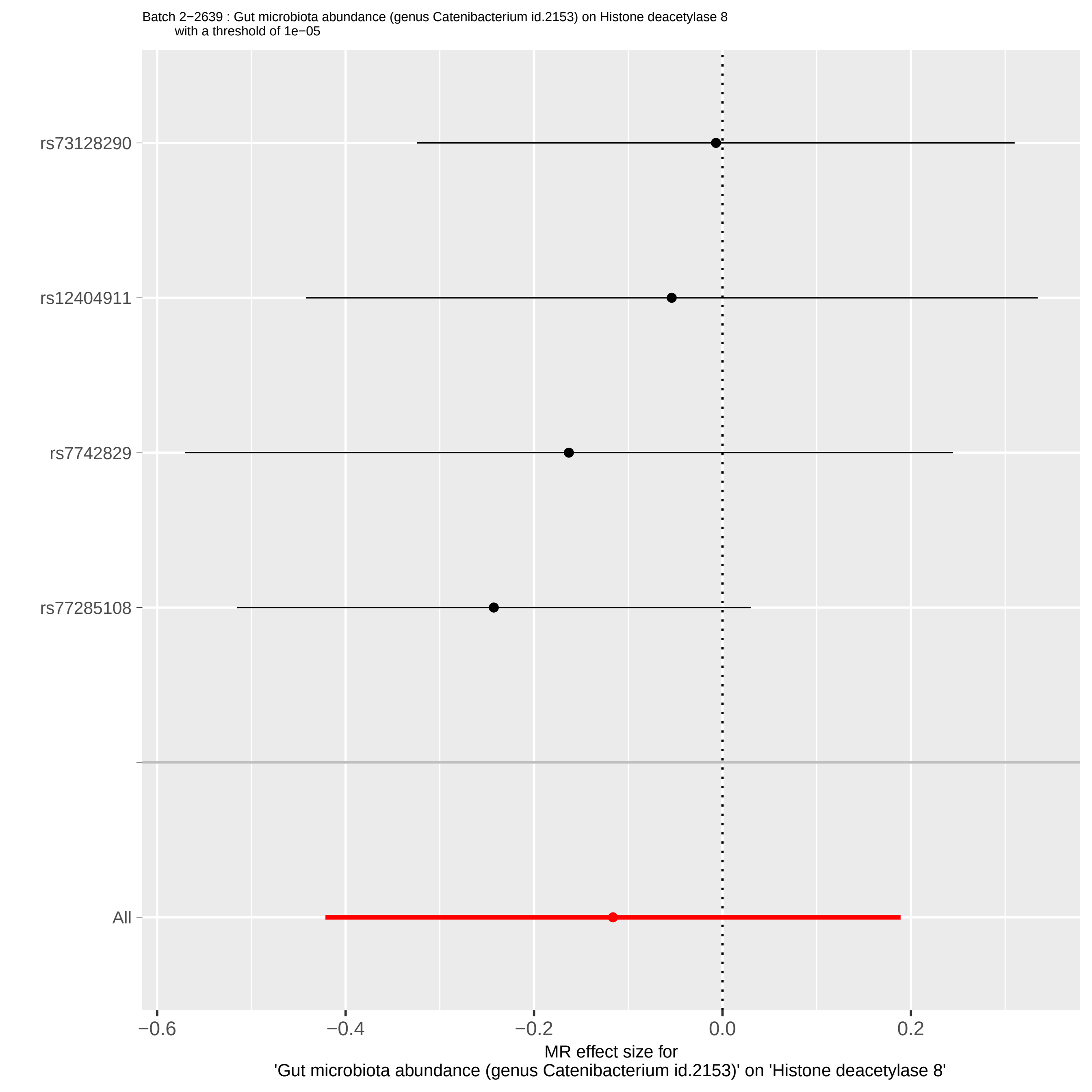

Batch 2−2639 : Gut microbiota abundance (genus Catenibacterium id.2153) on Histone deacetylase 8
with a threshold of 1e−05
rs73128290
rs12404911
rs7742829
rs77285108
All
−0.6
−0.4
−0.2
0.0
0.2
MR effect size for
'Gut microbiota abundance (genus Catenibacterium id.2153)' on 'Histone deacetylase 8'

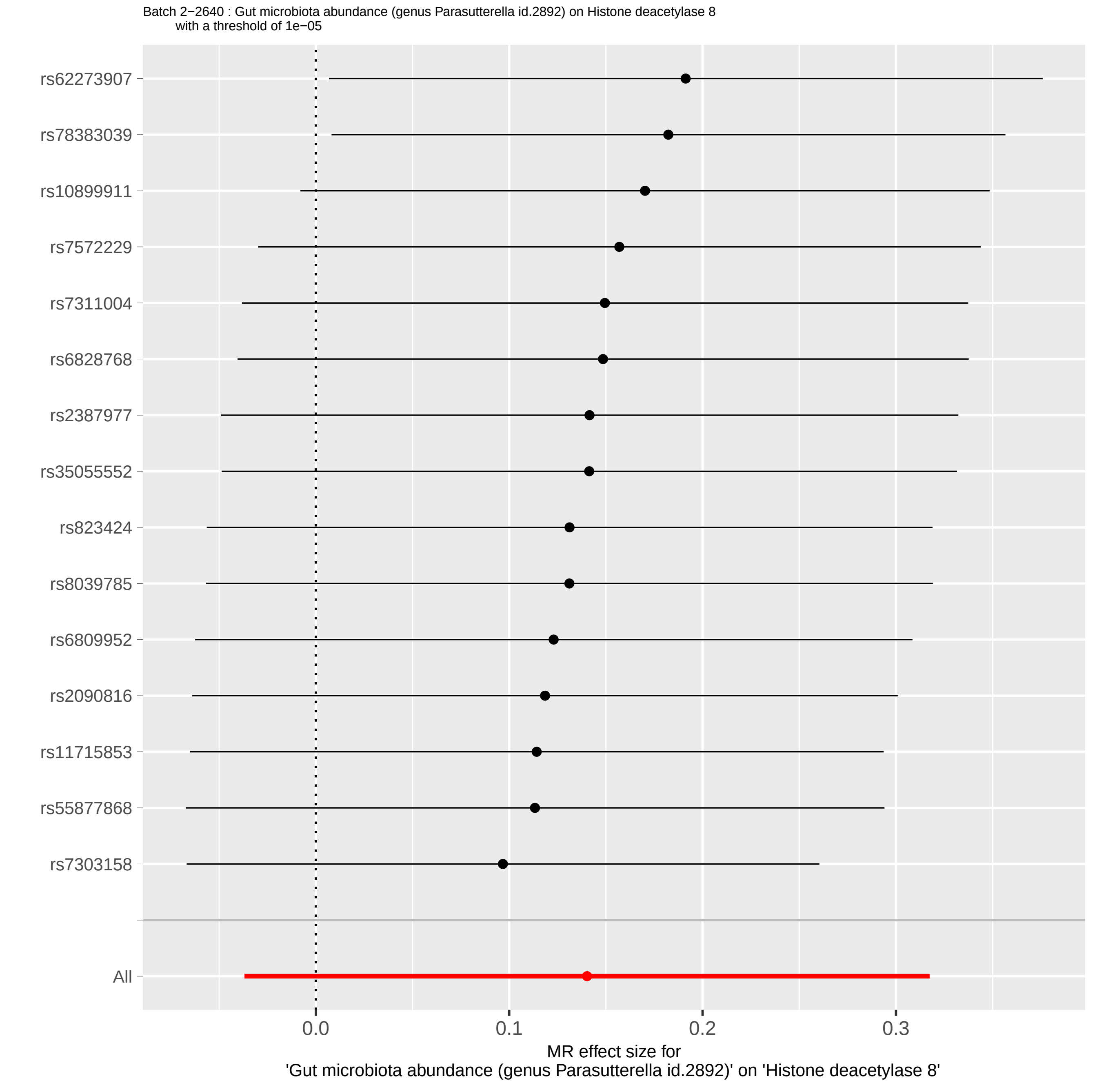

Batch 2−2640 : Gut microbiota abundance (genus Parasutterella id.2892) on Histone deacetylase 8
with a threshold of 1e−05
rs62273907
rs78383039
rs10899911
rs7572229
rs7311004
rs6828768
rs2387977
rs35055552
rs823424
rs8039785
rs6809952
rs2090816
rs11715853
rs55877868
rs7303158
All
0.0
0.1
0.2
0.3
MR effect size for
'Gut microbiota abundance (genus Parasutterella id.2892)' on 'Histone deacetylase 8'

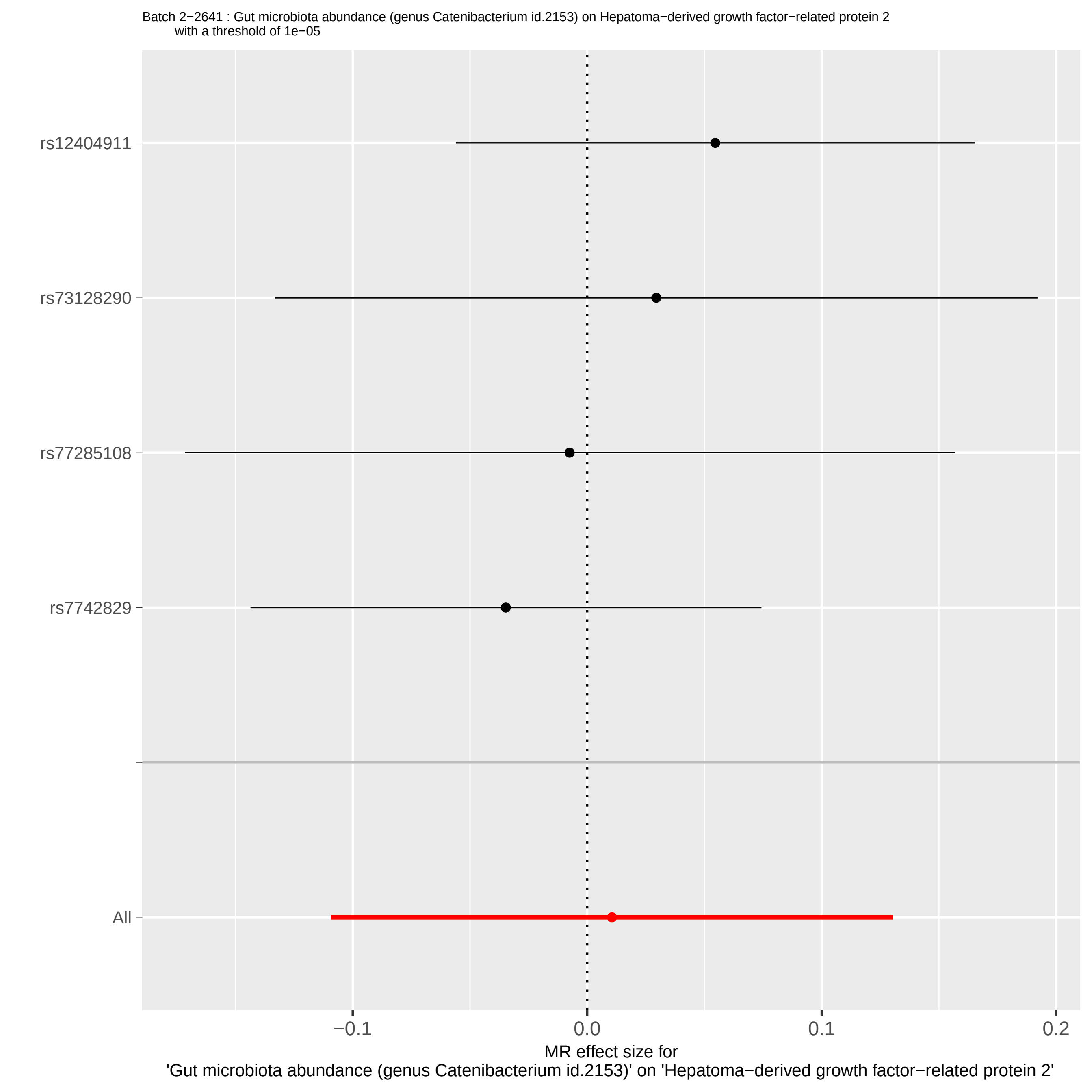

Batch 2−2641 : Gut microbiota abundance (genus Catenibacterium id.2153) on Hepatoma−derived growth factor−related protein 2
with a threshold of 1e−05
rs12404911
rs73128290
rs77285108
rs7742829
All
−0.1
0.0
0.1
0.2
MR effect size for
'Gut microbiota abundance (genus Catenibacterium id.2153)' on 'Hepatoma−derived growth factor−related protein 2'

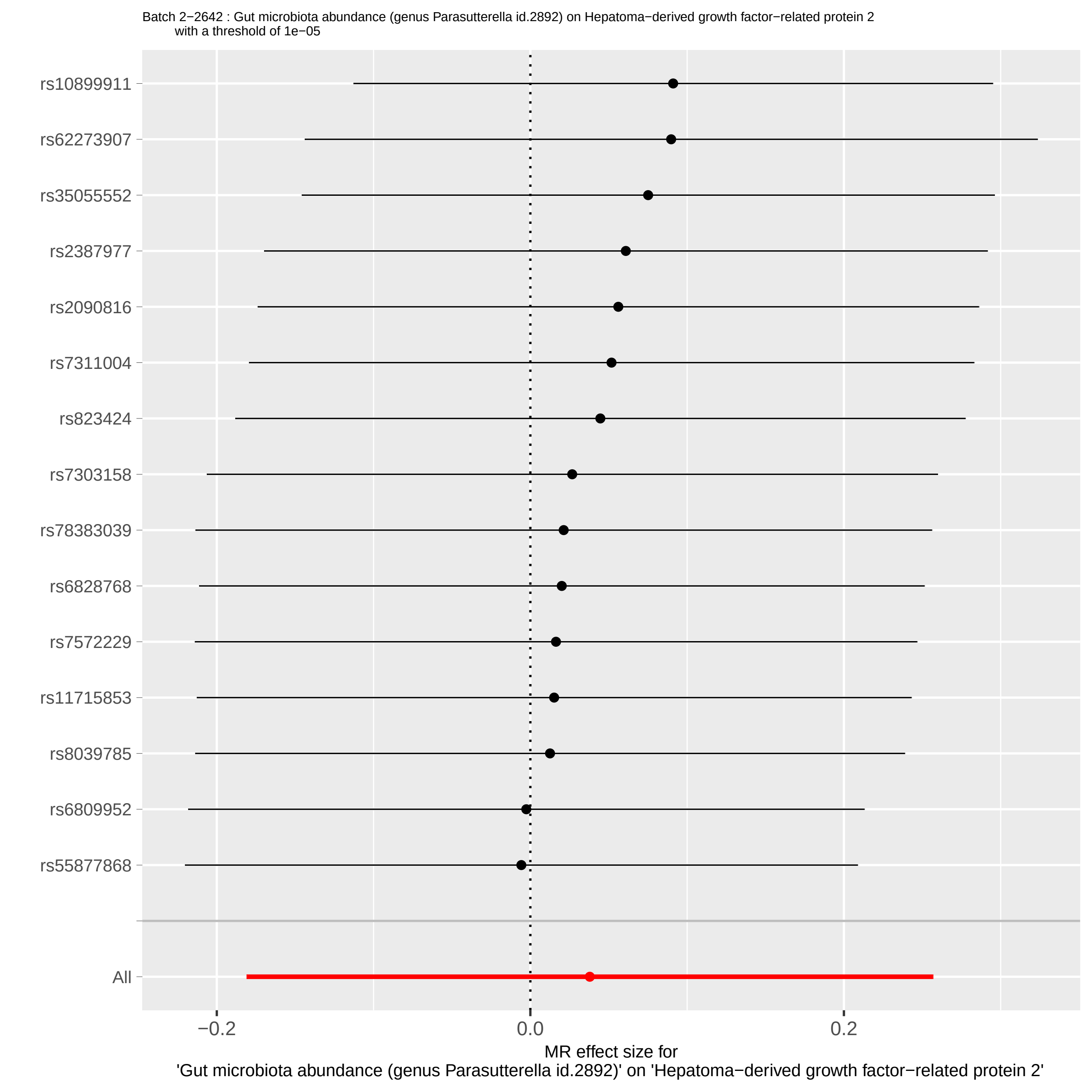
Batch 2−2642 : Gut microbiota abundance (genus Parasutterella id.2892) on Hepatoma−derived growth factor−related protein 2
with a threshold of 1e−05
rs10899911
rs62273907
rs35055552
rs2387977
rs2090816
rs7311004
rs823424
rs7303158
rs78383039
rs6828768
rs7572229
rs11715853
rs8039785
rs6809952
rs55877868
All
−0.2
0.0
0.2
MR effect size for
'Gut microbiota abundance (genus Parasutterella id.2892)' on 'Hepatoma−derived growth factor−related protein 2'

Batch 2−2643 : Gut microbiota abundance (genus Catenibacterium id.2153) on Haloacid dehalogenase−like hydrolase domain−containing protein 2 with a threshold of 1e−05

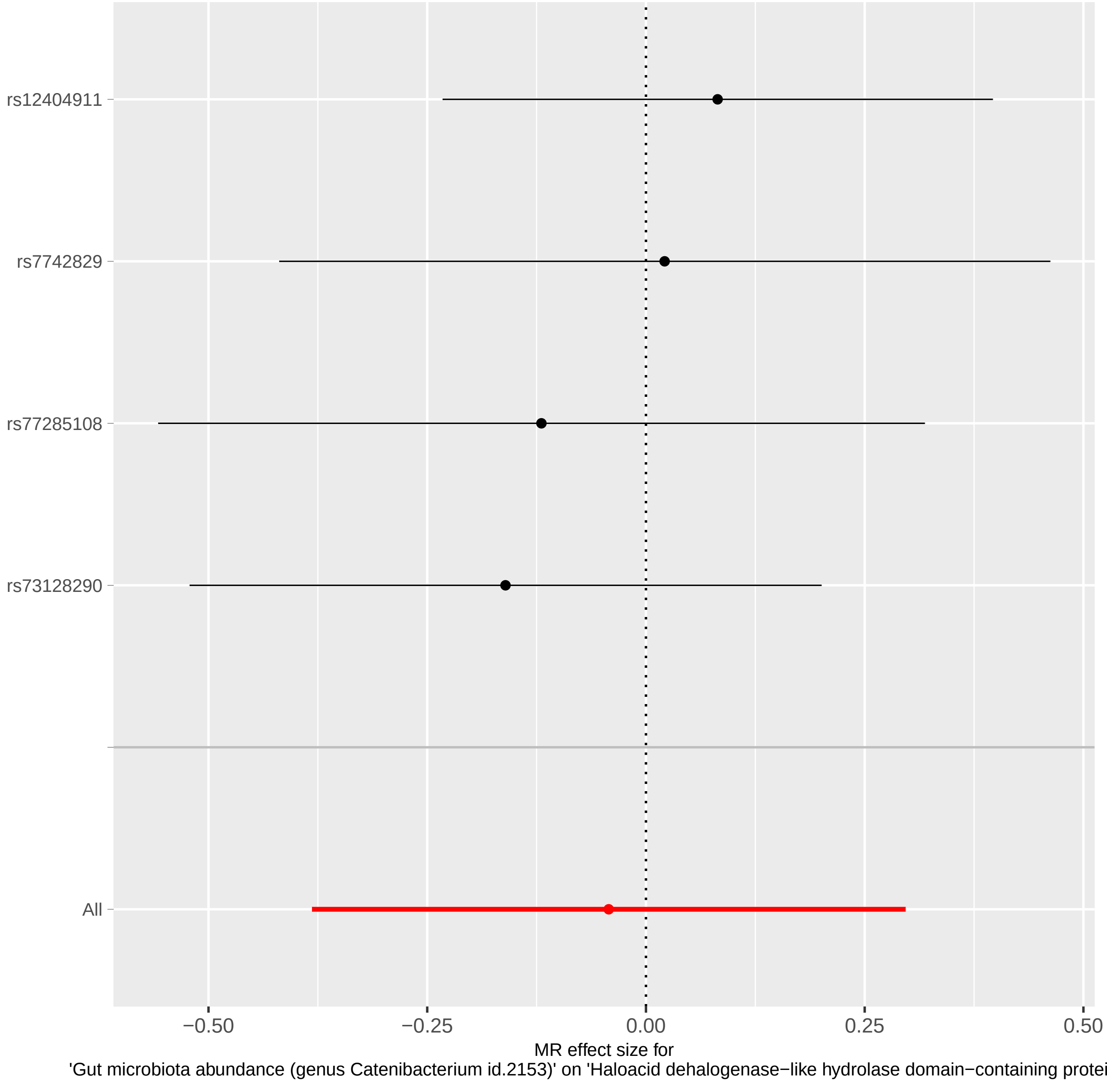

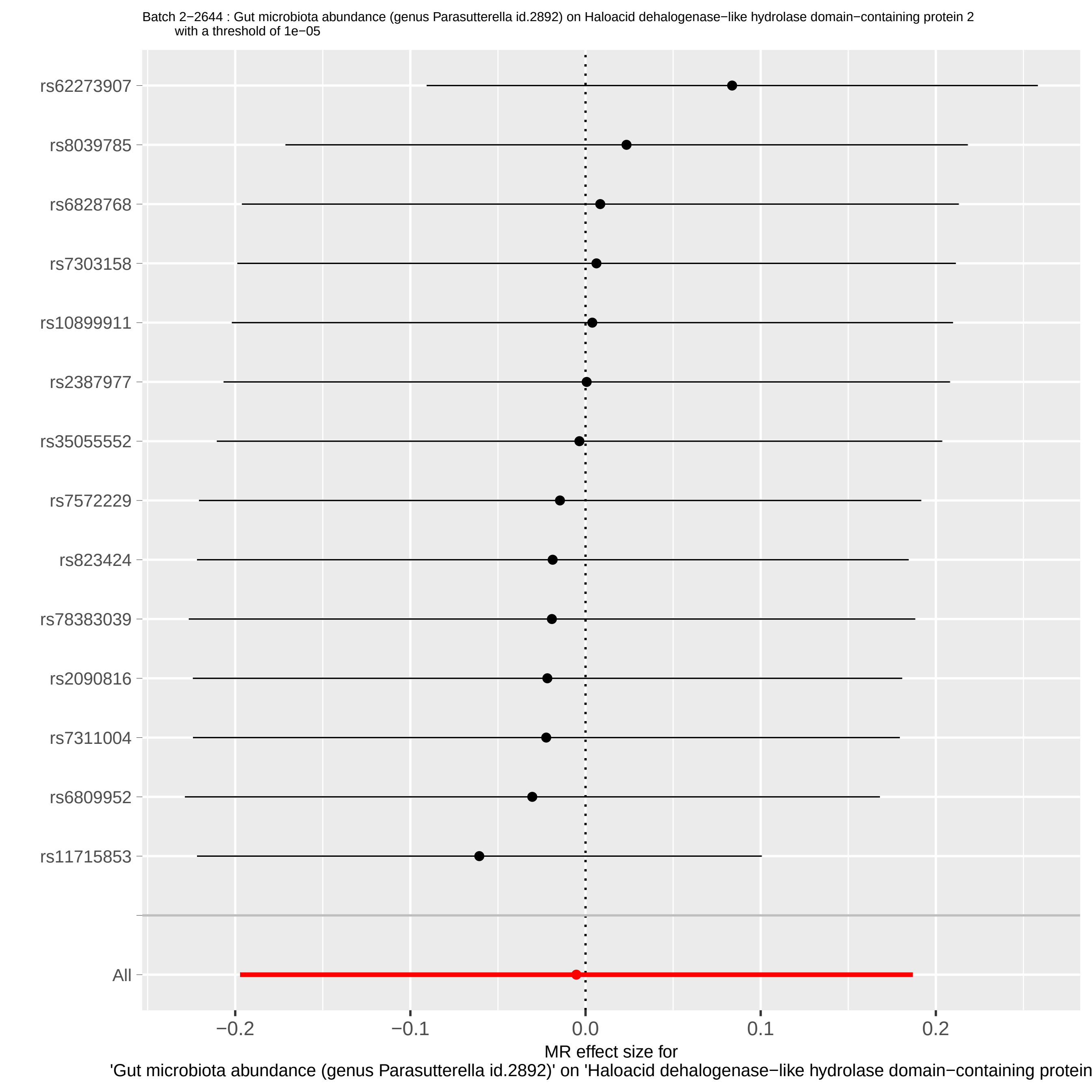
Batch 2−2644 : Gut microbiota abundance (genus Parasutterella id.2892) on Haloacid dehalogenase−like hydrolase domain−containing protein 2
with a threshold of 1e−05
rs62273907
rs8039785
rs6828768
rs7303158
rs10899911
rs2387977
rs35055552
rs7572229
rs823424
rs78383039
rs2090816
rs7311004
rs6809952
rs11715853
All
−0.2
−0.1
0.0
0.1
0.2
MR effect size for
'Gut microbiota abundance (genus Parasutterella id.2892)' on 'Haloacid dehalogenase−like hydrolase domain−containing protein

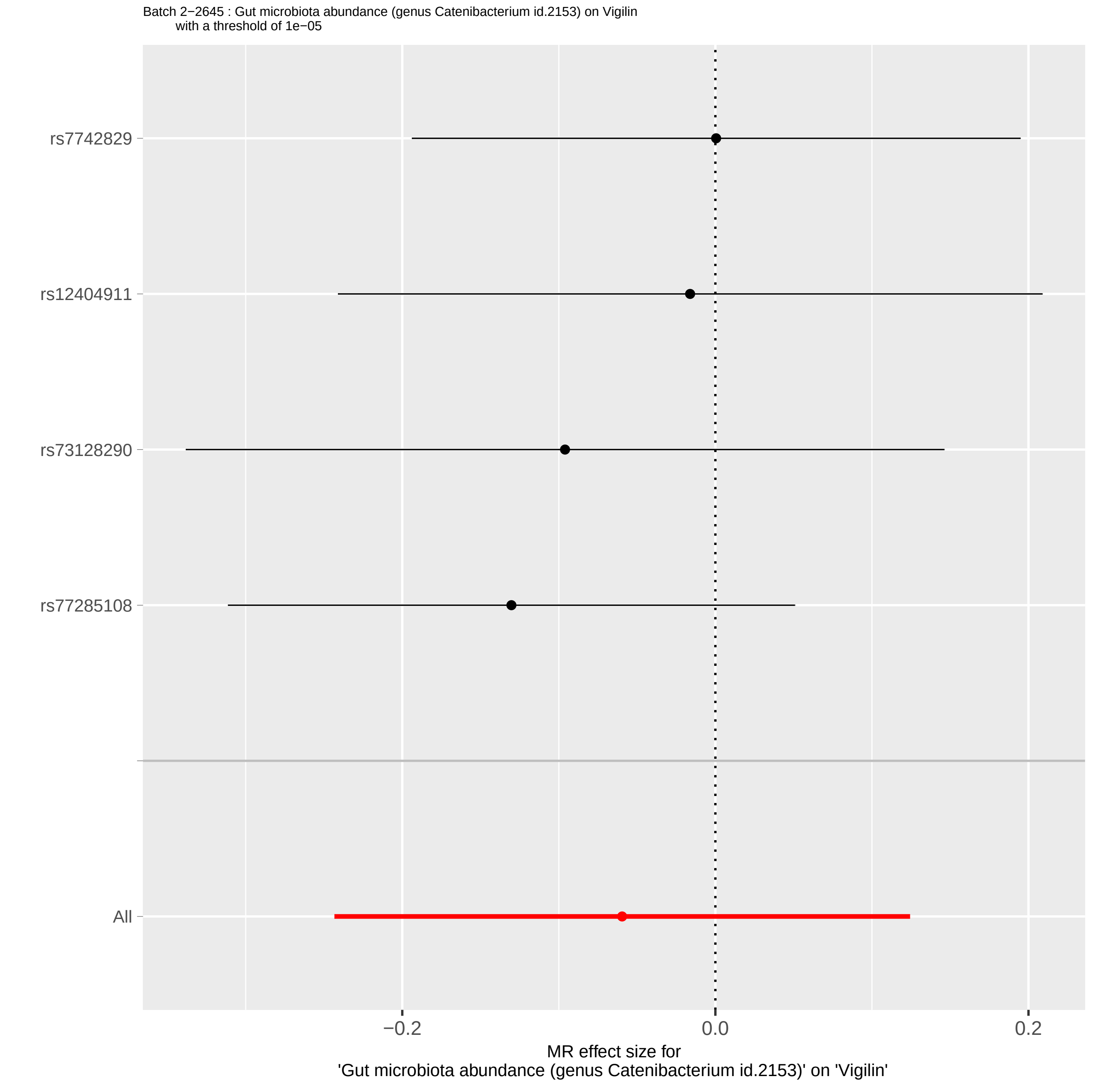

Batch 2−2645 : Gut microbiota abundance (genus Catenibacterium id.2153) on Vigilin
with a threshold of 1e−05
rs7742829
rs12404911
rs73128290
rs77285108
All
−0.2
0.0
0.2
MR effect size for
'Gut microbiota abundance (genus Catenibacterium id.2153)' on 'Vigilin'

Batch 2−2646 : Gut microbiota abundance (genus Parasutterella id.2892) on Vigilin
with a threshold of 1e−05

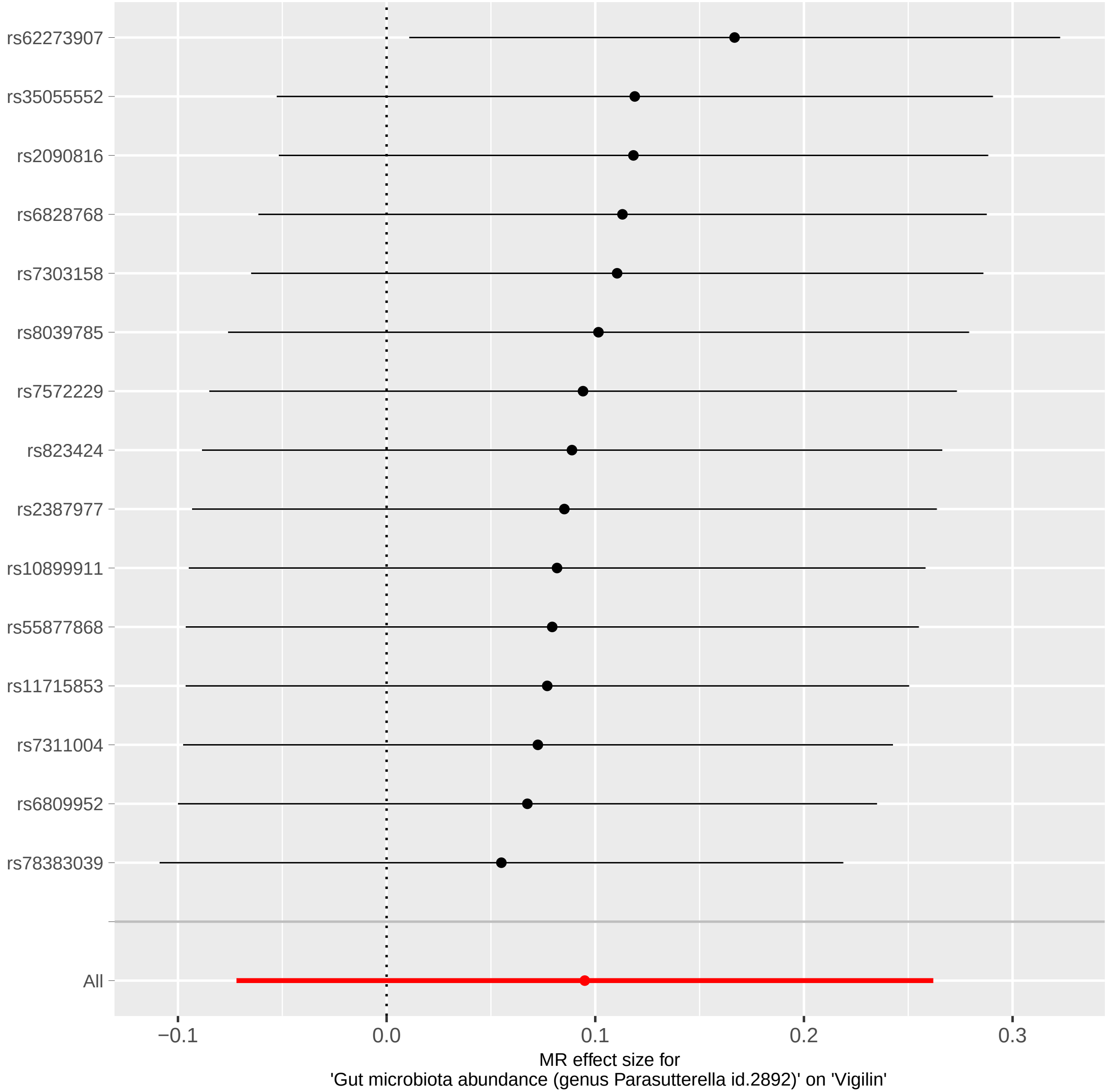

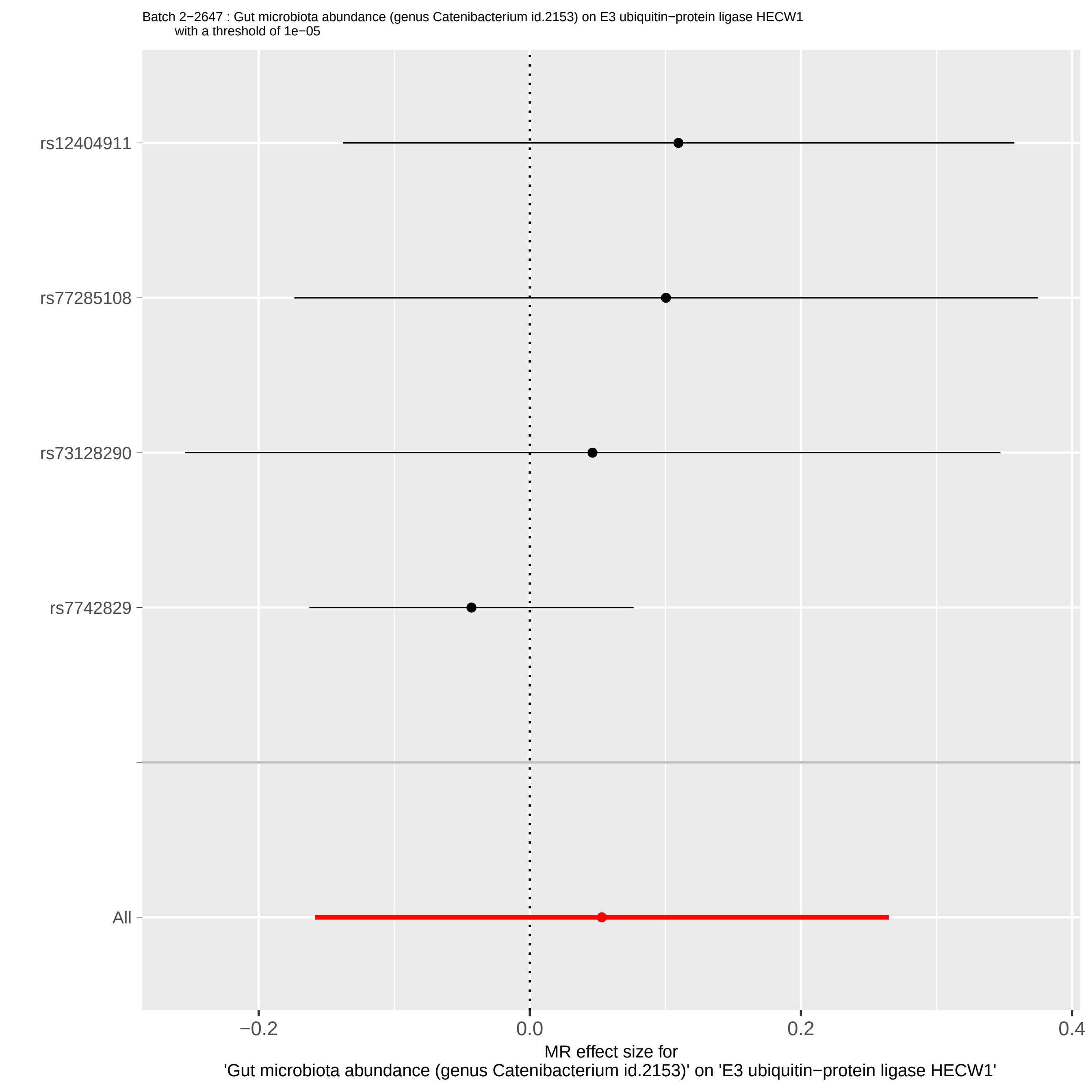

Batch 2−2647 : Gut microbiota abundance (genus Catenibacterium id.2153) on E3 ubiquitin−protein ligase HECW1
with a threshold of 1e−05
rs12404911
rs77285108
rs73128290
rs7742829
All
−0.2
0.0
0.2
0.4
MR effect size for
'Gut microbiota abundance (genus Catenibacterium id.2153)' on 'E3 ubiquitin−protein ligase HECW1'

Batch 2−2648 : Gut microbiota abundance (genus Parasutterella id.2892) on E3 ubiquitin−protein ligase HECW1
with a threshold of 1e−05

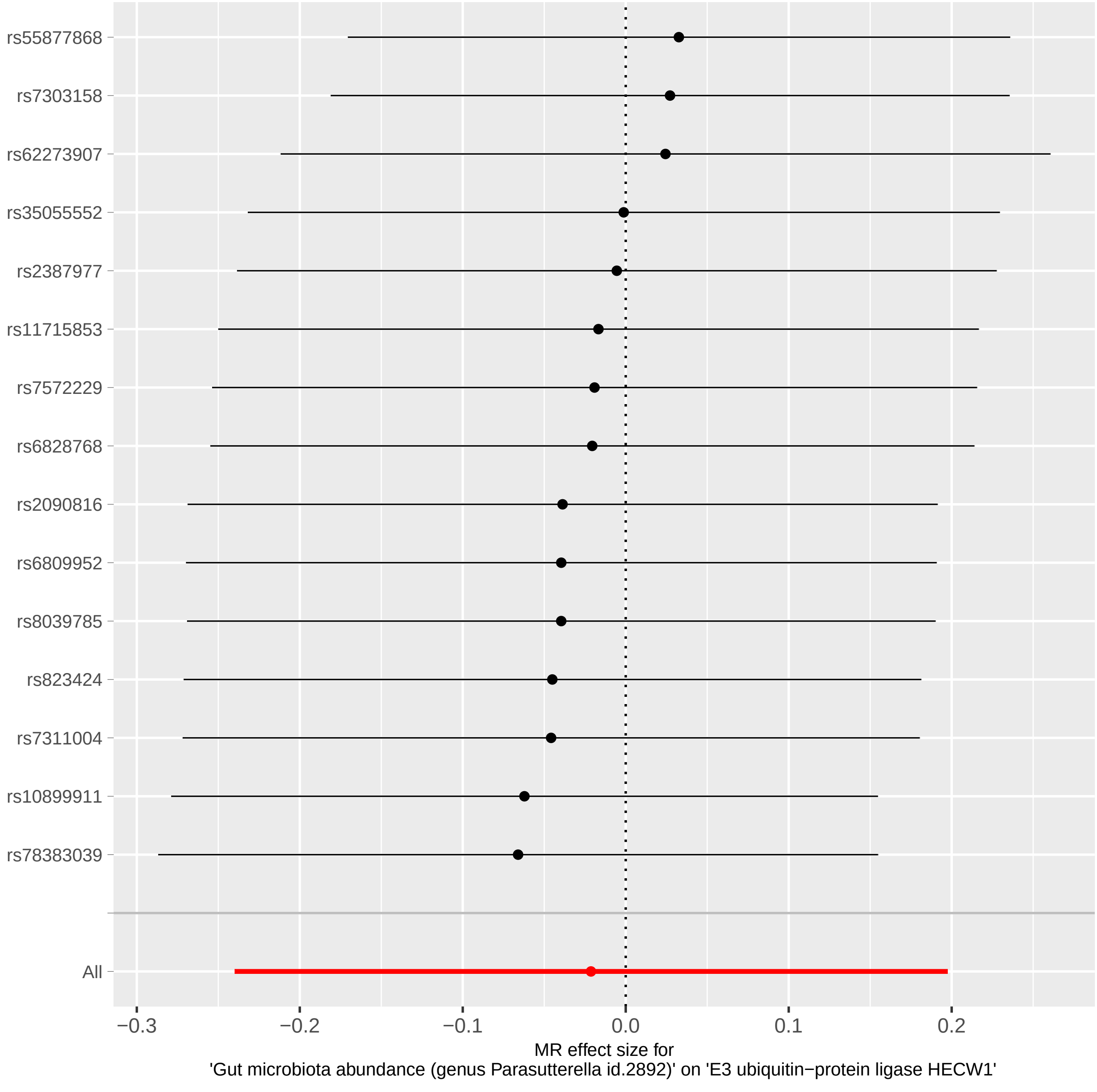

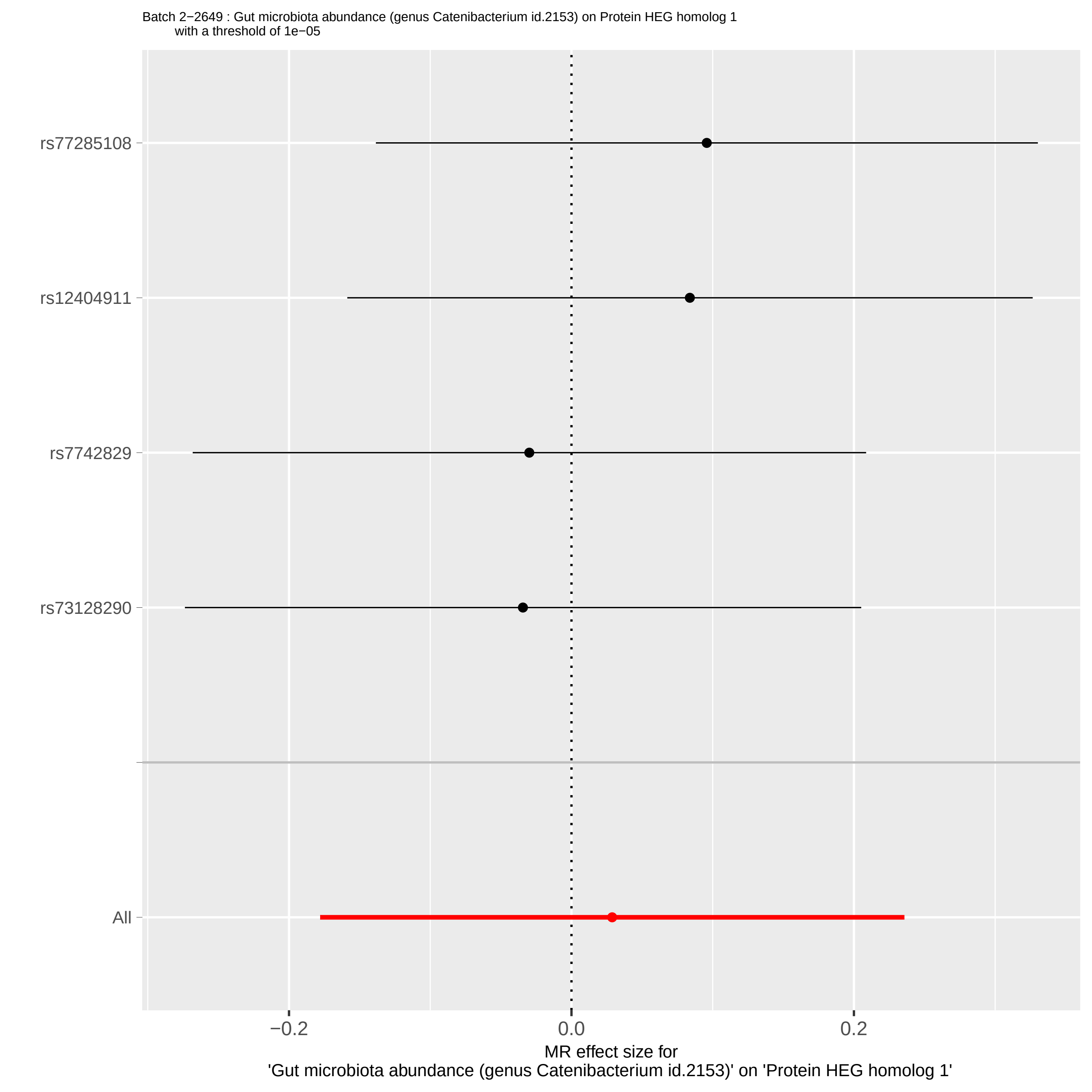
Batch 2−2649 : Gut microbiota abundance (genus Catenibacterium id.2153) on Protein HEG homolog 1
with a threshold of 1e−05
rs77285108
rs12404911
rs7742829
rs73128290
All
−0.2
0.0
0.2
MR effect size for
'Gut microbiota abundance (genus Catenibacterium id.2153)' on 'Protein HEG homolog 1'

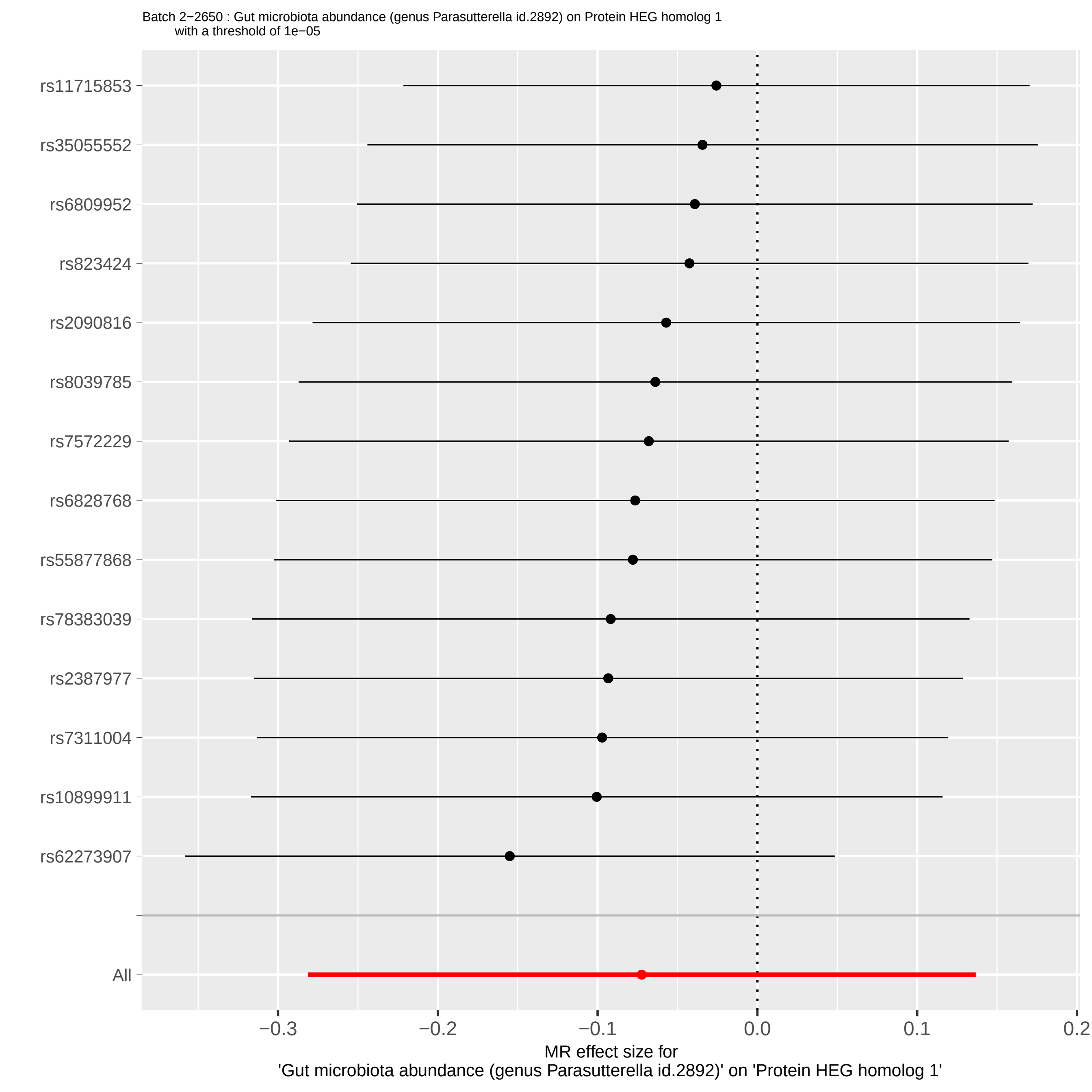
Batch 2−2650 : Gut microbiota abundance (genus Parasutterella id.2892) on Protein HEG homolog 1
with a threshold of 1e−05
rs11715853
rs35055552
rs6809952
rs823424
rs2090816
rs8039785
rs7572229
rs6828768
rs55877868
rs78383039
rs2387977
rs7311004
rs10899911
rs62273907
All
−0.3
−0.2
−0.1
0.0
0.1
0.2
MR effect size for
'Gut microbiota abundance (genus Parasutterella id.2892)' on 'Protein HEG homolog 1'

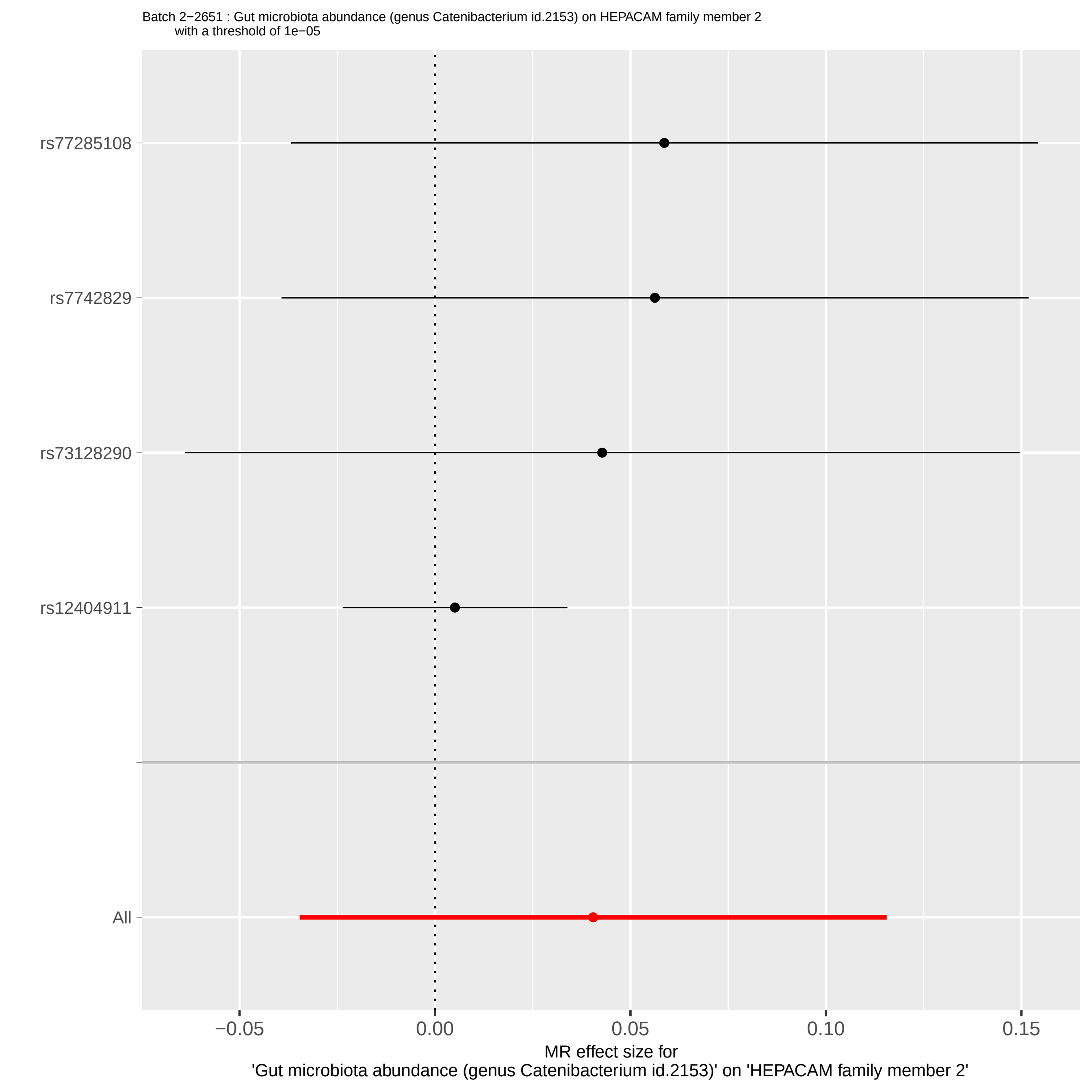

Batch 2−2651 : Gut microbiota abundance (genus Catenibacterium id.2153) on HEPACAM family member 2
with a threshold of 1e−05
rs77285108
rs7742829
rs73128290
rs12404911
All
−0.05
0.00
0.05
0.10
0.15
MR effect size for
'Gut microbiota abundance (genus Catenibacterium id.2153)' on 'HEPACAM family member 2'

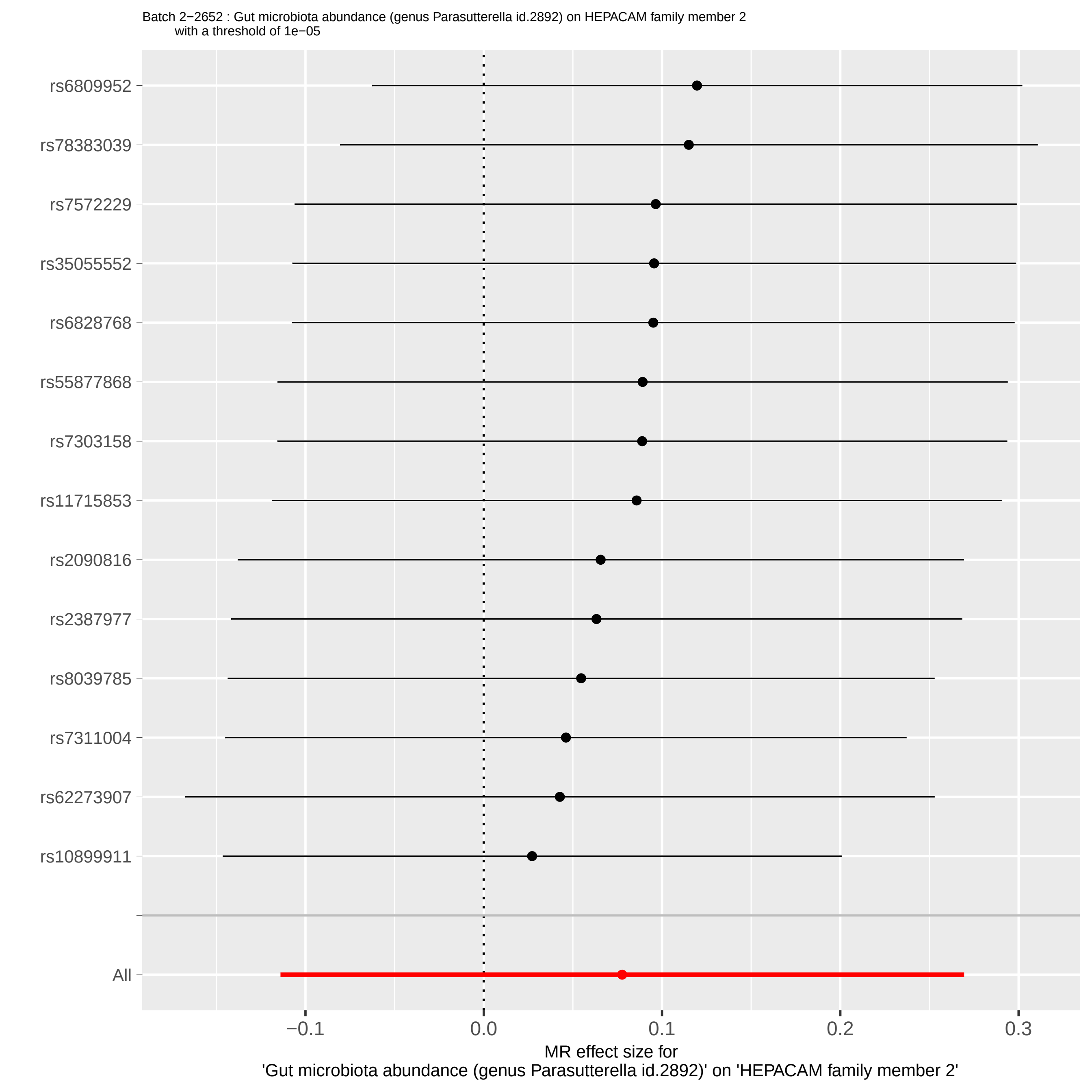
Batch 2−2652 : Gut microbiota abundance (genus Parasutterella id.2892) on HEPACAM family member 2
with a threshold of 1e−05
rs6809952
rs78383039
rs7572229
rs35055552
rs6828768
rs55877868
rs7303158
rs11715853
rs2090816
rs2387977
rs8039785
rs7311004
rs62273907
rs10899911
All
−0.1
0.0
0.1
0.2
0.3
MR effect size for
'Gut microbiota abundance (genus Parasutterella id.2892)' on 'HEPACAM family member 2'

Batch 2−2653 : Gut microbiota abundance (genus Catenibacterium id.2153) on HEPACAM family member 2
with a threshold of 1e−05

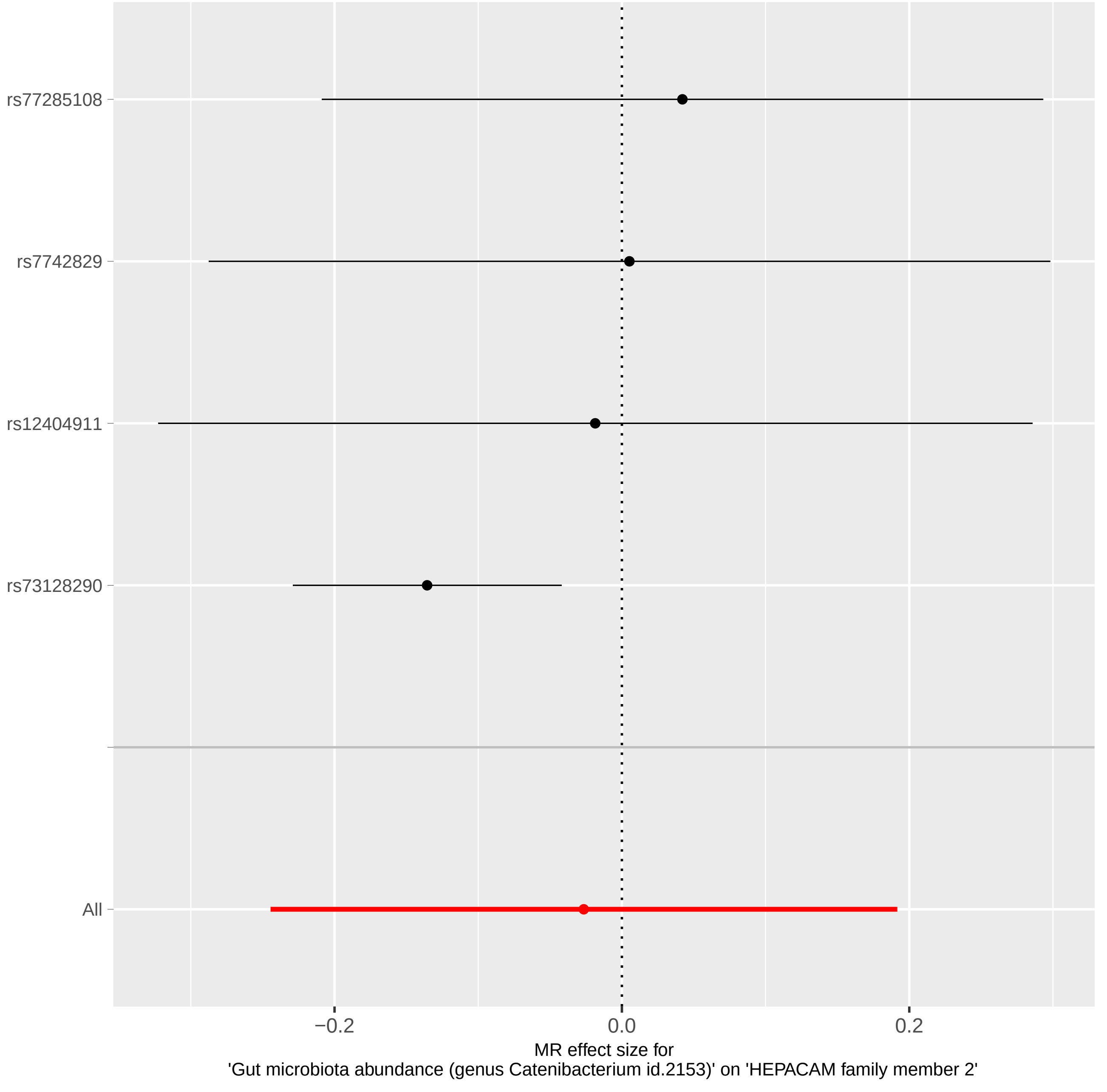

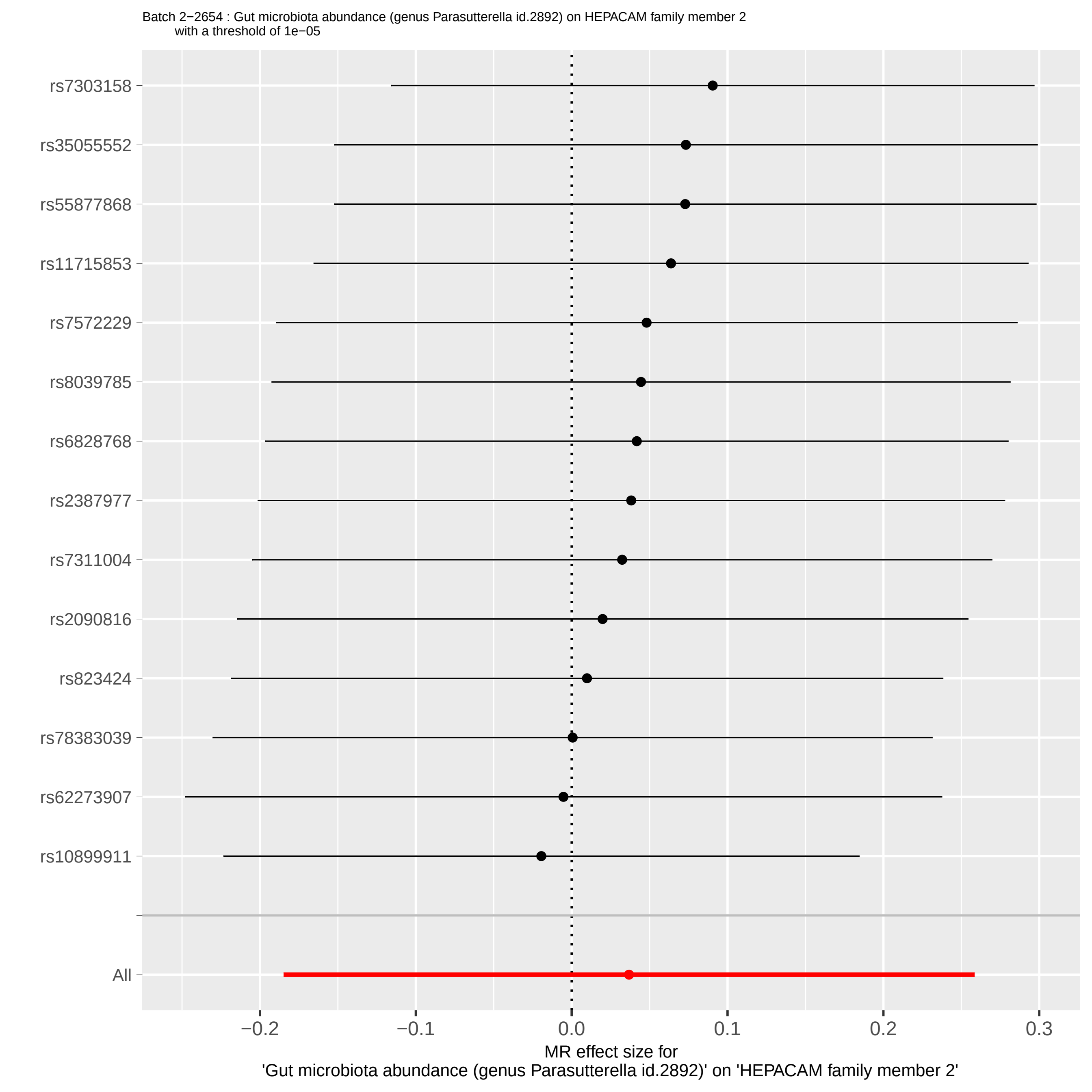
Batch 2−2654 : Gut microbiota abundance (genus Parasutterella id.2892) on HEPACAM family member 2
with a threshold of 1e−05
rs7303158
rs35055552
rs55877868
rs11715853
rs7572229
rs8039785
rs6828768
rs2387977
rs7311004
rs2090816
rs823424
rs78383039
rs62273907
rs10899911
All
−0.2
−0.1
0.0
0.1
0.2
0.3
MR effect size for
'Gut microbiota abundance (genus Parasutterella id.2892)' on 'HEPACAM family member 2'

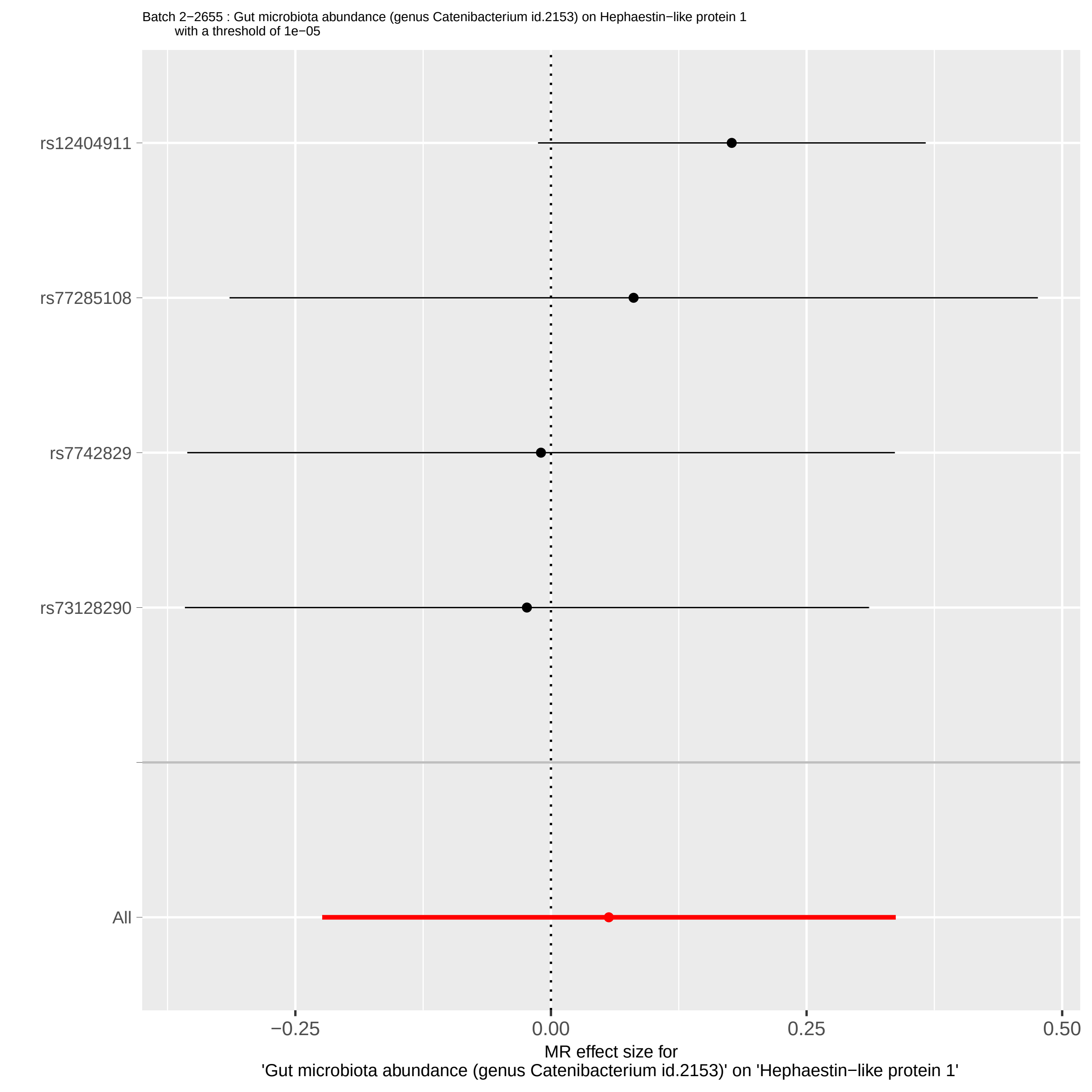

Batch 2−2655 : Gut microbiota abundance (genus Catenibacterium id.2153) on Hephaestin−like protein 1
with a threshold of 1e−05
rs12404911
rs77285108
rs7742829
rs73128290
All
−0.25
0.00
0.25
0.50
MR effect size for
'Gut microbiota abundance (genus Catenibacterium id.2153)' on 'Hephaestin−like protein 1'

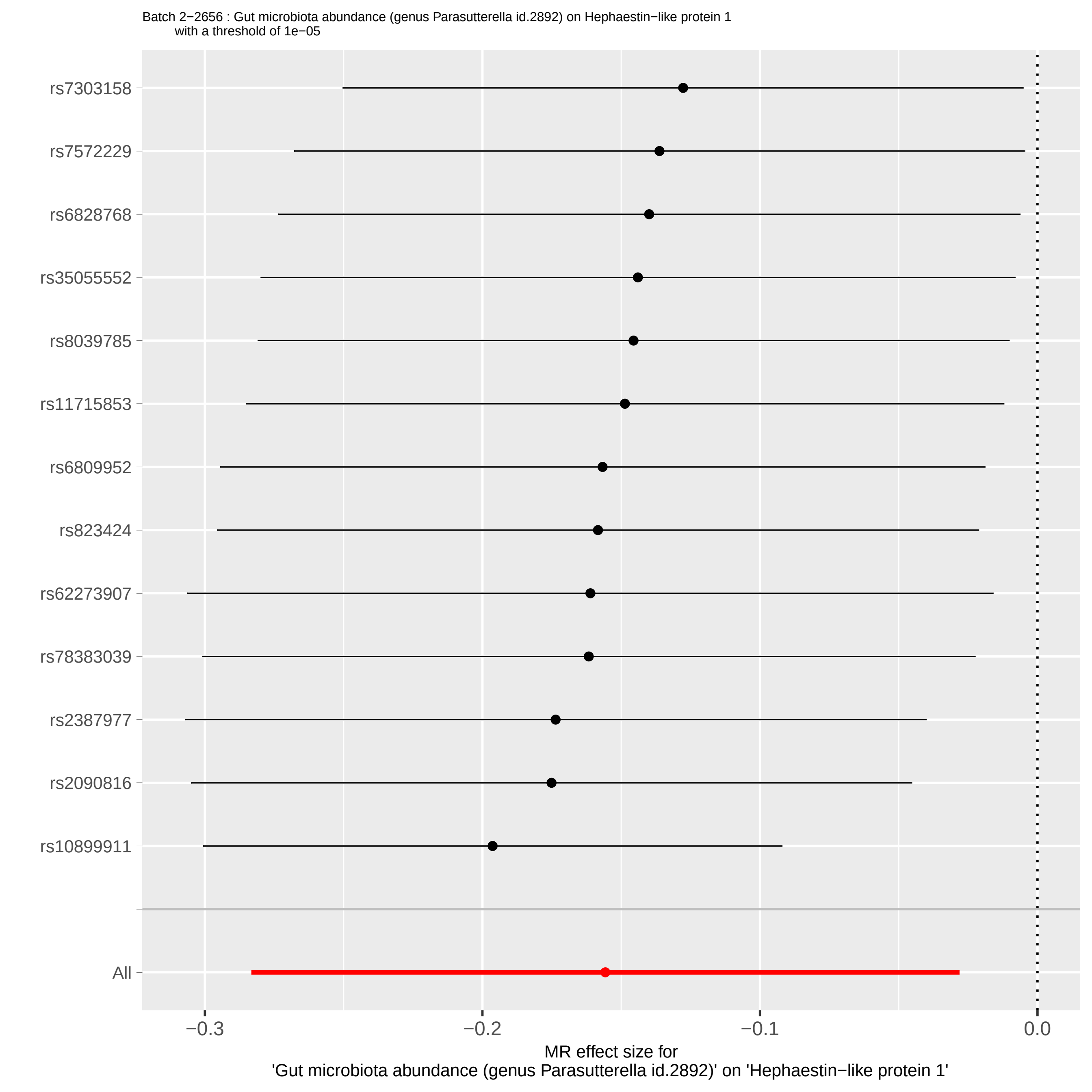

Batch 2−2656 : Gut microbiota abundance (genus Parasutterella id.2892) on Hephaestin−like protein 1
with a threshold of 1e−05
rs7303158
rs7572229
rs6828768
rs35055552
rs8039785
rs11715853
rs6809952
rs823424
rs62273907
rs78383039
rs2387977
rs2090816
rs10899911
All
−0.3
−0.2
−0.1
0.0
MR effect size for
'Gut microbiota abundance (genus Parasutterella id.2892)' on 'Hephaestin−like protein 1'

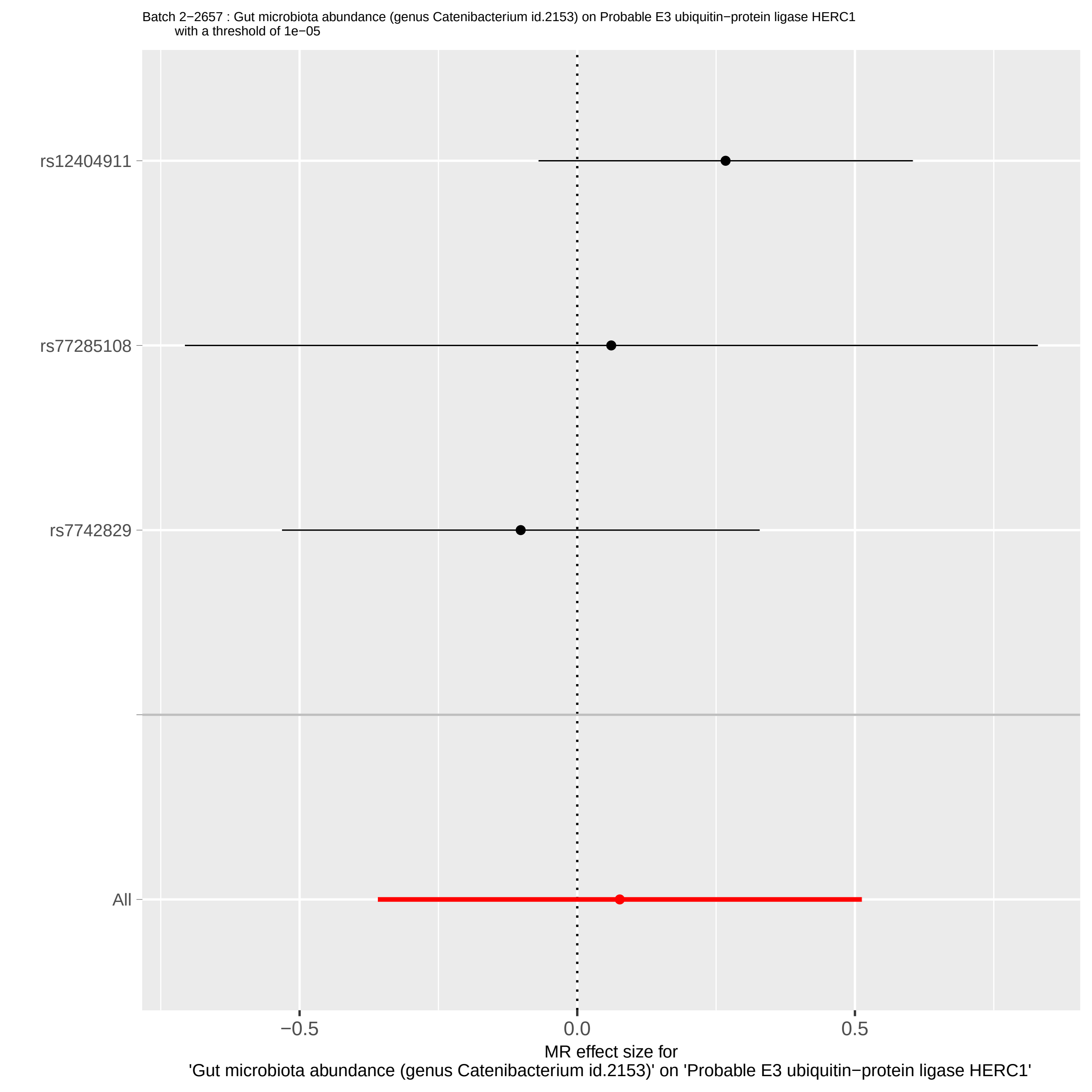
Batch 2−2657 : Gut microbiota abundance (genus Catenibacterium id.2153) on Probable E3 ubiquitin−protein ligase HERC1
with a threshold of 1e−05
rs12404911
rs77285108
rs7742829
All
−0.5
0.0
0.5
MR effect size for
'Gut microbiota abundance (genus Catenibacterium id.2153)' on 'Probable E3 ubiquitin−protein ligase HERC1'

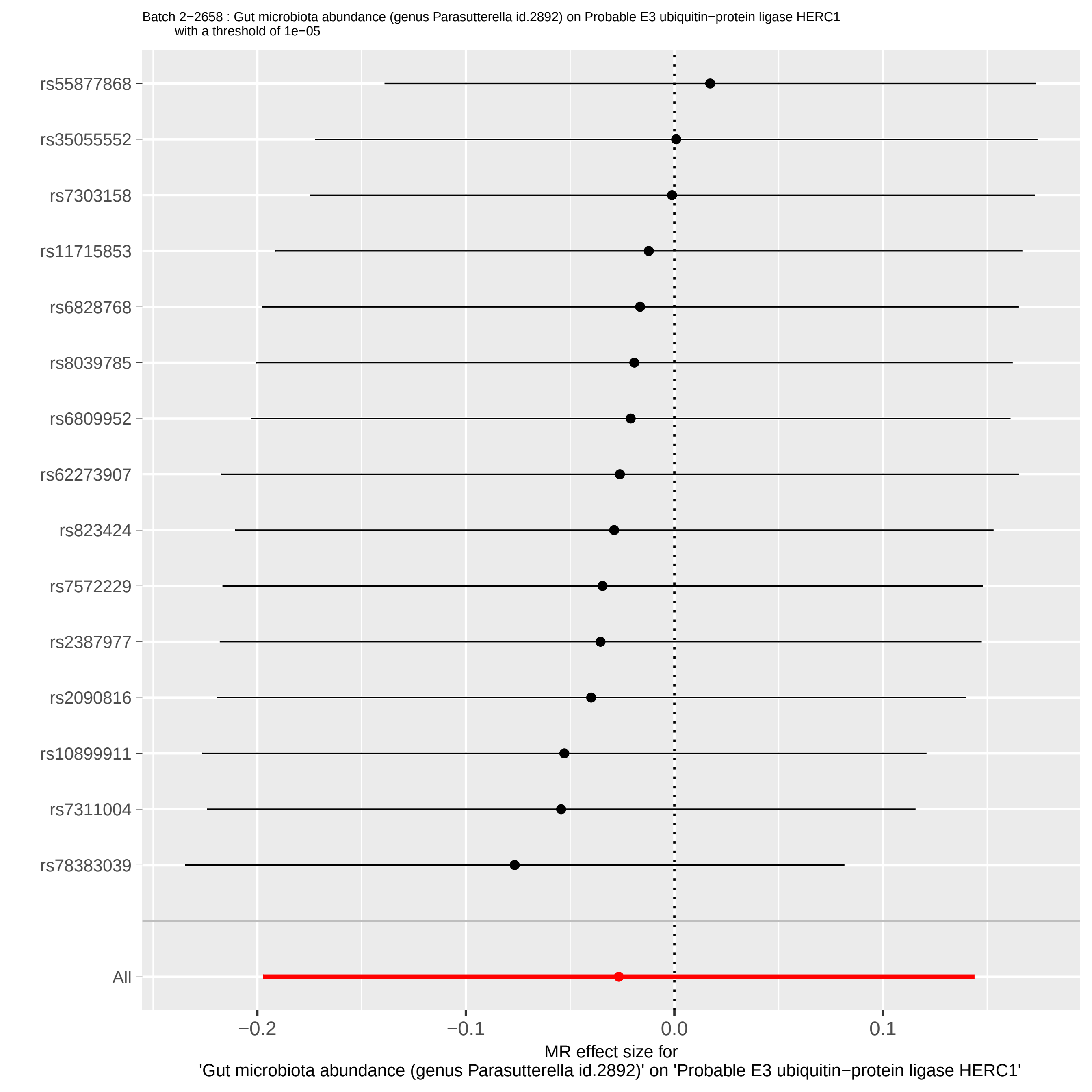

Batch 2−2658 : Gut microbiota abundance (genus Parasutterella id.2892) on Probable E3 ubiquitin−protein ligase HERC1
with a threshold of 1e−05
rs55877868
rs35055552
rs7303158
rs11715853
rs6828768
rs8039785
rs6809952
rs62273907
rs823424
rs7572229
rs2387977
rs2090816
rs10899911
rs7311004
rs78383039
All
−0.2
−0.1
0.0
0.1
MR effect size for
'Gut microbiota abundance (genus Parasutterella id.2892)' on 'Probable E3 ubiquitin−protein ligase HERC1'

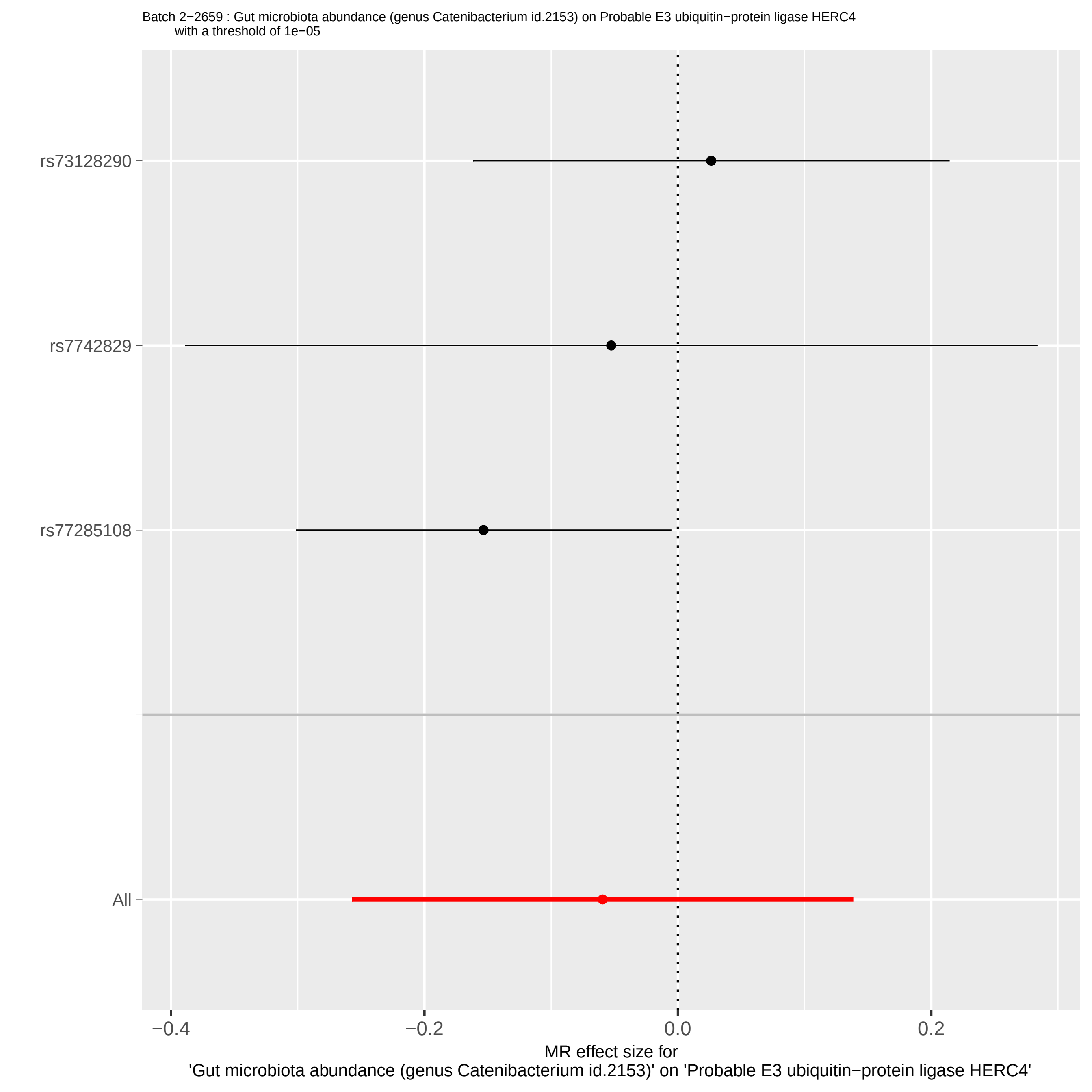

Batch 2−2659 : Gut microbiota abundance (genus Catenibacterium id.2153) on Probable E3 ubiquitin−protein ligase HERC4
with a threshold of 1e−05
rs73128290
rs7742829
rs77285108
All
−0.4
−0.2
0.0
0.2
MR effect size for
'Gut microbiota abundance (genus Catenibacterium id.2153)' on 'Probable E3 ubiquitin−protein ligase HERC4'

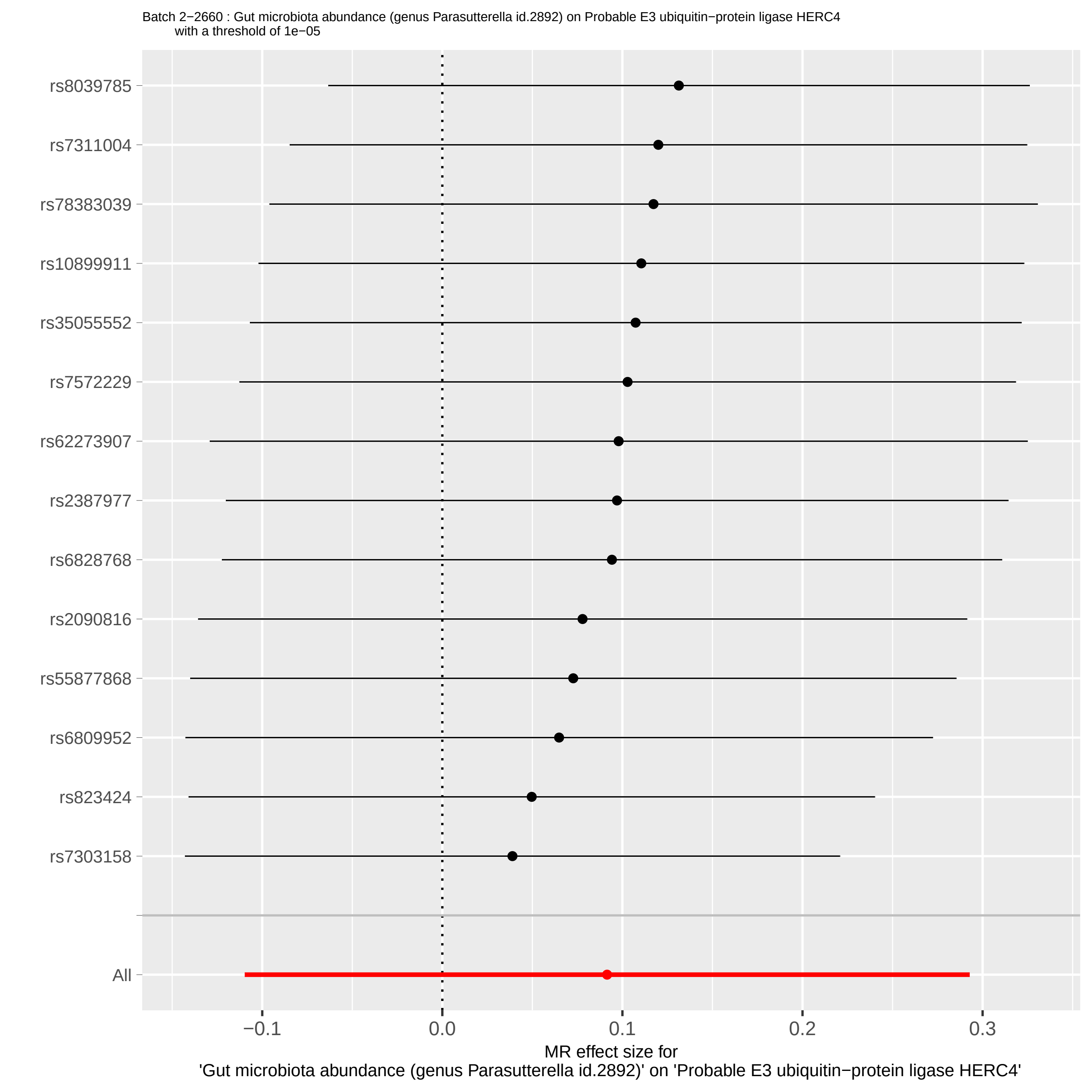
Batch 2−2660 : Gut microbiota abundance (genus Parasutterella id.2892) on Probable E3 ubiquitin−protein ligase HERC4
with a threshold of 1e−05
rs8039785
rs7311004
rs78383039
rs10899911
rs35055552
rs7572229
rs62273907
rs2387977
rs6828768
rs2090816
rs55877868
rs6809952
rs823424
rs7303158
All
−0.1
0.0
0.1
0.2
0.3
MR effect size for
'Gut microbiota abundance (genus Parasutterella id.2892)' on 'Probable E3 ubiquitin−protein ligase HERC4'

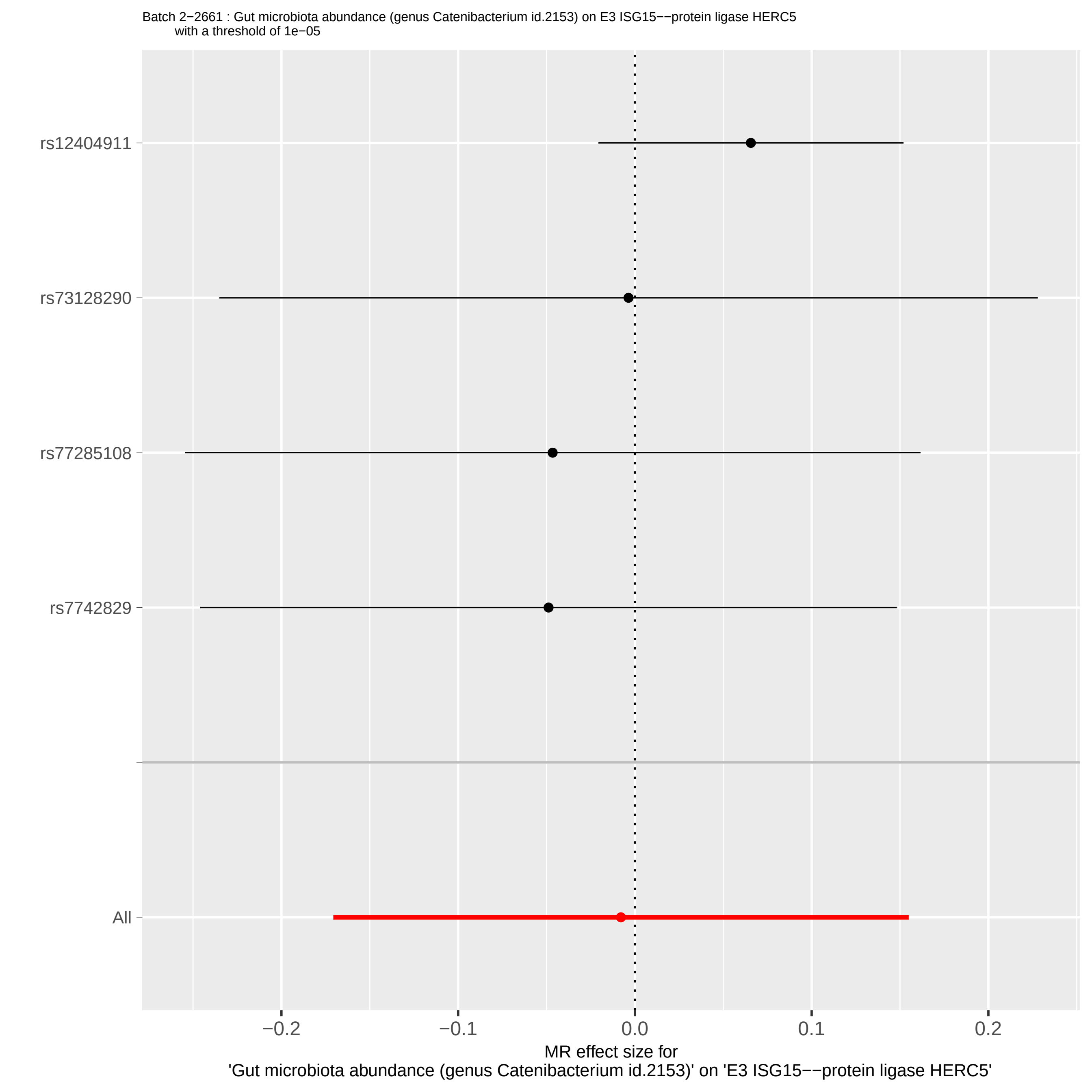
Batch 2−2661 : Gut microbiota abundance (genus Catenibacterium id.2153) on E3 ISG15−−protein ligase HERC5
with a threshold of 1e−05
rs12404911
rs73128290
rs77285108
rs7742829
All
−0.2
−0.1
0.0
0.1
0.2
MR effect size for
'Gut microbiota abundance (genus Catenibacterium id.2153)' on 'E3 ISG15−−protein ligase HERC5'

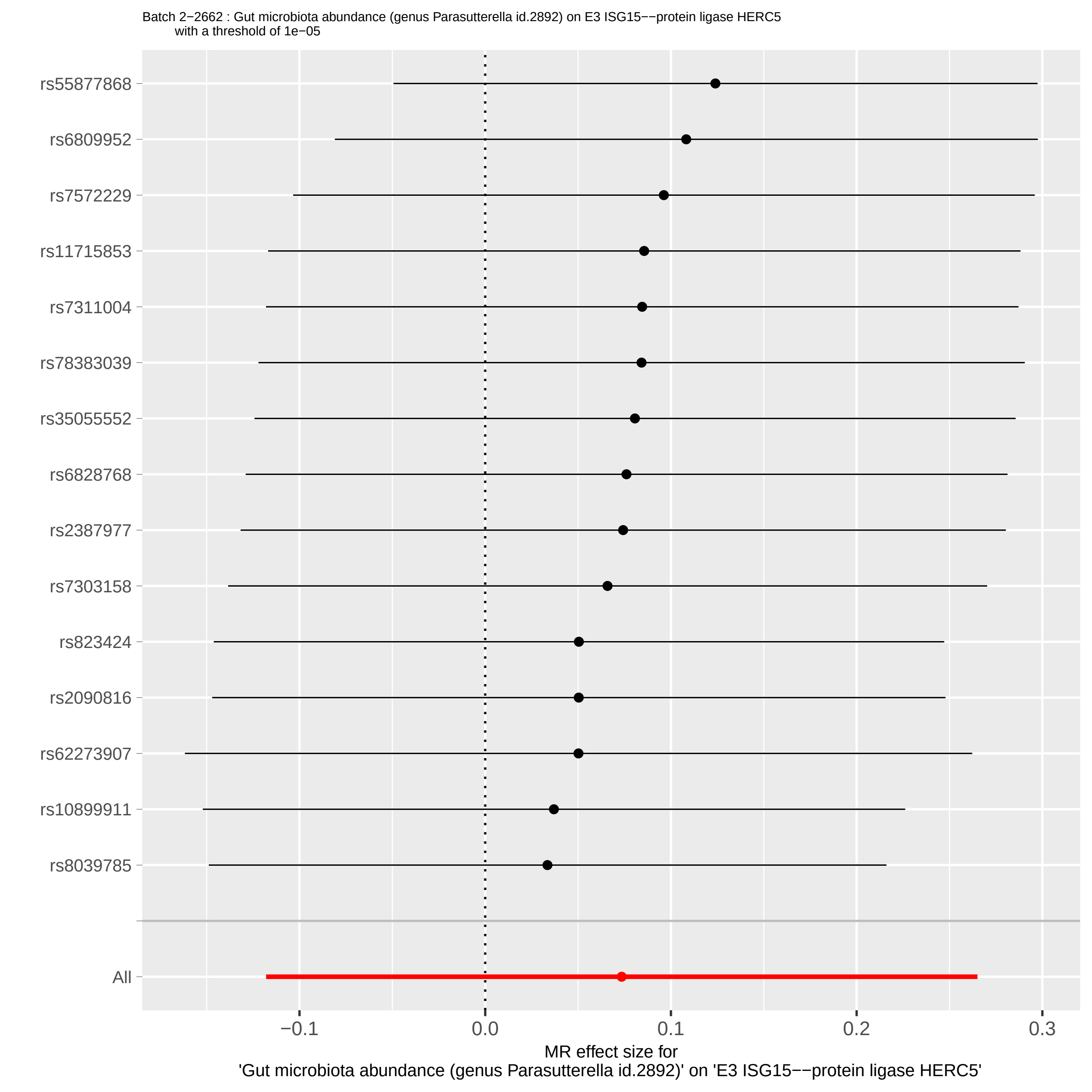
Batch 2−2662 : Gut microbiota abundance (genus Parasutterella id.2892) on E3 ISG15−−protein ligase HERC5
with a threshold of 1e−05
rs55877868
rs6809952
rs7572229
rs11715853
rs7311004
rs78383039
rs35055552
rs6828768
rs2387977
rs7303158
rs823424
rs2090816
rs62273907
rs10899911
rs8039785
All
−0.1
0.0
0.1
0.2
0.3
MR effect size for
'Gut microbiota abundance (genus Parasutterella id.2892)' on 'E3 ISG15−−protein ligase HERC5'

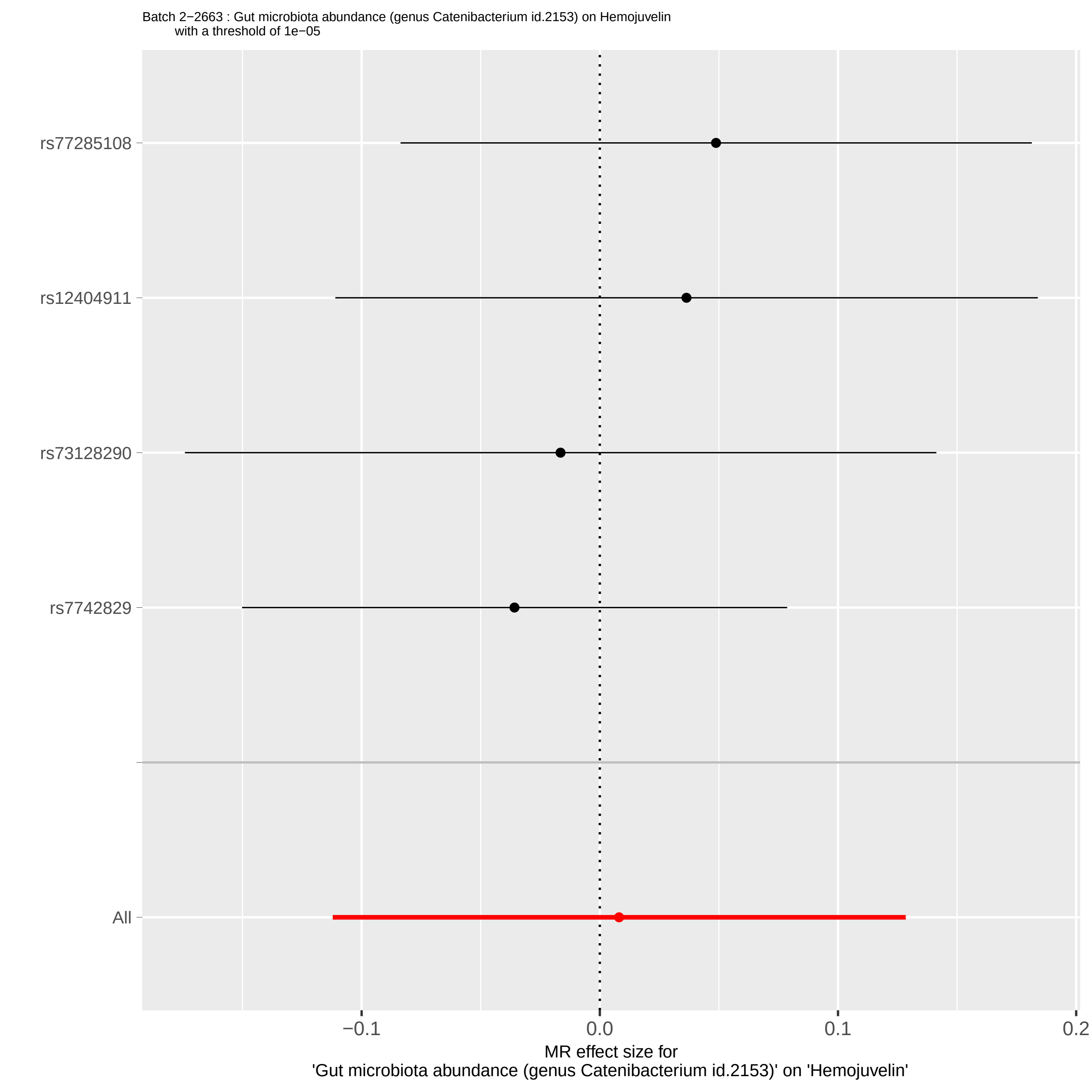
Batch 2−2663 : Gut microbiota abundance (genus Catenibacterium id.2153) on Hemojuvelin
with a threshold of 1e−05
rs77285108
rs12404911
rs73128290
rs7742829
All
−0.1
0.0
0.1
0.2
MR effect size for
'Gut microbiota abundance (genus Catenibacterium id.2153)' on 'Hemojuvelin'

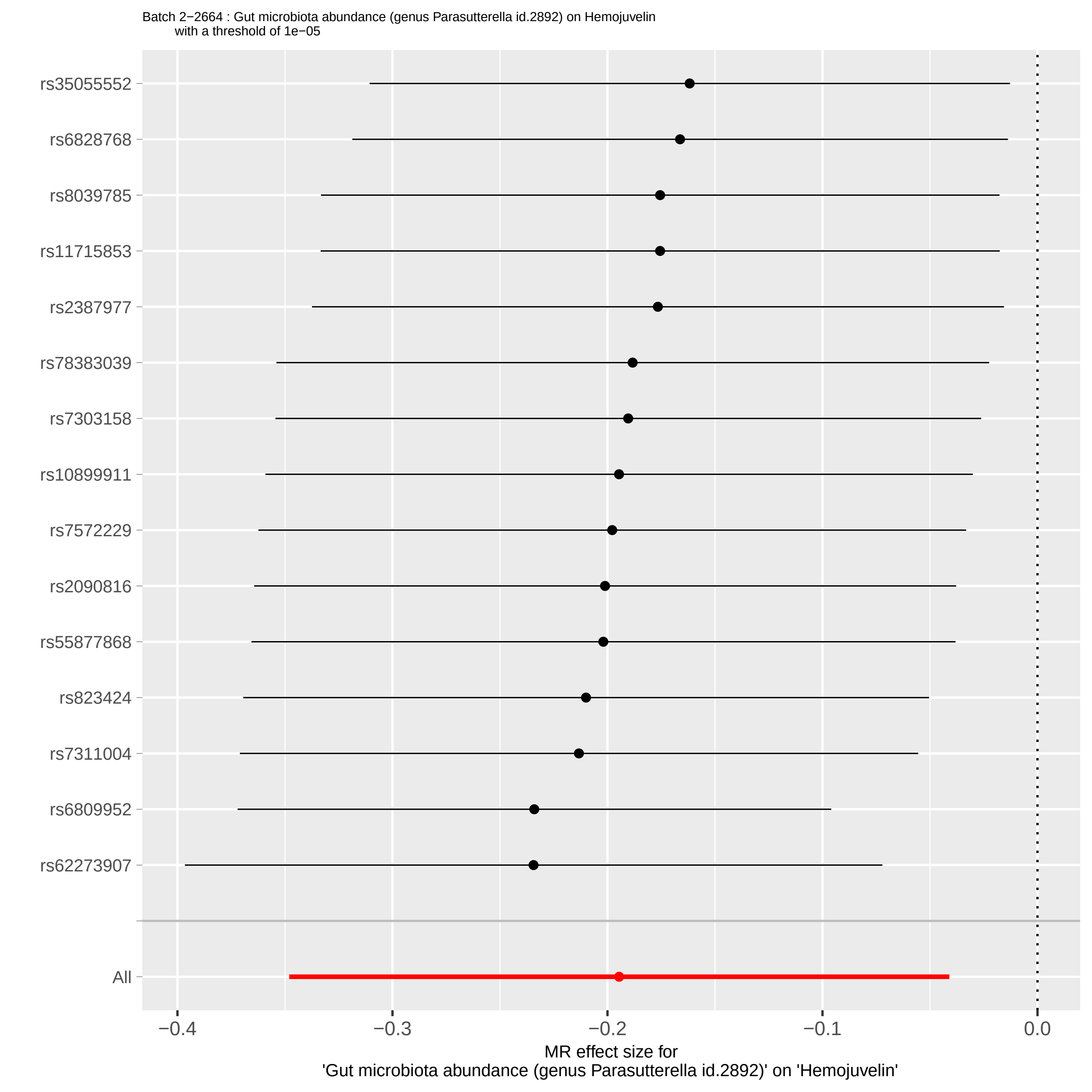
Batch 2−2664 : Gut microbiota abundance (genus Parasutterella id.2892) on Hemojuvelin
with a threshold of 1e−05
rs35055552
rs6828768
rs8039785
rs11715853
rs2387977
rs78383039
rs7303158
rs10899911
rs7572229
rs2090816
rs55877868
rs823424
rs7311004
rs6809952
rs62273907
All
−0.4
−0.3
−0.2
−0.1
0.0
MR effect size for
'Gut microbiota abundance (genus Parasutterella id.2892)' on 'Hemojuvelin'

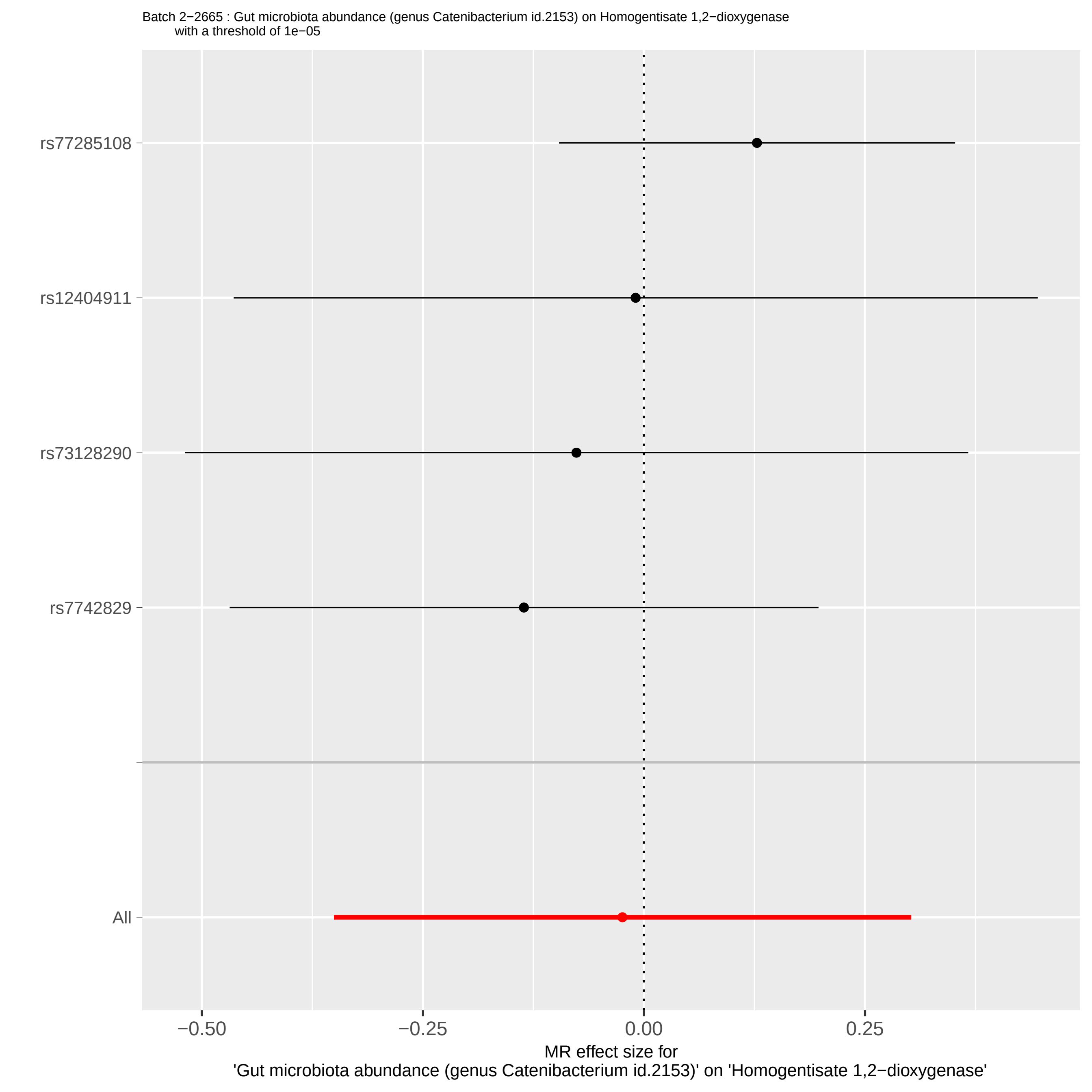
Batch 2−2665 : Gut microbiota abundance (genus Catenibacterium id.2153) on Homogentisate 1,2−dioxygenase
with a threshold of 1e−05
rs77285108
rs12404911
rs73128290
rs7742829
All
−0.50
−0.25
0.00
0.25
MR effect size for
'Gut microbiota abundance (genus Catenibacterium id.2153)' on 'Homogentisate 1,2−dioxygenase'

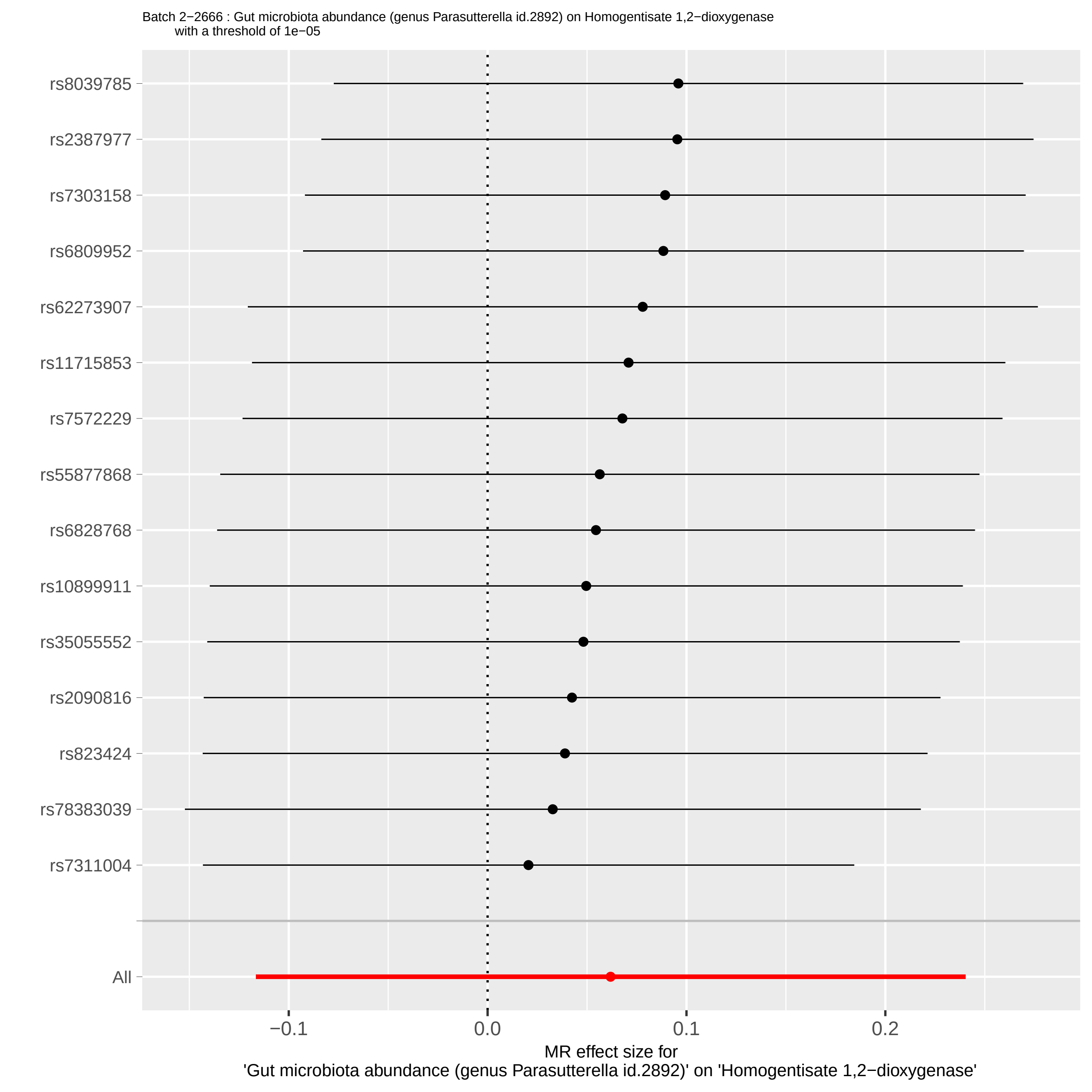

Batch 2−2666 : Gut microbiota abundance (genus Parasutterella id.2892) on Homogentisate 1,2−dioxygenase
with a threshold of 1e−05
rs8039785
rs2387977
rs7303158
rs6809952
rs62273907
rs11715853
rs7572229
rs55877868
rs6828768
rs10899911
rs35055552
rs2090816
rs823424
rs78383039
rs7311004
All
−0.1
0.0
0.1
0.2
MR effect size for
'Gut microbiota abundance (genus Parasutterella id.2892)' on 'Homogentisate 1,2−dioxygenase'

Batch 2−2667 : Gut microbiota abundance (genus Catenibacterium id.2153) on Hepatocyte growth factor
with a threshold of 1e−05

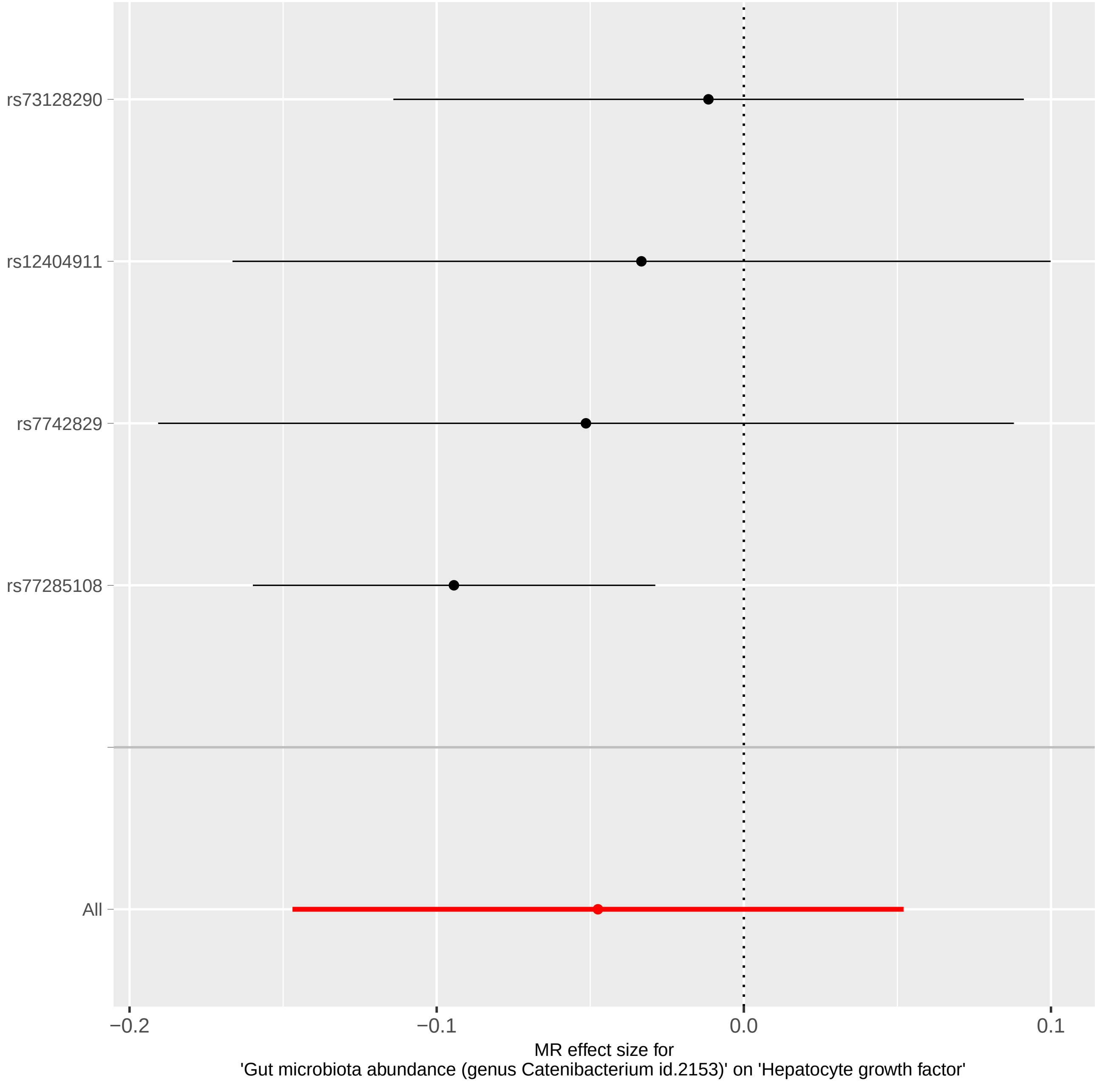

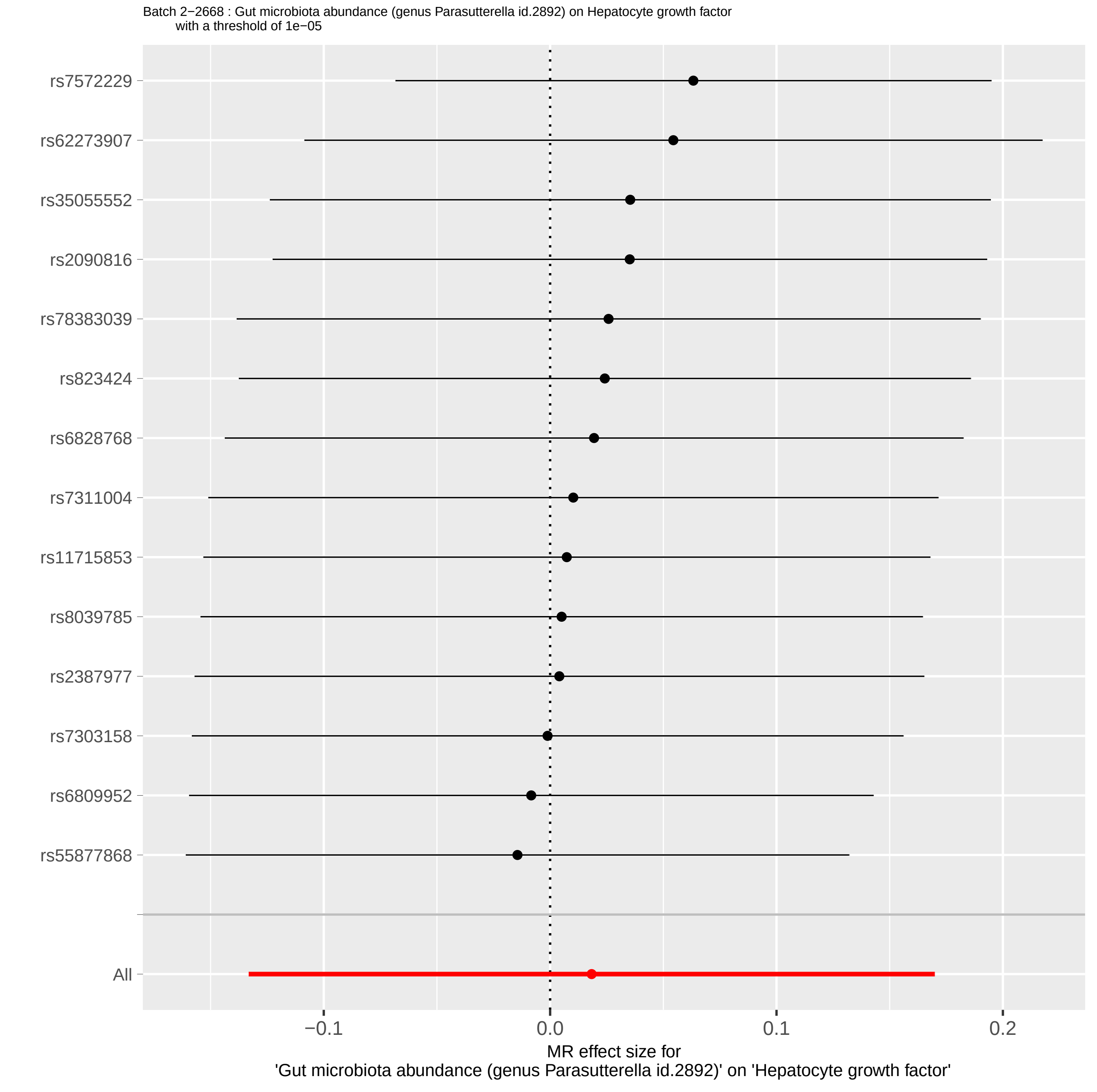

Batch 2−2668 : Gut microbiota abundance (genus Parasutterella id.2892) on Hepatocyte growth factor
with a threshold of 1e−05
rs7572229
rs62273907
rs35055552
rs2090816
rs78383039
rs823424
rs6828768
rs7311004
rs11715853
rs8039785
rs2387977
rs7303158
rs6809952
rs55877868
All
−0.1
0.0
0.1
0.2
MR effect size for
'Gut microbiota abundance (genus Parasutterella id.2892)' on 'Hepatocyte growth factor'

Batch 2−2669 : Gut microbiota abundance (genus Catenibacterium id.2153) on Hepatocyte growth factor−regulated tyrosine kinase substrate
with a threshold of 1e−05

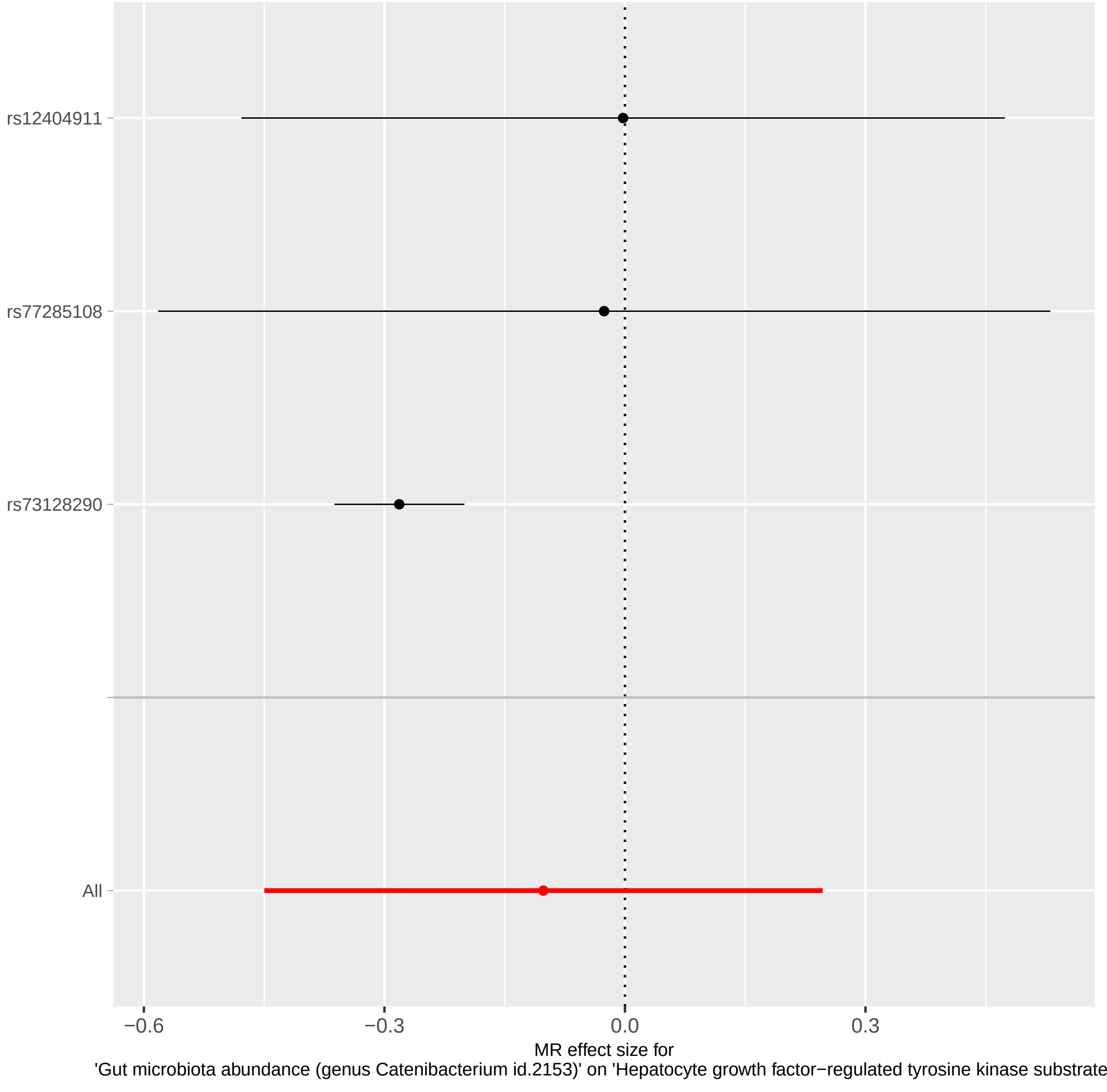

Batch 2−2670 : Gut microbiota abundance (genus Parasutterella id.2892) on Hepatocyte growth factor−regulated tyrosine kinase substrate
with a threshold of 1e−05

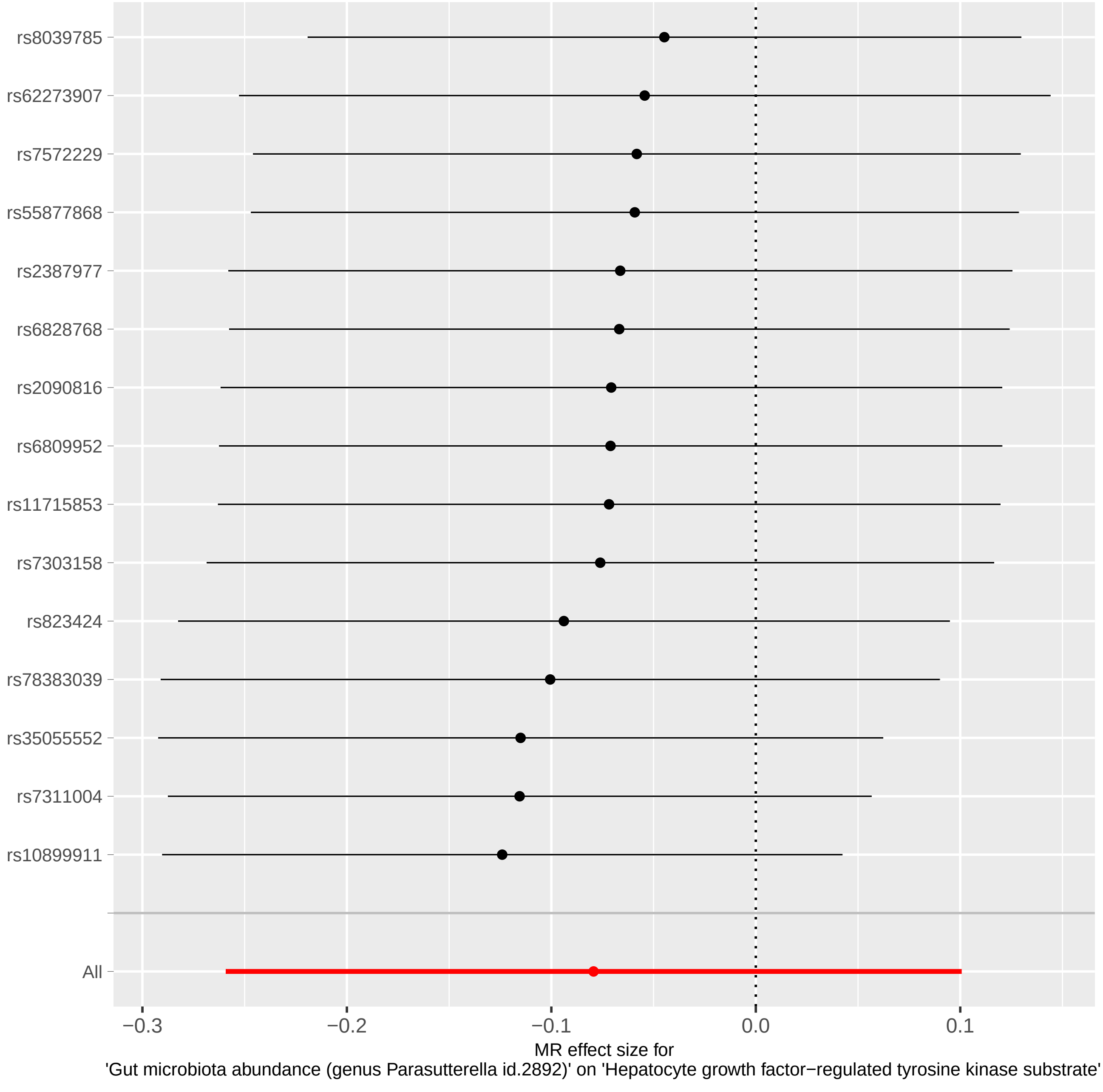

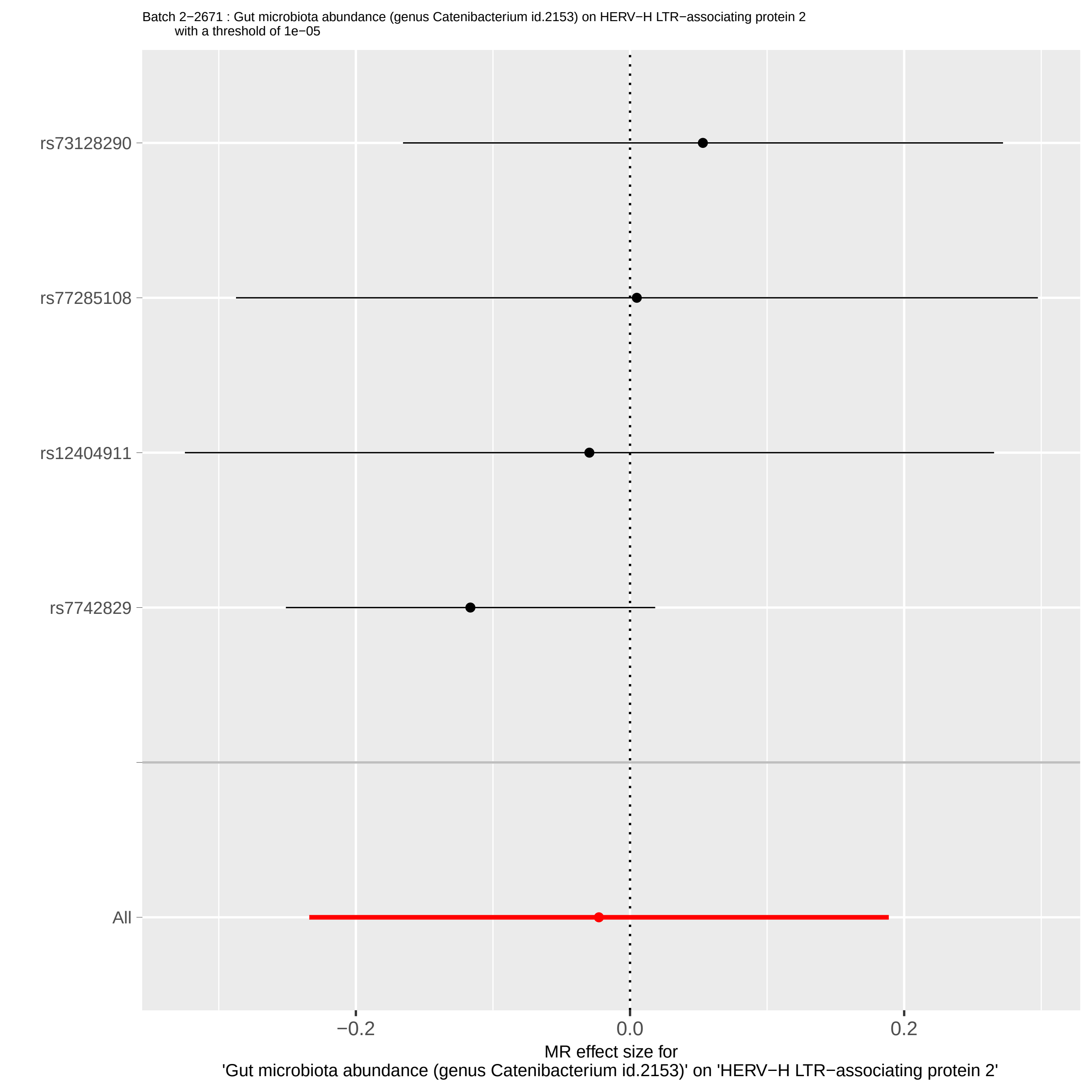
Batch 2−2671 : Gut microbiota abundance (genus Catenibacterium id.2153) on HERV−H LTR−associating protein 2
with a threshold of 1e−05
rs73128290
rs77285108
rs12404911
rs7742829
All
−0.2
0.0
0.2
MR effect size for
'Gut microbiota abundance (genus Catenibacterium id.2153)' on 'HERV−H LTR−associating protein 2'

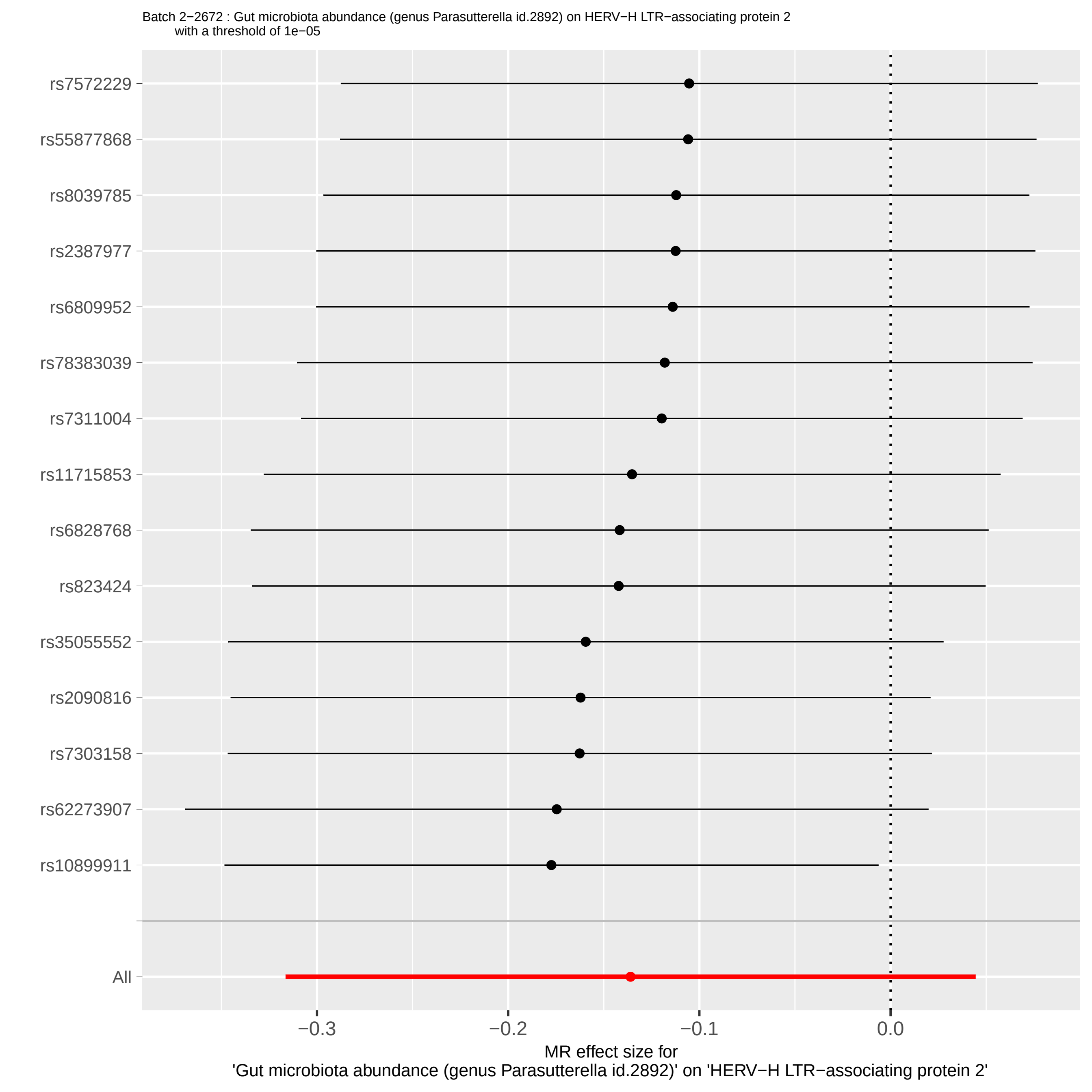

Batch 2−2672 : Gut microbiota abundance (genus Parasutterella id.2892) on HERV−H LTR−associating protein 2
with a threshold of 1e−05
rs7572229
rs55877868
rs8039785
rs2387977
rs6809952
rs78383039
rs7311004
rs11715853
rs6828768
rs823424
rs35055552
rs2090816
rs7303158
rs62273907
rs10899911
All
−0.3
−0.2
−0.1
0.0
MR effect size for
'Gut microbiota abundance (genus Parasutterella id.2892)' on 'HERV−H LTR−associating protein 2'

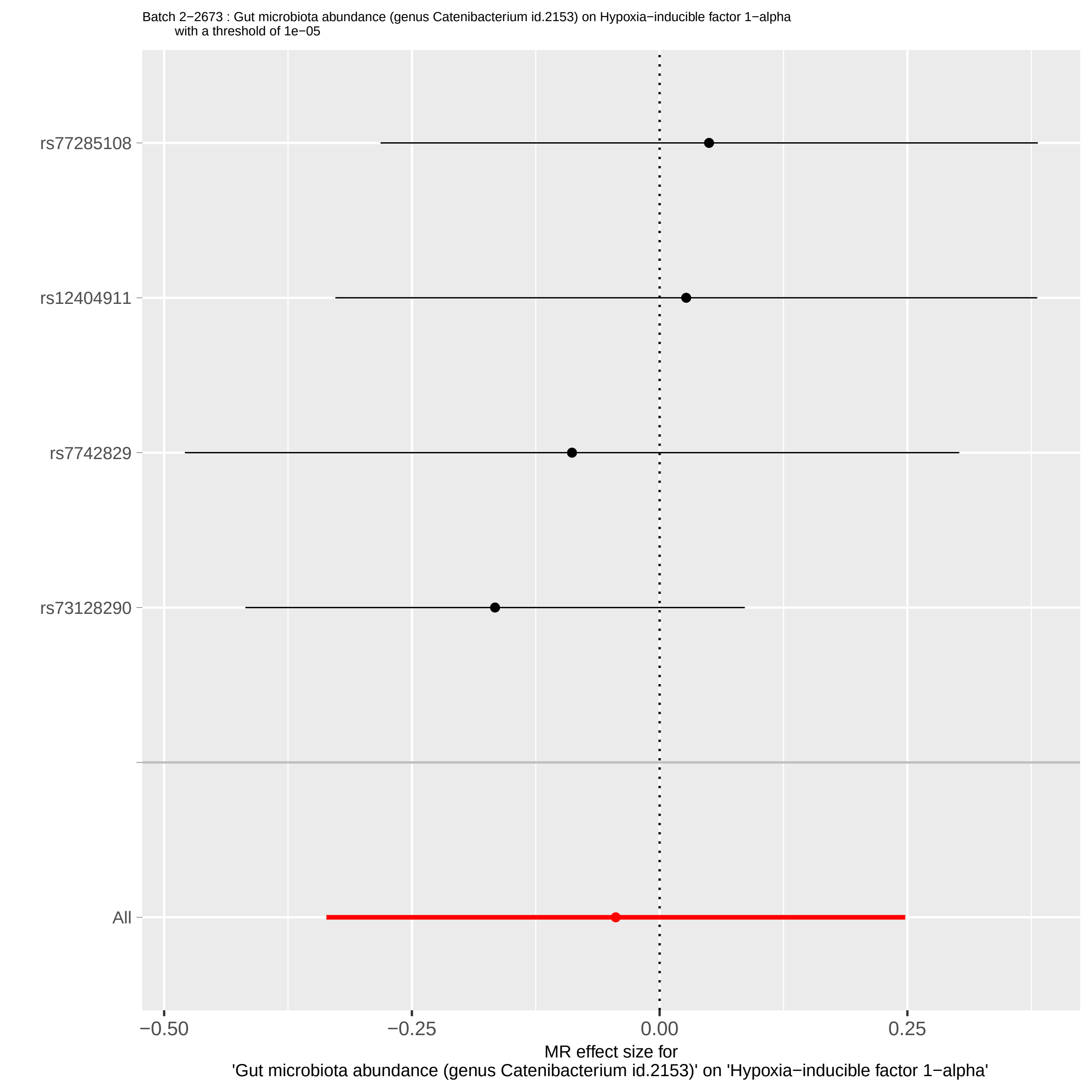
Batch 2−2673 : Gut microbiota abundance (genus Catenibacterium id.2153) on Hypoxia−inducible factor 1−alpha
with a threshold of 1e−05
rs77285108
rs12404911
rs7742829
rs73128290
All
−0.50
−0.25
0.00
0.25
MR effect size for
'Gut microbiota abundance (genus Catenibacterium id.2153)' on 'Hypoxia−inducible factor 1−alpha'

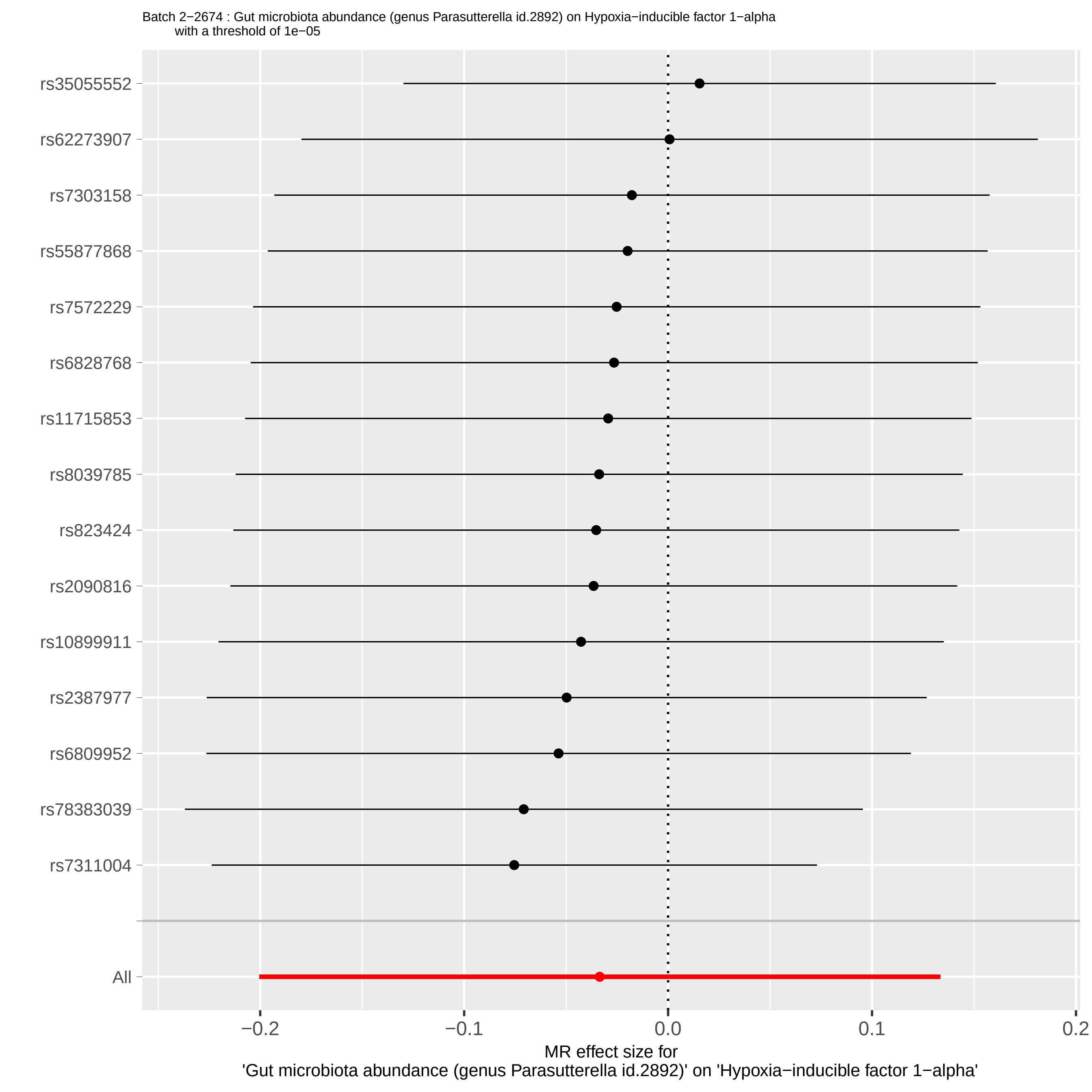

Batch 2−2674 : Gut microbiota abundance (genus Parasutterella id.2892) on Hypoxia−inducible factor 1−alpha
with a threshold of 1e−05
rs35055552
rs62273907
rs7303158
rs55877868
rs7572229
rs6828768
rs11715853
rs8039785
rs823424
rs2090816
rs10899911
rs2387977
rs6809952
rs78383039
rs7311004
All
−0.2
−0.1
0.0
0.1
0.2
MR effect size for
'Gut microbiota abundance (genus Parasutterella id.2892)' on 'Hypoxia−inducible factor 1−alpha'

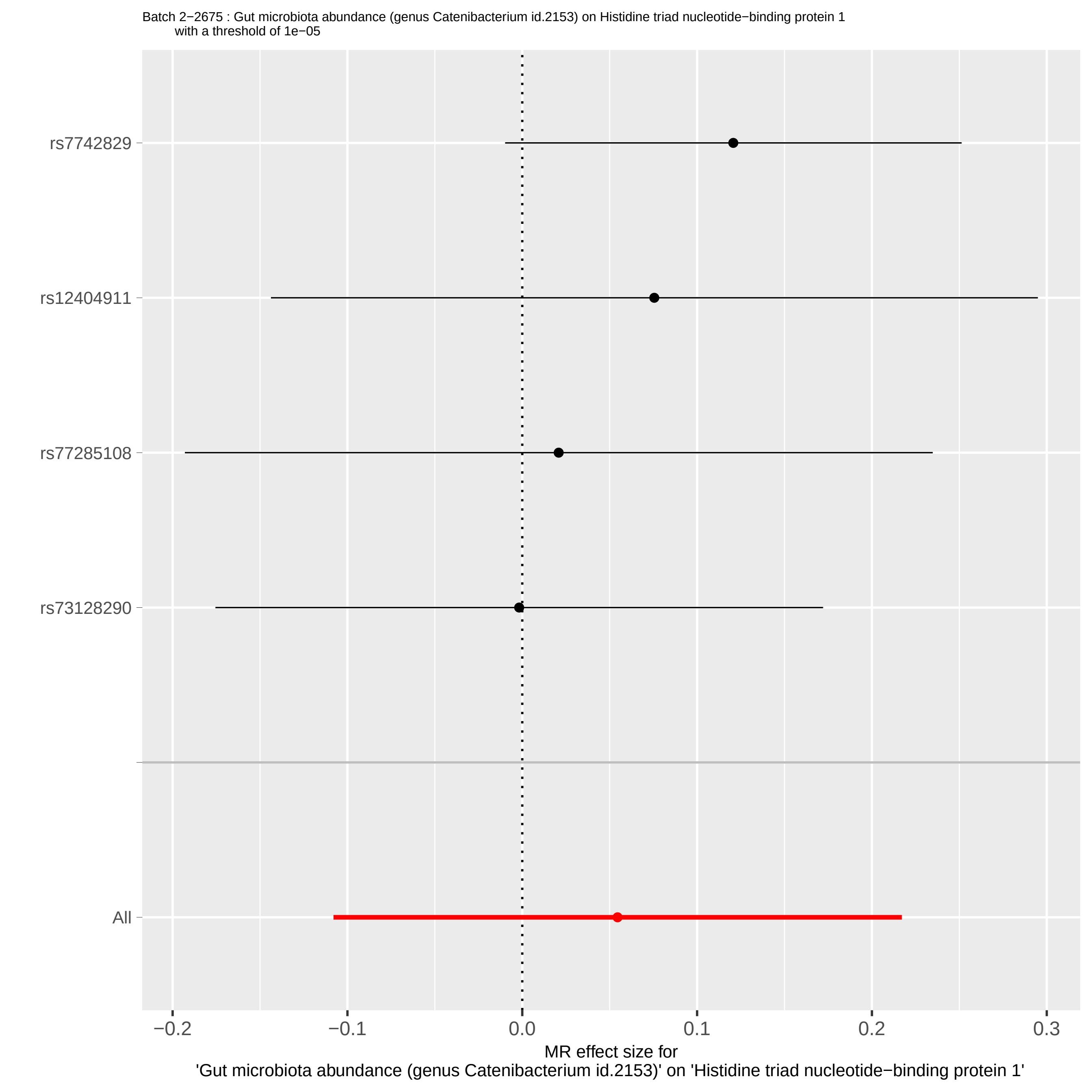
Batch 2−2675 : Gut microbiota abundance (genus Catenibacterium id.2153) on Histidine triad nucleotide−binding protein 1
with a threshold of 1e−05
rs7742829
rs12404911
rs77285108
rs73128290
All
−0.2
−0.1
0.0
0.1
0.2
0.3
MR effect size for
'Gut microbiota abundance (genus Catenibacterium id.2153)' on 'Histidine triad nucleotide−binding protein 1'

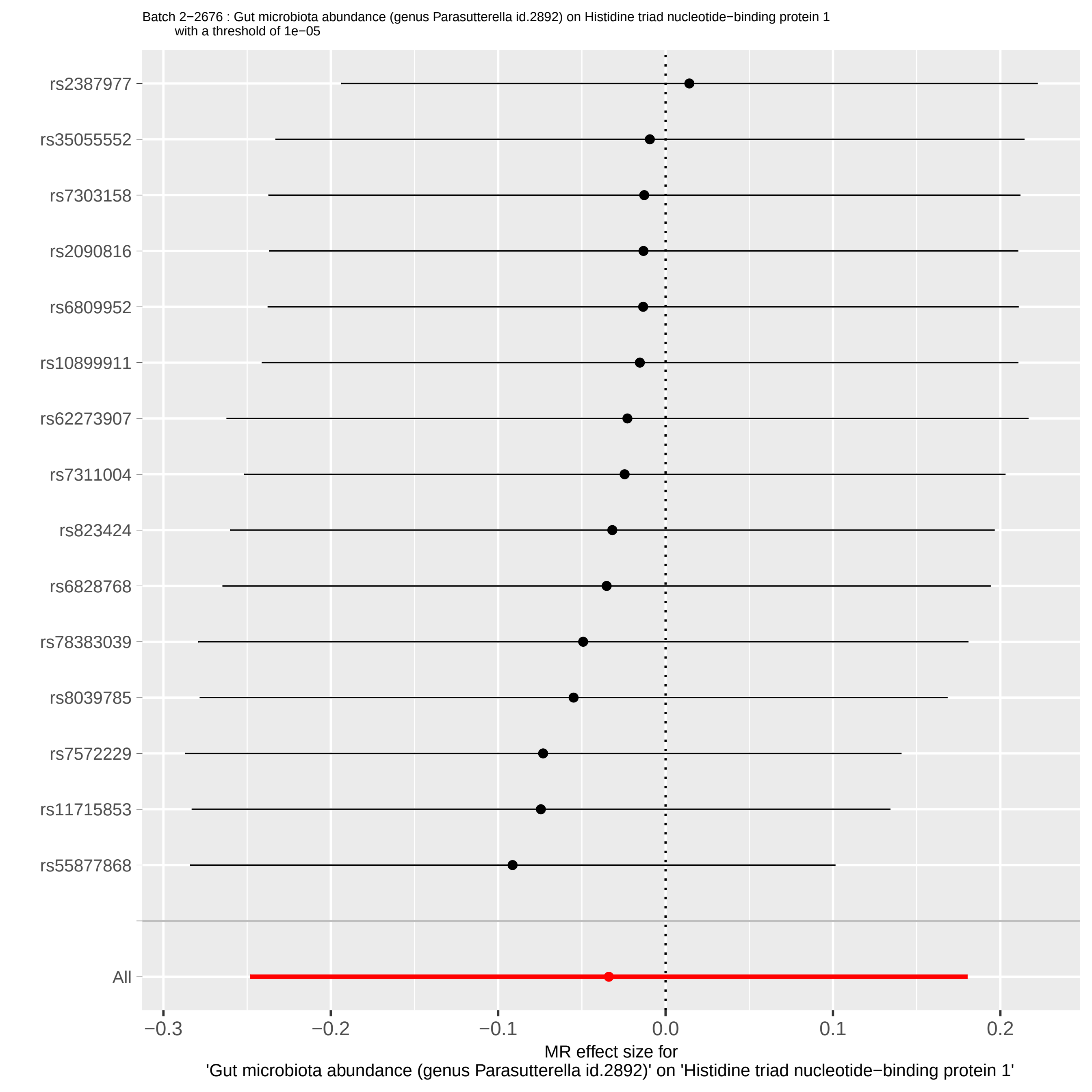
Batch 2−2676 : Gut microbiota abundance (genus Parasutterella id.2892) on Histidine triad nucleotide−binding protein 1
with a threshold of 1e−05
rs2387977
rs35055552
rs7303158
rs2090816
rs6809952
rs10899911
rs62273907
rs7311004
rs823424
rs6828768
rs78383039
rs8039785
rs7572229
rs11715853
rs55877868
All
−0.3
−0.2
−0.1
0.0
0.1
0.2
MR effect size for
'Gut microbiota abundance (genus Parasutterella id.2892)' on 'Histidine triad nucleotide−binding protein 1'

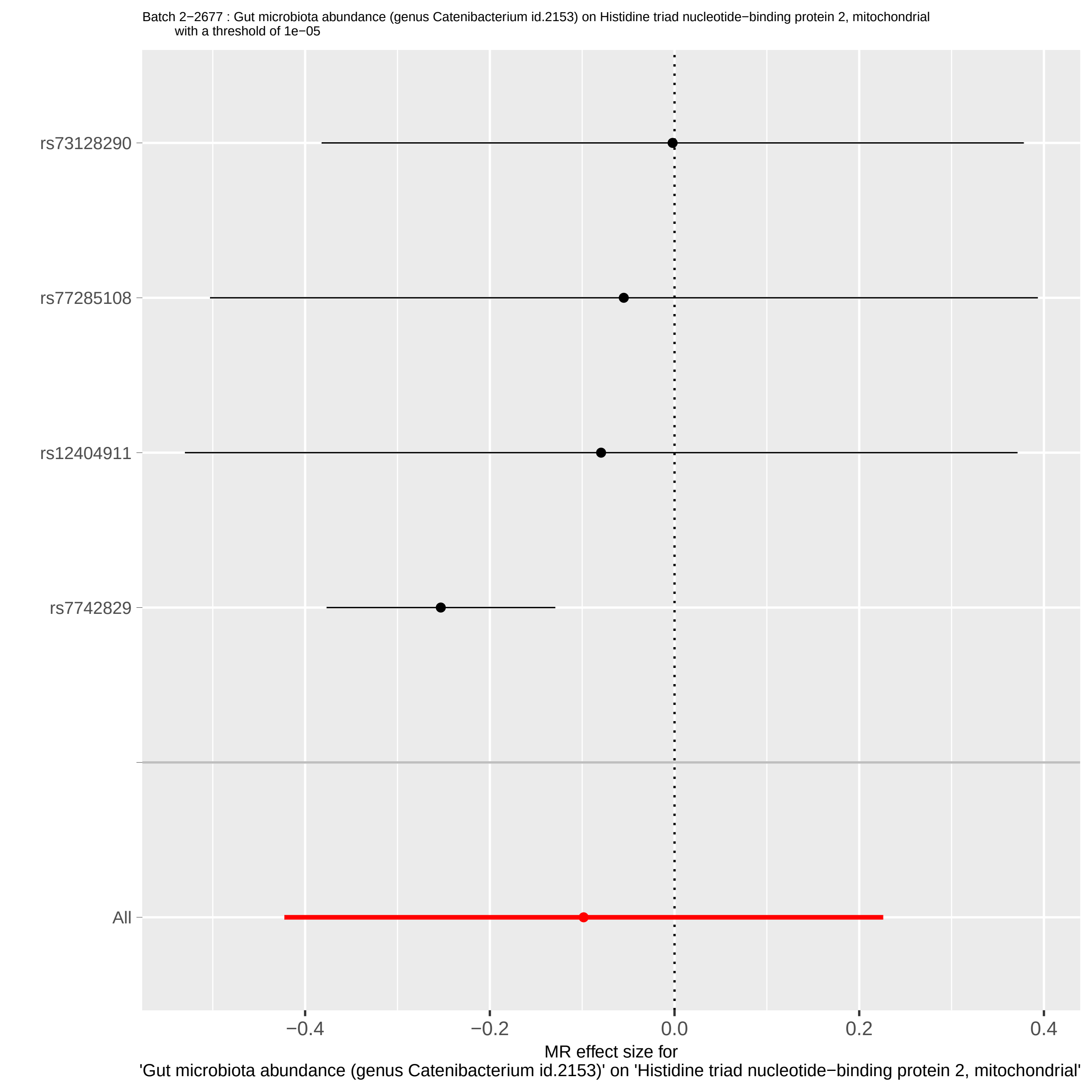
Batch 2−2677 : Gut microbiota abundance (genus Catenibacterium id.2153) on Histidine triad nucleotide−binding protein 2, mitochondrial
with a threshold of 1e−05
rs73128290
rs77285108
rs12404911
rs7742829
All
−0.4
−0.2
0.0
0.2
0.4
MR effect size for
'Gut microbiota abundance (genus Catenibacterium id.2153)' on 'Histidine triad nucleotide−binding protein 2, mitochondrial'

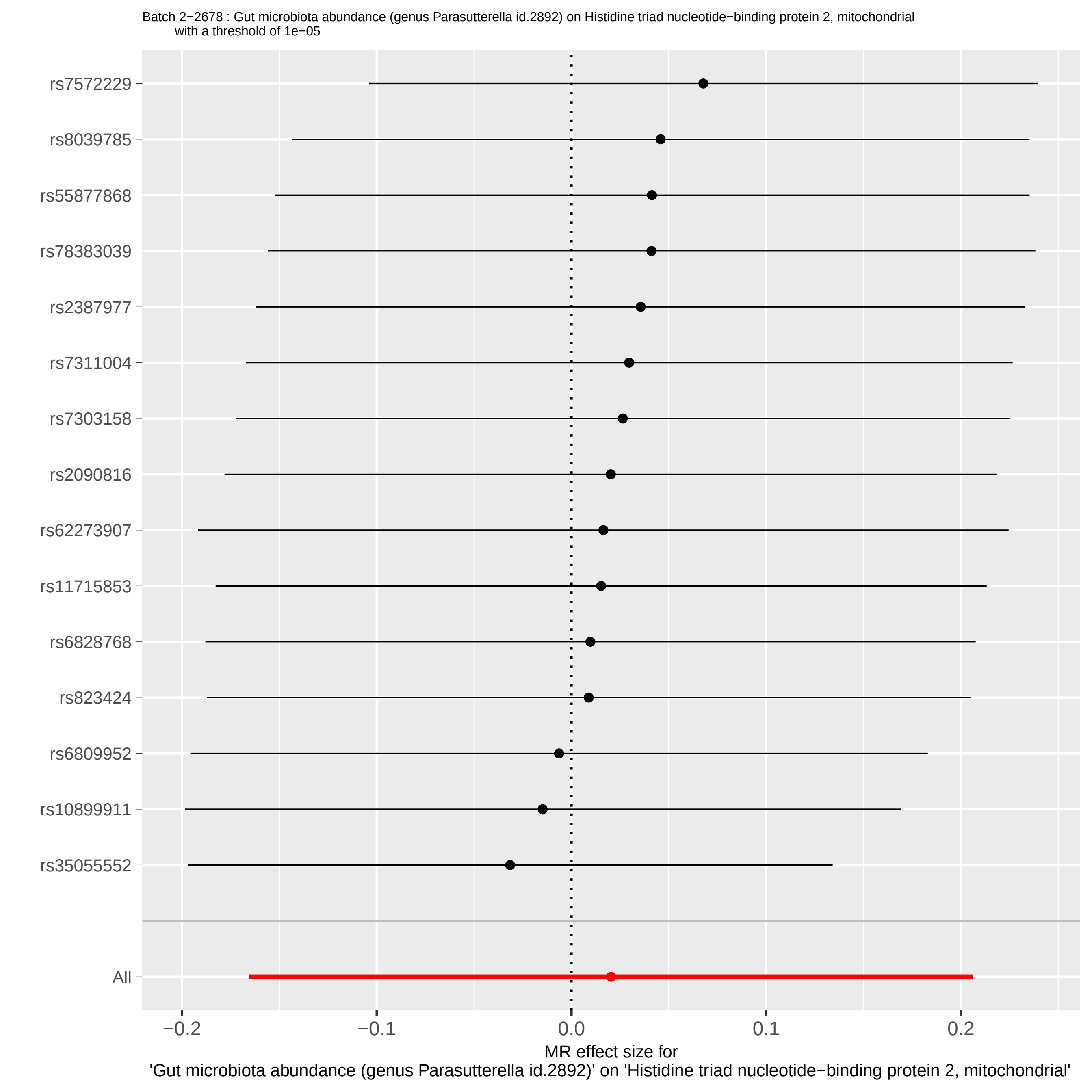
Batch 2−2678 : Gut microbiota abundance (genus Parasutterella id.2892) on Histidine triad nucleotide−binding protein 2, mitochondrial
with a threshold of 1e−05
rs7572229
rs8039785
rs55877868
rs78383039
rs2387977
rs7311004
rs7303158
rs2090816
rs62273907
rs11715853
rs6828768
rs823424
rs6809952
rs10899911
rs35055552
All
−0.2
−0.1
0.0
0.1
0.2
MR effect size for
'Gut microbiota abundance (genus Parasutterella id.2892)' on 'Histidine triad nucleotide−binding protein 2, mitochondrial'

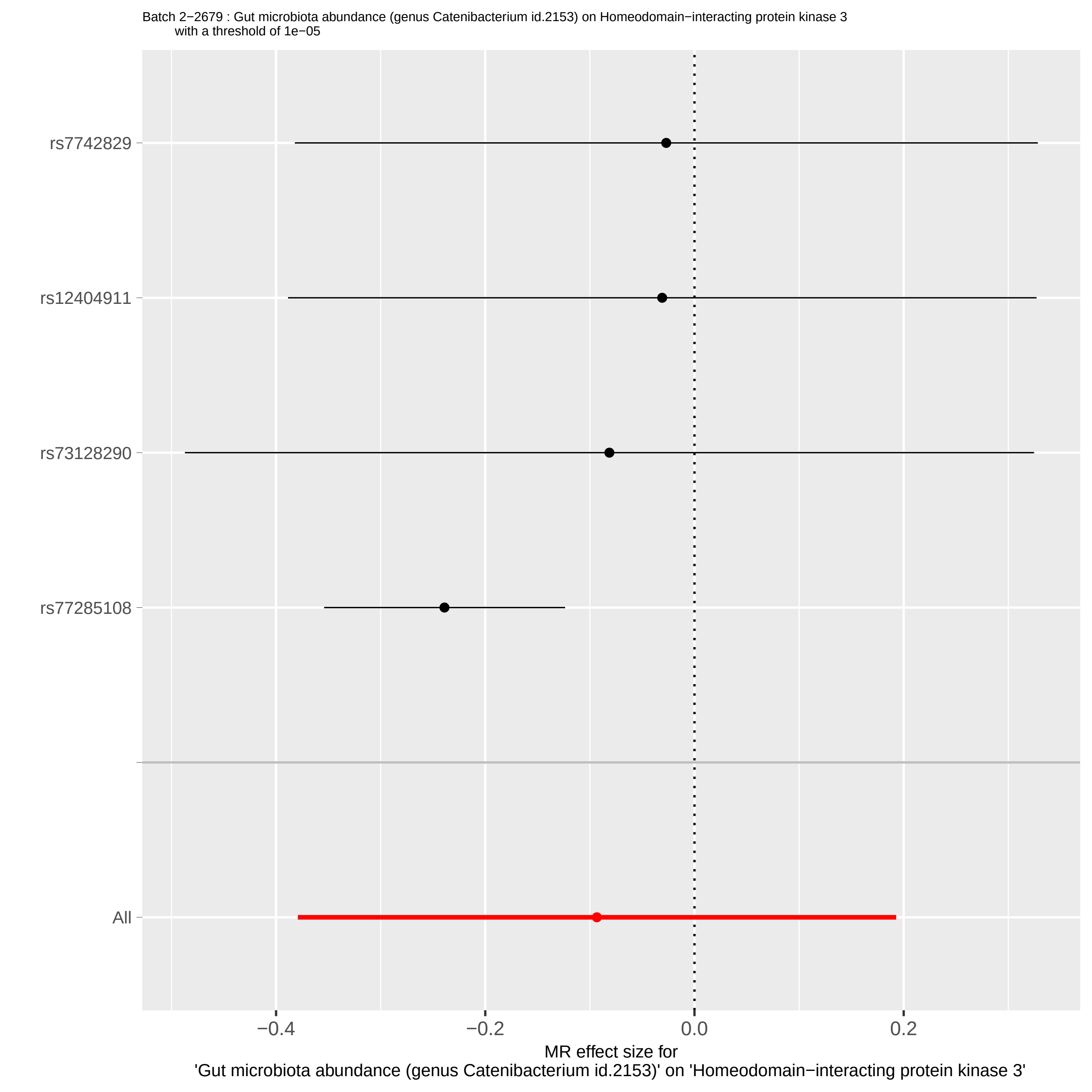
Batch 2−2679 : Gut microbiota abundance (genus Catenibacterium id.2153) on Homeodomain−interacting protein kinase 3
with a threshold of 1e−05
rs7742829
rs12404911
rs73128290
rs77285108
All
−0.4
−0.2
0.0
0.2
MR effect size for
'Gut microbiota abundance (genus Catenibacterium id.2153)' on 'Homeodomain−interacting protein kinase 3'

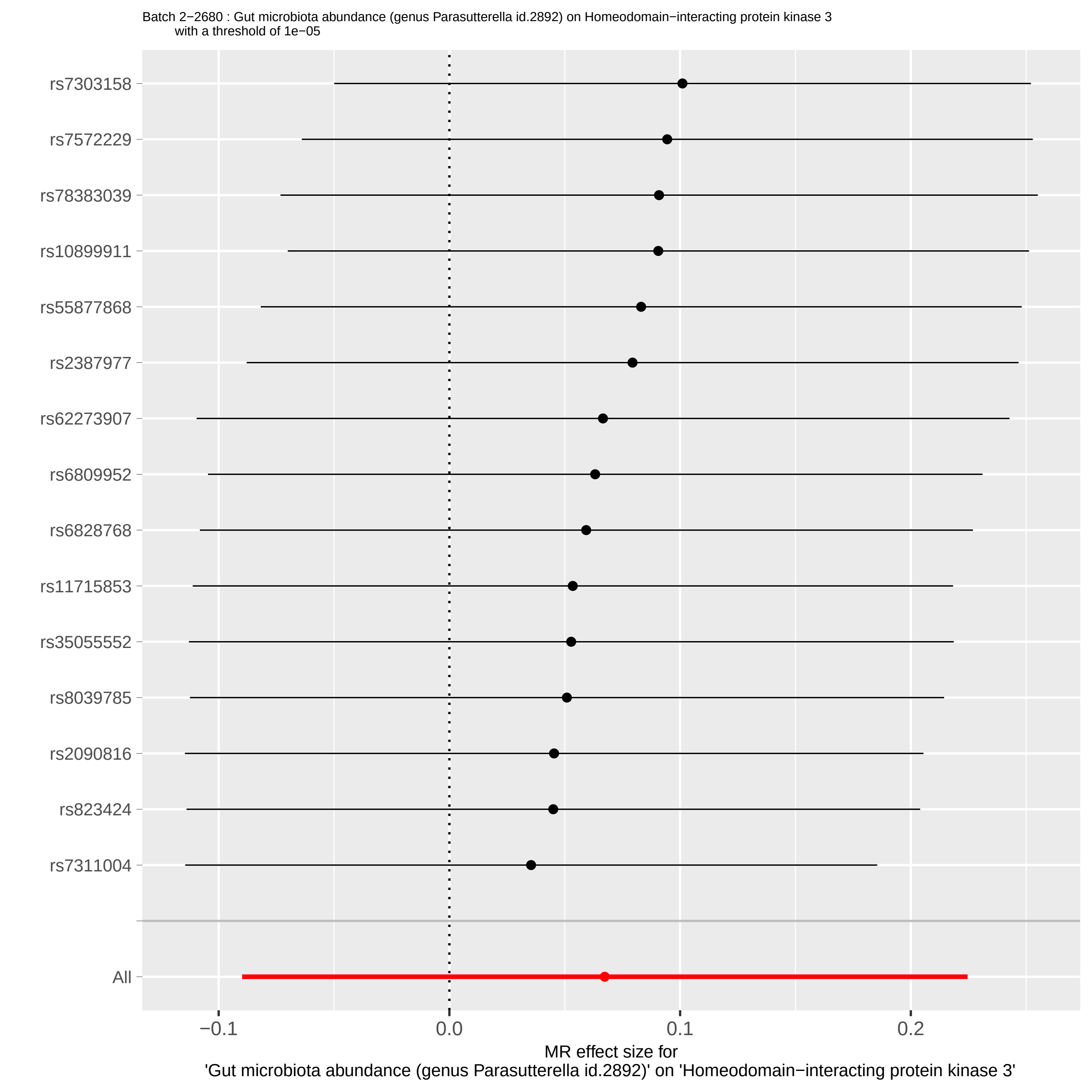

Batch 2−2680 : Gut microbiota abundance (genus Parasutterella id.2892) on Homeodomain−interacting protein kinase 3
with a threshold of 1e−05
rs7303158
rs7572229
rs78383039
rs10899911
rs55877868
rs2387977
rs62273907
rs6809952
rs6828768
rs11715853
rs35055552
rs8039785
rs2090816
rs823424
rs7311004
All
−0.1
0.0
0.1
0.2
MR effect size for
'Gut microbiota abundance (genus Parasutterella id.2892)' on 'Homeodomain−interacting protein kinase 3'

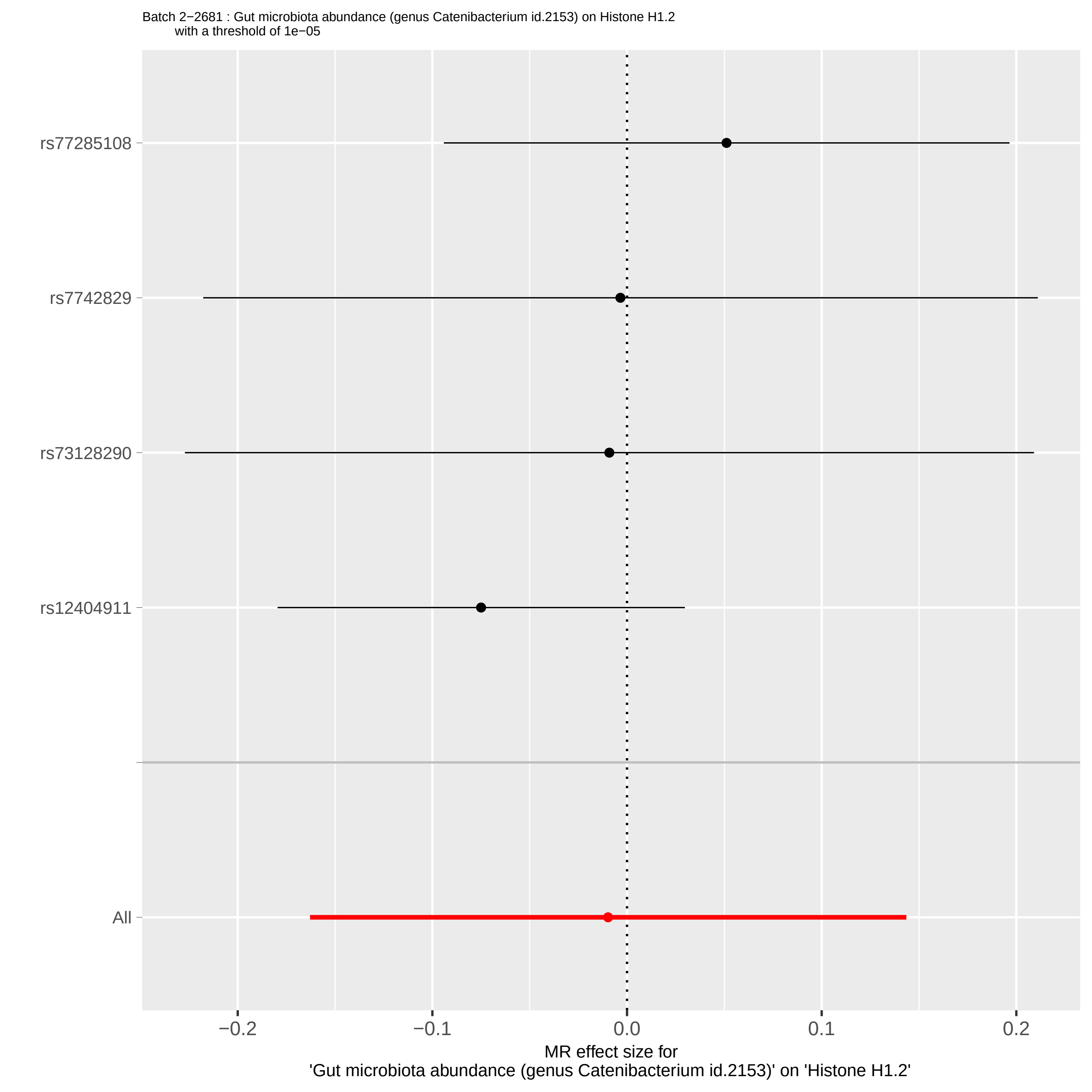
Batch 2−2681 : Gut microbiota abundance (genus Catenibacterium id.2153) on Histone H1.2
with a threshold of 1e−05
rs77285108
rs7742829
rs73128290
rs12404911
All
−0.2
−0.1
0.0
0.1
0.2
MR effect size for
'Gut microbiota abundance (genus Catenibacterium id.2153)' on 'Histone H1.2'

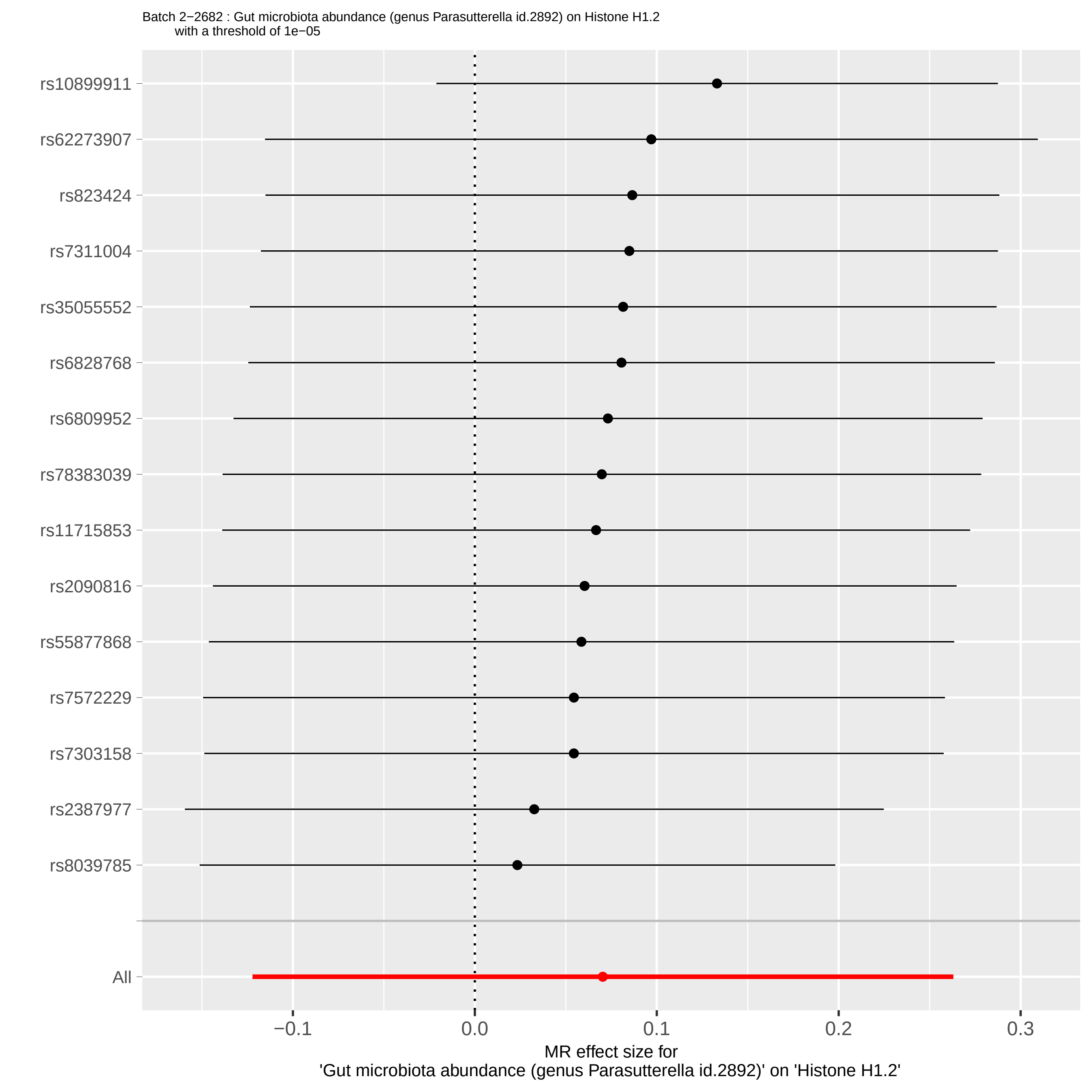
Batch 2−2682 : Gut microbiota abundance (genus Parasutterella id.2892) on Histone H1.2
with a threshold of 1e−05
rs10899911
rs62273907
rs823424
rs7311004
rs35055552
rs6828768
rs6809952
rs78383039
rs11715853
rs2090816
rs55877868
rs7572229
rs7303158
rs2387977
rs8039785
All
−0.1
0.0
0.1
0.2
0.3
MR effect size for
'Gut microbiota abundance (genus Parasutterella id.2892)' on 'Histone H1.2'

Batch 2−2683 : Gut microbiota abundance (genus Catenibacterium id.2153) on Histone H2A type 3
with a threshold of 1e−05

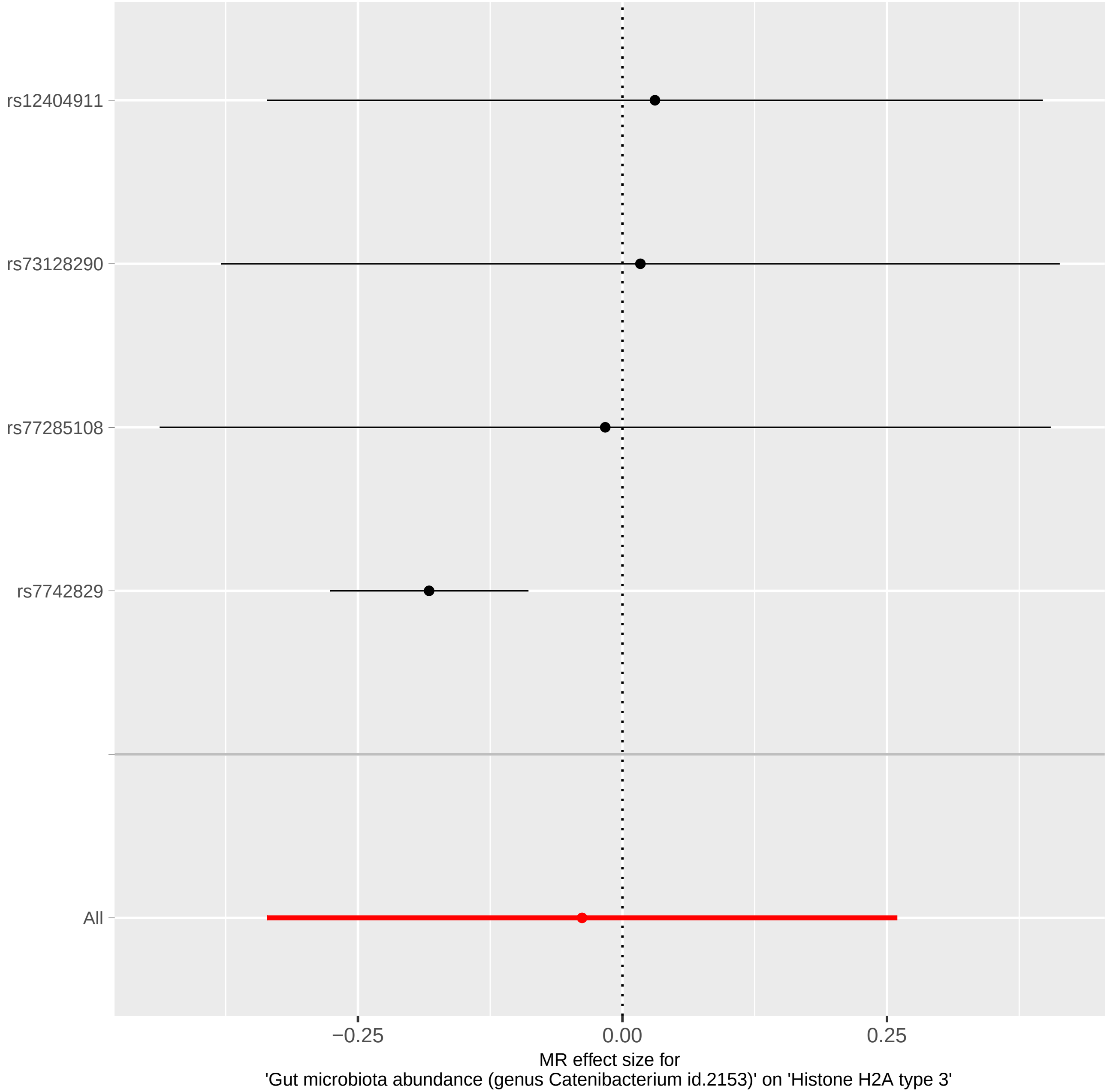

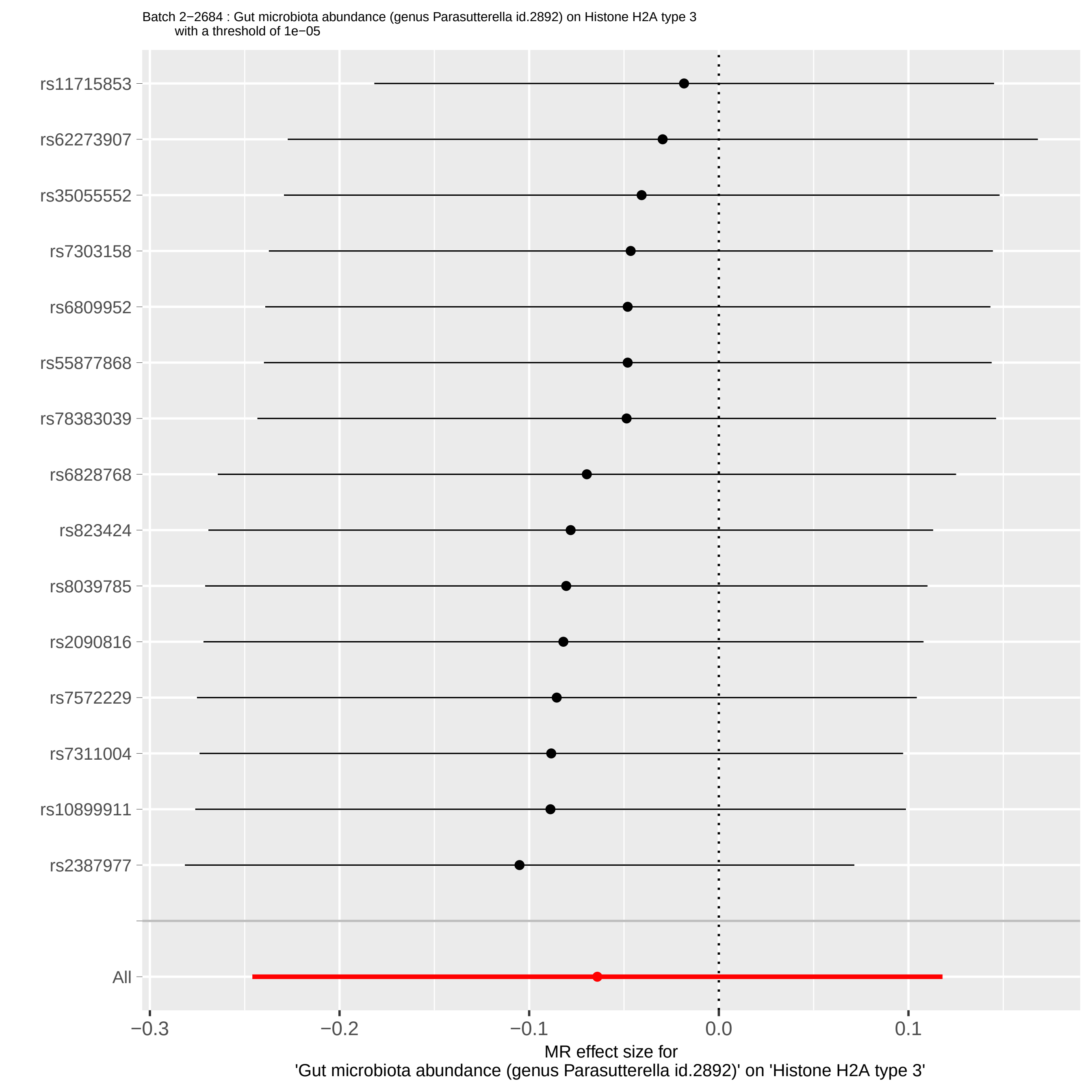

Batch 2−2684 : Gut microbiota abundance (genus Parasutterella id.2892) on Histone H2A type 3
with a threshold of 1e−05
rs11715853
rs62273907
rs35055552
rs7303158
rs6809952
rs55877868
rs78383039
rs6828768
rs823424
rs8039785
rs2090816
rs7572229
rs7311004
rs10899911
rs2387977
All
−0.3
−0.2
−0.1
0.0
0.1
MR effect size for
'Gut microbiota abundance (genus Parasutterella id.2892)' on 'Histone H2A type 3'

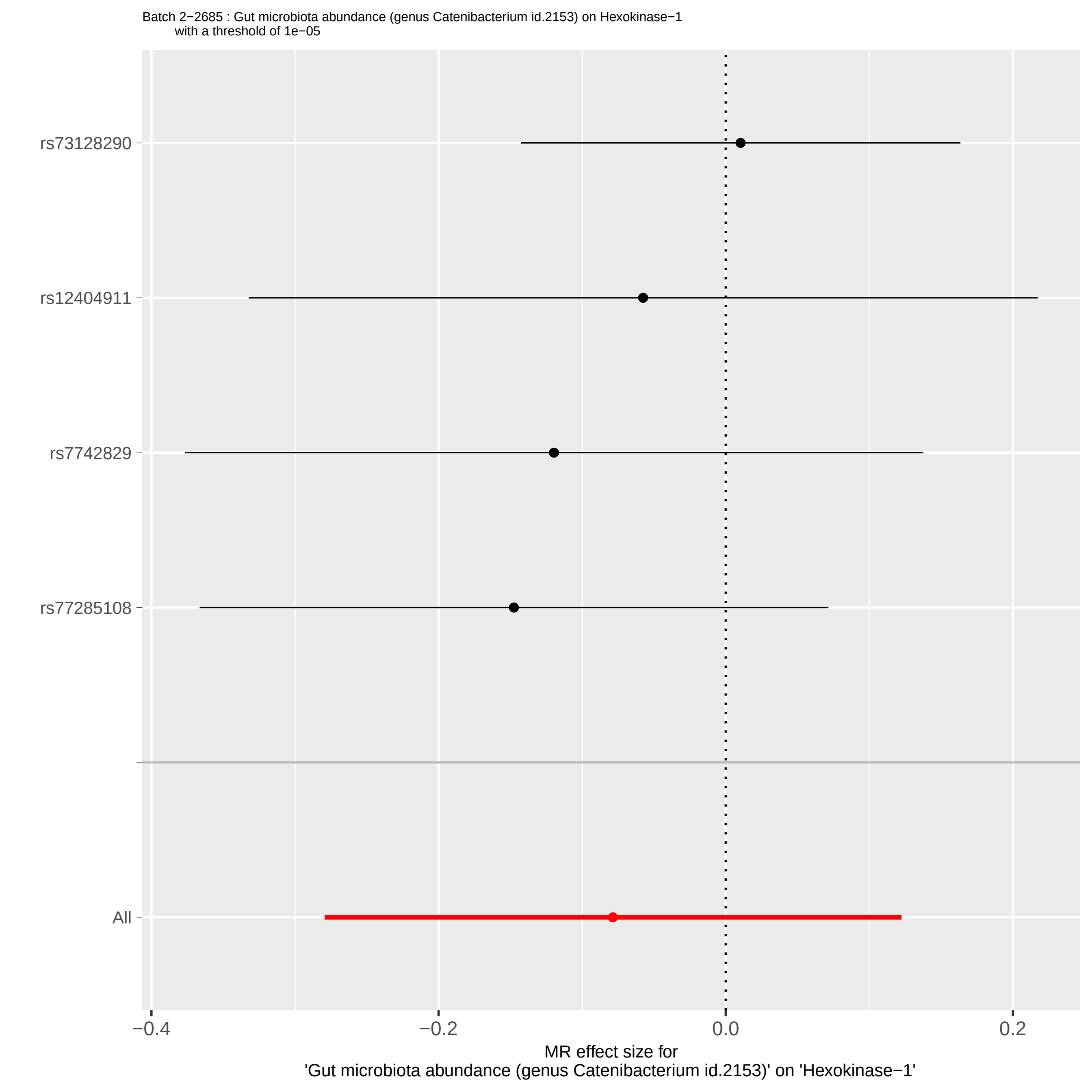

Batch 2−2685 : Gut microbiota abundance (genus Catenibacterium id.2153) on Hexokinase−1
with a threshold of 1e−05
rs73128290
rs12404911
rs7742829
rs77285108
All
−0.4
−0.2
0.0
0.2
MR effect size for
'Gut microbiota abundance (genus Catenibacterium id.2153)' on 'Hexokinase−1'

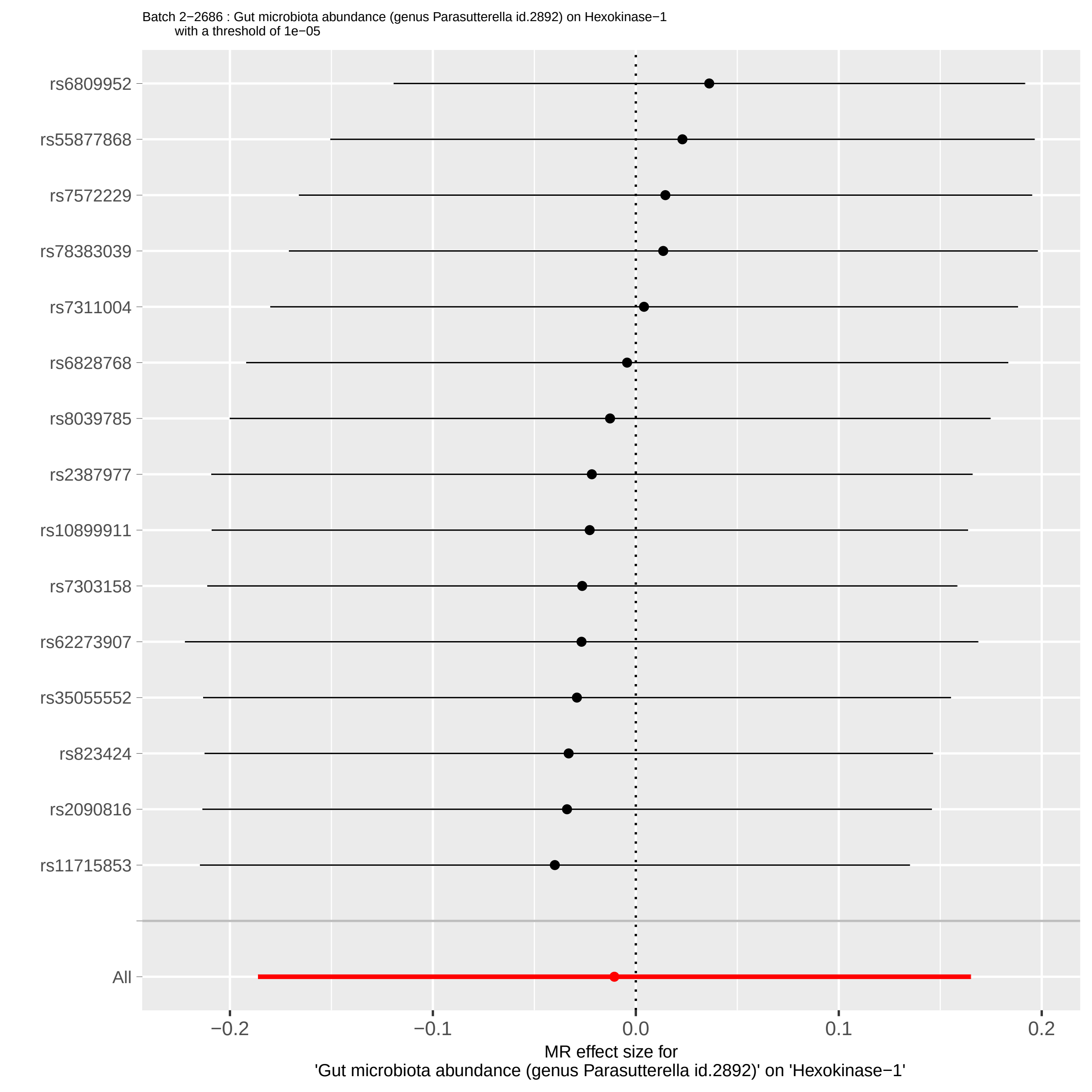

Batch 2−2686 : Gut microbiota abundance (genus Parasutterella id.2892) on Hexokinase−1
with a threshold of 1e−05
rs6809952
rs55877868
rs7572229
rs78383039
rs7311004
rs6828768
rs8039785
rs2387977
rs10899911
rs7303158
rs62273907
rs35055552
rs823424
rs2090816
rs11715853
All
−0.2
−0.1
0.0
0.1
0.2
MR effect size for
'Gut microbiota abundance (genus Parasutterella id.2892)' on 'Hexokinase−1'

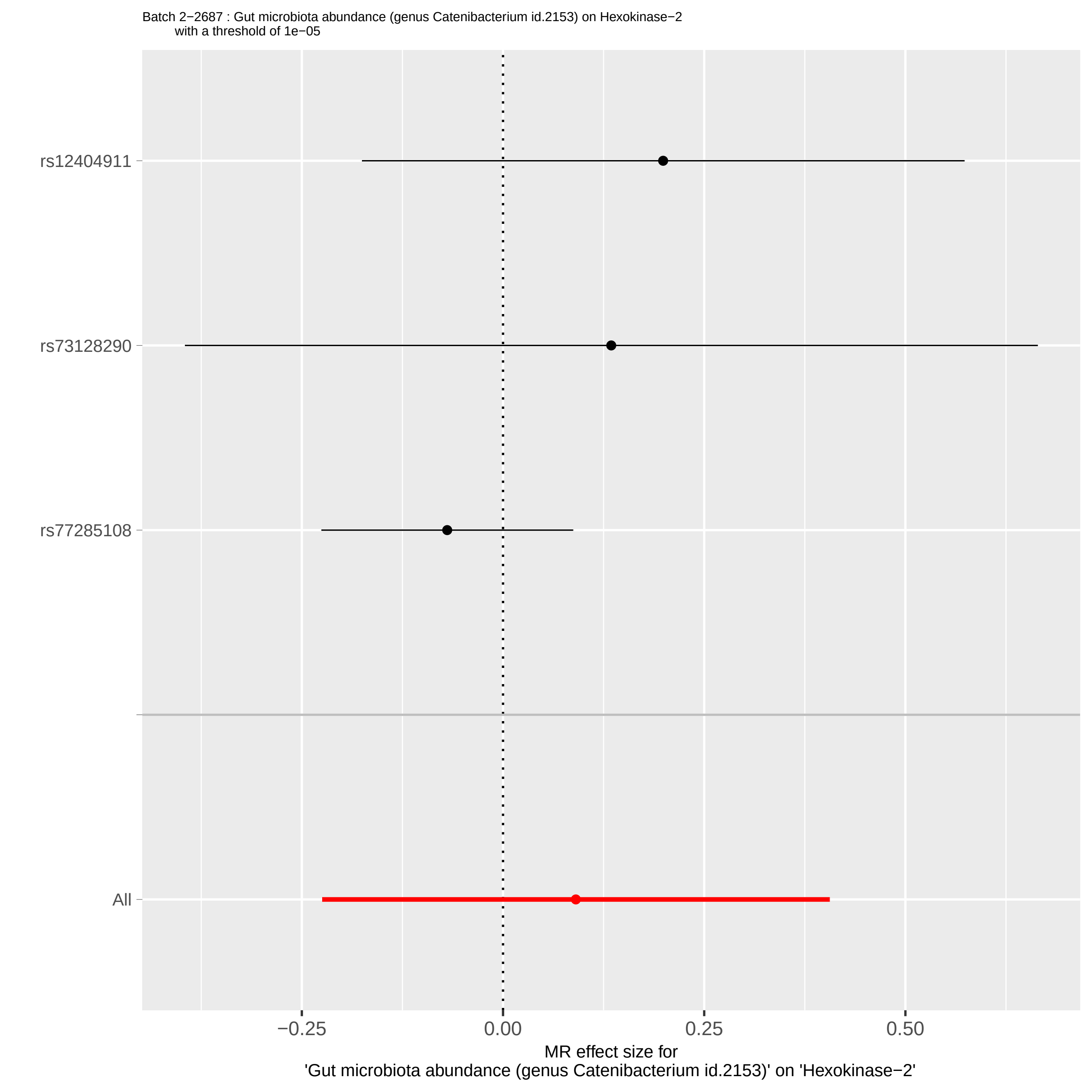
Batch 2−2687 : Gut microbiota abundance (genus Catenibacterium id.2153) on Hexokinase−2
with a threshold of 1e−05
rs12404911
rs73128290
rs77285108
All
−0.25
0.00
0.25
0.50
MR effect size for
'Gut microbiota abundance (genus Catenibacterium id.2153)' on 'Hexokinase−2'

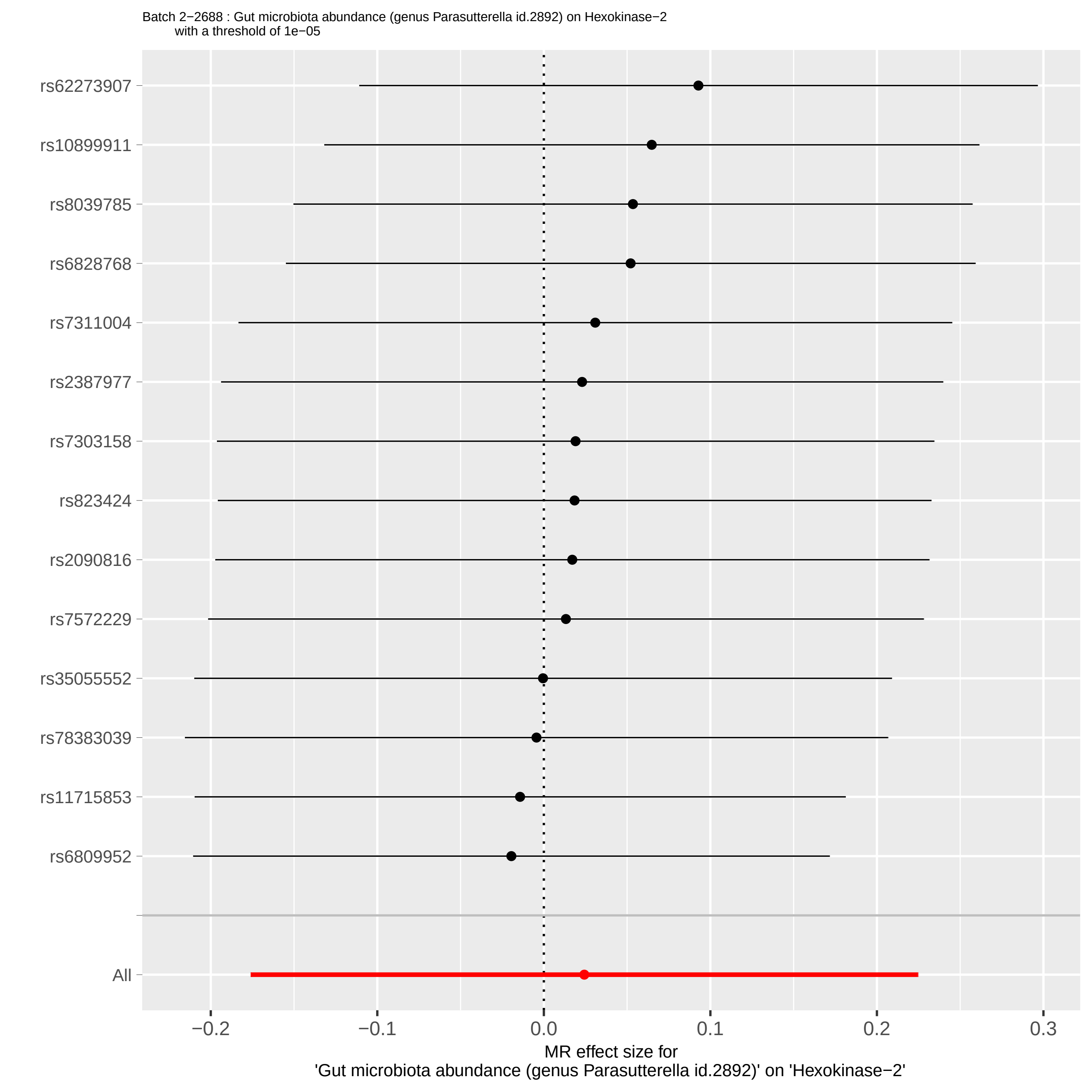

Batch 2−2688 : Gut microbiota abundance (genus Parasutterella id.2892) on Hexokinase−2
with a threshold of 1e−05
rs62273907
rs10899911
rs8039785
rs6828768
rs7311004
rs2387977
rs7303158
rs823424
rs2090816
rs7572229
rs35055552
rs78383039
rs11715853
rs6809952
All
−0.2
−0.1
0.0
0.1
0.2
0.3
MR effect size for
'Gut microbiota abundance (genus Parasutterella id.2892)' on 'Hexokinase−2'

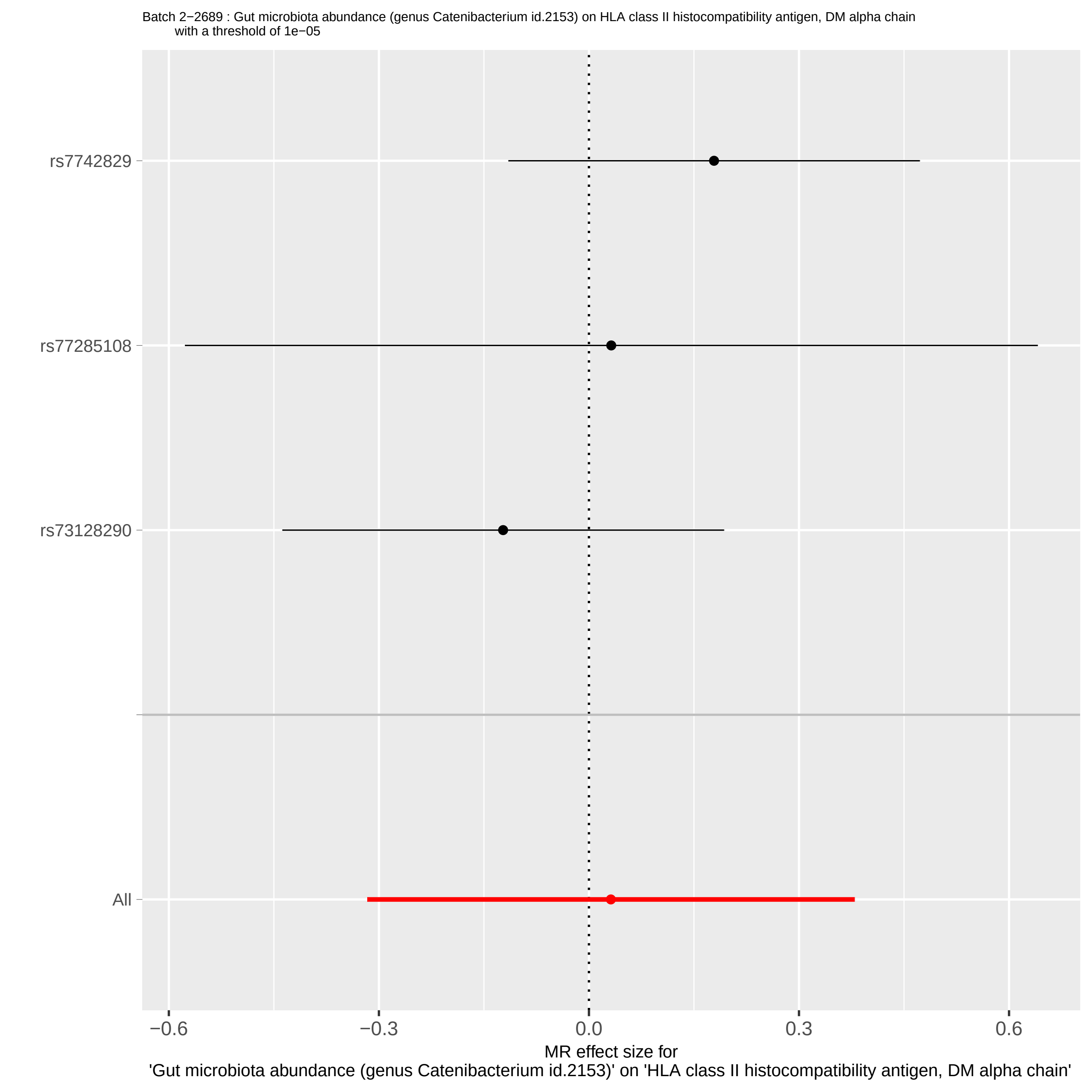
Batch 2−2689 : Gut microbiota abundance (genus Catenibacterium id.2153) on HLA class II histocompatibility antigen, DM alpha chain
with a threshold of 1e−05
rs7742829
rs77285108
rs73128290
All
−0.6
−0.3
0.0
0.3
0.6
MR effect size for
'Gut microbiota abundance (genus Catenibacterium id.2153)' on 'HLA class II histocompatibility antigen, DM alpha chain'

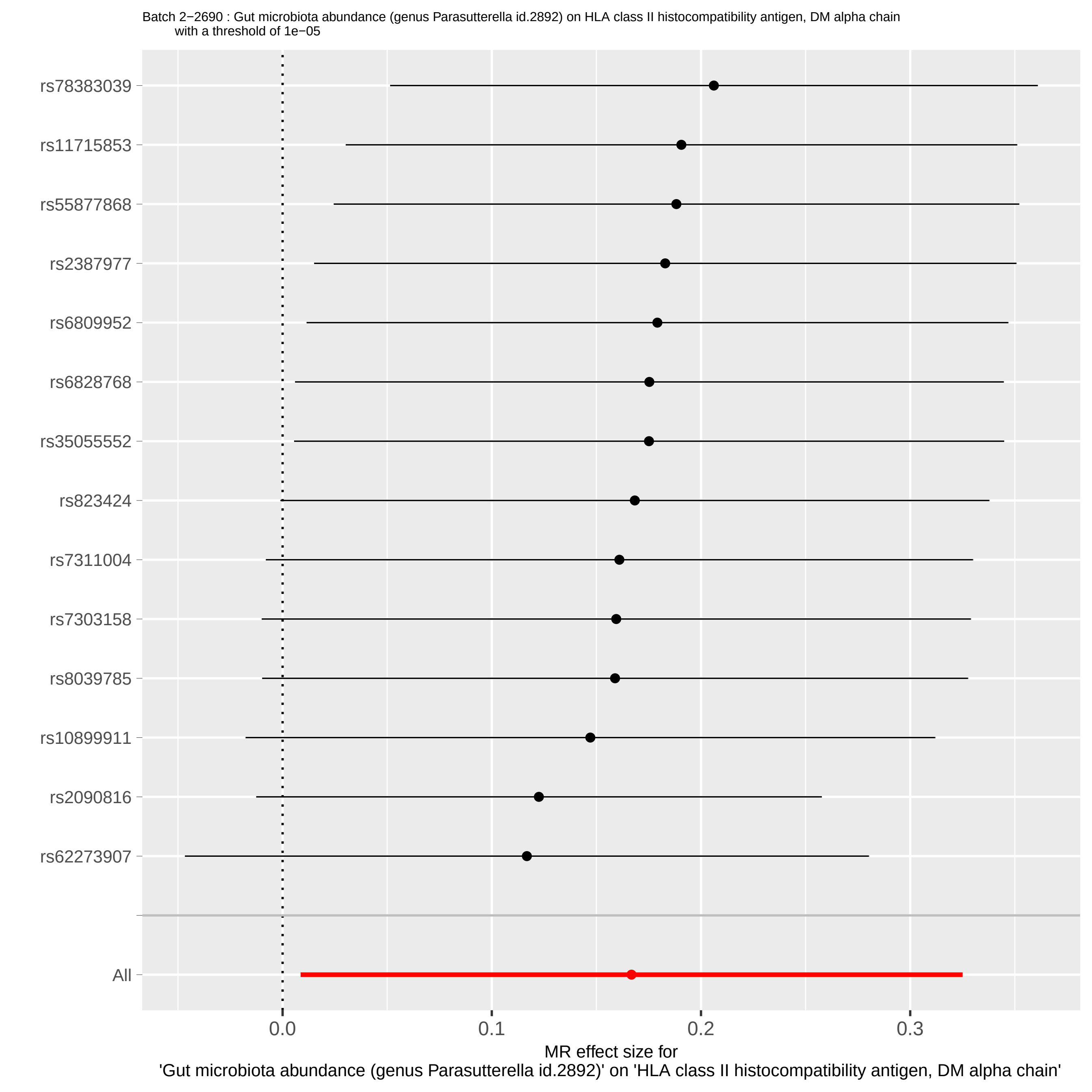
Batch 2−2690 : Gut microbiota abundance (genus Parasutterella id.2892) on HLA class II histocompatibility antigen, DM alpha chain
with a threshold of 1e−05
rs78383039
rs11715853
rs55877868
rs2387977
rs6809952
rs6828768
rs35055552
rs823424
rs7311004
rs7303158
rs8039785
rs10899911
rs2090816
rs62273907
All
0.0
0.1
0.2
0.3
MR effect size for
'Gut microbiota abundance (genus Parasutterella id.2892)' on 'HLA class II histocompatibility antigen, DM alpha chain'

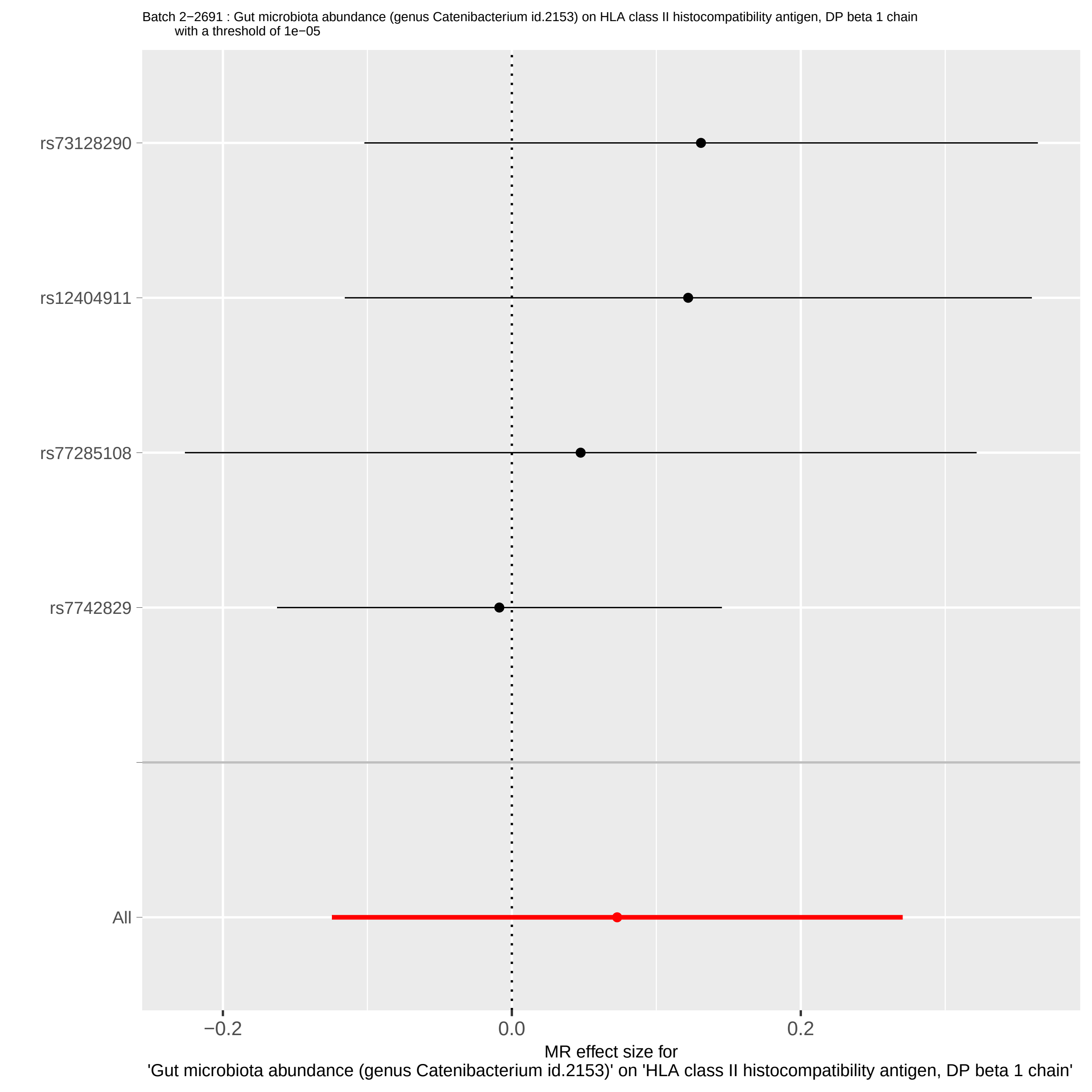
Batch 2−2691 : Gut microbiota abundance (genus Catenibacterium id.2153) on HLA class II histocompatibility antigen, DP beta 1 chain
with a threshold of 1e−05
rs73128290
rs12404911
rs77285108
rs7742829
All
−0.2
0.0
0.2
MR effect size for
'Gut microbiota abundance (genus Catenibacterium id.2153)' on 'HLA class II histocompatibility antigen, DP beta 1 chain'

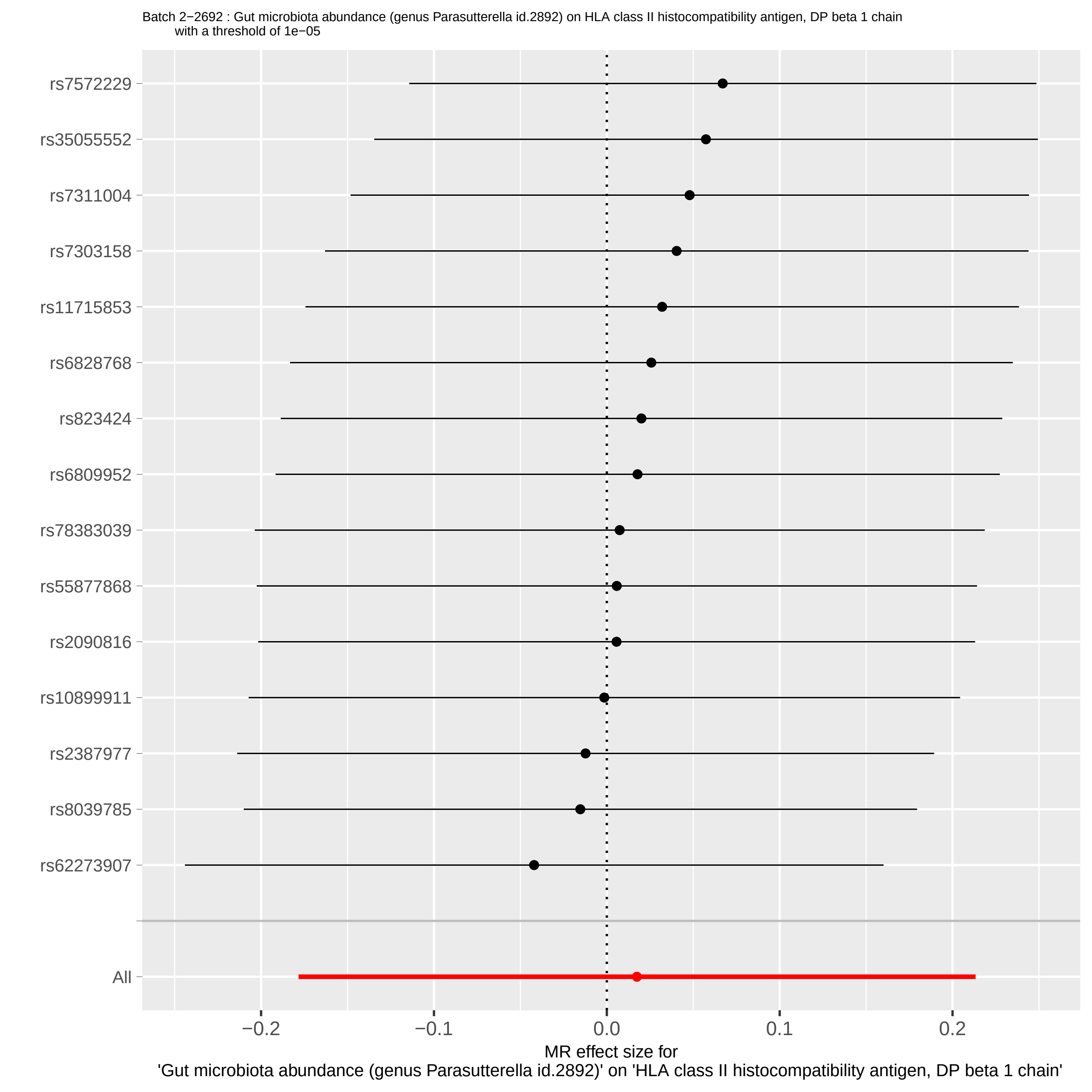
Batch 2−2692 : Gut microbiota abundance (genus Parasutterella id.2892) on HLA class II histocompatibility antigen, DP beta 1 chain
with a threshold of 1e−05
rs7572229
rs35055552
rs7311004
rs7303158
rs11715853
rs6828768
rs823424
rs6809952
rs78383039
rs55877868
rs2090816
rs10899911
rs2387977
rs8039785
rs62273907
All
−0.2
−0.1
0.0
0.1
0.2
MR effect size for
'Gut microbiota abundance (genus Parasutterella id.2892)' on 'HLA class II histocompatibility antigen, DP beta 1 chain'

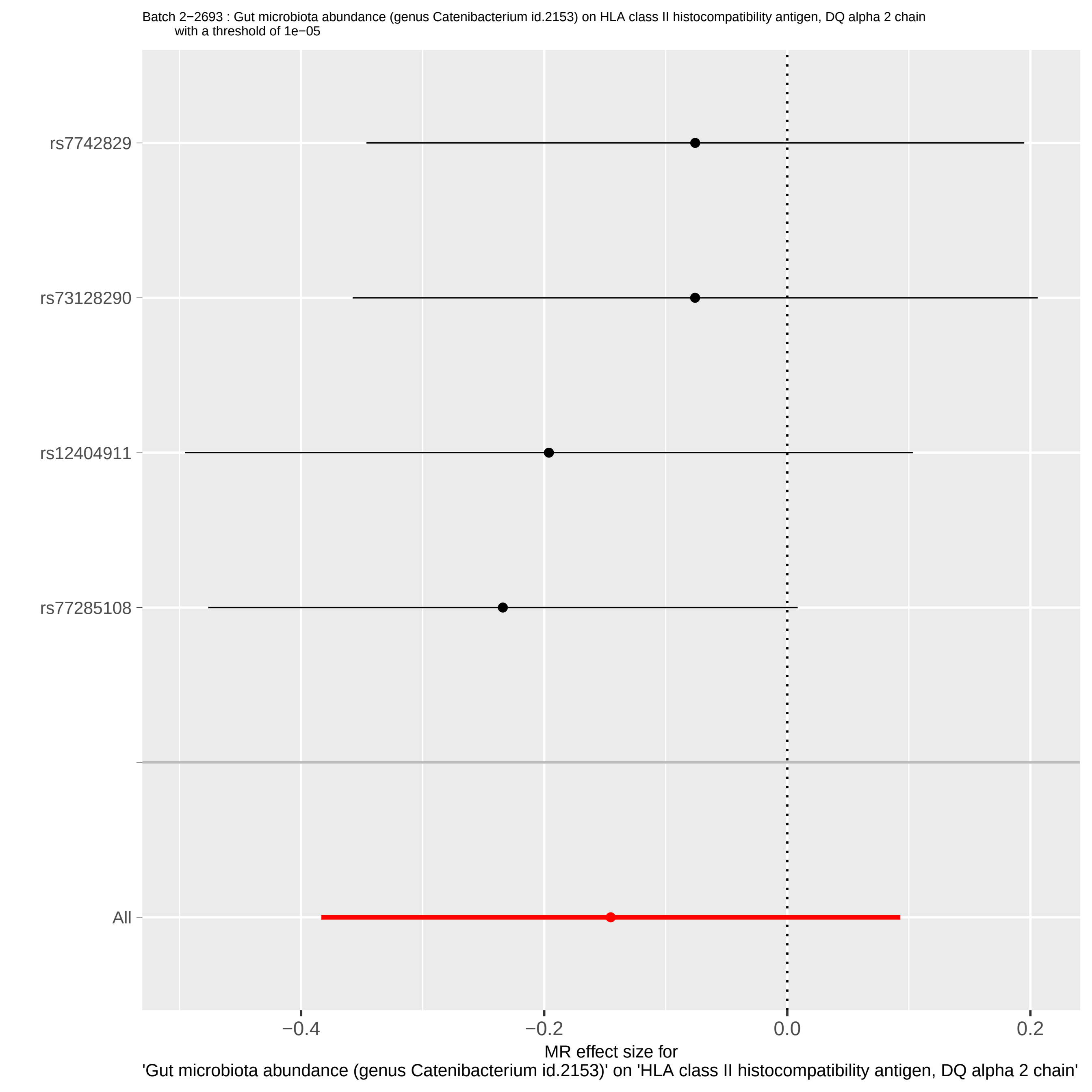
Batch 2−2693 : Gut microbiota abundance (genus Catenibacterium id.2153) on HLA class II histocompatibility antigen, DQ alpha 2 chain
with a threshold of 1e−05
rs7742829
rs73128290
rs12404911
rs77285108
All
−0.4
−0.2
0.0
0.2
MR effect size for
'Gut microbiota abundance (genus Catenibacterium id.2153)' on 'HLA class II histocompatibility antigen, DQ alpha 2 chain'

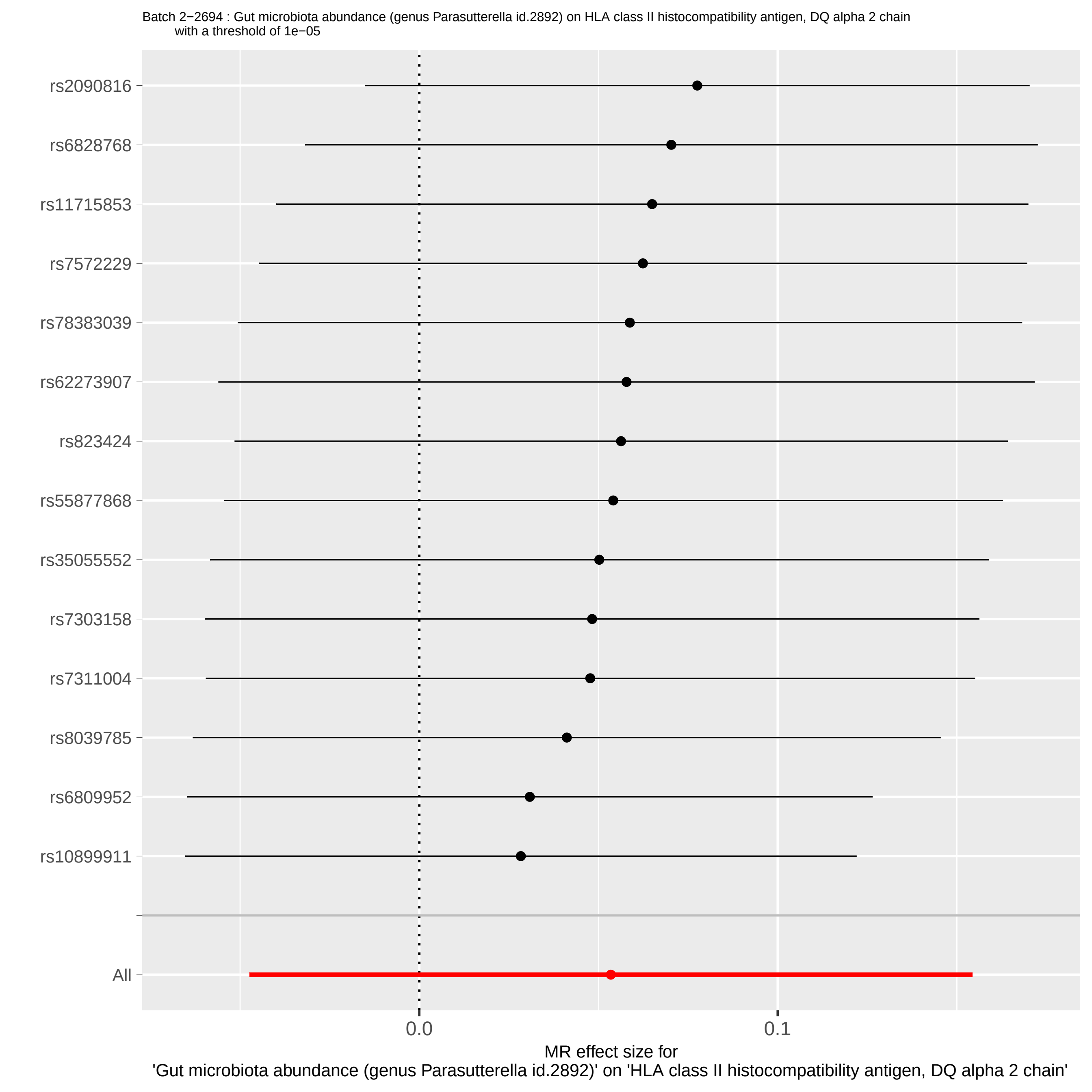

Batch 2−2694 : Gut microbiota abundance (genus Parasutterella id.2892) on HLA class II histocompatibility antigen, DQ alpha 2 chain
with a threshold of 1e−05
rs2090816
rs6828768
rs11715853
rs7572229
rs78383039
rs62273907
rs823424
rs55877868
rs35055552
rs7303158
rs7311004
rs8039785
rs6809952
rs10899911
All
0.0
0.1
MR effect size for
'Gut microbiota abundance (genus Parasutterella id.2892)' on 'HLA class II histocompatibility antigen, DQ alpha 2 chain'

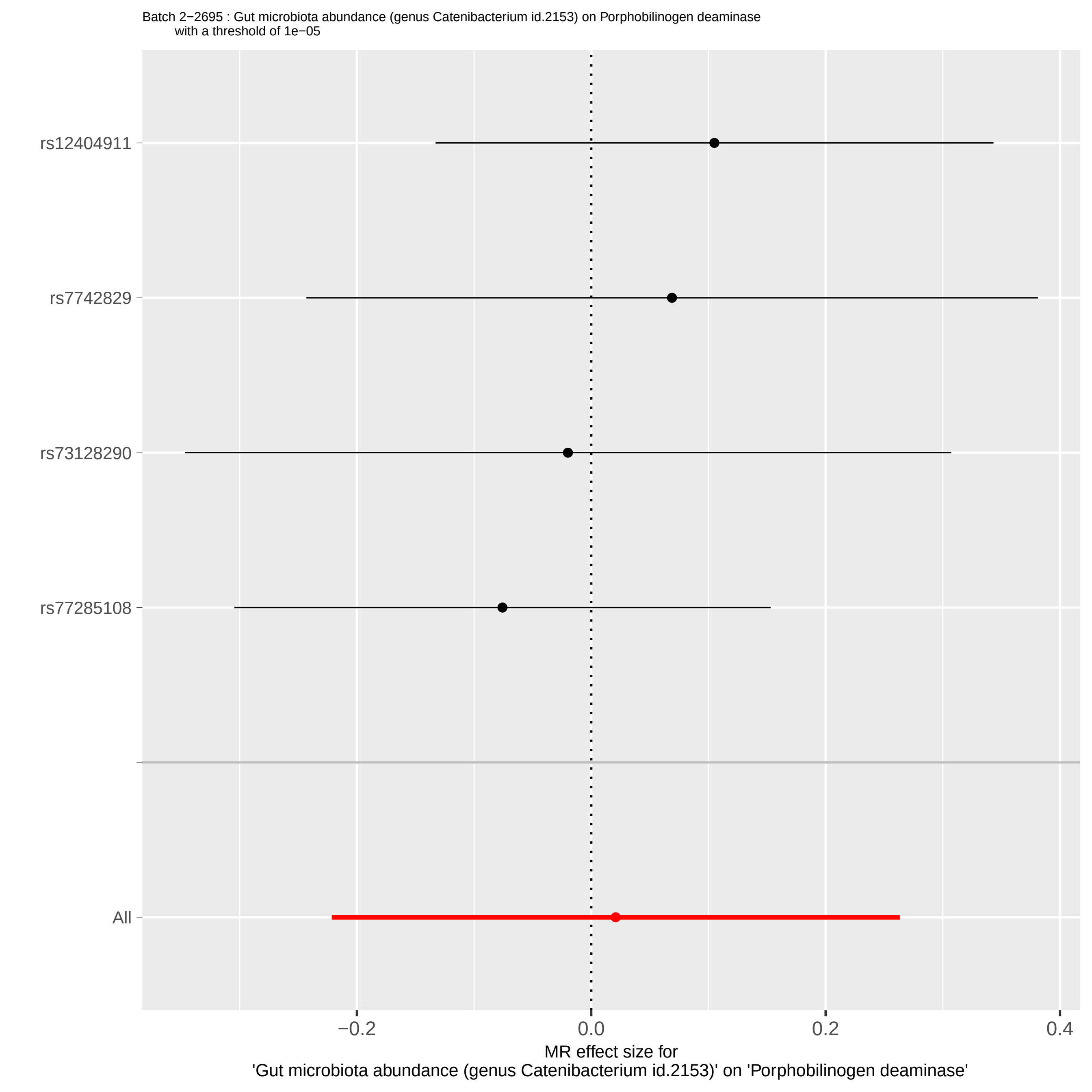
Batch 2−2695 : Gut microbiota abundance (genus Catenibacterium id.2153) on Porphobilinogen deaminase
with a threshold of 1e−05
rs12404911
rs7742829
rs73128290
rs77285108
All
−0.2
0.0
0.2
0.4
MR effect size for
'Gut microbiota abundance (genus Catenibacterium id.2153)' on 'Porphobilinogen deaminase'

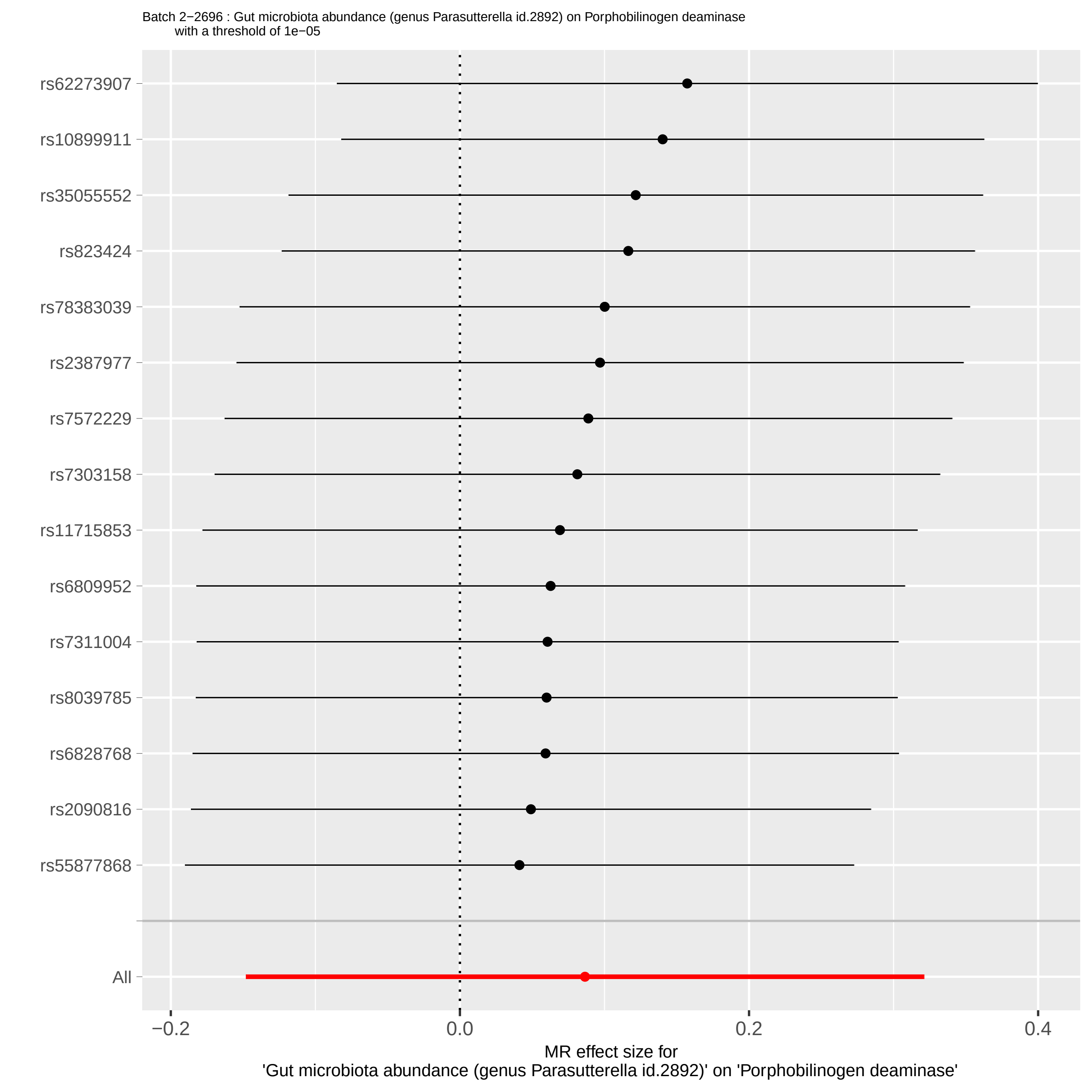
Batch 2−2696 : Gut microbiota abundance (genus Parasutterella id.2892) on Porphobilinogen deaminase
with a threshold of 1e−05
rs62273907
rs10899911
rs35055552
rs823424
rs78383039
rs2387977
rs7572229
rs7303158
rs11715853
rs6809952
rs7311004
rs8039785
rs6828768
rs2090816
rs55877868
All
−0.2
0.0
0.2
0.4
MR effect size for
'Gut microbiota abundance (genus Parasutterella id.2892)' on 'Porphobilinogen deaminase'

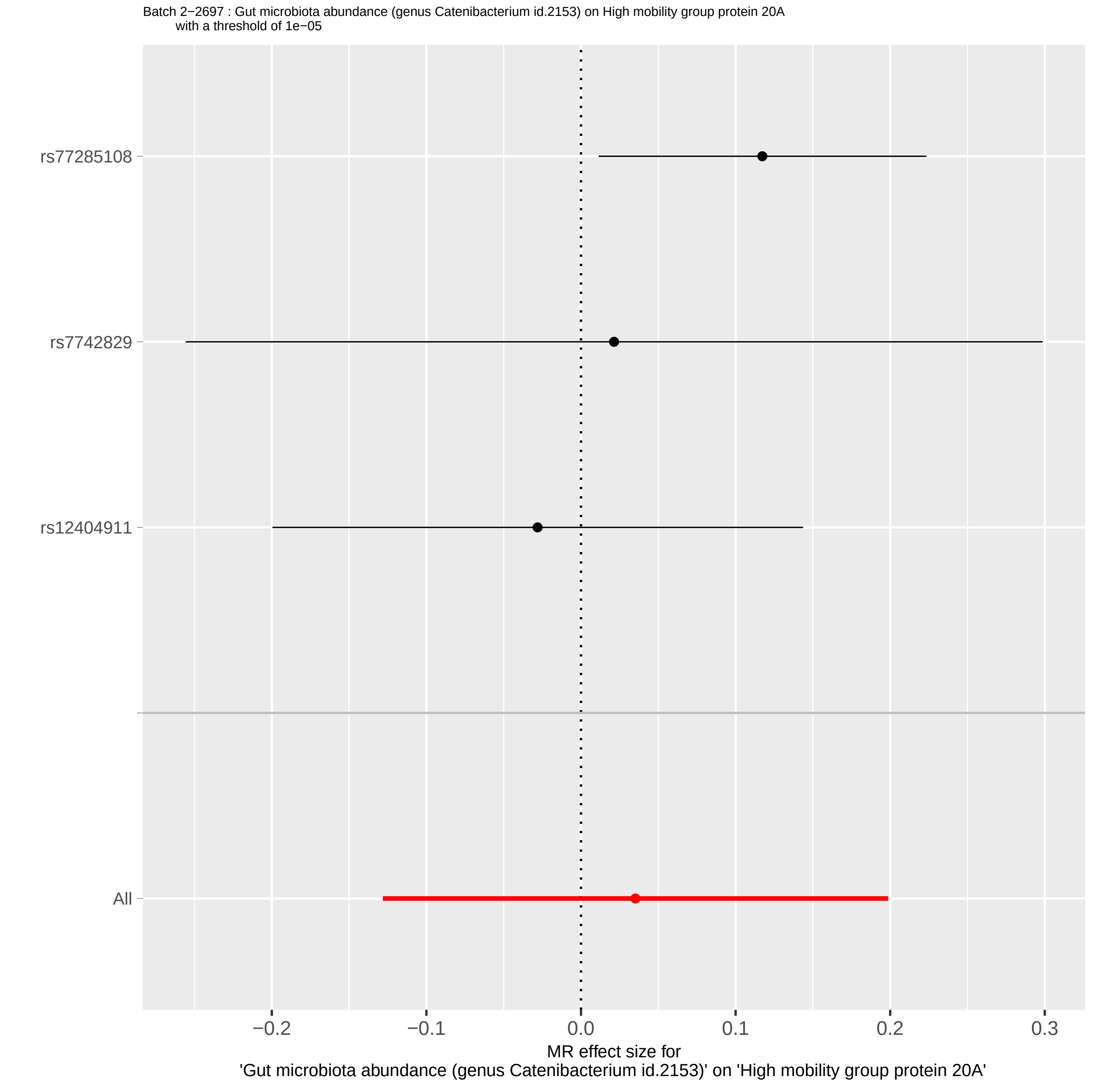
Batch 2−2697 : Gut microbiota abundance (genus Catenibacterium id.2153) on High mobility group protein 20A
with a threshold of 1e−05
rs77285108
rs7742829
rs12404911
All
−0.2
−0.1
0.0
0.1
0.2
0.3
MR effect size for
'Gut microbiota abundance (genus Catenibacterium id.2153)' on 'High mobility group protein 20A'

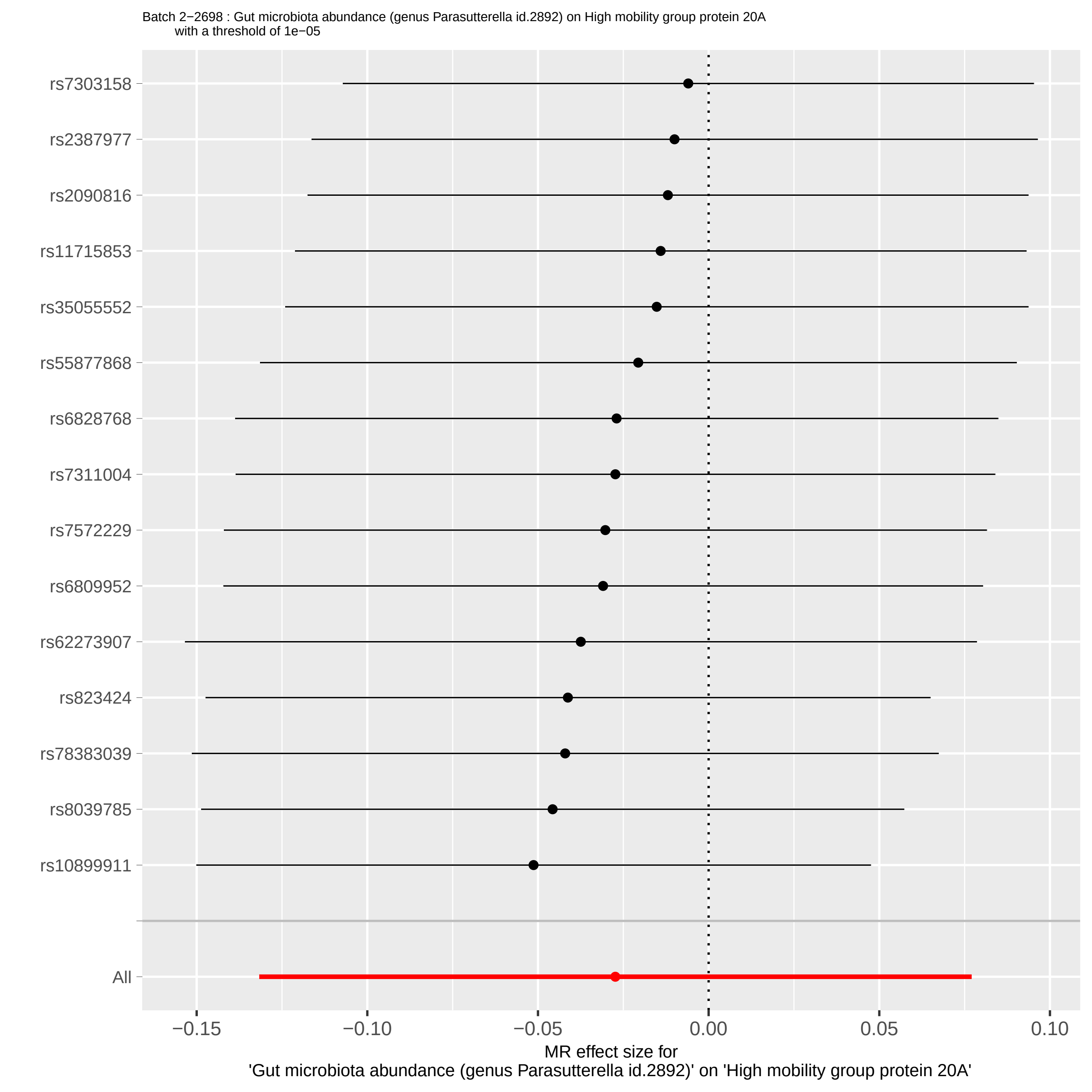

Batch 2−2698 : Gut microbiota abundance (genus Parasutterella id.2892) on High mobility group protein 20A
with a threshold of 1e−05
rs7303158
rs2387977
rs2090816
rs11715853
rs35055552
rs55877868
rs6828768
rs7311004
rs7572229
rs6809952
rs62273907
rs823424
rs78383039
rs8039785
rs10899911
All
−0.15
−0.10
−0.05
0.00
0.05
0.10
MR effect size for
'Gut microbiota abundance (genus Parasutterella id.2892)' on 'High mobility group protein 20A'

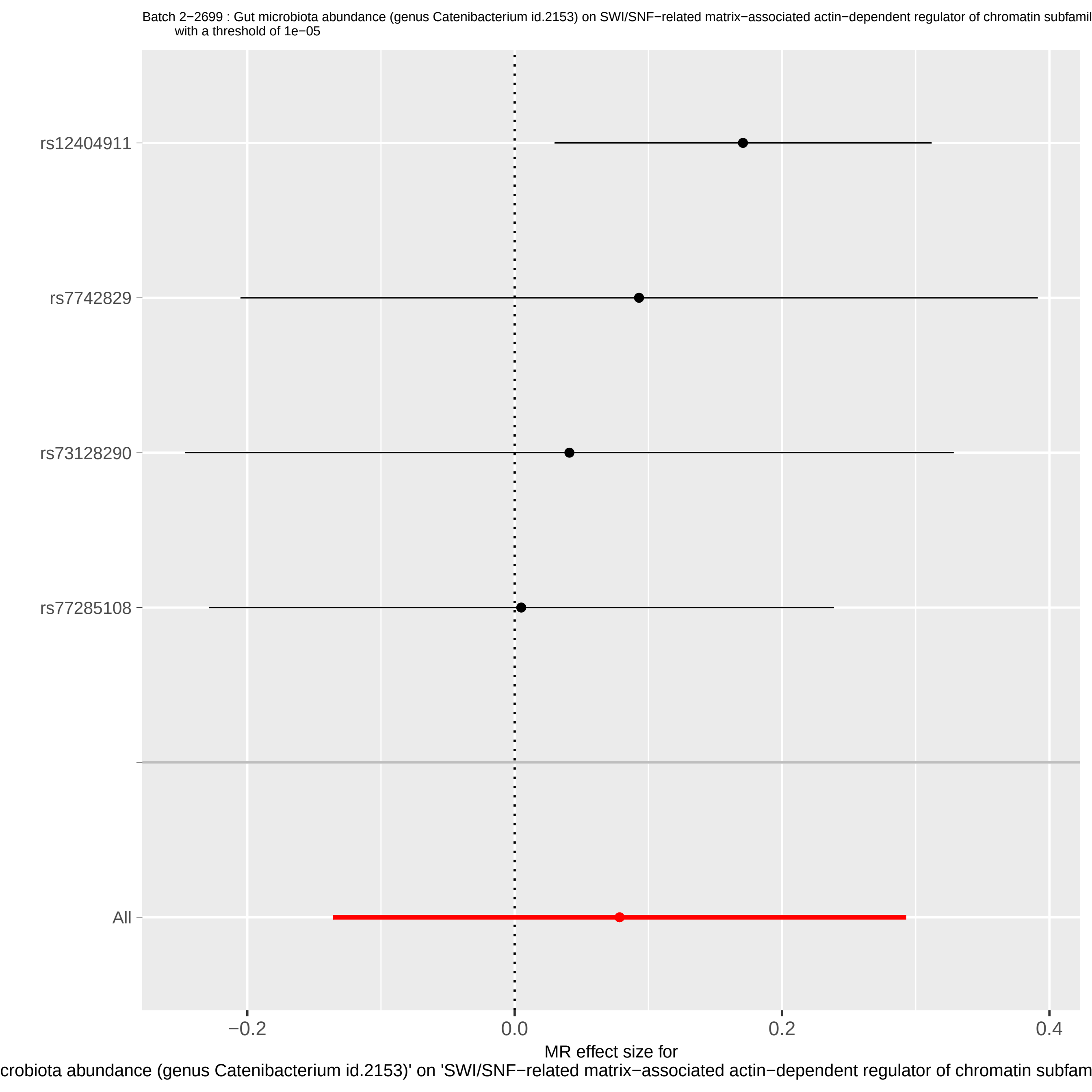
Batch 2−2699 : Gut microbiota abundance (genus Catenibacterium id.2153) on SWI/SNF−related matrix−associated actin−dependent regulator of chromatin subfamil
with a threshold of 1e−05
rs12404911
rs7742829
rs73128290
rs77285108
All
−0.2
0.0
0.2
0.4
MR effect size for
crobiota abundance (genus Catenibacterium id.2153)' on 'SWI/SNF−related matrix−associated actin−dependent regulator of chromatin subfam

Batch 2−2700 : Gut microbiota abundance (genus Parasutterella id.2892) on SWI/SNF−related matrix−associated actin−dependent regulator of chromatin subfamily B
with a threshold of 1e−05

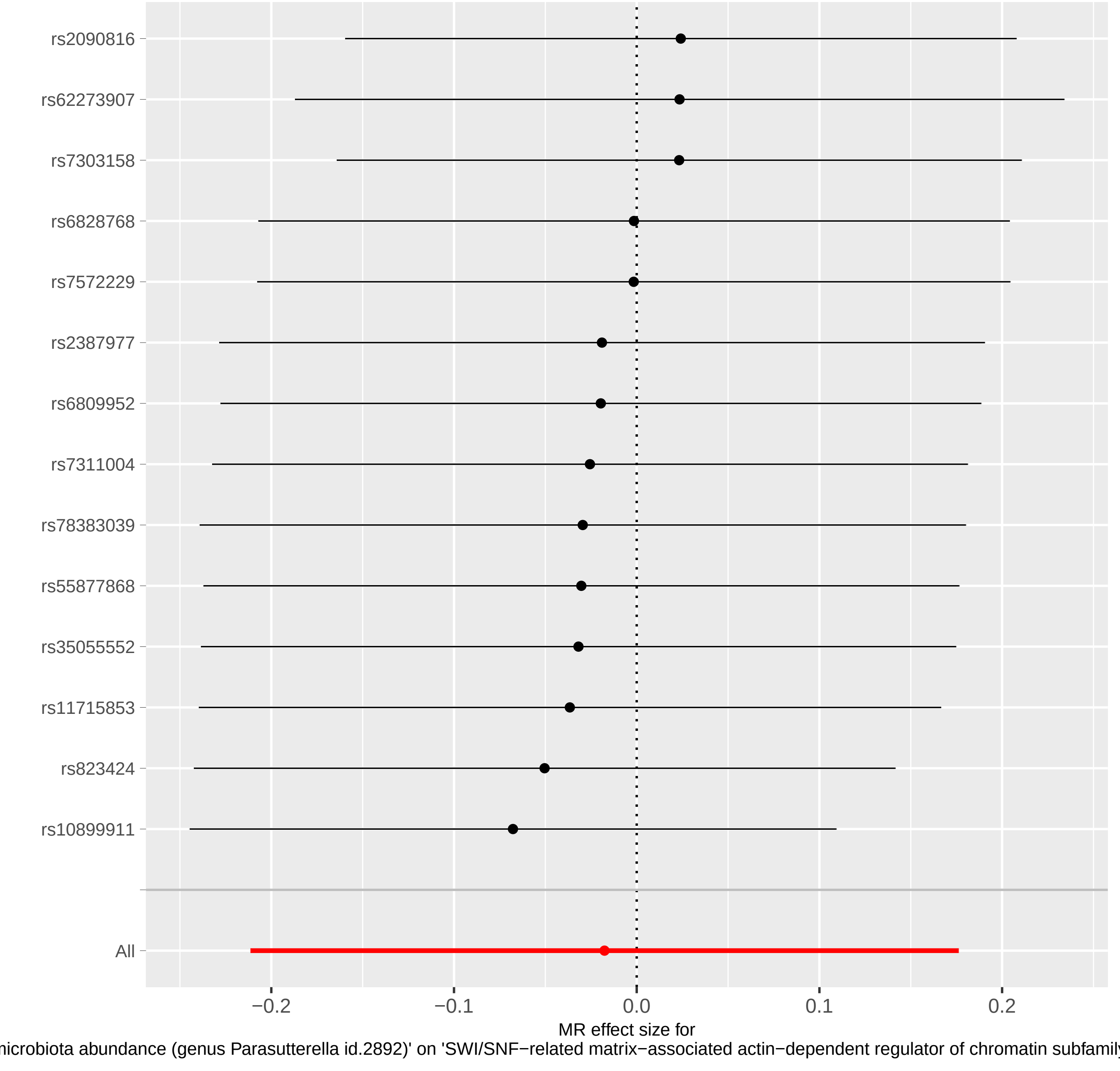

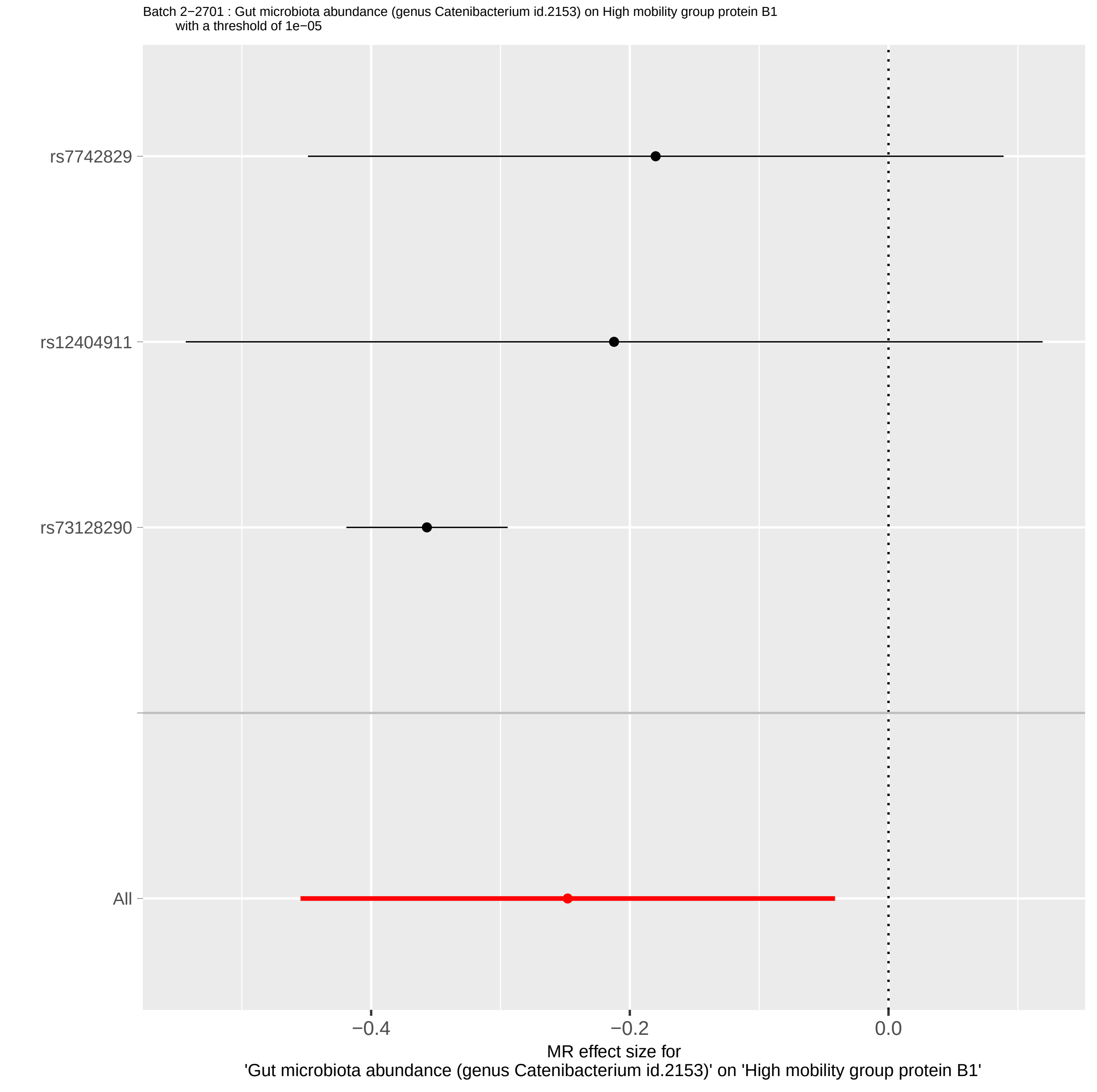
Batch 2−2701 : Gut microbiota abundance (genus Catenibacterium id.2153) on High mobility group protein B1
with a threshold of 1e−05
rs7742829
rs12404911
rs73128290
All
−0.4
−0.2
0.0
MR effect size for
'Gut microbiota abundance (genus Catenibacterium id.2153)' on 'High mobility group protein B1'

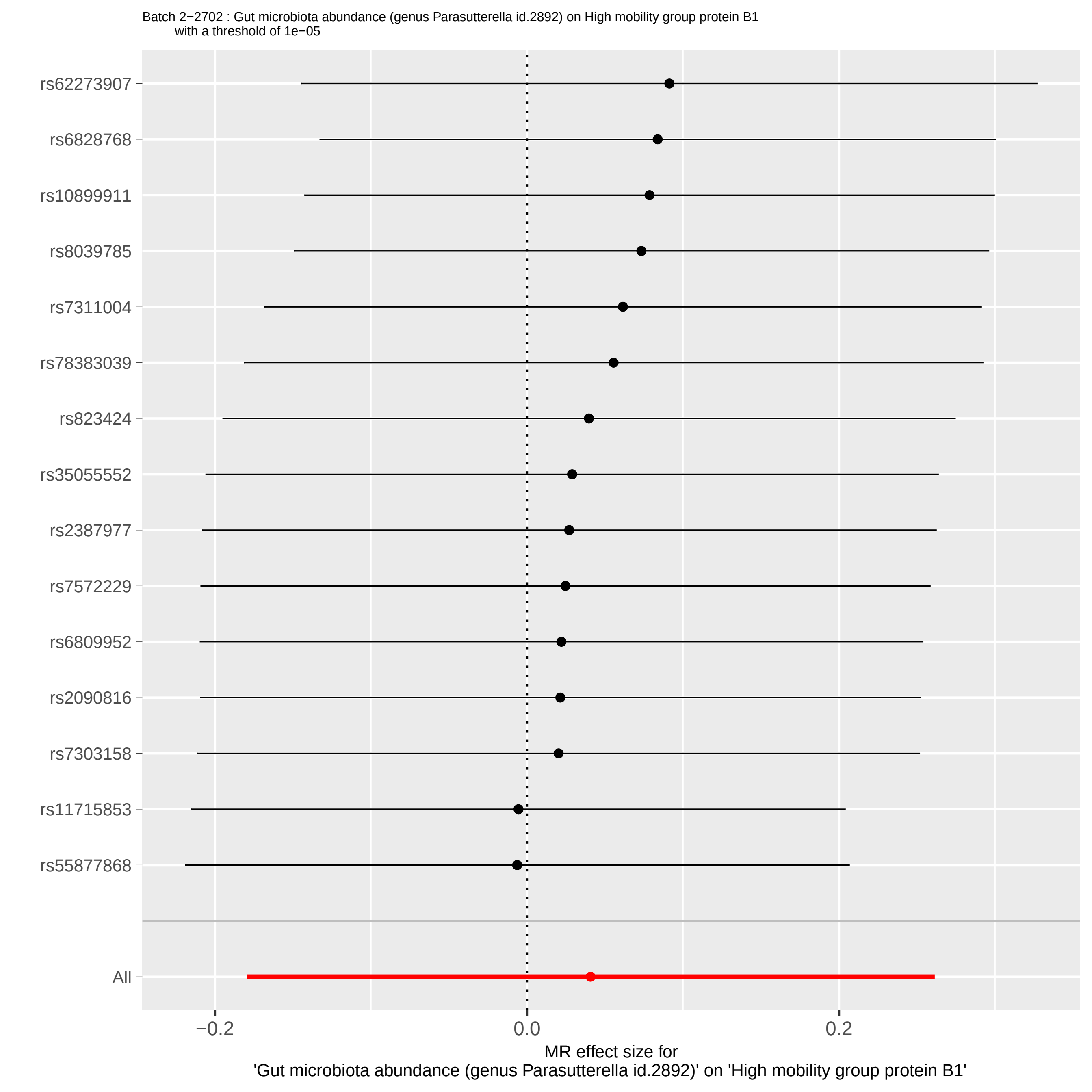
Batch 2−2702 : Gut microbiota abundance (genus Parasutterella id.2892) on High mobility group protein B1
with a threshold of 1e−05
rs62273907
rs6828768
rs10899911
rs8039785
rs7311004
rs78383039
rs823424
rs35055552
rs2387977
rs7572229
rs6809952
rs2090816
rs7303158
rs11715853
rs55877868
All
−0.2
0.0
0.2
MR effect size for
'Gut microbiota abundance (genus Parasutterella id.2892)' on 'High mobility group protein B1'

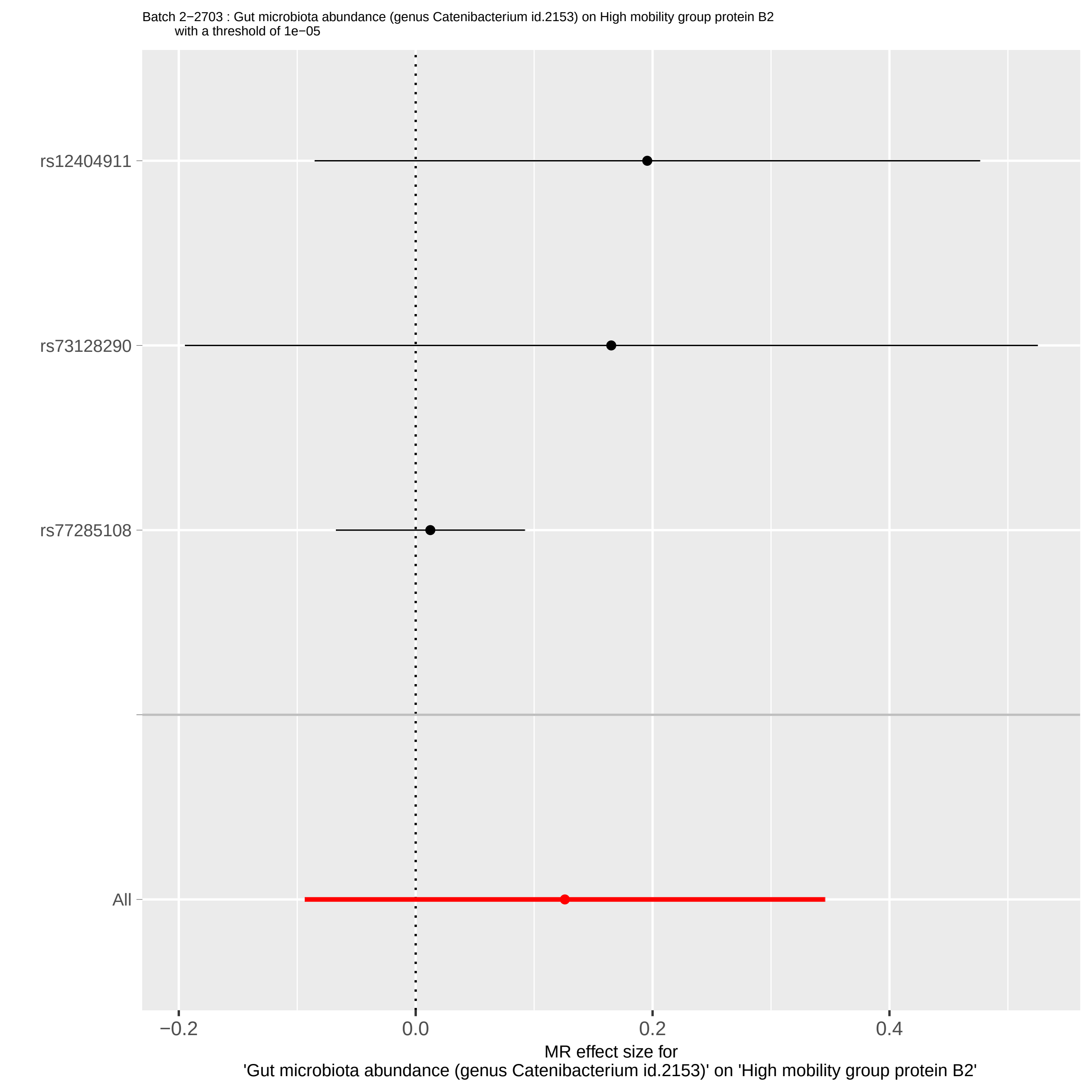
Batch 2−2703 : Gut microbiota abundance (genus Catenibacterium id.2153) on High mobility group protein B2
with a threshold of 1e−05
rs12404911
rs73128290
rs77285108
All
−0.2
0.0
0.2
0.4
MR effect size for
'Gut microbiota abundance (genus Catenibacterium id.2153)' on 'High mobility group protein B2'

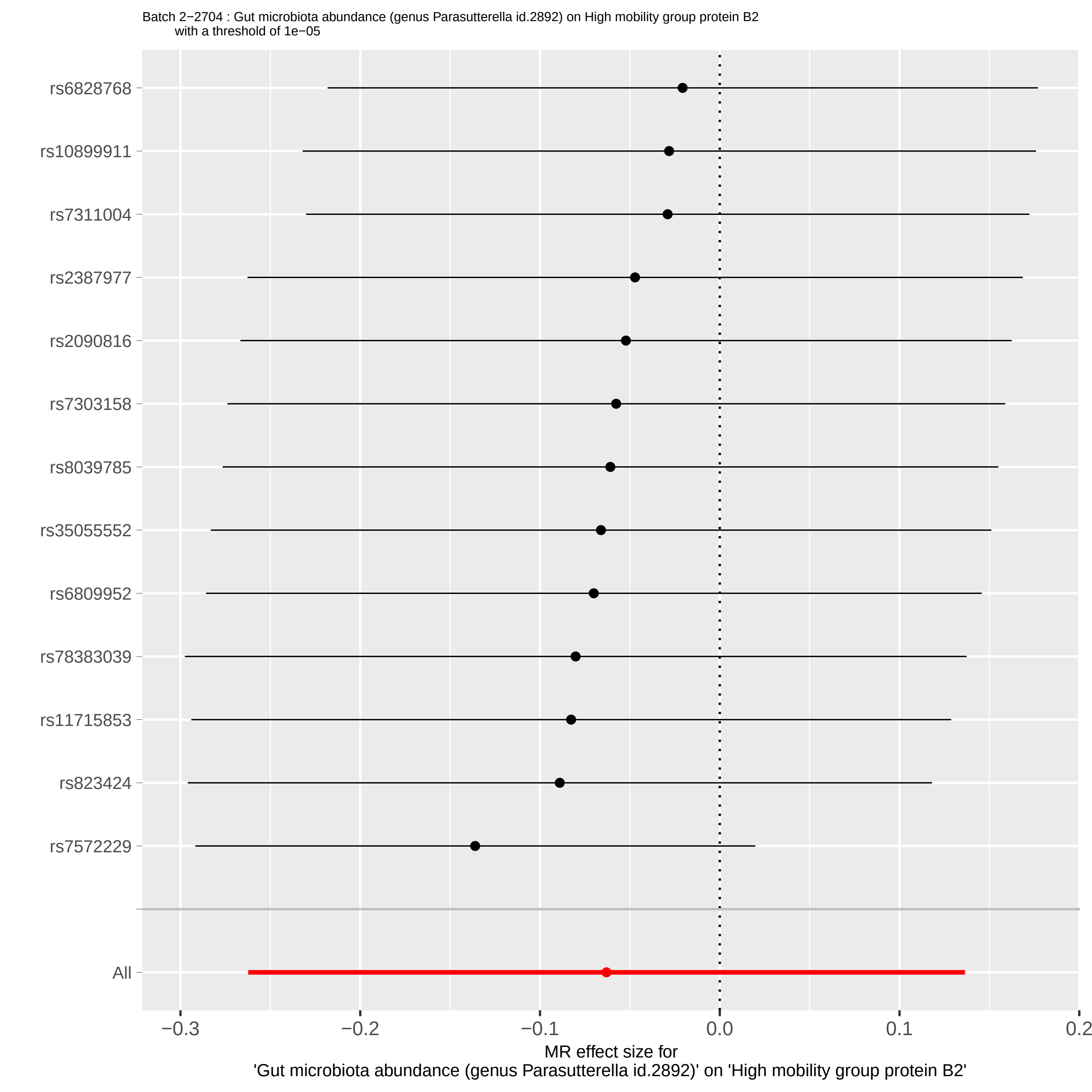

Batch 2−2704 : Gut microbiota abundance (genus Parasutterella id.2892) on High mobility group protein B2
with a threshold of 1e−05
rs6828768
rs10899911
rs7311004
rs2387977
rs2090816
rs7303158
rs8039785
rs35055552
rs6809952
rs78383039
rs11715853
rs823424
rs7572229
All
−0.3
−0.2
−0.1
0.0
0.1
0.2
MR effect size for
'Gut microbiota abundance (genus Parasutterella id.2892)' on 'High mobility group protein B2'

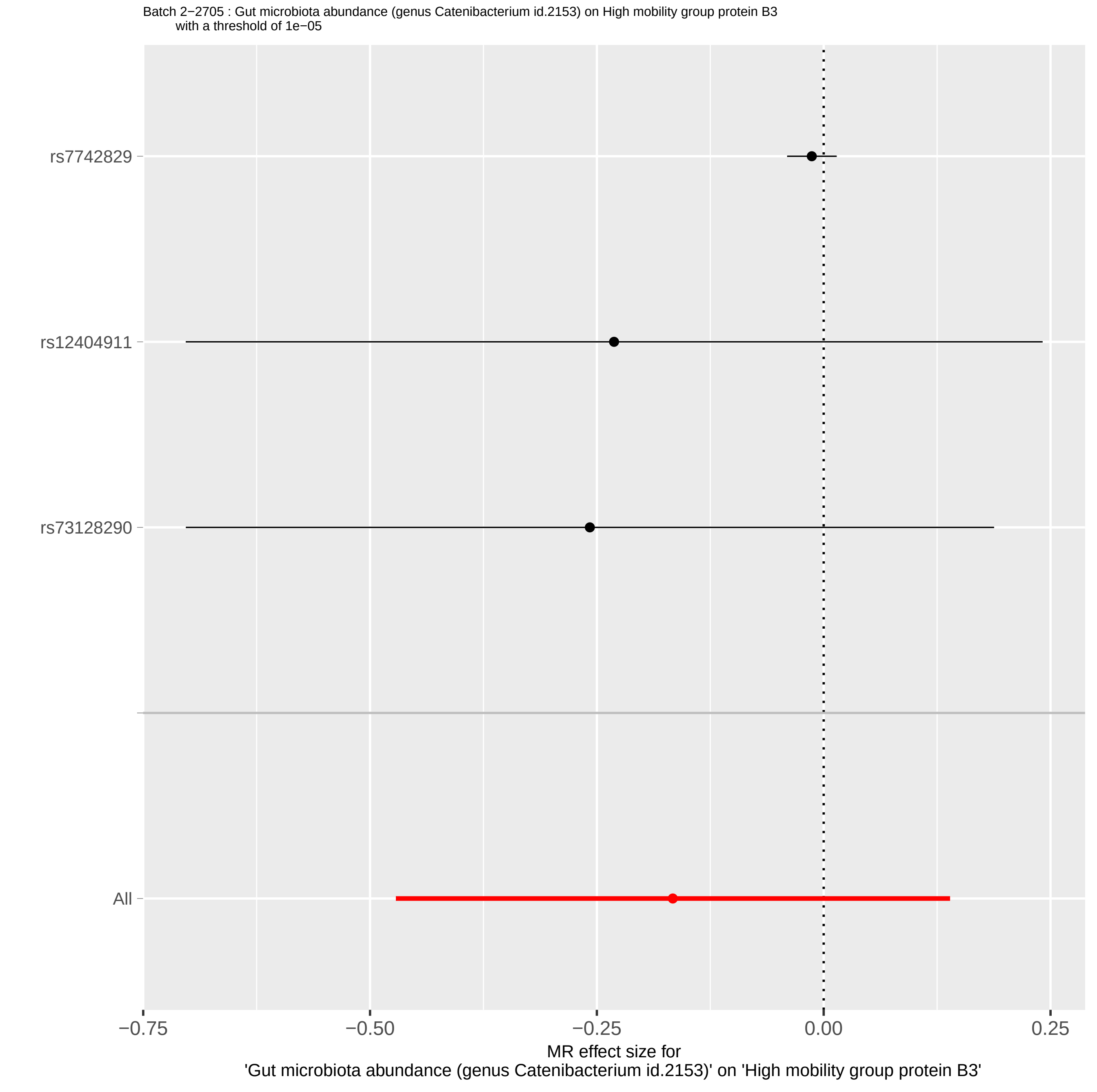

Batch 2−2705 : Gut microbiota abundance (genus Catenibacterium id.2153) on High mobility group protein B3
with a threshold of 1e−05
rs7742829
rs12404911
rs73128290
All
−0.75
−0.50
−0.25
0.00
0.25
MR effect size for
'Gut microbiota abundance (genus Catenibacterium id.2153)' on 'High mobility group protein B3'

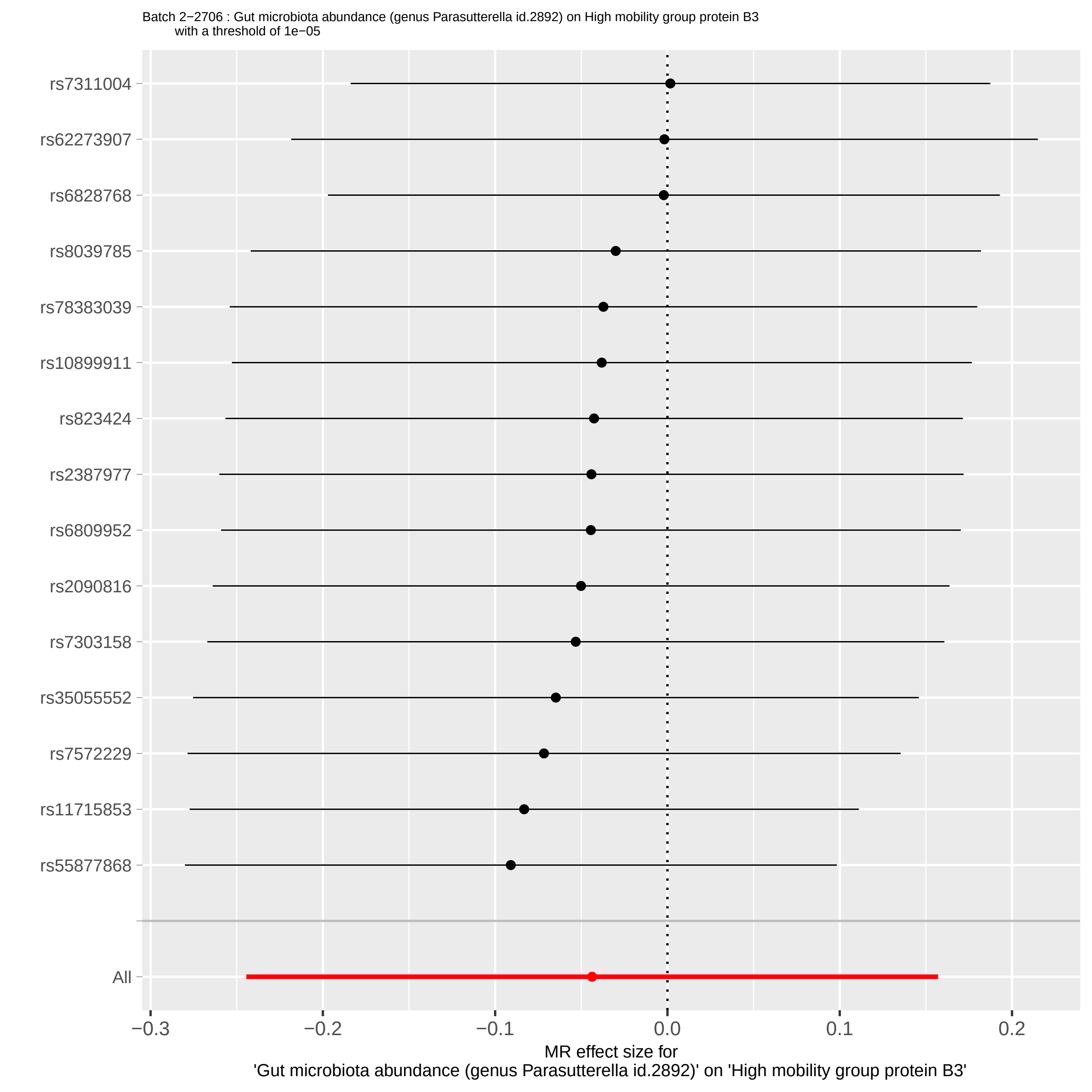

Batch 2−2706 : Gut microbiota abundance (genus Parasutterella id.2892) on High mobility group protein B3
with a threshold of 1e−05
rs7311004
rs62273907
rs6828768
rs8039785
rs78383039
rs10899911
rs823424
rs2387977
rs6809952
rs2090816
rs7303158
rs35055552
rs7572229
rs11715853
rs55877868
All
−0.3
−0.2
−0.1
0.0
0.1
0.2
MR effect size for
'Gut microbiota abundance (genus Parasutterella id.2892)' on 'High mobility group protein B3'

Batch 2−2707 : Gut microbiota abundance (genus Catenibacterium id.2153) on 3−hydroxy−3−methylglutaryl−coenzyme A reductase with a threshold of 1e−05

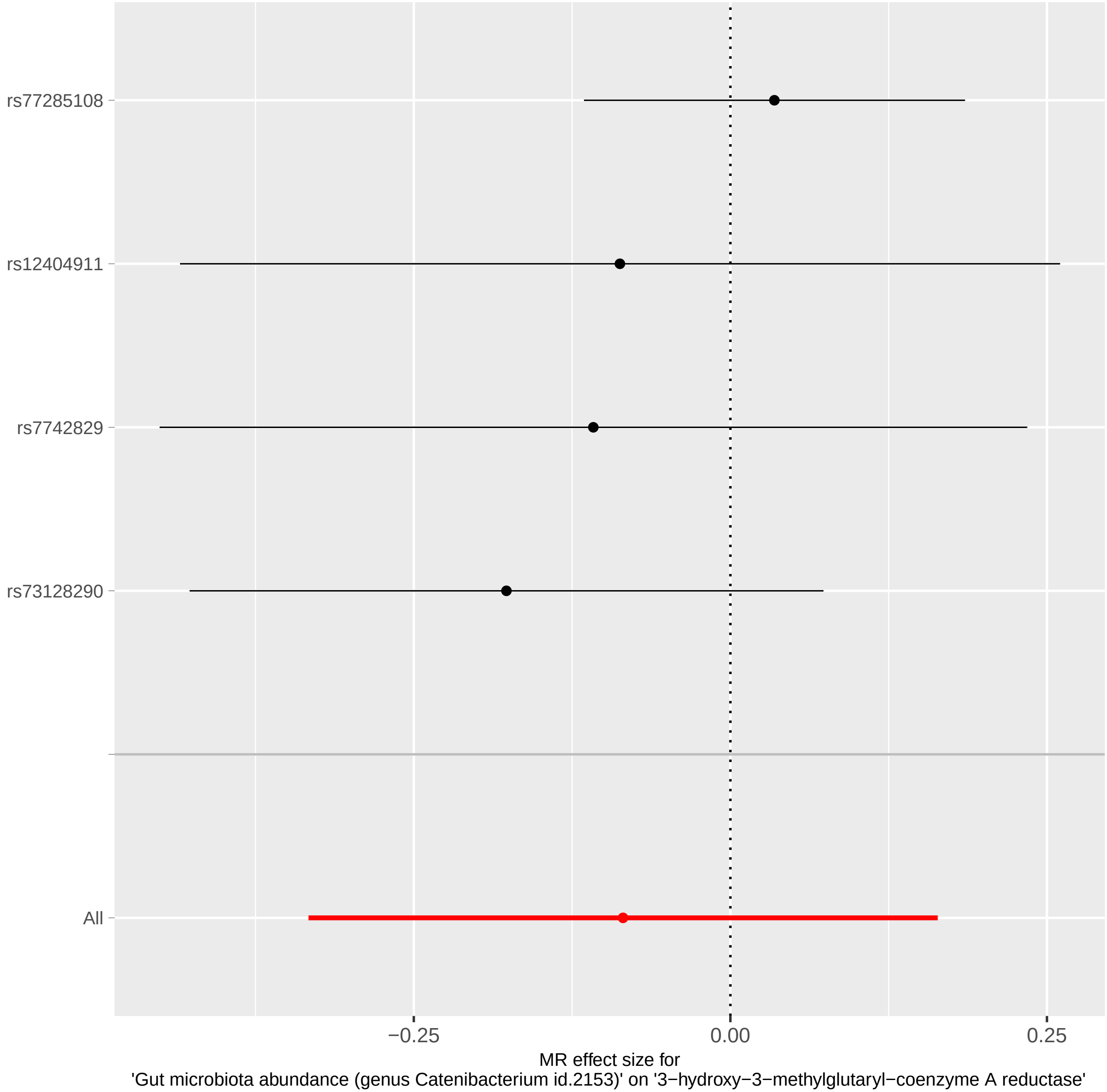

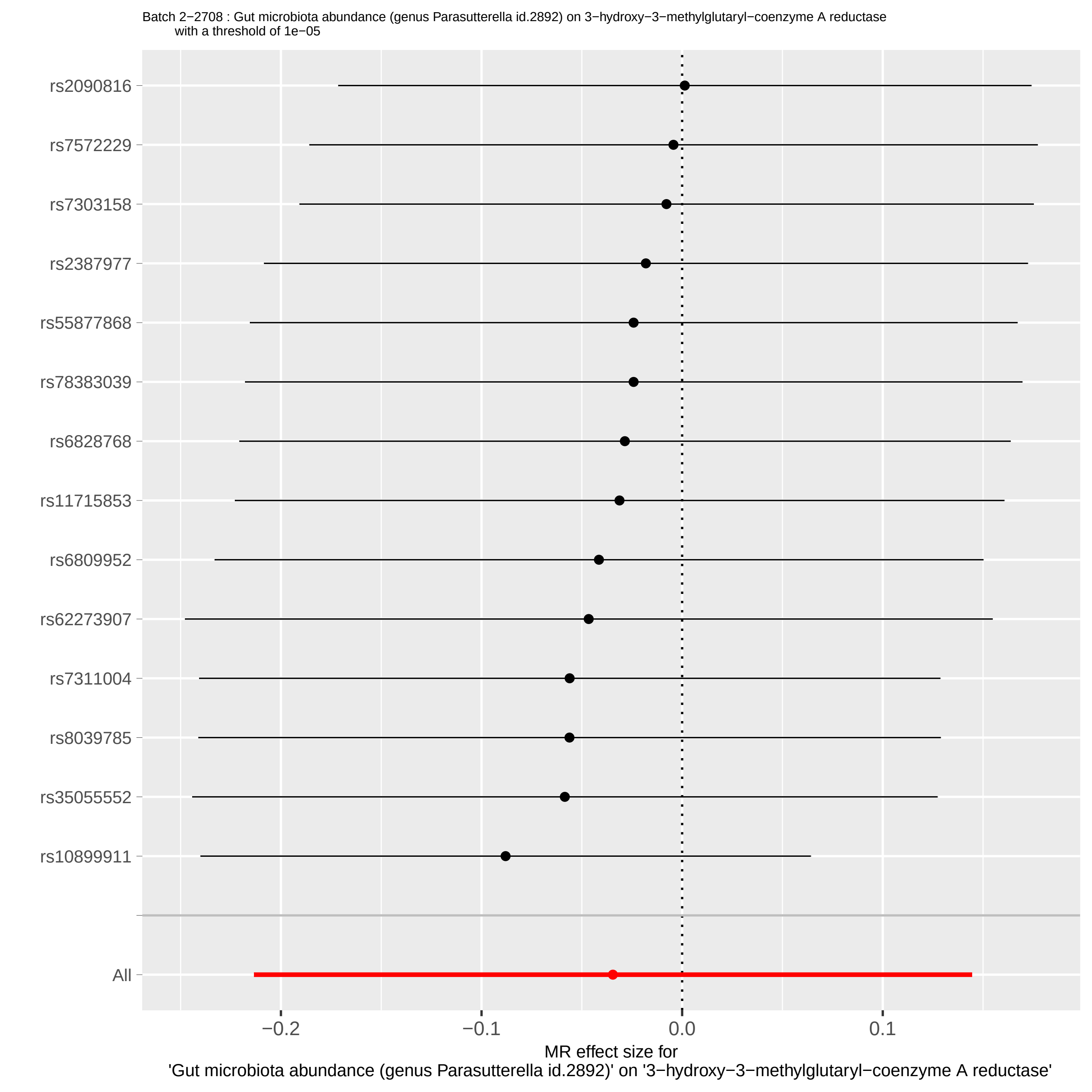
Batch 2−2708 : Gut microbiota abundance (genus Parasutterella id.2892) on 3−hydroxy−3−methylglutaryl−coenzyme A reductase
with a threshold of 1e−05
rs2090816
rs7572229
rs7303158
rs2387977
rs55877868
rs78383039
rs6828768
rs11715853
rs6809952
rs62273907
rs7311004
rs8039785
rs35055552
rs10899911
All
−0.2
−0.1
0.0
0.1
MR effect size for
'Gut microbiota abundance (genus Parasutterella id.2892)' on '3−hydroxy−3−methylglutaryl−coenzyme A reductase'

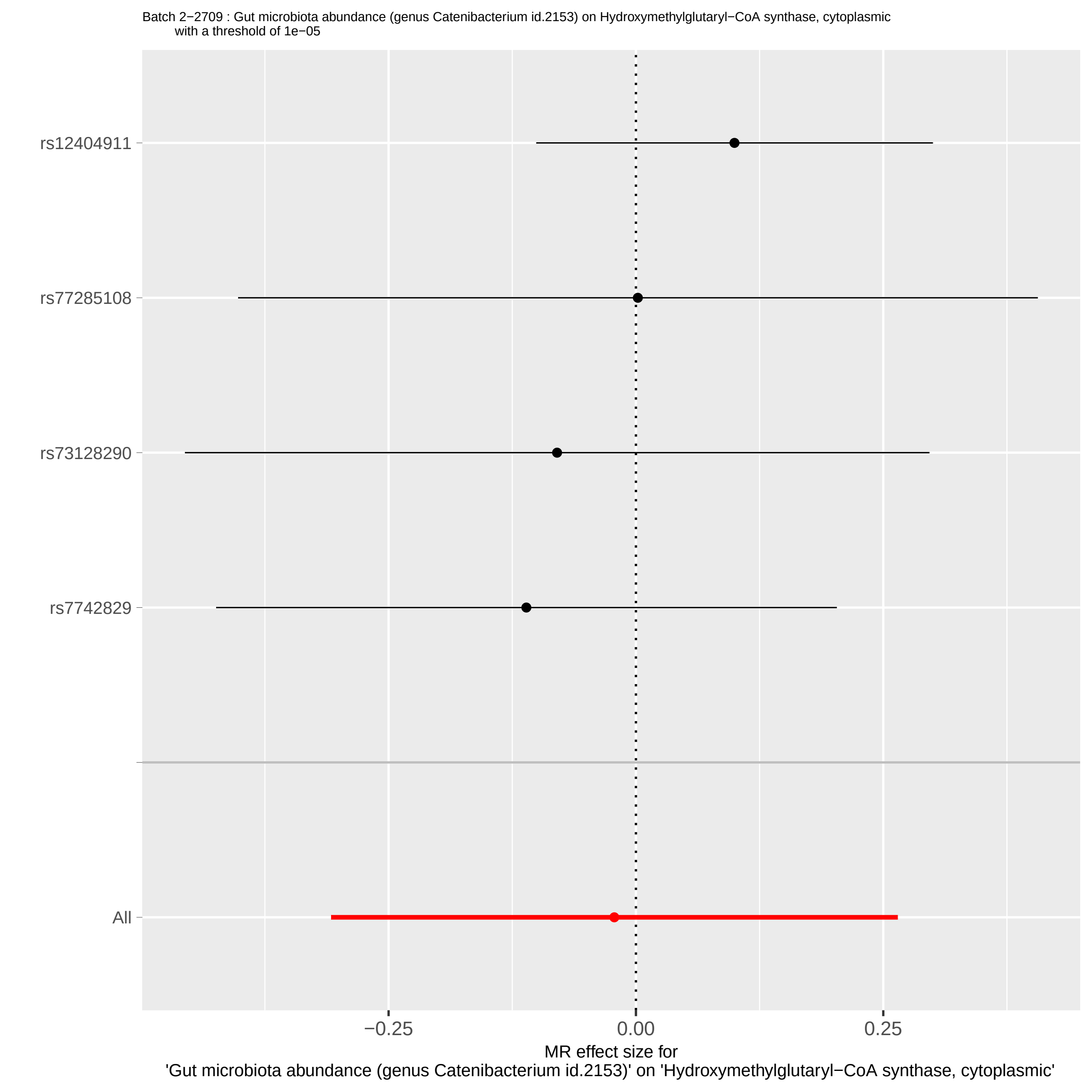
Batch 2−2709 : Gut microbiota abundance (genus Catenibacterium id.2153) on Hydroxymethylglutaryl−CoA synthase, cytoplasmic
with a threshold of 1e−05
rs12404911
rs77285108
rs73128290
rs7742829
All
−0.25
0.00
0.25
MR effect size for
'Gut microbiota abundance (genus Catenibacterium id.2153)' on 'Hydroxymethylglutaryl−CoA synthase, cytoplasmic'

Batch 2−2710 : Gut microbiota abundance (genus Parasutterella id.2892) on Hydroxymethylglutaryl−CoA synthase, cytoplasmic
with a threshold of 1e−05

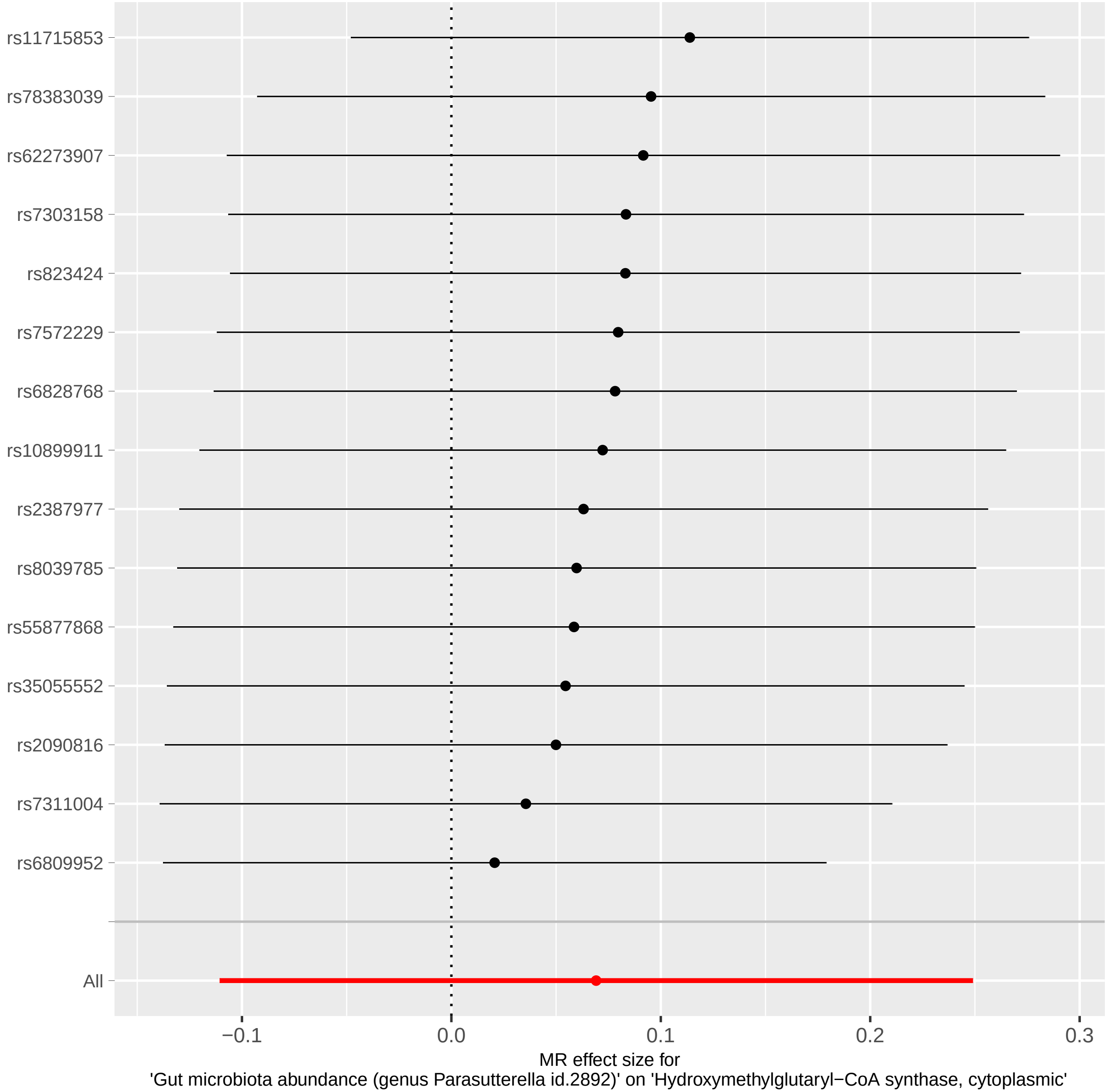

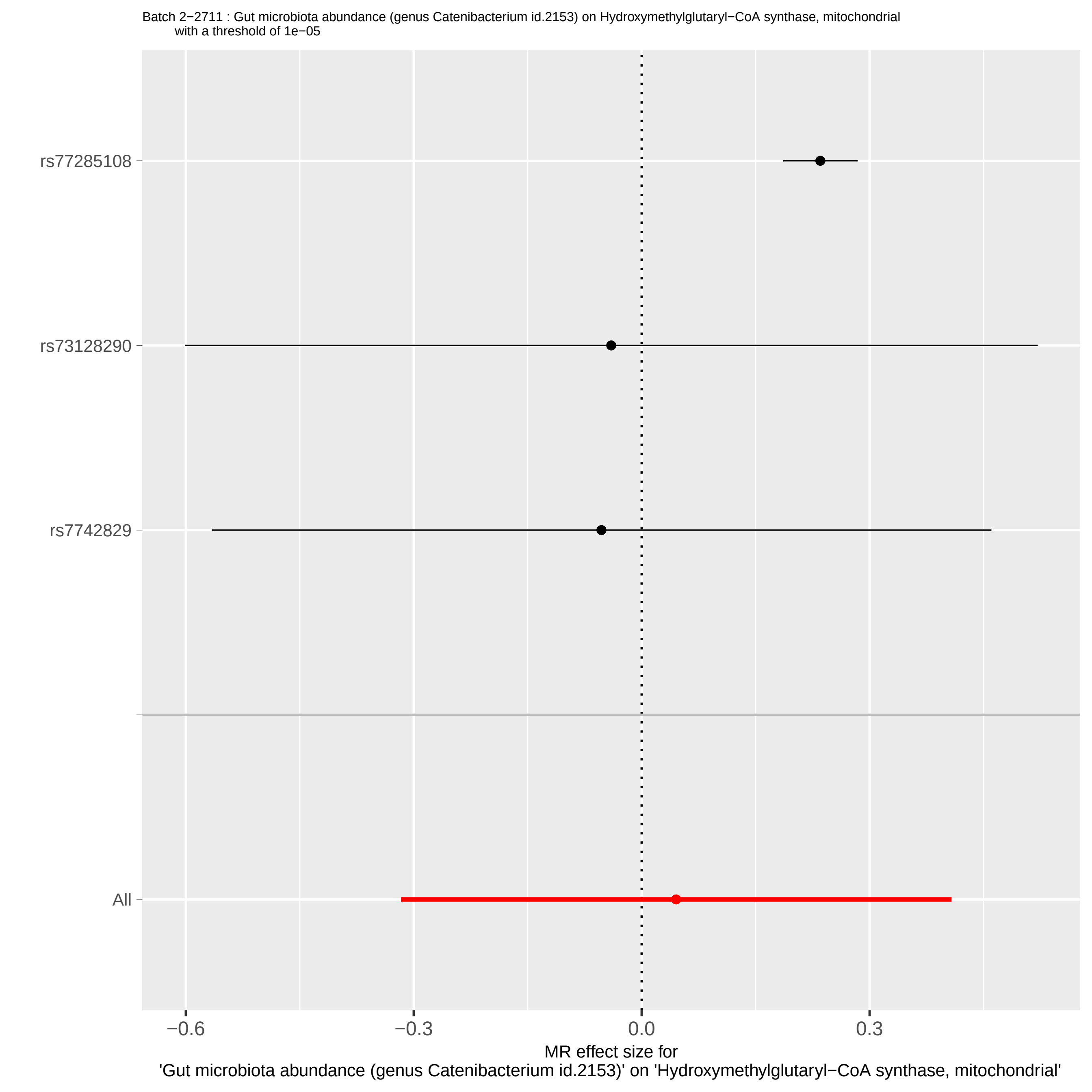
Batch 2−2711 : Gut microbiota abundance (genus Catenibacterium id.2153) on Hydroxymethylglutaryl−CoA synthase, mitochondrial
with a threshold of 1e−05
rs77285108
rs73128290
rs7742829
All
−0.6
−0.3
0.0
0.3
MR effect size for
'Gut microbiota abundance (genus Catenibacterium id.2153)' on 'Hydroxymethylglutaryl−CoA synthase, mitochondrial'

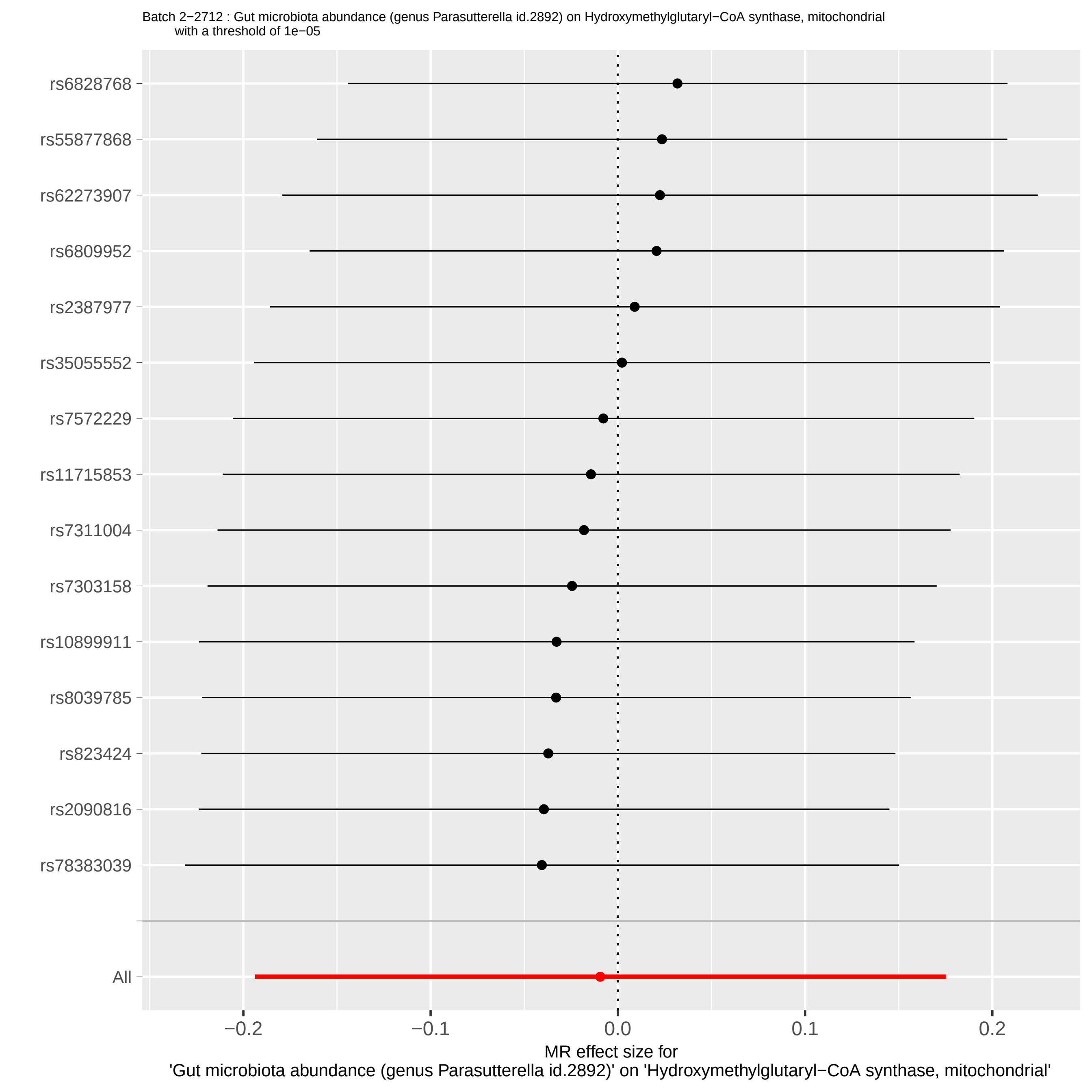

Batch 2−2712 : Gut microbiota abundance (genus Parasutterella id.2892) on Hydroxymethylglutaryl−CoA synthase, mitochondrial
with a threshold of 1e−05
rs6828768
rs55877868
rs62273907
rs6809952
rs2387977
rs35055552
rs7572229
rs11715853
rs7311004
rs7303158
rs10899911
rs8039785
rs823424
rs2090816
rs78383039
All
−0.2
−0.1
0.0
0.1
0.2
MR effect size for
'Gut microbiota abundance (genus Parasutterella id.2892)' on 'Hydroxymethylglutaryl−CoA synthase, mitochondrial'

Batch 2−2713 : Gut microbiota abundance (genus Catenibacterium id.2153) on Non−histone chromosomal protein HMG−14
with a threshold of 1e−05

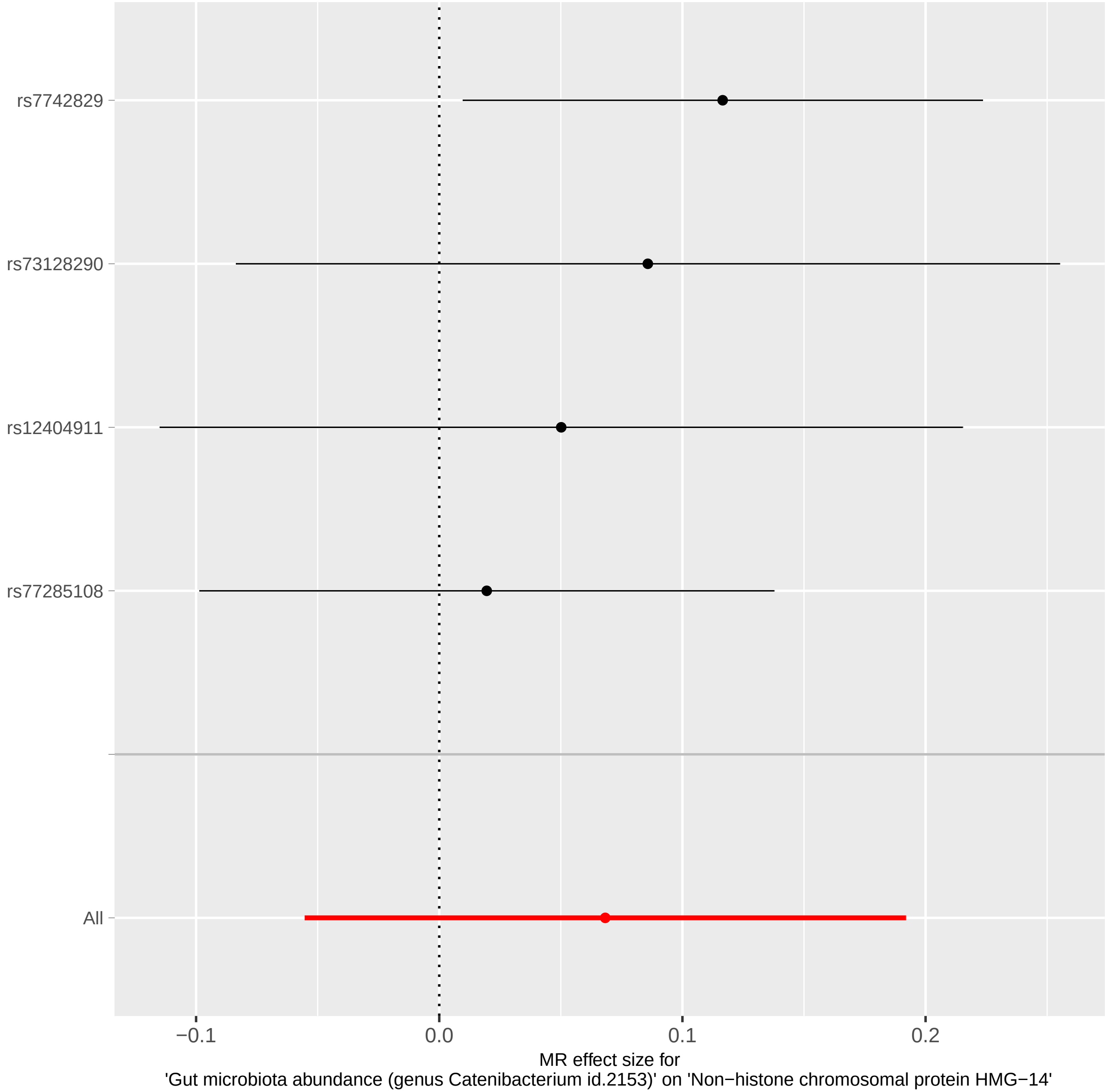

Batch 2−2714 : Gut microbiota abundance (genus Parasutterella id.2892) on Non−histone chromosomal protein HMG−14
with a threshold of 1e−05

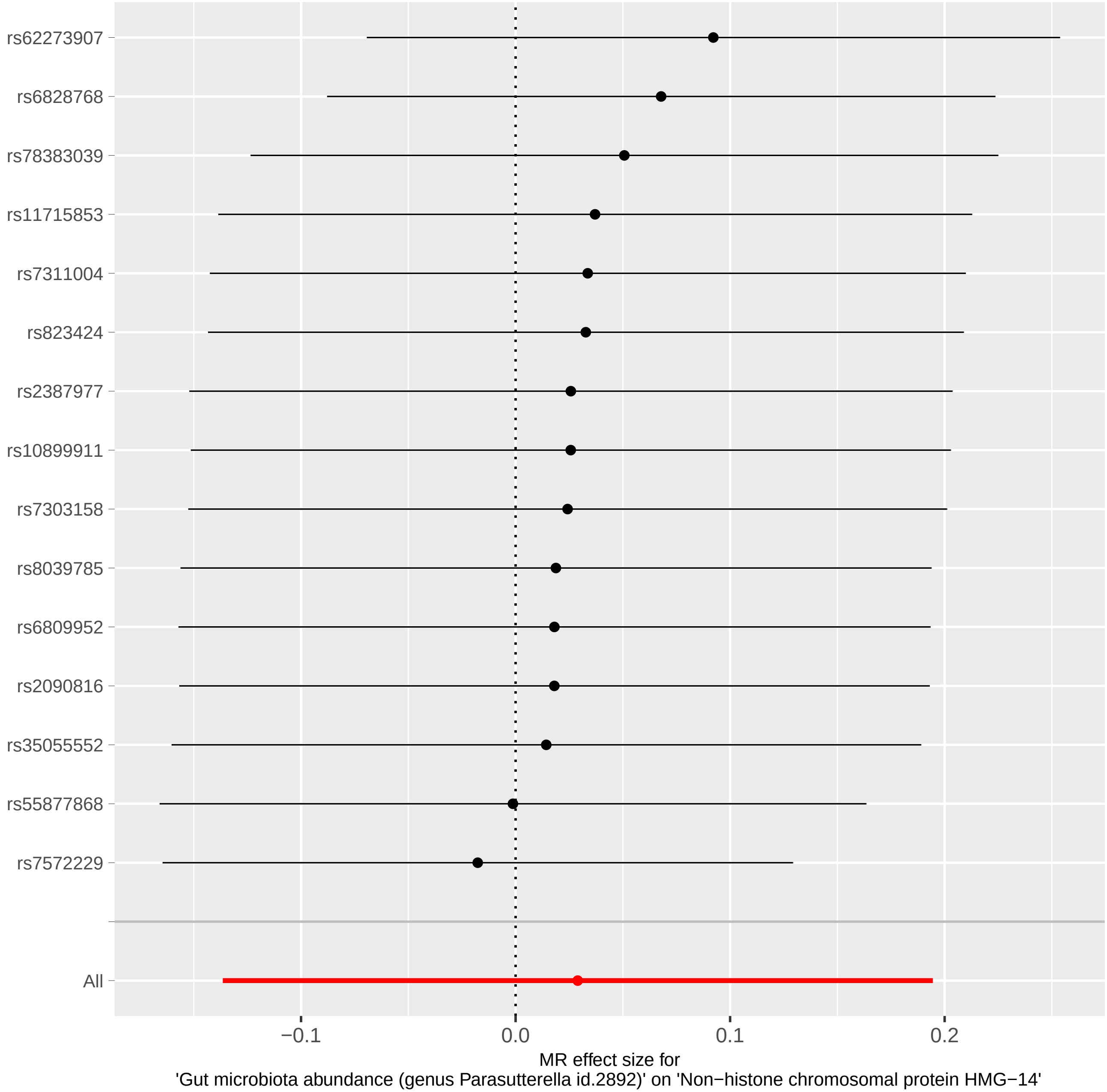

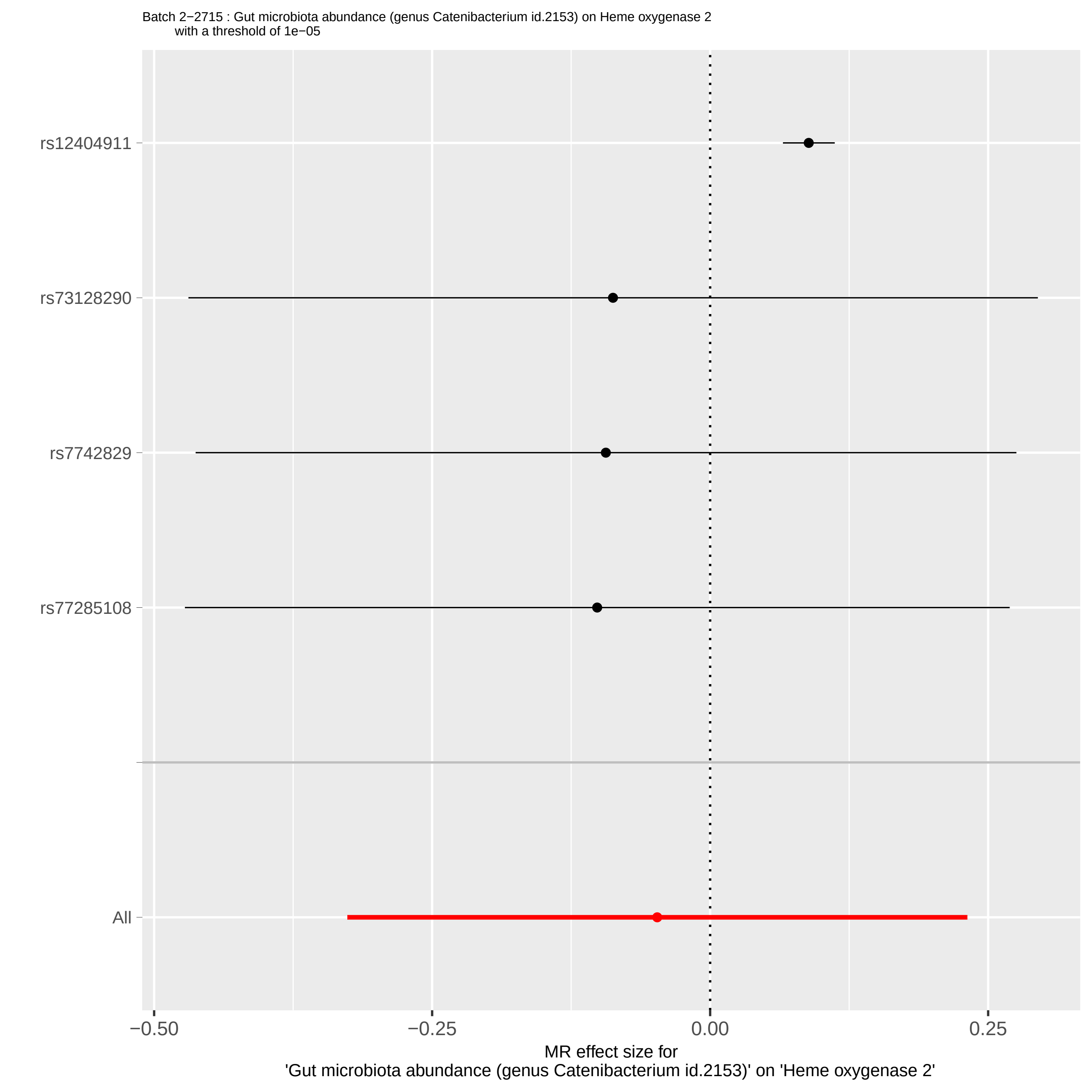

Batch 2−2715 : Gut microbiota abundance (genus Catenibacterium id.2153) on Heme oxygenase 2
with a threshold of 1e−05
rs12404911
rs73128290
rs7742829
rs77285108
All
−0.50
−0.25
0.00
0.25
MR effect size for
'Gut microbiota abundance (genus Catenibacterium id.2153)' on 'Heme oxygenase 2'

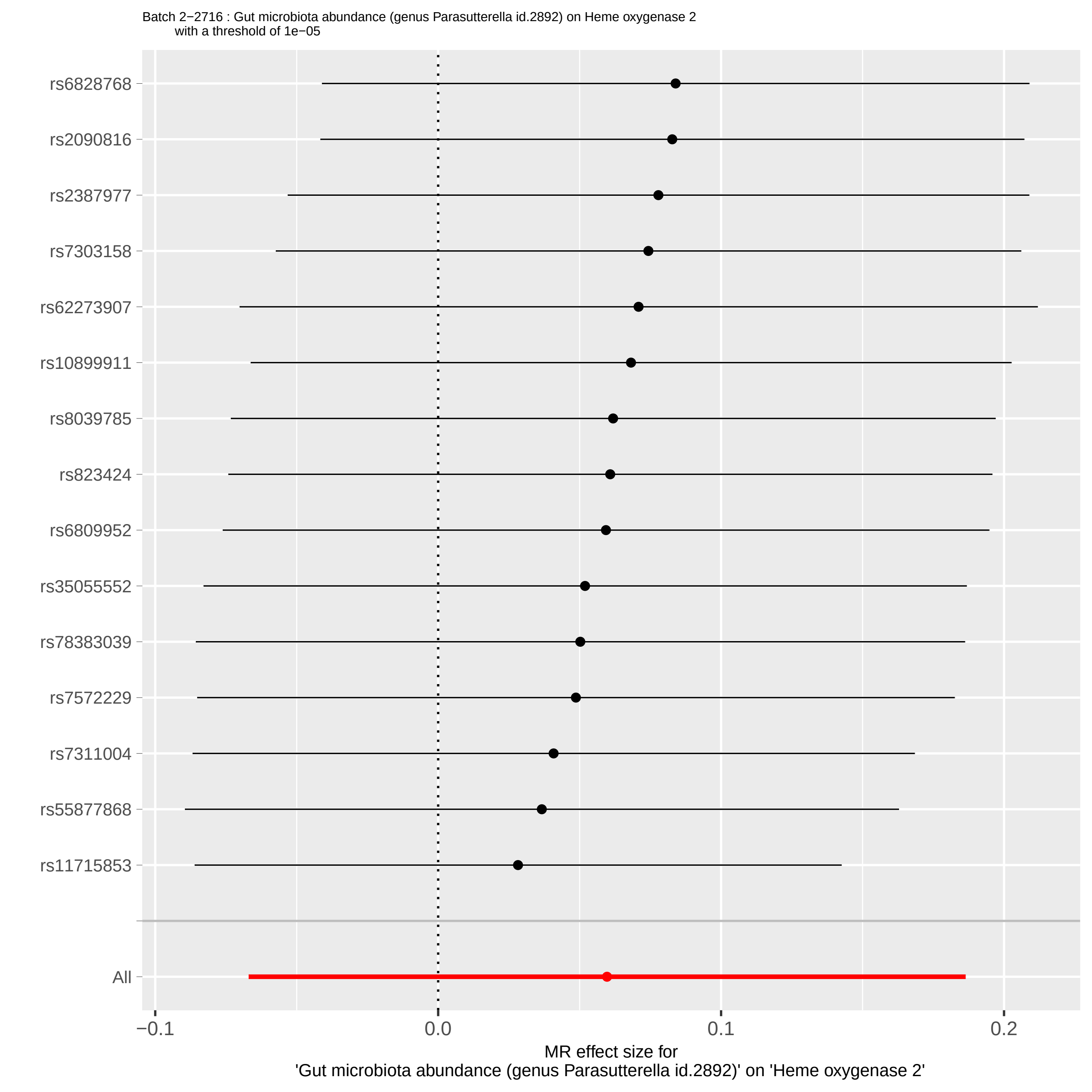

Batch 2−2716 : Gut microbiota abundance (genus Parasutterella id.2892) on Heme oxygenase 2
with a threshold of 1e−05
rs6828768
rs2090816
rs2387977
rs7303158
rs62273907
rs10899911
rs8039785
rs823424
rs6809952
rs35055552
rs78383039
rs7572229
rs7311004
rs55877868
rs11715853
All
−0.1
0.0
0.1
0.2
MR effect size for
'Gut microbiota abundance (genus Parasutterella id.2892)' on 'Heme oxygenase 2'

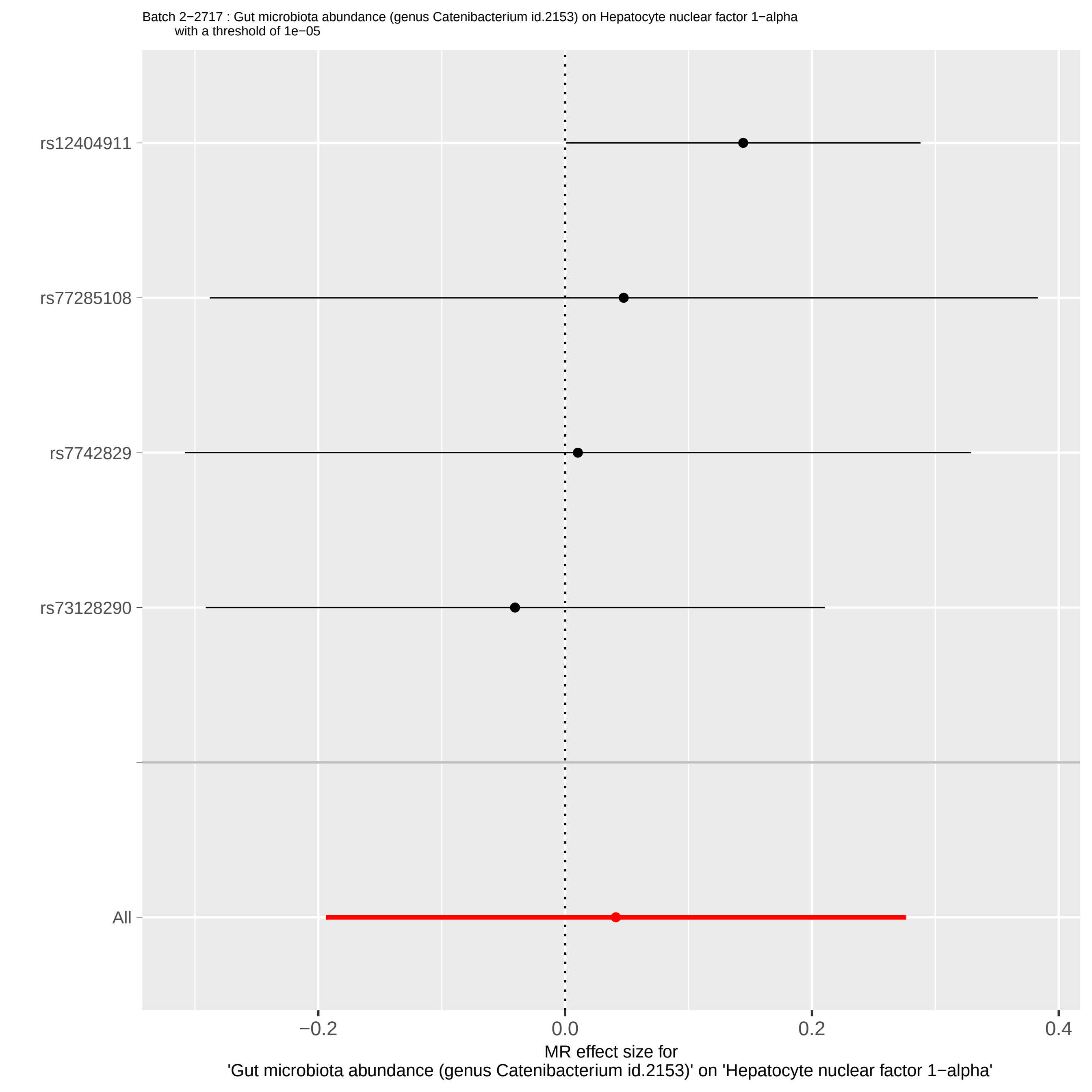

Batch 2−2717 : Gut microbiota abundance (genus Catenibacterium id.2153) on Hepatocyte nuclear factor 1−alpha
with a threshold of 1e−05
rs12404911
rs77285108
rs7742829
rs73128290
All
−0.2
0.0
0.2
0.4
MR effect size for
'Gut microbiota abundance (genus Catenibacterium id.2153)' on 'Hepatocyte nuclear factor 1−alpha'

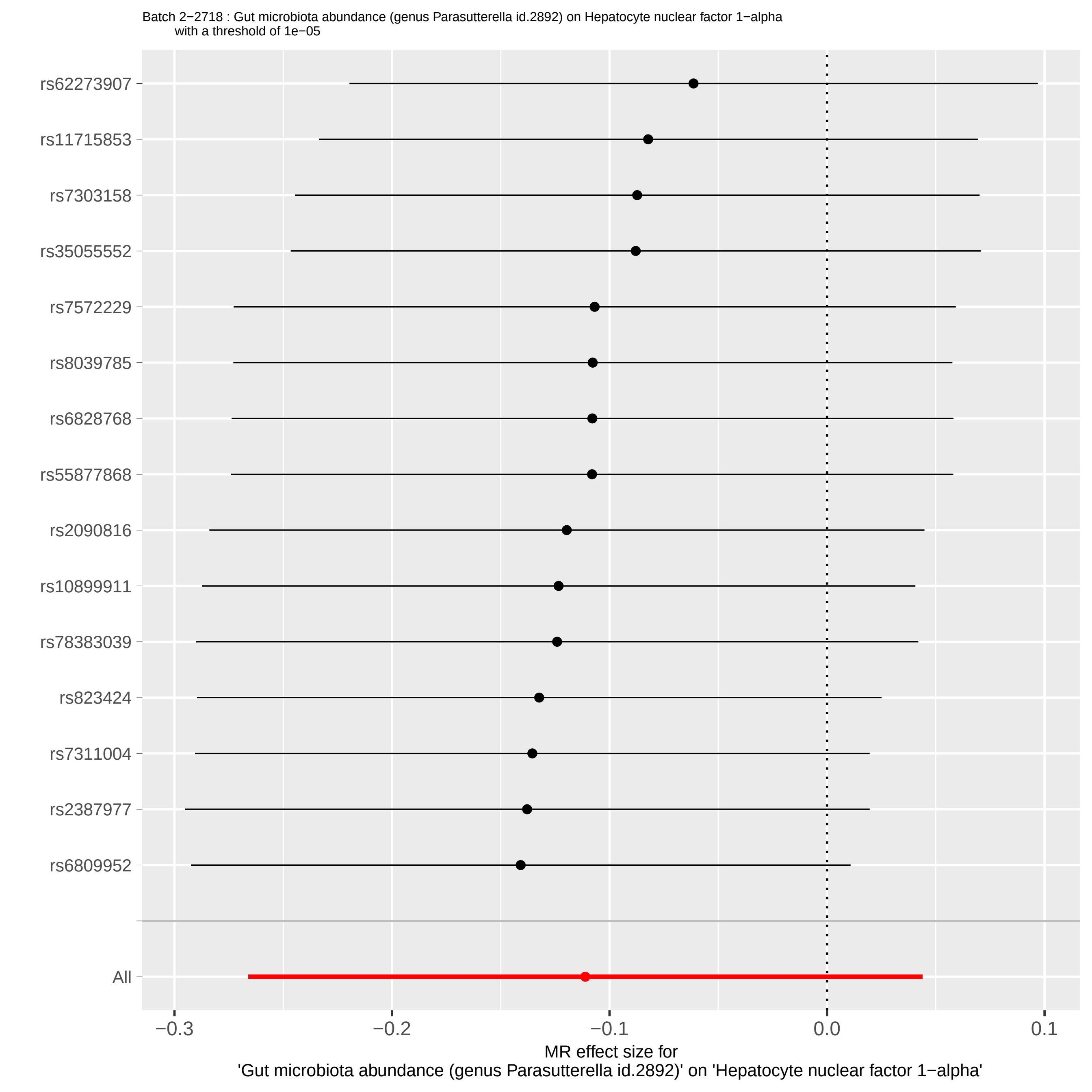

Batch 2−2718 : Gut microbiota abundance (genus Parasutterella id.2892) on Hepatocyte nuclear factor 1−alpha
with a threshold of 1e−05
rs62273907
rs11715853
rs7303158
rs35055552
rs7572229
rs8039785
rs6828768
rs55877868
rs2090816
rs10899911
rs78383039
rs823424
rs7311004
rs2387977
rs6809952
All
−0.3
−0.2
−0.1
0.0
0.1
MR effect size for
'Gut microbiota abundance (genus Parasutterella id.2892)' on 'Hepatocyte nuclear factor 1−alpha'

Batch 2−2719 : Gut microbiota abundance (genus Catenibacterium id.2153) on Hepatocyte nuclear factor 4−alpha
with a threshold of 1e−05

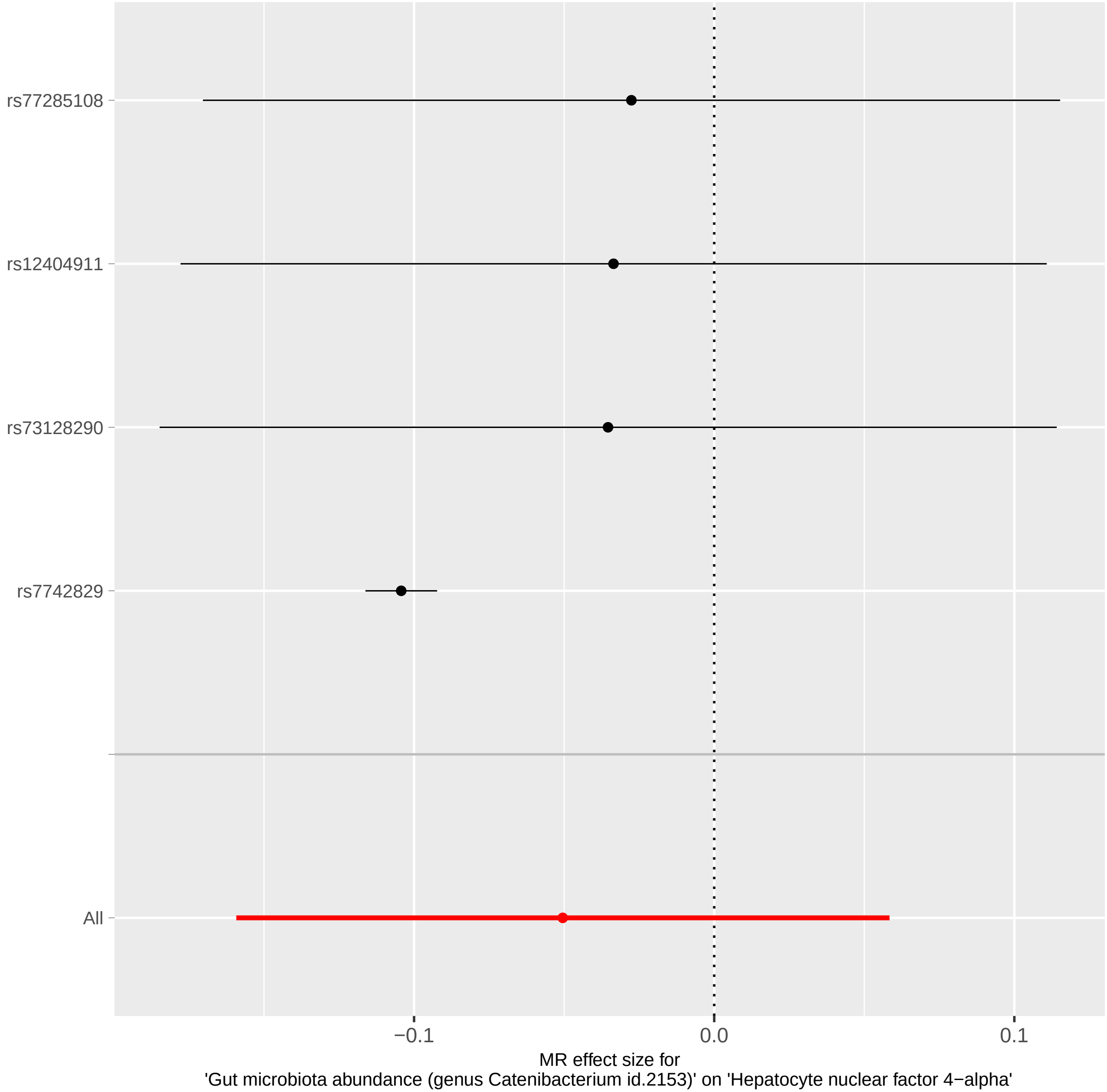

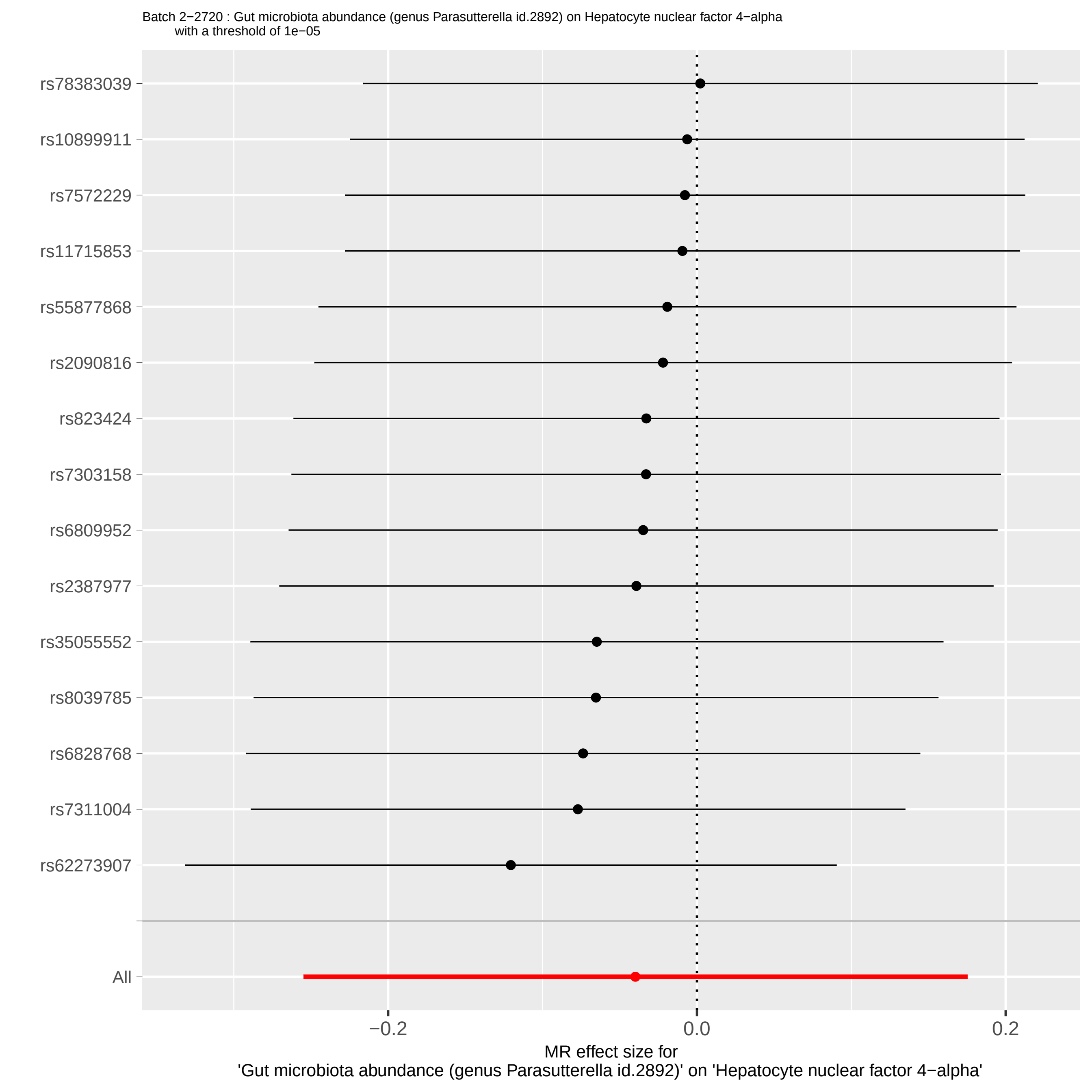

Batch 2−2720 : Gut microbiota abundance (genus Parasutterella id.2892) on Hepatocyte nuclear factor 4−alpha
with a threshold of 1e−05
rs78383039
rs10899911
rs7572229
rs11715853
rs55877868
rs2090816
rs823424
rs7303158
rs6809952
rs2387977
rs35055552
rs8039785
rs6828768
rs7311004
rs62273907
All
−0.2
0.0
0.2
MR effect size for
'Gut microbiota abundance (genus Parasutterella id.2892)' on 'Hepatocyte nuclear factor 4−alpha'

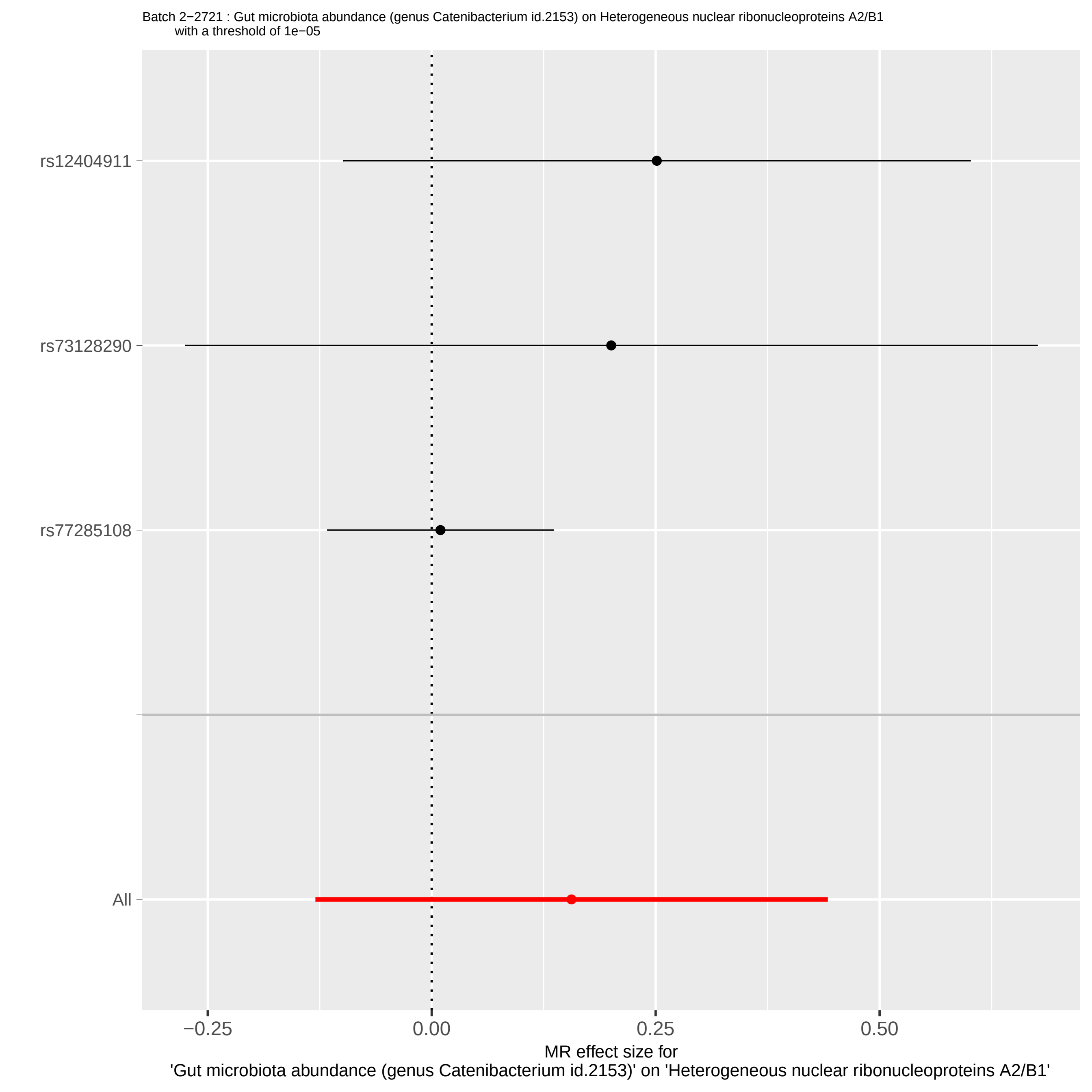
Batch 2−2721 : Gut microbiota abundance (genus Catenibacterium id.2153) on Heterogeneous nuclear ribonucleoproteins A2/B1
with a threshold of 1e−05
rs12404911
rs73128290
rs77285108
All
−0.25
0.00
0.25
0.50
MR effect size for
'Gut microbiota abundance (genus Catenibacterium id.2153)' on 'Heterogeneous nuclear ribonucleoproteins A2/B1'

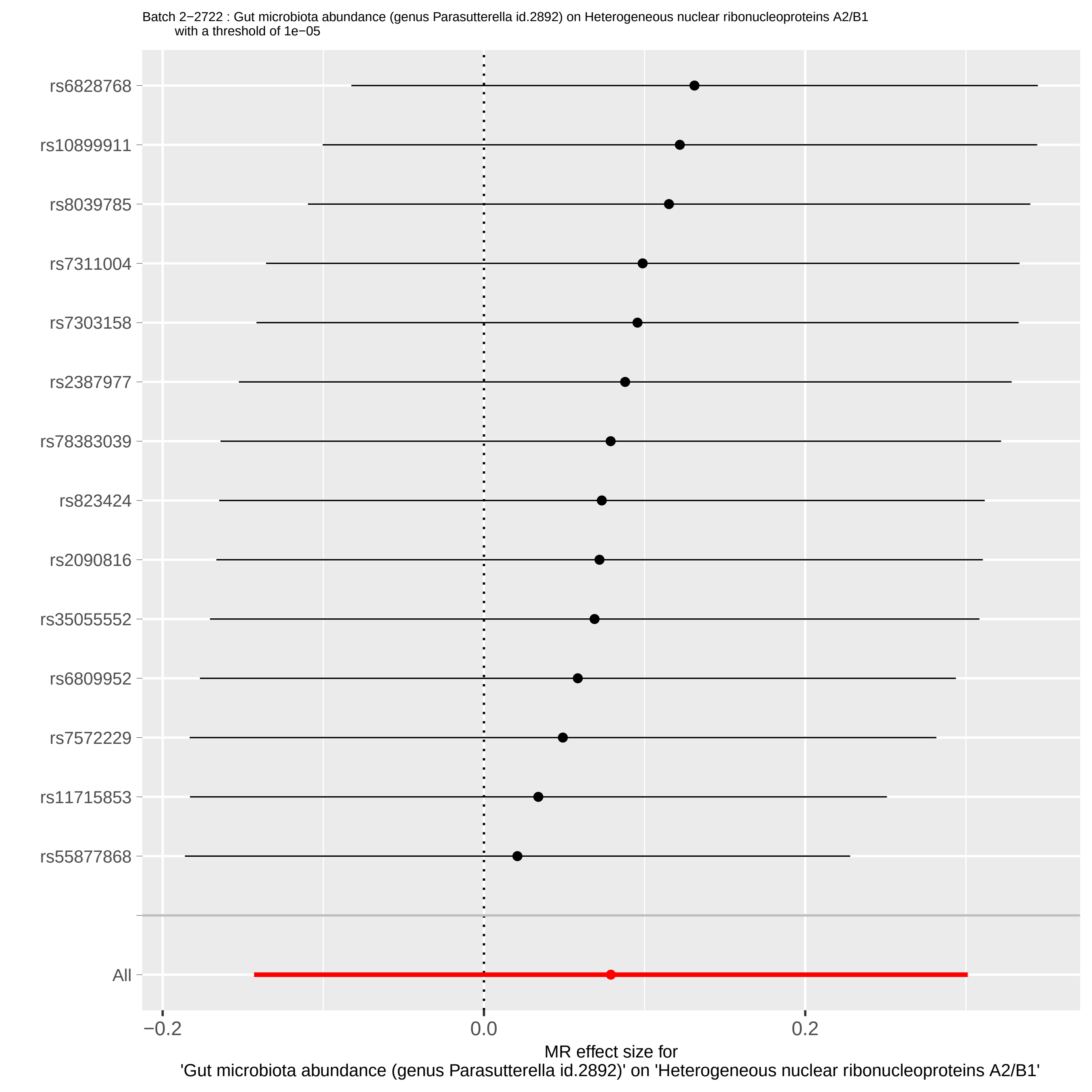

Batch 2−2722 : Gut microbiota abundance (genus Parasutterella id.2892) on Heterogeneous nuclear ribonucleoproteins A2/B1
with a threshold of 1e−05
rs6828768
rs10899911
rs8039785
rs7311004
rs7303158
rs2387977
rs78383039
rs823424
rs2090816
rs35055552
rs6809952
rs7572229
rs11715853
rs55877868
All
−0.2
0.0
0.2
MR effect size for
'Gut microbiota abundance (genus Parasutterella id.2892)' on 'Heterogeneous nuclear ribonucleoproteins A2/B1'

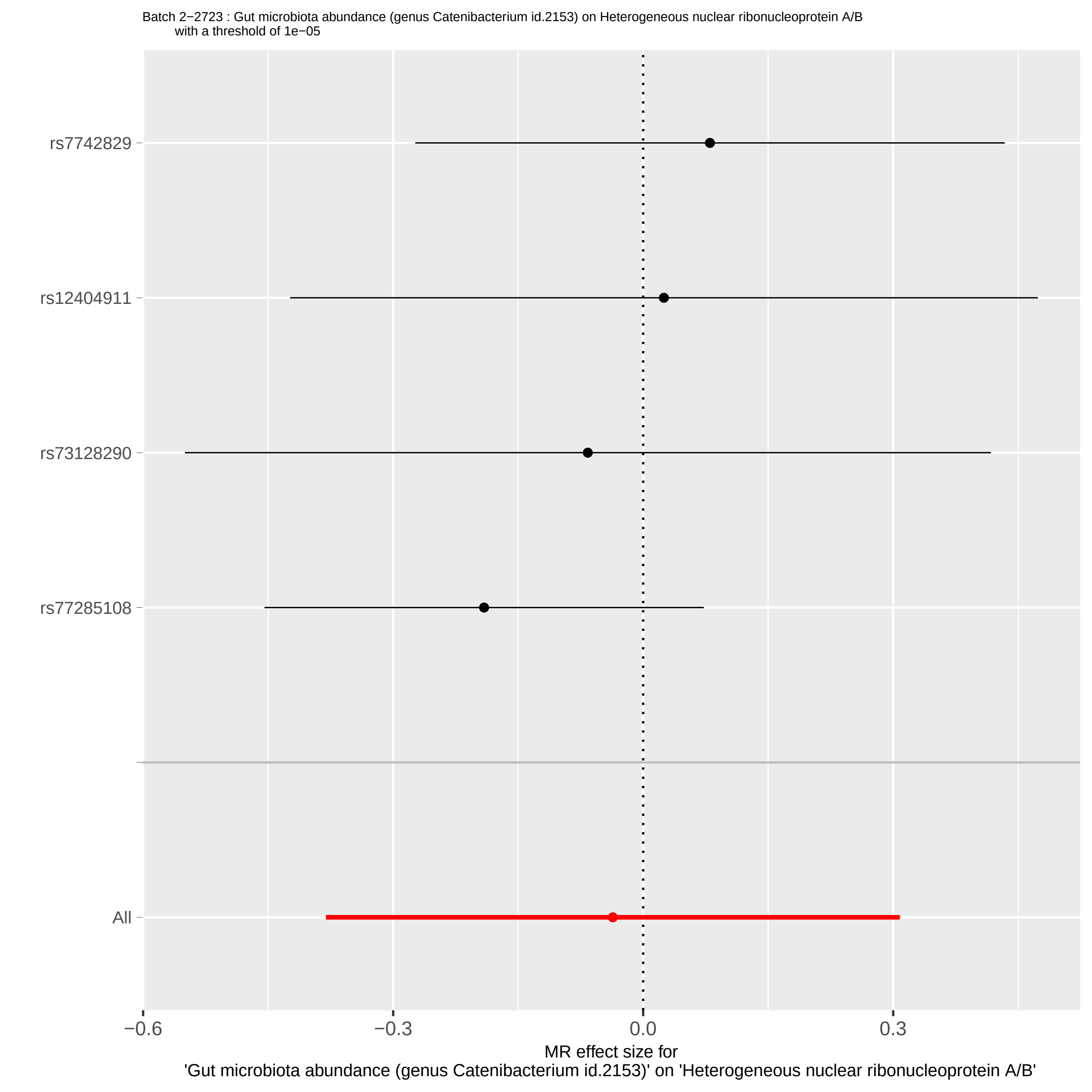
Batch 2−2723 : Gut microbiota abundance (genus Catenibacterium id.2153) on Heterogeneous nuclear ribonucleoprotein A/B
with a threshold of 1e−05
rs7742829
rs12404911
rs73128290
rs77285108
All
−0.6
−0.3
0.0
0.3
MR effect size for
'Gut microbiota abundance (genus Catenibacterium id.2153)' on 'Heterogeneous nuclear ribonucleoprotein A/B'

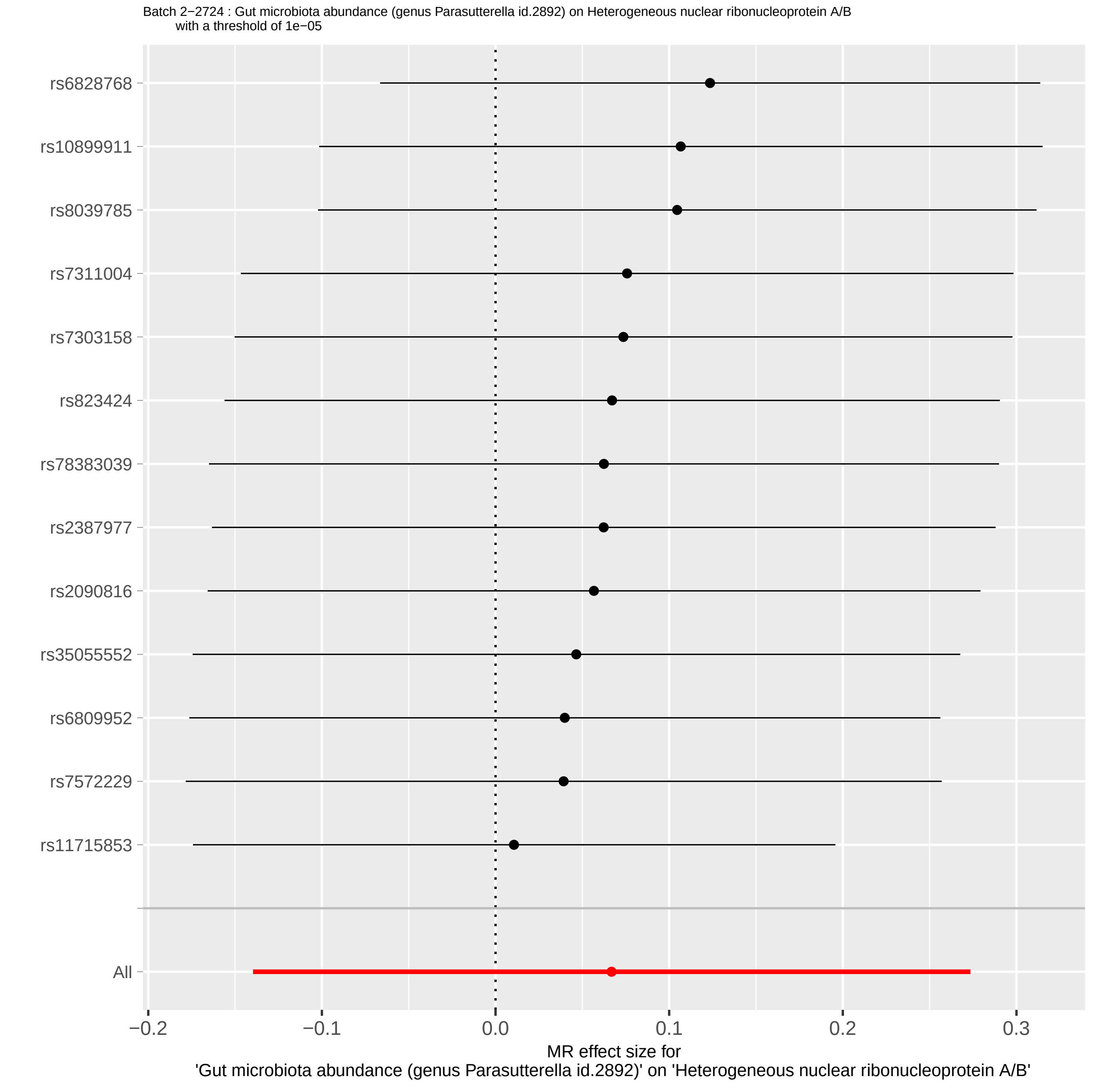
Batch 2−2724 : Gut microbiota abundance (genus Parasutterella id.2892) on Heterogeneous nuclear ribonucleoprotein A/B
with a threshold of 1e−05
rs6828768
rs10899911
rs8039785
rs7311004
rs7303158
rs823424
rs78383039
rs2387977
rs2090816
rs35055552
rs6809952
rs7572229
rs11715853
All
−0.2
−0.1
0.0
0.1
0.2
0.3
MR effect size for
'Gut microbiota abundance (genus Parasutterella id.2892)' on 'Heterogeneous nuclear ribonucleoprotein A/B'

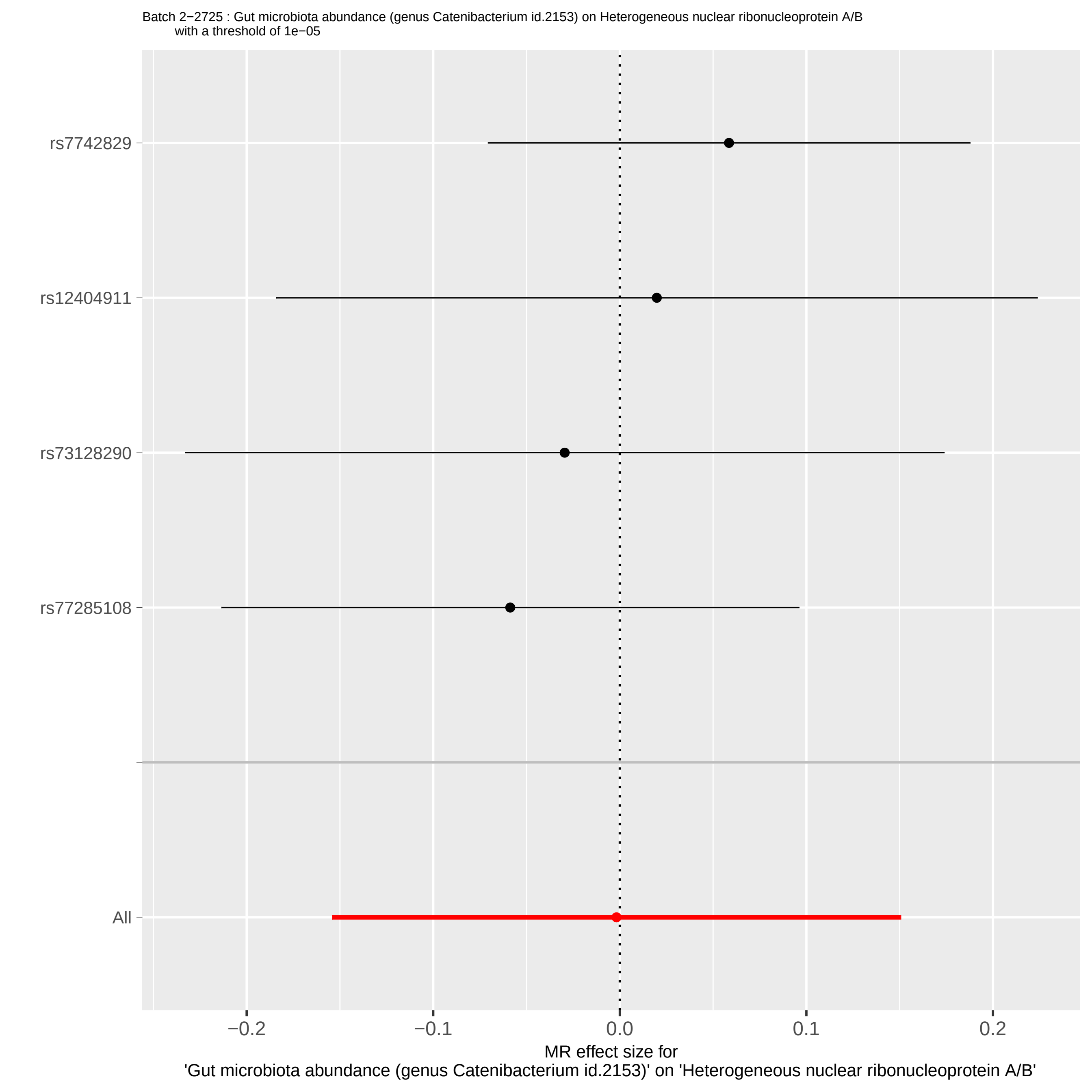

Batch 2−2725 : Gut microbiota abundance (genus Catenibacterium id.2153) on Heterogeneous nuclear ribonucleoprotein A/B
with a threshold of 1e−05
rs7742829
rs12404911
rs73128290
rs77285108
All
−0.2
−0.1
0.0
0.1
0.2
MR effect size for
'Gut microbiota abundance (genus Catenibacterium id.2153)' on 'Heterogeneous nuclear ribonucleoprotein A/B'

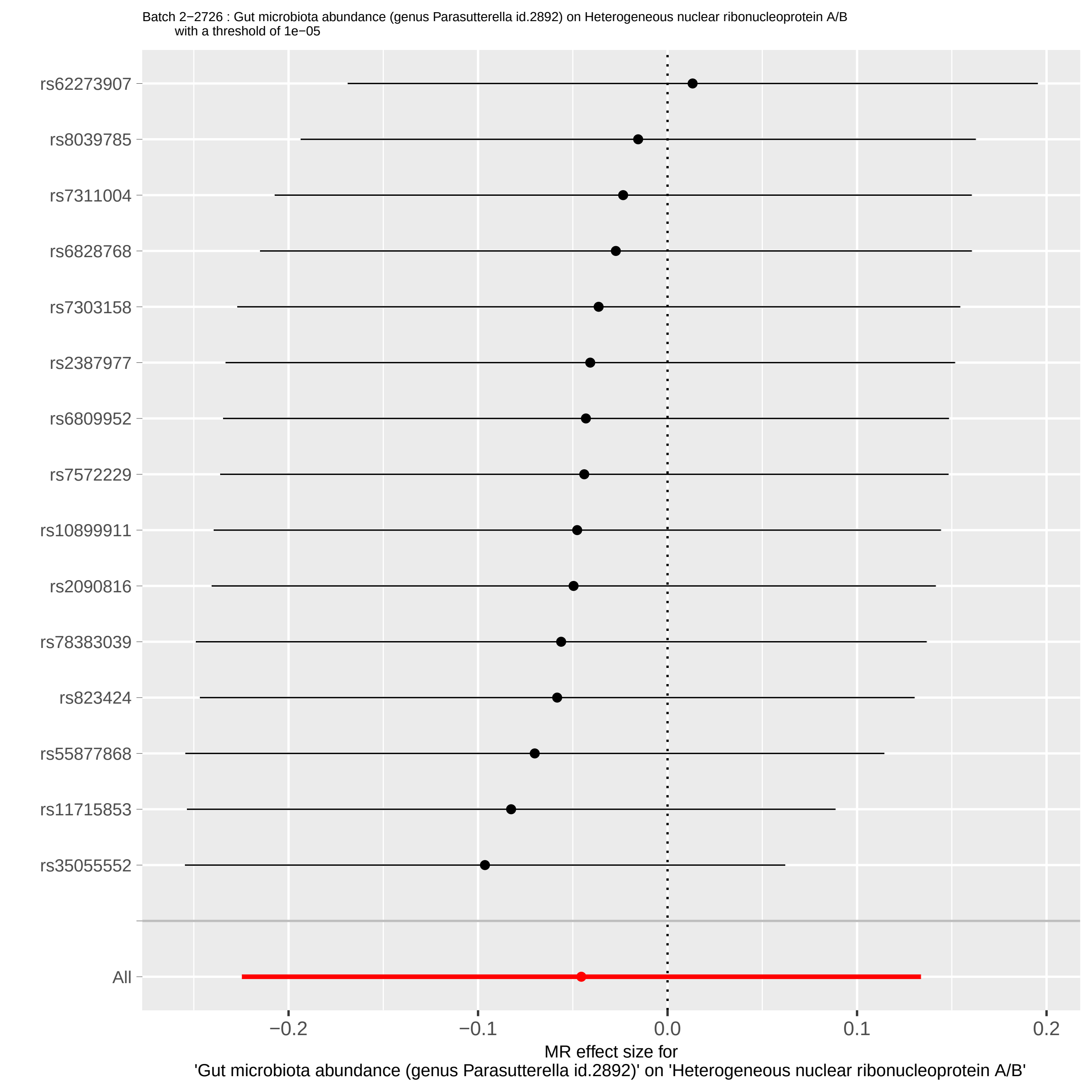
Batch 2−2726 : Gut microbiota abundance (genus Parasutterella id.2892) on Heterogeneous nuclear ribonucleoprotein A/B
with a threshold of 1e−05
rs62273907
rs8039785
rs7311004
rs6828768
rs7303158
rs2387977
rs6809952
rs7572229
rs10899911
rs2090816
rs78383039
rs823424
rs55877868
rs11715853
rs35055552
All
−0.2
−0.1
0.0
0.1
0.2
MR effect size for
'Gut microbiota abundance (genus Parasutterella id.2892)' on 'Heterogeneous nuclear ribonucleoprotein A/B'

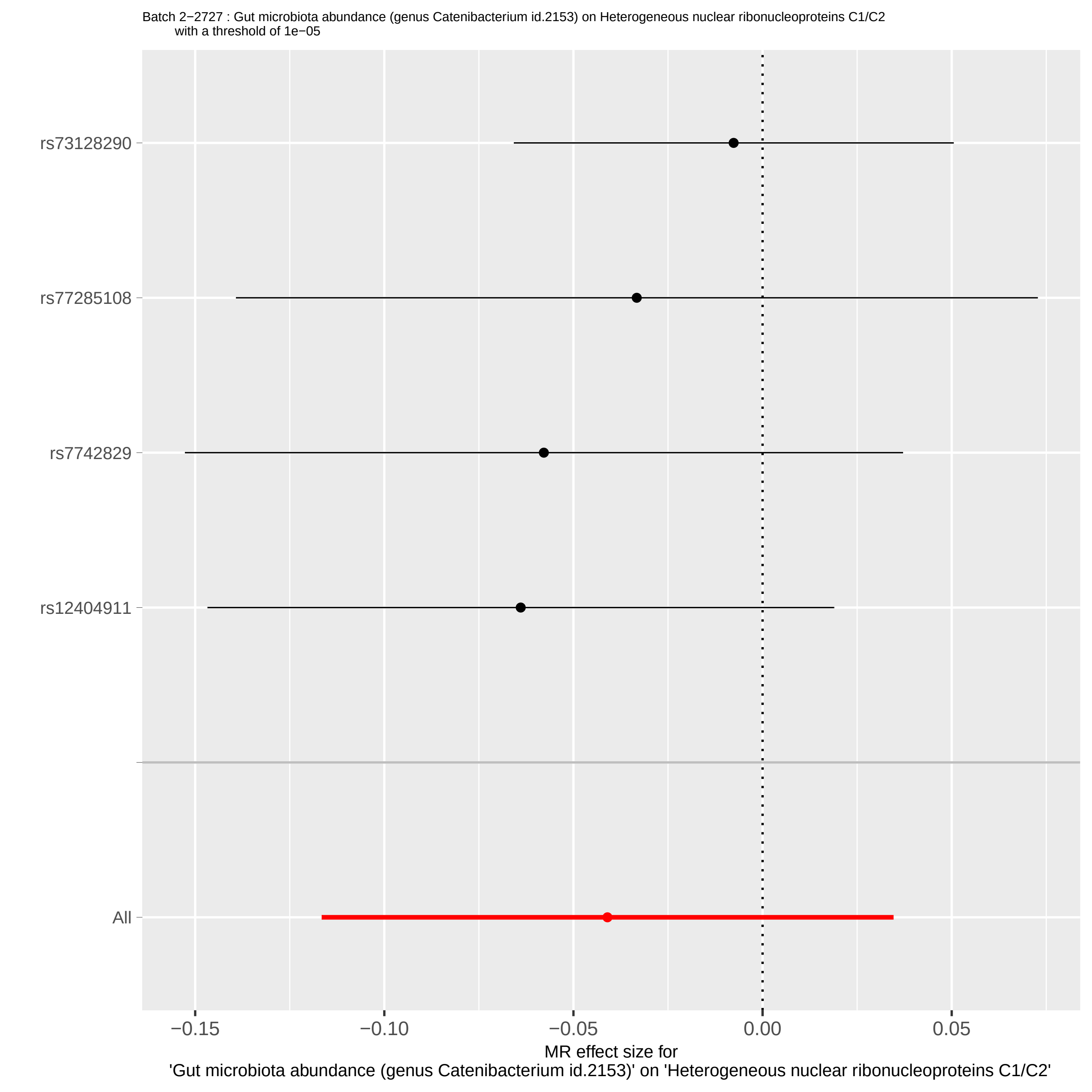
Batch 2−2727 : Gut microbiota abundance (genus Catenibacterium id.2153) on Heterogeneous nuclear ribonucleoproteins C1/C2
with a threshold of 1e−05
rs73128290
rs77285108
rs7742829
rs12404911
All
−0.15
−0.10
−0.05
0.00
0.05
MR effect size for
'Gut microbiota abundance (genus Catenibacterium id.2153)' on 'Heterogeneous nuclear ribonucleoproteins C1/C2'

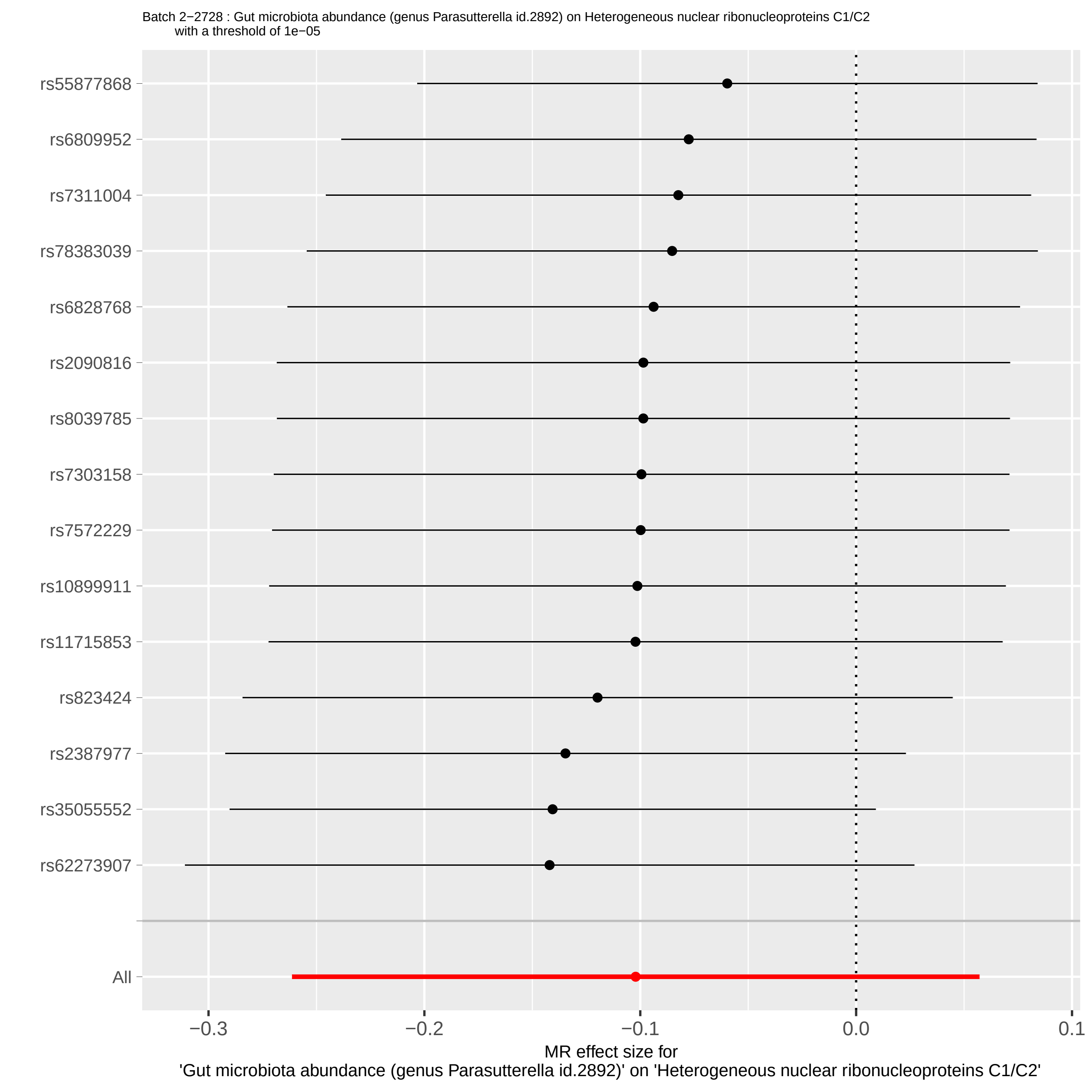
Batch 2−2728 : Gut microbiota abundance (genus Parasutterella id.2892) on Heterogeneous nuclear ribonucleoproteins C1/C2
with a threshold of 1e−05
rs55877868
rs6809952
rs7311004
rs78383039
rs6828768
rs2090816
rs8039785
rs7303158
rs7572229
rs10899911
rs11715853
rs823424
rs2387977
rs35055552
rs62273907
All
−0.3
−0.2
−0.1
0.0
0.1
MR effect size for
'Gut microbiota abundance (genus Parasutterella id.2892)' on 'Heterogeneous nuclear ribonucleoproteins C1/C2'

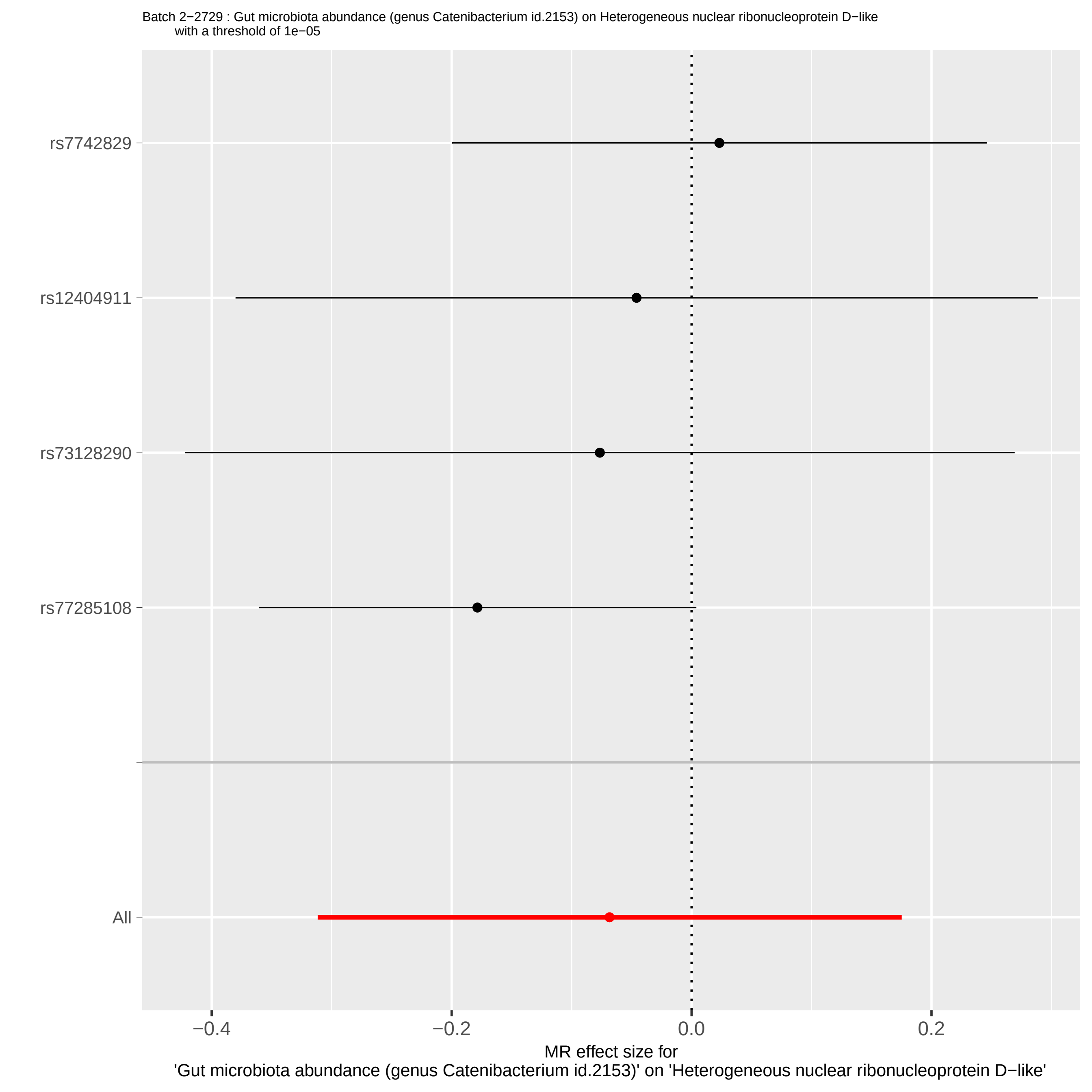
Batch 2−2729 : Gut microbiota abundance (genus Catenibacterium id.2153) on Heterogeneous nuclear ribonucleoprotein D−like
with a threshold of 1e−05
rs7742829
rs12404911
rs73128290
rs77285108
All
−0.4
−0.2
0.0
0.2
MR effect size for
'Gut microbiota abundance (genus Catenibacterium id.2153)' on 'Heterogeneous nuclear ribonucleoprotein D−like'

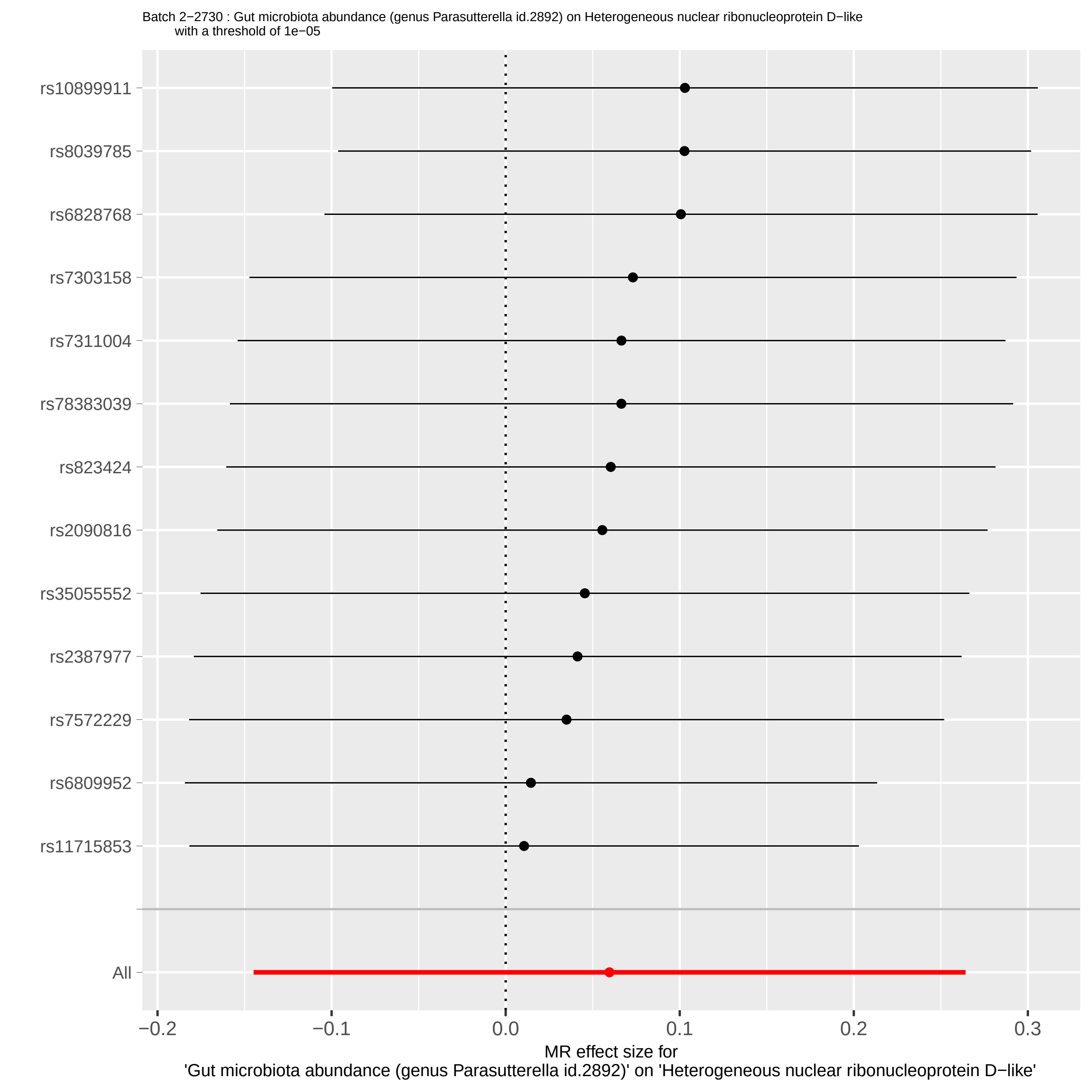

Batch 2−2730 : Gut microbiota abundance (genus Parasutterella id.2892) on Heterogeneous nuclear ribonucleoprotein D−like
with a threshold of 1e−05
rs10899911
rs8039785
rs6828768
rs7303158
rs7311004
rs78383039
rs823424
rs2090816
rs35055552
rs2387977
rs7572229
rs6809952
rs11715853
All
−0.2
−0.1
0.0
0.1
0.2
0.3
MR effect size for
'Gut microbiota abundance (genus Parasutterella id.2892)' on 'Heterogeneous nuclear ribonucleoprotein D−like'

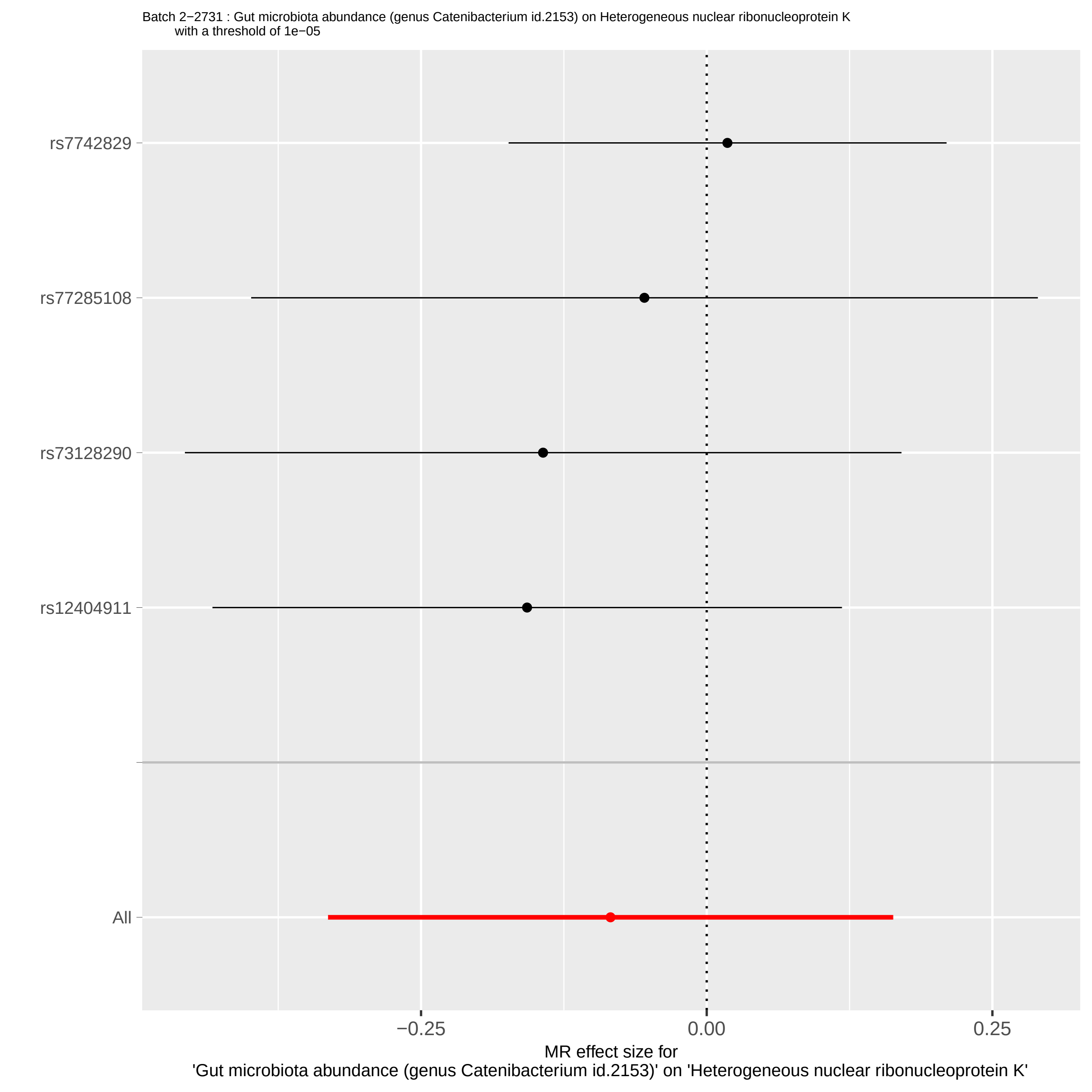

Batch 2−2731 : Gut microbiota abundance (genus Catenibacterium id.2153) on Heterogeneous nuclear ribonucleoprotein K
with a threshold of 1e−05
rs7742829
rs77285108
rs73128290
rs12404911
All
−0.25
0.00
0.25
MR effect size for
'Gut microbiota abundance (genus Catenibacterium id.2153)' on 'Heterogeneous nuclear ribonucleoprotein K'

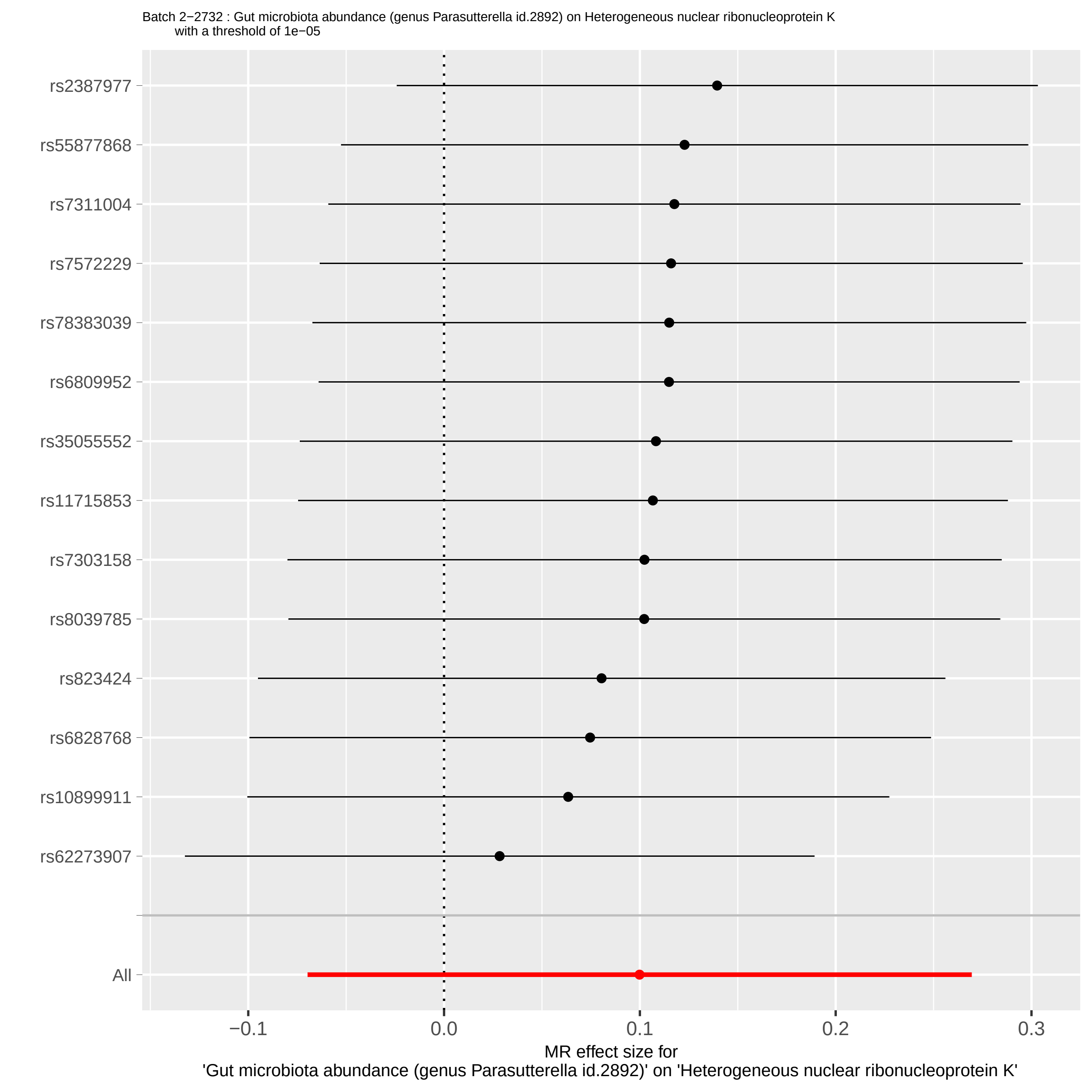

Batch 2−2732 : Gut microbiota abundance (genus Parasutterella id.2892) on Heterogeneous nuclear ribonucleoprotein K
with a threshold of 1e−05
rs2387977
rs55877868
rs7311004
rs7572229
rs78383039
rs6809952
rs35055552
rs11715853
rs7303158
rs8039785
rs823424
rs6828768
rs10899911
rs62273907
All
−0.1
0.0
0.1
0.2
0.3
MR effect size for
'Gut microbiota abundance (genus Parasutterella id.2892)' on 'Heterogeneous nuclear ribonucleoprotein K'

Batch 2−2733 : Gut microbiota abundance (genus Catenibacterium id.2153) on Heterogeneous nuclear ribonucleoprotein M
with a threshold of 1e−05

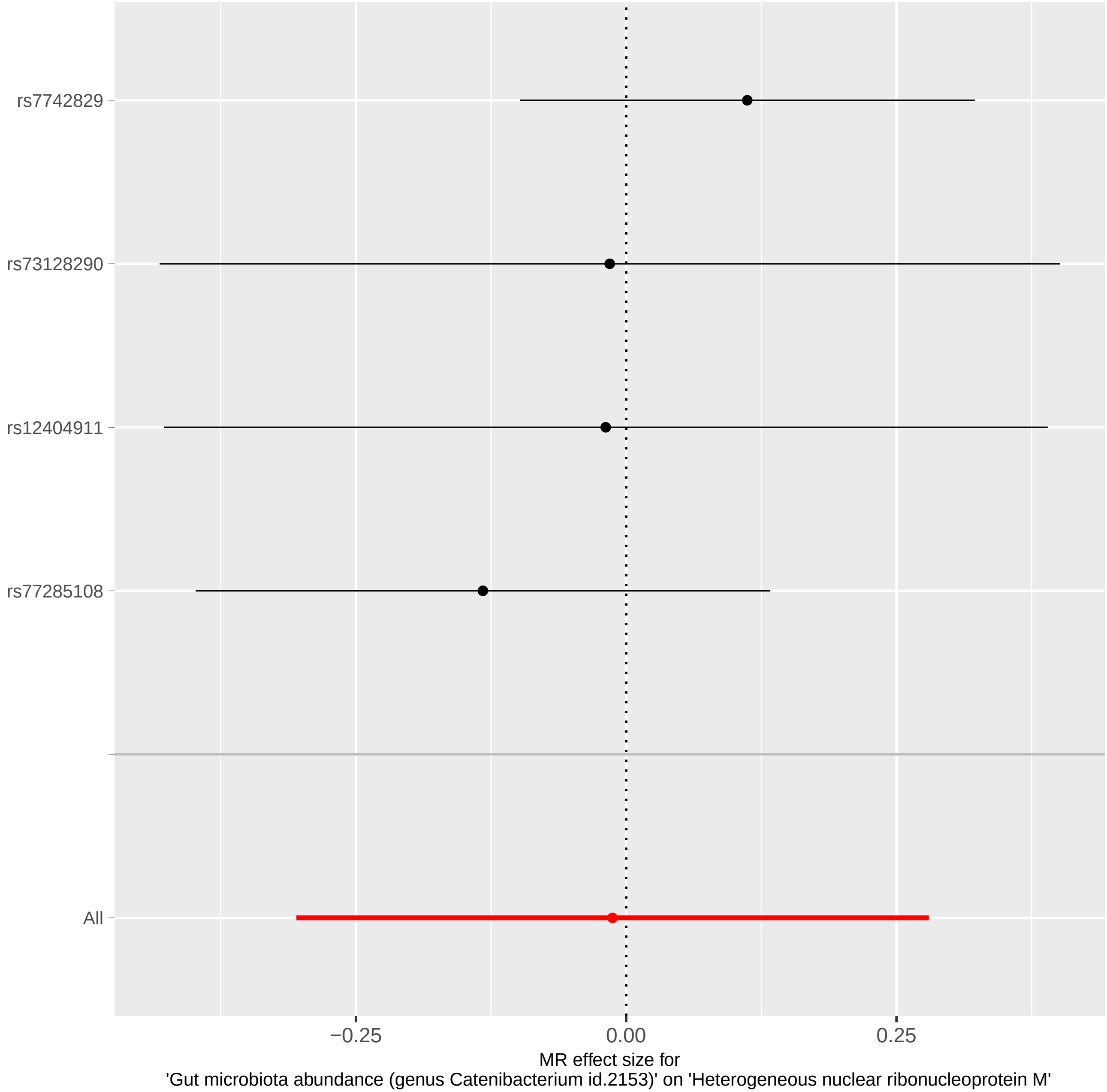

Batch 2−2734 : Gut microbiota abundance (genus Parasutterella id.2892) on Heterogeneous nuclear ribonucleoprotein M
with a threshold of 1e−05

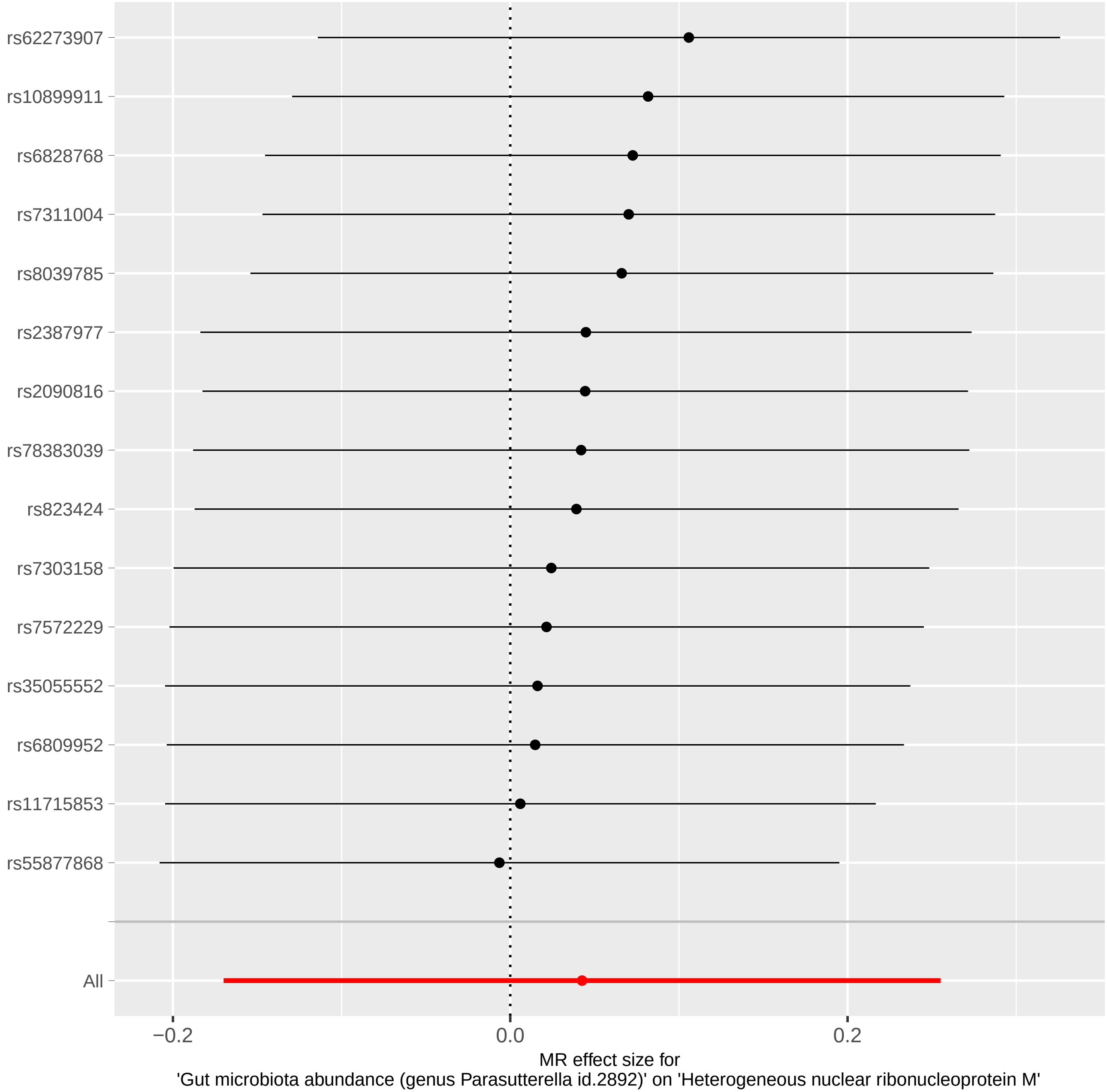

Batch 2−2735 : Gut microbiota abundance (genus Catenibacterium id.2153) on 4−hydroxy−2−oxoglutarate aldolase, mitochondrial
with a threshold of 1e−05

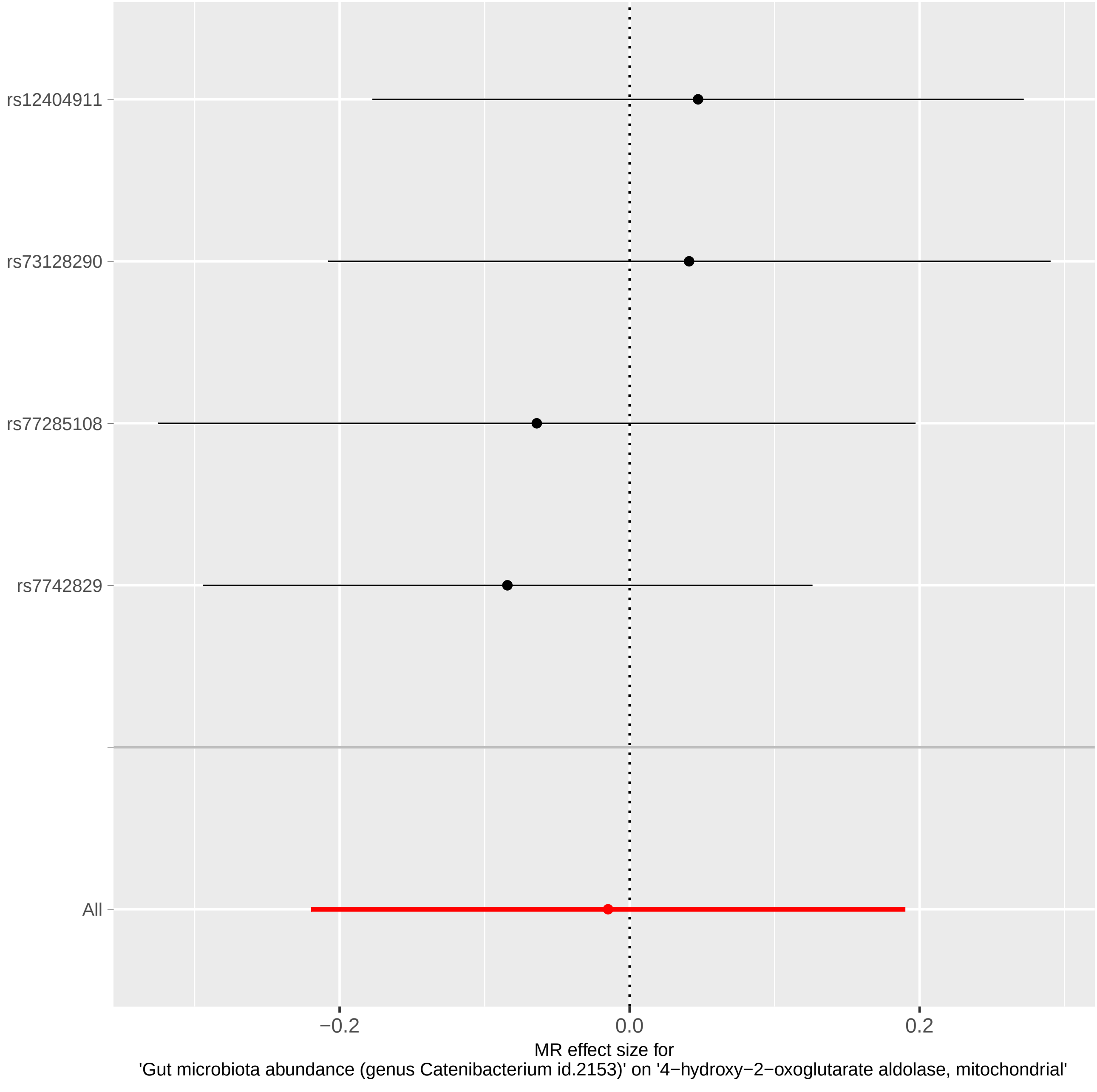

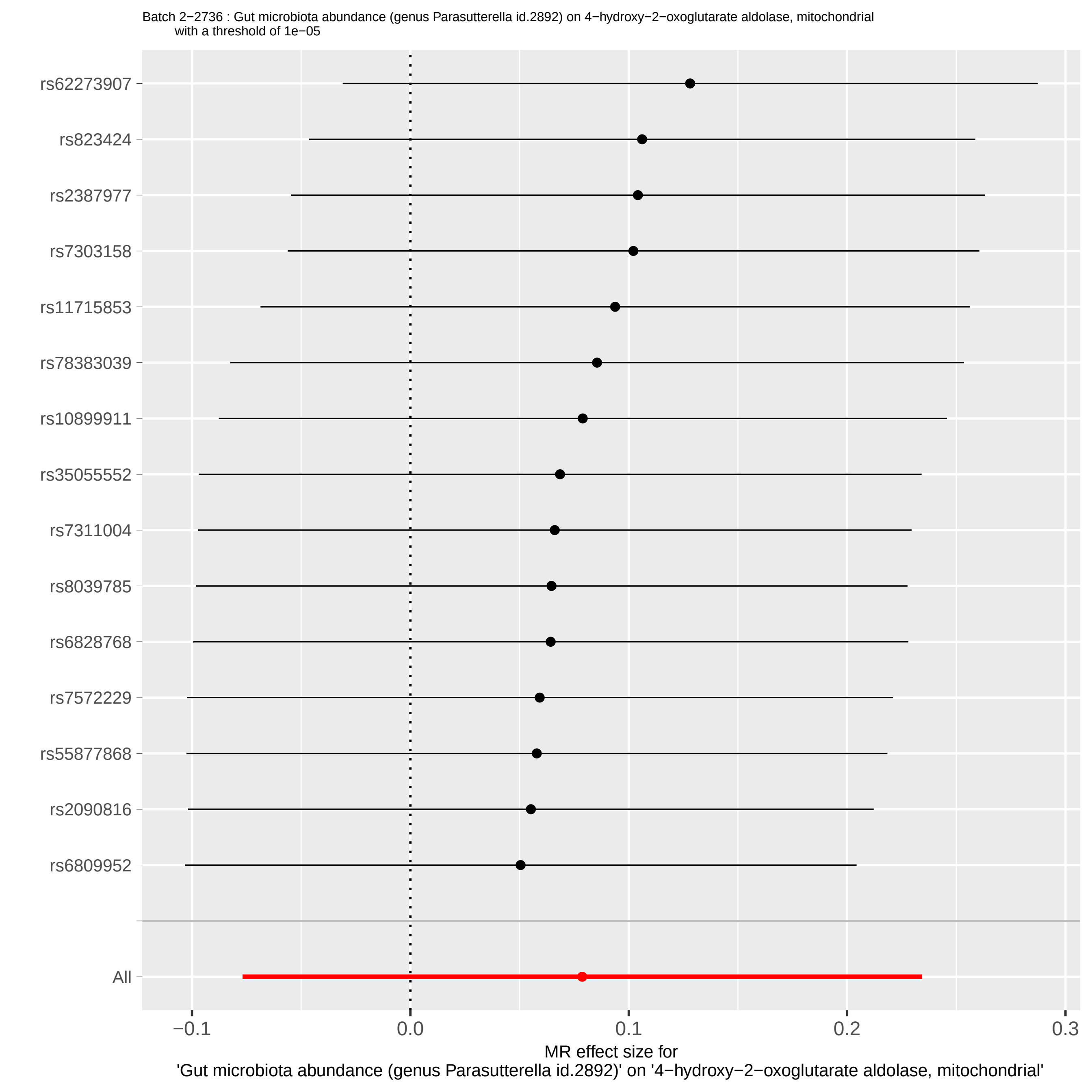
Batch 2−2736 : Gut microbiota abundance (genus Parasutterella id.2892) on 4−hydroxy−2−oxoglutarate aldolase, mitochondrial
with a threshold of 1e−05
rs62273907
rs823424
rs2387977
rs7303158
rs11715853
rs78383039
rs10899911
rs35055552
rs7311004
rs8039785
rs6828768
rs7572229
rs55877868
rs2090816
rs6809952
All
−0.1
0.0
0.1
0.2
0.3
MR effect size for
'Gut microbiota abundance (genus Parasutterella id.2892)' on '4−hydroxy−2−oxoglutarate aldolase, mitochondrial'

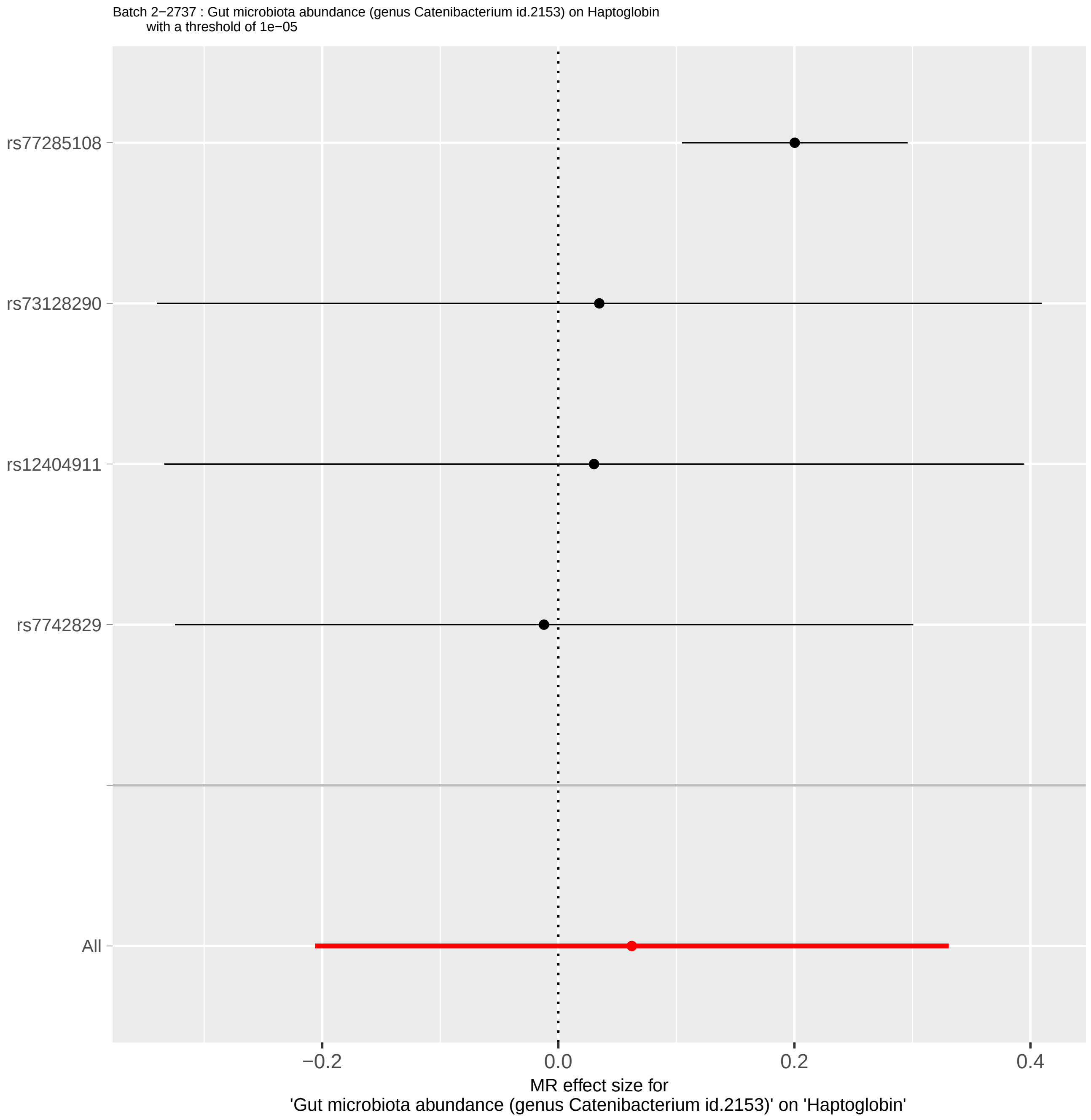
Batch 2−2737 : Gut microbiota abundance (genus Catenibacterium id.2153) on Haptoglobin
with a threshold of 1e−05
rs77285108
rs73128290
rs12404911
rs7742829
All
−0.2
0.0
0.2
0.4
MR effect size for
'Gut microbiota abundance (genus Catenibacterium id.2153)' on 'Haptoglobin'

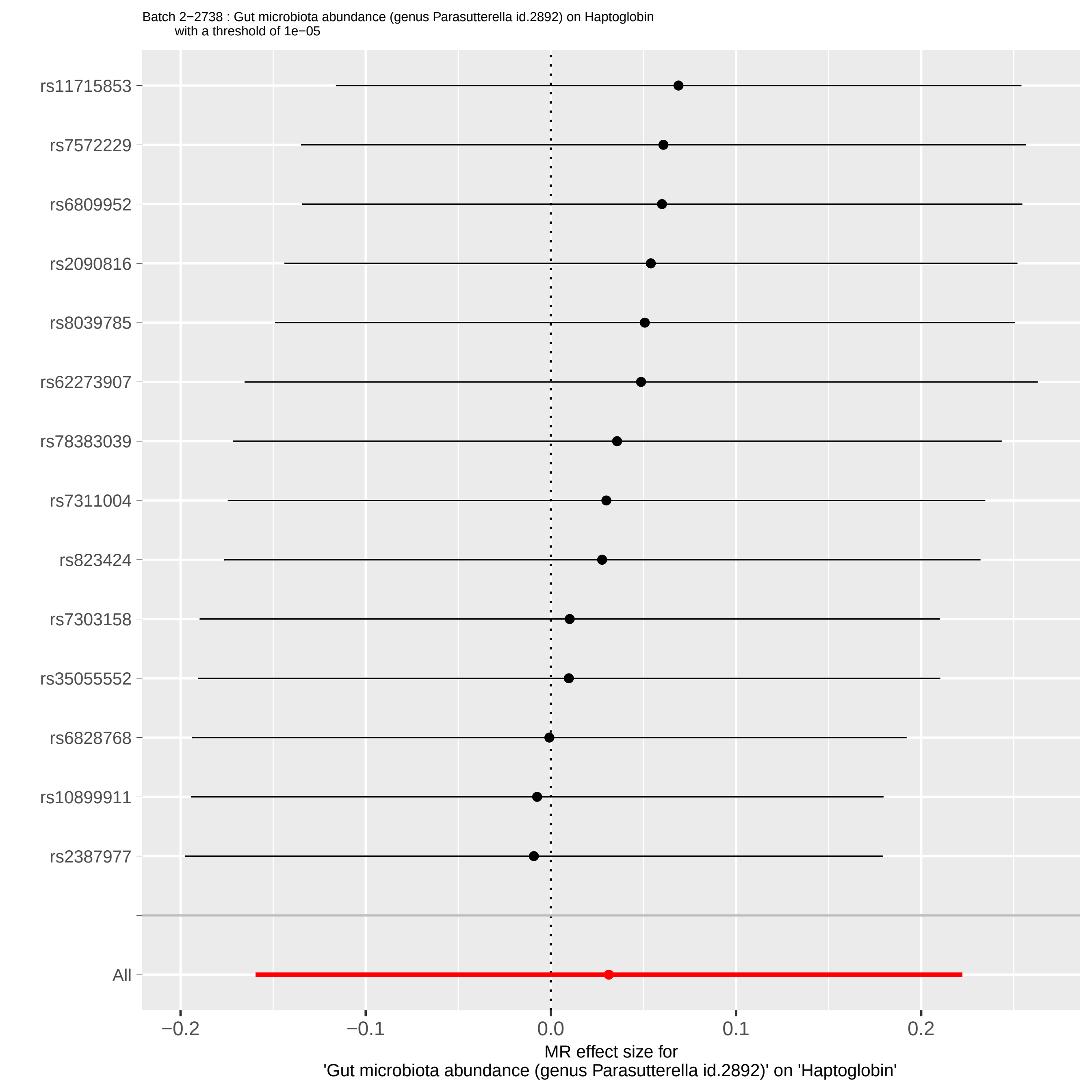

Batch 2−2738 : Gut microbiota abundance (genus Parasutterella id.2892) on Haptoglobin
with a threshold of 1e−05
rs11715853
rs7572229
rs6809952
rs2090816
rs8039785
rs62273907
rs78383039
rs7311004
rs823424
rs7303158
rs35055552
rs6828768
rs10899911
rs2387977
All
−0.2
−0.1
0.0
0.1
0.2
MR effect size for
'Gut microbiota abundance (genus Parasutterella id.2892)' on 'Haptoglobin'

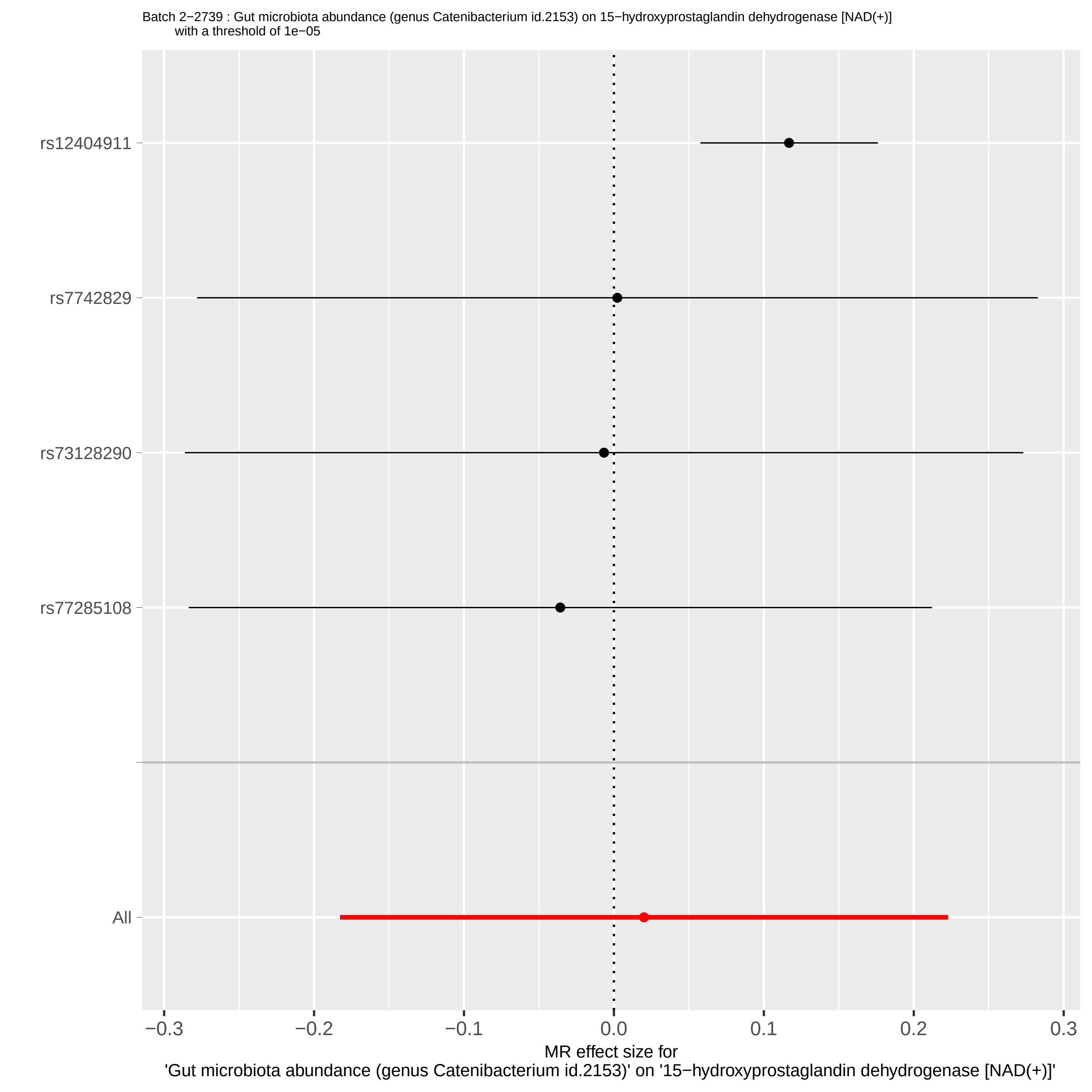

Batch 2−2739 : Gut microbiota abundance (genus Catenibacterium id.2153) on 15−hydroxyprostaglandin dehydrogenase [NAD(+)]
with a threshold of 1e−05
rs12404911
rs7742829
rs73128290
rs77285108
All
−0.3
−0.2
−0.1
0.0
0.1
0.2
0.3
MR effect size for
'Gut microbiota abundance (genus Catenibacterium id.2153)' on '15−hydroxyprostaglandin dehydrogenase [NAD(+)]'

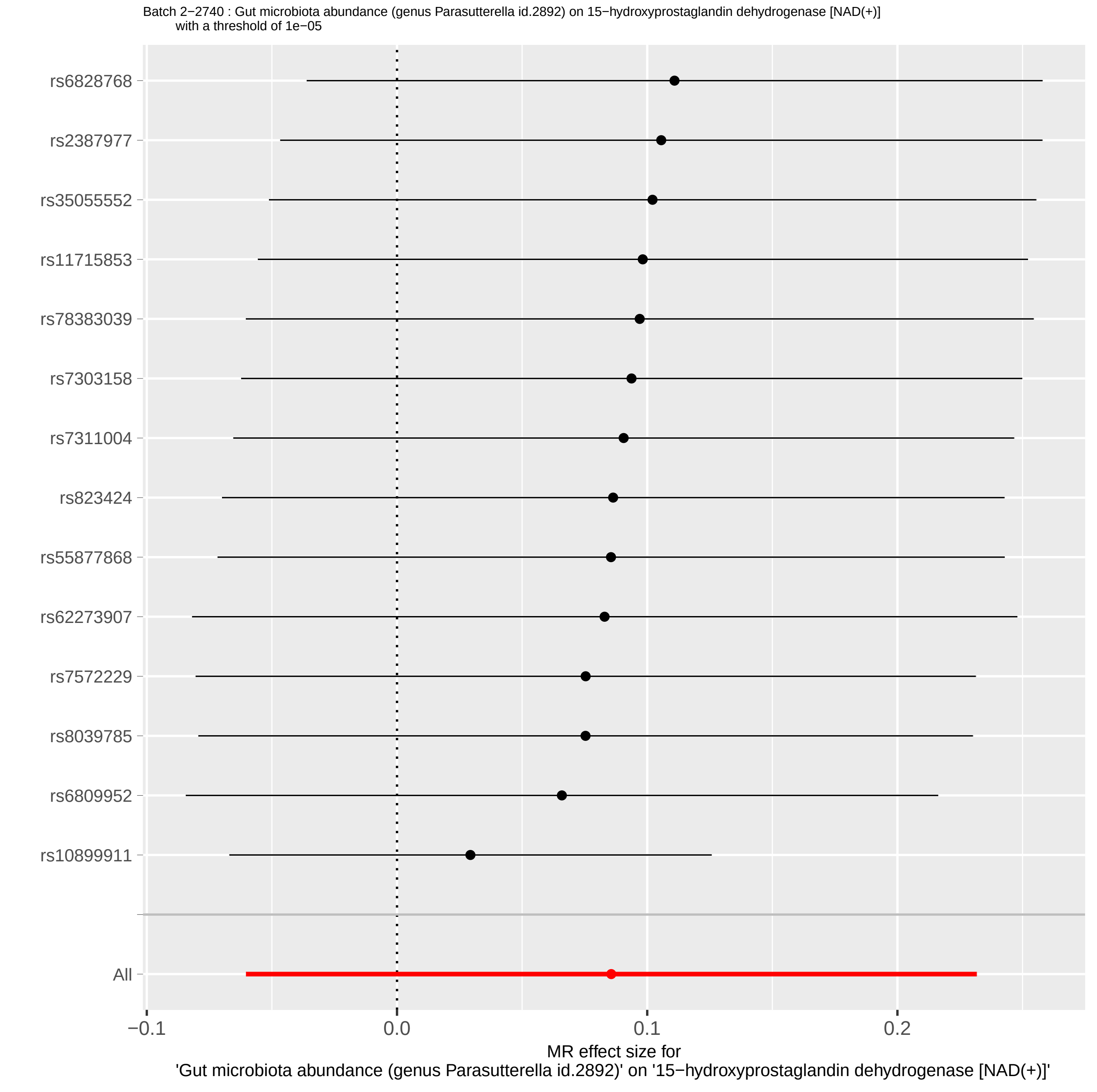
Batch 2−2740 : Gut microbiota abundance (genus Parasutterella id.2892) on 15−hydroxyprostaglandin dehydrogenase [NAD(+)]
with a threshold of 1e−05
rs6828768
rs2387977
rs35055552
rs11715853
rs78383039
rs7303158
rs7311004
rs823424
rs55877868
rs62273907
rs7572229
rs8039785
rs6809952
rs10899911
All
−0.1
0.0
0.1
0.2
MR effect size for
'Gut microbiota abundance (genus Parasutterella id.2892)' on '15−hydroxyprostaglandin dehydrogenase [NAD(+)]'

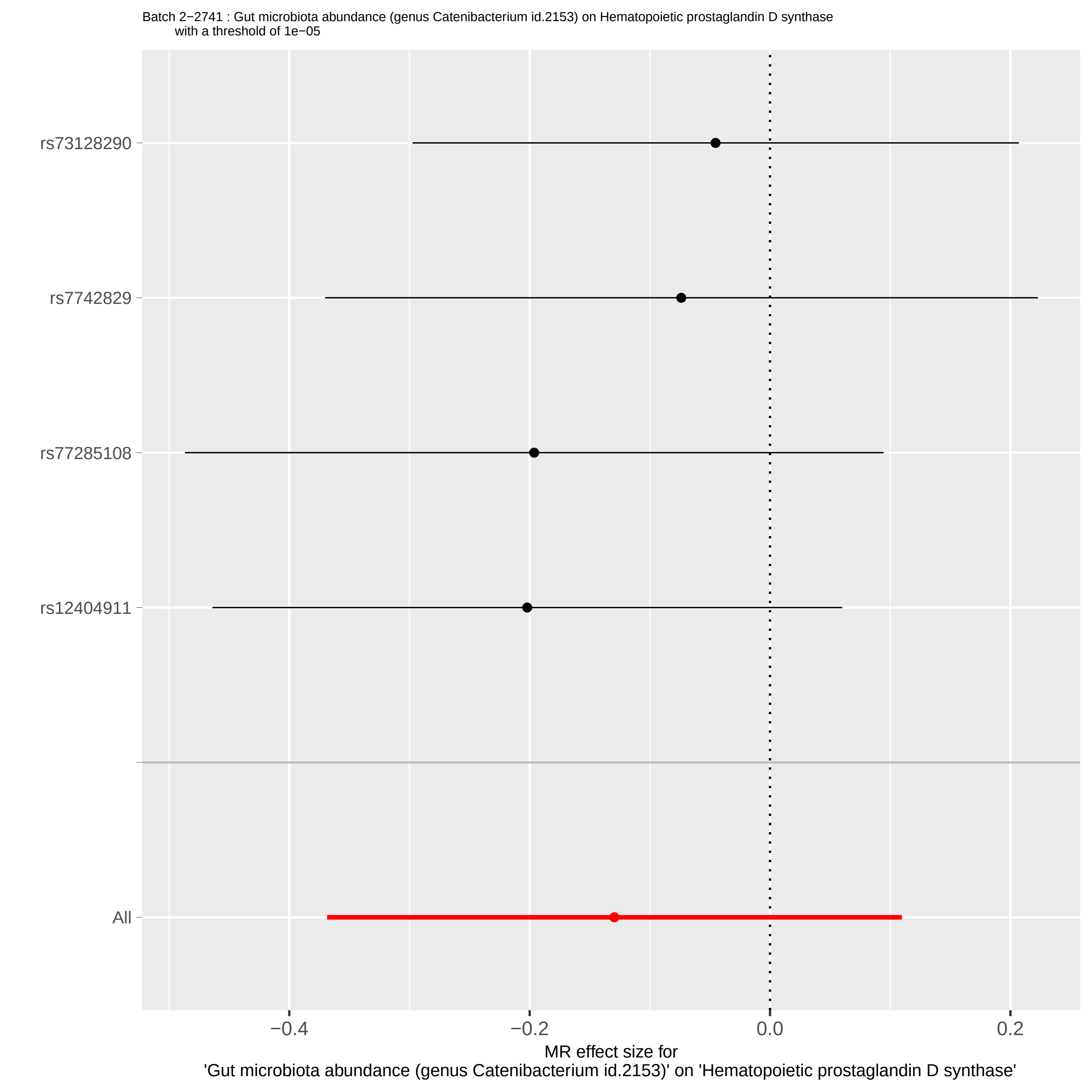

Batch 2−2741 : Gut microbiota abundance (genus Catenibacterium id.2153) on Hematopoietic prostaglandin D synthase
with a threshold of 1e−05
rs73128290
rs7742829
rs77285108
rs12404911
All
−0.4
−0.2
0.0
0.2
MR effect size for
'Gut microbiota abundance (genus Catenibacterium id.2153)' on 'Hematopoietic prostaglandin D synthase'

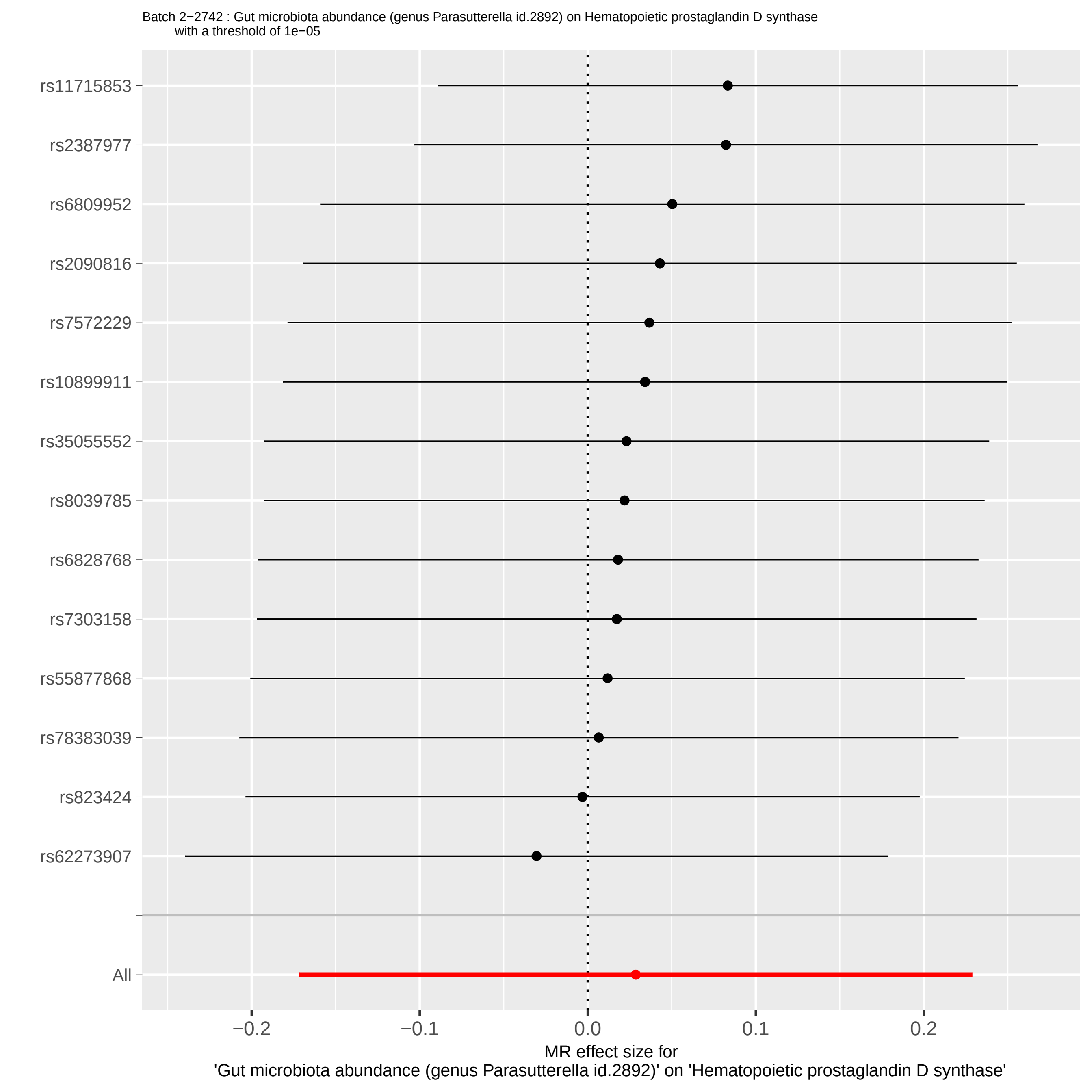

Batch 2−2742 : Gut microbiota abundance (genus Parasutterella id.2892) on Hematopoietic prostaglandin D synthase
with a threshold of 1e−05
rs11715853
rs2387977
rs6809952
rs2090816
rs7572229
rs10899911
rs35055552
rs8039785
rs6828768
rs7303158
rs55877868
rs78383039
rs823424
rs62273907
All
−0.2
−0.1
0.0
0.1
0.2
MR effect size for
'Gut microbiota abundance (genus Parasutterella id.2892)' on 'Hematopoietic prostaglandin D synthase'

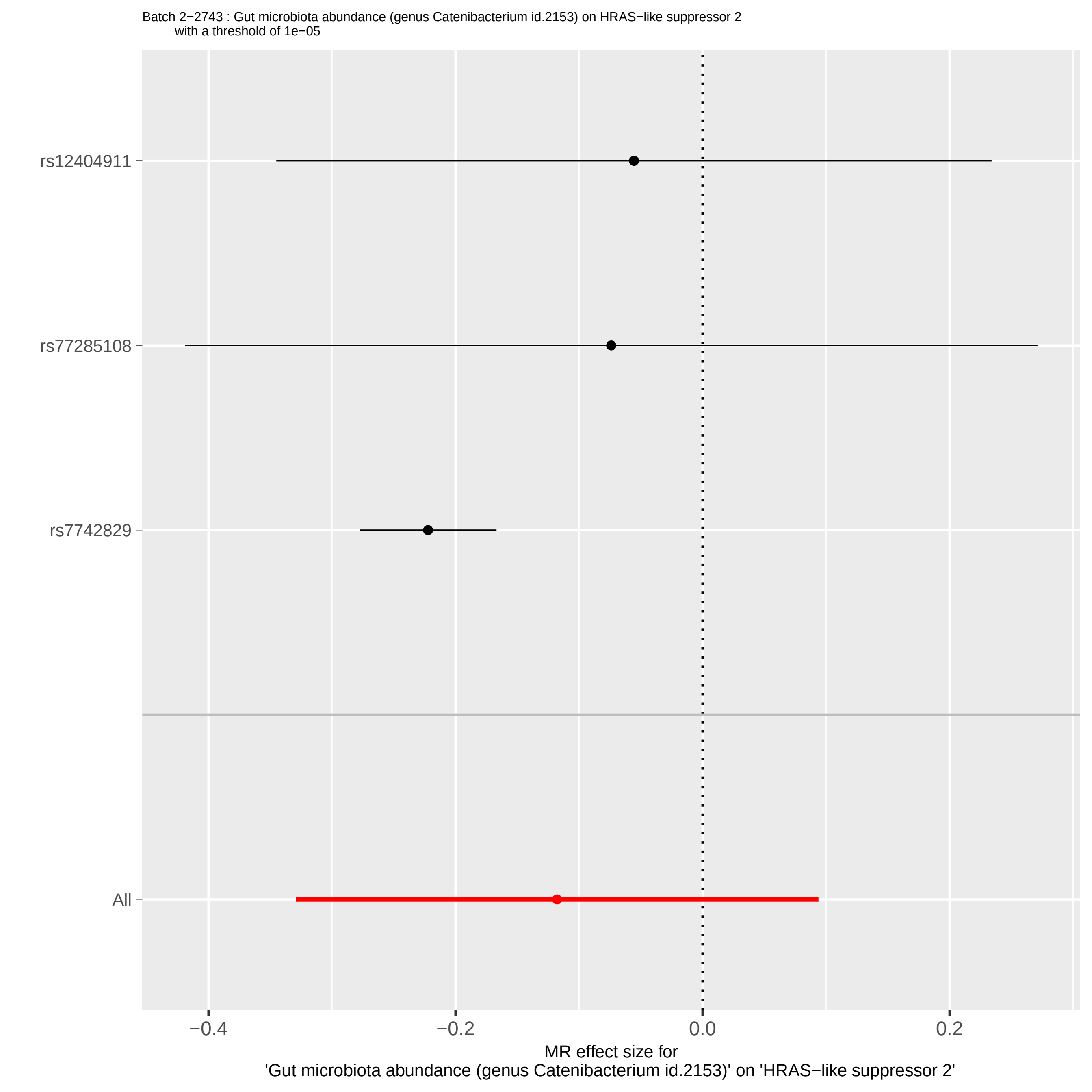
Batch 2−2743 : Gut microbiota abundance (genus Catenibacterium id.2153) on HRAS−like suppressor 2
with a threshold of 1e−05
rs12404911
rs77285108
rs7742829
All
−0.4
−0.2
0.0
0.2
MR effect size for
'Gut microbiota abundance (genus Catenibacterium id.2153)' on 'HRAS−like suppressor 2'

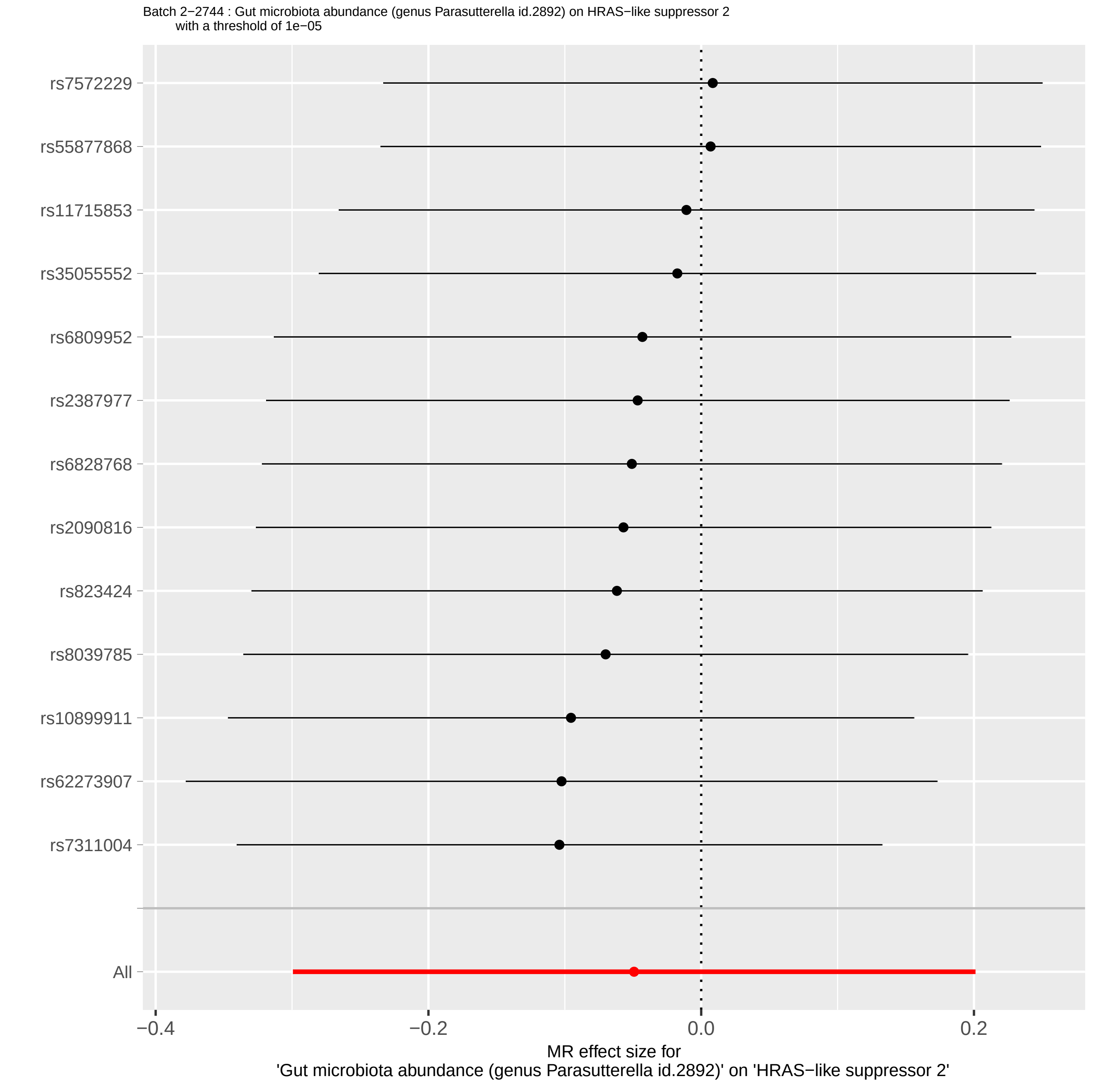
Batch 2−2744 : Gut microbiota abundance (genus Parasutterella id.2892) on HRAS−like suppressor 2
with a threshold of 1e−05
rs7572229
rs55877868
rs11715853
rs35055552
rs6809952
rs2387977
rs6828768
rs2090816
rs823424
rs8039785
rs10899911
rs62273907
rs7311004
All
−0.4
−0.2
0.0
0.2
MR effect size for
'Gut microbiota abundance (genus Parasutterella id.2892)' on 'HRAS−like suppressor 2'

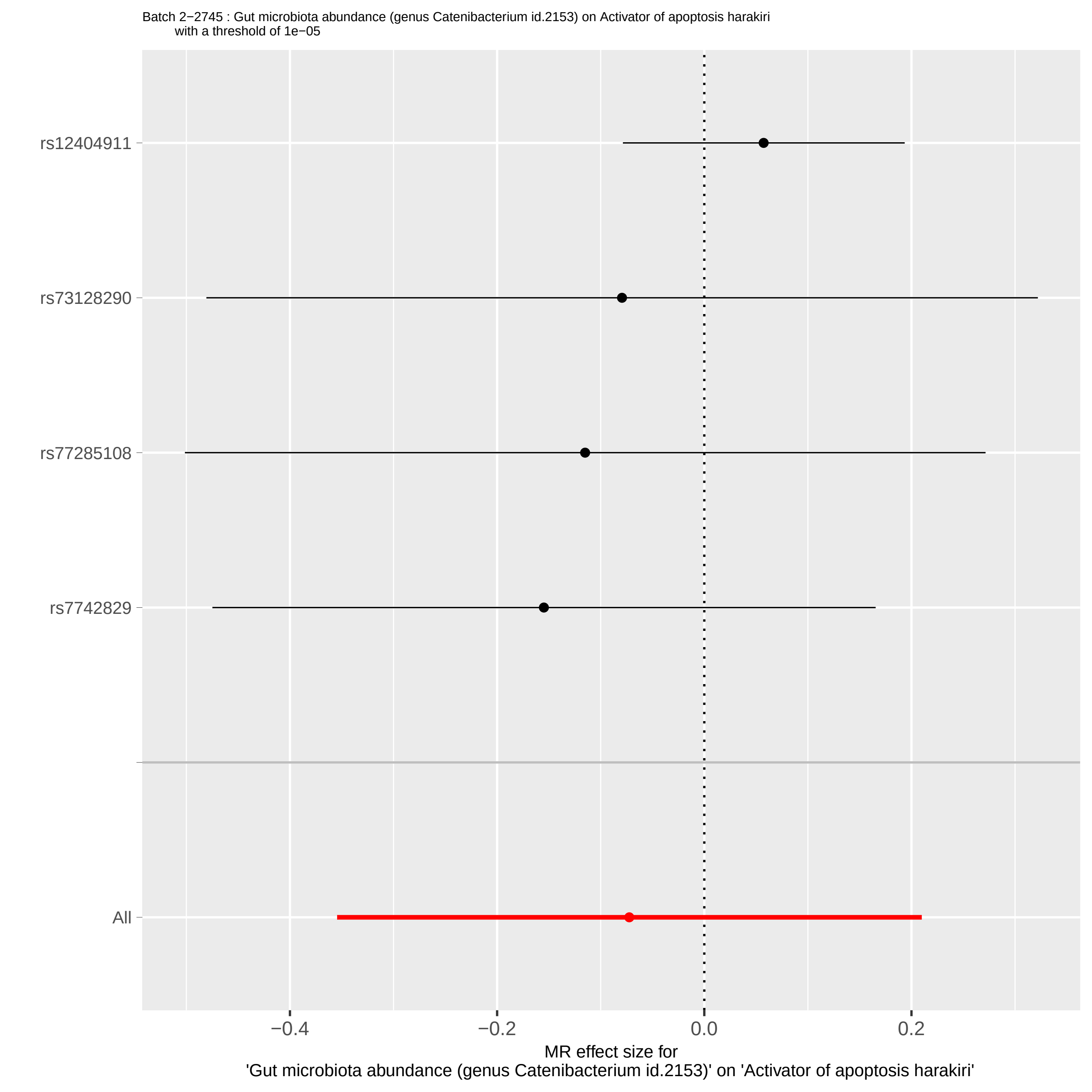
Batch 2−2745 : Gut microbiota abundance (genus Catenibacterium id.2153) on Activator of apoptosis harakiri
with a threshold of 1e−05
rs12404911
rs73128290
rs77285108
rs7742829
All
−0.4
−0.2
0.0
0.2
MR effect size for
'Gut microbiota abundance (genus Catenibacterium id.2153)' on 'Activator of apoptosis harakiri'

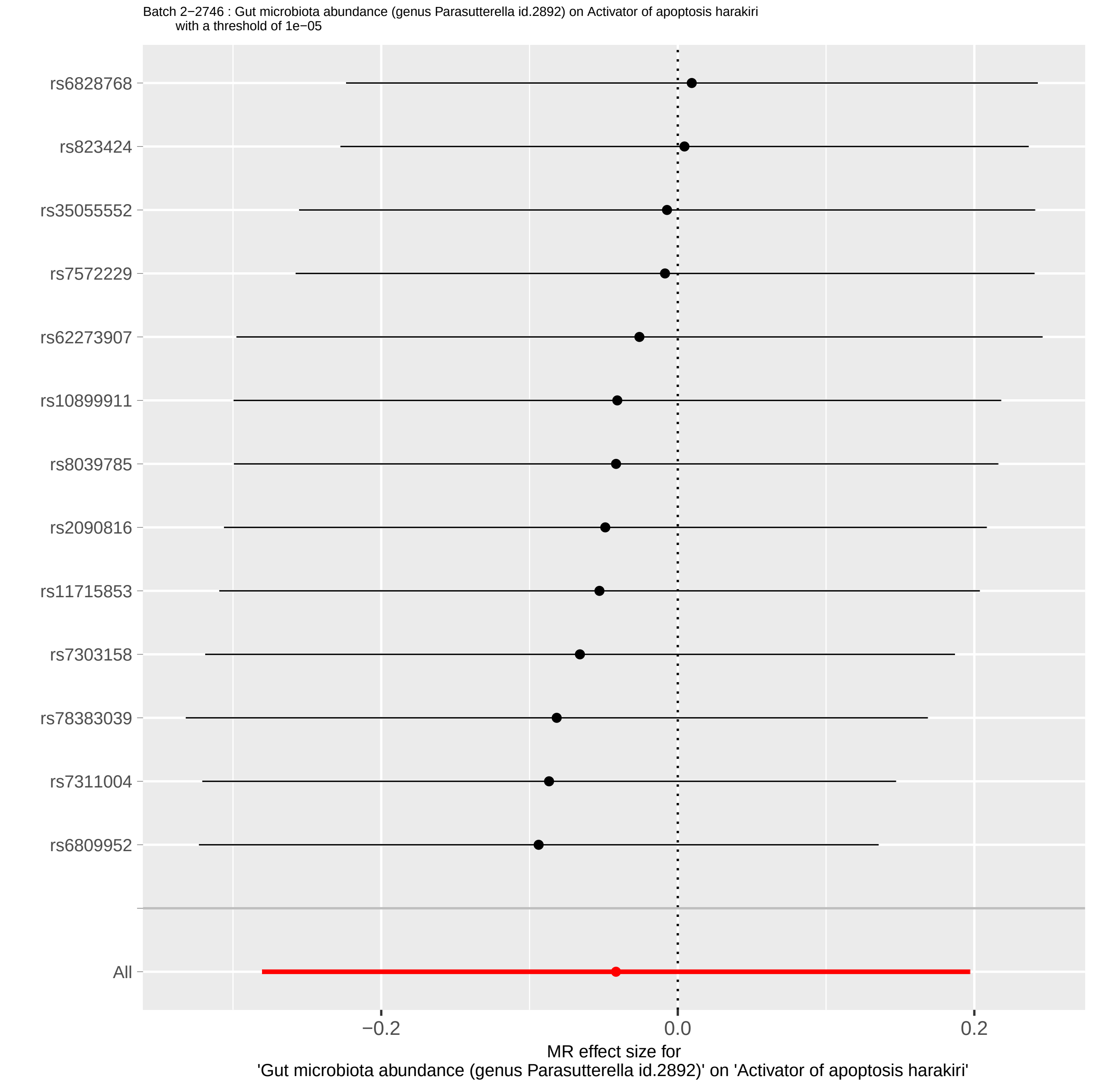
Batch 2−2746 : Gut microbiota abundance (genus Parasutterella id.2892) on Activator of apoptosis harakiri
with a threshold of 1e−05
rs6828768
rs823424
rs35055552
rs7572229
rs62273907
rs10899911
rs8039785
rs2090816
rs11715853
rs7303158
rs78383039
rs7311004
rs6809952
All
−0.2
0.0
0.2
MR effect size for
'Gut microbiota abundance (genus Parasutterella id.2892)' on 'Activator of apoptosis harakiri'

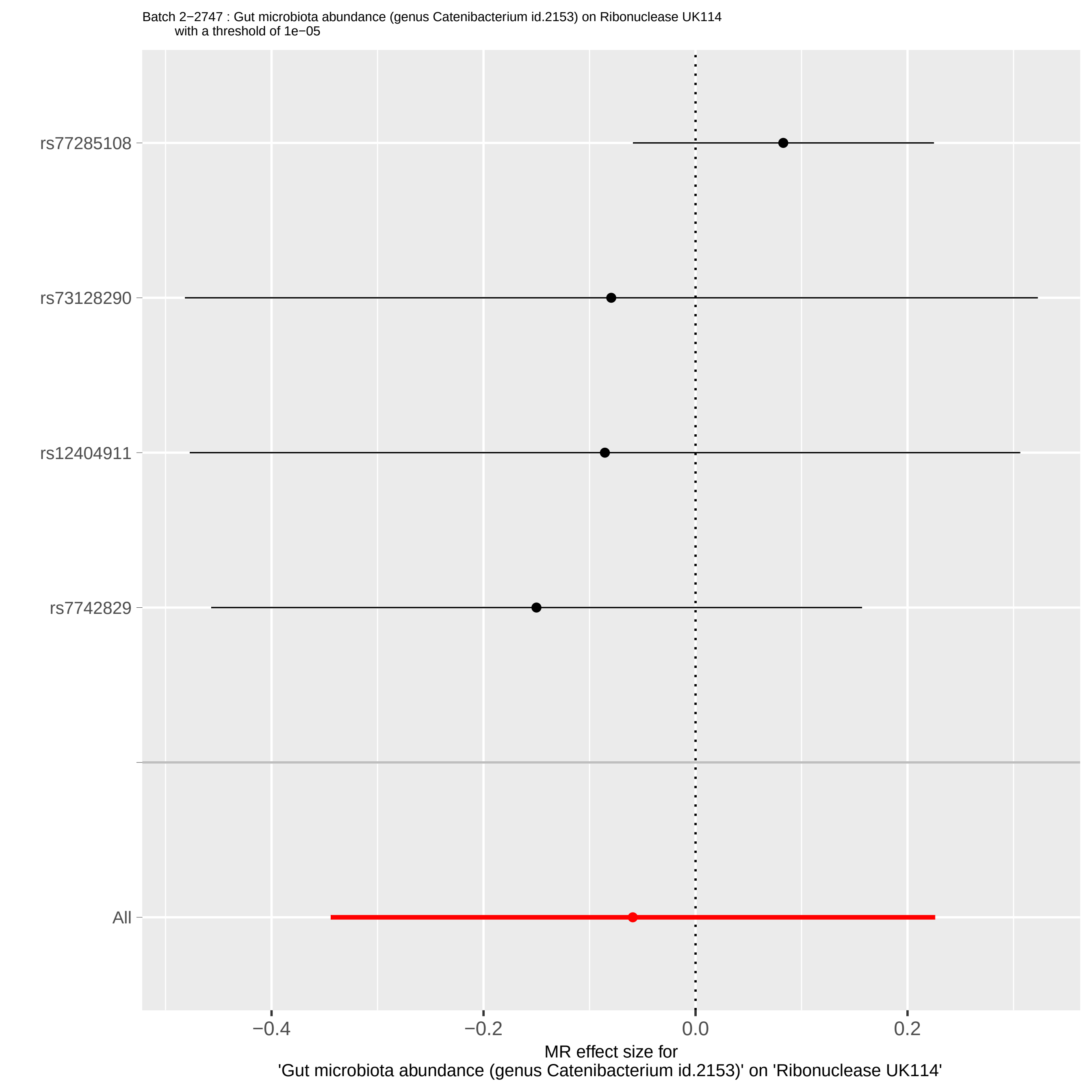
Batch 2−2747 : Gut microbiota abundance (genus Catenibacterium id.2153) on Ribonuclease UK114
with a threshold of 1e−05
rs77285108
rs73128290
rs12404911
rs7742829
All
−0.4
−0.2
0.0
0.2
MR effect size for
'Gut microbiota abundance (genus Catenibacterium id.2153)' on 'Ribonuclease UK114'

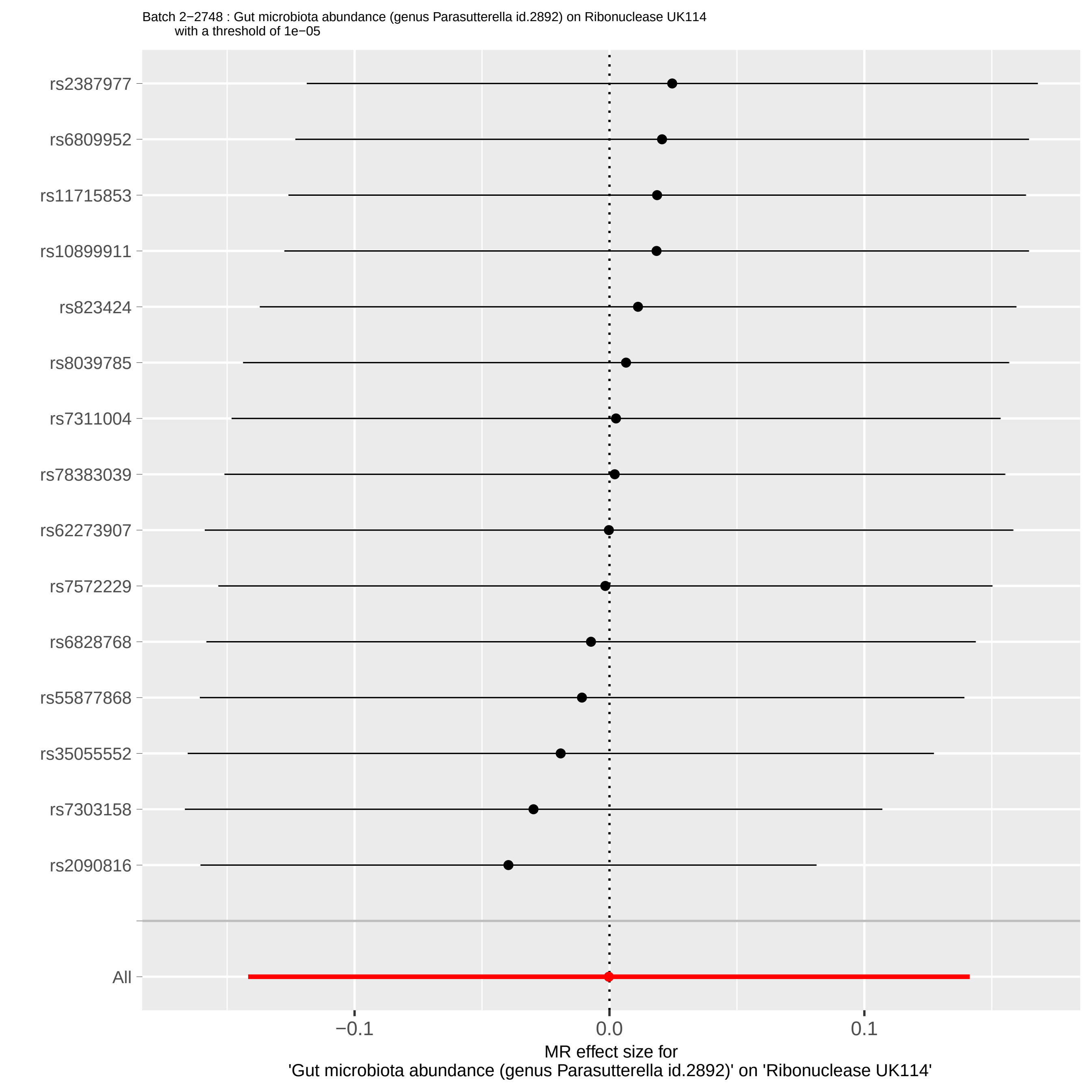
Batch 2−2748 : Gut microbiota abundance (genus Parasutterella id.2892) on Ribonuclease UK114
with a threshold of 1e−05
rs2387977
rs6809952
rs11715853
rs10899911
rs823424
rs8039785
rs7311004
rs78383039
rs62273907
rs7572229
rs6828768
rs55877868
rs35055552
rs7303158
rs2090816
All
−0.1
0.0
0.1
MR effect size for
'Gut microbiota abundance (genus Parasutterella id.2892)' on 'Ribonuclease UK114'

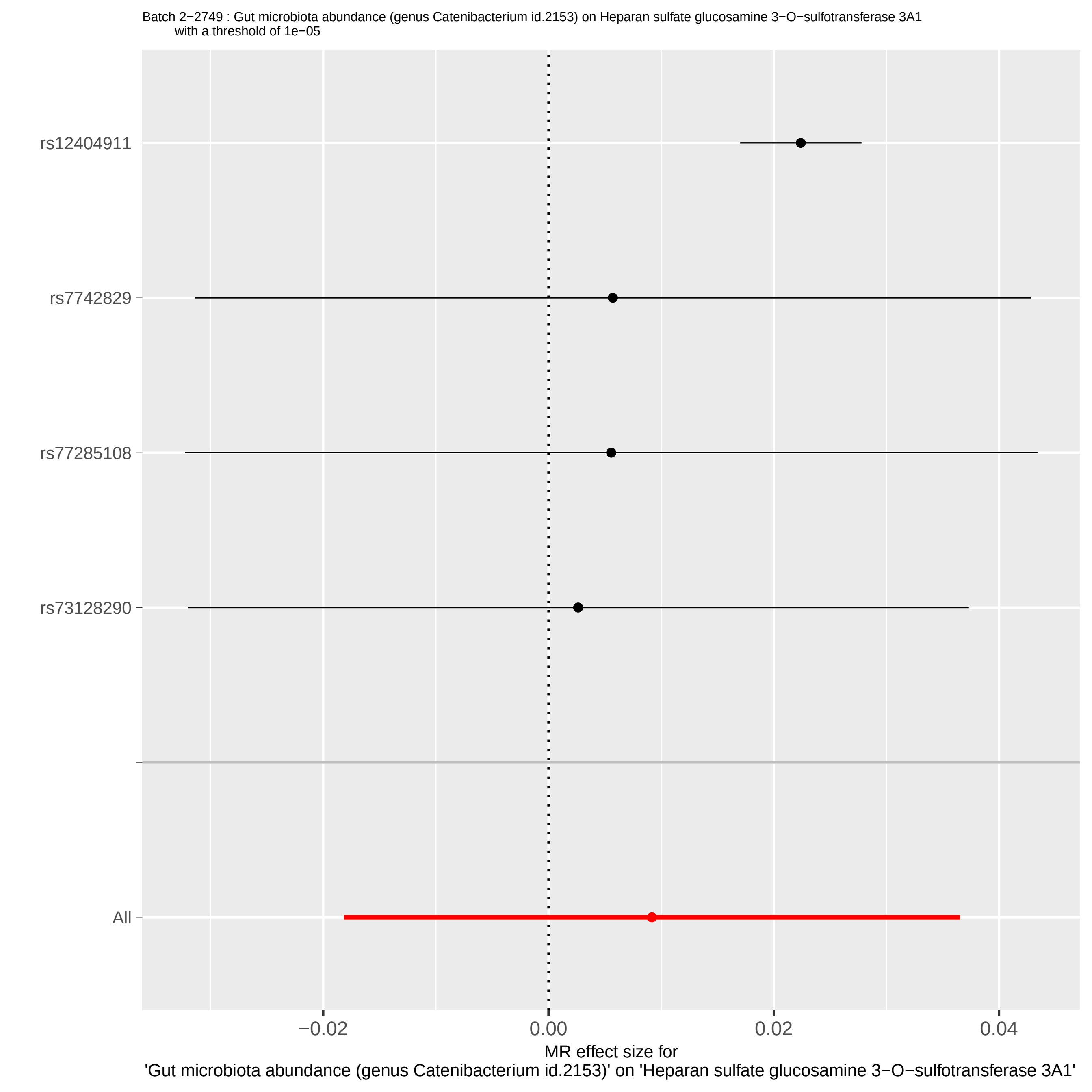
Batch 2−2749 : Gut microbiota abundance (genus Catenibacterium id.2153) on Heparan sulfate glucosamine 3−O−sulfotransferase 3A1
with a threshold of 1e−05
rs12404911
rs7742829
rs77285108
rs73128290
All
−0.02
0.00
0.02
0.04
MR effect size for
'Gut microbiota abundance (genus Catenibacterium id.2153)' on 'Heparan sulfate glucosamine 3−O−sulfotransferase 3A1'

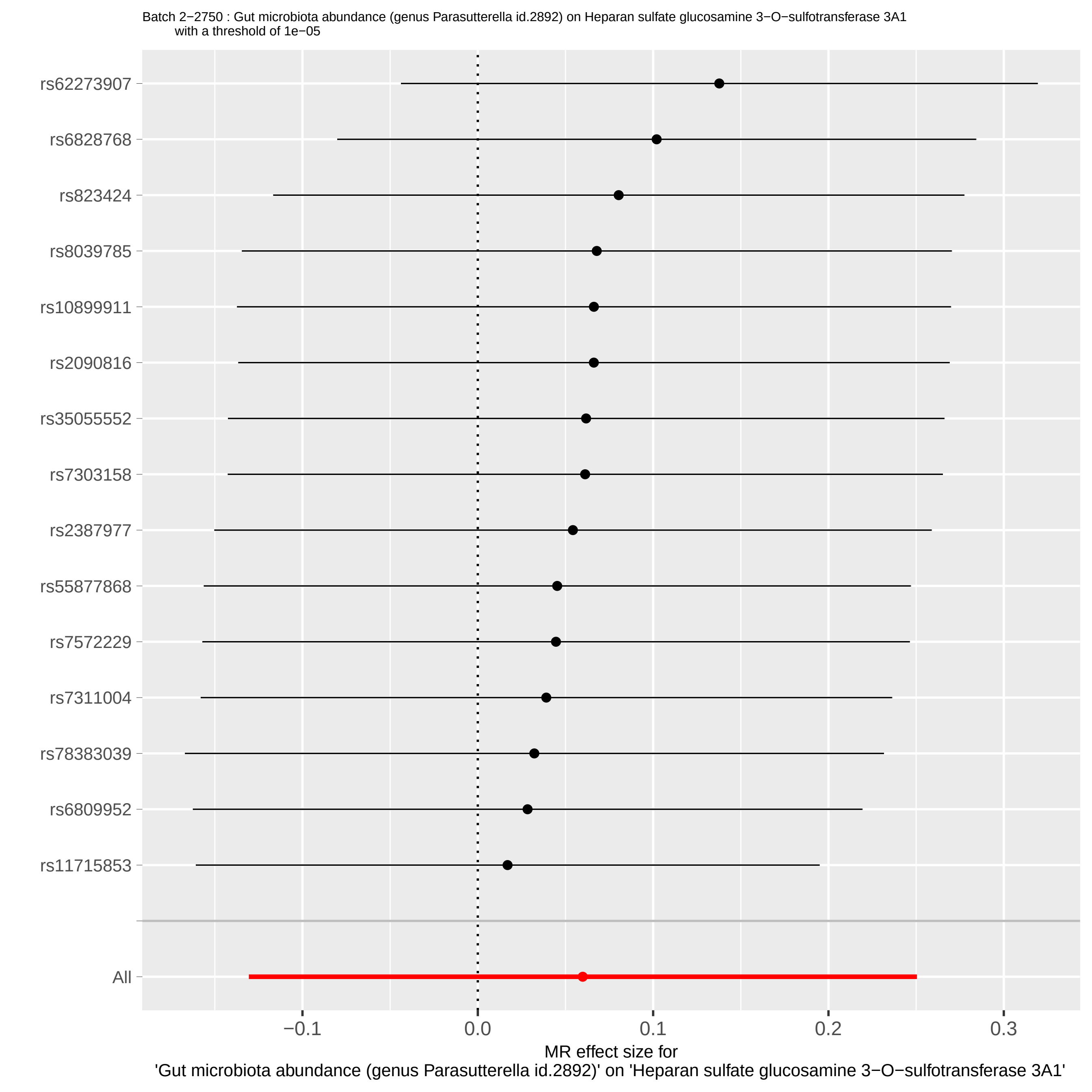

Batch 2−2750 : Gut microbiota abundance (genus Parasutterella id.2892) on Heparan sulfate glucosamine 3−O−sulfotransferase 3A1
with a threshold of 1e−05
rs62273907
rs6828768
rs823424
rs8039785
rs10899911
rs2090816
rs35055552
rs7303158
rs2387977
rs55877868
rs7572229
rs7311004
rs78383039
rs6809952
rs11715853
All
−0.1
0.0
0.1
0.2
0.3
MR effect size for
'Gut microbiota abundance (genus Parasutterella id.2892)' on 'Heparan sulfate glucosamine 3−O−sulfotransferase 3A1'

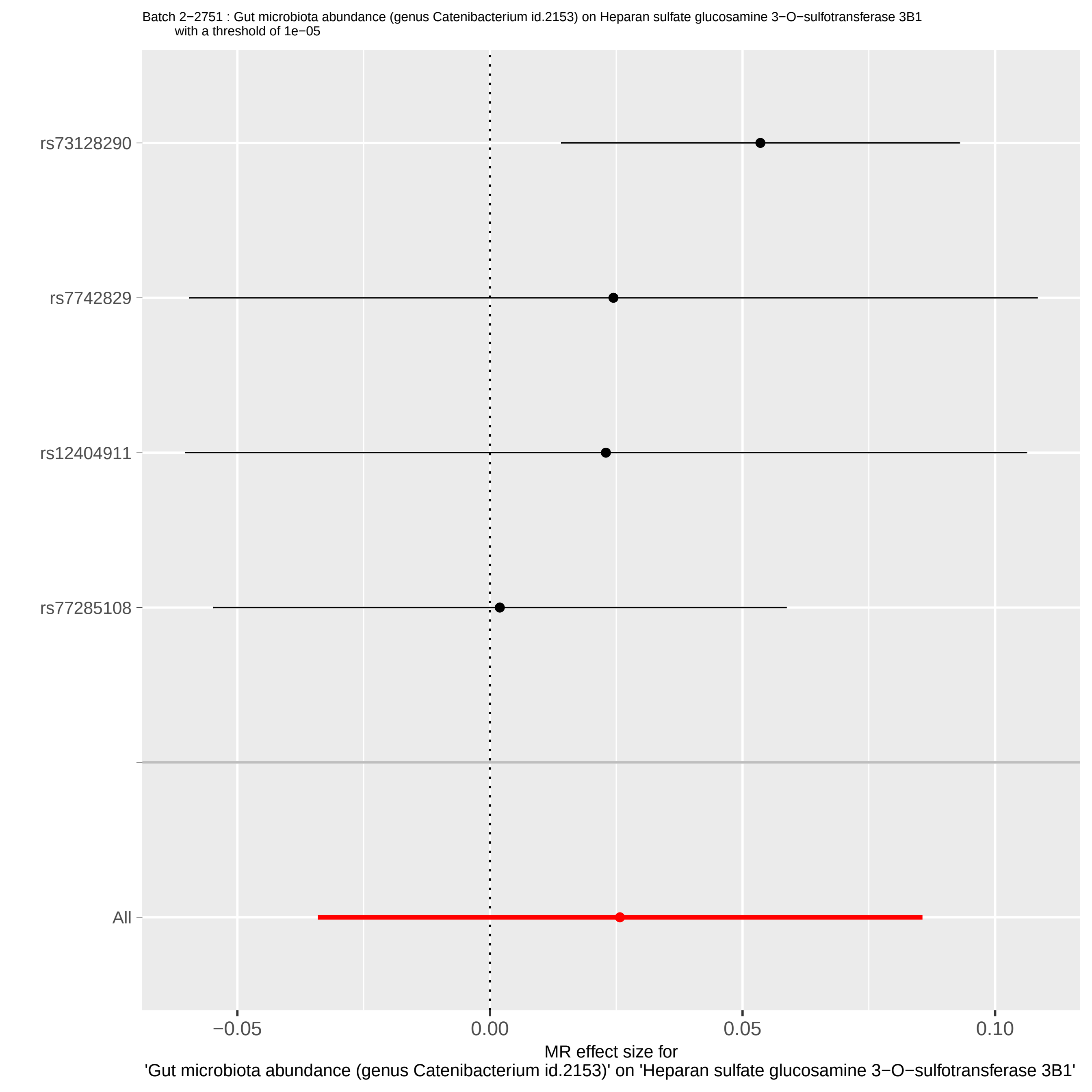
Batch 2−2751 : Gut microbiota abundance (genus Catenibacterium id.2153) on Heparan sulfate glucosamine 3−O−sulfotransferase 3B1
with a threshold of 1e−05
rs73128290
rs7742829
rs12404911
rs77285108
All
−0.05
0.00
0.05
0.10
MR effect size for
'Gut microbiota abundance (genus Catenibacterium id.2153)' on 'Heparan sulfate glucosamine 3−O−sulfotransferase 3B1'

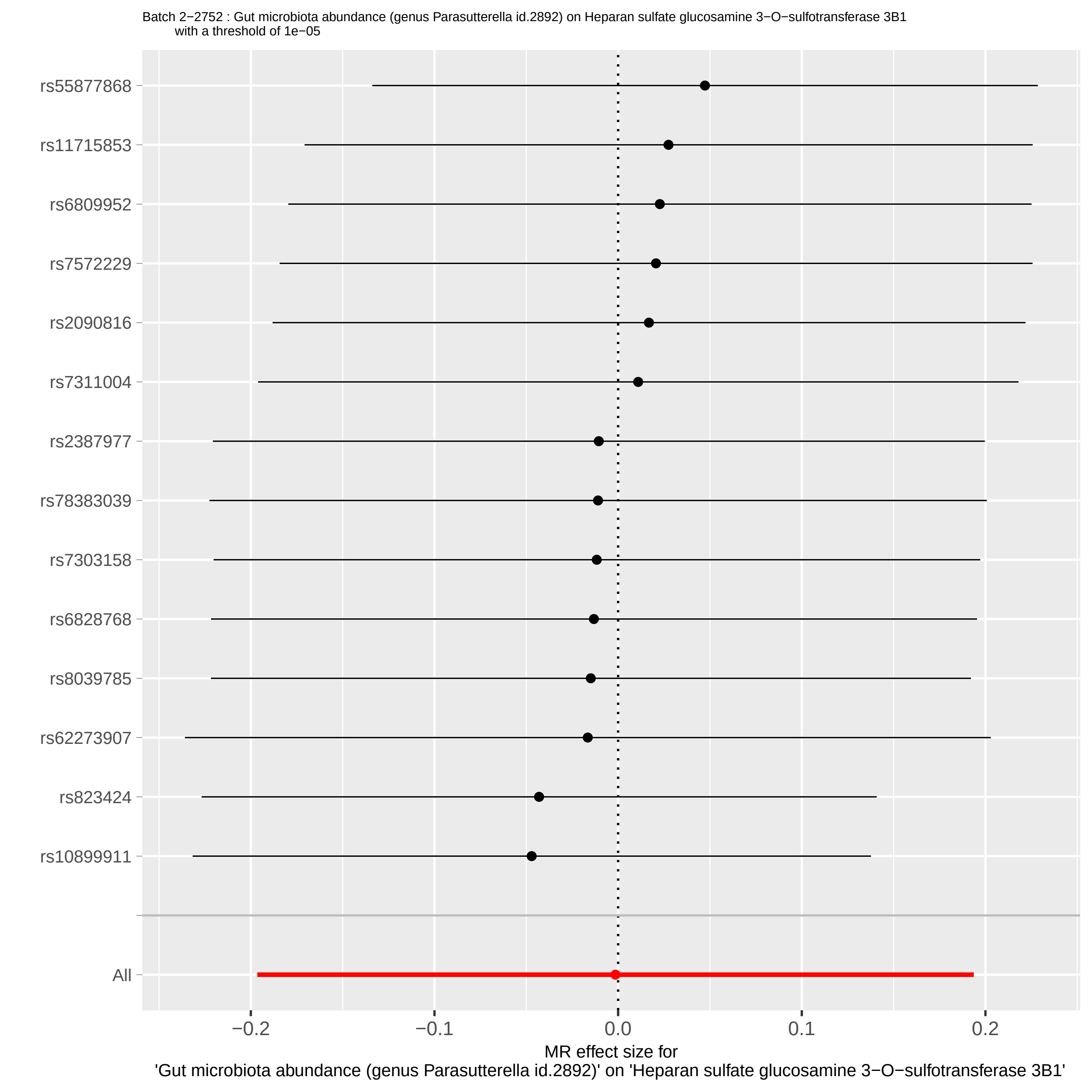
Batch 2−2752 : Gut microbiota abundance (genus Parasutterella id.2892) on Heparan sulfate glucosamine 3−O−sulfotransferase 3B1
with a threshold of 1e−05
rs55877868
rs11715853
rs6809952
rs7572229
rs2090816
rs7311004
rs2387977
rs78383039
rs7303158
rs6828768
rs8039785
rs62273907
rs823424
rs10899911
All
−0.2
−0.1
0.0
0.1
0.2
MR effect size for
'Gut microbiota abundance (genus Parasutterella id.2892)' on 'Heparan sulfate glucosamine 3−O−sulfotransferase 3B1'

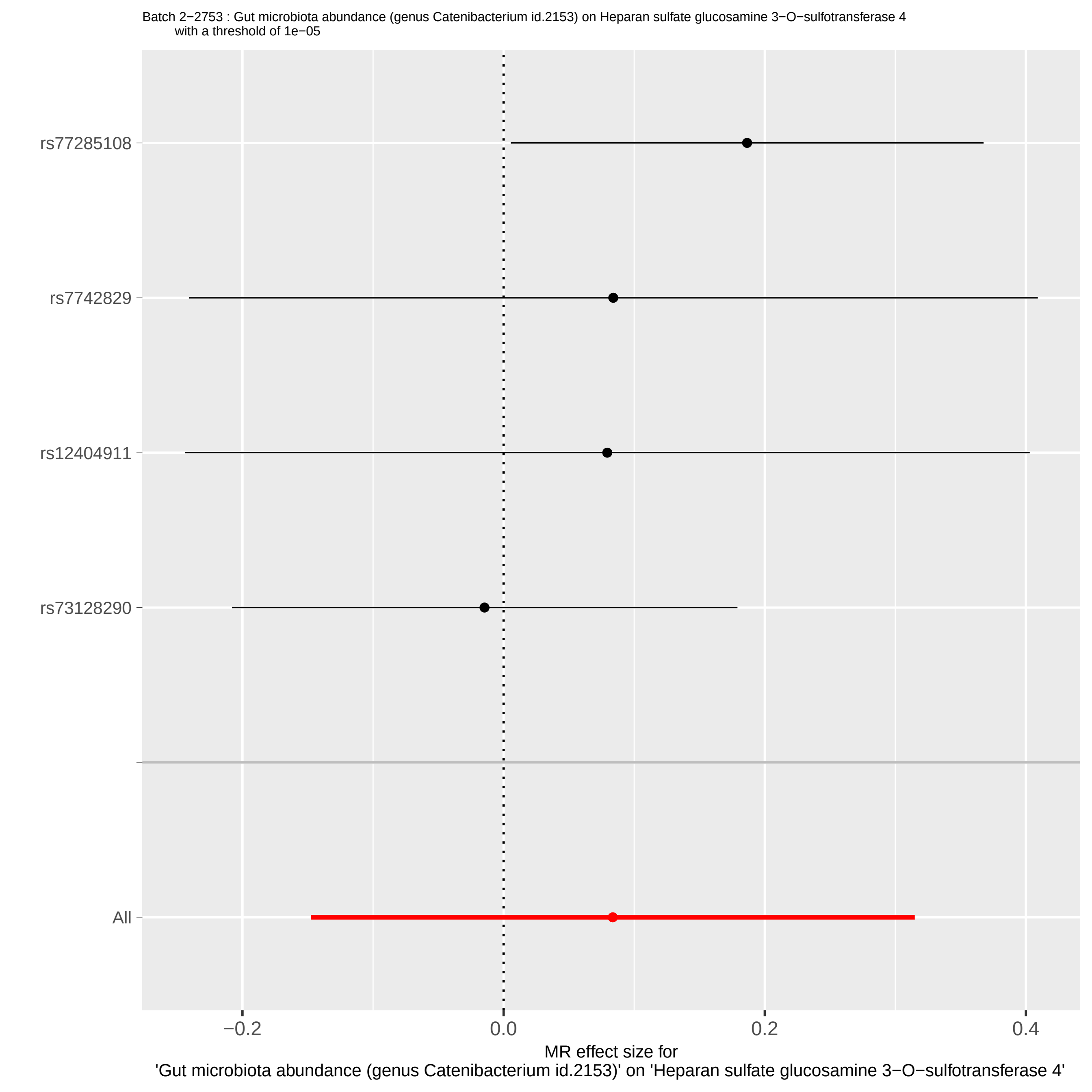

Batch 2−2753 : Gut microbiota abundance (genus Catenibacterium id.2153) on Heparan sulfate glucosamine 3−O−sulfotransferase 4
with a threshold of 1e−05
rs77285108
rs7742829
rs12404911
rs73128290
All
−0.2
0.0
0.2
0.4
MR effect size for
'Gut microbiota abundance (genus Catenibacterium id.2153)' on 'Heparan sulfate glucosamine 3−O−sulfotransferase 4'

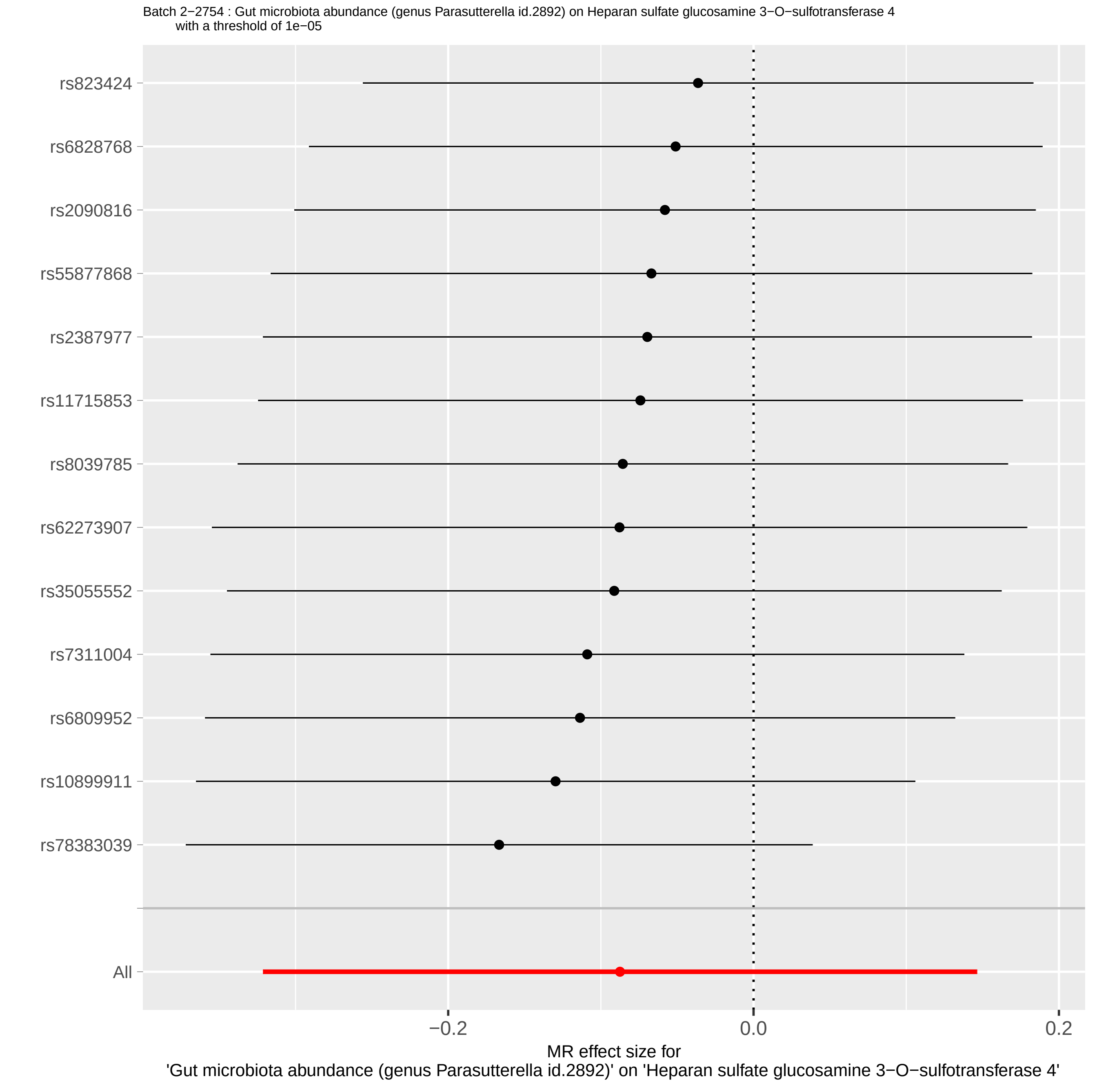
Batch 2−2754 : Gut microbiota abundance (genus Parasutterella id.2892) on Heparan sulfate glucosamine 3−O−sulfotransferase 4
with a threshold of 1e−05
rs823424
rs6828768
rs2090816
rs55877868
rs2387977
rs11715853
rs8039785
rs62273907
rs35055552
rs7311004
rs6809952
rs10899911
rs78383039
All
−0.2
0.0
0.2
MR effect size for
'Gut microbiota abundance (genus Parasutterella id.2892)' on 'Heparan sulfate glucosamine 3−O−sulfotransferase 4'

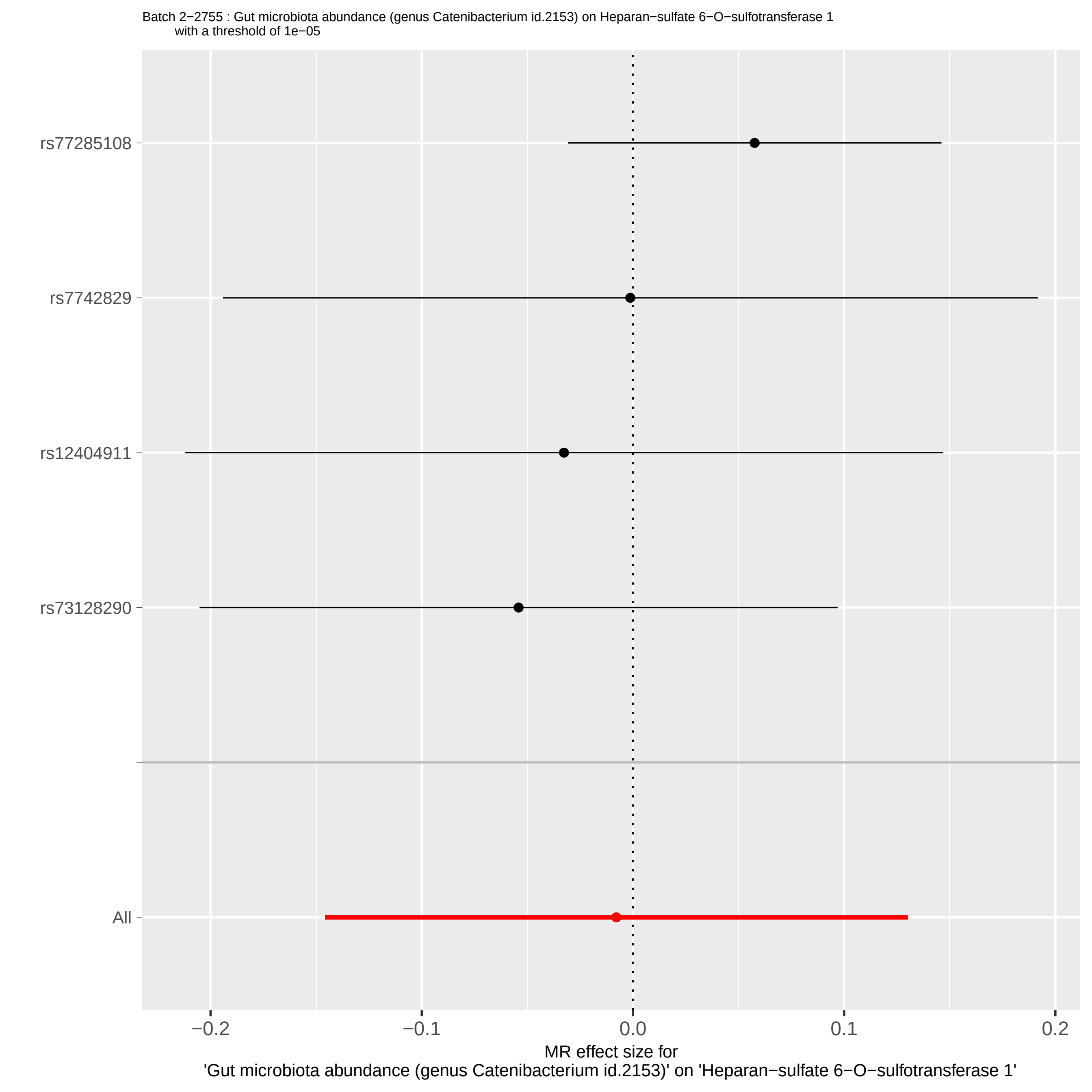

Batch 2−2755 : Gut microbiota abundance (genus Catenibacterium id.2153) on Heparan−sulfate 6−O−sulfotransferase 1
with a threshold of 1e−05
rs77285108
rs7742829
rs12404911
rs73128290
All
−0.2
−0.1
0.0
0.1
0.2
MR effect size for
'Gut microbiota abundance (genus Catenibacterium id.2153)' on 'Heparan−sulfate 6−O−sulfotransferase 1'

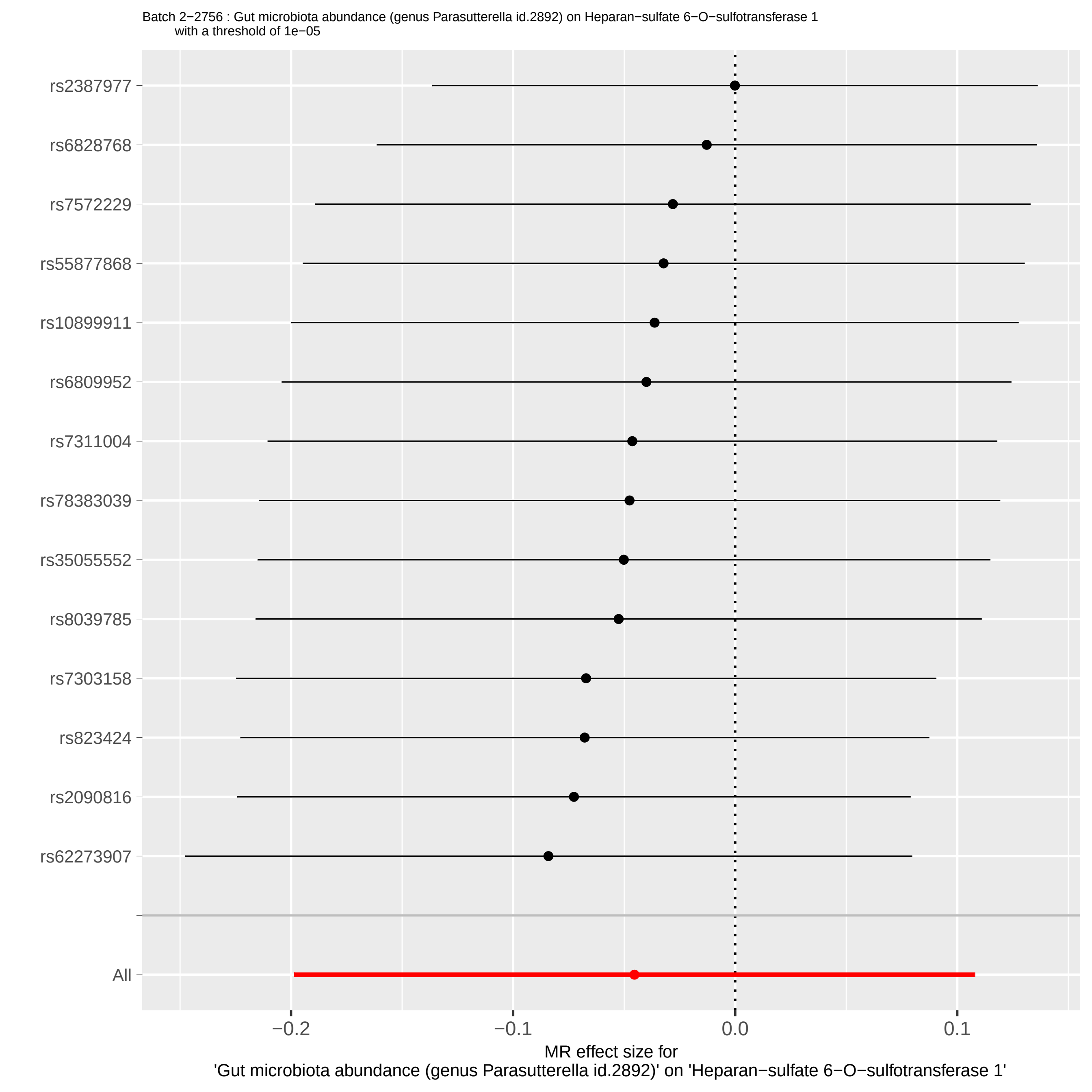
Batch 2−2756 : Gut microbiota abundance (genus Parasutterella id.2892) on Heparan−sulfate 6−O−sulfotransferase 1
with a threshold of 1e−05
rs2387977
rs6828768
rs7572229
rs55877868
rs10899911
rs6809952
rs7311004
rs78383039
rs35055552
rs8039785
rs7303158
rs823424
rs2090816
rs62273907
All
−0.2
−0.1
0.0
0.1
MR effect size for
'Gut microbiota abundance (genus Parasutterella id.2892)' on 'Heparan−sulfate 6−O−sulfotransferase 1'

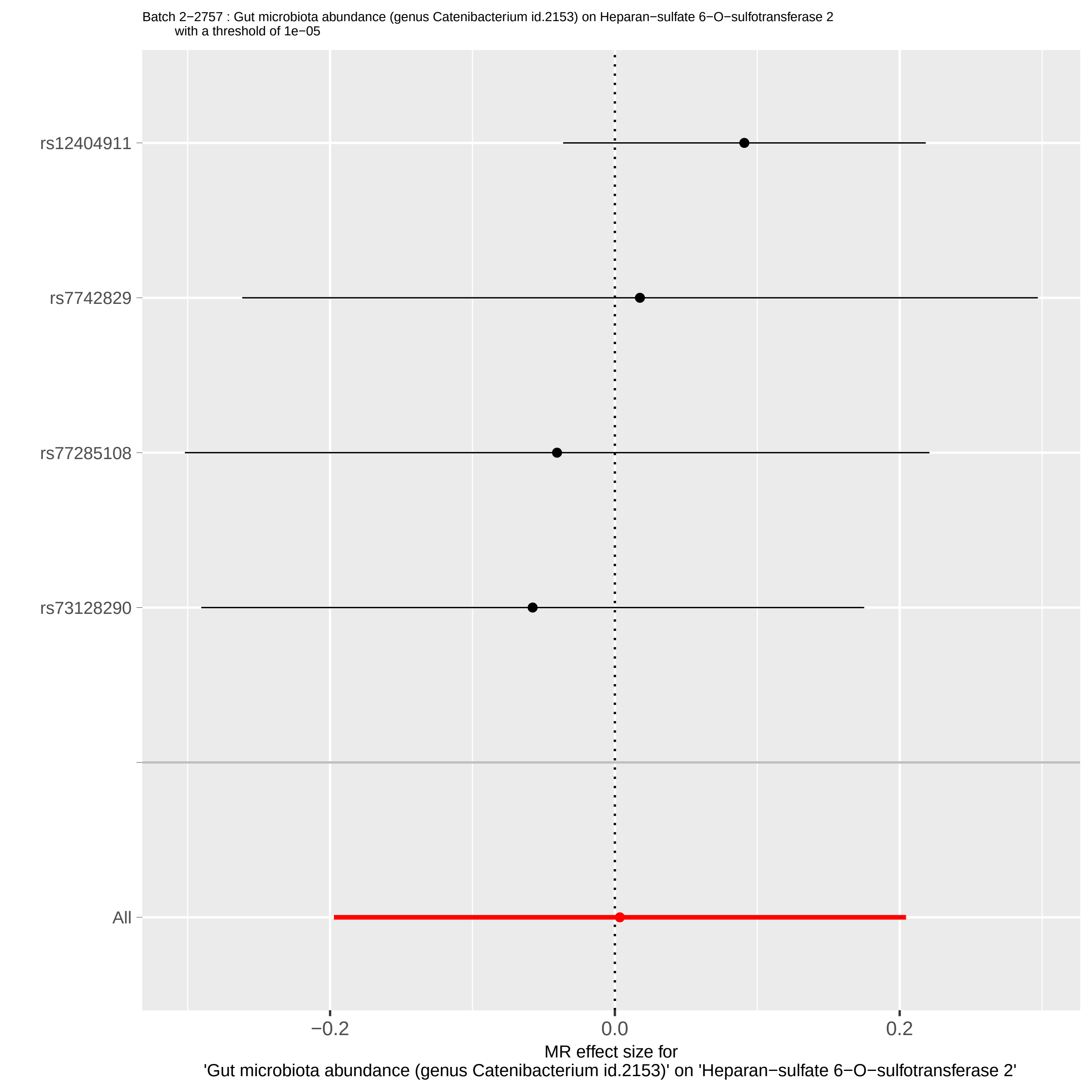

Batch 2−2757 : Gut microbiota abundance (genus Catenibacterium id.2153) on Heparan−sulfate 6−O−sulfotransferase 2
with a threshold of 1e−05
rs12404911
rs7742829
rs77285108
rs73128290
All
−0.2
0.0
0.2
MR effect size for
'Gut microbiota abundance (genus Catenibacterium id.2153)' on 'Heparan−sulfate 6−O−sulfotransferase 2'

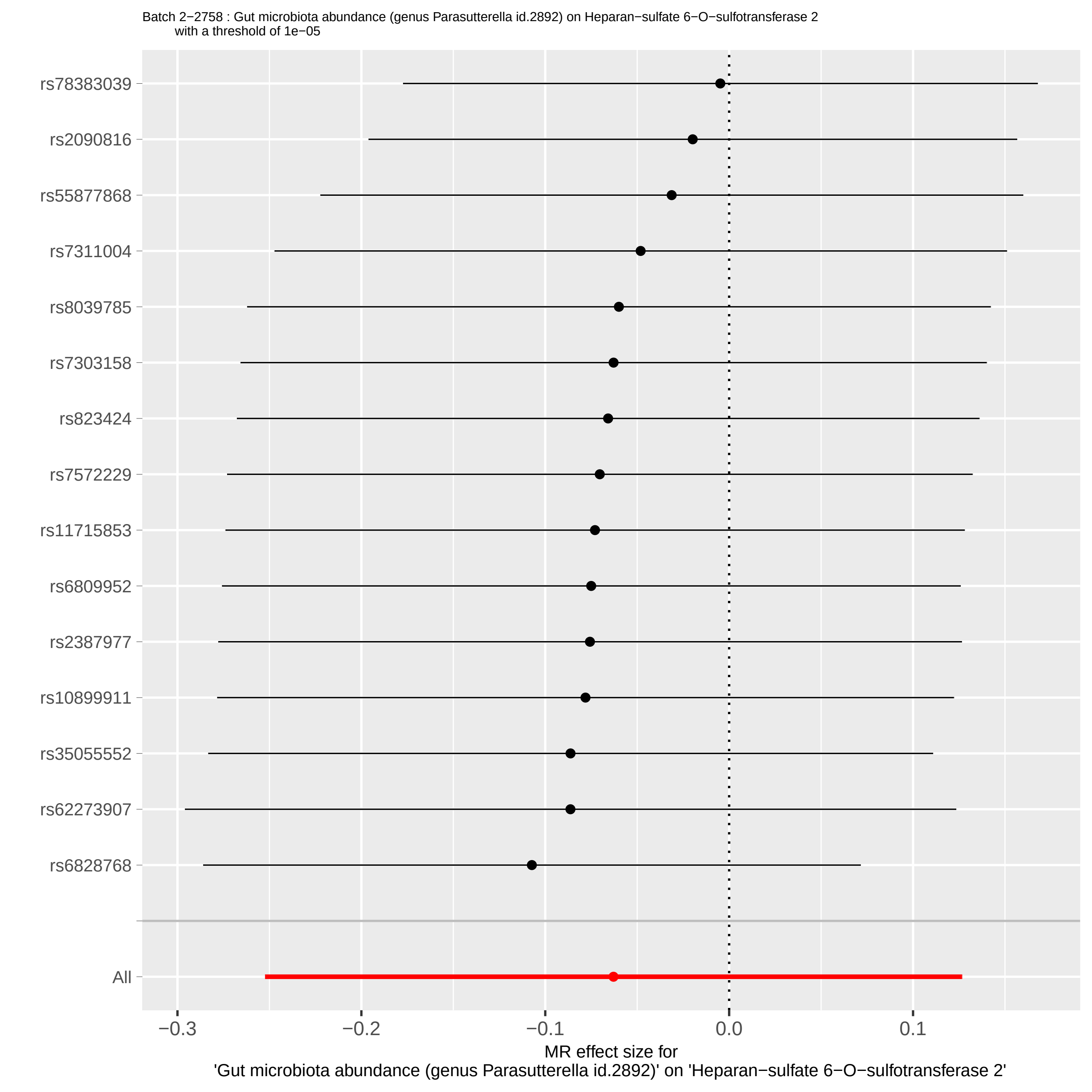
Batch 2−2758 : Gut microbiota abundance (genus Parasutterella id.2892) on Heparan−sulfate 6−O−sulfotransferase 2
with a threshold of 1e−05
rs78383039
rs2090816
rs55877868
rs7311004
rs8039785
rs7303158
rs823424
rs7572229
rs11715853
rs6809952
rs2387977
rs10899911
rs35055552
rs62273907
rs6828768
All
−0.3
−0.2
−0.1
0.0
0.1
MR effect size for
'Gut microbiota abundance (genus Parasutterella id.2892)' on 'Heparan−sulfate 6−O−sulfotransferase 2'

Batch 2−2759 : Gut microbiota abundance (genus Catenibacterium id.2153) on 3−hydroxyacyl−CoA dehydrogenase type−2
with a threshold of 1e−05

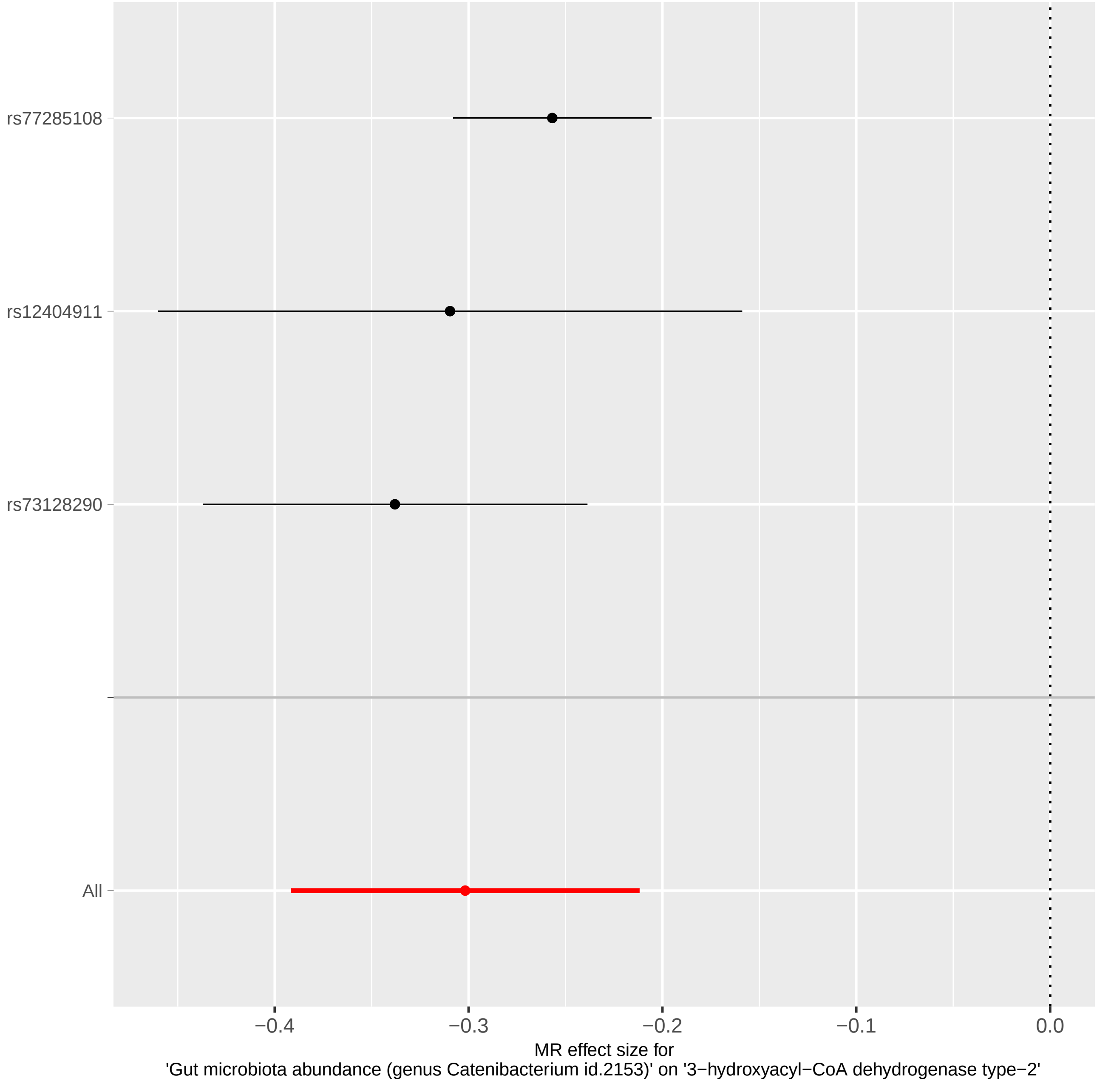

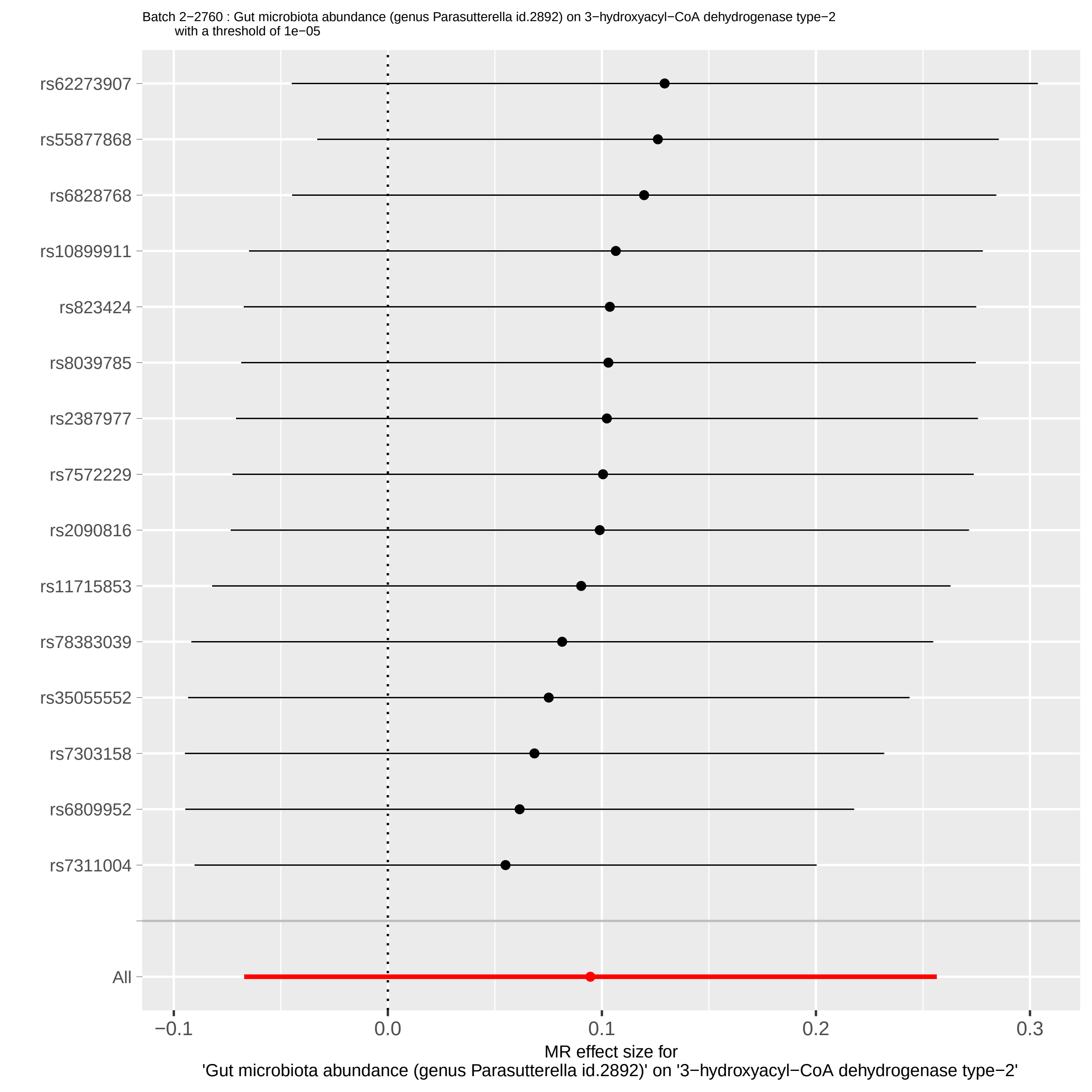

Batch 2−2760 : Gut microbiota abundance (genus Parasutterella id.2892) on 3−hydroxyacyl−CoA dehydrogenase type−2
with a threshold of 1e−05
rs62273907
rs55877868
rs6828768
rs10899911
rs823424
rs8039785
rs2387977
rs7572229
rs2090816
rs11715853
rs78383039
rs35055552
rs7303158
rs6809952
rs7311004
All
−0.1
0.0
0.1
0.2
0.3
MR effect size for
'Gut microbiota abundance (genus Parasutterella id.2892)' on '3−hydroxyacyl−CoA dehydrogenase type−2'

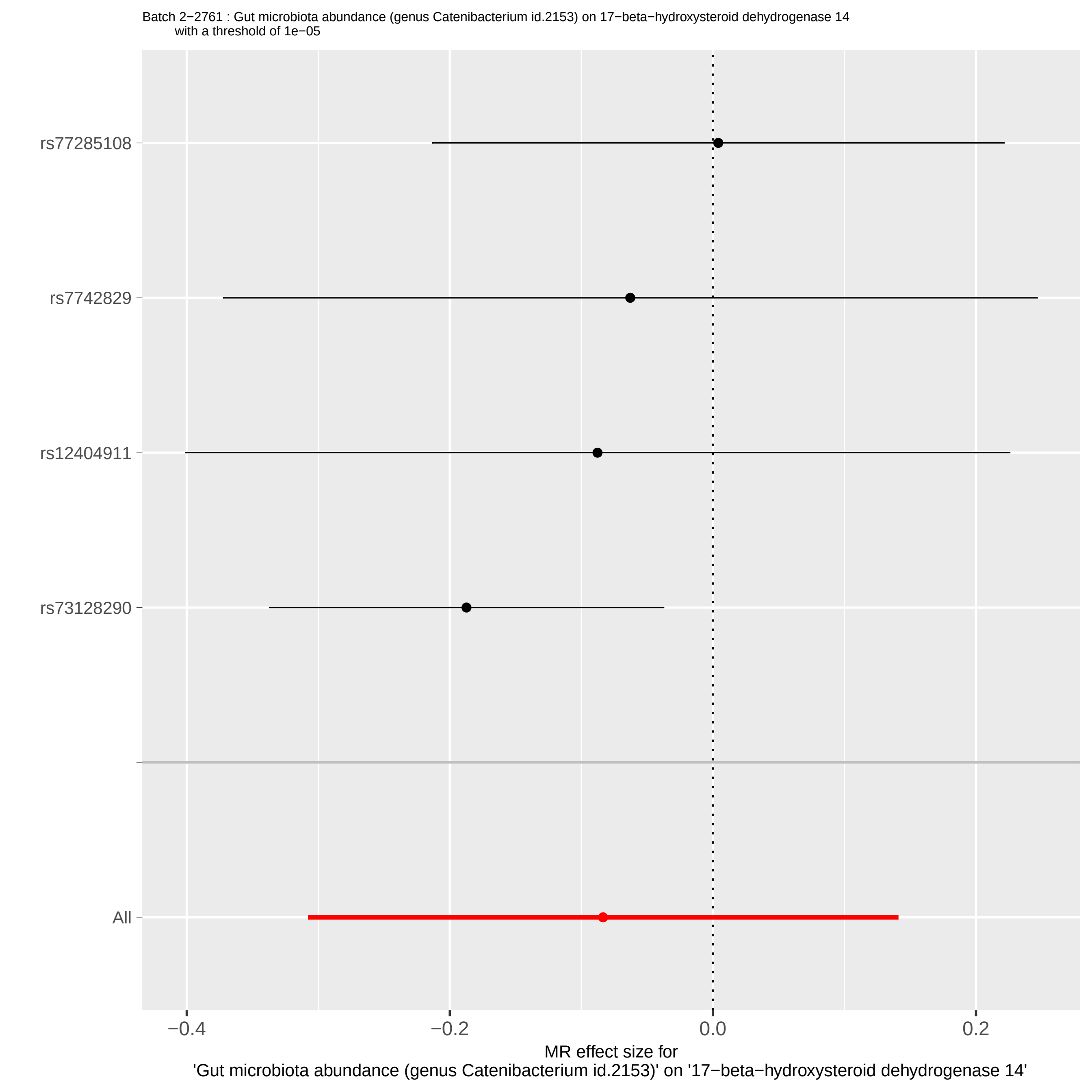
Batch 2−2761 : Gut microbiota abundance (genus Catenibacterium id.2153) on 17−beta−hydroxysteroid dehydrogenase 14
with a threshold of 1e−05
rs77285108
rs7742829
rs12404911
rs73128290
All
−0.4
−0.2
0.0
0.2
MR effect size for
'Gut microbiota abundance (genus Catenibacterium id.2153)' on '17−beta−hydroxysteroid dehydrogenase 14'

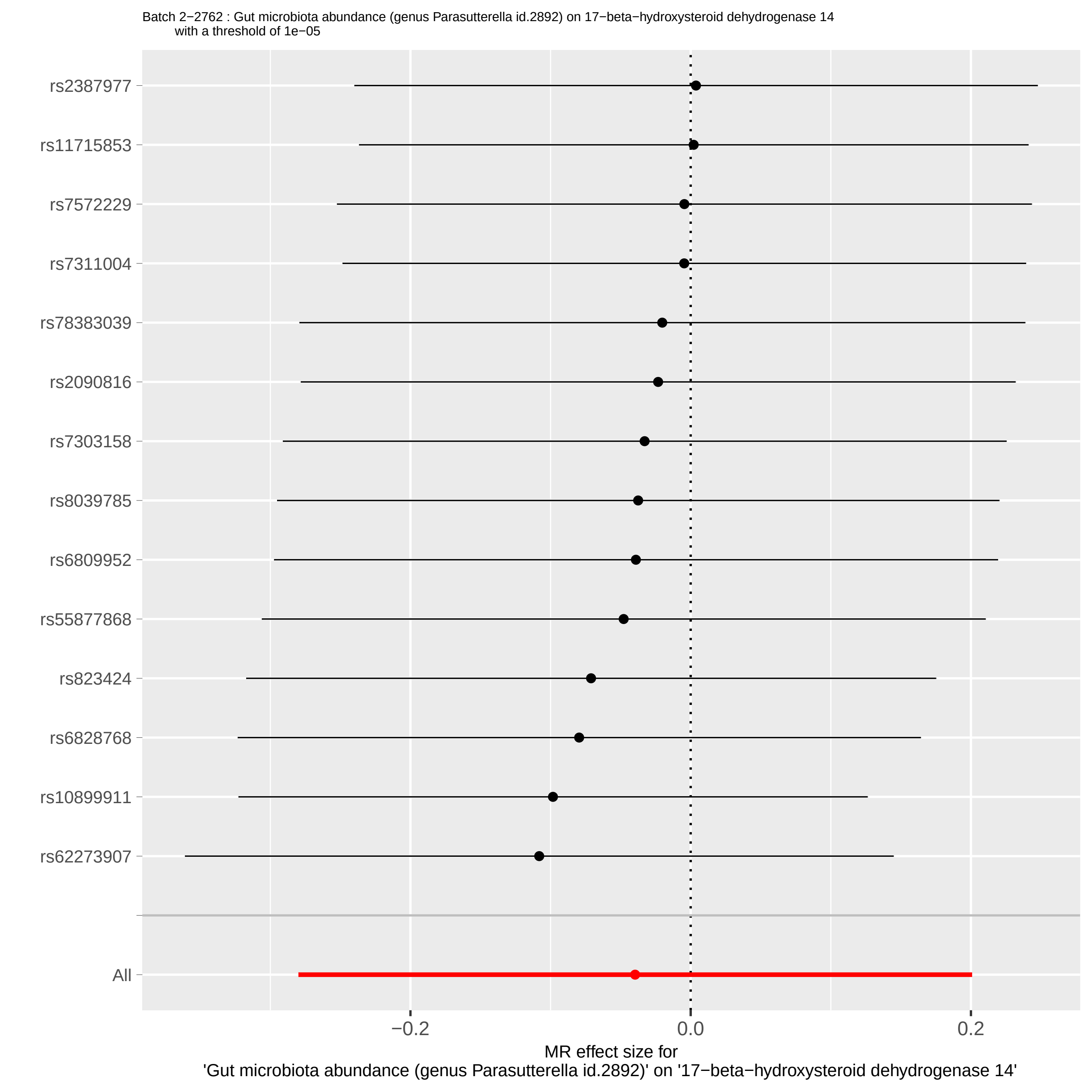

Batch 2−2762 : Gut microbiota abundance (genus Parasutterella id.2892) on 17−beta−hydroxysteroid dehydrogenase 14
with a threshold of 1e−05
rs2387977
rs11715853
rs7572229
rs7311004
rs78383039
rs2090816
rs7303158
rs8039785
rs6809952
rs55877868
rs823424
rs6828768
rs10899911
rs62273907
All
−0.2
0.0
0.2
MR effect size for
'Gut microbiota abundance (genus Parasutterella id.2892)' on '17−beta−hydroxysteroid dehydrogenase 14'

Batch 2−2763 : Gut microbiota abundance (genus Catenibacterium id.2153) on Estradiol 17−beta−dehydrogenase 1
with a threshold of 1e−05

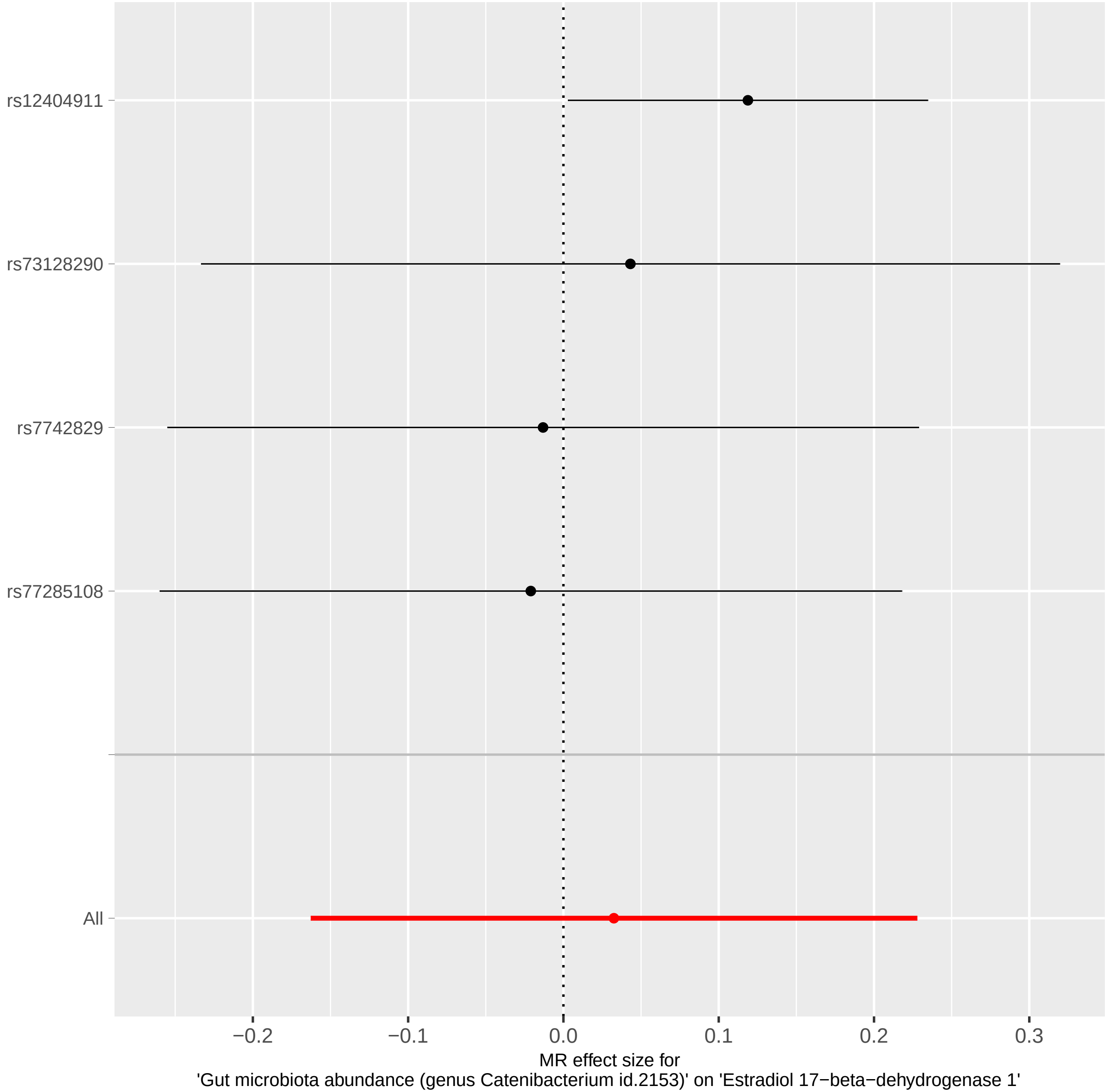

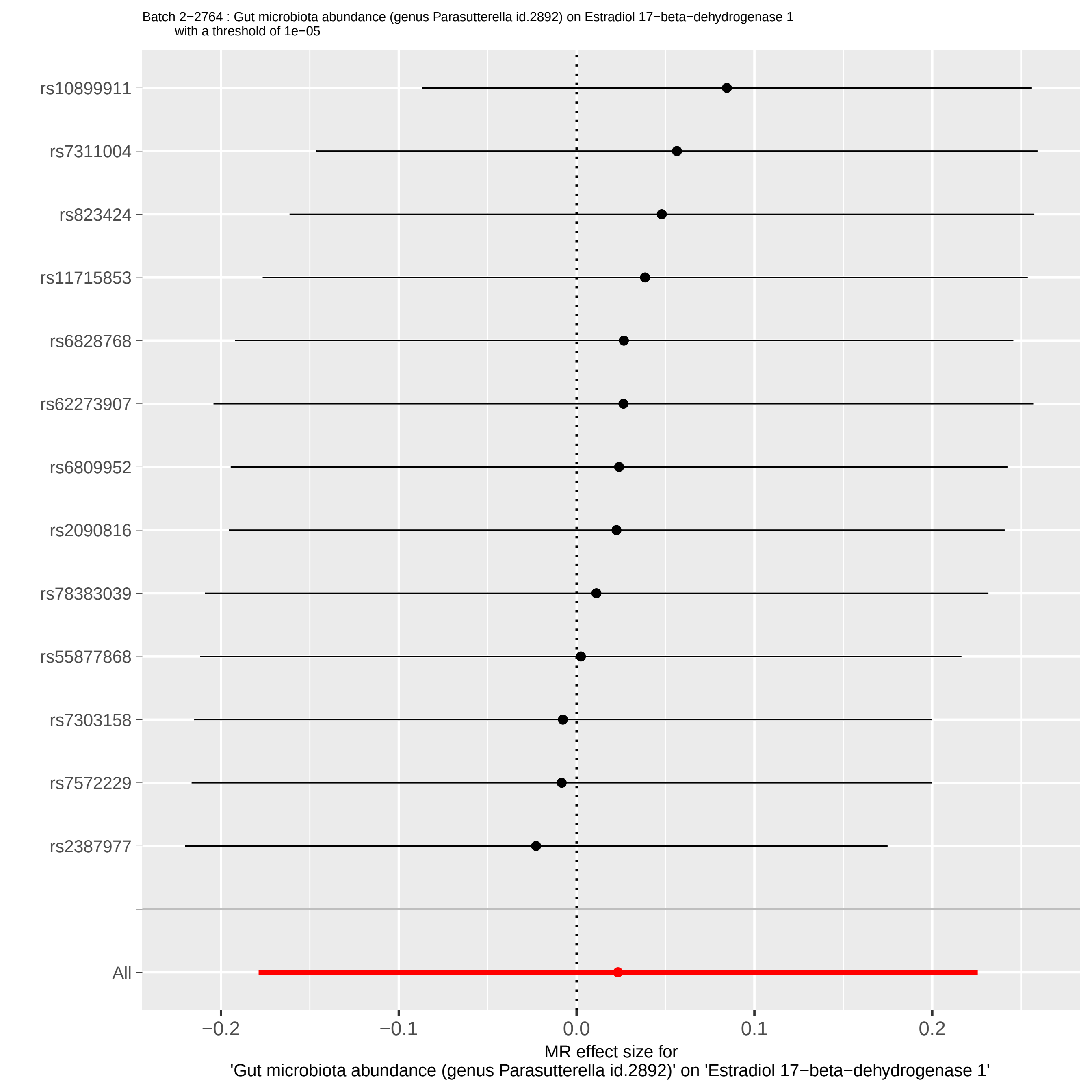
Batch 2−2764 : Gut microbiota abundance (genus Parasutterella id.2892) on Estradiol 17−beta−dehydrogenase 1
with a threshold of 1e−05
rs10899911
rs7311004
rs823424
rs11715853
rs6828768
rs62273907
rs6809952
rs2090816
rs78383039
rs55877868
rs7303158
rs7572229
rs2387977
All
−0.2
−0.1
0.0
0.1
0.2
MR effect size for
'Gut microbiota abundance (genus Parasutterella id.2892)' on 'Estradiol 17−beta−dehydrogenase 1'

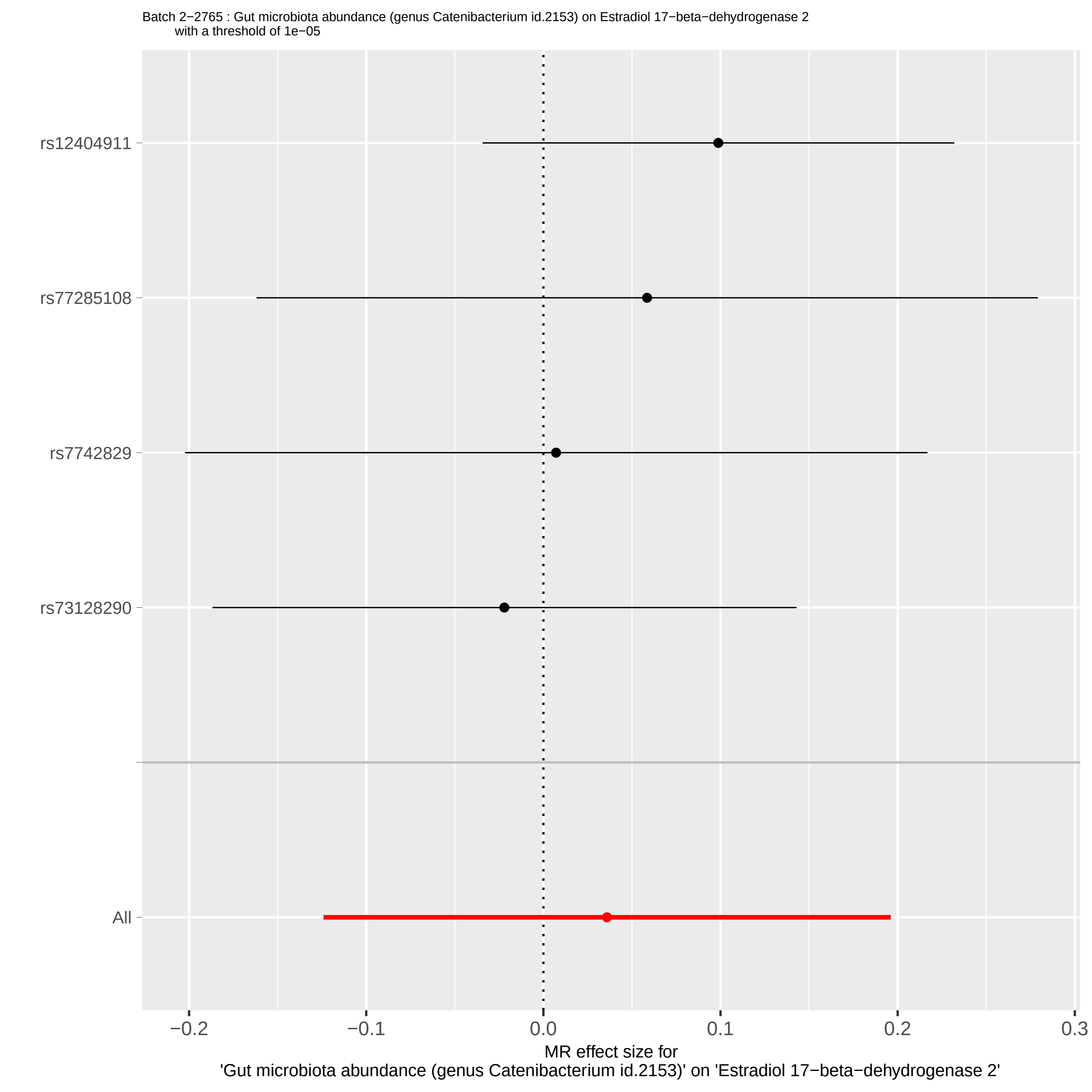
Batch 2−2765 : Gut microbiota abundance (genus Catenibacterium id.2153) on Estradiol 17−beta−dehydrogenase 2
with a threshold of 1e−05
rs12404911
rs77285108
rs7742829
rs73128290
All
−0.2
−0.1
0.0
0.1
0.2
0.3
MR effect size for
'Gut microbiota abundance (genus Catenibacterium id.2153)' on 'Estradiol 17−beta−dehydrogenase 2'

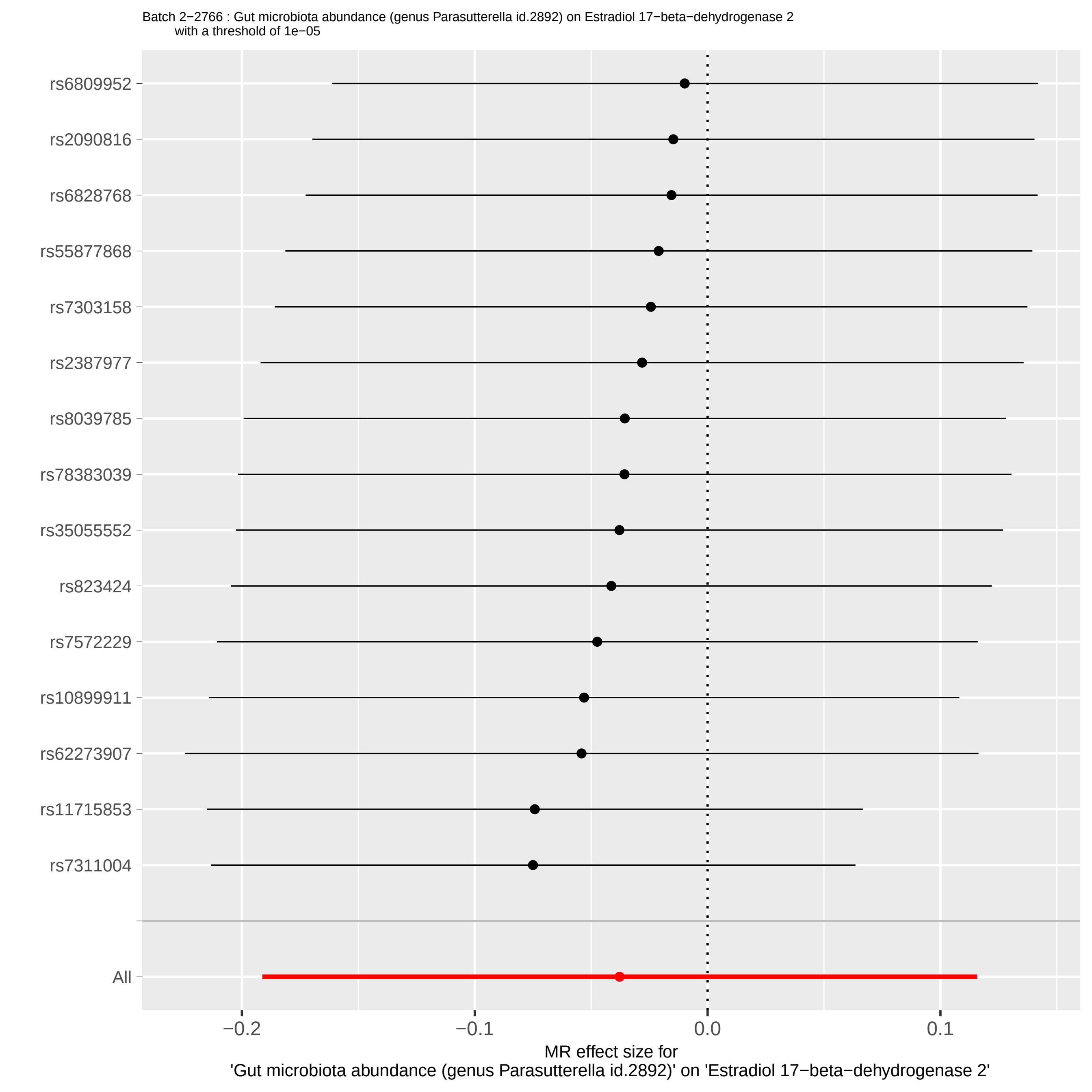

Batch 2−2766 : Gut microbiota abundance (genus Parasutterella id.2892) on Estradiol 17−beta−dehydrogenase 2
with a threshold of 1e−05
rs6809952
rs2090816
rs6828768
rs55877868
rs7303158
rs2387977
rs8039785
rs78383039
rs35055552
rs823424
rs7572229
rs10899911
rs62273907
rs11715853
rs7311004
All
−0.2
−0.1
0.0
0.1
MR effect size for
'Gut microbiota abundance (genus Parasutterella id.2892)' on 'Estradiol 17−beta−dehydrogenase 2'

Batch 2−2767 : Gut microbiota abundance (genus Catenibacterium id.2153) on Heat shock protein HSP 90−alpha/beta
with a threshold of 1e−05

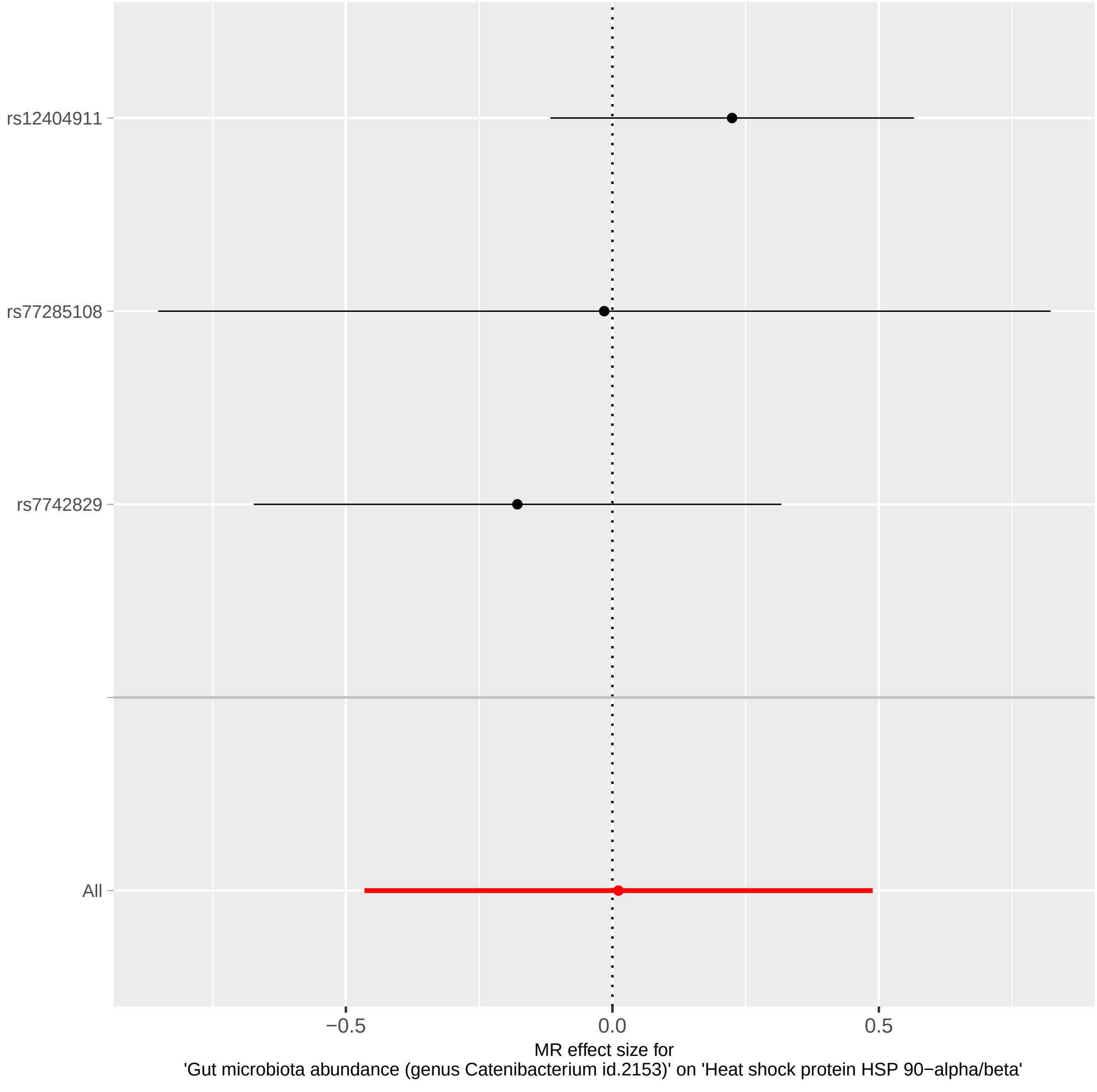

Batch 2−2768 : Gut microbiota abundance (genus Parasutterella id.2892) on Heat shock protein HSP 90−alpha/beta
with a threshold of 1e−05

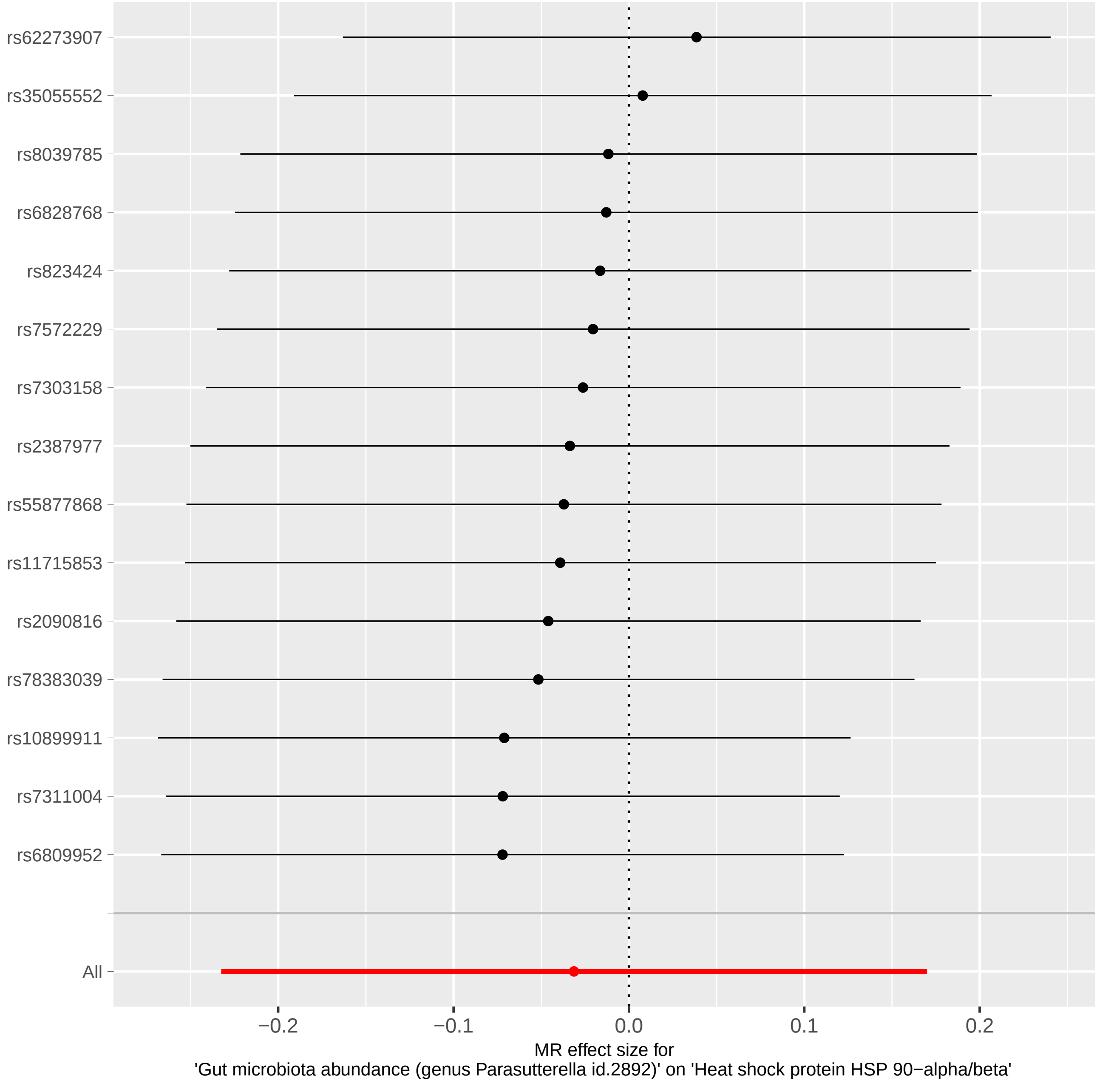

Batch 2−2769 : Gut microbiota abundance (genus Catenibacterium id.2153) on Heat shock protein HSP 90−beta
with a threshold of 1e−05

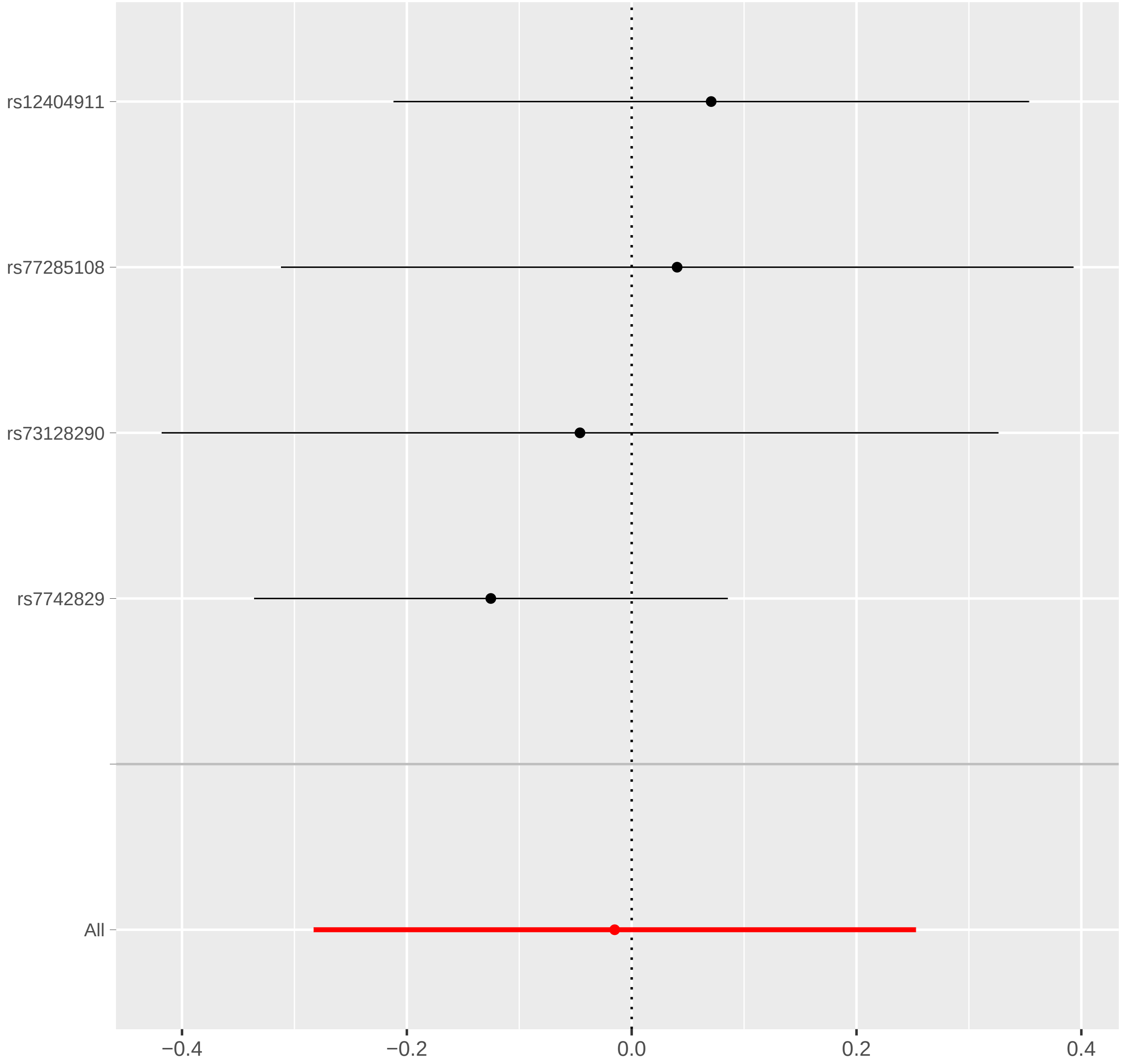

MR effect size for
'Gut microbiota abundance (genus Catenibacterium id.2153)' on 'Heat shock protein HSP 90−beta'

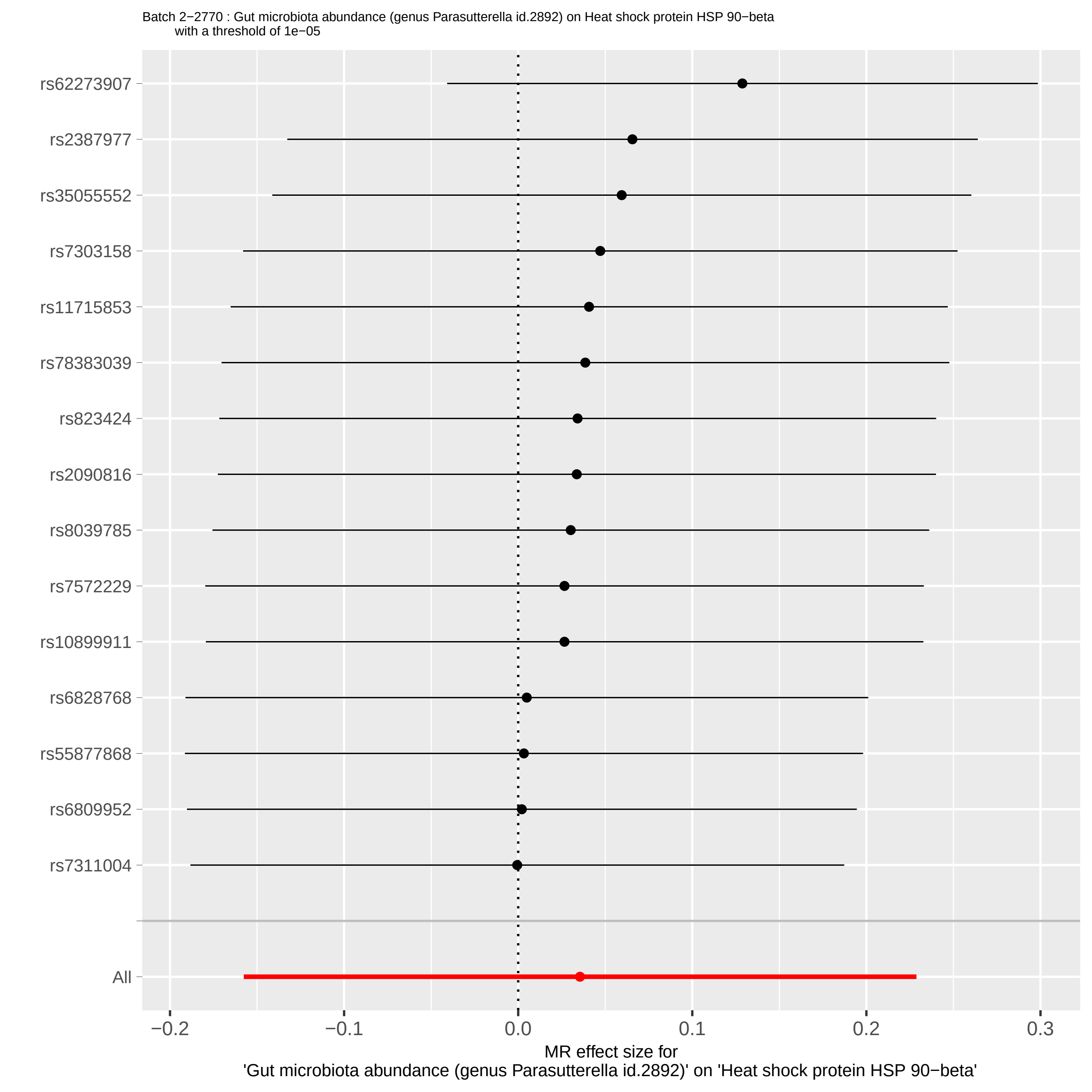
Batch 2−2770 : Gut microbiota abundance (genus Parasutterella id.2892) on Heat shock protein HSP 90−beta
with a threshold of 1e−05
rs62273907
rs2387977
rs35055552
rs7303158
rs11715853
rs78383039
rs823424
rs2090816
rs8039785
rs7572229
rs10899911
rs6828768
rs55877868
rs6809952
rs7311004
All
−0.2
−0.1
0.0
0.1
0.2
0.3
MR effect size for
'Gut microbiota abundance (genus Parasutterella id.2892)' on 'Heat shock protein HSP 90−beta'

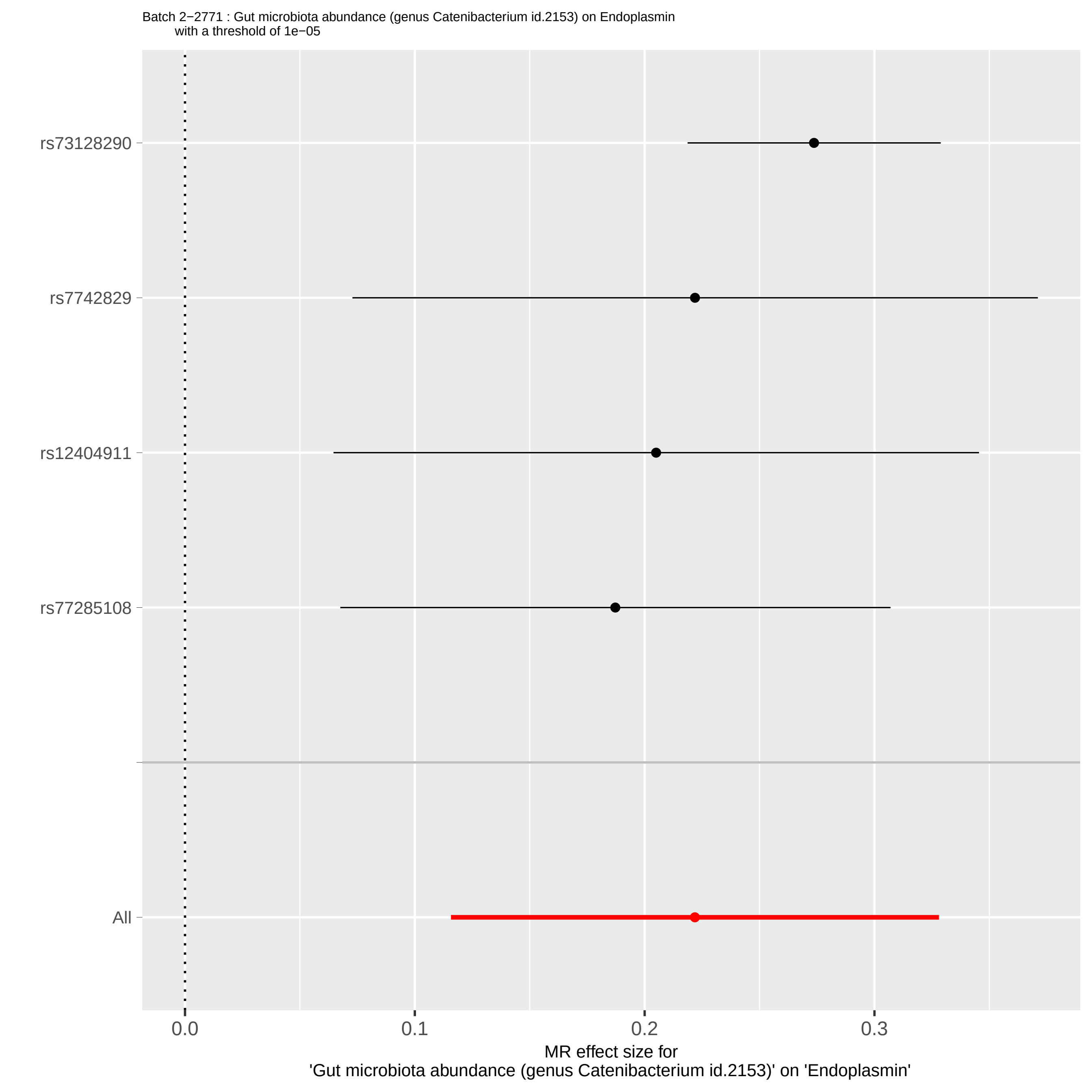

Batch 2−2771 : Gut microbiota abundance (genus Catenibacterium id.2153) on Endoplasmin
with a threshold of 1e−05
rs73128290
rs7742829
rs12404911
rs77285108
All
0.0
0.1
0.2
0.3
MR effect size for
'Gut microbiota abundance (genus Catenibacterium id.2153)' on 'Endoplasmin'

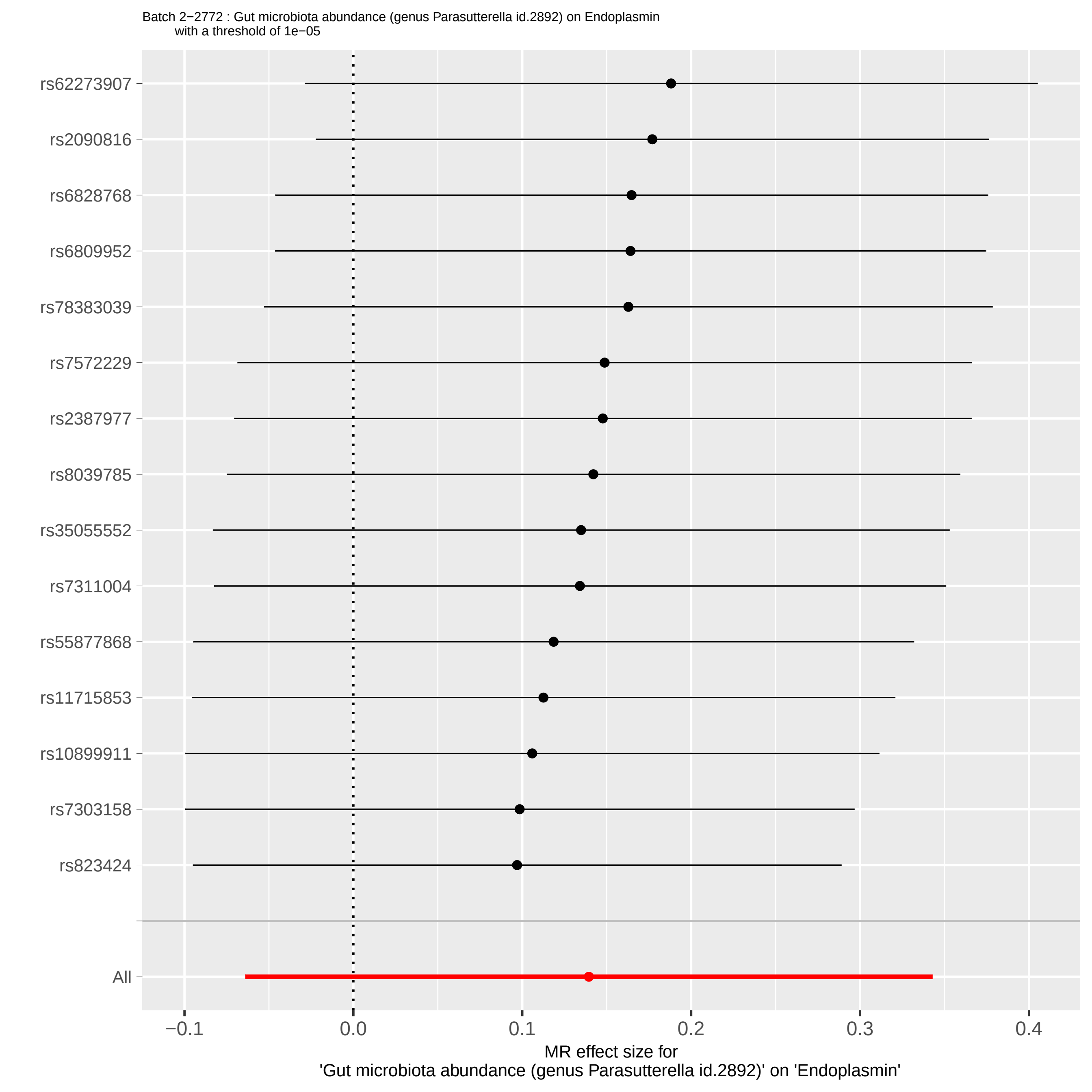
Batch 2−2772 : Gut microbiota abundance (genus Parasutterella id.2892) on Endoplasmin
with a threshold of 1e−05
rs62273907
rs2090816
rs6828768
rs6809952
rs78383039
rs7572229
rs2387977
rs8039785
rs35055552
rs7311004
rs55877868
rs11715853
rs10899911
rs7303158
rs823424
All
−0.1
0.0
0.1
0.2
0.3
0.4
MR effect size for
'Gut microbiota abundance (genus Parasutterella id.2892)' on 'Endoplasmin'

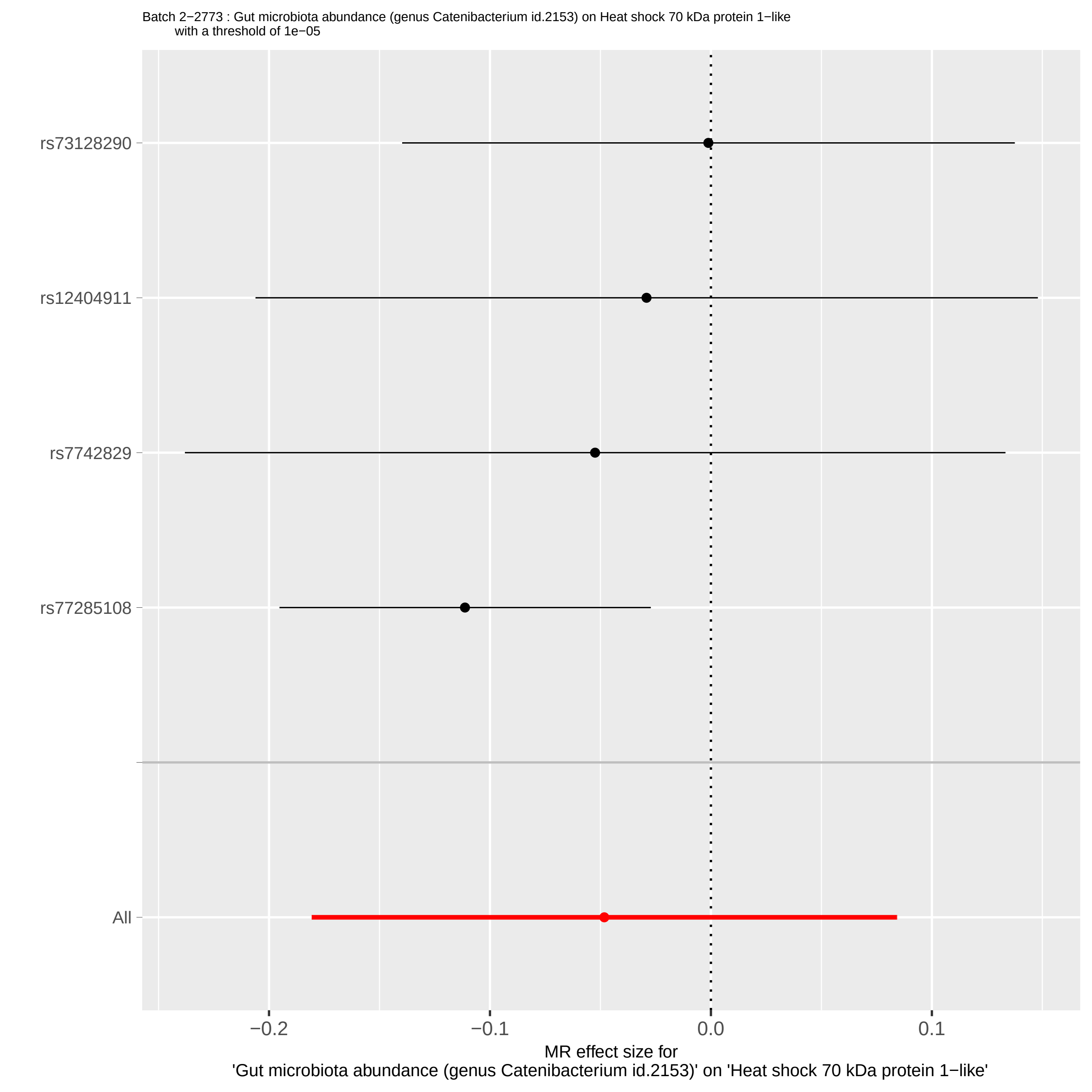
Batch 2−2773 : Gut microbiota abundance (genus Catenibacterium id.2153) on Heat shock 70 kDa protein 1−like
with a threshold of 1e−05
rs73128290
rs12404911
rs7742829
rs77285108
All
−0.2
−0.1
0.0
0.1
MR effect size for
'Gut microbiota abundance (genus Catenibacterium id.2153)' on 'Heat shock 70 kDa protein 1−like'

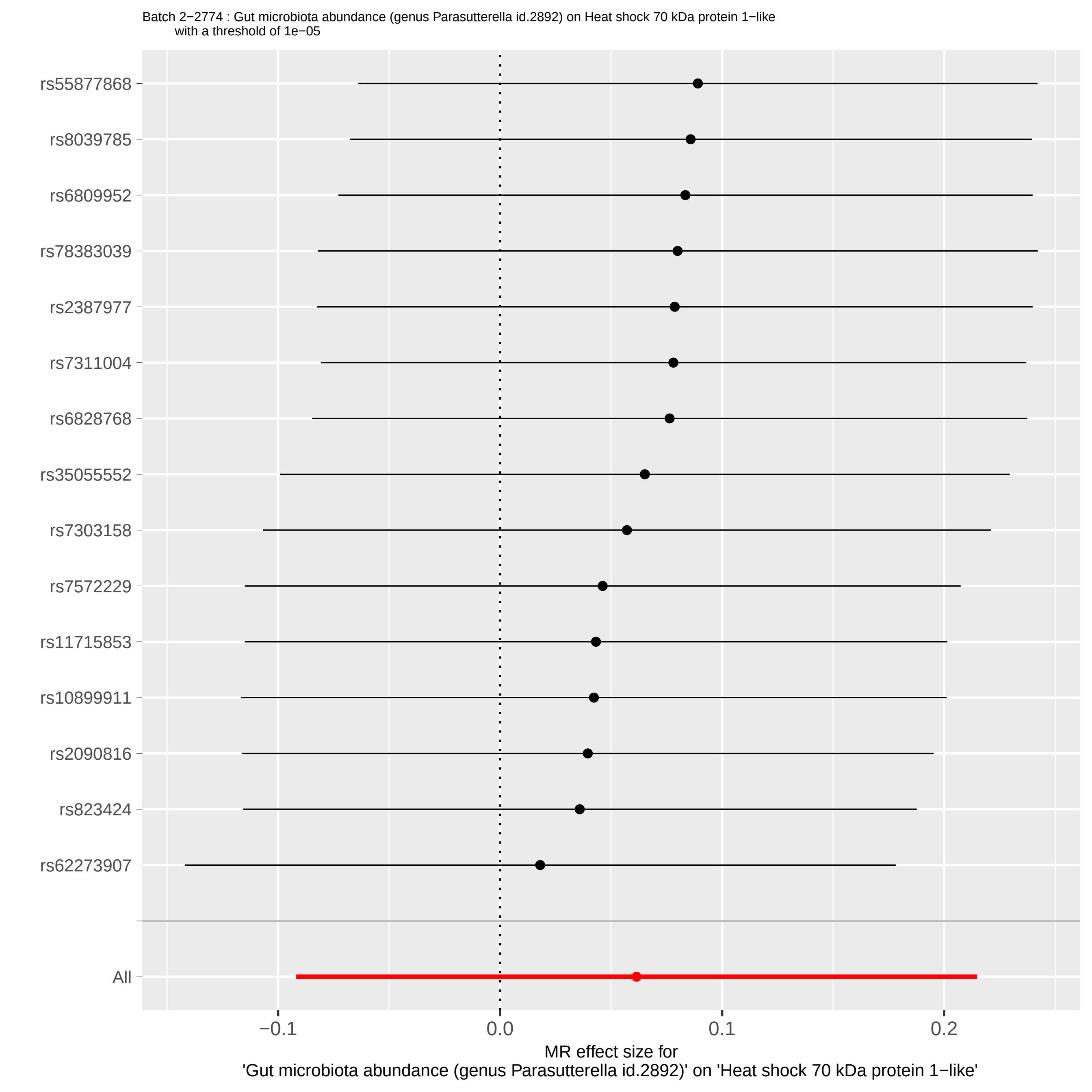

Batch 2−2774 : Gut microbiota abundance (genus Parasutterella id.2892) on Heat shock 70 kDa protein 1−like
with a threshold of 1e−05
rs55877868
rs8039785
rs6809952
rs78383039
rs2387977
rs7311004
rs6828768
rs35055552
rs7303158
rs7572229
rs11715853
rs10899911
rs2090816
rs823424
rs62273907
All
−0.1
0.0
0.1
0.2
MR effect size for
'Gut microbiota abundance (genus Parasutterella id.2892)' on 'Heat shock 70 kDa protein 1−like'

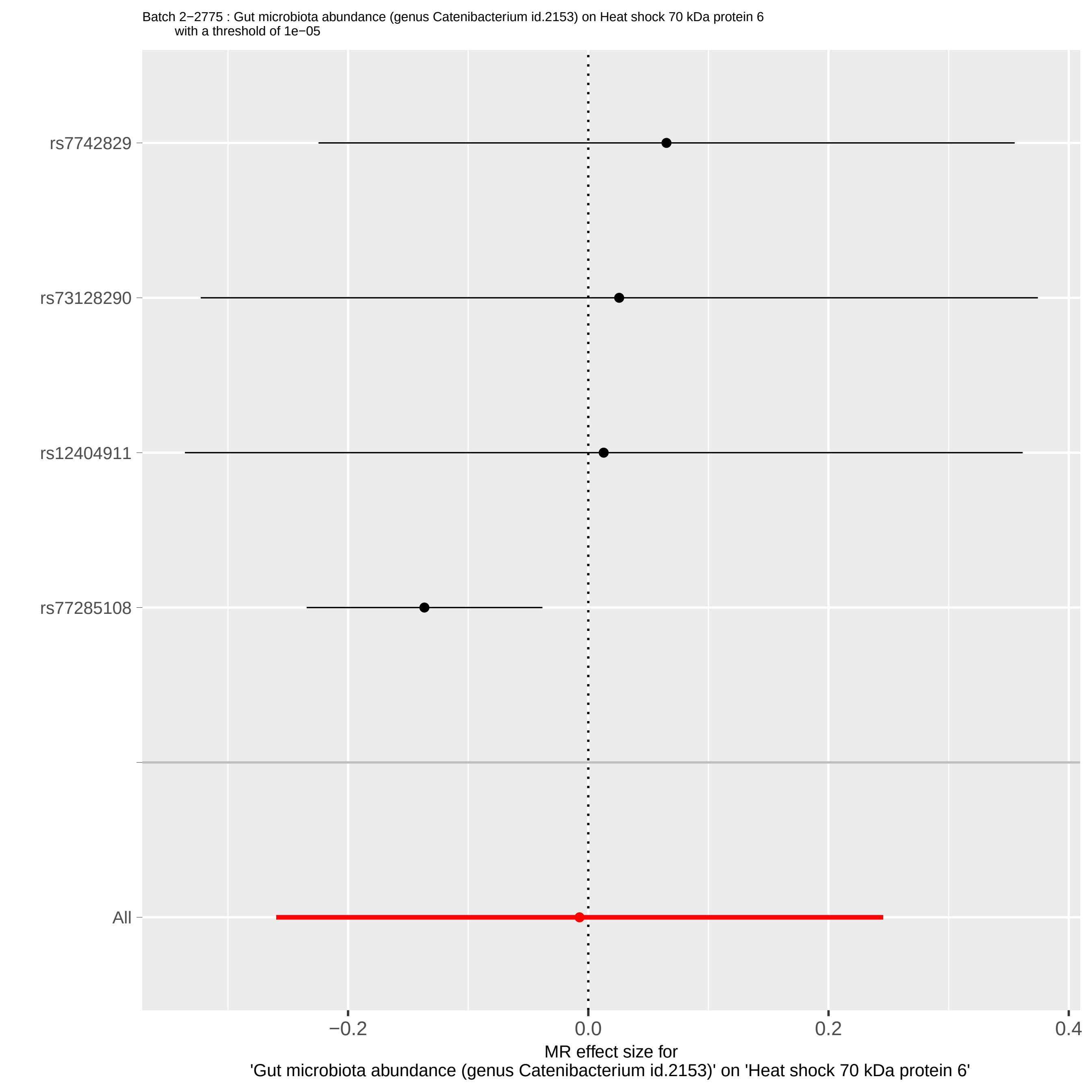
Batch 2−2775 : Gut microbiota abundance (genus Catenibacterium id.2153) on Heat shock 70 kDa protein 6
with a threshold of 1e−05
rs7742829
rs73128290
rs12404911
rs77285108
All
−0.2
0.0
0.2
0.4
MR effect size for
'Gut microbiota abundance (genus Catenibacterium id.2153)' on 'Heat shock 70 kDa protein 6'

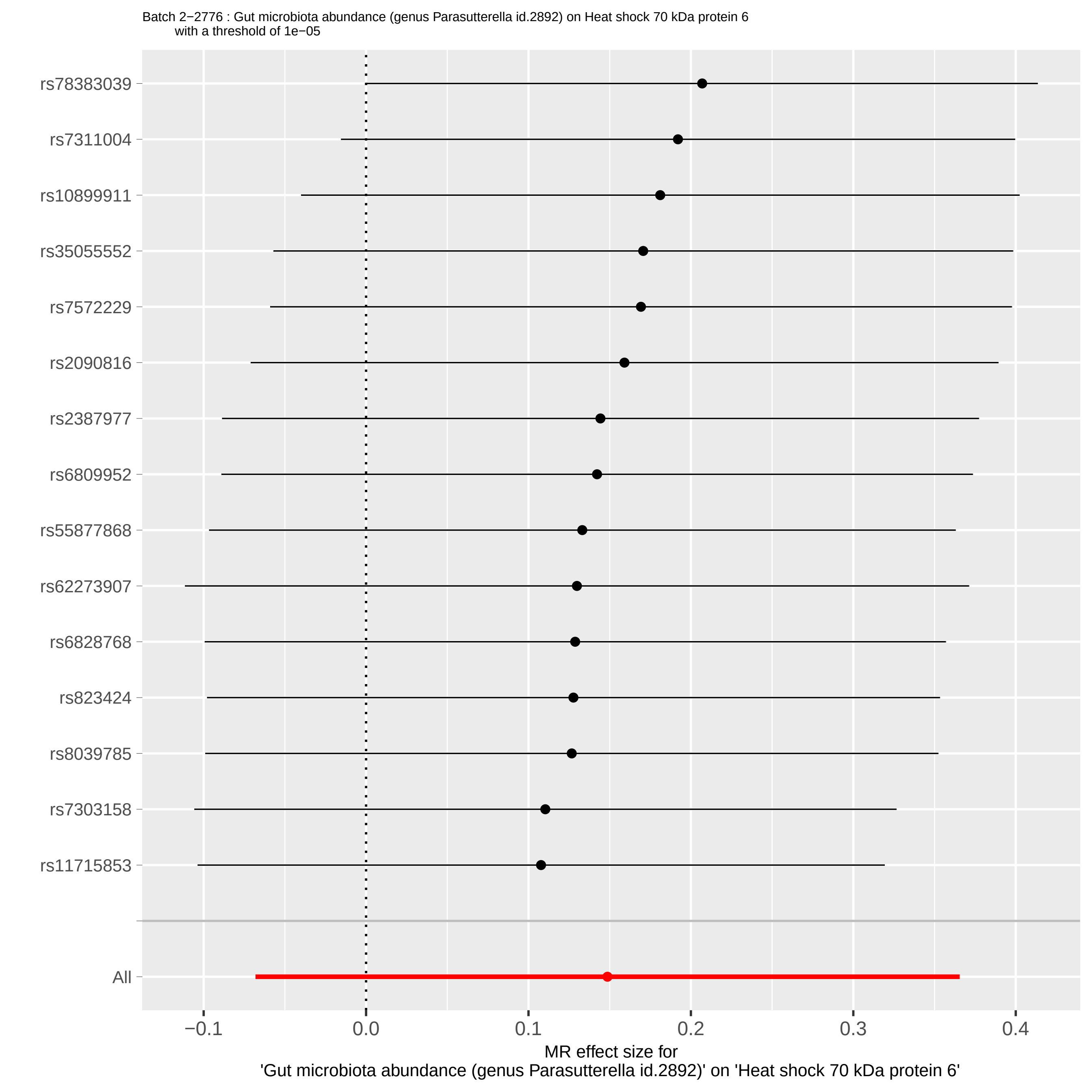
Batch 2−2776 : Gut microbiota abundance (genus Parasutterella id.2892) on Heat shock 70 kDa protein 6
with a threshold of 1e−05
rs78383039
rs7311004
rs10899911
rs35055552
rs7572229
rs2090816
rs2387977
rs6809952
rs55877868
rs62273907
rs6828768
rs823424
rs8039785
rs7303158
rs11715853
All
−0.1
0.0
0.1
0.2
0.3
0.4
MR effect size for
'Gut microbiota abundance (genus Parasutterella id.2892)' on 'Heat shock 70 kDa protein 6'

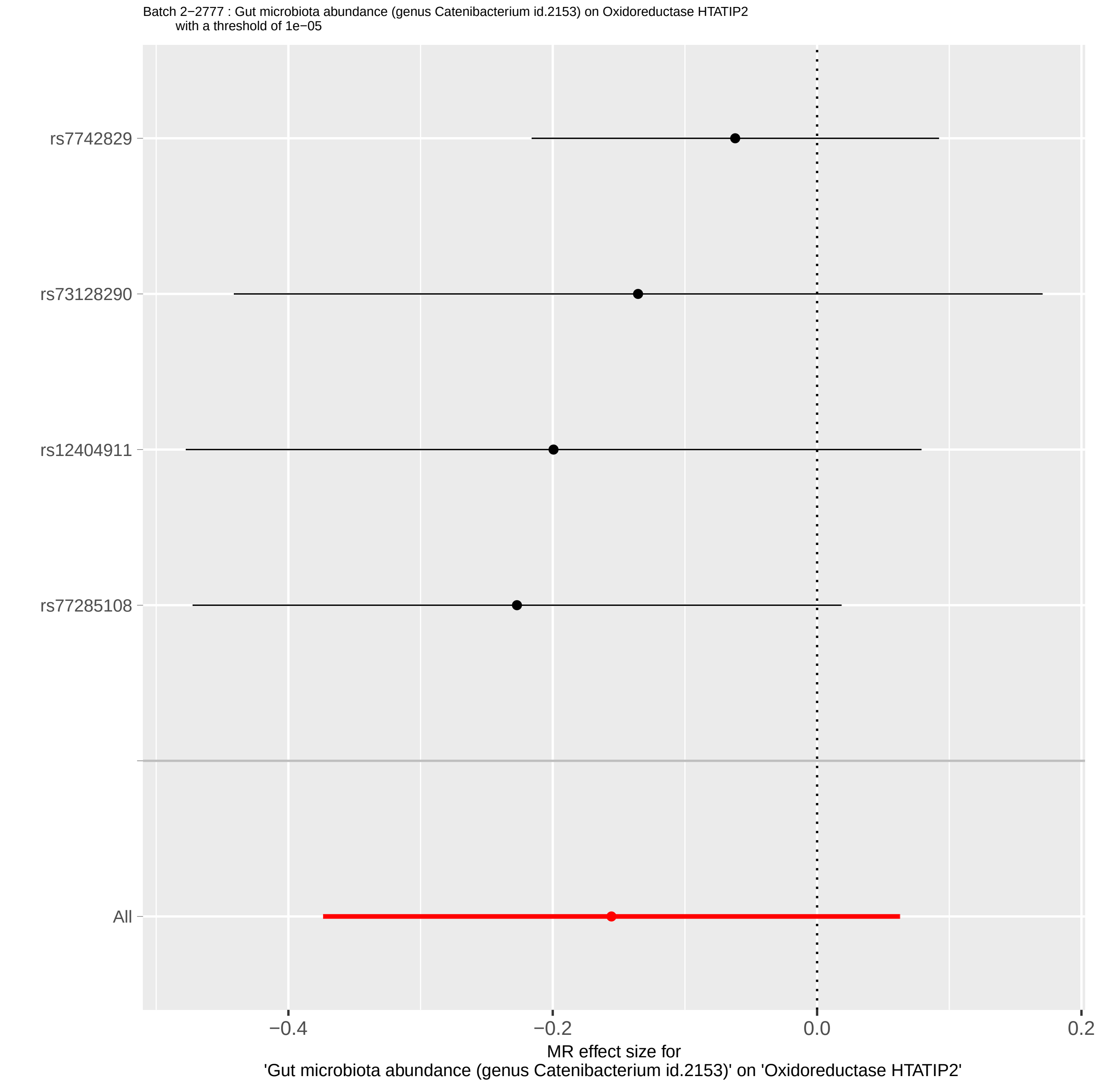
Batch 2−2777 : Gut microbiota abundance (genus Catenibacterium id.2153) on Oxidoreductase HTATIP2
with a threshold of 1e−05
rs7742829
rs73128290
rs12404911
rs77285108
All
−0.4
−0.2
0.0
0.2
MR effect size for
'Gut microbiota abundance (genus Catenibacterium id.2153)' on 'Oxidoreductase HTATIP2'

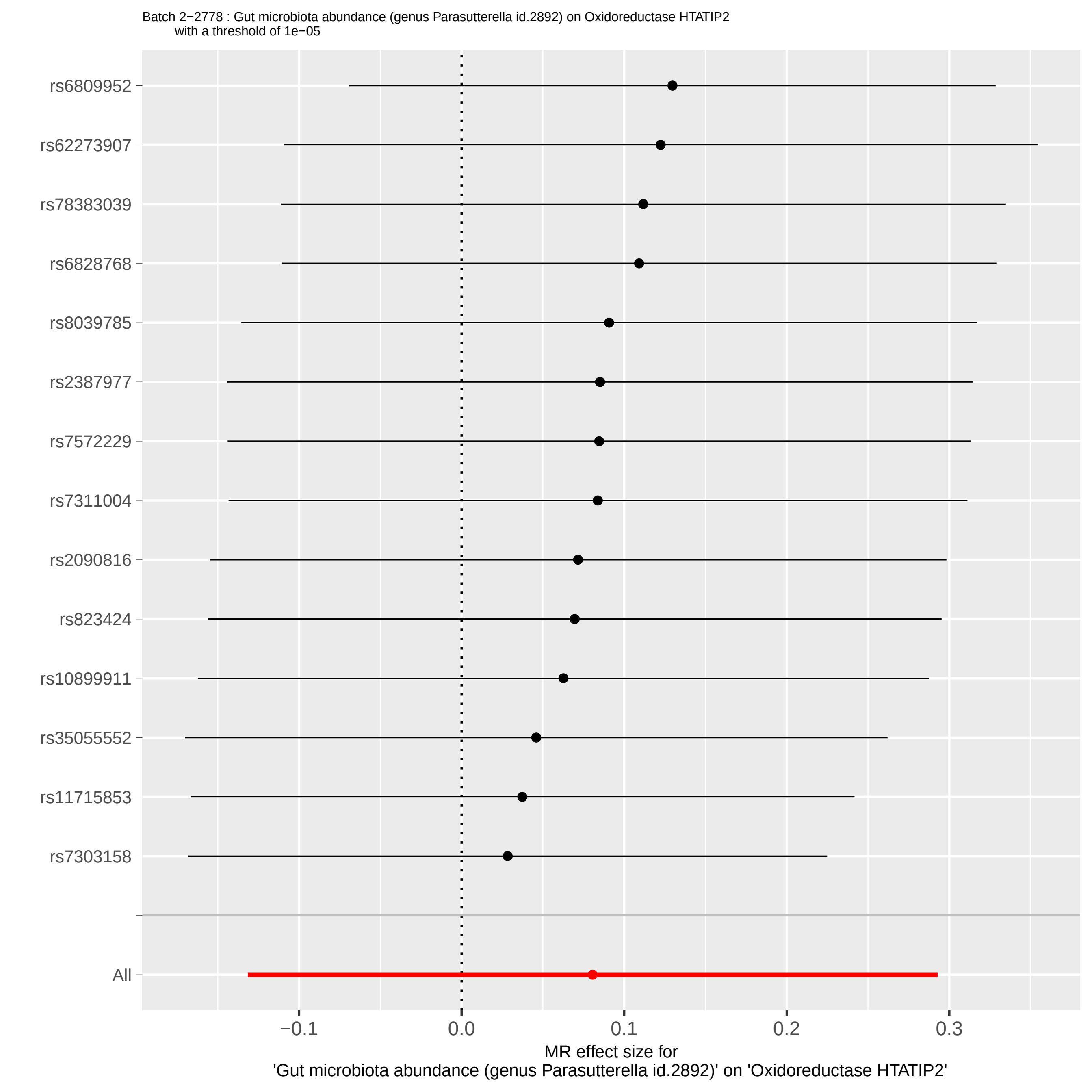

Batch 2−2778 : Gut microbiota abundance (genus Parasutterella id.2892) on Oxidoreductase HTATIP2
with a threshold of 1e−05
rs6809952
rs62273907
rs78383039
rs6828768
rs8039785
rs2387977
rs7572229
rs7311004
rs2090816
rs823424
rs10899911
rs35055552
rs11715853
rs7303158
All
−0.1
0.0
0.1
0.2
0.3
MR effect size for
'Gut microbiota abundance (genus Parasutterella id.2892)' on 'Oxidoreductase HTATIP2'

Batch 2−2779 : Gut microbiota abundance (genus Catenibacterium id.2153) on Oxidoreductase HTATIP2
with a threshold of 1e−05

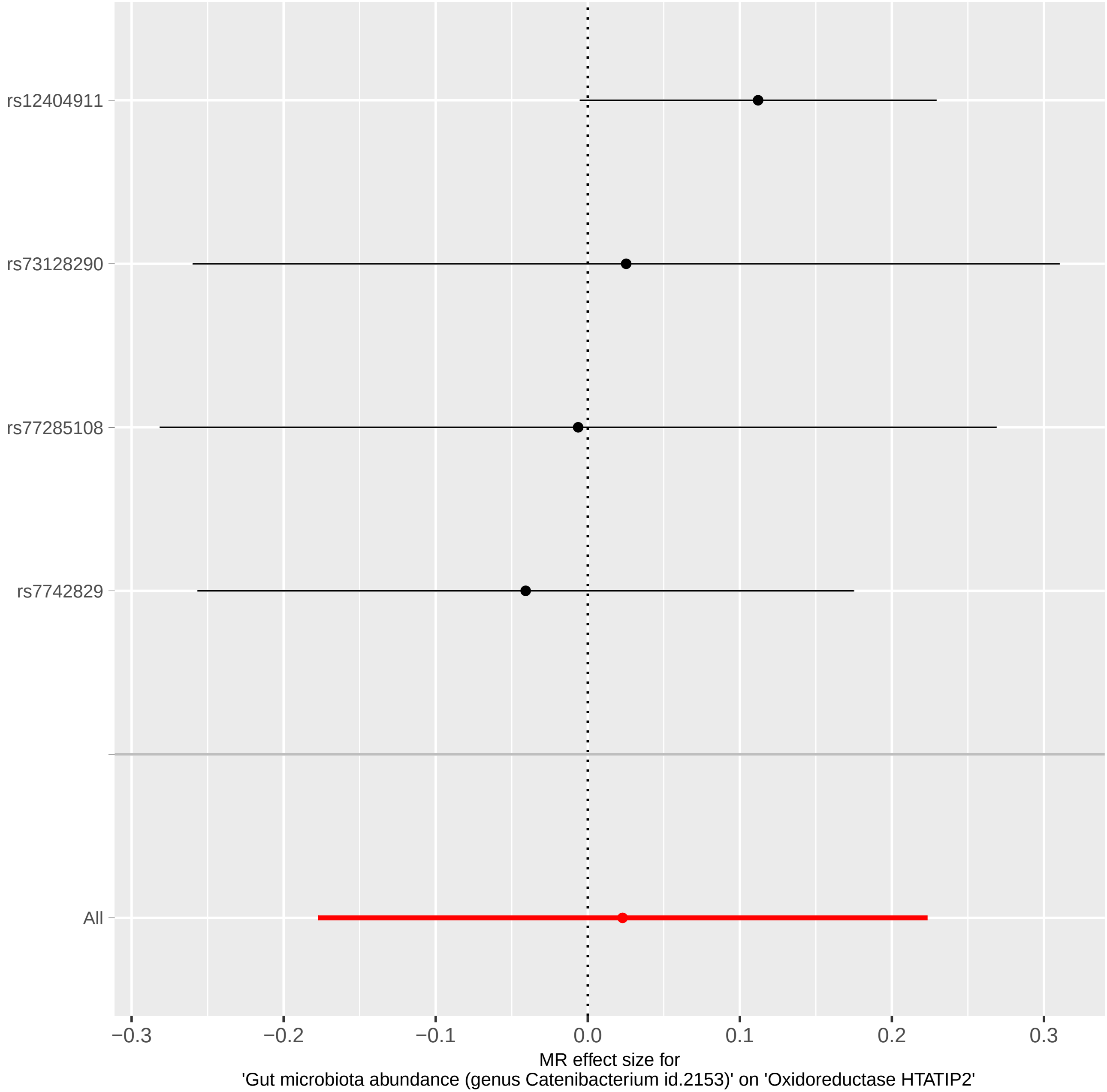

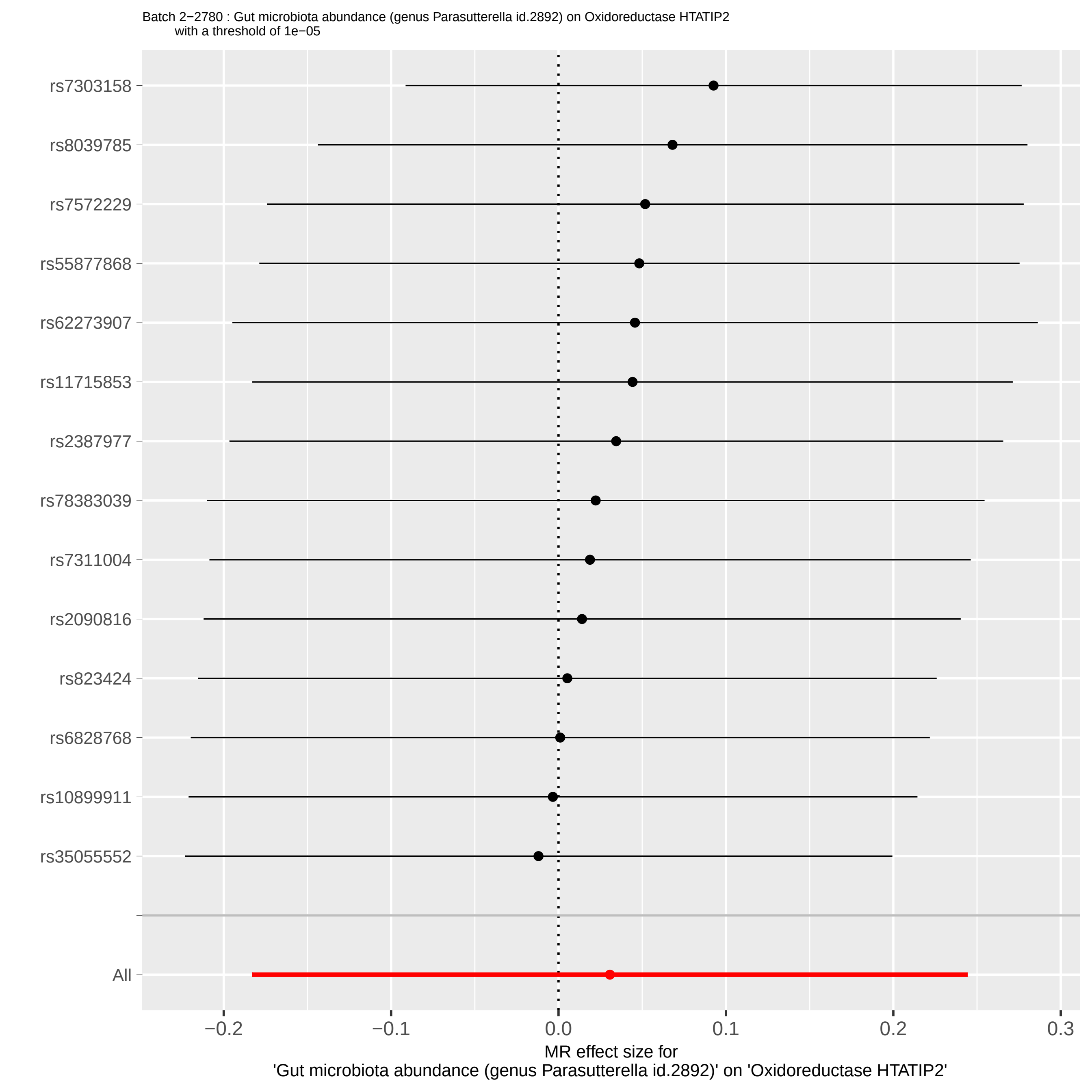
Batch 2−2780 : Gut microbiota abundance (genus Parasutterella id.2892) on Oxidoreductase HTATIP2
with a threshold of 1e−05
rs7303158
rs8039785
rs7572229
rs55877868
rs62273907
rs11715853
rs2387977
rs78383039
rs7311004
rs2090816
rs823424
rs6828768
rs10899911
rs35055552
All
−0.2
−0.1
0.0
0.1
0.2
0.3
MR effect size for
'Gut microbiota abundance (genus Parasutterella id.2892)' on 'Oxidoreductase HTATIP2'

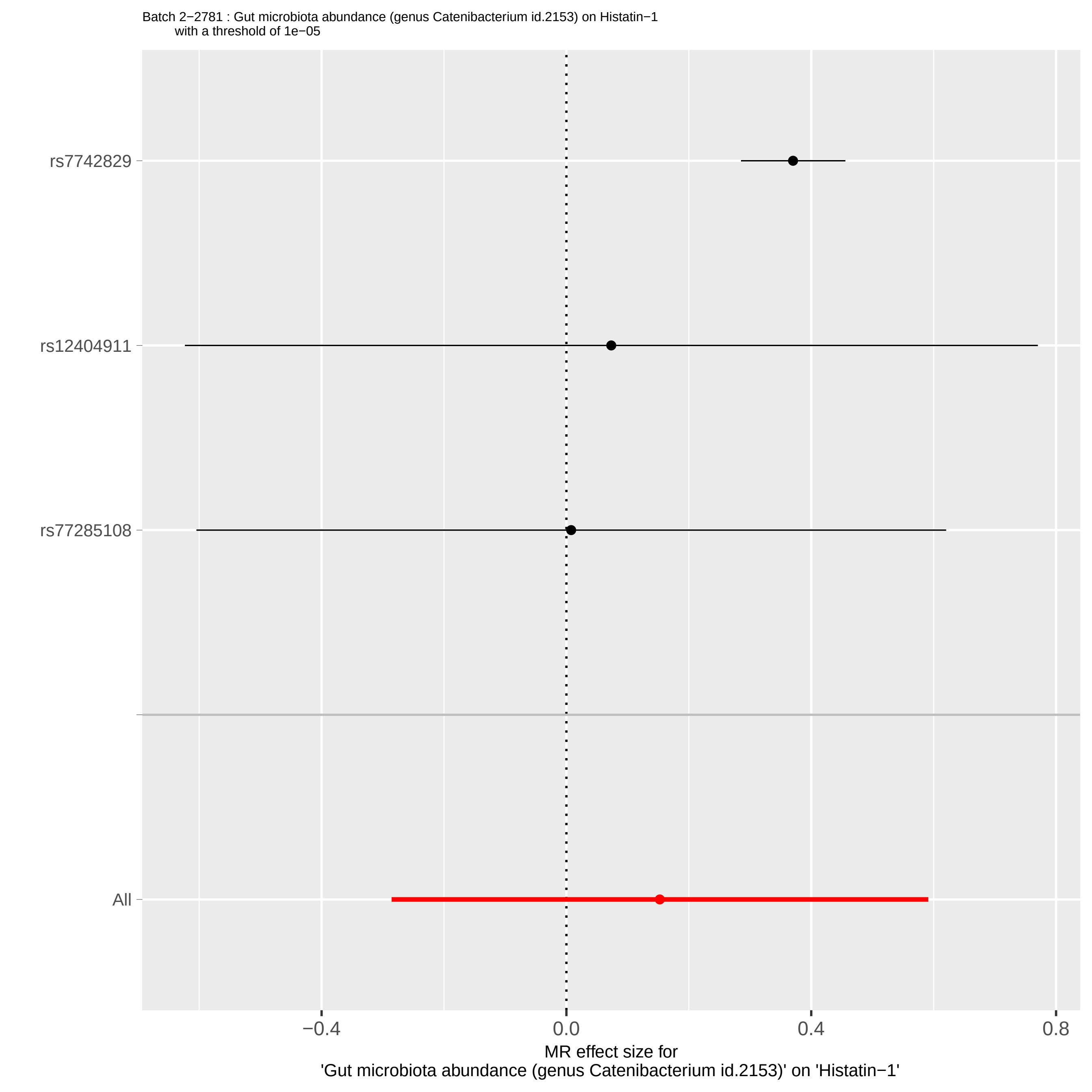
Batch 2−2781 : Gut microbiota abundance (genus Catenibacterium id.2153) on Histatin−1
with a threshold of 1e−05
rs7742829
rs12404911
rs77285108
All
−0.4
0.0
0.4
0.8
MR effect size for
'Gut microbiota abundance (genus Catenibacterium id.2153)' on 'Histatin−1'

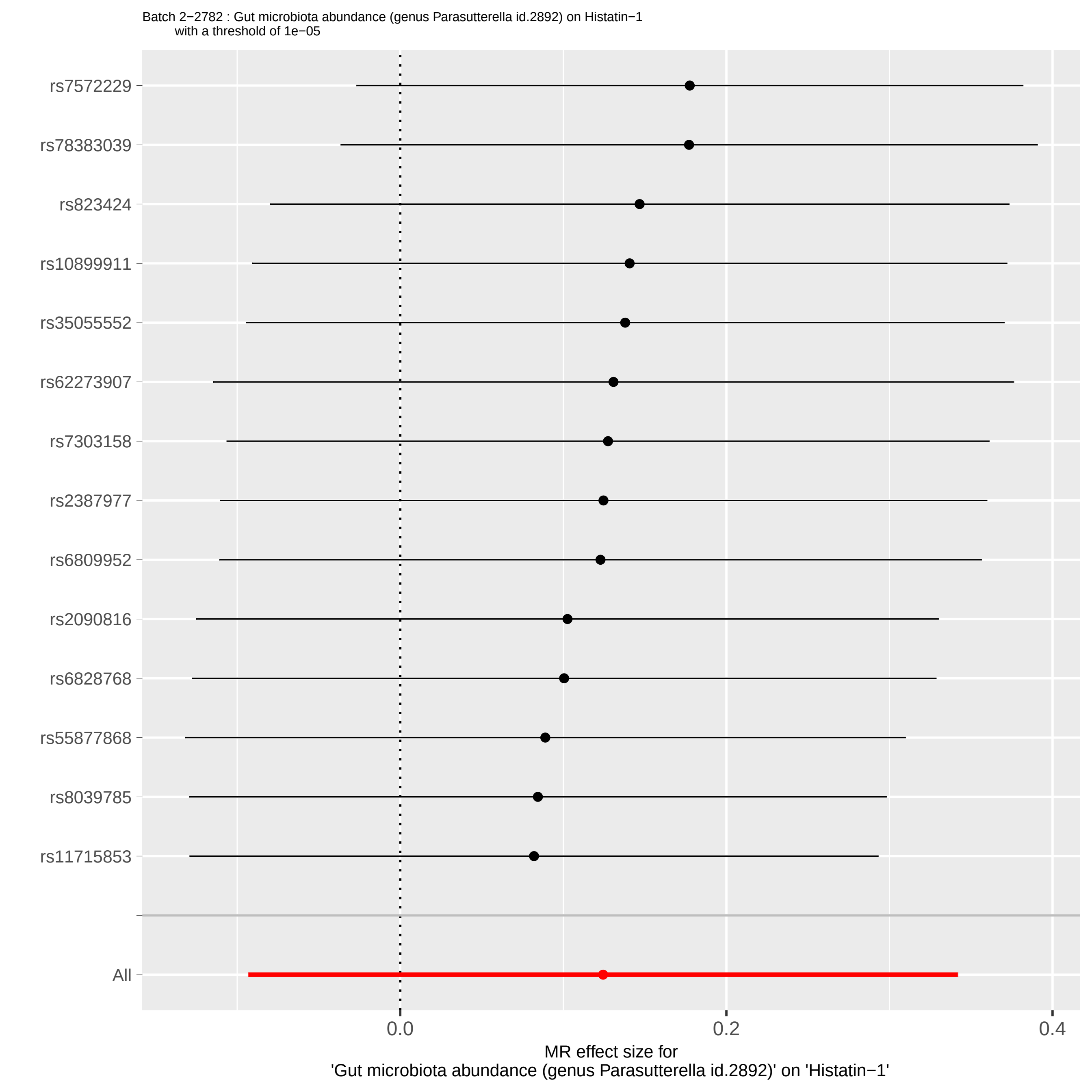

Batch 2−2782 : Gut microbiota abundance (genus Parasutterella id.2892) on Histatin−1
with a threshold of 1e−05
rs7572229
rs78383039
rs823424
rs10899911
rs35055552
rs62273907
rs7303158
rs2387977
rs6809952
rs2090816
rs6828768
rs55877868
rs8039785
rs11715853
All
0.0
0.2
0.4
MR effect size for
'Gut microbiota abundance (genus Parasutterella id.2892)' on 'Histatin−1'

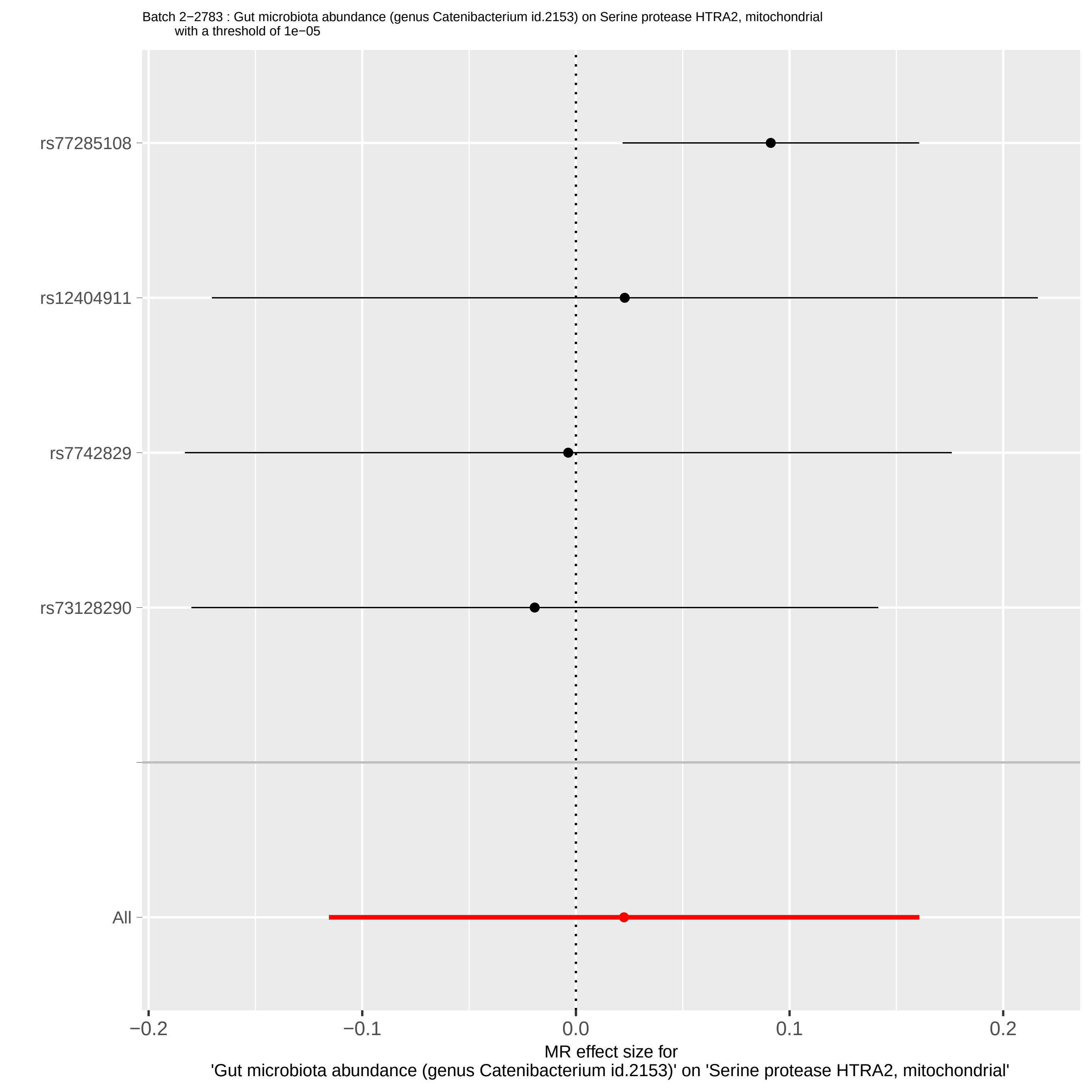
Batch 2−2783 : Gut microbiota abundance (genus Catenibacterium id.2153) on Serine protease HTRA2, mitochondrial
with a threshold of 1e−05
rs77285108
rs12404911
rs7742829
rs73128290
All
−0.2
−0.1
0.0
0.1
0.2
MR effect size for
'Gut microbiota abundance (genus Catenibacterium id.2153)' on 'Serine protease HTRA2, mitochondrial'

Batch 2−2784 : Gut microbiota abundance (genus Parasutterella id.2892) on Serine protease HTRA2, mitochondrial
with a threshold of 1e−05

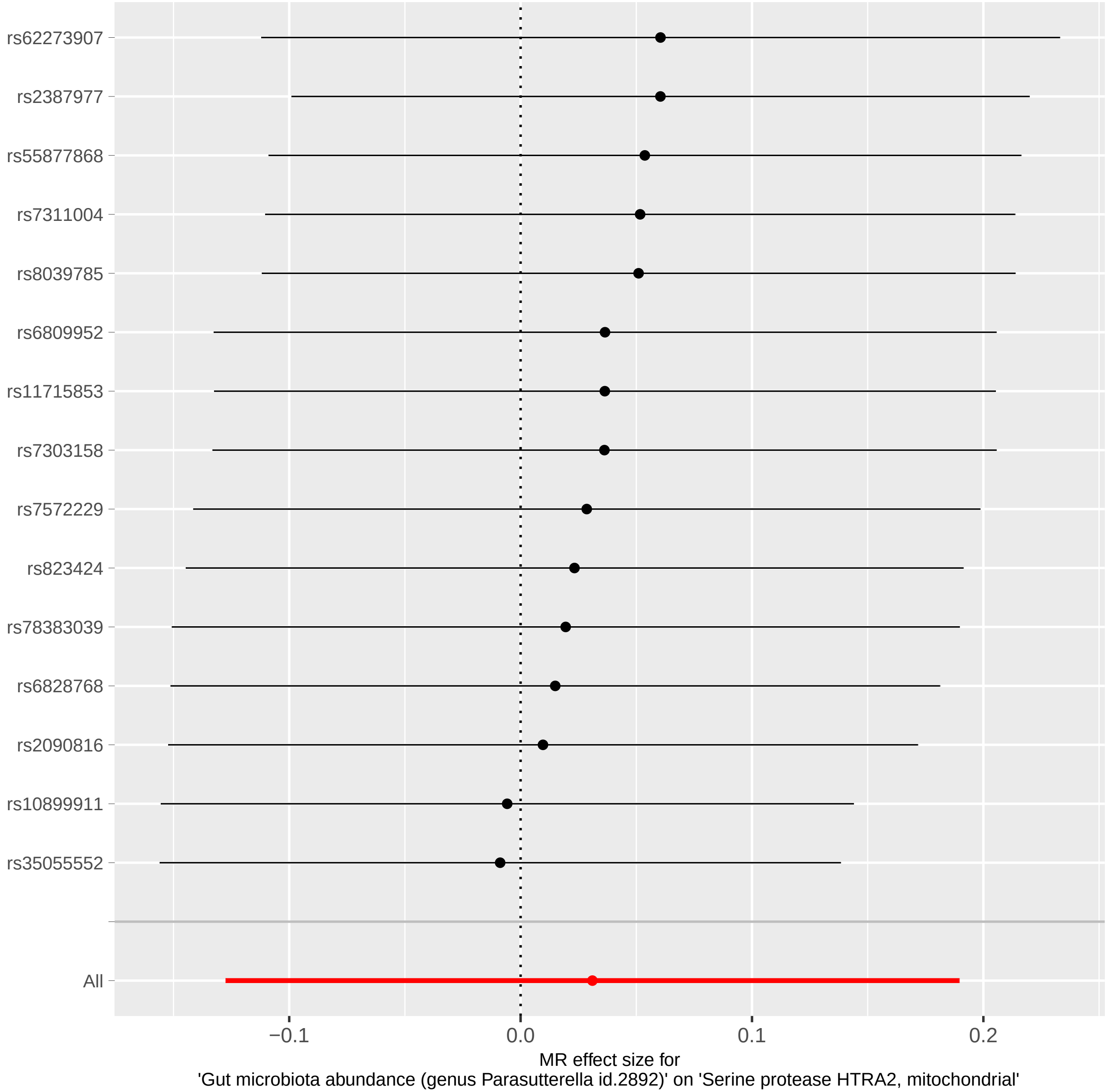

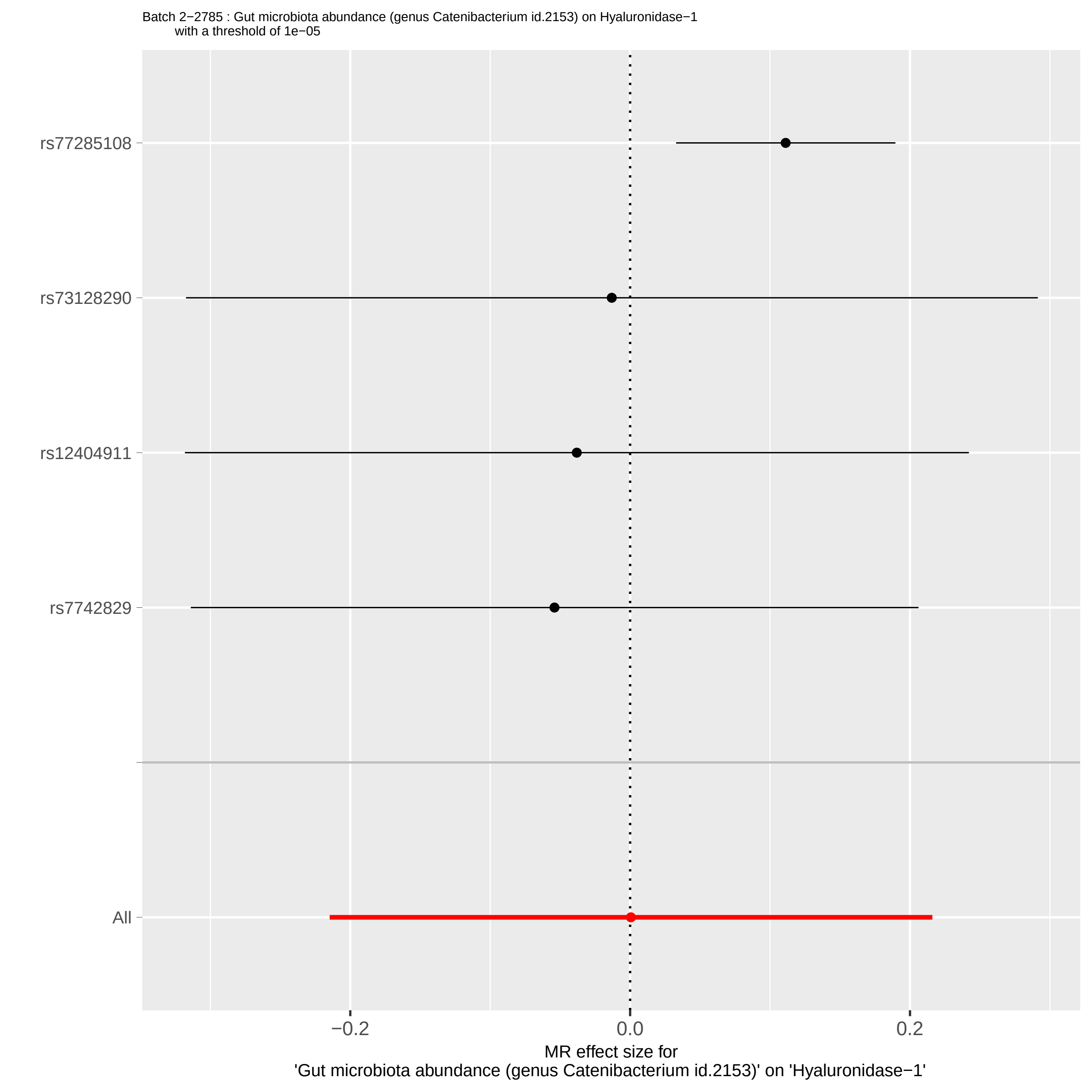
Batch 2−2785 : Gut microbiota abundance (genus Catenibacterium id.2153) on Hyaluronidase−1
with a threshold of 1e−05
rs77285108
rs73128290
rs12404911
rs7742829
All
−0.2
0.0
0.2
MR effect size for
'Gut microbiota abundance (genus Catenibacterium id.2153)' on 'Hyaluronidase−1'

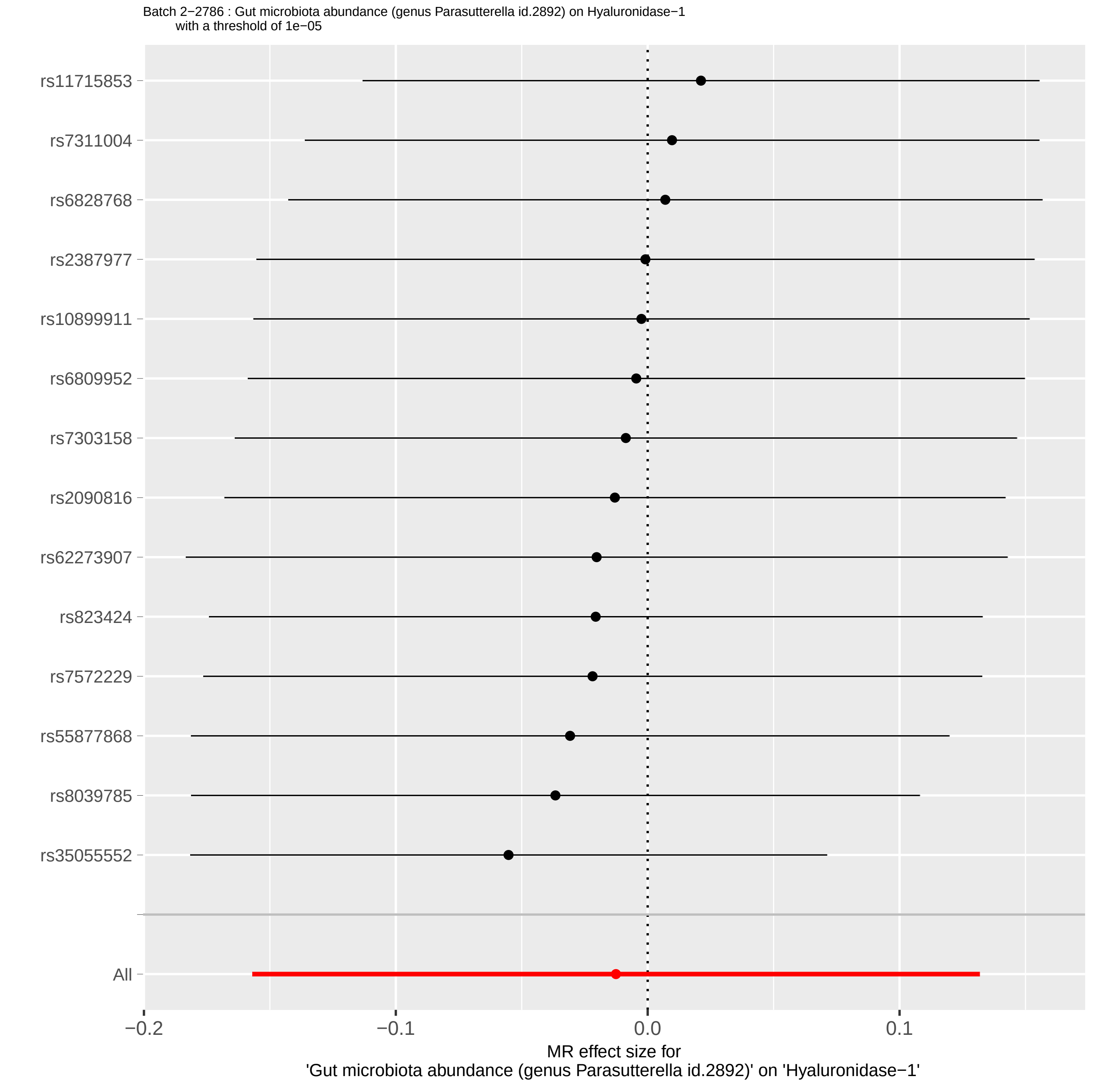
Batch 2−2786 : Gut microbiota abundance (genus Parasutterella id.2892) on Hyaluronidase−1
with a threshold of 1e−05
rs11715853
rs7311004
rs6828768
rs2387977
rs10899911
rs6809952
rs7303158
rs2090816
rs62273907
rs823424
rs7572229
rs55877868
rs8039785
rs35055552
All
−0.2
−0.1
0.0
0.1
MR effect size for
'Gut microbiota abundance (genus Parasutterella id.2892)' on 'Hyaluronidase−1'

Batch 2−2787 : Gut microbiota abundance (genus Catenibacterium id.2153) on Isoleucine−−tRNA ligase, cytoplasmic
with a threshold of 1e−05

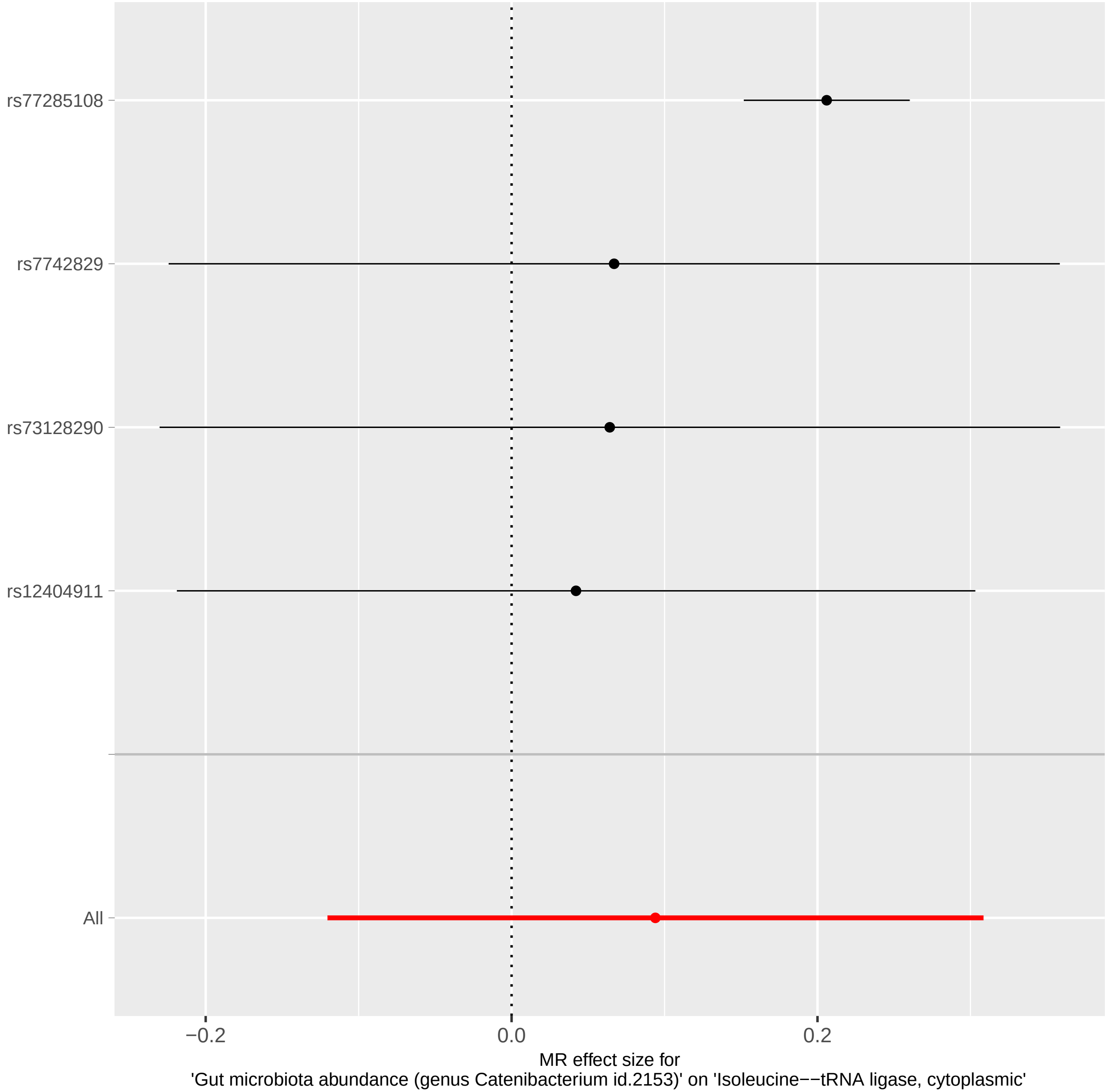

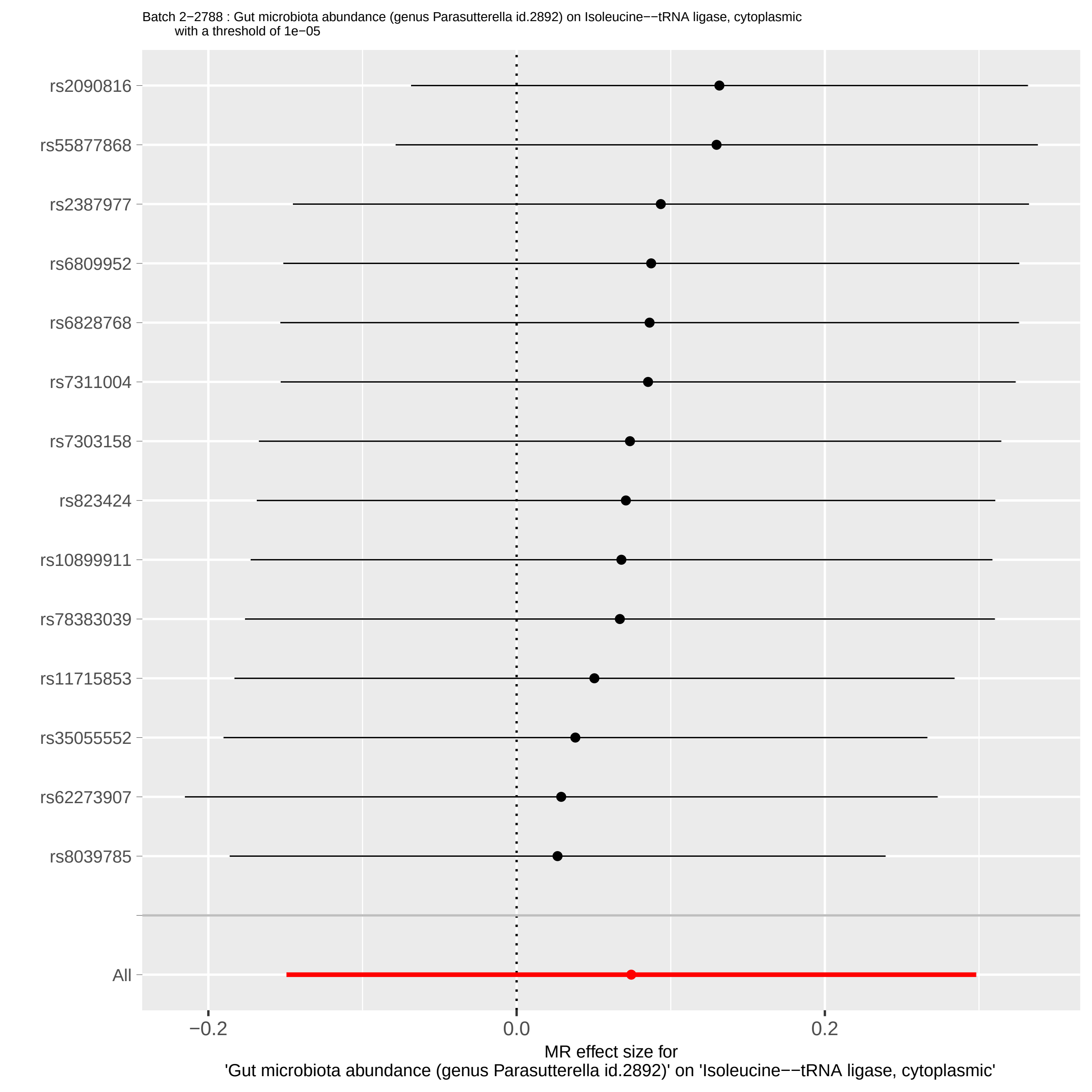
Batch 2−2788 : Gut microbiota abundance (genus Parasutterella id.2892) on Isoleucine−−tRNA ligase, cytoplasmic
with a threshold of 1e−05
rs2090816
rs55877868
rs2387977
rs6809952
rs6828768
rs7311004
rs7303158
rs823424
rs10899911
rs78383039
rs11715853
rs35055552
rs62273907
rs8039785
All
−0.2
0.0
0.2
MR effect size for
'Gut microbiota abundance (genus Parasutterella id.2892)' on 'Isoleucine−−tRNA ligase, cytoplasmic'

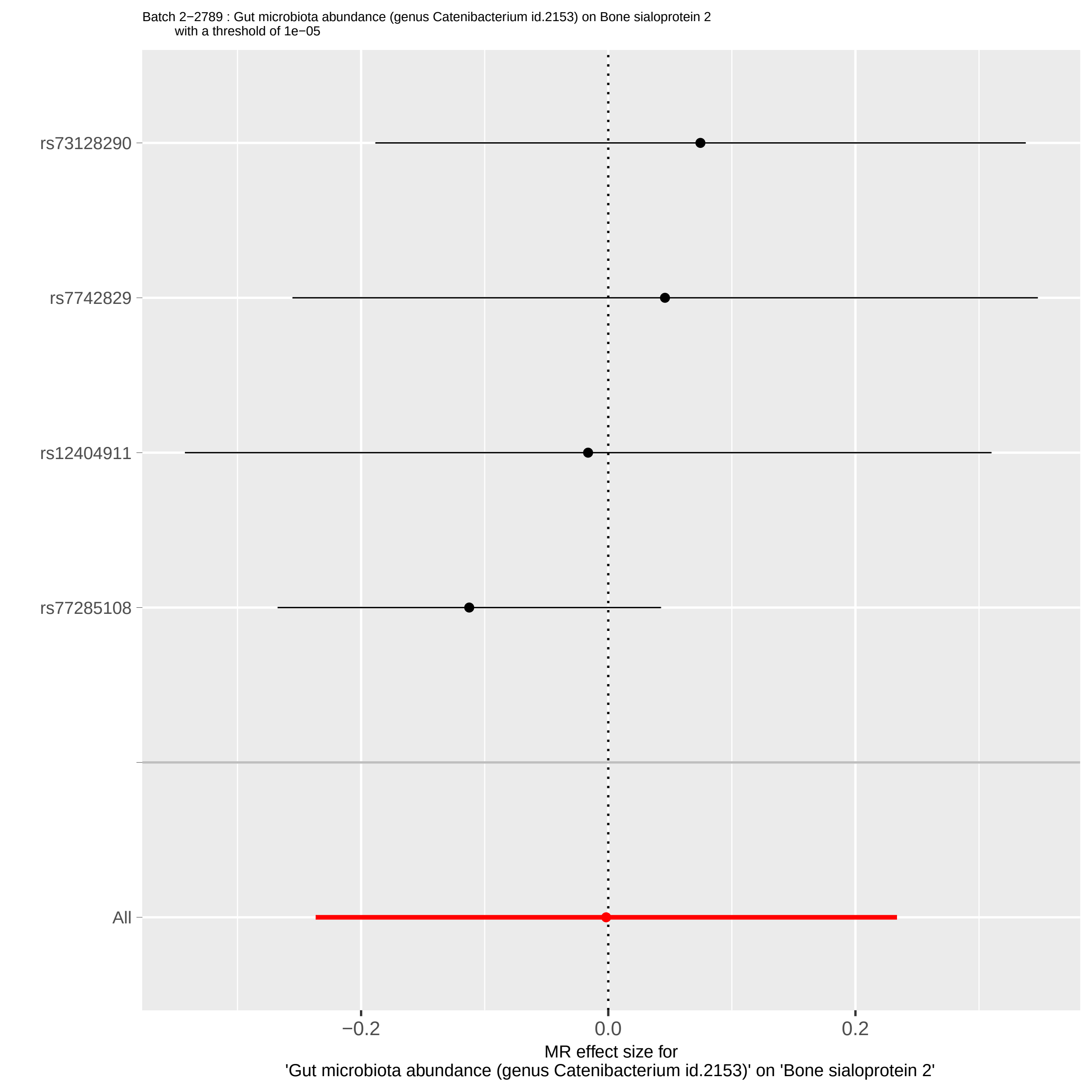

Batch 2−2789 : Gut microbiota abundance (genus Catenibacterium id.2153) on Bone sialoprotein 2
with a threshold of 1e−05
rs73128290
rs7742829
rs12404911
rs77285108
All
−0.2
0.0
0.2
MR effect size for
'Gut microbiota abundance (genus Catenibacterium id.2153)' on 'Bone sialoprotein 2'

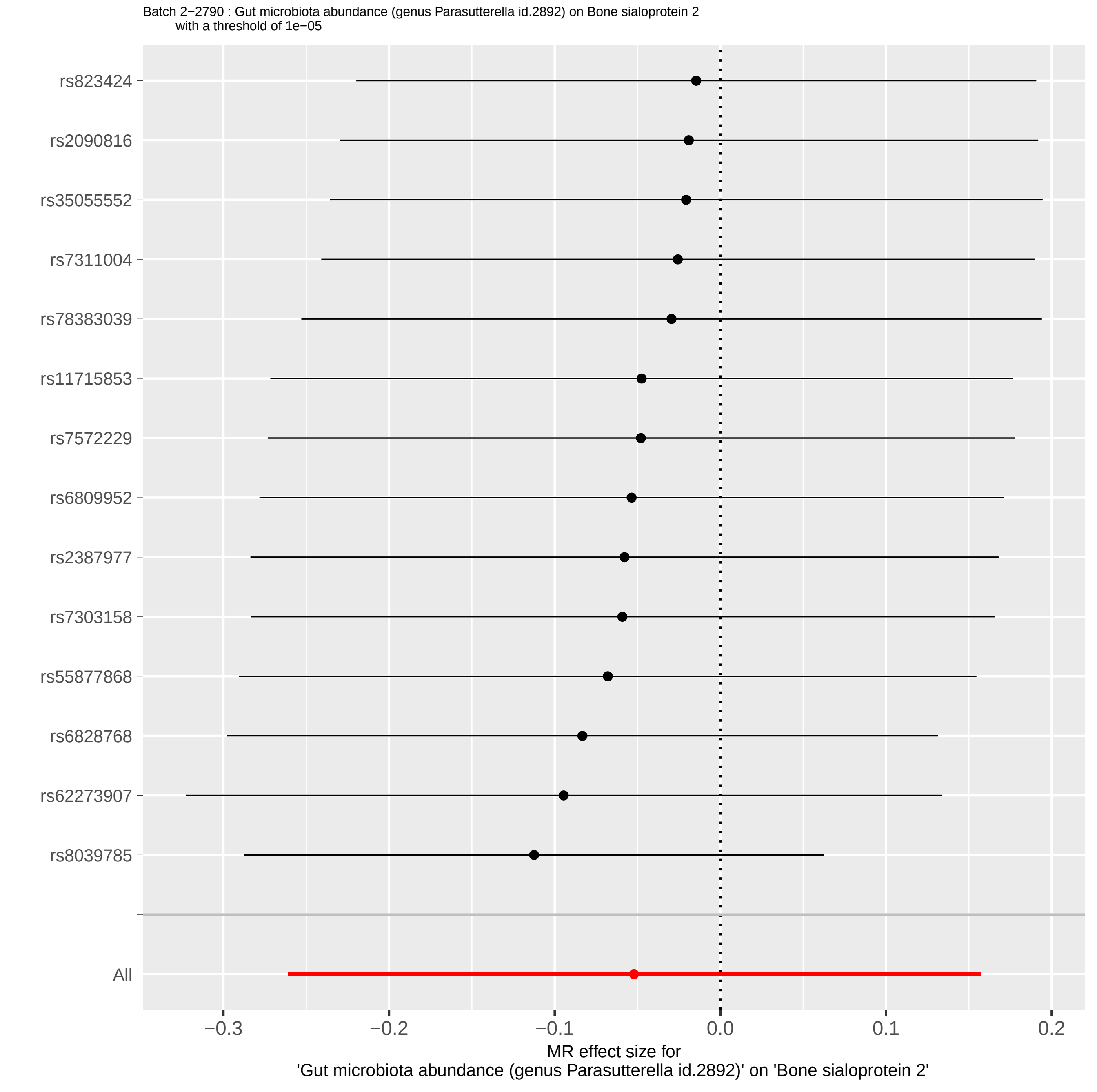

Batch 2−2790 : Gut microbiota abundance (genus Parasutterella id.2892) on Bone sialoprotein 2
with a threshold of 1e−05
rs823424
rs2090816
rs35055552
rs7311004
rs78383039
rs11715853
rs7572229
rs6809952
rs2387977
rs7303158
rs55877868
rs6828768
rs62273907
rs8039785
All
−0.3
−0.2
−0.1
0.0
0.1
0.2
MR effect size for
'Gut microbiota abundance (genus Parasutterella id.2892)' on 'Bone sialoprotein 2'

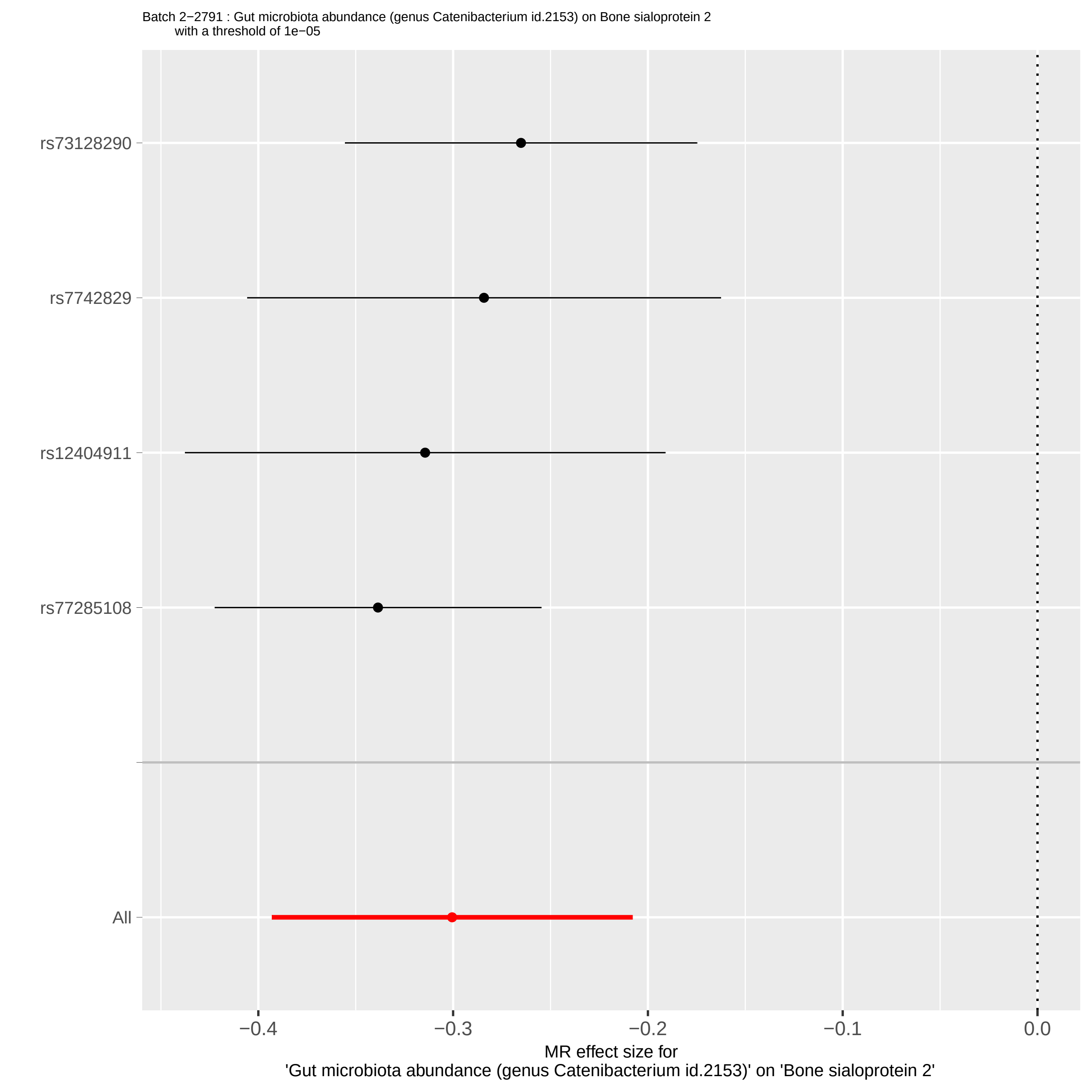
Batch 2−2791 : Gut microbiota abundance (genus Catenibacterium id.2153) on Bone sialoprotein 2
with a threshold of 1e−05
rs73128290
rs7742829
rs12404911
rs77285108
All
−0.4
−0.3
−0.2
−0.1
0.0
MR effect size for
'Gut microbiota abundance (genus Catenibacterium id.2153)' on 'Bone sialoprotein 2'

Batch 2−2792 : Gut microbiota abundance (genus Parasutterella id.2892) on Bone sialoprotein 2
with a threshold of 1e−05

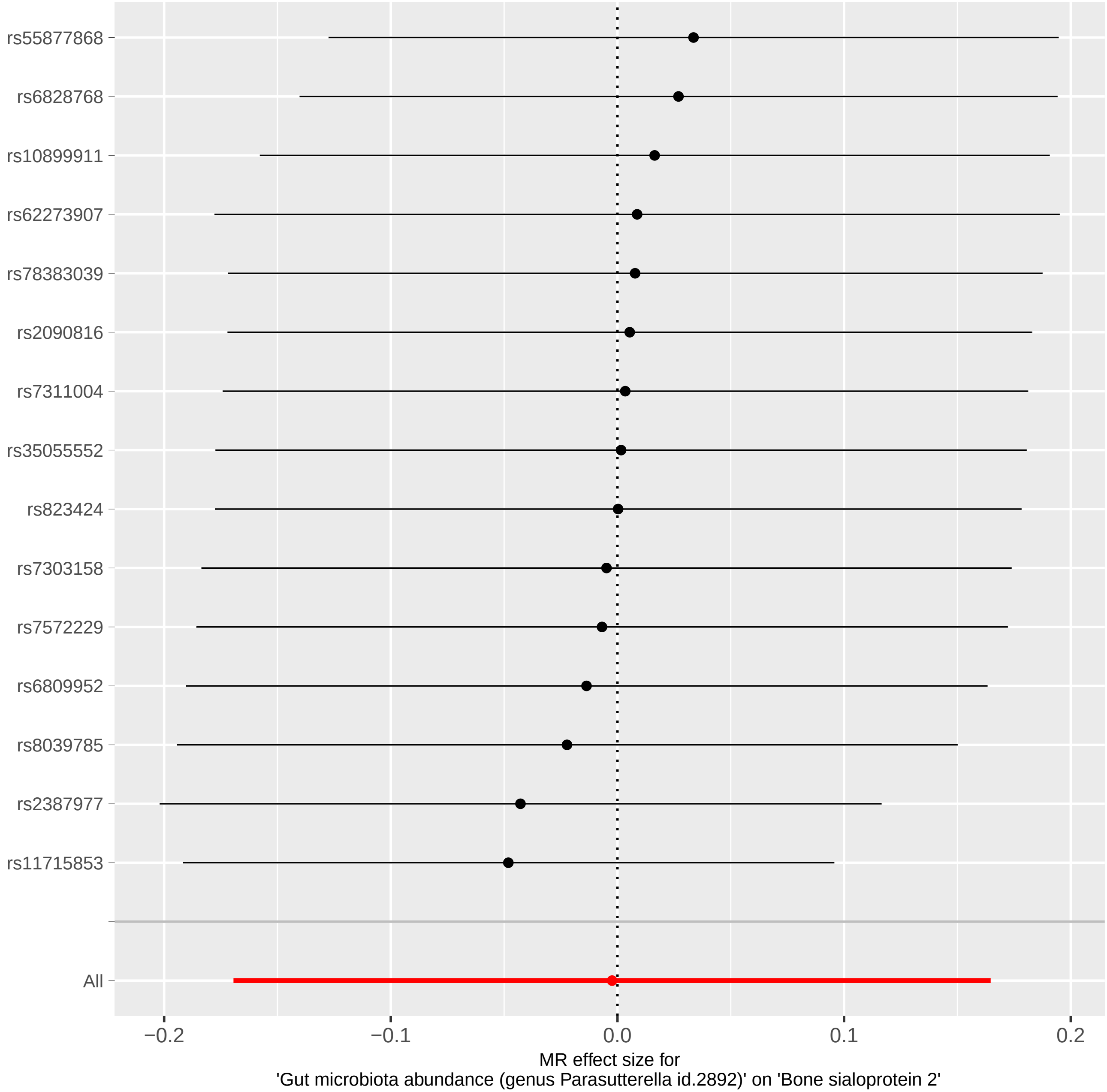

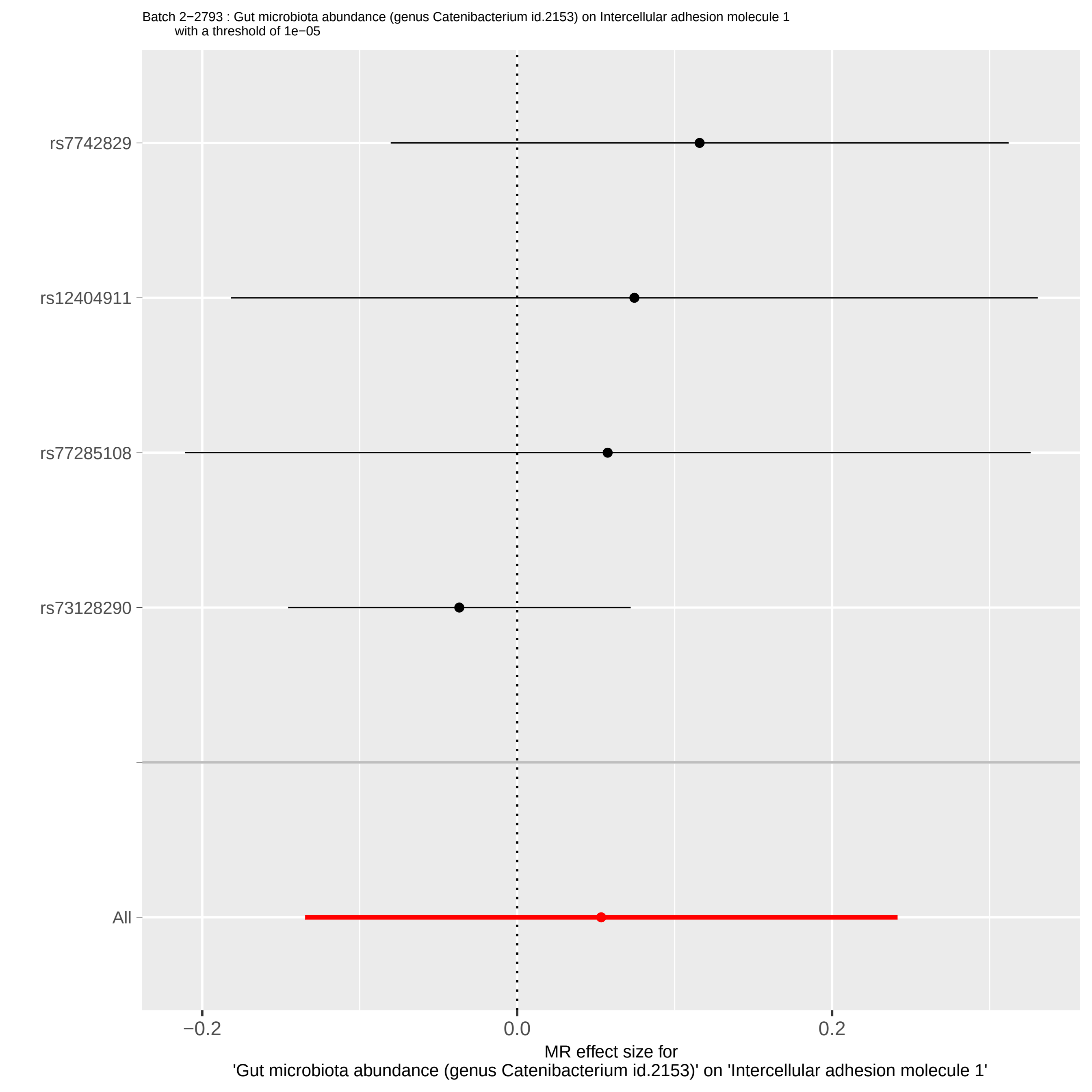

Batch 2−2793 : Gut microbiota abundance (genus Catenibacterium id.2153) on Intercellular adhesion molecule 1
with a threshold of 1e−05
rs7742829
rs12404911
rs77285108
rs73128290
All
−0.2
0.0
0.2
MR effect size for
'Gut microbiota abundance (genus Catenibacterium id.2153)' on 'Intercellular adhesion molecule 1'

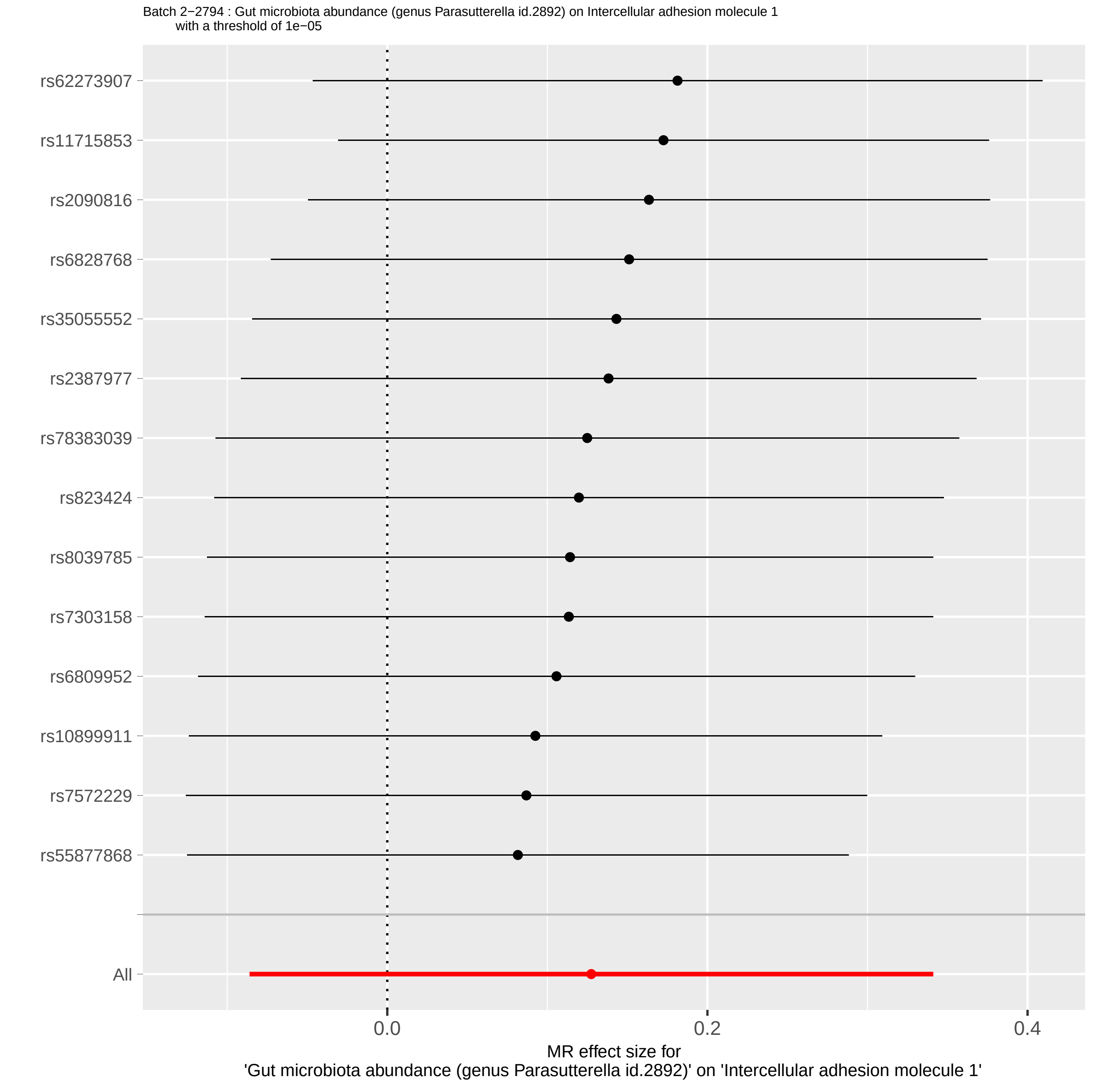
Batch 2−2794 : Gut microbiota abundance (genus Parasutterella id.2892) on Intercellular adhesion molecule 1
with a threshold of 1e−05
rs62273907
rs11715853
rs2090816
rs6828768
rs35055552
rs2387977
rs78383039
rs823424
rs8039785
rs7303158
rs6809952
rs10899911
rs7572229
rs55877868
All
0.0
0.2
0.4
MR effect size for
'Gut microbiota abundance (genus Parasutterella id.2892)' on 'Intercellular adhesion molecule 1'

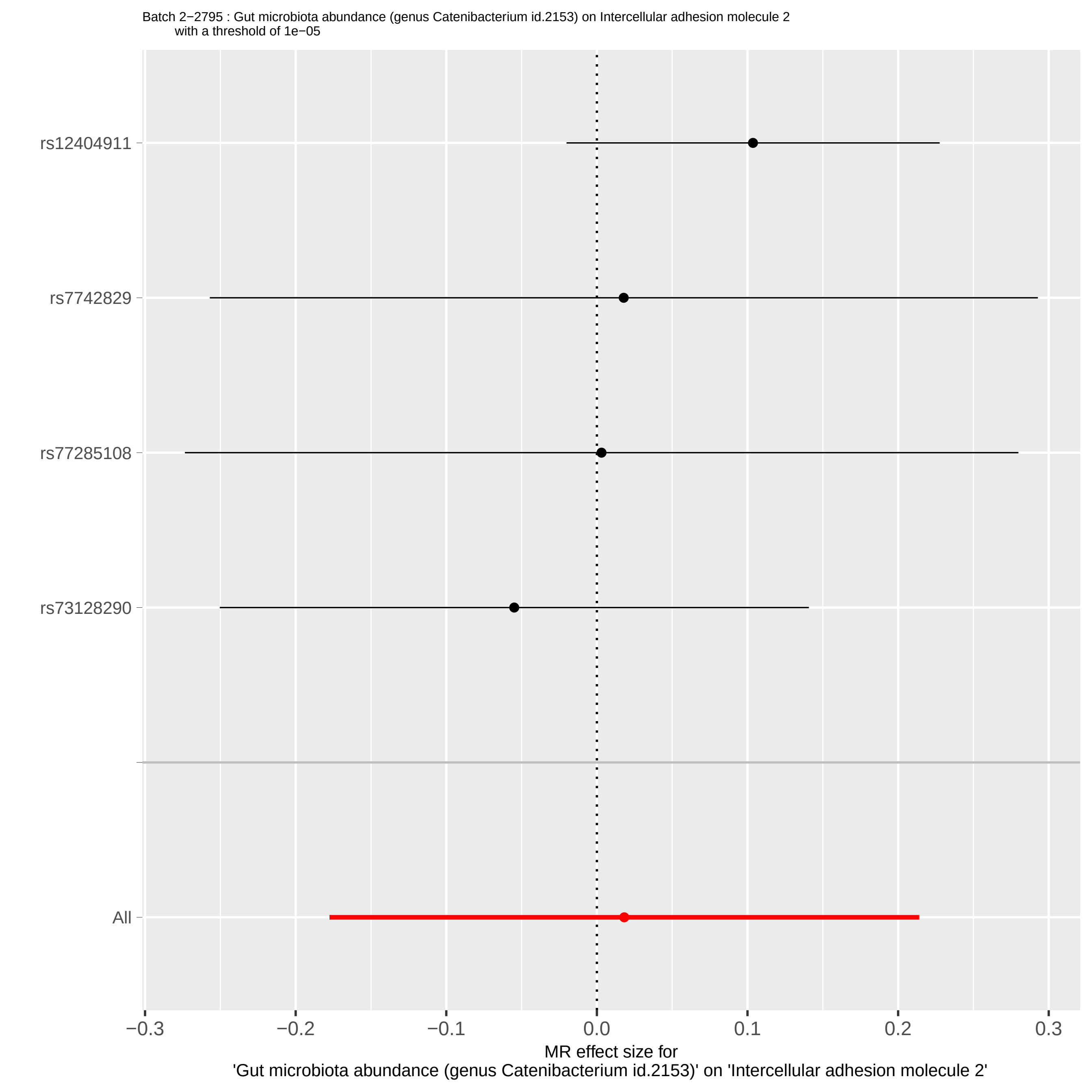
Batch 2−2795 : Gut microbiota abundance (genus Catenibacterium id.2153) on Intercellular adhesion molecule 2
with a threshold of 1e−05
rs12404911
rs7742829
rs77285108
rs73128290
All
−0.3
−0.2
−0.1
0.0
0.1
0.2
0.3
MR effect size for
'Gut microbiota abundance (genus Catenibacterium id.2153)' on 'Intercellular adhesion molecule 2'

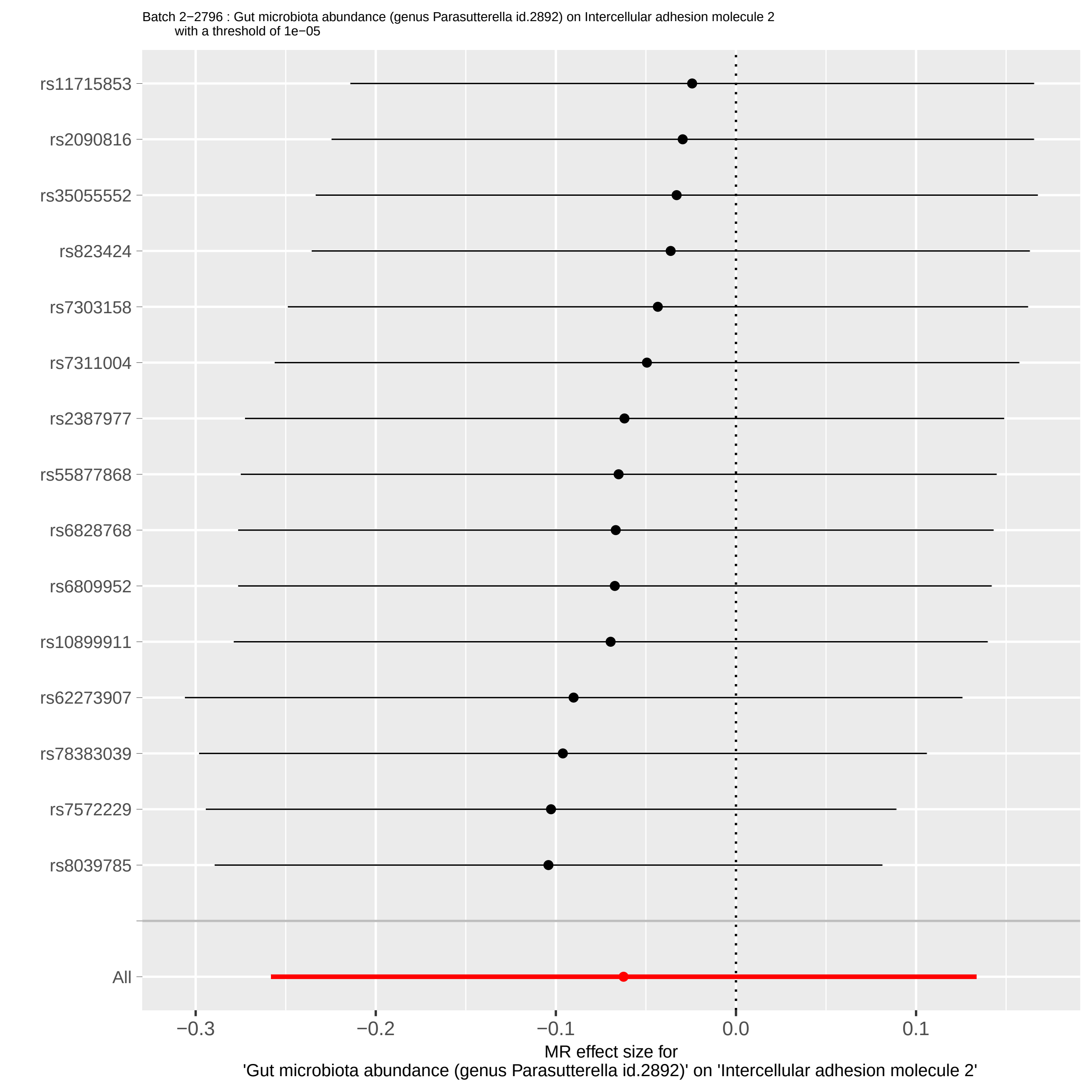
Batch 2−2796 : Gut microbiota abundance (genus Parasutterella id.2892) on Intercellular adhesion molecule 2
with a threshold of 1e−05
rs11715853
rs2090816
rs35055552
rs823424
rs7303158
rs7311004
rs2387977
rs55877868
rs6828768
rs6809952
rs10899911
rs62273907
rs78383039
rs7572229
rs8039785
All
−0.3
−0.2
−0.1
0.0
0.1
MR effect size for
'Gut microbiota abundance (genus Parasutterella id.2892)' on 'Intercellular adhesion molecule 2'

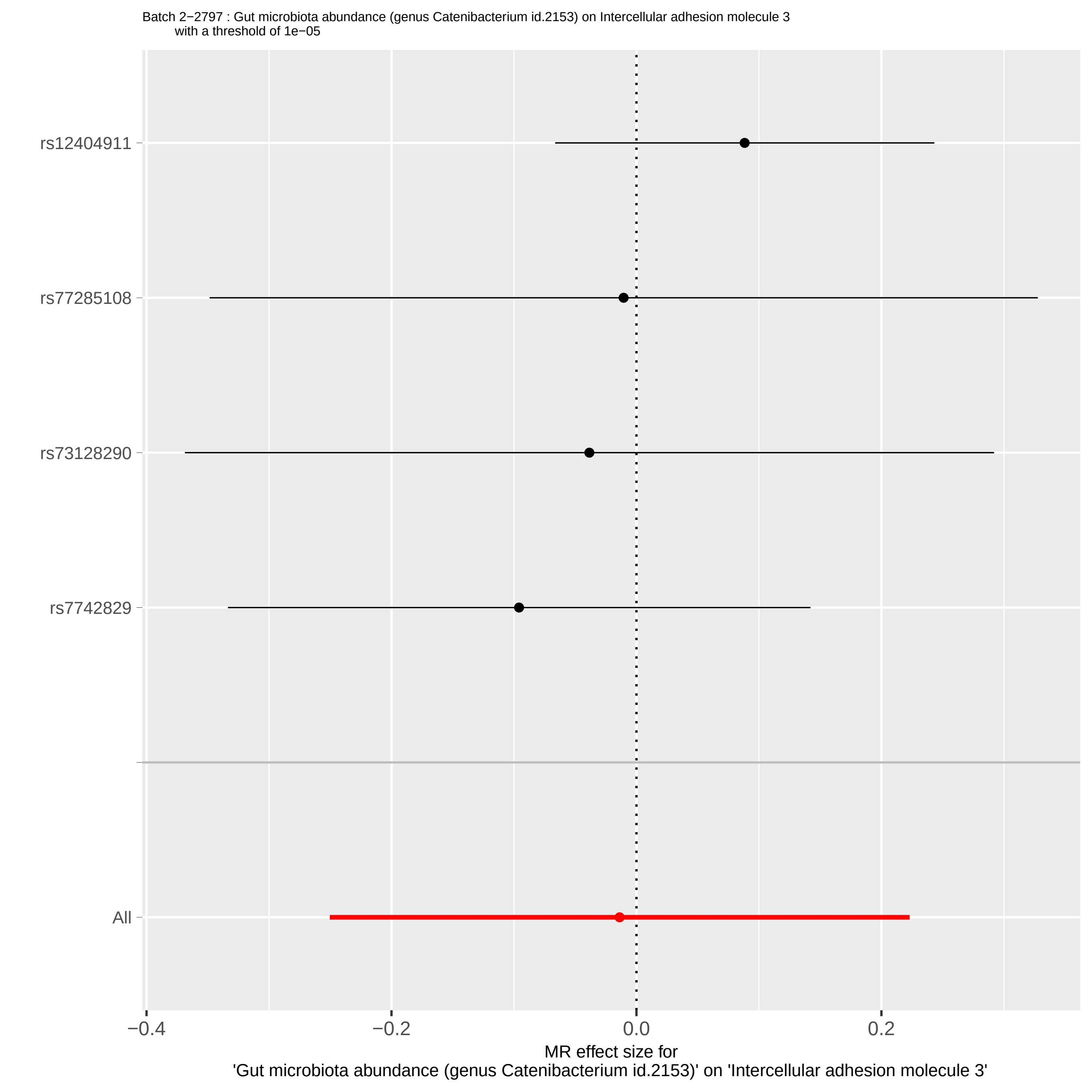
Batch 2−2797 : Gut microbiota abundance (genus Catenibacterium id.2153) on Intercellular adhesion molecule 3
with a threshold of 1e−05
rs12404911
rs77285108
rs73128290
rs7742829
All
−0.4
−0.2
0.0
0.2
MR effect size for
'Gut microbiota abundance (genus Catenibacterium id.2153)' on 'Intercellular adhesion molecule 3'

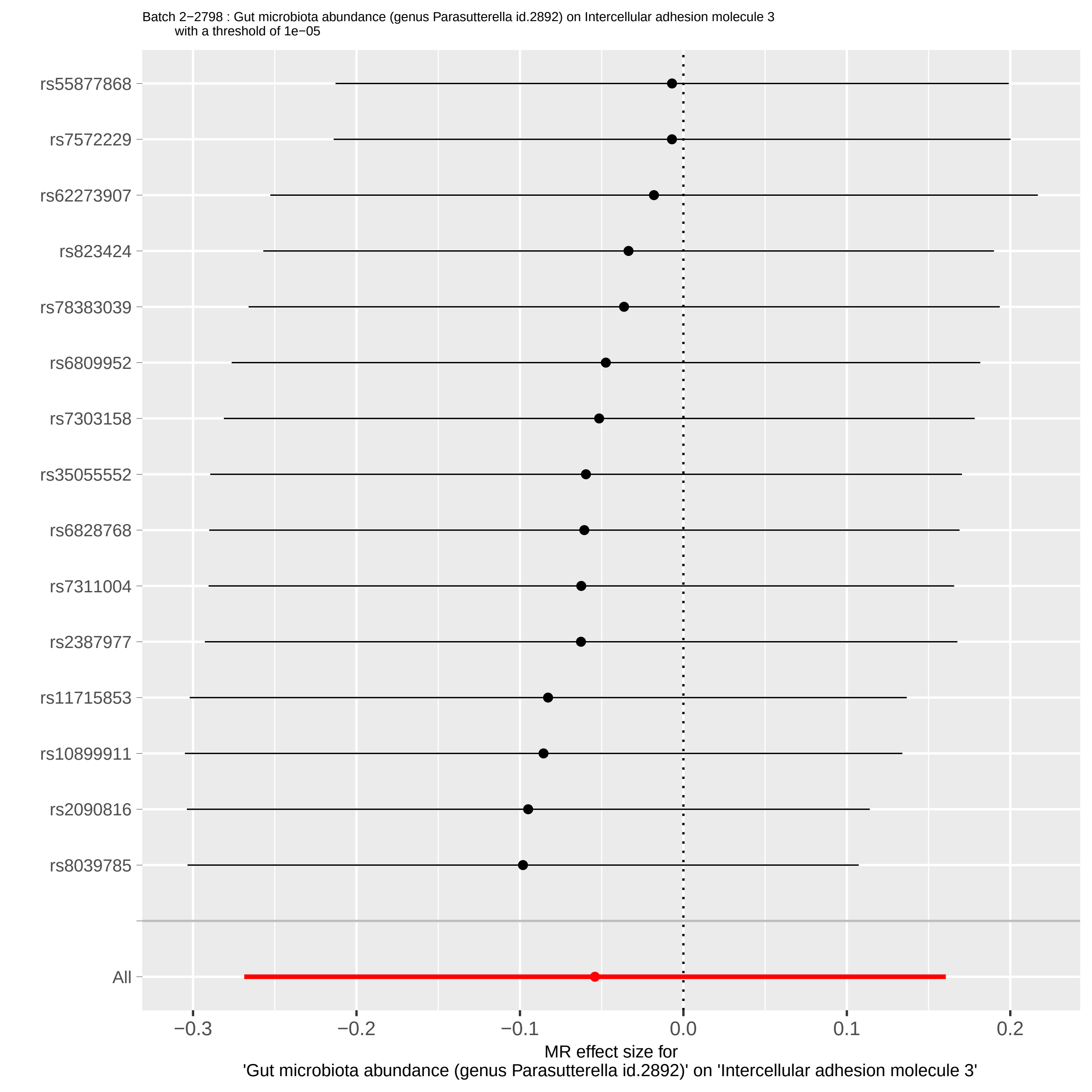
Batch 2−2798 : Gut microbiota abundance (genus Parasutterella id.2892) on Intercellular adhesion molecule 3
with a threshold of 1e−05
rs55877868
rs7572229
rs62273907
rs823424
rs78383039
rs6809952
rs7303158
rs35055552
rs6828768
rs7311004
rs2387977
rs11715853
rs10899911
rs2090816
rs8039785
All
−0.3
−0.2
−0.1
0.0
0.1
0.2
MR effect size for
'Gut microbiota abundance (genus Parasutterella id.2892)' on 'Intercellular adhesion molecule 3'

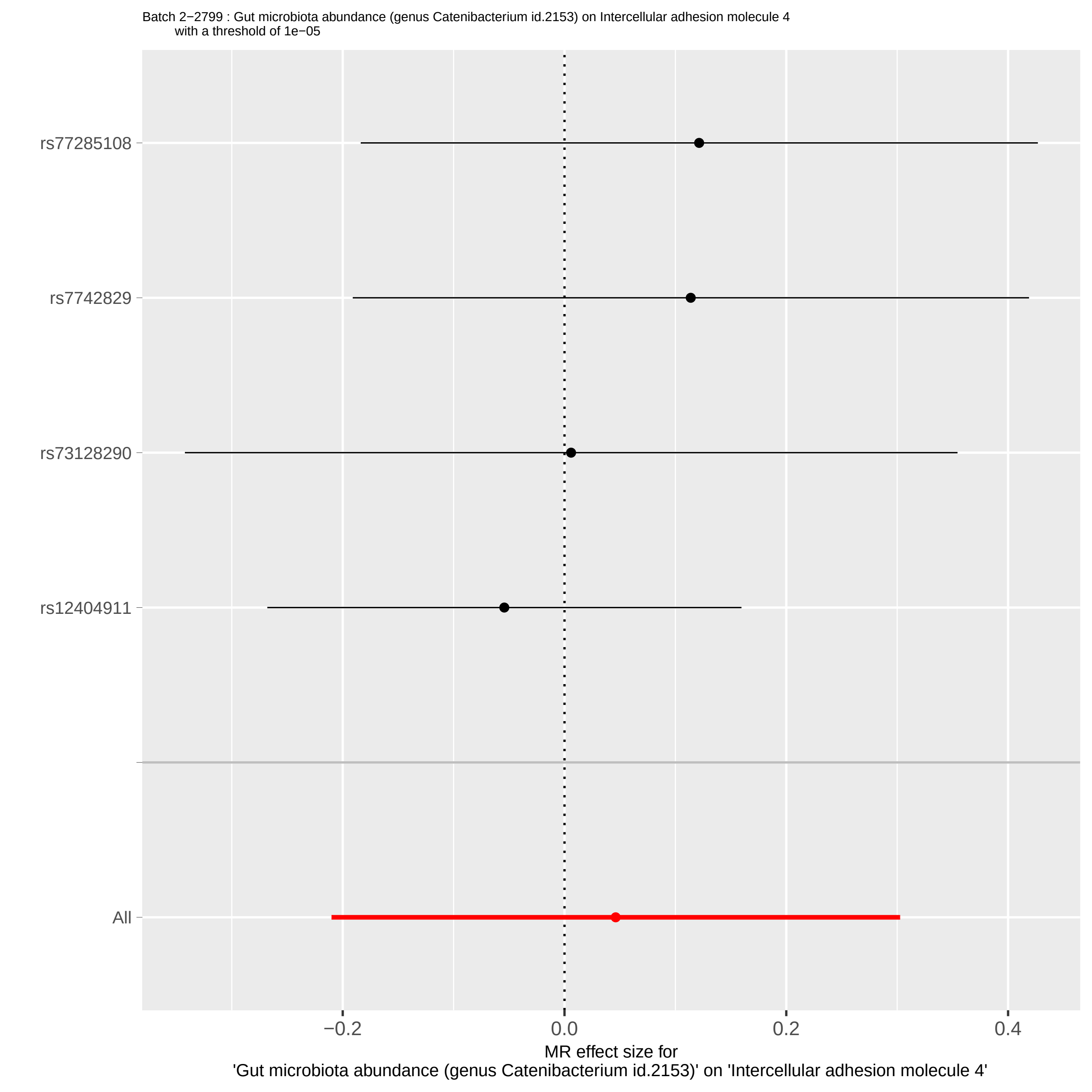
Batch 2−2799 : Gut microbiota abundance (genus Catenibacterium id.2153) on Intercellular adhesion molecule 4
with a threshold of 1e−05
rs77285108
rs7742829
rs73128290
rs12404911
All
−0.2
0.0
0.2
0.4
MR effect size for
'Gut microbiota abundance (genus Catenibacterium id.2153)' on 'Intercellular adhesion molecule 4'

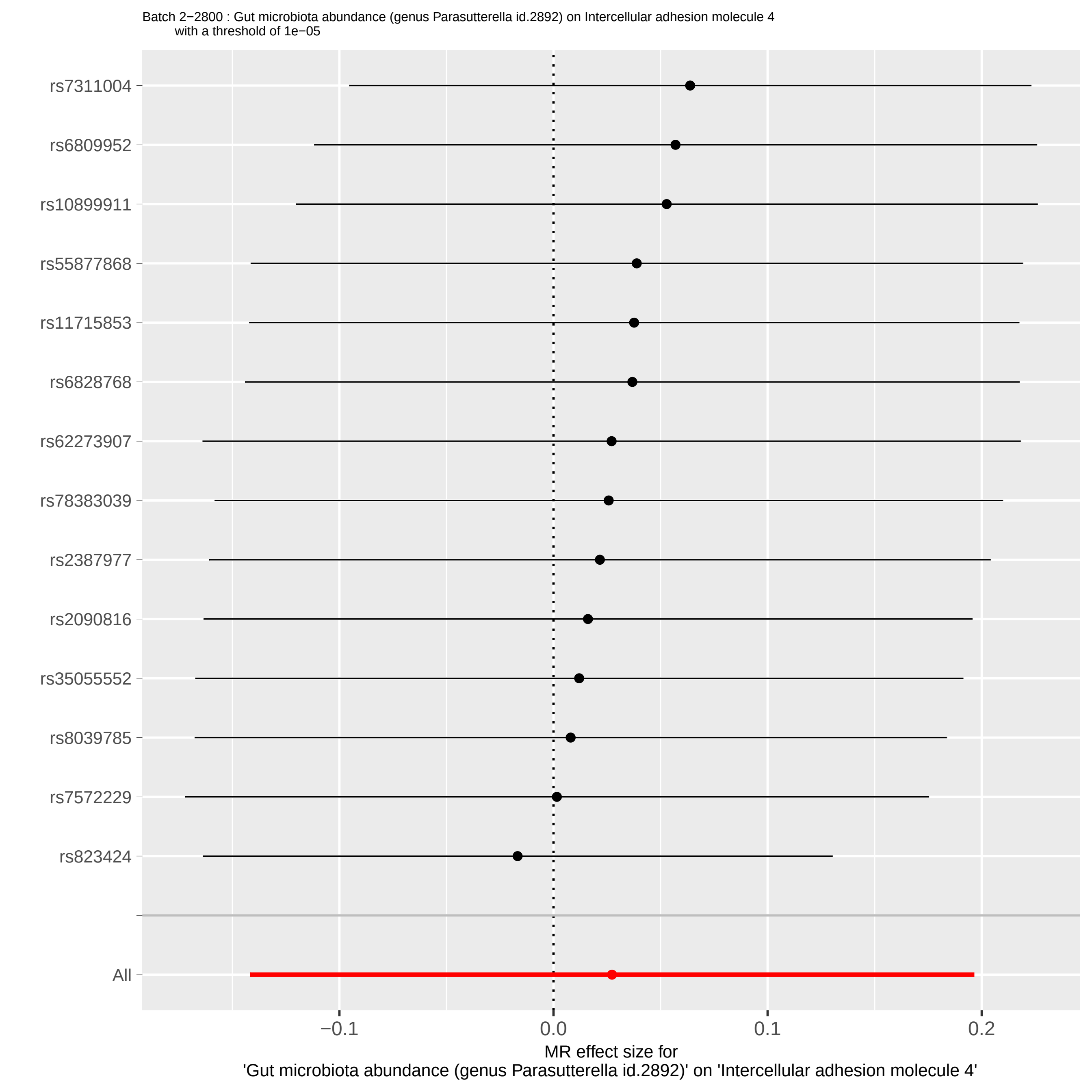

Batch 2−2800 : Gut microbiota abundance (genus Parasutterella id.2892) on Intercellular adhesion molecule 4
with a threshold of 1e−05
rs7311004
rs6809952
rs10899911
rs55877868
rs11715853
rs6828768
rs62273907
rs78383039
rs2387977
rs2090816
rs35055552
rs8039785
rs7572229
rs823424
All
−0.1
0.0
0.1
0.2
MR effect size for
'Gut microbiota abundance (genus Parasutterella id.2892)' on 'Intercellular adhesion molecule 4'

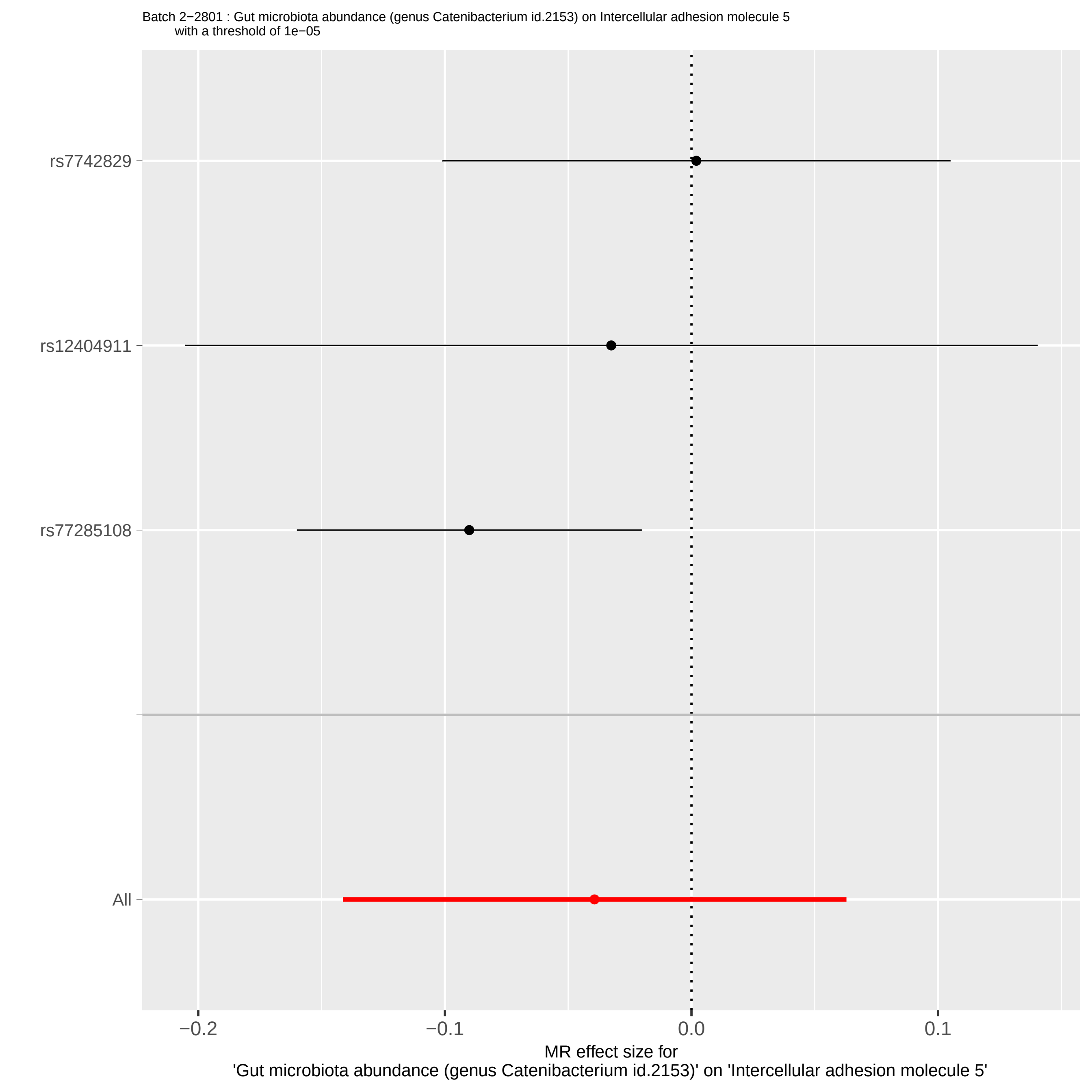
Batch 2−2801 : Gut microbiota abundance (genus Catenibacterium id.2153) on Intercellular adhesion molecule 5
with a threshold of 1e−05
rs7742829
rs12404911
rs77285108
All
−0.2
−0.1
0.0
0.1
MR effect size for
'Gut microbiota abundance (genus Catenibacterium id.2153)' on 'Intercellular adhesion molecule 5'

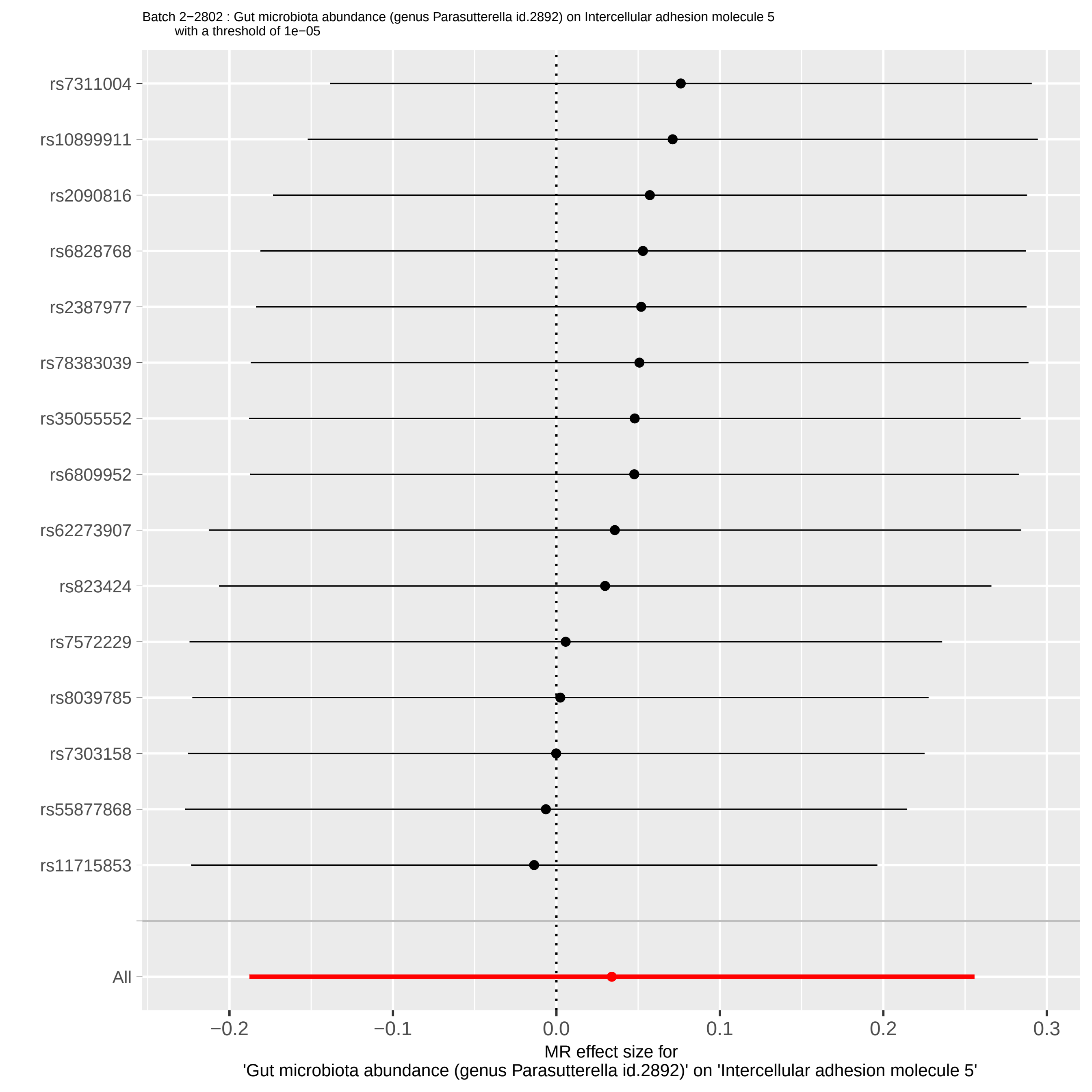

Batch 2−2802 : Gut microbiota abundance (genus Parasutterella id.2892) on Intercellular adhesion molecule 5
with a threshold of 1e−05
rs7311004
rs10899911
rs2090816
rs6828768
rs2387977
rs78383039
rs35055552
rs6809952
rs62273907
rs823424
rs7572229
rs8039785
rs7303158
rs55877868
rs11715853
All
−0.2
−0.1
0.0
0.1
0.2
0.3
MR effect size for
'Gut microbiota abundance (genus Parasutterella id.2892)' on 'Intercellular adhesion molecule 5'

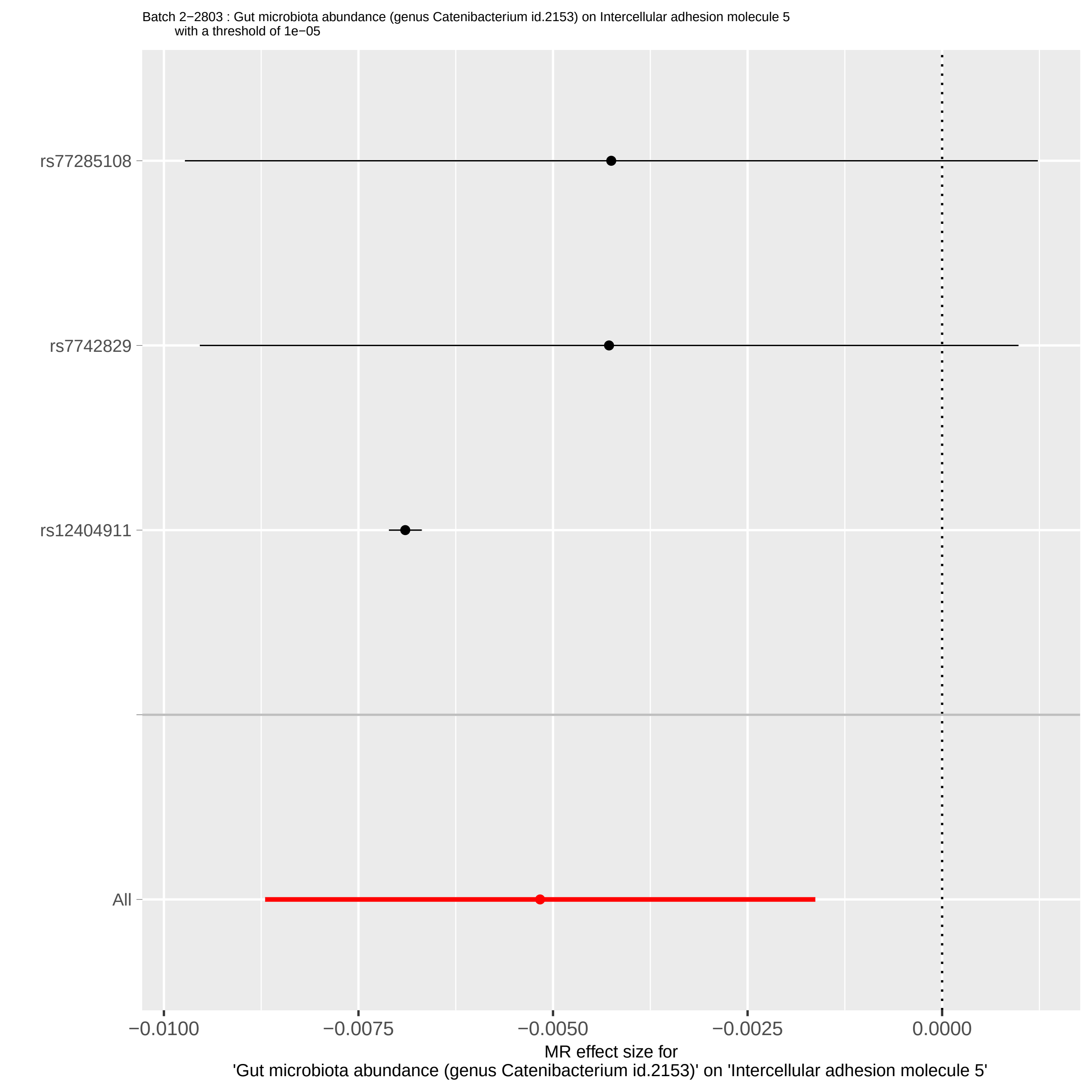
Batch 2−2803 : Gut microbiota abundance (genus Catenibacterium id.2153) on Intercellular adhesion molecule 5
with a threshold of 1e−05
rs77285108
rs7742829
rs12404911
All
−0.0100
−0.0075
−0.0050
−0.0025
0.0000
MR effect size for
'Gut microbiota abundance (genus Catenibacterium id.2153)' on 'Intercellular adhesion molecule 5'

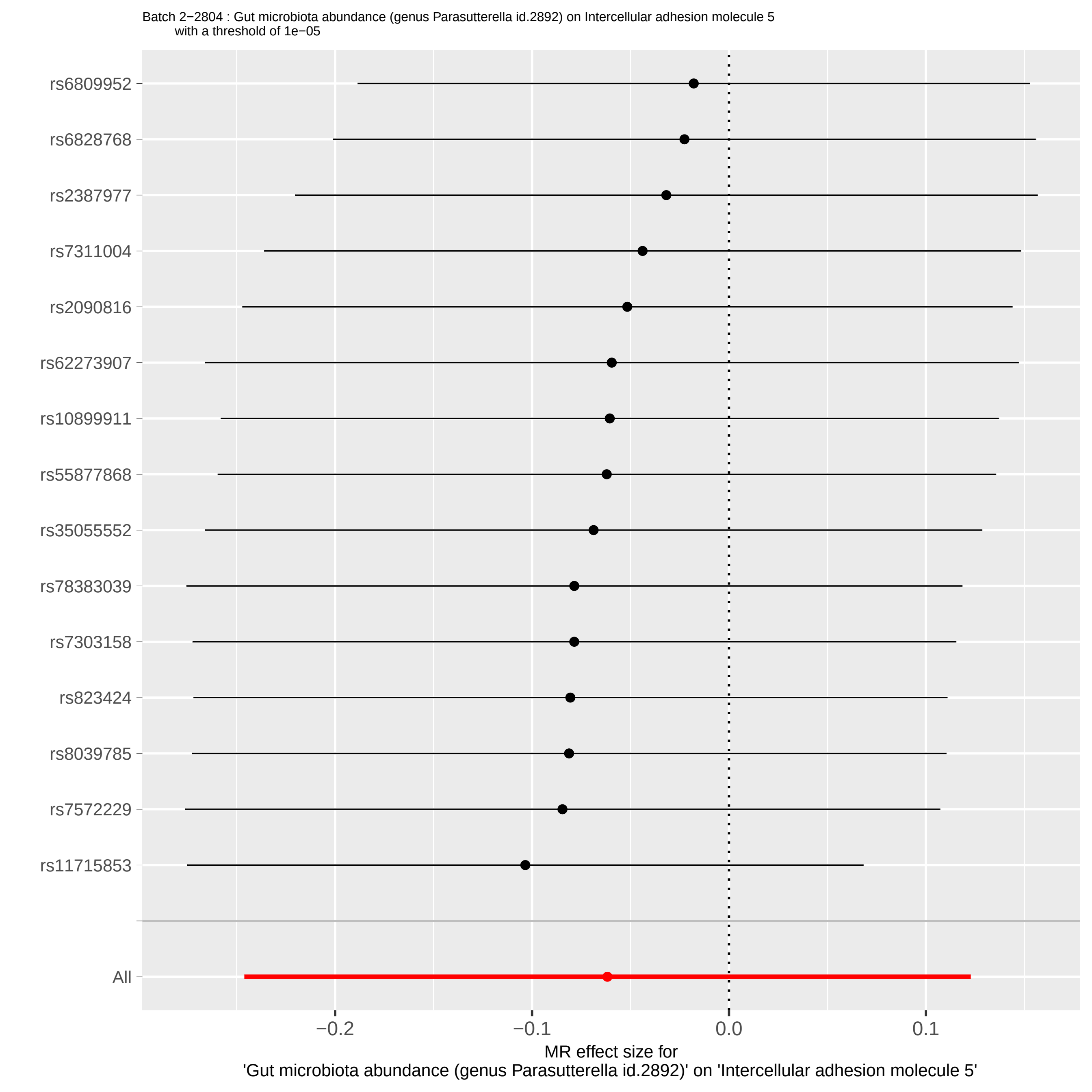

Batch 2−2804 : Gut microbiota abundance (genus Parasutterella id.2892) on Intercellular adhesion molecule 5
with a threshold of 1e−05
rs6809952
rs6828768
rs2387977
rs7311004
rs2090816
rs62273907
rs10899911
rs55877868
rs35055552
rs78383039
rs7303158
rs823424
rs8039785
rs7572229
rs11715853
All
−0.2
−0.1
0.0
0.1
MR effect size for
'Gut microbiota abundance (genus Parasutterella id.2892)' on 'Intercellular adhesion molecule 5'

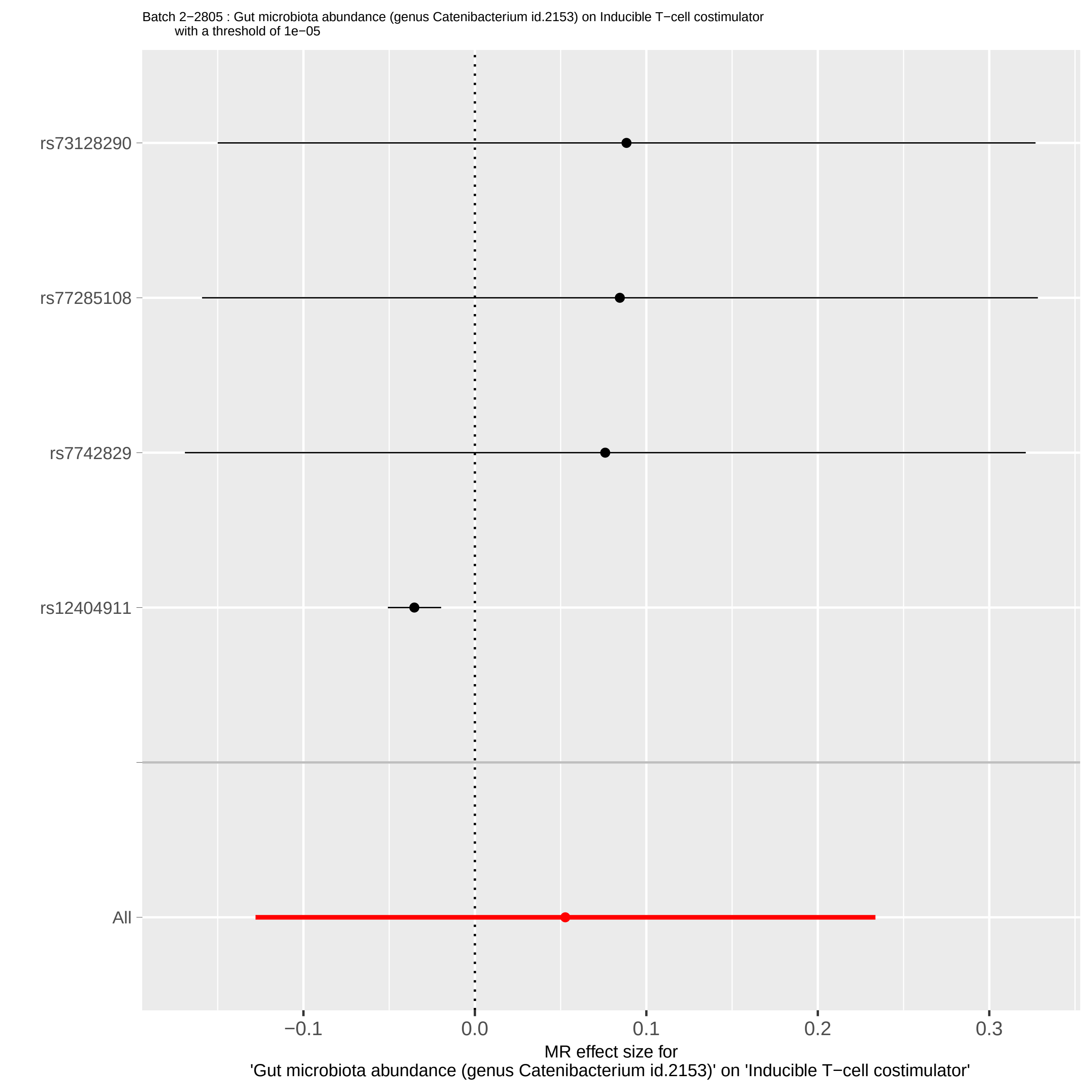
Batch 2−2805 : Gut microbiota abundance (genus Catenibacterium id.2153) on Inducible T−cell costimulator
with a threshold of 1e−05
rs73128290
rs77285108
rs7742829
rs12404911
All
−0.1
0.0
0.1
0.2
0.3
MR effect size for
'Gut microbiota abundance (genus Catenibacterium id.2153)' on 'Inducible T−cell costimulator'

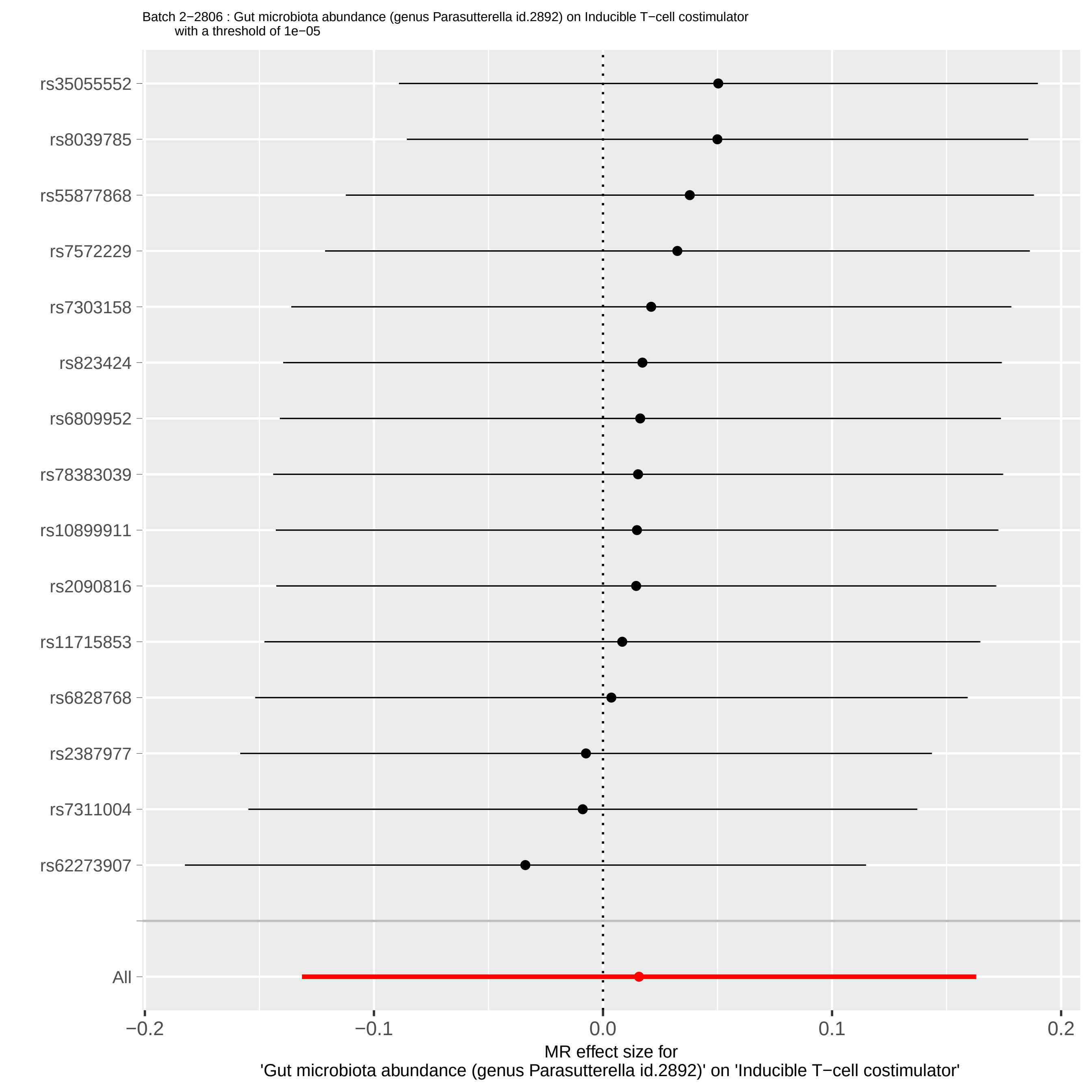
Batch 2−2806 : Gut microbiota abundance (genus Parasutterella id.2892) on Inducible T−cell costimulator
with a threshold of 1e−05
rs35055552
rs8039785
rs55877868
rs7572229
rs7303158
rs823424
rs6809952
rs78383039
rs10899911
rs2090816
rs11715853
rs6828768
rs2387977
rs7311004
rs62273907
All
−0.2
−0.1
0.0
0.1
0.2
MR effect size for
'Gut microbiota abundance (genus Parasutterella id.2892)' on 'Inducible T−cell costimulator'

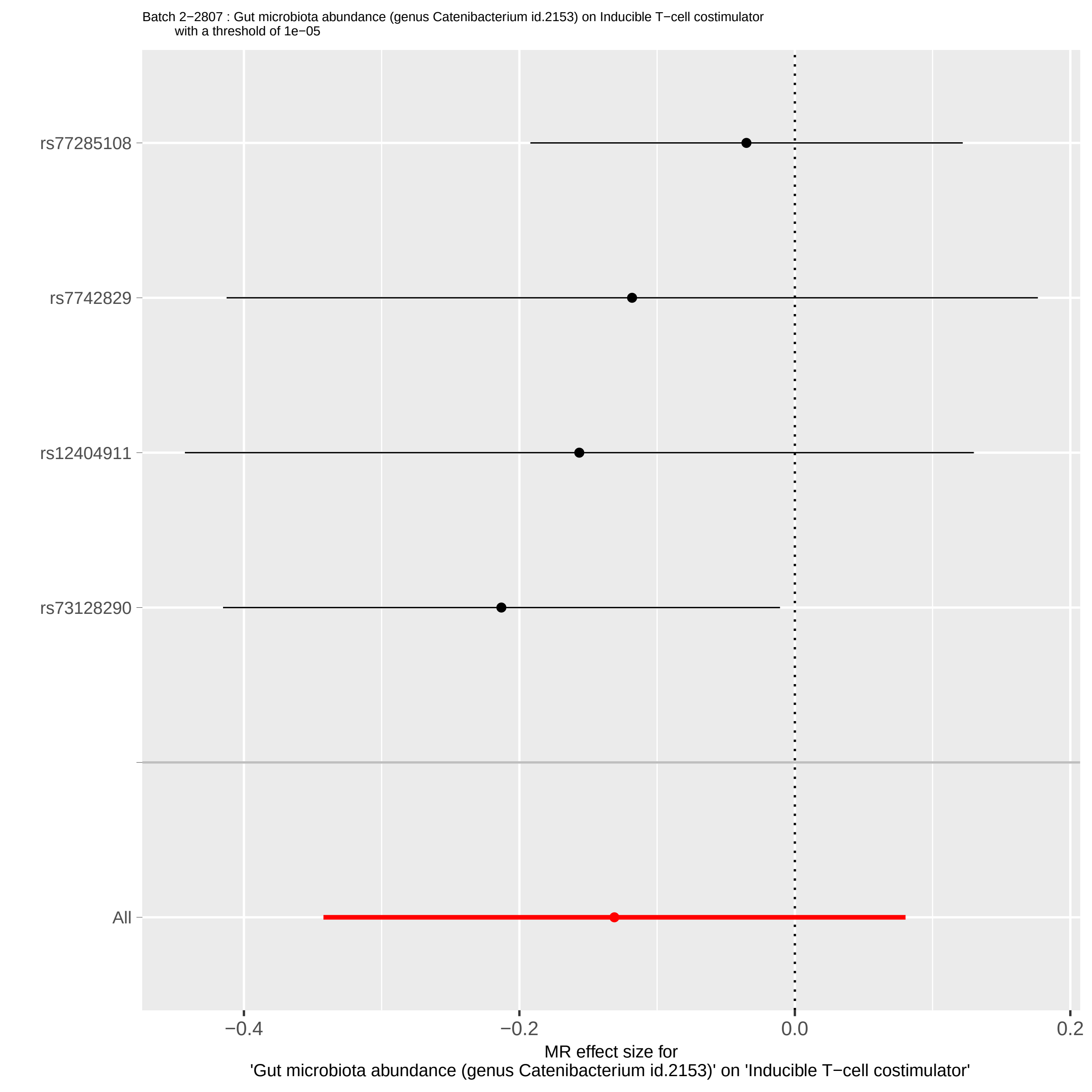

Batch 2−2807 : Gut microbiota abundance (genus Catenibacterium id.2153) on Inducible T−cell costimulator
with a threshold of 1e−05
rs77285108
rs7742829
rs12404911
rs73128290
All
−0.4
−0.2
0.0
0.2
MR effect size for
'Gut microbiota abundance (genus Catenibacterium id.2153)' on 'Inducible T−cell costimulator'

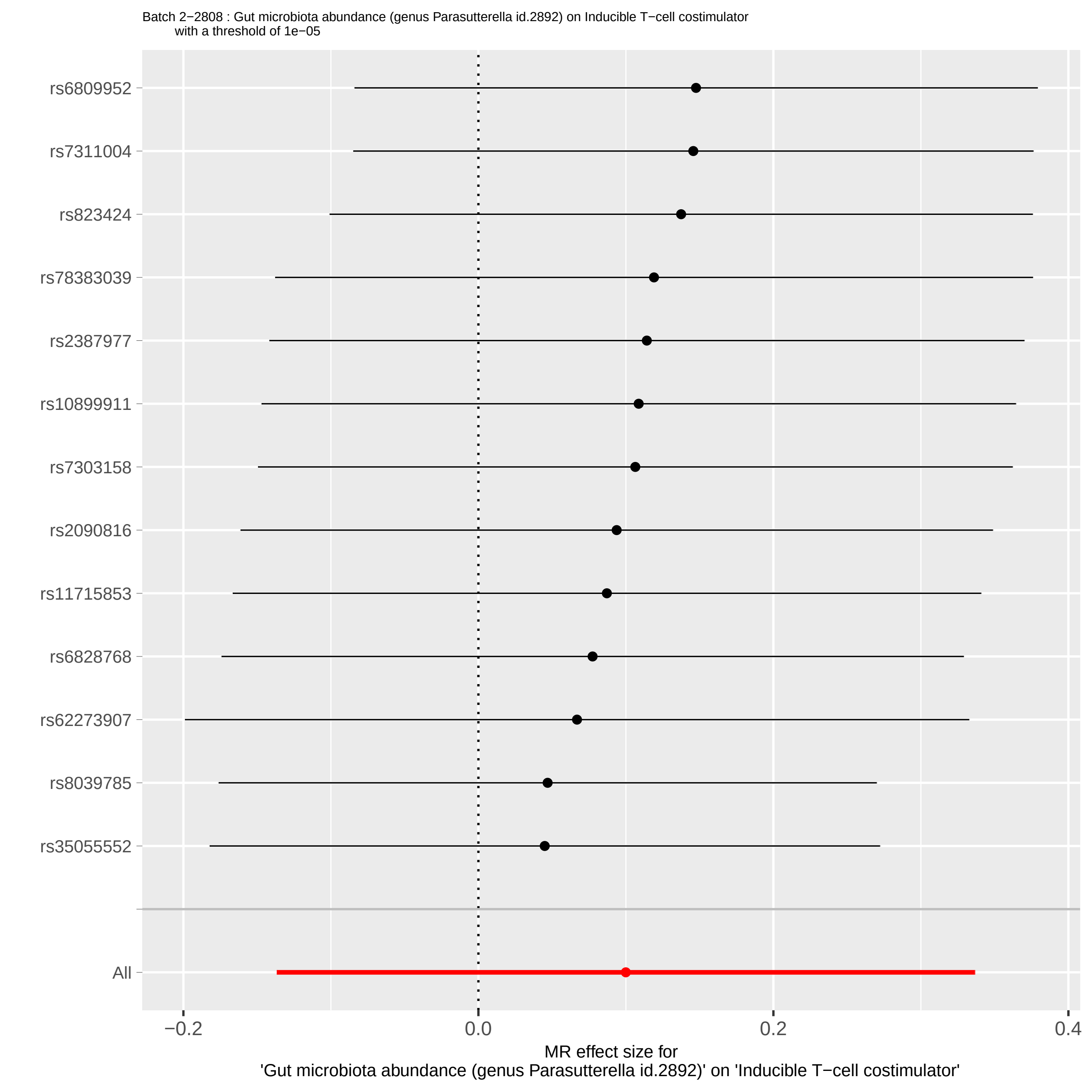
Batch 2−2808 : Gut microbiota abundance (genus Parasutterella id.2892) on Inducible T−cell costimulator
with a threshold of 1e−05
rs6809952
rs7311004
rs823424
rs78383039
rs2387977
rs10899911
rs7303158
rs2090816
rs11715853
rs6828768
rs62273907
rs8039785
rs35055552
All
−0.2
0.0
0.2
0.4
MR effect size for
'Gut microbiota abundance (genus Parasutterella id.2892)' on 'Inducible T−cell costimulator'

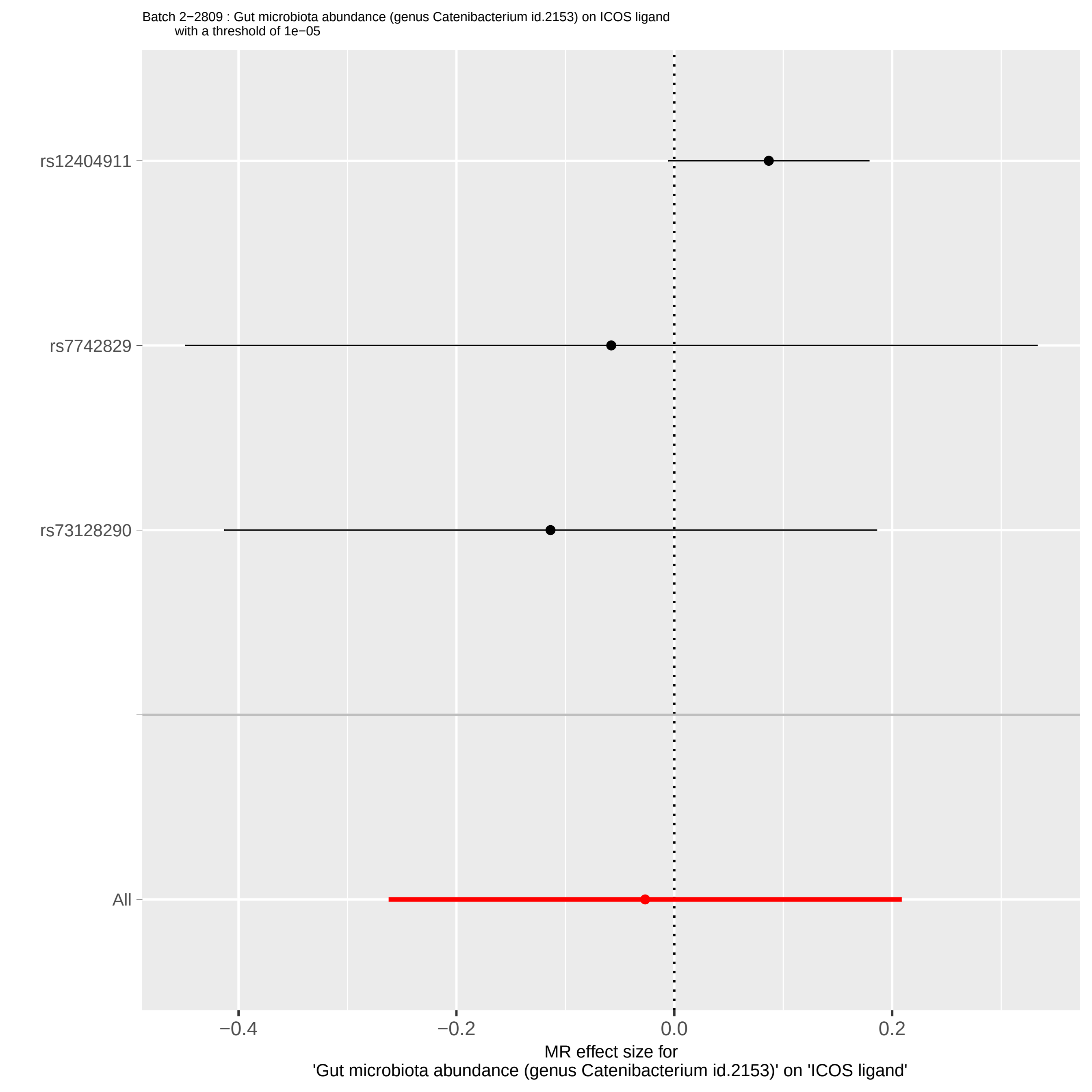
Batch 2−2809 : Gut microbiota abundance (genus Catenibacterium id.2153) on ICOS ligand
with a threshold of 1e−05
rs12404911
rs7742829
rs73128290
All
−0.4
−0.2
0.0
0.2
MR effect size for
'Gut microbiota abundance (genus Catenibacterium id.2153)' on 'ICOS ligand'

Batch 2−2810 : Gut microbiota abundance (genus Parasutterella id.2892) on ICOS ligand
with a threshold of 1e−05

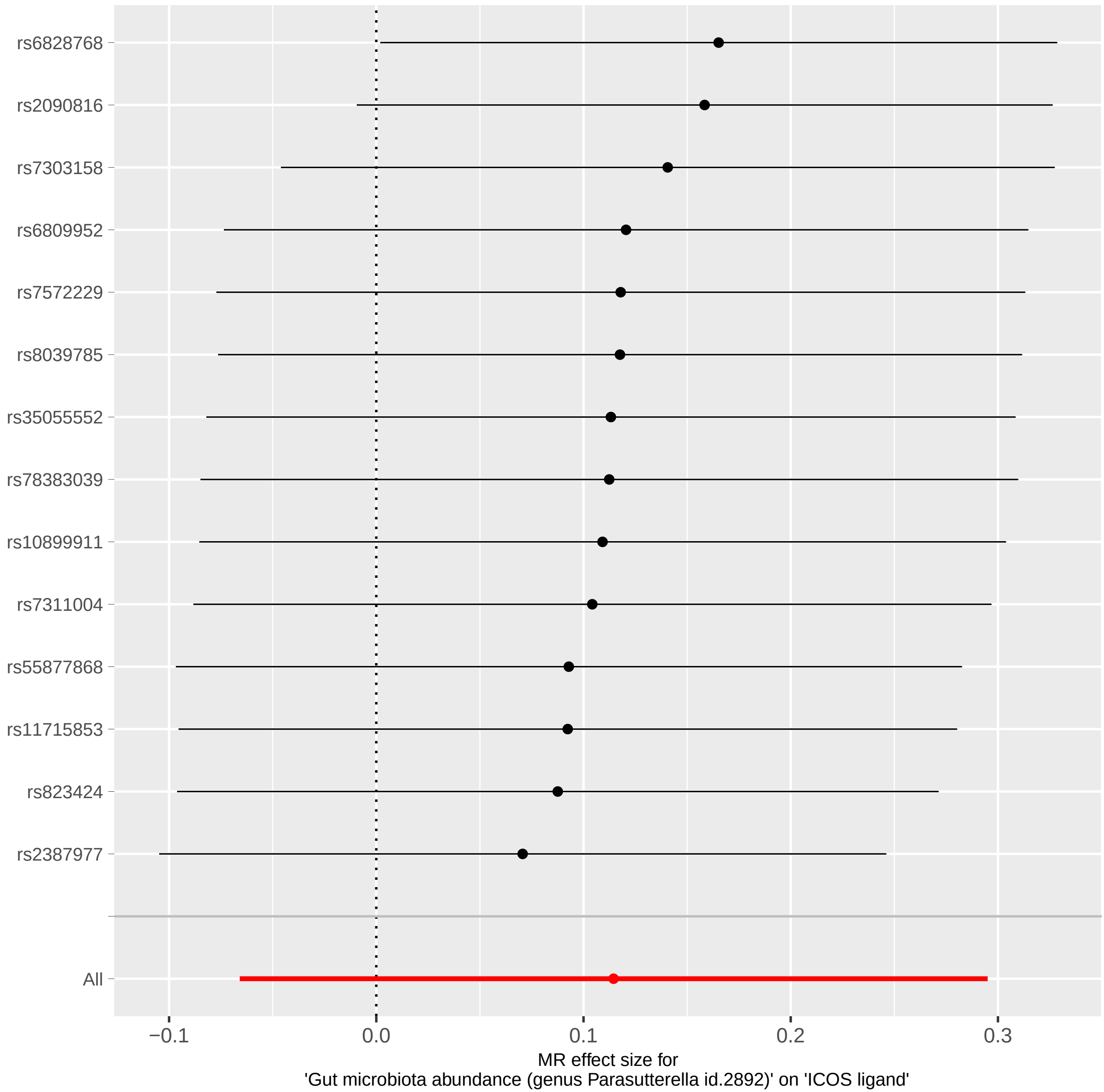

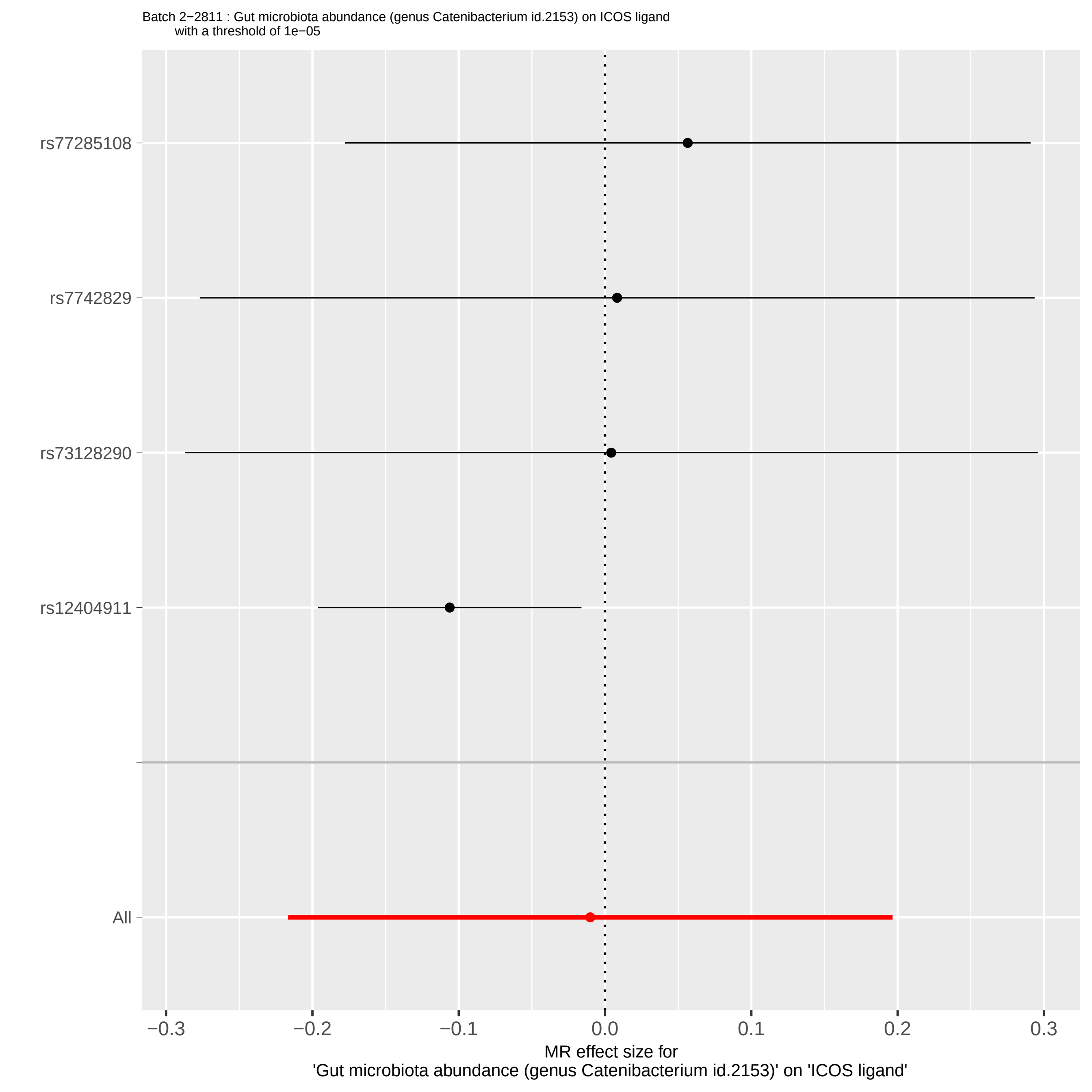

Batch 2−2811 : Gut microbiota abundance (genus Catenibacterium id.2153) on ICOS ligand
with a threshold of 1e−05
rs77285108
rs7742829
rs73128290
rs12404911
All
−0.3
−0.2
−0.1
0.0
0.1
0.2
0.3
MR effect size for
'Gut microbiota abundance (genus Catenibacterium id.2153)' on 'ICOS ligand'

Batch 2−2812 : Gut microbiota abundance (genus Parasutterella id.2892) on ICOS ligand
with a threshold of 1e−05

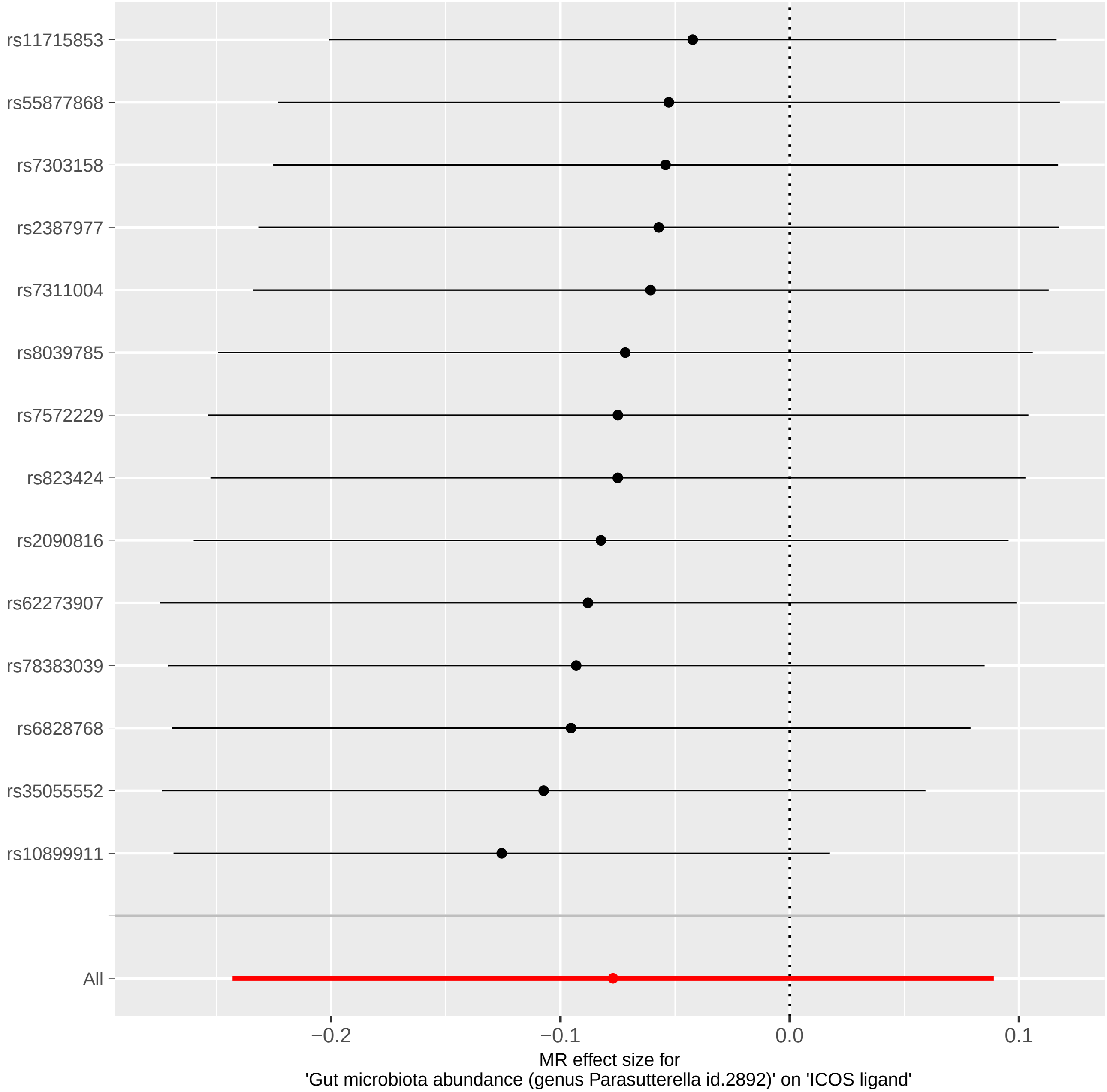

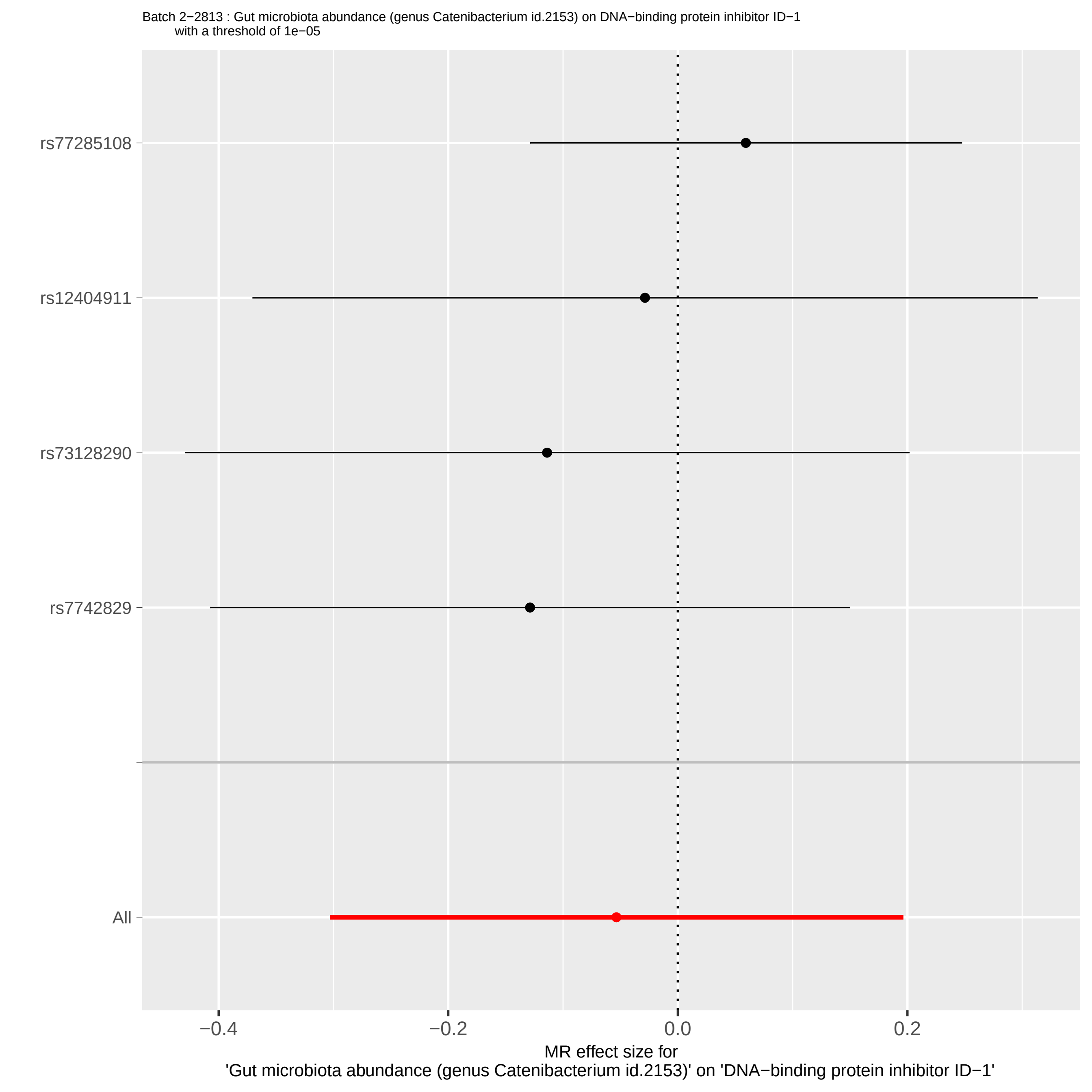

Batch 2−2813 : Gut microbiota abundance (genus Catenibacterium id.2153) on DNA−binding protein inhibitor ID−1
with a threshold of 1e−05
rs77285108
rs12404911
rs73128290
rs7742829
All
−0.4
−0.2
0.0
0.2
MR effect size for
'Gut microbiota abundance (genus Catenibacterium id.2153)' on 'DNA−binding protein inhibitor ID−1'

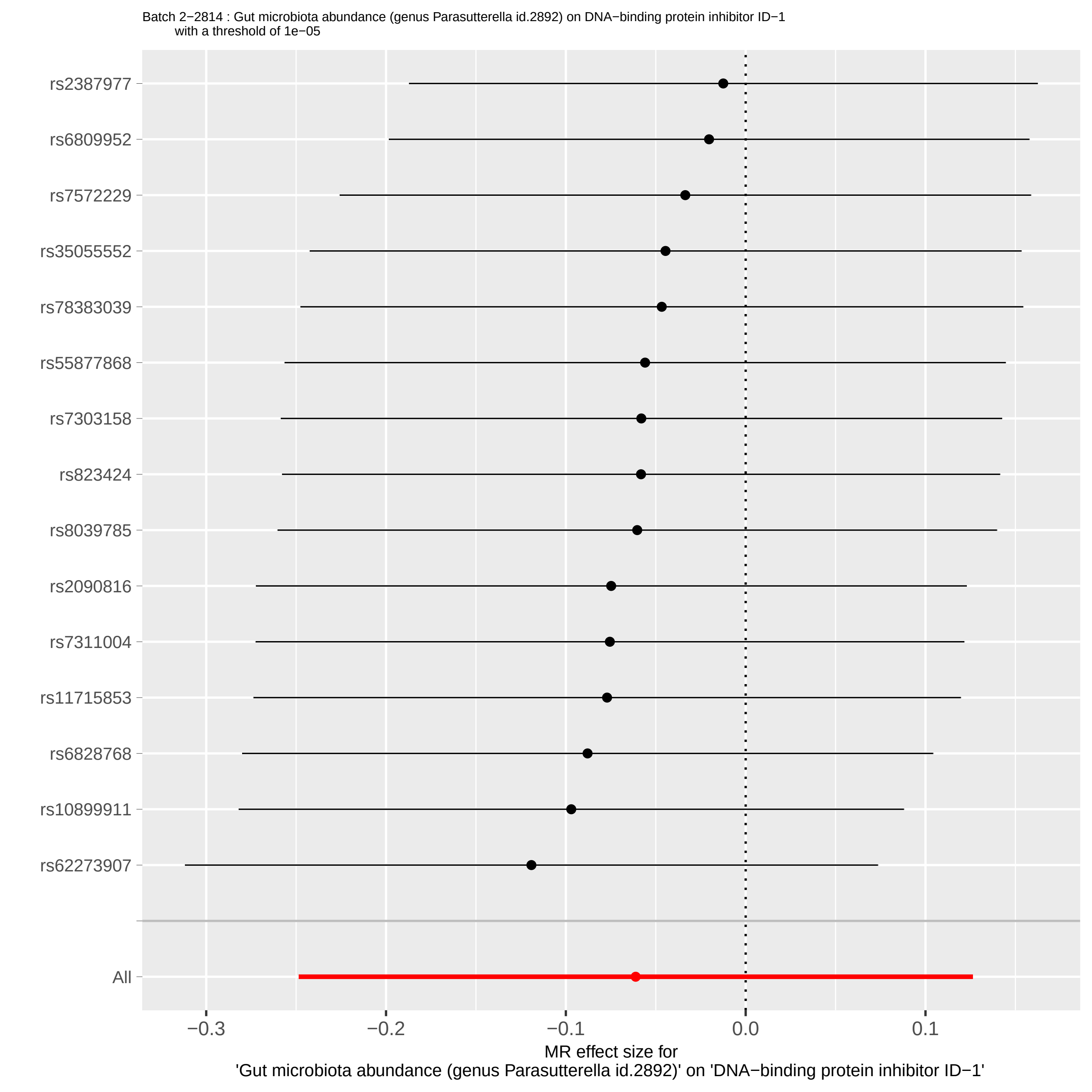

Batch 2−2814 : Gut microbiota abundance (genus Parasutterella id.2892) on DNA−binding protein inhibitor ID−1
with a threshold of 1e−05
rs2387977
rs6809952
rs7572229
rs35055552
rs78383039
rs55877868
rs7303158
rs823424
rs8039785
rs2090816
rs7311004
rs11715853
rs6828768
rs10899911
rs62273907
All
−0.3
−0.2
−0.1
0.0
0.1
MR effect size for
'Gut microbiota abundance (genus Parasutterella id.2892)' on 'DNA−binding protein inhibitor ID−1'

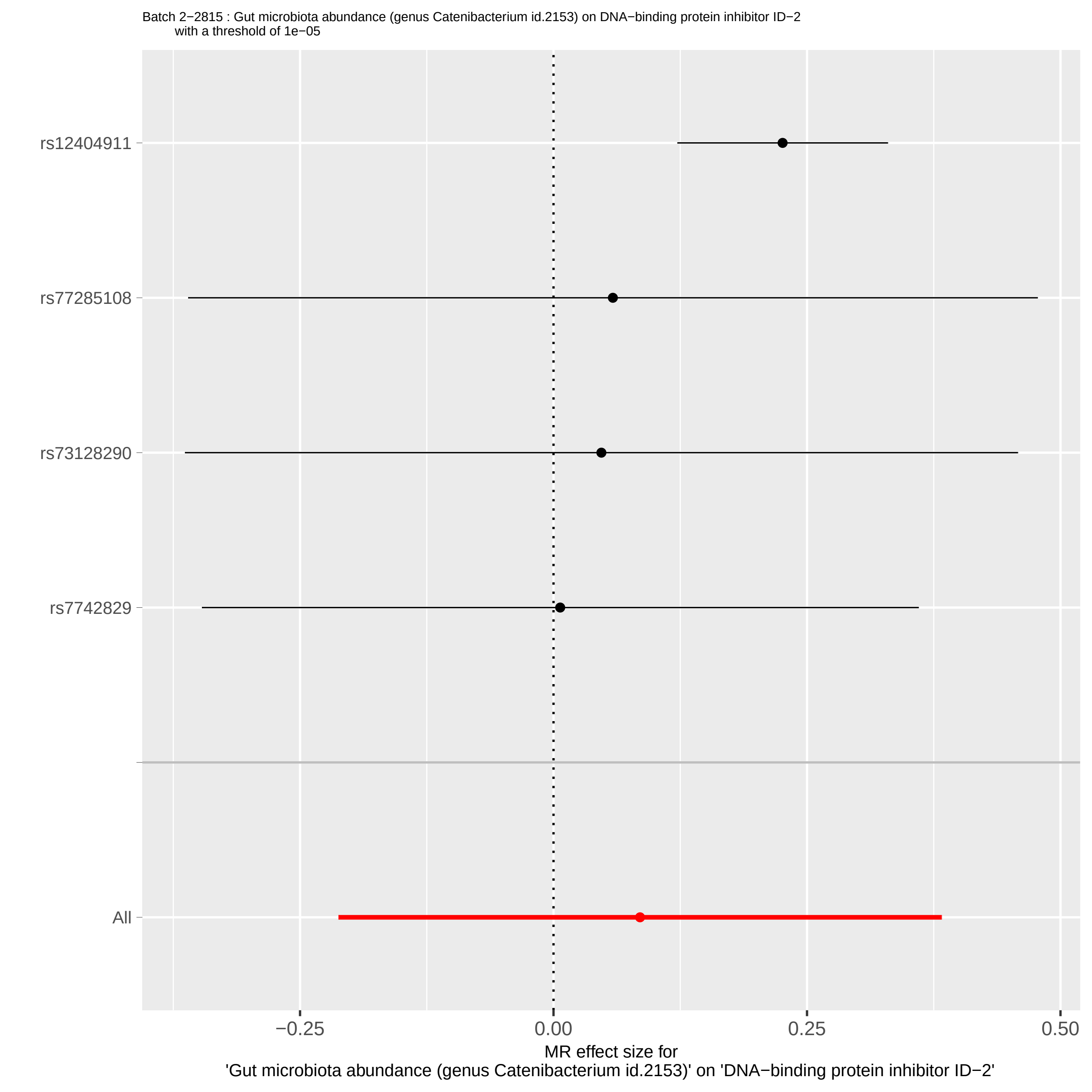
Batch 2−2815 : Gut microbiota abundance (genus Catenibacterium id.2153) on DNA−binding protein inhibitor ID−2
with a threshold of 1e−05
rs12404911
rs77285108
rs73128290
rs7742829
All
−0.25
0.00
0.25
0.50
MR effect size for
'Gut microbiota abundance (genus Catenibacterium id.2153)' on 'DNA−binding protein inhibitor ID−2'

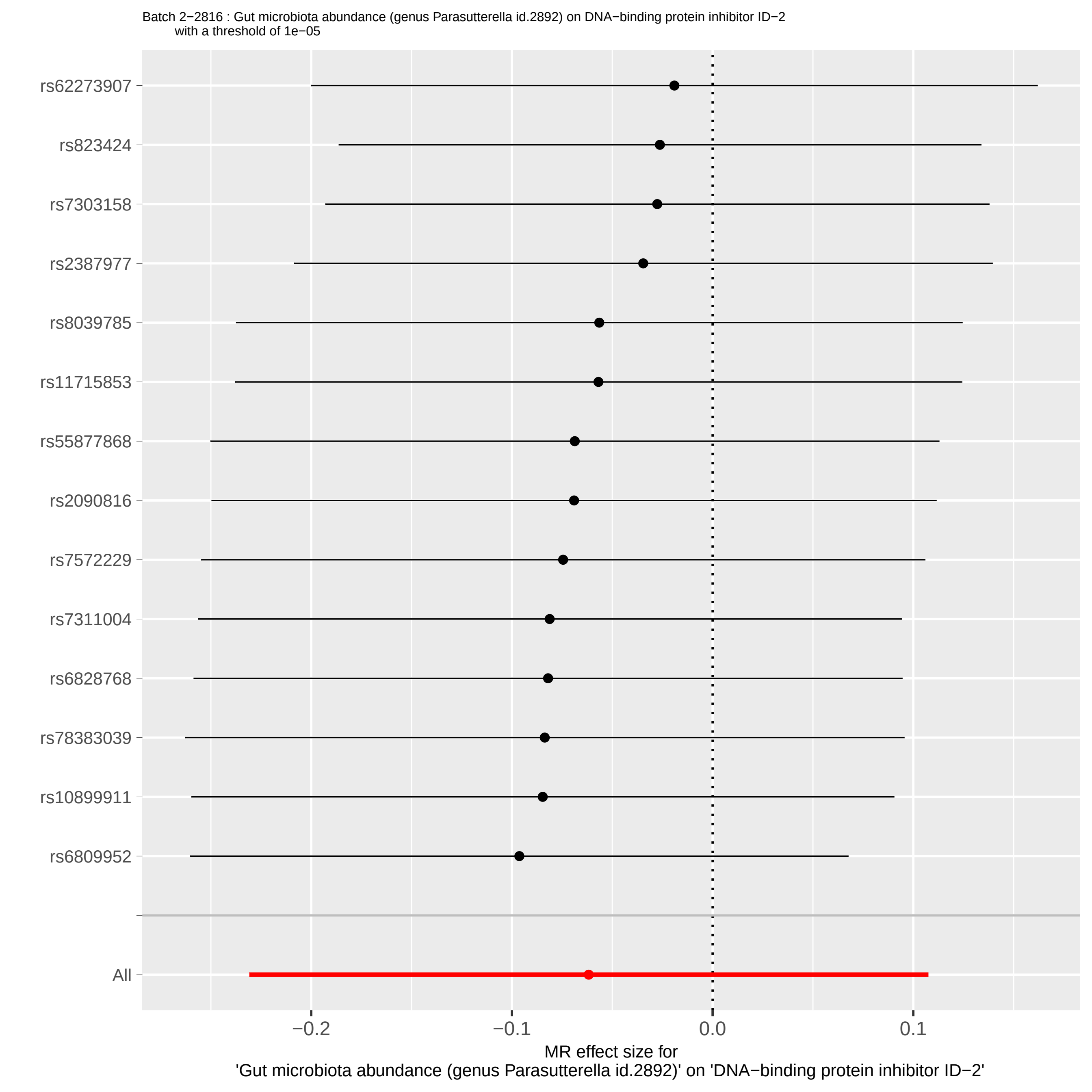
Batch 2−2816 : Gut microbiota abundance (genus Parasutterella id.2892) on DNA−binding protein inhibitor ID−2
with a threshold of 1e−05
rs62273907
rs823424
rs7303158
rs2387977
rs8039785
rs11715853
rs55877868
rs2090816
rs7572229
rs7311004
rs6828768
rs78383039
rs10899911
rs6809952
All
−0.2
−0.1
0.0
0.1
MR effect size for
'Gut microbiota abundance (genus Parasutterella id.2892)' on 'DNA−binding protein inhibitor ID−2'

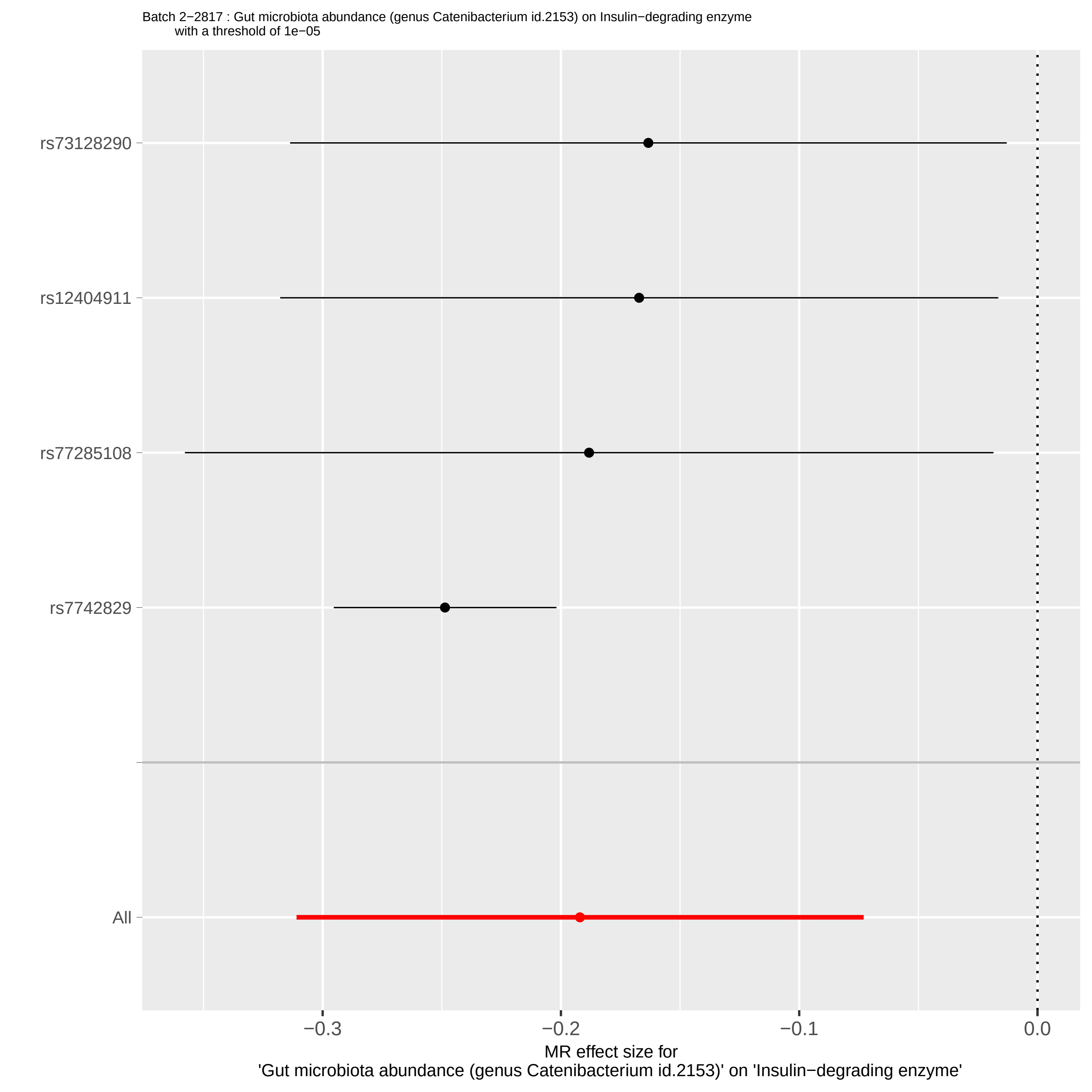
Batch 2−2817 : Gut microbiota abundance (genus Catenibacterium id.2153) on Insulin−degrading enzyme
with a threshold of 1e−05
rs73128290
rs12404911
rs77285108
rs7742829
All
−0.3
−0.2
−0.1
0.0
MR effect size for
'Gut microbiota abundance (genus Catenibacterium id.2153)' on 'Insulin−degrading enzyme'

Batch 2−2818 : Gut microbiota abundance (genus Parasutterella id.2892) on Insulin−degrading enzyme
with a threshold of 1e−05

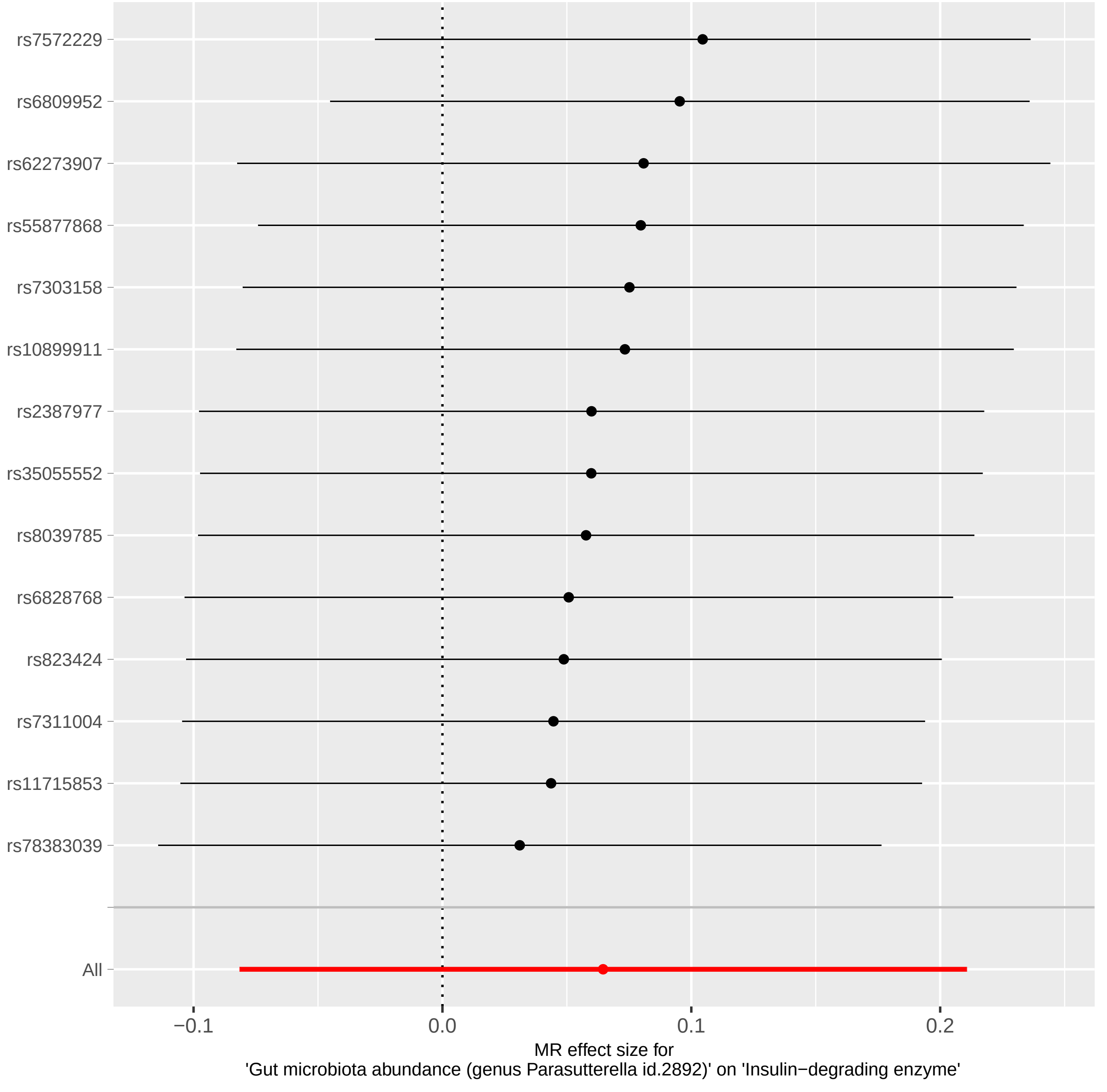

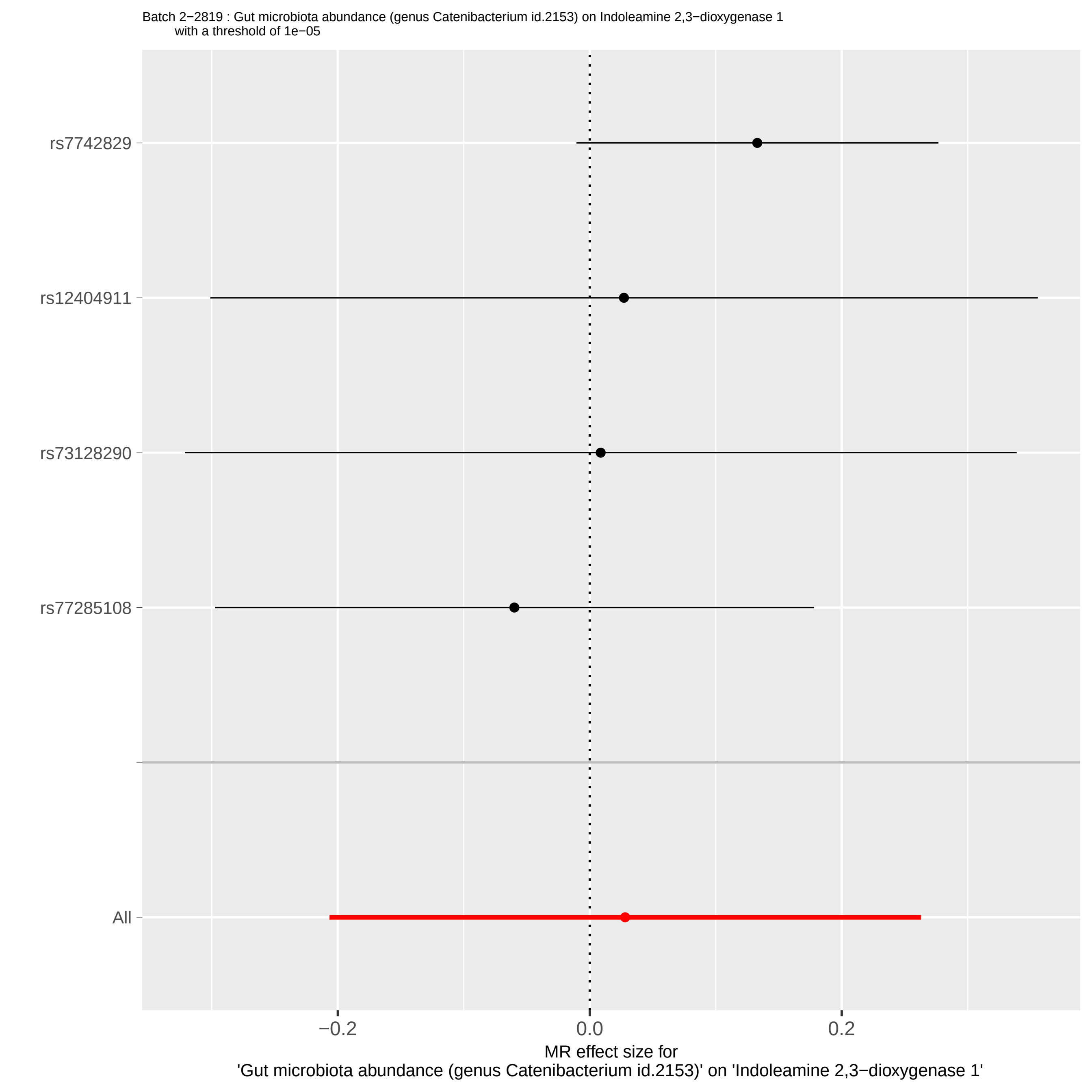

Batch 2−2819 : Gut microbiota abundance (genus Catenibacterium id.2153) on Indoleamine 2,3−dioxygenase 1
with a threshold of 1e−05
rs7742829
rs12404911
rs73128290
rs77285108
All
−0.2
0.0
0.2
MR effect size for
'Gut microbiota abundance (genus Catenibacterium id.2153)' on 'Indoleamine 2,3−dioxygenase 1'

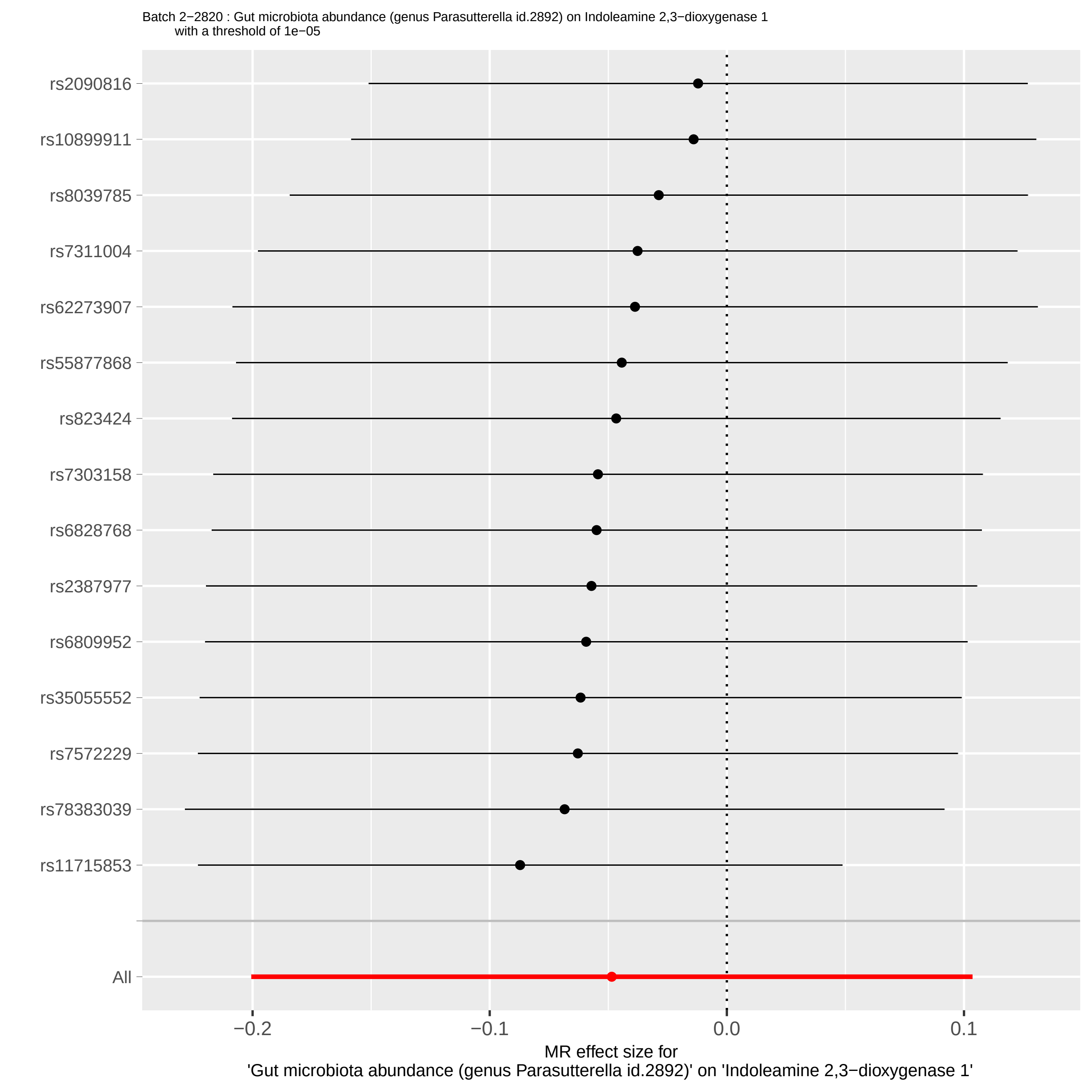

Batch 2−2820 : Gut microbiota abundance (genus Parasutterella id.2892) on Indoleamine 2,3−dioxygenase 1
with a threshold of 1e−05
rs2090816
rs10899911
rs8039785
rs7311004
rs62273907
rs55877868
rs823424
rs7303158
rs6828768
rs2387977
rs6809952
rs35055552
rs7572229
rs78383039
rs11715853
All
−0.2
−0.1
0.0
0.1
MR effect size for
'Gut microbiota abundance (genus Parasutterella id.2892)' on 'Indoleamine 2,3−dioxygenase 1'

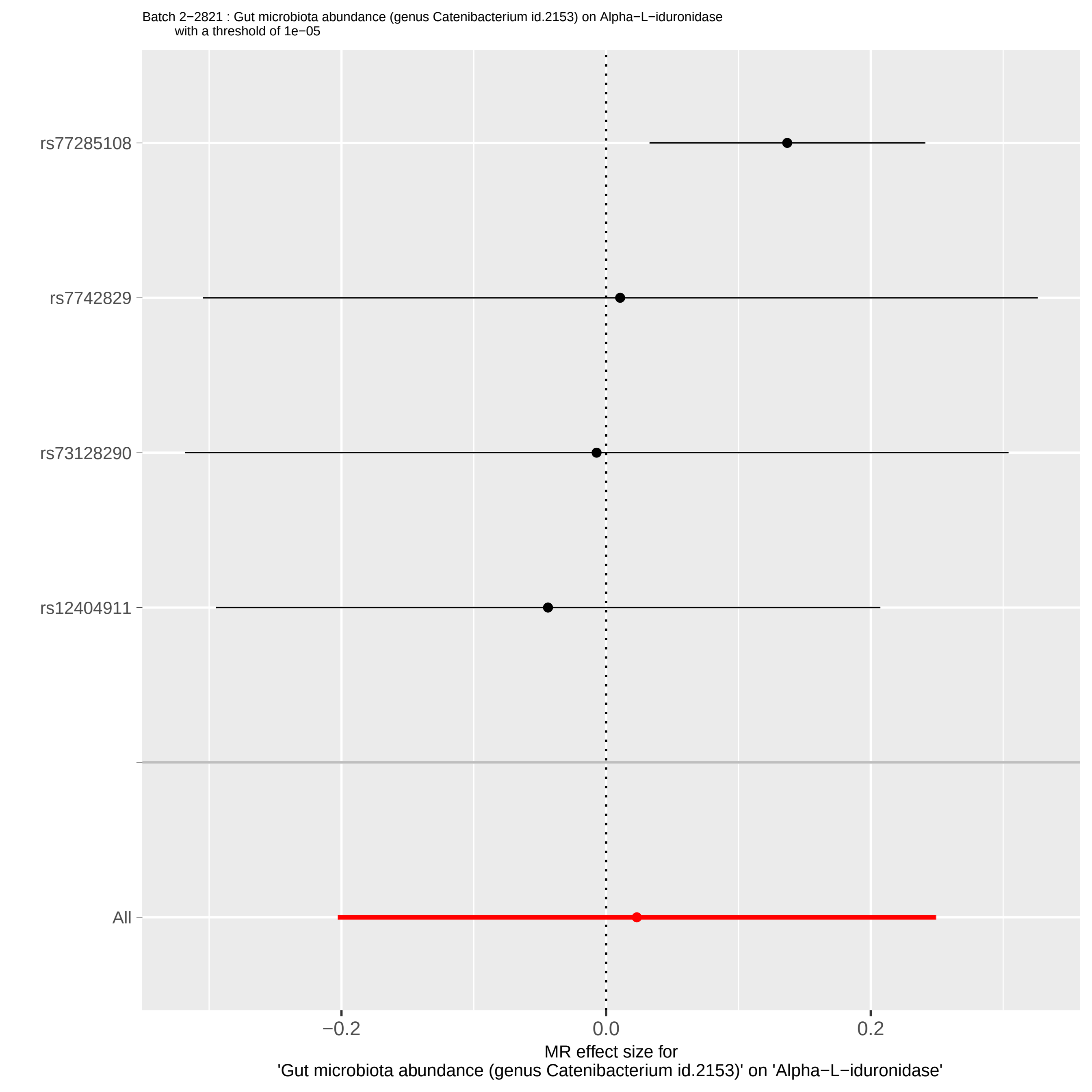

Batch 2−2821 : Gut microbiota abundance (genus Catenibacterium id.2153) on Alpha−L−iduronidase
with a threshold of 1e−05
rs77285108
rs7742829
rs73128290
rs12404911
All
−0.2
0.0
0.2
MR effect size for
'Gut microbiota abundance (genus Catenibacterium id.2153)' on 'Alpha−L−iduronidase'

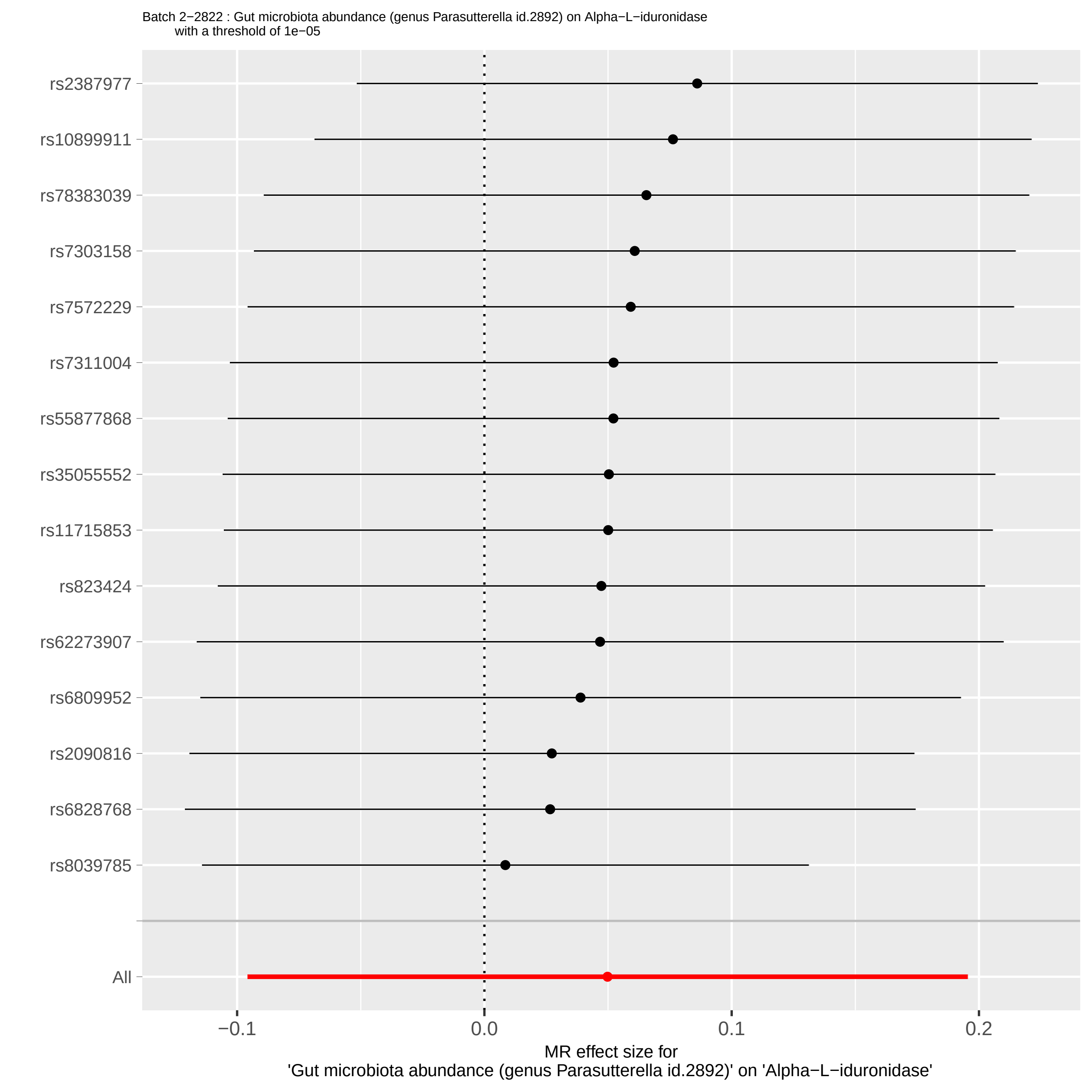

Batch 2−2822 : Gut microbiota abundance (genus Parasutterella id.2892) on Alpha−L−iduronidase
with a threshold of 1e−05
rs2387977
rs10899911
rs78383039
rs7303158
rs7572229
rs7311004
rs55877868
rs35055552
rs11715853
rs823424
rs62273907
rs6809952
rs2090816
rs6828768
rs8039785
All
−0.1
0.0
0.1
0.2
MR effect size for
'Gut microbiota abundance (genus Parasutterella id.2892)' on 'Alpha−L−iduronidase'

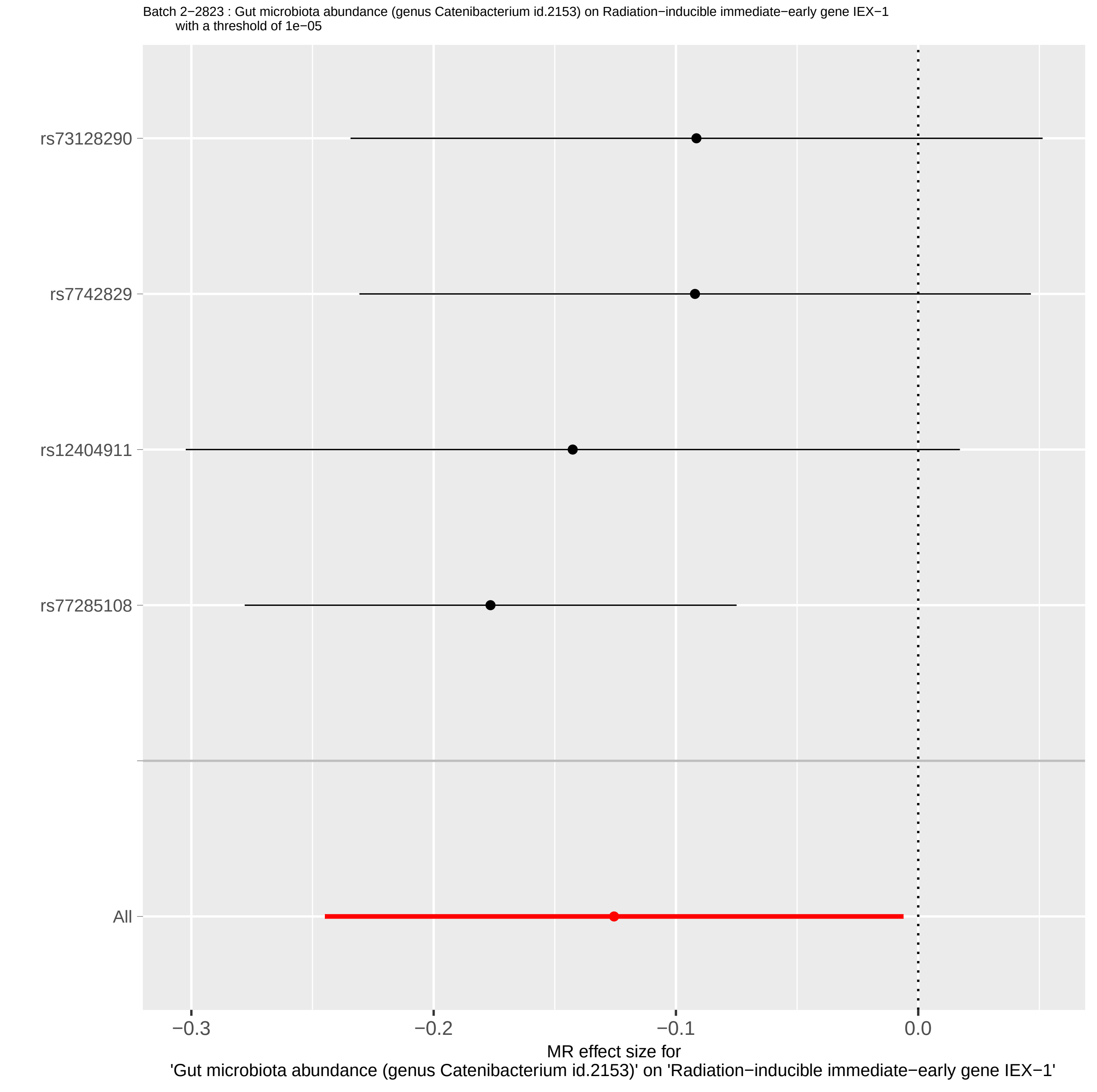
Batch 2−2823 : Gut microbiota abundance (genus Catenibacterium id.2153) on Radiation−inducible immediate−early gene IEX−1
with a threshold of 1e−05
rs73128290
rs7742829
rs12404911
rs77285108
All
−0.3
−0.2
−0.1
0.0
MR effect size for
'Gut microbiota abundance (genus Catenibacterium id.2153)' on 'Radiation−inducible immediate−early gene IEX−1'

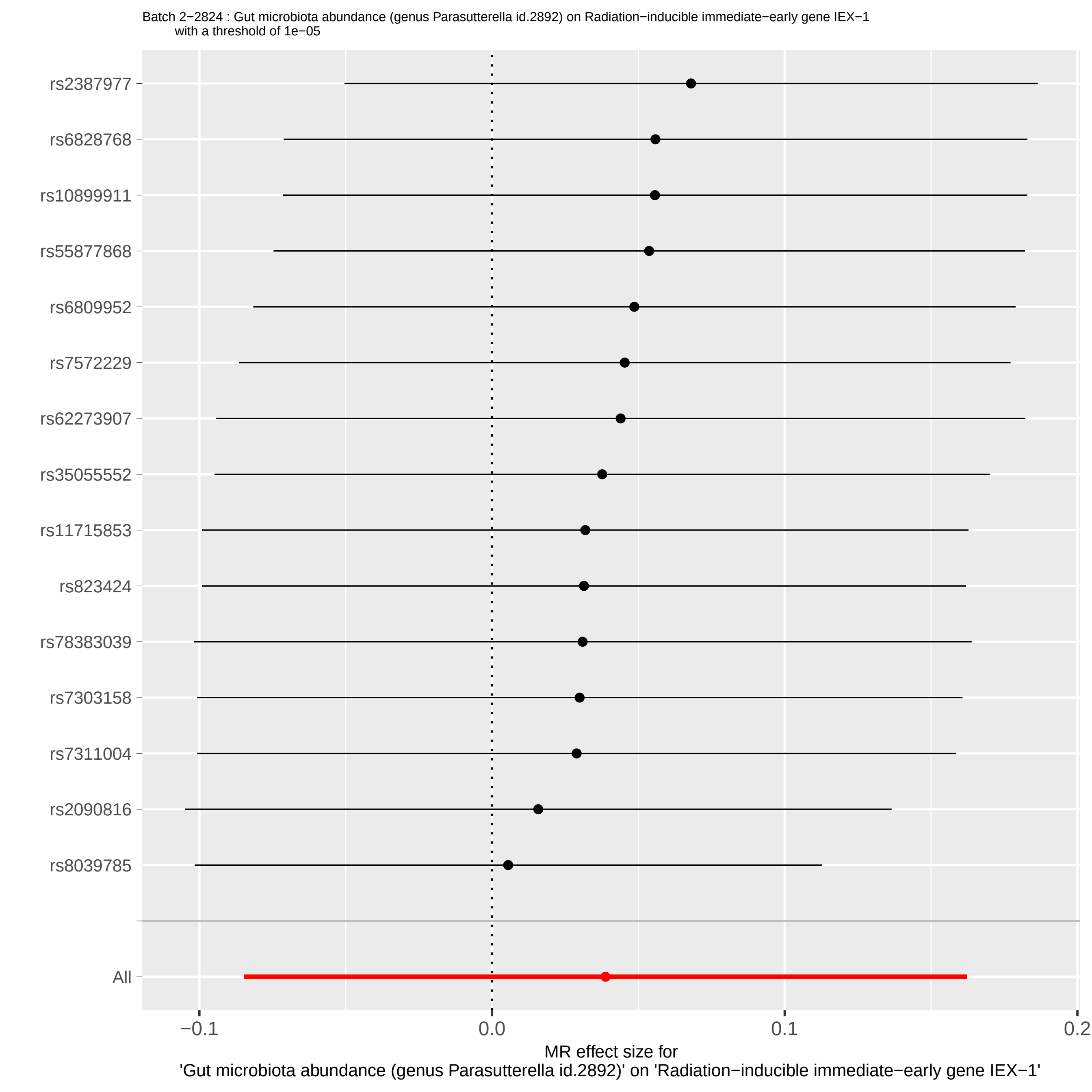
Batch 2−2824 : Gut microbiota abundance (genus Parasutterella id.2892) on Radiation−inducible immediate−early gene IEX−1
with a threshold of 1e−05
rs2387977
rs6828768
rs10899911
rs55877868
rs6809952
rs7572229
rs62273907
rs35055552
rs11715853
rs823424
rs78383039
rs7303158
rs7311004
rs2090816
rs8039785
All
−0.1
0.0
0.1
0.2
MR effect size for
'Gut microbiota abundance (genus Parasutterella id.2892)' on 'Radiation−inducible immediate−early gene IEX−1'

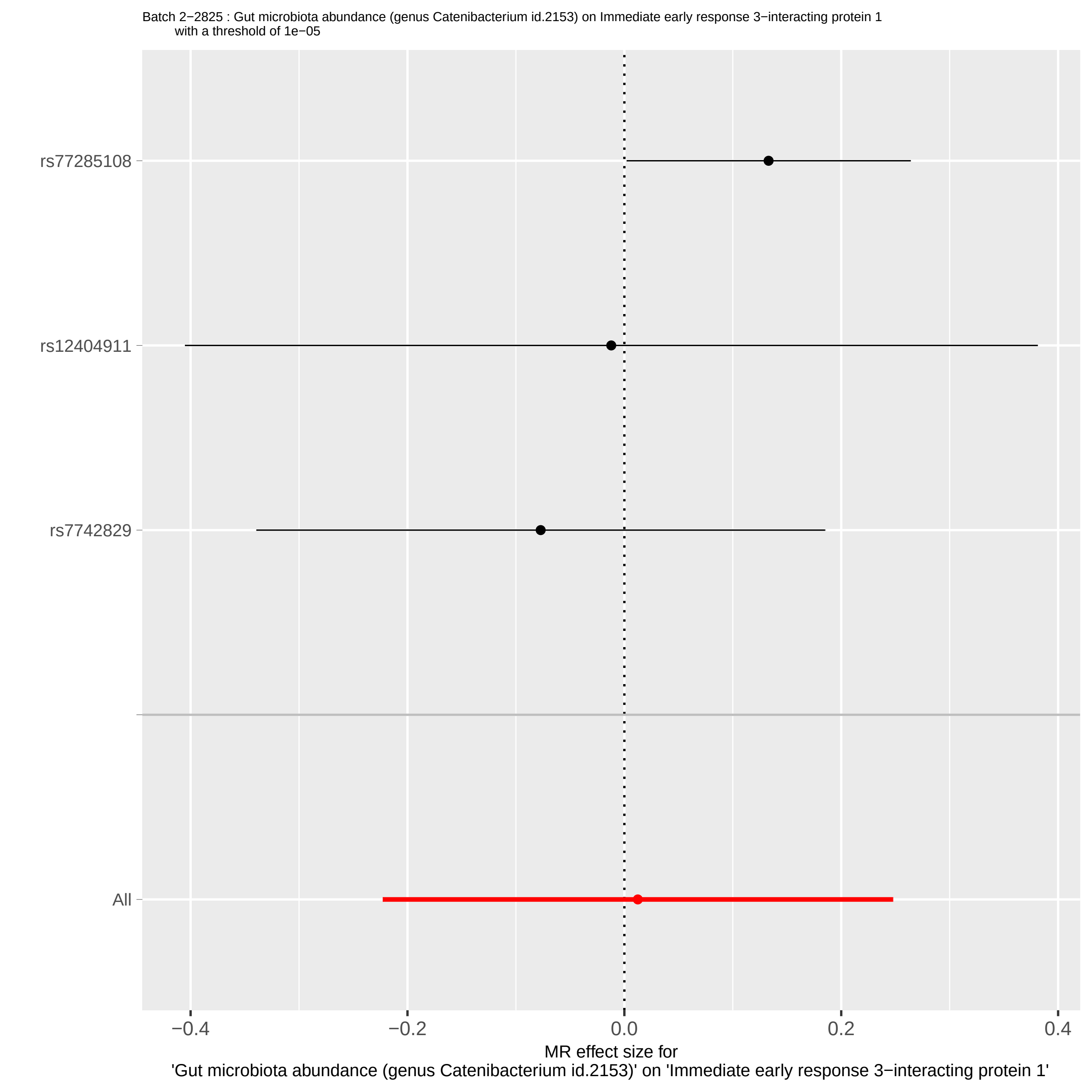

Batch 2−2825 : Gut microbiota abundance (genus Catenibacterium id.2153) on Immediate early response 3−interacting protein 1
with a threshold of 1e−05
rs77285108
rs12404911
rs7742829
All
−0.4
−0.2
0.0
0.2
0.4
MR effect size for
'Gut microbiota abundance (genus Catenibacterium id.2153)' on 'Immediate early response 3−interacting protein 1'

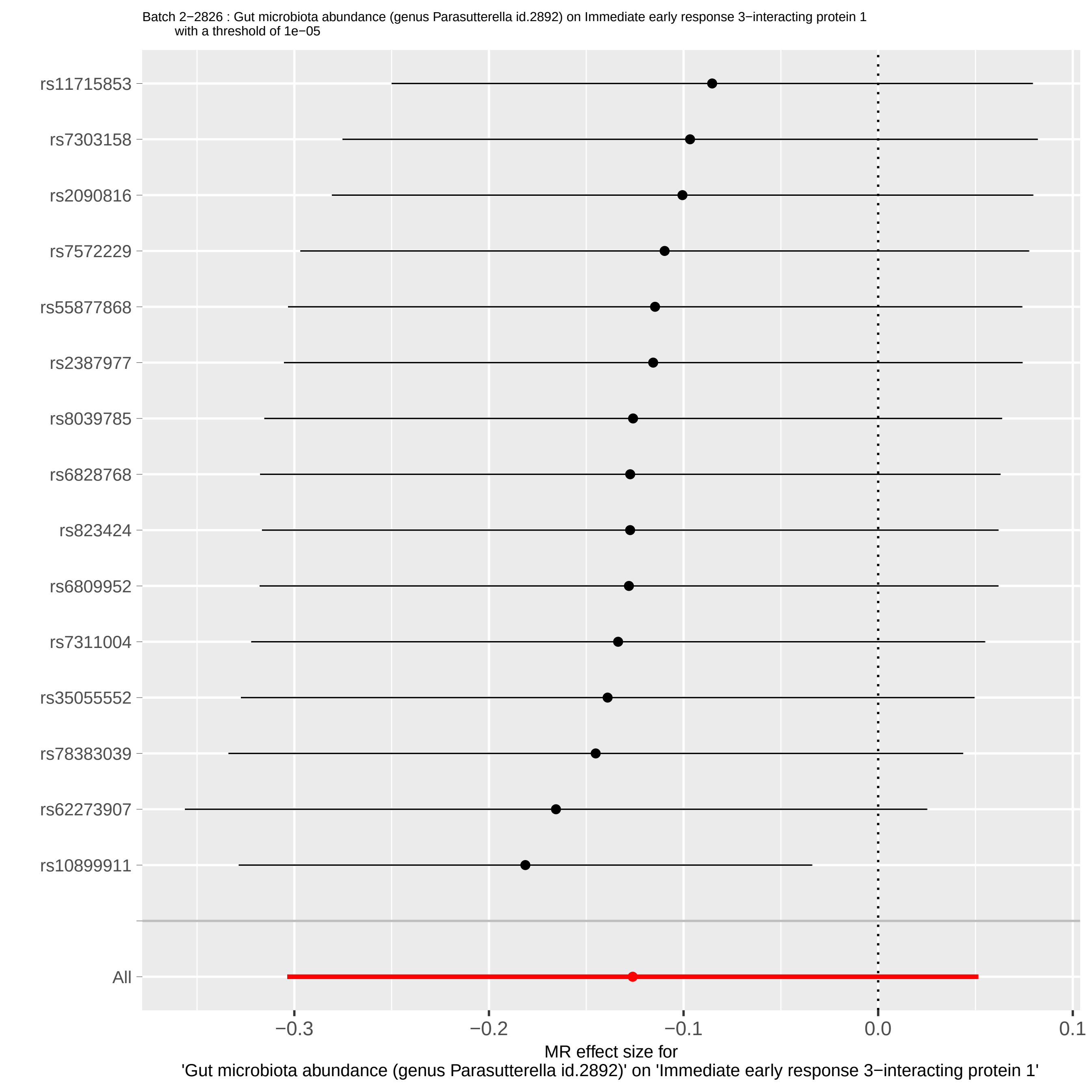

Batch 2−2826 : Gut microbiota abundance (genus Parasutterella id.2892) on Immediate early response 3−interacting protein 1
with a threshold of 1e−05
rs11715853
rs7303158
rs2090816
rs7572229
rs55877868
rs2387977
rs8039785
rs6828768
rs823424
rs6809952
rs7311004
rs35055552
rs78383039
rs62273907
rs10899911
All
−0.3
−0.2
−0.1
0.0
0.1
MR effect size for
'Gut microbiota abundance (genus Parasutterella id.2892)' on 'Immediate early response 3−interacting protein 1'

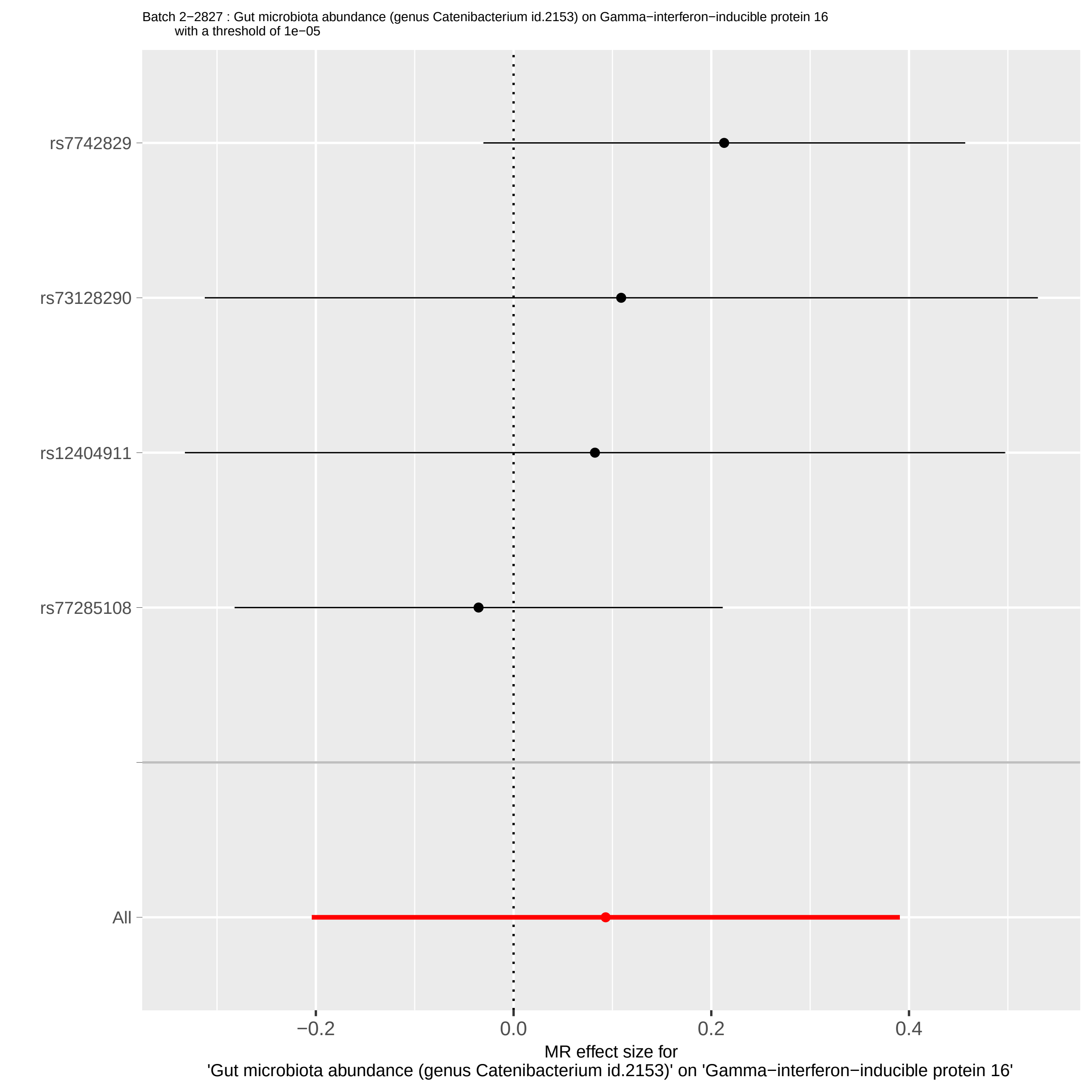

Batch 2−2827 : Gut microbiota abundance (genus Catenibacterium id.2153) on Gamma−interferon−inducible protein 16
with a threshold of 1e−05
rs7742829
rs73128290
rs12404911
rs77285108
All
−0.2
0.0
0.2
0.4
MR effect size for
'Gut microbiota abundance (genus Catenibacterium id.2153)' on 'Gamma−interferon−inducible protein 16'

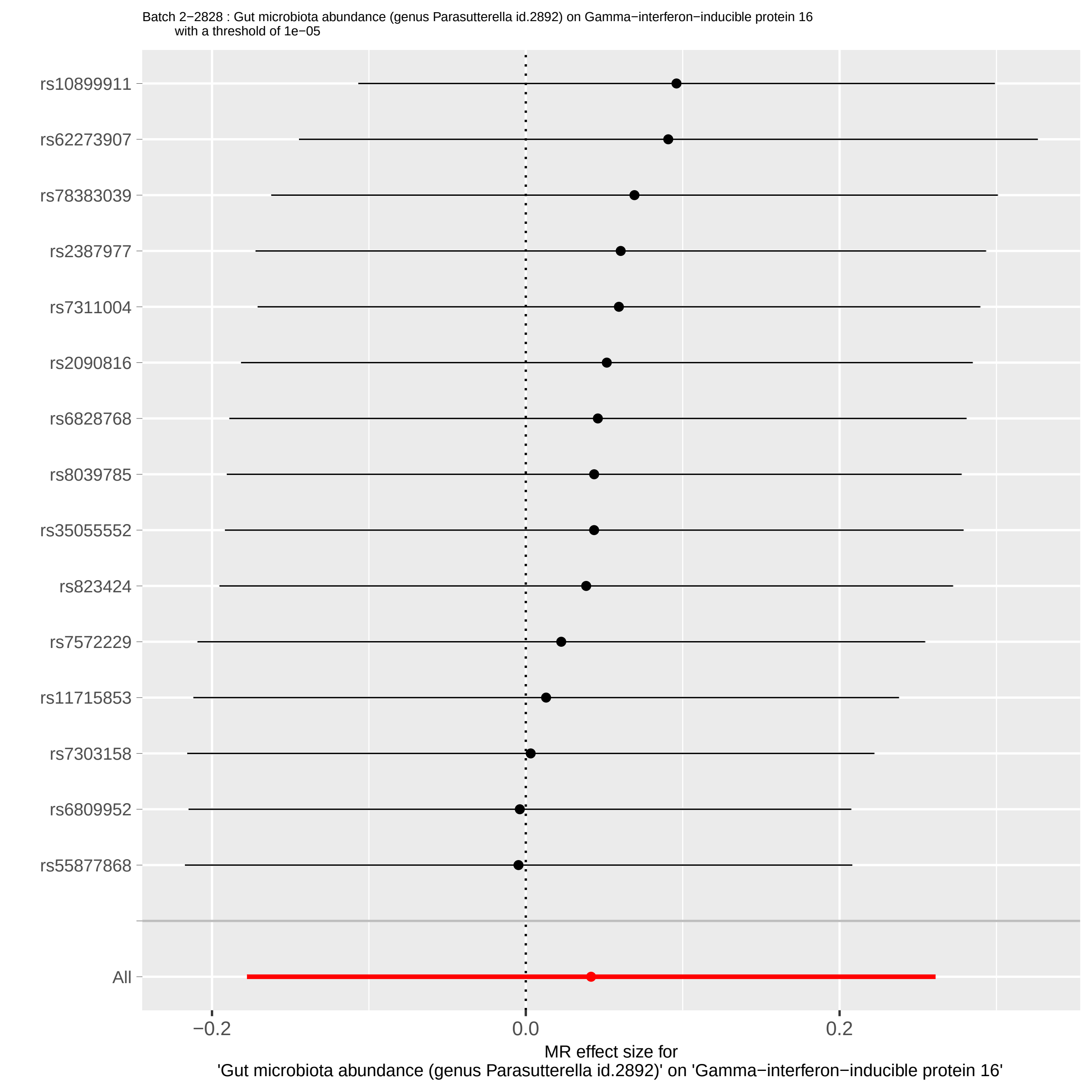

Batch 2−2828 : Gut microbiota abundance (genus Parasutterella id.2892) on Gamma−interferon−inducible protein 16
with a threshold of 1e−05
rs10899911
rs62273907
rs78383039
rs2387977
rs7311004
rs2090816
rs6828768
rs8039785
rs35055552
rs823424
rs7572229
rs11715853
rs7303158
rs6809952
rs55877868
All
−0.2
0.0
0.2
MR effect size for
'Gut microbiota abundance (genus Parasutterella id.2892)' on 'Gamma−interferon−inducible protein 16'

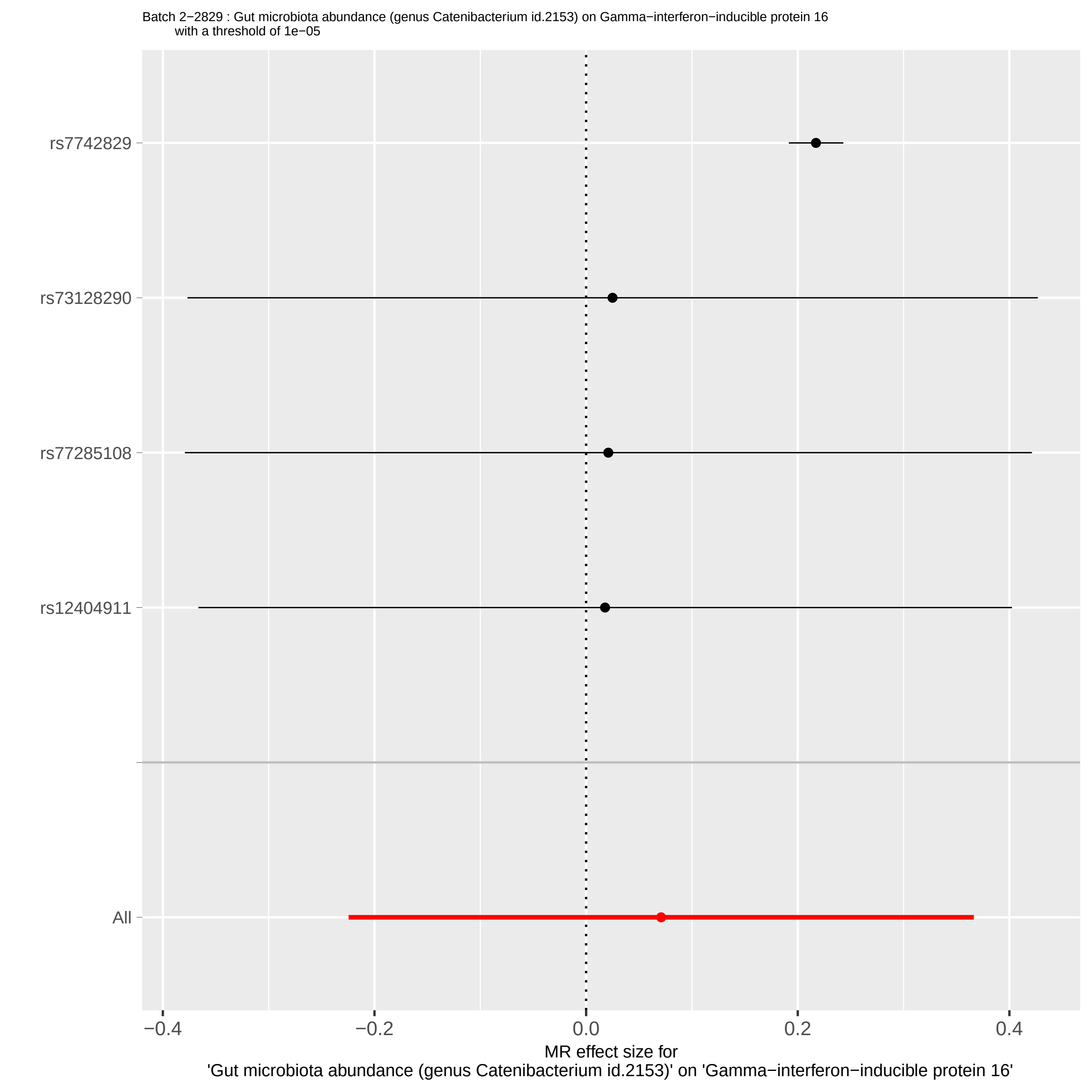
Batch 2−2829 : Gut microbiota abundance (genus Catenibacterium id.2153) on Gamma−interferon−inducible protein 16
with a threshold of 1e−05
rs7742829
rs73128290
rs77285108
rs12404911
All
−0.4
−0.2
0.0
0.2
0.4
MR effect size for
'Gut microbiota abundance (genus Catenibacterium id.2153)' on 'Gamma−interferon−inducible protein 16'

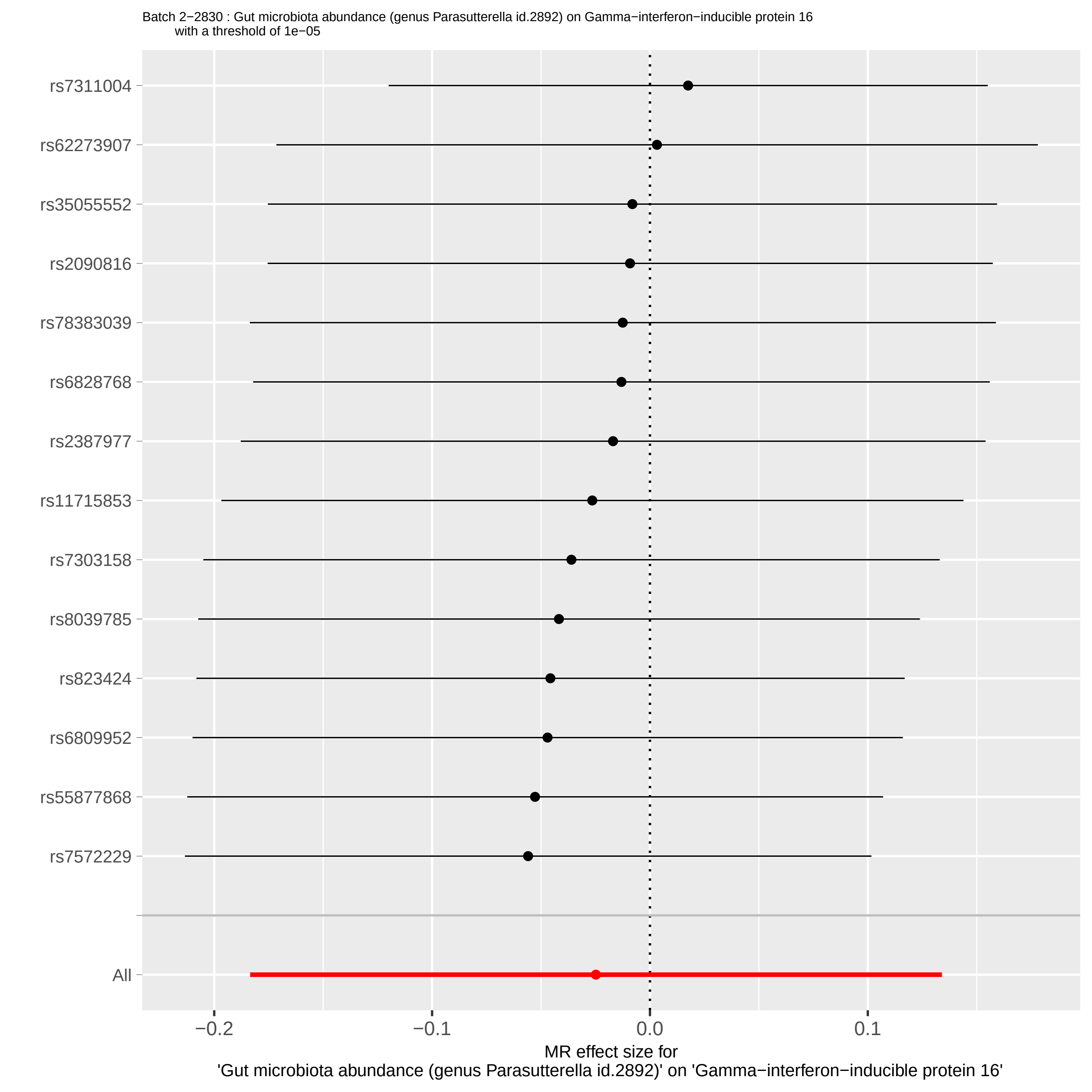

Batch 2−2830 : Gut microbiota abundance (genus Parasutterella id.2892) on Gamma−interferon−inducible protein 16
with a threshold of 1e−05
rs7311004
rs62273907
rs35055552
rs2090816
rs78383039
rs6828768
rs2387977
rs11715853
rs7303158
rs8039785
rs823424
rs6809952
rs55877868
rs7572229
All
−0.2
−0.1
0.0
0.1
MR effect size for
'Gut microbiota abundance (genus Parasutterella id.2892)' on 'Gamma−interferon−inducible protein 16'

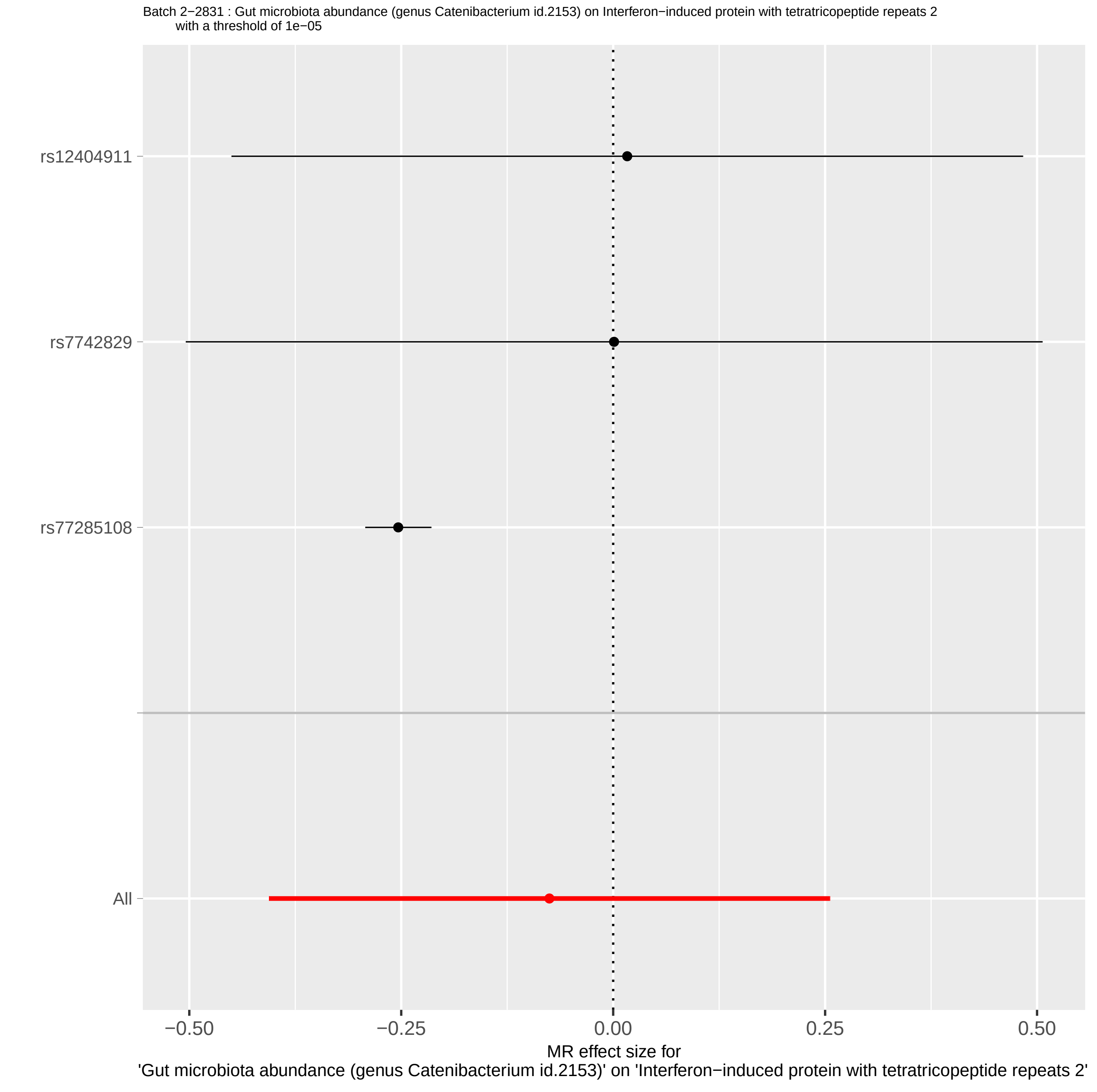
Batch 2−2831 : Gut microbiota abundance (genus Catenibacterium id.2153) on Interferon−induced protein with tetratricopeptide repeats 2
with a threshold of 1e−05
rs12404911
rs7742829
rs77285108
All
−0.50
−0.25
0.00
0.25
0.50
MR effect size for
'Gut microbiota abundance (genus Catenibacterium id.2153)' on 'Interferon−induced protein with tetratricopeptide repeats 2'

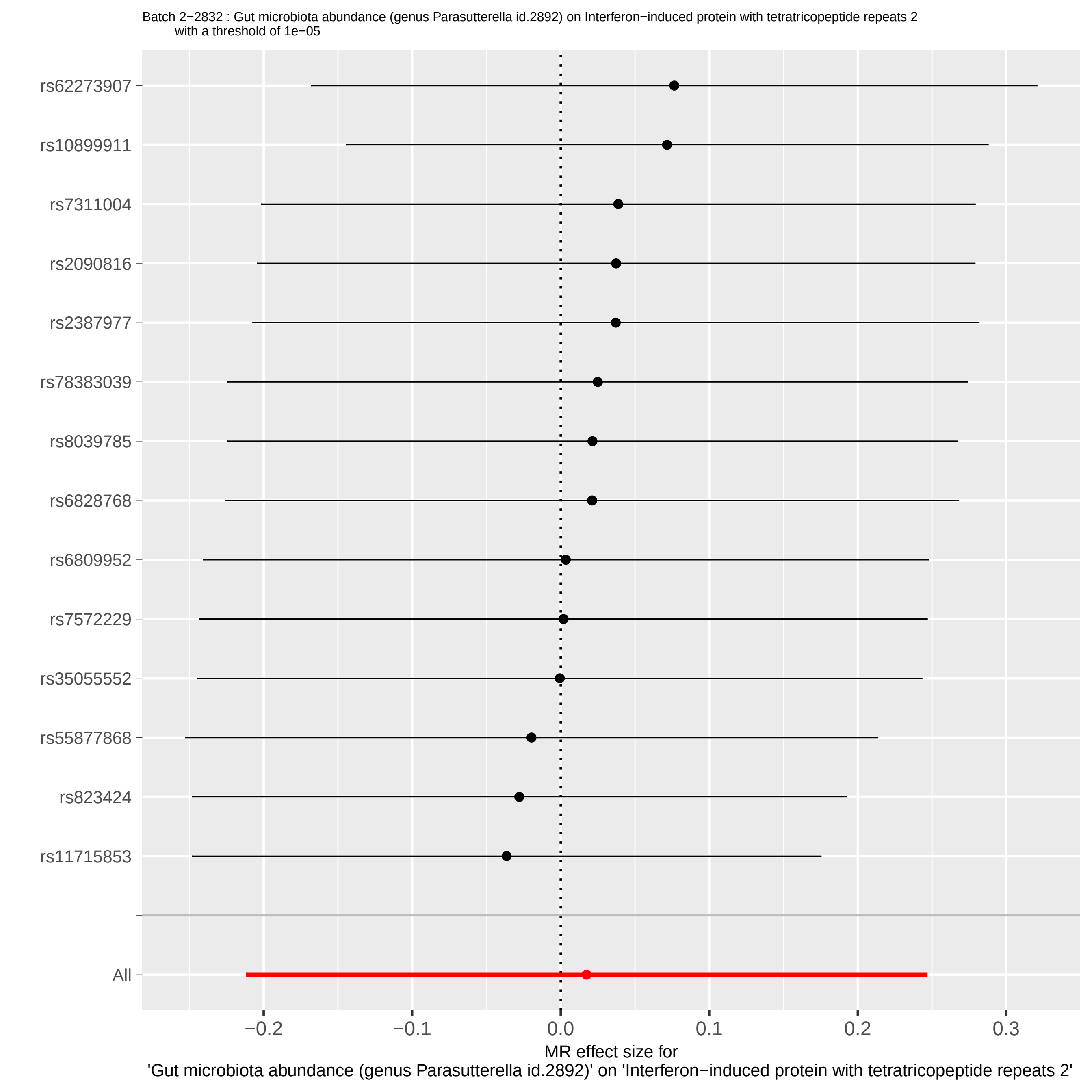
Batch 2−2832 : Gut microbiota abundance (genus Parasutterella id.2892) on Interferon−induced protein with tetratricopeptide repeats 2
with a threshold of 1e−05
rs62273907
rs10899911
rs7311004
rs2090816
rs2387977
rs78383039
rs8039785
rs6828768
rs6809952
rs7572229
rs35055552
rs55877868
rs823424
rs11715853
All
−0.2
−0.1
0.0
0.1
0.2
0.3
MR effect size for
'Gut microbiota abundance (genus Parasutterella id.2892)' on 'Interferon−induced protein with tetratricopeptide repeats 2'

Batch 2−2833 : Gut microbiota abundance (genus Catenibacterium id.2153) on Interferon−induced protein with tetratricopeptide repeats 3
with a threshold of 1e−05

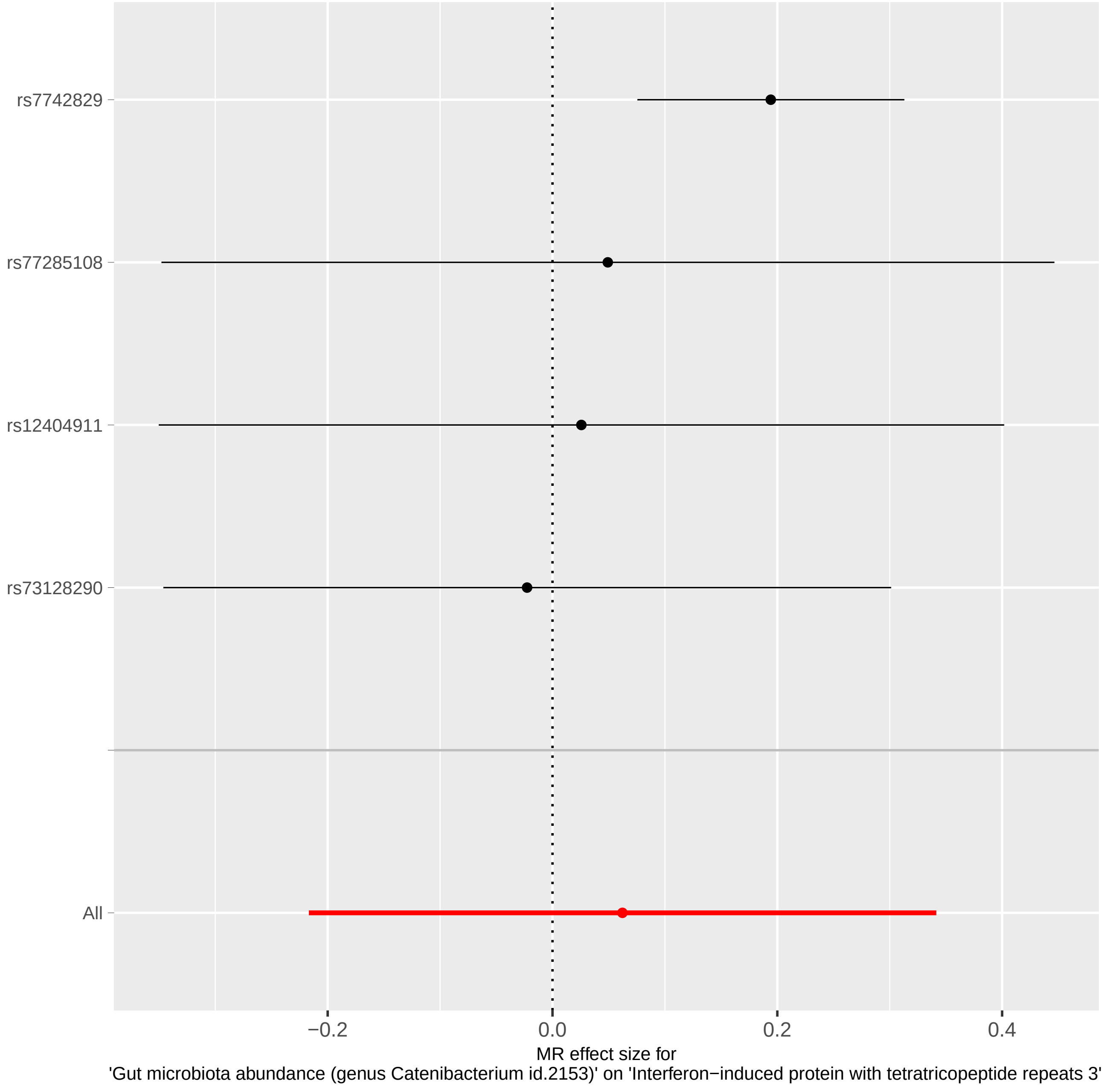

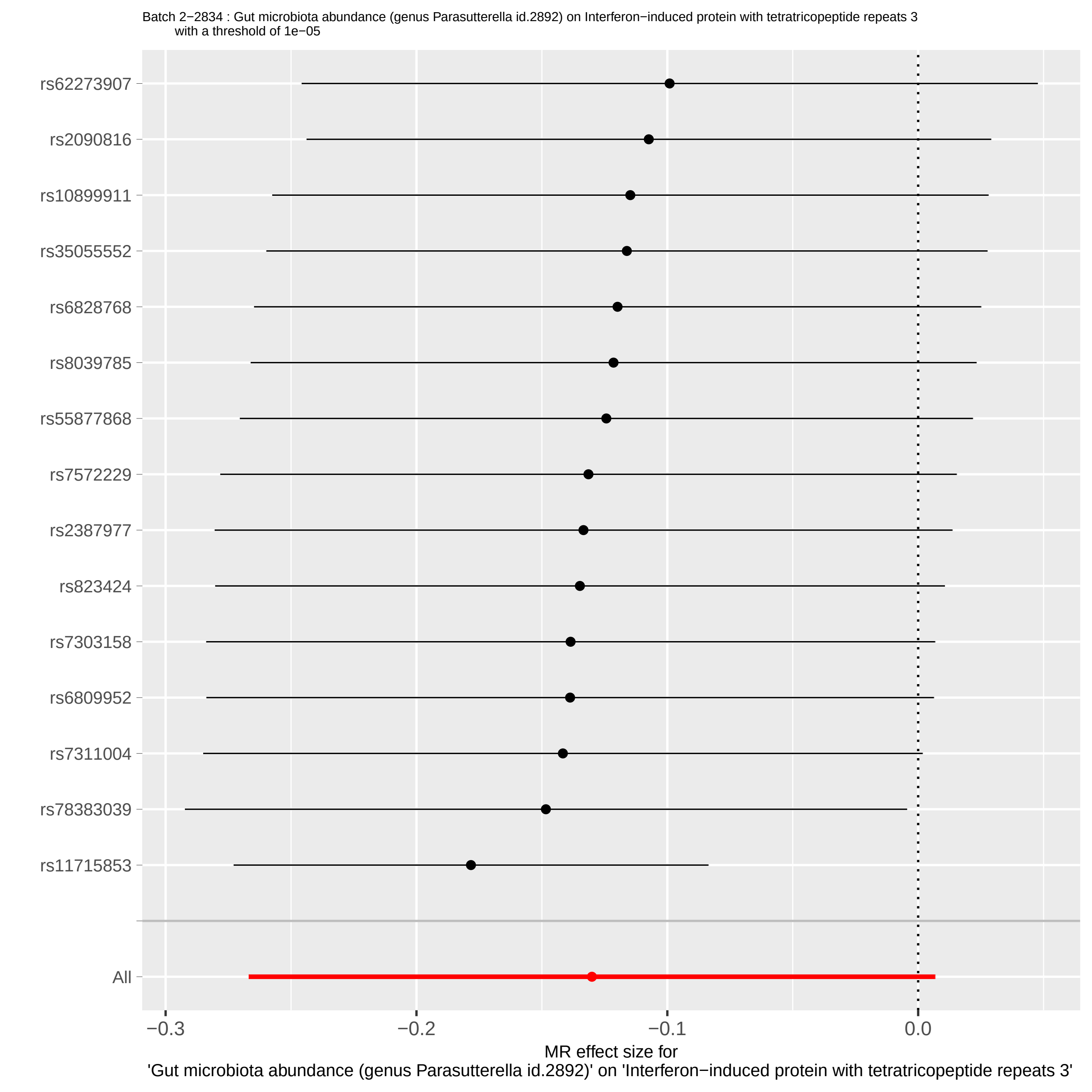

Batch 2−2834 : Gut microbiota abundance (genus Parasutterella id.2892) on Interferon−induced protein with tetratricopeptide repeats 3
with a threshold of 1e−05
rs62273907
rs2090816
rs10899911
rs35055552
rs6828768
rs8039785
rs55877868
rs7572229
rs2387977
rs823424
rs7303158
rs6809952
rs7311004
rs78383039
rs11715853
All
−0.3
−0.2
−0.1
0.0
MR effect size for
'Gut microbiota abundance (genus Parasutterella id.2892)' on 'Interferon−induced protein with tetratricopeptide repeats 3'

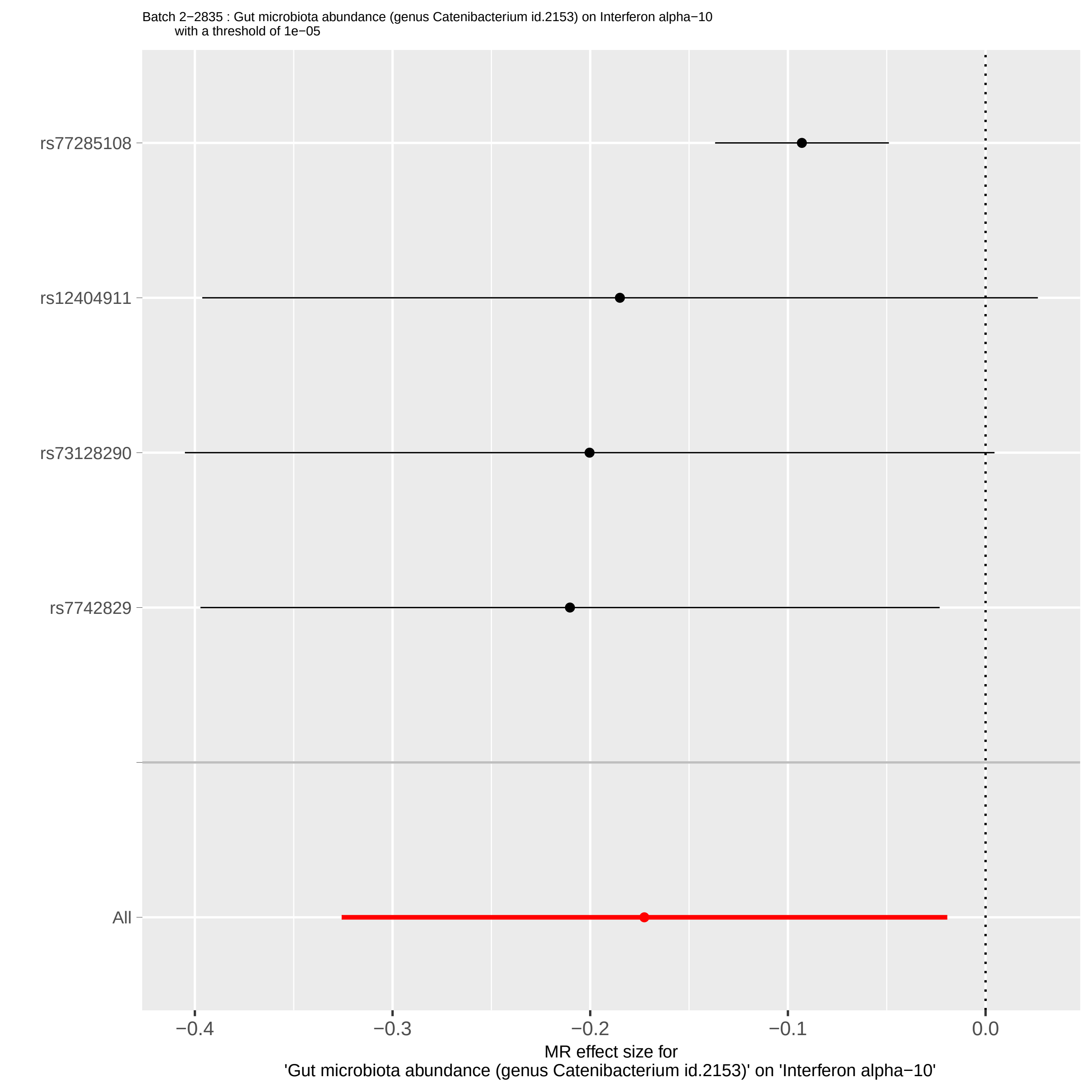

Batch 2−2835 : Gut microbiota abundance (genus Catenibacterium id.2153) on Interferon alpha−10
with a threshold of 1e−05
rs77285108
rs12404911
rs73128290
rs7742829
All
−0.4
−0.3
−0.2
−0.1
0.0
MR effect size for
'Gut microbiota abundance (genus Catenibacterium id.2153)' on 'Interferon alpha−10'

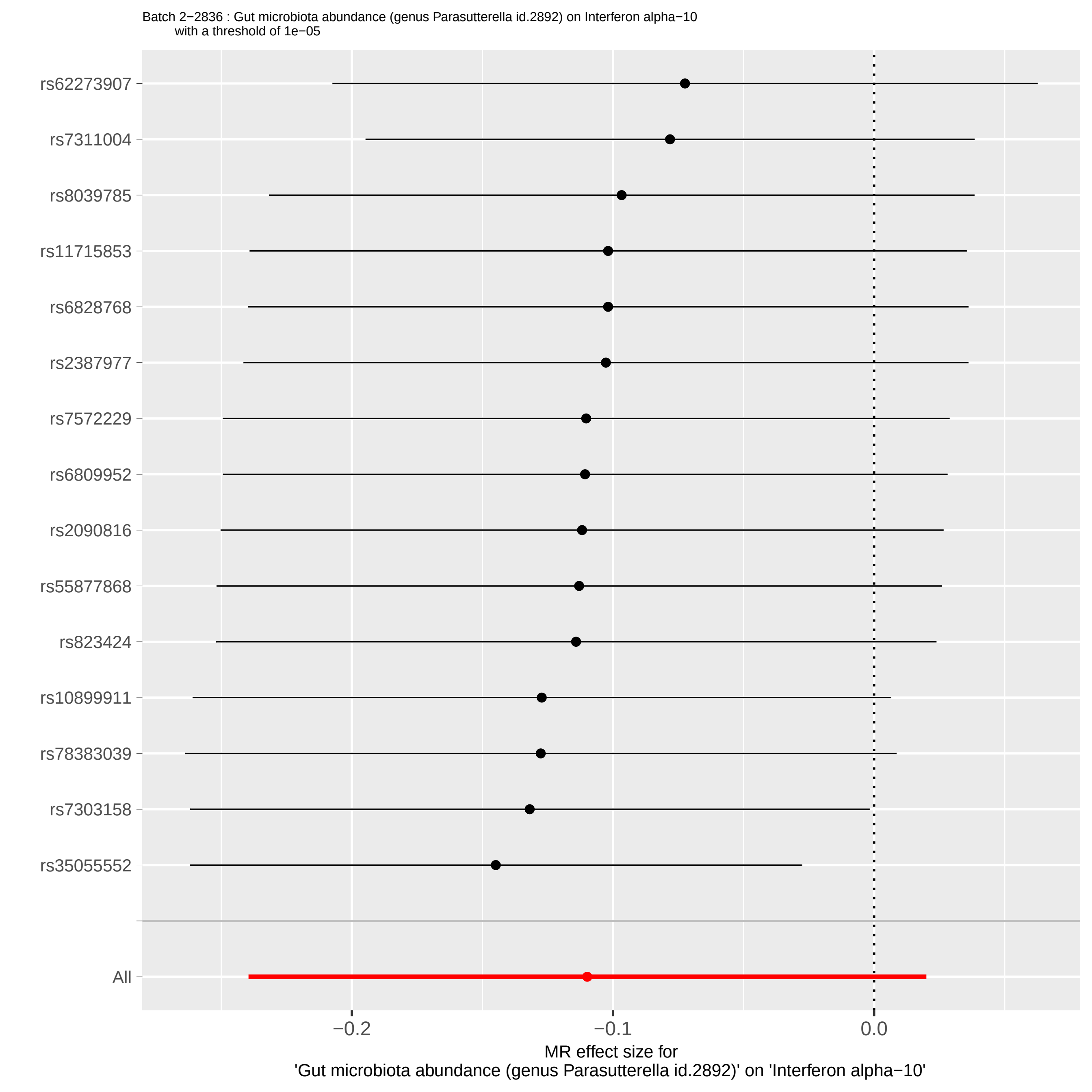
Batch 2−2836 : Gut microbiota abundance (genus Parasutterella id.2892) on Interferon alpha−10
with a threshold of 1e−05
rs62273907
rs7311004
rs8039785
rs11715853
rs6828768
rs2387977
rs7572229
rs6809952
rs2090816
rs55877868
rs823424
rs10899911
rs78383039
rs7303158
rs35055552
All
−0.2
−0.1
0.0
MR effect size for
'Gut microbiota abundance (genus Parasutterella id.2892)' on 'Interferon alpha−10'

Batch 2−2837 : Gut microbiota abundance (genus Catenibacterium id.2153) on Interferon alpha−10
with a threshold of 1e−05

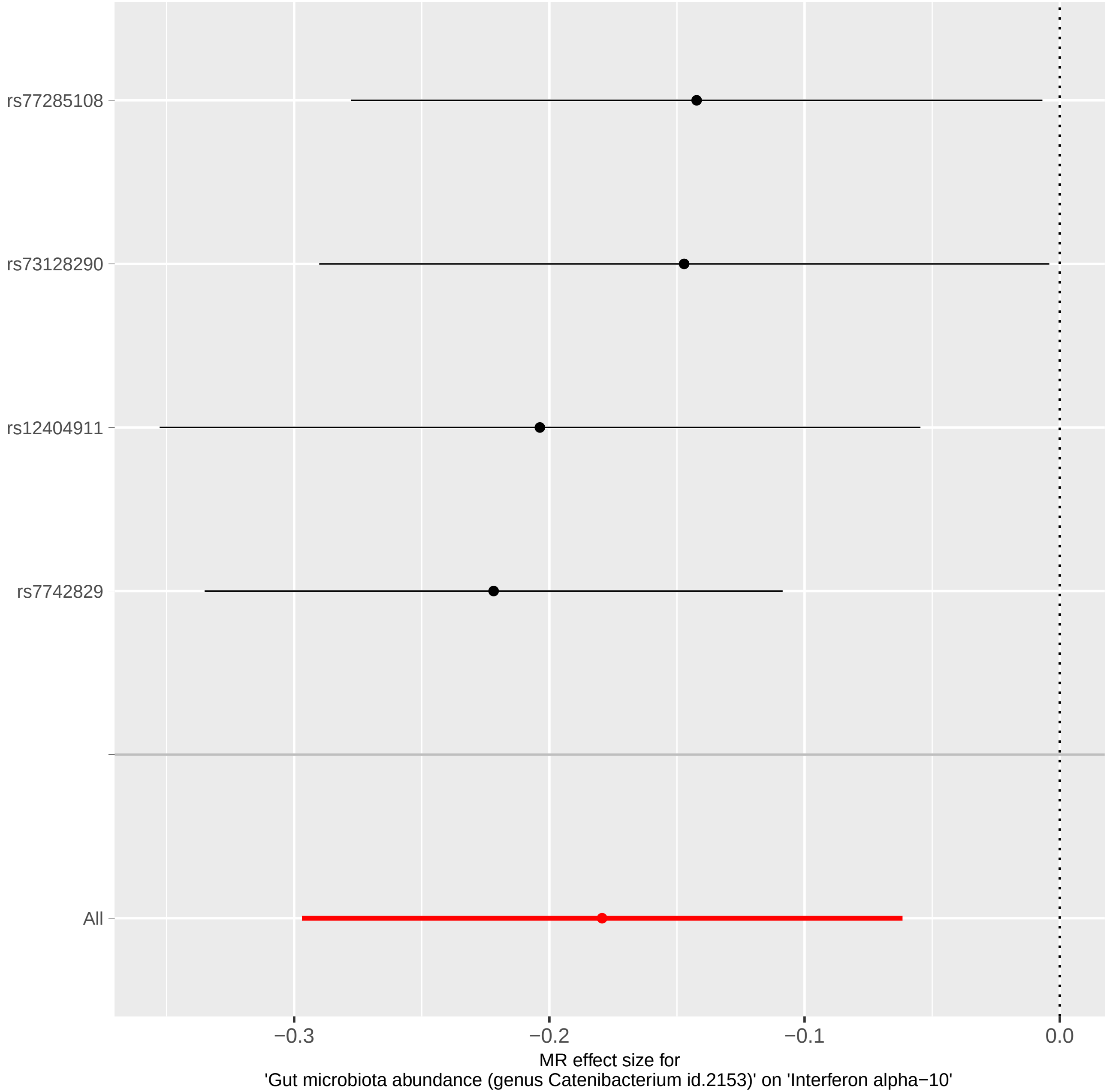

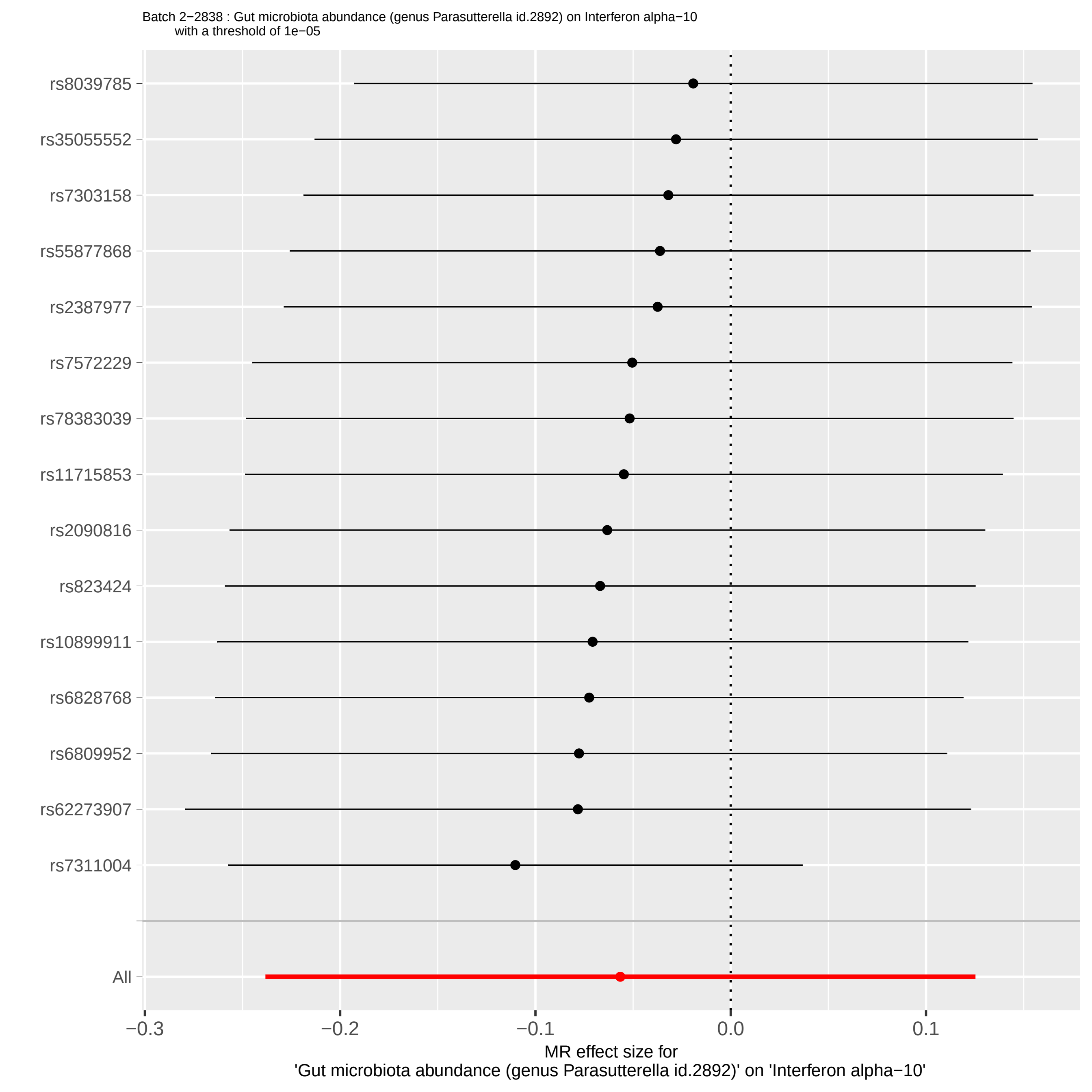

Batch 2−2838 : Gut microbiota abundance (genus Parasutterella id.2892) on Interferon alpha−10
with a threshold of 1e−05
rs8039785
rs35055552
rs7303158
rs55877868
rs2387977
rs7572229
rs78383039
rs11715853
rs2090816
rs823424
rs10899911
rs6828768
rs6809952
rs62273907
rs7311004
All
−0.3
−0.2
−0.1
0.0
0.1
MR effect size for
'Gut microbiota abundance (genus Parasutterella id.2892)' on 'Interferon alpha−10'

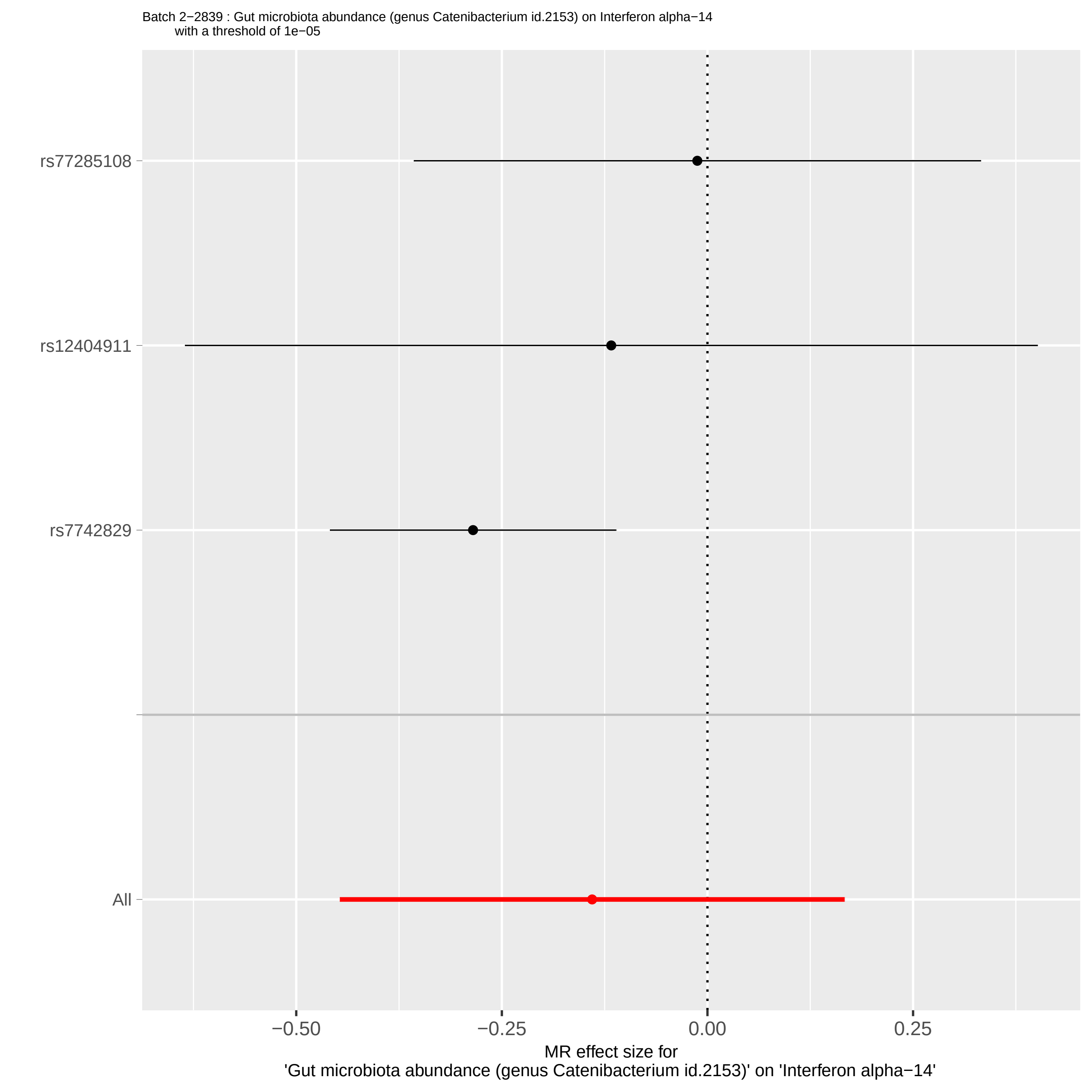
Batch 2−2839 : Gut microbiota abundance (genus Catenibacterium id.2153) on Interferon alpha−14
with a threshold of 1e−05
rs77285108
rs12404911
rs7742829
All
−0.50
−0.25
0.00
0.25
MR effect size for
'Gut microbiota abundance (genus Catenibacterium id.2153)' on 'Interferon alpha−14'

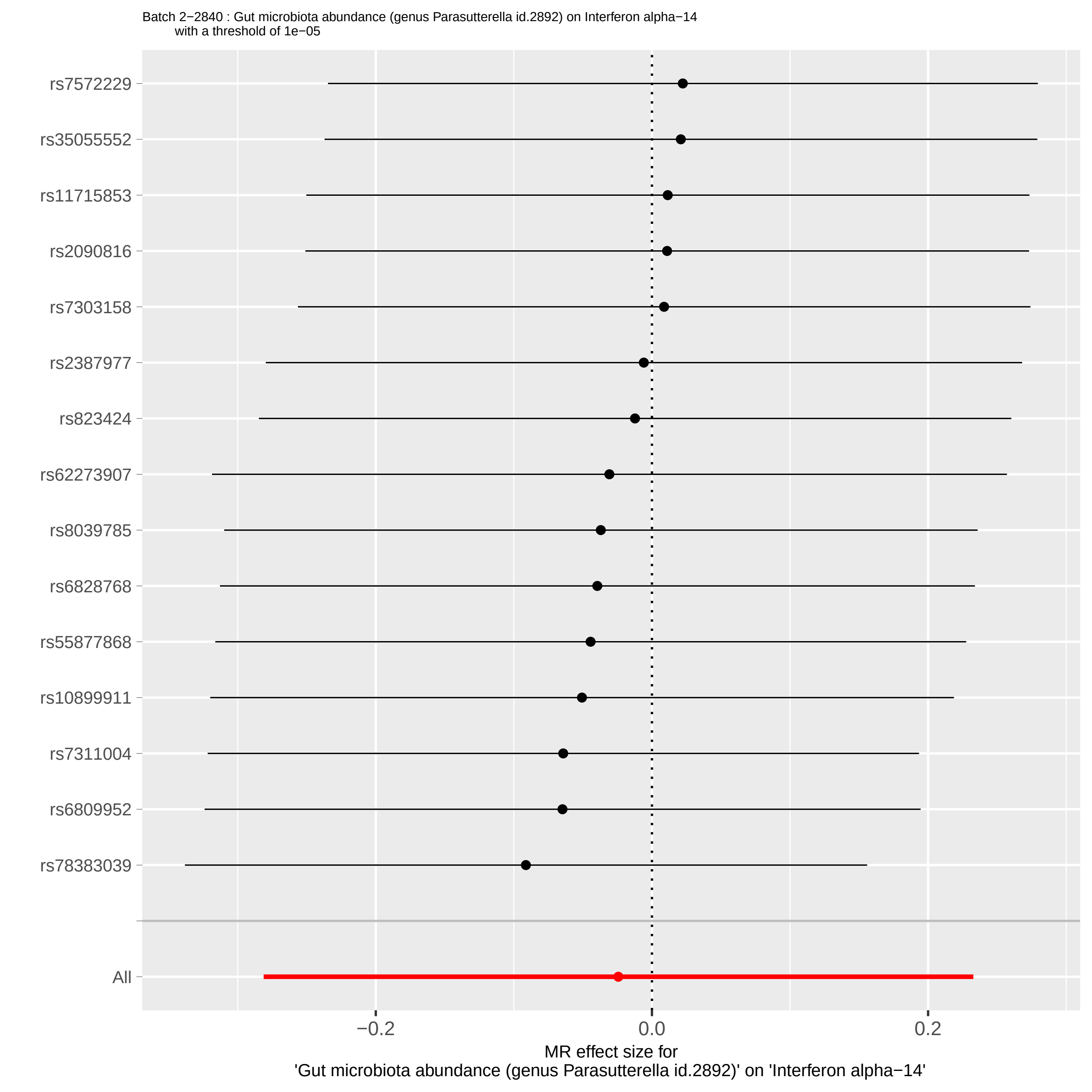
Batch 2−2840 : Gut microbiota abundance (genus Parasutterella id.2892) on Interferon alpha−14
with a threshold of 1e−05
rs7572229
rs35055552
rs11715853
rs2090816
rs7303158
rs2387977
rs823424
rs62273907
rs8039785
rs6828768
rs55877868
rs10899911
rs7311004
rs6809952
rs78383039
All
−0.2
0.0
0.2
MR effect size for
'Gut microbiota abundance (genus Parasutterella id.2892)' on 'Interferon alpha−14'

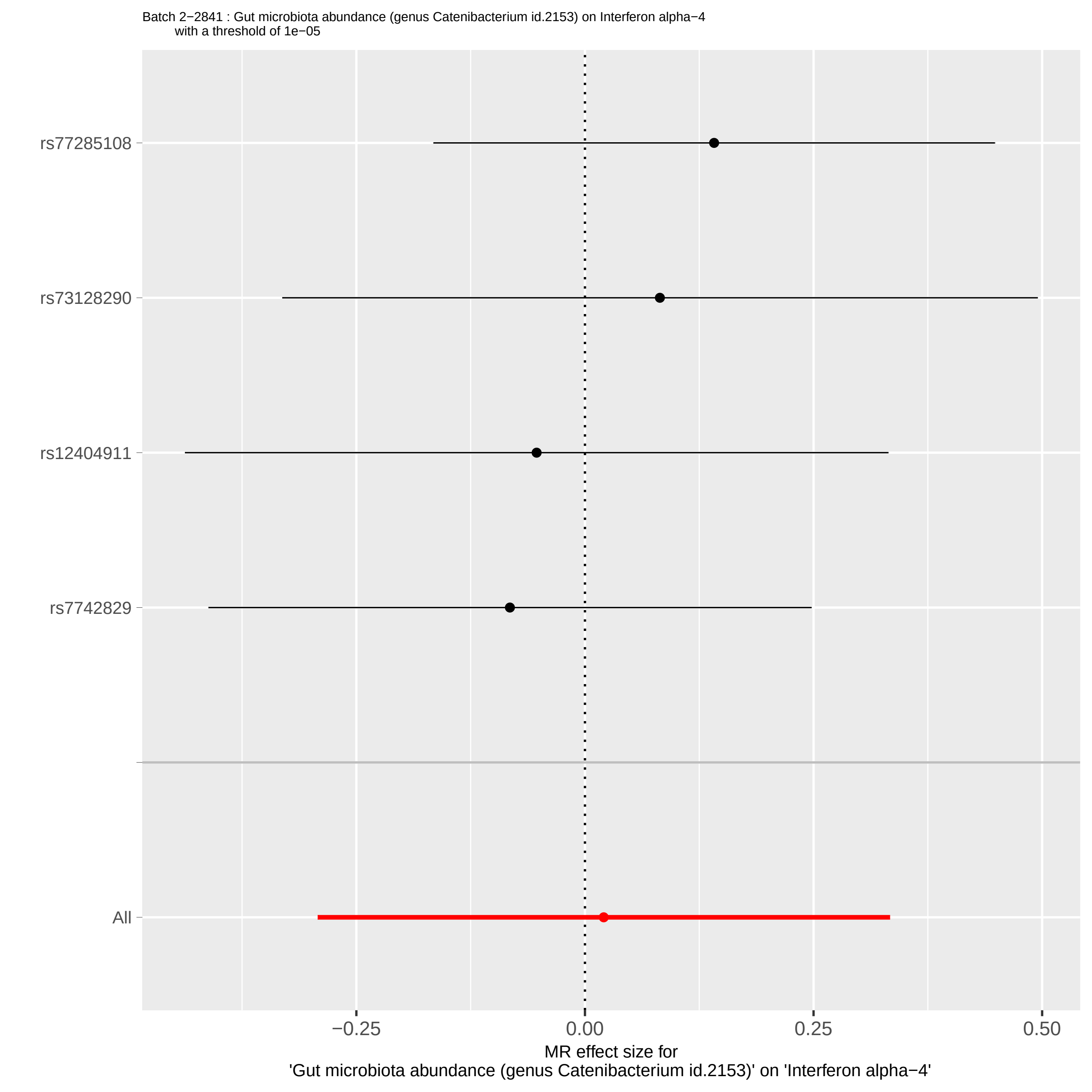
Batch 2−2841 : Gut microbiota abundance (genus Catenibacterium id.2153) on Interferon alpha−4
with a threshold of 1e−05
rs77285108
rs73128290
rs12404911
rs7742829
All
−0.25
0.00
0.25
0.50
MR effect size for
'Gut microbiota abundance (genus Catenibacterium id.2153)' on 'Interferon alpha−4'

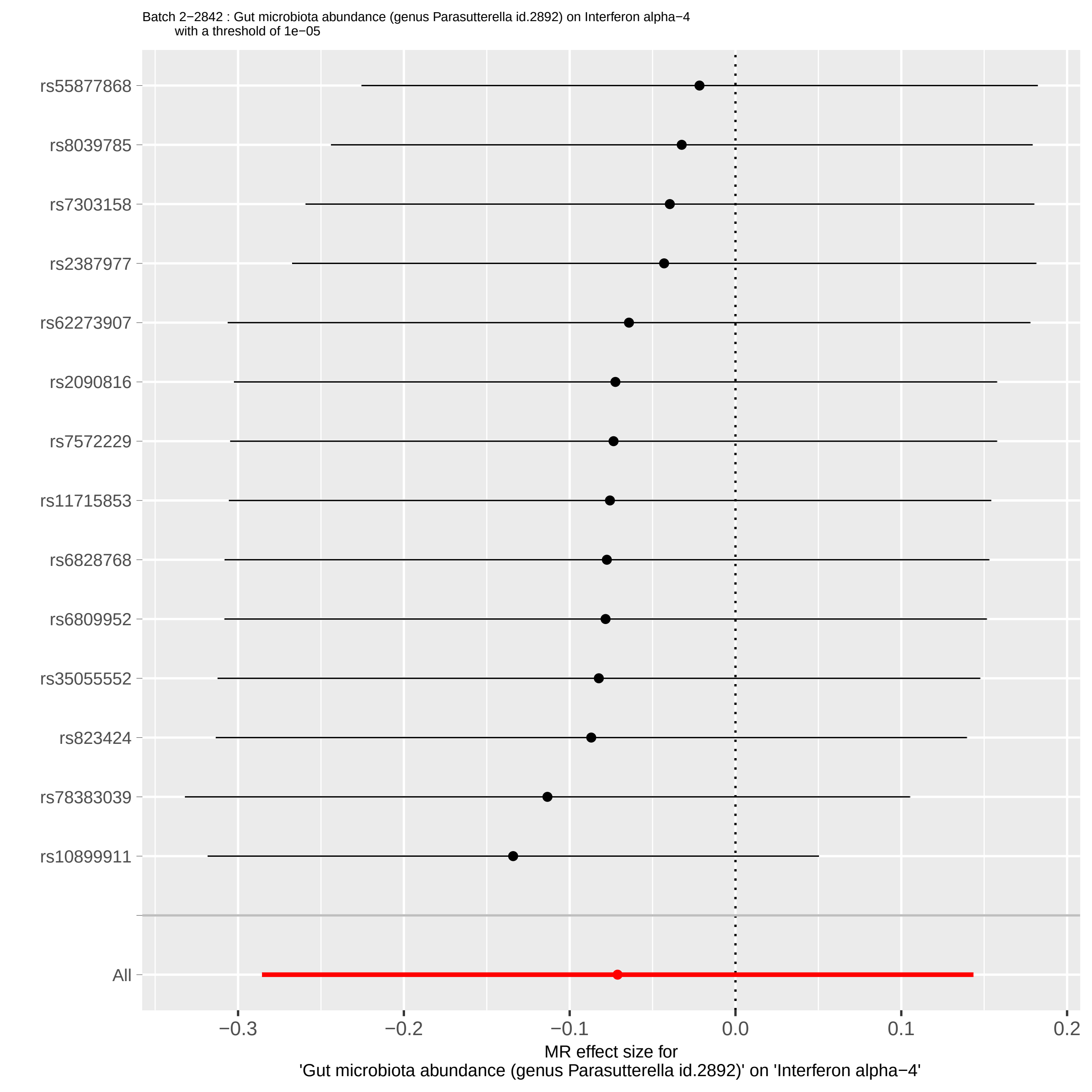
Batch 2−2842 : Gut microbiota abundance (genus Parasutterella id.2892) on Interferon alpha−4
with a threshold of 1e−05
rs55877868
rs8039785
rs7303158
rs2387977
rs62273907
rs2090816
rs7572229
rs11715853
rs6828768
rs6809952
rs35055552
rs823424
rs78383039
rs10899911
All
−0.3
−0.2
−0.1
0.0
0.1
0.2
MR effect size for
'Gut microbiota abundance (genus Parasutterella id.2892)' on 'Interferon alpha−4'

Batch 2−2843 : Gut microbiota abundance (genus Catenibacterium id.2153) on Interferon alpha−5
with a threshold of 1e−05

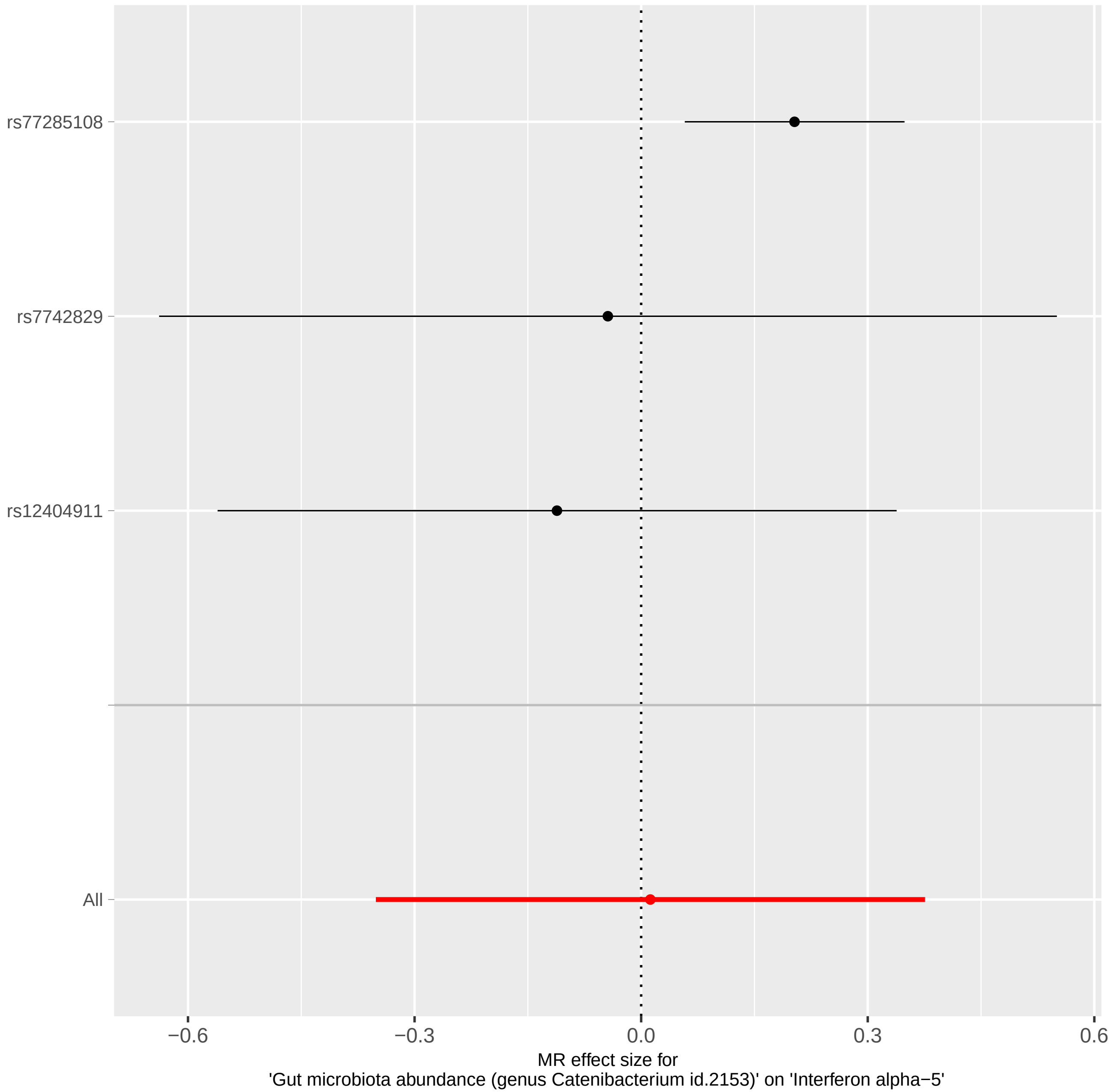

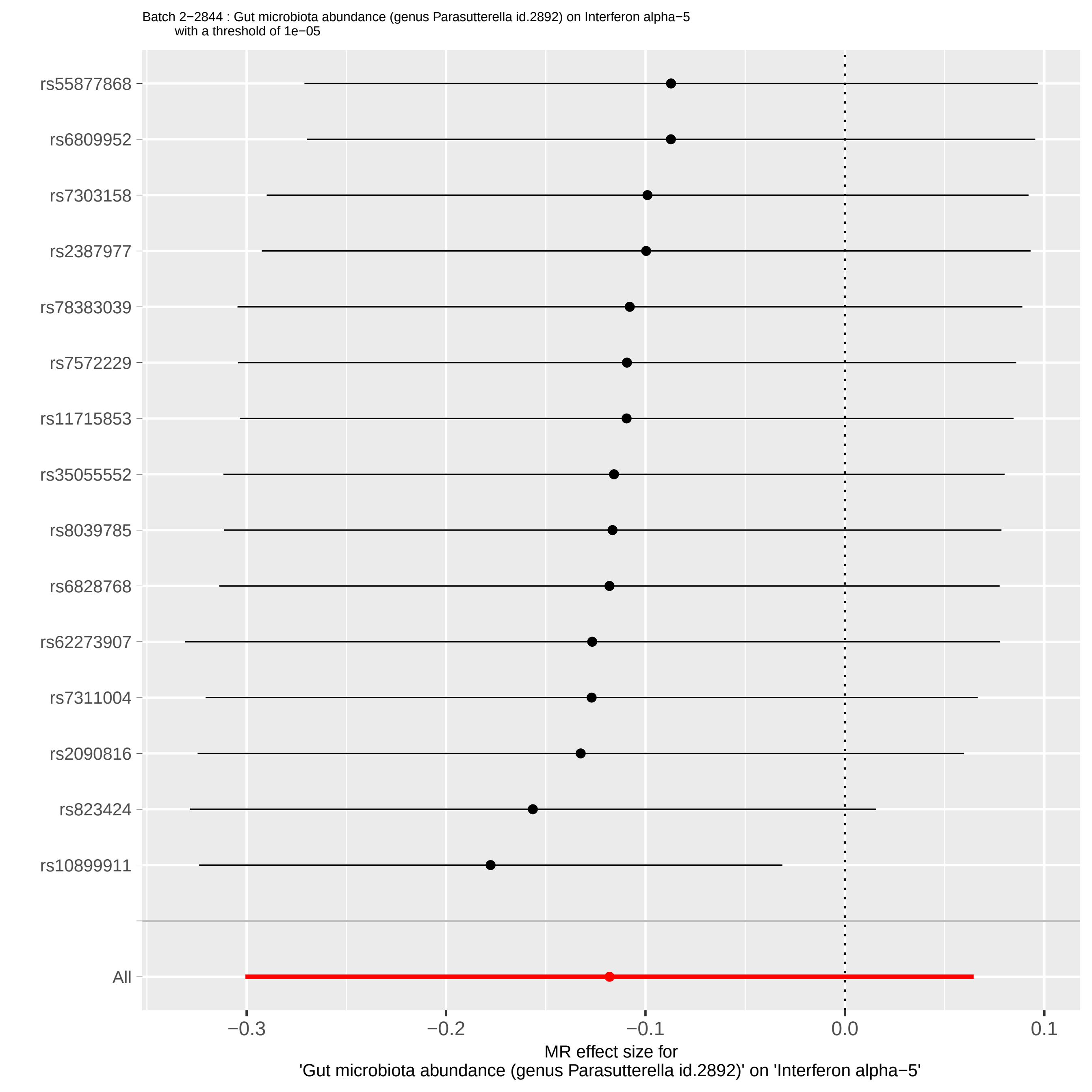

Batch 2−2844 : Gut microbiota abundance (genus Parasutterella id.2892) on Interferon alpha−5
with a threshold of 1e−05
rs55877868
rs6809952
rs7303158
rs2387977
rs78383039
rs7572229
rs11715853
rs35055552
rs8039785
rs6828768
rs62273907
rs7311004
rs2090816
rs823424
rs10899911
All
−0.3
−0.2
−0.1
0.0
0.1
MR effect size for
'Gut microbiota abundance (genus Parasutterella id.2892)' on 'Interferon alpha−5'

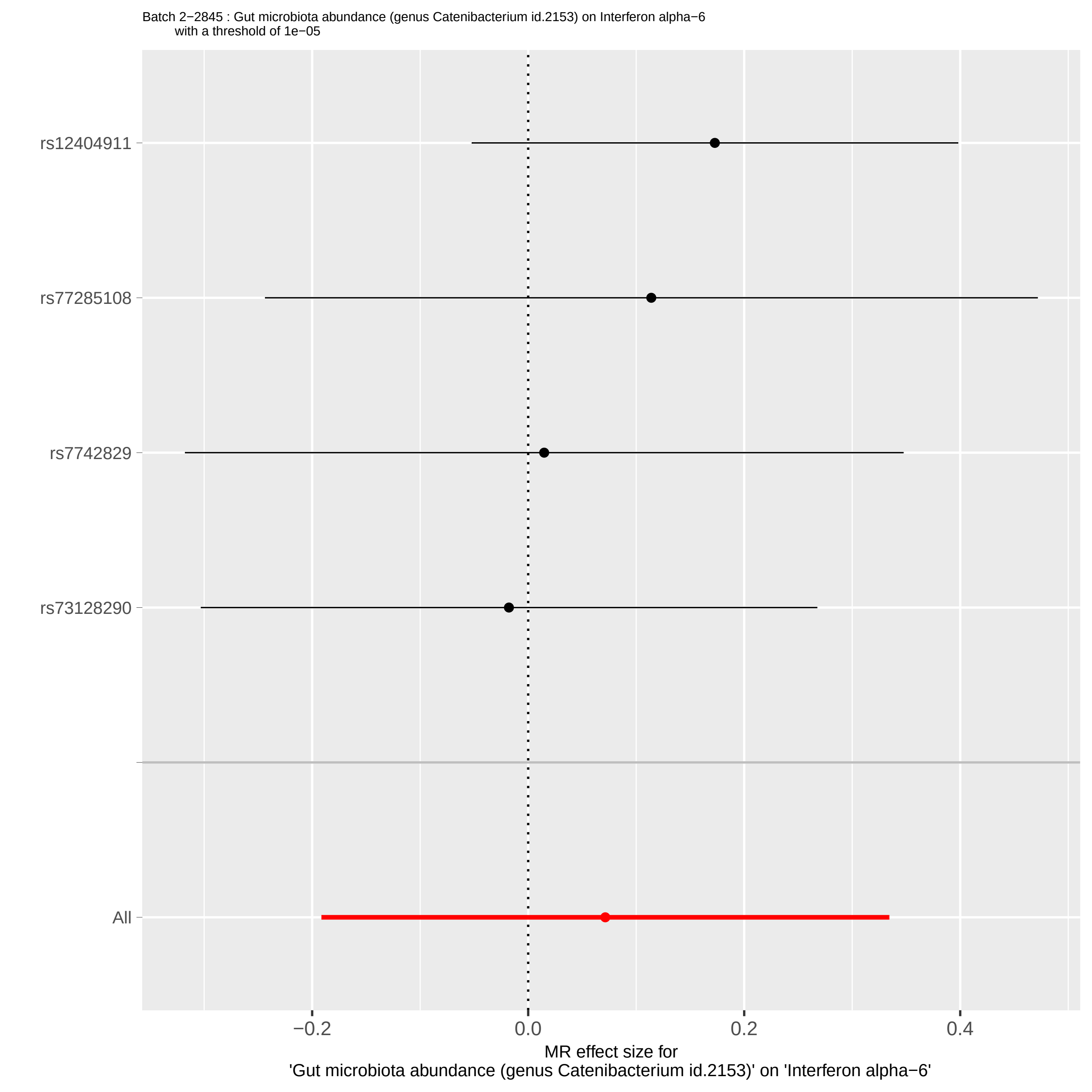
Batch 2−2845 : Gut microbiota abundance (genus Catenibacterium id.2153) on Interferon alpha−6
with a threshold of 1e−05
rs12404911
rs77285108
rs7742829
rs73128290
All
−0.2
0.0
0.2
0.4
MR effect size for
'Gut microbiota abundance (genus Catenibacterium id.2153)' on 'Interferon alpha−6'

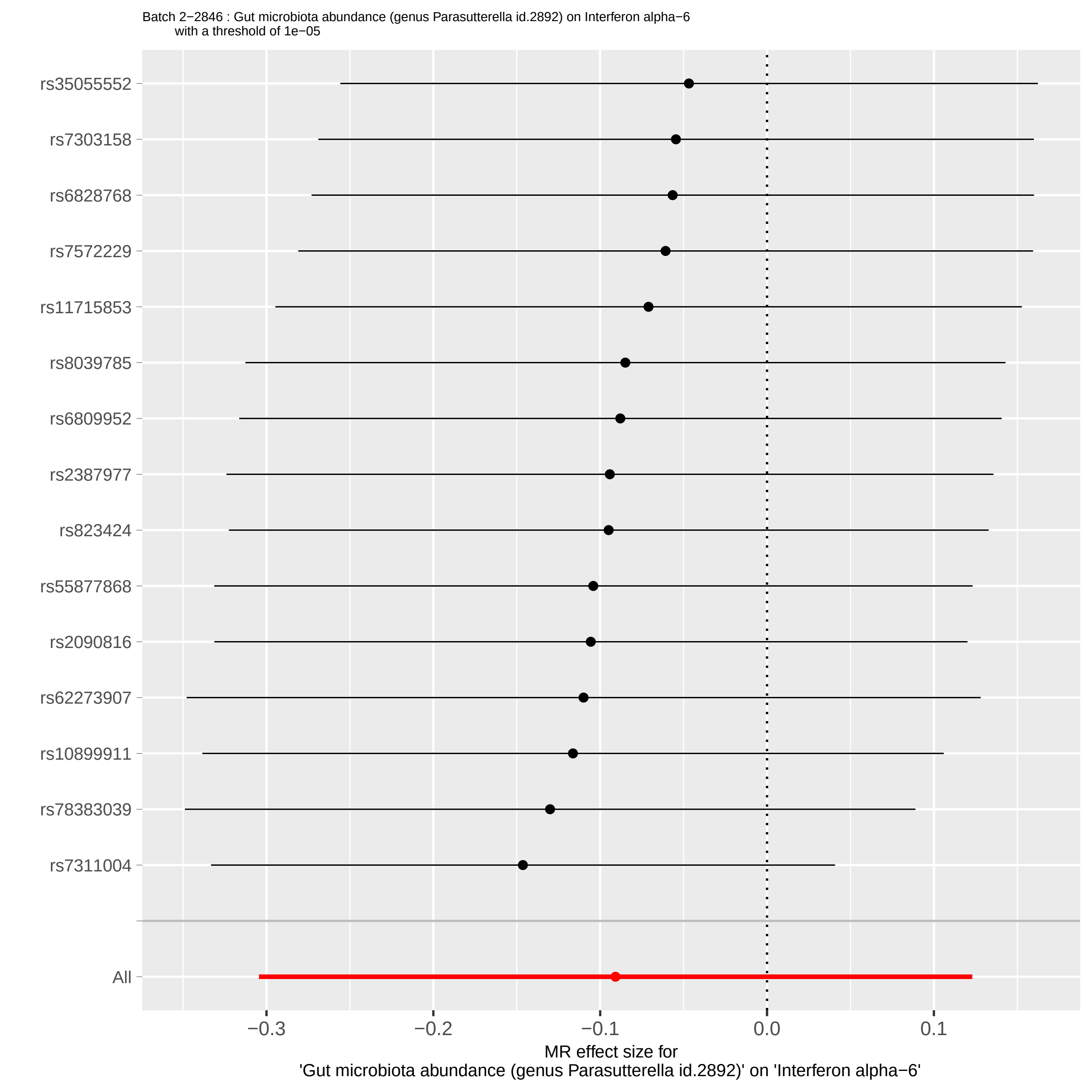

Batch 2−2846 : Gut microbiota abundance (genus Parasutterella id.2892) on Interferon alpha−6
with a threshold of 1e−05
rs35055552
rs7303158
rs6828768
rs7572229
rs11715853
rs8039785
rs6809952
rs2387977
rs823424
rs55877868
rs2090816
rs62273907
rs10899911
rs78383039
rs7311004
All
−0.3
−0.2
−0.1
0.0
0.1
MR effect size for
'Gut microbiota abundance (genus Parasutterella id.2892)' on 'Interferon alpha−6'

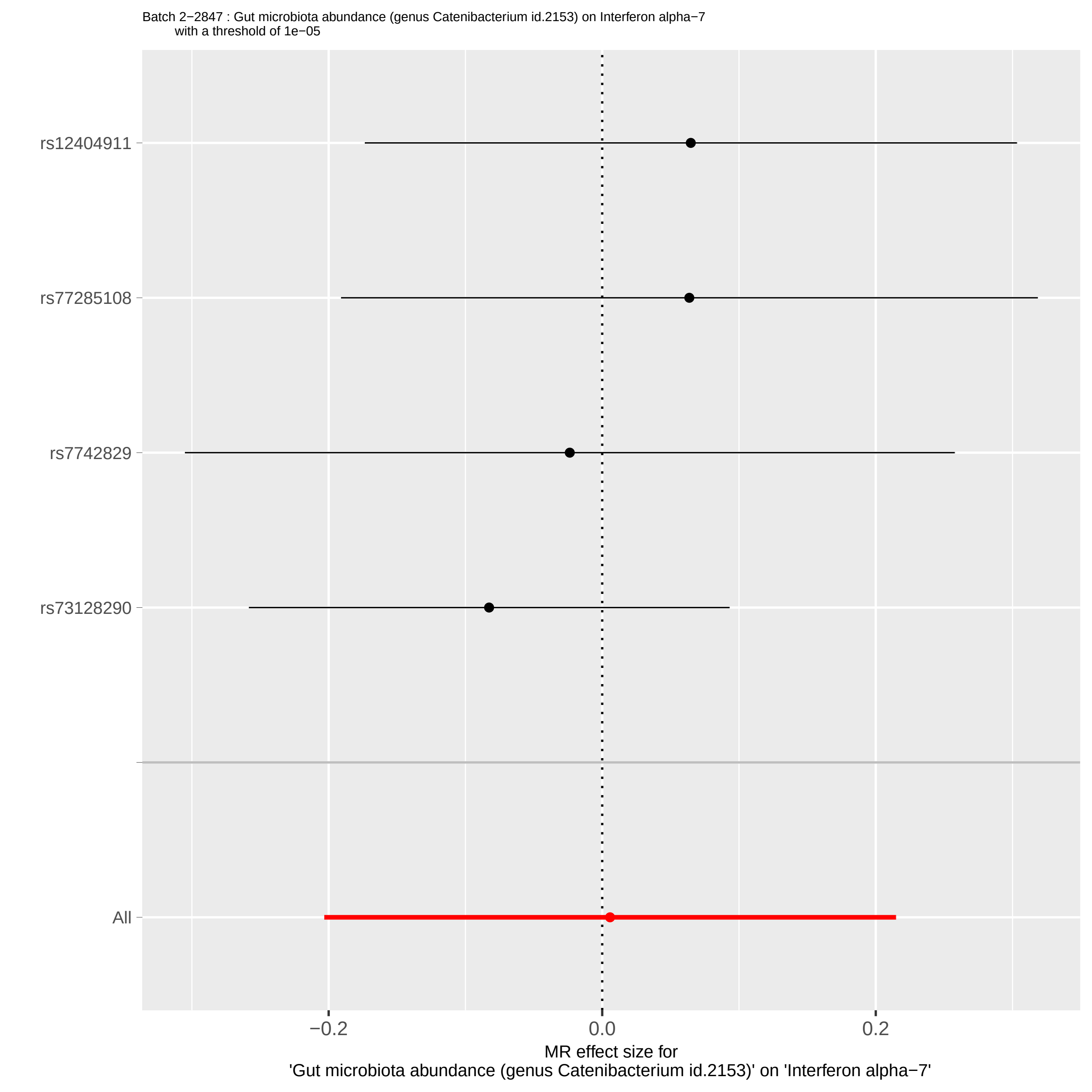

Batch 2−2847 : Gut microbiota abundance (genus Catenibacterium id.2153) on Interferon alpha−7
with a threshold of 1e−05
rs12404911
rs77285108
rs7742829
rs73128290
All
−0.2
0.0
0.2
MR effect size for
'Gut microbiota abundance (genus Catenibacterium id.2153)' on 'Interferon alpha−7'

Batch 2−2848 : Gut microbiota abundance (genus Parasutterella id.2892) on Interferon alpha−7
with a threshold of 1e−05

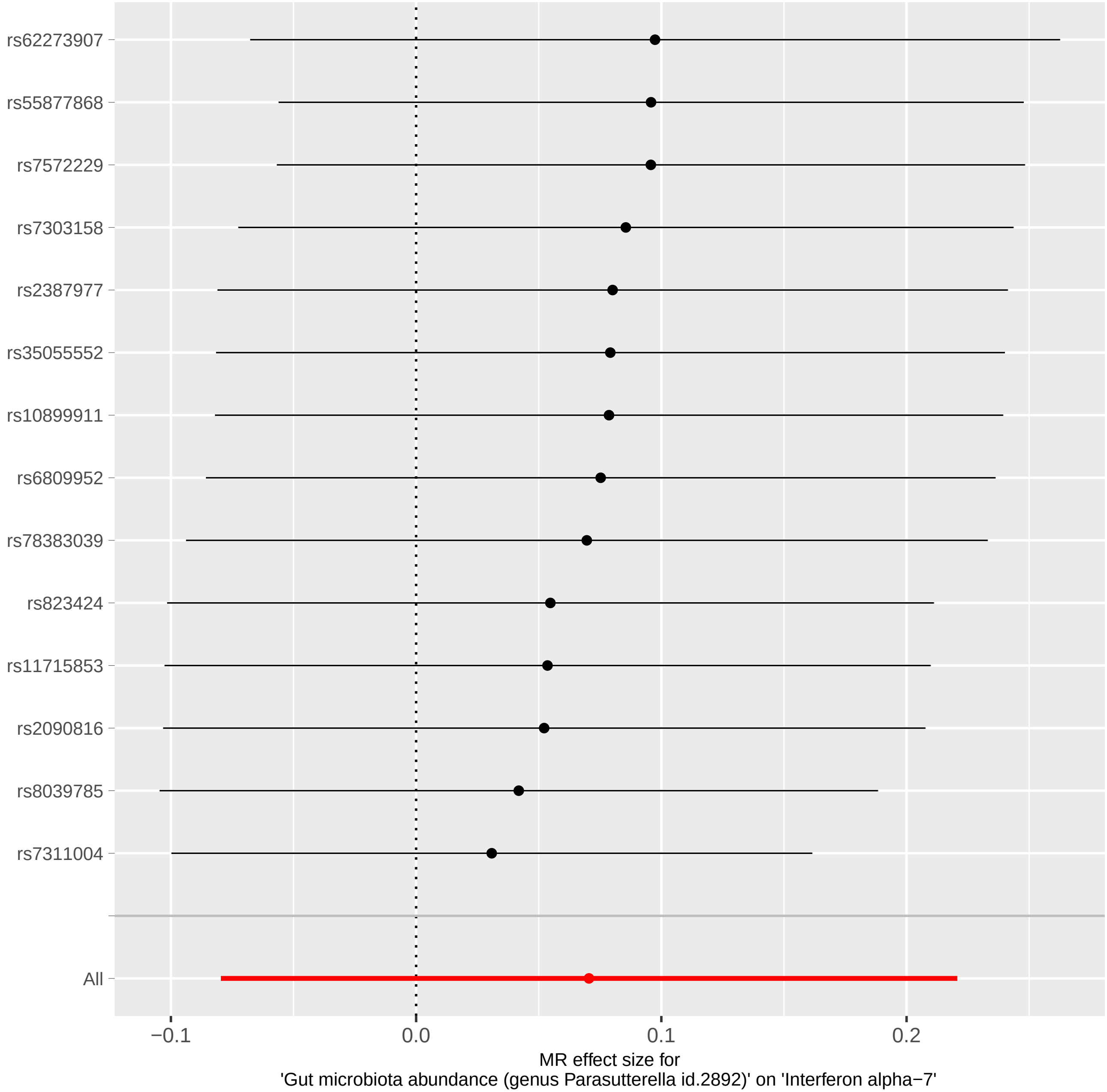

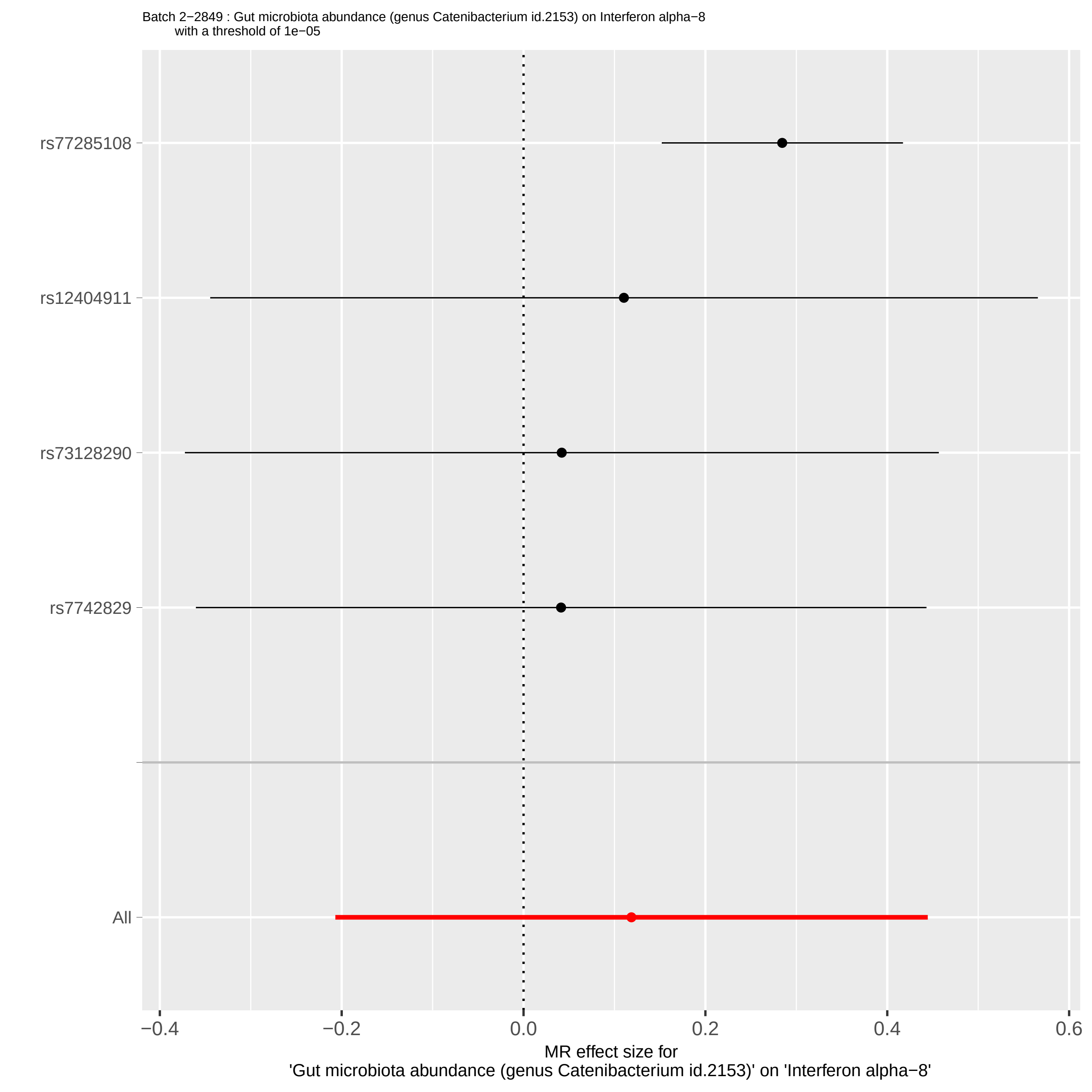

Batch 2−2849 : Gut microbiota abundance (genus Catenibacterium id.2153) on Interferon alpha−8
with a threshold of 1e−05
rs77285108
rs12404911
rs73128290
rs7742829
All
−0.4
−0.2
0.0
0.2
0.4
0.6
MR effect size for
'Gut microbiota abundance (genus Catenibacterium id.2153)' on 'Interferon alpha−8'

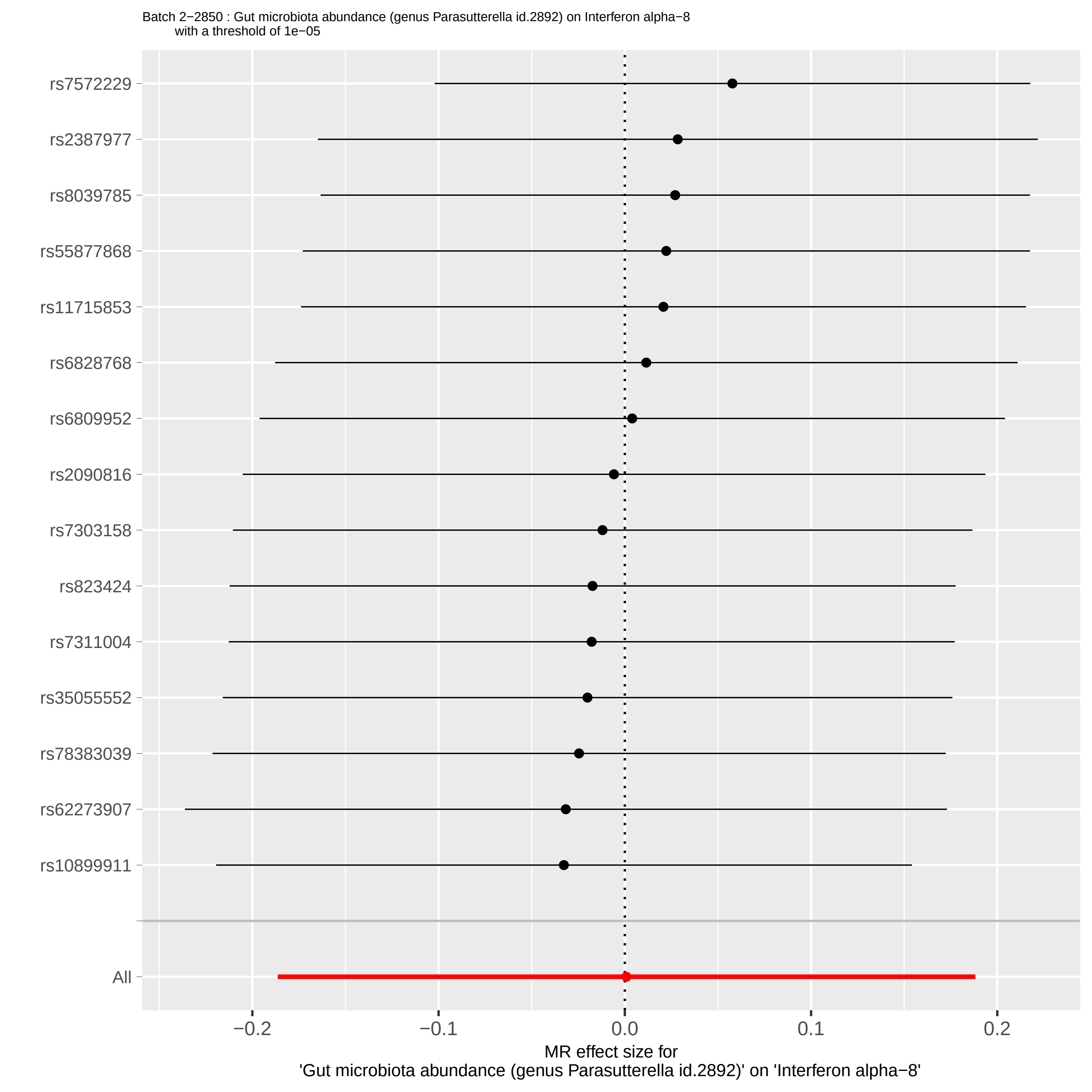

Batch 2−2850 : Gut microbiota abundance (genus Parasutterella id.2892) on Interferon alpha−8
with a threshold of 1e−05
rs7572229
rs2387977
rs8039785
rs55877868
rs11715853
rs6828768
rs6809952
rs2090816
rs7303158
rs823424
rs7311004
rs35055552
rs78383039
rs62273907
rs10899911
All
−0.2
−0.1
0.0
0.1
0.2
MR effect size for
'Gut microbiota abundance (genus Parasutterella id.2892)' on 'Interferon alpha−8'

Batch 2−2851 : Gut microbiota abundance (genus Catenibacterium id.2153) on Interferon alpha/beta receptor 1 with a threshold of 1e−05

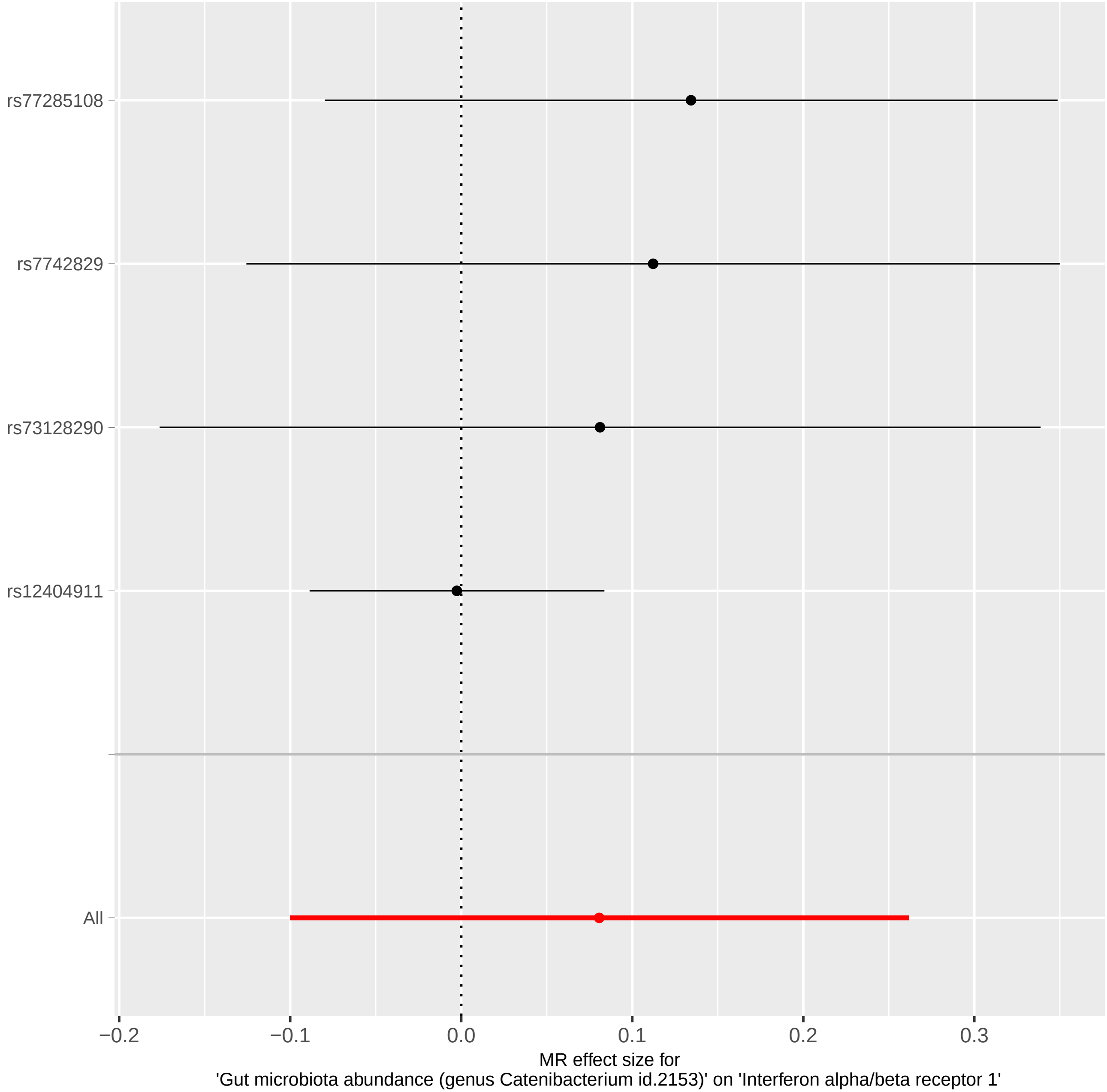

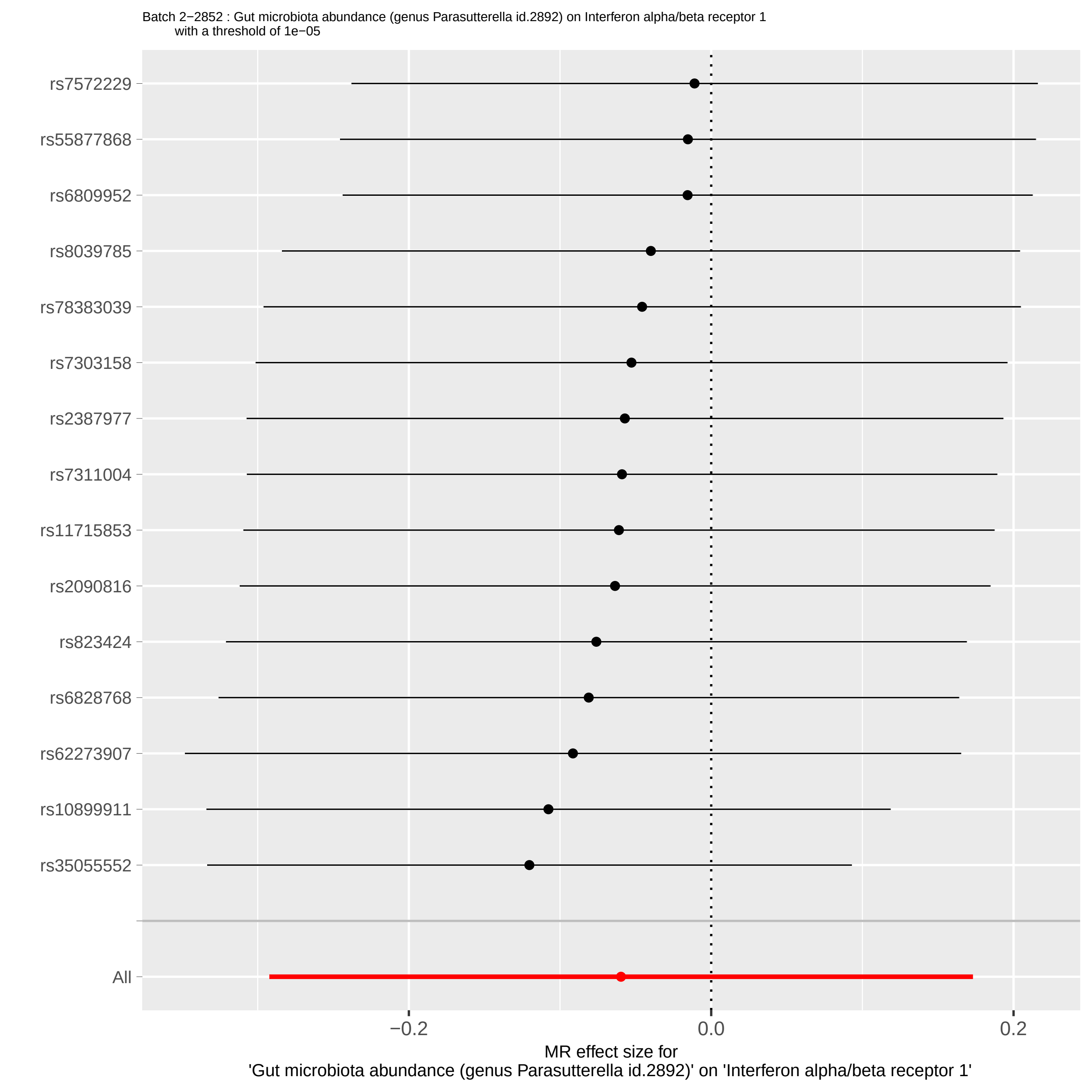
Batch 2−2852 : Gut microbiota abundance (genus Parasutterella id.2892) on Interferon alpha/beta receptor 1
with a threshold of 1e−05
rs7572229
rs55877868
rs6809952
rs8039785
rs78383039
rs7303158
rs2387977
rs7311004
rs11715853
rs2090816
rs823424
rs6828768
rs62273907
rs10899911
rs35055552
All
−0.2
0.0
0.2
MR effect size for
'Gut microbiota abundance (genus Parasutterella id.2892)' on 'Interferon alpha/beta receptor 1'

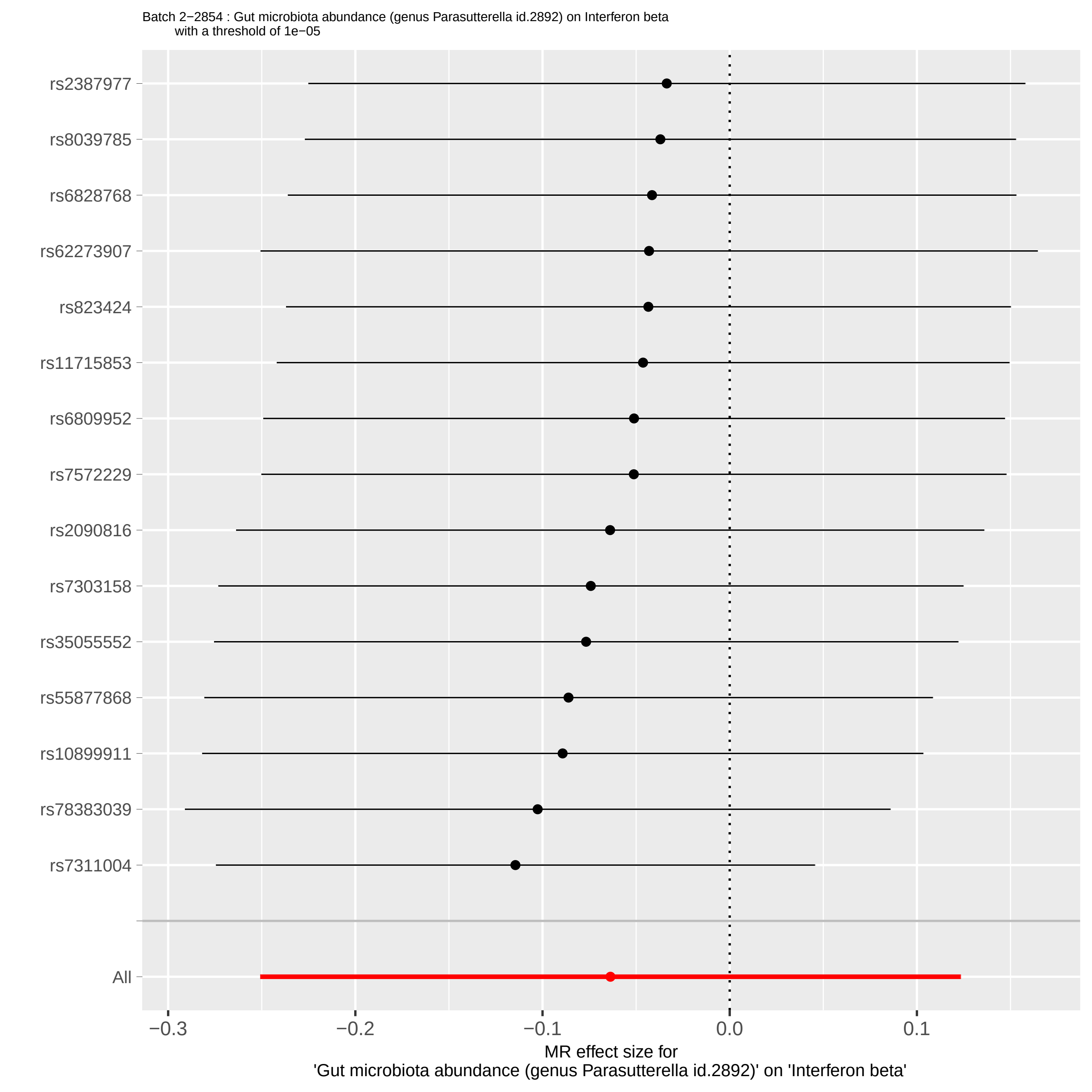
Batch 2−2854 : Gut microbiota abundance (genus Parasutterella id.2892) on Interferon beta
with a threshold of 1e−05
rs2387977
rs8039785
rs6828768
rs62273907
rs823424
rs11715853
rs6809952
rs7572229
rs2090816
rs7303158
rs35055552
rs55877868
rs10899911
rs78383039
rs7311004
All
−0.3
−0.2
−0.1
0.0
0.1
MR effect size for
'Gut microbiota abundance (genus Parasutterella id.2892)' on 'Interferon beta'

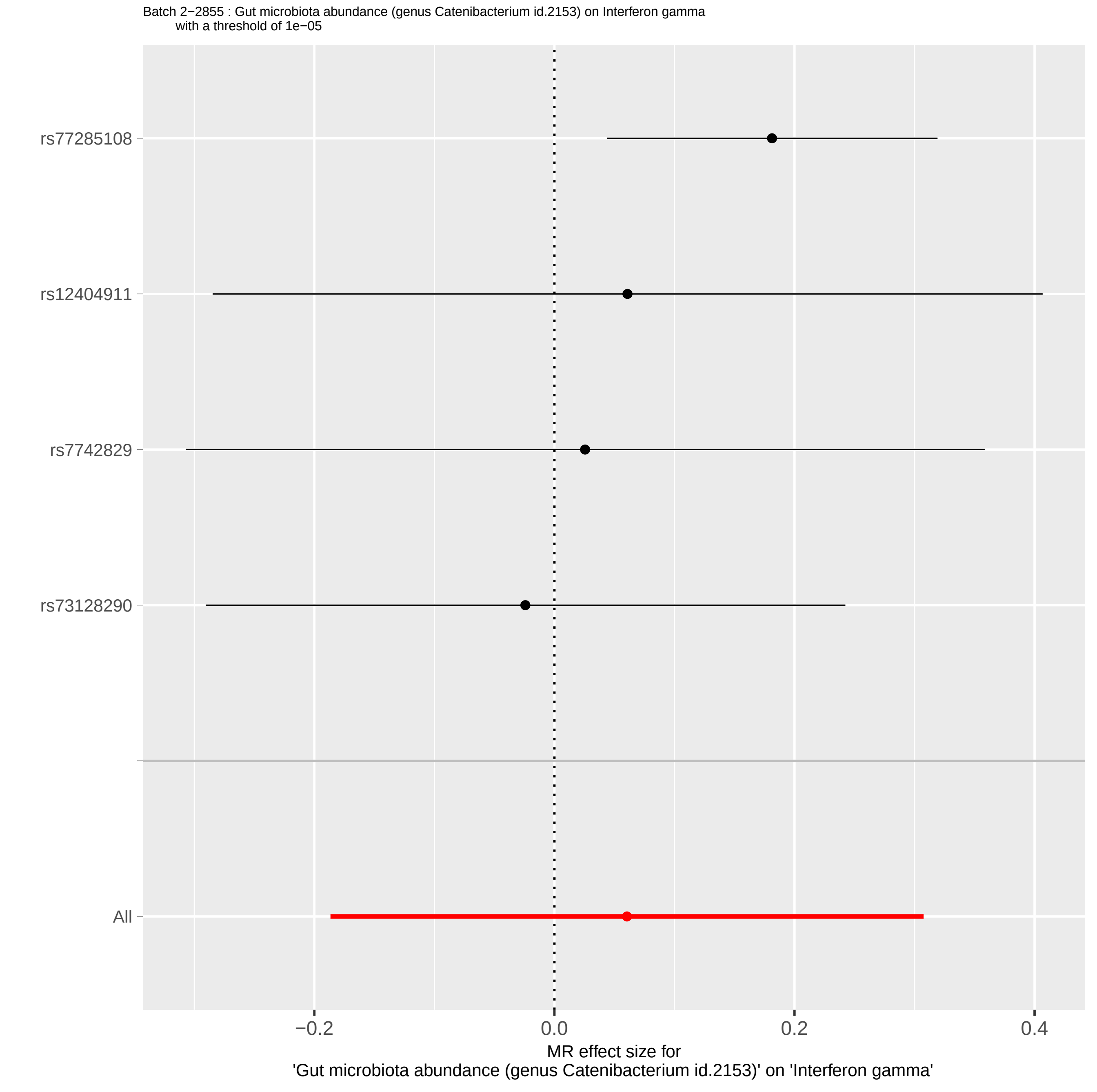

Batch 2−2855 : Gut microbiota abundance (genus Catenibacterium id.2153) on Interferon gamma
with a threshold of 1e−05
rs77285108
rs12404911
rs7742829
rs73128290
All
−0.2
0.0
0.2
0.4
MR effect size for
'Gut microbiota abundance (genus Catenibacterium id.2153)' on 'Interferon gamma'

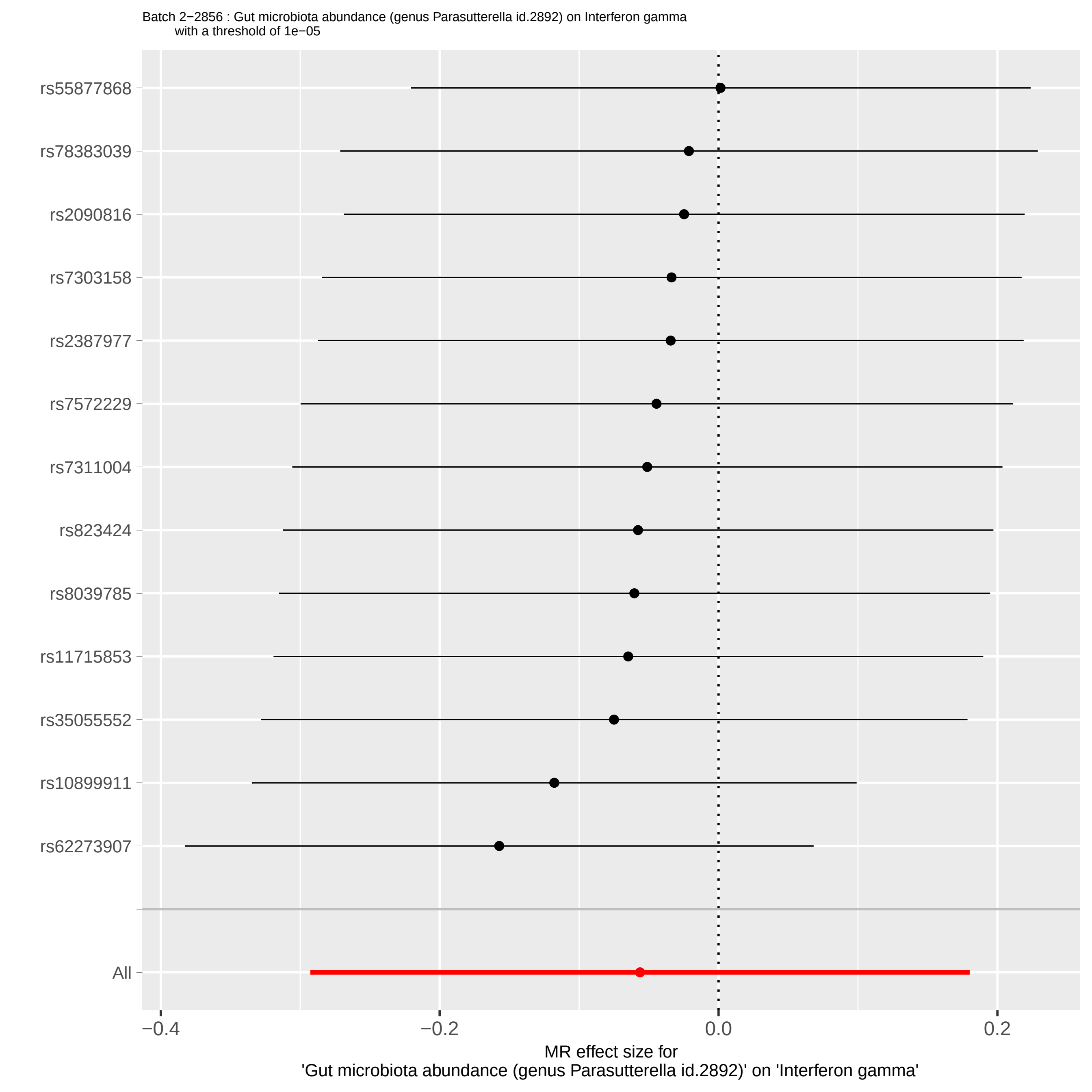

Batch 2−2856 : Gut microbiota abundance (genus Parasutterella id.2892) on Interferon gamma
with a threshold of 1e−05
rs55877868
rs78383039
rs2090816
rs7303158
rs2387977
rs7572229
rs7311004
rs823424
rs8039785
rs11715853
rs35055552
rs10899911
rs62273907
All
−0.4
−0.2
0.0
0.2
MR effect size for
'Gut microbiota abundance (genus Parasutterella id.2892)' on 'Interferon gamma'

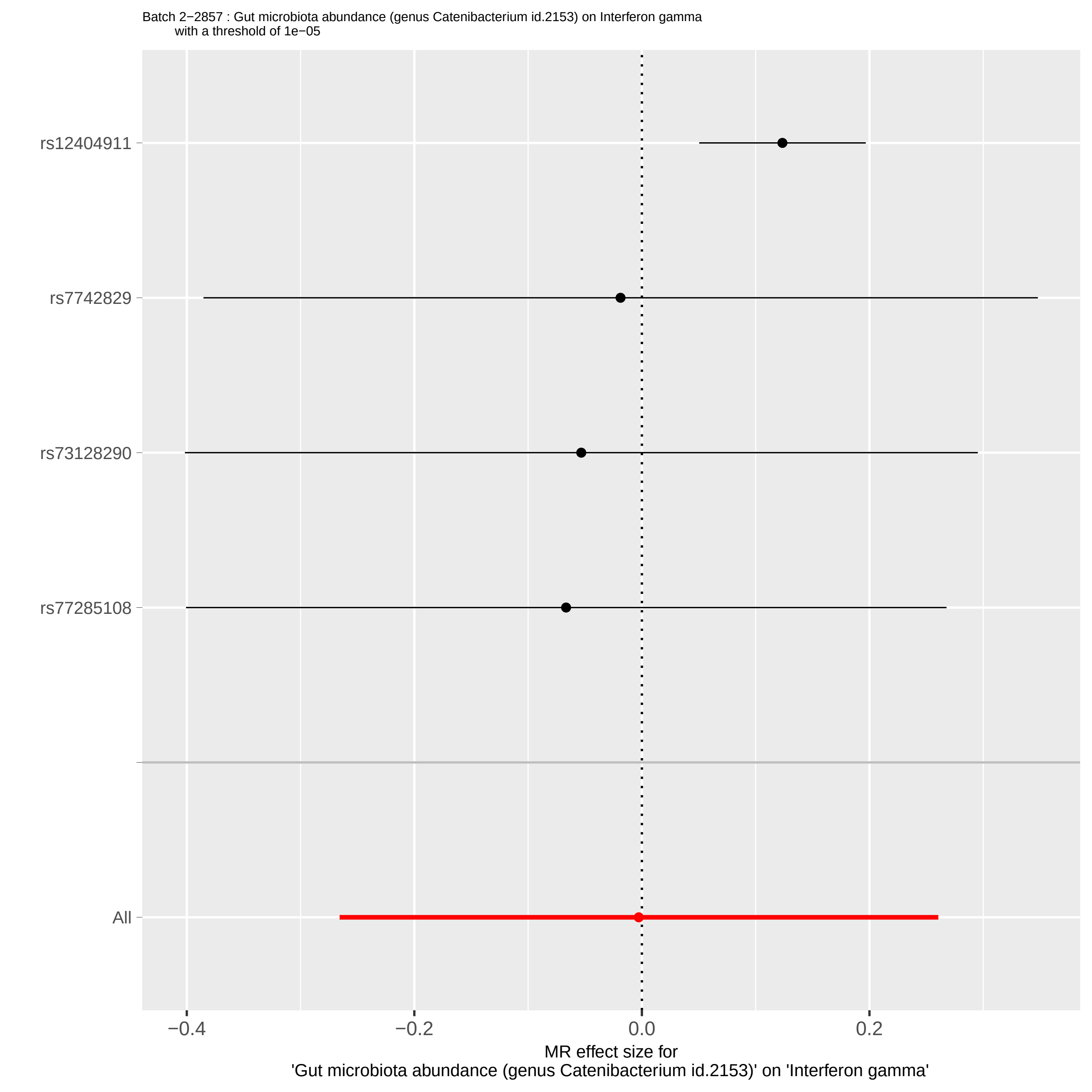
Batch 2−2857 : Gut microbiota abundance (genus Catenibacterium id.2153) on Interferon gamma
with a threshold of 1e−05
rs12404911
rs7742829
rs73128290
rs77285108
All
−0.4
−0.2
0.0
0.2
MR effect size for
'Gut microbiota abundance (genus Catenibacterium id.2153)' on 'Interferon gamma'

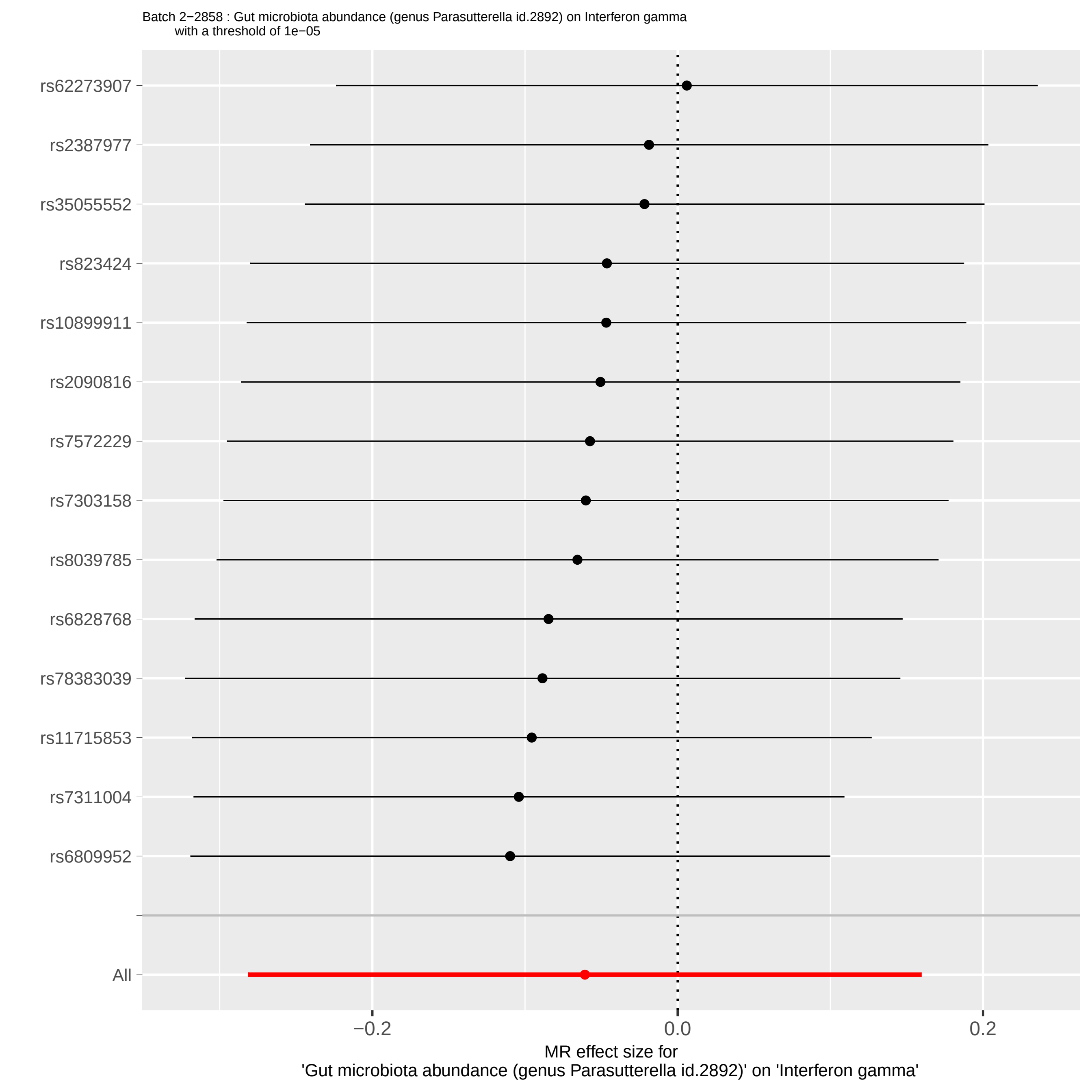

Batch 2−2858 : Gut microbiota abundance (genus Parasutterella id.2892) on Interferon gamma
with a threshold of 1e−05
rs62273907
rs2387977
rs35055552
rs823424
rs10899911
rs2090816
rs7572229
rs7303158
rs8039785
rs6828768
rs78383039
rs11715853
rs7311004
rs6809952
All
−0.2
0.0
0.2
MR effect size for
'Gut microbiota abundance (genus Parasutterella id.2892)' on 'Interferon gamma'

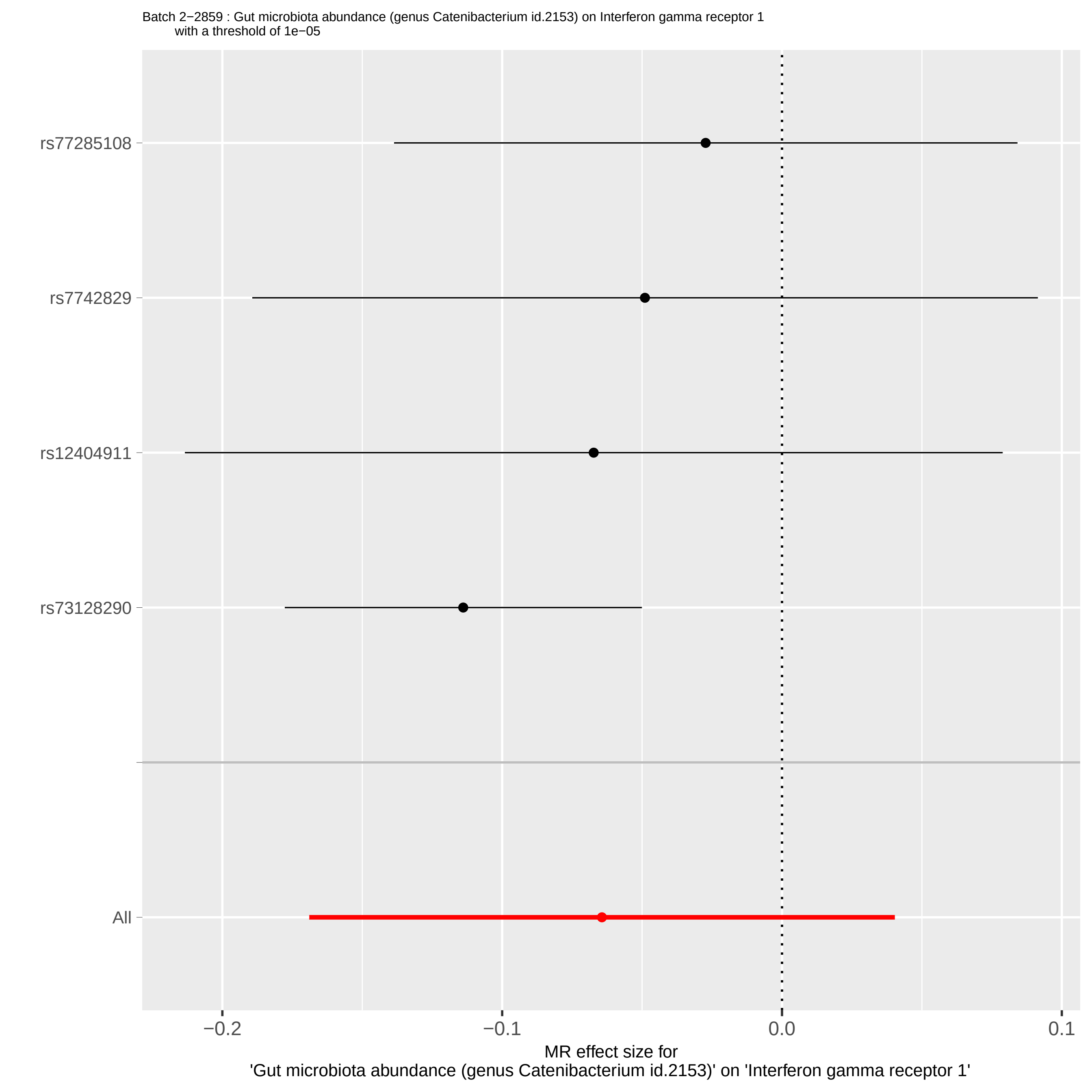
Batch 2−2859 : Gut microbiota abundance (genus Catenibacterium id.2153) on Interferon gamma receptor 1
with a threshold of 1e−05
rs77285108
rs7742829
rs12404911
rs73128290
All
−0.2
−0.1
0.0
0.1
MR effect size for
'Gut microbiota abundance (genus Catenibacterium id.2153)' on 'Interferon gamma receptor 1'

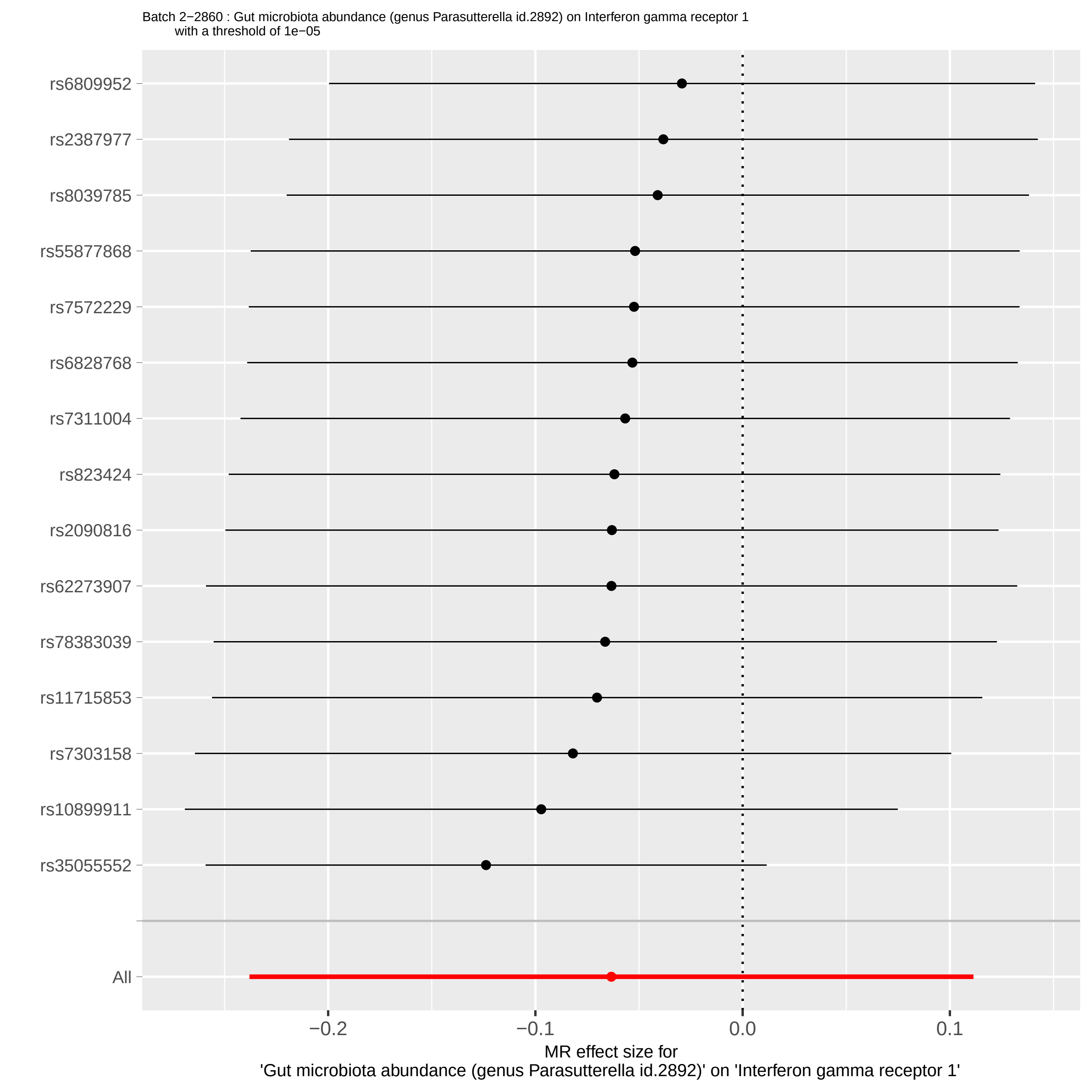
Batch 2−2860 : Gut microbiota abundance (genus Parasutterella id.2892) on Interferon gamma receptor 1
with a threshold of 1e−05
rs6809952
rs2387977
rs8039785
rs55877868
rs7572229
rs6828768
rs7311004
rs823424
rs2090816
rs62273907
rs78383039
rs11715853
rs7303158
rs10899911
rs35055552
All
−0.2
−0.1
0.0
0.1
MR effect size for
'Gut microbiota abundance (genus Parasutterella id.2892)' on 'Interferon gamma receptor 1'

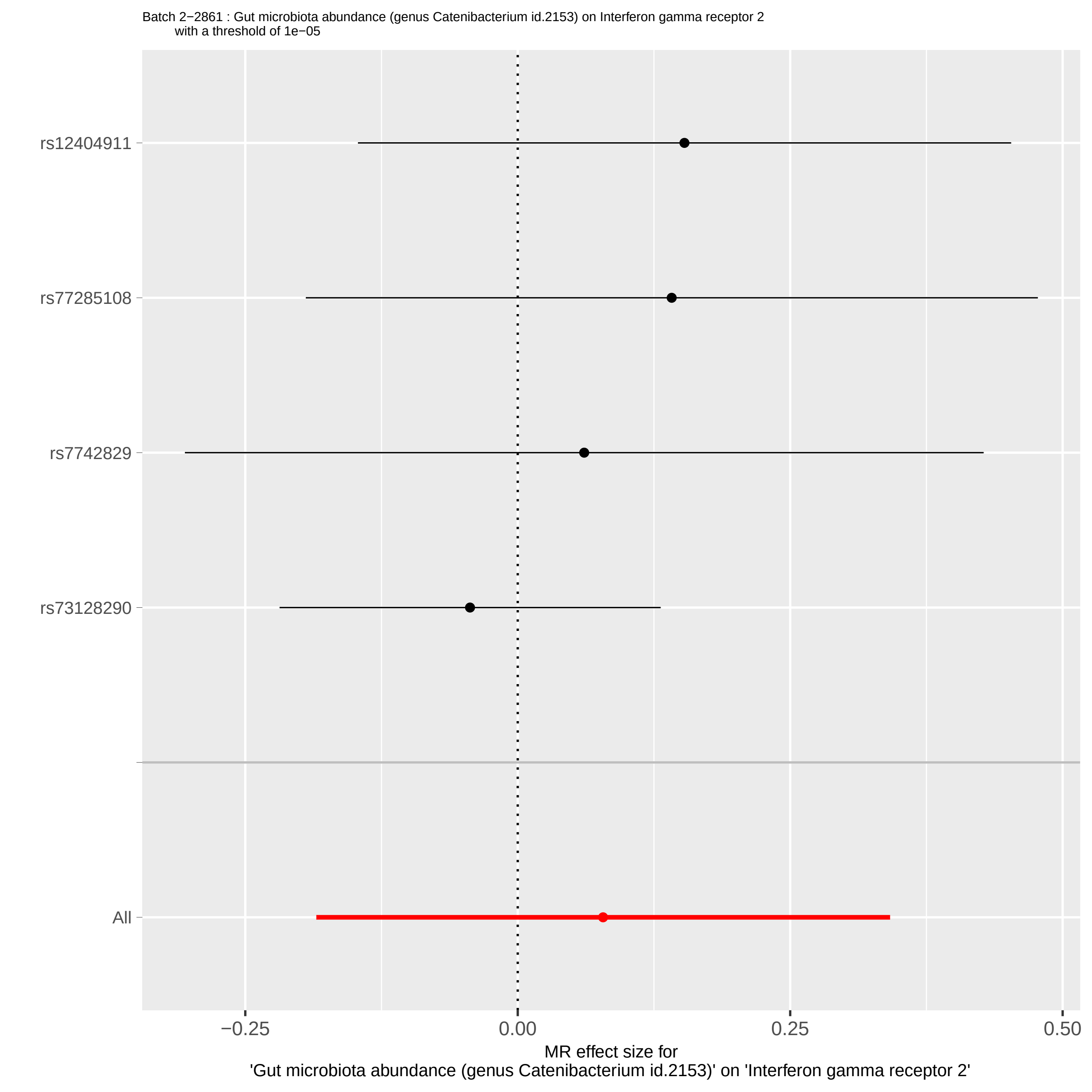
Batch 2−2861 : Gut microbiota abundance (genus Catenibacterium id.2153) on Interferon gamma receptor 2
with a threshold of 1e−05
rs12404911
rs77285108
rs7742829
rs73128290
All
−0.25
0.00
0.25
0.50
MR effect size for
'Gut microbiota abundance (genus Catenibacterium id.2153)' on 'Interferon gamma receptor 2'

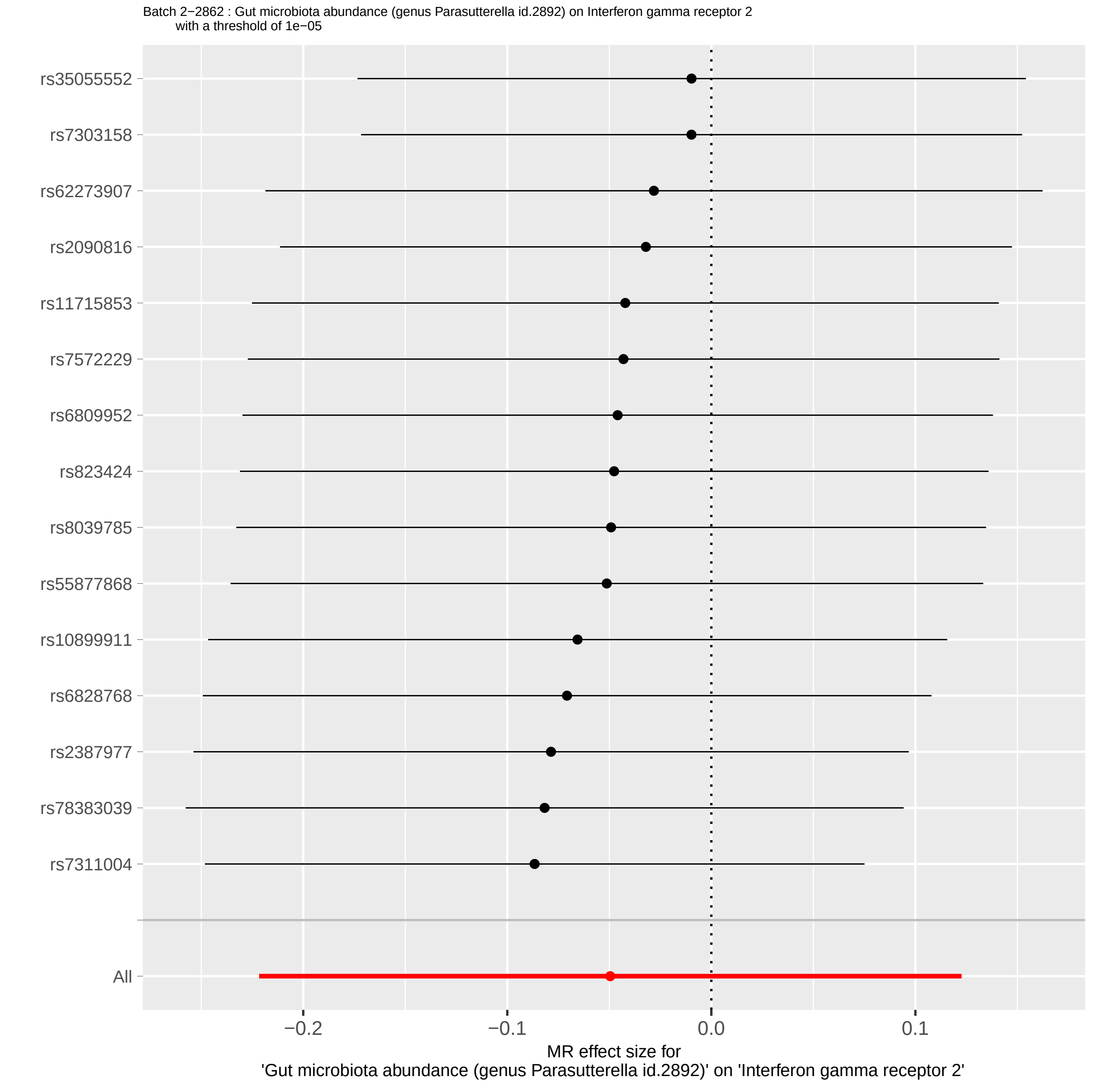
Batch 2−2862 : Gut microbiota abundance (genus Parasutterella id.2892) on Interferon gamma receptor 2
with a threshold of 1e−05
rs35055552
rs7303158
rs62273907
rs2090816
rs11715853
rs7572229
rs6809952
rs823424
rs8039785
rs55877868
rs10899911
rs6828768
rs2387977
rs78383039
rs7311004
All
−0.2
−0.1
0.0
0.1
MR effect size for
'Gut microbiota abundance (genus Parasutterella id.2892)' on 'Interferon gamma receptor 2'

Batch 2−2863 : Gut microbiota abundance (genus Catenibacterium id.2153) on Interferon gamma receptor 2
with a threshold of 1e−05

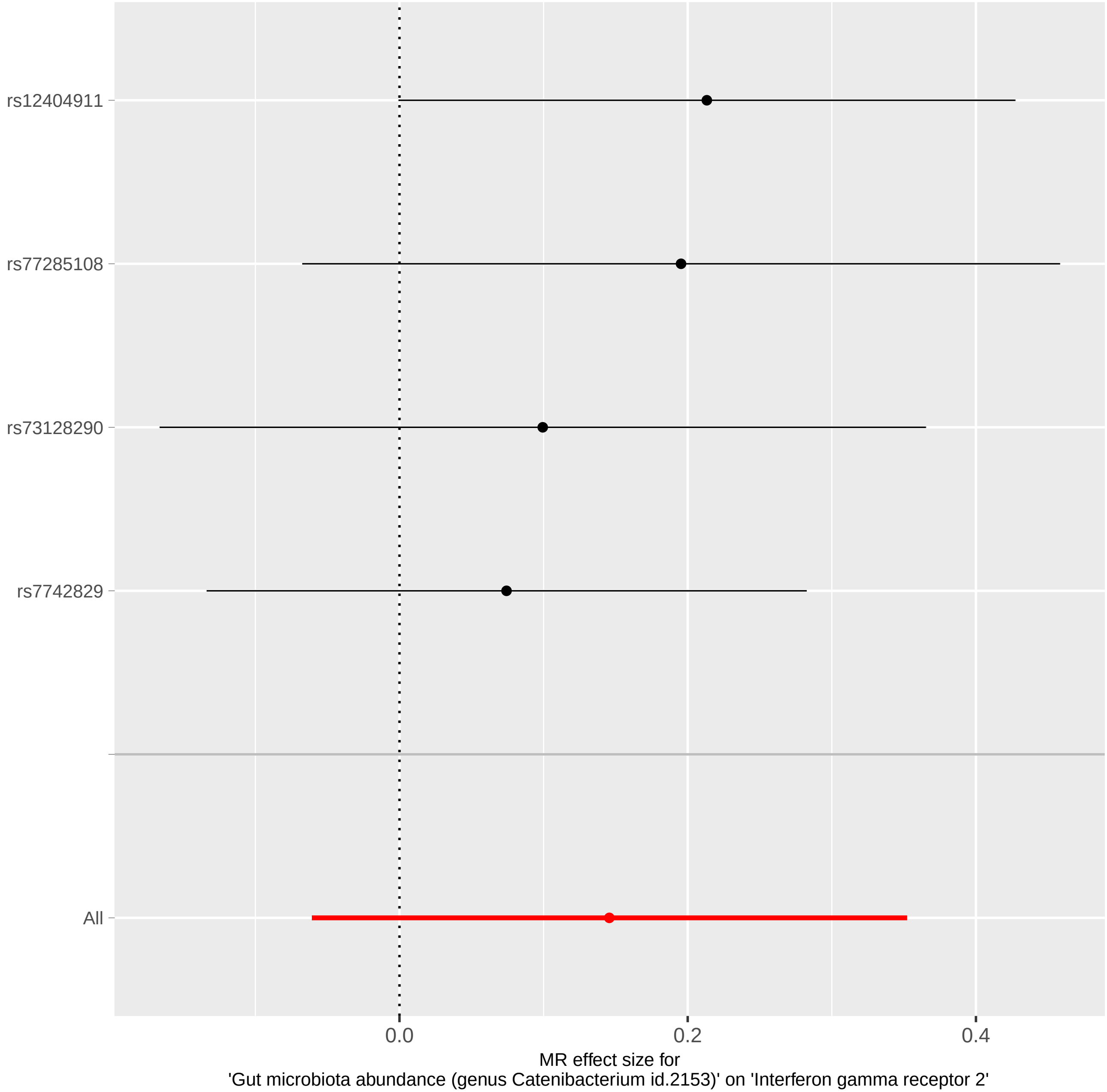

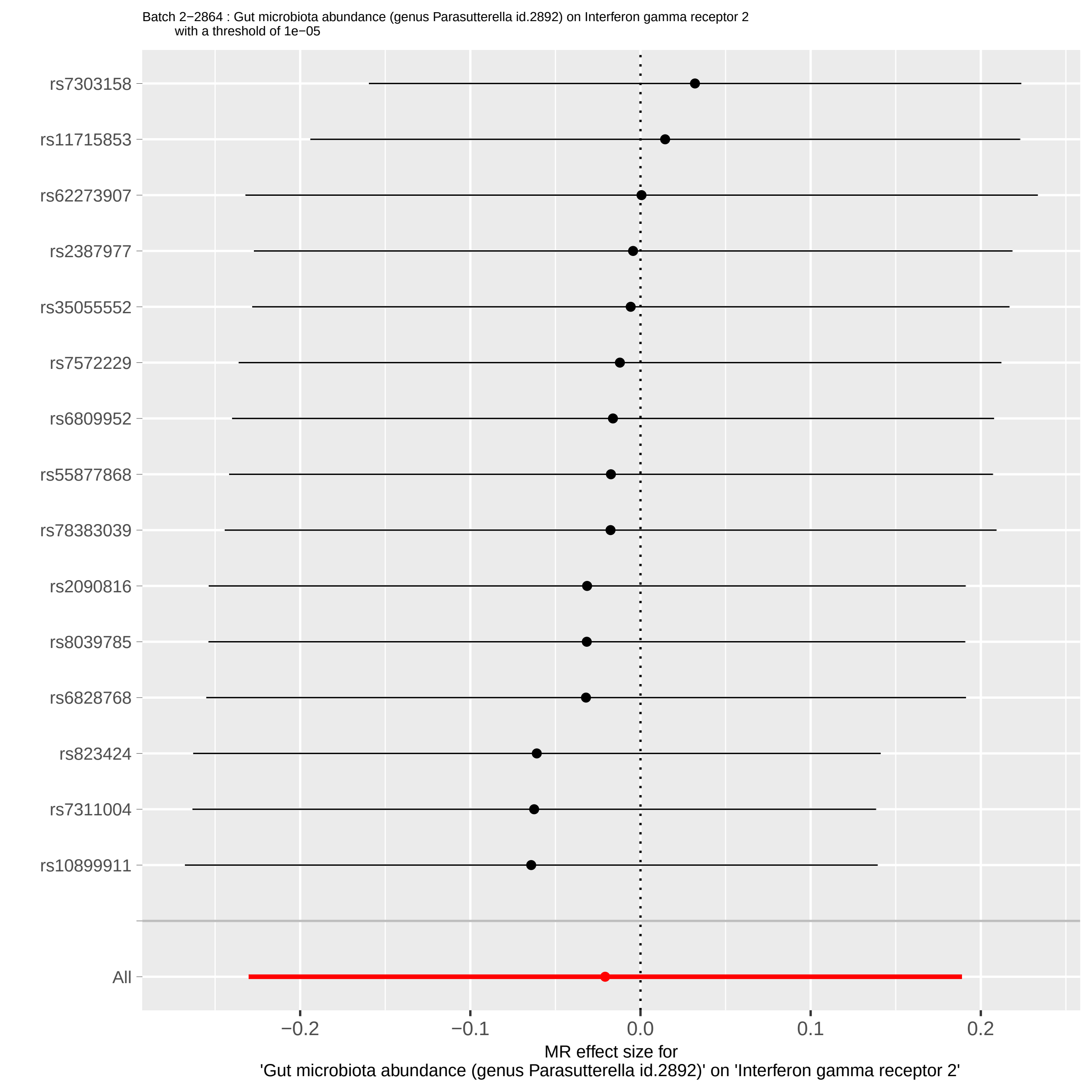

Batch 2−2864 : Gut microbiota abundance (genus Parasutterella id.2892) on Interferon gamma receptor 2
with a threshold of 1e−05
rs7303158
rs11715853
rs62273907
rs2387977
rs35055552
rs7572229
rs6809952
rs55877868
rs78383039
rs2090816
rs8039785
rs6828768
rs823424
rs7311004
rs10899911
All
−0.2
−0.1
0.0
0.1
0.2
MR effect size for
'Gut microbiota abundance (genus Parasutterella id.2892)' on 'Interferon gamma receptor 2'

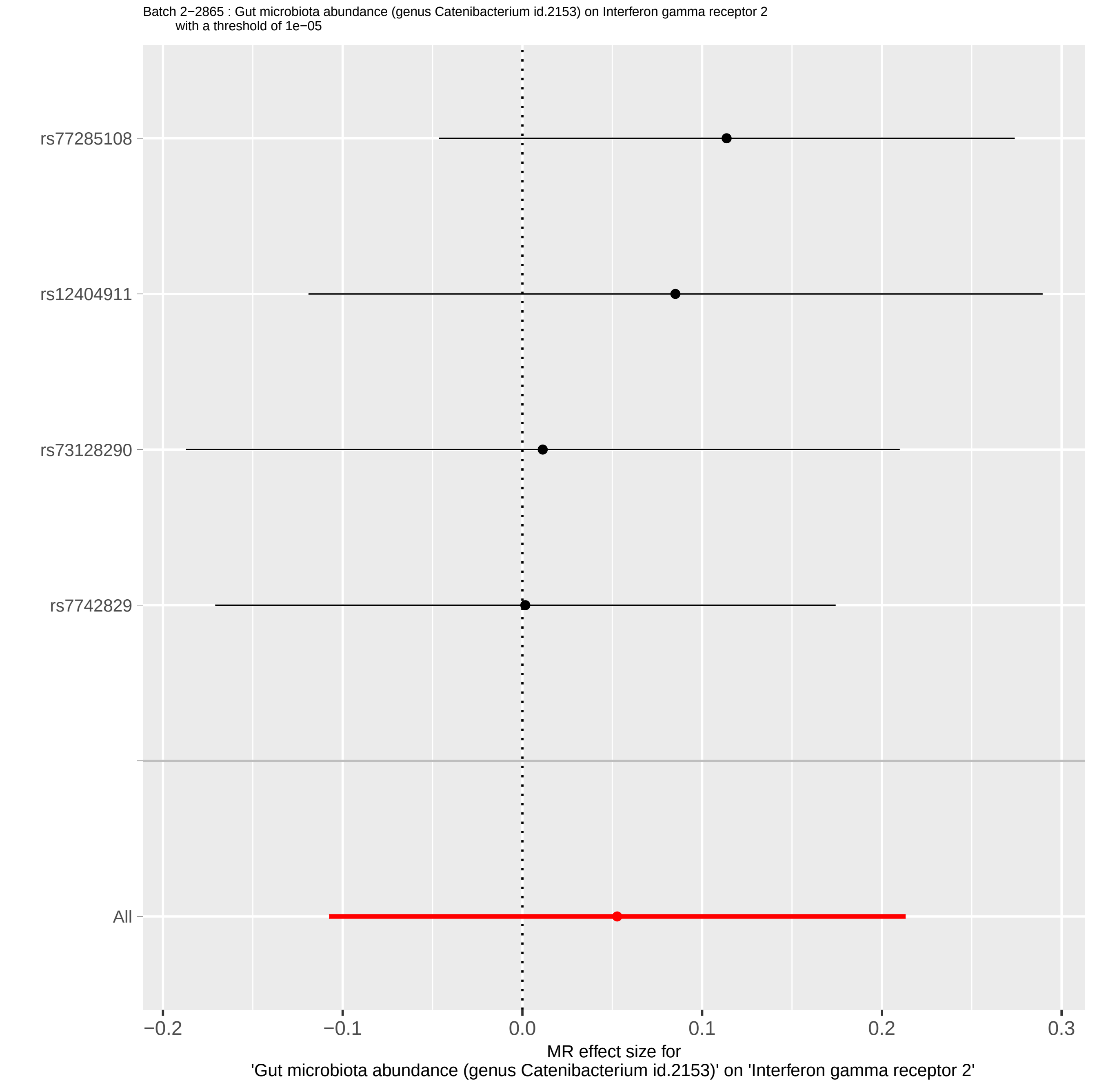

Batch 2−2865 : Gut microbiota abundance (genus Catenibacterium id.2153) on Interferon gamma receptor 2
with a threshold of 1e−05
rs77285108
rs12404911
rs73128290
rs7742829
All
−0.2
−0.1
0.0
0.1
0.2
0.3
MR effect size for
'Gut microbiota abundance (genus Catenibacterium id.2153)' on 'Interferon gamma receptor 2'

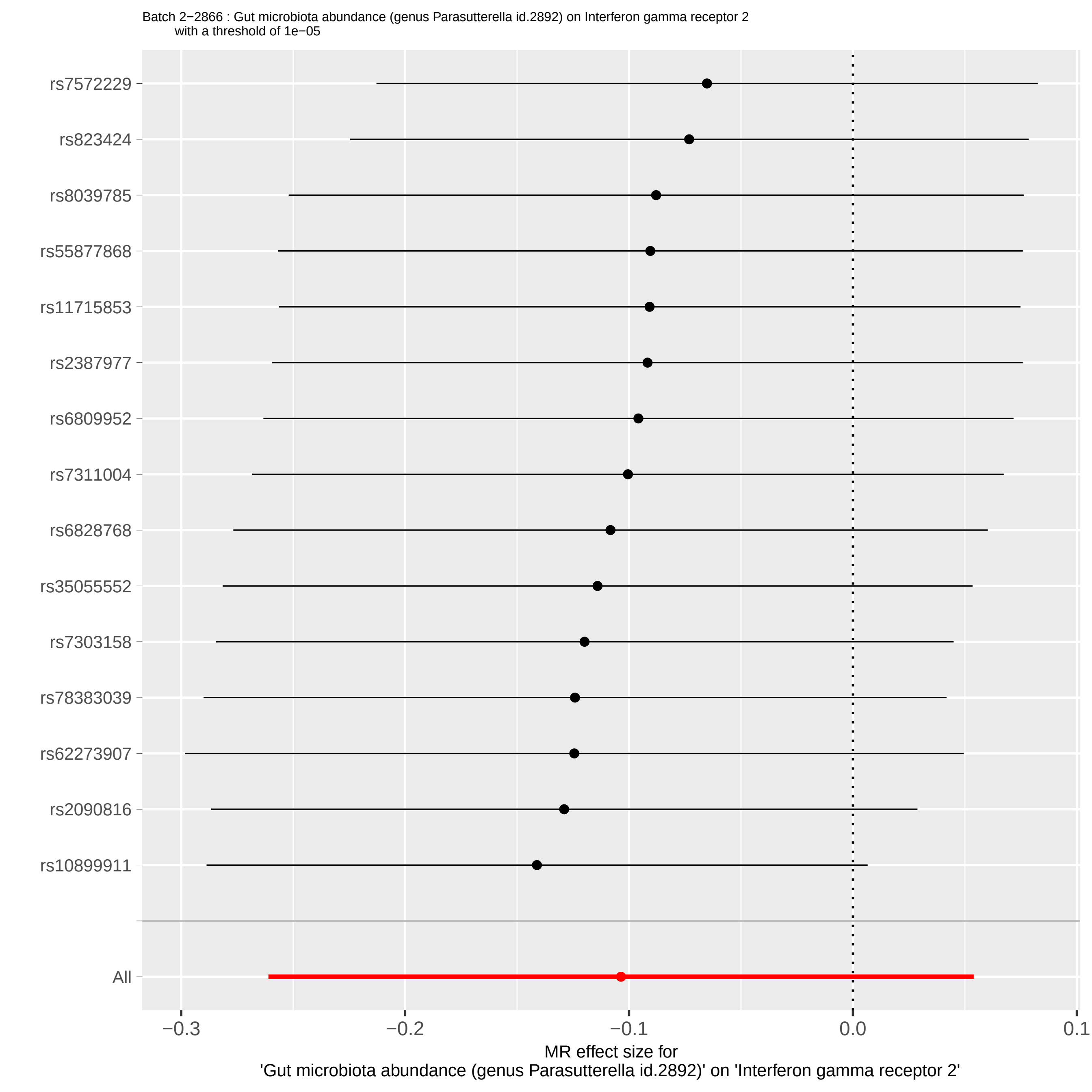

Batch 2−2866 : Gut microbiota abundance (genus Parasutterella id.2892) on Interferon gamma receptor 2
with a threshold of 1e−05
rs7572229
rs823424
rs8039785
rs55877868
rs11715853
rs2387977
rs6809952
rs7311004
rs6828768
rs35055552
rs7303158
rs78383039
rs62273907
rs2090816
rs10899911
All
−0.3
−0.2
−0.1
0.0
0.1
MR effect size for
'Gut microbiota abundance (genus Parasutterella id.2892)' on 'Interferon gamma receptor 2'

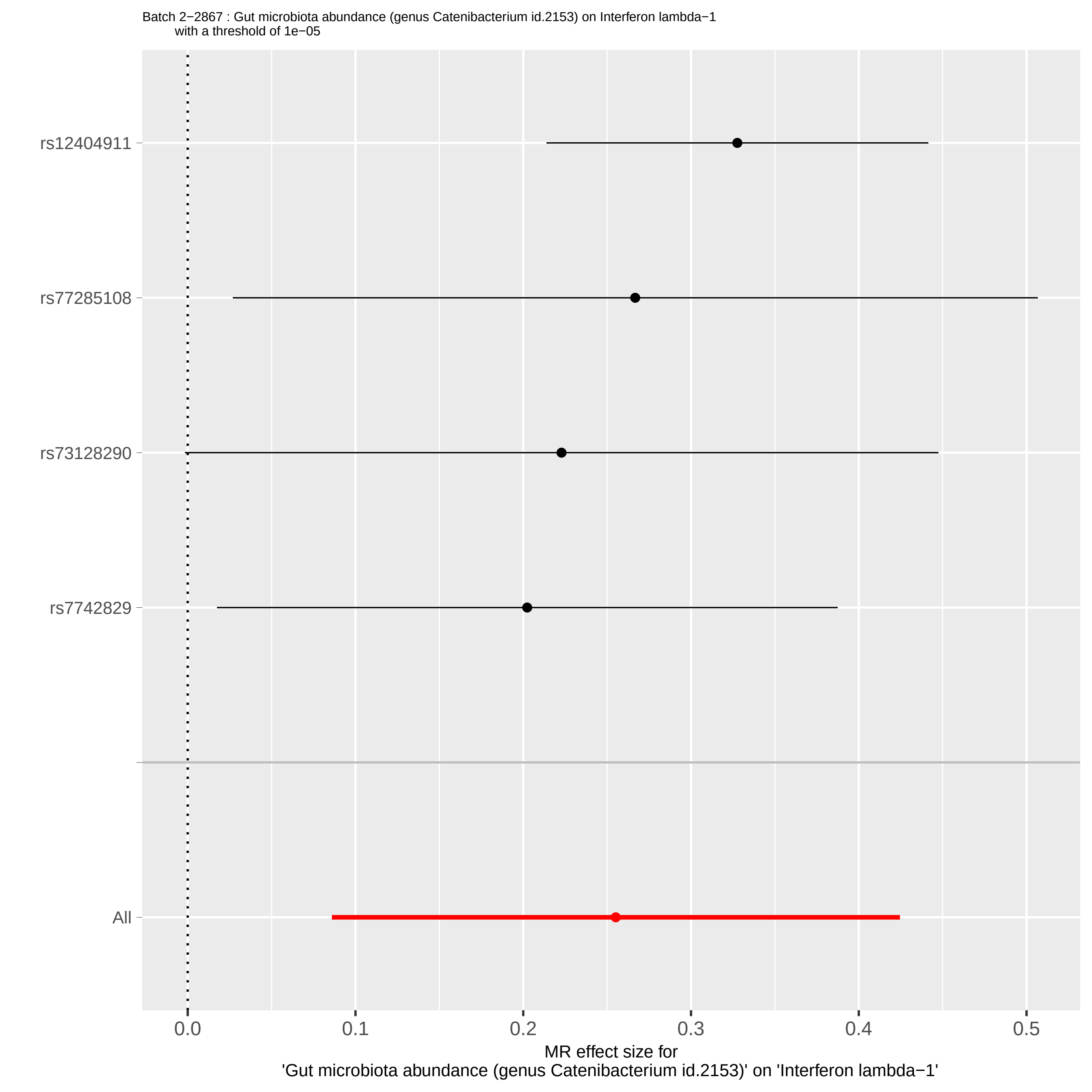
Batch 2−2867 : Gut microbiota abundance (genus Catenibacterium id.2153) on Interferon lambda−1
with a threshold of 1e−05
rs12404911
rs77285108
rs73128290
rs7742829
All
0.0
0.1
0.2
0.3
0.4
0.5
MR effect size for
'Gut microbiota abundance (genus Catenibacterium id.2153)' on 'Interferon lambda−1'

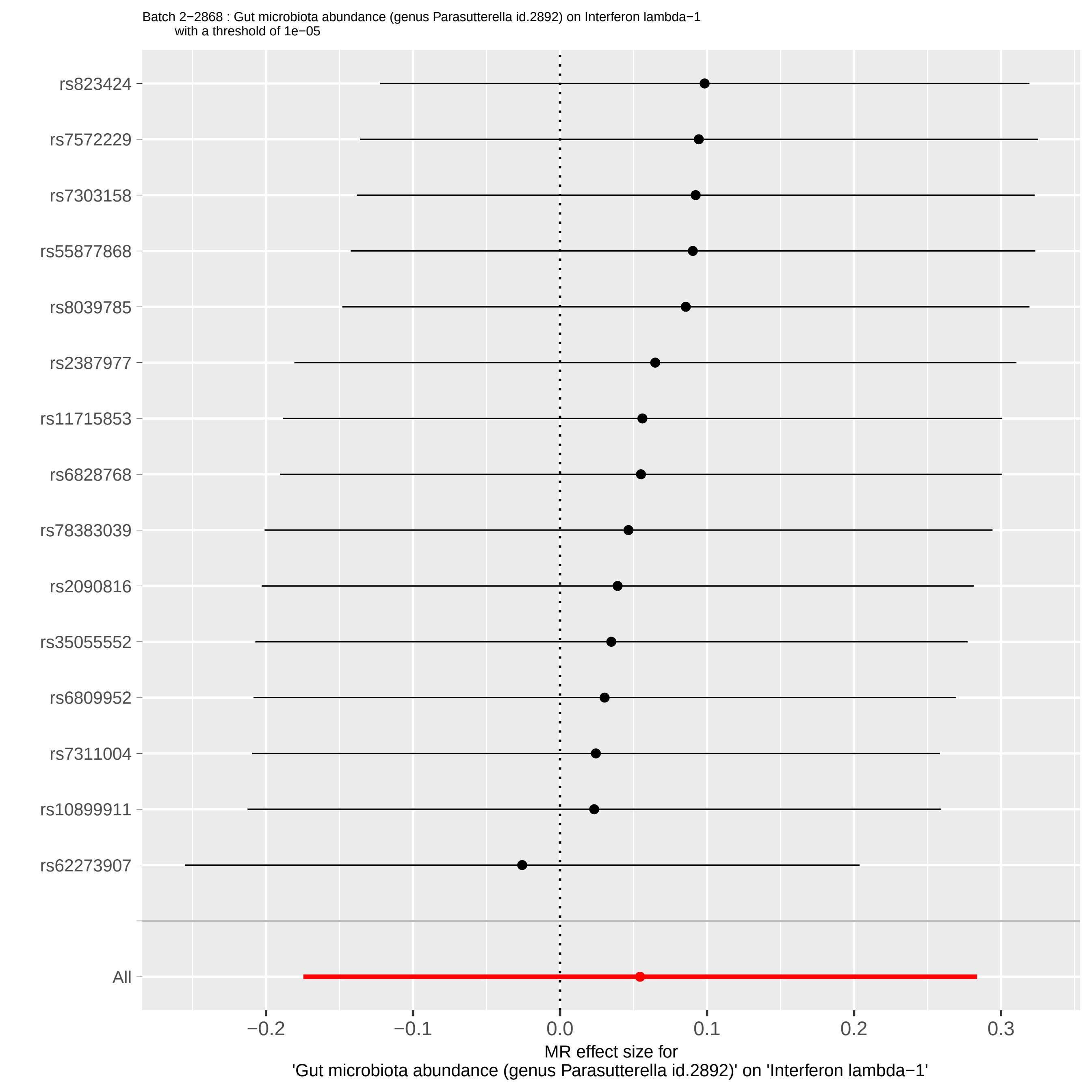

Batch 2−2868 : Gut microbiota abundance (genus Parasutterella id.2892) on Interferon lambda−1
with a threshold of 1e−05
rs823424
rs7572229
rs7303158
rs55877868
rs8039785
rs2387977
rs11715853
rs6828768
rs78383039
rs2090816
rs35055552
rs6809952
rs7311004
rs10899911
rs62273907
All
−0.2
−0.1
0.0
0.1
0.2
0.3
MR effect size for
'Gut microbiota abundance (genus Parasutterella id.2892)' on 'Interferon lambda−1'

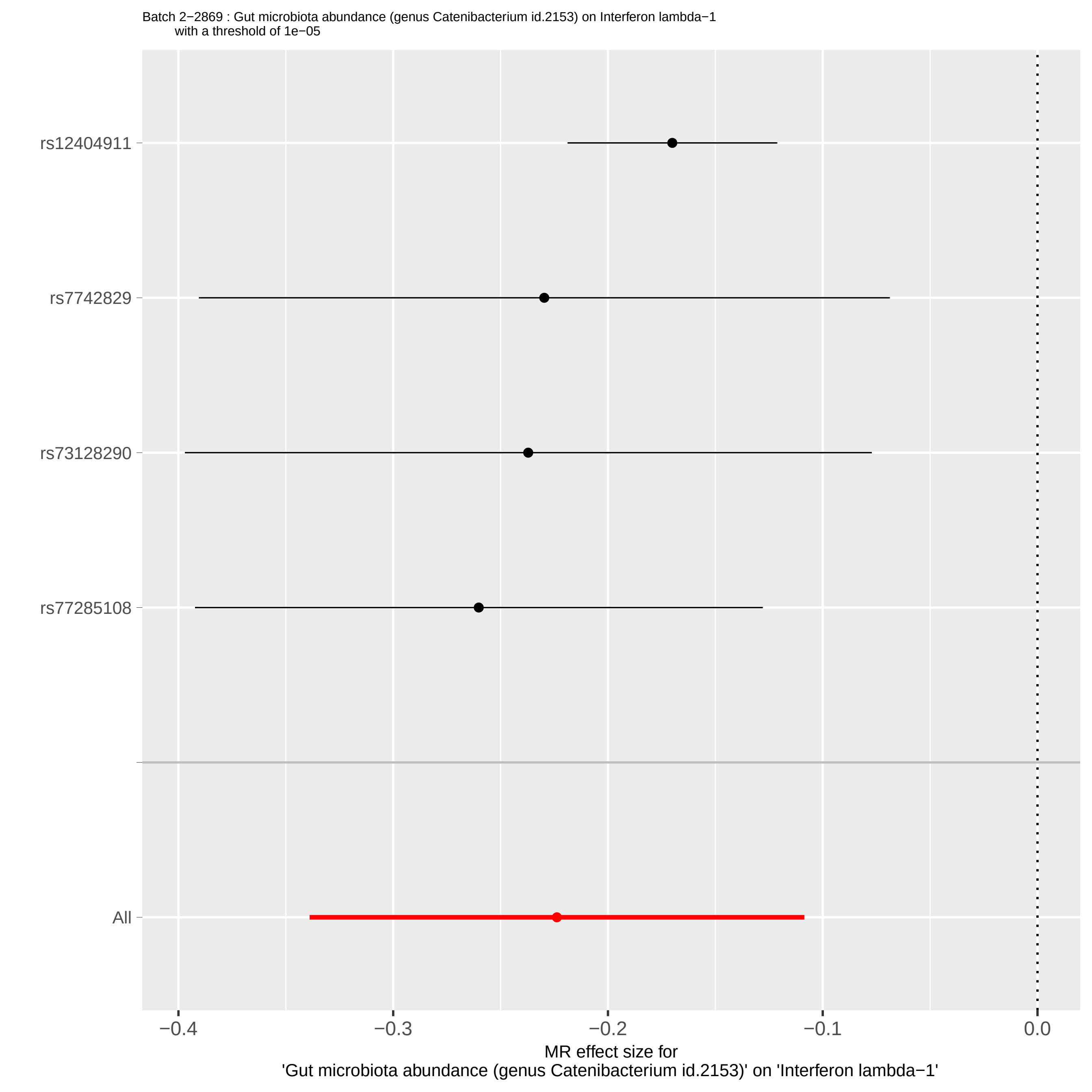

Batch 2−2869 : Gut microbiota abundance (genus Catenibacterium id.2153) on Interferon lambda−1
with a threshold of 1e−05
rs12404911
rs7742829
rs73128290
rs77285108
All
−0.4
−0.3
−0.2
−0.1
0.0
MR effect size for
'Gut microbiota abundance (genus Catenibacterium id.2153)' on 'Interferon lambda−1'

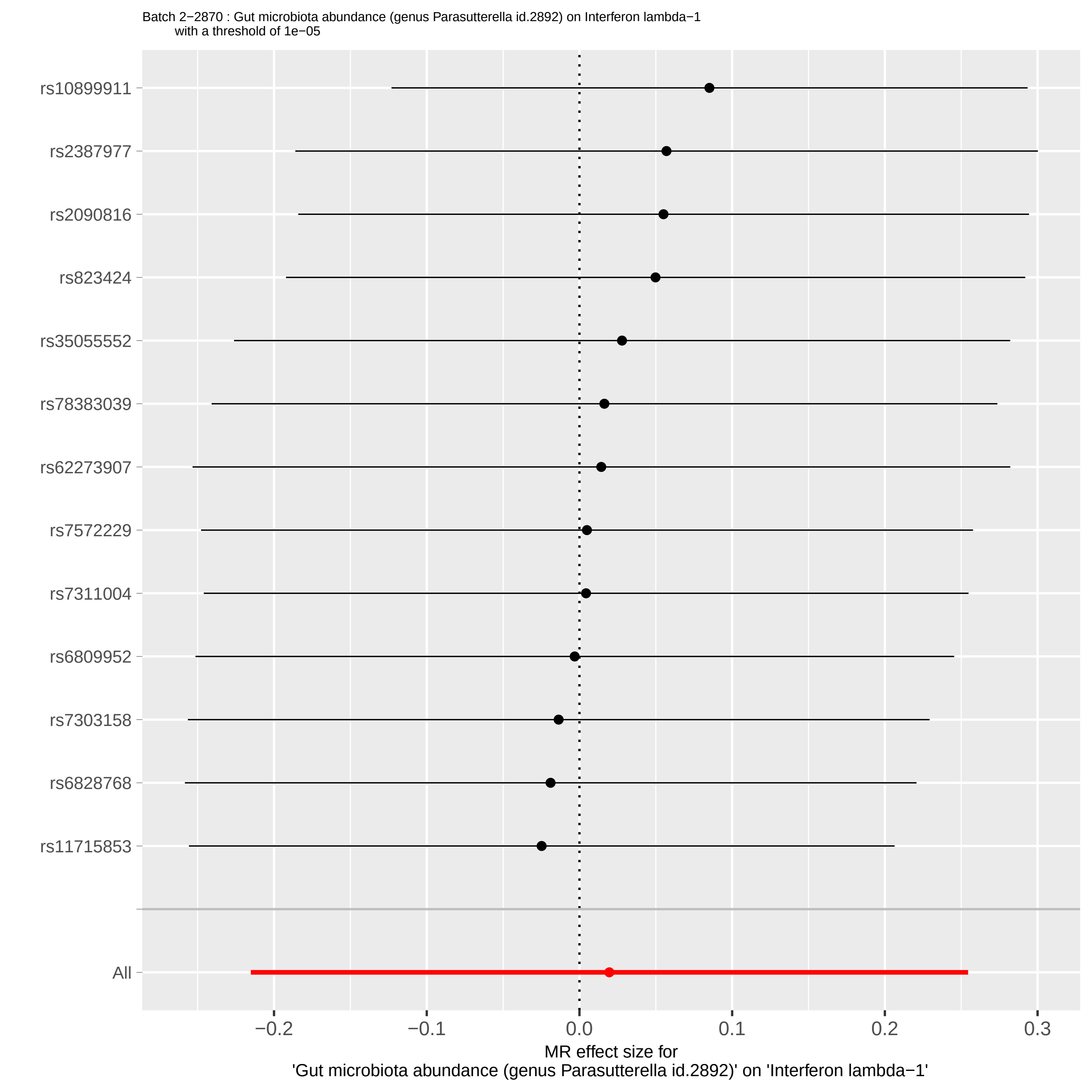
Batch 2−2870 : Gut microbiota abundance (genus Parasutterella id.2892) on Interferon lambda−1
with a threshold of 1e−05
rs10899911
rs2387977
rs2090816
rs823424
rs35055552
rs78383039
rs62273907
rs7572229
rs7311004
rs6809952
rs7303158
rs6828768
rs11715853
All
−0.2
−0.1
0.0
0.1
0.2
0.3
MR effect size for
'Gut microbiota abundance (genus Parasutterella id.2892)' on 'Interferon lambda−1'

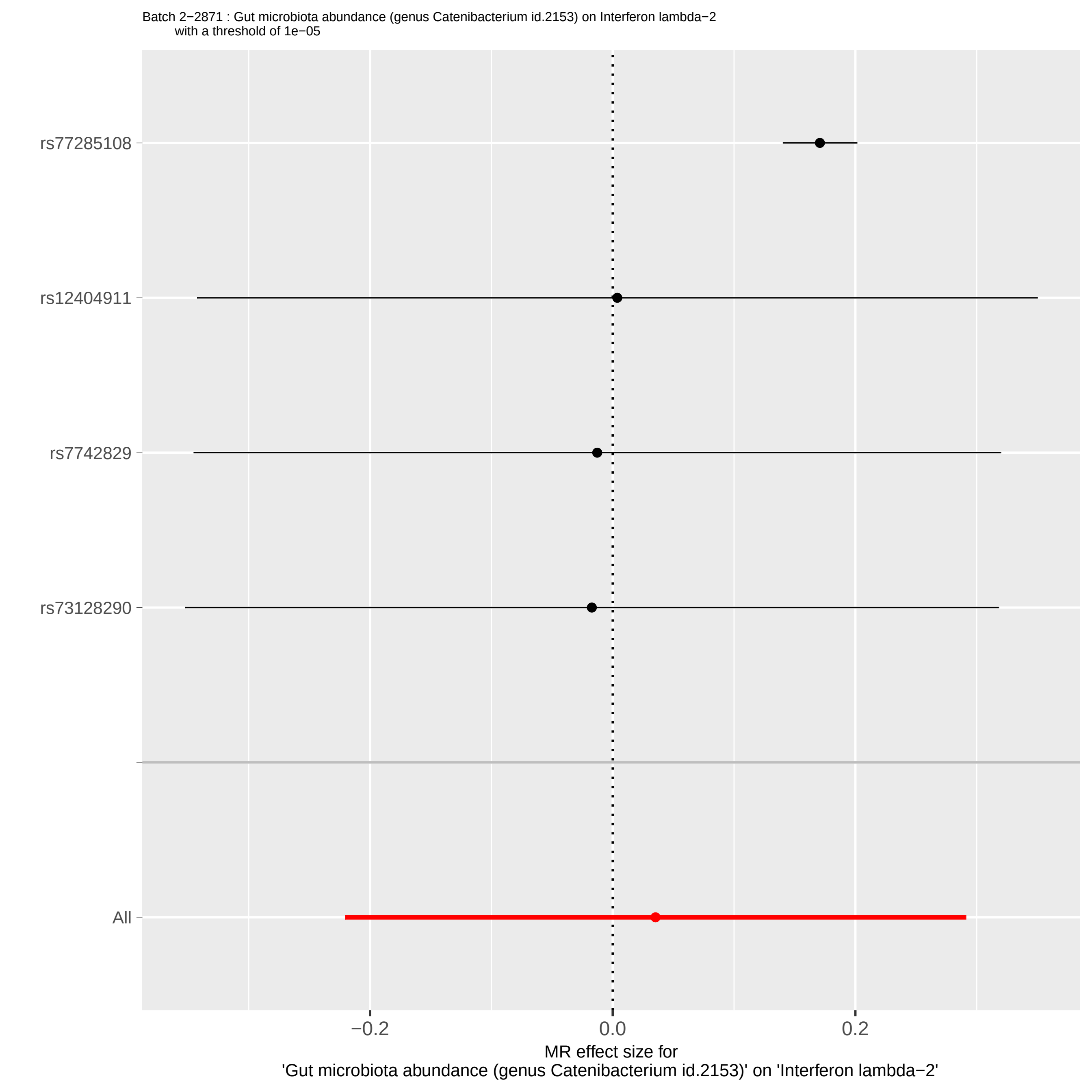

Batch 2−2871 : Gut microbiota abundance (genus Catenibacterium id.2153) on Interferon lambda−2
with a threshold of 1e−05
rs77285108
rs12404911
rs7742829
rs73128290
All
−0.2
0.0
0.2
MR effect size for
'Gut microbiota abundance (genus Catenibacterium id.2153)' on 'Interferon lambda−2'

Batch 2−2872 : Gut microbiota abundance (genus Parasutterella id.2892) on Interferon lambda−2
with a threshold of 1e−05

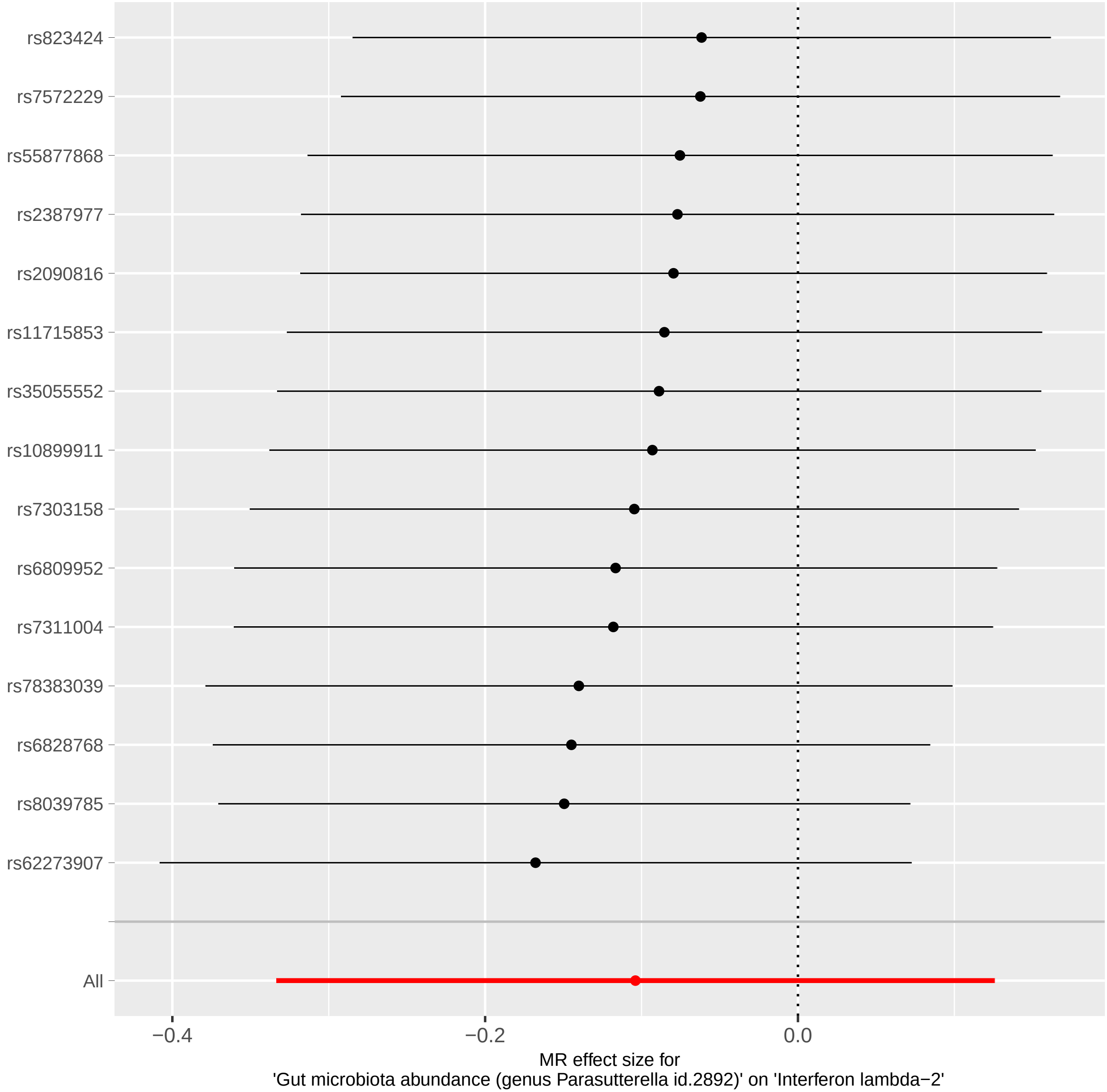

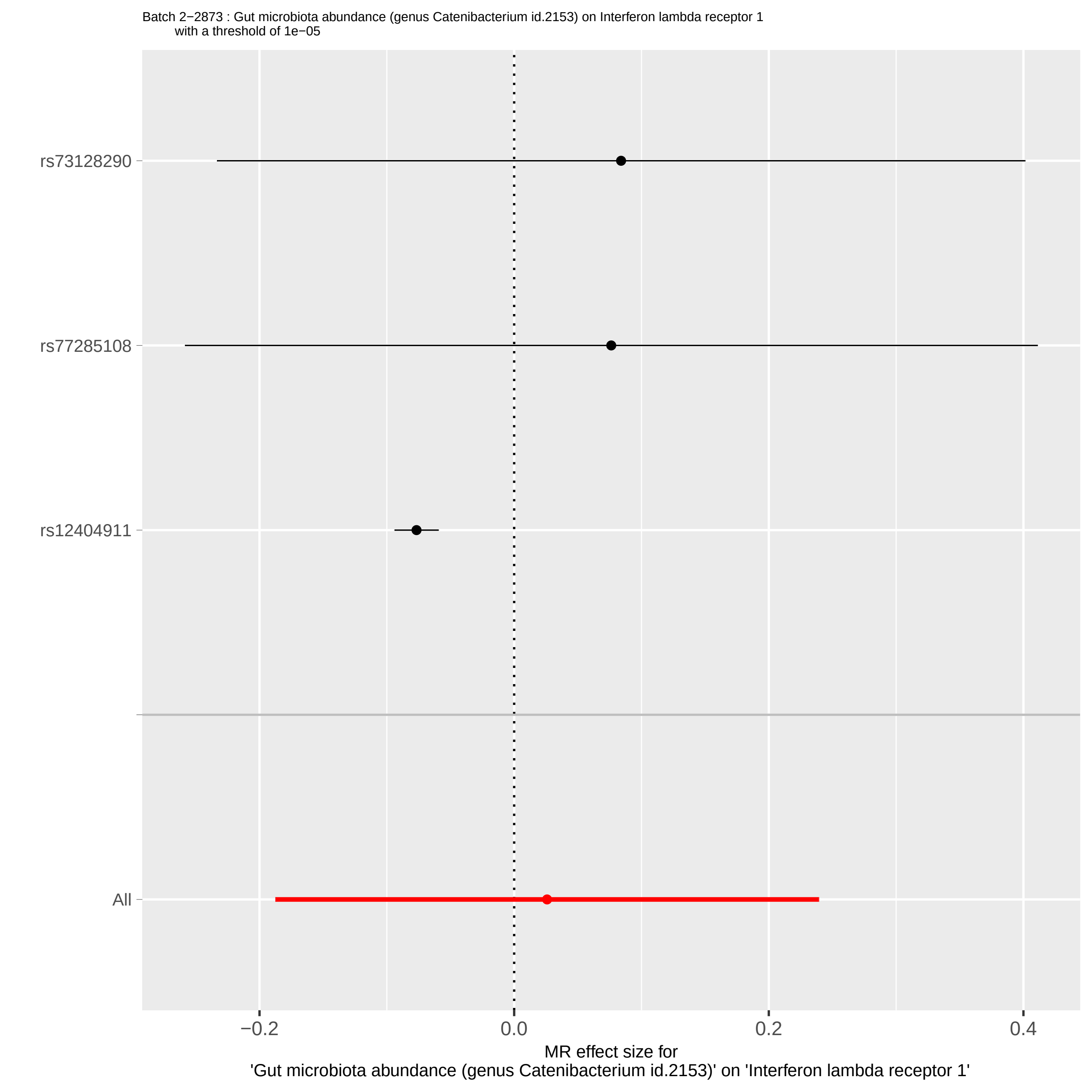
Batch 2−2873 : Gut microbiota abundance (genus Catenibacterium id.2153) on Interferon lambda receptor 1
with a threshold of 1e−05
rs73128290
rs77285108
rs12404911
All
−0.2
0.0
0.2
0.4
MR effect size for
'Gut microbiota abundance (genus Catenibacterium id.2153)' on 'Interferon lambda receptor 1'

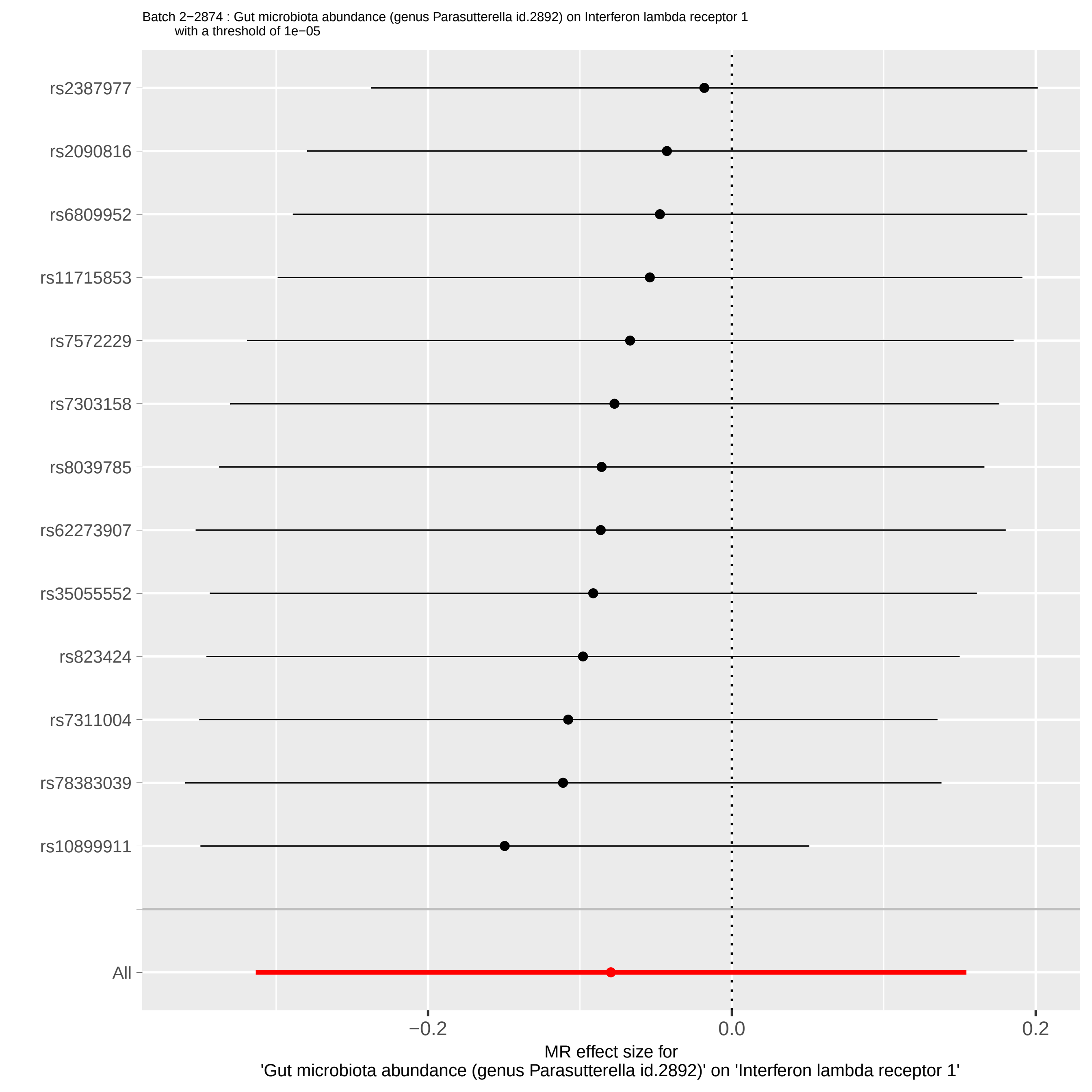

Batch 2−2874 : Gut microbiota abundance (genus Parasutterella id.2892) on Interferon lambda receptor 1
with a threshold of 1e−05
rs2387977
rs2090816
rs6809952
rs11715853
rs7572229
rs7303158
rs8039785
rs62273907
rs35055552
rs823424
rs7311004
rs78383039
rs10899911
All
−0.2
0.0
0.2
MR effect size for
'Gut microbiota abundance (genus Parasutterella id.2892)' on 'Interferon lambda receptor 1'

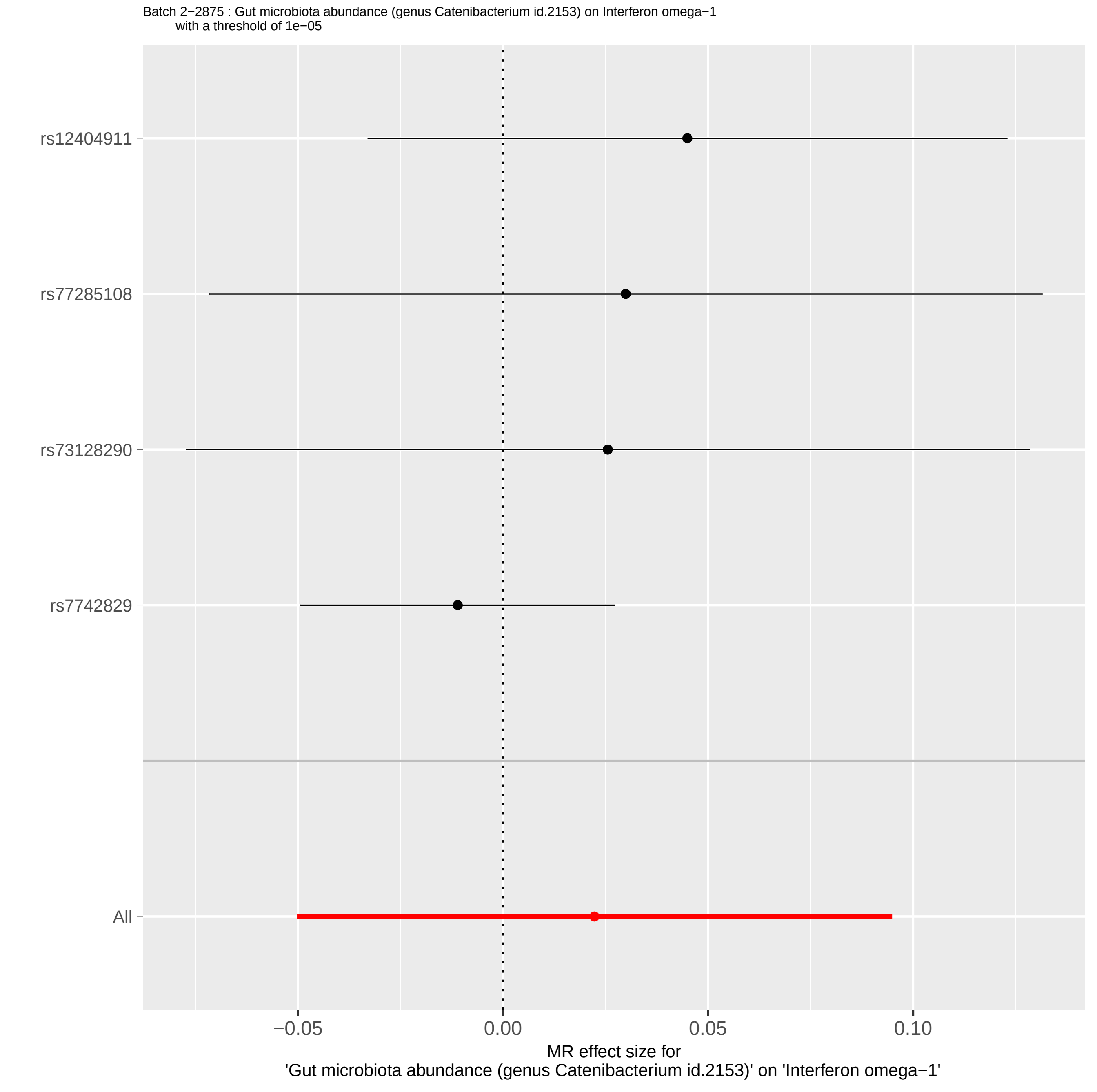
Batch 2−2875 : Gut microbiota abundance (genus Catenibacterium id.2153) on Interferon omega−1
with a threshold of 1e−05
rs12404911
rs77285108
rs73128290
rs7742829
All
−0.05
0.00
0.05
0.10
MR effect size for
'Gut microbiota abundance (genus Catenibacterium id.2153)' on 'Interferon omega−1'

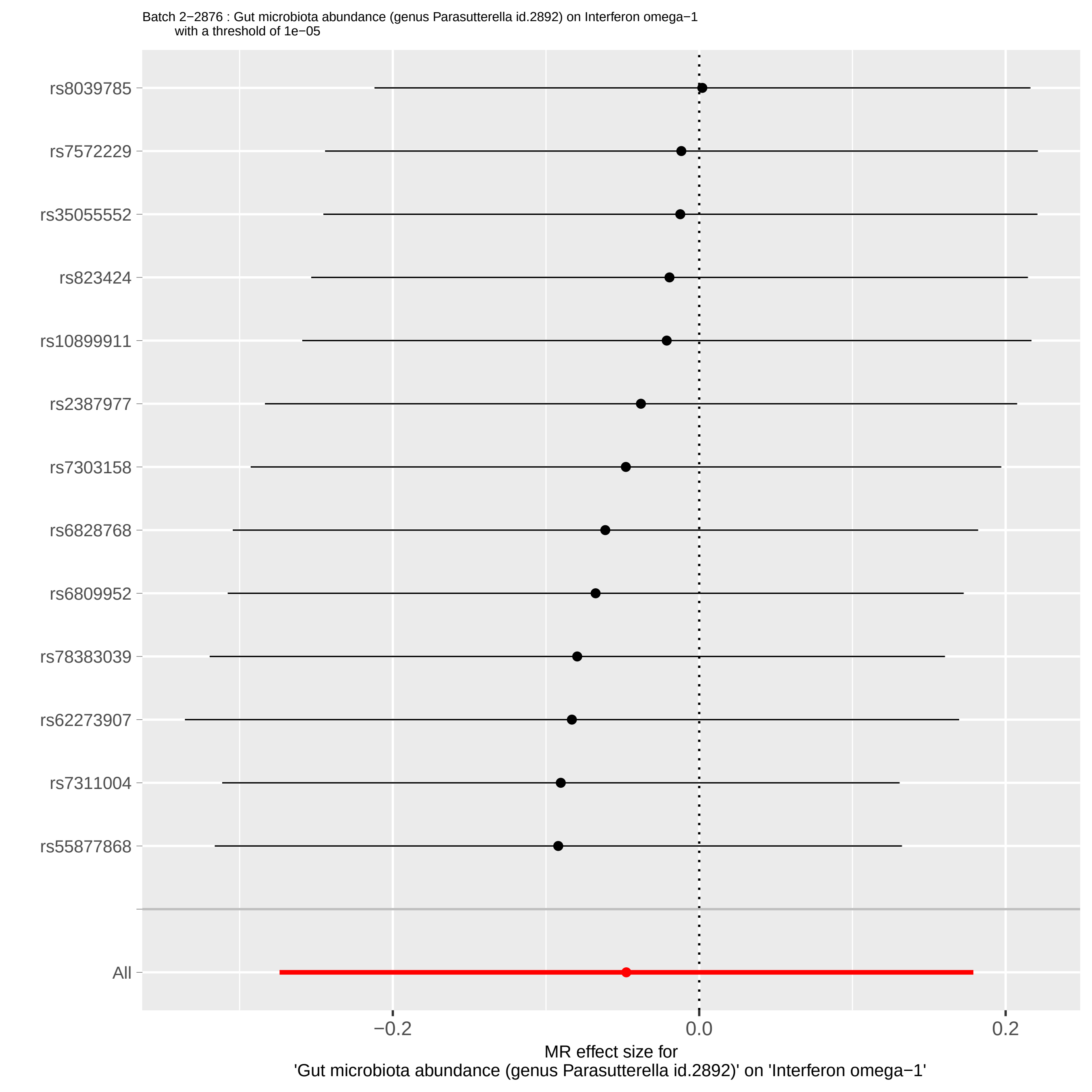
Batch 2−2876 : Gut microbiota abundance (genus Parasutterella id.2892) on Interferon omega−1
with a threshold of 1e−05
rs8039785
rs7572229
rs35055552
rs823424
rs10899911
rs2387977
rs7303158
rs6828768
rs6809952
rs78383039
rs62273907
rs7311004
rs55877868
All
−0.2
0.0
0.2
MR effect size for
'Gut microbiota abundance (genus Parasutterella id.2892)' on 'Interferon omega−1'

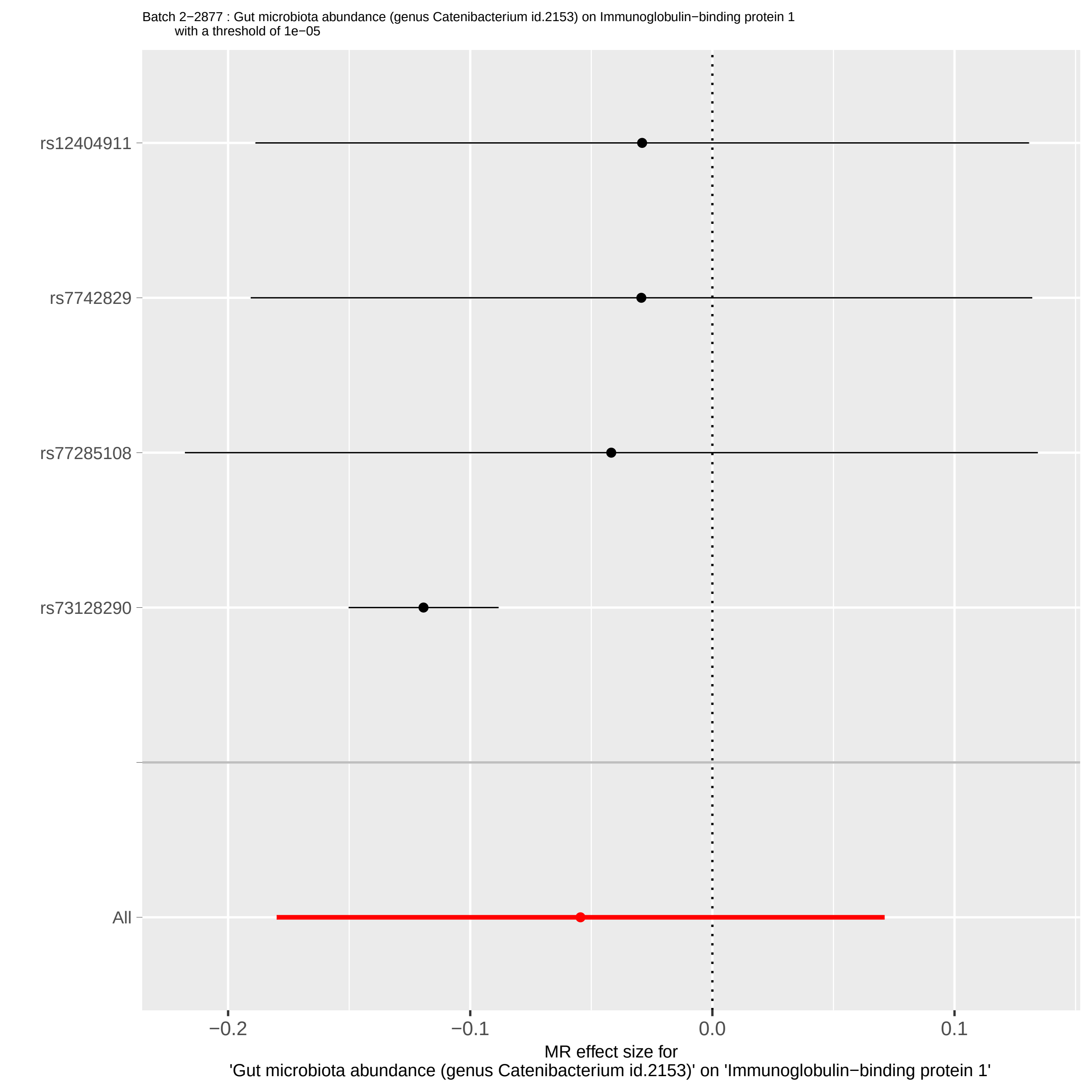
Batch 2−2877 : Gut microbiota abundance (genus Catenibacterium id.2153) on Immunoglobulin−binding protein 1
with a threshold of 1e−05
rs12404911
rs7742829
rs77285108
rs73128290
All
−0.2
−0.1
0.0
0.1
MR effect size for
'Gut microbiota abundance (genus Catenibacterium id.2153)' on 'Immunoglobulin−binding protein 1'

Batch 2−2878 : Gut microbiota abundance (genus Parasutterella id.2892) on Immunoglobulin−binding protein 1
with a threshold of 1e−05

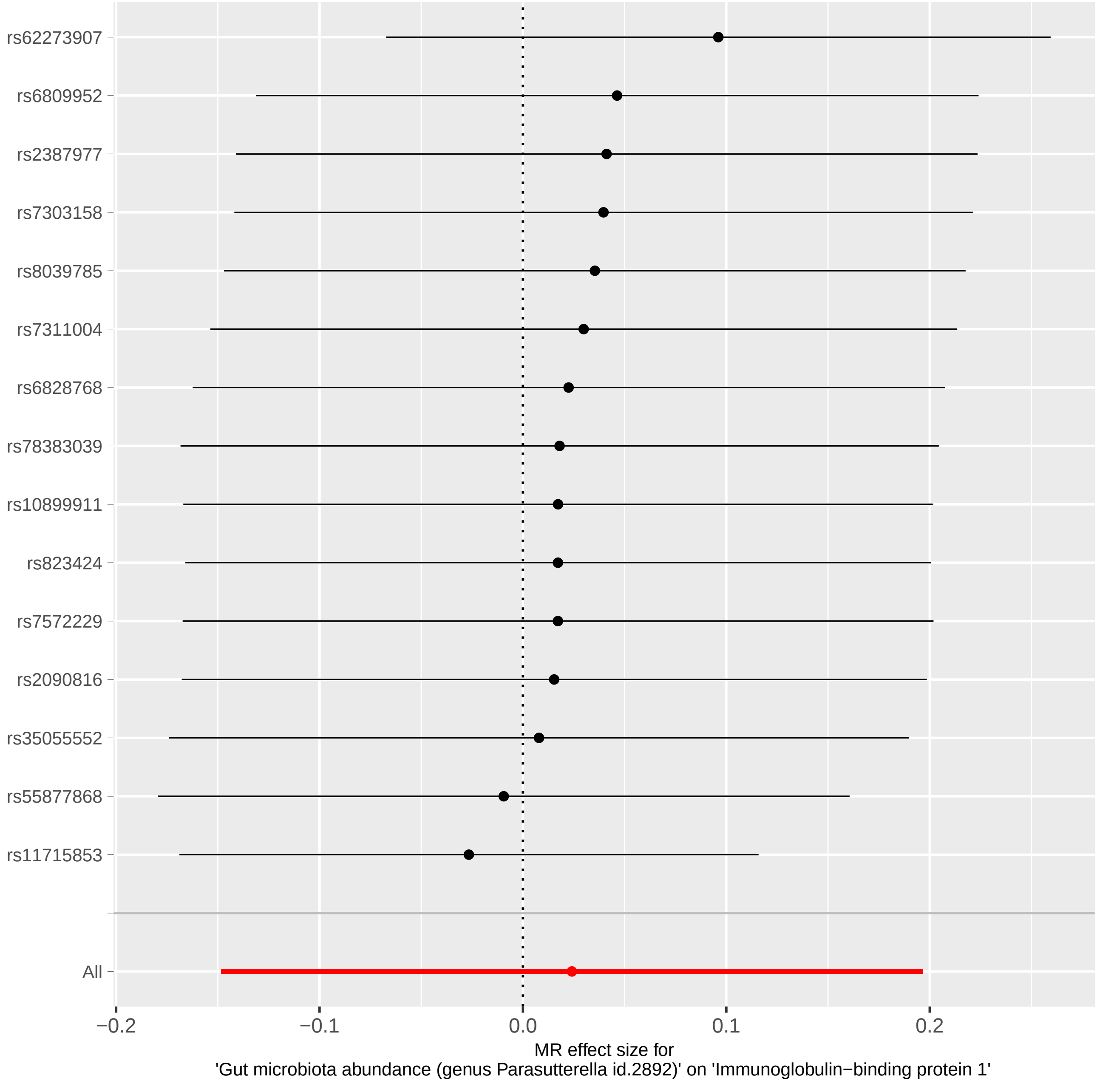

Batch 2−2879 : Gut microbiota abundance (genus Catenibacterium id.2153) on Immunoglobulin superfamily DCC subclass member 3
with a threshold of 1e−05

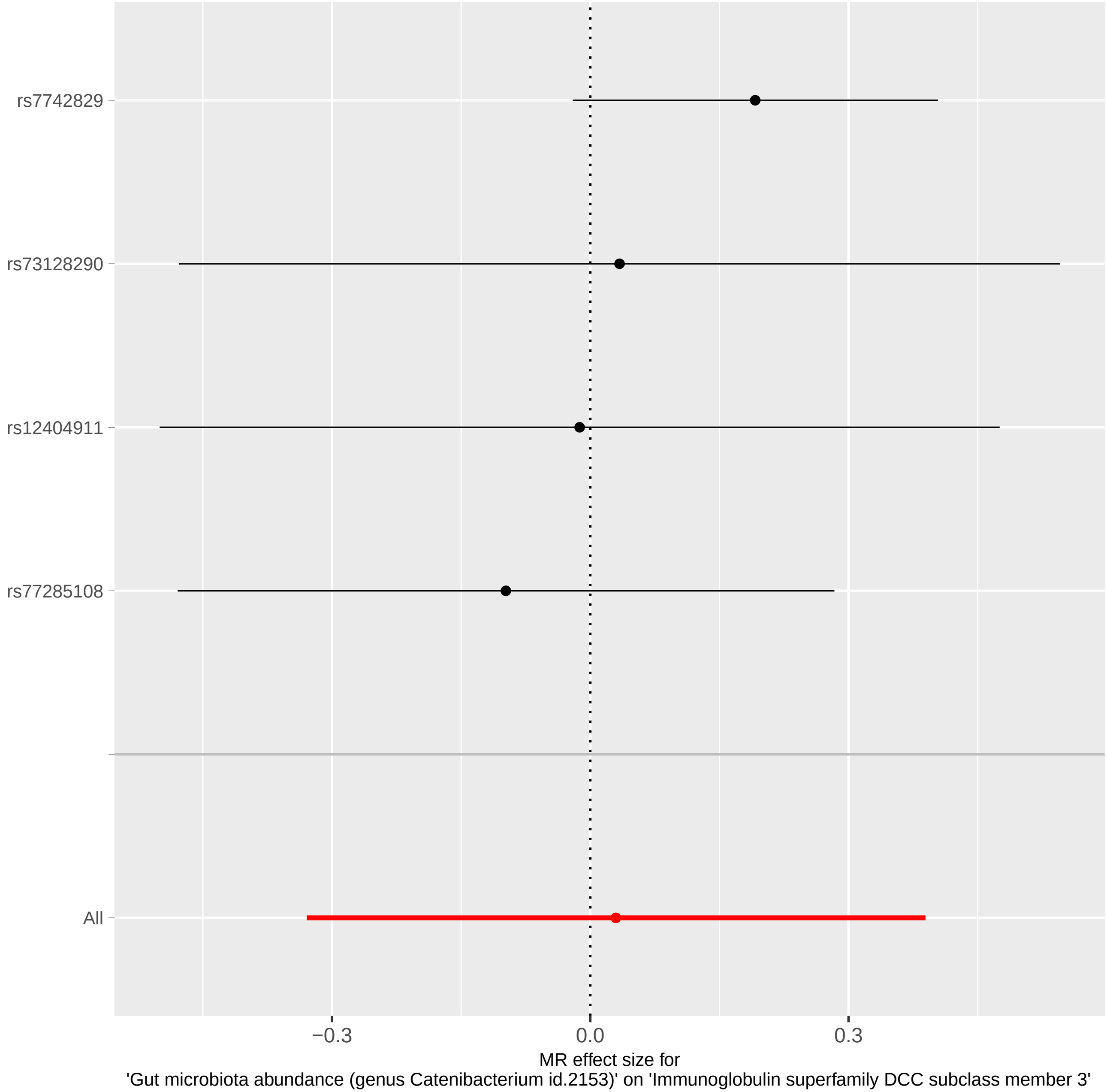

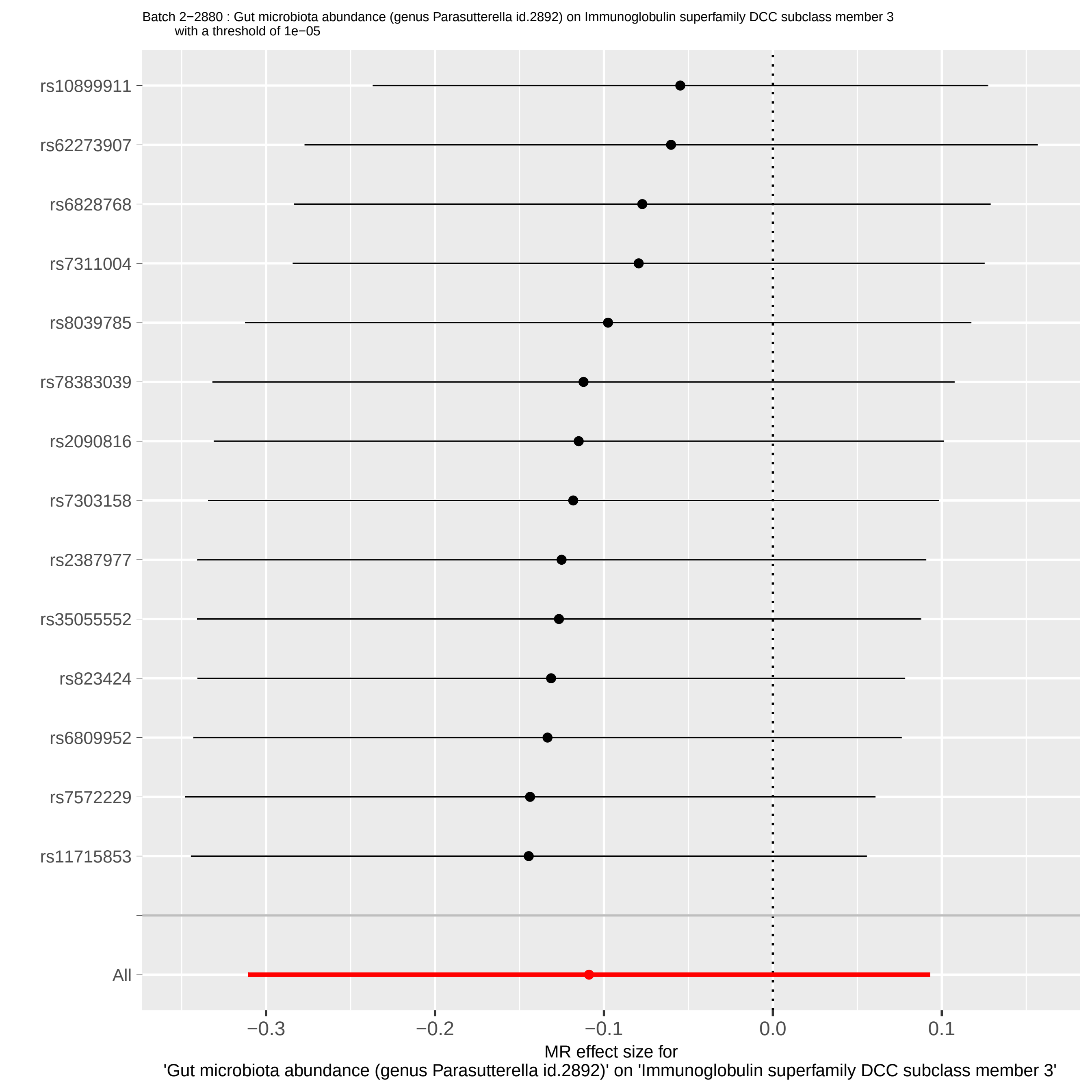
Batch 2−2880 : Gut microbiota abundance (genus Parasutterella id.2892) on Immunoglobulin superfamily DCC subclass member 3
with a threshold of 1e−05
rs10899911
rs62273907
rs6828768
rs7311004
rs8039785
rs78383039
rs2090816
rs7303158
rs2387977
rs35055552
rs823424
rs6809952
rs7572229
rs11715853
All
−0.3
−0.2
−0.1
0.0
0.1
MR effect size for
'Gut microbiota abundance (genus Parasutterella id.2892)' on 'Immunoglobulin superfamily DCC subclass member 3'

Batch 2−2881 : Gut microbiota abundance (genus Catenibacterium id.2153) on Immunoglobulin superfamily DCC subclass member 3 with a threshold of 1e−05

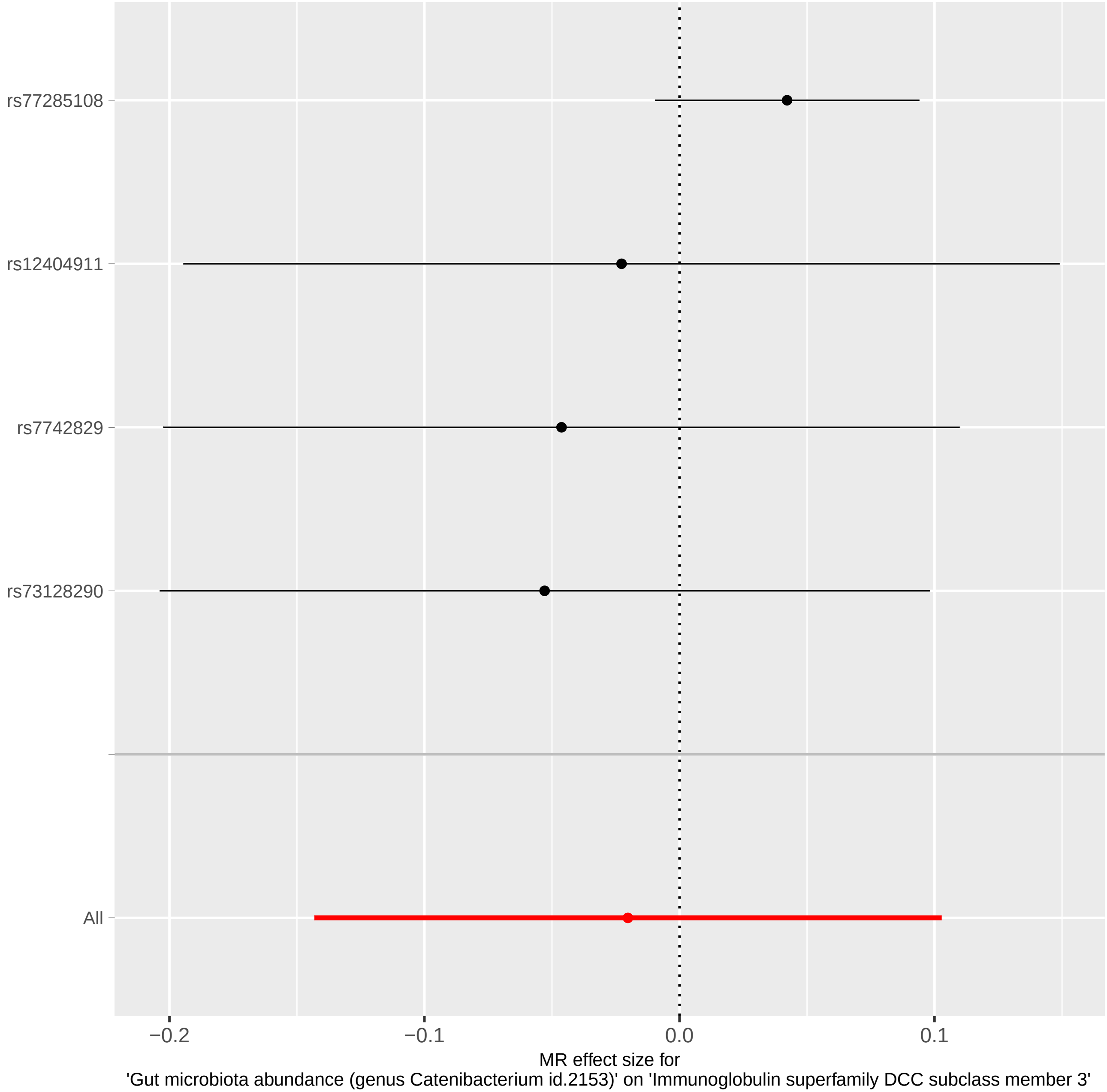

Batch 2−2882 : Gut microbiota abundance (genus Parasutterella id.2892) on Immunoglobulin superfamily DCC subclass member 3 with a threshold of 1e−05

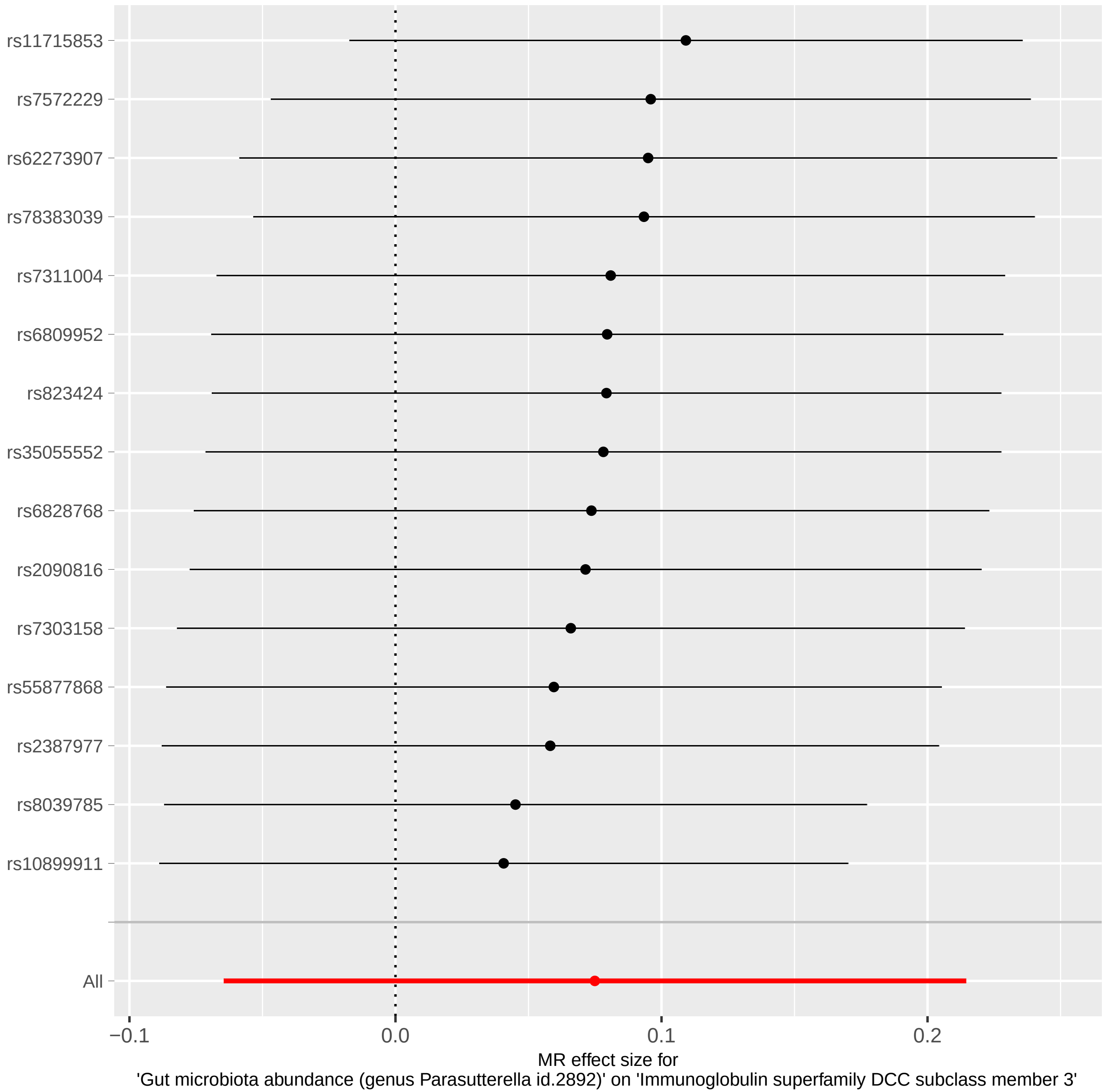

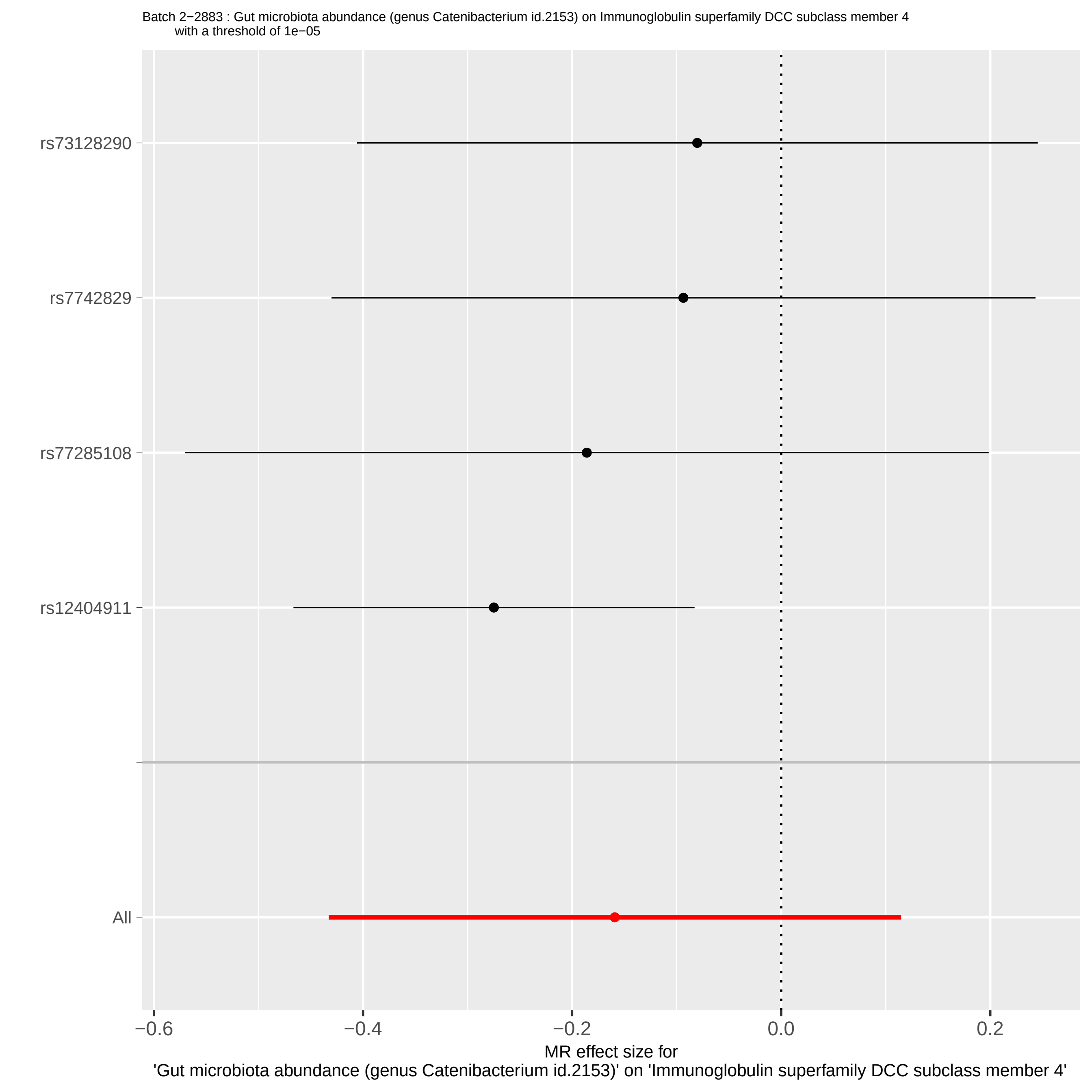
Batch 2−2883 : Gut microbiota abundance (genus Catenibacterium id.2153) on Immunoglobulin superfamily DCC subclass member 4
with a threshold of 1e−05
rs73128290
rs7742829
rs77285108
rs12404911
All
−0.6
−0.4
−0.2
0.0
0.2
MR effect size for
'Gut microbiota abundance (genus Catenibacterium id.2153)' on 'Immunoglobulin superfamily DCC subclass member 4'

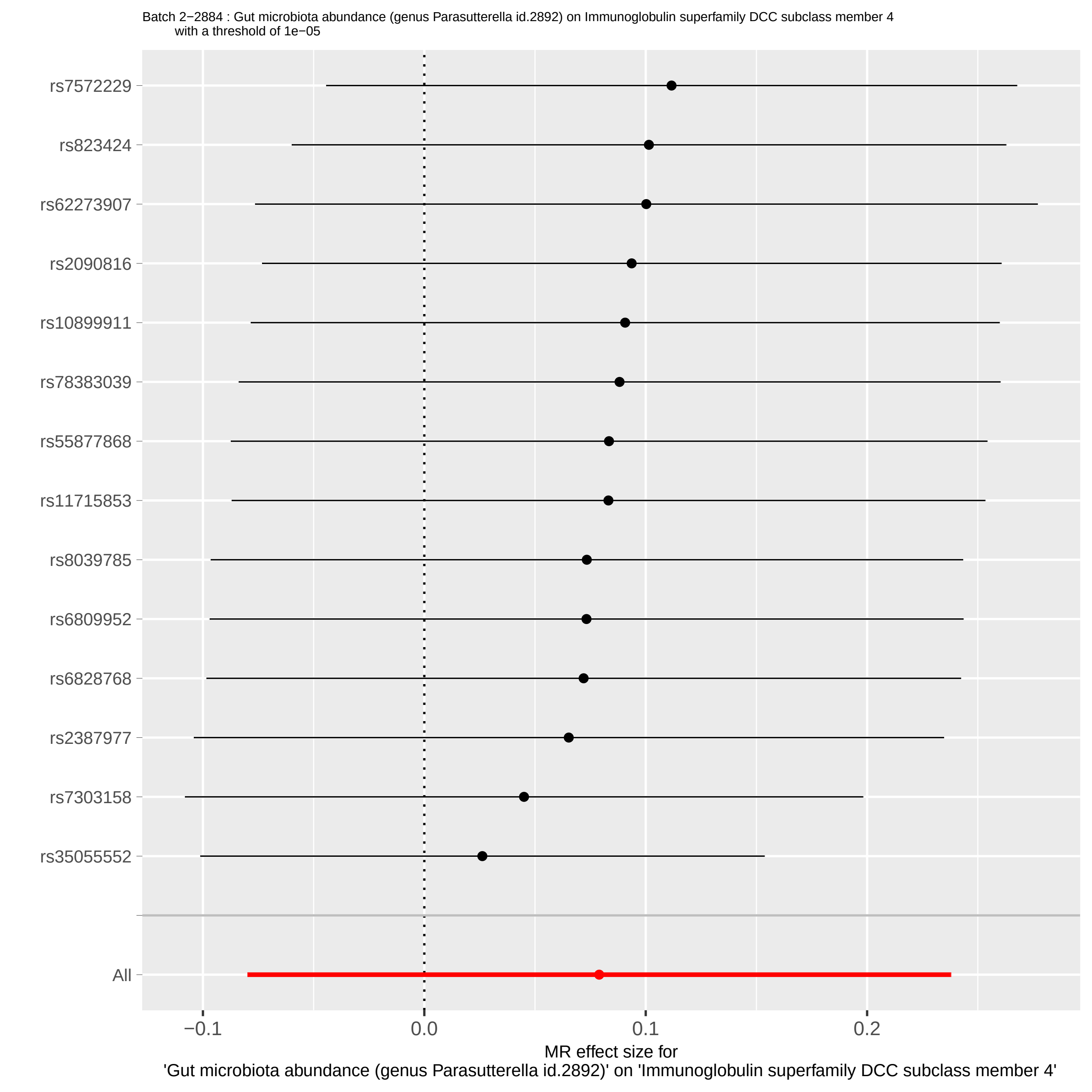

Batch 2−2884 : Gut microbiota abundance (genus Parasutterella id.2892) on Immunoglobulin superfamily DCC subclass member 4
with a threshold of 1e−05
rs7572229
rs823424
rs62273907
rs2090816
rs10899911
rs78383039
rs55877868
rs11715853
rs8039785
rs6809952
rs6828768
rs2387977
rs7303158
rs35055552
All
−0.1
0.0
0.1
0.2
MR effect size for
'Gut microbiota abundance (genus Parasutterella id.2892)' on 'Immunoglobulin superfamily DCC subclass member 4'

Batch 2−2885 : Gut microbiota abundance (genus Catenibacterium id.2153) on Insulin−like growth factor I
with a threshold of 1e−05

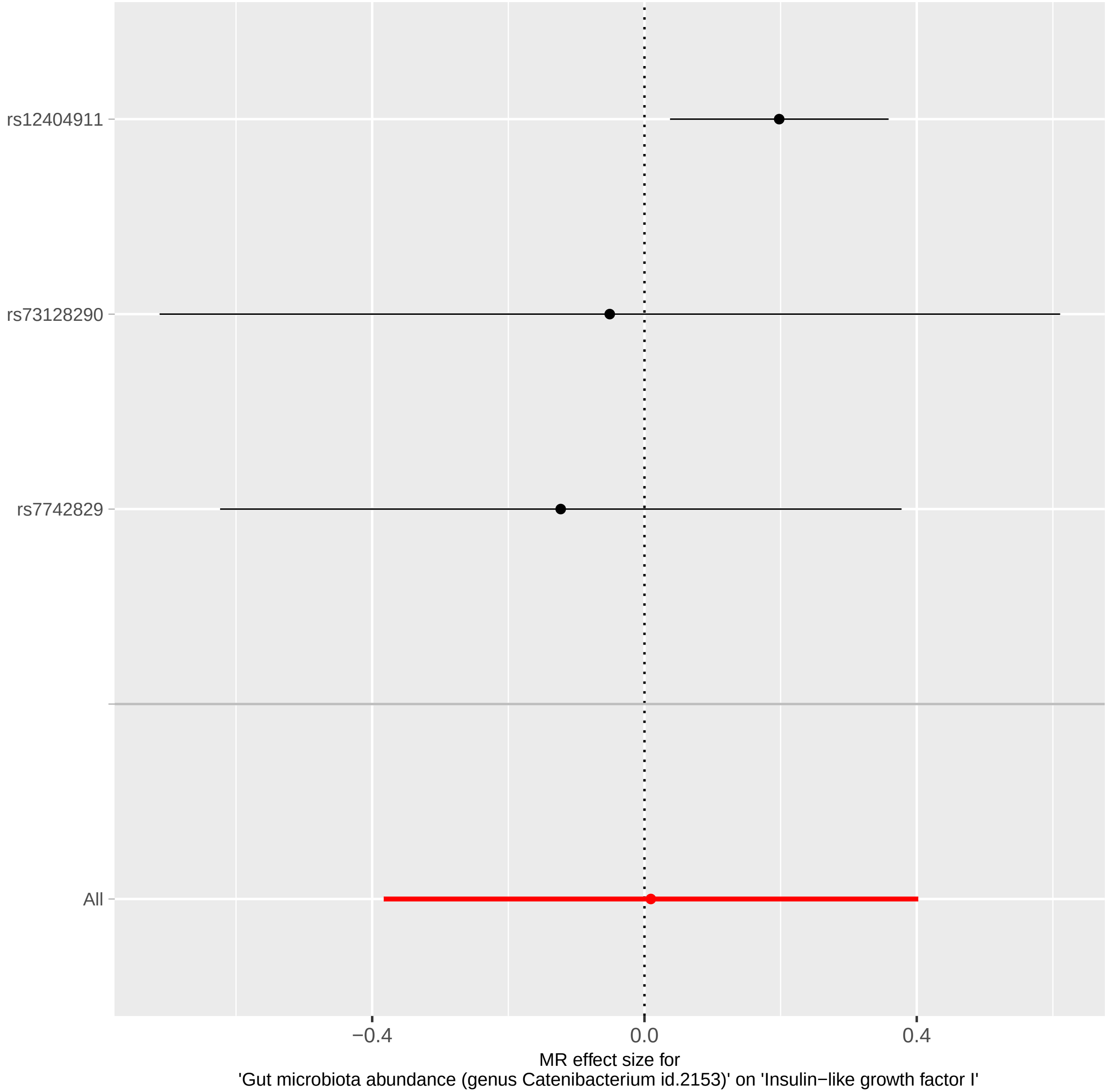

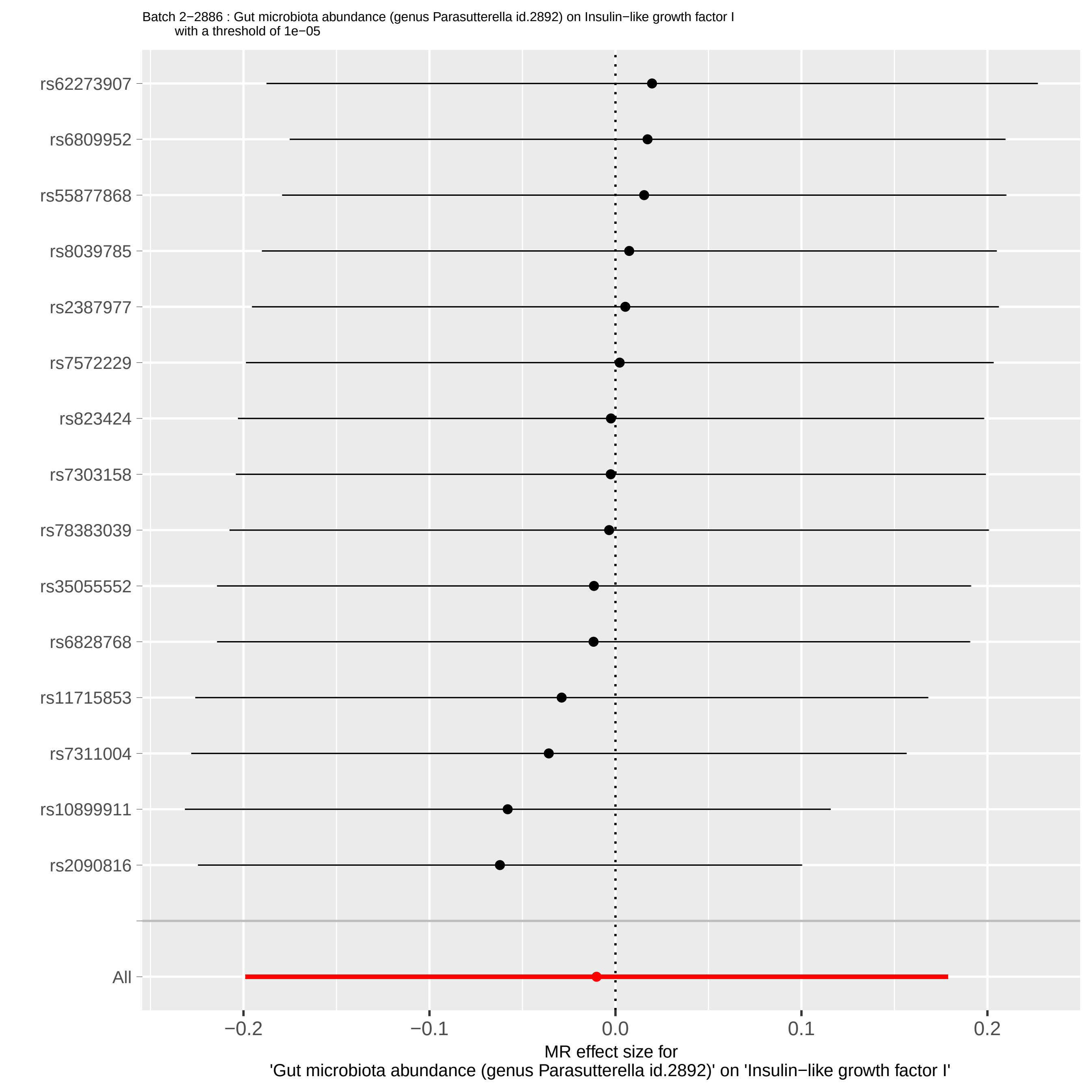
Batch 2−2886 : Gut microbiota abundance (genus Parasutterella id.2892) on Insulin−like growth factor I
with a threshold of 1e−05
rs62273907
rs6809952
rs55877868
rs8039785
rs2387977
rs7572229
rs823424
rs7303158
rs78383039
rs35055552
rs6828768
rs11715853
rs7311004
rs10899911
rs2090816
All
−0.2
−0.1
0.0
0.1
0.2
MR effect size for
'Gut microbiota abundance (genus Parasutterella id.2892)' on 'Insulin−like growth factor I'

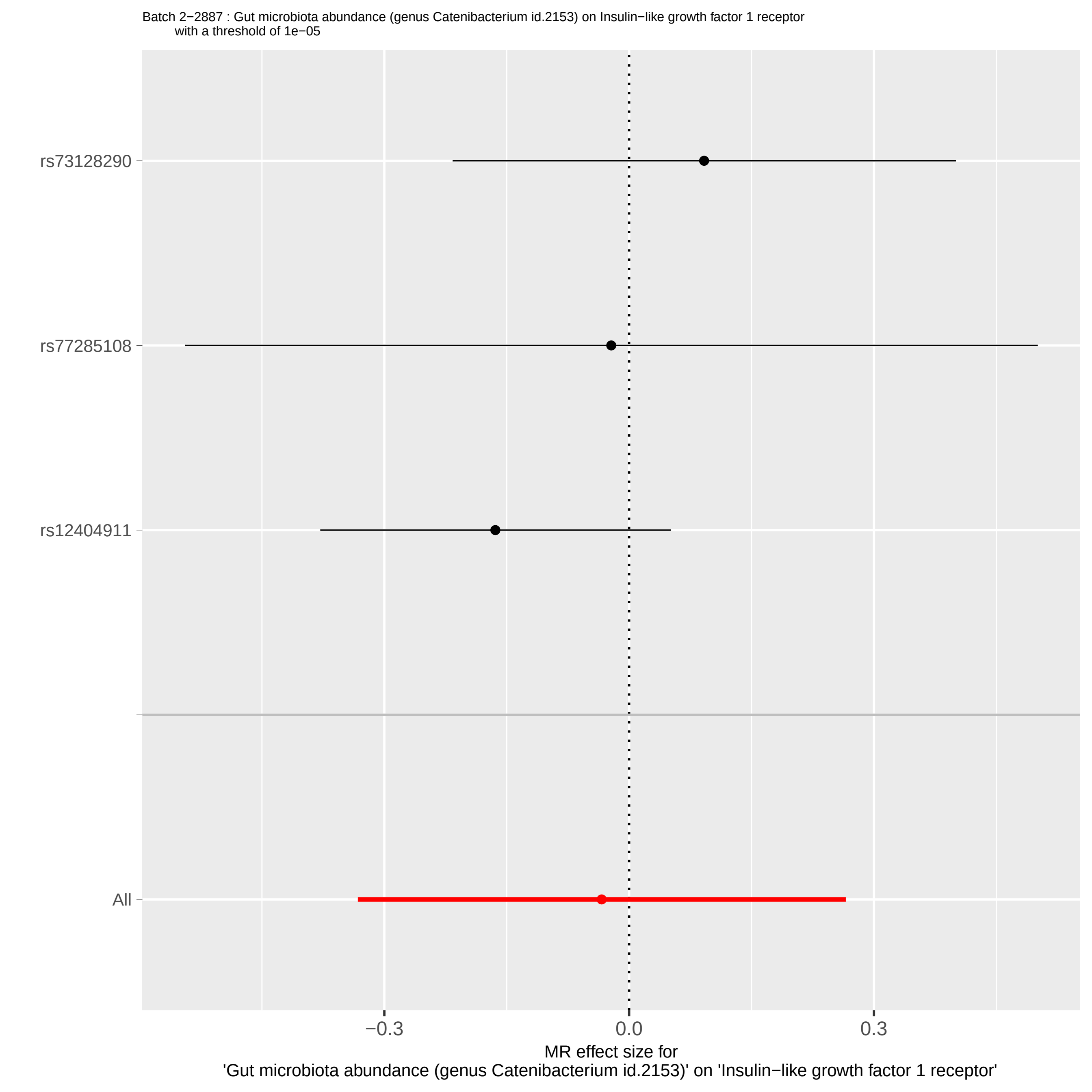
Batch 2−2887 : Gut microbiota abundance (genus Catenibacterium id.2153) on Insulin−like growth factor 1 receptor
with a threshold of 1e−05
rs73128290
rs77285108
rs12404911
All
−0.3
0.0
0.3
MR effect size for
'Gut microbiota abundance (genus Catenibacterium id.2153)' on 'Insulin−like growth factor 1 receptor'

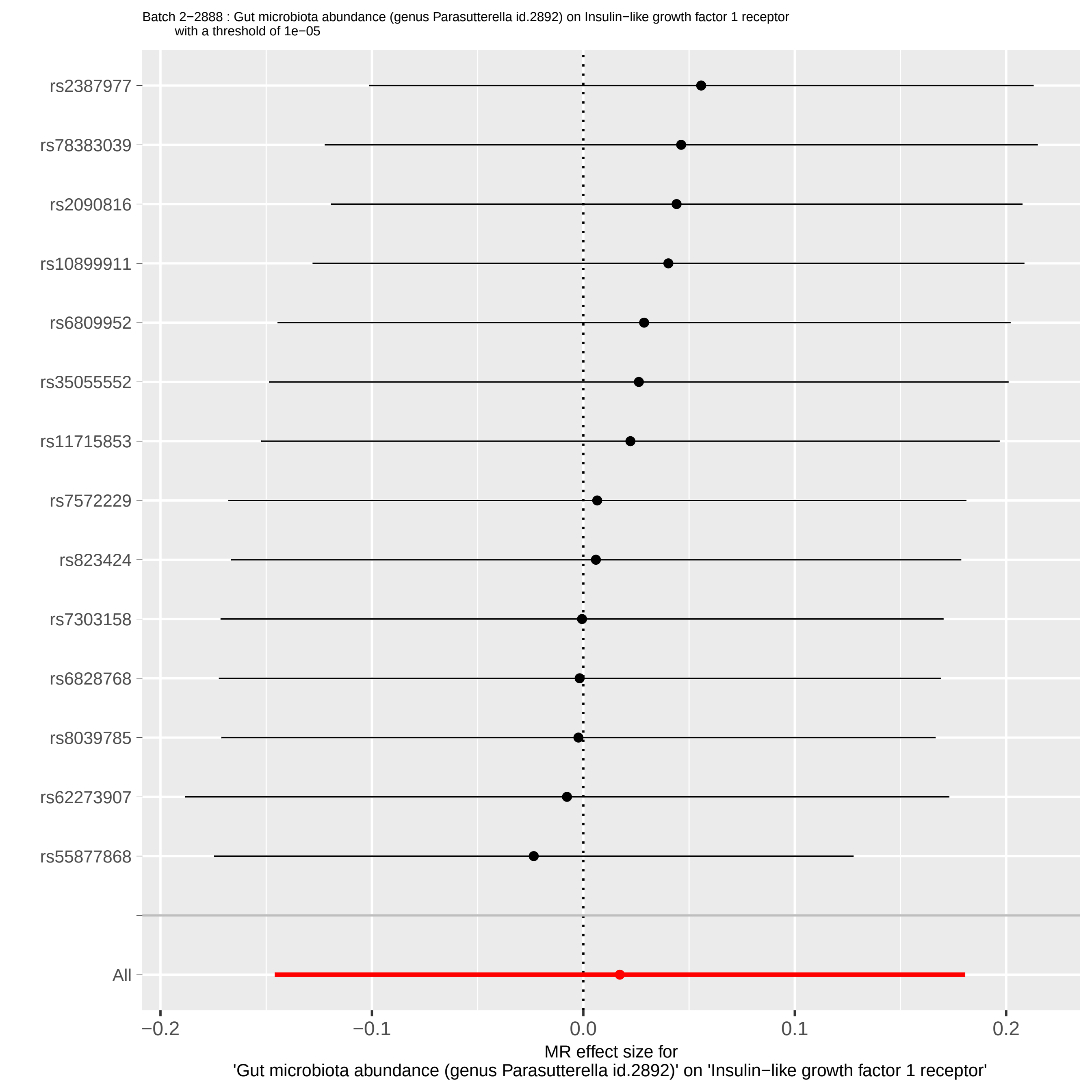

Batch 2−2888 : Gut microbiota abundance (genus Parasutterella id.2892) on Insulin−like growth factor 1 receptor
with a threshold of 1e−05
rs2387977
rs78383039
rs2090816
rs10899911
rs6809952
rs35055552
rs11715853
rs7572229
rs823424
rs7303158
rs6828768
rs8039785
rs62273907
rs55877868
All
−0.2
−0.1
0.0
0.1
0.2
MR effect size for
'Gut microbiota abundance (genus Parasutterella id.2892)' on 'Insulin−like growth factor 1 receptor'

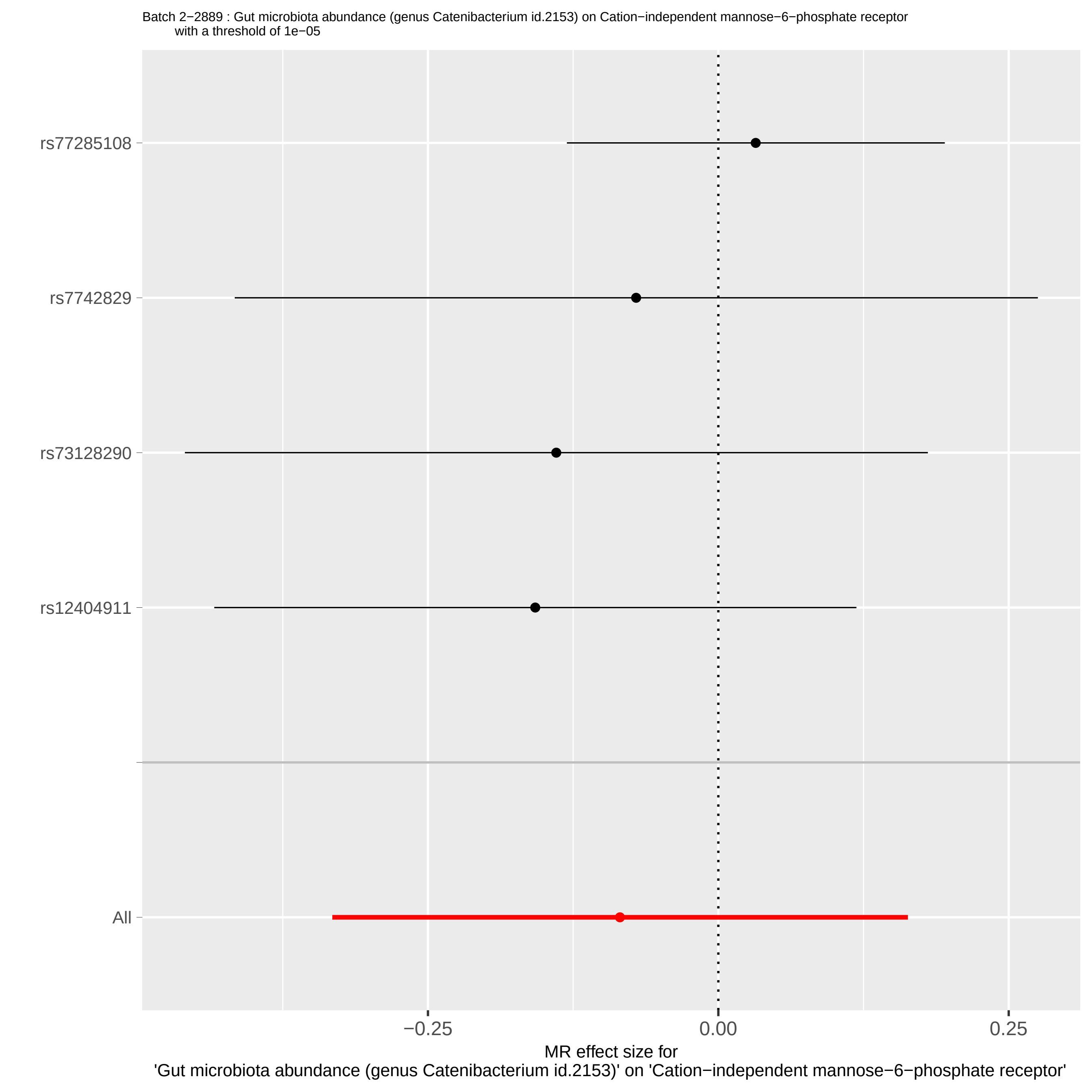
Batch 2−2889 : Gut microbiota abundance (genus Catenibacterium id.2153) on Cation−independent mannose−6−phosphate receptor
with a threshold of 1e−05
rs77285108
rs7742829
rs73128290
rs12404911
All
−0.25
0.00
0.25
MR effect size for
'Gut microbiota abundance (genus Catenibacterium id.2153)' on 'Cation−independent mannose−6−phosphate receptor'

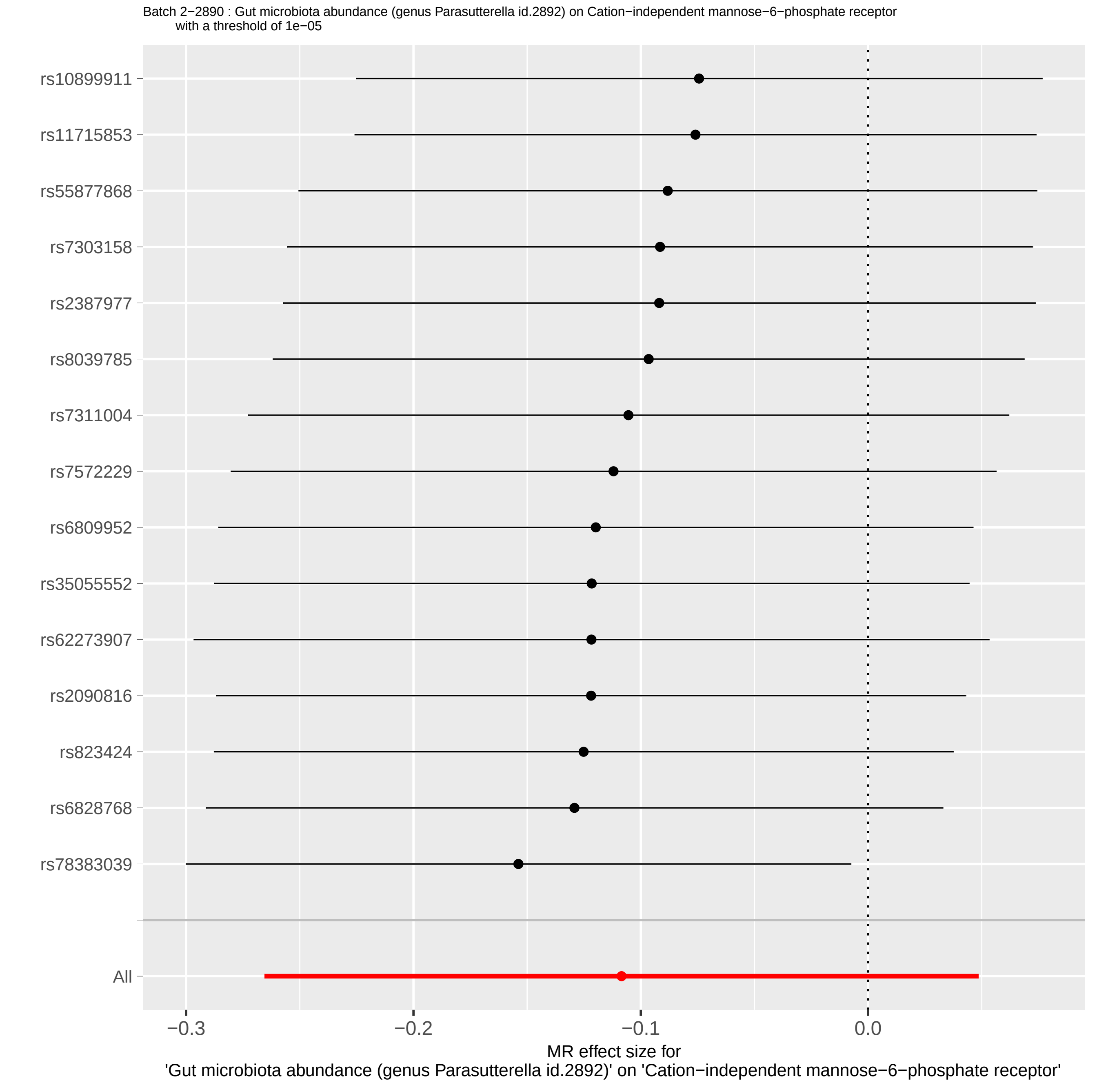
Batch 2−2890 : Gut microbiota abundance (genus Parasutterella id.2892) on Cation−independent mannose−6−phosphate receptor
with a threshold of 1e−05
rs10899911
rs11715853
rs55877868
rs7303158
rs2387977
rs8039785
rs7311004
rs7572229
rs6809952
rs35055552
rs62273907
rs2090816
rs823424
rs6828768
rs78383039
All
−0.3
−0.2
−0.1
0.0
MR effect size for
'Gut microbiota abundance (genus Parasutterella id.2892)' on 'Cation−independent mannose−6−phosphate receptor'

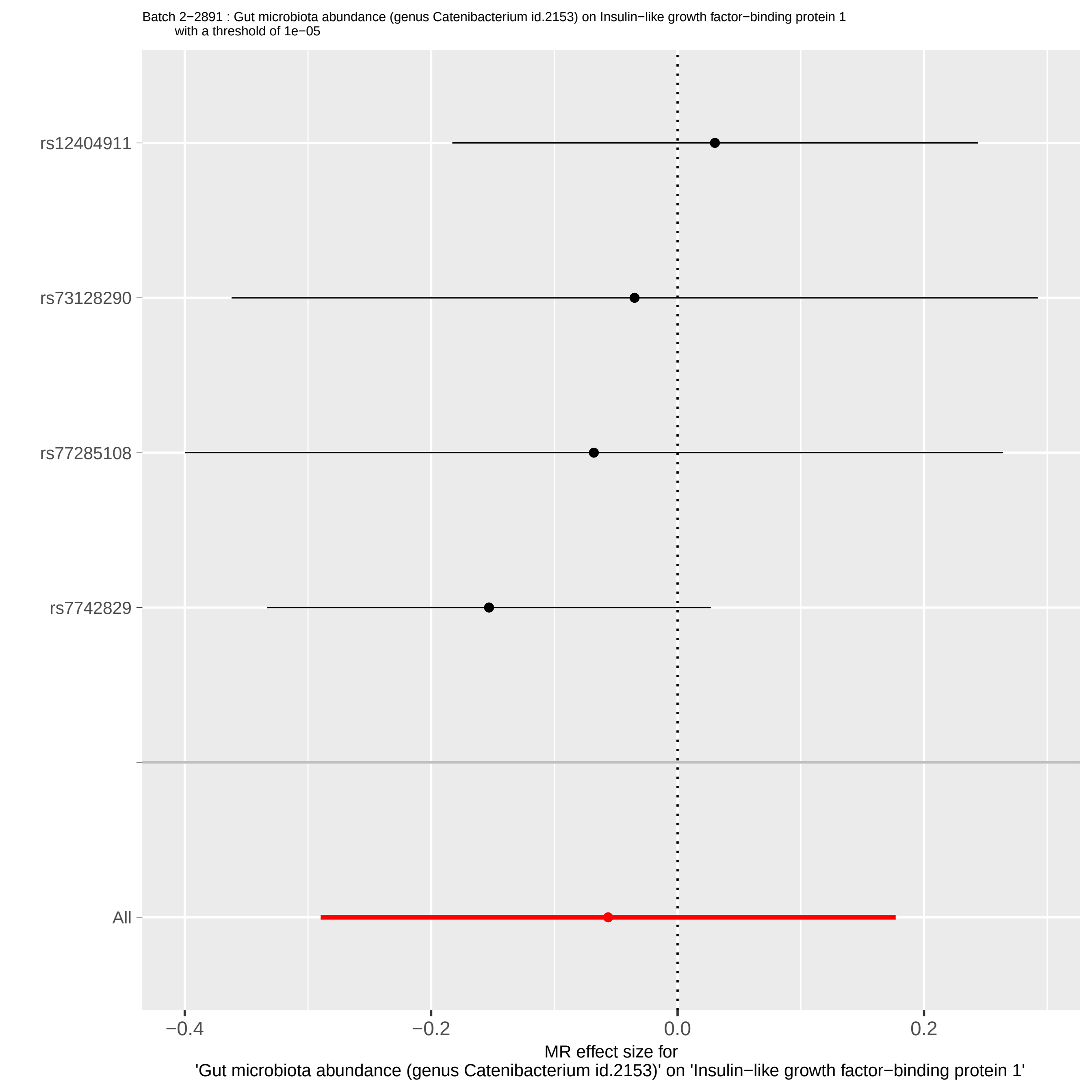
Batch 2−2891 : Gut microbiota abundance (genus Catenibacterium id.2153) on Insulin−like growth factor−binding protein 1
with a threshold of 1e−05
rs12404911
rs73128290
rs77285108
rs7742829
All
−0.4
−0.2
0.0
0.2
MR effect size for
'Gut microbiota abundance (genus Catenibacterium id.2153)' on 'Insulin−like growth factor−binding protein 1'

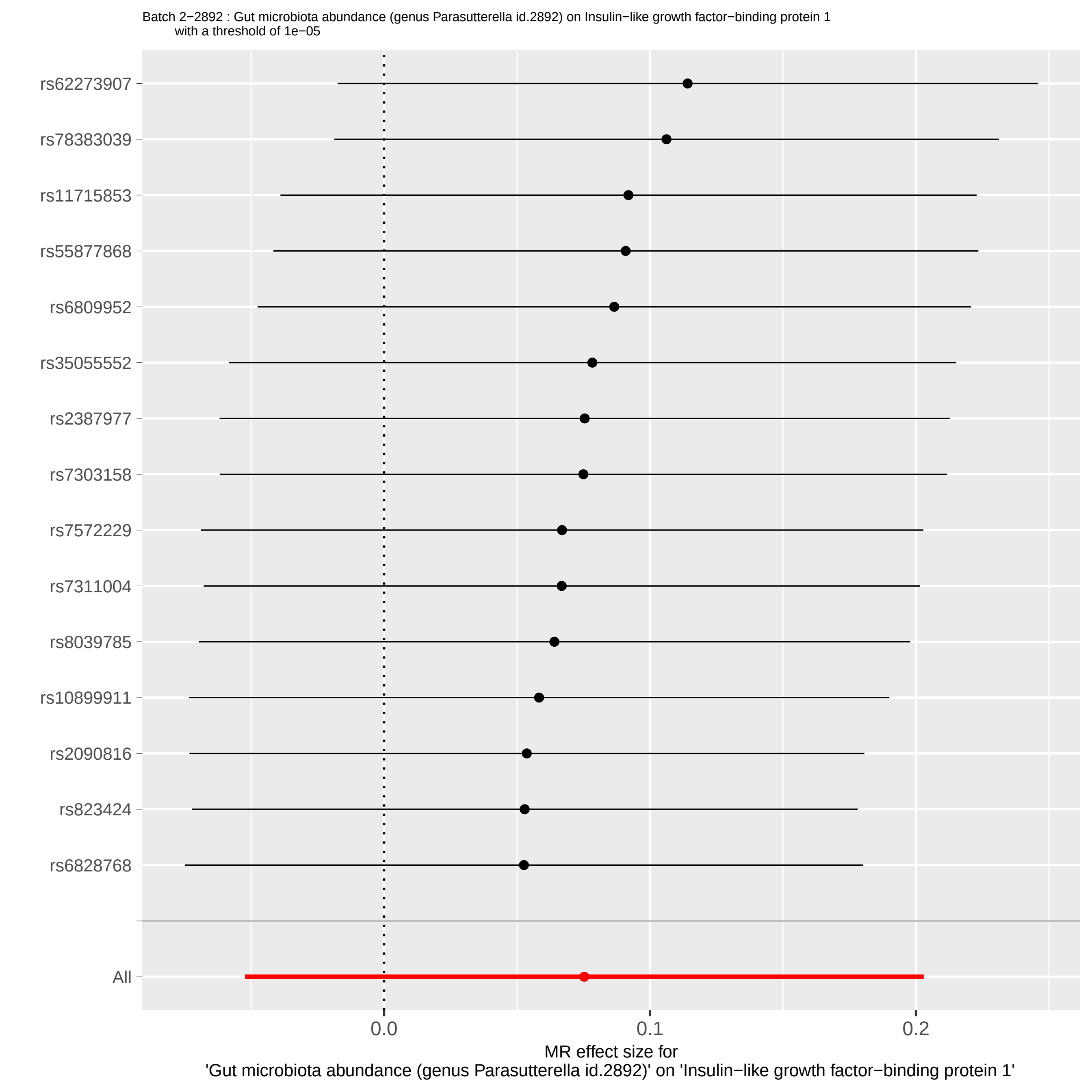
Batch 2−2892 : Gut microbiota abundance (genus Parasutterella id.2892) on Insulin−like growth factor−binding protein 1
with a threshold of 1e−05
rs62273907
rs78383039
rs11715853
rs55877868
rs6809952
rs35055552
rs2387977
rs7303158
rs7572229
rs7311004
rs8039785
rs10899911
rs2090816
rs823424
rs6828768
All
0.0
0.1
0.2
MR effect size for
'Gut microbiota abundance (genus Parasutterella id.2892)' on 'Insulin−like growth factor−binding protein 1'

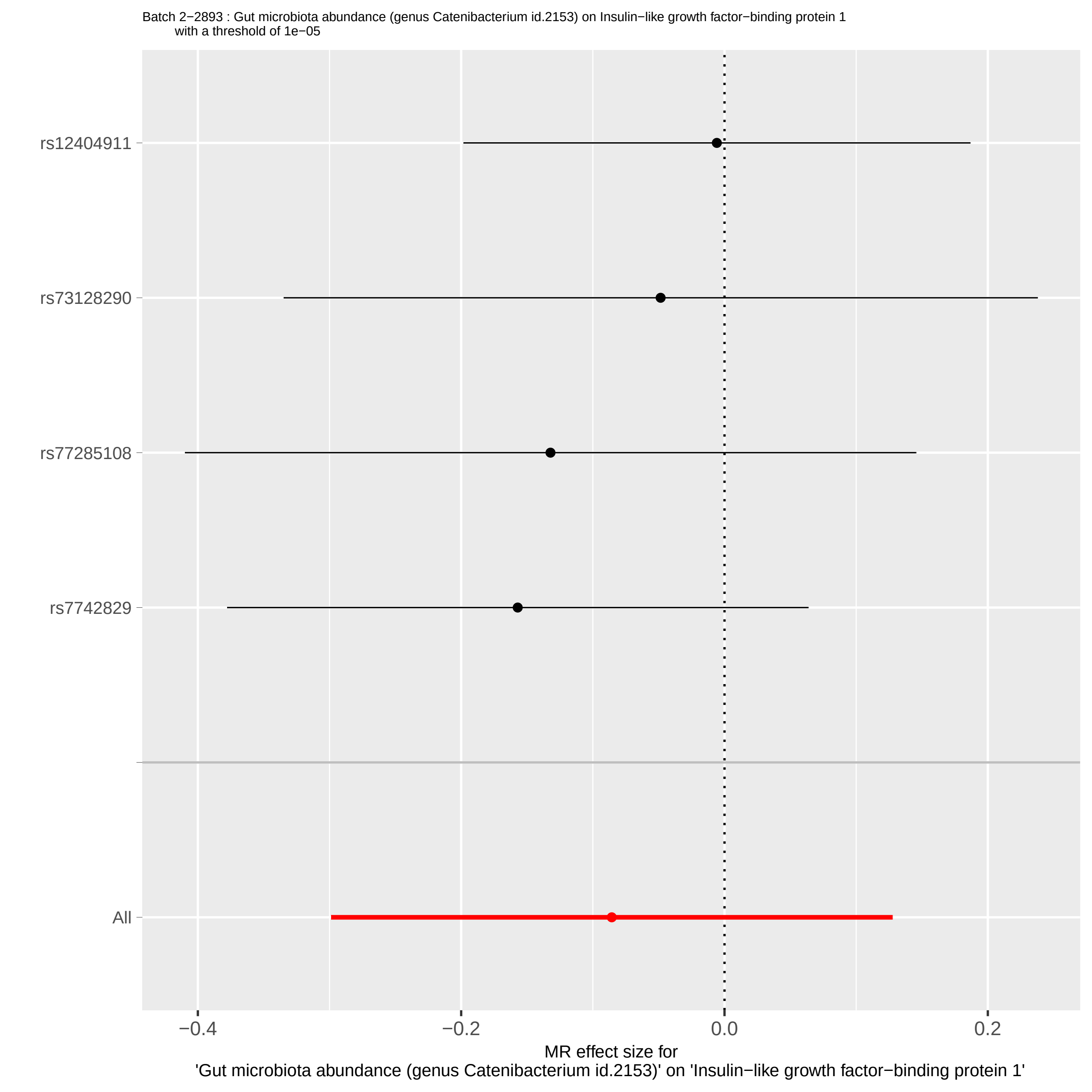
Batch 2−2893 : Gut microbiota abundance (genus Catenibacterium id.2153) on Insulin−like growth factor−binding protein 1
with a threshold of 1e−05
rs12404911
rs73128290
rs77285108
rs7742829
All
−0.4
−0.2
0.0
0.2
MR effect size for
'Gut microbiota abundance (genus Catenibacterium id.2153)' on 'Insulin−like growth factor−binding protein 1'

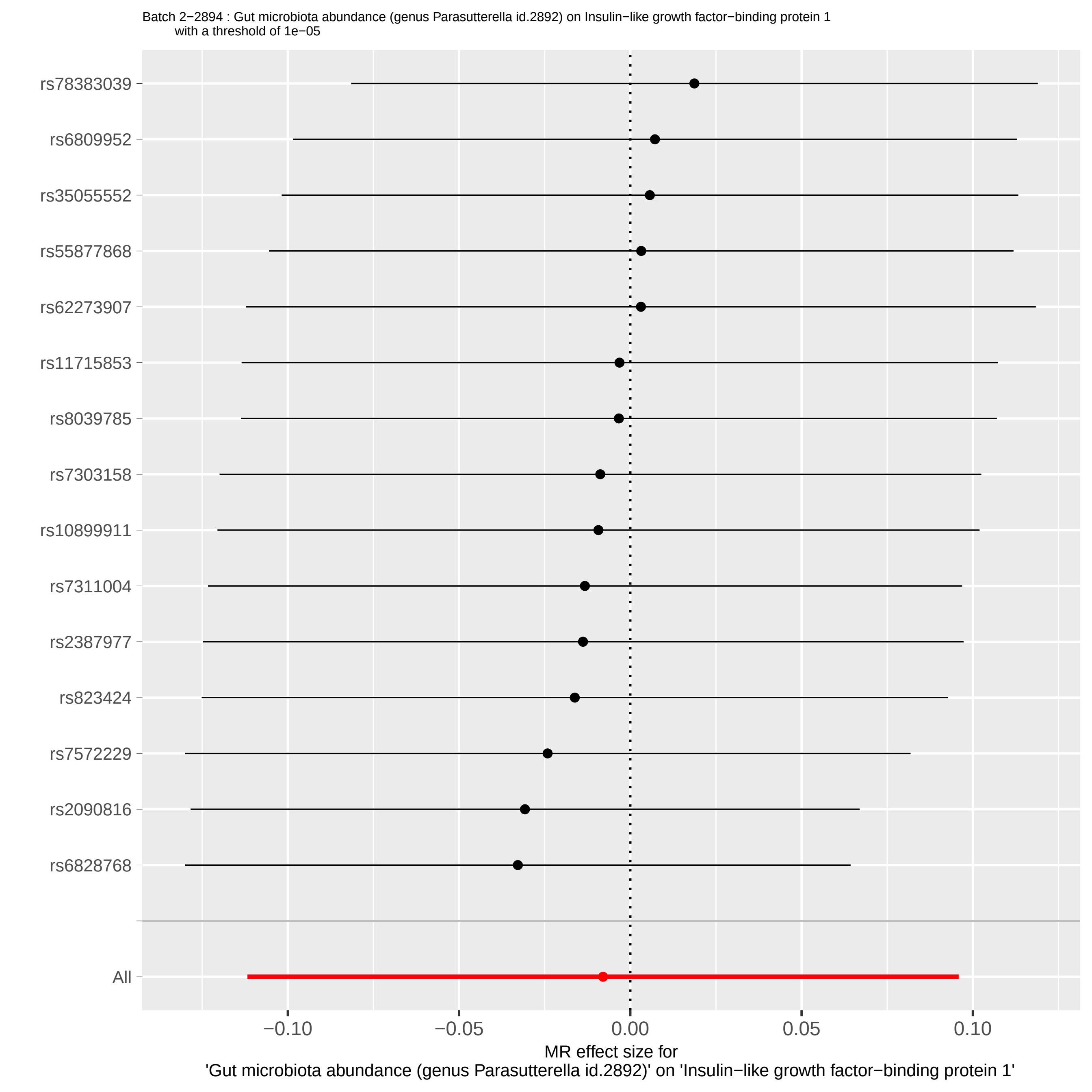

Batch 2−2894 : Gut microbiota abundance (genus Parasutterella id.2892) on Insulin−like growth factor−binding protein 1
with a threshold of 1e−05
rs78383039
rs6809952
rs35055552
rs55877868
rs62273907
rs11715853
rs8039785
rs7303158
rs10899911
rs7311004
rs2387977
rs823424
rs7572229
rs2090816
rs6828768
All
−0.10
−0.05
0.00
0.05
0.10
MR effect size for
'Gut microbiota abundance (genus Parasutterella id.2892)' on 'Insulin−like growth factor−binding protein 1'

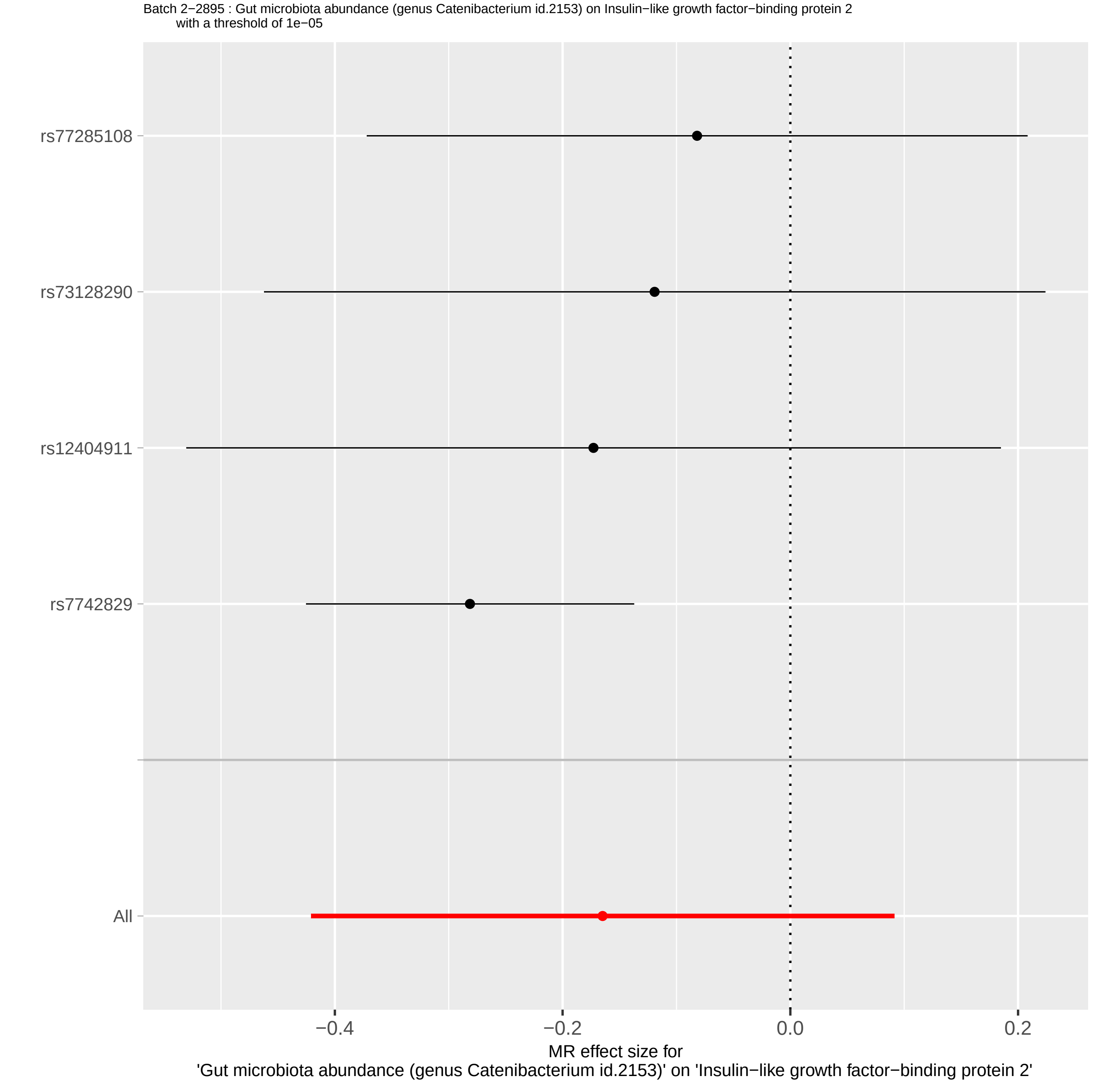

Batch 2−2895 : Gut microbiota abundance (genus Catenibacterium id.2153) on Insulin−like growth factor−binding protein 2
with a threshold of 1e−05
rs77285108
rs73128290
rs12404911
rs7742829
All
−0.4
−0.2
0.0
0.2
MR effect size for
'Gut microbiota abundance (genus Catenibacterium id.2153)' on 'Insulin−like growth factor−binding protein 2'

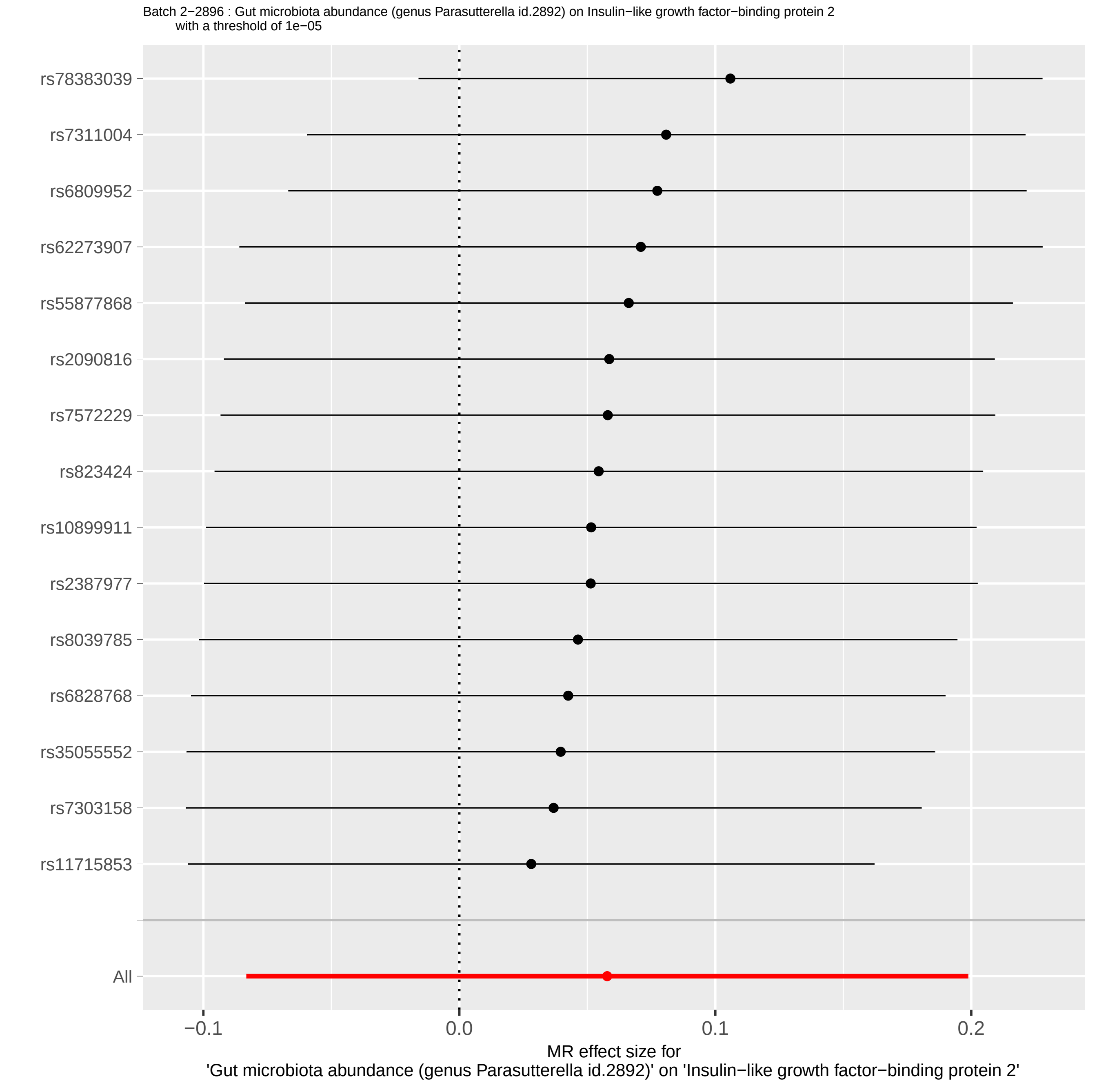

Batch 2−2896 : Gut microbiota abundance (genus Parasutterella id.2892) on Insulin−like growth factor−binding protein 2
with a threshold of 1e−05
rs78383039
rs7311004
rs6809952
rs62273907
rs55877868
rs2090816
rs7572229
rs823424
rs10899911
rs2387977
rs8039785
rs6828768
rs35055552
rs7303158
rs11715853
All
−0.1
0.0
0.1
0.2
MR effect size for
'Gut microbiota abundance (genus Parasutterella id.2892)' on 'Insulin−like growth factor−binding protein 2'

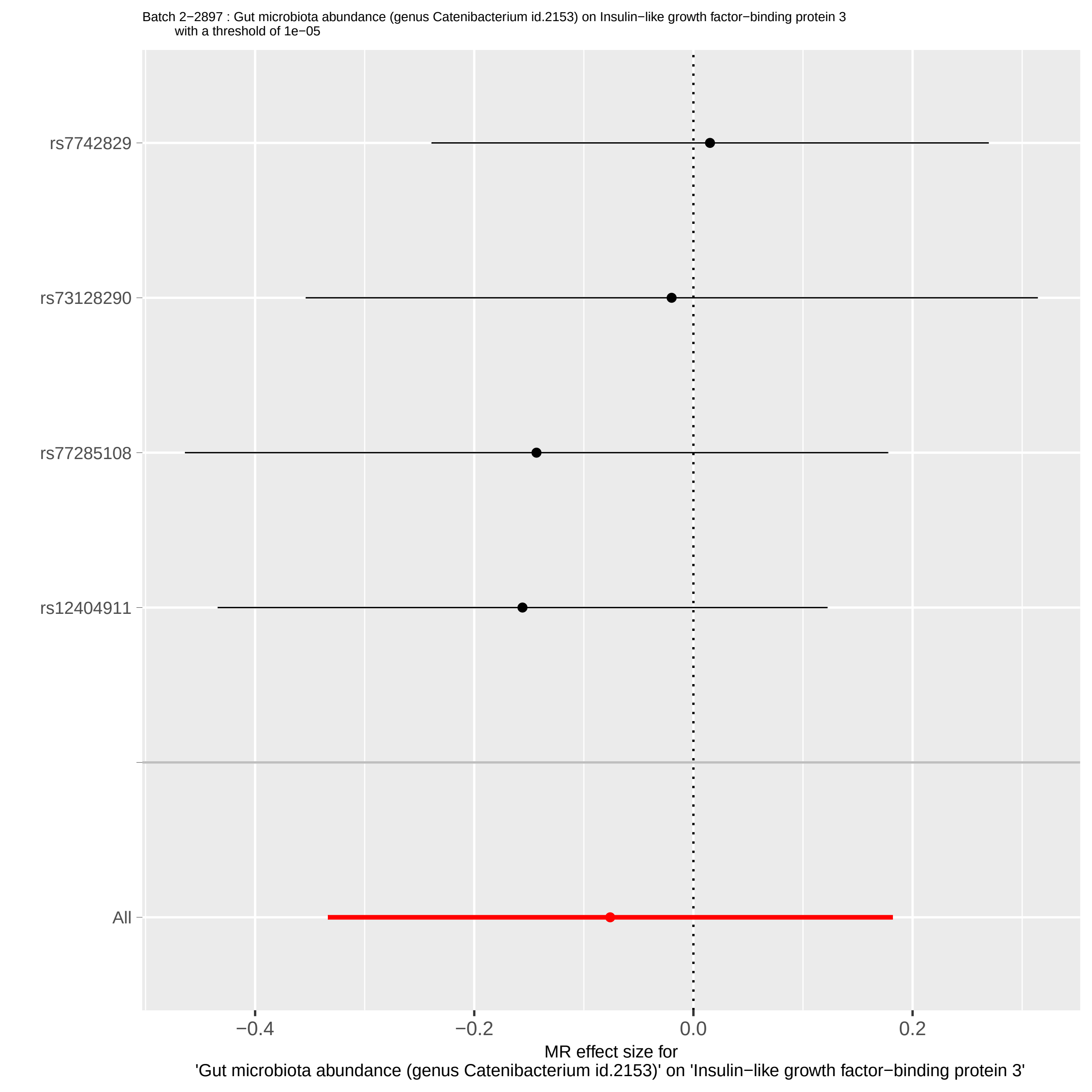
Batch 2−2897 : Gut microbiota abundance (genus Catenibacterium id.2153) on Insulin−like growth factor−binding protein 3
with a threshold of 1e−05
rs7742829
rs73128290
rs77285108
rs12404911
All
−0.4
−0.2
0.0
0.2
MR effect size for
'Gut microbiota abundance (genus Catenibacterium id.2153)' on 'Insulin−like growth factor−binding protein 3'

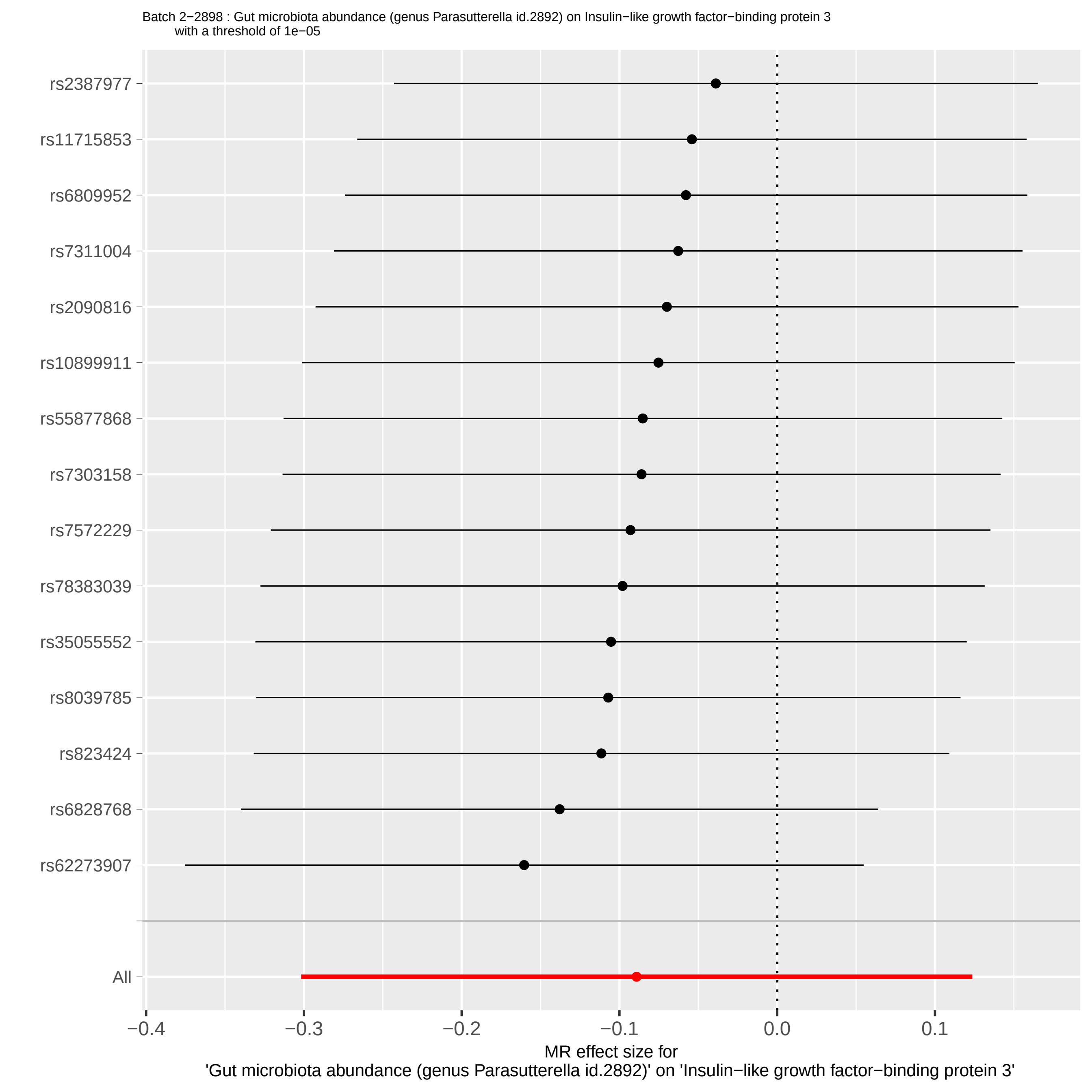
Batch 2−2898 : Gut microbiota abundance (genus Parasutterella id.2892) on Insulin−like growth factor−binding protein 3
with a threshold of 1e−05
rs2387977
rs11715853
rs6809952
rs7311004
rs2090816
rs10899911
rs55877868
rs7303158
rs7572229
rs78383039
rs35055552
rs8039785
rs823424
rs6828768
rs62273907
All
−0.4
−0.3
−0.2
−0.1
0.0
0.1
MR effect size for
'Gut microbiota abundance (genus Parasutterella id.2892)' on 'Insulin−like growth factor−binding protein 3'

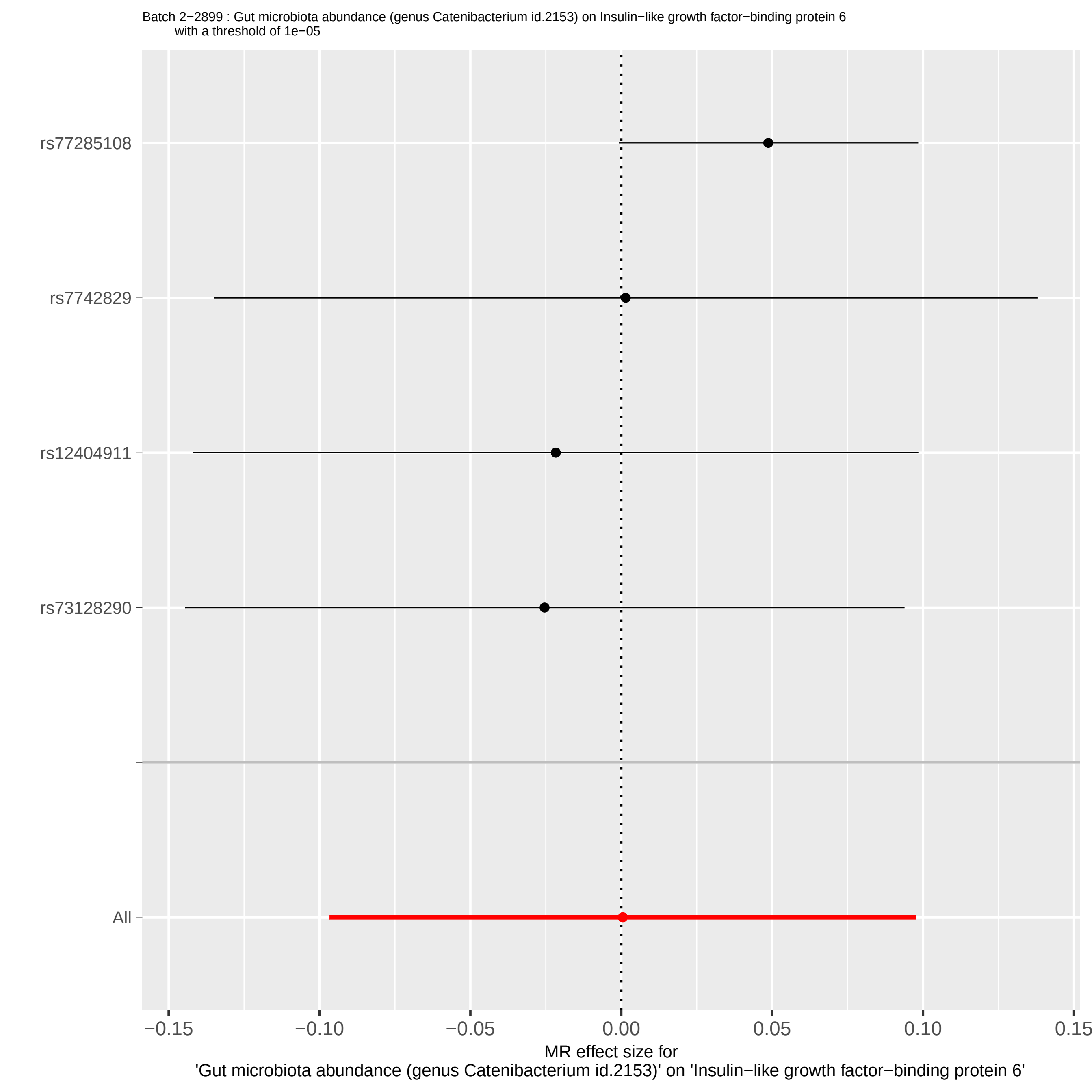
Batch 2−2899 : Gut microbiota abundance (genus Catenibacterium id.2153) on Insulin−like growth factor−binding protein 6
with a threshold of 1e−05
rs77285108
rs7742829
rs12404911
rs73128290
All
−0.15
−0.10
−0.05
0.00
0.05
0.10
0.15
MR effect size for
'Gut microbiota abundance (genus Catenibacterium id.2153)' on 'Insulin−like growth factor−binding protein 6'

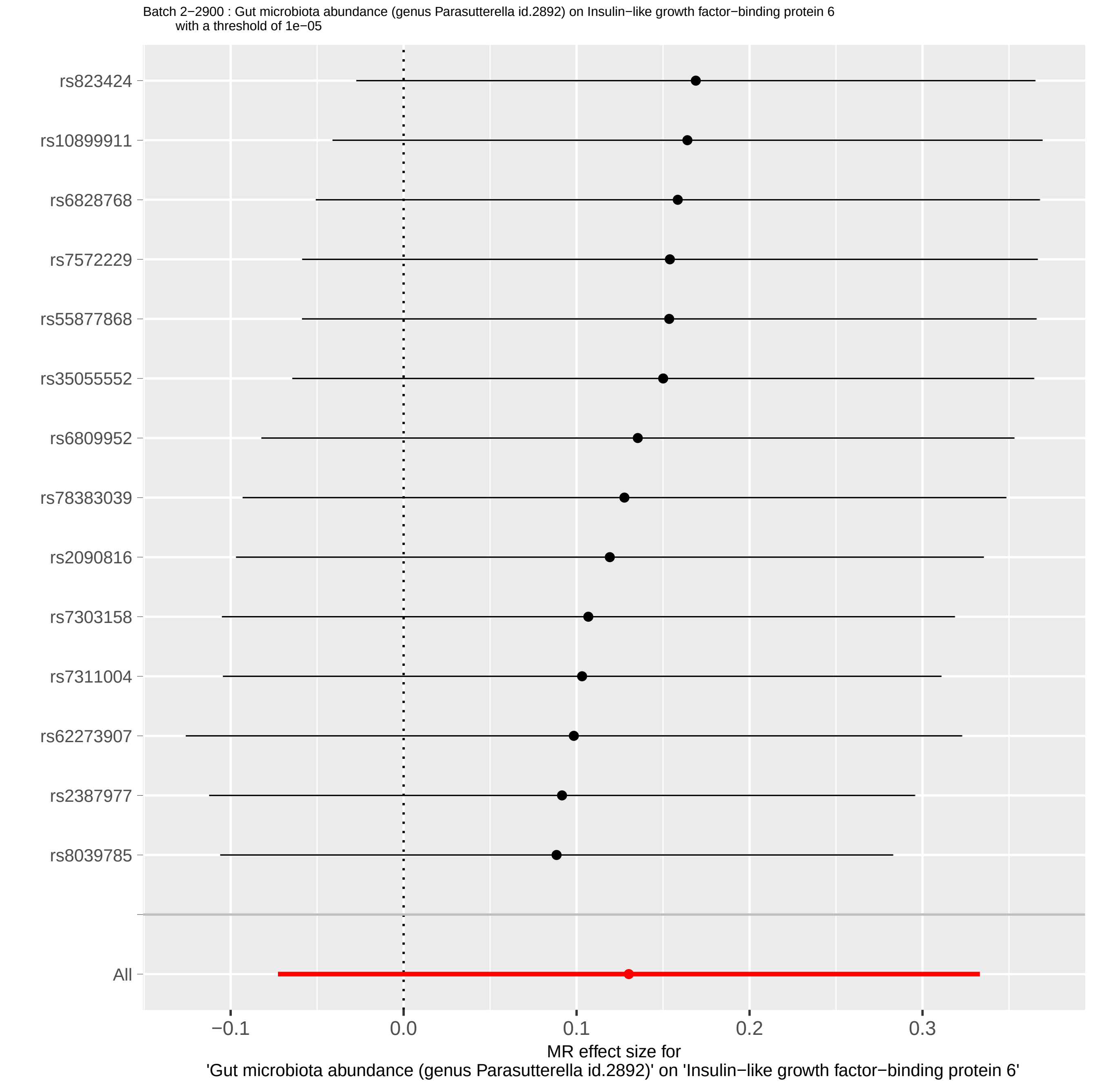

Batch 2−2900 : Gut microbiota abundance (genus Parasutterella id.2892) on Insulin−like growth factor−binding protein 6
with a threshold of 1e−05
rs823424
rs10899911
rs6828768
rs7572229
rs55877868
rs35055552
rs6809952
rs78383039
rs2090816
rs7303158
rs7311004
rs62273907
rs2387977
rs8039785
All
−0.1
0.0
0.1
0.2
0.3
MR effect size for
'Gut microbiota abundance (genus Parasutterella id.2892)' on 'Insulin−like growth factor−binding protein 6'

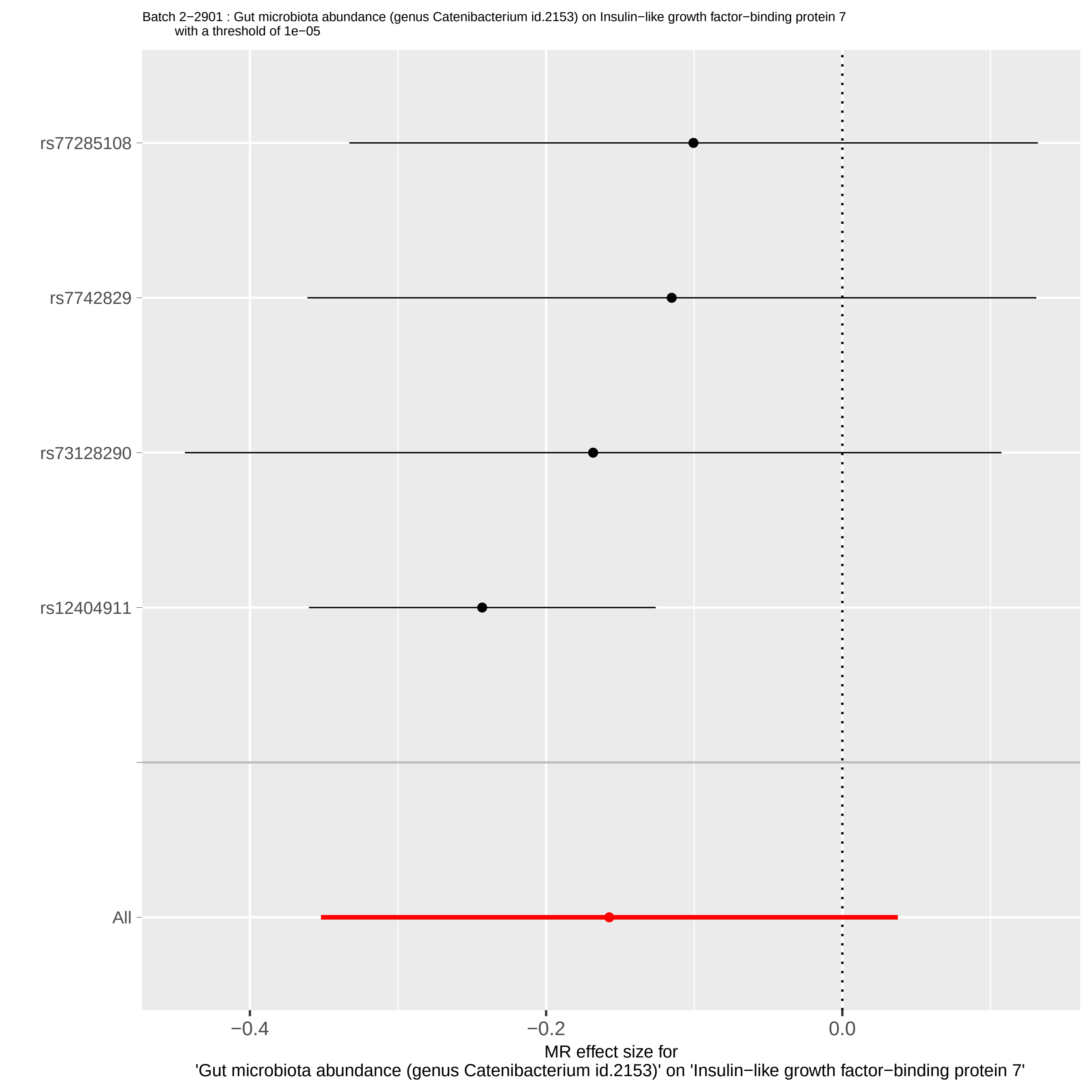
Batch 2−2901 : Gut microbiota abundance (genus Catenibacterium id.2153) on Insulin−like growth factor−binding protein 7
with a threshold of 1e−05
rs77285108
rs7742829
rs73128290
rs12404911
All
−0.4
−0.2
0.0
MR effect size for
'Gut microbiota abundance (genus Catenibacterium id.2153)' on 'Insulin−like growth factor−binding protein 7'

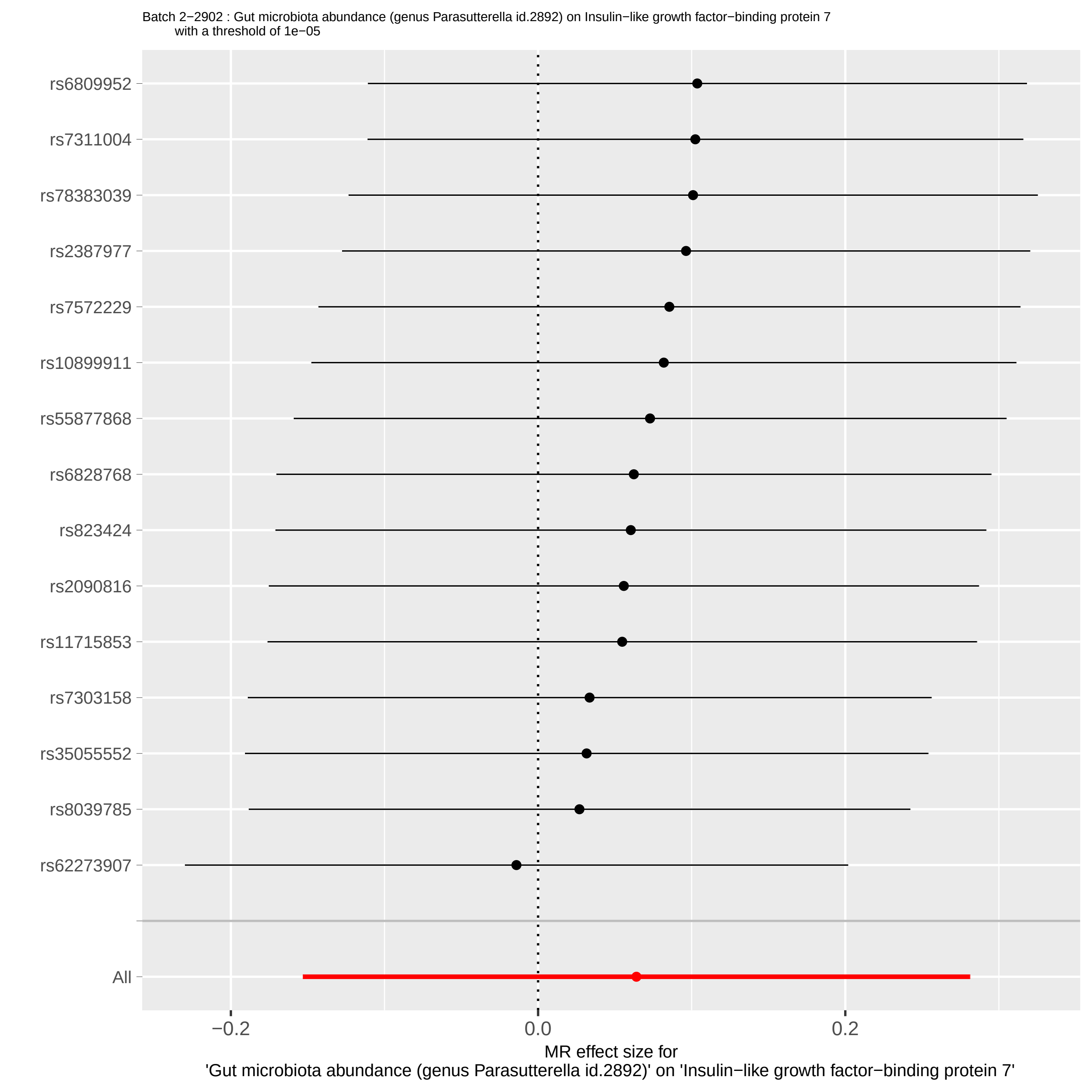

Batch 2−2902 : Gut microbiota abundance (genus Parasutterella id.2892) on Insulin−like growth factor−binding protein 7
with a threshold of 1e−05
rs6809952
rs7311004
rs78383039
rs2387977
rs7572229
rs10899911
rs55877868
rs6828768
rs823424
rs2090816
rs11715853
rs7303158
rs35055552
rs8039785
rs62273907
All
−0.2
0.0
0.2
MR effect size for
'Gut microbiota abundance (genus Parasutterella id.2892)' on 'Insulin−like growth factor−binding protein 7'

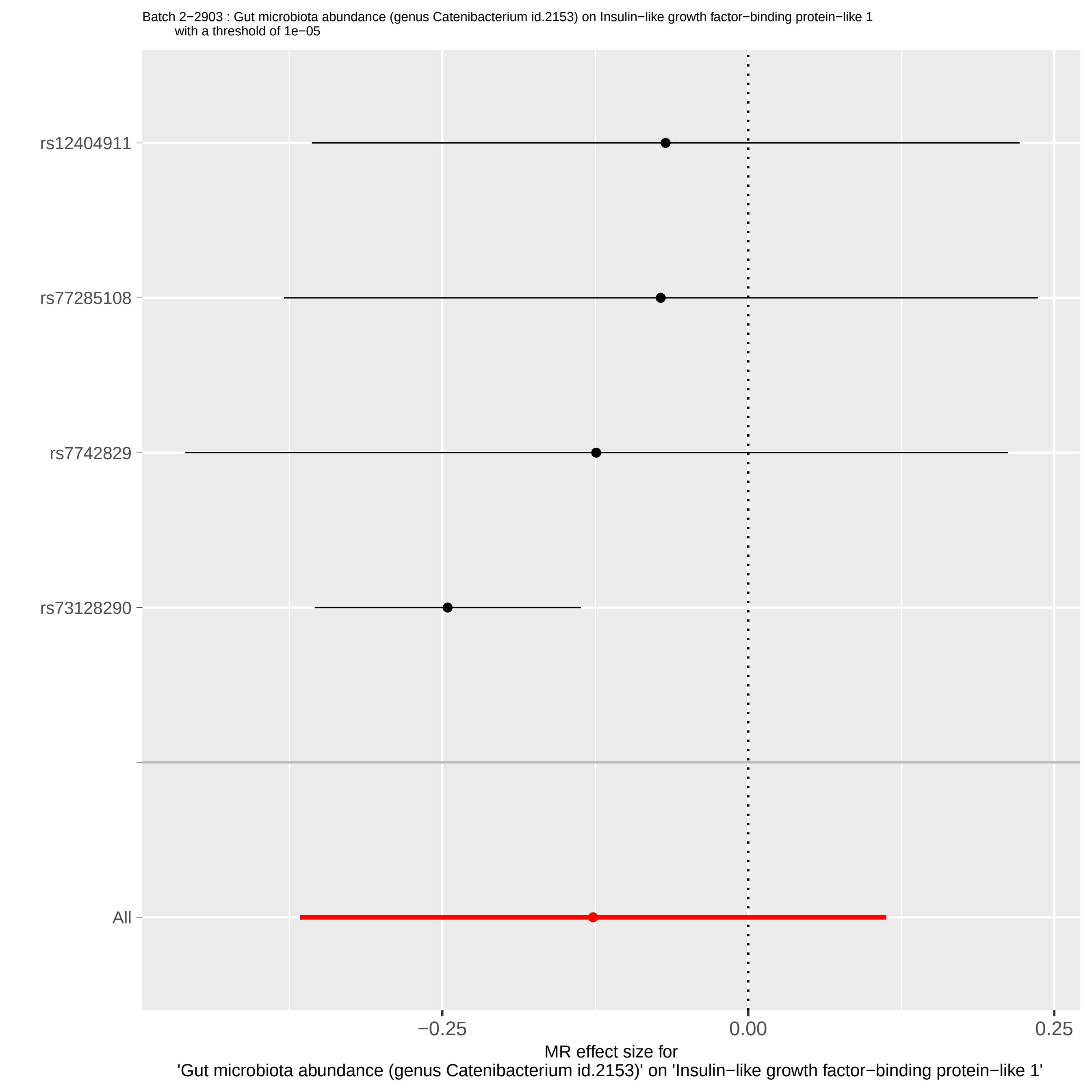
Batch 2−2903 : Gut microbiota abundance (genus Catenibacterium id.2153) on Insulin−like growth factor−binding protein−like 1
with a threshold of 1e−05
rs12404911
rs77285108
rs7742829
rs73128290
All
−0.25
0.00
0.25
MR effect size for
'Gut microbiota abundance (genus Catenibacterium id.2153)' on 'Insulin−like growth factor−binding protein−like 1'

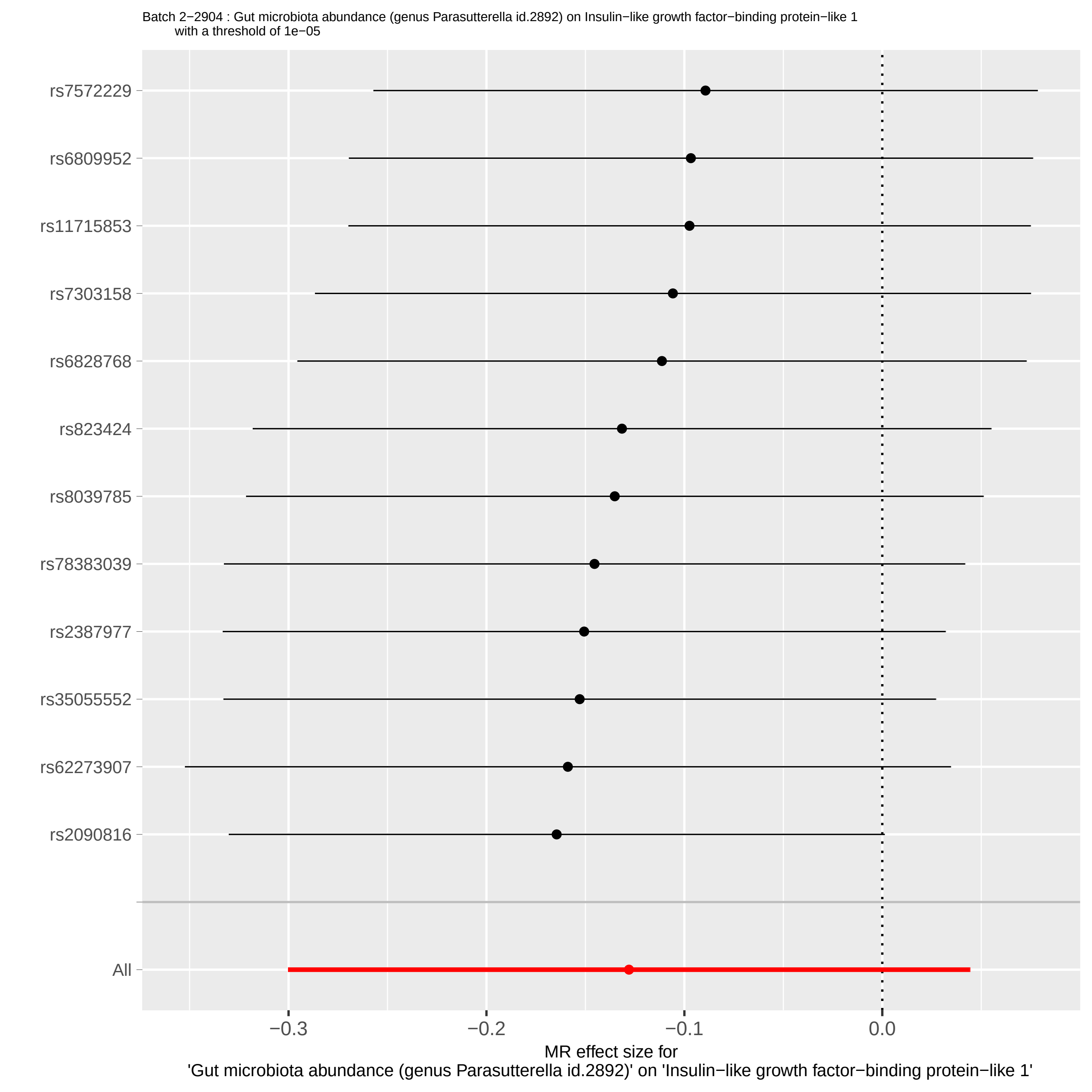

Batch 2−2904 : Gut microbiota abundance (genus Parasutterella id.2892) on Insulin−like growth factor−binding protein−like 1
with a threshold of 1e−05
rs7572229
rs6809952
rs11715853
rs7303158
rs6828768
rs823424
rs8039785
rs78383039
rs2387977
rs35055552
rs62273907
rs2090816
All
−0.3
−0.2
−0.1
0.0
MR effect size for
'Gut microbiota abundance (genus Parasutterella id.2892)' on 'Insulin−like growth factor−binding protein−like 1'

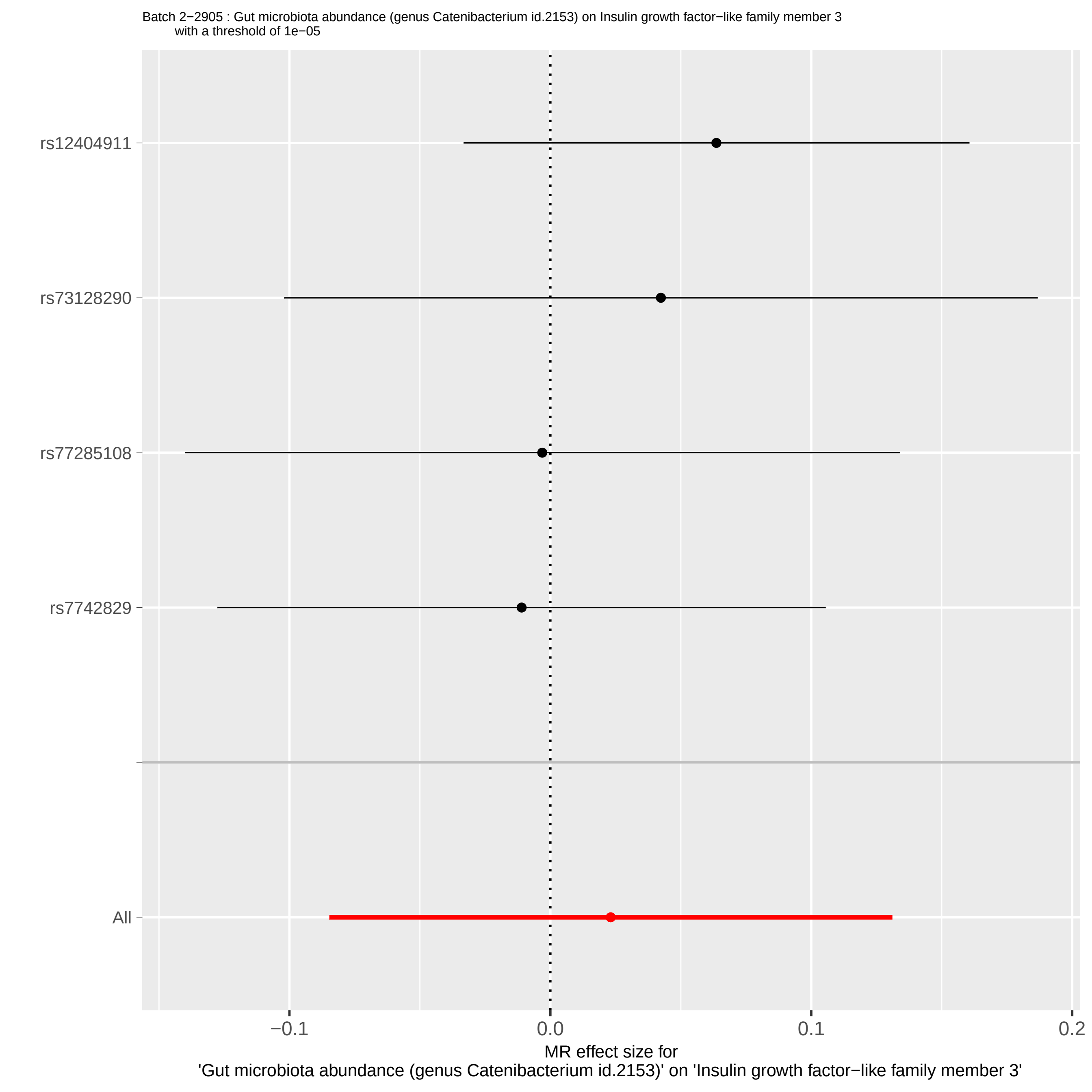
Batch 2−2905 : Gut microbiota abundance (genus Catenibacterium id.2153) on Insulin growth factor−like family member 3
with a threshold of 1e−05
rs12404911
rs73128290
rs77285108
rs7742829
All
−0.1
0.0
0.1
0.2
MR effect size for
'Gut microbiota abundance (genus Catenibacterium id.2153)' on 'Insulin growth factor−like family member 3'

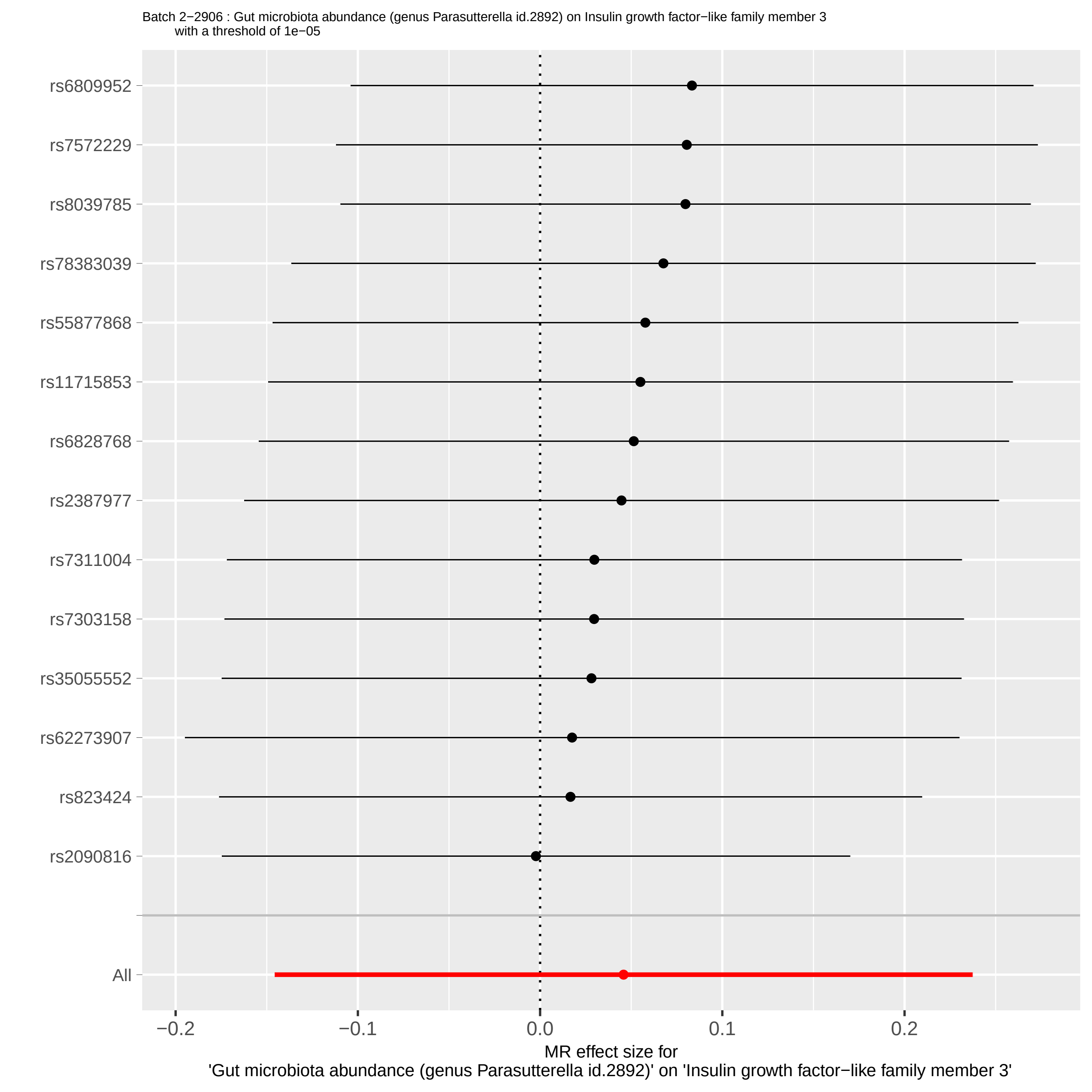
Batch 2−2906 : Gut microbiota abundance (genus Parasutterella id.2892) on Insulin growth factor−like family member 3
with a threshold of 1e−05
rs6809952
rs7572229
rs8039785
rs78383039
rs55877868
rs11715853
rs6828768
rs2387977
rs7311004
rs7303158
rs35055552
rs62273907
rs823424
rs2090816
All
−0.2
−0.1
0.0
0.1
0.2
MR effect size for
'Gut microbiota abundance (genus Parasutterella id.2892)' on 'Insulin growth factor−like family member 3'

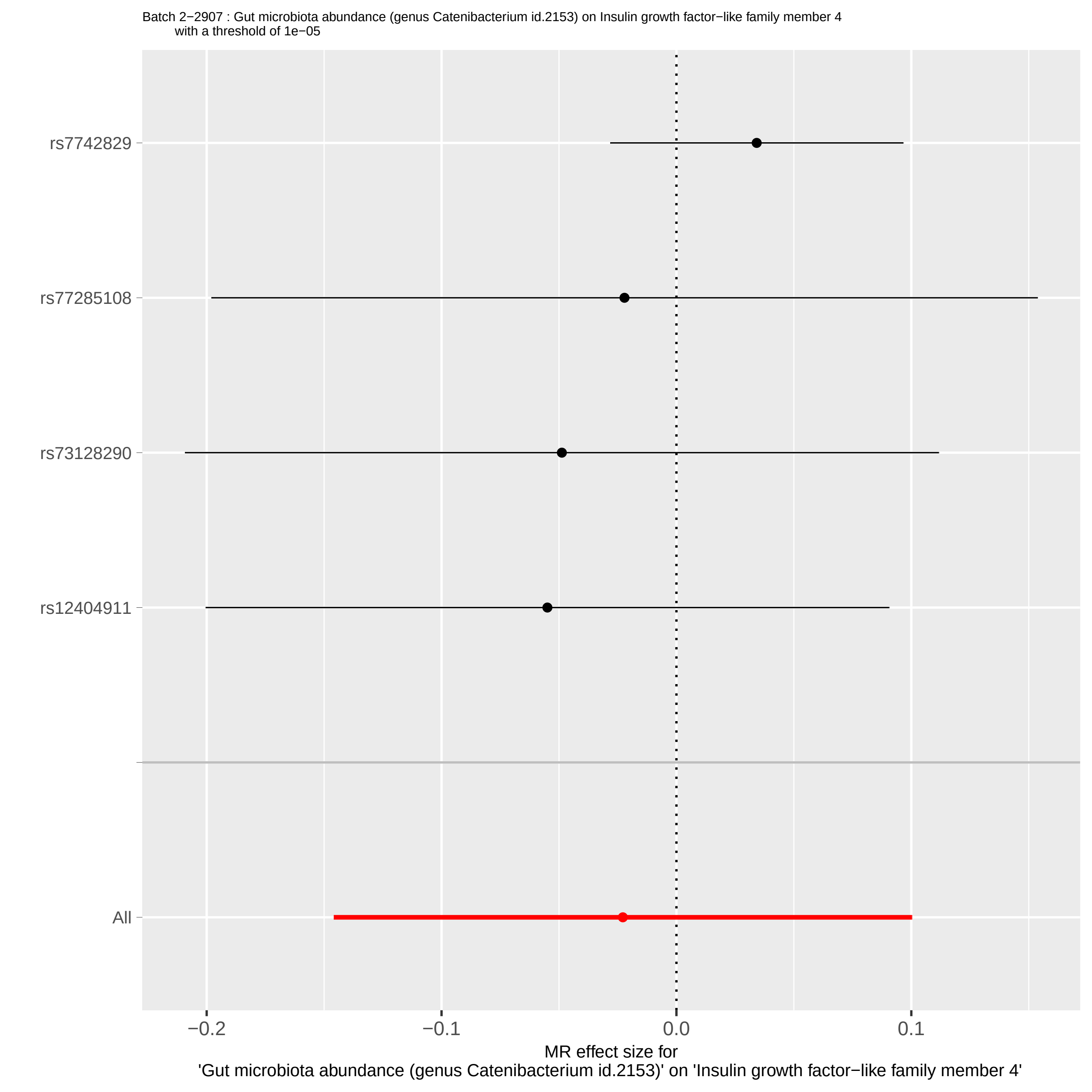
Batch 2−2907 : Gut microbiota abundance (genus Catenibacterium id.2153) on Insulin growth factor−like family member 4
with a threshold of 1e−05
rs7742829
rs77285108
rs73128290
rs12404911
All
−0.2
−0.1
0.0
0.1
MR effect size for
'Gut microbiota abundance (genus Catenibacterium id.2153)' on 'Insulin growth factor−like family member 4'

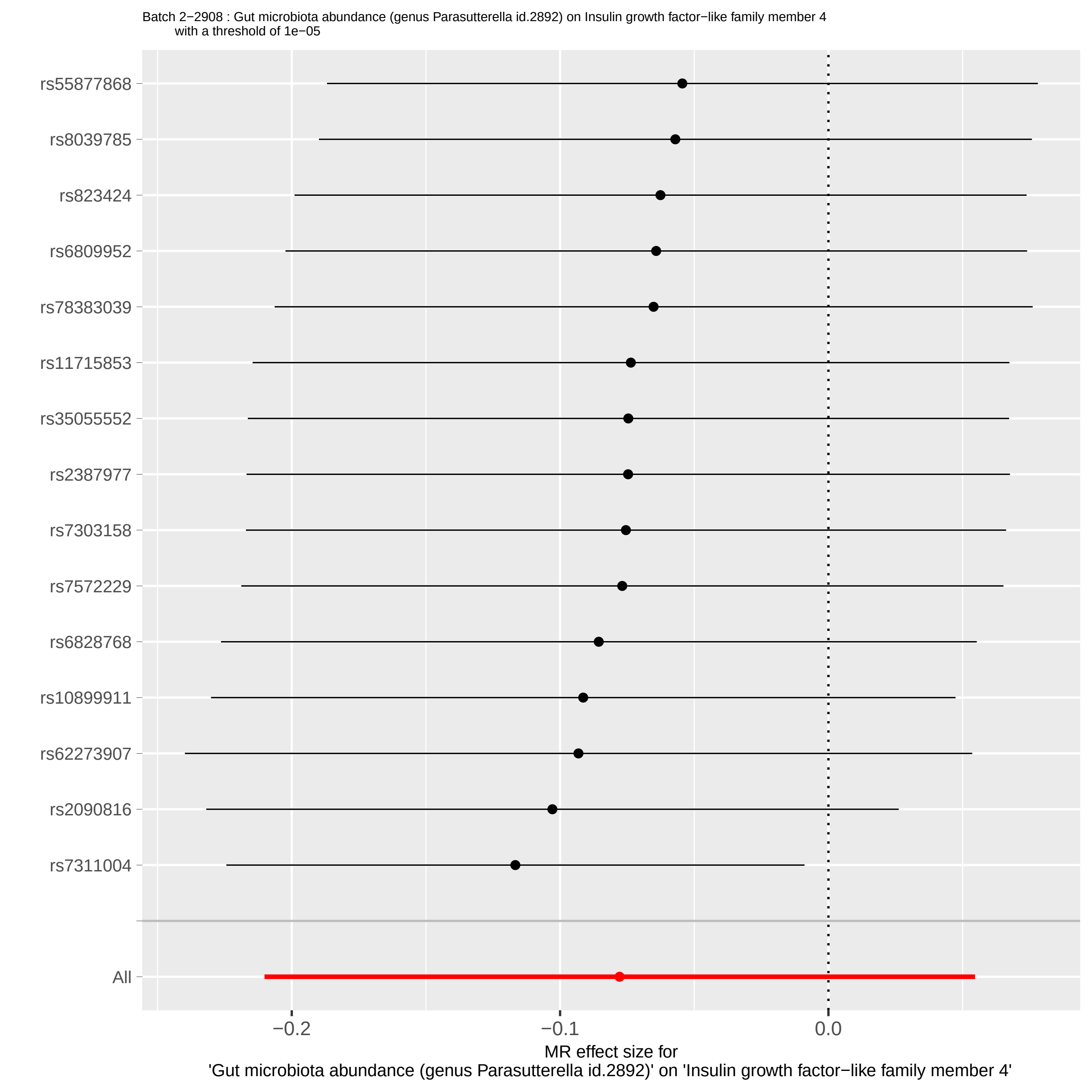

Batch 2−2908 : Gut microbiota abundance (genus Parasutterella id.2892) on Insulin growth factor−like family member 4
with a threshold of 1e−05
rs55877868
rs8039785
rs823424
rs6809952
rs78383039
rs11715853
rs35055552
rs2387977
rs7303158
rs7572229
rs6828768
rs10899911
rs62273907
rs2090816
rs7311004
All
−0.2
−0.1
0.0
MR effect size for
'Gut microbiota abundance (genus Parasutterella id.2892)' on 'Insulin growth factor−like family member 4'

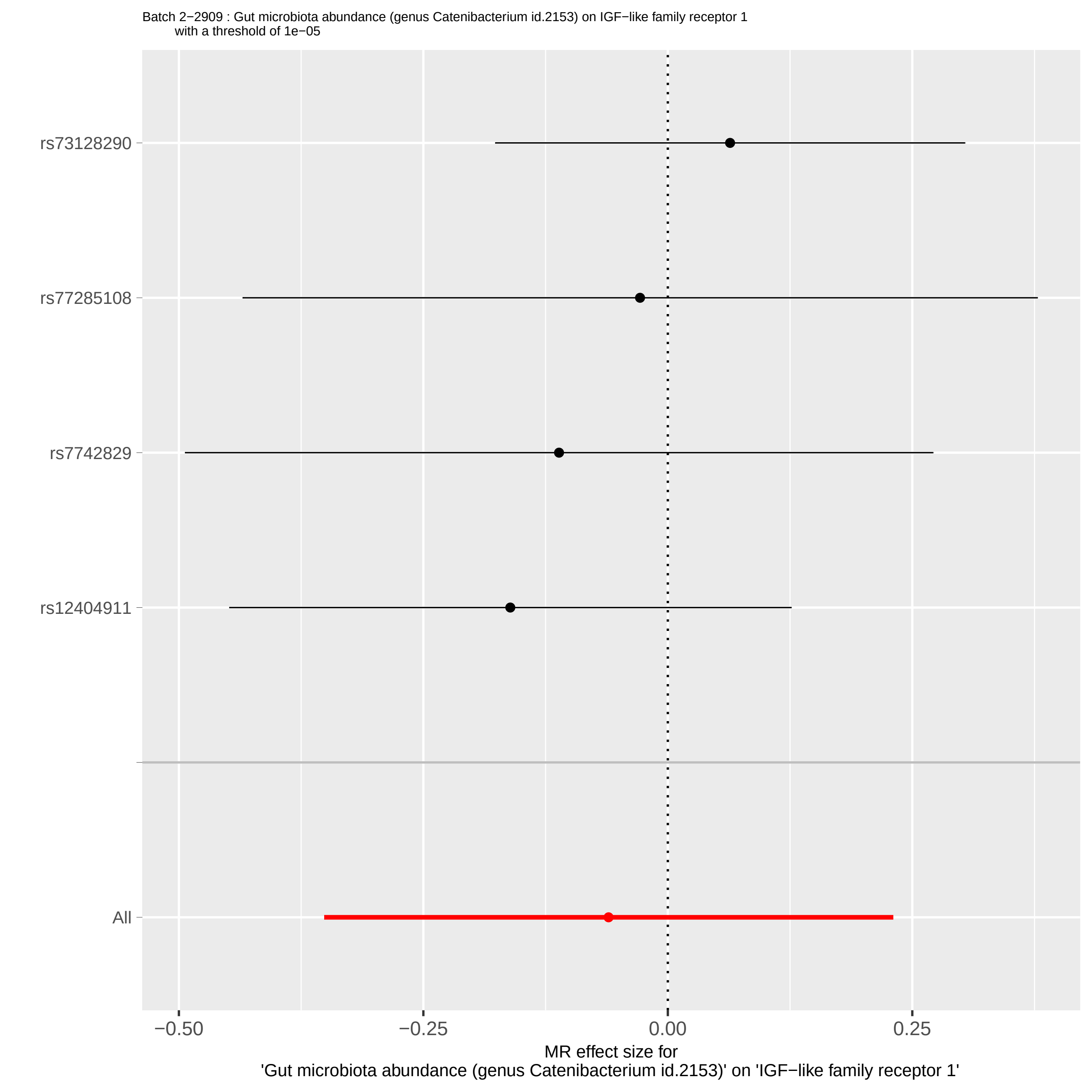
Batch 2−2909 : Gut microbiota abundance (genus Catenibacterium id.2153) on IGF−like family receptor 1
with a threshold of 1e−05
rs73128290
rs77285108
rs7742829
rs12404911
All
−0.50
−0.25
0.00
0.25
MR effect size for
'Gut microbiota abundance (genus Catenibacterium id.2153)' on 'IGF−like family receptor 1'

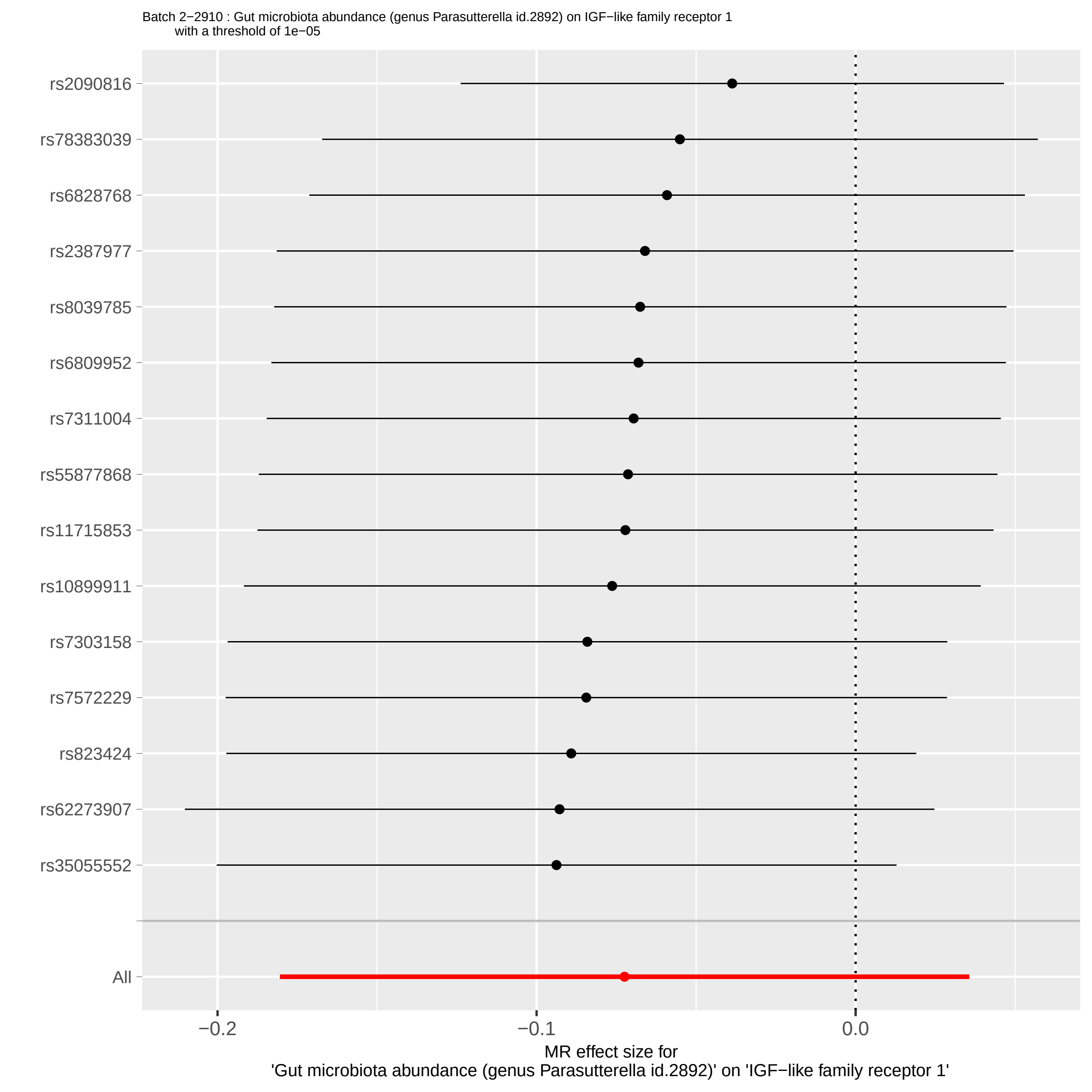

Batch 2−2910 : Gut microbiota abundance (genus Parasutterella id.2892) on IGF−like family receptor 1
with a threshold of 1e−05
rs2090816
rs78383039
rs6828768
rs2387977
rs8039785
rs6809952
rs7311004
rs55877868
rs11715853
rs10899911
rs7303158
rs7572229
rs823424
rs62273907
rs35055552
All
−0.2
−0.1
0.0
MR effect size for
'Gut microbiota abundance (genus Parasutterella id.2892)' on 'IGF−like family receptor 1'

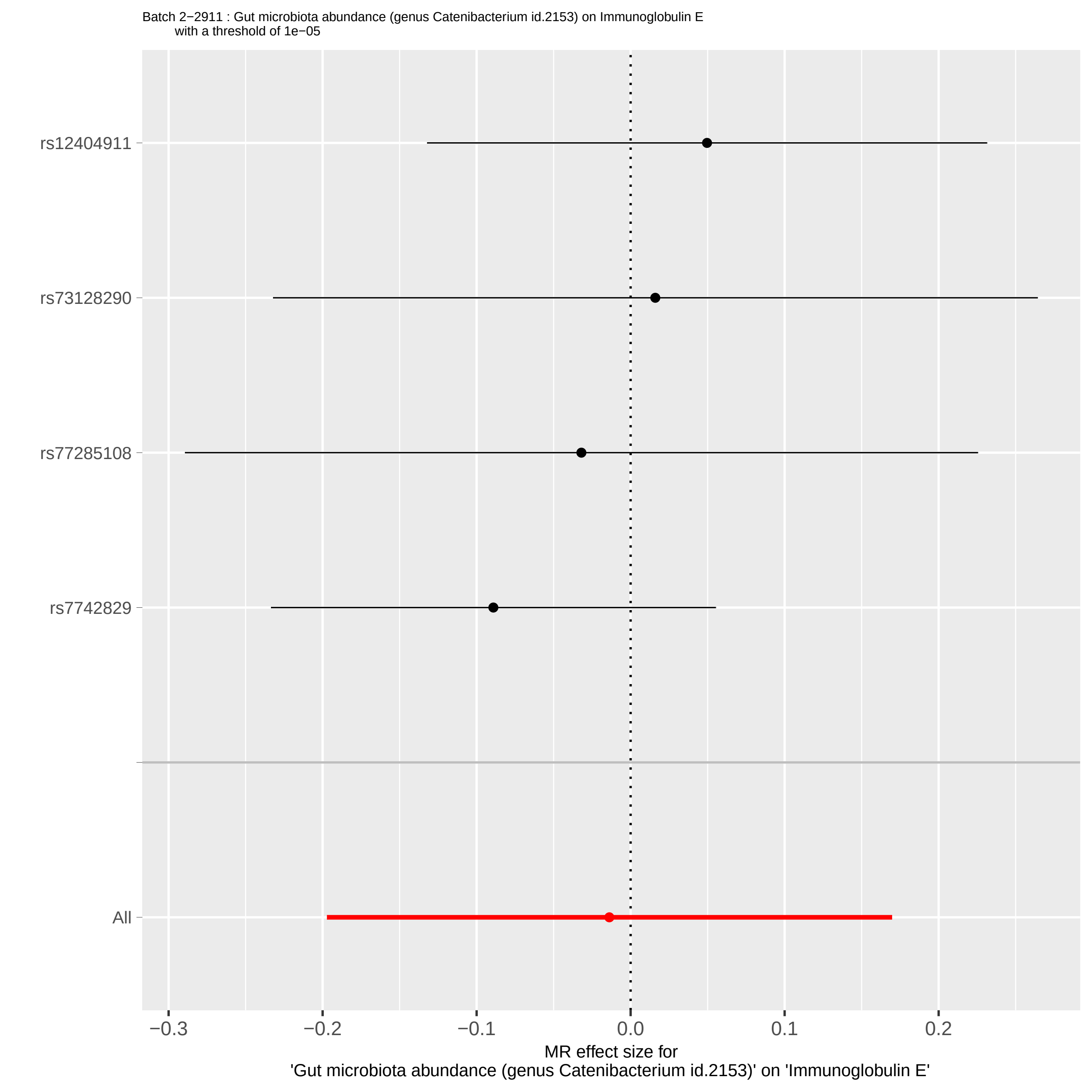
Batch 2−2911 : Gut microbiota abundance (genus Catenibacterium id.2153) on Immunoglobulin E
with a threshold of 1e−05
rs12404911
rs73128290
rs77285108
rs7742829
All
−0.3
−0.2
−0.1
0.0
0.1
0.2
MR effect size for
'Gut microbiota abundance (genus Catenibacterium id.2153)' on 'Immunoglobulin E'

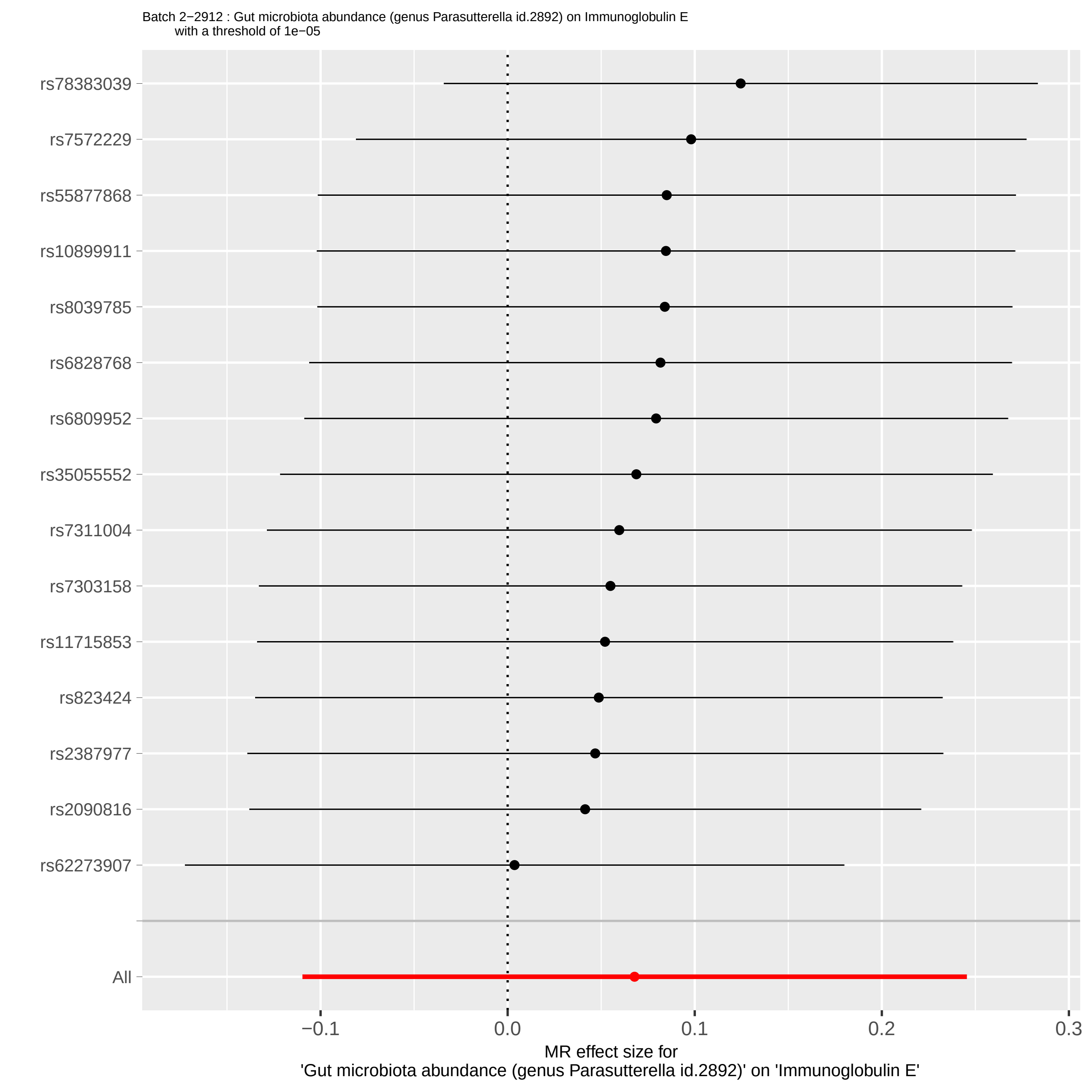

Batch 2−2912 : Gut microbiota abundance (genus Parasutterella id.2892) on Immunoglobulin E
with a threshold of 1e−05
rs78383039
rs7572229
rs55877868
rs10899911
rs8039785
rs6828768
rs6809952
rs35055552
rs7311004
rs7303158
rs11715853
rs823424
rs2387977
rs2090816
rs62273907
All
−0.1
0.0
0.1
0.2
0.3
MR effect size for
'Gut microbiota abundance (genus Parasutterella id.2892)' on 'Immunoglobulin E'

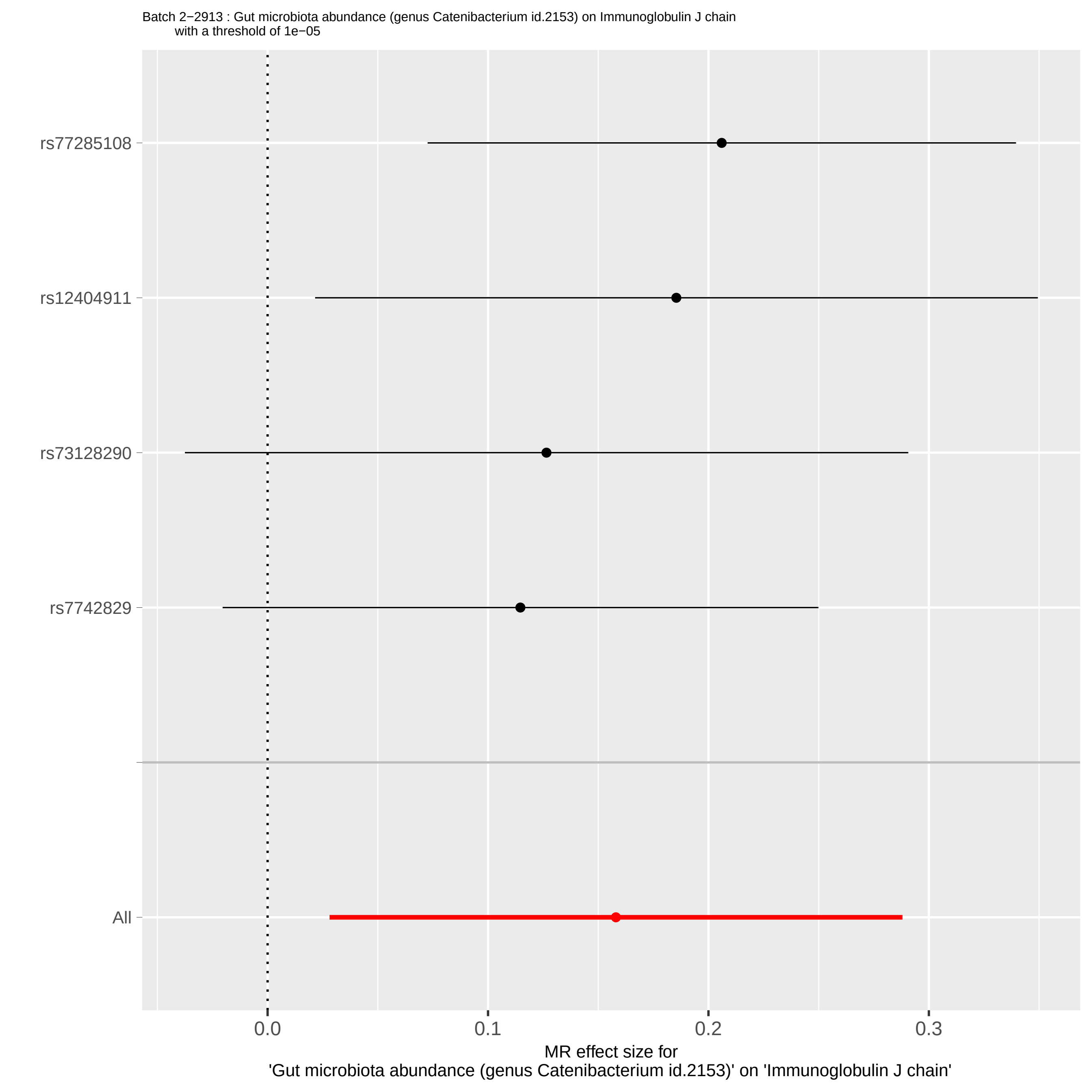
Batch 2−2913 : Gut microbiota abundance (genus Catenibacterium id.2153) on Immunoglobulin J chain
with a threshold of 1e−05
rs77285108
rs12404911
rs73128290
rs7742829
All
0.0
0.1
0.2
0.3
MR effect size for
'Gut microbiota abundance (genus Catenibacterium id.2153)' on 'Immunoglobulin J chain'

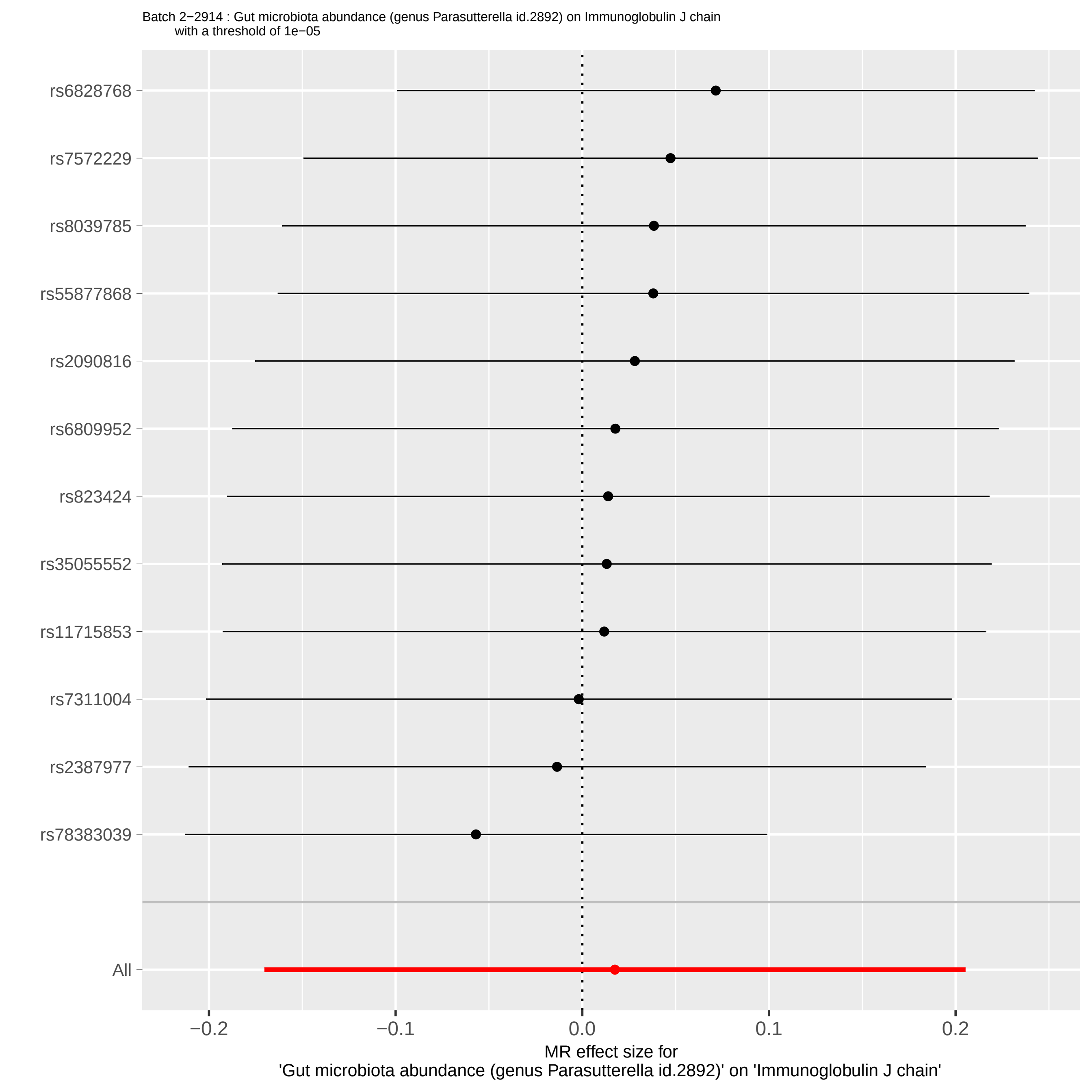
Batch 2−2914 : Gut microbiota abundance (genus Parasutterella id.2892) on Immunoglobulin J chain
with a threshold of 1e−05
rs6828768
rs7572229
rs8039785
rs55877868
rs2090816
rs6809952
rs823424
rs35055552
rs11715853
rs7311004
rs2387977
rs78383039
All
−0.2
−0.1
0.0
0.1
0.2
MR effect size for
'Gut microbiota abundance (genus Parasutterella id.2892)' on 'Immunoglobulin J chain'

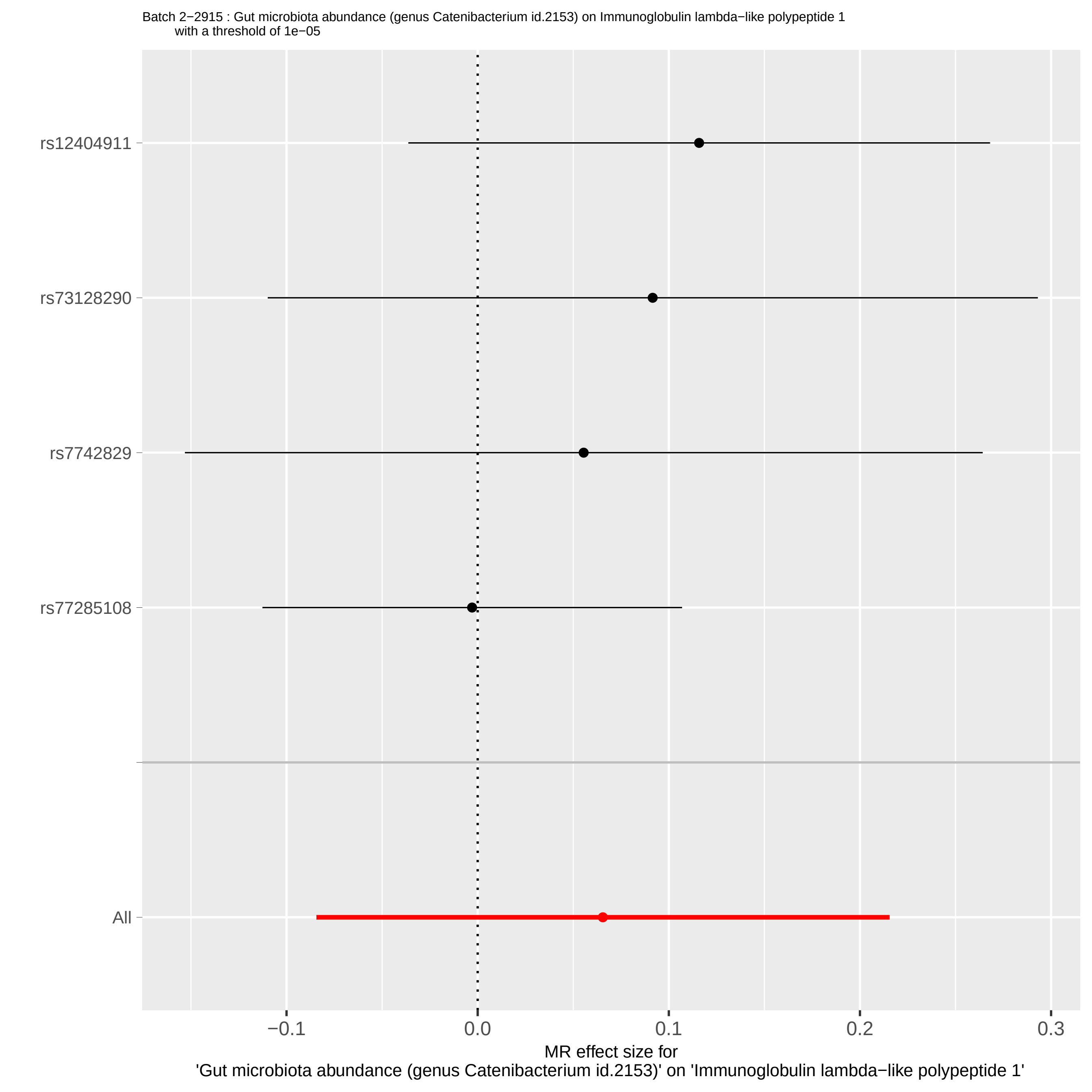
Batch 2−2915 : Gut microbiota abundance (genus Catenibacterium id.2153) on Immunoglobulin lambda−like polypeptide 1
with a threshold of 1e−05
rs12404911
rs73128290
rs7742829
rs77285108
All
−0.1
0.0
0.1
0.2
0.3
MR effect size for
'Gut microbiota abundance (genus Catenibacterium id.2153)' on 'Immunoglobulin lambda−like polypeptide 1'

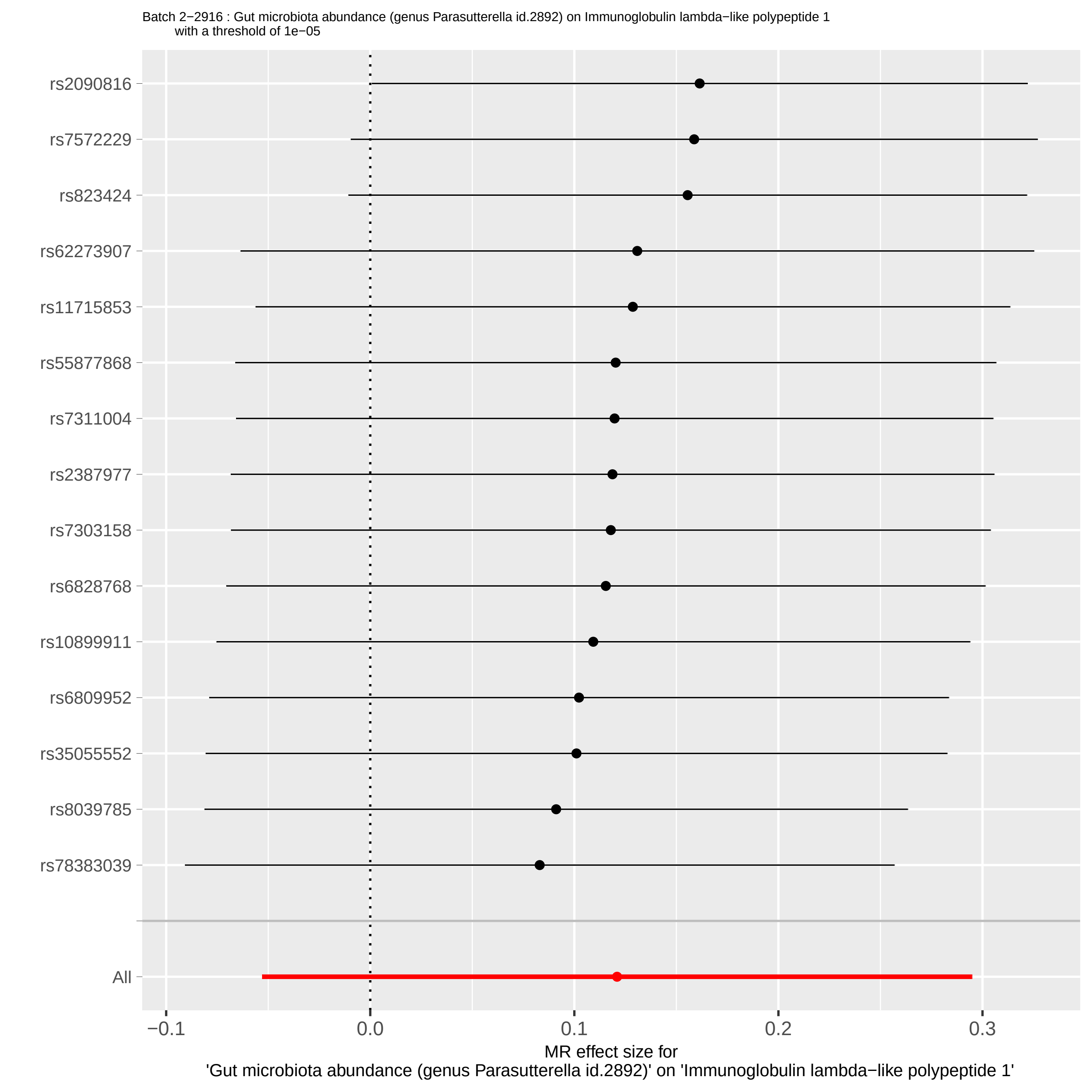
Batch 2−2916 : Gut microbiota abundance (genus Parasutterella id.2892) on Immunoglobulin lambda−like polypeptide 1
with a threshold of 1e−05
rs2090816
rs7572229
rs823424
rs62273907
rs11715853
rs55877868
rs7311004
rs2387977
rs7303158
rs6828768
rs10899911
rs6809952
rs35055552
rs8039785
rs78383039
All
−0.1
0.0
0.1
0.2
0.3
MR effect size for
'Gut microbiota abundance (genus Parasutterella id.2892)' on 'Immunoglobulin lambda−like polypeptide 1'

Batch 2−2917 : Gut microbiota abundance (genus Catenibacterium id.2153) on IgLON family member 5
with a threshold of 1e−05

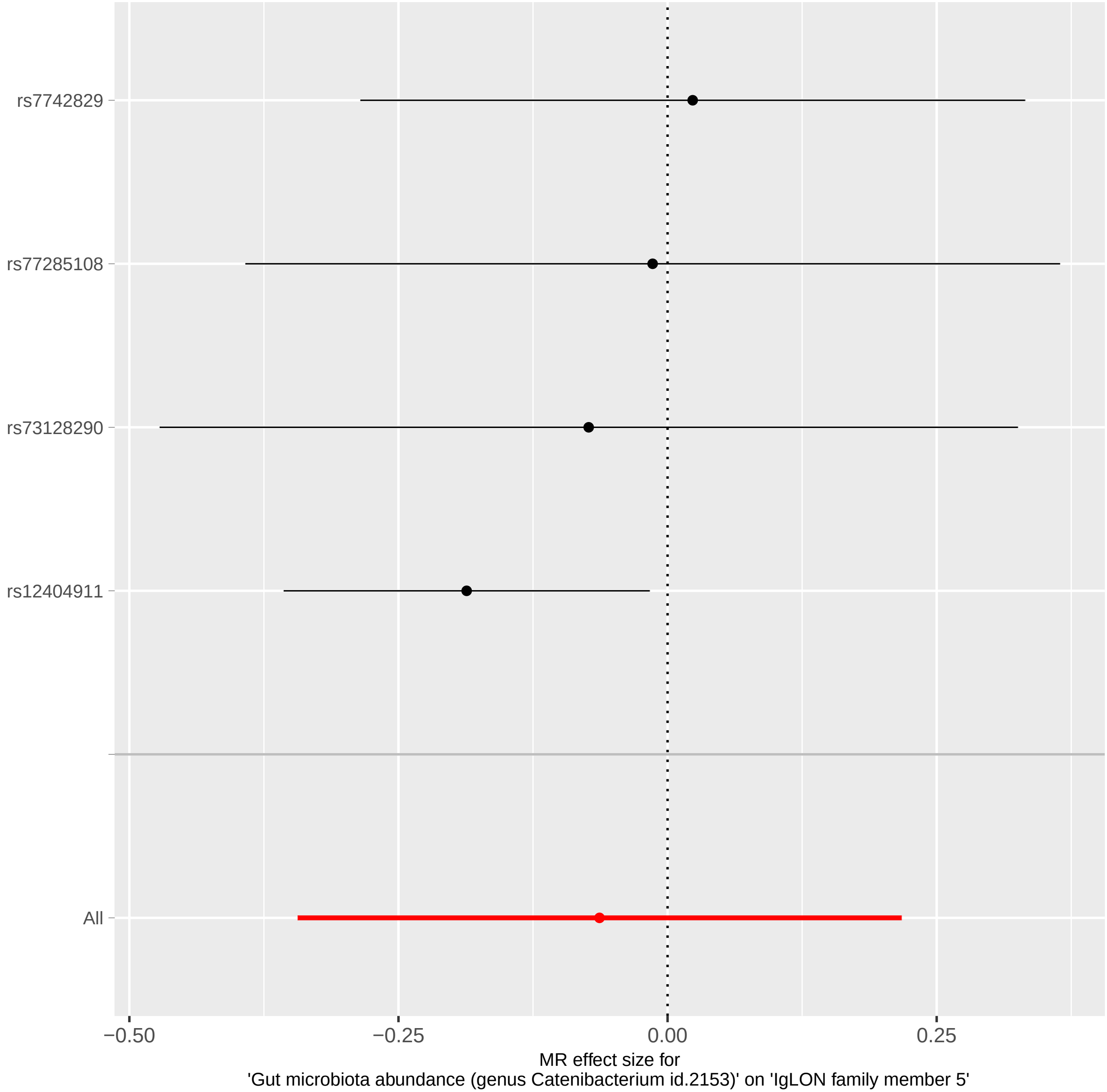

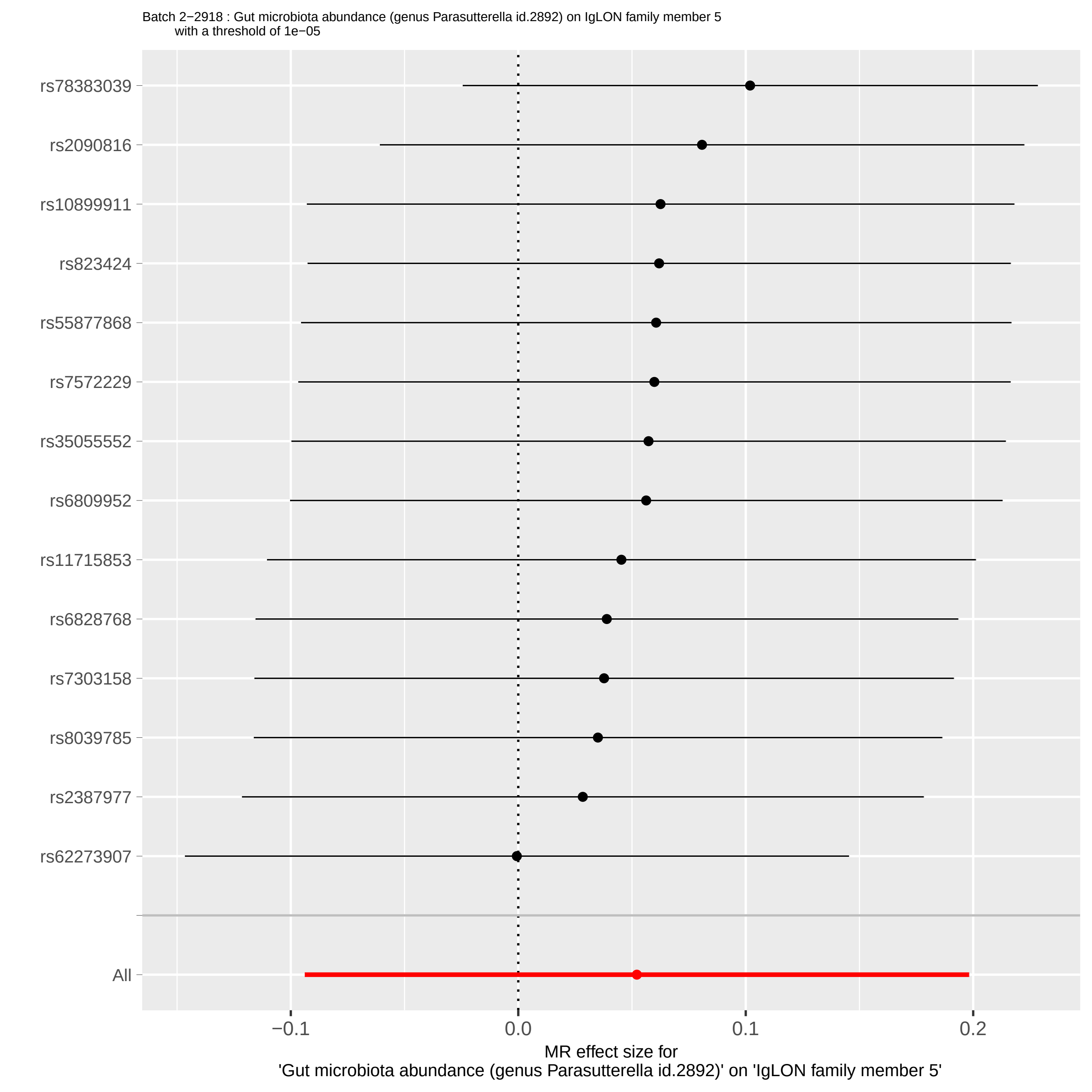

Batch 2−2918 : Gut microbiota abundance (genus Parasutterella id.2892) on IgLON family member 5
with a threshold of 1e−05
rs78383039
rs2090816
rs10899911
rs823424
rs55877868
rs7572229
rs35055552
rs6809952
rs11715853
rs6828768
rs7303158
rs8039785
rs2387977
rs62273907
All
−0.1
0.0
0.1
0.2
MR effect size for
'Gut microbiota abundance (genus Parasutterella id.2892)' on 'IgLON family member 5'

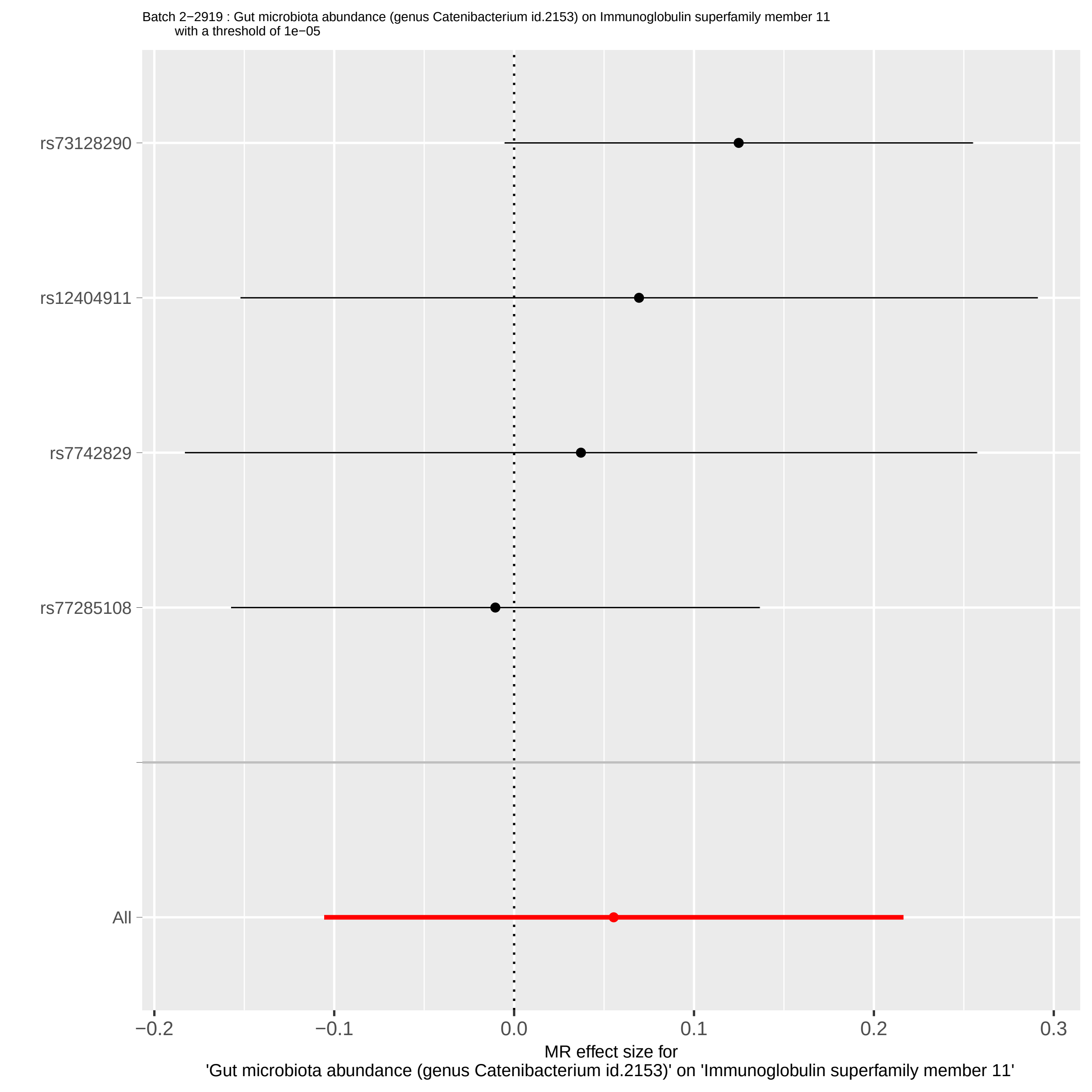
Batch 2−2919 : Gut microbiota abundance (genus Catenibacterium id.2153) on Immunoglobulin superfamily member 11
with a threshold of 1e−05
rs73128290
rs12404911
rs7742829
rs77285108
All
−0.2
−0.1
0.0
0.1
0.2
0.3
MR effect size for
'Gut microbiota abundance (genus Catenibacterium id.2153)' on 'Immunoglobulin superfamily member 11'

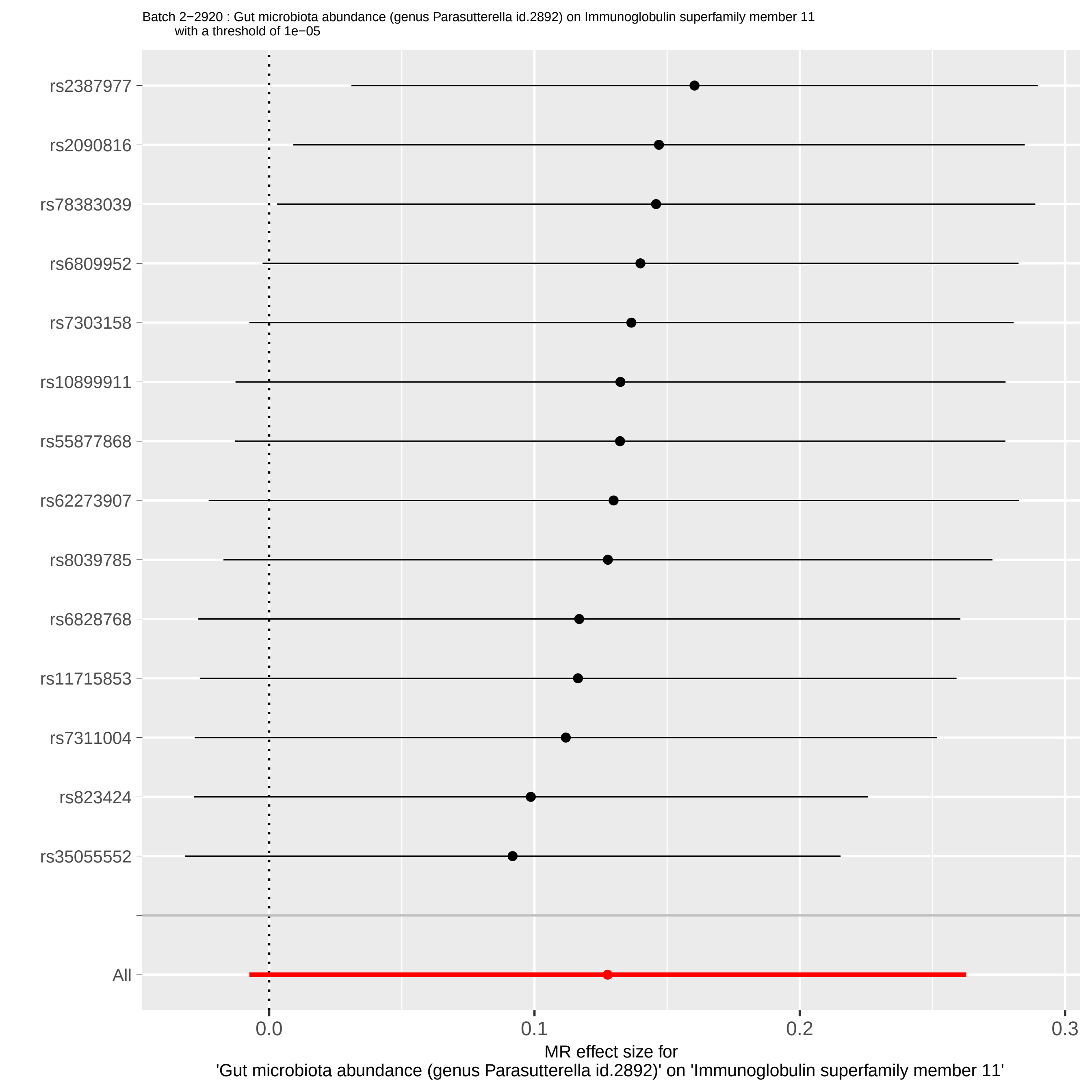
Batch 2−2920 : Gut microbiota abundance (genus Parasutterella id.2892) on Immunoglobulin superfamily member 11
with a threshold of 1e−05
rs2387977
rs2090816
rs78383039
rs6809952
rs7303158
rs10899911
rs55877868
rs62273907
rs8039785
rs6828768
rs11715853
rs7311004
rs823424
rs35055552
All
0.0
0.1
0.2
0.3
MR effect size for
'Gut microbiota abundance (genus Parasutterella id.2892)' on 'Immunoglobulin superfamily member 11'

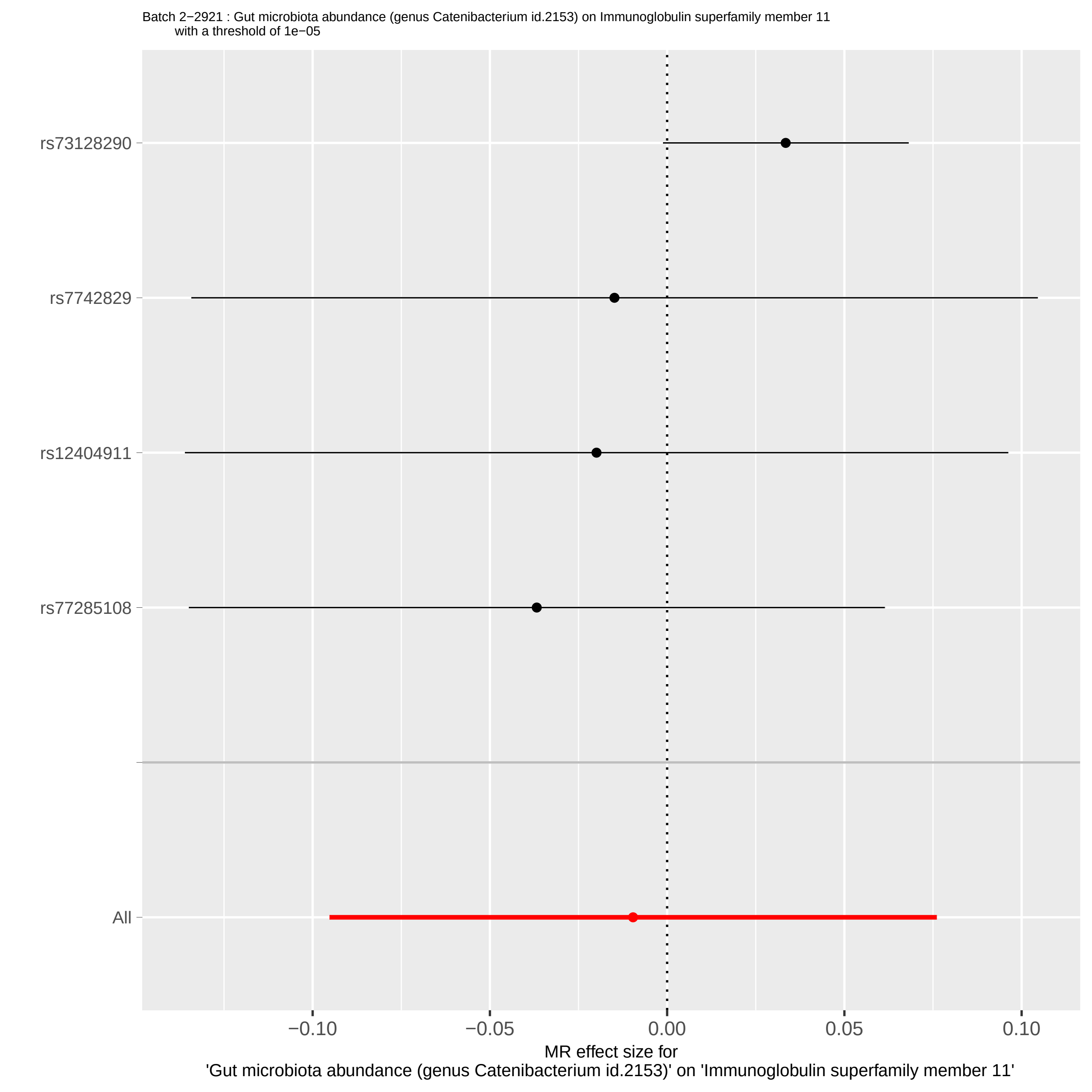

Batch 2−2921 : Gut microbiota abundance (genus Catenibacterium id.2153) on Immunoglobulin superfamily member 11
with a threshold of 1e−05
rs73128290
rs7742829
rs12404911
rs77285108
All
−0.10
−0.05
0.00
0.05
0.10
MR effect size for
'Gut microbiota abundance (genus Catenibacterium id.2153)' on 'Immunoglobulin superfamily member 11'

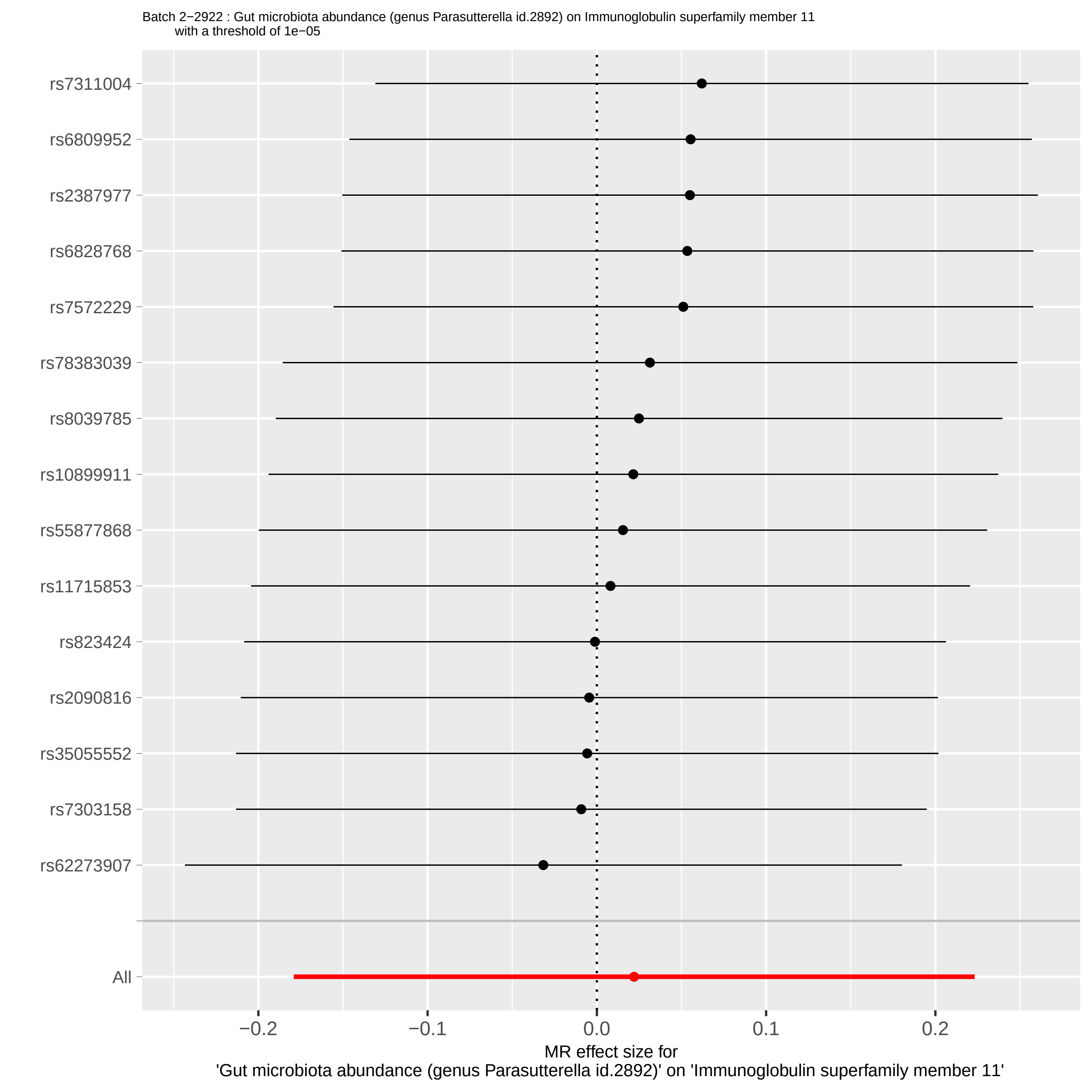
Batch 2−2922 : Gut microbiota abundance (genus Parasutterella id.2892) on Immunoglobulin superfamily member 11
with a threshold of 1e−05
rs7311004
rs6809952
rs2387977
rs6828768
rs7572229
rs78383039
rs8039785
rs10899911
rs55877868
rs11715853
rs823424
rs2090816
rs35055552
rs7303158
rs62273907
All
−0.2
−0.1
0.0
0.1
0.2
MR effect size for
'Gut microbiota abundance (genus Parasutterella id.2892)' on 'Immunoglobulin superfamily member 11'

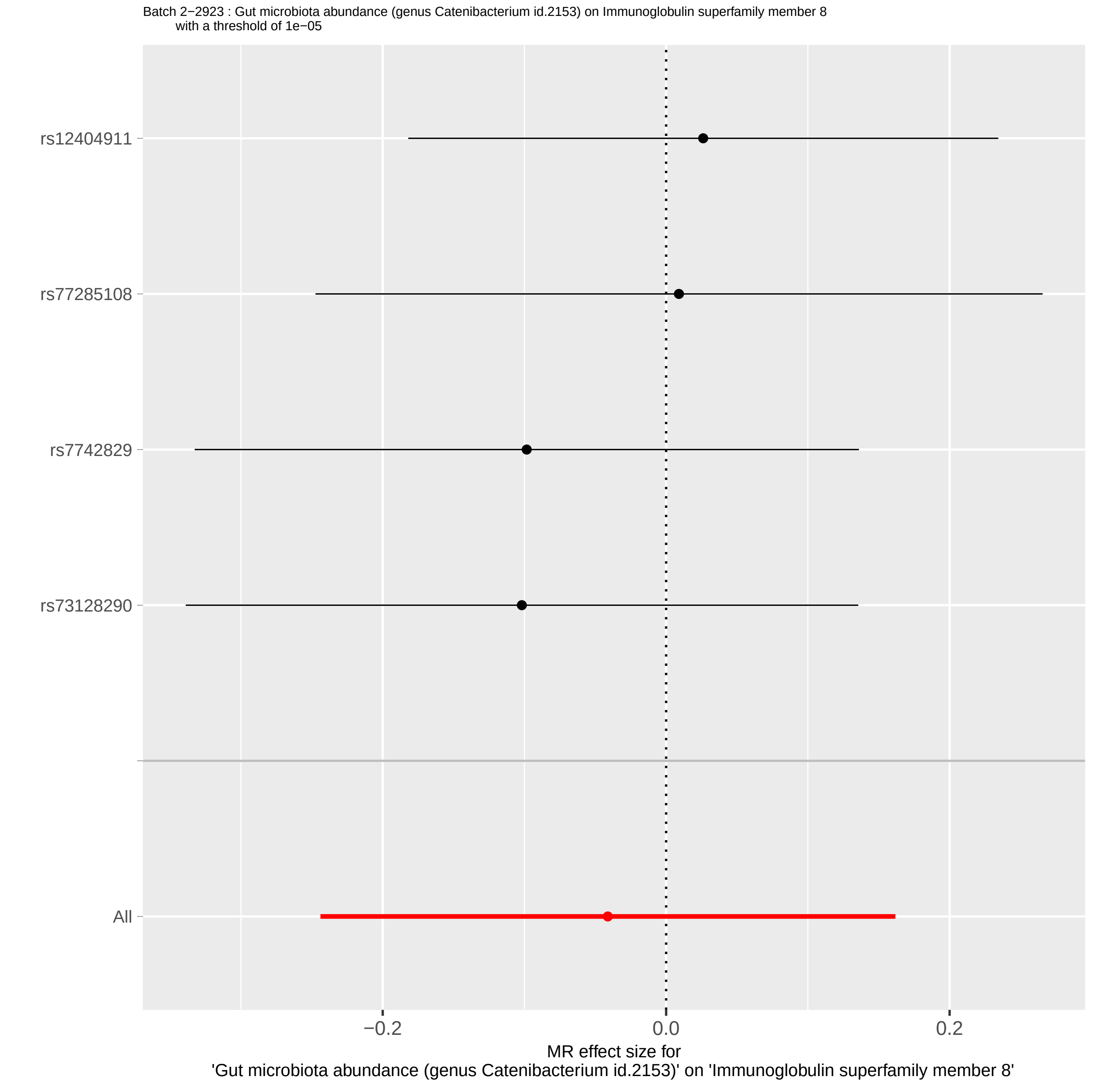
Batch 2−2923 : Gut microbiota abundance (genus Catenibacterium id.2153) on Immunoglobulin superfamily member 8
with a threshold of 1e−05
rs12404911
rs77285108
rs7742829
rs73128290
All
−0.2
0.0
0.2
MR effect size for
'Gut microbiota abundance (genus Catenibacterium id.2153)' on 'Immunoglobulin superfamily member 8'

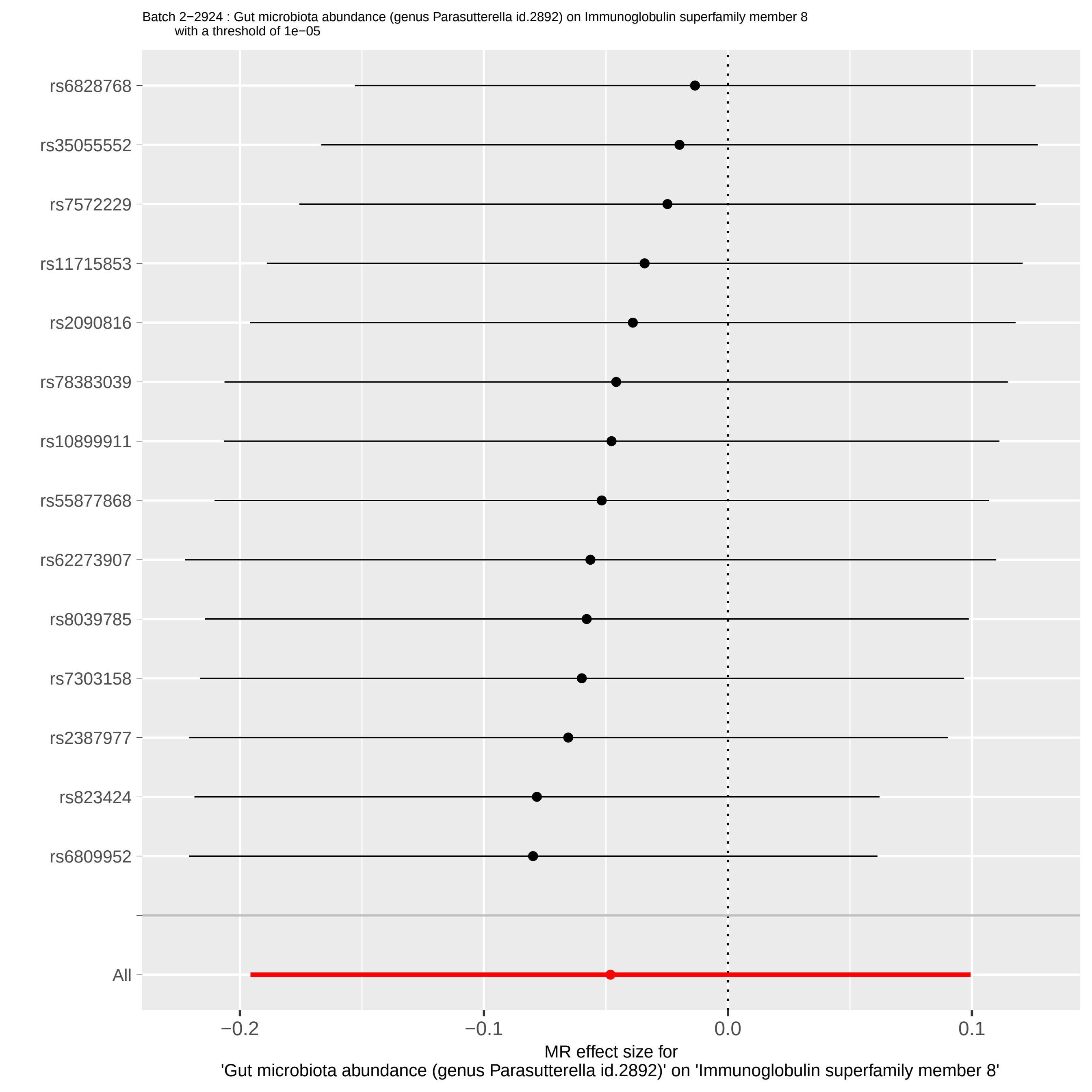
Batch 2−2924 : Gut microbiota abundance (genus Parasutterella id.2892) on Immunoglobulin superfamily member 8
with a threshold of 1e−05
rs6828768
rs35055552
rs7572229
rs11715853
rs2090816
rs78383039
rs10899911
rs55877868
rs62273907
rs8039785
rs7303158
rs2387977
rs823424
rs6809952
All
−0.2
−0.1
0.0
0.1
MR effect size for
'Gut microbiota abundance (genus Parasutterella id.2892)' on 'Immunoglobulin superfamily member 8'

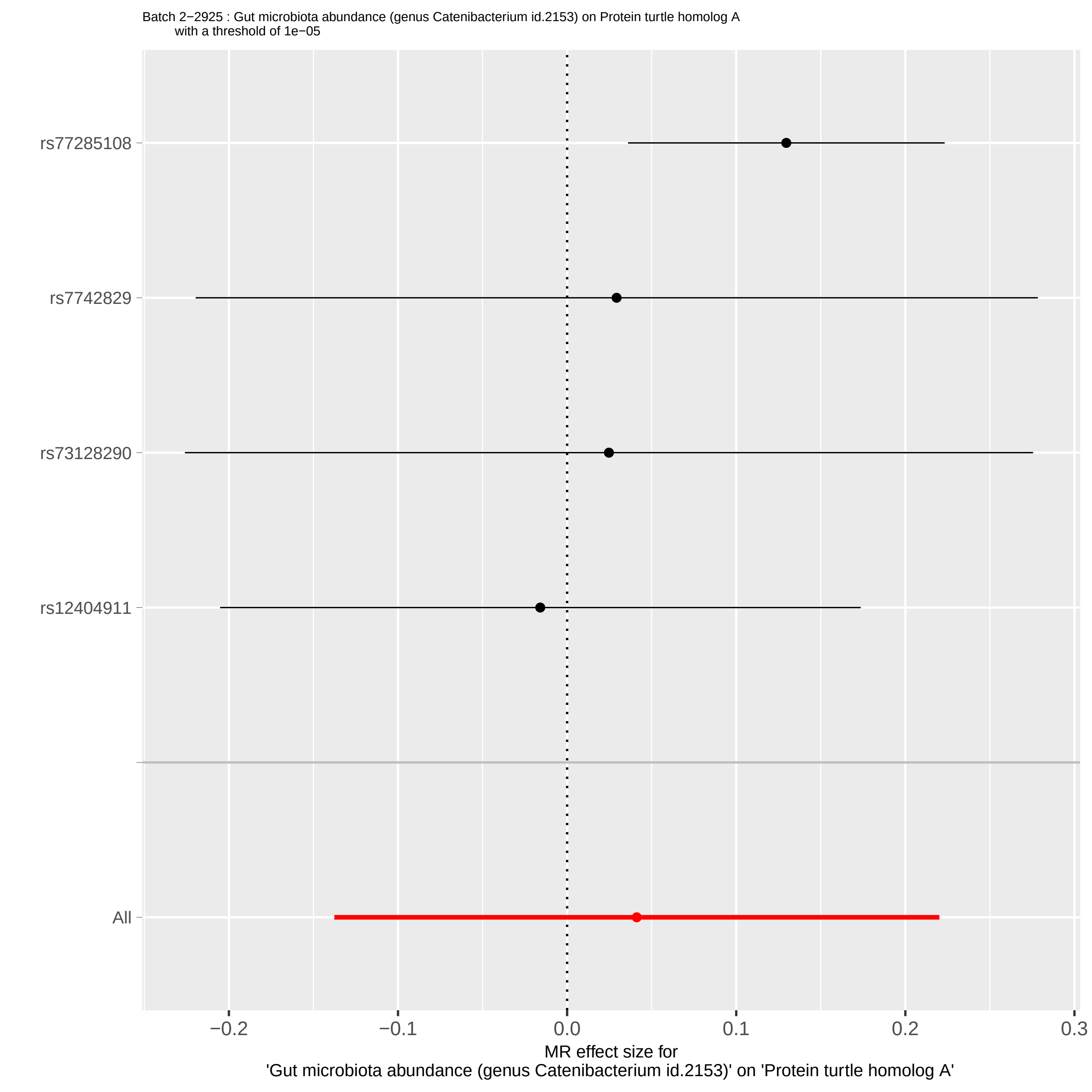
Batch 2−2925 : Gut microbiota abundance (genus Catenibacterium id.2153) on Protein turtle homolog A
with a threshold of 1e−05
rs77285108
rs7742829
rs73128290
rs12404911
All
−0.2
−0.1
0.0
0.1
0.2
0.3
MR effect size for
'Gut microbiota abundance (genus Catenibacterium id.2153)' on 'Protein turtle homolog A'

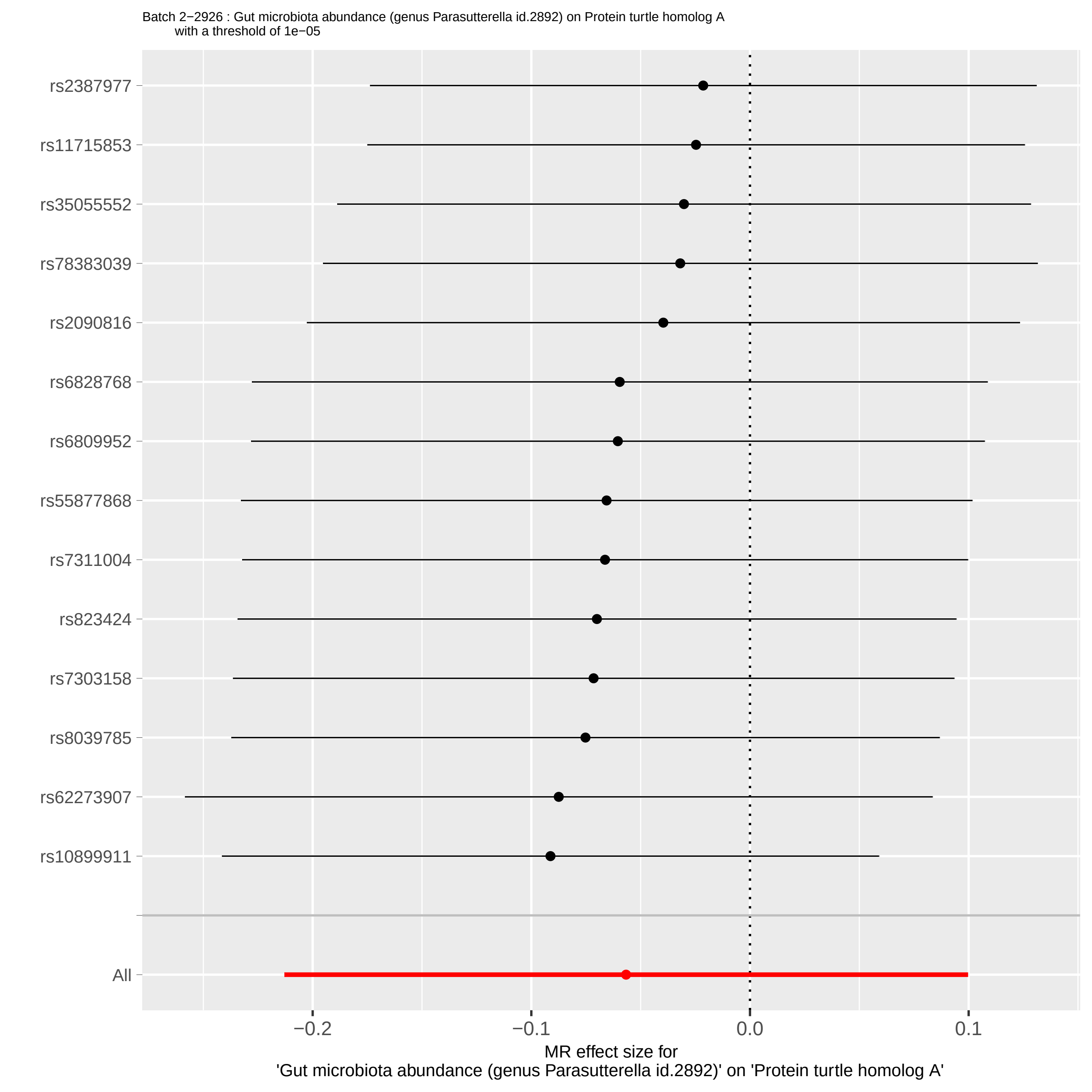

Batch 2−2926 : Gut microbiota abundance (genus Parasutterella id.2892) on Protein turtle homolog A
with a threshold of 1e−05
rs2387977
rs11715853
rs35055552
rs78383039
rs2090816
rs6828768
rs6809952
rs55877868
rs7311004
rs823424
rs7303158
rs8039785
rs62273907
rs10899911
All
−0.2
−0.1
0.0
0.1
MR effect size for
'Gut microbiota abundance (genus Parasutterella id.2892)' on 'Protein turtle homolog A'

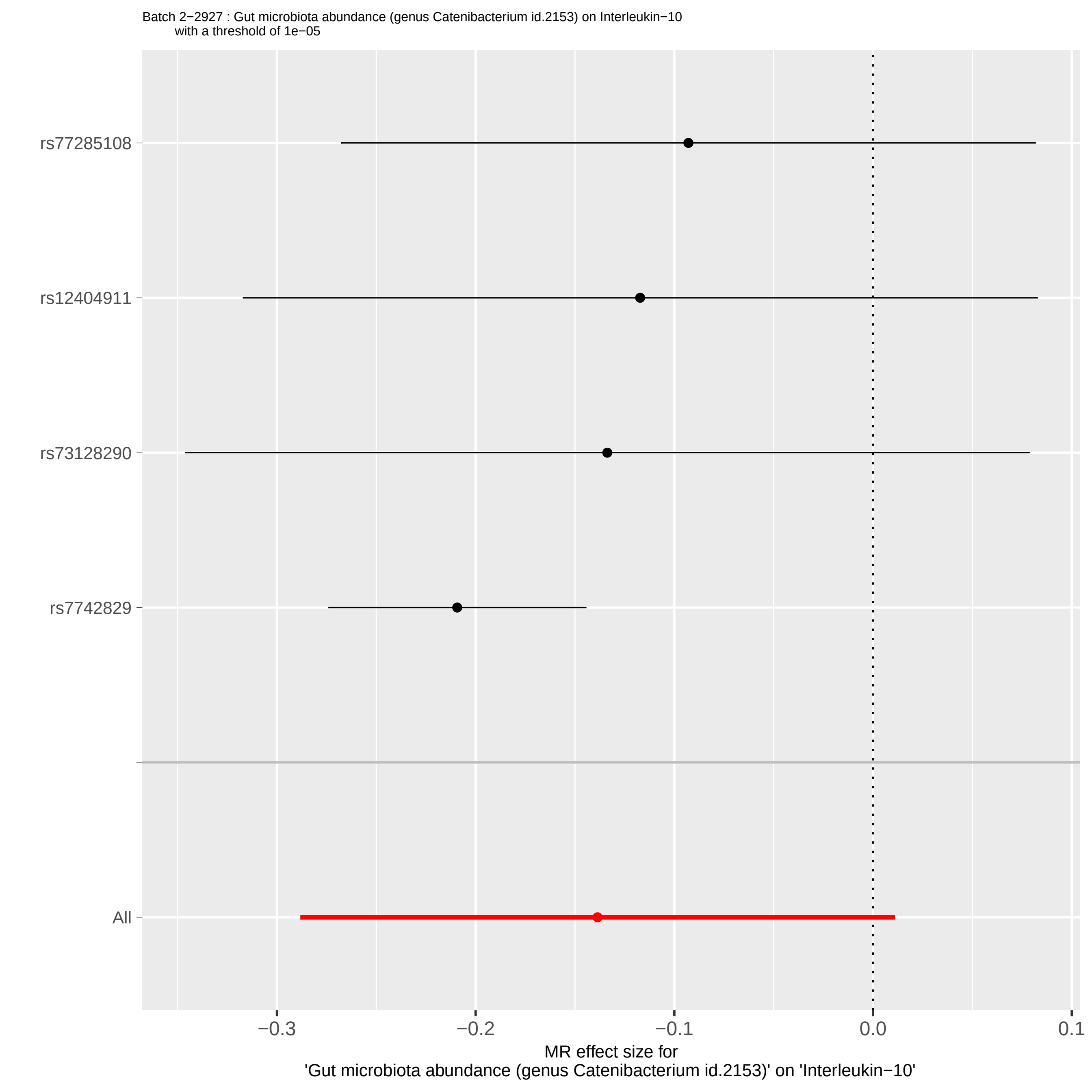

Batch 2−2927 : Gut microbiota abundance (genus Catenibacterium id.2153) on Interleukin−10
with a threshold of 1e−05
rs77285108
rs12404911
rs73128290
rs7742829
All
−0.3
−0.2
−0.1
0.0
0.1
MR effect size for
'Gut microbiota abundance (genus Catenibacterium id.2153)' on 'Interleukin−10'

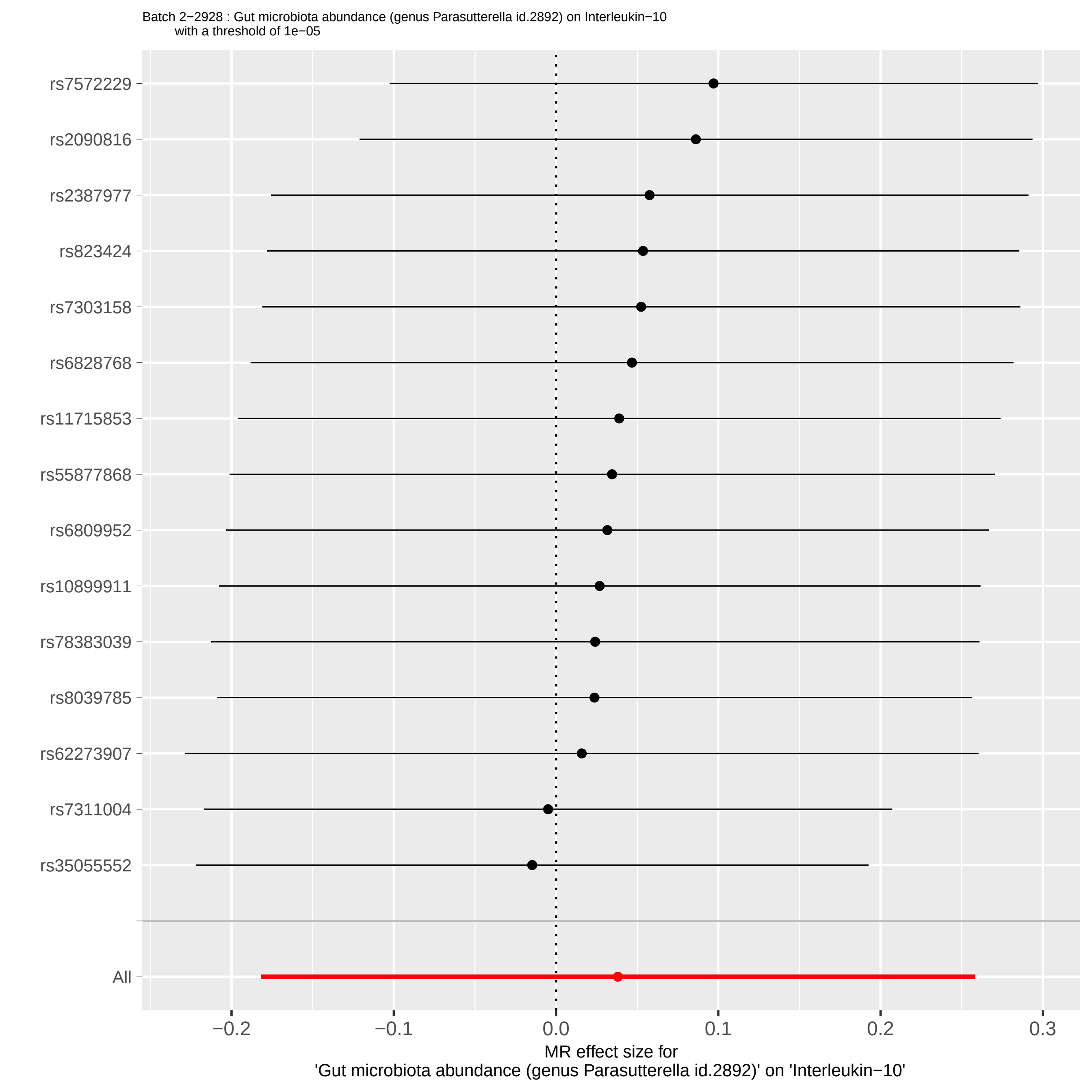
Batch 2−2928 : Gut microbiota abundance (genus Parasutterella id.2892) on Interleukin−10
with a threshold of 1e−05
rs7572229
rs2090816
rs2387977
rs823424
rs7303158
rs6828768
rs11715853
rs55877868
rs6809952
rs10899911
rs78383039
rs8039785
rs62273907
rs7311004
rs35055552
All
−0.2
−0.1
0.0
0.1
0.2
0.3
MR effect size for
'Gut microbiota abundance (genus Parasutterella id.2892)' on 'Interleukin−10'

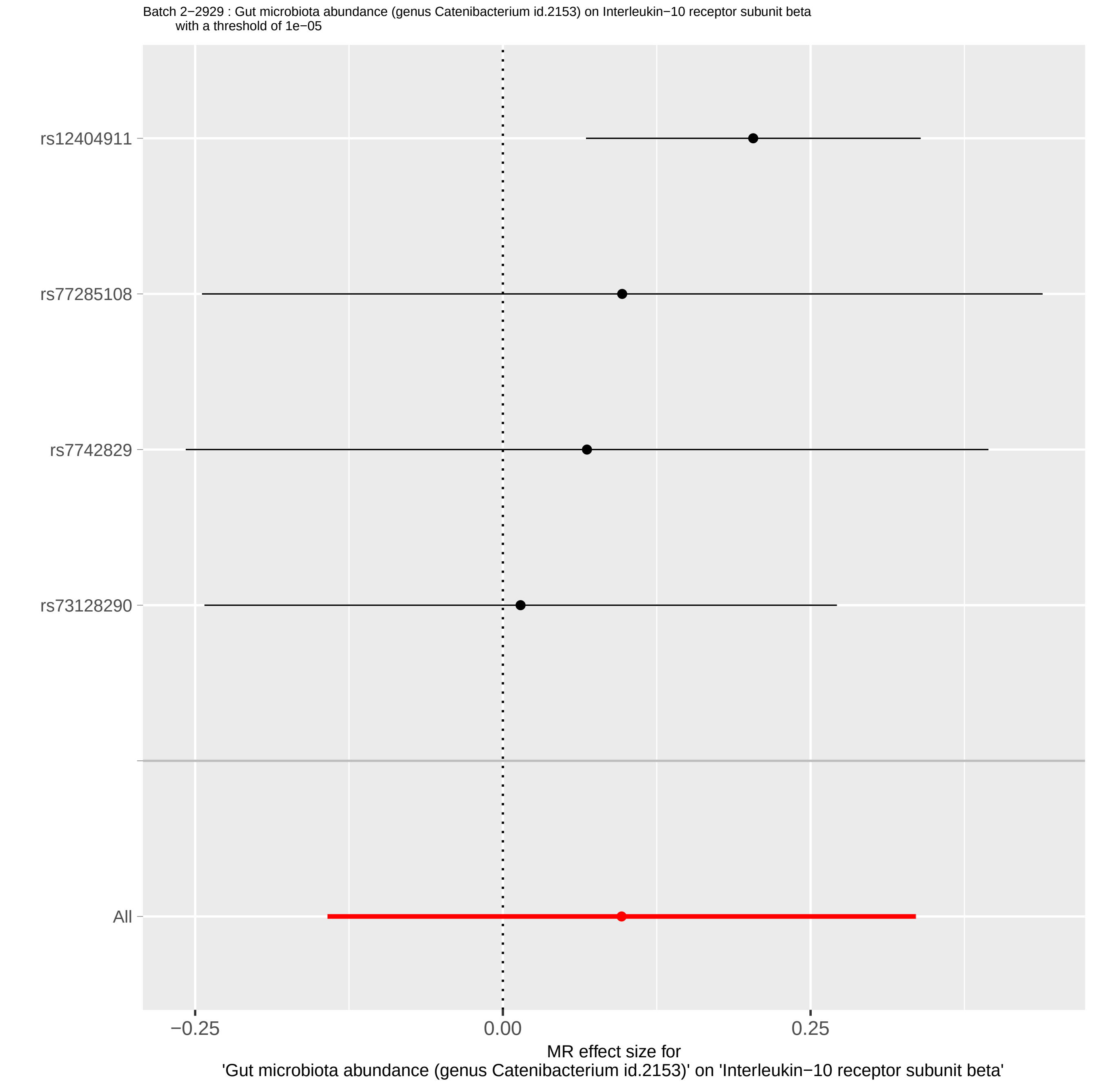
Batch 2−2929 : Gut microbiota abundance (genus Catenibacterium id.2153) on Interleukin−10 receptor subunit beta
with a threshold of 1e−05
rs12404911
rs77285108
rs7742829
rs73128290
All
−0.25
0.00
0.25
MR effect size for
'Gut microbiota abundance (genus Catenibacterium id.2153)' on 'Interleukin−10 receptor subunit beta'

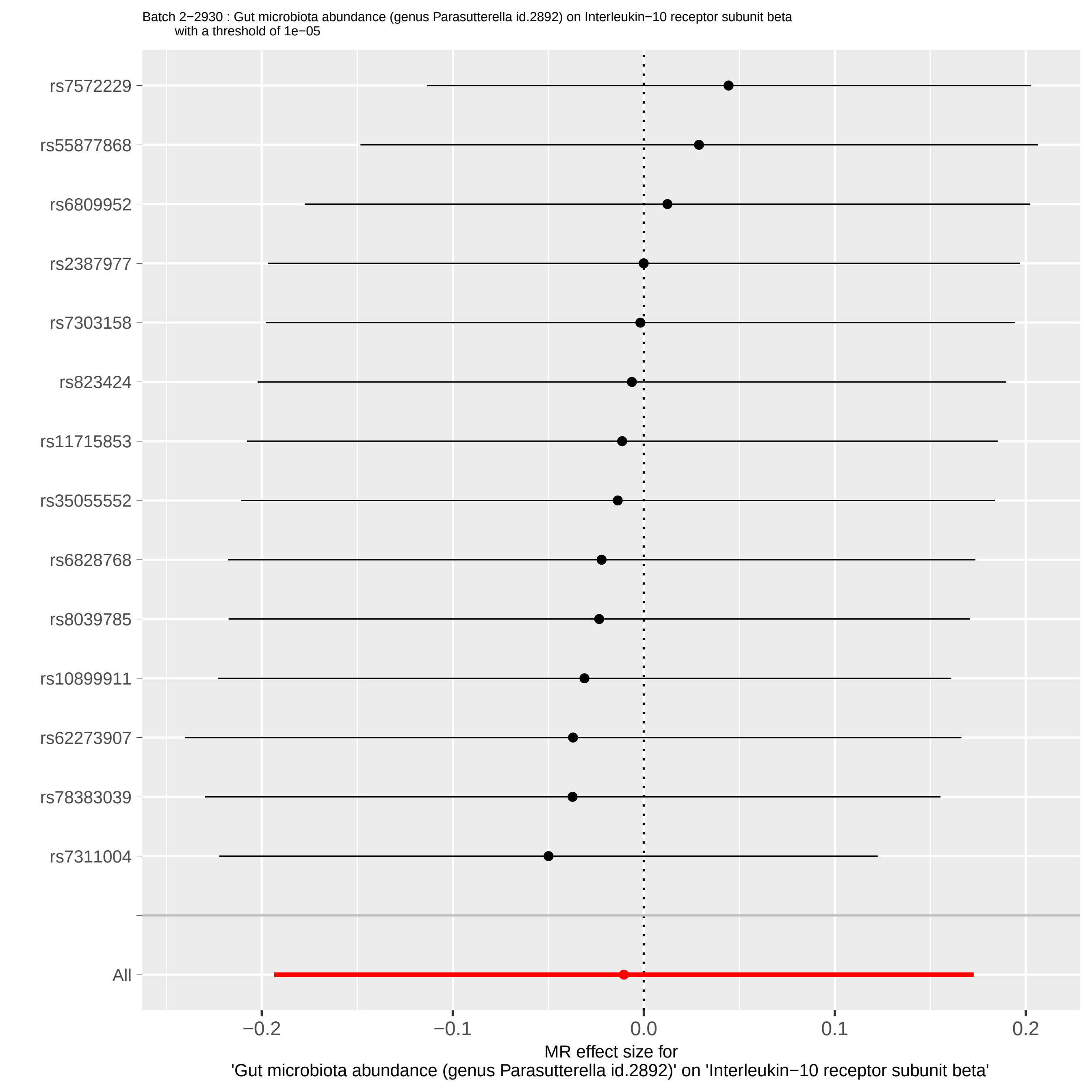
Batch 2−2930 : Gut microbiota abundance (genus Parasutterella id.2892) on Interleukin−10 receptor subunit beta
with a threshold of 1e−05
rs7572229
rs55877868
rs6809952
rs2387977
rs7303158
rs823424
rs11715853
rs35055552
rs6828768
rs8039785
rs10899911
rs62273907
rs78383039
rs7311004
All
−0.2
−0.1
0.0
0.1
0.2
MR effect size for
'Gut microbiota abundance (genus Parasutterella id.2892)' on 'Interleukin−10 receptor subunit beta'

Batch 2−2931 : Gut microbiota abundance (genus Catenibacterium id.2153) on Interleukin−11
with a threshold of 1e−05

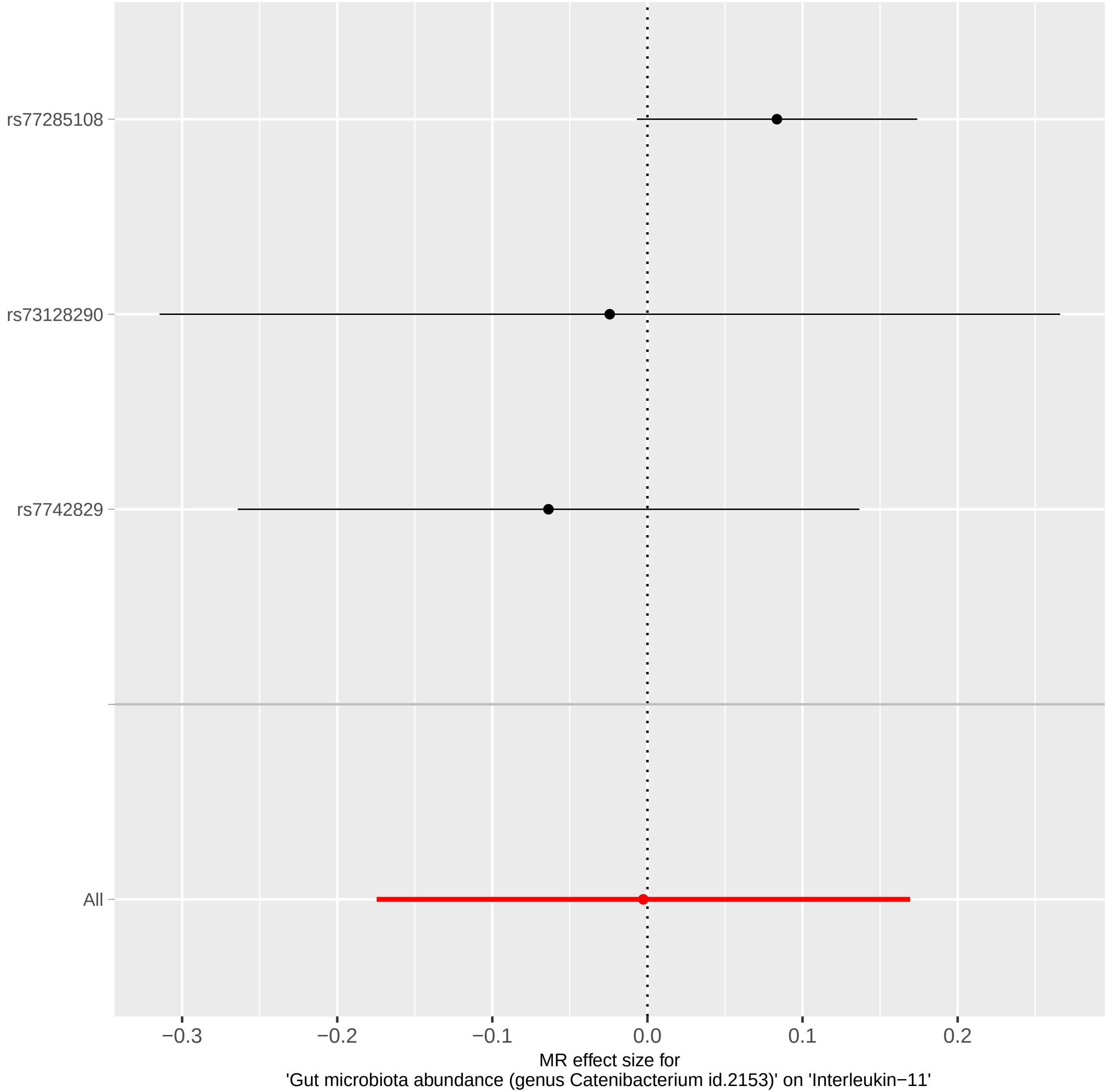

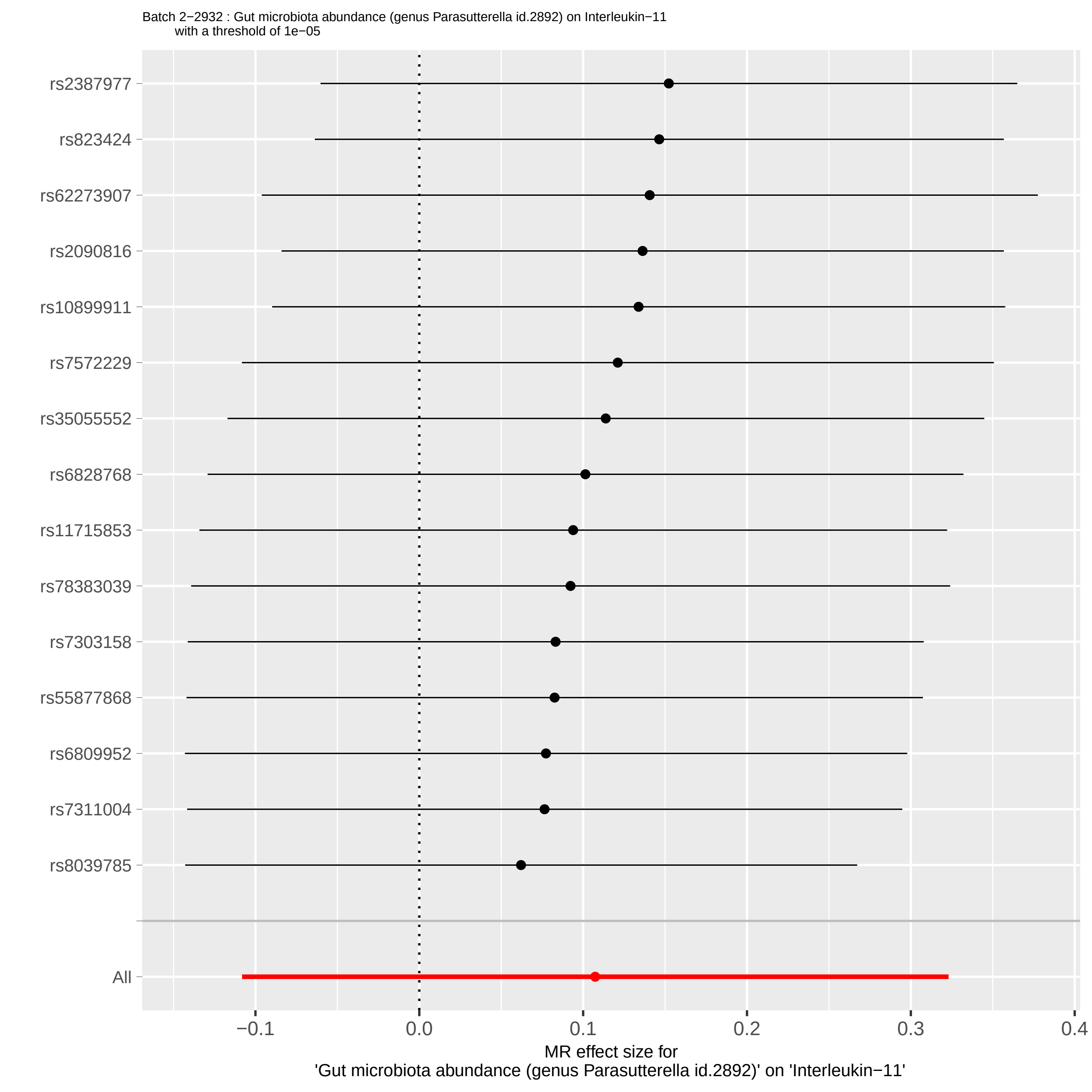
Batch 2−2932 : Gut microbiota abundance (genus Parasutterella id.2892) on Interleukin−11
with a threshold of 1e−05
rs2387977
rs823424
rs62273907
rs2090816
rs10899911
rs7572229
rs35055552
rs6828768
rs11715853
rs78383039
rs7303158
rs55877868
rs6809952
rs7311004
rs8039785
All
−0.1
0.0
0.1
0.2
0.3
0.4
MR effect size for
'Gut microbiota abundance (genus Parasutterella id.2892)' on 'Interleukin−11'

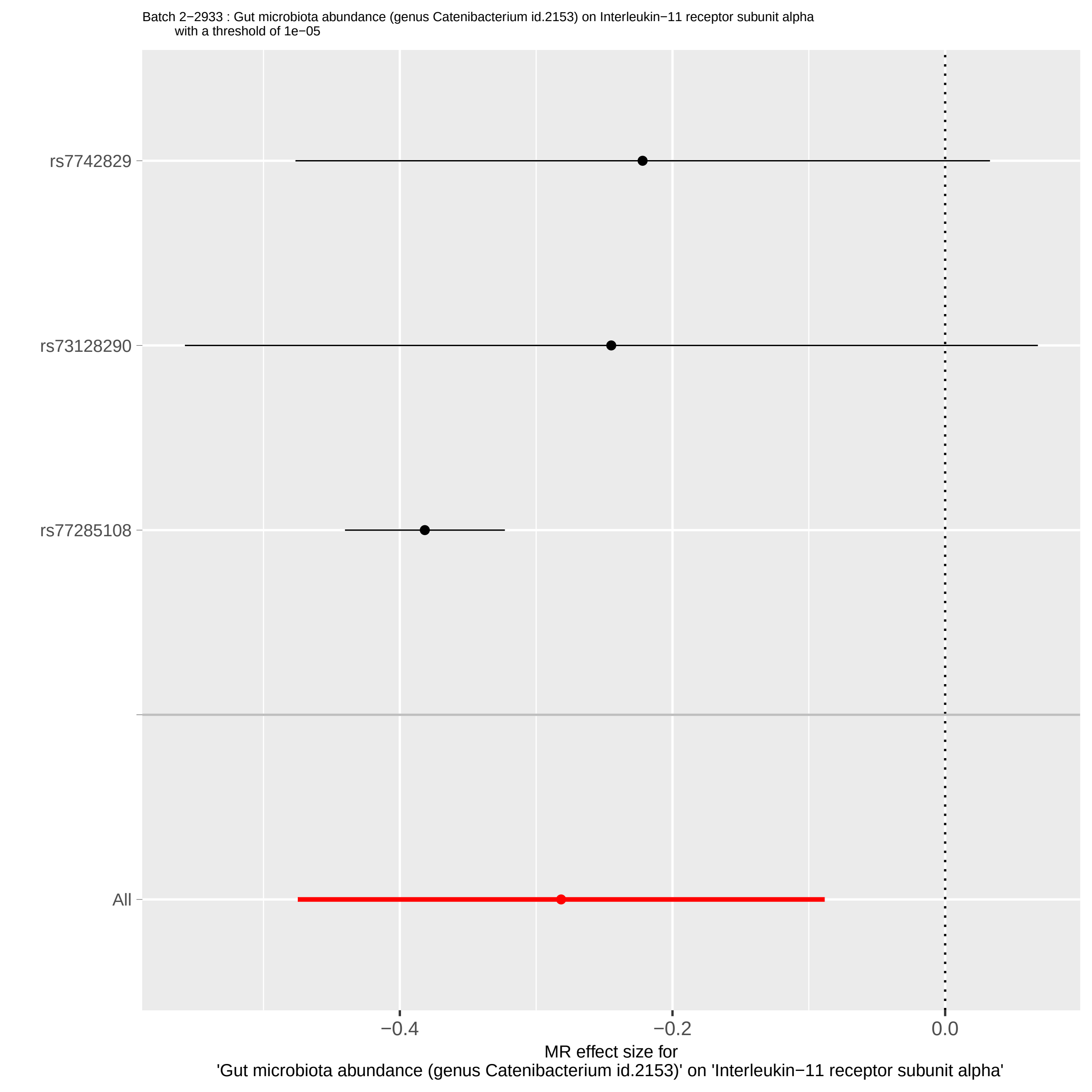
Batch 2−2933 : Gut microbiota abundance (genus Catenibacterium id.2153) on Interleukin−11 receptor subunit alpha
with a threshold of 1e−05
rs7742829
rs73128290
rs77285108
All
−0.4
−0.2
0.0
MR effect size for
'Gut microbiota abundance (genus Catenibacterium id.2153)' on 'Interleukin−11 receptor subunit alpha'

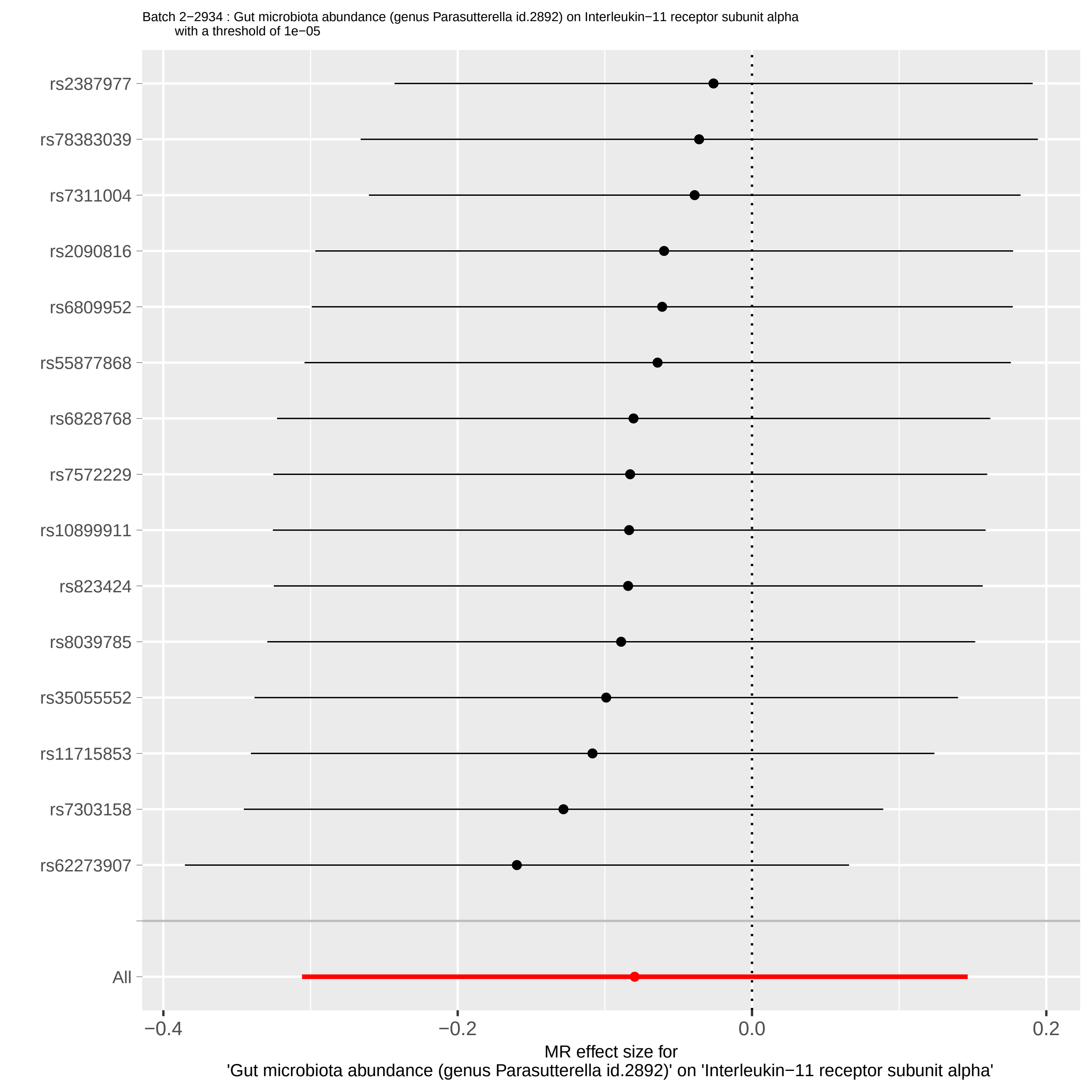

Batch 2−2934 : Gut microbiota abundance (genus Parasutterella id.2892) on Interleukin−11 receptor subunit alpha
with a threshold of 1e−05
rs2387977
rs78383039
rs7311004
rs2090816
rs6809952
rs55877868
rs6828768
rs7572229
rs10899911
rs823424
rs8039785
rs35055552
rs11715853
rs7303158
rs62273907
All
−0.4
−0.2
0.0
0.2
MR effect size for
'Gut microbiota abundance (genus Parasutterella id.2892)' on 'Interleukin−11 receptor subunit alpha'

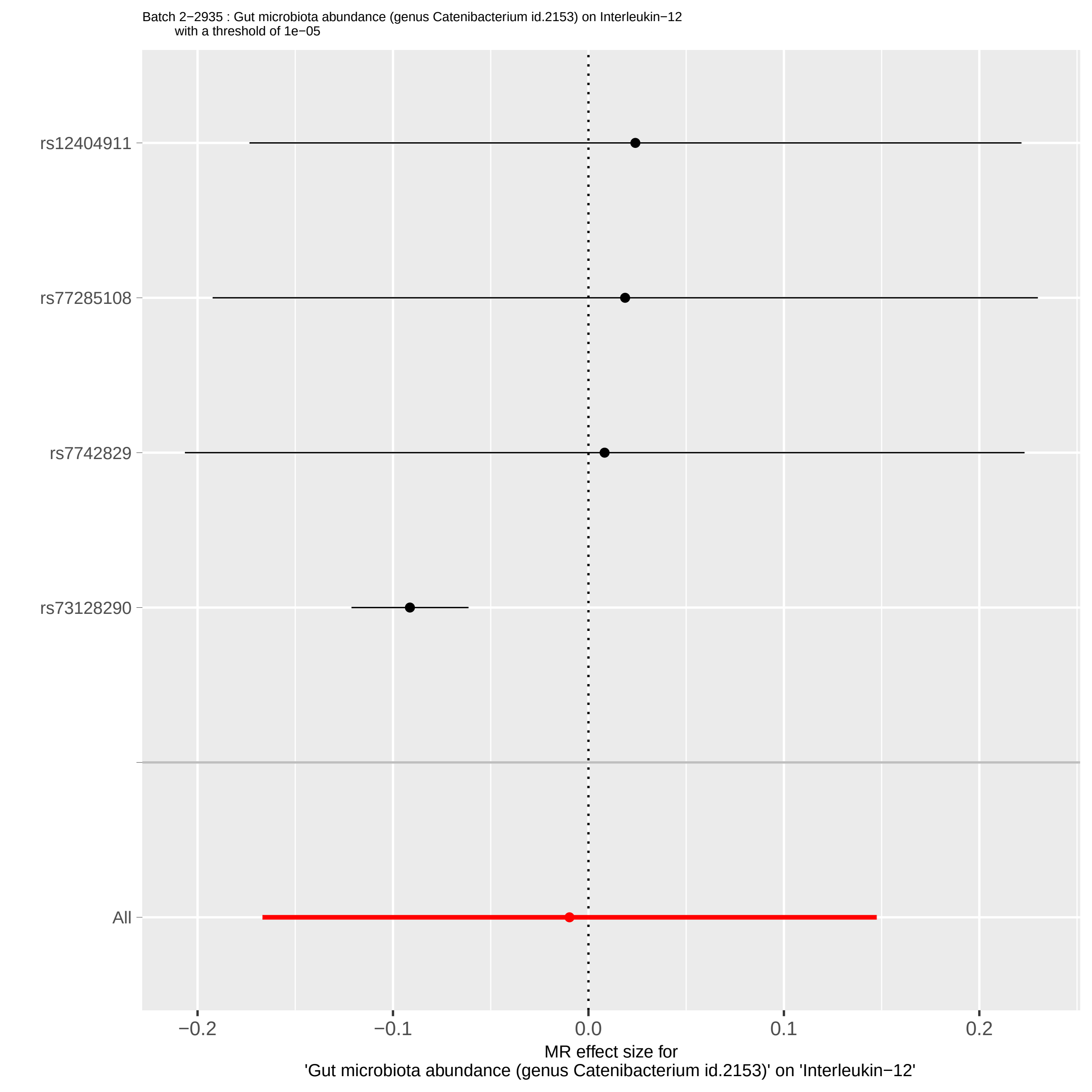
Batch 2−2935 : Gut microbiota abundance (genus Catenibacterium id.2153) on Interleukin−12
with a threshold of 1e−05
rs12404911
rs77285108
rs7742829
rs73128290
All
−0.2
−0.1
0.0
0.1
0.2
MR effect size for
'Gut microbiota abundance (genus Catenibacterium id.2153)' on 'Interleukin−12'

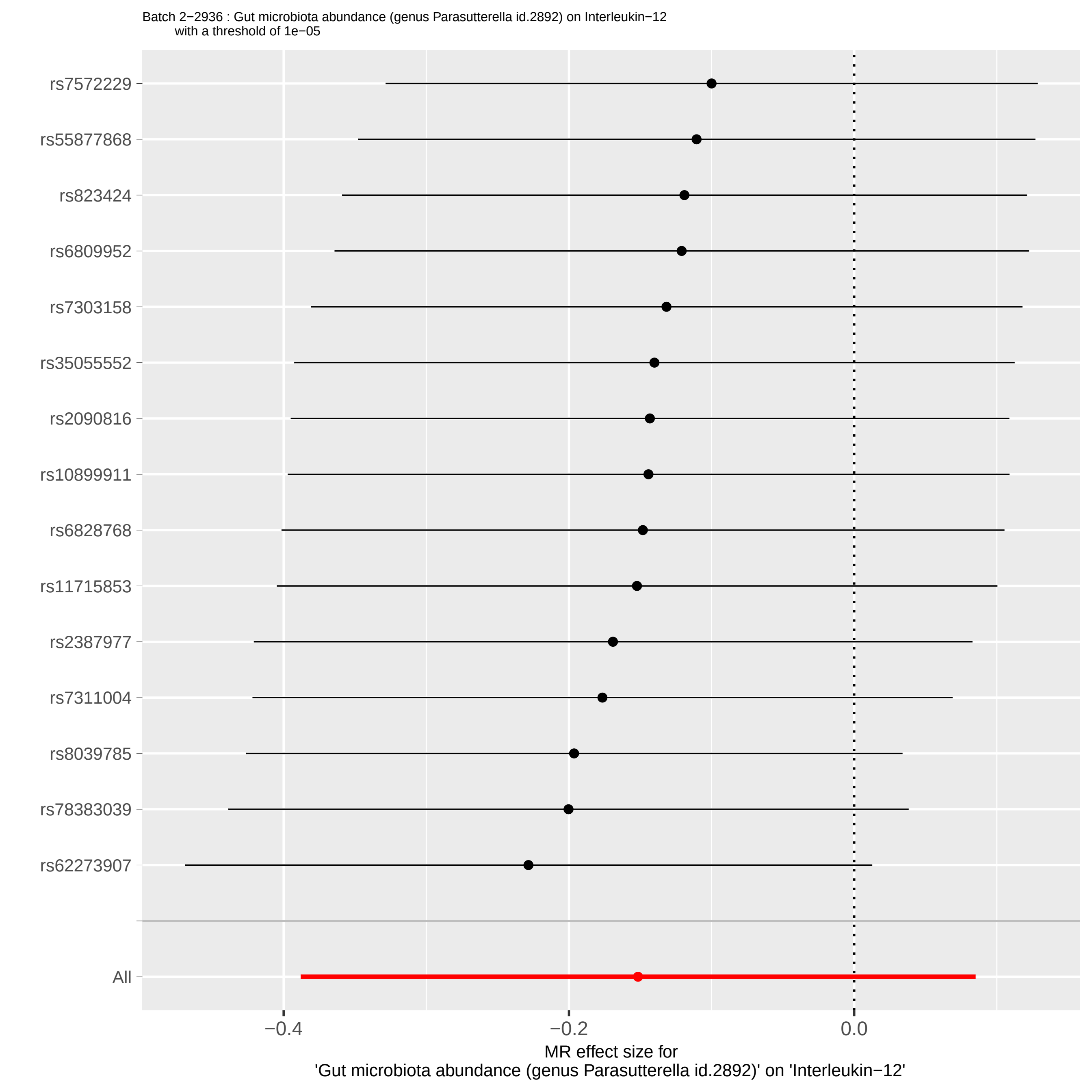
Batch 2−2936 : Gut microbiota abundance (genus Parasutterella id.2892) on Interleukin−12
with a threshold of 1e−05
rs7572229
rs55877868
rs823424
rs6809952
rs7303158
rs35055552
rs2090816
rs10899911
rs6828768
rs11715853
rs2387977
rs7311004
rs8039785
rs78383039
rs62273907
All
−0.4
−0.2
0.0
MR effect size for
'Gut microbiota abundance (genus Parasutterella id.2892)' on 'Interleukin−12'

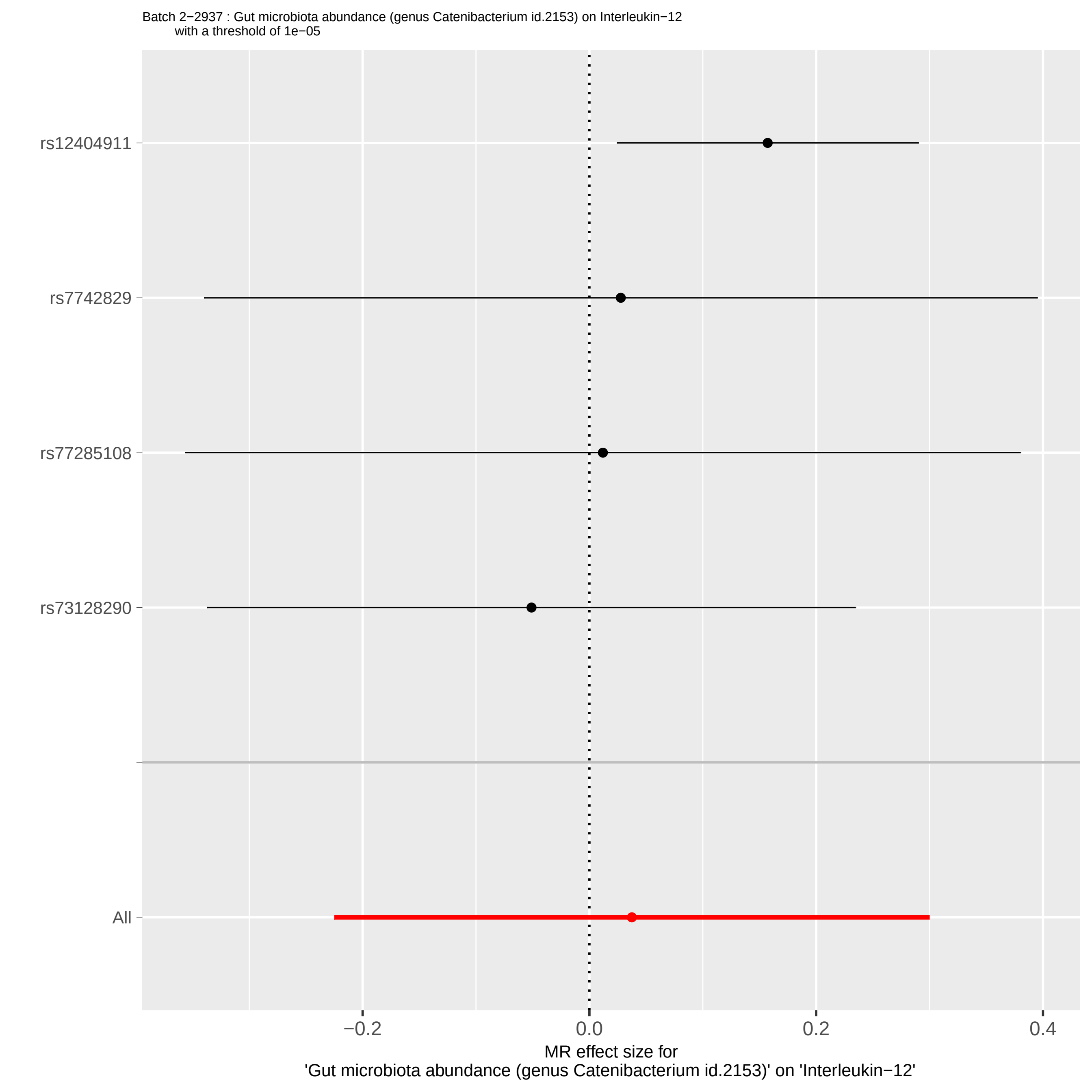
Batch 2−2937 : Gut microbiota abundance (genus Catenibacterium id.2153) on Interleukin−12
with a threshold of 1e−05
rs12404911
rs7742829
rs77285108
rs73128290
All
−0.2
0.0
0.2
0.4
MR effect size for
'Gut microbiota abundance (genus Catenibacterium id.2153)' on 'Interleukin−12'

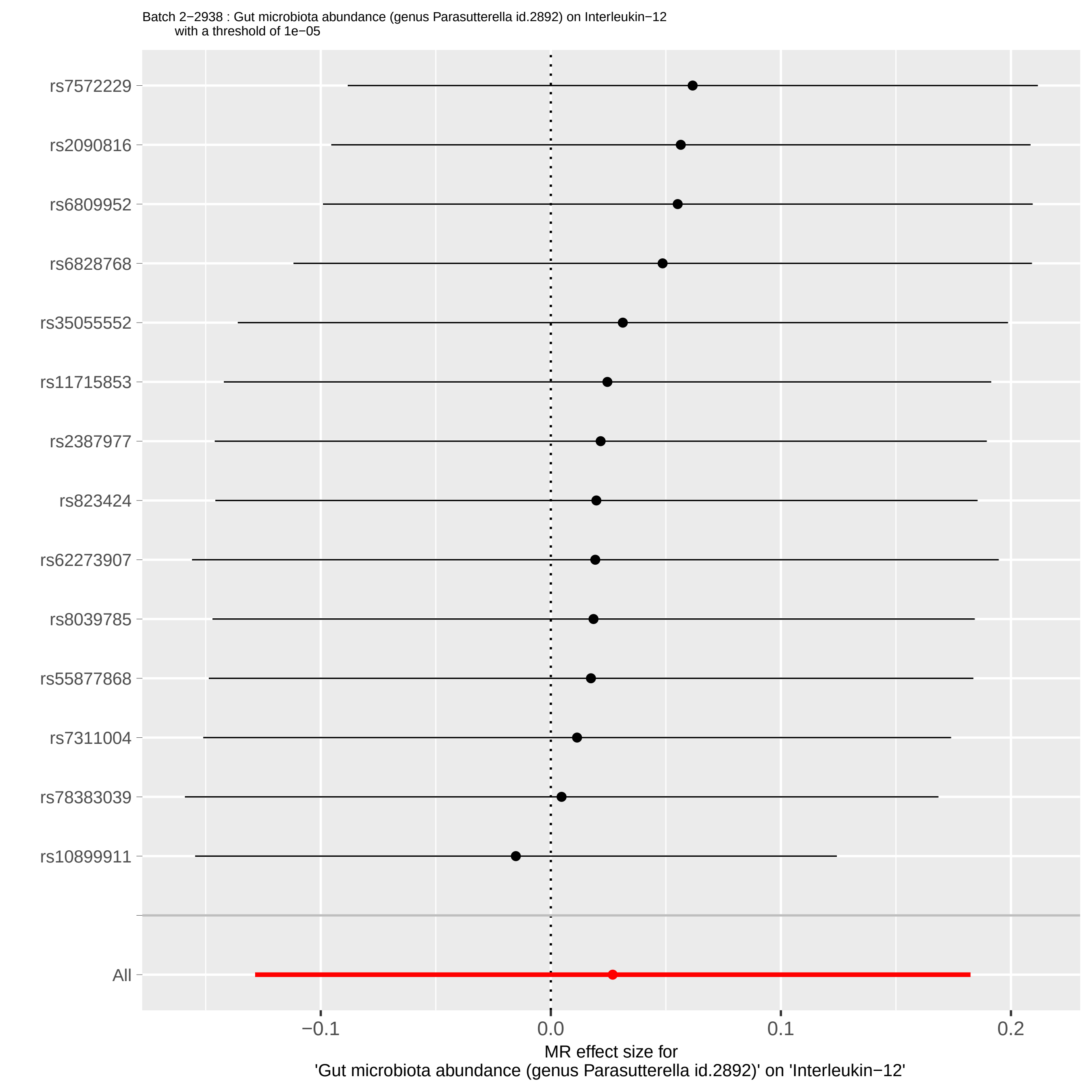
Batch 2−2938 : Gut microbiota abundance (genus Parasutterella id.2892) on Interleukin−12
with a threshold of 1e−05
rs7572229
rs2090816
rs6809952
rs6828768
rs35055552
rs11715853
rs2387977
rs823424
rs62273907
rs8039785
rs55877868
rs7311004
rs78383039
rs10899911
All
−0.1
0.0
0.1
0.2
MR effect size for
'Gut microbiota abundance (genus Parasutterella id.2892)' on 'Interleukin−12'

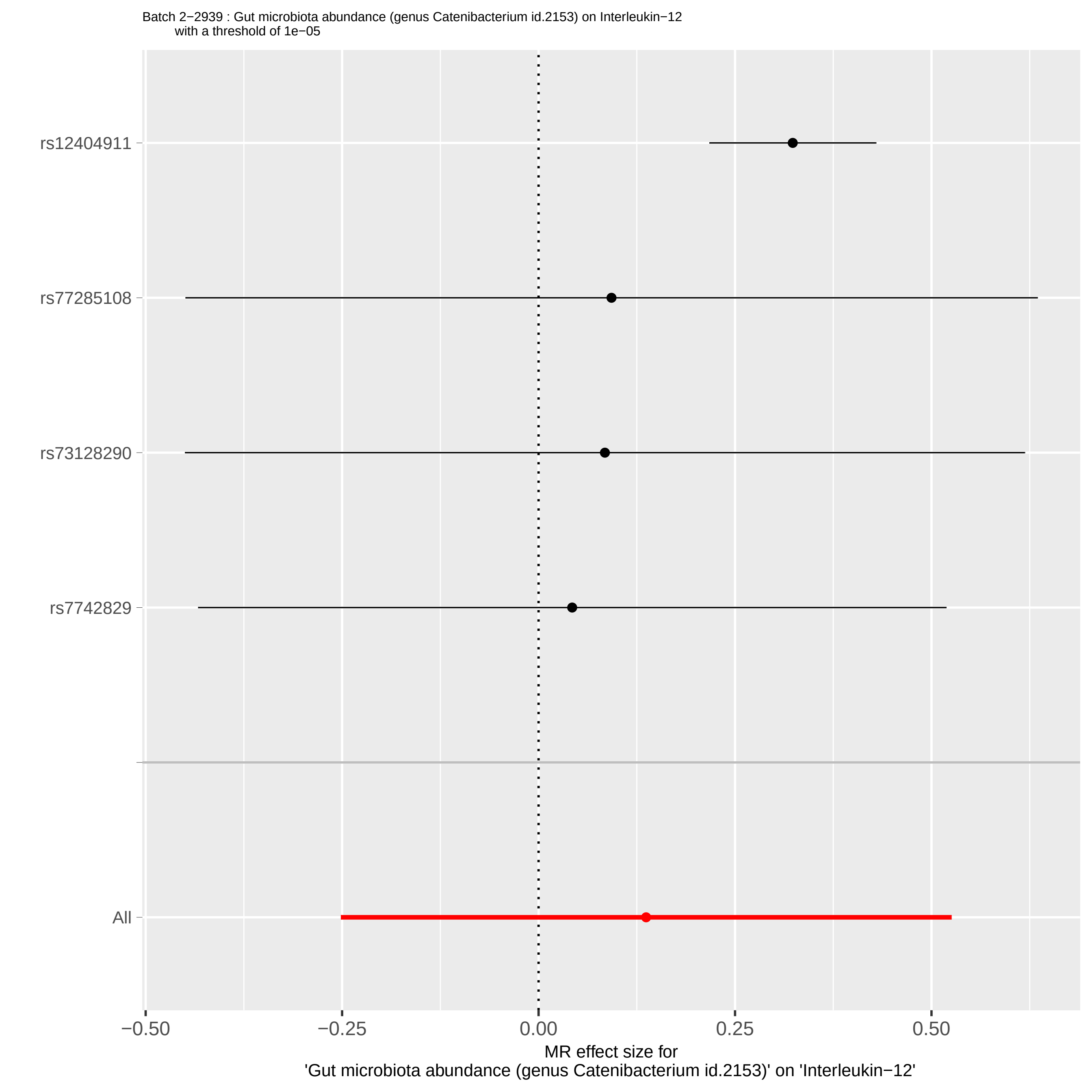

Batch 2−2939 : Gut microbiota abundance (genus Catenibacterium id.2153) on Interleukin−12
with a threshold of 1e−05
rs12404911
rs77285108
rs73128290
rs7742829
All
−0.50
−0.25
0.00
0.25
0.50
MR effect size for
'Gut microbiota abundance (genus Catenibacterium id.2153)' on 'Interleukin−12'

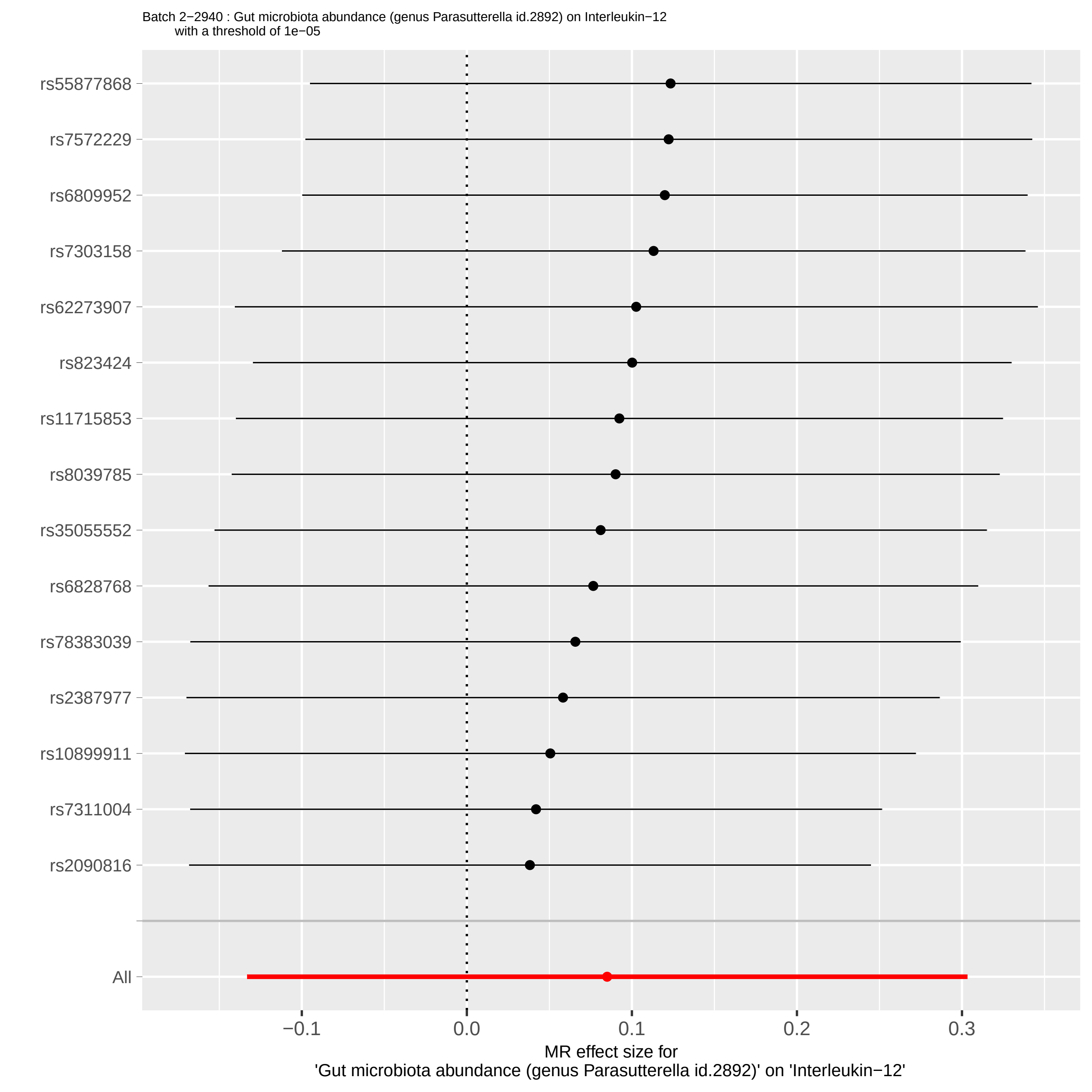
Batch 2−2940 : Gut microbiota abundance (genus Parasutterella id.2892) on Interleukin−12
with a threshold of 1e−05
rs55877868
rs7572229
rs6809952
rs7303158
rs62273907
rs823424
rs11715853
rs8039785
rs35055552
rs6828768
rs78383039
rs2387977
rs10899911
rs7311004
rs2090816
All
−0.1
0.0
0.1
0.2
0.3
MR effect size for
'Gut microbiota abundance (genus Parasutterella id.2892)' on 'Interleukin−12'

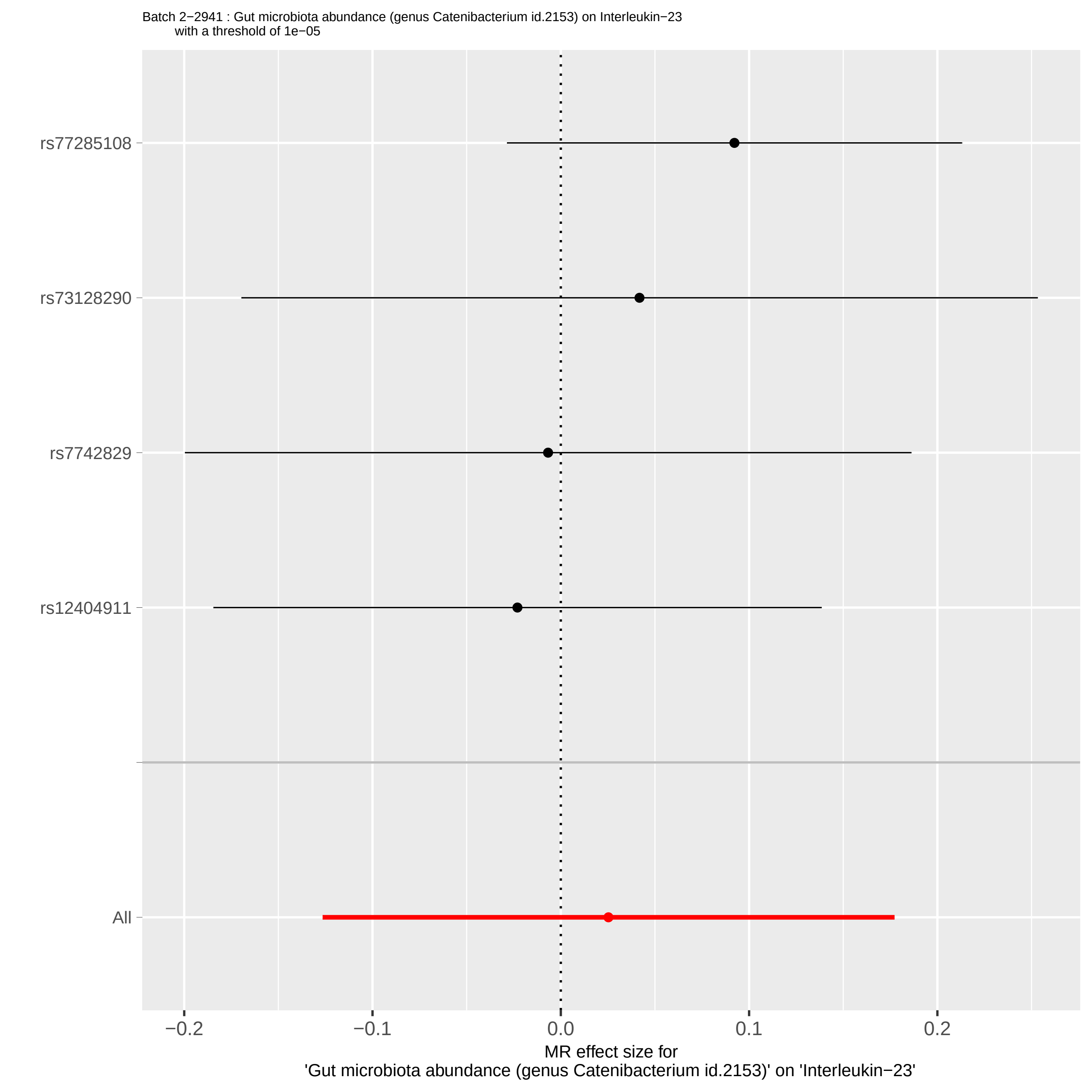

Batch 2−2941 : Gut microbiota abundance (genus Catenibacterium id.2153) on Interleukin−23
with a threshold of 1e−05
rs77285108
rs73128290
rs7742829
rs12404911
All
−0.2
−0.1
0.0
0.1
0.2
MR effect size for
'Gut microbiota abundance (genus Catenibacterium id.2153)' on 'Interleukin−23'

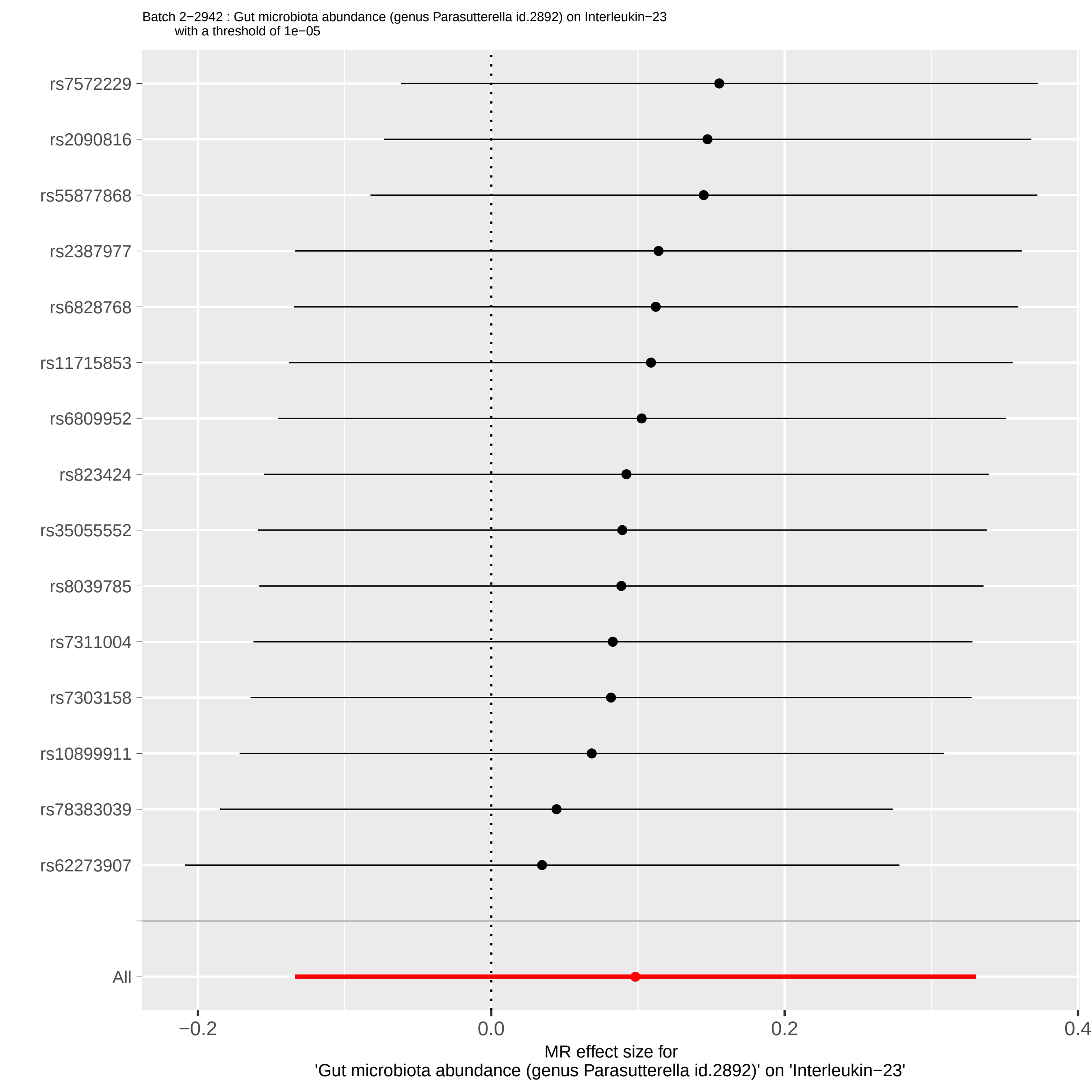
Batch 2−2942 : Gut microbiota abundance (genus Parasutterella id.2892) on Interleukin−23
with a threshold of 1e−05
rs7572229
rs2090816
rs55877868
rs2387977
rs6828768
rs11715853
rs6809952
rs823424
rs35055552
rs8039785
rs7311004
rs7303158
rs10899911
rs78383039
rs62273907
All
−0.2
0.0
0.2
0.4
MR effect size for
'Gut microbiota abundance (genus Parasutterella id.2892)' on 'Interleukin−23'

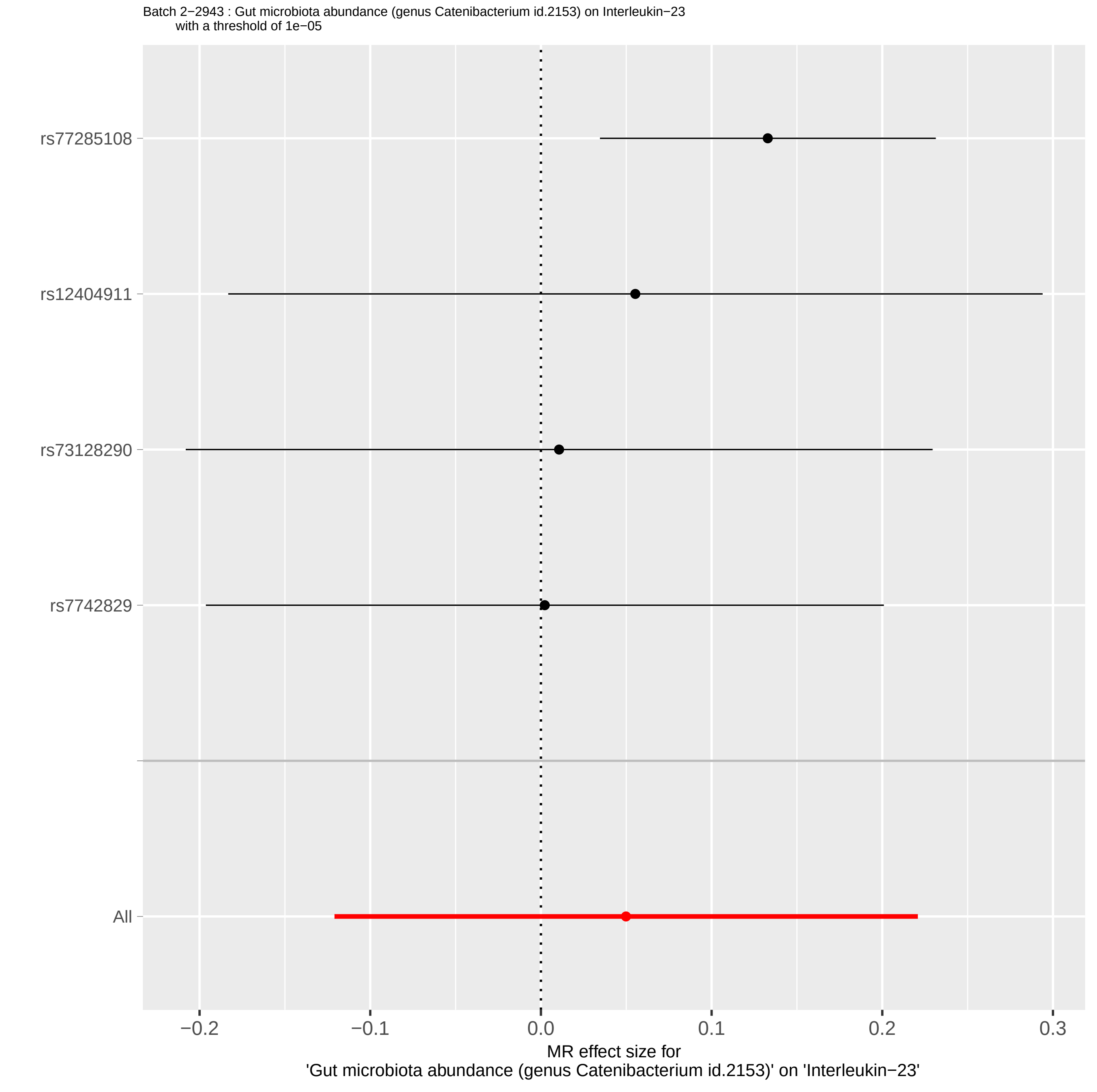
Batch 2−2943 : Gut microbiota abundance (genus Catenibacterium id.2153) on Interleukin−23
with a threshold of 1e−05
rs77285108
rs12404911
rs73128290
rs7742829
All
−0.2
−0.1
0.0
0.1
0.2
0.3
MR effect size for
'Gut microbiota abundance (genus Catenibacterium id.2153)' on 'Interleukin−23'

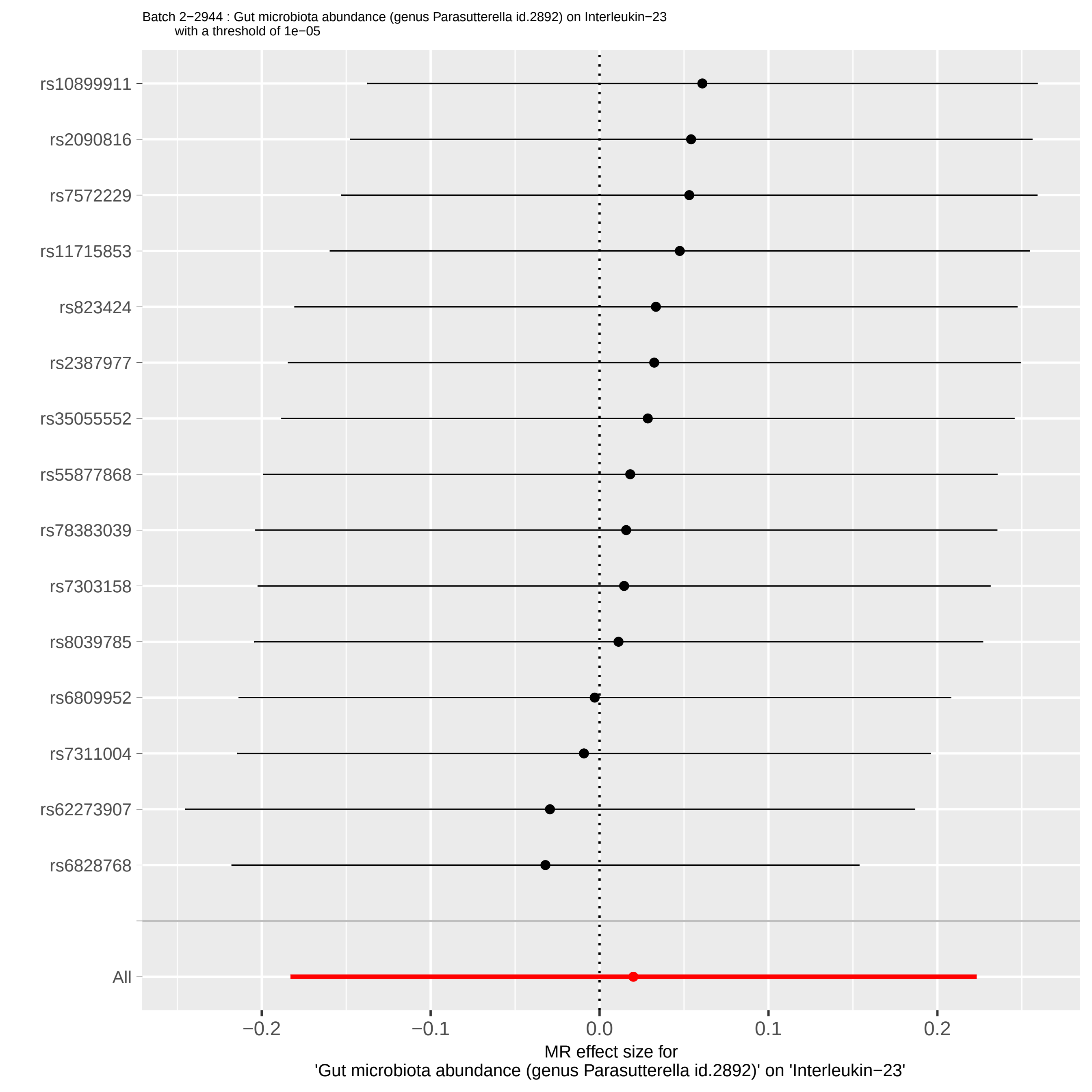
Batch 2−2944 : Gut microbiota abundance (genus Parasutterella id.2892) on Interleukin−23
with a threshold of 1e−05
rs10899911
rs2090816
rs7572229
rs11715853
rs823424
rs2387977
rs35055552
rs55877868
rs78383039
rs7303158
rs8039785
rs6809952
rs7311004
rs62273907
rs6828768
All
−0.2
−0.1
0.0
0.1
0.2
MR effect size for
'Gut microbiota abundance (genus Parasutterella id.2892)' on 'Interleukin−23'

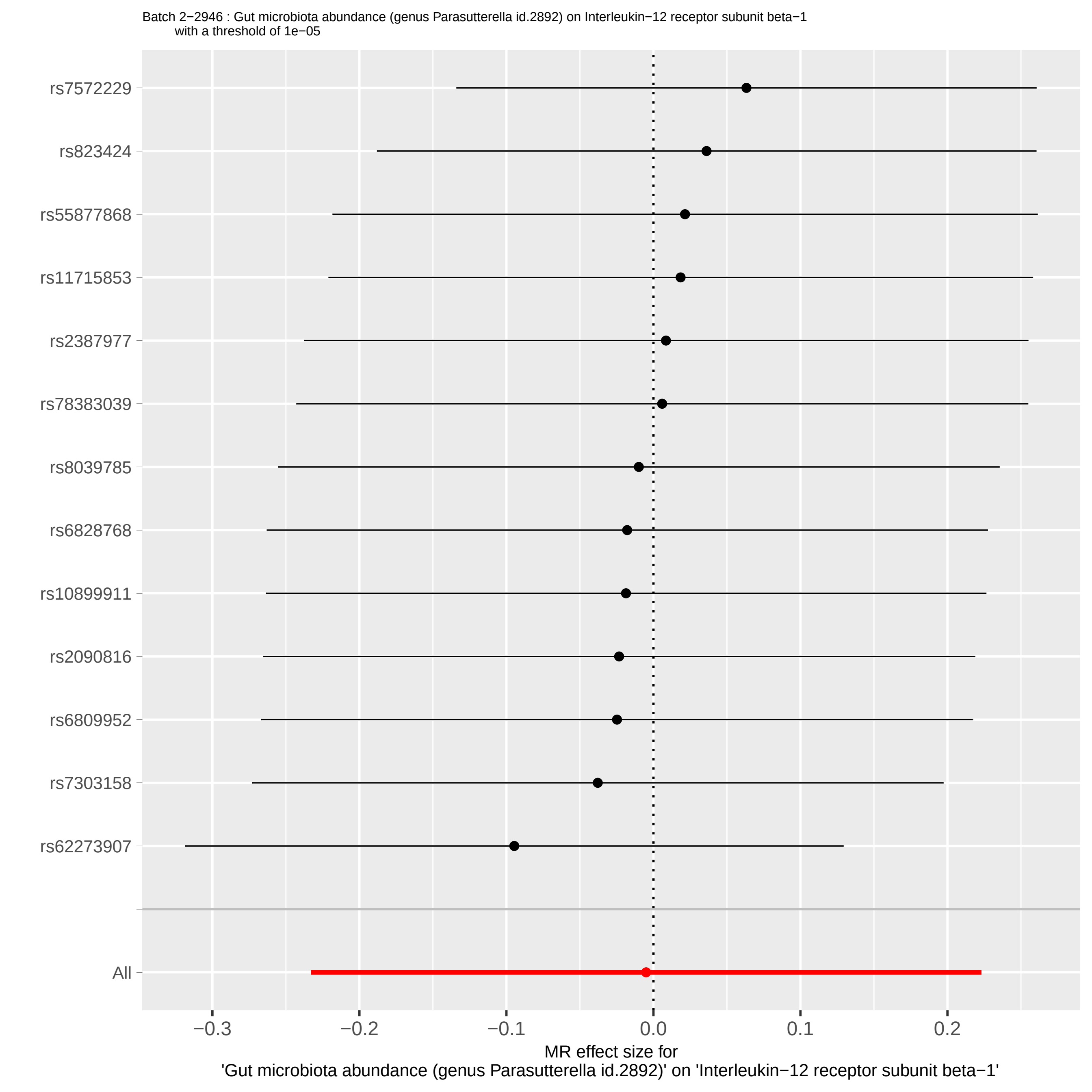
Batch 2−2946 : Gut microbiota abundance (genus Parasutterella id.2892) on Interleukin−12 receptor subunit beta−1
with a threshold of 1e−05
rs7572229
rs823424
rs55877868
rs11715853
rs2387977
rs78383039
rs8039785
rs6828768
rs10899911
rs2090816
rs6809952
rs7303158
rs62273907
All
−0.3
−0.2
−0.1
0.0
0.1
0.2
MR effect size for
'Gut microbiota abundance (genus Parasutterella id.2892)' on 'Interleukin−12 receptor subunit beta−1'

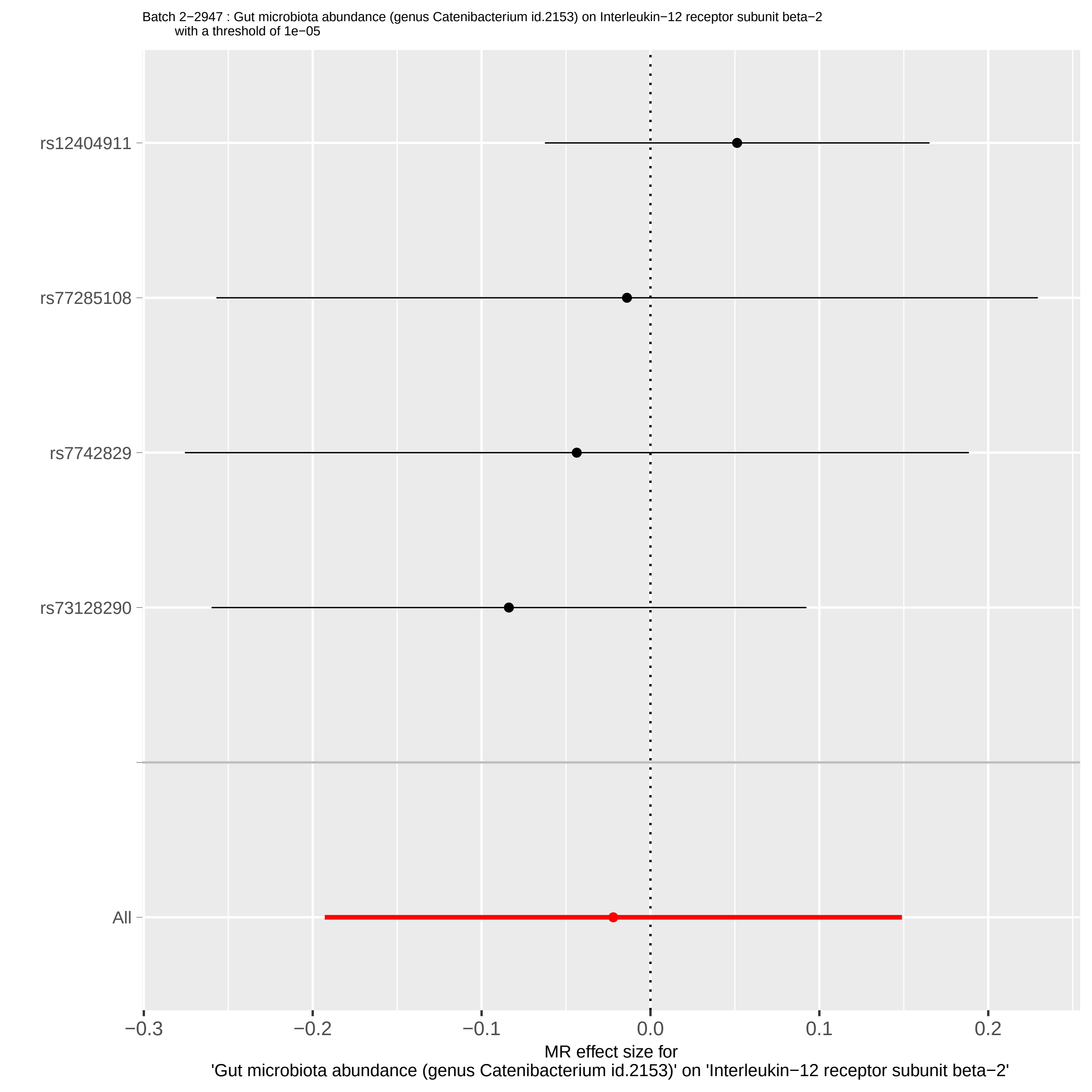
Batch 2−2947 : Gut microbiota abundance (genus Catenibacterium id.2153) on Interleukin−12 receptor subunit beta−2
with a threshold of 1e−05
rs12404911
rs77285108
rs7742829
rs73128290
All
−0.3
−0.2
−0.1
0.0
0.1
0.2
MR effect size for
'Gut microbiota abundance (genus Catenibacterium id.2153)' on 'Interleukin−12 receptor subunit beta−2'

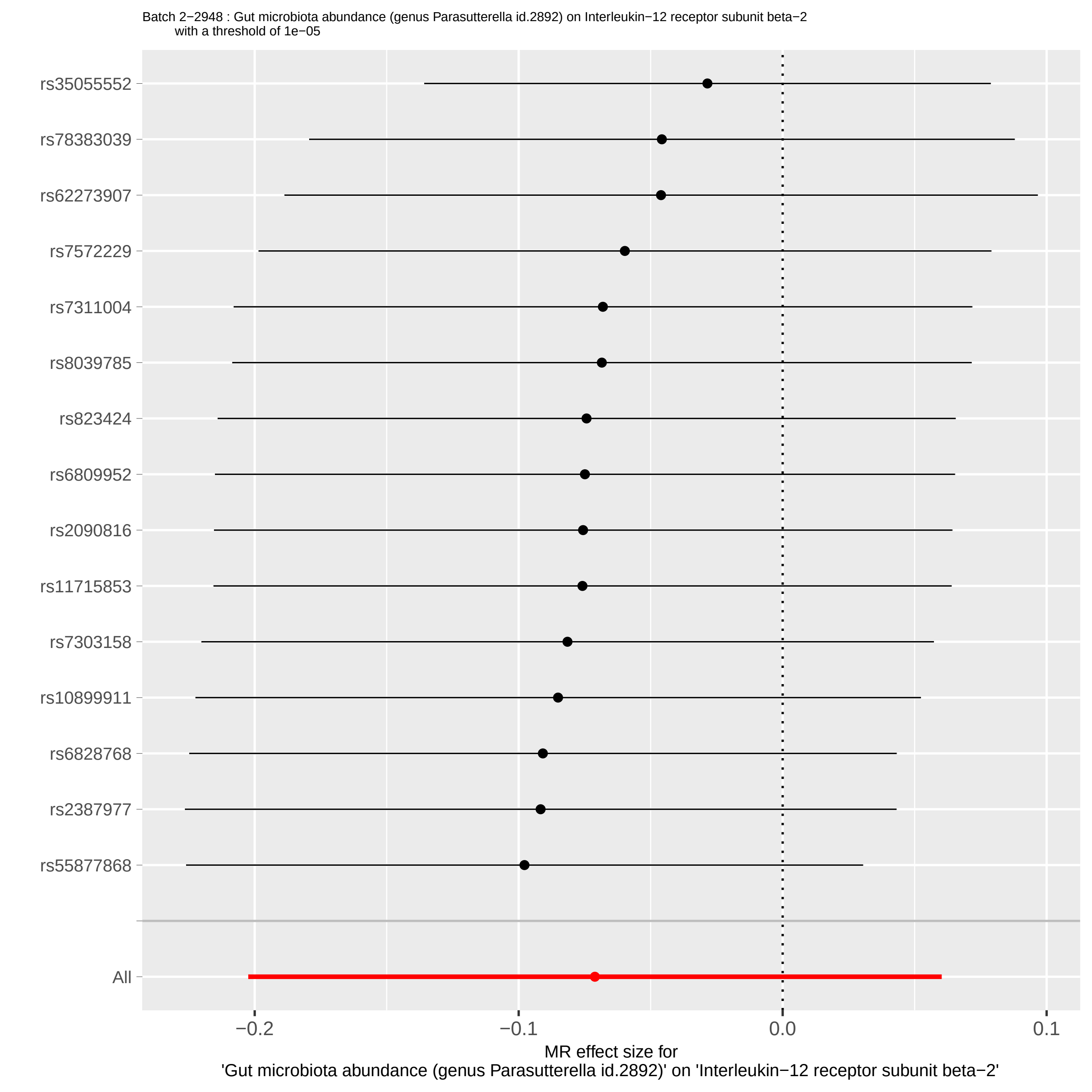

Batch 2−2948 : Gut microbiota abundance (genus Parasutterella id.2892) on Interleukin−12 receptor subunit beta−2
with a threshold of 1e−05
rs35055552
rs78383039
rs62273907
rs7572229
rs7311004
rs8039785
rs823424
rs6809952
rs2090816
rs11715853
rs7303158
rs10899911
rs6828768
rs2387977
rs55877868
All
−0.2
−0.1
0.0
0.1
MR effect size for
'Gut microbiota abundance (genus Parasutterella id.2892)' on 'Interleukin−12 receptor subunit beta−2'

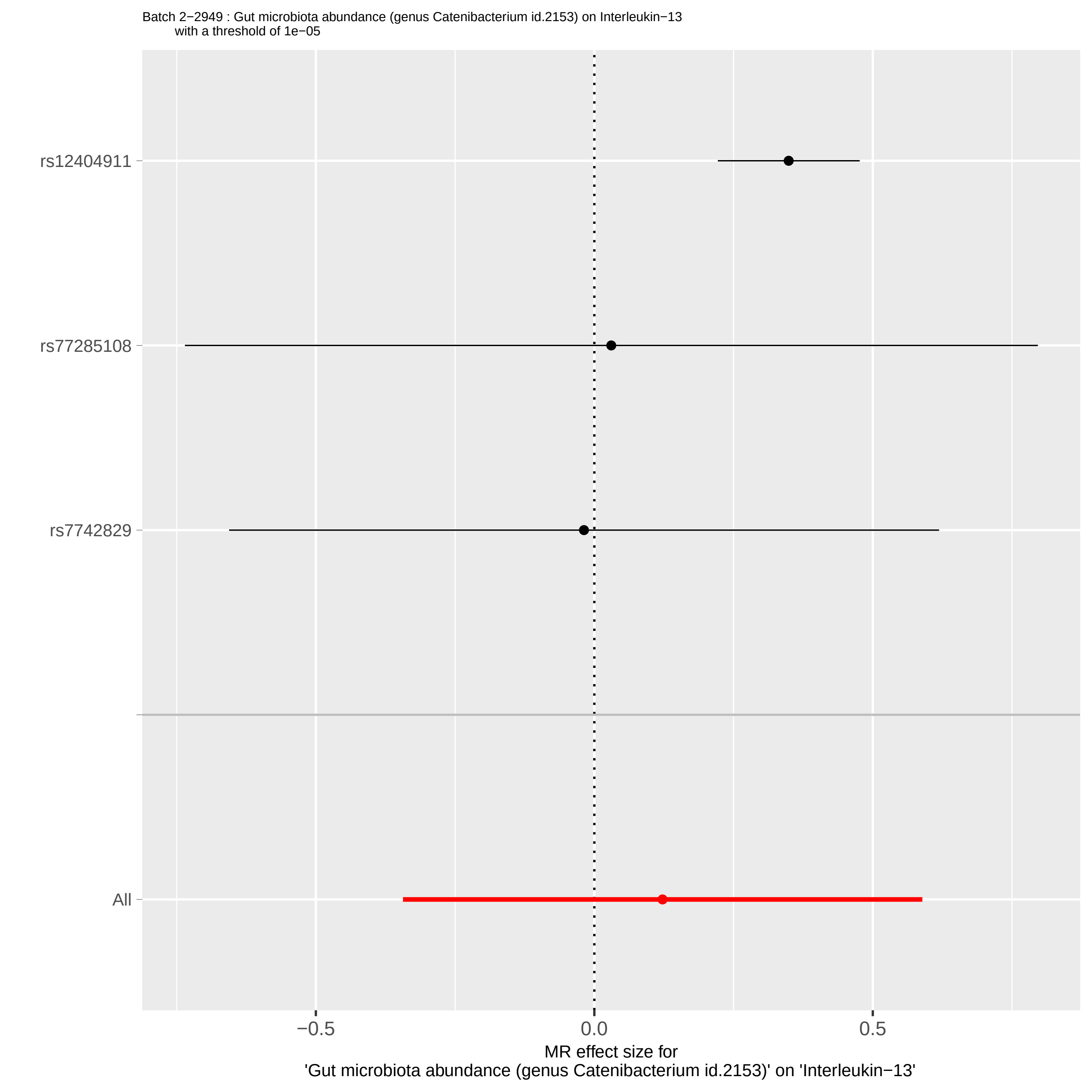

Batch 2−2949 : Gut microbiota abundance (genus Catenibacterium id.2153) on Interleukin−13
with a threshold of 1e−05
rs12404911
rs77285108
rs7742829
All
−0.5
0.0
0.5
MR effect size for
'Gut microbiota abundance (genus Catenibacterium id.2153)' on 'Interleukin−13'

Batch 2−2950 : Gut microbiota abundance (genus Parasutterella id.2892) on Interleukin−13
with a threshold of 1e−05

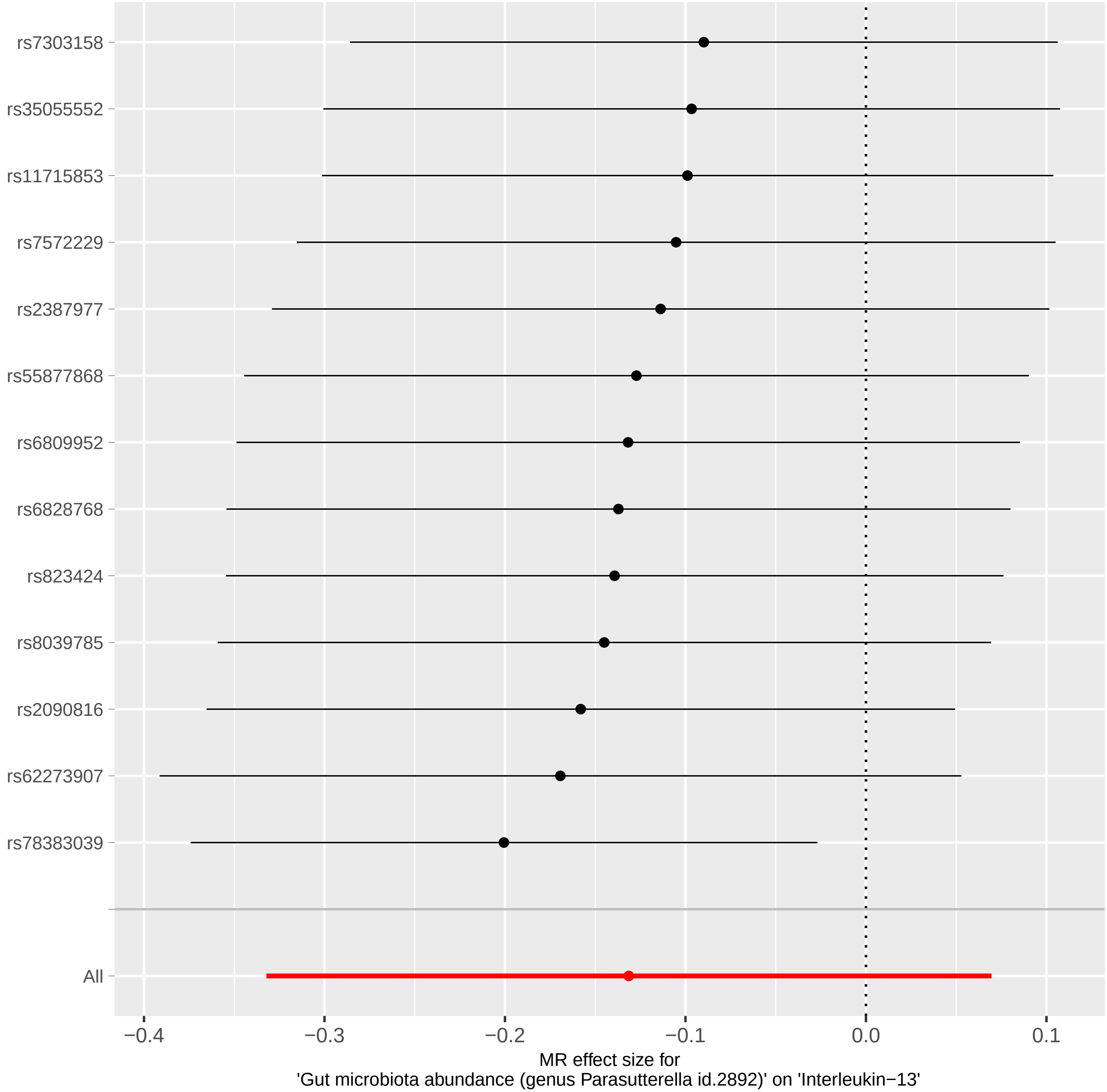

Batch 2−2951 : Gut microbiota abundance (genus Catenibacterium id.2153) on Interleukin−13 receptor subunit alpha−1
with a threshold of 1e−05

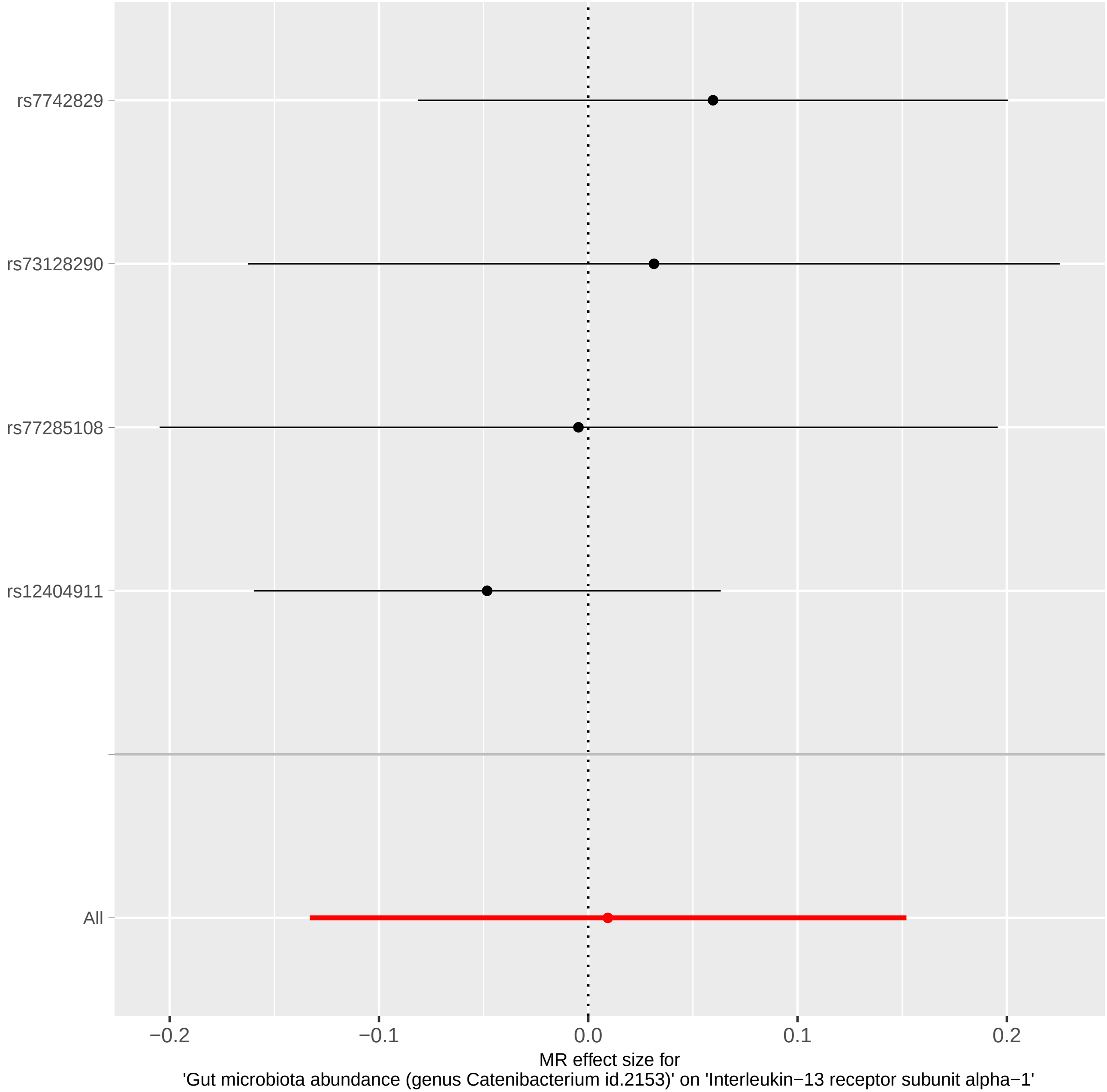

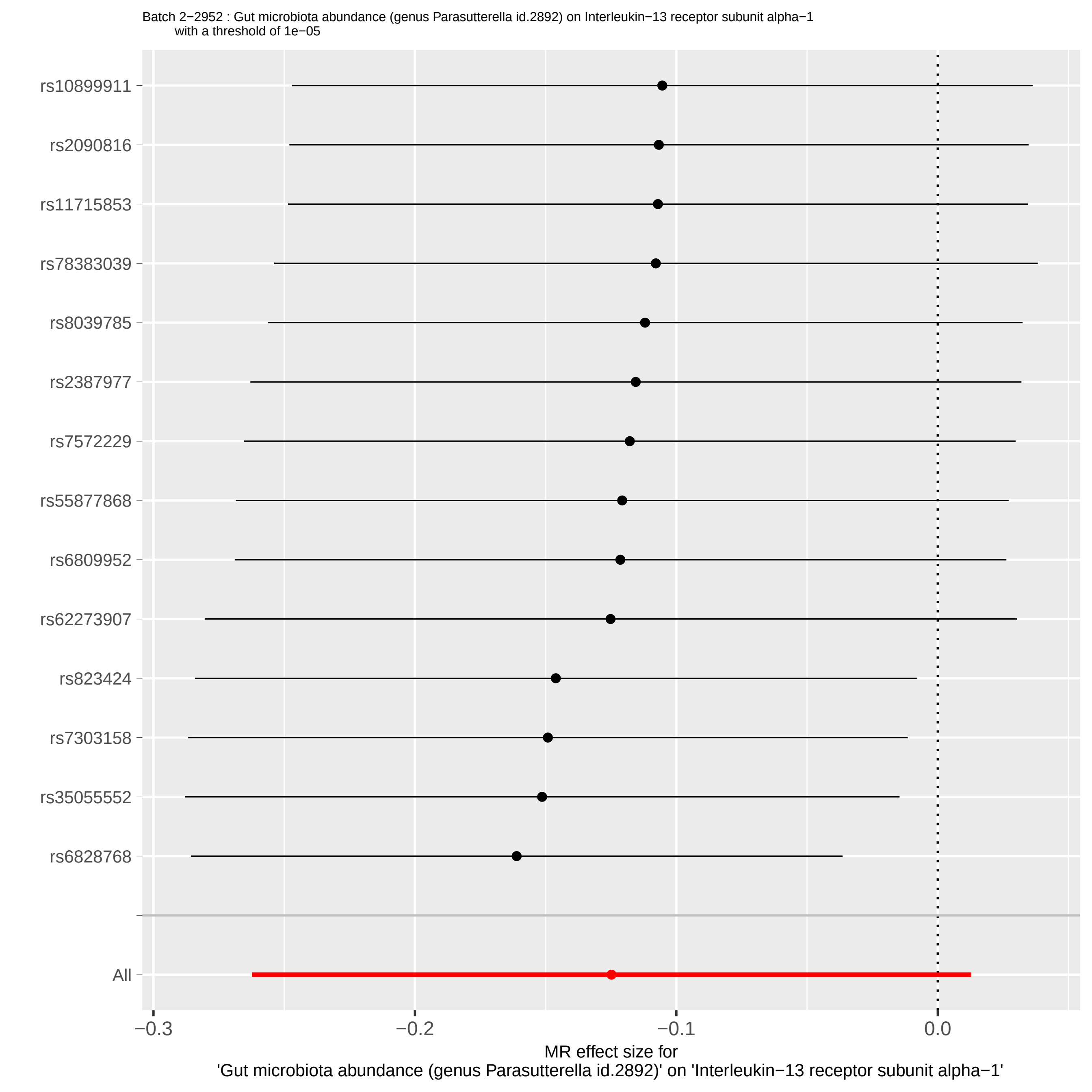
Batch 2−2952 : Gut microbiota abundance (genus Parasutterella id.2892) on Interleukin−13 receptor subunit alpha−1
with a threshold of 1e−05
rs10899911
rs2090816
rs11715853
rs78383039
rs8039785
rs2387977
rs7572229
rs55877868
rs6809952
rs62273907
rs823424
rs7303158
rs35055552
rs6828768
All
−0.3
−0.2
−0.1
0.0
MR effect size for
'Gut microbiota abundance (genus Parasutterella id.2892)' on 'Interleukin−13 receptor subunit alpha−1'

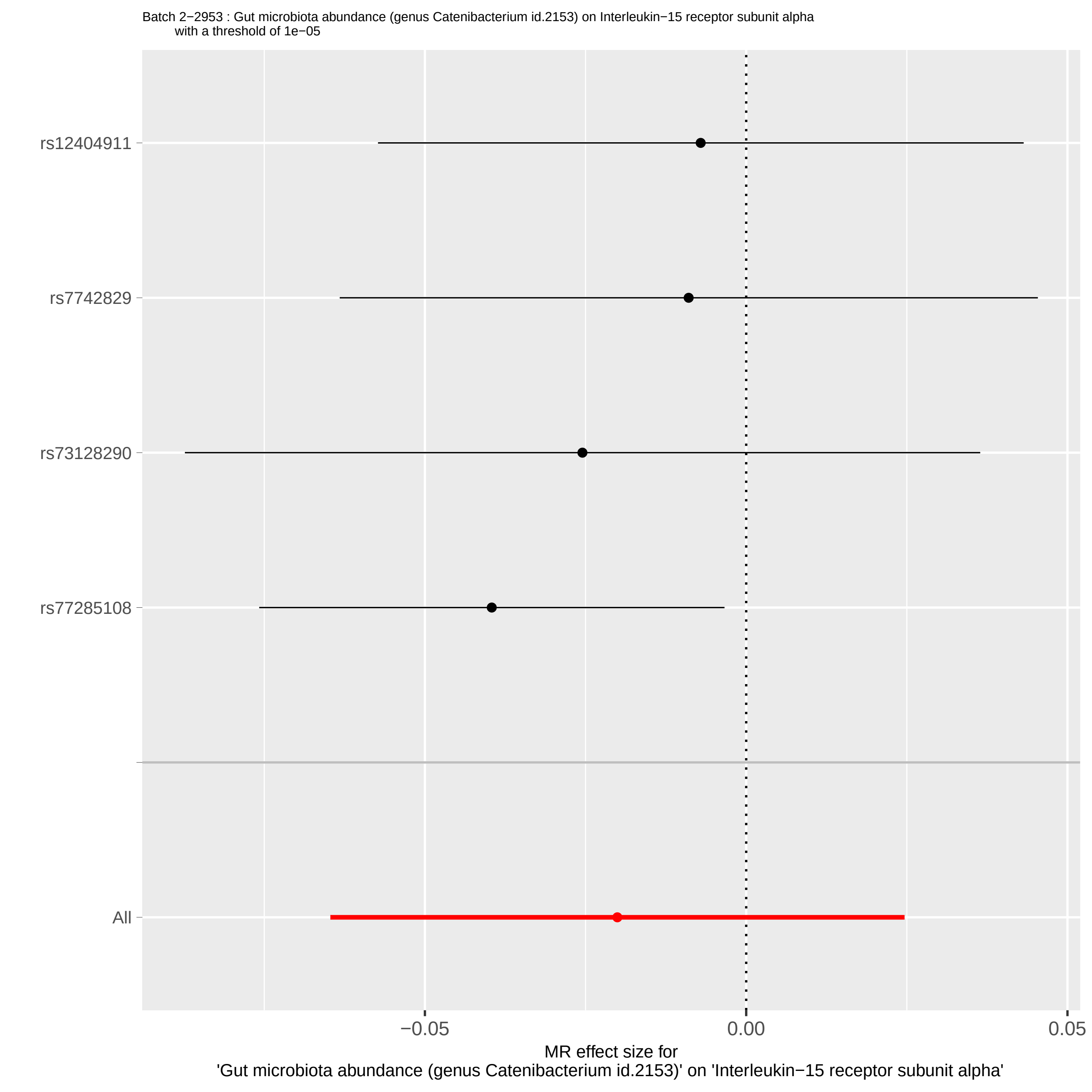
Batch 2−2953 : Gut microbiota abundance (genus Catenibacterium id.2153) on Interleukin−15 receptor subunit alpha
with a threshold of 1e−05
rs12404911
rs7742829
rs73128290
rs77285108
All
−0.05
0.00
0.05
MR effect size for
'Gut microbiota abundance (genus Catenibacterium id.2153)' on 'Interleukin−15 receptor subunit alpha'

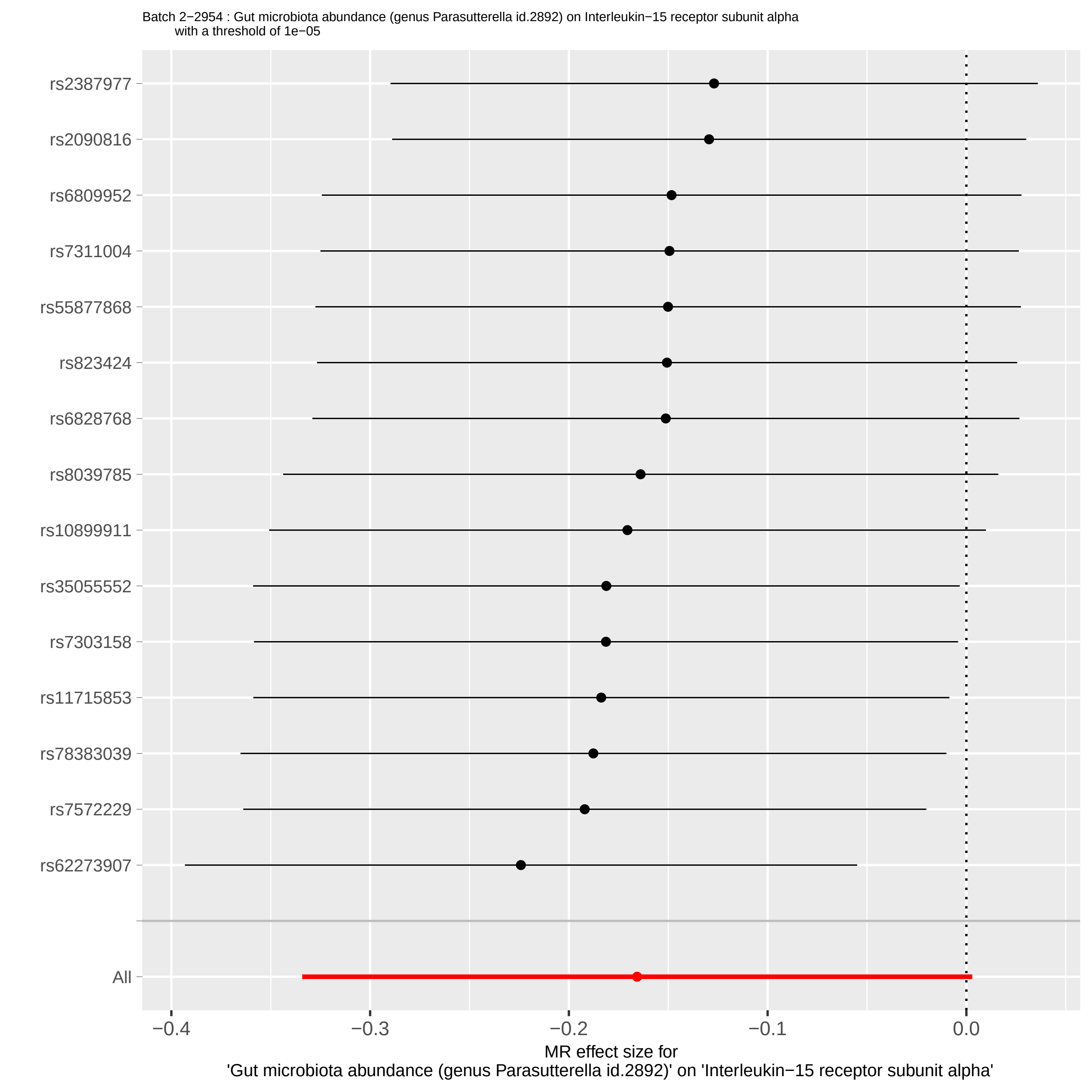

Batch 2−2954 : Gut microbiota abundance (genus Parasutterella id.2892) on Interleukin−15 receptor subunit alpha
with a threshold of 1e−05
rs2387977
rs2090816
rs6809952
rs7311004
rs55877868
rs823424
rs6828768
rs8039785
rs10899911
rs35055552
rs7303158
rs11715853
rs78383039
rs7572229
rs62273907
All
−0.4
−0.3
−0.2
−0.1
0.0
MR effect size for
'Gut microbiota abundance (genus Parasutterella id.2892)' on 'Interleukin−15 receptor subunit alpha'

Batch 2−2955 : Gut microbiota abundance (genus Catenibacterium id.2153) on Interleukin−15 receptor subunit alpha
with a threshold of 1e−05

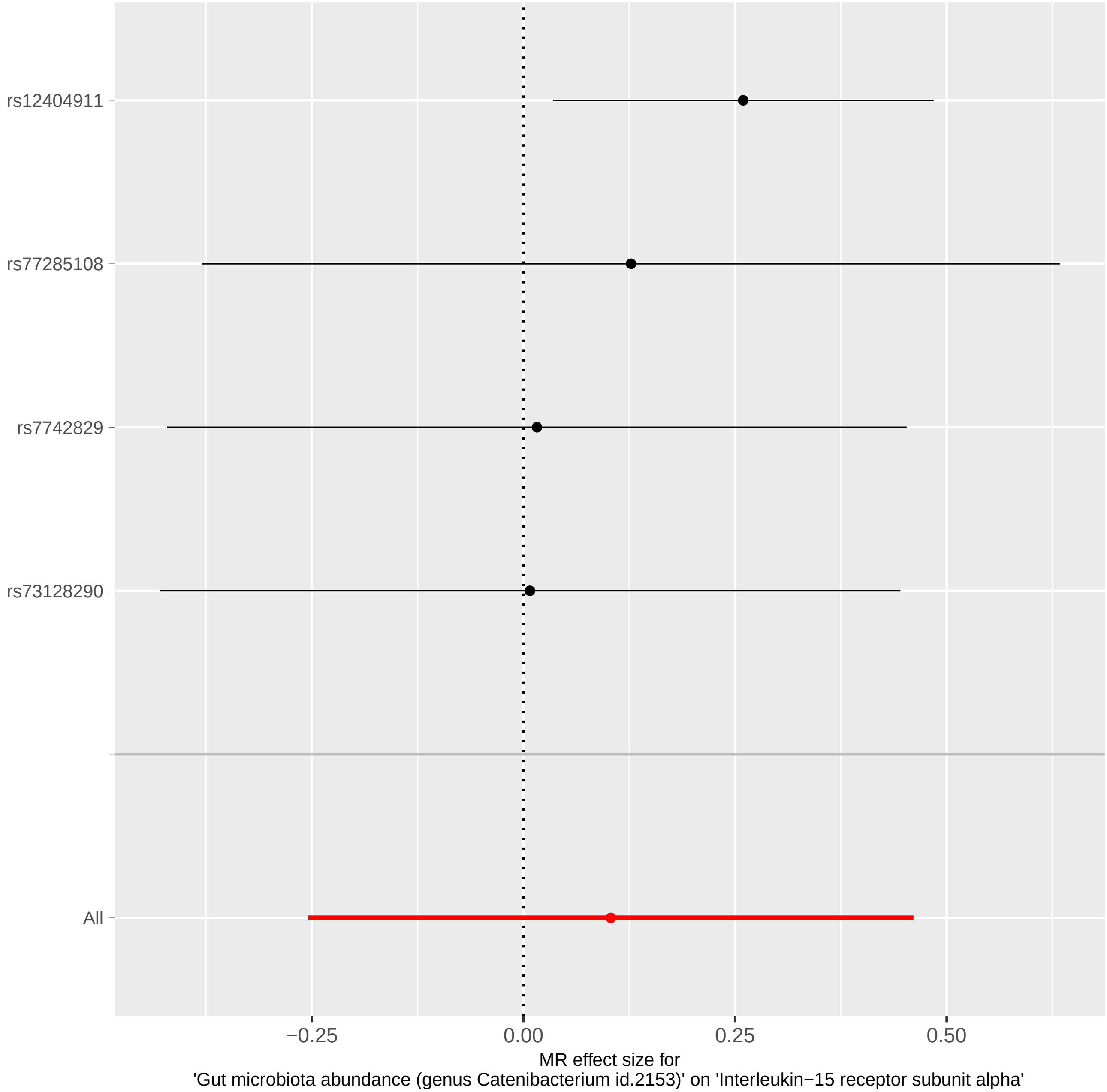

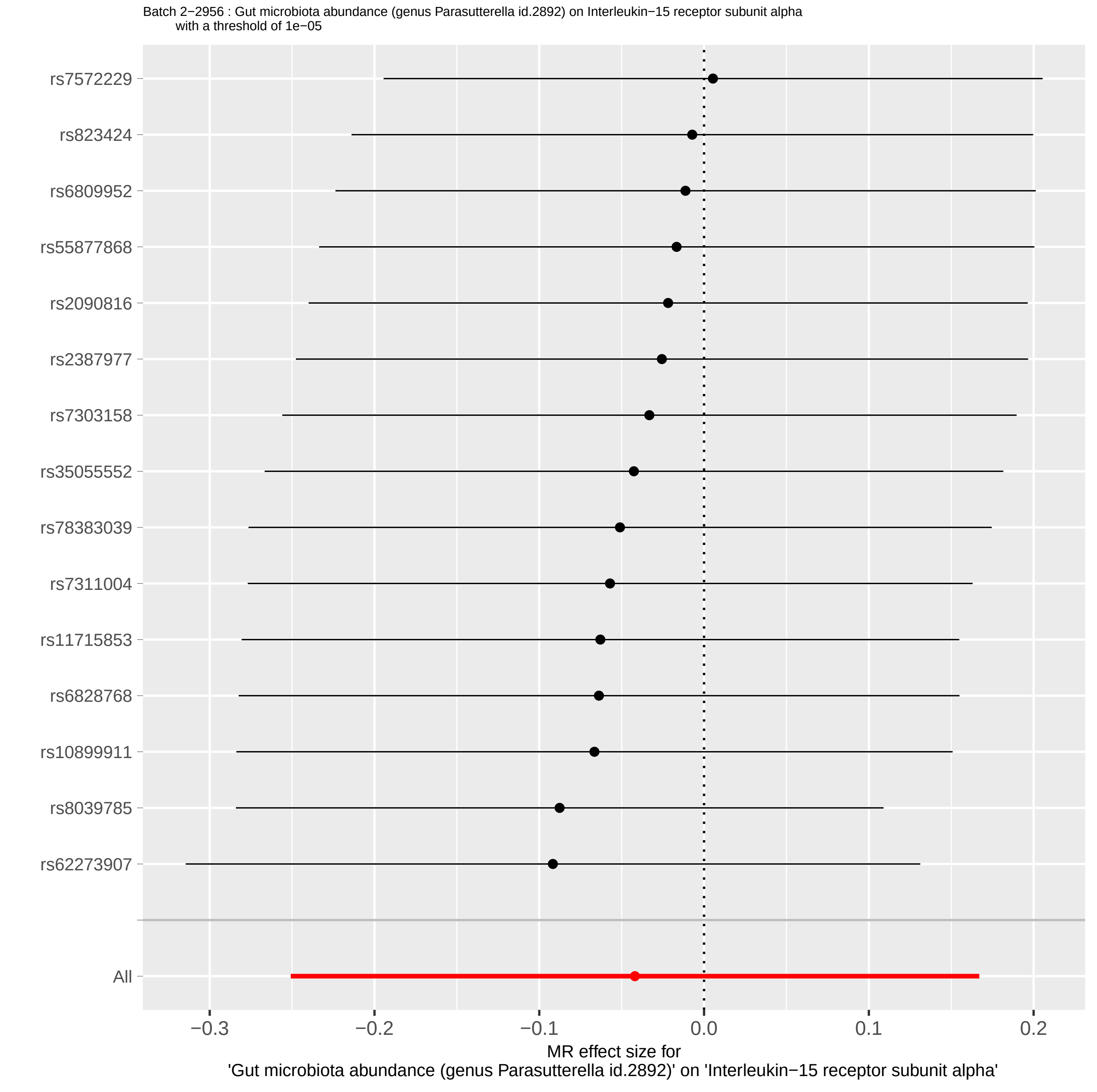

Batch 2−2956 : Gut microbiota abundance (genus Parasutterella id.2892) on Interleukin−15 receptor subunit alpha
with a threshold of 1e−05
rs7572229
rs823424
rs6809952
rs55877868
rs2090816
rs2387977
rs7303158
rs35055552
rs78383039
rs7311004
rs11715853
rs6828768
rs10899911
rs8039785
rs62273907
All
−0.3
−0.2
−0.1
0.0
0.1
0.2
MR effect size for
'Gut microbiota abundance (genus Parasutterella id.2892)' on 'Interleukin−15 receptor subunit alpha'

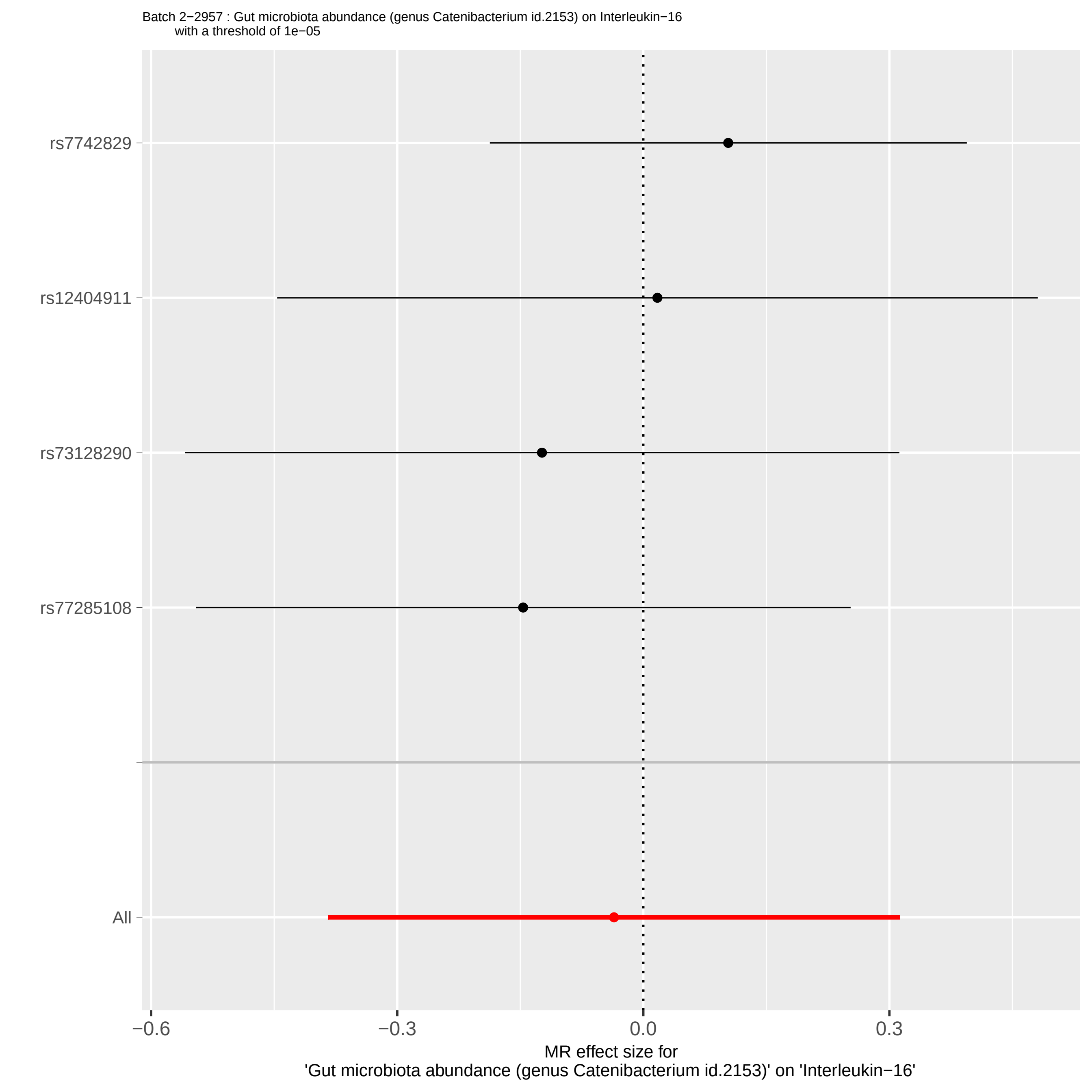
Batch 2−2957 : Gut microbiota abundance (genus Catenibacterium id.2153) on Interleukin−16
with a threshold of 1e−05
rs7742829
rs12404911
rs73128290
rs77285108
All
−0.6
−0.3
0.0
0.3
MR effect size for
'Gut microbiota abundance (genus Catenibacterium id.2153)' on 'Interleukin−16'

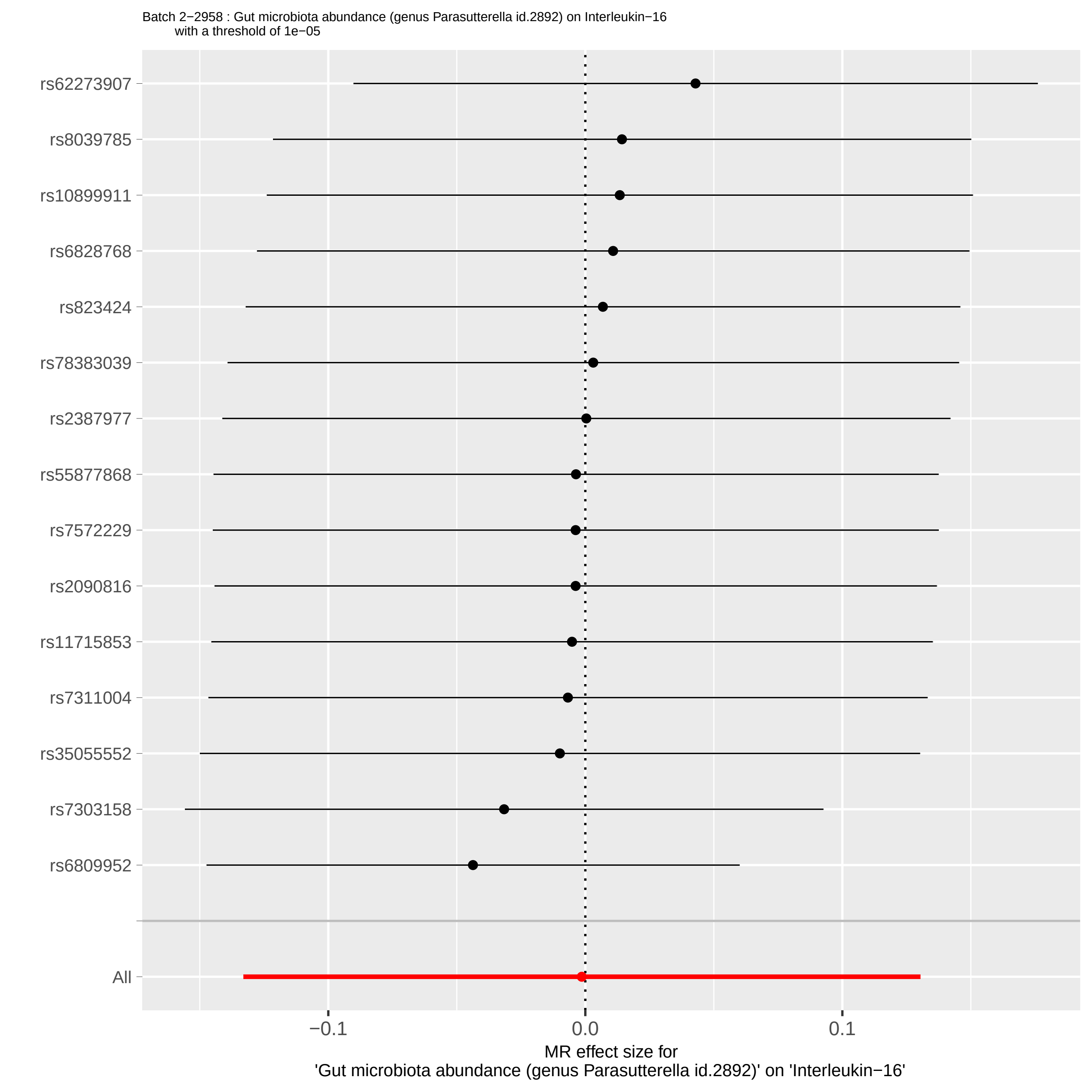

Batch 2−2958 : Gut microbiota abundance (genus Parasutterella id.2892) on Interleukin−16
with a threshold of 1e−05
rs62273907
rs8039785
rs10899911
rs6828768
rs823424
rs78383039
rs2387977
rs55877868
rs7572229
rs2090816
rs11715853
rs7311004
rs35055552
rs7303158
rs6809952
All
−0.1
0.0
0.1
MR effect size for
'Gut microbiota abundance (genus Parasutterella id.2892)' on 'Interleukin−16'

Batch 2−2959 : Gut microbiota abundance (genus Catenibacterium id.2153) on Interleukin−17A
with a threshold of 1e−05

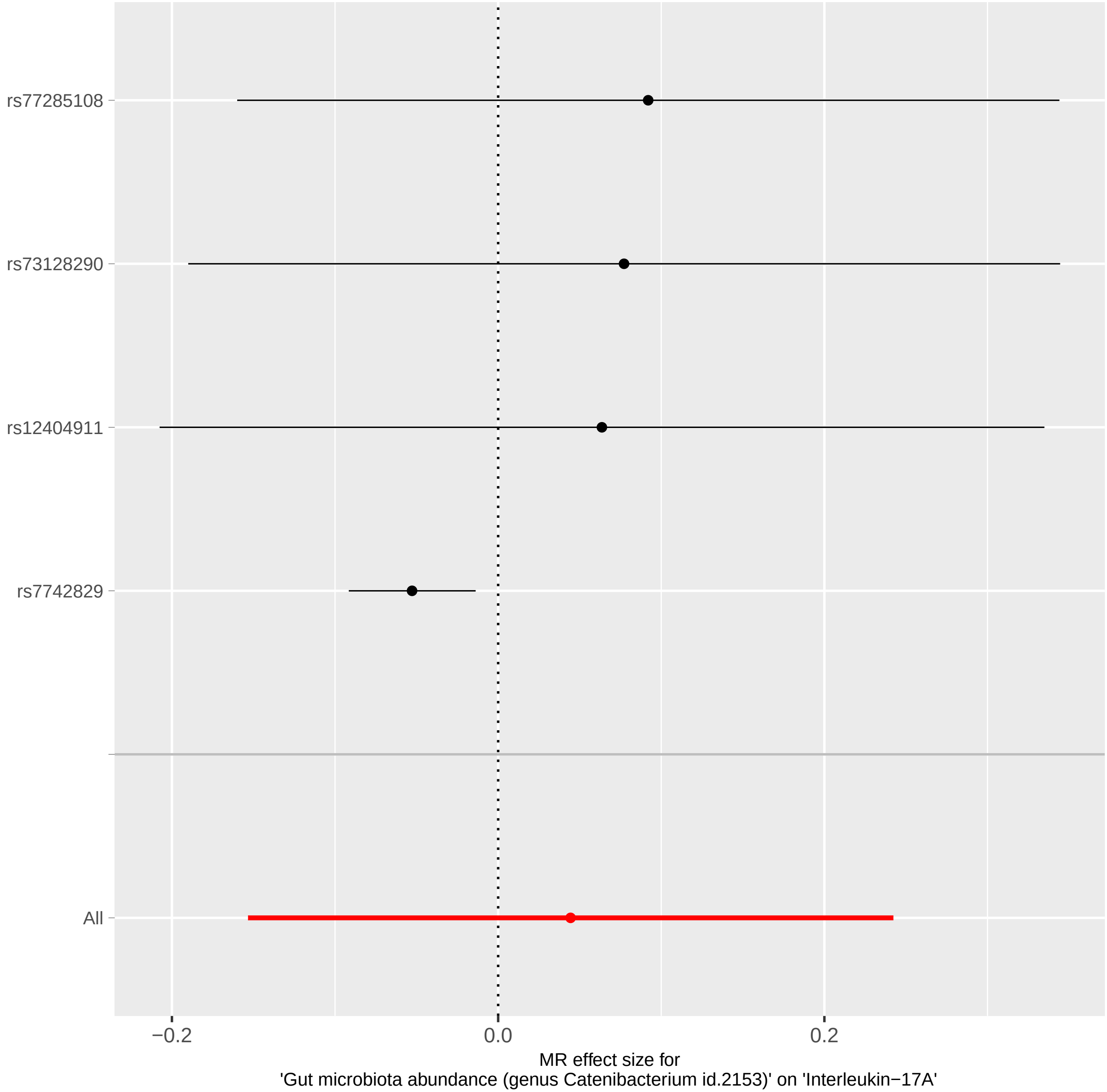

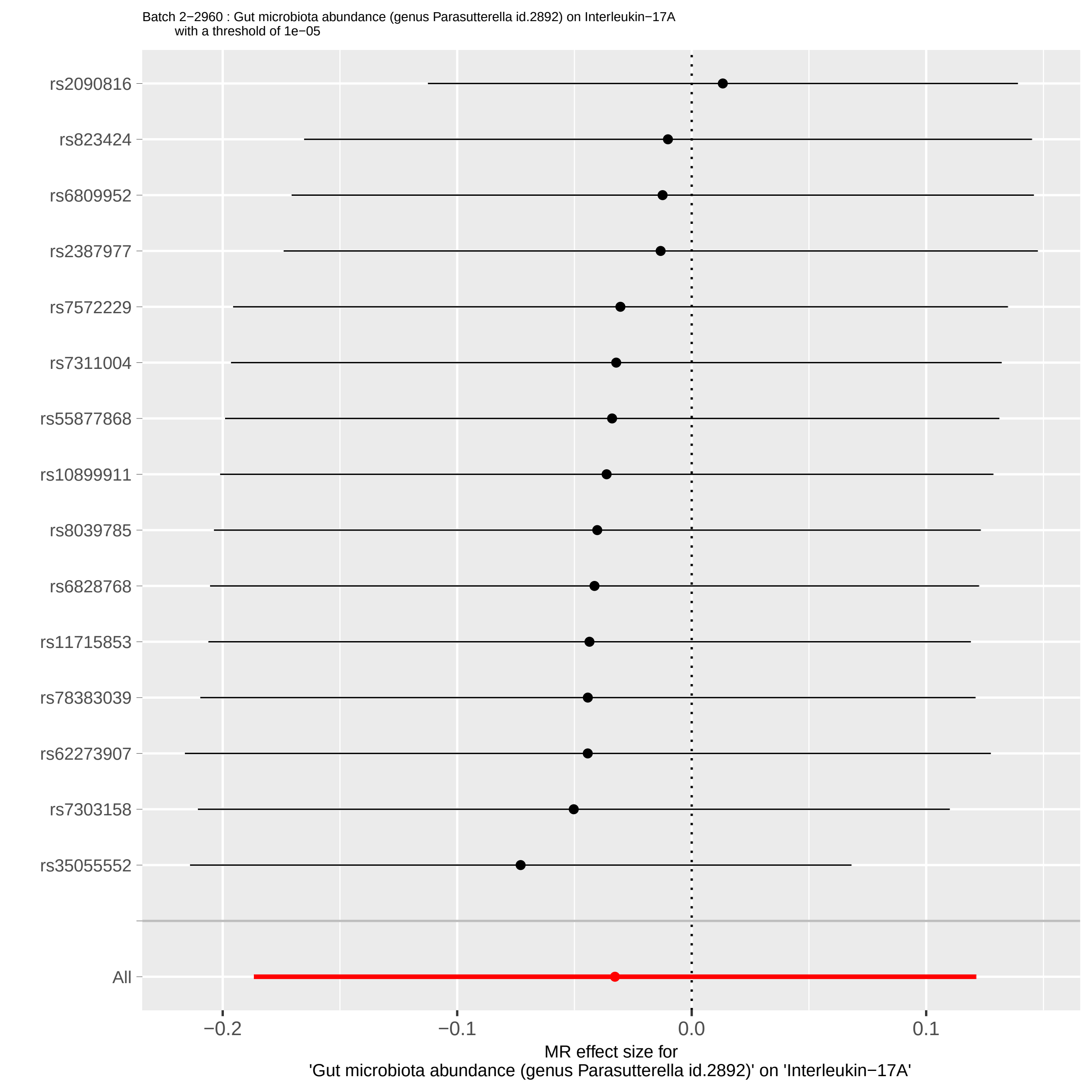
Batch 2−2960 : Gut microbiota abundance (genus Parasutterella id.2892) on Interleukin−17A
with a threshold of 1e−05
rs2090816
rs823424
rs6809952
rs2387977
rs7572229
rs7311004
rs55877868
rs10899911
rs8039785
rs6828768
rs11715853
rs78383039
rs62273907
rs7303158
rs35055552
All
−0.2
−0.1
0.0
0.1
MR effect size for
'Gut microbiota abundance (genus Parasutterella id.2892)' on 'Interleukin−17A'

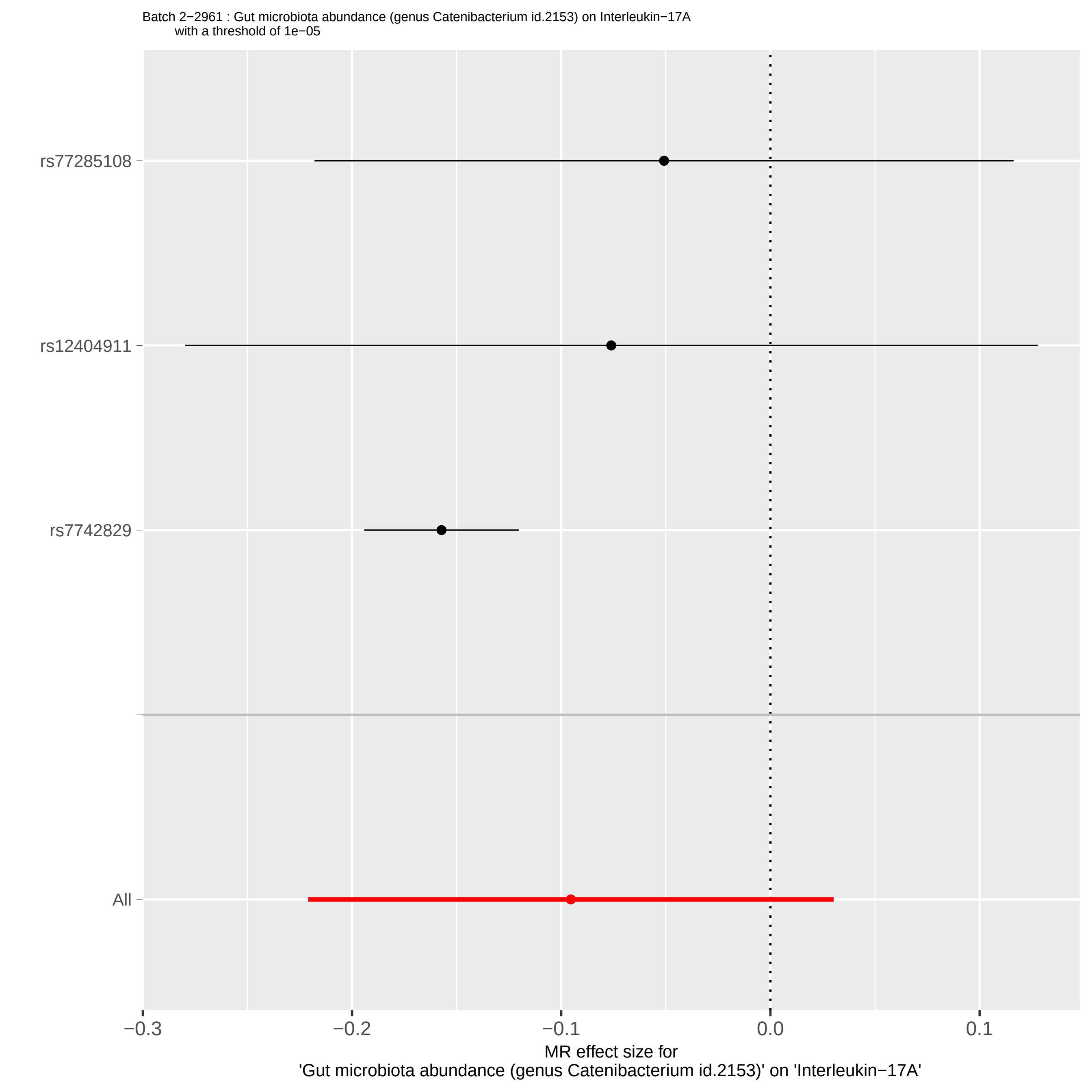

Batch 2−2961 : Gut microbiota abundance (genus Catenibacterium id.2153) on Interleukin−17A
with a threshold of 1e−05
rs77285108
rs12404911
rs7742829
All
−0.3
−0.2
−0.1
0.0
0.1
MR effect size for
'Gut microbiota abundance (genus Catenibacterium id.2153)' on 'Interleukin−17A'

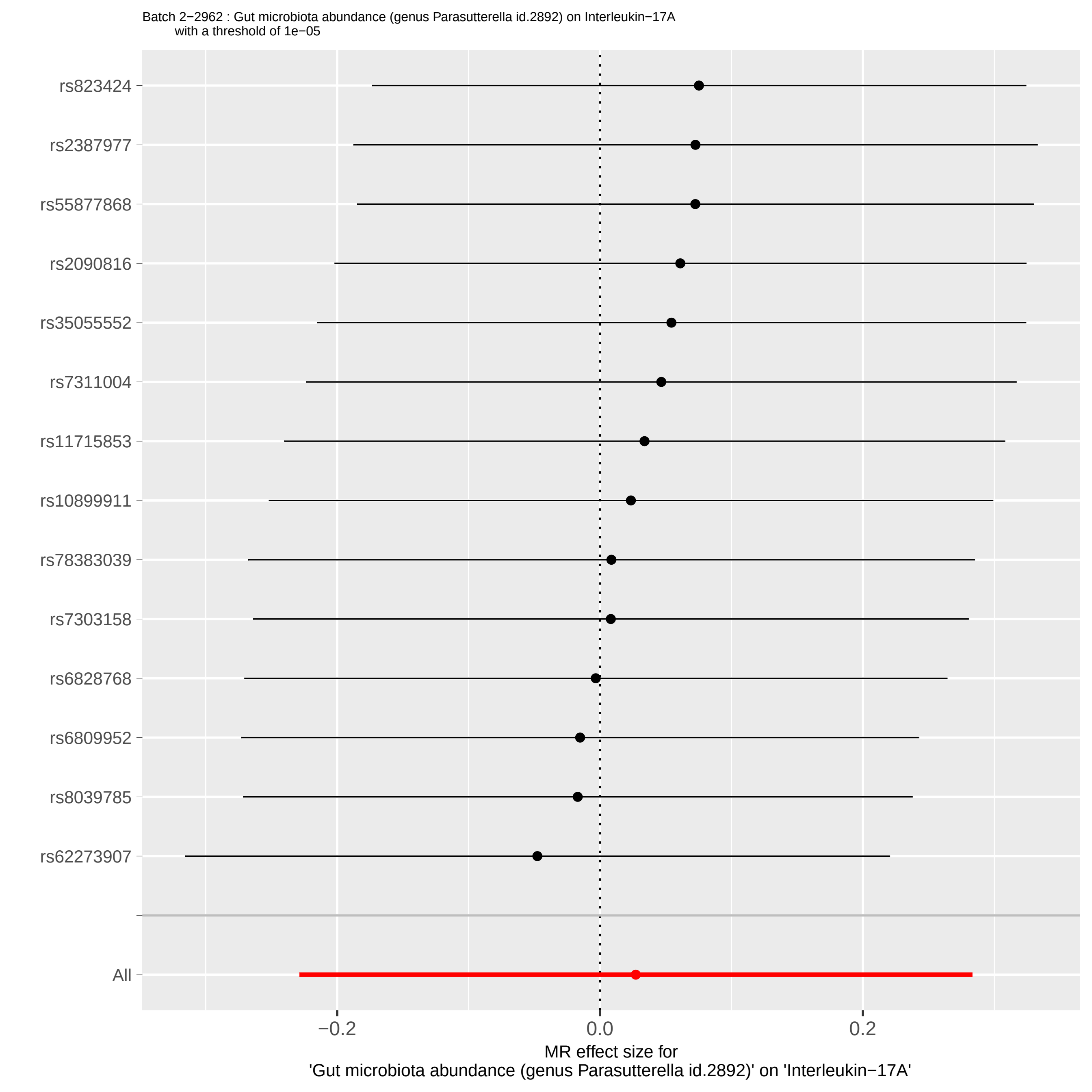

Batch 2−2962 : Gut microbiota abundance (genus Parasutterella id.2892) on Interleukin−17A
with a threshold of 1e−05
rs823424
rs2387977
rs55877868
rs2090816
rs35055552
rs7311004
rs11715853
rs10899911
rs78383039
rs7303158
rs6828768
rs6809952
rs8039785
rs62273907
All
−0.2
0.0
0.2
MR effect size for
'Gut microbiota abundance (genus Parasutterella id.2892)' on 'Interleukin−17A'

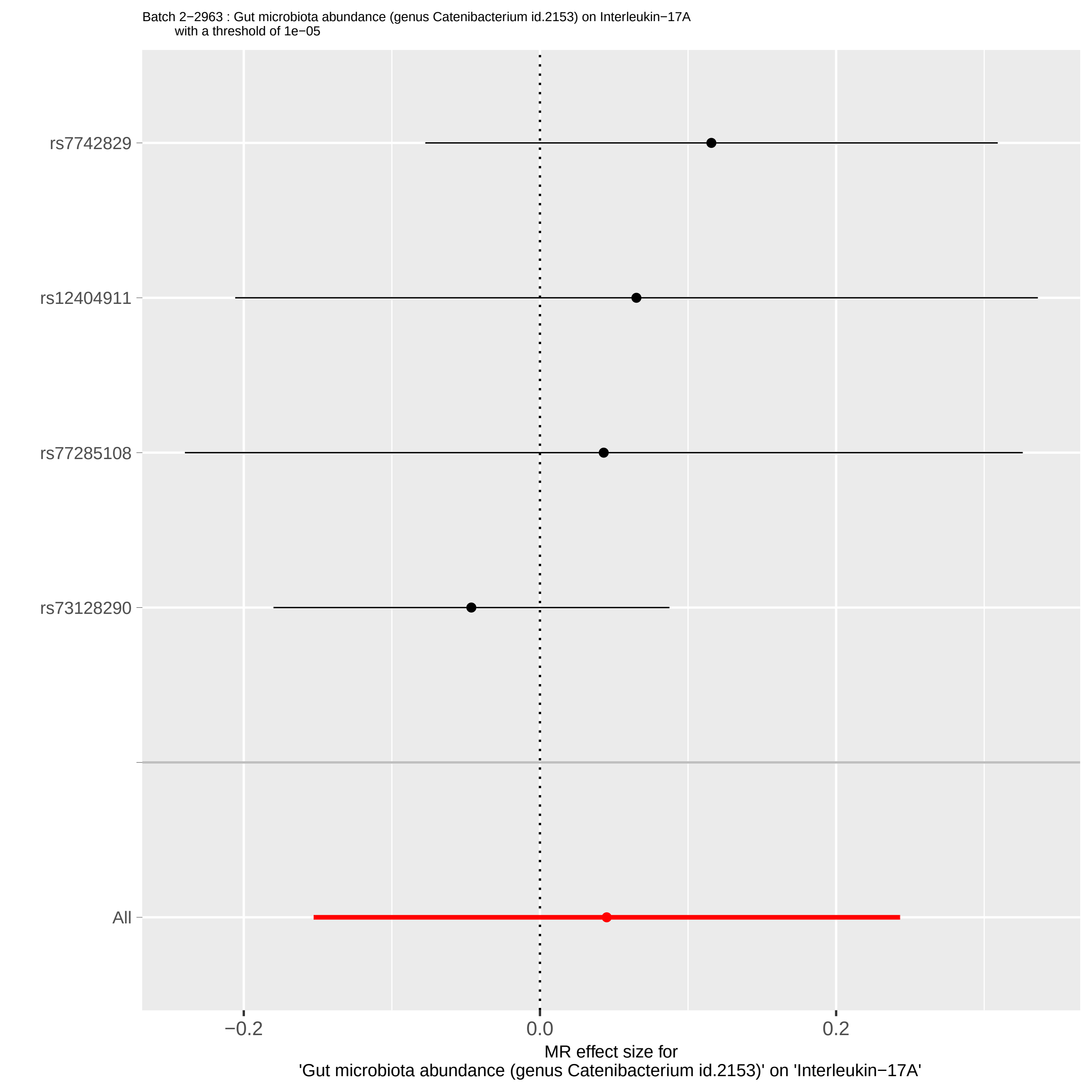

Batch 2−2963 : Gut microbiota abundance (genus Catenibacterium id.2153) on Interleukin−17A
with a threshold of 1e−05
rs7742829
rs12404911
rs77285108
rs73128290
All
−0.2
0.0
0.2
MR effect size for
'Gut microbiota abundance (genus Catenibacterium id.2153)' on 'Interleukin−17A'

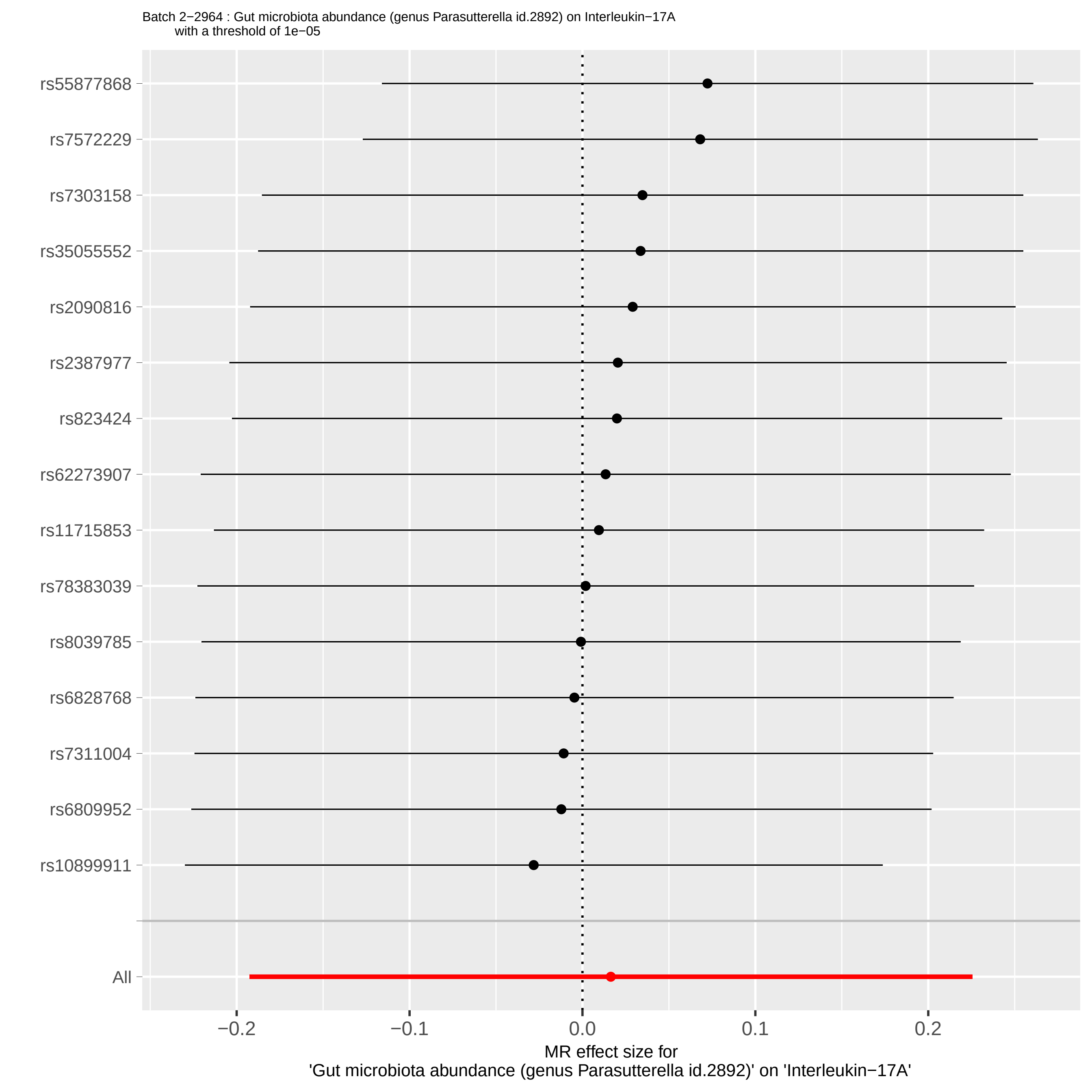

Batch 2−2964 : Gut microbiota abundance (genus Parasutterella id.2892) on Interleukin−17A
with a threshold of 1e−05
rs55877868
rs7572229
rs7303158
rs35055552
rs2090816
rs2387977
rs823424
rs62273907
rs11715853
rs78383039
rs8039785
rs6828768
rs7311004
rs6809952
rs10899911
All
−0.2
−0.1
0.0
0.1
0.2
MR effect size for
'Gut microbiota abundance (genus Parasutterella id.2892)' on 'Interleukin−17A'

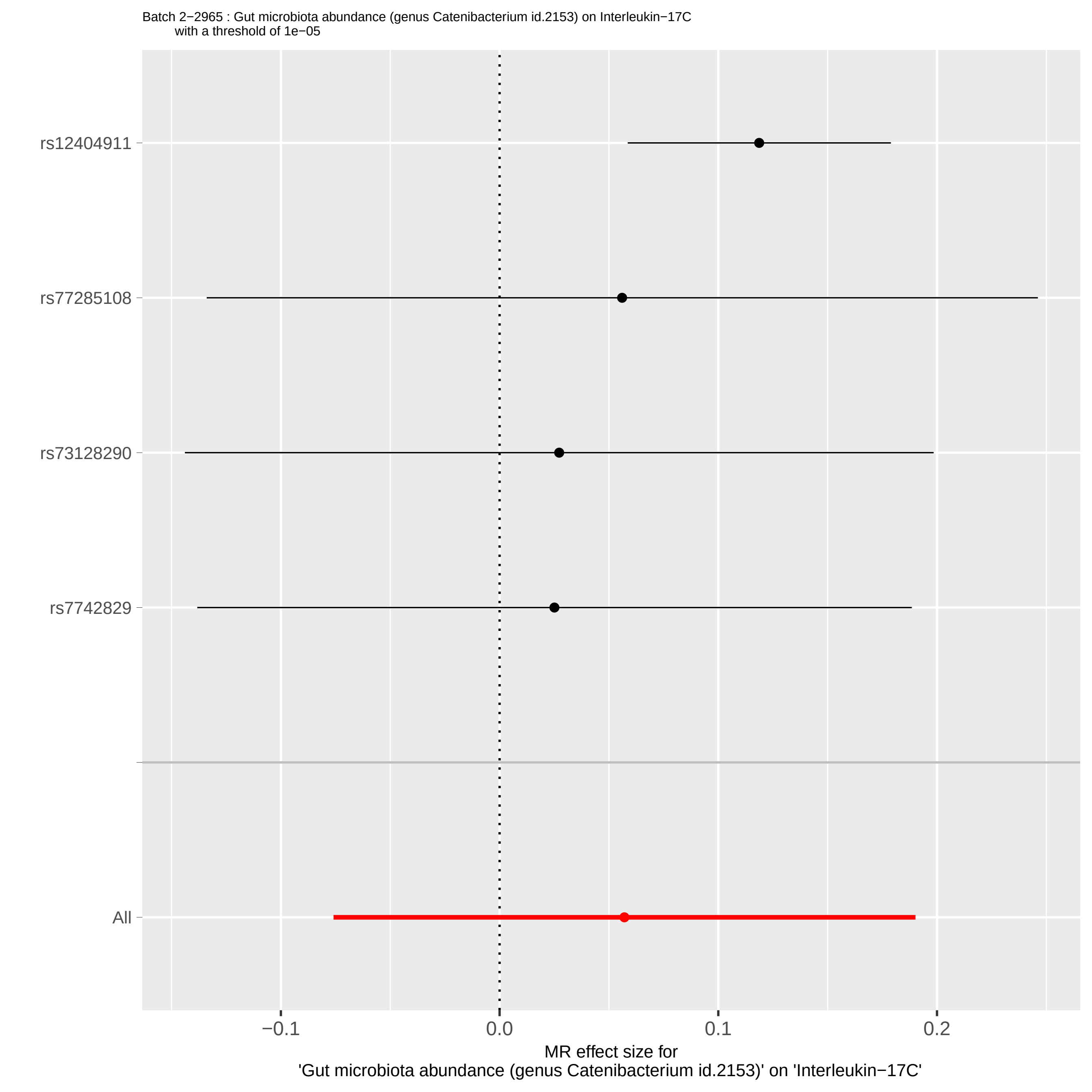
Batch 2−2965 : Gut microbiota abundance (genus Catenibacterium id.2153) on Interleukin−17C
with a threshold of 1e−05
rs12404911
rs77285108
rs73128290
rs7742829
All
−0.1
0.0
0.1
0.2
MR effect size for
'Gut microbiota abundance (genus Catenibacterium id.2153)' on 'Interleukin−17C'

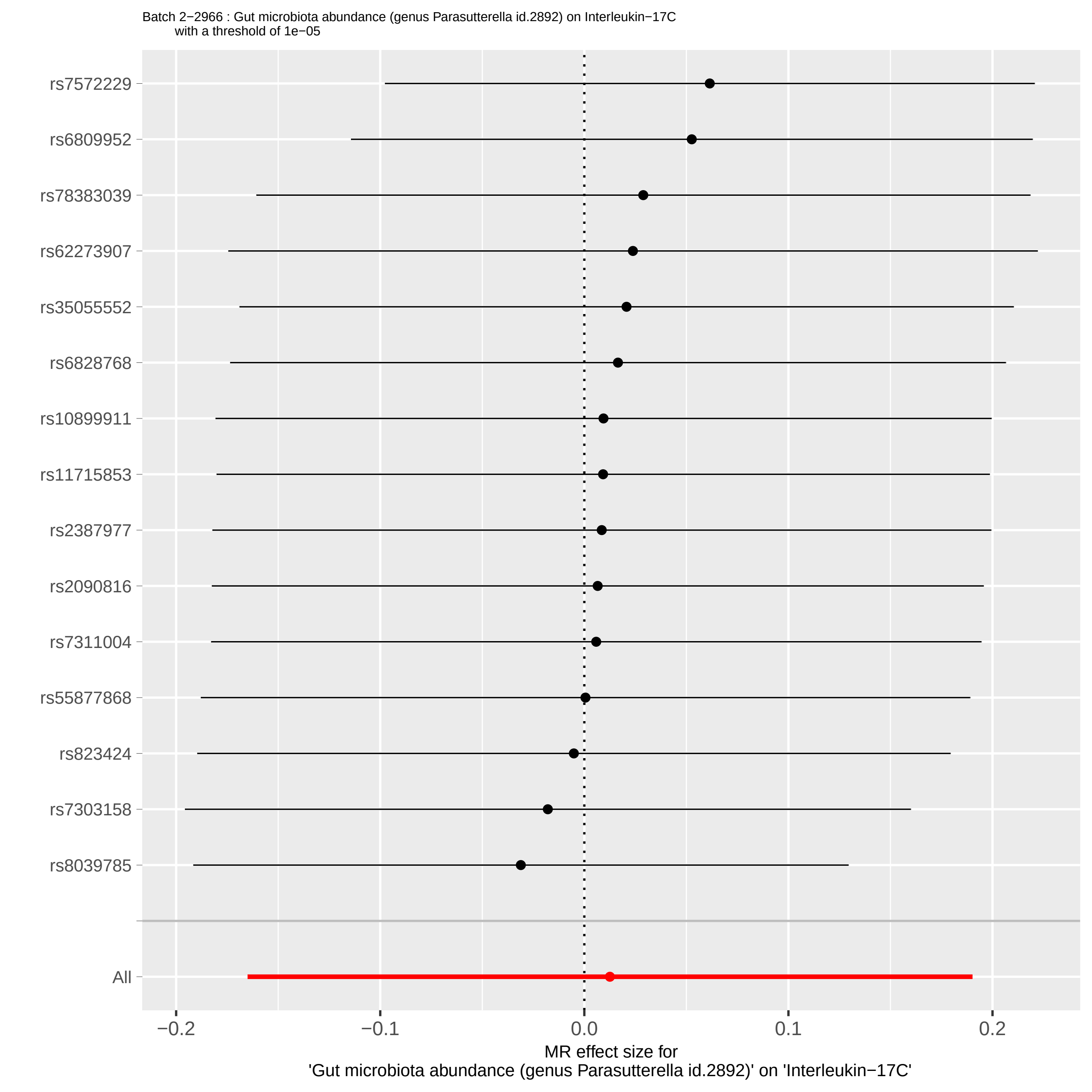
Batch 2−2966 : Gut microbiota abundance (genus Parasutterella id.2892) on Interleukin−17C
with a threshold of 1e−05
rs7572229
rs6809952
rs78383039
rs62273907
rs35055552
rs6828768
rs10899911
rs11715853
rs2387977
rs2090816
rs7311004
rs55877868
rs823424
rs7303158
rs8039785
All
−0.2
−0.1
0.0
0.1
0.2
MR effect size for
'Gut microbiota abundance (genus Parasutterella id.2892)' on 'Interleukin−17C'

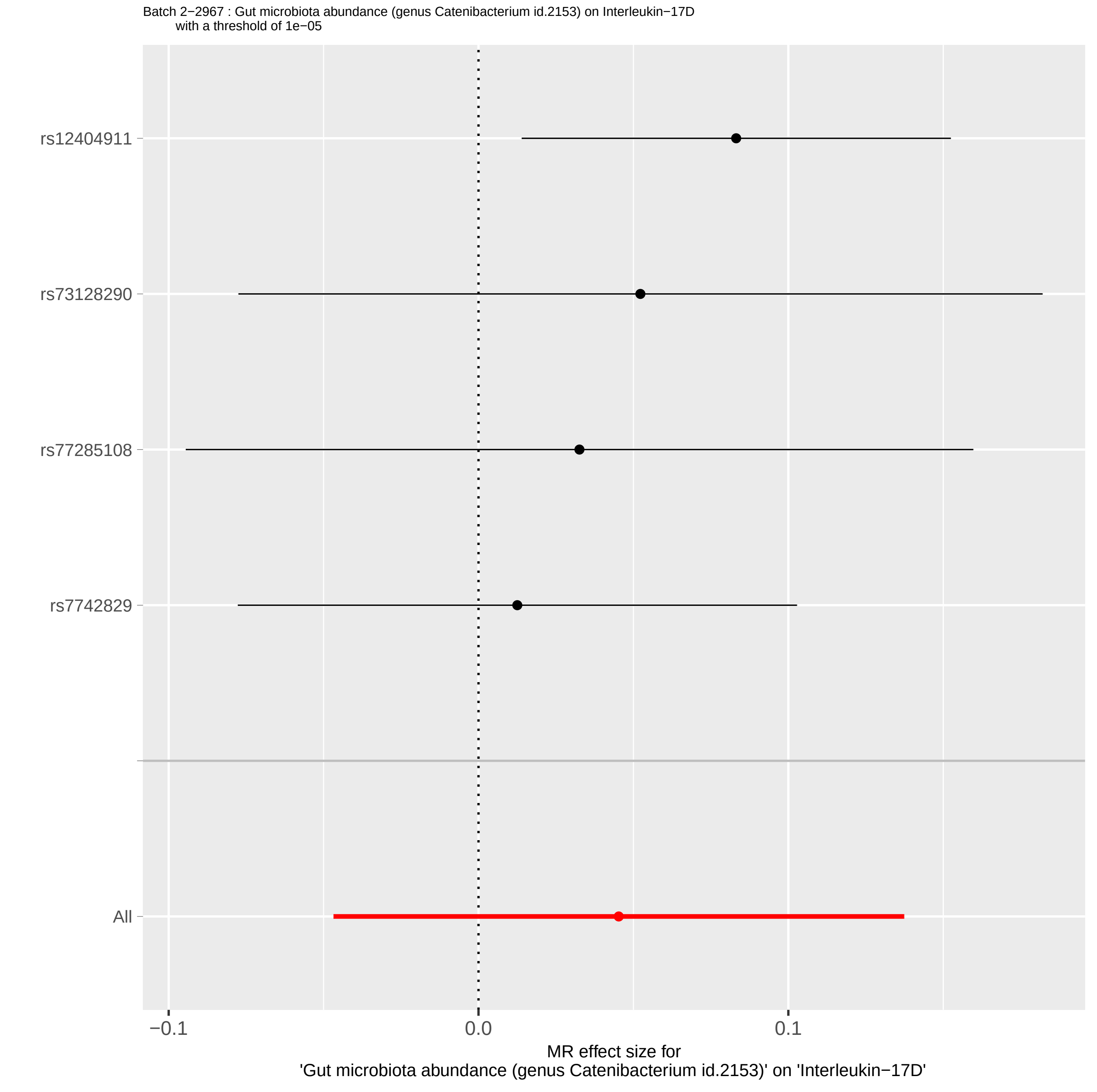
Batch 2−2967 : Gut microbiota abundance (genus Catenibacterium id.2153) on Interleukin−17D
with a threshold of 1e−05
rs12404911
rs73128290
rs77285108
rs7742829
All
−0.1
0.0
0.1
MR effect size for
'Gut microbiota abundance (genus Catenibacterium id.2153)' on 'Interleukin−17D'

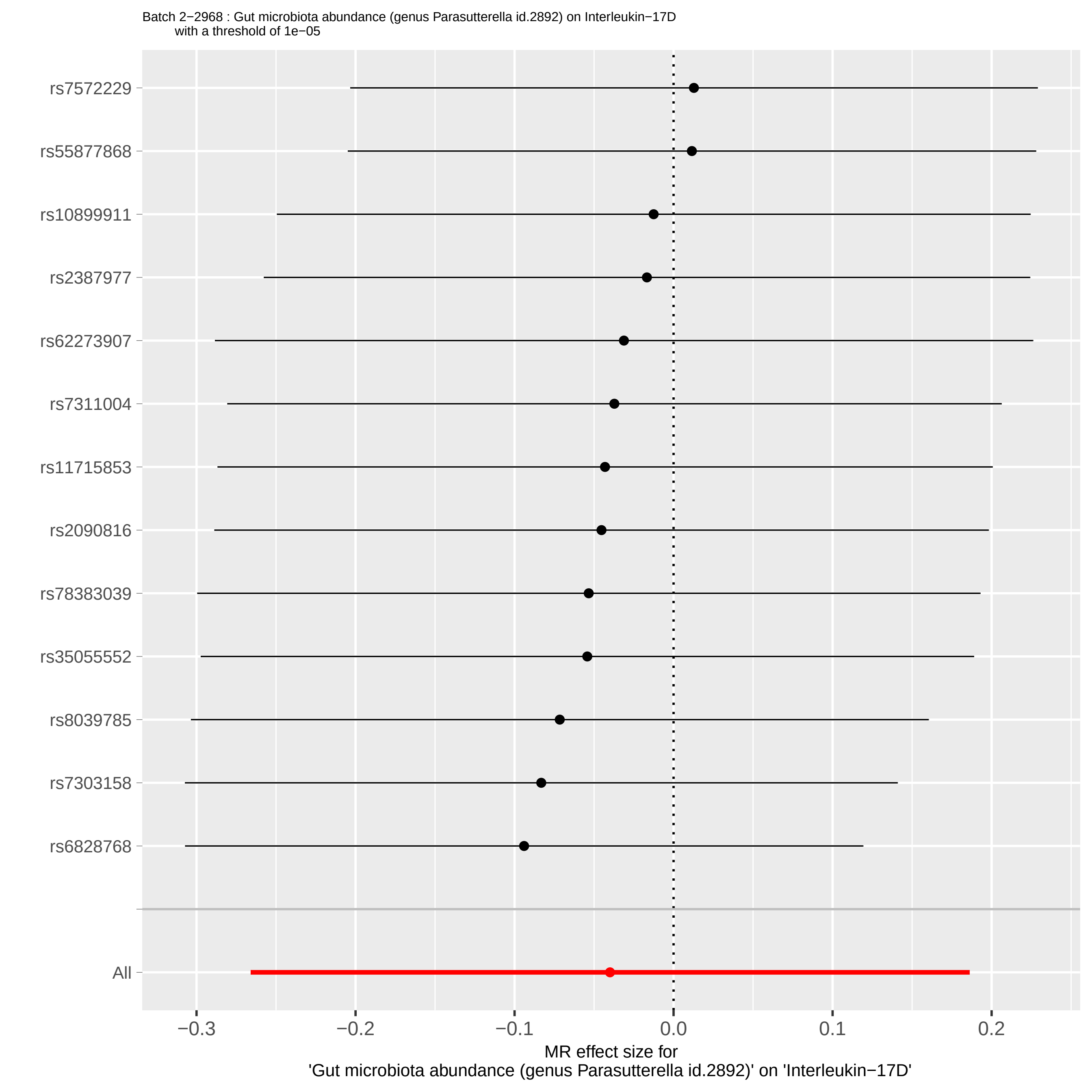
Batch 2−2968 : Gut microbiota abundance (genus Parasutterella id.2892) on Interleukin−17D
with a threshold of 1e−05
rs7572229
rs55877868
rs10899911
rs2387977
rs62273907
rs7311004
rs11715853
rs2090816
rs78383039
rs35055552
rs8039785
rs7303158
rs6828768
All
−0.3
−0.2
−0.1
0.0
0.1
0.2
MR effect size for
'Gut microbiota abundance (genus Parasutterella id.2892)' on 'Interleukin−17D'

Batch 2−2969 : Gut microbiota abundance (genus Catenibacterium id.2153) on Interleukin−17F
with a threshold of 1e−05

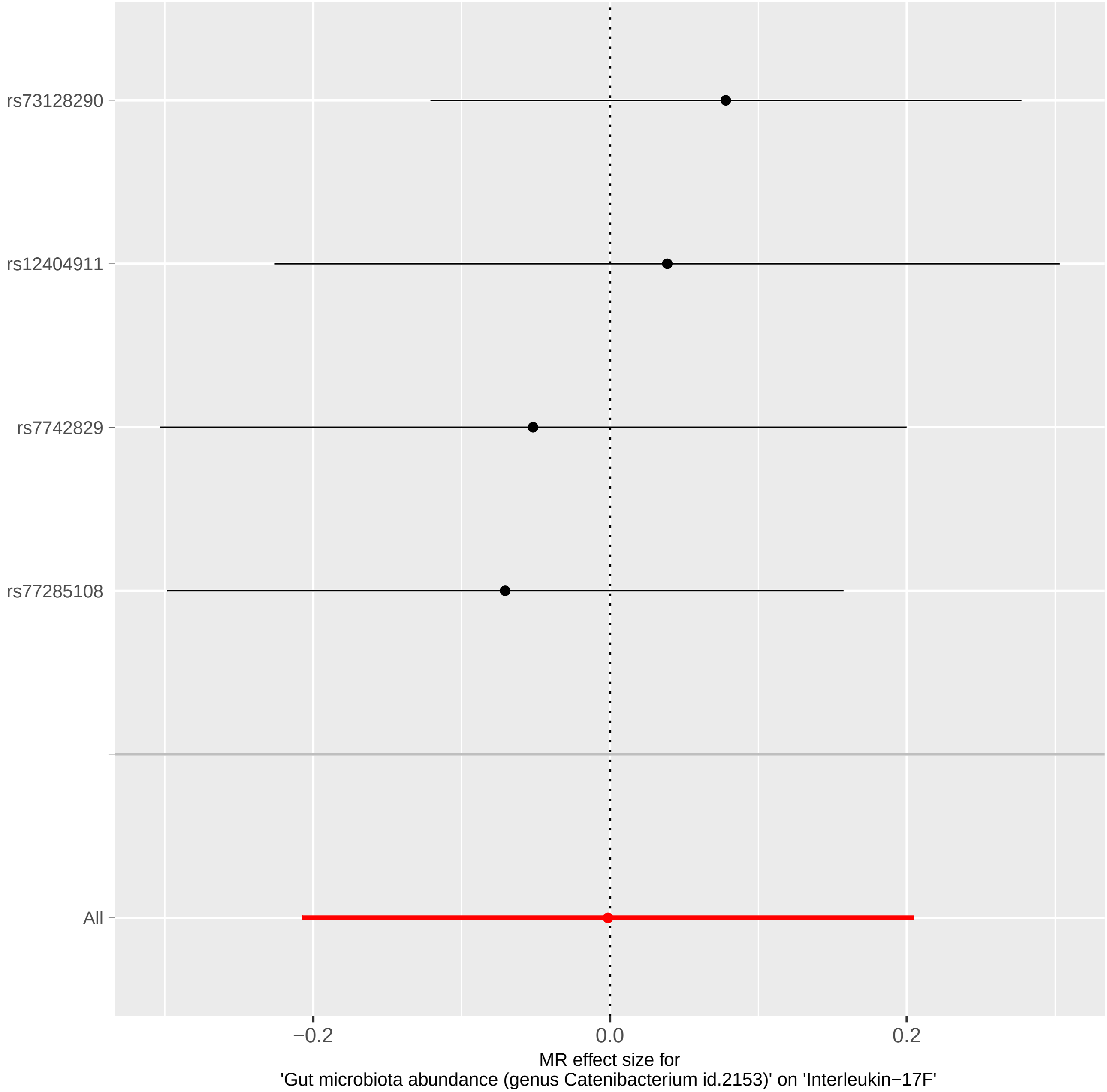

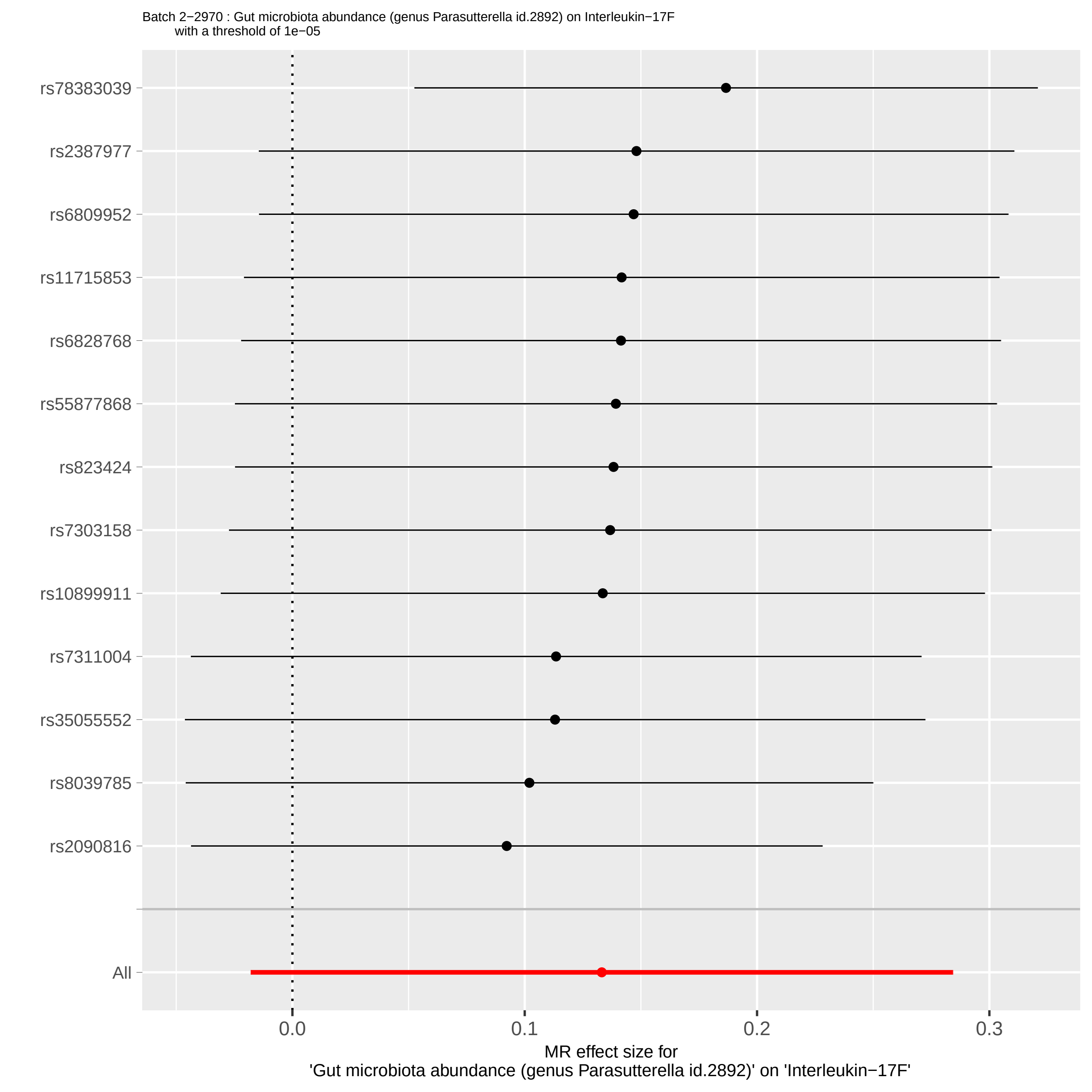

Batch 2−2970 : Gut microbiota abundance (genus Parasutterella id.2892) on Interleukin−17F
with a threshold of 1e−05
rs78383039
rs2387977
rs6809952
rs11715853
rs6828768
rs55877868
rs823424
rs7303158
rs10899911
rs7311004
rs35055552
rs8039785
rs2090816
All
0.0
0.1
0.2
0.3
MR effect size for
'Gut microbiota abundance (genus Parasutterella id.2892)' on 'Interleukin−17F'

Batch 2−2971 : Gut microbiota abundance (genus Catenibacterium id.2153) on Interleukin−17 receptor A
with a threshold of 1e−05

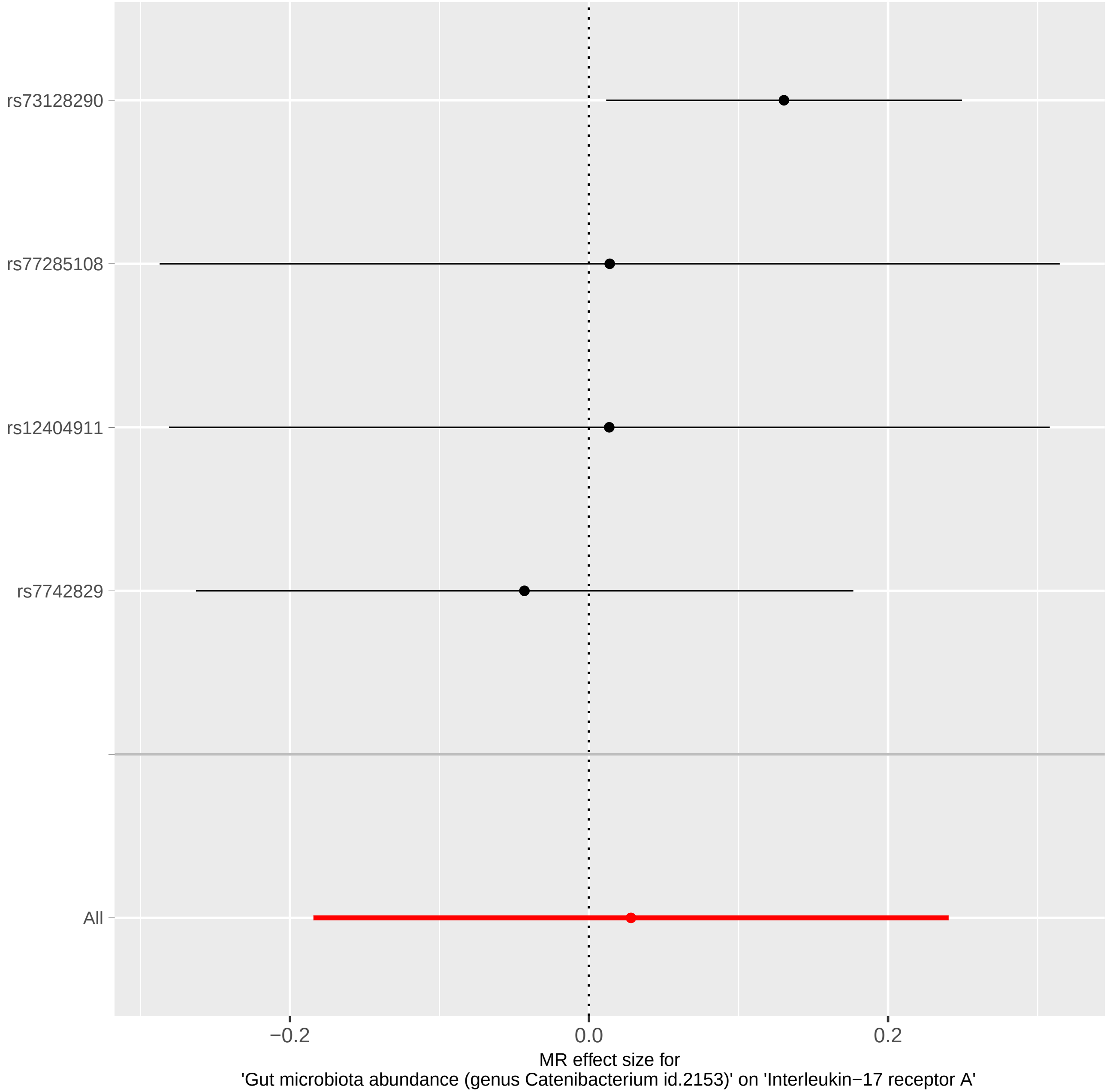

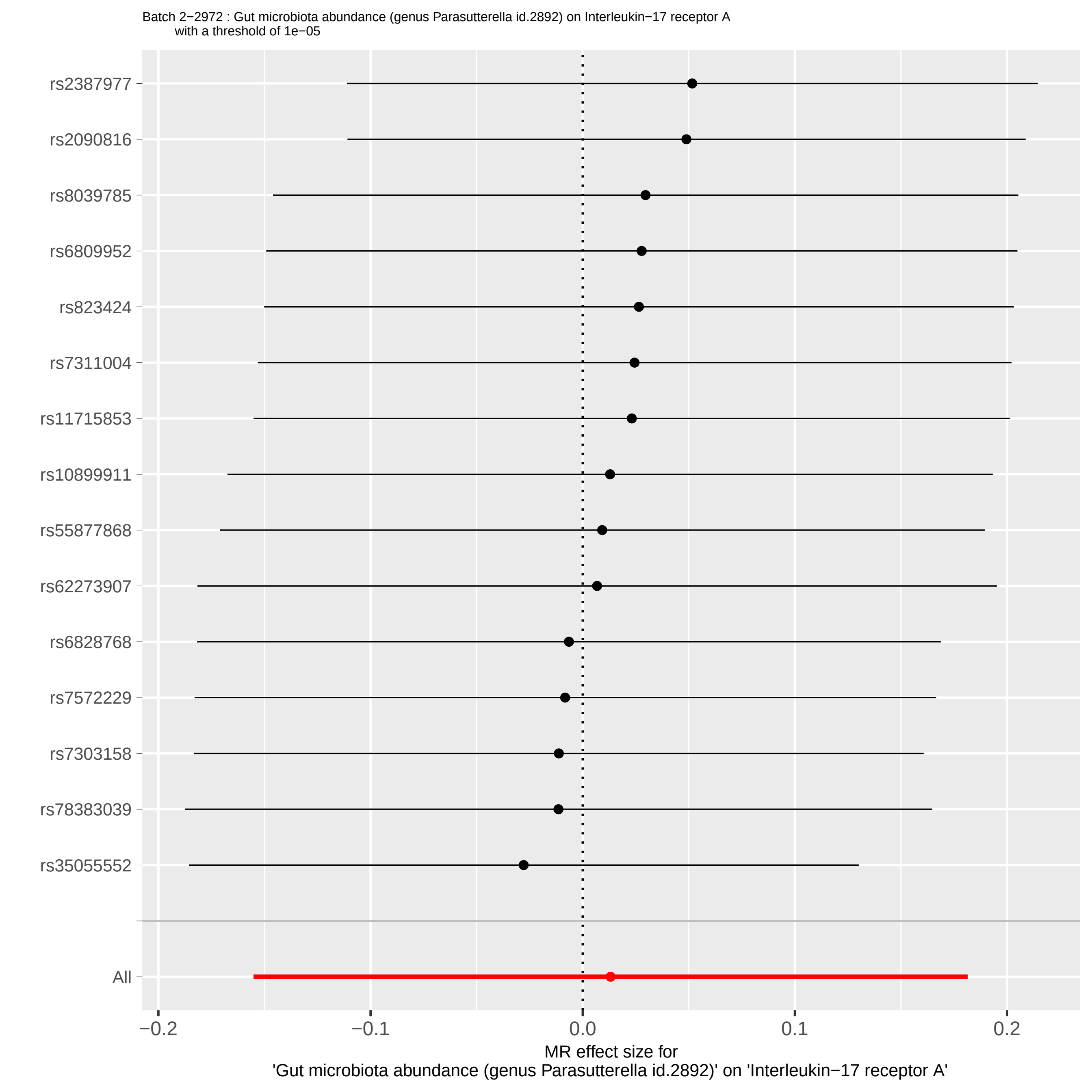

Batch 2−2972 : Gut microbiota abundance (genus Parasutterella id.2892) on Interleukin−17 receptor A
with a threshold of 1e−05
rs2387977
rs2090816
rs8039785
rs6809952
rs823424
rs7311004
rs11715853
rs10899911
rs55877868
rs62273907
rs6828768
rs7572229
rs7303158
rs78383039
rs35055552
All
−0.2
−0.1
0.0
0.1
0.2
MR effect size for
'Gut microbiota abundance (genus Parasutterella id.2892)' on 'Interleukin−17 receptor A'

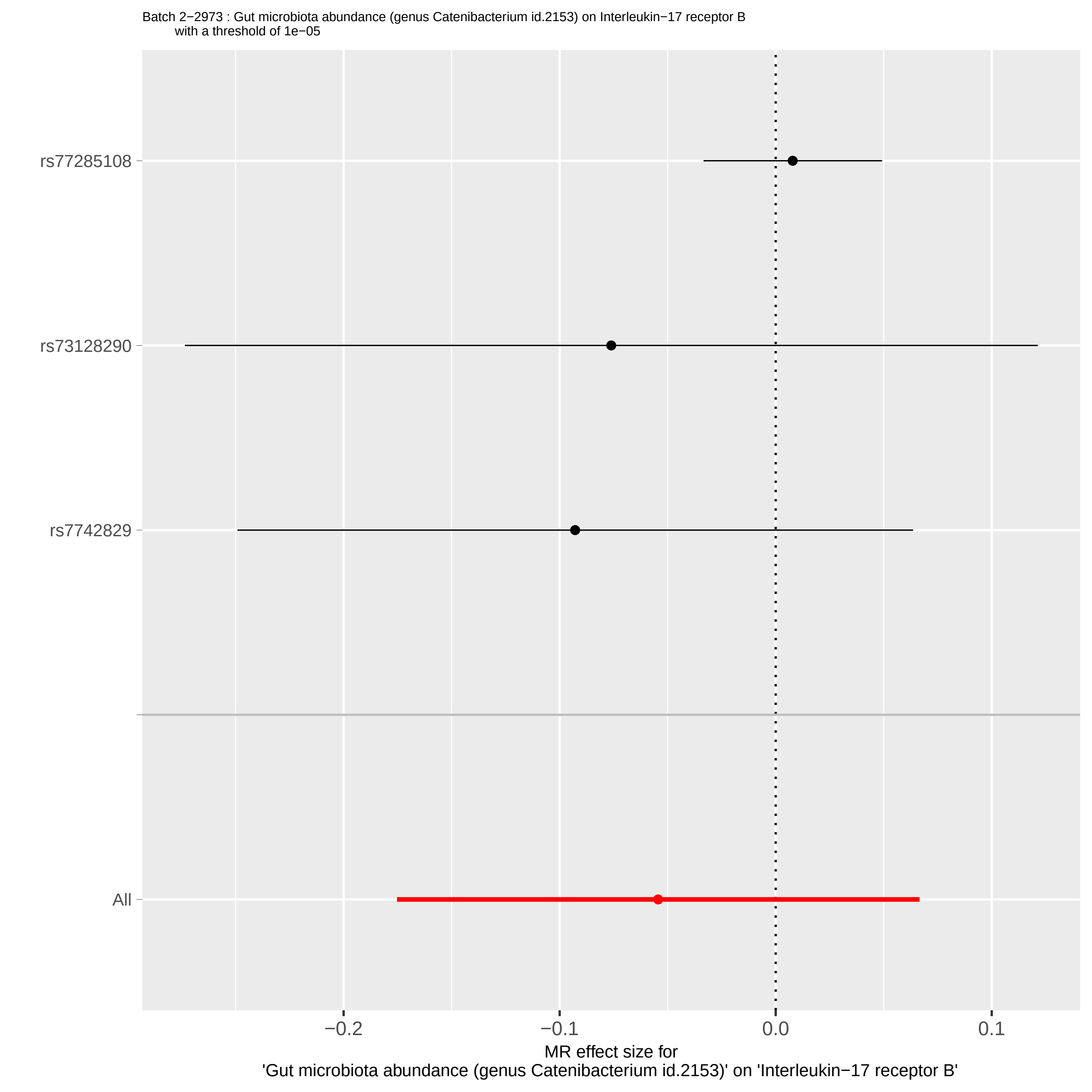
Batch 2−2973 : Gut microbiota abundance (genus Catenibacterium id.2153) on Interleukin−17 receptor B
with a threshold of 1e−05
rs77285108
rs73128290
rs7742829
All
−0.2
−0.1
0.0
0.1
MR effect size for
'Gut microbiota abundance (genus Catenibacterium id.2153)' on 'Interleukin−17 receptor B'

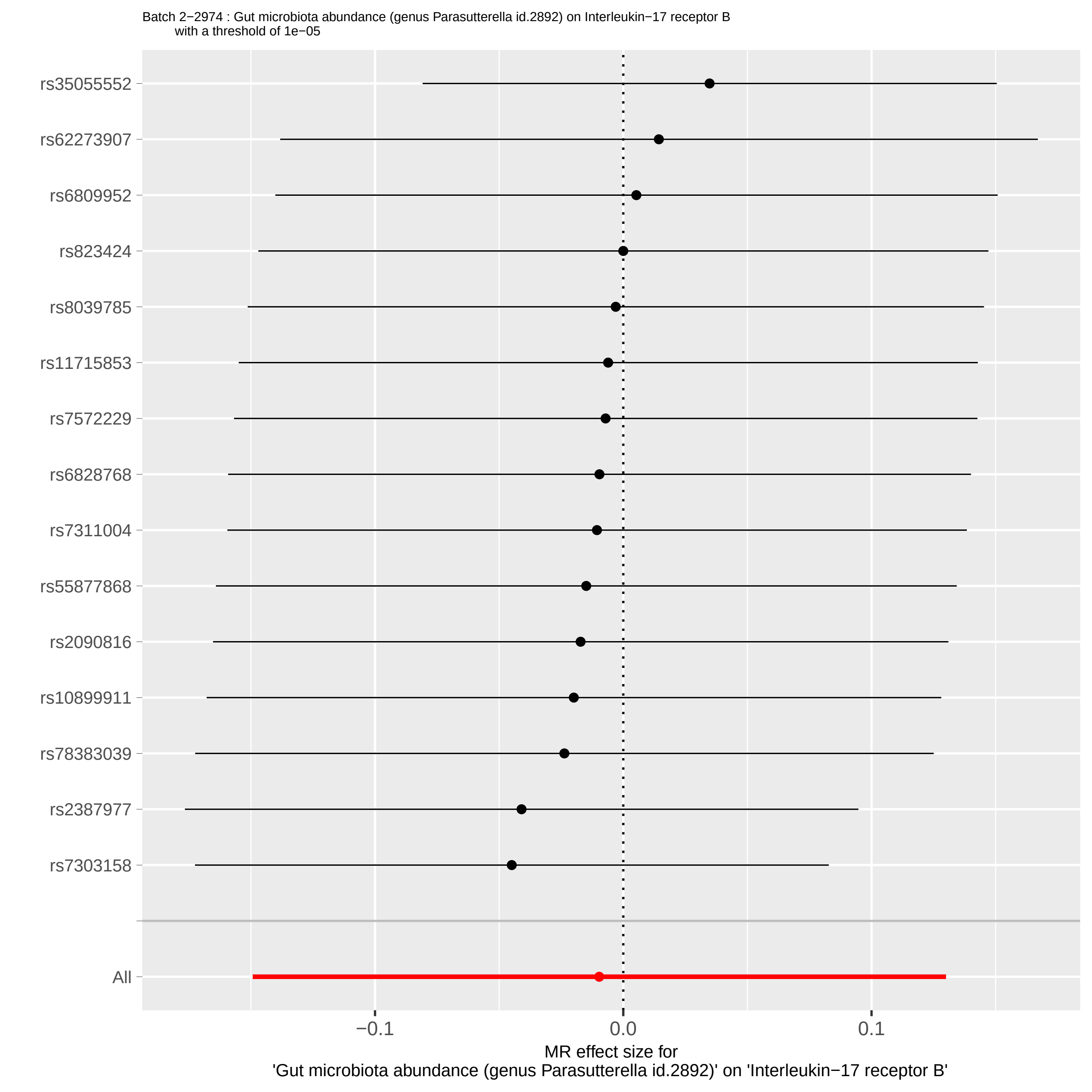
Batch 2−2974 : Gut microbiota abundance (genus Parasutterella id.2892) on Interleukin−17 receptor B
with a threshold of 1e−05
rs35055552
rs62273907
rs6809952
rs823424
rs8039785
rs11715853
rs7572229
rs6828768
rs7311004
rs55877868
rs2090816
rs10899911
rs78383039
rs2387977
rs7303158
All
−0.1
0.0
0.1
MR effect size for
'Gut microbiota abundance (genus Parasutterella id.2892)' on 'Interleukin−17 receptor B'

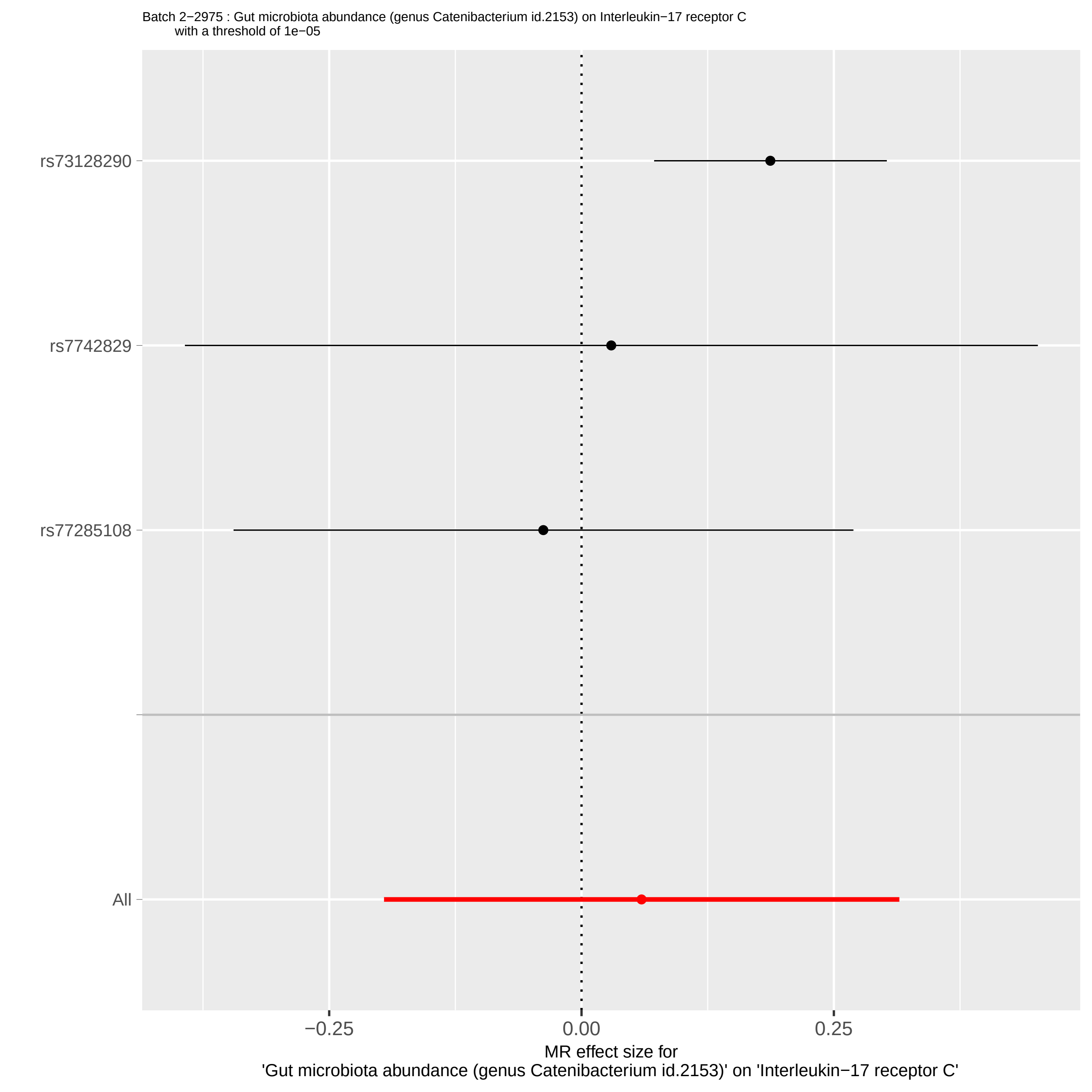
Batch 2−2975 : Gut microbiota abundance (genus Catenibacterium id.2153) on Interleukin−17 receptor C
with a threshold of 1e−05
rs73128290
rs7742829
rs77285108
All
−0.25
0.00
0.25
MR effect size for
'Gut microbiota abundance (genus Catenibacterium id.2153)' on 'Interleukin−17 receptor C'

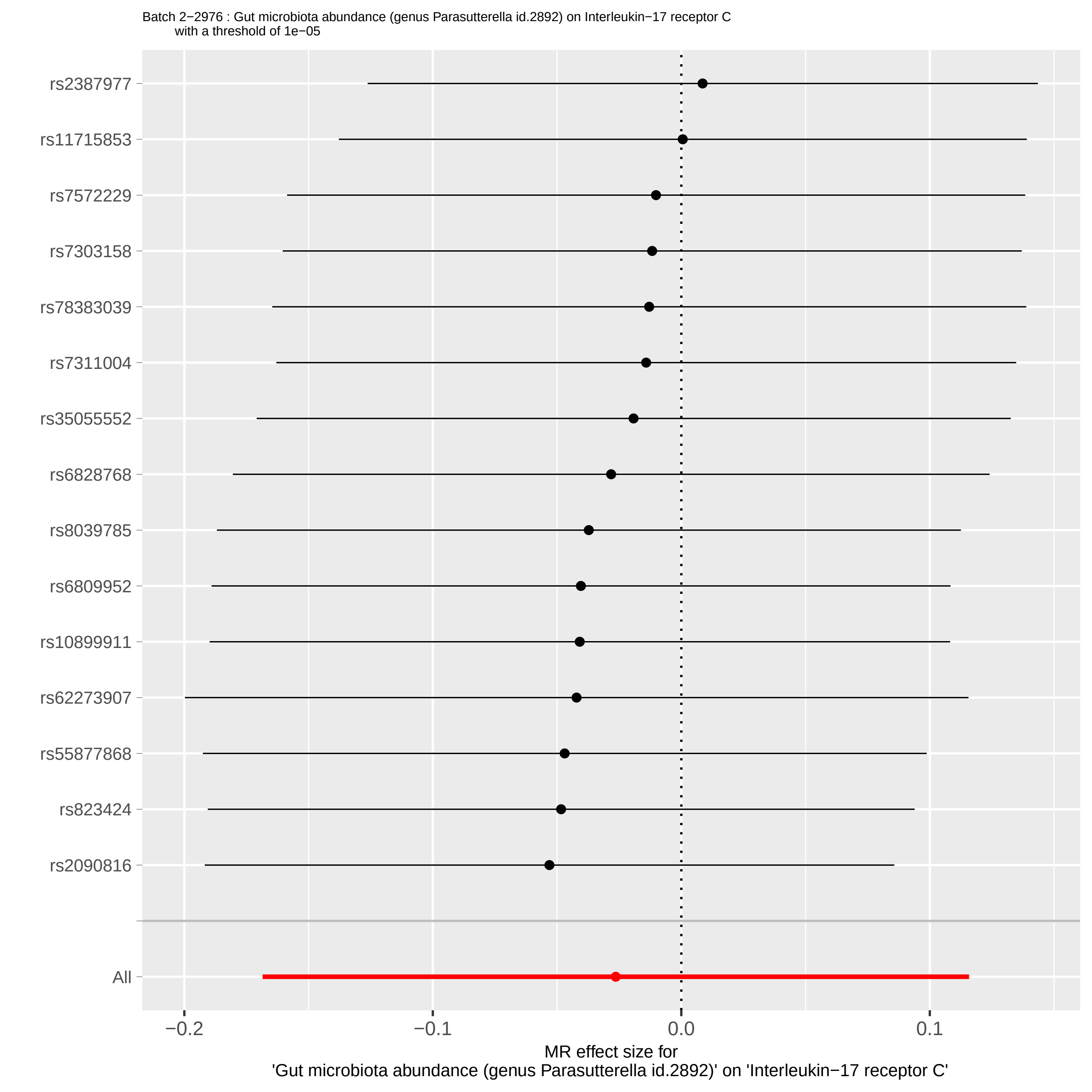
Batch 2−2976 : Gut microbiota abundance (genus Parasutterella id.2892) on Interleukin−17 receptor C
with a threshold of 1e−05
rs2387977
rs11715853
rs7572229
rs7303158
rs78383039
rs7311004
rs35055552
rs6828768
rs8039785
rs6809952
rs10899911
rs62273907
rs55877868
rs823424
rs2090816
All
−0.2
−0.1
0.0
0.1
MR effect size for
'Gut microbiota abundance (genus Parasutterella id.2892)' on 'Interleukin−17 receptor C'

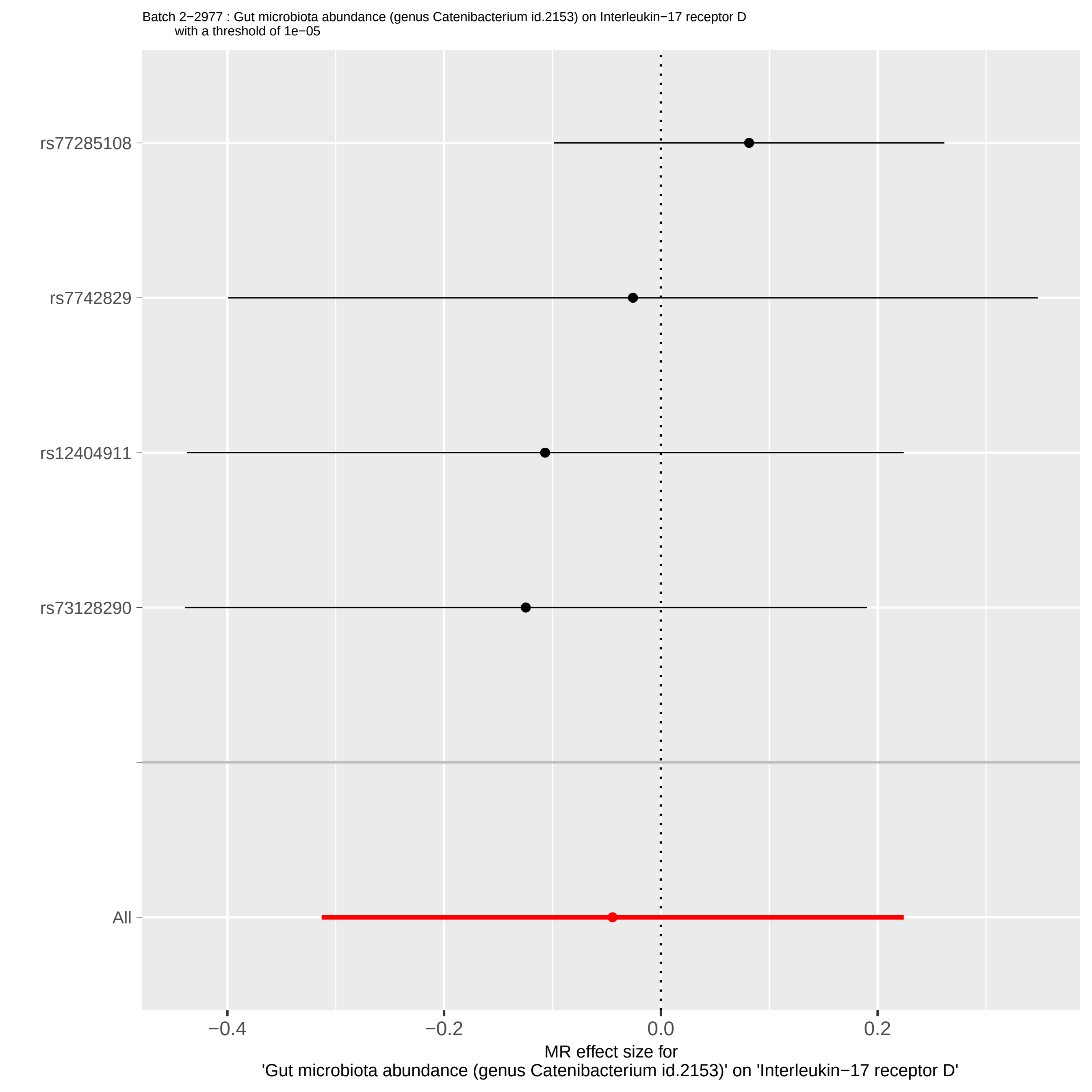
Batch 2−2977 : Gut microbiota abundance (genus Catenibacterium id.2153) on Interleukin−17 receptor D
with a threshold of 1e−05
rs77285108
rs7742829
rs12404911
rs73128290
All
−0.4
−0.2
0.0
0.2
MR effect size for
'Gut microbiota abundance (genus Catenibacterium id.2153)' on 'Interleukin−17 receptor D'

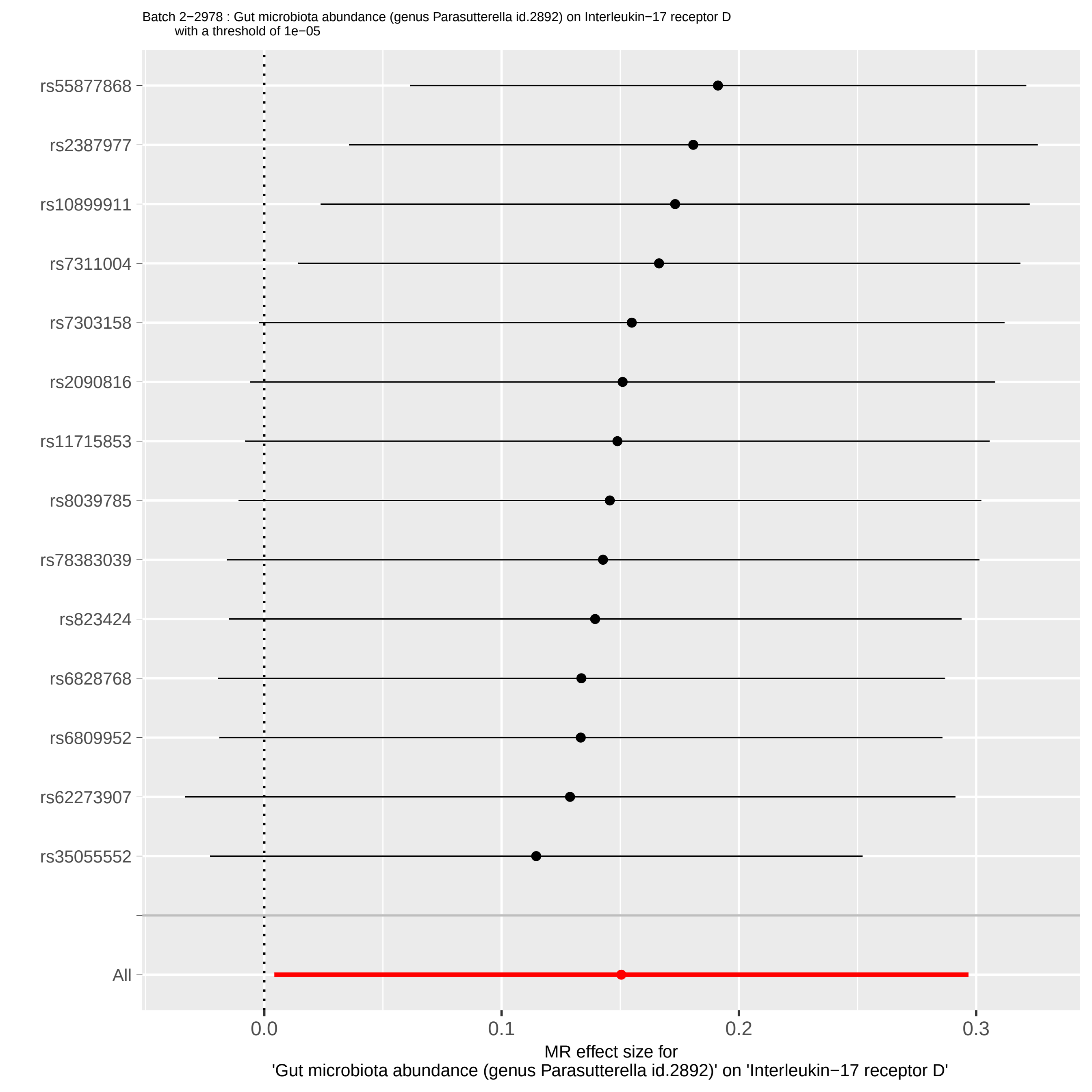
Batch 2−2978 : Gut microbiota abundance (genus Parasutterella id.2892) on Interleukin−17 receptor D
with a threshold of 1e−05
rs55877868
rs2387977
rs10899911
rs7311004
rs7303158
rs2090816
rs11715853
rs8039785
rs78383039
rs823424
rs6828768
rs6809952
rs62273907
rs35055552
All
0.0
0.1
0.2
0.3
MR effect size for
'Gut microbiota abundance (genus Parasutterella id.2892)' on 'Interleukin−17 receptor D'

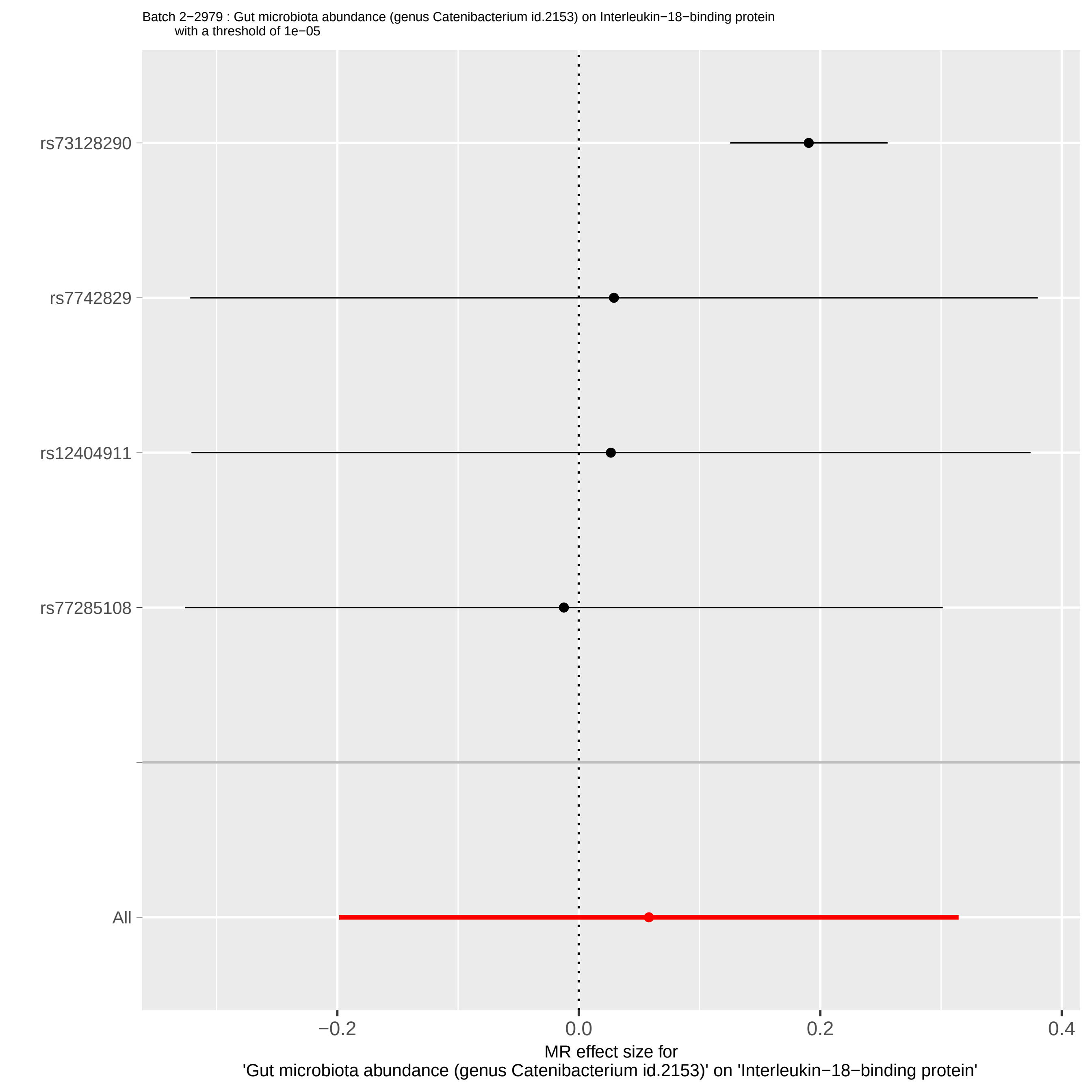
Batch 2−2979 : Gut microbiota abundance (genus Catenibacterium id.2153) on Interleukin−18−binding protein
with a threshold of 1e−05
rs73128290
rs7742829
rs12404911
rs77285108
All
−0.2
0.0
0.2
0.4
MR effect size for
'Gut microbiota abundance (genus Catenibacterium id.2153)' on 'Interleukin−18−binding protein'

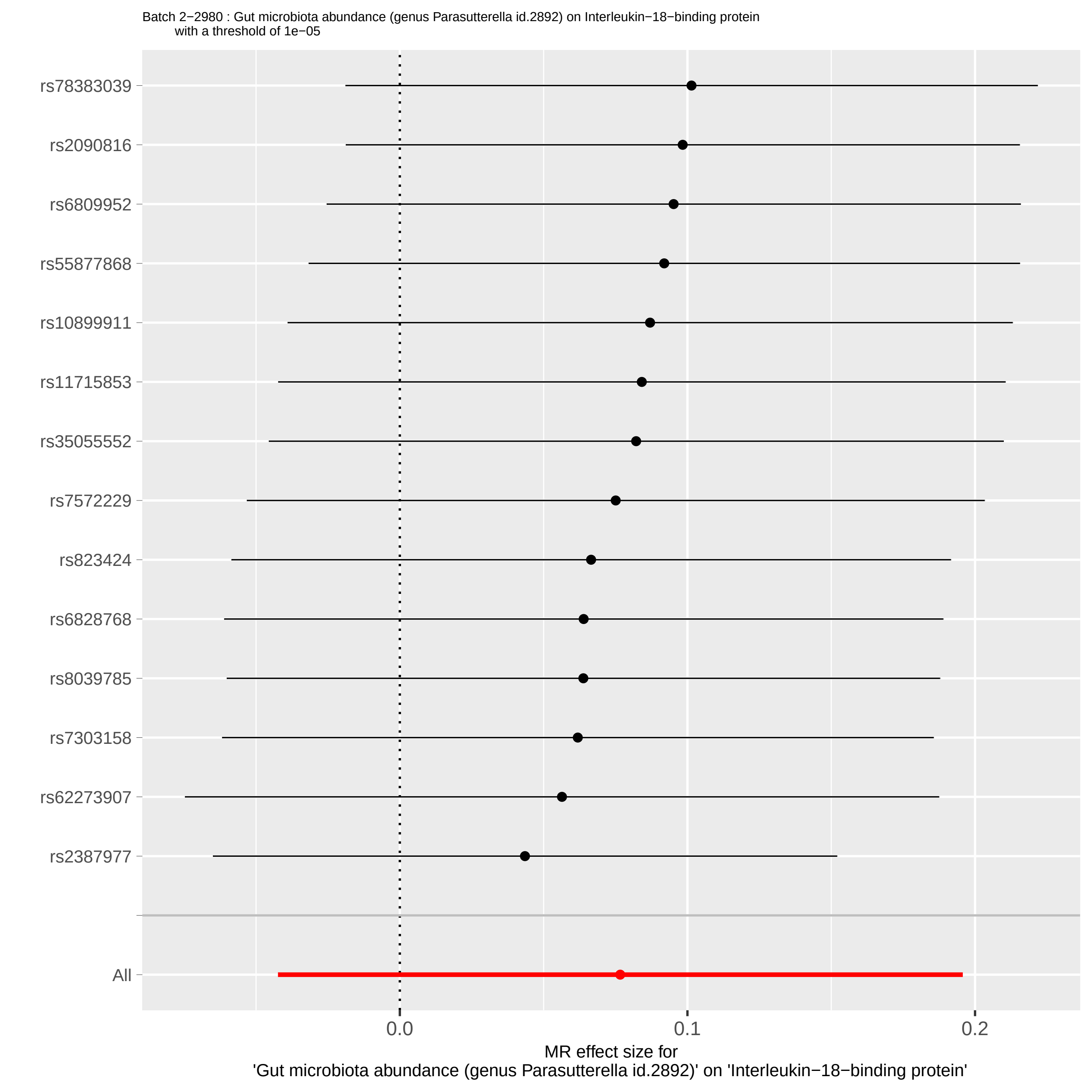

Batch 2−2980 : Gut microbiota abundance (genus Parasutterella id.2892) on Interleukin−18−binding protein
with a threshold of 1e−05
rs78383039
rs2090816
rs6809952
rs55877868
rs10899911
rs11715853
rs35055552
rs7572229
rs823424
rs6828768
rs8039785
rs7303158
rs62273907
rs2387977
All
0.0
0.1
0.2
MR effect size for
'Gut microbiota abundance (genus Parasutterella id.2892)' on 'Interleukin−18−binding protein'

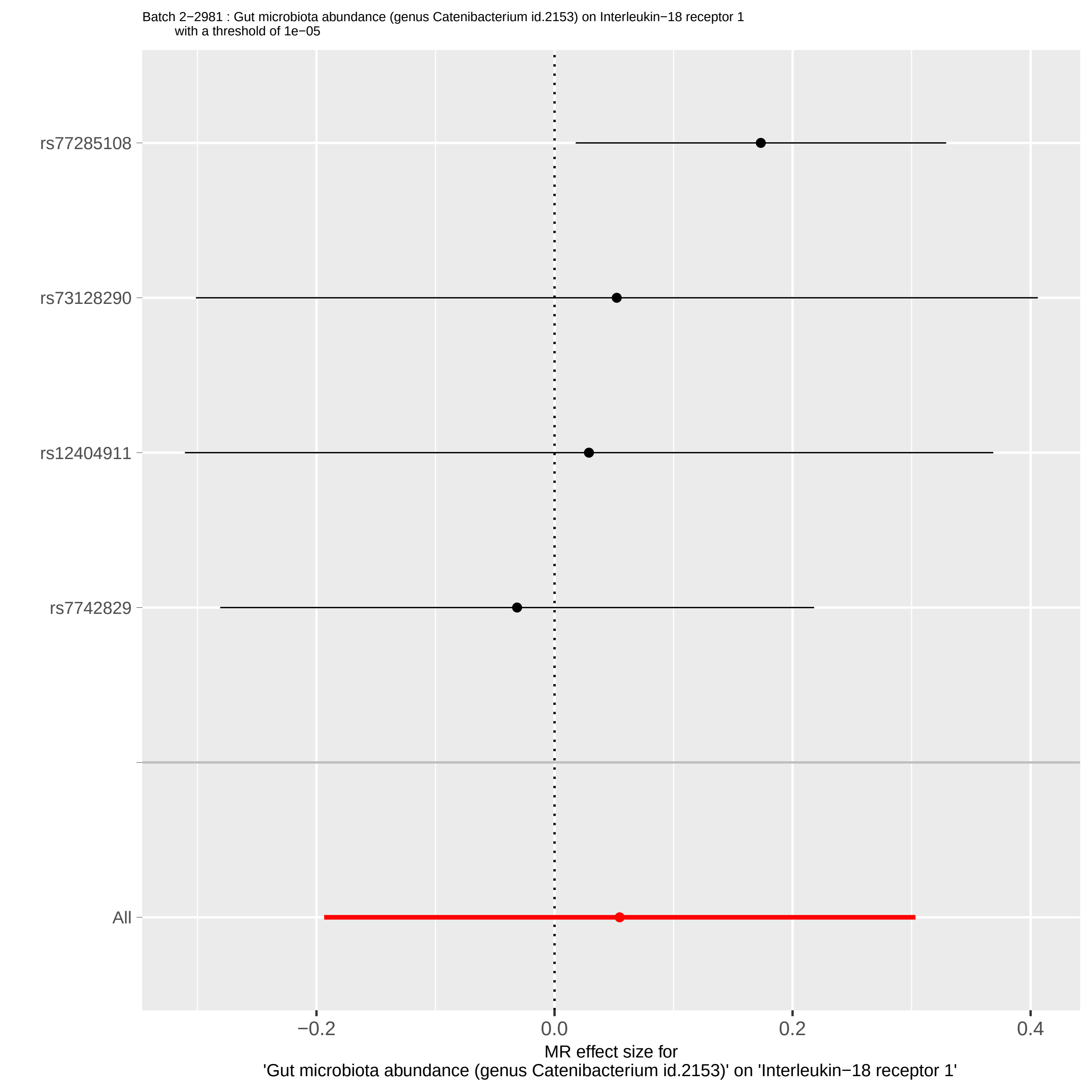
Batch 2−2981 : Gut microbiota abundance (genus Catenibacterium id.2153) on Interleukin−18 receptor 1
with a threshold of 1e−05
rs77285108
rs73128290
rs12404911
rs7742829
All
−0.2
0.0
0.2
0.4
MR effect size for
'Gut microbiota abundance (genus Catenibacterium id.2153)' on 'Interleukin−18 receptor 1'

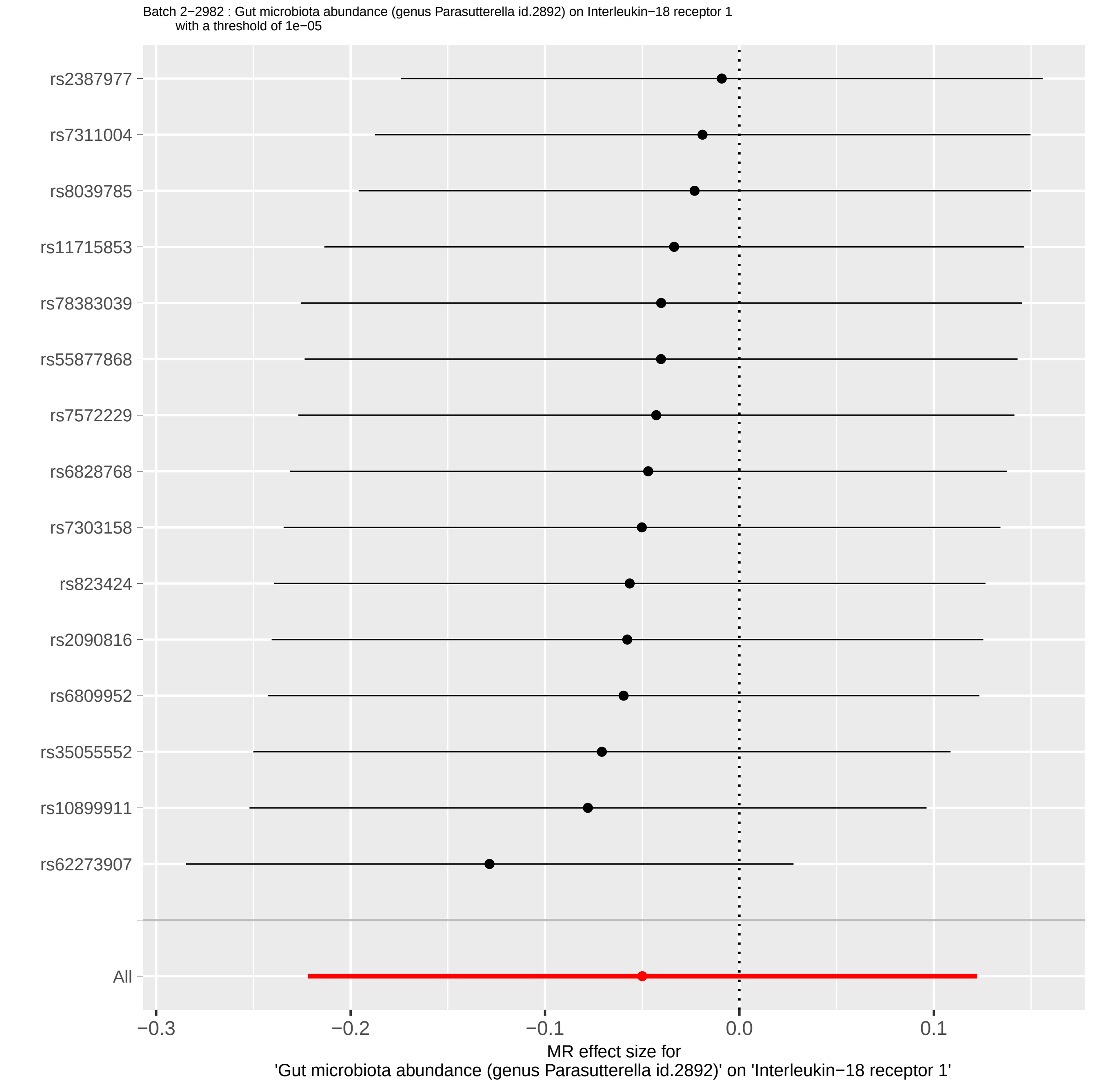

Batch 2−2982 : Gut microbiota abundance (genus Parasutterella id.2892) on Interleukin−18 receptor 1
with a threshold of 1e−05
rs2387977
rs7311004
rs8039785
rs11715853
rs78383039
rs55877868
rs7572229
rs6828768
rs7303158
rs823424
rs2090816
rs6809952
rs35055552
rs10899911
rs62273907
All
−0.3
−0.2
−0.1
0.0
0.1
MR effect size for
'Gut microbiota abundance (genus Parasutterella id.2892)' on 'Interleukin−18 receptor 1'

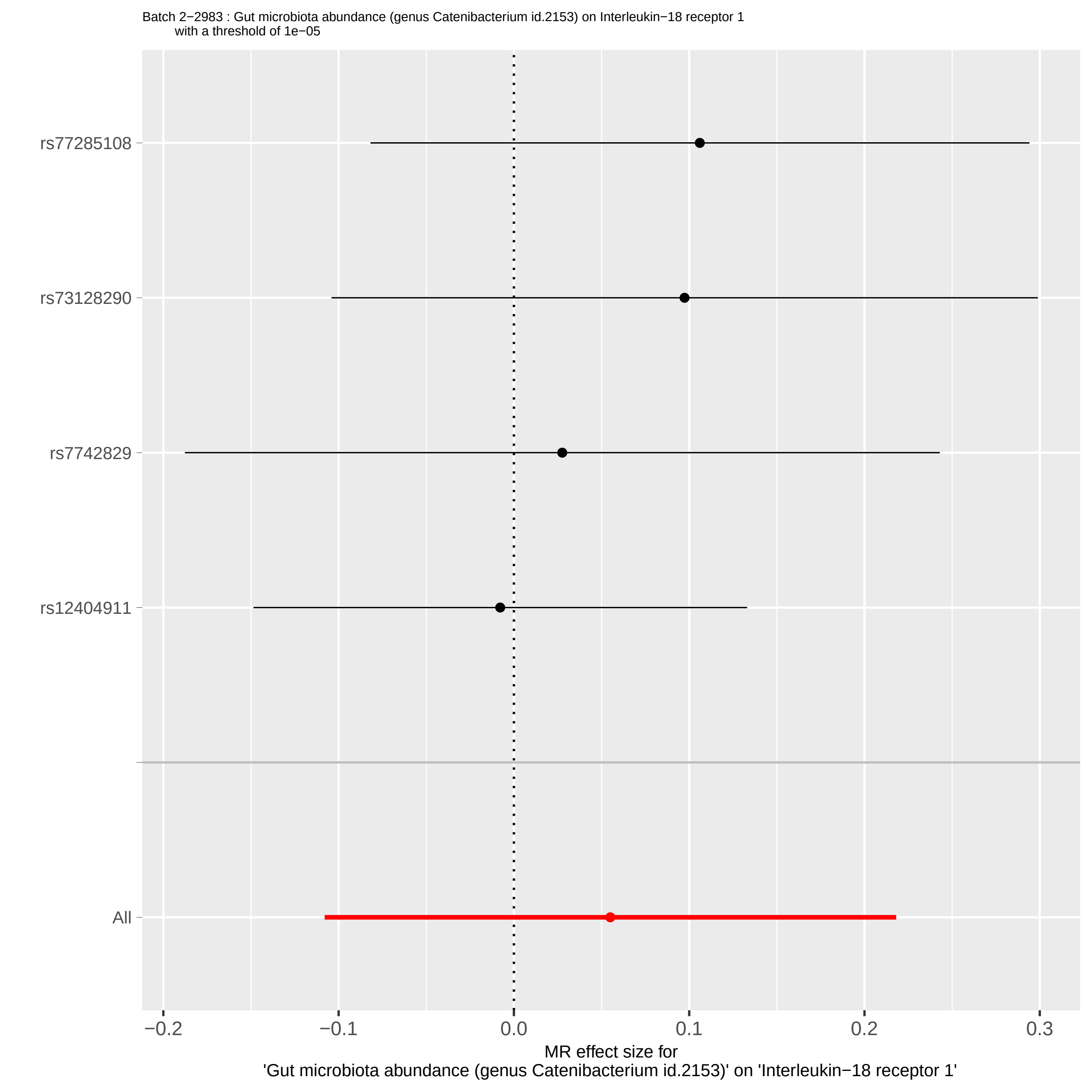
Batch 2−2983 : Gut microbiota abundance (genus Catenibacterium id.2153) on Interleukin−18 receptor 1
with a threshold of 1e−05
rs77285108
rs73128290
rs7742829
rs12404911
All
−0.2
−0.1
0.0
0.1
0.2
0.3
MR effect size for
'Gut microbiota abundance (genus Catenibacterium id.2153)' on 'Interleukin−18 receptor 1'

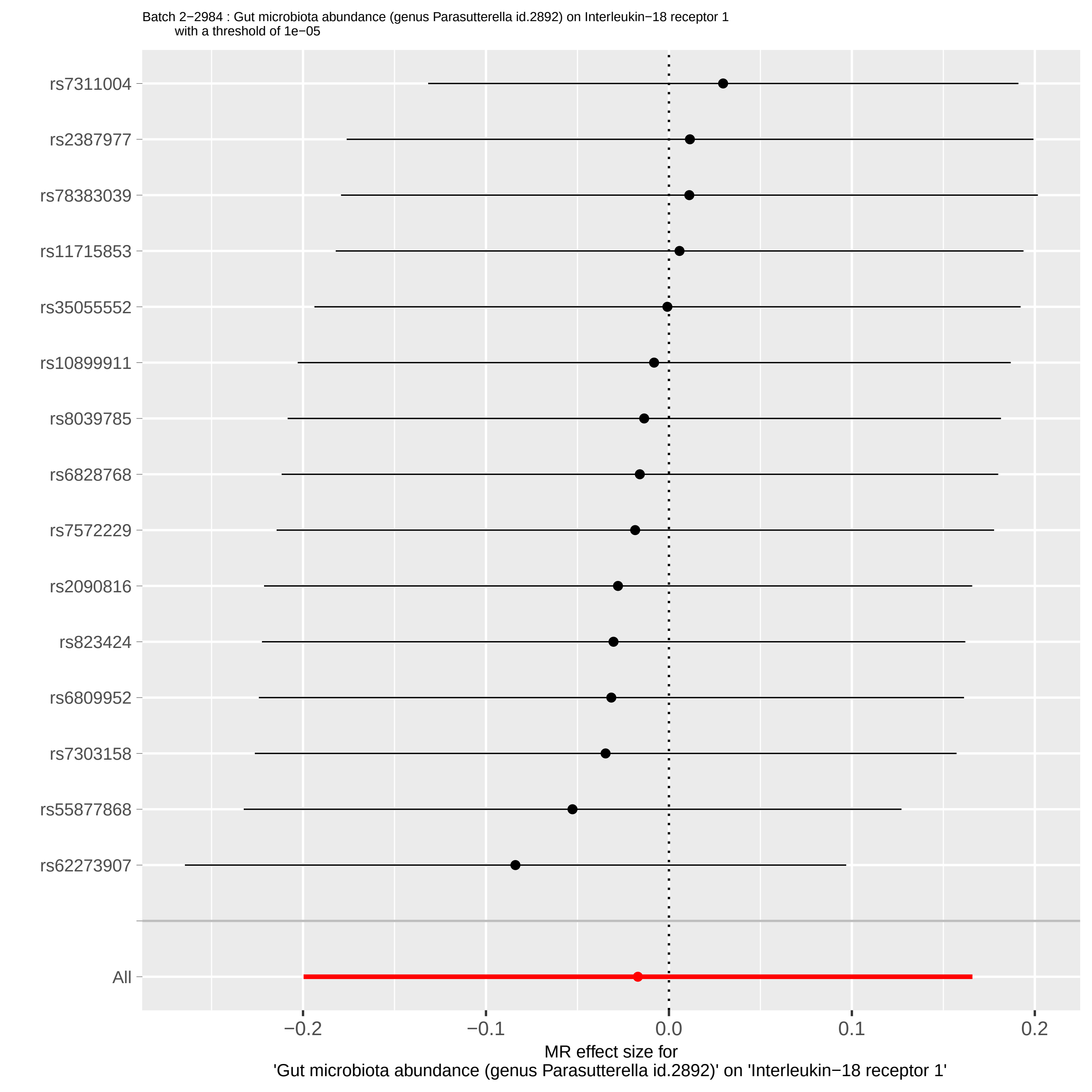

Batch 2−2984 : Gut microbiota abundance (genus Parasutterella id.2892) on Interleukin−18 receptor 1
with a threshold of 1e−05
rs7311004
rs2387977
rs78383039
rs11715853
rs35055552
rs10899911
rs8039785
rs6828768
rs7572229
rs2090816
rs823424
rs6809952
rs7303158
rs55877868
rs62273907
All
−0.2
−0.1
0.0
0.1
0.2
MR effect size for
'Gut microbiota abundance (genus Parasutterella id.2892)' on 'Interleukin−18 receptor 1'

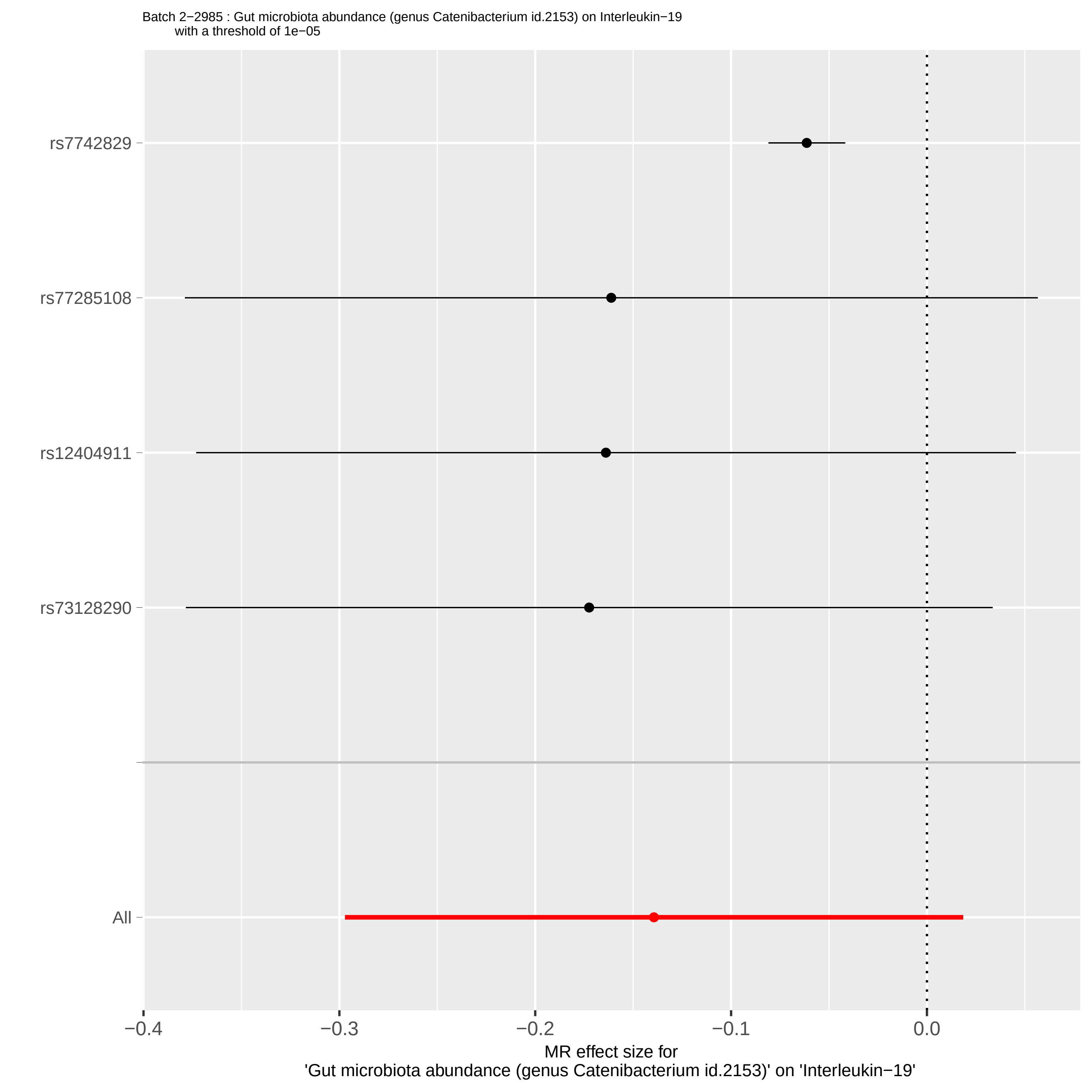
Batch 2−2985 : Gut microbiota abundance (genus Catenibacterium id.2153) on Interleukin−19
with a threshold of 1e−05
rs7742829
rs77285108
rs12404911
rs73128290
All
−0.4
−0.3
−0.2
−0.1
0.0
MR effect size for
'Gut microbiota abundance (genus Catenibacterium id.2153)' on 'Interleukin−19'

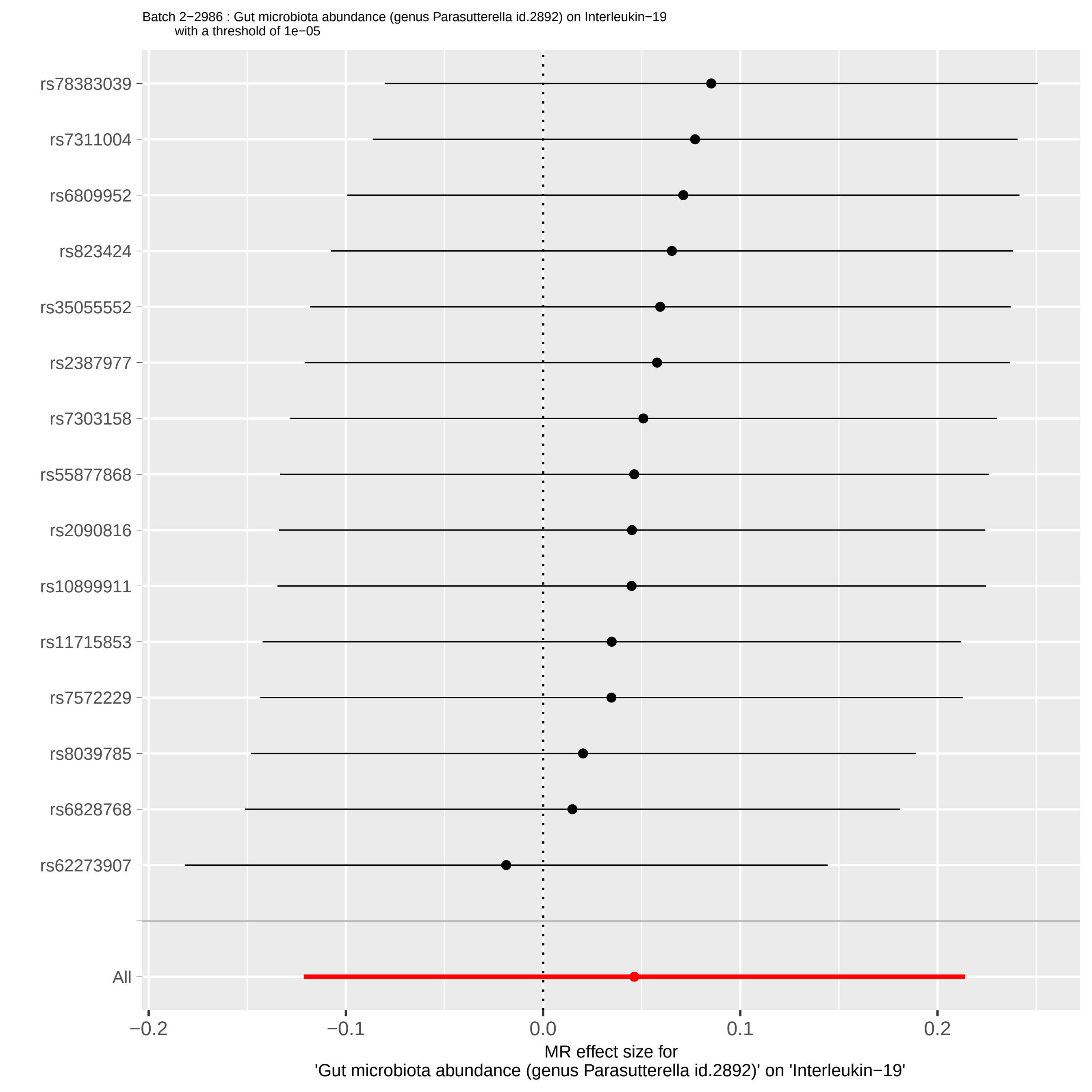
Batch 2−2986 : Gut microbiota abundance (genus Parasutterella id.2892) on Interleukin−19
with a threshold of 1e−05
rs78383039
rs7311004
rs6809952
rs823424
rs35055552
rs2387977
rs7303158
rs55877868
rs2090816
rs10899911
rs11715853
rs7572229
rs8039785
rs6828768
rs62273907
All
−0.2
−0.1
0.0
0.1
0.2
MR effect size for
'Gut microbiota abundance (genus Parasutterella id.2892)' on 'Interleukin−19'

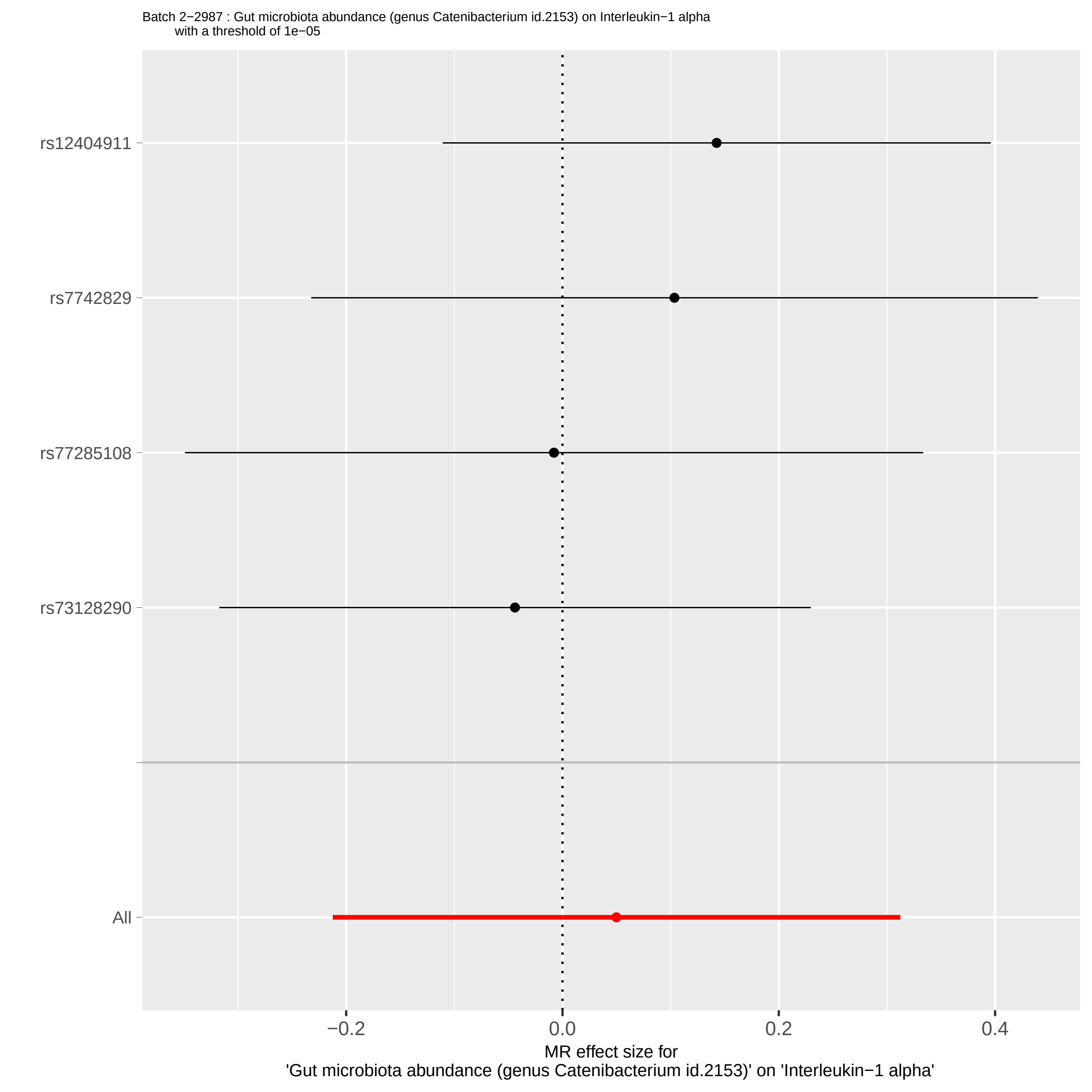

Batch 2−2987 : Gut microbiota abundance (genus Catenibacterium id.2153) on Interleukin−1 alpha
with a threshold of 1e−05
rs12404911
rs7742829
rs77285108
rs73128290
All
−0.2
0.0
0.2
0.4
MR effect size for
'Gut microbiota abundance (genus Catenibacterium id.2153)' on 'Interleukin−1 alpha'

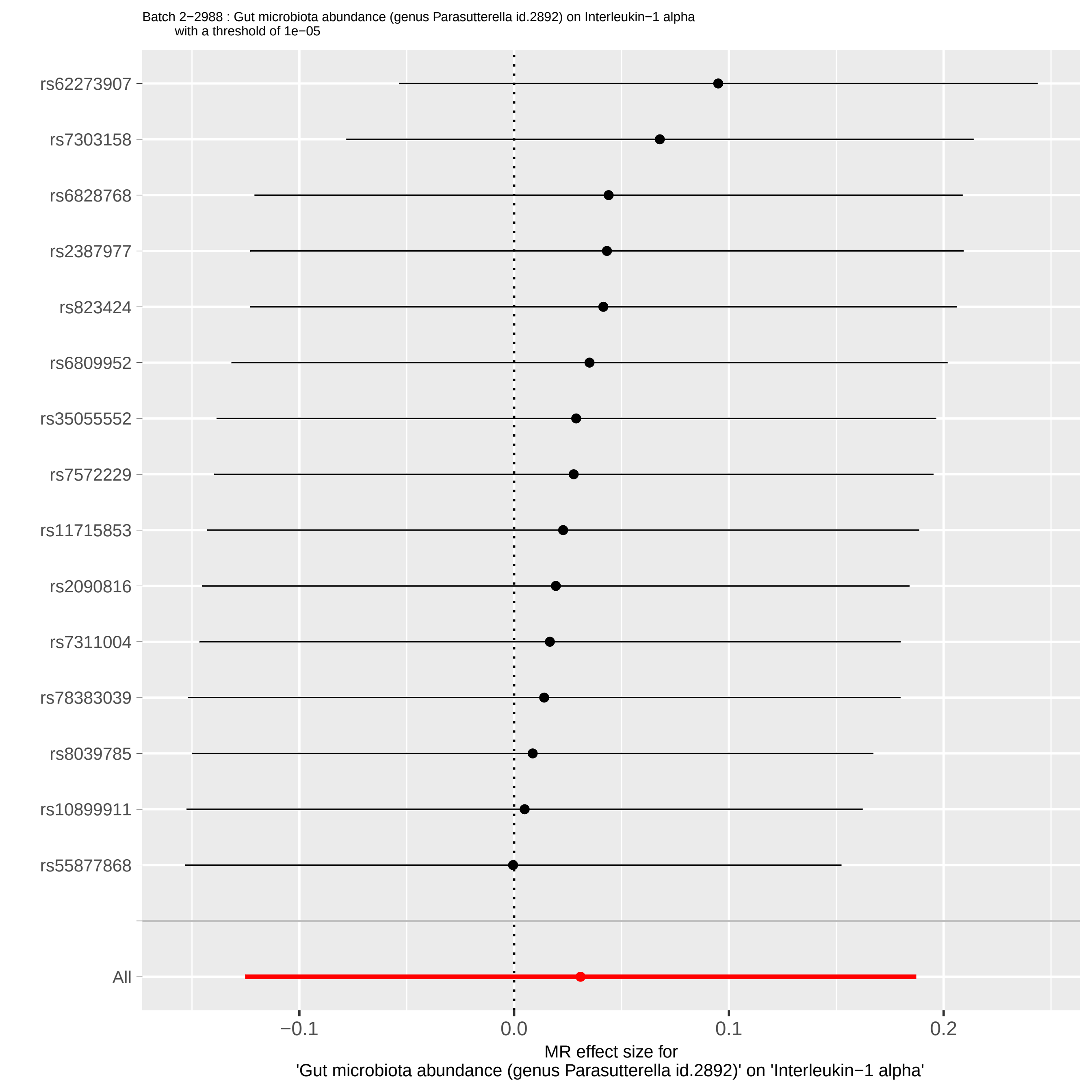
Batch 2−2988 : Gut microbiota abundance (genus Parasutterella id.2892) on Interleukin−1 alpha
with a threshold of 1e−05
rs62273907
rs7303158
rs6828768
rs2387977
rs823424
rs6809952
rs35055552
rs7572229
rs11715853
rs2090816
rs7311004
rs78383039
rs8039785
rs10899911
rs55877868
All
−0.1
0.0
0.1
0.2
MR effect size for
'Gut microbiota abundance (genus Parasutterella id.2892)' on 'Interleukin−1 alpha'

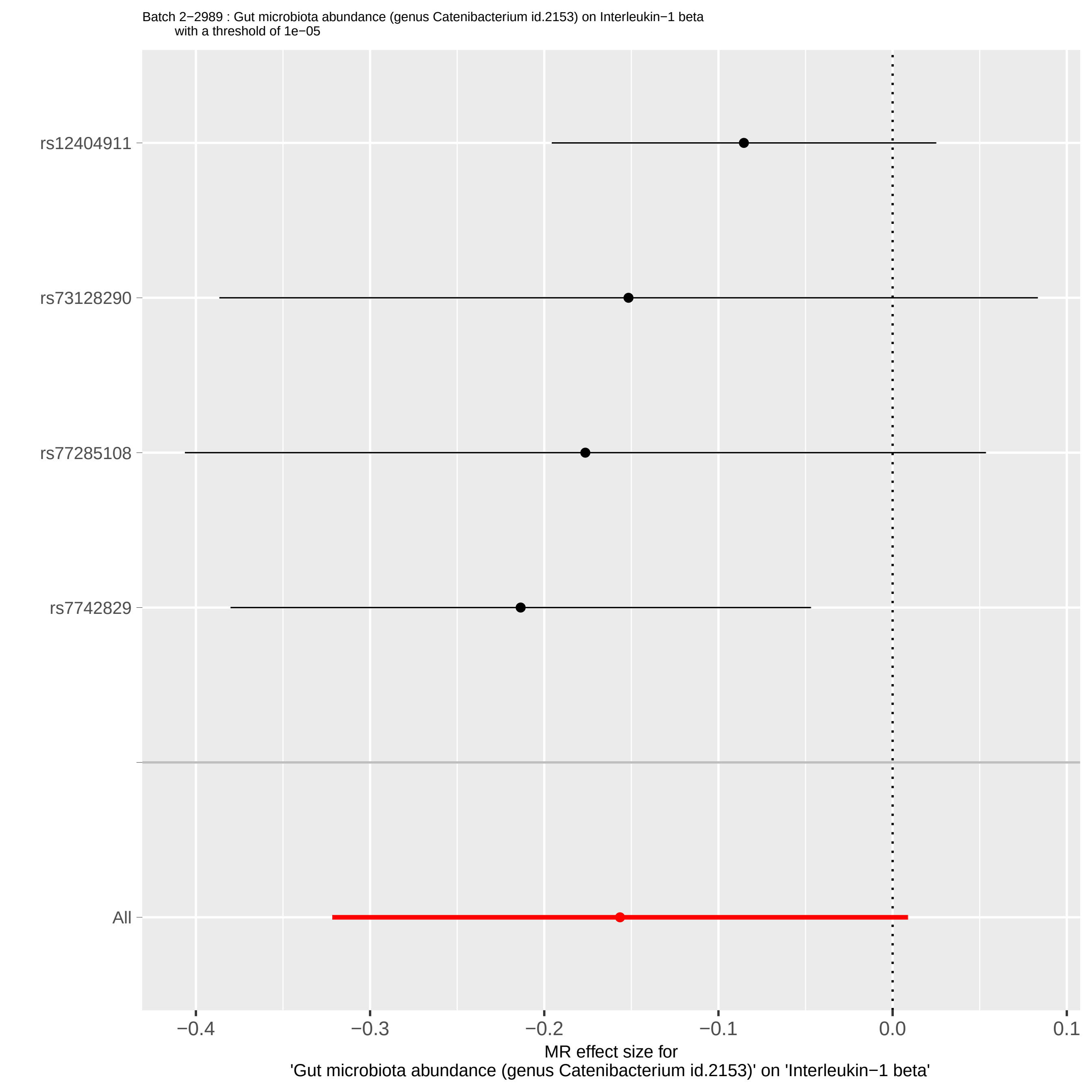

Batch 2−2989 : Gut microbiota abundance (genus Catenibacterium id.2153) on Interleukin−1 beta
with a threshold of 1e−05
rs12404911
rs73128290
rs77285108
rs7742829
All
−0.4
−0.3
−0.2
−0.1
0.0
0.1
MR effect size for
'Gut microbiota abundance (genus Catenibacterium id.2153)' on 'Interleukin−1 beta'

Batch 2−2990 : Gut microbiota abundance (genus Parasutterella id.2892) on Interleukin−1 beta
with a threshold of 1e−05

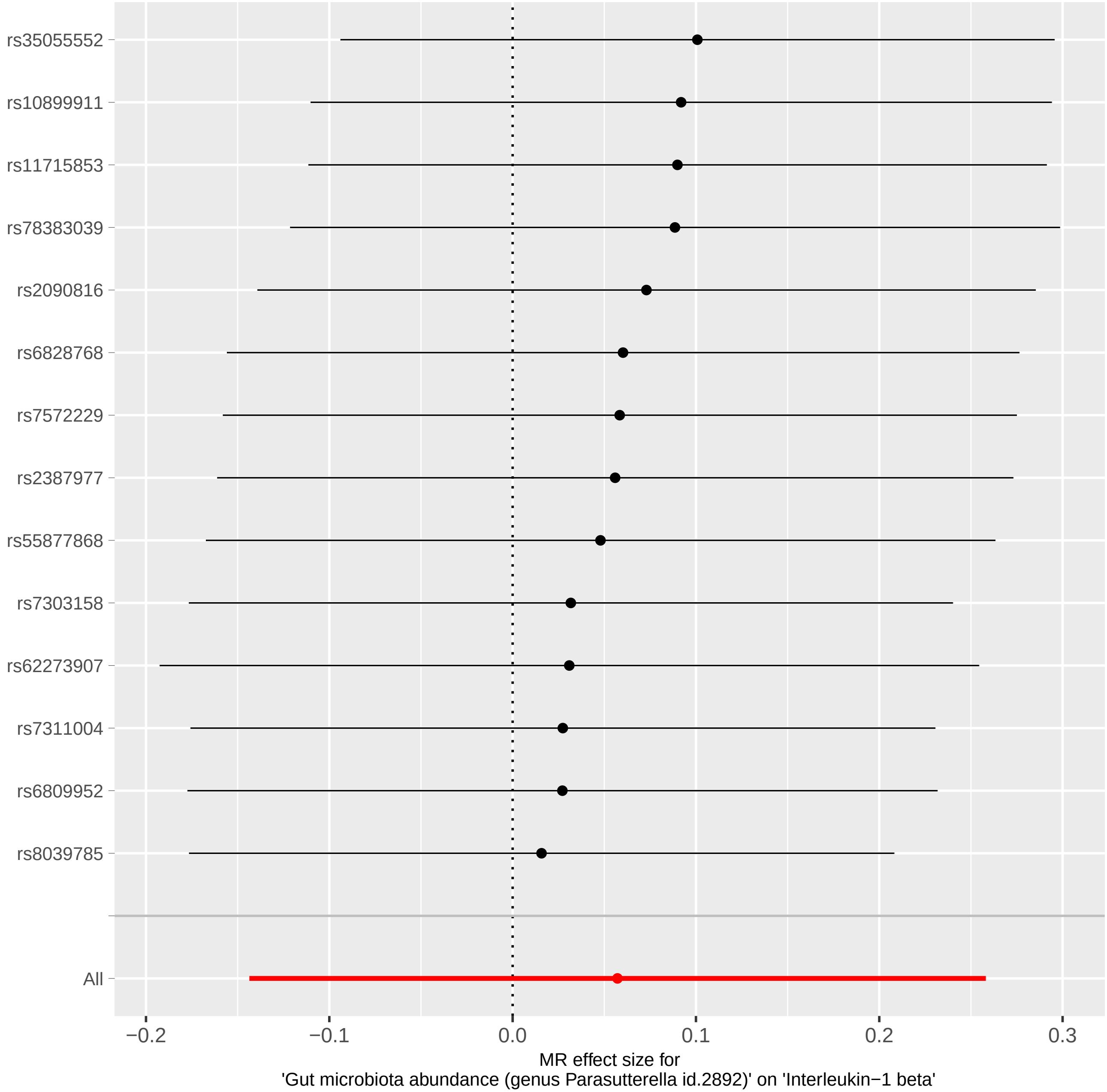

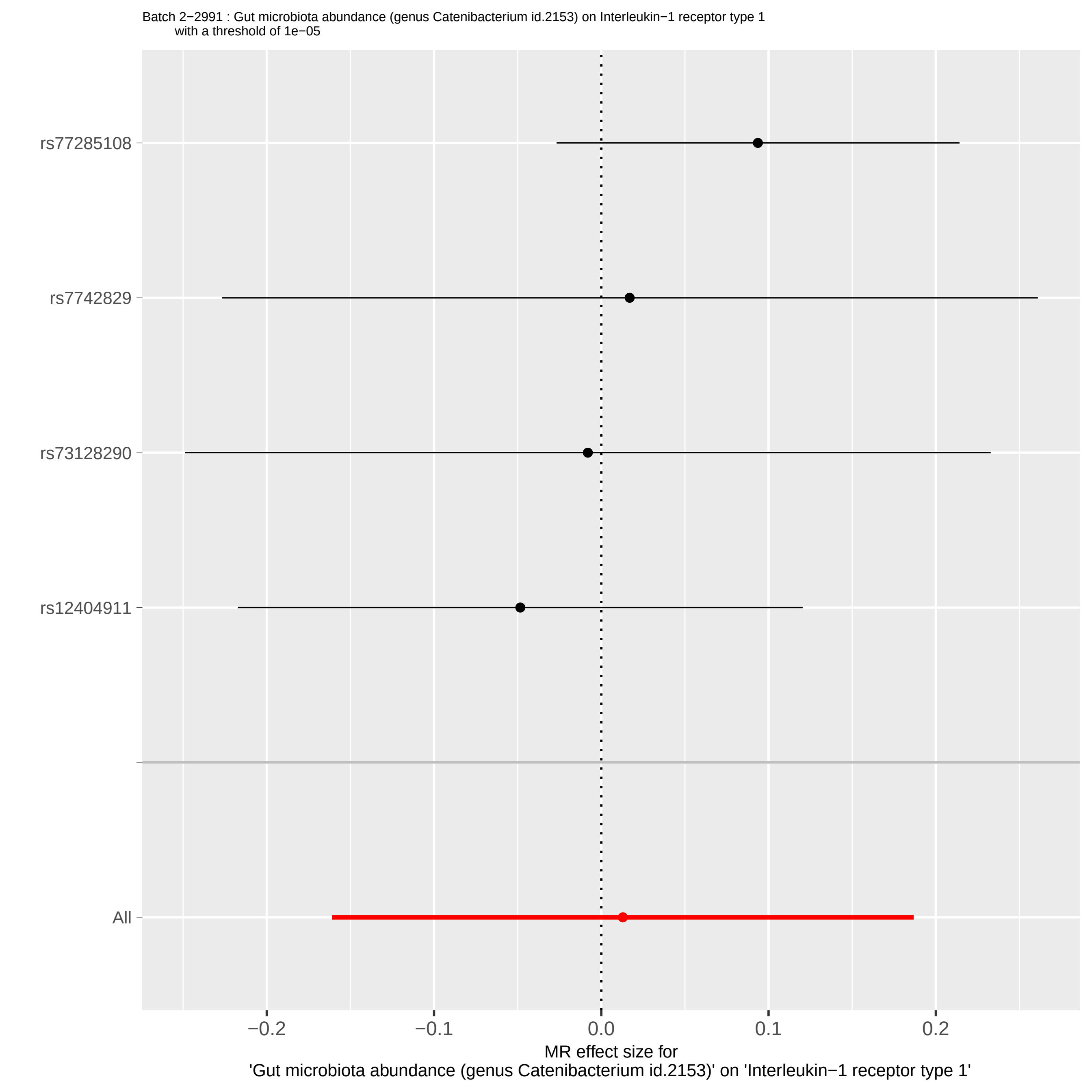
Batch 2−2991 : Gut microbiota abundance (genus Catenibacterium id.2153) on Interleukin−1 receptor type 1
with a threshold of 1e−05
rs77285108
rs7742829
rs73128290
rs12404911
All
−0.2
−0.1
0.0
0.1
0.2
MR effect size for
'Gut microbiota abundance (genus Catenibacterium id.2153)' on 'Interleukin−1 receptor type 1'

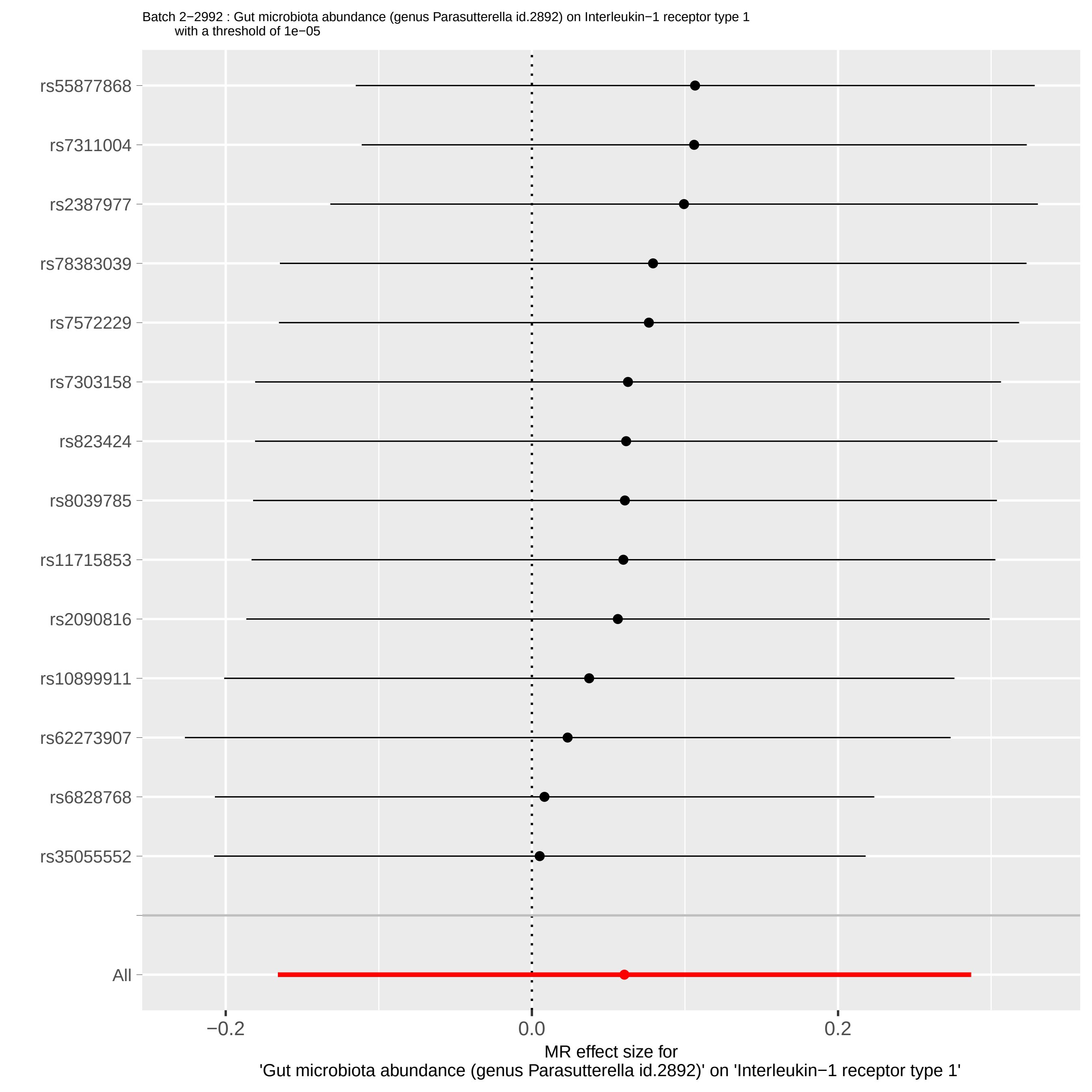

Batch 2−2992 : Gut microbiota abundance (genus Parasutterella id.2892) on Interleukin−1 receptor type 1
with a threshold of 1e−05
rs55877868
rs7311004
rs2387977
rs78383039
rs7572229
rs7303158
rs823424
rs8039785
rs11715853
rs2090816
rs10899911
rs62273907
rs6828768
rs35055552
All
−0.2
0.0
0.2
MR effect size for
'Gut microbiota abundance (genus Parasutterella id.2892)' on 'Interleukin−1 receptor type 1'

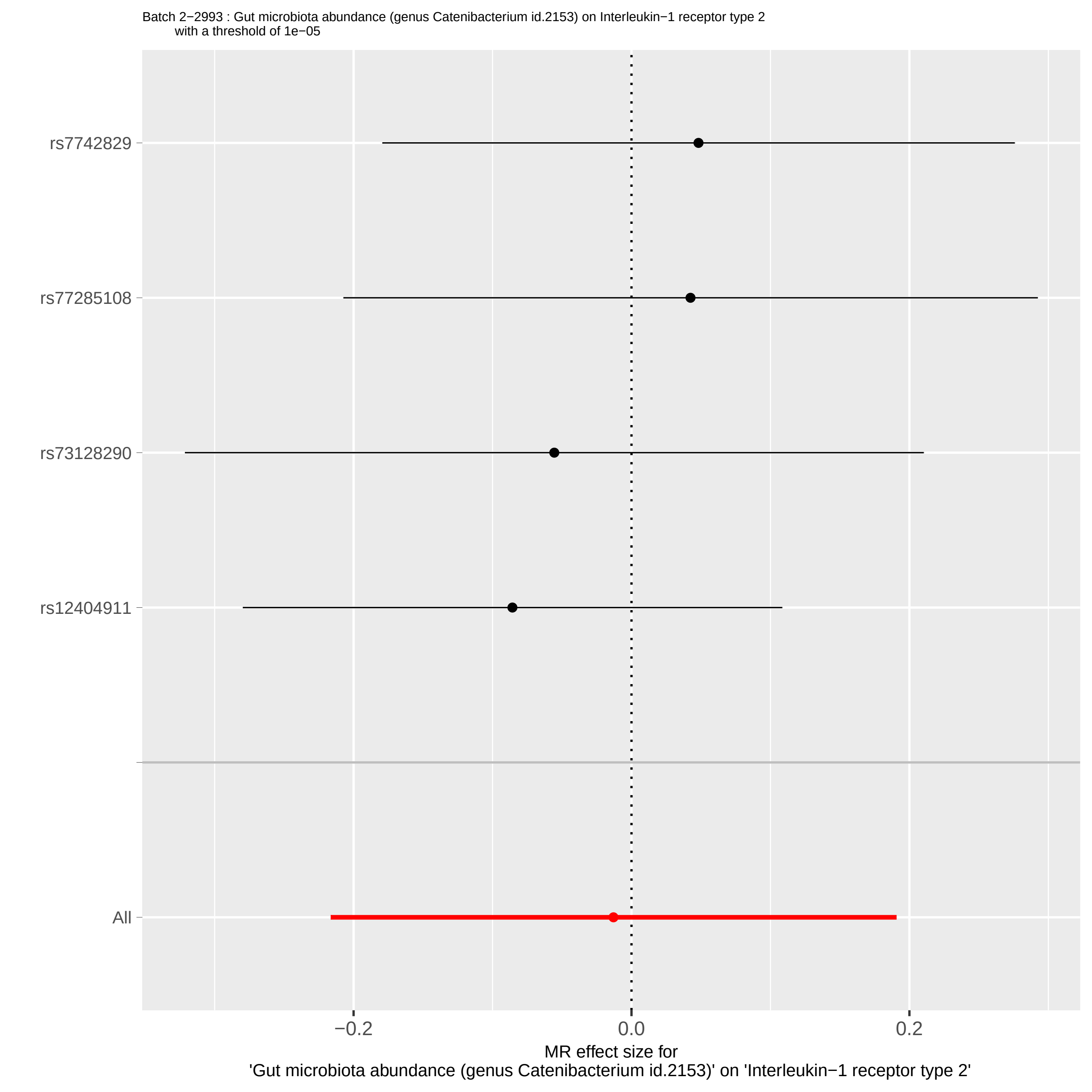
Batch 2−2993 : Gut microbiota abundance (genus Catenibacterium id.2153) on Interleukin−1 receptor type 2
with a threshold of 1e−05
rs7742829
rs77285108
rs73128290
rs12404911
All
−0.2
0.0
0.2
MR effect size for
'Gut microbiota abundance (genus Catenibacterium id.2153)' on 'Interleukin−1 receptor type 2'

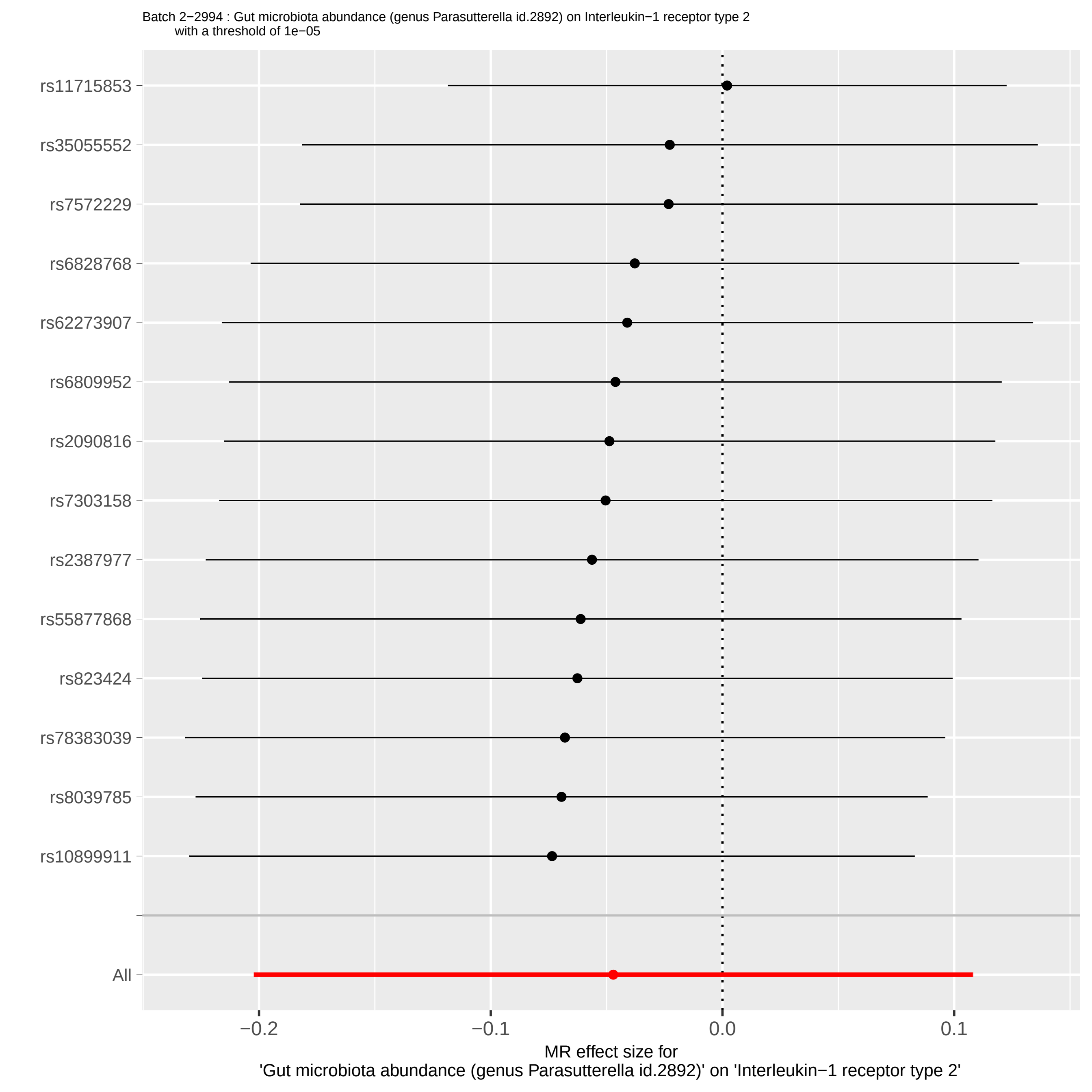
Batch 2−2994 : Gut microbiota abundance (genus Parasutterella id.2892) on Interleukin−1 receptor type 2
with a threshold of 1e−05
rs11715853
rs35055552
rs7572229
rs6828768
rs62273907
rs6809952
rs2090816
rs7303158
rs2387977
rs55877868
rs823424
rs78383039
rs8039785
rs10899911
All
−0.2
−0.1
0.0
0.1
MR effect size for
'Gut microbiota abundance (genus Parasutterella id.2892)' on 'Interleukin−1 receptor type 2'

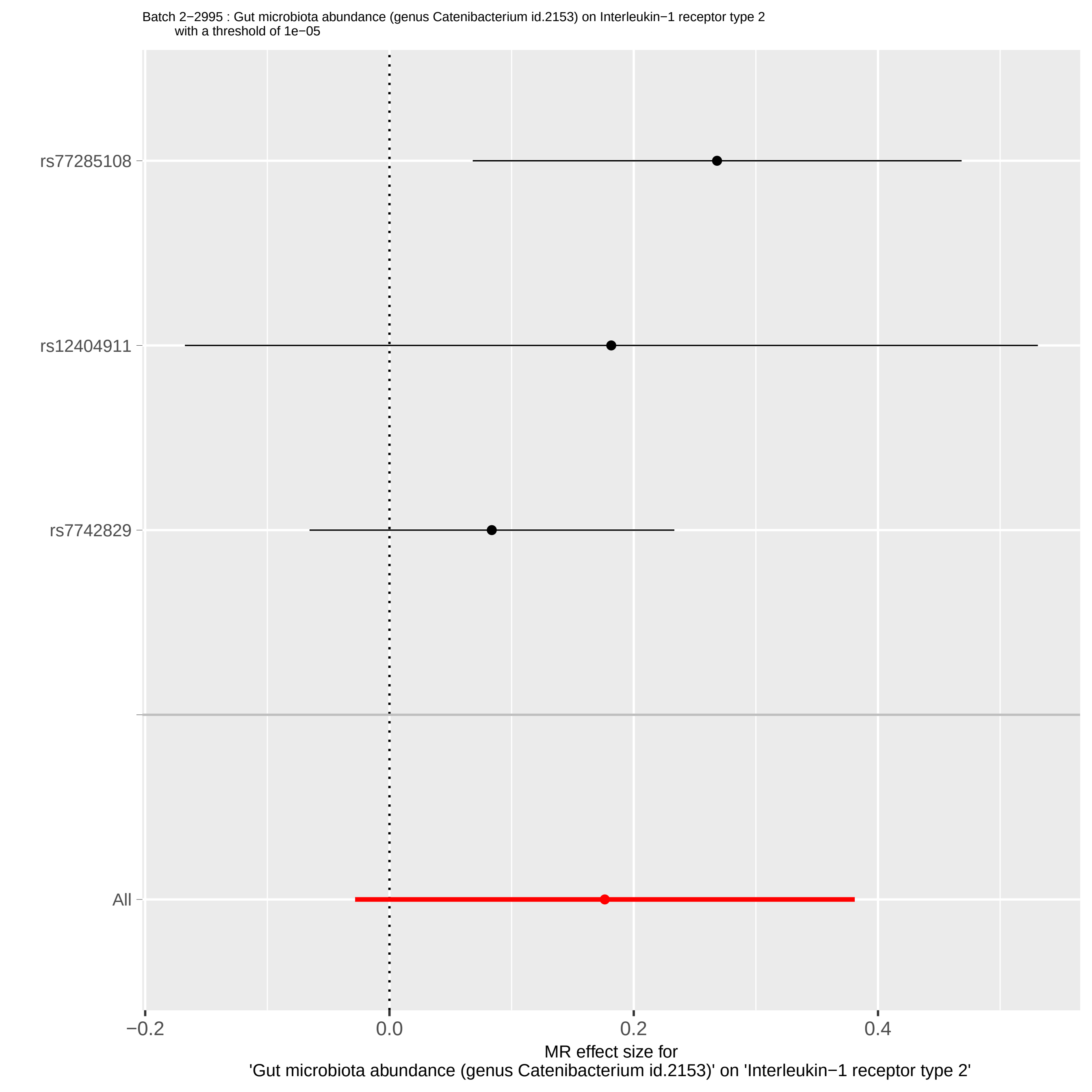
Batch 2−2995 : Gut microbiota abundance (genus Catenibacterium id.2153) on Interleukin−1 receptor type 2
with a threshold of 1e−05
rs77285108
rs12404911
rs7742829
All
−0.2
0.0
0.2
0.4
MR effect size for
'Gut microbiota abundance (genus Catenibacterium id.2153)' on 'Interleukin−1 receptor type 2'

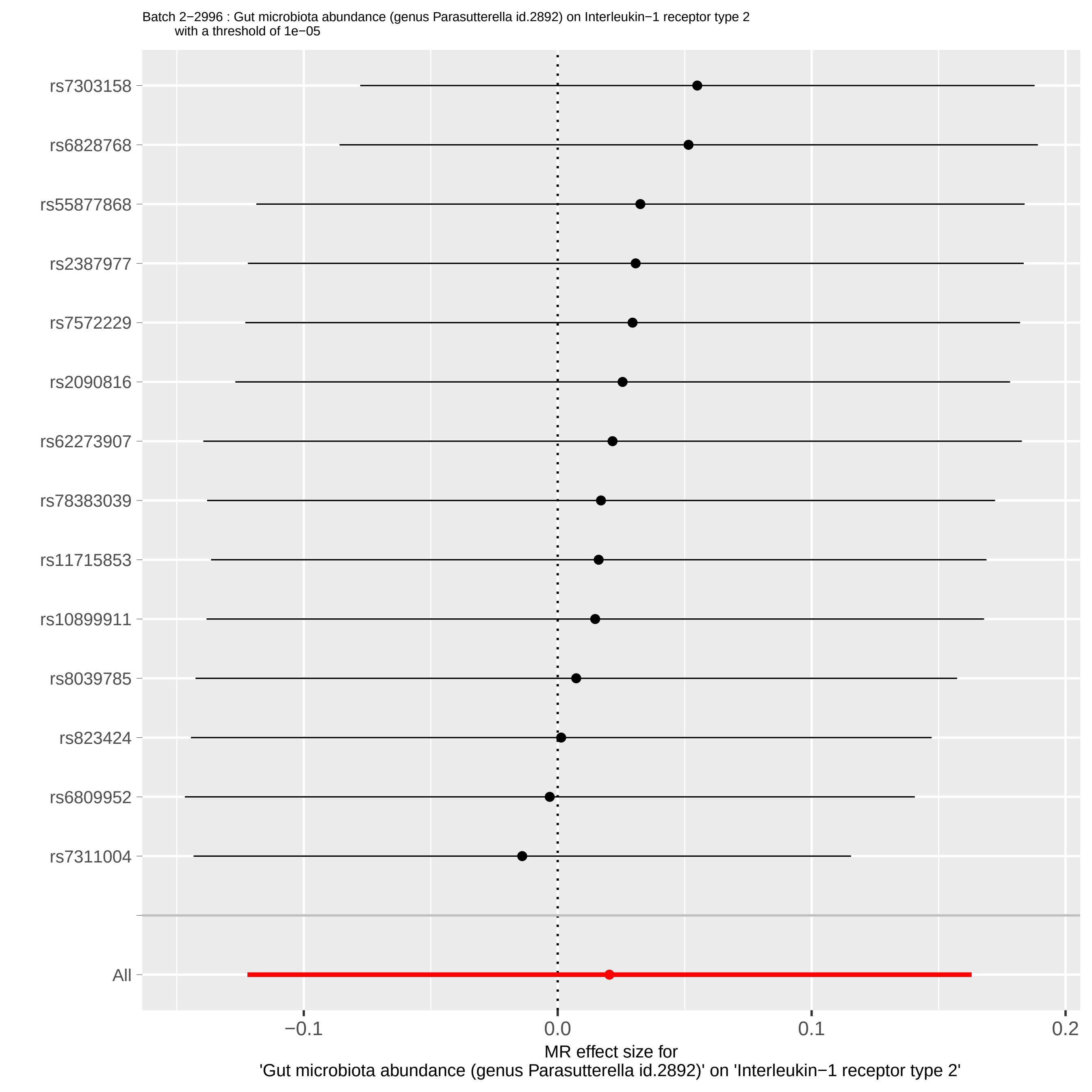
Batch 2−2996 : Gut microbiota abundance (genus Parasutterella id.2892) on Interleukin−1 receptor type 2
with a threshold of 1e−05
rs7303158
rs6828768
rs55877868
rs2387977
rs7572229
rs2090816
rs62273907
rs78383039
rs11715853
rs10899911
rs8039785
rs823424
rs6809952
rs7311004
All
−0.1
0.0
0.1
0.2
MR effect size for
'Gut microbiota abundance (genus Parasutterella id.2892)' on 'Interleukin−1 receptor type 2'

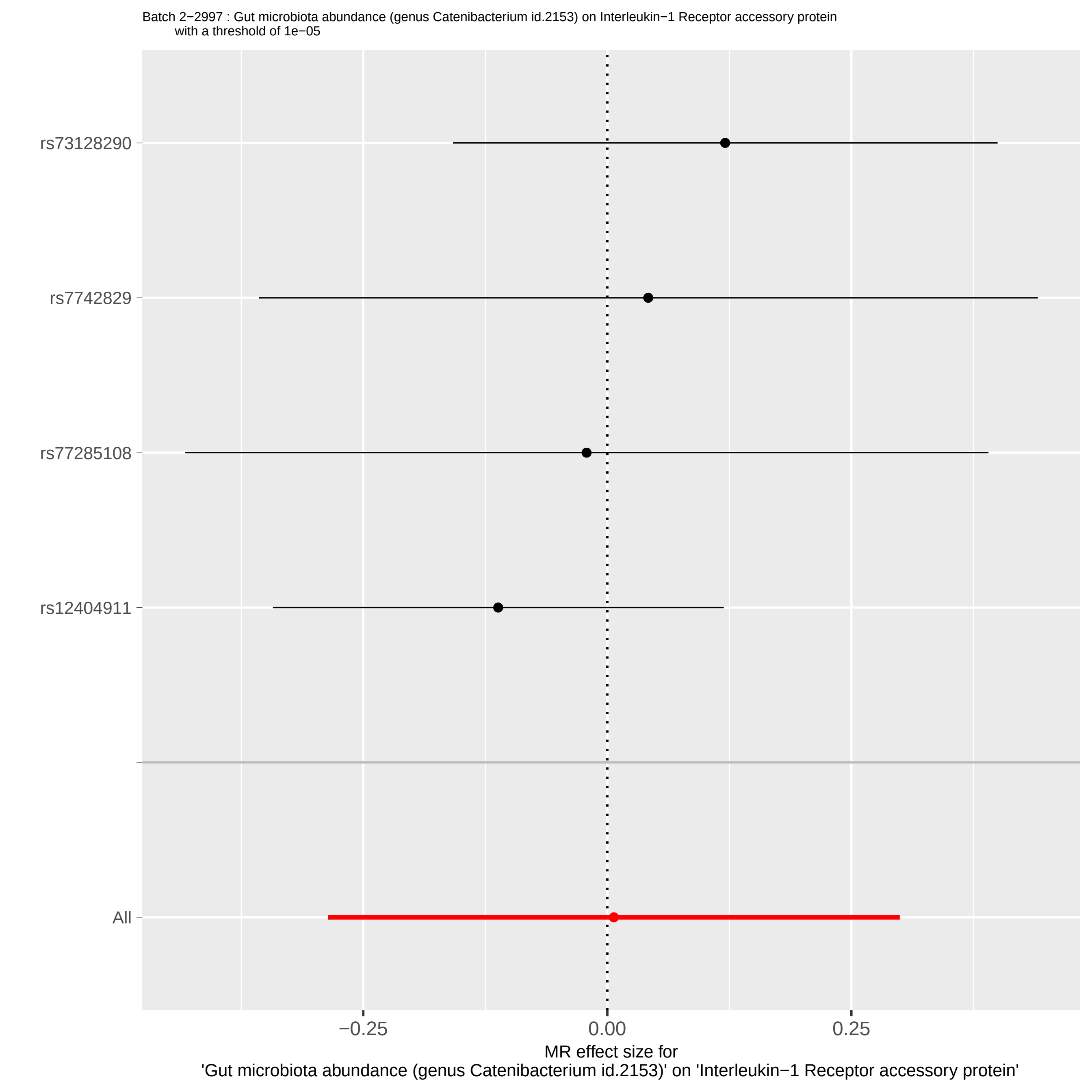
Batch 2−2997 : Gut microbiota abundance (genus Catenibacterium id.2153) on Interleukin−1 Receptor accessory protein
with a threshold of 1e−05
rs73128290
rs7742829
rs77285108
rs12404911
All
−0.25
0.00
0.25
MR effect size for
'Gut microbiota abundance (genus Catenibacterium id.2153)' on 'Interleukin−1 Receptor accessory protein'

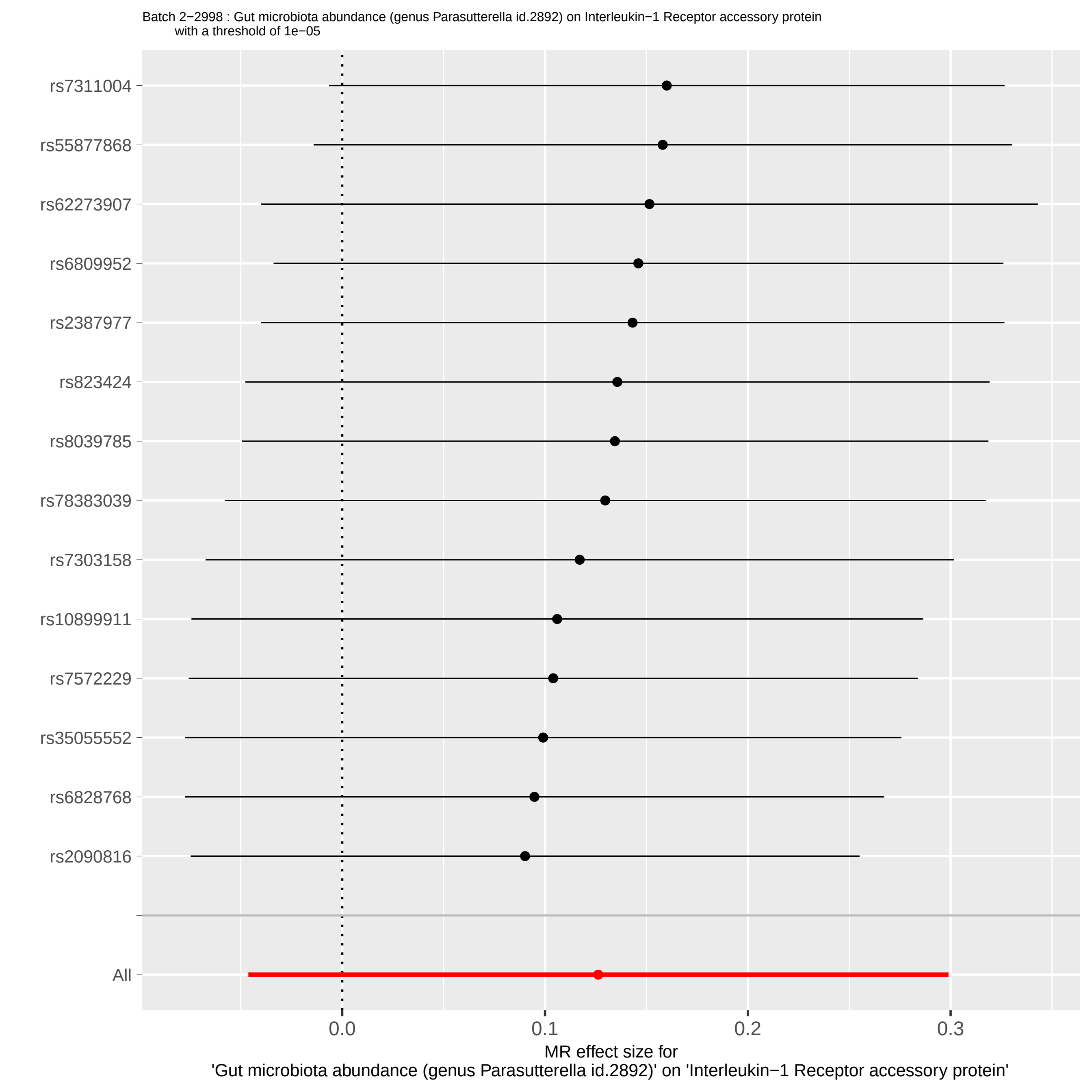

Batch 2−2998 : Gut microbiota abundance (genus Parasutterella id.2892) on Interleukin−1 Receptor accessory protein
with a threshold of 1e−05
rs7311004
rs55877868
rs62273907
rs6809952
rs2387977
rs823424
rs8039785
rs78383039
rs7303158
rs10899911
rs7572229
rs35055552
rs6828768
rs2090816
All
0.0
0.1
0.2
0.3
MR effect size for
'Gut microbiota abundance (genus Parasutterella id.2892)' on 'Interleukin−1 Receptor accessory protein'

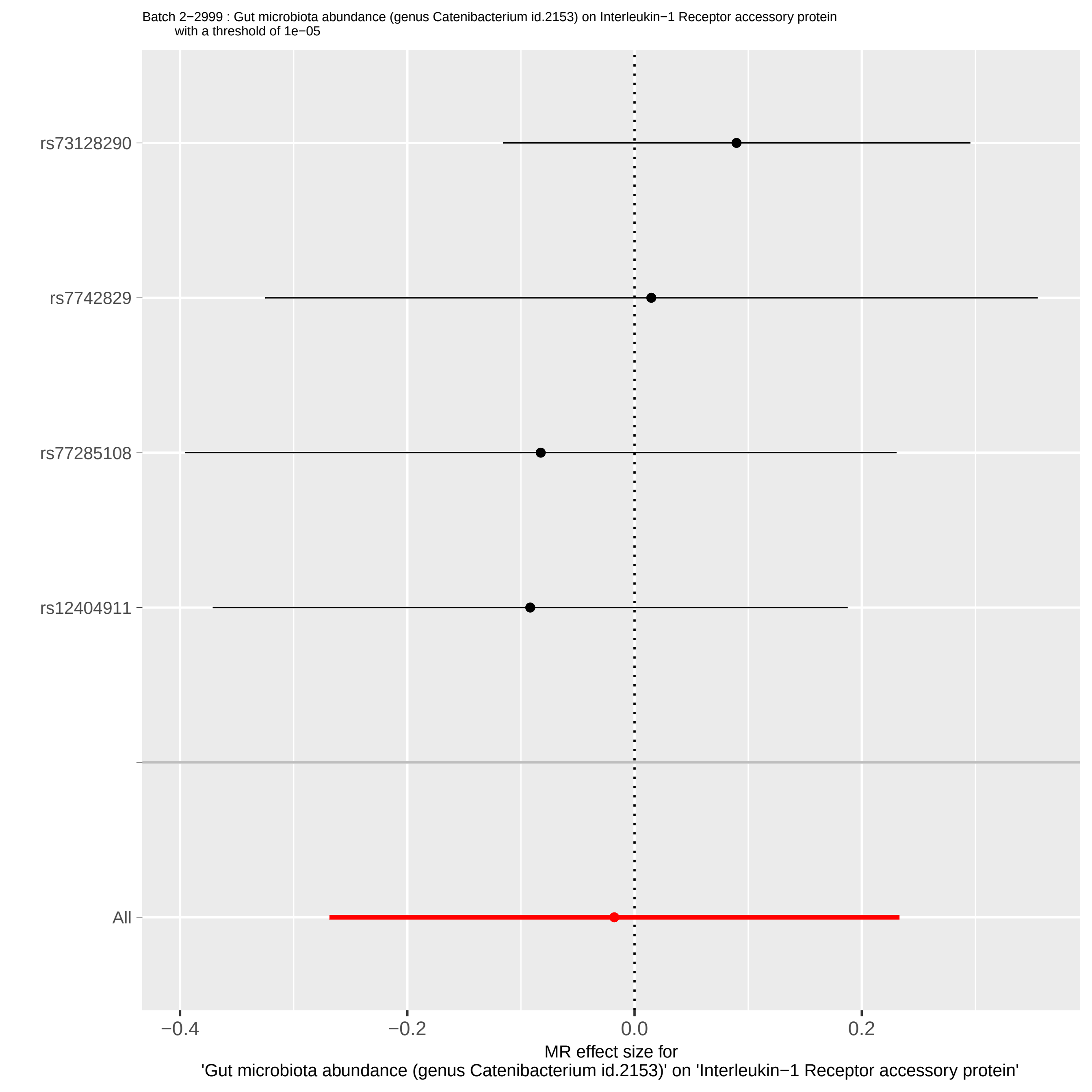

Batch 2−2999 : Gut microbiota abundance (genus Catenibacterium id.2153) on Interleukin−1 Receptor accessory protein
with a threshold of 1e−05
rs73128290
rs7742829
rs77285108
rs12404911
All
−0.4
−0.2
0.0
0.2
MR effect size for
'Gut microbiota abundance (genus Catenibacterium id.2153)' on 'Interleukin−1 Receptor accessory protein'

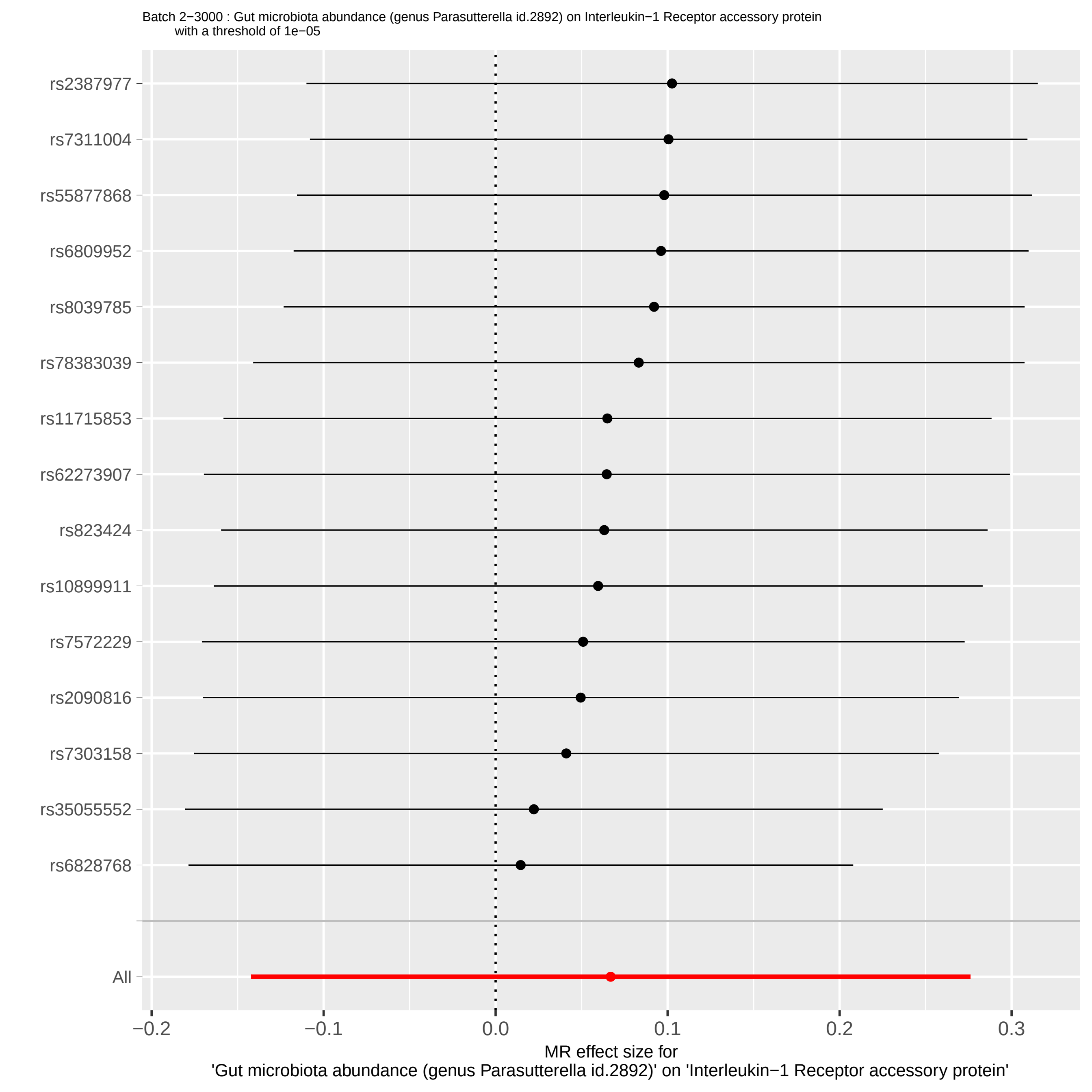

Batch 2−3000 : Gut microbiota abundance (genus Parasutterella id.2892) on Interleukin−1 Receptor accessory protein
with a threshold of 1e−05
rs2387977
rs7311004
rs55877868
rs6809952
rs8039785
rs78383039
rs11715853
rs62273907
rs823424
rs10899911
rs7572229
rs2090816
rs7303158
rs35055552
rs6828768
All
−0.2
−0.1
0.0
0.1
0.2
0.3
MR effect size for
'Gut microbiota abundance (genus Parasutterella id.2892)' on 'Interleukin−1 Receptor accessory protein'

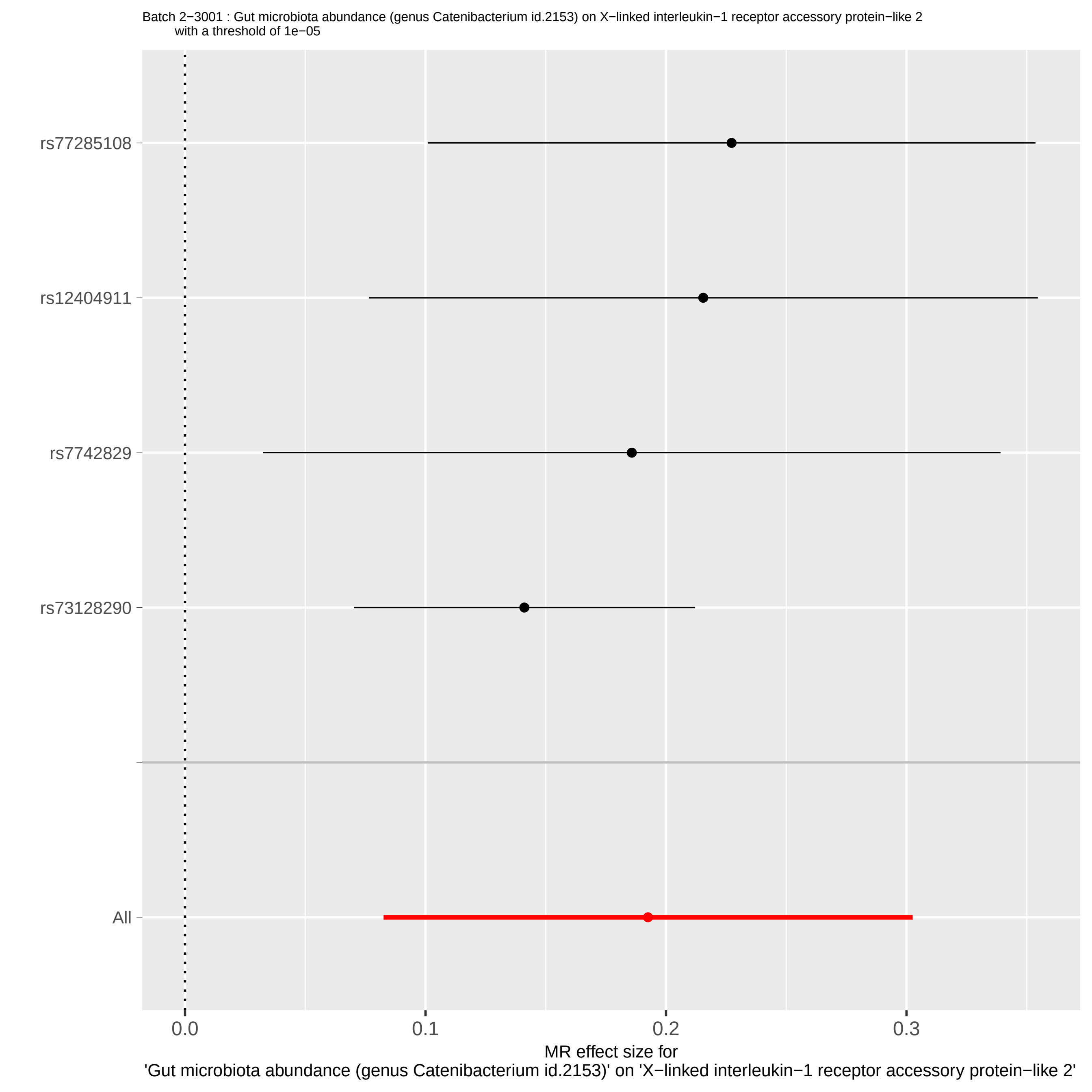
Batch 2−3001 : Gut microbiota abundance (genus Catenibacterium id.2153) on X−linked interleukin−1 receptor accessory protein−like 2
with a threshold of 1e−05
rs77285108
rs12404911
rs7742829
rs73128290
All
0.0
0.1
0.2
0.3
MR effect size for
'Gut microbiota abundance (genus Catenibacterium id.2153)' on 'X−linked interleukin−1 receptor accessory protein−like 2'

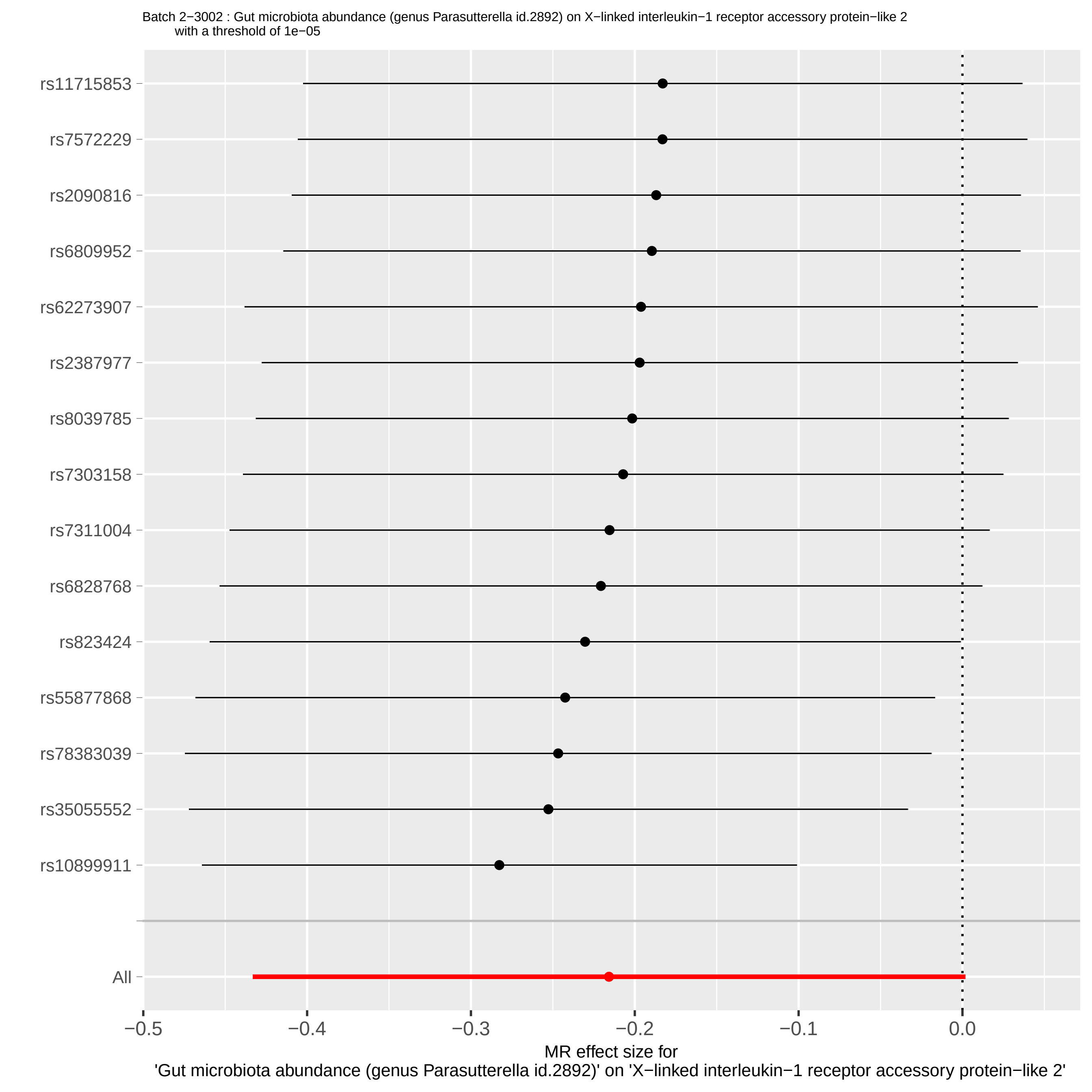

Batch 2−3002 : Gut microbiota abundance (genus Parasutterella id.2892) on X−linked interleukin−1 receptor accessory protein−like 2
with a threshold of 1e−05
rs11715853
rs7572229
rs2090816
rs6809952
rs62273907
rs2387977
rs8039785
rs7303158
rs7311004
rs6828768
rs823424
rs55877868
rs78383039
rs35055552
rs10899911
All
−0.5
−0.4
−0.3
−0.2
−0.1
0.0
MR effect size for
'Gut microbiota abundance (genus Parasutterella id.2892)' on 'X−linked interleukin−1 receptor accessory protein−like 2'

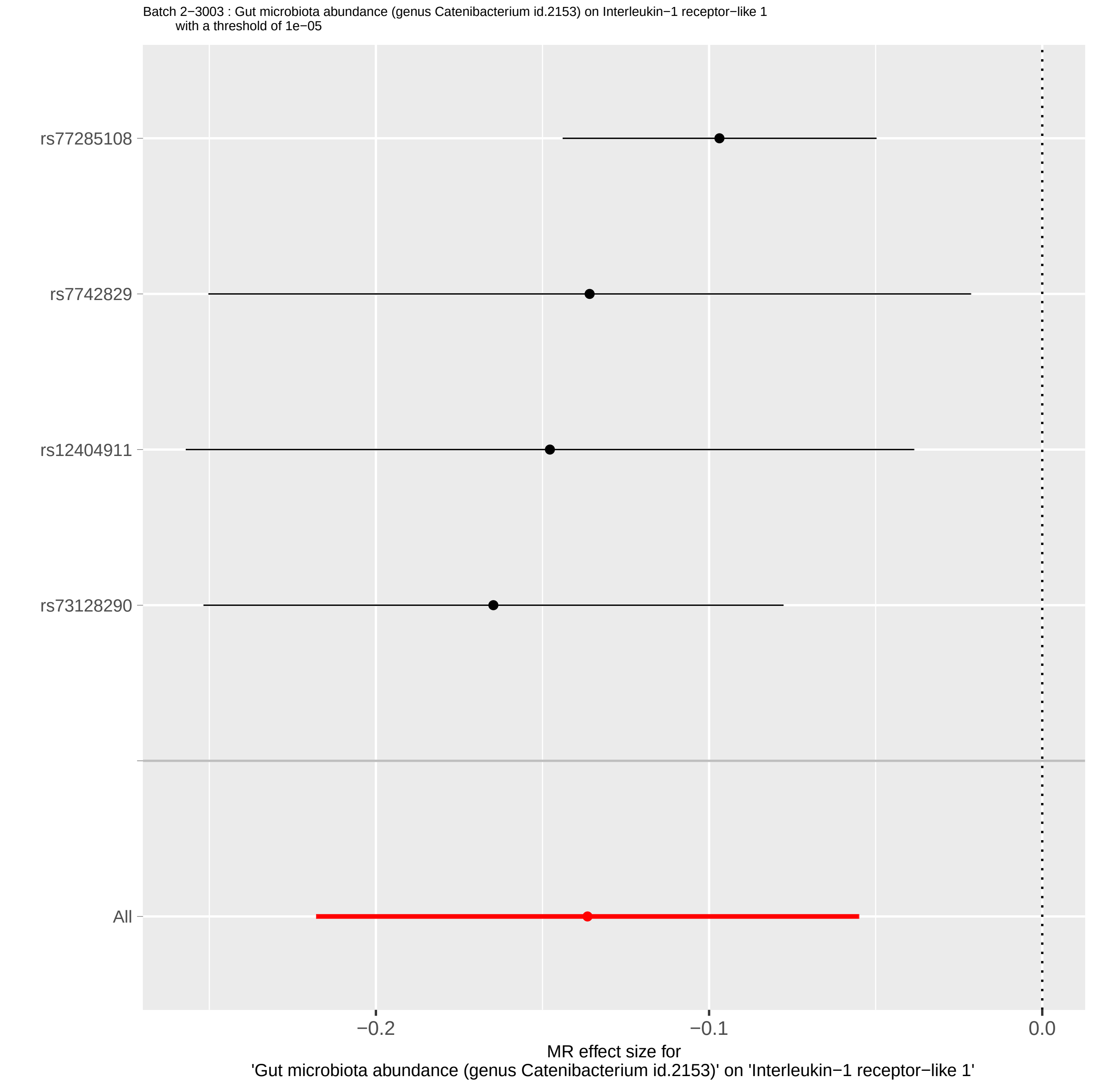
Batch 2−3003 : Gut microbiota abundance (genus Catenibacterium id.2153) on Interleukin−1 receptor−like 1
with a threshold of 1e−05
rs77285108
rs7742829
rs12404911
rs73128290
All
−0.2
−0.1
0.0
MR effect size for
'Gut microbiota abundance (genus Catenibacterium id.2153)' on 'Interleukin−1 receptor−like 1'

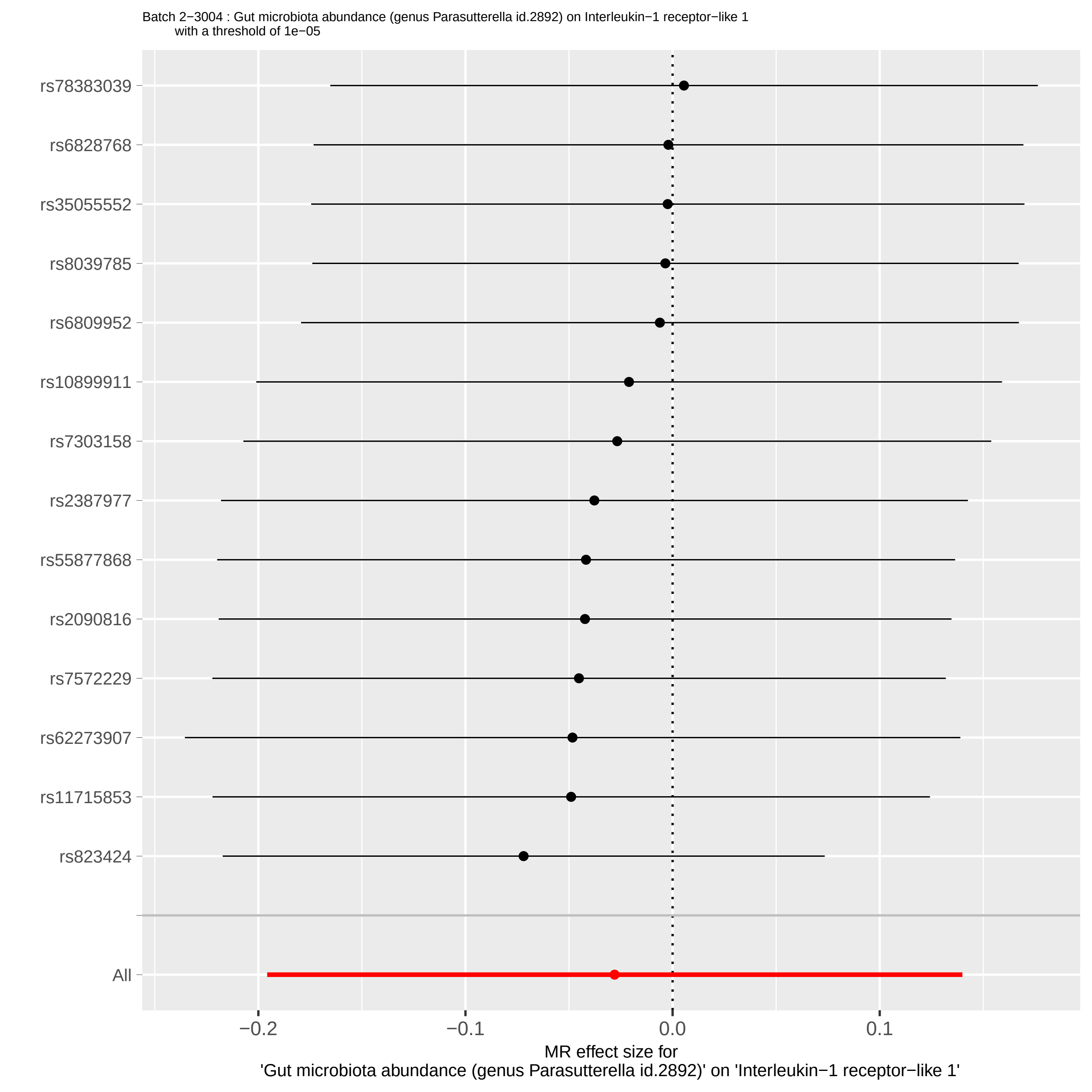

Batch 2−3004 : Gut microbiota abundance (genus Parasutterella id.2892) on Interleukin−1 receptor−like 1
with a threshold of 1e−05
rs78383039
rs6828768
rs35055552
rs8039785
rs6809952
rs10899911
rs7303158
rs2387977
rs55877868
rs2090816
rs7572229
rs62273907
rs11715853
rs823424
All
−0.2
−0.1
0.0
0.1
MR effect size for
'Gut microbiota abundance (genus Parasutterella id.2892)' on 'Interleukin−1 receptor−like 1'

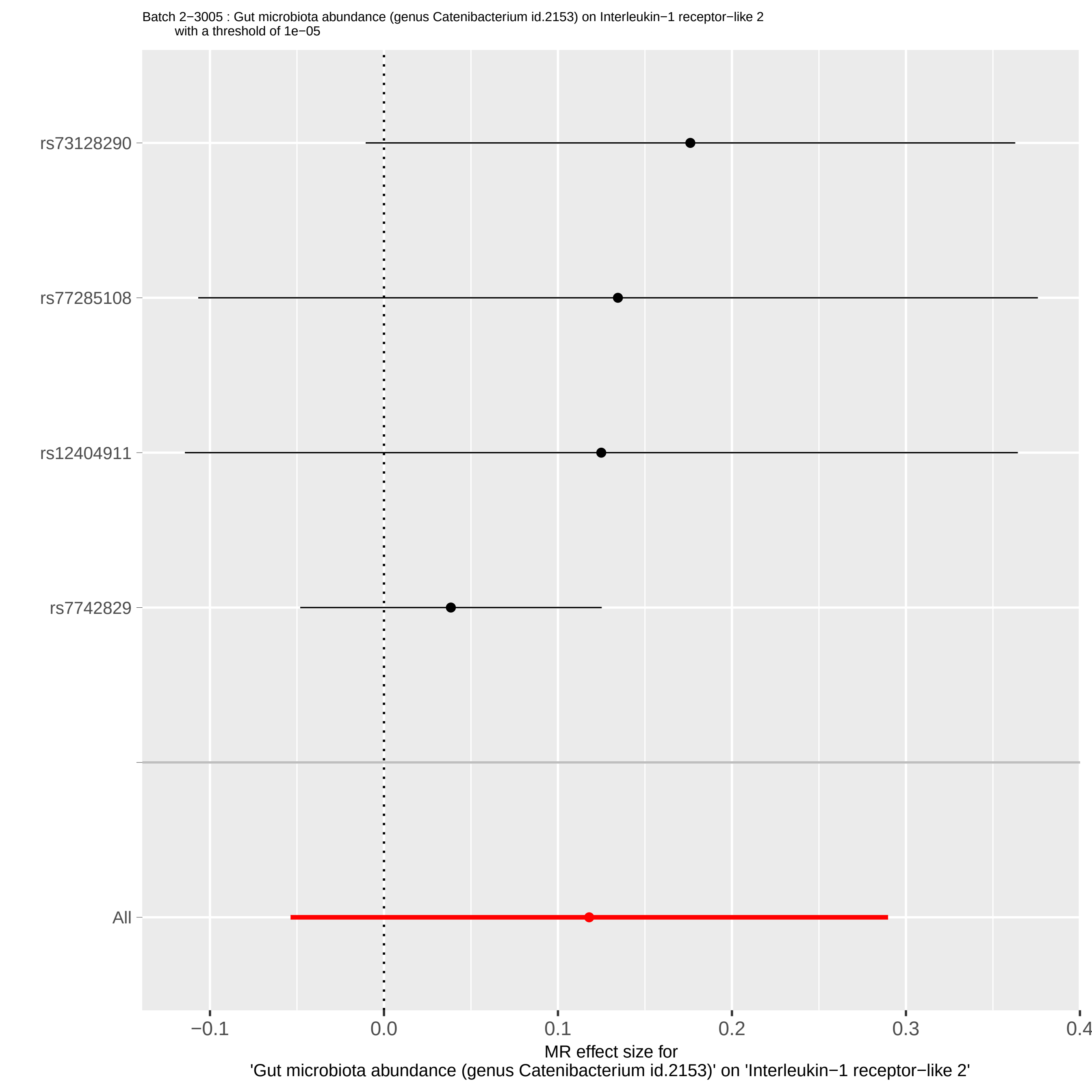
Batch 2−3005 : Gut microbiota abundance (genus Catenibacterium id.2153) on Interleukin−1 receptor−like 2
with a threshold of 1e−05
rs73128290
rs77285108
rs12404911
rs7742829
All
−0.1
0.0
0.1
0.2
0.3
0.4
MR effect size for
'Gut microbiota abundance (genus Catenibacterium id.2153)' on 'Interleukin−1 receptor−like 2'

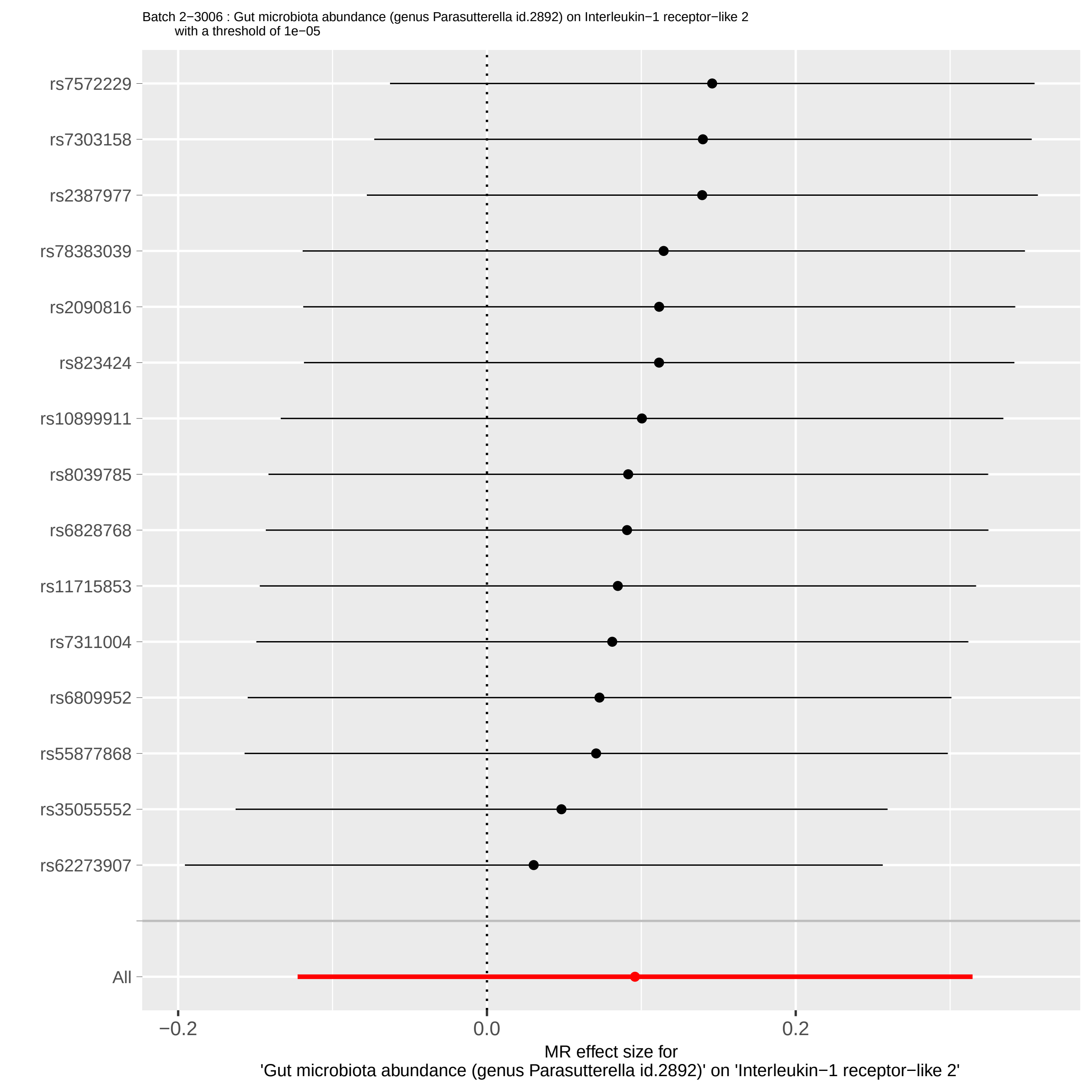

Batch 2−3006 : Gut microbiota abundance (genus Parasutterella id.2892) on Interleukin−1 receptor−like 2
with a threshold of 1e−05
rs7572229
rs7303158
rs2387977
rs78383039
rs2090816
rs823424
rs10899911
rs8039785
rs6828768
rs11715853
rs7311004
rs6809952
rs55877868
rs35055552
rs62273907
All
−0.2
0.0
0.2
MR effect size for
'Gut microbiota abundance (genus Parasutterella id.2892)' on 'Interleukin−1 receptor−like 2'

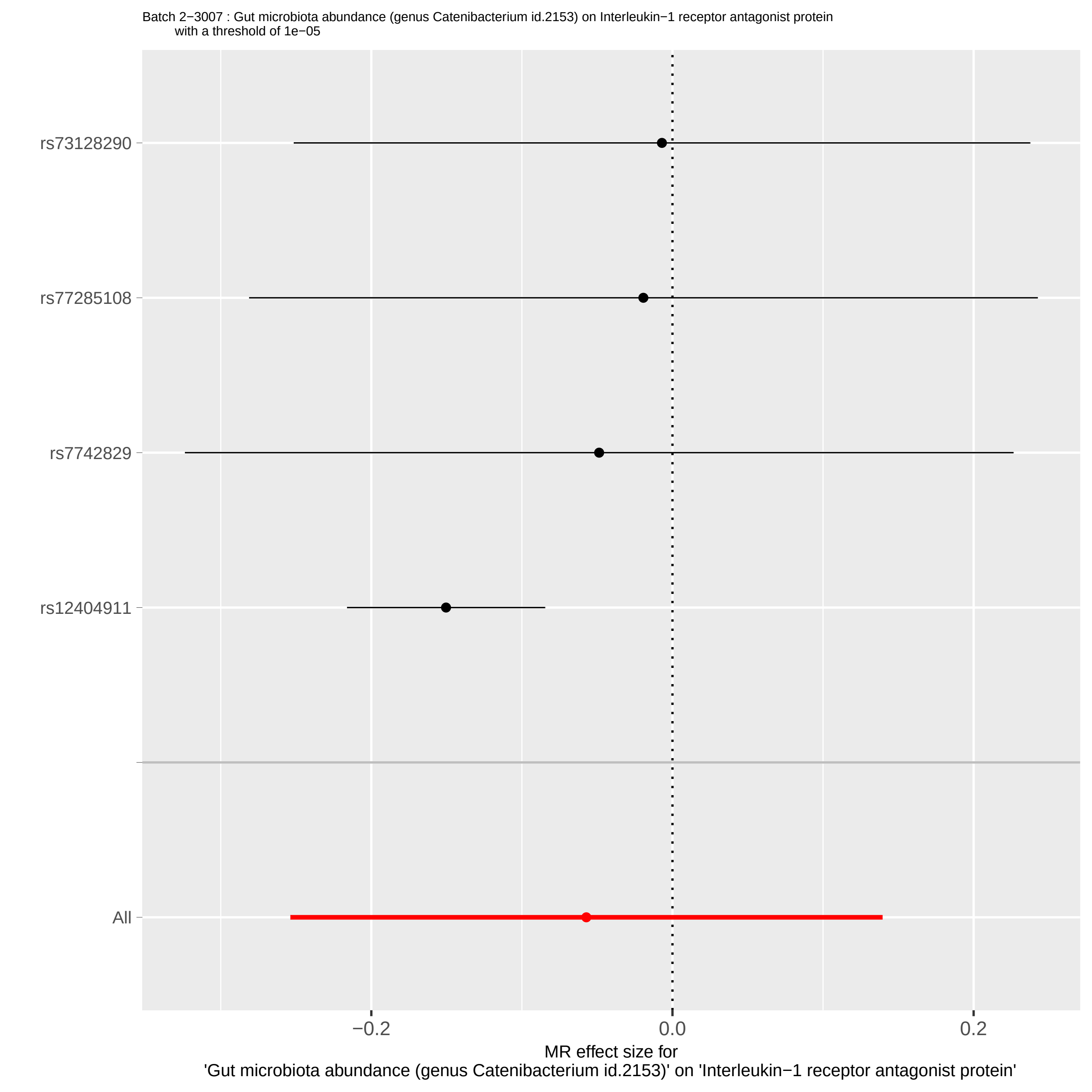
Batch 2−3007 : Gut microbiota abundance (genus Catenibacterium id.2153) on Interleukin−1 receptor antagonist protein
with a threshold of 1e−05
rs73128290
rs77285108
rs7742829
rs12404911
All
−0.2
0.0
0.2
MR effect size for
'Gut microbiota abundance (genus Catenibacterium id.2153)' on 'Interleukin−1 receptor antagonist protein'

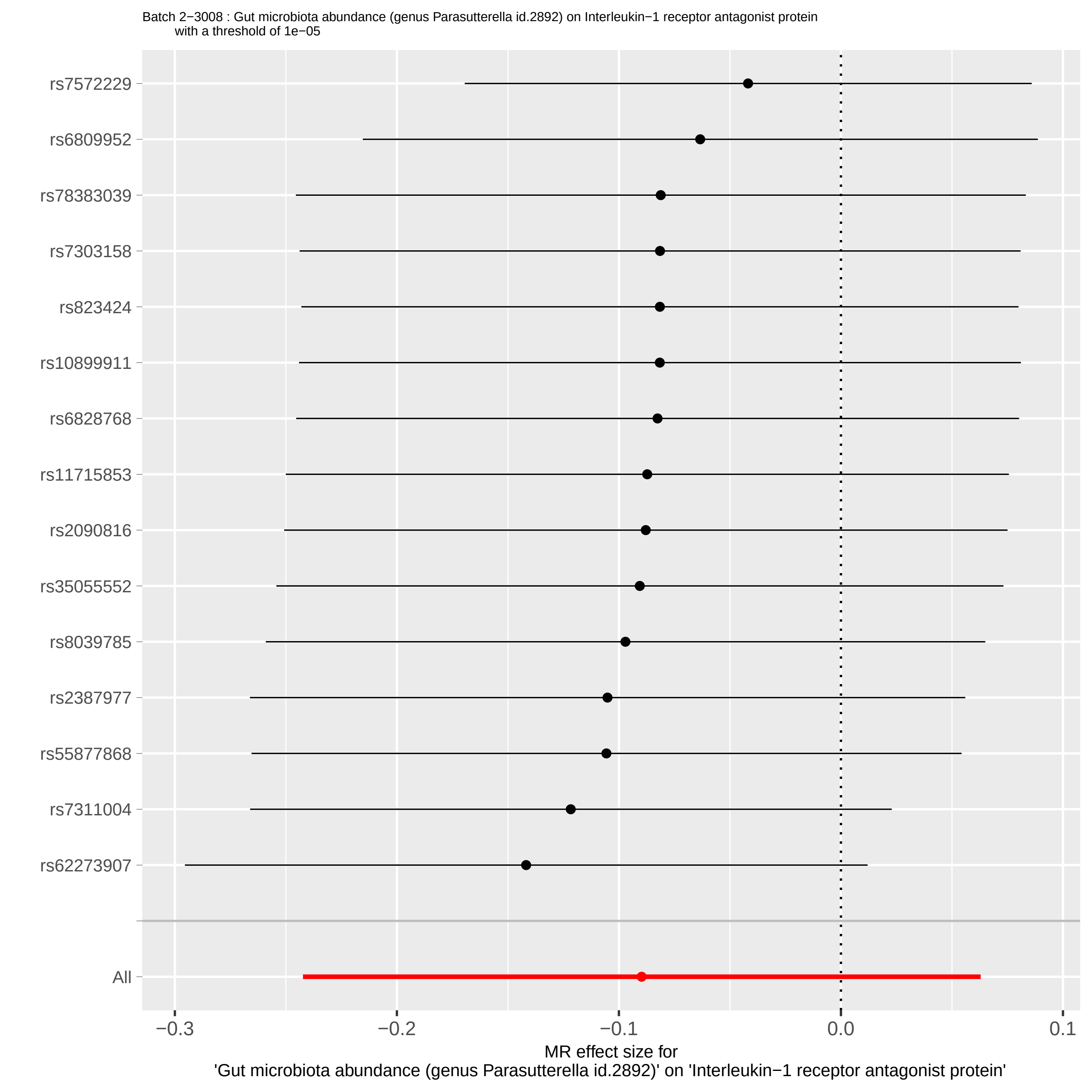

Batch 2−3008 : Gut microbiota abundance (genus Parasutterella id.2892) on Interleukin−1 receptor antagonist protein
with a threshold of 1e−05
rs7572229
rs6809952
rs78383039
rs7303158
rs823424
rs10899911
rs6828768
rs11715853
rs2090816
rs35055552
rs8039785
rs2387977
rs55877868
rs7311004
rs62273907
All
−0.3
−0.2
−0.1
0.0
0.1
MR effect size for
'Gut microbiota abundance (genus Parasutterella id.2892)' on 'Interleukin−1 receptor antagonist protein'

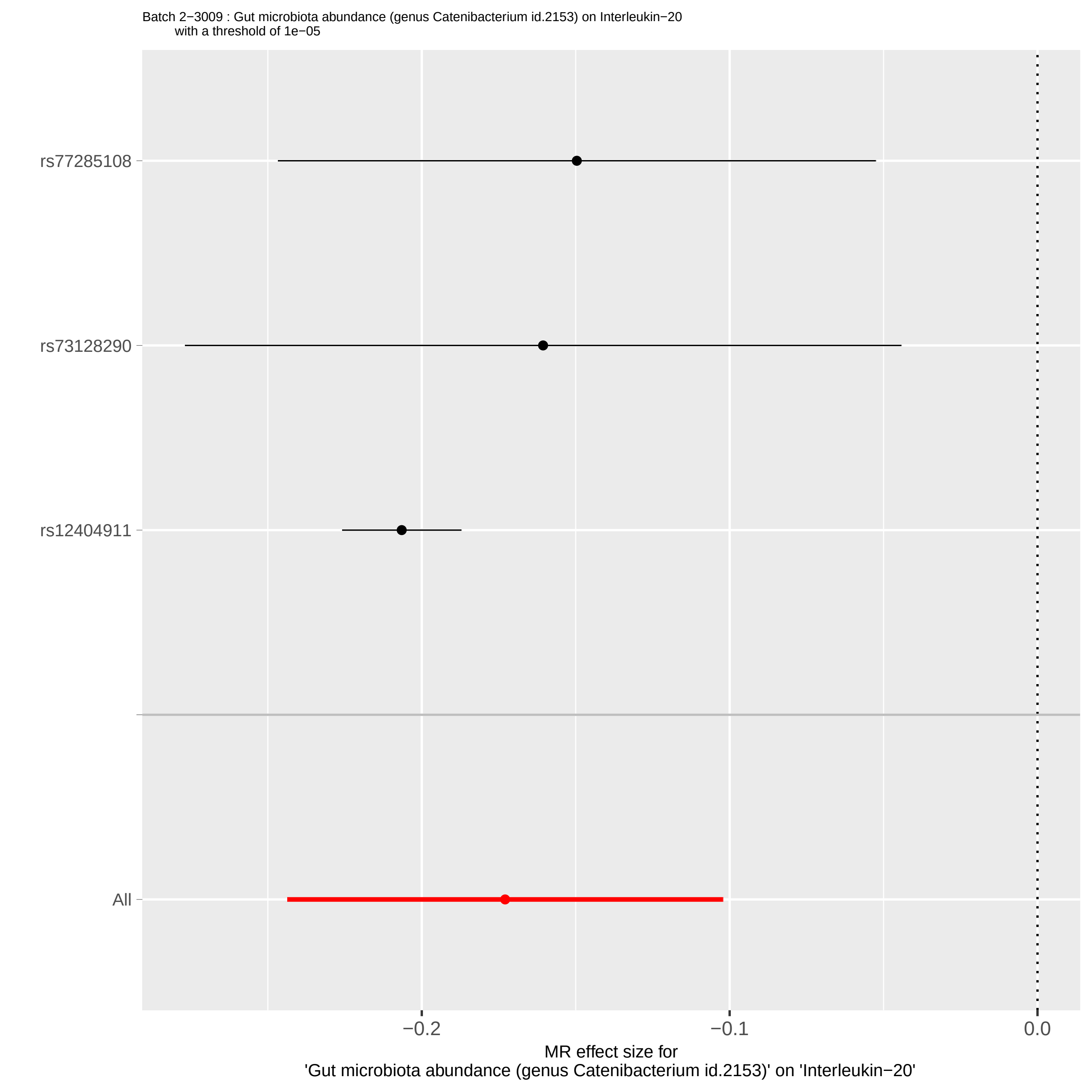

Batch 2−3009 : Gut microbiota abundance (genus Catenibacterium id.2153) on Interleukin−20
with a threshold of 1e−05
rs77285108
rs73128290
rs12404911
All
−0.2
−0.1
0.0
MR effect size for
'Gut microbiota abundance (genus Catenibacterium id.2153)' on 'Interleukin−20'

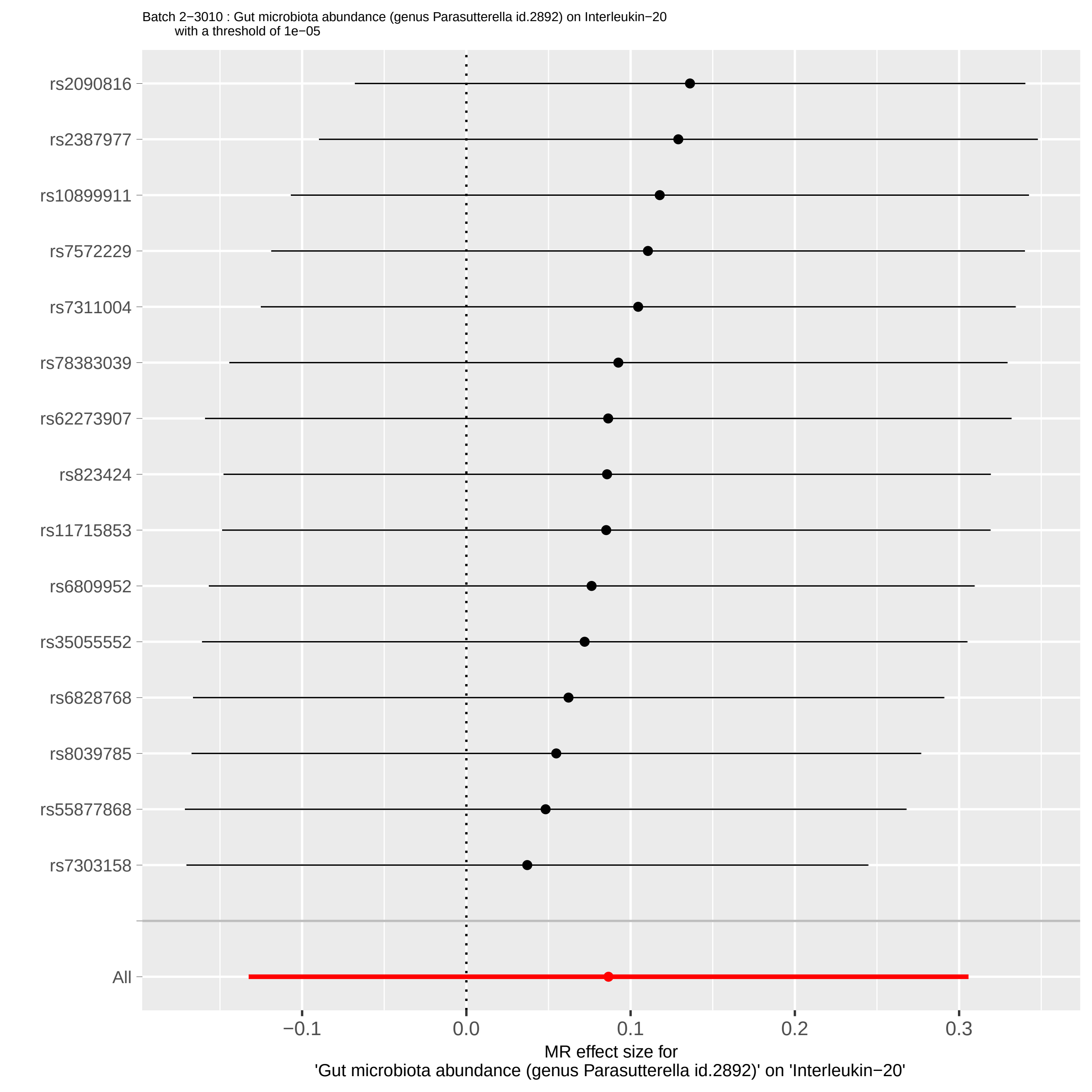
Batch 2−3010 : Gut microbiota abundance (genus Parasutterella id.2892) on Interleukin−20
with a threshold of 1e−05
rs2090816
rs2387977
rs10899911
rs7572229
rs7311004
rs78383039
rs62273907
rs823424
rs11715853
rs6809952
rs35055552
rs6828768
rs8039785
rs55877868
rs7303158
All
−0.1
0.0
0.1
0.2
0.3
MR effect size for
'Gut microbiota abundance (genus Parasutterella id.2892)' on 'Interleukin−20'

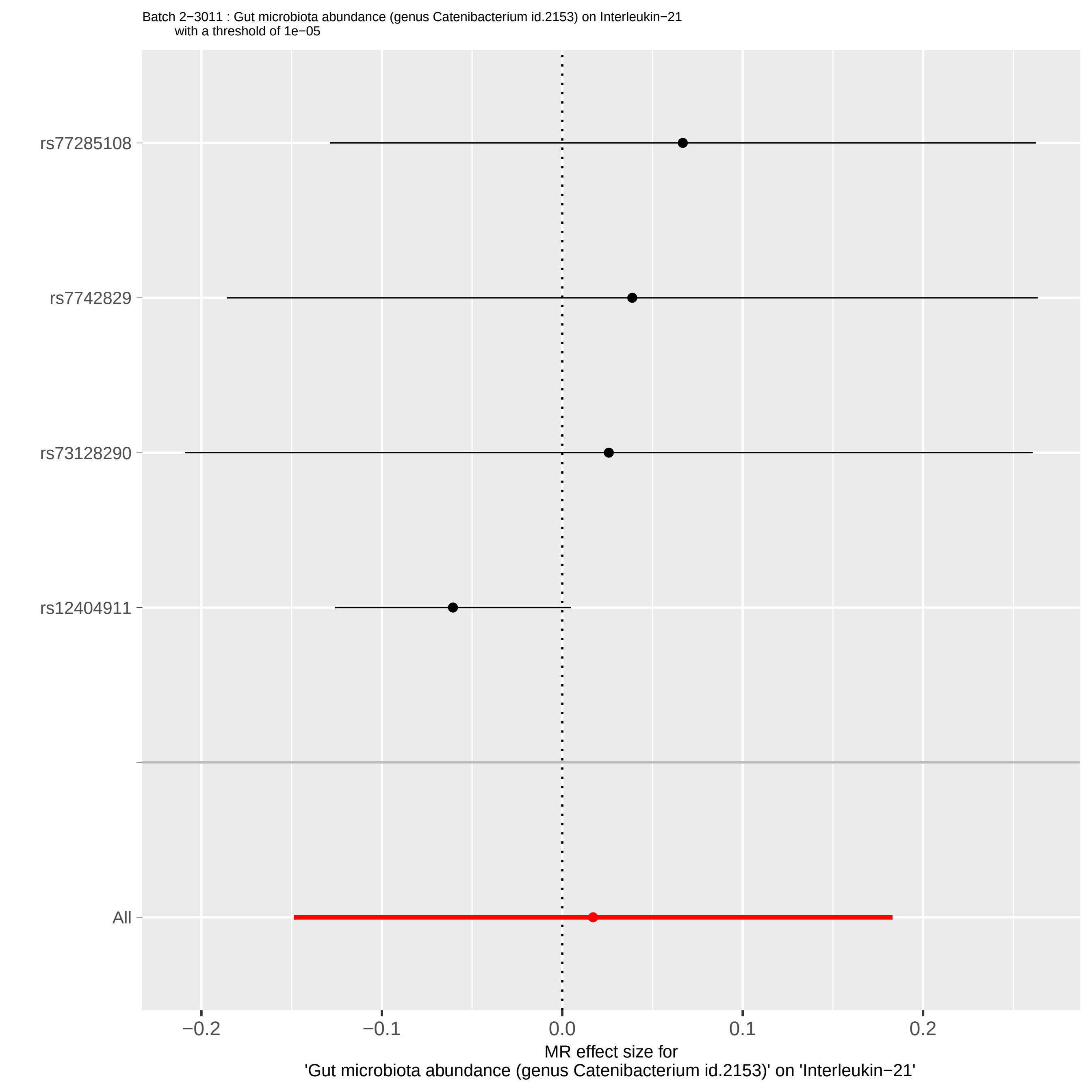

Batch 2−3011 : Gut microbiota abundance (genus Catenibacterium id.2153) on Interleukin−21
with a threshold of 1e−05
rs77285108
rs7742829
rs73128290
rs12404911
All
−0.2
−0.1
0.0
0.1
0.2
MR effect size for
'Gut microbiota abundance (genus Catenibacterium id.2153)' on 'Interleukin−21'

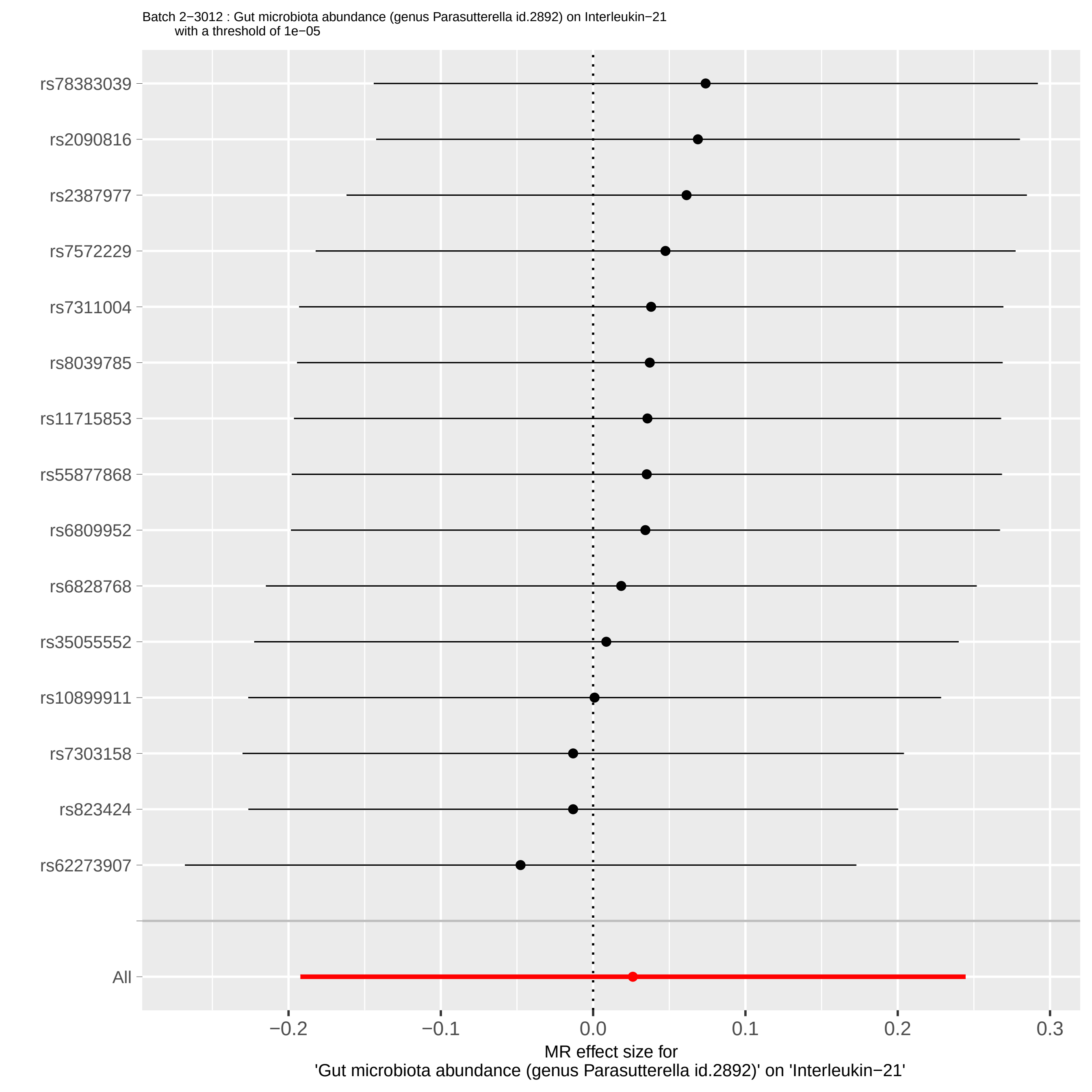

Batch 2−3012 : Gut microbiota abundance (genus Parasutterella id.2892) on Interleukin−21
with a threshold of 1e−05
rs78383039
rs2090816
rs2387977
rs7572229
rs7311004
rs8039785
rs11715853
rs55877868
rs6809952
rs6828768
rs35055552
rs10899911
rs7303158
rs823424
rs62273907
All
−0.2
−0.1
0.0
0.1
0.2
0.3
MR effect size for
'Gut microbiota abundance (genus Parasutterella id.2892)' on 'Interleukin−21'

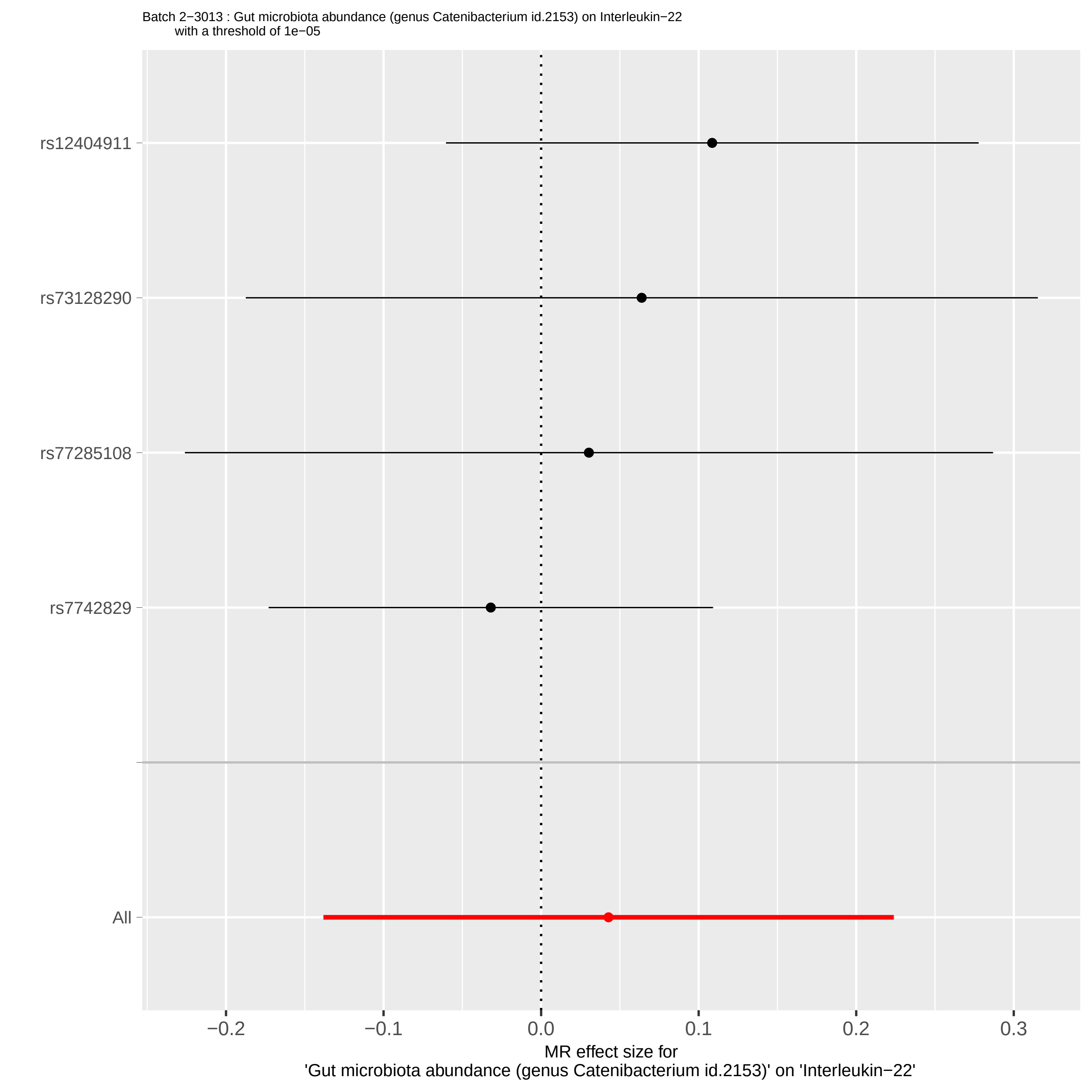

Batch 2−3013 : Gut microbiota abundance (genus Catenibacterium id.2153) on Interleukin−22
with a threshold of 1e−05
rs12404911
rs73128290
rs77285108
rs7742829
All
−0.2
−0.1
0.0
0.1
0.2
0.3
MR effect size for
'Gut microbiota abundance (genus Catenibacterium id.2153)' on 'Interleukin−22'

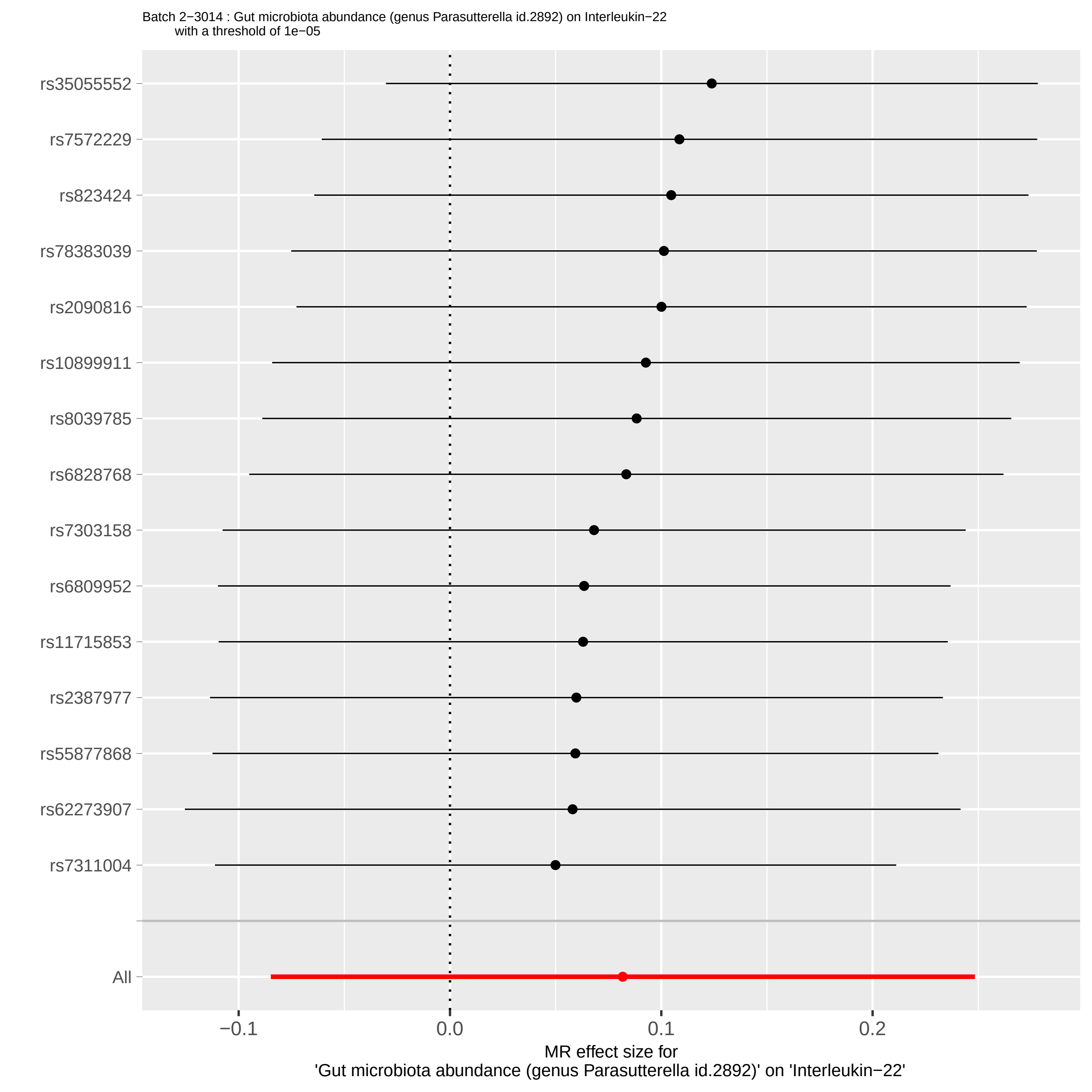

Batch 2−3014 : Gut microbiota abundance (genus Parasutterella id.2892) on Interleukin−22
with a threshold of 1e−05
rs35055552
rs7572229
rs823424
rs78383039
rs2090816
rs10899911
rs8039785
rs6828768
rs7303158
rs6809952
rs11715853
rs2387977
rs55877868
rs62273907
rs7311004
All
−0.1
0.0
0.1
0.2
MR effect size for
'Gut microbiota abundance (genus Parasutterella id.2892)' on 'Interleukin−22'

Batch 2−3015 : Gut microbiota abundance (genus Catenibacterium id.2153) on Interleukin−22 receptor subunit alpha−1
with a threshold of 1e−05

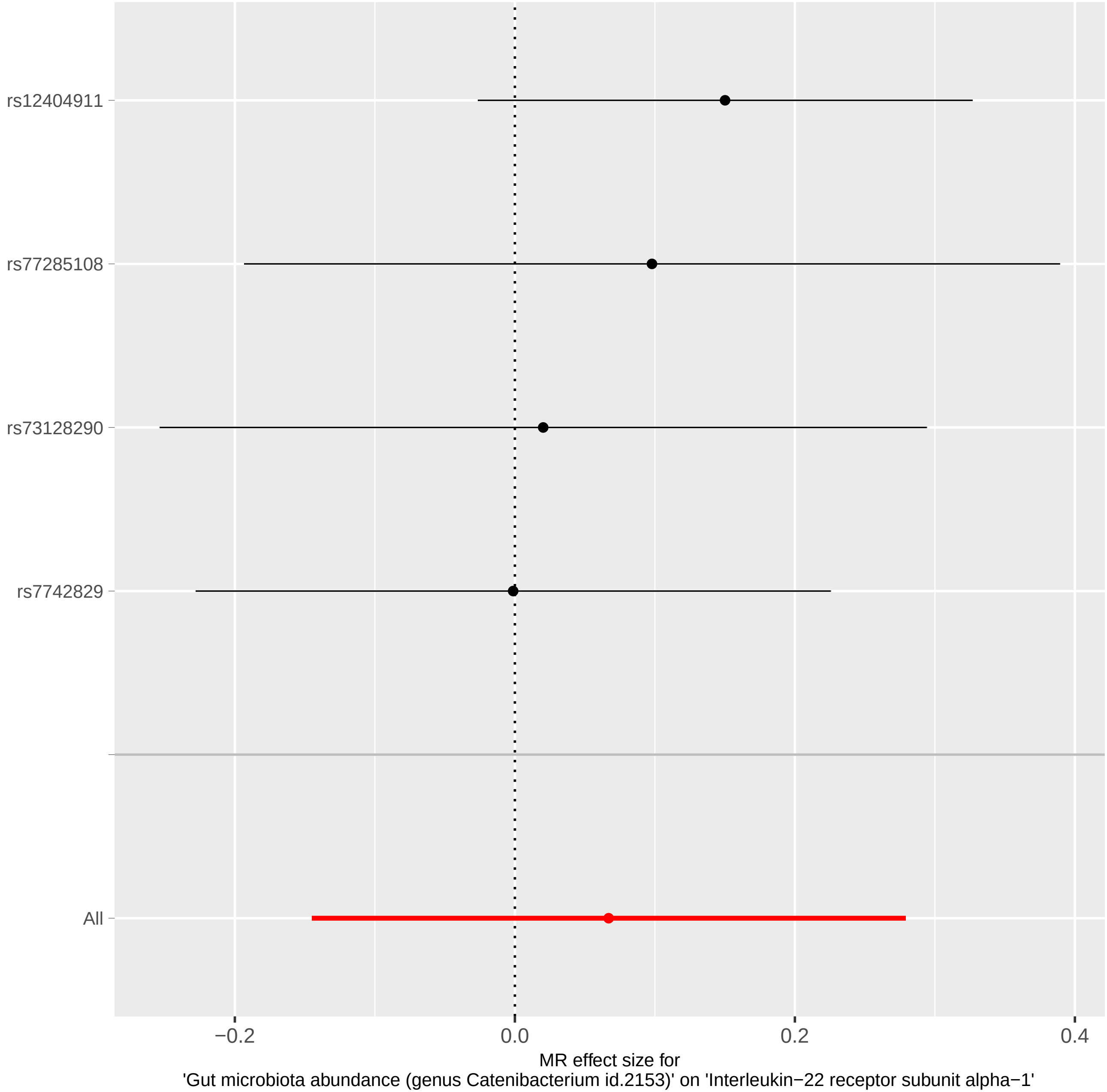

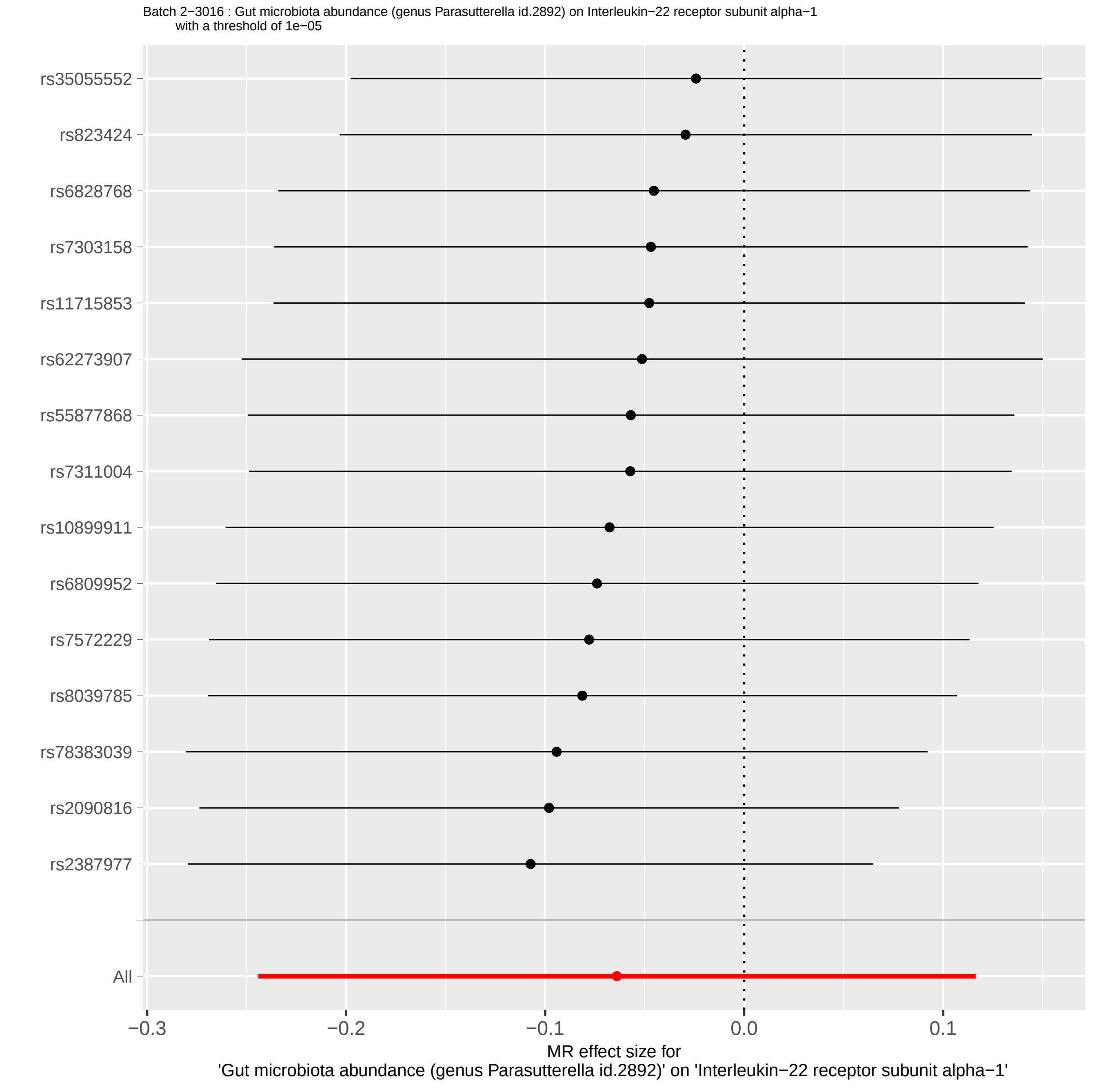
Batch 2−3016 : Gut microbiota abundance (genus Parasutterella id.2892) on Interleukin−22 receptor subunit alpha−1
with a threshold of 1e−05
rs35055552
rs823424
rs6828768
rs7303158
rs11715853
rs62273907
rs55877868
rs7311004
rs10899911
rs6809952
rs7572229
rs8039785
rs78383039
rs2090816
rs2387977
All
−0.3
−0.2
−0.1
0.0
0.1
MR effect size for
'Gut microbiota abundance (genus Parasutterella id.2892)' on 'Interleukin−22 receptor subunit alpha−1'

Batch 2−3017 : Gut microbiota abundance (genus Catenibacterium id.2153) on Interleukin−22 receptor subunit alpha−1
with a threshold of 1e−05

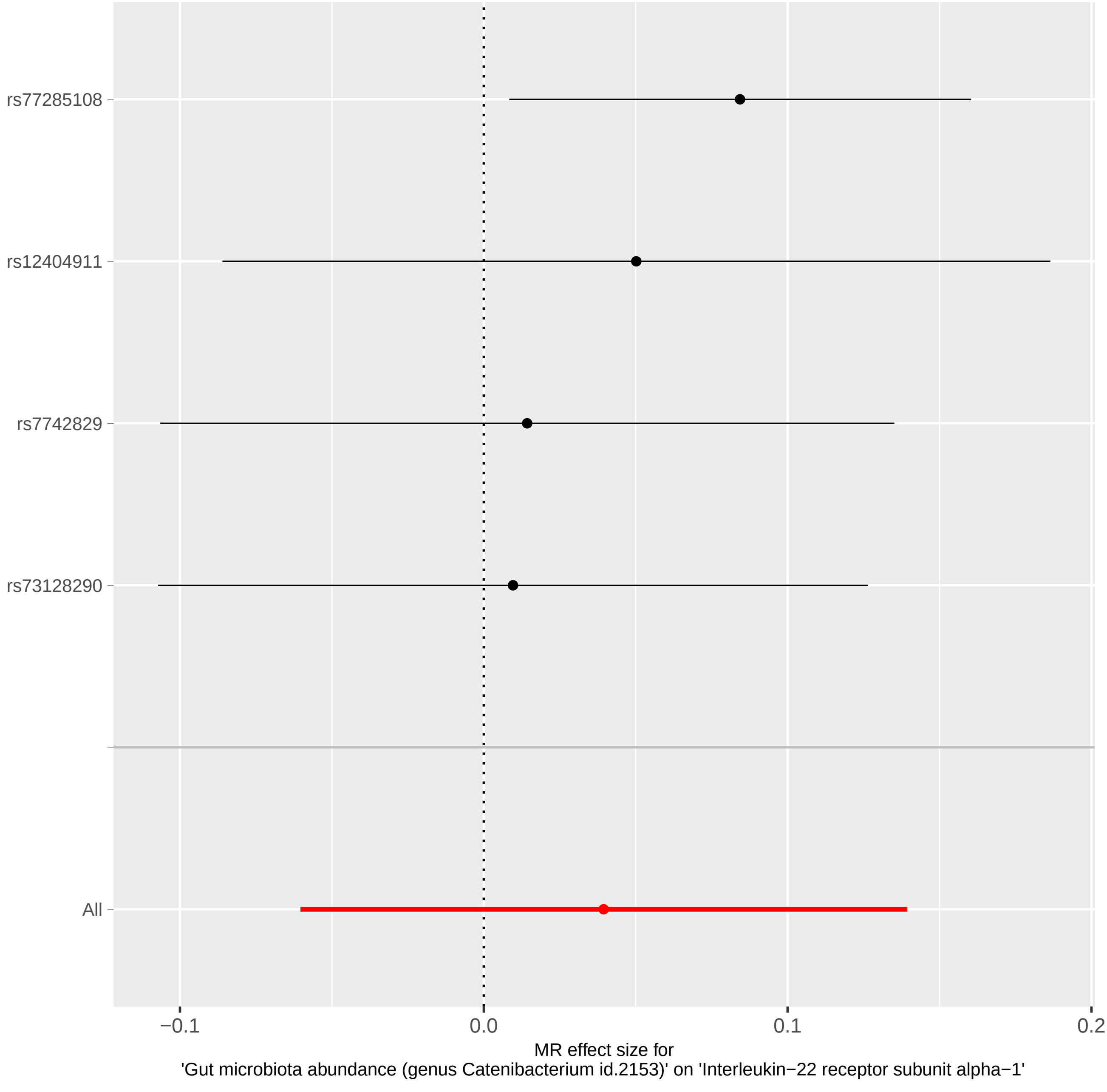

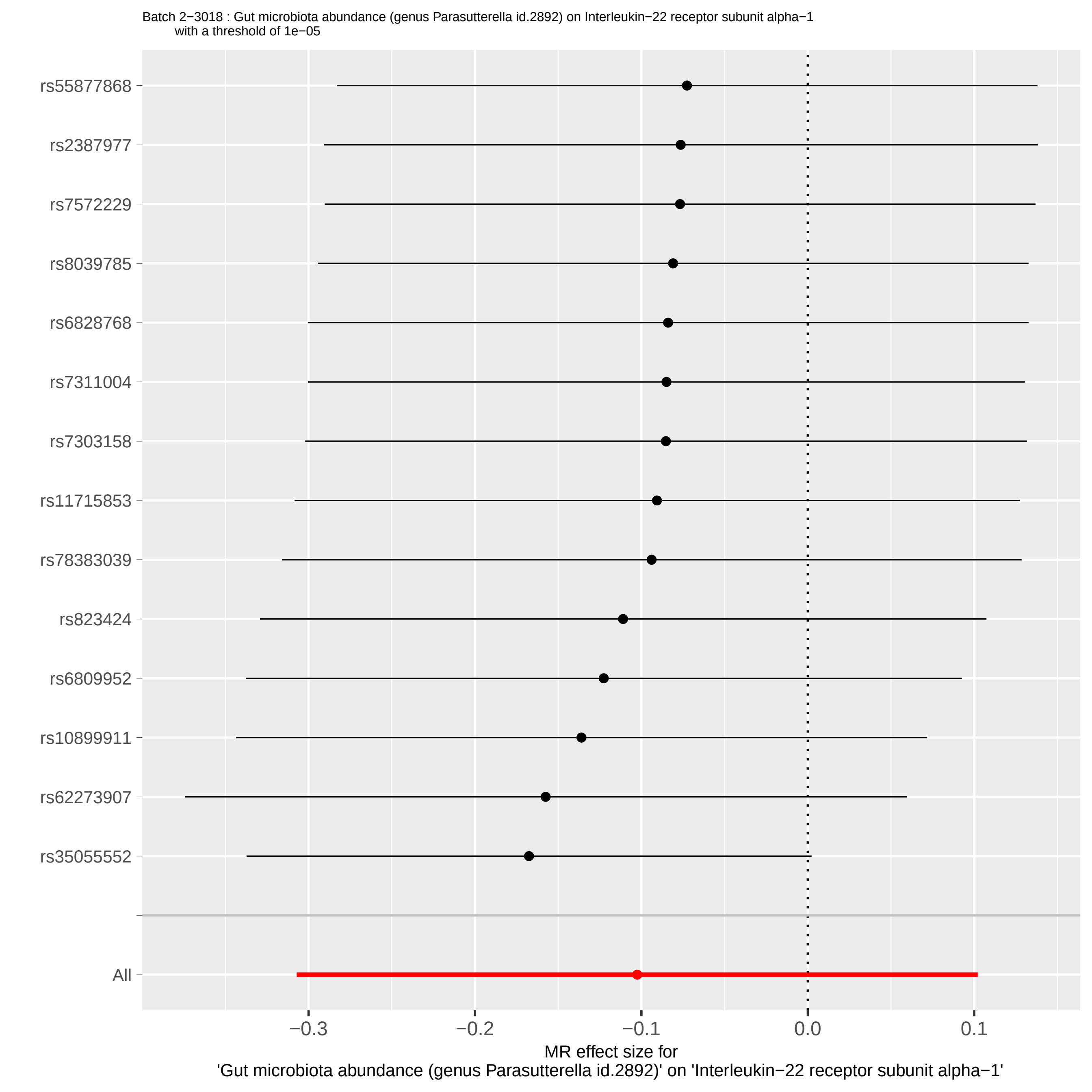
Batch 2−3018 : Gut microbiota abundance (genus Parasutterella id.2892) on Interleukin−22 receptor subunit alpha−1
with a threshold of 1e−05
rs55877868
rs2387977
rs7572229
rs8039785
rs6828768
rs7311004
rs7303158
rs11715853
rs78383039
rs823424
rs6809952
rs10899911
rs62273907
rs35055552
All
−0.3
−0.2
−0.1
0.0
0.1
MR effect size for
'Gut microbiota abundance (genus Parasutterella id.2892)' on 'Interleukin−22 receptor subunit alpha−1'

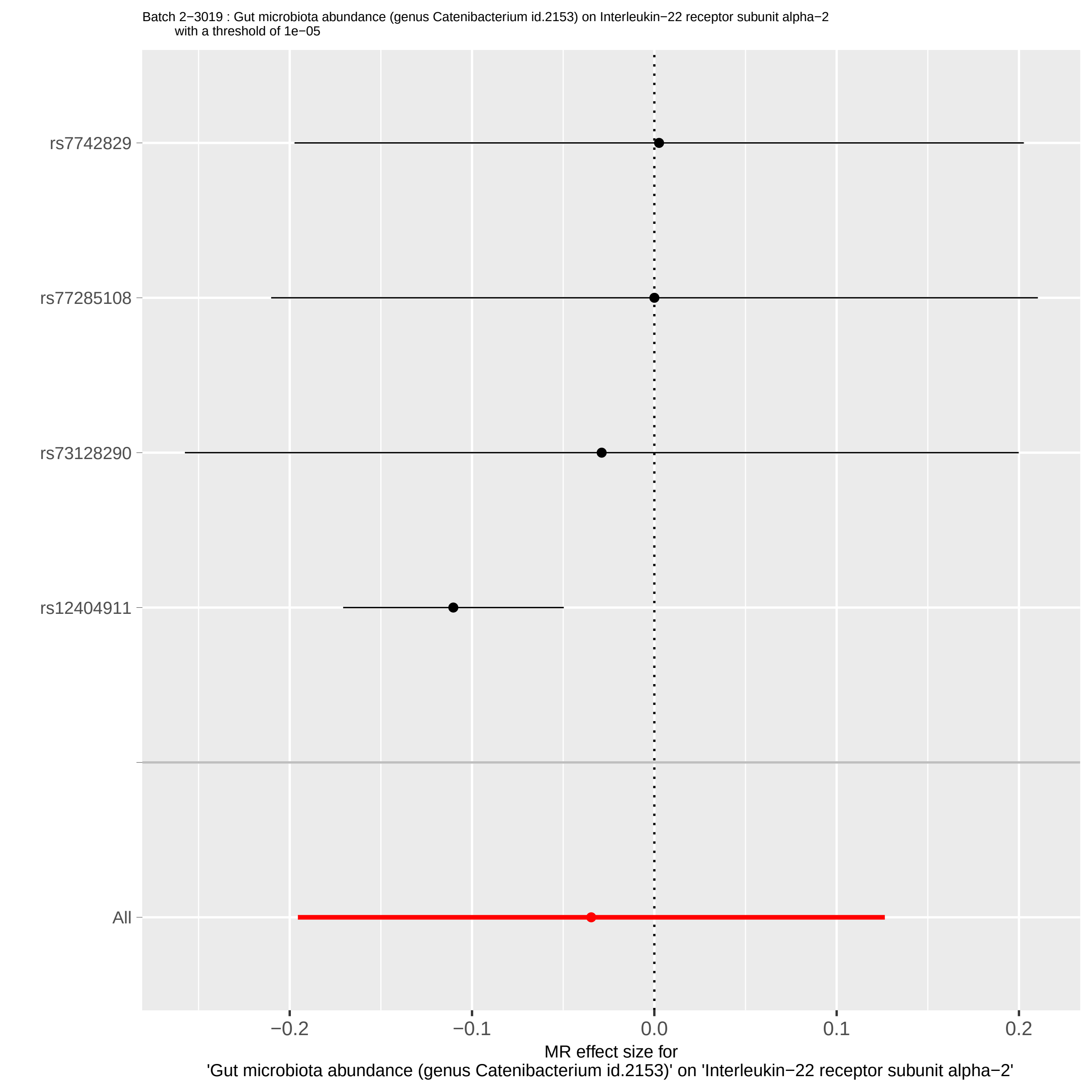

Batch 2−3019 : Gut microbiota abundance (genus Catenibacterium id.2153) on Interleukin−22 receptor subunit alpha−2
with a threshold of 1e−05
rs7742829
rs77285108
rs73128290
rs12404911
All
−0.2
−0.1
0.0
0.1
0.2
MR effect size for
'Gut microbiota abundance (genus Catenibacterium id.2153)' on 'Interleukin−22 receptor subunit alpha−2'

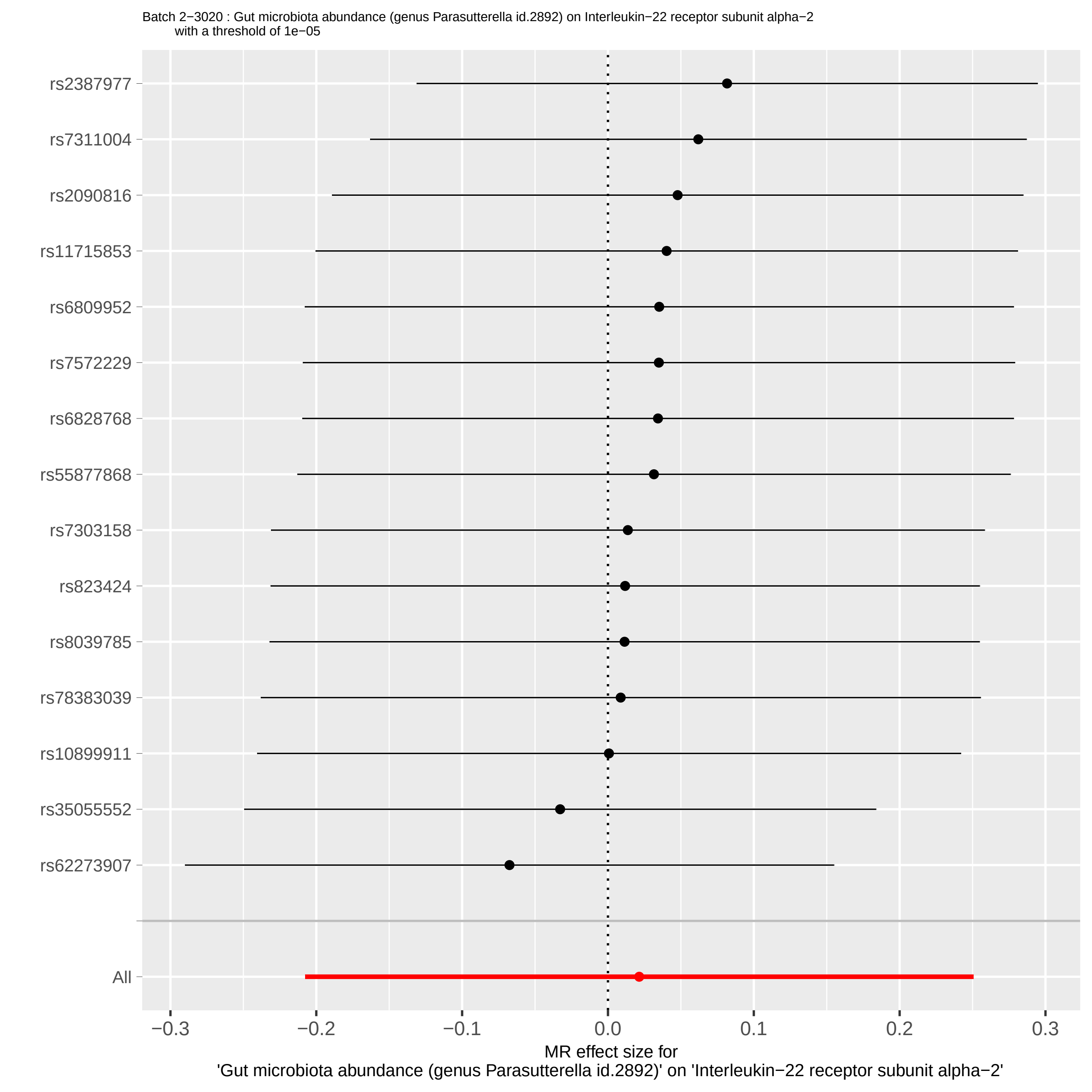

Batch 2−3020 : Gut microbiota abundance (genus Parasutterella id.2892) on Interleukin−22 receptor subunit alpha−2
with a threshold of 1e−05
rs2387977
rs7311004
rs2090816
rs11715853
rs6809952
rs7572229
rs6828768
rs55877868
rs7303158
rs823424
rs8039785
rs78383039
rs10899911
rs35055552
rs62273907
All
−0.3
−0.2
−0.1
0.0
0.1
0.2
0.3
MR effect size for
'Gut microbiota abundance (genus Parasutterella id.2892)' on 'Interleukin−22 receptor subunit alpha−2'

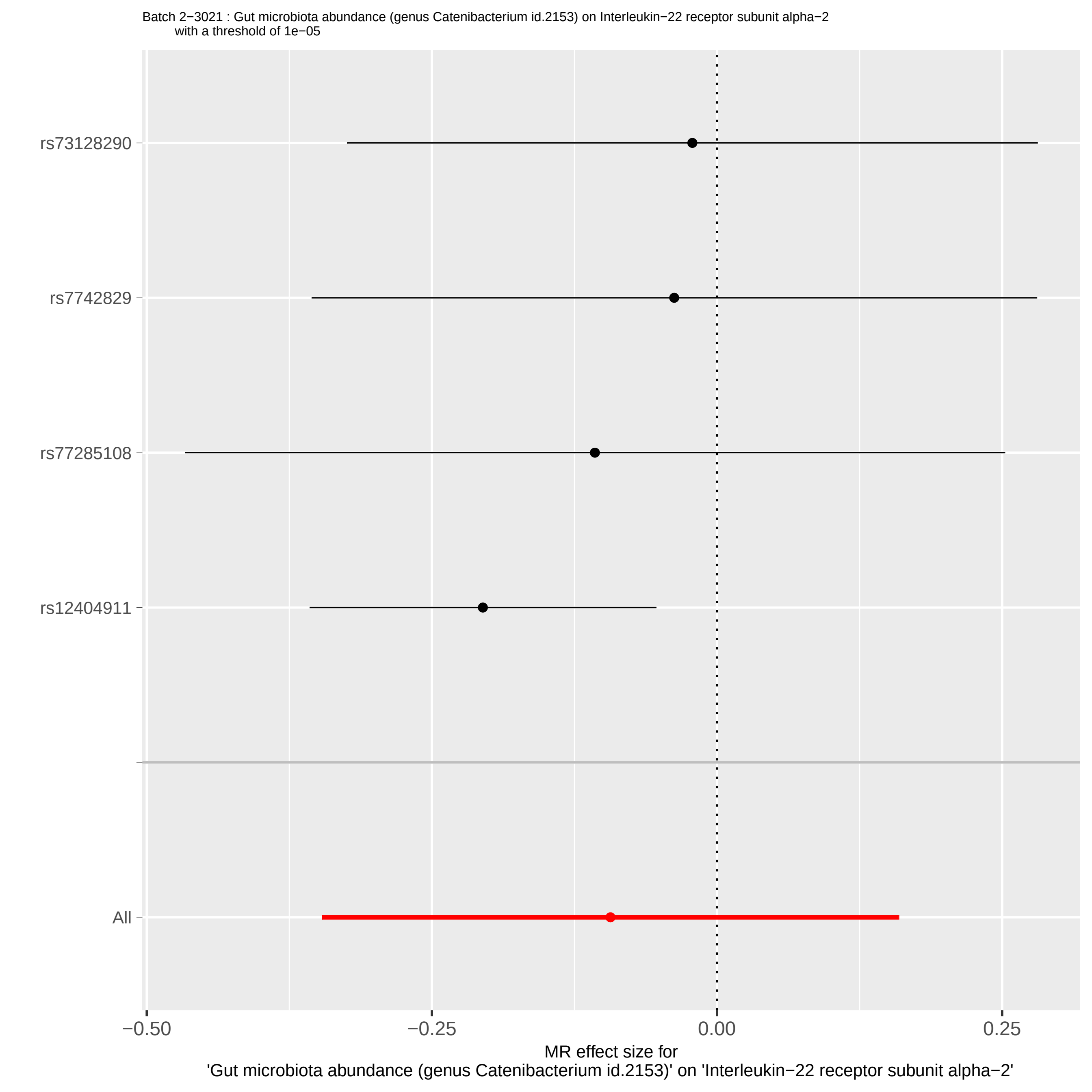
Batch 2−3021 : Gut microbiota abundance (genus Catenibacterium id.2153) on Interleukin−22 receptor subunit alpha−2
with a threshold of 1e−05
rs73128290
rs7742829
rs77285108
rs12404911
All
−0.50
−0.25
0.00
0.25
MR effect size for
'Gut microbiota abundance (genus Catenibacterium id.2153)' on 'Interleukin−22 receptor subunit alpha−2'

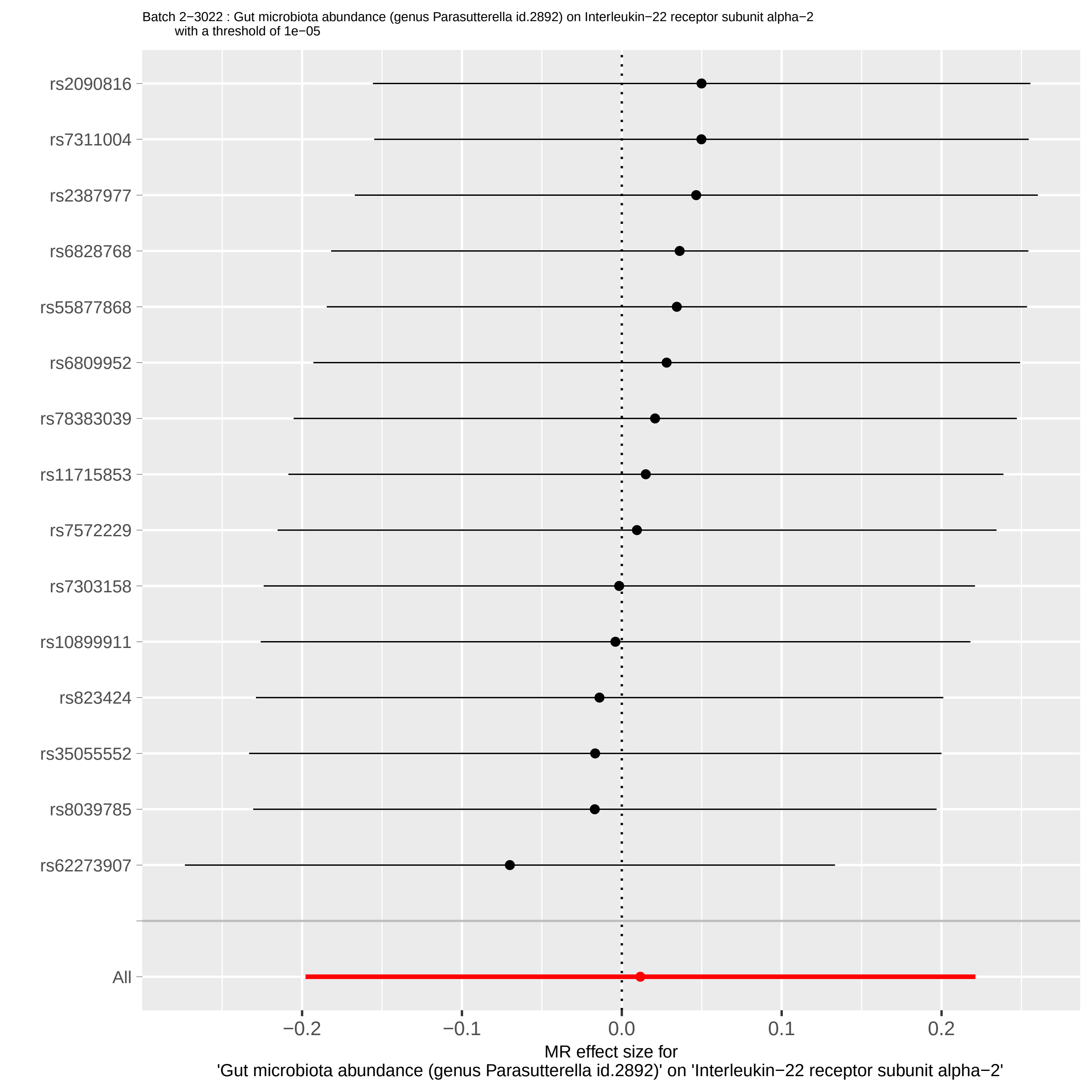
Batch 2−3022 : Gut microbiota abundance (genus Parasutterella id.2892) on Interleukin−22 receptor subunit alpha−2
with a threshold of 1e−05
rs2090816
rs7311004
rs2387977
rs6828768
rs55877868
rs6809952
rs78383039
rs11715853
rs7572229
rs7303158
rs10899911
rs823424
rs35055552
rs8039785
rs62273907
All
−0.2
−0.1
0.0
0.1
0.2
MR effect size for
'Gut microbiota abundance (genus Parasutterella id.2892)' on 'Interleukin−22 receptor subunit alpha−2'

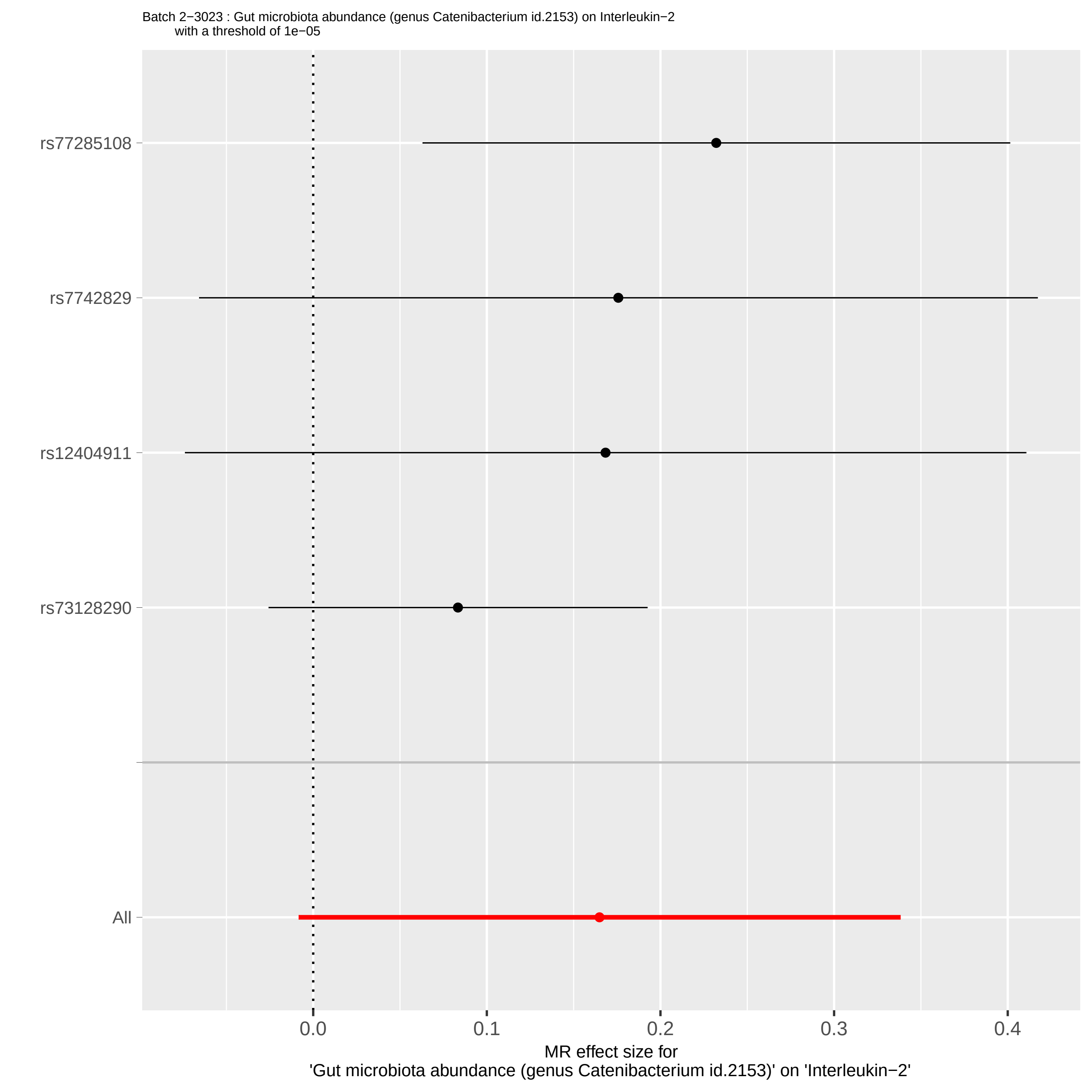

Batch 2−3023 : Gut microbiota abundance (genus Catenibacterium id.2153) on Interleukin−2
with a threshold of 1e−05
rs77285108
rs7742829
rs12404911
rs73128290
All
0.0
0.1
0.2
0.3
0.4
MR effect size for
'Gut microbiota abundance (genus Catenibacterium id.2153)' on 'Interleukin−2'

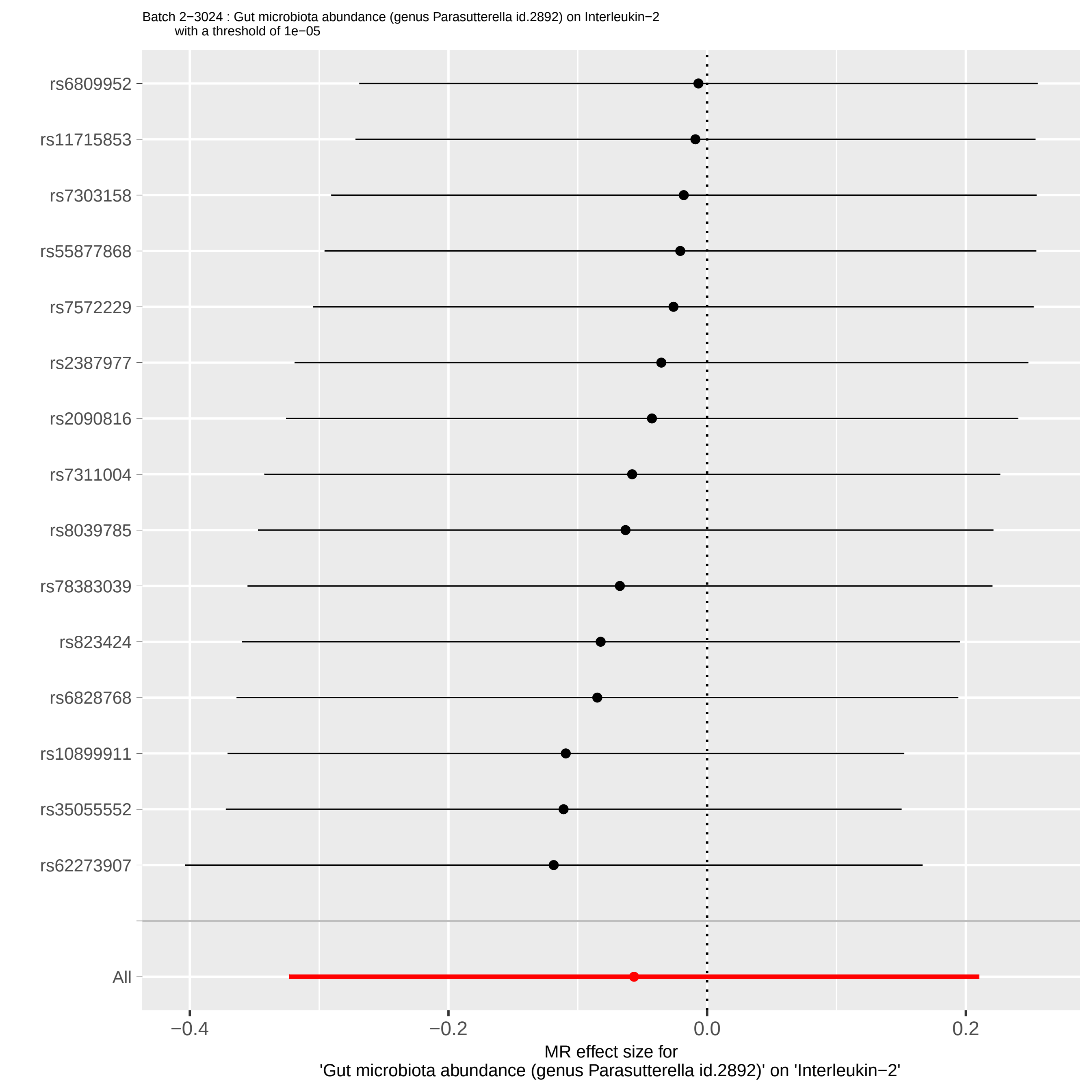
Batch 2−3024 : Gut microbiota abundance (genus Parasutterella id.2892) on Interleukin−2
with a threshold of 1e−05
rs6809952
rs11715853
rs7303158
rs55877868
rs7572229
rs2387977
rs2090816
rs7311004
rs8039785
rs78383039
rs823424
rs6828768
rs10899911
rs35055552
rs62273907
All
−0.4
−0.2
0.0
0.2
MR effect size for
'Gut microbiota abundance (genus Parasutterella id.2892)' on 'Interleukin−2'

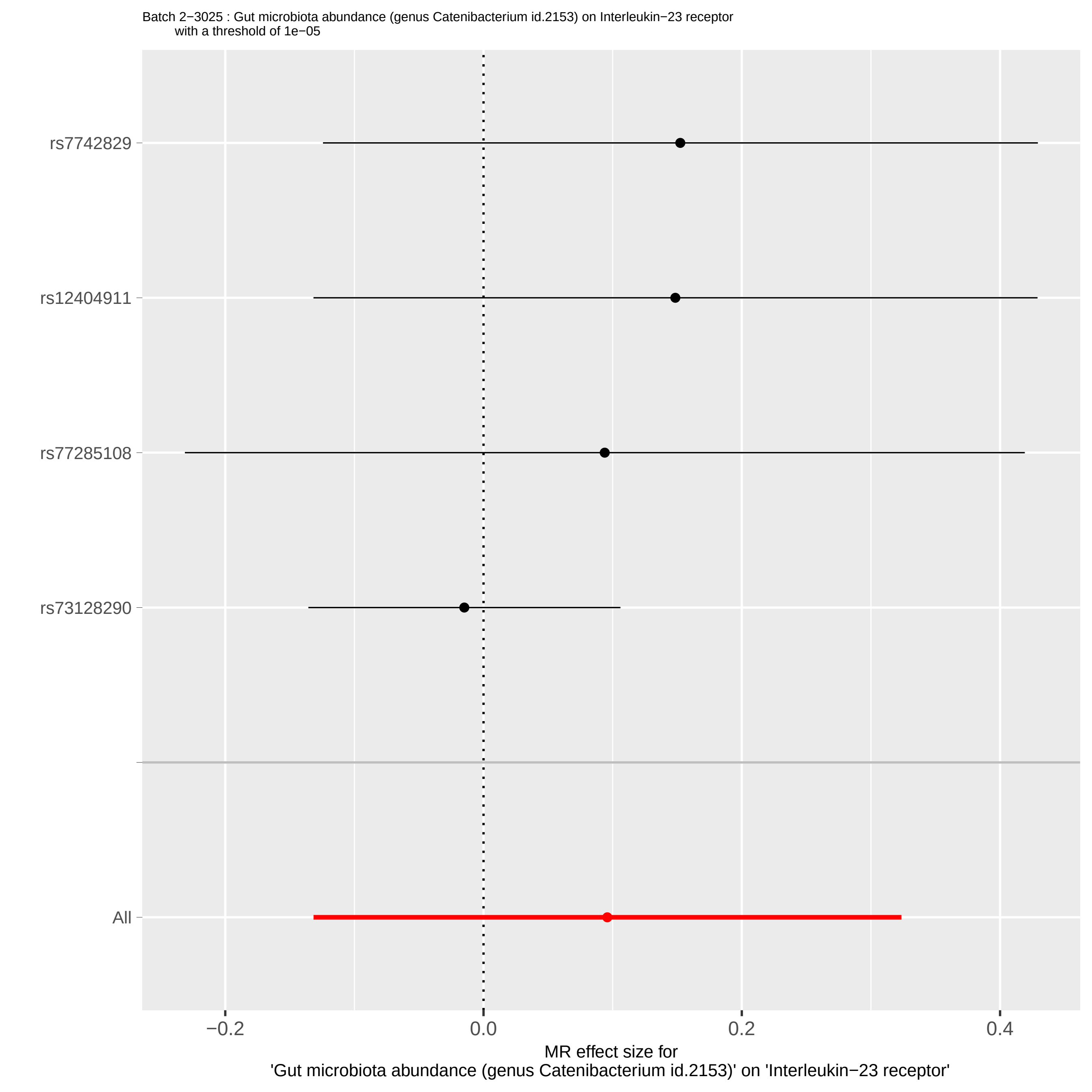
Batch 2−3025 : Gut microbiota abundance (genus Catenibacterium id.2153) on Interleukin−23 receptor
with a threshold of 1e−05
rs7742829
rs12404911
rs77285108
rs73128290
All
−0.2
0.0
0.2
0.4
MR effect size for
'Gut microbiota abundance (genus Catenibacterium id.2153)' on 'Interleukin−23 receptor'

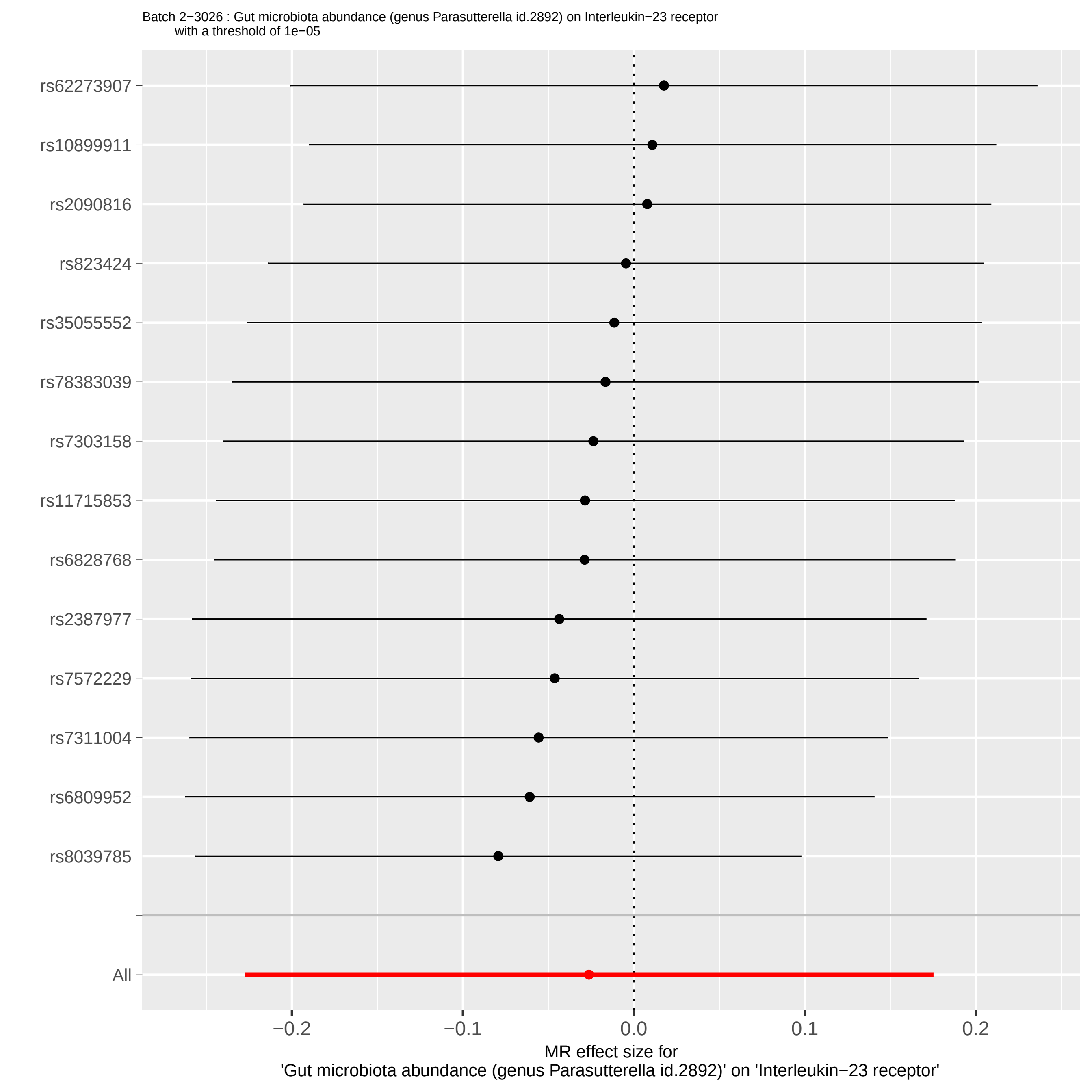
Batch 2−3026 : Gut microbiota abundance (genus Parasutterella id.2892) on Interleukin−23 receptor
with a threshold of 1e−05
rs62273907
rs10899911
rs2090816
rs823424
rs35055552
rs78383039
rs7303158
rs11715853
rs6828768
rs2387977
rs7572229
rs7311004
rs6809952
rs8039785
All
−0.2
−0.1
0.0
0.1
0.2
MR effect size for
'Gut microbiota abundance (genus Parasutterella id.2892)' on 'Interleukin−23 receptor'

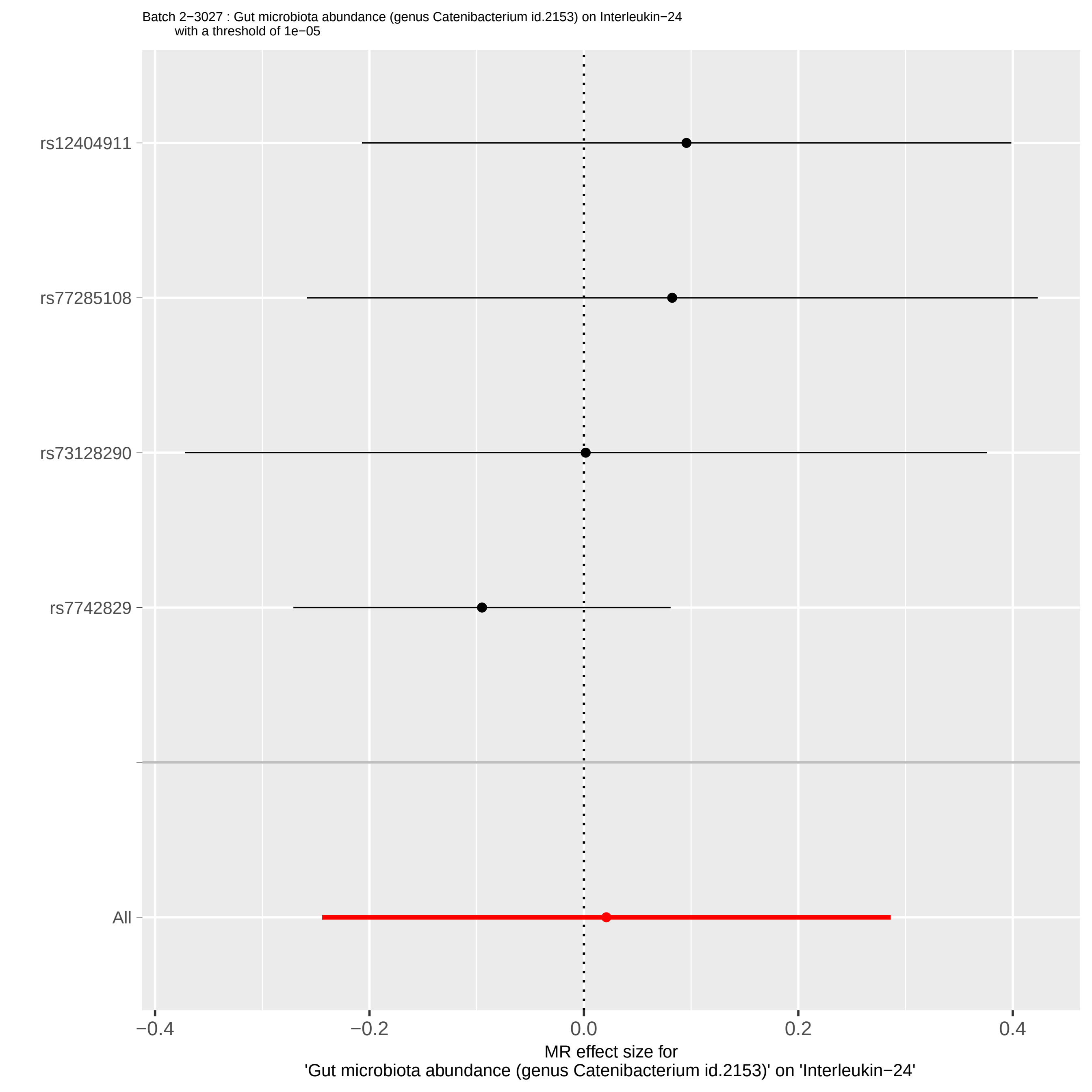

Batch 2−3027 : Gut microbiota abundance (genus Catenibacterium id.2153) on Interleukin−24
with a threshold of 1e−05
rs12404911
rs77285108
rs73128290
rs7742829
All
−0.4
−0.2
0.0
0.2
0.4
MR effect size for
'Gut microbiota abundance (genus Catenibacterium id.2153)' on 'Interleukin−24'

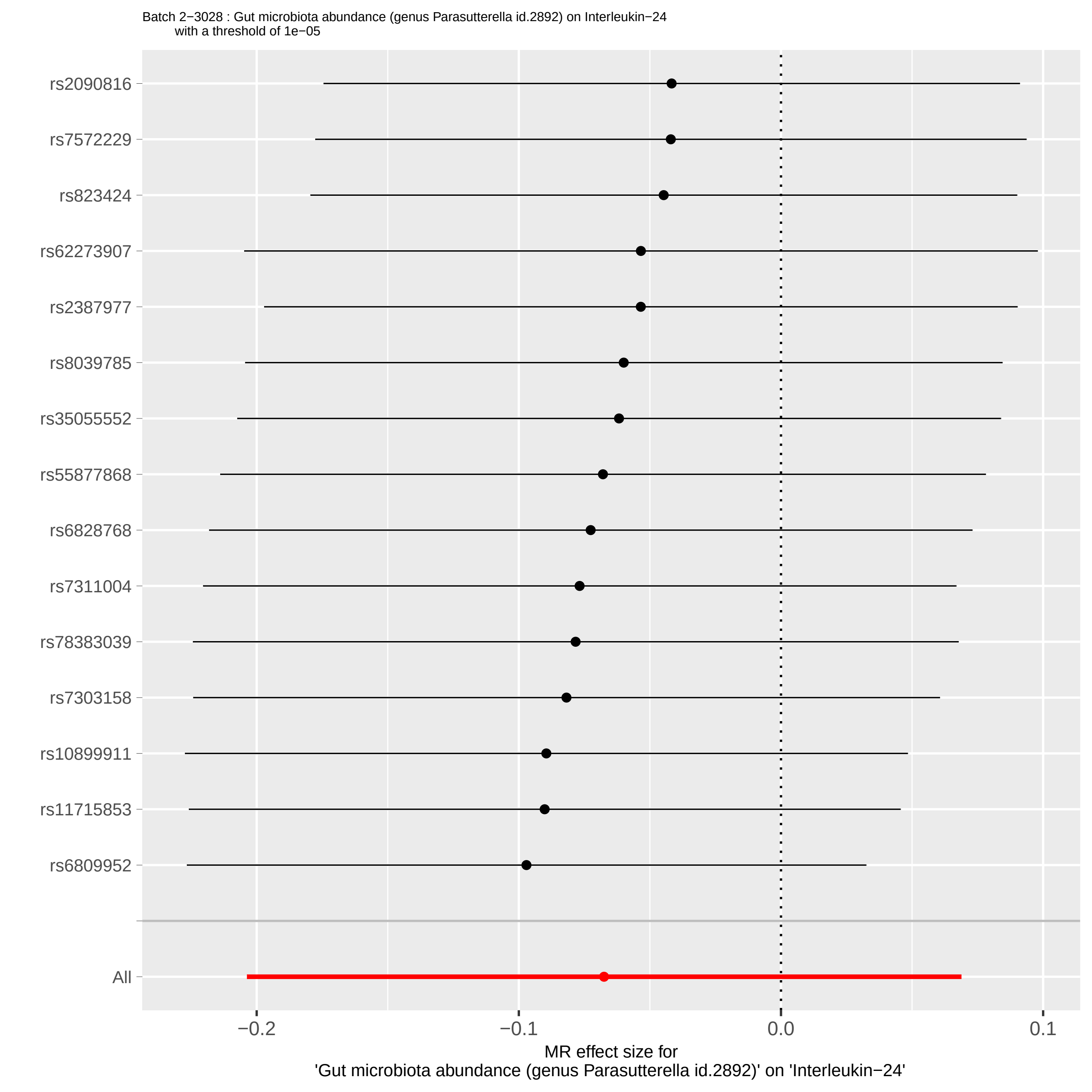

Batch 2−3028 : Gut microbiota abundance (genus Parasutterella id.2892) on Interleukin−24
with a threshold of 1e−05
rs2090816
rs7572229
rs823424
rs62273907
rs2387977
rs8039785
rs35055552
rs55877868
rs6828768
rs7311004
rs78383039
rs7303158
rs10899911
rs11715853
rs6809952
All
−0.2
−0.1
0.0
0.1
MR effect size for
'Gut microbiota abundance (genus Parasutterella id.2892)' on 'Interleukin−24'

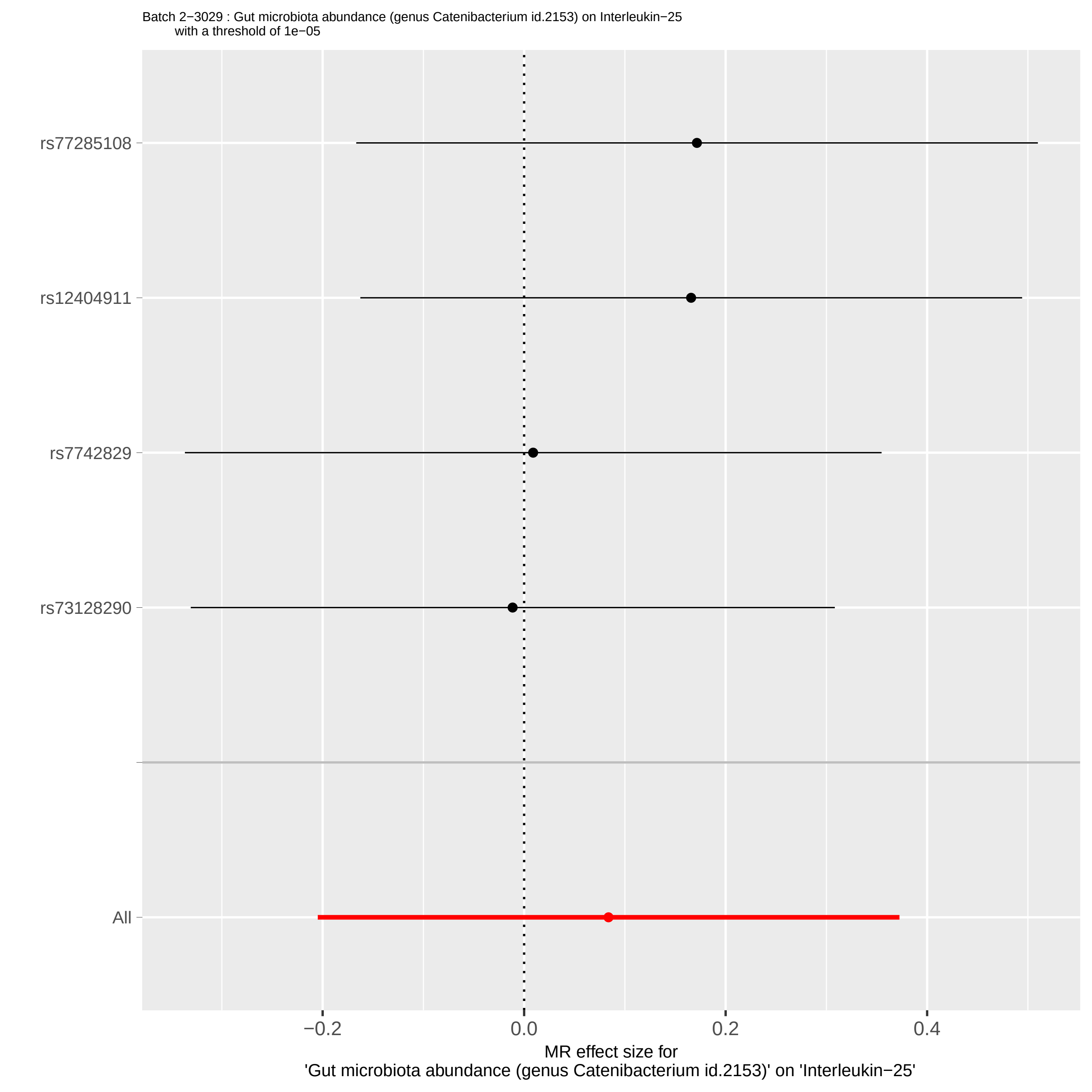
Batch 2−3029 : Gut microbiota abundance (genus Catenibacterium id.2153) on Interleukin−25
with a threshold of 1e−05
rs77285108
rs12404911
rs7742829
rs73128290
All
−0.2
0.0
0.2
0.4
MR effect size for
'Gut microbiota abundance (genus Catenibacterium id.2153)' on 'Interleukin−25'

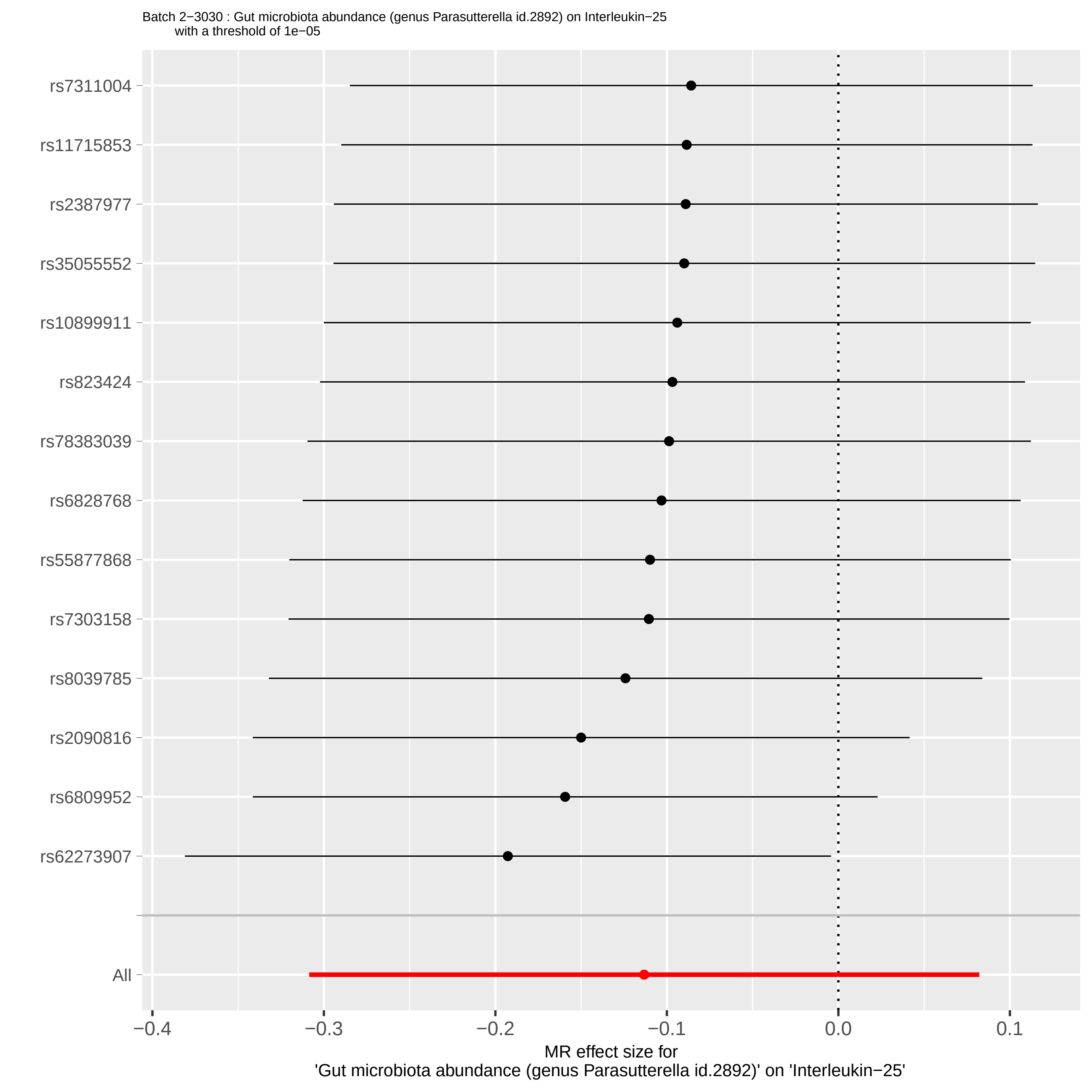
Batch 2−3030 : Gut microbiota abundance (genus Parasutterella id.2892) on Interleukin−25
with a threshold of 1e−05
rs7311004
rs11715853
rs2387977
rs35055552
rs10899911
rs823424
rs78383039
rs6828768
rs55877868
rs7303158
rs8039785
rs2090816
rs6809952
rs62273907
All
−0.4
−0.3
−0.2
−0.1
0.0
0.1
MR effect size for
'Gut microbiota abundance (genus Parasutterella id.2892)' on 'Interleukin−25'

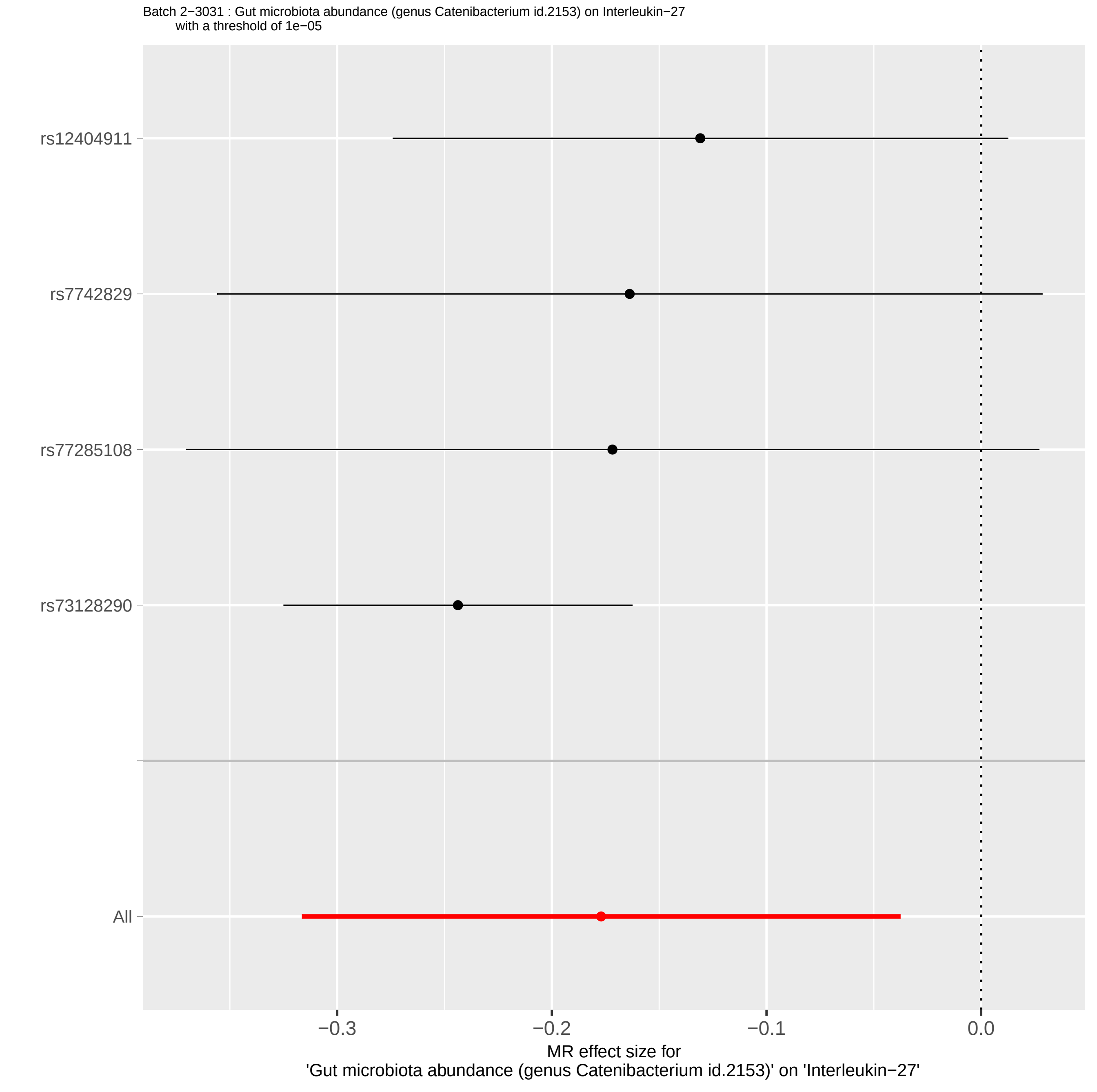

Batch 2−3031 : Gut microbiota abundance (genus Catenibacterium id.2153) on Interleukin−27
with a threshold of 1e−05
rs12404911
rs7742829
rs77285108
rs73128290
All
−0.3
−0.2
−0.1
0.0
MR effect size for
'Gut microbiota abundance (genus Catenibacterium id.2153)' on 'Interleukin−27'

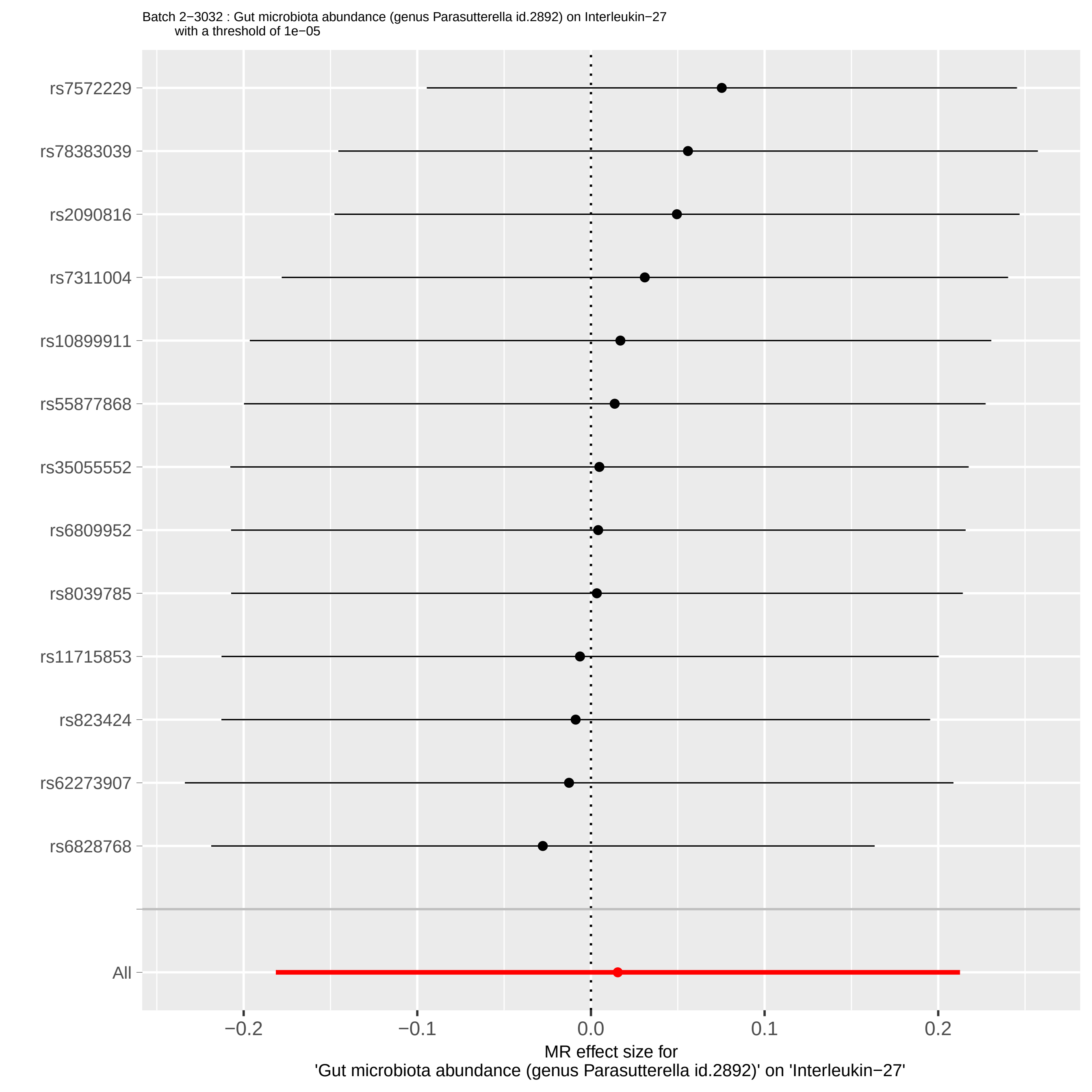
Batch 2−3032 : Gut microbiota abundance (genus Parasutterella id.2892) on Interleukin−27
with a threshold of 1e−05
rs7572229
rs78383039
rs2090816
rs7311004
rs10899911
rs55877868
rs35055552
rs6809952
rs8039785
rs11715853
rs823424
rs62273907
rs6828768
All
−0.2
−0.1
0.0
0.1
0.2
MR effect size for
'Gut microbiota abundance (genus Parasutterella id.2892)' on 'Interleukin−27'

Batch 2−3033 : Gut microbiota abundance (genus Catenibacterium id.2153) on Interleukin−27 receptor subunit alpha
with a threshold of 1e−05

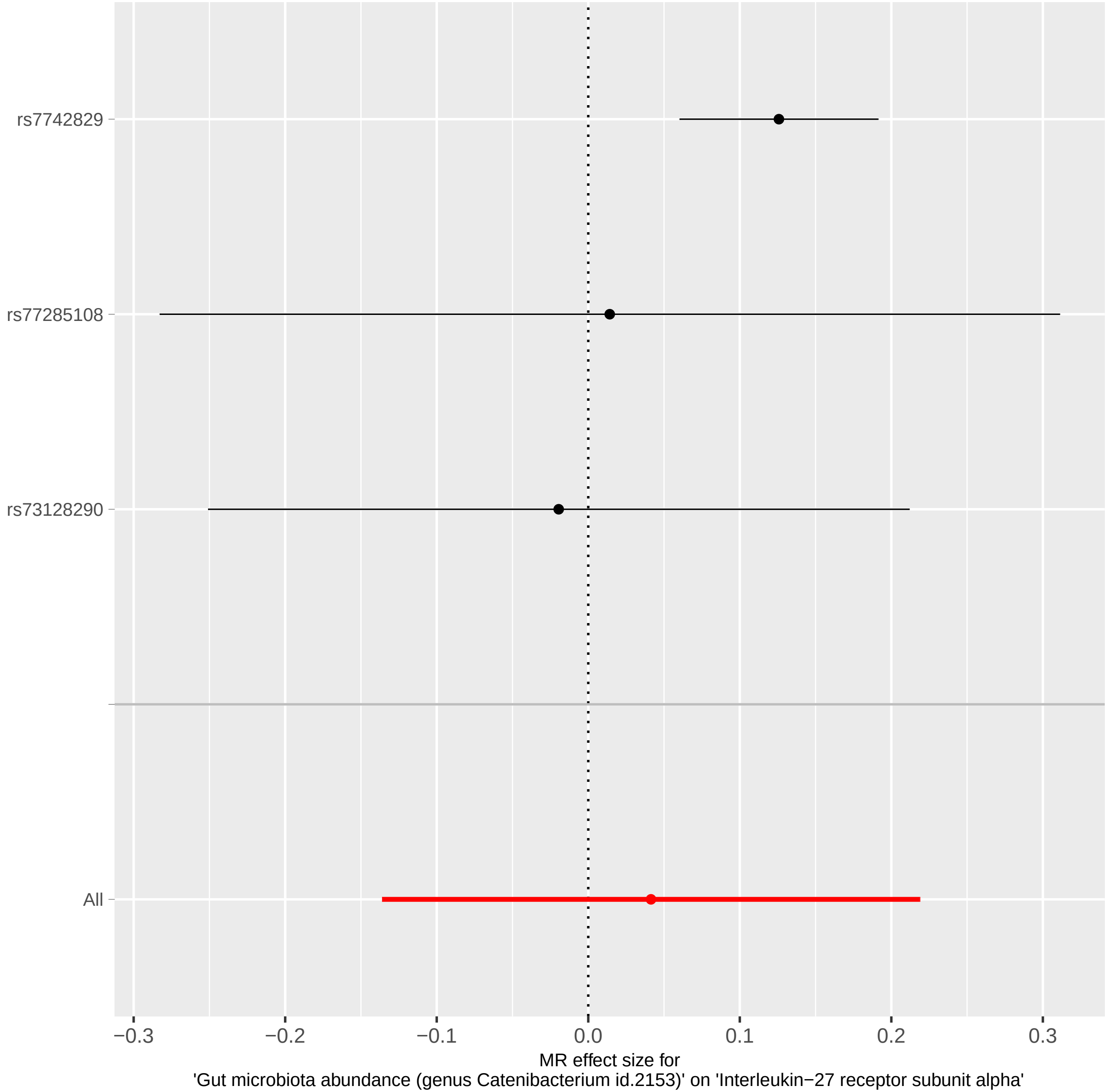

Batch 2−3034 : Gut microbiota abundance (genus Parasutterella id.2892) on Interleukin−27 receptor subunit alpha
with a threshold of 1e−05

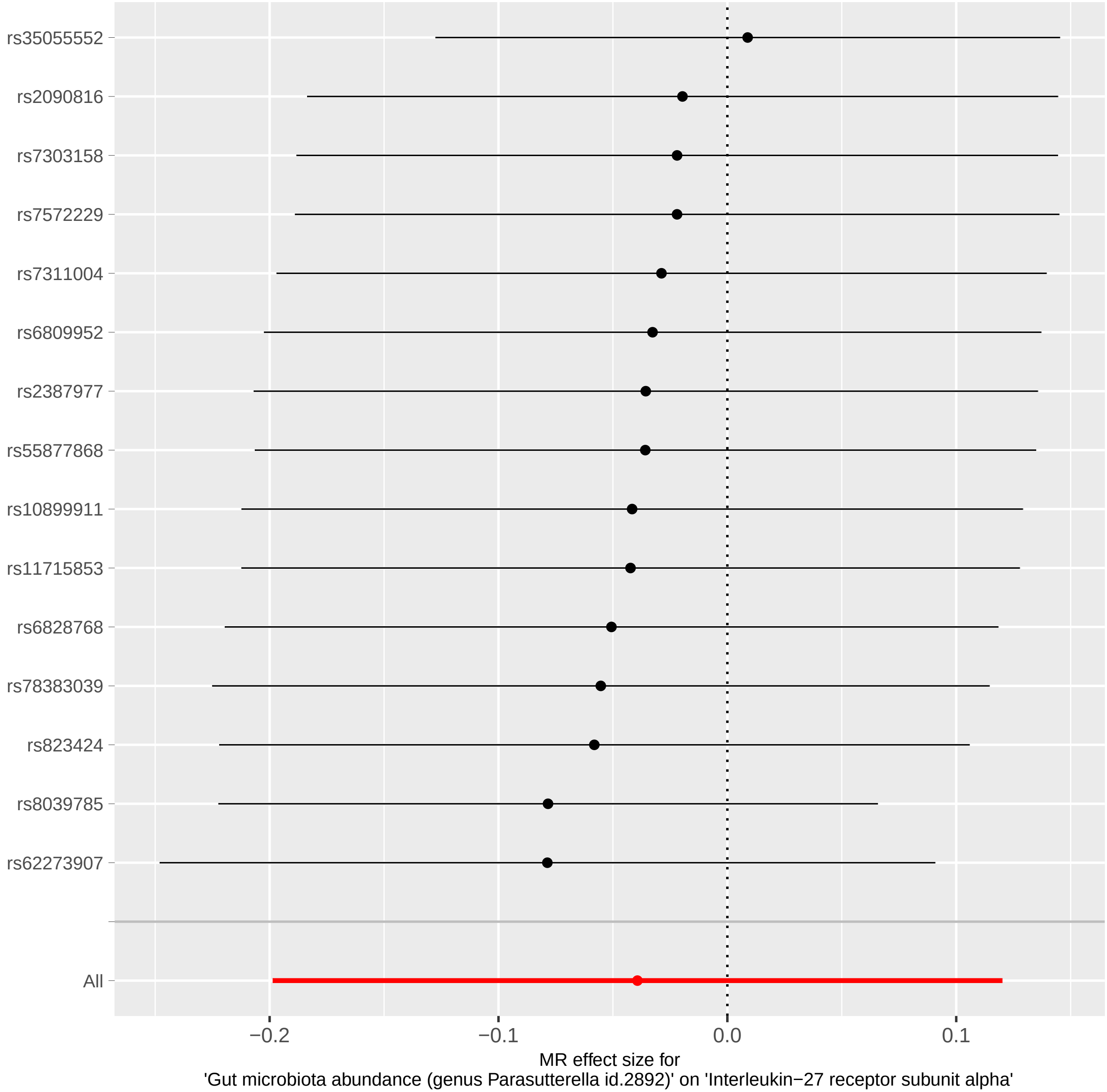

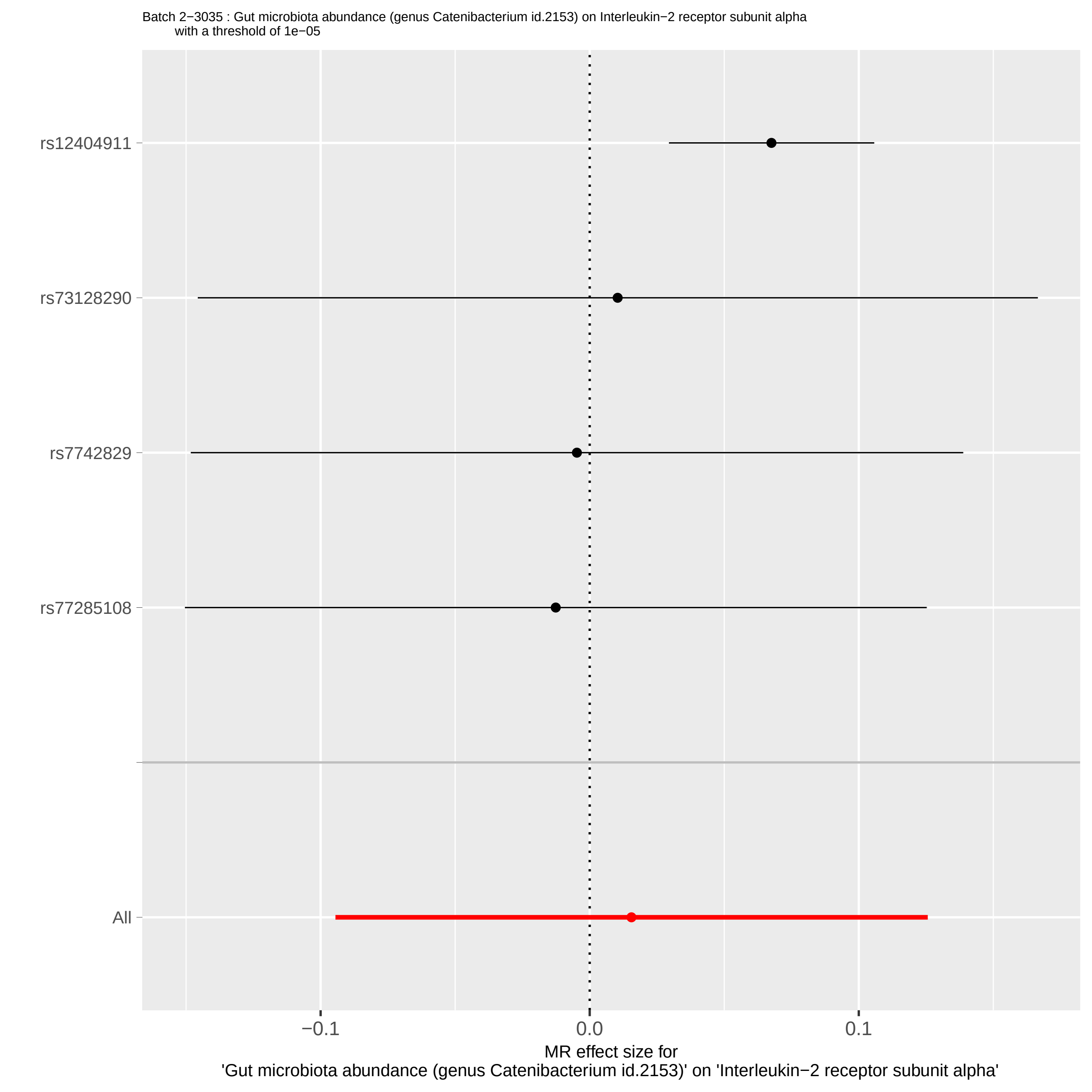

Batch 2−3035 : Gut microbiota abundance (genus Catenibacterium id.2153) on Interleukin−2 receptor subunit alpha
with a threshold of 1e−05
rs12404911
rs73128290
rs7742829
rs77285108
All
−0.1
0.0
0.1
MR effect size for
'Gut microbiota abundance (genus Catenibacterium id.2153)' on 'Interleukin−2 receptor subunit alpha'

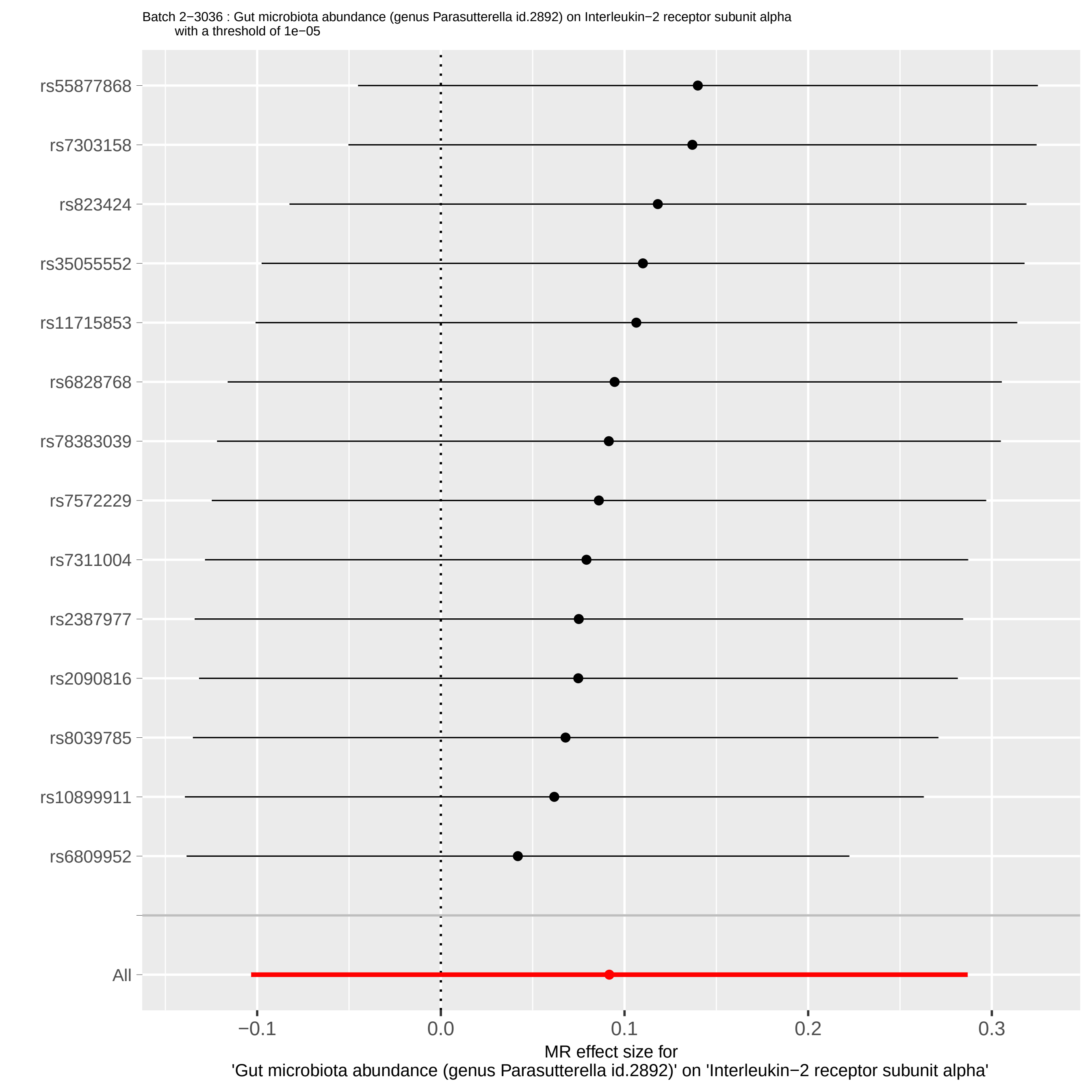
Batch 2−3036 : Gut microbiota abundance (genus Parasutterella id.2892) on Interleukin−2 receptor subunit alpha
with a threshold of 1e−05
rs55877868
rs7303158
rs823424
rs35055552
rs11715853
rs6828768
rs78383039
rs7572229
rs7311004
rs2387977
rs2090816
rs8039785
rs10899911
rs6809952
All
−0.1
0.0
0.1
0.2
0.3
MR effect size for
'Gut microbiota abundance (genus Parasutterella id.2892)' on 'Interleukin−2 receptor subunit alpha'

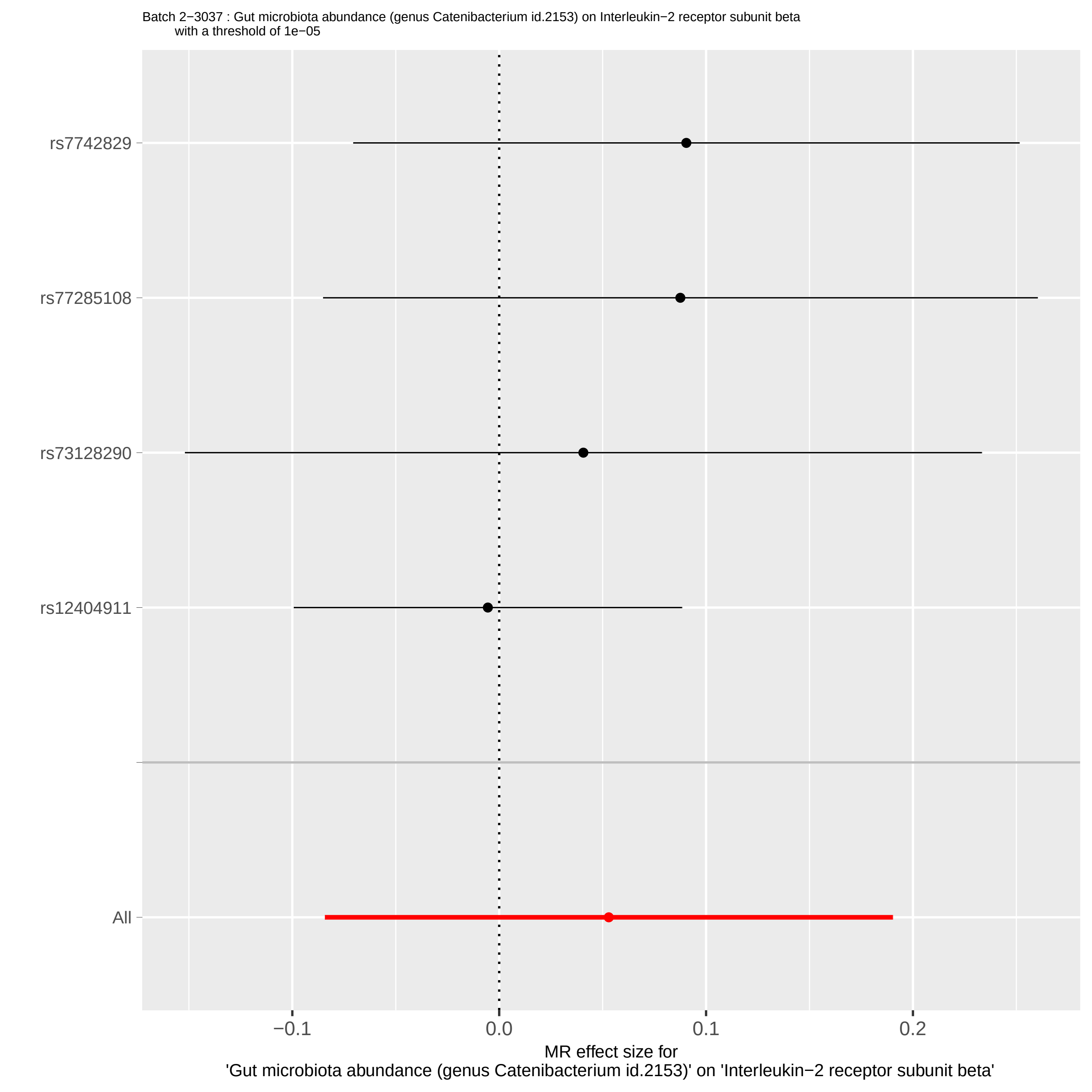
Batch 2−3037 : Gut microbiota abundance (genus Catenibacterium id.2153) on Interleukin−2 receptor subunit beta
with a threshold of 1e−05
rs7742829
rs77285108
rs73128290
rs12404911
All
−0.1
0.0
0.1
0.2
MR effect size for
'Gut microbiota abundance (genus Catenibacterium id.2153)' on 'Interleukin−2 receptor subunit beta'

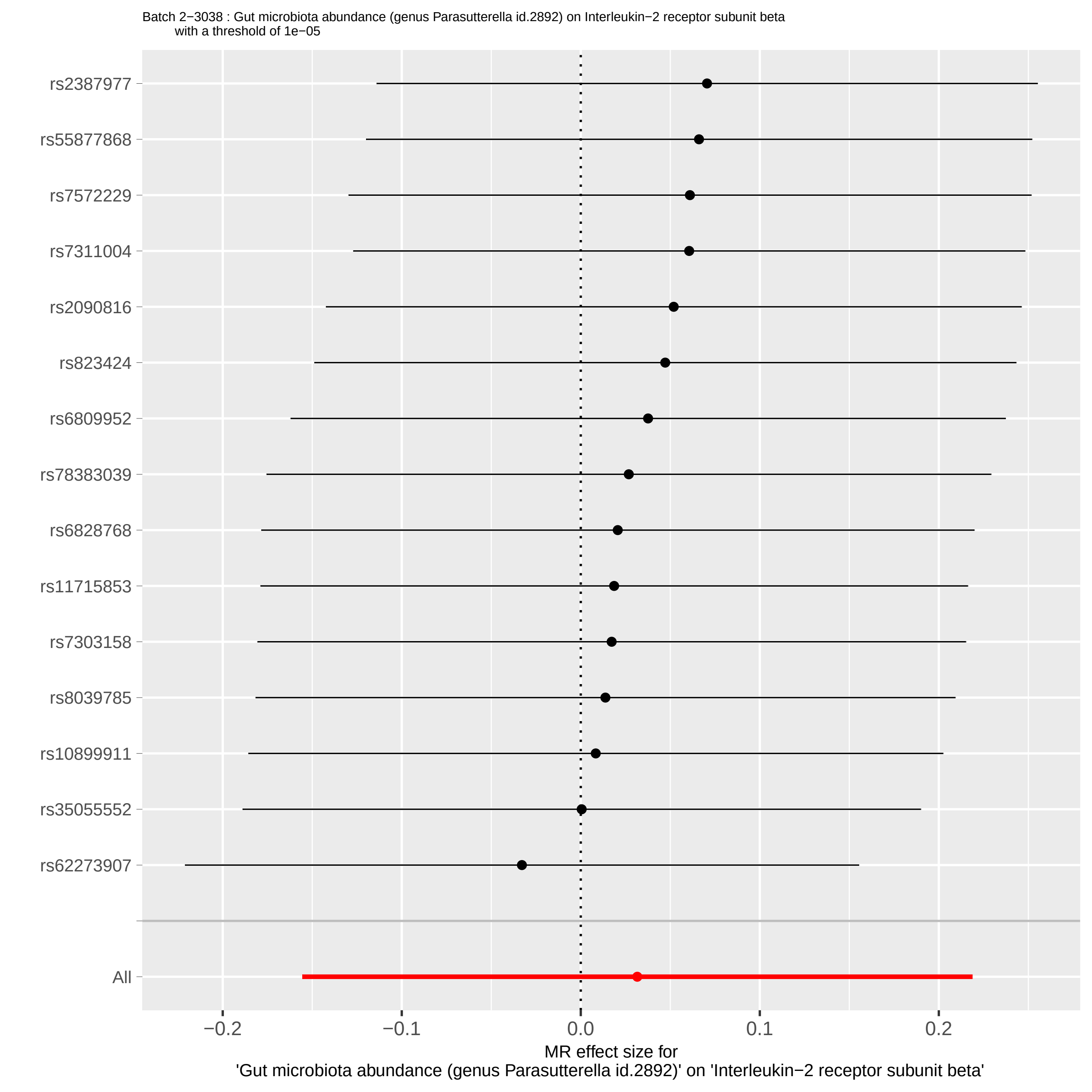

Batch 2−3038 : Gut microbiota abundance (genus Parasutterella id.2892) on Interleukin−2 receptor subunit beta
with a threshold of 1e−05
rs2387977
rs55877868
rs7572229
rs7311004
rs2090816
rs823424
rs6809952
rs78383039
rs6828768
rs11715853
rs7303158
rs8039785
rs10899911
rs35055552
rs62273907
All
−0.2
−0.1
0.0
0.1
0.2
MR effect size for
'Gut microbiota abundance (genus Parasutterella id.2892)' on 'Interleukin−2 receptor subunit beta'

Batch 2−3039 : Gut microbiota abundance (genus Catenibacterium id.2153) on Cytokine receptor common subunit gamma
with a threshold of 1e−05

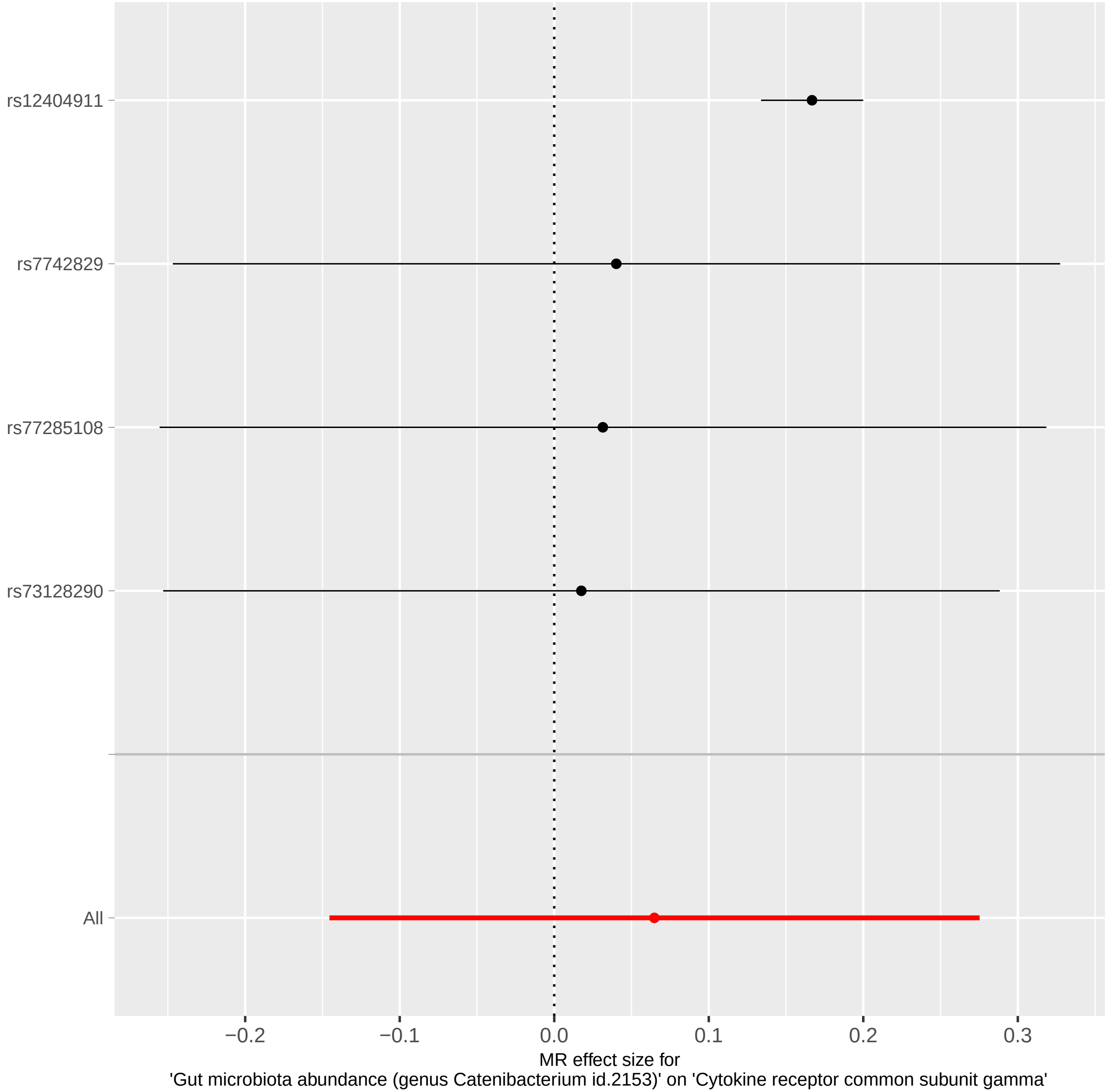

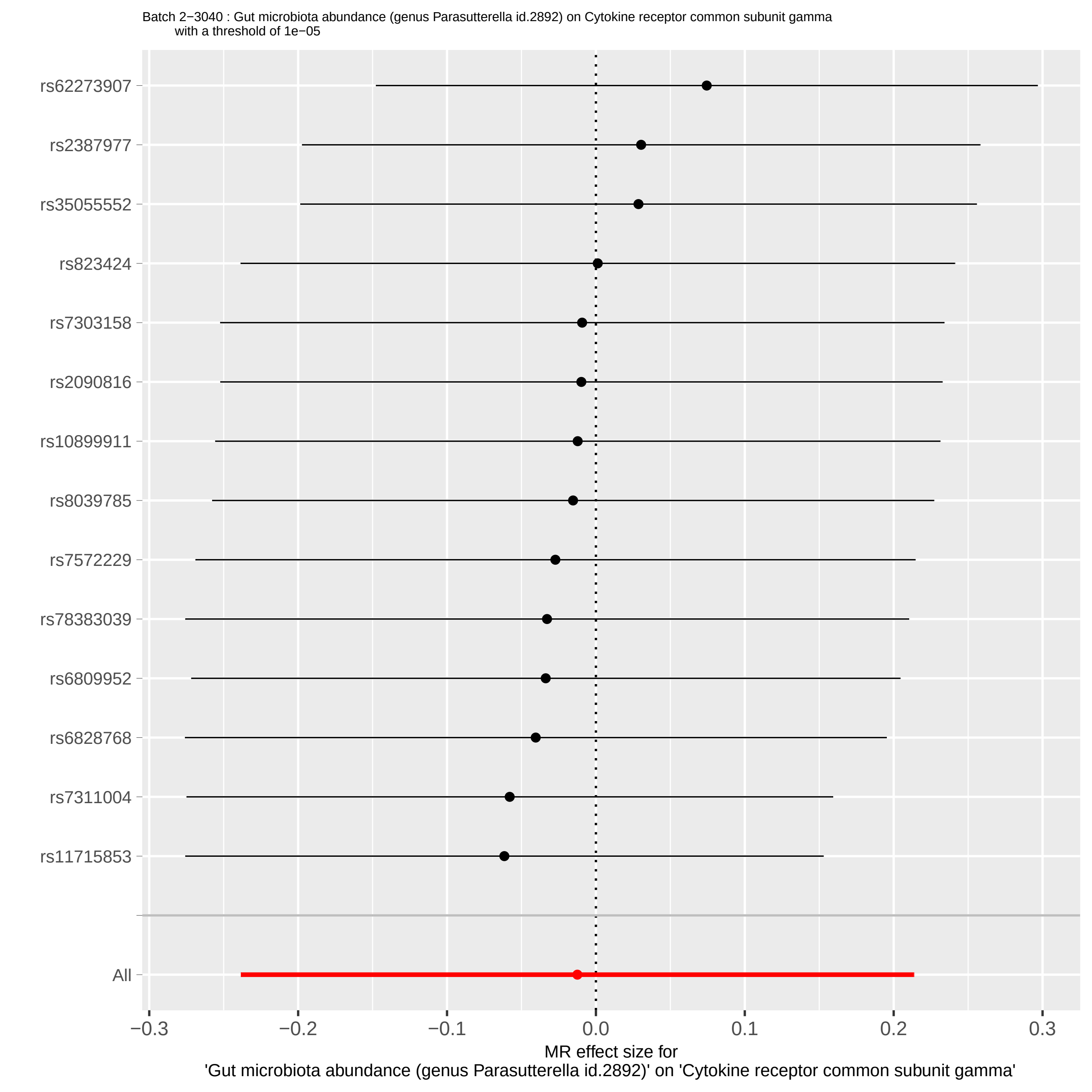

Batch 2−3040 : Gut microbiota abundance (genus Parasutterella id.2892) on Cytokine receptor common subunit gamma
with a threshold of 1e−05
rs62273907
rs2387977
rs35055552
rs823424
rs7303158
rs2090816
rs10899911
rs8039785
rs7572229
rs78383039
rs6809952
rs6828768
rs7311004
rs11715853
All
−0.3
−0.2
−0.1
0.0
0.1
0.2
0.3
MR effect size for
'Gut microbiota abundance (genus Parasutterella id.2892)' on 'Cytokine receptor common subunit gamma'

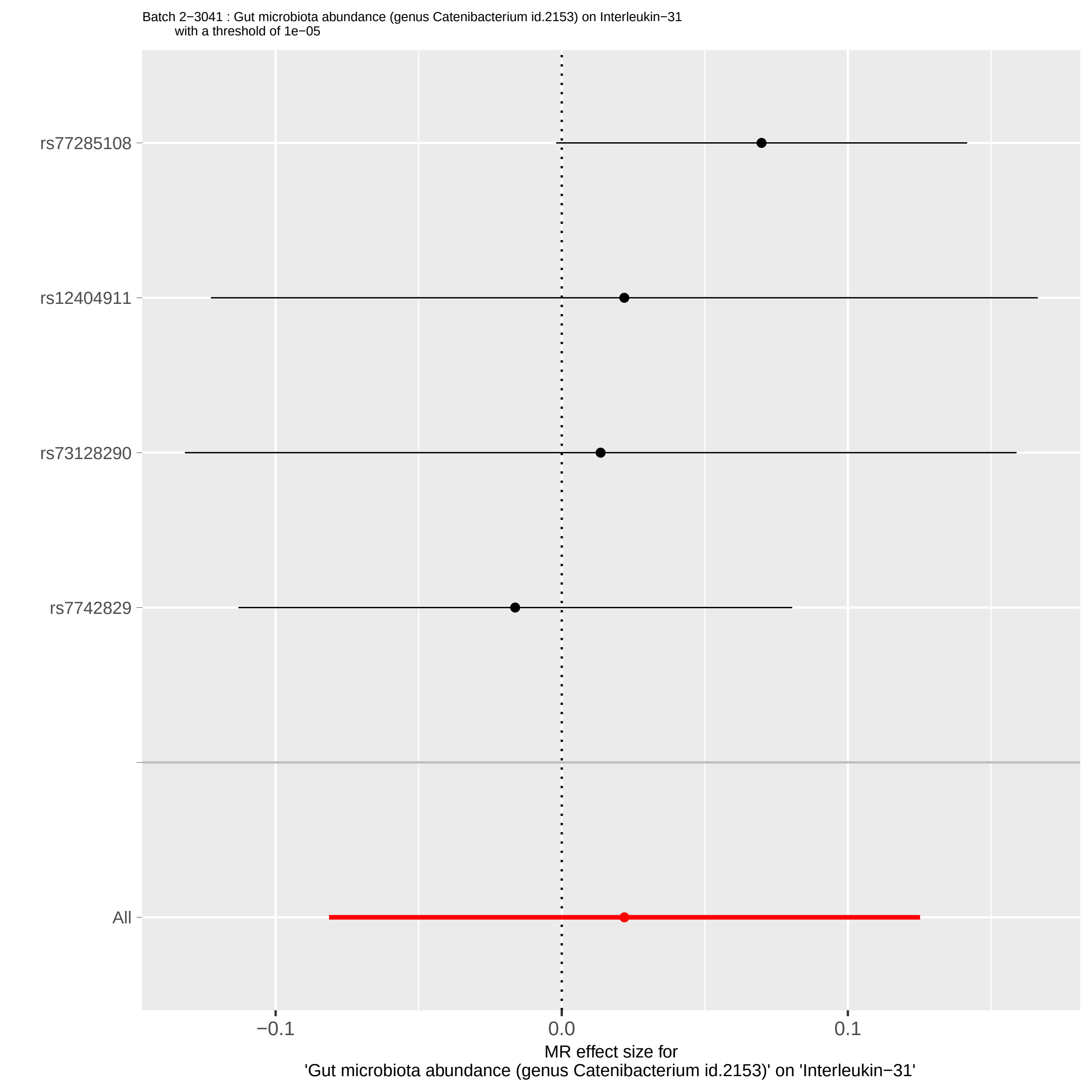
Batch 2−3041 : Gut microbiota abundance (genus Catenibacterium id.2153) on Interleukin−31
with a threshold of 1e−05
rs77285108
rs12404911
rs73128290
rs7742829
All
−0.1
0.0
0.1
MR effect size for
'Gut microbiota abundance (genus Catenibacterium id.2153)' on 'Interleukin−31'

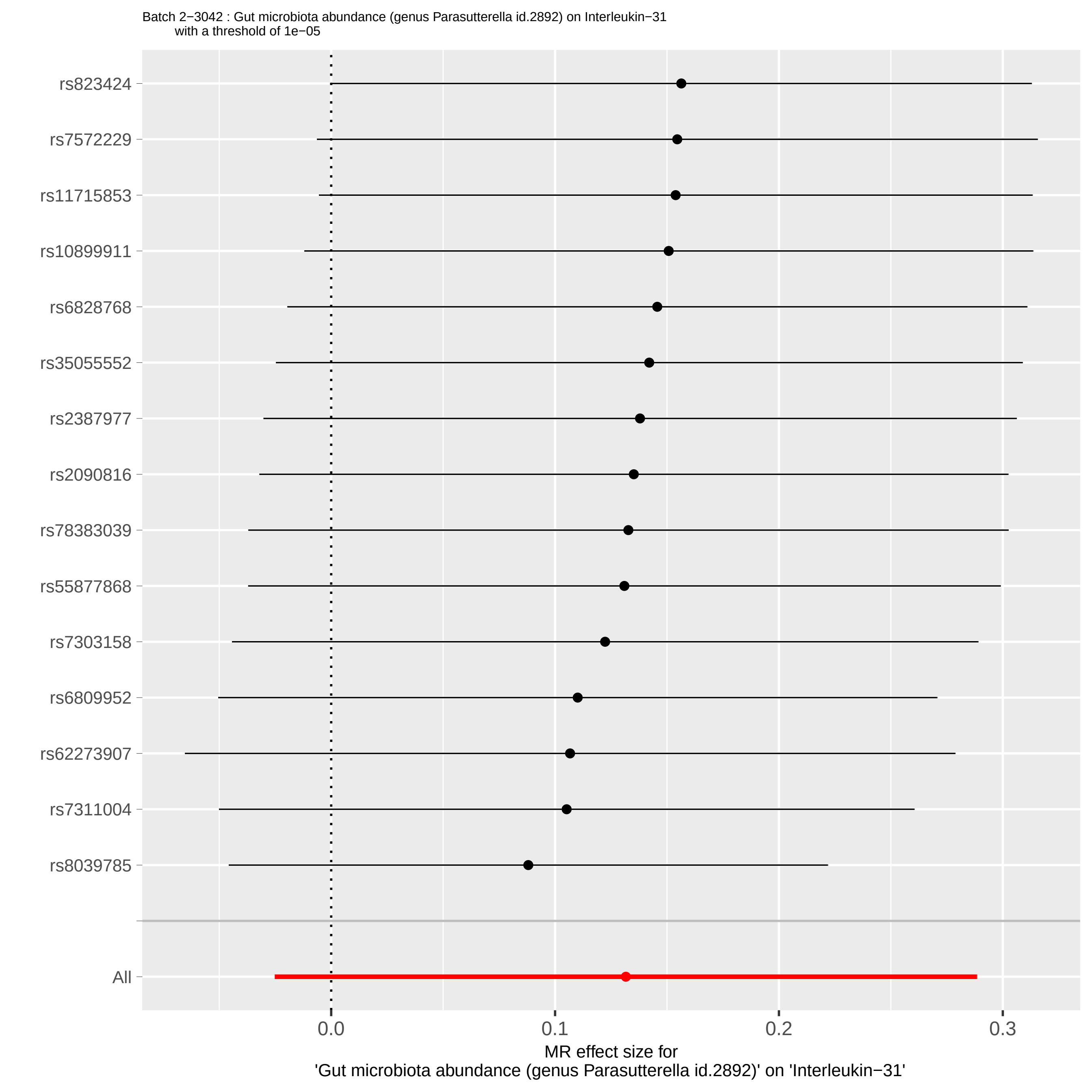

Batch 2−3042 : Gut microbiota abundance (genus Parasutterella id.2892) on Interleukin−31
with a threshold of 1e−05
rs823424
rs7572229
rs11715853
rs10899911
rs6828768
rs35055552
rs2387977
rs2090816
rs78383039
rs55877868
rs7303158
rs6809952
rs62273907
rs7311004
rs8039785
All
0.0
0.1
0.2
0.3
MR effect size for
'Gut microbiota abundance (genus Parasutterella id.2892)' on 'Interleukin−31'

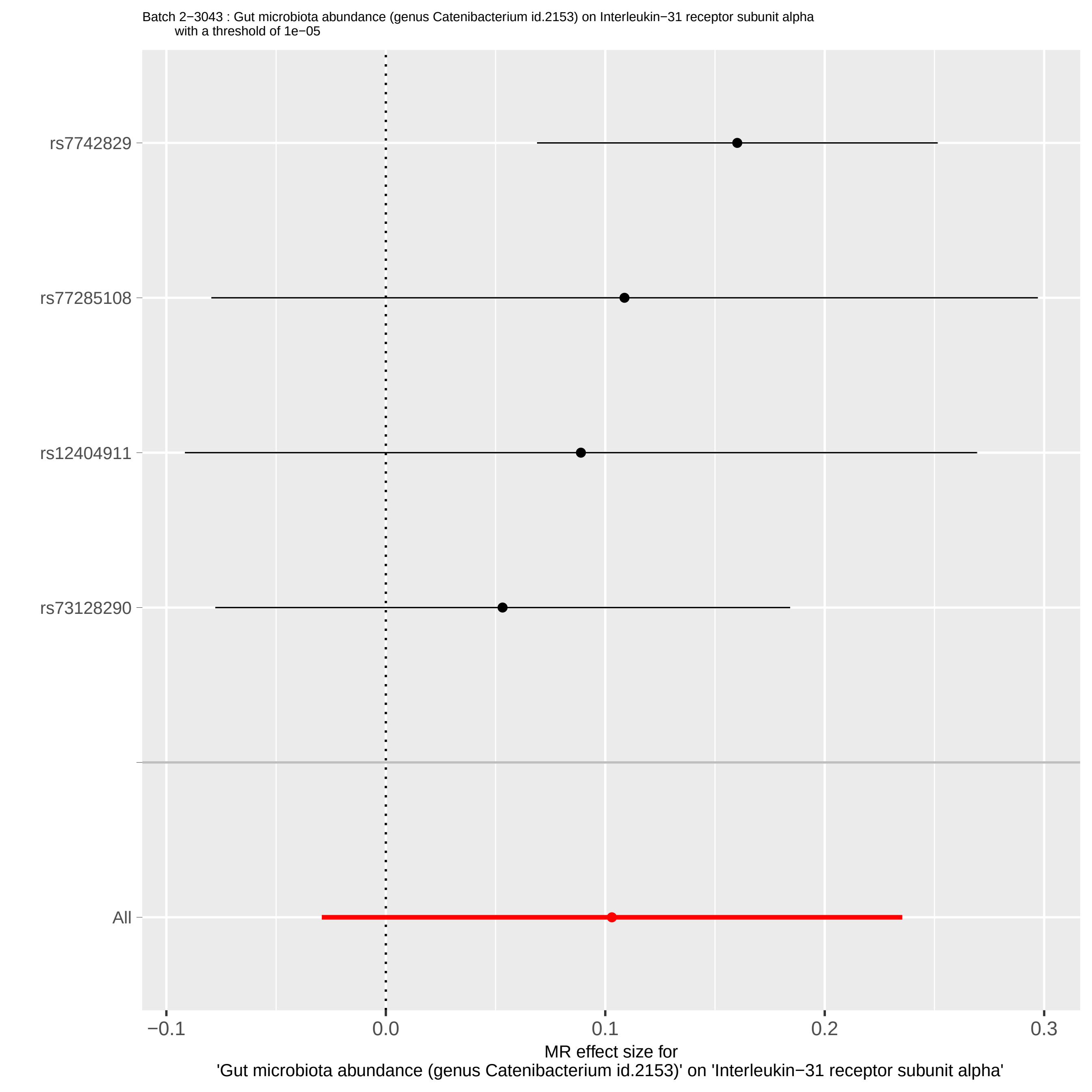

Batch 2−3043 : Gut microbiota abundance (genus Catenibacterium id.2153) on Interleukin−31 receptor subunit alpha
with a threshold of 1e−05
rs7742829
rs77285108
rs12404911
rs73128290
All
−0.1
0.0
0.1
0.2
0.3
MR effect size for
'Gut microbiota abundance (genus Catenibacterium id.2153)' on 'Interleukin−31 receptor subunit alpha'

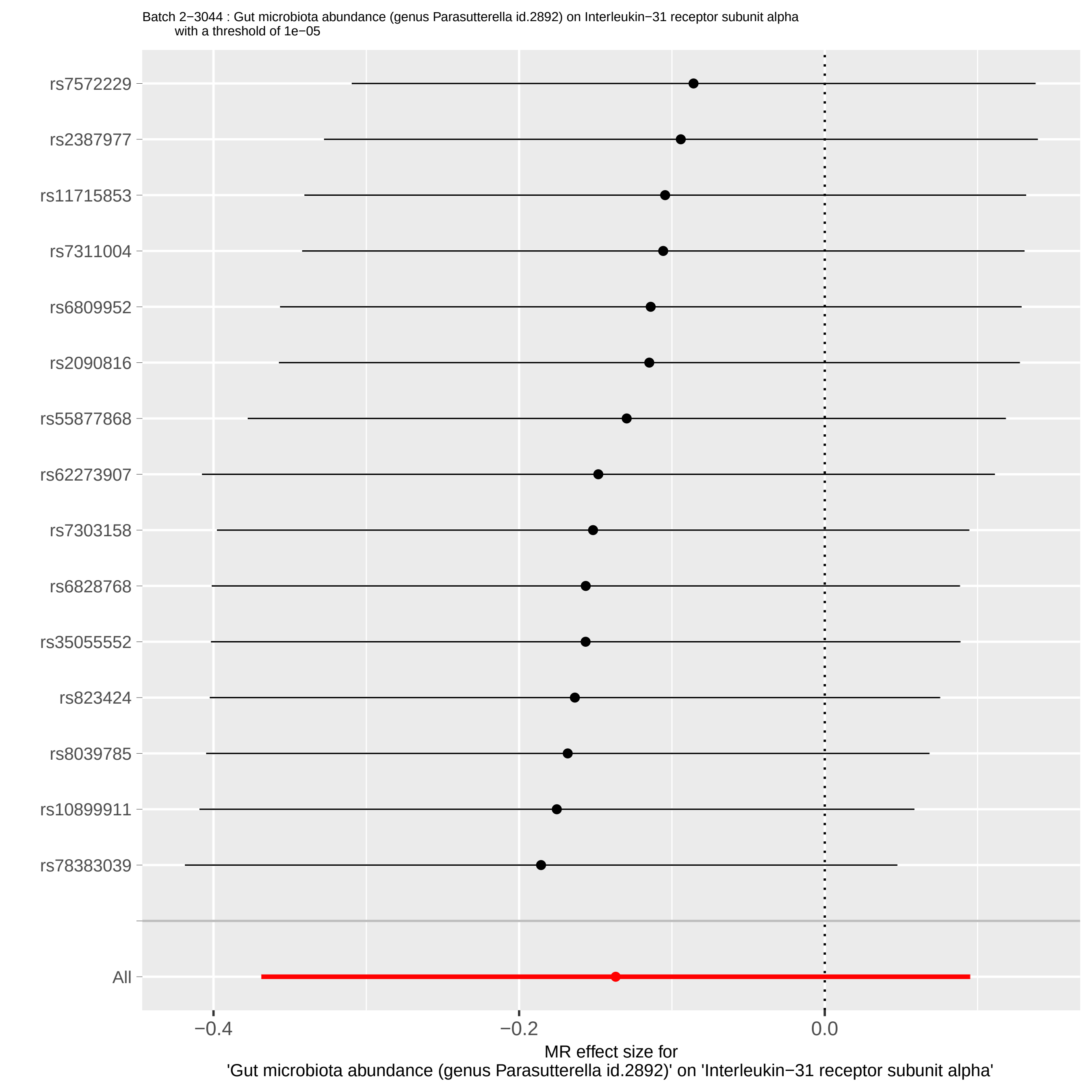

Batch 2−3044 : Gut microbiota abundance (genus Parasutterella id.2892) on Interleukin−31 receptor subunit alpha
with a threshold of 1e−05
rs7572229
rs2387977
rs11715853
rs7311004
rs6809952
rs2090816
rs55877868
rs62273907
rs7303158
rs6828768
rs35055552
rs823424
rs8039785
rs10899911
rs78383039
All
−0.4
−0.2
0.0
MR effect size for
'Gut microbiota abundance (genus Parasutterella id.2892)' on 'Interleukin−31 receptor subunit alpha'

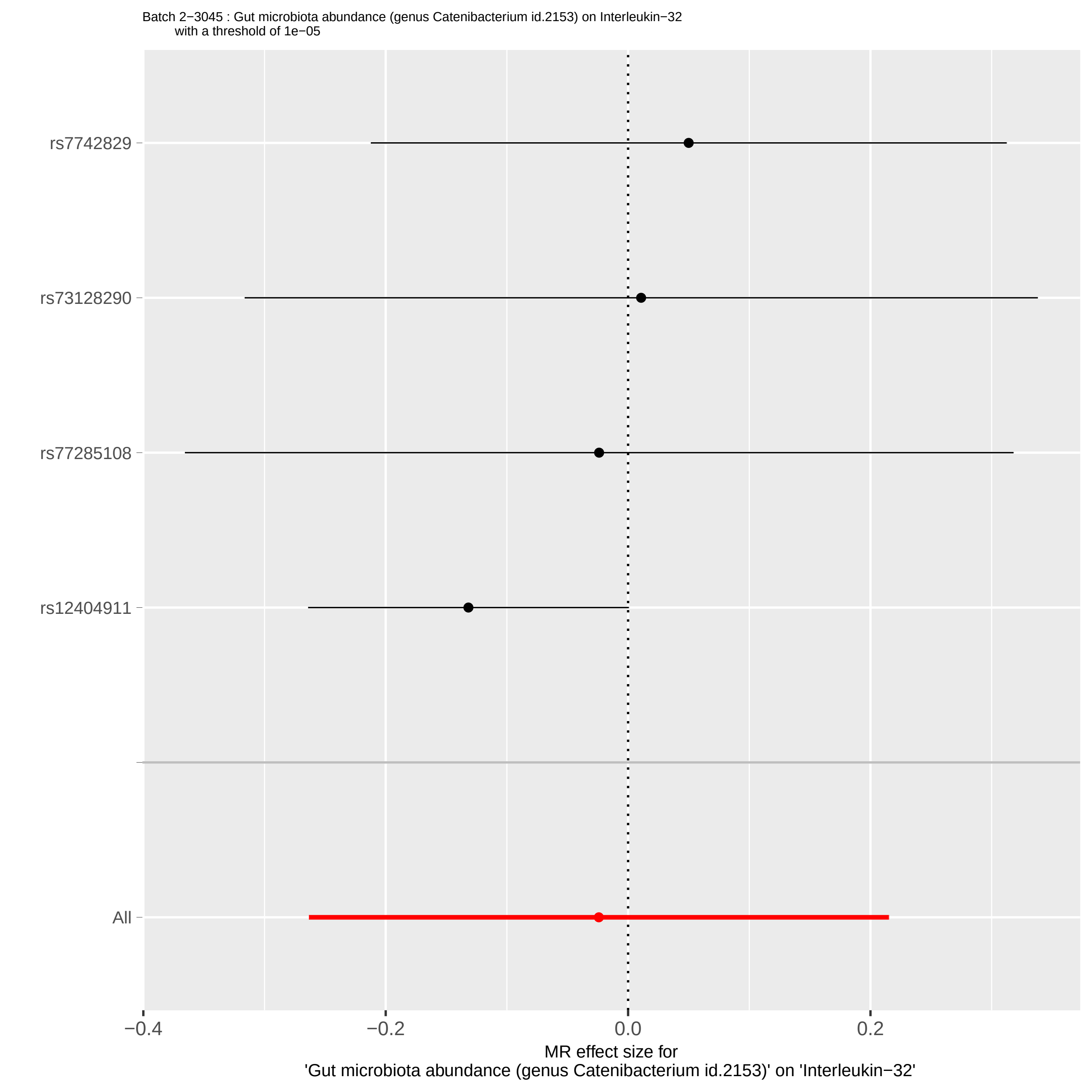
Batch 2−3045 : Gut microbiota abundance (genus Catenibacterium id.2153) on Interleukin−32
with a threshold of 1e−05
rs7742829
rs73128290
rs77285108
rs12404911
All
−0.4
−0.2
0.0
0.2
MR effect size for
'Gut microbiota abundance (genus Catenibacterium id.2153)' on 'Interleukin−32'

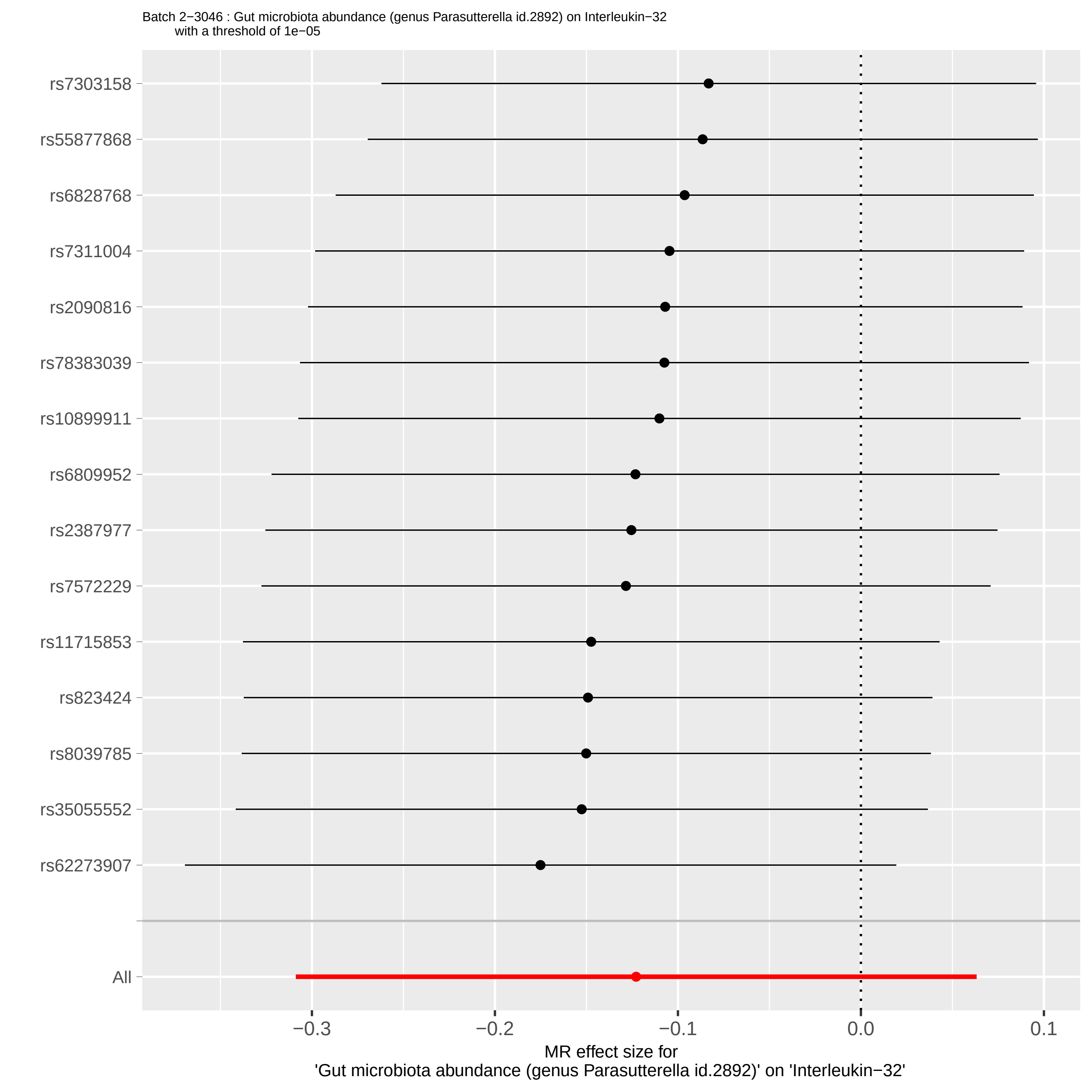

Batch 2−3046 : Gut microbiota abundance (genus Parasutterella id.2892) on Interleukin−32
with a threshold of 1e−05
rs7303158
rs55877868
rs6828768
rs7311004
rs2090816
rs78383039
rs10899911
rs6809952
rs2387977
rs7572229
rs11715853
rs823424
rs8039785
rs35055552
rs62273907
All
−0.3
−0.2
−0.1
0.0
0.1
MR effect size for
'Gut microbiota abundance (genus Parasutterella id.2892)' on 'Interleukin−32'

Batch 2−3047 : Gut microbiota abundance (genus Catenibacterium id.2153) on Interleukin−34
with a threshold of 1e−05

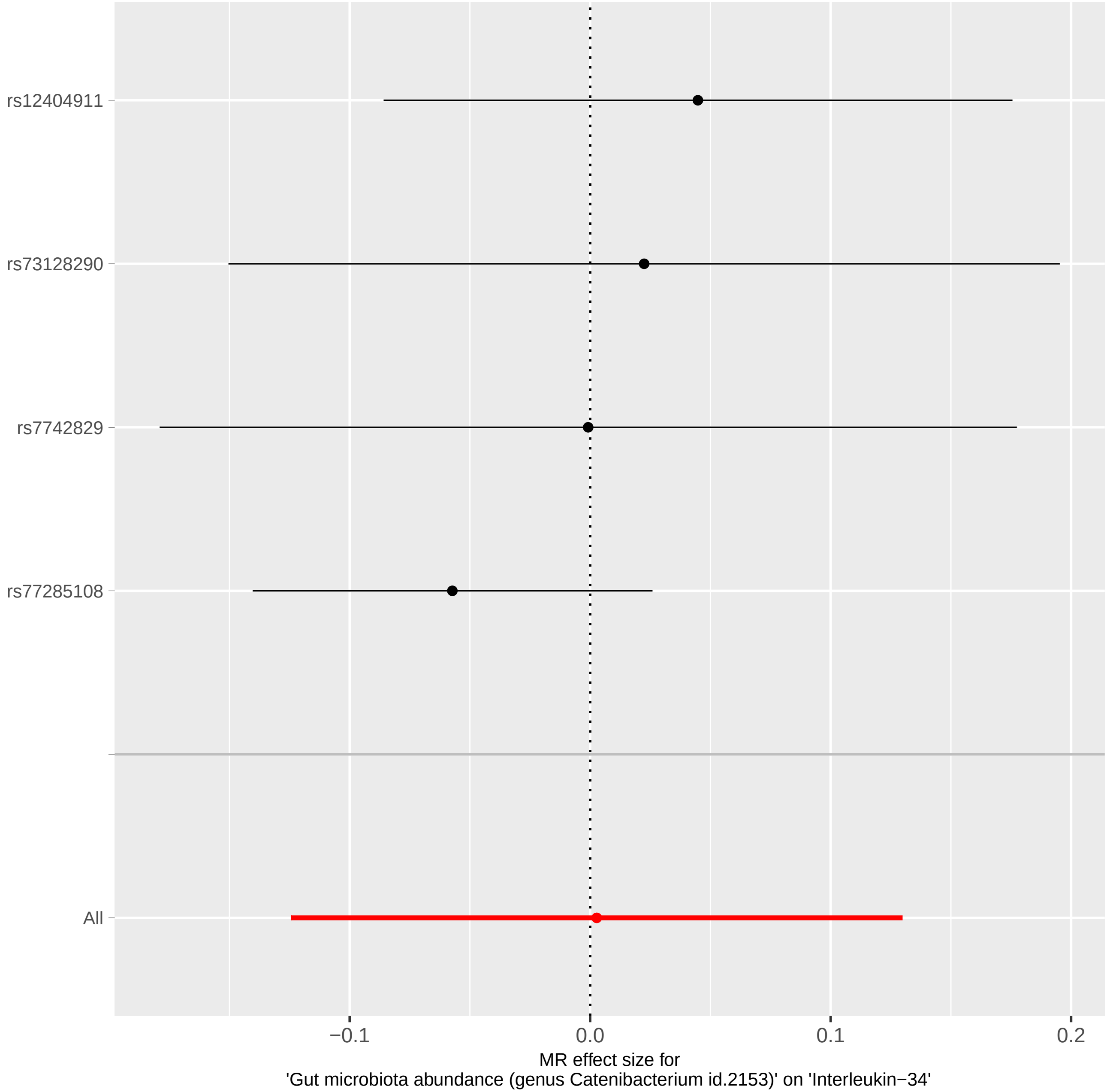

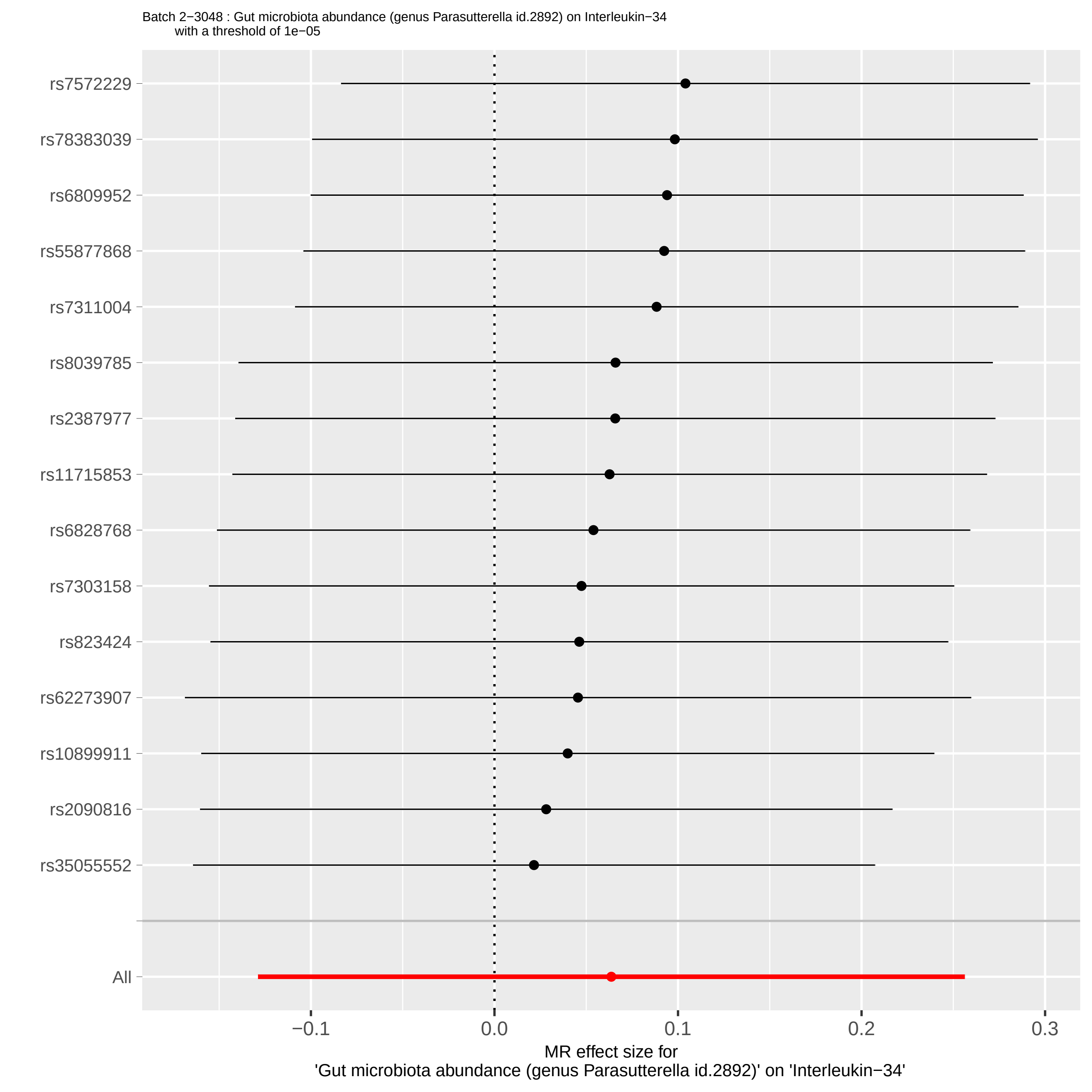

Batch 2−3048 : Gut microbiota abundance (genus Parasutterella id.2892) on Interleukin−34
with a threshold of 1e−05
rs7572229
rs78383039
rs6809952
rs55877868
rs7311004
rs8039785
rs2387977
rs11715853
rs6828768
rs7303158
rs823424
rs62273907
rs10899911
rs2090816
rs35055552
All
−0.1
0.0
0.1
0.2
0.3
MR effect size for
'Gut microbiota abundance (genus Parasutterella id.2892)' on 'Interleukin−34'

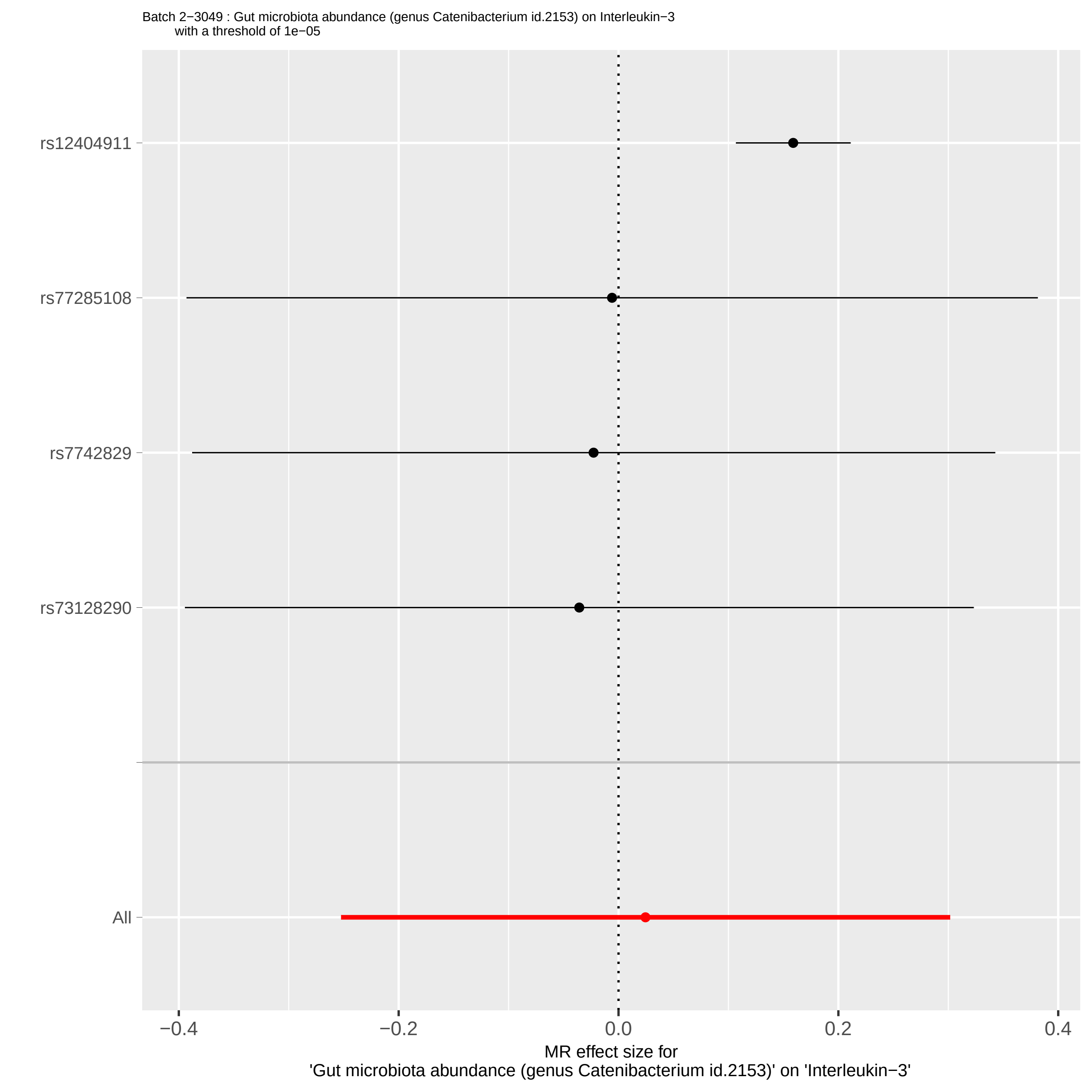

Batch 2−3049 : Gut microbiota abundance (genus Catenibacterium id.2153) on Interleukin−3
with a threshold of 1e−05
rs12404911
rs77285108
rs7742829
rs73128290
All
−0.4
−0.2
0.0
0.2
0.4
MR effect size for
'Gut microbiota abundance (genus Catenibacterium id.2153)' on 'Interleukin−3'

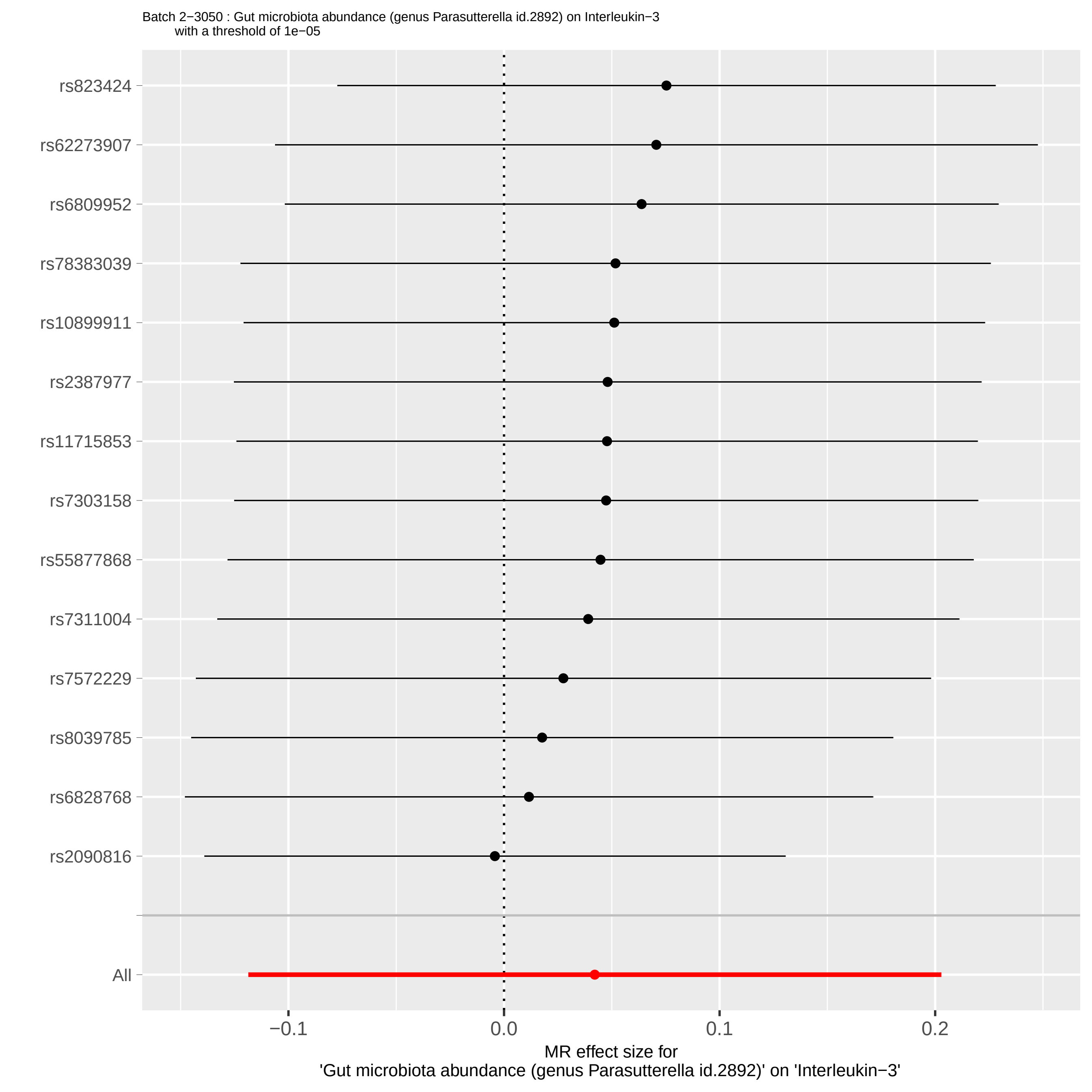
Batch 2−3050 : Gut microbiota abundance (genus Parasutterella id.2892) on Interleukin−3
with a threshold of 1e−05
rs823424
rs62273907
rs6809952
rs78383039
rs10899911
rs2387977
rs11715853
rs7303158
rs55877868
rs7311004
rs7572229
rs8039785
rs6828768
rs2090816
All
−0.1
0.0
0.1
0.2
MR effect size for
'Gut microbiota abundance (genus Parasutterella id.2892)' on 'Interleukin−3'

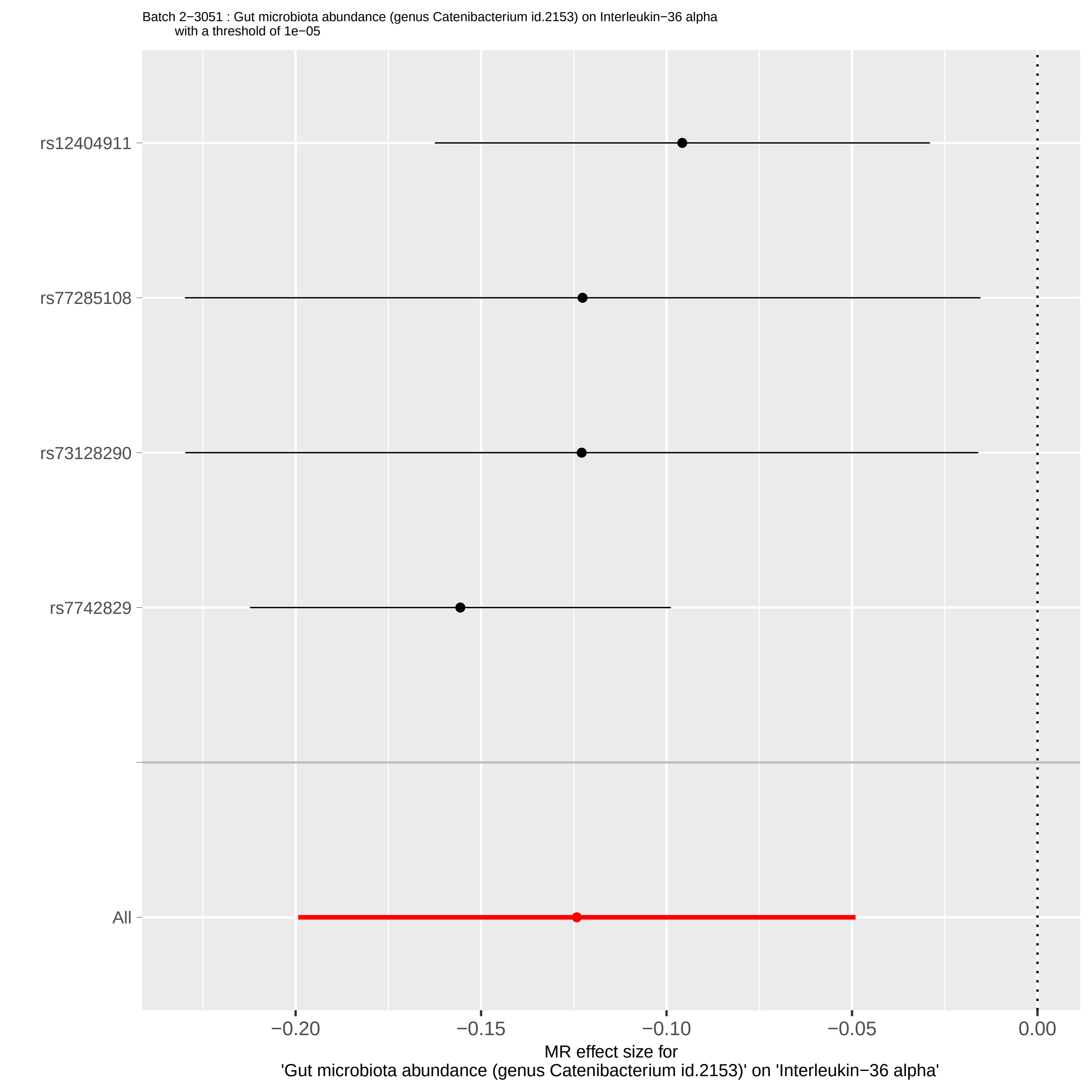

Batch 2−3051 : Gut microbiota abundance (genus Catenibacterium id.2153) on Interleukin−36 alpha
with a threshold of 1e−05
rs12404911
rs77285108
rs73128290
rs7742829
All
−0.20
−0.15
−0.10
−0.05
0.00
MR effect size for
'Gut microbiota abundance (genus Catenibacterium id.2153)' on 'Interleukin−36 alpha'

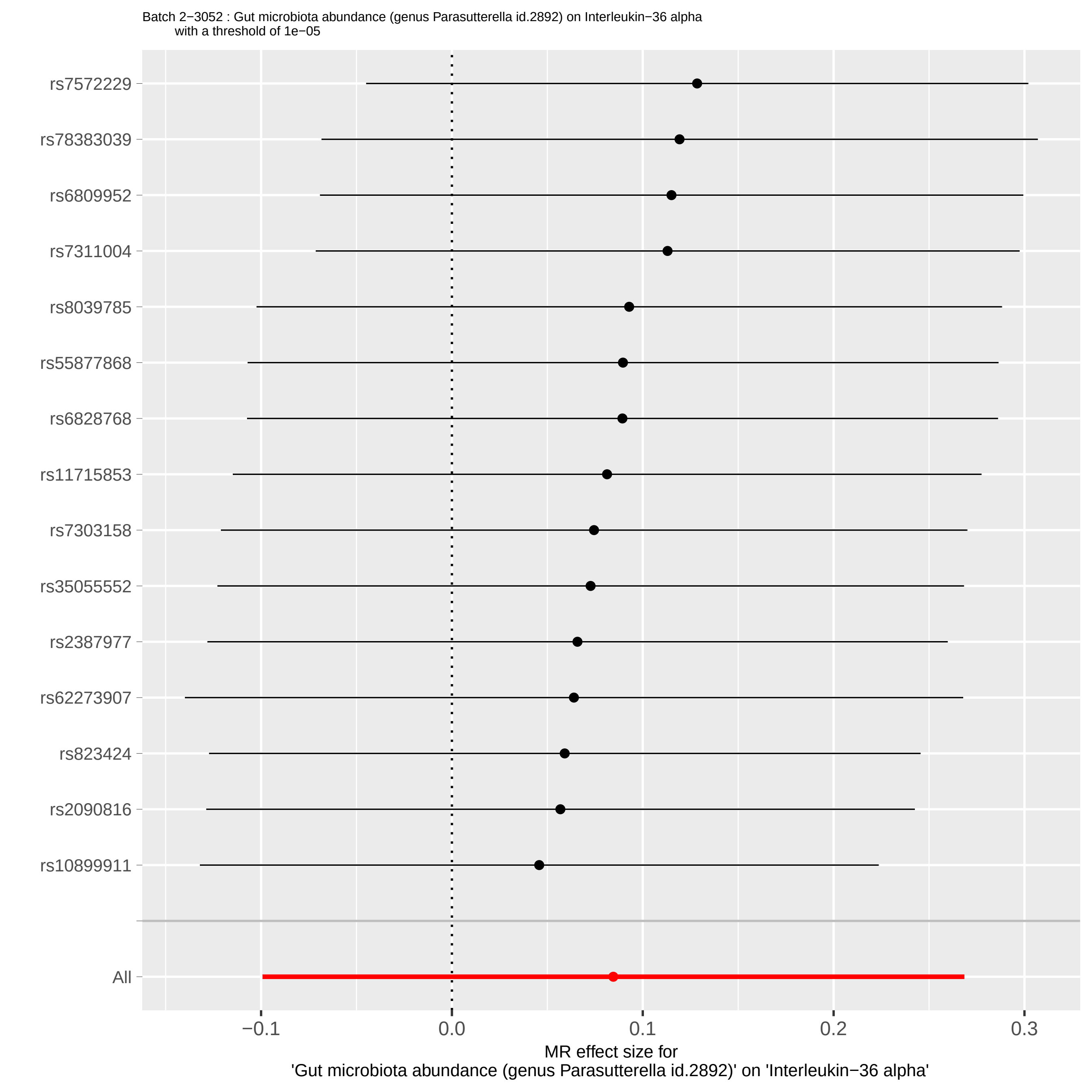
Batch 2−3052 : Gut microbiota abundance (genus Parasutterella id.2892) on Interleukin−36 alpha
with a threshold of 1e−05
rs7572229
rs78383039
rs6809952
rs7311004
rs8039785
rs55877868
rs6828768
rs11715853
rs7303158
rs35055552
rs2387977
rs62273907
rs823424
rs2090816
rs10899911
All
−0.1
0.0
0.1
0.2
0.3
MR effect size for
'Gut microbiota abundance (genus Parasutterella id.2892)' on 'Interleukin−36 alpha'

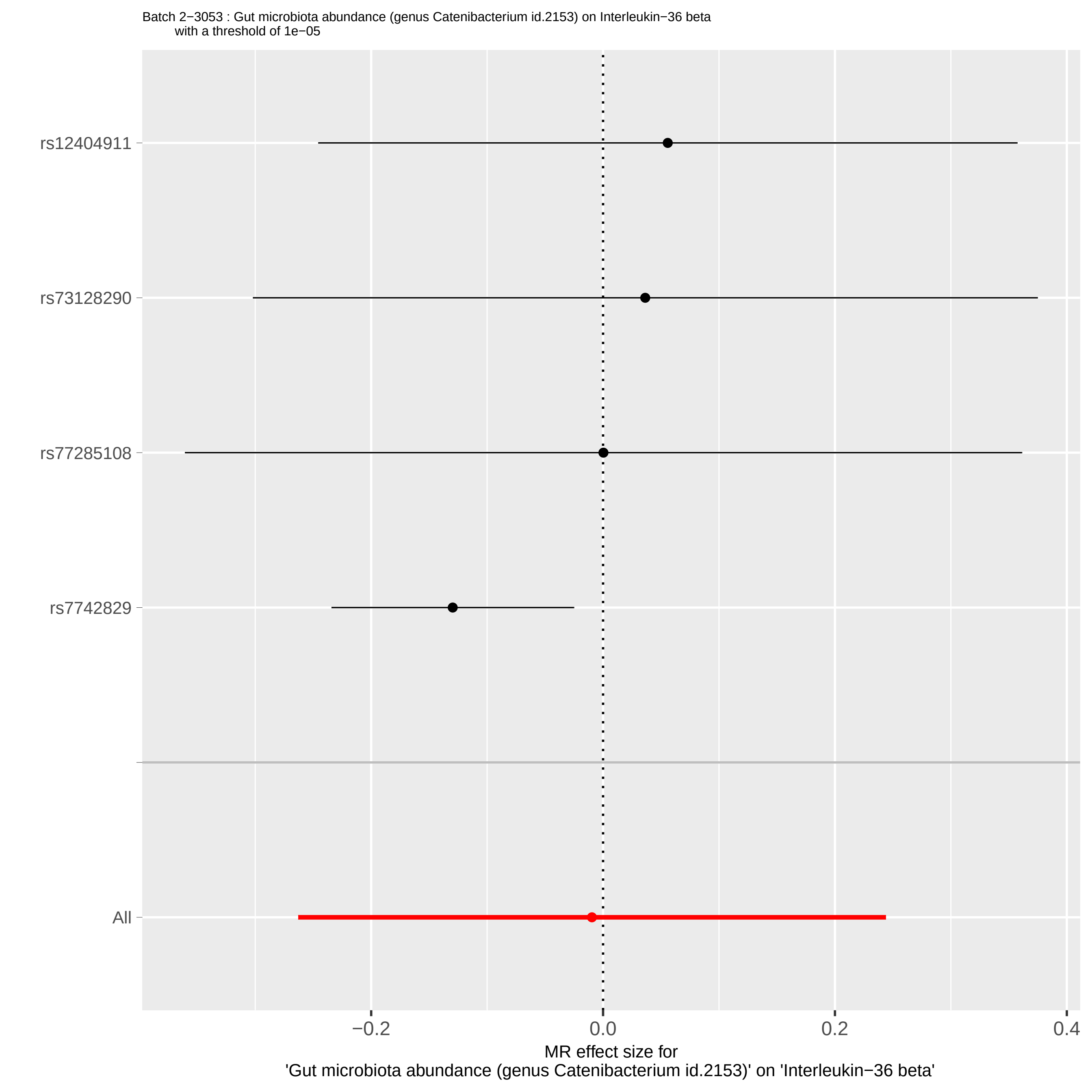
Batch 2−3053 : Gut microbiota abundance (genus Catenibacterium id.2153) on Interleukin−36 beta
with a threshold of 1e−05
rs12404911
rs73128290
rs77285108
rs7742829
All
−0.2
0.0
0.2
0.4
MR effect size for
'Gut microbiota abundance (genus Catenibacterium id.2153)' on 'Interleukin−36 beta'

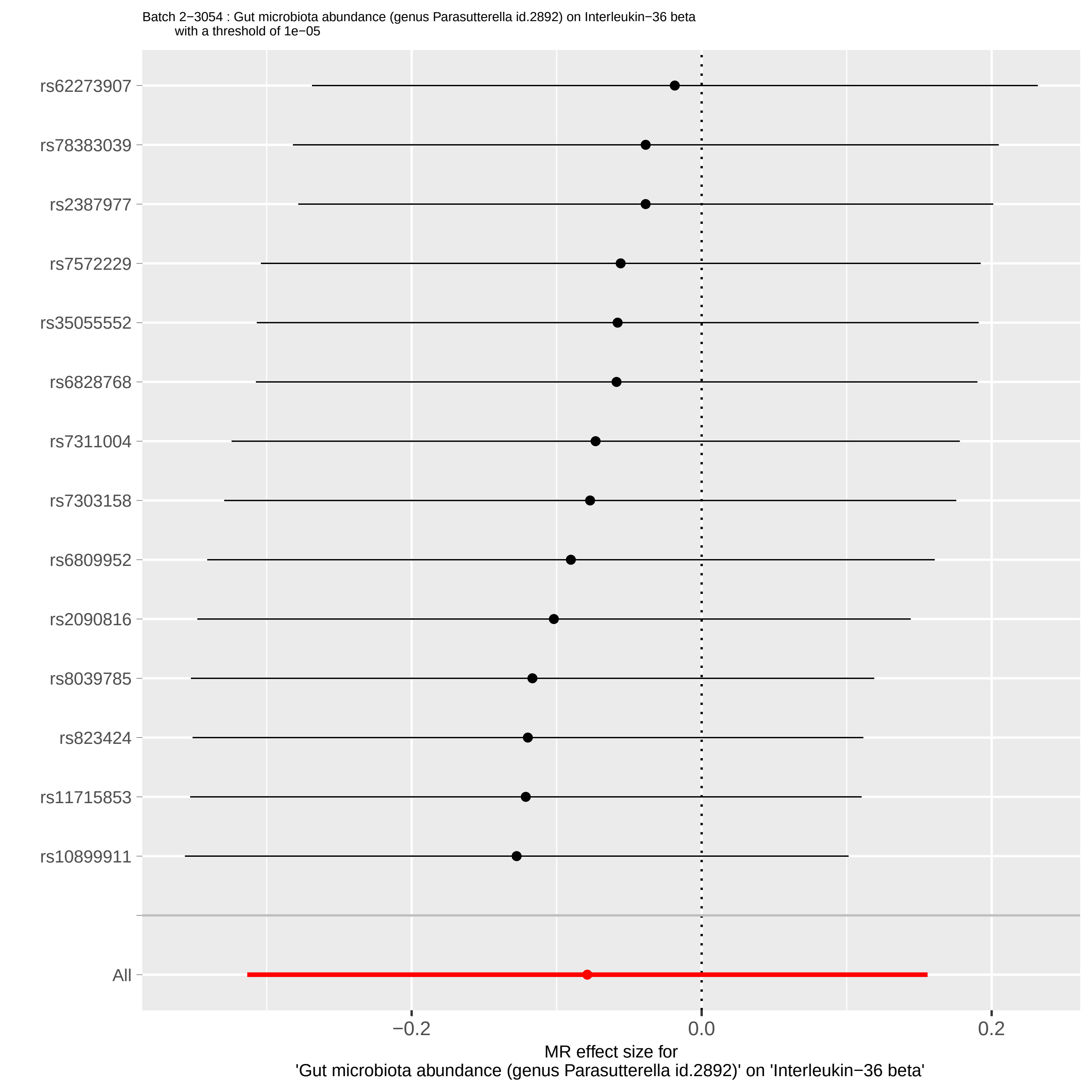
Batch 2−3054 : Gut microbiota abundance (genus Parasutterella id.2892) on Interleukin−36 beta
with a threshold of 1e−05
rs62273907
rs78383039
rs2387977
rs7572229
rs35055552
rs6828768
rs7311004
rs7303158
rs6809952
rs2090816
rs8039785
rs823424
rs11715853
rs10899911
All
−0.2
0.0
0.2
MR effect size for
'Gut microbiota abundance (genus Parasutterella id.2892)' on 'Interleukin−36 beta'

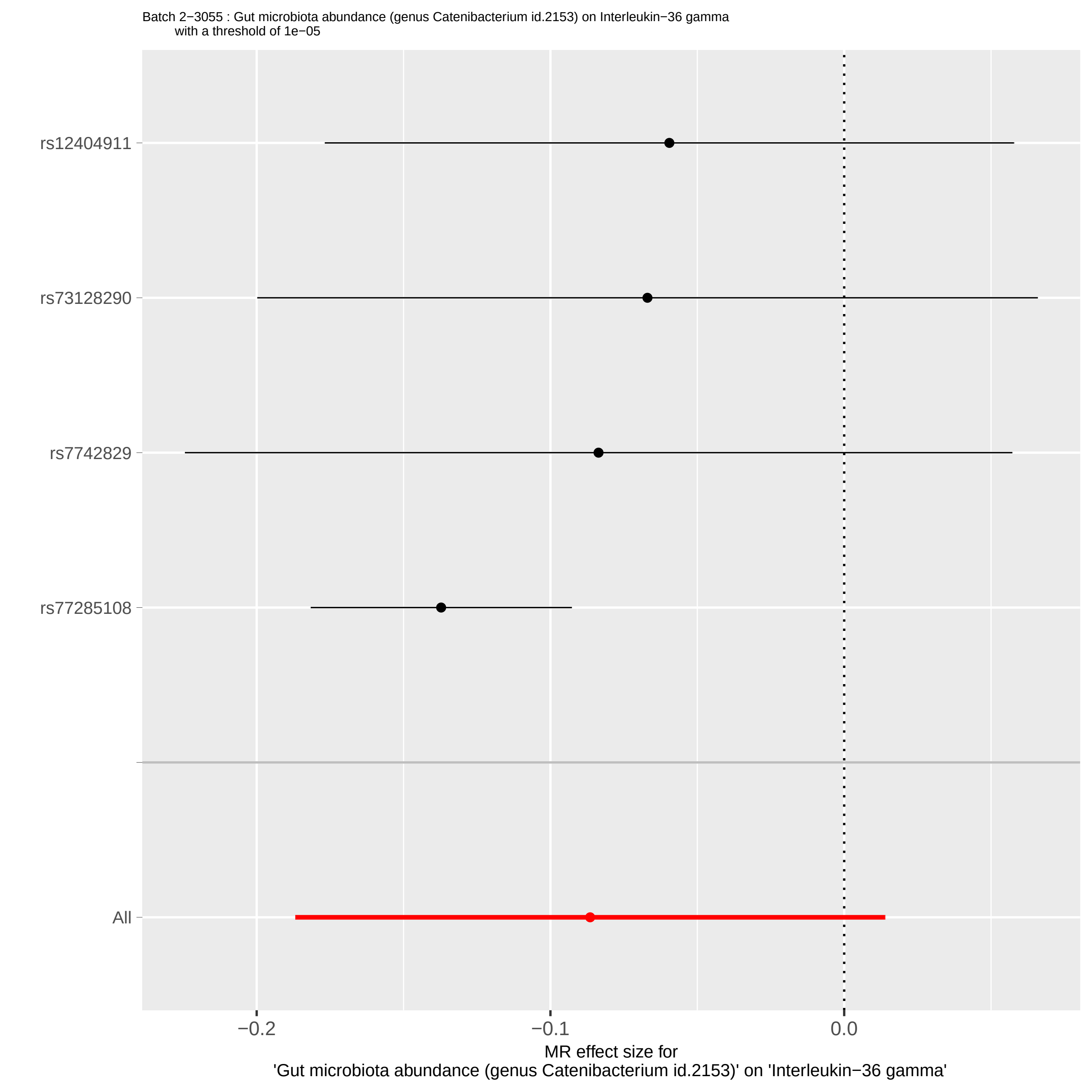

Batch 2−3055 : Gut microbiota abundance (genus Catenibacterium id.2153) on Interleukin−36 gamma
with a threshold of 1e−05
rs12404911
rs73128290
rs7742829
rs77285108
All
−0.2
−0.1
0.0
MR effect size for
'Gut microbiota abundance (genus Catenibacterium id.2153)' on 'Interleukin−36 gamma'

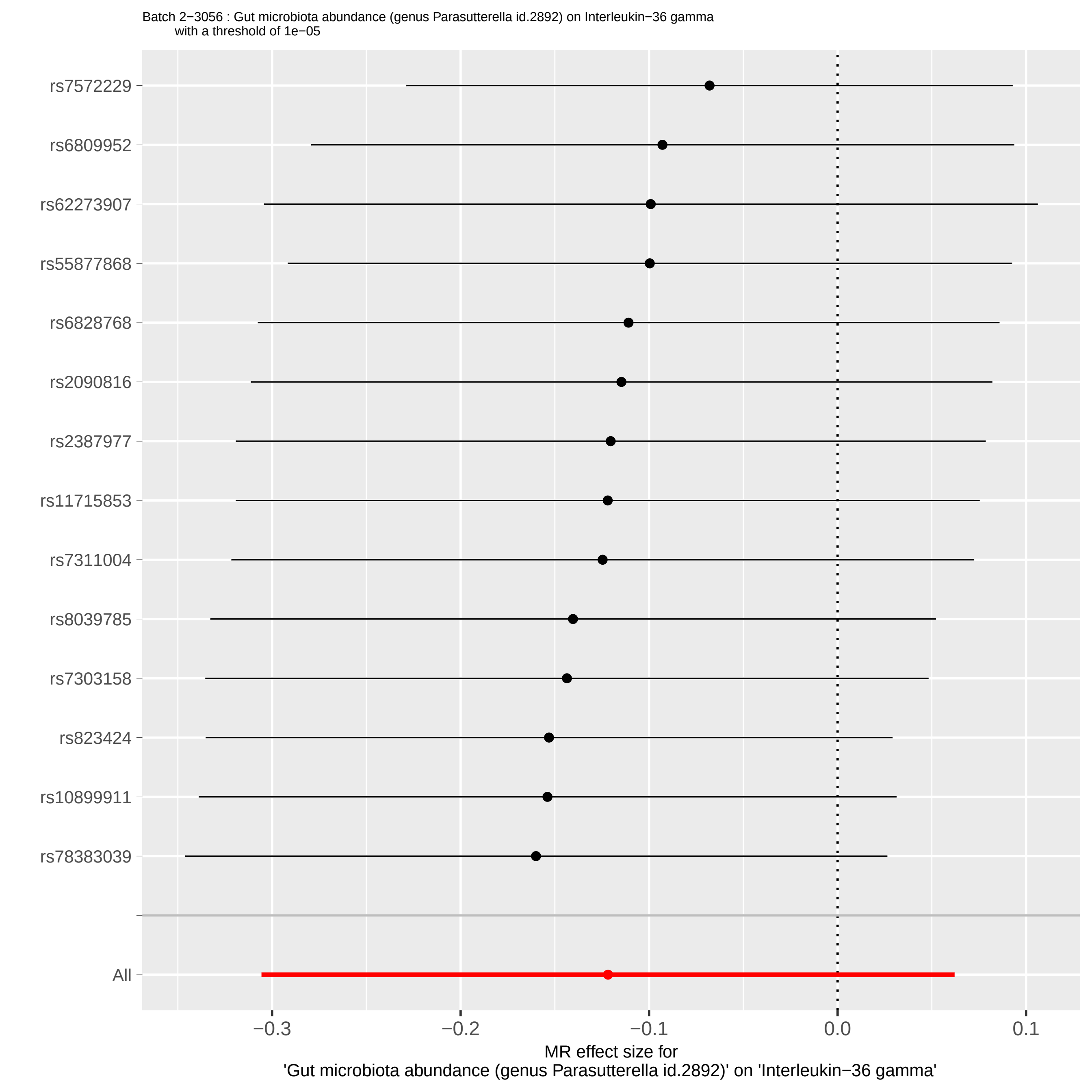
Batch 2−3056 : Gut microbiota abundance (genus Parasutterella id.2892) on Interleukin−36 gamma
with a threshold of 1e−05
rs7572229
rs6809952
rs62273907
rs55877868
rs6828768
rs2090816
rs2387977
rs11715853
rs7311004
rs8039785
rs7303158
rs823424
rs10899911
rs78383039
All
−0.3
−0.2
−0.1
0.0
0.1
MR effect size for
'Gut microbiota abundance (genus Parasutterella id.2892)' on 'Interleukin−36 gamma'

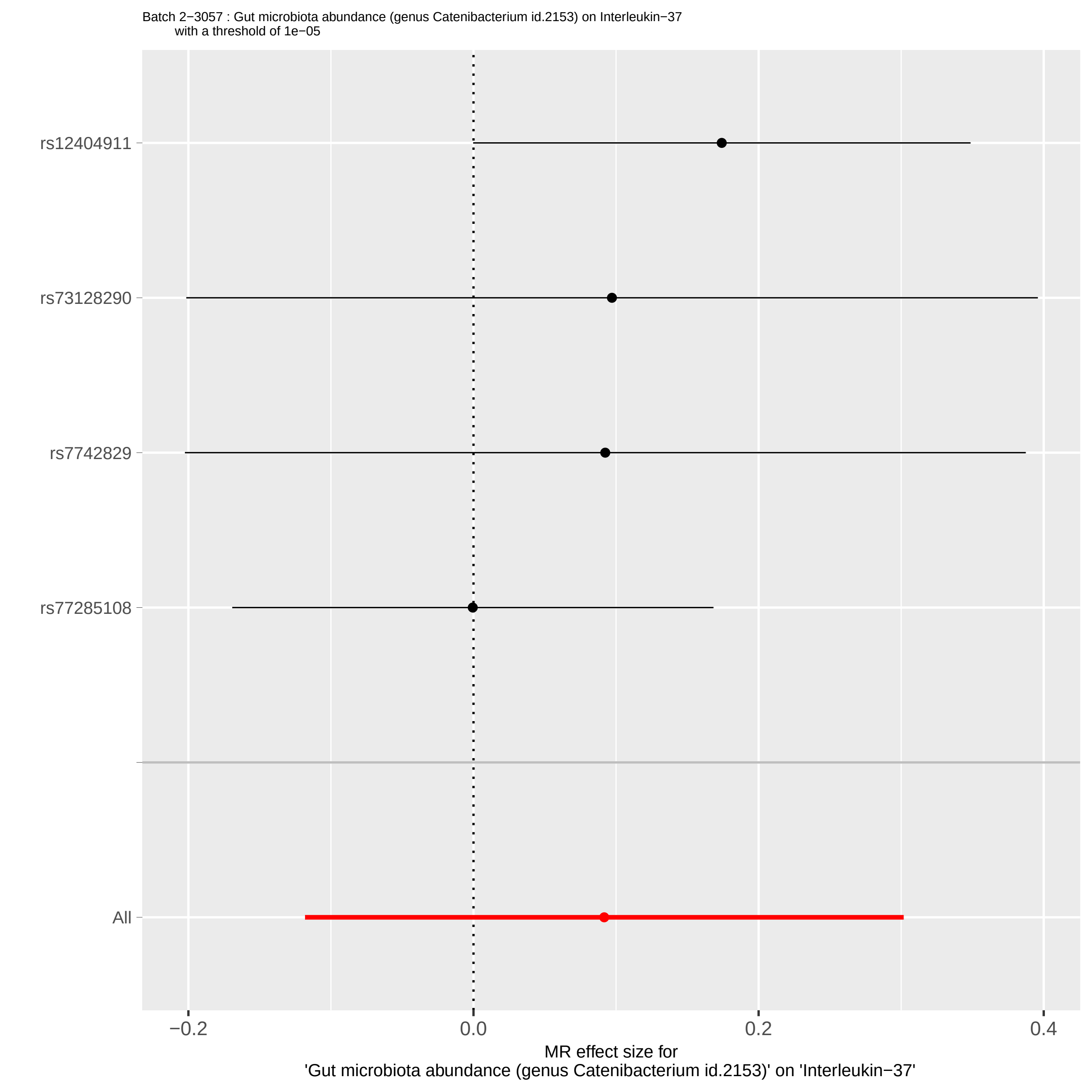
Batch 2−3057 : Gut microbiota abundance (genus Catenibacterium id.2153) on Interleukin−37
with a threshold of 1e−05
rs12404911
rs73128290
rs7742829
rs77285108
All
−0.2
0.0
0.2
0.4
MR effect size for
'Gut microbiota abundance (genus Catenibacterium id.2153)' on 'Interleukin−37'

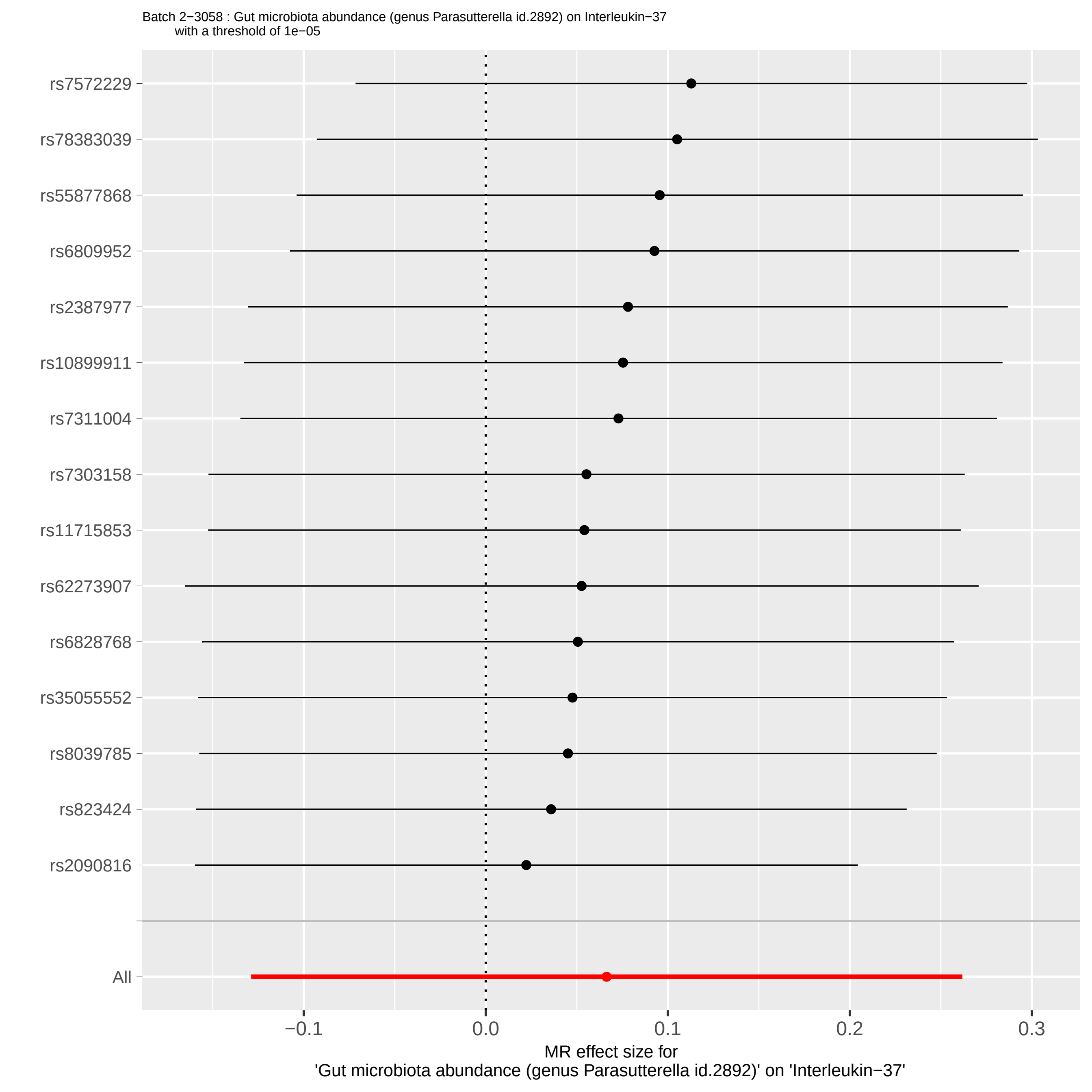

Batch 2−3058 : Gut microbiota abundance (genus Parasutterella id.2892) on Interleukin−37
with a threshold of 1e−05
rs7572229
rs78383039
rs55877868
rs6809952
rs2387977
rs10899911
rs7311004
rs7303158
rs11715853
rs62273907
rs6828768
rs35055552
rs8039785
rs823424
rs2090816
All
−0.1
0.0
0.1
0.2
0.3
MR effect size for
'Gut microbiota abundance (genus Parasutterella id.2892)' on 'Interleukin−37'

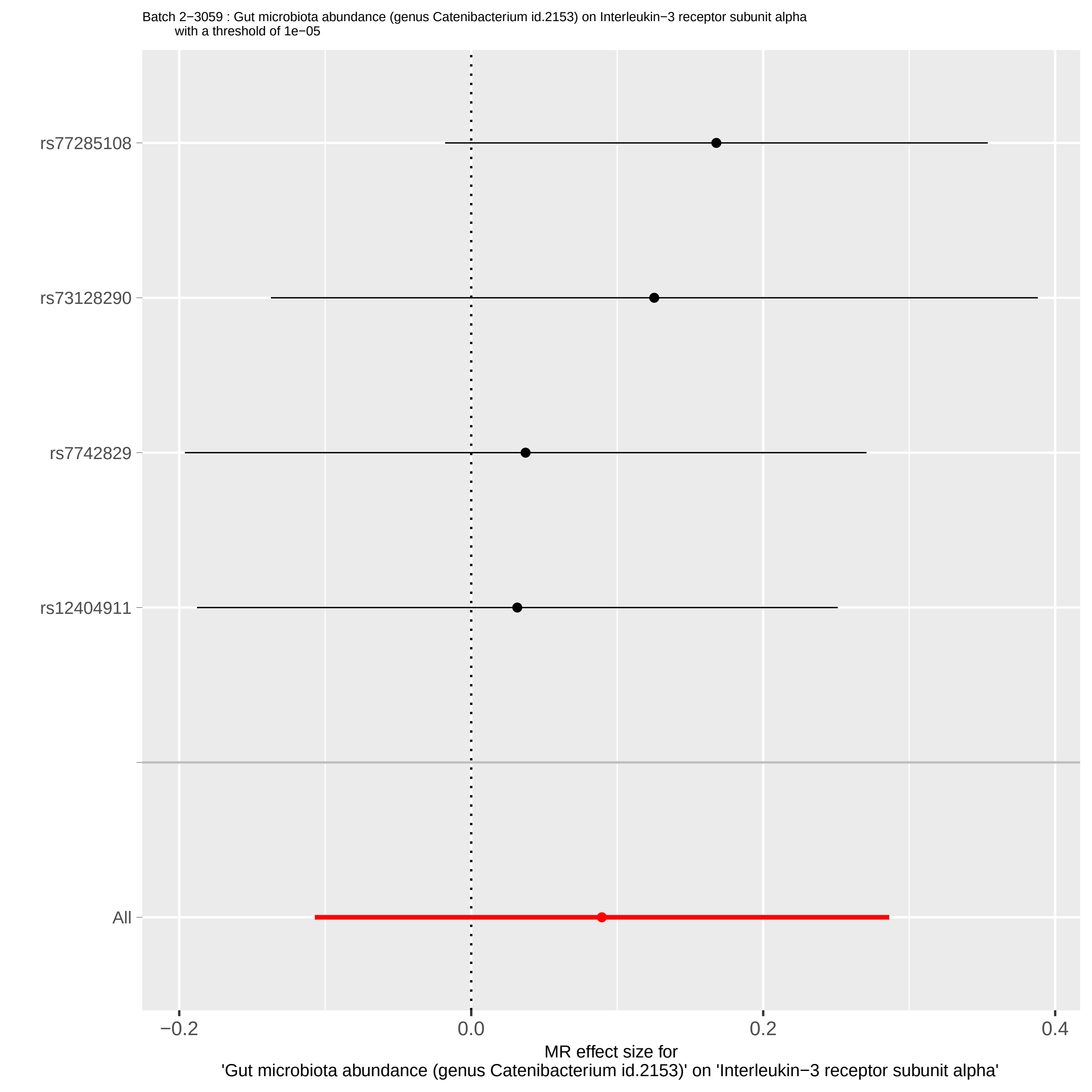
Batch 2−3059 : Gut microbiota abundance (genus Catenibacterium id.2153) on Interleukin−3 receptor subunit alpha
with a threshold of 1e−05
rs77285108
rs73128290
rs7742829
rs12404911
All
−0.2
0.0
0.2
0.4
MR effect size for
'Gut microbiota abundance (genus Catenibacterium id.2153)' on 'Interleukin−3 receptor subunit alpha'

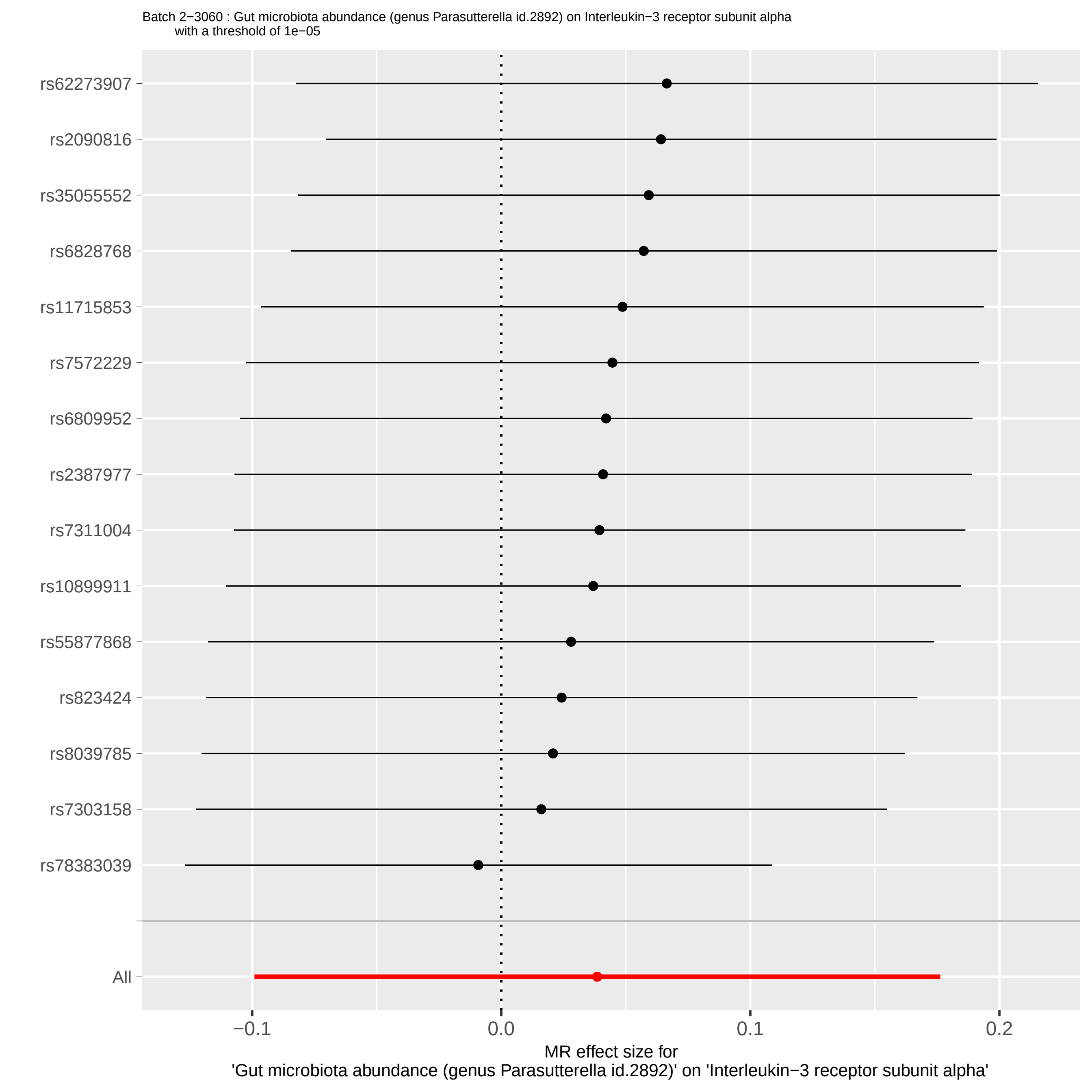
Batch 2−3060 : Gut microbiota abundance (genus Parasutterella id.2892) on Interleukin−3 receptor subunit alpha
with a threshold of 1e−05
rs62273907
rs2090816
rs35055552
rs6828768
rs11715853
rs7572229
rs6809952
rs2387977
rs7311004
rs10899911
rs55877868
rs823424
rs8039785
rs7303158
rs78383039
All
−0.1
0.0
0.1
0.2
MR effect size for
'Gut microbiota abundance (genus Parasutterella id.2892)' on 'Interleukin−3 receptor subunit alpha'

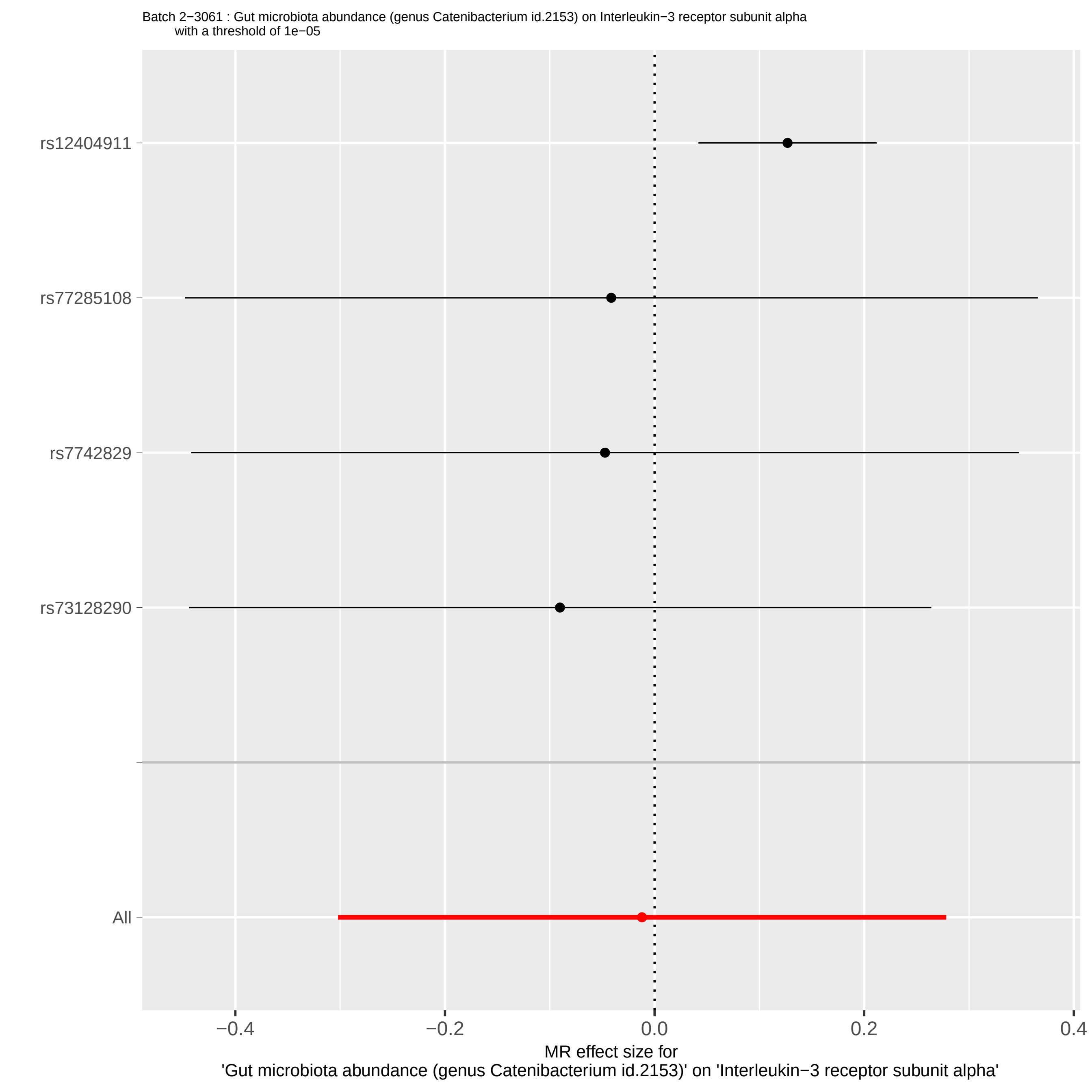

Batch 2−3061 : Gut microbiota abundance (genus Catenibacterium id.2153) on Interleukin−3 receptor subunit alpha
with a threshold of 1e−05
rs12404911
rs77285108
rs7742829
rs73128290
All
−0.4
−0.2
0.0
0.2
0.4
MR effect size for
'Gut microbiota abundance (genus Catenibacterium id.2153)' on 'Interleukin−3 receptor subunit alpha'

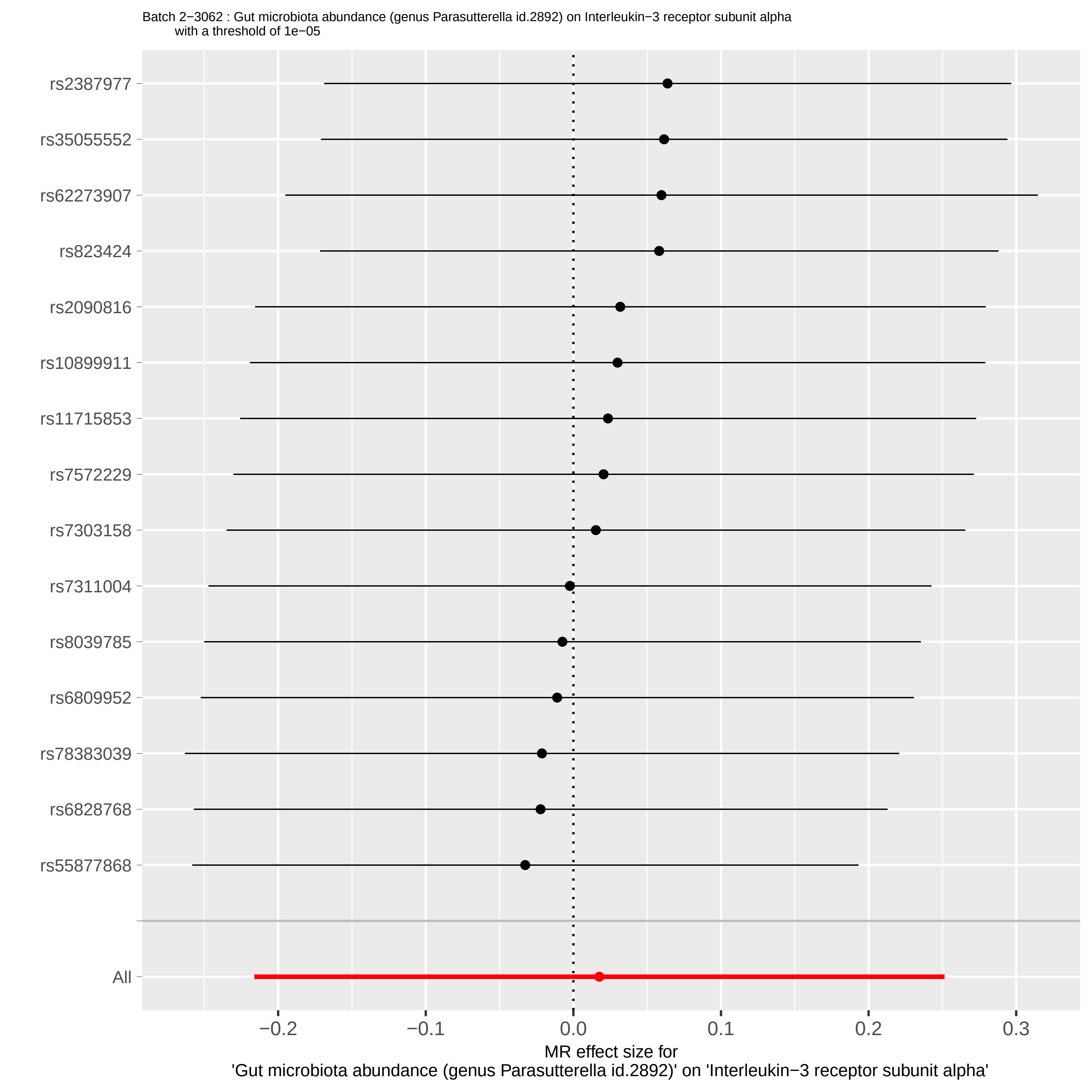
Batch 2−3062 : Gut microbiota abundance (genus Parasutterella id.2892) on Interleukin−3 receptor subunit alpha
with a threshold of 1e−05
rs2387977
rs35055552
rs62273907
rs823424
rs2090816
rs10899911
rs11715853
rs7572229
rs7303158
rs7311004
rs8039785
rs6809952
rs78383039
rs6828768
rs55877868
All
−0.2
−0.1
0.0
0.1
0.2
0.3
MR effect size for
'Gut microbiota abundance (genus Parasutterella id.2892)' on 'Interleukin−3 receptor subunit alpha'

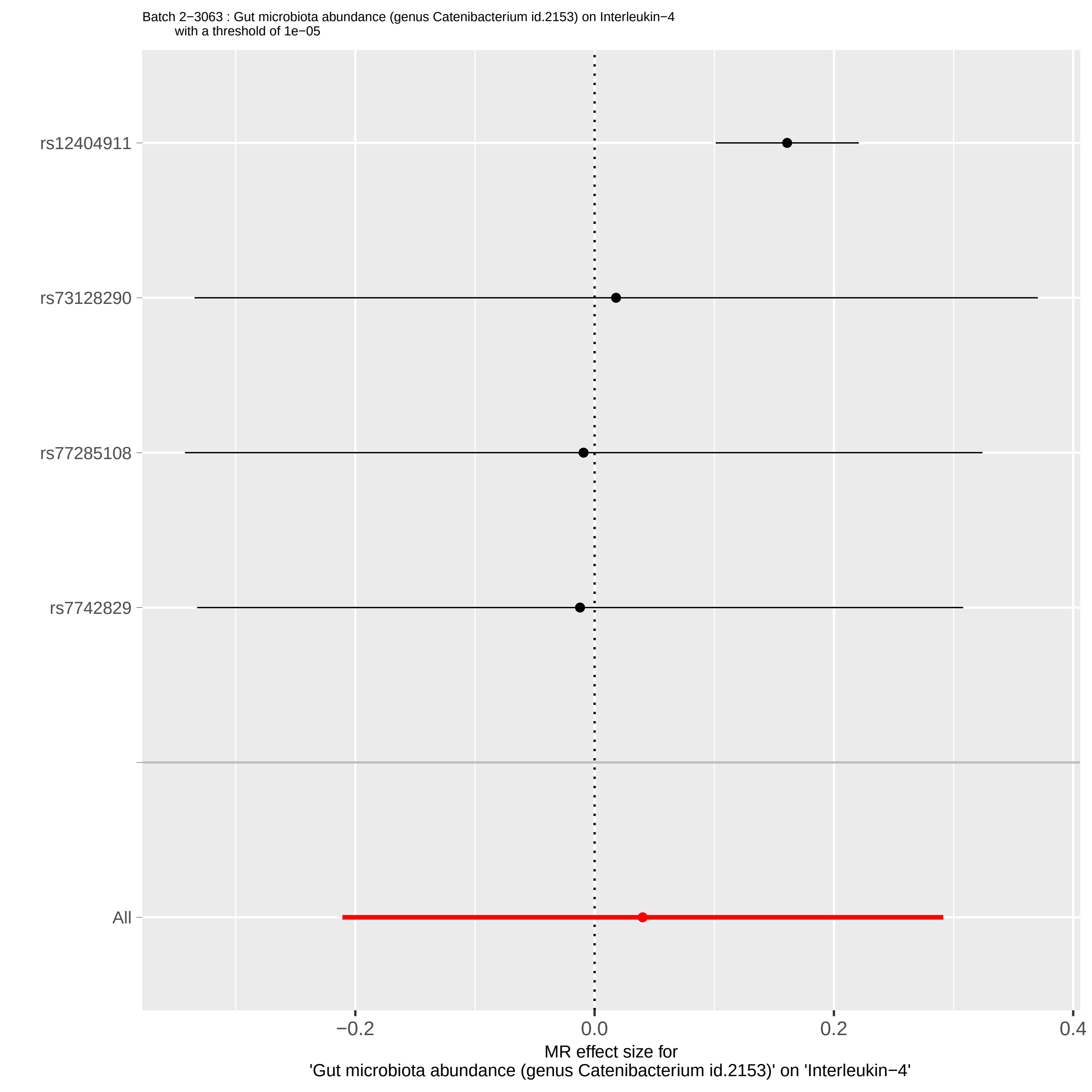

Batch 2−3063 : Gut microbiota abundance (genus Catenibacterium id.2153) on Interleukin−4
with a threshold of 1e−05
rs12404911
rs73128290
rs77285108
rs7742829
All
−0.2
0.0
0.2
0.4
MR effect size for
'Gut microbiota abundance (genus Catenibacterium id.2153)' on 'Interleukin−4'

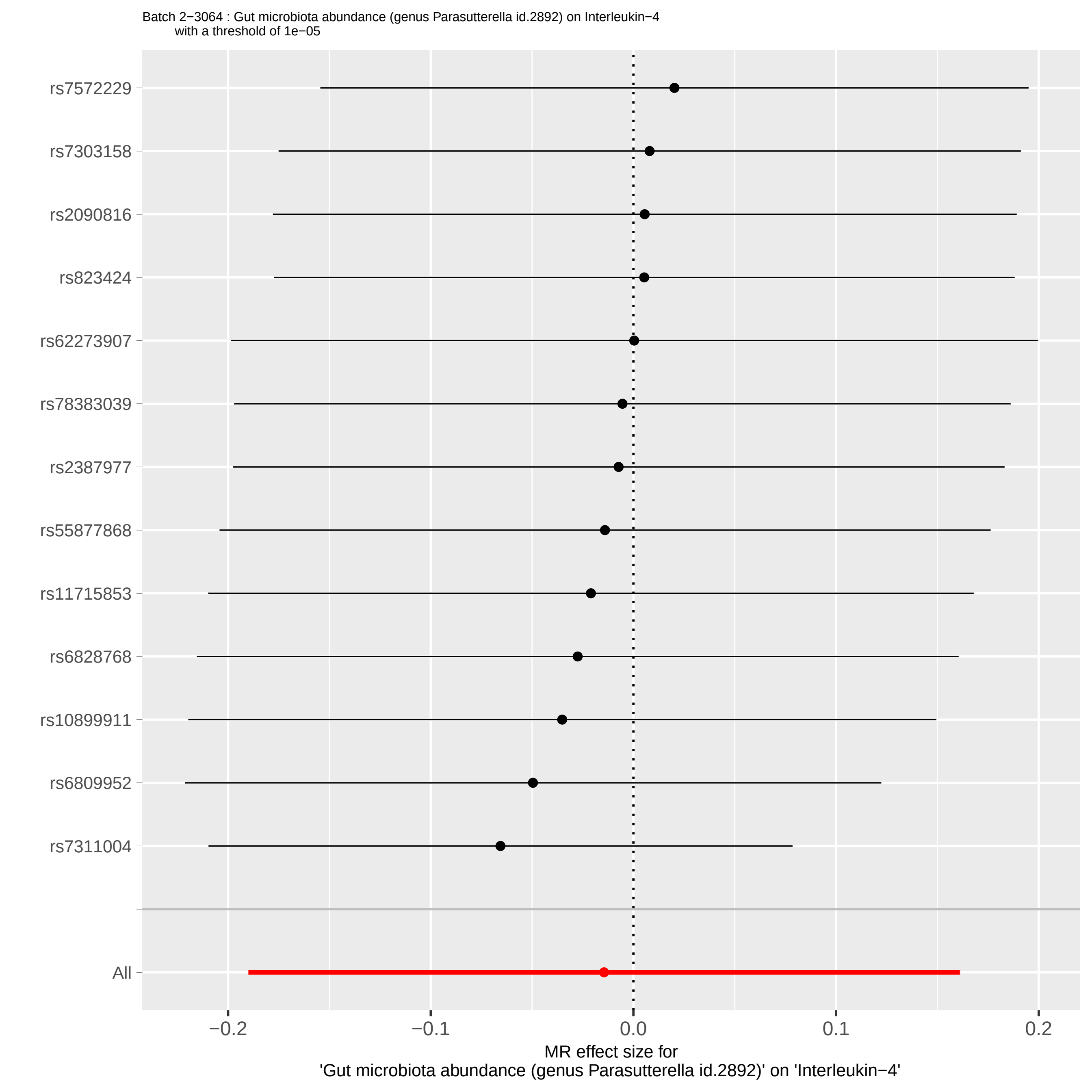

Batch 2−3064 : Gut microbiota abundance (genus Parasutterella id.2892) on Interleukin−4
with a threshold of 1e−05
rs7572229
rs7303158
rs2090816
rs823424
rs62273907
rs78383039
rs2387977
rs55877868
rs11715853
rs6828768
rs10899911
rs6809952
rs7311004
All
−0.2
−0.1
0.0
0.1
0.2
MR effect size for
'Gut microbiota abundance (genus Parasutterella id.2892)' on 'Interleukin−4'

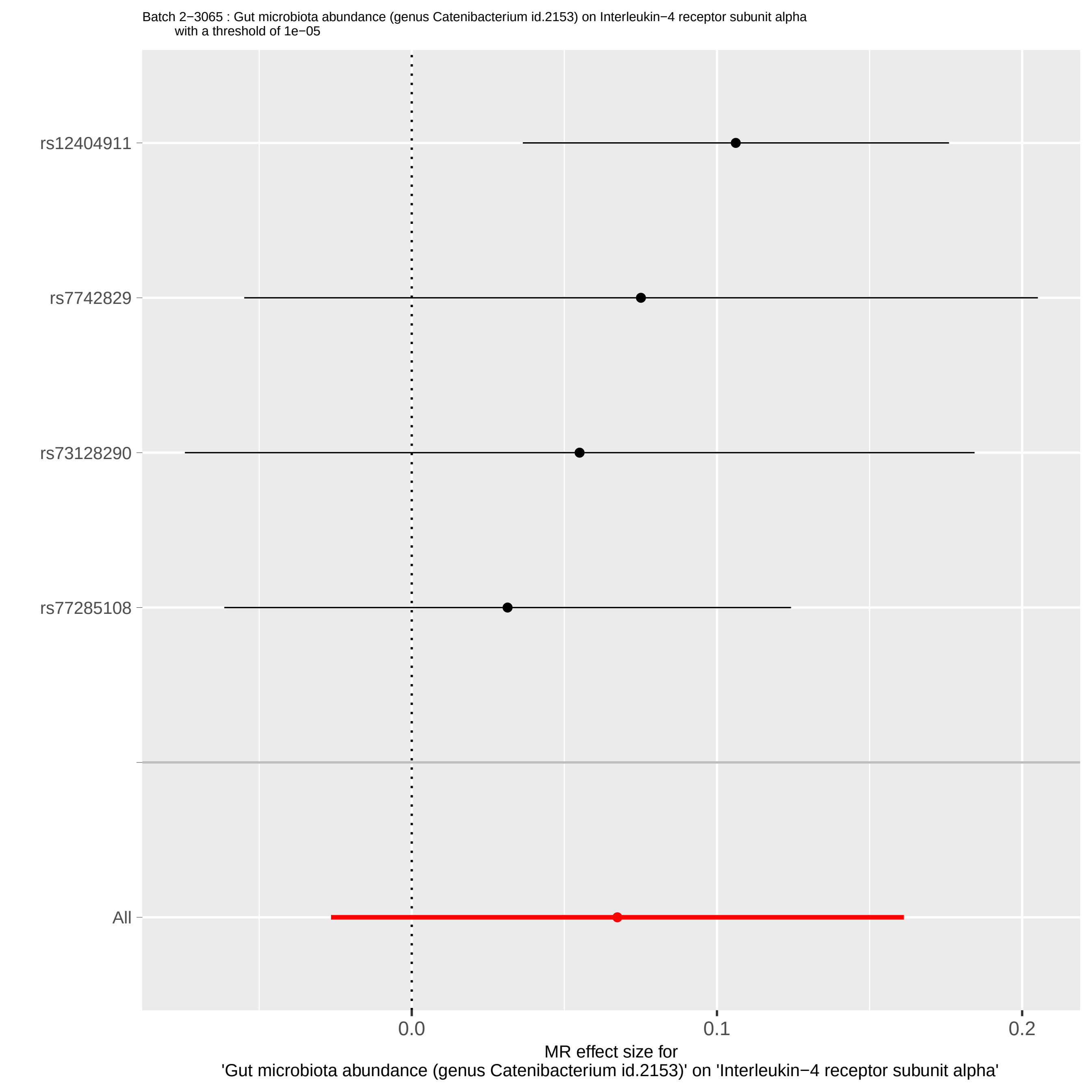
Batch 2−3065 : Gut microbiota abundance (genus Catenibacterium id.2153) on Interleukin−4 receptor subunit alpha
with a threshold of 1e−05
rs12404911
rs7742829
rs73128290
rs77285108
All
0.0
0.1
0.2
MR effect size for
'Gut microbiota abundance (genus Catenibacterium id.2153)' on 'Interleukin−4 receptor subunit alpha'

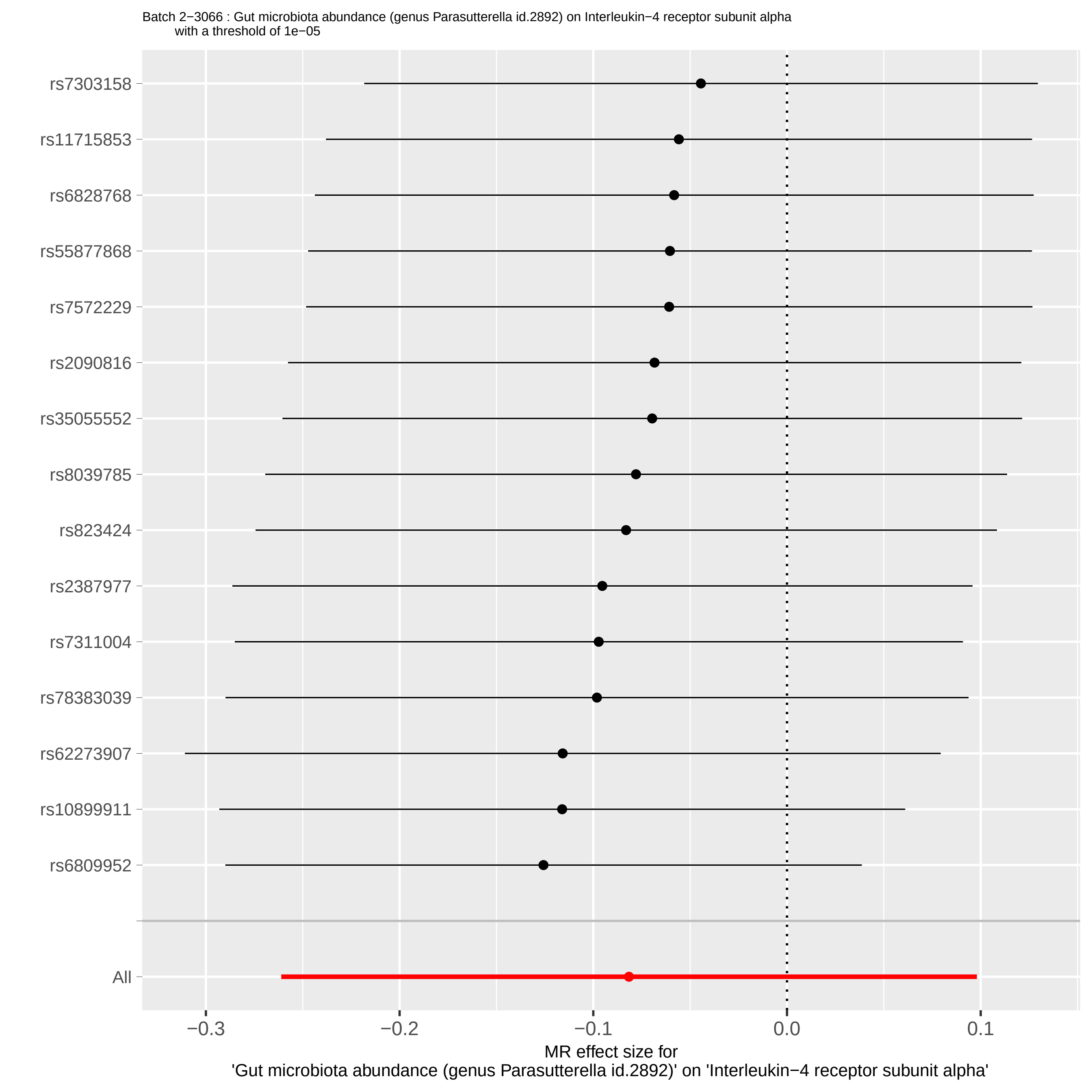
Batch 2−3066 : Gut microbiota abundance (genus Parasutterella id.2892) on Interleukin−4 receptor subunit alpha
with a threshold of 1e−05
rs7303158
rs11715853
rs6828768
rs55877868
rs7572229
rs2090816
rs35055552
rs8039785
rs823424
rs2387977
rs7311004
rs78383039
rs62273907
rs10899911
rs6809952
All
−0.3
−0.2
−0.1
0.0
0.1
MR effect size for
'Gut microbiota abundance (genus Parasutterella id.2892)' on 'Interleukin−4 receptor subunit alpha

Batch 2−3067 : Gut microbiota abundance (genus Catenibacterium id.2153) on Interleukin−5
with a threshold of 1e−05

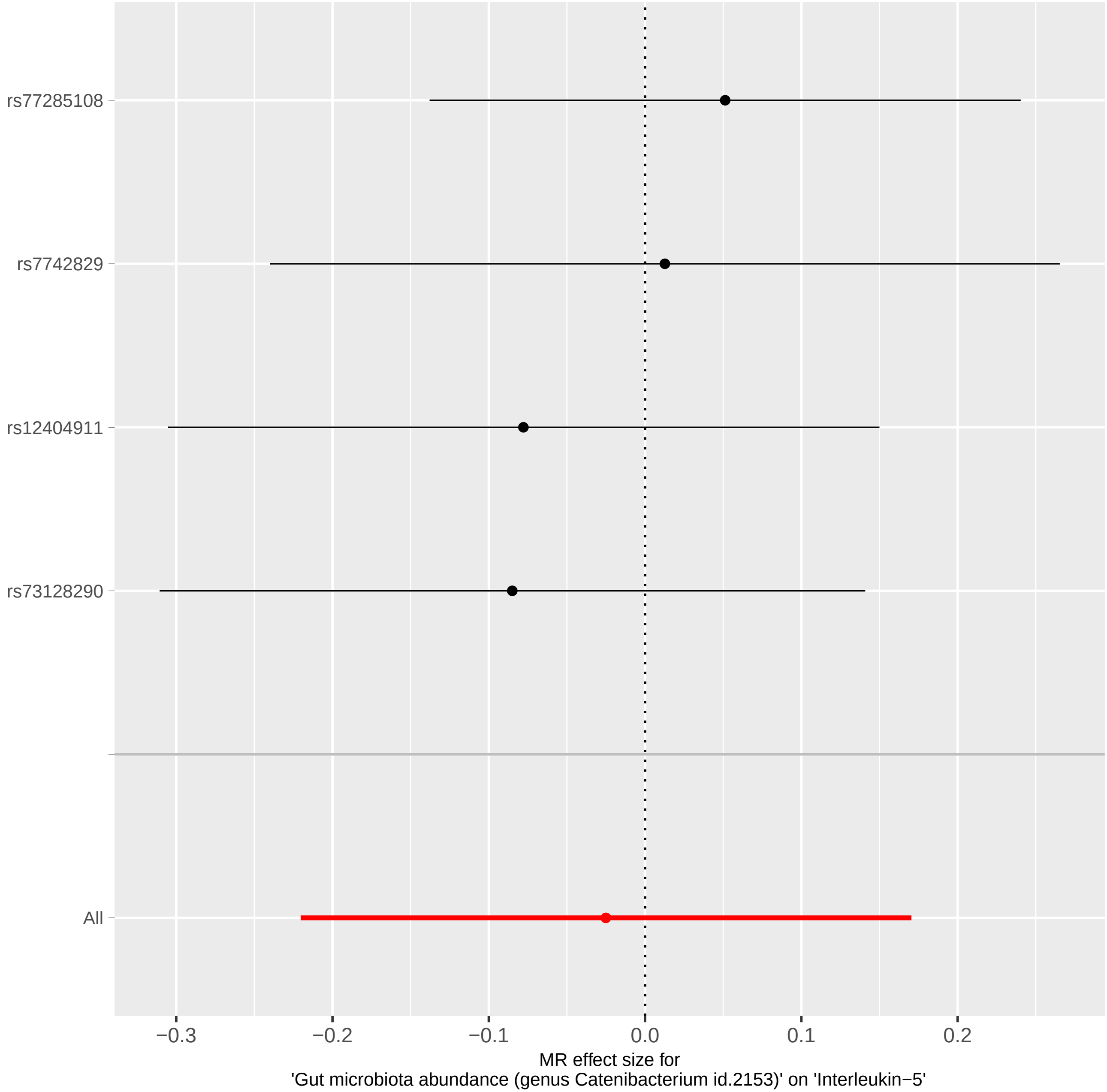

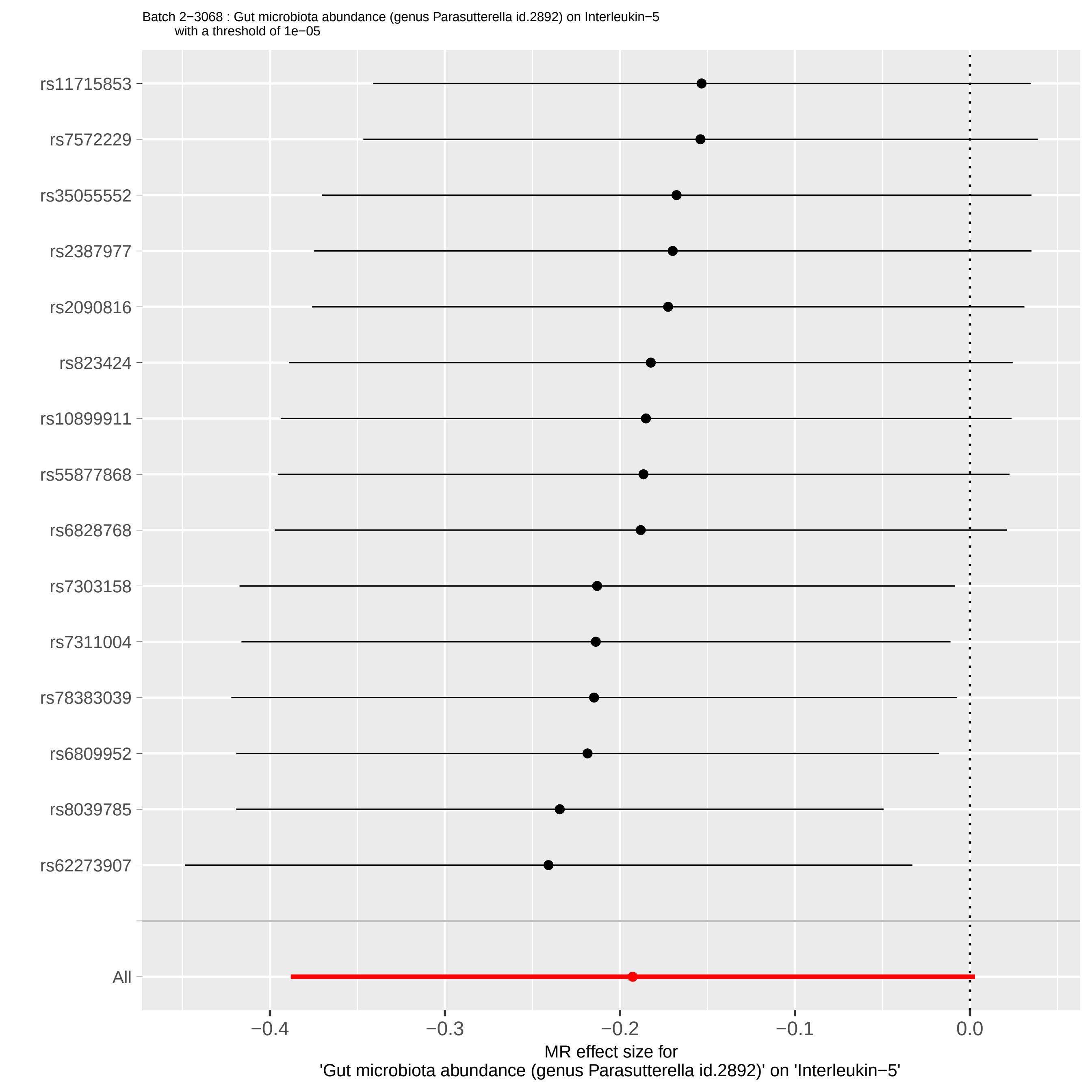
Batch 2−3068 : Gut microbiota abundance (genus Parasutterella id.2892) on Interleukin−5
with a threshold of 1e−05
rs11715853
rs7572229
rs35055552
rs2387977
rs2090816
rs823424
rs10899911
rs55877868
rs6828768
rs7303158
rs7311004
rs78383039
rs6809952
rs8039785
rs62273907
All
−0.4
−0.3
−0.2
−0.1
0.0
MR effect size for
'Gut microbiota abundance (genus Parasutterella id.2892)' on 'Interleukin−5'

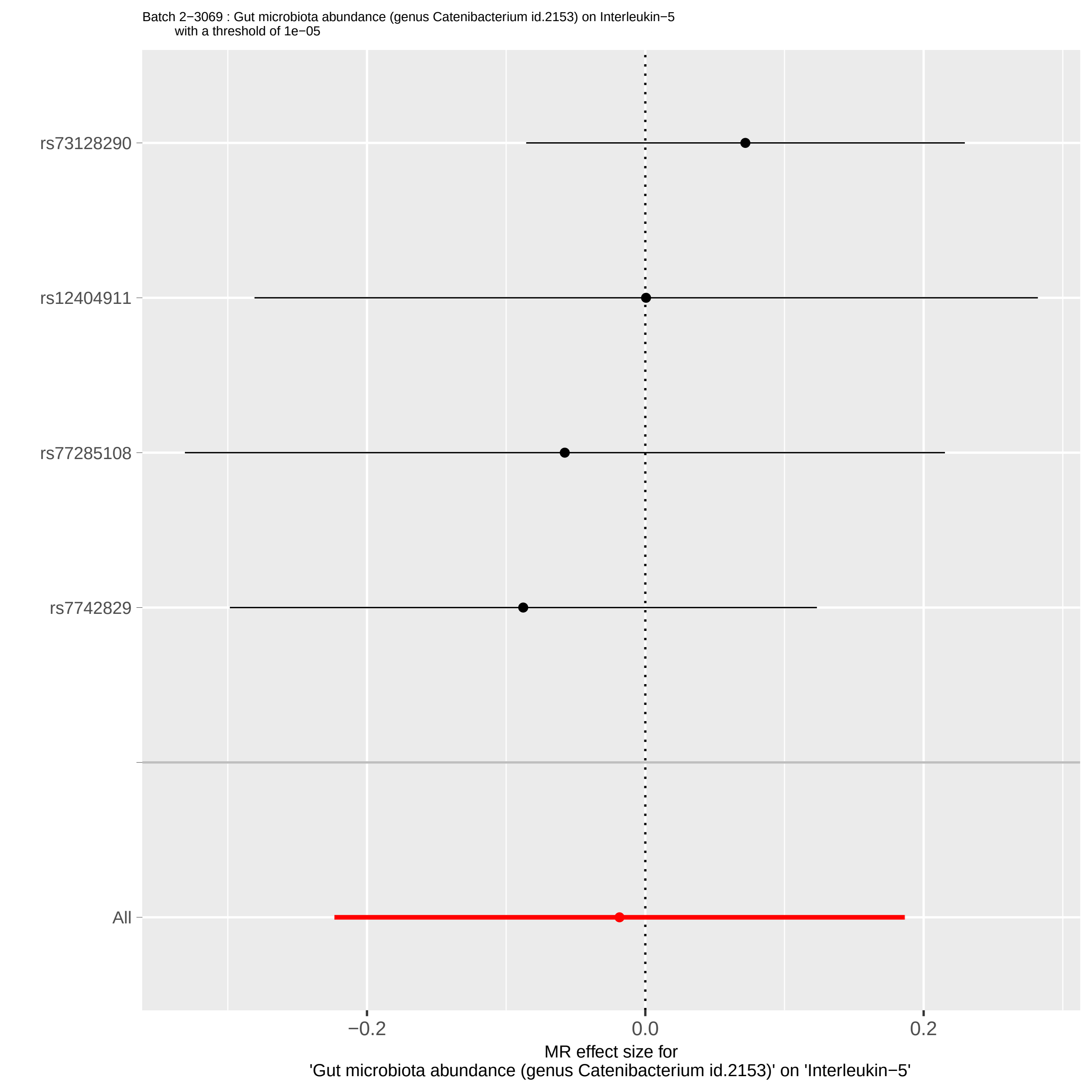
Batch 2−3069 : Gut microbiota abundance (genus Catenibacterium id.2153) on Interleukin−5
with a threshold of 1e−05
rs73128290
rs12404911
rs77285108
rs7742829
All
−0.2
0.0
0.2
MR effect size for
'Gut microbiota abundance (genus Catenibacterium id.2153)' on 'Interleukin−5'

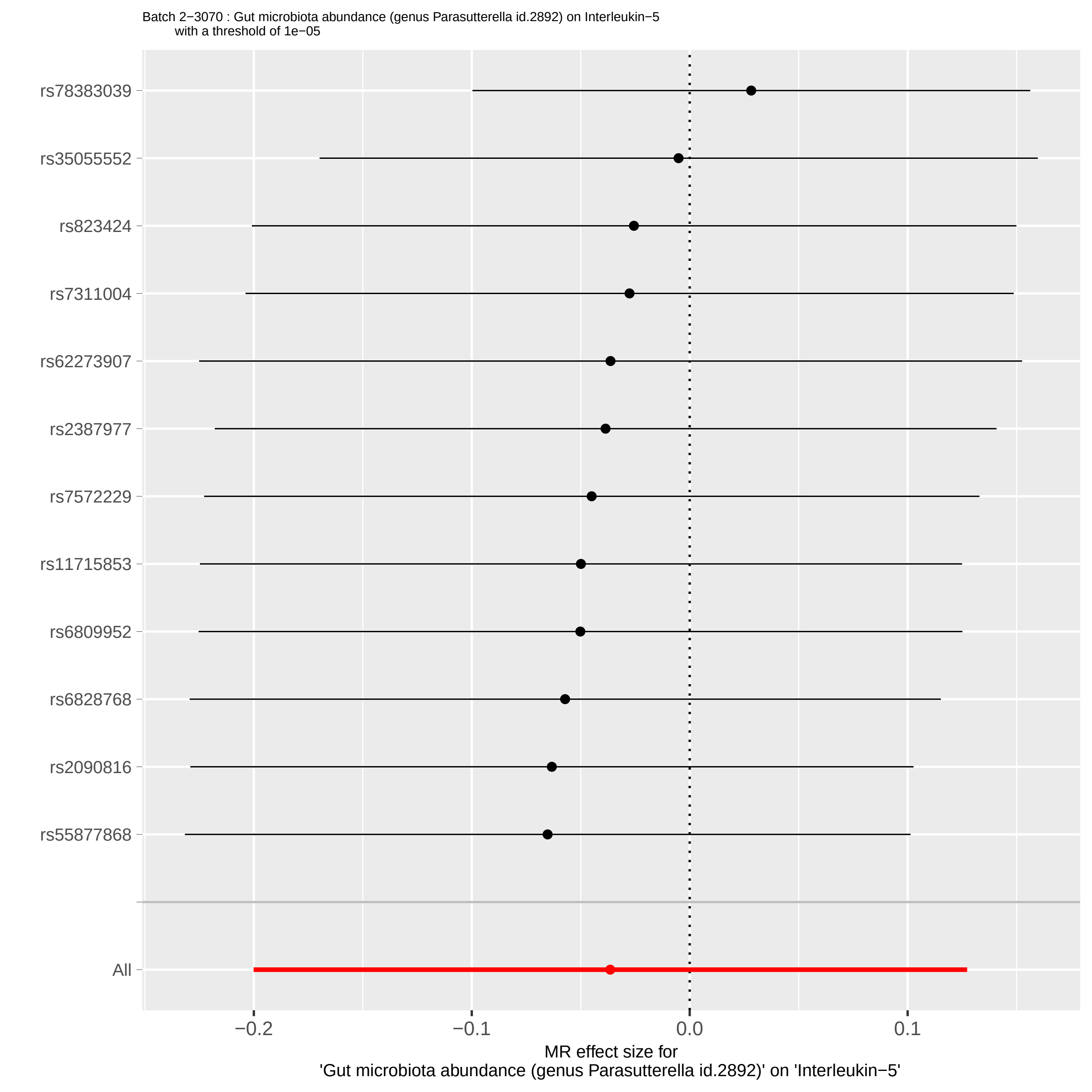

Batch 2−3070 : Gut microbiota abundance (genus Parasutterella id.2892) on Interleukin−5
with a threshold of 1e−05
rs78383039
rs35055552
rs823424
rs7311004
rs62273907
rs2387977
rs7572229
rs11715853
rs6809952
rs6828768
rs2090816
rs55877868
All
−0.2
−0.1
0.0
0.1
MR effect size for
'Gut microbiota abundance (genus Parasutterella id.2892)' on 'Interleukin−5'

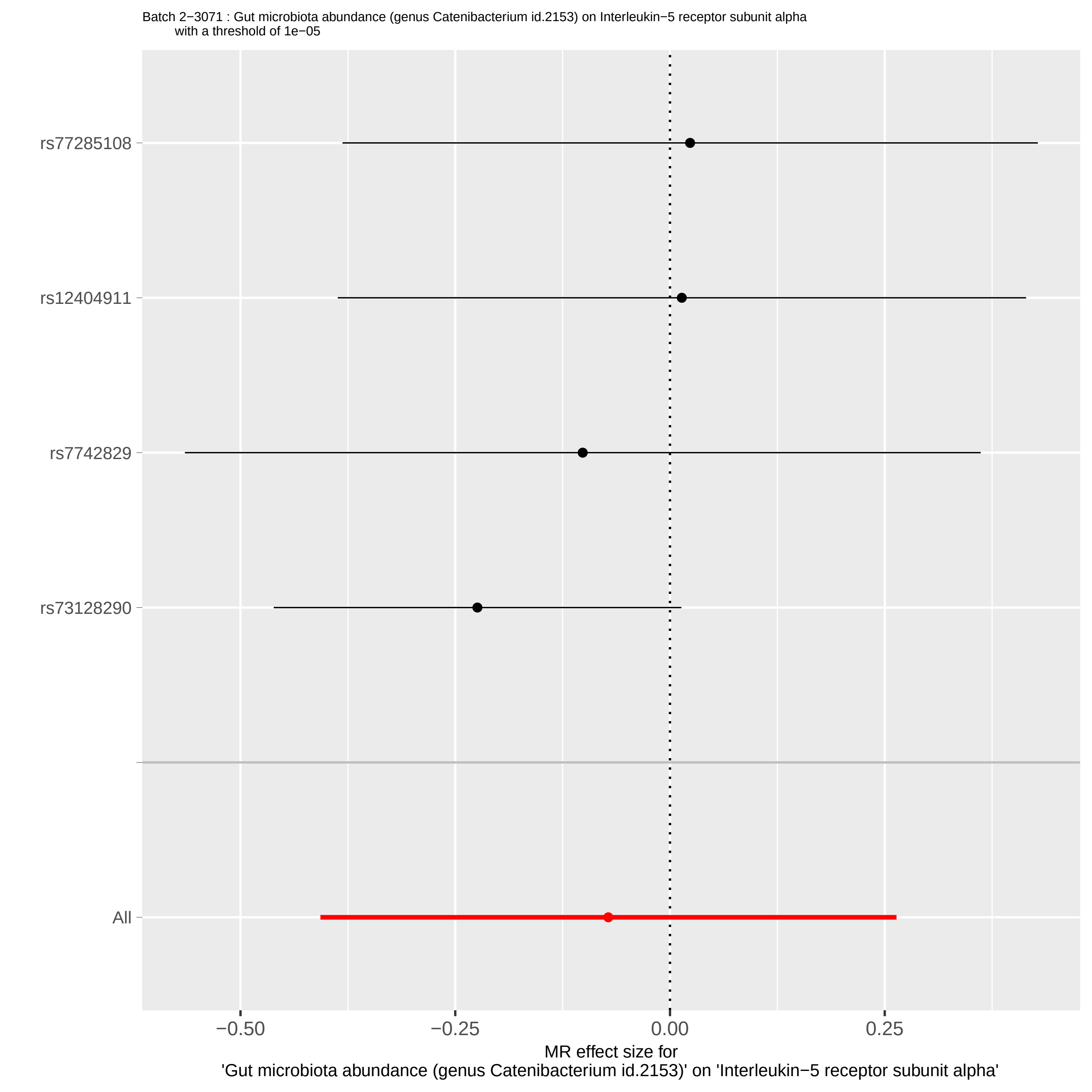
Batch 2−3071 : Gut microbiota abundance (genus Catenibacterium id.2153) on Interleukin−5 receptor subunit alpha
with a threshold of 1e−05
rs77285108
rs12404911
rs7742829
rs73128290
All
−0.50
−0.25
0.00
0.25
MR effect size for
'Gut microbiota abundance (genus Catenibacterium id.2153)' on 'Interleukin−5 receptor subunit alpha'

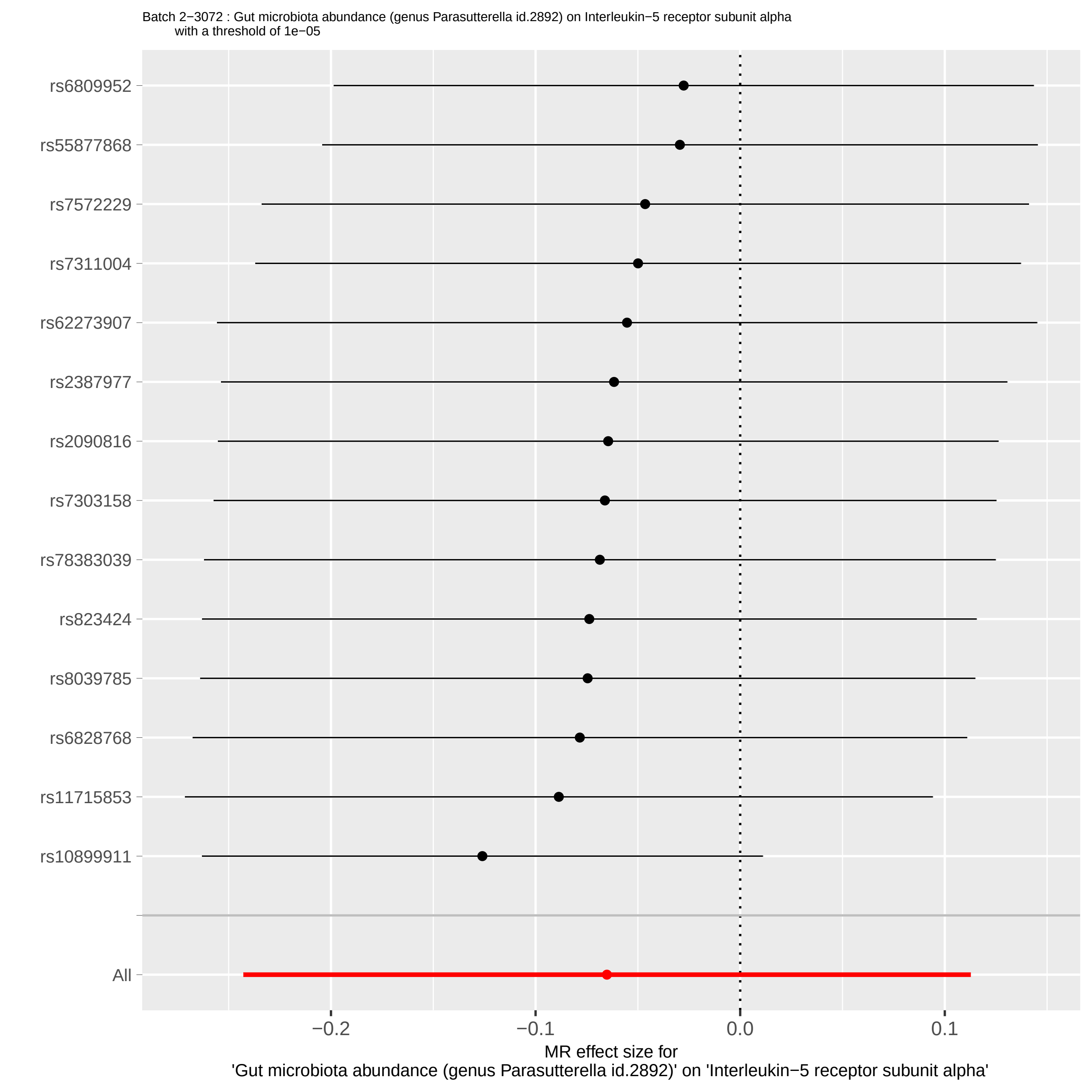

Batch 2−3072 : Gut microbiota abundance (genus Parasutterella id.2892) on Interleukin−5 receptor subunit alpha
with a threshold of 1e−05
rs6809952
rs55877868
rs7572229
rs7311004
rs62273907
rs2387977
rs2090816
rs7303158
rs78383039
rs823424
rs8039785
rs6828768
rs11715853
rs10899911
All
−0.2
−0.1
0.0
0.1
MR effect size for
'Gut microbiota abundance (genus Parasutterella id.2892)' on 'Interleukin−5 receptor subunit alpha'

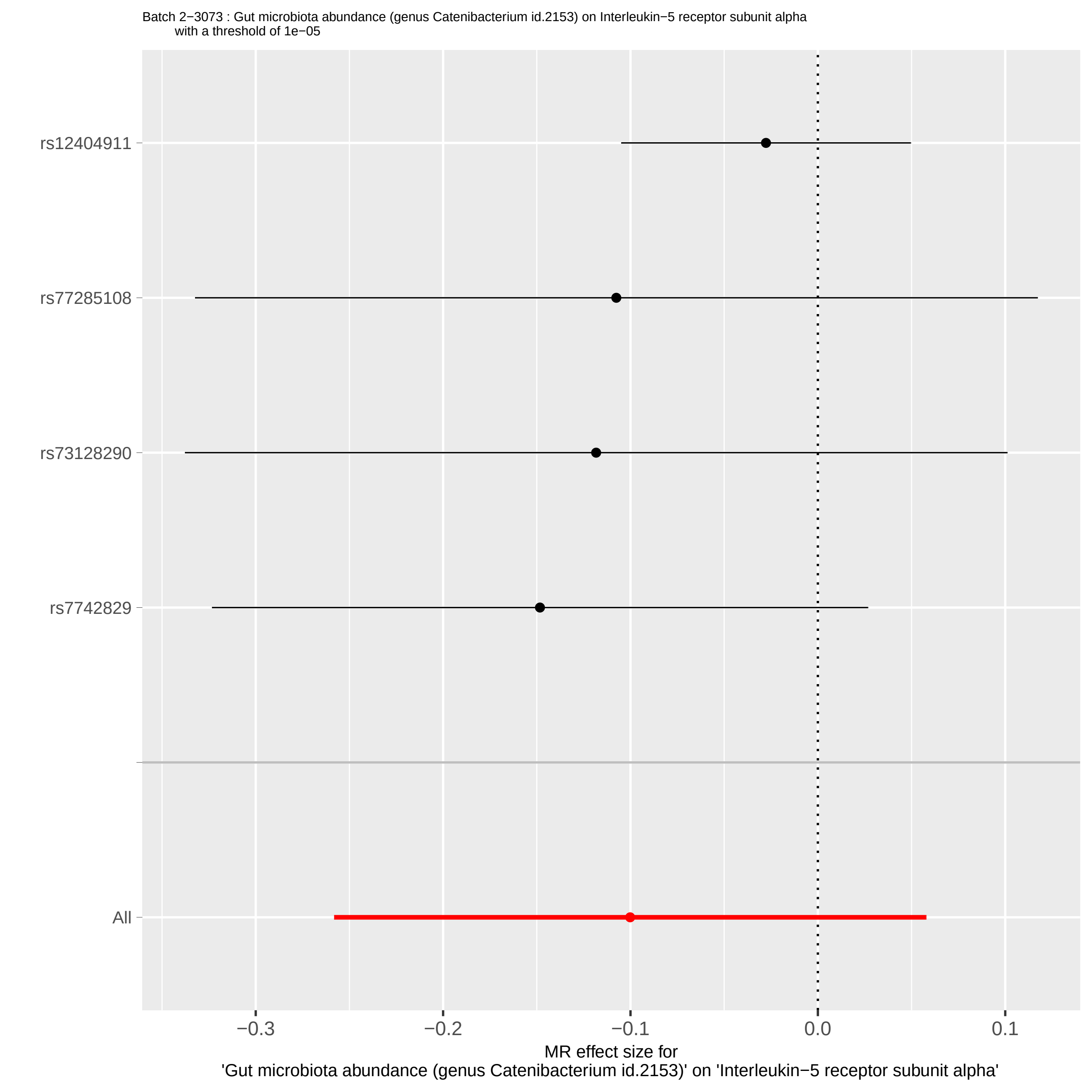
Batch 2−3073 : Gut microbiota abundance (genus Catenibacterium id.2153) on Interleukin−5 receptor subunit alpha
with a threshold of 1e−05
rs12404911
rs77285108
rs73128290
rs7742829
All
−0.3
−0.2
−0.1
0.0
0.1
MR effect size for
'Gut microbiota abundance (genus Catenibacterium id.2153)' on 'Interleukin−5 receptor subunit alpha'

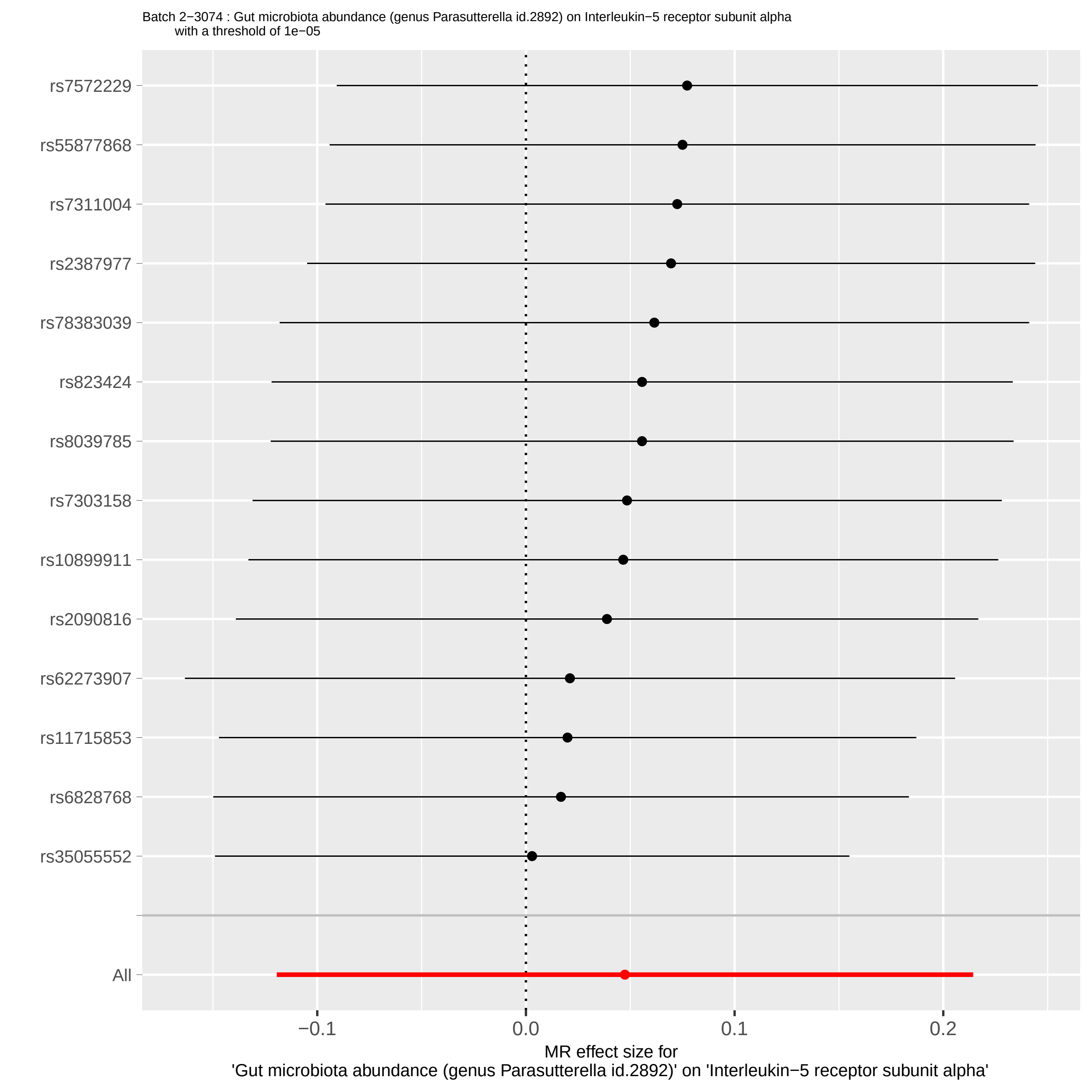
Batch 2−3074 : Gut microbiota abundance (genus Parasutterella id.2892) on Interleukin−5 receptor subunit alpha
with a threshold of 1e−05
rs7572229
rs55877868
rs7311004
rs2387977
rs78383039
rs823424
rs8039785
rs7303158
rs10899911
rs2090816
rs62273907
rs11715853
rs6828768
rs35055552
All
−0.1
0.0
0.1
0.2
MR effect size for
'Gut microbiota abundance (genus Parasutterella id.2892)' on 'Interleukin−5 receptor subunit alpha'

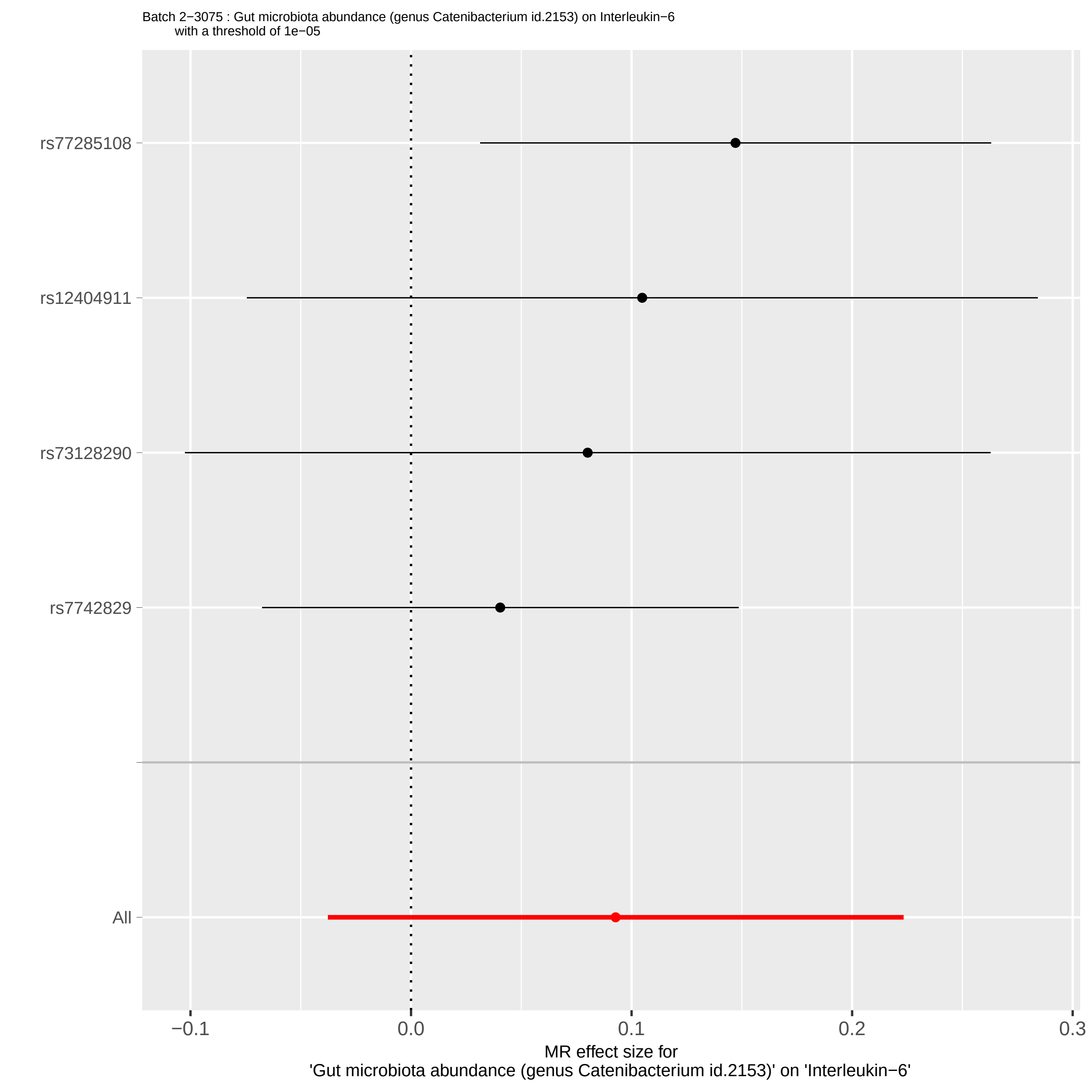

Batch 2−3075 : Gut microbiota abundance (genus Catenibacterium id.2153) on Interleukin−6
with a threshold of 1e−05
rs77285108
rs12404911
rs73128290
rs7742829
All
−0.1
0.0
0.1
0.2
0.3
MR effect size for
'Gut microbiota abundance (genus Catenibacterium id.2153)' on 'Interleukin−6'

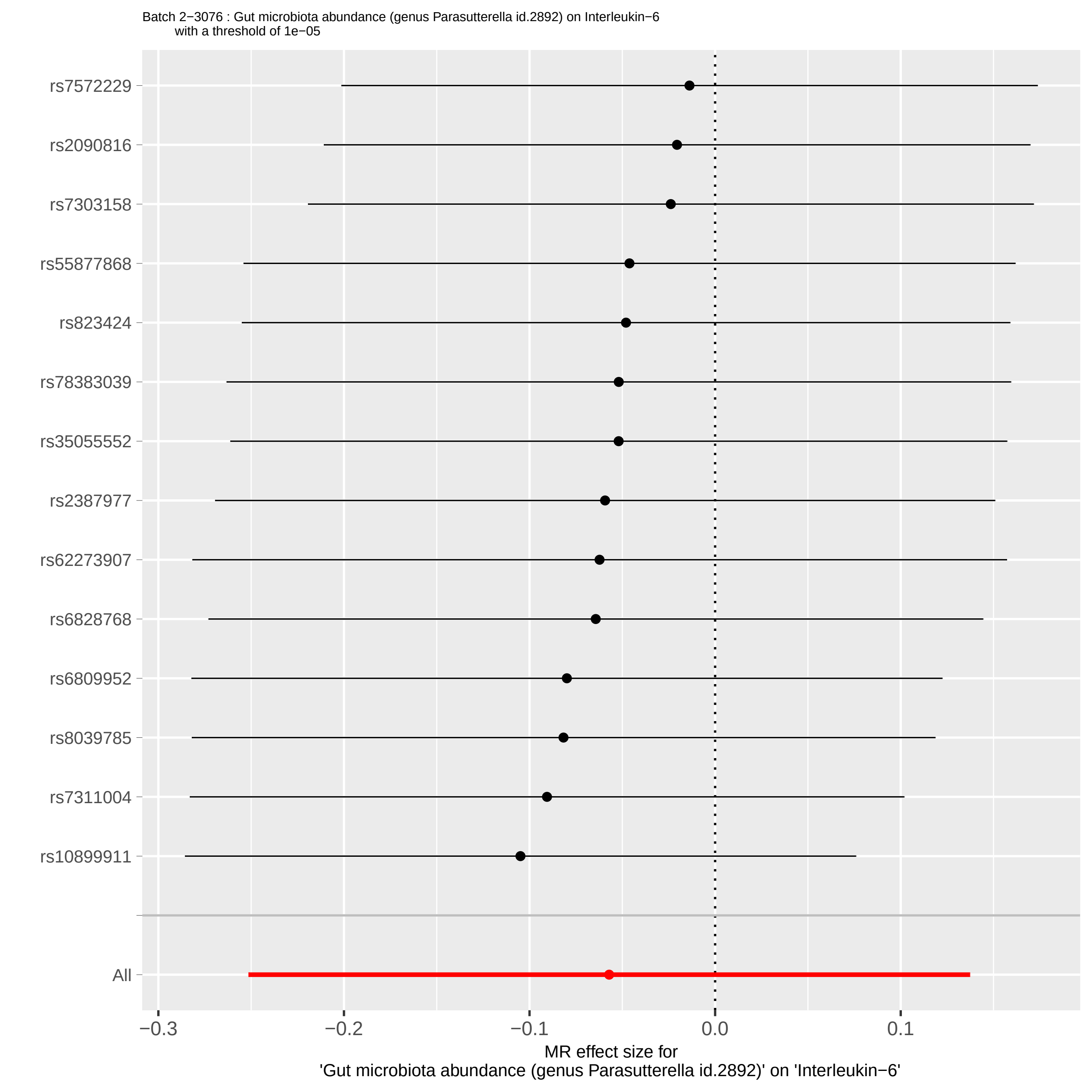
Batch 2−3076 : Gut microbiota abundance (genus Parasutterella id.2892) on Interleukin−6
with a threshold of 1e−05
rs7572229
rs2090816
rs7303158
rs55877868
rs823424
rs78383039
rs35055552
rs2387977
rs62273907
rs6828768
rs6809952
rs8039785
rs7311004
rs10899911
All
−0.3
−0.2
−0.1
0.0
0.1
MR effect size for
'Gut microbiota abundance (genus Parasutterella id.2892)' on 'Interleukin−6'

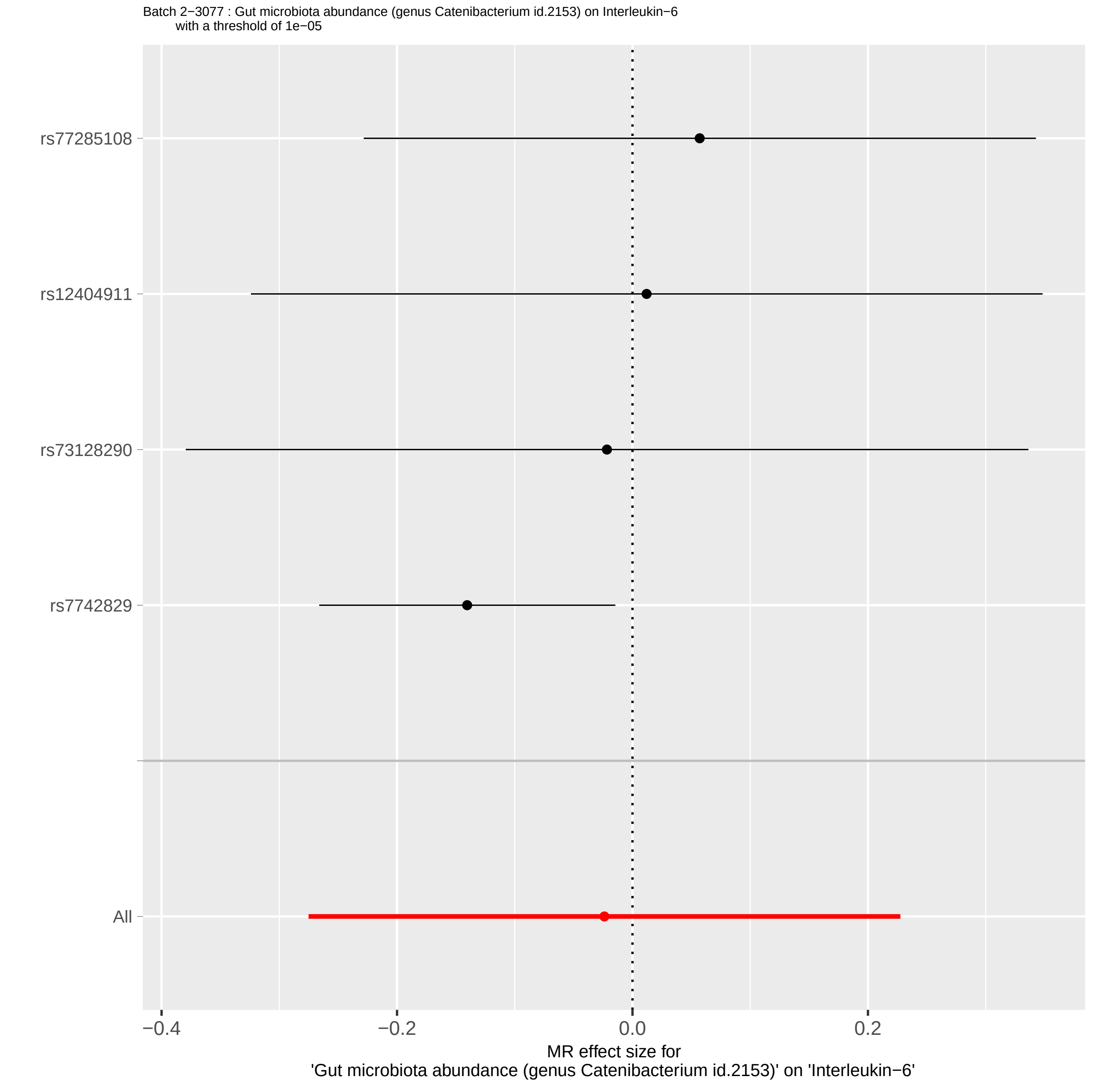
Batch 2−3077 : Gut microbiota abundance (genus Catenibacterium id.2153) on Interleukin−6
with a threshold of 1e−05
rs77285108
rs12404911
rs73128290
rs7742829
All
−0.4
−0.2
0.0
0.2
MR effect size for
'Gut microbiota abundance (genus Catenibacterium id.2153)' on 'Interleukin−6'

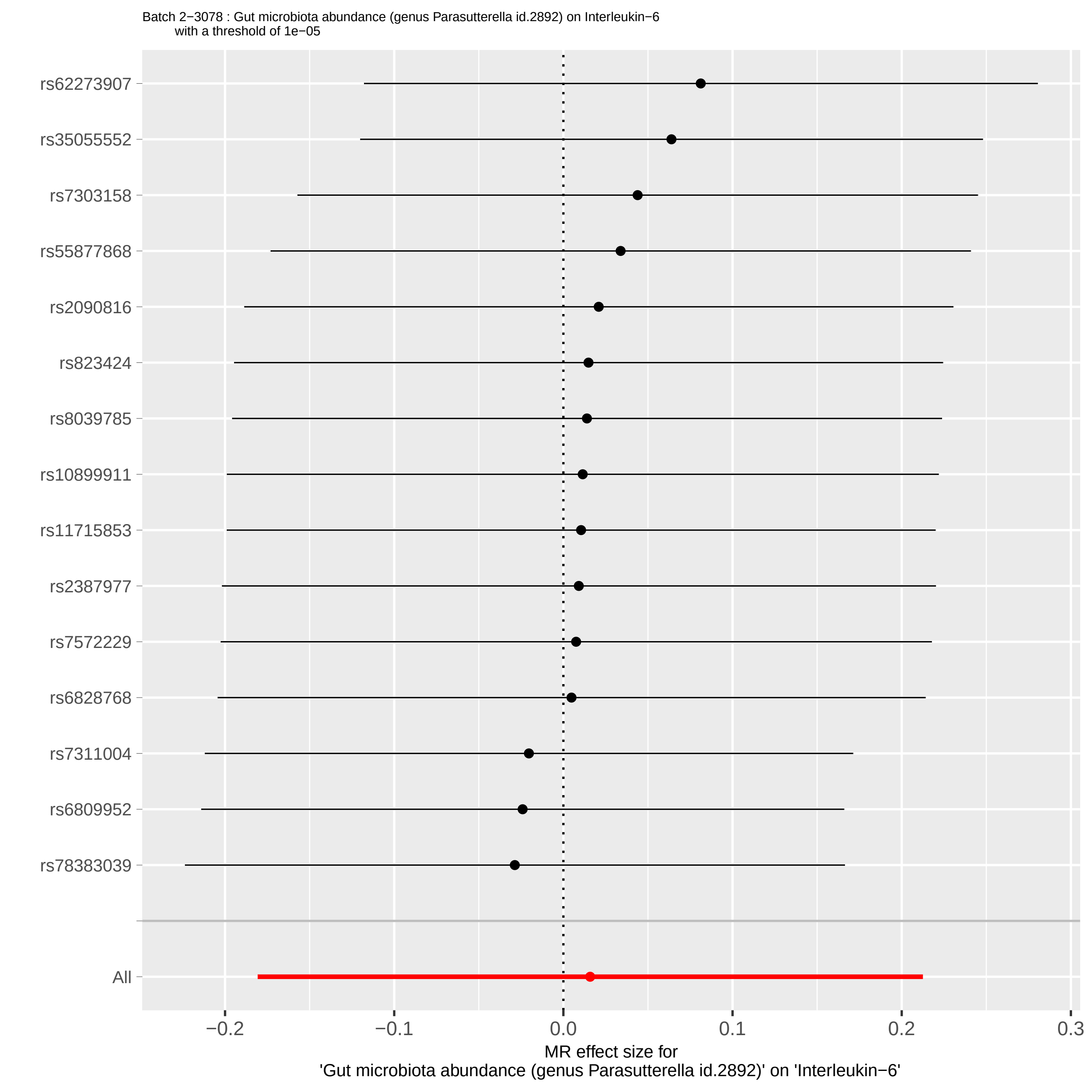
Batch 2−3078 : Gut microbiota abundance (genus Parasutterella id.2892) on Interleukin−6
with a threshold of 1e−05
rs62273907
rs35055552
rs7303158
rs55877868
rs2090816
rs823424
rs8039785
rs10899911
rs11715853
rs2387977
rs7572229
rs6828768
rs7311004
rs6809952
rs78383039
All
−0.2
−0.1
0.0
0.1
0.2
0.3
MR effect size for
'Gut microbiota abundance (genus Parasutterella id.2892)' on 'Interleukin−6'

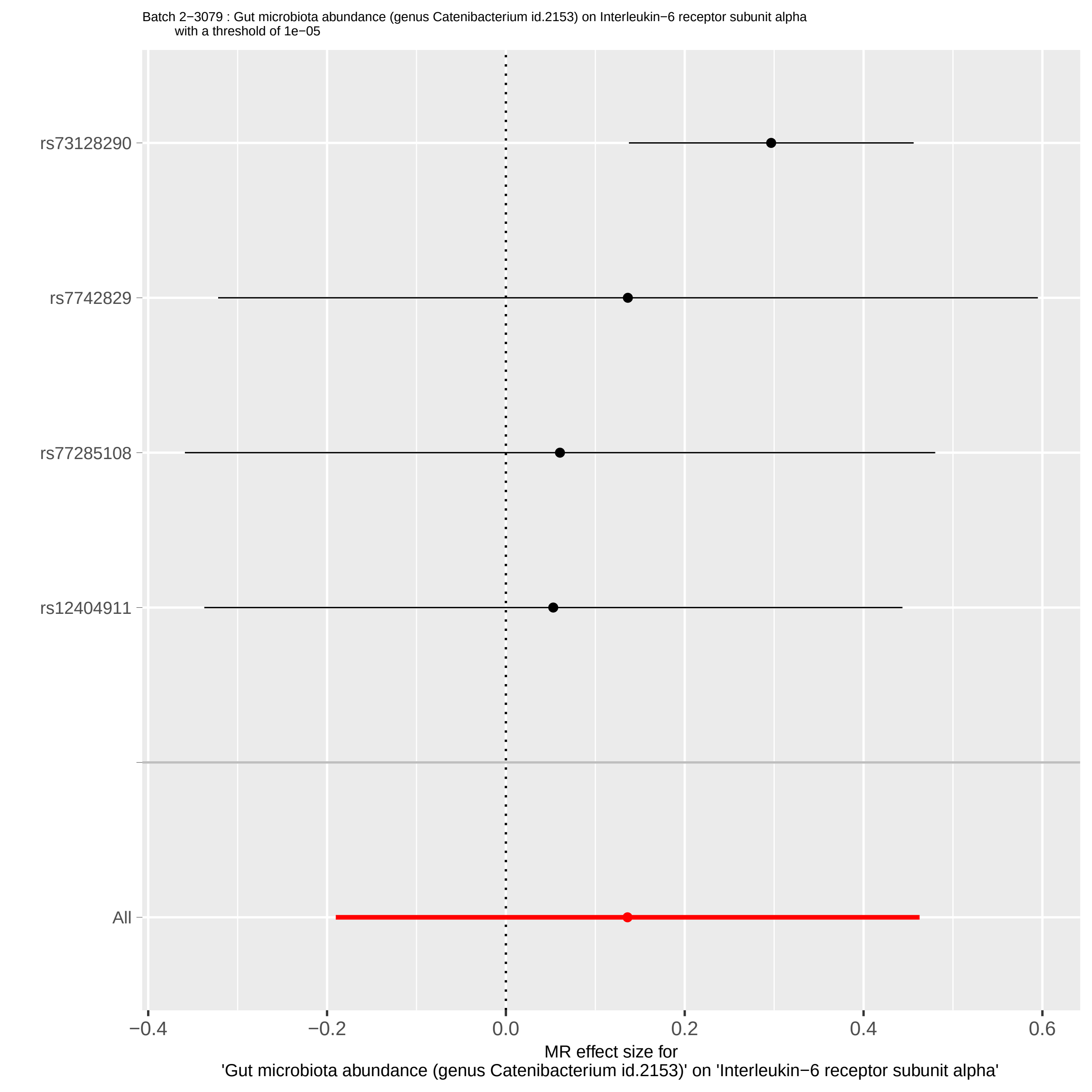

Batch 2−3079 : Gut microbiota abundance (genus Catenibacterium id.2153) on Interleukin−6 receptor subunit alpha
with a threshold of 1e−05
rs73128290
rs7742829
rs77285108
rs12404911
All
−0.4
−0.2
0.0
0.2
0.4
0.6
MR effect size for
'Gut microbiota abundance (genus Catenibacterium id.2153)' on 'Interleukin−6 receptor subunit alpha'

Batch 2−3080 : Gut microbiota abundance (genus Parasutterella id.2892) on Interleukin−6 receptor subunit alpha
with a threshold of 1e−05

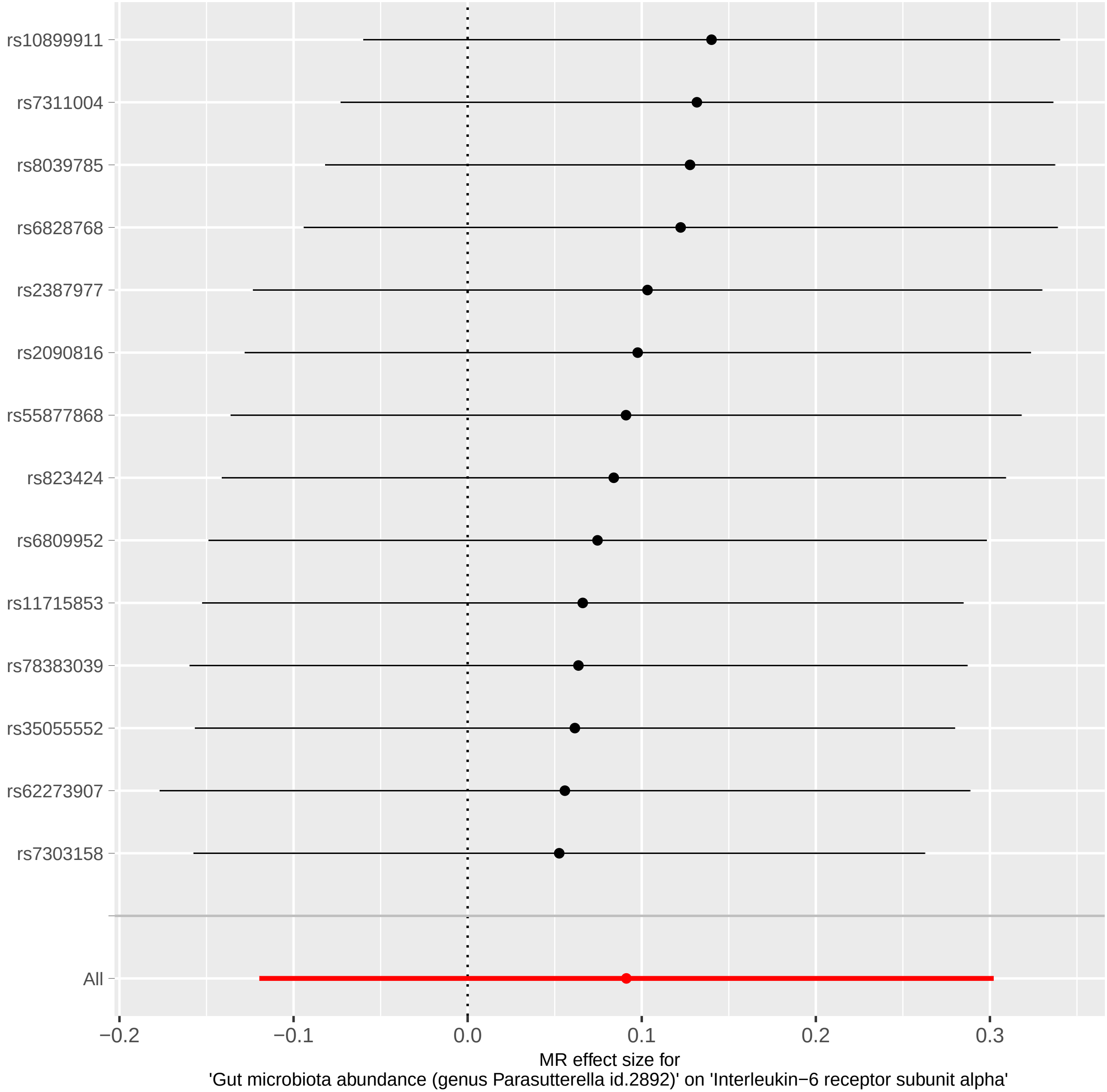

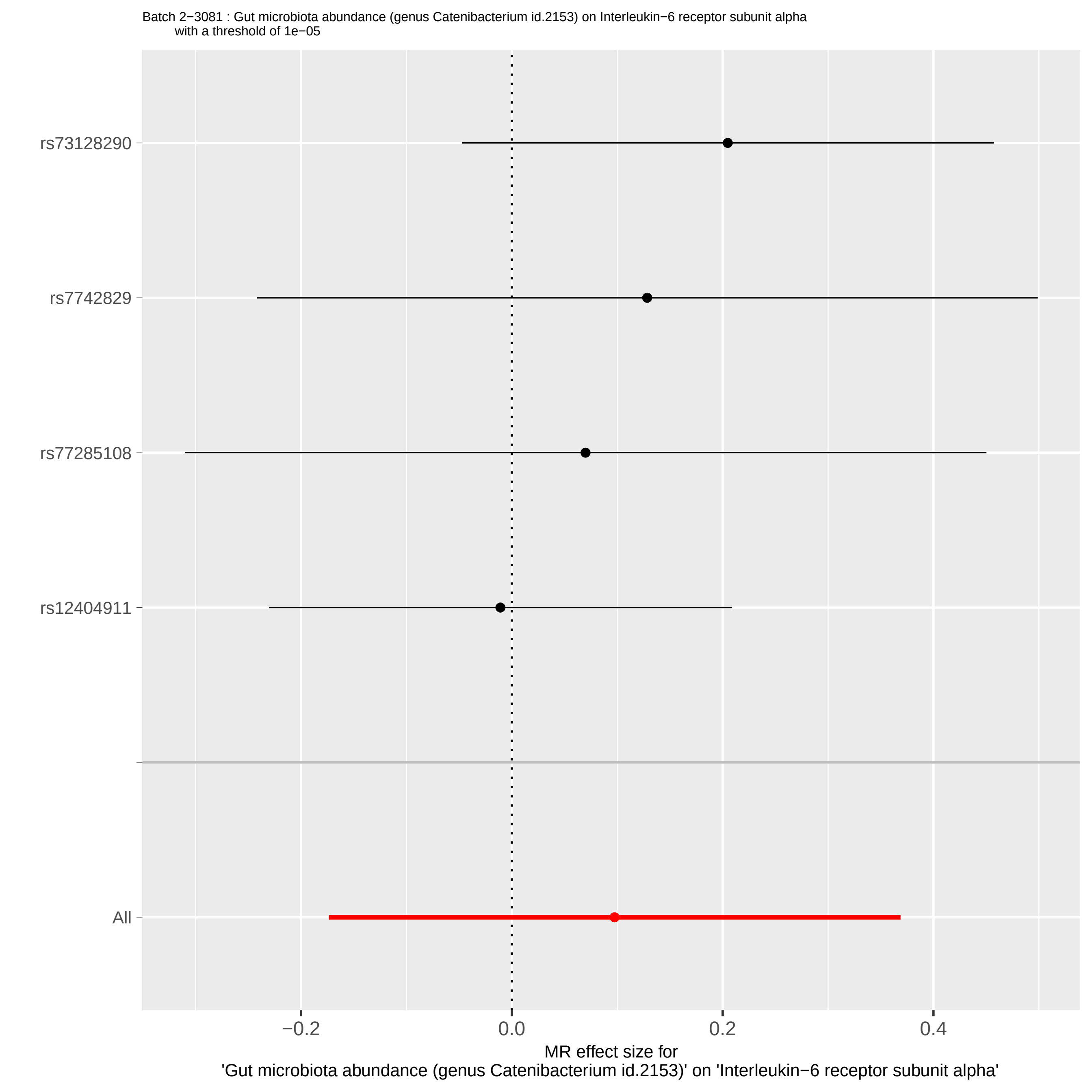
Batch 2−3081 : Gut microbiota abundance (genus Catenibacterium id.2153) on Interleukin−6 receptor subunit alpha
with a threshold of 1e−05
rs73128290
rs7742829
rs77285108
rs12404911
All
−0.2
0.0
0.2
0.4
MR effect size for
'Gut microbiota abundance (genus Catenibacterium id.2153)' on 'Interleukin−6 receptor subunit alpha'

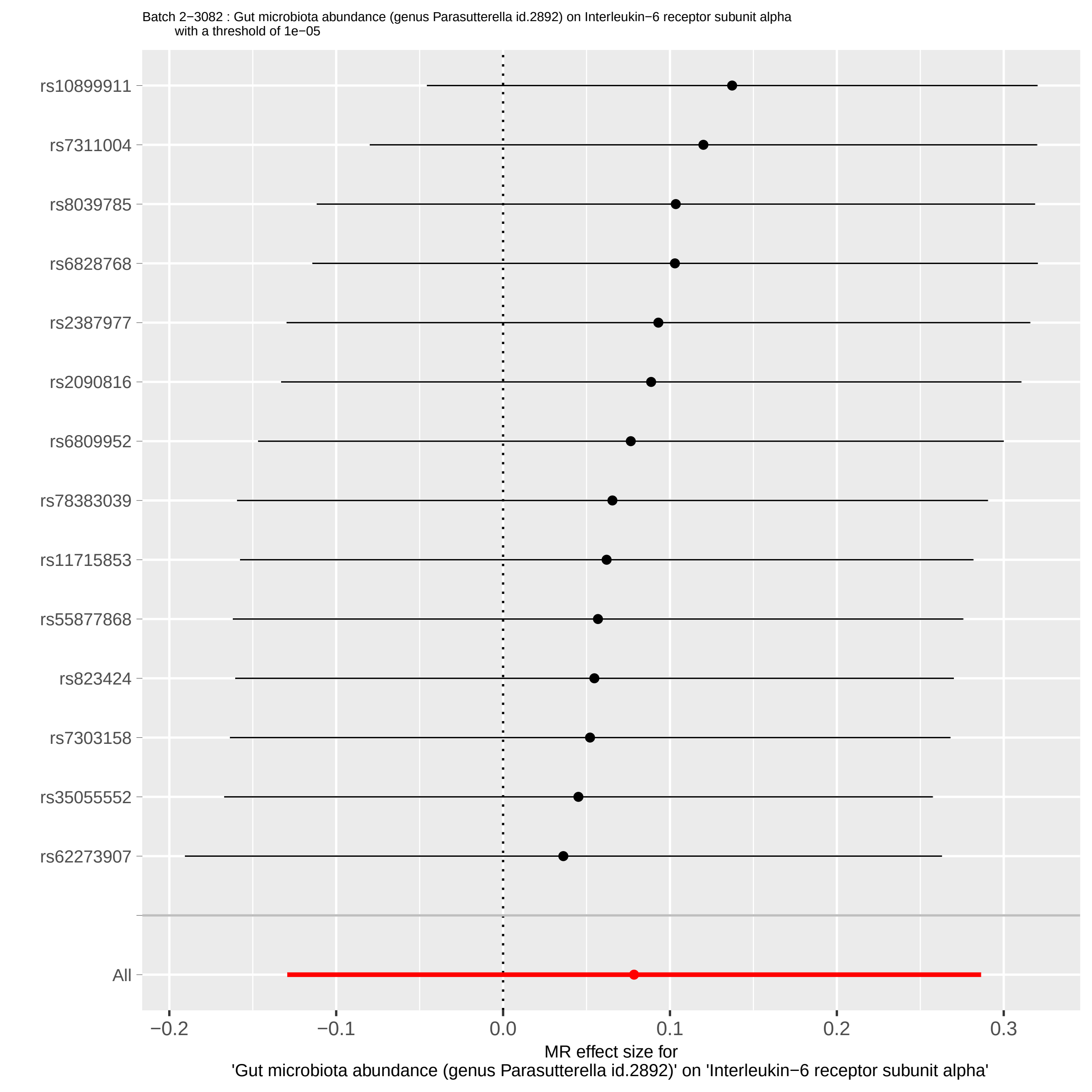

Batch 2−3082 : Gut microbiota abundance (genus Parasutterella id.2892) on Interleukin−6 receptor subunit alpha
with a threshold of 1e−05
rs10899911
rs7311004
rs8039785
rs6828768
rs2387977
rs2090816
rs6809952
rs78383039
rs11715853
rs55877868
rs823424
rs7303158
rs35055552
rs62273907
All
−0.2
−0.1
0.0
0.1
0.2
0.3
MR effect size for
'Gut microbiota abundance (genus Parasutterella id.2892)' on 'Interleukin−6 receptor subunit alpha'

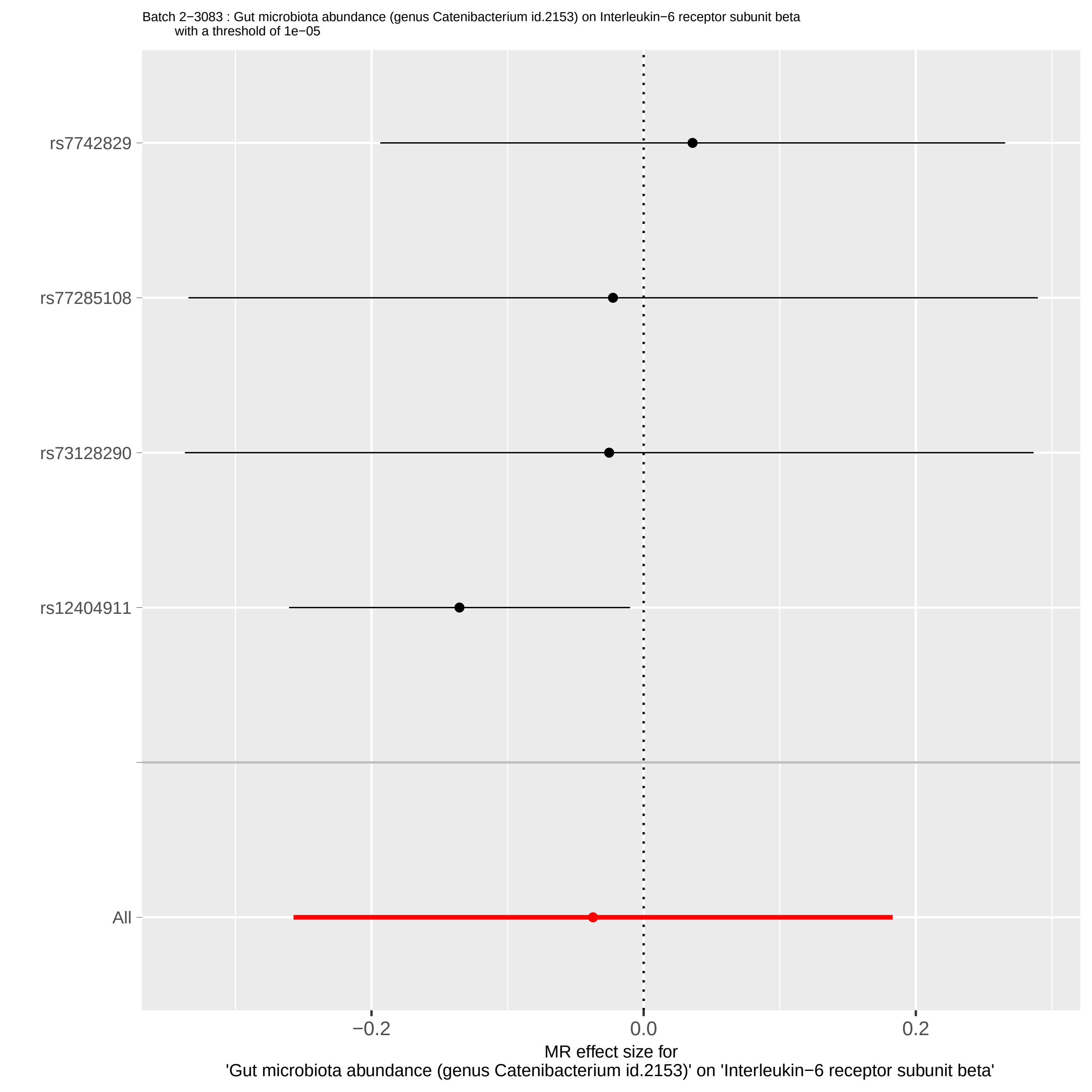

Batch 2−3083 : Gut microbiota abundance (genus Catenibacterium id.2153) on Interleukin−6 receptor subunit beta
with a threshold of 1e−05
rs7742829
rs77285108
rs73128290
rs12404911
All
−0.2
0.0
0.2
MR effect size for
'Gut microbiota abundance (genus Catenibacterium id.2153)' on 'Interleukin−6 receptor subunit beta'

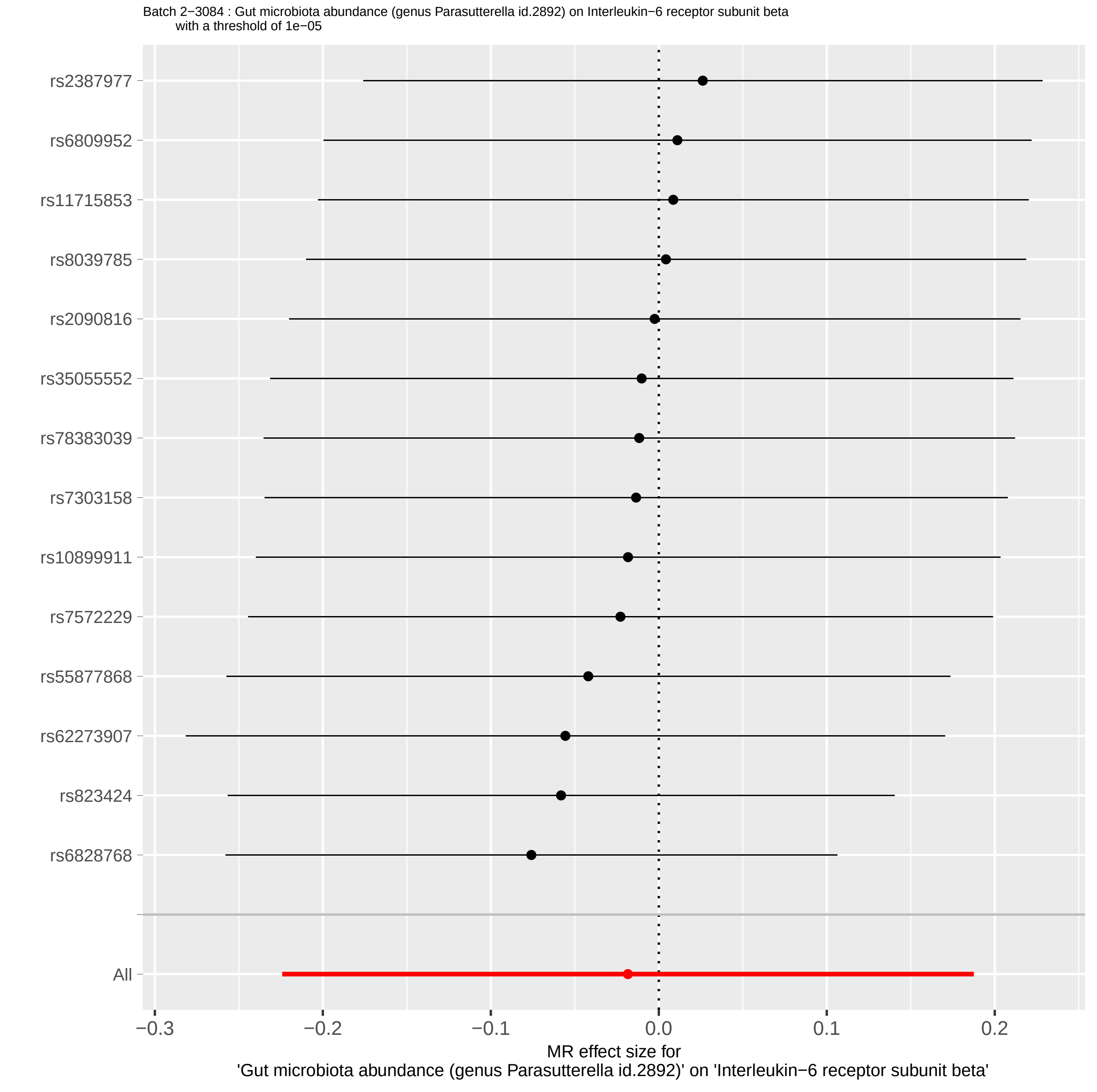
Batch 2−3084 : Gut microbiota abundance (genus Parasutterella id.2892) on Interleukin−6 receptor subunit beta
with a threshold of 1e−05
rs2387977
rs6809952
rs11715853
rs8039785
rs2090816
rs35055552
rs78383039
rs7303158
rs10899911
rs7572229
rs55877868
rs62273907
rs823424
rs6828768
All
−0.3
−0.2
−0.1
0.0
0.1
0.2
MR effect size for
'Gut microbiota abundance (genus Parasutterella id.2892)' on 'Interleukin−6 receptor subunit beta'

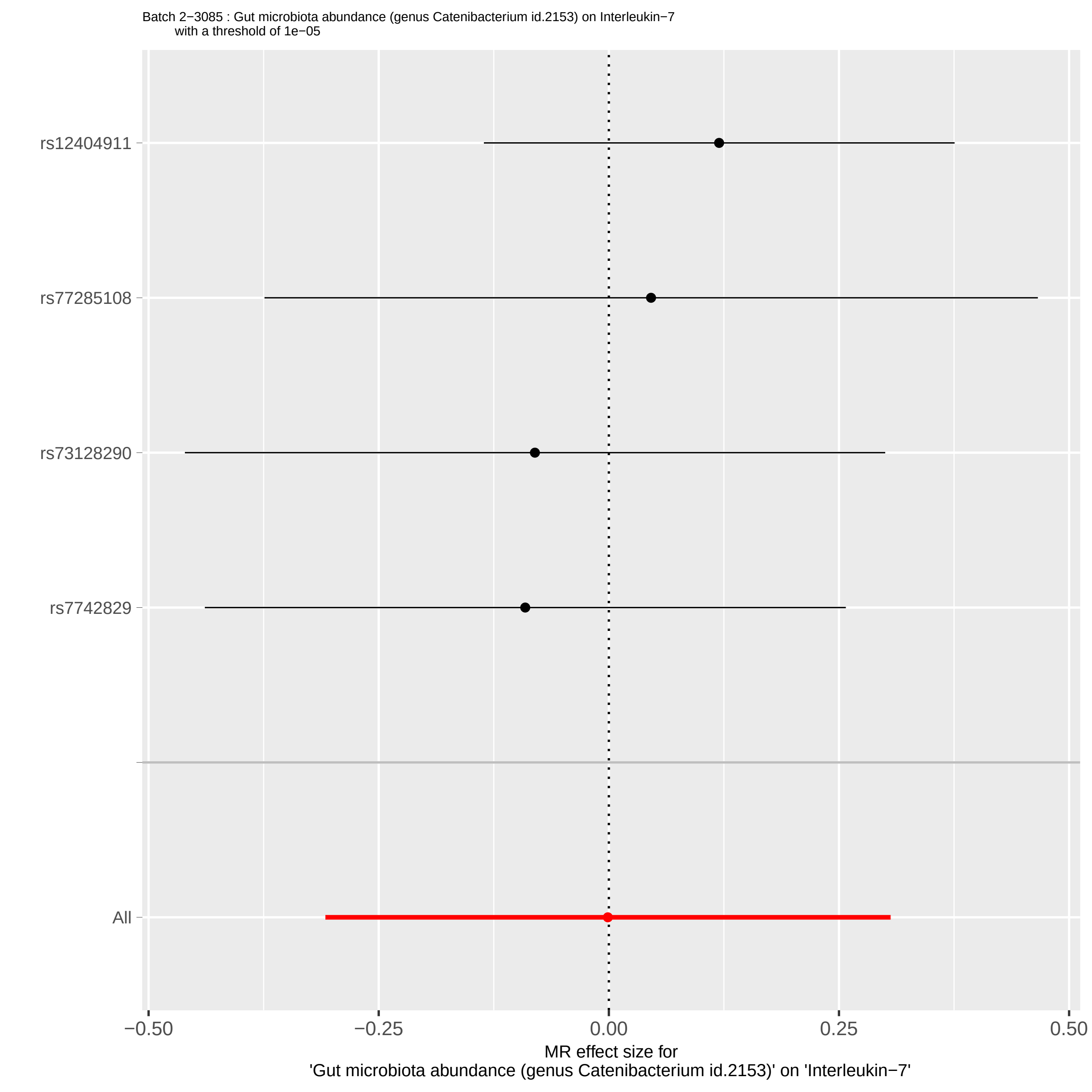

Batch 2−3085 : Gut microbiota abundance (genus Catenibacterium id.2153) on Interleukin−7
with a threshold of 1e−05
rs12404911
rs77285108
rs73128290
rs7742829
All
−0.50
−0.25
0.00
0.25
0.50
MR effect size for
'Gut microbiota abundance (genus Catenibacterium id.2153)' on 'Interleukin−7'

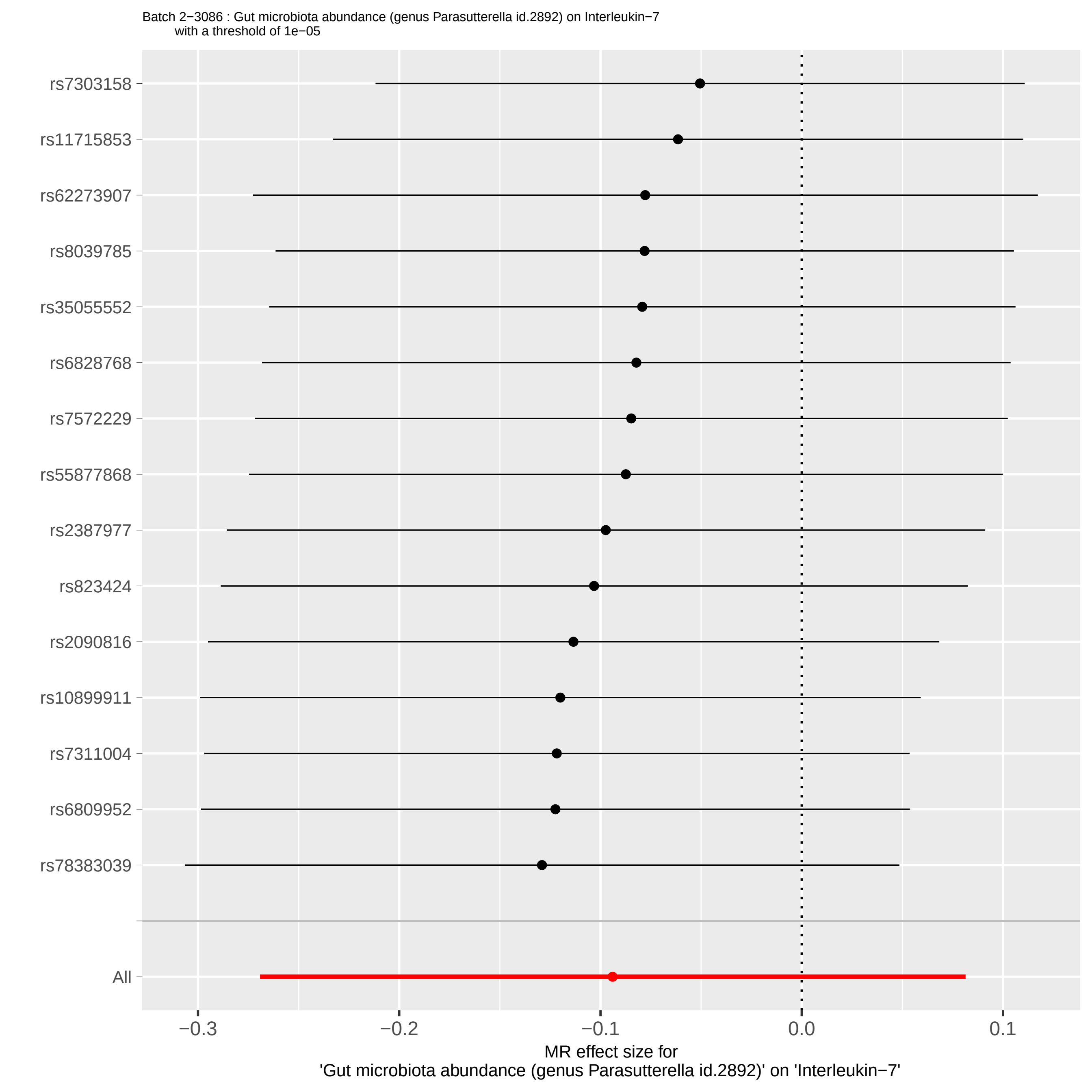

Batch 2−3086 : Gut microbiota abundance (genus Parasutterella id.2892) on Interleukin−7
with a threshold of 1e−05
rs7303158
rs11715853
rs62273907
rs8039785
rs35055552
rs6828768
rs7572229
rs55877868
rs2387977
rs823424
rs2090816
rs10899911
rs7311004
rs6809952
rs78383039
All
−0.3
−0.2
−0.1
0.0
0.1
MR effect size for
'Gut microbiota abundance (genus Parasutterella id.2892)' on 'Interleukin−7'

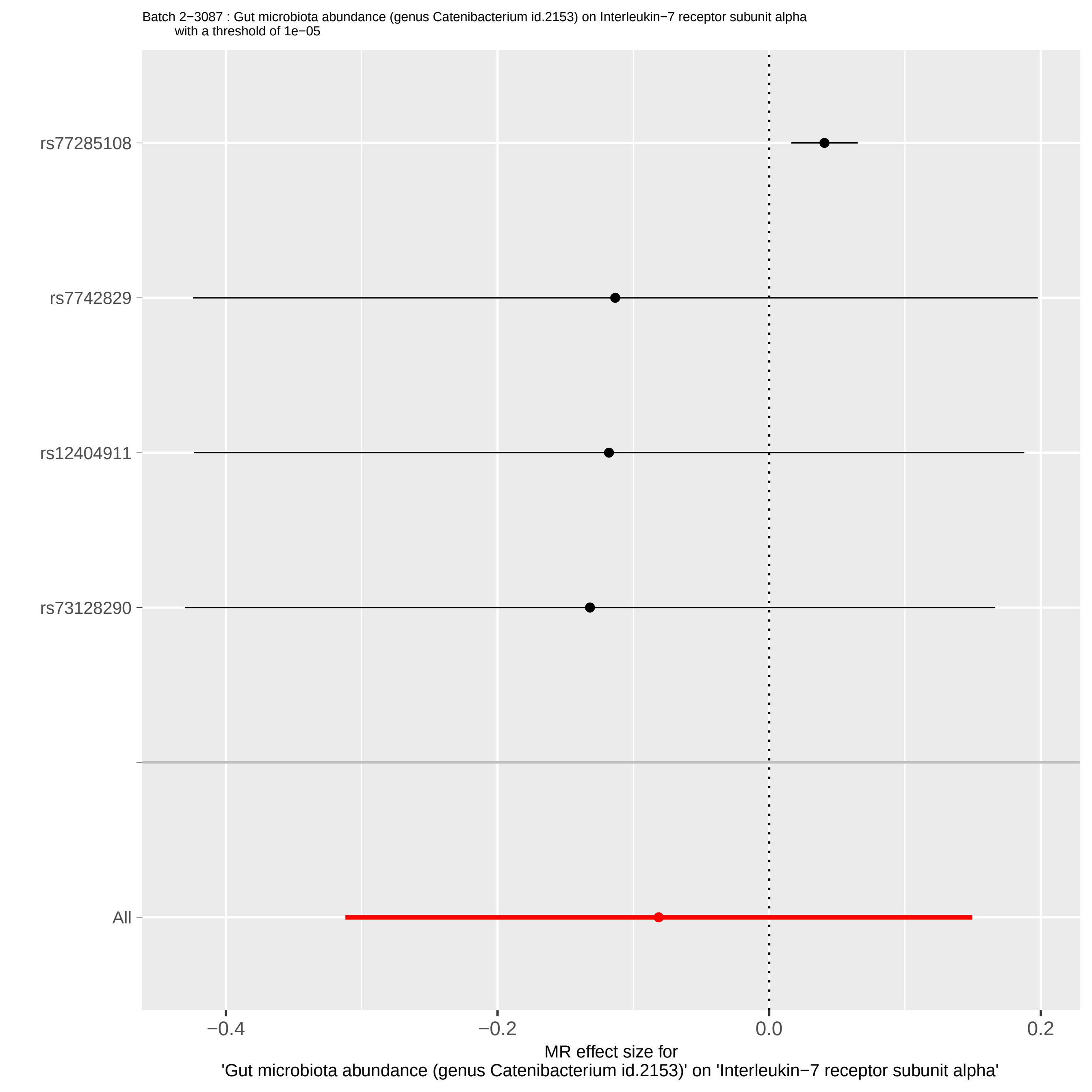
Batch 2−3087 : Gut microbiota abundance (genus Catenibacterium id.2153) on Interleukin−7 receptor subunit alpha
with a threshold of 1e−05
rs77285108
rs7742829
rs12404911
rs73128290
All
−0.4
−0.2
0.0
0.2
MR effect size for
'Gut microbiota abundance (genus Catenibacterium id.2153)' on 'Interleukin−7 receptor subunit alpha'

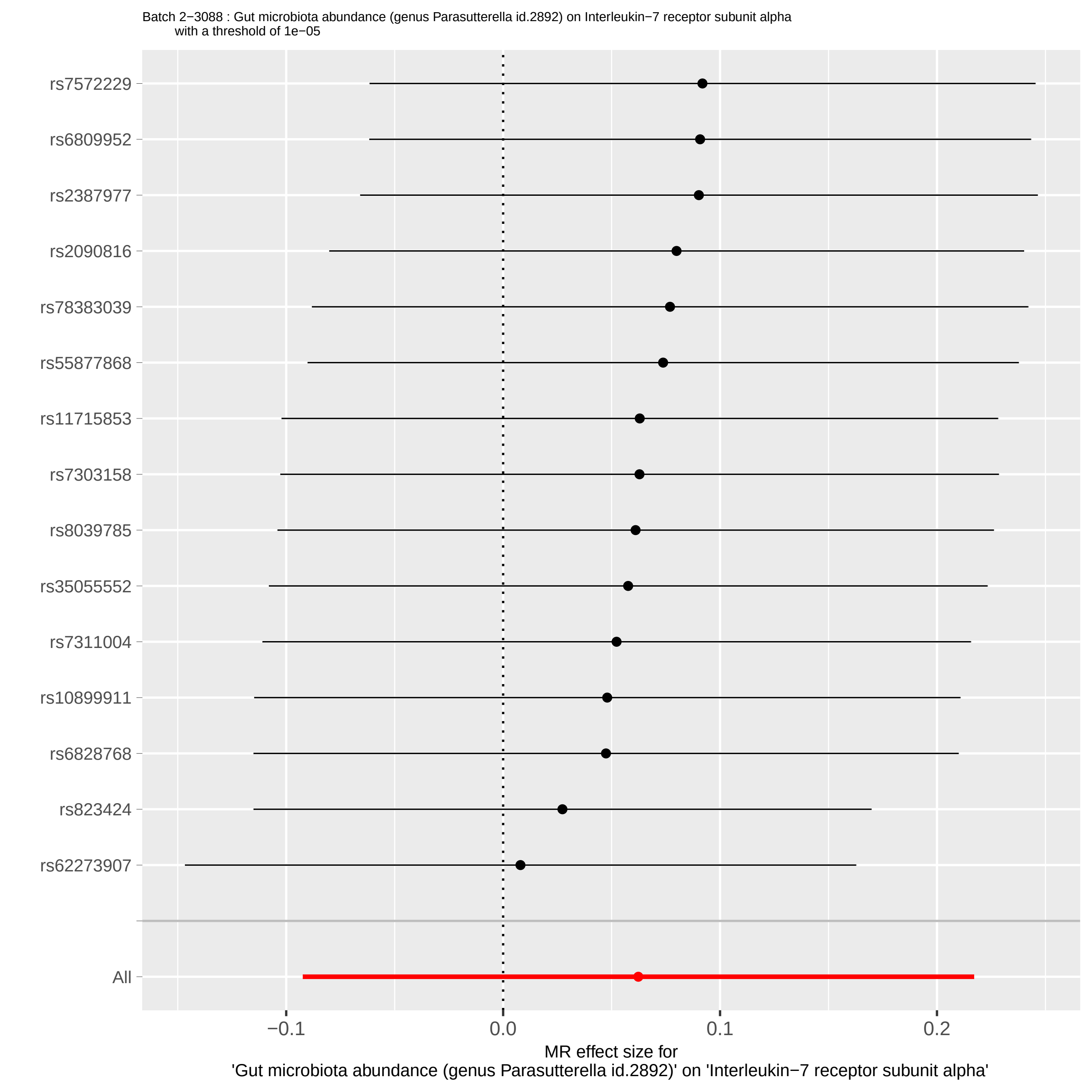

Batch 2−3088 : Gut microbiota abundance (genus Parasutterella id.2892) on Interleukin−7 receptor subunit alpha
with a threshold of 1e−05
rs7572229
rs6809952
rs2387977
rs2090816
rs78383039
rs55877868
rs11715853
rs7303158
rs8039785
rs35055552
rs7311004
rs10899911
rs6828768
rs823424
rs62273907
All
−0.1
0.0
0.1
0.2
MR effect size for
'Gut microbiota abundance (genus Parasutterella id.2892)' on 'Interleukin−7 receptor subunit alpha'

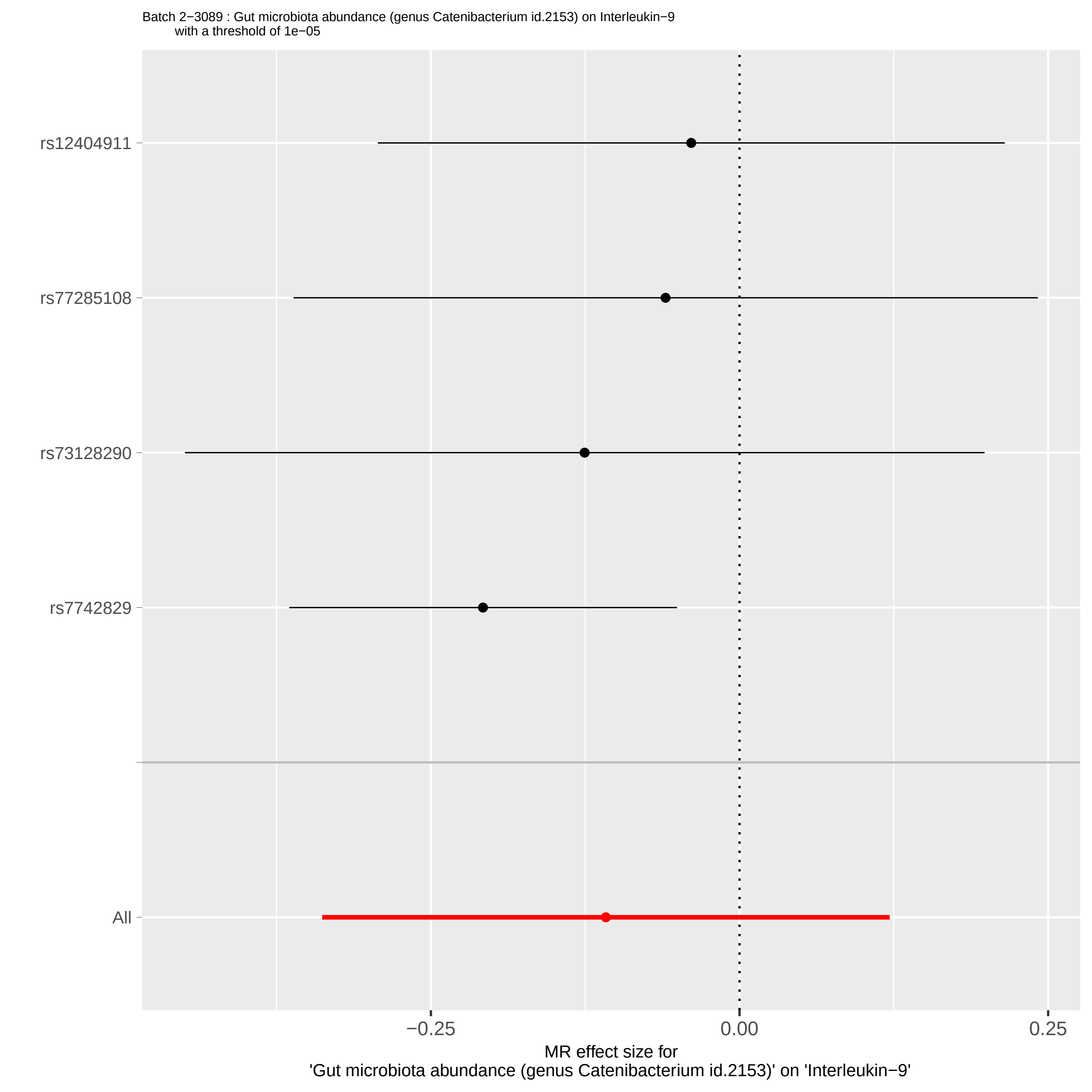
Batch 2−3089 : Gut microbiota abundance (genus Catenibacterium id.2153) on Interleukin−9
with a threshold of 1e−05
rs12404911
rs77285108
rs73128290
rs7742829
All
−0.25
0.00
0.25
MR effect size for
'Gut microbiota abundance (genus Catenibacterium id.2153)' on 'Interleukin−9'

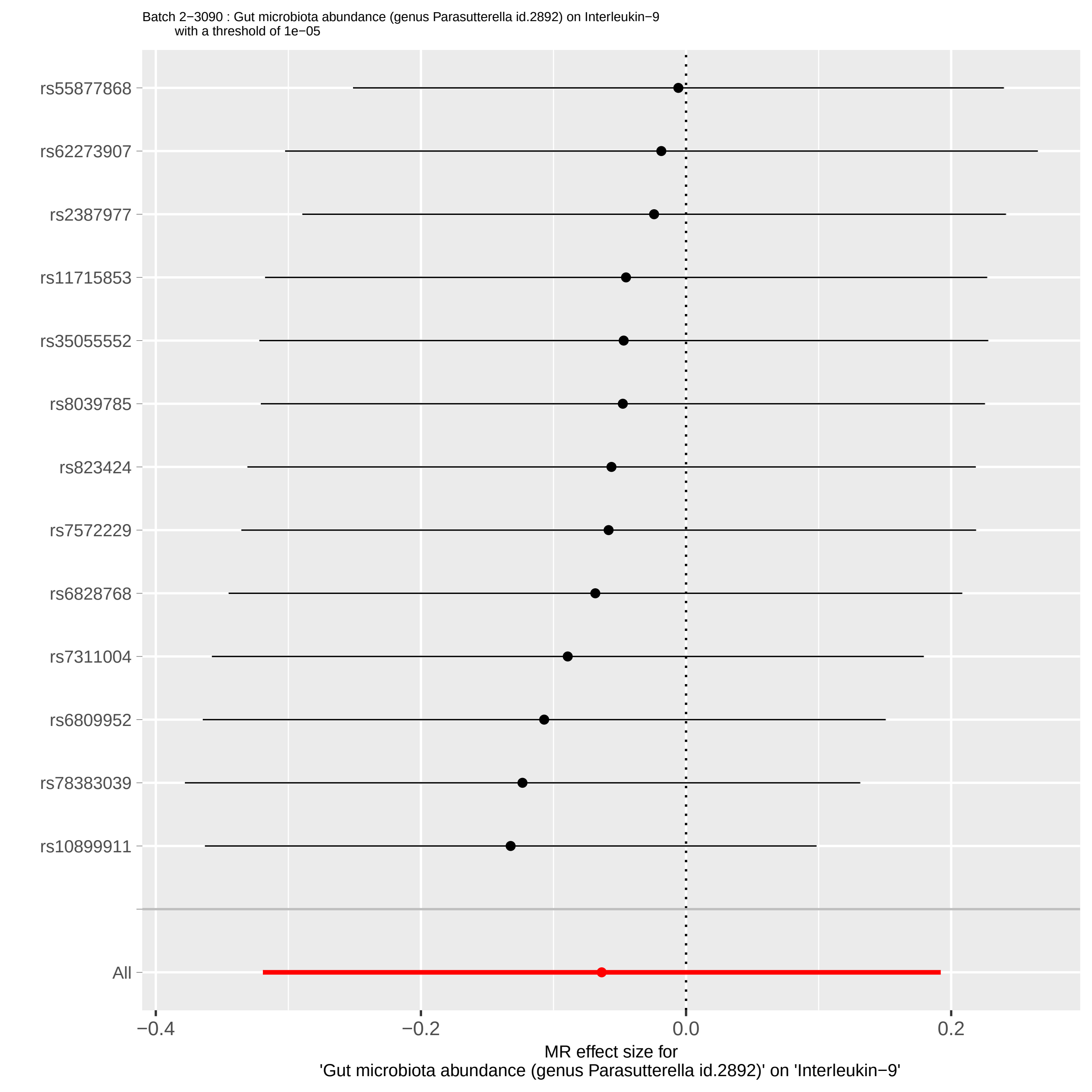
Batch 2−3090 : Gut microbiota abundance (genus Parasutterella id.2892) on Interleukin−9
with a threshold of 1e−05
rs55877868
rs62273907
rs2387977
rs11715853
rs35055552
rs8039785
rs823424
rs7572229
rs6828768
rs7311004
rs6809952
rs78383039
rs10899911
All
−0.4
−0.2
0.0
0.2
MR effect size for
'Gut microbiota abundance (genus Parasutterella id.2892)' on 'Interleukin−9'

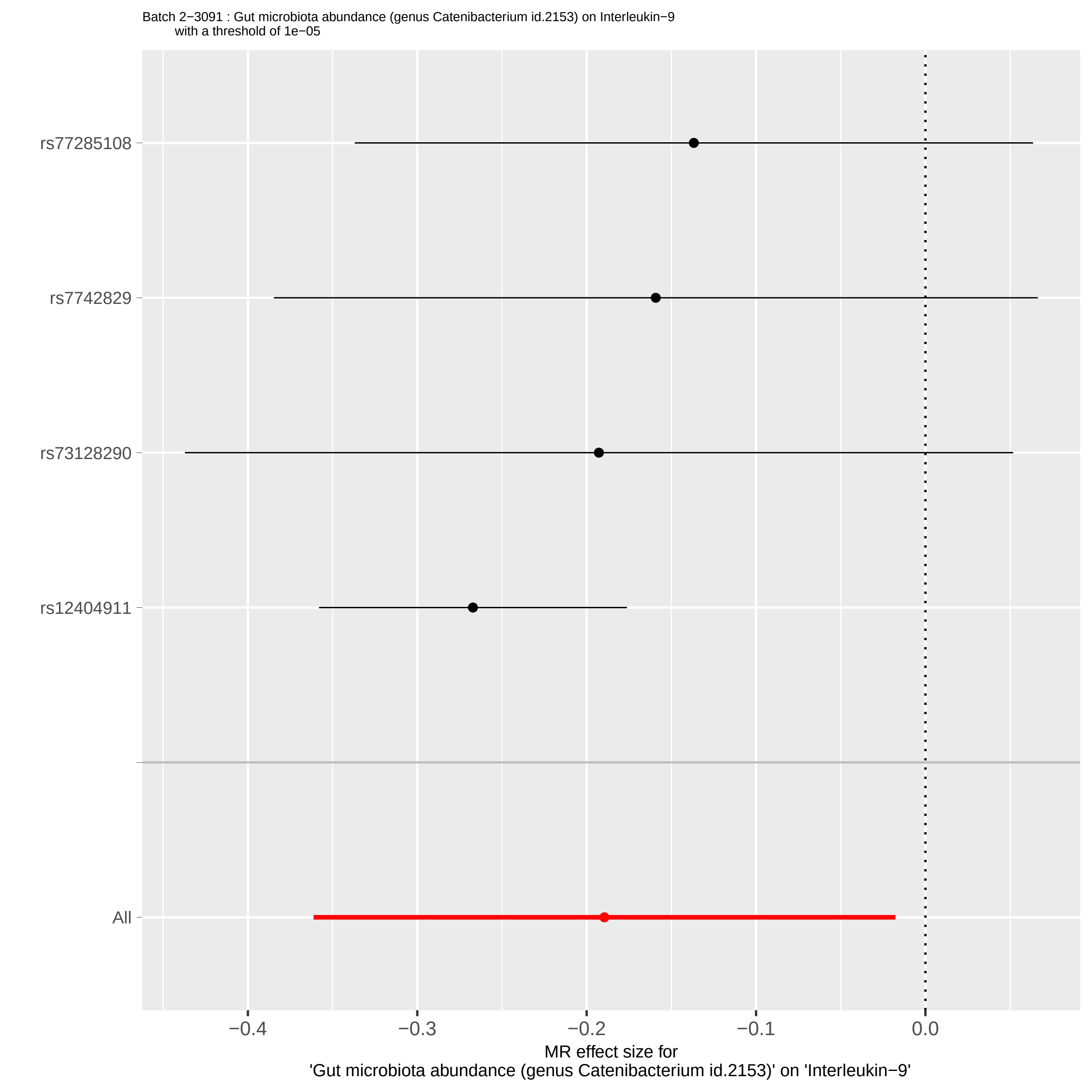
Batch 2−3091 : Gut microbiota abundance (genus Catenibacterium id.2153) on Interleukin−9
with a threshold of 1e−05
rs77285108
rs7742829
rs73128290
rs12404911
All
−0.4
−0.3
−0.2
−0.1
0.0
MR effect size for
'Gut microbiota abundance (genus Catenibacterium id.2153)' on 'Interleukin−9'

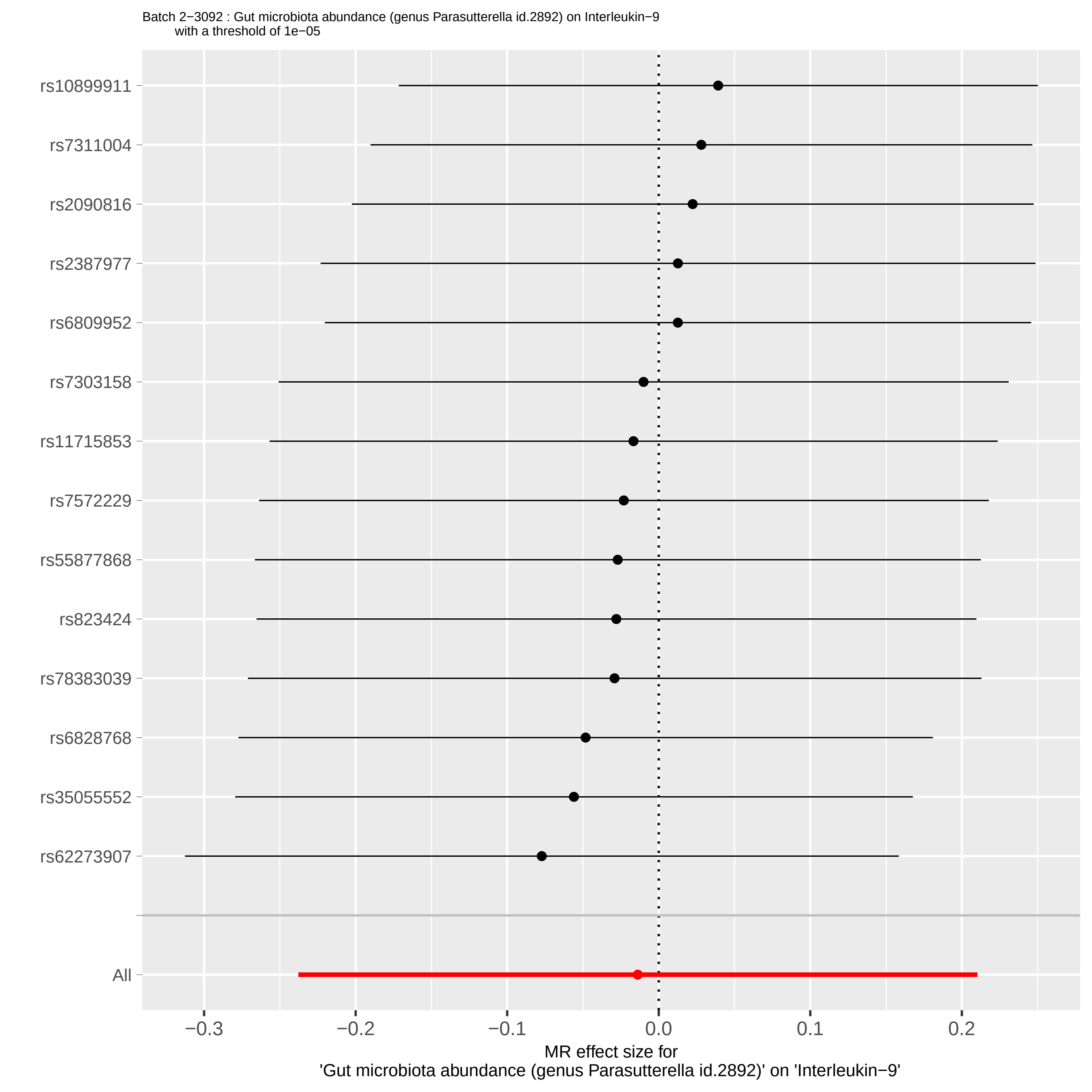

Batch 2−3092 : Gut microbiota abundance (genus Parasutterella id.2892) on Interleukin−9
with a threshold of 1e−05
rs10899911
rs7311004
rs2090816
rs2387977
rs6809952
rs7303158
rs11715853
rs7572229
rs55877868
rs823424
rs78383039
rs6828768
rs35055552
rs62273907
All
−0.3
−0.2
−0.1
0.0
0.1
0.2
MR effect size for
'Gut microbiota abundance (genus Parasutterella id.2892)' on 'Interleukin−9'

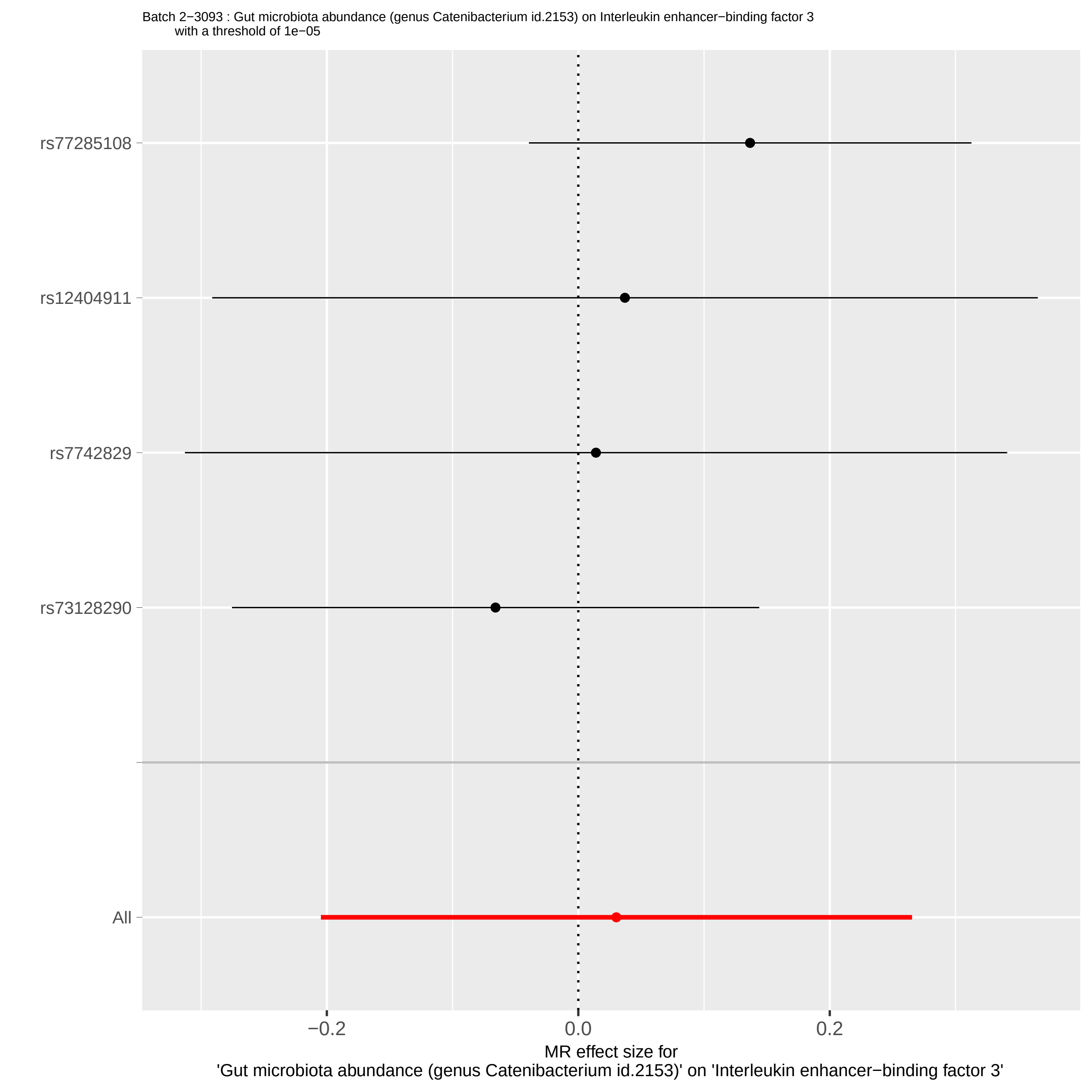
Batch 2−3093 : Gut microbiota abundance (genus Catenibacterium id.2153) on Interleukin enhancer−binding factor 3
with a threshold of 1e−05
rs77285108
rs12404911
rs7742829
rs73128290
All
−0.2
0.0
0.2
MR effect size for
'Gut microbiota abundance (genus Catenibacterium id.2153)' on 'Interleukin enhancer−binding factor 3'

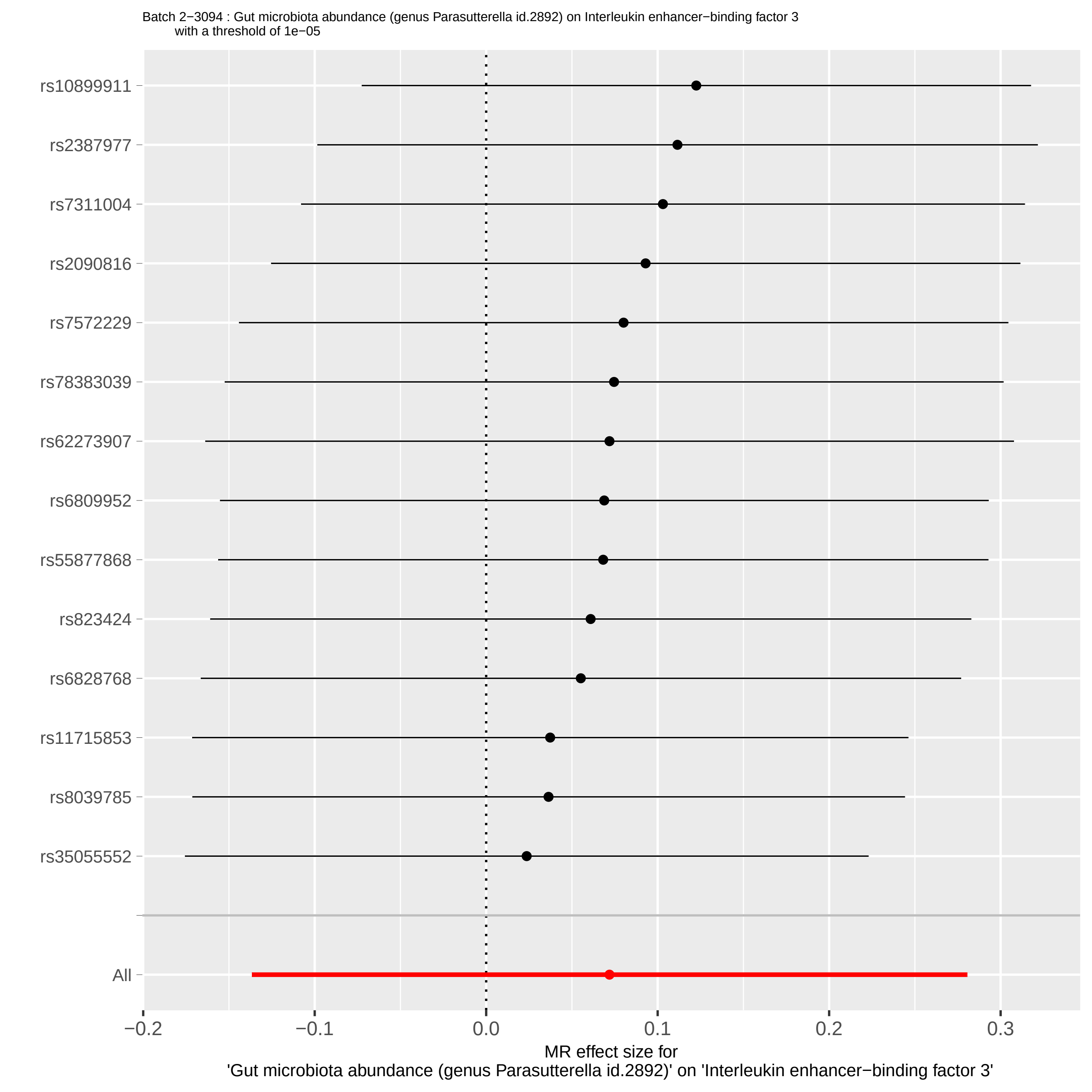
Batch 2−3094 : Gut microbiota abundance (genus Parasutterella id.2892) on Interleukin enhancer−binding factor 3
with a threshold of 1e−05
rs10899911
rs2387977
rs7311004
rs2090816
rs7572229
rs78383039
rs62273907
rs6809952
rs55877868
rs823424
rs6828768
rs11715853
rs8039785
rs35055552
All
−0.2
−0.1
0.0
0.1
0.2
0.3
MR effect size for
'Gut microbiota abundance (genus Parasutterella id.2892)' on 'Interleukin enhancer−binding factor 3'

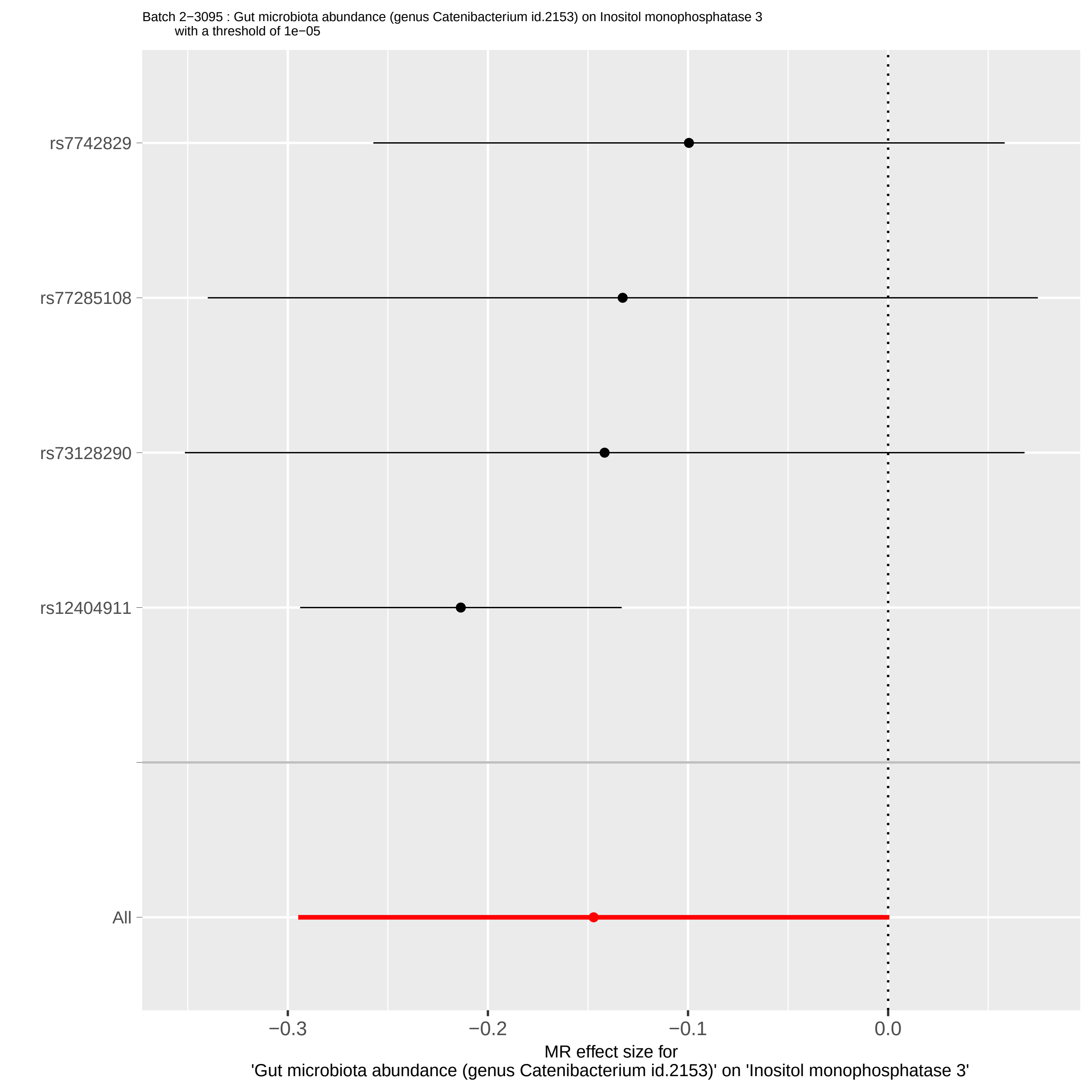

Batch 2−3095 : Gut microbiota abundance (genus Catenibacterium id.2153) on Inositol monophosphatase 3
with a threshold of 1e−05
rs7742829
rs77285108
rs73128290
rs12404911
All
−0.3
−0.2
−0.1
0.0
MR effect size for
'Gut microbiota abundance (genus Catenibacterium id.2153)' on 'Inositol monophosphatase 3'

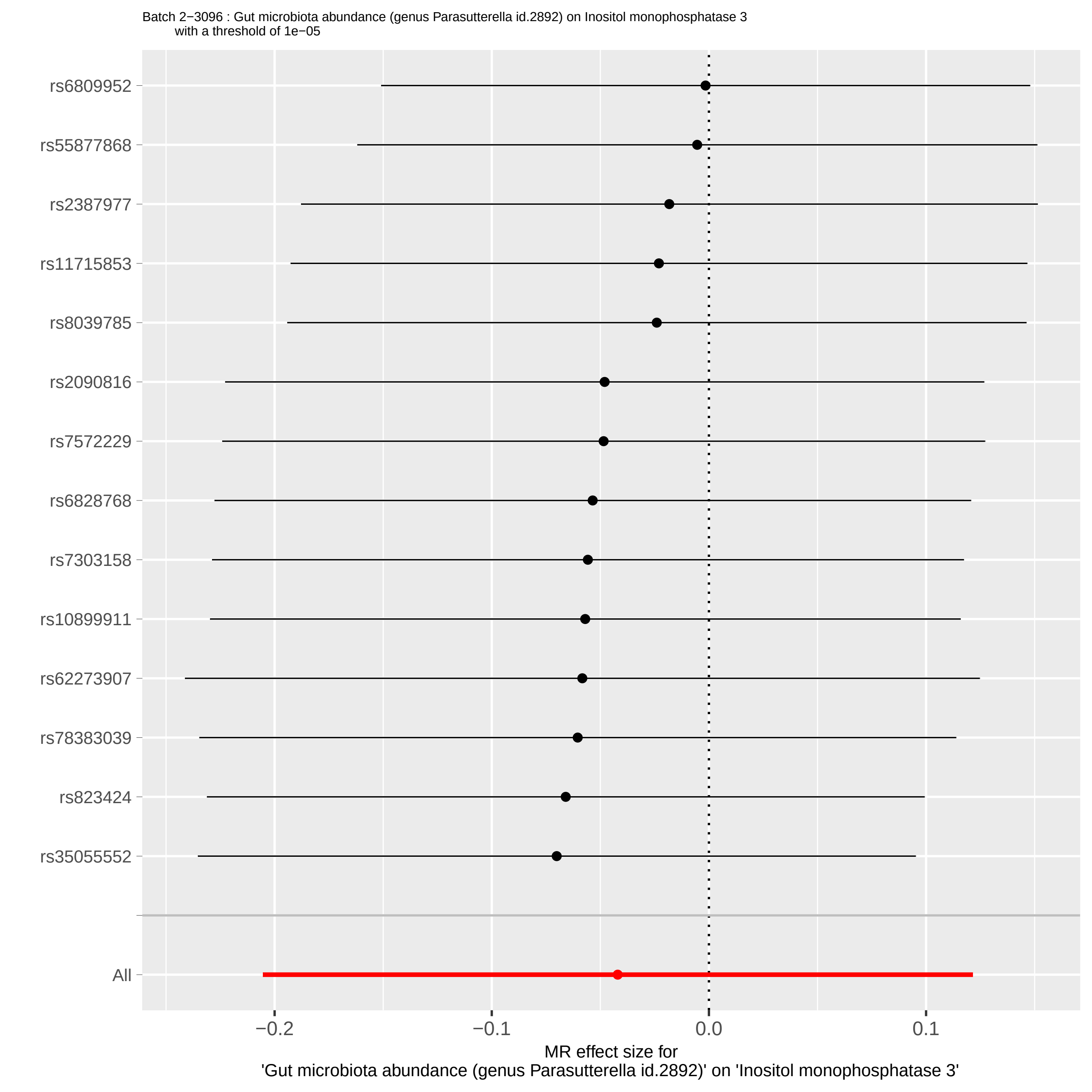

Batch 2−3096 : Gut microbiota abundance (genus Parasutterella id.2892) on Inositol monophosphatase 3
with a threshold of 1e−05
rs6809952
rs55877868
rs2387977
rs11715853
rs8039785
rs2090816
rs7572229
rs6828768
rs7303158
rs10899911
rs62273907
rs78383039
rs823424
rs35055552
All
−0.2
−0.1
0.0
0.1
MR effect size for
'Gut microbiota abundance (genus Parasutterella id.2892)' on 'Inositol monophosphatase 3'

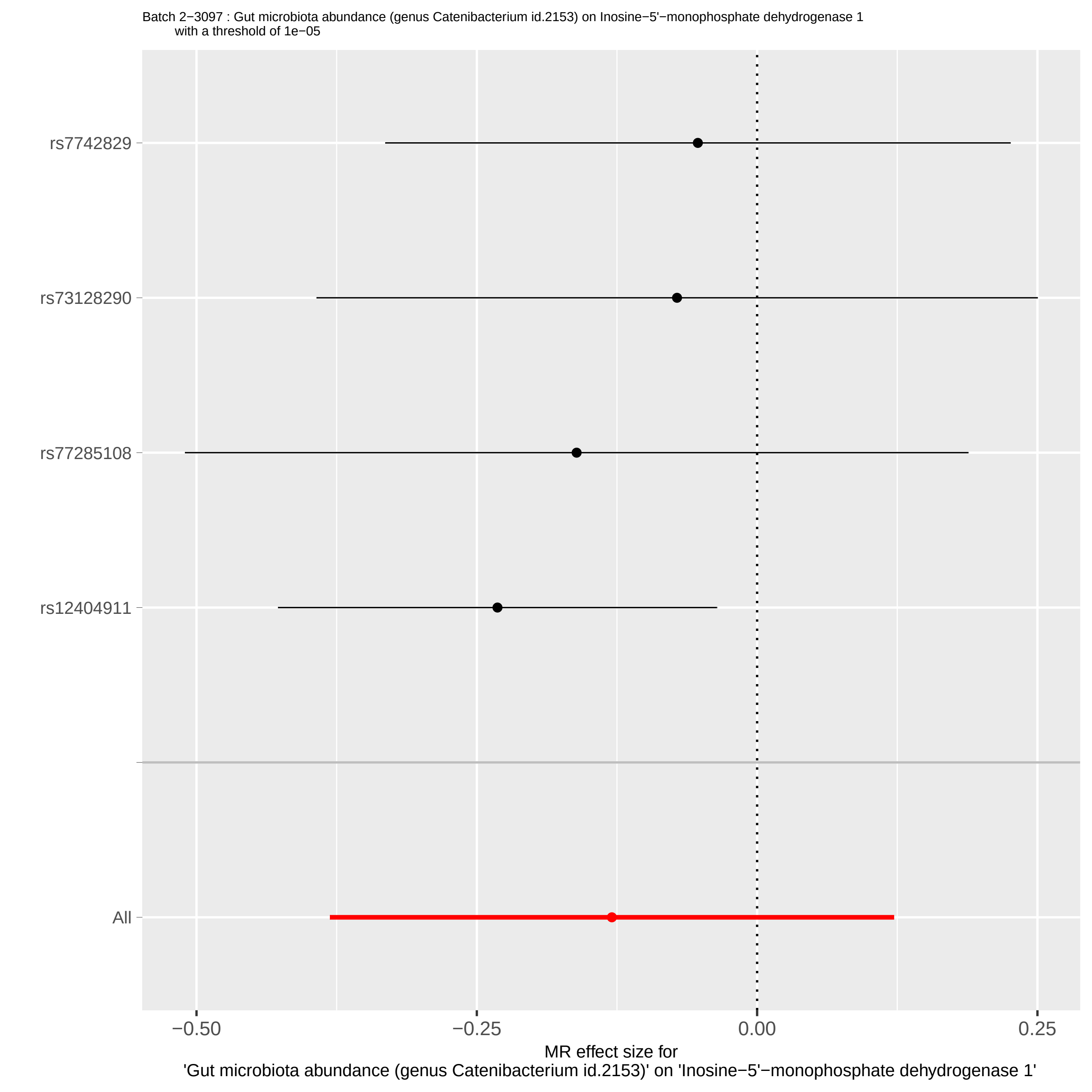
Batch 2−3097 : Gut microbiota abundance (genus Catenibacterium id.2153) on Inosine−5'−monophosphate dehydrogenase 1
with a threshold of 1e−05
rs7742829
rs73128290
rs77285108
rs12404911
All
−0.50
−0.25
0.00
0.25
MR effect size for
'Gut microbiota abundance (genus Catenibacterium id.2153)' on 'Inosine−5'−monophosphate dehydrogenase 1'

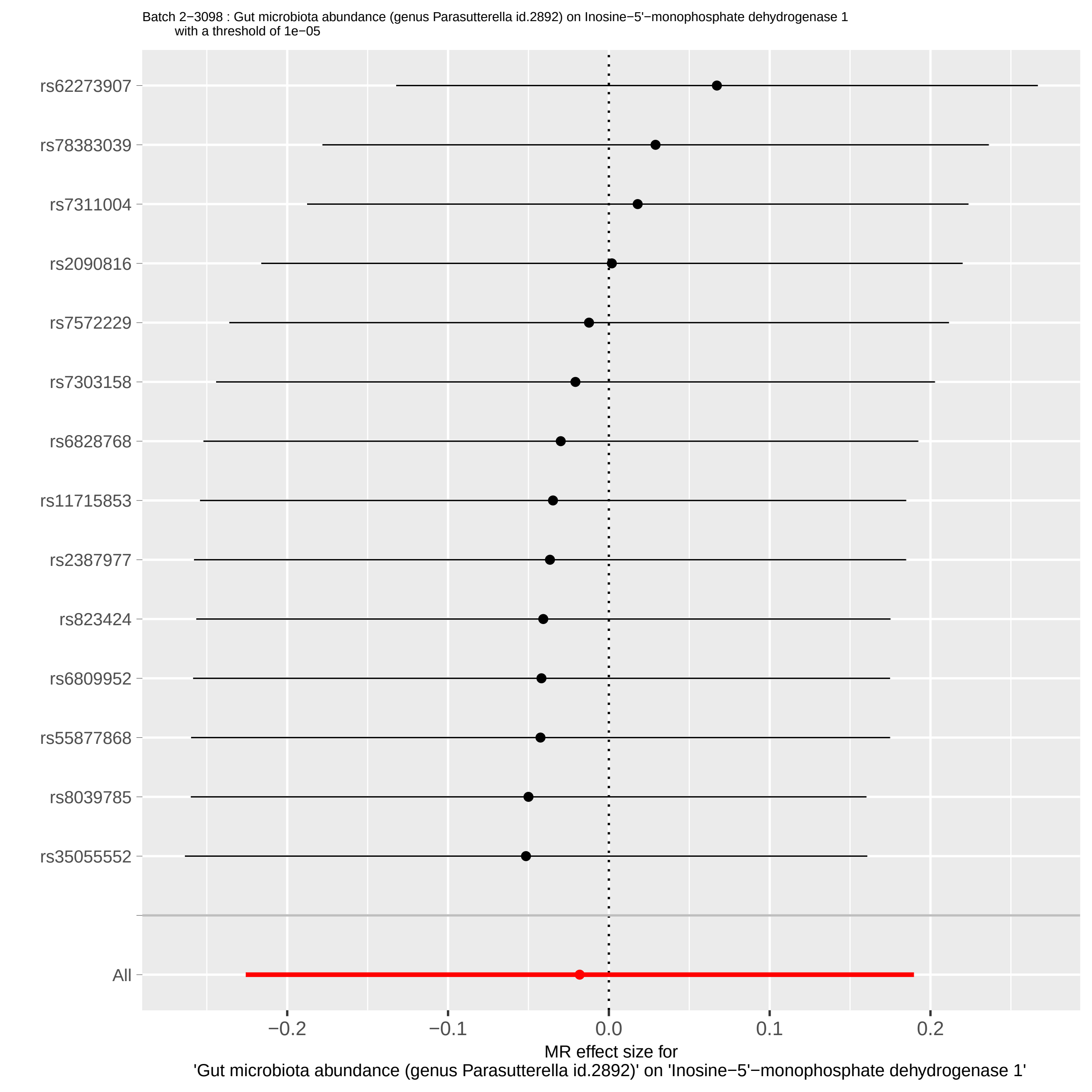

Batch 2−3098 : Gut microbiota abundance (genus Parasutterella id.2892) on Inosine−5'−monophosphate dehydrogenase 1
with a threshold of 1e−05
rs62273907
rs78383039
rs7311004
rs2090816
rs7572229
rs7303158
rs6828768
rs11715853
rs2387977
rs823424
rs6809952
rs55877868
rs8039785
rs35055552
All
−0.2
−0.1
0.0
0.1
0.2
MR effect size for
'Gut microbiota abundance (genus Parasutterella id.2892)' on 'Inosine−5'−monophosphate dehydrogenase 1'

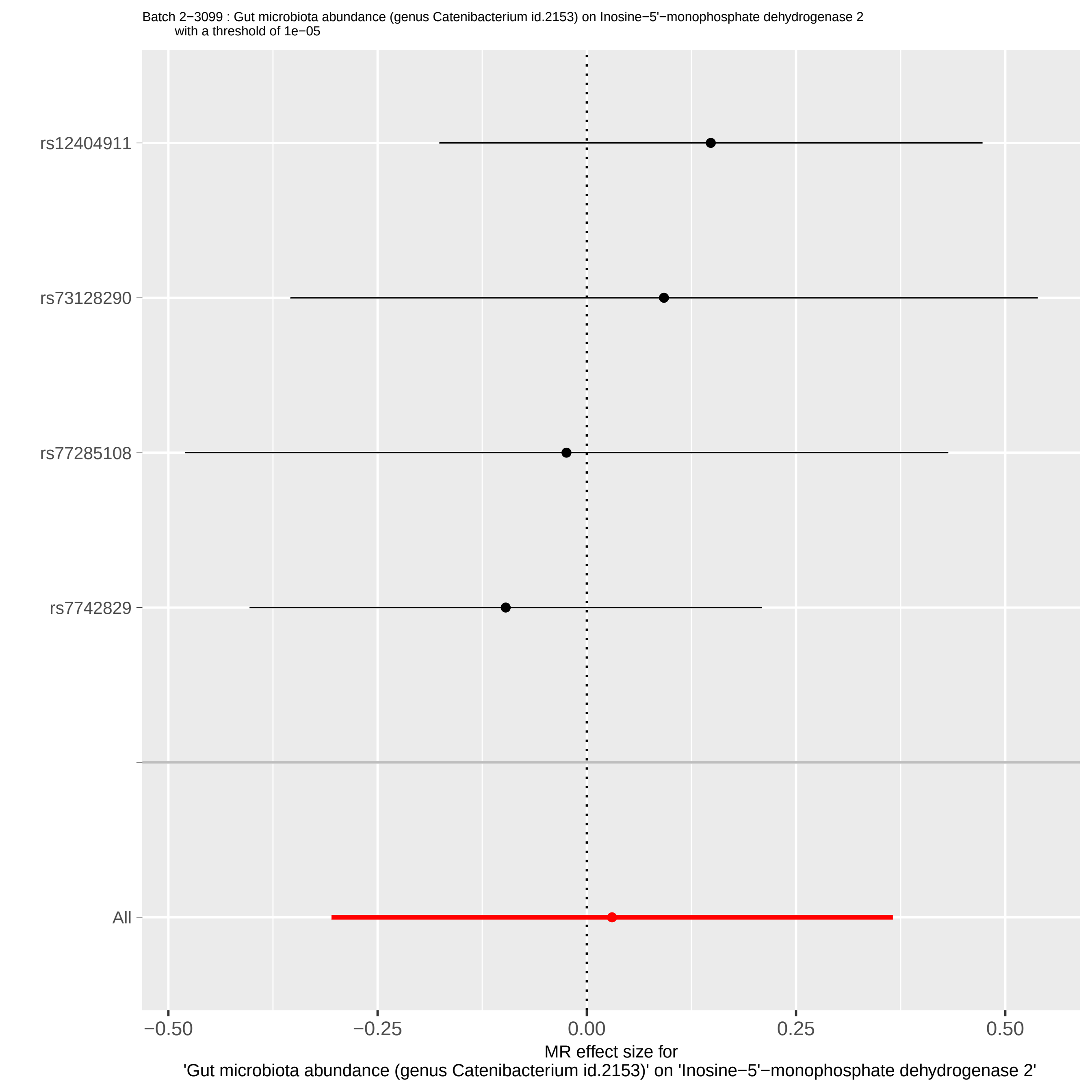

Batch 2−3099 : Gut microbiota abundance (genus Catenibacterium id.2153) on Inosine−5'−monophosphate dehydrogenase 2
with a threshold of 1e−05
rs12404911
rs73128290
rs77285108
rs7742829
All
−0.50
−0.25
0.00
0.25
0.50
MR effect size for
'Gut microbiota abundance (genus Catenibacterium id.2153)' on 'Inosine−5'−monophosphate dehydrogenase 2'

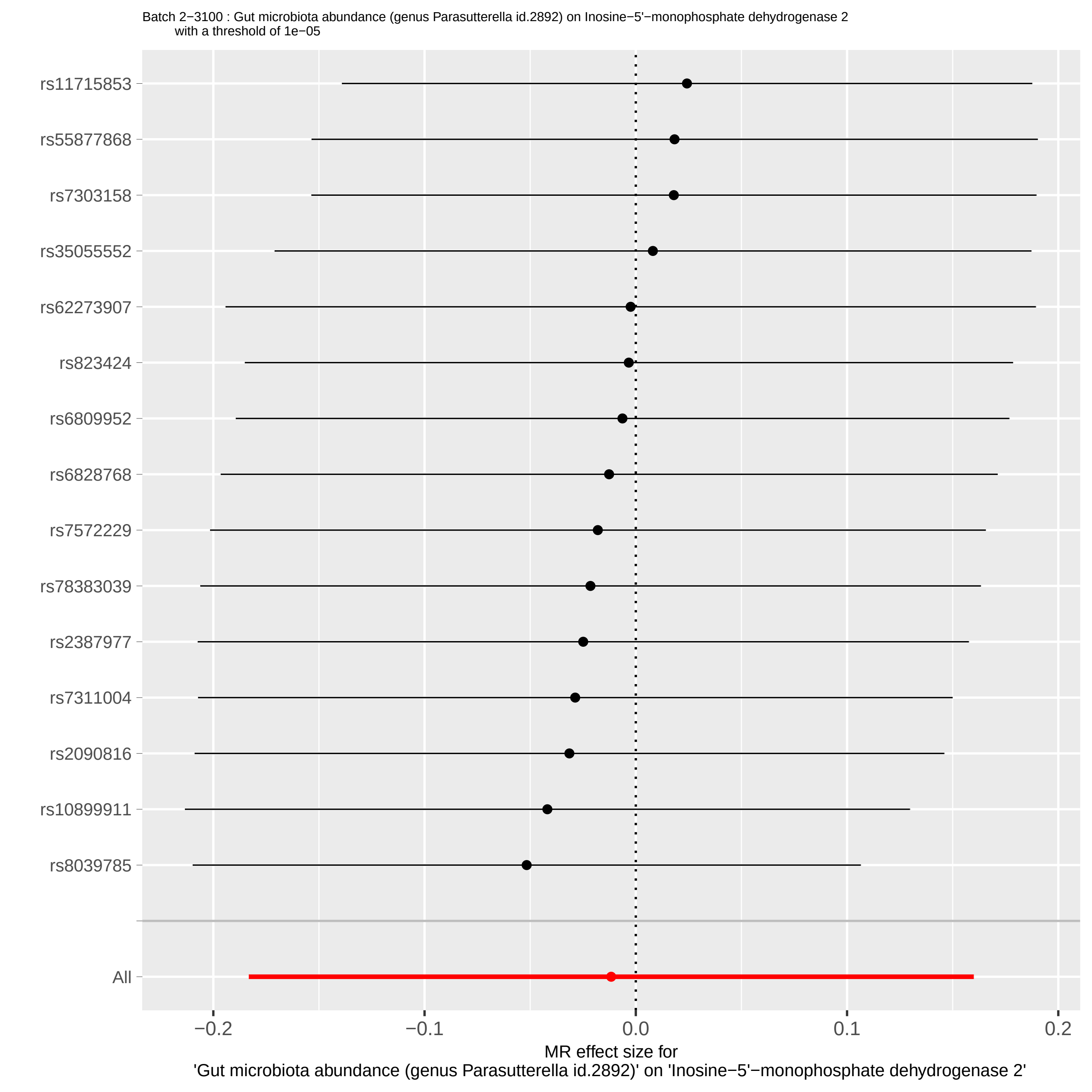

Batch 2−3100 : Gut microbiota abundance (genus Parasutterella id.2892) on Inosine−5'−monophosphate dehydrogenase 2
with a threshold of 1e−05
rs11715853
rs55877868
rs7303158
rs35055552
rs62273907
rs823424
rs6809952
rs6828768
rs7572229
rs78383039
rs2387977
rs7311004
rs2090816
rs10899911
rs8039785
All
−0.2
−0.1
0.0
0.1
0.2
MR effect size for
'Gut microbiota abundance (genus Parasutterella id.2892)' on 'Inosine−5'−monophosphate dehydrogenase 2'

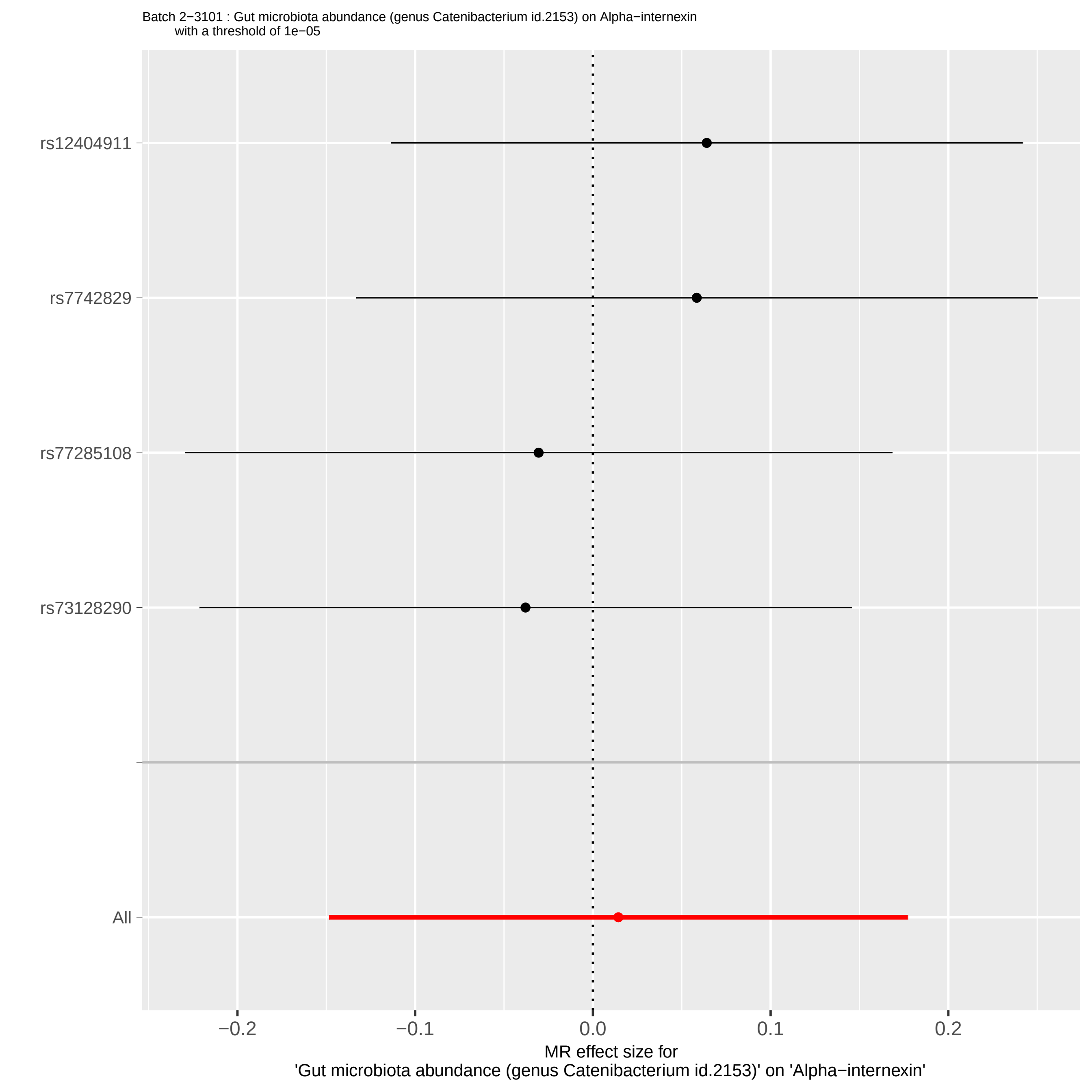
Batch 2−3101 : Gut microbiota abundance (genus Catenibacterium id.2153) on Alpha−internexin
with a threshold of 1e−05
rs12404911
rs7742829
rs77285108
rs73128290
All
−0.2
−0.1
0.0
0.1
0.2
MR effect size for
'Gut microbiota abundance (genus Catenibacterium id.2153)' on 'Alpha−internexin'

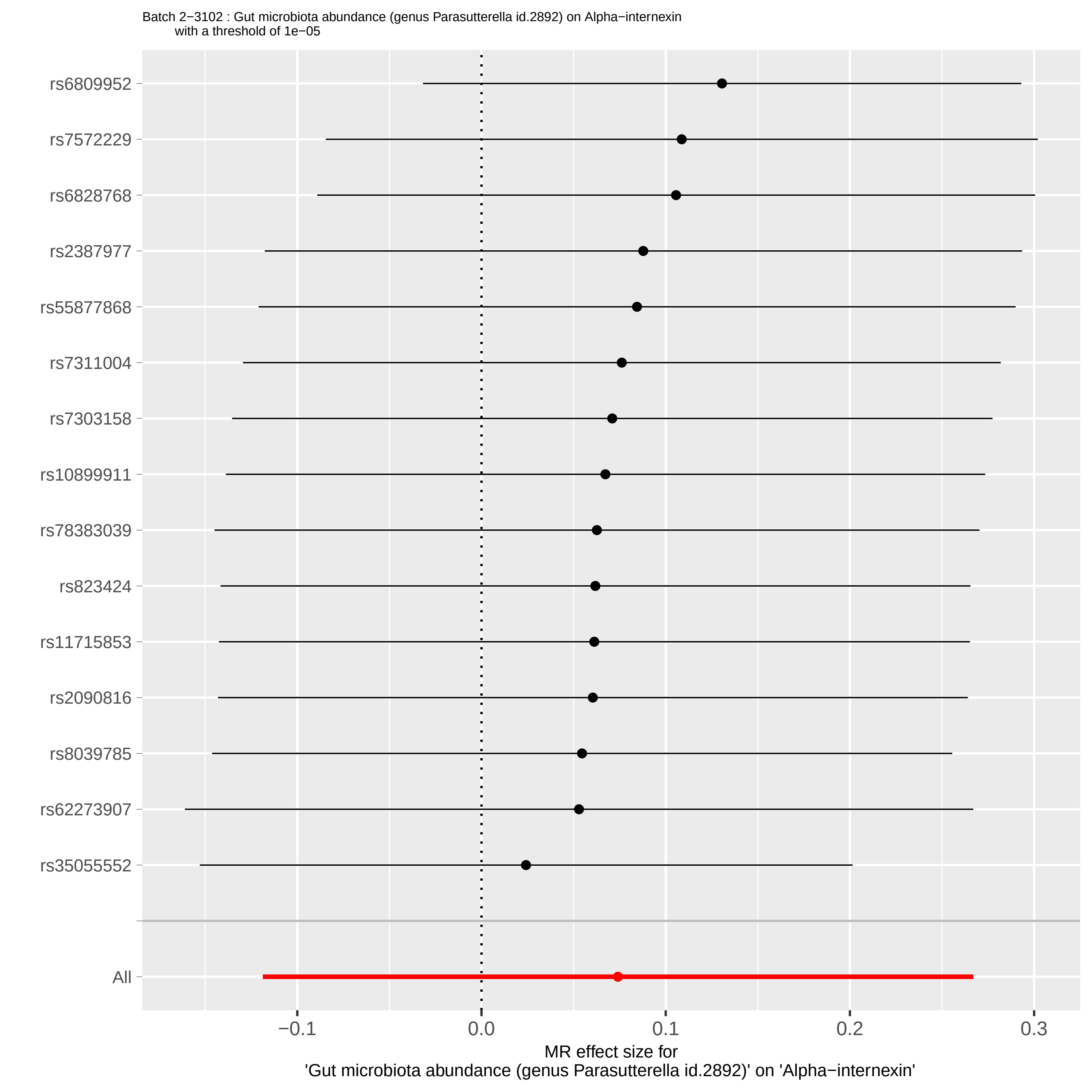

Batch 2−3102 : Gut microbiota abundance (genus Parasutterella id.2892) on Alpha−internexin
with a threshold of 1e−05
rs6809952
rs7572229
rs6828768
rs2387977
rs55877868
rs7311004
rs7303158
rs10899911
rs78383039
rs823424
rs11715853
rs2090816
rs8039785
rs62273907
rs35055552
All
−0.1
0.0
0.1
0.2
0.3
MR effect size for
'Gut microbiota abundance (genus Parasutterella id.2892)' on 'Alpha−internexin'

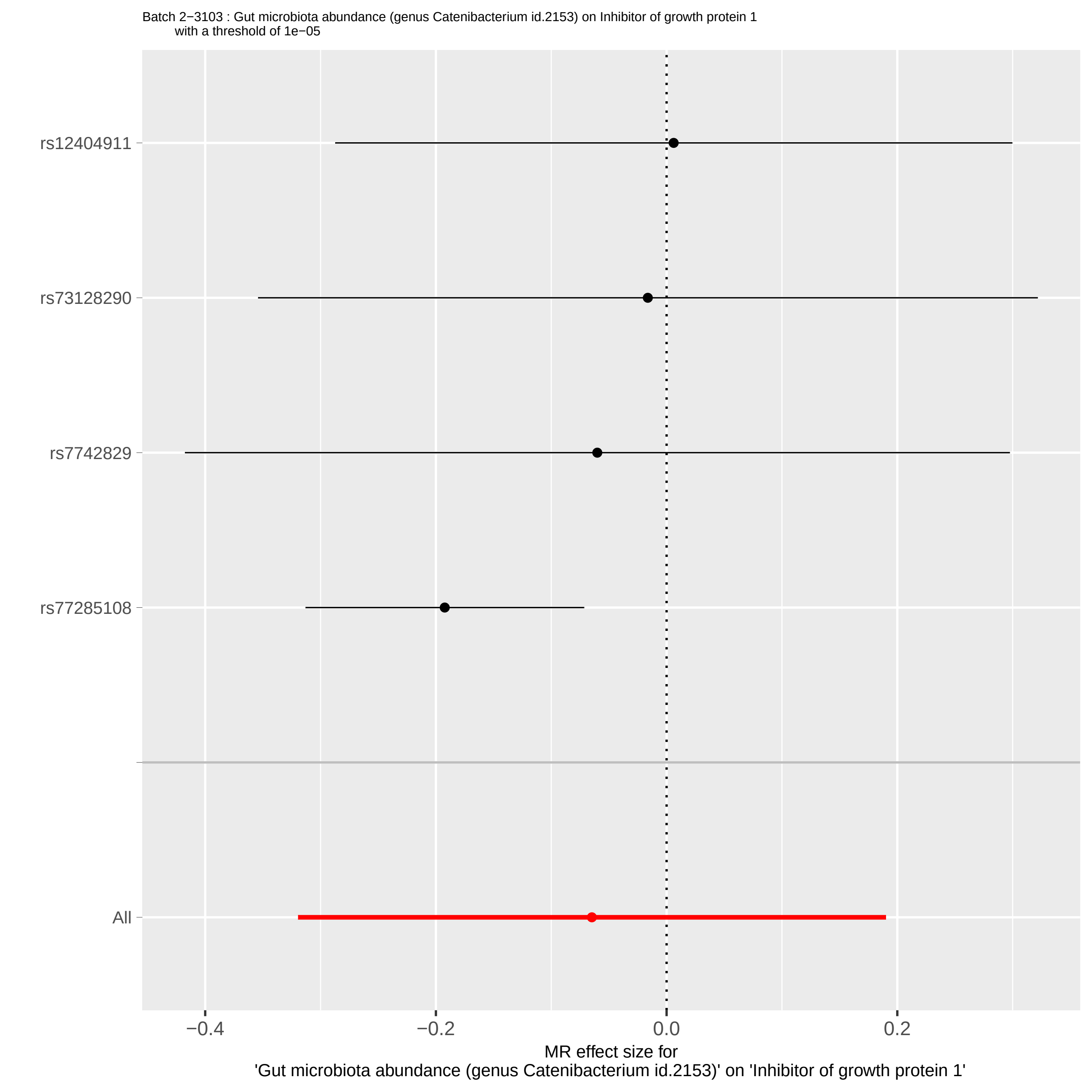
Batch 2−3103 : Gut microbiota abundance (genus Catenibacterium id.2153) on Inhibitor of growth protein 1
with a threshold of 1e−05
rs12404911
rs73128290
rs7742829
rs77285108
All
−0.4
−0.2
0.0
0.2
MR effect size for
'Gut microbiota abundance (genus Catenibacterium id.2153)' on 'Inhibitor of growth protein 1'

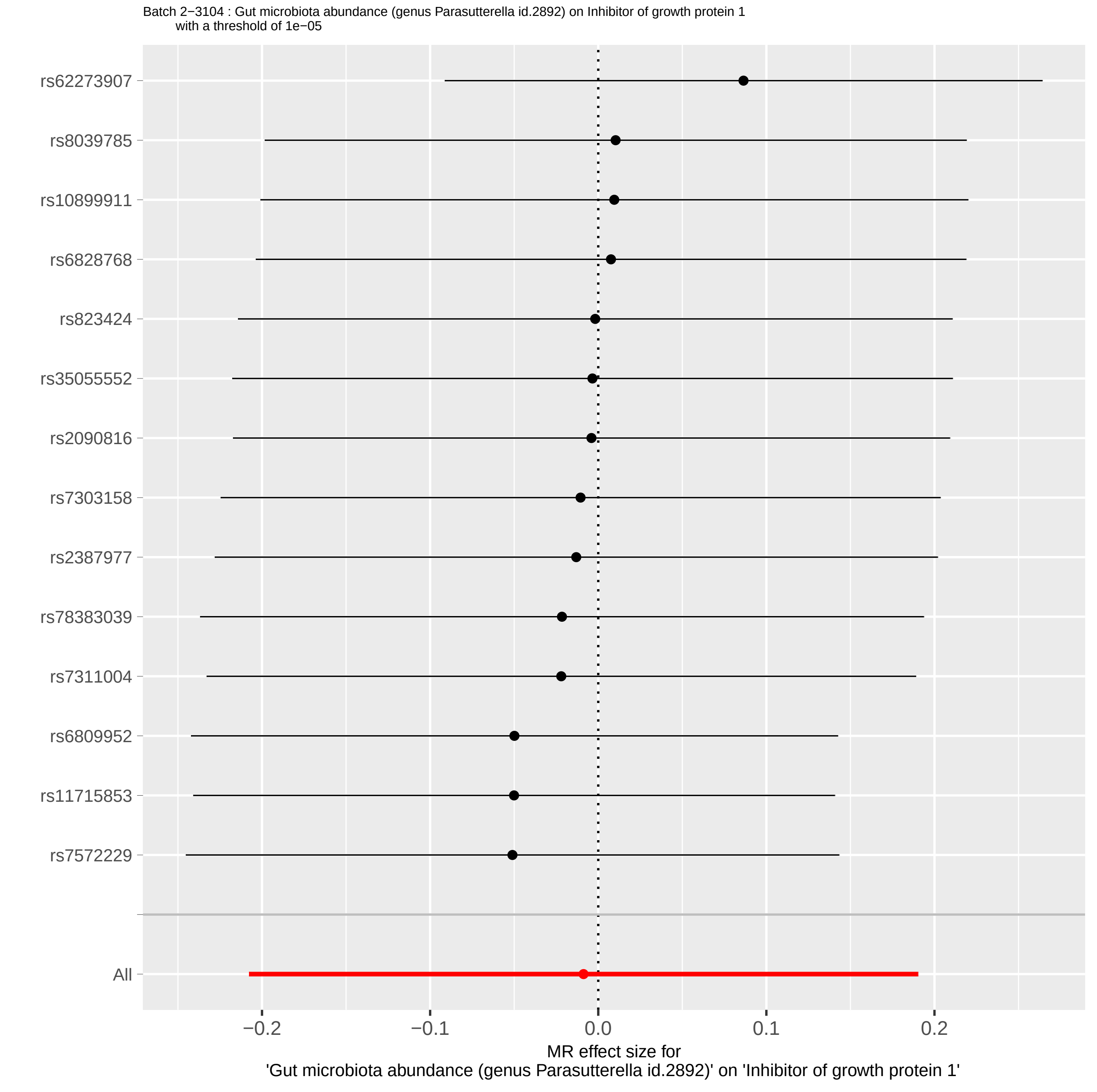

Batch 2−3104 : Gut microbiota abundance (genus Parasutterella id.2892) on Inhibitor of growth protein 1
with a threshold of 1e−05
rs62273907
rs8039785
rs10899911
rs6828768
rs823424
rs35055552
rs2090816
rs7303158
rs2387977
rs78383039
rs7311004
rs6809952
rs11715853
rs7572229
All
−0.2
−0.1
0.0
0.1
0.2
MR effect size for
'Gut microbiota abundance (genus Parasutterella id.2892)' on 'Inhibitor of growth protein 1'

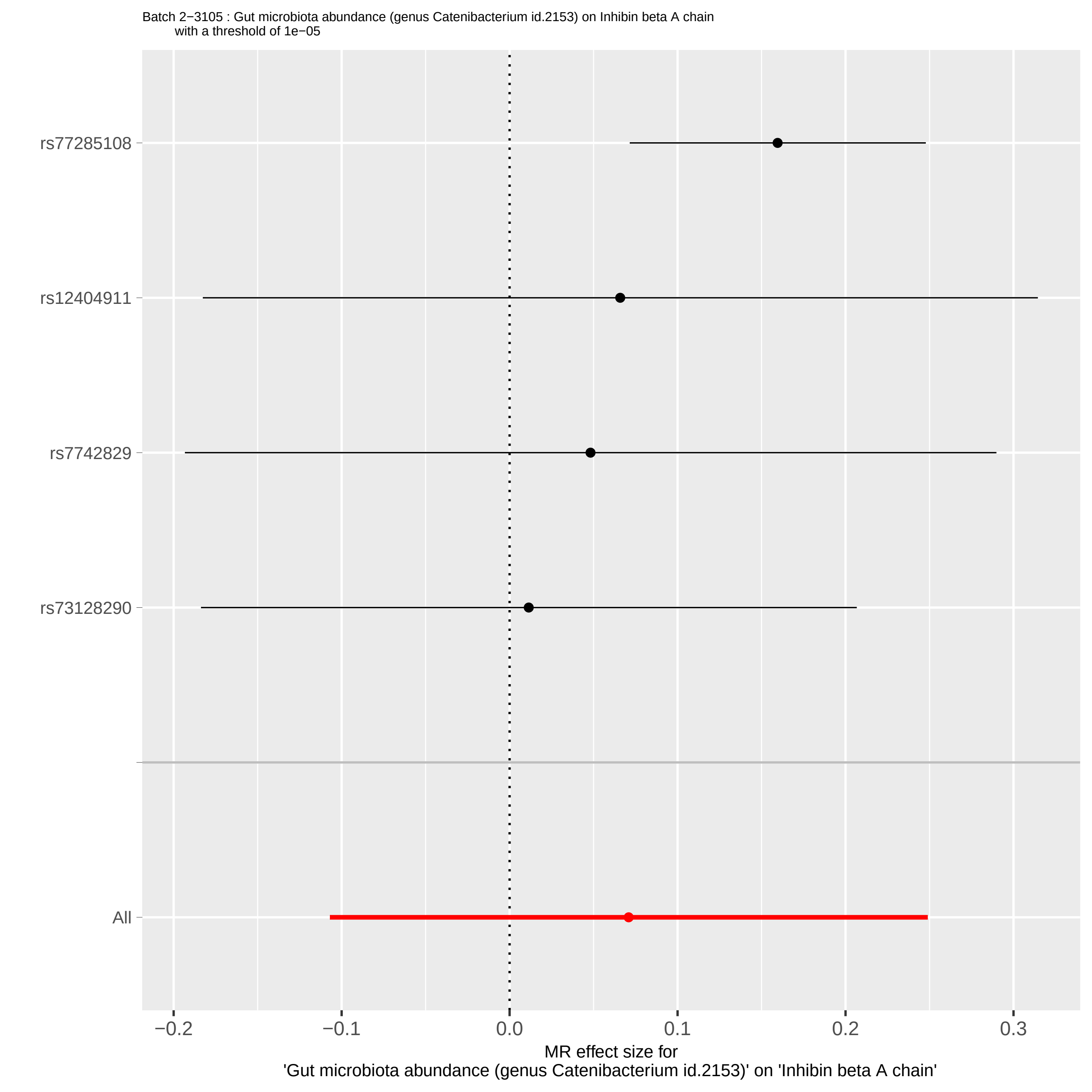
Batch 2−3105 : Gut microbiota abundance (genus Catenibacterium id.2153) on Inhibin beta A chain
with a threshold of 1e−05
rs77285108
rs12404911
rs7742829
rs73128290
All
−0.2
−0.1
0.0
0.1
0.2
0.3
MR effect size for
'Gut microbiota abundance (genus Catenibacterium id.2153)' on 'Inhibin beta A chain'

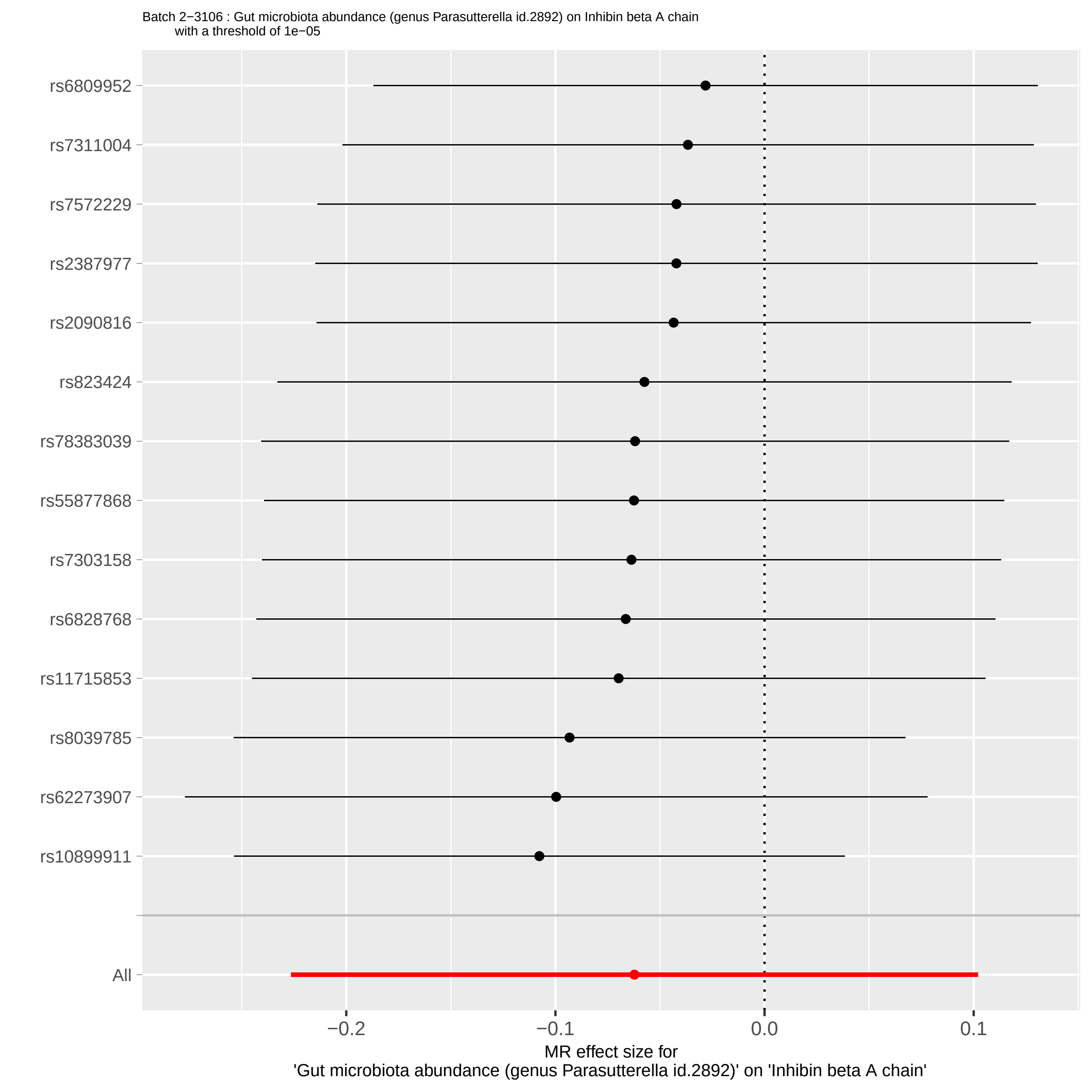

Batch 2−3106 : Gut microbiota abundance (genus Parasutterella id.2892) on Inhibin beta A chain
with a threshold of 1e−05
rs6809952
rs7311004
rs7572229
rs2387977
rs2090816
rs823424
rs78383039
rs55877868
rs7303158
rs6828768
rs11715853
rs8039785
rs62273907
rs10899911
All
−0.2
−0.1
0.0
0.1
MR effect size for
'Gut microbiota abundance (genus Parasutterella id.2892)' on 'Inhibin beta A chain'

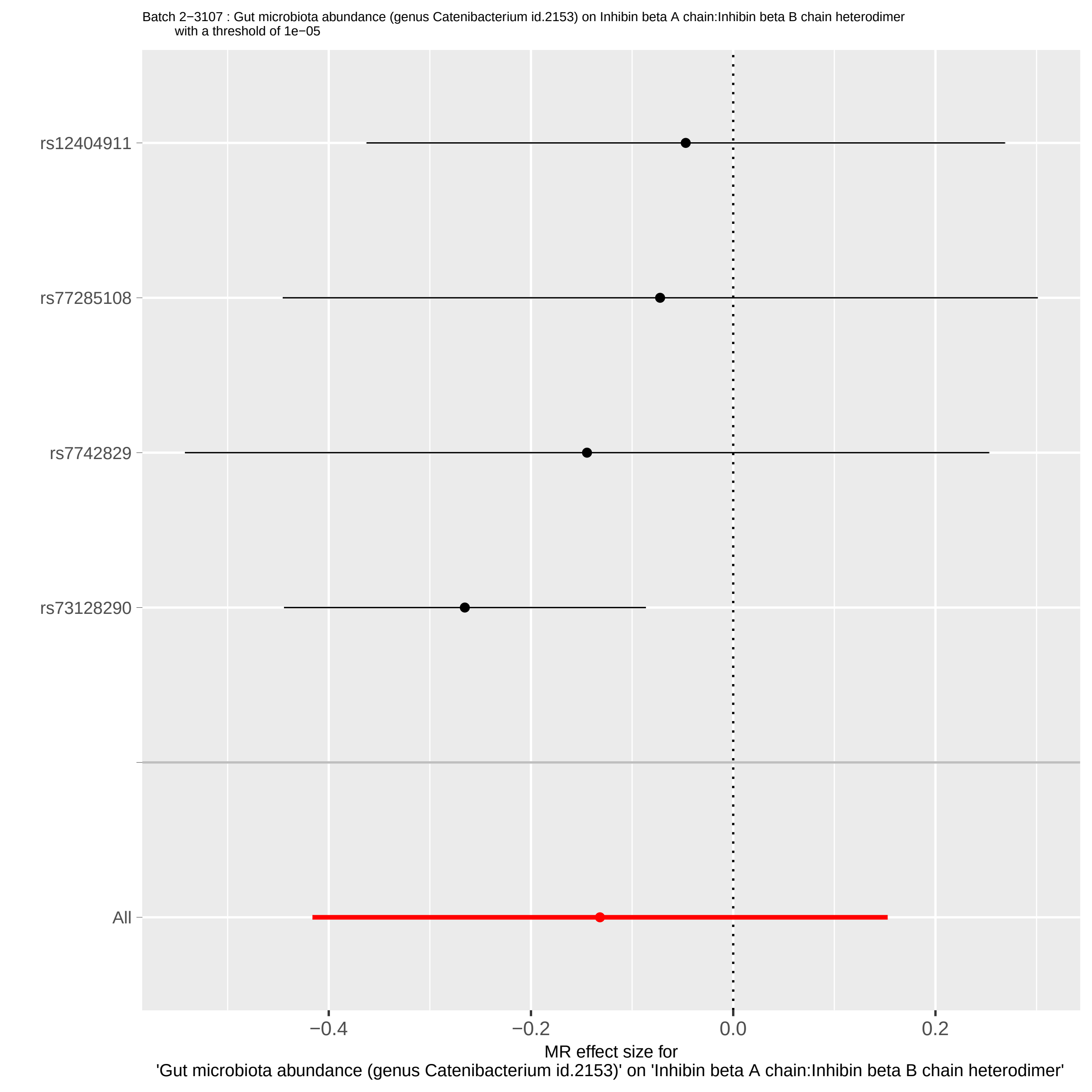

Batch 2−3107 : Gut microbiota abundance (genus Catenibacterium id.2153) on Inhibin beta A chain:Inhibin beta B chain heterodimer
with a threshold of 1e−05
rs12404911
rs77285108
rs7742829
rs73128290
All
−0.4
−0.2
0.0
0.2
MR effect size for
'Gut microbiota abundance (genus Catenibacterium id.2153)' on 'Inhibin beta A chain:Inhibin beta B chain heterodimer'

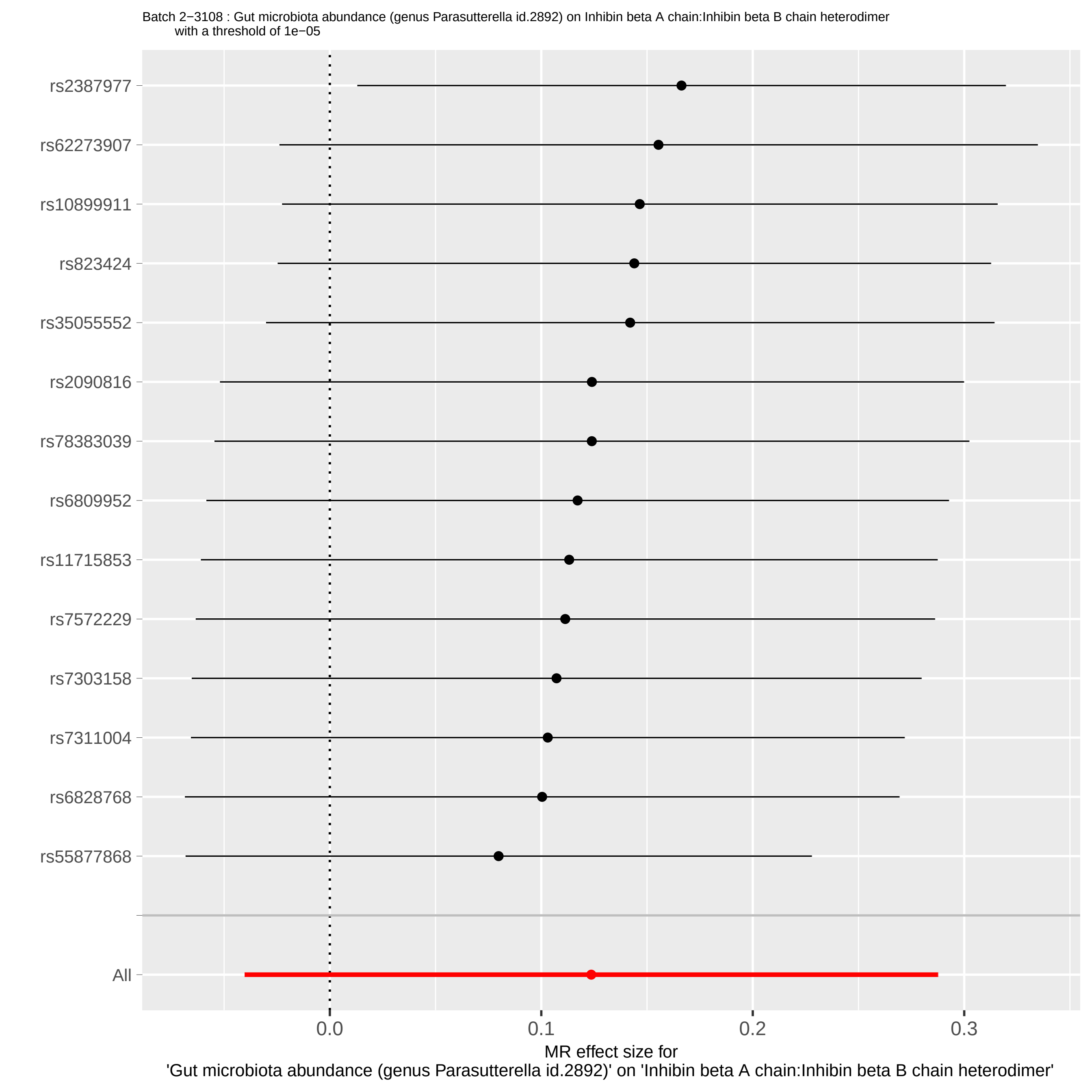

Batch 2−3108 : Gut microbiota abundance (genus Parasutterella id.2892) on Inhibin beta A chain:Inhibin beta B chain heterodimer
with a threshold of 1e−05
rs2387977
rs62273907
rs10899911
rs823424
rs35055552
rs2090816
rs78383039
rs6809952
rs11715853
rs7572229
rs7303158
rs7311004
rs6828768
rs55877868
All
0.0
0.1
0.2
0.3
MR effect size for
'Gut microbiota abundance (genus Parasutterella id.2892)' on 'Inhibin beta A chain:Inhibin beta B chain heterodimer'

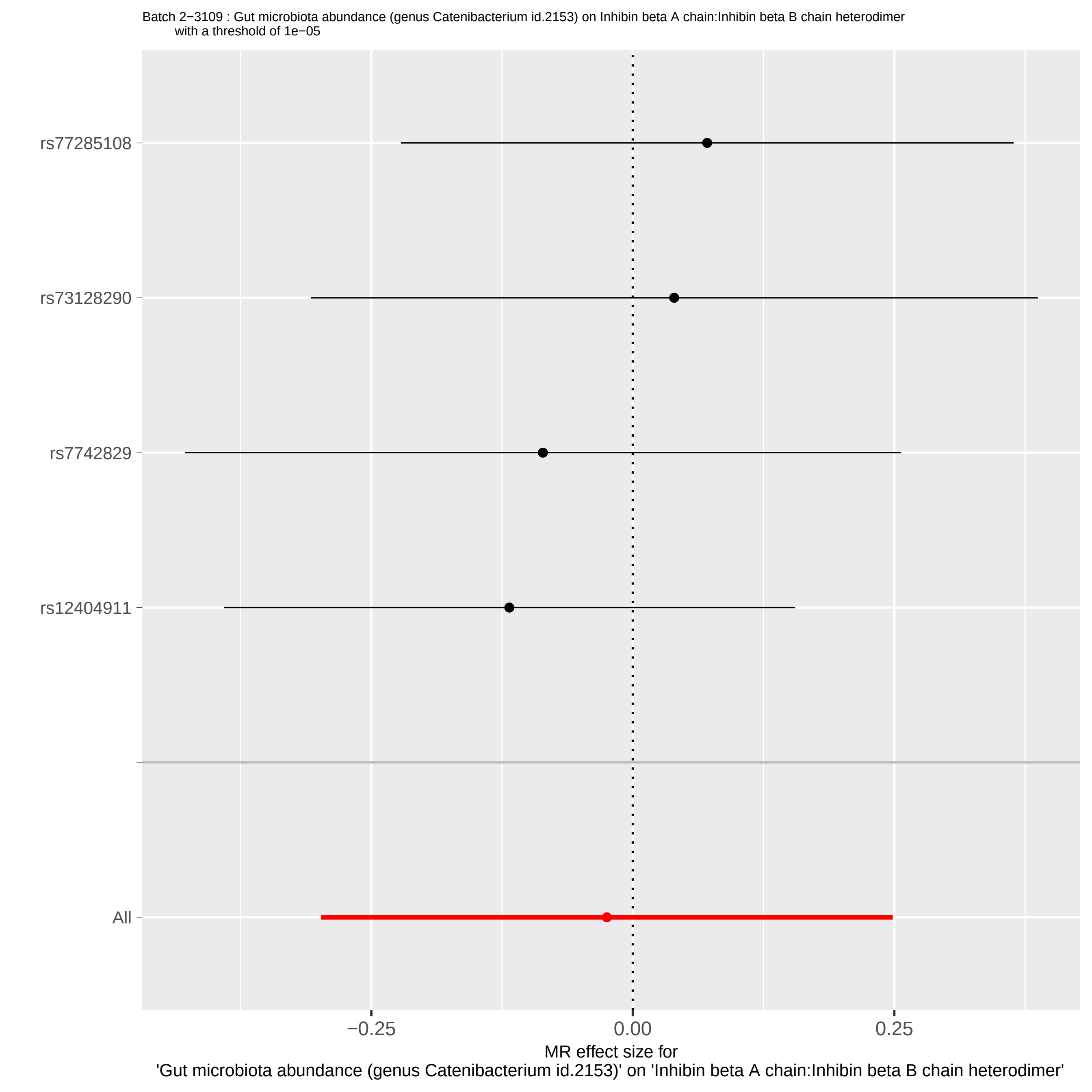
Batch 2−3109 : Gut microbiota abundance (genus Catenibacterium id.2153) on Inhibin beta A chain:Inhibin beta B chain heterodimer
with a threshold of 1e−05
rs77285108
rs73128290
rs7742829
rs12404911
All
−0.25
0.00
0.25
MR effect size for
'Gut microbiota abundance (genus Catenibacterium id.2153)' on 'Inhibin beta A chain:Inhibin beta B chain heterodimer'

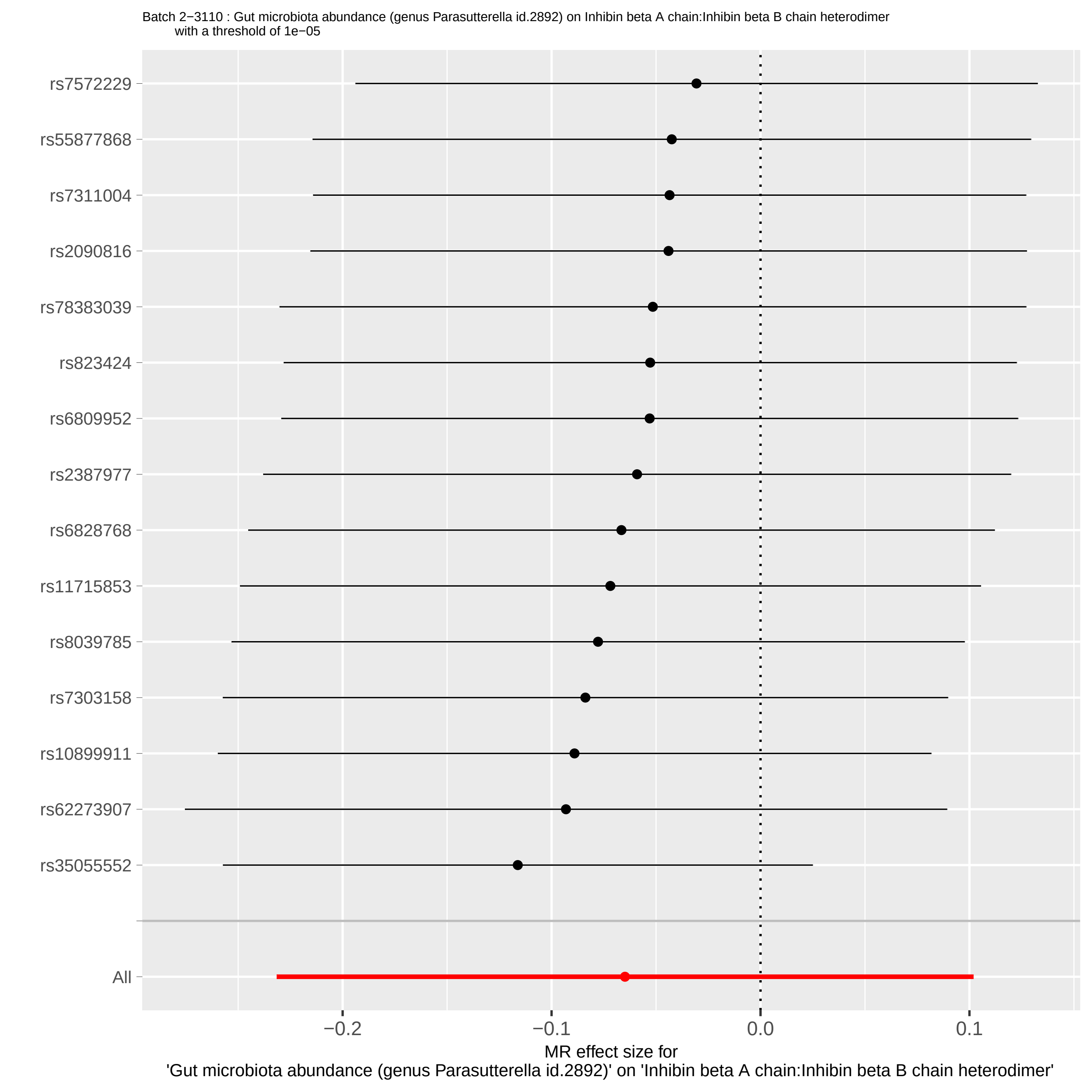
Batch 2−3110 : Gut microbiota abundance (genus Parasutterella id.2892) on Inhibin beta A chain:Inhibin beta B chain heterodimer
with a threshold of 1e−05
rs7572229
rs55877868
rs7311004
rs2090816
rs78383039
rs823424
rs6809952
rs2387977
rs6828768
rs11715853
rs8039785
rs7303158
rs10899911
rs62273907
rs35055552
All
−0.2
−0.1
0.0
0.1
MR effect size for
'Gut microbiota abundance (genus Parasutterella id.2892)' on 'Inhibin beta A chain:Inhibin beta B chain heterodimer'

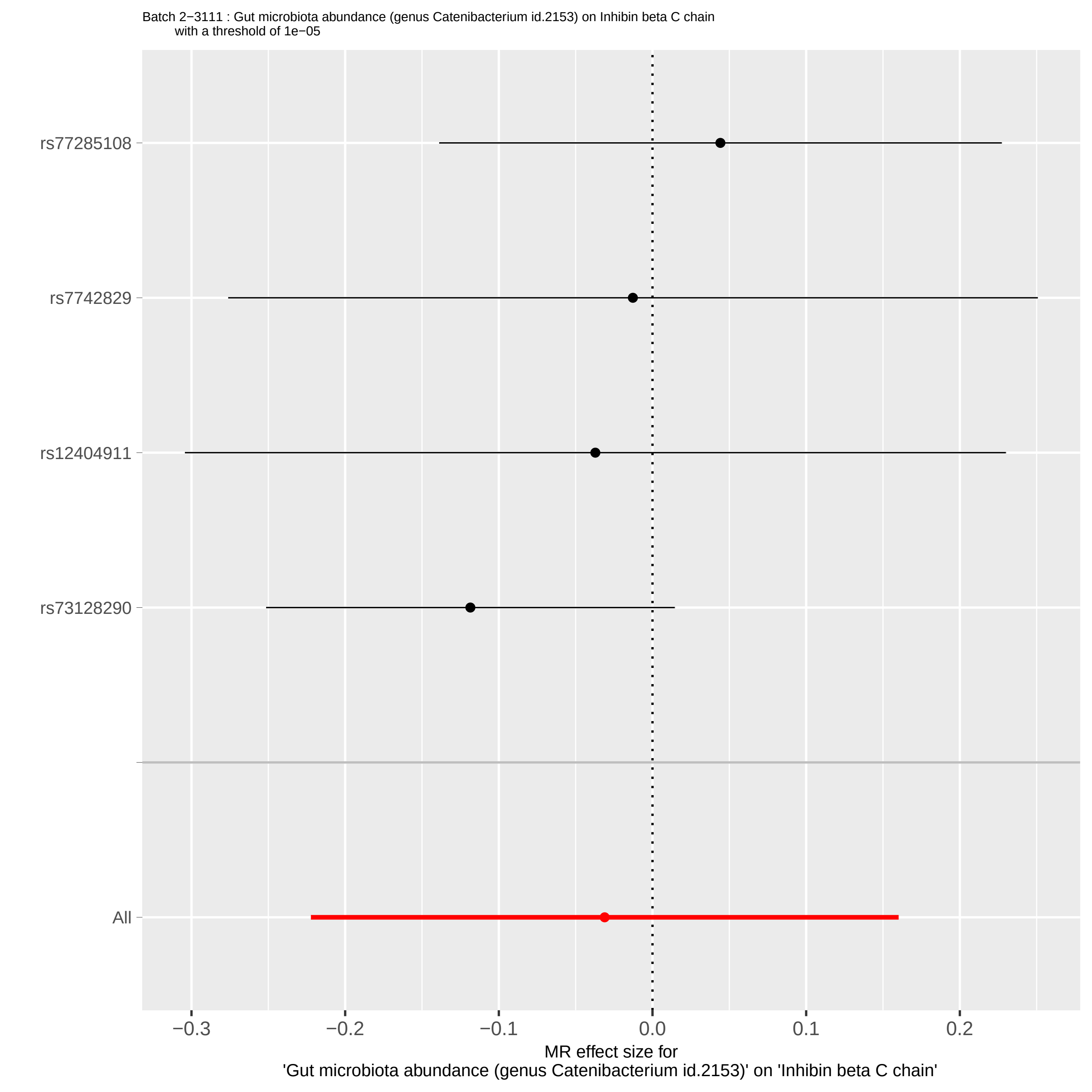
Batch 2−3111 : Gut microbiota abundance (genus Catenibacterium id.2153) on Inhibin beta C chain
with a threshold of 1e−05
rs77285108
rs7742829
rs12404911
rs73128290
All
−0.3
−0.2
−0.1
0.0
0.1
0.2
MR effect size for
'Gut microbiota abundance (genus Catenibacterium id.2153)' on 'Inhibin beta C chain'

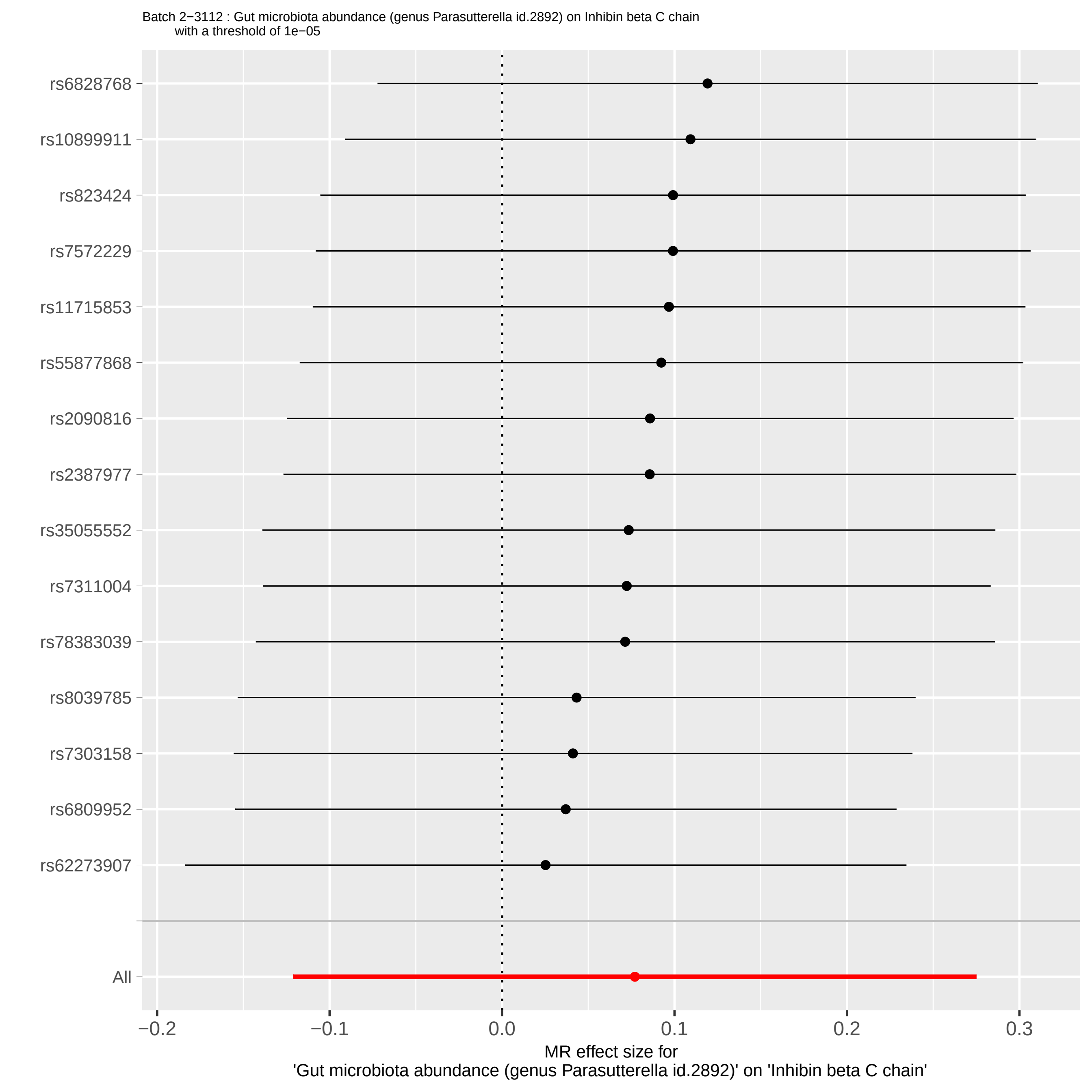

Batch 2−3112 : Gut microbiota abundance (genus Parasutterella id.2892) on Inhibin beta C chain
with a threshold of 1e−05
rs6828768
rs10899911
rs823424
rs7572229
rs11715853
rs55877868
rs2090816
rs2387977
rs35055552
rs7311004
rs78383039
rs8039785
rs7303158
rs6809952
rs62273907
All
−0.2
−0.1
0.0
0.1
0.2
0.3
MR effect size for
'Gut microbiota abundance (genus Parasutterella id.2892)' on 'Inhibin beta C chain'

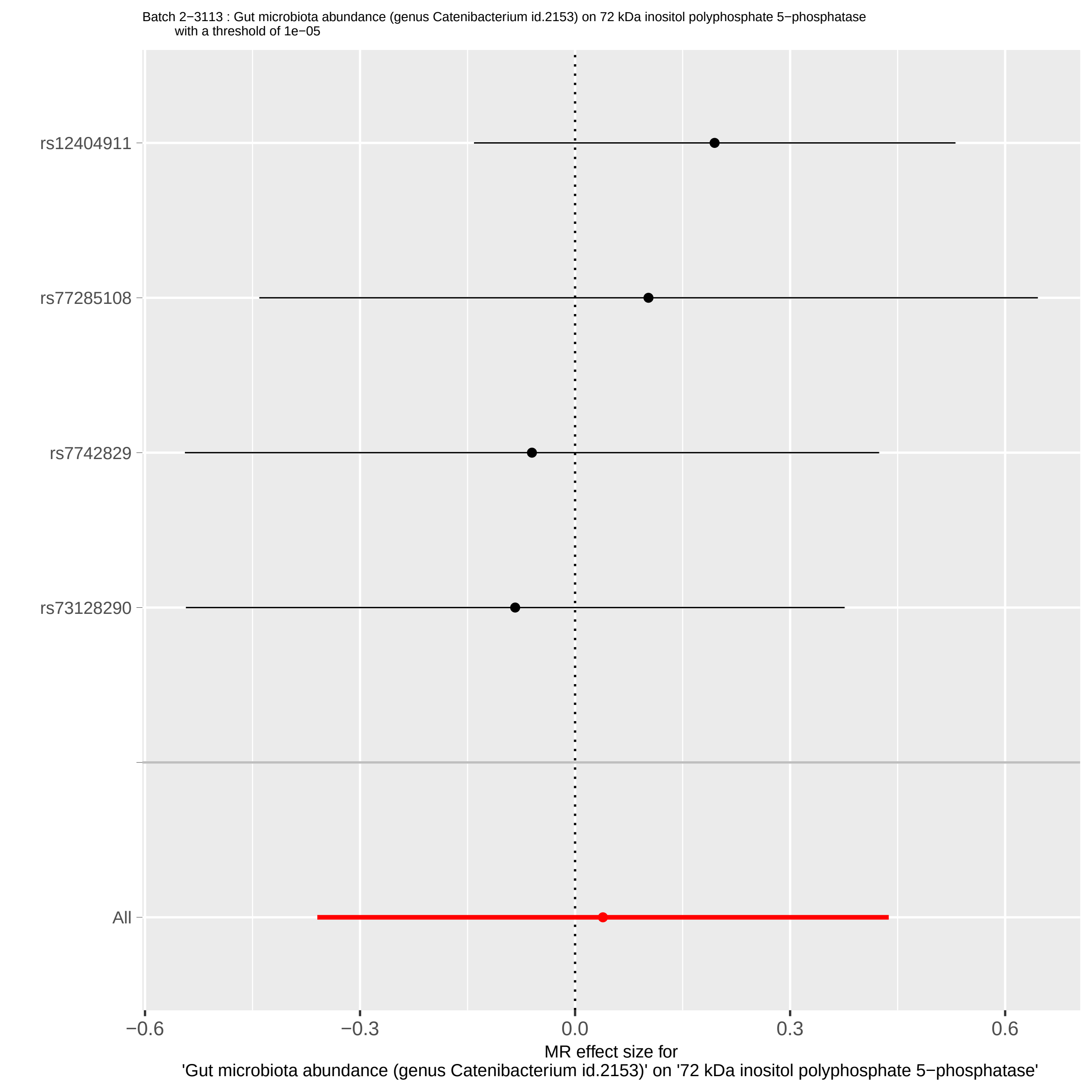

Batch 2−3113 : Gut microbiota abundance (genus Catenibacterium id.2153) on 72 kDa inositol polyphosphate 5−phosphatase
with a threshold of 1e−05
rs12404911
rs77285108
rs7742829
rs73128290
All
−0.6
−0.3
0.0
0.3
0.6
MR effect size for
'Gut microbiota abundance (genus Catenibacterium id.2153)' on '72 kDa inositol polyphosphate 5−phosphatase'

Batch 2−3114 : Gut microbiota abundance (genus Parasutterella id.2892) on 72 kDa inositol polyphosphate 5−phosphatase with a threshold of 1e−05

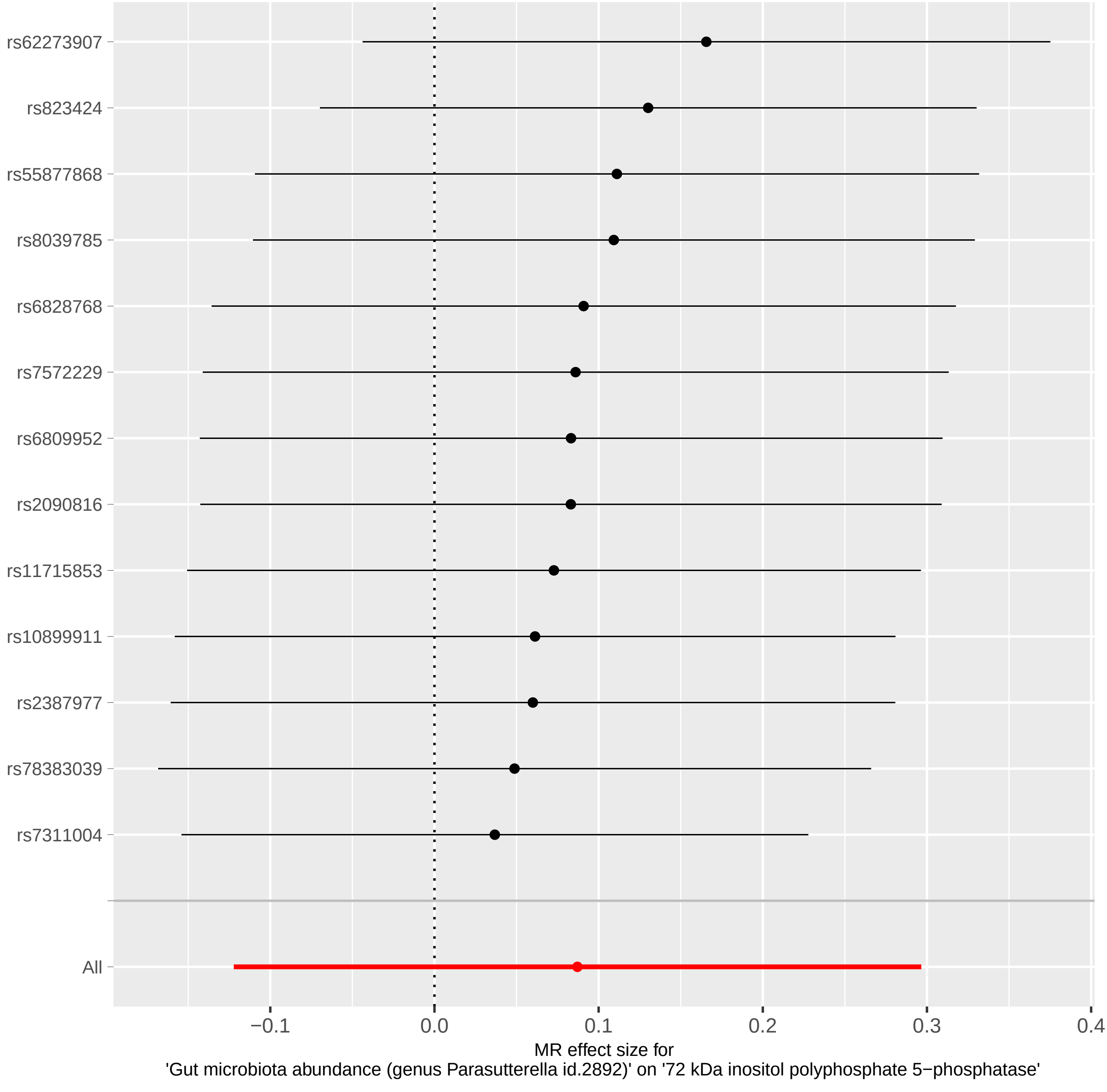

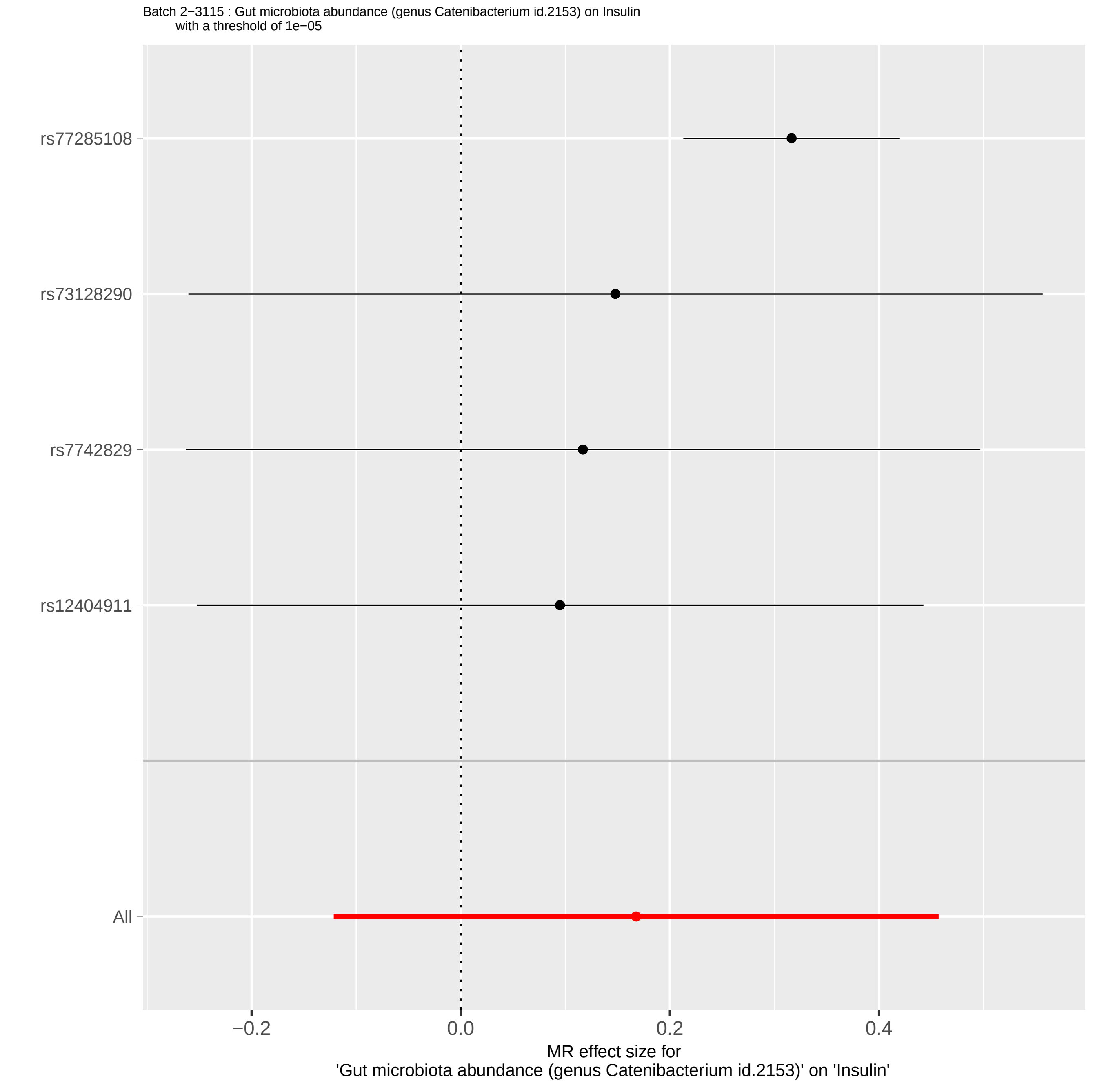

Batch 2−3115 : Gut microbiota abundance (genus Catenibacterium id.2153) on Insulin
with a threshold of 1e−05
rs77285108
rs73128290
rs7742829
rs12404911
All
−0.2
0.0
0.2
0.4
MR effect size for
'Gut microbiota abundance (genus Catenibacterium id.2153)' on 'Insulin'

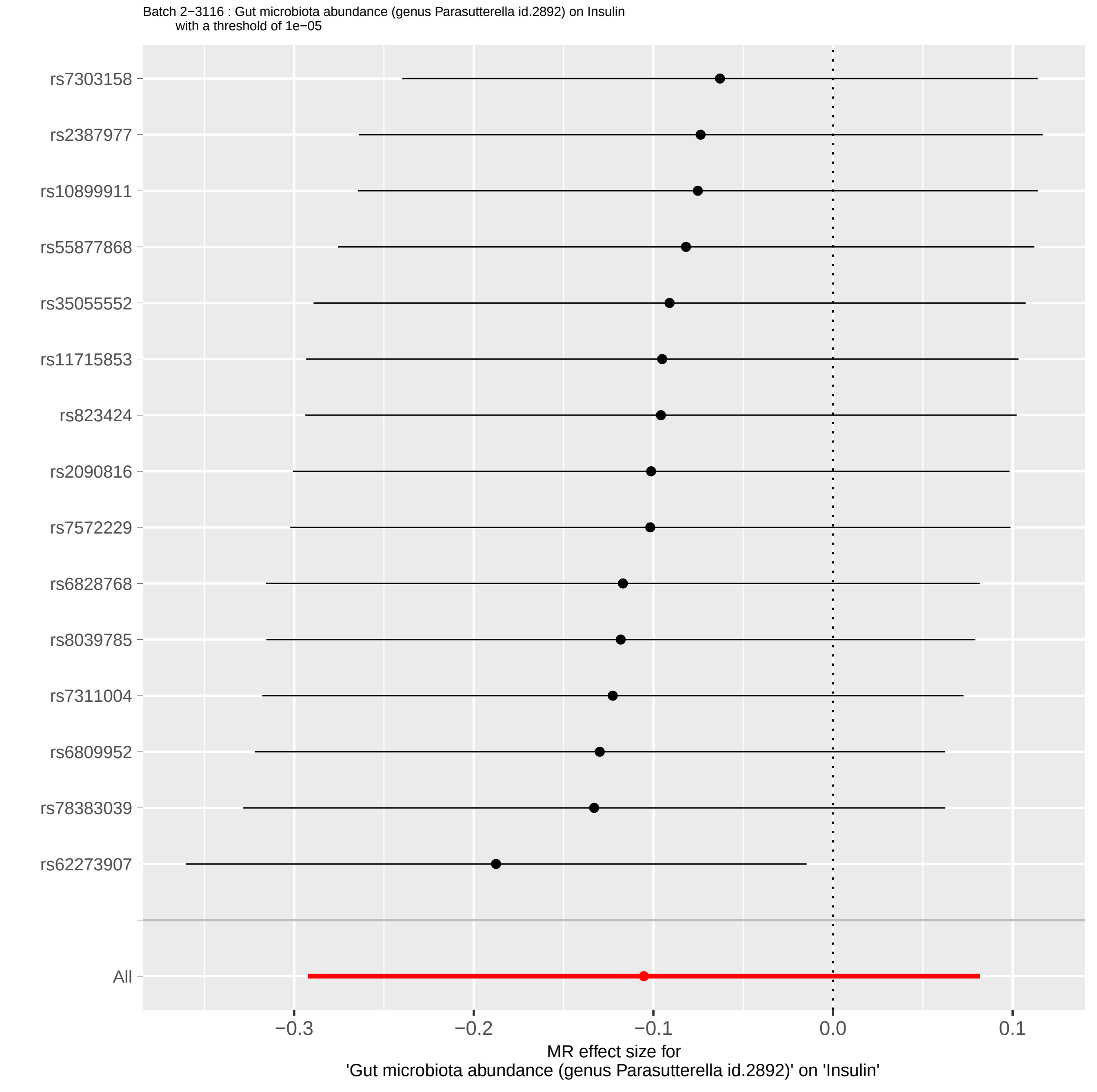
Batch 2−3116 : Gut microbiota abundance (genus Parasutterella id.2892) on Insulin
with a threshold of 1e−05
rs7303158
rs2387977
rs10899911
rs55877868
rs35055552
rs11715853
rs823424
rs2090816
rs7572229
rs6828768
rs8039785
rs7311004
rs6809952
rs78383039
rs62273907
All
−0.3
−0.2
−0.1
0.0
0.1
MR effect size for
'Gut microbiota abundance (genus Parasutterella id.2892)' on 'Insulin'

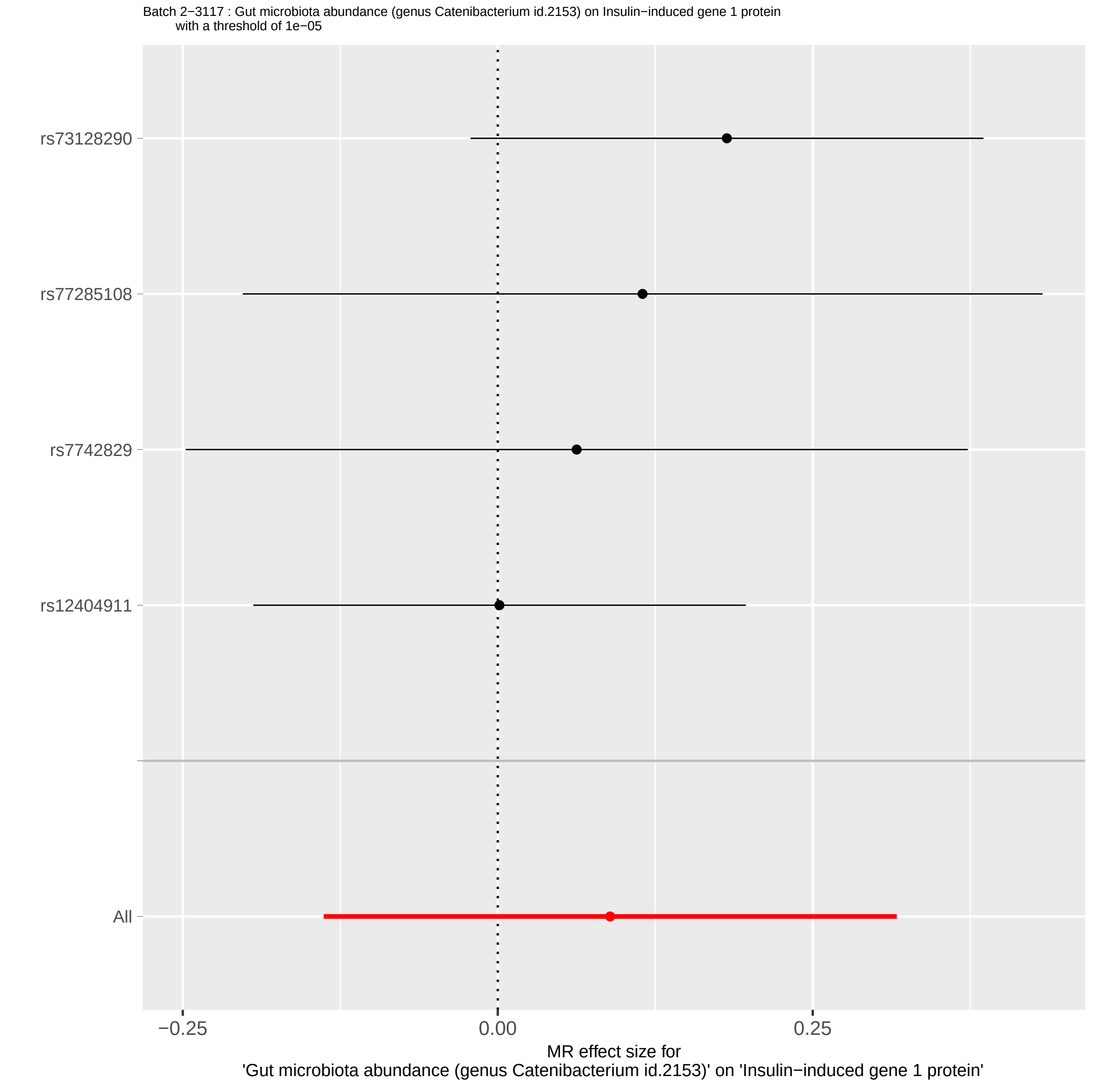
Batch 2−3117 : Gut microbiota abundance (genus Catenibacterium id.2153) on Insulin−induced gene 1 protein
with a threshold of 1e−05
rs73128290
rs77285108
rs7742829
rs12404911
All
−0.25
0.00
0.25
MR effect size for
'Gut microbiota abundance (genus Catenibacterium id.2153)' on 'Insulin−induced gene 1 protein'

Batch 2−3118 : Gut microbiota abundance (genus Parasutterella id.2892) on Insulin−induced gene 1 protein
with a threshold of 1e−05

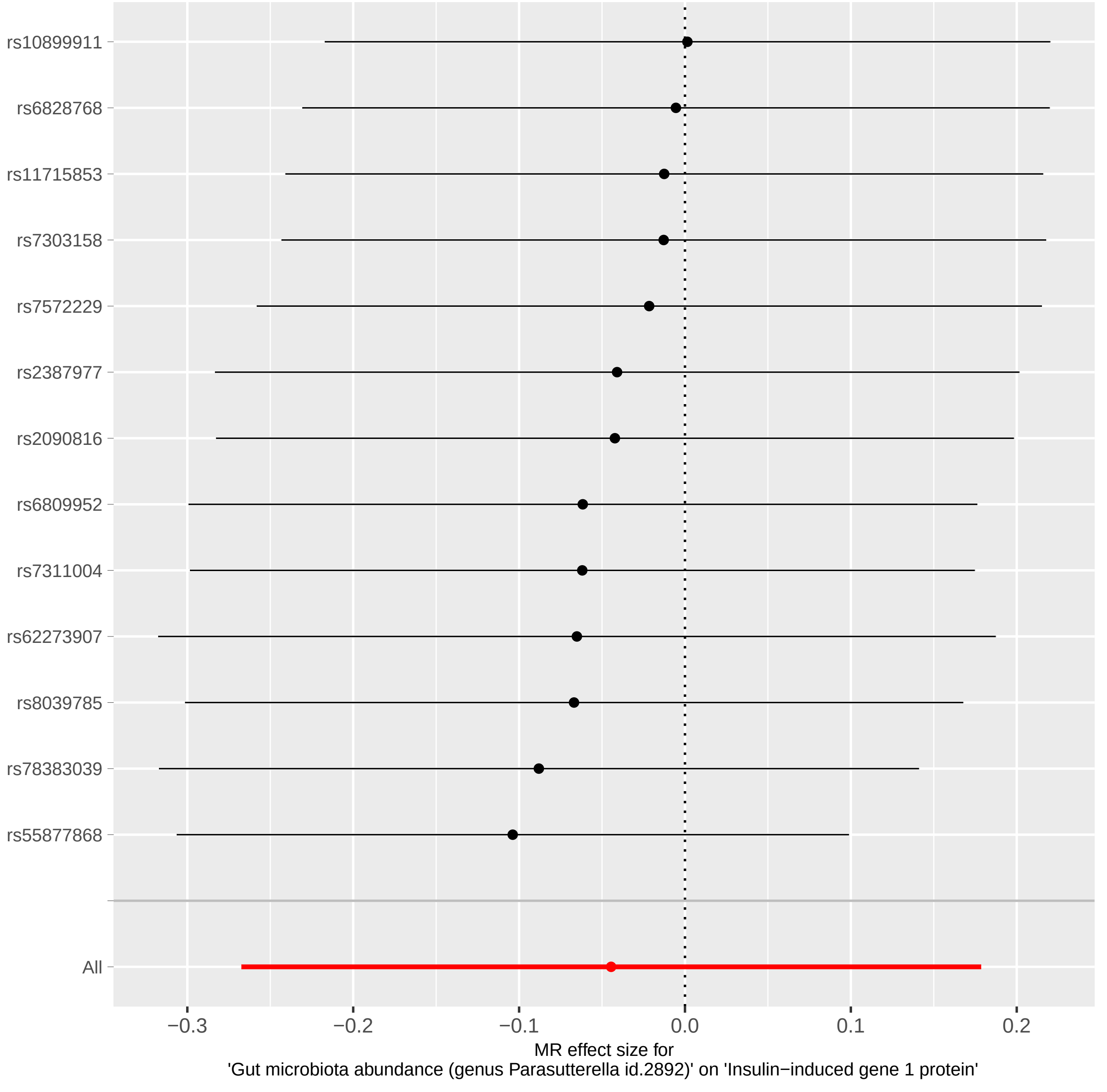

Batch 2−3119 : Gut microbiota abundance (genus Catenibacterium id.2153) on Insulin−like 3
with a threshold of 1e−05

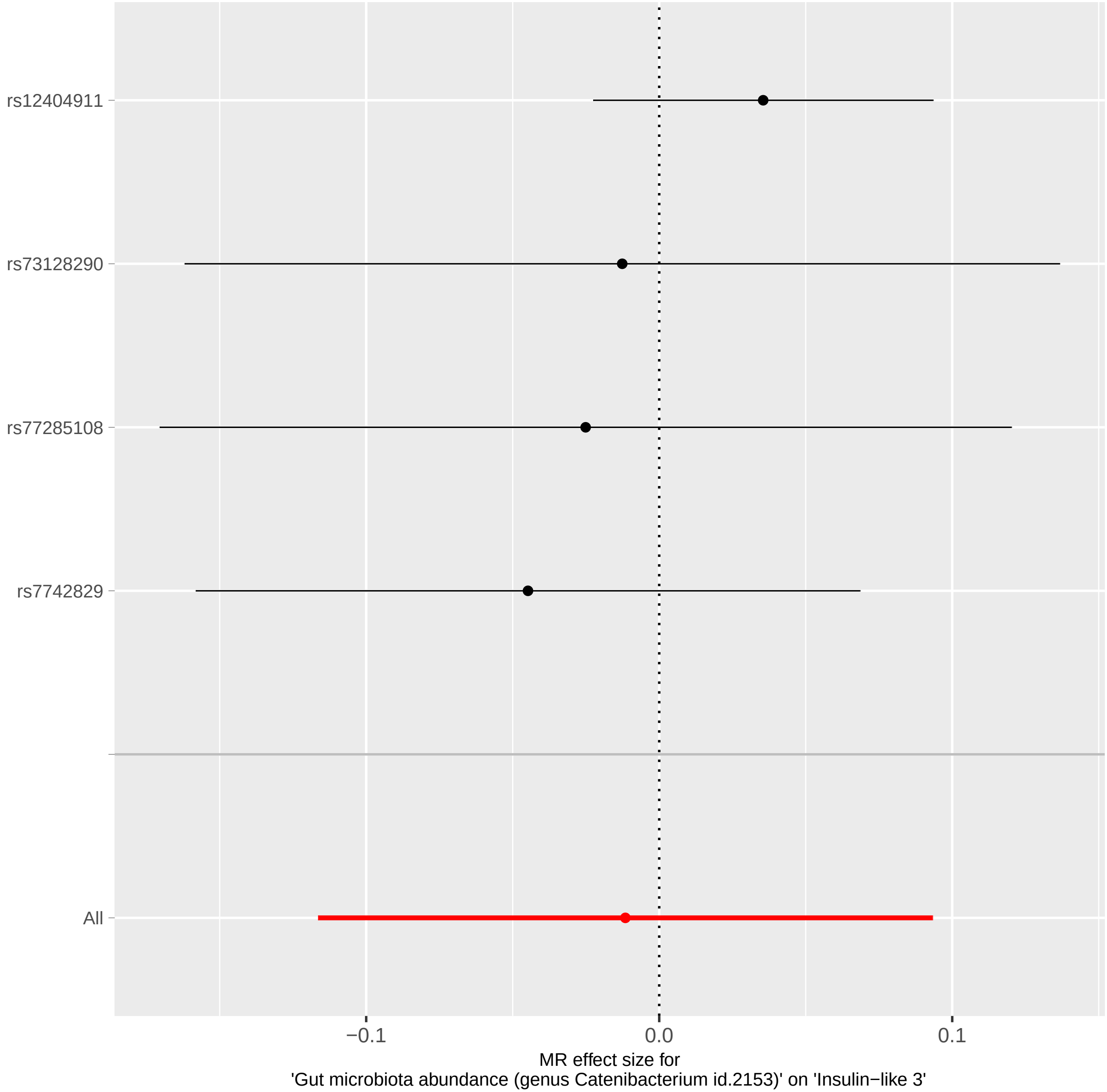

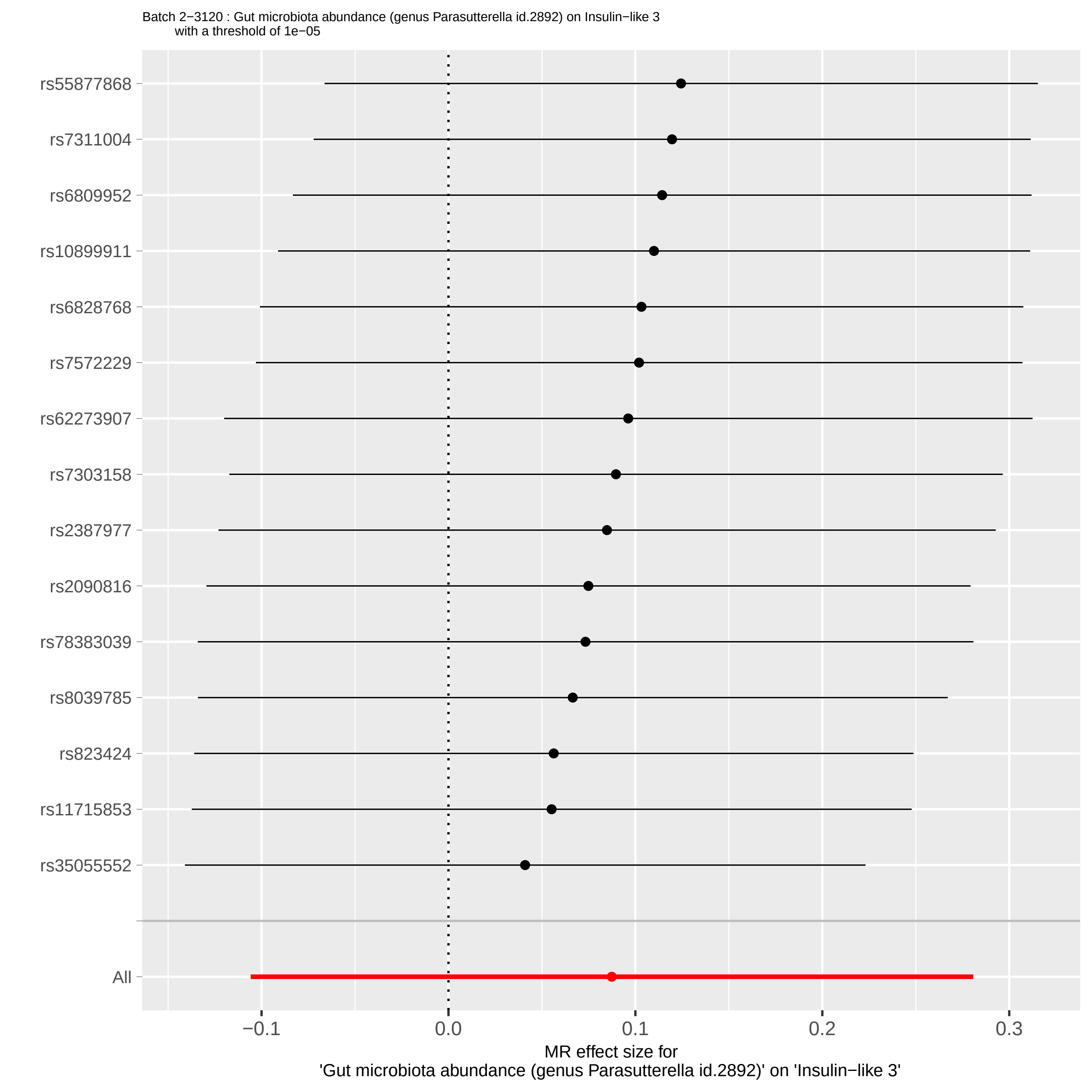
Batch 2−3120 : Gut microbiota abundance (genus Parasutterella id.2892) on Insulin−like 3
with a threshold of 1e−05
rs55877868
rs7311004
rs6809952
rs10899911
rs6828768
rs7572229
rs62273907
rs7303158
rs2387977
rs2090816
rs78383039
rs8039785
rs823424
rs11715853
rs35055552
All
−0.1
0.0
0.1
0.2
0.3
MR effect size for
'Gut microbiota abundance (genus Parasutterella id.2892)' on 'Insulin−like 3'

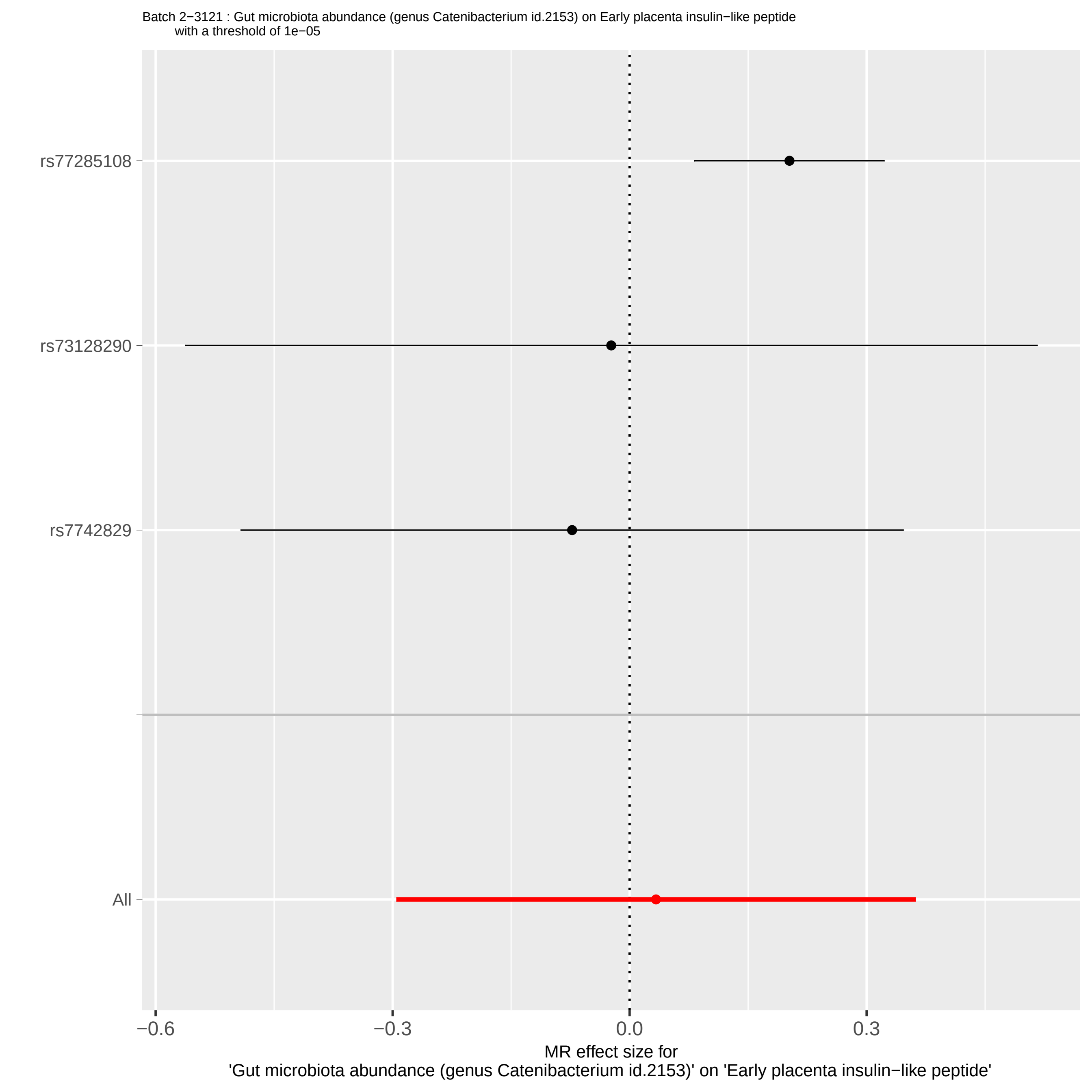
Batch 2−3121 : Gut microbiota abundance (genus Catenibacterium id.2153) on Early placenta insulin−like peptide
with a threshold of 1e−05
rs77285108
rs73128290
rs7742829
All
−0.6
−0.3
0.0
0.3
MR effect size for
'Gut microbiota abundance (genus Catenibacterium id.2153)' on 'Early placenta insulin−like peptide'

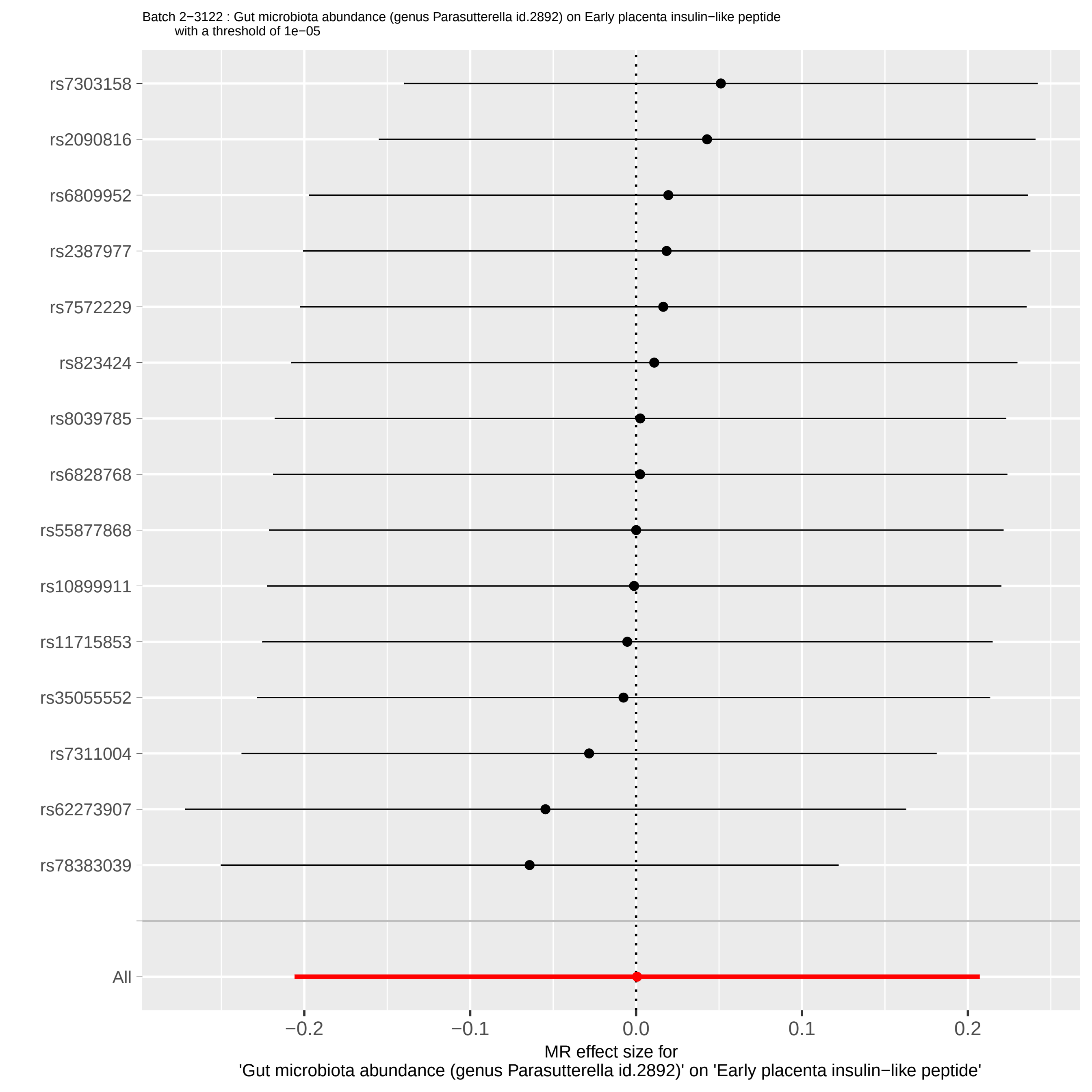

Batch 2−3122 : Gut microbiota abundance (genus Parasutterella id.2892) on Early placenta insulin−like peptide
with a threshold of 1e−05
rs7303158
rs2090816
rs6809952
rs2387977
rs7572229
rs823424
rs8039785
rs6828768
rs55877868
rs10899911
rs11715853
rs35055552
rs7311004
rs62273907
rs78383039
All
−0.2
−0.1
0.0
0.1
0.2
MR effect size for
'Gut microbiota abundance (genus Parasutterella id.2892)' on 'Early placenta insulin−like peptide'

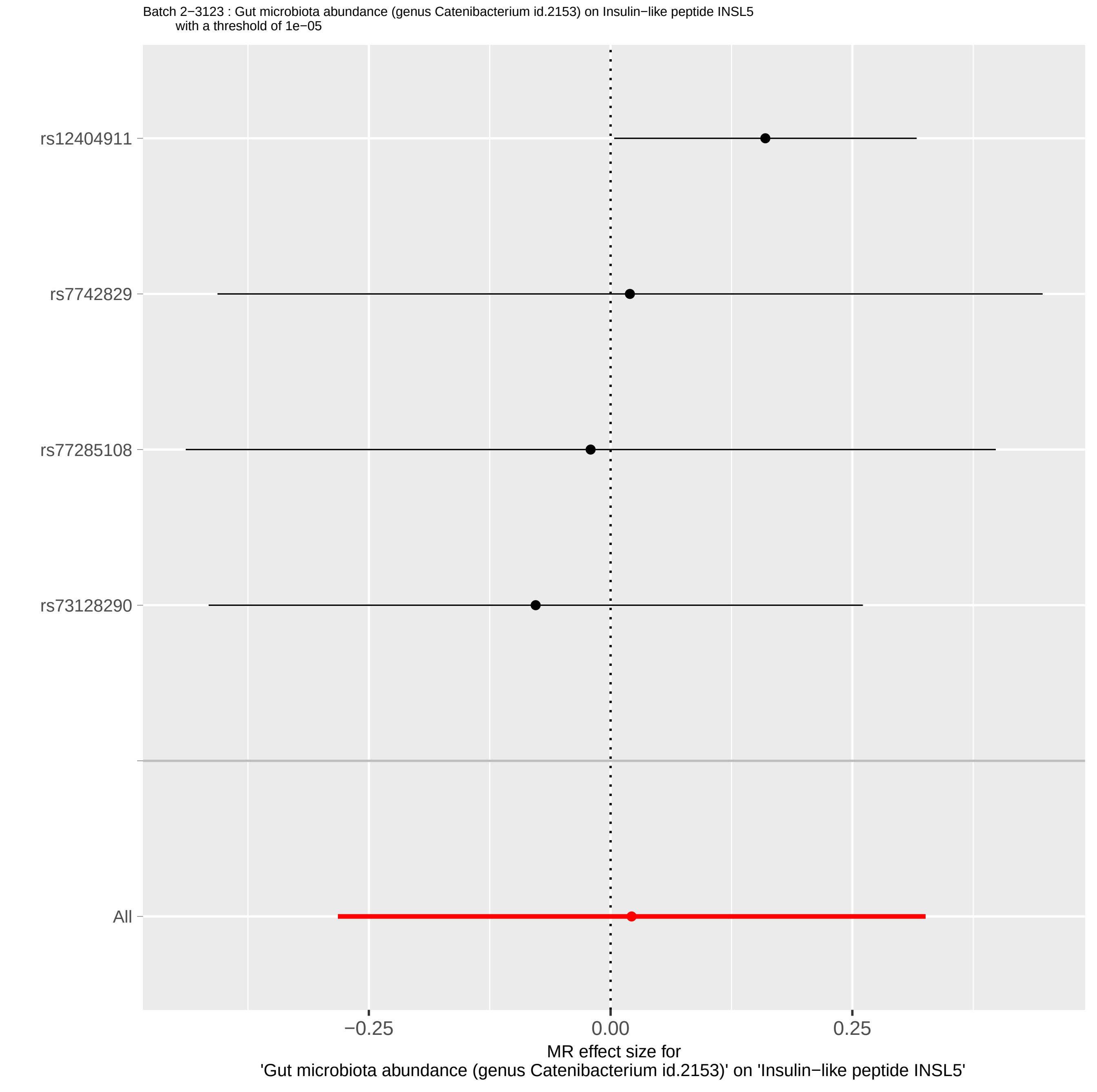
Batch 2−3123 : Gut microbiota abundance (genus Catenibacterium id.2153) on Insulin−like peptide INSL5
with a threshold of 1e−05
rs12404911
rs7742829
rs77285108
rs73128290
All
−0.25
0.00
0.25
MR effect size for
'Gut microbiota abundance (genus Catenibacterium id.2153)' on 'Insulin−like peptide INSL5'

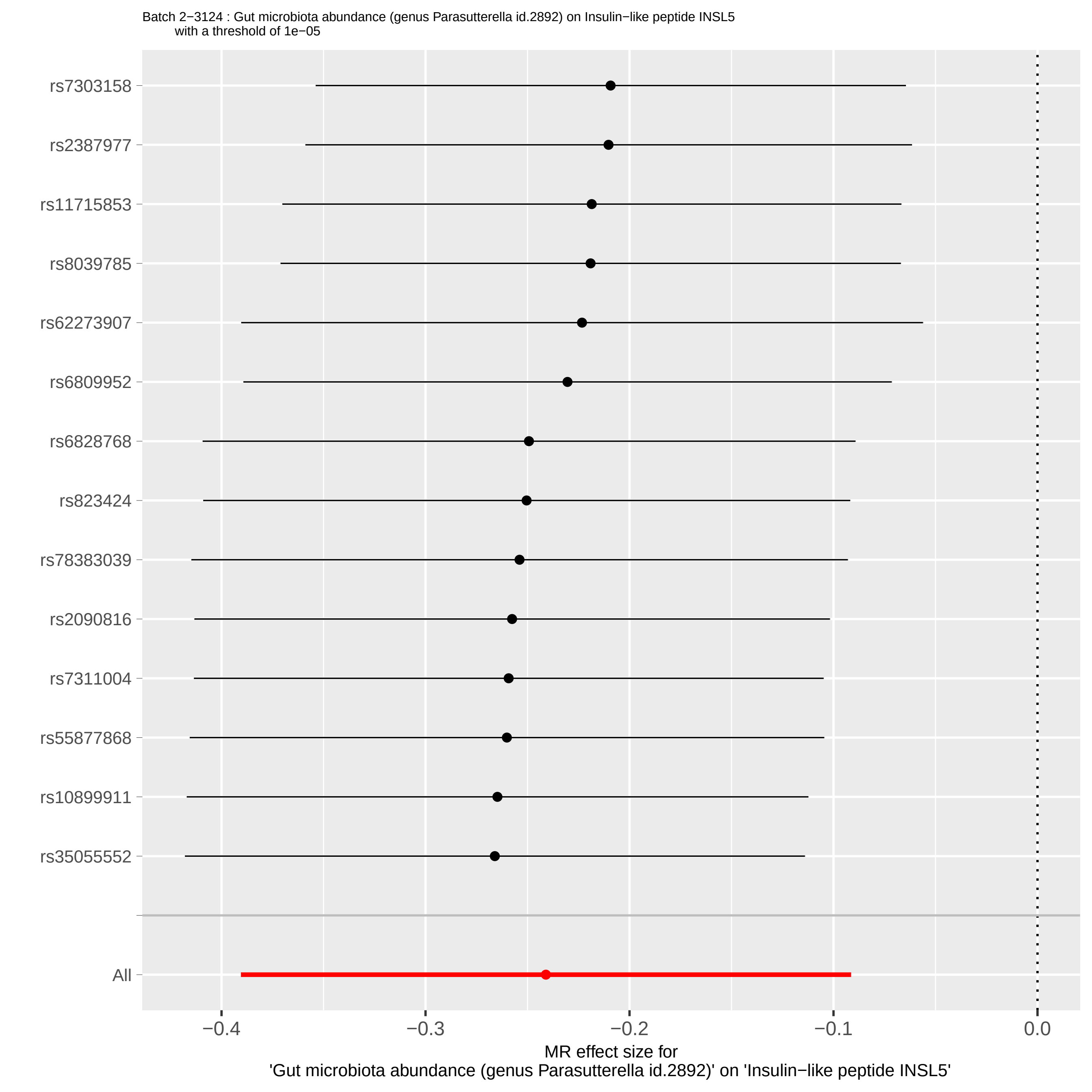

Batch 2−3124 : Gut microbiota abundance (genus Parasutterella id.2892) on Insulin−like peptide INSL5
with a threshold of 1e−05
rs7303158
rs2387977
rs11715853
rs8039785
rs62273907
rs6809952
rs6828768
rs823424
rs78383039
rs2090816
rs7311004
rs55877868
rs10899911
rs35055552
All
−0.4
−0.3
−0.2
−0.1
0.0
MR effect size for
'Gut microbiota abundance (genus Parasutterella id.2892)' on 'Insulin−like peptide INSL5'

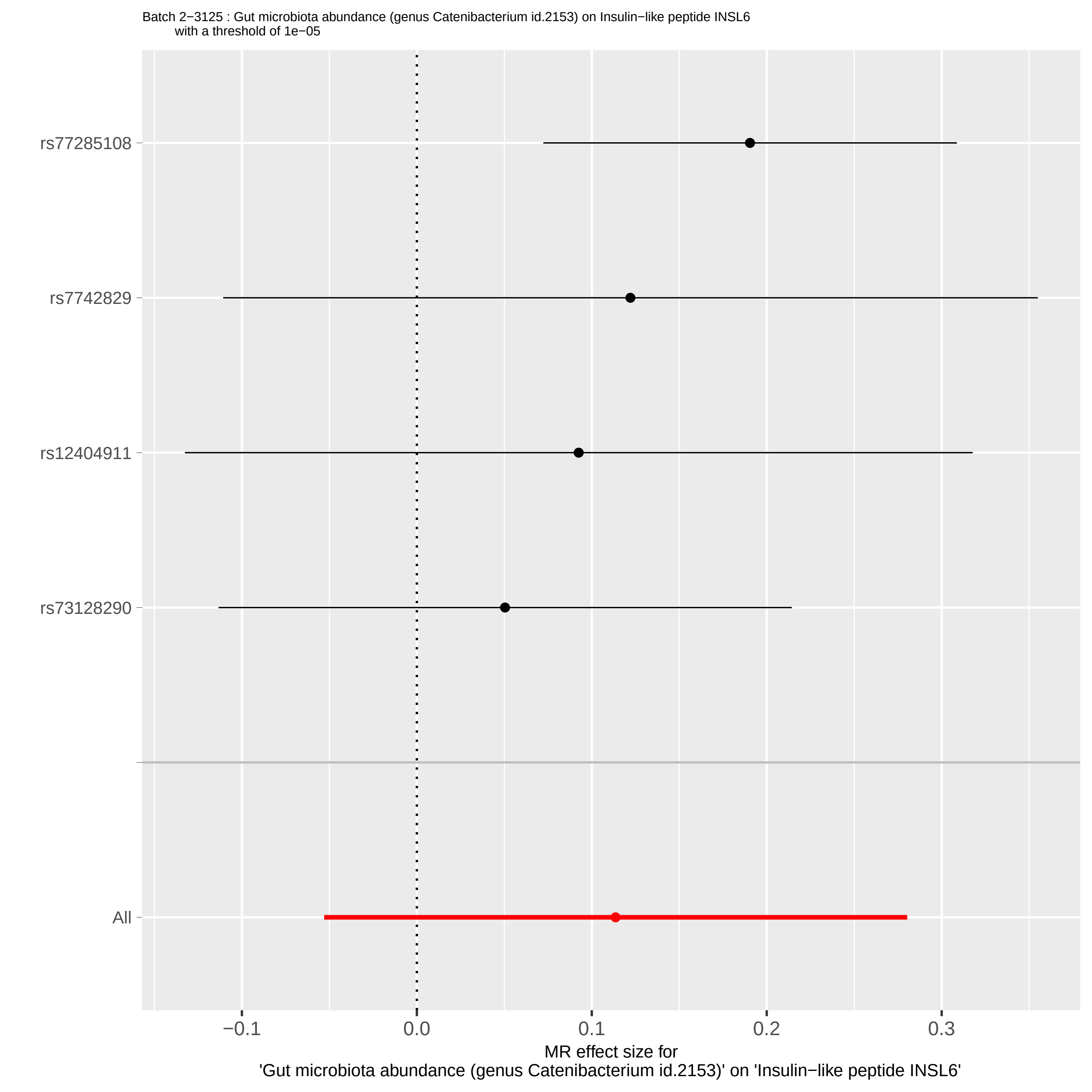
Batch 2−3125 : Gut microbiota abundance (genus Catenibacterium id.2153) on Insulin−like peptide INSL6
with a threshold of 1e−05
rs77285108
rs7742829
rs12404911
rs73128290
All
−0.1
0.0
0.1
0.2
0.3
MR effect size for
'Gut microbiota abundance (genus Catenibacterium id.2153)' on 'Insulin−like peptide INSL6'

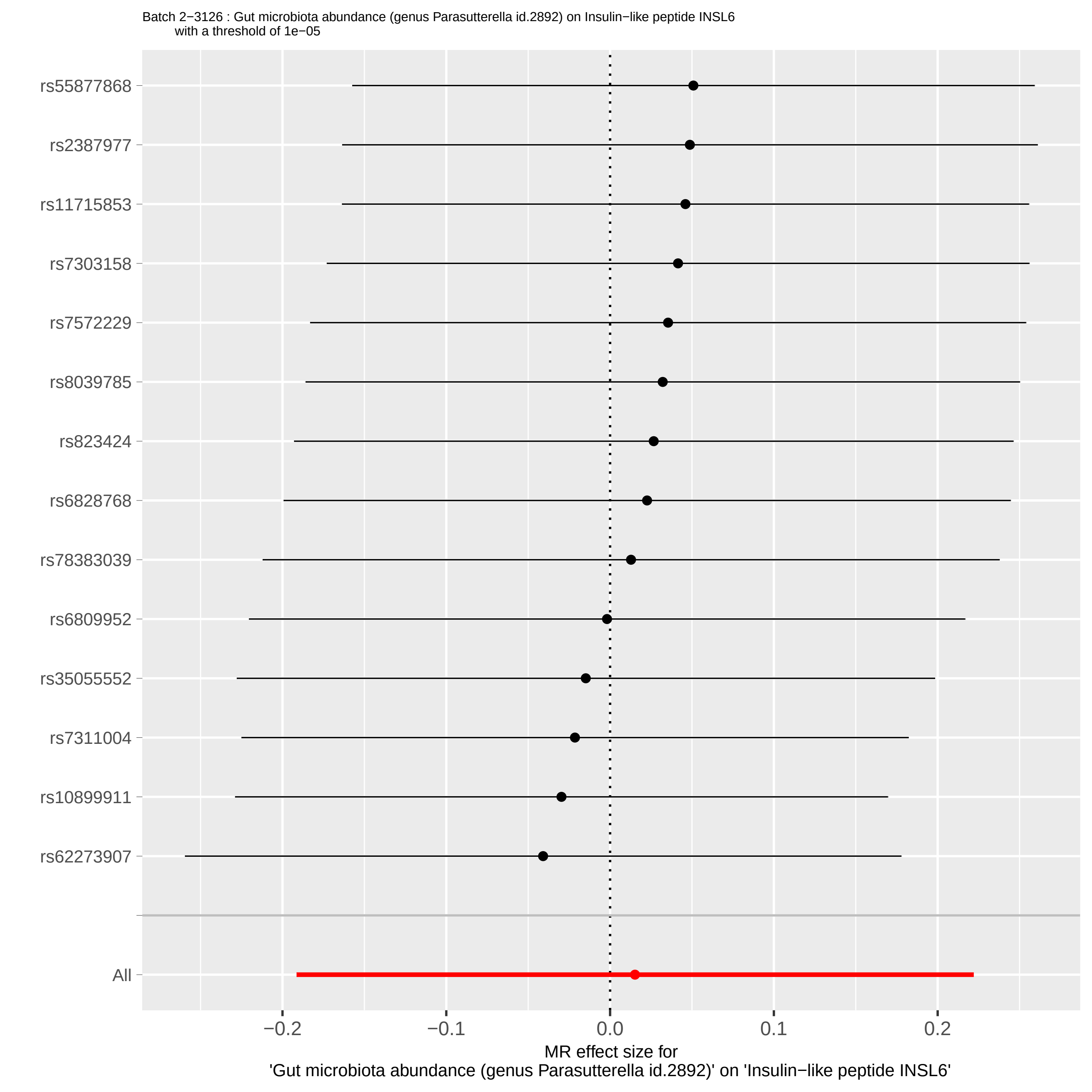

Batch 2−3126 : Gut microbiota abundance (genus Parasutterella id.2892) on Insulin−like peptide INSL6
with a threshold of 1e−05
rs55877868
rs2387977
rs11715853
rs7303158
rs7572229
rs8039785
rs823424
rs6828768
rs78383039
rs6809952
rs35055552
rs7311004
rs10899911
rs62273907
All
−0.2
−0.1
0.0
0.1
0.2
MR effect size for
'Gut microbiota abundance (genus Parasutterella id.2892)' on 'Insulin−like peptide INSL6'

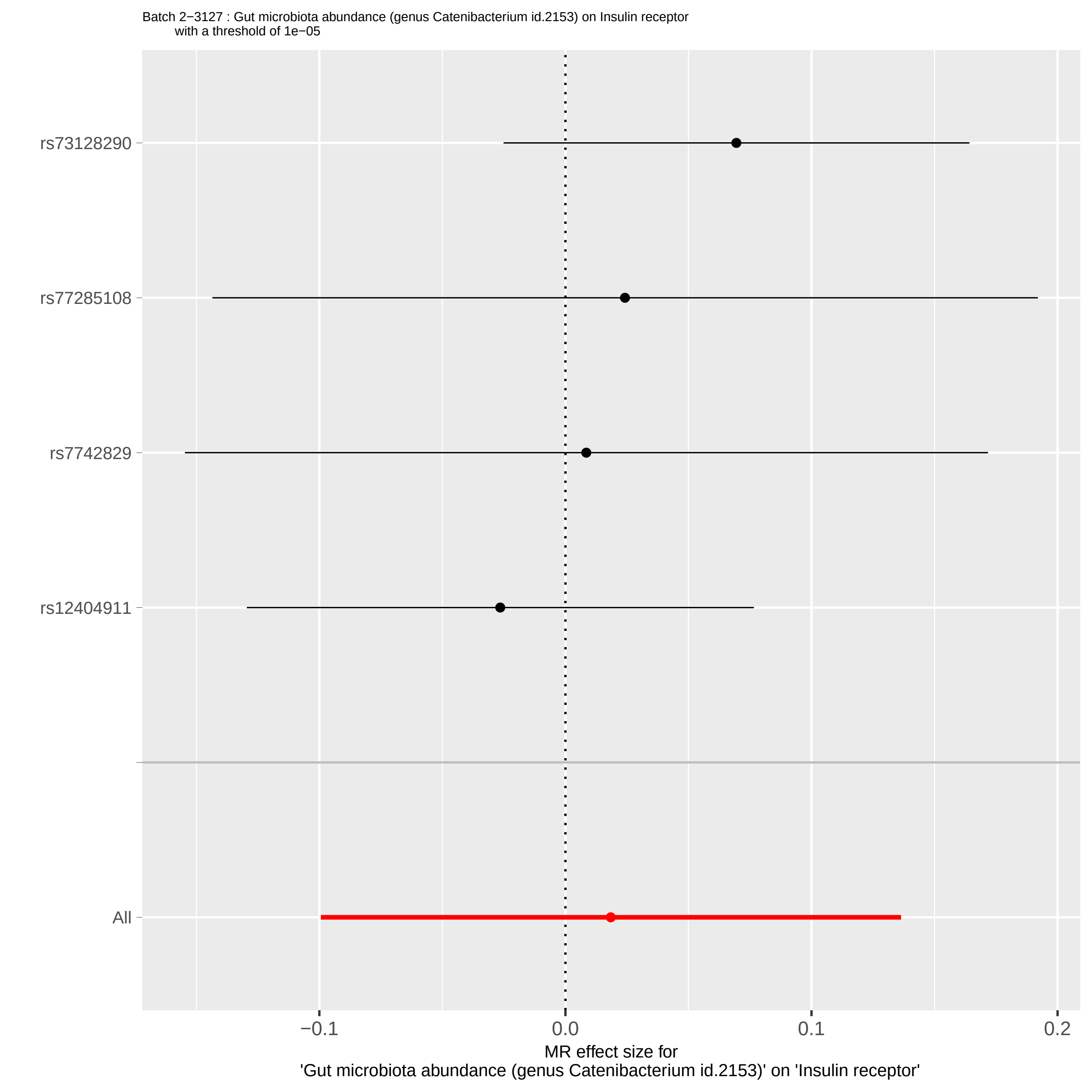
Batch 2−3127 : Gut microbiota abundance (genus Catenibacterium id.2153) on Insulin receptor
with a threshold of 1e−05
rs73128290
rs77285108
rs7742829
rs12404911
All
−0.1
0.0
0.1
0.2
MR effect size for
'Gut microbiota abundance (genus Catenibacterium id.2153)' on 'Insulin receptor'

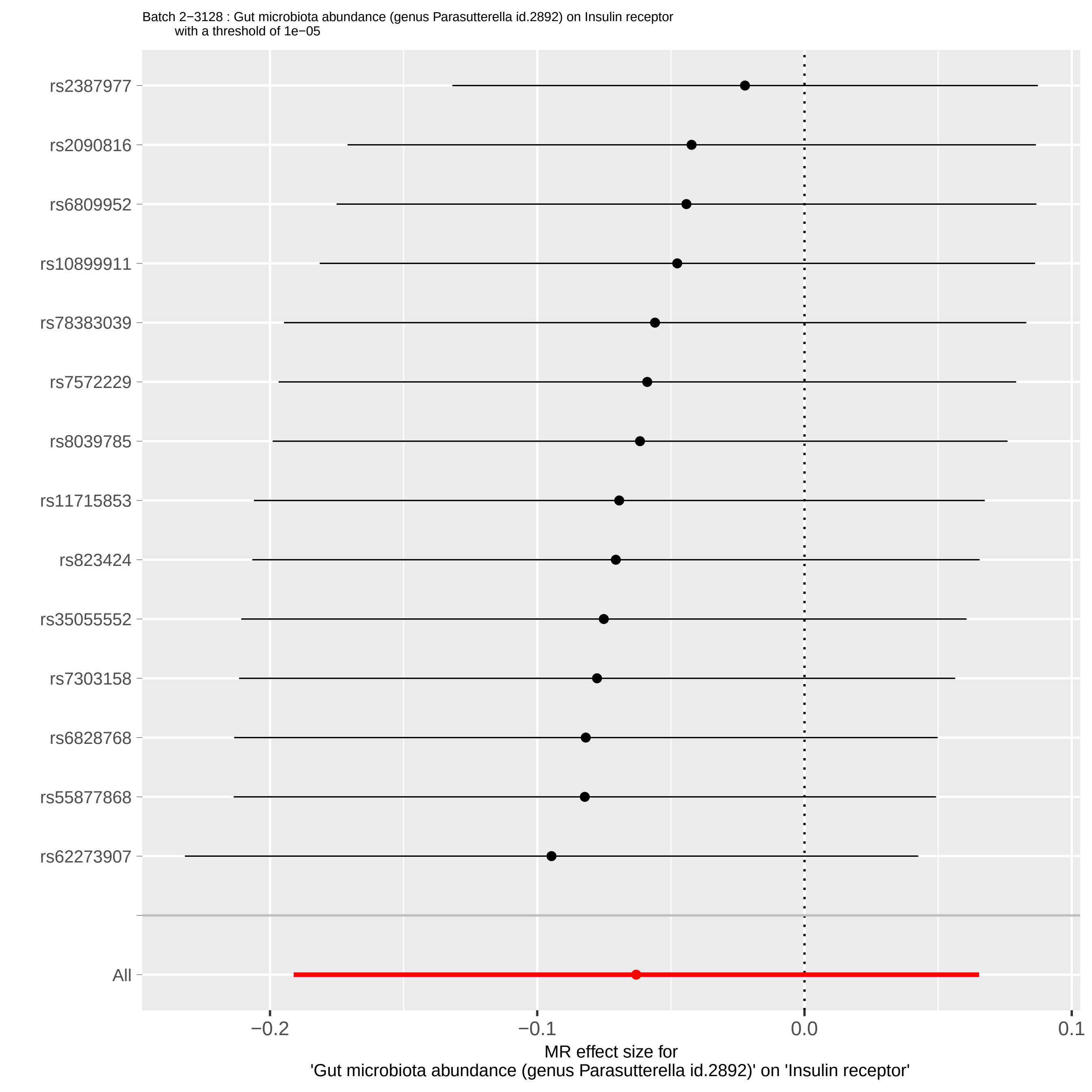

Batch 2−3128 : Gut microbiota abundance (genus Parasutterella id.2892) on Insulin receptor
with a threshold of 1e−05
rs2387977
rs2090816
rs6809952
rs10899911
rs78383039
rs7572229
rs8039785
rs11715853
rs823424
rs35055552
rs7303158
rs6828768
rs55877868
rs62273907
All
−0.2
−0.1
0.0
0.1
MR effect size for
'Gut microbiota abundance (genus Parasutterella id.2892)' on 'Insulin receptor'

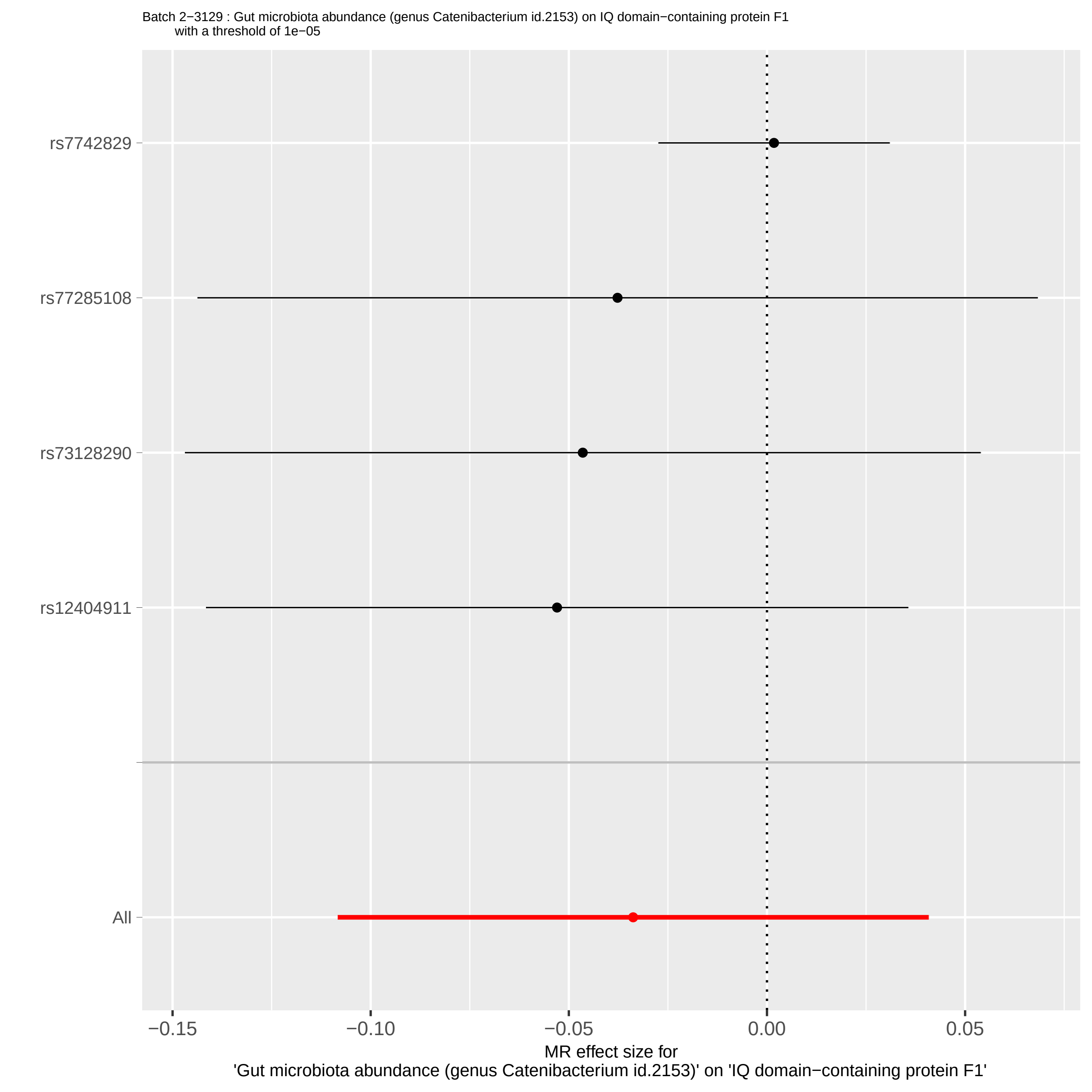
Batch 2−3129 : Gut microbiota abundance (genus Catenibacterium id.2153) on IQ domain−containing protein F1
with a threshold of 1e−05
rs7742829
rs77285108
rs73128290
rs12404911
All
−0.15
−0.10
−0.05
0.00
0.05
MR effect size for
'Gut microbiota abundance (genus Catenibacterium id.2153)' on 'IQ domain−containing protein F1'

Batch 2−3130 : Gut microbiota abundance (genus Parasutterella id.2892) on IQ domain−containing protein F1
with a threshold of 1e−05

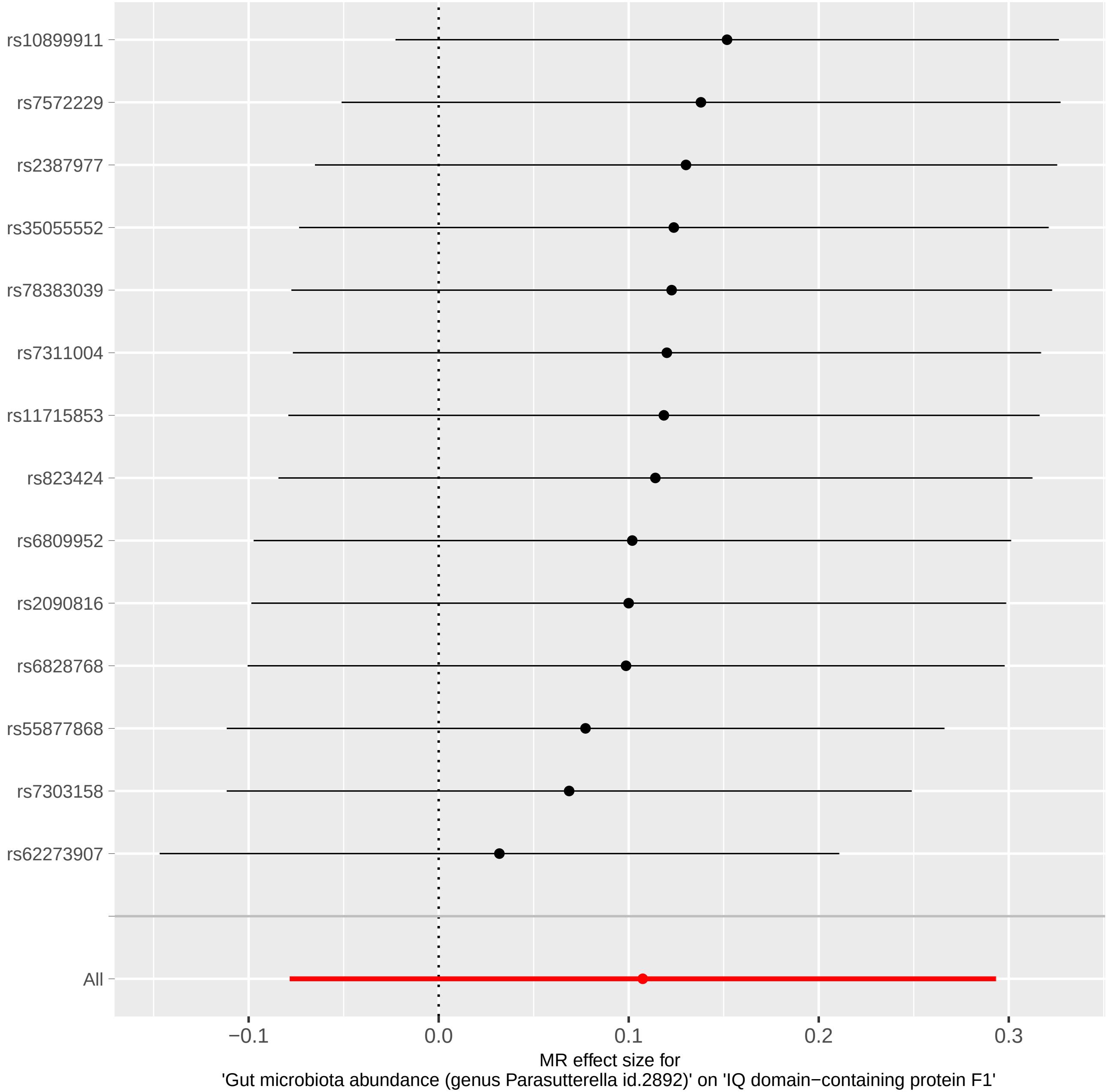

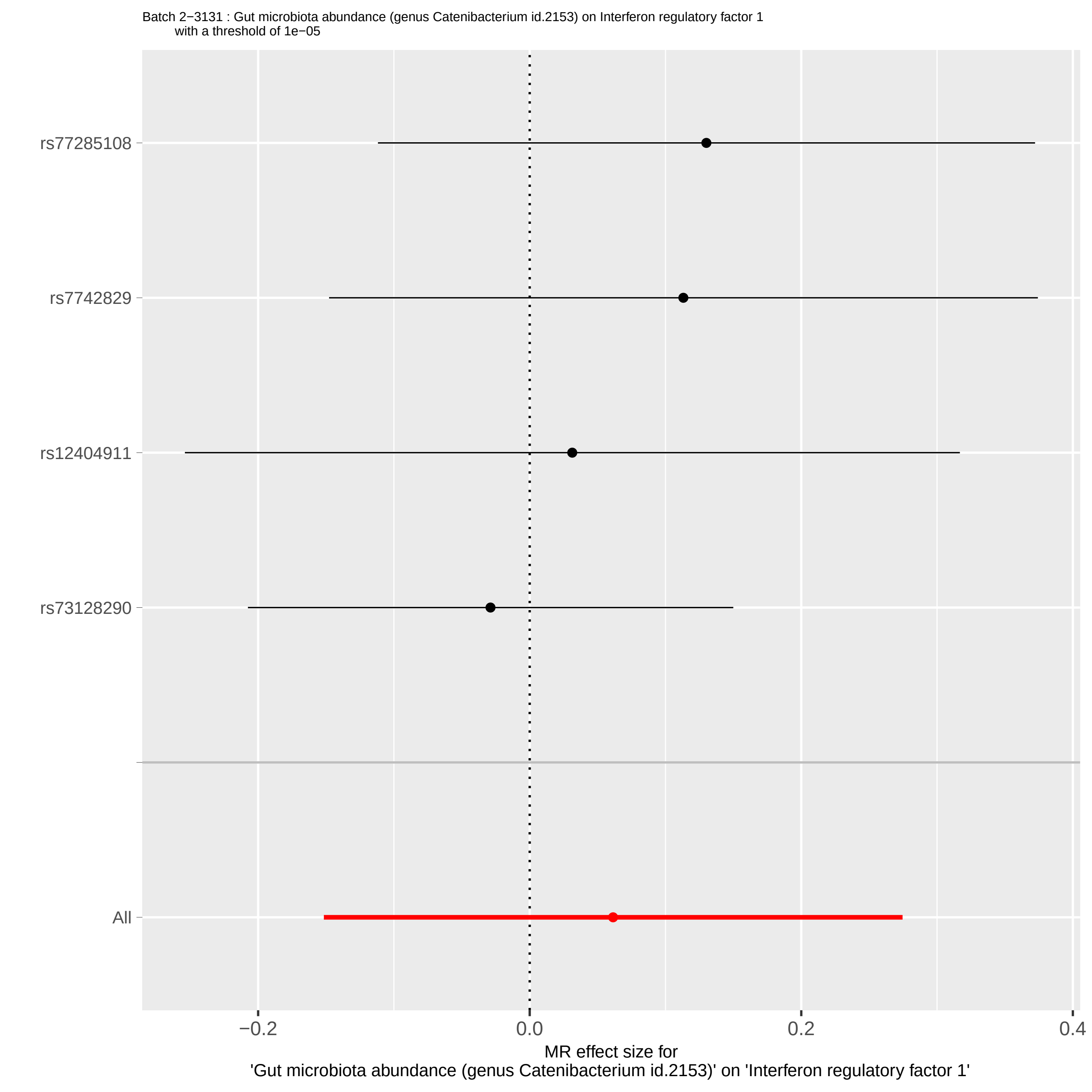

Batch 2−3131 : Gut microbiota abundance (genus Catenibacterium id.2153) on Interferon regulatory factor 1
with a threshold of 1e−05
rs77285108
rs7742829
rs12404911
rs73128290
All
−0.2
0.0
0.2
0.4
MR effect size for
'Gut microbiota abundance (genus Catenibacterium id.2153)' on 'Interferon regulatory factor 1'

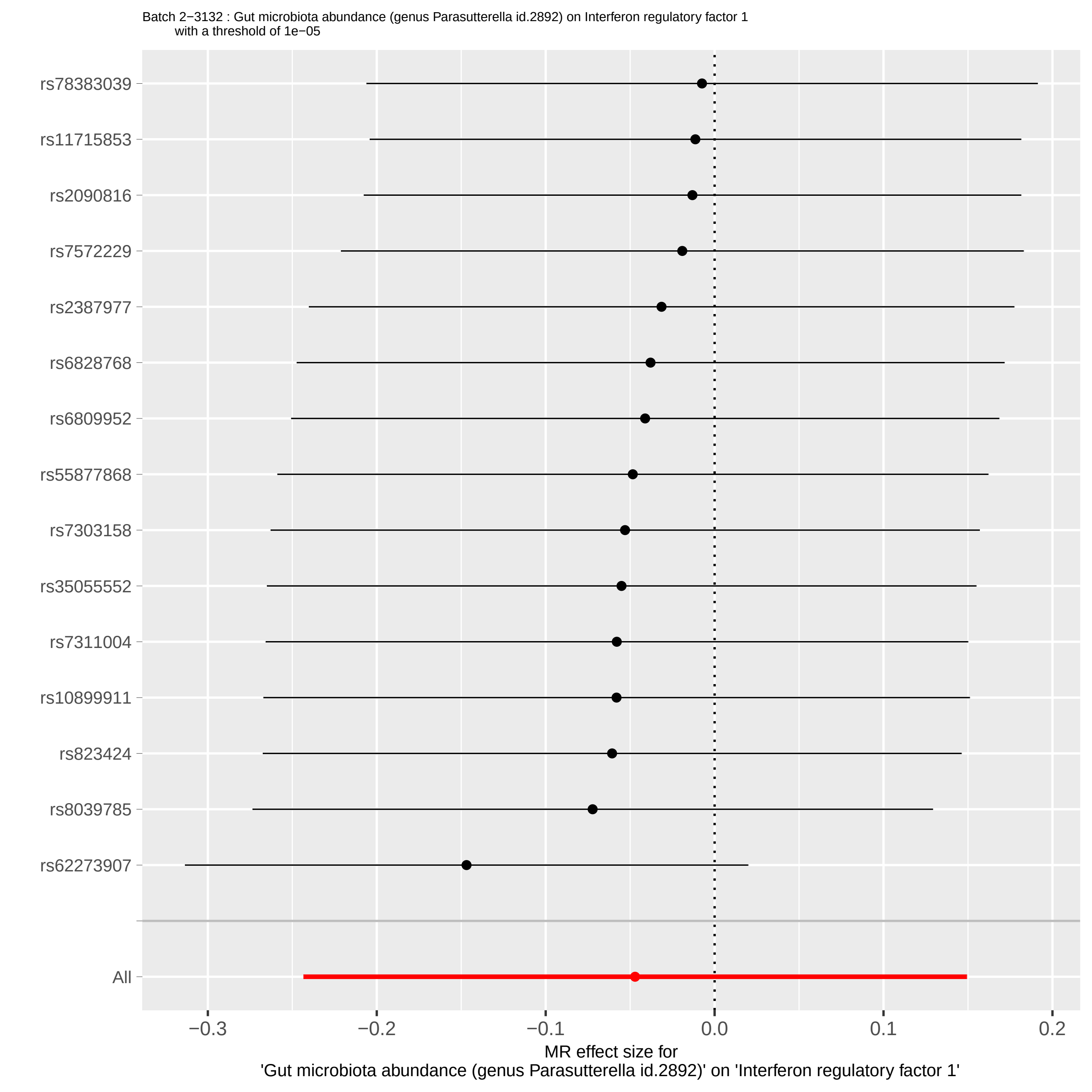

Batch 2−3132 : Gut microbiota abundance (genus Parasutterella id.2892) on Interferon regulatory factor 1 with a threshold of 1e−05
rs78383039
rs11715853
rs2090816
rs7572229
rs2387977
rs6828768
rs6809952
rs55877868
rs7303158
rs35055552
rs7311004
rs10899911
rs823424
rs8039785
rs62273907
All
−0.3
−0.2
−0.1
0.0
0.1
0.2
MR effect size for
'Gut microbiota abundance (genus Parasutterella id.2892)' on 'Interferon regulatory factor 1'

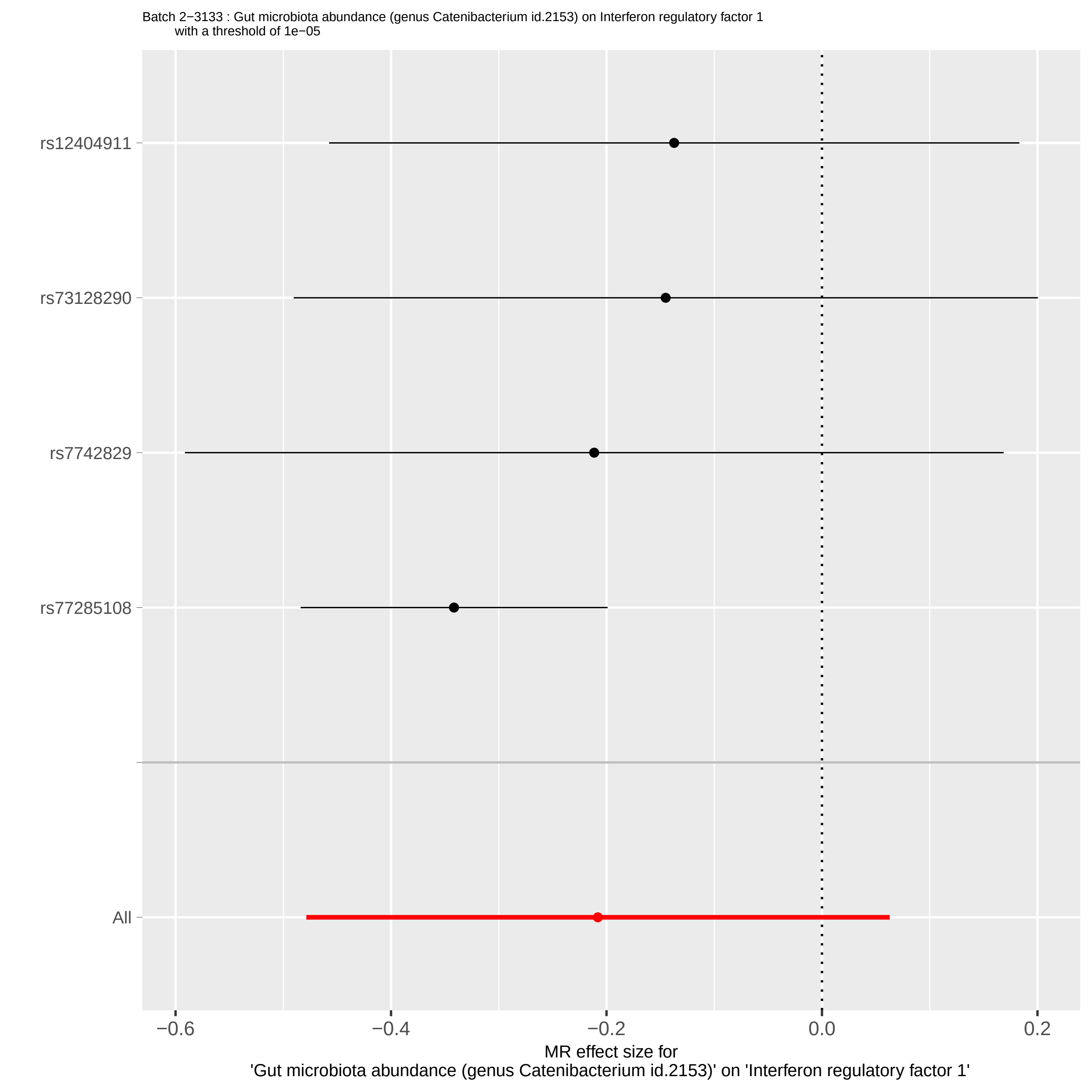
Batch 2−3133 : Gut microbiota abundance (genus Catenibacterium id.2153) on Interferon regulatory factor 1
with a threshold of 1e−05
rs12404911
rs73128290
rs7742829
rs77285108
All
−0.6
−0.4
−0.2
0.0
0.2
MR effect size for
'Gut microbiota abundance (genus Catenibacterium id.2153)' on 'Interferon regulatory factor 1'

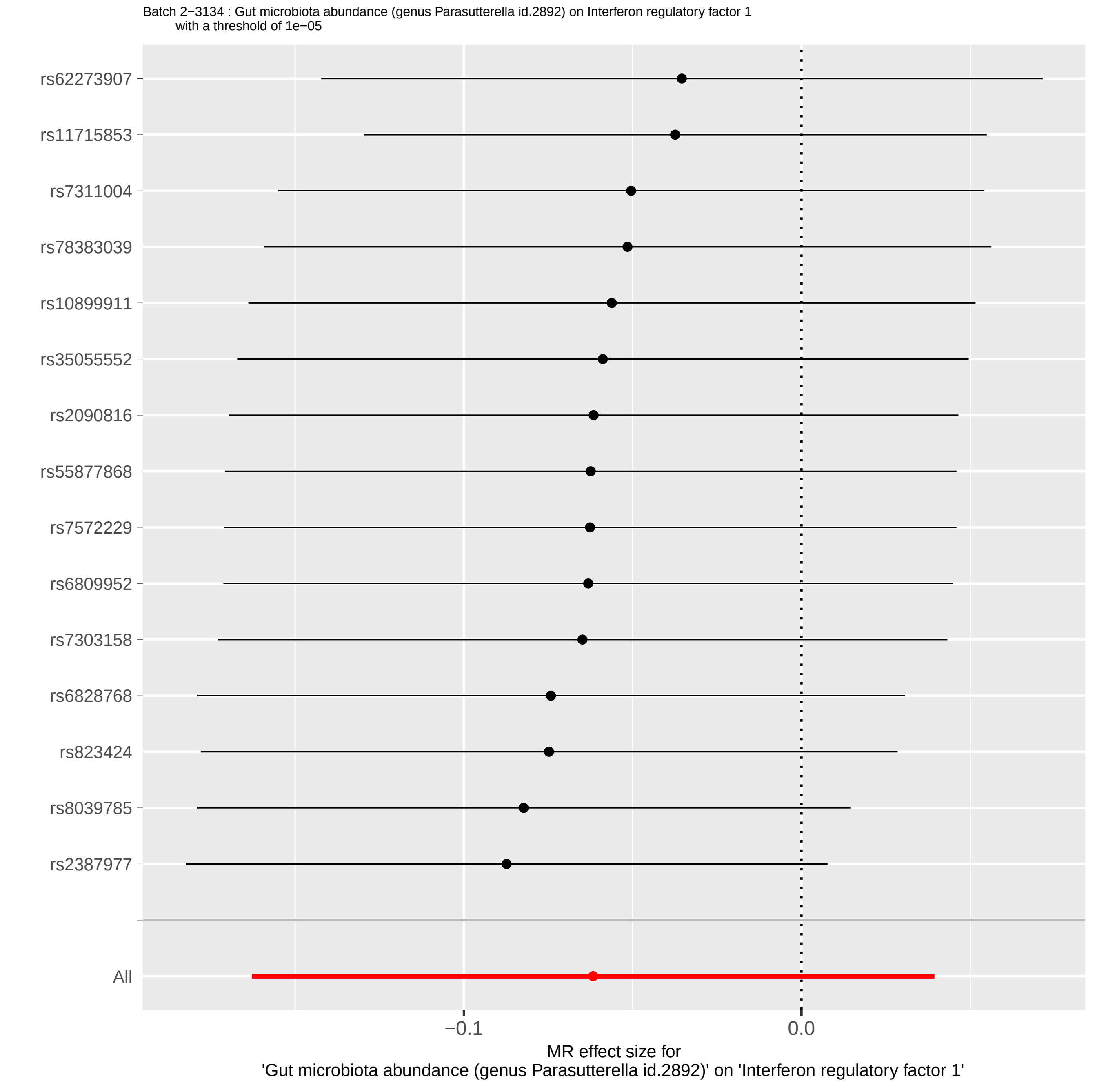

Batch 2−3134 : Gut microbiota abundance (genus Parasutterella id.2892) on Interferon regulatory factor 1
with a threshold of 1e−05
rs62273907
rs11715853
rs7311004
rs78383039
rs10899911
rs35055552
rs2090816
rs55877868
rs7572229
rs6809952
rs7303158
rs6828768
rs823424
rs8039785
rs2387977
All
−0.1
0.0
MR effect size for
'Gut microbiota abundance (genus Parasutterella id.2892)' on 'Interferon regulatory factor 1'

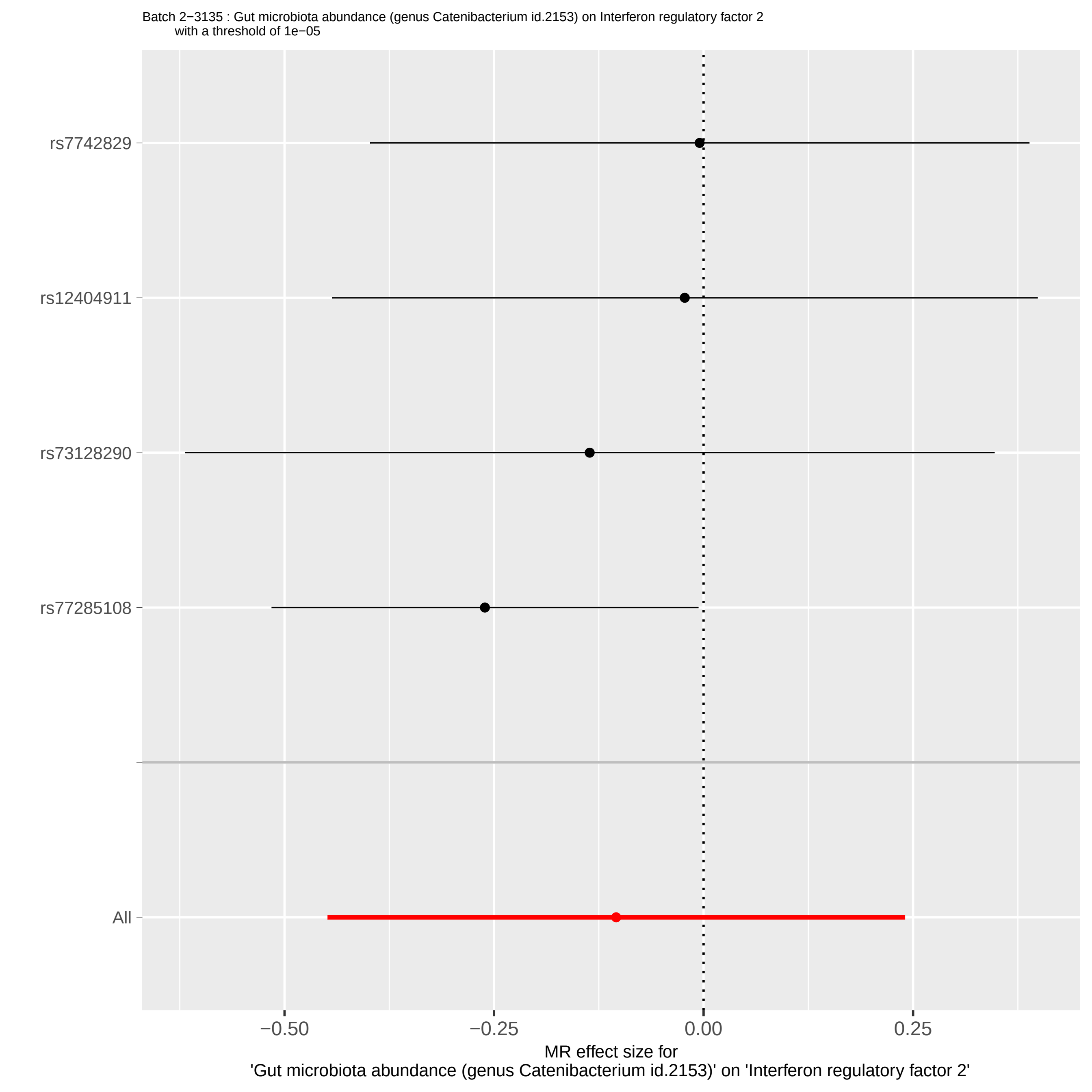
Batch 2−3135 : Gut microbiota abundance (genus Catenibacterium id.2153) on Interferon regulatory factor 2
with a threshold of 1e−05
rs7742829
rs12404911
rs73128290
rs77285108
All
−0.50
−0.25
0.00
0.25
MR effect size for
'Gut microbiota abundance (genus Catenibacterium id.2153)' on 'Interferon regulatory factor 2'

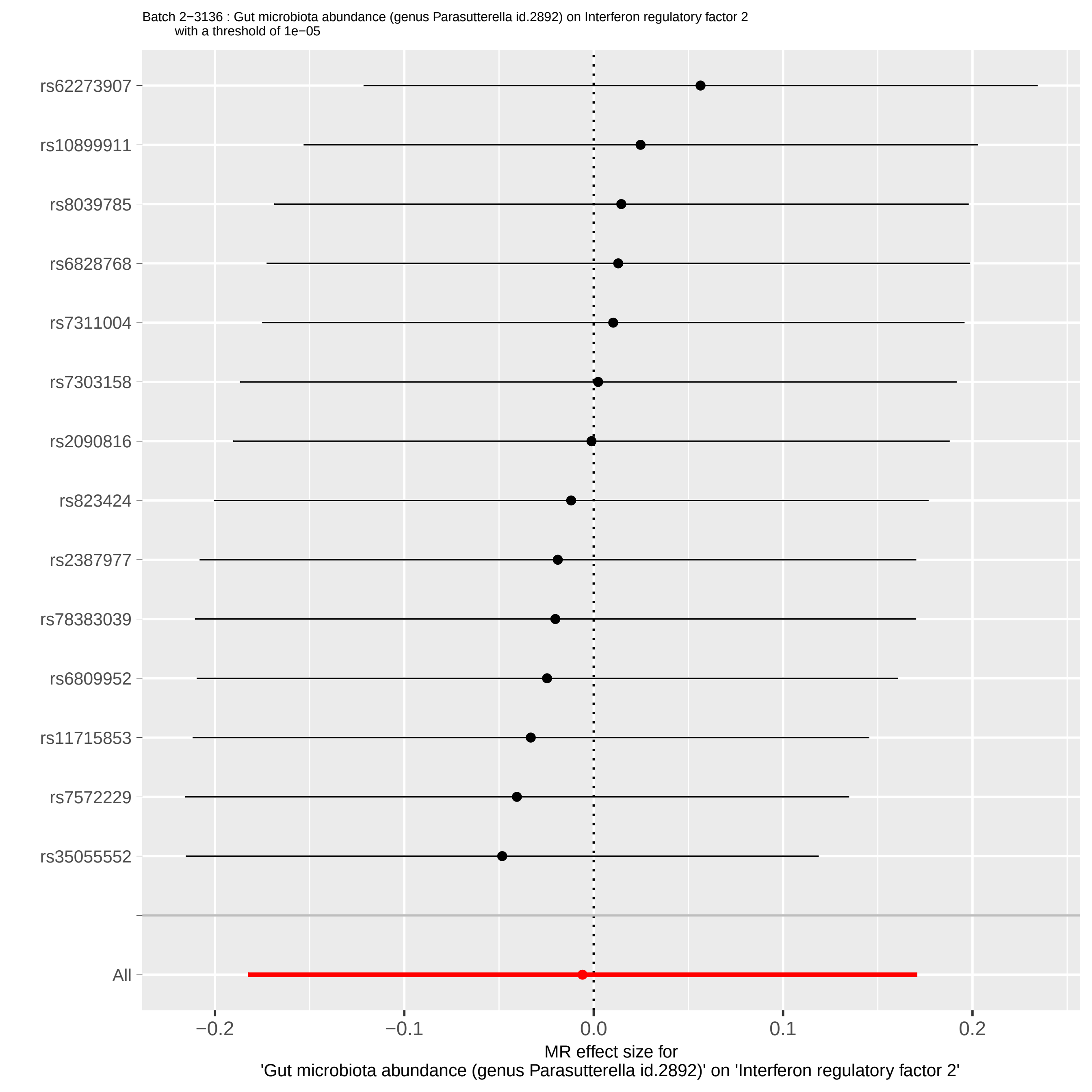
Batch 2−3136 : Gut microbiota abundance (genus Parasutterella id.2892) on Interferon regulatory factor 2
with a threshold of 1e−05
rs62273907
rs10899911
rs8039785
rs6828768
rs7311004
rs7303158
rs2090816
rs823424
rs2387977
rs78383039
rs6809952
rs11715853
rs7572229
rs35055552
All
−0.2
−0.1
0.0
0.1
0.2
MR effect size for
'Gut microbiota abundance (genus Parasutterella id.2892)' on 'Interferon regulatory factor 2'

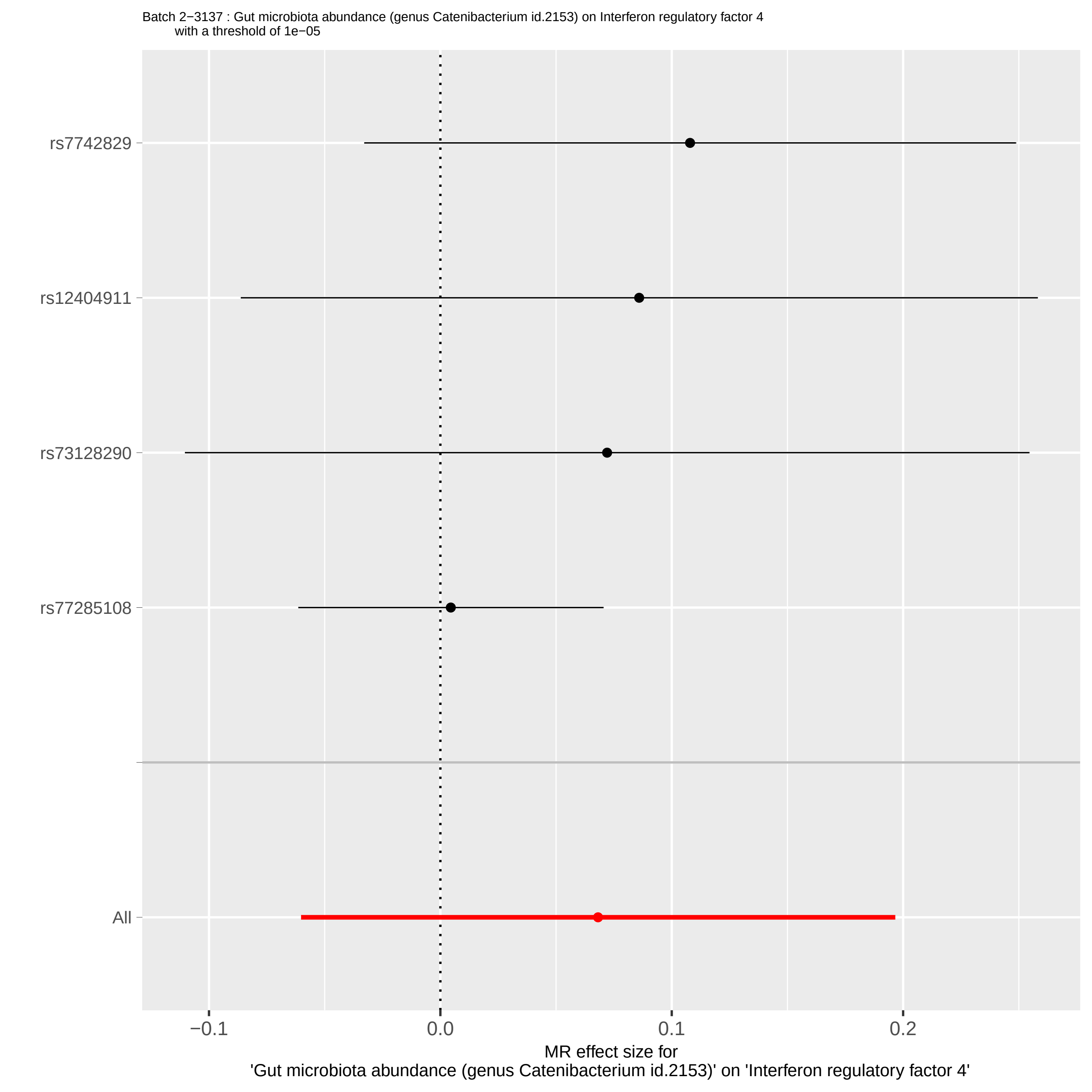
Batch 2−3137 : Gut microbiota abundance (genus Catenibacterium id.2153) on Interferon regulatory factor 4
with a threshold of 1e−05
rs7742829
rs12404911
rs73128290
rs77285108
All
−0.1
0.0
0.1
0.2
MR effect size for
'Gut microbiota abundance (genus Catenibacterium id.2153)' on 'Interferon regulatory factor 4'

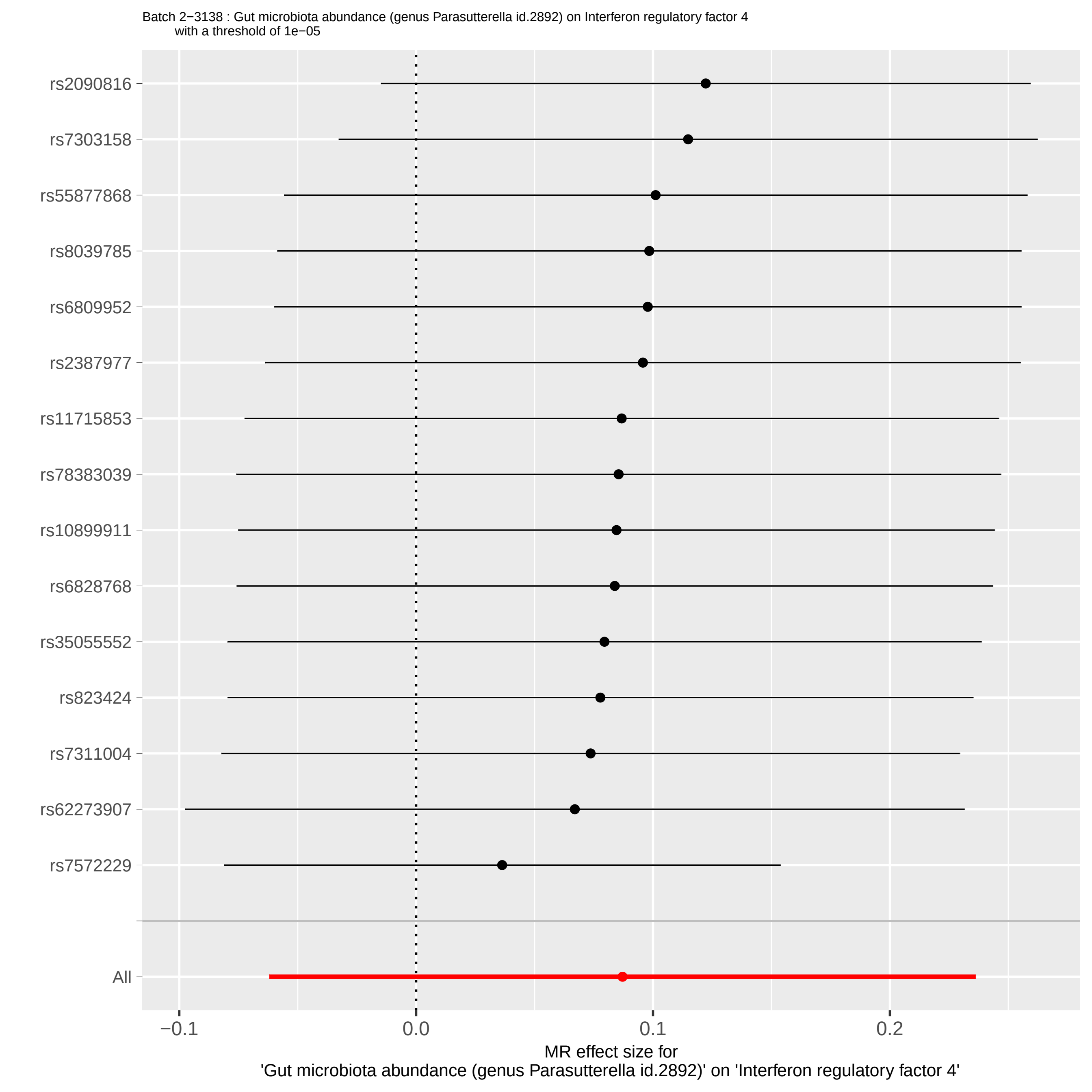
Batch 2−3138 : Gut microbiota abundance (genus Parasutterella id.2892) on Interferon regulatory factor 4
with a threshold of 1e−05
rs2090816
rs7303158
rs55877868
rs8039785
rs6809952
rs2387977
rs11715853
rs78383039
rs10899911
rs6828768
rs35055552
rs823424
rs7311004
rs62273907
rs7572229
All
−0.1
0.0
0.1
0.2
MR effect size for
'Gut microbiota abundance (genus Parasutterella id.2892)' on 'Interferon regulatory factor 4'

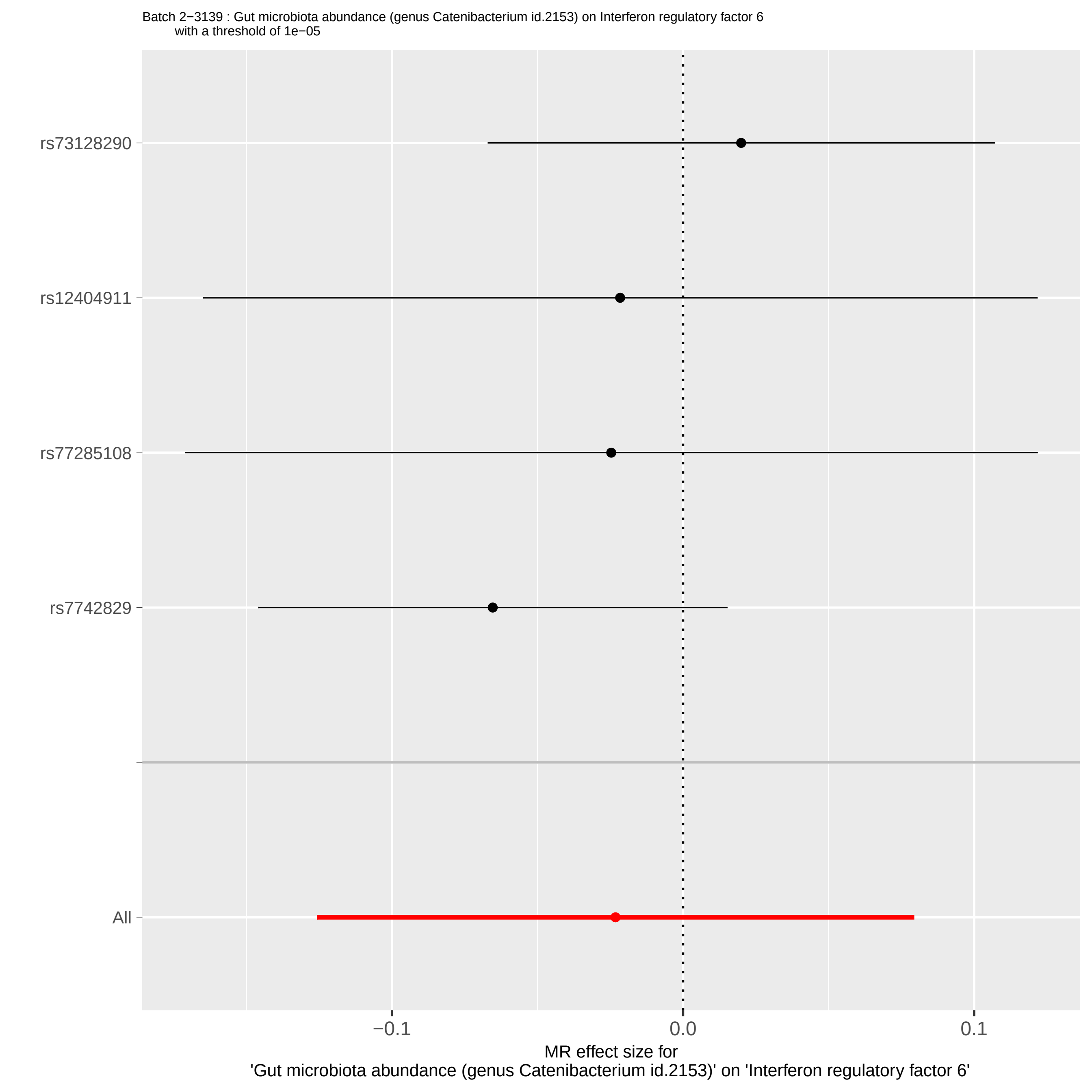

Batch 2−3139 : Gut microbiota abundance (genus Catenibacterium id.2153) on Interferon regulatory factor 6
with a threshold of 1e−05
rs73128290
rs12404911
rs77285108
rs7742829
All
−0.1
0.0
0.1
MR effect size for
'Gut microbiota abundance (genus Catenibacterium id.2153)' on 'Interferon regulatory factor 6'

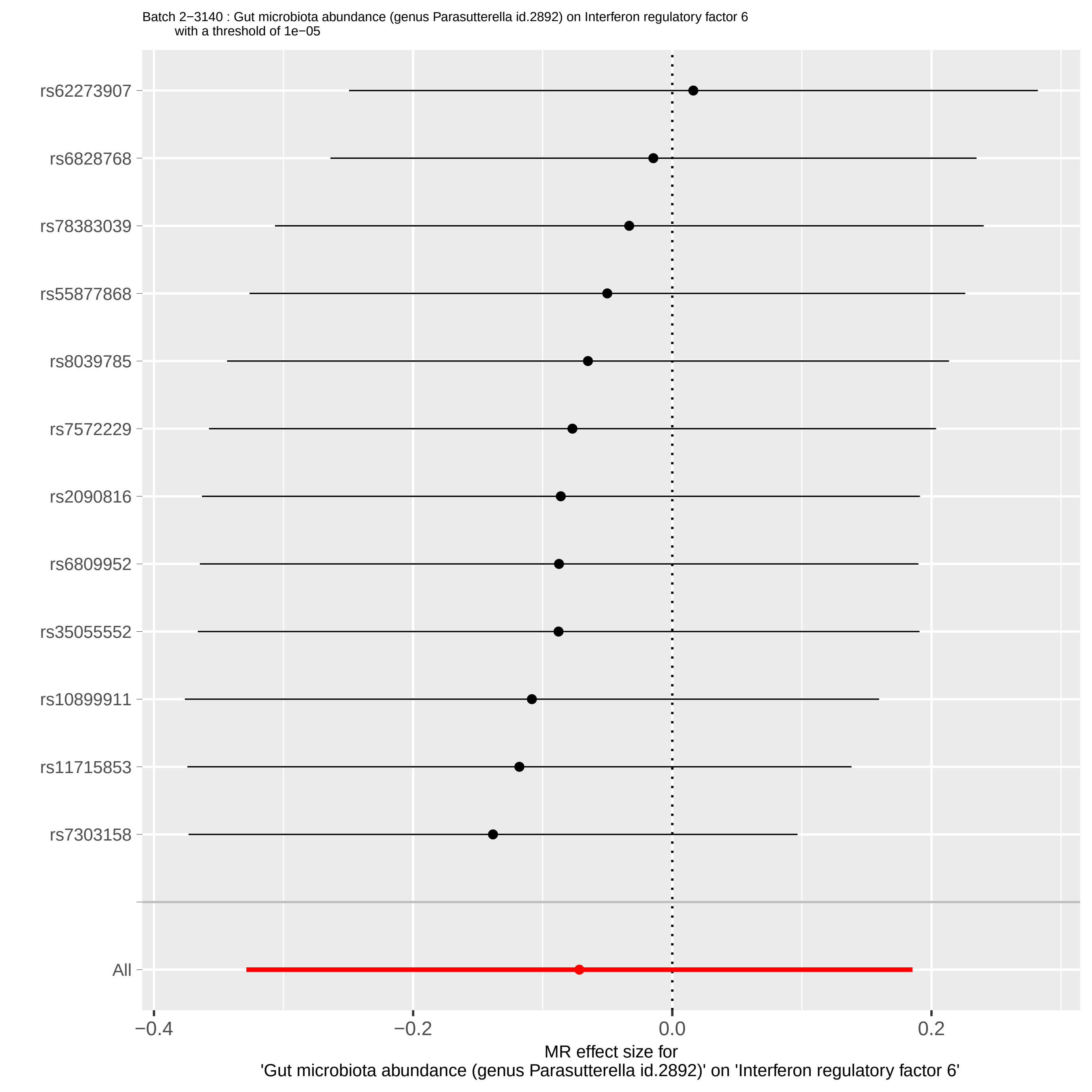

Batch 2−3140 : Gut microbiota abundance (genus Parasutterella id.2892) on Interferon regulatory factor 6
with a threshold of 1e−05
rs62273907
rs6828768
rs78383039
rs55877868
rs8039785
rs7572229
rs2090816
rs6809952
rs35055552
rs10899911
rs11715853
rs7303158
All
−0.4
−0.2
0.0
0.2
MR effect size for
'Gut microbiota abundance (genus Parasutterella id.2892)' on 'Interferon regulatory factor 6'

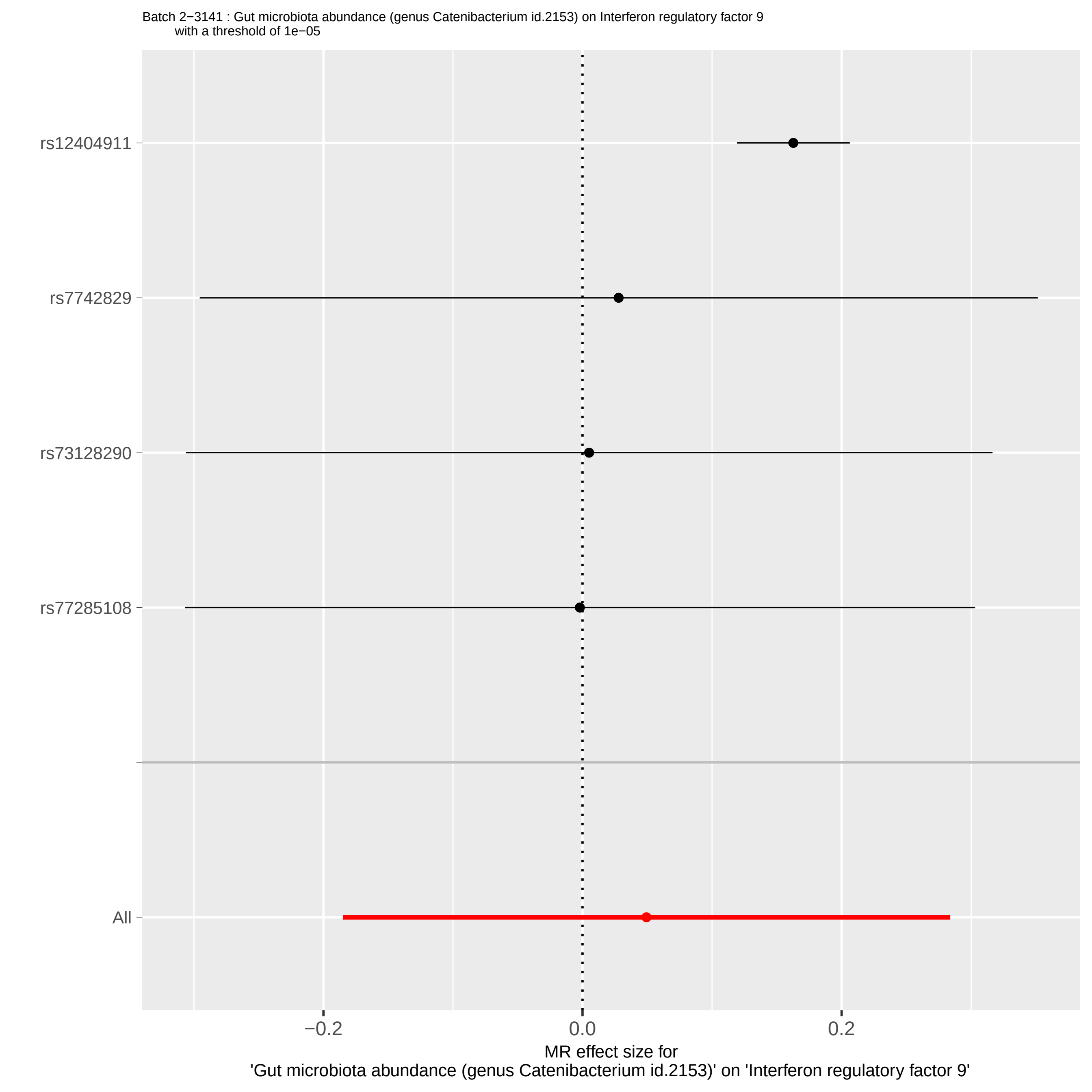
Batch 2−3141 : Gut microbiota abundance (genus Catenibacterium id.2153) on Interferon regulatory factor 9
with a threshold of 1e−05
rs12404911
rs7742829
rs73128290
rs77285108
All
−0.2
0.0
0.2
MR effect size for
'Gut microbiota abundance (genus Catenibacterium id.2153)' on 'Interferon regulatory factor 9'

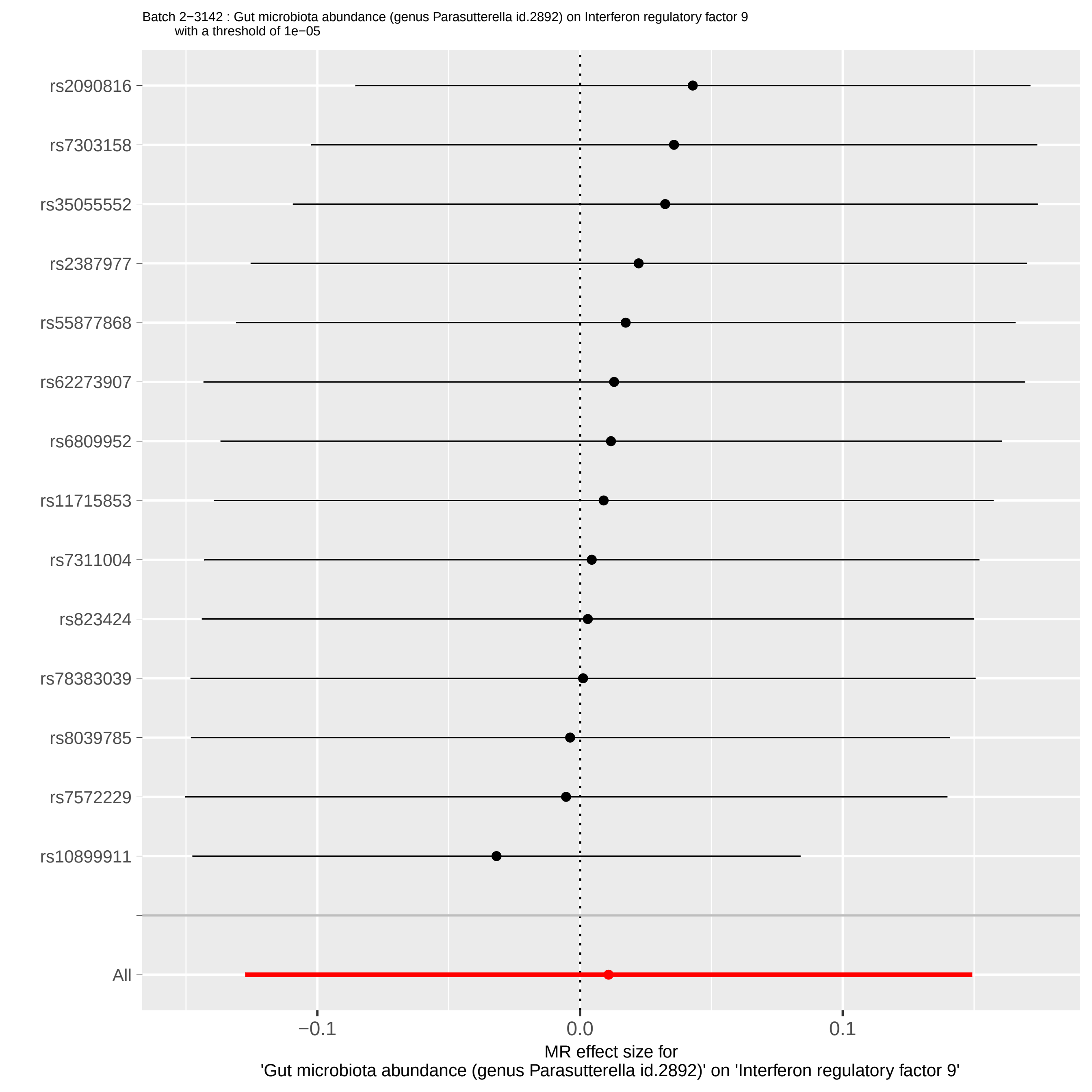

Batch 2−3142 : Gut microbiota abundance (genus Parasutterella id.2892) on Interferon regulatory factor 9
with a threshold of 1e−05
rs2090816
rs7303158
rs35055552
rs2387977
rs55877868
rs62273907
rs6809952
rs11715853
rs7311004
rs823424
rs78383039
rs8039785
rs7572229
rs10899911
All
−0.1
0.0
0.1
MR effect size for
'Gut microbiota abundance (genus Parasutterella id.2892)' on 'Interferon regulatory factor 9'

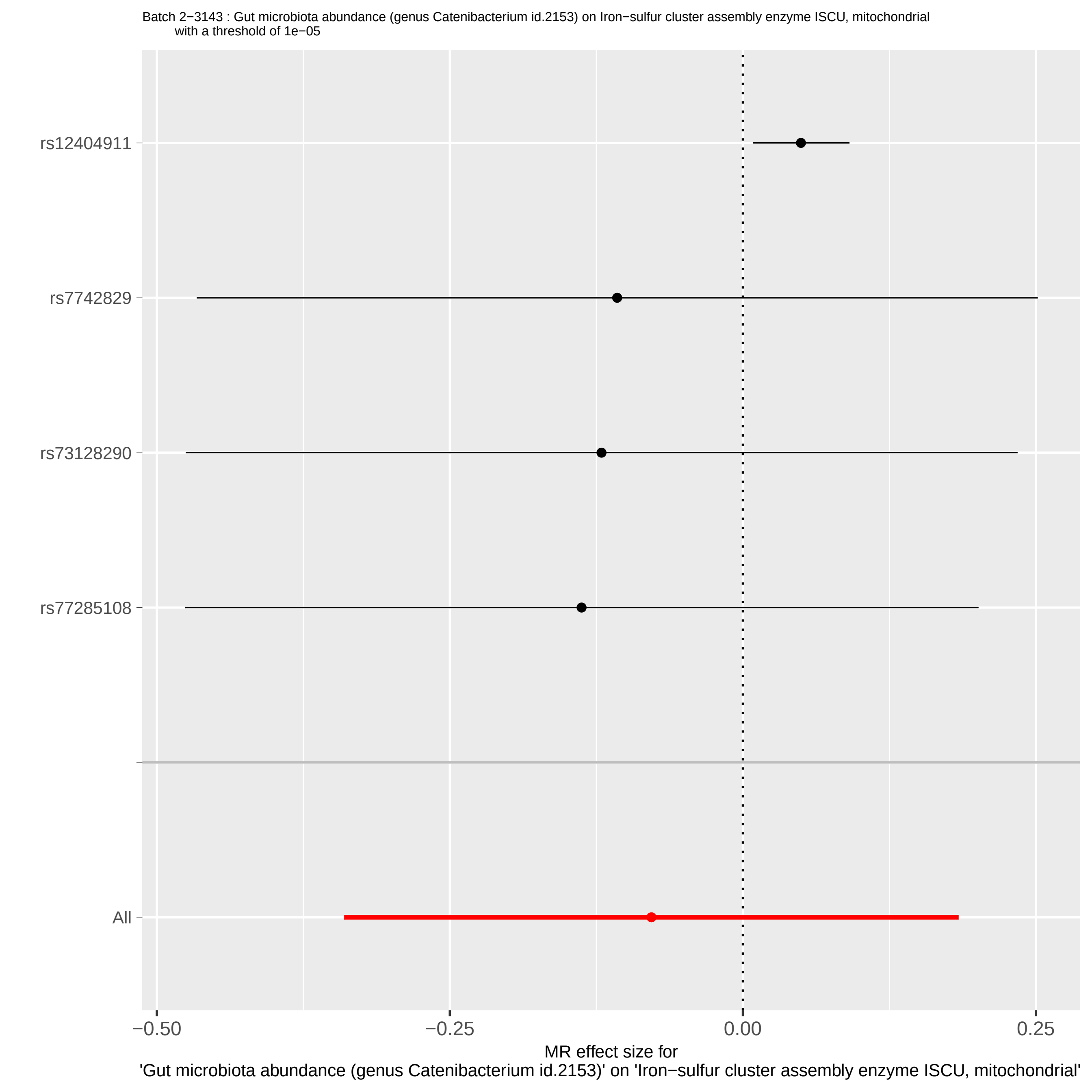
Batch 2−3143 : Gut microbiota abundance (genus Catenibacterium id.2153) on Iron−sulfur cluster assembly enzyme ISCU, mitochondrial
with a threshold of 1e−05
rs12404911
rs7742829
rs73128290
rs77285108
All
−0.50
−0.25
0.00
0.25
MR effect size for
'Gut microbiota abundance (genus Catenibacterium id.2153)' on 'Iron−sulfur cluster assembly enzyme ISCU, mitochondrial'

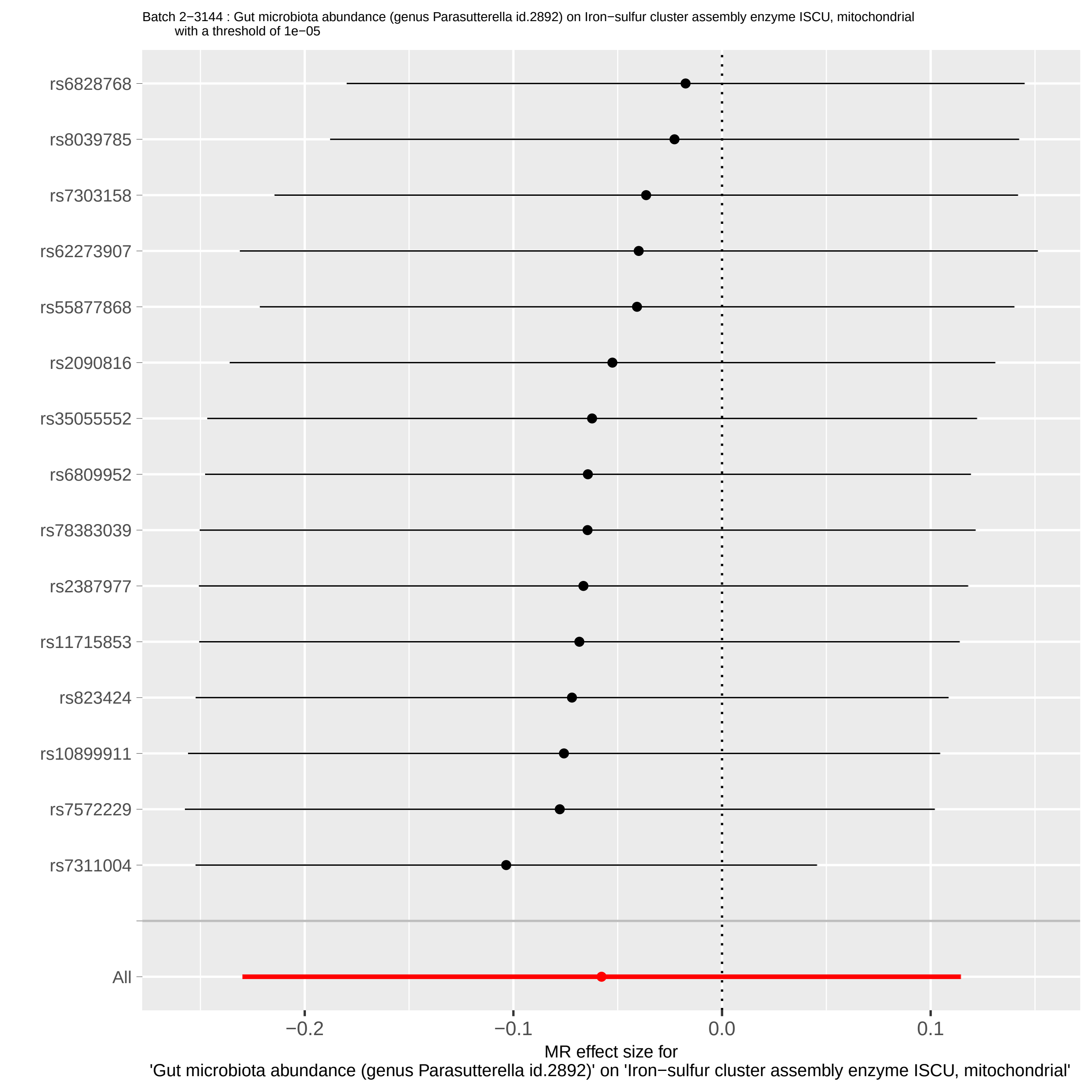

Batch 2−3144 : Gut microbiota abundance (genus Parasutterella id.2892) on Iron−sulfur cluster assembly enzyme ISCU, mitochondrial
with a threshold of 1e−05
rs6828768
rs8039785
rs7303158
rs62273907
rs55877868
rs2090816
rs35055552
rs6809952
rs78383039
rs2387977
rs11715853
rs823424
rs10899911
rs7572229
rs7311004
All
−0.2
−0.1
0.0
0.1
MR effect size for
'Gut microbiota abundance (genus Parasutterella id.2892)' on 'Iron−sulfur cluster assembly enzyme ISCU, mitochondrial'

Batch 2−3145 : Gut microbiota abundance (genus Catenibacterium id.2153) on Ubiquitin−like protein ISG15
with a threshold of 1e−05

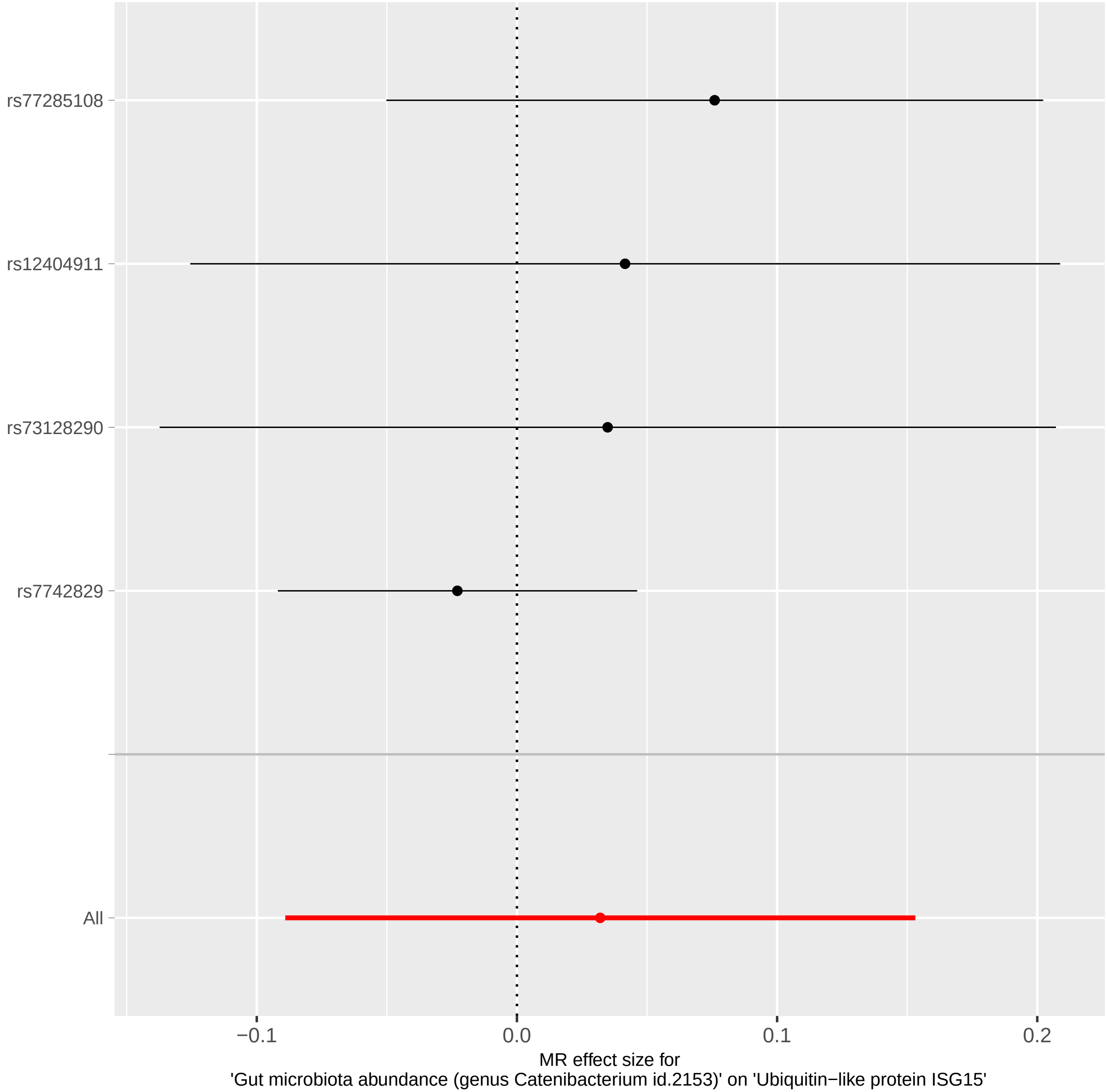

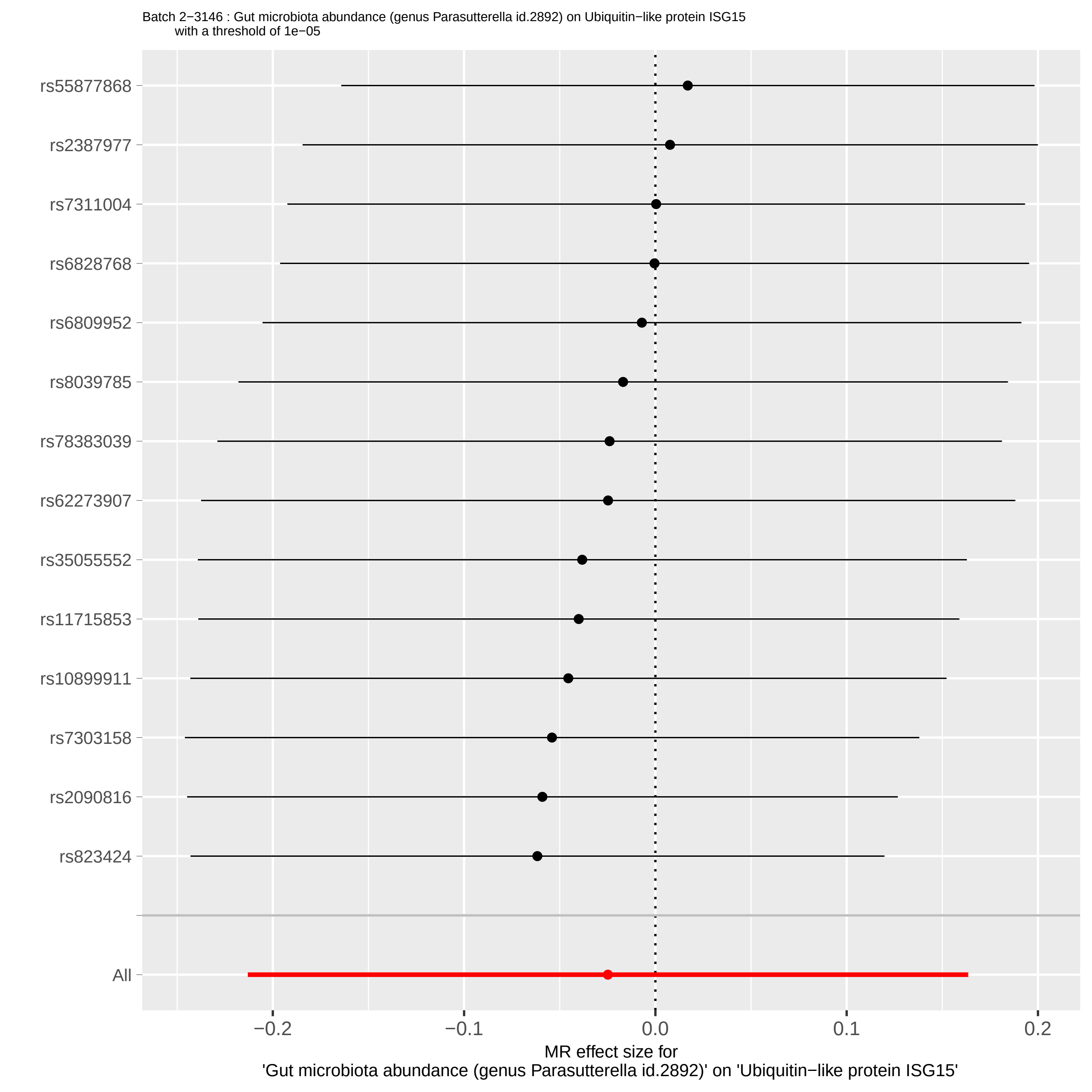

Batch 2−3146 : Gut microbiota abundance (genus Parasutterella id.2892) on Ubiquitin−like protein ISG15
with a threshold of 1e−05
rs55877868
rs2387977
rs7311004
rs6828768
rs6809952
rs8039785
rs78383039
rs62273907
rs35055552
rs11715853
rs10899911
rs7303158
rs2090816
rs823424
All
−0.2
−0.1
0.0
0.1
0.2
MR effect size for
'Gut microbiota abundance (genus Parasutterella id.2892)' on 'Ubiquitin−like protein ISG15'

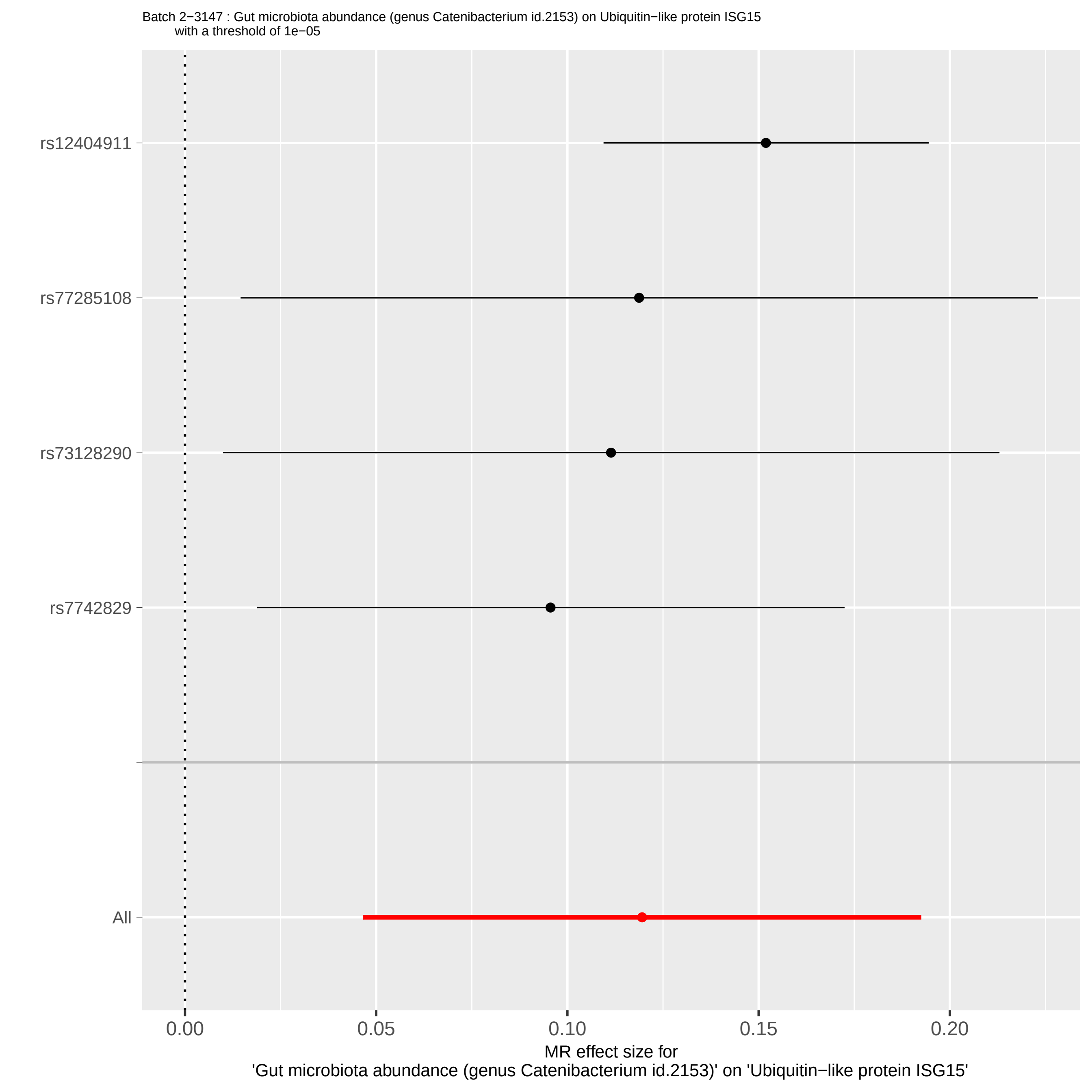

Batch 2−3147 : Gut microbiota abundance (genus Catenibacterium id.2153) on Ubiquitin−like protein ISG15
with a threshold of 1e−05
rs12404911
rs77285108
rs73128290
rs7742829
All
0.00
0.05
0.10
0.15
0.20
MR effect size for
'Gut microbiota abundance (genus Catenibacterium id.2153)' on 'Ubiquitin−like protein ISG15'

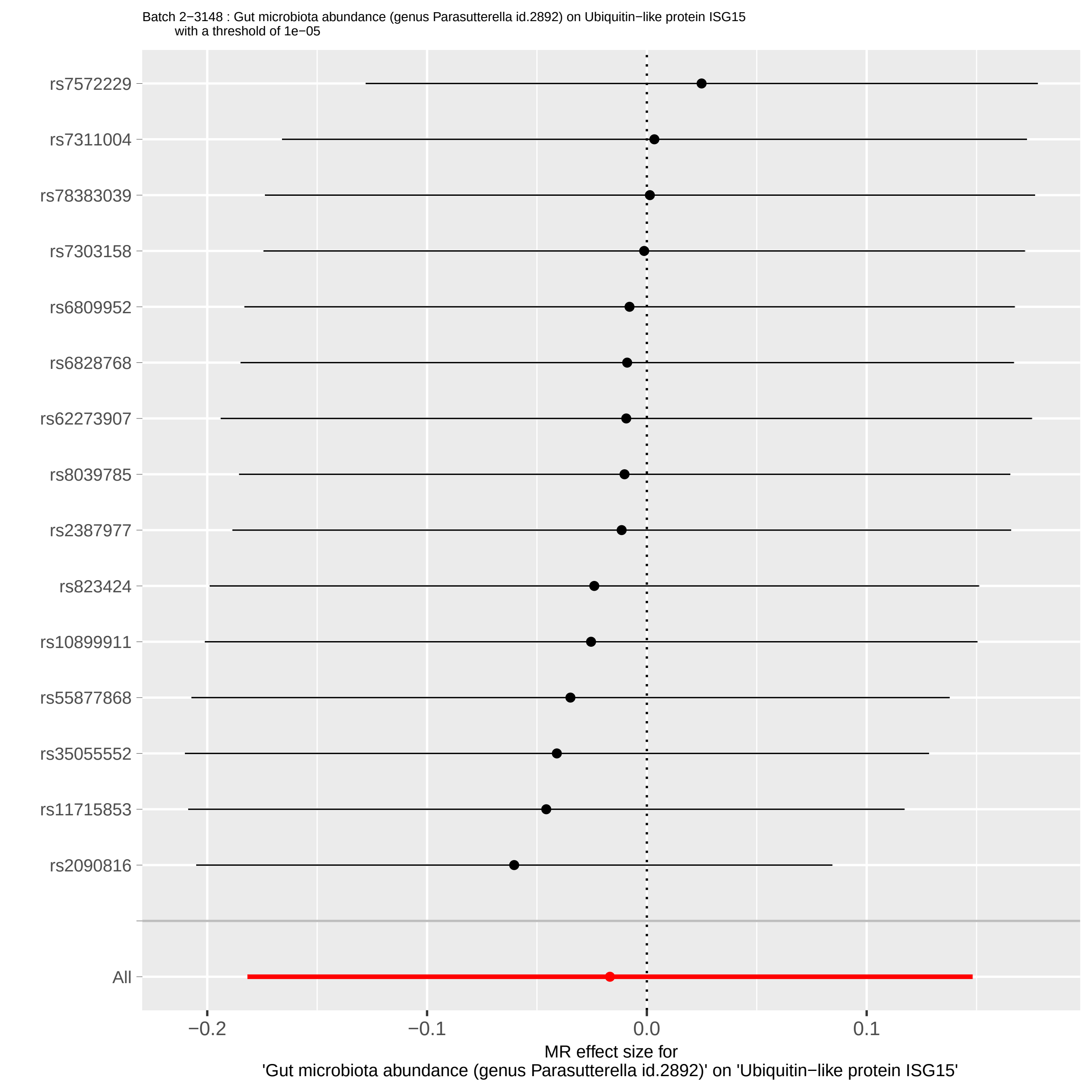

Batch 2−3148 : Gut microbiota abundance (genus Parasutterella id.2892) on Ubiquitin−like protein ISG15
with a threshold of 1e−05
rs7572229
rs7311004
rs78383039
rs7303158
rs6809952
rs6828768
rs62273907
rs8039785
rs2387977
rs823424
rs10899911
rs55877868
rs35055552
rs11715853
rs2090816
All
−0.2
−0.1
0.0
0.1
MR effect size for
'Gut microbiota abundance (genus Parasutterella id.2892)' on 'Ubiquitin−like protein ISG15'

Batch 2−3149 : Gut microbiota abundance (genus Catenibacterium id.2153) on Insulin gene enhancer protein ISL−1
with a threshold of 1e−05

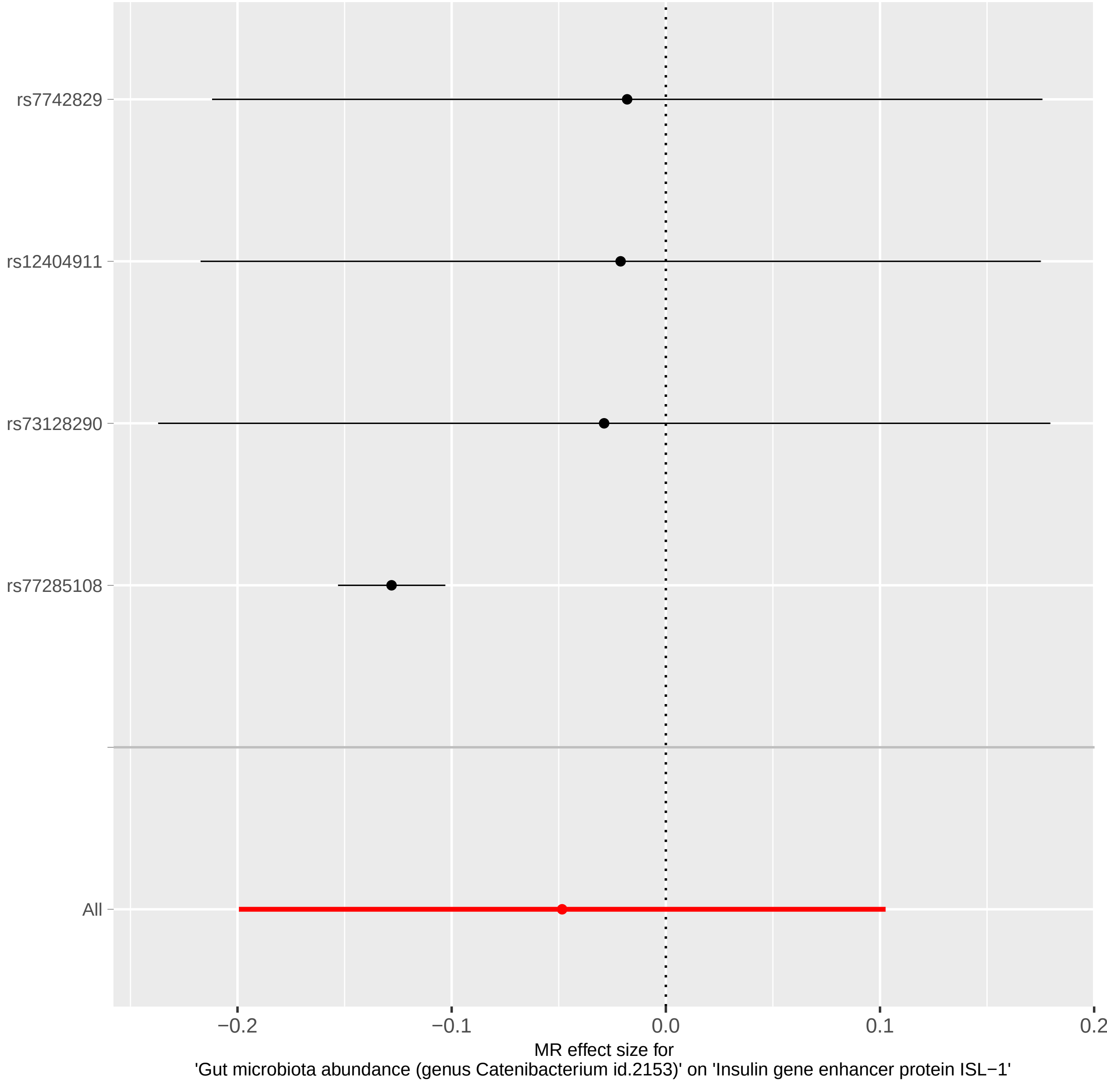

Batch 2−3150 : Gut microbiota abundance (genus Parasutterella id.2892) on Insulin gene enhancer protein ISL−1
with a threshold of 1e−05

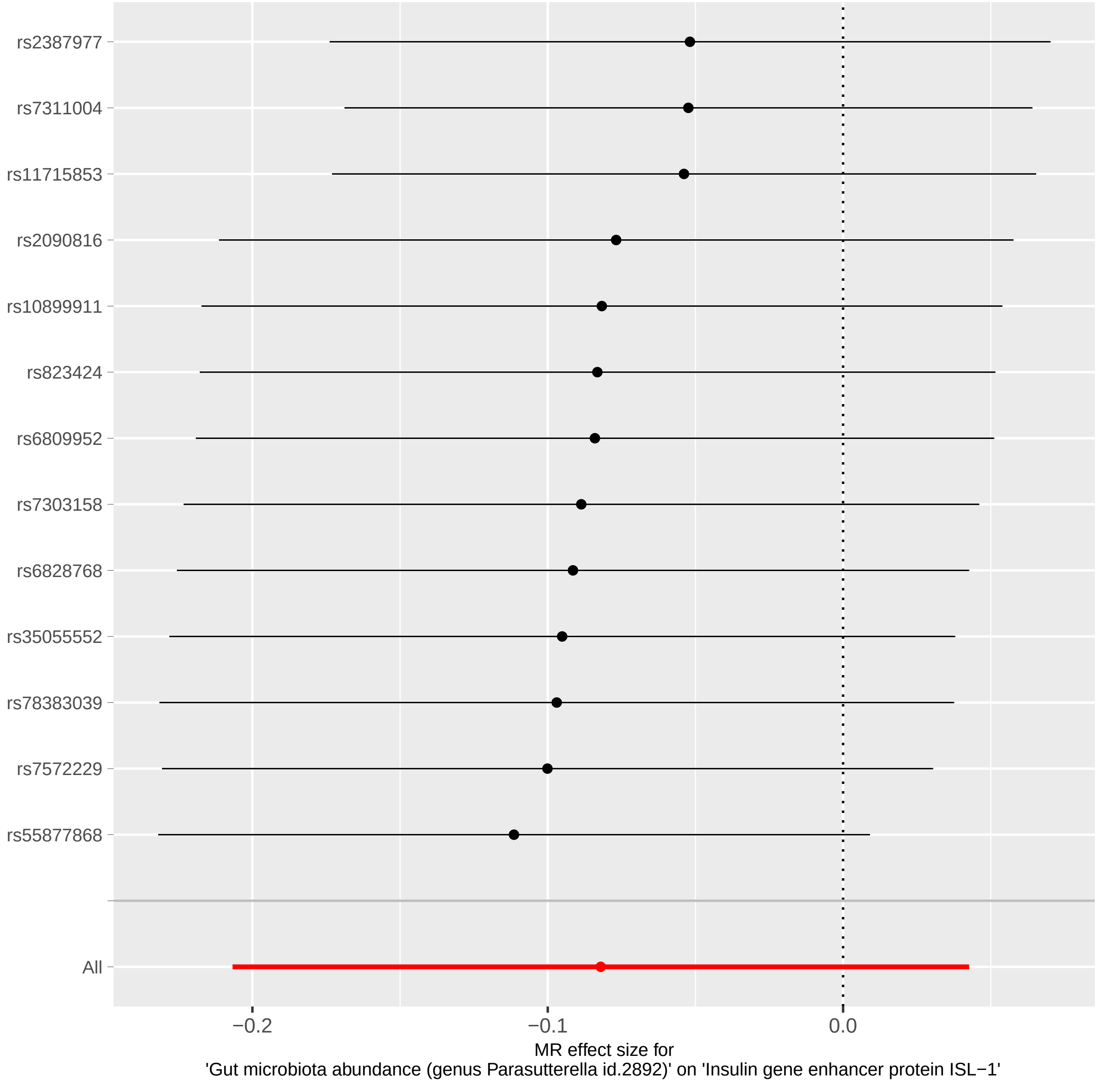

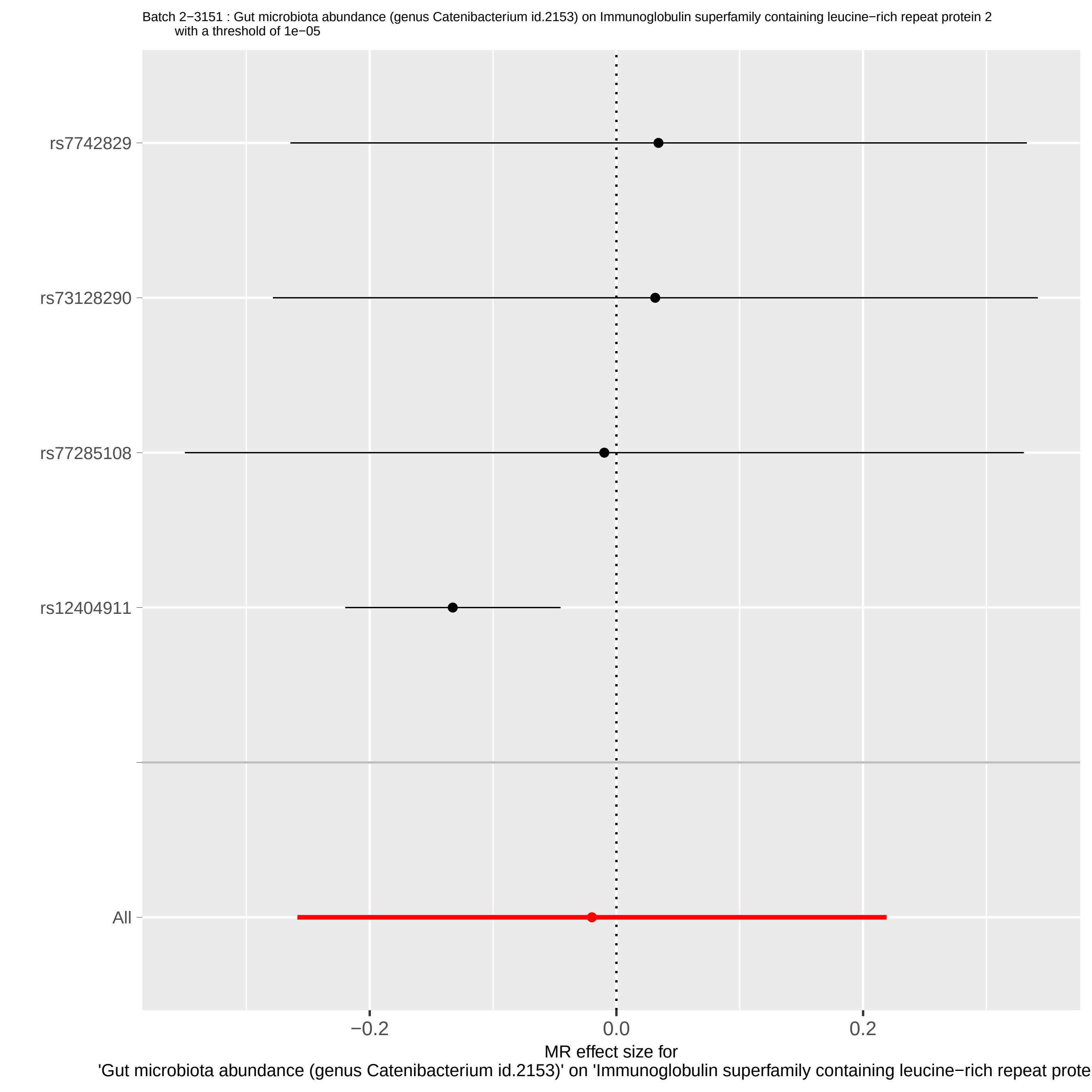
Batch 2−3151 : Gut microbiota abundance (genus Catenibacterium id.2153) on Immunoglobulin superfamily containing leucine−rich repeat protein 2
with a threshold of 1e−05
rs7742829
rs73128290
rs77285108
rs12404911
All
−0.2
0.0
0.2
MR effect size for
'Gut microbiota abundance (genus Catenibacterium id.2153)' on 'Immunoglobulin superfamily containing leucine−rich repeat prote

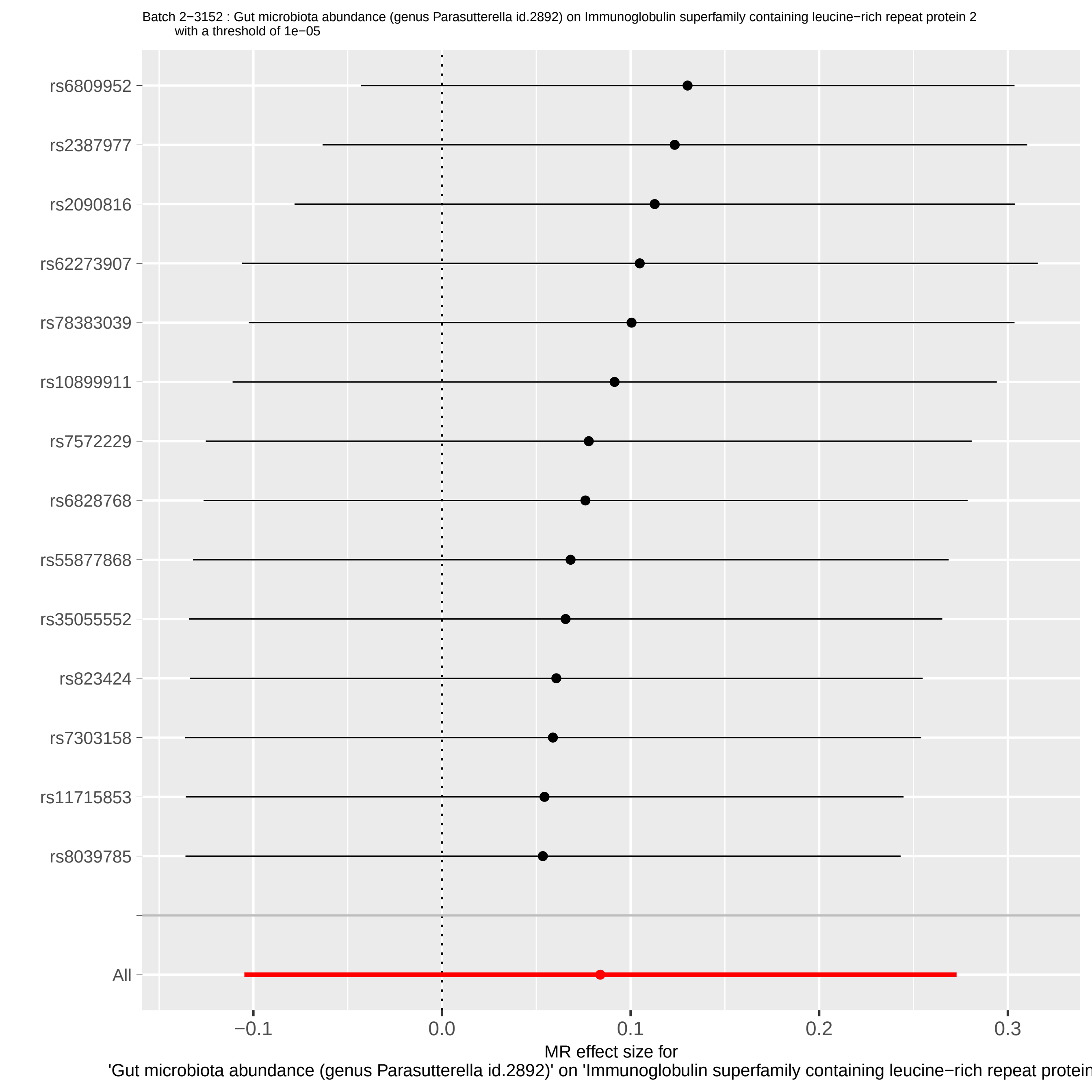
Batch 2−3152 : Gut microbiota abundance (genus Parasutterella id.2892) on Immunoglobulin superfamily containing leucine−rich repeat protein 2
with a threshold of 1e−05
rs6809952
rs2387977
rs2090816
rs62273907
rs78383039
rs10899911
rs7572229
rs6828768
rs55877868
rs35055552
rs823424
rs7303158
rs11715853
rs8039785
All
−0.1
0.0
0.1
0.2
0.3
MR effect size for
'Gut microbiota abundance (genus Parasutterella id.2892)' on 'Immunoglobulin superfamily containing leucine−rich repeat protein

Batch 2−3153 : Gut microbiota abundance (genus Catenibacterium id.2153) on Isthmin−1
with a threshold of 1e−05

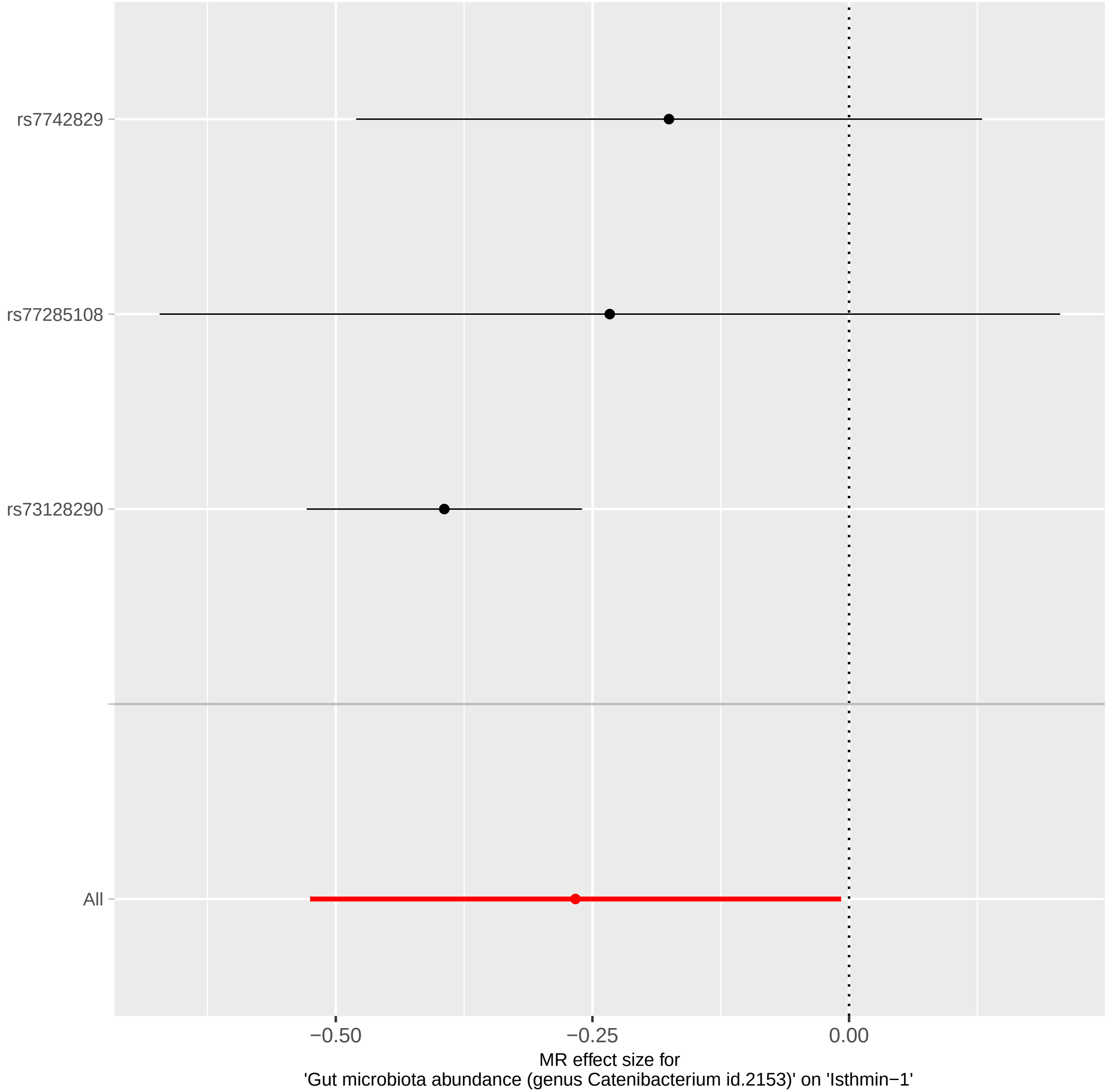

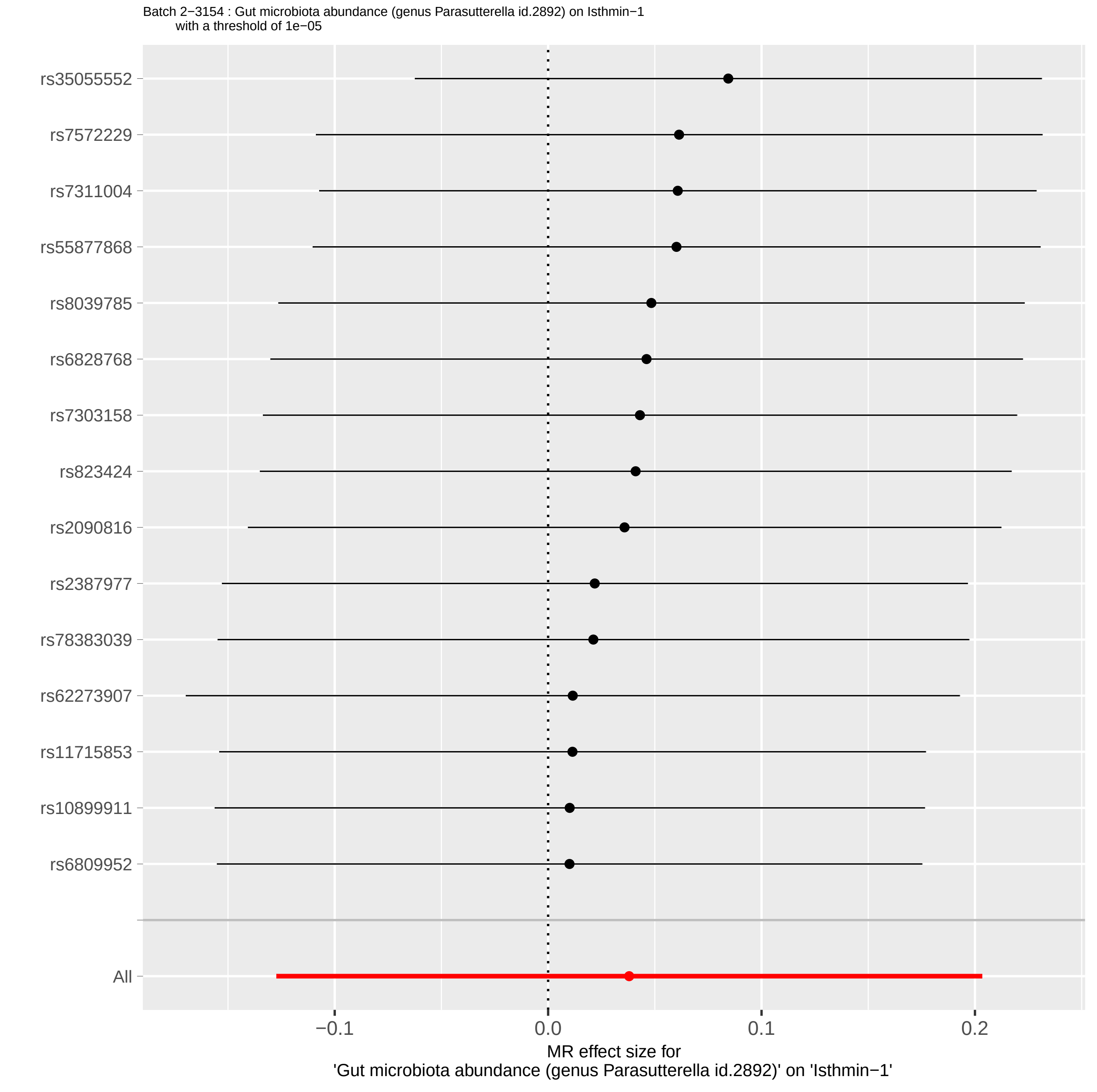

Batch 2−3154 : Gut microbiota abundance (genus Parasutterella id.2892) on Isthmin−1
with a threshold of 1e−05
rs35055552
rs7572229
rs7311004
rs55877868
rs8039785
rs6828768
rs7303158
rs823424
rs2090816
rs2387977
rs78383039
rs62273907
rs11715853
rs10899911
rs6809952
All
−0.1
0.0
0.1
0.2
MR effect size for
'Gut microbiota abundance (genus Parasutterella id.2892)' on 'Isthmin−1'

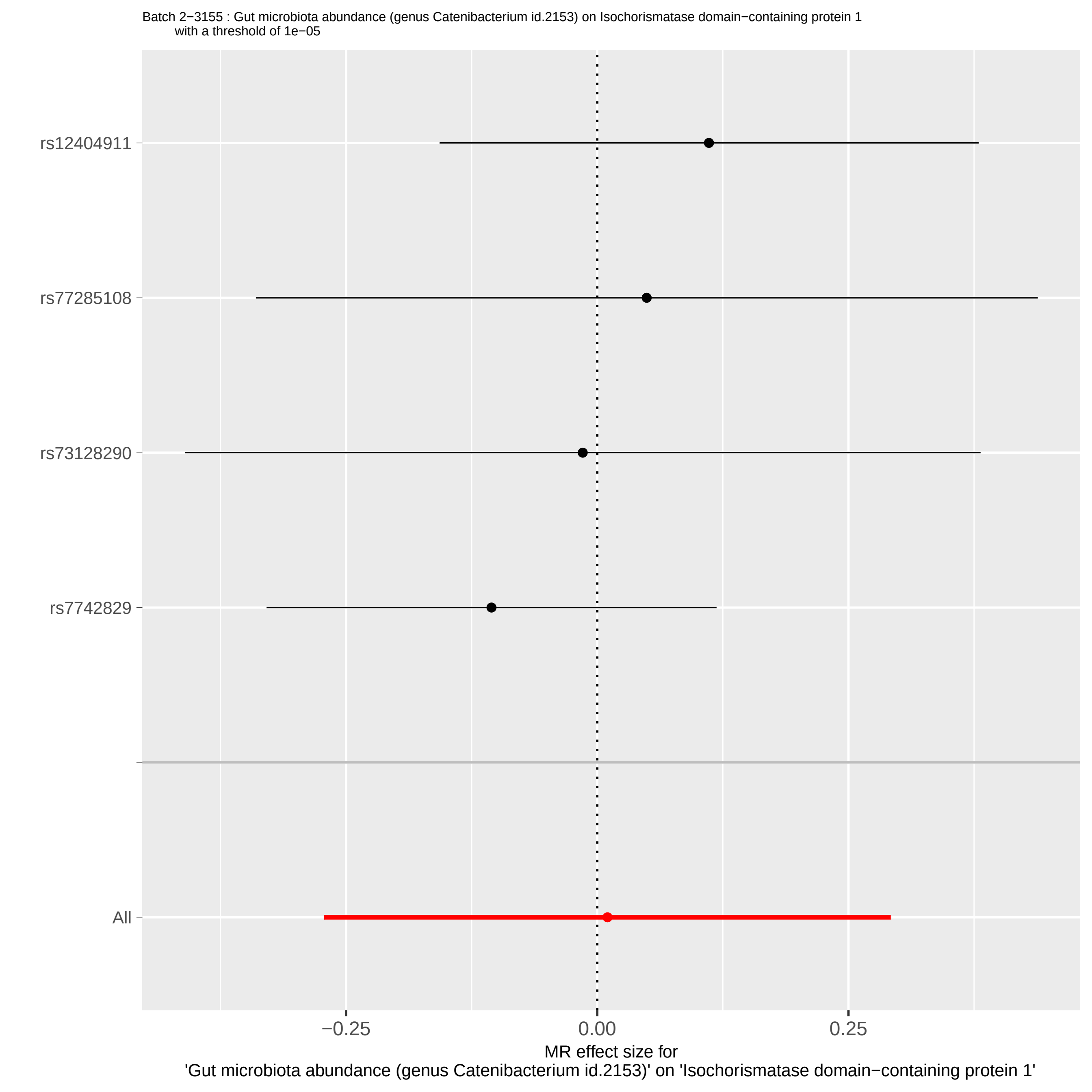

Batch 2−3155 : Gut microbiota abundance (genus Catenibacterium id.2153) on Isochorismatase domain−containing protein 1
with a threshold of 1e−05
rs12404911
rs77285108
rs73128290
rs7742829
All
−0.25
0.00
0.25
MR effect size for
'Gut microbiota abundance (genus Catenibacterium id.2153)' on 'Isochorismatase domain−containing protein 1'

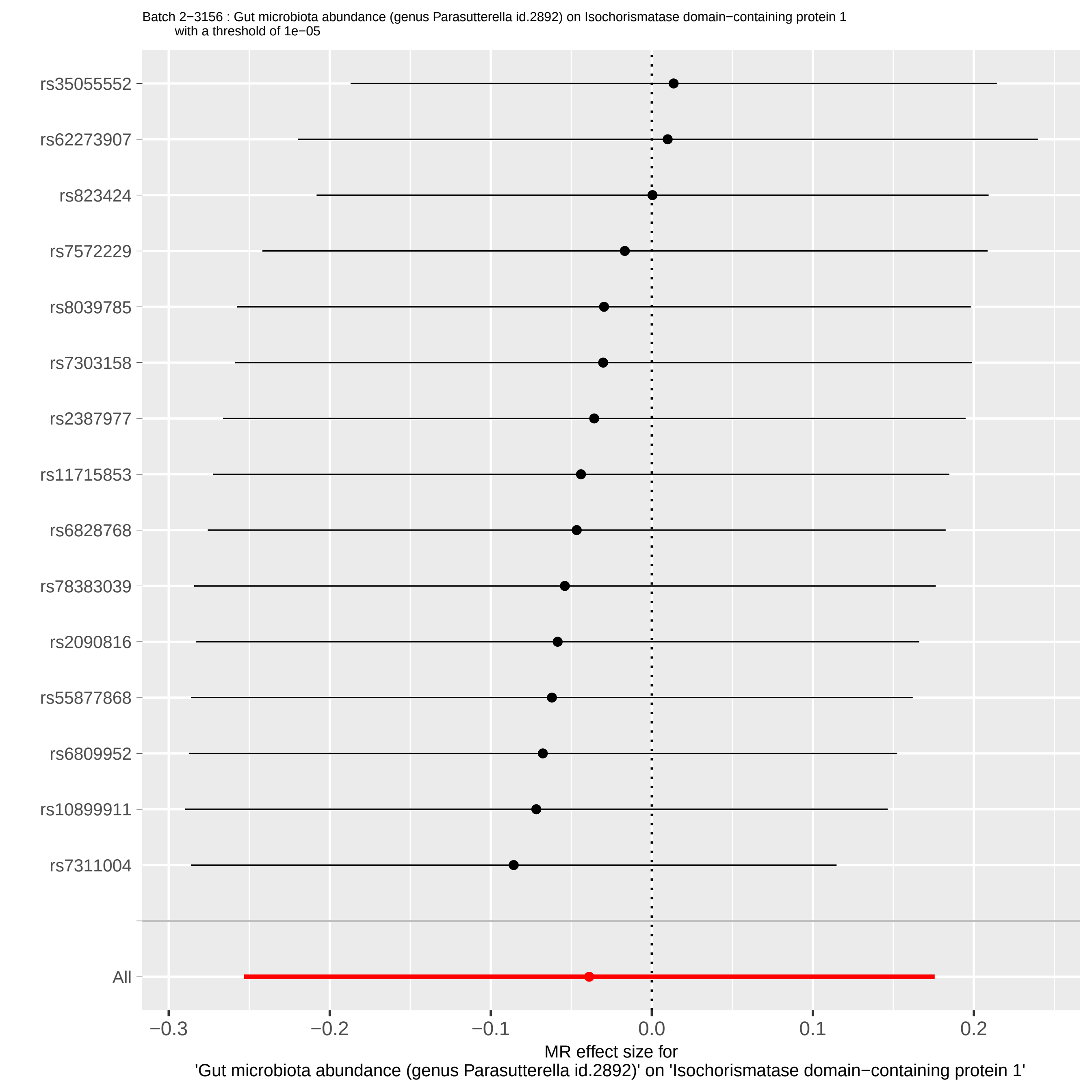
Batch 2−3156 : Gut microbiota abundance (genus Parasutterella id.2892) on Isochorismatase domain−containing protein 1
with a threshold of 1e−05
rs35055552
rs62273907
rs823424
rs7572229
rs8039785
rs7303158
rs2387977
rs11715853
rs6828768
rs78383039
rs2090816
rs55877868
rs6809952
rs10899911
rs7311004
All
−0.3
−0.2
−0.1
0.0
0.1
0.2
MR effect size for
'Gut microbiota abundance (genus Parasutterella id.2892)' on 'Isochorismatase domain−containing protein 1'

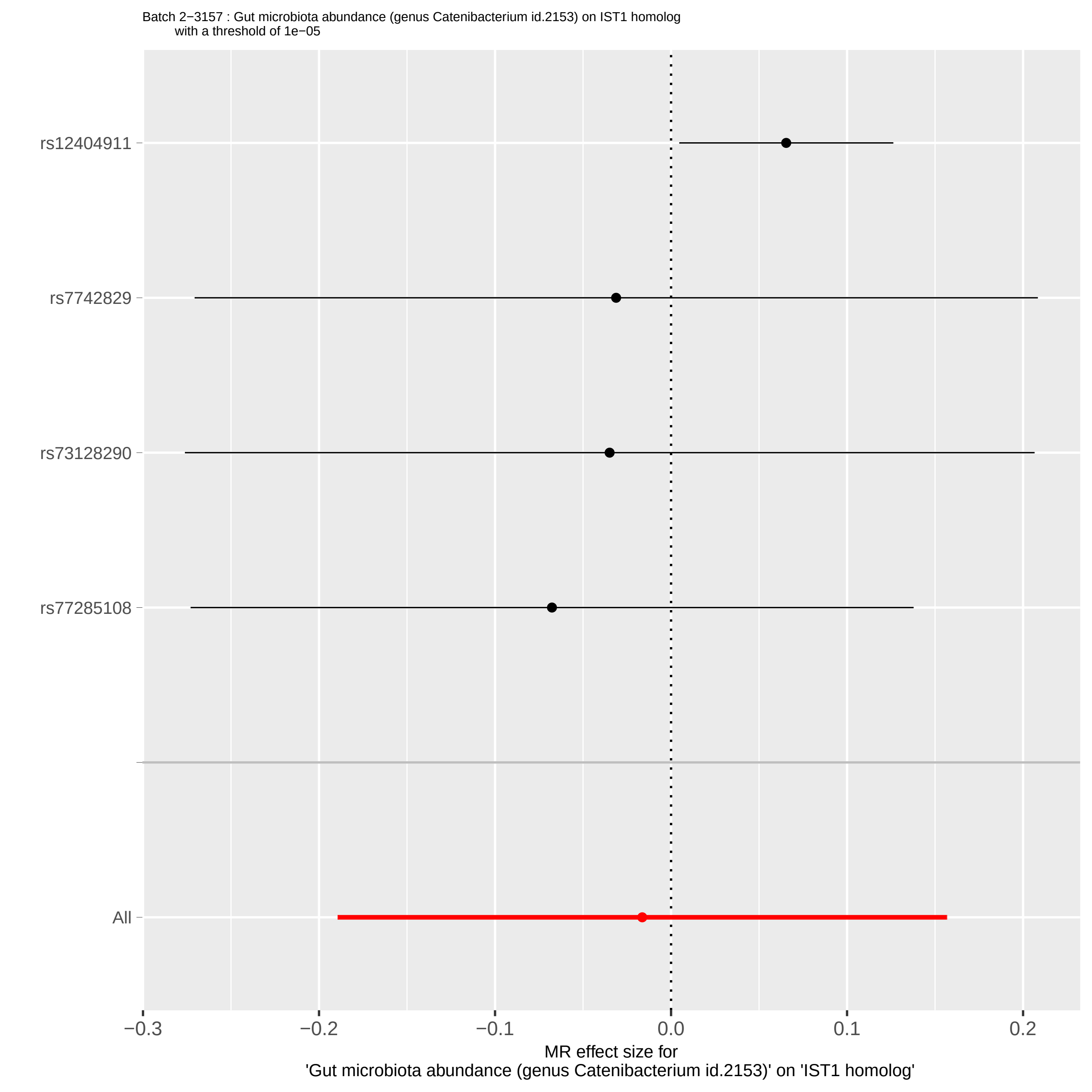

Batch 2−3157 : Gut microbiota abundance (genus Catenibacterium id.2153) on IST1 homolog
with a threshold of 1e−05
rs12404911
rs7742829
rs73128290
rs77285108
All
−0.3
−0.2
−0.1
0.0
0.1
0.2
MR effect size for
'Gut microbiota abundance (genus Catenibacterium id.2153)' on 'IST1 homolog'

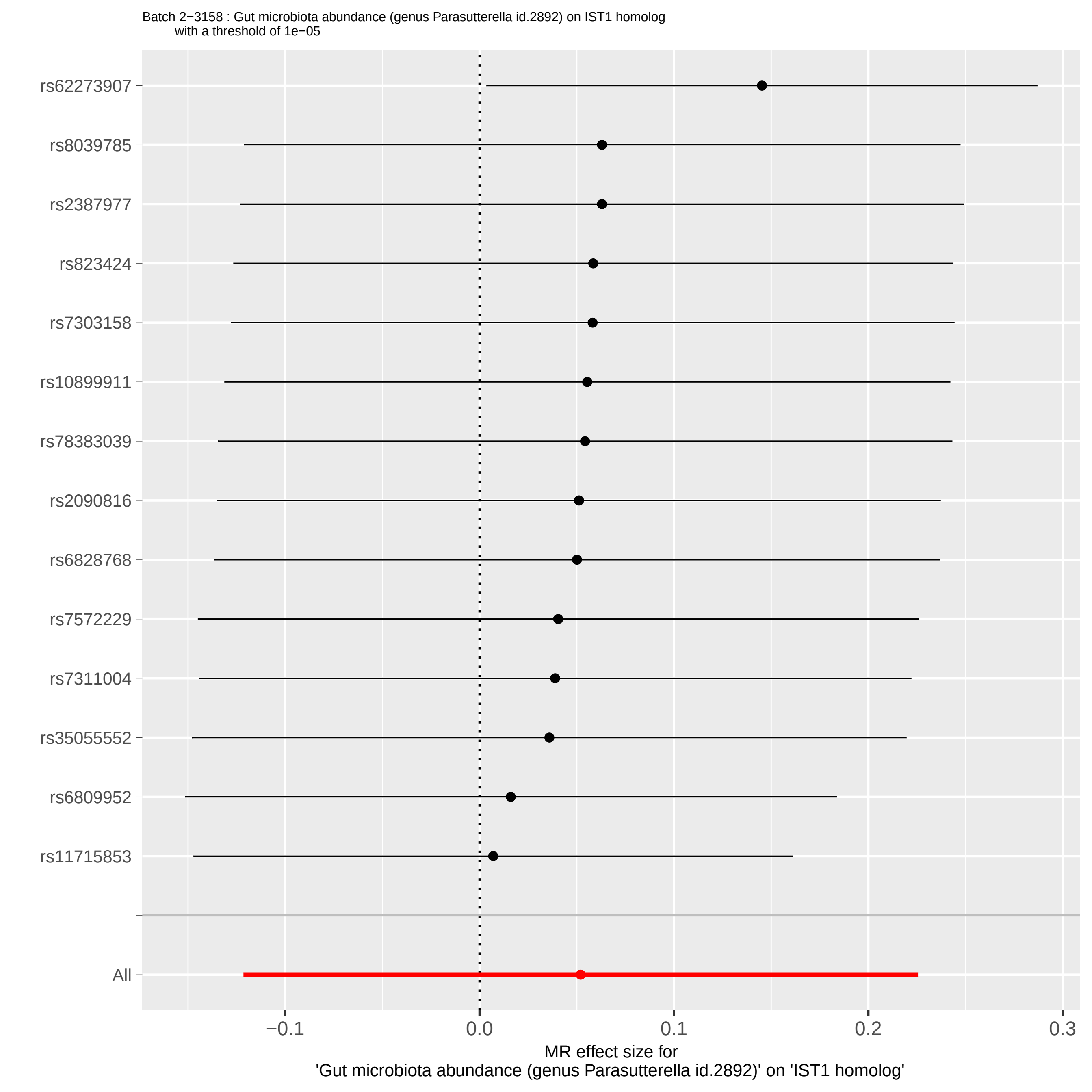

Batch 2−3158 : Gut microbiota abundance (genus Parasutterella id.2892) on IST1 homolog
with a threshold of 1e−05
rs62273907
rs8039785
rs2387977
rs823424
rs7303158
rs10899911
rs78383039
rs2090816
rs6828768
rs7572229
rs7311004
rs35055552
rs6809952
rs11715853
All
−0.1
0.0
0.1
0.2
0.3
MR effect size for
'Gut microbiota abundance (genus Parasutterella id.2892)' on 'IST1 homolog'

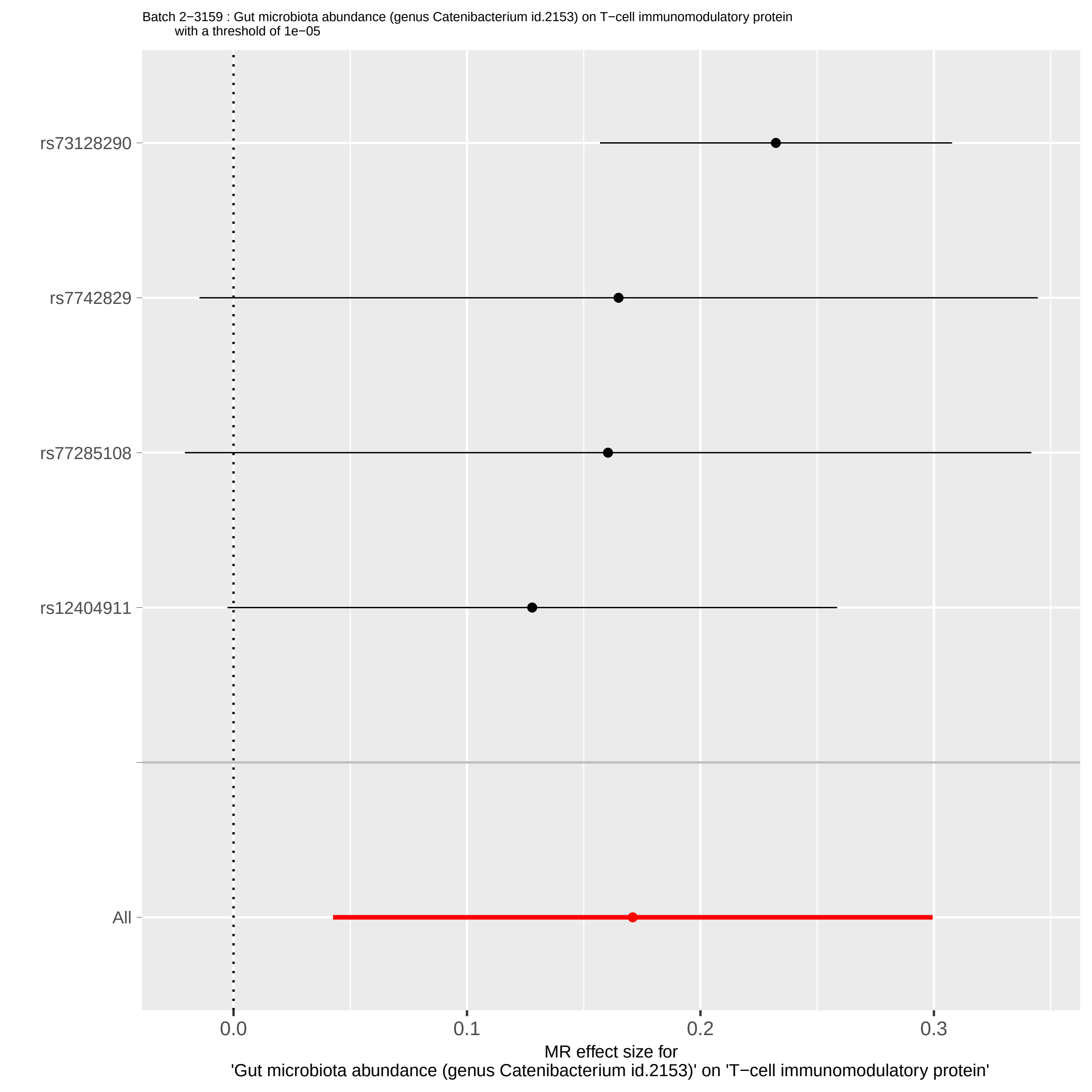

Batch 2−3159 : Gut microbiota abundance (genus Catenibacterium id.2153) on T−cell immunomodulatory protein
with a threshold of 1e−05
rs73128290
rs7742829
rs77285108
rs12404911
All
0.0
0.1
0.2
0.3
MR effect size for
'Gut microbiota abundance (genus Catenibacterium id.2153)' on 'T−cell immunomodulatory protein'

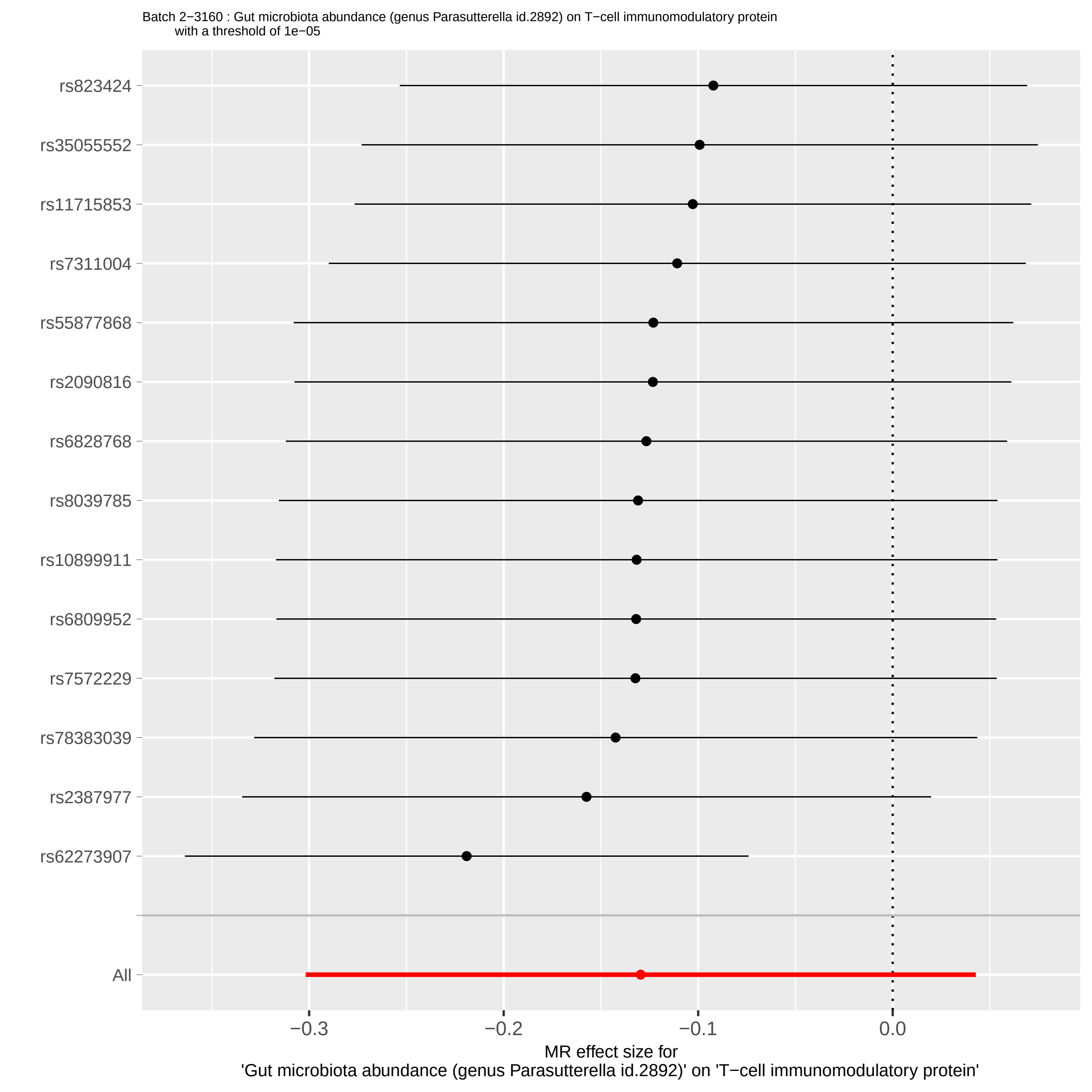

Batch 2−3160 : Gut microbiota abundance (genus Parasutterella id.2892) on T−cell immunomodulatory protein
with a threshold of 1e−05
rs823424
rs35055552
rs11715853
rs7311004
rs55877868
rs2090816
rs6828768
rs8039785
rs10899911
rs6809952
rs7572229
rs78383039
rs2387977
rs62273907
All
−0.3
−0.2
−0.1
0.0
MR effect size for
'Gut microbiota abundance (genus Parasutterella id.2892)' on 'T−cell immunomodulatory protein'

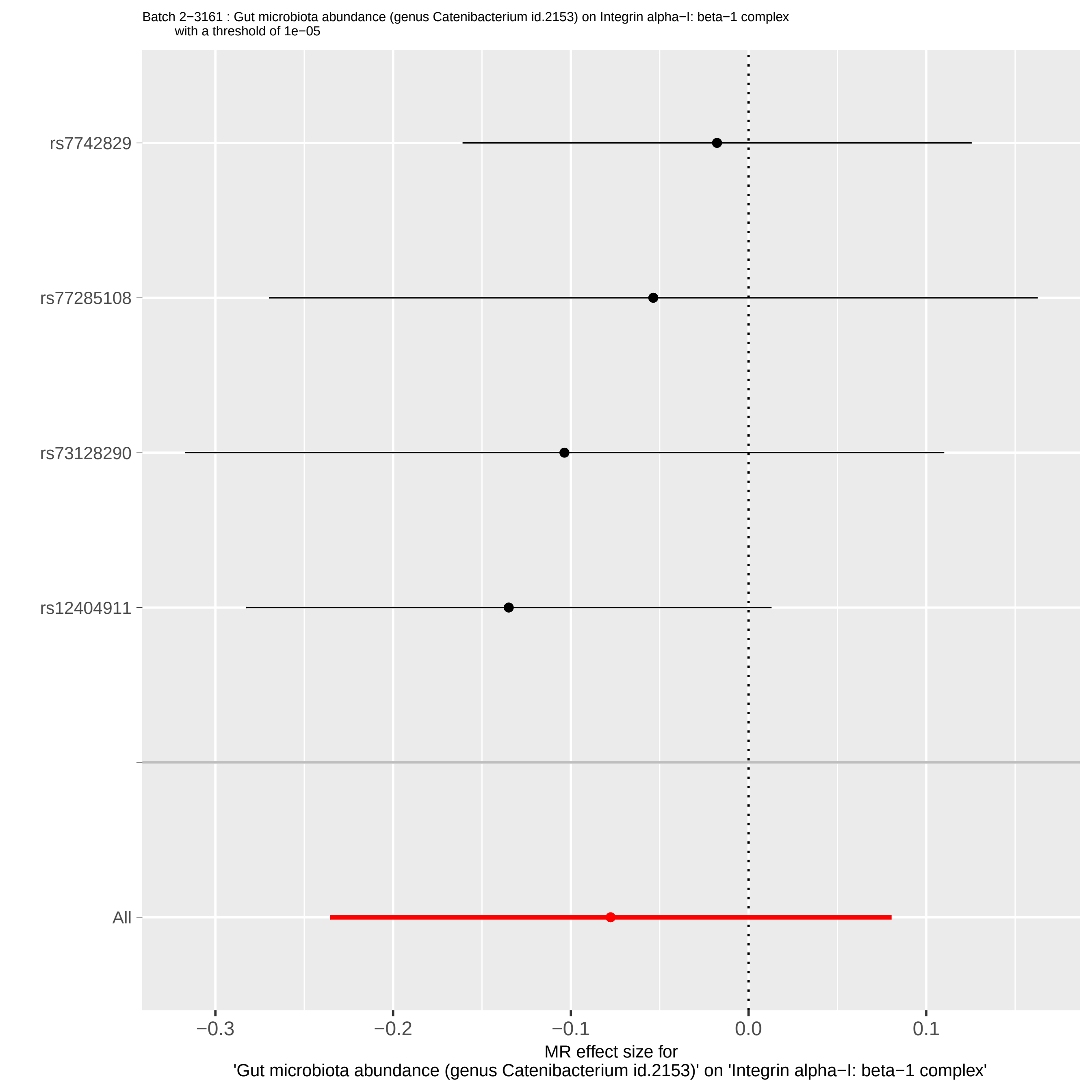
Batch 2−3161 : Gut microbiota abundance (genus Catenibacterium id.2153) on Integrin alpha−I: beta−1 complex
with a threshold of 1e−05
rs7742829
rs77285108
rs73128290
rs12404911
All
−0.3
−0.2
−0.1
0.0
0.1
MR effect size for
'Gut microbiota abundance (genus Catenibacterium id.2153)' on 'Integrin alpha−I: beta−1 complex'

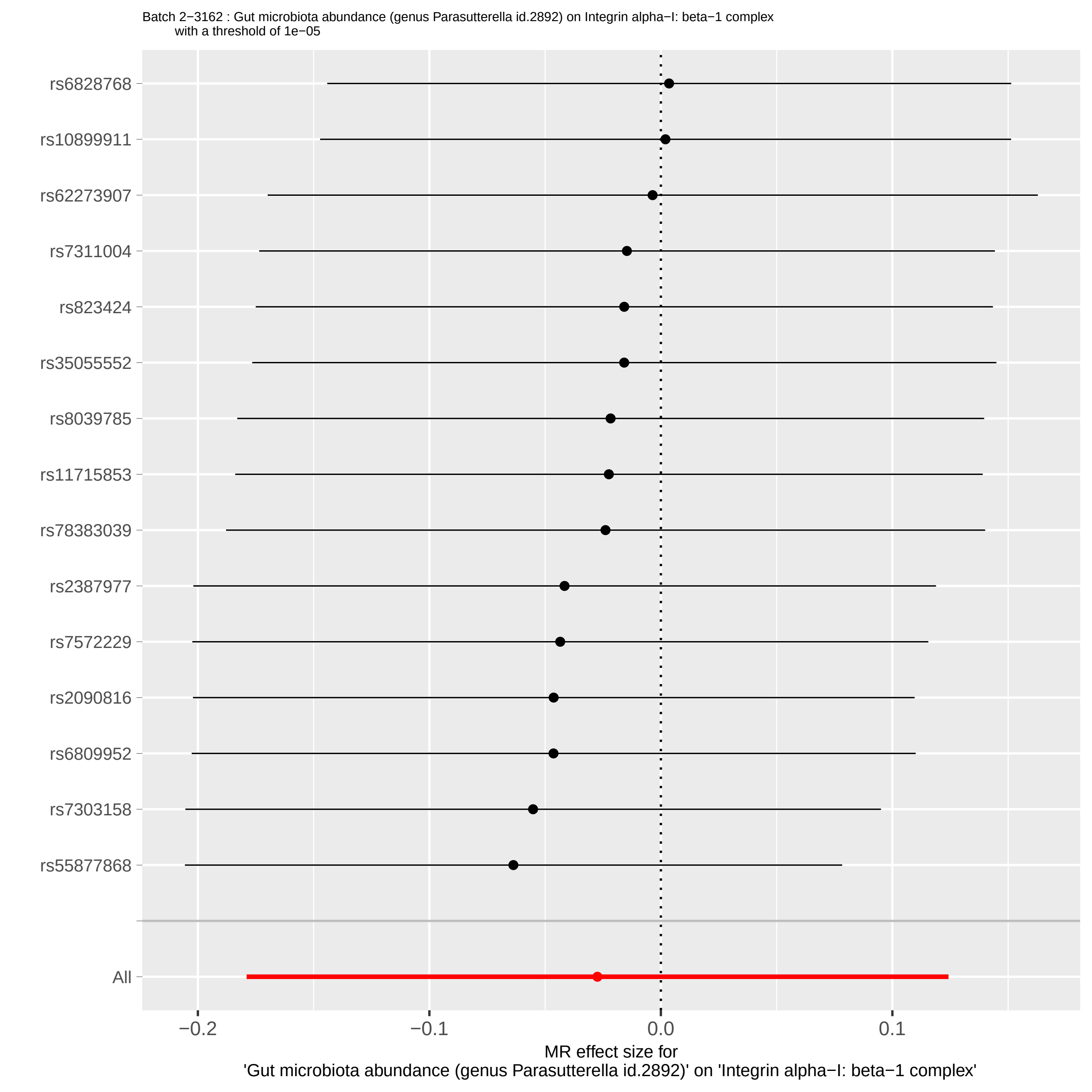

Batch 2−3162 : Gut microbiota abundance (genus Parasutterella id.2892) on Integrin alpha−I: beta−1 complex
with a threshold of 1e−05
rs6828768
rs10899911
rs62273907
rs7311004
rs823424
rs35055552
rs8039785
rs11715853
rs78383039
rs2387977
rs7572229
rs2090816
rs6809952
rs7303158
rs55877868
All
−0.2
−0.1
0.0
0.1
MR effect size for
'Gut microbiota abundance (genus Parasutterella id.2892)' on 'Integrin alpha−I: beta−1 complex'

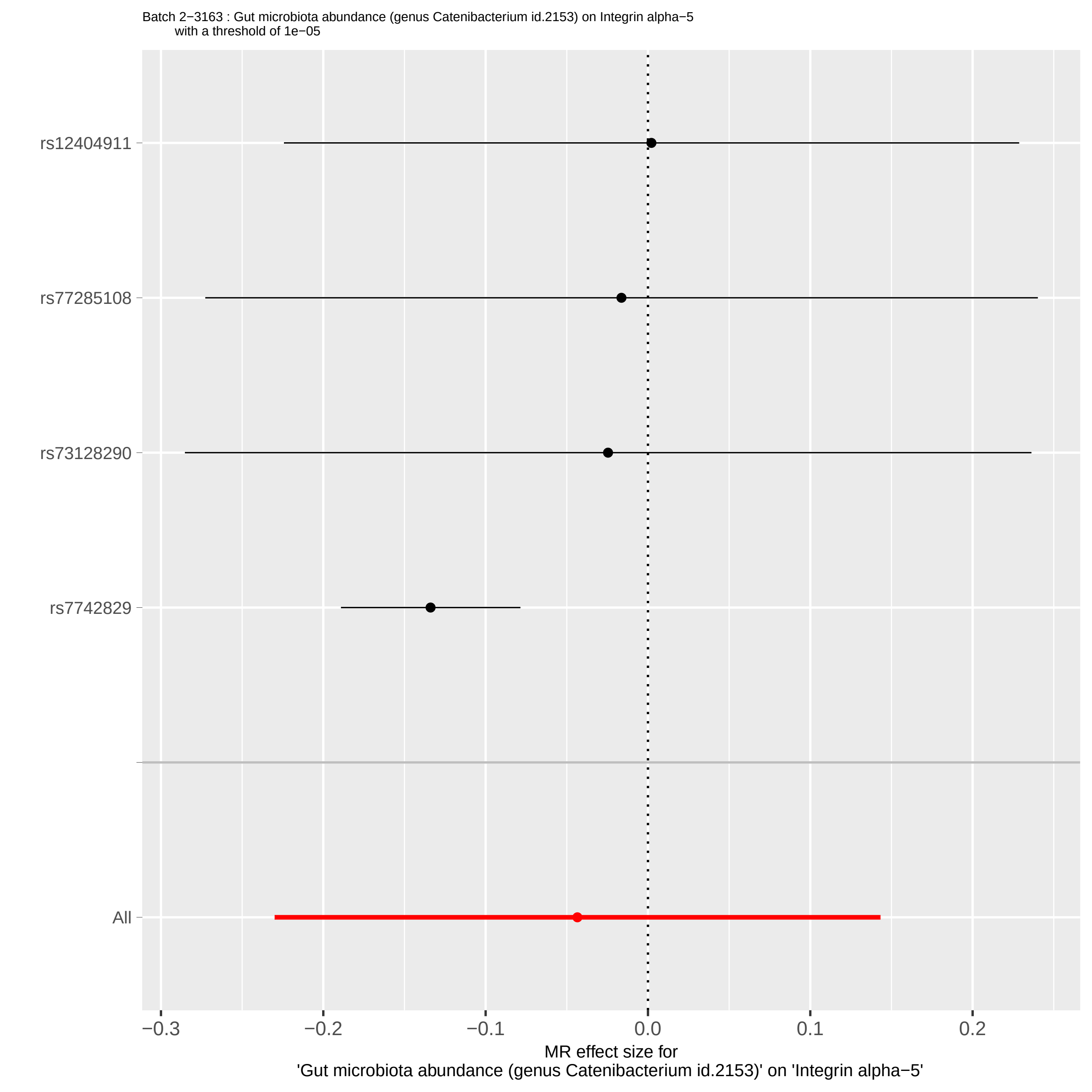

Batch 2−3163 : Gut microbiota abundance (genus Catenibacterium id.2153) on Integrin alpha−5
with a threshold of 1e−05
rs12404911
rs77285108
rs73128290
rs7742829
All
−0.3
−0.2
−0.1
0.0
0.1
0.2
MR effect size for
'Gut microbiota abundance (genus Catenibacterium id.2153)' on 'Integrin alpha−5'

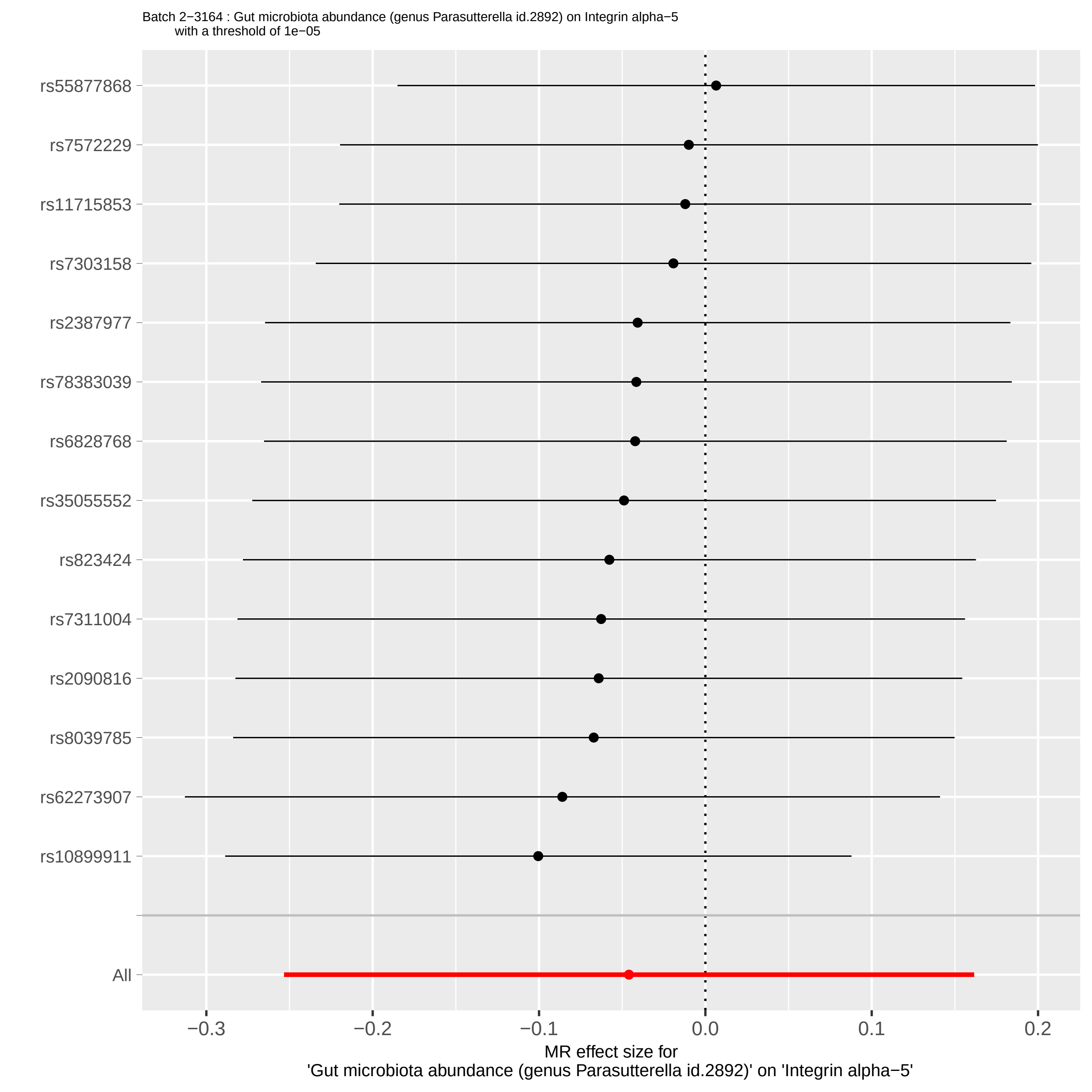

Batch 2−3164 : Gut microbiota abundance (genus Parasutterella id.2892) on Integrin alpha−5
with a threshold of 1e−05
rs55877868
rs7572229
rs11715853
rs7303158
rs2387977
rs78383039
rs6828768
rs35055552
rs823424
rs7311004
rs2090816
rs8039785
rs62273907
rs10899911
All
−0.3
−0.2
−0.1
0.0
0.1
0.2
MR effect size for
'Gut microbiota abundance (genus Parasutterella id.2892)' on 'Integrin alpha−5'

Batch 2−3165 : Gut microbiota abundance (genus Catenibacterium id.2153) on Integrin alpha−L
with a threshold of 1e−05

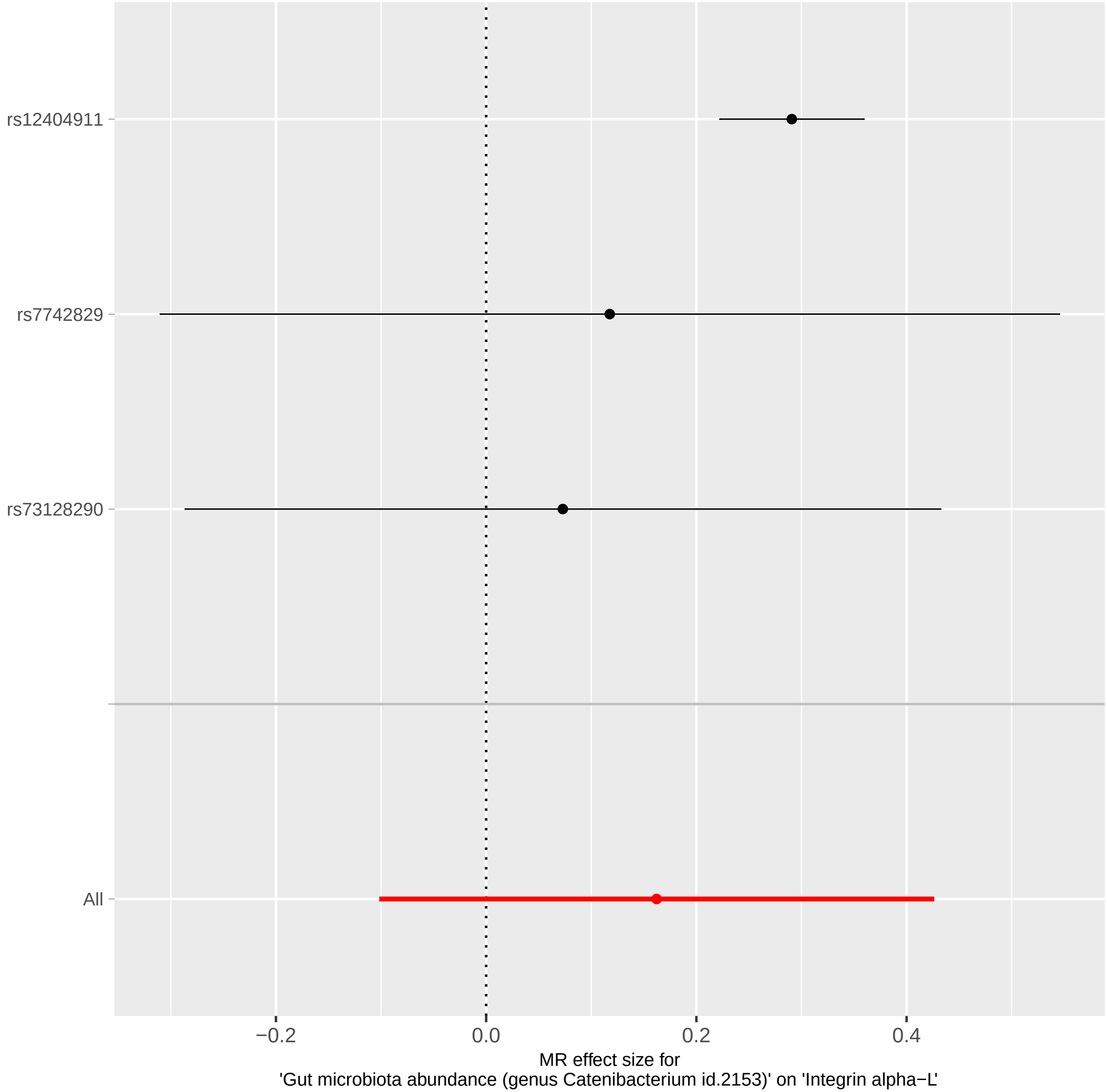

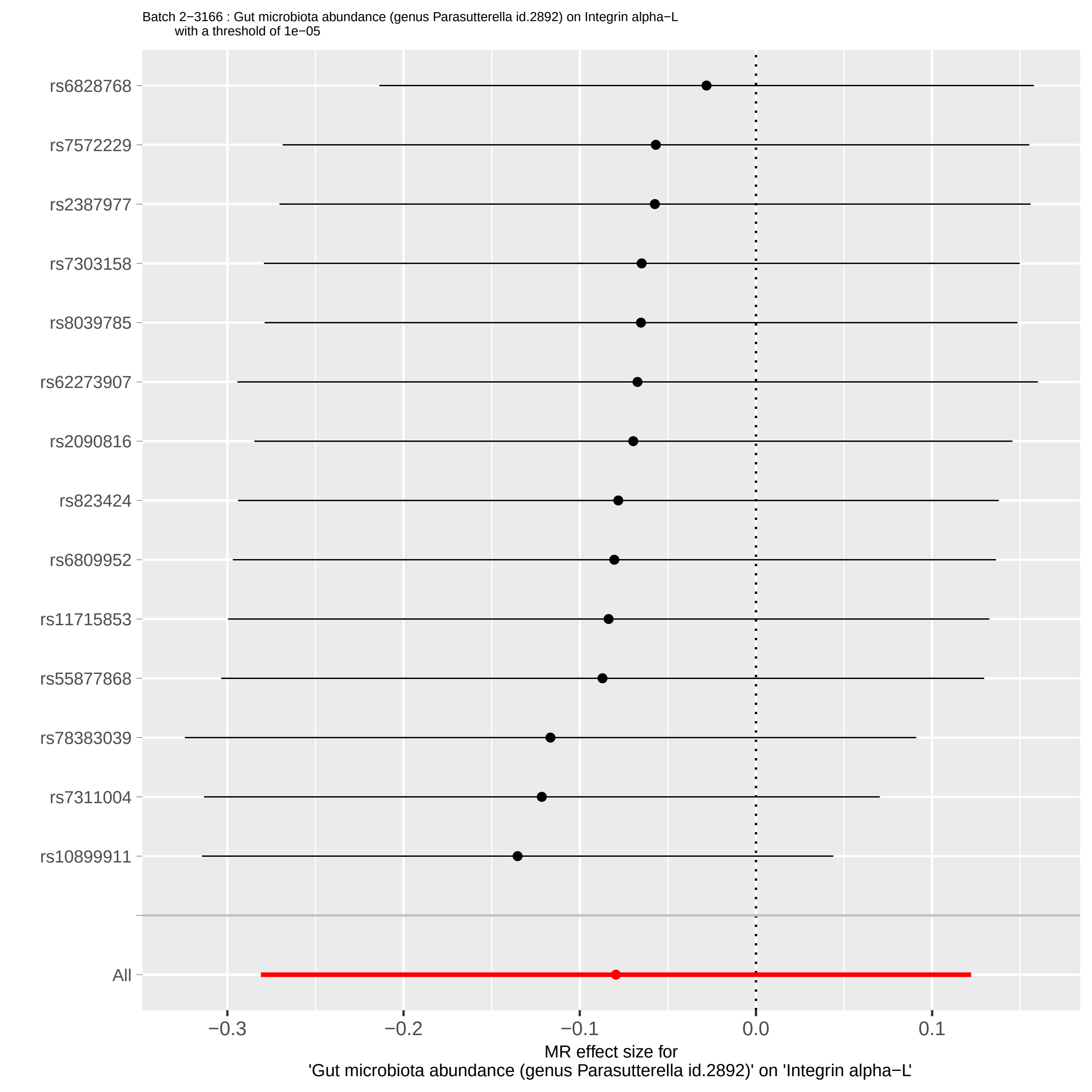
Batch 2−3166 : Gut microbiota abundance (genus Parasutterella id.2892) on Integrin alpha−L
with a threshold of 1e−05
rs6828768
rs7572229
rs2387977
rs7303158
rs8039785
rs62273907
rs2090816
rs823424
rs6809952
rs11715853
rs55877868
rs78383039
rs7311004
rs10899911
All
−0.3
−0.2
−0.1
0.0
0.1
MR effect size for
'Gut microbiota abundance (genus Parasutterella id.2892)' on 'Integrin alpha−L'

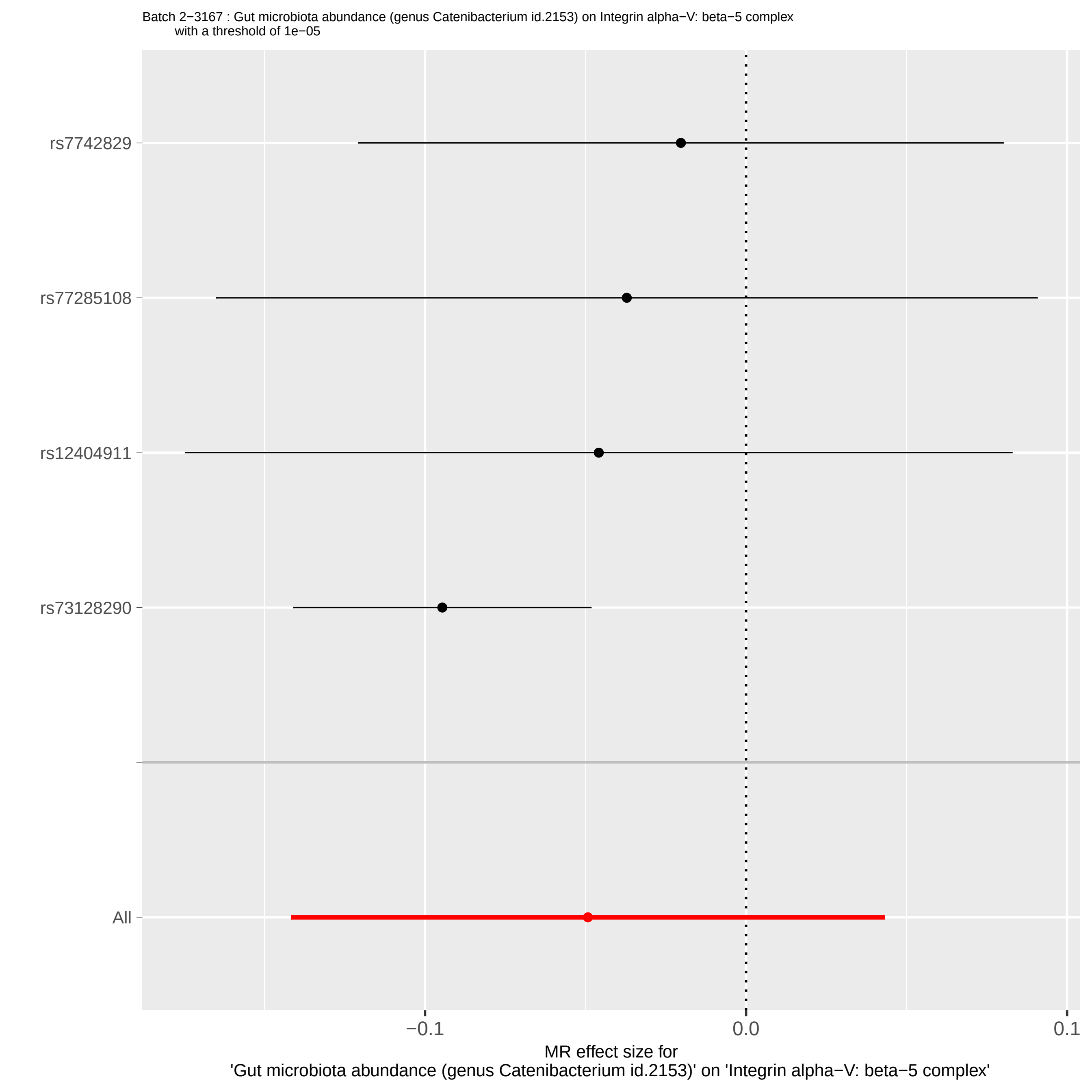
Batch 2−3167 : Gut microbiota abundance (genus Catenibacterium id.2153) on Integrin alpha−V: beta−5 complex
with a threshold of 1e−05
rs7742829
rs77285108
rs12404911
rs73128290
All
−0.1
0.0
0.1
MR effect size for
'Gut microbiota abundance (genus Catenibacterium id.2153)' on 'Integrin alpha−V: beta−5 complex'

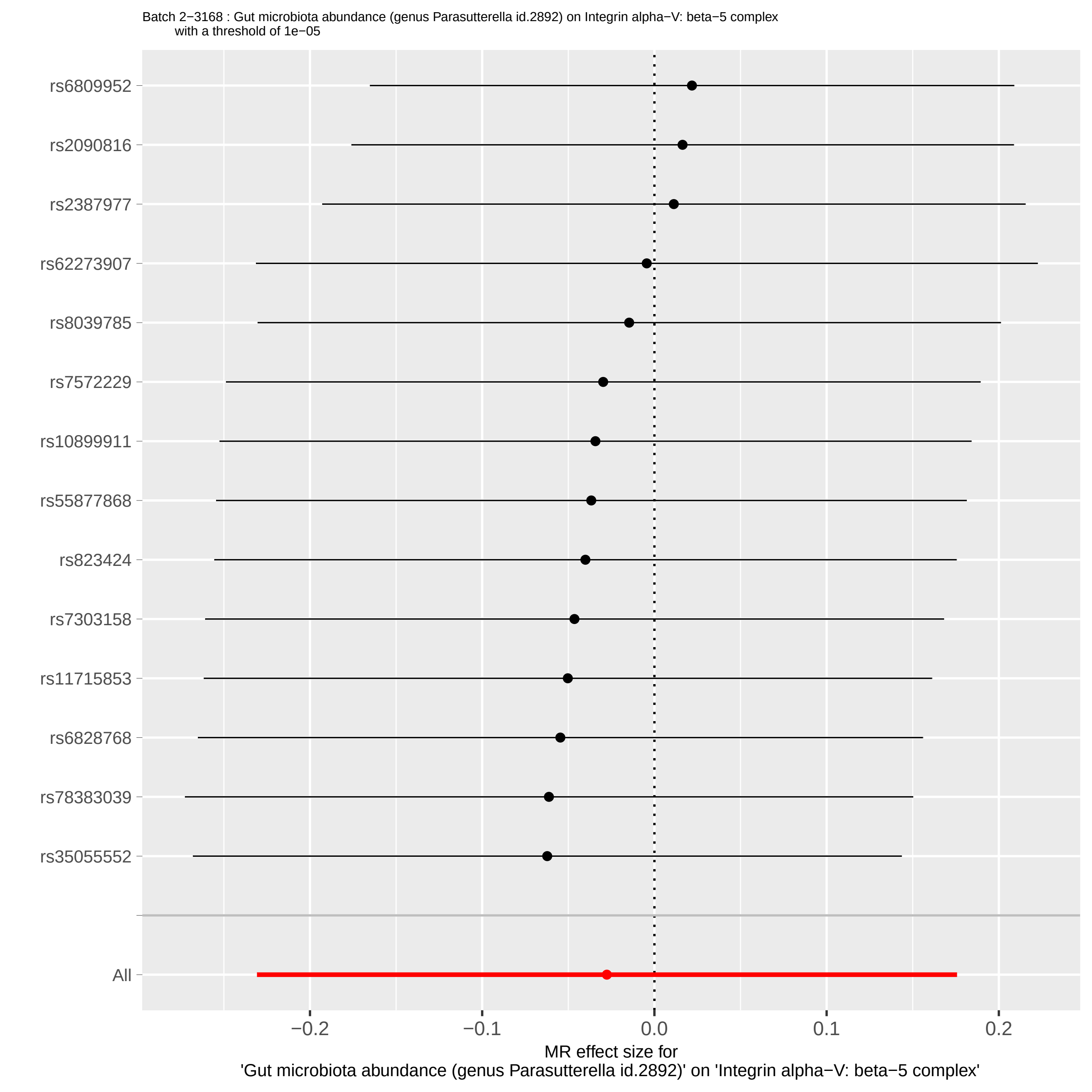

Batch 2−3168 : Gut microbiota abundance (genus Parasutterella id.2892) on Integrin alpha−V: beta−5 complex
with a threshold of 1e−05
rs6809952
rs2090816
rs2387977
rs62273907
rs8039785
rs7572229
rs10899911
rs55877868
rs823424
rs7303158
rs11715853
rs6828768
rs78383039
rs35055552
All
−0.2
−0.1
0.0
0.1
0.2
MR effect size for
'Gut microbiota abundance (genus Parasutterella id.2892)' on 'Integrin alpha−V: beta−5 complex'

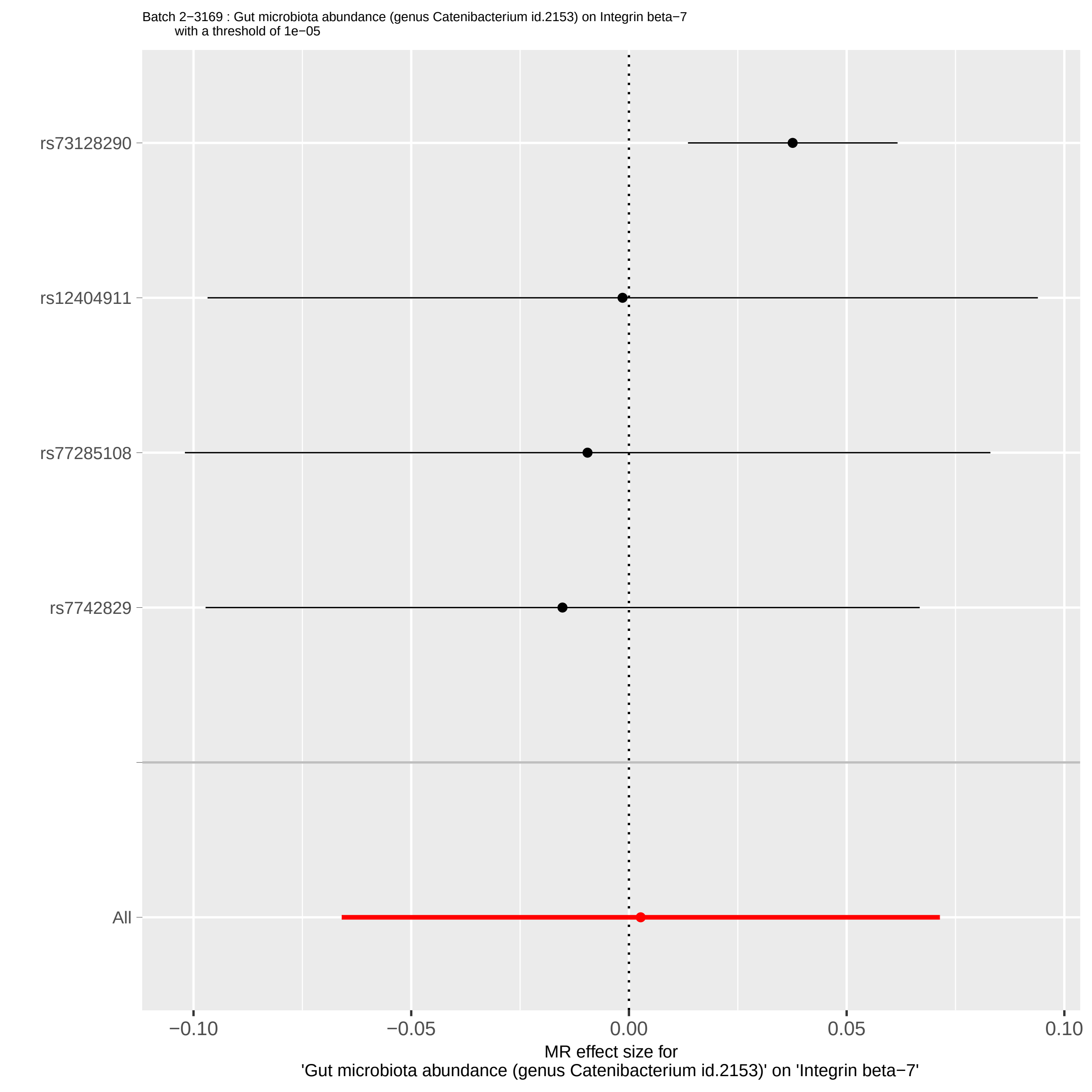

Batch 2−3169 : Gut microbiota abundance (genus Catenibacterium id.2153) on Integrin beta−7
with a threshold of 1e−05
rs73128290
rs12404911
rs77285108
rs7742829
All
−0.10
−0.05
0.00
0.05
0.10
MR effect size for
'Gut microbiota abundance (genus Catenibacterium id.2153)' on 'Integrin beta−7'

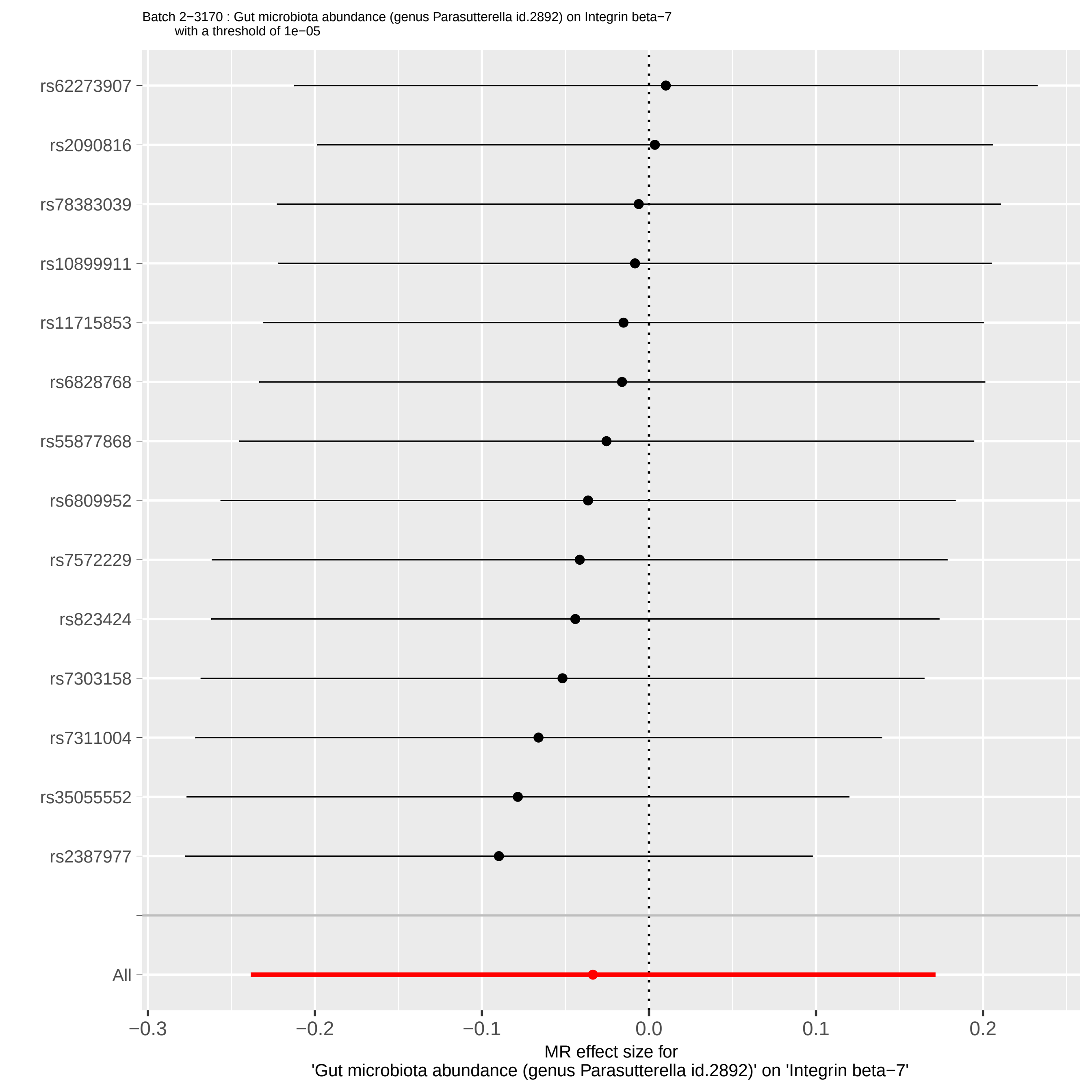
Batch 2−3170 : Gut microbiota abundance (genus Parasutterella id.2892) on Integrin beta−7
with a threshold of 1e−05
rs62273907
rs2090816
rs78383039
rs10899911
rs11715853
rs6828768
rs55877868
rs6809952
rs7572229
rs823424
rs7303158
rs7311004
rs35055552
rs2387977
All
−0.3
−0.2
−0.1
0.0
0.1
0.2
MR effect size for
'Gut microbiota abundance (genus Parasutterella id.2892)' on 'Integrin beta−7'

Batch 2−3171 : Gut microbiota abundance (genus Catenibacterium id.2153) on Inter−alpha−trypsin inhibitor heavy chain H1
with a threshold of 1e−05

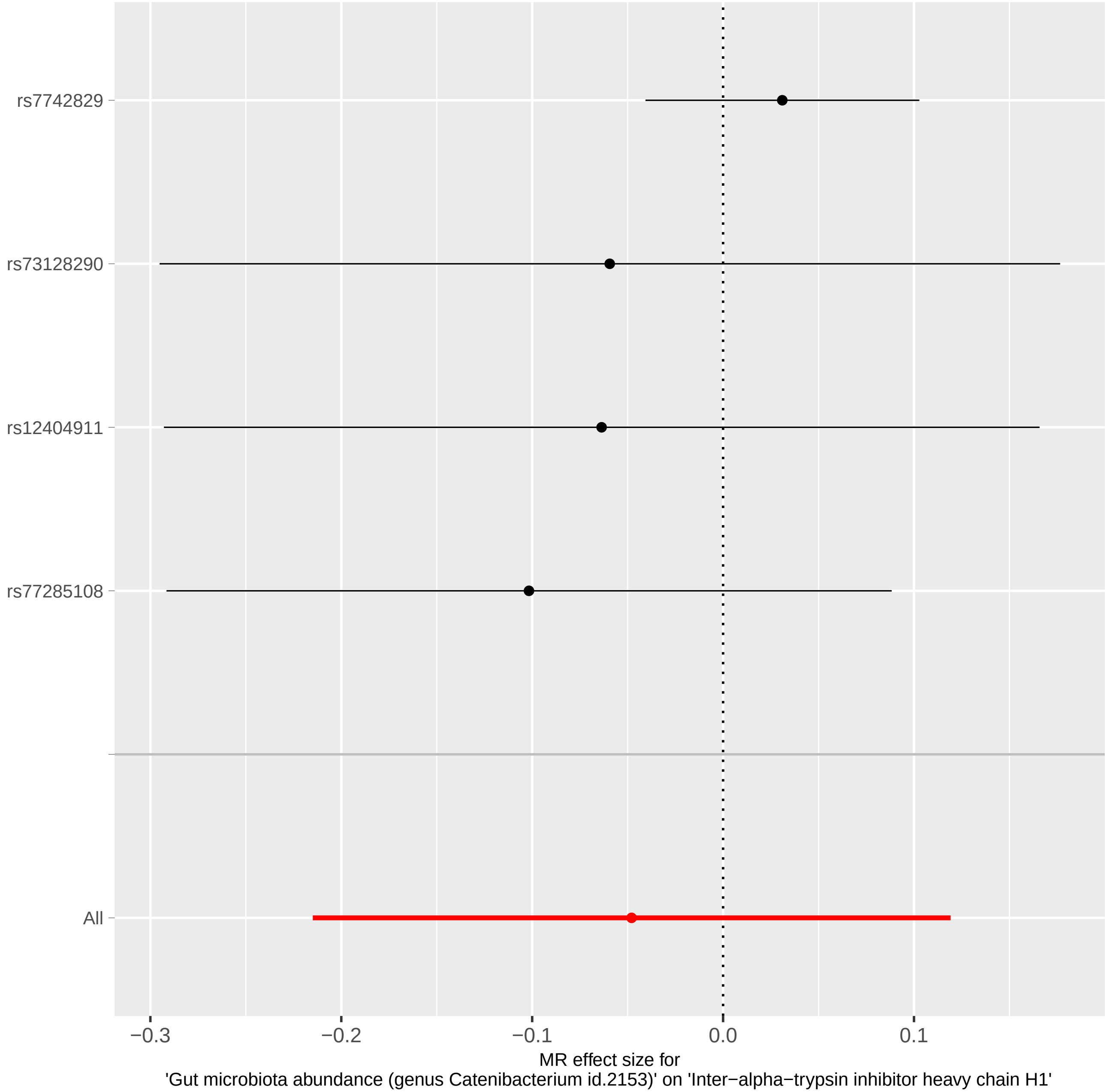

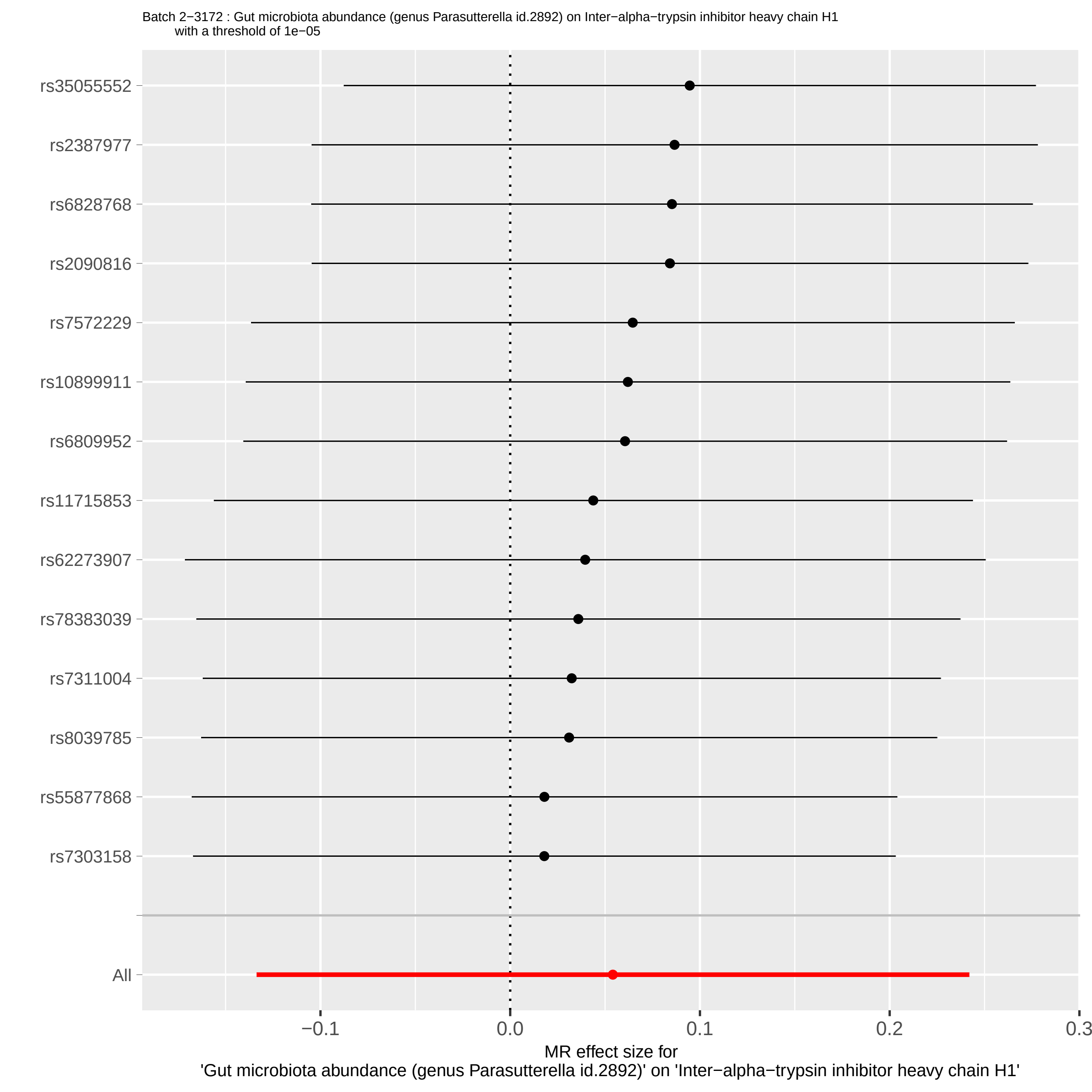

Batch 2−3172 : Gut microbiota abundance (genus Parasutterella id.2892) on Inter−alpha−trypsin inhibitor heavy chain H1
with a threshold of 1e−05
rs35055552
rs2387977
rs6828768
rs2090816
rs7572229
rs10899911
rs6809952
rs11715853
rs62273907
rs78383039
rs7311004
rs8039785
rs55877868
rs7303158
All
−0.1
0.0
0.1
0.2
0.3
MR effect size for
'Gut microbiota abundance (genus Parasutterella id.2892)' on 'Inter−alpha−trypsin inhibitor heavy chain H1'

Batch 2−3173 : Gut microbiota abundance (genus Catenibacterium id.2153) on Inter−alpha−trypsin inhibitor heavy chain H5
with a threshold of 1e−05

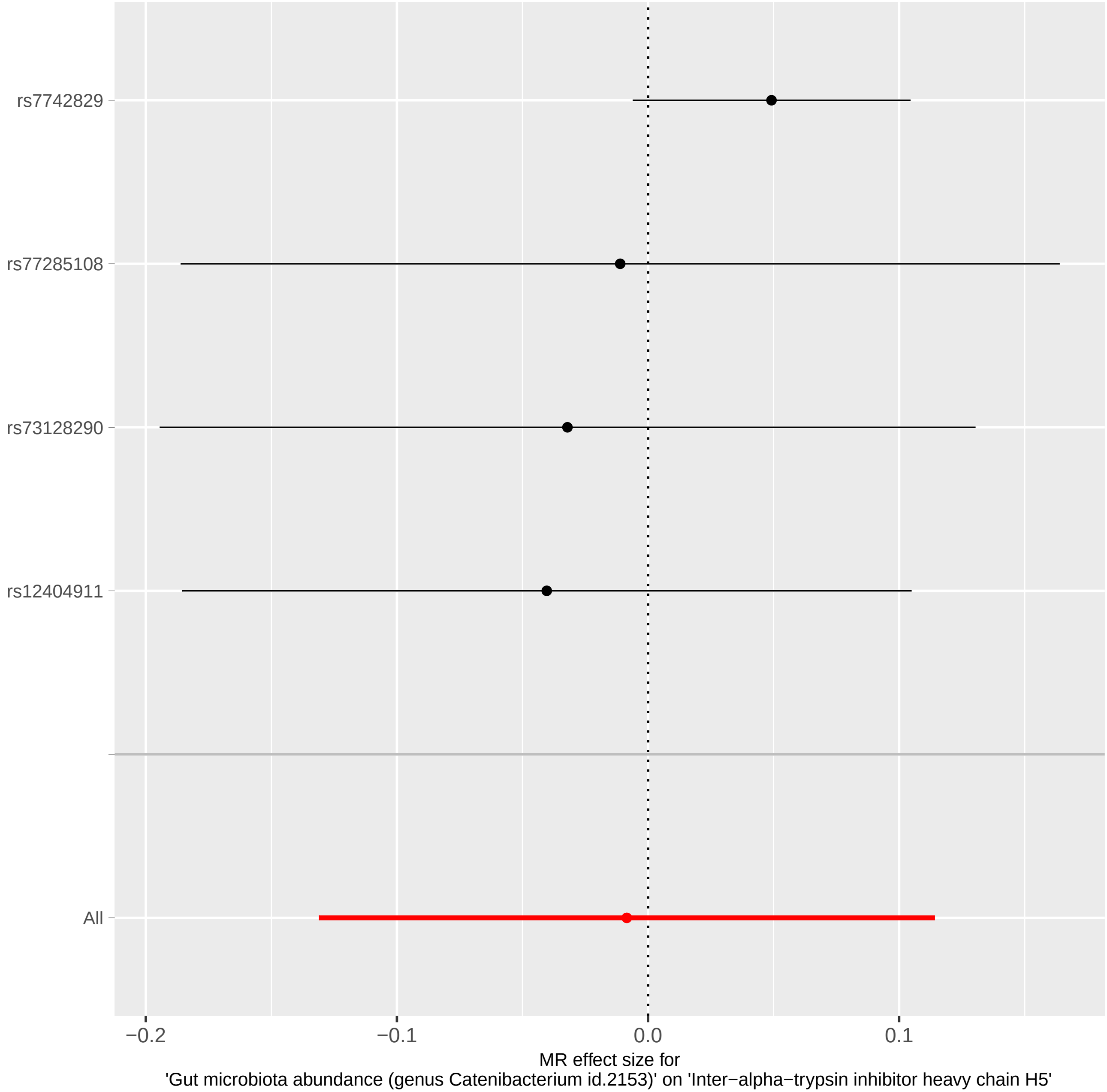

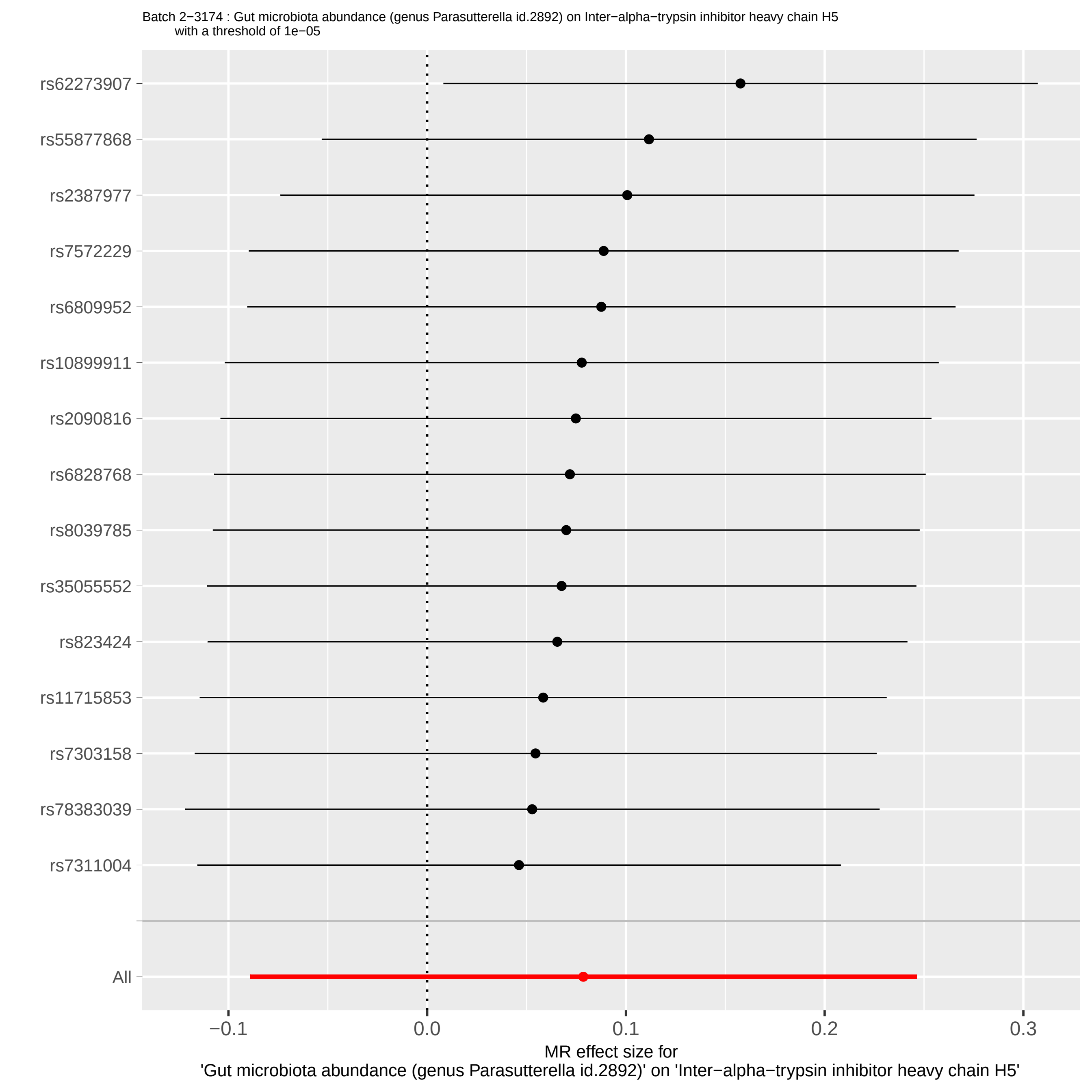
Batch 2−3174 : Gut microbiota abundance (genus Parasutterella id.2892) on Inter−alpha−trypsin inhibitor heavy chain H5
with a threshold of 1e−05
rs62273907
rs55877868
rs2387977
rs7572229
rs6809952
rs10899911
rs2090816
rs6828768
rs8039785
rs35055552
rs823424
rs11715853
rs7303158
rs78383039
rs7311004
All
−0.1
0.0
0.1
0.2
0.3
MR effect size for
'Gut microbiota abundance (genus Parasutterella id.2892)' on 'Inter−alpha−trypsin inhibitor heavy chain H5'

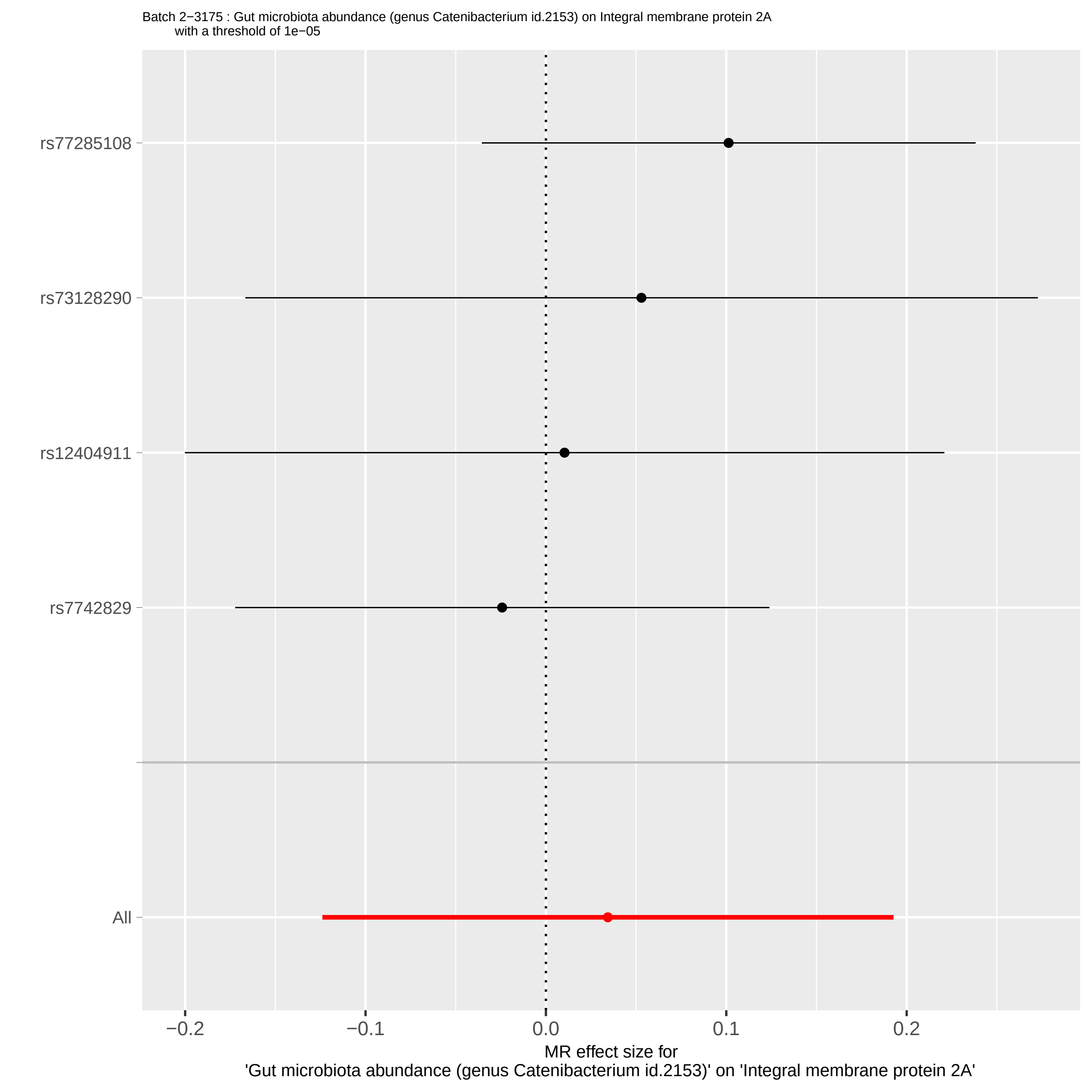
Batch 2−3175 : Gut microbiota abundance (genus Catenibacterium id.2153) on Integral membrane protein 2A
with a threshold of 1e−05
rs77285108
rs73128290
rs12404911
rs7742829
All
−0.2
−0.1
0.0
0.1
0.2
MR effect size for
'Gut microbiota abundance (genus Catenibacterium id.2153)' on 'Integral membrane protein 2A'

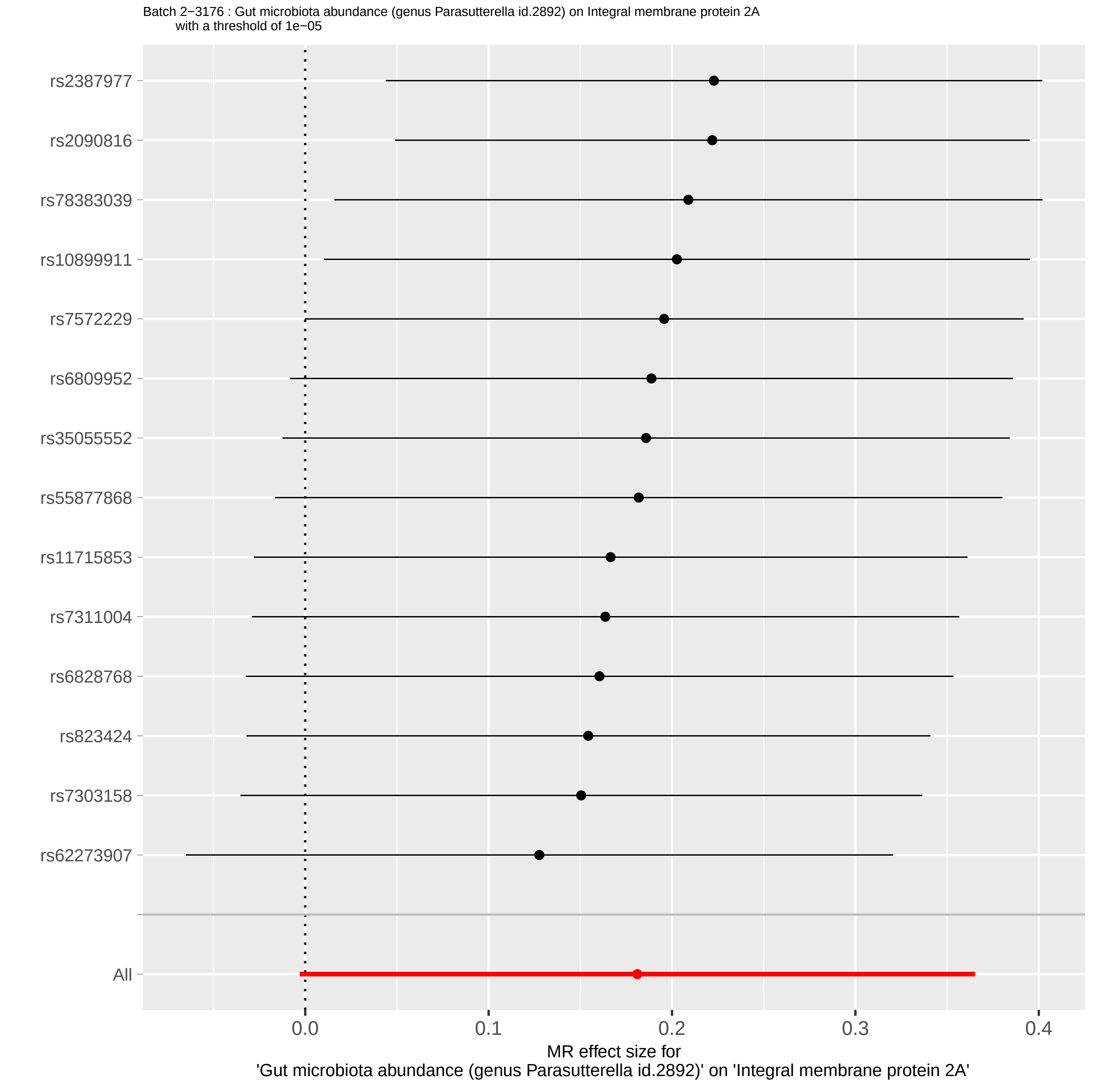

Batch 2−3176 : Gut microbiota abundance (genus Parasutterella id.2892) on Integral membrane protein 2A
with a threshold of 1e−05
rs2387977
rs2090816
rs78383039
rs10899911
rs7572229
rs6809952
rs35055552
rs55877868
rs11715853
rs7311004
rs6828768
rs823424
rs7303158
rs62273907
All
0.0
0.1
0.2
0.3
0.4
MR effect size for
'Gut microbiota abundance (genus Parasutterella id.2892)' on 'Integral membrane protein 2A'

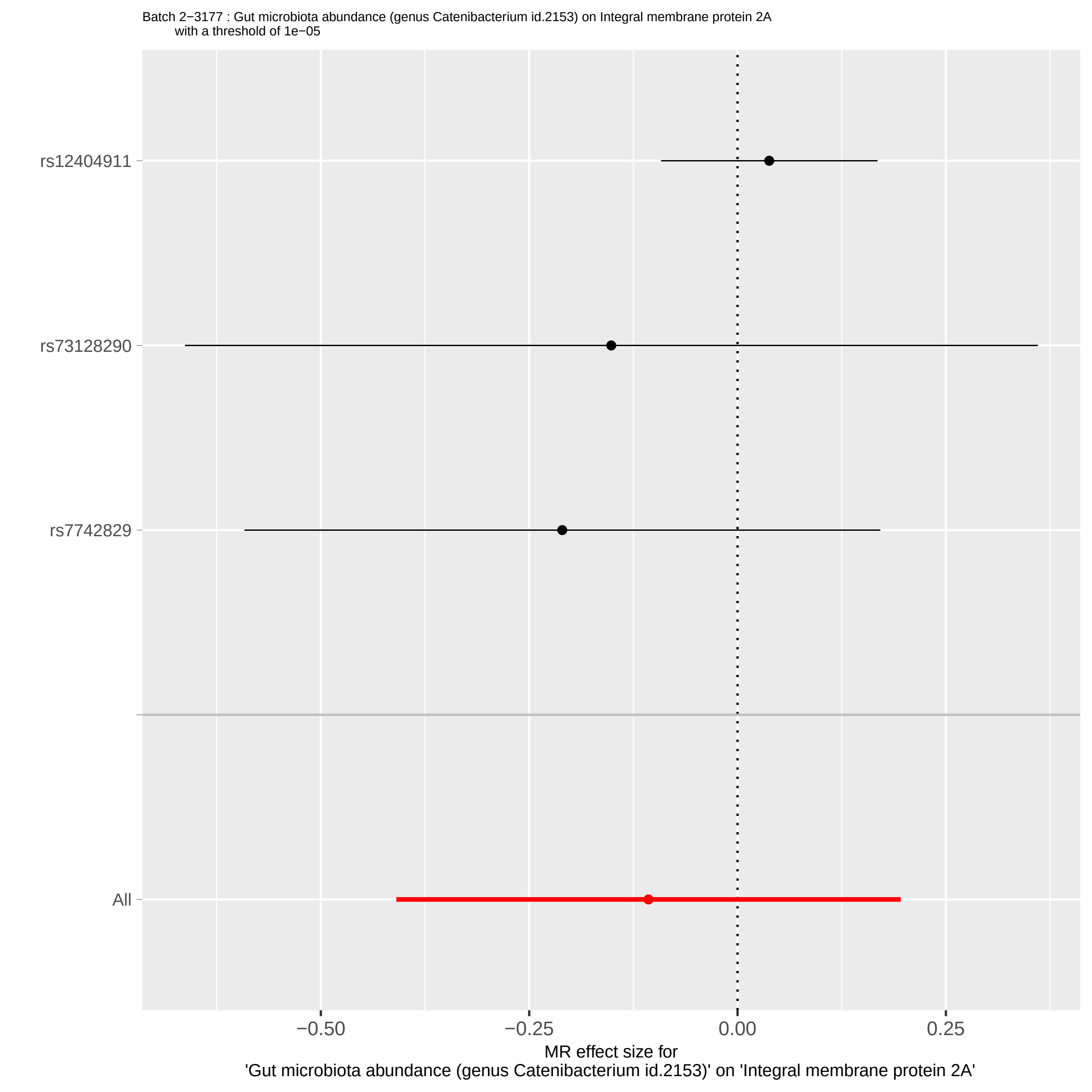
Batch 2−3177 : Gut microbiota abundance (genus Catenibacterium id.2153) on Integral membrane protein 2A
with a threshold of 1e−05
rs12404911
rs73128290
rs7742829
All
−0.50
−0.25
0.00
0.25
MR effect size for
'Gut microbiota abundance (genus Catenibacterium id.2153)' on 'Integral membrane protein 2A'

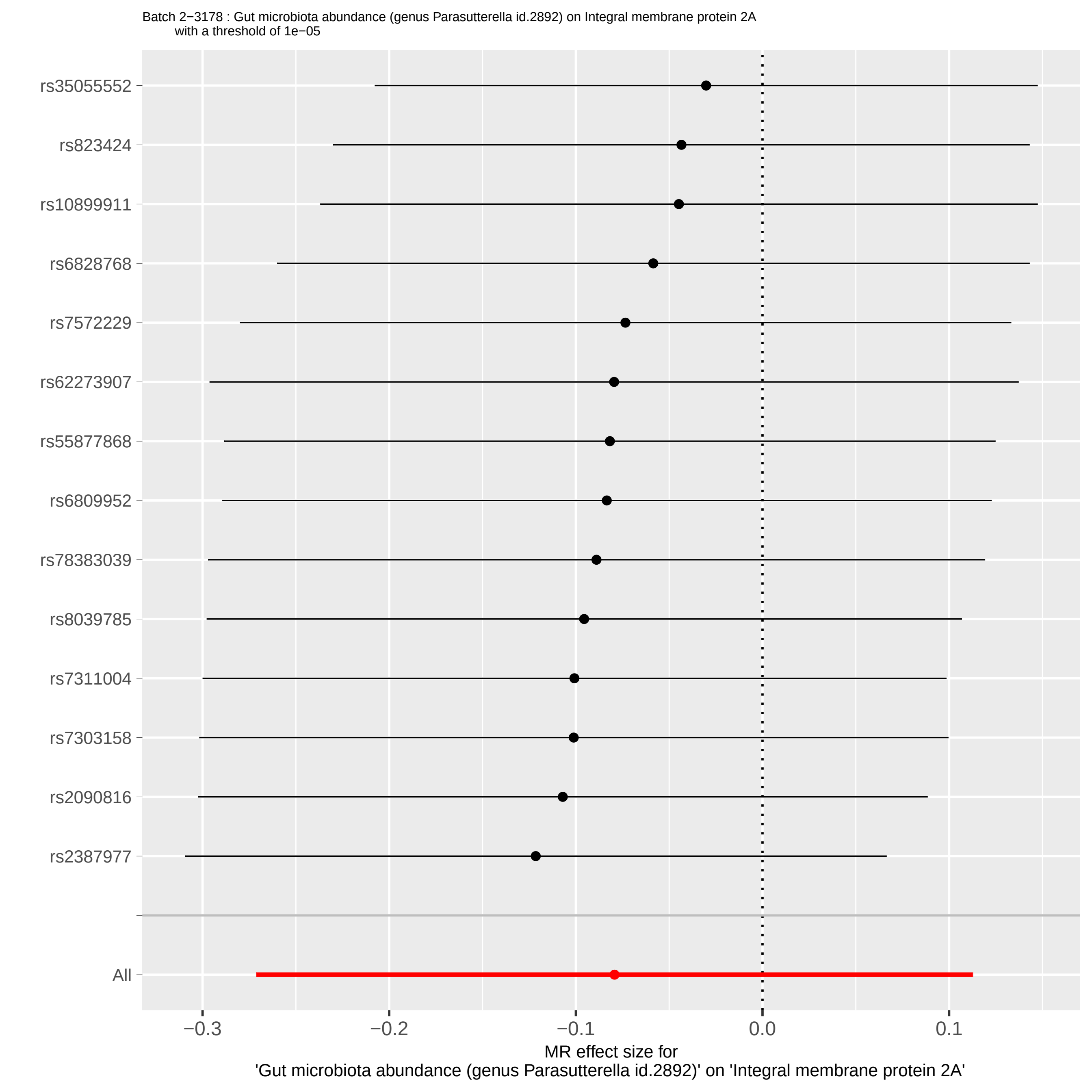
Batch 2−3178 : Gut microbiota abundance (genus Parasutterella id.2892) on Integral membrane protein 2A
with a threshold of 1e−05
rs35055552
rs823424
rs10899911
rs6828768
rs7572229
rs62273907
rs55877868
rs6809952
rs78383039
rs8039785
rs7311004
rs7303158
rs2090816
rs2387977
All
−0.3
−0.2
−0.1
0.0
0.1
MR effect size for
'Gut microbiota abundance (genus Parasutterella id.2892)' on 'Integral membrane protein 2A'

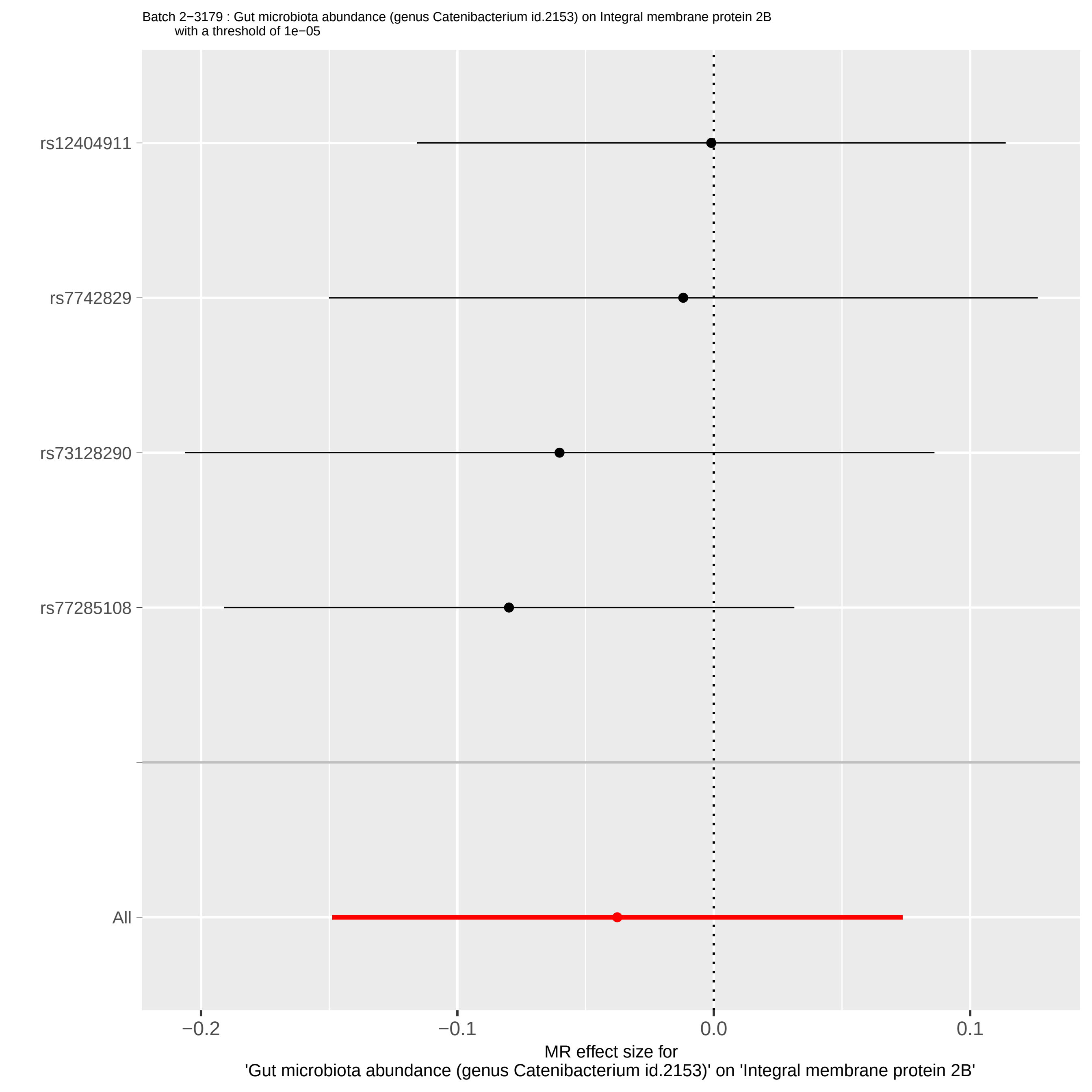

Batch 2−3179 : Gut microbiota abundance (genus Catenibacterium id.2153) on Integral membrane protein 2B
with a threshold of 1e−05
rs12404911
rs7742829
rs73128290
rs77285108
All
−0.2
−0.1
0.0
0.1
MR effect size for
'Gut microbiota abundance (genus Catenibacterium id.2153)' on 'Integral membrane protein 2B'

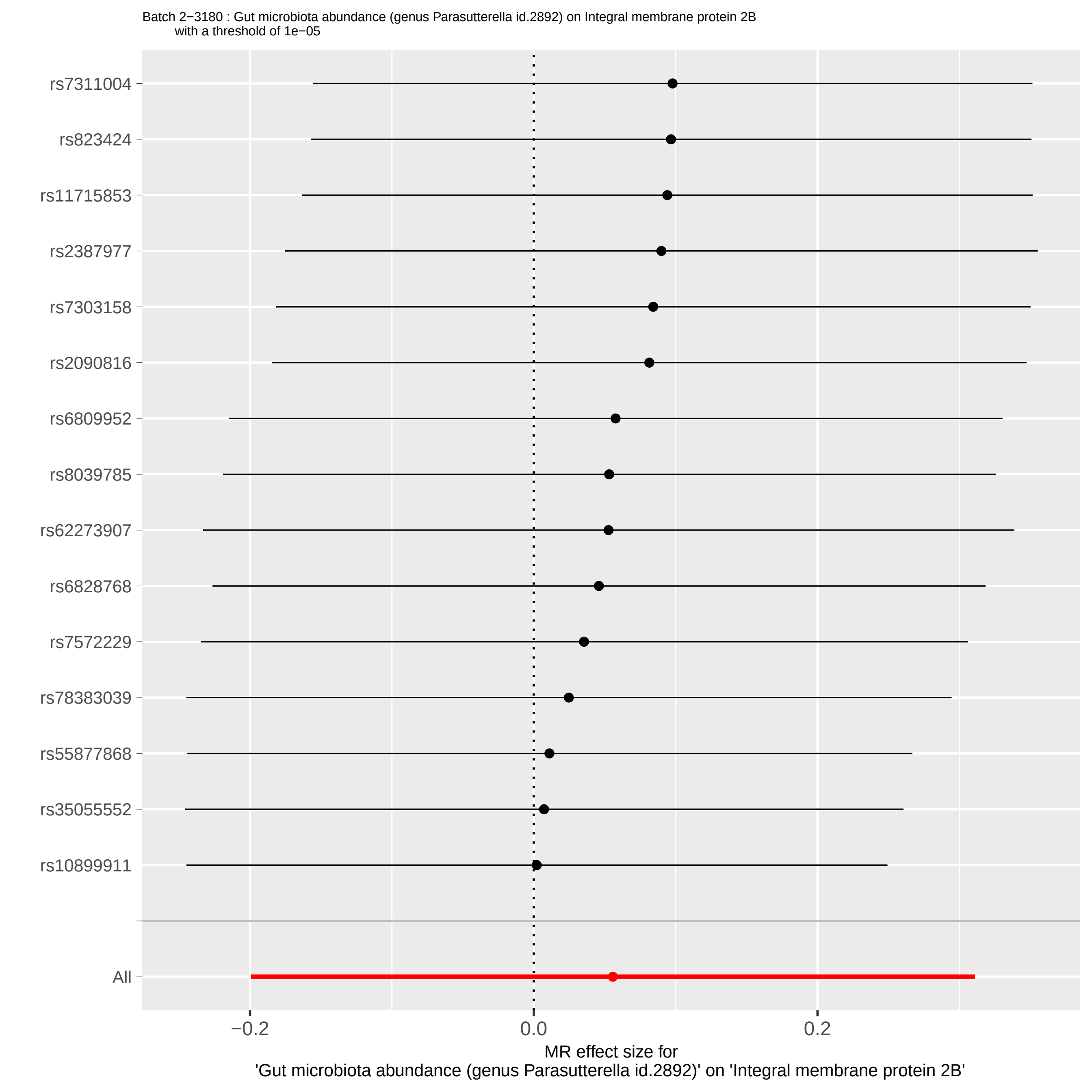

Batch 2−3180 : Gut microbiota abundance (genus Parasutterella id.2892) on Integral membrane protein 2B
with a threshold of 1e−05
rs7311004
rs823424
rs11715853
rs2387977
rs7303158
rs2090816
rs6809952
rs8039785
rs62273907
rs6828768
rs7572229
rs78383039
rs55877868
rs35055552
rs10899911
All
−0.2
0.0
0.2
MR effect size for
'Gut microbiota abundance (genus Parasutterella id.2892)' on 'Integral membrane protein 2B'

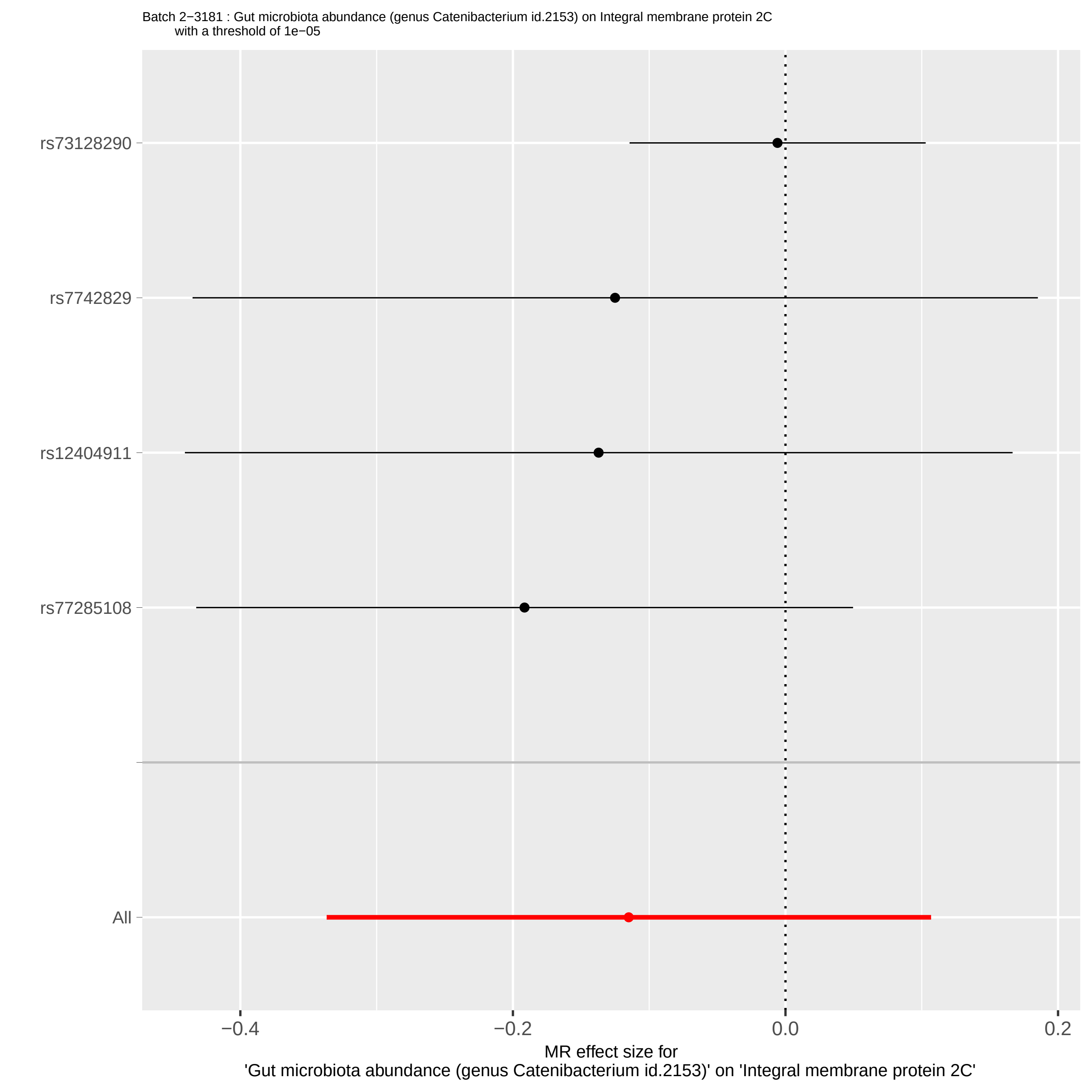
Batch 2−3181 : Gut microbiota abundance (genus Catenibacterium id.2153) on Integral membrane protein 2C
with a threshold of 1e−05
rs73128290
rs7742829
rs12404911
rs77285108
All
−0.4
−0.2
0.0
0.2
MR effect size for
'Gut microbiota abundance (genus Catenibacterium id.2153)' on 'Integral membrane protein 2C'

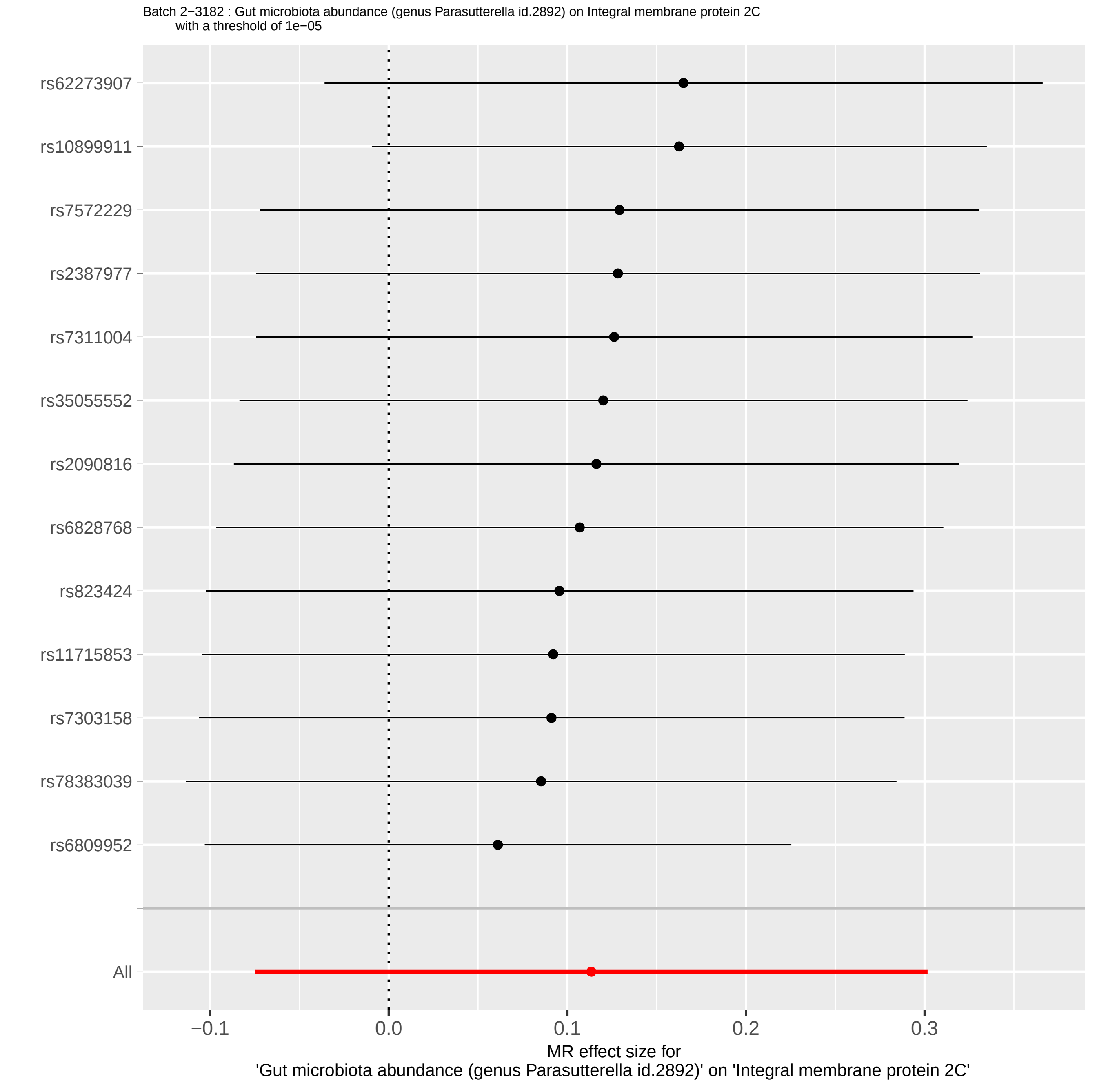
Batch 2−3182 : Gut microbiota abundance (genus Parasutterella id.2892) on Integral membrane protein 2C
with a threshold of 1e−05
rs62273907
rs10899911
rs7572229
rs2387977
rs7311004
rs35055552
rs2090816
rs6828768
rs823424
rs11715853
rs7303158
rs78383039
rs6809952
All
−0.1
0.0
0.1
0.2
0.3
MR effect size for
'Gut microbiota abundance (genus Parasutterella id.2892)' on 'Integral membrane protein 2C'

Batch 2−3183 : Gut microbiota abundance (genus Catenibacterium id.2153) on Integral membrane protein 2C
with a threshold of 1e−05

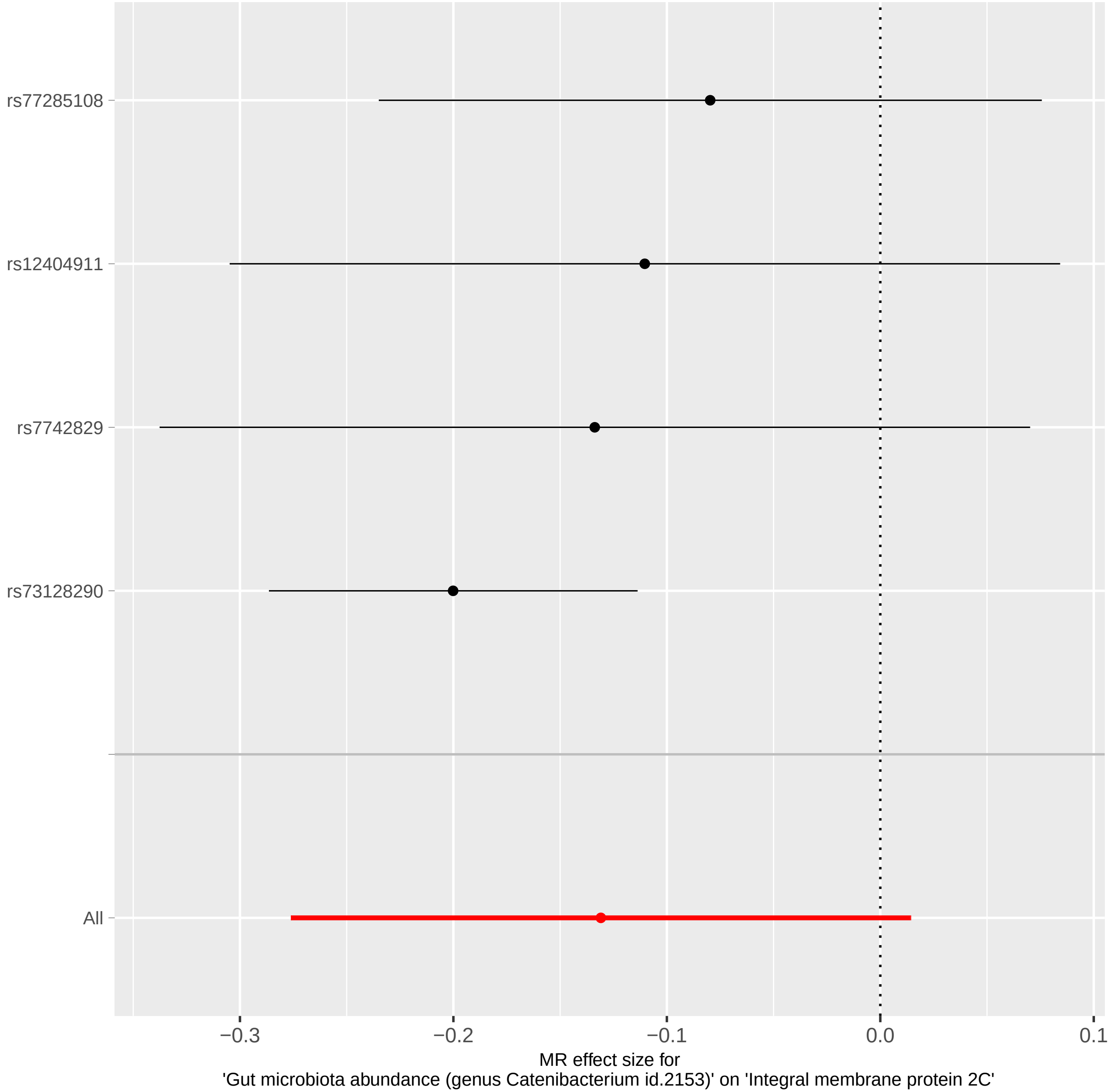

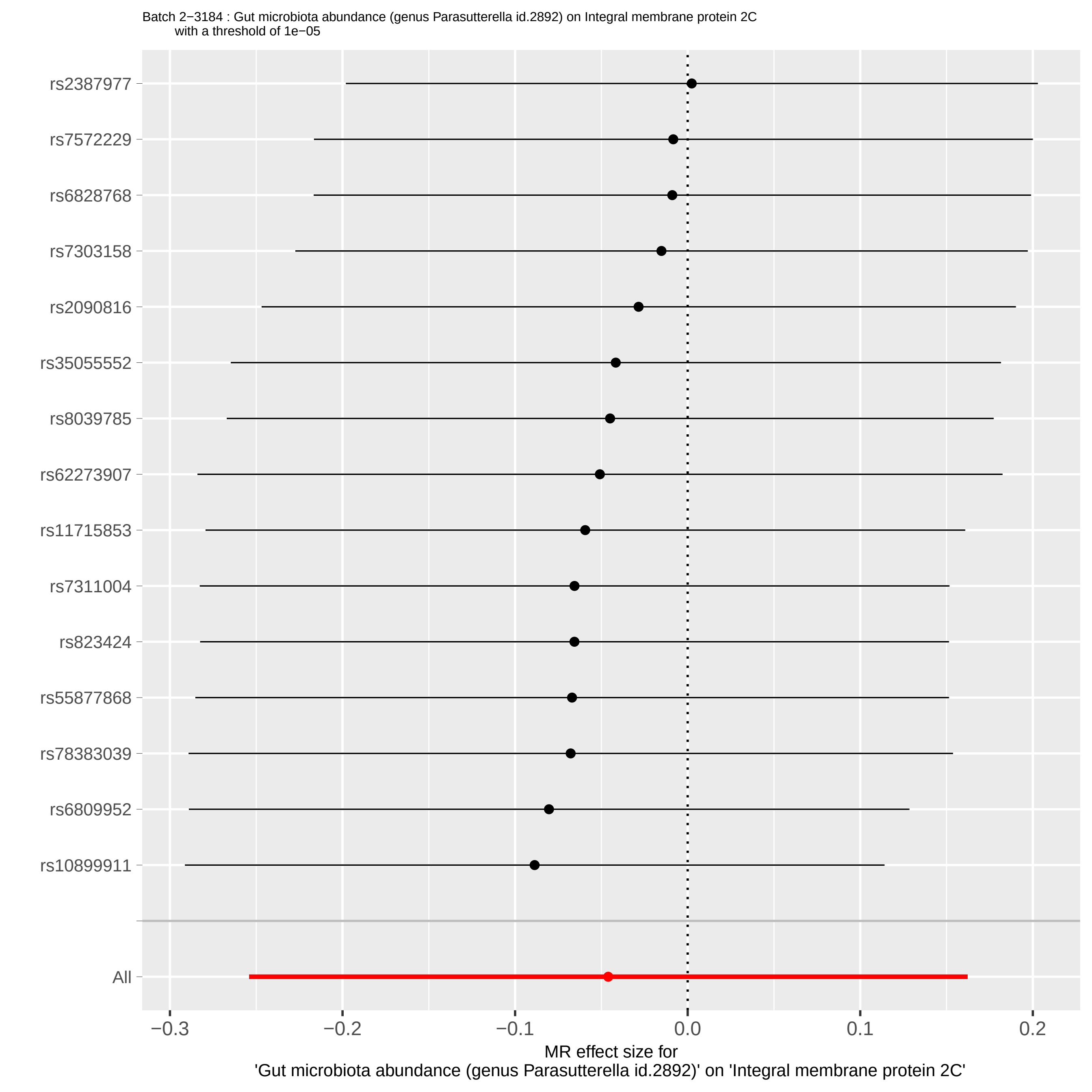
Batch 2−3184 : Gut microbiota abundance (genus Parasutterella id.2892) on Integral membrane protein 2C
with a threshold of 1e−05
rs2387977
rs7572229
rs6828768
rs7303158
rs2090816
rs35055552
rs8039785
rs62273907
rs11715853
rs7311004
rs823424
rs55877868
rs78383039
rs6809952
rs10899911
All
−0.3
−0.2
−0.1
0.0
0.1
0.2
MR effect size for
'Gut microbiota abundance (genus Parasutterella id.2892)' on 'Integral membrane protein 2C'

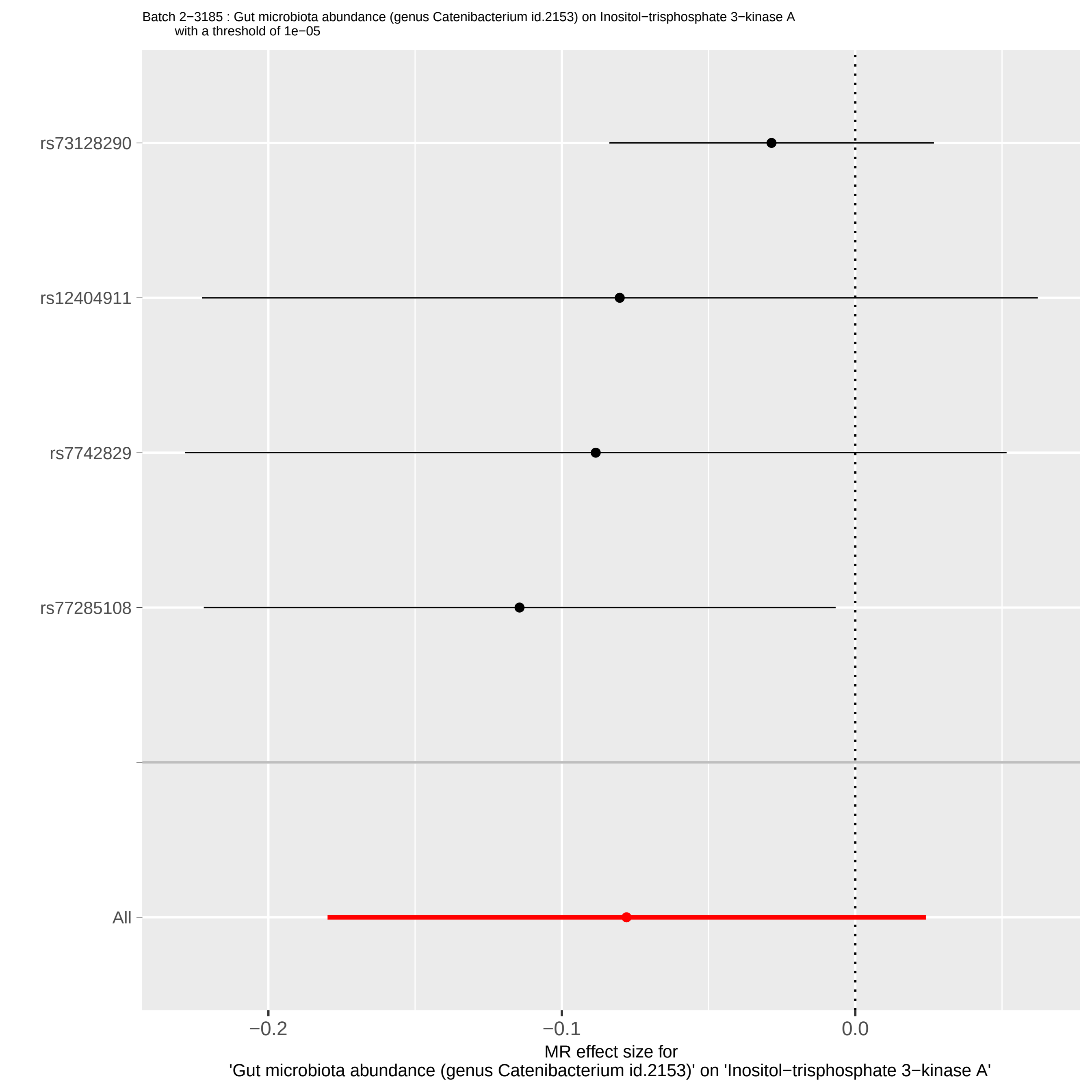

Batch 2−3185 : Gut microbiota abundance (genus Catenibacterium id.2153) on Inositol−trisphosphate 3−kinase A
with a threshold of 1e−05
rs73128290
rs12404911
rs7742829
rs77285108
All
−0.2
−0.1
0.0
MR effect size for
'Gut microbiota abundance (genus Catenibacterium id.2153)' on 'Inositol−trisphosphate 3−kinase A'

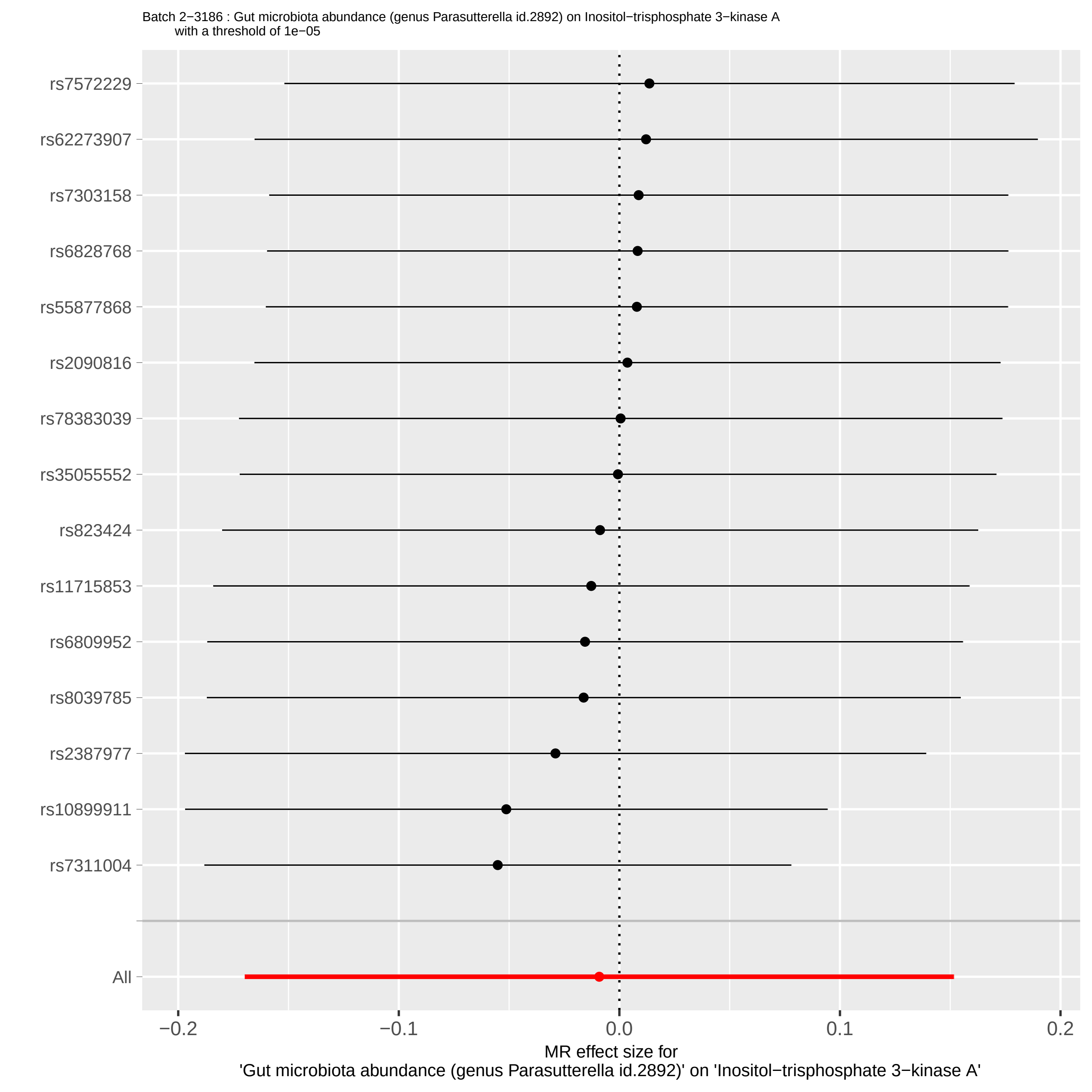

Batch 2−3186 : Gut microbiota abundance (genus Parasutterella id.2892) on Inositol−trisphosphate 3−kinase A
with a threshold of 1e−05
rs7572229
rs62273907
rs7303158
rs6828768
rs55877868
rs2090816
rs78383039
rs35055552
rs823424
rs11715853
rs6809952
rs8039785
rs2387977
rs10899911
rs7311004
All
−0.2
−0.1
0.0
0.1
0.2
MR effect size for
'Gut microbiota abundance (genus Parasutterella id.2892)' on 'Inositol−trisphosphate 3−kinase A'

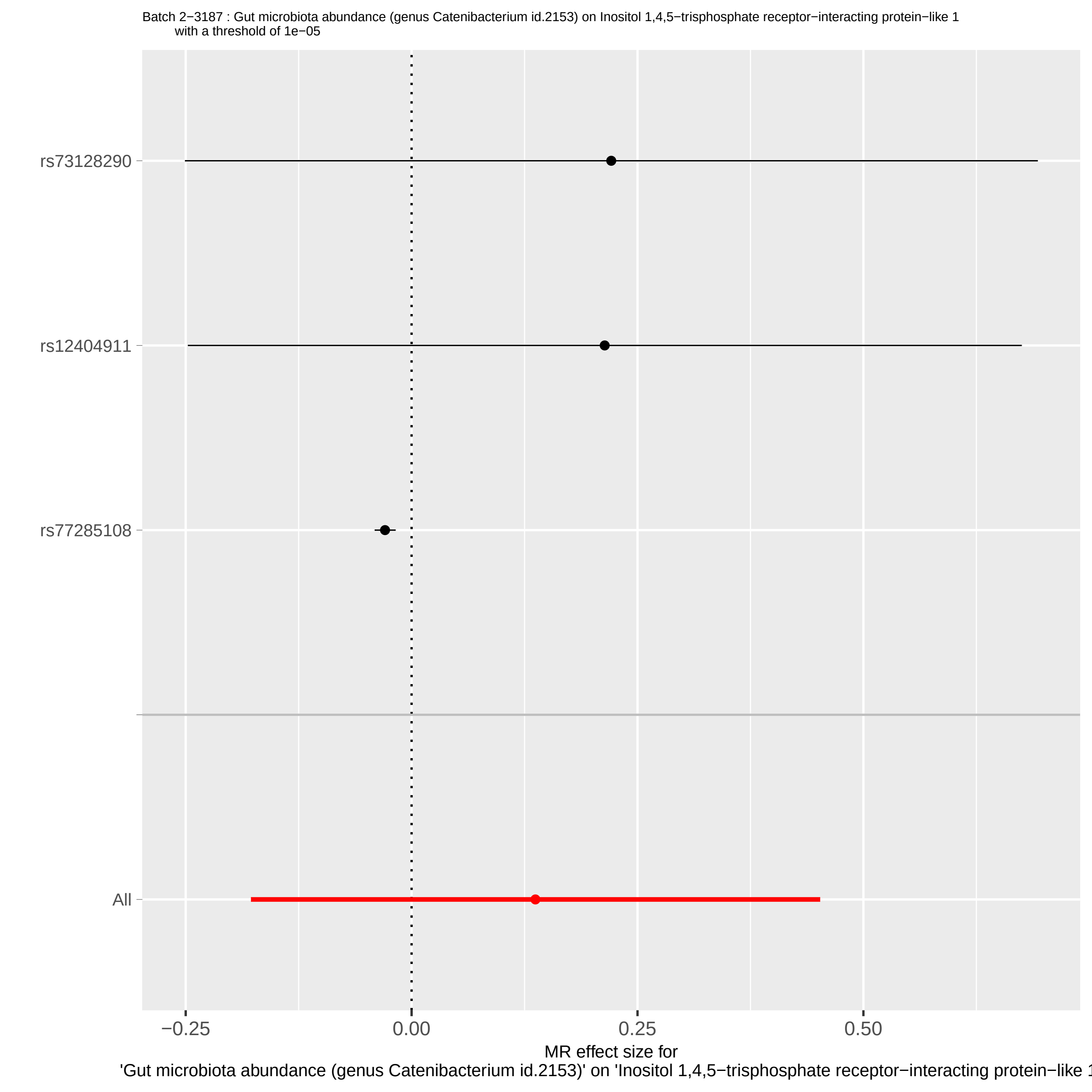
Batch 2−3187 : Gut microbiota abundance (genus Catenibacterium id.2153) on Inositol 1,4,5−trisphosphate receptor−interacting protein−like 1
with a threshold of 1e−05
rs73128290
rs12404911
rs77285108
All
−0.25
0.00
0.25
0.50
MR effect size for
'Gut microbiota abundance (genus Catenibacterium id.2153)' on 'Inositol 1,4,5−trisphosphate receptor−interacting protein−like 1

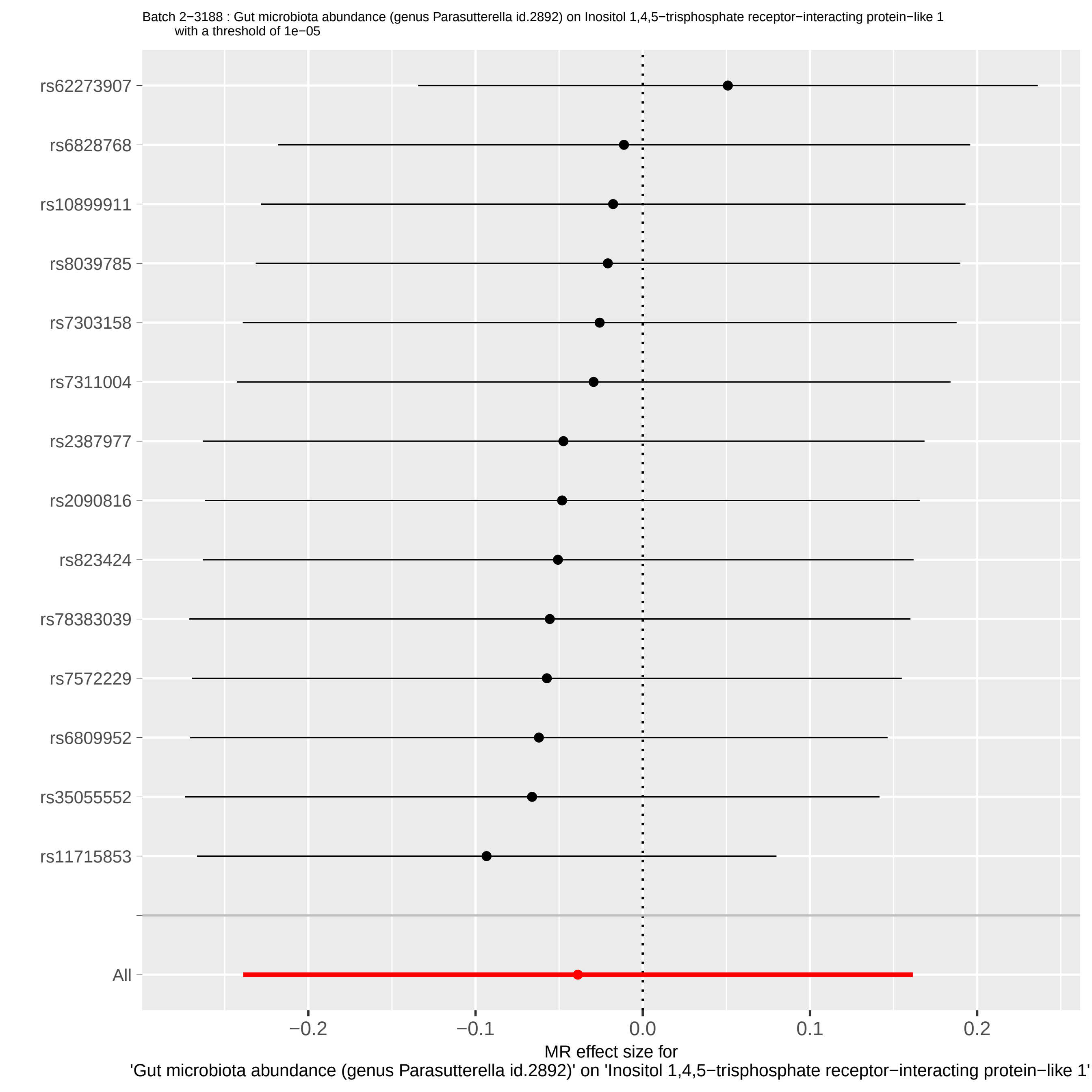

Batch 2−3188 : Gut microbiota abundance (genus Parasutterella id.2892) on Inositol 1,4,5−trisphosphate receptor−interacting protein−like 1
with a threshold of 1e−05
rs62273907
rs6828768
rs10899911
rs8039785
rs7303158
rs7311004
rs2387977
rs2090816
rs823424
rs78383039
rs7572229
rs6809952
rs35055552
rs11715853
All
−0.2
−0.1
0.0
0.1
0.2
MR effect size for
'Gut microbiota abundance (genus Parasutterella id.2892)' on 'Inositol 1,4,5−trisphosphate receptor−interacting protein−like 1'

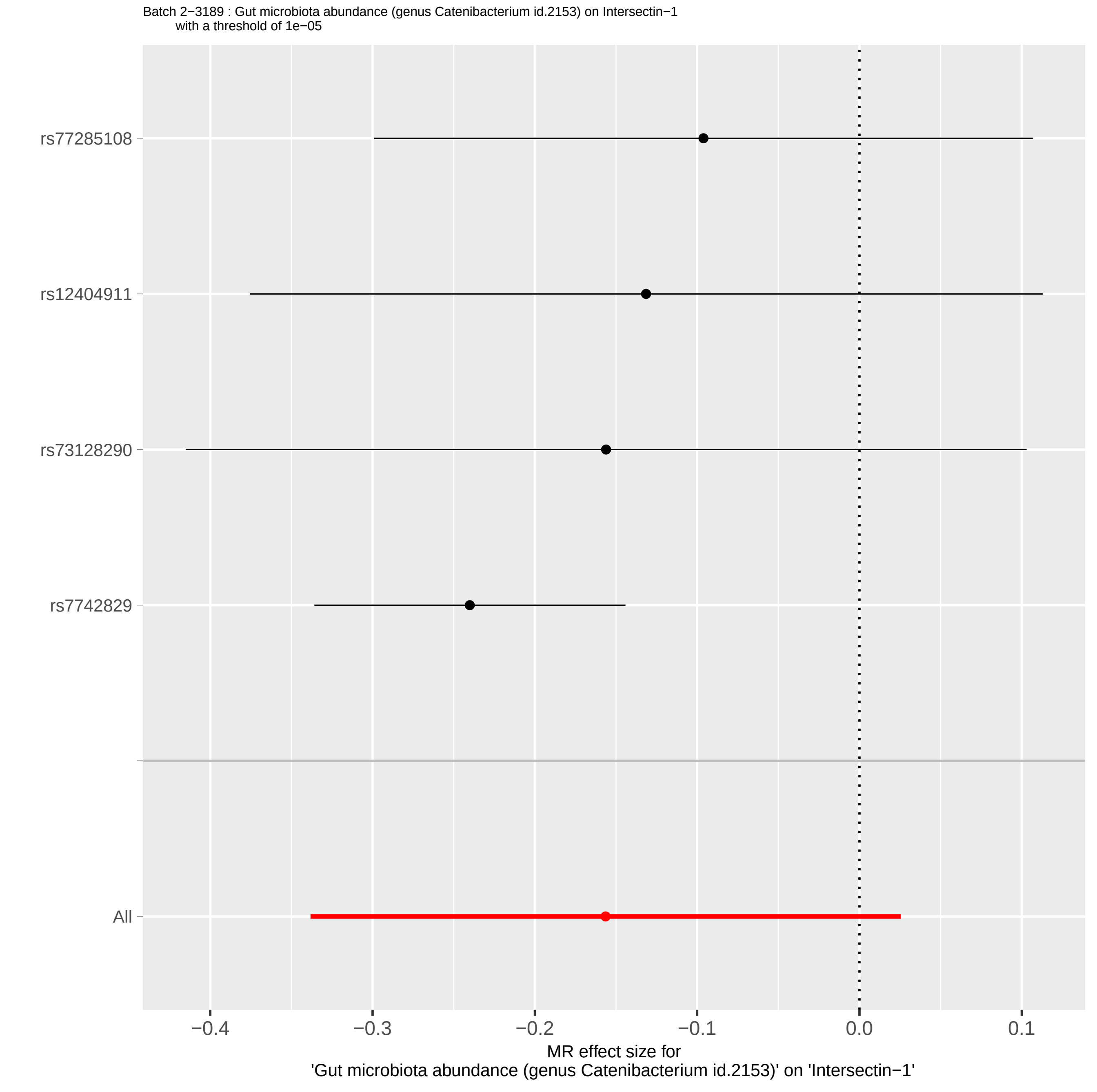

Batch 2−3189 : Gut microbiota abundance (genus Catenibacterium id.2153) on Intersectin−1
with a threshold of 1e−05
rs77285108
rs12404911
rs73128290
rs7742829
All
−0.4
−0.3
−0.2
−0.1
0.0
0.1
MR effect size for
'Gut microbiota abundance (genus Catenibacterium id.2153)' on 'Intersectin−1'

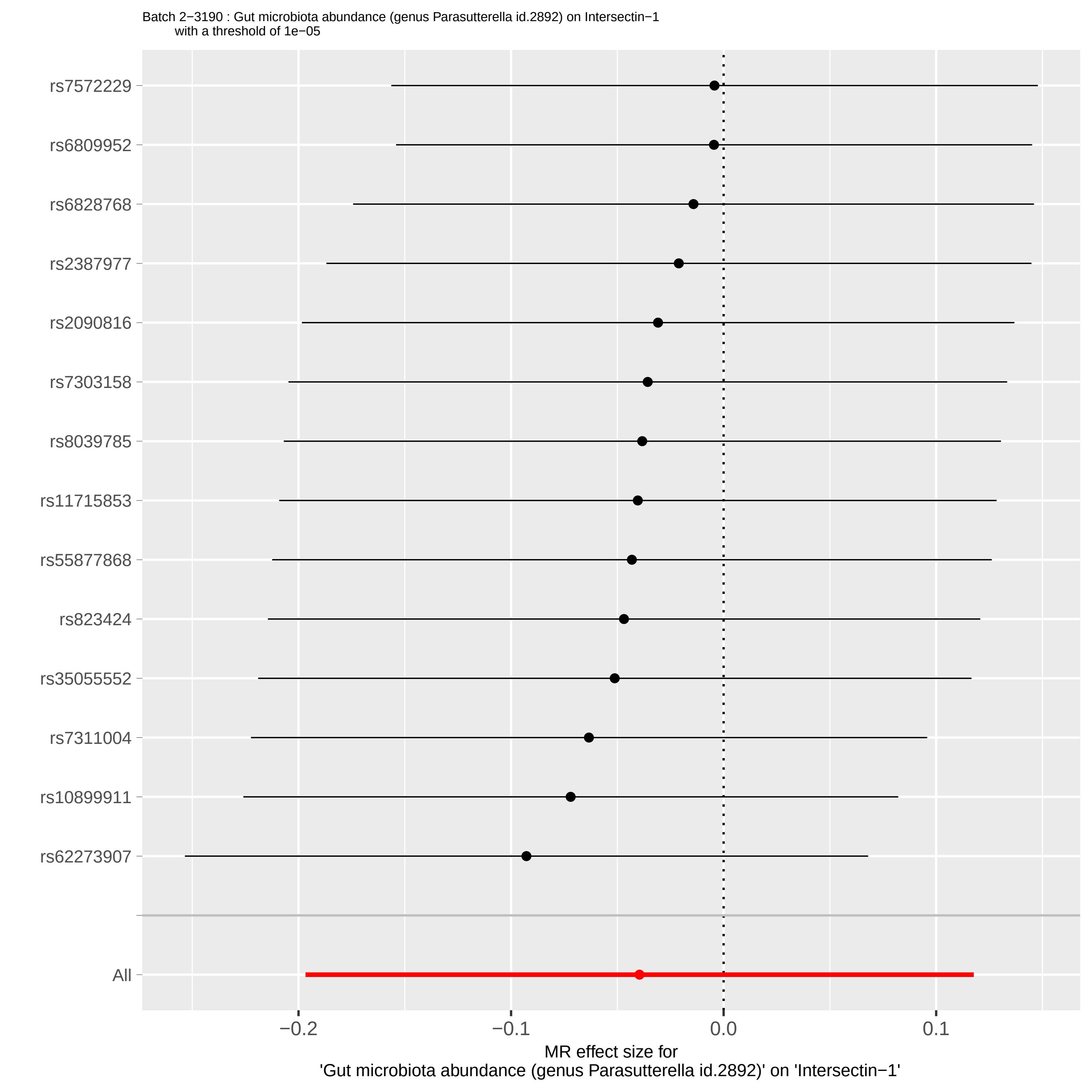

Batch 2−3190 : Gut microbiota abundance (genus Parasutterella id.2892) on Intersectin−1
with a threshold of 1e−05
rs7572229
rs6809952
rs6828768
rs2387977
rs2090816
rs7303158
rs8039785
rs11715853
rs55877868
rs823424
rs35055552
rs7311004
rs10899911
rs62273907
All
−0.2
−0.1
0.0
0.1
MR effect size for
'Gut microbiota abundance (genus Parasutterella id.2892)' on 'Intersectin−1'

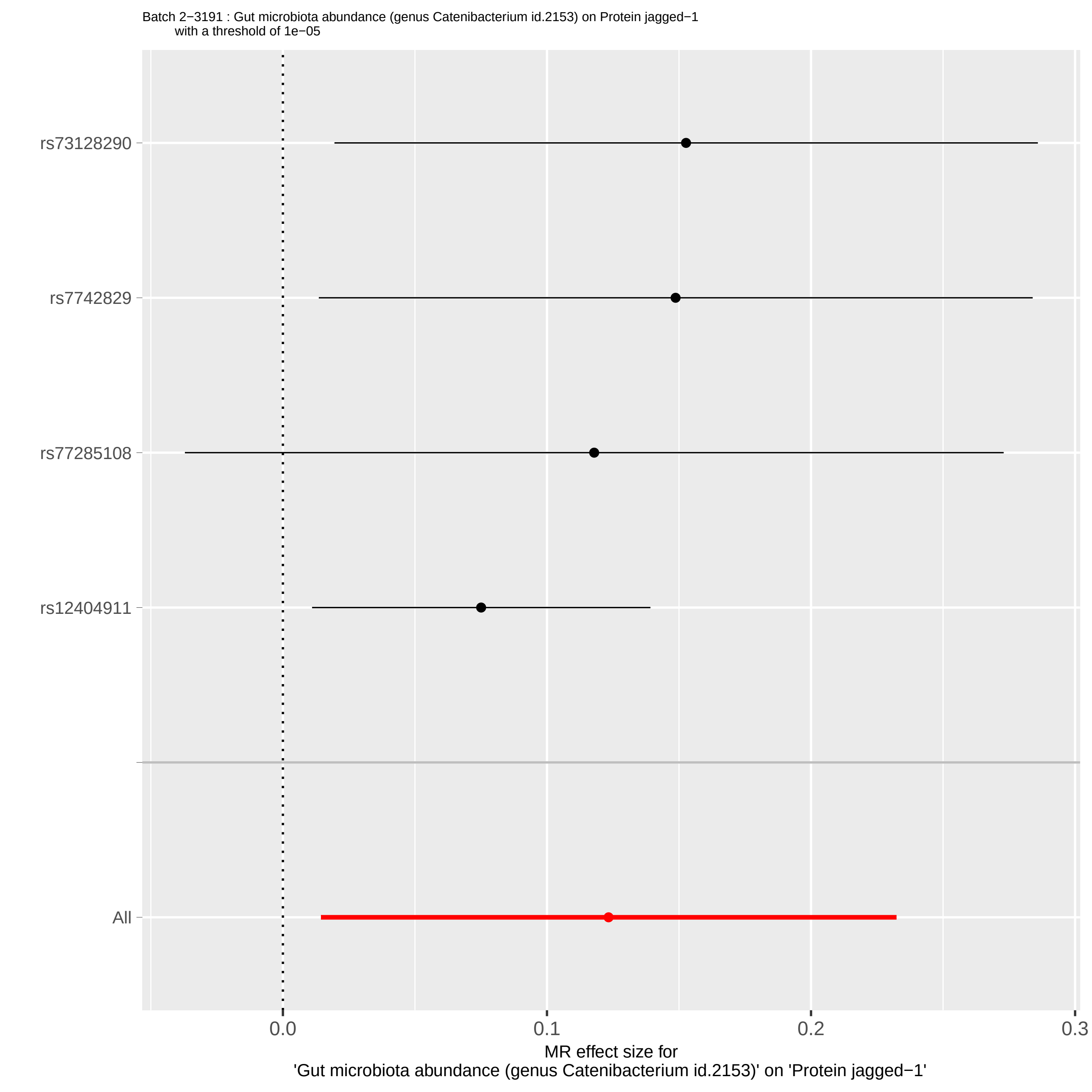
Batch 2−3191 : Gut microbiota abundance (genus Catenibacterium id.2153) on Protein jagged−1
with a threshold of 1e−05
rs73128290
rs7742829
rs77285108
rs12404911
All
0.0
0.1
0.2
0.3
MR effect size for
'Gut microbiota abundance (genus Catenibacterium id.2153)' on 'Protein jagged−1'

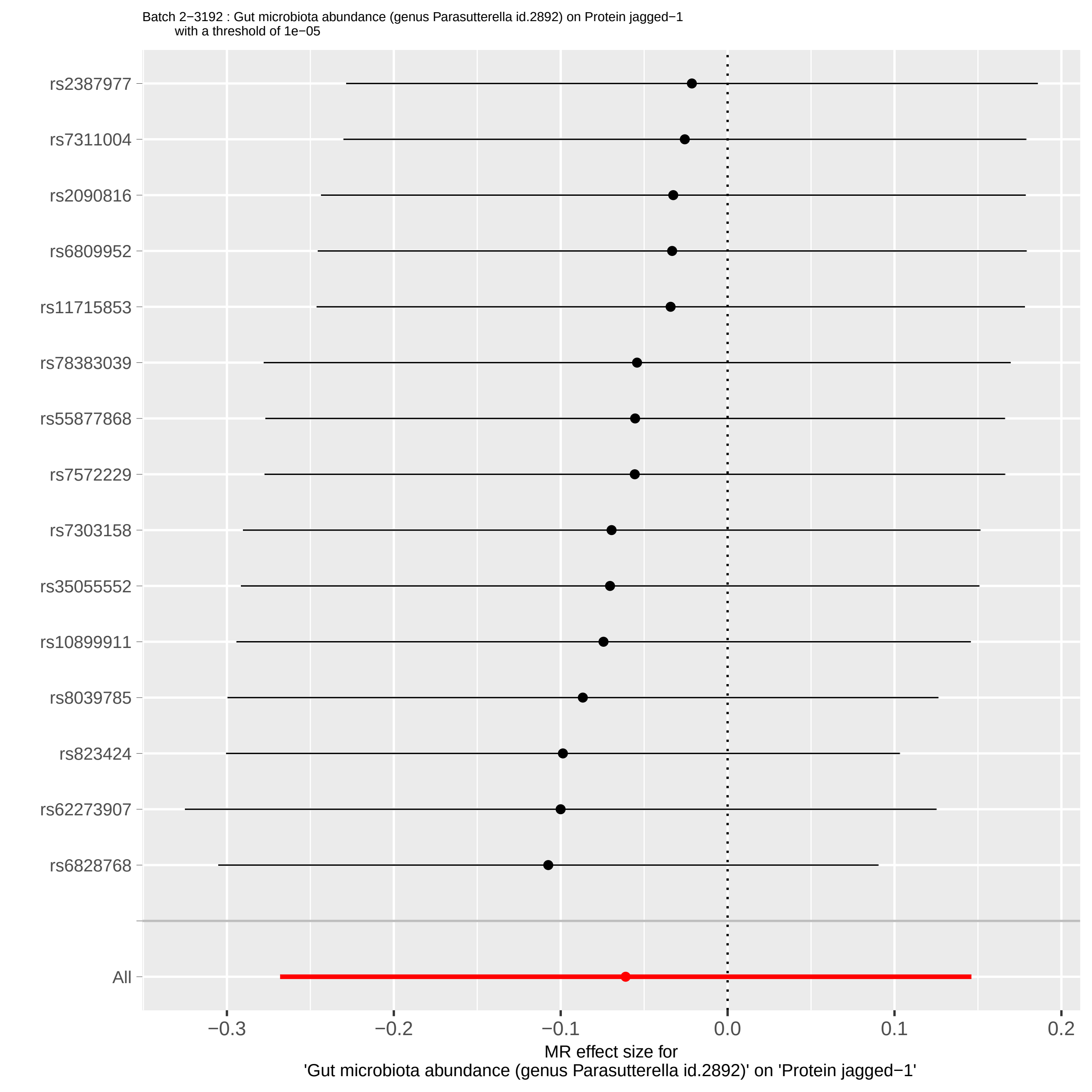

Batch 2−3192 : Gut microbiota abundance (genus Parasutterella id.2892) on Protein jagged−1
with a threshold of 1e−05
rs2387977
rs7311004
rs2090816
rs6809952
rs11715853
rs78383039
rs55877868
rs7572229
rs7303158
rs35055552
rs10899911
rs8039785
rs823424
rs62273907
rs6828768
All
−0.3
−0.2
−0.1
0.0
0.1
0.2
MR effect size for
'Gut microbiota abundance (genus Parasutterella id.2892)' on 'Protein jagged−1'

Batch 2−3193 : Gut microbiota abundance (genus Catenibacterium id.2153) on Protein jagged−2
with a threshold of 1e−05

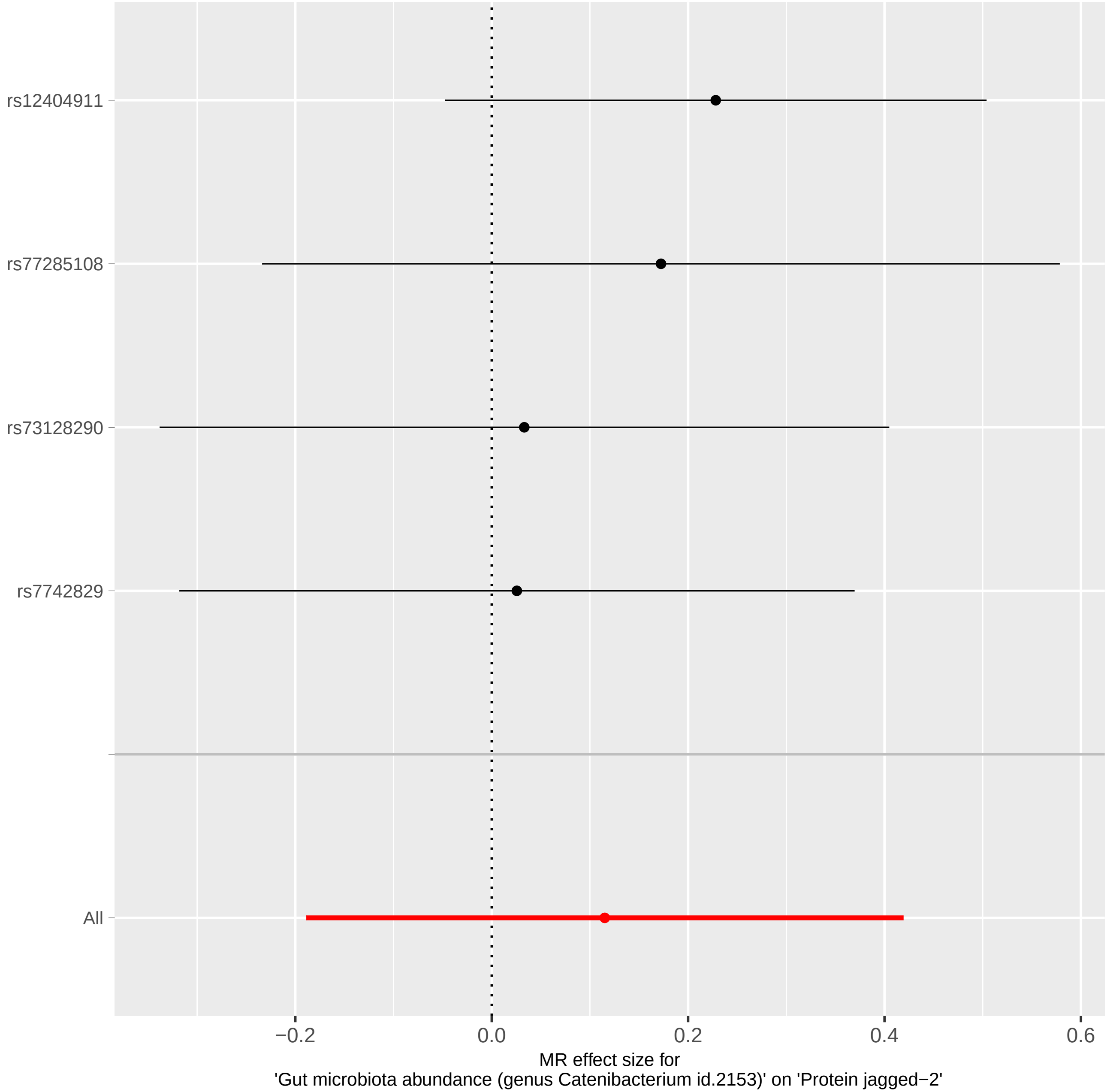

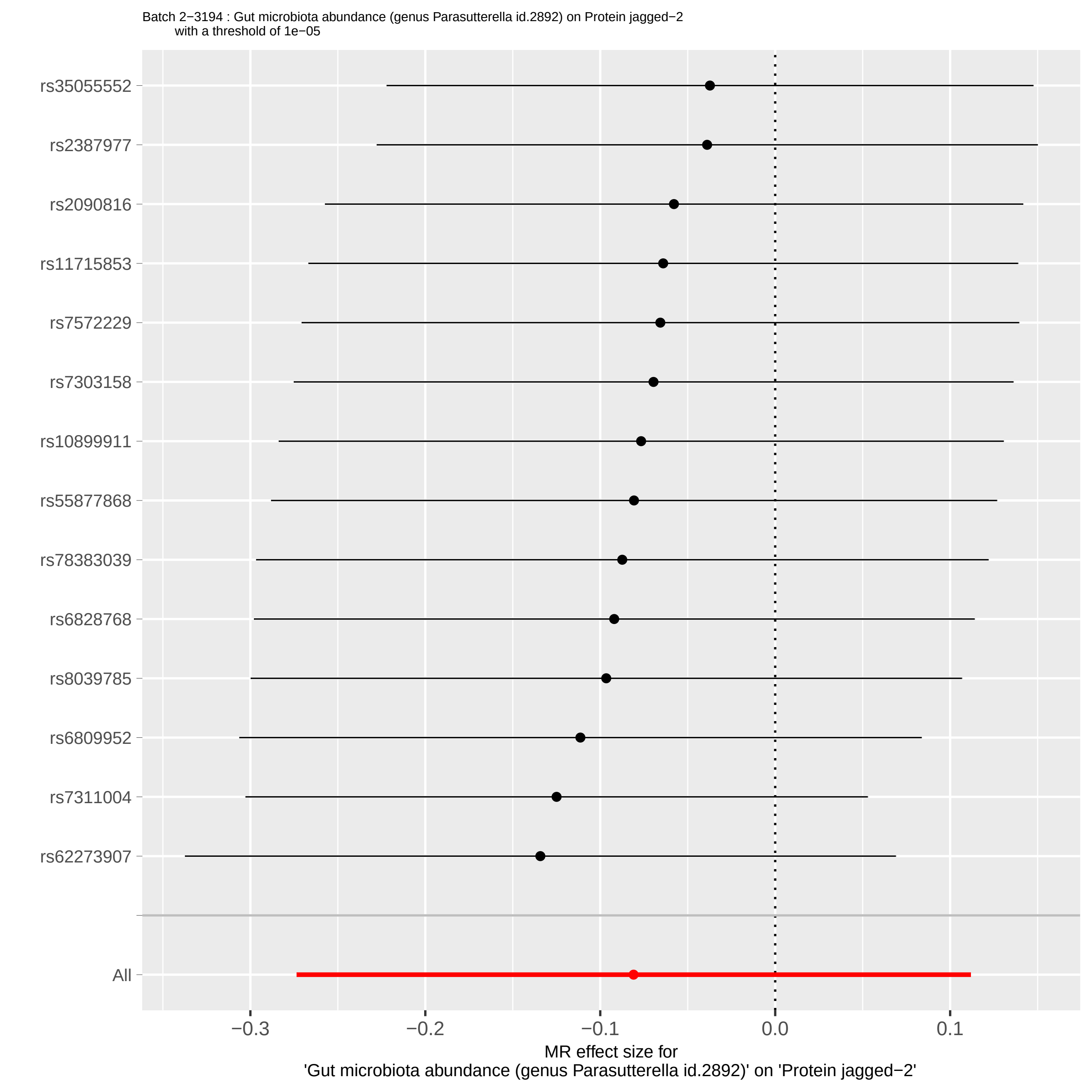

Batch 2−3194 : Gut microbiota abundance (genus Parasutterella id.2892) on Protein jagged−2
with a threshold of 1e−05
rs35055552
rs2387977
rs2090816
rs11715853
rs7572229
rs7303158
rs10899911
rs55877868
rs78383039
rs6828768
rs8039785
rs6809952
rs7311004
rs62273907
All
−0.3
−0.2
−0.1
0.0
0.1
MR effect size for
'Gut microbiota abundance (genus Parasutterella id.2892)' on 'Protein jagged−2'

Batch 2−3195 : Gut microbiota abundance (genus Catenibacterium id.2153) on Tyrosine−protein kinase JAK2
with a threshold of 1e−05

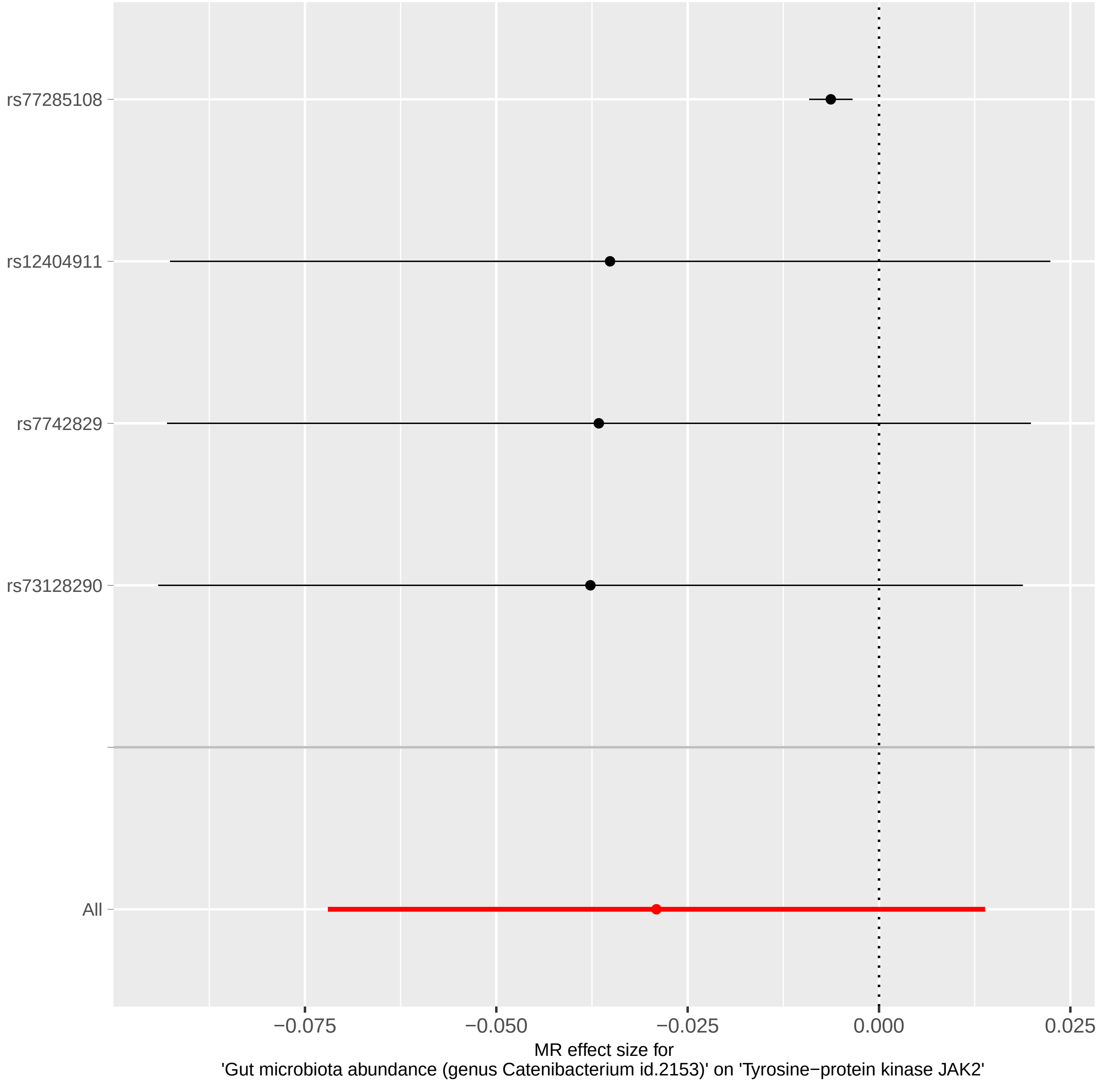

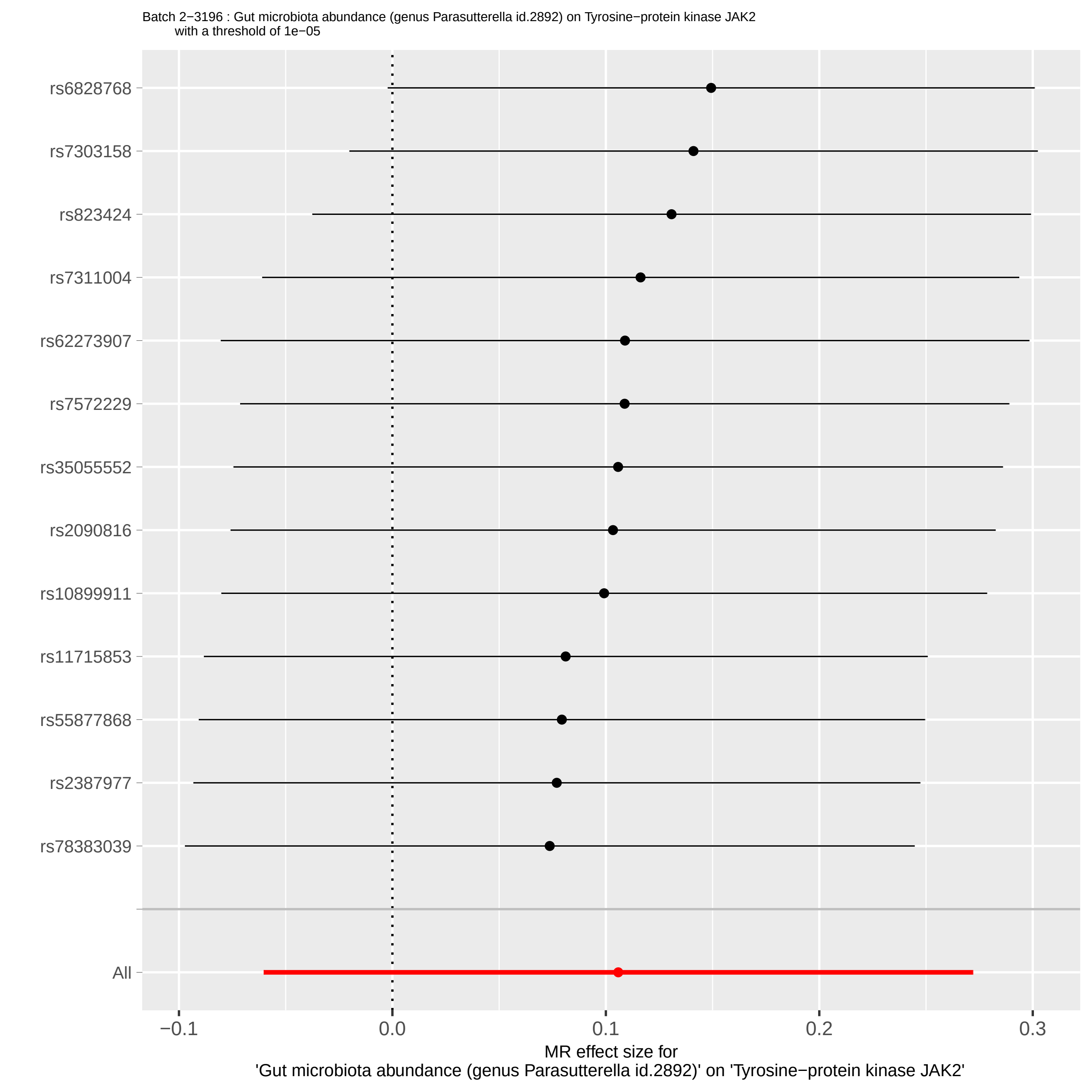

Batch 2−3196 : Gut microbiota abundance (genus Parasutterella id.2892) on Tyrosine−protein kinase JAK2
with a threshold of 1e−05
rs6828768
rs7303158
rs823424
rs7311004
rs62273907
rs7572229
rs35055552
rs2090816
rs10899911
rs11715853
rs55877868
rs2387977
rs78383039
All
−0.1
0.0
0.1
0.2
0.3
MR effect size for
'Gut microbiota abundance (genus Parasutterella id.2892)' on 'Tyrosine−protein kinase JAK2'

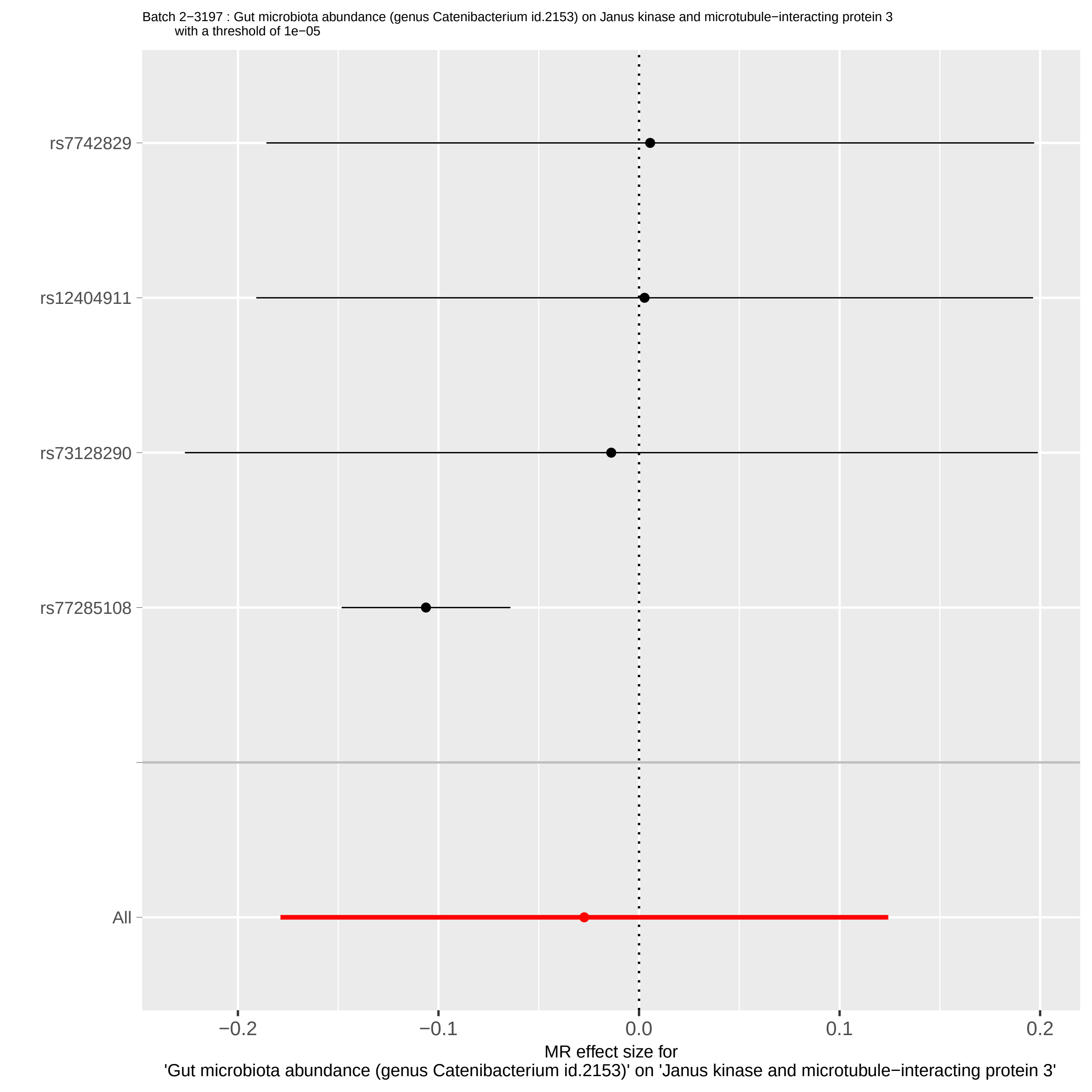

Batch 2−3197 : Gut microbiota abundance (genus Catenibacterium id.2153) on Janus kinase and microtubule−interacting protein 3
with a threshold of 1e−05
rs7742829
rs12404911
rs73128290
rs77285108
All
−0.2
−0.1
0.0
0.1
0.2
MR effect size for
'Gut microbiota abundance (genus Catenibacterium id.2153)' on 'Janus kinase and microtubule−interacting protein 3'

Batch 2−3198 : Gut microbiota abundance (genus Parasutterella id.2892) on Janus kinase and microtubule−interacting protein 3
with a threshold of 1e−05

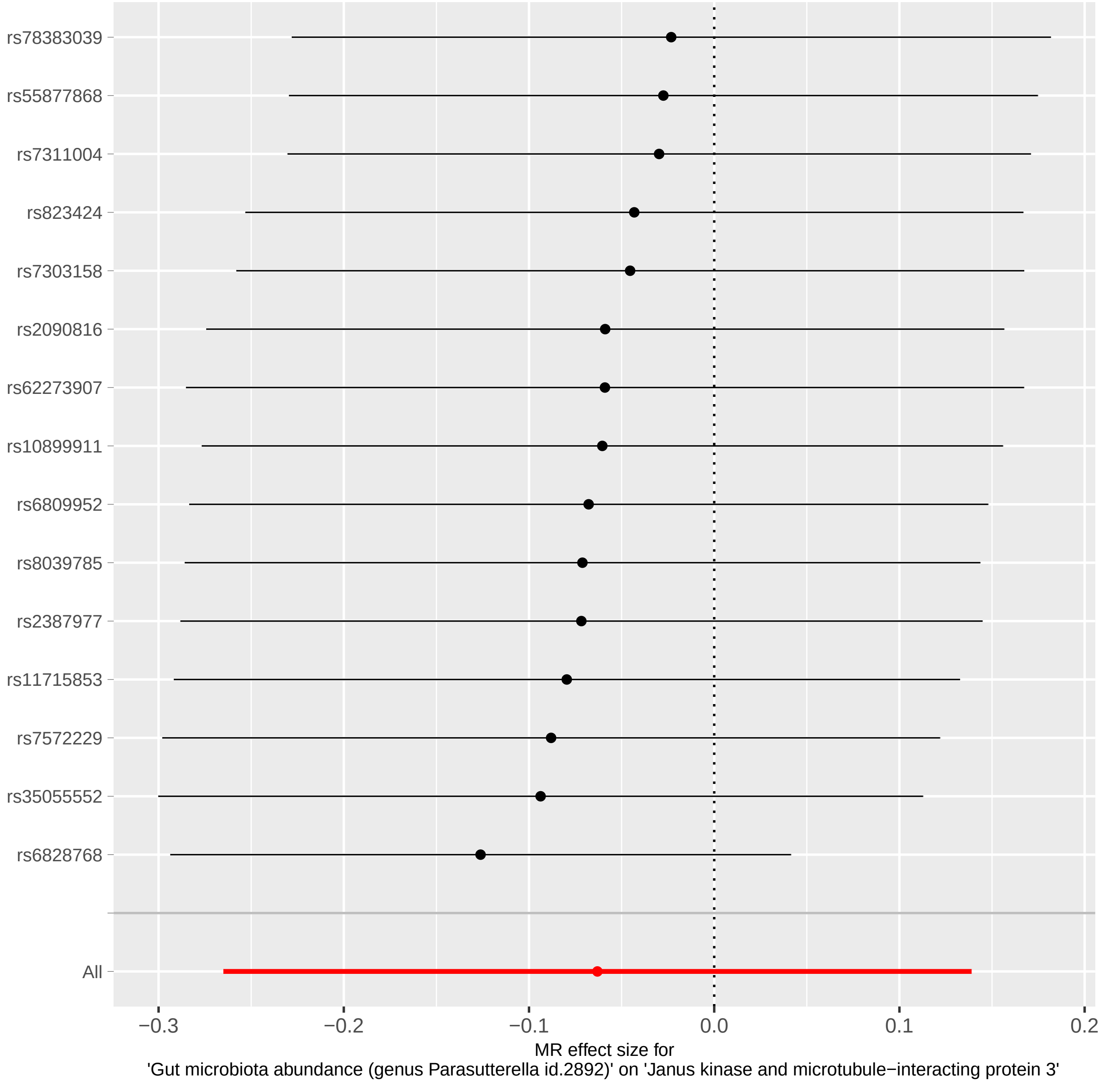

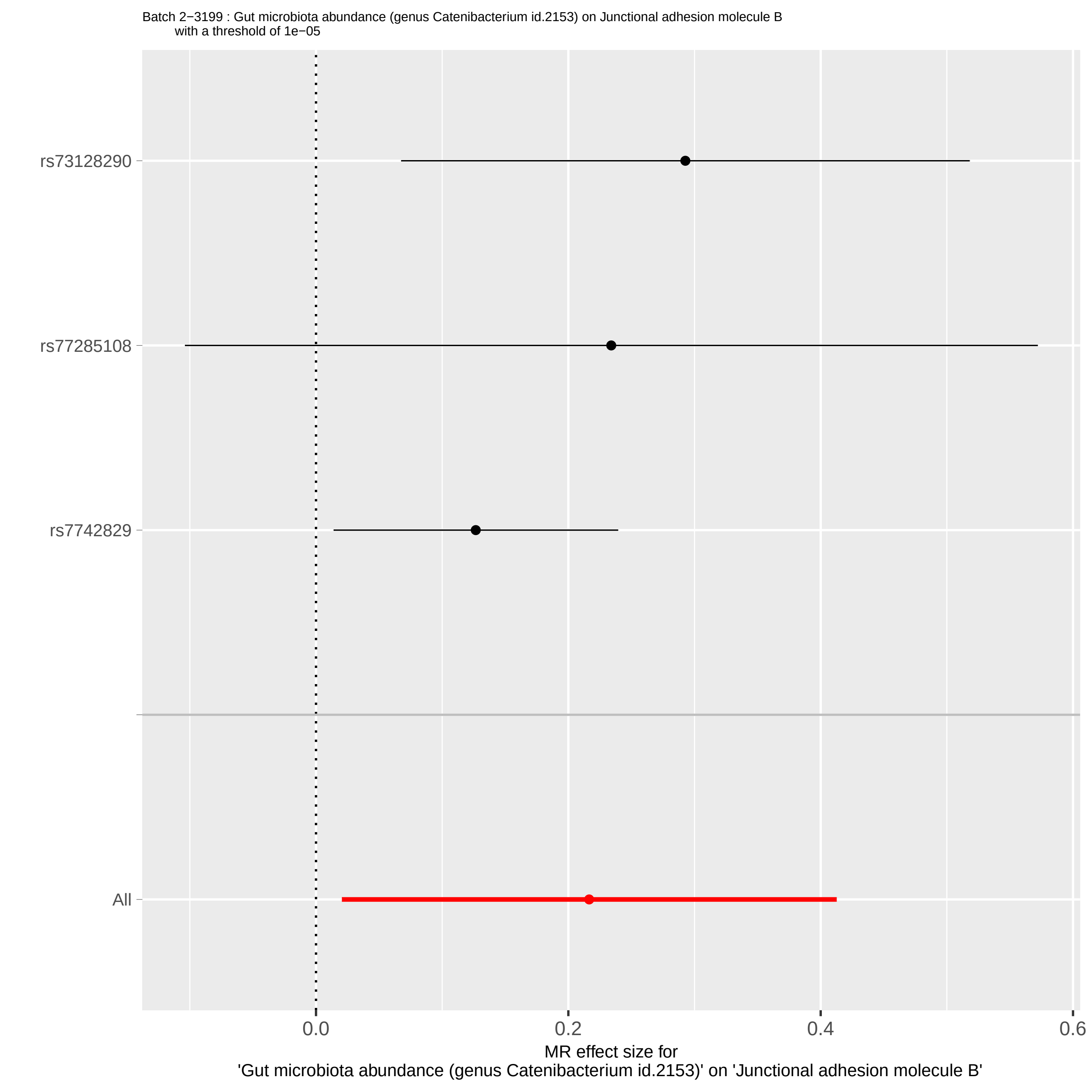
Batch 2−3199 : Gut microbiota abundance (genus Catenibacterium id.2153) on Junctional adhesion molecule B
with a threshold of 1e−05
rs73128290
rs77285108
rs7742829
All
0.0
0.2
0.4
0.6
MR effect size for
'Gut microbiota abundance (genus Catenibacterium id.2153)' on 'Junctional adhesion molecule B'

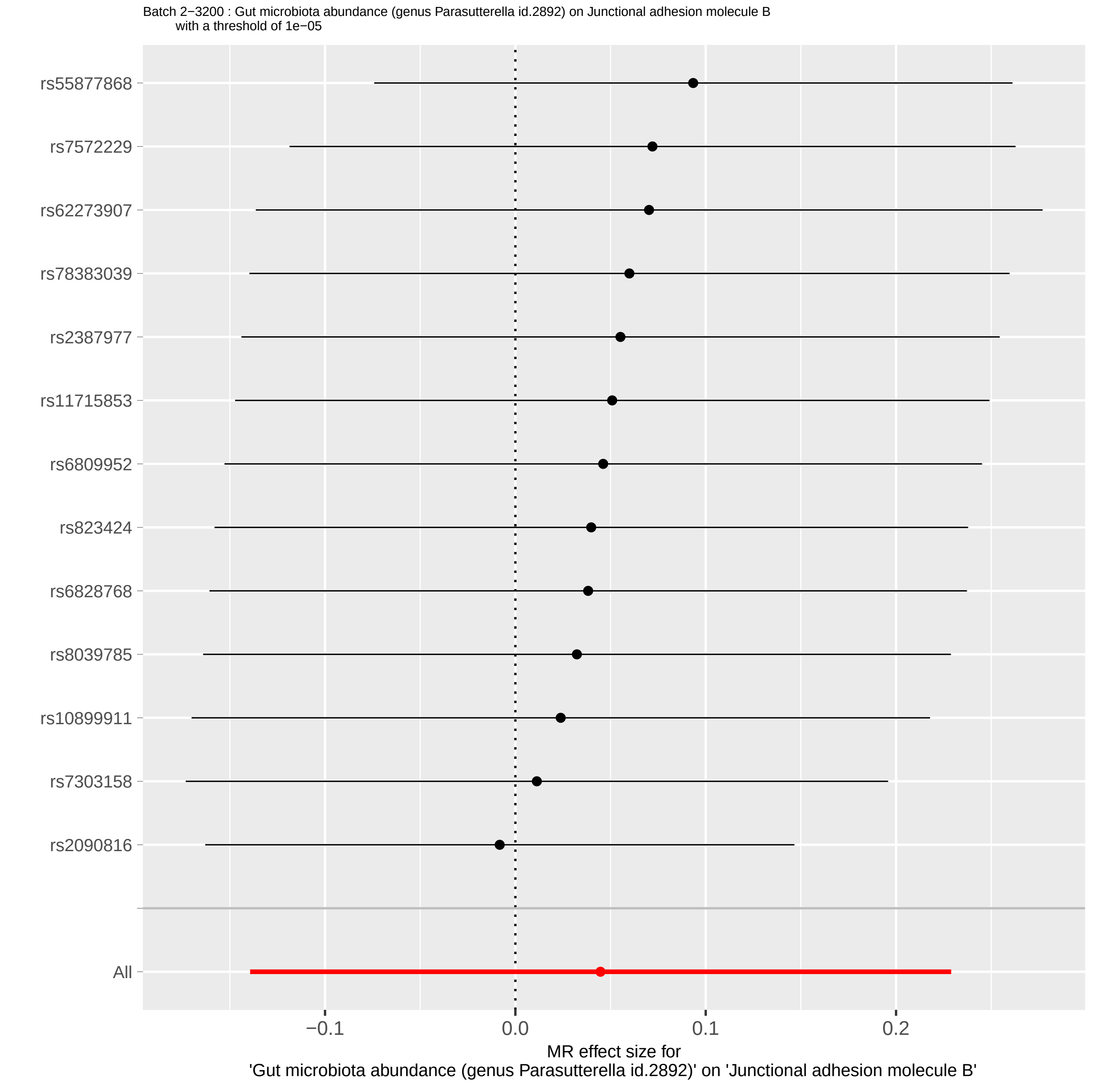
Batch 2−3200 : Gut microbiota abundance (genus Parasutterella id.2892) on Junctional adhesion molecule B
with a threshold of 1e−05
rs55877868
rs7572229
rs62273907
rs78383039
rs2387977
rs11715853
rs6809952
rs823424
rs6828768
rs8039785
rs10899911
rs7303158
rs2090816
All
−0.1
0.0
0.1
0.2
MR effect size for
'Gut microbiota abundance (genus Parasutterella id.2892)' on 'Junctional adhesion molecule B'

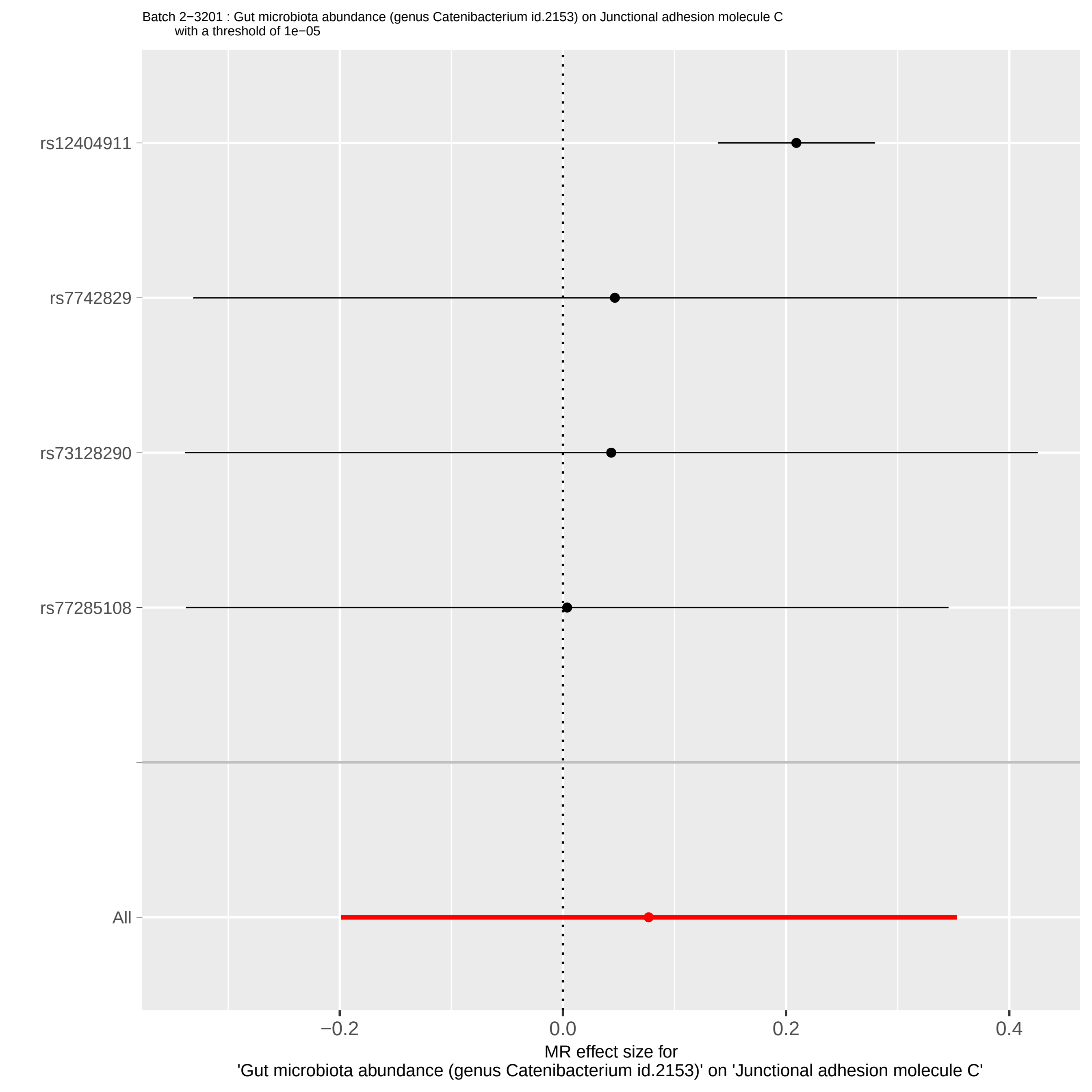
Batch 2−3201 : Gut microbiota abundance (genus Catenibacterium id.2153) on Junctional adhesion molecule C
with a threshold of 1e−05
rs12404911
rs7742829
rs73128290
rs77285108
All
−0.2
0.0
0.2
0.4
MR effect size for
'Gut microbiota abundance (genus Catenibacterium id.2153)' on 'Junctional adhesion molecule C'

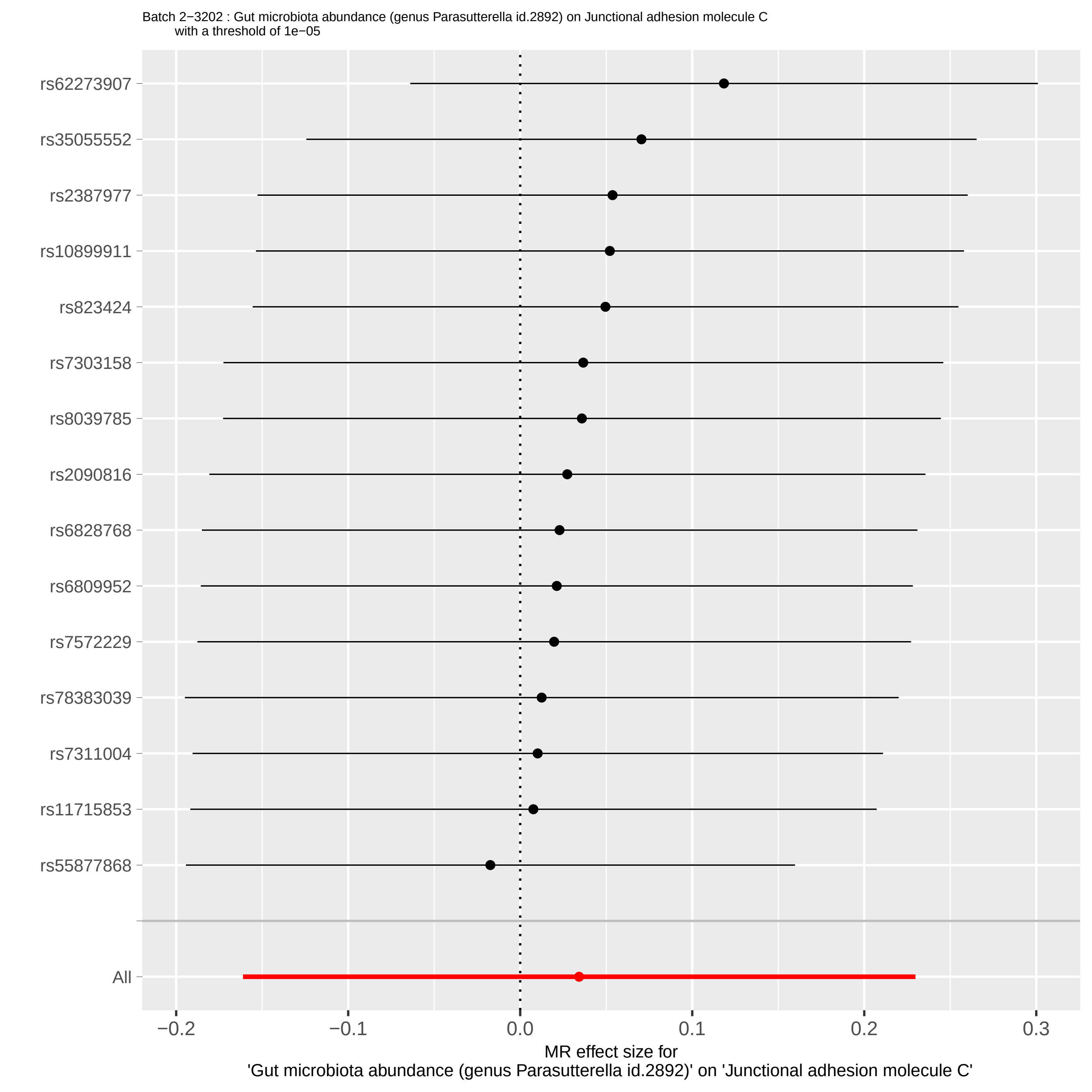
Batch 2−3202 : Gut microbiota abundance (genus Parasutterella id.2892) on Junctional adhesion molecule C
with a threshold of 1e−05
rs62273907
rs35055552
rs2387977
rs10899911
rs823424
rs7303158
rs8039785
rs2090816
rs6828768
rs6809952
rs7572229
rs78383039
rs7311004
rs11715853
rs55877868
All
−0.2
−0.1
0.0
0.1
0.2
0.3
MR effect size for
'Gut microbiota abundance (genus Parasutterella id.2892)' on 'Junctional adhesion molecule C'

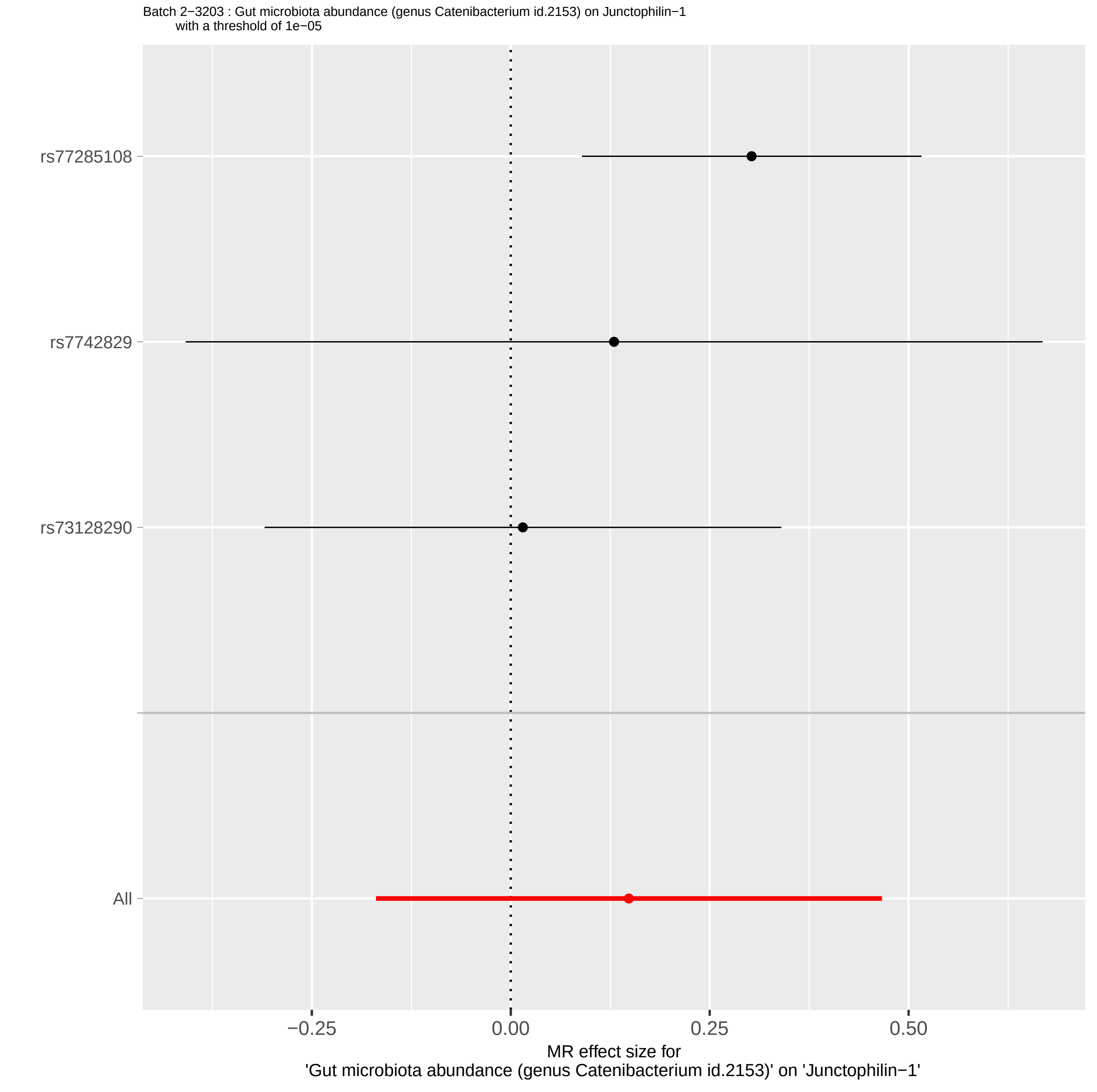
Batch 2−3203 : Gut microbiota abundance (genus Catenibacterium id.2153) on Junctophilin−1
with a threshold of 1e−05
rs77285108
rs7742829
rs73128290
All
−0.25
0.00
0.25
0.50
MR effect size for
'Gut microbiota abundance (genus Catenibacterium id.2153)' on 'Junctophilin−1'

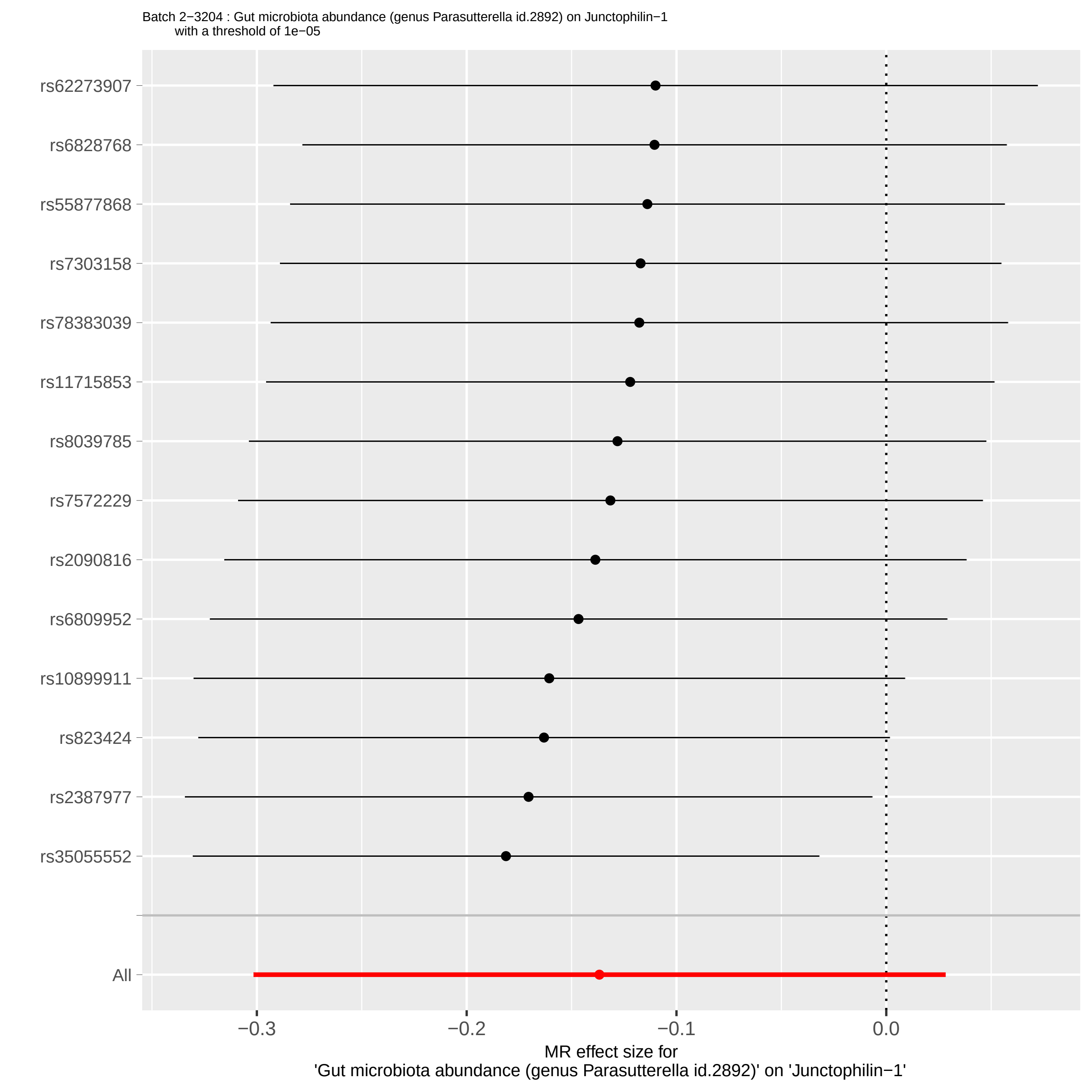

Batch 2−3204 : Gut microbiota abundance (genus Parasutterella id.2892) on Junctophilin−1
with a threshold of 1e−05
rs62273907
rs6828768
rs55877868
rs7303158
rs78383039
rs11715853
rs8039785
rs7572229
rs2090816
rs6809952
rs10899911
rs823424
rs2387977
rs35055552
All
−0.3
−0.2
−0.1
0.0
MR effect size for
'Gut microbiota abundance (genus Parasutterella id.2892)' on 'Junctophilin−1'

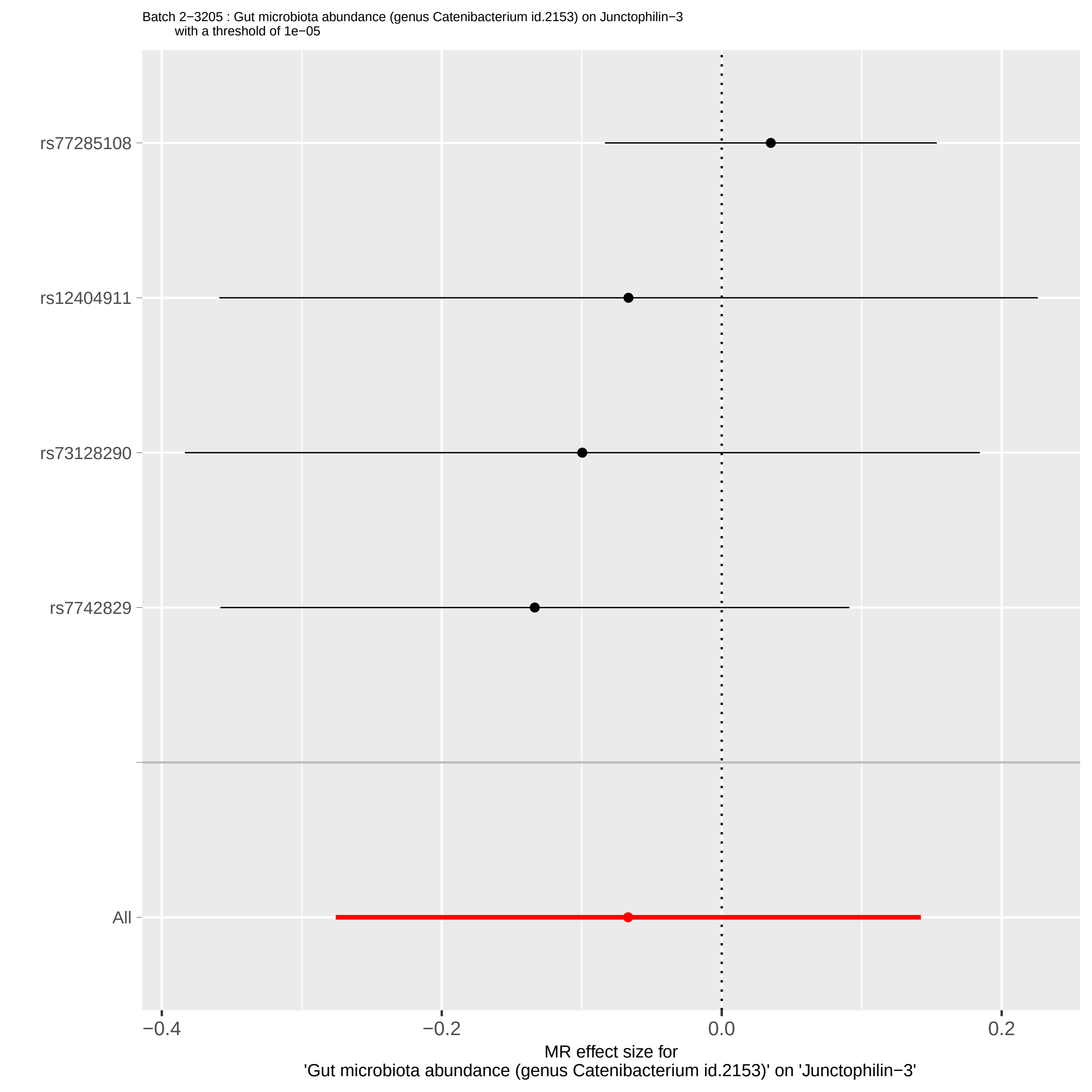
Batch 2−3205 : Gut microbiota abundance (genus Catenibacterium id.2153) on Junctophilin−3
with a threshold of 1e−05
rs77285108
rs12404911
rs73128290
rs7742829
All
−0.4
−0.2
0.0
0.2
MR effect size for
'Gut microbiota abundance (genus Catenibacterium id.2153)' on 'Junctophilin−3'

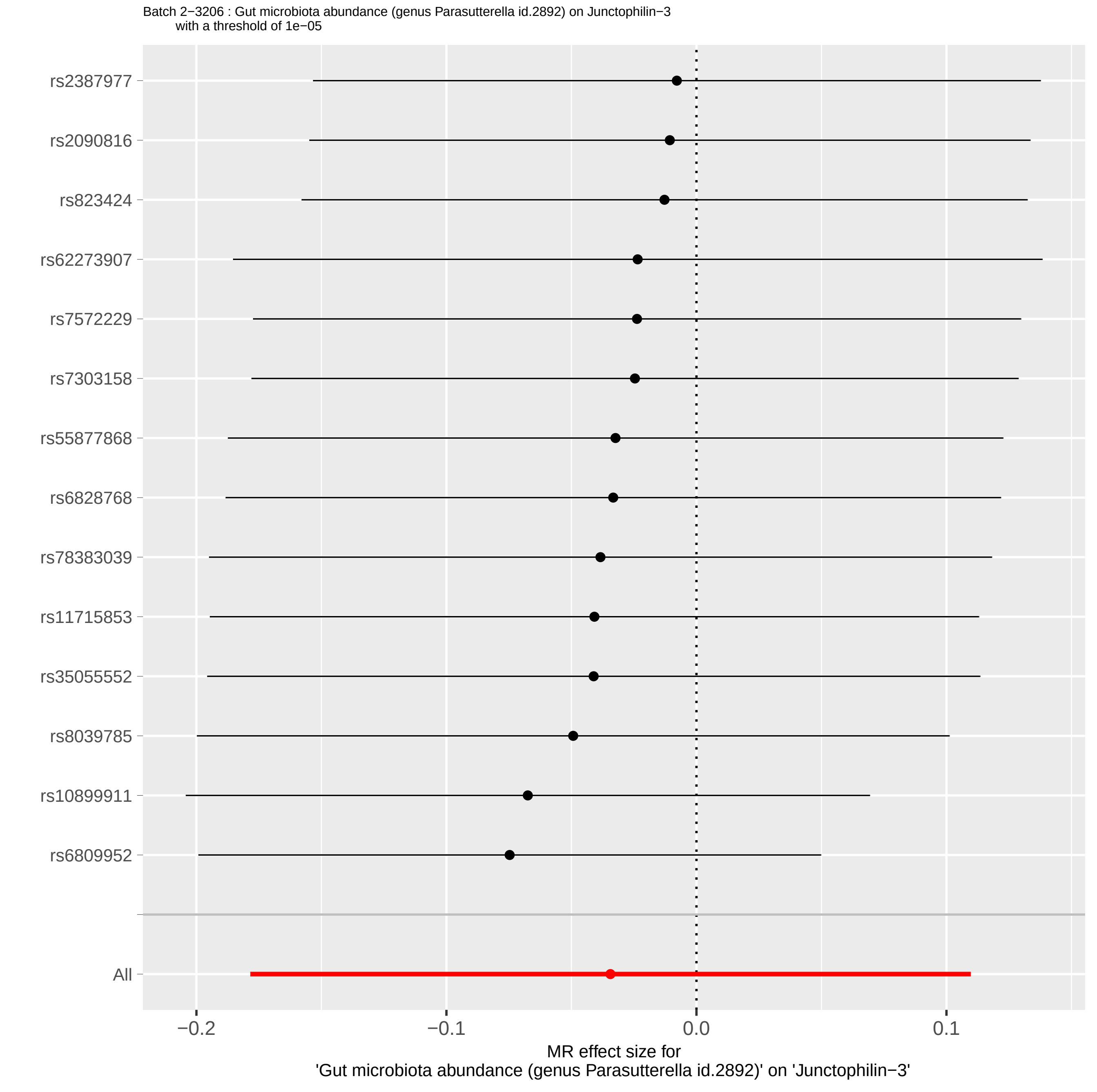

Batch 2−3206 : Gut microbiota abundance (genus Parasutterella id.2892) on Junctophilin−3
with a threshold of 1e−05
rs2387977
rs2090816
rs823424
rs62273907
rs7572229
rs7303158
rs55877868
rs6828768
rs78383039
rs11715853
rs35055552
rs8039785
rs10899911
rs6809952
All
−0.2
−0.1
0.0
0.1
MR effect size for
'Gut microbiota abundance (genus Parasutterella id.2892)' on 'Junctophilin−3'

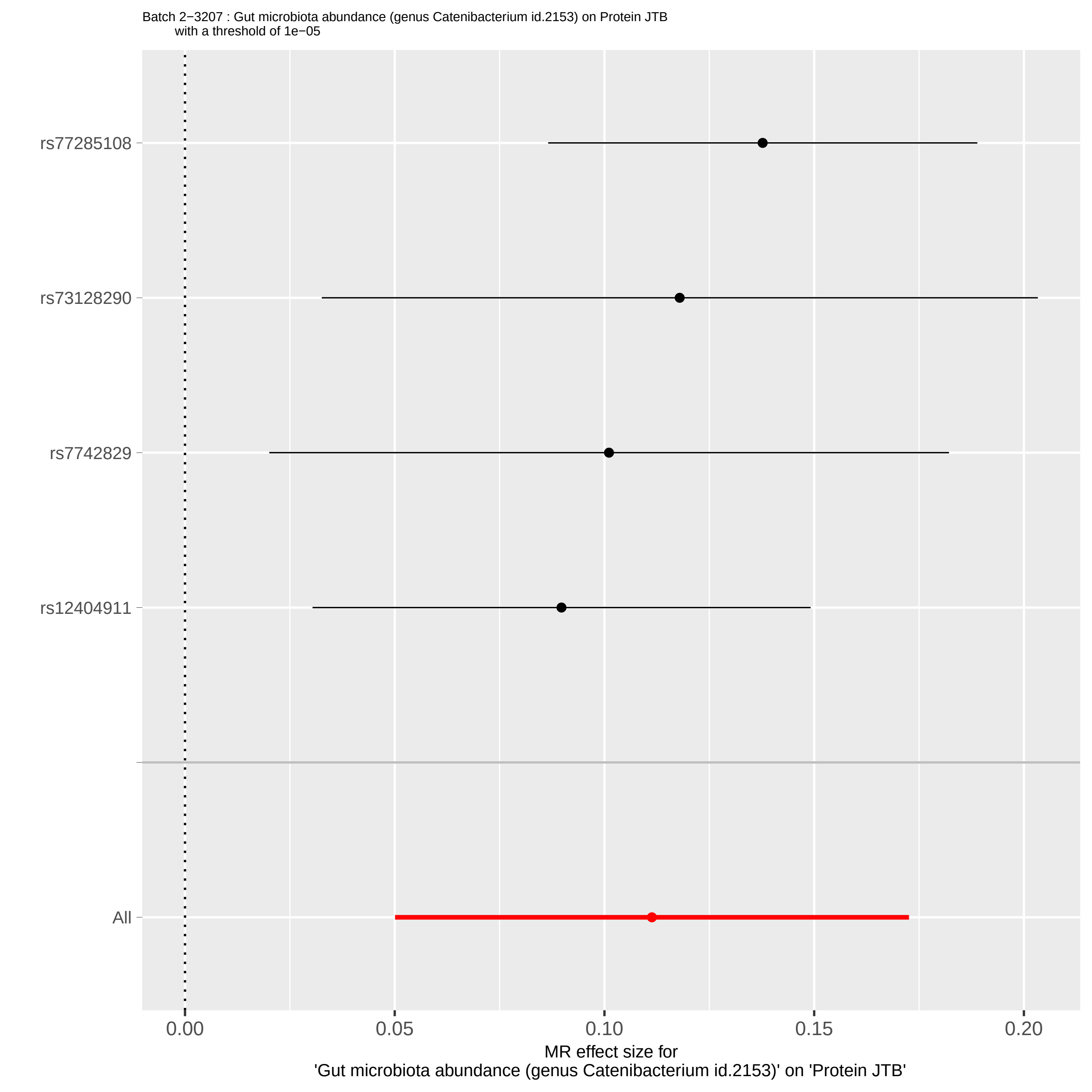
Batch 2−3207 : Gut microbiota abundance (genus Catenibacterium id.2153) on Protein JTB
with a threshold of 1e−05
rs77285108
rs73128290
rs7742829
rs12404911
All
0.00
0.05
0.10
0.15
0.20
MR effect size for
'Gut microbiota abundance (genus Catenibacterium id.2153)' on 'Protein JTB'

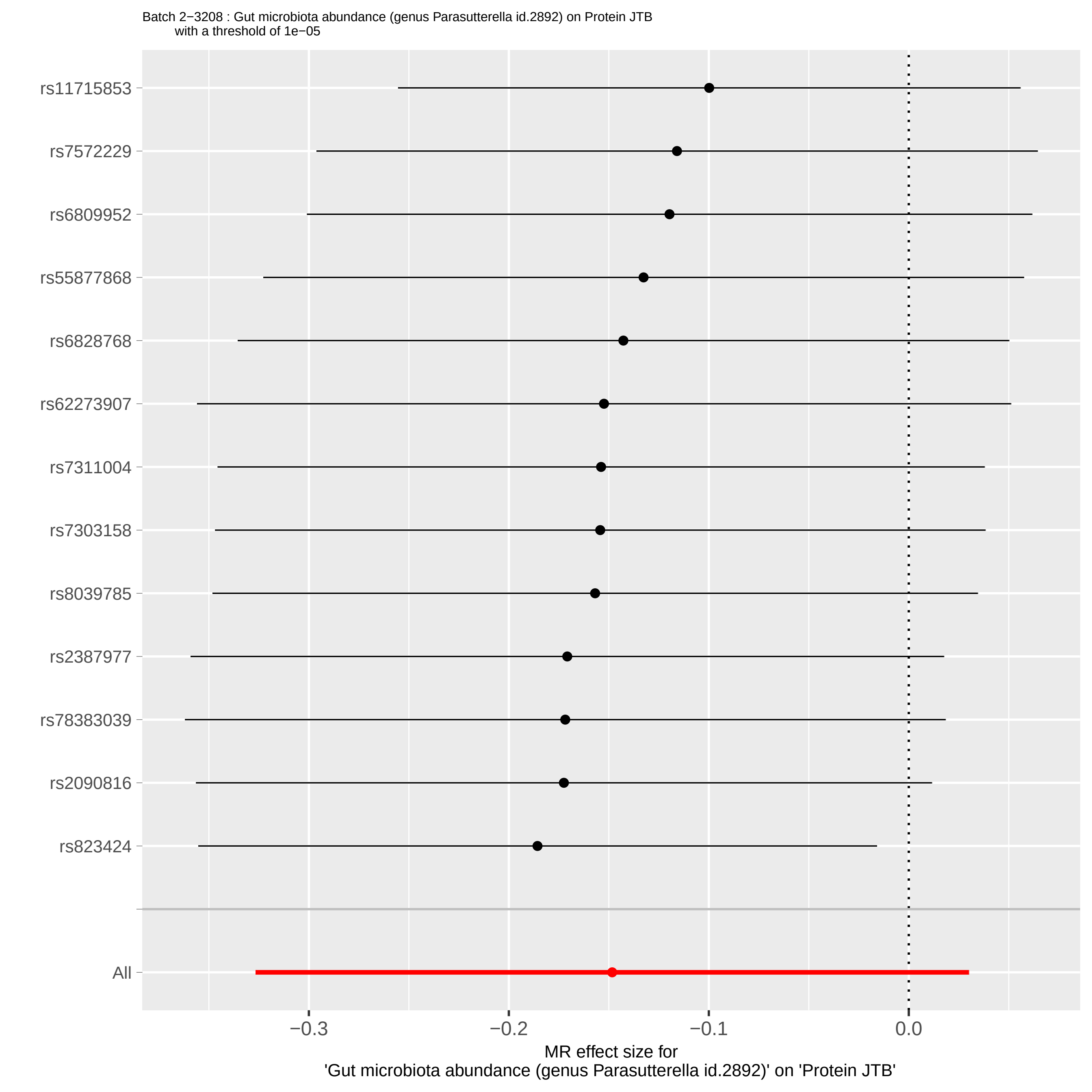

Batch 2−3208 : Gut microbiota abundance (genus Parasutterella id.2892) on Protein JTB
with a threshold of 1e−05
rs11715853
rs7572229
rs6809952
rs55877868
rs6828768
rs62273907
rs7311004
rs7303158
rs8039785
rs2387977
rs78383039
rs2090816
rs823424
All
−0.3
−0.2
−0.1
0.0
MR effect size for
'Gut microbiota abundance (genus Parasutterella id.2892)' on 'Protein JTB'

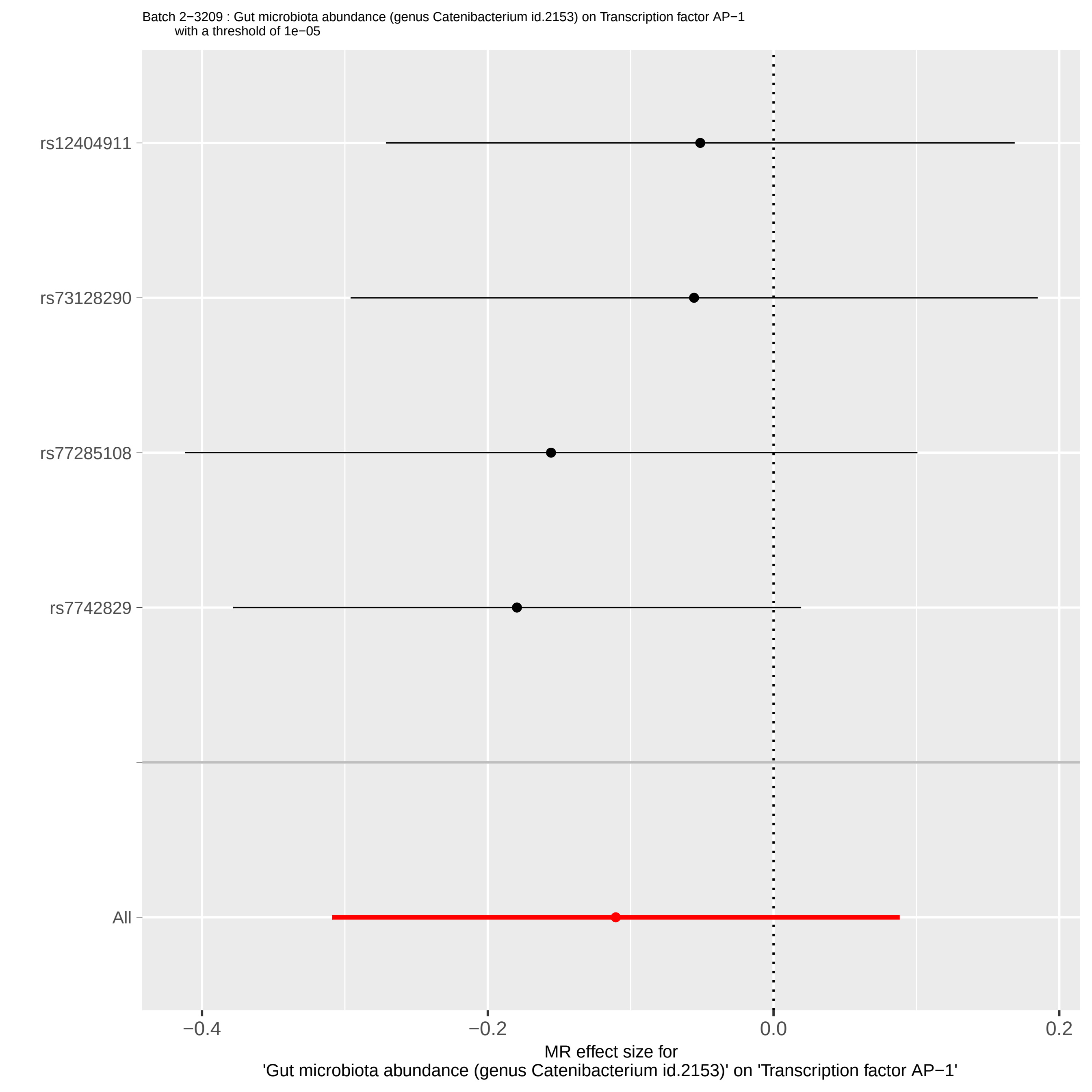
Batch 2−3209 : Gut microbiota abundance (genus Catenibacterium id.2153) on Transcription factor AP−1
with a threshold of 1e−05
rs12404911
rs73128290
rs77285108
rs7742829
All
−0.4
−0.2
0.0
0.2
MR effect size for
'Gut microbiota abundance (genus Catenibacterium id.2153)' on 'Transcription factor AP−1'

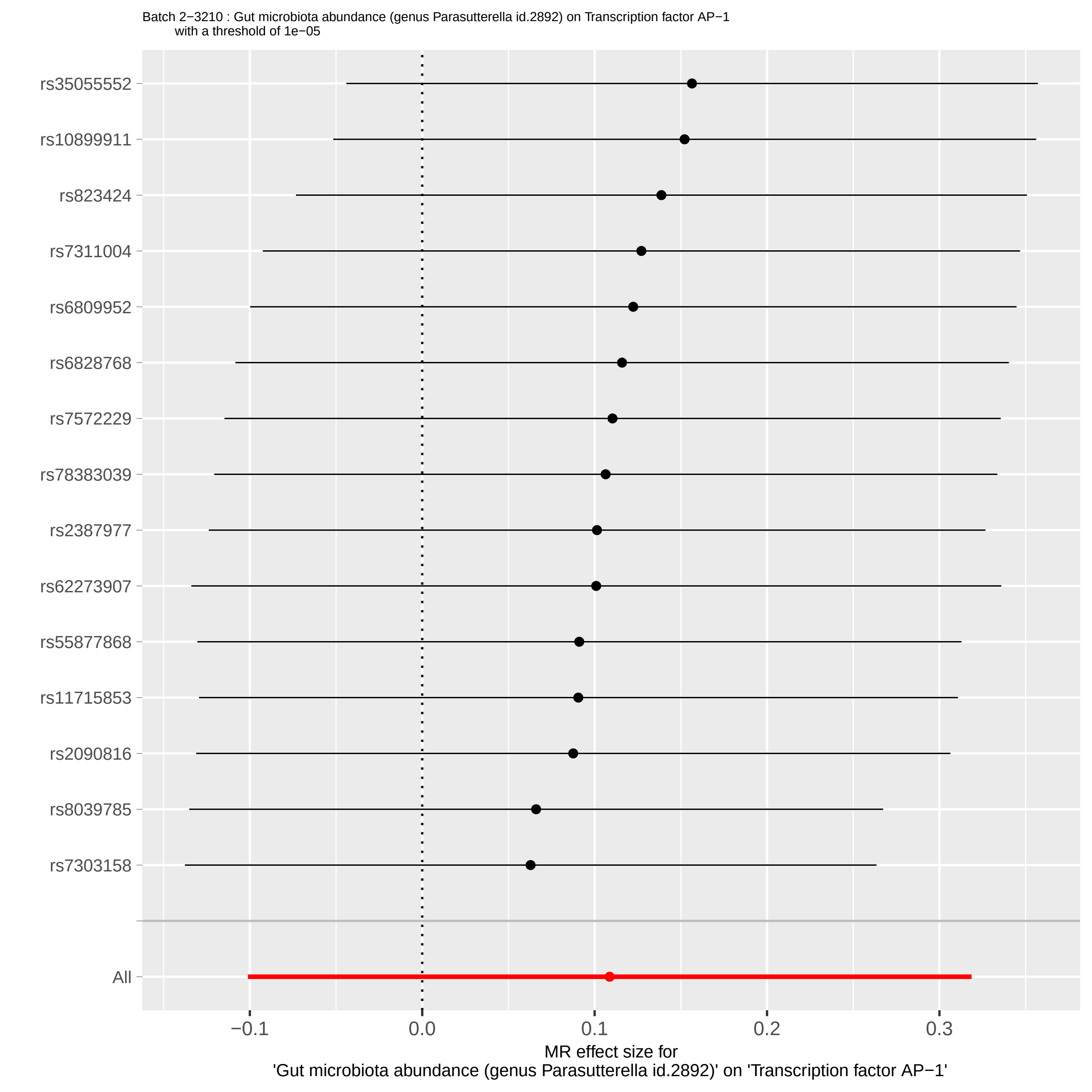
Batch 2−3210 : Gut microbiota abundance (genus Parasutterella id.2892) on Transcription factor AP−1
with a threshold of 1e−05
rs35055552
rs10899911
rs823424
rs7311004
rs6809952
rs6828768
rs7572229
rs78383039
rs2387977
rs62273907
rs55877868
rs11715853
rs2090816
rs8039785
rs7303158
All
−0.1
0.0
0.1
0.2
0.3
MR effect size for
'Gut microbiota abundance (genus Parasutterella id.2892)' on 'Transcription factor AP−1'

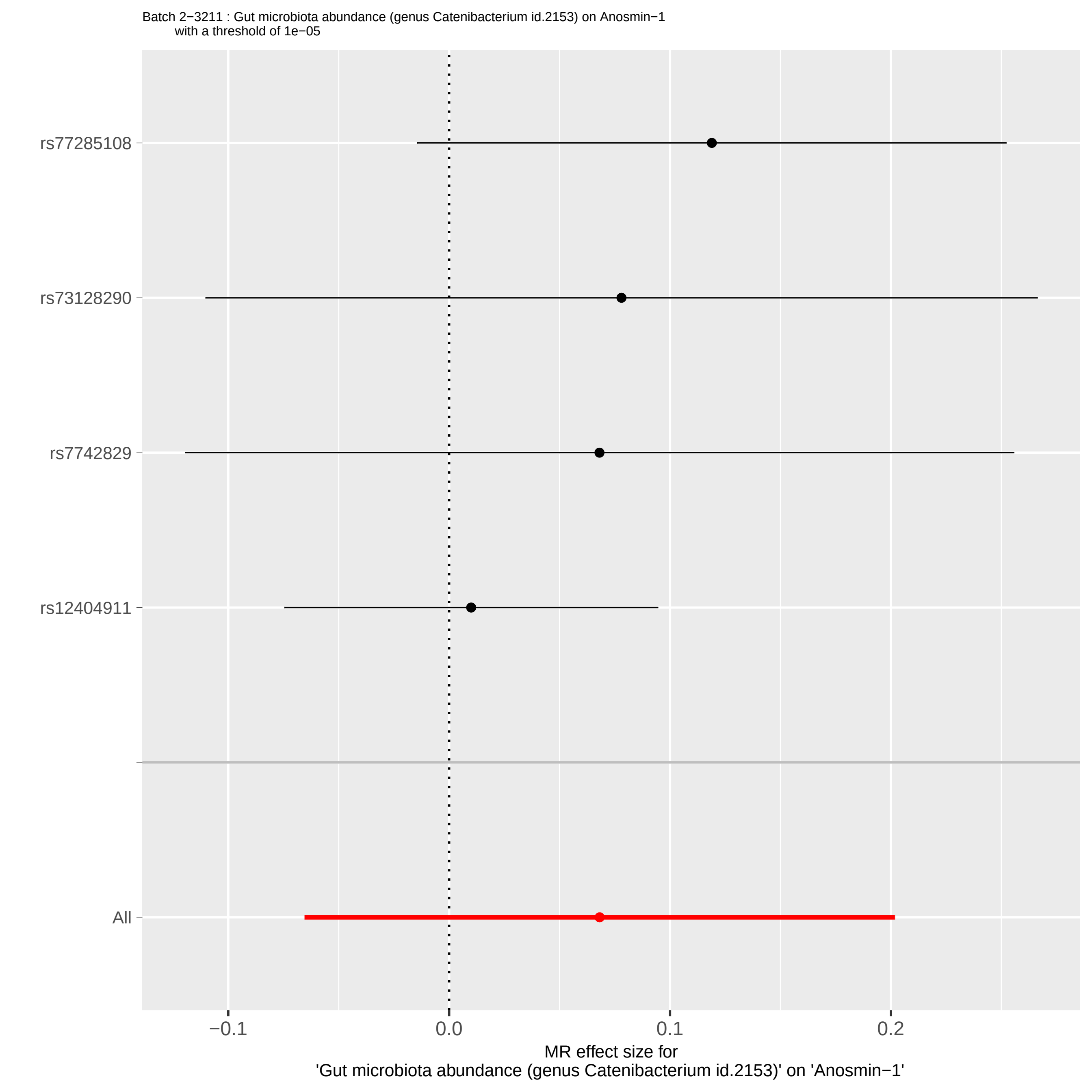

Batch 2−3211 : Gut microbiota abundance (genus Catenibacterium id.2153) on Anosmin−1
with a threshold of 1e−05
rs77285108
rs73128290
rs7742829
rs12404911
All
−0.1
0.0
0.1
0.2
MR effect size for
'Gut microbiota abundance (genus Catenibacterium id.2153)' on 'Anosmin−1'

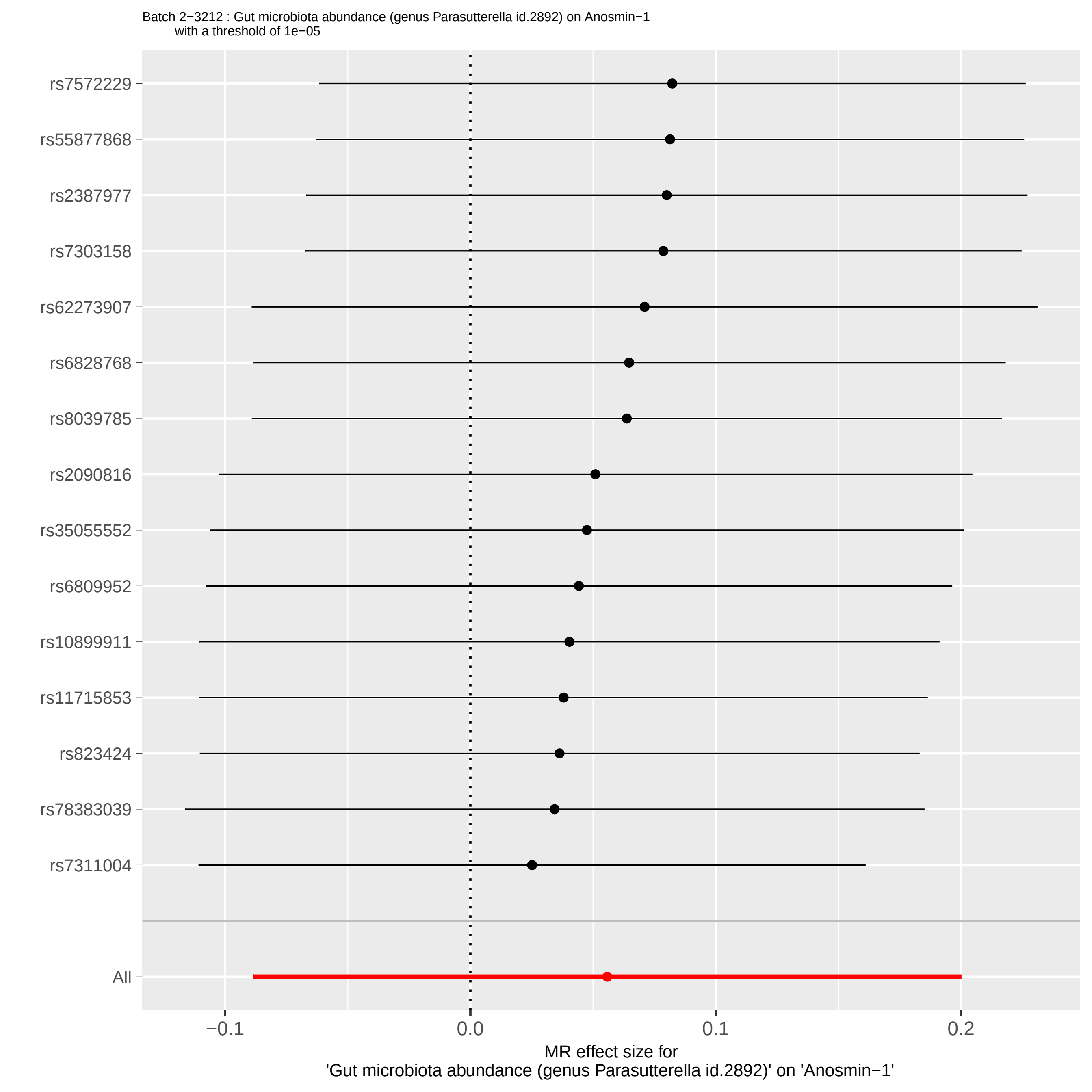

Batch 2−3212 : Gut microbiota abundance (genus Parasutterella id.2892) on Anosmin−1
with a threshold of 1e−05
rs7572229
rs55877868
rs2387977
rs7303158
rs62273907
rs6828768
rs8039785
rs2090816
rs35055552
rs6809952
rs10899911
rs11715853
rs823424
rs78383039
rs7311004
All
−0.1
0.0
0.1
0.2
MR effect size for
'Gut microbiota abundance (genus Parasutterella id.2892)' on 'Anosmin−1'

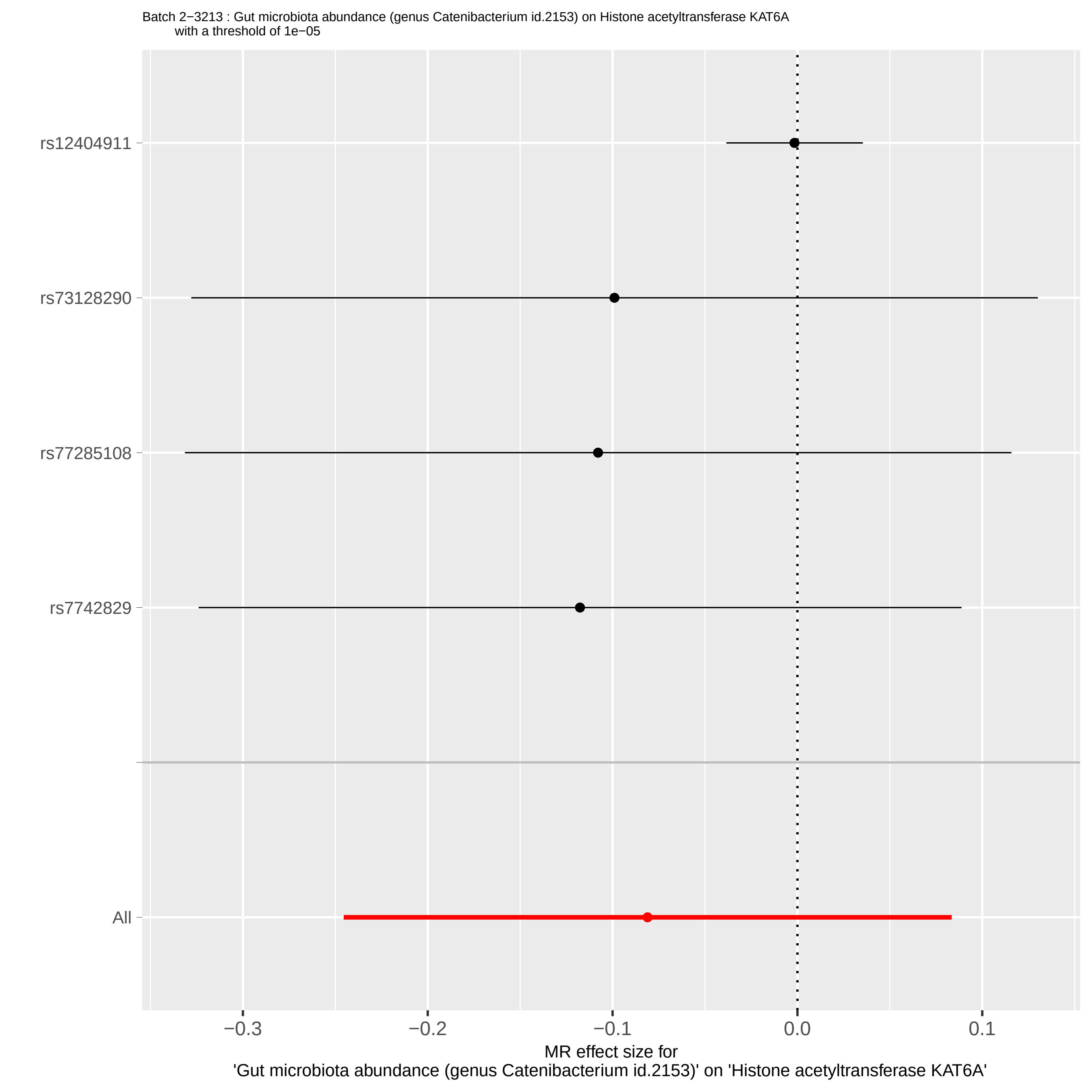
Batch 2−3213 : Gut microbiota abundance (genus Catenibacterium id.2153) on Histone acetyltransferase KAT6A
with a threshold of 1e−05
rs12404911
rs73128290
rs77285108
rs7742829
All
−0.3
−0.2
−0.1
0.0
0.1
MR effect size for
'Gut microbiota abundance (genus Catenibacterium id.2153)' on 'Histone acetyltransferase KAT6A'

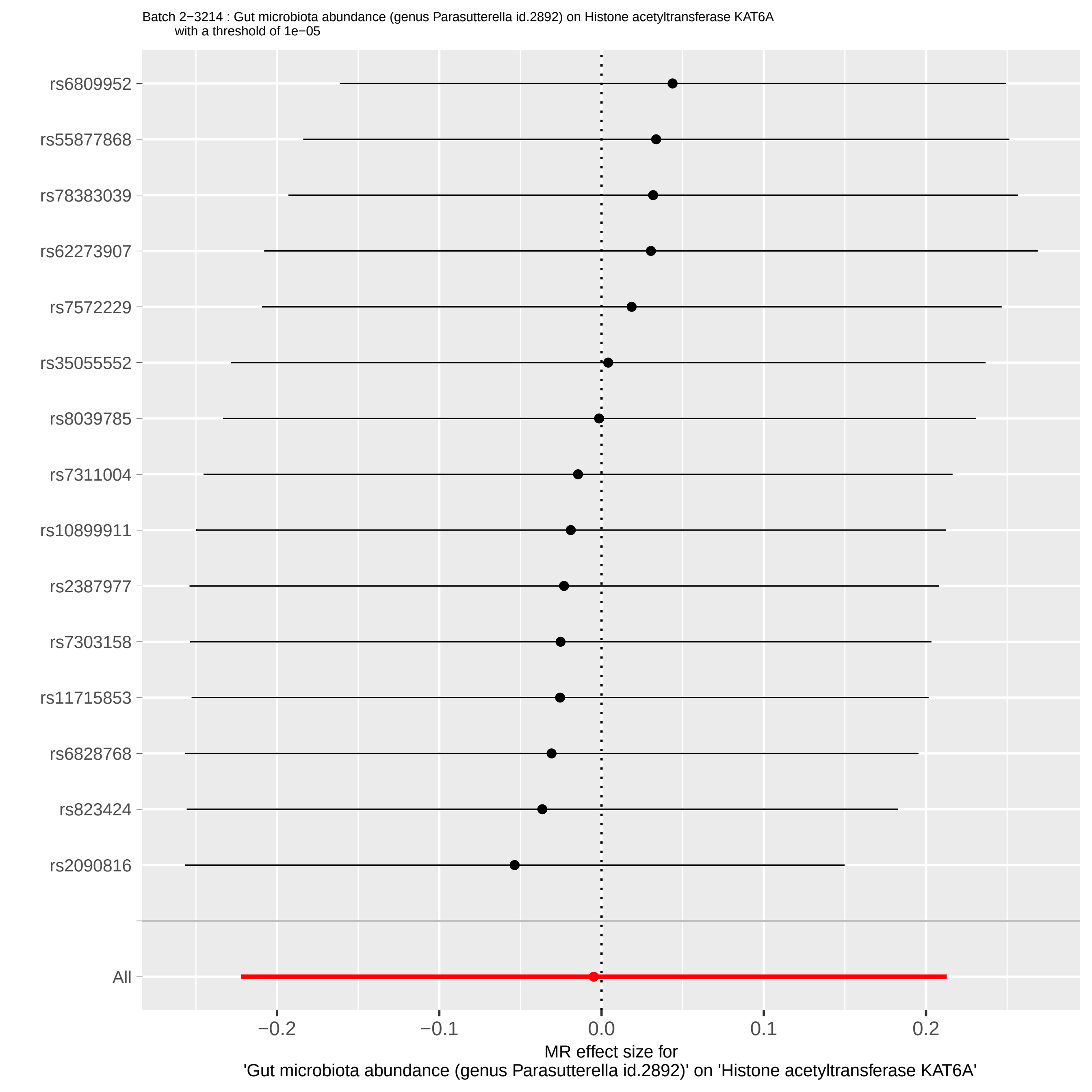

Batch 2−3214 : Gut microbiota abundance (genus Parasutterella id.2892) on Histone acetyltransferase KAT6A
with a threshold of 1e−05
rs6809952
rs55877868
rs78383039
rs62273907
rs7572229
rs35055552
rs8039785
rs7311004
rs10899911
rs2387977
rs7303158
rs11715853
rs6828768
rs823424
rs2090816
All
−0.2
−0.1
0.0
0.1
0.2
MR effect size for
'Gut microbiota abundance (genus Parasutterella id.2892)' on 'Histone acetyltransferase KAT6A'

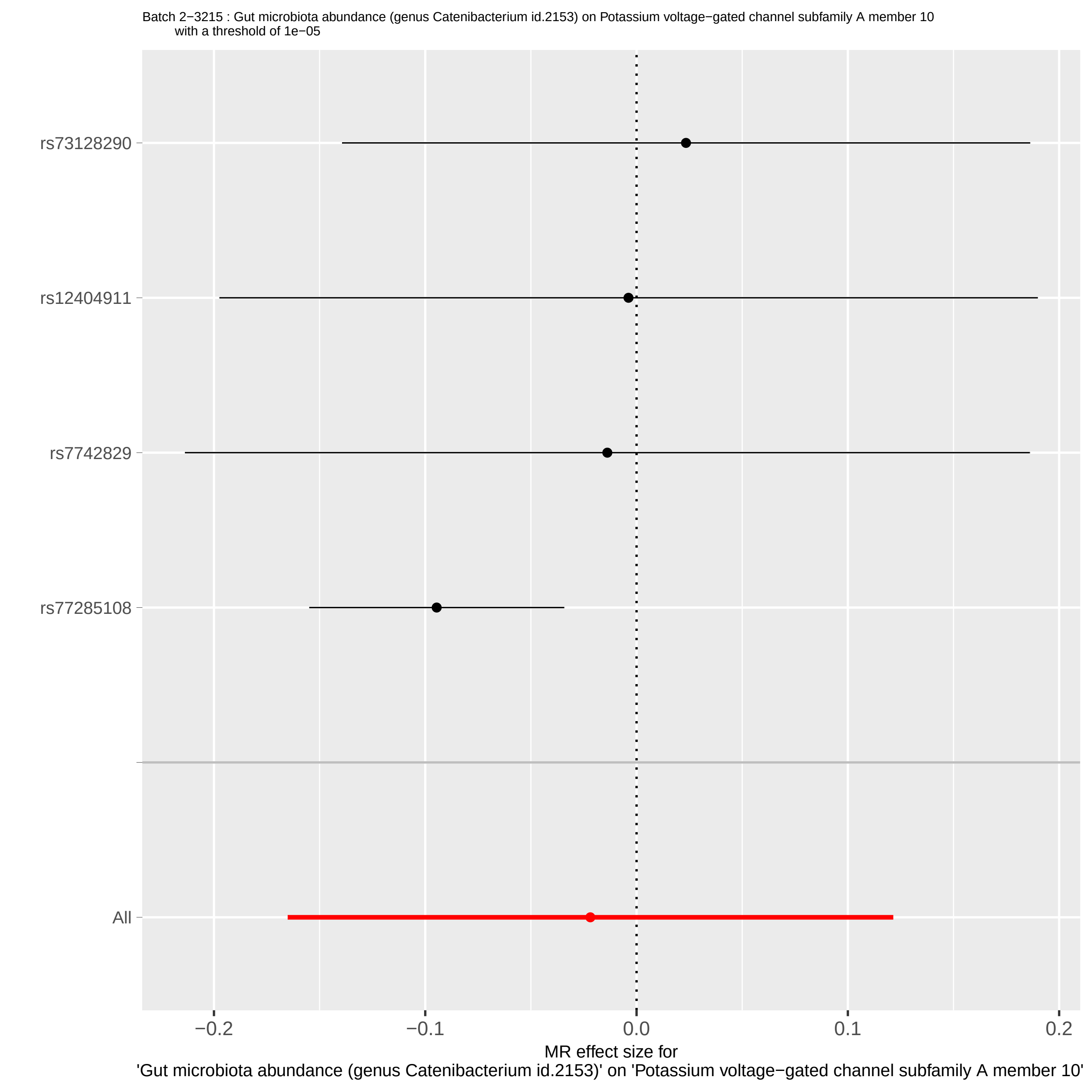
Batch 2−3215 : Gut microbiota abundance (genus Catenibacterium id.2153) on Potassium voltage−gated channel subfamily A member 10
with a threshold of 1e−05
rs73128290
rs12404911
rs7742829
rs77285108
All
−0.2
−0.1
0.0
0.1
0.2
MR effect size for
'Gut microbiota abundance (genus Catenibacterium id.2153)' on 'Potassium voltage−gated channel subfamily A member 10'

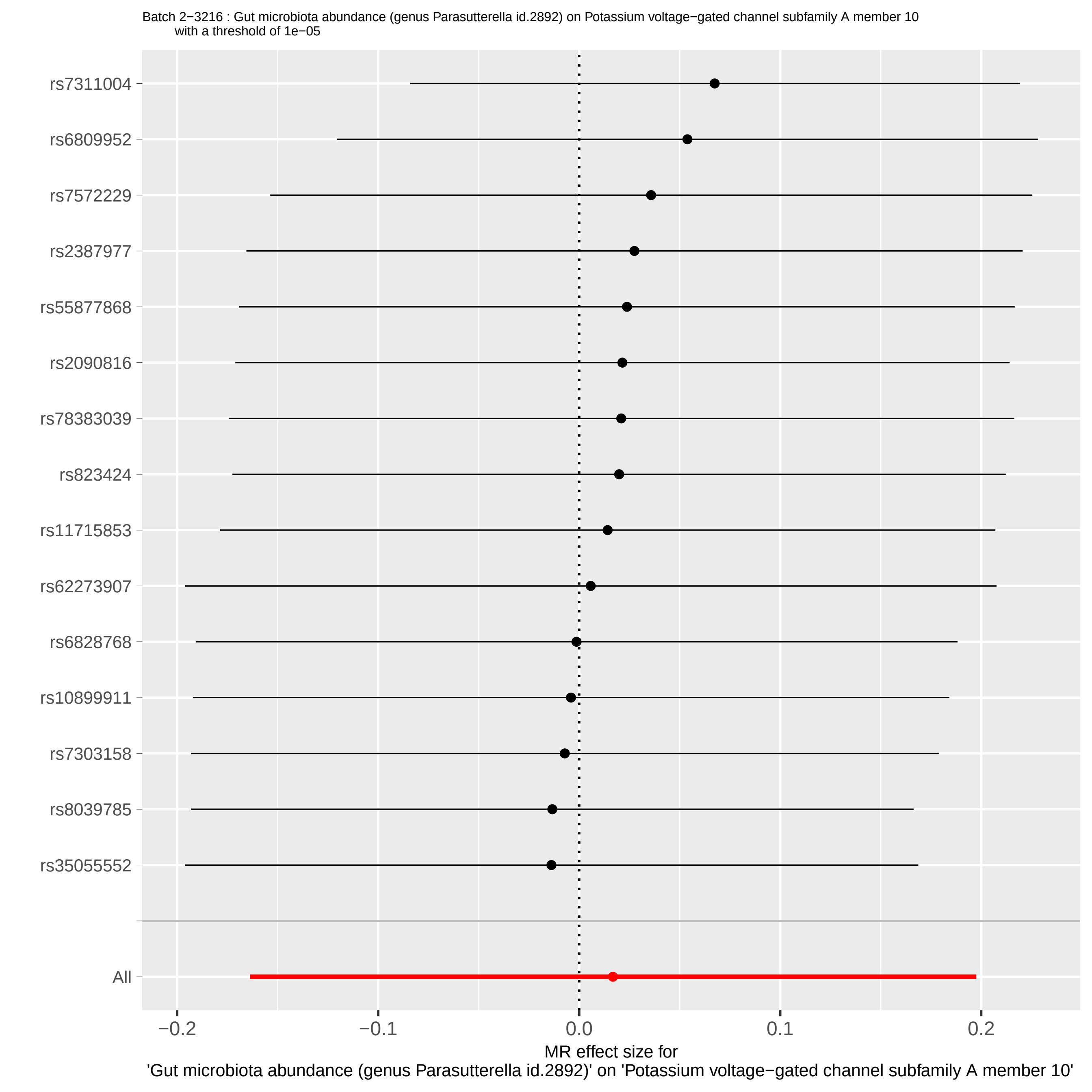
Batch 2−3216 : Gut microbiota abundance (genus Parasutterella id.2892) on Potassium voltage−gated channel subfamily A member 10
with a threshold of 1e−05
rs7311004
rs6809952
rs7572229
rs2387977
rs55877868
rs2090816
rs78383039
rs823424
rs11715853
rs62273907
rs6828768
rs10899911
rs7303158
rs8039785
rs35055552
All
−0.2
−0.1
0.0
0.1
0.2
MR effect size for
'Gut microbiota abundance (genus Parasutterella id.2892)' on 'Potassium voltage−gated channel subfamily A member 10'

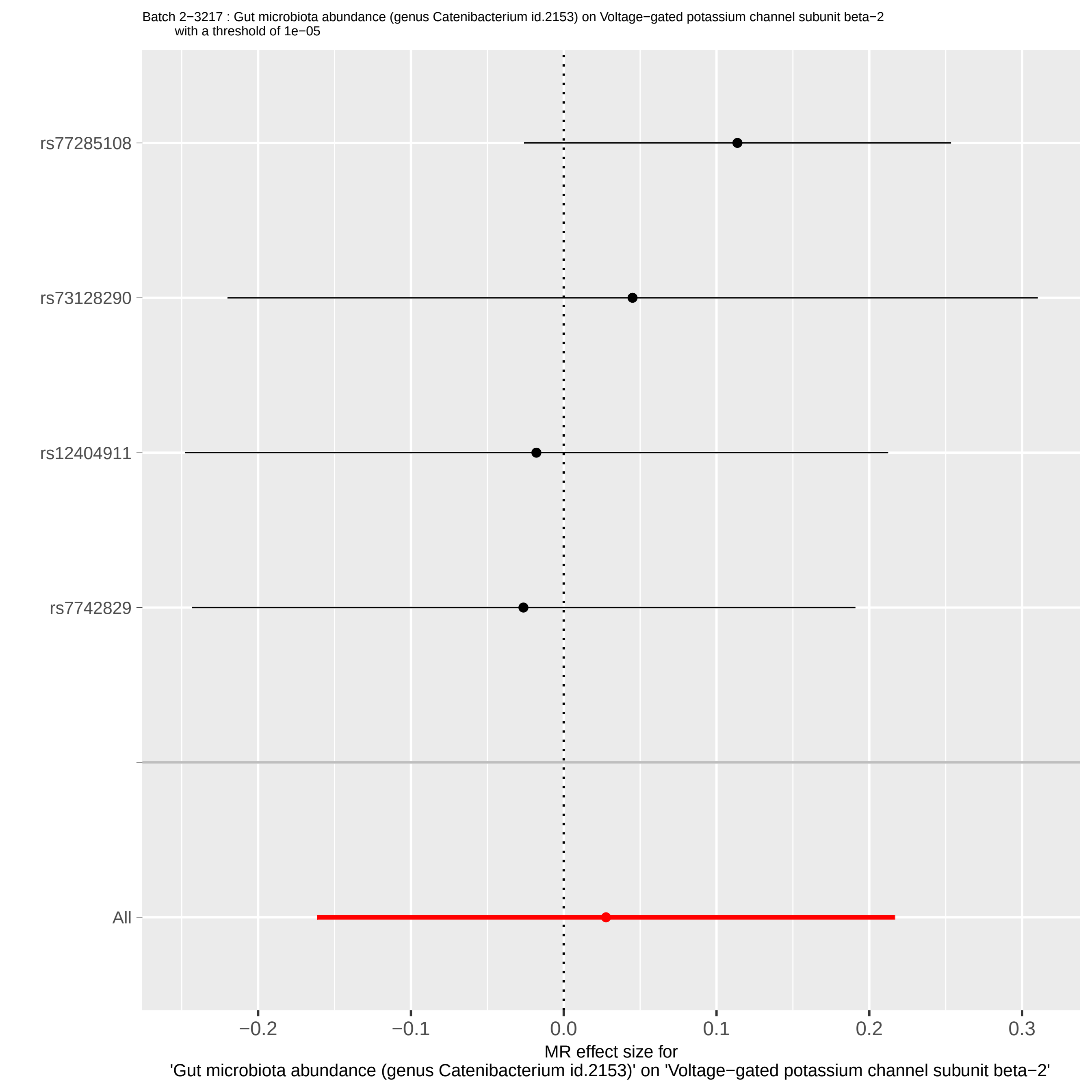
Batch 2−3217 : Gut microbiota abundance (genus Catenibacterium id.2153) on Voltage−gated potassium channel subunit beta−2
with a threshold of 1e−05
rs77285108
rs73128290
rs12404911
rs7742829
All
−0.2
−0.1
0.0
0.1
0.2
0.3
MR effect size for
'Gut microbiota abundance (genus Catenibacterium id.2153)' on 'Voltage−gated potassium channel subunit beta−2'

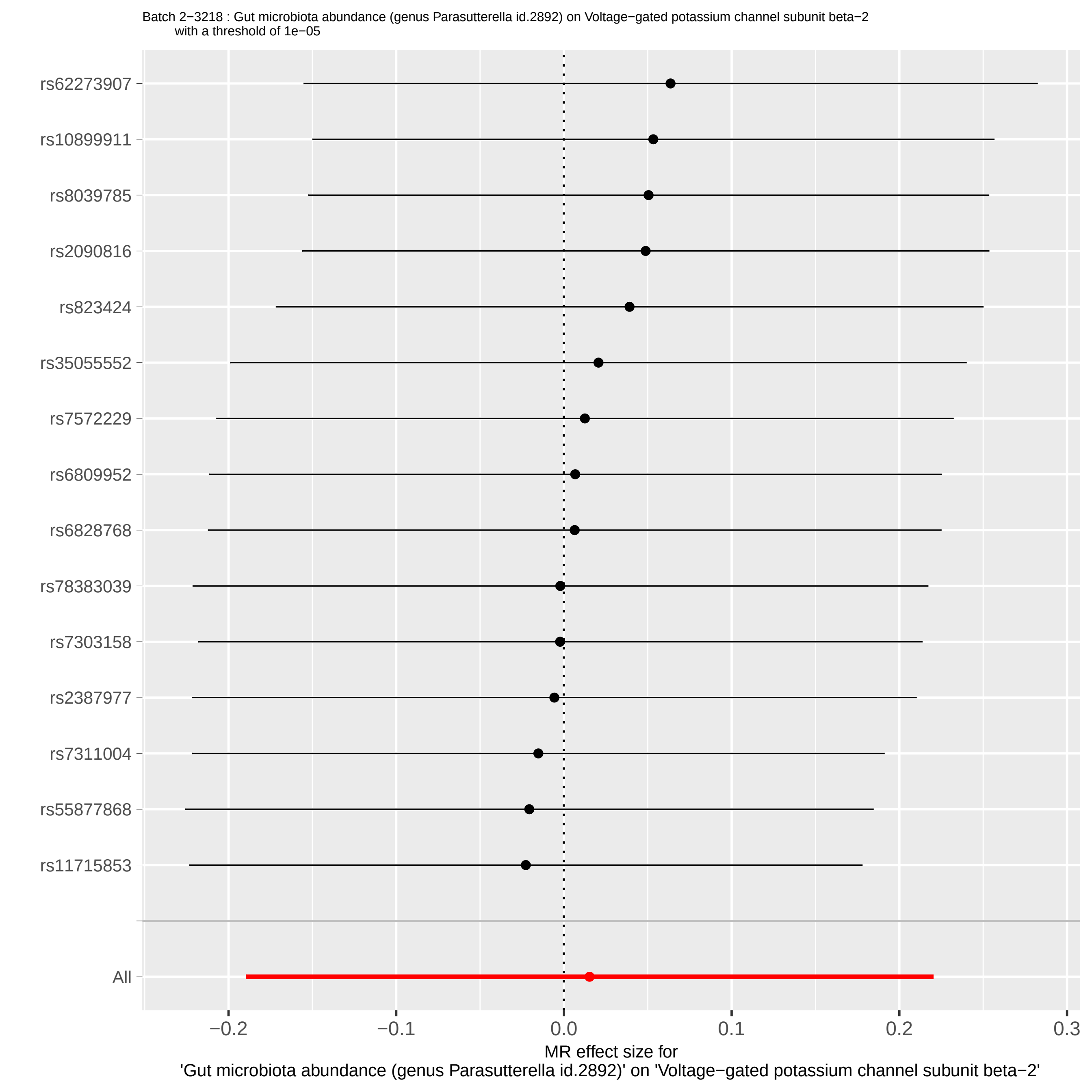
Batch 2−3218 : Gut microbiota abundance (genus Parasutterella id.2892) on Voltage−gated potassium channel subunit beta−2
with a threshold of 1e−05
rs62273907
rs10899911
rs8039785
rs2090816
rs823424
rs35055552
rs7572229
rs6809952
rs6828768
rs78383039
rs7303158
rs2387977
rs7311004
rs55877868
rs11715853
All
−0.2
−0.1
0.0
0.1
0.2
0.3
MR effect size for
'Gut microbiota abundance (genus Parasutterella id.2892)' on 'Voltage−gated potassium channel subunit beta−2'

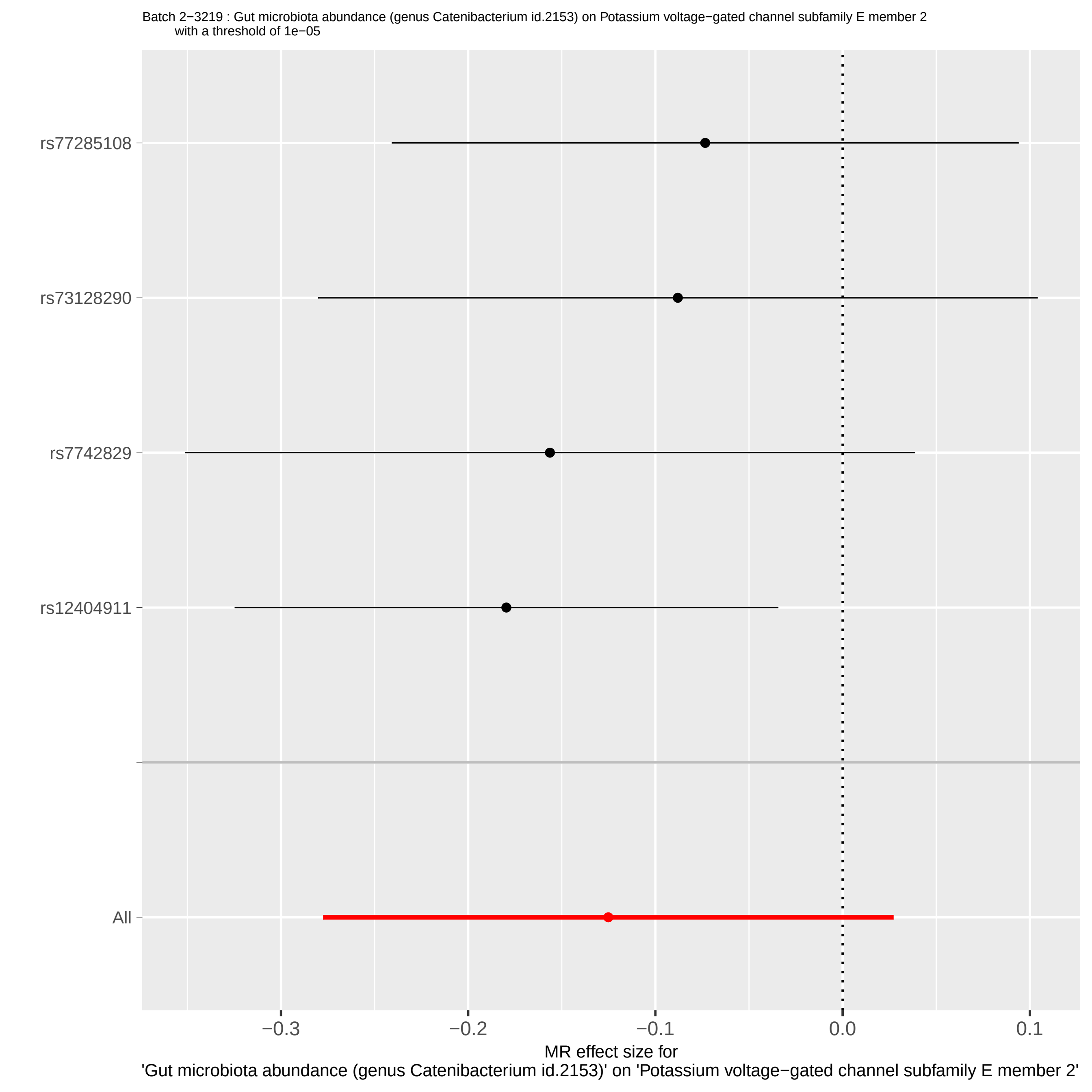
Batch 2−3219 : Gut microbiota abundance (genus Catenibacterium id.2153) on Potassium voltage−gated channel subfamily E member 2
with a threshold of 1e−05
rs77285108
rs73128290
rs7742829
rs12404911
All
−0.3
−0.2
−0.1
0.0
0.1
MR effect size for
'Gut microbiota abundance (genus Catenibacterium id.2153)' on 'Potassium voltage−gated channel subfamily E member 2'

Batch 2−3220 : Gut microbiota abundance (genus Parasutterella id.2892) on Potassium voltage−gated channel subfamily E member 2
with a threshold of 1e−05

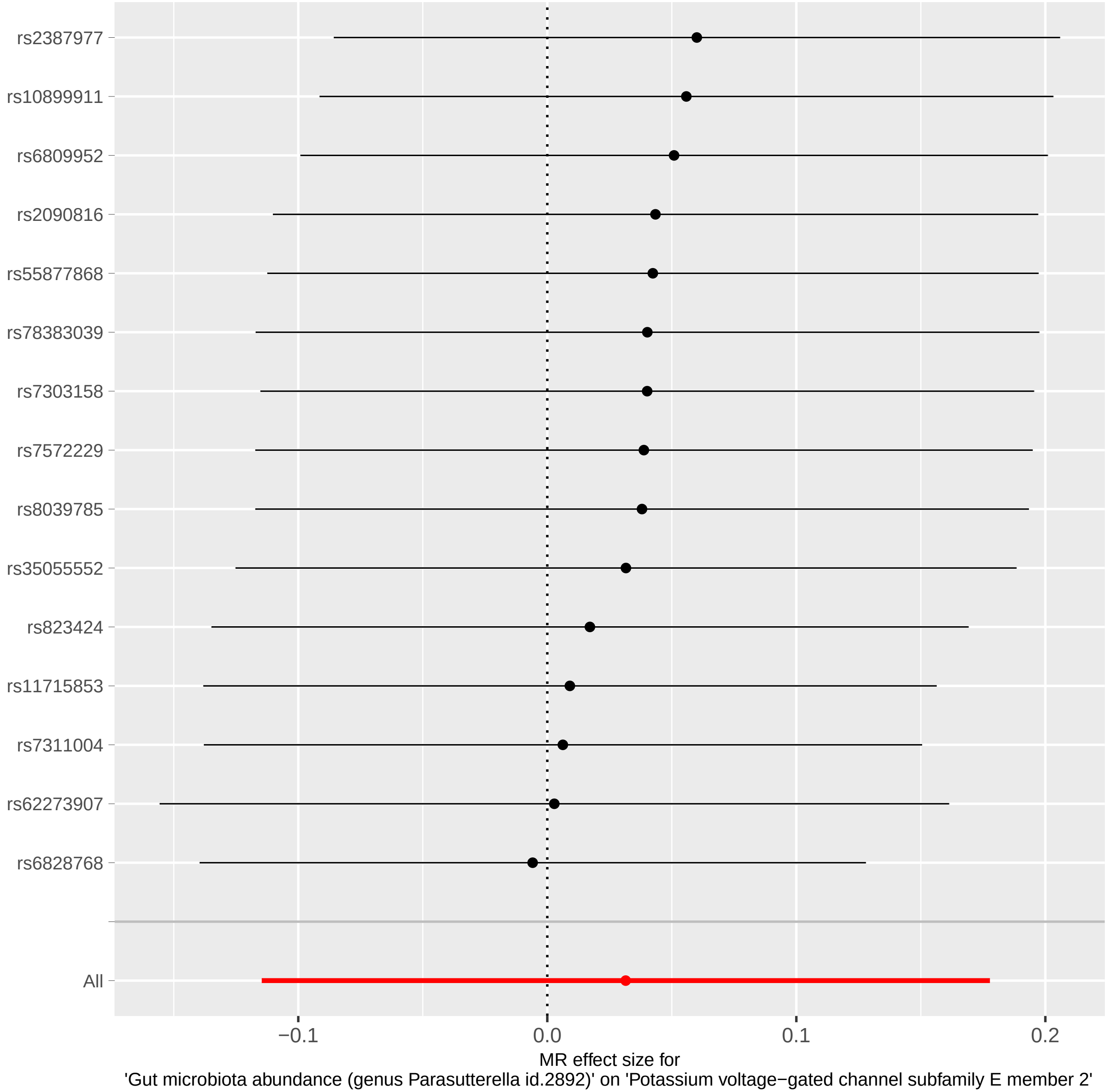

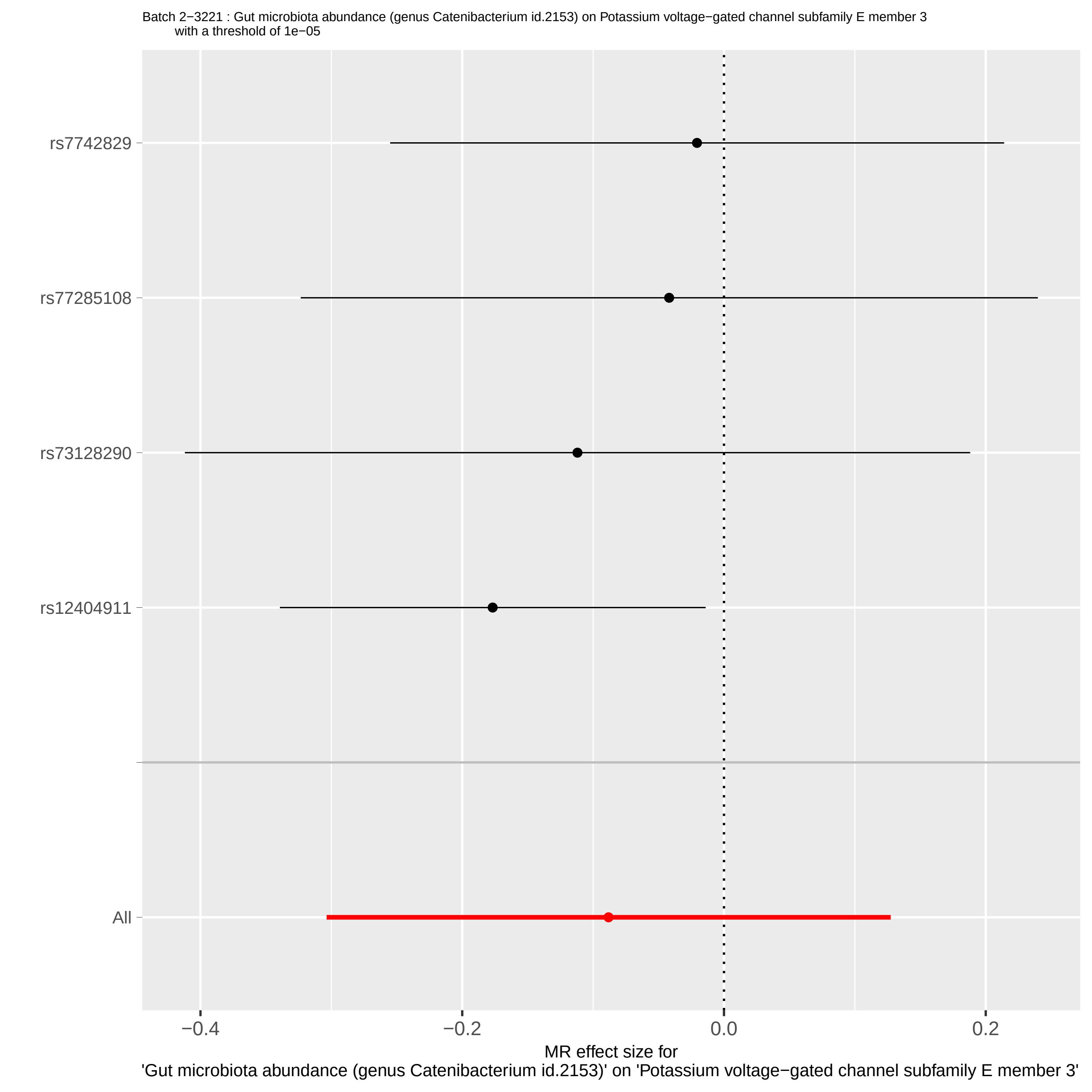
Batch 2−3221 : Gut microbiota abundance (genus Catenibacterium id.2153) on Potassium voltage−gated channel subfamily E member 3
with a threshold of 1e−05
rs7742829
rs77285108
rs73128290
rs12404911
All
−0.4
−0.2
0.0
0.2
MR effect size for
'Gut microbiota abundance (genus Catenibacterium id.2153)' on 'Potassium voltage−gated channel subfamily E member 3'

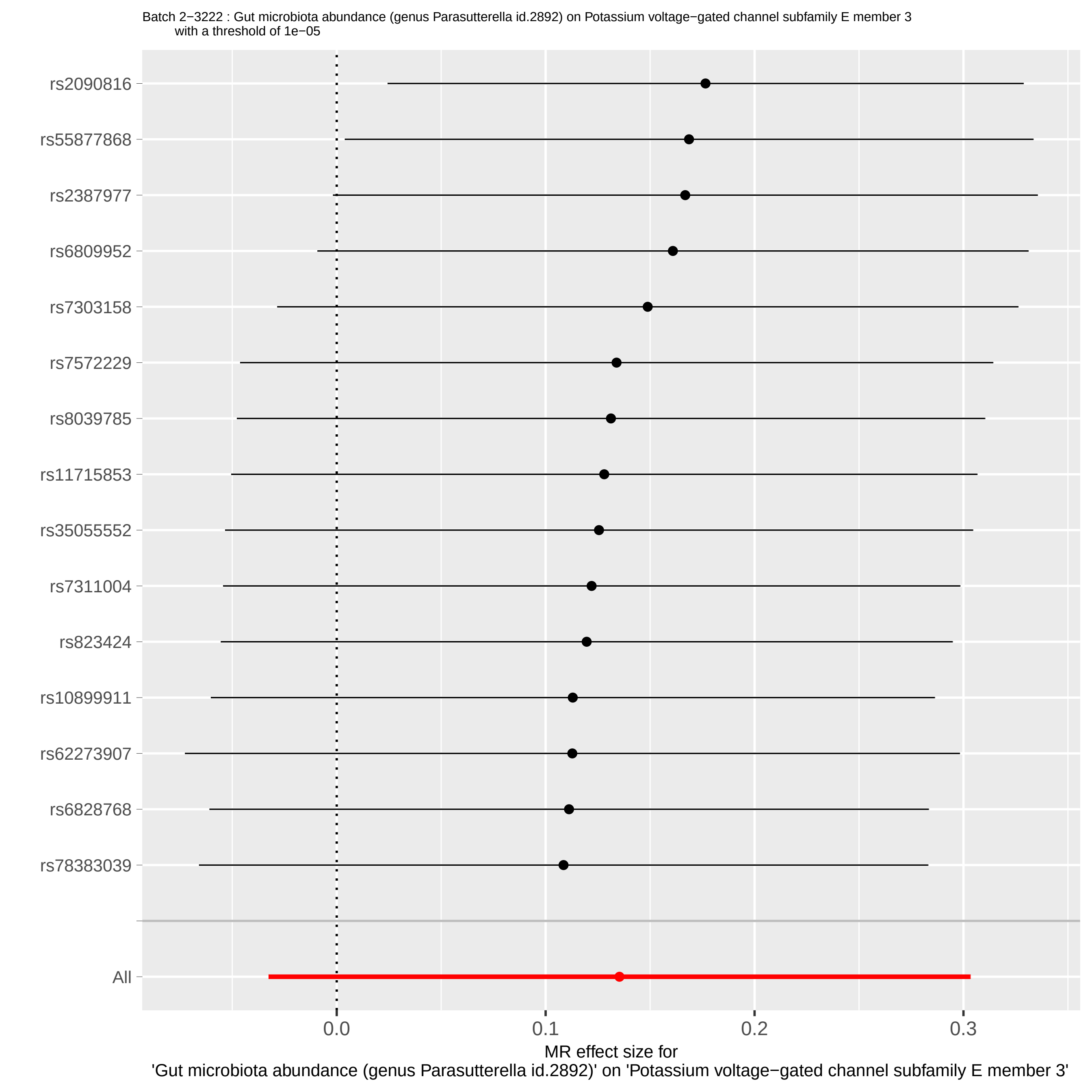
Batch 2−3222 : Gut microbiota abundance (genus Parasutterella id.2892) on Potassium voltage−gated channel subfamily E member 3
with a threshold of 1e−05
rs2090816
rs55877868
rs2387977
rs6809952
rs7303158
rs7572229
rs8039785
rs11715853
rs35055552
rs7311004
rs823424
rs10899911
rs62273907
rs6828768
rs78383039
All
0.0
0.1
0.2
0.3
MR effect size for
'Gut microbiota abundance (genus Parasutterella id.2892)' on 'Potassium voltage−gated channel subfamily E member 3'

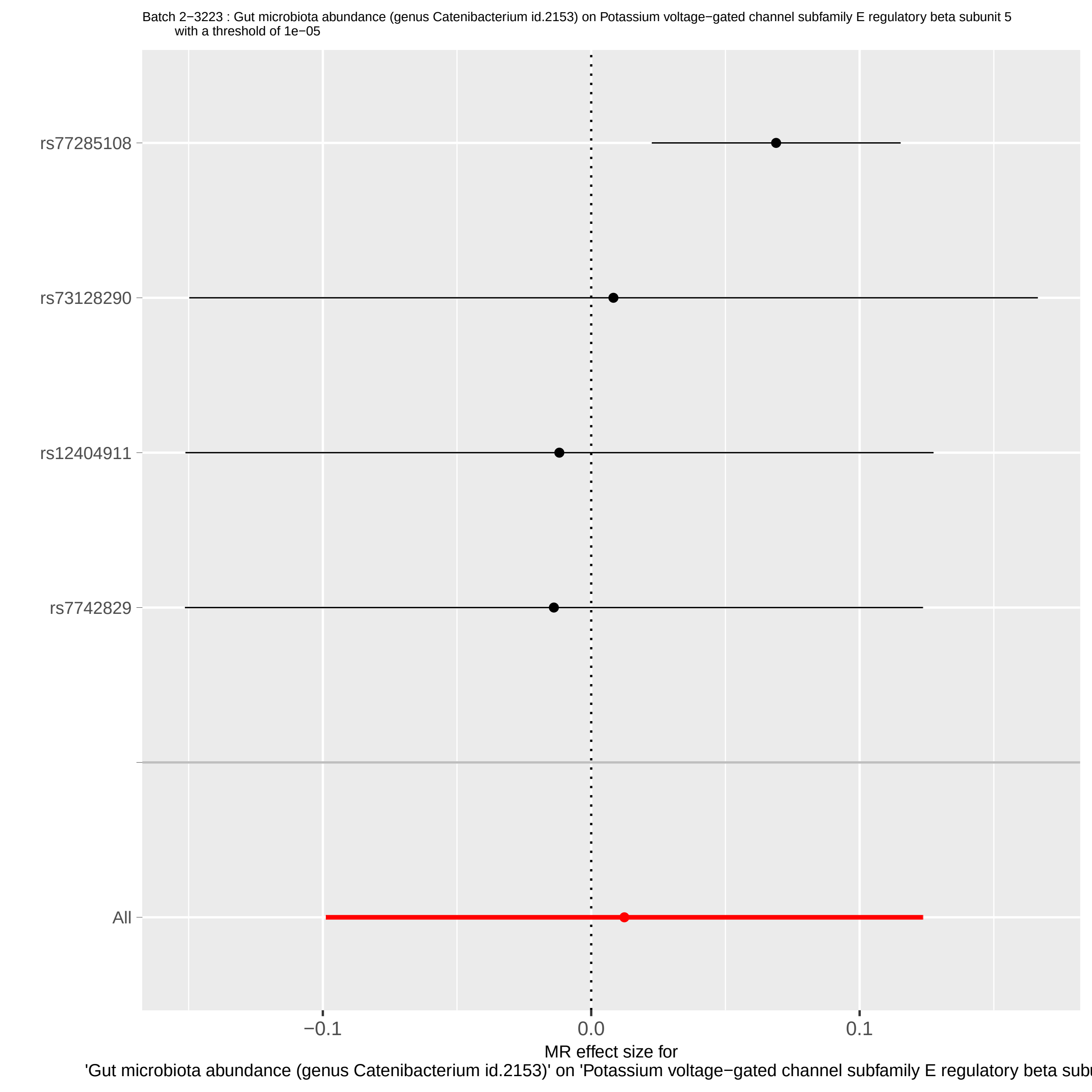
Batch 2−3223 : Gut microbiota abundance (genus Catenibacterium id.2153) on Potassium voltage−gated channel subfamily E regulatory beta subunit 5
with a threshold of 1e−05
rs77285108
rs73128290
rs12404911
rs7742829
All
−0.1
0.0
0.1
MR effect size for
'Gut microbiota abundance (genus Catenibacterium id.2153)' on 'Potassium voltage−gated channel subfamily E regulatory beta subu

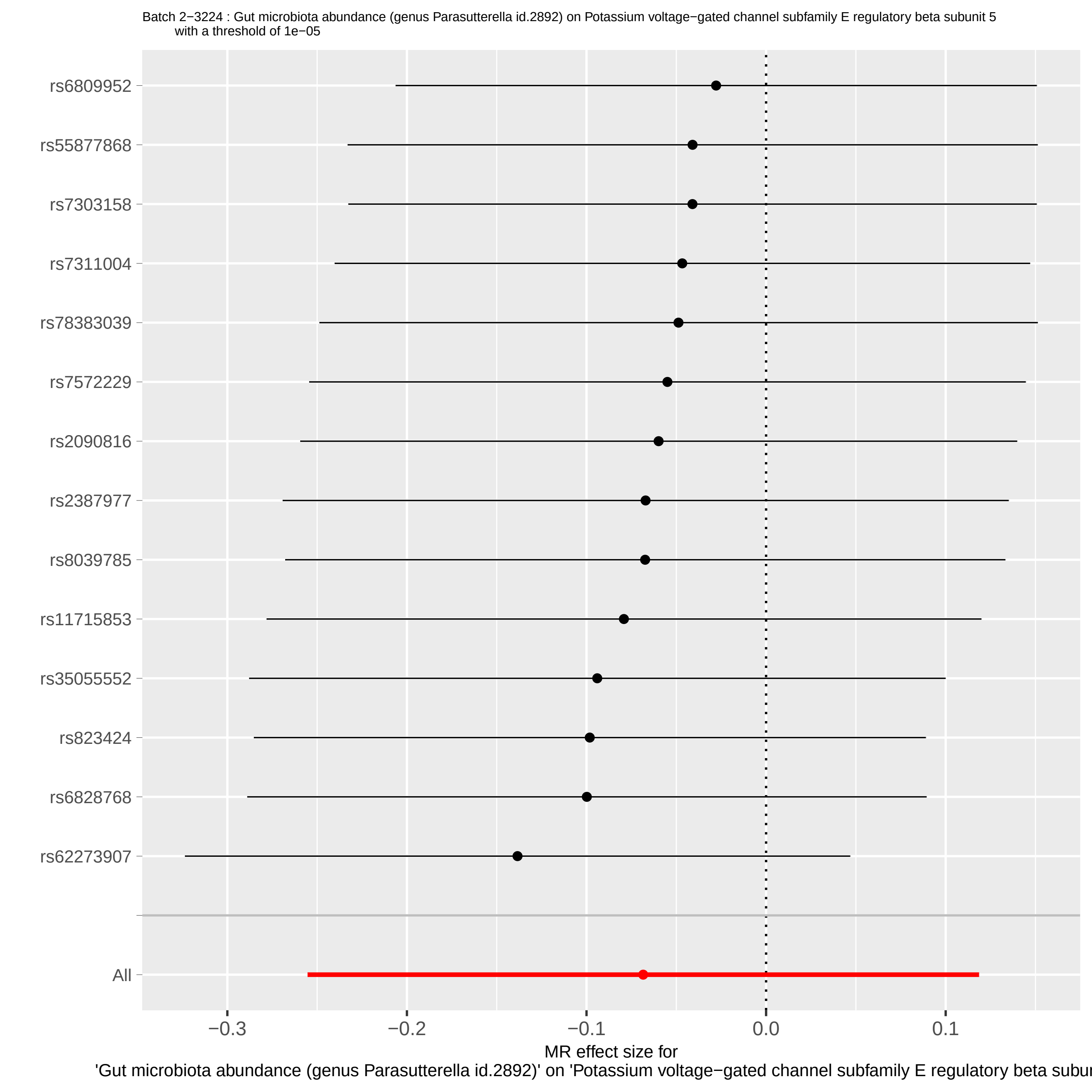
Batch 2−3224 : Gut microbiota abundance (genus Parasutterella id.2892) on Potassium voltage−gated channel subfamily E regulatory beta subunit 5
with a threshold of 1e−05
rs6809952
rs55877868
rs7303158
rs7311004
rs78383039
rs7572229
rs2090816
rs2387977
rs8039785
rs11715853
rs35055552
rs823424
rs6828768
rs62273907
All
−0.3
−0.2
−0.1
0.0
0.1
MR effect size for
'Gut microbiota abundance (genus Parasutterella id.2892)' on 'Potassium voltage−gated channel subfamily E regulatory beta subun

Batch 2−3225 : Gut microbiota abundance (genus Catenibacterium id.2153) on Potassium voltage−gated channel subfamily E regulatory beta subunit 5 with a threshold of 1e−05

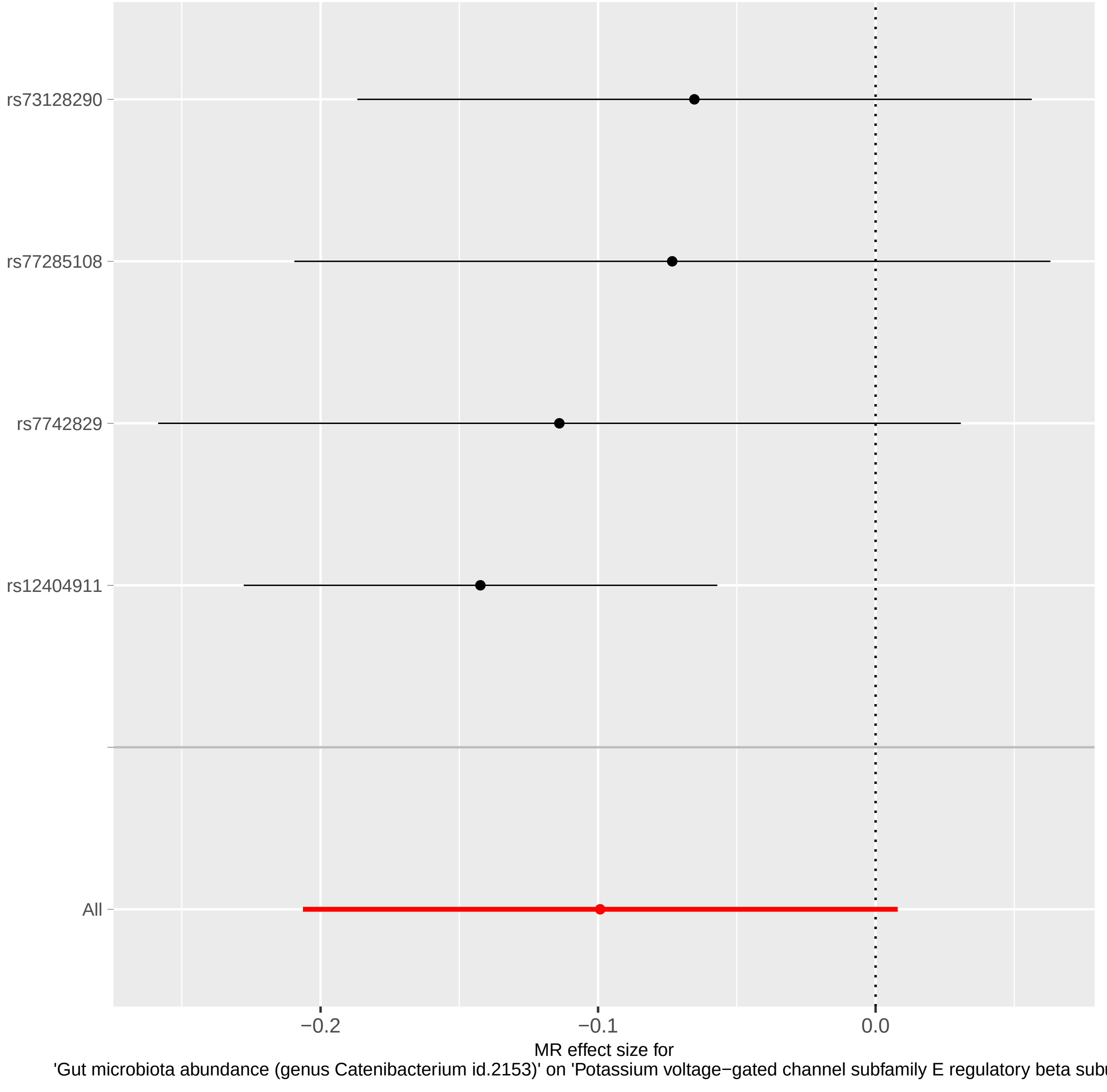

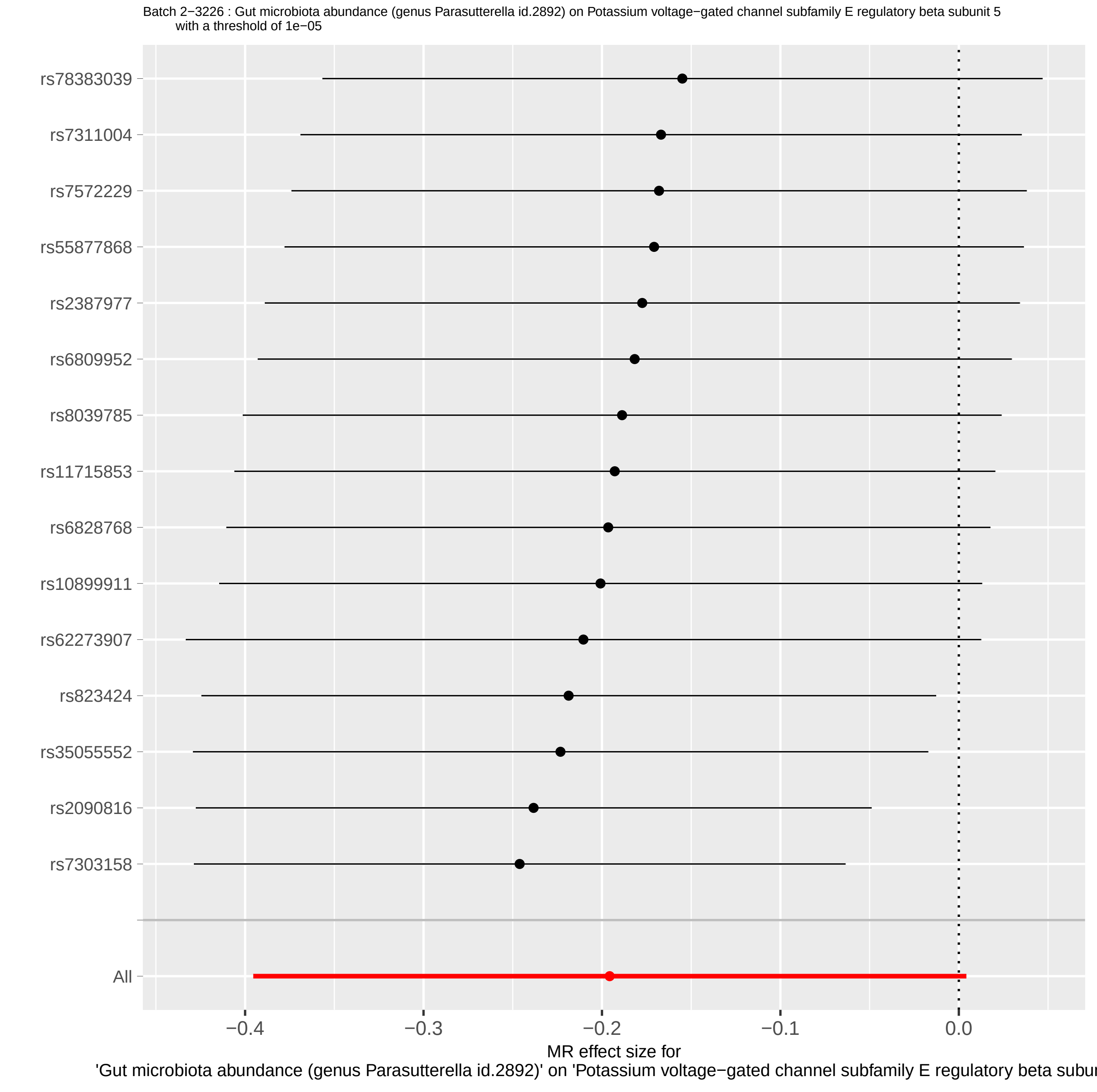
Batch 2−3226 : Gut microbiota abundance (genus Parasutterella id.2892) on Potassium voltage−gated channel subfamily E regulatory beta subunit 5
with a threshold of 1e−05
rs78383039
rs7311004
rs7572229
rs55877868
rs2387977
rs6809952
rs8039785
rs11715853
rs6828768
rs10899911
rs62273907
rs823424
rs35055552
rs2090816
rs7303158
All
−0.4
−0.3
−0.2
−0.1
0.0
MR effect size for
'Gut microbiota abundance (genus Parasutterella id.2892)' on 'Potassium voltage−gated channel subfamily E regulatory beta subun

Batch 2−3227 : Gut microbiota abundance (genus Catenibacterium id.2153) on Potassium voltage−gated channel subfamily F member 1 with a threshold of 1e−05

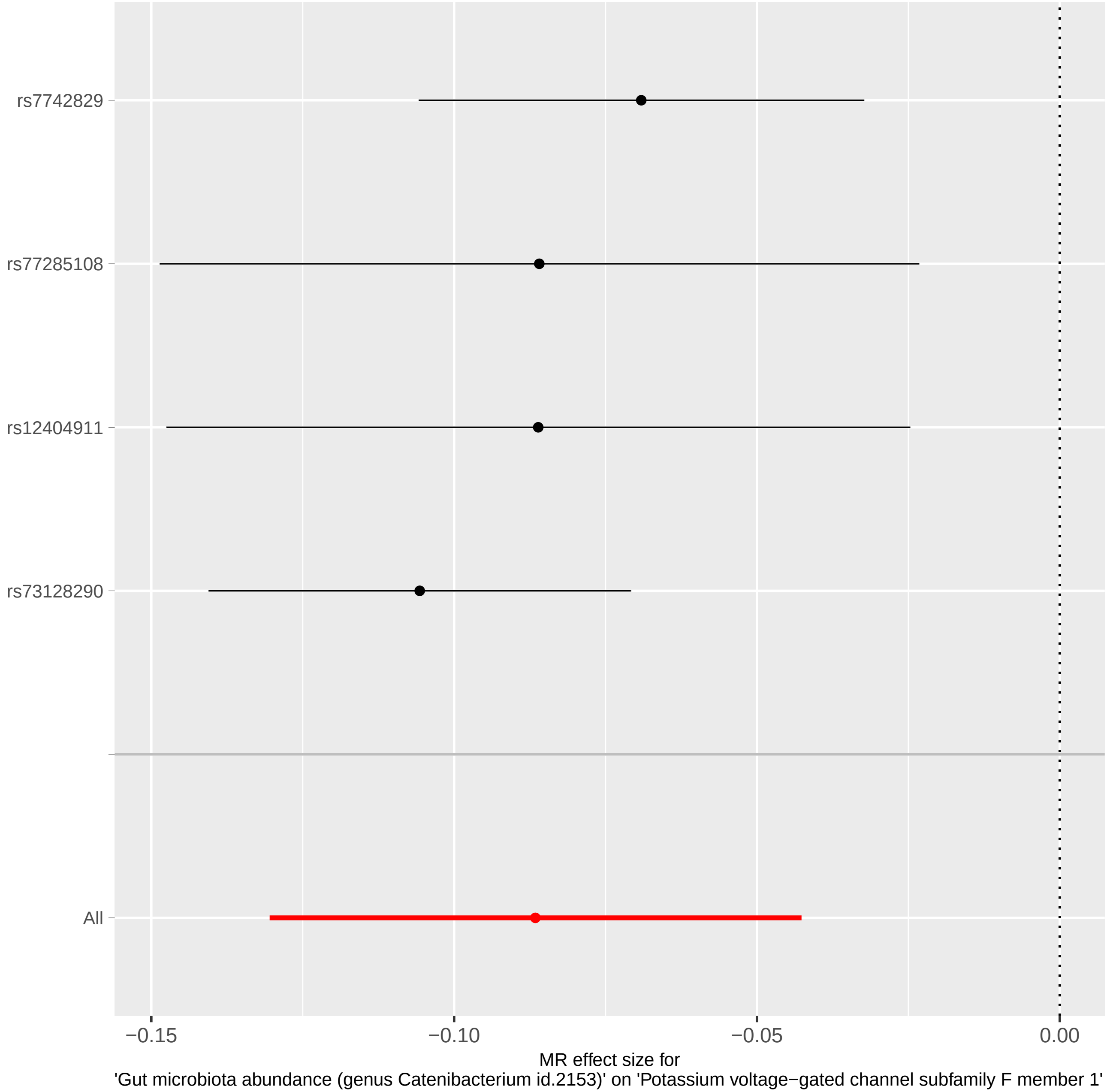

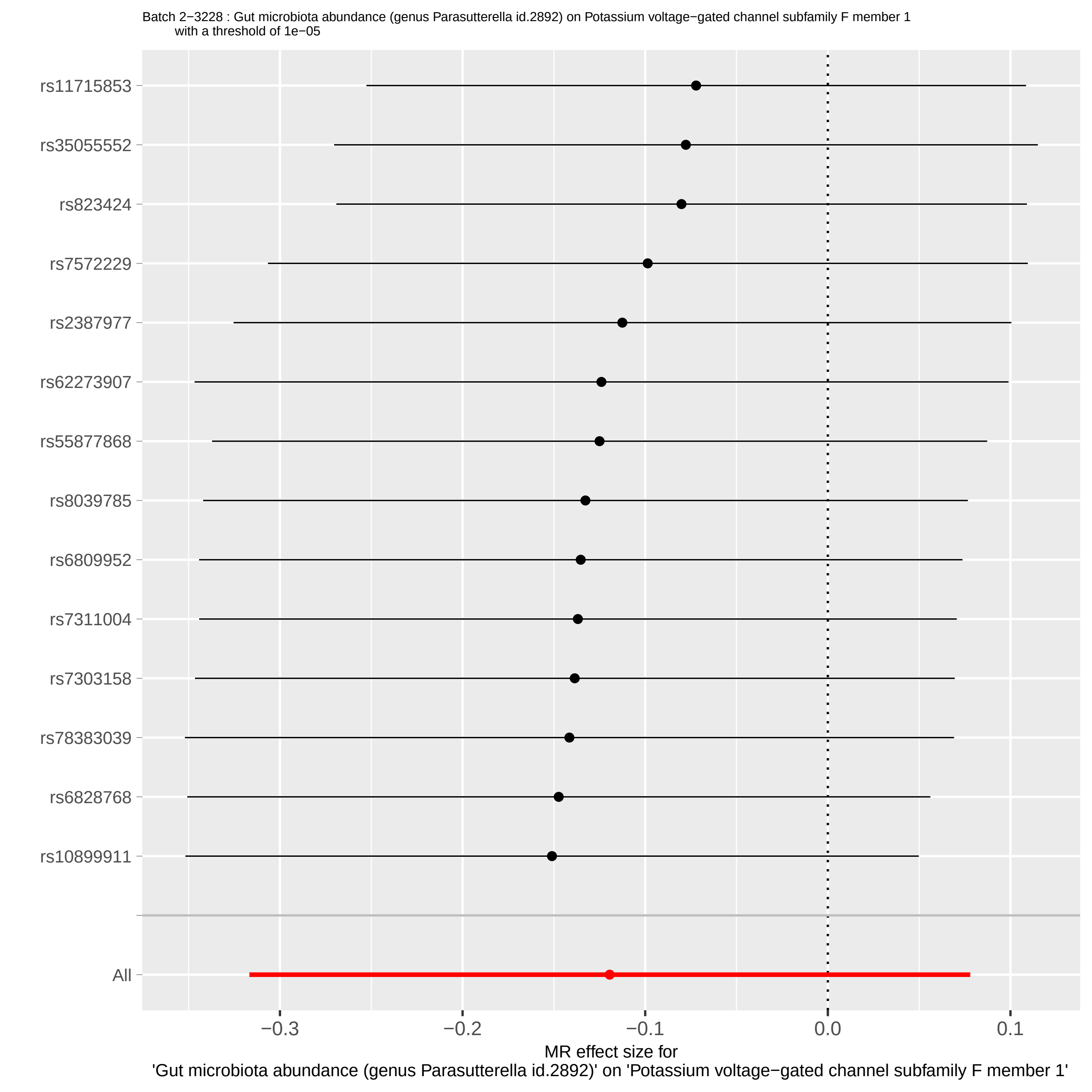
Batch 2−3228 : Gut microbiota abundance (genus Parasutterella id.2892) on Potassium voltage−gated channel subfamily F member 1 with a threshold of 1e−05
rs11715853
rs35055552
rs823424
rs7572229
rs2387977
rs62273907
rs55877868
rs8039785
rs6809952
rs7311004
rs7303158
rs78383039
rs6828768
rs10899911
All
−0.3
−0.2
−0.1
0.0
0.1
MR effect size for
'Gut microbiota abundance (genus Parasutterella id.2892)' on 'Potassium voltage−gated channel subfamily F member 1'

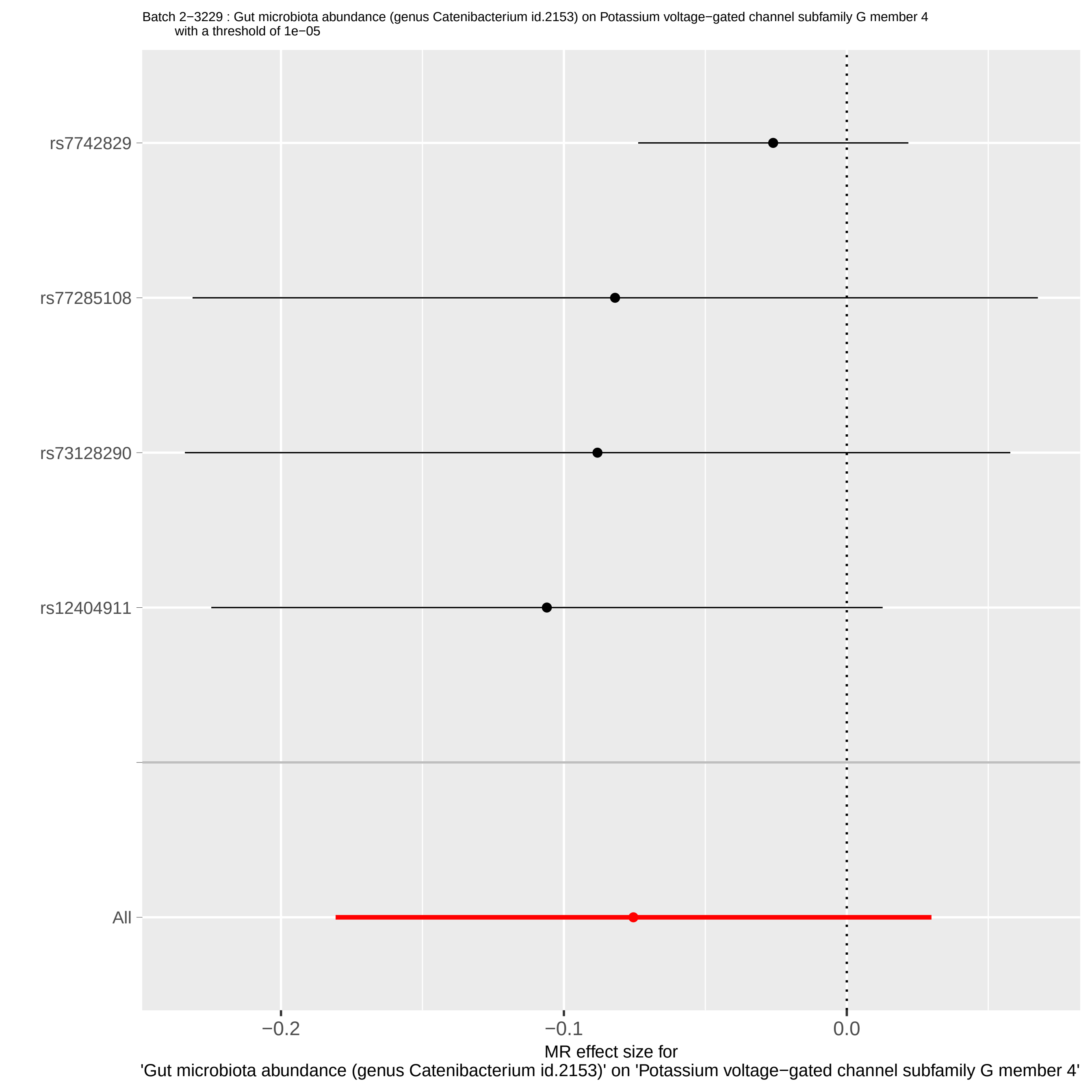

Batch 2−3229 : Gut microbiota abundance (genus Catenibacterium id.2153) on Potassium voltage−gated channel subfamily G member 4
with a threshold of 1e−05
rs7742829
rs77285108
rs73128290
rs12404911
All
−0.2
−0.1
0.0
MR effect size for
'Gut microbiota abundance (genus Catenibacterium id.2153)' on 'Potassium voltage−gated channel subfamily G member 4'

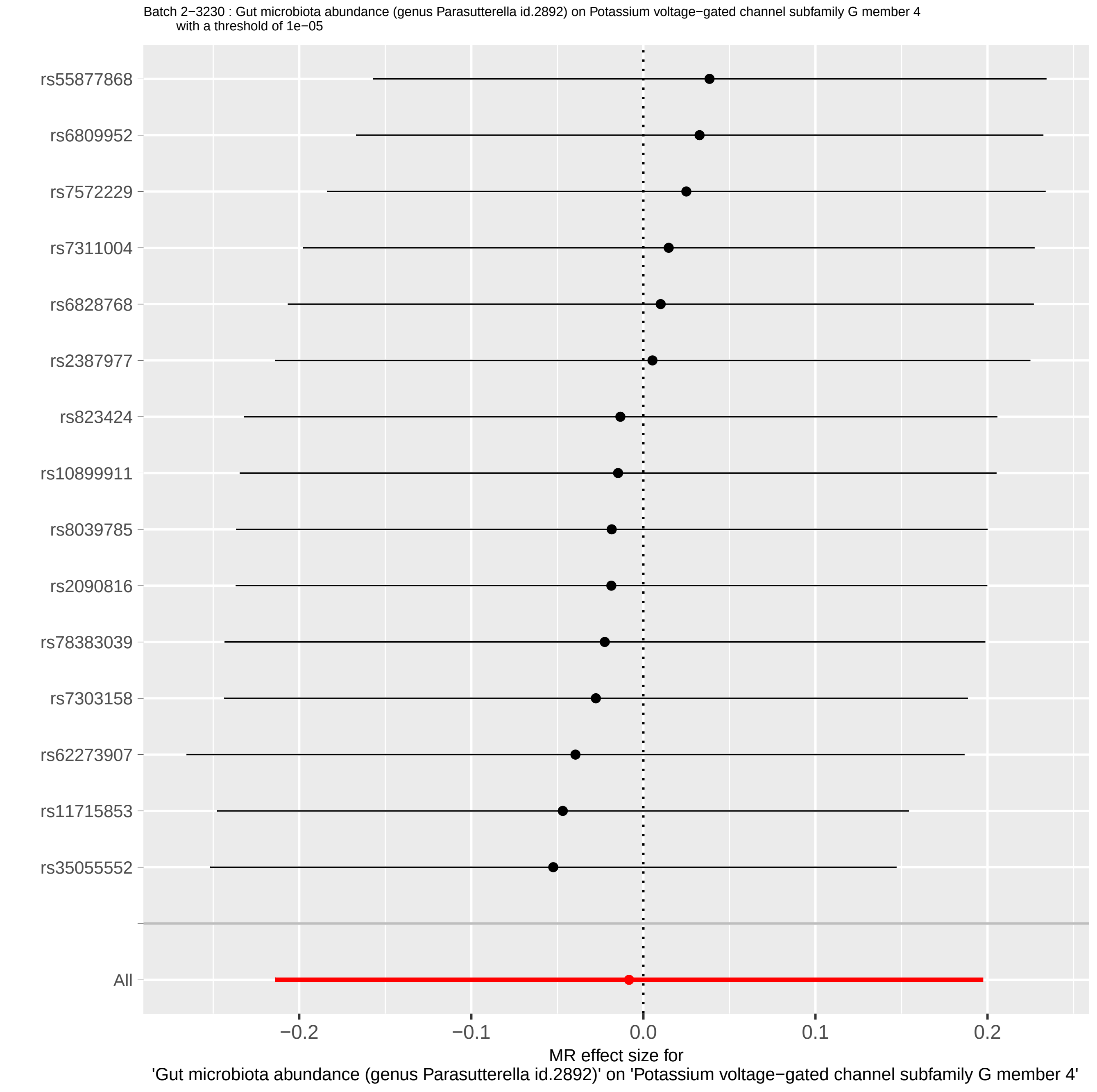

Batch 2−3230 : Gut microbiota abundance (genus Parasutterella id.2892) on Potassium voltage−gated channel subfamily G member 4 with a threshold of 1e−05
rs55877868
rs6809952
rs7572229
rs7311004
rs6828768
rs2387977
rs823424
rs10899911
rs8039785
rs2090816
rs78383039
rs7303158
rs62273907
rs11715853
rs35055552
All
−0.2
−0.1
0.0
0.1
0.2
MR effect size for
'Gut microbiota abundance (genus Parasutterella id.2892)' on 'Potassium voltage−gated channel subfamily G member 4'

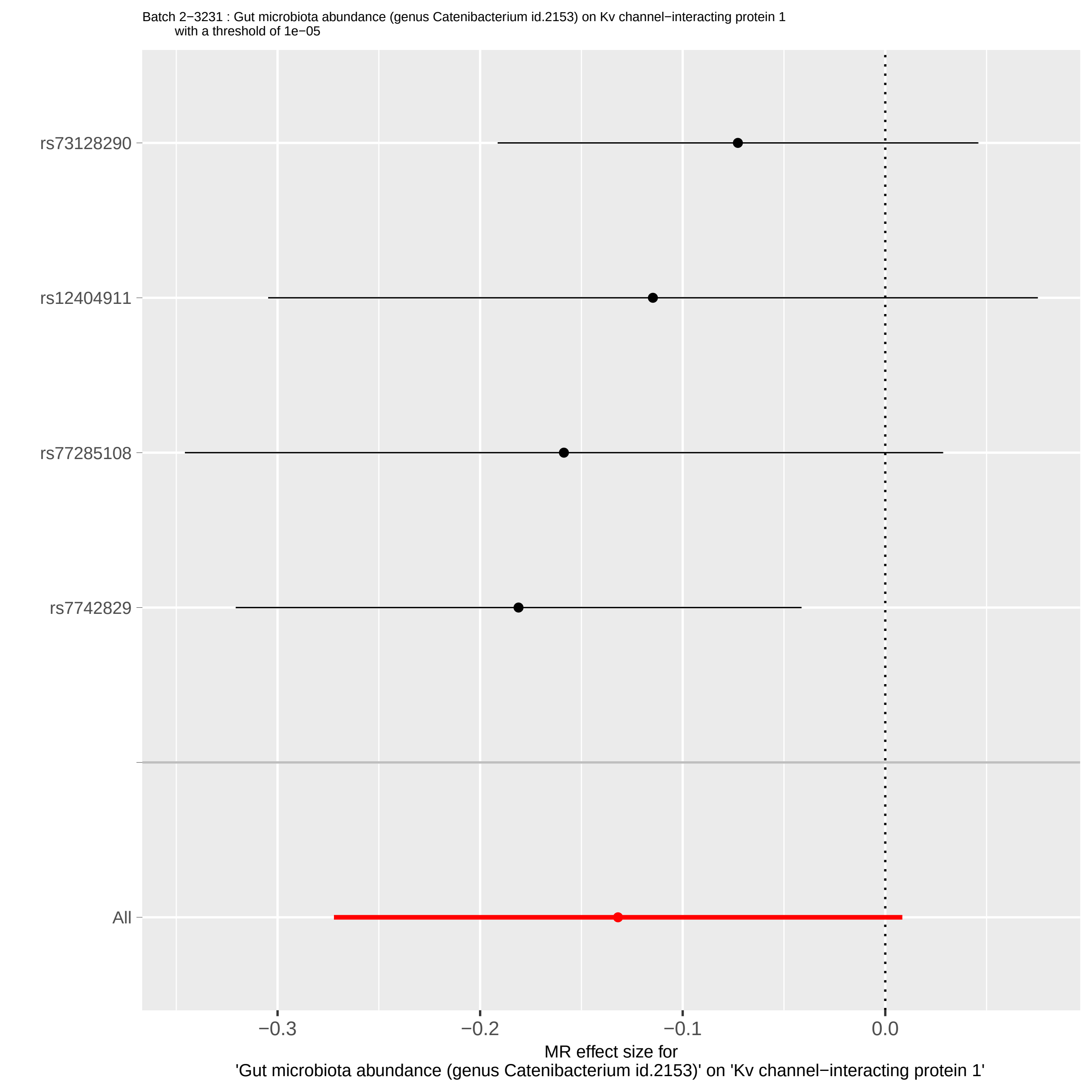

Batch 2−3231 : Gut microbiota abundance (genus Catenibacterium id.2153) on Kv channel−interacting protein 1
with a threshold of 1e−05
rs73128290
rs12404911
rs77285108
rs7742829
All
−0.3
−0.2
−0.1
0.0
MR effect size for
'Gut microbiota abundance (genus Catenibacterium id.2153)' on 'Kv channel−interacting protein 1'

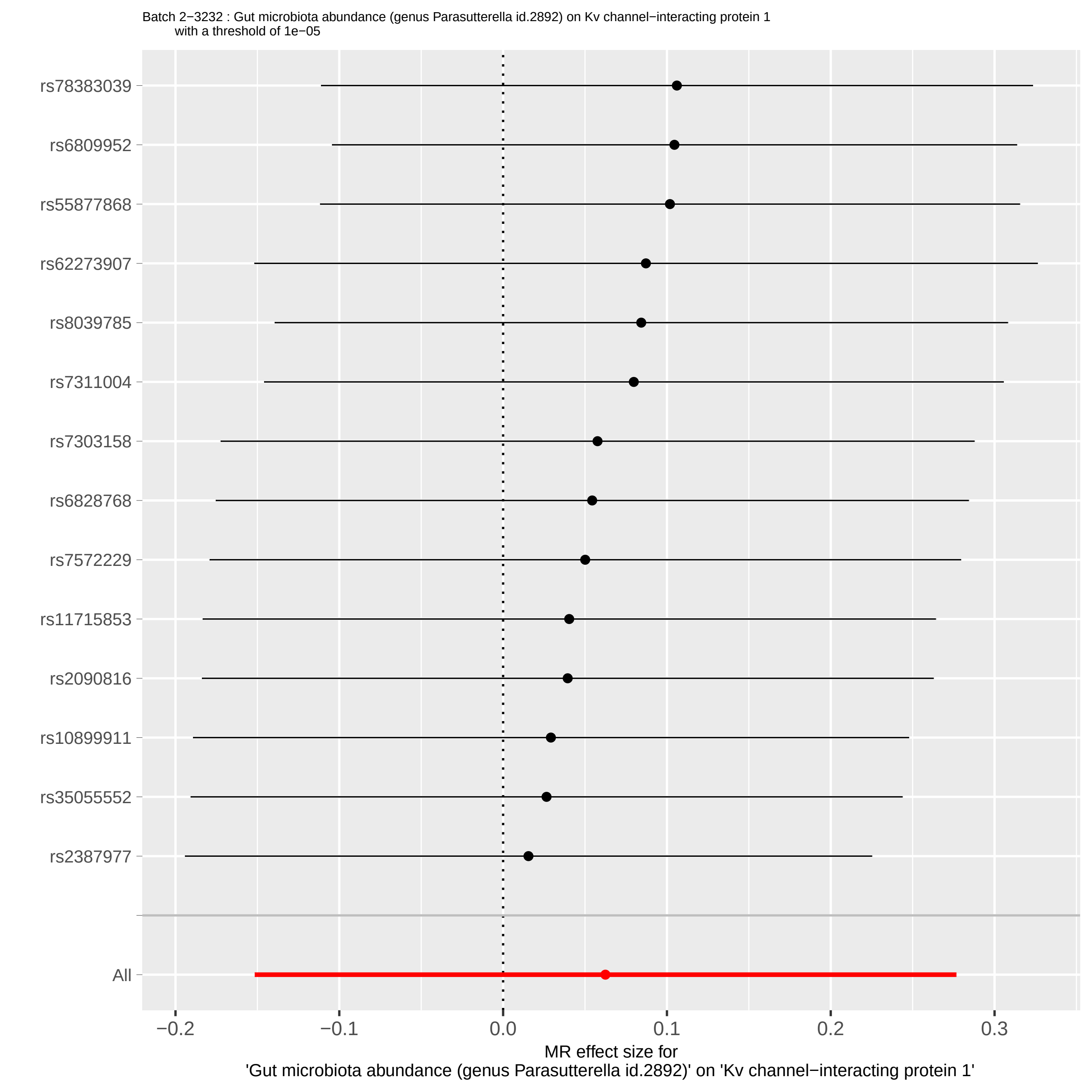

Batch 2−3232 : Gut microbiota abundance (genus Parasutterella id.2892) on Kv channel−interacting protein 1
with a threshold of 1e−05
rs78383039
rs6809952
rs55877868
rs62273907
rs8039785
rs7311004
rs7303158
rs6828768
rs7572229
rs11715853
rs2090816
rs10899911
rs35055552
rs2387977
All
−0.2
−0.1
0.0
0.1
0.2
0.3
MR effect size for
'Gut microbiota abundance (genus Parasutterella id.2892)' on 'Kv channel−interacting protein 1'

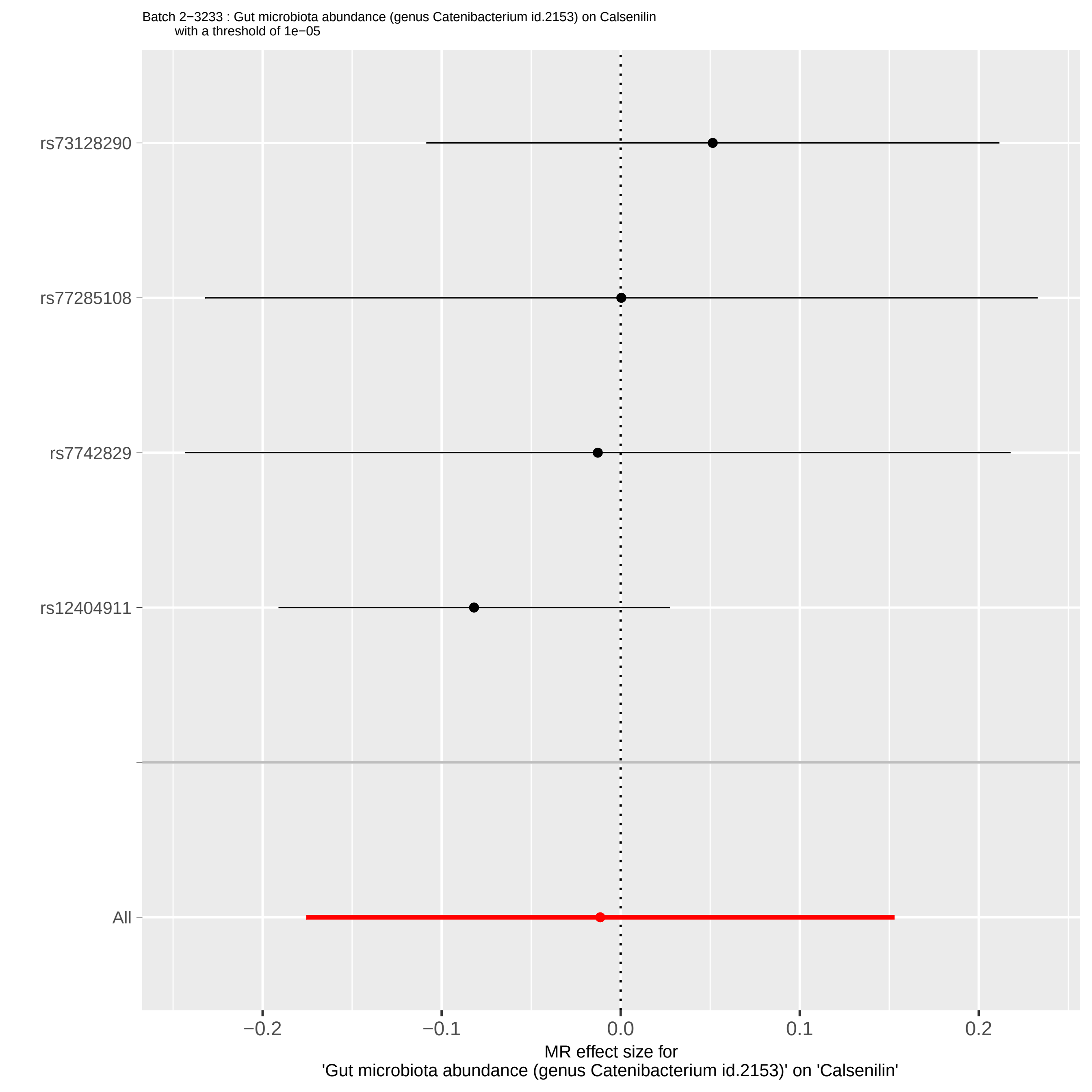
Batch 2−3233 : Gut microbiota abundance (genus Catenibacterium id.2153) on Calsenilin
with a threshold of 1e−05
rs73128290
rs77285108
rs7742829
rs12404911
All
−0.2
−0.1
0.0
0.1
0.2
MR effect size for
'Gut microbiota abundance (genus Catenibacterium id.2153)' on 'Calsenilin'

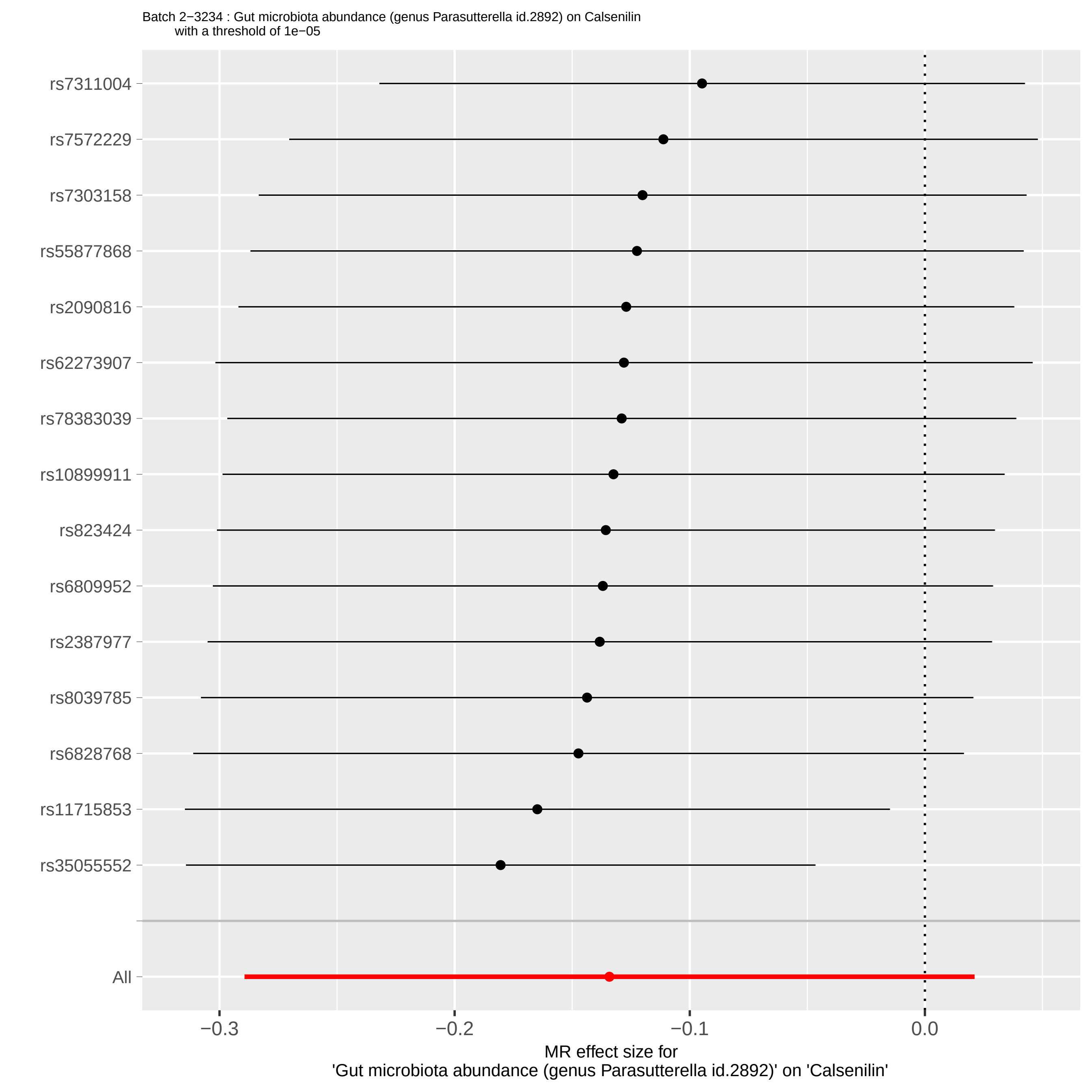
Batch 2−3234 : Gut microbiota abundance (genus Parasutterella id.2892) on Calsenilin
with a threshold of 1e−05
rs7311004
rs7572229
rs7303158
rs55877868
rs2090816
rs62273907
rs78383039
rs10899911
rs823424
rs6809952
rs2387977
rs8039785
rs6828768
rs11715853
rs35055552
All
−0.3
−0.2
−0.1
0.0
MR effect size for
'Gut microbiota abundance (genus Parasutterella id.2892)' on 'Calsenilin'

Batch 2−3235 : Gut microbiota abundance (genus Catenibacterium id.2153) on Small conductance calcium−activated potassium channel protein 1 with a threshold of 1e−05

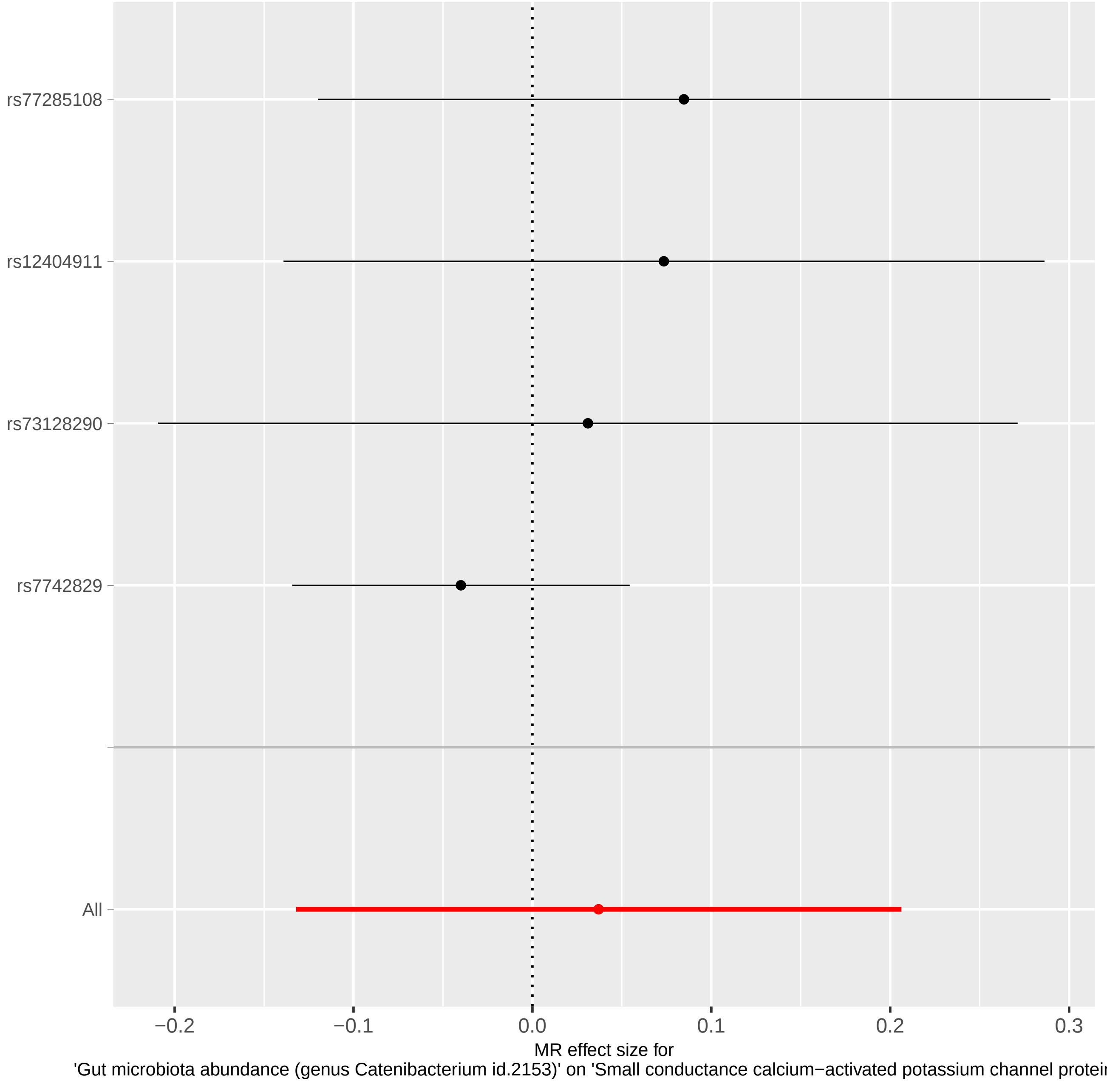

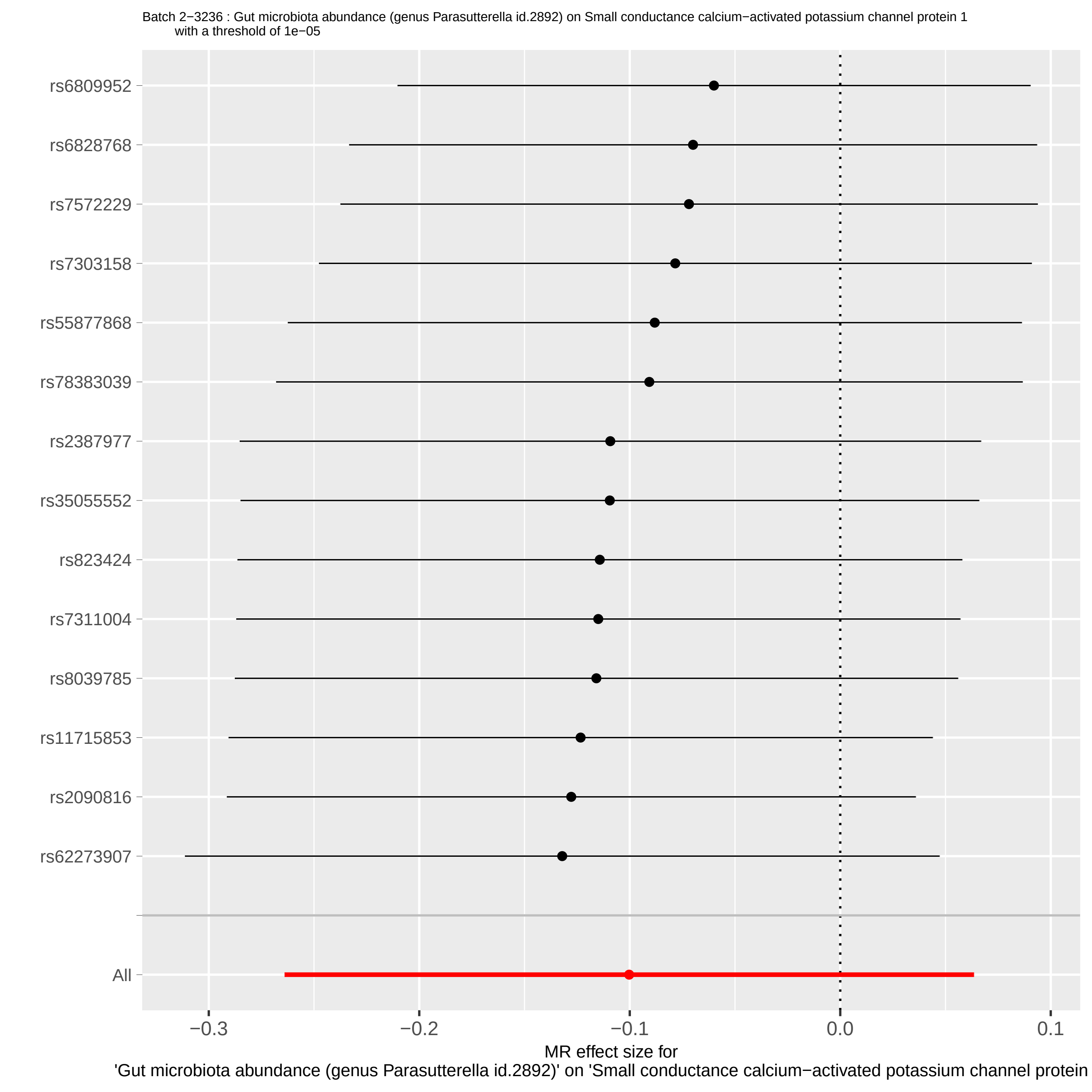
Batch 2−3236 : Gut microbiota abundance (genus Parasutterella id.2892) on Small conductance calcium−activated potassium channel protein 1
with a threshold of 1e−05
rs6809952
rs6828768
rs7572229
rs7303158
rs55877868
rs78383039
rs2387977
rs35055552
rs823424
rs7311004
rs8039785
rs11715853
rs2090816
rs62273907
All
−0.3
−0.2
−0.1
0.0
0.1
MR effect size for
'Gut microbiota abundance (genus Parasutterella id.2892)' on 'Small conductance calcium−activated potassium channel protein

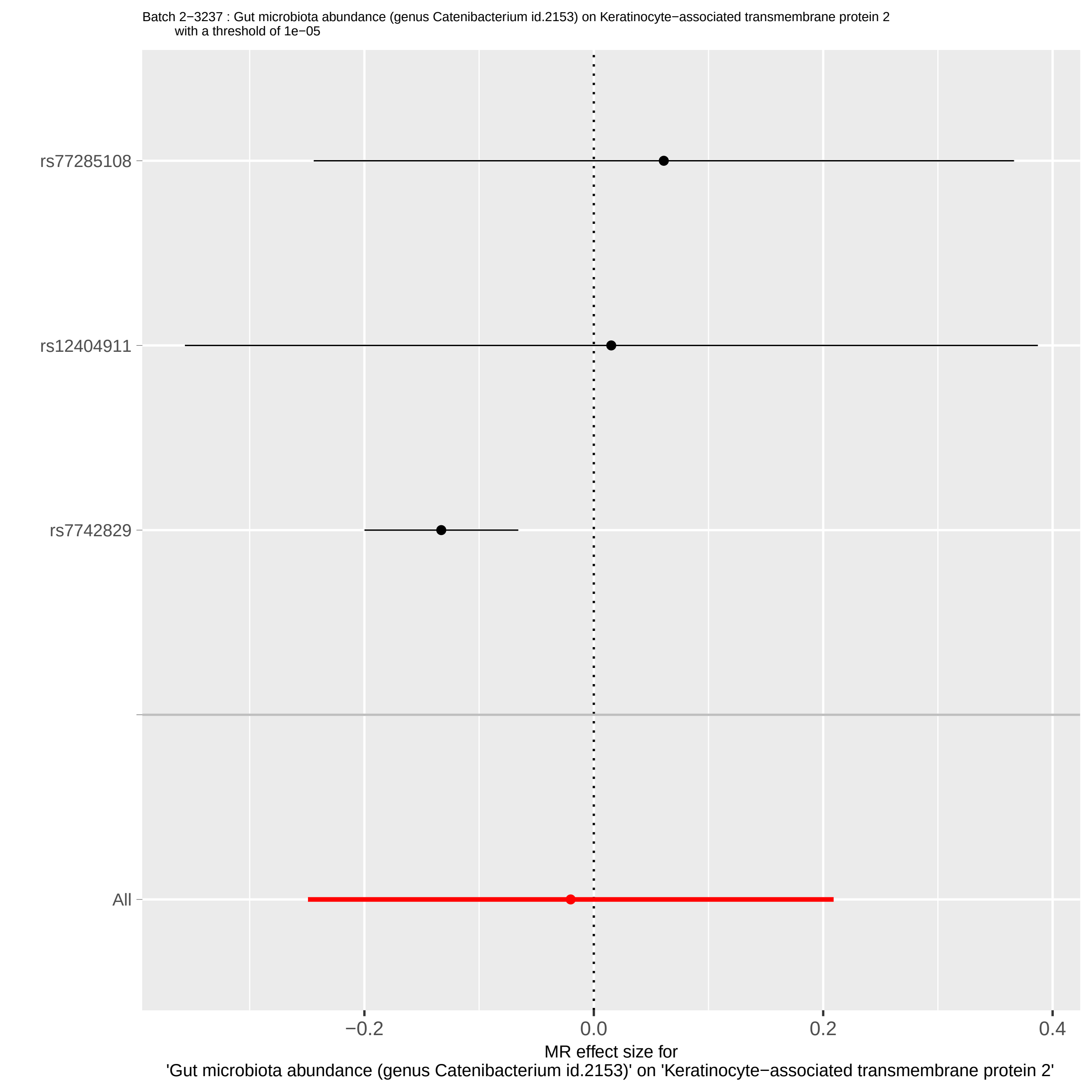
Batch 2−3237 : Gut microbiota abundance (genus Catenibacterium id.2153) on Keratinocyte−associated transmembrane protein 2
with a threshold of 1e−05
rs77285108
rs12404911
rs7742829
All
−0.2
0.0
0.2
0.4
MR effect size for
'Gut microbiota abundance (genus Catenibacterium id.2153)' on 'Keratinocyte−associated transmembrane protein 2'

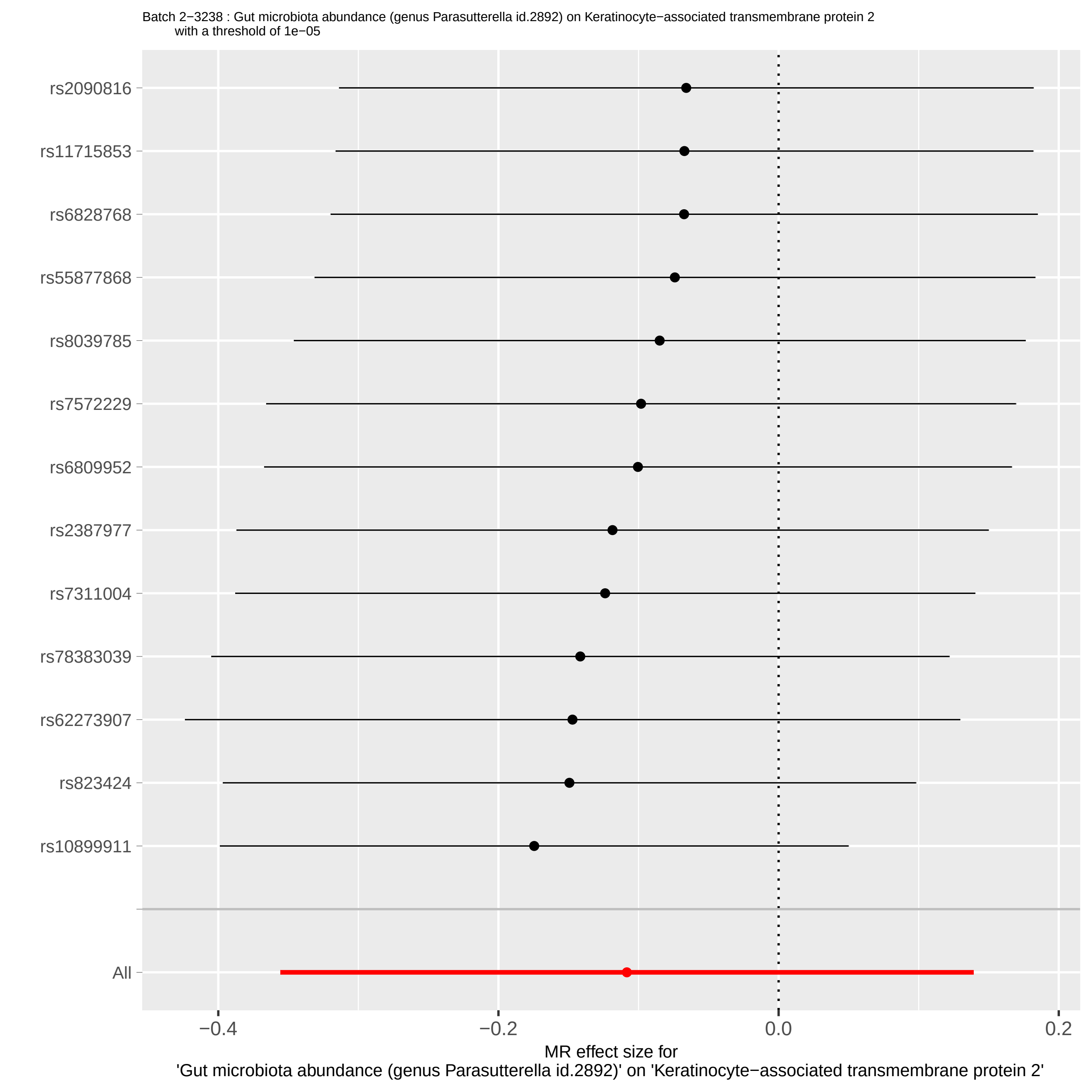
Batch 2−3238 : Gut microbiota abundance (genus Parasutterella id.2892) on Keratinocyte−associated transmembrane protein 2
with a threshold of 1e−05
rs2090816
rs11715853
rs6828768
rs55877868
rs8039785
rs7572229
rs6809952
rs2387977
rs7311004
rs78383039
rs62273907
rs823424
rs10899911
All
−0.4
−0.2
0.0
0.2
MR effect size for
'Gut microbiota abundance (genus Parasutterella id.2892)' on 'Keratinocyte−associated transmembrane protein 2'

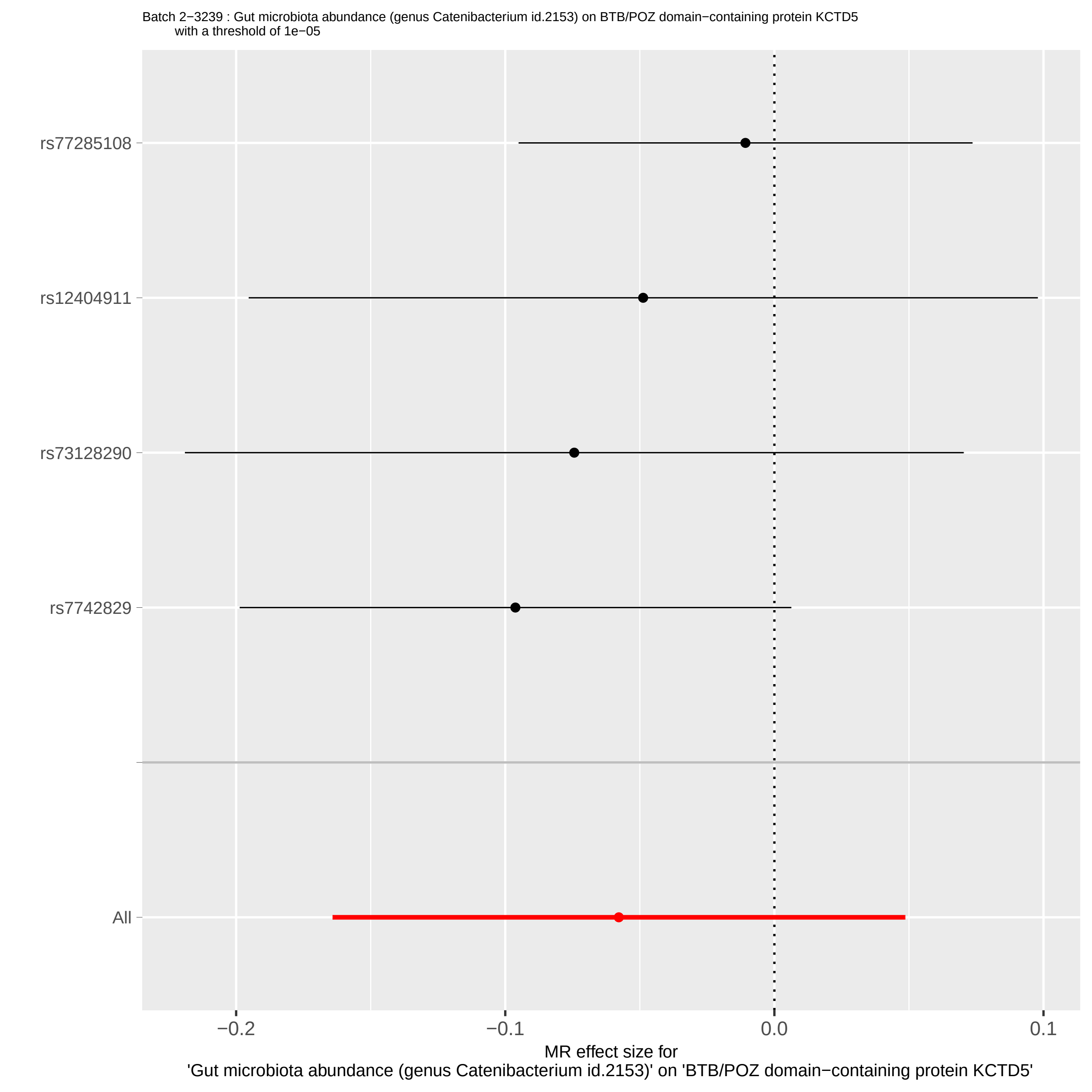
Batch 2−3239 : Gut microbiota abundance (genus Catenibacterium id.2153) on BTB/POZ domain−containing protein KCTD5
with a threshold of 1e−05
rs77285108
rs12404911
rs73128290
rs7742829
All
−0.2
−0.1
0.0
0.1
MR effect size for
'Gut microbiota abundance (genus Catenibacterium id.2153)' on 'BTB/POZ domain−containing protein KCTD5'

Batch 2−3240 : Gut microbiota abundance (genus Parasutterella id.2892) on BTB/POZ domain−containing protein KCTD5
with a threshold of 1e−05

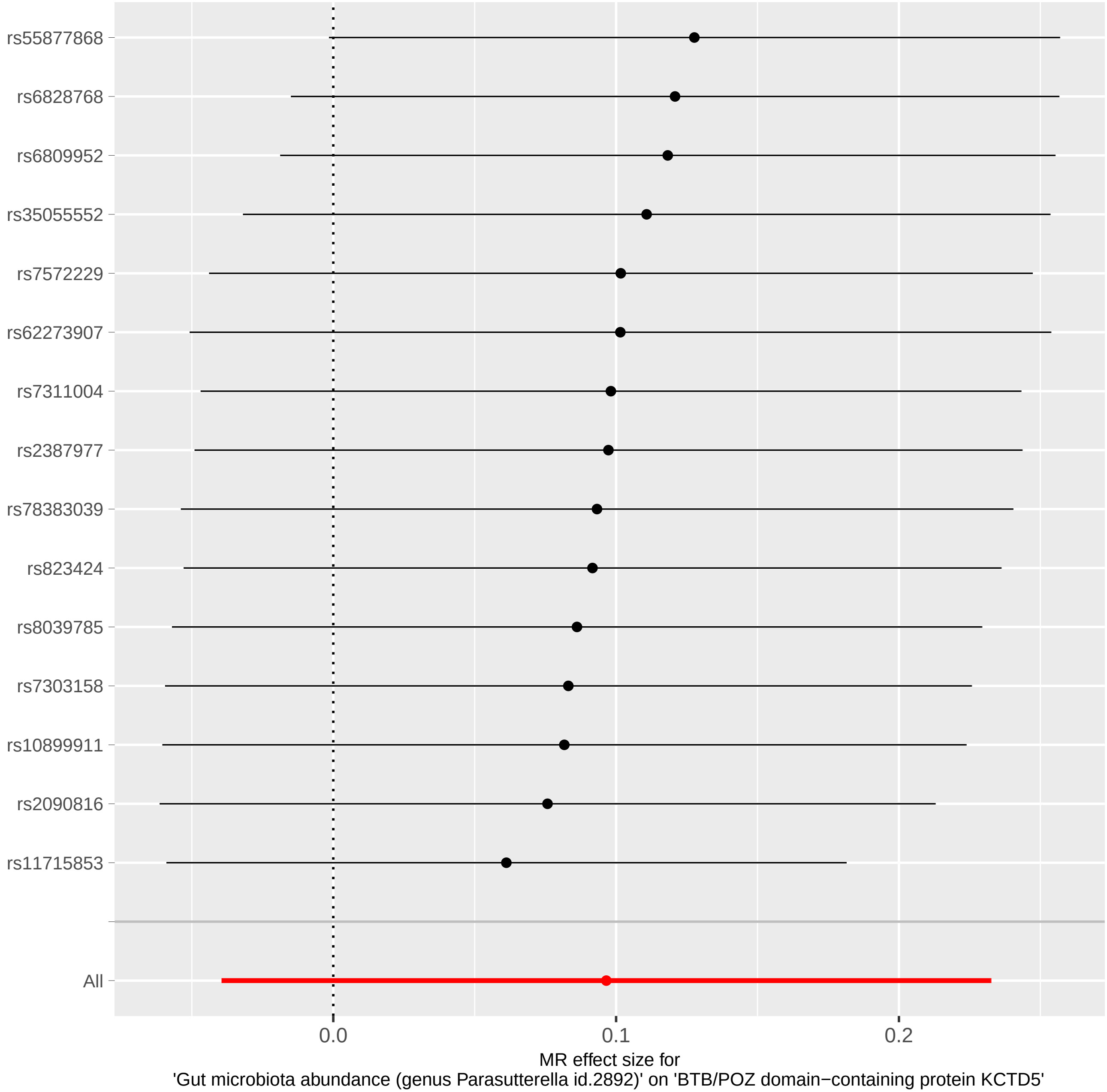

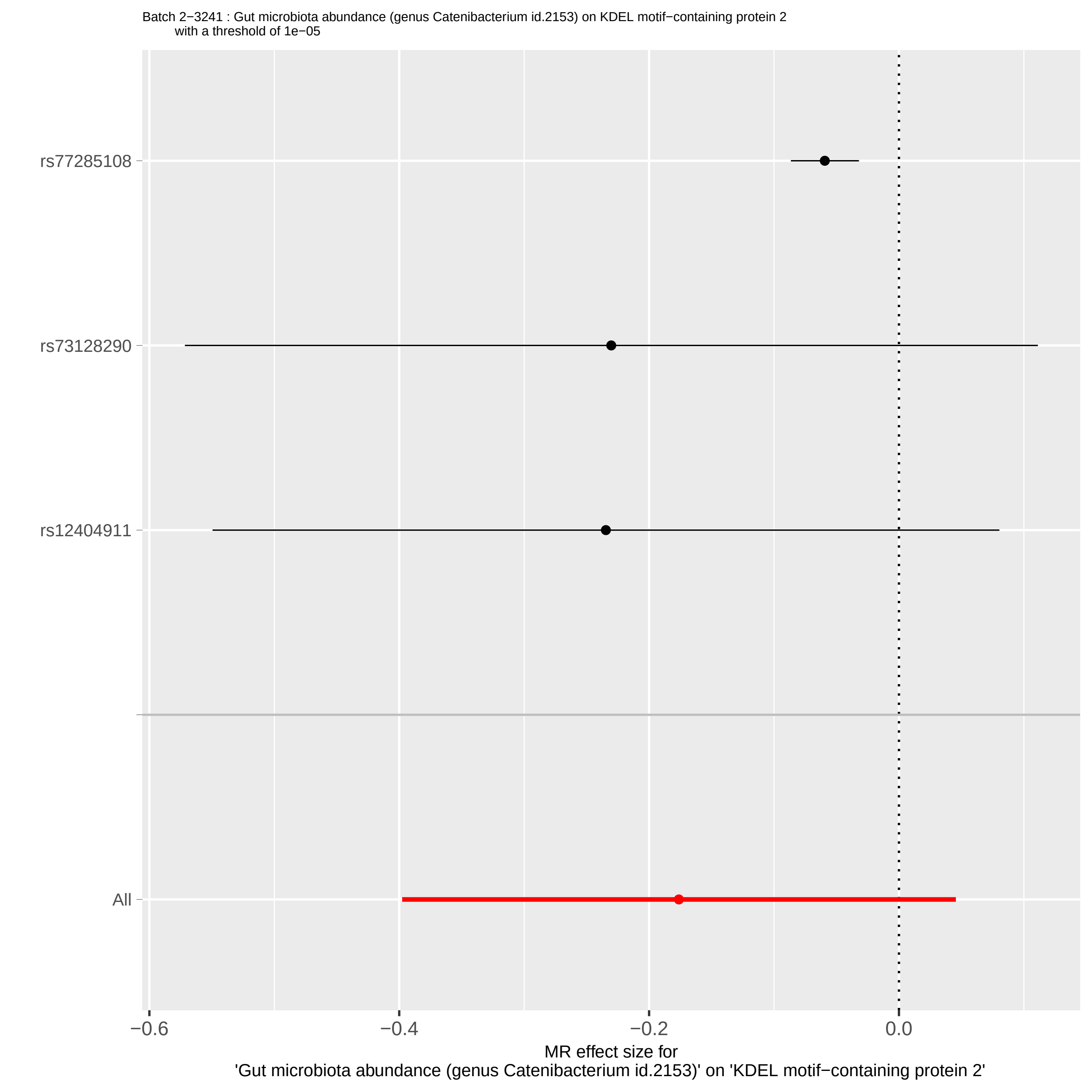

Batch 2−3241 : Gut microbiota abundance (genus Catenibacterium id.2153) on KDEL motif−containing protein 2
with a threshold of 1e−05
rs77285108
rs73128290
rs12404911
All
−0.6
−0.4
−0.2
0.0
MR effect size for
'Gut microbiota abundance (genus Catenibacterium id.2153)' on 'KDEL motif−containing protein 2'

Batch 2−3242 : Gut microbiota abundance (genus Parasutterella id.2892) on KDEL motif−containing protein 2
with a threshold of 1e−05

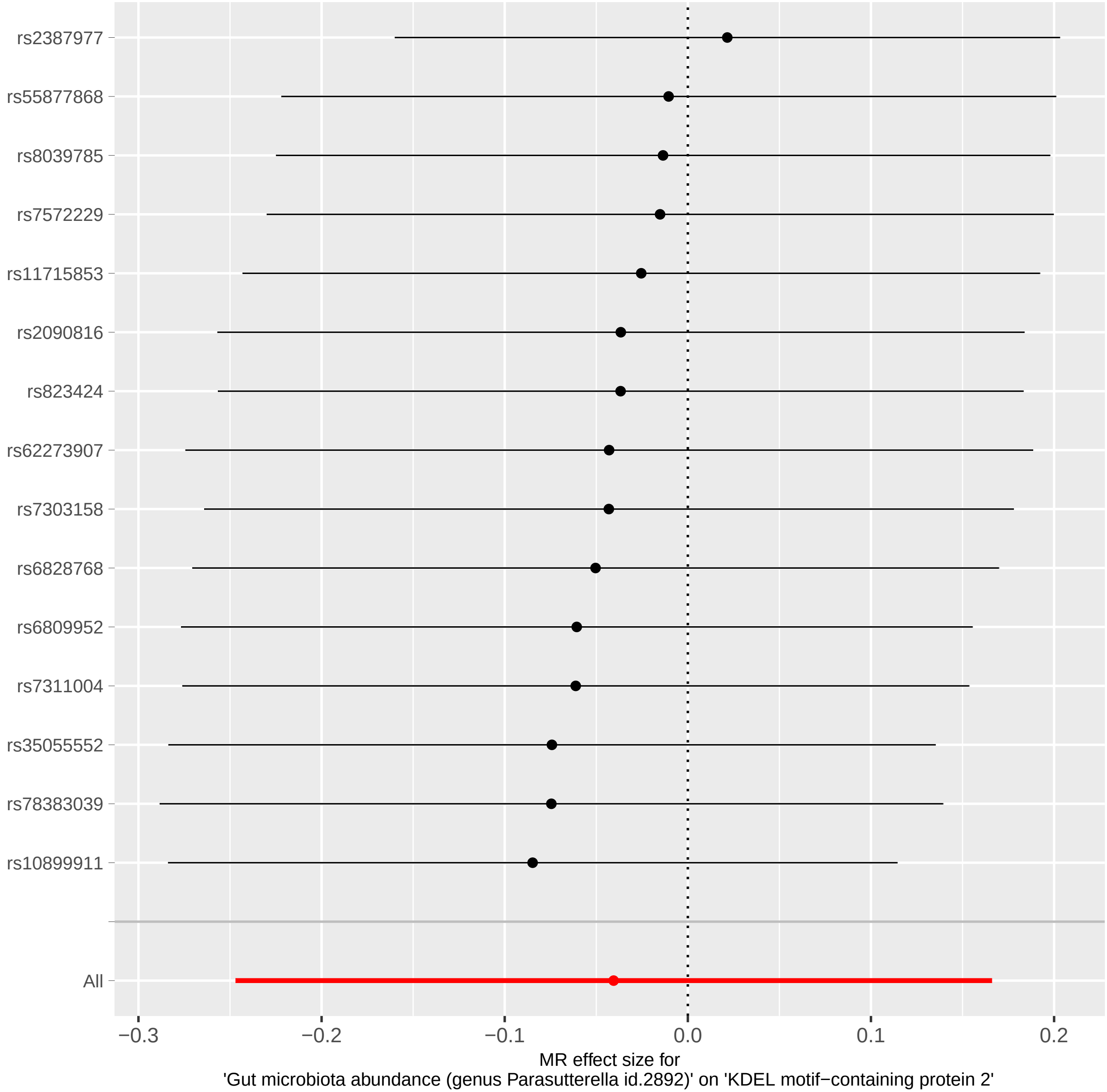

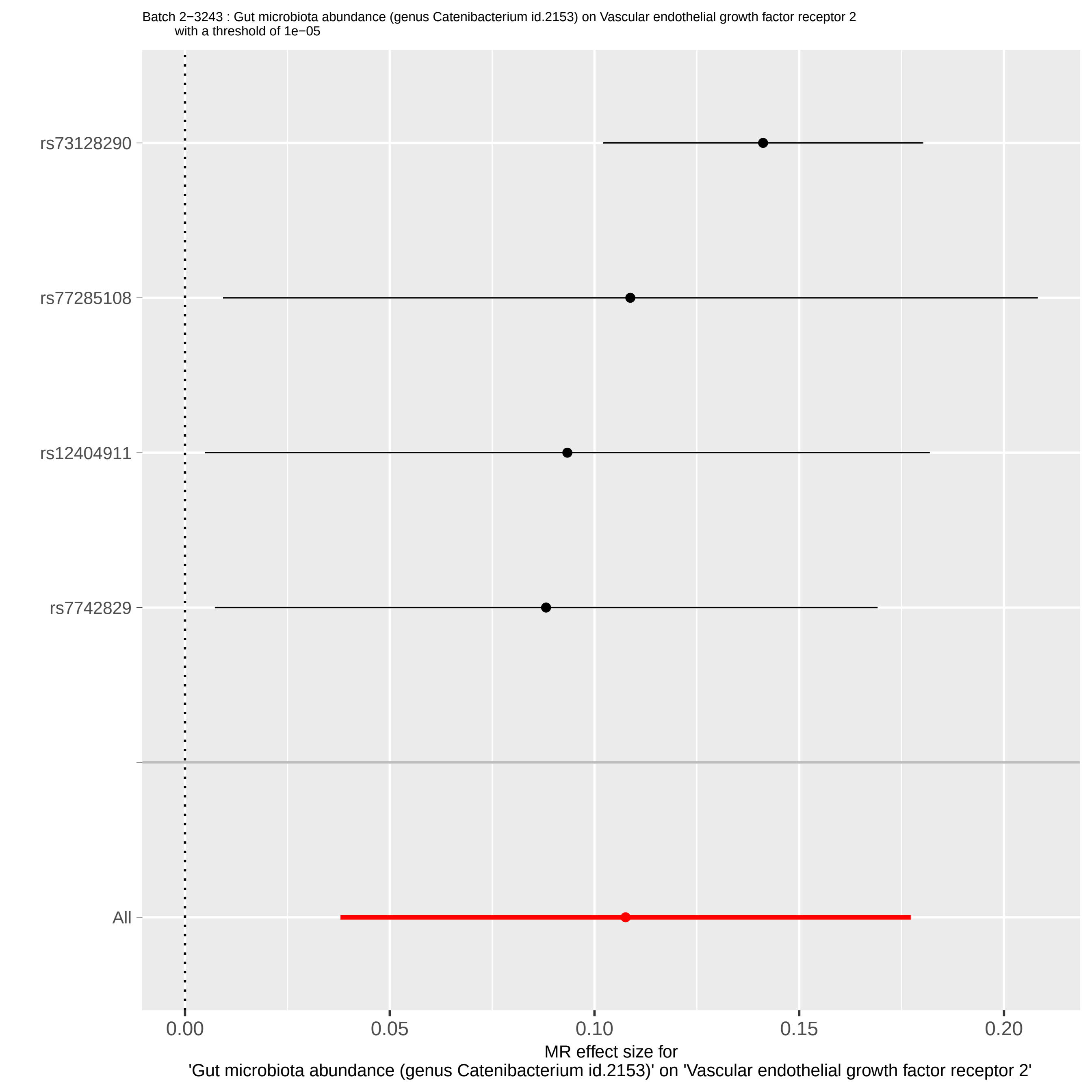
Batch 2−3243 : Gut microbiota abundance (genus Catenibacterium id.2153) on Vascular endothelial growth factor receptor 2
with a threshold of 1e−05
rs73128290
rs77285108
rs12404911
rs7742829
All
0.00
0.05
0.10
0.15
0.20
MR effect size for
'Gut microbiota abundance (genus Catenibacterium id.2153)' on 'Vascular endothelial growth factor receptor 2'

Batch 2−3244 : Gut microbiota abundance (genus Parasutterella id.2892) on Vascular endothelial growth factor receptor 2
with a threshold of 1e−05

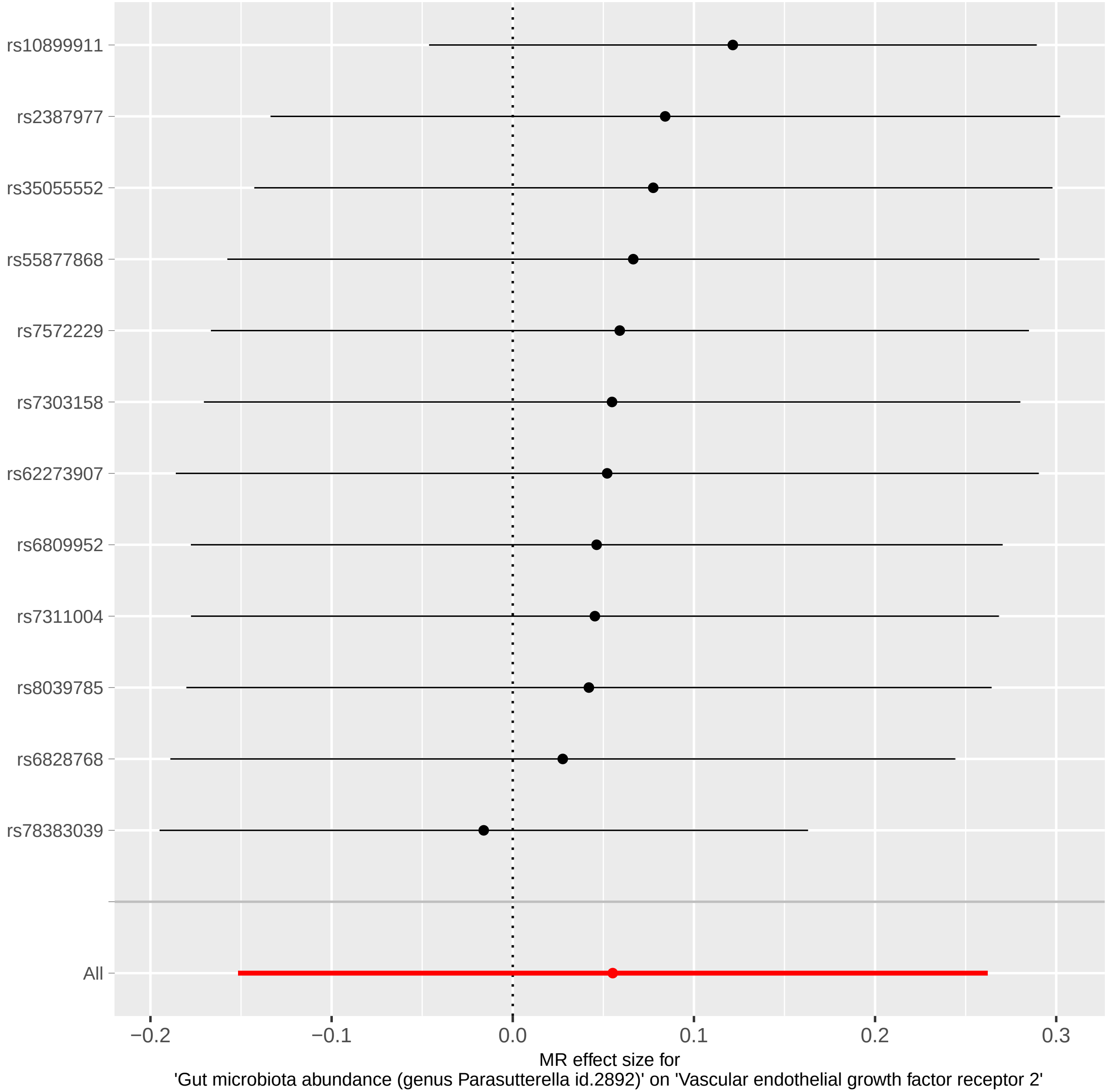

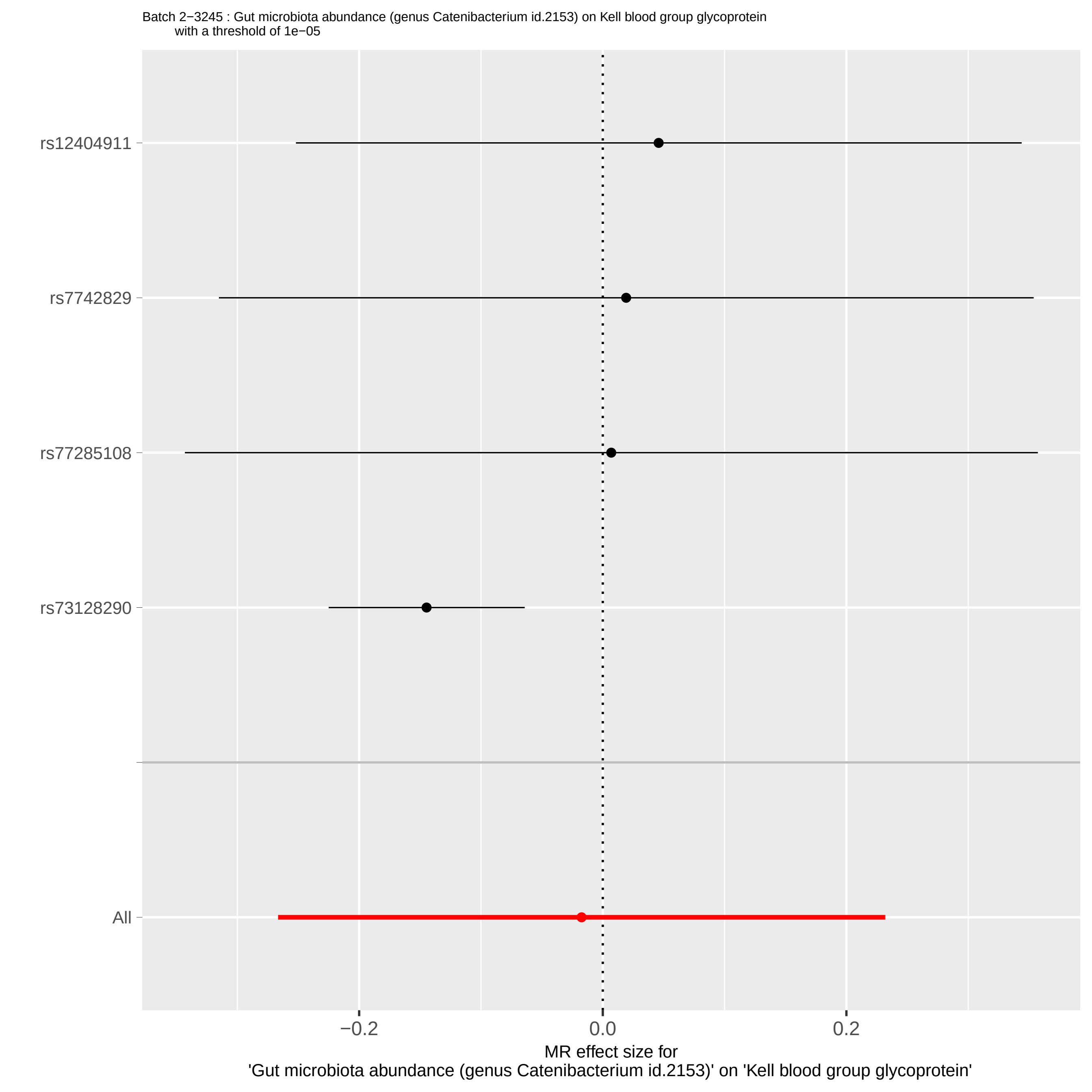

Batch 2−3245 : Gut microbiota abundance (genus Catenibacterium id.2153) on Kell blood group glycoprotein
with a threshold of 1e−05
rs12404911
rs7742829
rs77285108
rs73128290
All
−0.2
0.0
0.2
MR effect size for
'Gut microbiota abundance (genus Catenibacterium id.2153)' on 'Kell blood group glycoprotein'

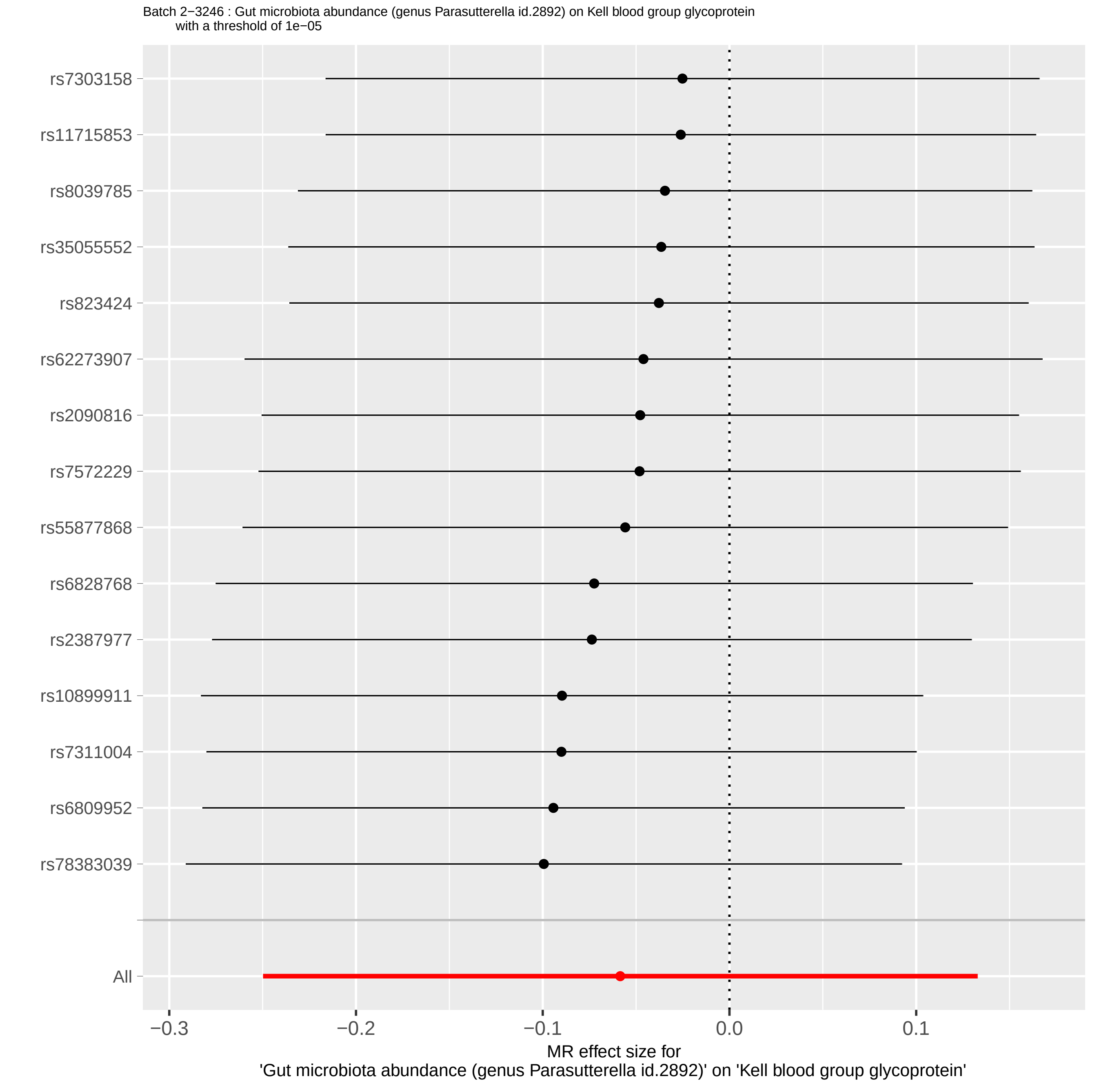

Batch 2−3246 : Gut microbiota abundance (genus Parasutterella id.2892) on Kell blood group glycoprotein
with a threshold of 1e−05
rs7303158
rs11715853
rs8039785
rs35055552
rs823424
rs62273907
rs2090816
rs7572229
rs55877868
rs6828768
rs2387977
rs10899911
rs7311004
rs6809952
rs78383039
All
−0.3
−0.2
−0.1
0.0
0.1
MR effect size for
'Gut microbiota abundance (genus Parasutterella id.2892)' on 'Kell blood group glycoprotein'

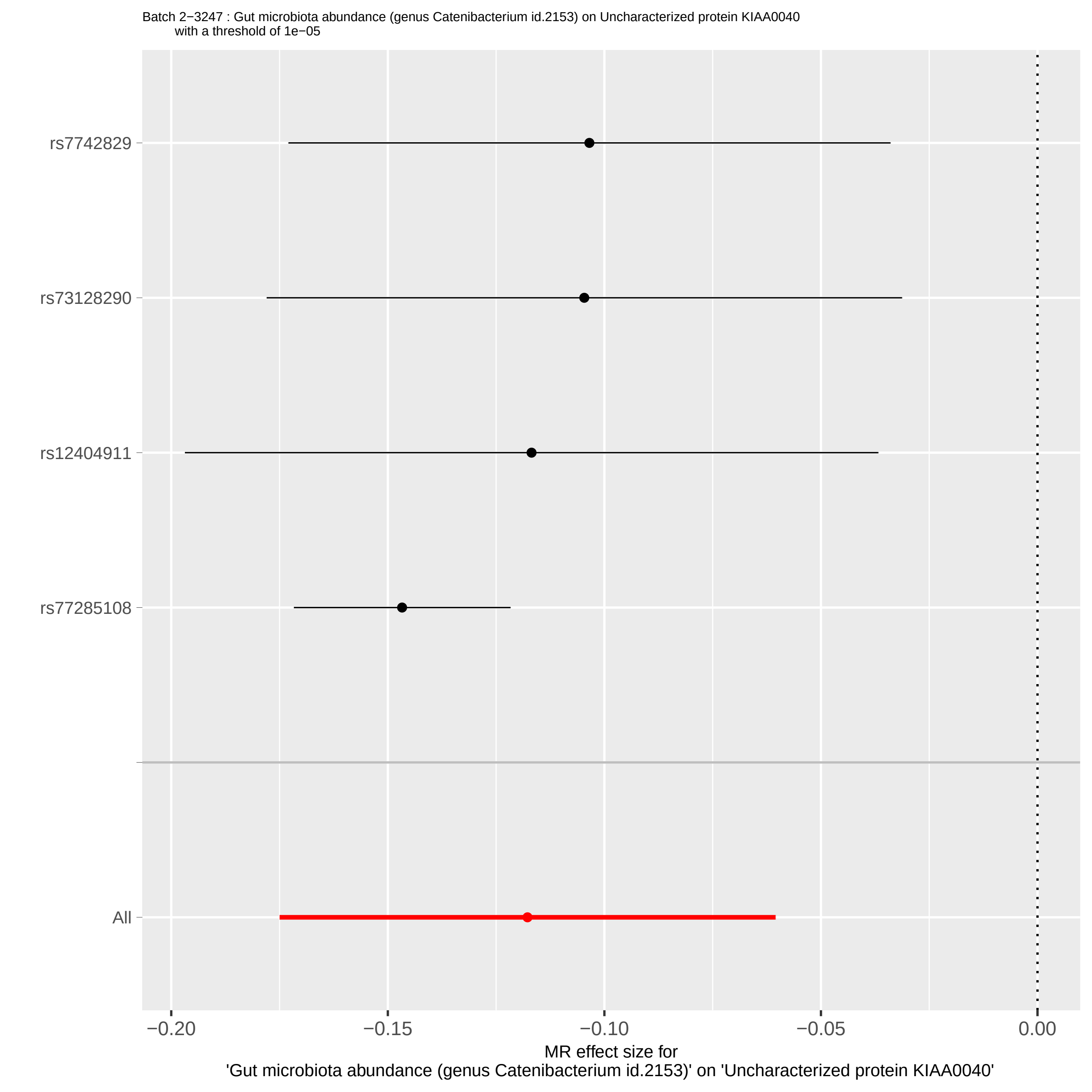
Batch 2−3247 : Gut microbiota abundance (genus Catenibacterium id.2153) on Uncharacterized protein KIAA0040
with a threshold of 1e−05
rs7742829
rs73128290
rs12404911
rs77285108
All
−0.20
−0.15
−0.10
−0.05
0.00
MR effect size for
'Gut microbiota abundance (genus Catenibacterium id.2153)' on 'Uncharacterized protein KIAA0040'

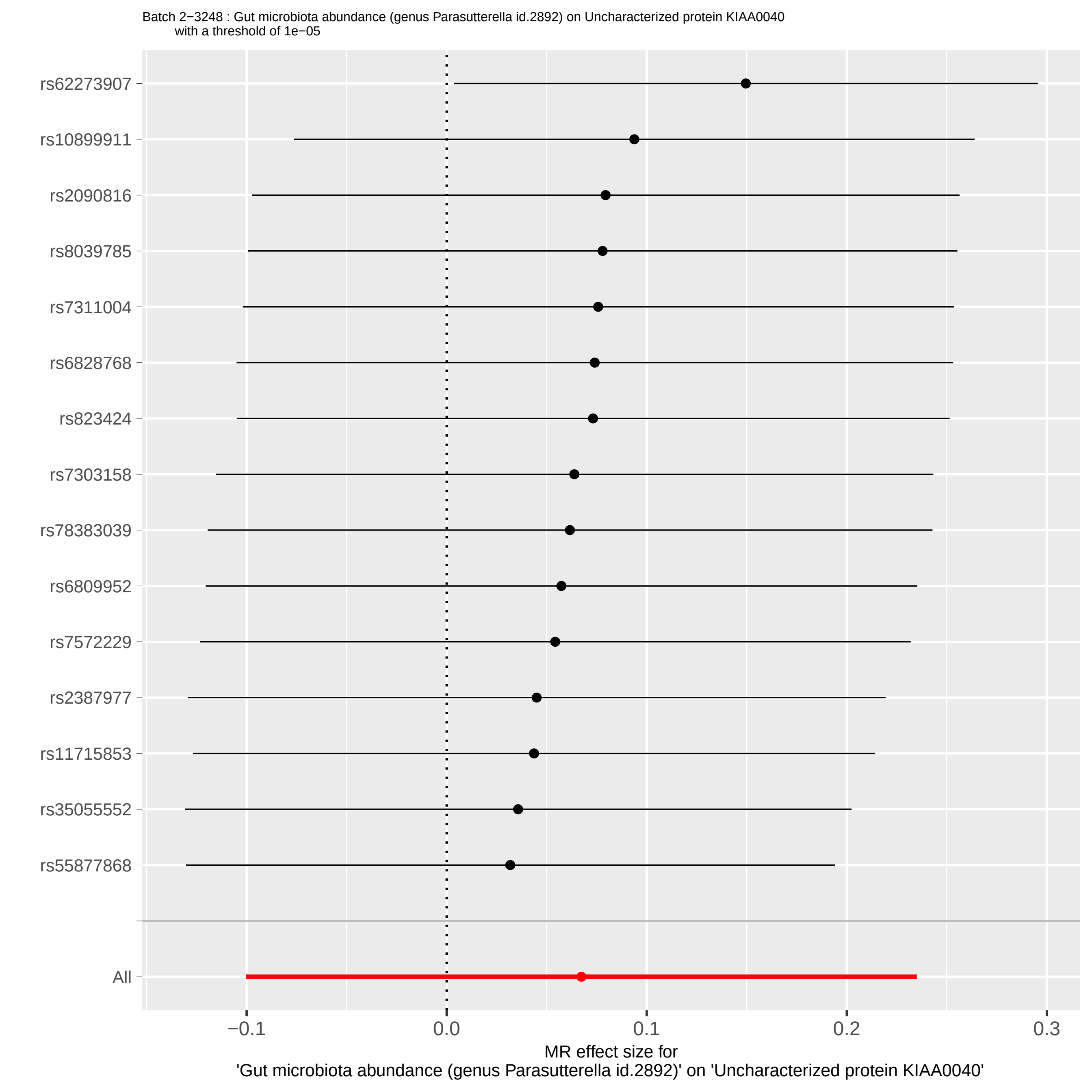
Batch 2−3248 : Gut microbiota abundance (genus Parasutterella id.2892) on Uncharacterized protein KIAA0040
with a threshold of 1e−05
rs62273907
rs10899911
rs2090816
rs8039785
rs7311004
rs6828768
rs823424
rs7303158
rs78383039
rs6809952
rs7572229
rs2387977
rs11715853
rs35055552
rs55877868
All
−0.1
0.0
0.1
0.2
0.3
MR effect size for
'Gut microbiota abundance (genus Parasutterella id.2892)' on 'Uncharacterized protein KIAA0040'

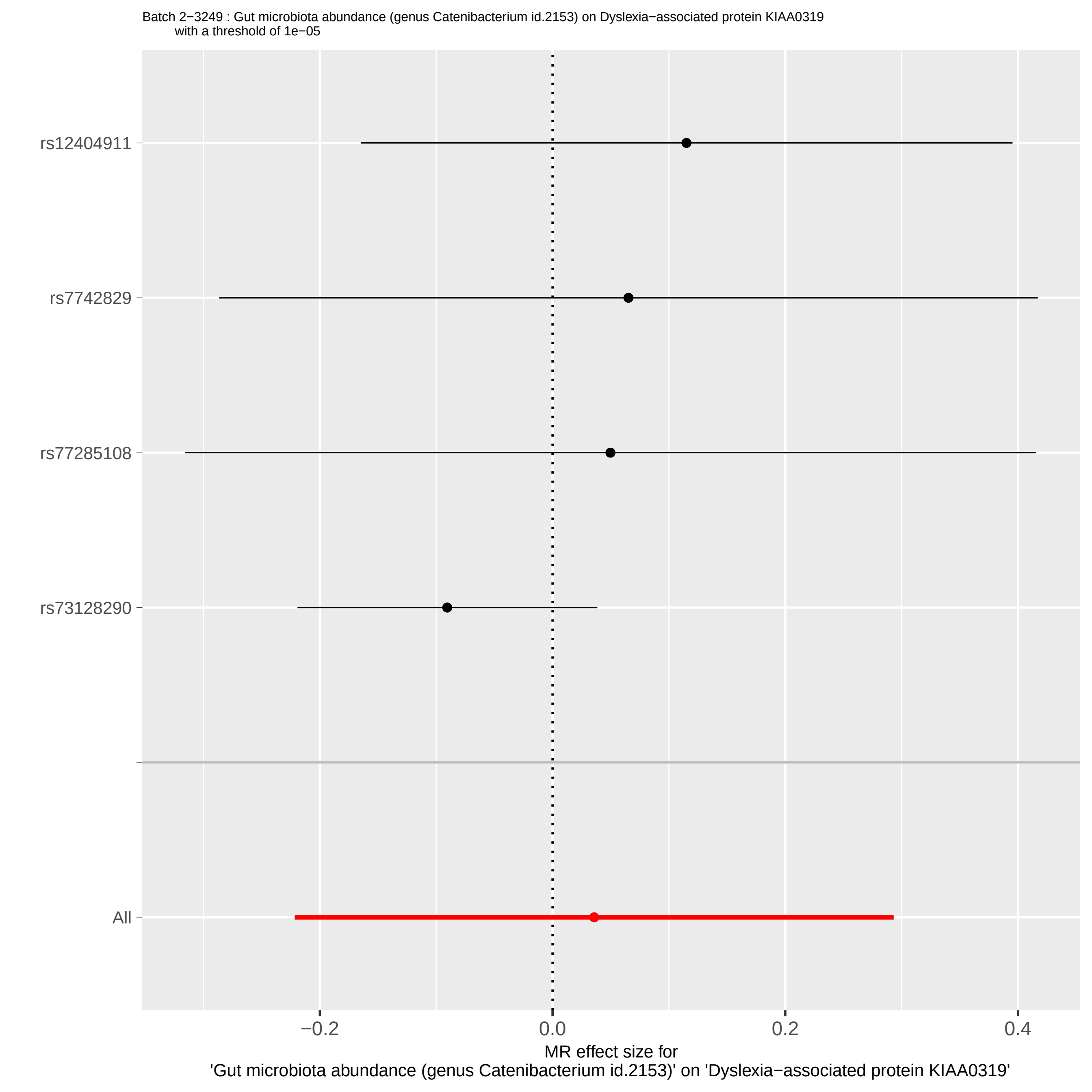
Batch 2−3249 : Gut microbiota abundance (genus Catenibacterium id.2153) on Dyslexia−associated protein KIAA0319
with a threshold of 1e−05
rs12404911
rs7742829
rs77285108
rs73128290
All
−0.2
0.0
0.2
0.4
MR effect size for
'Gut microbiota abundance (genus Catenibacterium id.2153)' on 'Dyslexia−associated protein KIAA0319'

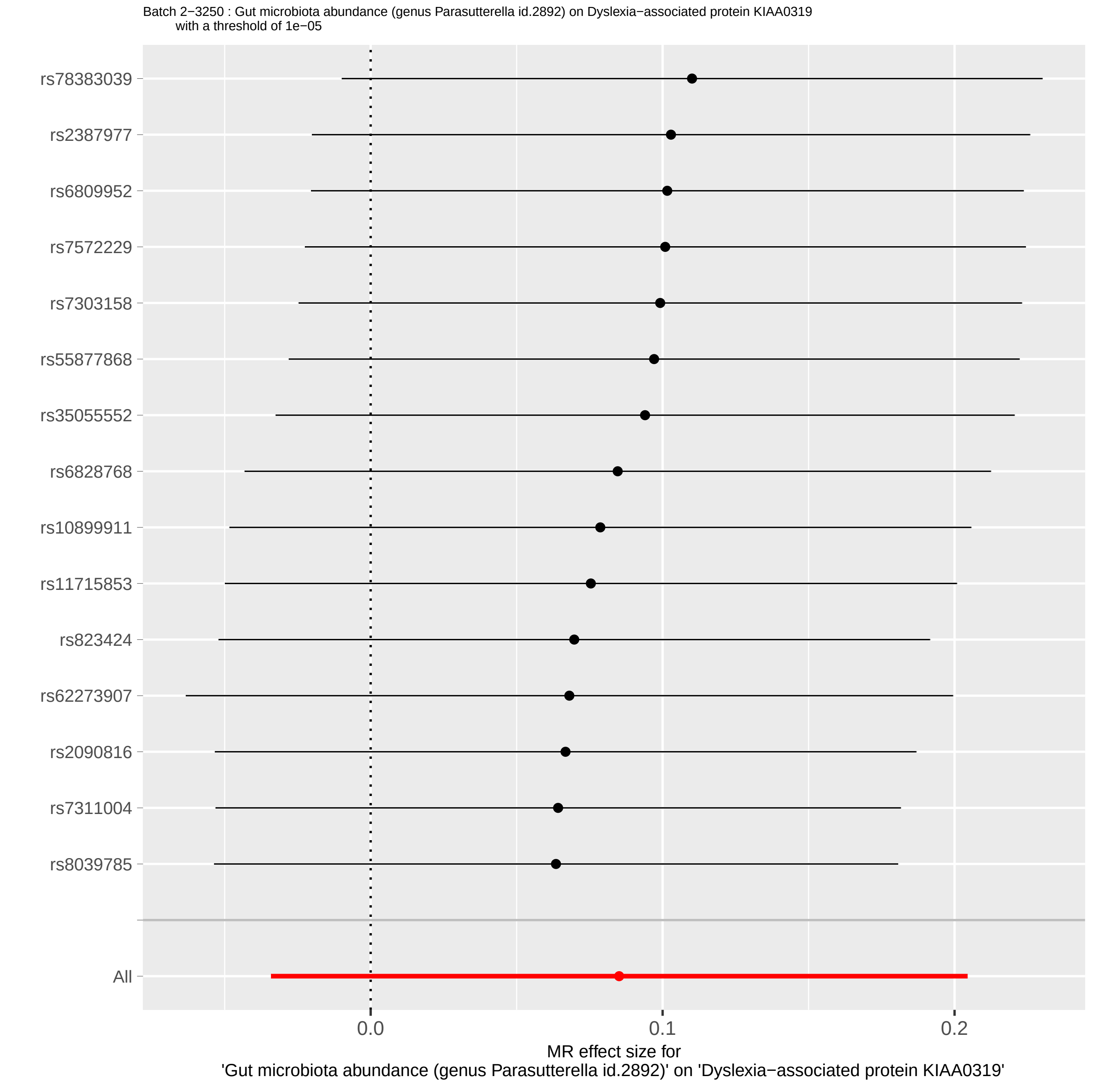
Batch 2−3250 : Gut microbiota abundance (genus Parasutterella id.2892) on Dyslexia−associated protein KIAA0319
with a threshold of 1e−05
rs78383039
rs2387977
rs6809952
rs7572229
rs7303158
rs55877868
rs35055552
rs6828768
rs10899911
rs11715853
rs823424
rs62273907
rs2090816
rs7311004
rs8039785
All
0.0
0.1
0.2
MR effect size for
'Gut microbiota abundance (genus Parasutterella id.2892)' on 'Dyslexia−associated protein KIAA0319'

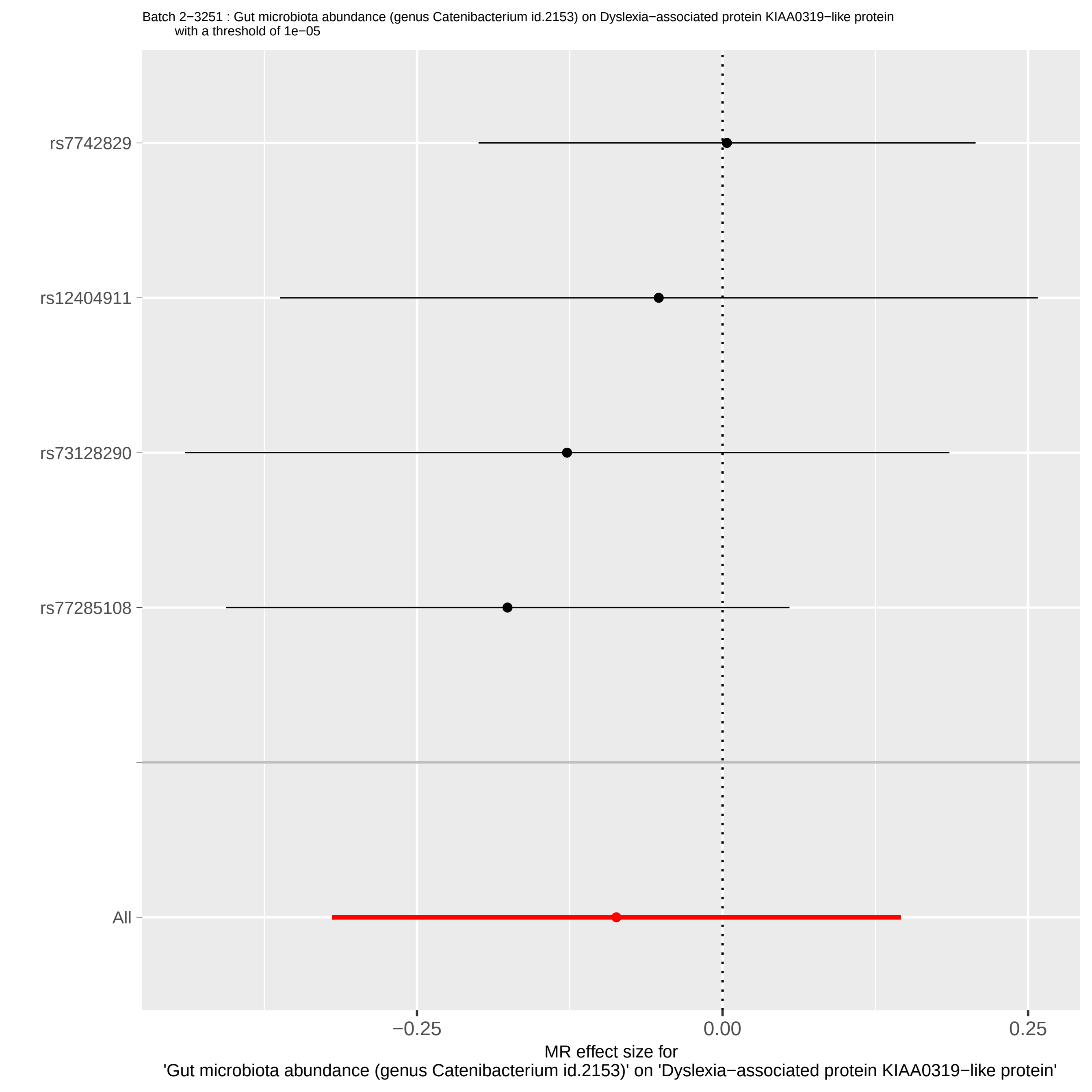

Batch 2−3251 : Gut microbiota abundance (genus Catenibacterium id.2153) on Dyslexia−associated protein KIAA0319−like protein
with a threshold of 1e−05
rs7742829
rs12404911
rs73128290
rs77285108
All
−0.25
0.00
0.25
MR effect size for
'Gut microbiota abundance (genus Catenibacterium id.2153)' on 'Dyslexia−associated protein KIAA0319−like protein'

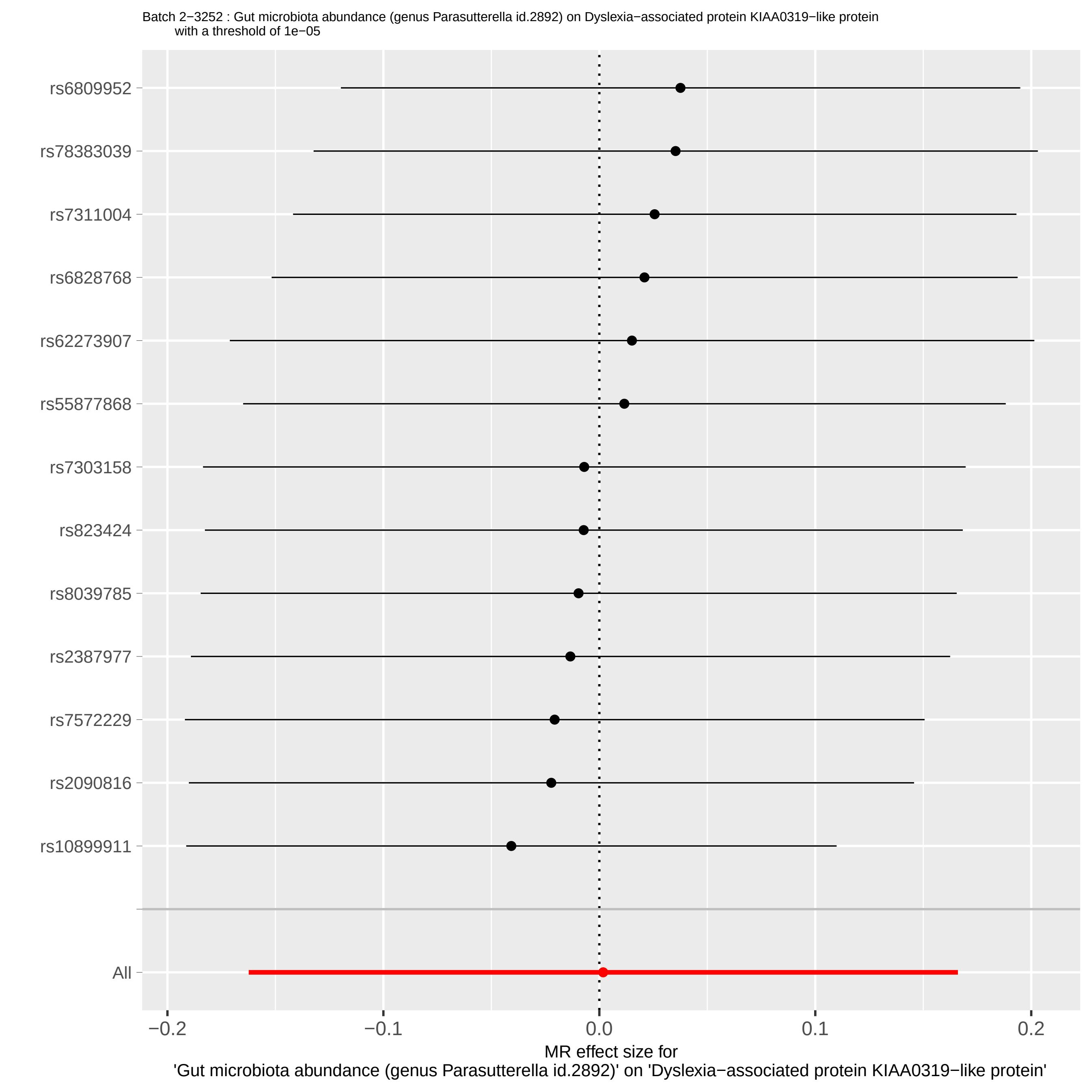

Batch 2−3252 : Gut microbiota abundance (genus Parasutterella id.2892) on Dyslexia−associated protein KIAA0319−like protein
with a threshold of 1e−05
rs6809952
rs78383039
rs7311004
rs6828768
rs62273907
rs55877868
rs7303158
rs823424
rs8039785
rs2387977
rs7572229
rs2090816
rs10899911
All
−0.2
−0.1
0.0
0.1
0.2
MR effect size for
'Gut microbiota abundance (genus Parasutterella id.2892)' on 'Dyslexia−associated protein KIAA0319−like protein'

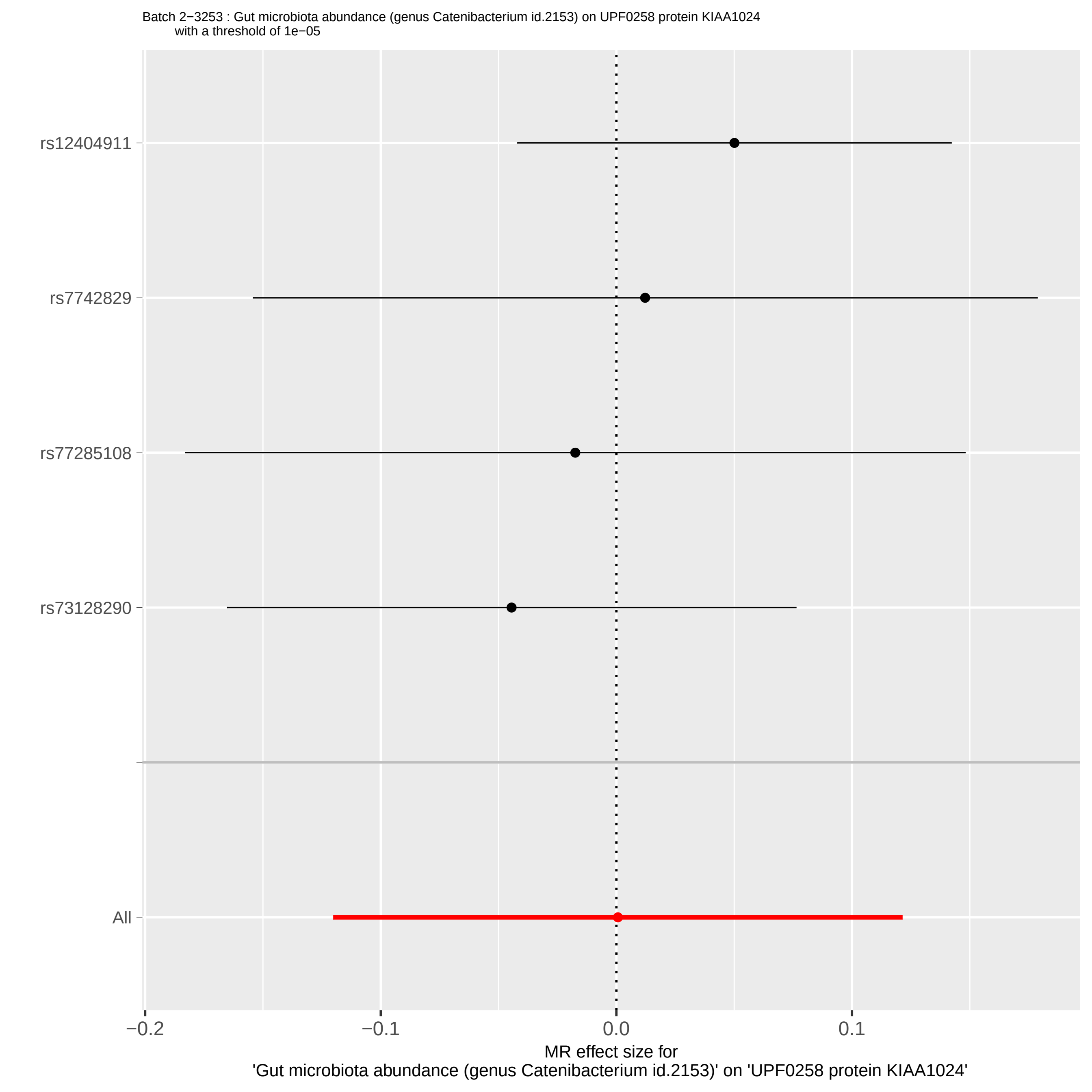

Batch 2−3253 : Gut microbiota abundance (genus Catenibacterium id.2153) on UPF0258 protein KIAA1024
with a threshold of 1e−05
rs12404911
rs7742829
rs77285108
rs73128290
All
−0.2
−0.1
0.0
0.1
MR effect size for
'Gut microbiota abundance (genus Catenibacterium id.2153)' on 'UPF0258 protein KIAA1024'

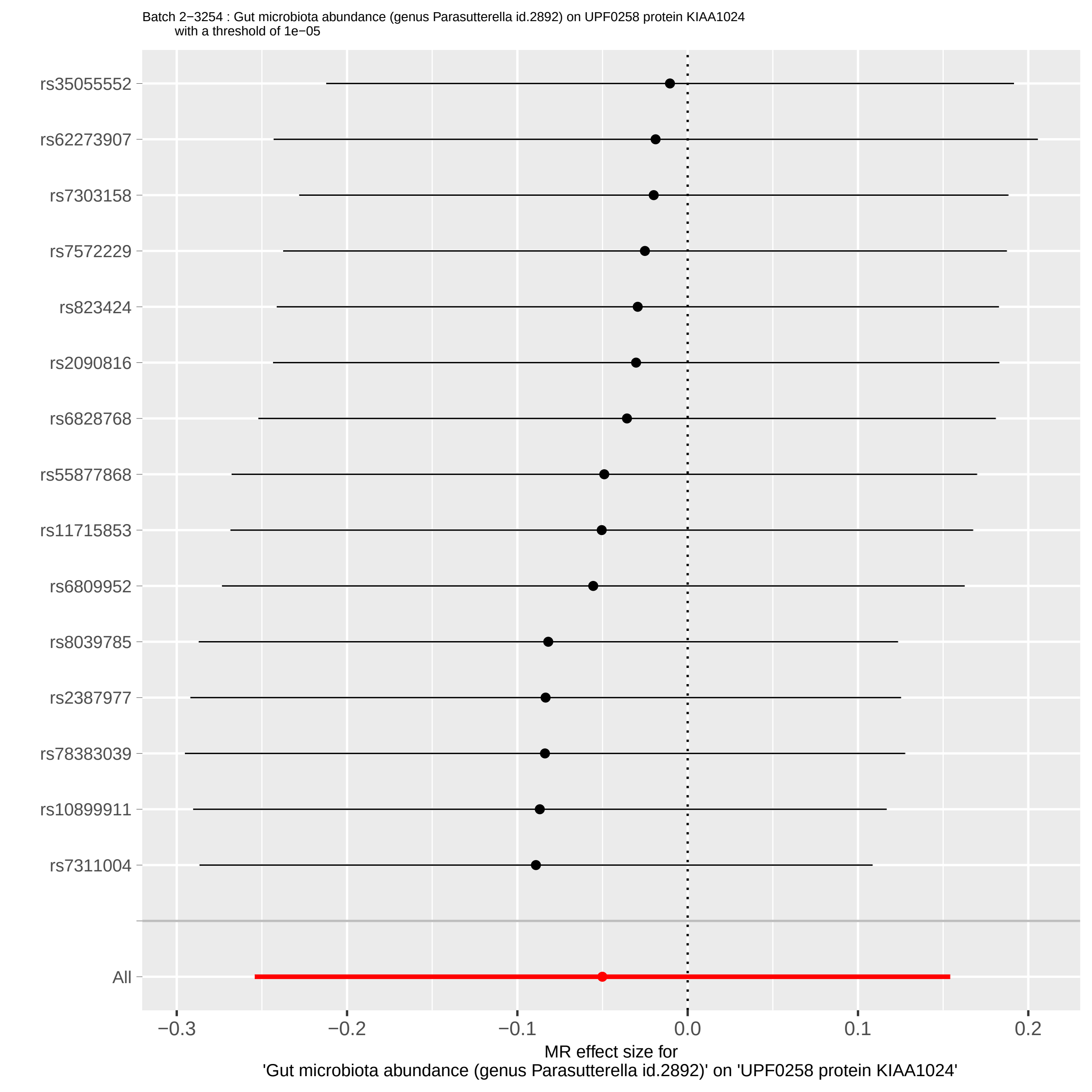

Batch 2−3254 : Gut microbiota abundance (genus Parasutterella id.2892) on UPF0258 protein KIAA1024
with a threshold of 1e−05
rs35055552
rs62273907
rs7303158
rs7572229
rs823424
rs2090816
rs6828768
rs55877868
rs11715853
rs6809952
rs8039785
rs2387977
rs78383039
rs10899911
rs7311004
All
−0.3
−0.2
−0.1
0.0
0.1
0.2
MR effect size for
'Gut microbiota abundance (genus Parasutterella id.2892)' on 'UPF0258 protein KIAA1024'

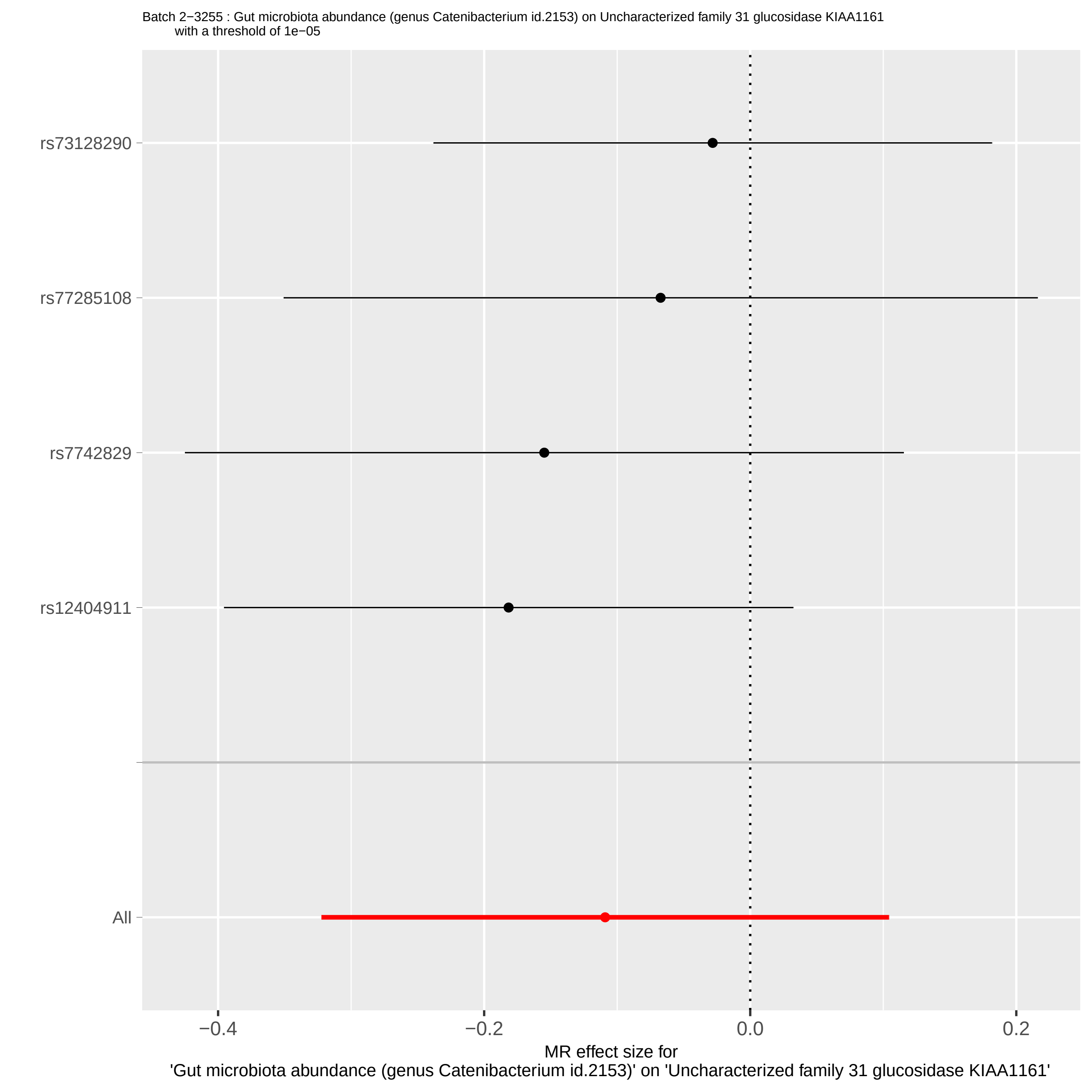
Batch 2−3255 : Gut microbiota abundance (genus Catenibacterium id.2153) on Uncharacterized family 31 glucosidase KIAA1161
with a threshold of 1e−05
rs73128290
rs77285108
rs7742829
rs12404911
All
−0.4
−0.2
0.0
0.2
MR effect size for
'Gut microbiota abundance (genus Catenibacterium id.2153)' on 'Uncharacterized family 31 glucosidase KIAA1161'

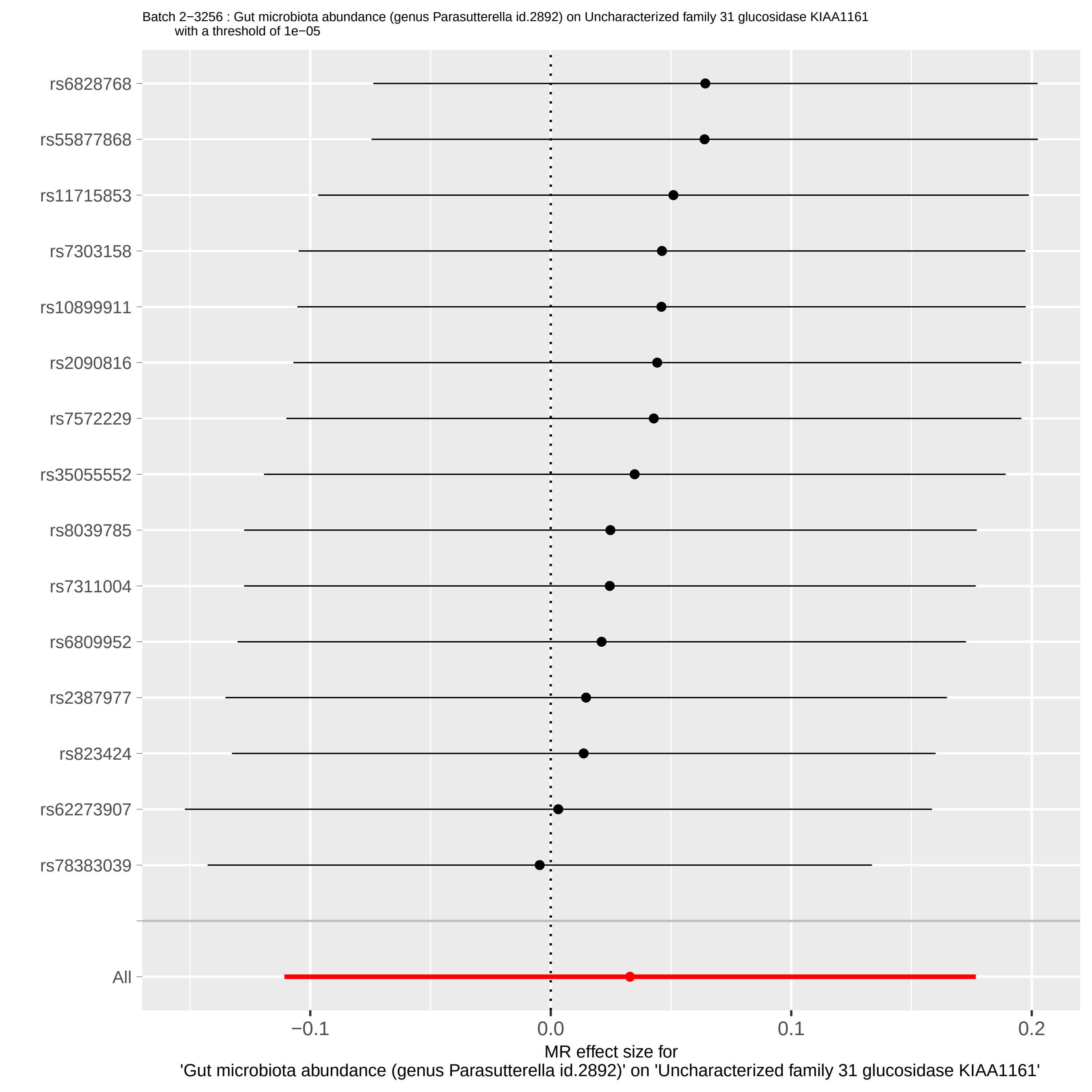

Batch 2−3256 : Gut microbiota abundance (genus Parasutterella id.2892) on Uncharacterized family 31 glucosidase KIAA1161
with a threshold of 1e−05
rs6828768
rs55877868
rs11715853
rs7303158
rs10899911
rs2090816
rs7572229
rs35055552
rs8039785
rs7311004
rs6809952
rs2387977
rs823424
rs62273907
rs78383039
All
−0.1
0.0
0.1
0.2
MR effect size for
'Gut microbiota abundance (genus Parasutterella id.2892)' on 'Uncharacterized family 31 glucosidase KIAA1161'

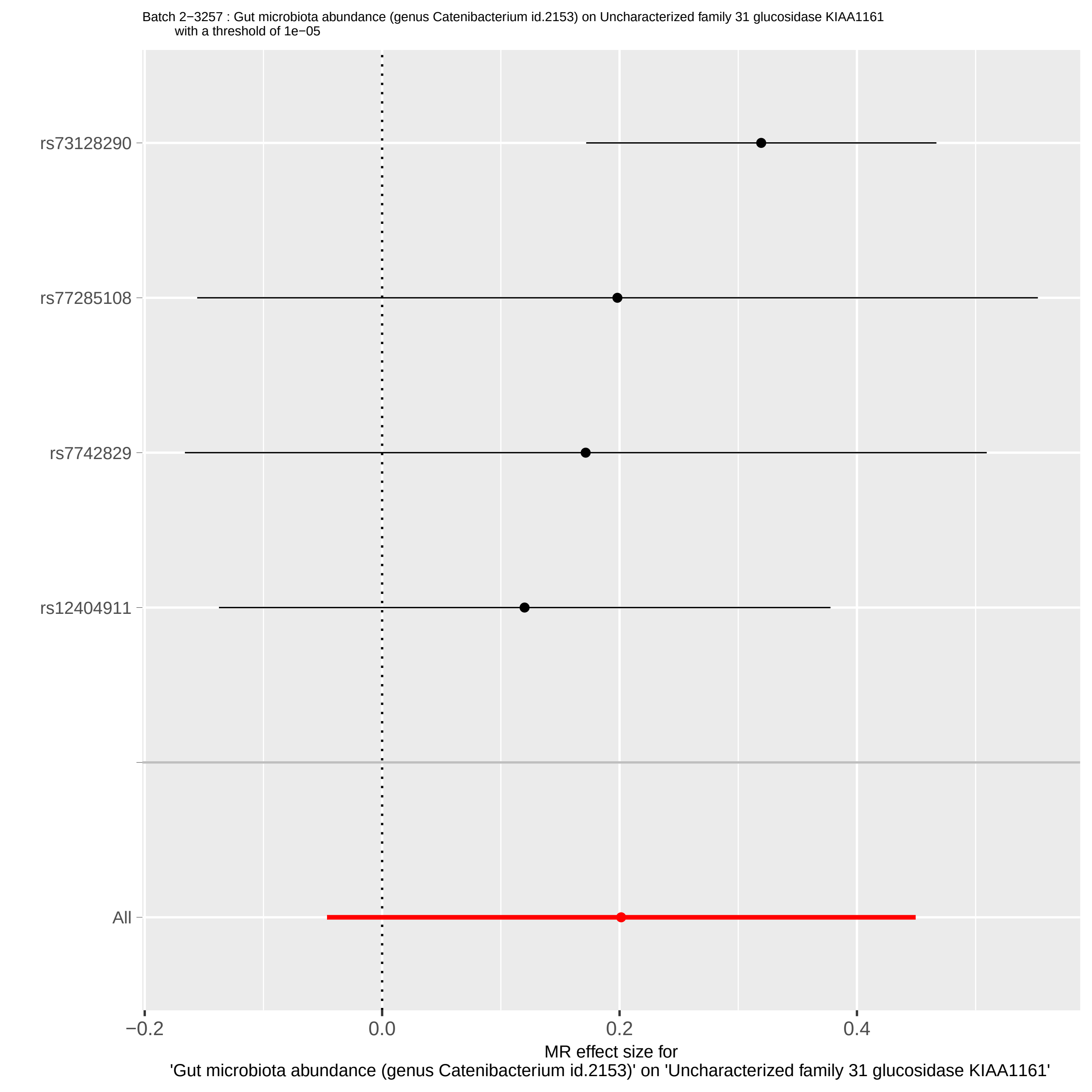
Batch 2−3257 : Gut microbiota abundance (genus Catenibacterium id.2153) on Uncharacterized family 31 glucosidase KIAA1161
with a threshold of 1e−05
rs73128290
rs77285108
rs7742829
rs12404911
All
−0.2
0.0
0.2
0.4
MR effect size for
'Gut microbiota abundance (genus Catenibacterium id.2153)' on 'Uncharacterized family 31 glucosidase KIAA1161'

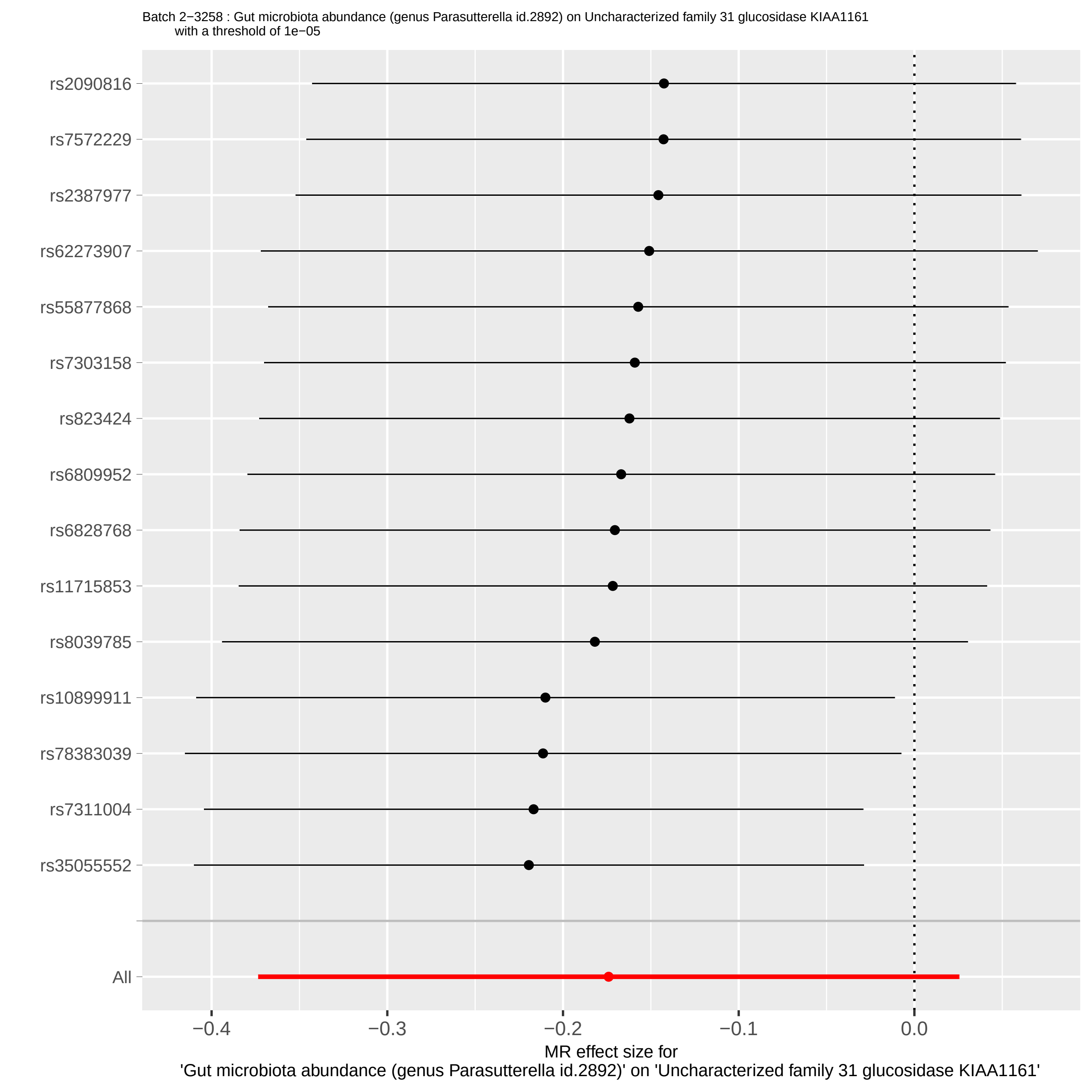
Batch 2−3258 : Gut microbiota abundance (genus Parasutterella id.2892) on Uncharacterized family 31 glucosidase KIAA1161
with a threshold of 1e−05
rs2090816
rs7572229
rs2387977
rs62273907
rs55877868
rs7303158
rs823424
rs6809952
rs6828768
rs11715853
rs8039785
rs10899911
rs78383039
rs7311004
rs35055552
All
−0.4
−0.3
−0.2
−0.1
0.0
MR effect size for
'Gut microbiota abundance (genus Parasutterella id.2892)' on 'Uncharacterized family 31 glucosidase KIAA1161'

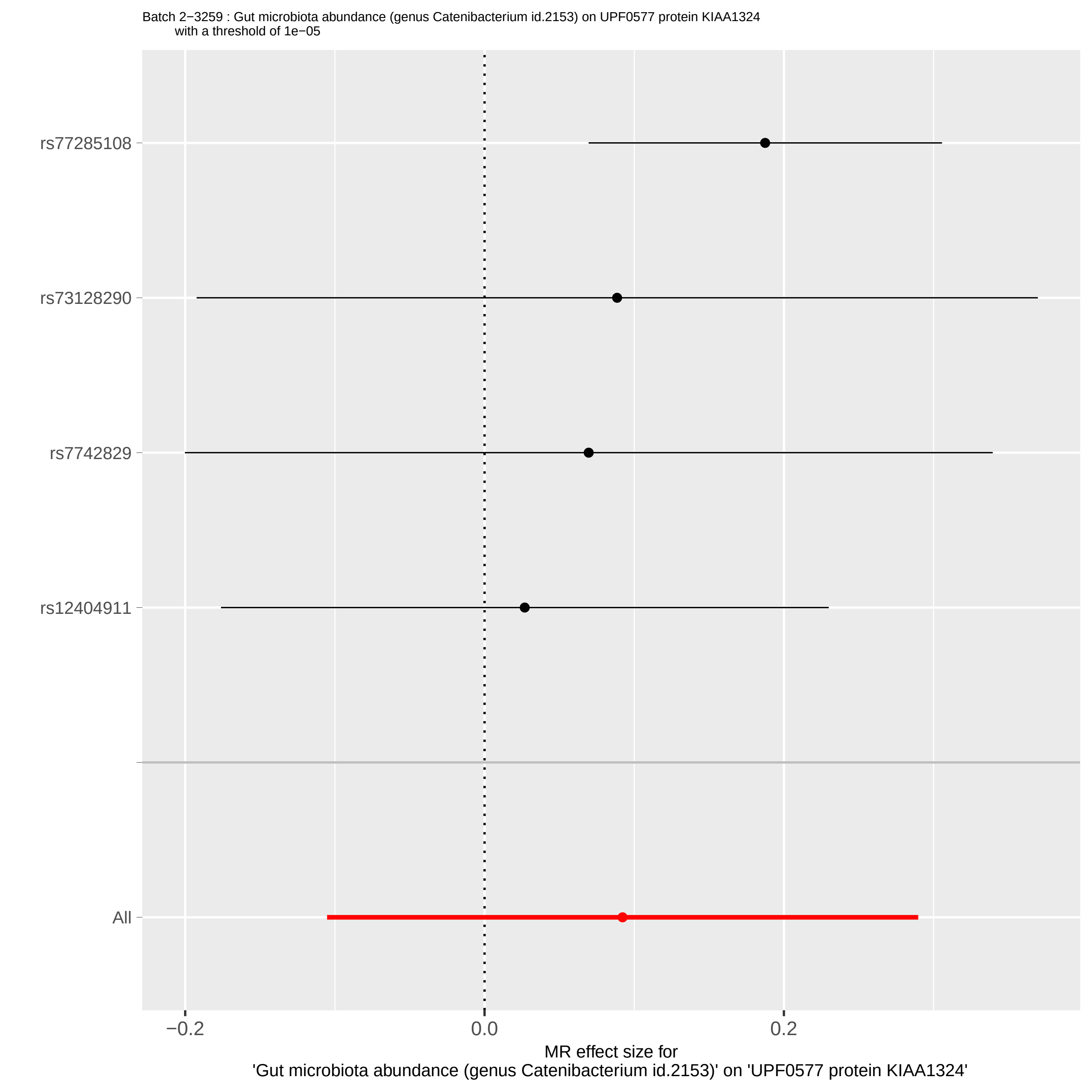
Batch 2−3259 : Gut microbiota abundance (genus Catenibacterium id.2153) on UPF0577 protein KIAA1324
with a threshold of 1e−05
rs77285108
rs73128290
rs7742829
rs12404911
All
−0.2
0.0
0.2
MR effect size for
'Gut microbiota abundance (genus Catenibacterium id.2153)' on 'UPF0577 protein KIAA1324'

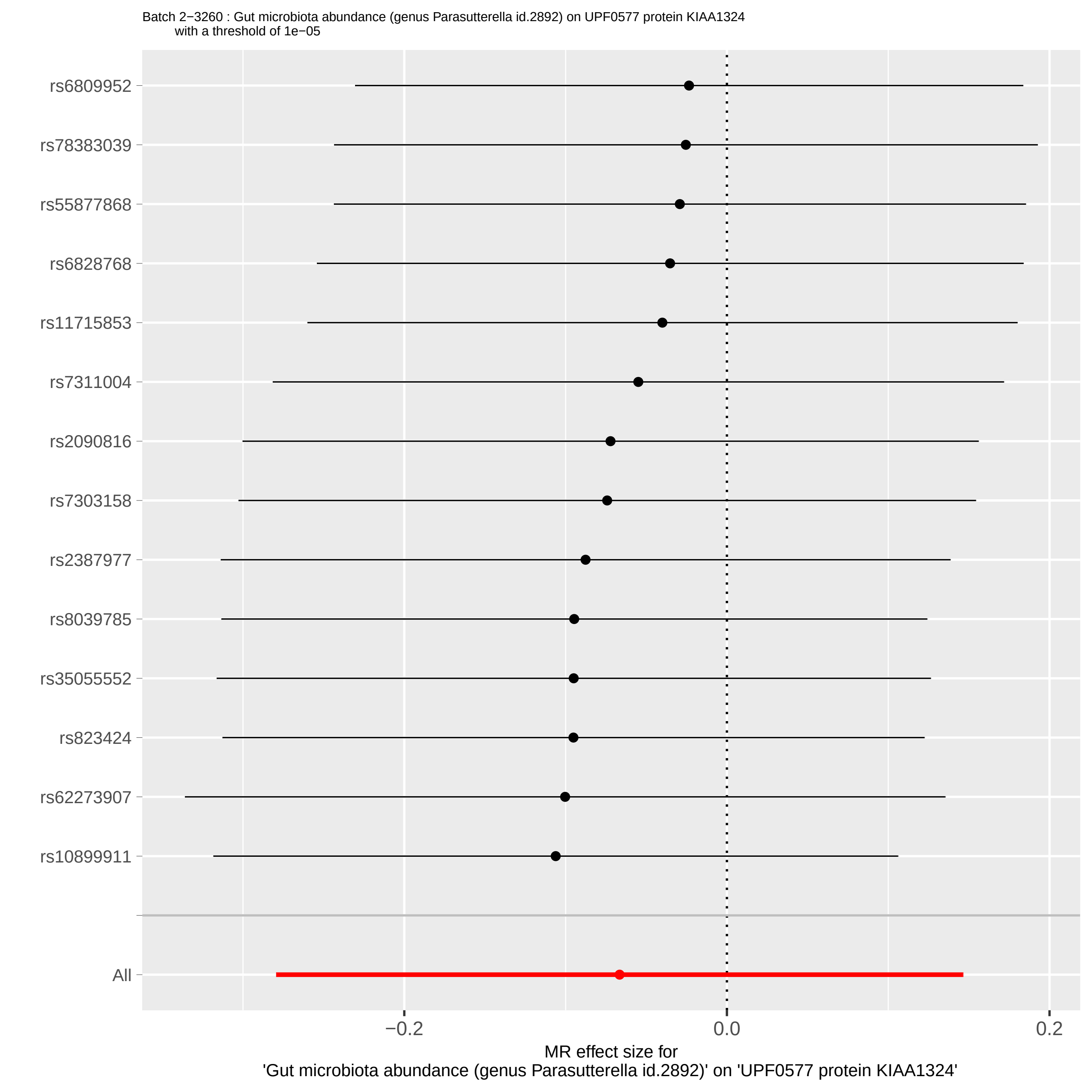
Batch 2−3260 : Gut microbiota abundance (genus Parasutterella id.2892) on UPF0577 protein KIAA1324
with a threshold of 1e−05
rs6809952
rs78383039
rs55877868
rs6828768
rs11715853
rs7311004
rs2090816
rs7303158
rs2387977
rs8039785
rs35055552
rs823424
rs62273907
rs10899911
All
−0.2
0.0
0.2
MR effect size for
'Gut microbiota abundance (genus Parasutterella id.2892)' on 'UPF0577 protein KIAA1324'

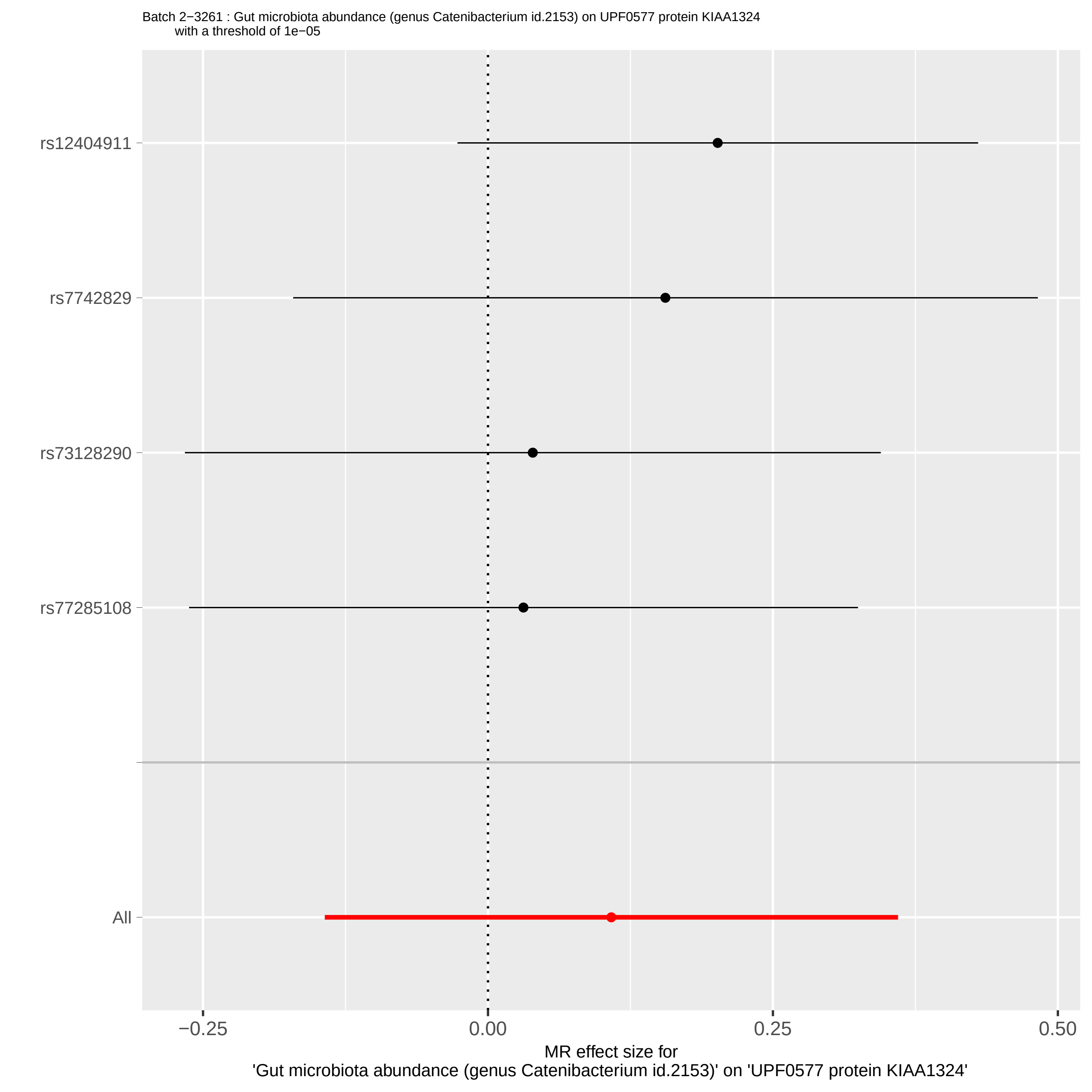
Batch 2−3261 : Gut microbiota abundance (genus Catenibacterium id.2153) on UPF0577 protein KIAA1324
with a threshold of 1e−05
rs12404911
rs7742829
rs73128290
rs77285108
All
−0.25
0.00
0.25
0.50
MR effect size for
'Gut microbiota abundance (genus Catenibacterium id.2153)' on 'UPF0577 protein KIAA1324'

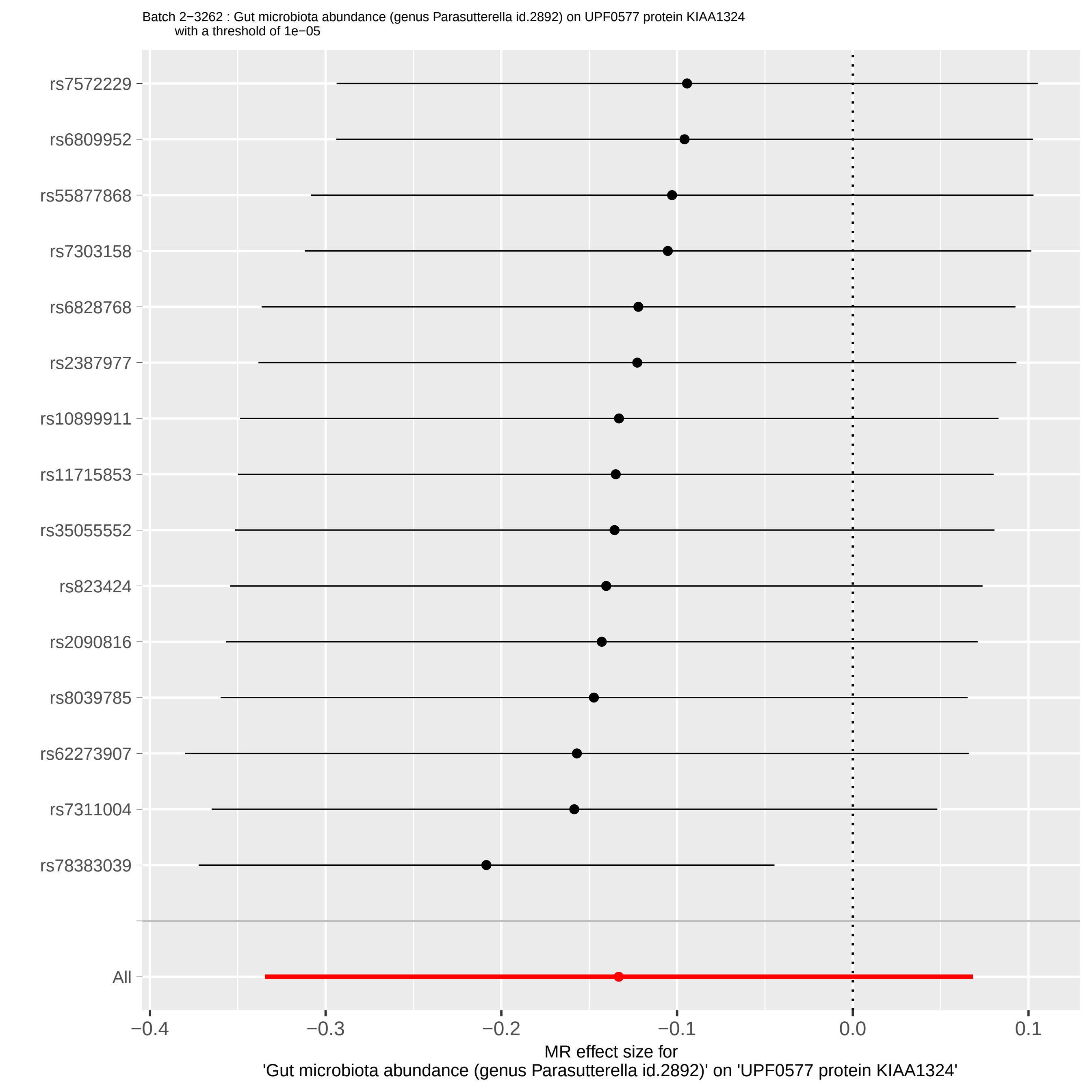
Batch 2−3262 : Gut microbiota abundance (genus Parasutterella id.2892) on UPF0577 protein KIAA1324
with a threshold of 1e−05
rs7572229
rs6809952
rs55877868
rs7303158
rs6828768
rs2387977
rs10899911
rs11715853
rs35055552
rs823424
rs2090816
rs8039785
rs62273907
rs7311004
rs78383039
All
−0.4
−0.3
−0.2
−0.1
0.0
0.1
MR effect size for
'Gut microbiota abundance (genus Parasutterella id.2892)' on 'UPF0577 protein KIAA1324'

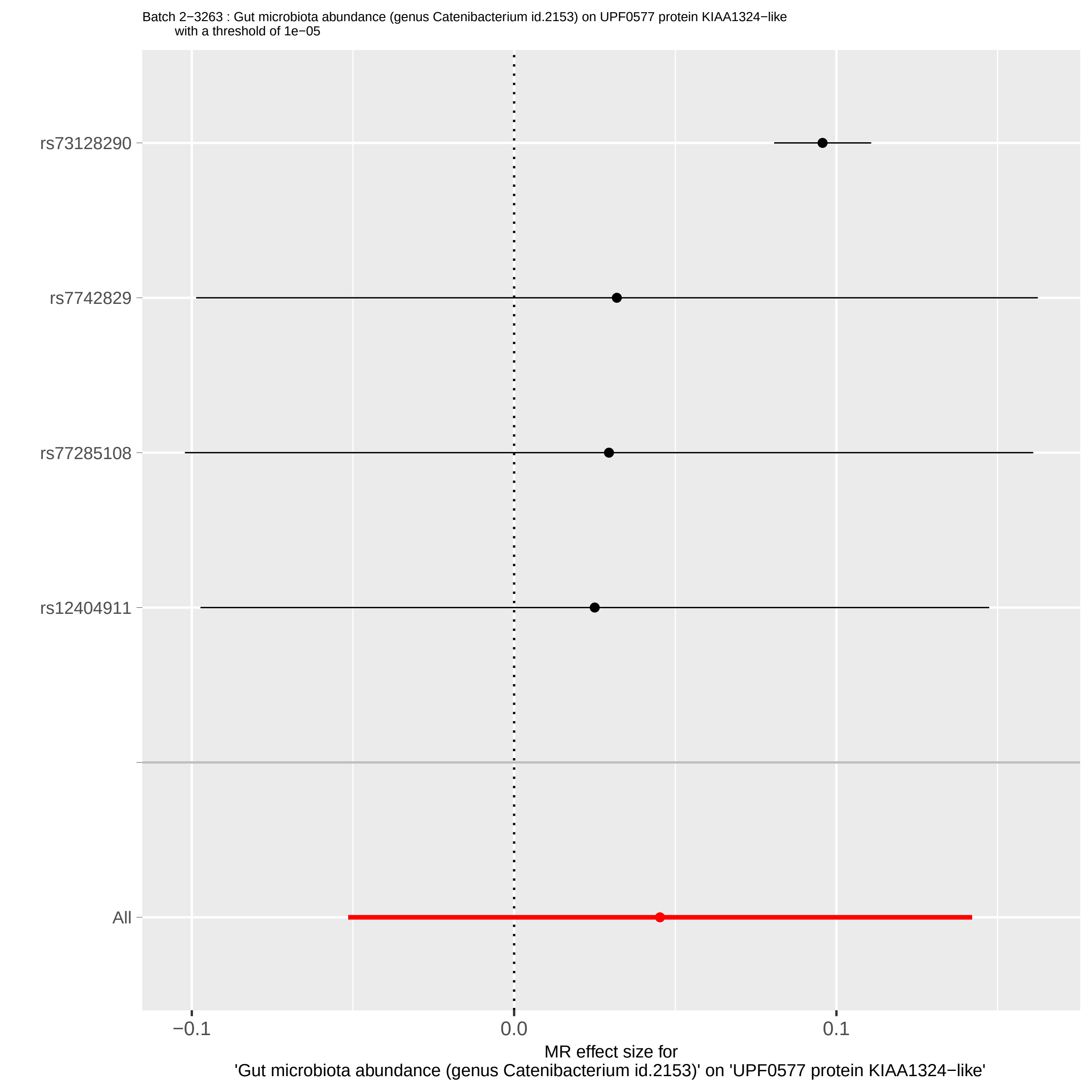
Batch 2−3263 : Gut microbiota abundance (genus Catenibacterium id.2153) on UPF0577 protein KIAA1324−like
with a threshold of 1e−05
rs73128290
rs7742829
rs77285108
rs12404911
All
−0.1
0.0
0.1
MR effect size for
'Gut microbiota abundance (genus Catenibacterium id.2153)' on 'UPF0577 protein KIAA1324−like'

Batch 2−3264 : Gut microbiota abundance (genus Parasutterella id.2892) on UPF0577 protein KIAA1324−like
with a threshold of 1e−05

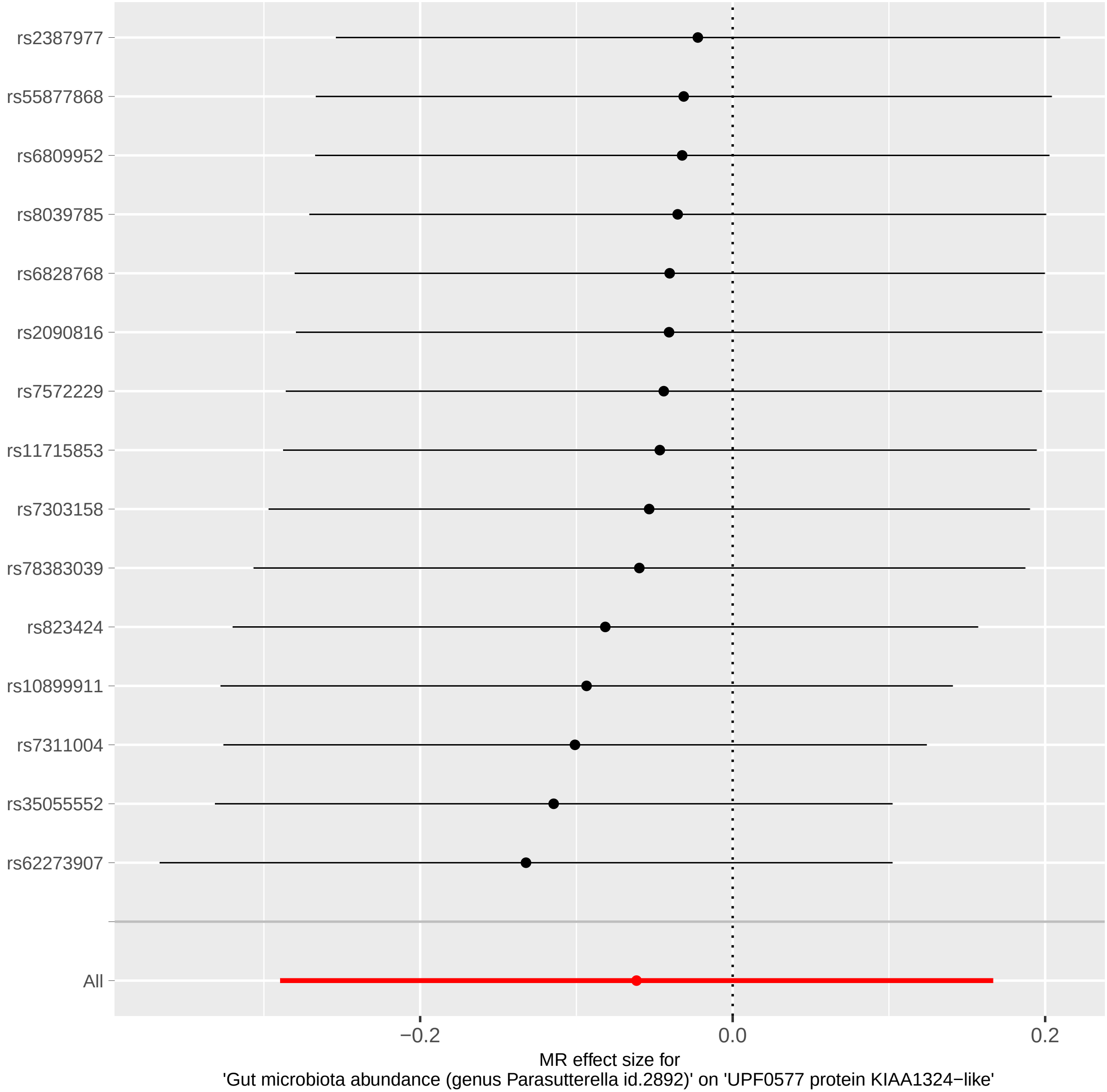

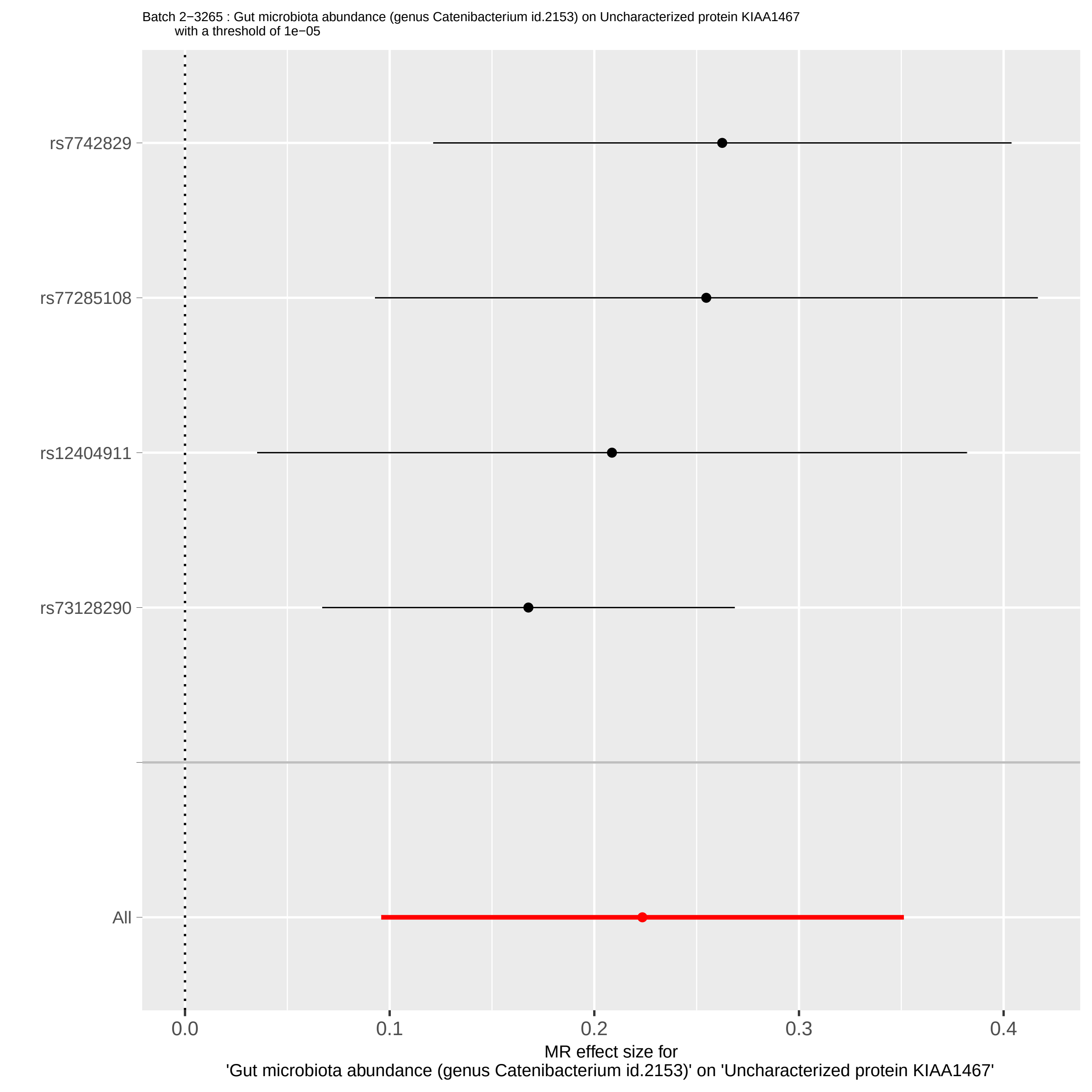
Batch 2−3265 : Gut microbiota abundance (genus Catenibacterium id.2153) on Uncharacterized protein KIAA1467
with a threshold of 1e−05
rs7742829
rs77285108
rs12404911
rs73128290
All
0.0
0.1
0.2
0.3
0.4
MR effect size for
'Gut microbiota abundance (genus Catenibacterium id.2153)' on 'Uncharacterized protein KIAA1467'

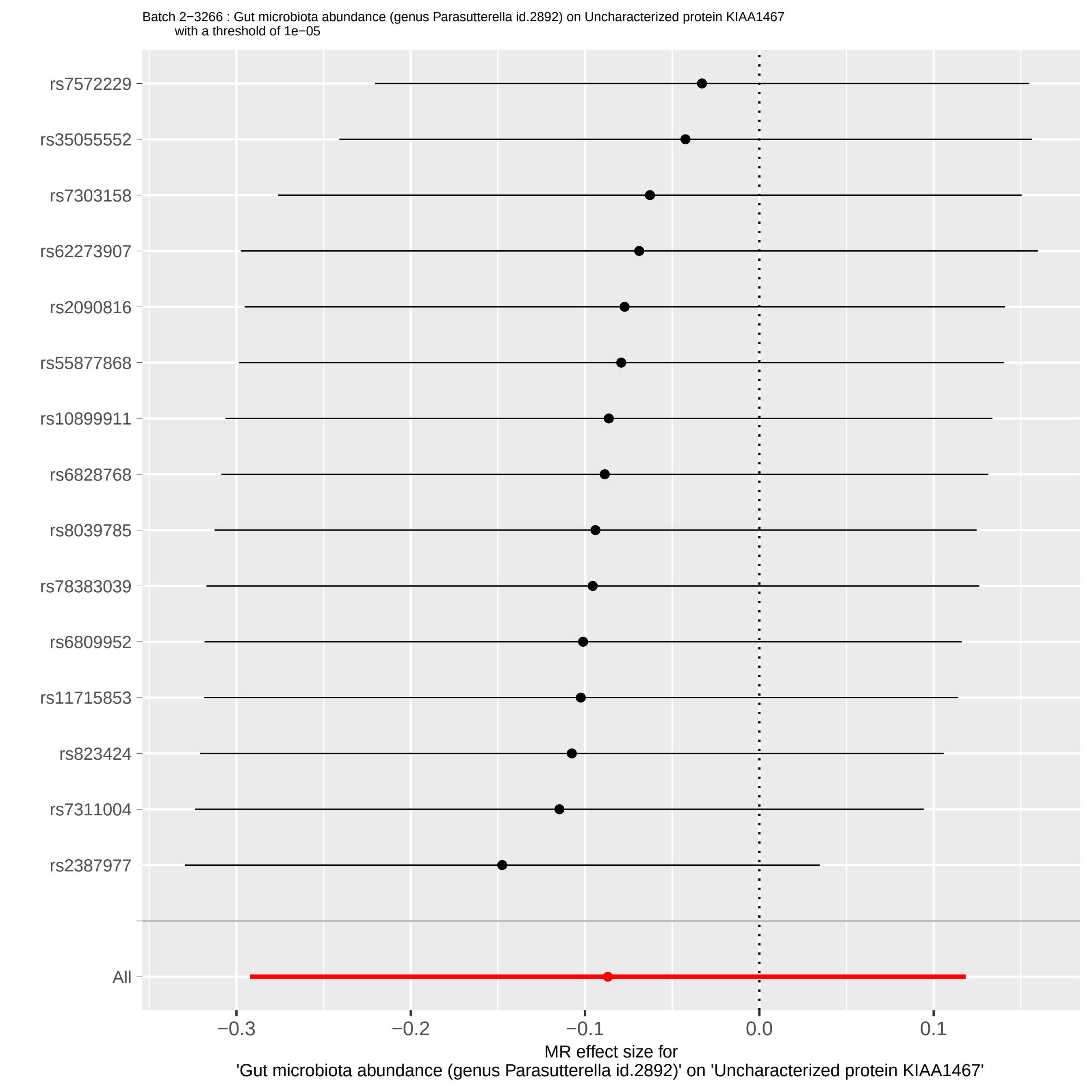
Batch 2−3266 : Gut microbiota abundance (genus Parasutterella id.2892) on Uncharacterized protein KIAA1467
with a threshold of 1e−05
rs7572229
rs35055552
rs7303158
rs62273907
rs2090816
rs55877868
rs10899911
rs6828768
rs8039785
rs78383039
rs6809952
rs11715853
rs823424
rs7311004
rs2387977
All
−0.3
−0.2
−0.1
0.0
0.1
MR effect size for
'Gut microbiota abundance (genus Parasutterella id.2892)' on 'Uncharacterized protein KIAA1467'

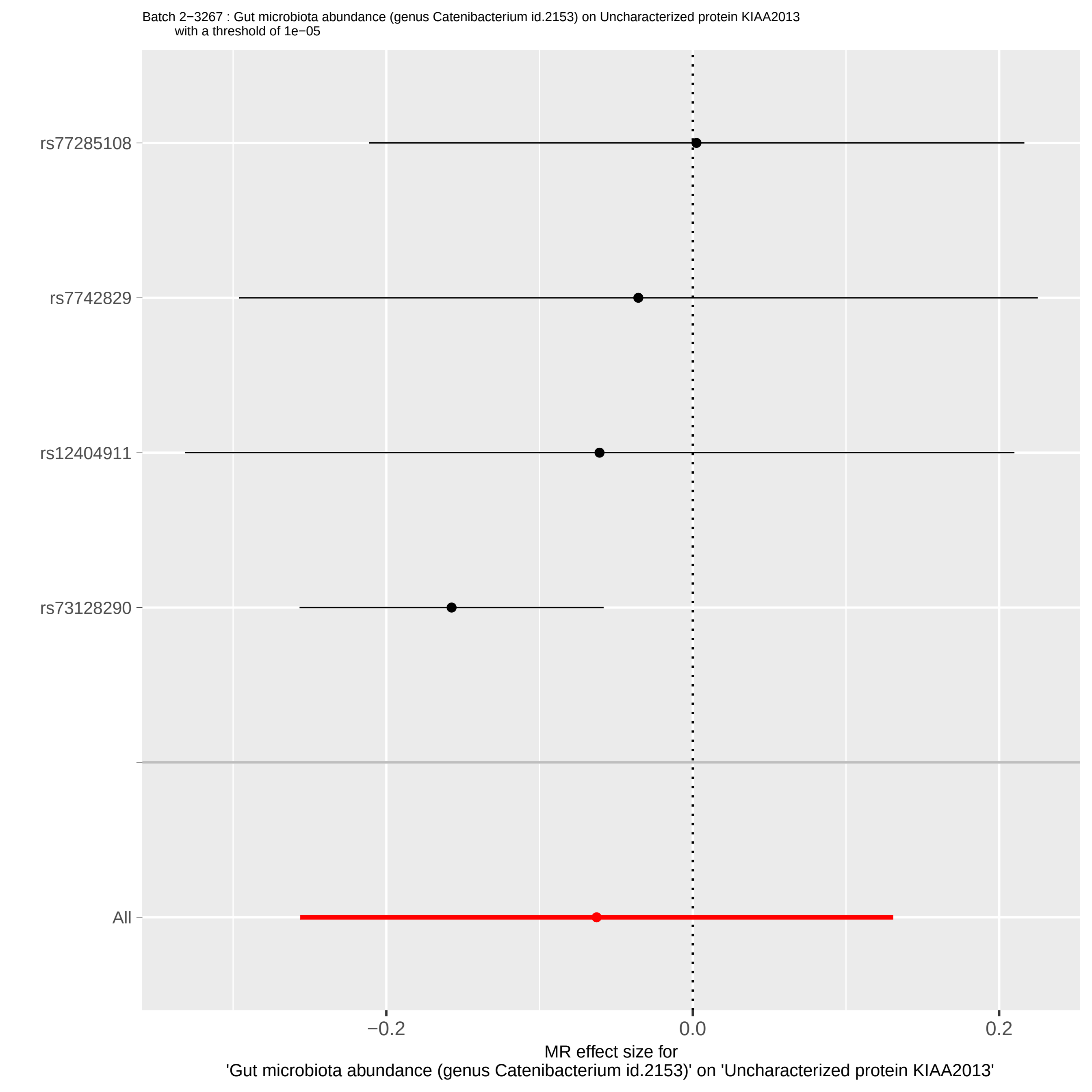
Batch 2−3267 : Gut microbiota abundance (genus Catenibacterium id.2153) on Uncharacterized protein KIAA2013
with a threshold of 1e−05
rs77285108
rs7742829
rs12404911
rs73128290
All
−0.2
0.0
0.2
MR effect size for
'Gut microbiota abundance (genus Catenibacterium id.2153)' on 'Uncharacterized protein KIAA2013'

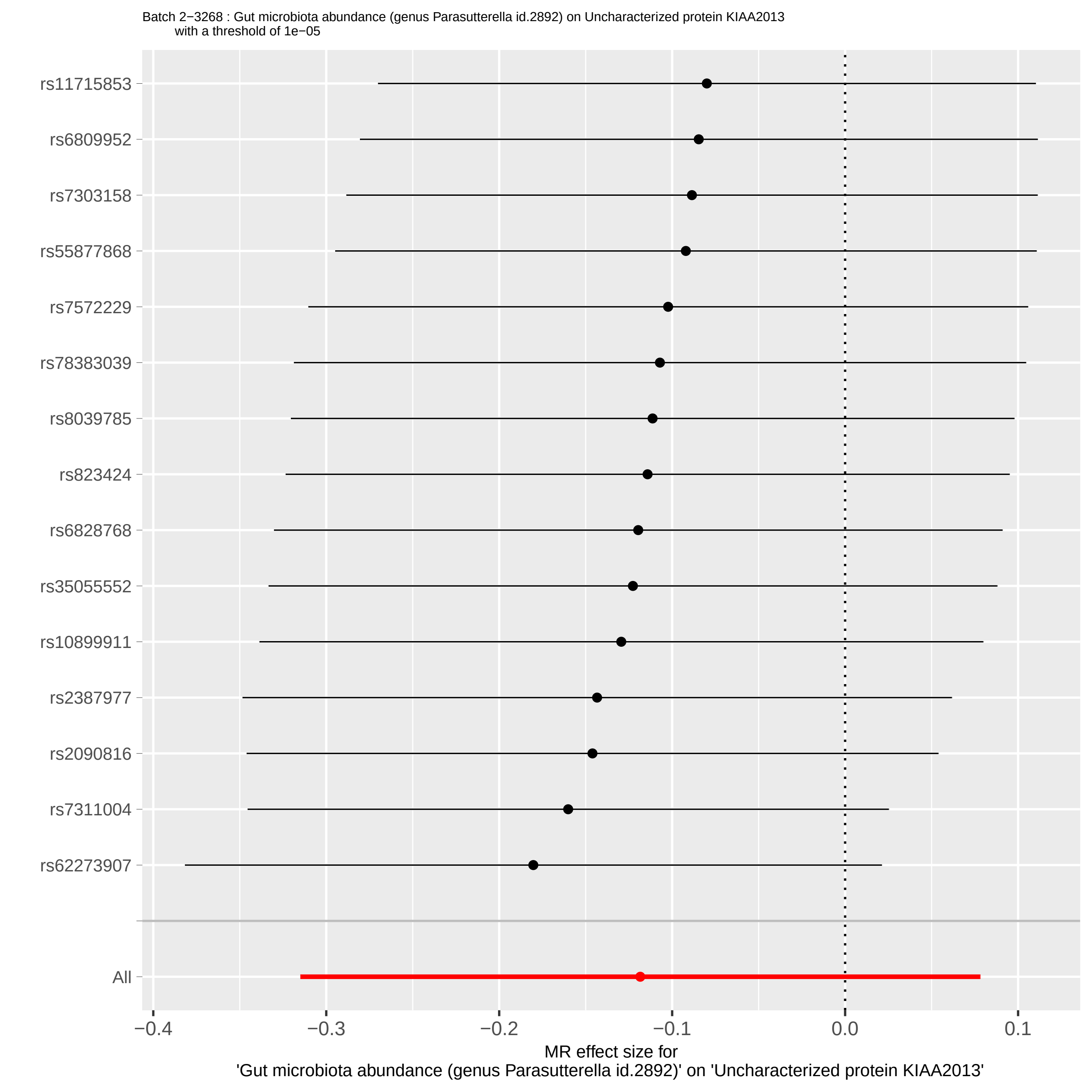
Batch 2−3268 : Gut microbiota abundance (genus Parasutterella id.2892) on Uncharacterized protein KIAA2013
with a threshold of 1e−05
rs11715853
rs6809952
rs7303158
rs55877868
rs7572229
rs78383039
rs8039785
rs823424
rs6828768
rs35055552
rs10899911
rs2387977
rs2090816
rs7311004
rs62273907
All
−0.4
−0.3
−0.2
−0.1
0.0
0.1
MR effect size for
'Gut microbiota abundance (genus Parasutterella id.2892)' on 'Uncharacterized protein KIAA2013'

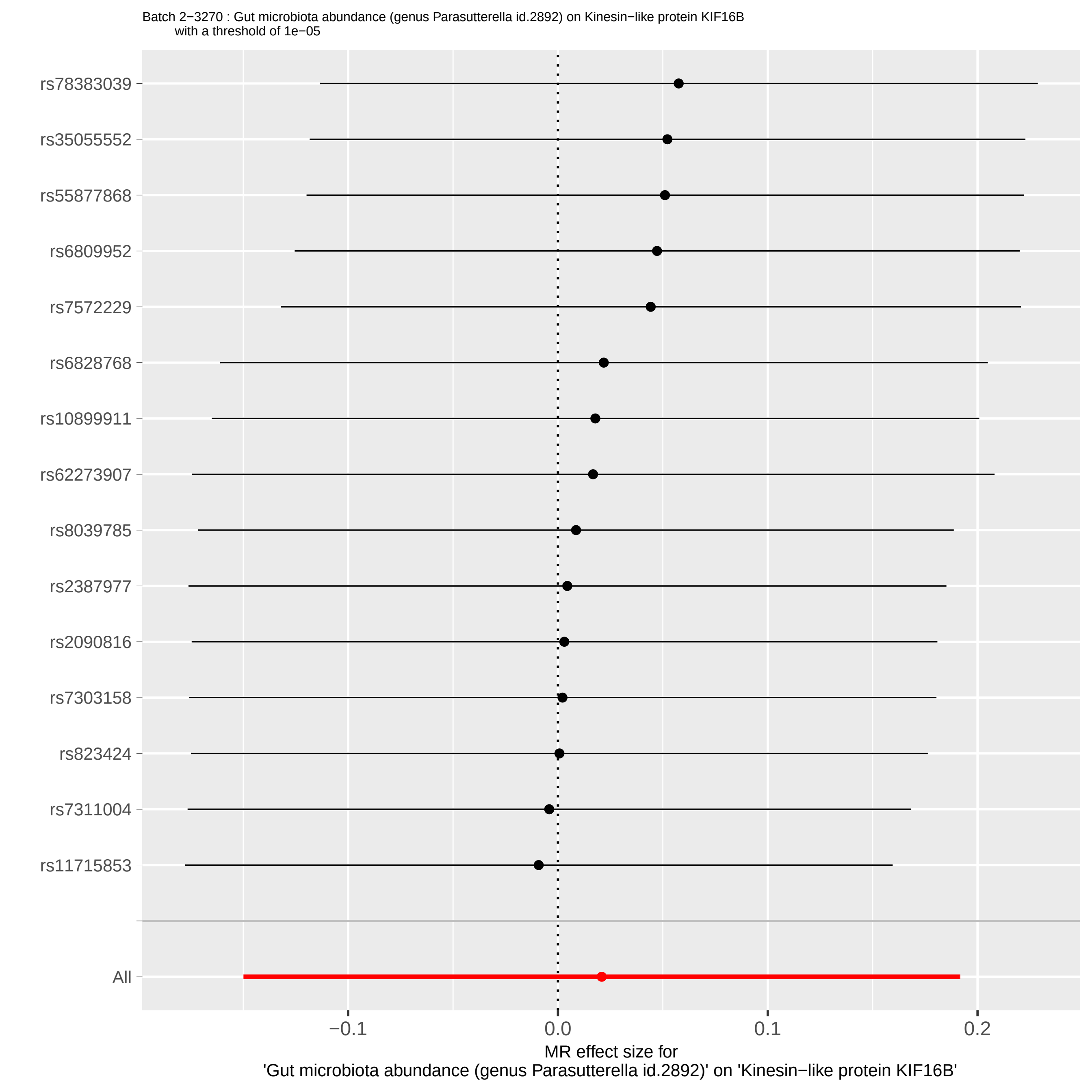
Batch 2−3270 : Gut microbiota abundance (genus Parasutterella id.2892) on Kinesin−like protein KIF16B
with a threshold of 1e−05
rs78383039
rs35055552
rs55877868
rs6809952
rs7572229
rs6828768
rs10899911
rs62273907
rs8039785
rs2387977
rs2090816
rs7303158
rs823424
rs7311004
rs11715853
All
−0.1
0.0
0.1
0.2
MR effect size for
'Gut microbiota abundance (genus Parasutterella id.2892)' on 'Kinesin−like protein KIF16B'

Batch 2−3271 : Gut microbiota abundance (genus Catenibacterium id.2153) on Kinesin−like protein KIF1C
with a threshold of 1e−05

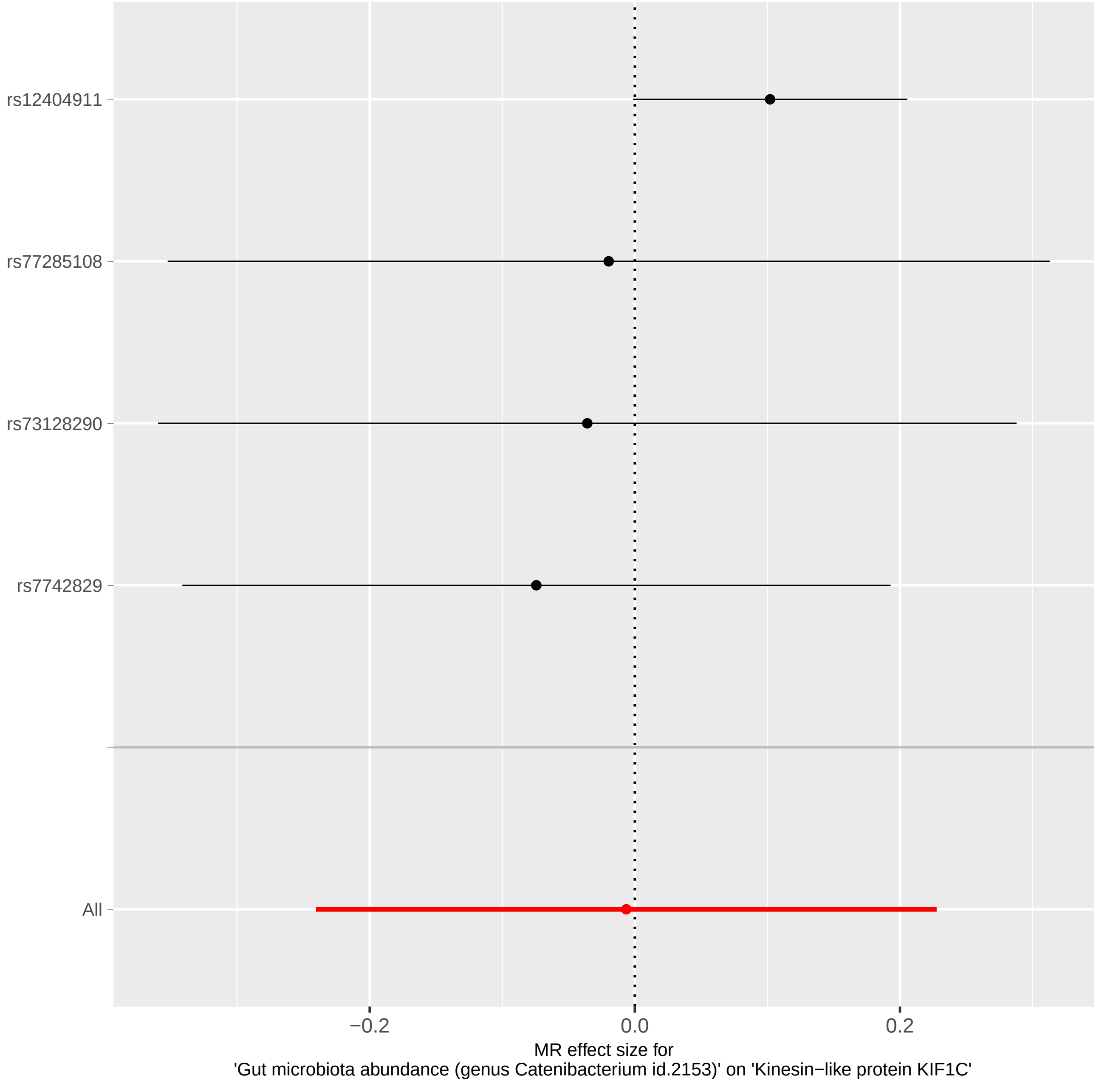

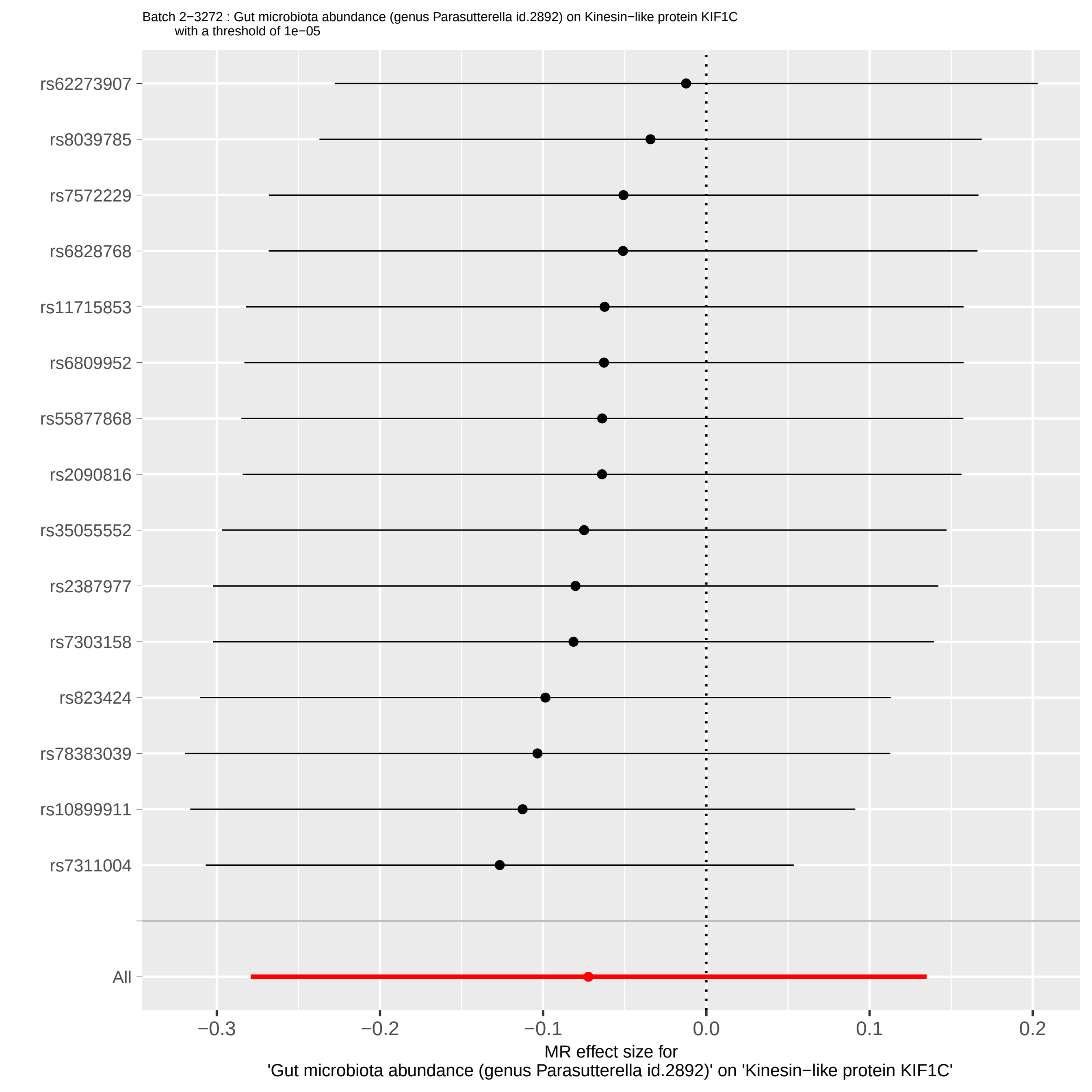
Batch 2−3272 : Gut microbiota abundance (genus Parasutterella id.2892) on Kinesin−like protein KIF1C
with a threshold of 1e−05
rs62273907
rs8039785
rs7572229
rs6828768
rs11715853
rs6809952
rs55877868
rs2090816
rs35055552
rs2387977
rs7303158
rs823424
rs78383039
rs10899911
rs7311004
All
−0.3
−0.2
−0.1
0.0
0.1
0.2
MR effect size for
'Gut microbiota abundance (genus Parasutterella id.2892)' on 'Kinesin−like protein KIF1C'

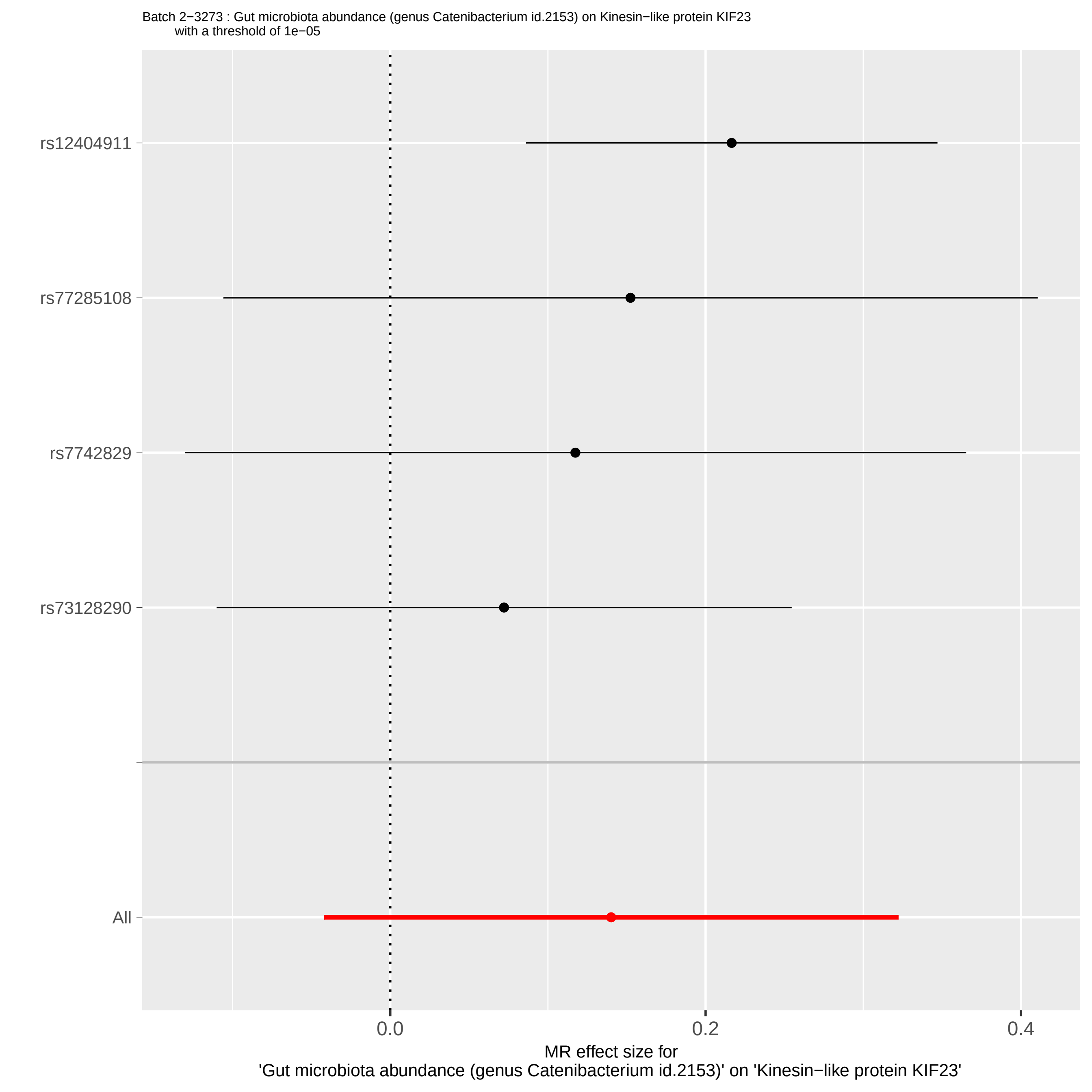
Batch 2−3273 : Gut microbiota abundance (genus Catenibacterium id.2153) on Kinesin−like protein KIF23
with a threshold of 1e−05
rs12404911
rs77285108
rs7742829
rs73128290
All
0.0
0.2
0.4
MR effect size for
'Gut microbiota abundance (genus Catenibacterium id.2153)' on 'Kinesin−like protein KIF23'

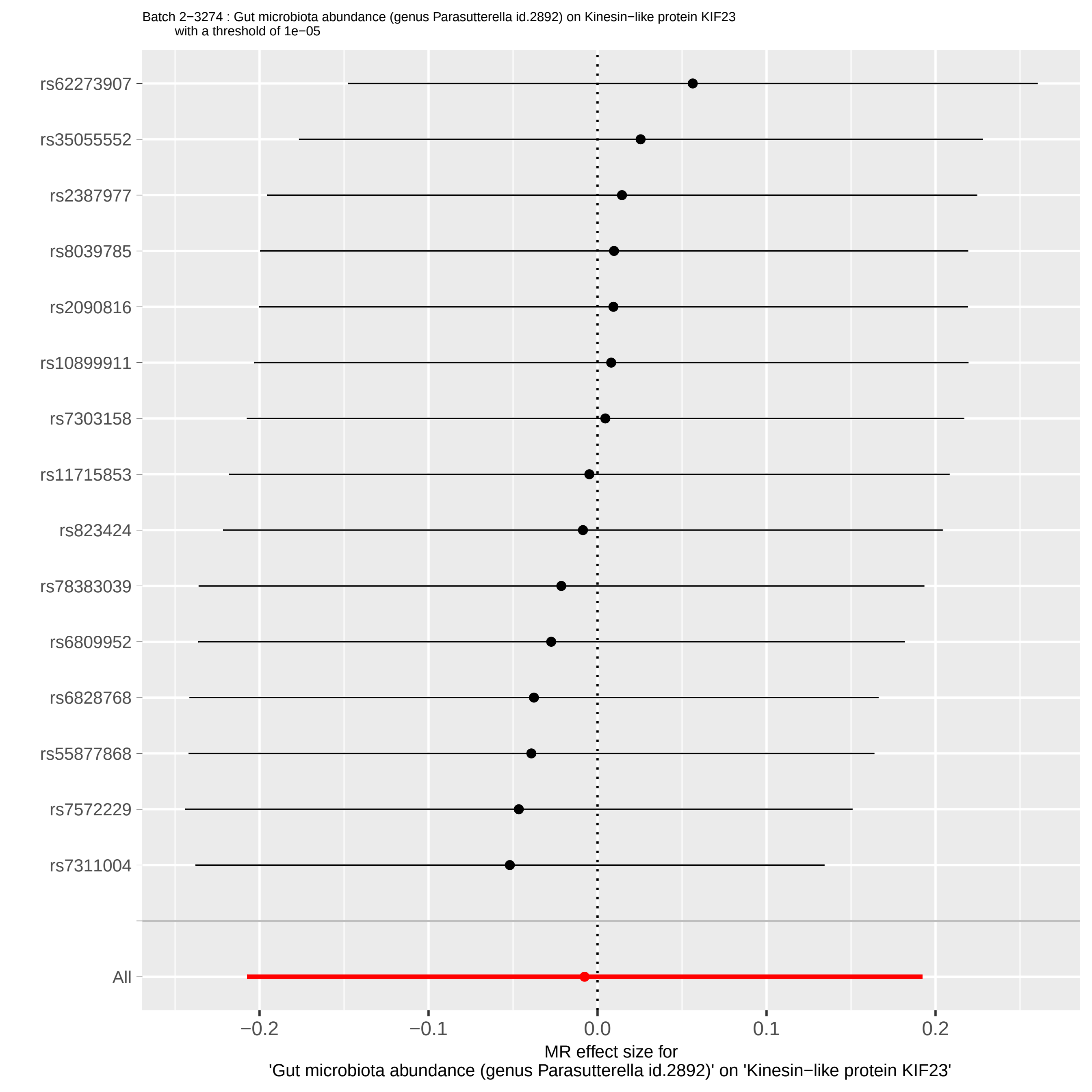

Batch 2−3274 : Gut microbiota abundance (genus Parasutterella id.2892) on Kinesin−like protein KIF23
with a threshold of 1e−05
rs62273907
rs35055552
rs2387977
rs8039785
rs2090816
rs10899911
rs7303158
rs11715853
rs823424
rs78383039
rs6809952
rs6828768
rs55877868
rs7572229
rs7311004
All
−0.2
−0.1
0.0
0.1
0.2
MR effect size for
'Gut microbiota abundance (genus Parasutterella id.2892)' on 'Kinesin−like protein KIF23'

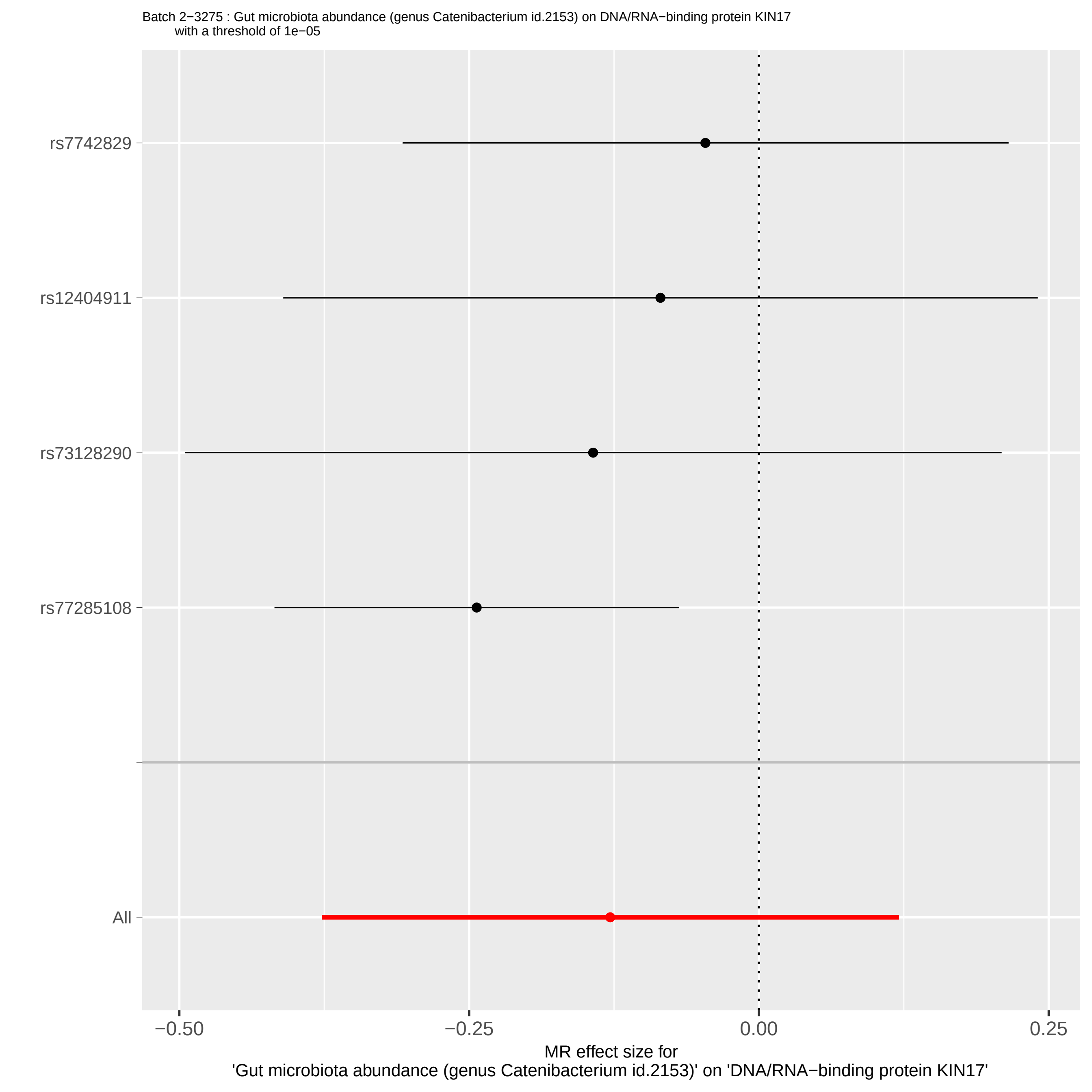
Batch 2−3275 : Gut microbiota abundance (genus Catenibacterium id.2153) on DNA/RNA−binding protein KIN17
with a threshold of 1e−05
rs7742829
rs12404911
rs73128290
rs77285108
All
−0.50
−0.25
0.00
0.25
MR effect size for
'Gut microbiota abundance (genus Catenibacterium id.2153)' on 'DNA/RNA−binding protein KIN17'

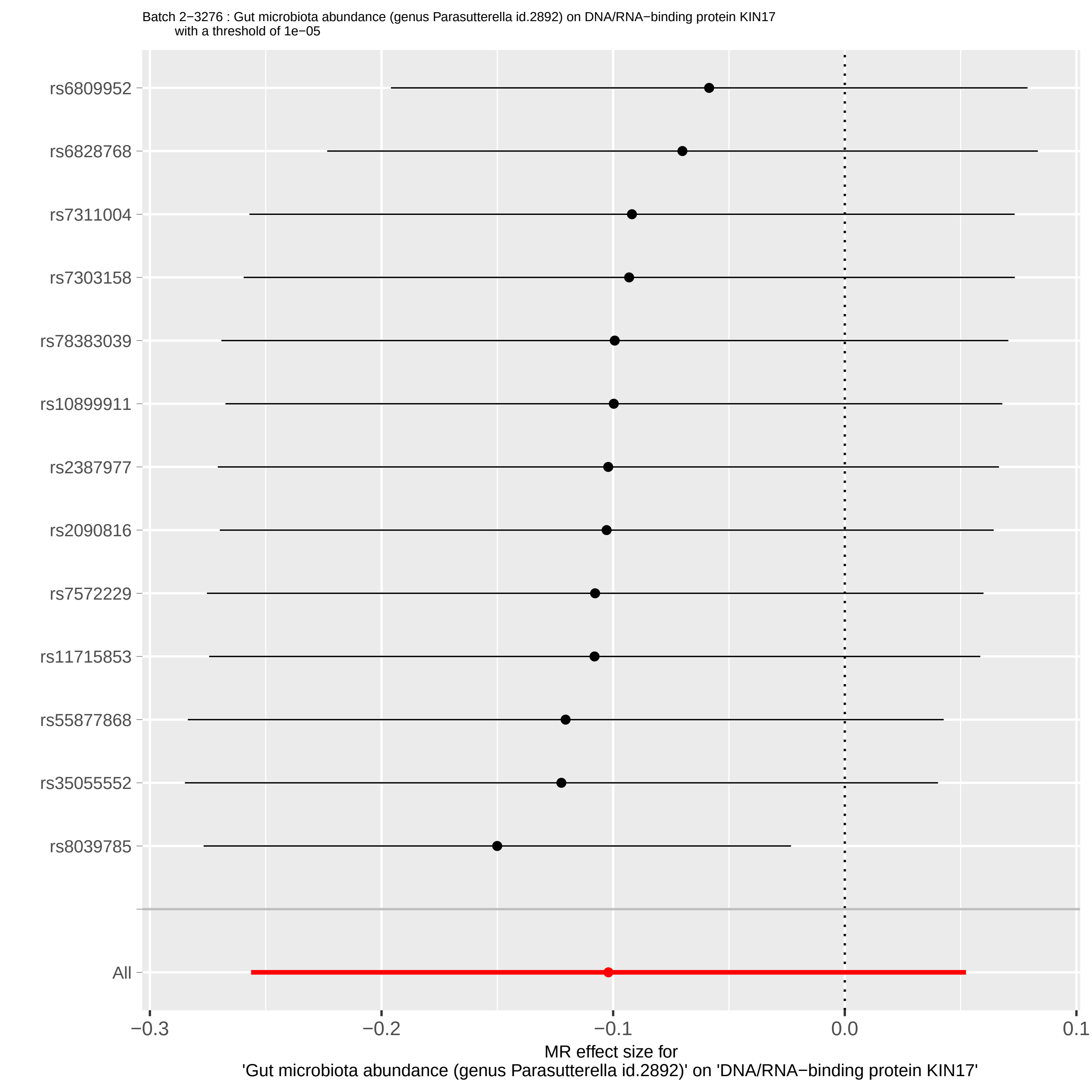
Batch 2−3276 : Gut microbiota abundance (genus Parasutterella id.2892) on DNA/RNA−binding protein KIN17
with a threshold of 1e−05
rs6809952
rs6828768
rs7311004
rs7303158
rs78383039
rs10899911
rs2387977
rs2090816
rs7572229
rs11715853
rs55877868
rs35055552
rs8039785
All
−0.3
−0.2
−0.1
0.0
0.1
MR effect size for
'Gut microbiota abundance (genus Parasutterella id.2892)' on 'DNA/RNA−binding protein KIN17'

Batch 2−3277 : Gut microbiota abundance (genus Catenibacterium id.2153) on DNA/RNA−binding protein KIN17
with a threshold of 1e−05

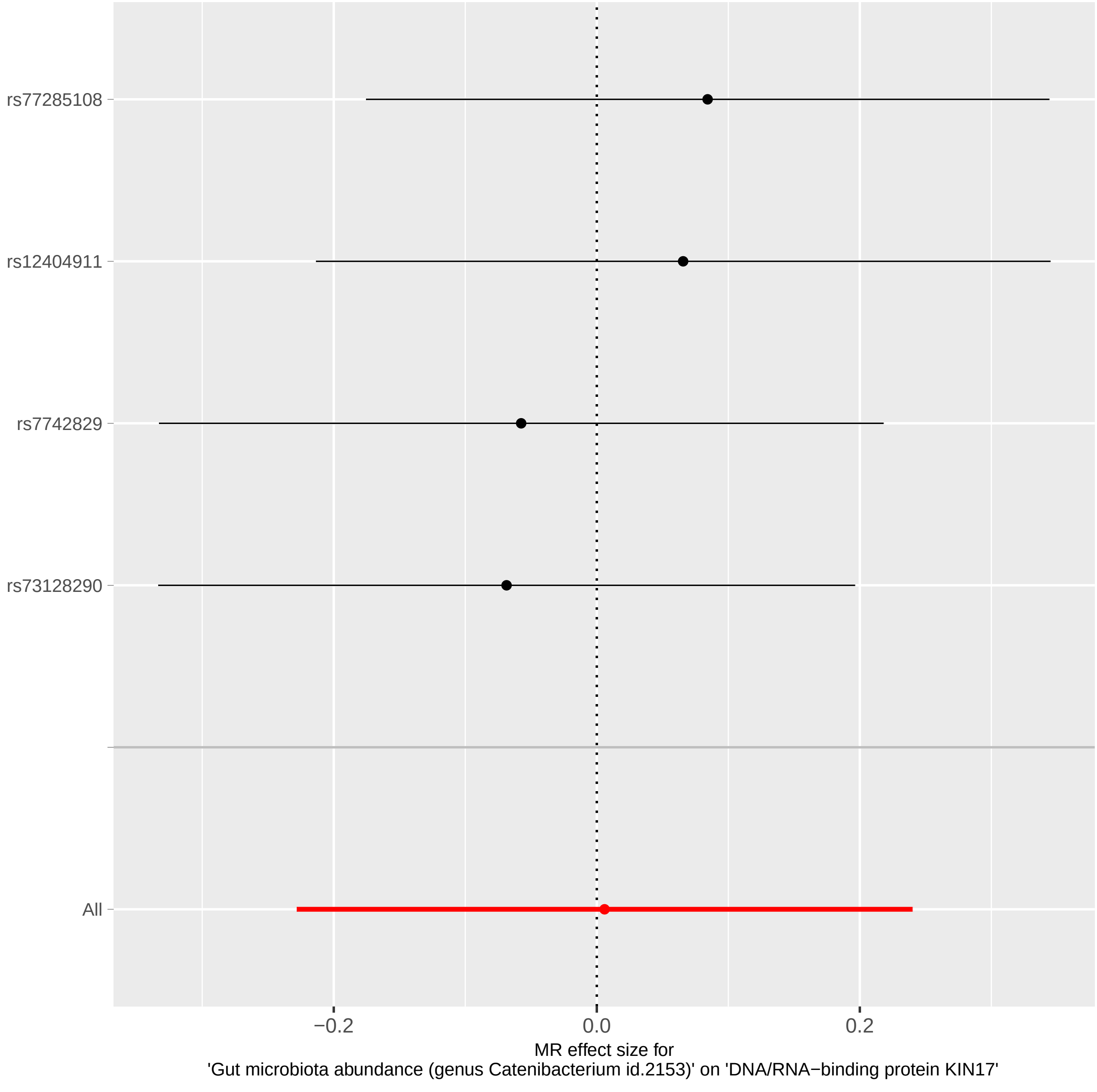

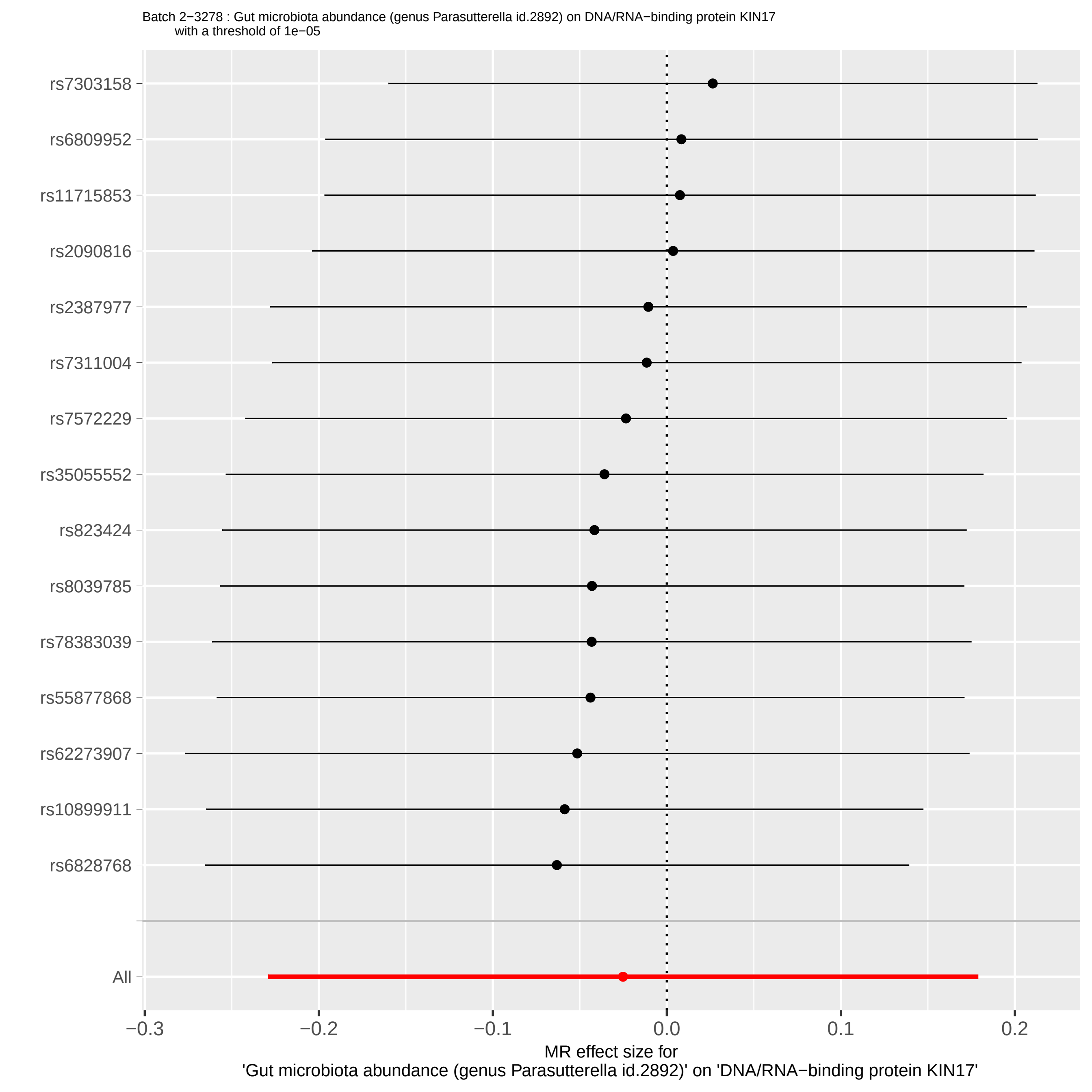

Batch 2−3278 : Gut microbiota abundance (genus Parasutterella id.2892) on DNA/RNA−binding protein KIN17
with a threshold of 1e−05
rs7303158
rs6809952
rs11715853
rs2090816
rs2387977
rs7311004
rs7572229
rs35055552
rs823424
rs8039785
rs78383039
rs55877868
rs62273907
rs10899911
rs6828768
All
−0.3
−0.2
−0.1
0.0
0.1
0.2
MR effect size for
'Gut microbiota abundance (genus Parasutterella id.2892)' on 'DNA/RNA−binding protein KIN17'

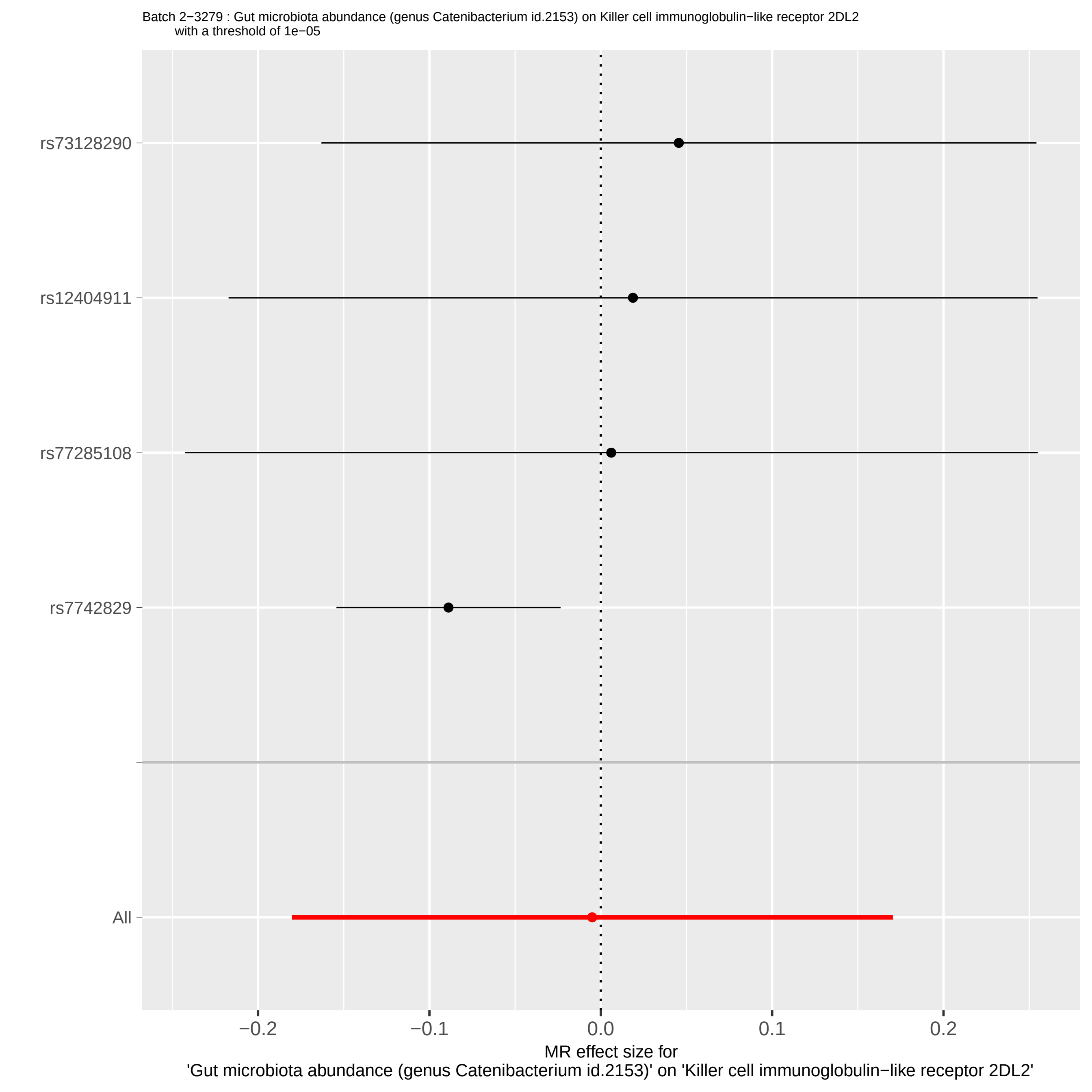

Batch 2−3279 : Gut microbiota abundance (genus Catenibacterium id.2153) on Killer cell immunoglobulin−like receptor 2DL2
with a threshold of 1e−05
rs73128290
rs12404911
rs77285108
rs7742829
All
−0.2
−0.1
0.0
0.1
0.2
MR effect size for
'Gut microbiota abundance (genus Catenibacterium id.2153)' on 'Killer cell immunoglobulin−like receptor 2DL2'

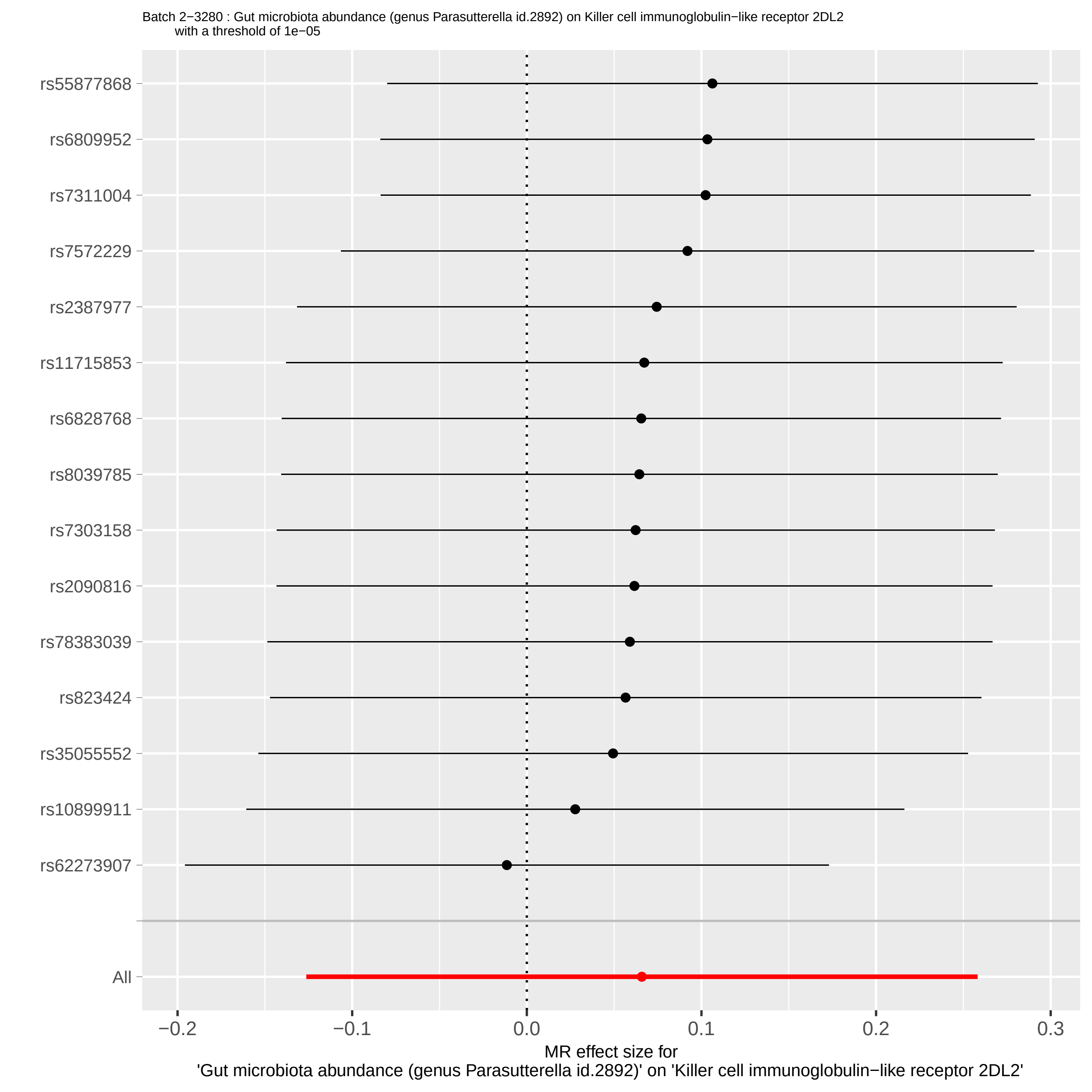
Batch 2−3280 : Gut microbiota abundance (genus Parasutterella id.2892) on Killer cell immunoglobulin−like receptor 2DL2
with a threshold of 1e−05
rs55877868
rs6809952
rs7311004
rs7572229
rs2387977
rs11715853
rs6828768
rs8039785
rs7303158
rs2090816
rs78383039
rs823424
rs35055552
rs10899911
rs62273907
All
−0.2
−0.1
0.0
0.1
0.2
0.3
MR effect size for
'Gut microbiota abundance (genus Parasutterella id.2892)' on 'Killer cell immunoglobulin−like receptor 2DL2'

Batch 2−3281 : Gut microbiota abundance (genus Catenibacterium id.2153) on Killer cell immunoglobulin−like receptor 2DL4
with a threshold of 1e−05

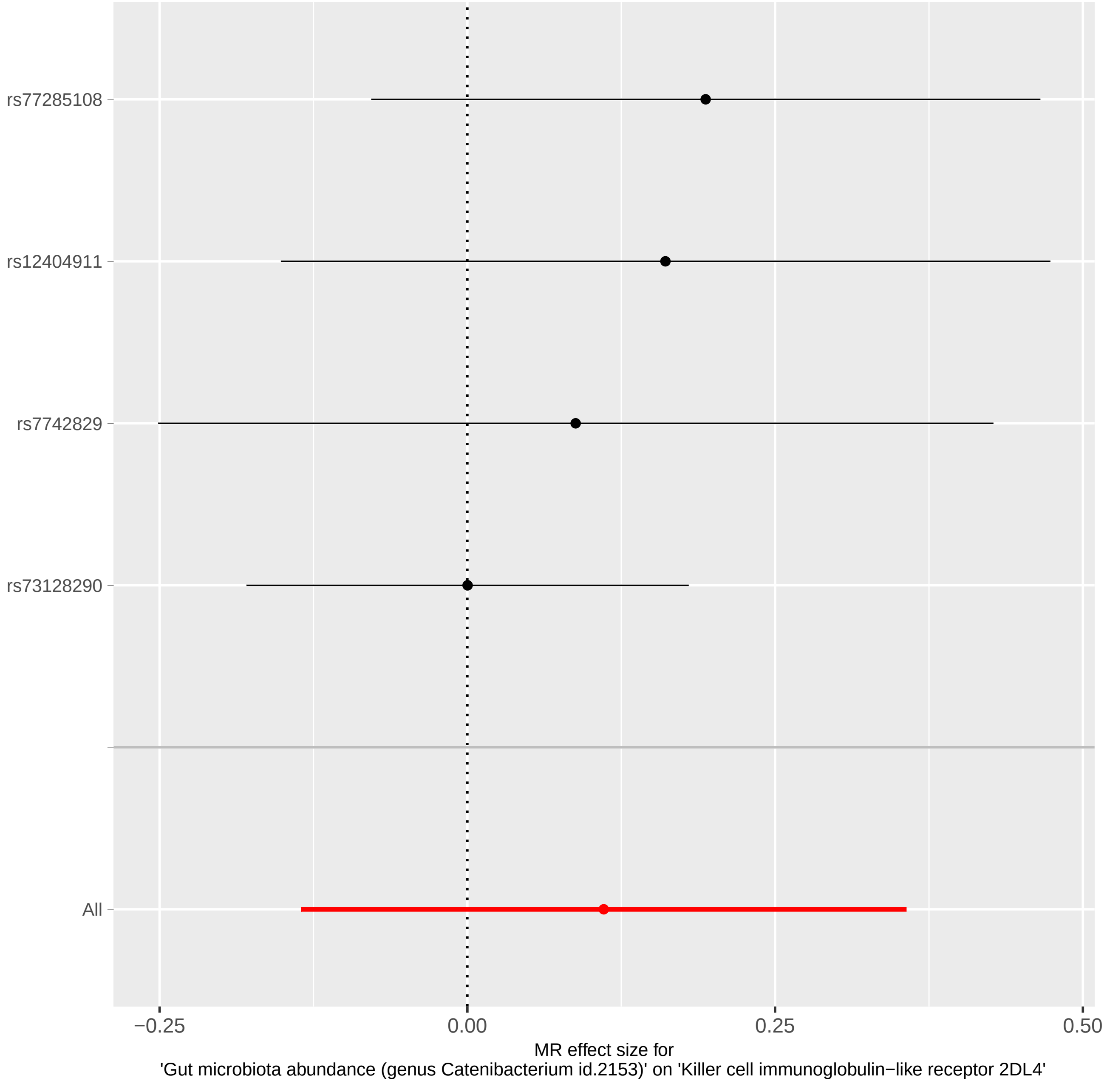

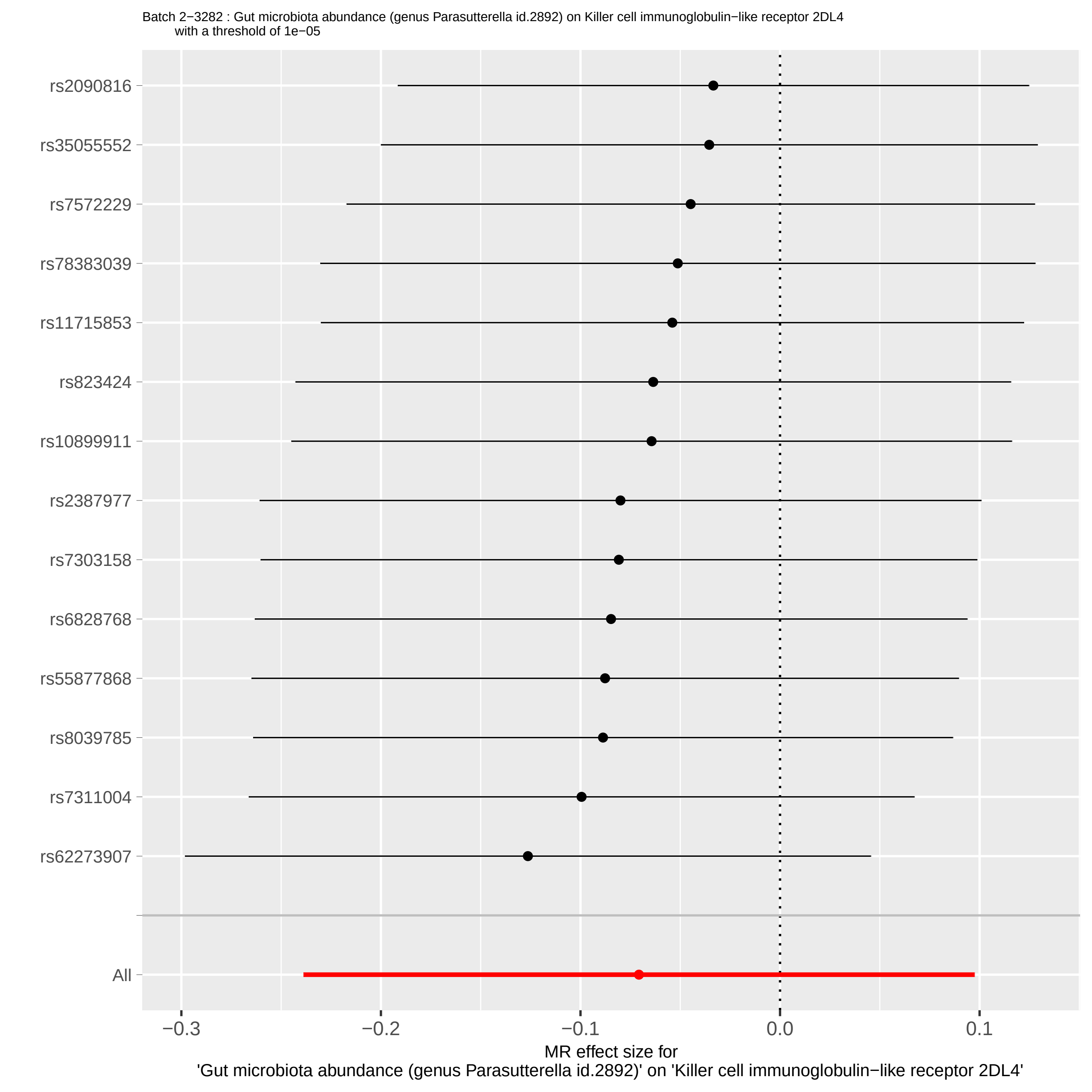
Batch 2−3282 : Gut microbiota abundance (genus Parasutterella id.2892) on Killer cell immunoglobulin−like receptor 2DL4
with a threshold of 1e−05
rs2090816
rs35055552
rs7572229
rs78383039
rs11715853
rs823424
rs10899911
rs2387977
rs7303158
rs6828768
rs55877868
rs8039785
rs7311004
rs62273907
All
−0.3
−0.2
−0.1
0.0
0.1
MR effect size for
'Gut microbiota abundance (genus Parasutterella id.2892)' on 'Killer cell immunoglobulin−like receptor 2DL4'

Batch 2−3283 : Gut microbiota abundance (genus Catenibacterium id.2153) on Killer cell immunoglobulin−like receptor 2DL4
with a threshold of 1e−05

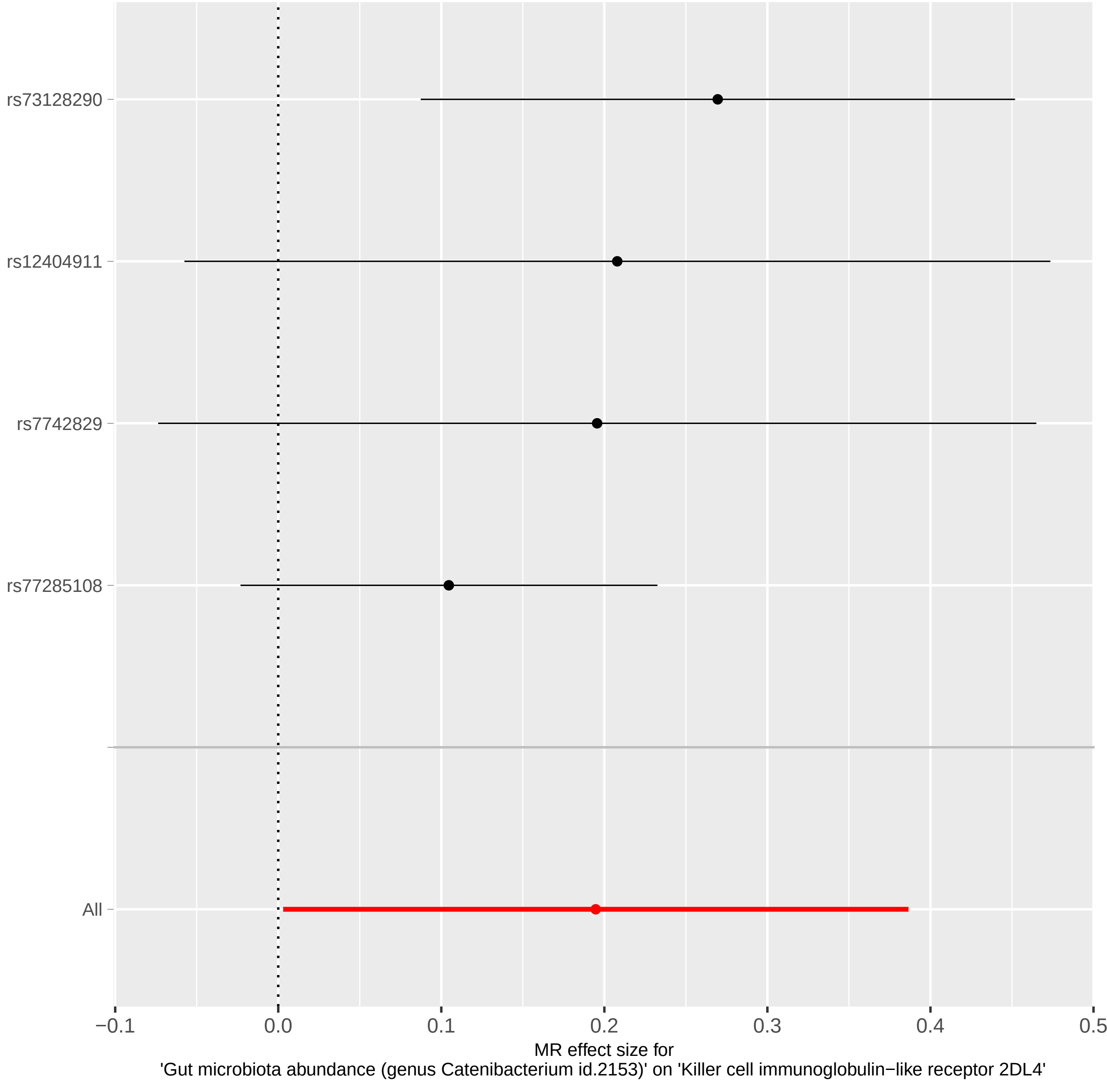

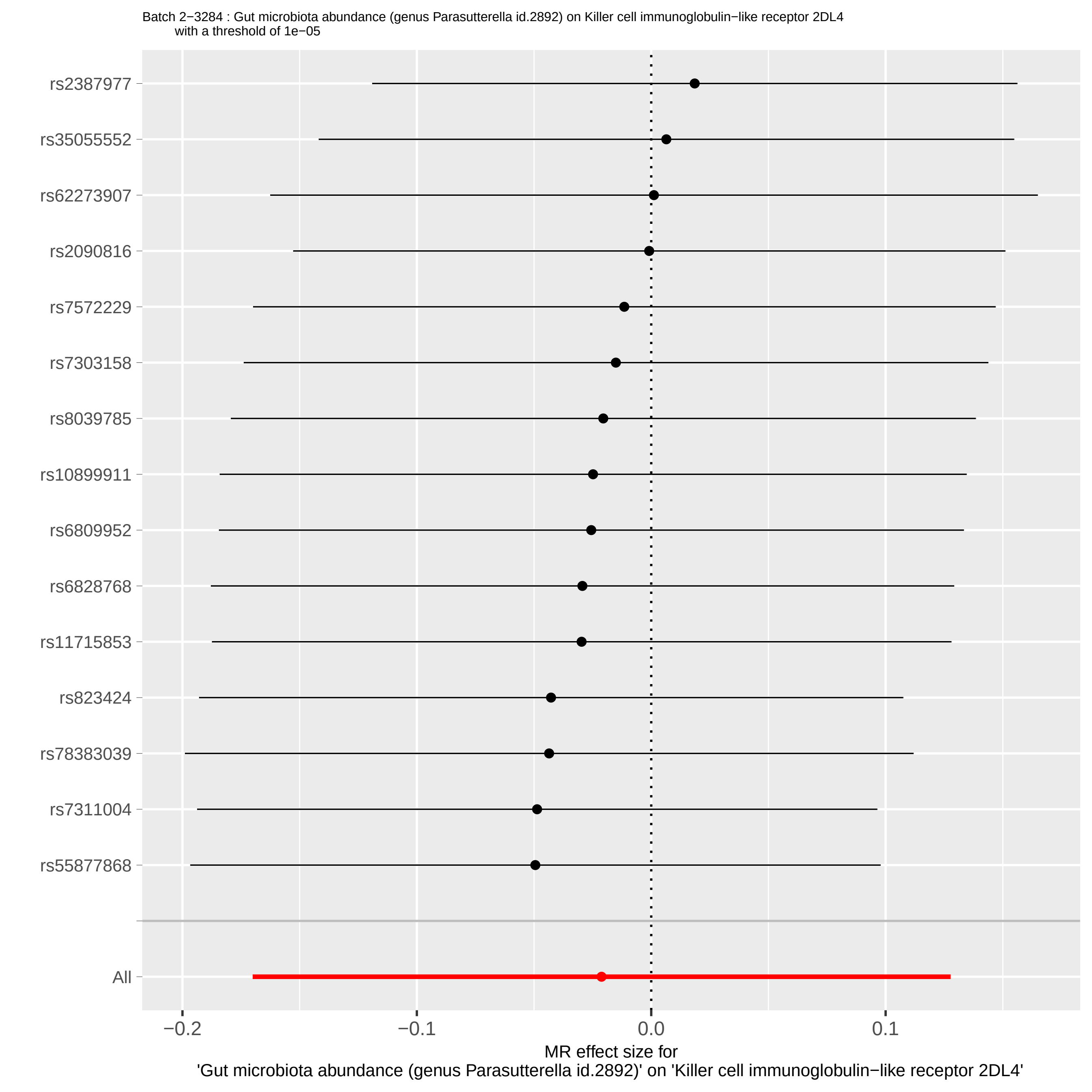

Batch 2−3284 : Gut microbiota abundance (genus Parasutterella id.2892) on Killer cell immunoglobulin−like receptor 2DL4
with a threshold of 1e−05
rs2387977
rs35055552
rs62273907
rs2090816
rs7572229
rs7303158
rs8039785
rs10899911
rs6809952
rs6828768
rs11715853
rs823424
rs78383039
rs7311004
rs55877868
All
−0.2
−0.1
0.0
0.1
MR effect size for
'Gut microbiota abundance (genus Parasutterella id.2892)' on 'Killer cell immunoglobulin−like receptor 2DL4'

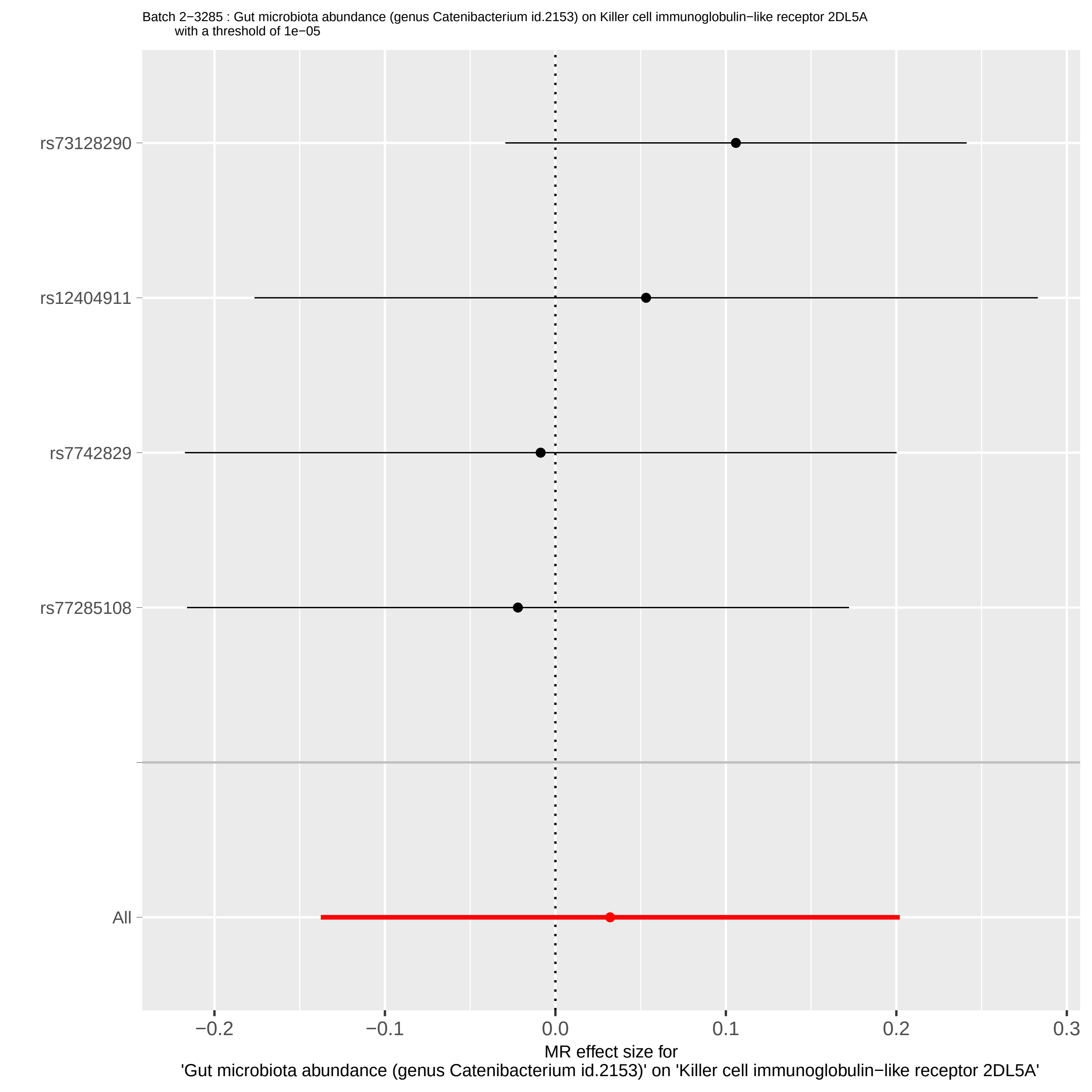
Batch 2−3285 : Gut microbiota abundance (genus Catenibacterium id.2153) on Killer cell immunoglobulin−like receptor 2DL5A
with a threshold of 1e−05
rs73128290
rs12404911
rs7742829
rs77285108
All
−0.2
−0.1
0.0
0.1
0.2
0.3
MR effect size for
'Gut microbiota abundance (genus Catenibacterium id.2153)' on 'Killer cell immunoglobulin−like receptor 2DL5A'

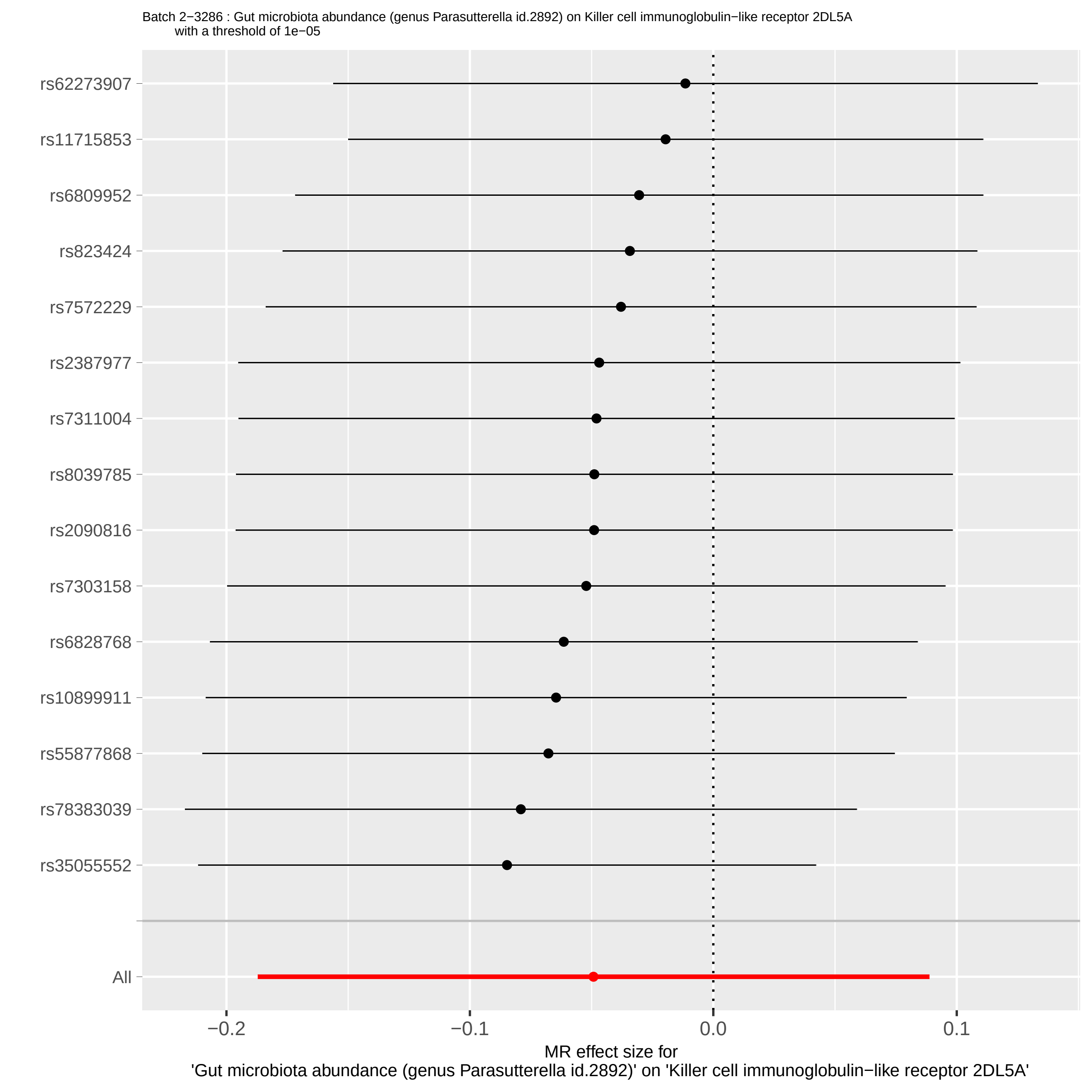

Batch 2−3286 : Gut microbiota abundance (genus Parasutterella id.2892) on Killer cell immunoglobulin−like receptor 2DL5A
with a threshold of 1e−05
rs62273907
rs11715853
rs6809952
rs823424
rs7572229
rs2387977
rs7311004
rs8039785
rs2090816
rs7303158
rs6828768
rs10899911
rs55877868
rs78383039
rs35055552
All
−0.2
−0.1
0.0
0.1
MR effect size for
'Gut microbiota abundance (genus Parasutterella id.2892)' on 'Killer cell immunoglobulin−like receptor 2DL5A'

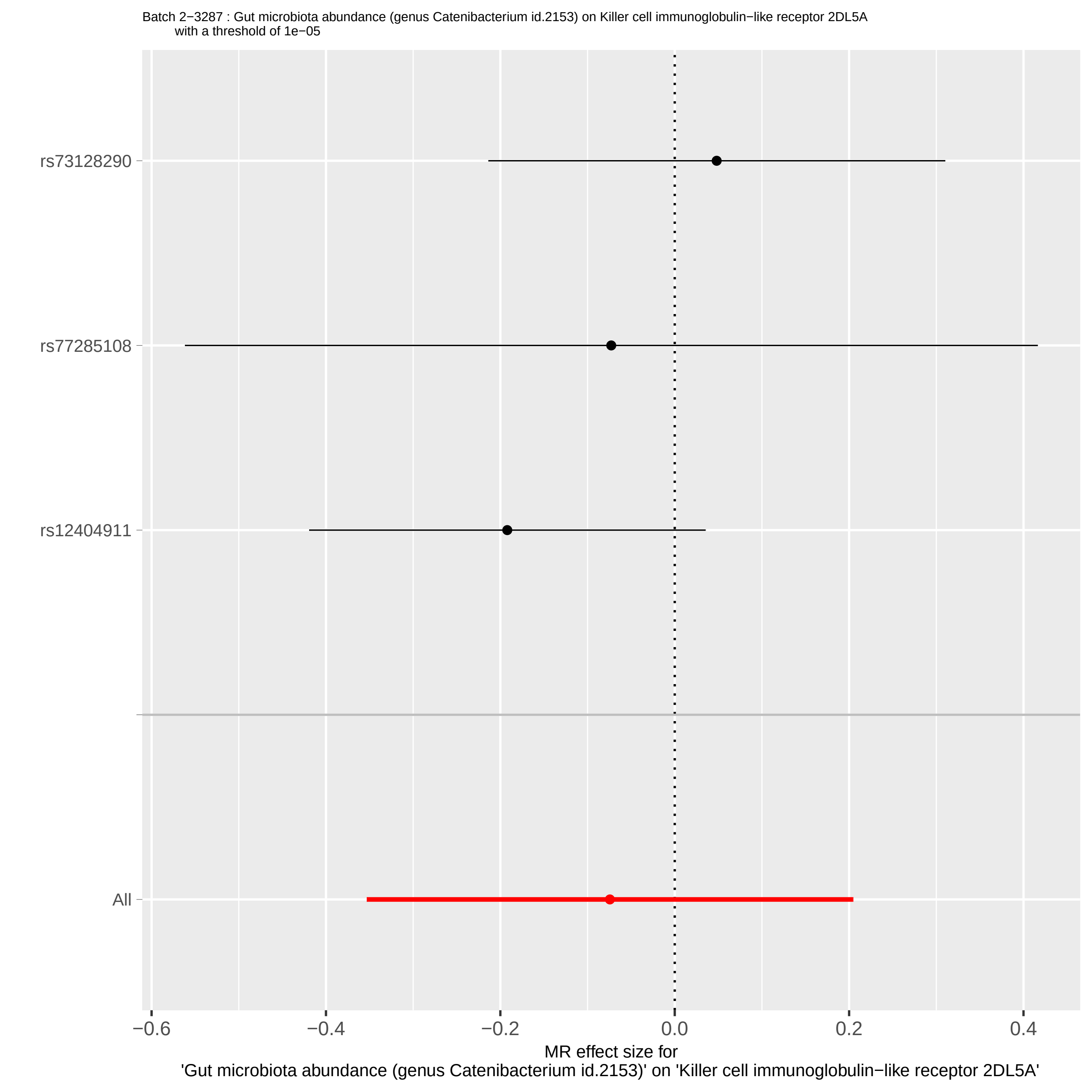
Batch 2−3287 : Gut microbiota abundance (genus Catenibacterium id.2153) on Killer cell immunoglobulin−like receptor 2DL5A
with a threshold of 1e−05
rs73128290
rs77285108
rs12404911
All
−0.6
−0.4
−0.2
0.0
0.2
0.4
MR effect size for
'Gut microbiota abundance (genus Catenibacterium id.2153)' on 'Killer cell immunoglobulin−like receptor 2DL5A'

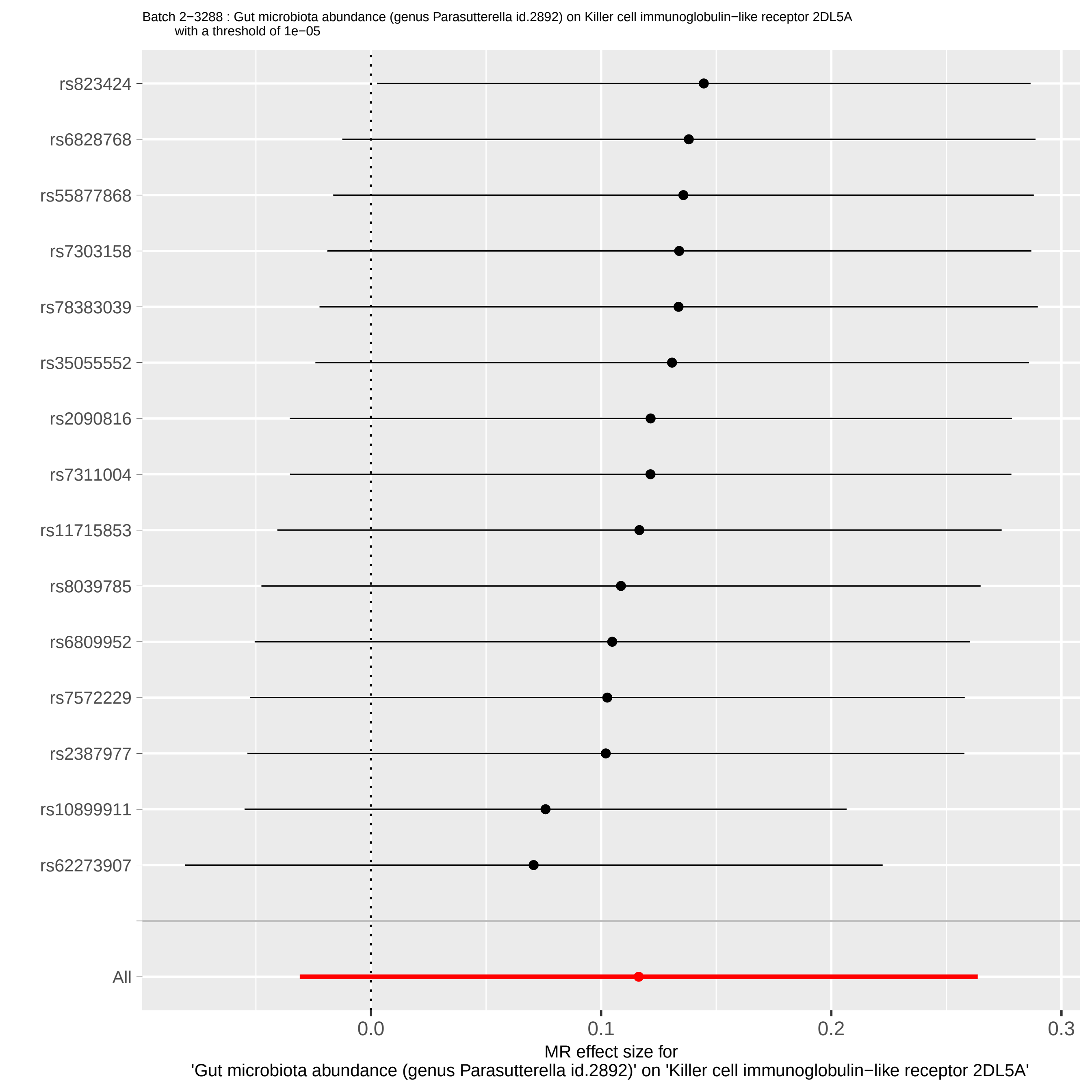

Batch 2−3288 : Gut microbiota abundance (genus Parasutterella id.2892) on Killer cell immunoglobulin−like receptor 2DL5A
with a threshold of 1e−05
rs823424
rs6828768
rs55877868
rs7303158
rs78383039
rs35055552
rs2090816
rs7311004
rs11715853
rs8039785
rs6809952
rs7572229
rs2387977
rs10899911
rs62273907
All
0.0
0.1
0.2
0.3
MR effect size for
'Gut microbiota abundance (genus Parasutterella id.2892)' on 'Killer cell immunoglobulin−like receptor 2DL5A'

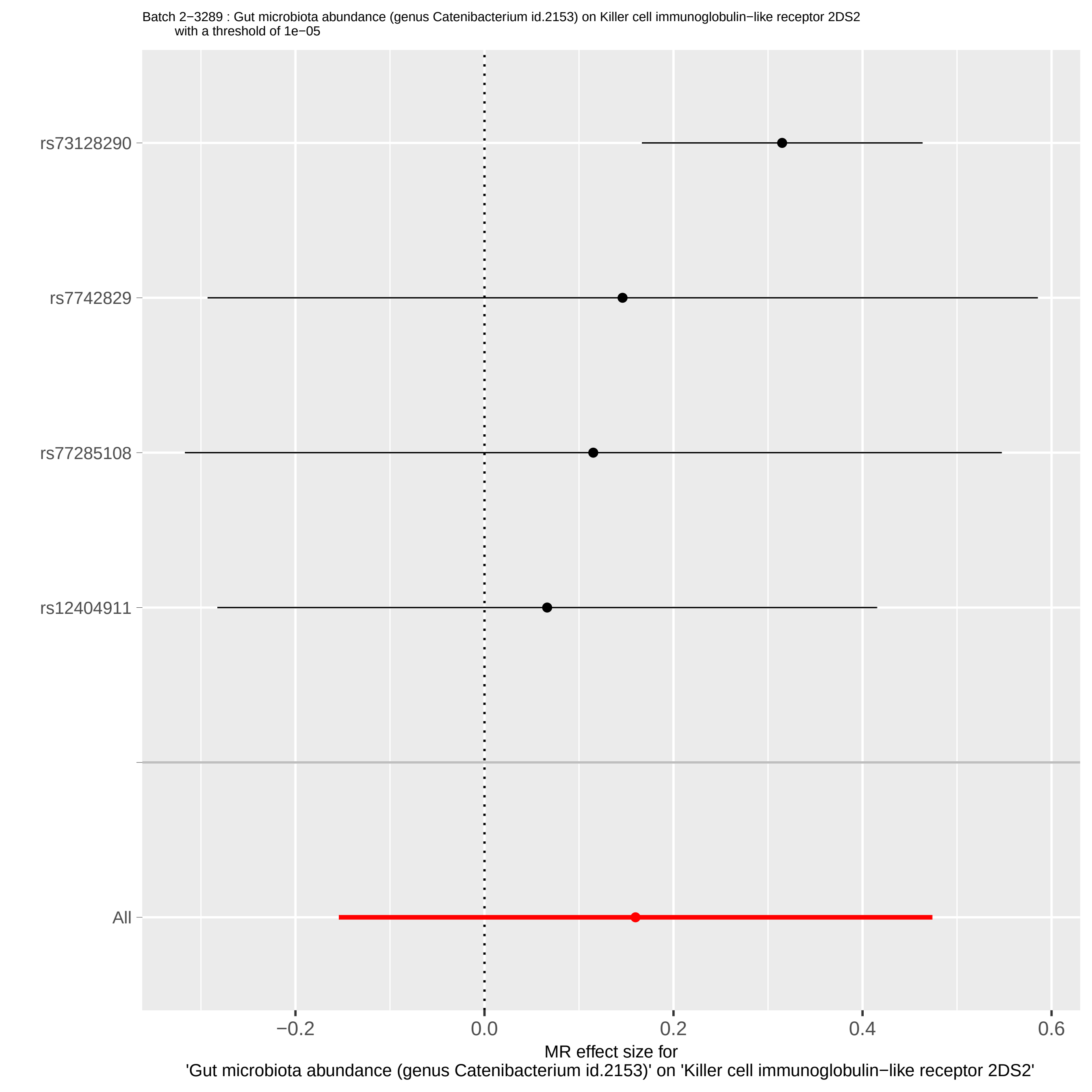

Batch 2−3289 : Gut microbiota abundance (genus Catenibacterium id.2153) on Killer cell immunoglobulin−like receptor 2DS2
with a threshold of 1e−05
rs73128290
rs7742829
rs77285108
rs12404911
All
−0.2
0.0
0.2
0.4
0.6
MR effect size for
'Gut microbiota abundance (genus Catenibacterium id.2153)' on 'Killer cell immunoglobulin−like receptor 2DS2'

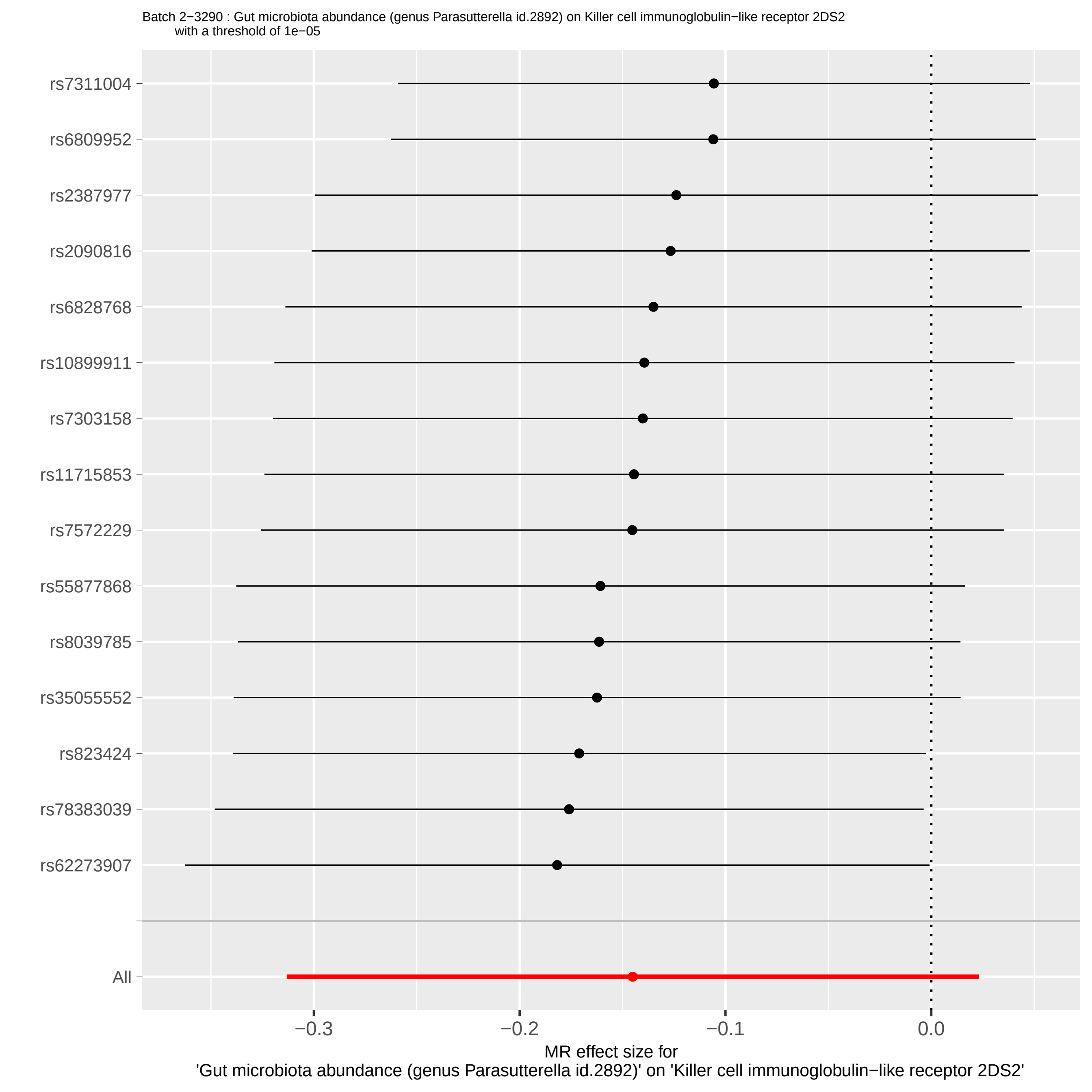
Batch 2−3290 : Gut microbiota abundance (genus Parasutterella id.2892) on Killer cell immunoglobulin−like receptor 2DS2
with a threshold of 1e−05
rs7311004
rs6809952
rs2387977
rs2090816
rs6828768
rs10899911
rs7303158
rs11715853
rs7572229
rs55877868
rs8039785
rs35055552
rs823424
rs78383039
rs62273907
All
−0.3
−0.2
−0.1
0.0
MR effect size for
'Gut microbiota abundance (genus Parasutterella id.2892)' on 'Killer cell immunoglobulin−like receptor 2DS2'

Batch 2−3291 : Gut microbiota abundance (genus Catenibacterium id.2153) on Killer cell immunoglobulin−like receptor 3DL2
with a threshold of 1e−05

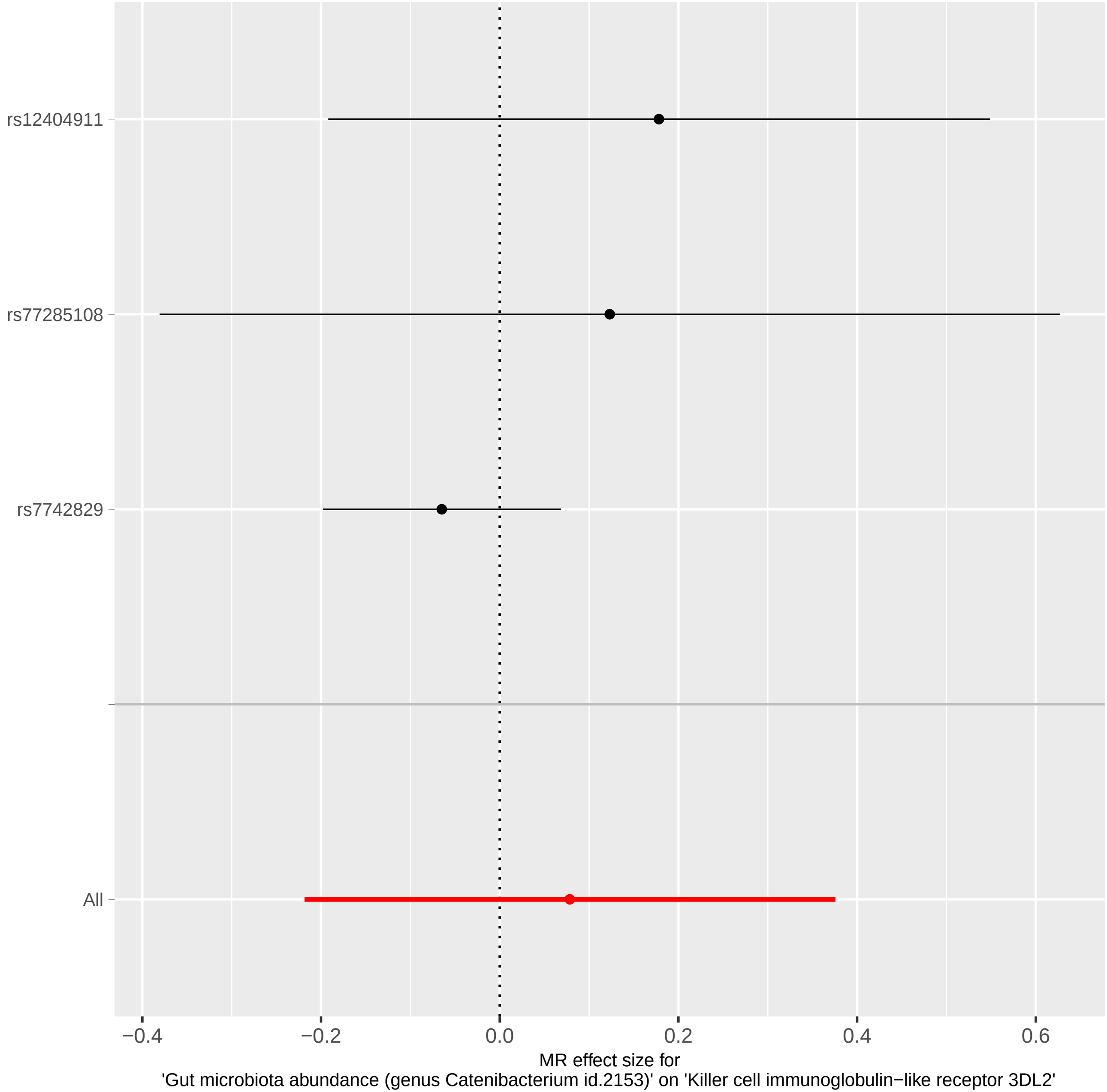

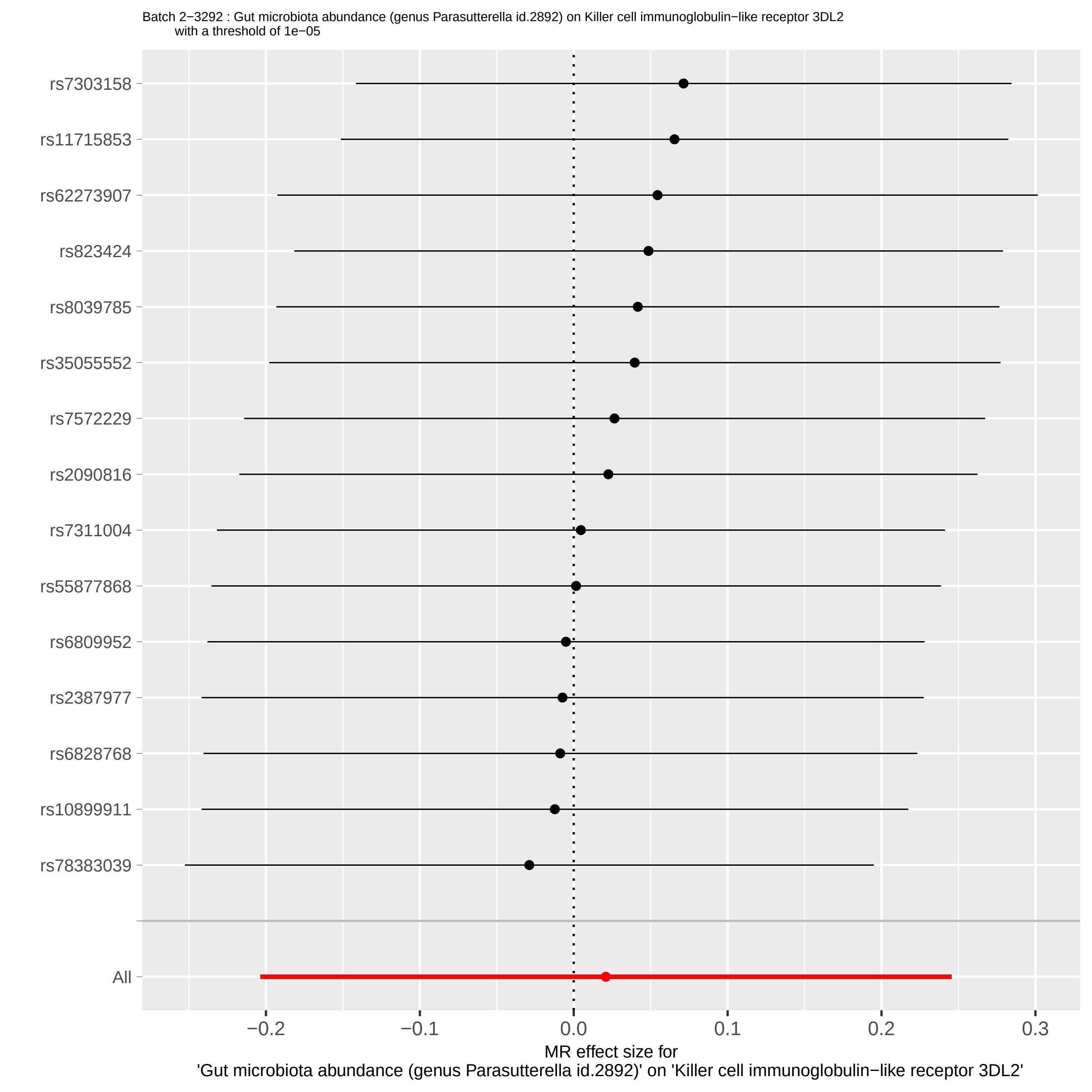

Batch 2−3292 : Gut microbiota abundance (genus Parasutterella id.2892) on Killer cell immunoglobulin−like receptor 3DL2
with a threshold of 1e−05
rs7303158
rs11715853
rs62273907
rs823424
rs8039785
rs35055552
rs7572229
rs2090816
rs7311004
rs55877868
rs6809952
rs2387977
rs6828768
rs10899911
rs78383039
All
−0.2
−0.1
0.0
0.1
0.2
0.3
MR effect size for
'Gut microbiota abundance (genus Parasutterella id.2892)' on 'Killer cell immunoglobulin−like receptor 3DL2'

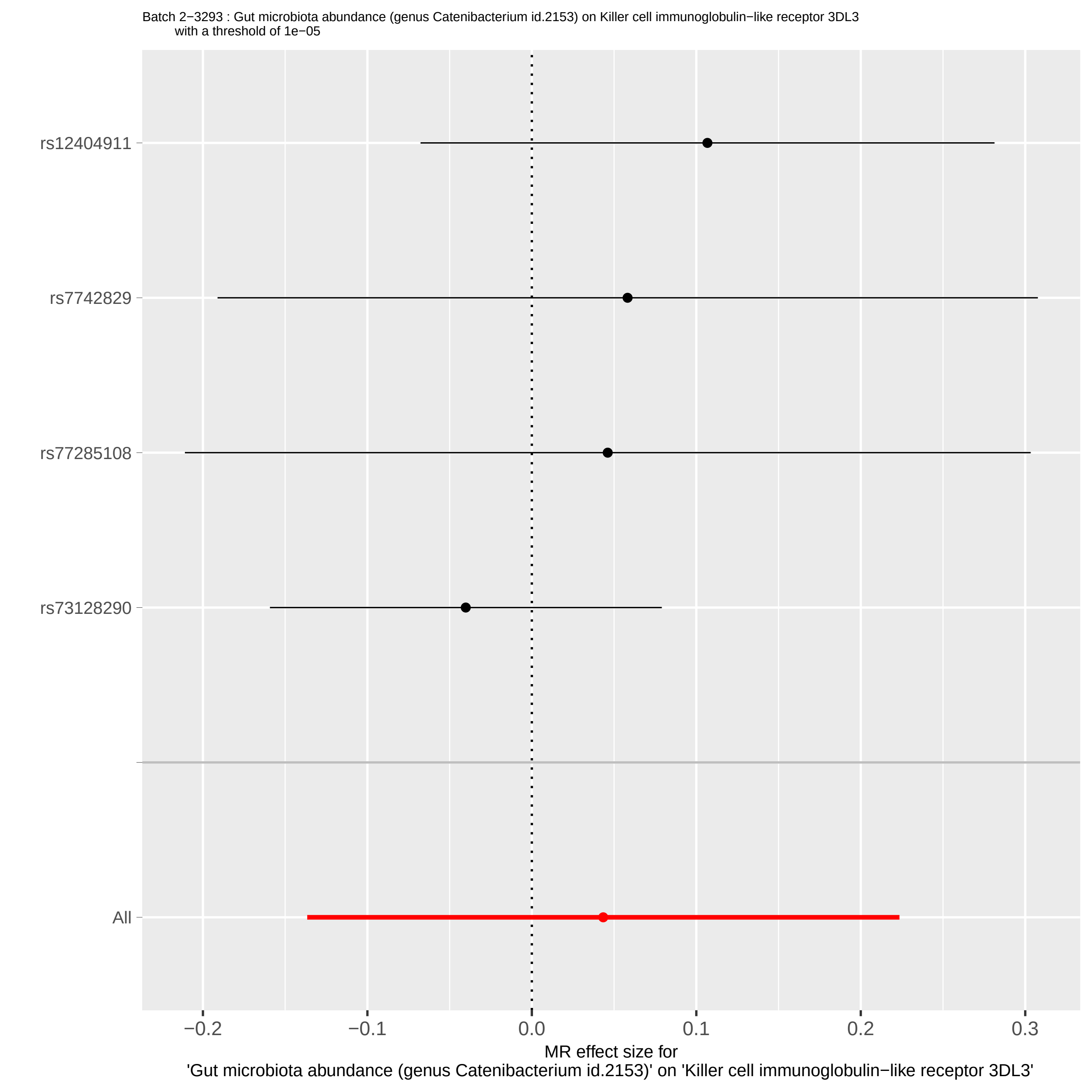
Batch 2−3293 : Gut microbiota abundance (genus Catenibacterium id.2153) on Killer cell immunoglobulin−like receptor 3DL3
with a threshold of 1e−05
rs12404911
rs7742829
rs77285108
rs73128290
All
−0.2
−0.1
0.0
0.1
0.2
0.3
MR effect size for
'Gut microbiota abundance (genus Catenibacterium id.2153)' on 'Killer cell immunoglobulin−like receptor 3DL3'

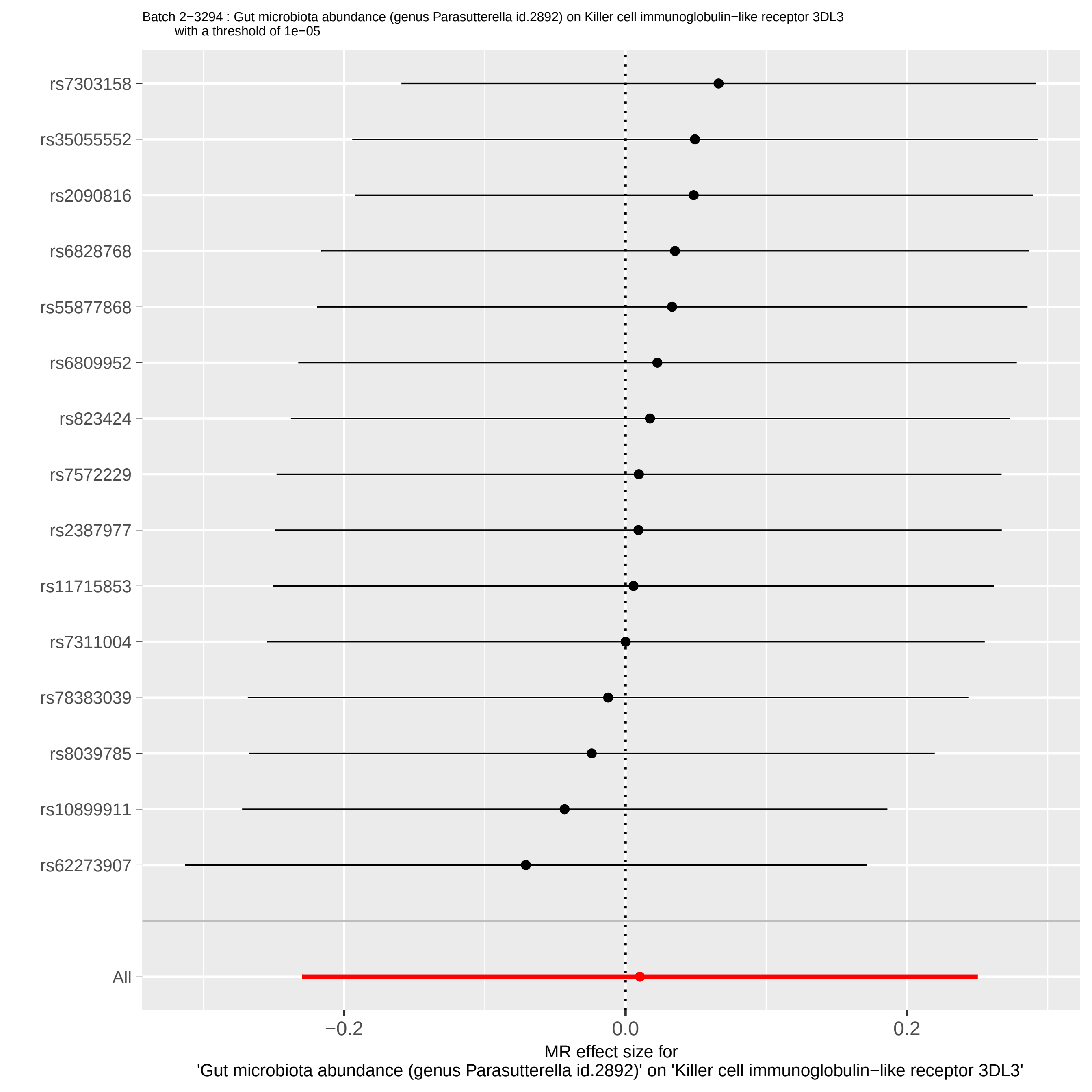
Batch 2−3294 : Gut microbiota abundance (genus Parasutterella id.2892) on Killer cell immunoglobulin−like receptor 3DL3
with a threshold of 1e−05
rs7303158
rs35055552
rs2090816
rs6828768
rs55877868
rs6809952
rs823424
rs7572229
rs2387977
rs11715853
rs7311004
rs78383039
rs8039785
rs10899911
rs62273907
All
−0.2
0.0
0.2
MR effect size for
'Gut microbiota abundance (genus Parasutterella id.2892)' on 'Killer cell immunoglobulin−like receptor 3DL3'

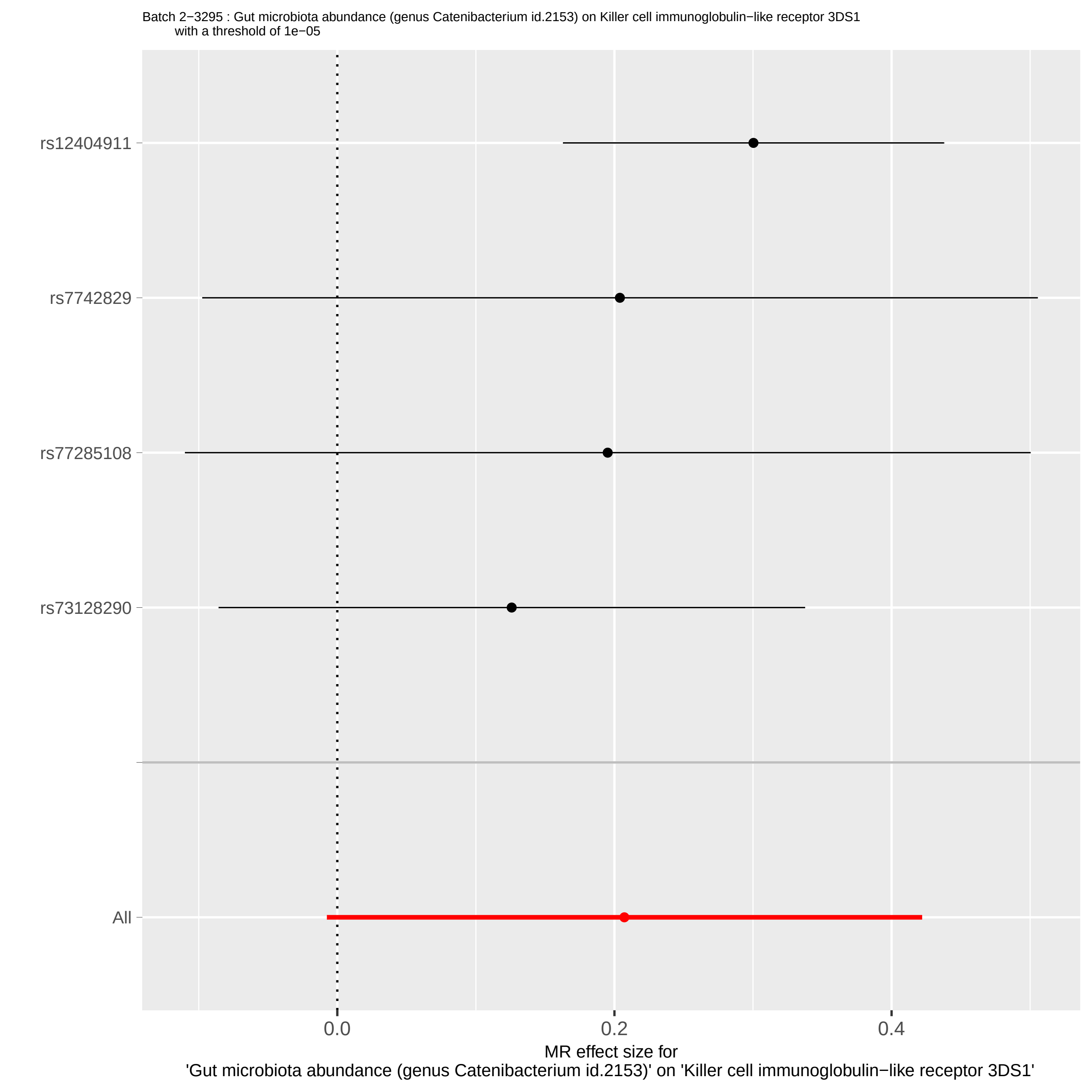
Batch 2−3295 : Gut microbiota abundance (genus Catenibacterium id.2153) on Killer cell immunoglobulin−like receptor 3DS1
with a threshold of 1e−05
rs12404911
rs7742829
rs77285108
rs73128290
All
0.0
0.2
0.4
MR effect size for
'Gut microbiota abundance (genus Catenibacterium id.2153)' on 'Killer cell immunoglobulin−like receptor 3DS1'

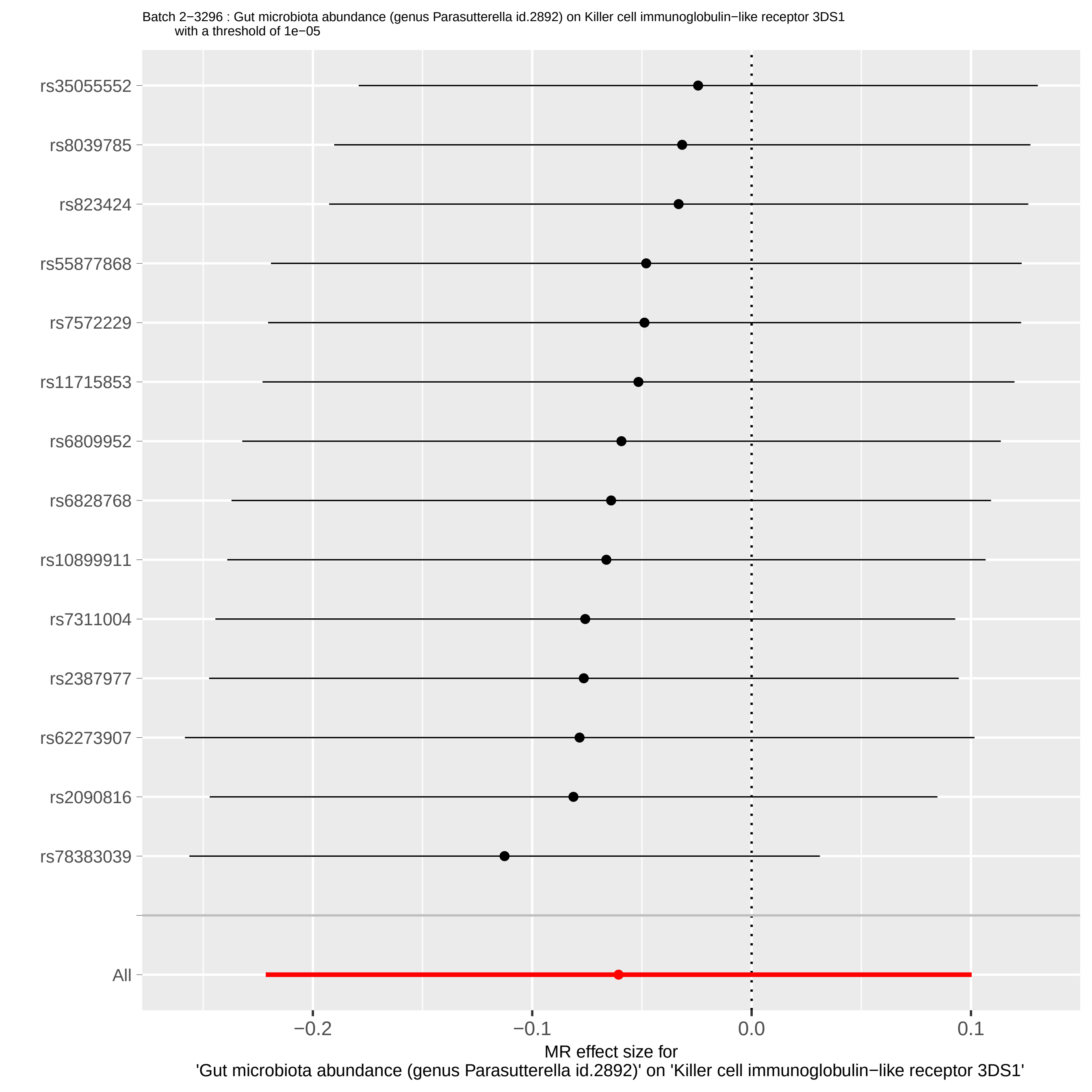
Batch 2−3296 : Gut microbiota abundance (genus Parasutterella id.2892) on Killer cell immunoglobulin−like receptor 3DS1
with a threshold of 1e−05
rs35055552
rs8039785
rs823424
rs55877868
rs7572229
rs11715853
rs6809952
rs6828768
rs10899911
rs7311004
rs2387977
rs62273907
rs2090816
rs78383039
All
−0.2
−0.1
0.0
0.1
MR effect size for
'Gut microbiota abundance (genus Parasutterella id.2892)' on 'Killer cell immunoglobulin−like receptor 3DS1'

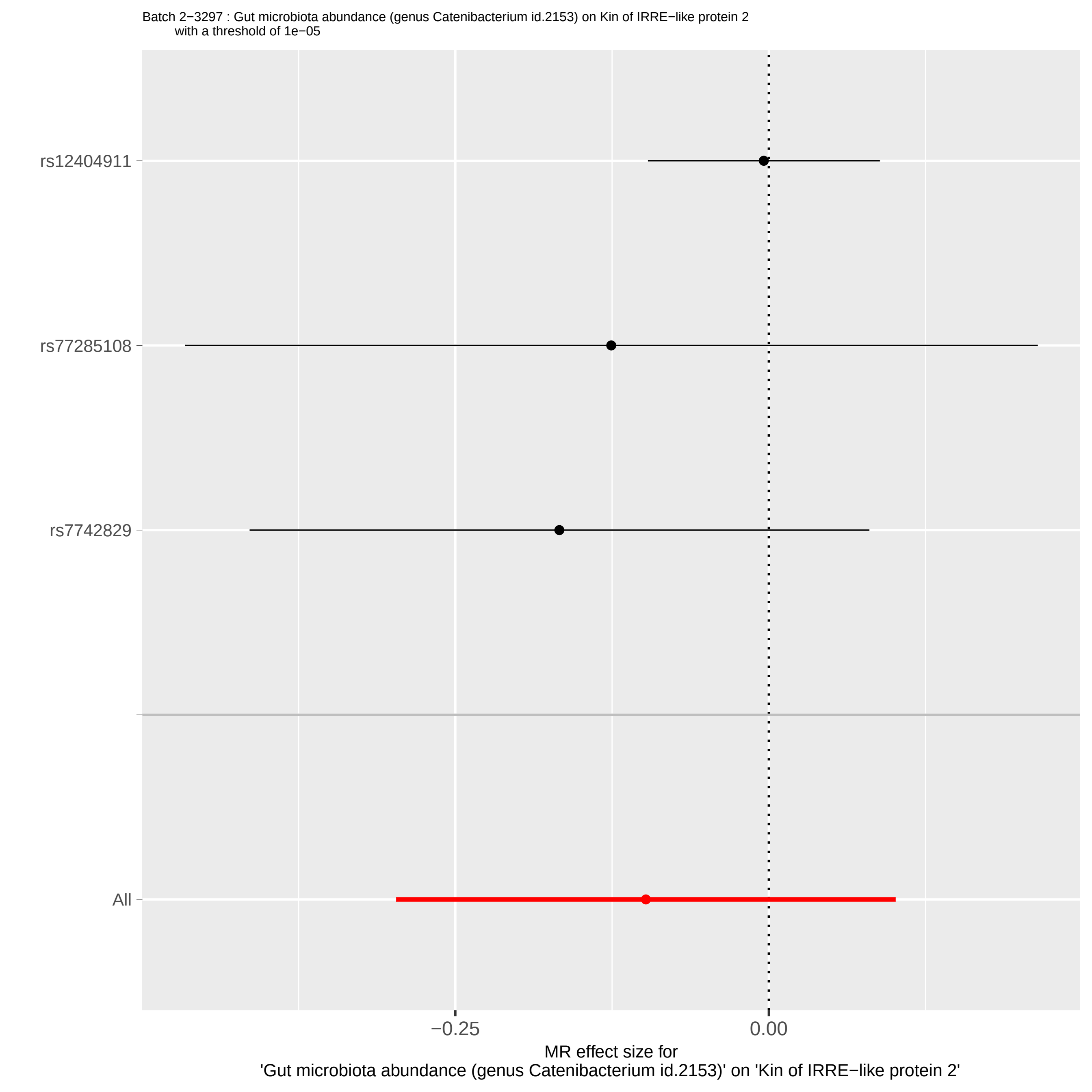
Batch 2−3297 : Gut microbiota abundance (genus Catenibacterium id.2153) on Kin of IRRE−like protein 2
with a threshold of 1e−05
rs12404911
rs77285108
rs7742829
All
−0.25
0.00
MR effect size for
'Gut microbiota abundance (genus Catenibacterium id.2153)' on 'Kin of IRRE−like protein 2'

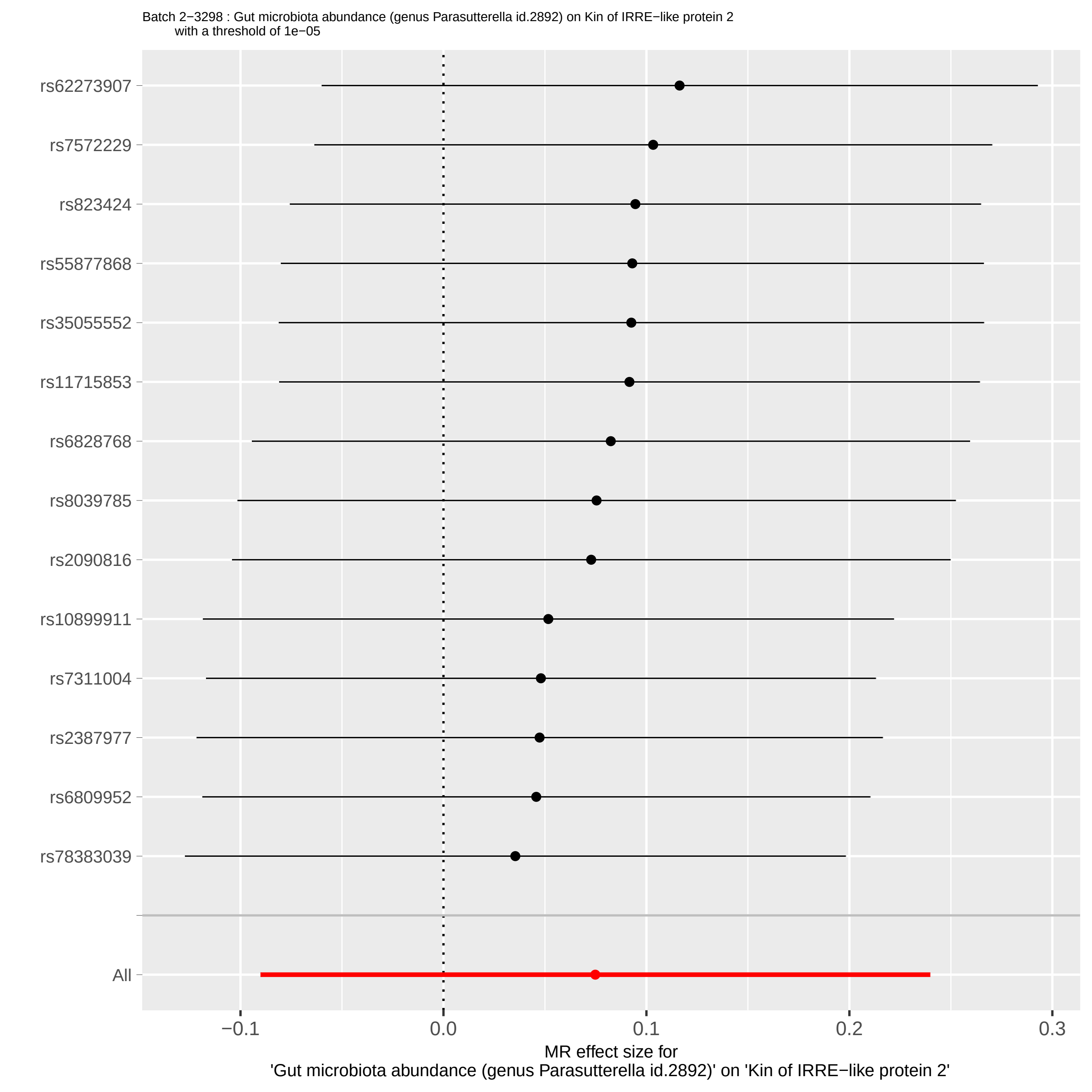

Batch 2−3298 : Gut microbiota abundance (genus Parasutterella id.2892) on Kin of IRRE−like protein 2
with a threshold of 1e−05
rs62273907
rs7572229
rs823424
rs55877868
rs35055552
rs11715853
rs6828768
rs8039785
rs2090816
rs10899911
rs7311004
rs2387977
rs6809952
rs78383039
All
−0.1
0.0
0.1
0.2
0.3
MR effect size for
'Gut microbiota abundance (genus Parasutterella id.2892)' on 'Kin of IRRE−like protein 2'

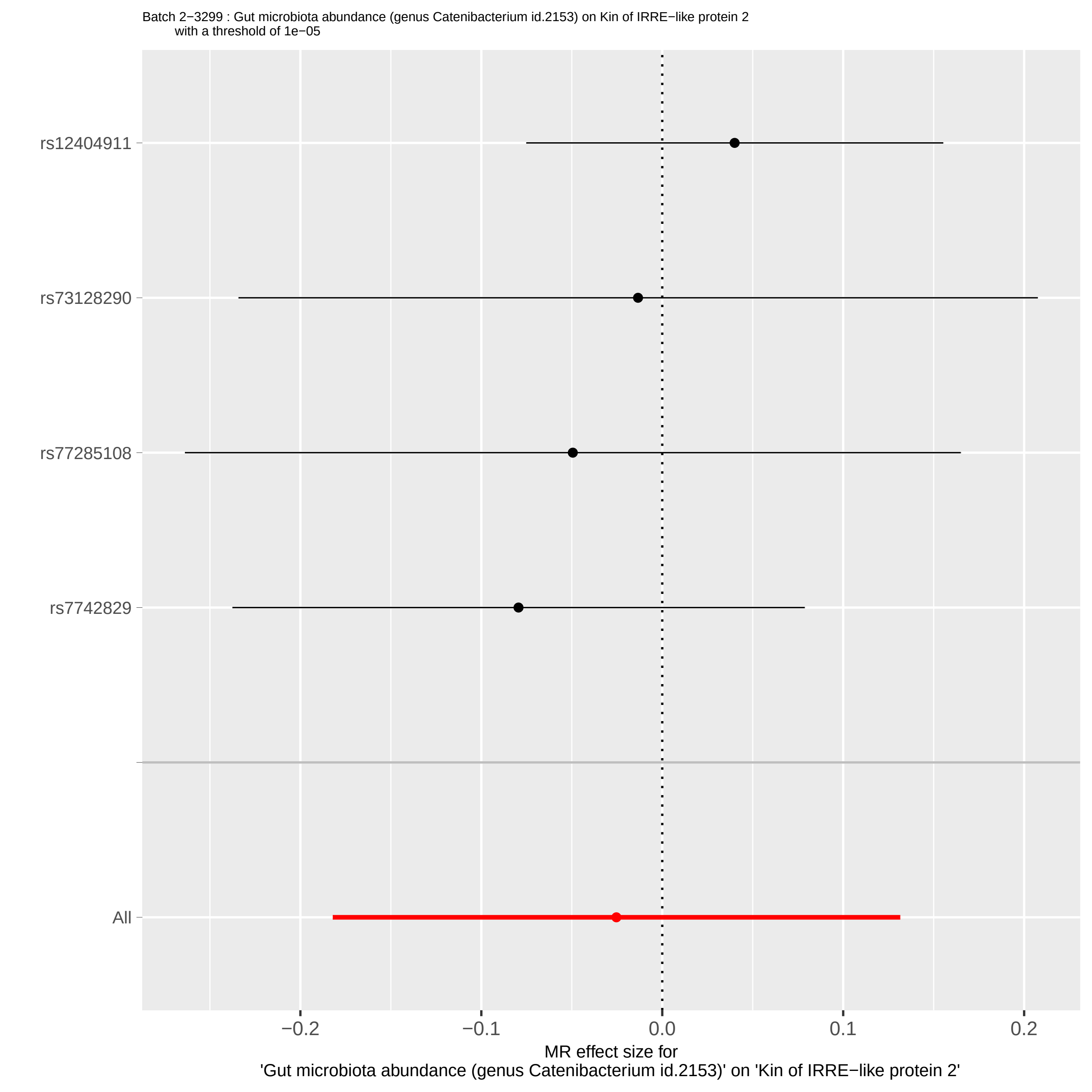
Batch 2−3299 : Gut microbiota abundance (genus Catenibacterium id.2153) on Kin of IRRE−like protein 2
with a threshold of 1e−05
rs12404911
rs73128290
rs77285108
rs7742829
All
−0.2
−0.1
0.0
0.1
0.2
MR effect size for
'Gut microbiota abundance (genus Catenibacterium id.2153)' on 'Kin of IRRE−like protein 2'

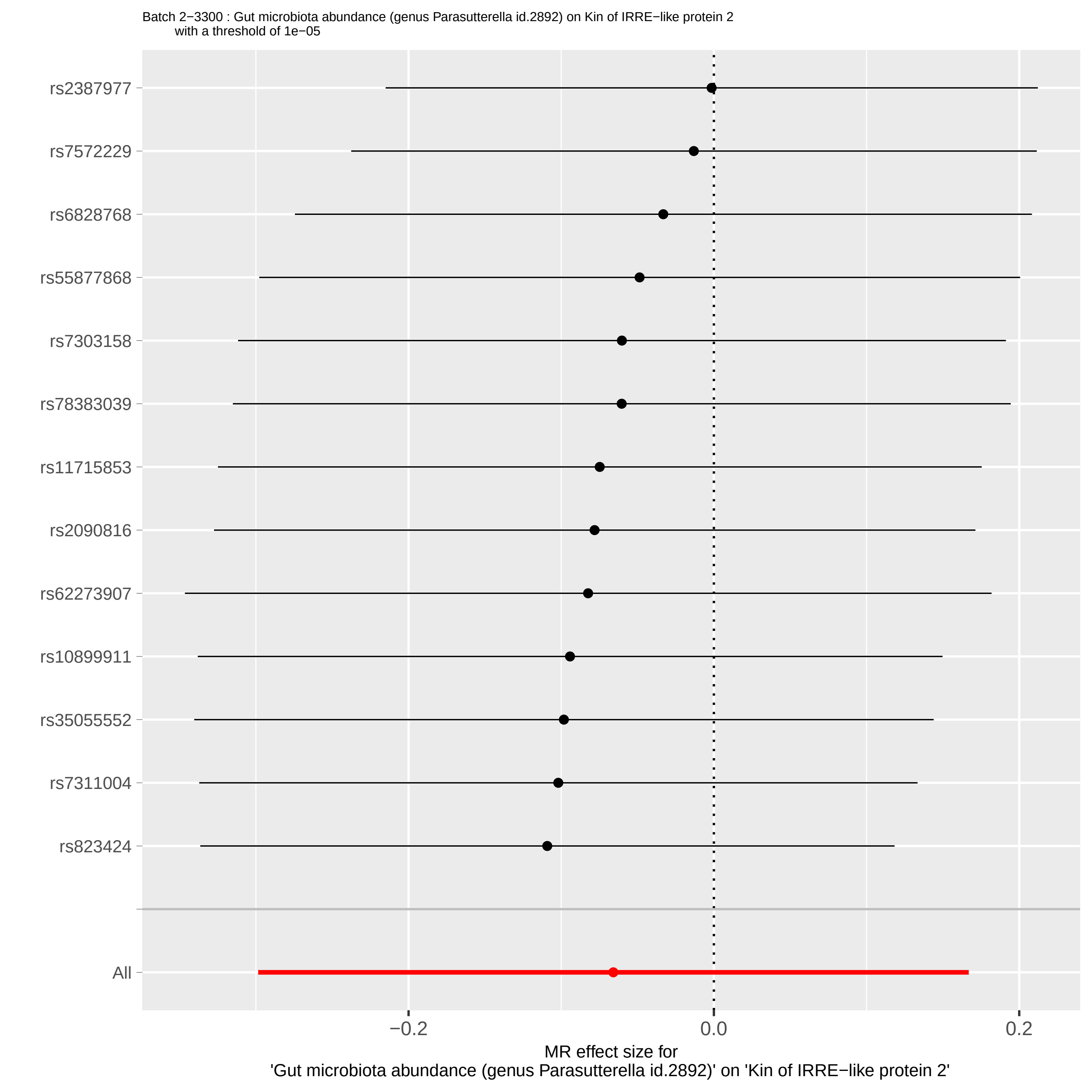
Batch 2−3300 : Gut microbiota abundance (genus Parasutterella id.2892) on Kin of IRRE−like protein 2
with a threshold of 1e−05
rs2387977
rs7572229
rs6828768
rs55877868
rs7303158
rs78383039
rs11715853
rs2090816
rs62273907
rs10899911
rs35055552
rs7311004
rs823424
All
−0.2
0.0
0.2
MR effect size for
'Gut microbiota abundance (genus Parasutterella id.2892)' on 'Kin of IRRE−like protein 2'

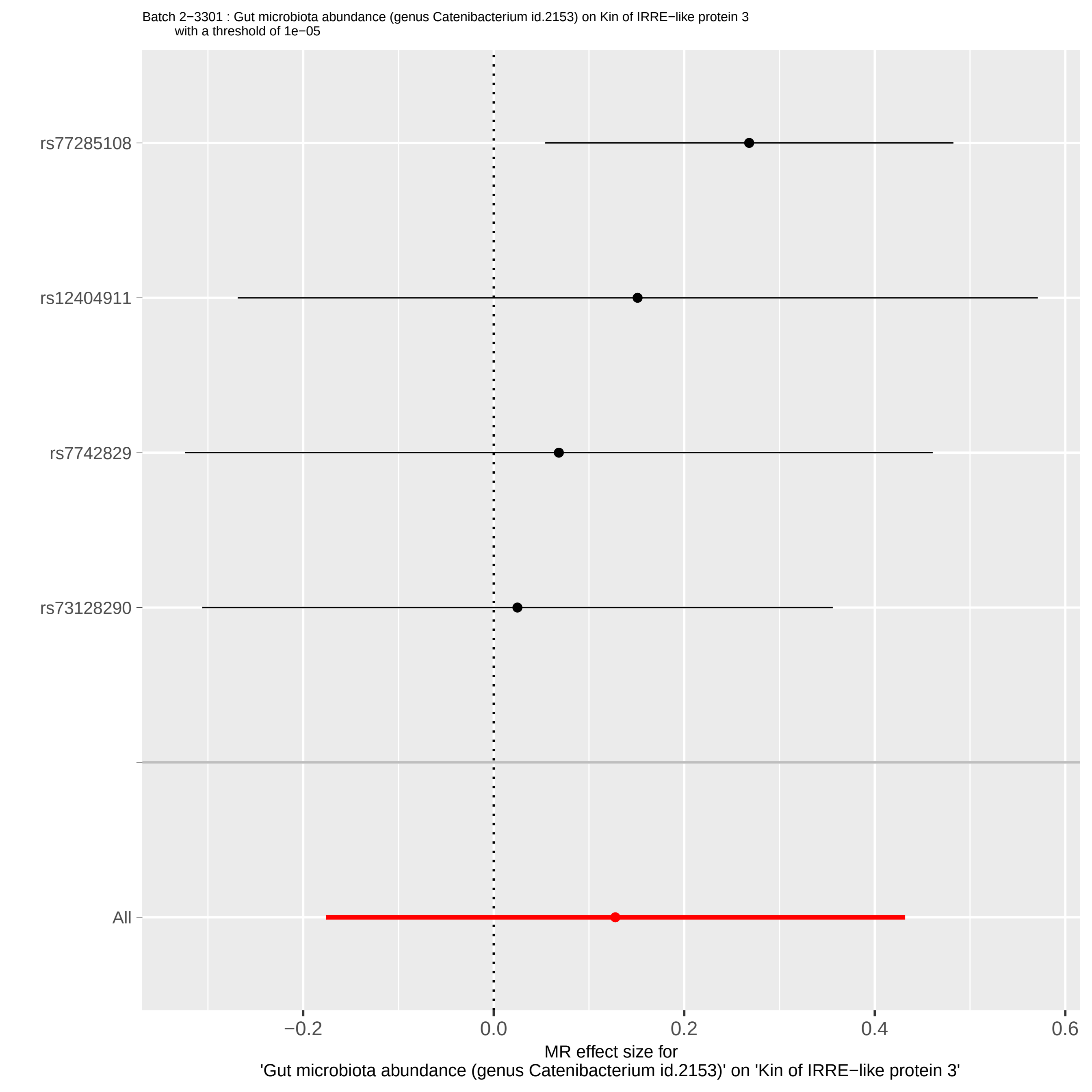

Batch 2−3301 : Gut microbiota abundance (genus Catenibacterium id.2153) on Kin of IRRE−like protein 3
with a threshold of 1e−05
rs77285108
rs12404911
rs7742829
rs73128290
All
−0.2
0.0
0.2
0.4
0.6
MR effect size for
'Gut microbiota abundance (genus Catenibacterium id.2153)' on 'Kin of IRRE−like protein 3'

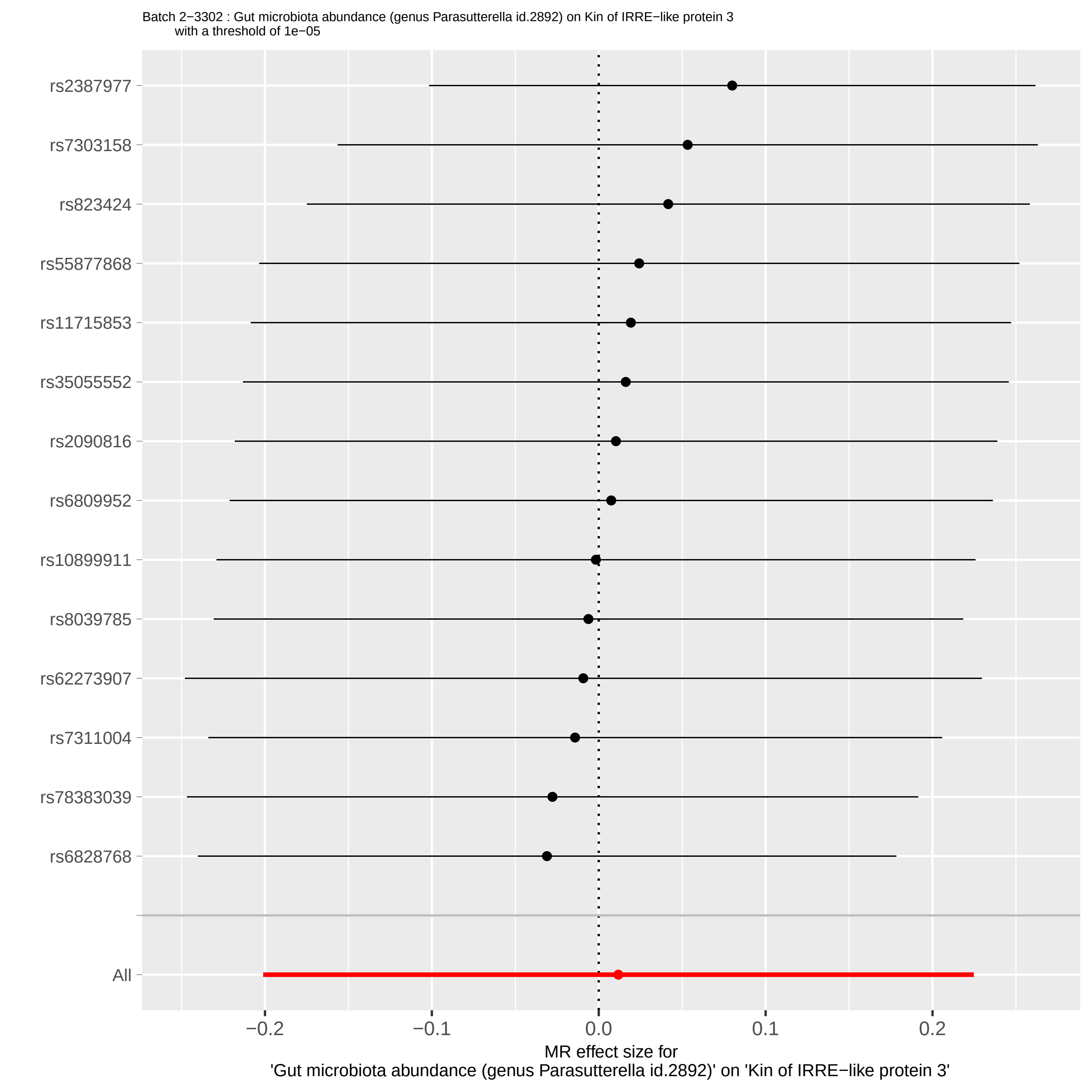

Batch 2−3302 : Gut microbiota abundance (genus Parasutterella id.2892) on Kin of IRRE−like protein 3
with a threshold of 1e−05
rs2387977
rs7303158
rs823424
rs55877868
rs11715853
rs35055552
rs2090816
rs6809952
rs10899911
rs8039785
rs62273907
rs7311004
rs78383039
rs6828768
All
−0.2
−0.1
0.0
0.1
0.2
MR effect size for
'Gut microbiota abundance (genus Parasutterella id.2892)' on 'Kin of IRRE−like protein 3'

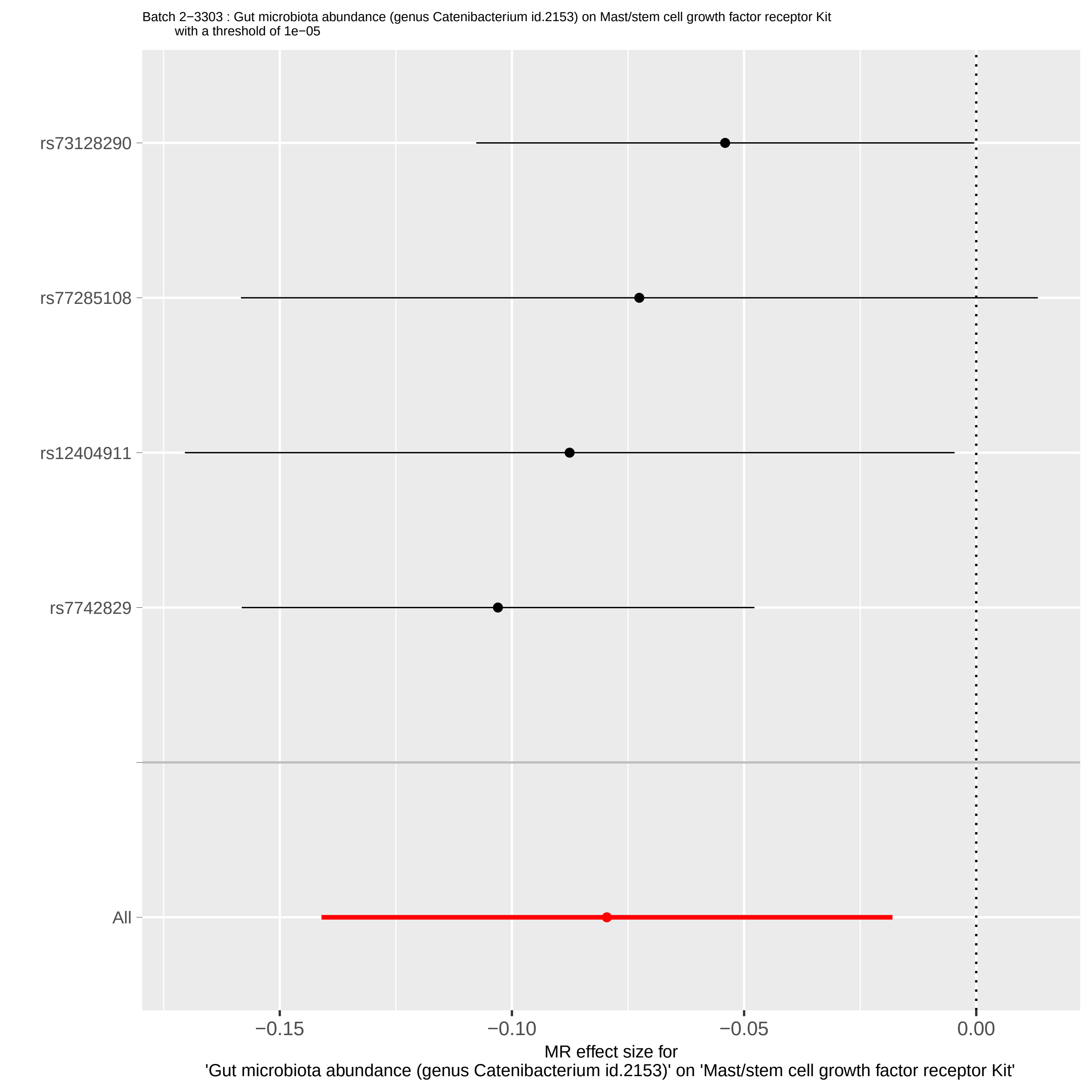

Batch 2−3303 : Gut microbiota abundance (genus Catenibacterium id.2153) on Mast/stem cell growth factor receptor Kit
with a threshold of 1e−05
rs73128290
rs77285108
rs12404911
rs7742829
All
−0.15
−0.10
−0.05
0.00
MR effect size for
'Gut microbiota abundance (genus Catenibacterium id.2153)' on 'Mast/stem cell growth factor receptor Kit'

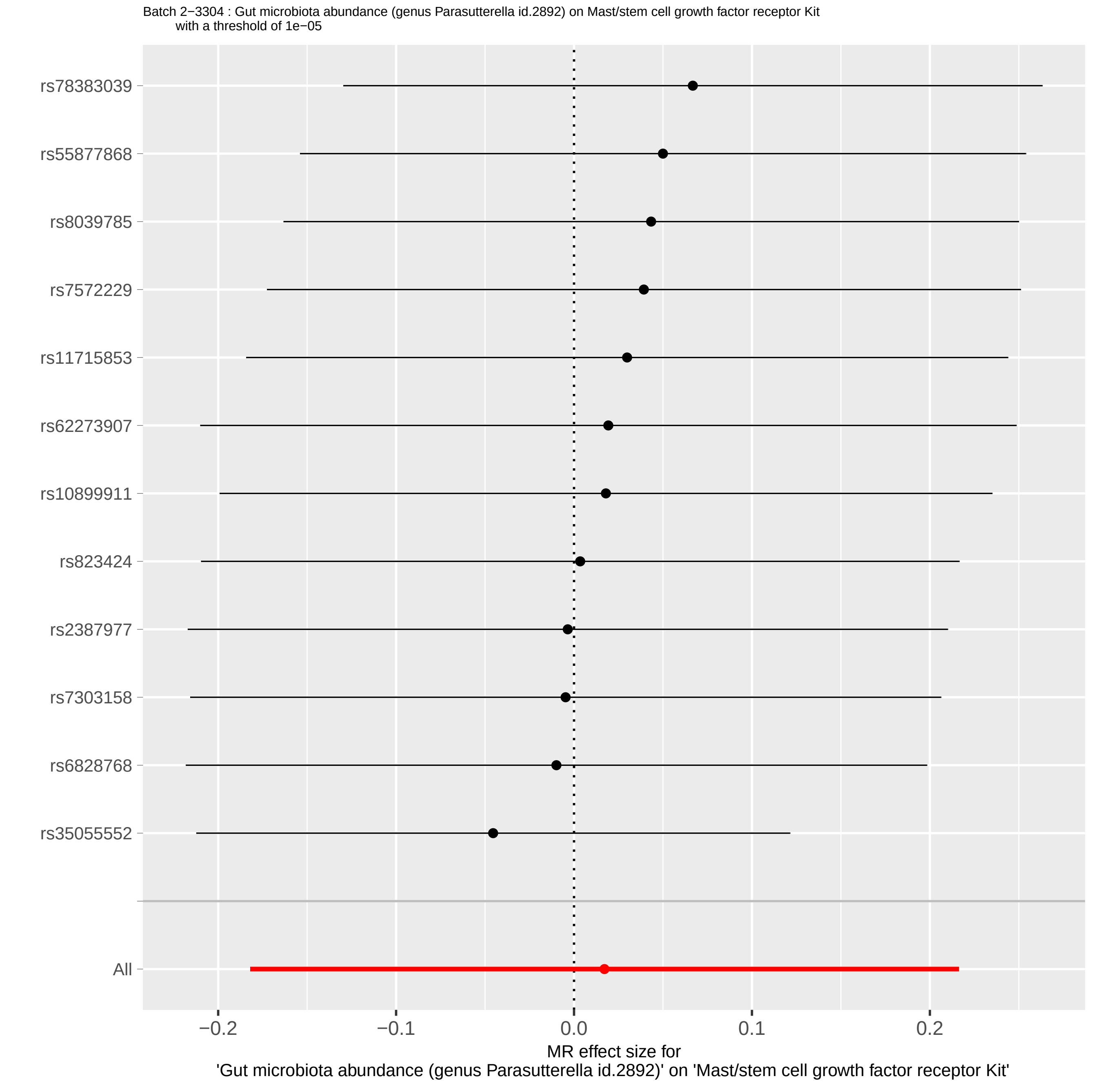
Batch 2−3304 : Gut microbiota abundance (genus Parasutterella id.2892) on Mast/stem cell growth factor receptor Kit
with a threshold of 1e−05
rs78383039
rs55877868
rs8039785
rs7572229
rs11715853
rs62273907
rs10899911
rs823424
rs2387977
rs7303158
rs6828768
rs35055552
All
−0.2
−0.1
0.0
0.1
0.2
MR effect size for
'Gut microbiota abundance (genus Parasutterella id.2892)' on 'Mast/stem cell growth factor receptor Kit'

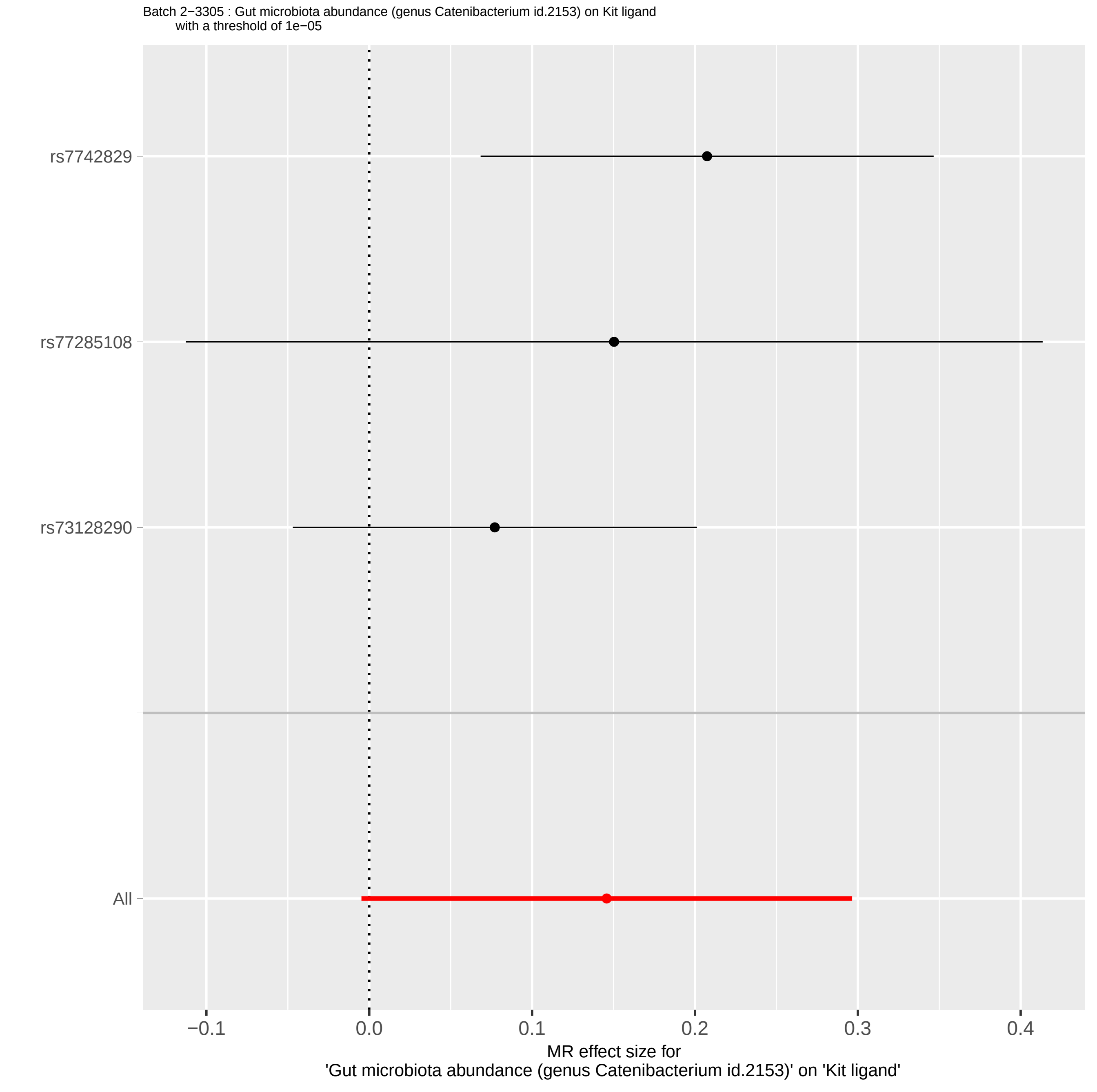

Batch 2−3305 : Gut microbiota abundance (genus Catenibacterium id.2153) on Kit ligand
with a threshold of 1e−05
rs7742829
rs77285108
rs73128290
All
−0.1
0.0
0.1
0.2
0.3
0.4
MR effect size for
'Gut microbiota abundance (genus Catenibacterium id.2153)' on 'Kit ligand'

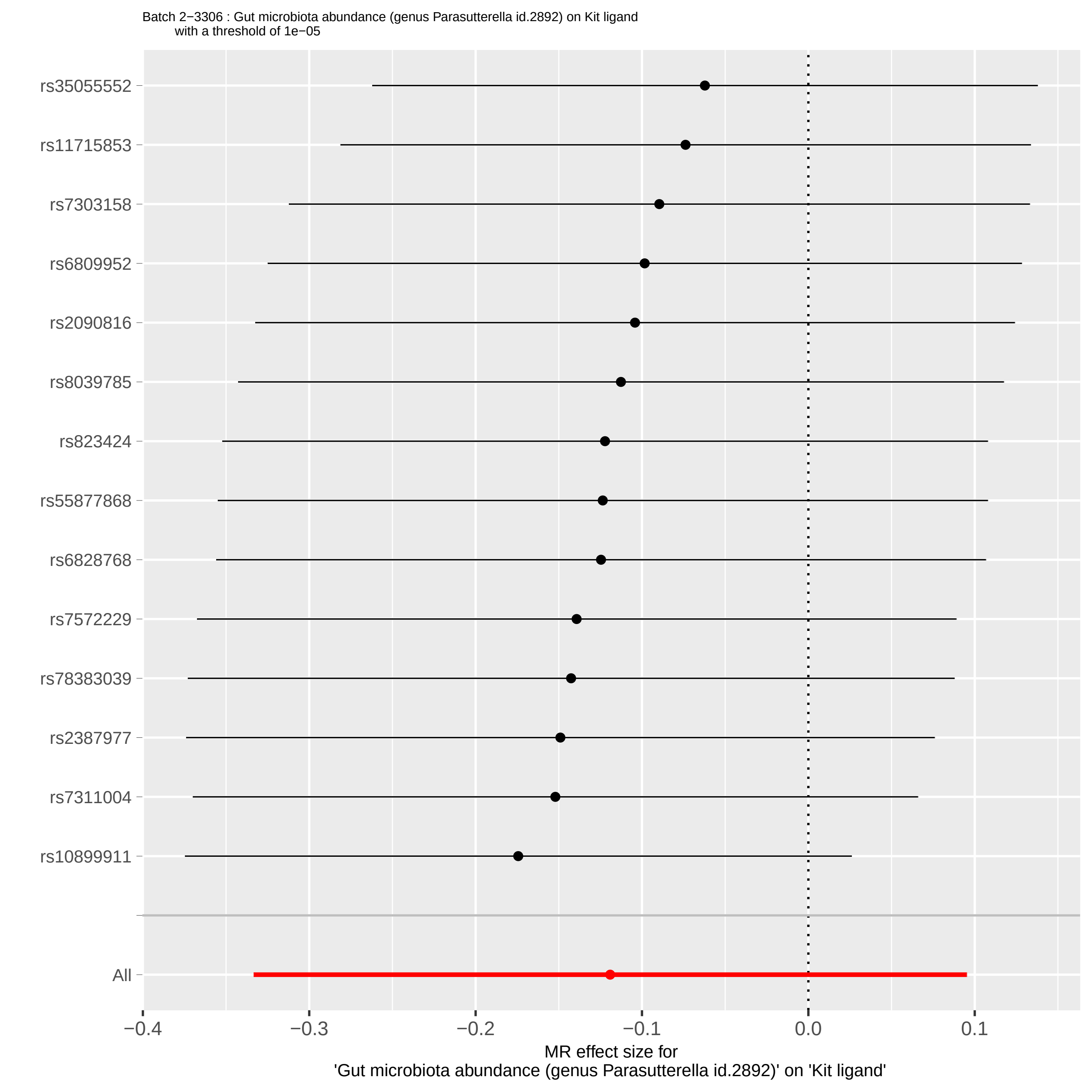
Batch 2−3306 : Gut microbiota abundance (genus Parasutterella id.2892) on Kit ligand
with a threshold of 1e−05
rs35055552
rs11715853
rs7303158
rs6809952
rs2090816
rs8039785
rs823424
rs55877868
rs6828768
rs7572229
rs78383039
rs2387977
rs7311004
rs10899911
All
−0.4
−0.3
−0.2
−0.1
0.0
0.1
MR effect size for
'Gut microbiota abundance (genus Parasutterella id.2892)' on 'Kit ligand'

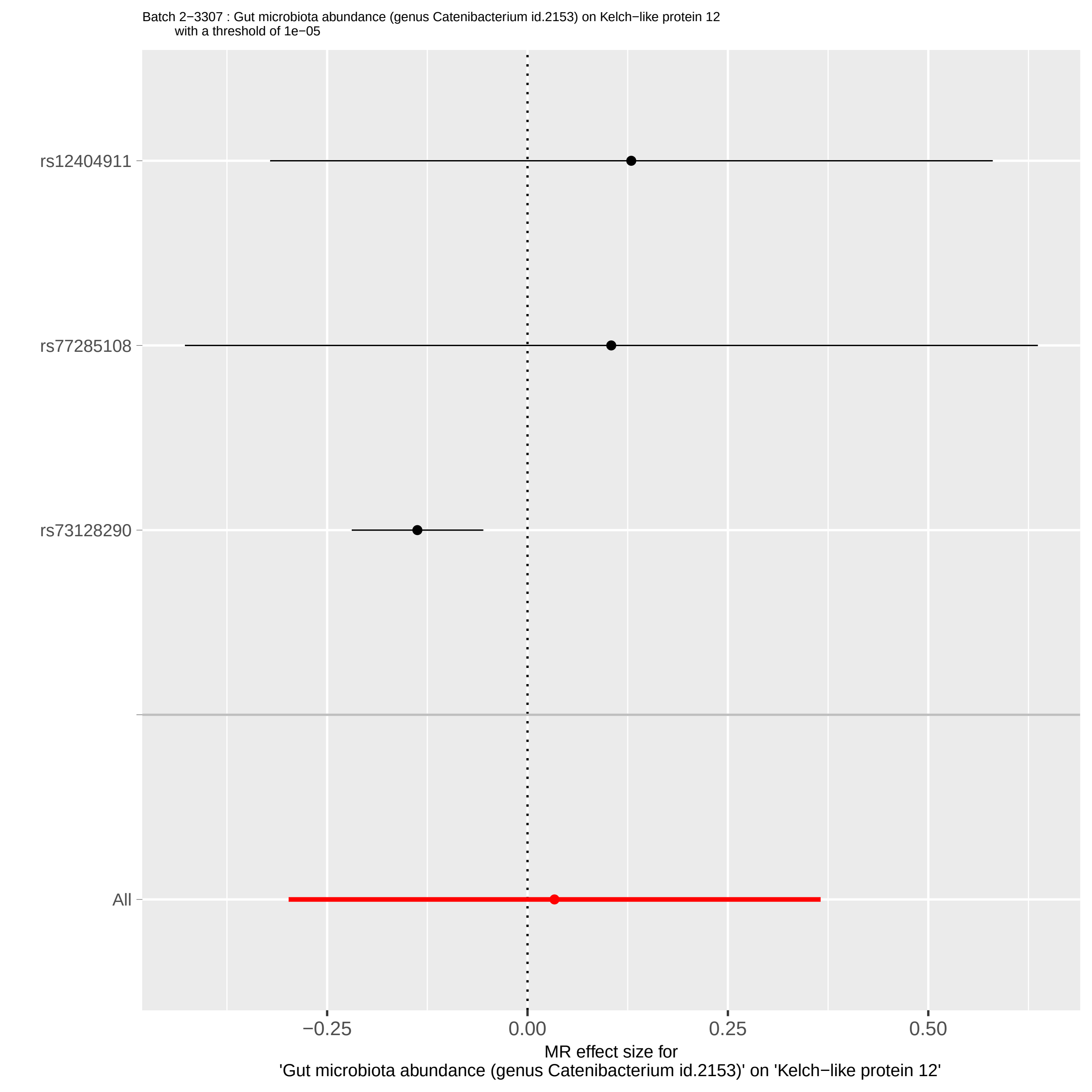
Batch 2−3307 : Gut microbiota abundance (genus Catenibacterium id.2153) on Kelch−like protein 12
with a threshold of 1e−05
rs12404911
rs77285108
rs73128290
All
−0.25
0.00
0.25
0.50
MR effect size for
'Gut microbiota abundance (genus Catenibacterium id.2153)' on 'Kelch−like protein 12'

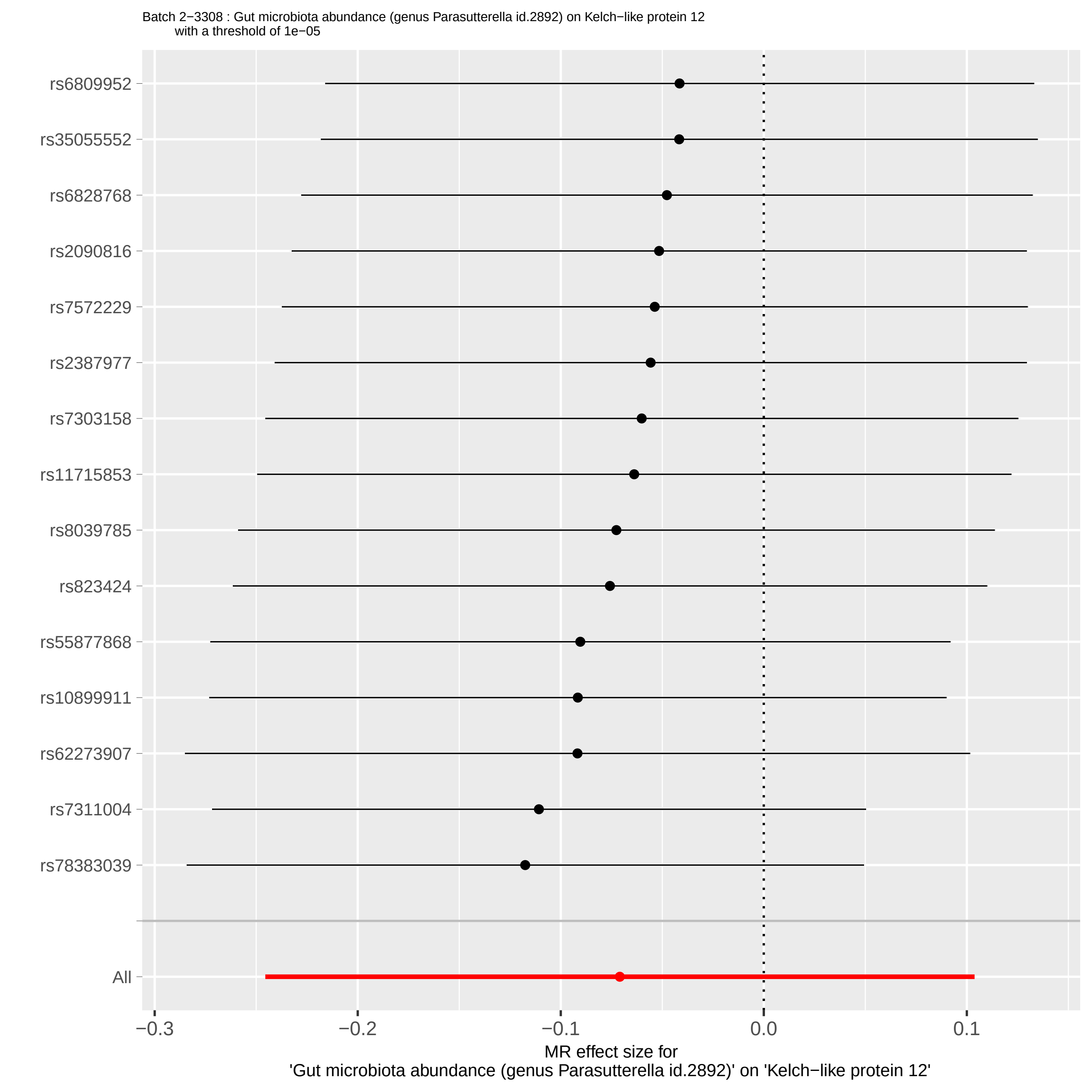

Batch 2−3308 : Gut microbiota abundance (genus Parasutterella id.2892) on Kelch−like protein 12
with a threshold of 1e−05
rs6809952
rs35055552
rs6828768
rs2090816
rs7572229
rs2387977
rs7303158
rs11715853
rs8039785
rs823424
rs55877868
rs10899911
rs62273907
rs7311004
rs78383039
All
−0.3
−0.2
−0.1
0.0
0.1
MR effect size for
'Gut microbiota abundance (genus Parasutterella id.2892)' on 'Kelch−like protein 12'

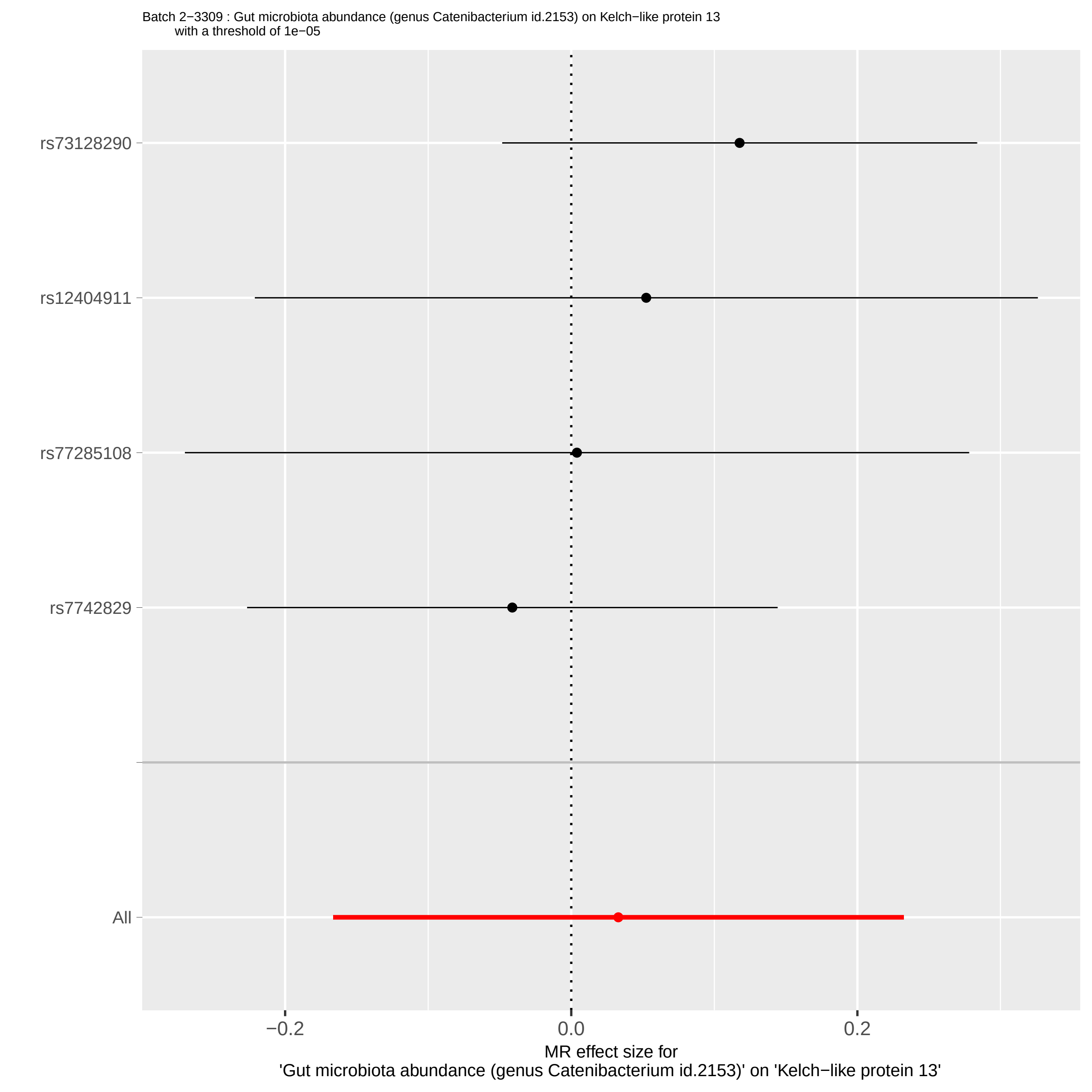

Batch 2−3309 : Gut microbiota abundance (genus Catenibacterium id.2153) on Kelch−like protein 13
with a threshold of 1e−05
rs73128290
rs12404911
rs77285108
rs7742829
All
−0.2
0.0
0.2
MR effect size for
'Gut microbiota abundance (genus Catenibacterium id.2153)' on 'Kelch−like protein 13'

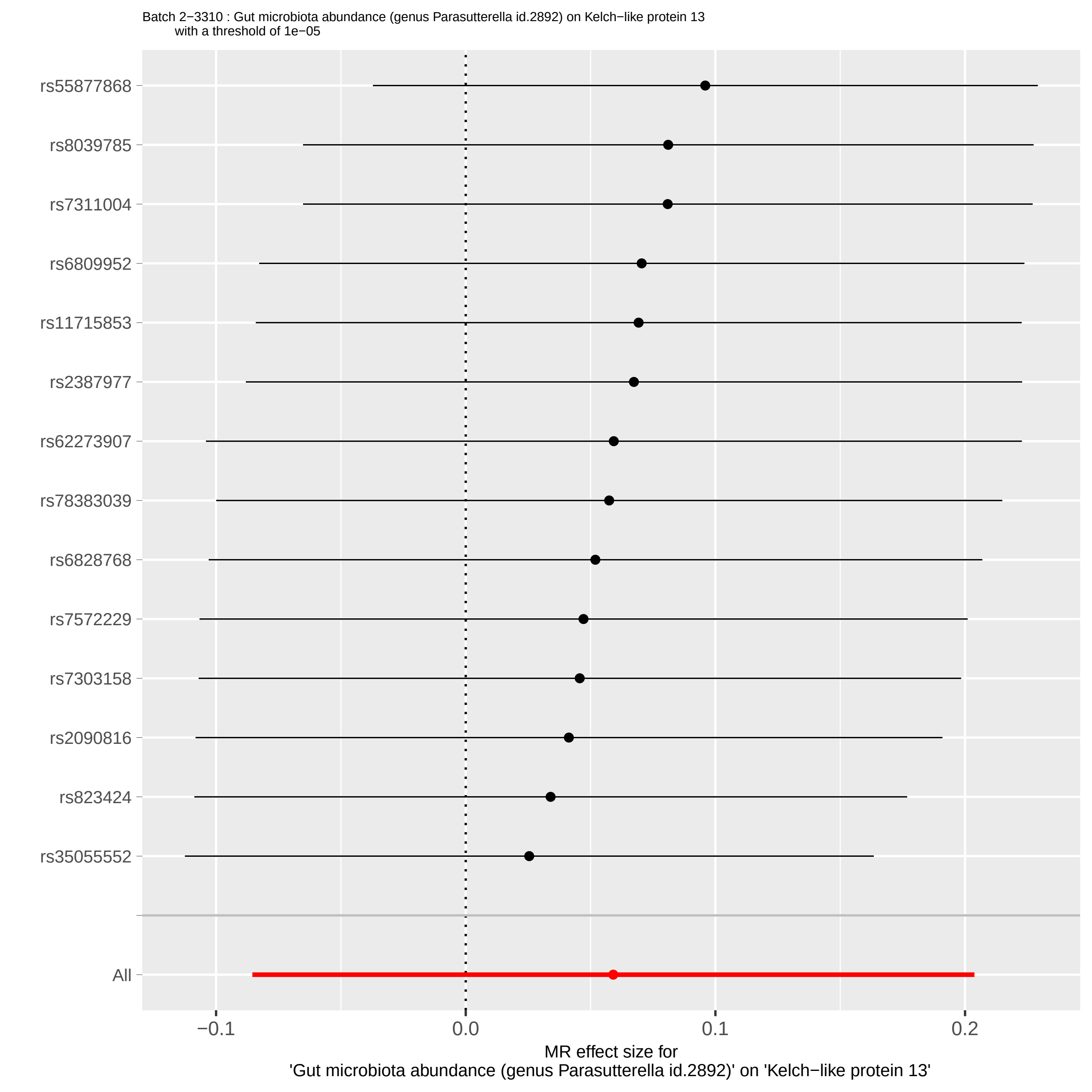

Batch 2−3310 : Gut microbiota abundance (genus Parasutterella id.2892) on Kelch−like protein 13
with a threshold of 1e−05
rs55877868
rs8039785
rs7311004
rs6809952
rs11715853
rs2387977
rs62273907
rs78383039
rs6828768
rs7572229
rs7303158
rs2090816
rs823424
rs35055552
All
−0.1
0.0
0.1
0.2
MR effect size for
'Gut microbiota abundance (genus Parasutterella id.2892)' on 'Kelch−like protein 13'

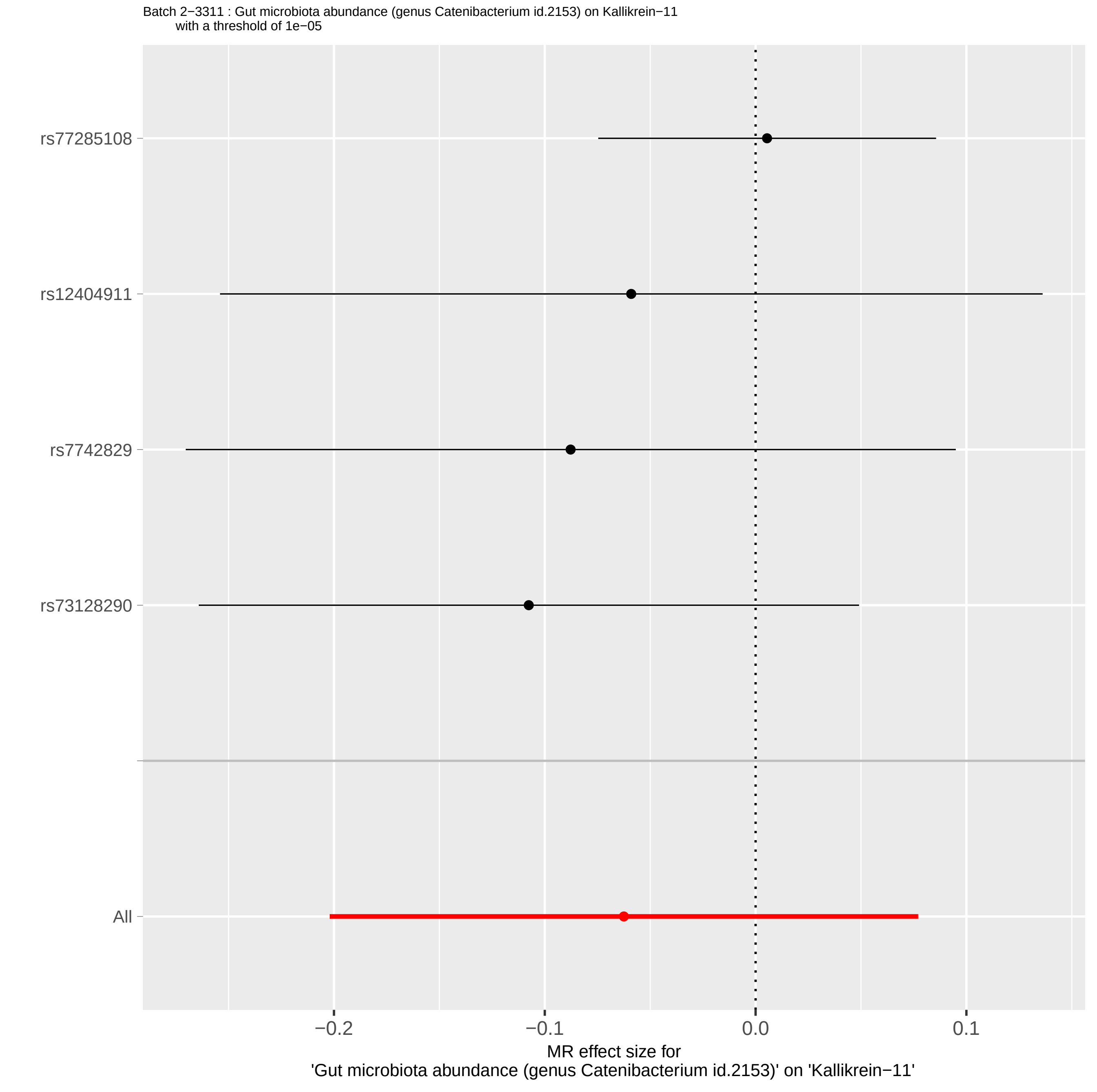
Batch 2−3311 : Gut microbiota abundance (genus Catenibacterium id.2153) on Kallikrein−11
with a threshold of 1e−05
rs77285108
rs12404911
rs7742829
rs73128290
All
−0.2
−0.1
0.0
0.1
MR effect size for
'Gut microbiota abundance (genus Catenibacterium id.2153)' on 'Kallikrein−11'

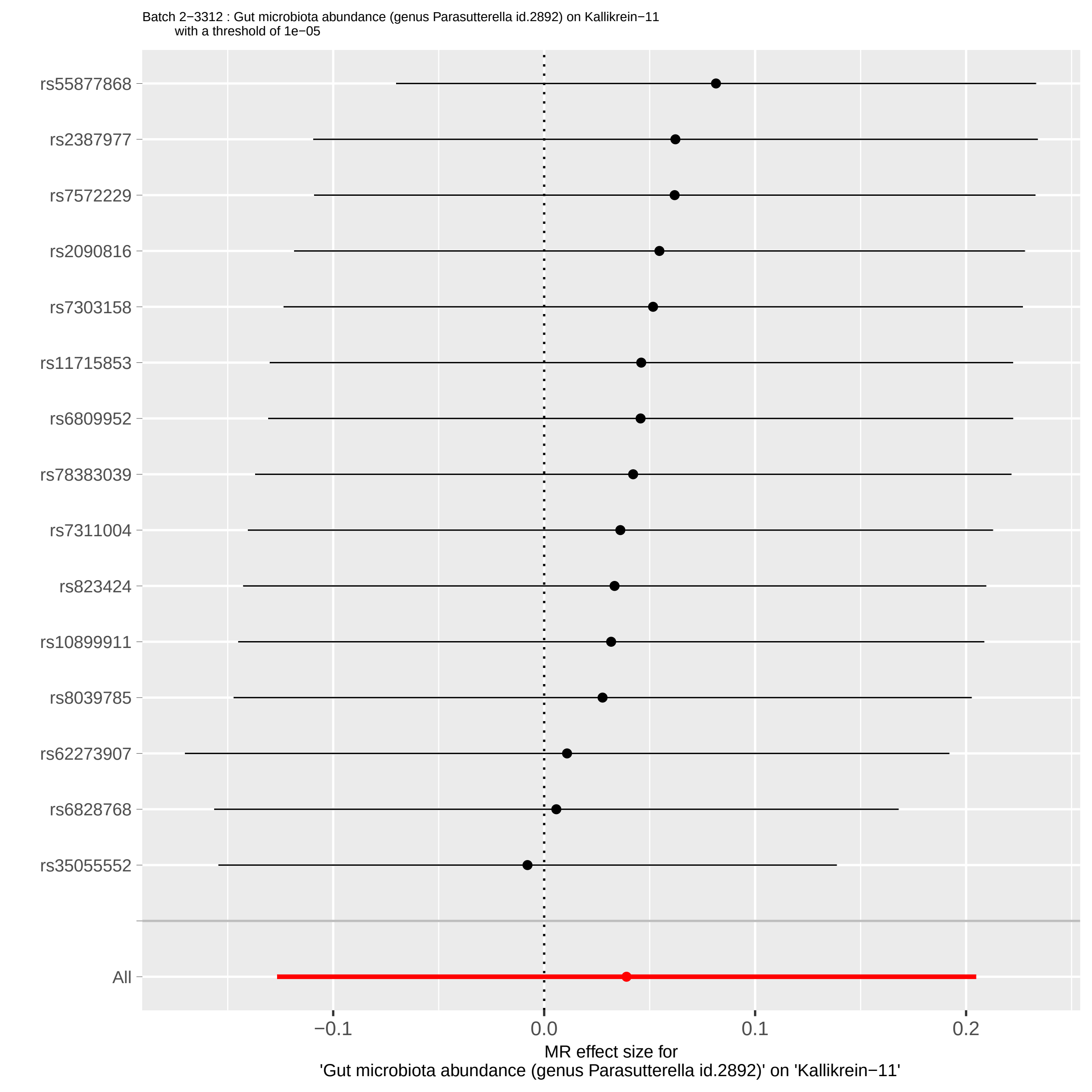

Batch 2−3312 : Gut microbiota abundance (genus Parasutterella id.2892) on Kallikrein−11
with a threshold of 1e−05
rs55877868
rs2387977
rs7572229
rs2090816
rs7303158
rs11715853
rs6809952
rs78383039
rs7311004
rs823424
rs10899911
rs8039785
rs62273907
rs6828768
rs35055552
All
−0.1
0.0
0.1
0.2
MR effect size for
'Gut microbiota abundance (genus Parasutterella id.2892)' on 'Kallikrein−11'

Batch 2−3313 : Gut microbiota abundance (genus Catenibacterium id.2153) on Kallikrein−12
with a threshold of 1e−05

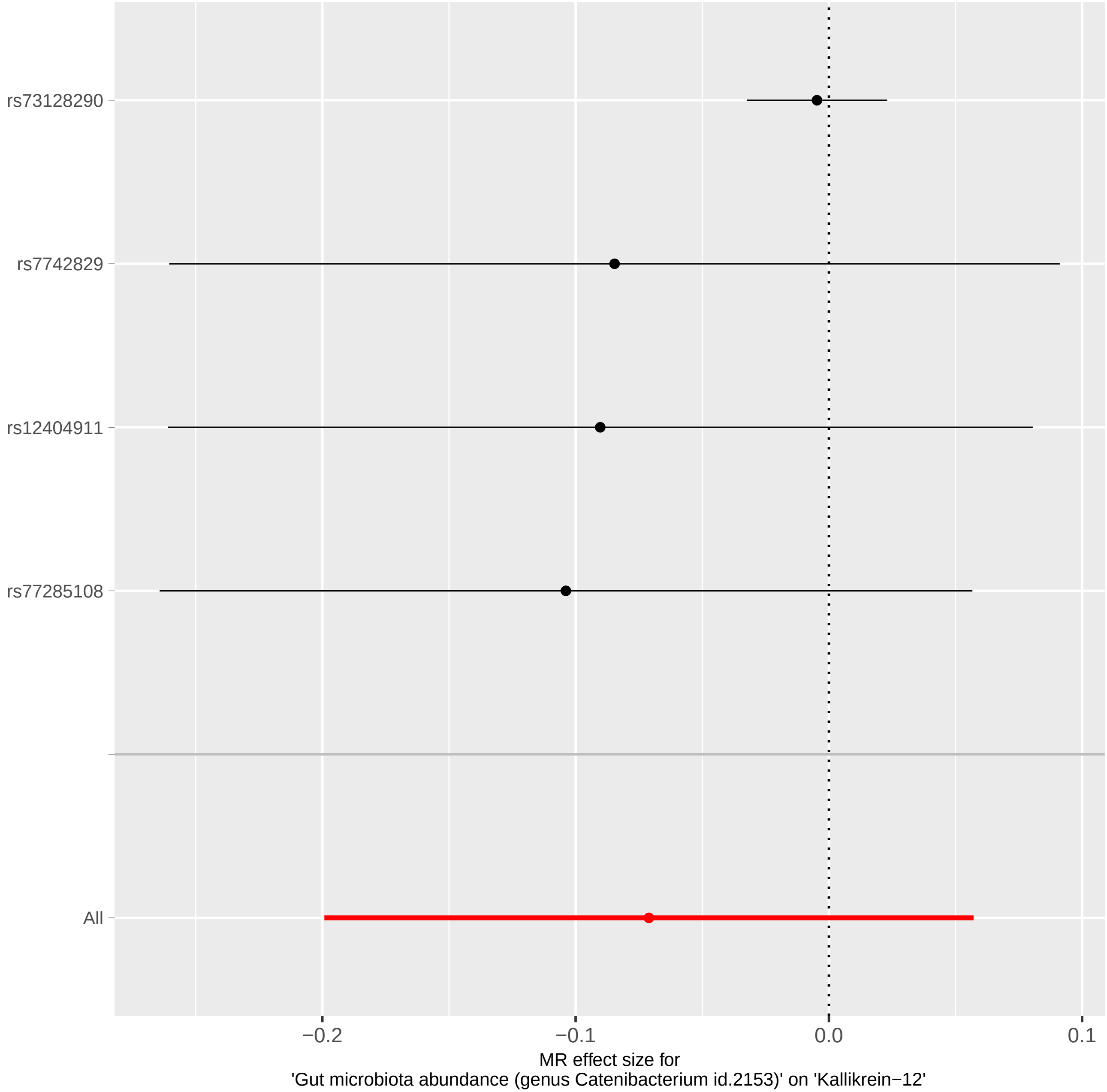

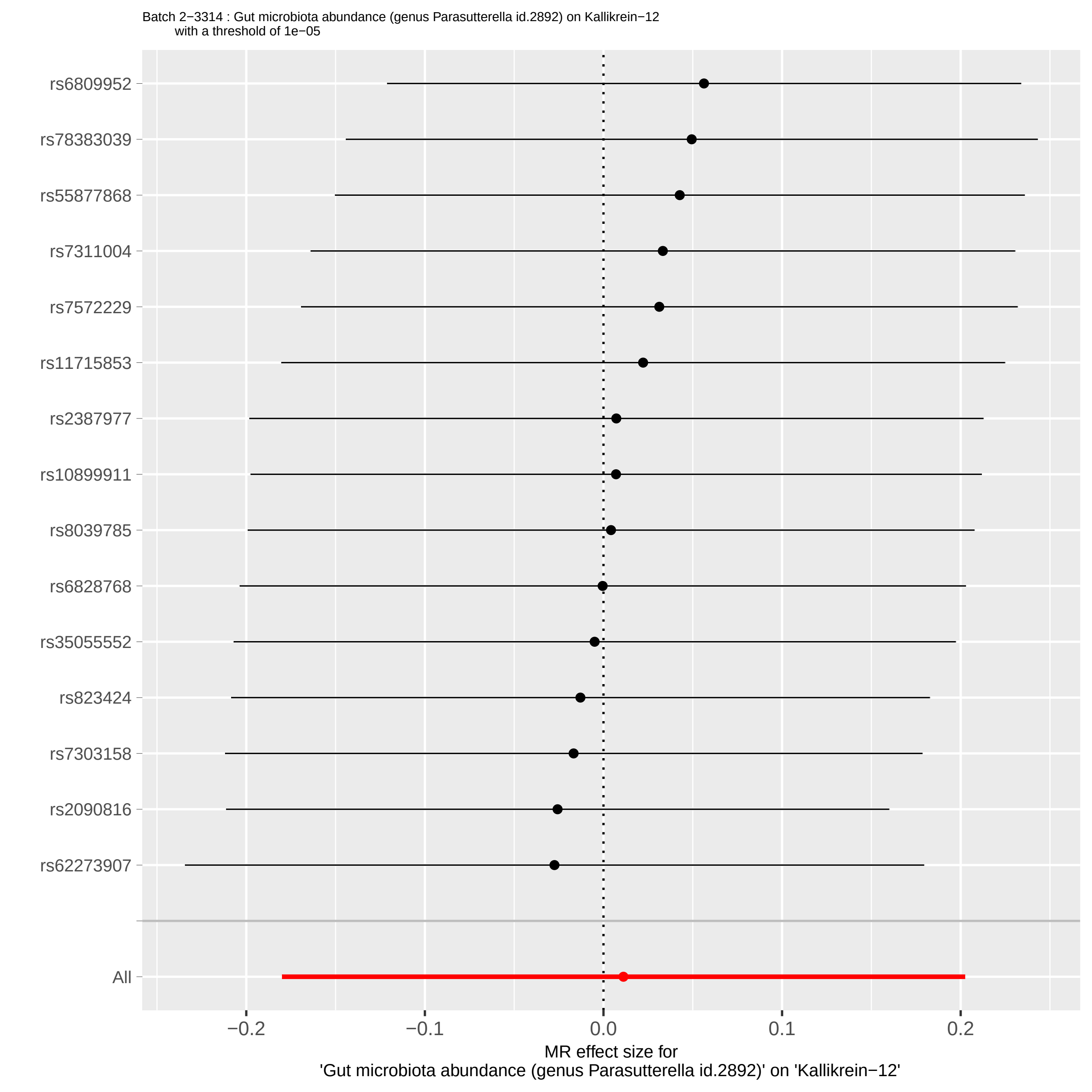

Batch 2−3314 : Gut microbiota abundance (genus Parasutterella id.2892) on Kallikrein−12
with a threshold of 1e−05
rs6809952
rs78383039
rs55877868
rs7311004
rs7572229
rs11715853
rs2387977
rs10899911
rs8039785
rs6828768
rs35055552
rs823424
rs7303158
rs2090816
rs62273907
All
−0.2
−0.1
0.0
0.1
0.2
MR effect size for
'Gut microbiota abundance (genus Parasutterella id.2892)' on 'Kallikrein−12'

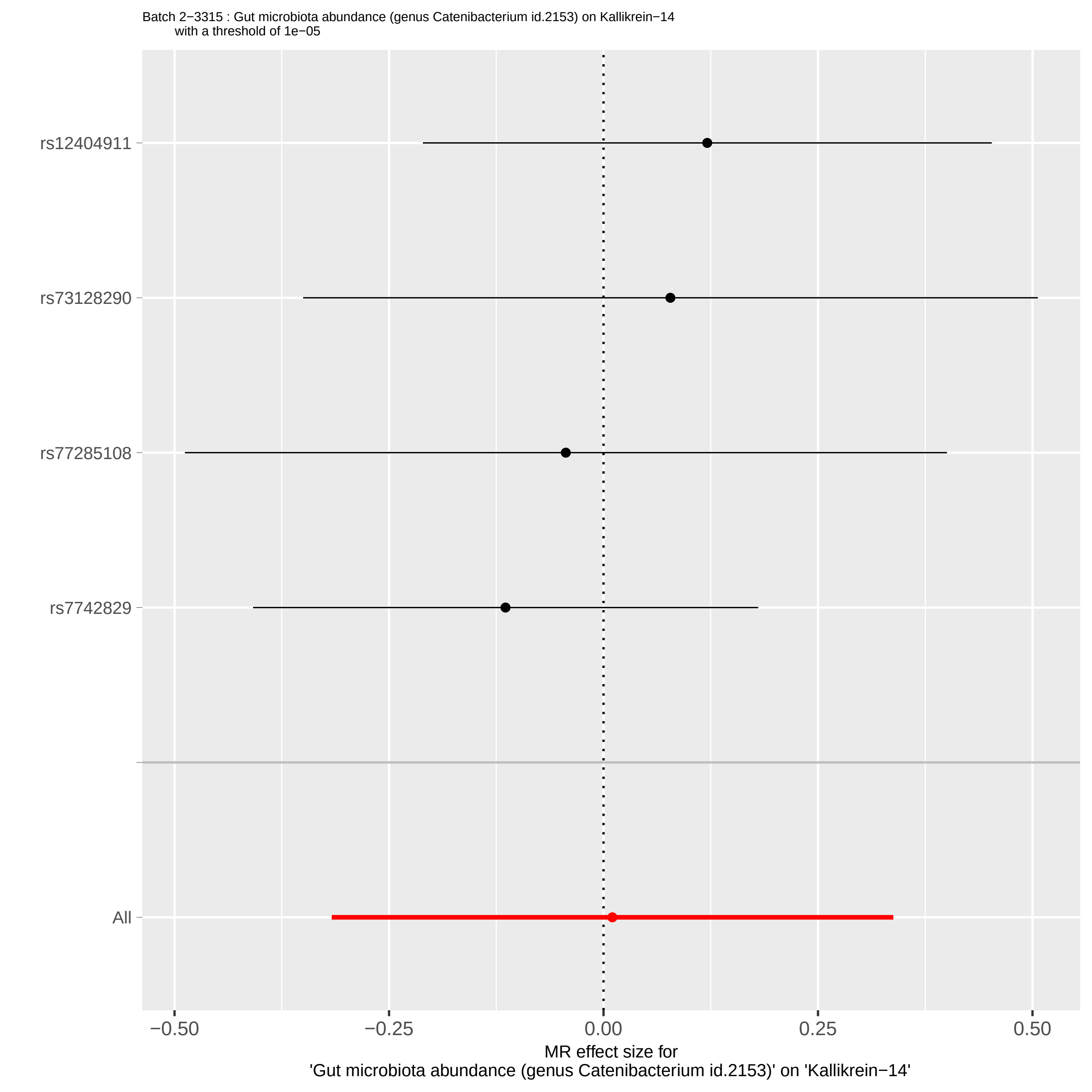

Batch 2−3315 : Gut microbiota abundance (genus Catenibacterium id.2153) on Kallikrein−14
with a threshold of 1e−05
rs12404911
rs73128290
rs77285108
rs7742829
All
−0.50
−0.25
0.00
0.25
0.50
MR effect size for
'Gut microbiota abundance (genus Catenibacterium id.2153)' on 'Kallikrein−14'

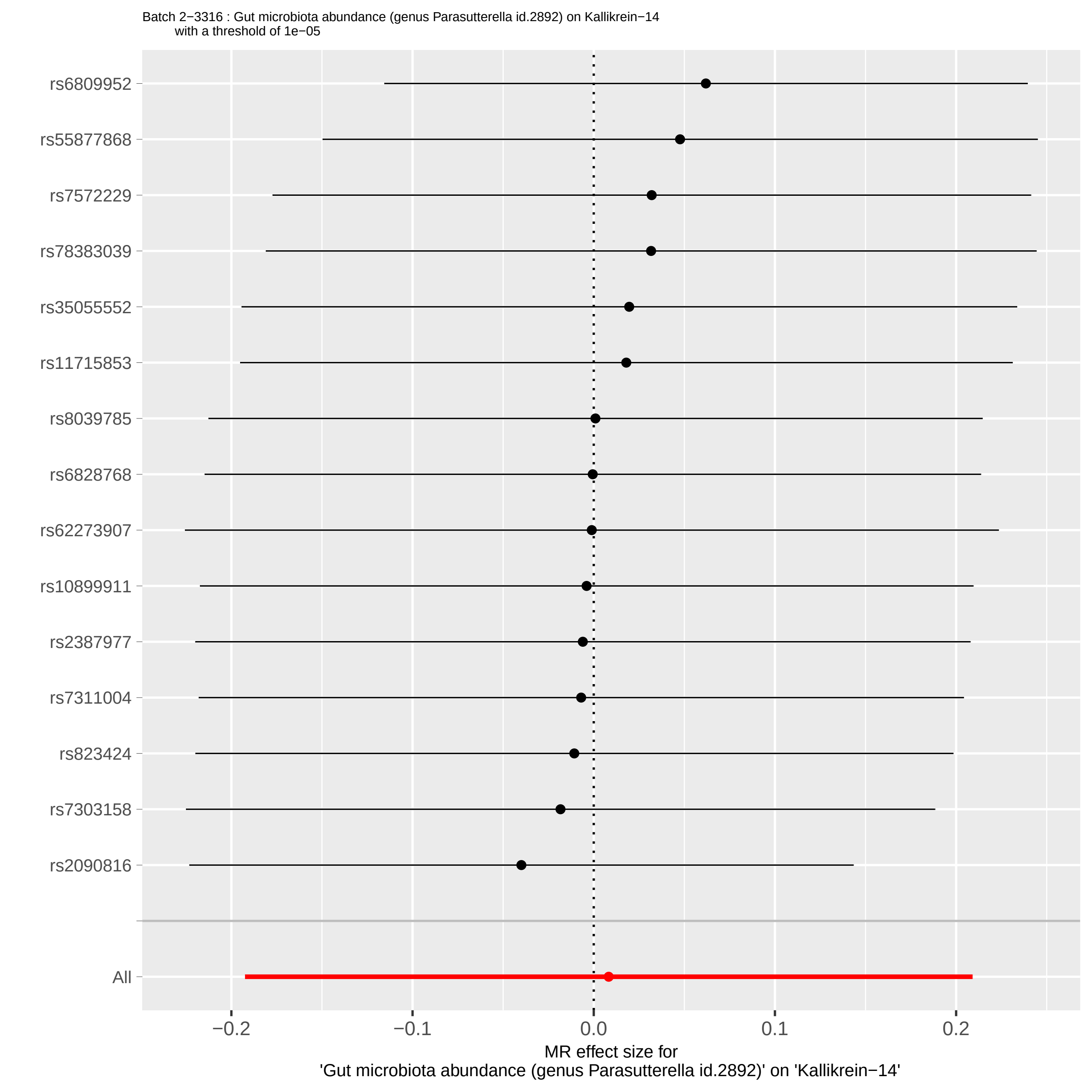

Batch 2−3316 : Gut microbiota abundance (genus Parasutterella id.2892) on Kallikrein−14
with a threshold of 1e−05
rs6809952
rs55877868
rs7572229
rs78383039
rs35055552
rs11715853
rs8039785
rs6828768
rs62273907
rs10899911
rs2387977
rs7311004
rs823424
rs7303158
rs2090816
All
−0.2
−0.1
0.0
0.1
0.2
MR effect size for
'Gut microbiota abundance (genus Parasutterella id.2892)' on 'Kallikrein−14'

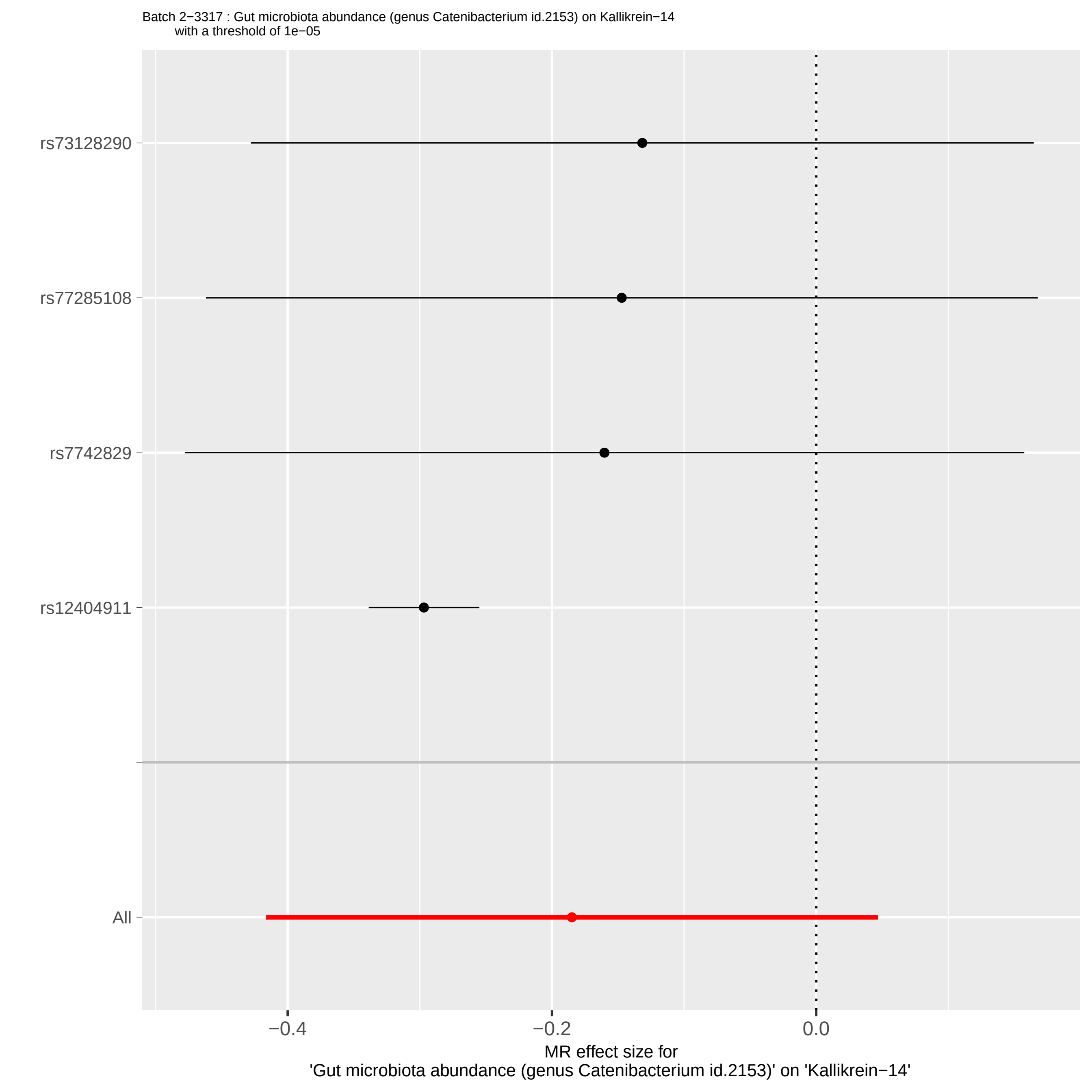
Batch 2−3317 : Gut microbiota abundance (genus Catenibacterium id.2153) on Kallikrein−14
with a threshold of 1e−05
rs73128290
rs77285108
rs7742829
rs12404911
All
−0.4
−0.2
0.0
MR effect size for
'Gut microbiota abundance (genus Catenibacterium id.2153)' on 'Kallikrein−14'

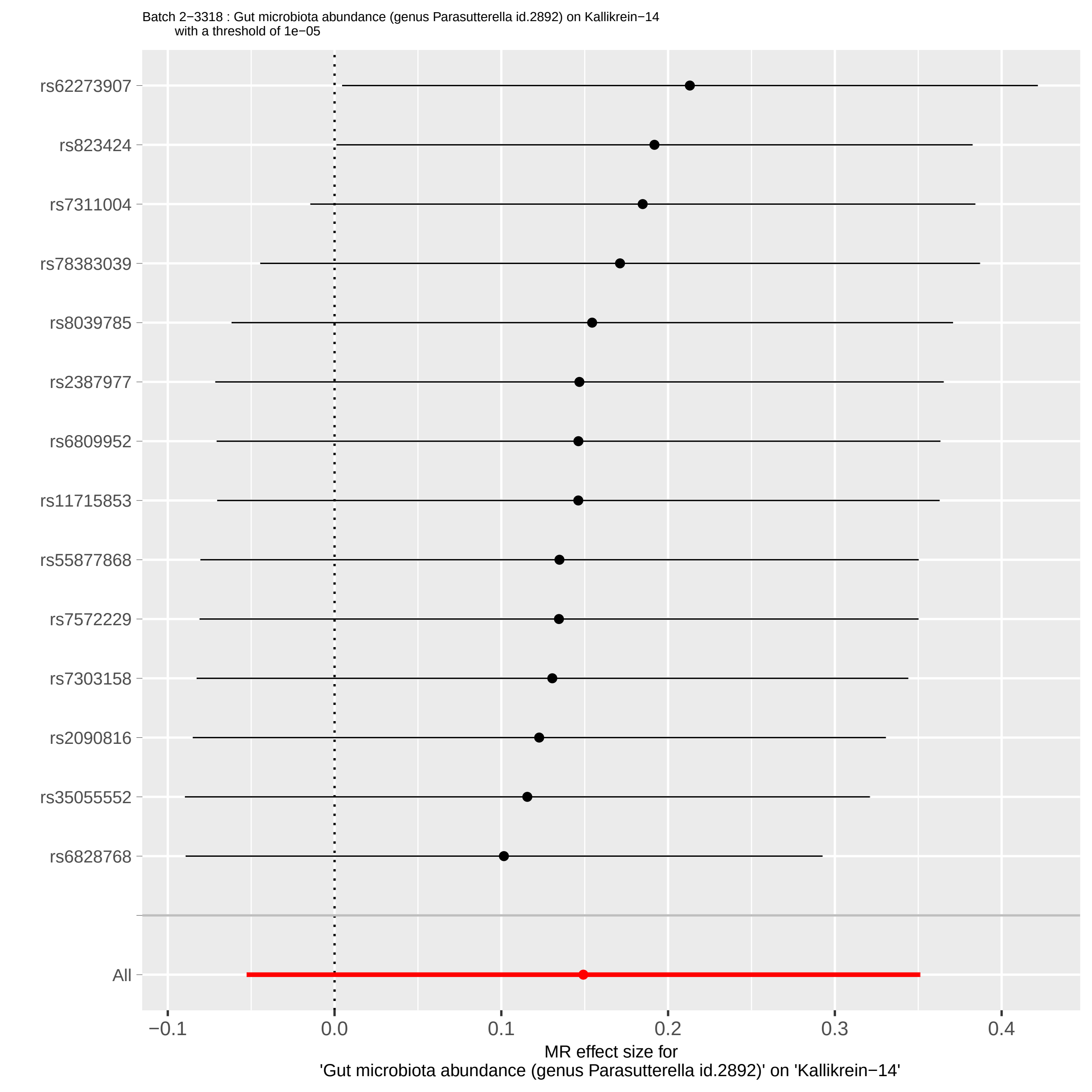
Batch 2−3318 : Gut microbiota abundance (genus Parasutterella id.2892) on Kallikrein−14
with a threshold of 1e−05
rs62273907
rs823424
rs7311004
rs78383039
rs8039785
rs2387977
rs6809952
rs11715853
rs55877868
rs7572229
rs7303158
rs2090816
rs35055552
rs6828768
All
−0.1
0.0
0.1
0.2
0.3
0.4
MR effect size for
'Gut microbiota abundance (genus Parasutterella id.2892)' on 'Kallikrein−14

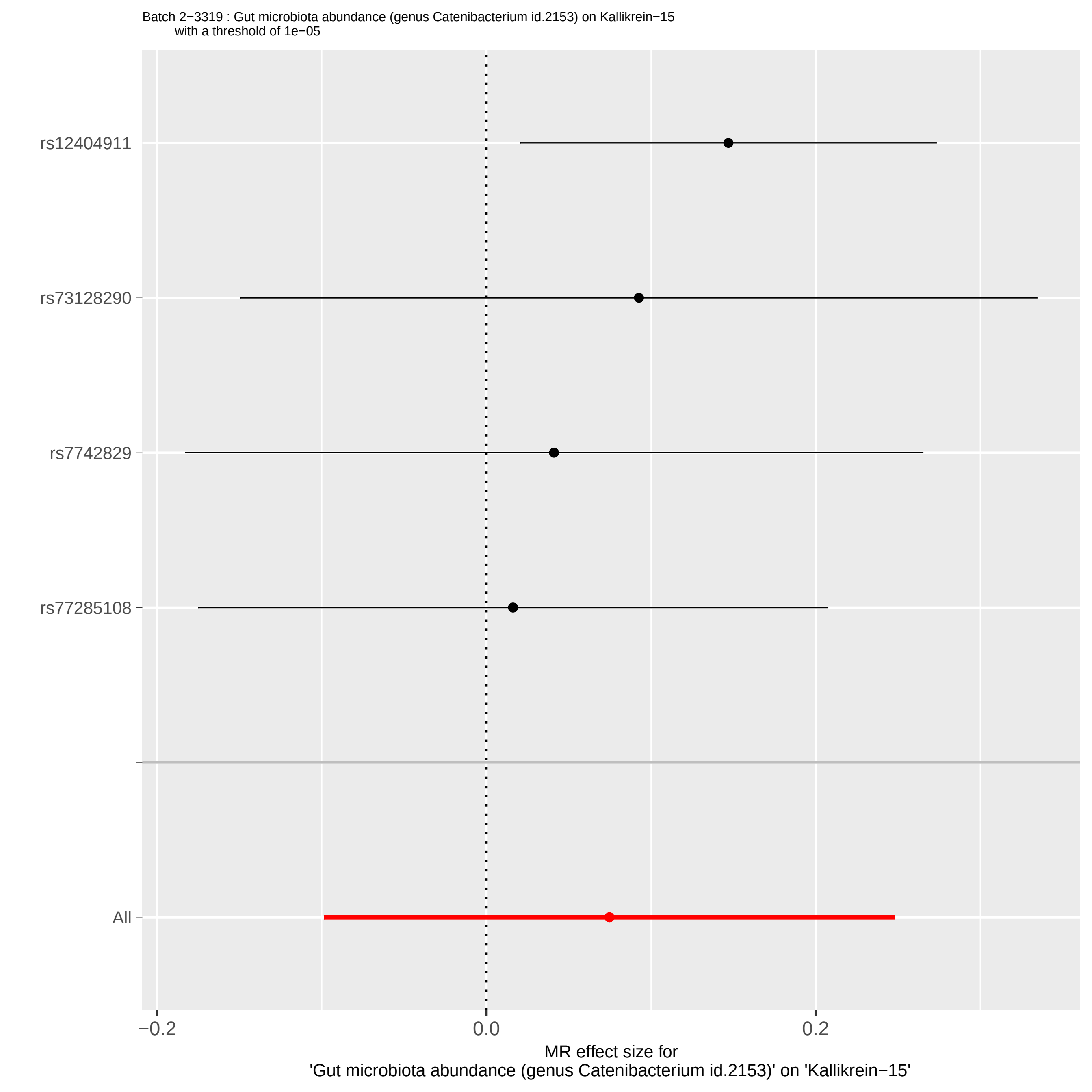

Batch 2−3319 : Gut microbiota abundance (genus Catenibacterium id.2153) on Kallikrein−15
with a threshold of 1e−05
rs12404911
rs73128290
rs7742829
rs77285108
All
−0.2
0.0
0.2
MR effect size for
'Gut microbiota abundance (genus Catenibacterium id.2153)' on 'Kallikrein−15'

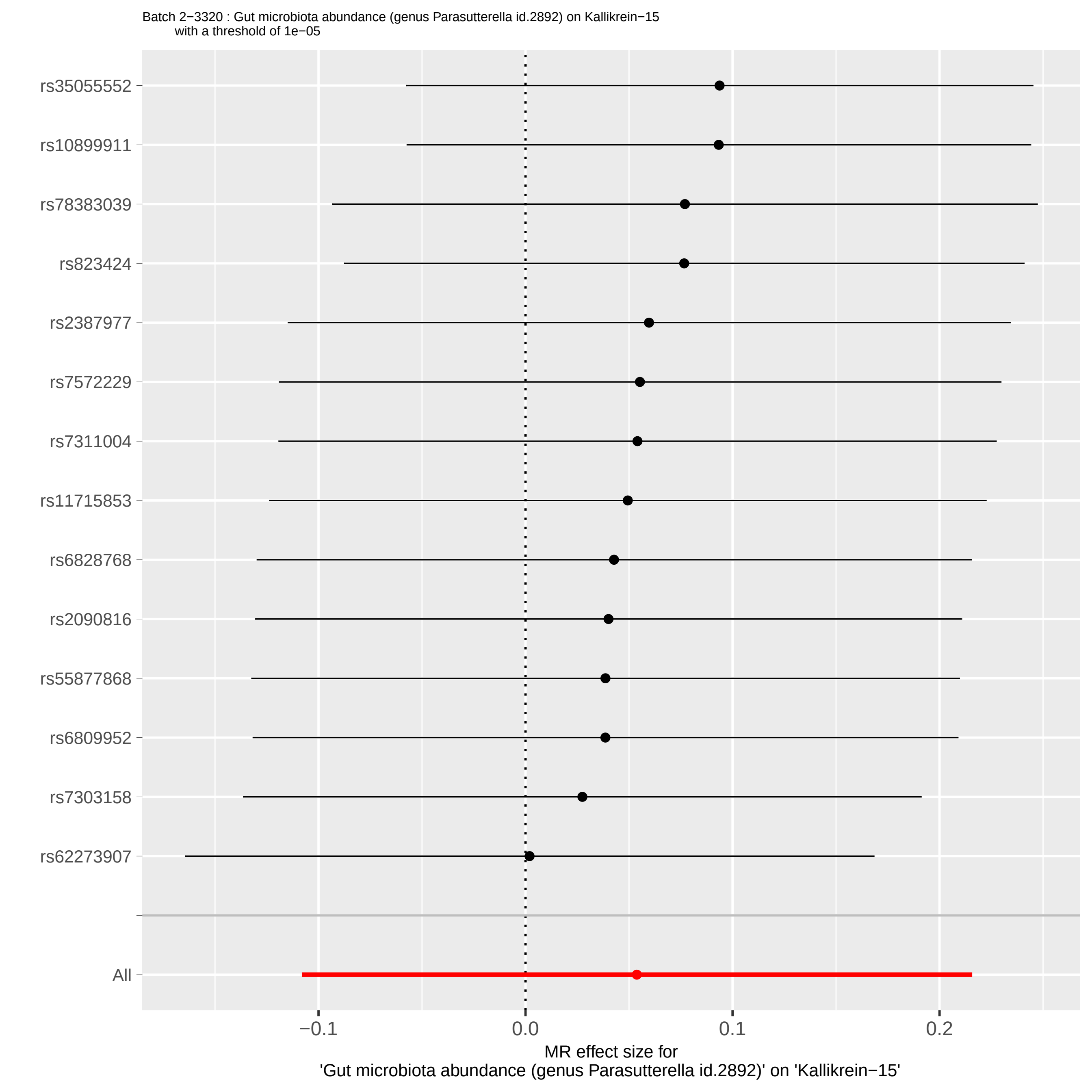

Batch 2−3320 : Gut microbiota abundance (genus Parasutterella id.2892) on Kallikrein−15
with a threshold of 1e−05
rs35055552
rs10899911
rs78383039
rs823424
rs2387977
rs7572229
rs7311004
rs11715853
rs6828768
rs2090816
rs55877868
rs6809952
rs7303158
rs62273907
All
−0.1
0.0
0.1
0.2
MR effect size for
'Gut microbiota abundance (genus Parasutterella id.2892)' on 'Kallikrein−15'

Batch 2−3321 : Gut microbiota abundance (genus Catenibacterium id.2153) on Prostate−specific antigen
with a threshold of 1e−05

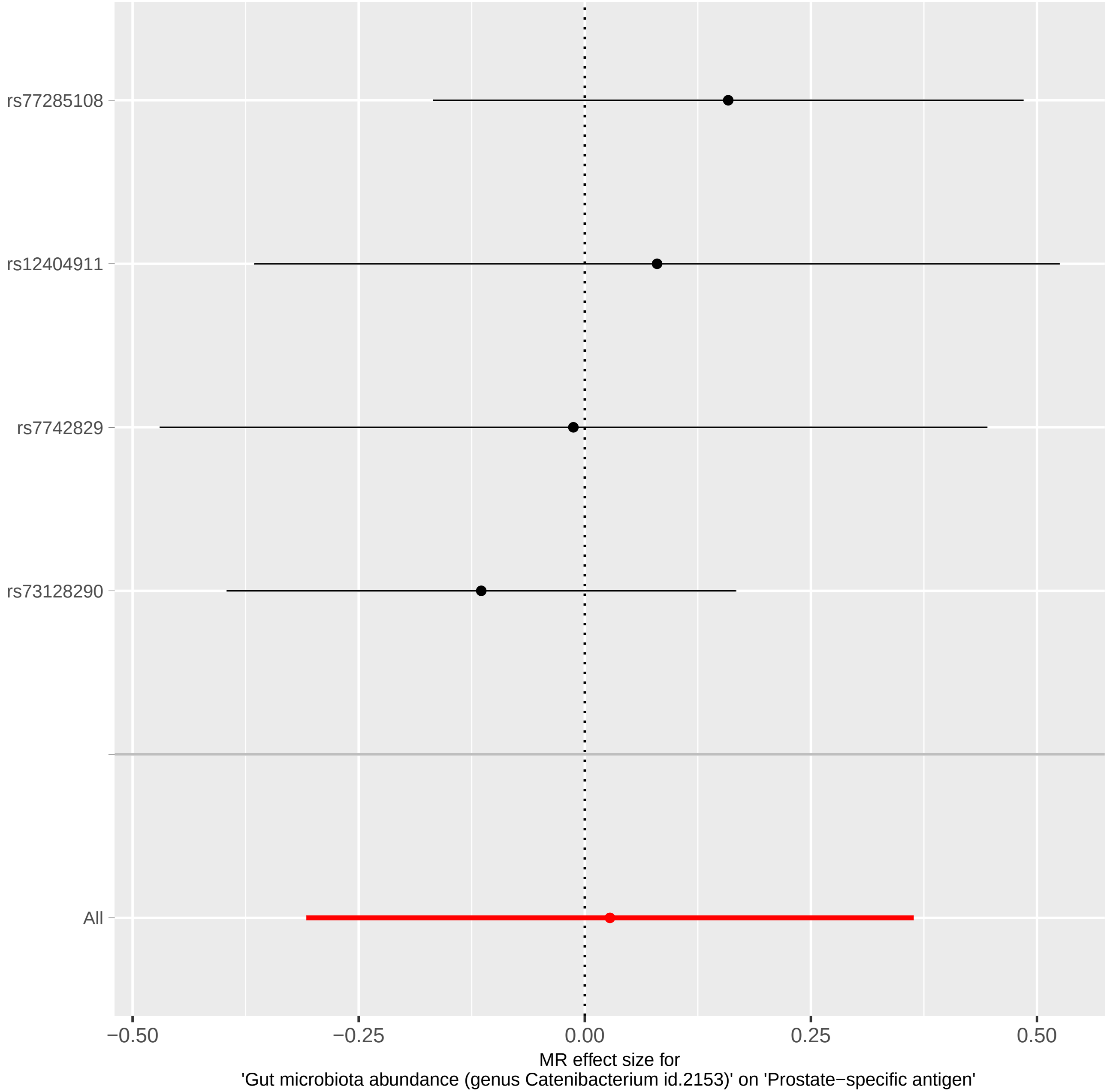

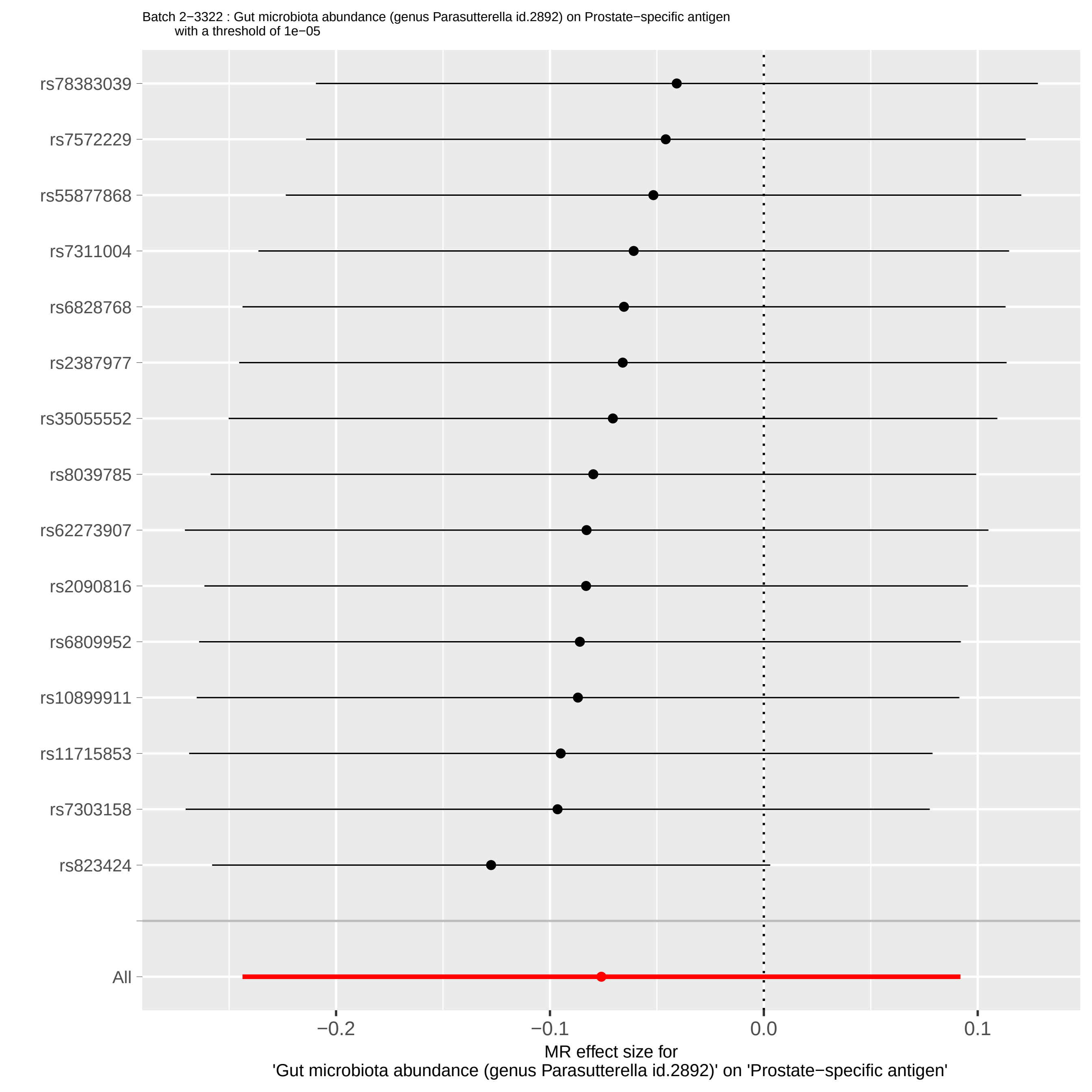

Batch 2−3322 : Gut microbiota abundance (genus Parasutterella id.2892) on Prostate−specific antigen
with a threshold of 1e−05
rs78383039
rs7572229
rs55877868
rs7311004
rs6828768
rs2387977
rs35055552
rs8039785
rs62273907
rs2090816
rs6809952
rs10899911
rs11715853
rs7303158
rs823424
All
−0.2
−0.1
0.0
0.1
MR effect size for
'Gut microbiota abundance (genus Parasutterella id.2892)' on 'Prostate−specific antigen'

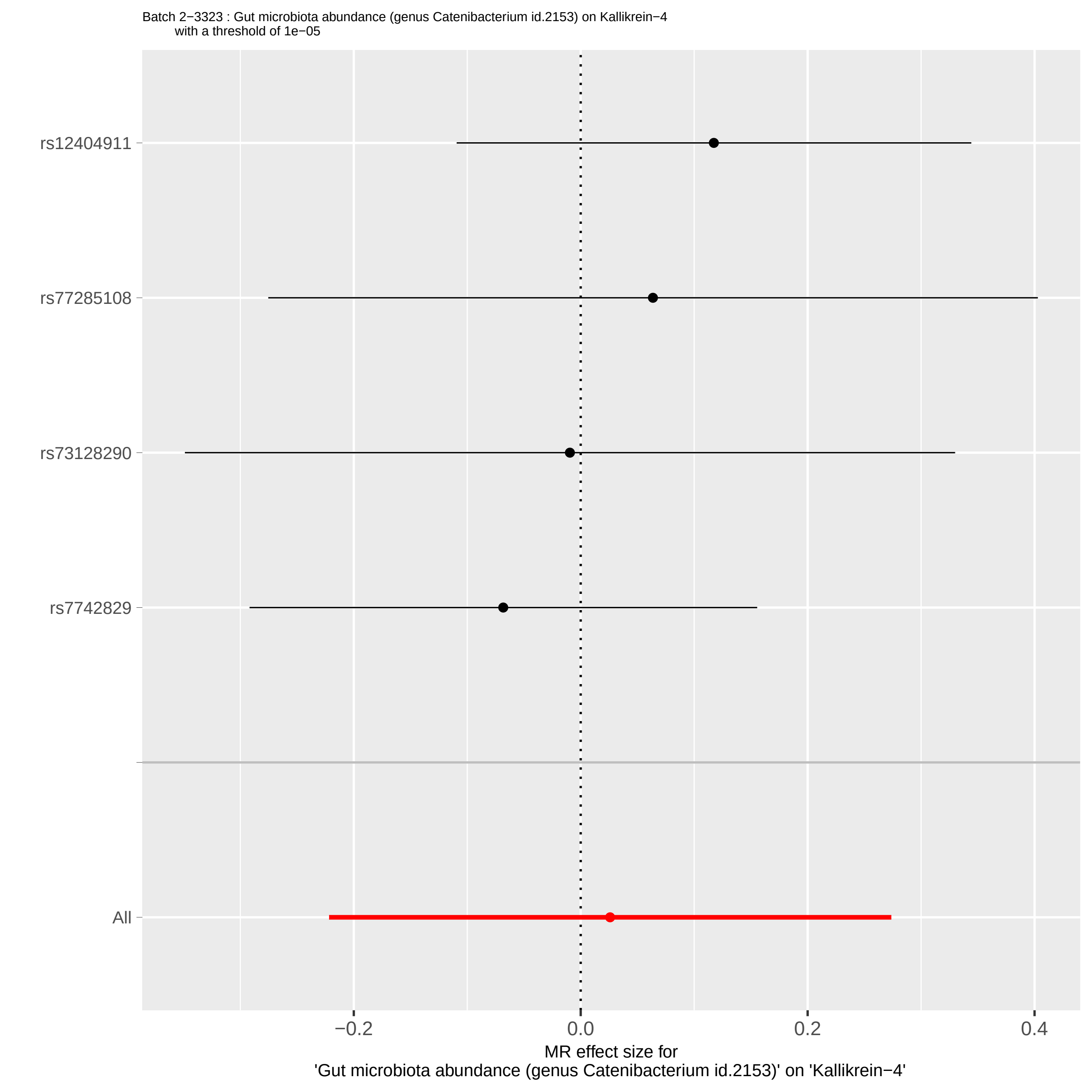
Batch 2−3323 : Gut microbiota abundance (genus Catenibacterium id.2153) on Kallikrein−4
with a threshold of 1e−05
rs12404911
rs77285108
rs73128290
rs7742829
All
−0.2
0.0
0.2
0.4
MR effect size for
'Gut microbiota abundance (genus Catenibacterium id.2153)' on 'Kallikrein−4'

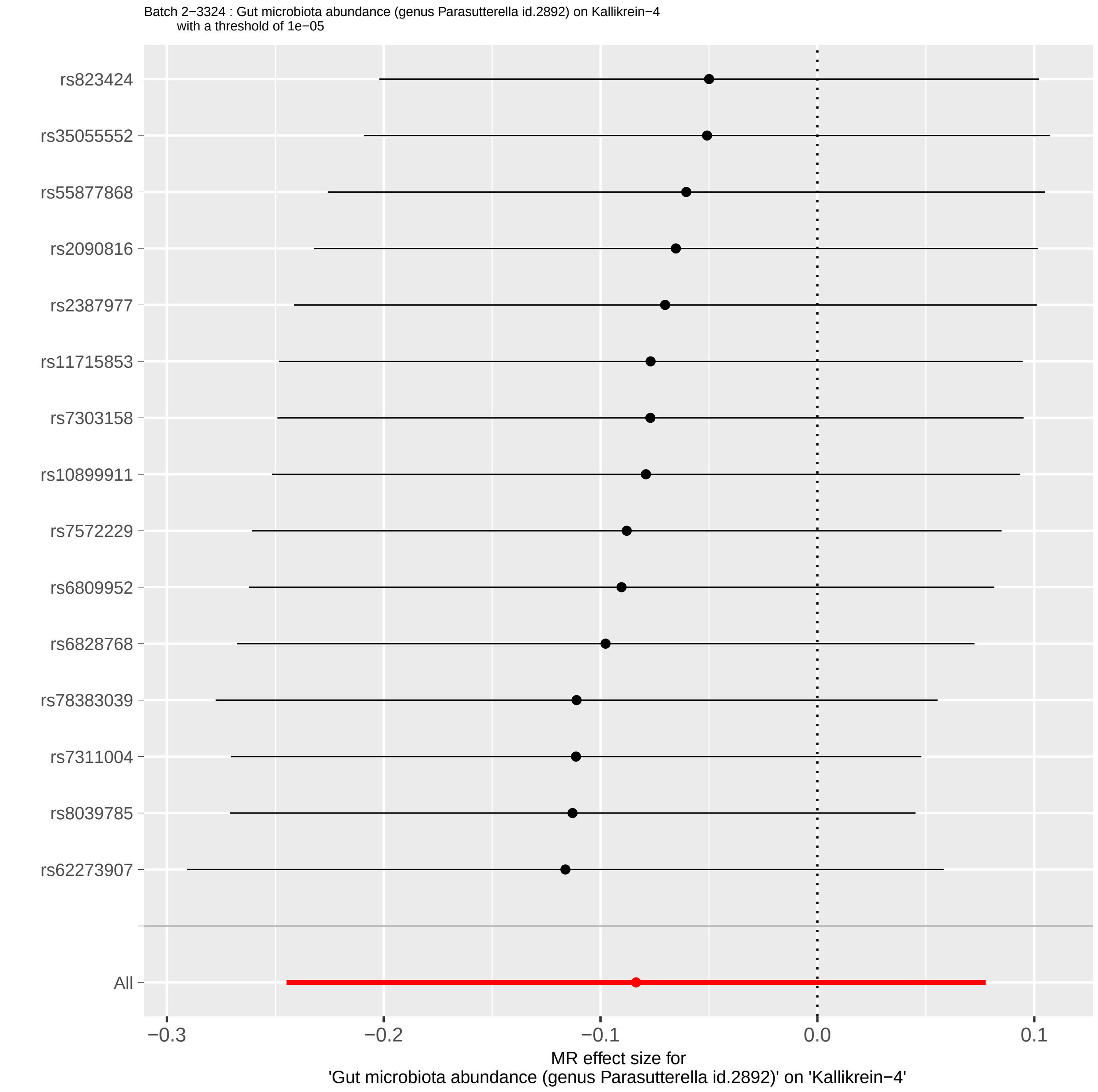
Batch 2−3324 : Gut microbiota abundance (genus Parasutterella id.2892) on Kallikrein−4
with a threshold of 1e−05
rs823424
rs35055552
rs55877868
rs2090816
rs2387977
rs11715853
rs7303158
rs10899911
rs7572229
rs6809952
rs6828768
rs78383039
rs7311004
rs8039785
rs62273907
All
−0.3
−0.2
−0.1
0.0
0.1
MR effect size for
'Gut microbiota abundance (genus Parasutterella id.2892)' on 'Kallikrein−4'

Batch 2−3325 : Gut microbiota abundance (genus Catenibacterium id.2153) on Kallikrein−5
with a threshold of 1e−05

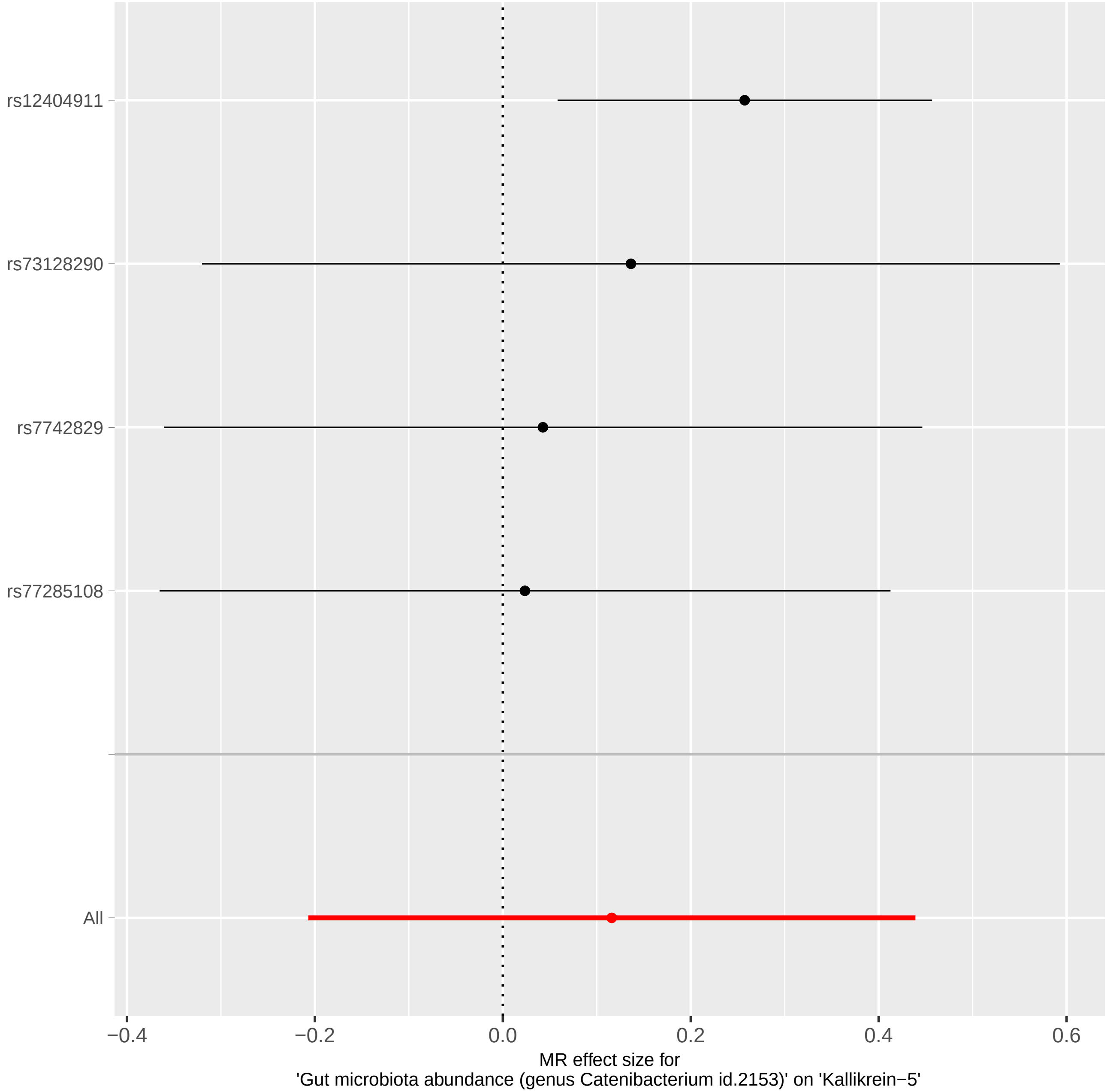

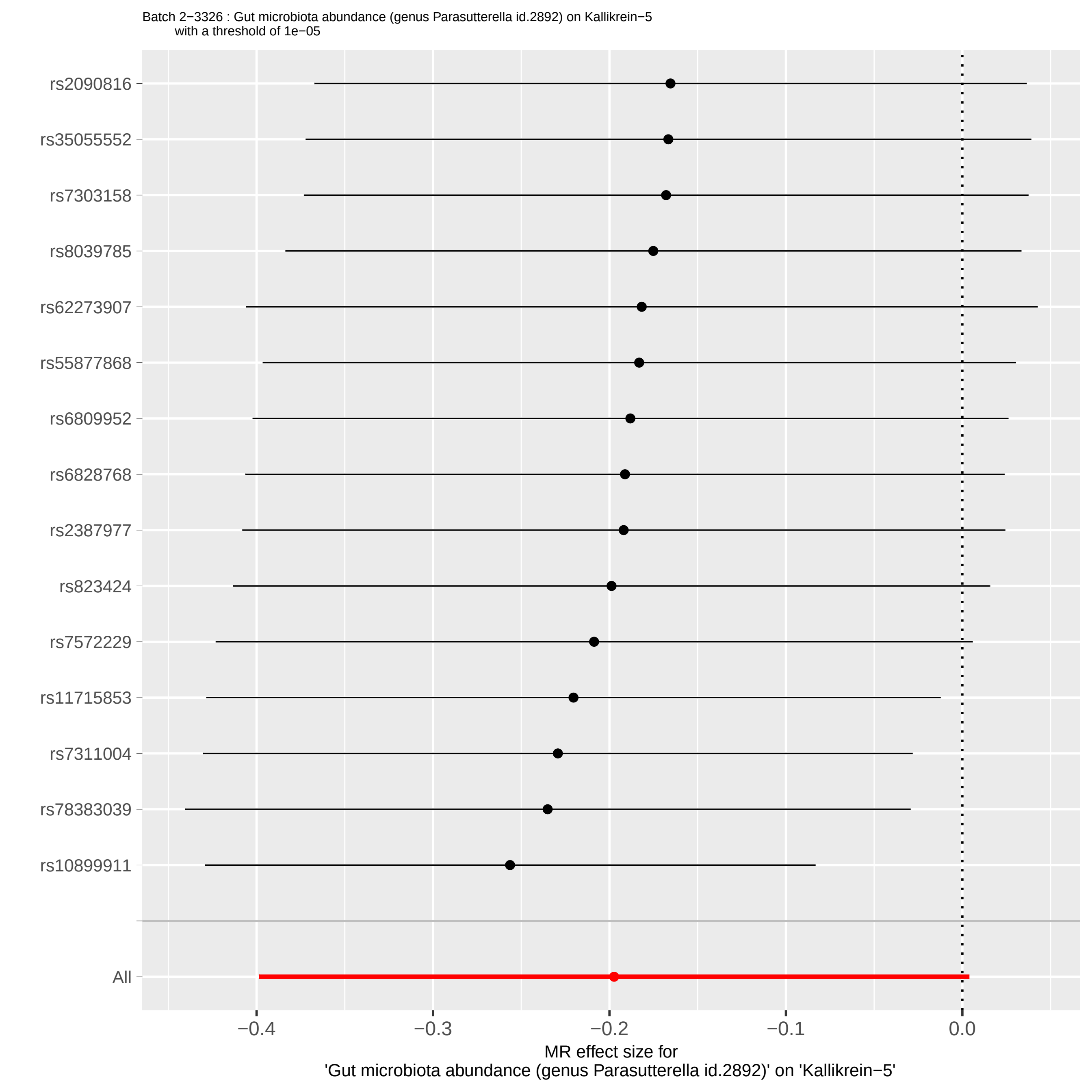

Batch 2−3326 : Gut microbiota abundance (genus Parasutterella id.2892) on Kallikrein−5
with a threshold of 1e−05
rs2090816
rs35055552
rs7303158
rs8039785
rs62273907
rs55877868
rs6809952
rs6828768
rs2387977
rs823424
rs7572229
rs11715853
rs7311004
rs78383039
rs10899911
All
−0.4
−0.3
−0.2
−0.1
0.0
MR effect size for
'Gut microbiota abundance (genus Parasutterella id.2892)' on 'Kallikrein−5'

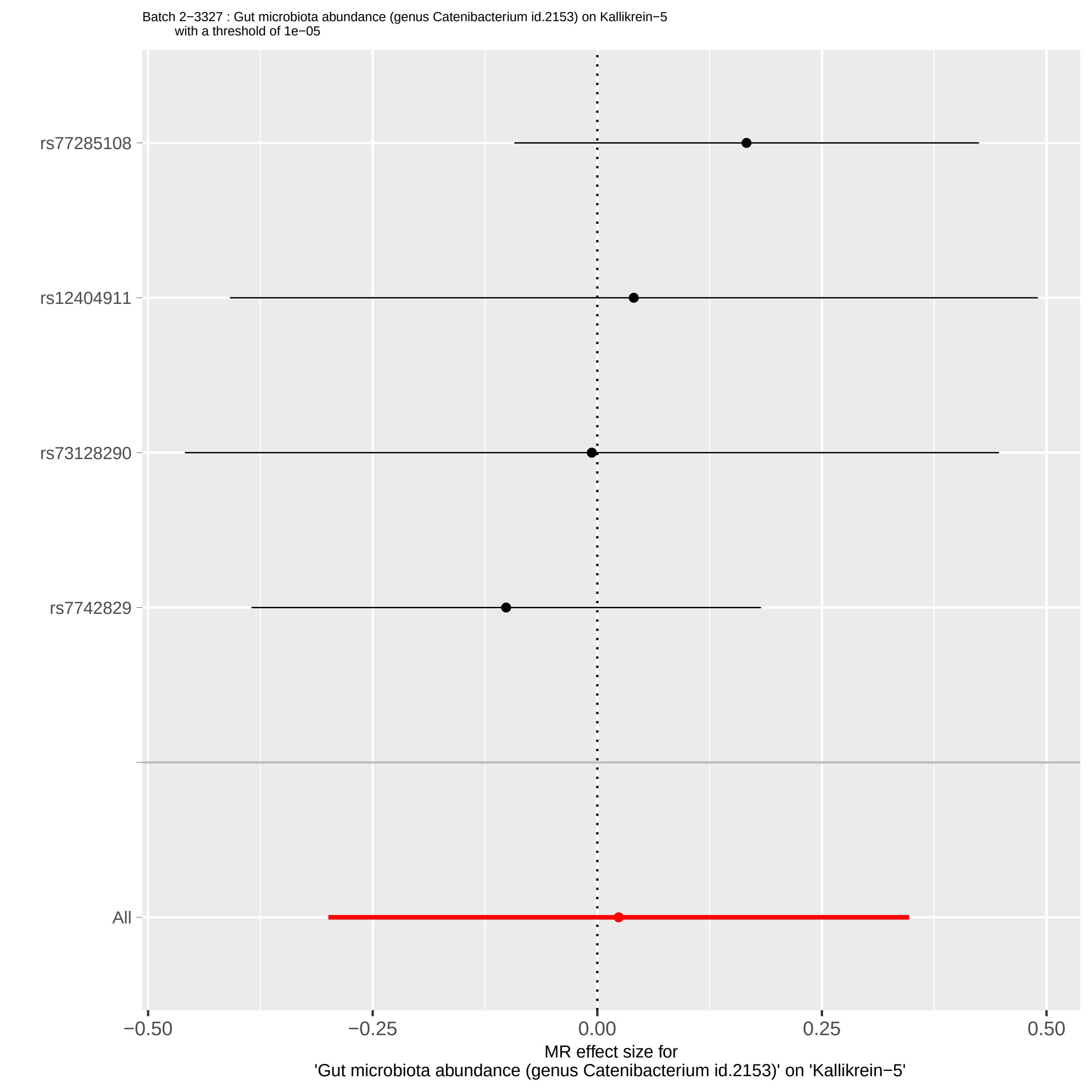

Batch 2−3327 : Gut microbiota abundance (genus Catenibacterium id.2153) on Kallikrein−5
with a threshold of 1e−05
rs77285108
rs12404911
rs73128290
rs7742829
All
−0.50
−0.25
0.00
0.25
0.50
MR effect size for
'Gut microbiota abundance (genus Catenibacterium id.2153)' on 'Kallikrein−5'

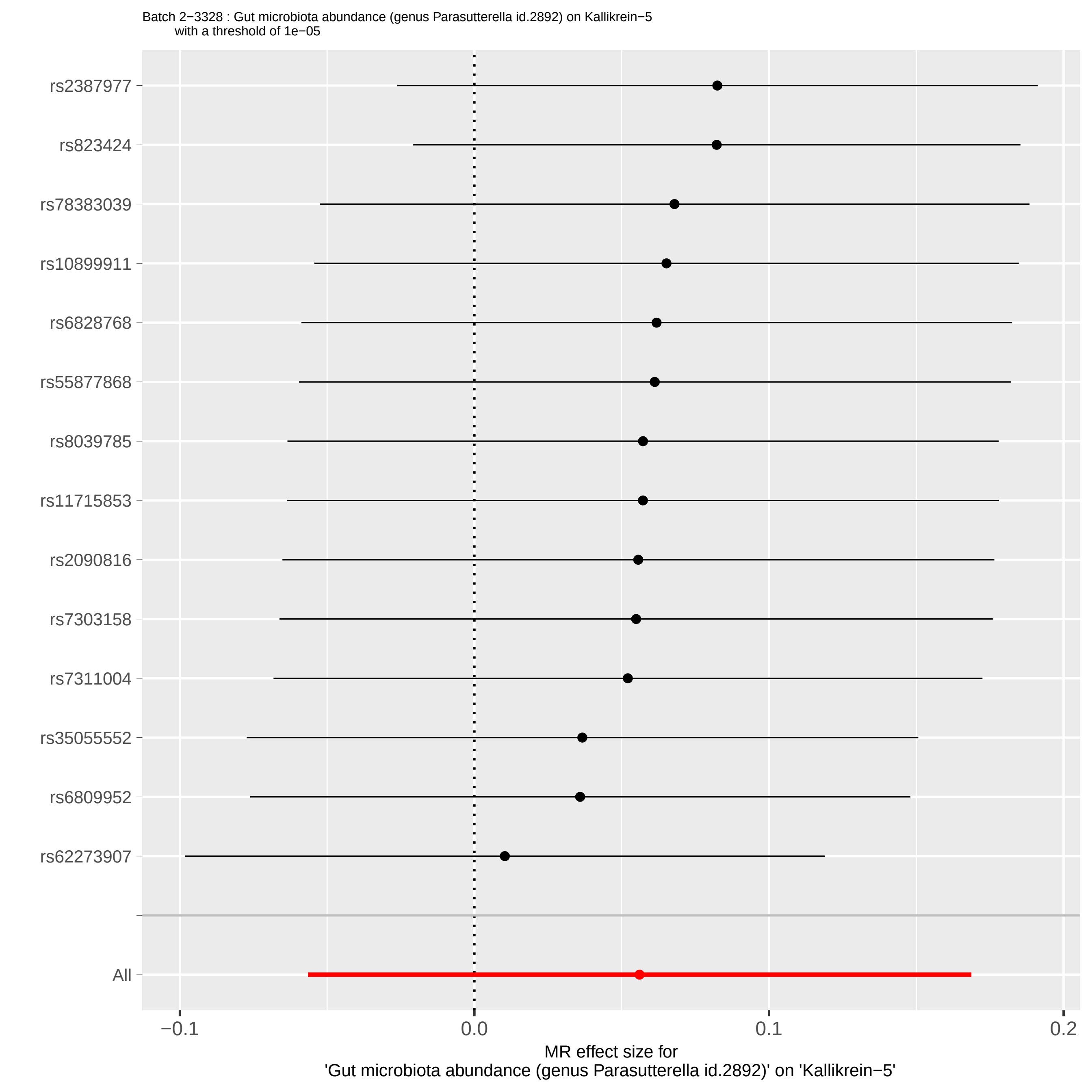

Batch 2−3328 : Gut microbiota abundance (genus Parasutterella id.2892) on Kallikrein−5
with a threshold of 1e−05
rs2387977
rs823424
rs78383039
rs10899911
rs6828768
rs55877868
rs8039785
rs11715853
rs2090816
rs7303158
rs7311004
rs35055552
rs6809952
rs62273907
All
−0.1
0.0
0.1
0.2
MR effect size for
'Gut microbiota abundance (genus Parasutterella id.2892)' on 'Kallikrein−5'

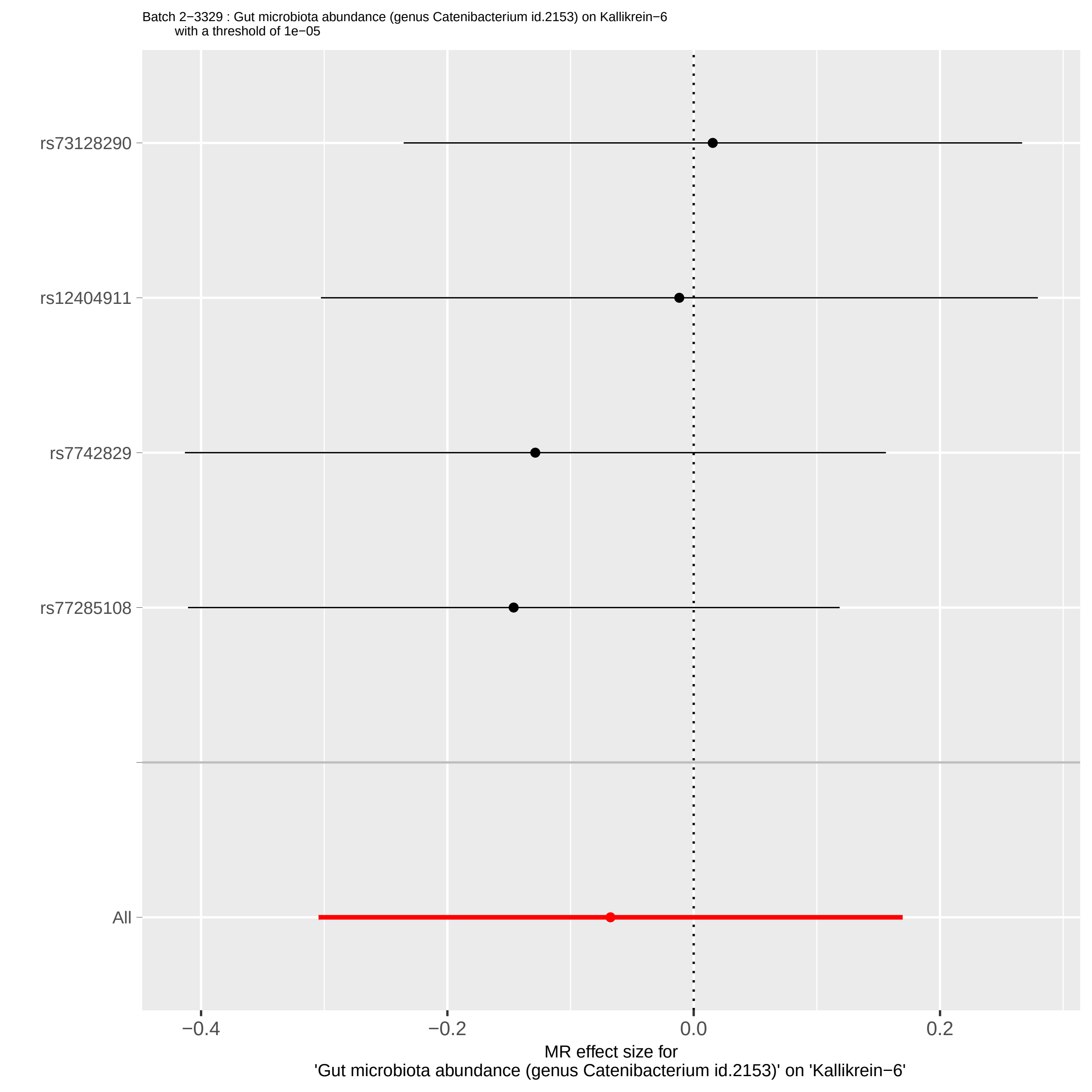
Batch 2−3329 : Gut microbiota abundance (genus Catenibacterium id.2153) on Kallikrein−6
with a threshold of 1e−05
rs73128290
rs12404911
rs7742829
rs77285108
All
−0.4
−0.2
0.0
0.2
MR effect size for
'Gut microbiota abundance (genus Catenibacterium id.2153)' on 'Kallikrein−6'

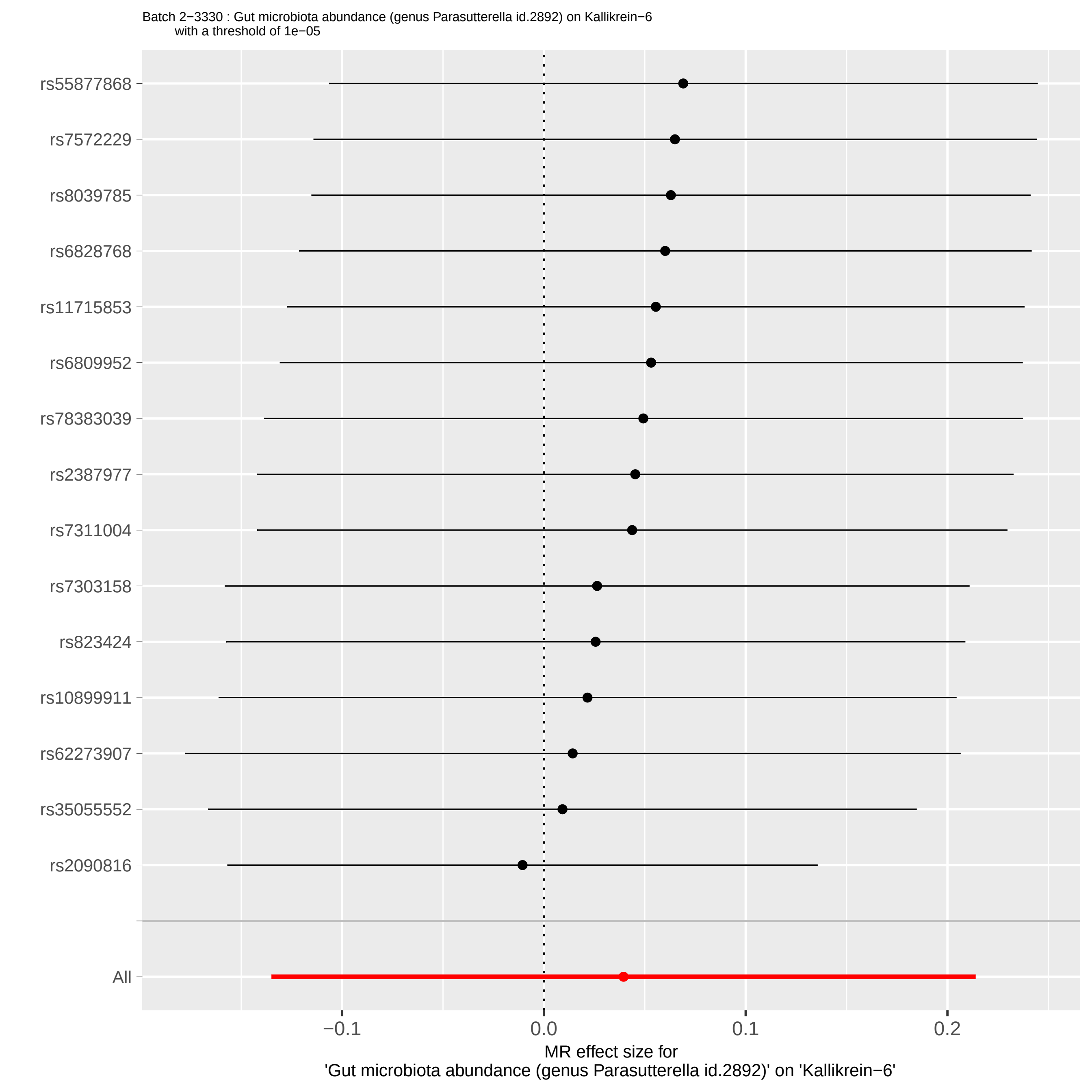
Batch 2−3330 : Gut microbiota abundance (genus Parasutterella id.2892) on Kallikrein−6
with a threshold of 1e−05
rs55877868
rs7572229
rs8039785
rs6828768
rs11715853
rs6809952
rs78383039
rs2387977
rs7311004
rs7303158
rs823424
rs10899911
rs62273907
rs35055552
rs2090816
All
−0.1
0.0
0.1
0.2
MR effect size for
'Gut microbiota abundance (genus Parasutterella id.2892)' on 'Kallikrein−6'

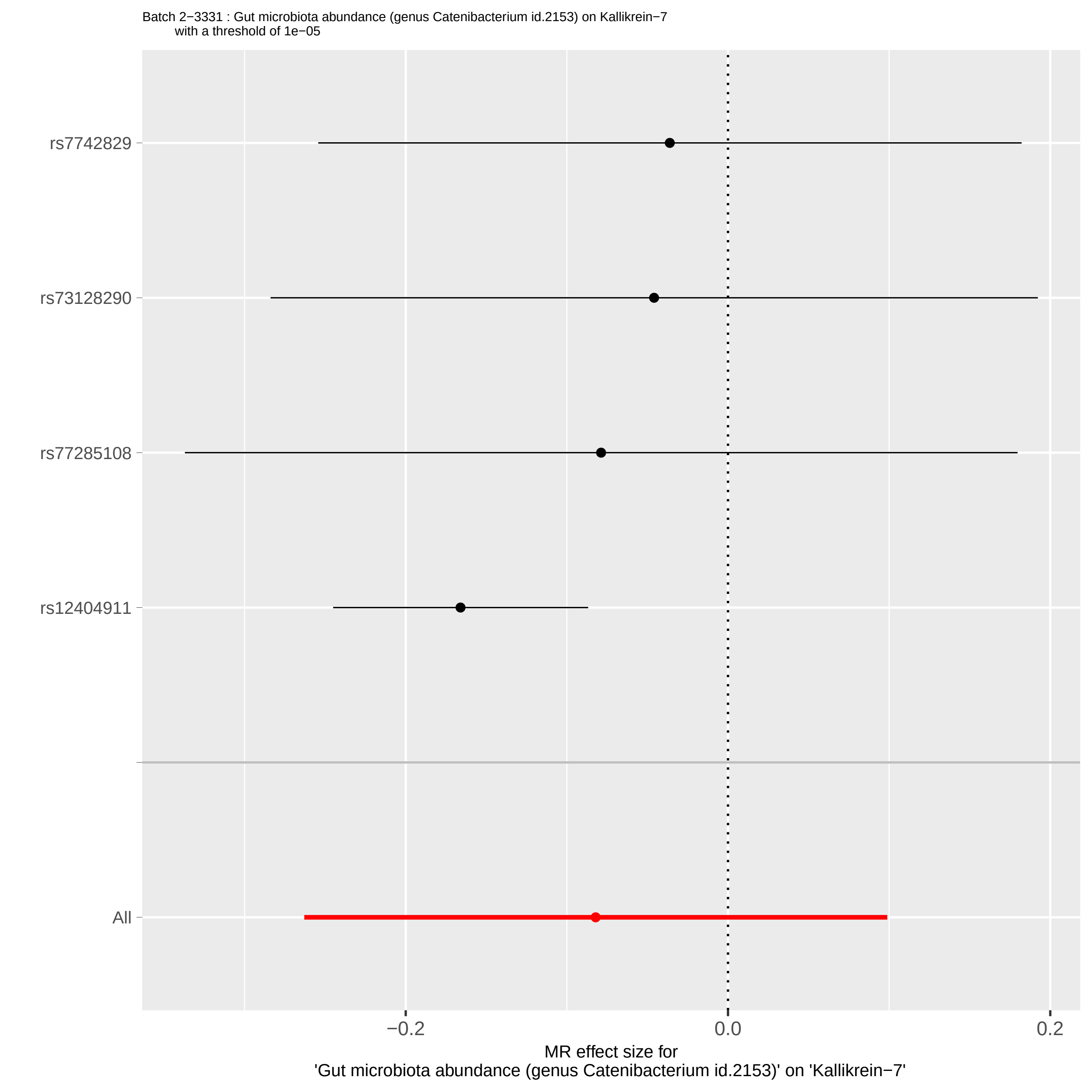
Batch 2−3331 : Gut microbiota abundance (genus Catenibacterium id.2153) on Kallikrein−7
with a threshold of 1e−05
rs7742829
rs73128290
rs77285108
rs12404911
All
−0.2
0.0
0.2
MR effect size for
'Gut microbiota abundance (genus Catenibacterium id.2153)' on 'Kallikrein−7'

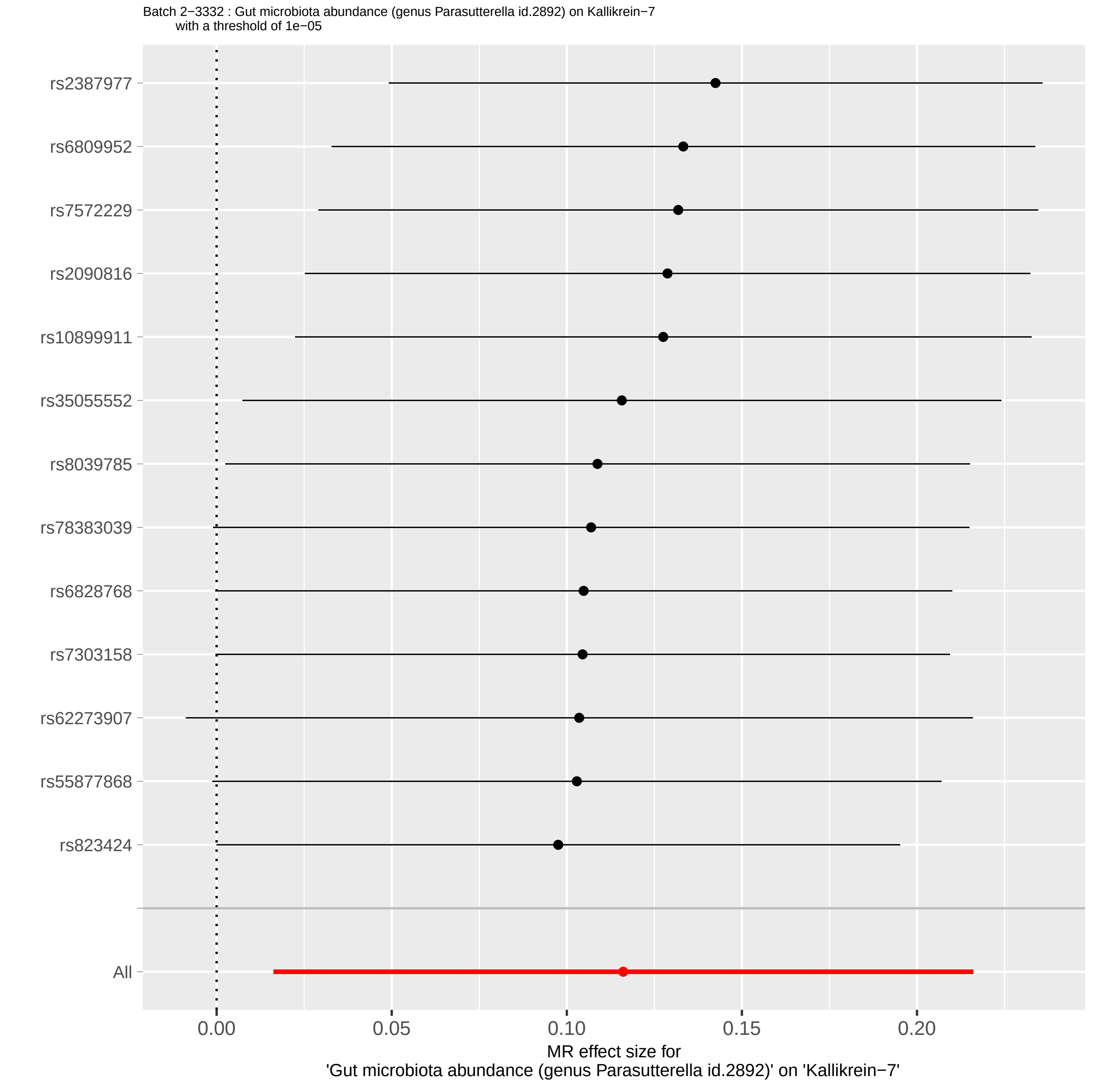

Batch 2−3332 : Gut microbiota abundance (genus Parasutterella id.2892) on Kallikrein−7
with a threshold of 1e−05
rs2387977
rs6809952
rs7572229
rs2090816
rs10899911
rs35055552
rs8039785
rs78383039
rs6828768
rs7303158
rs62273907
rs55877868
rs823424
All
0.00
0.05
0.10
0.15
0.20
MR effect size for
'Gut microbiota abundance (genus Parasutterella id.2892)' on 'Kallikrein−7'

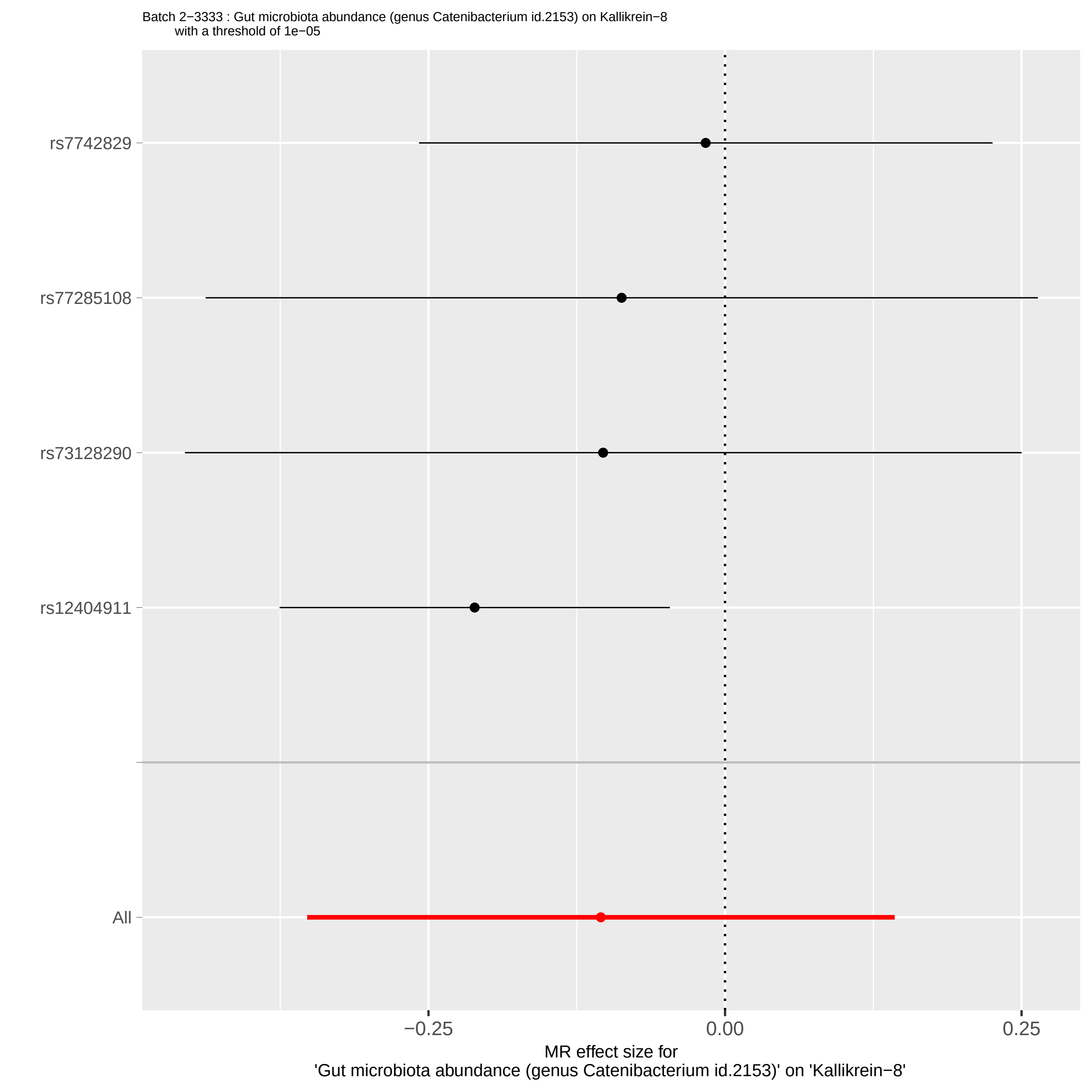

Batch 2−3333 : Gut microbiota abundance (genus Catenibacterium id.2153) on Kallikrein−8
with a threshold of 1e−05
rs7742829
rs77285108
rs73128290
rs12404911
All
−0.25
0.00
0.25
MR effect size for
'Gut microbiota abundance (genus Catenibacterium id.2153)' on 'Kallikrein−8'

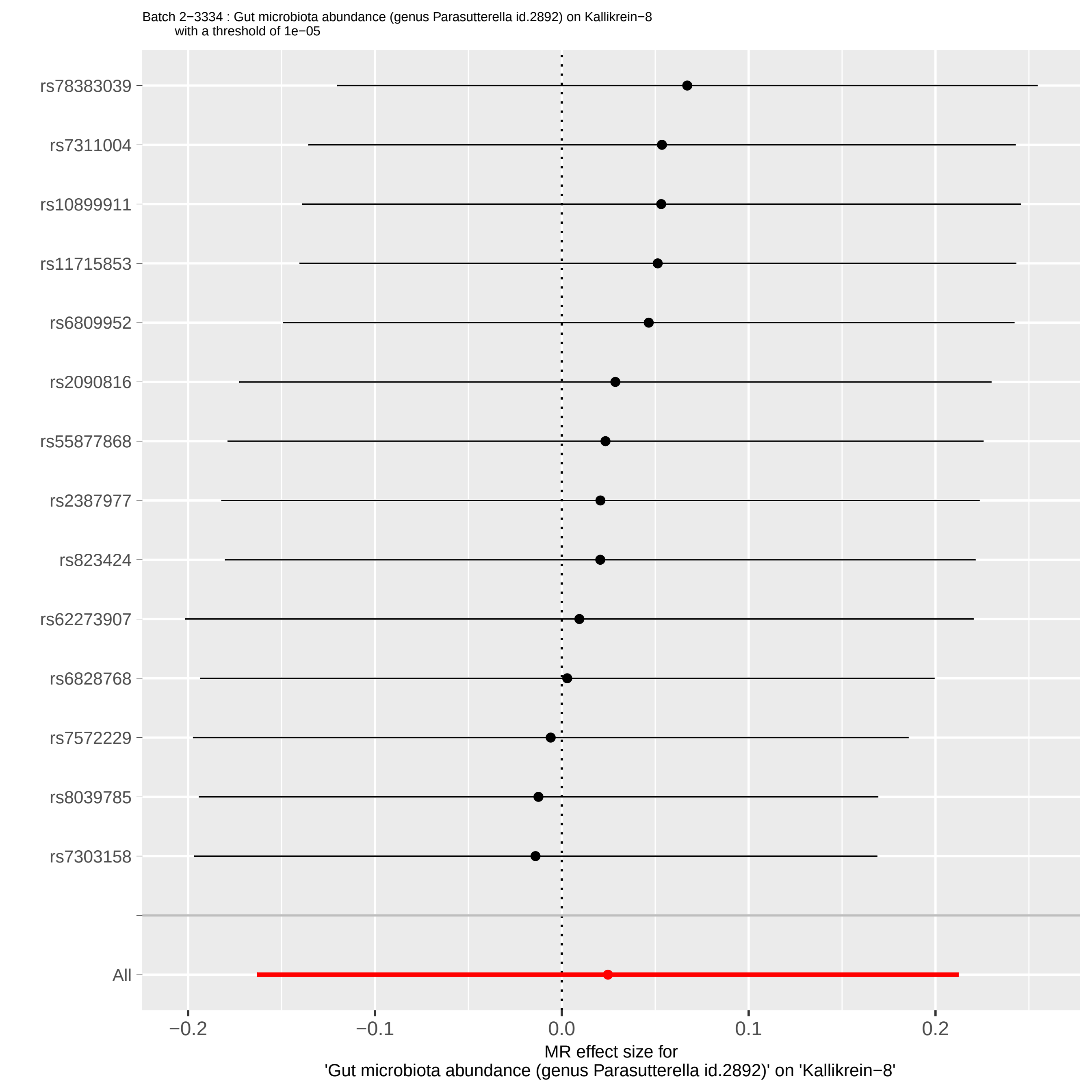

Batch 2−3334 : Gut microbiota abundance (genus Parasutterella id.2892) on Kallikrein−8
with a threshold of 1e−05
rs78383039
rs7311004
rs10899911
rs11715853
rs6809952
rs2090816
rs55877868
rs2387977
rs823424
rs62273907
rs6828768
rs7572229
rs8039785
rs7303158
All
−0.2
−0.1
0.0
0.1
0.2
MR effect size for
'Gut microbiota abundance (genus Parasutterella id.2892)' on 'Kallikrein−8'

Batch 2−3335 : Gut microbiota abundance (genus Catenibacterium id.2153) on Kallikrein−8
with a threshold of 1e−05

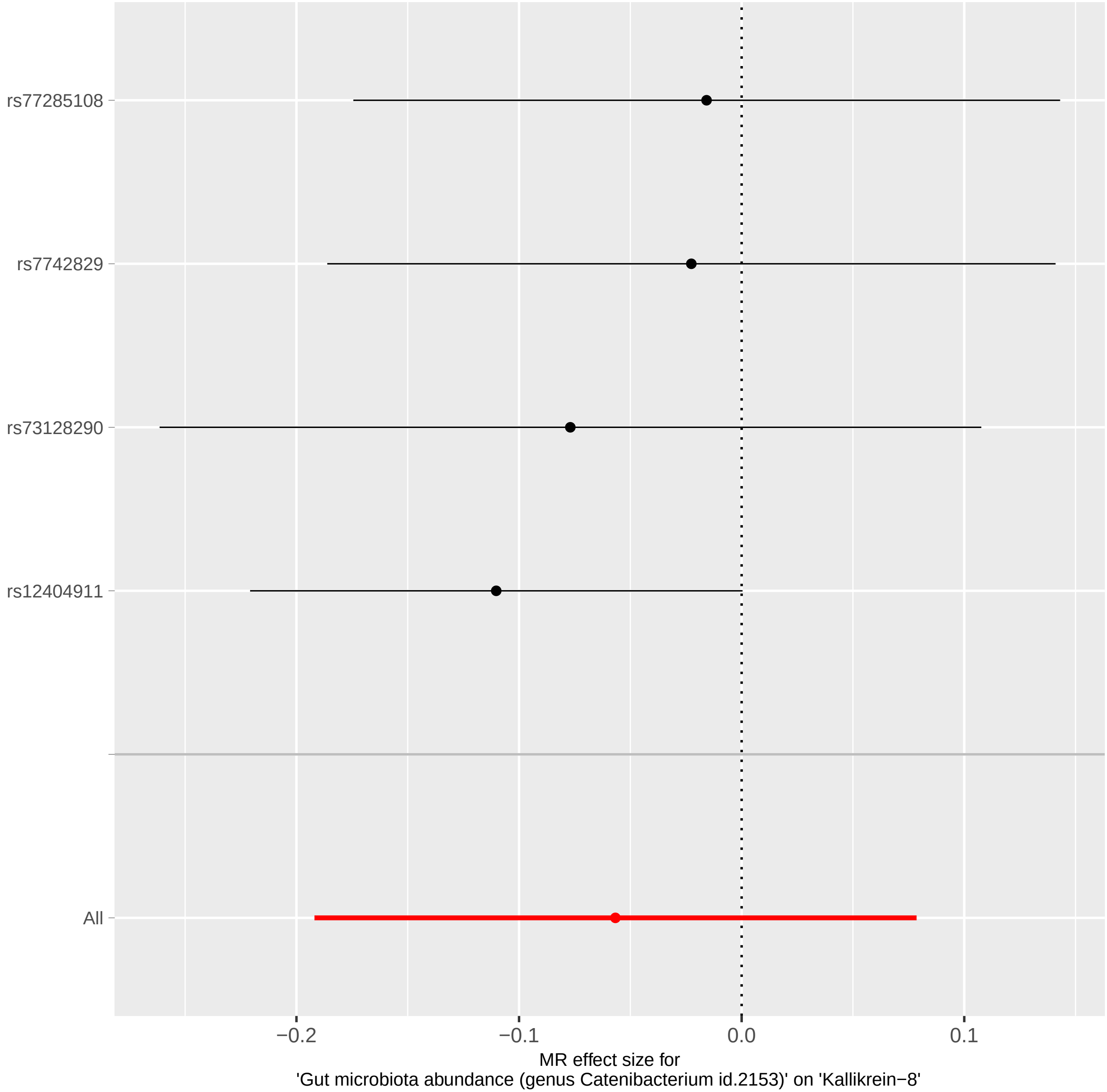

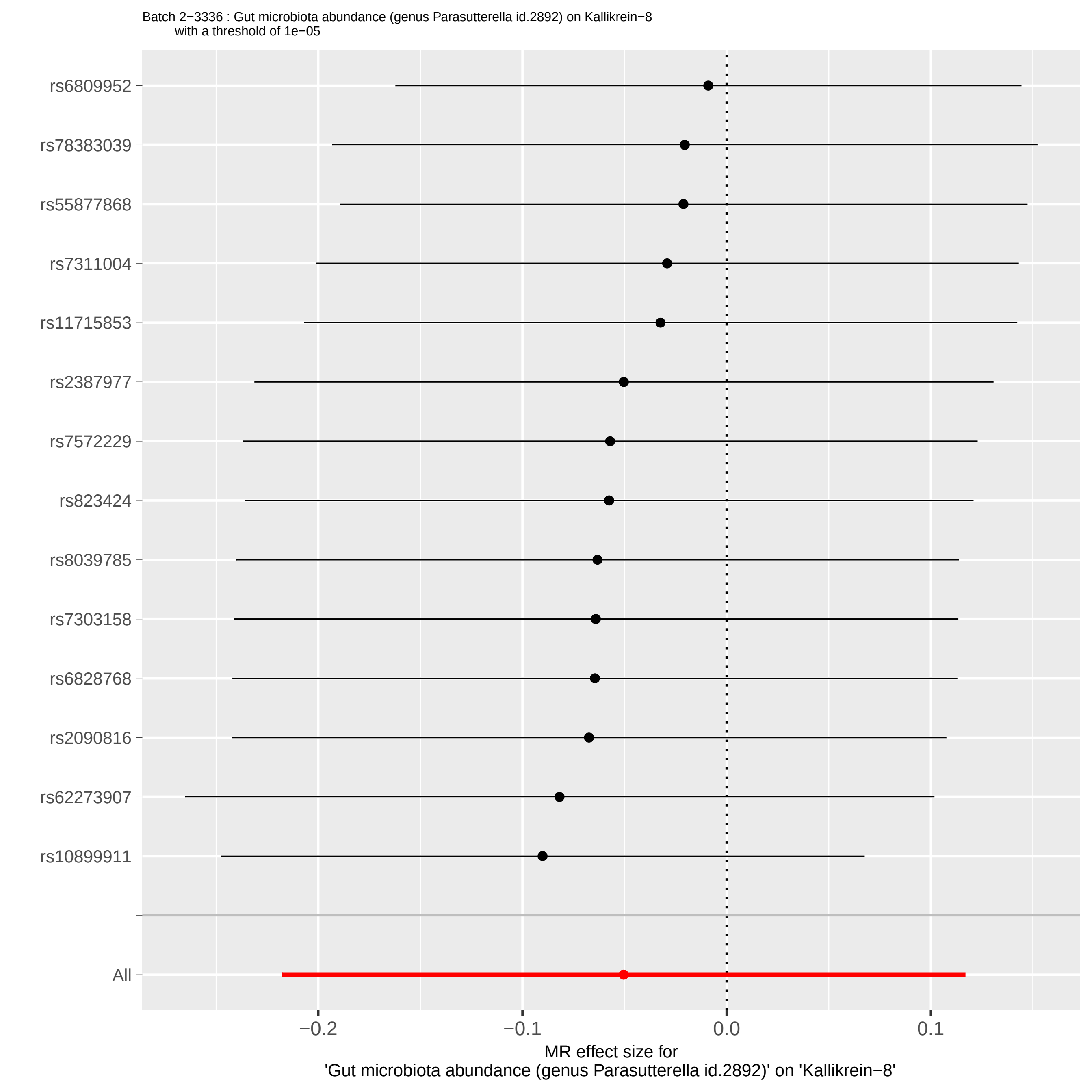

Batch 2−3336 : Gut microbiota abundance (genus Parasutterella id.2892) on Kallikrein−8
with a threshold of 1e−05
rs6809952
rs78383039
rs55877868
rs7311004
rs11715853
rs2387977
rs7572229
rs823424
rs8039785
rs7303158
rs6828768
rs2090816
rs62273907
rs10899911
All
−0.2
−0.1
0.0
0.1
MR effect size for
'Gut microbiota abundance (genus Parasutterella id.2892)' on 'Kallikrein−8'

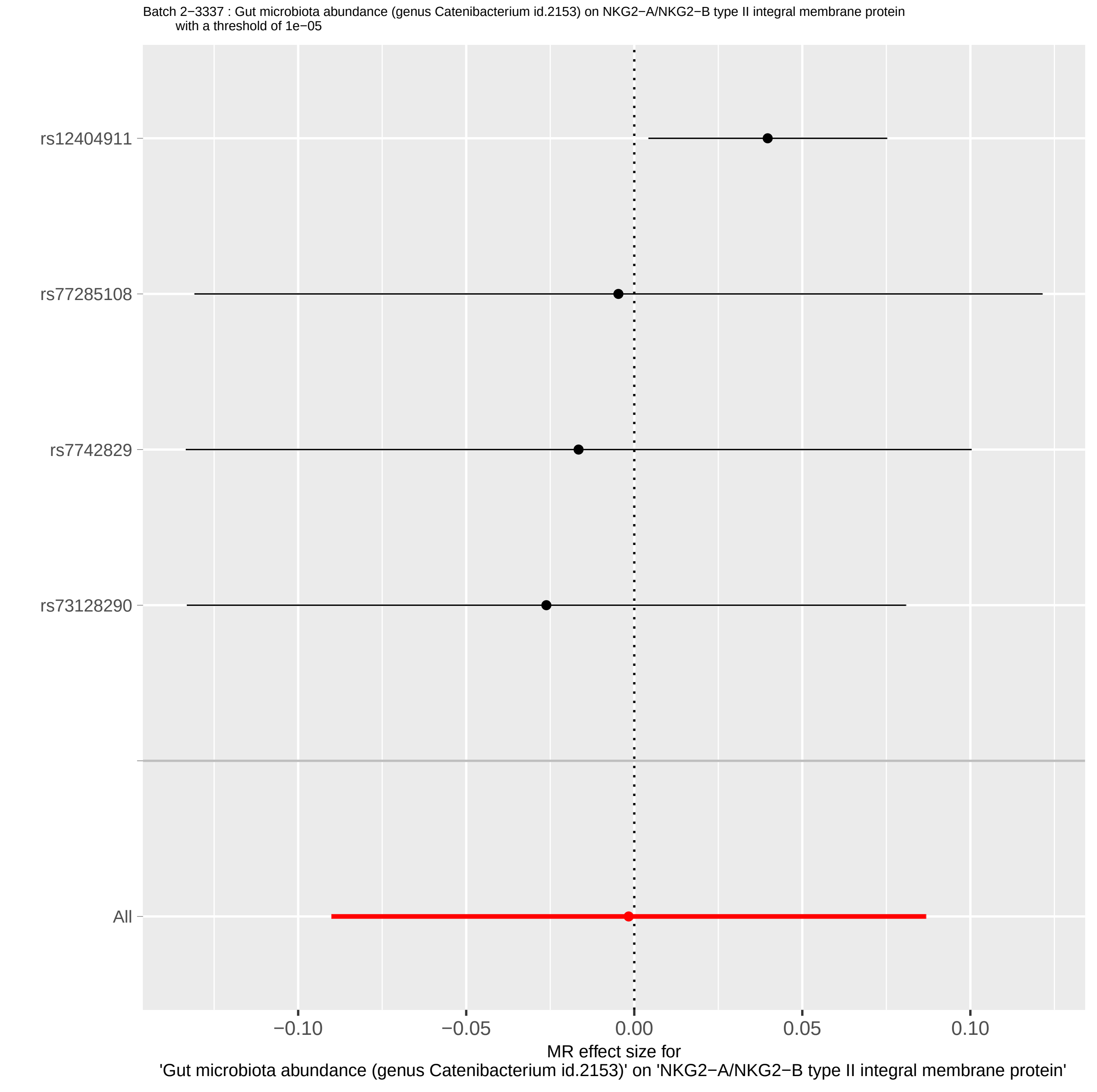
Batch 2−3337 : Gut microbiota abundance (genus Catenibacterium id.2153) on NKG2−A/NKG2−B type II integral membrane protein
with a threshold of 1e−05
rs12404911
rs77285108
rs7742829
rs73128290
All
−0.10
−0.05
0.00
0.05
0.10
MR effect size for
'Gut microbiota abundance (genus Catenibacterium id.2153)' on 'NKG2−A/NKG2−B type II integral membrane protein'

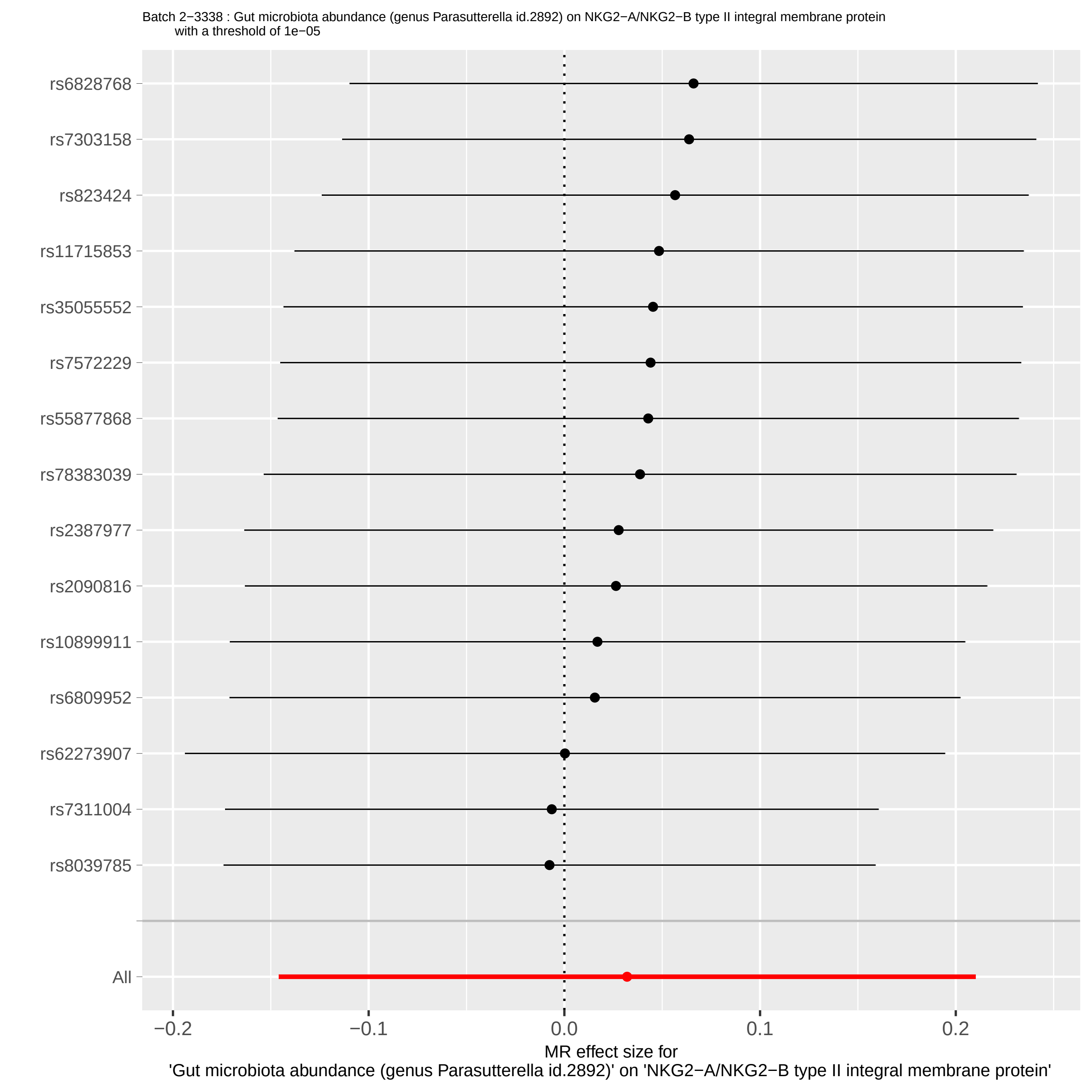
Batch 2−3338 : Gut microbiota abundance (genus Parasutterella id.2892) on NKG2−A/NKG2−B type II integral membrane protein
with a threshold of 1e−05
rs6828768
rs7303158
rs823424
rs11715853
rs35055552
rs7572229
rs55877868
rs78383039
rs2387977
rs2090816
rs10899911
rs6809952
rs62273907
rs7311004
rs8039785
All
−0.2
−0.1
0.0
0.1
0.2
MR effect size for
'Gut microbiota abundance (genus Parasutterella id.2892)' on 'NKG2−A/NKG2−B type II integral membrane protein'

Batch 2−3339 : Gut microbiota abundance (genus Catenibacterium id.2153) on NKG2−E type II integral membrane protein
with a threshold of 1e−05

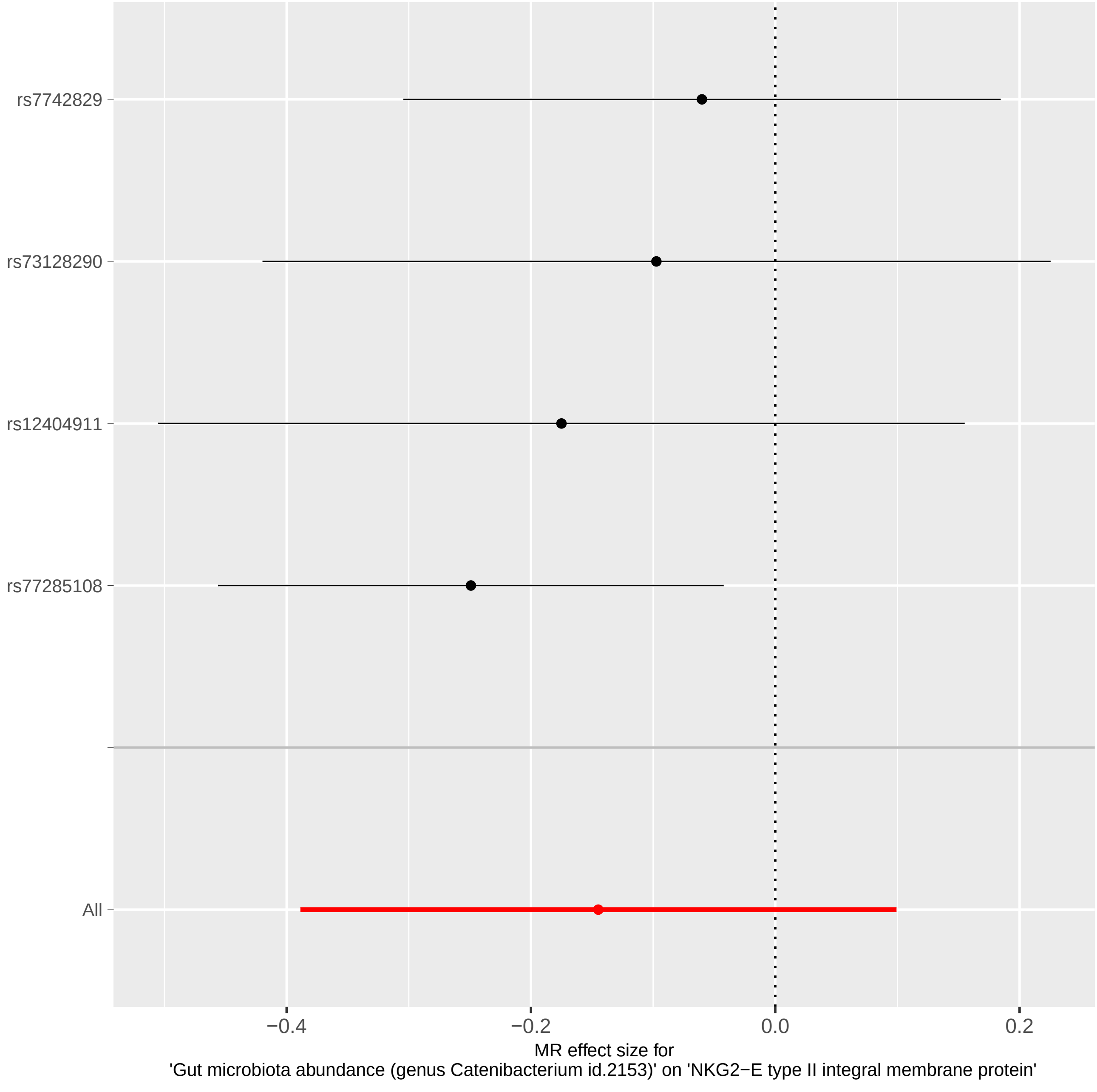

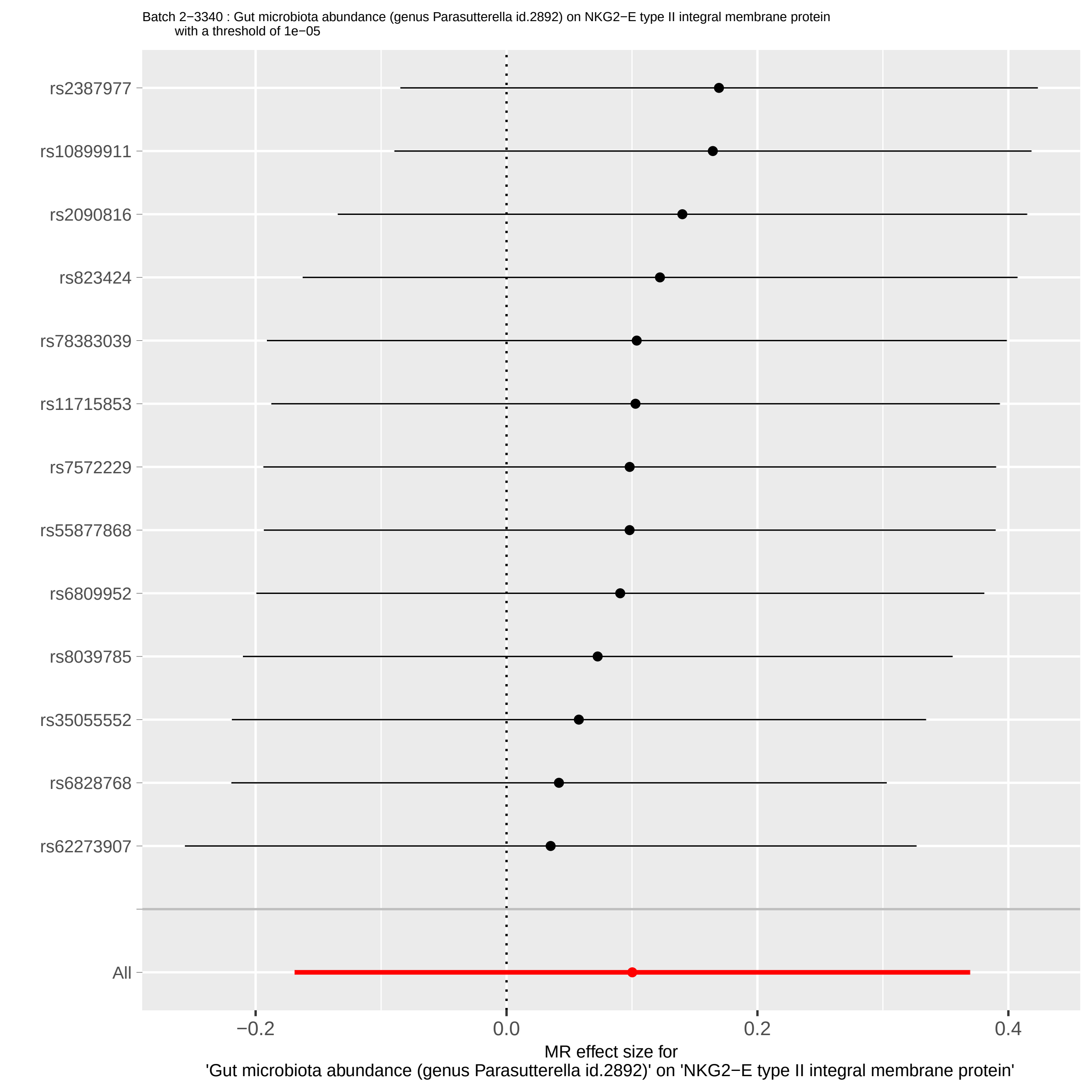

Batch 2−3340 : Gut microbiota abundance (genus Parasutterella id.2892) on NKG2−E type II integral membrane protein
with a threshold of 1e−05
rs2387977
rs10899911
rs2090816
rs823424
rs78383039
rs11715853
rs7572229
rs55877868
rs6809952
rs8039785
rs35055552
rs6828768
rs62273907
All
−0.2
0.0
0.2
0.4
MR effect size for
'Gut microbiota abundance (genus Parasutterella id.2892)' on 'NKG2−E type II integral membrane protein'

Batch 2−3341 : Gut microbiota abundance (genus Catenibacterium id.2153) on NKG2−E type II integral membrane protein
with a threshold of 1e−05

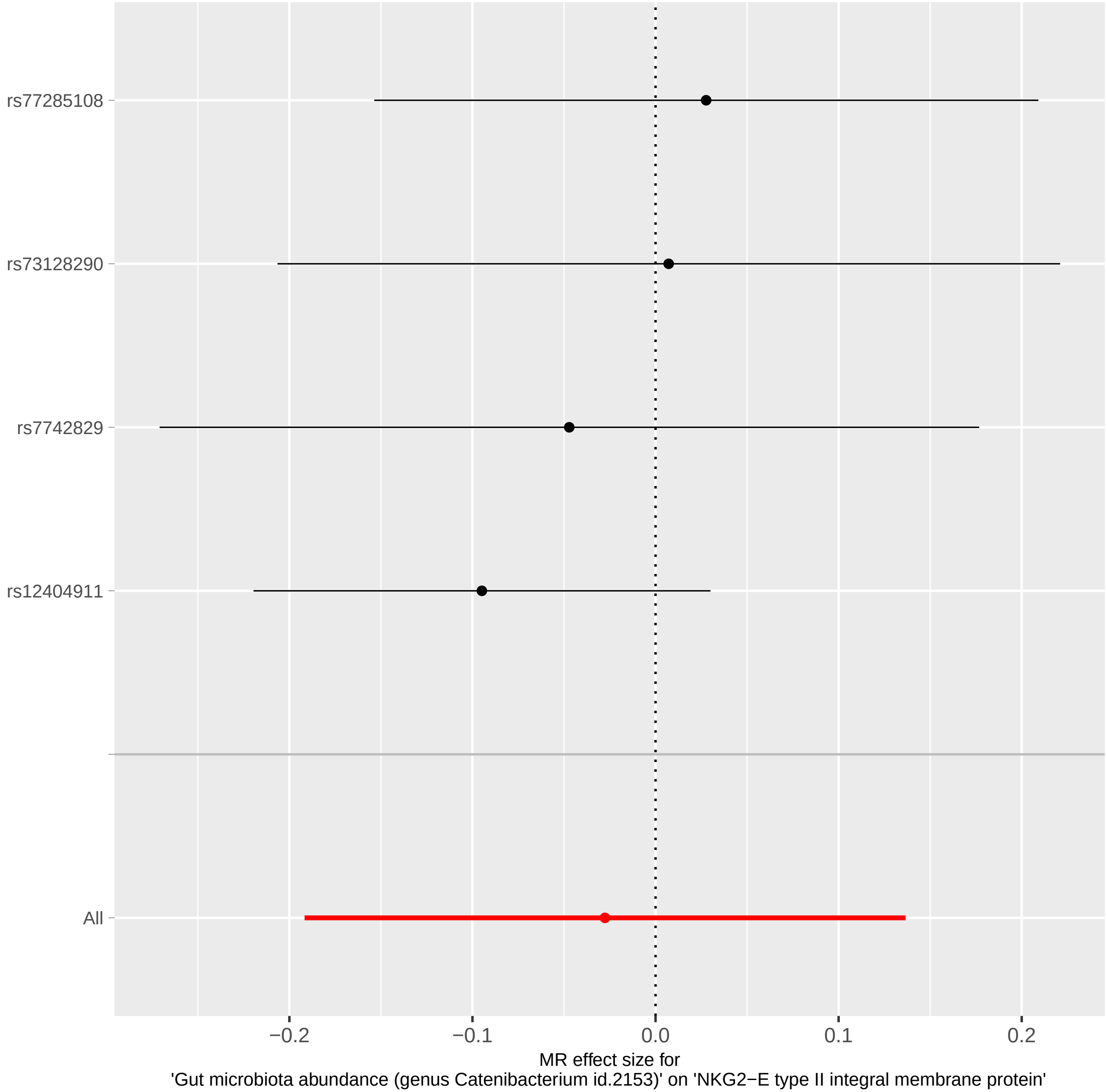

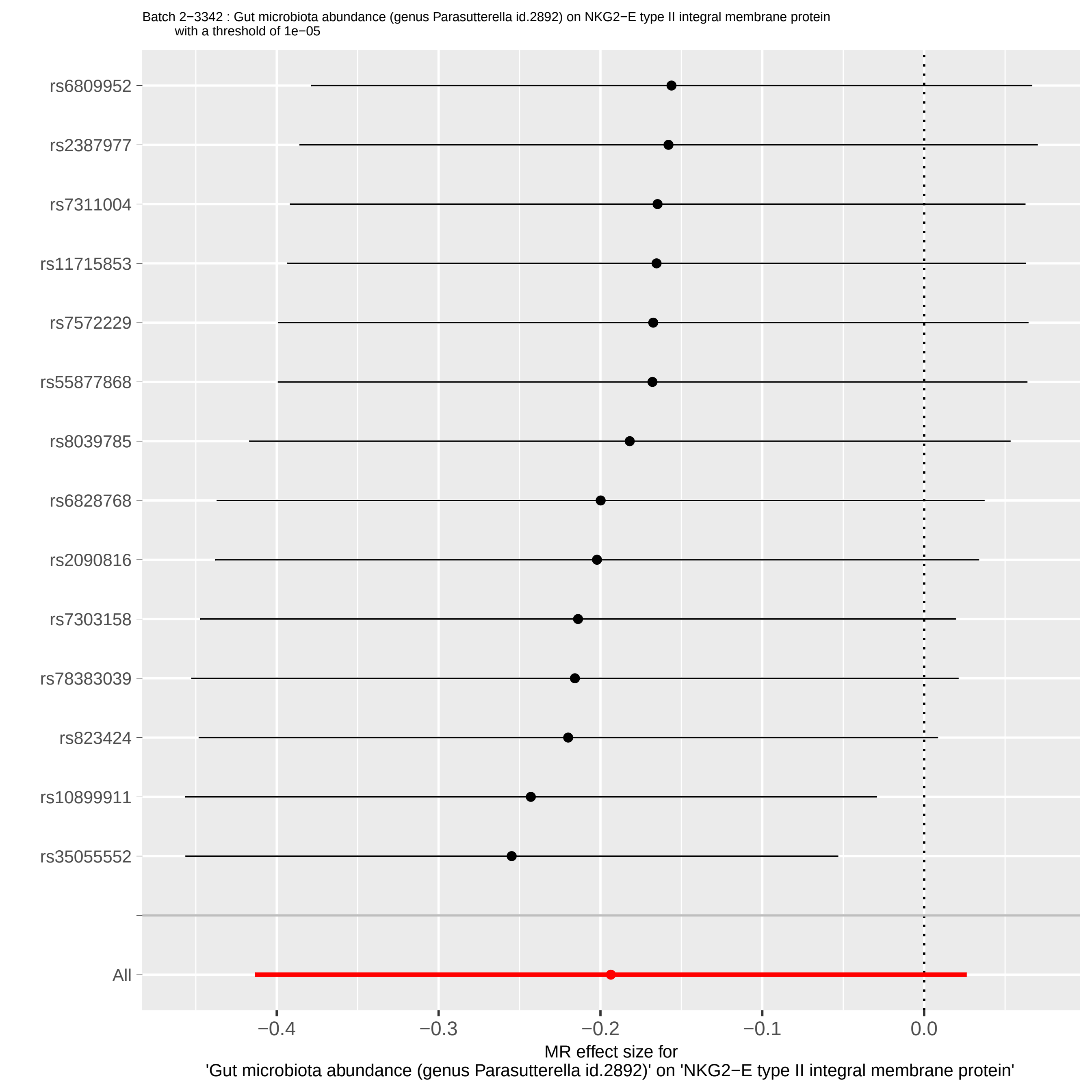

Batch 2−3342 : Gut microbiota abundance (genus Parasutterella id.2892) on NKG2−E type II integral membrane protein
with a threshold of 1e−05
rs6809952
rs2387977
rs7311004
rs11715853
rs7572229
rs55877868
rs8039785
rs6828768
rs2090816
rs7303158
rs78383039
rs823424
rs10899911
rs35055552
All
−0.4
−0.3
−0.2
−0.1
0.0
MR effect size for
'Gut microbiota abundance (genus Parasutterella id.2892)' on 'NKG2−E type II integral membrane protein'

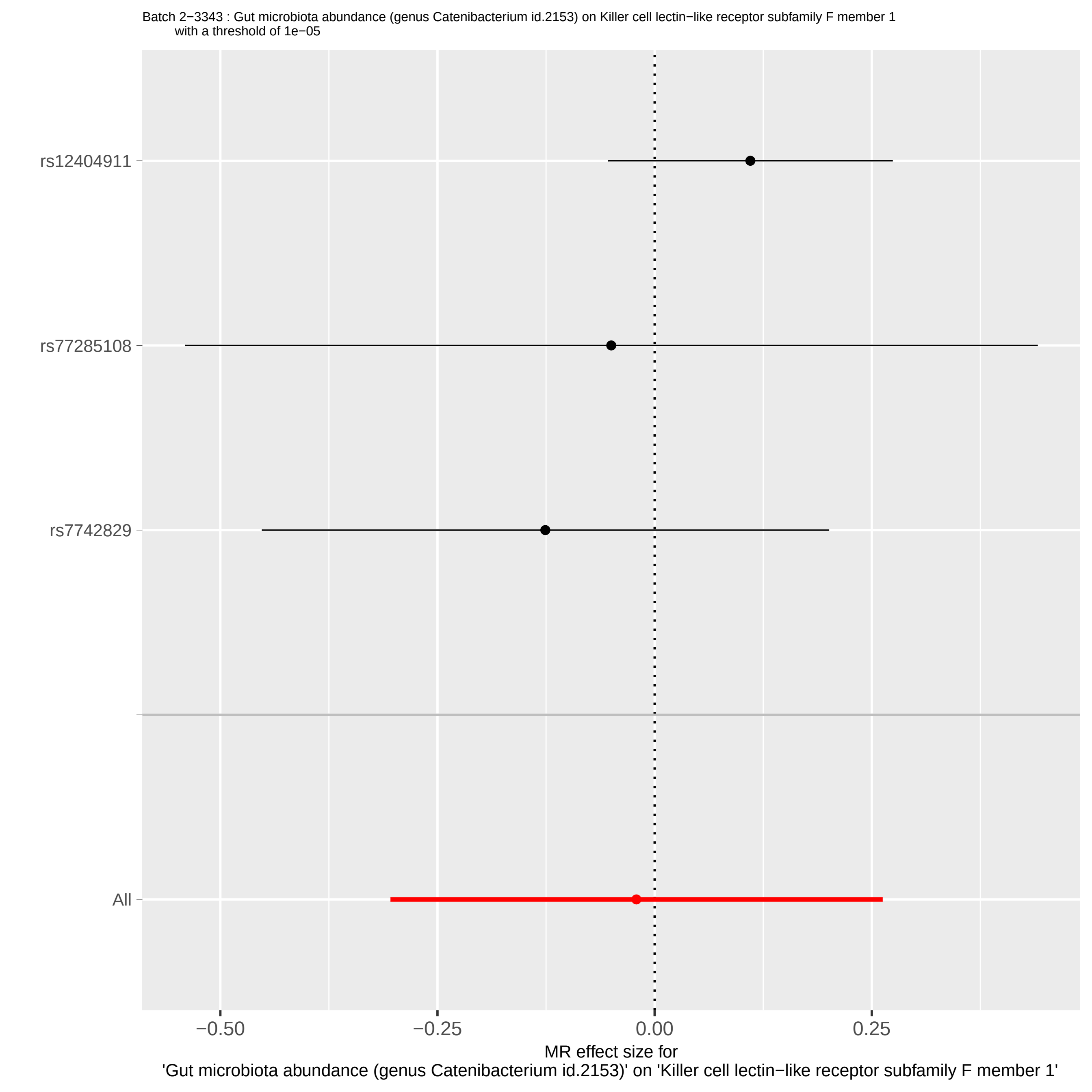
Batch 2−3343 : Gut microbiota abundance (genus Catenibacterium id.2153) on Killer cell lectin−like receptor subfamily F member 1
with a threshold of 1e−05
rs12404911
rs77285108
rs7742829
All
−0.50
−0.25
0.00
0.25
MR effect size for
'Gut microbiota abundance (genus Catenibacterium id.2153)' on 'Killer cell lectin−like receptor subfamily F member 1'

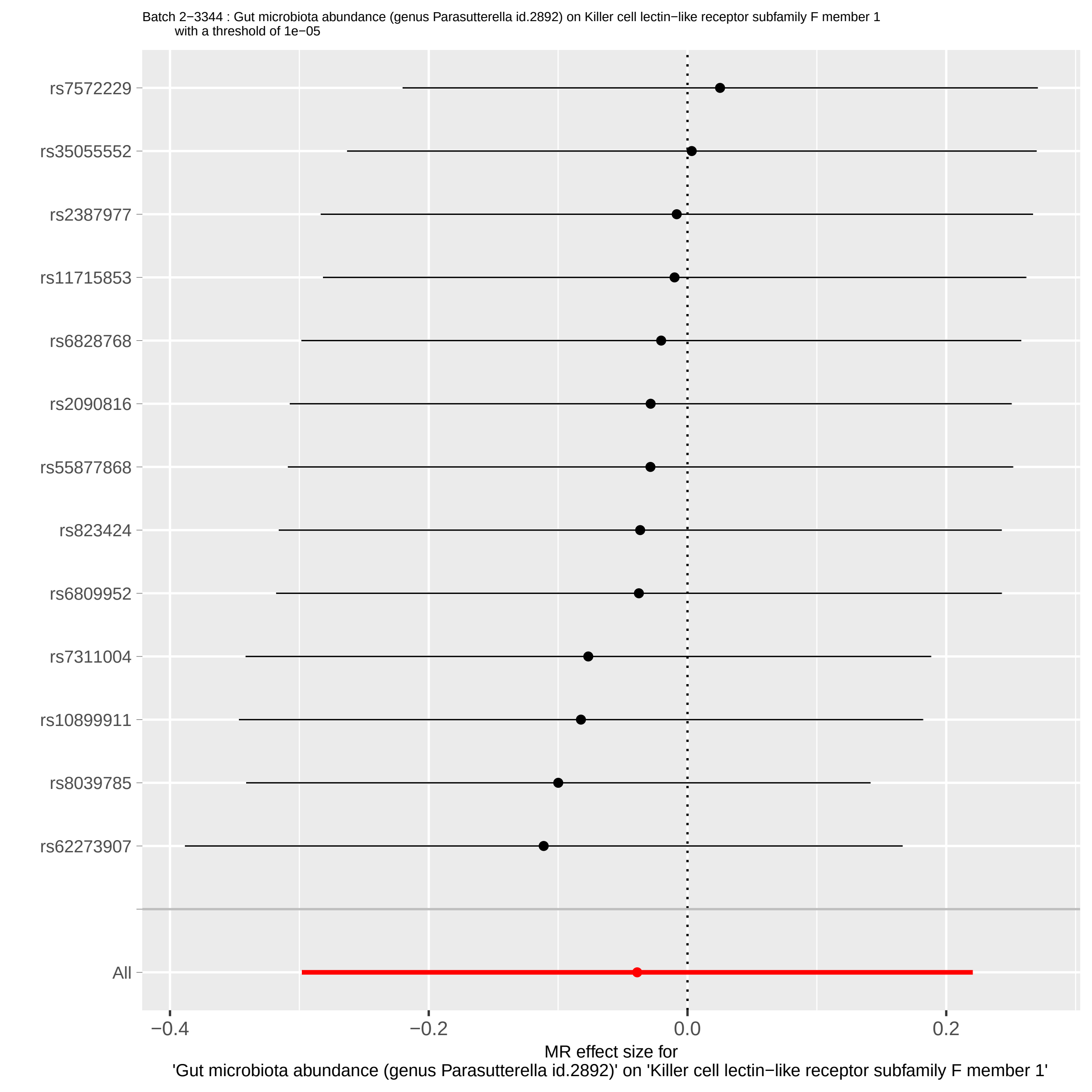
Batch 2−3344 : Gut microbiota abundance (genus Parasutterella id.2892) on Killer cell lectin−like receptor subfamily F member 1
with a threshold of 1e−05
rs7572229
rs35055552
rs2387977
rs11715853
rs6828768
rs2090816
rs55877868
rs823424
rs6809952
rs7311004
rs10899911
rs8039785
rs62273907
All
−0.4
−0.2
0.0
0.2
MR effect size for
'Gut microbiota abundance (genus Parasutterella id.2892)' on 'Killer cell lectin−like receptor subfamily F member 1'

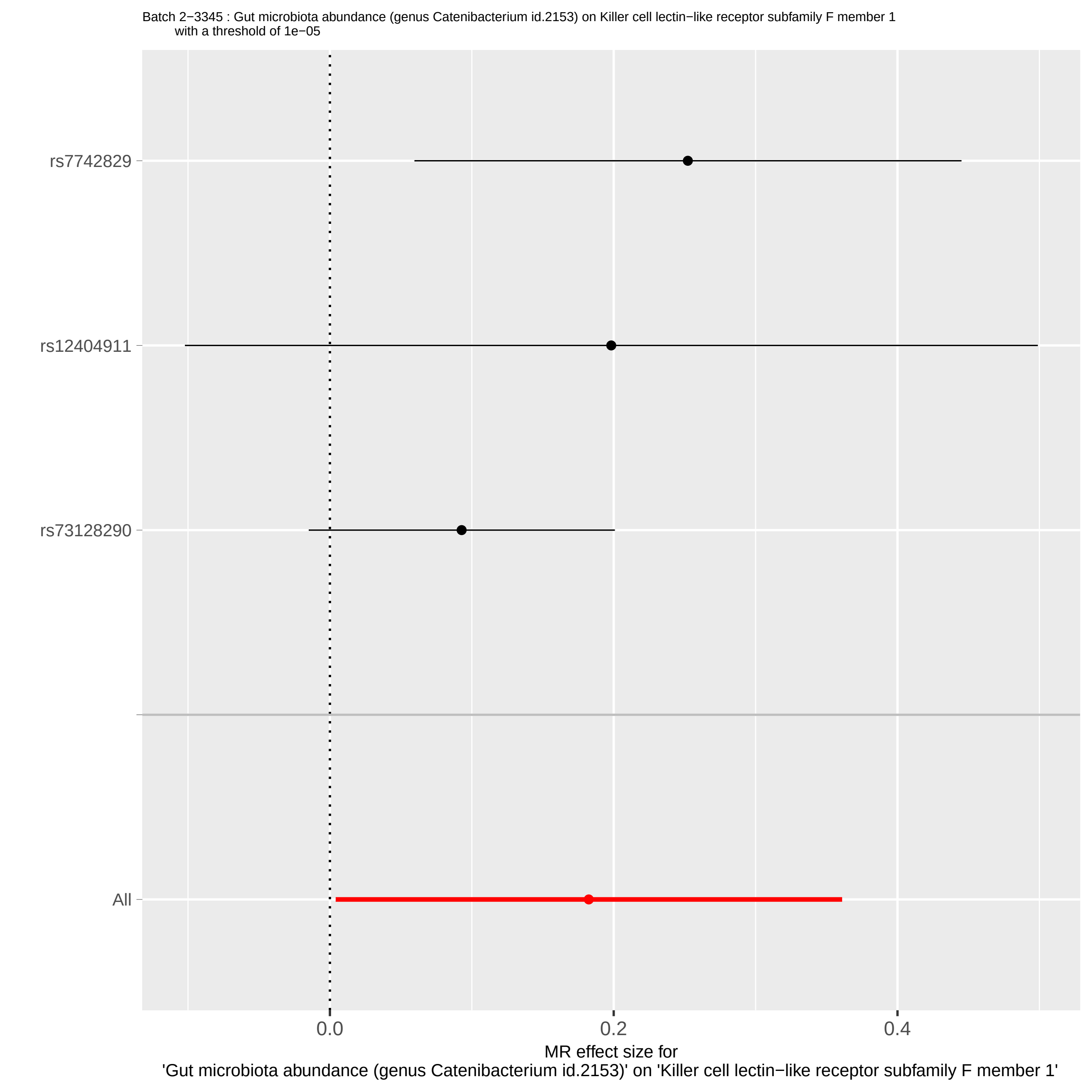
Batch 2−3345 : Gut microbiota abundance (genus Catenibacterium id.2153) on Killer cell lectin−like receptor subfamily F member 1
with a threshold of 1e−05
rs7742829
rs12404911
rs73128290
All
0.0
0.2
0.4
MR effect size for
'Gut microbiota abundance (genus Catenibacterium id.2153)' on 'Killer cell lectin−like receptor subfamily F member 1'

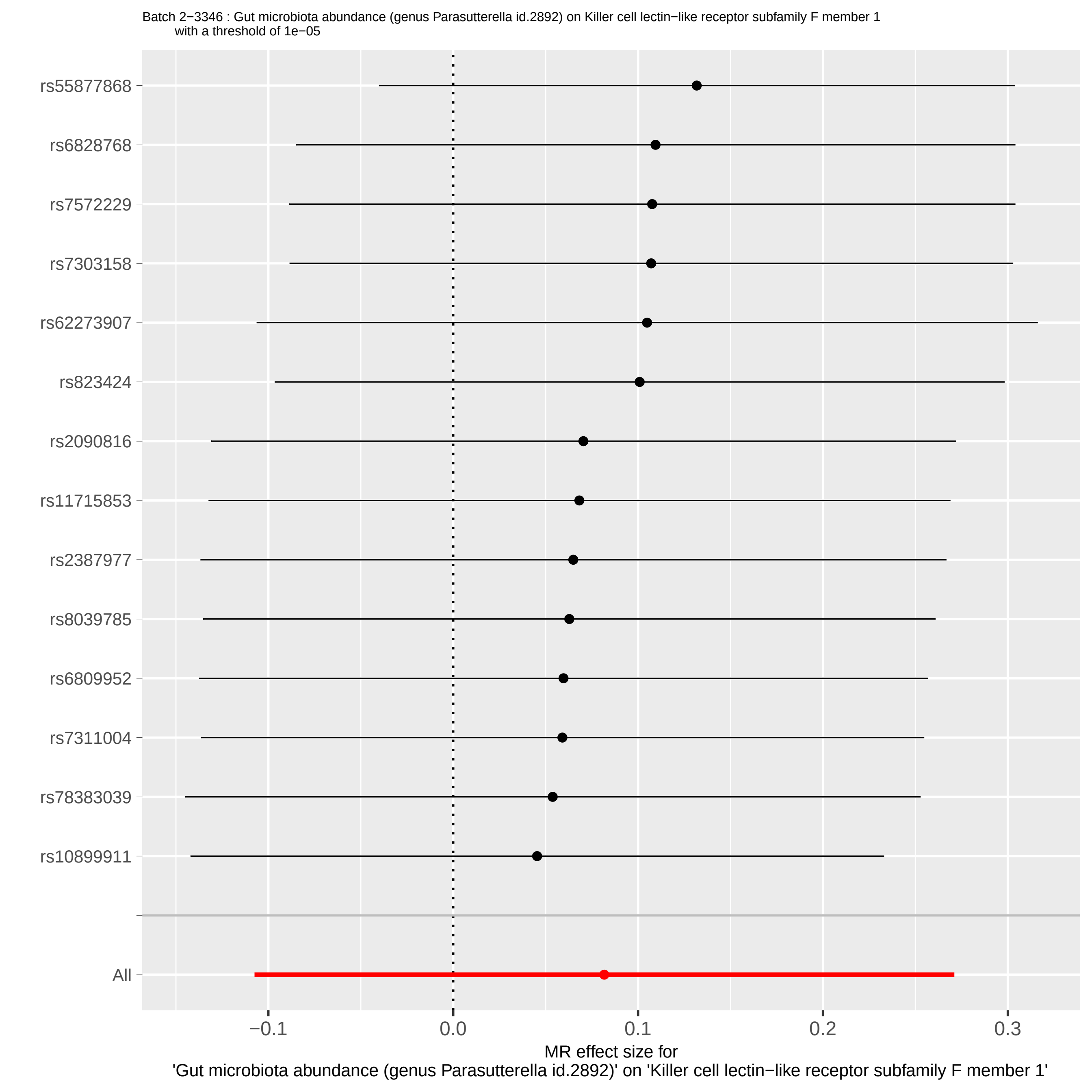

Batch 2−3346 : Gut microbiota abundance (genus Parasutterella id.2892) on Killer cell lectin−like receptor subfamily F member 1
with a threshold of 1e−05
rs55877868
rs6828768
rs7572229
rs7303158
rs62273907
rs823424
rs2090816
rs11715853
rs2387977
rs8039785
rs6809952
rs7311004
rs78383039
rs10899911
All
−0.1
0.0
0.1
0.2
0.3
MR effect size for
'Gut microbiota abundance (genus Parasutterella id.2892)' on 'Killer cell lectin−like receptor subfamily F member 1'

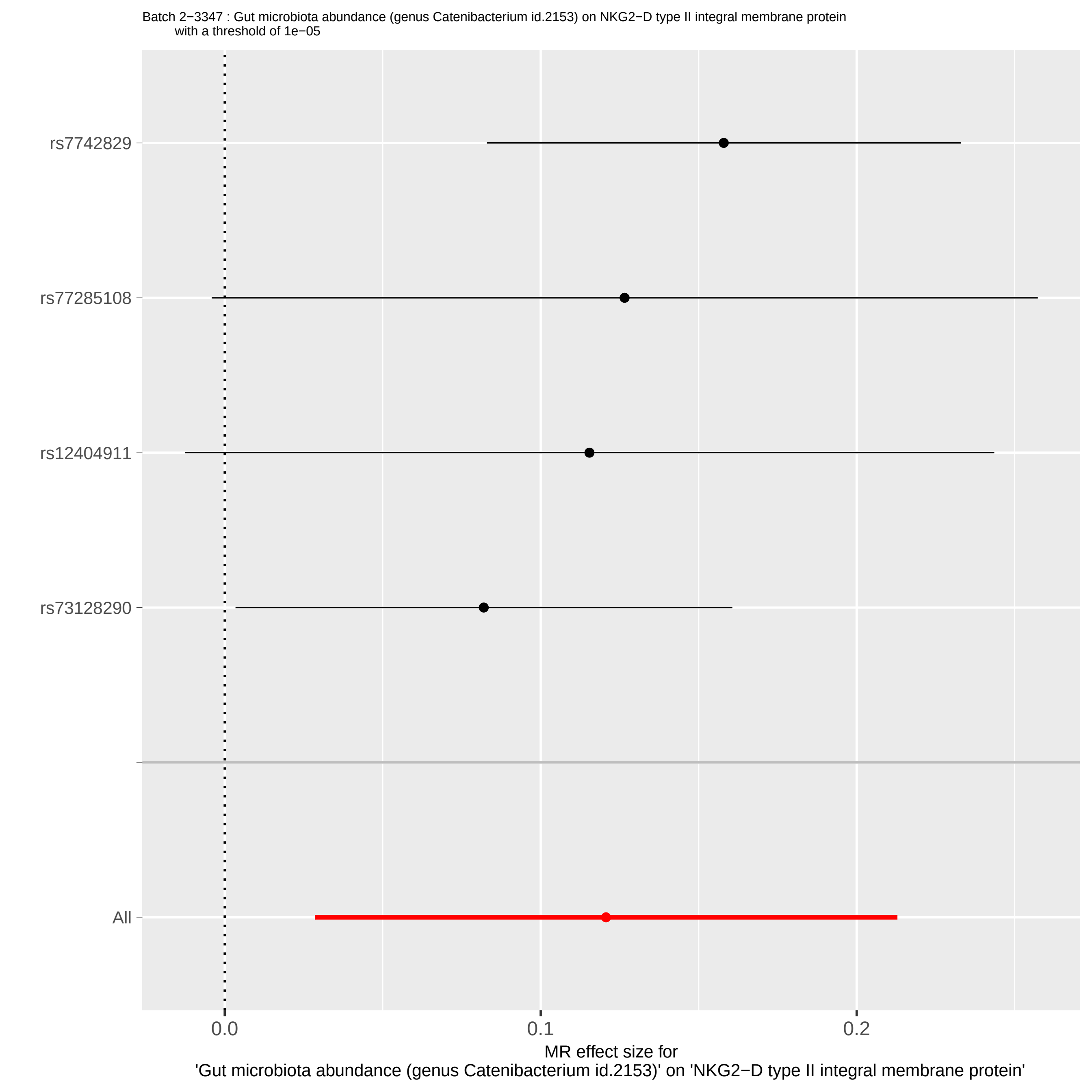
Batch 2−3347 : Gut microbiota abundance (genus Catenibacterium id.2153) on NKG2−D type II integral membrane protein
with a threshold of 1e−05
rs7742829
rs77285108
rs12404911
rs73128290
All
0.0
0.1
0.2
MR effect size for
'Gut microbiota abundance (genus Catenibacterium id.2153)' on 'NKG2−D type II integral membrane protein'

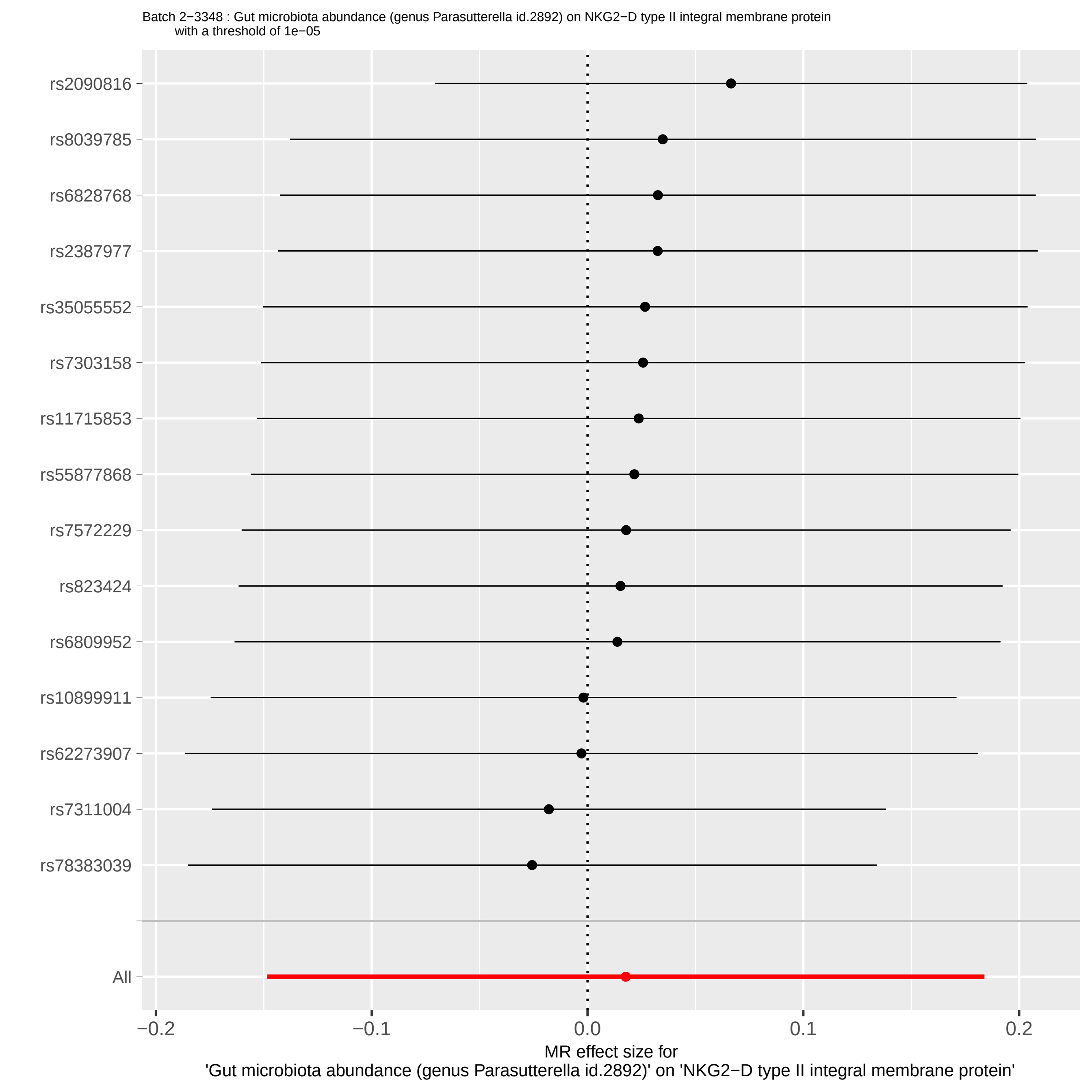

Batch 2−3348 : Gut microbiota abundance (genus Parasutterella id.2892) on NKG2−D type II integral membrane protein
with a threshold of 1e−05
rs2090816
rs8039785
rs6828768
rs2387977
rs35055552
rs7303158
rs11715853
rs55877868
rs7572229
rs823424
rs6809952
rs10899911
rs62273907
rs7311004
rs78383039
All
−0.2
−0.1
0.0
0.1
0.2
MR effect size for
'Gut microbiota abundance (genus Parasutterella id.2892)' on 'NKG2−D type II integral membrane protein'

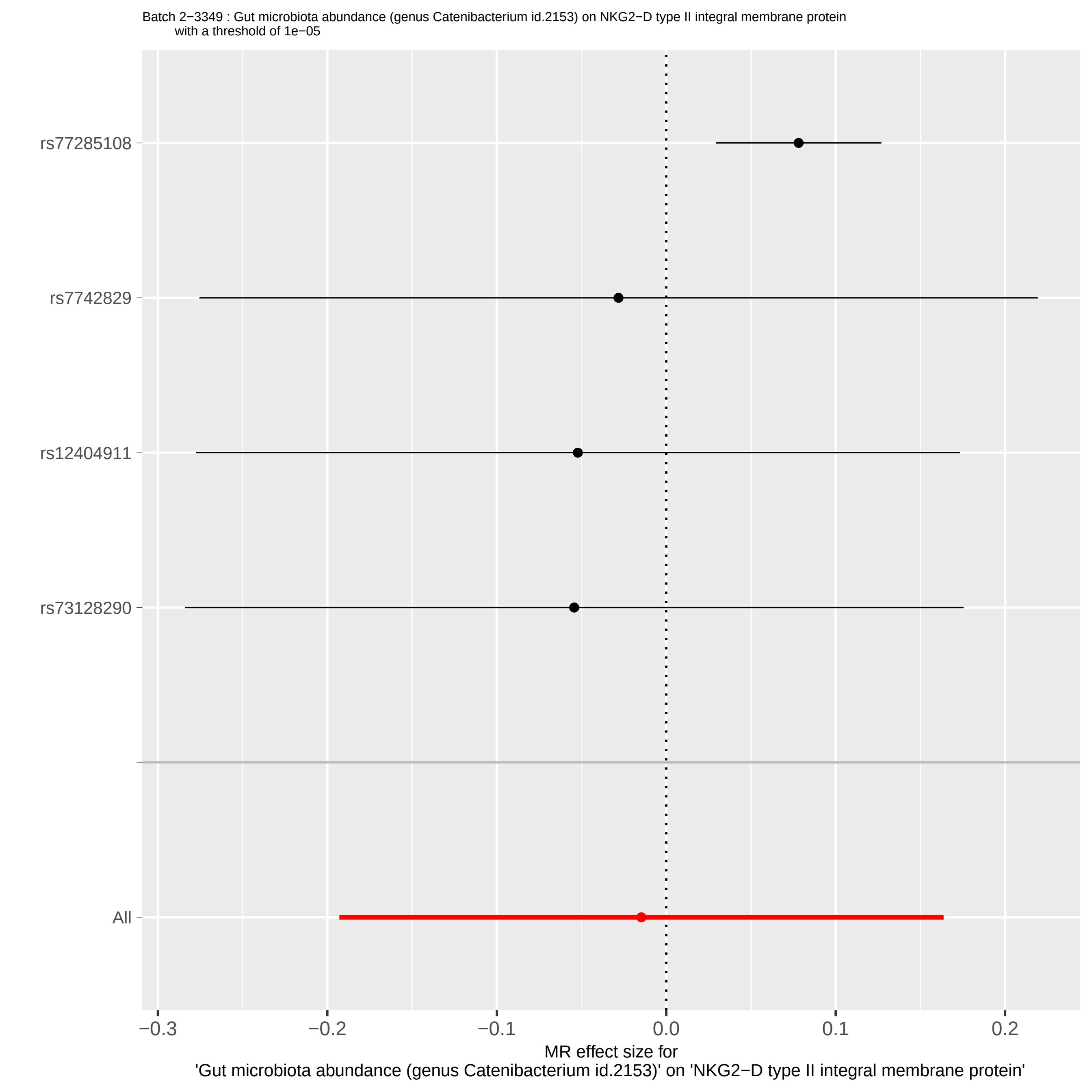
Batch 2−3349 : Gut microbiota abundance (genus Catenibacterium id.2153) on NKG2−D type II integral membrane protein
with a threshold of 1e−05
rs77285108
rs7742829
rs12404911
rs73128290
All
−0.3
−0.2
−0.1
0.0
0.1
0.2
MR effect size for
'Gut microbiota abundance (genus Catenibacterium id.2153)' on 'NKG2−D type II integral membrane protein'

Batch 2−3350 : Gut microbiota abundance (genus Parasutterella id.2892) on NKG2−D type II integral membrane protein
with a threshold of 1e−05

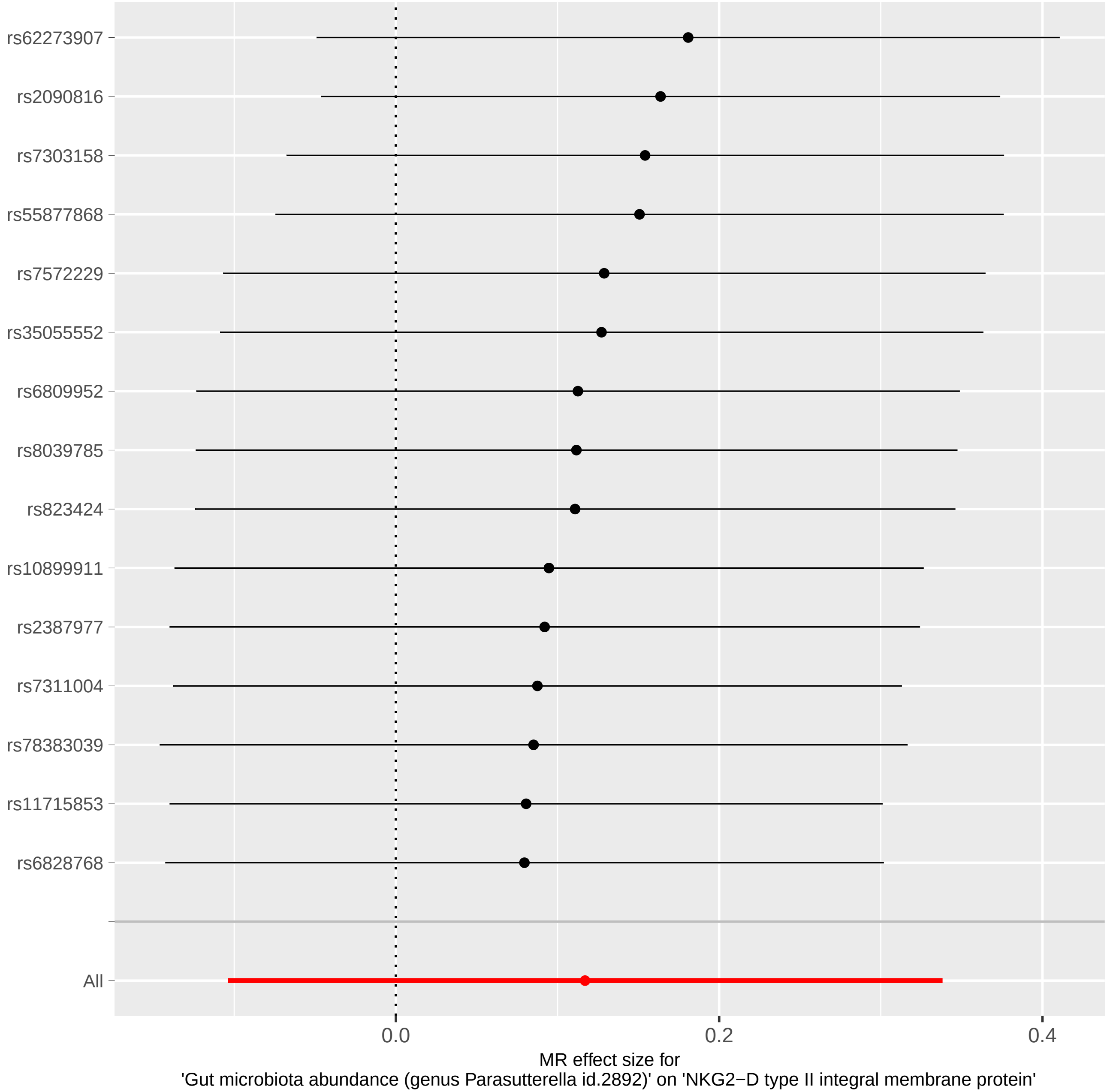

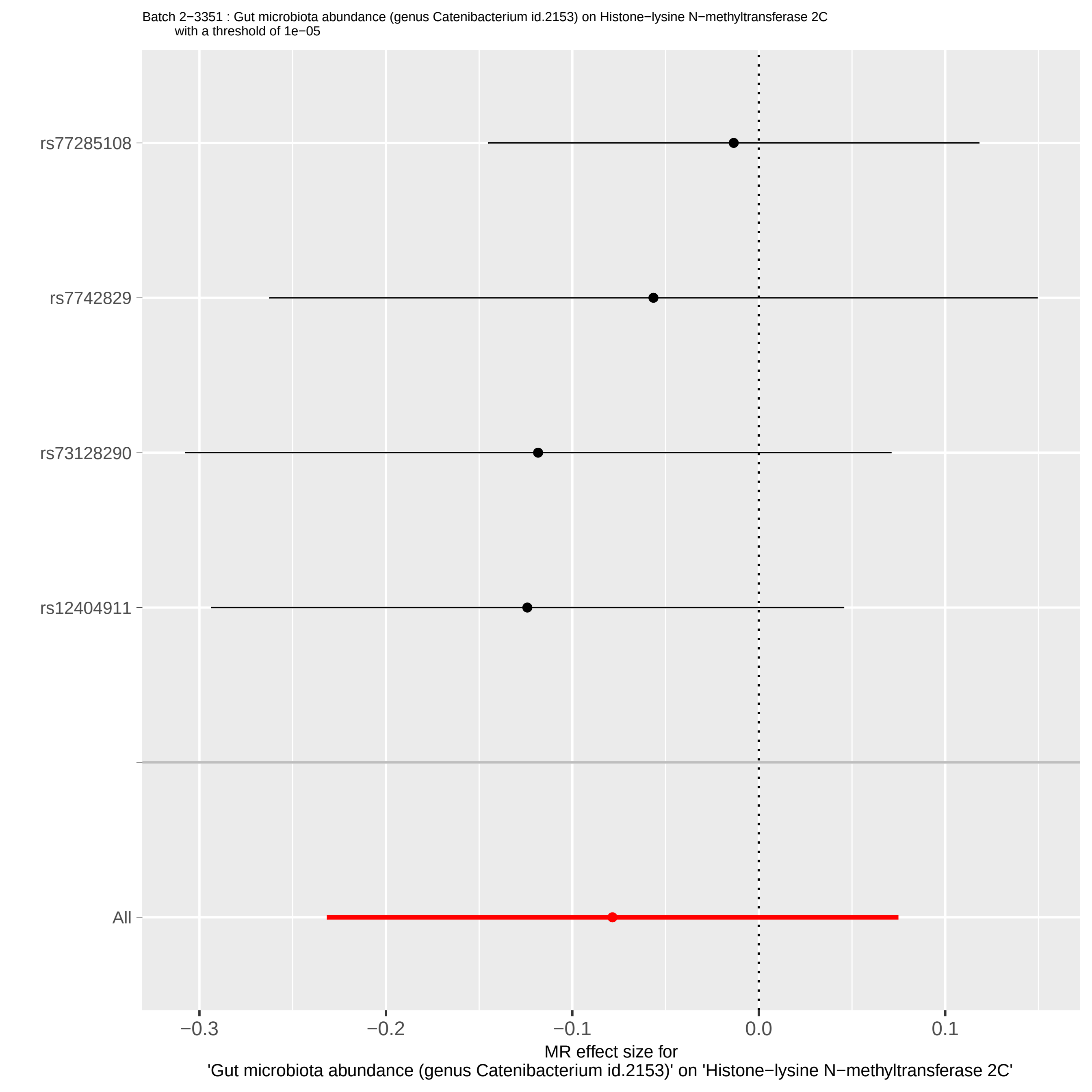
Batch 2−3351 : Gut microbiota abundance (genus Catenibacterium id.2153) on Histone−lysine N−methyltransferase 2C
with a threshold of 1e−05
rs77285108
rs7742829
rs73128290
rs12404911
All
−0.3
−0.2
−0.1
0.0
0.1
MR effect size for
'Gut microbiota abundance (genus Catenibacterium id.2153)' on 'Histone−lysine N−methyltransferase 2C'

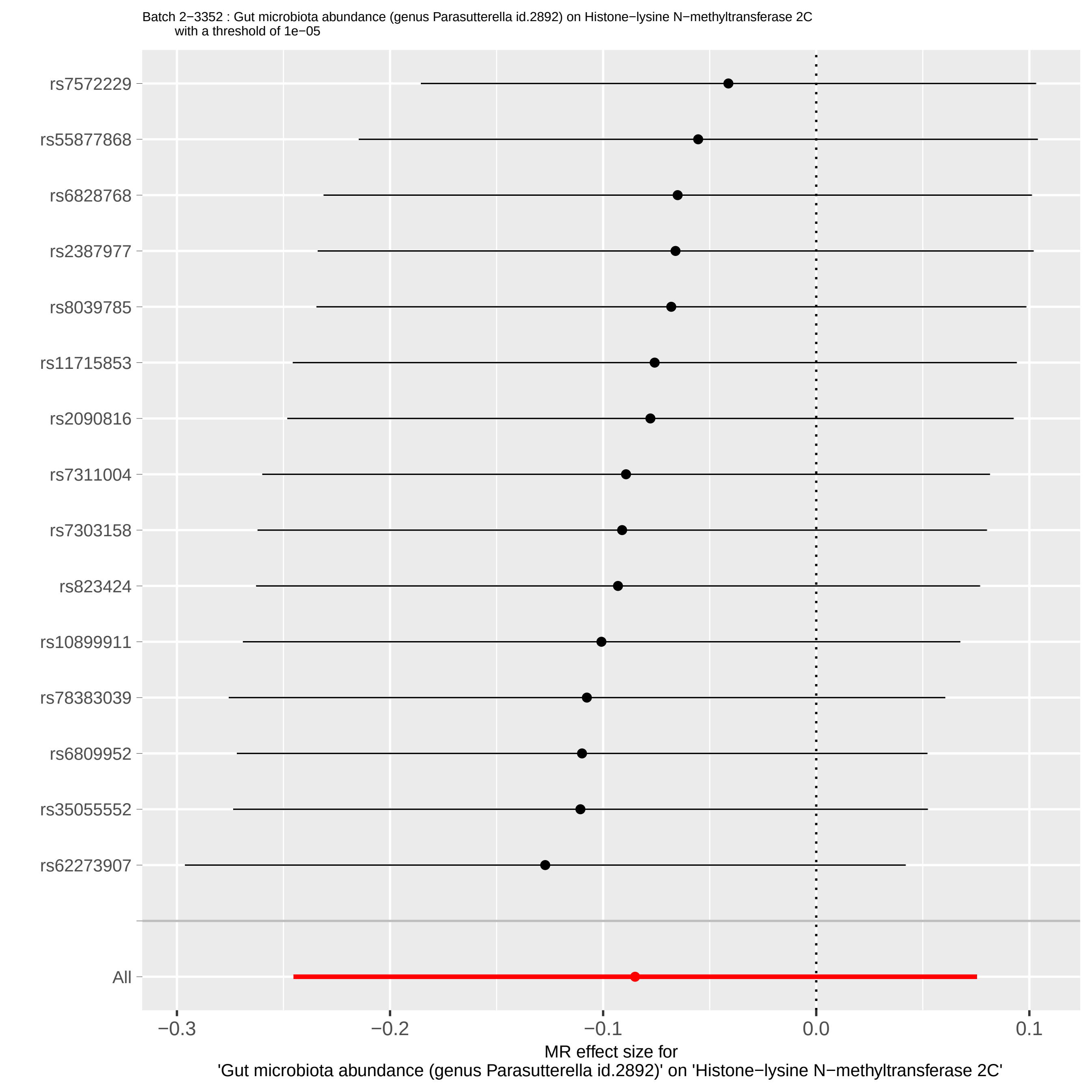
Batch 2−3352 : Gut microbiota abundance (genus Parasutterella id.2892) on Histone−lysine N−methyltransferase 2C
with a threshold of 1e−05
rs7572229
rs55877868
rs6828768
rs2387977
rs8039785
rs11715853
rs2090816
rs7311004
rs7303158
rs823424
rs10899911
rs78383039
rs6809952
rs35055552
rs62273907
All
−0.3
−0.2
−0.1
0.0
0.1
MR effect size for
'Gut microbiota abundance (genus Parasutterella id.2892)' on 'Histone−lysine N−methyltransferase 2C'

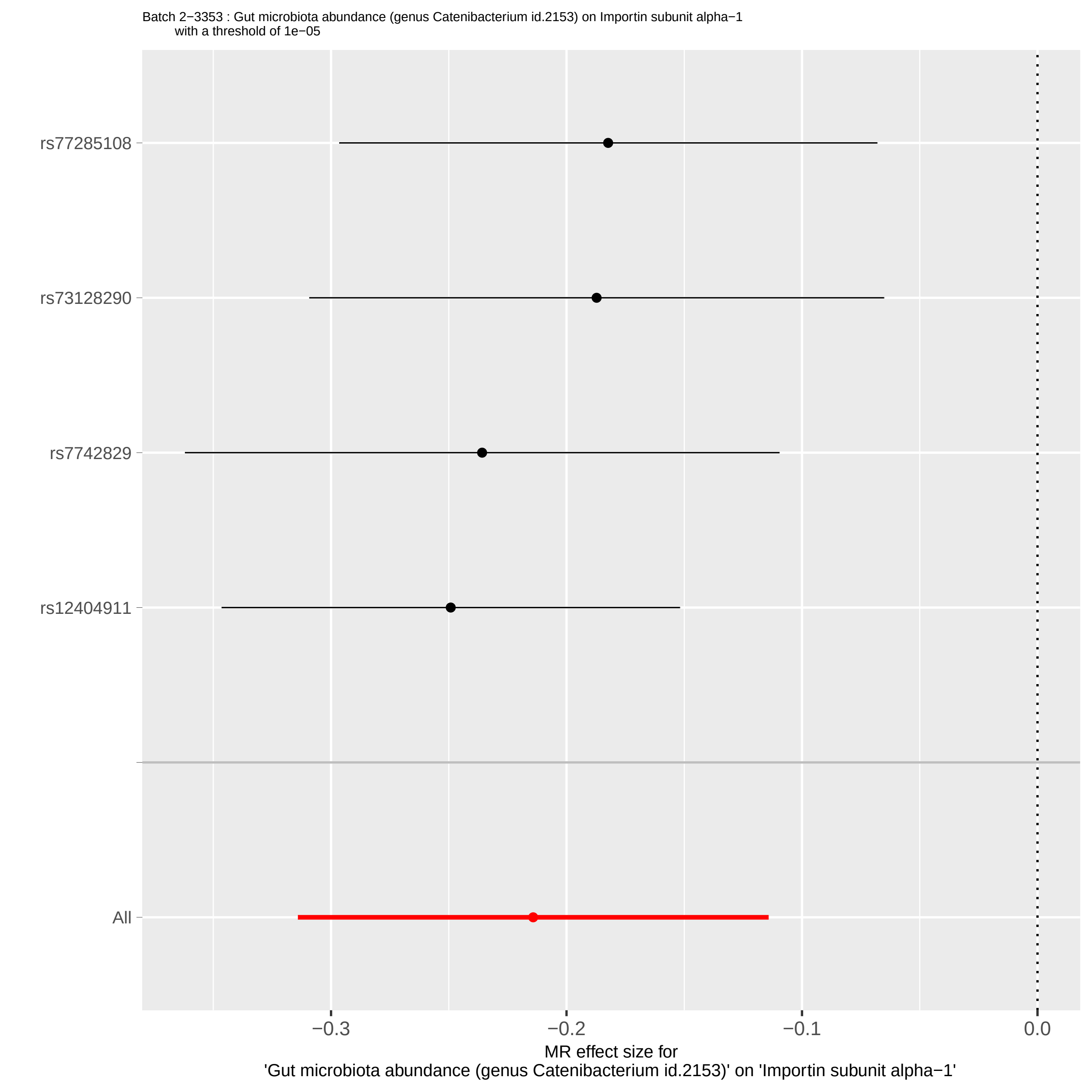

Batch 2−3353 : Gut microbiota abundance (genus Catenibacterium id.2153) on Importin subunit alpha−1
with a threshold of 1e−05
rs77285108
rs73128290
rs7742829
rs12404911
All
−0.3
−0.2
−0.1
0.0
MR effect size for
'Gut microbiota abundance (genus Catenibacterium id.2153)' on 'Importin subunit alpha−1'

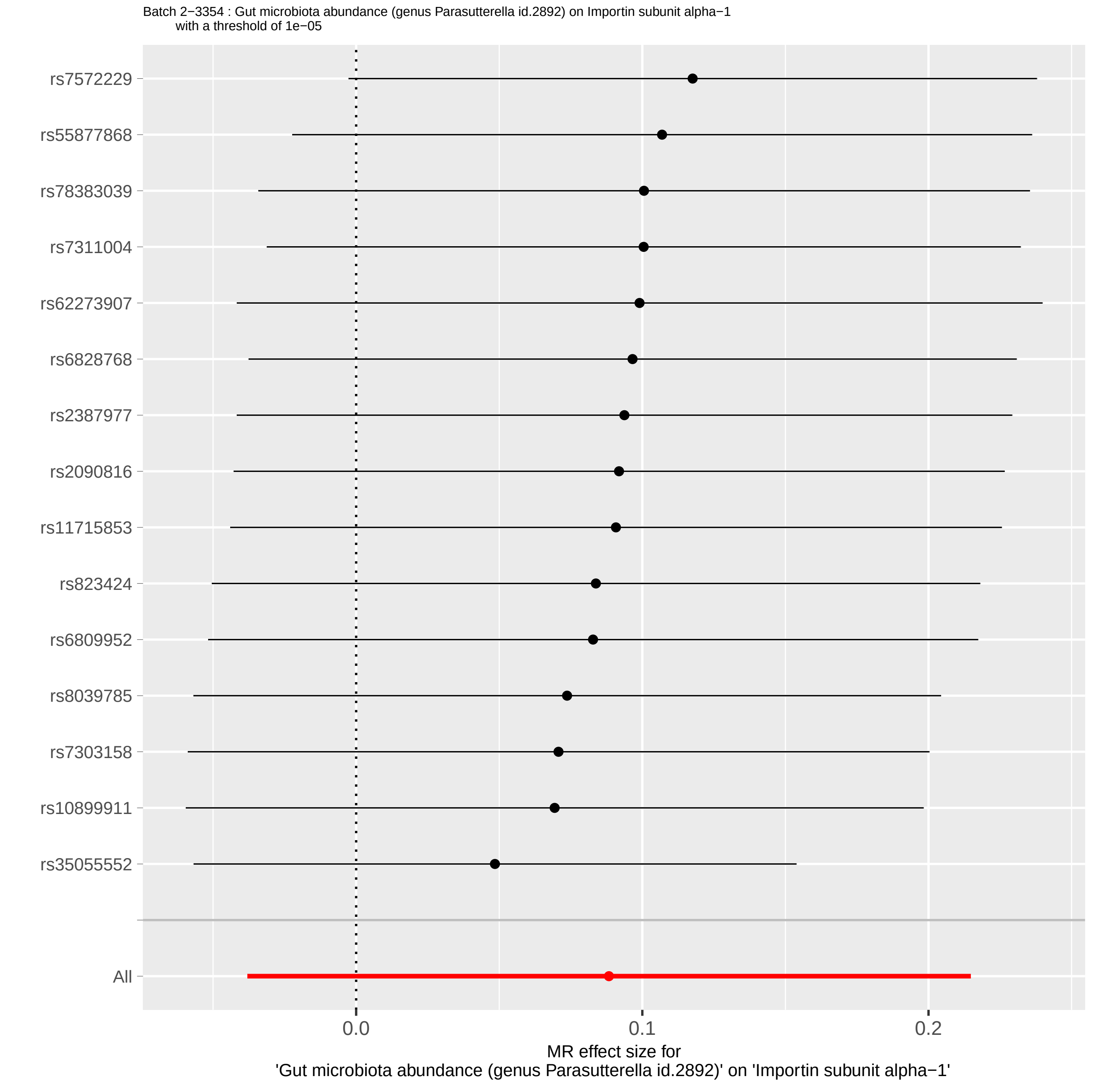

Batch 2−3354 : Gut microbiota abundance (genus Parasutterella id.2892) on Importin subunit alpha−1
with a threshold of 1e−05
rs7572229
rs55877868
rs78383039
rs7311004
rs62273907
rs6828768
rs2387977
rs2090816
rs11715853
rs823424
rs6809952
rs8039785
rs7303158
rs10899911
rs35055552
All
0.0
0.1
0.2
MR effect size for
'Gut microbiota abundance (genus Parasutterella id.2892)' on 'Importin subunit alpha−1'

Batch 2−3355 : Gut microbiota abundance (genus Catenibacterium id.2153) on Importin subunit alpha−3
with a threshold of 1e−05

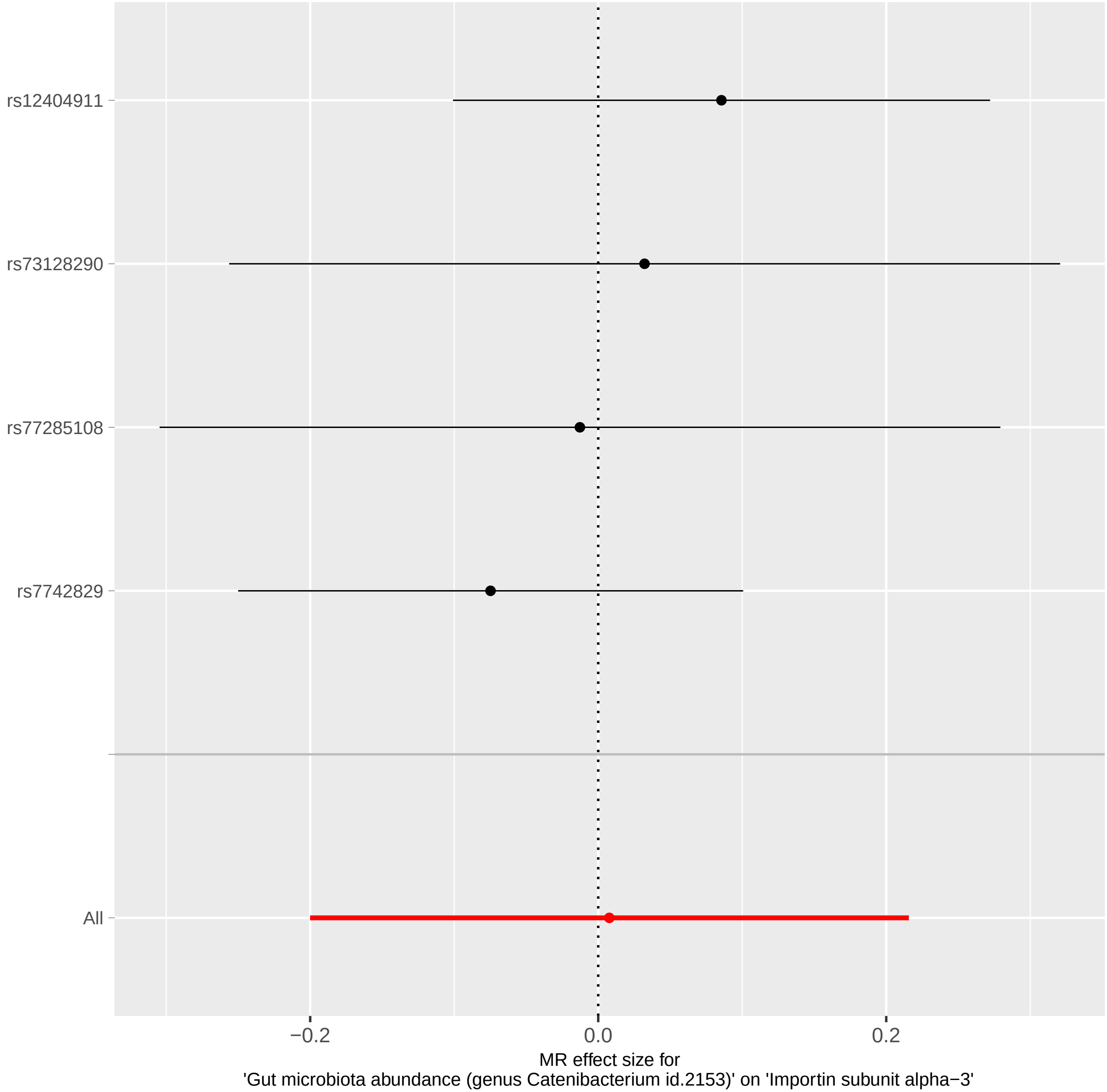

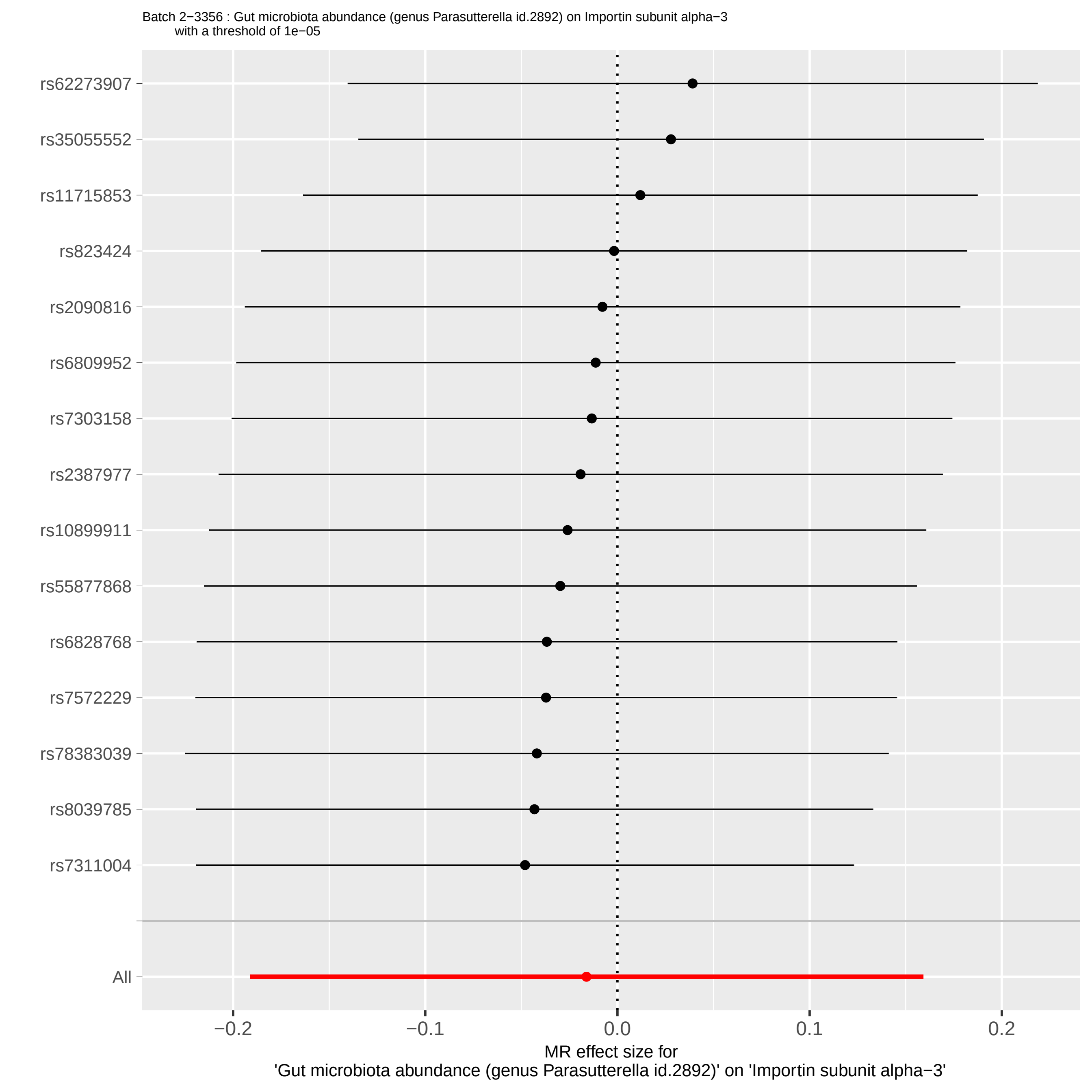

Batch 2−3356 : Gut microbiota abundance (genus Parasutterella id.2892) on Importin subunit alpha−3
with a threshold of 1e−05
rs62273907
rs35055552
rs11715853
rs823424
rs2090816
rs6809952
rs7303158
rs2387977
rs10899911
rs55877868
rs6828768
rs7572229
rs78383039
rs8039785
rs7311004
All
−0.2
−0.1
0.0
0.1
0.2
MR effect size for
'Gut microbiota abundance (genus Parasutterella id.2892)' on 'Importin subunit alpha−3'

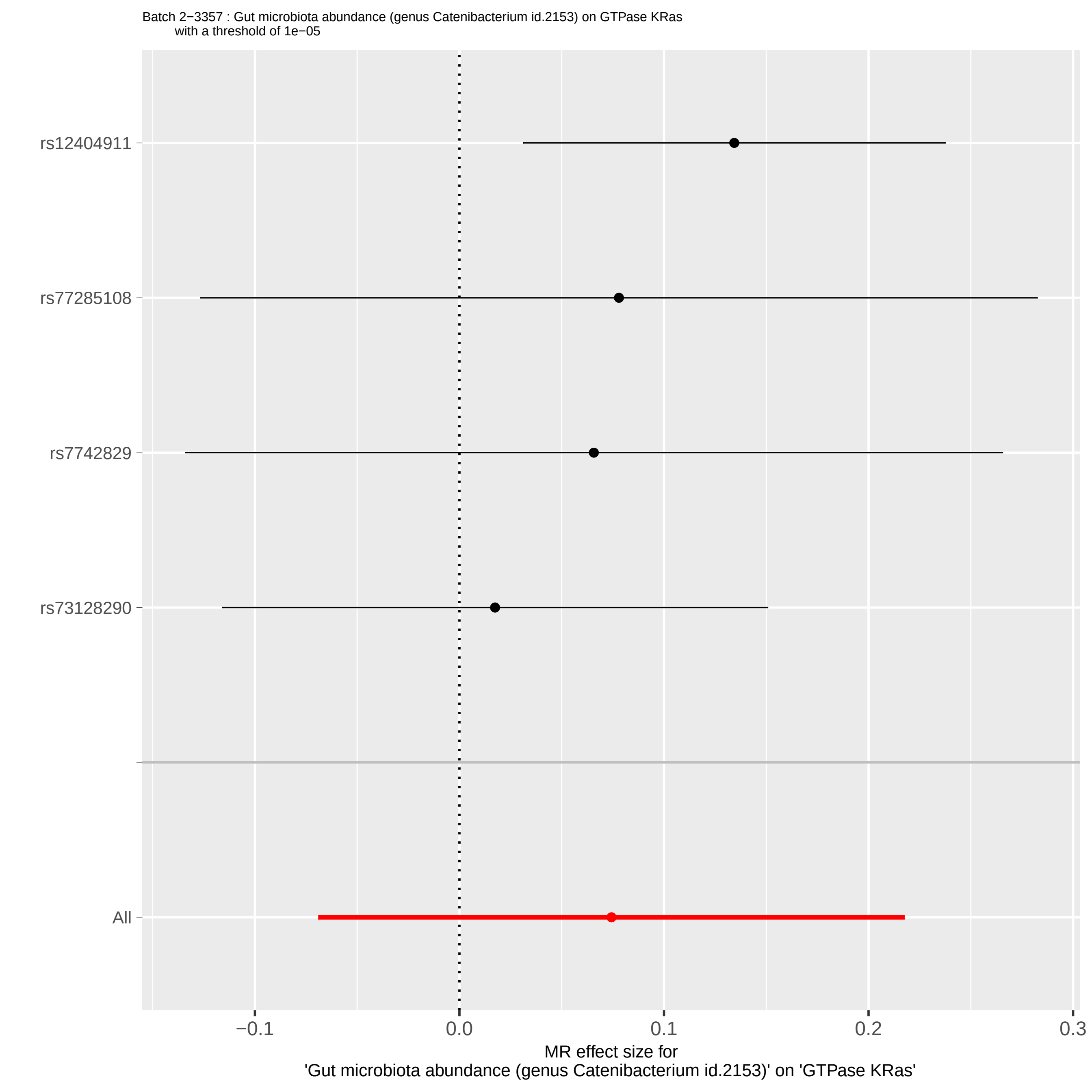
Batch 2−3357 : Gut microbiota abundance (genus Catenibacterium id.2153) on GTPase KRas
with a threshold of 1e−05
rs12404911
rs77285108
rs7742829
rs73128290
All
−0.1
0.0
0.1
0.2
0.3
MR effect size for
'Gut microbiota abundance (genus Catenibacterium id.2153)' on 'GTPase KRas'

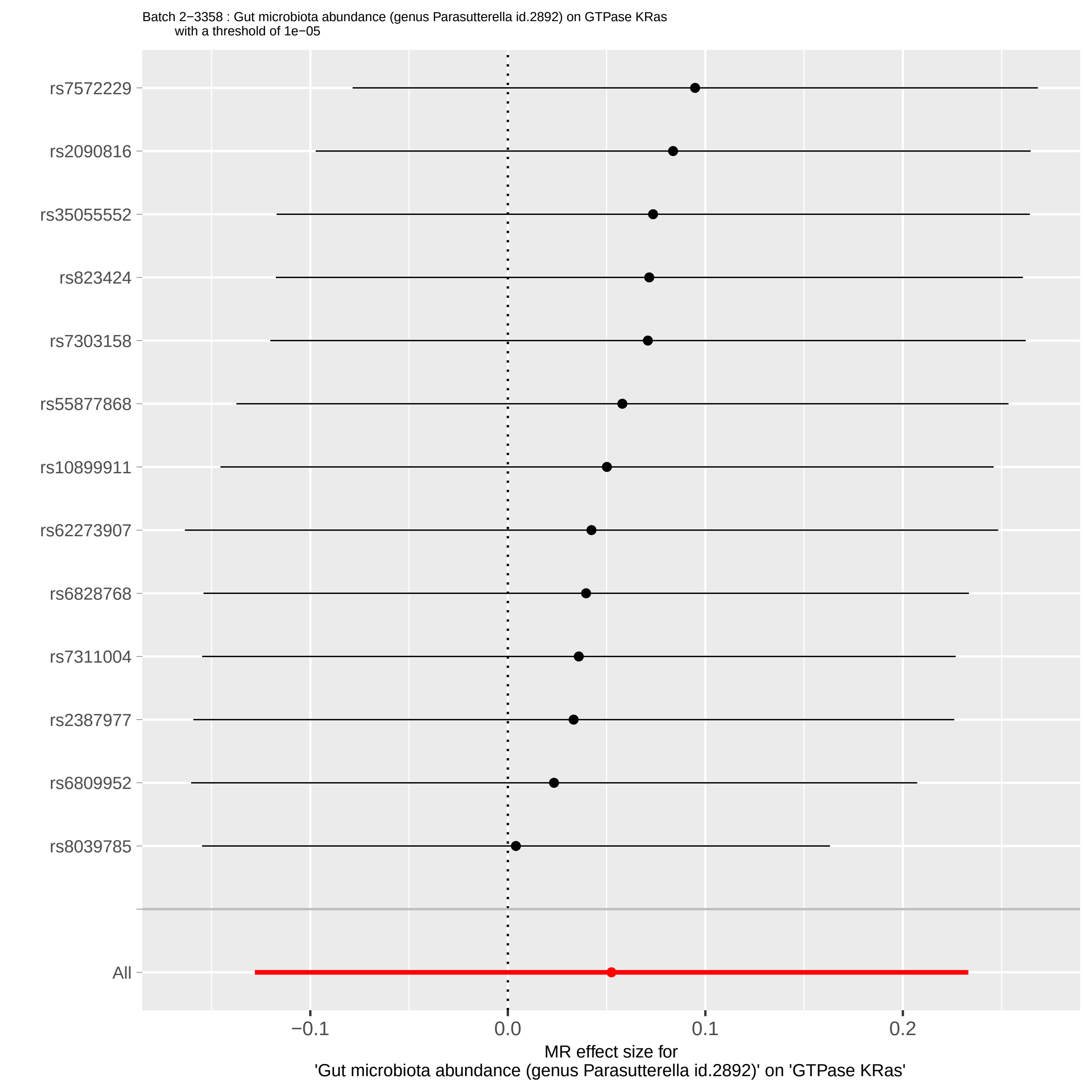

Batch 2−3358 : Gut microbiota abundance (genus Parasutterella id.2892) on GTPase KRas
with a threshold of 1e−05
rs7572229
rs2090816
rs35055552
rs823424
rs7303158
rs55877868
rs10899911
rs62273907
rs6828768
rs7311004
rs2387977
rs6809952
rs8039785
All
−0.1
0.0
0.1
0.2
MR effect size for
'Gut microbiota abundance (genus Parasutterella id.2892)' on 'GTPase KRas'

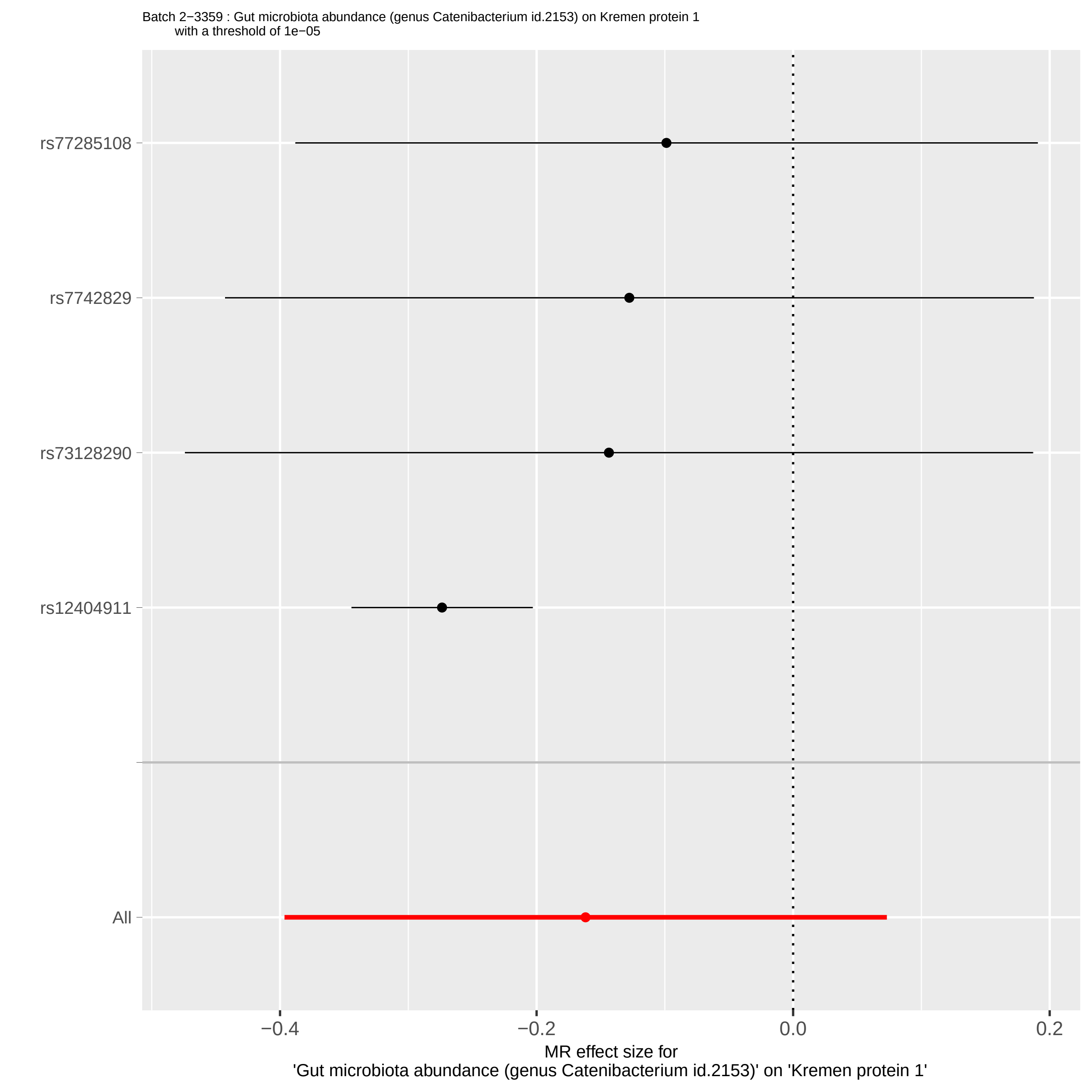

Batch 2−3359 : Gut microbiota abundance (genus Catenibacterium id.2153) on Kremen protein 1
with a threshold of 1e−05
rs77285108
rs7742829
rs73128290
rs12404911
All
−0.4
−0.2
0.0
0.2
MR effect size for
'Gut microbiota abundance (genus Catenibacterium id.2153)' on 'Kremen protein 1'

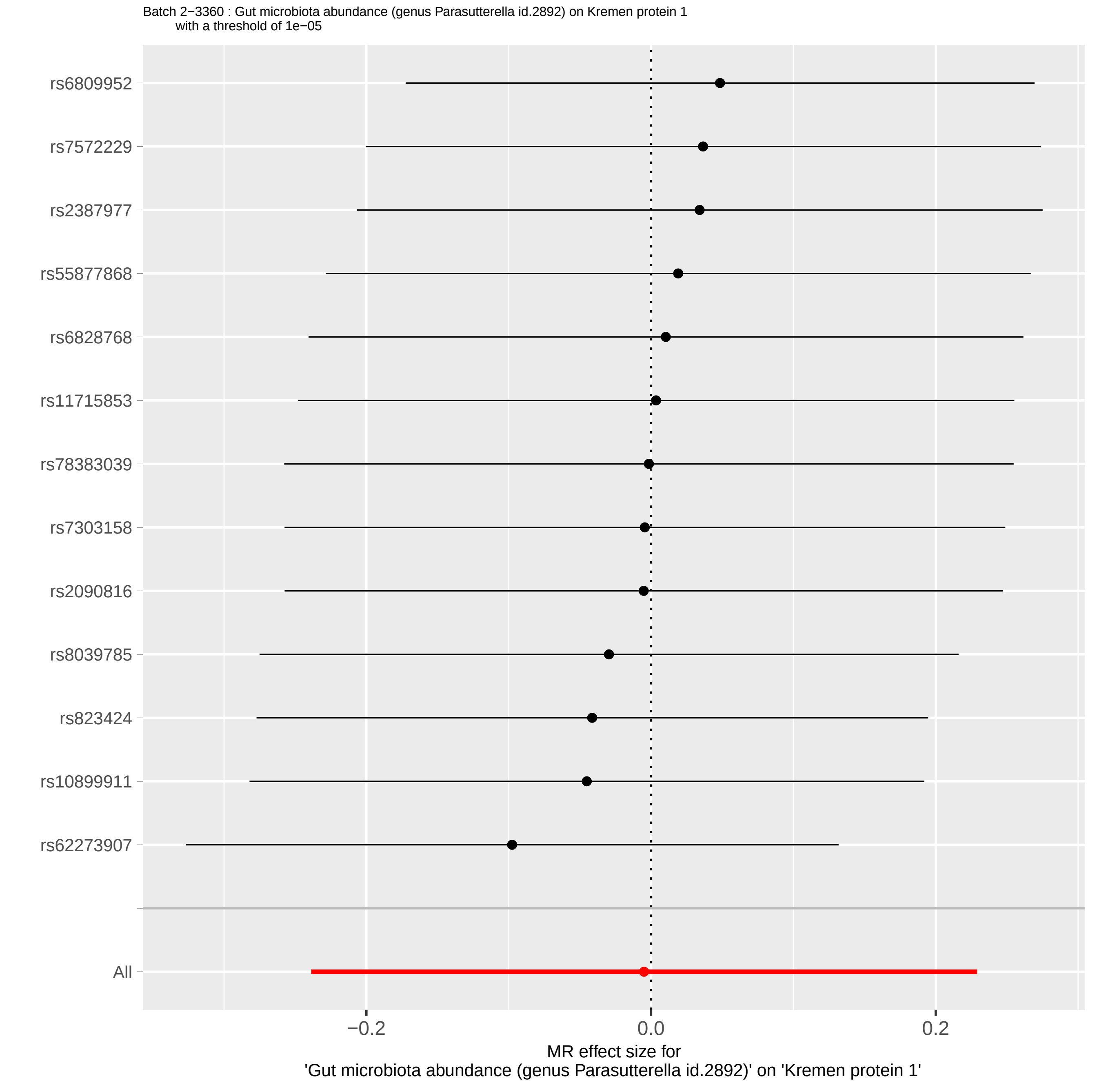
Batch 2−3360 : Gut microbiota abundance (genus Parasutterella id.2892) on Kremen protein 1
with a threshold of 1e−05
rs6809952
rs7572229
rs2387977
rs55877868
rs6828768
rs11715853
rs78383039
rs7303158
rs2090816
rs8039785
rs823424
rs10899911
rs62273907
All
−0.2
0.0
0.2
MR effect size for
'Gut microbiota abundance (genus Parasutterella id.2892)' on 'Kremen protein 1'

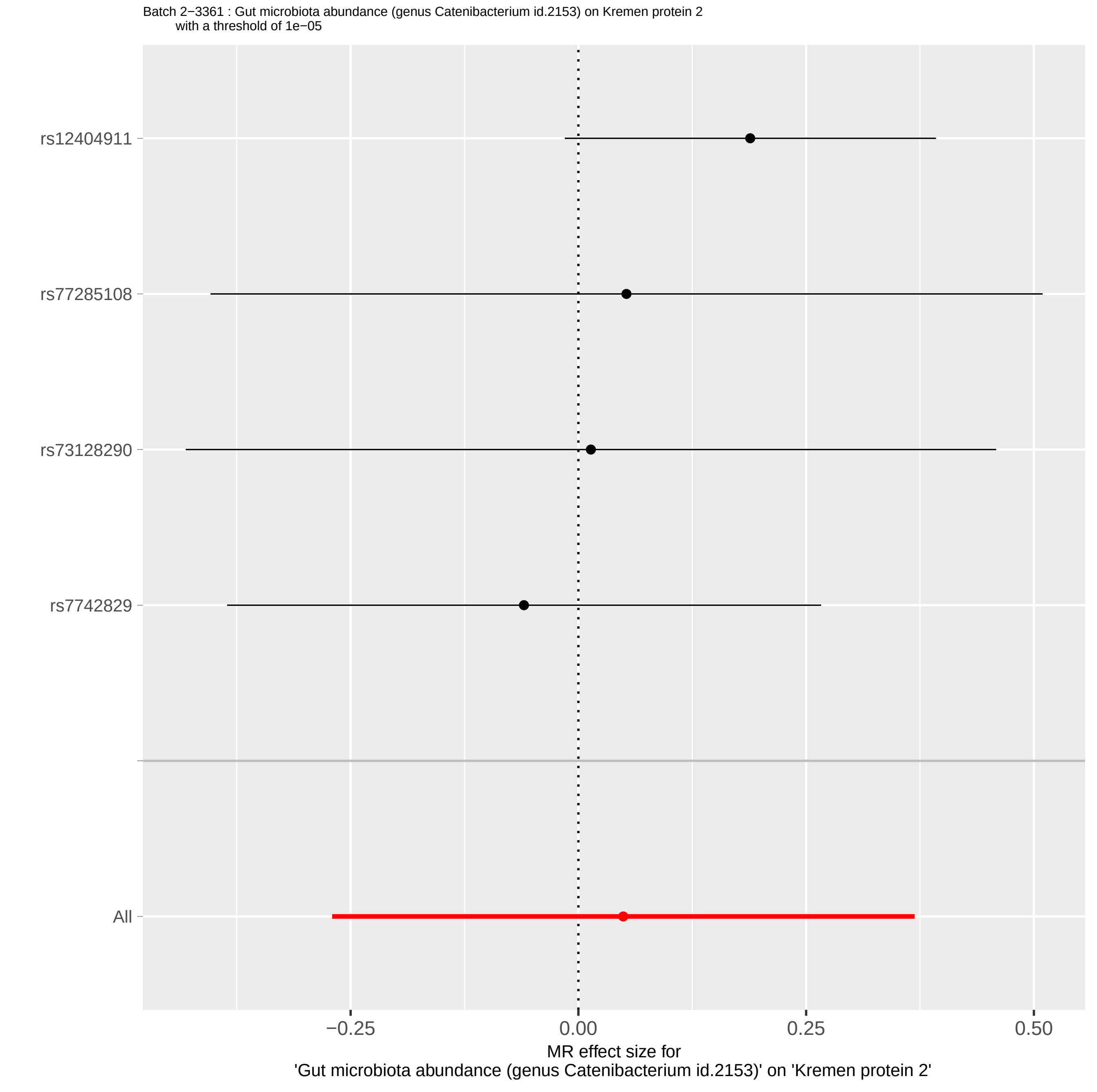
Batch 2−3361 : Gut microbiota abundance (genus Catenibacterium id.2153) on Kremen protein 2
with a threshold of 1e−05
rs12404911
rs77285108
rs73128290
rs7742829
All
−0.25
0.00
0.25
0.50
MR effect size for
'Gut microbiota abundance (genus Catenibacterium id.2153)' on 'Kremen protein 2'

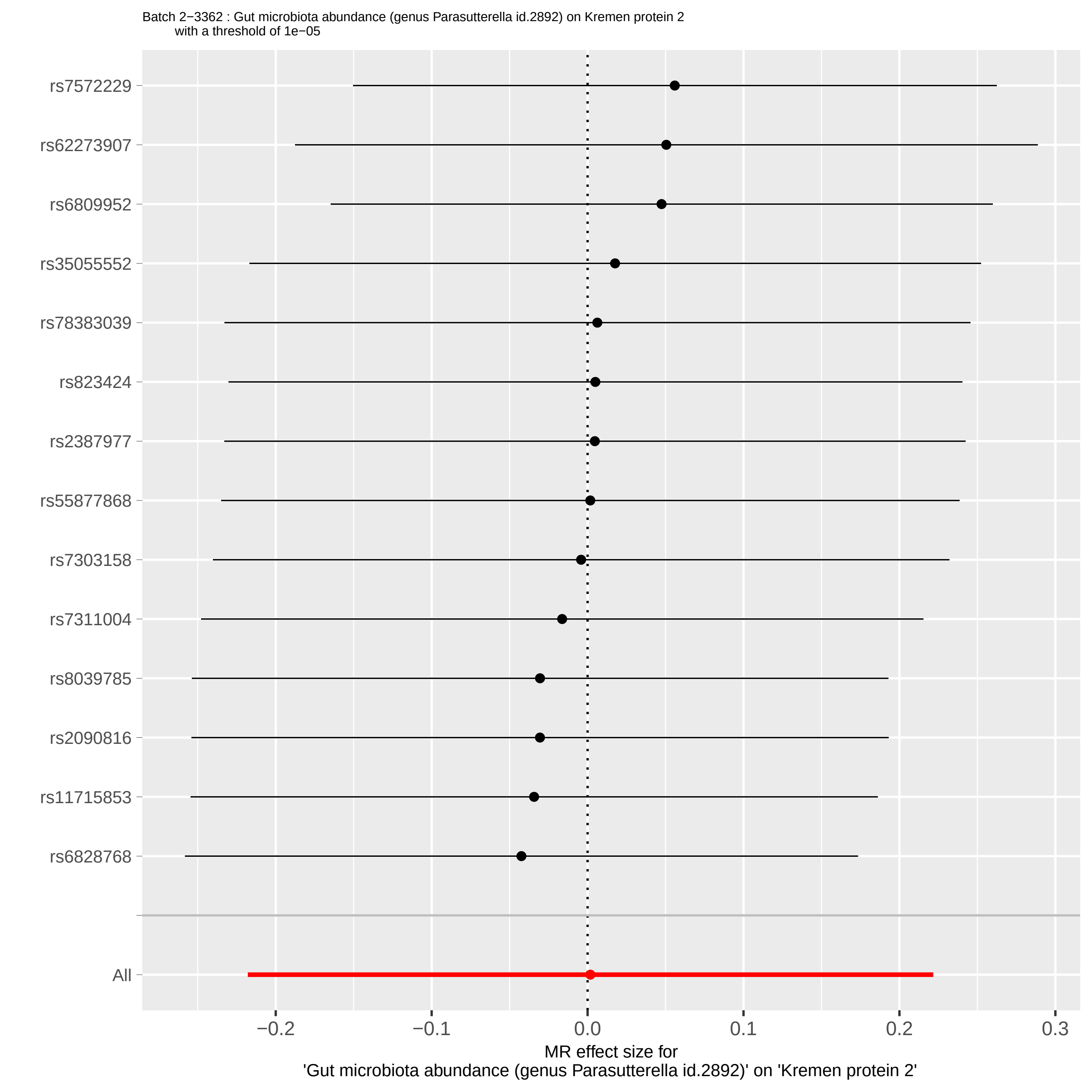

Batch 2−3362 : Gut microbiota abundance (genus Parasutterella id.2892) on Kremen protein 2
with a threshold of 1e−05
rs7572229
rs62273907
rs6809952
rs35055552
rs78383039
rs823424
rs2387977
rs55877868
rs7303158
rs7311004
rs8039785
rs2090816
rs11715853
rs6828768
All
−0.2
−0.1
0.0
0.1
0.2
0.3
MR effect size for
'Gut microbiota abundance (genus Parasutterella id.2892)' on 'Kremen protein 2'

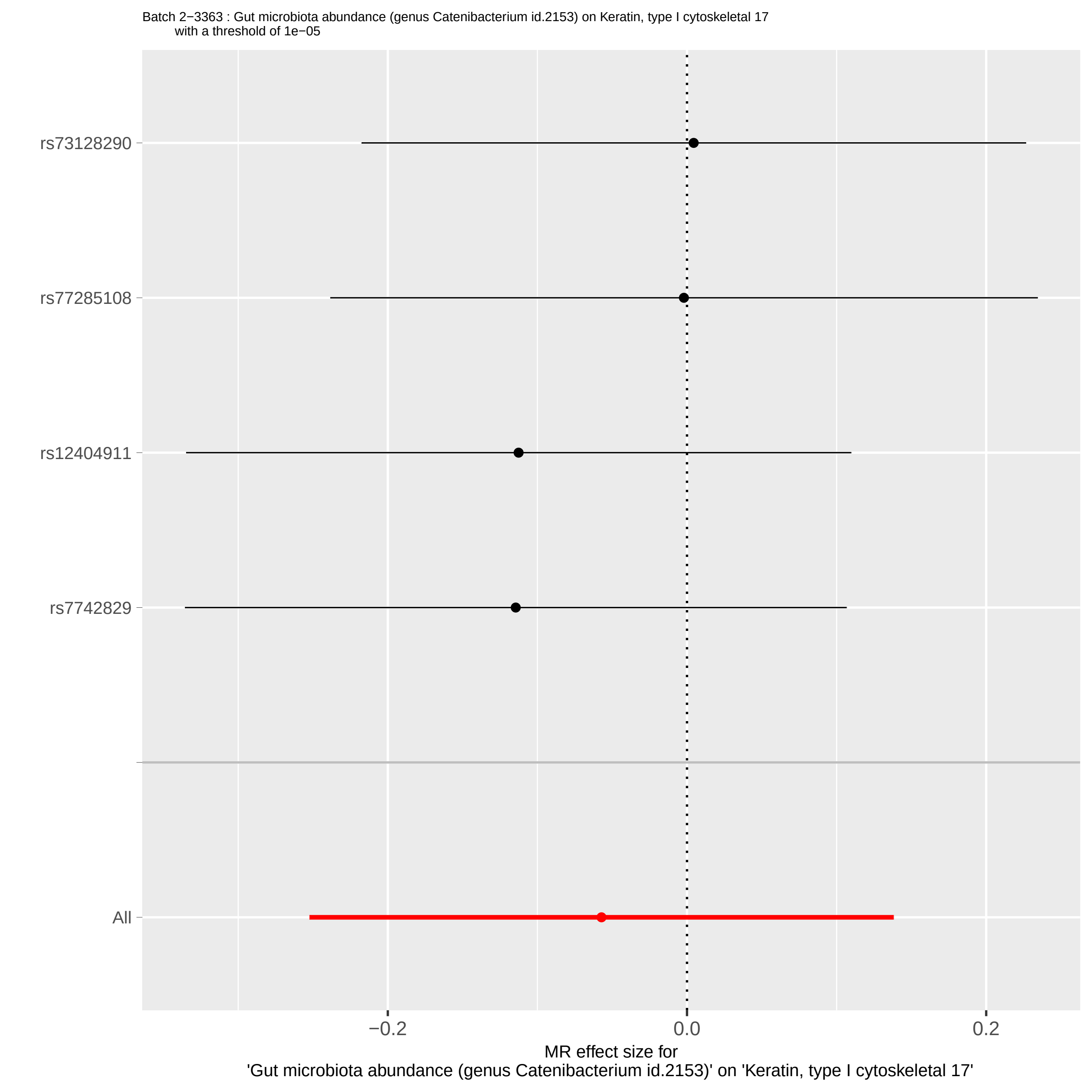

Batch 2−3363 : Gut microbiota abundance (genus Catenibacterium id.2153) on Keratin, type I cytoskeletal 17
with a threshold of 1e−05
rs73128290
rs77285108
rs12404911
rs7742829
All
−0.2
0.0
0.2
MR effect size for
'Gut microbiota abundance (genus Catenibacterium id.2153)' on 'Keratin, type I cytoskeletal 17'

Batch 2−3364 : Gut microbiota abundance (genus Parasutterella id.2892) on Keratin, type I cytoskeletal 17
with a threshold of 1e−05

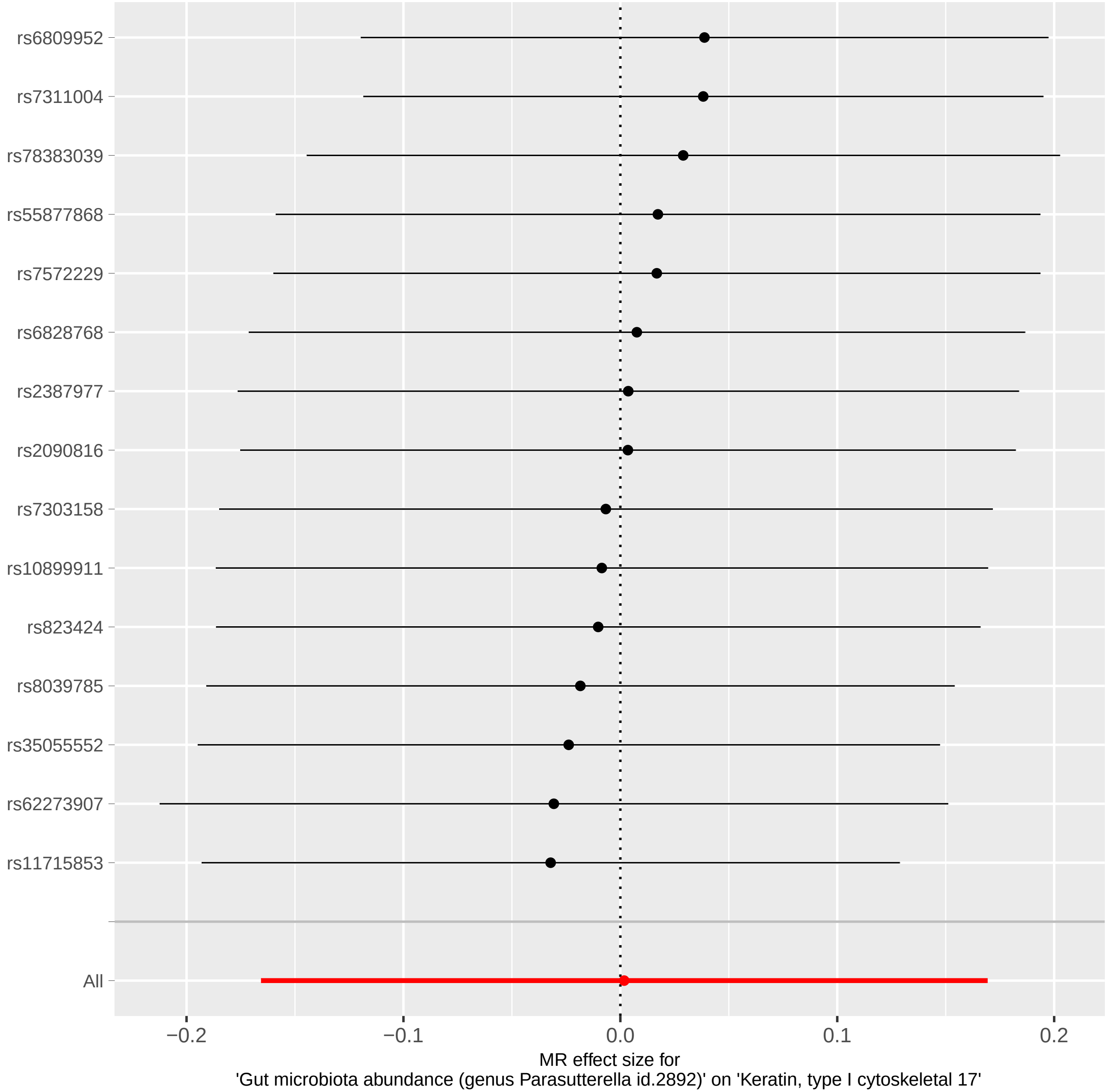

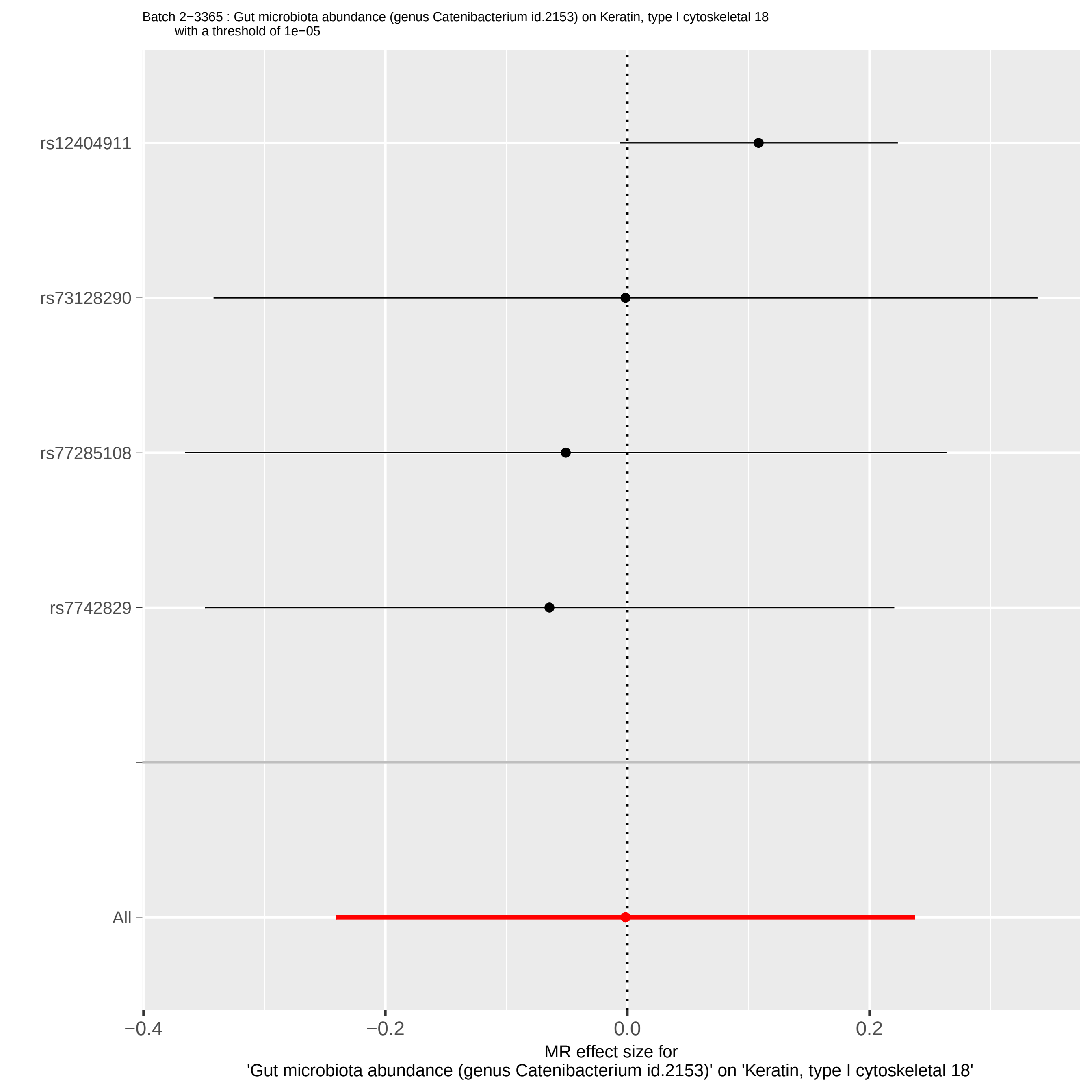
Batch 2−3365 : Gut microbiota abundance (genus Catenibacterium id.2153) on Keratin, type I cytoskeletal 18
with a threshold of 1e−05
rs12404911
rs73128290
rs77285108
rs7742829
All
−0.4
−0.2
0.0
0.2
MR effect size for
'Gut microbiota abundance (genus Catenibacterium id.2153)' on 'Keratin, type I cytoskeletal 18'

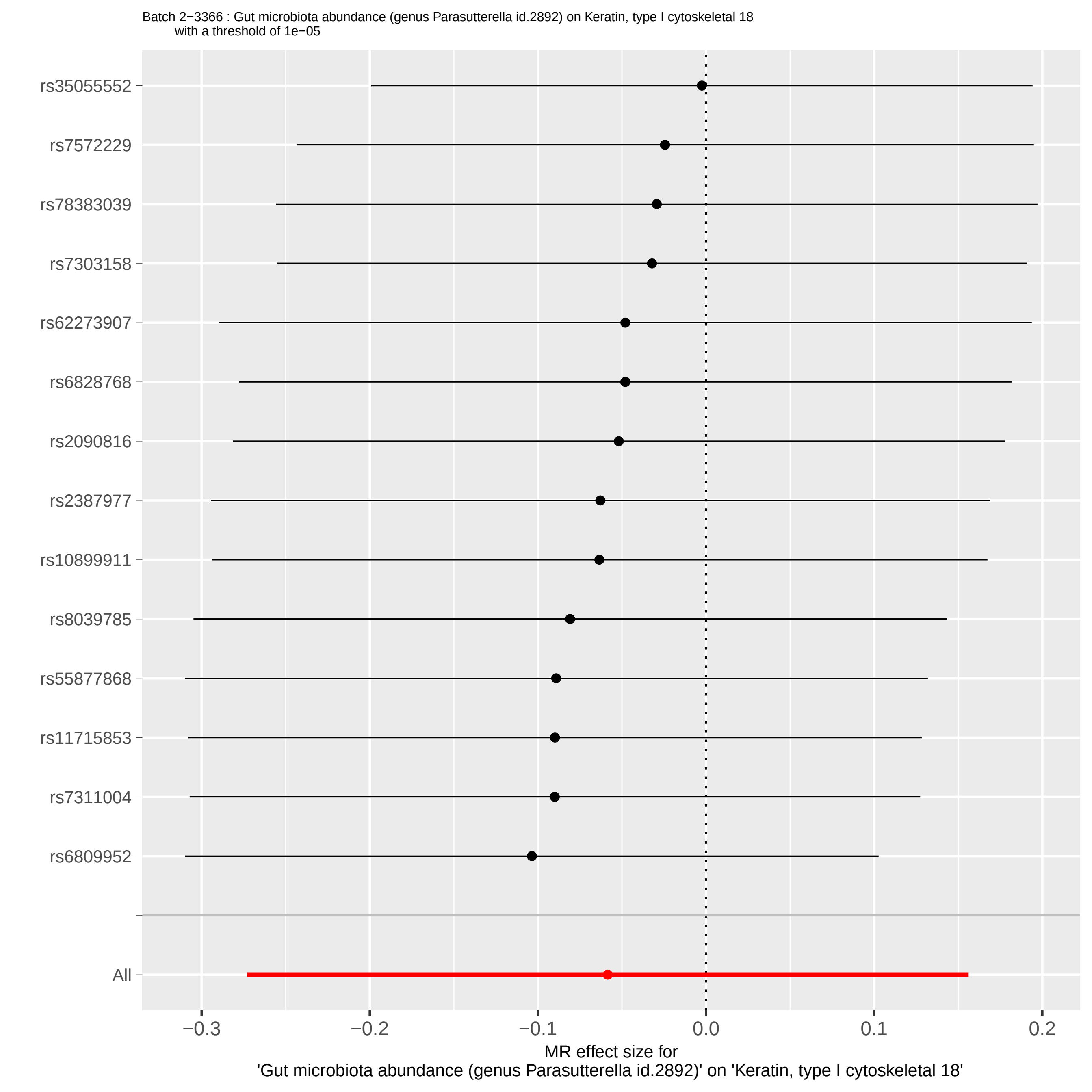

Batch 2−3366 : Gut microbiota abundance (genus Parasutterella id.2892) on Keratin, type I cytoskeletal 18
with a threshold of 1e−05
rs35055552
rs7572229
rs78383039
rs7303158
rs62273907
rs6828768
rs2090816
rs2387977
rs10899911
rs8039785
rs55877868
rs11715853
rs7311004
rs6809952
All
−0.3
−0.2
−0.1
0.0
0.1
0.2
MR effect size for
'Gut microbiota abundance (genus Parasutterella id.2892)' on 'Keratin, type I cytoskeletal 18'

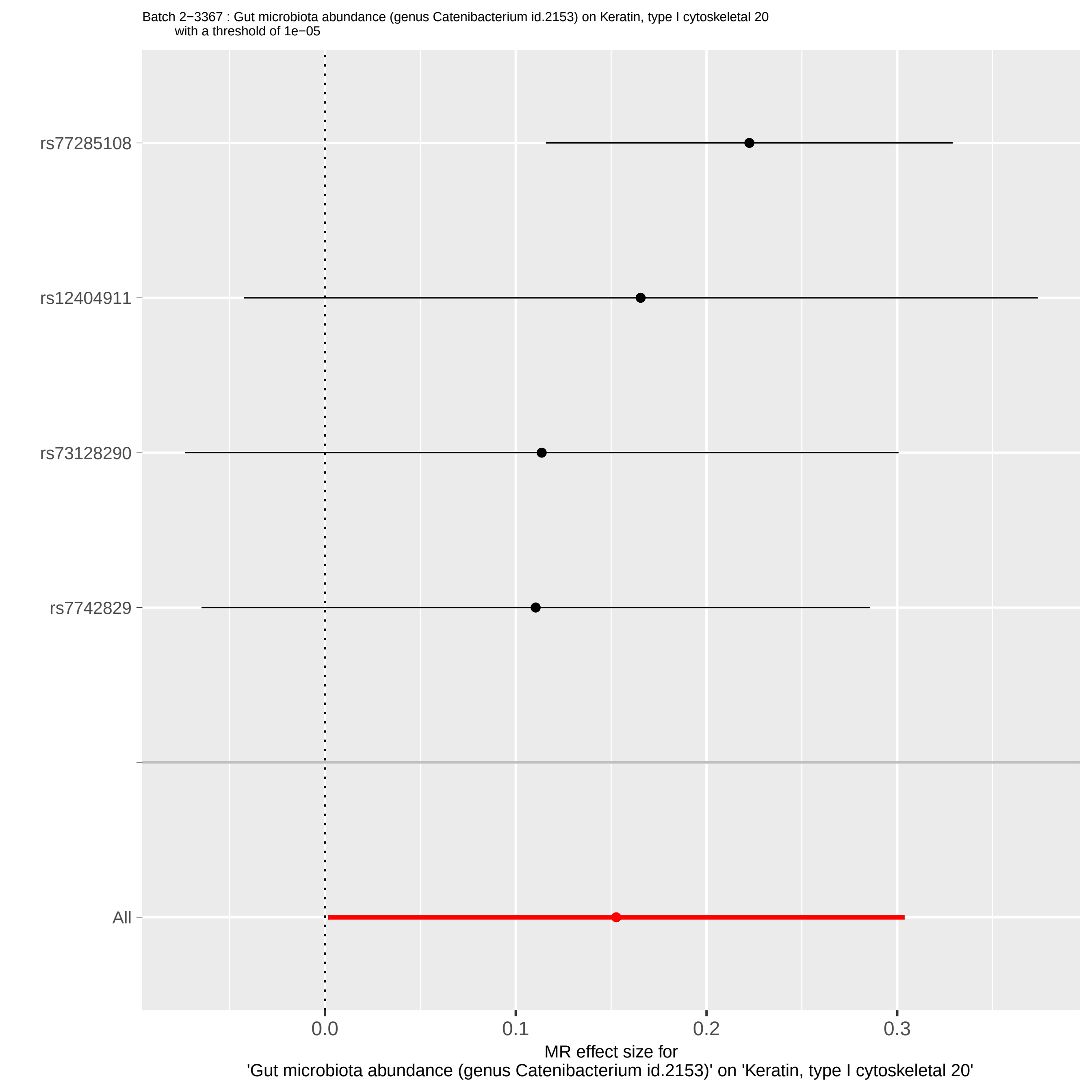

Batch 2−3367 : Gut microbiota abundance (genus Catenibacterium id.2153) on Keratin, type I cytoskeletal 20
with a threshold of 1e−05
rs77285108
rs12404911
rs73128290
rs7742829
All
0.0
0.1
0.2
0.3
MR effect size for
'Gut microbiota abundance (genus Catenibacterium id.2153)' on 'Keratin, type I cytoskeletal 20'

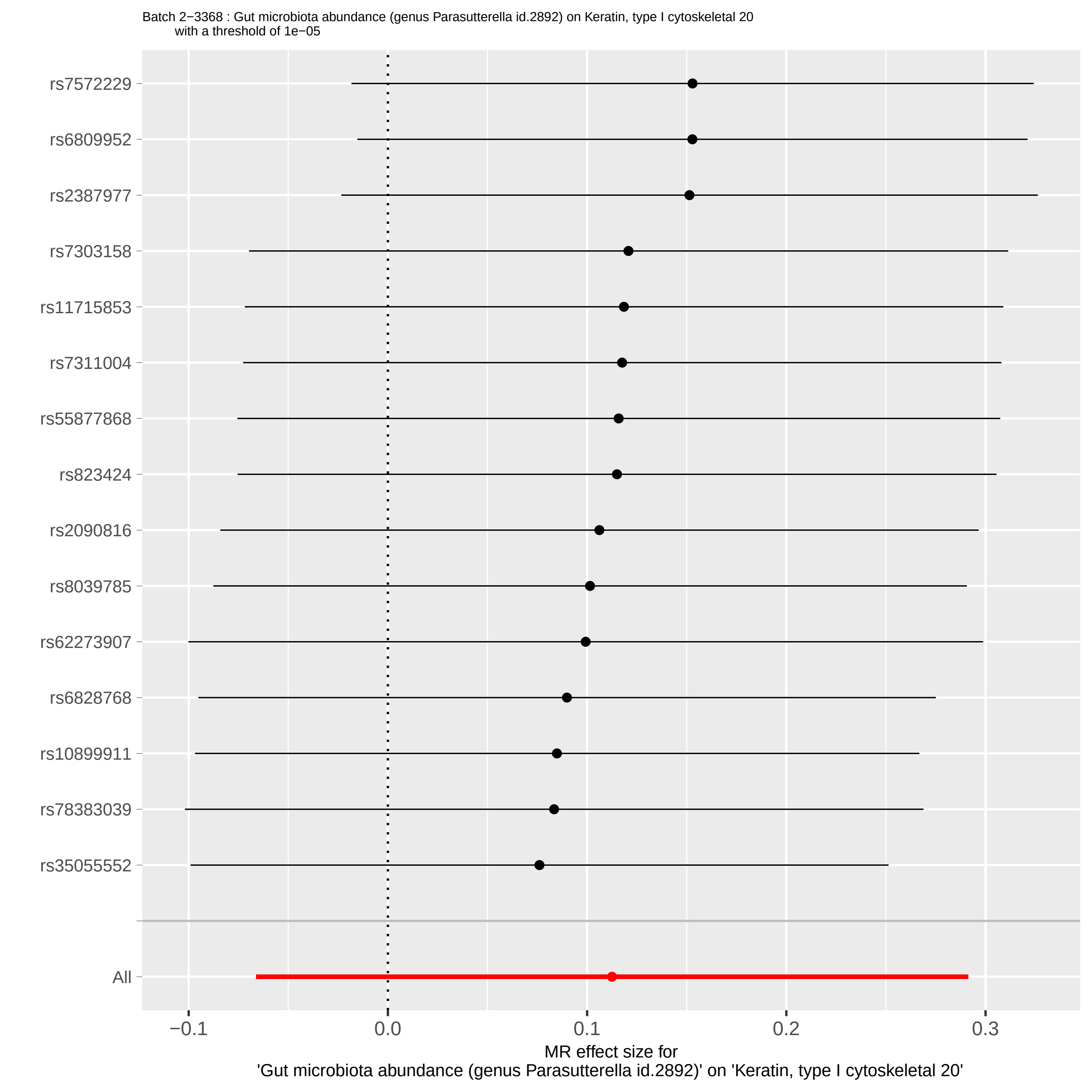
Batch 2−3368 : Gut microbiota abundance (genus Parasutterella id.2892) on Keratin, type I cytoskeletal 20
with a threshold of 1e−05
rs7572229
rs6809952
rs2387977
rs7303158
rs11715853
rs7311004
rs55877868
rs823424
rs2090816
rs8039785
rs62273907
rs6828768
rs10899911
rs78383039
rs35055552
All
−0.1
0.0
0.1
0.2
0.3
MR effect size for
'Gut microbiota abundance (genus Parasutterella id.2892)' on 'Keratin, type I cytoskeletal 20'

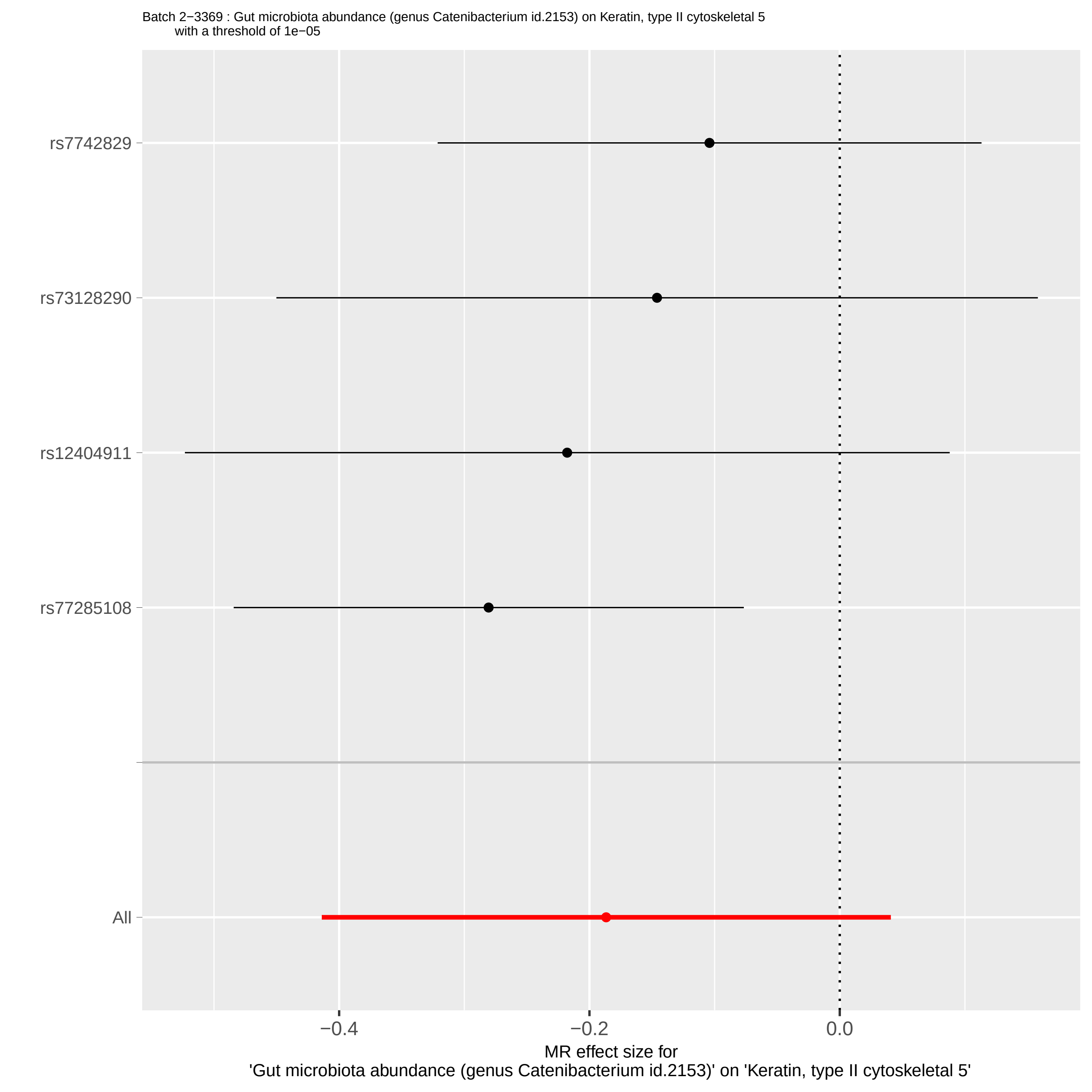
Batch 2−3369 : Gut microbiota abundance (genus Catenibacterium id.2153) on Keratin, type II cytoskeletal 5
with a threshold of 1e−05
rs7742829
rs73128290
rs12404911
rs77285108
All
−0.4
−0.2
0.0
MR effect size for
'Gut microbiota abundance (genus Catenibacterium id.2153)' on 'Keratin, type II cytoskeletal 5'

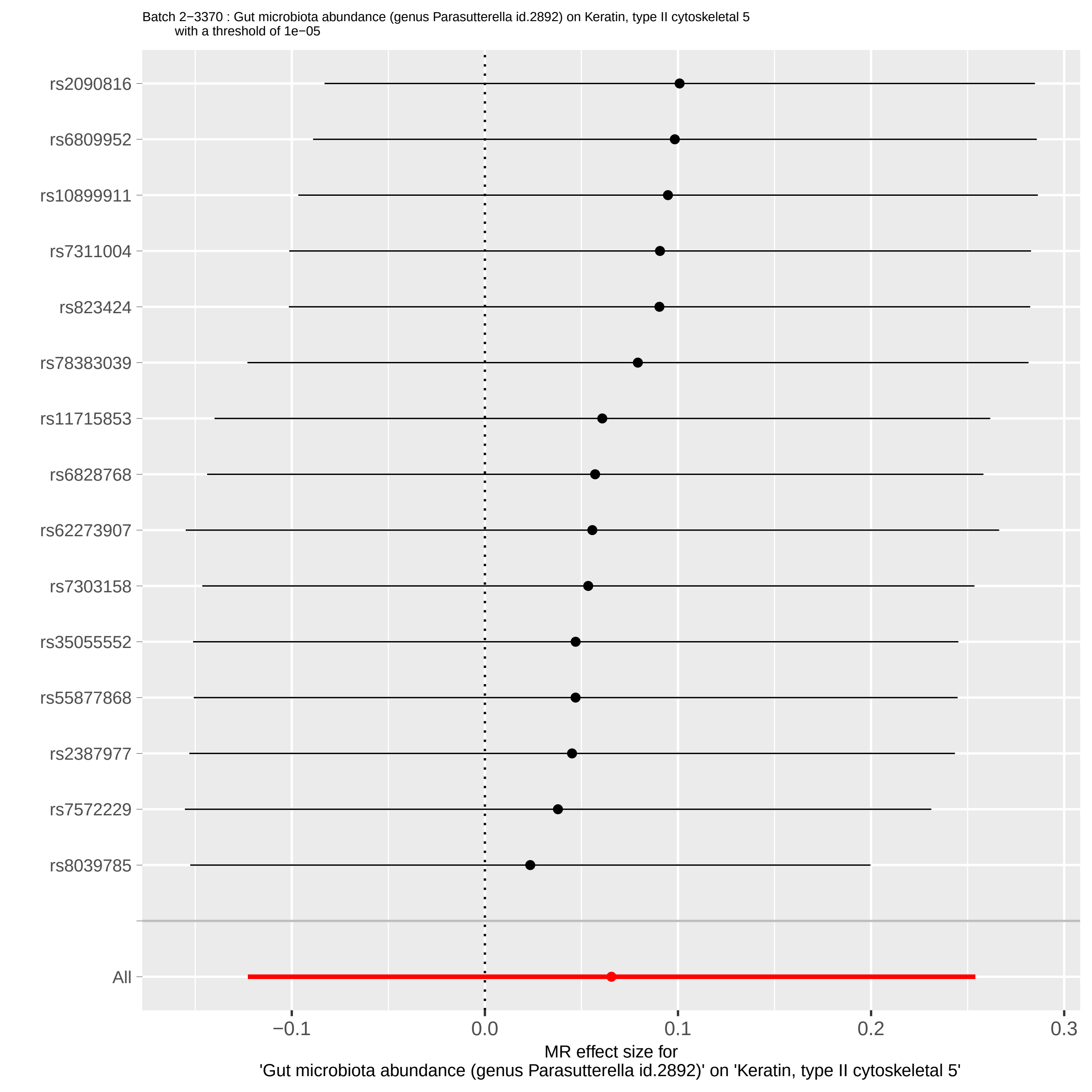

Batch 2−3370 : Gut microbiota abundance (genus Parasutterella id.2892) on Keratin, type II cytoskeletal 5
with a threshold of 1e−05
rs2090816
rs6809952
rs10899911
rs7311004
rs823424
rs78383039
rs11715853
rs6828768
rs62273907
rs7303158
rs35055552
rs55877868
rs2387977
rs7572229
rs8039785
All
−0.1
0.0
0.1
0.2
0.3
MR effect size for
'Gut microbiota abundance (genus Parasutterella id.2892)' on 'Keratin, type II cytoskeletal 5'

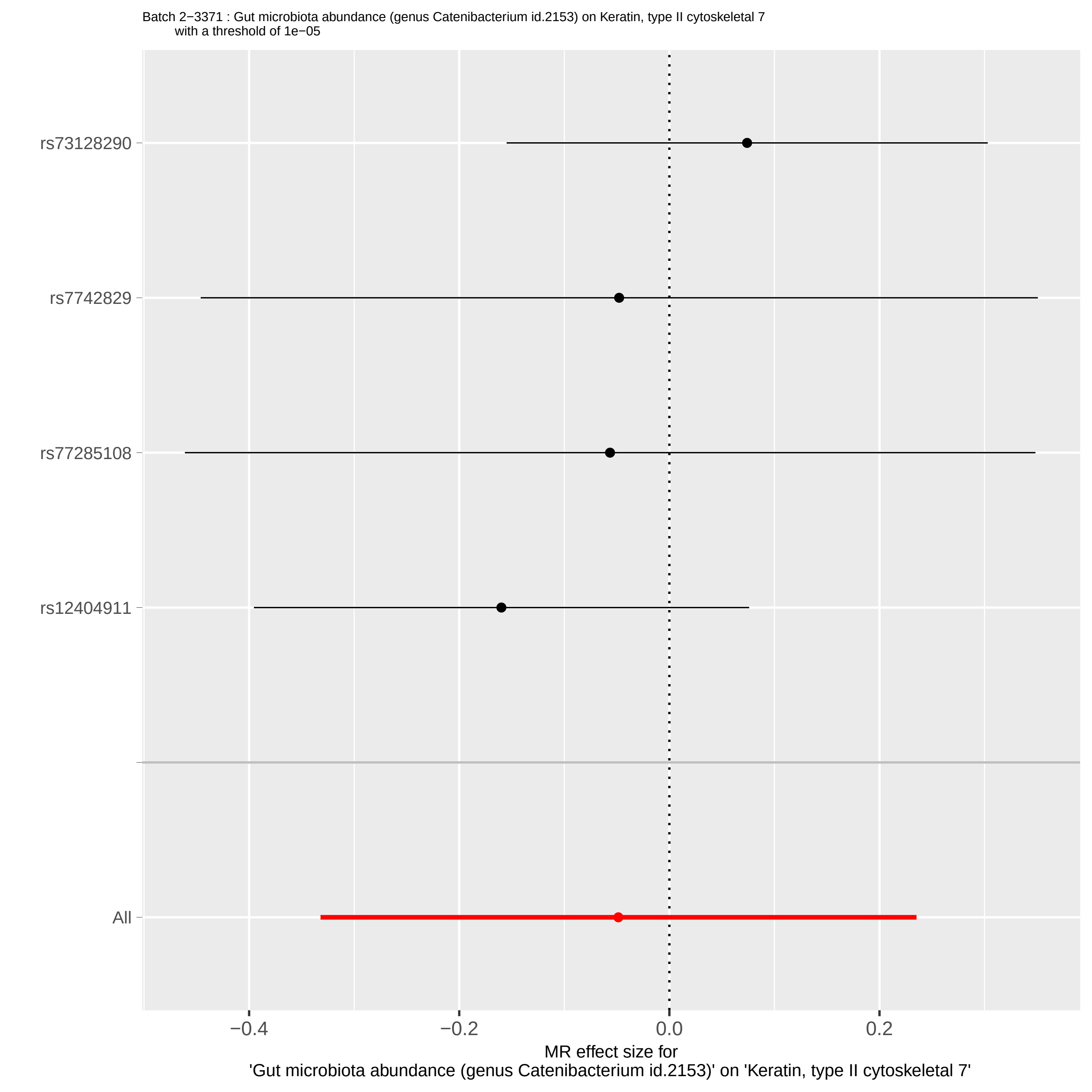
Batch 2−3371 : Gut microbiota abundance (genus Catenibacterium id.2153) on Keratin, type II cytoskeletal 7
with a threshold of 1e−05
rs73128290
rs7742829
rs77285108
rs12404911
All
−0.4
−0.2
0.0
0.2
MR effect size for
'Gut microbiota abundance (genus Catenibacterium id.2153)' on 'Keratin, type II cytoskeletal 7'

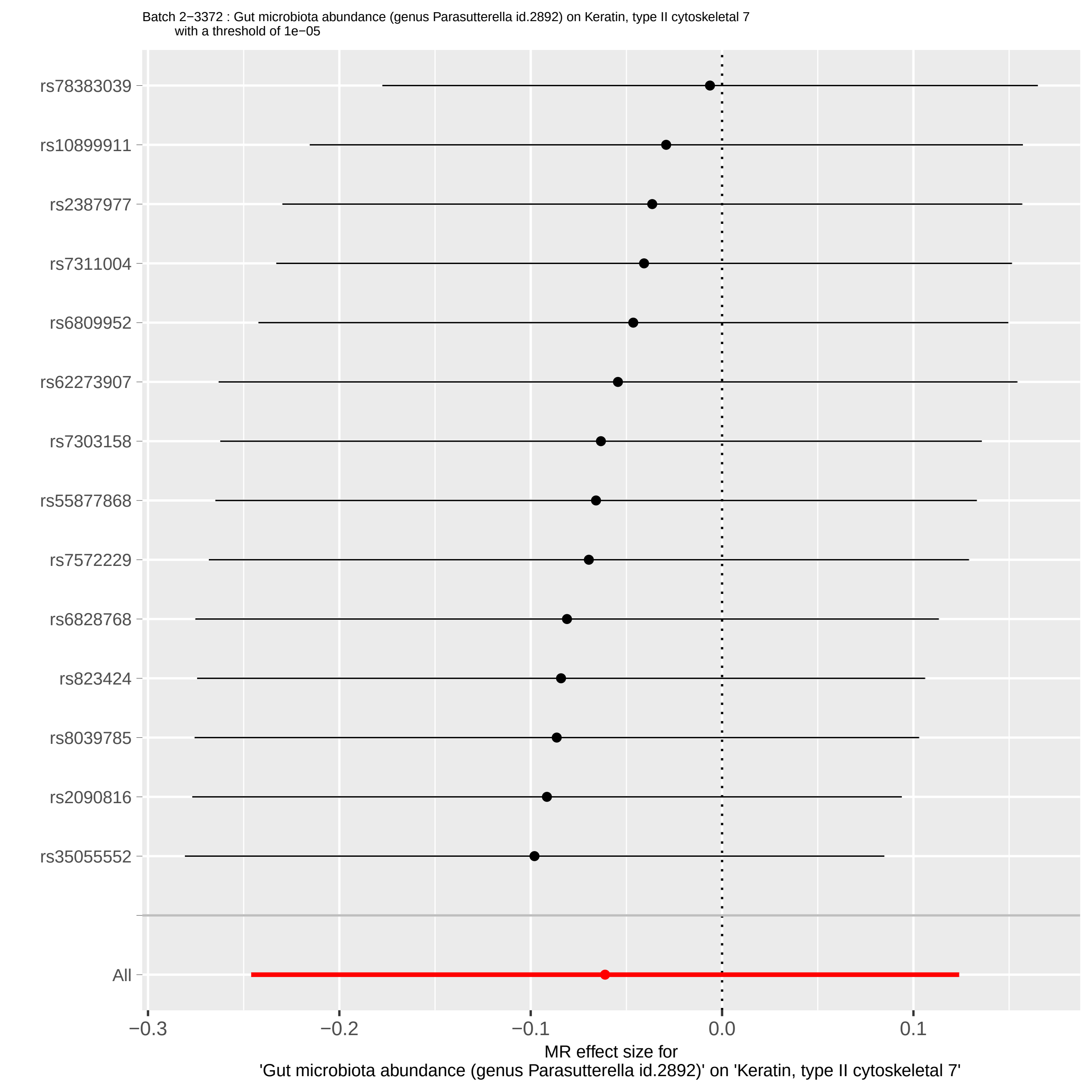

Batch 2−3372 : Gut microbiota abundance (genus Parasutterella id.2892) on Keratin, type II cytoskeletal 7
with a threshold of 1e−05
rs78383039
rs10899911
rs2387977
rs7311004
rs6809952
rs62273907
rs7303158
rs55877868
rs7572229
rs6828768
rs823424
rs8039785
rs2090816
rs35055552
All
−0.3
−0.2
−0.1
0.0
0.1
MR effect size for
'Gut microbiota abundance (genus Parasutterella id.2892)' on 'Keratin, type II cytoskeletal 7'

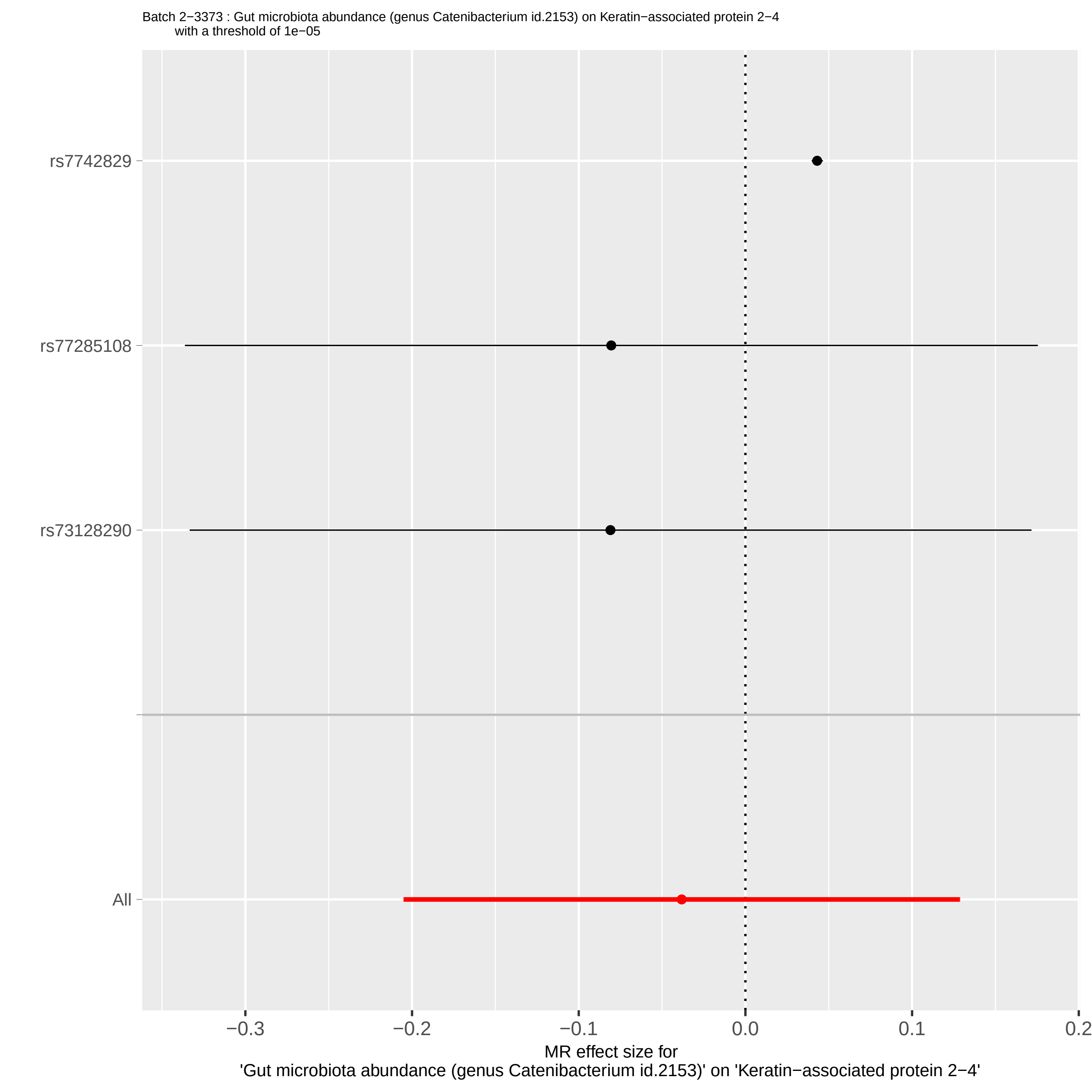
Batch 2−3373 : Gut microbiota abundance (genus Catenibacterium id.2153) on Keratin−associated protein 2−4
with a threshold of 1e−05
rs7742829
rs77285108
rs73128290
All
−0.3
−0.2
−0.1
0.0
0.1
0.2
MR effect size for
'Gut microbiota abundance (genus Catenibacterium id.2153)' on 'Keratin−associated protein 2−4'

Batch 2−3374 : Gut microbiota abundance (genus Parasutterella id.2892) on Keratin−associated protein 2−4
with a threshold of 1e−05

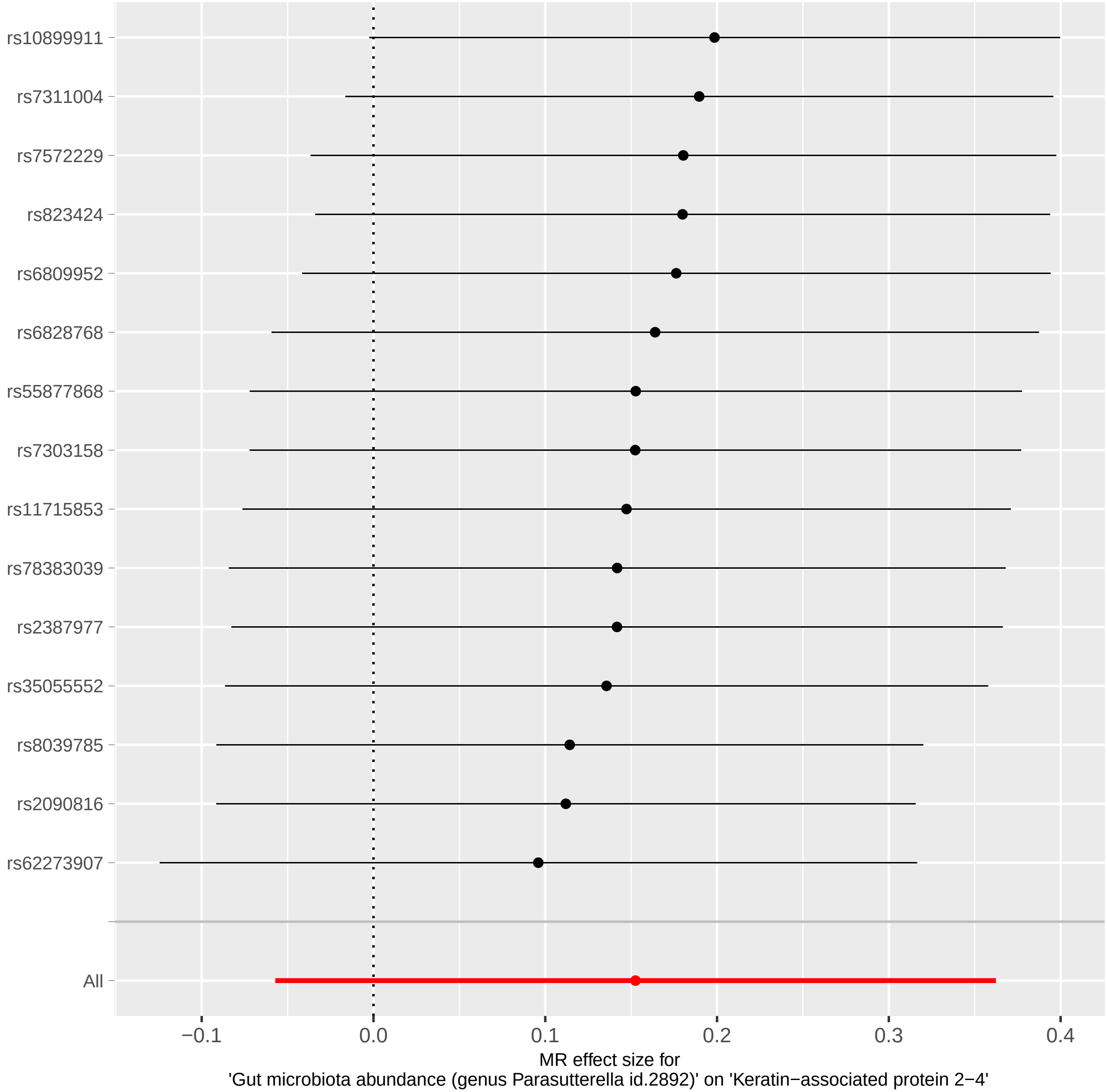

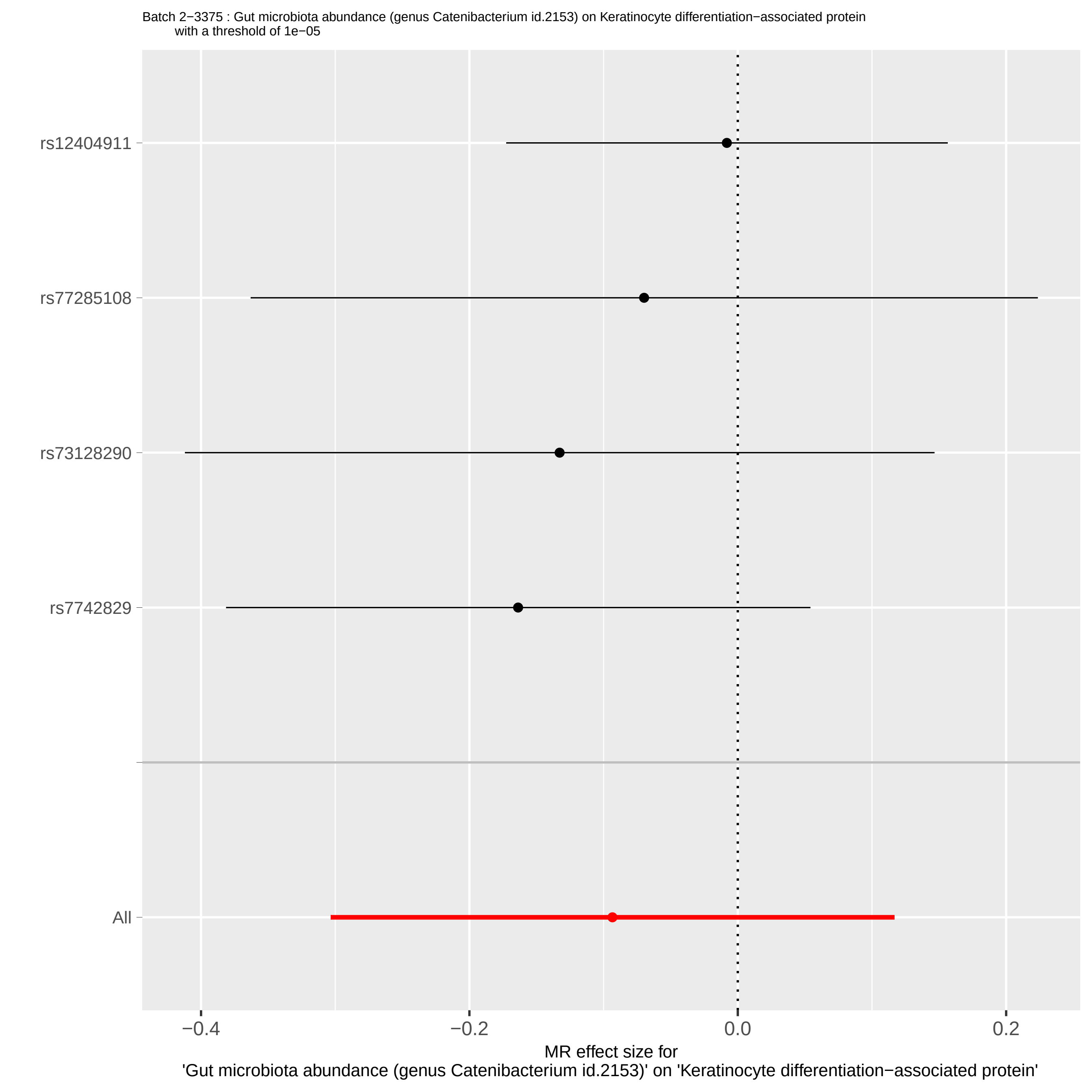
Batch 2−3375 : Gut microbiota abundance (genus Catenibacterium id.2153) on Keratinocyte differentiation−associated protein
with a threshold of 1e−05
rs12404911
rs77285108
rs73128290
rs7742829
All
−0.4
−0.2
0.0
0.2
MR effect size for
'Gut microbiota abundance (genus Catenibacterium id.2153)' on 'Keratinocyte differentiation−associated protein'

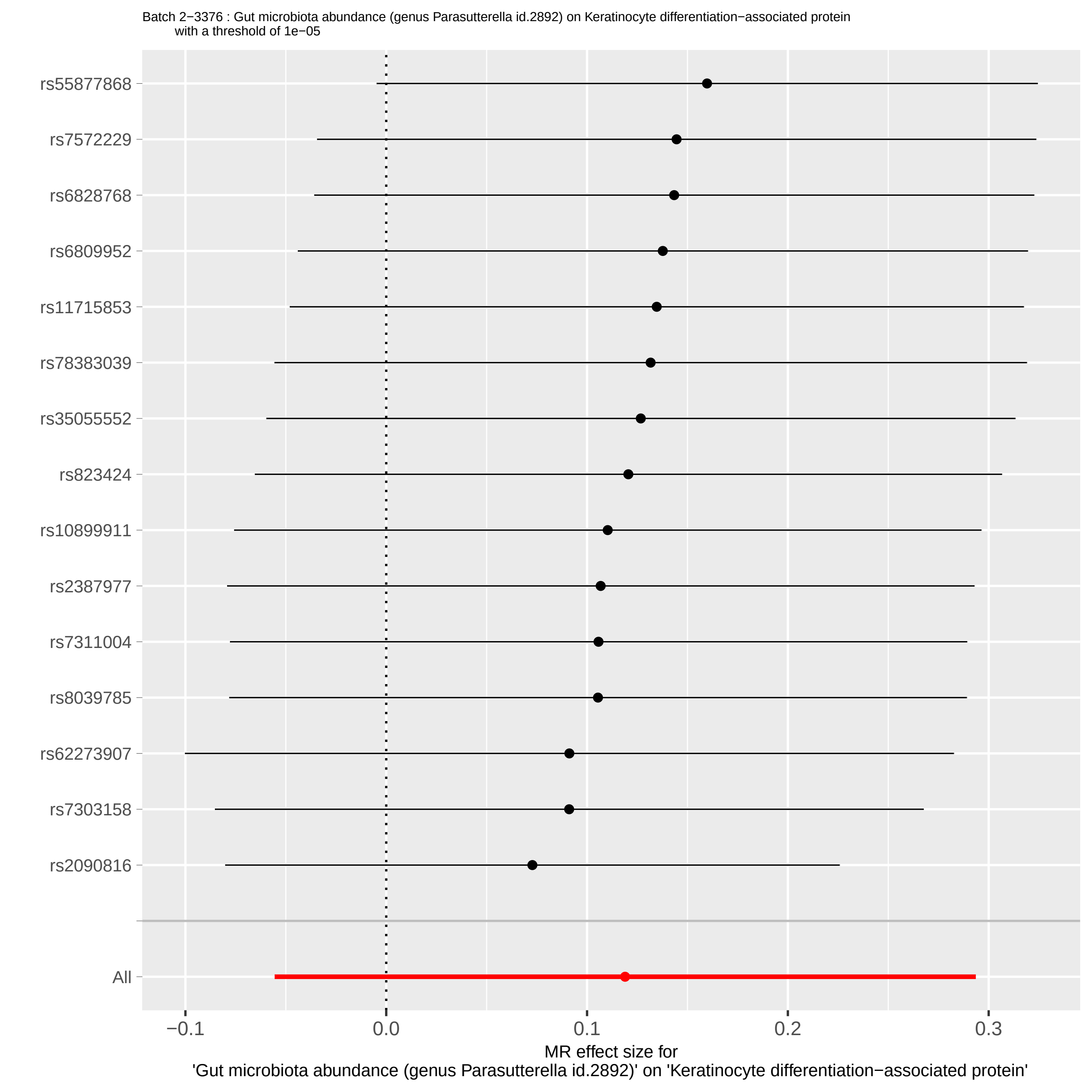

Batch 2−3376 : Gut microbiota abundance (genus Parasutterella id.2892) on Keratinocyte differentiation−associated protein
with a threshold of 1e−05
rs55877868
rs7572229
rs6828768
rs6809952
rs11715853
rs78383039
rs35055552
rs823424
rs10899911
rs2387977
rs7311004
rs8039785
rs62273907
rs7303158
rs2090816
All
−0.1
0.0
0.1
0.2
0.3
MR effect size for
'Gut microbiota abundance (genus Parasutterella id.2892)' on 'Keratinocyte differentiation−associated protein'

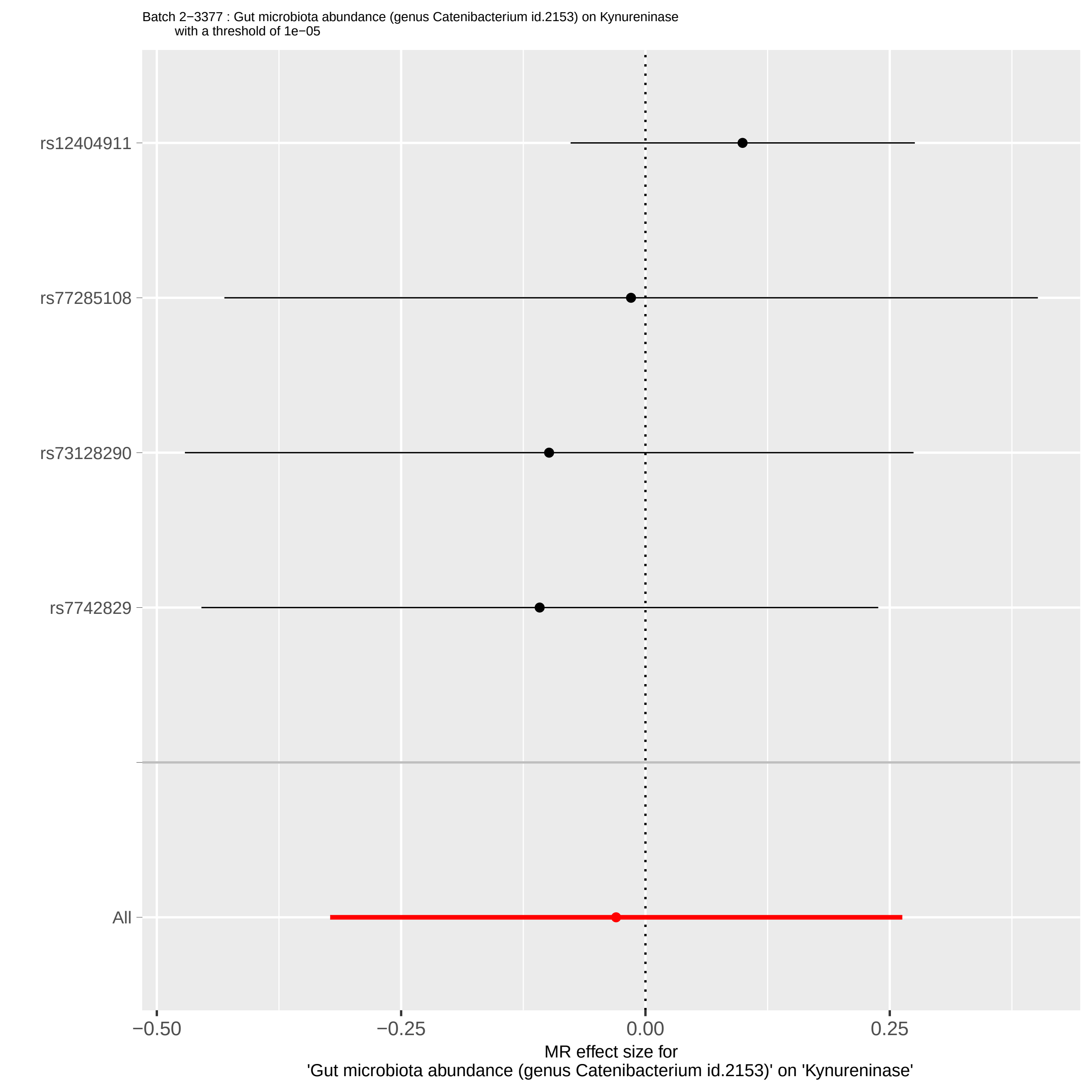
Batch 2−3377 : Gut microbiota abundance (genus Catenibacterium id.2153) on Kynureninase
with a threshold of 1e−05
rs12404911
rs77285108
rs73128290
rs7742829
All
−0.50
−0.25
0.00
0.25
MR effect size for
'Gut microbiota abundance (genus Catenibacterium id.2153)' on 'Kynureninase'

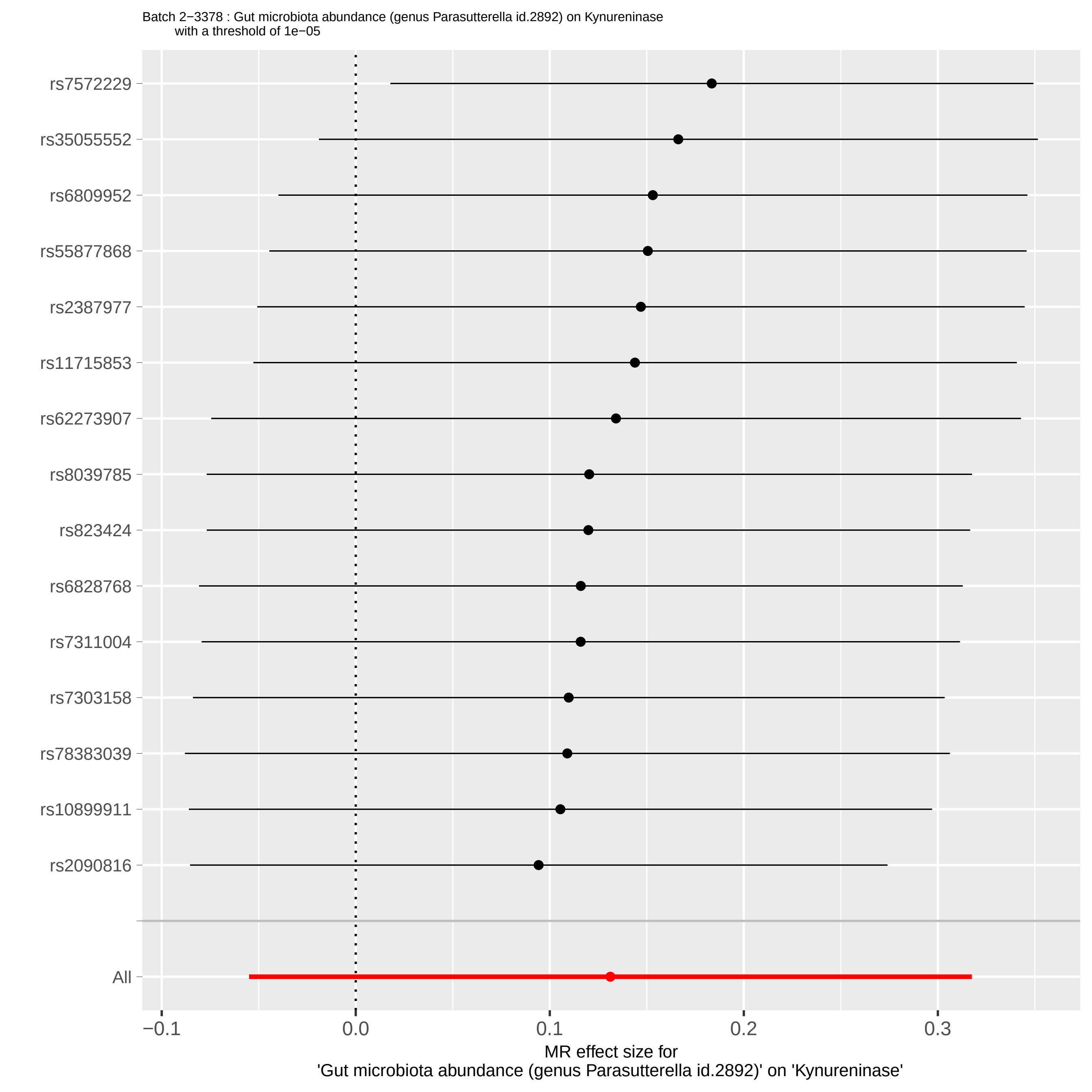
Batch 2−3378 : Gut microbiota abundance (genus Parasutterella id.2892) on Kynureninase
with a threshold of 1e−05
rs7572229
rs35055552
rs6809952
rs55877868
rs2387977
rs11715853
rs62273907
rs8039785
rs823424
rs6828768
rs7311004
rs7303158
rs78383039
rs10899911
rs2090816
All
−0.1
0.0
0.1
0.2
0.3
MR effect size for
'Gut microbiota abundance (genus Parasutterella id.2892)' on 'Kynureninase'

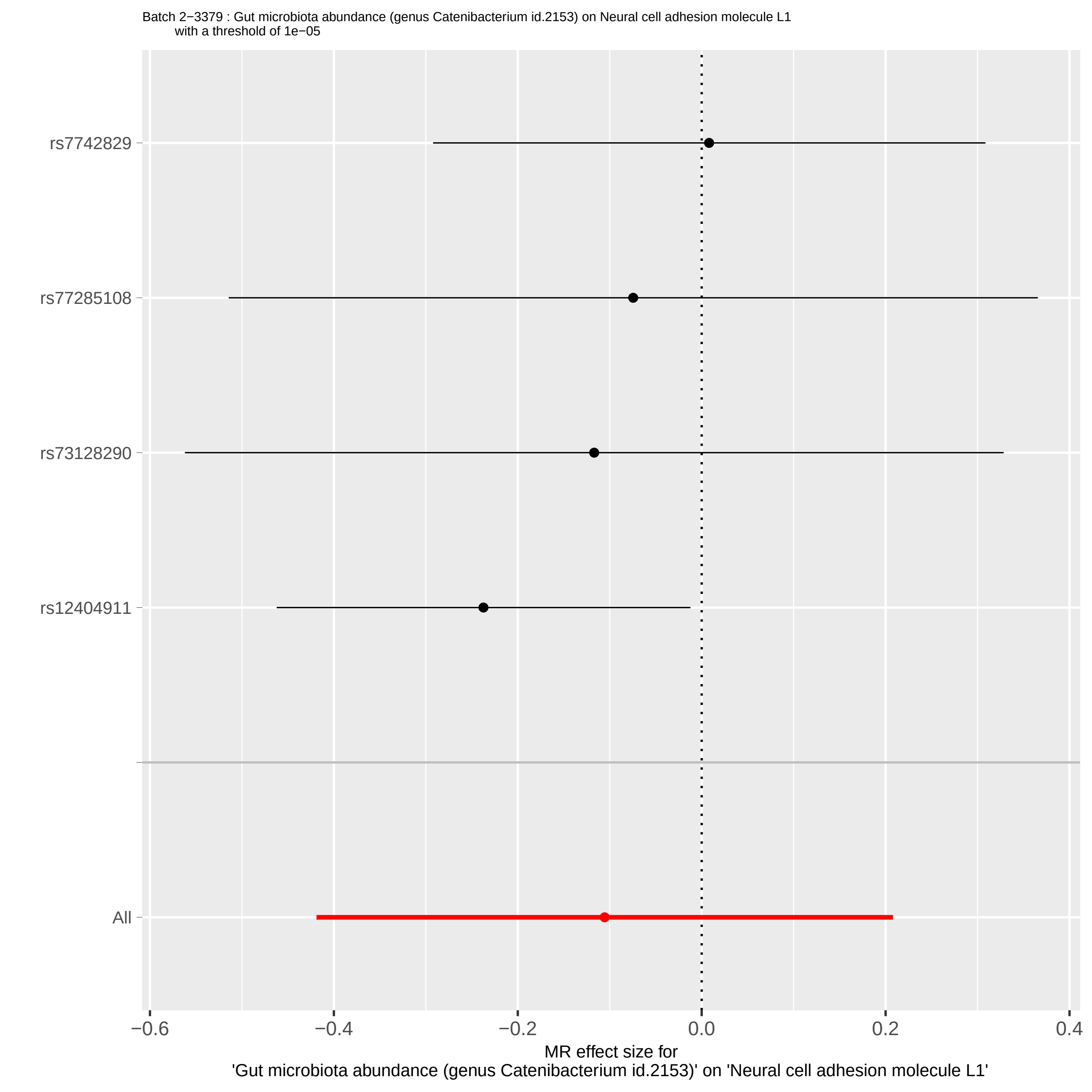

Batch 2−3379 : Gut microbiota abundance (genus Catenibacterium id.2153) on Neural cell adhesion molecule L1
with a threshold of 1e−05
rs7742829
rs77285108
rs73128290
rs12404911
All
−0.6
−0.4
−0.2
0.0
0.2
0.4
MR effect size for
'Gut microbiota abundance (genus Catenibacterium id.2153)' on 'Neural cell adhesion molecule L1'

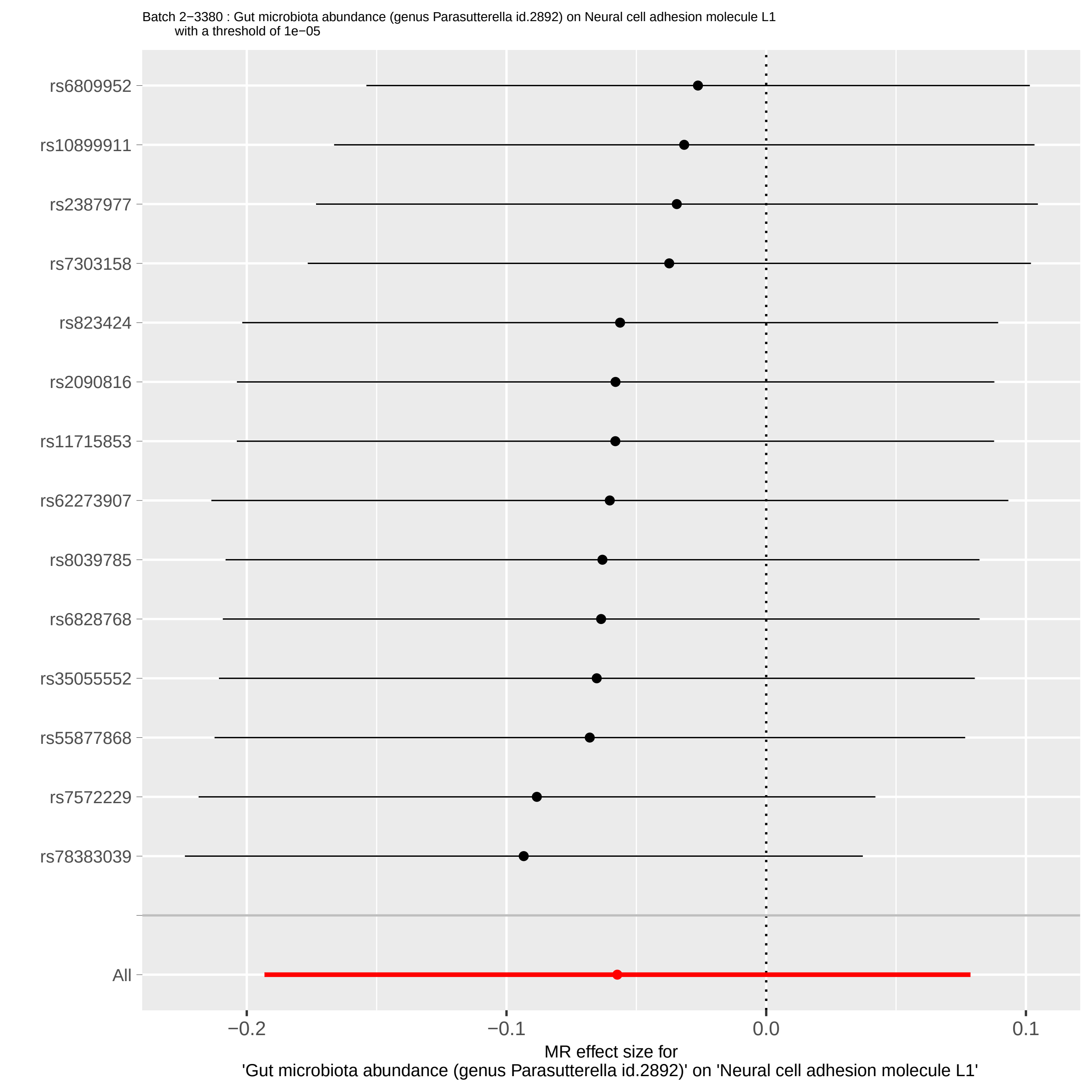
Batch 2−3380 : Gut microbiota abundance (genus Parasutterella id.2892) on Neural cell adhesion molecule L1
with a threshold of 1e−05
rs6809952
rs10899911
rs2387977
rs7303158
rs823424
rs2090816
rs11715853
rs62273907
rs8039785
rs6828768
rs35055552
rs55877868
rs7572229
rs78383039
All
−0.2
−0.1
0.0
0.1
MR effect size for
'Gut microbiota abundance (genus Parasutterella id.2892)' on 'Neural cell adhesion molecule L1'

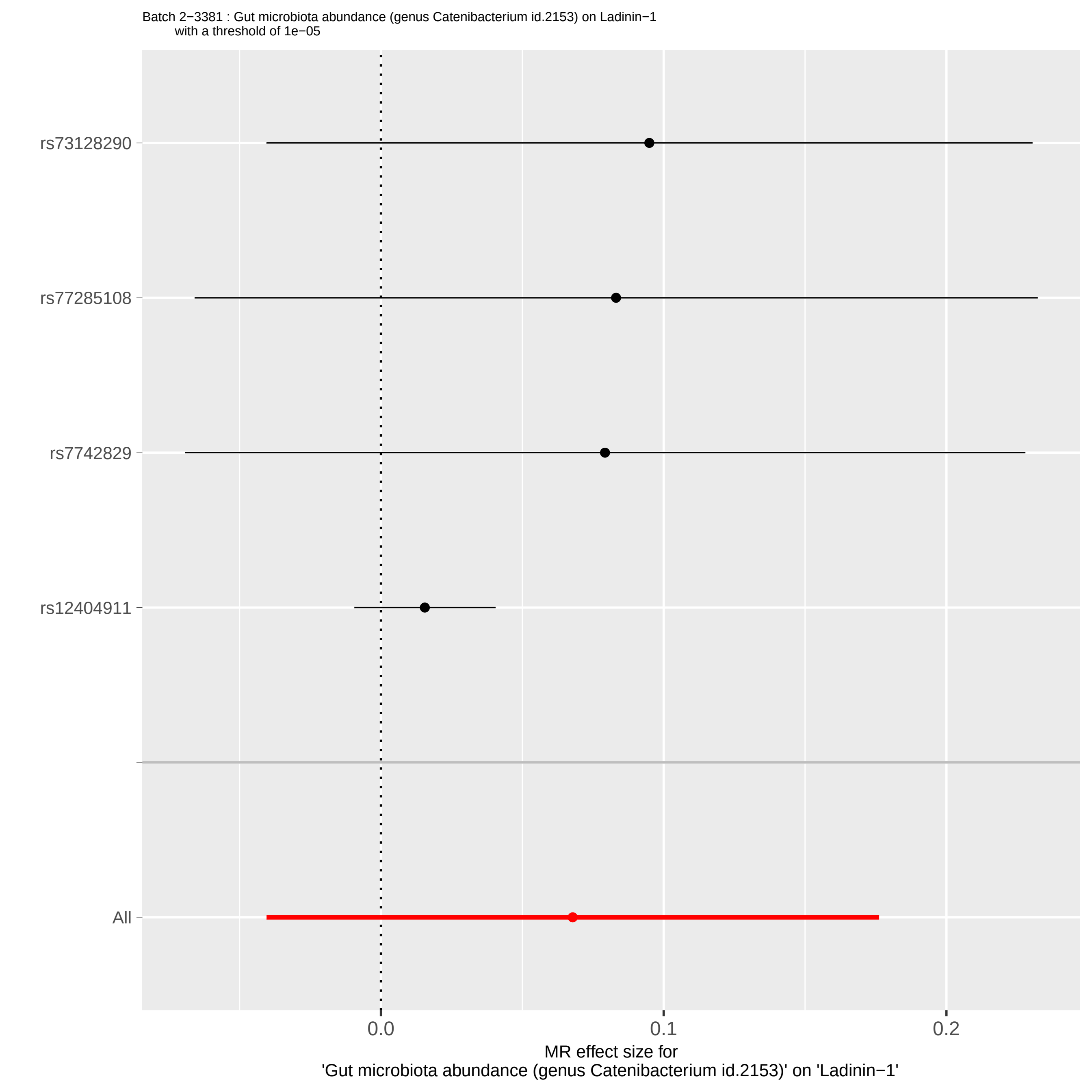
Batch 2−3381 : Gut microbiota abundance (genus Catenibacterium id.2153) on Ladinin−1
with a threshold of 1e−05
rs73128290
rs77285108
rs7742829
rs12404911
All
0.0
0.1
0.2
MR effect size for
'Gut microbiota abundance (genus Catenibacterium id.2153)' on 'Ladinin−1'

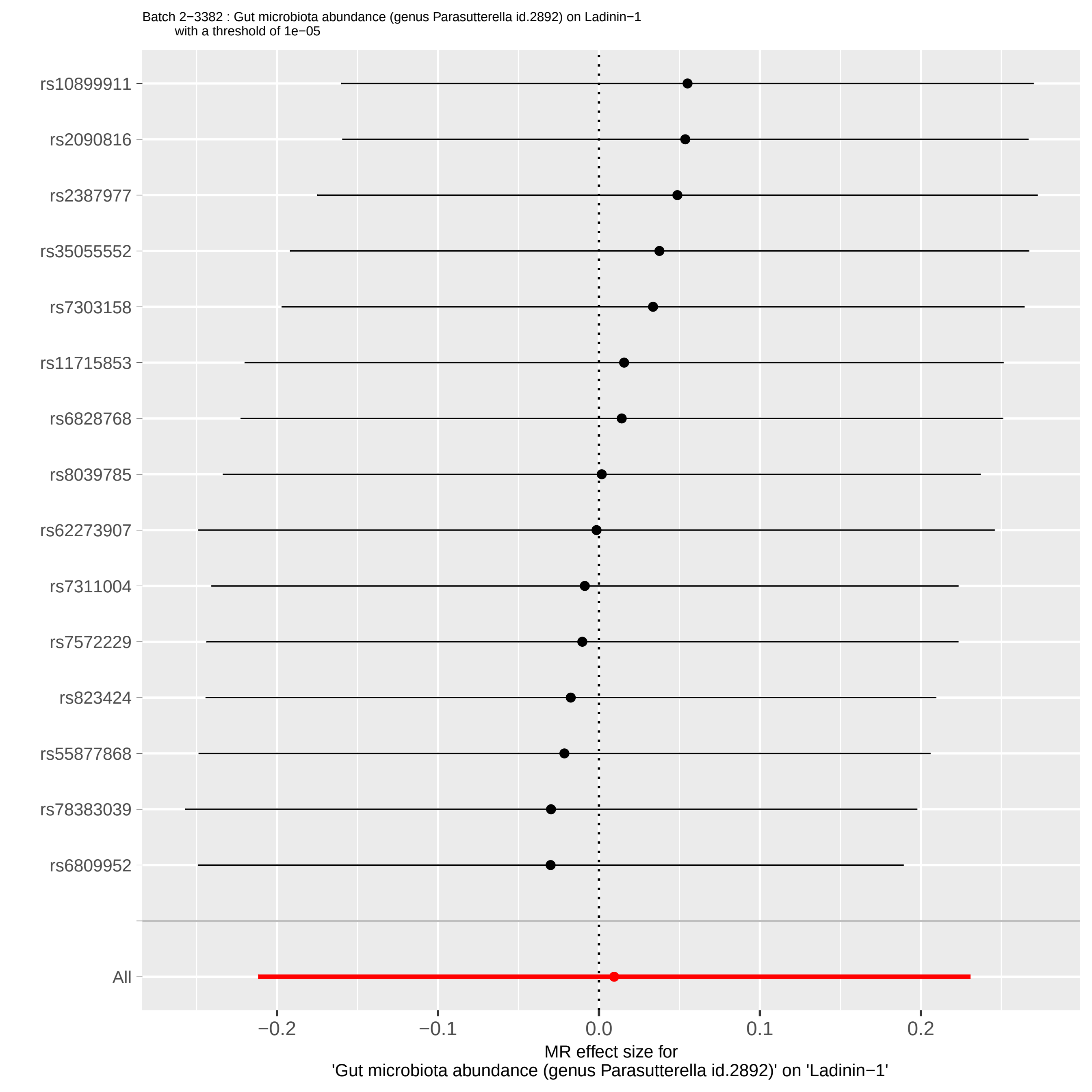
Batch 2−3382 : Gut microbiota abundance (genus Parasutterella id.2892) on Ladinin−1
with a threshold of 1e−05
rs10899911
rs2090816
rs2387977
rs35055552
rs7303158
rs11715853
rs6828768
rs8039785
rs62273907
rs7311004
rs7572229
rs823424
rs55877868
rs78383039
rs6809952
All
−0.2
−0.1
0.0
0.1
0.2
MR effect size for
'Gut microbiota abundance (genus Parasutterella id.2892)' on 'Ladinin−1'

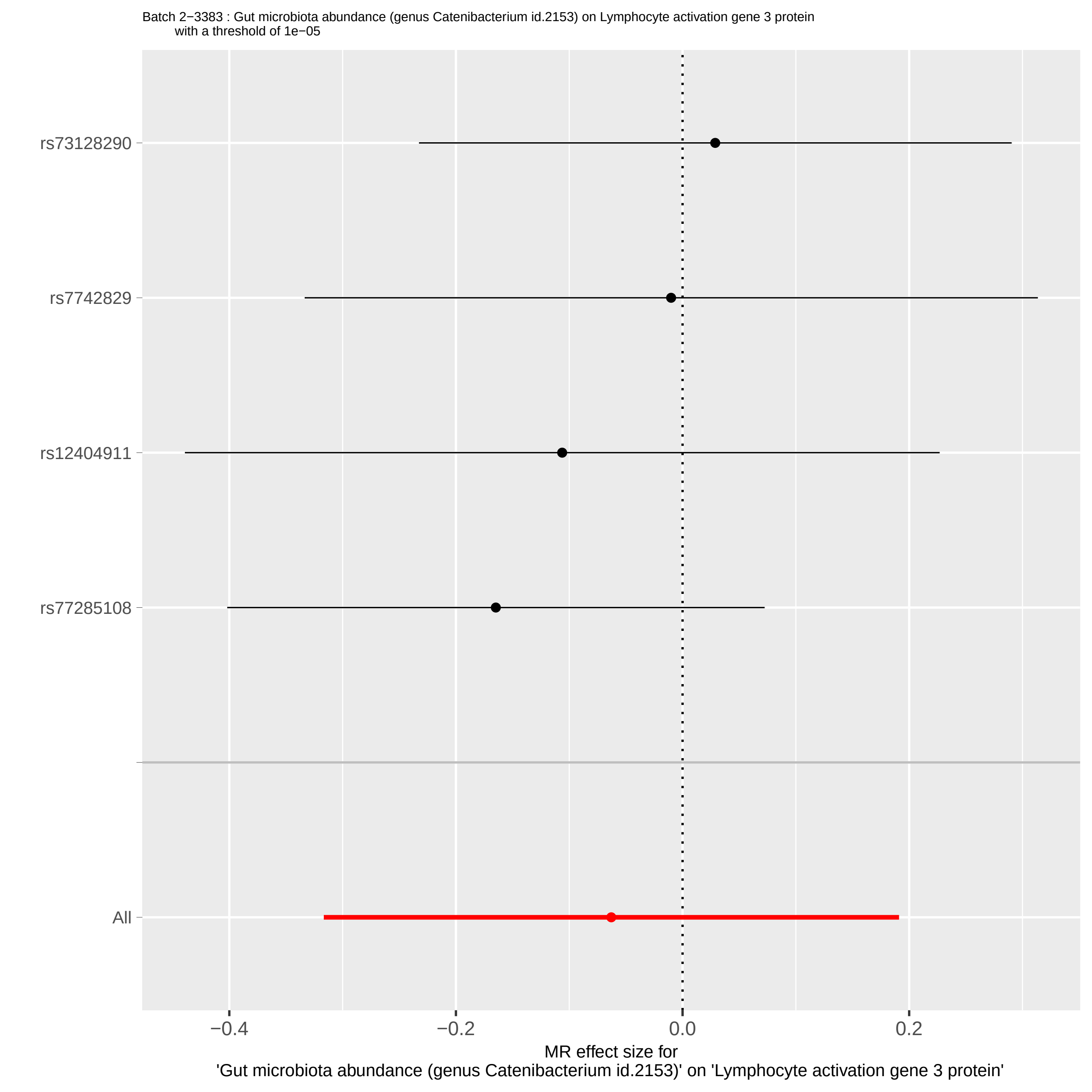

Batch 2−3383 : Gut microbiota abundance (genus Catenibacterium id.2153) on Lymphocyte activation gene 3 protein
with a threshold of 1e−05
rs73128290
rs7742829
rs12404911
rs77285108
All
−0.4
−0.2
0.0
0.2
MR effect size for
'Gut microbiota abundance (genus Catenibacterium id.2153)' on 'Lymphocyte activation gene 3 protein'

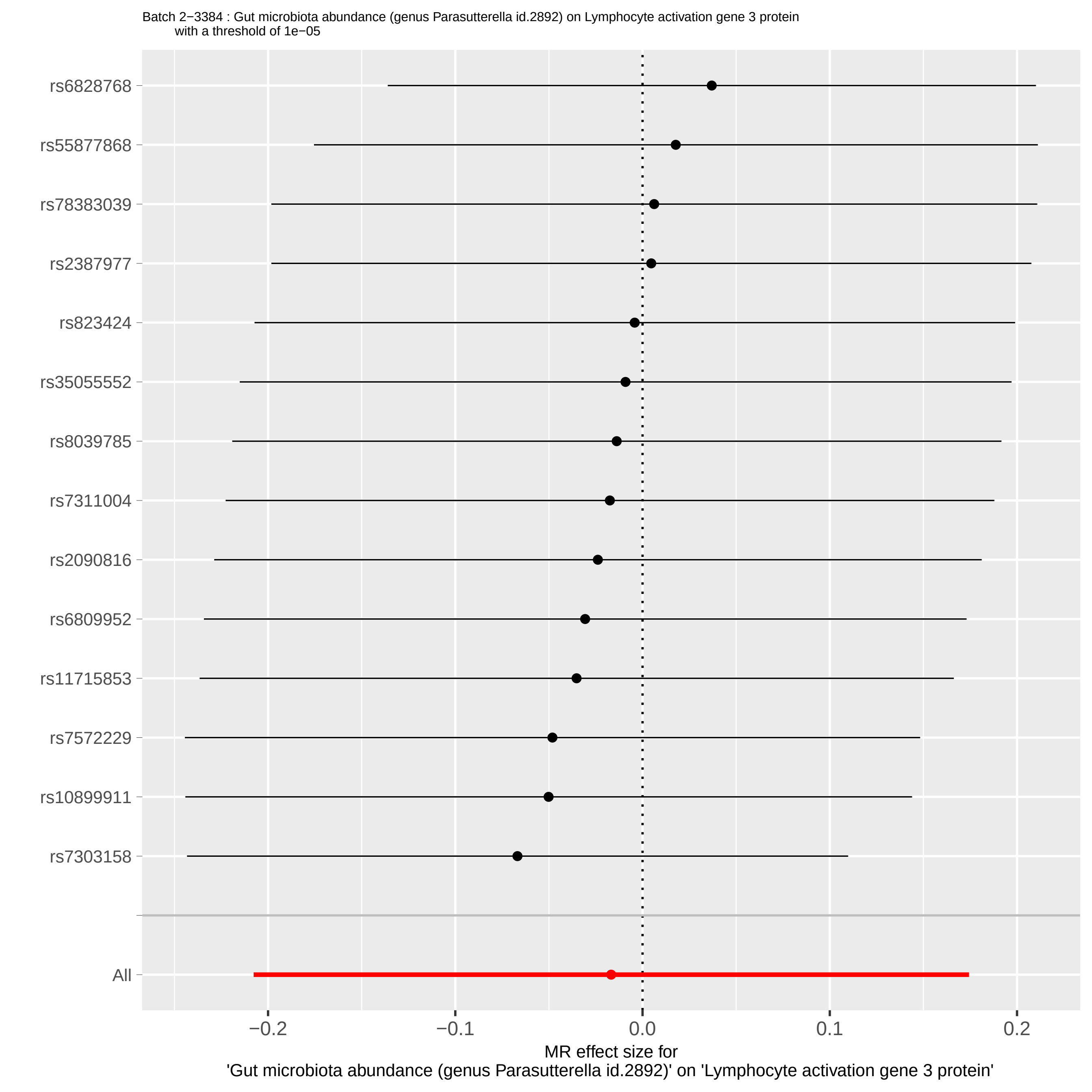
Batch 2−3384 : Gut microbiota abundance (genus Parasutterella id.2892) on Lymphocyte activation gene 3 protein
with a threshold of 1e−05
rs6828768
rs55877868
rs78383039
rs2387977
rs823424
rs35055552
rs8039785
rs7311004
rs2090816
rs6809952
rs11715853
rs7572229
rs10899911
rs7303158
All
−0.2
−0.1
0.0
0.1
0.2
MR effect size for
'Gut microbiota abundance (genus Parasutterella id.2892)' on 'Lymphocyte activation gene 3 protein'

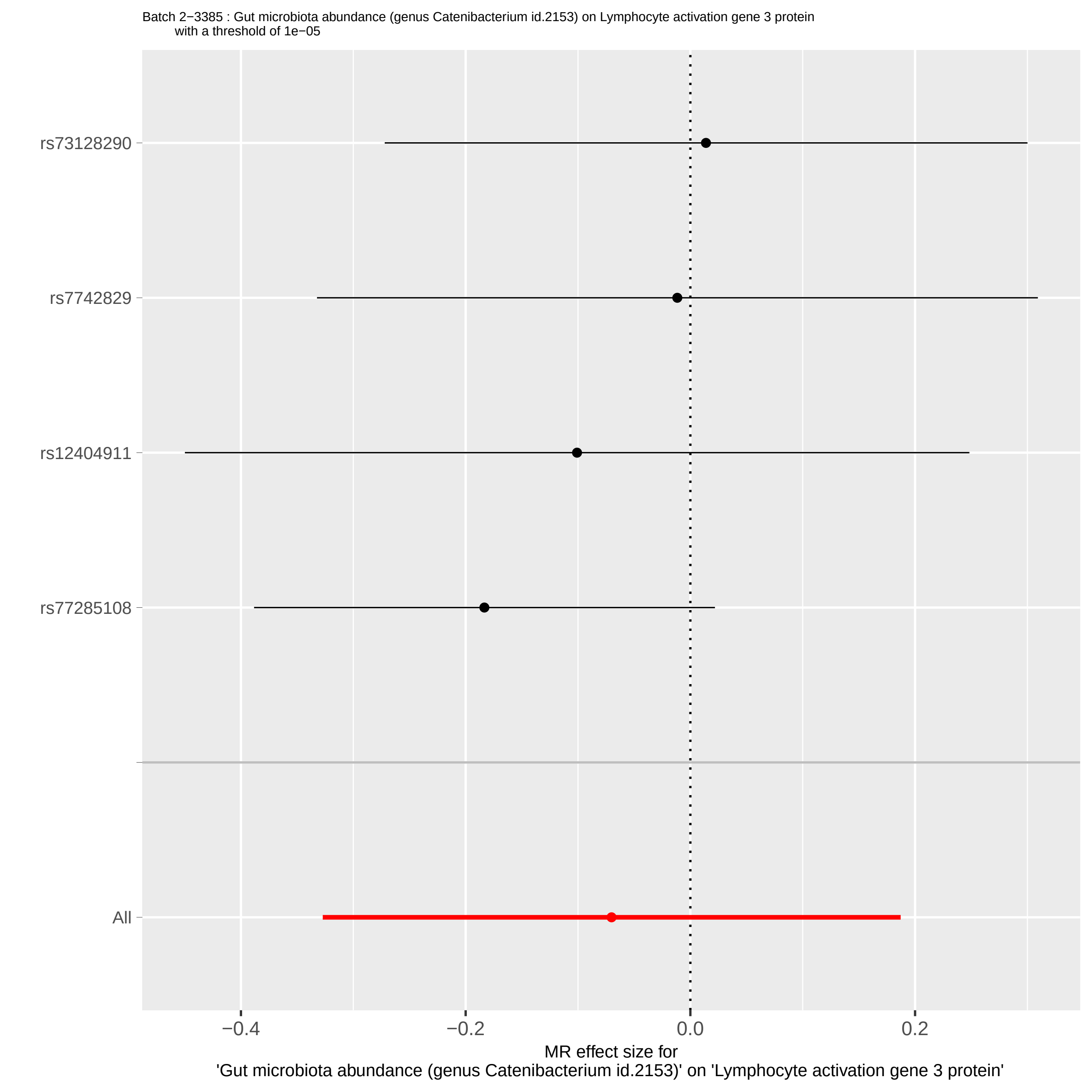

Batch 2−3385 : Gut microbiota abundance (genus Catenibacterium id.2153) on Lymphocyte activation gene 3 protein
with a threshold of 1e−05
rs73128290
rs7742829
rs12404911
rs77285108
All
−0.4
−0.2
0.0
0.2
MR effect size for
'Gut microbiota abundance (genus Catenibacterium id.2153)' on 'Lymphocyte activation gene 3 protein'

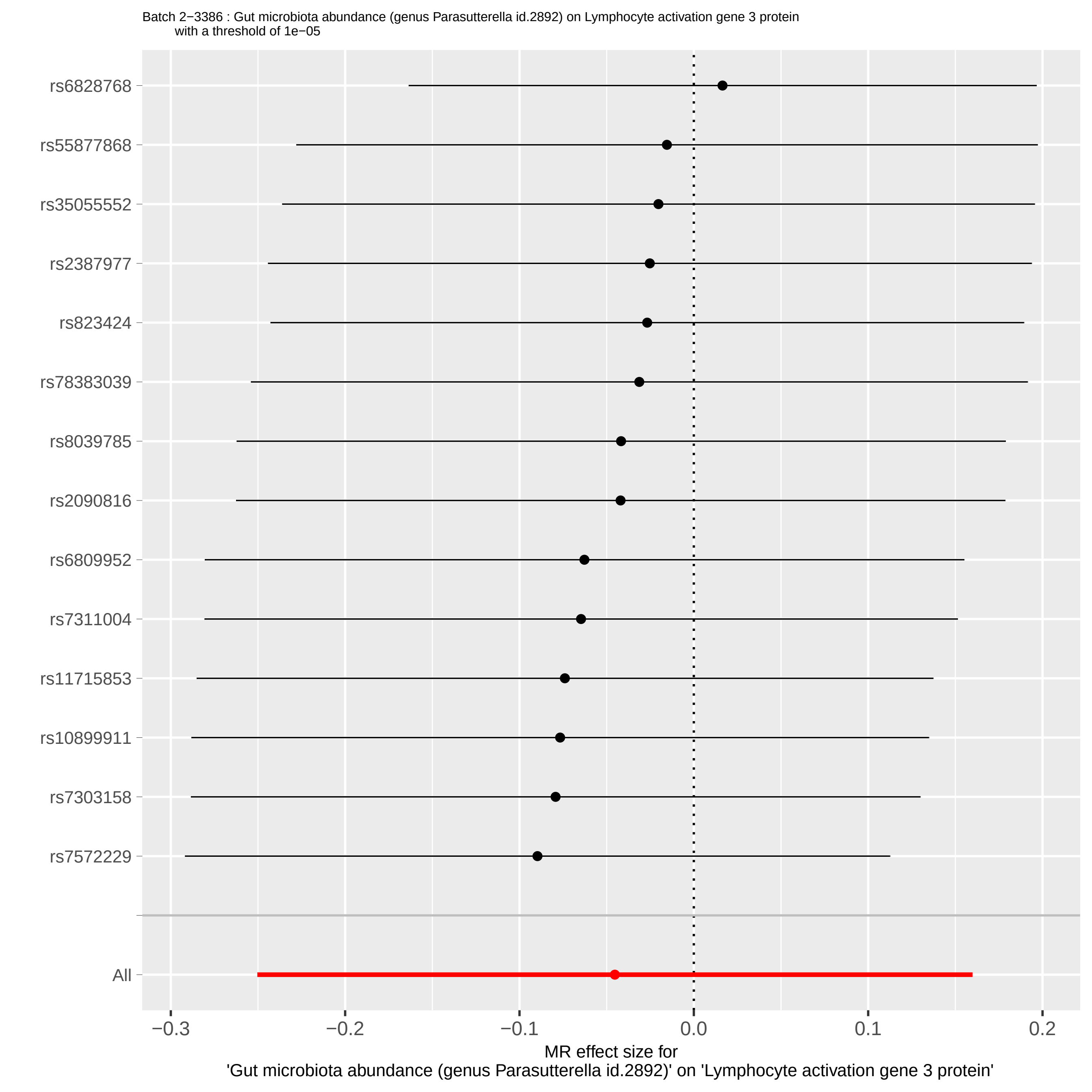

Batch 2−3386 : Gut microbiota abundance (genus Parasutterella id.2892) on Lymphocyte activation gene 3 protein
with a threshold of 1e−05
rs6828768
rs55877868
rs35055552
rs2387977
rs823424
rs78383039
rs8039785
rs2090816
rs6809952
rs7311004
rs11715853
rs10899911
rs7303158
rs7572229
All
−0.3
−0.2
−0.1
0.0
0.1
0.2
MR effect size for
'Gut microbiota abundance (genus Parasutterella id.2892)' on 'Lymphocyte activation gene 3 protein'

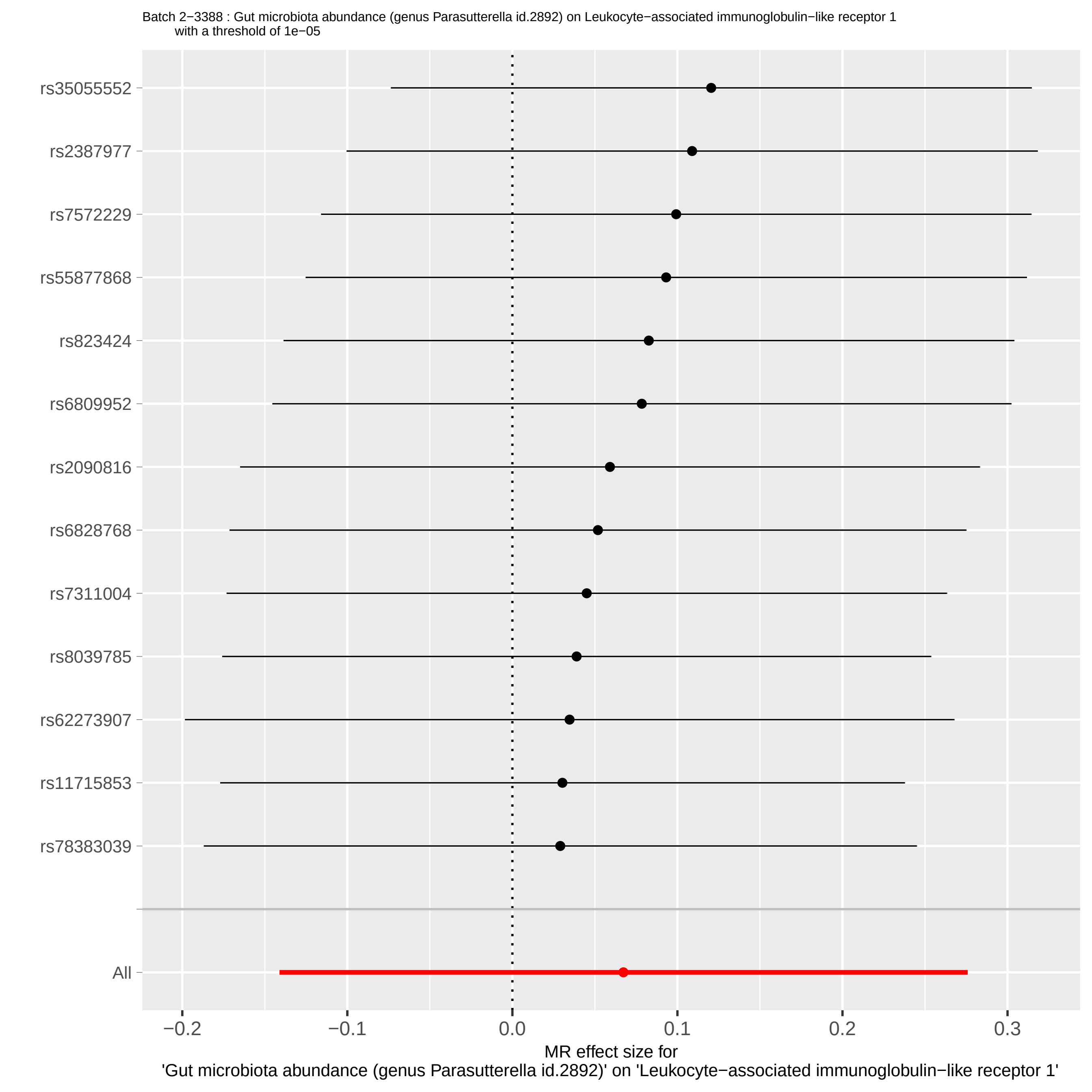

Batch 2−3388 : Gut microbiota abundance (genus Parasutterella id.2892) on Leukocyte−associated immunoglobulin−like receptor 1
with a threshold of 1e−05
rs35055552
rs2387977
rs7572229
rs55877868
rs823424
rs6809952
rs2090816
rs6828768
rs7311004
rs8039785
rs62273907
rs11715853
rs78383039
All
−0.2
−0.1
0.0
0.1
0.2
0.3
MR effect size for
'Gut microbiota abundance (genus Parasutterella id.2892)' on 'Leukocyte−associated immunoglobulin−like receptor 1'

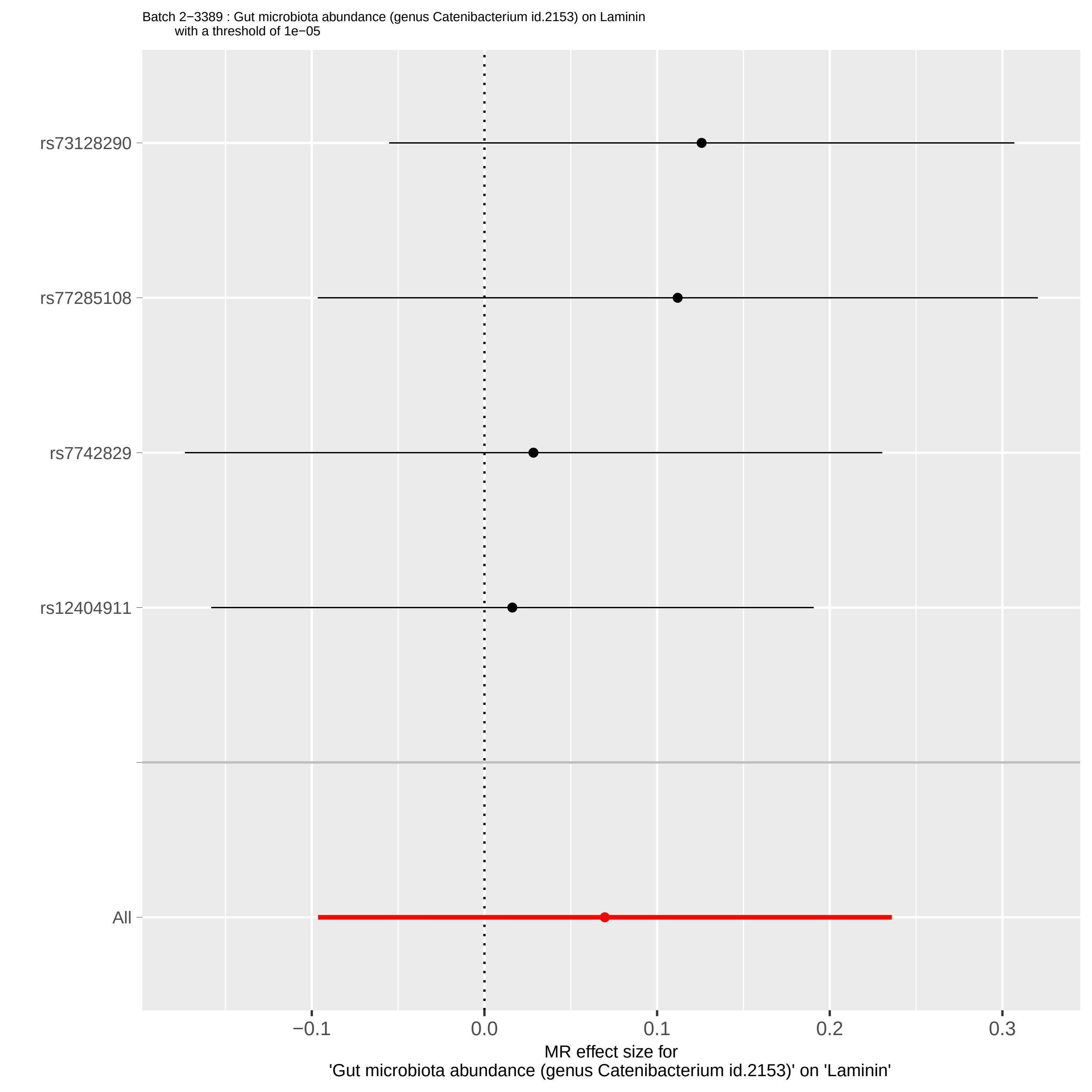
Batch 2−3389 : Gut microbiota abundance (genus Catenibacterium id.2153) on Laminin
with a threshold of 1e−05
rs73128290
rs77285108
rs7742829
rs12404911
All
−0.1
0.0
0.1
0.2
0.3
MR effect size for
'Gut microbiota abundance (genus Catenibacterium id.2153)' on 'Laminin'

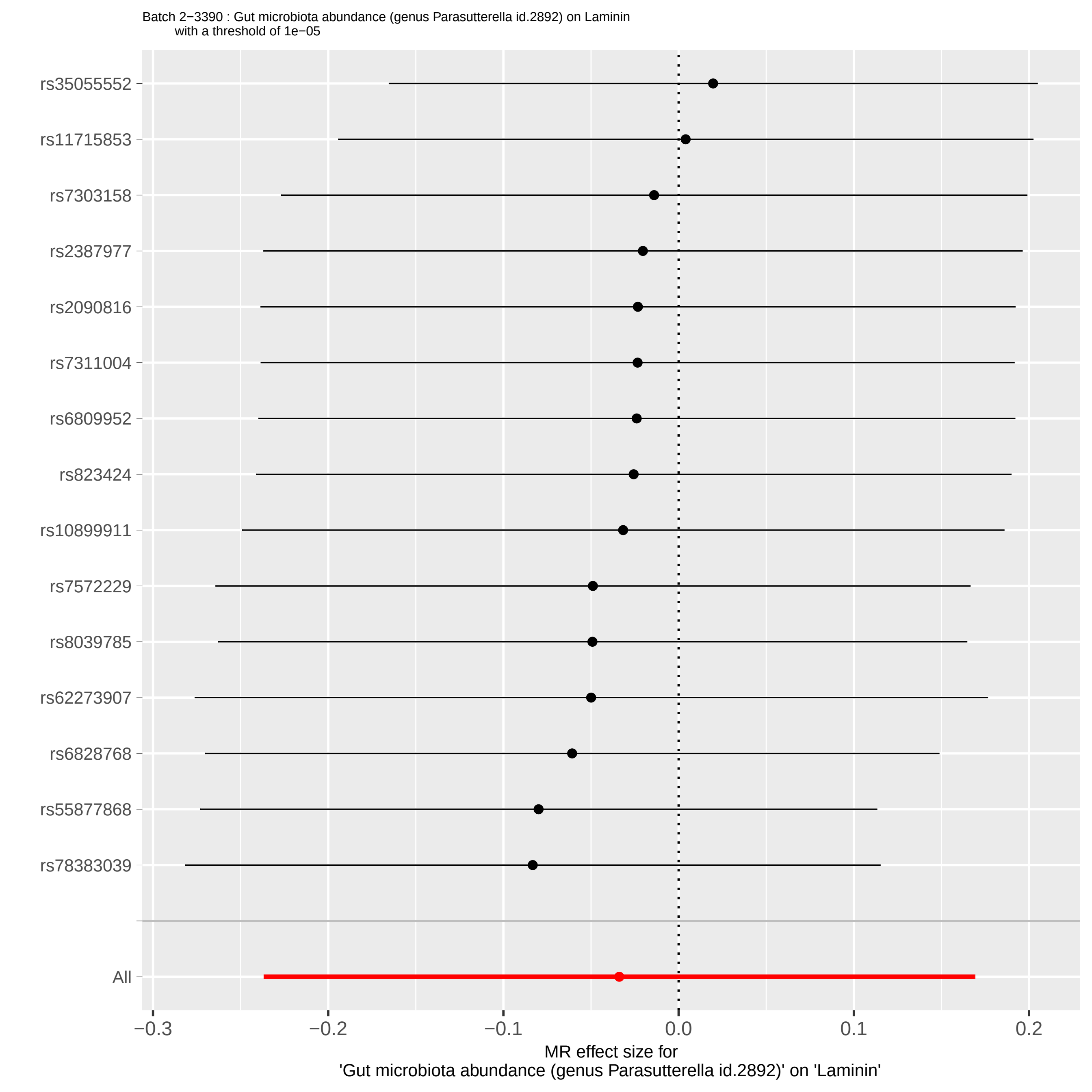
Batch 2−3390 : Gut microbiota abundance (genus Parasutterella id.2892) on Laminin
with a threshold of 1e−05
rs35055552
rs11715853
rs7303158
rs2387977
rs2090816
rs7311004
rs6809952
rs823424
rs10899911
rs7572229
rs8039785
rs62273907
rs6828768
rs55877868
rs78383039
All
−0.3
−0.2
−0.1
0.0
0.1
0.2
MR effect size for
'Gut microbiota abundance (genus Parasutterella id.2892)' on 'Laminin'

Batch 2−3391 : Gut microbiota abundance (genus Catenibacterium id.2153) on Laminin subunit alpha−4
with a threshold of 1e−05

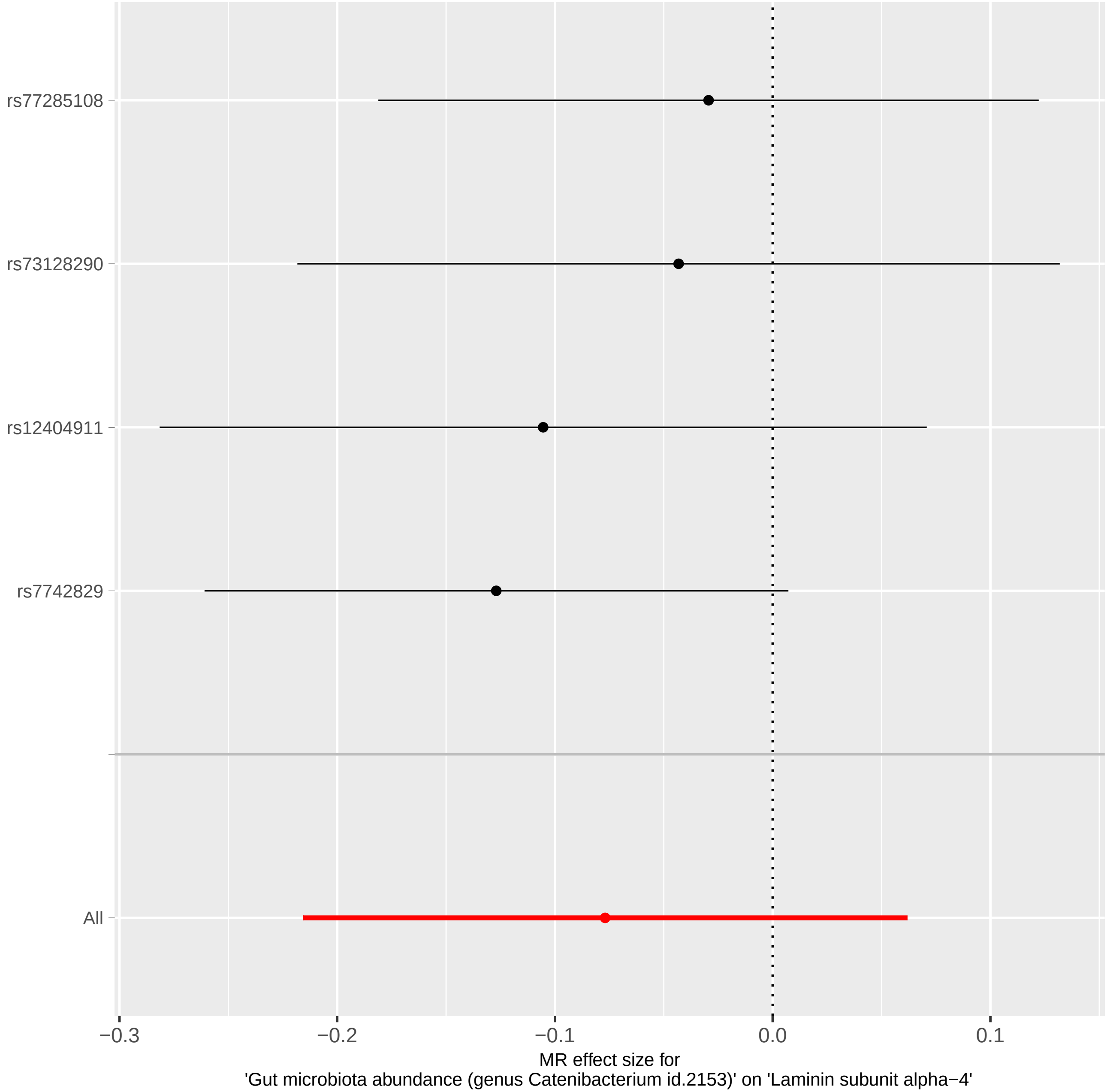

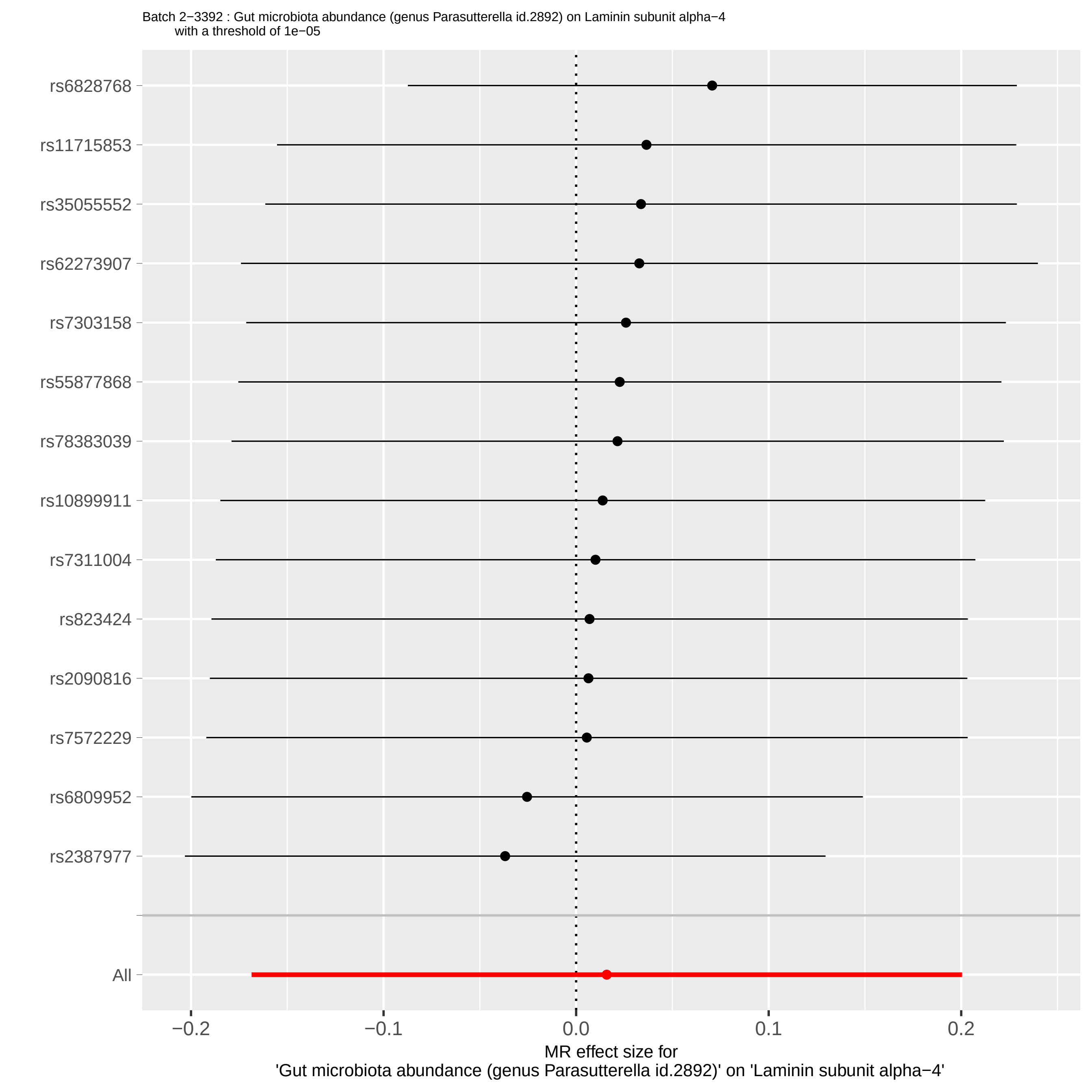
Batch 2−3392 : Gut microbiota abundance (genus Parasutterella id.2892) on Laminin subunit alpha−4
with a threshold of 1e−05
rs6828768
rs11715853
rs35055552
rs62273907
rs7303158
rs55877868
rs78383039
rs10899911
rs7311004
rs823424
rs2090816
rs7572229
rs6809952
rs2387977
All
−0.2
−0.1
0.0
0.1
0.2
MR effect size for
'Gut microbiota abundance (genus Parasutterella id.2892)' on 'Laminin subunit alpha−4'

Batch 2−3393 : Gut microbiota abundance (genus Catenibacterium id.2153) on Laminin subunit gamma−2
with a threshold of 1e−05

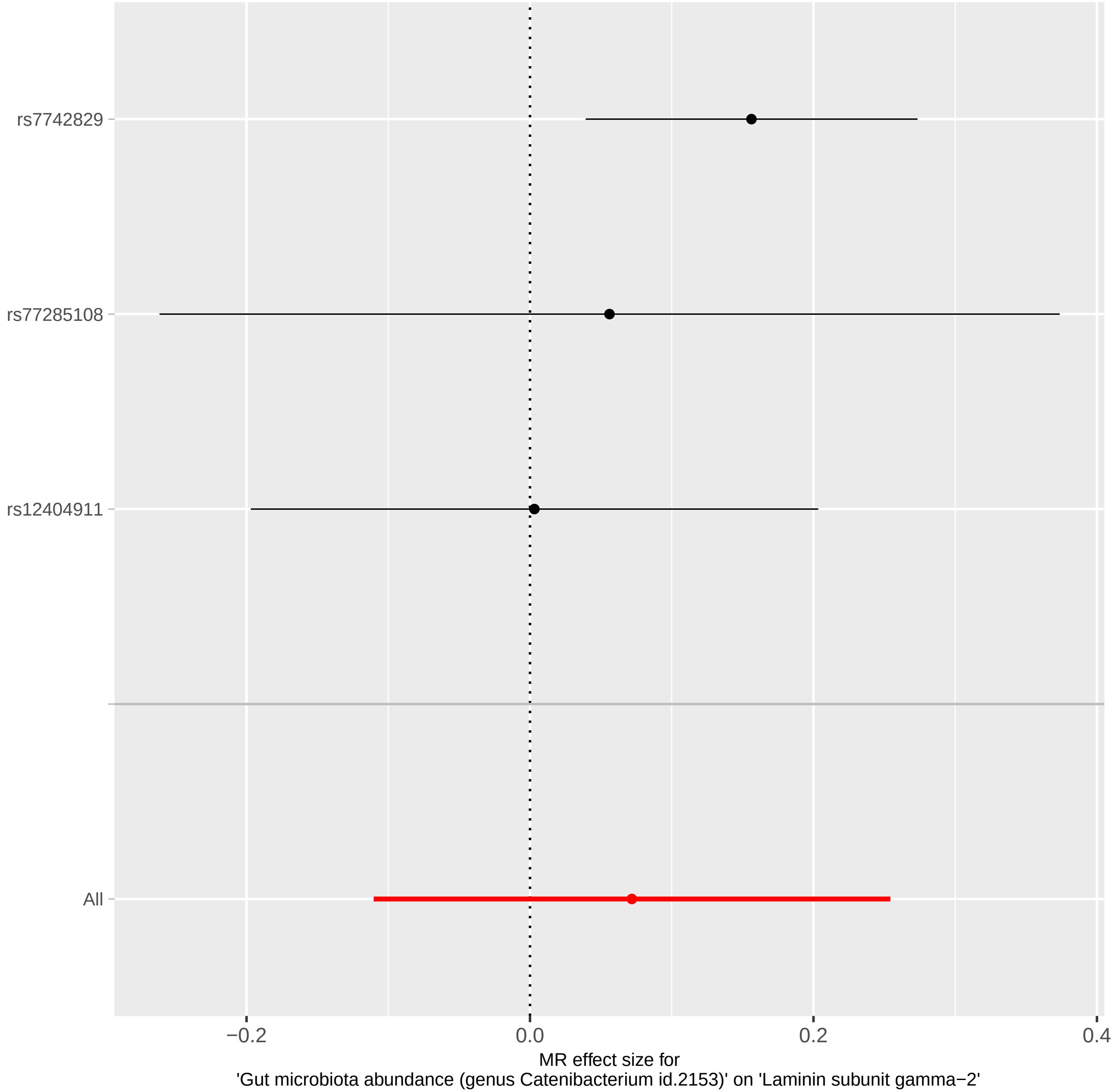

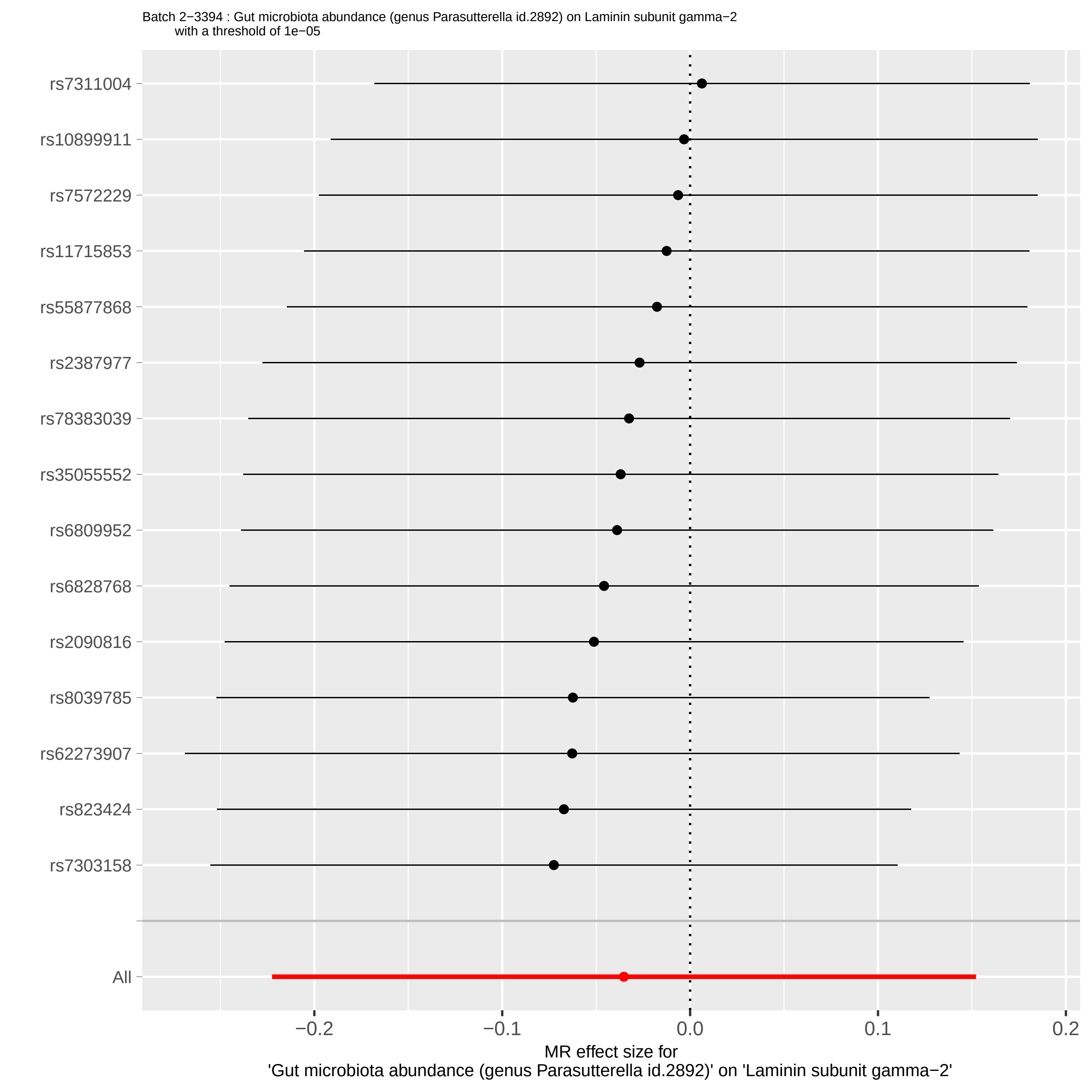
Batch 2−3394 : Gut microbiota abundance (genus Parasutterella id.2892) on Laminin subunit gamma−2
with a threshold of 1e−05
rs7311004
rs10899911
rs7572229
rs11715853
rs55877868
rs2387977
rs78383039
rs35055552
rs6809952
rs6828768
rs2090816
rs8039785
rs62273907
rs823424
rs7303158
All
−0.2
−0.1
0.0
0.1
0.2
MR effect size for
'Gut microbiota abundance (genus Parasutterella id.2892)' on 'Laminin subunit gamma−2'

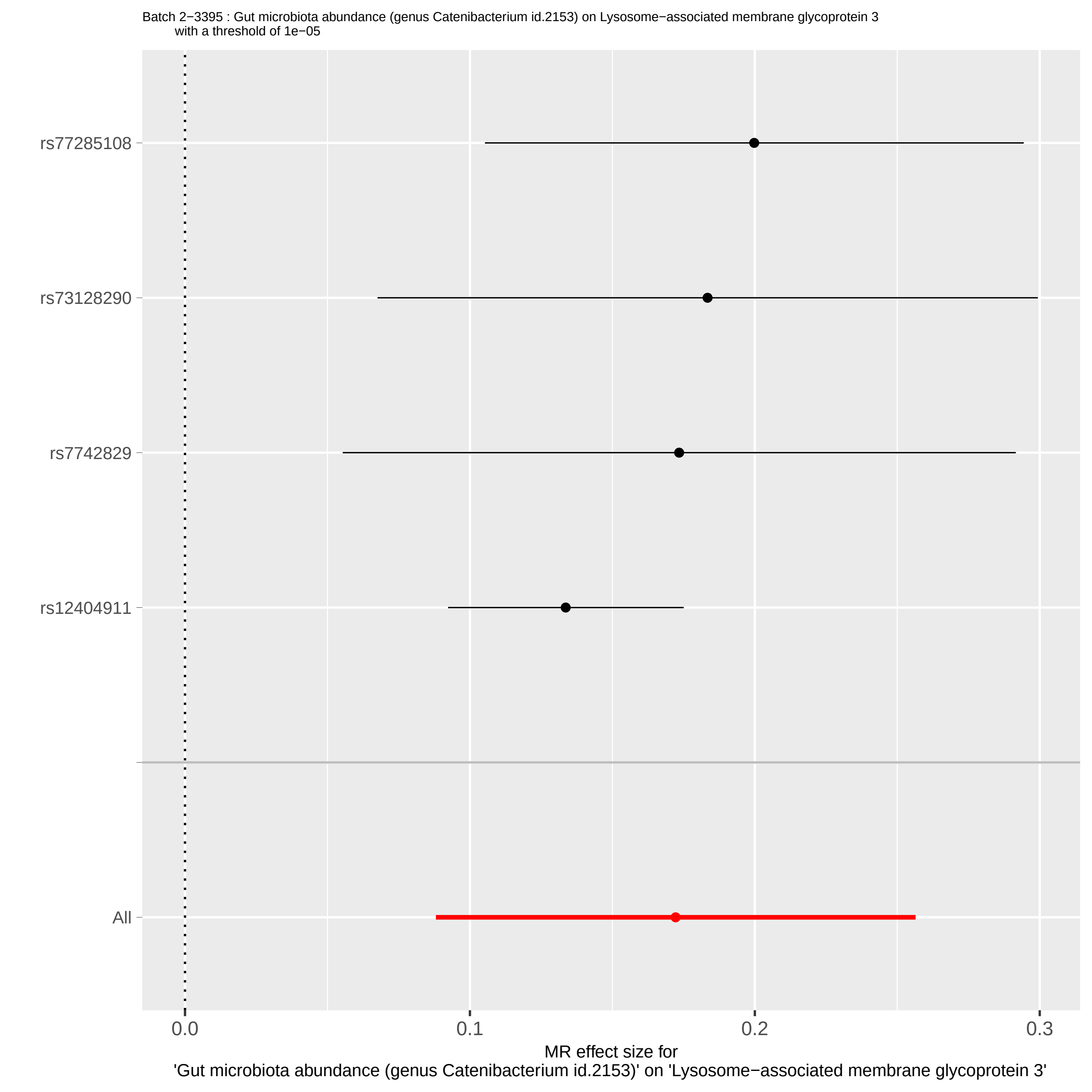

Batch 2−3395 : Gut microbiota abundance (genus Catenibacterium id.2153) on Lysosome−associated membrane glycoprotein 3
with a threshold of 1e−05
rs77285108
rs73128290
rs7742829
rs12404911
All
0.0
0.1
0.2
0.3
MR effect size for
'Gut microbiota abundance (genus Catenibacterium id.2153)' on 'Lysosome−associated membrane glycoprotein 3'

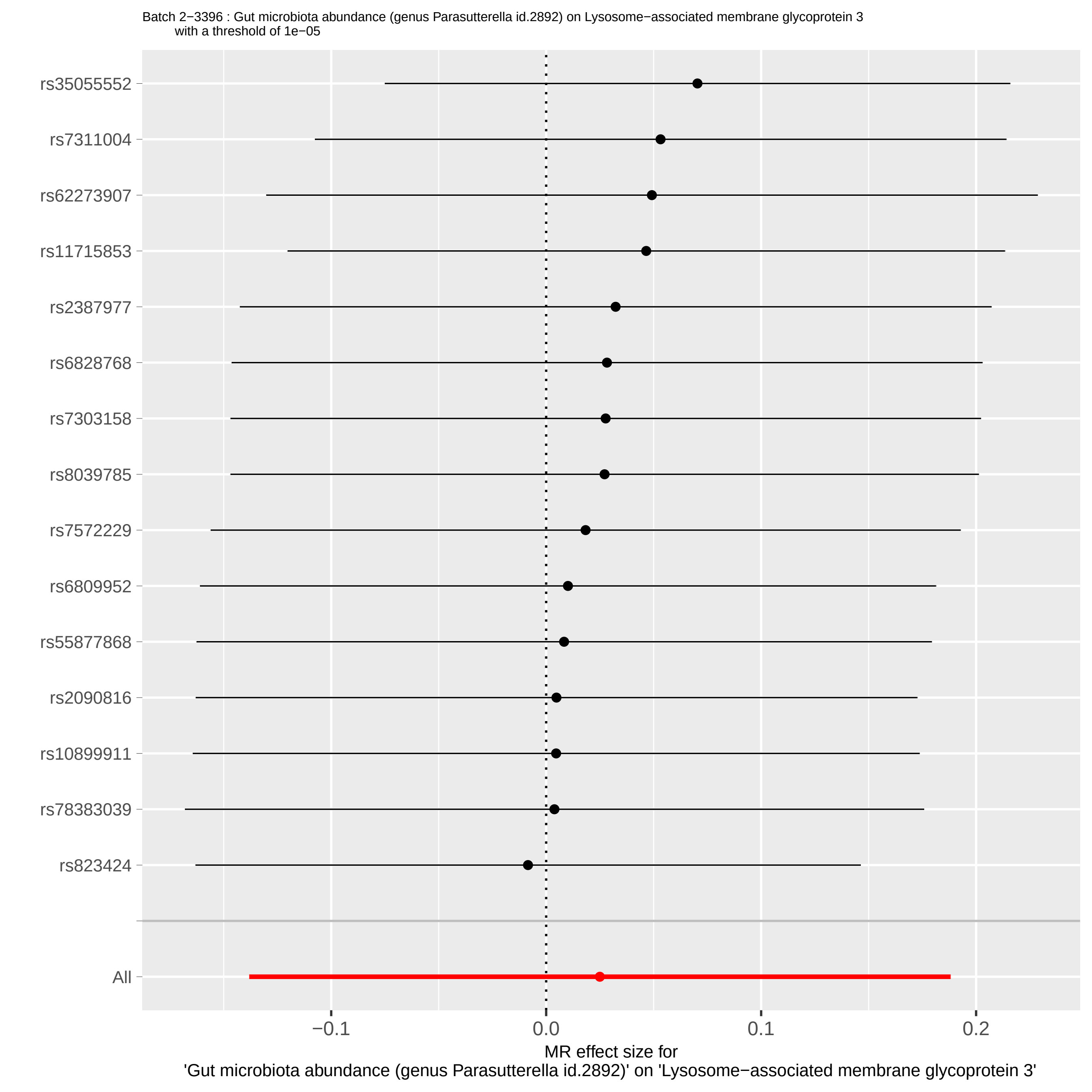

Batch 2−3396 : Gut microbiota abundance (genus Parasutterella id.2892) on Lysosome−associated membrane glycoprotein 3
with a threshold of 1e−05
rs35055552
rs7311004
rs62273907
rs11715853
rs2387977
rs6828768
rs7303158
rs8039785
rs7572229
rs6809952
rs55877868
rs2090816
rs10899911
rs78383039
rs823424
All
−0.1
0.0
0.1
0.2
MR effect size for
'Gut microbiota abundance (genus Parasutterella id.2892)' on 'Lysosome−associated membrane glycoprotein 3'

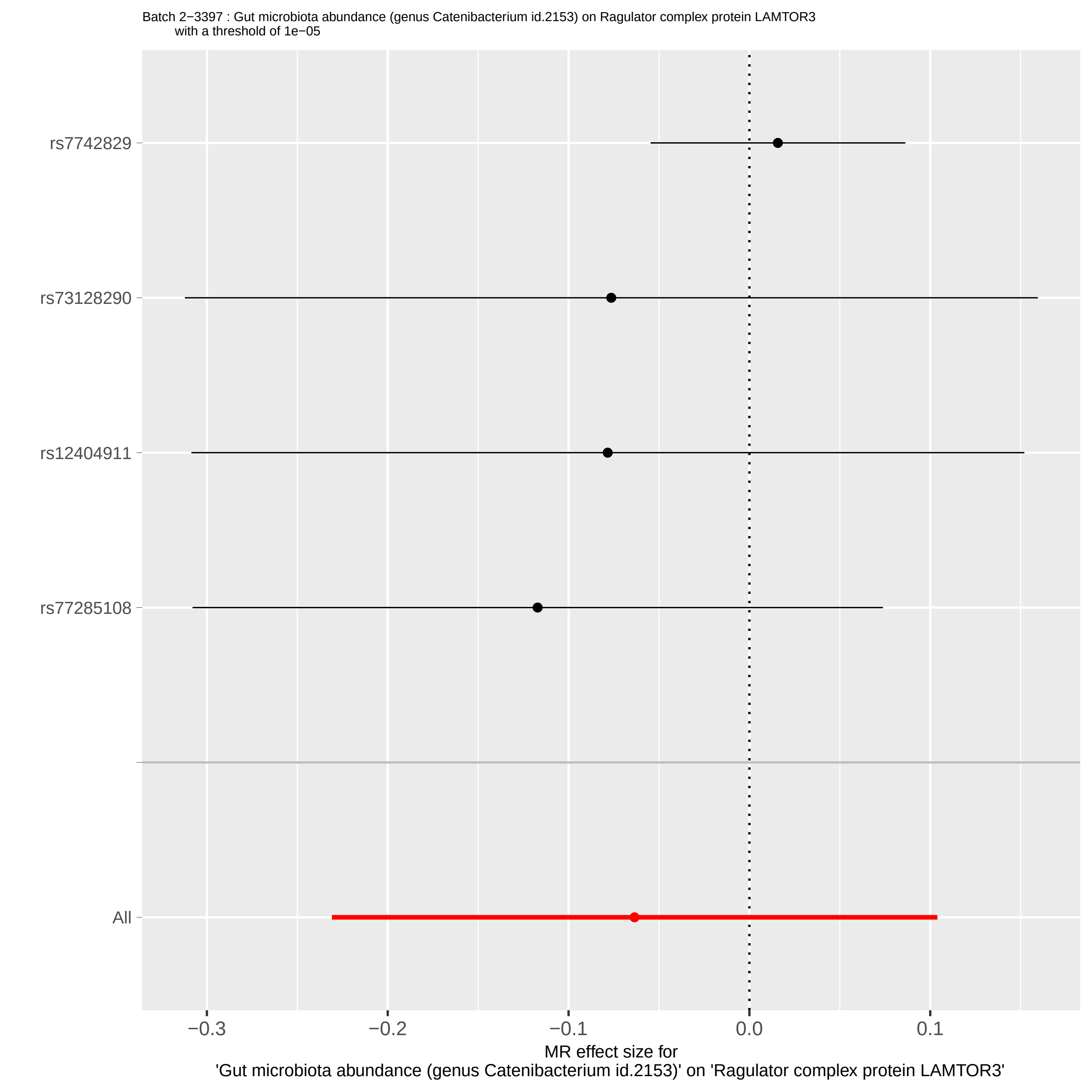

Batch 2−3397 : Gut microbiota abundance (genus Catenibacterium id.2153) on Ragulator complex protein LAMTOR3
with a threshold of 1e−05
rs7742829
rs73128290
rs12404911
rs77285108
All
−0.3
−0.2
−0.1
0.0
0.1
MR effect size for
'Gut microbiota abundance (genus Catenibacterium id.2153)' on 'Ragulator complex protein LAMTOR3'

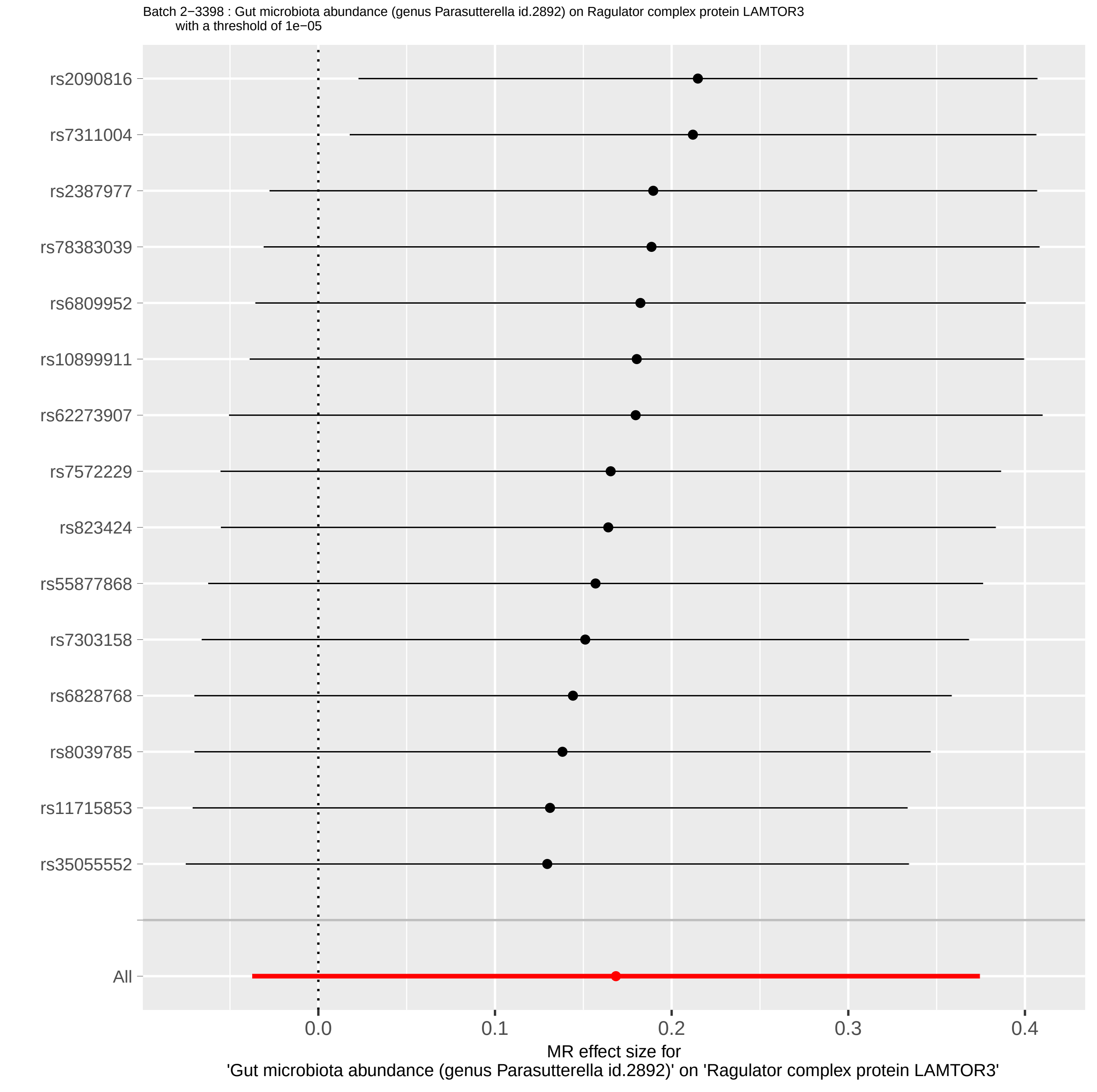

Batch 2−3398 : Gut microbiota abundance (genus Parasutterella id.2892) on Ragulator complex protein LAMTOR3
with a threshold of 1e−05
rs2090816
rs7311004
rs2387977
rs78383039
rs6809952
rs10899911
rs62273907
rs7572229
rs823424
rs55877868
rs7303158
rs6828768
rs8039785
rs11715853
rs35055552
All
0.0
0.1
0.2
0.3
0.4
MR effect size for
'Gut microbiota abundance (genus Parasutterella id.2892)' on 'Ragulator complex protein LAMTOR3'

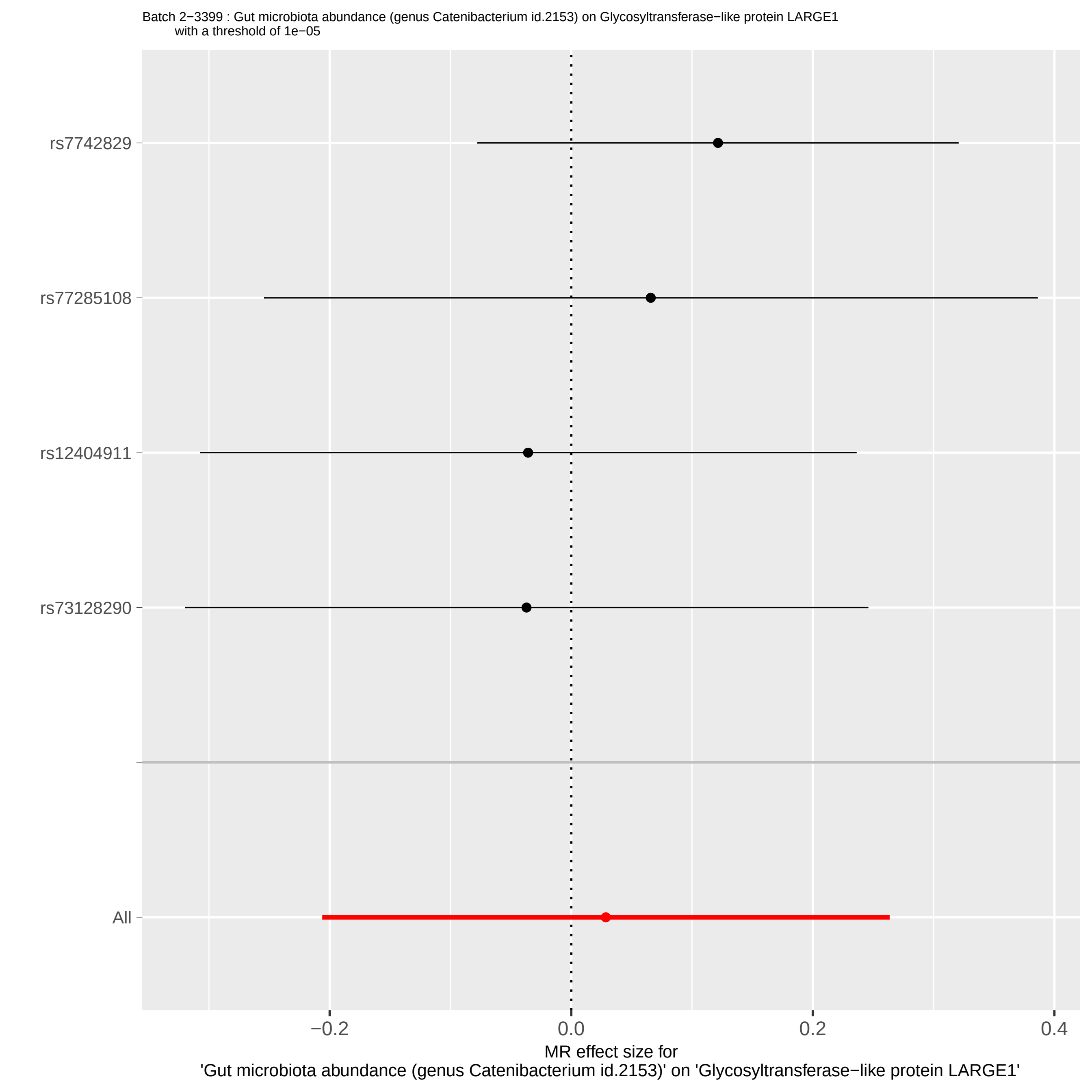
Batch 2−3399 : Gut microbiota abundance (genus Catenibacterium id.2153) on Glycosyltransferase−like protein LARGE1
with a threshold of 1e−05
rs7742829
rs77285108
rs12404911
rs73128290
All
−0.2
0.0
0.2
0.4
MR effect size for
'Gut microbiota abundance (genus Catenibacterium id.2153)' on 'Glycosyltransferase−like protein LARGE1'

Batch 2−3400 : Gut microbiota abundance (genus Parasutterella id.2892) on Glycosyltransferase−like protein LARGE1
with a threshold of 1e−05

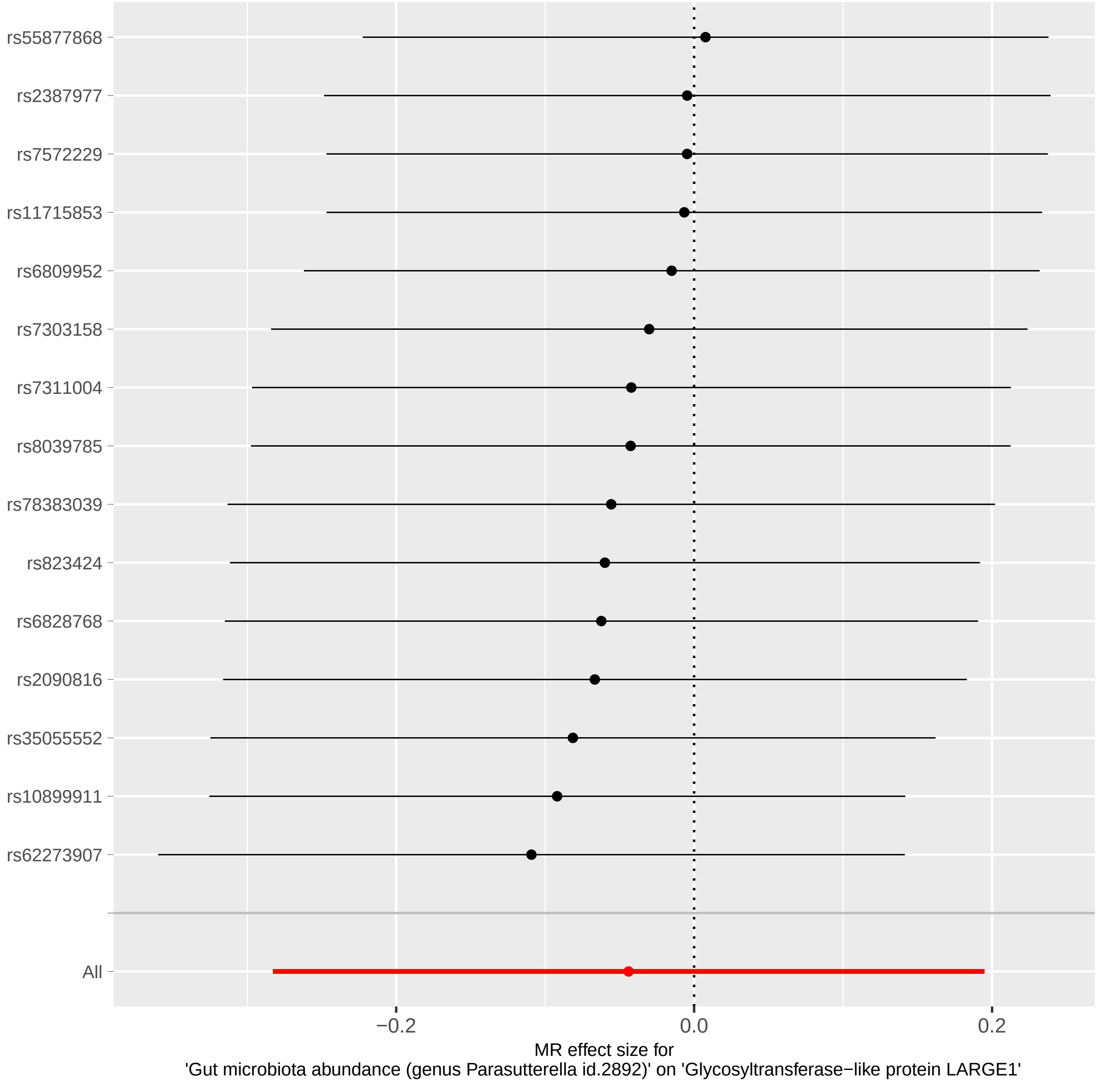

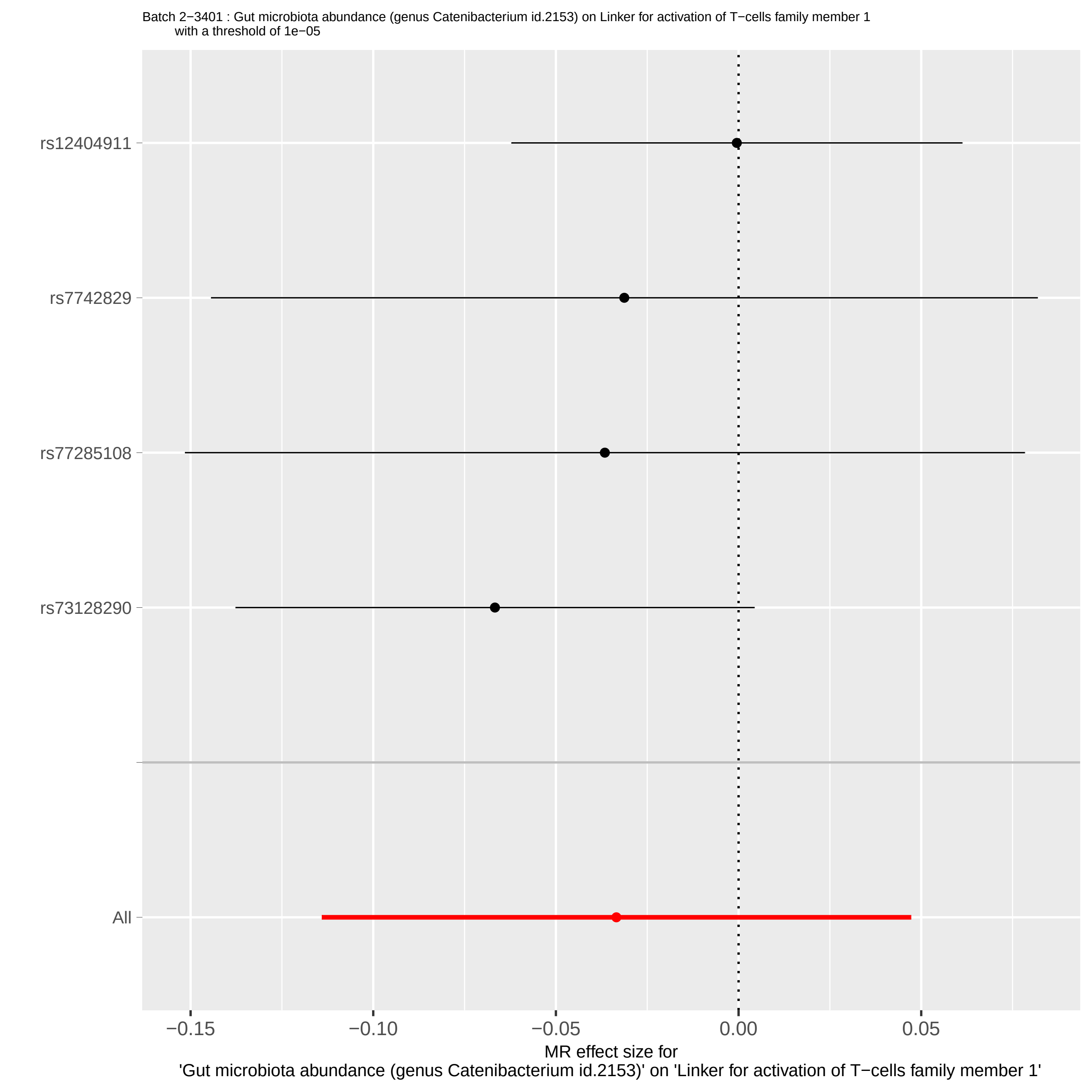

Batch 2−3401 : Gut microbiota abundance (genus Catenibacterium id.2153) on Linker for activation of T−cells family member 1
with a threshold of 1e−05
rs12404911
rs7742829
rs77285108
rs73128290
All
−0.15
−0.10
−0.05
0.00
0.05
MR effect size for
'Gut microbiota abundance (genus Catenibacterium id.2153)' on 'Linker for activation of T−cells family member 1'

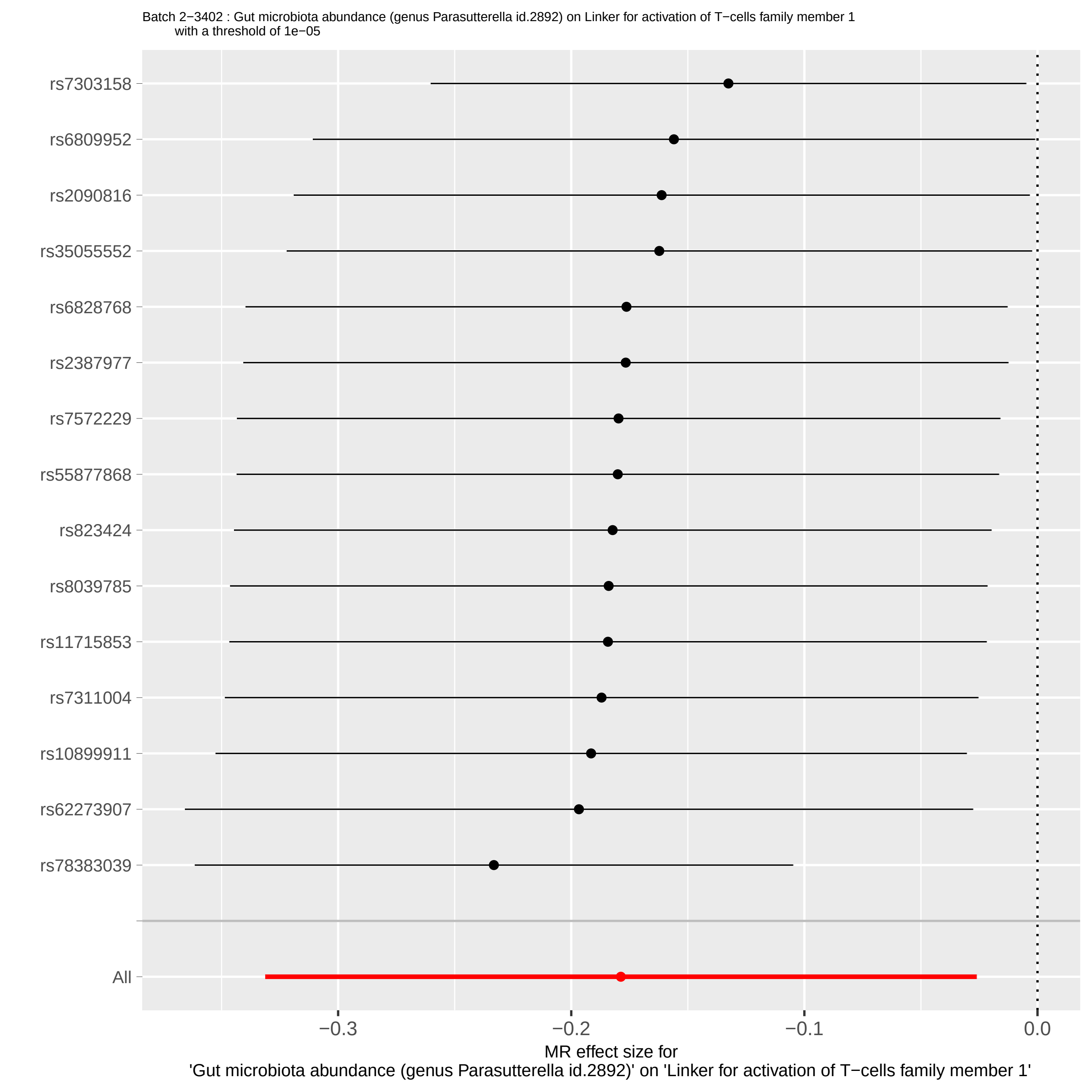

Batch 2−3402 : Gut microbiota abundance (genus Parasutterella id.2892) on Linker for activation of T−cells family member 1
with a threshold of 1e−05
rs7303158
rs6809952
rs2090816
rs35055552
rs6828768
rs2387977
rs7572229
rs55877868
rs823424
rs8039785
rs11715853
rs7311004
rs10899911
rs62273907
rs78383039
All
−0.3
−0.2
−0.1
0.0
MR effect size for
'Gut microbiota abundance (genus Parasutterella id.2892)' on 'Linker for activation of T−cells family member 1'

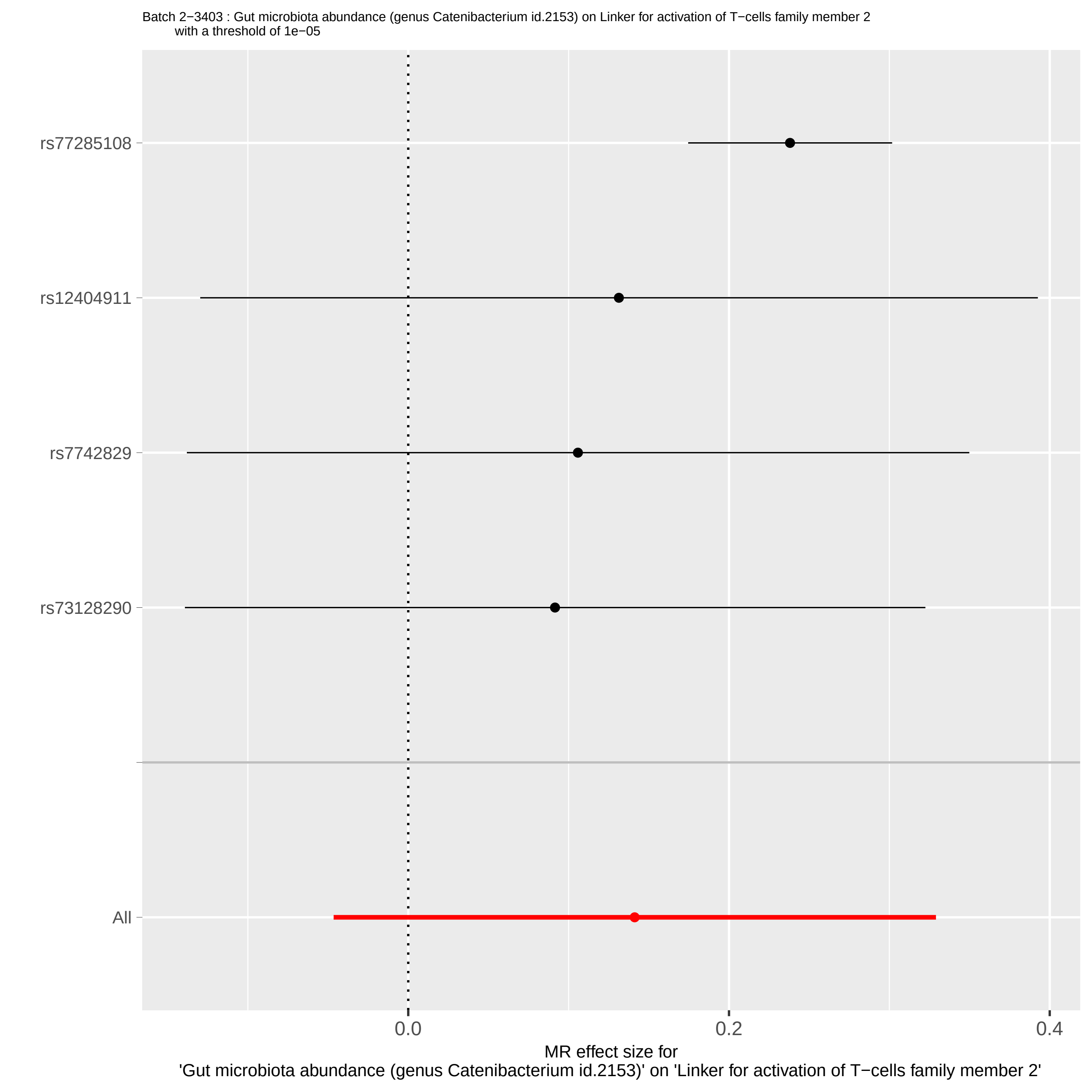
Batch 2−3403 : Gut microbiota abundance (genus Catenibacterium id.2153) on Linker for activation of T−cells family member 2
with a threshold of 1e−05
rs77285108
rs12404911
rs7742829
rs73128290
All
0.0
0.2
0.4
MR effect size for
'Gut microbiota abundance (genus Catenibacterium id.2153)' on 'Linker for activation of T−cells family member 2'

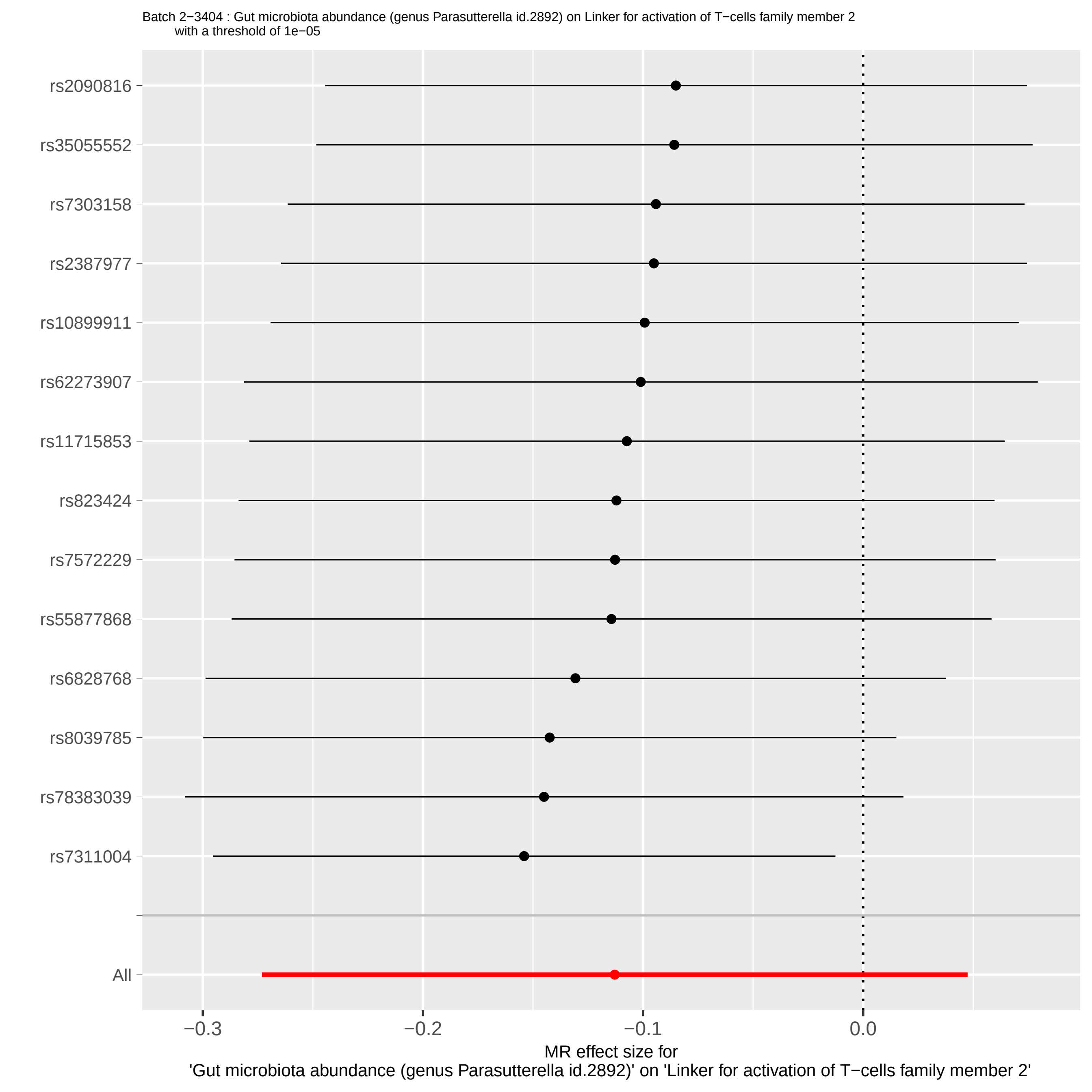

Batch 2−3404 : Gut microbiota abundance (genus Parasutterella id.2892) on Linker for activation of T−cells family member 2
with a threshold of 1e−05
rs2090816
rs35055552
rs7303158
rs2387977
rs10899911
rs62273907
rs11715853
rs823424
rs7572229
rs55877868
rs6828768
rs8039785
rs78383039
rs7311004
All
−0.3
−0.2
−0.1
0.0
MR effect size for
'Gut microbiota abundance (genus Parasutterella id.2892)' on 'Linker for activation of T−cells family member 2'

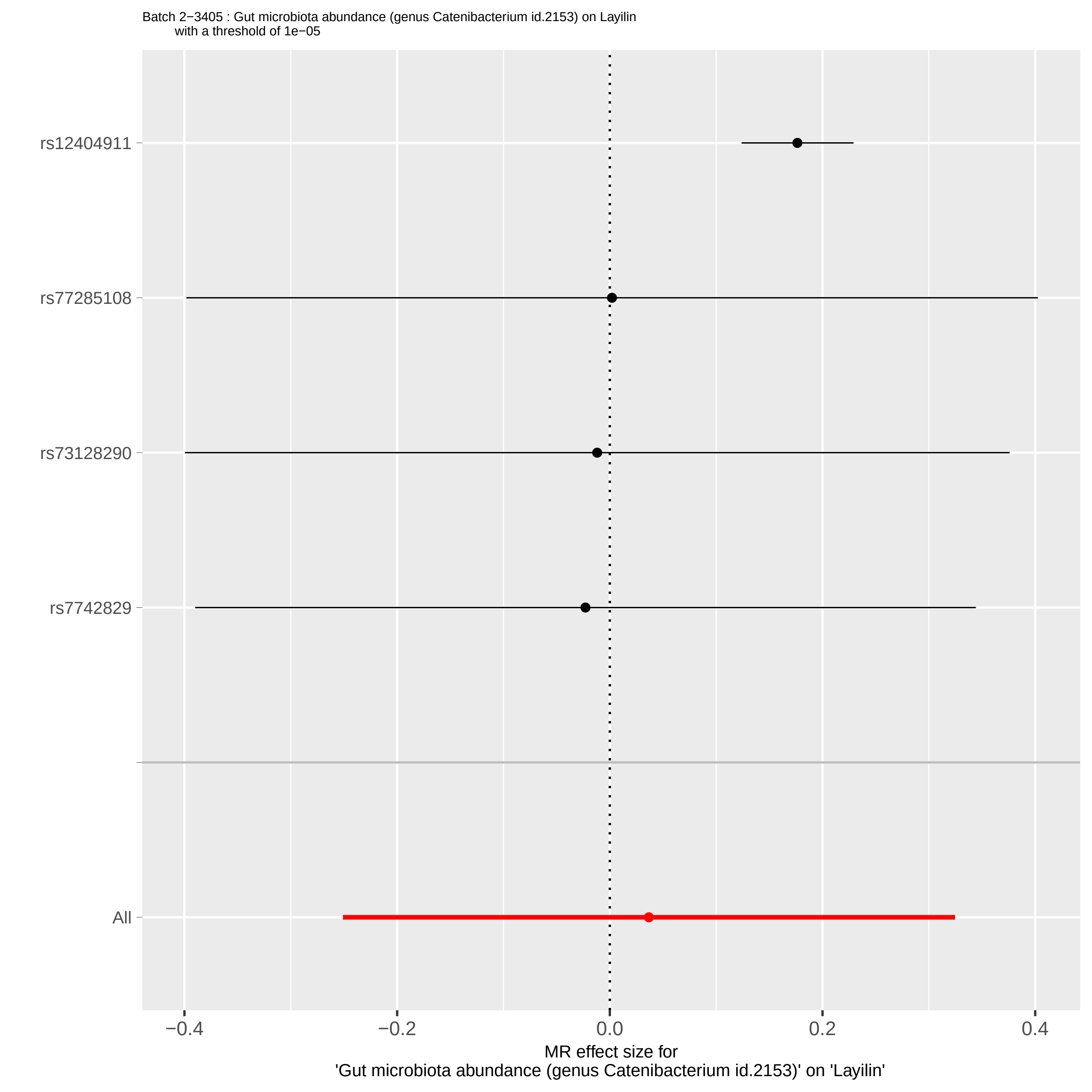
Batch 2−3405 : Gut microbiota abundance (genus Catenibacterium id.2153) on Layilin
with a threshold of 1e−05
rs12404911
rs77285108
rs73128290
rs7742829
All
−0.4
−0.2
0.0
0.2
0.4
MR effect size for
'Gut microbiota abundance (genus Catenibacterium id.2153)' on 'Layilin'

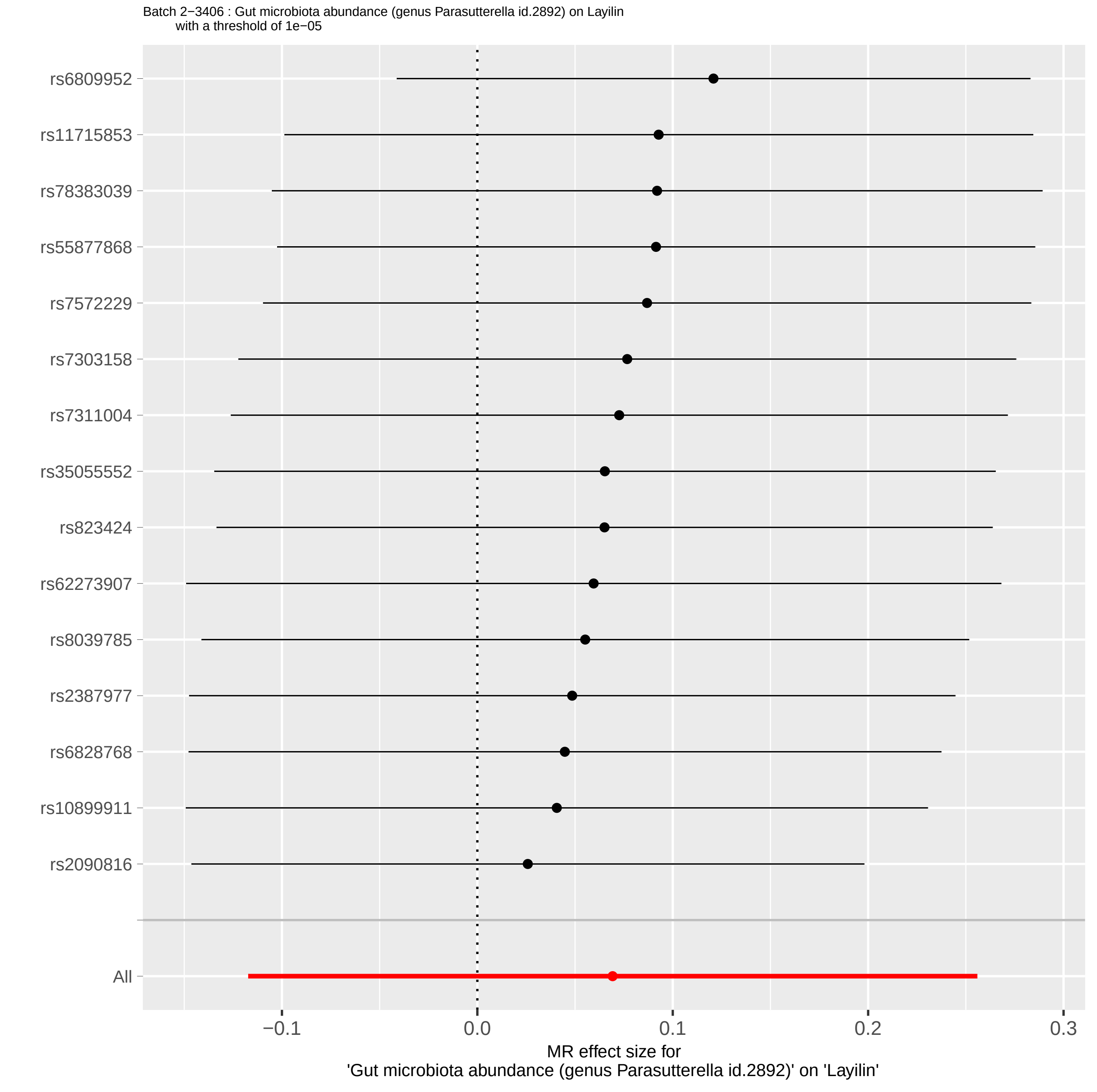
Batch 2−3406 : Gut microbiota abundance (genus Parasutterella id.2892) on Layilin
with a threshold of 1e−05
rs6809952
rs11715853
rs78383039
rs55877868
rs7572229
rs7303158
rs7311004
rs35055552
rs823424
rs62273907
rs8039785
rs2387977
rs6828768
rs10899911
rs2090816
All
−0.1
0.0
0.1
0.2
0.3
MR effect size for
'Gut microbiota abundance (genus Parasutterella id.2892)' on 'Layilin'

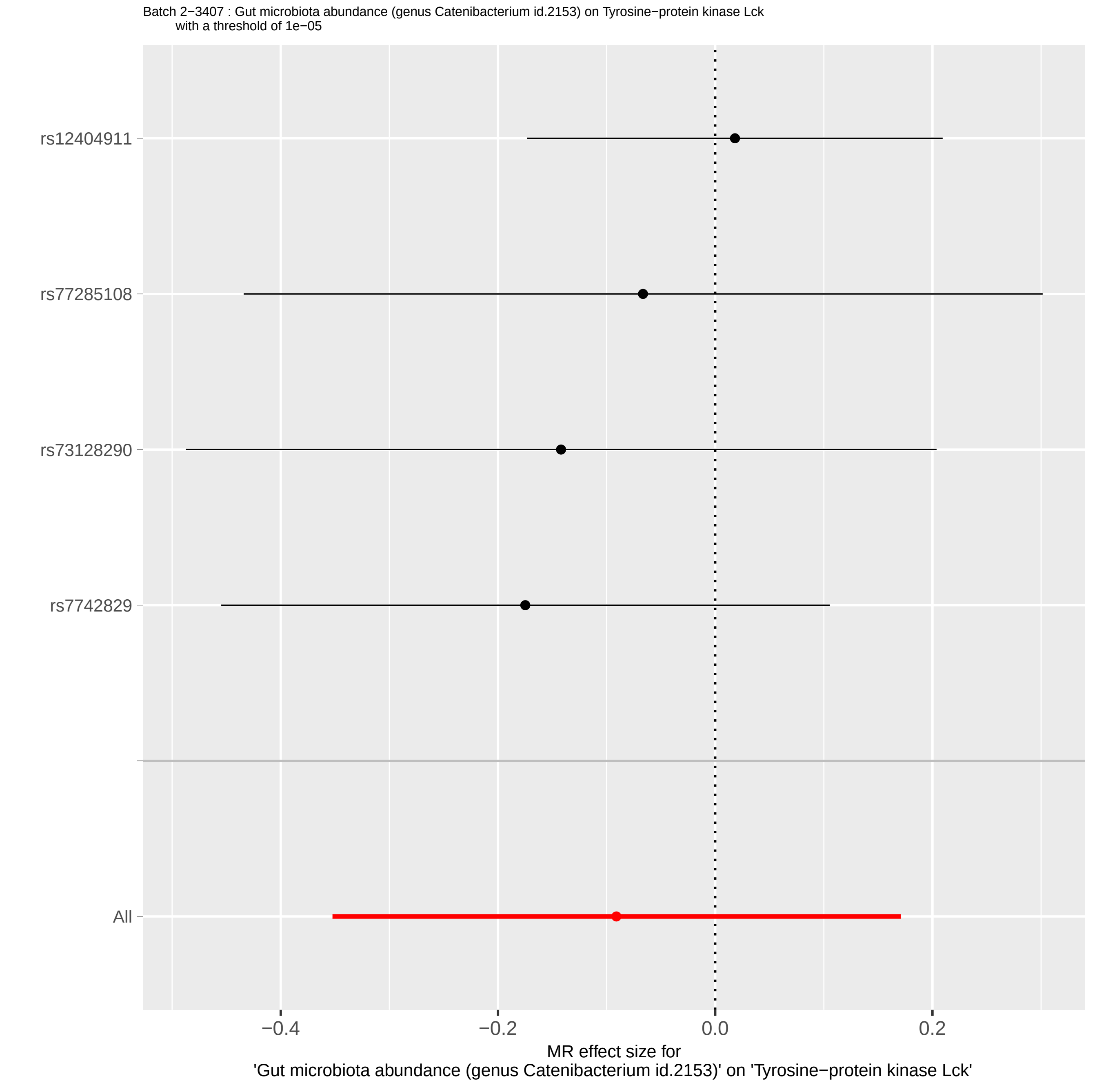

Batch 2−3407 : Gut microbiota abundance (genus Catenibacterium id.2153) on Tyrosine−protein kinase Lck
with a threshold of 1e−05
rs12404911
rs77285108
rs73128290
rs7742829
All
−0.4
−0.2
0.0
0.2
MR effect size for
'Gut microbiota abundance (genus Catenibacterium id.2153)' on 'Tyrosine−protein kinase Lck'

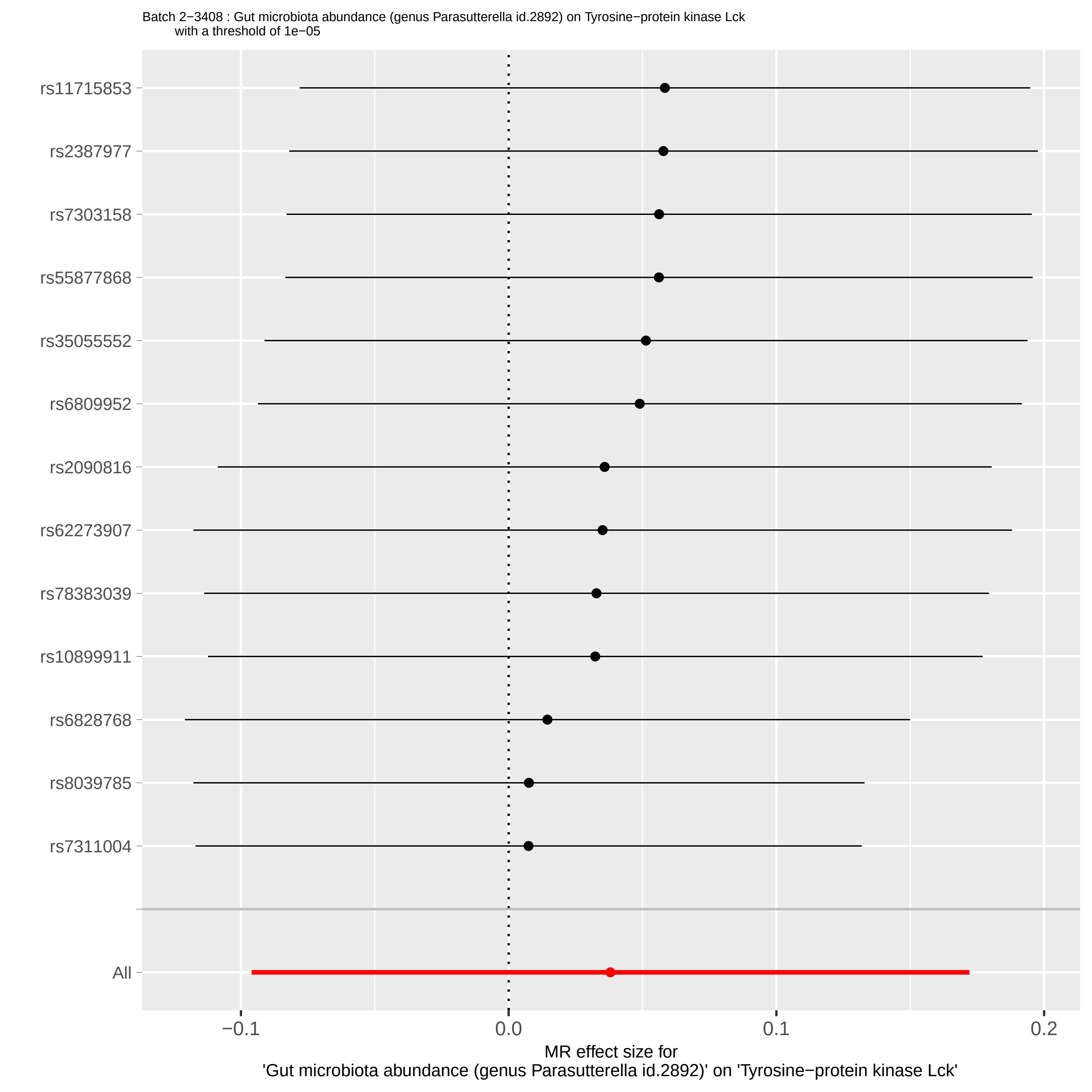

Batch 2−3408 : Gut microbiota abundance (genus Parasutterella id.2892) on Tyrosine−protein kinase Lck
with a threshold of 1e−05
rs11715853
rs2387977
rs7303158
rs55877868
rs35055552
rs6809952
rs2090816
rs62273907
rs78383039
rs10899911
rs6828768
rs8039785
rs7311004
All
−0.1
0.0
0.1
0.2
MR effect size for
'Gut microbiota abundance (genus Parasutterella id.2892)' on 'Tyrosine−protein kinase Lck

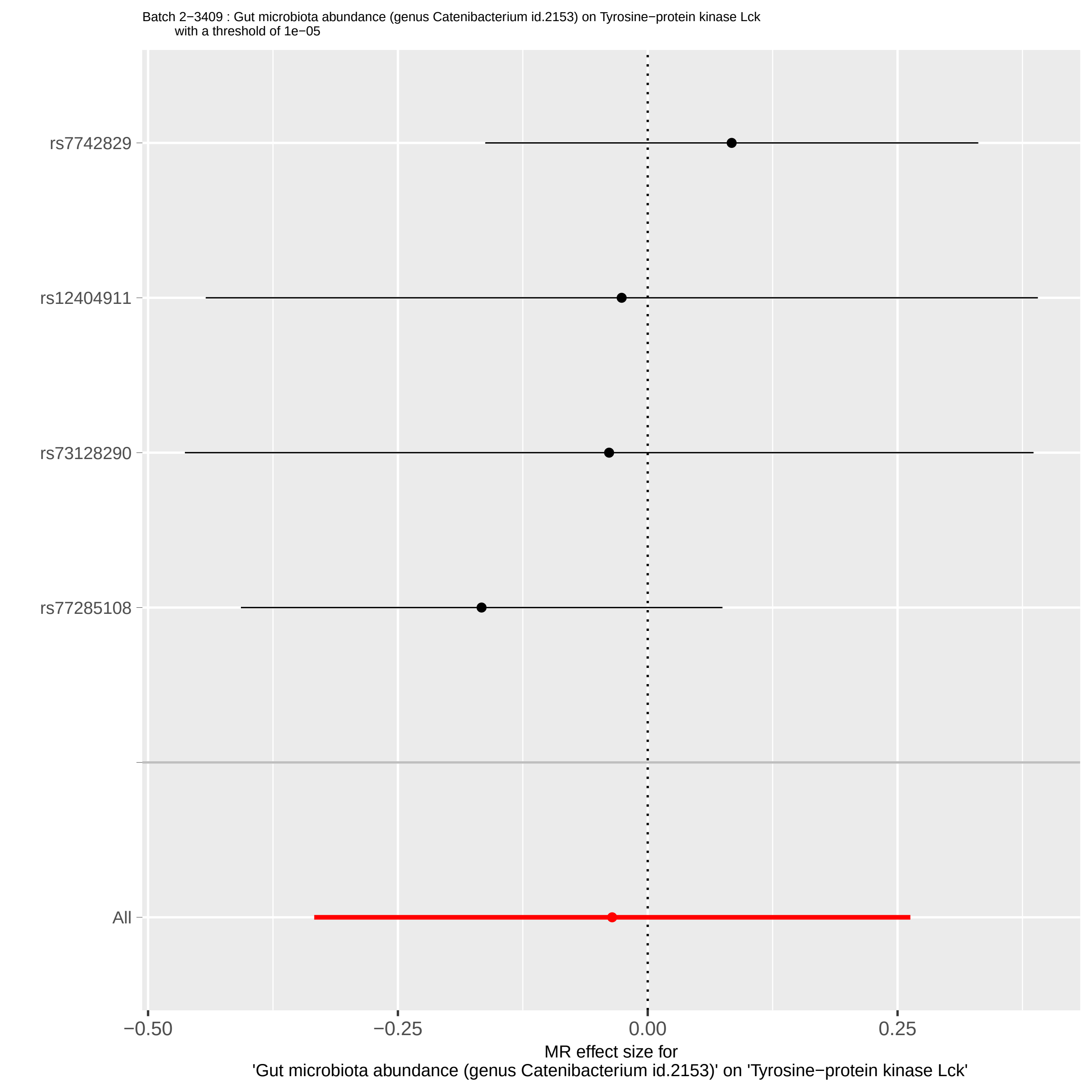
Batch 2−3409 : Gut microbiota abundance (genus Catenibacterium id.2153) on Tyrosine−protein kinase Lck
with a threshold of 1e−05
rs7742829
rs12404911
rs73128290
rs77285108
All
−0.50
−0.25
0.00
0.25
MR effect size for
'Gut microbiota abundance (genus Catenibacterium id.2153)' on 'Tyrosine−protein kinase Lck'

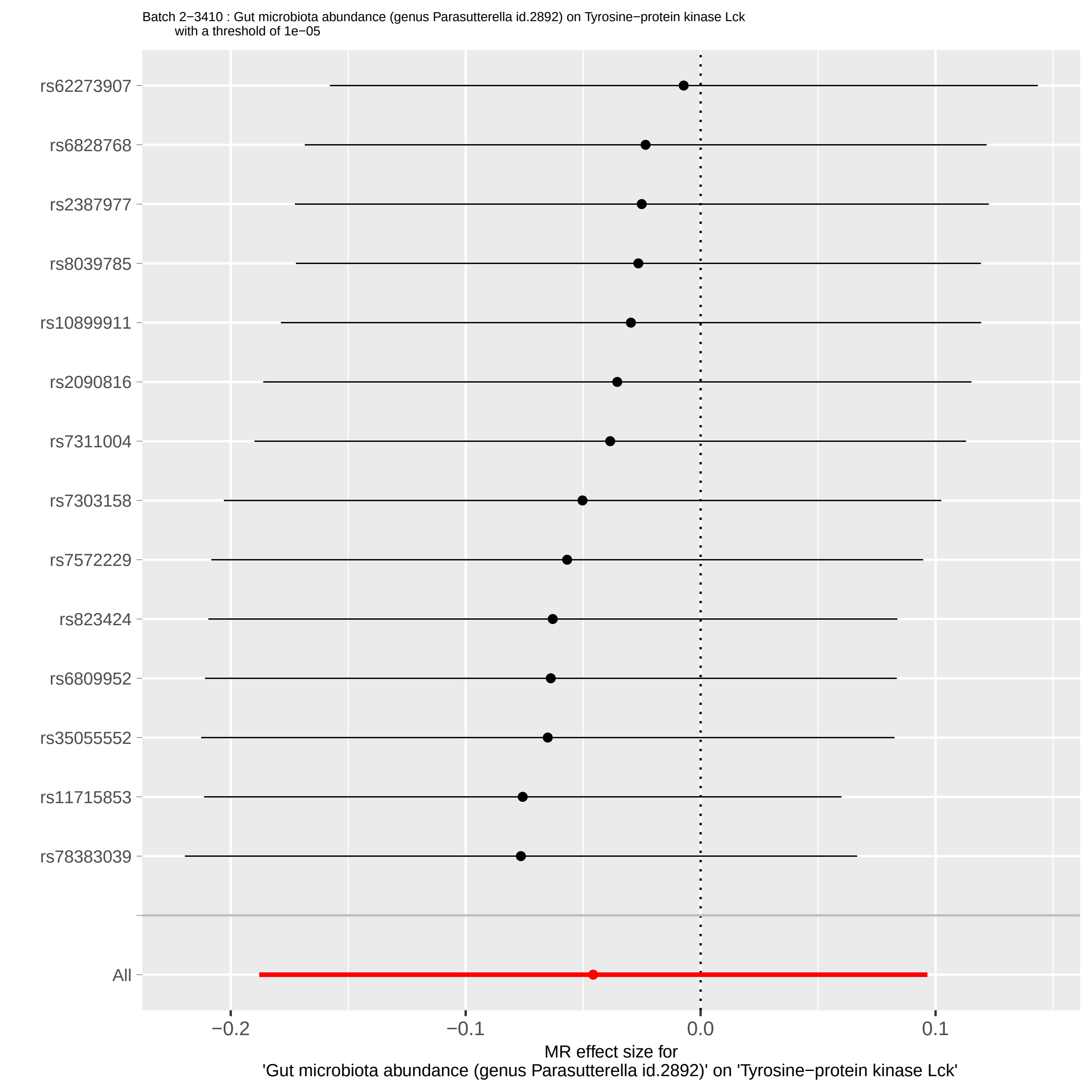
Batch 2−3410 : Gut microbiota abundance (genus Parasutterella id.2892) on Tyrosine−protein kinase Lck
with a threshold of 1e−05
rs62273907
rs6828768
rs2387977
rs8039785
rs10899911
rs2090816
rs7311004
rs7303158
rs7572229
rs823424
rs6809952
rs35055552
rs11715853
rs78383039
All
−0.2
−0.1
0.0
0.1
MR effect size for
'Gut microbiota abundance (genus Parasutterella id.2892)' on 'Tyrosine−protein kinase Lck'

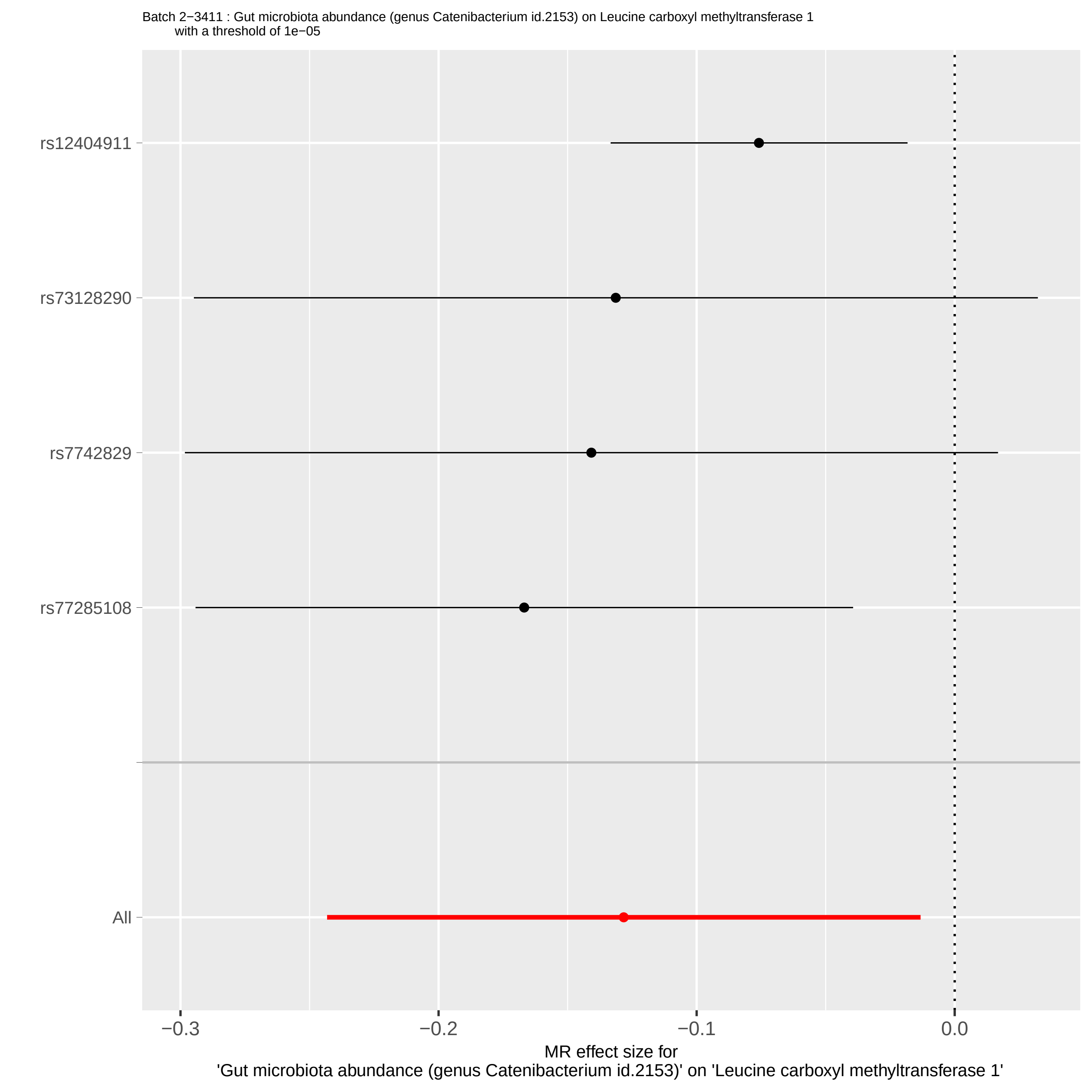

Batch 2−3411 : Gut microbiota abundance (genus Catenibacterium id.2153) on Leucine carboxyl methyltransferase 1
with a threshold of 1e−05
rs12404911
rs73128290
rs7742829
rs77285108
All
−0.3
−0.2
−0.1
0.0
MR effect size for
'Gut microbiota abundance (genus Catenibacterium id.2153)' on 'Leucine carboxyl methyltransferase 1'

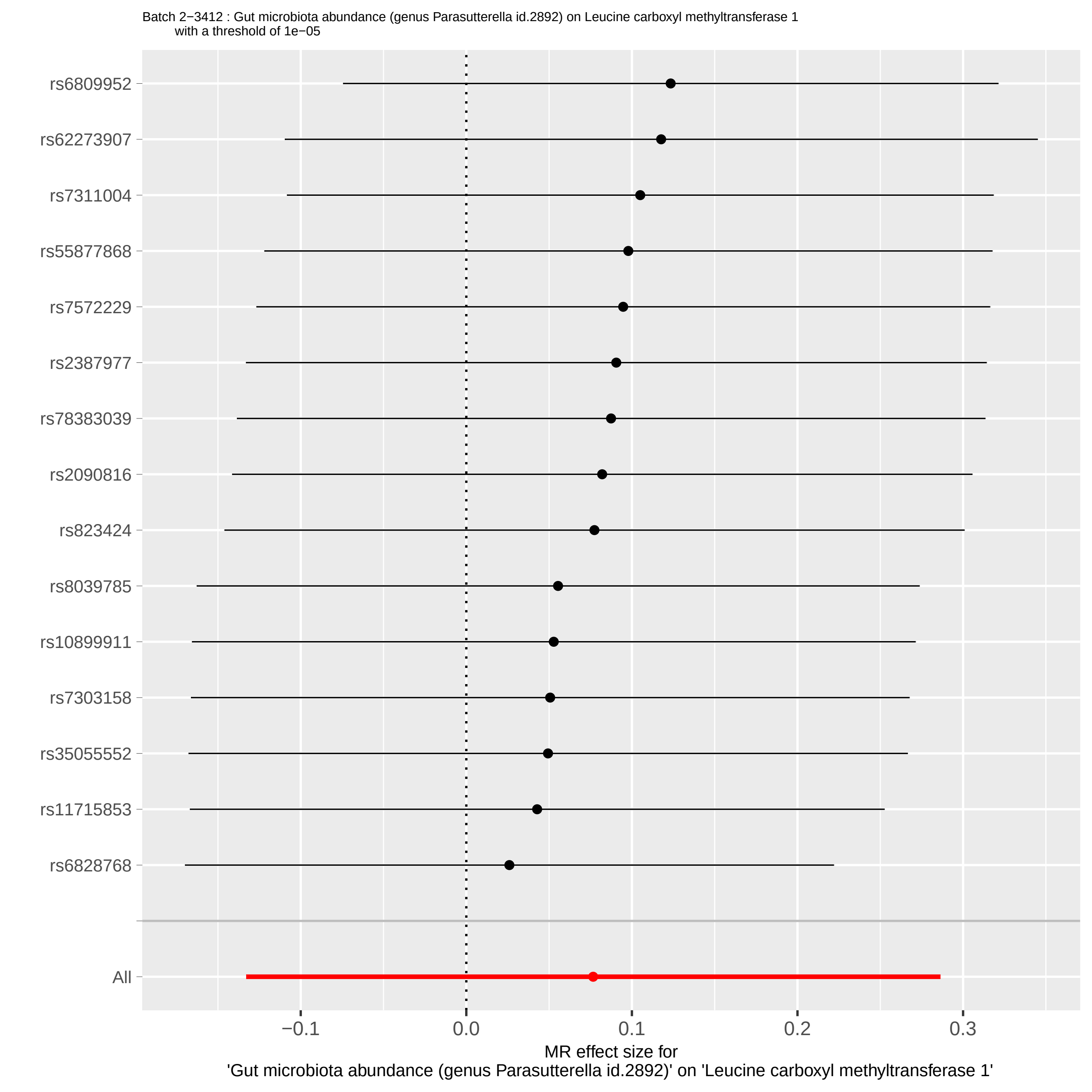
Batch 2−3412 : Gut microbiota abundance (genus Parasutterella id.2892) on Leucine carboxyl methyltransferase 1
with a threshold of 1e−05
rs6809952
rs62273907
rs7311004
rs55877868
rs7572229
rs2387977
rs78383039
rs2090816
rs823424
rs8039785
rs10899911
rs7303158
rs35055552
rs11715853
rs6828768
All
−0.1
0.0
0.1
0.2
0.3
MR effect size for
'Gut microbiota abundance (genus Parasutterella id.2892)' on 'Leucine carboxyl methyltransferase 1'

Batch 2−3413 : Gut microbiota abundance (genus Catenibacterium id.2153) on Epididymal−specific lipocalin−10
with a threshold of 1e−05

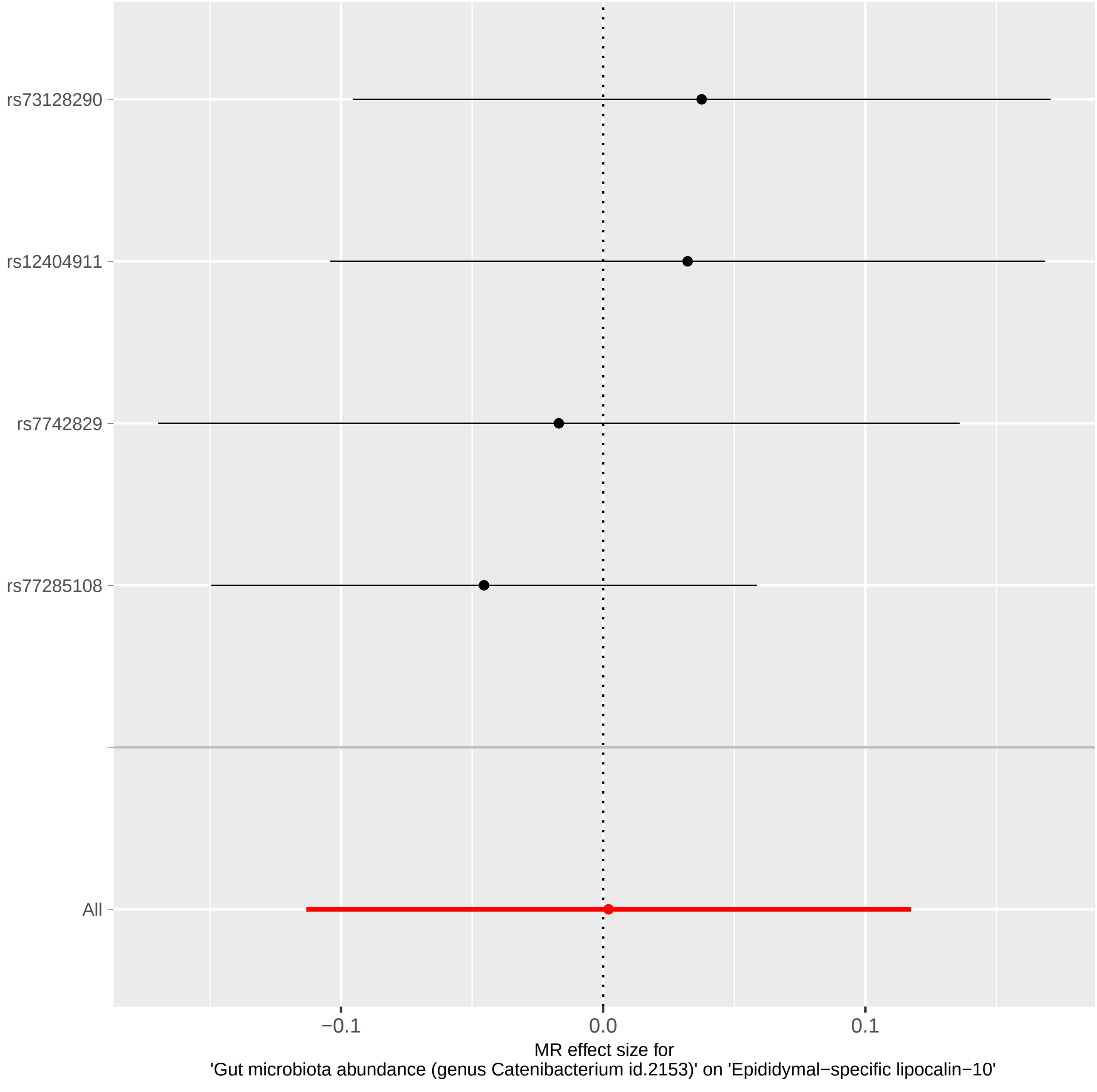

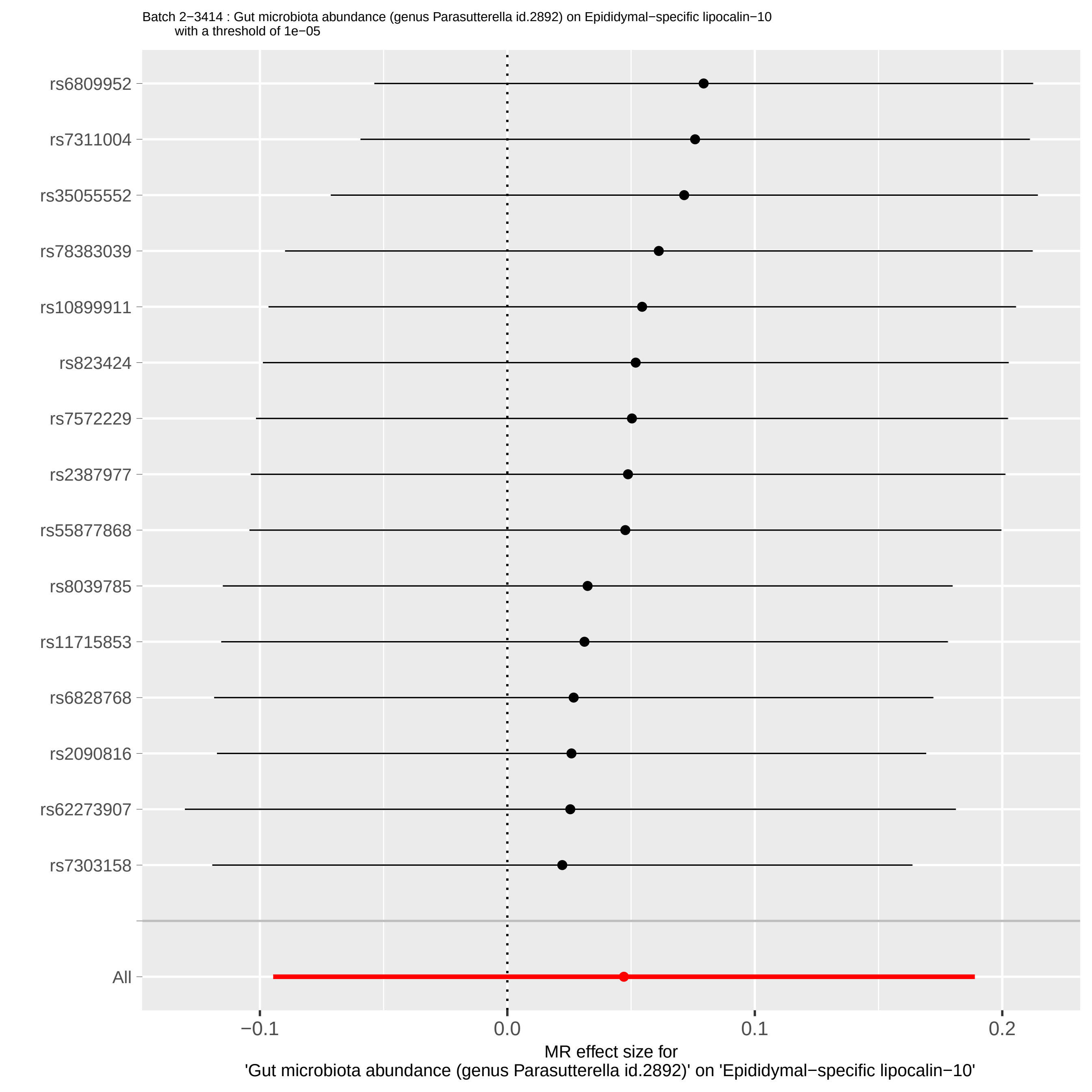
Batch 2−3414 : Gut microbiota abundance (genus Parasutterella id.2892) on Epididymal−specific lipocalin−10
with a threshold of 1e−05
rs6809952
rs7311004
rs35055552
rs78383039
rs10899911
rs823424
rs7572229
rs2387977
rs55877868
rs8039785
rs11715853
rs6828768
rs2090816
rs62273907
rs7303158
All
−0.1
0.0
0.1
0.2
MR effect size for
'Gut microbiota abundance (genus Parasutterella id.2892)' on 'Epididymal−specific lipocalin−10'

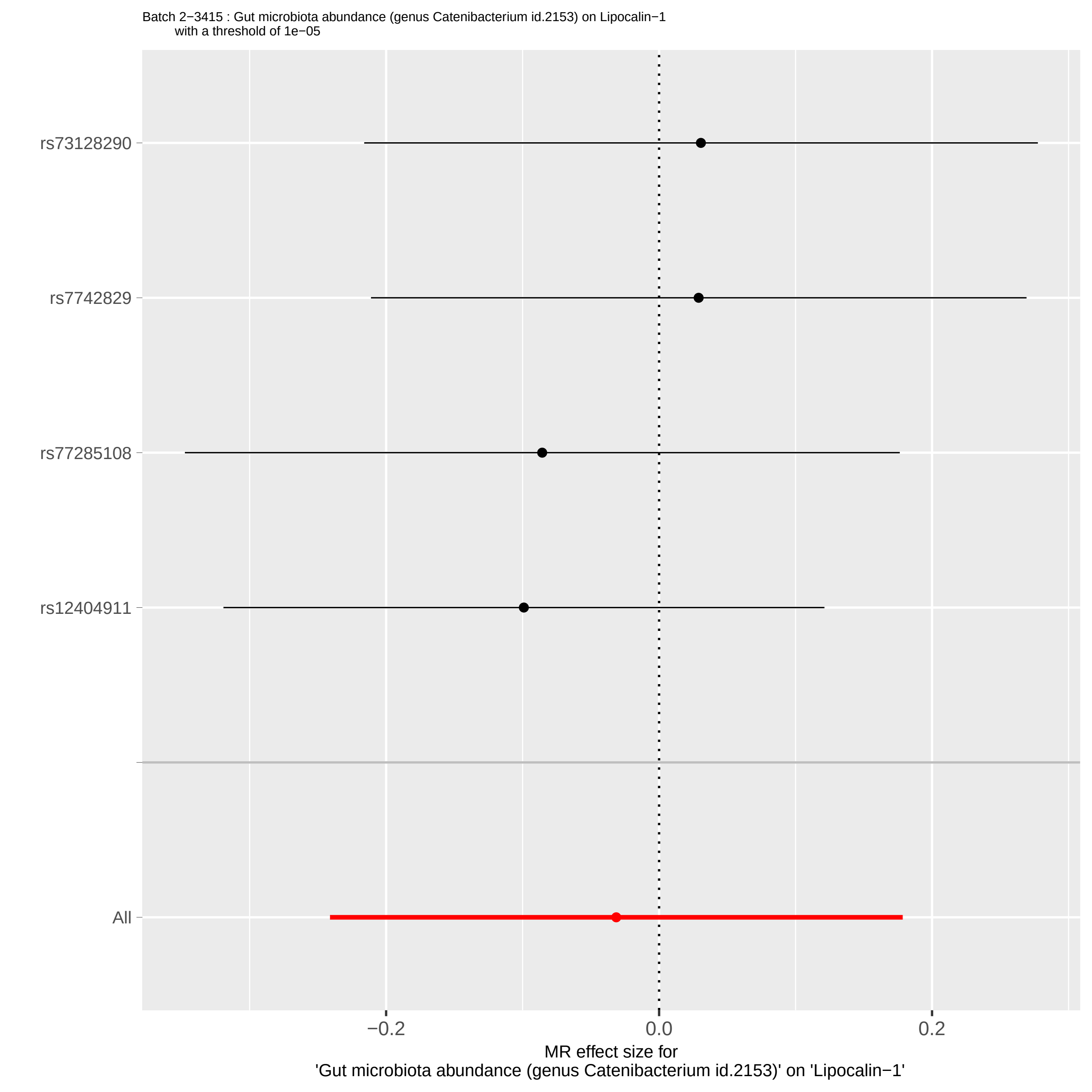

Batch 2−3415 : Gut microbiota abundance (genus Catenibacterium id.2153) on Lipocalin−1
with a threshold of 1e−05
rs73128290
rs7742829
rs77285108
rs12404911
All
−0.2
0.0
0.2
MR effect size for
'Gut microbiota abundance (genus Catenibacterium id.2153)' on 'Lipocalin−1'

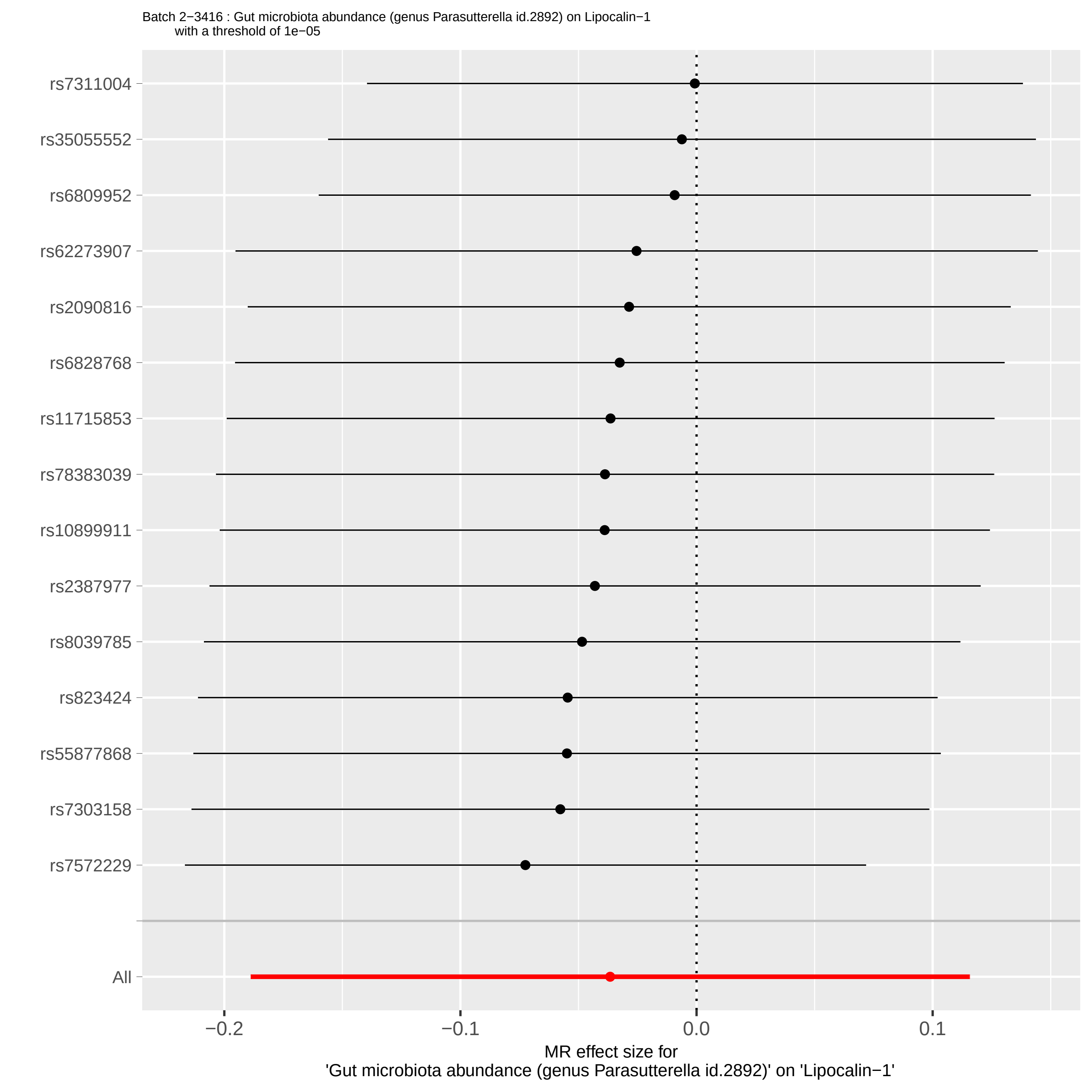
Batch 2−3416 : Gut microbiota abundance (genus Parasutterella id.2892) on Lipocalin−1
with a threshold of 1e−05
rs7311004
rs35055552
rs6809952
rs62273907
rs2090816
rs6828768
rs11715853
rs78383039
rs10899911
rs2387977
rs8039785
rs823424
rs55877868
rs7303158
rs7572229
All
−0.2
−0.1
0.0
0.1
MR effect size for
'Gut microbiota abundance (genus Parasutterella id.2892)' on 'Lipocalin−1'

Batch 2−3417 : Gut microbiota abundance (genus Catenibacterium id.2153) on Neutrophil gelatinase−associated lipocalin
with a threshold of 1e−05

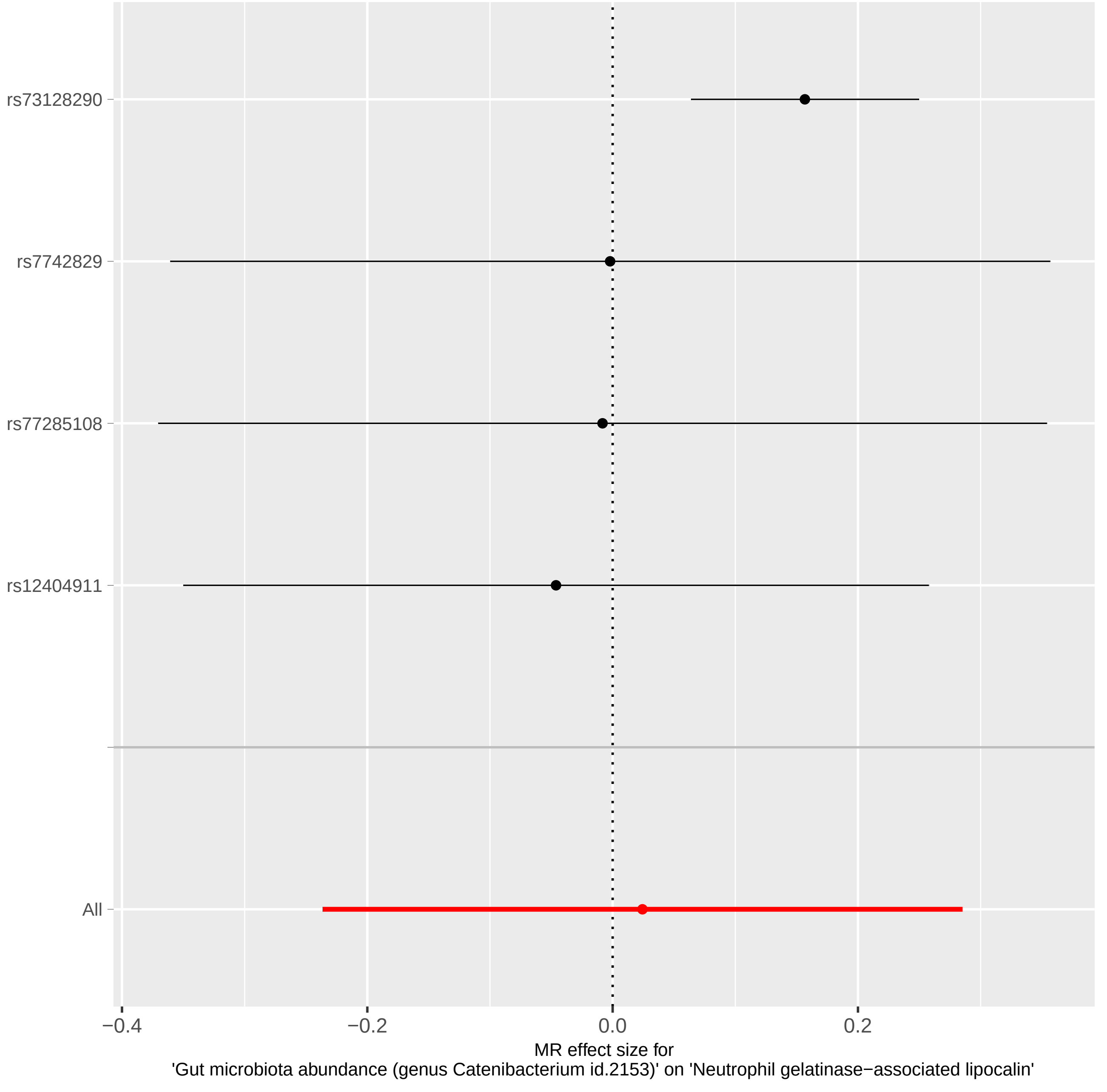

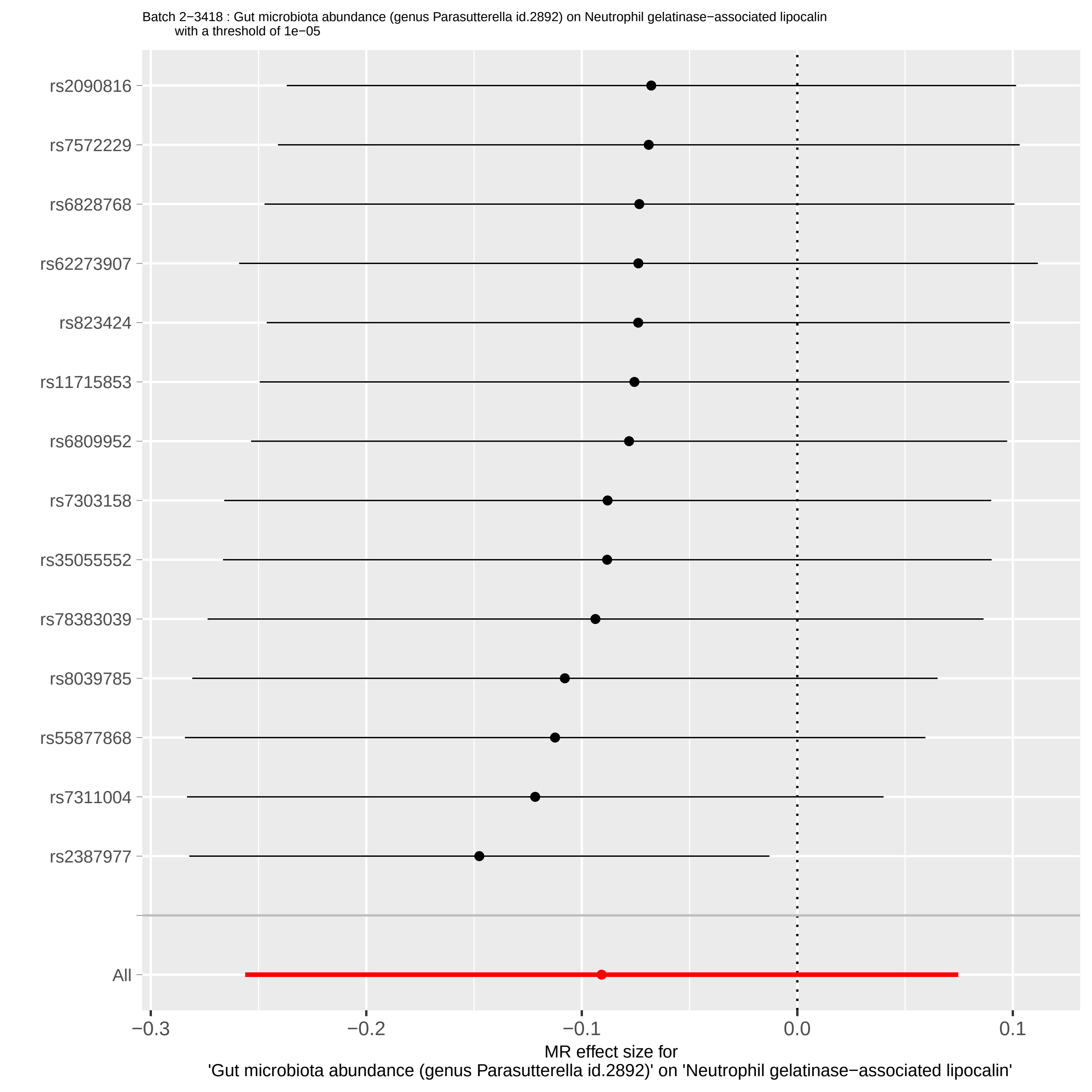
Batch 2−3418 : Gut microbiota abundance (genus Parasutterella id.2892) on Neutrophil gelatinase−associated lipocalin
with a threshold of 1e−05
rs2090816
rs7572229
rs6828768
rs62273907
rs823424
rs11715853
rs6809952
rs7303158
rs35055552
rs78383039
rs8039785
rs55877868
rs7311004
rs2387977
All
−0.3
−0.2
−0.1
0.0
0.1
MR effect size for
'Gut microbiota abundance (genus Parasutterella id.2892)' on 'Neutrophil gelatinase−associated lipocalin'

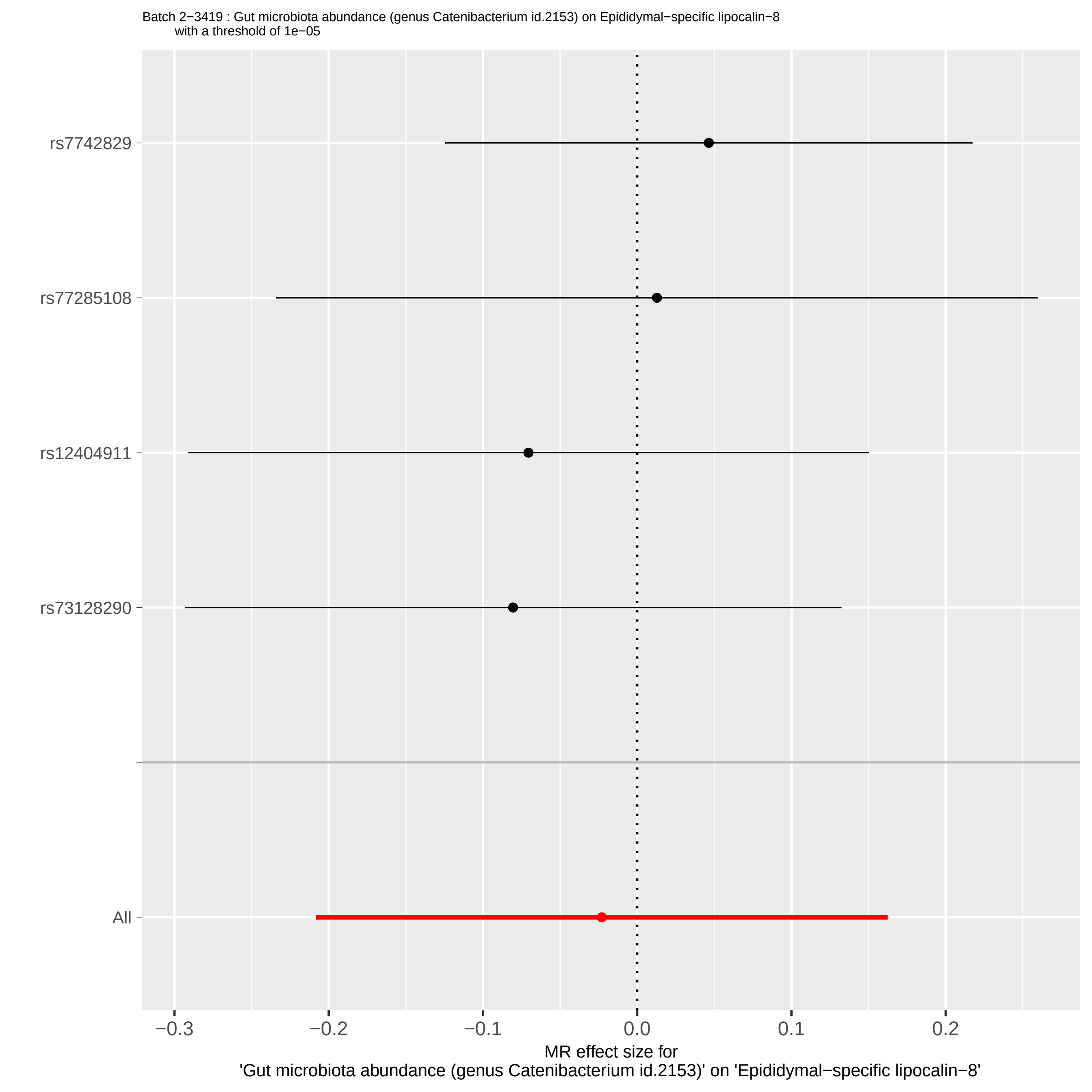

Batch 2−3419 : Gut microbiota abundance (genus Catenibacterium id.2153) on Epididymal−specific lipocalin−8
with a threshold of 1e−05
rs7742829
rs77285108
rs12404911
rs73128290
All
−0.3
−0.2
−0.1
0.0
0.1
0.2
MR effect size for
'Gut microbiota abundance (genus Catenibacterium id.2153)' on 'Epididymal−specific lipocalin−8'

Batch 2−3420 : Gut microbiota abundance (genus Parasutterella id.2892) on Epididymal−specific lipocalin−8
with a threshold of 1e−05

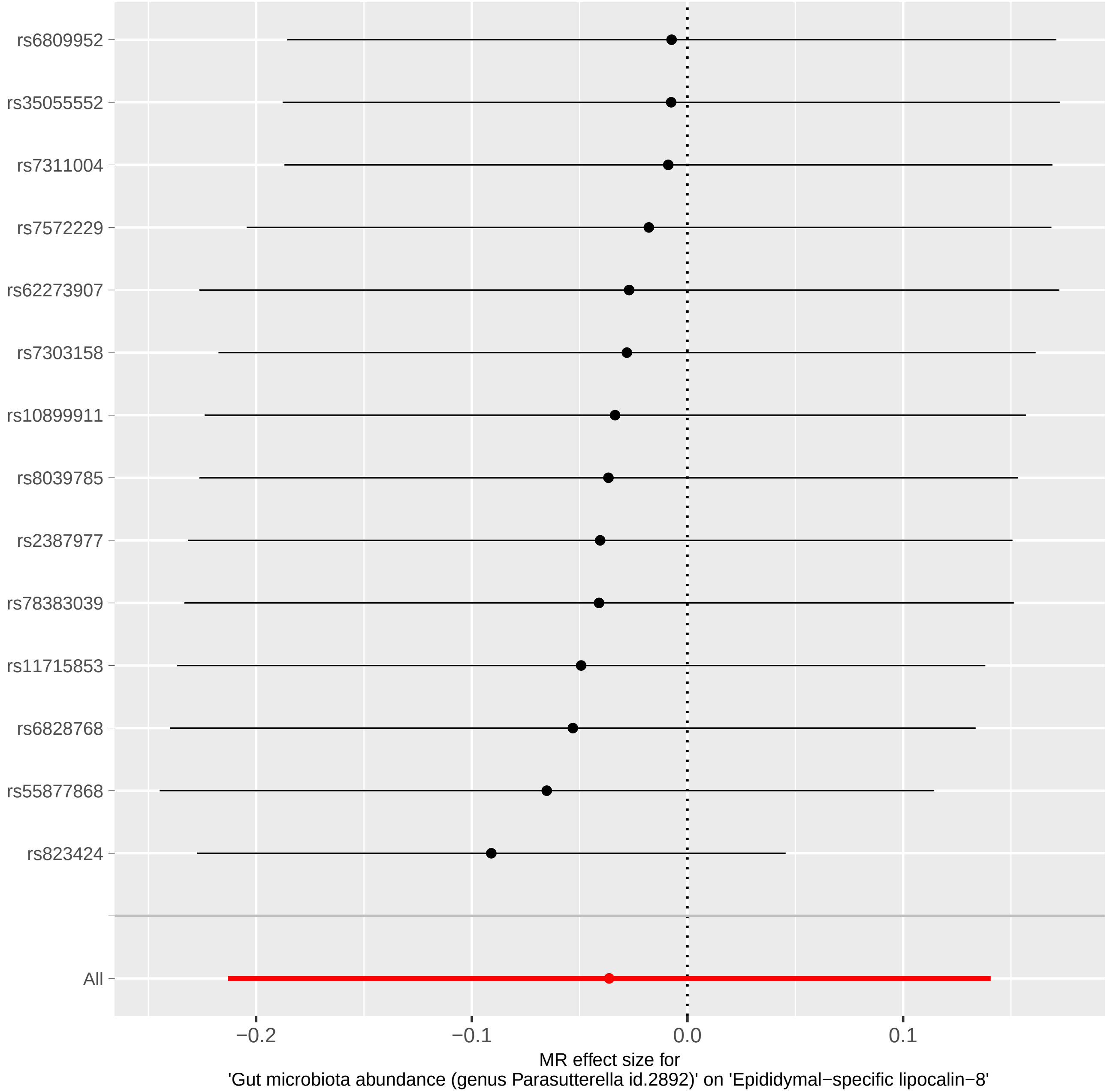

Batch 2−3421 : Gut microbiota abundance (genus Catenibacterium id.2153) on Ligand−dependent nuclear receptor corepressor−like protein
with a threshold of 1e−05

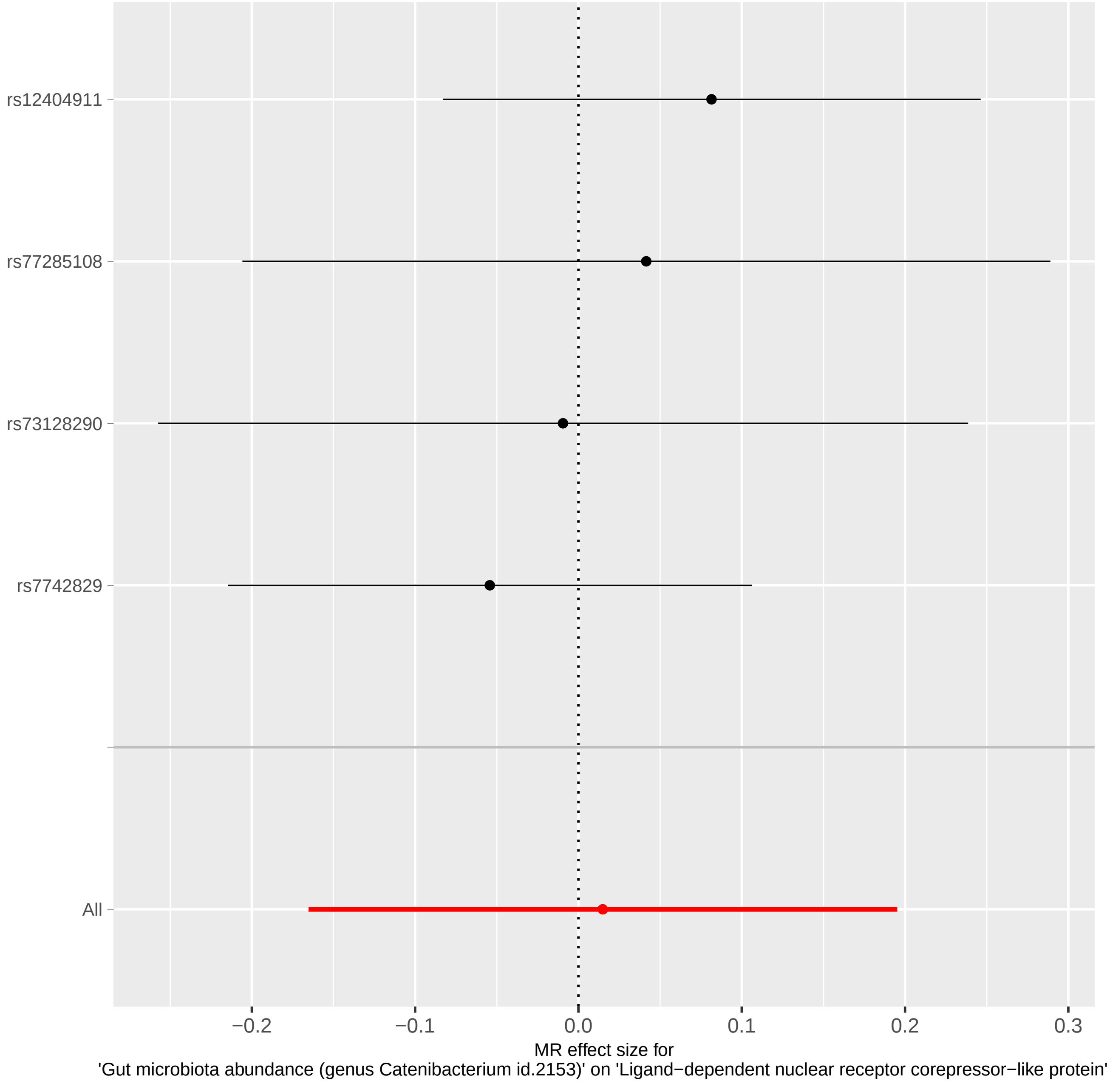

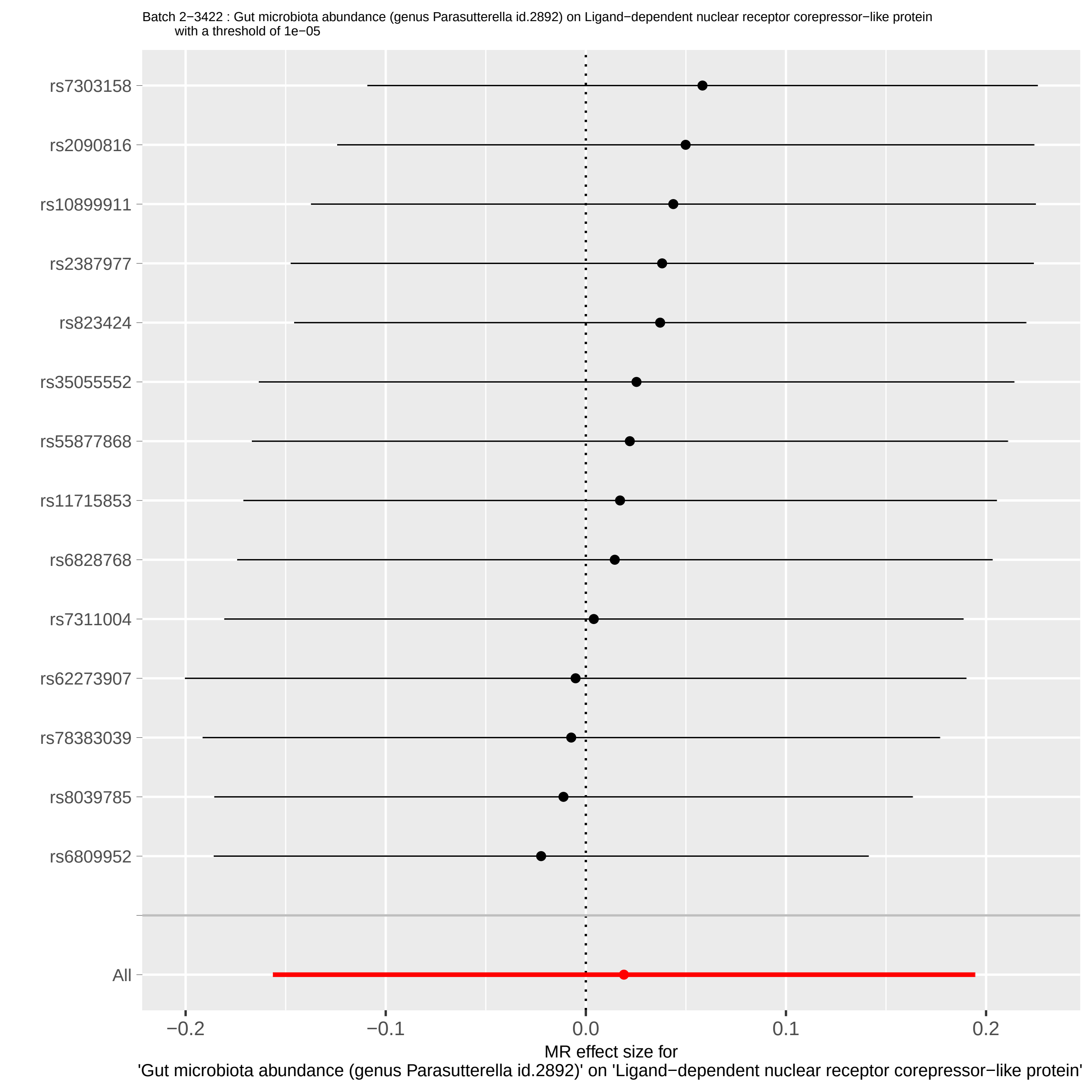
Batch 2−3422 : Gut microbiota abundance (genus Parasutterella id.2892) on Ligand−dependent nuclear receptor corepressor−like protein
with a threshold of 1e−05
rs7303158
rs2090816
rs10899911
rs2387977
rs823424
rs35055552
rs55877868
rs11715853
rs6828768
rs7311004
rs62273907
rs78383039
rs8039785
rs6809952
All
−0.2
−0.1
0.0
0.1
0.2
MR effect size for
'Gut microbiota abundance (genus Parasutterella id.2892)' on 'Ligand−dependent nuclear receptor corepressor−like protein'

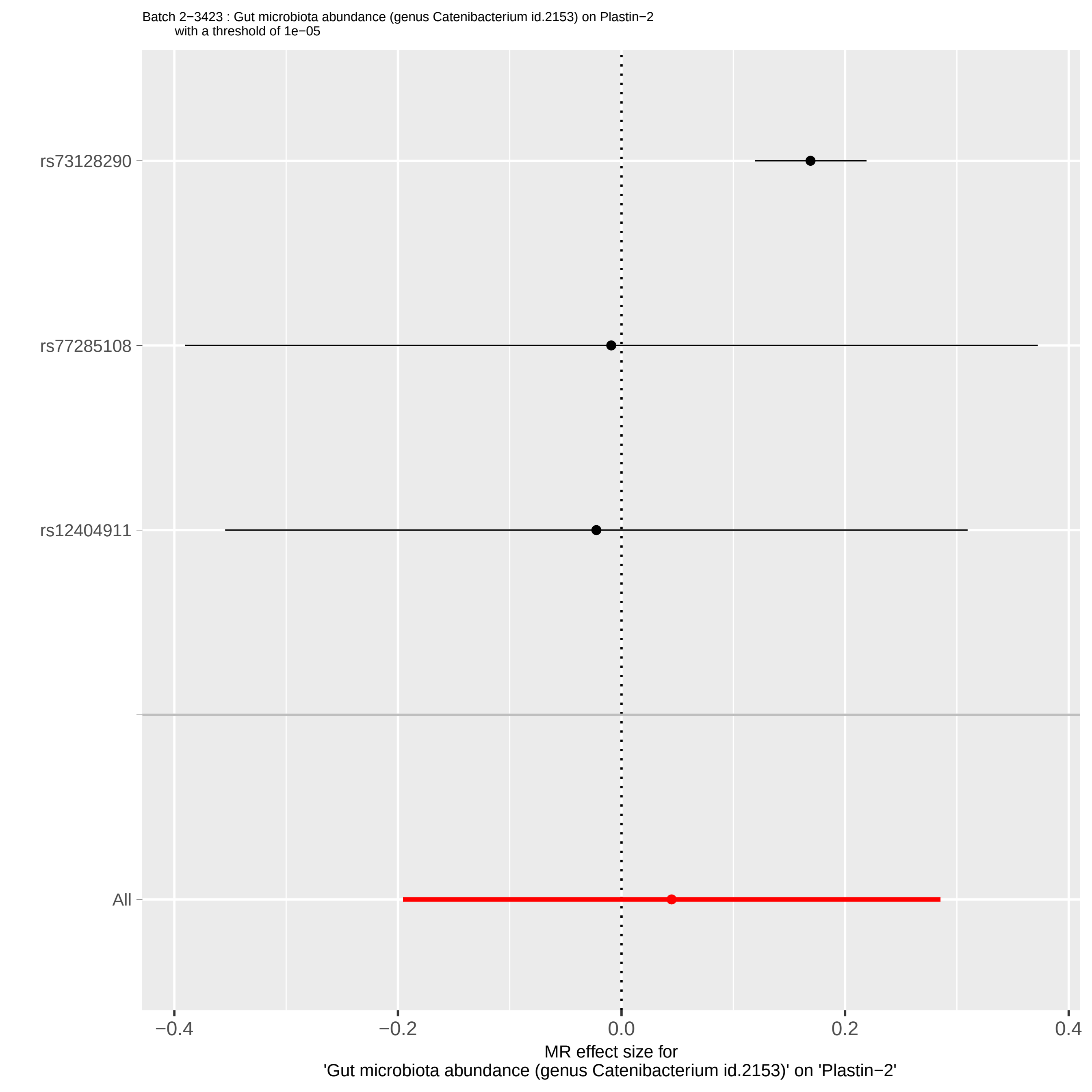
Batch 2−3423 : Gut microbiota abundance (genus Catenibacterium id.2153) on Plastin−2
with a threshold of 1e−05
rs73128290
rs77285108
rs12404911
All
−0.4
−0.2
0.0
0.2
0.4
MR effect size for
'Gut microbiota abundance (genus Catenibacterium id.2153)' on 'Plastin−2'

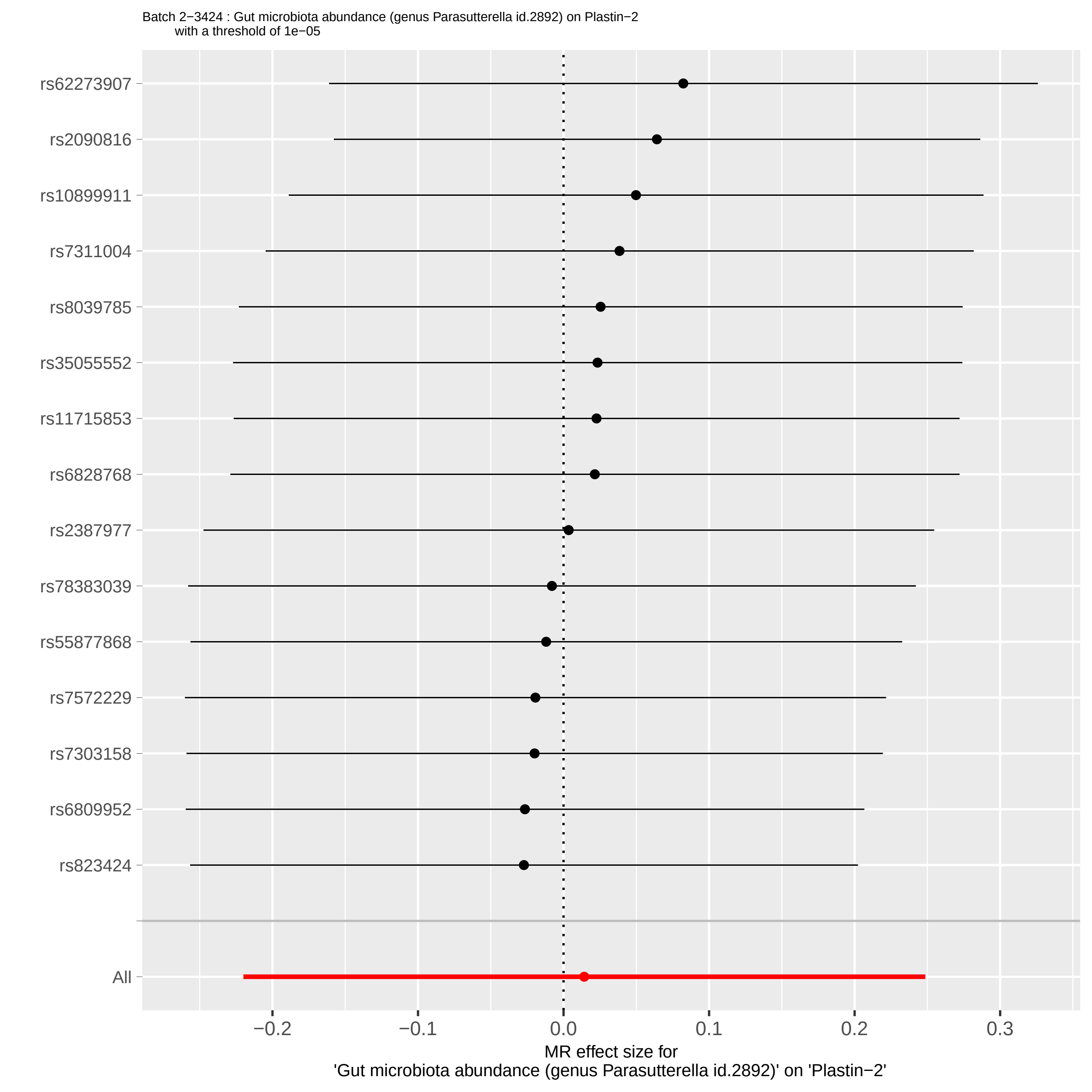

Batch 2−3424 : Gut microbiota abundance (genus Parasutterella id.2892) on Plastin−2
with a threshold of 1e−05
rs62273907
rs2090816
rs10899911
rs7311004
rs8039785
rs35055552
rs11715853
rs6828768
rs2387977
rs78383039
rs55877868
rs7572229
rs7303158
rs6809952
rs823424
All
−0.2
−0.1
0.0
0.1
0.2
0.3
MR effect size for
'Gut microbiota abundance (genus Parasutterella id.2892)' on 'Plastin−2'

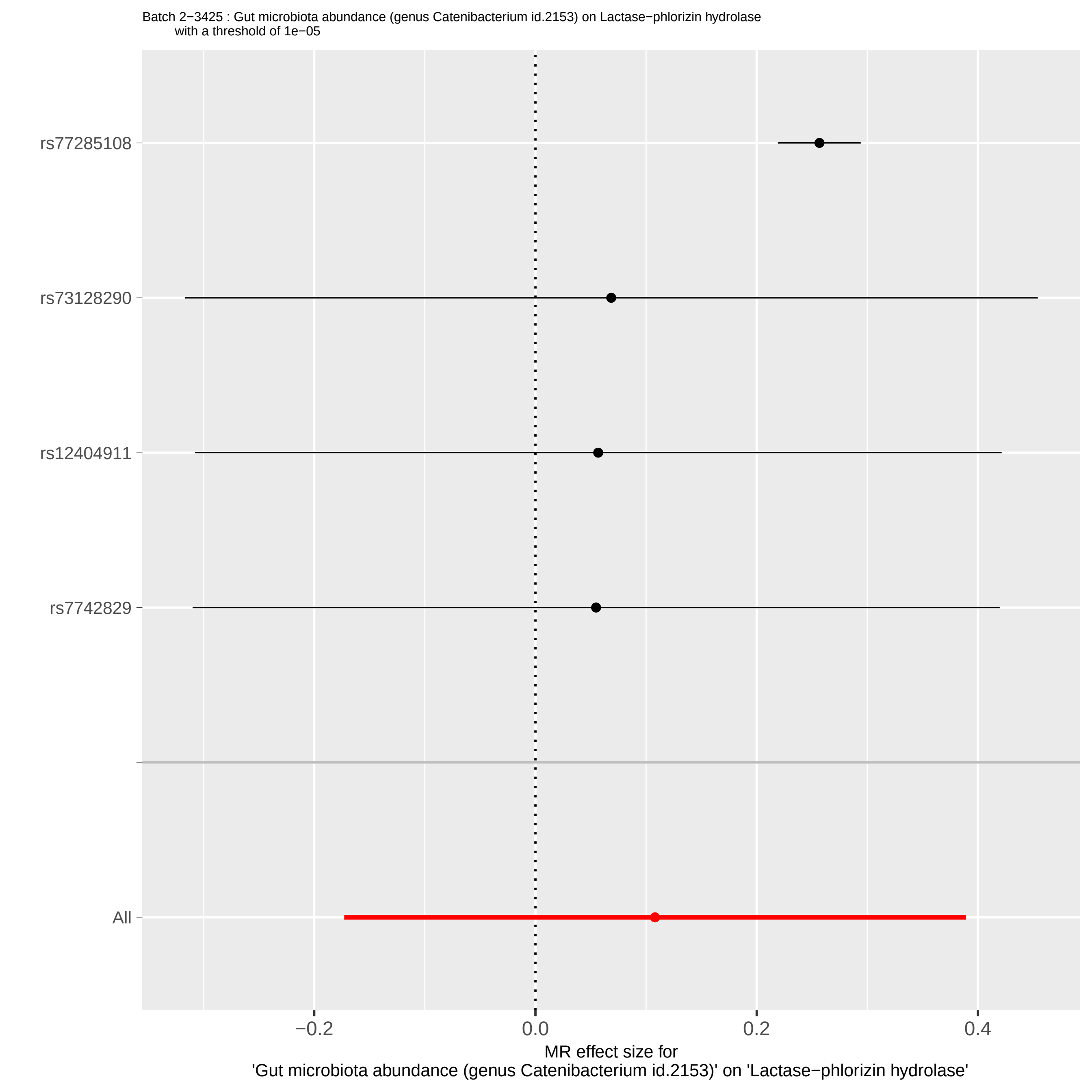

Batch 2−3425 : Gut microbiota abundance (genus Catenibacterium id.2153) on Lactase−phlorizin hydrolase
with a threshold of 1e−05
rs77285108
rs73128290
rs12404911
rs7742829
All
−0.2
0.0
0.2
0.4
MR effect size for
'Gut microbiota abundance (genus Catenibacterium id.2153)' on 'Lactase−phlorizin hydrolase'

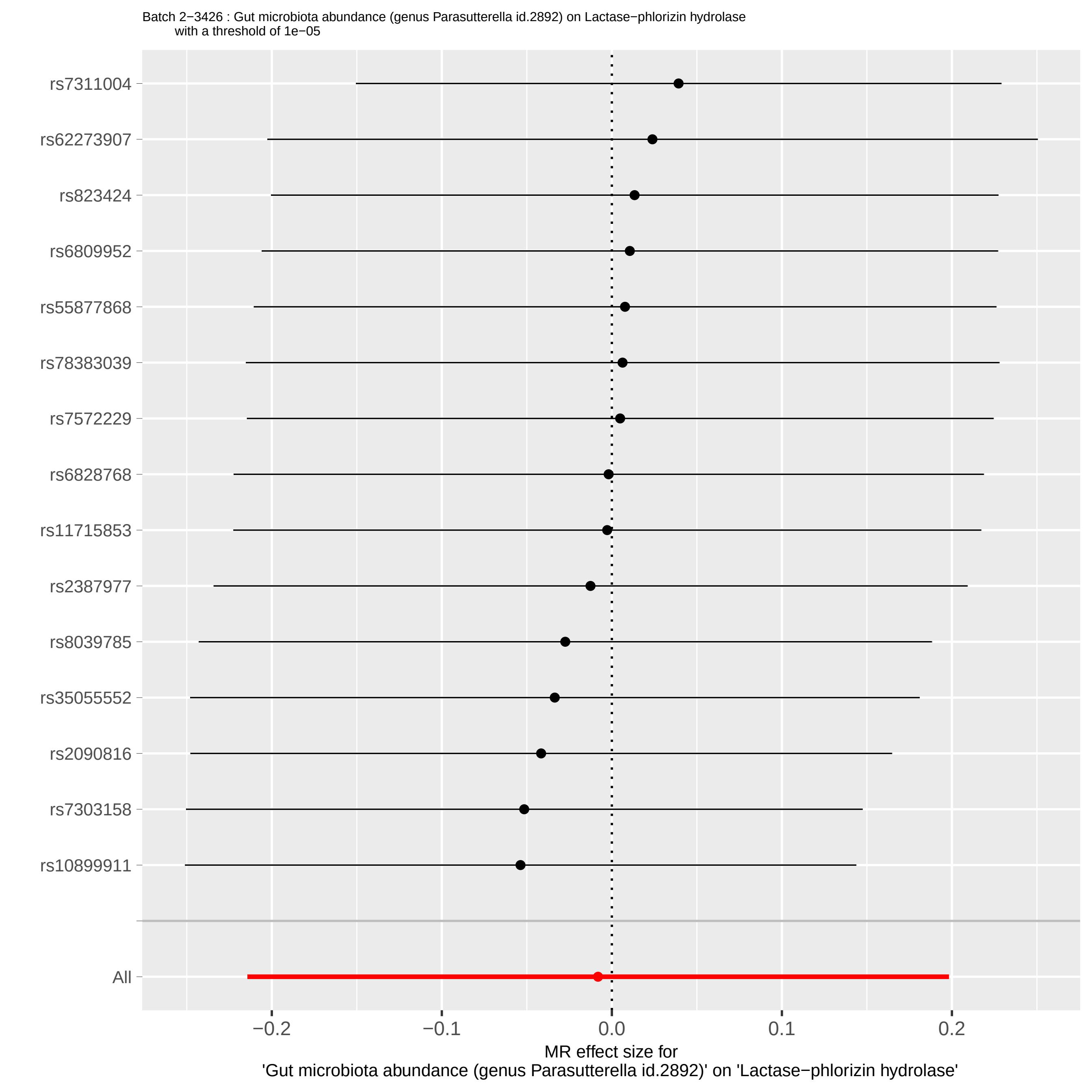
Batch 2−3426 : Gut microbiota abundance (genus Parasutterella id.2892) on Lactase−phlorizin hydrolase
with a threshold of 1e−05
rs7311004
rs62273907
rs823424
rs6809952
rs55877868
rs78383039
rs7572229
rs6828768
rs11715853
rs2387977
rs8039785
rs35055552
rs2090816
rs7303158
rs10899911
All
−0.2
−0.1
0.0
0.1
0.2
MR effect size for
'Gut microbiota abundance (genus Parasutterella id.2892)' on 'Lactase−phlorizin hydrolase'

Batch 2−3427 : Gut microbiota abundance (genus Catenibacterium id.2153) on Lactase−like protein
with a threshold of 1e−05

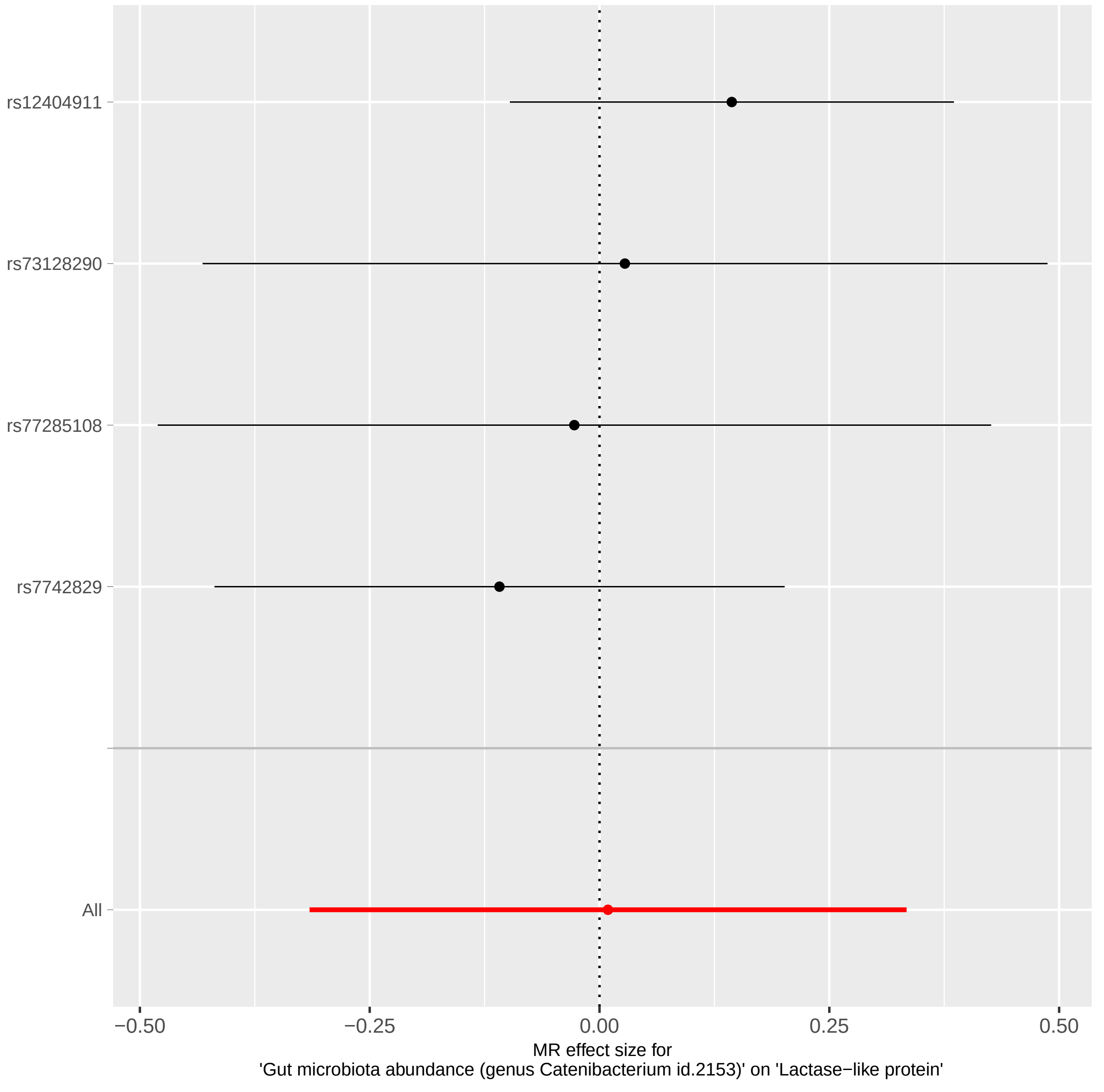

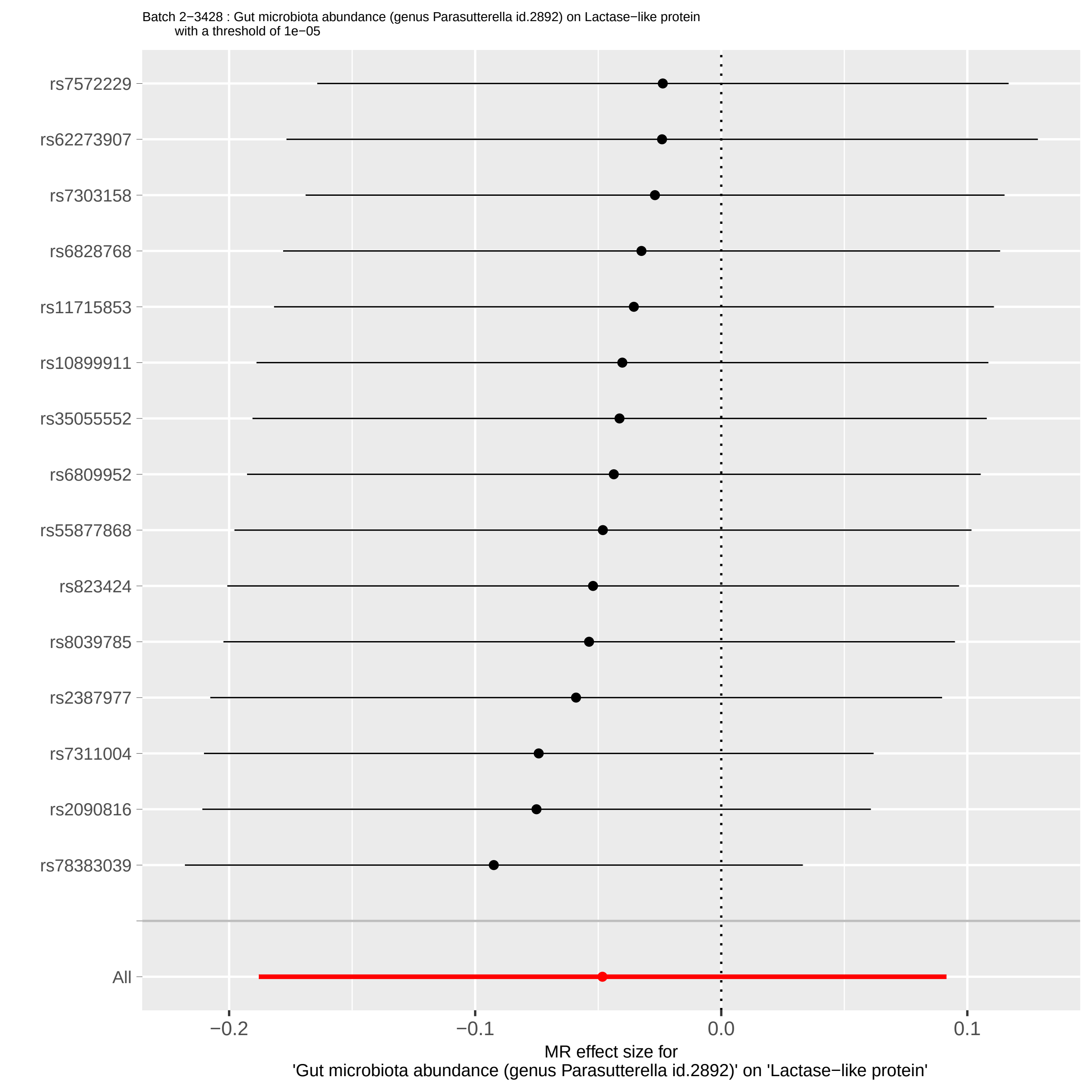

Batch 2−3428 : Gut microbiota abundance (genus Parasutterella id.2892) on Lactase−like protein
with a threshold of 1e−05
rs7572229
rs62273907
rs7303158
rs6828768
rs11715853
rs10899911
rs35055552
rs6809952
rs55877868
rs823424
rs8039785
rs2387977
rs7311004
rs2090816
rs78383039
All
−0.2
−0.1
0.0
0.1
MR effect size for
'Gut microbiota abundance (genus Parasutterella id.2892)' on 'Lactase−like protein'

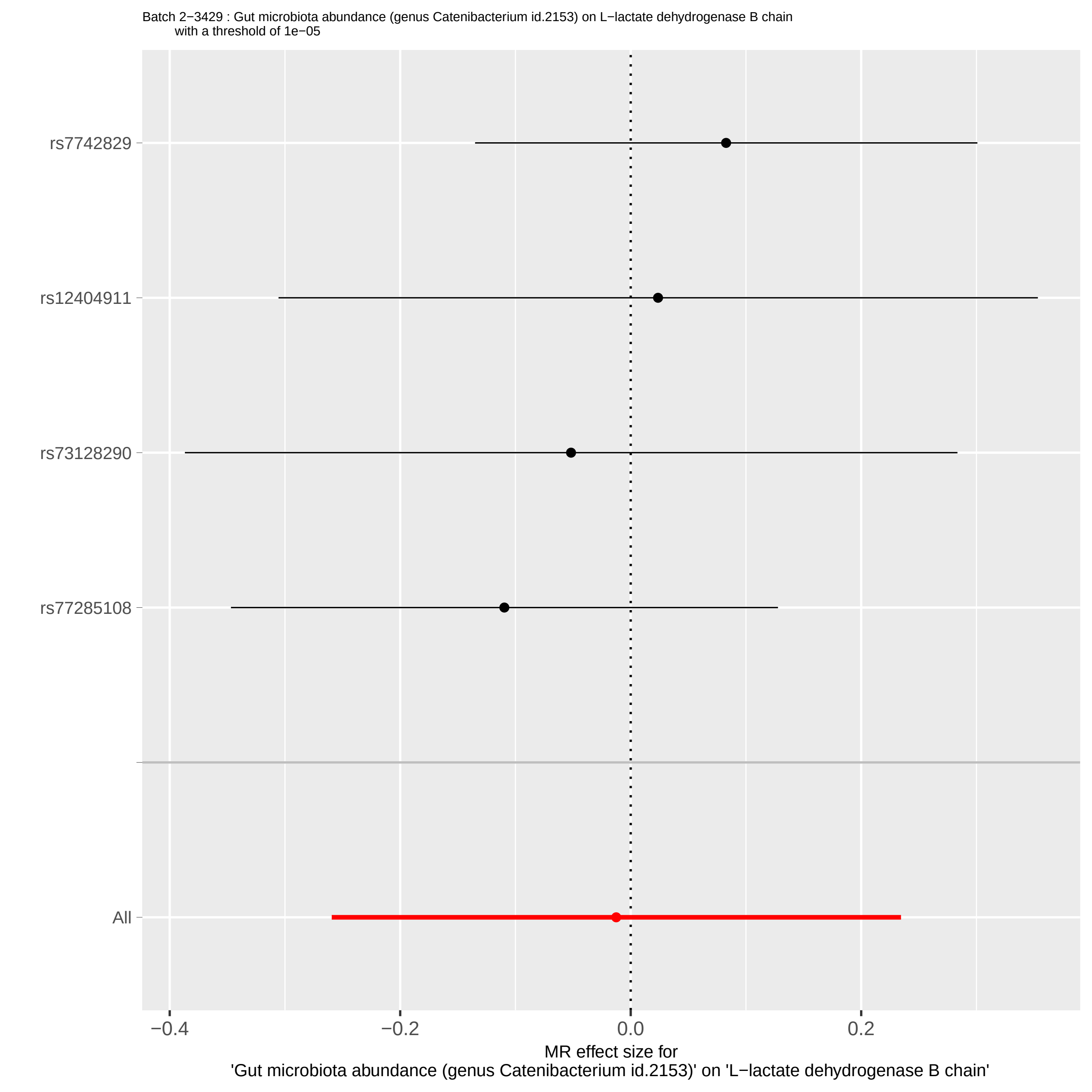

Batch 2−3429 : Gut microbiota abundance (genus Catenibacterium id.2153) on L−lactate dehydrogenase B chain
with a threshold of 1e−05
rs7742829
rs12404911
rs73128290
rs77285108
All
−0.4
−0.2
0.0
0.2
MR effect size for
'Gut microbiota abundance (genus Catenibacterium id.2153)' on 'L−lactate dehydrogenase B chain'

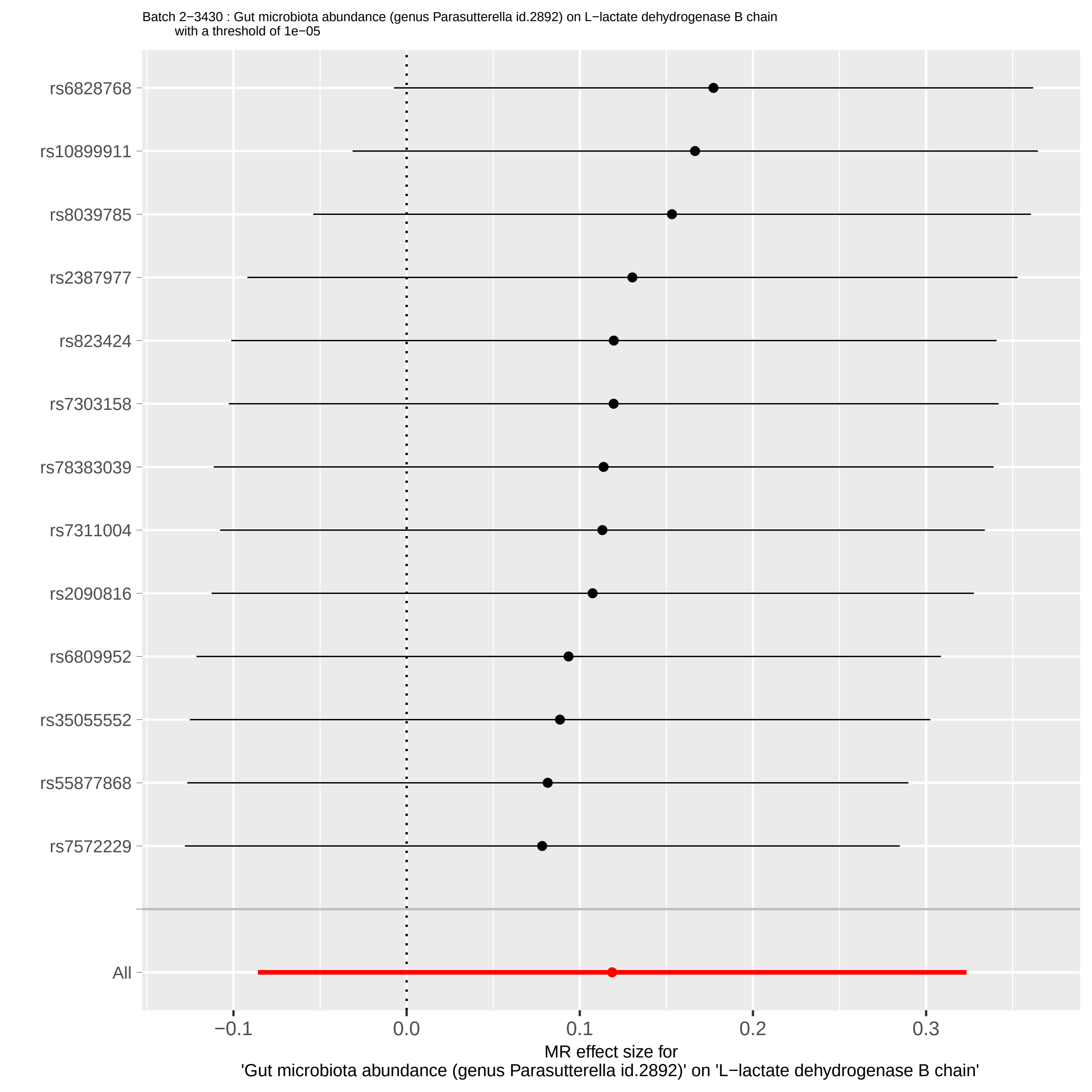

Batch 2−3430 : Gut microbiota abundance (genus Parasutterella id.2892) on L−lactate dehydrogenase B chain
with a threshold of 1e−05
rs6828768
rs10899911
rs8039785
rs2387977
rs823424
rs7303158
rs78383039
rs7311004
rs2090816
rs6809952
rs35055552
rs55877868
rs7572229
All
−0.1
0.0
0.1
0.2
0.3
MR effect size for
'Gut microbiota abundance (genus Parasutterella id.2892)' on 'L−lactate dehydrogenase B chain'

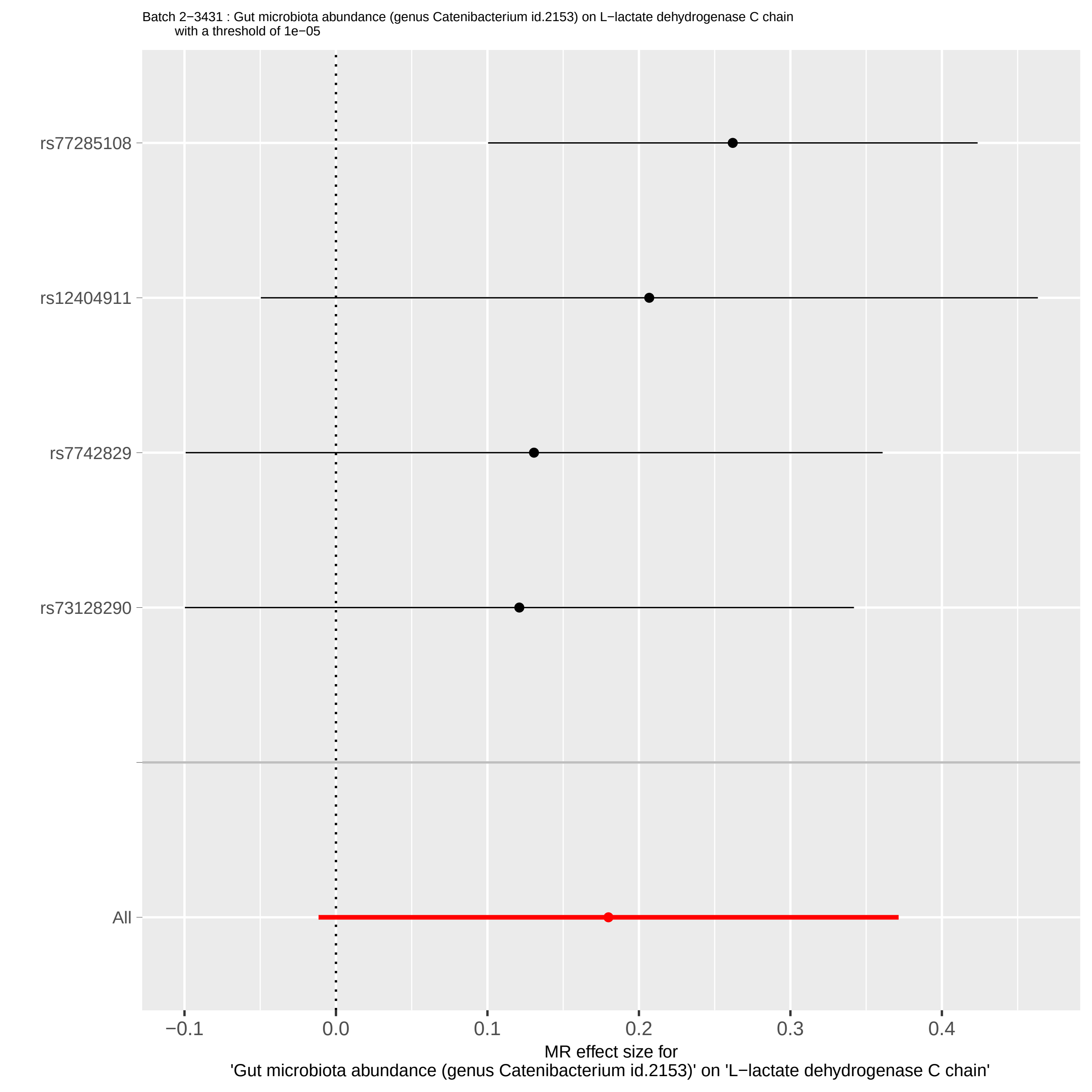

Batch 2−3431 : Gut microbiota abundance (genus Catenibacterium id.2153) on L−lactate dehydrogenase C chain
with a threshold of 1e−05
rs77285108
rs12404911
rs7742829
rs73128290
All
−0.1
0.0
0.1
0.2
0.3
0.4
MR effect size for
'Gut microbiota abundance (genus Catenibacterium id.2153)' on 'L−lactate dehydrogenase C chain'

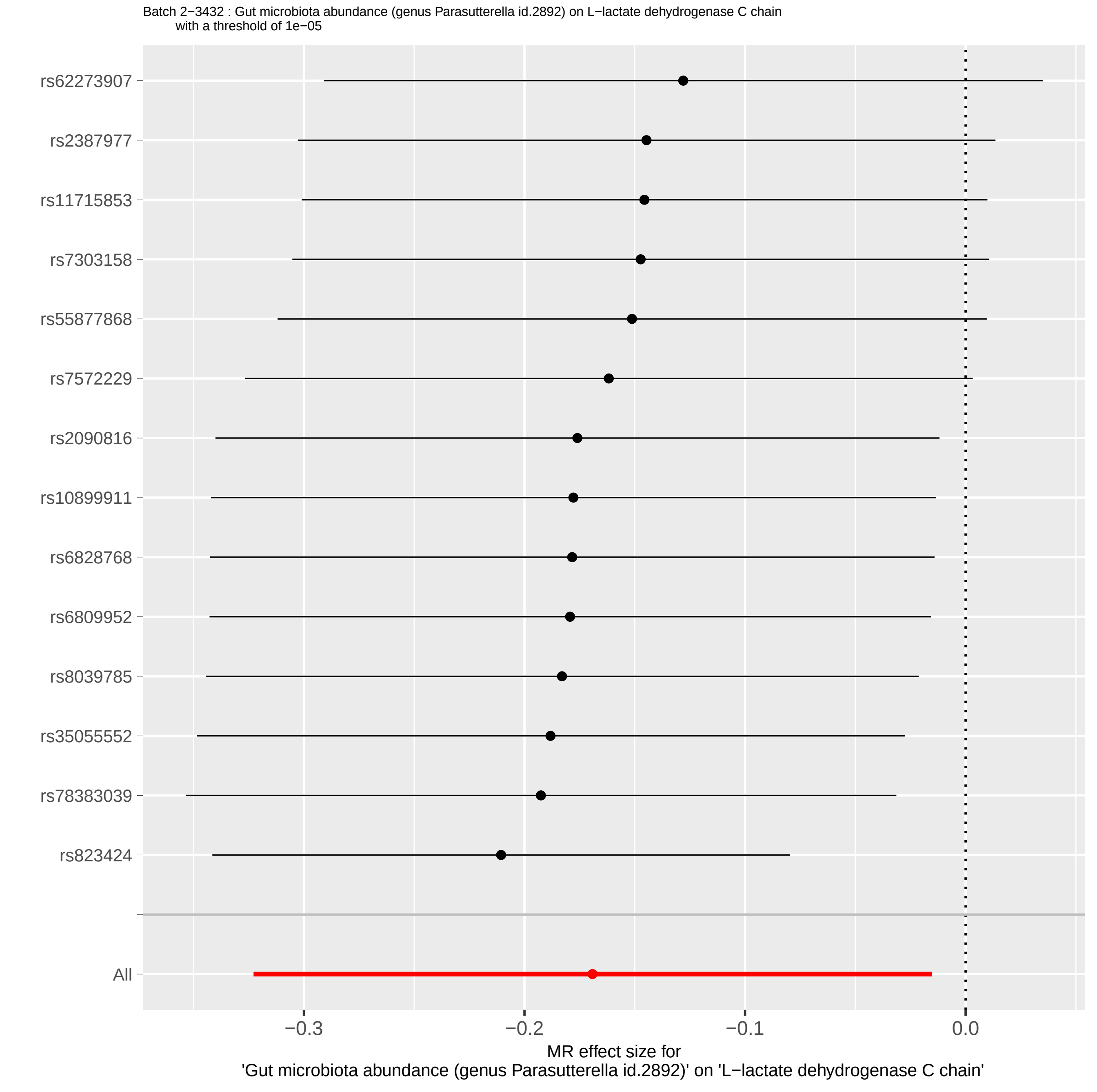
Batch 2−3432 : Gut microbiota abundance (genus Parasutterella id.2892) on L−lactate dehydrogenase C chain
with a threshold of 1e−05
rs62273907
rs2387977
rs11715853
rs7303158
rs55877868
rs7572229
rs2090816
rs10899911
rs6828768
rs6809952
rs8039785
rs35055552
rs78383039
rs823424
All
−0.3
−0.2
−0.1
0.0
MR effect size for
'Gut microbiota abundance (genus Parasutterella id.2892)' on 'L−lactate dehydrogenase C chain'

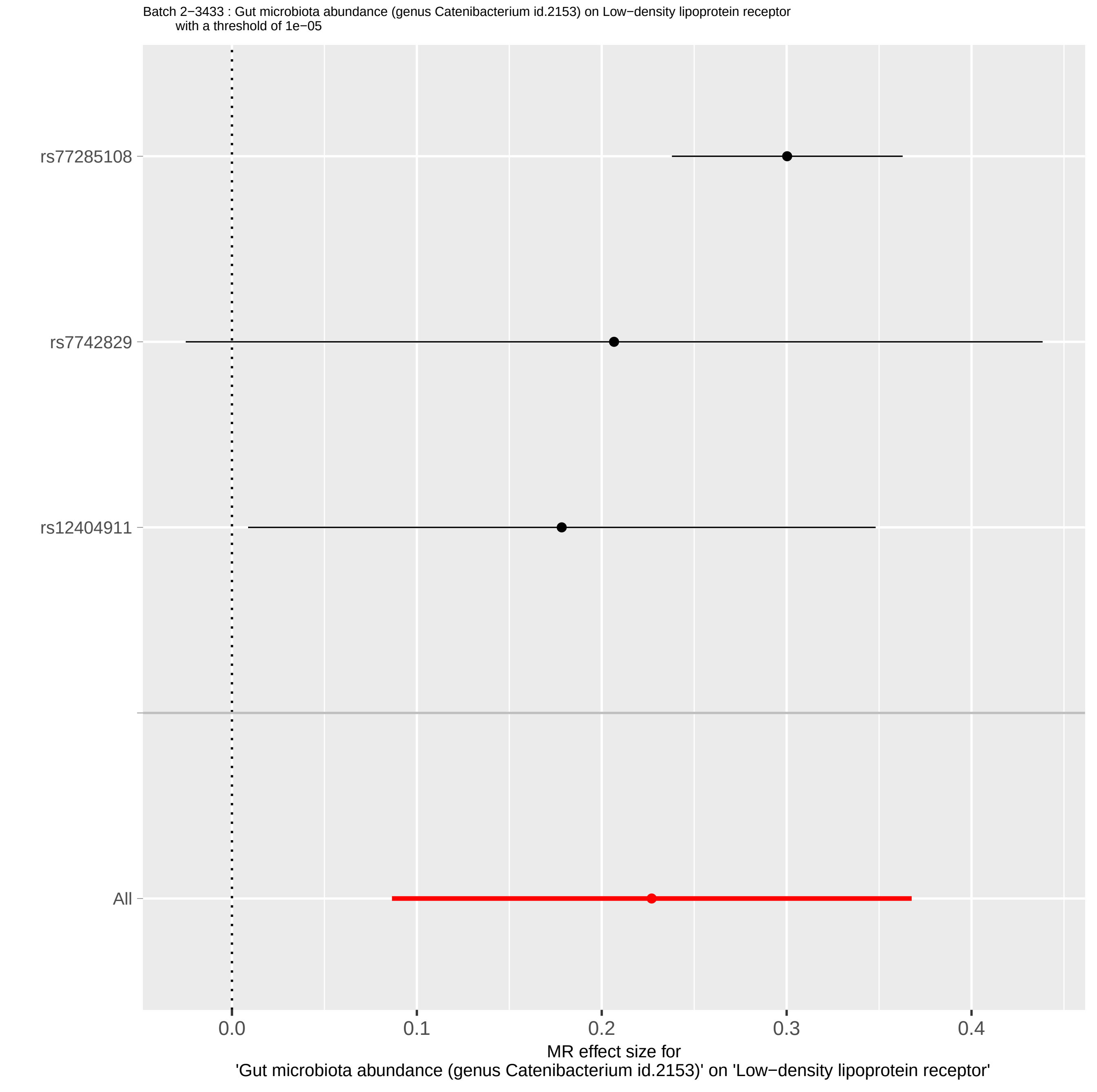
Batch 2−3433 : Gut microbiota abundance (genus Catenibacterium id.2153) on Low−density lipoprotein receptor
with a threshold of 1e−05
rs77285108
rs7742829
rs12404911
All
0.0
0.1
0.2
0.3
0.4
MR effect size for
'Gut microbiota abundance (genus Catenibacterium id.2153)' on 'Low−density lipoprotein receptor'

Batch 2−3434 : Gut microbiota abundance (genus Parasutterella id.2892) on Low−density lipoprotein receptor
with a threshold of 1e−05

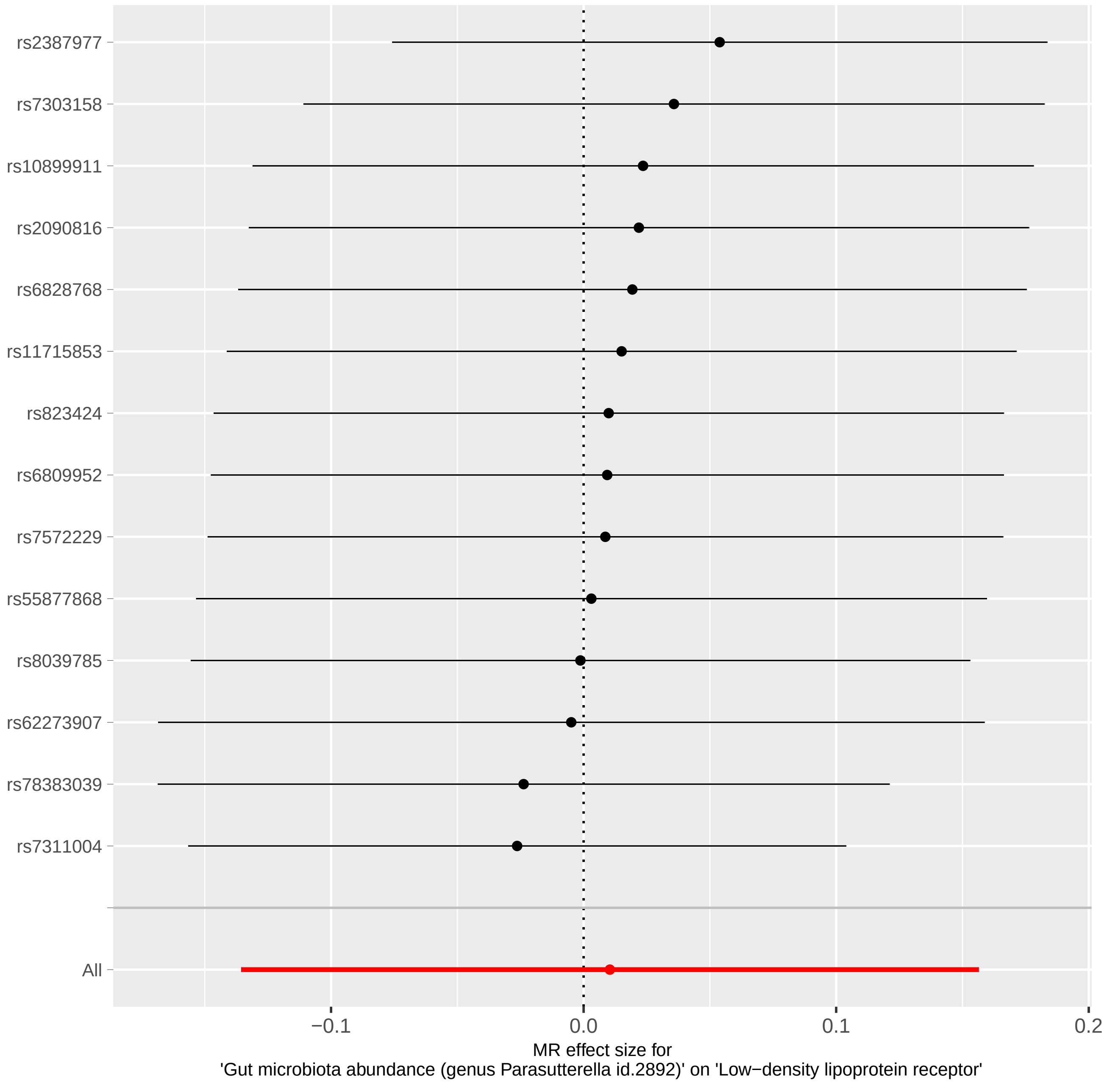

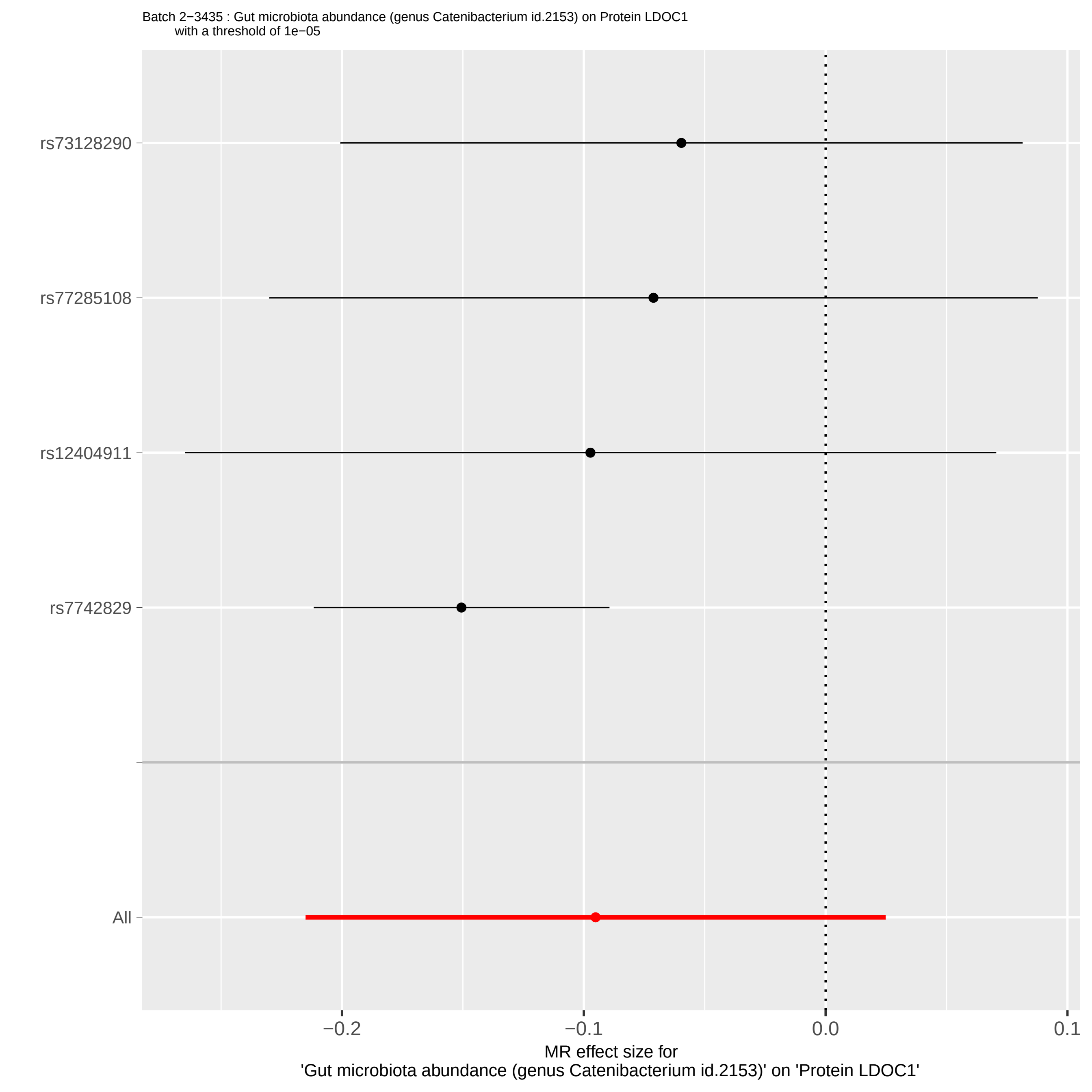
Batch 2−3435 : Gut microbiota abundance (genus Catenibacterium id.2153) on Protein LDOC1
with a threshold of 1e−05
rs73128290
rs77285108
rs12404911
rs7742829
All
−0.2
−0.1
0.0
0.1
MR effect size for
'Gut microbiota abundance (genus Catenibacterium id.2153)' on 'Protein LDOC1'

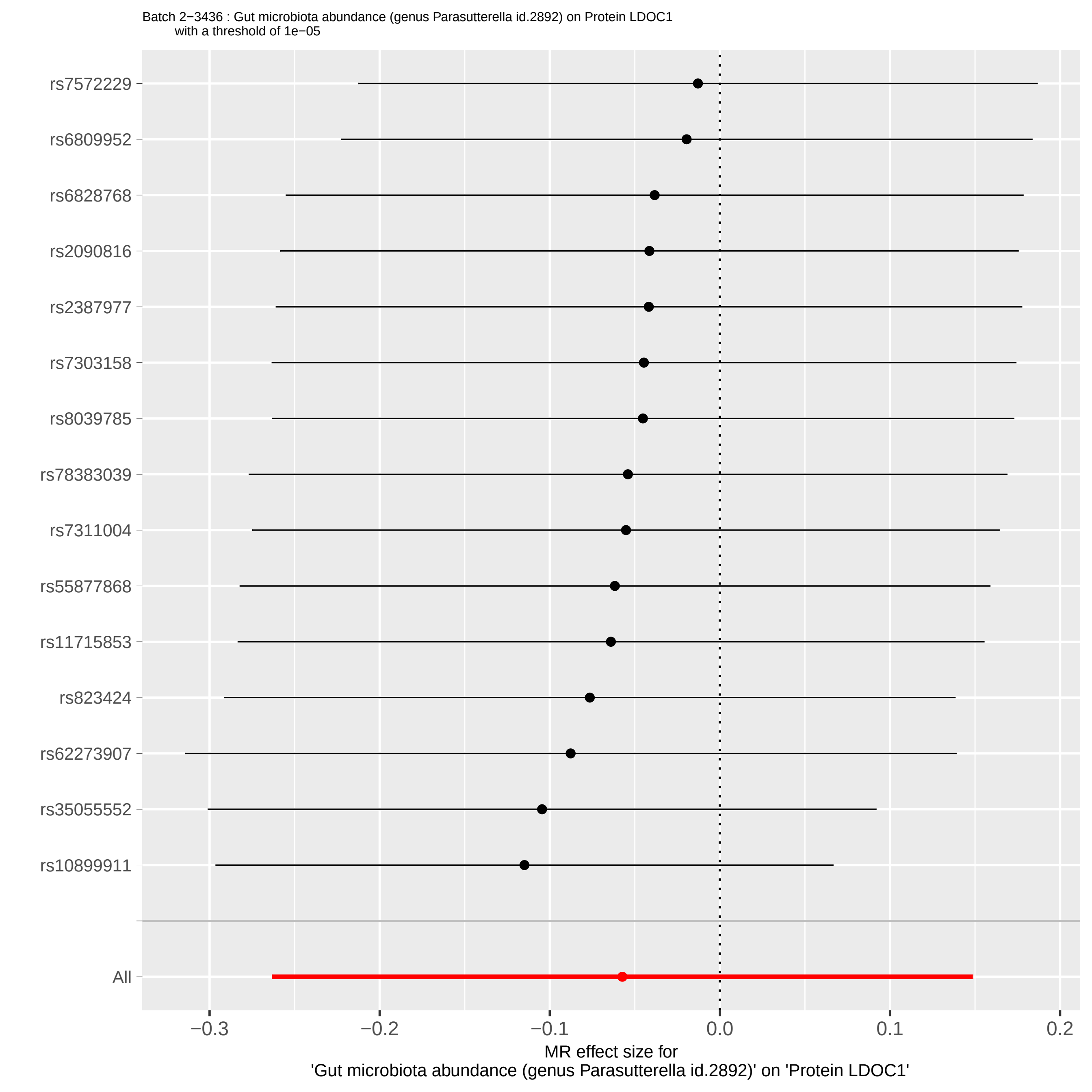

Batch 2−3436 : Gut microbiota abundance (genus Parasutterella id.2892) on Protein LDOC1
with a threshold of 1e−05
rs7572229
rs6809952
rs6828768
rs2090816
rs2387977
rs7303158
rs8039785
rs78383039
rs7311004
rs55877868
rs11715853
rs823424
rs62273907
rs35055552
rs10899911
All
−0.3
−0.2
−0.1
0.0
0.1
0.2
MR effect size for
'Gut microbiota abundance (genus Parasutterella id.2892)' on 'Protein LDOC1'

Batch 2−3437 : Gut microbiota abundance (genus Catenibacterium id.2153) on Leukocyte cell−derived chemotaxin 1
with a threshold of 1e−05

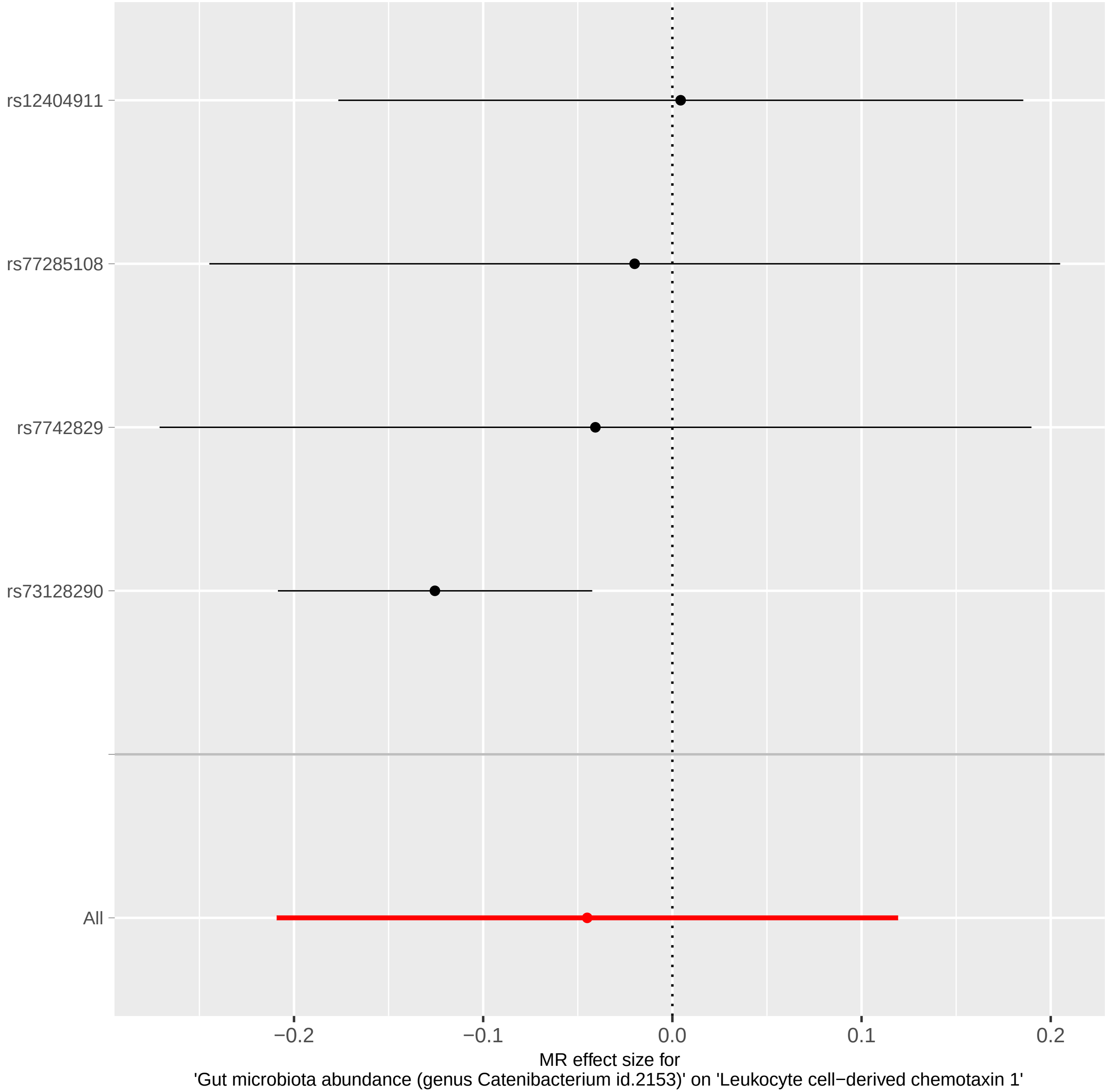

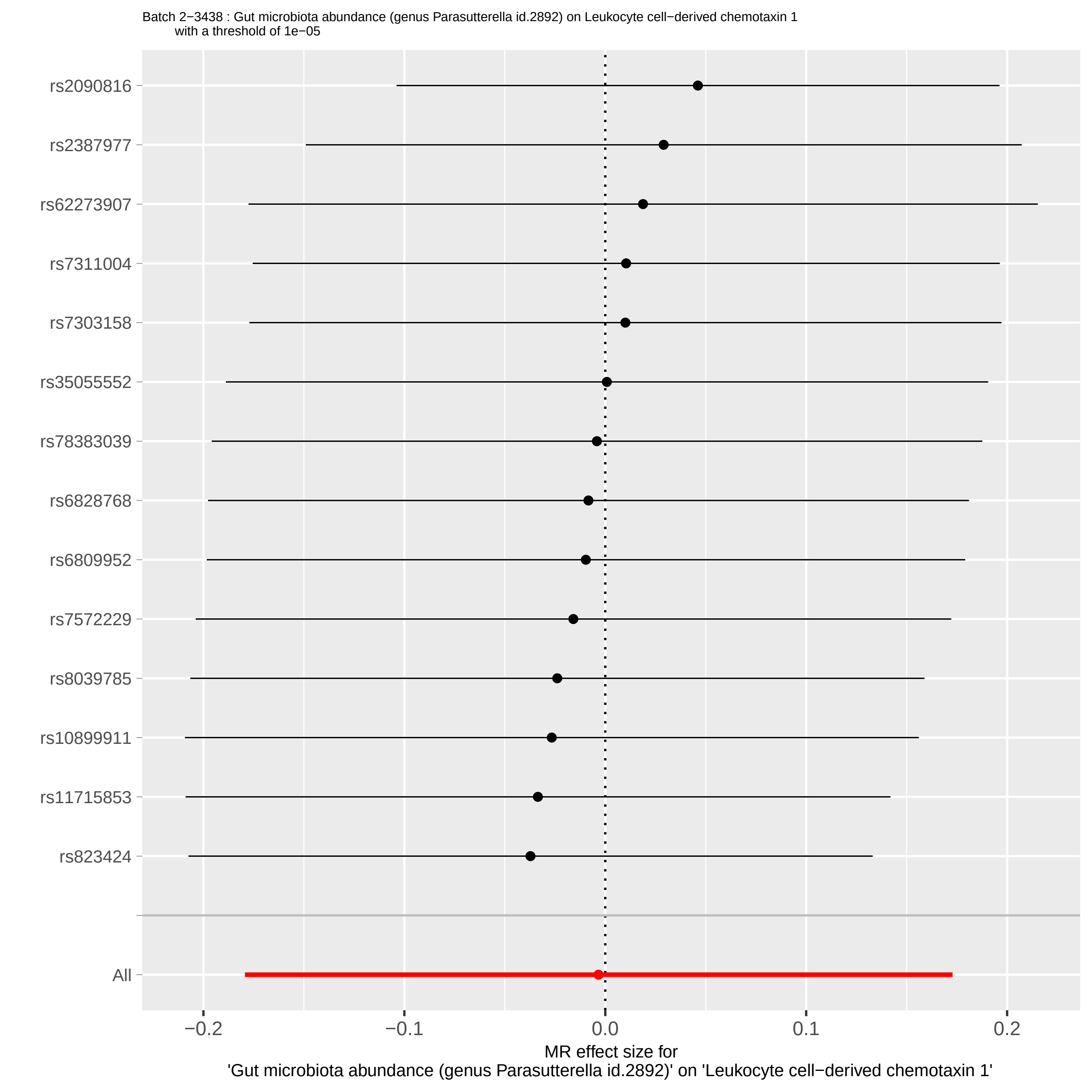
Batch 2−3438 : Gut microbiota abundance (genus Parasutterella id.2892) on Leukocyte cell−derived chemotaxin 1
with a threshold of 1e−05
rs2090816
rs2387977
rs62273907
rs7311004
rs7303158
rs35055552
rs78383039
rs6828768
rs6809952
rs7572229
rs8039785
rs10899911
rs11715853
rs823424
All
−0.2
−0.1
0.0
0.1
0.2
MR effect size for
'Gut microbiota abundance (genus Parasutterella id.2892)' on 'Leukocyte cell−derived chemotaxin 1'

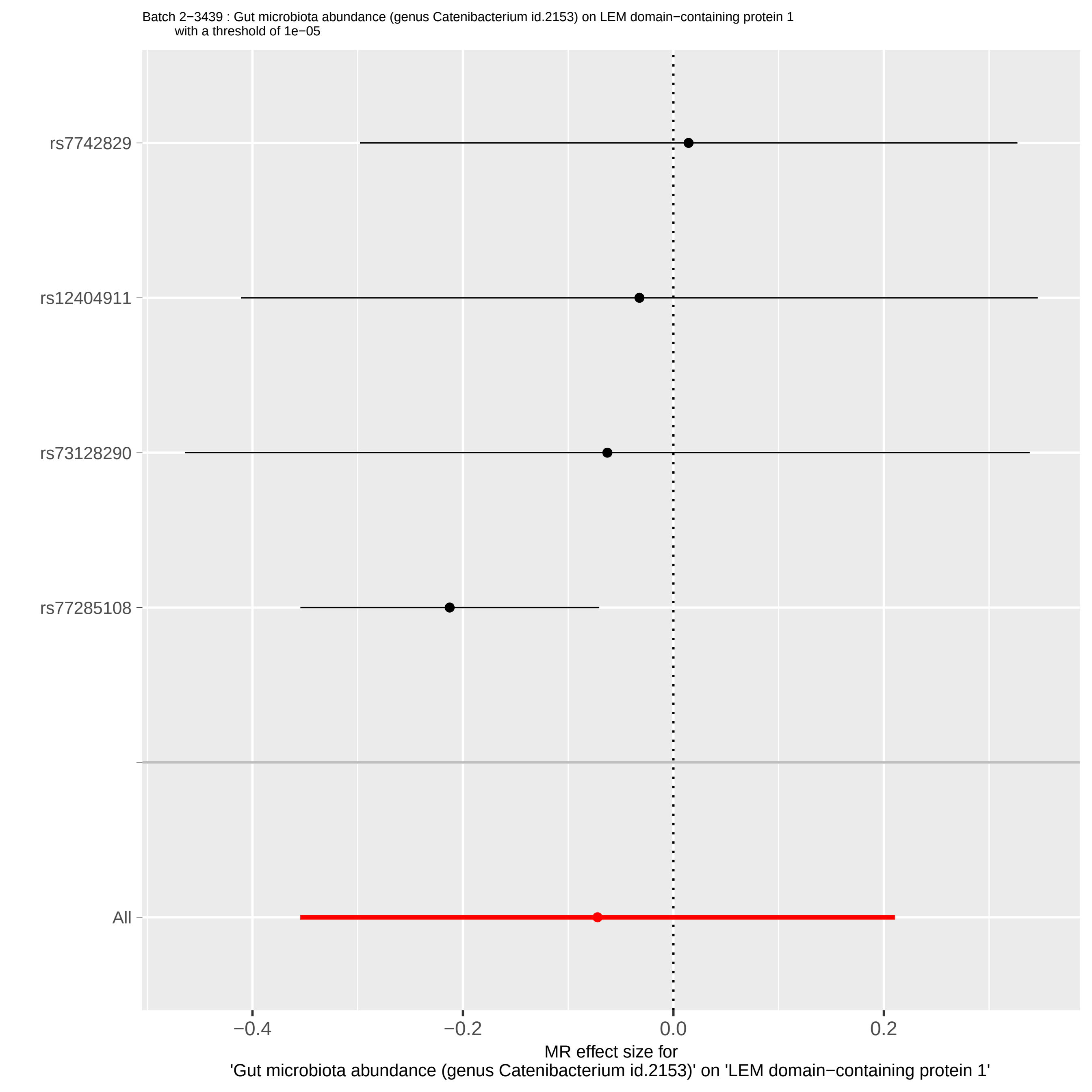

Batch 2−3439 : Gut microbiota abundance (genus Catenibacterium id.2153) on LEM domain−containing protein 1
with a threshold of 1e−05
rs7742829
rs12404911
rs73128290
rs77285108
All
−0.4
−0.2
0.0
0.2
MR effect size for
'Gut microbiota abundance (genus Catenibacterium id.2153)' on 'LEM domain−containing protein 1'

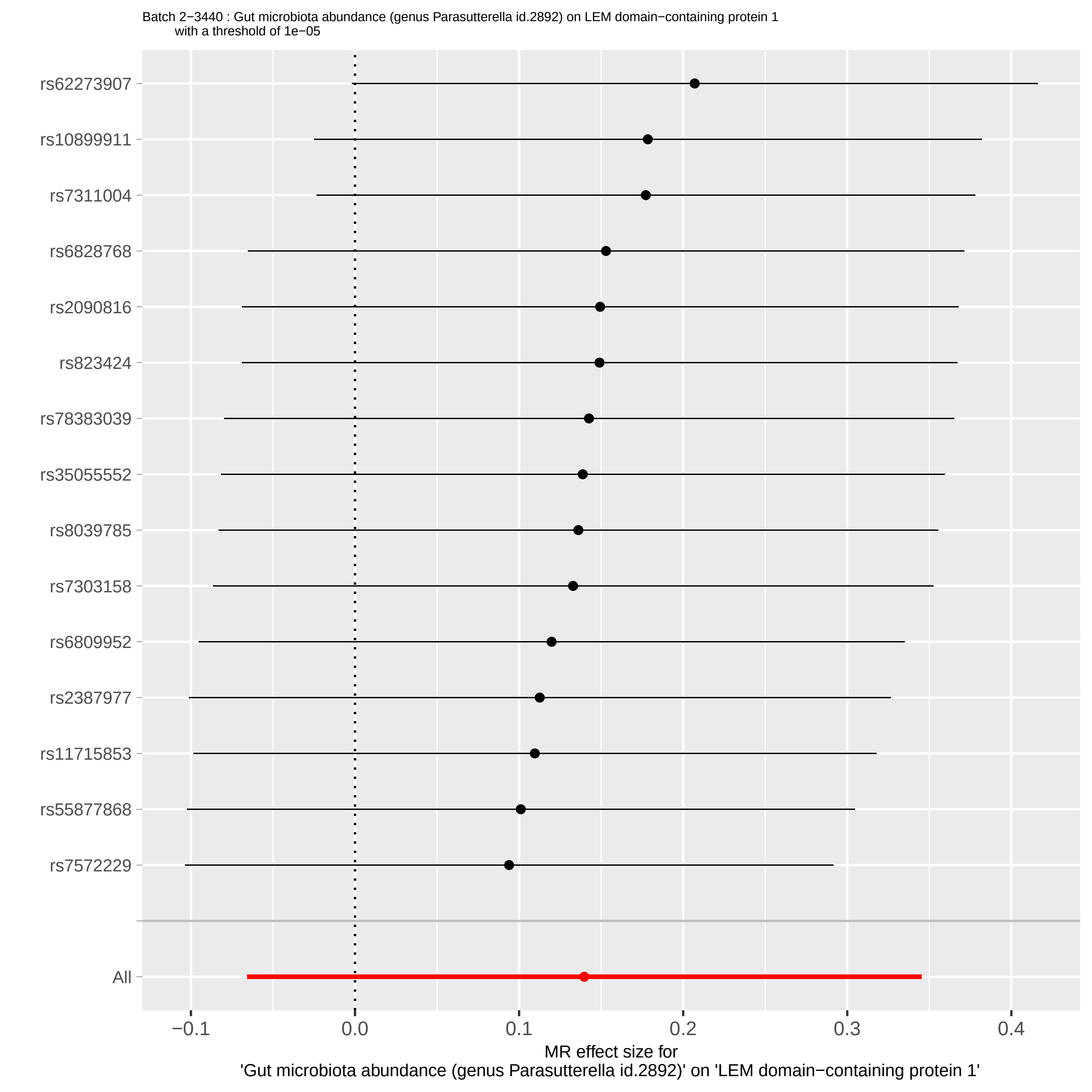

Batch 2−3440 : Gut microbiota abundance (genus Parasutterella id.2892) on LEM domain−containing protein 1 with a threshold of 1e−05
rs62273907
rs10899911
rs7311004
rs6828768
rs2090816
rs823424
rs78383039
rs35055552
rs8039785
rs7303158
rs6809952
rs2387977
rs11715853
rs55877868
rs7572229
All
−0.1
0.0
0.1
0.2
0.3
0.4
MR effect size for
'Gut microbiota abundance (genus Parasutterella id.2892)' on 'LEM domain−containing protein 1'

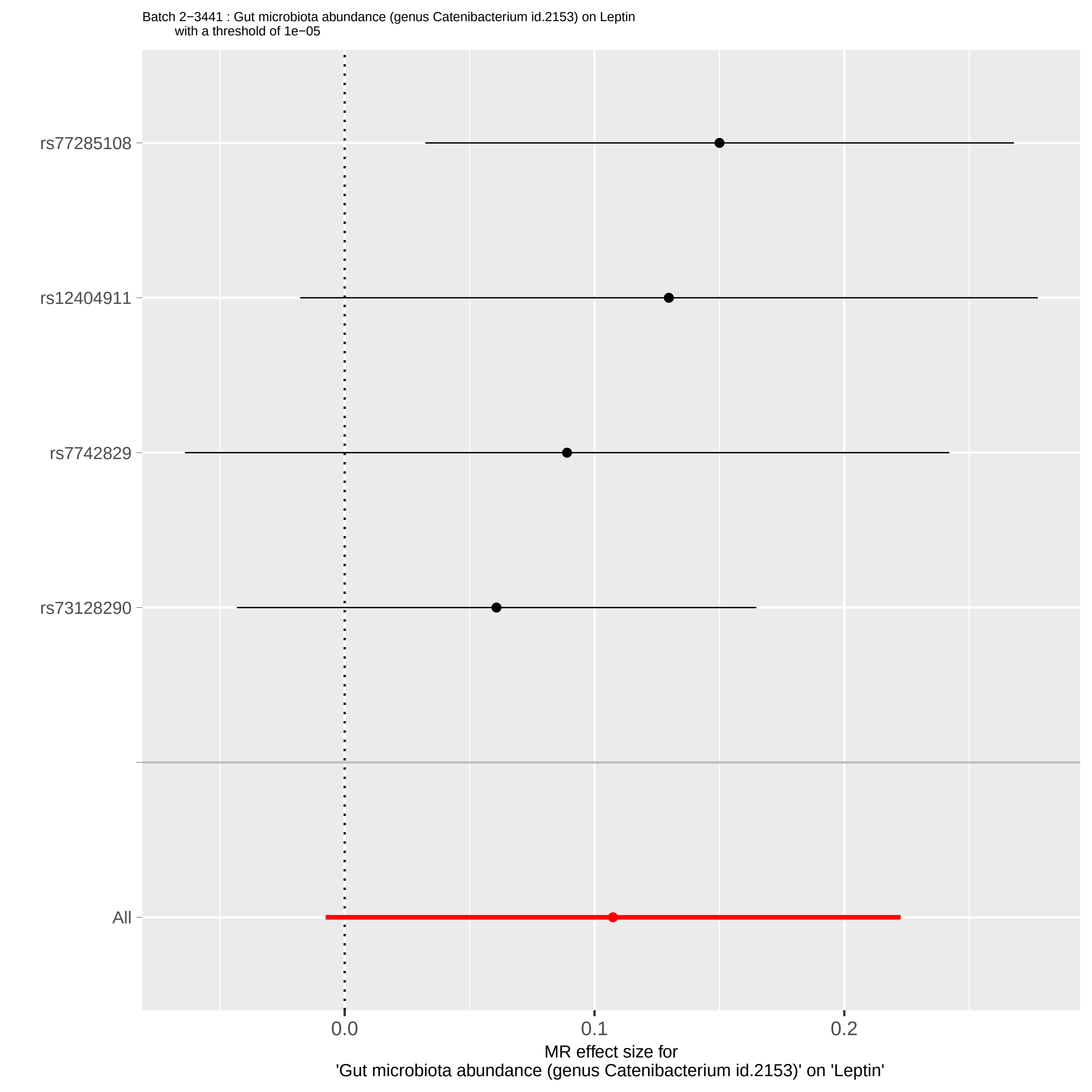
Batch 2−3441 : Gut microbiota abundance (genus Catenibacterium id.2153) on Leptin
with a threshold of 1e−05
rs77285108
rs12404911
rs7742829
rs73128290
All
0.0
0.1
0.2
MR effect size for
'Gut microbiota abundance (genus Catenibacterium id.2153)' on 'Leptin'

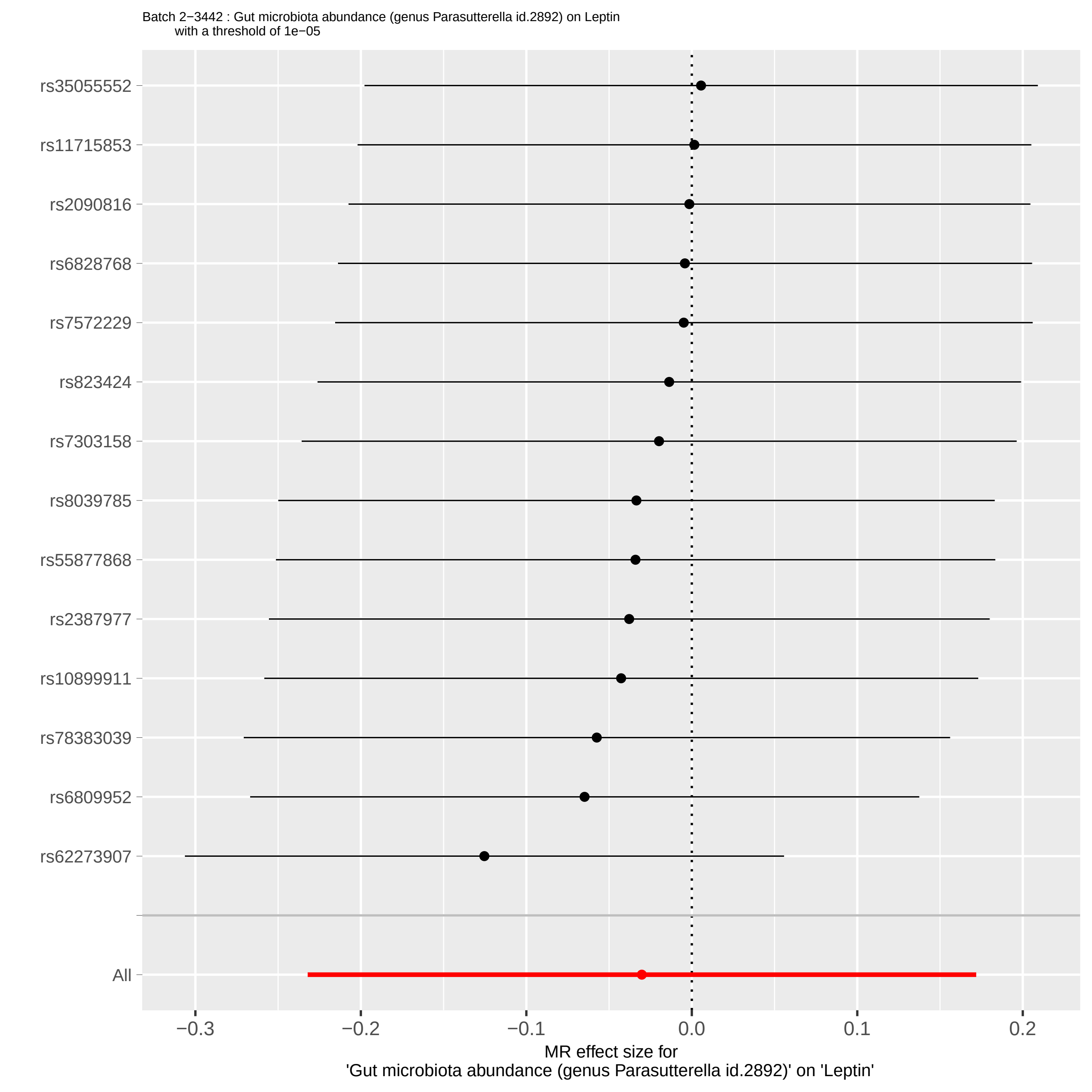
Batch 2−3442 : Gut microbiota abundance (genus Parasutterella id.2892) on Leptin
with a threshold of 1e−05
rs35055552
rs11715853
rs2090816
rs6828768
rs7572229
rs823424
rs7303158
rs8039785
rs55877868
rs2387977
rs10899911
rs78383039
rs6809952
rs62273907
All
−0.3
−0.2
−0.1
0.0
0.1
0.2
MR effect size for
'Gut microbiota abundance (genus Parasutterella id.2892)' on 'Leptin'

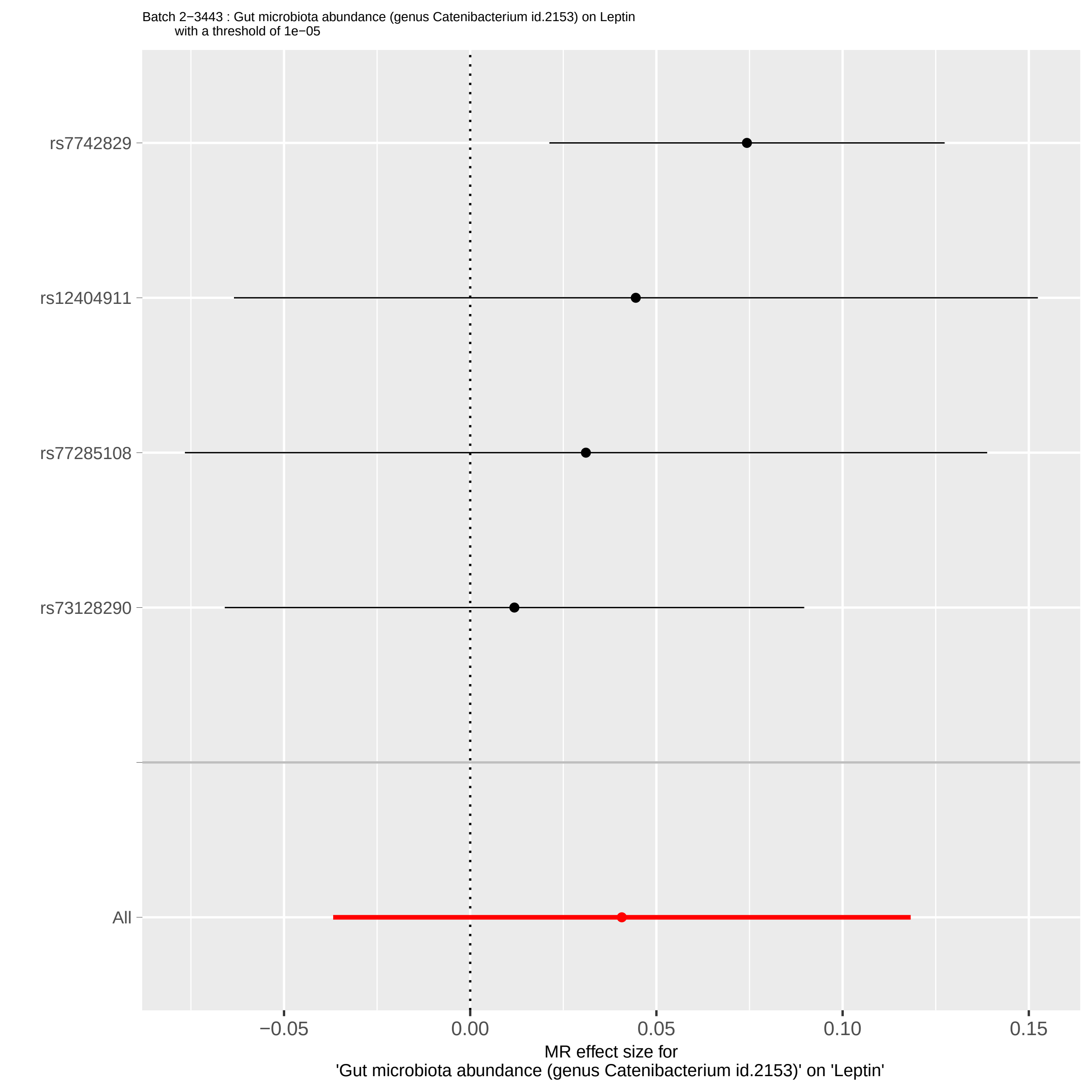
Batch 2−3443 : Gut microbiota abundance (genus Catenibacterium id.2153) on Leptin
with a threshold of 1e−05
rs7742829
rs12404911
rs77285108
rs73128290
All
−0.05
0.00
0.05
0.10
0.15
MR effect size for
'Gut microbiota abundance (genus Catenibacterium id.2153)' on 'Leptin'

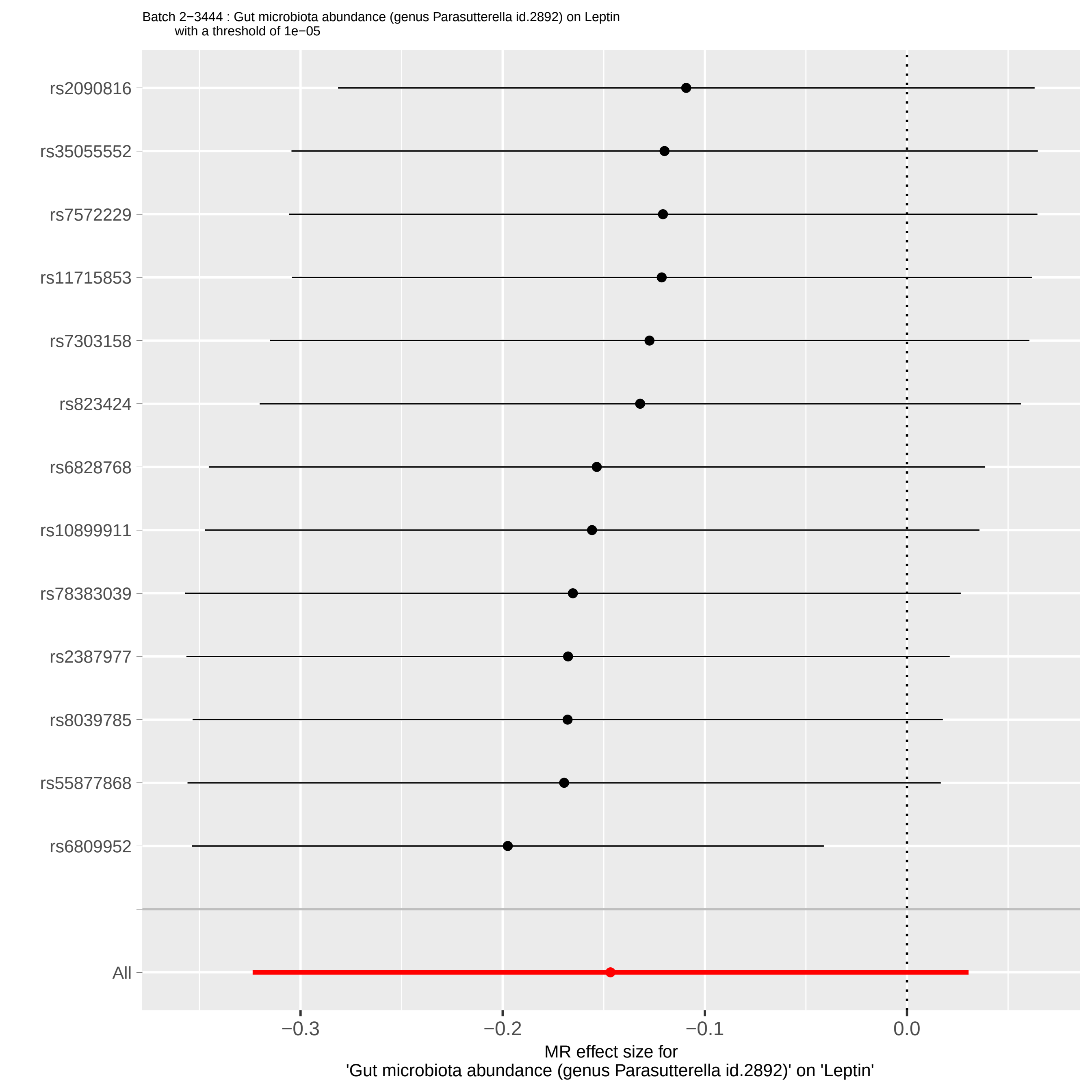
Batch 2−3444 : Gut microbiota abundance (genus Parasutterella id.2892) on Leptin
with a threshold of 1e−05
rs2090816
rs35055552
rs7572229
rs11715853
rs7303158
rs823424
rs6828768
rs10899911
rs78383039
rs2387977
rs8039785
rs55877868
rs6809952
All
−0.3
−0.2
−0.1
0.0
MR effect size for
'Gut microbiota abundance (genus Parasutterella id.2892)' on 'Leptin'

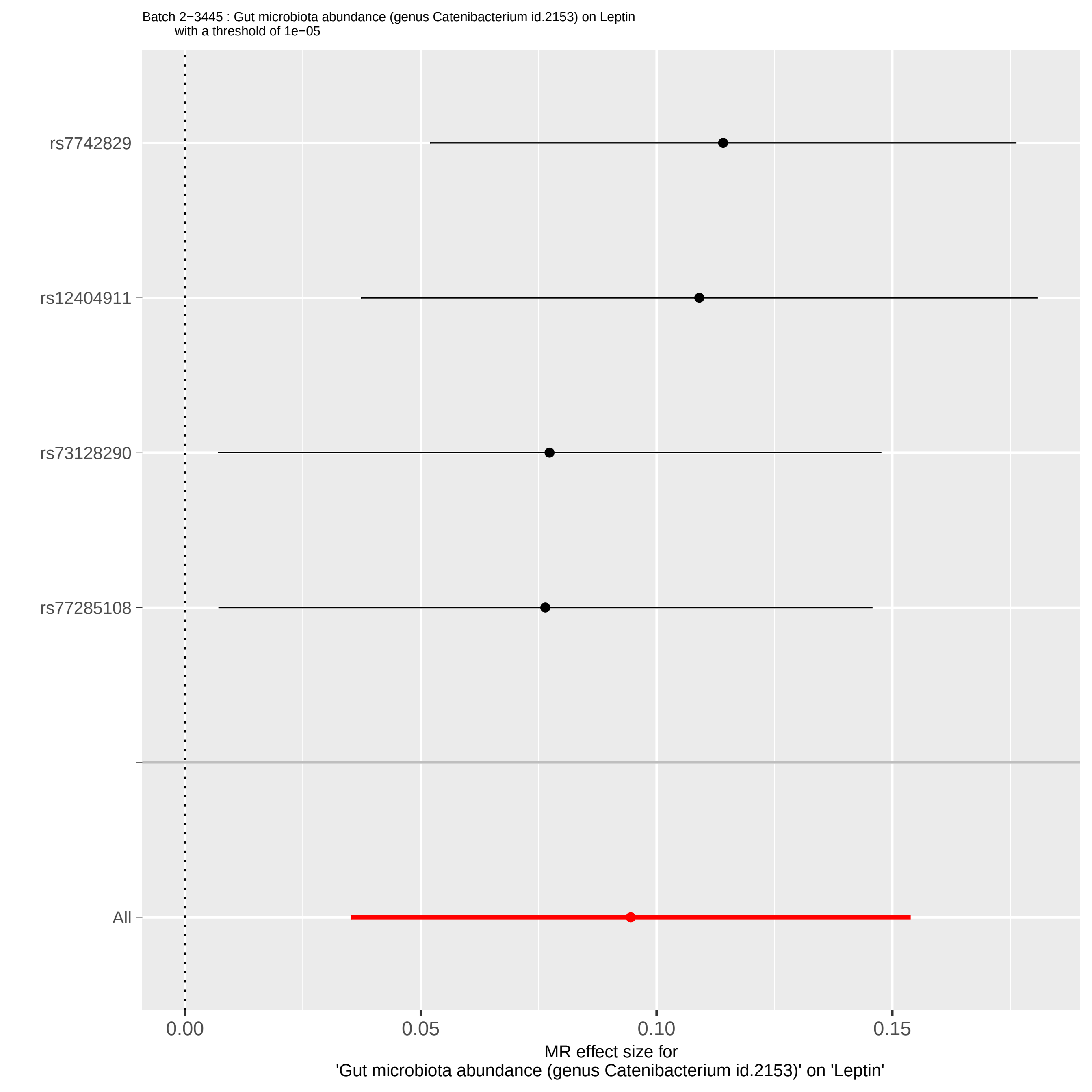
Batch 2−3445 : Gut microbiota abundance (genus Catenibacterium id.2153) on Leptin
with a threshold of 1e−05
rs7742829
rs12404911
rs73128290
rs77285108
All
0.00
0.05
0.10
0.15
MR effect size for
'Gut microbiota abundance (genus Catenibacterium id.2153)' on 'Leptin'

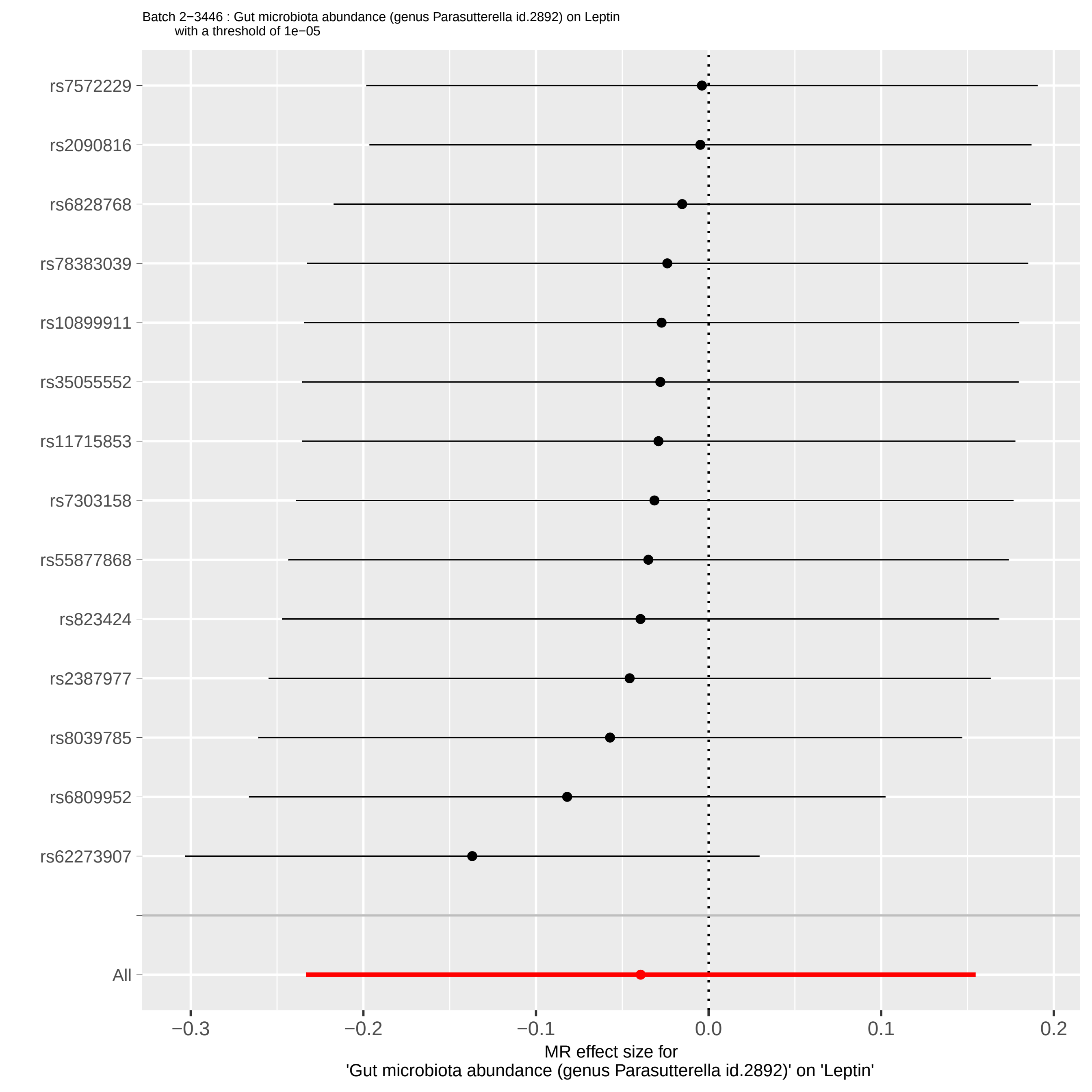

Batch 2−3446 : Gut microbiota abundance (genus Parasutterella id.2892) on Leptin
with a threshold of 1e−05
rs7572229
rs2090816
rs6828768
rs78383039
rs10899911
rs35055552
rs11715853
rs7303158
rs55877868
rs823424
rs2387977
rs8039785
rs6809952
rs62273907
All
−0.3
−0.2
−0.1
0.0
0.1
0.2
MR effect size for
'Gut microbiota abundance (genus Parasutterella id.2892)' on 'Leptin'

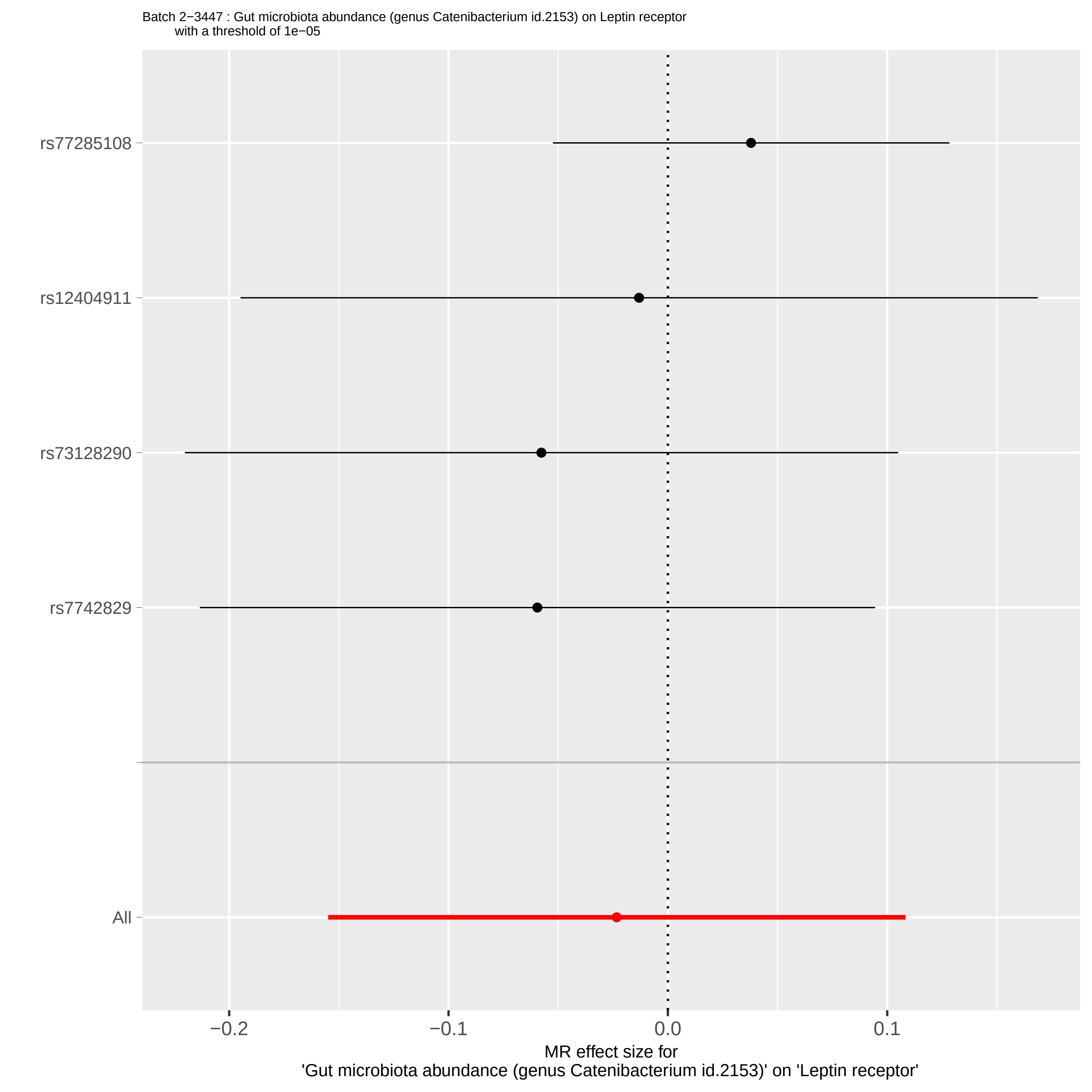
Batch 2−3447 : Gut microbiota abundance (genus Catenibacterium id.2153) on Leptin receptor
with a threshold of 1e−05
rs77285108
rs12404911
rs73128290
rs7742829
All
−0.2
−0.1
0.0
0.1
MR effect size for
'Gut microbiota abundance (genus Catenibacterium id.2153)' on 'Leptin receptor'

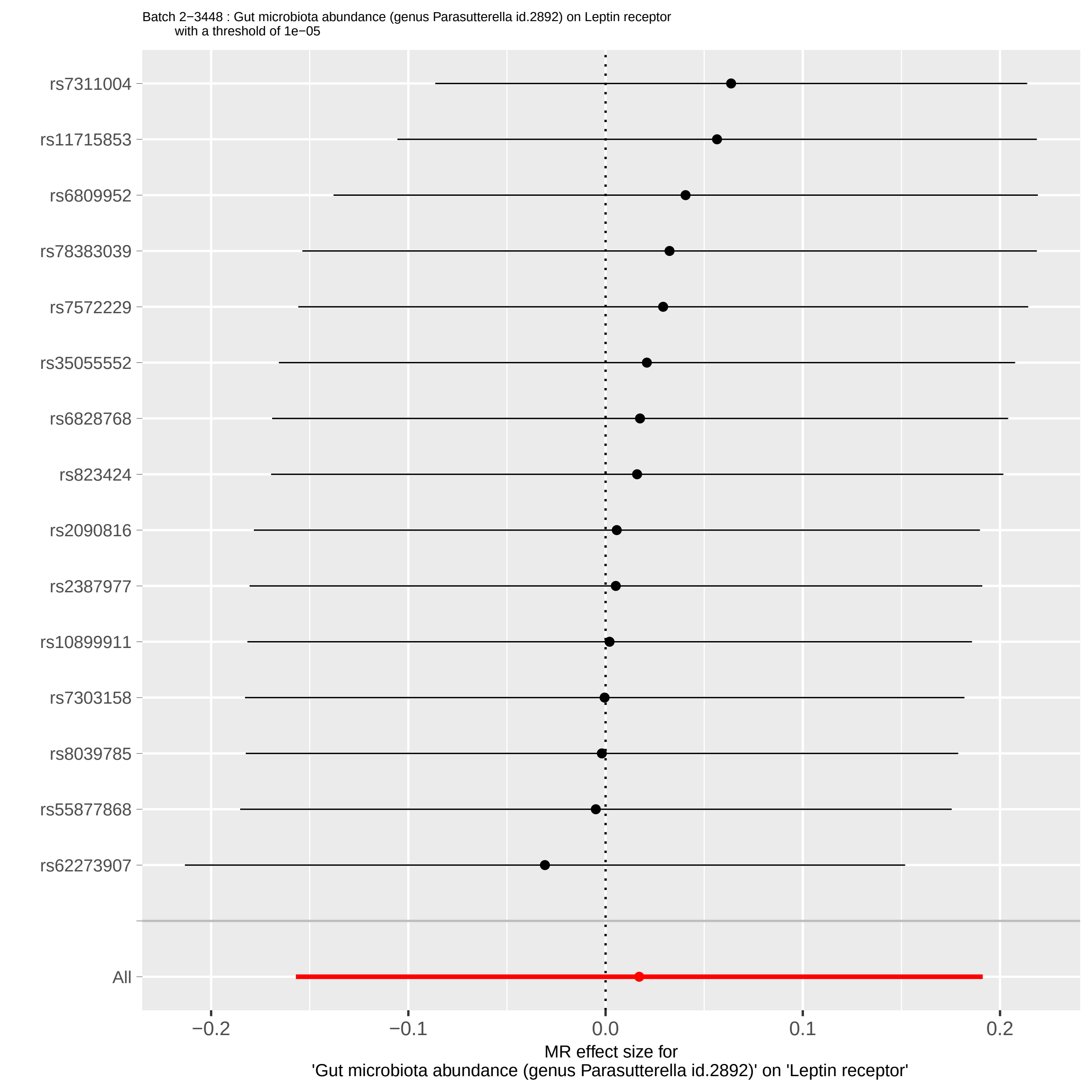

Batch 2−3448 : Gut microbiota abundance (genus Parasutterella id.2892) on Leptin receptor
with a threshold of 1e−05
rs7311004
rs11715853
rs6809952
rs78383039
rs7572229
rs35055552
rs6828768
rs823424
rs2090816
rs2387977
rs10899911
rs7303158
rs8039785
rs55877868
rs62273907
All
−0.2
−0.1
0.0
0.1
0.2
MR effect size for
'Gut microbiota abundance (genus Parasutterella id.2892)' on 'Leptin receptor'

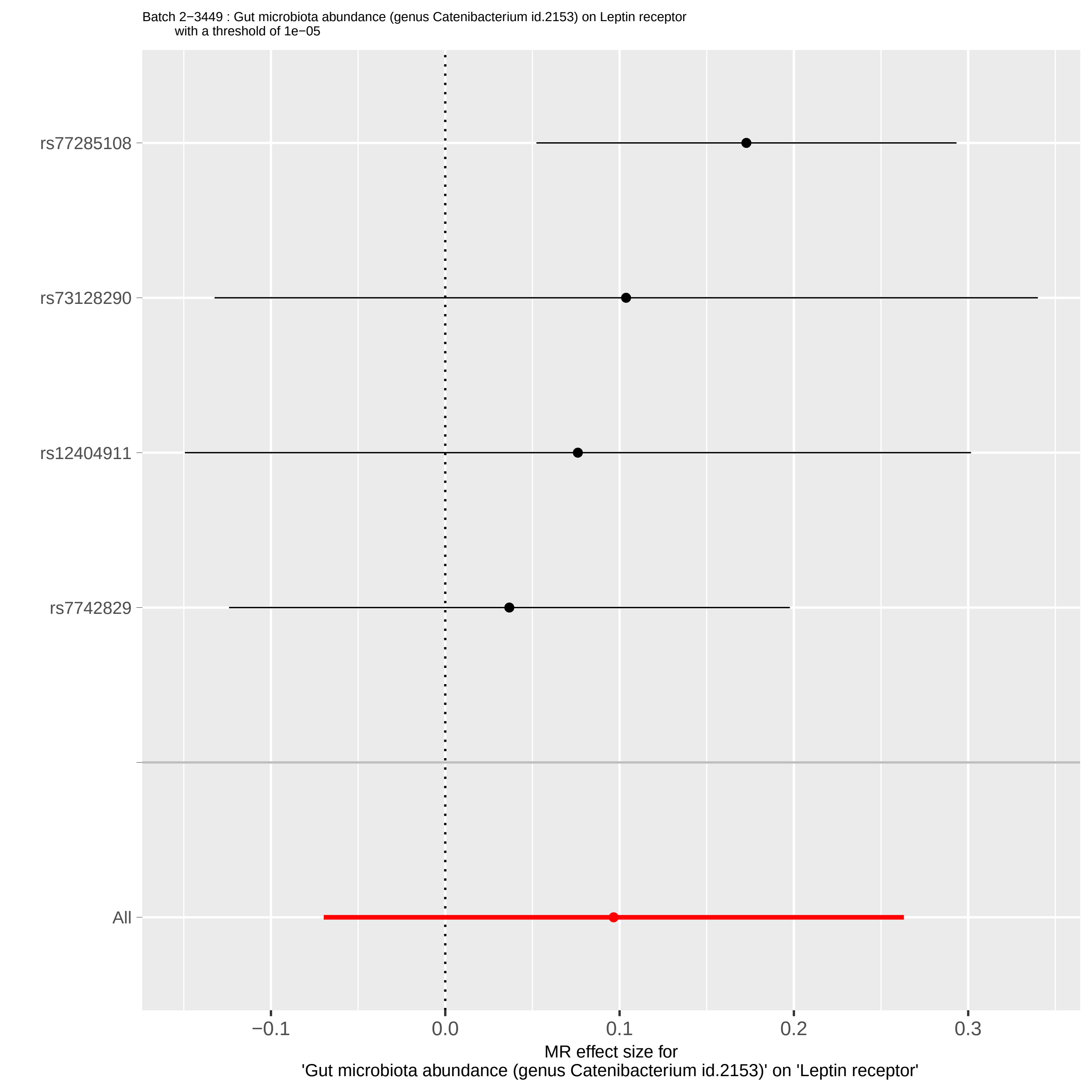
Batch 2−3449 : Gut microbiota abundance (genus Catenibacterium id.2153) on Leptin receptor
with a threshold of 1e−05
rs77285108
rs73128290
rs12404911
rs7742829
All
−0.1
0.0
0.1
0.2
0.3
MR effect size for
'Gut microbiota abundance (genus Catenibacterium id.2153)' on 'Leptin receptor'

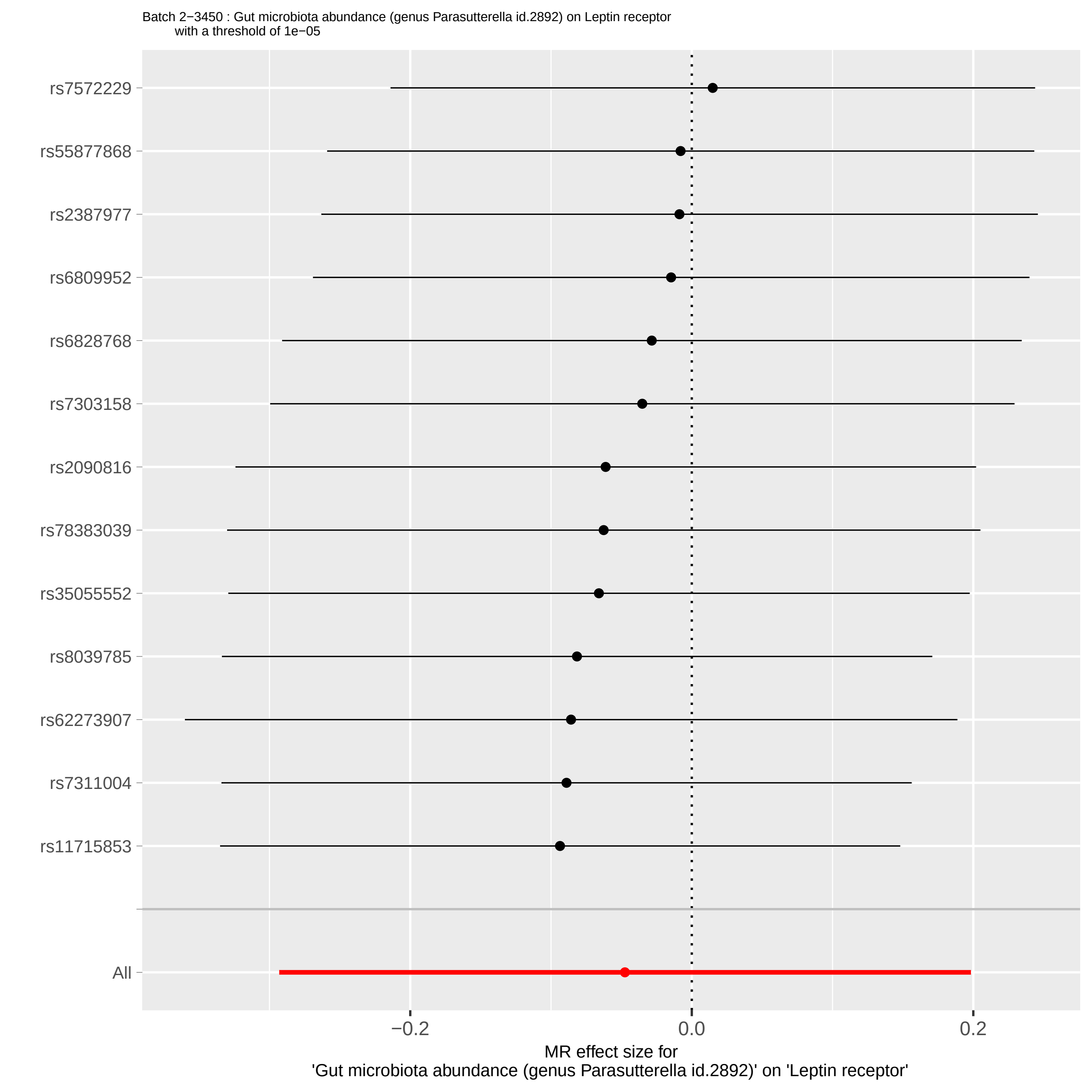
Batch 2−3450 : Gut microbiota abundance (genus Parasutterella id.2892) on Leptin receptor
with a threshold of 1e−05
rs7572229
rs55877868
rs2387977
rs6809952
rs6828768
rs7303158
rs2090816
rs78383039
rs35055552
rs8039785
rs62273907
rs7311004
rs11715853
All
−0.2
0.0
0.2
MR effect size for
'Gut microbiota abundance (genus Parasutterella id.2892)' on 'Leptin receptor'

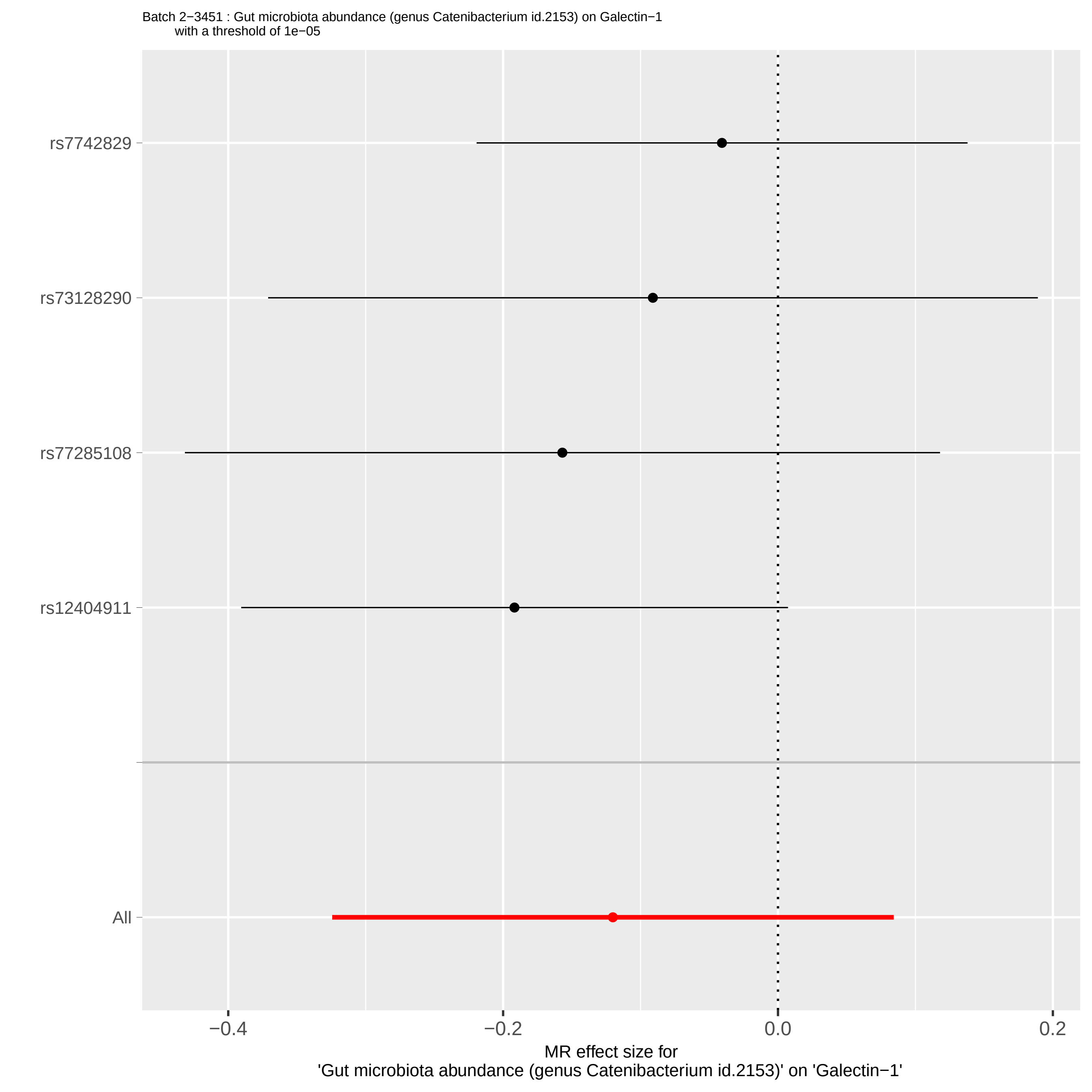
Batch 2−3451 : Gut microbiota abundance (genus Catenibacterium id.2153) on Galectin−1
with a threshold of 1e−05
rs7742829
rs73128290
rs77285108
rs12404911
All
−0.4
−0.2
0.0
0.2
MR effect size for
'Gut microbiota abundance (genus Catenibacterium id.2153)' on 'Galectin−1'

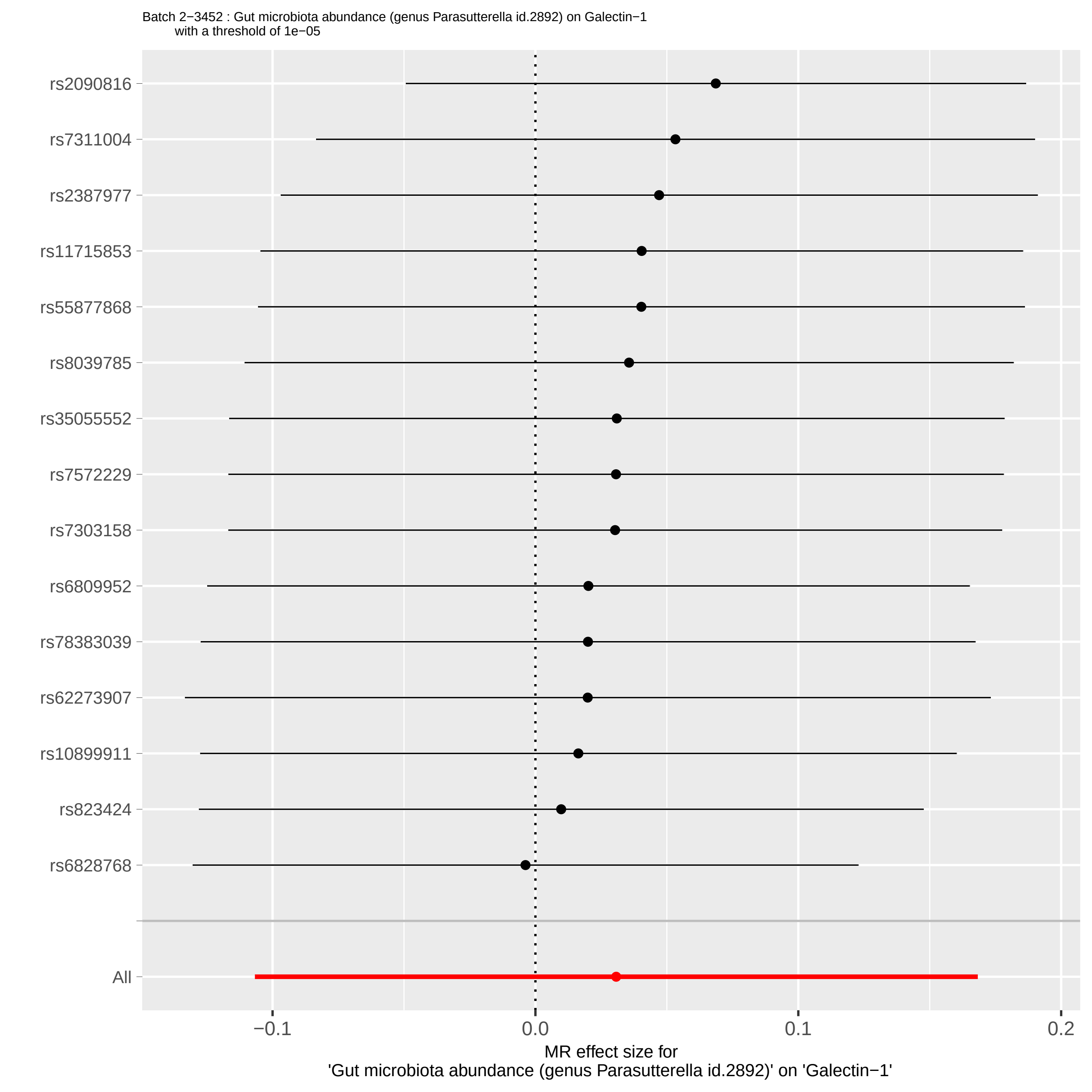

Batch 2−3452 : Gut microbiota abundance (genus Parasutterella id.2892) on Galectin−1
with a threshold of 1e−05
rs2090816
rs7311004
rs2387977
rs11715853
rs55877868
rs8039785
rs35055552
rs7572229
rs7303158
rs6809952
rs78383039
rs62273907
rs10899911
rs823424
rs6828768
All
−0.1
0.0
0.1
0.2
MR effect size for
'Gut microbiota abundance (genus Parasutterella id.2892)' on 'Galectin−1'

Batch 2−3453 : Gut microbiota abundance (genus Catenibacterium id.2153) on Galectin−2
with a threshold of 1e−05

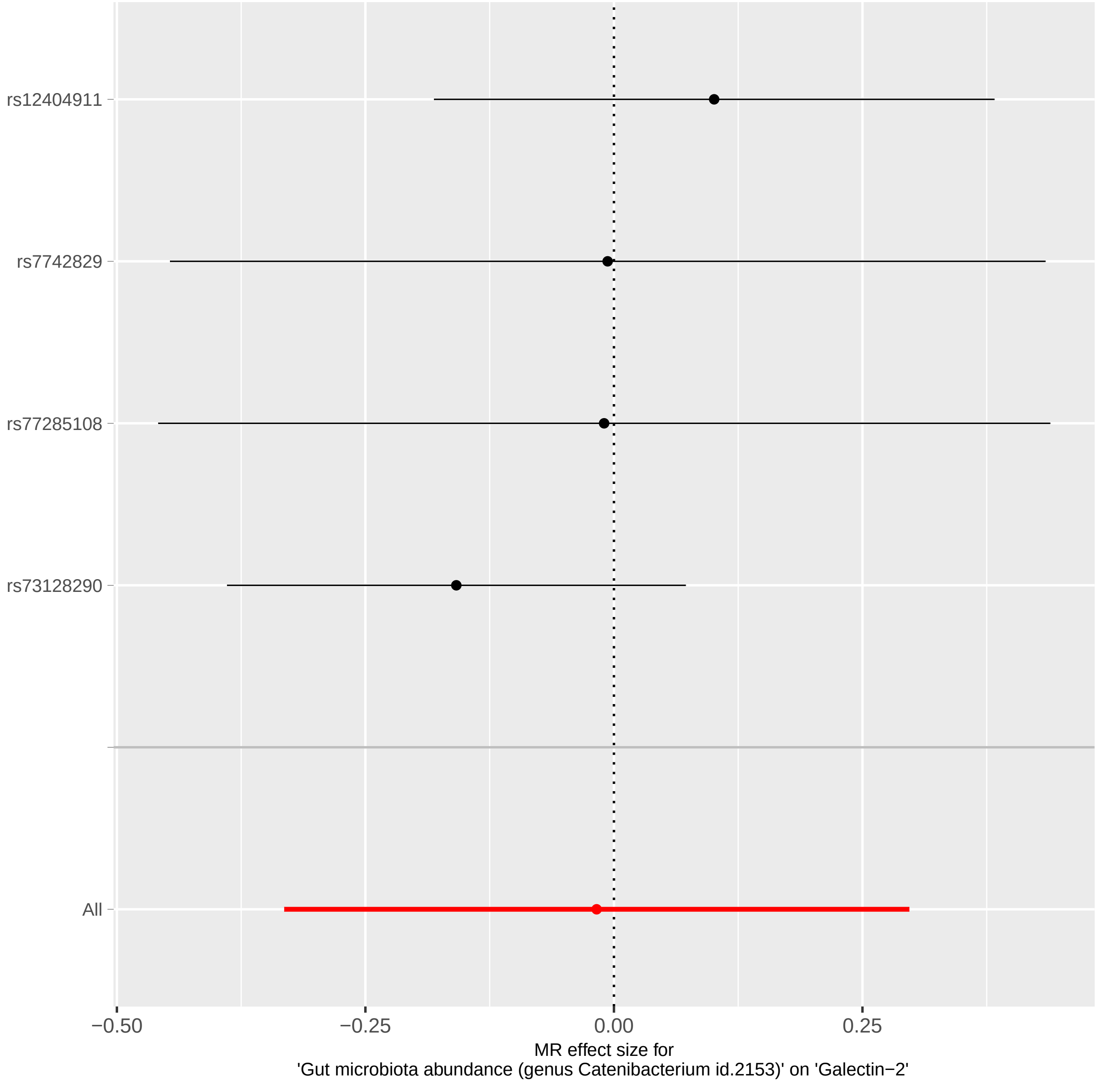

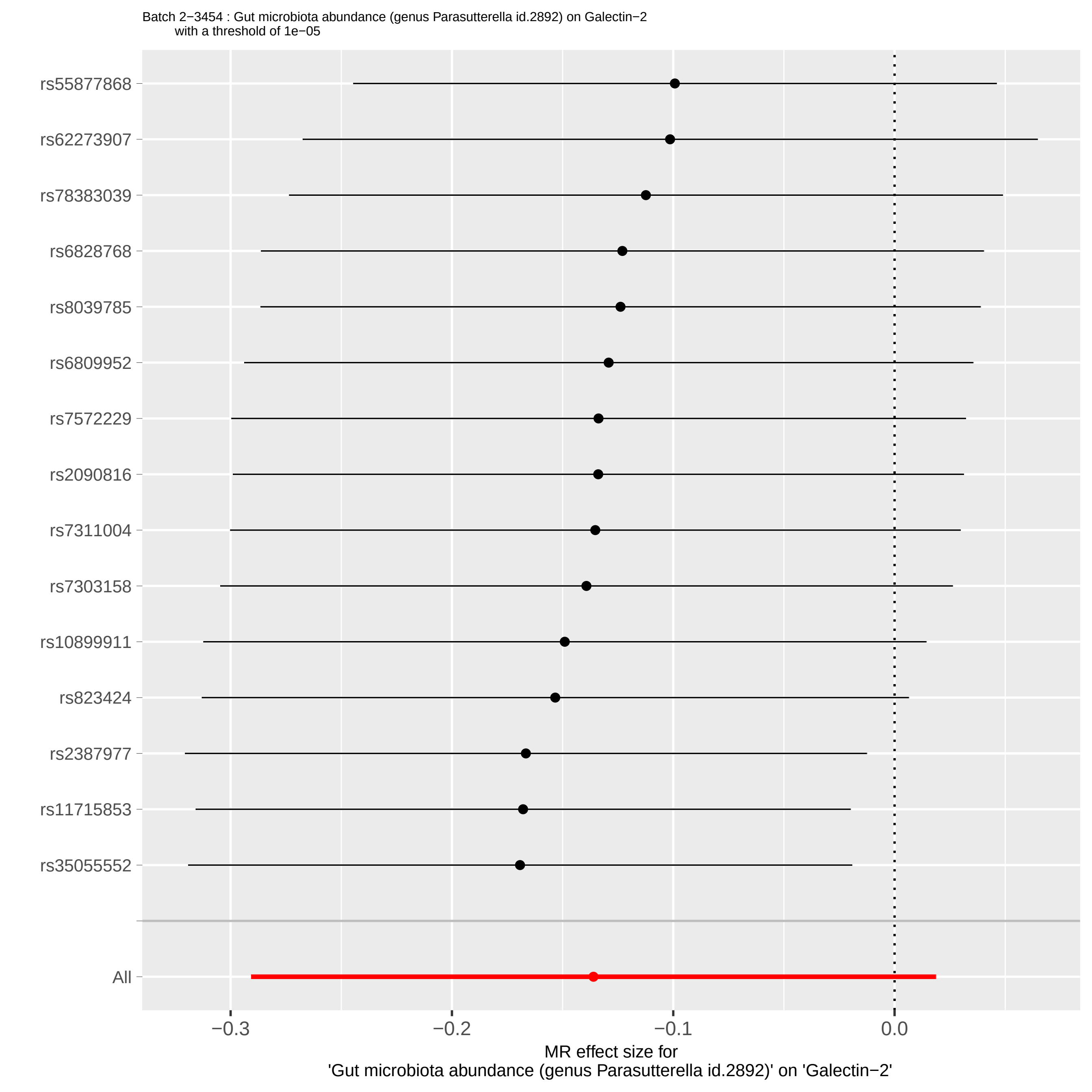
Batch 2−3454 : Gut microbiota abundance (genus Parasutterella id.2892) on Galectin−2
with a threshold of 1e−05
rs55877868
rs62273907
rs78383039
rs6828768
rs8039785
rs6809952
rs7572229
rs2090816
rs7311004
rs7303158
rs10899911
rs823424
rs2387977
rs11715853
rs35055552
All
−0.3
−0.2
−0.1
0.0
MR effect size for
'Gut microbiota abundance (genus Parasutterella id.2892)' on 'Galectin−2'

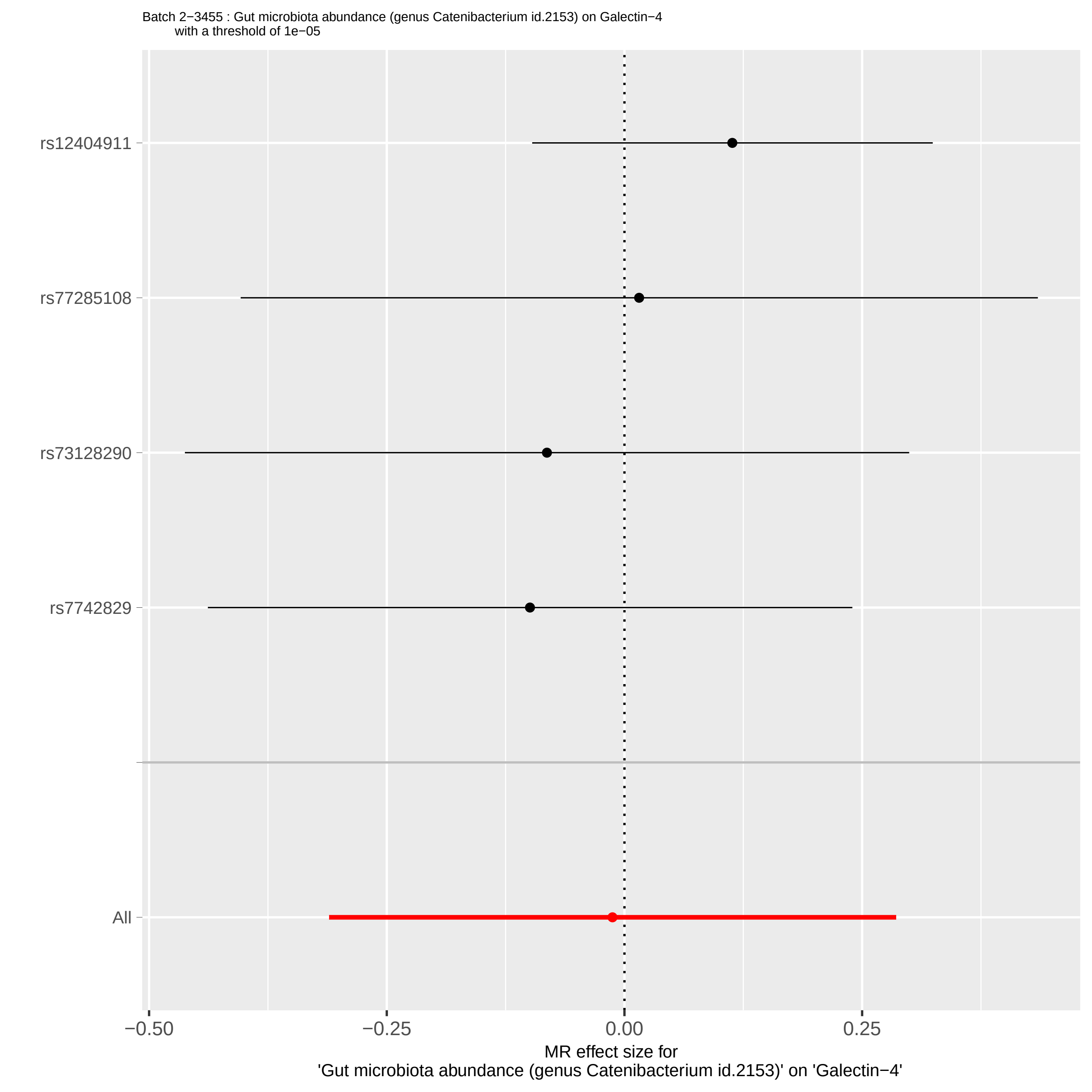
Batch 2−3455 : Gut microbiota abundance (genus Catenibacterium id.2153) on Galectin−4
with a threshold of 1e−05
rs12404911
rs77285108
rs73128290
rs7742829
All
−0.50
−0.25
0.00
0.25
MR effect size for
'Gut microbiota abundance (genus Catenibacterium id.2153)' on 'Galectin−4'

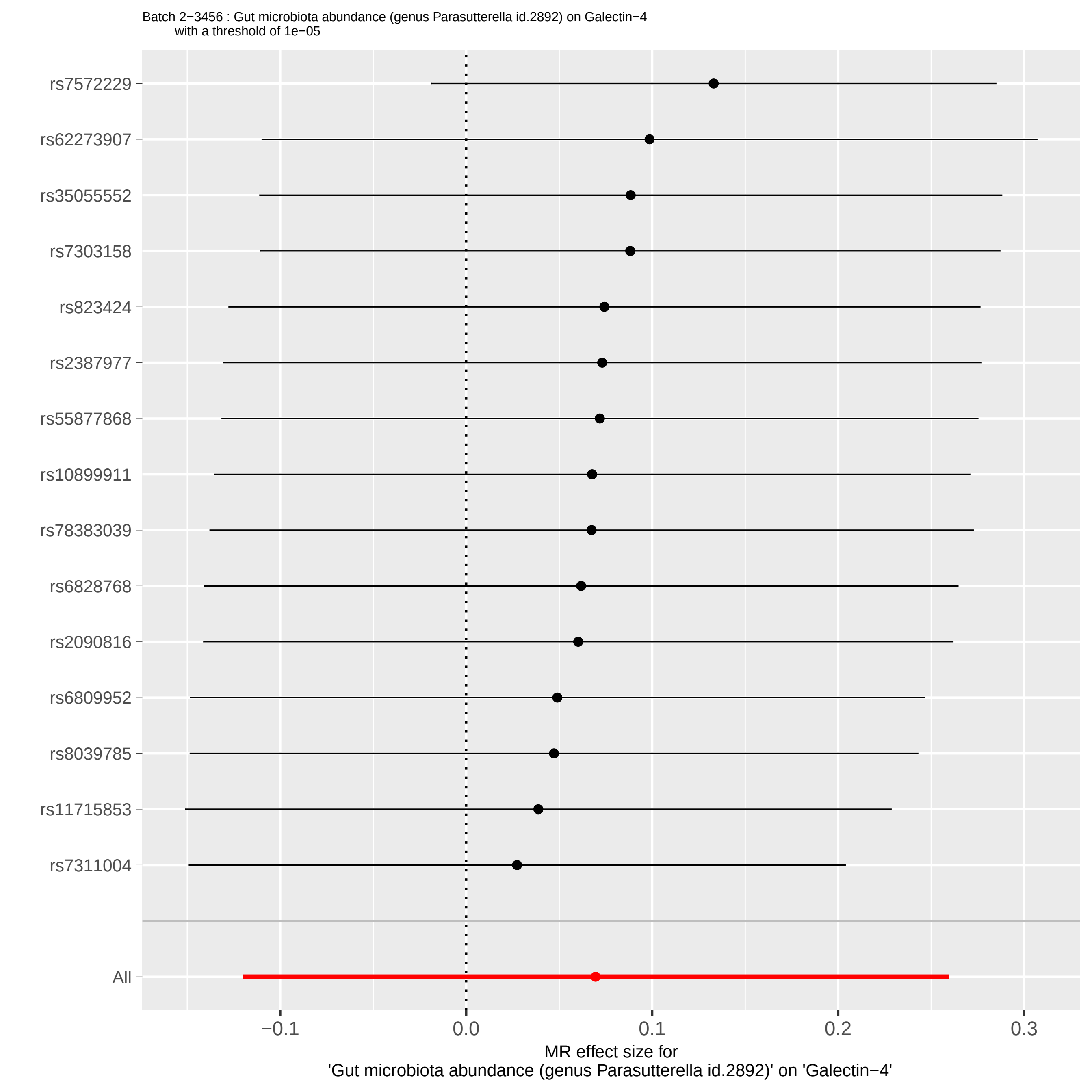
Batch 2−3456 : Gut microbiota abundance (genus Parasutterella id.2892) on Galectin−4
with a threshold of 1e−05
rs7572229
rs62273907
rs35055552
rs7303158
rs823424
rs2387977
rs55877868
rs10899911
rs78383039
rs6828768
rs2090816
rs6809952
rs8039785
rs11715853
rs7311004
All
−0.1
0.0
0.1
0.2
0.3
MR effect size for
'Gut microbiota abundance (genus Parasutterella id.2892)' on 'Galectin−4'

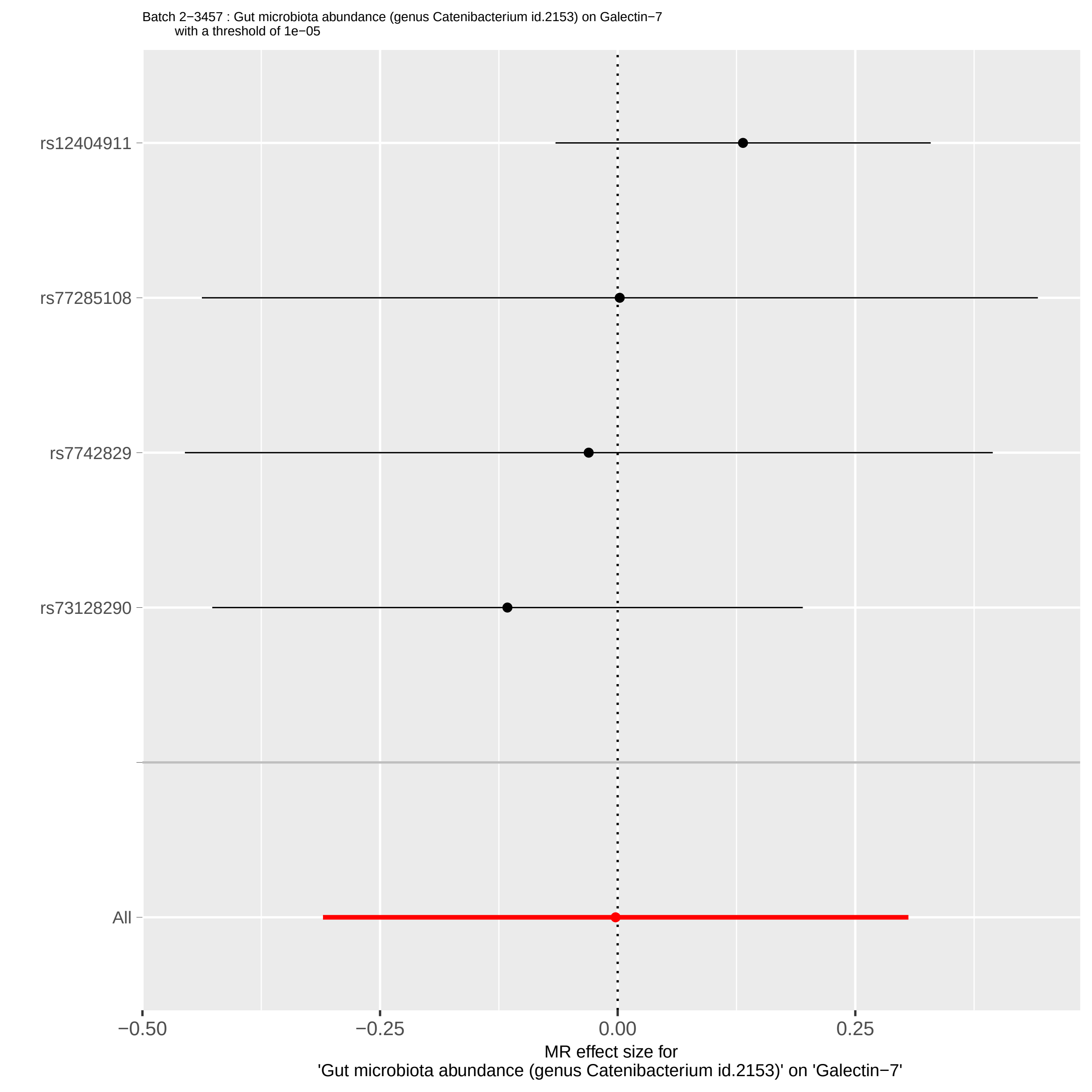

Batch 2−3457 : Gut microbiota abundance (genus Catenibacterium id.2153) on Galectin−7
with a threshold of 1e−05
rs12404911
rs77285108
rs7742829
rs73128290
All
−0.50
−0.25
0.00
0.25
MR effect size for
'Gut microbiota abundance (genus Catenibacterium id.2153)' on 'Galectin−7'

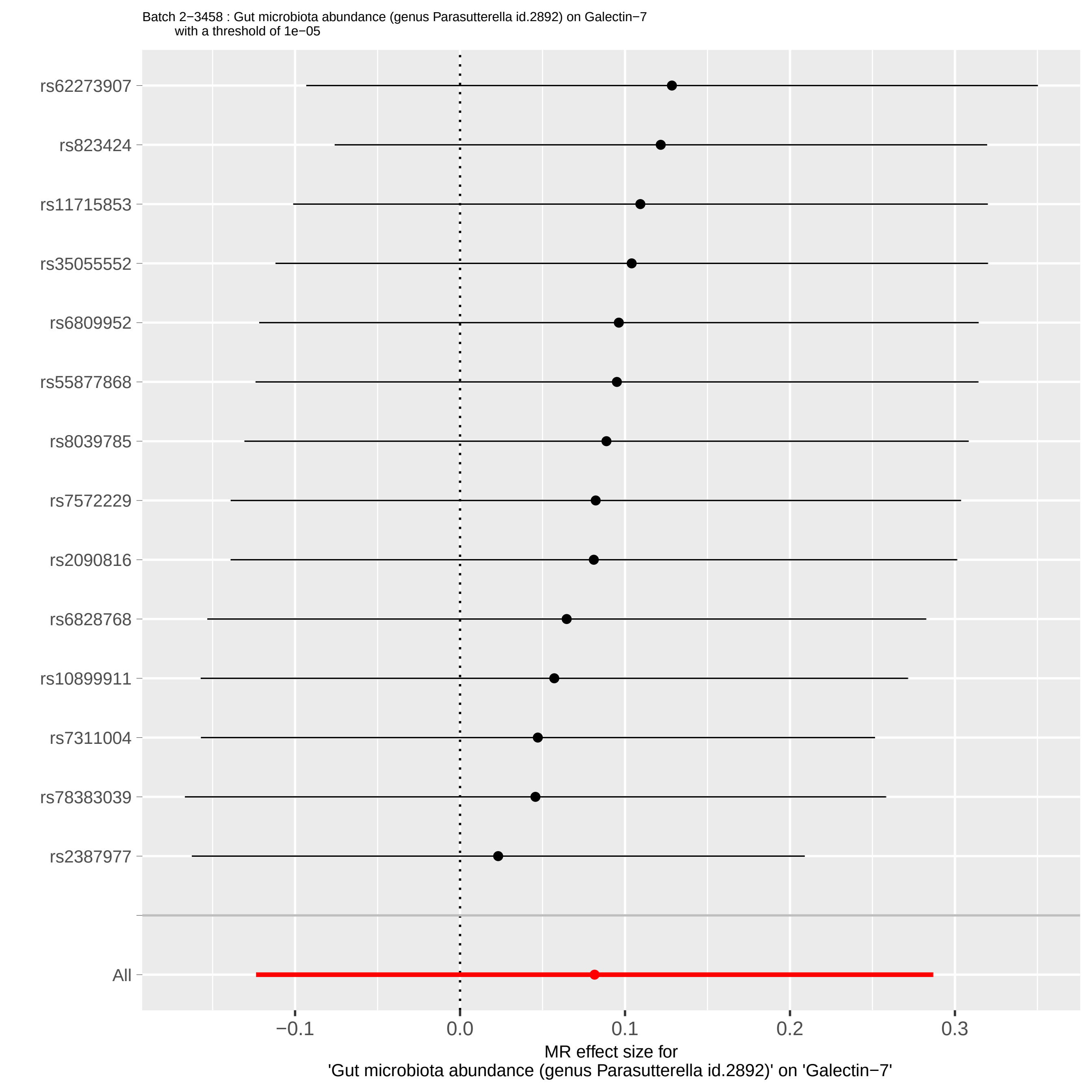

Batch 2−3458 : Gut microbiota abundance (genus Parasutterella id.2892) on Galectin−7
with a threshold of 1e−05
rs62273907
rs823424
rs11715853
rs35055552
rs6809952
rs55877868
rs8039785
rs7572229
rs2090816
rs6828768
rs10899911
rs7311004
rs78383039
rs2387977
All
−0.1
0.0
0.1
0.2
0.3
MR effect size for
'Gut microbiota abundance (genus Parasutterella id.2892)' on 'Galectin−7'

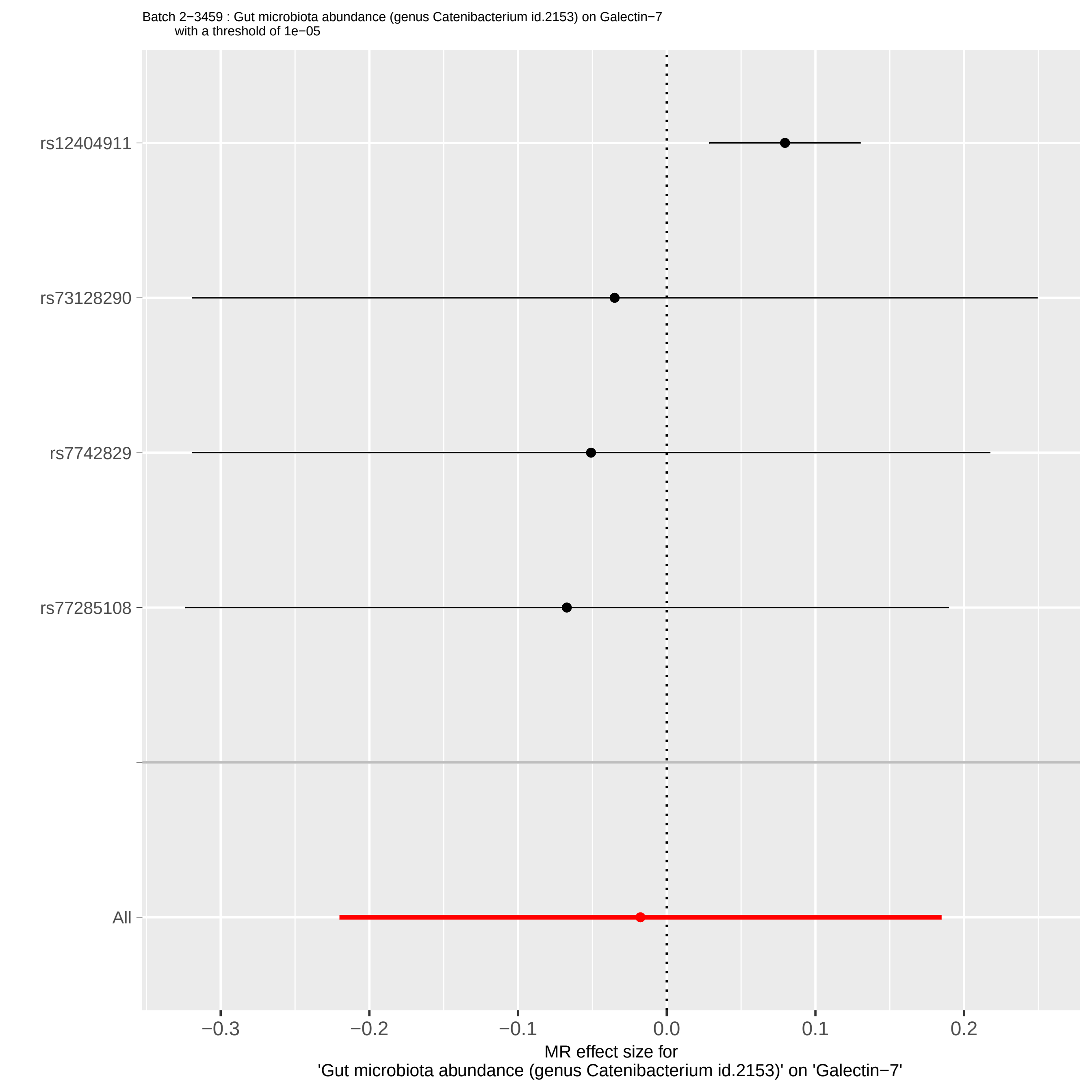
Batch 2−3459 : Gut microbiota abundance (genus Catenibacterium id.2153) on Galectin−7
with a threshold of 1e−05
rs12404911
rs73128290
rs7742829
rs77285108
All
−0.3
−0.2
−0.1
0.0
0.1
0.2
MR effect size for
'Gut microbiota abundance (genus Catenibacterium id.2153)' on 'Galectin−7'

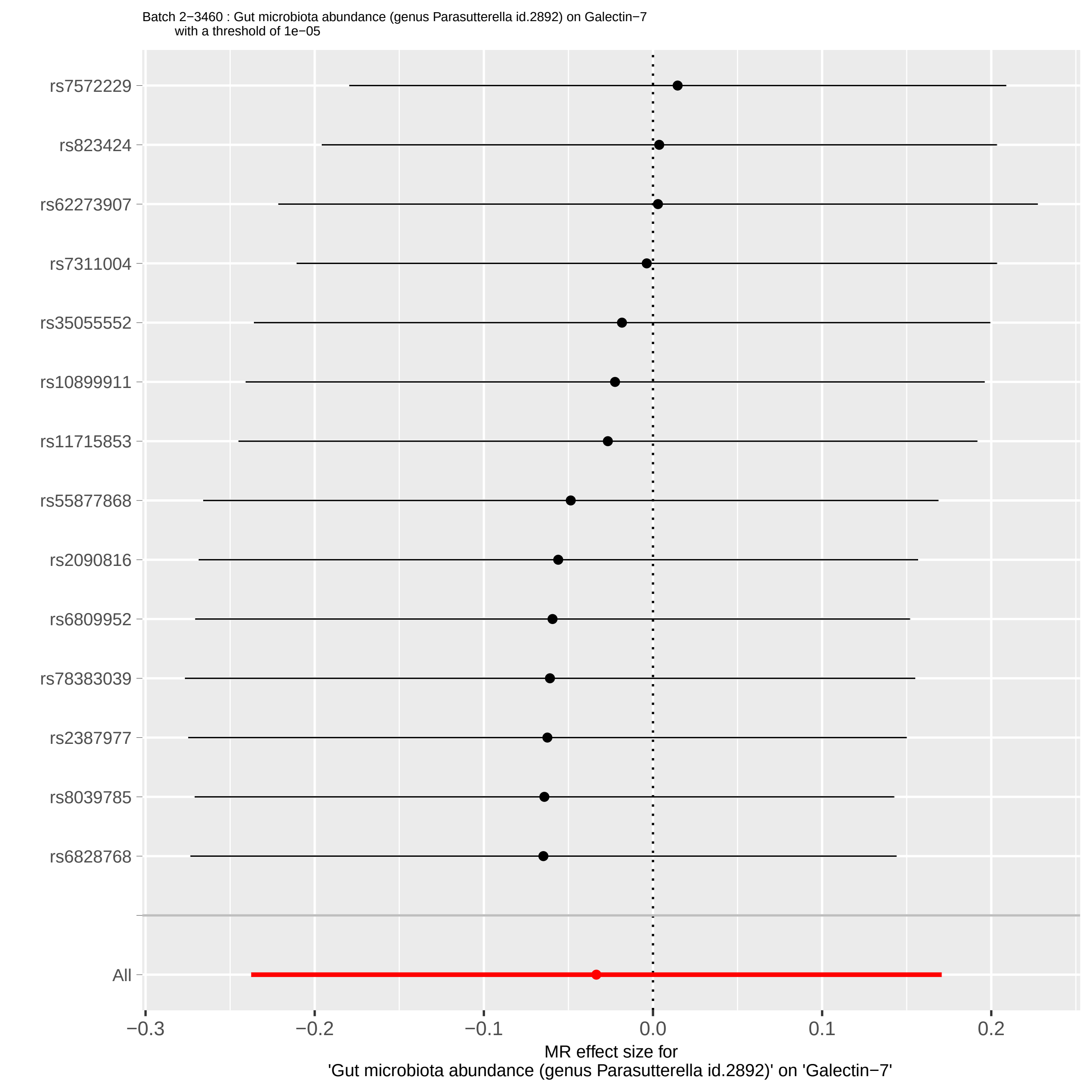
Batch 2−3460 : Gut microbiota abundance (genus Parasutterella id.2892) on Galectin−7
with a threshold of 1e−05
rs7572229
rs823424
rs62273907
rs7311004
rs35055552
rs10899911
rs11715853
rs55877868
rs2090816
rs6809952
rs78383039
rs2387977
rs8039785
rs6828768
All
−0.3
−0.2
−0.1
0.0
0.1
0.2
MR effect size for
'Gut microbiota abundance (genus Parasutterella id.2892)' on 'Galectin−7'

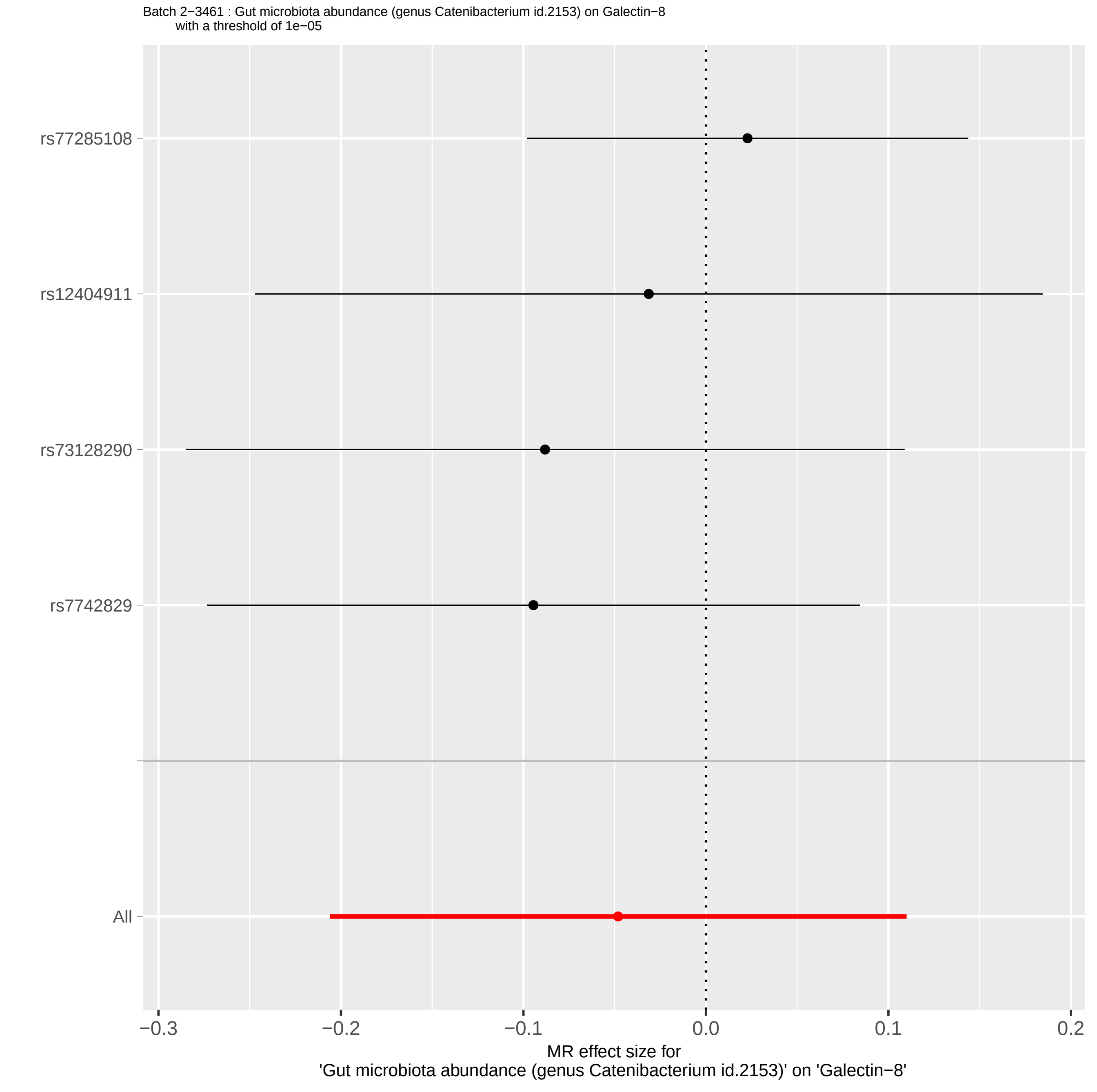
Batch 2−3461 : Gut microbiota abundance (genus Catenibacterium id.2153) on Galectin−8
with a threshold of 1e−05
rs77285108
rs12404911
rs73128290
rs7742829
All
−0.3
−0.2
−0.1
0.0
0.1
0.2
MR effect size for
'Gut microbiota abundance (genus Catenibacterium id.2153)' on 'Galectin−8'

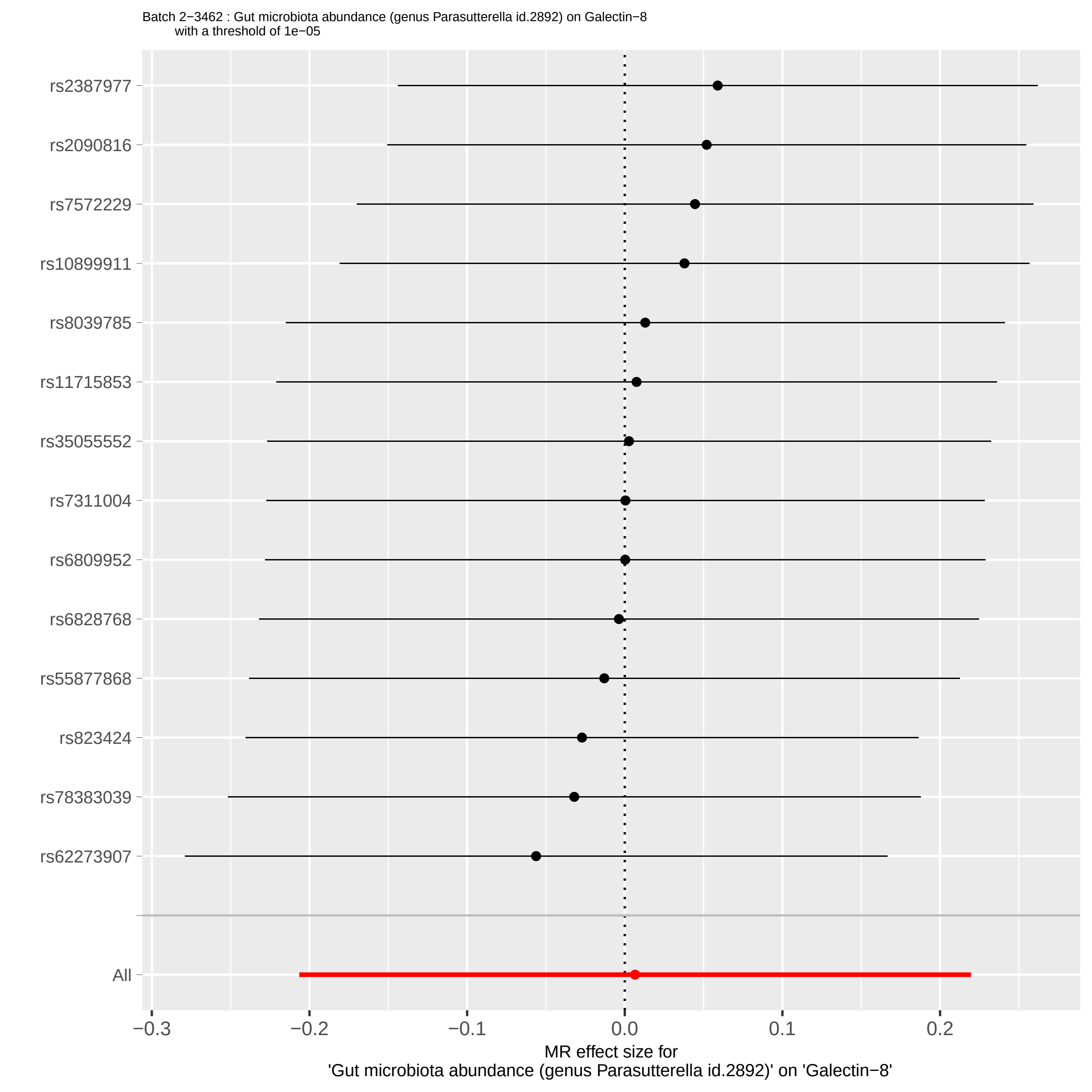
Batch 2−3462 : Gut microbiota abundance (genus Parasutterella id.2892) on Galectin−8
with a threshold of 1e−05
rs2387977
rs2090816
rs7572229
rs10899911
rs8039785
rs11715853
rs35055552
rs7311004
rs6809952
rs6828768
rs55877868
rs823424
rs78383039
rs62273907
All
−0.3
−0.2
−0.1
0.0
0.1
0.2
MR effect size for
'Gut microbiota abundance (genus Parasutterella id.2892)' on 'Galectin−8'

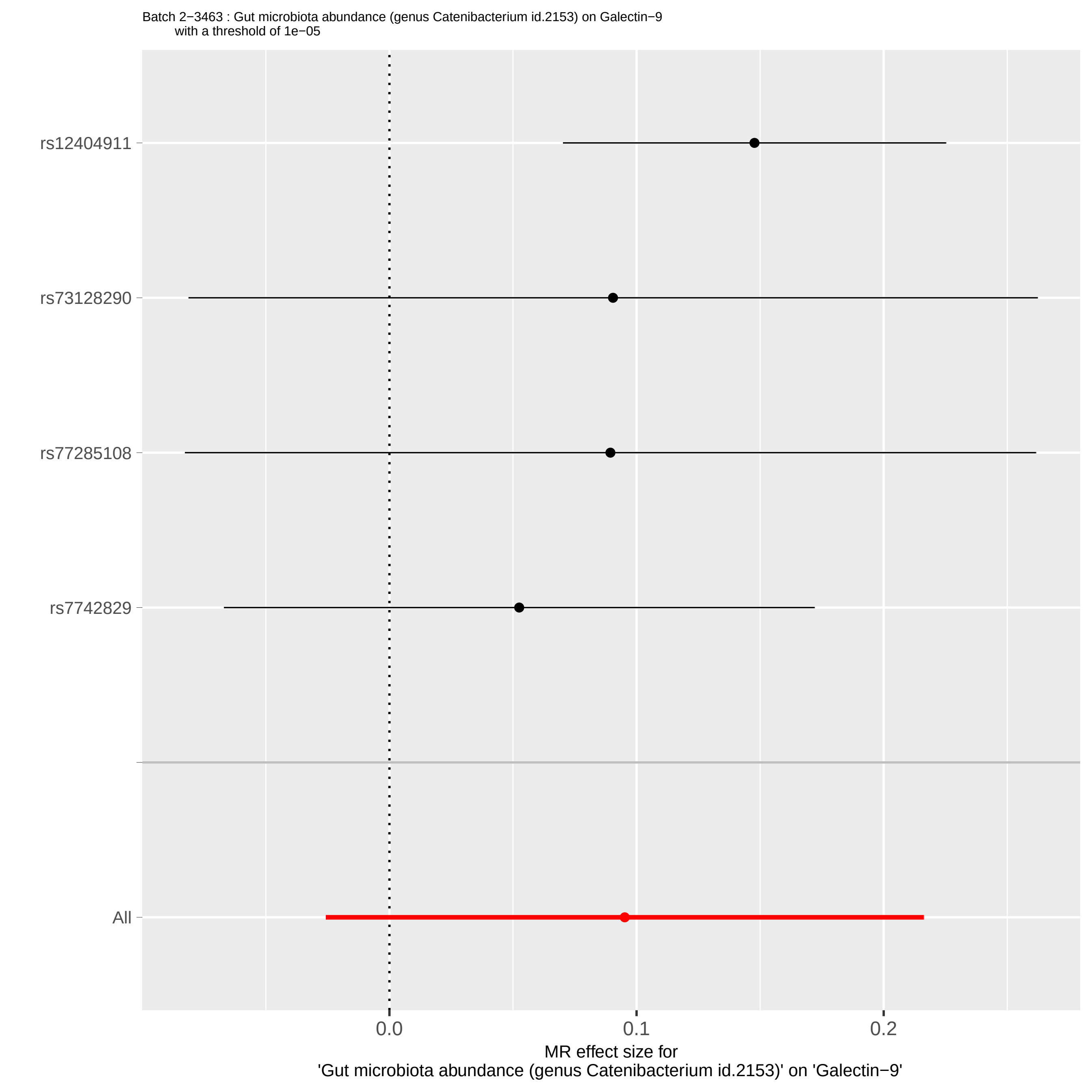

Batch 2−3463 : Gut microbiota abundance (genus Catenibacterium id.2153) on Galectin−9
with a threshold of 1e−05
rs12404911
rs73128290
rs77285108
rs7742829
All
0.0
0.1
0.2
MR effect size for
'Gut microbiota abundance (genus Catenibacterium id.2153)' on 'Galectin−9'

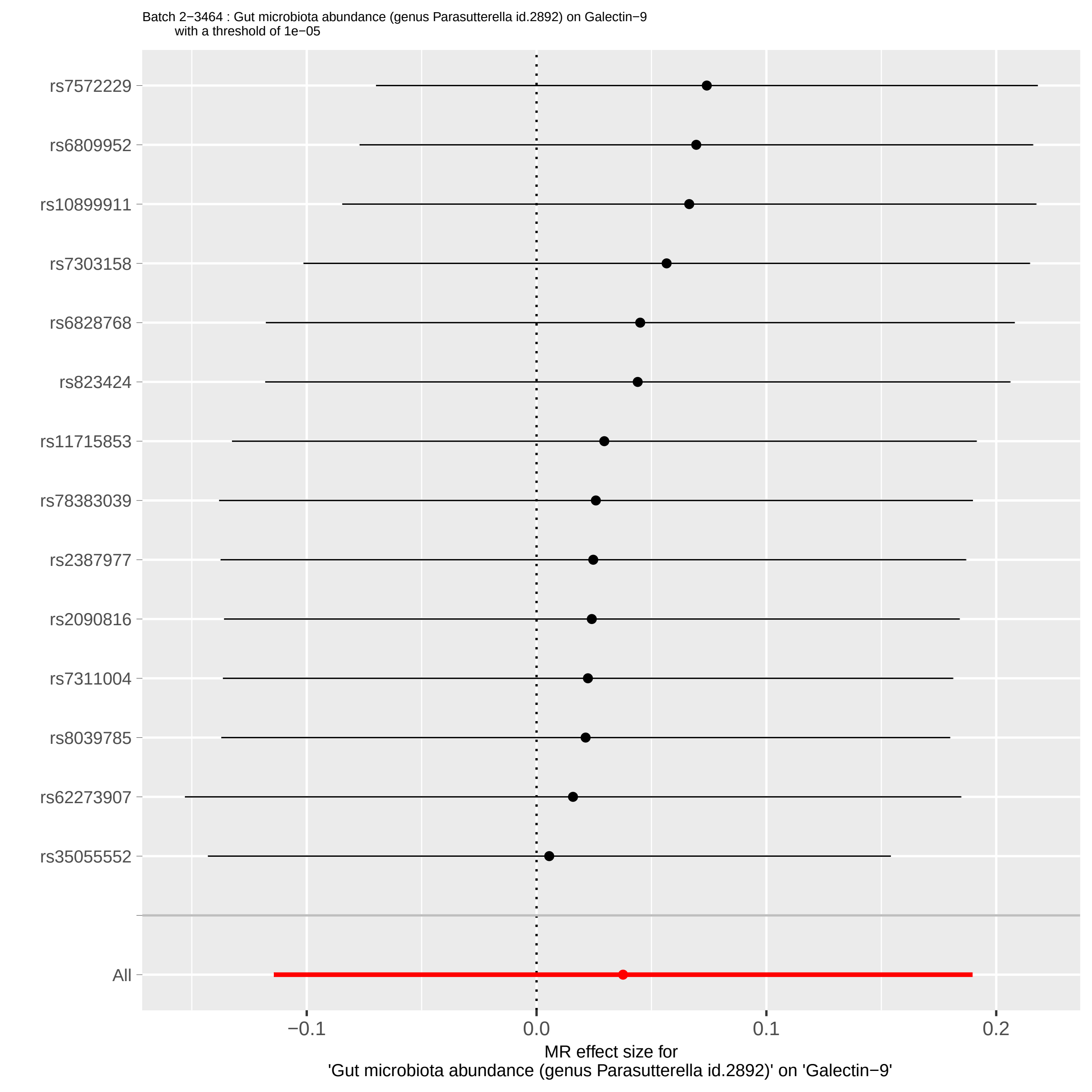
Batch 2−3464 : Gut microbiota abundance (genus Parasutterella id.2892) on Galectin−9
with a threshold of 1e−05
rs7572229
rs6809952
rs10899911
rs7303158
rs6828768
rs823424
rs11715853
rs78383039
rs2387977
rs2090816
rs7311004
rs8039785
rs62273907
rs35055552
All
−0.1
0.0
0.1
0.2
MR effect size for
'Gut microbiota abundance (genus Parasutterella id.2892)' on 'Galectin−9'

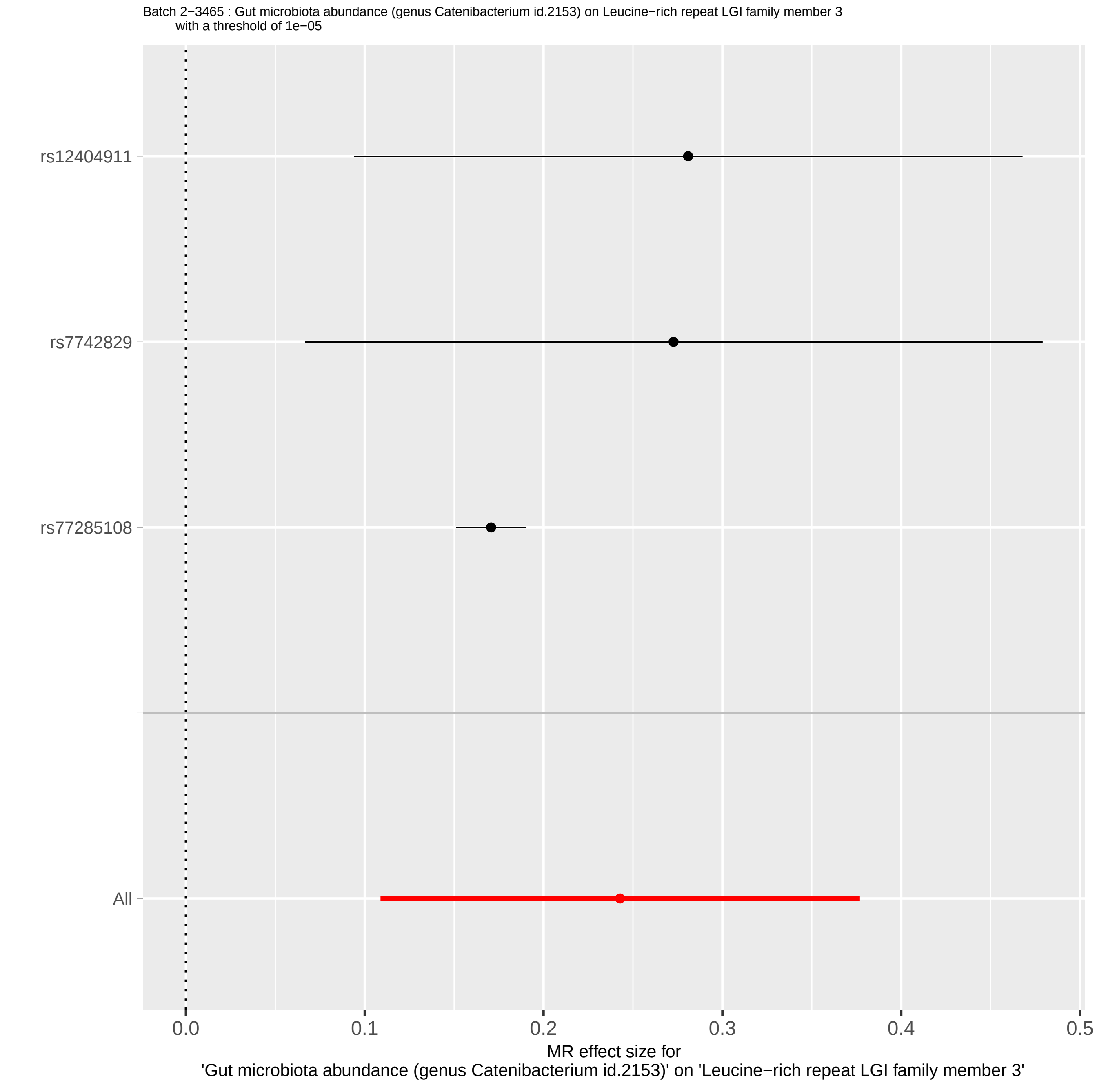

Batch 2−3465 : Gut microbiota abundance (genus Catenibacterium id.2153) on Leucine−rich repeat LGI family member 3
with a threshold of 1e−05
rs12404911
rs7742829
rs77285108
All
0.0
0.1
0.2
0.3
0.4
0.5
MR effect size for
'Gut microbiota abundance (genus Catenibacterium id.2153)' on 'Leucine−rich repeat LGI family member 3'

Batch 2−3466 : Gut microbiota abundance (genus Parasutterella id.2892) on Leucine−rich repeat LGI family member 3
with a threshold of 1e−05

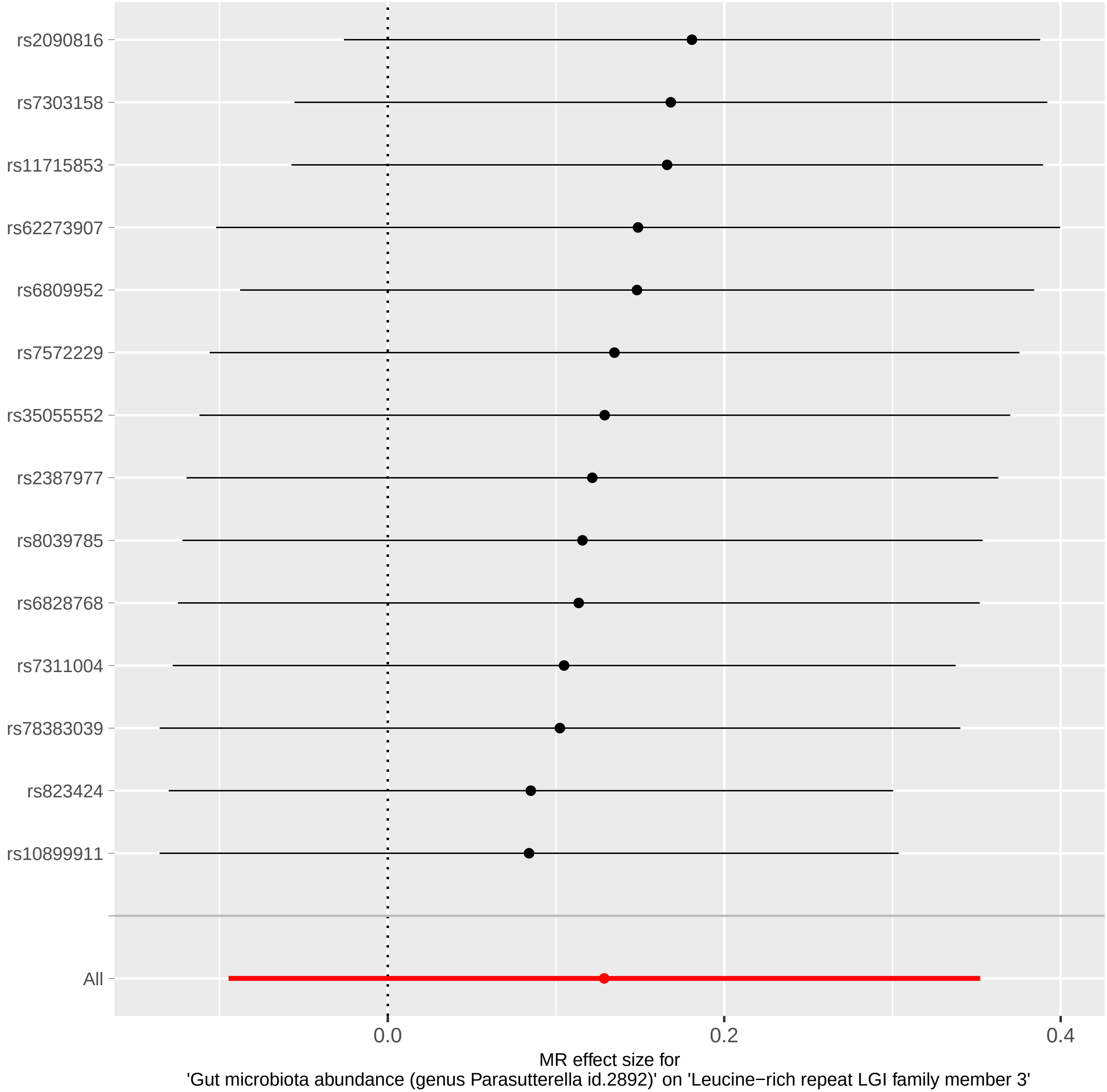

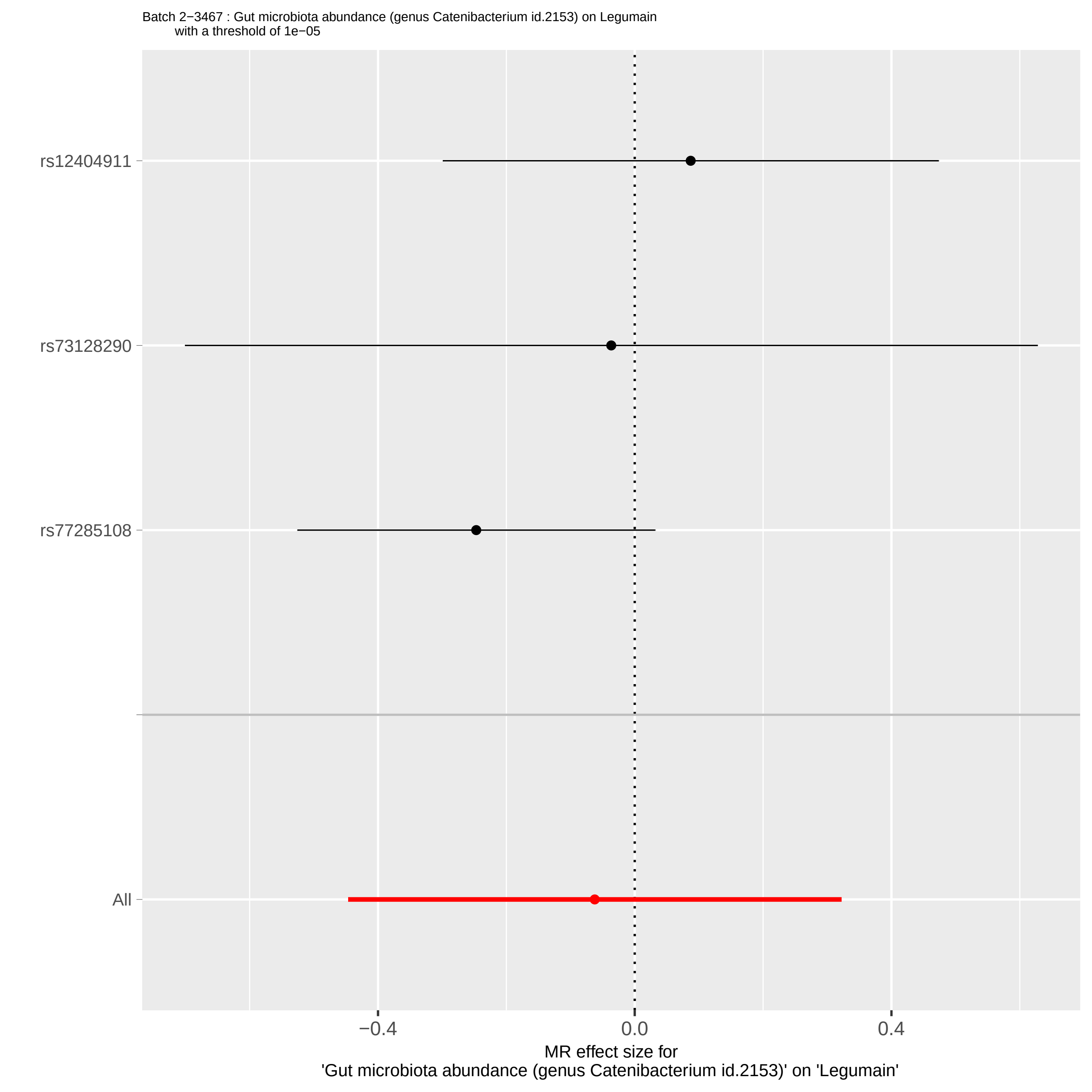
Batch 2−3467 : Gut microbiota abundance (genus Catenibacterium id.2153) on Legumain
with a threshold of 1e−05
rs12404911
rs73128290
rs77285108
All
−0.4
0.0
0.4
MR effect size for
'Gut microbiota abundance (genus Catenibacterium id.2153)' on 'Legumain'

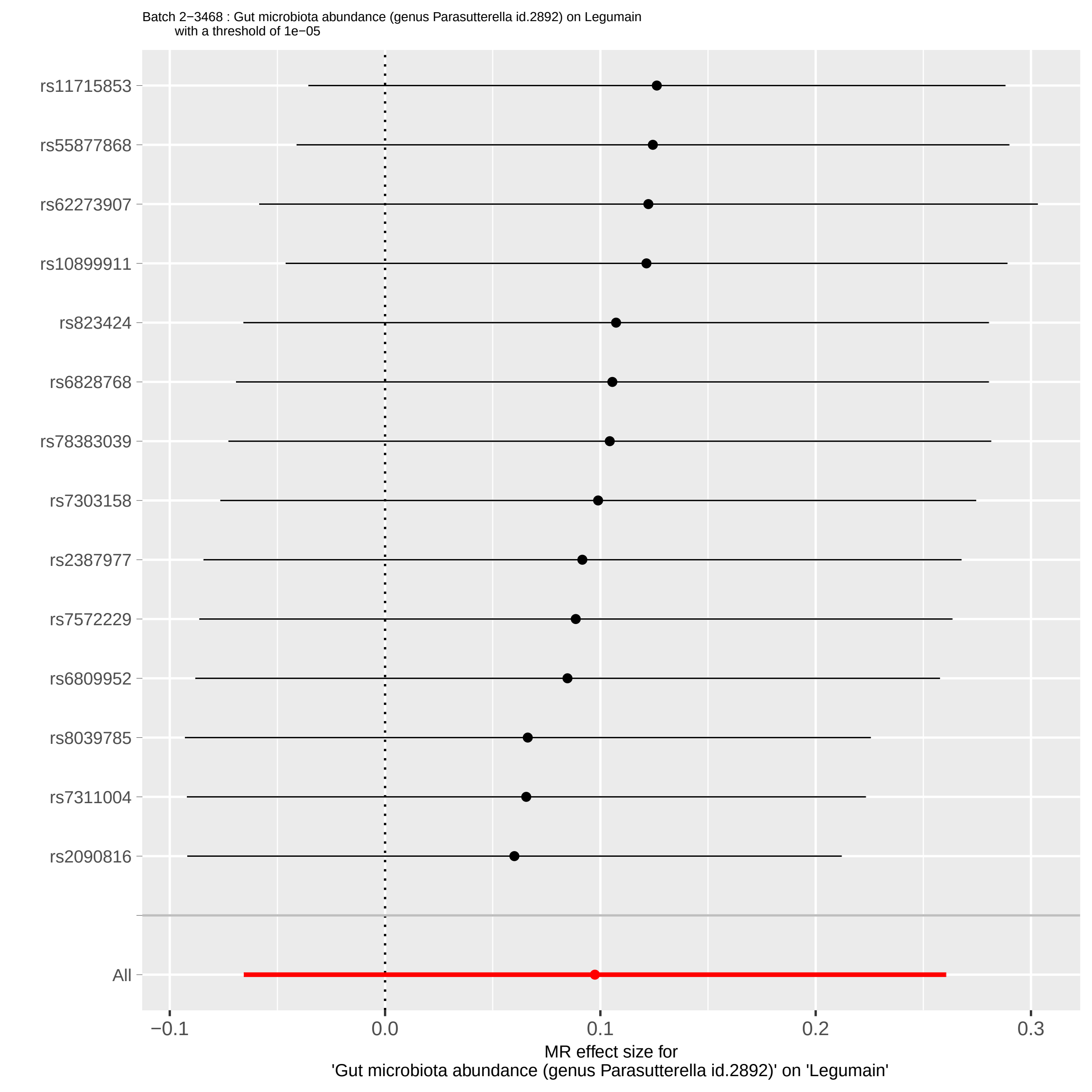
Batch 2−3468 : Gut microbiota abundance (genus Parasutterella id.2892) on Legumain
with a threshold of 1e−05
rs11715853
rs55877868
rs62273907
rs10899911
rs823424
rs6828768
rs78383039
rs7303158
rs2387977
rs7572229
rs6809952
rs8039785
rs7311004
rs2090816
All
−0.1
0.0
0.1
0.2
0.3
MR effect size for
'Gut microbiota abundance (genus Parasutterella id.2892)' on 'Legumain'

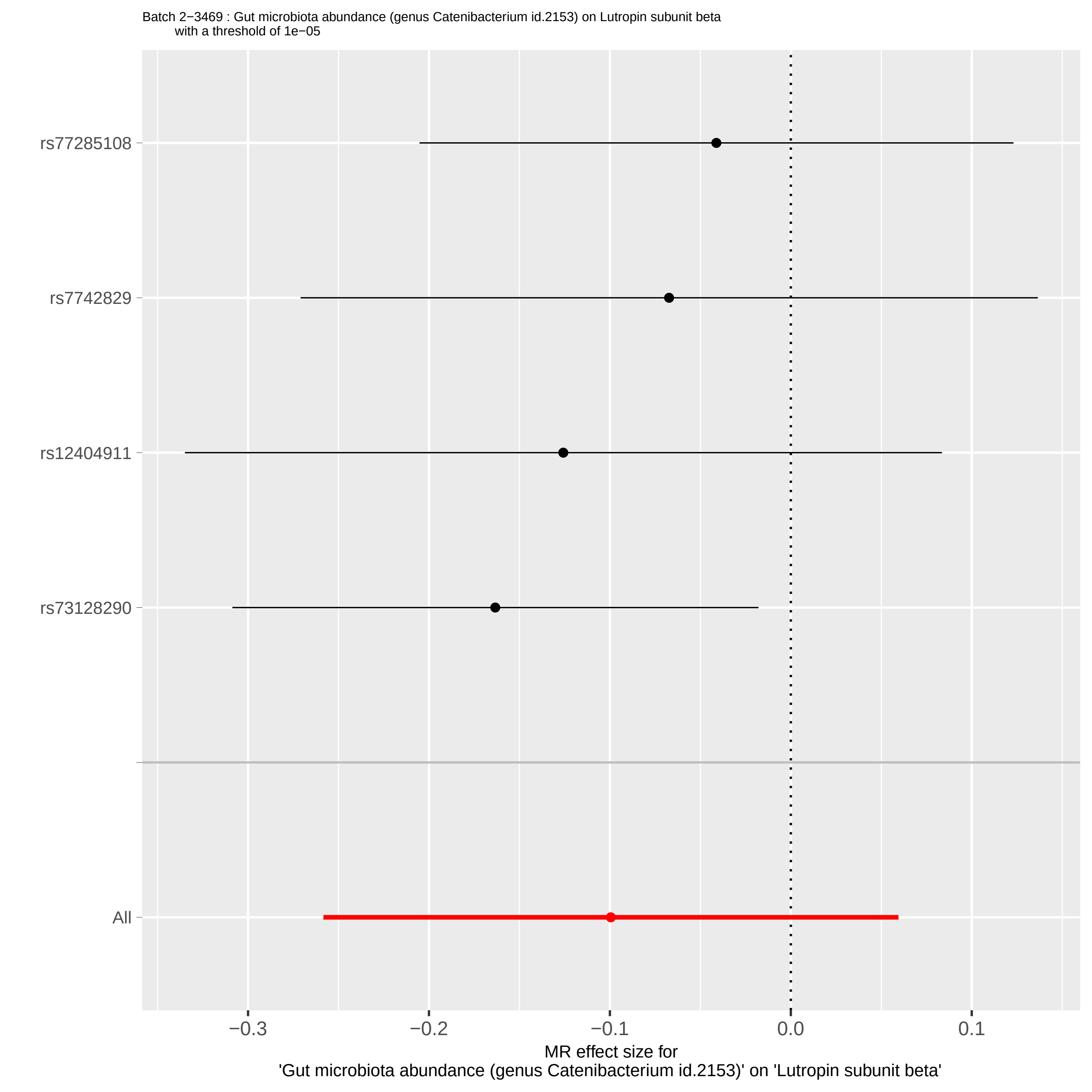
Batch 2−3469 : Gut microbiota abundance (genus Catenibacterium id.2153) on Lutropin subunit beta
with a threshold of 1e−05
rs77285108
rs7742829
rs12404911
rs73128290
All
−0.3
−0.2
−0.1
0.0
0.1
MR effect size for
'Gut microbiota abundance (genus Catenibacterium id.2153)' on 'Lutropin subunit beta'

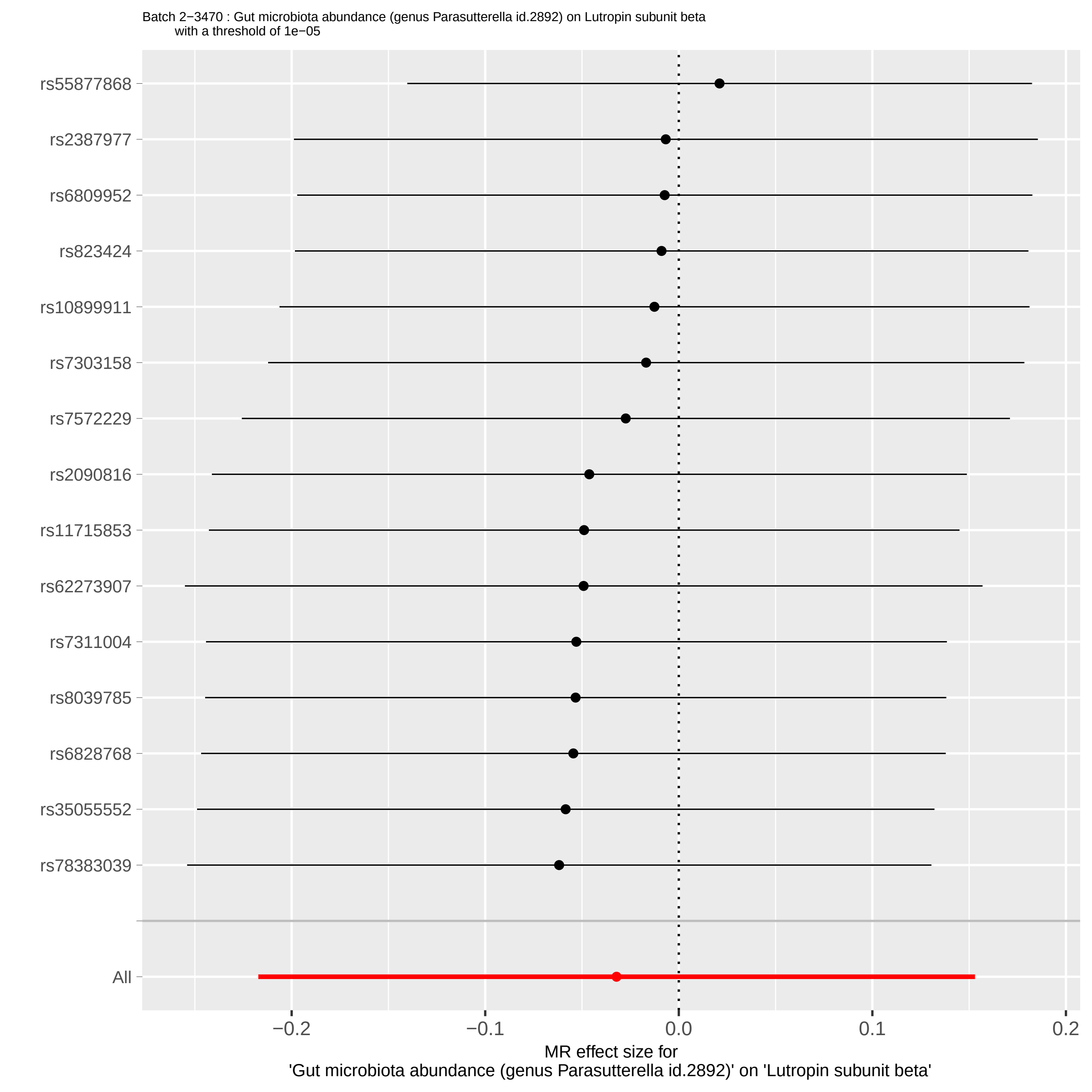

Batch 2−3470 : Gut microbiota abundance (genus Parasutterella id.2892) on Lutropin subunit beta
with a threshold of 1e−05
rs55877868
rs2387977
rs6809952
rs823424
rs10899911
rs7303158
rs7572229
rs2090816
rs11715853
rs62273907
rs7311004
rs8039785
rs6828768
rs35055552
rs78383039
All
−0.2
−0.1
0.0
0.1
0.2
MR effect size for
'Gut microbiota abundance (genus Parasutterella id.2892)' on 'Lutropin subunit beta'

Batch 2−3471 : Gut microbiota abundance (genus Catenibacterium id.2153) on Leukemia inhibitory factor
with a threshold of 1e−05

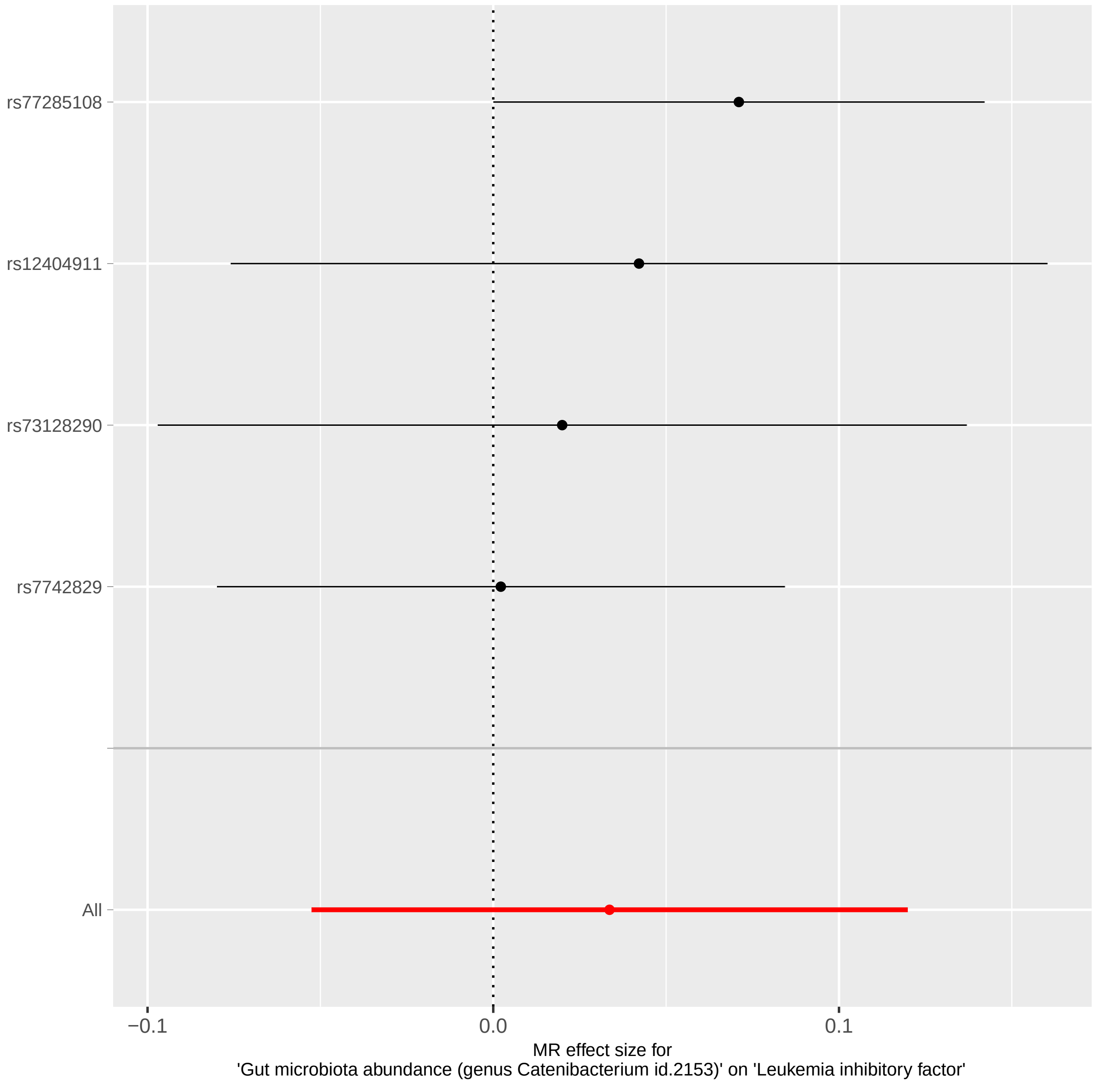

Batch 2−3472 : Gut microbiota abundance (genus Parasutterella id.2892) on Leukemia inhibitory factor
with a threshold of 1e−05

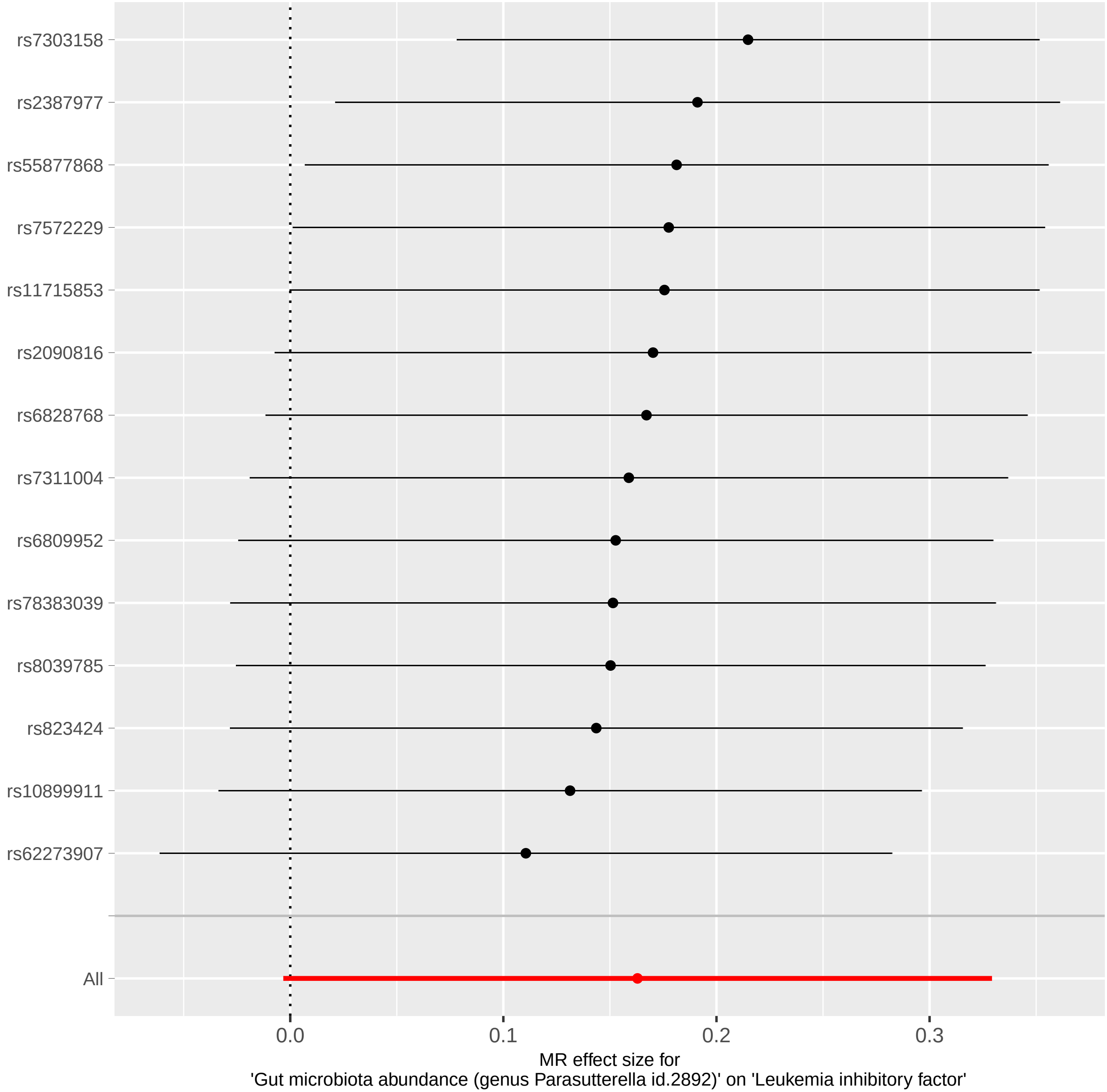

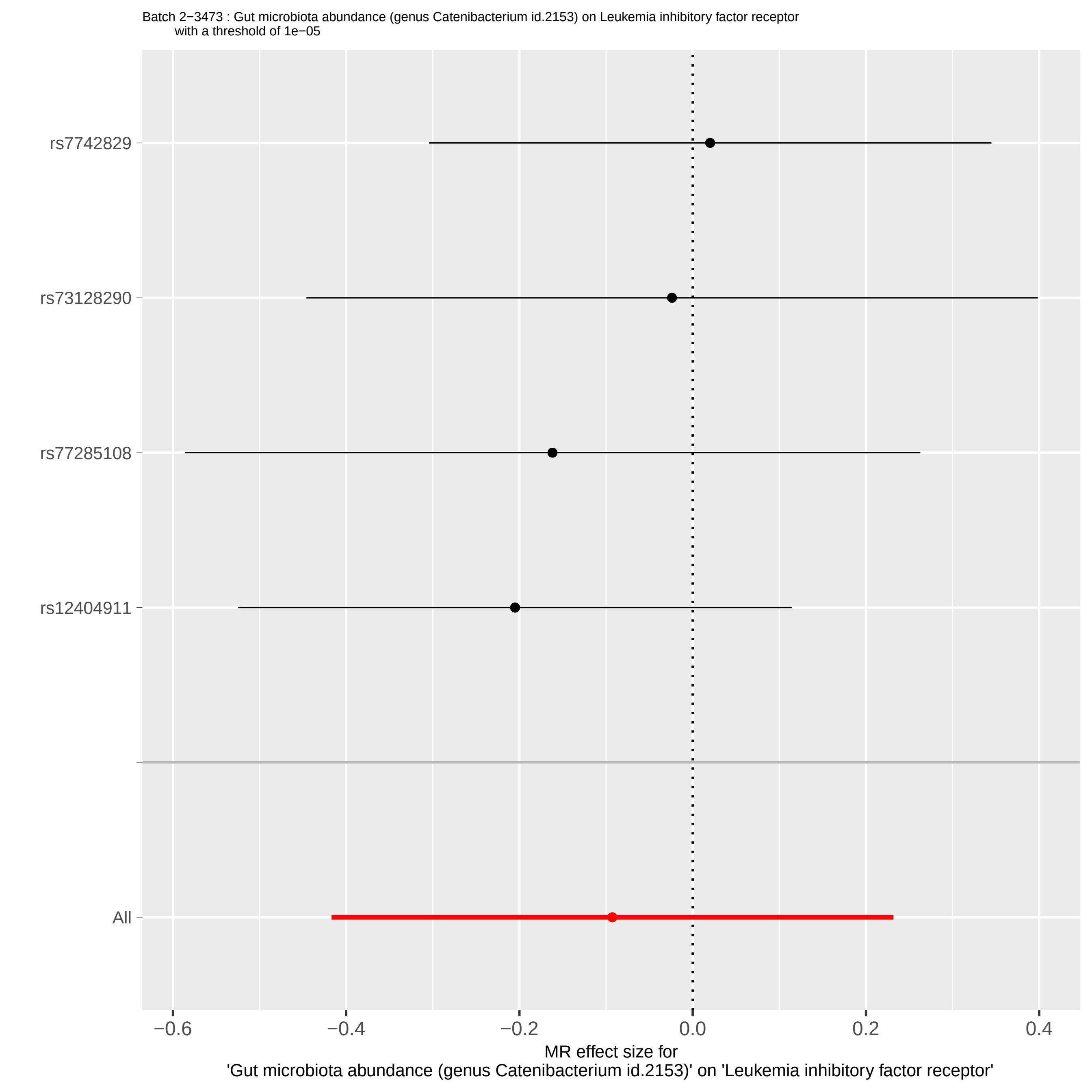
Batch 2−3473 : Gut microbiota abundance (genus Catenibacterium id.2153) on Leukemia inhibitory factor receptor
with a threshold of 1e−05
rs7742829
rs73128290
rs77285108
rs12404911
All
−0.6
−0.4
−0.2
0.0
0.2
0.4
MR effect size for
'Gut microbiota abundance (genus Catenibacterium id.2153)' on 'Leukemia inhibitory factor receptor'

Batch 2−3474 : Gut microbiota abundance (genus Parasutterella id.2892) on Leukemia inhibitory factor receptor
with a threshold of 1e−05

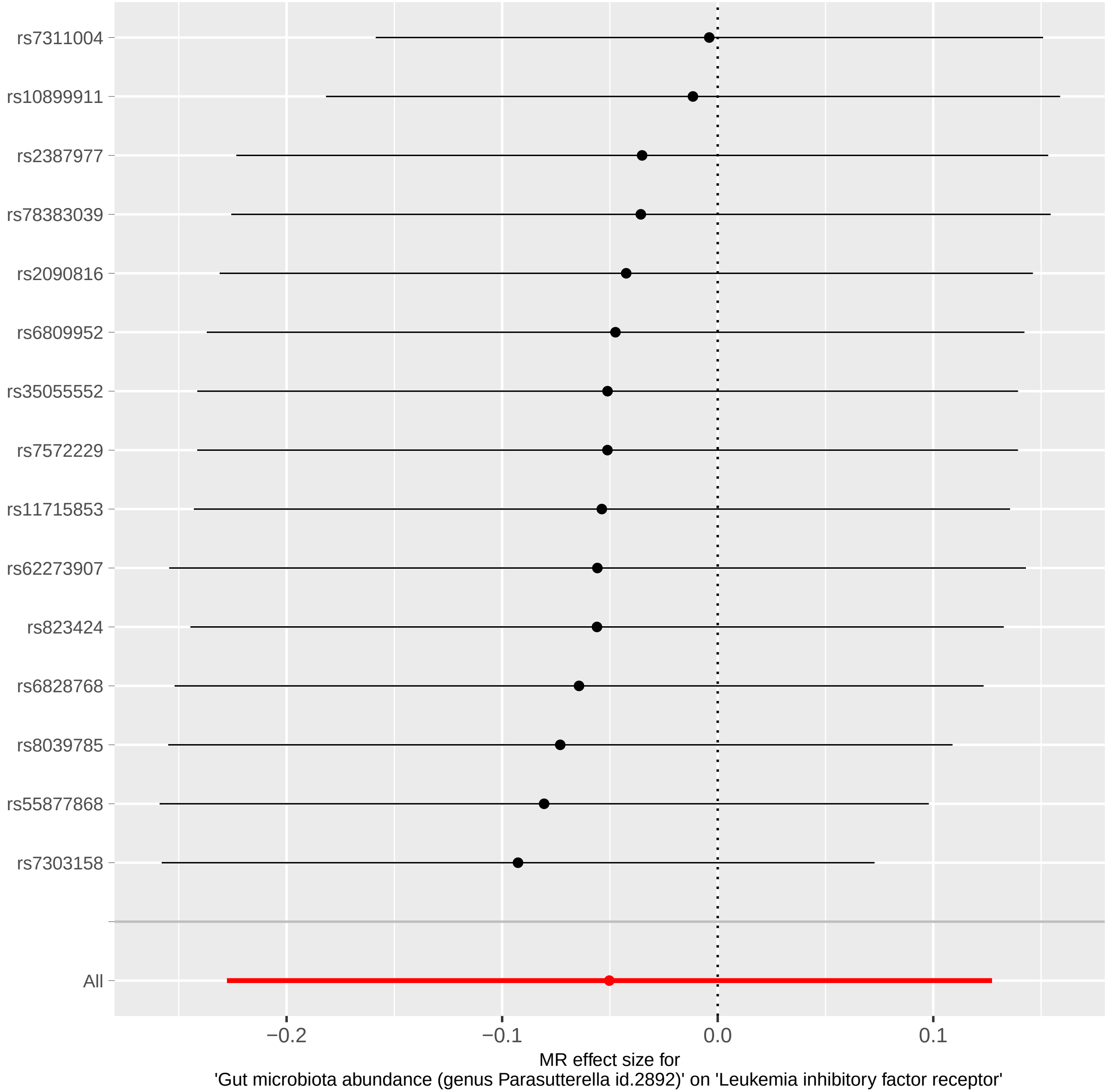

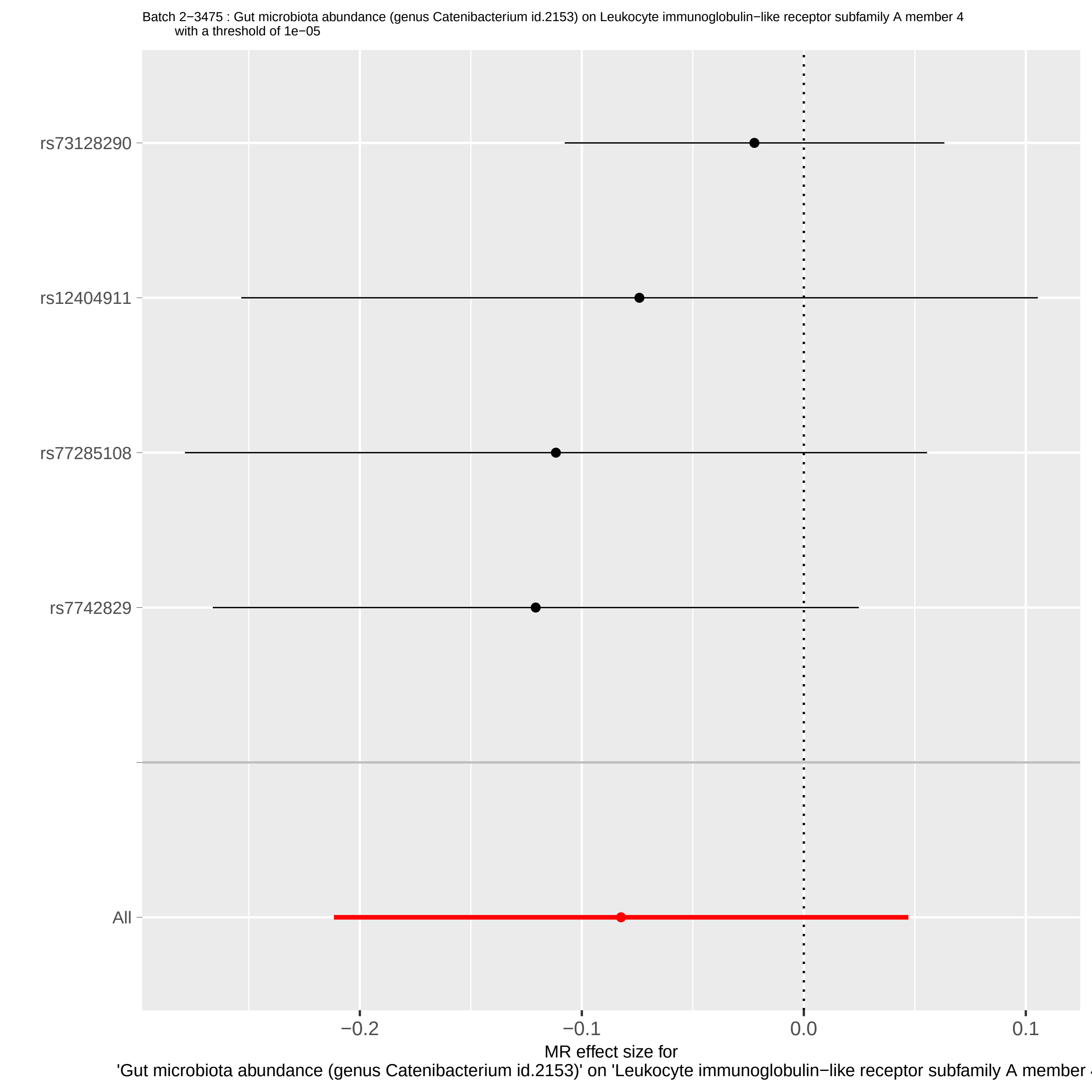

Batch 2−3475 : Gut microbiota abundance (genus Catenibacterium id.2153) on Leukocyte immunoglobulin−like receptor subfamily A member 4
with a threshold of 1e−05
rs73128290
rs12404911
rs77285108
rs7742829
All
−0.2
−0.1
0.0
0.1
MR effect size for
'Gut microbiota abundance (genus Catenibacterium id.2153)' on 'Leukocyte immunoglobulin−like receptor subfamily A member 4

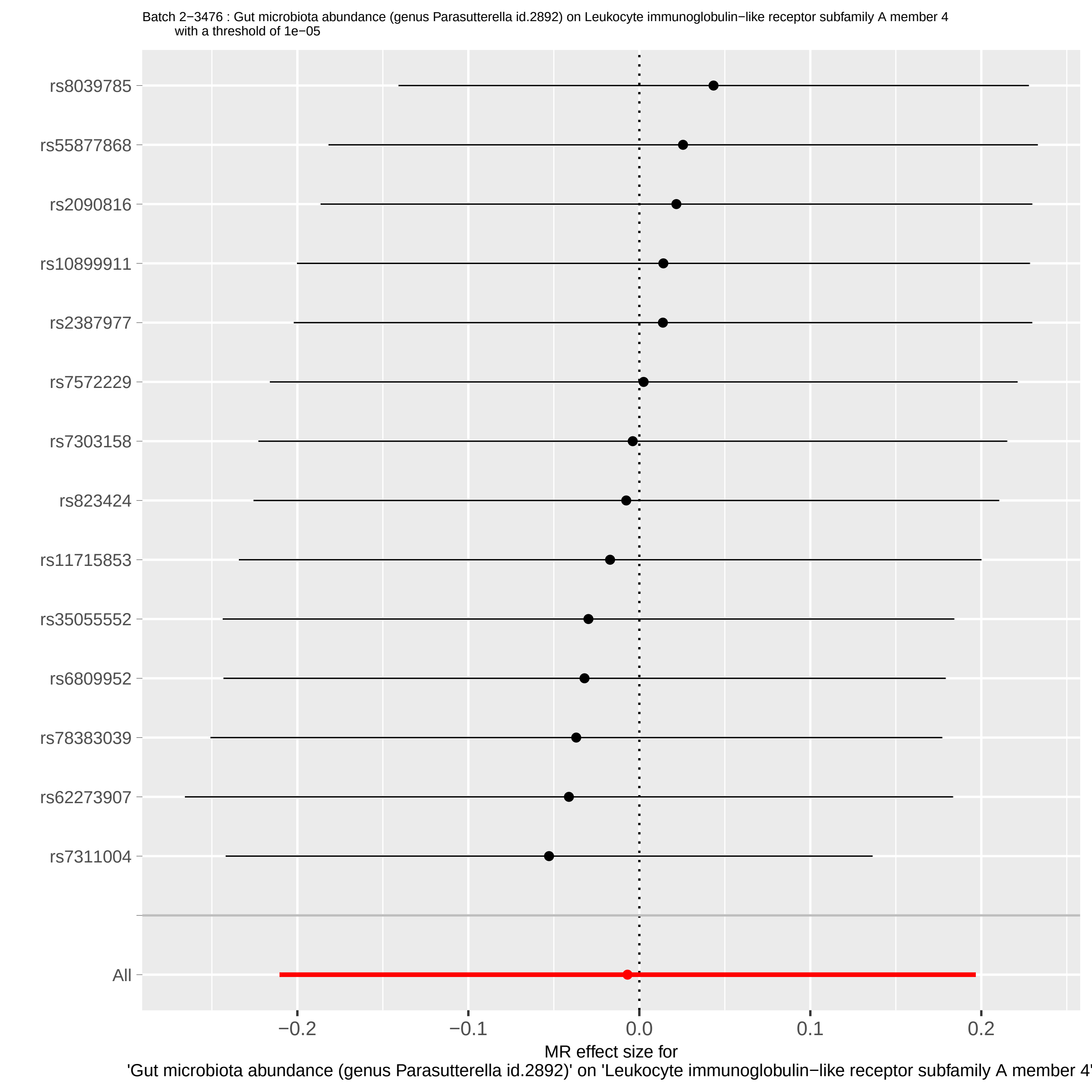
Batch 2−3476 : Gut microbiota abundance (genus Parasutterella id.2892) on Leukocyte immunoglobulin−like receptor subfamily A member 4
with a threshold of 1e−05
rs8039785
rs55877868
rs2090816
rs10899911
rs2387977
rs7572229
rs7303158
rs823424
rs11715853
rs35055552
rs6809952
rs78383039
rs62273907
rs7311004
All
−0.2
−0.1
0.0
0.1
0.2
MR effect size for
'Gut microbiota abundance (genus Parasutterella id.2892)' on 'Leukocyte immunoglobulin−like receptor subfamily A member 4'

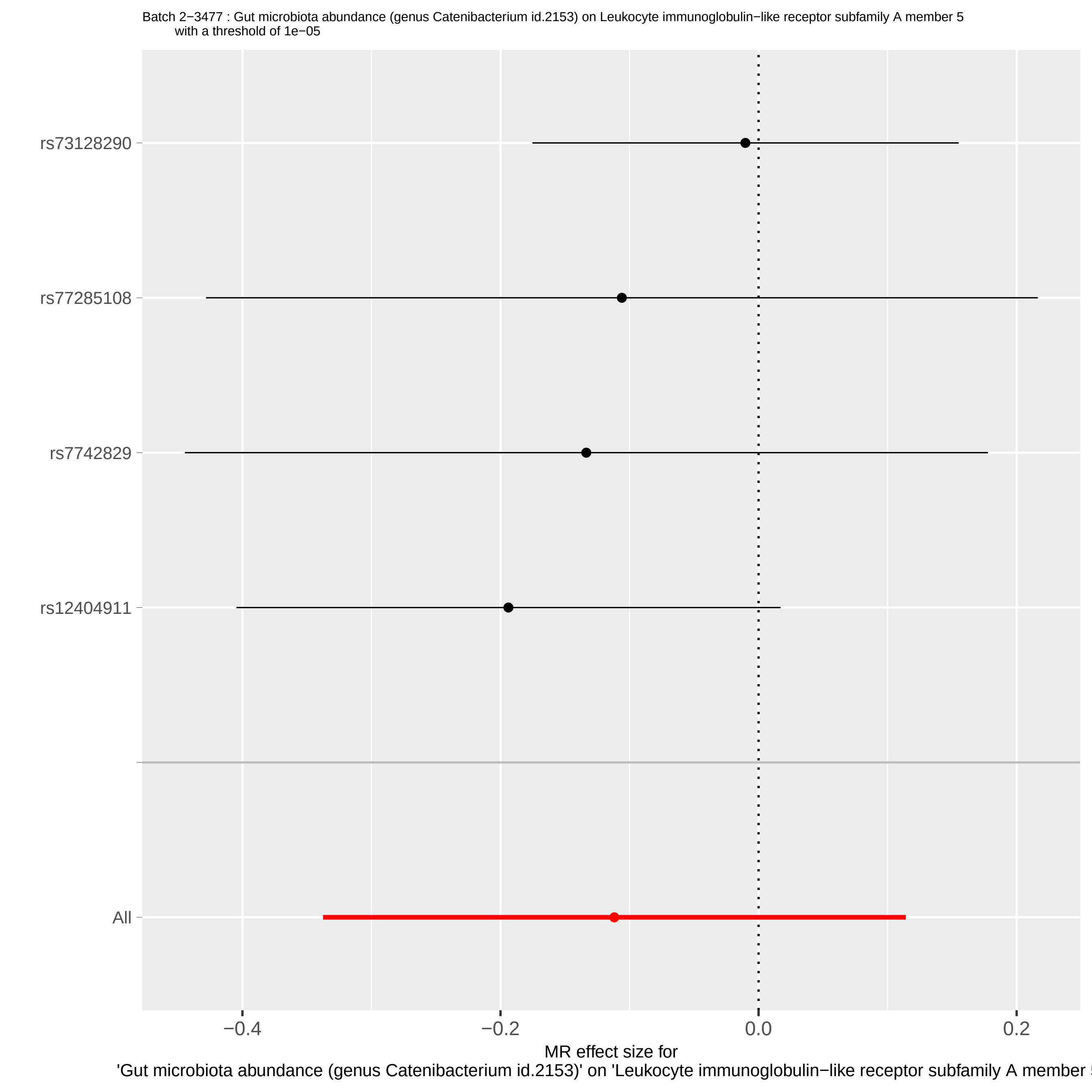
Batch 2−3477 : Gut microbiota abundance (genus Catenibacterium id.2153) on Leukocyte immunoglobulin−like receptor subfamily A member 5
with a threshold of 1e−05
rs73128290
rs77285108
rs7742829
rs12404911
All
−0.4
−0.2
0.0
0.2
MR effect size for
'Gut microbiota abundance (genus Catenibacterium id.2153)' on 'Leukocyte immunoglobulin−like receptor subfamily A member

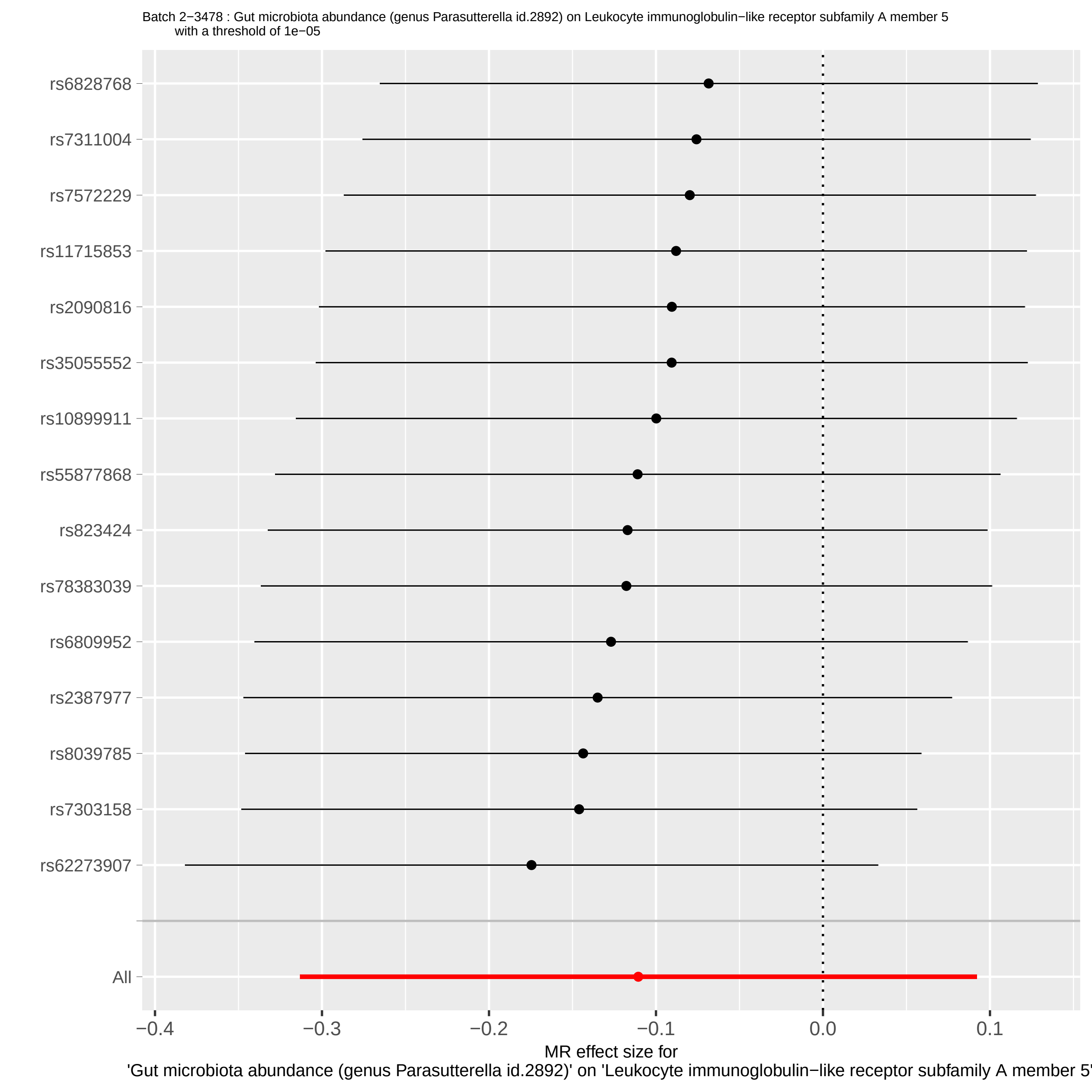

Batch 2−3478 : Gut microbiota abundance (genus Parasutterella id.2892) on Leukocyte immunoglobulin−like receptor subfamily A member 5
with a threshold of 1e−05
rs6828768
rs7311004
rs7572229
rs11715853
rs2090816
rs35055552
rs10899911
rs55877868
rs823424
rs78383039
rs6809952
rs2387977
rs8039785
rs7303158
rs62273907
All
−0.4
−0.3
−0.2
−0.1
0.0
0.1
MR effect size for
'Gut microbiota abundance (genus Parasutterella id.2892)' on 'Leukocyte immunoglobulin−like receptor subfamily A member 5'

Batch 2−3479 : Gut microbiota abundance (genus Catenibacterium id.2153) on Leukocyte immunoglobulin−like receptor subfamily A member 5 with a threshold of 1e−05

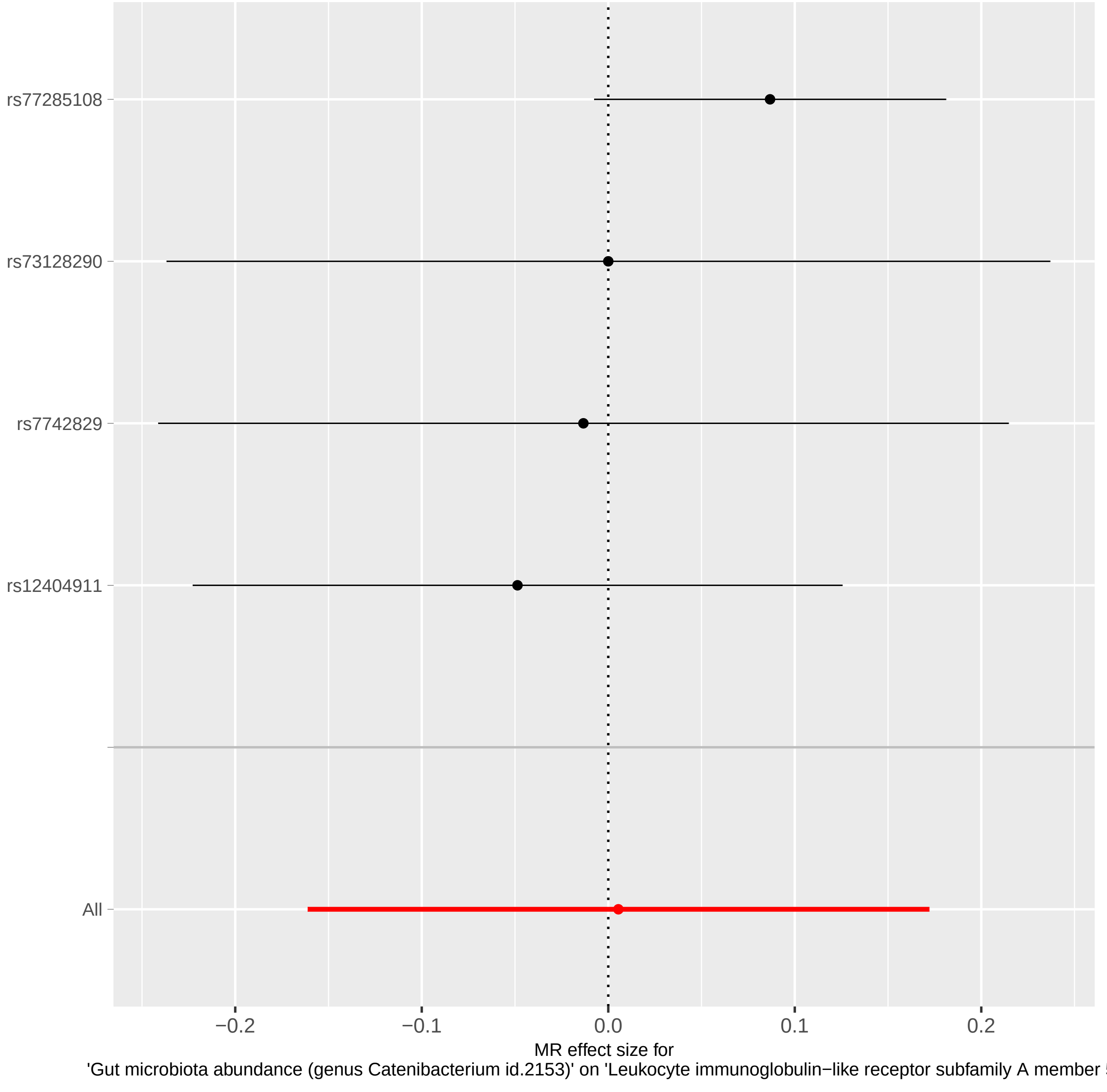

Batch 2−3480 : Gut microbiota abundance (genus Parasutterella id.2892) on Leukocyte immunoglobulin−like receptor subfamily A member 5
with a threshold of 1e−05

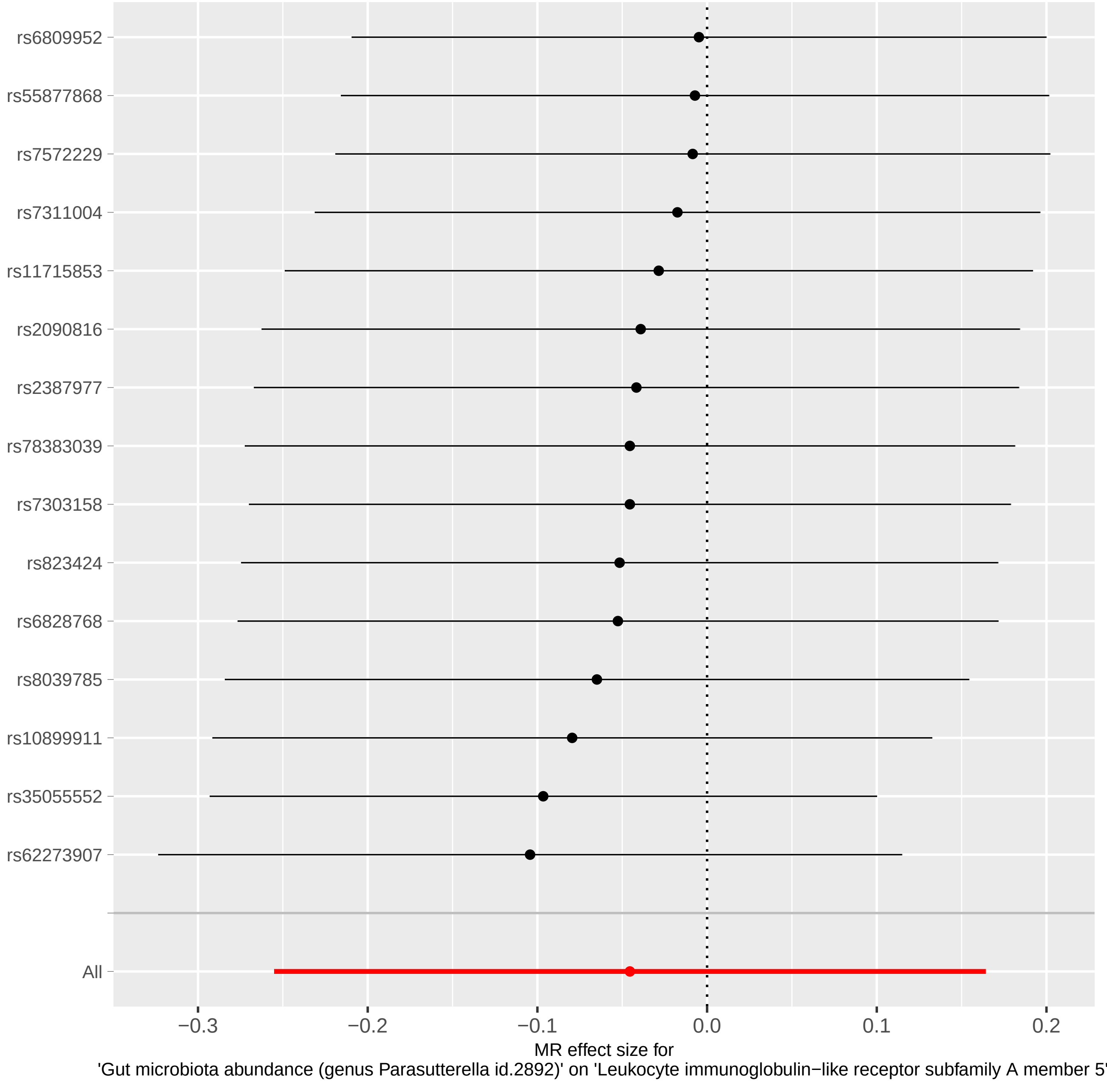

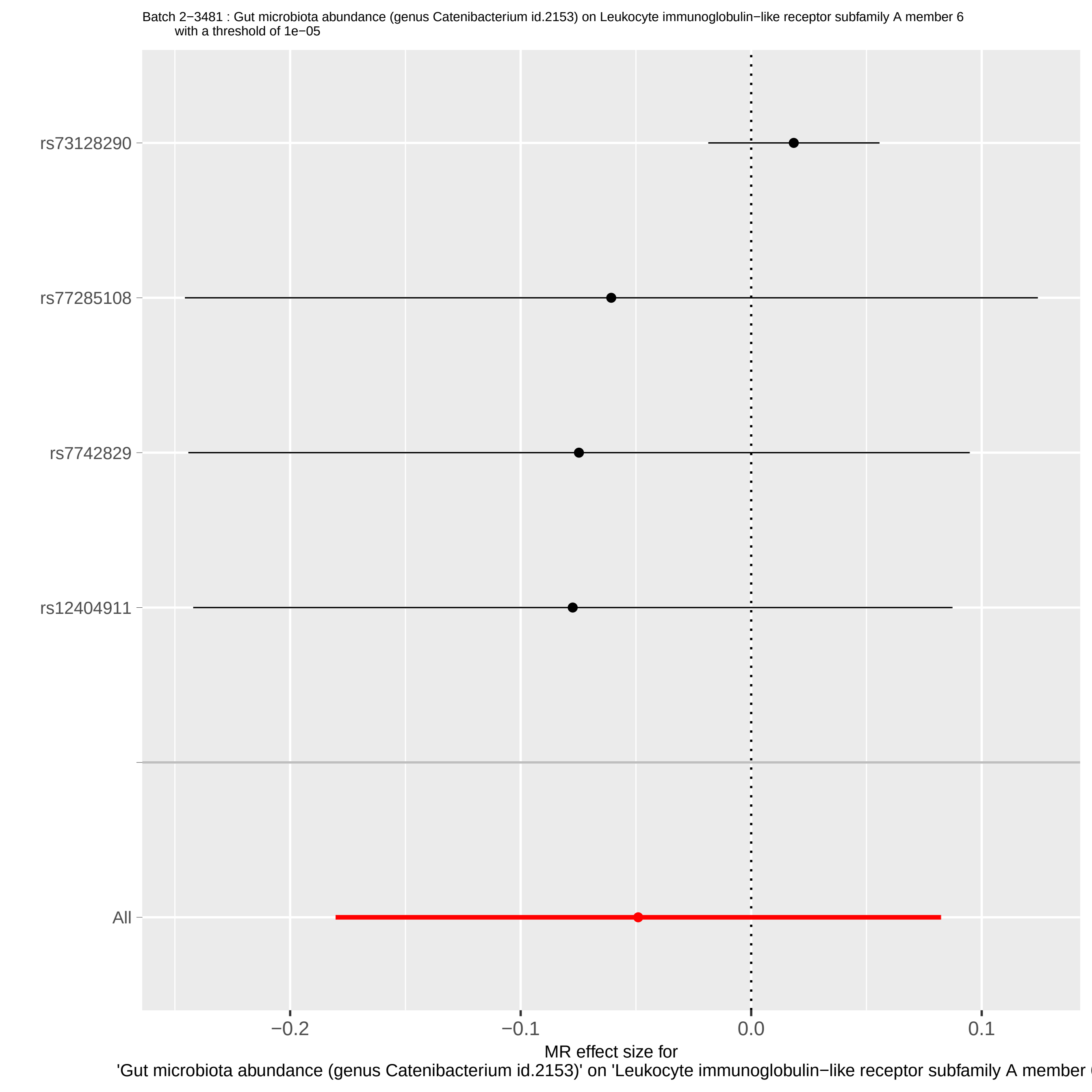
Batch 2−3481 : Gut microbiota abundance (genus Catenibacterium id.2153) on Leukocyte immunoglobulin−like receptor subfamily A member 6
with a threshold of 1e−05
rs73128290
rs77285108
rs7742829
rs12404911
All
−0.2
−0.1
0.0
0.1
MR effect size for
'Gut microbiota abundance (genus Catenibacterium id.2153)' on 'Leukocyte immunoglobulin−like receptor subfamily A member

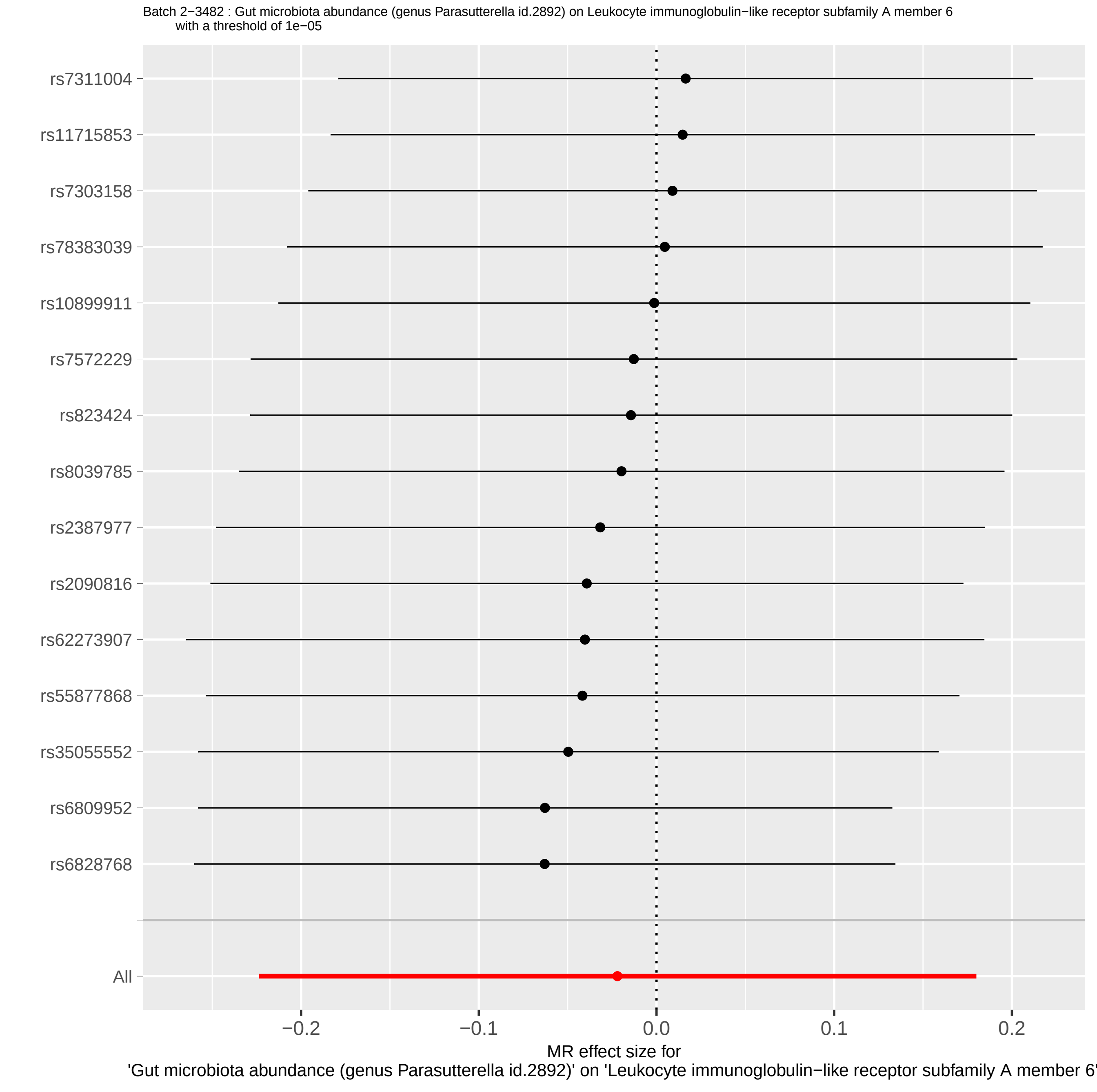

Batch 2−3482 : Gut microbiota abundance (genus Parasutterella id.2892) on Leukocyte immunoglobulin−like receptor subfamily A member 6 with a threshold of 1e−05
rs7311004
rs11715853
rs7303158
rs78383039
rs10899911
rs7572229
rs823424
rs8039785
rs2387977
rs2090816
rs62273907
rs55877868
rs35055552
rs6809952
rs6828768
All
−0.2
−0.1
0.0
0.1
0.2
MR effect size for
'Gut microbiota abundance (genus Parasutterella id.2892)' on 'Leukocyte immunoglobulin−like receptor subfamily A member 6'

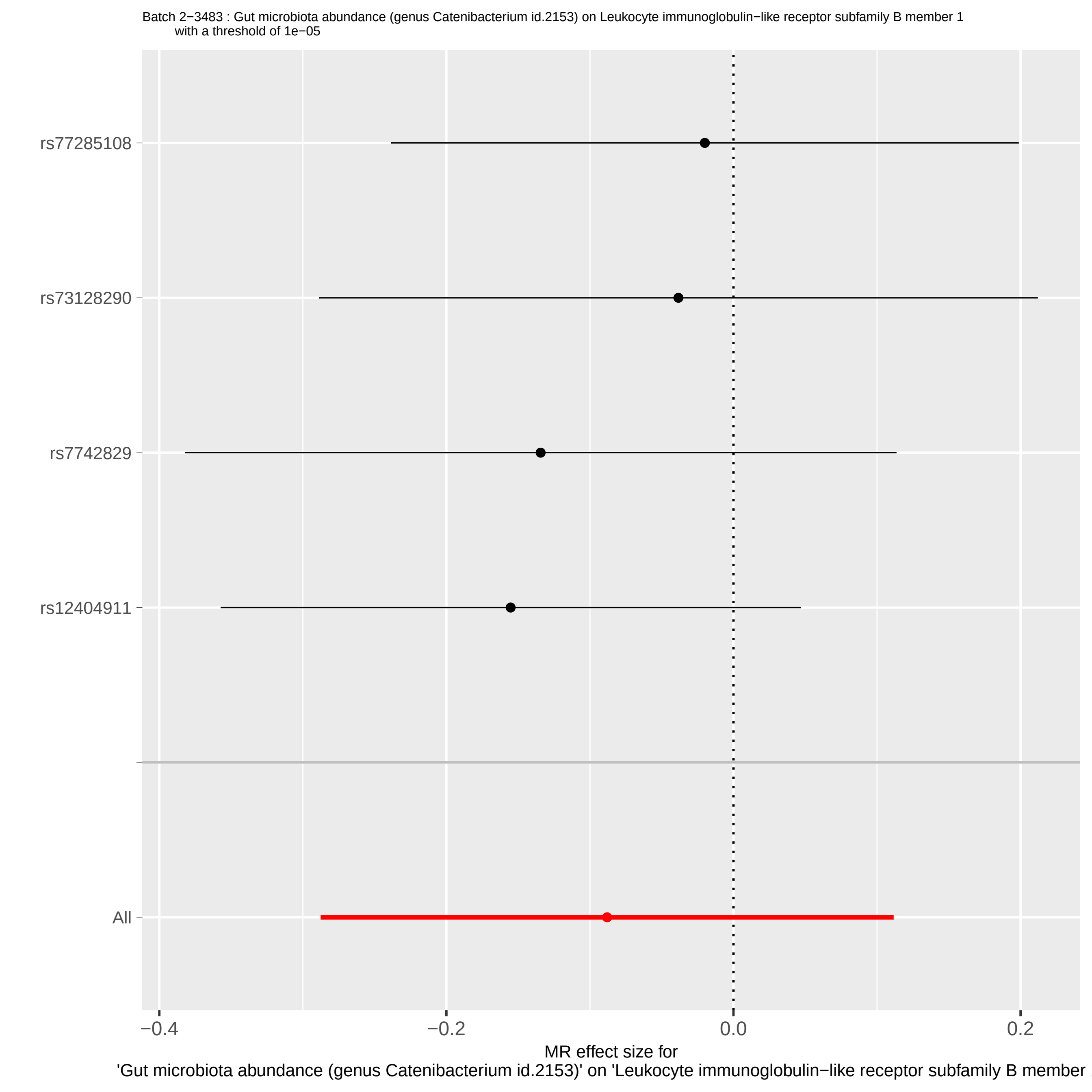

Batch 2−3483 : Gut microbiota abundance (genus Catenibacterium id.2153) on Leukocyte immunoglobulin−like receptor subfamily B member 1
with a threshold of 1e−05
rs77285108
rs73128290
rs7742829
rs12404911
All
−0.4
−0.2
0.0
0.2
MR effect size for
'Gut microbiota abundance (genus Catenibacterium id.2153)' on 'Leukocyte immunoglobulin−like receptor subfamily B member

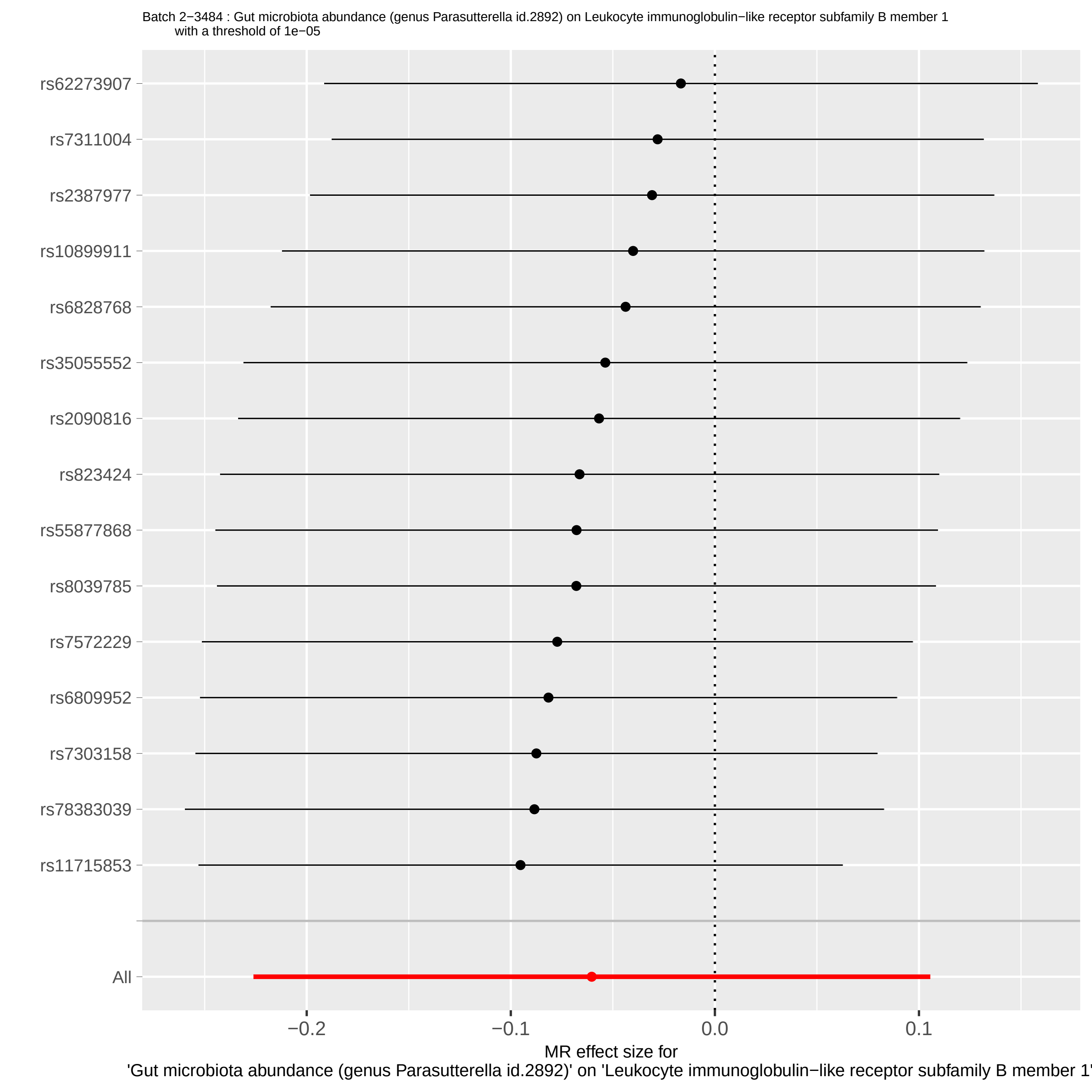
Batch 2−3484 : Gut microbiota abundance (genus Parasutterella id.2892) on Leukocyte immunoglobulin−like receptor subfamily B member 1
with a threshold of 1e−05
rs62273907
rs7311004
rs2387977
rs10899911
rs6828768
rs35055552
rs2090816
rs823424
rs55877868
rs8039785
rs7572229
rs6809952
rs7303158
rs78383039
rs11715853
All
−0.2
−0.1
0.0
0.1
MR effect size for
'Gut microbiota abundance (genus Parasutterella id.2892)' on 'Leukocyte immunoglobulin−like receptor subfamily B member 1'

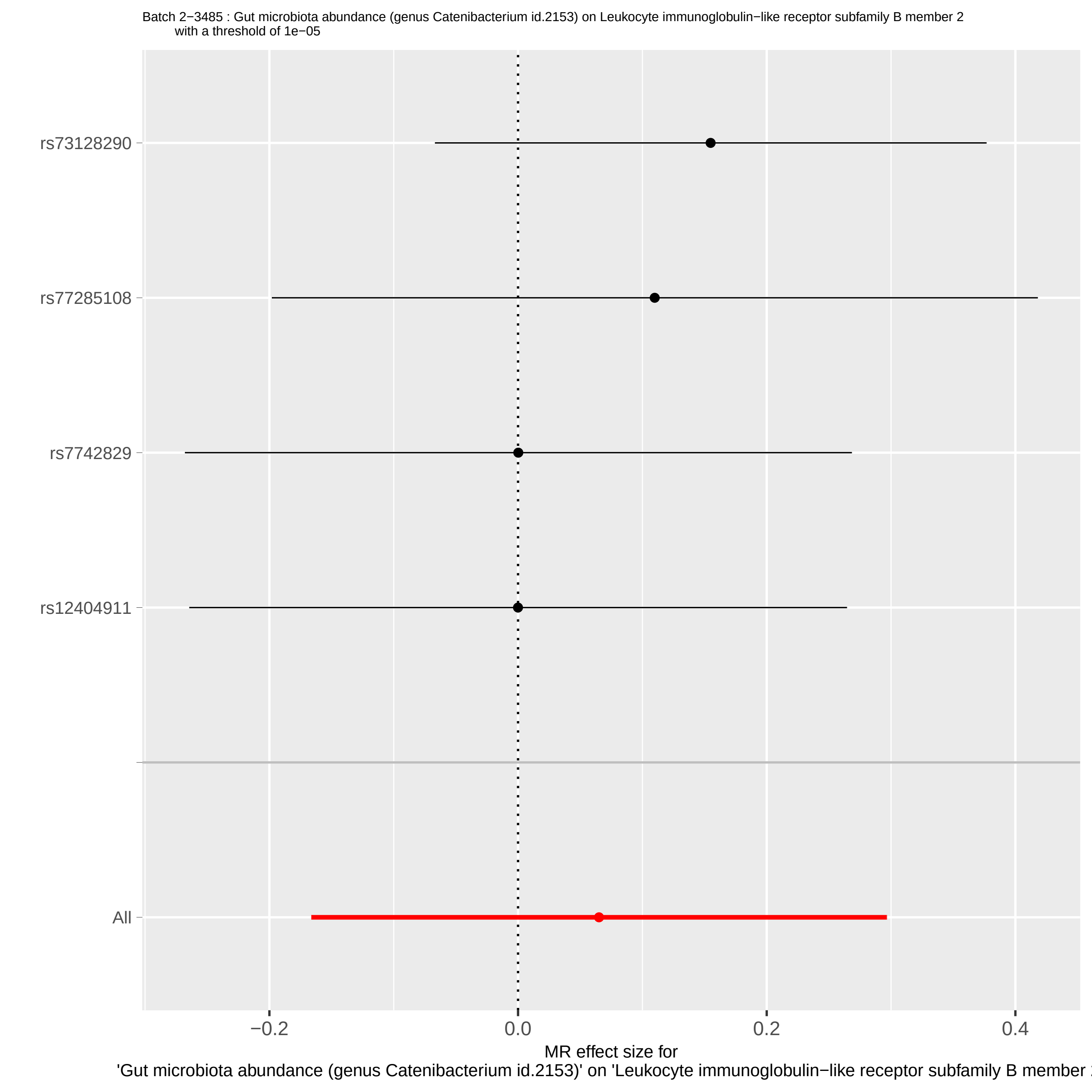
Batch 2−3485 : Gut microbiota abundance (genus Catenibacterium id.2153) on Leukocyte immunoglobulin−like receptor subfamily B member 2
with a threshold of 1e−05
rs73128290
rs77285108
rs7742829
rs12404911
All
−0.2
0.0
0.2
0.4
MR effect size for
'Gut microbiota abundance (genus Catenibacterium id.2153)' on 'Leukocyte immunoglobulin−like receptor subfamily B member

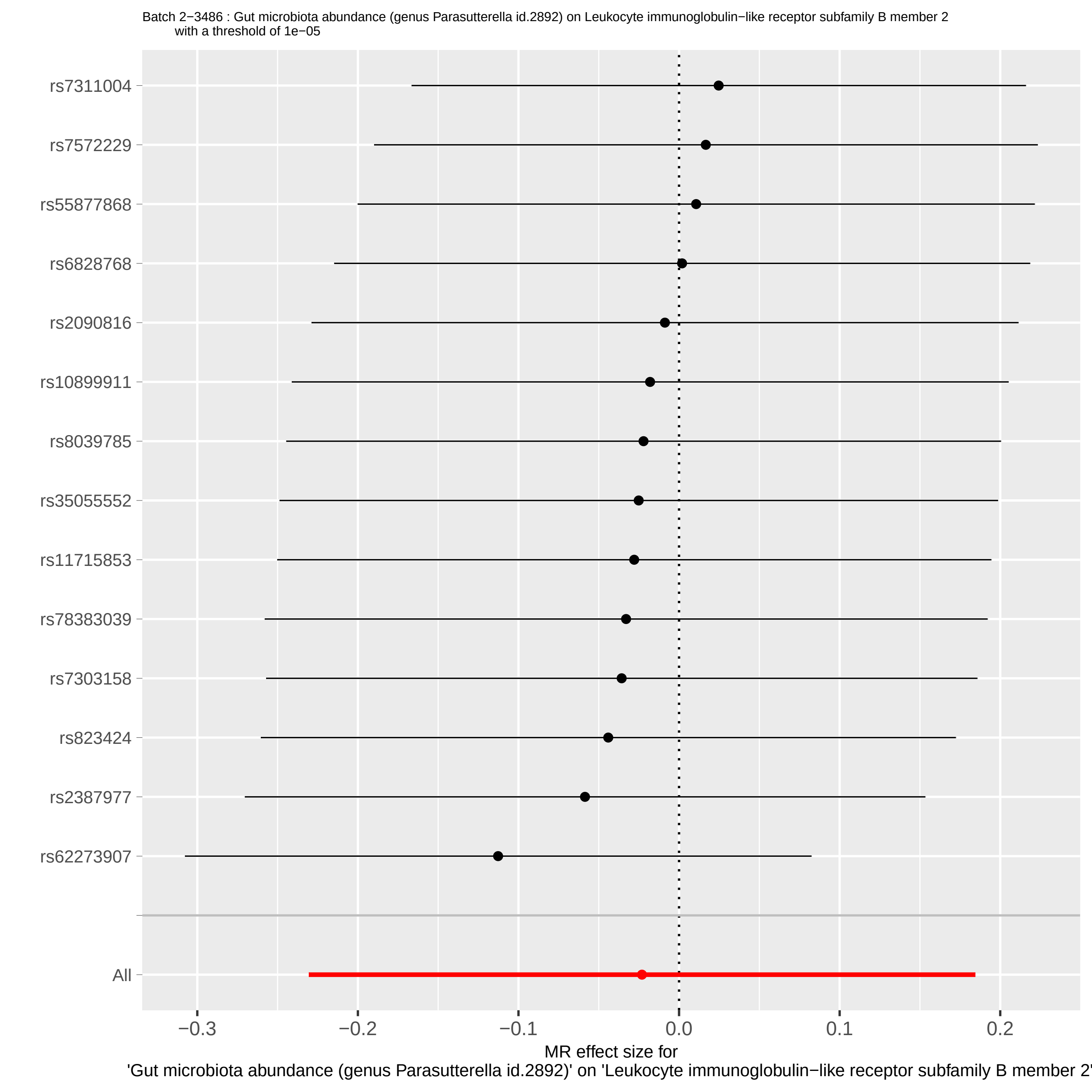

Batch 2−3486 : Gut microbiota abundance (genus Parasutterella id.2892) on Leukocyte immunoglobulin−like receptor subfamily B member 2
with a threshold of 1e−05
rs7311004
rs7572229
rs55877868
rs6828768
rs2090816
rs10899911
rs8039785
rs35055552
rs11715853
rs78383039
rs7303158
rs823424
rs2387977
rs62273907
All
−0.3
−0.2
−0.1
0.0
0.1
0.2
MR effect size for
'Gut microbiota abundance (genus Parasutterella id.2892)' on 'Leukocyte immunoglobulin−like receptor subfamily B member 2'

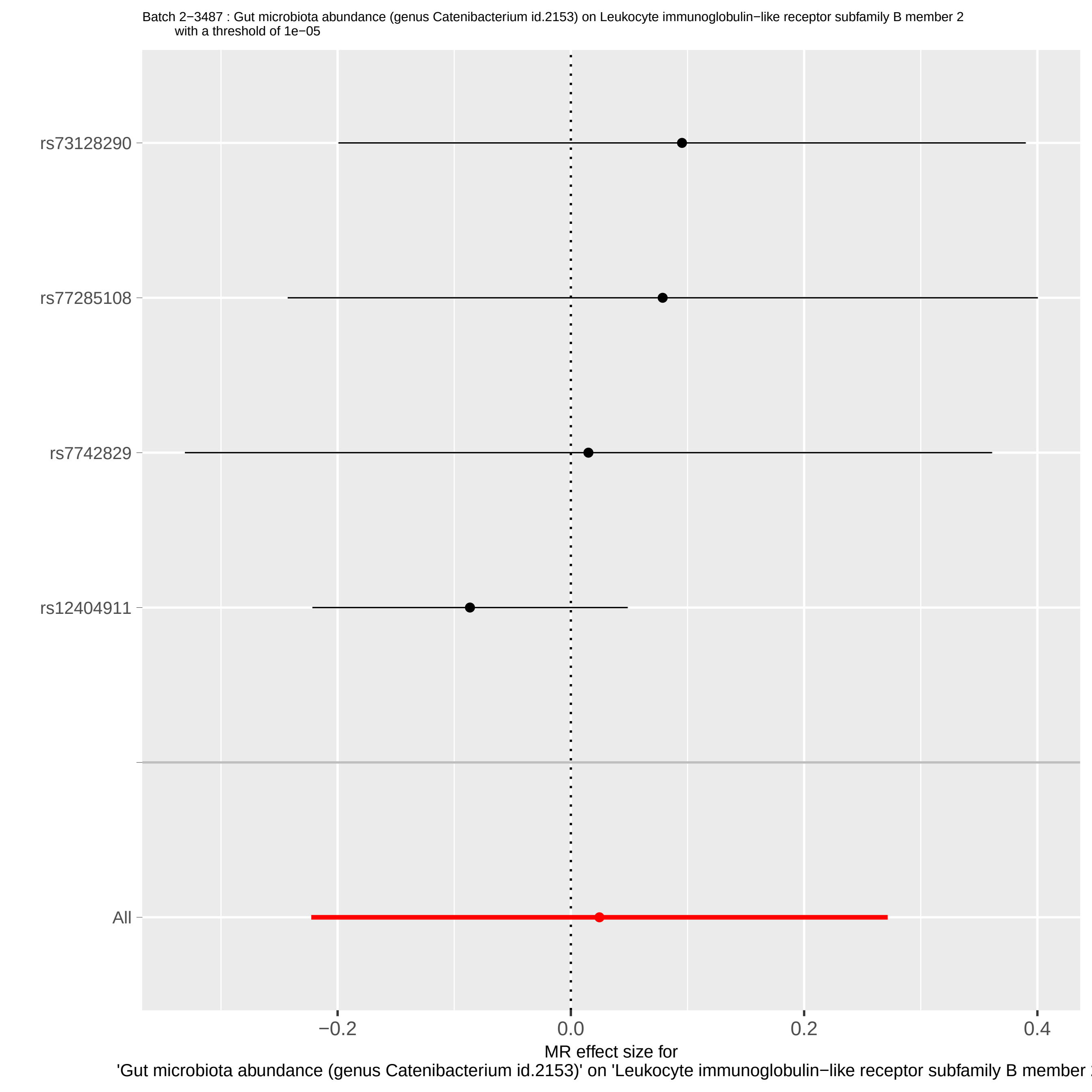

Batch 2−3487 : Gut microbiota abundance (genus Catenibacterium id.2153) on Leukocyte immunoglobulin−like receptor subfamily B member 2
with a threshold of 1e−05
rs73128290
rs77285108
rs7742829
rs12404911
All
−0.2
0.0
0.2
0.4
MR effect size for
'Gut microbiota abundance (genus Catenibacterium id.2153)' on 'Leukocyte immunoglobulin−like receptor subfamily B member

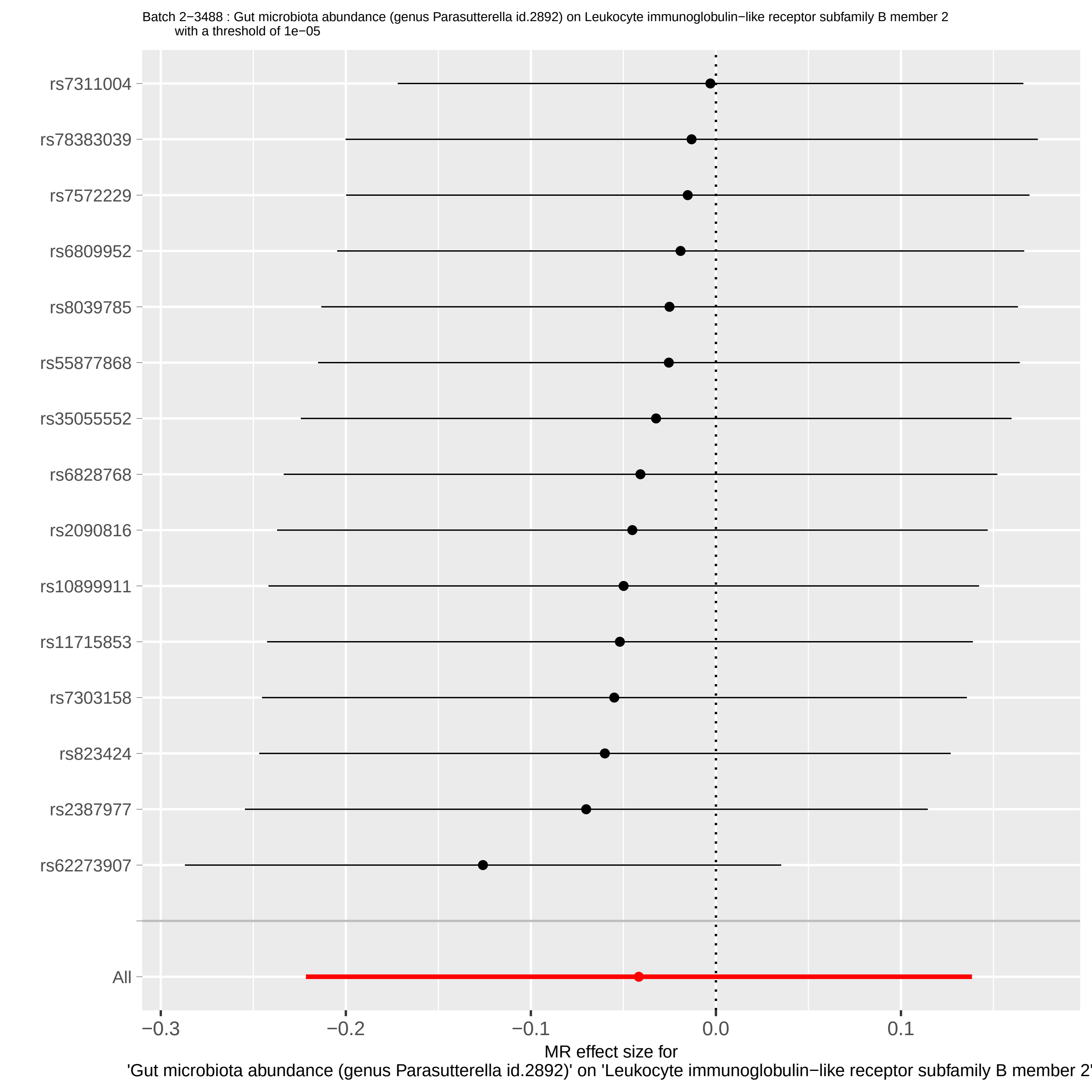

Batch 2−3488 : Gut microbiota abundance (genus Parasutterella id.2892) on Leukocyte immunoglobulin−like receptor subfamily B member 2
with a threshold of 1e−05
rs7311004
rs78383039
rs7572229
rs6809952
rs8039785
rs55877868
rs35055552
rs6828768
rs2090816
rs10899911
rs11715853
rs7303158
rs823424
rs2387977
rs62273907
All
−0.3
−0.2
−0.1
0.0
0.1
MR effect size for
'Gut microbiota abundance (genus Parasutterella id.2892)' on 'Leukocyte immunoglobulin−like receptor subfamily B member 2'

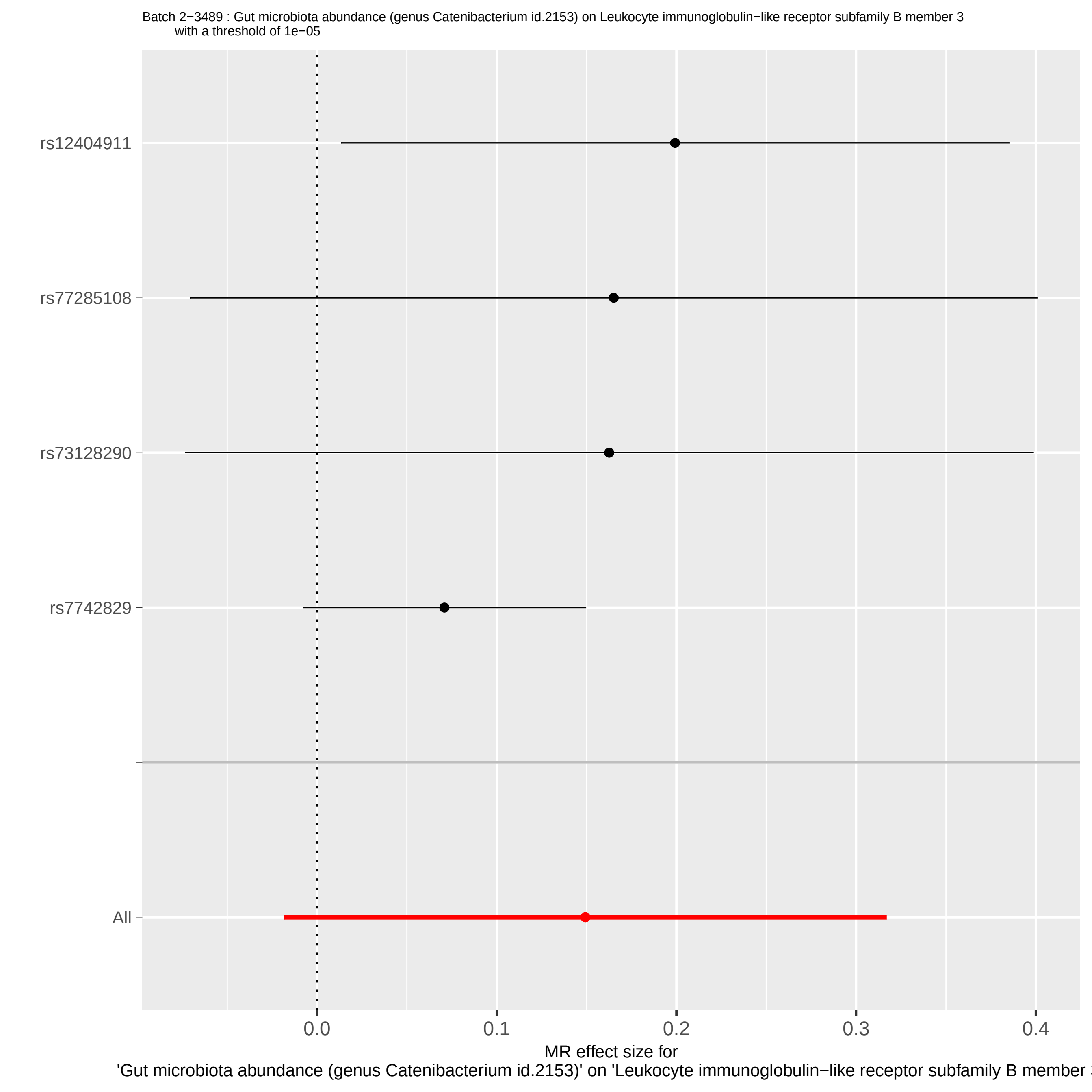
Batch 2−3489 : Gut microbiota abundance (genus Catenibacterium id.2153) on Leukocyte immunoglobulin−like receptor subfamily B member 3
with a threshold of 1e−05
rs12404911
rs77285108
rs73128290
rs7742829
All
0.0
0.1
0.2
0.3
0.4
MR effect size for
'Gut microbiota abundance (genus Catenibacterium id.2153)' on 'Leukocyte immunoglobulin−like receptor subfamily B member 3

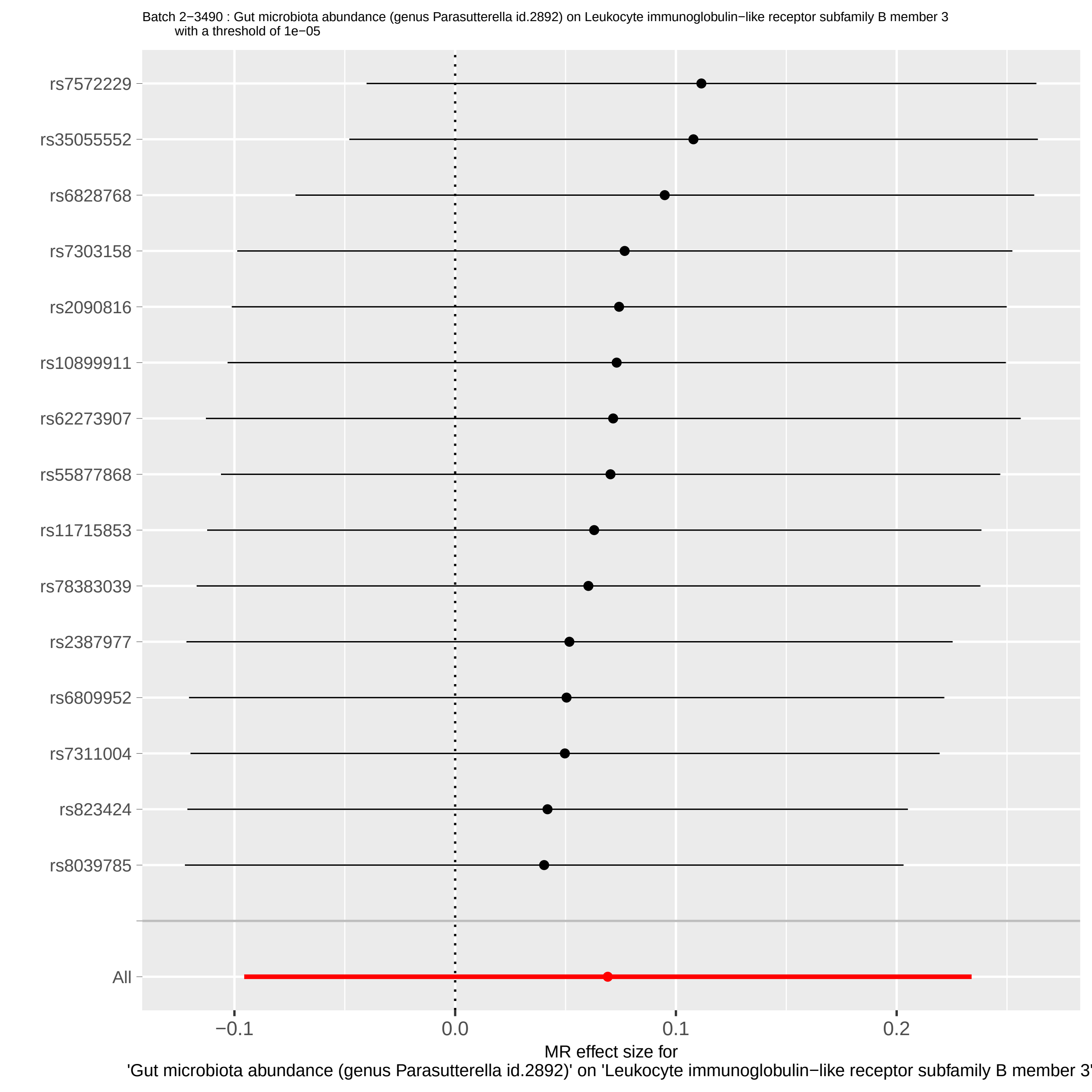
Batch 2−3490 : Gut microbiota abundance (genus Parasutterella id.2892) on Leukocyte immunoglobulin−like receptor subfamily B member 3
with a threshold of 1e−05
rs7572229
rs35055552
rs6828768
rs7303158
rs2090816
rs10899911
rs62273907
rs55877868
rs11715853
rs78383039
rs2387977
rs6809952
rs7311004
rs823424
rs8039785
All
−0.1
0.0
0.1
0.2
MR effect size for
'Gut microbiota abundance (genus Parasutterella id.2892)' on 'Leukocyte immunoglobulin−like receptor subfamily B member 3'

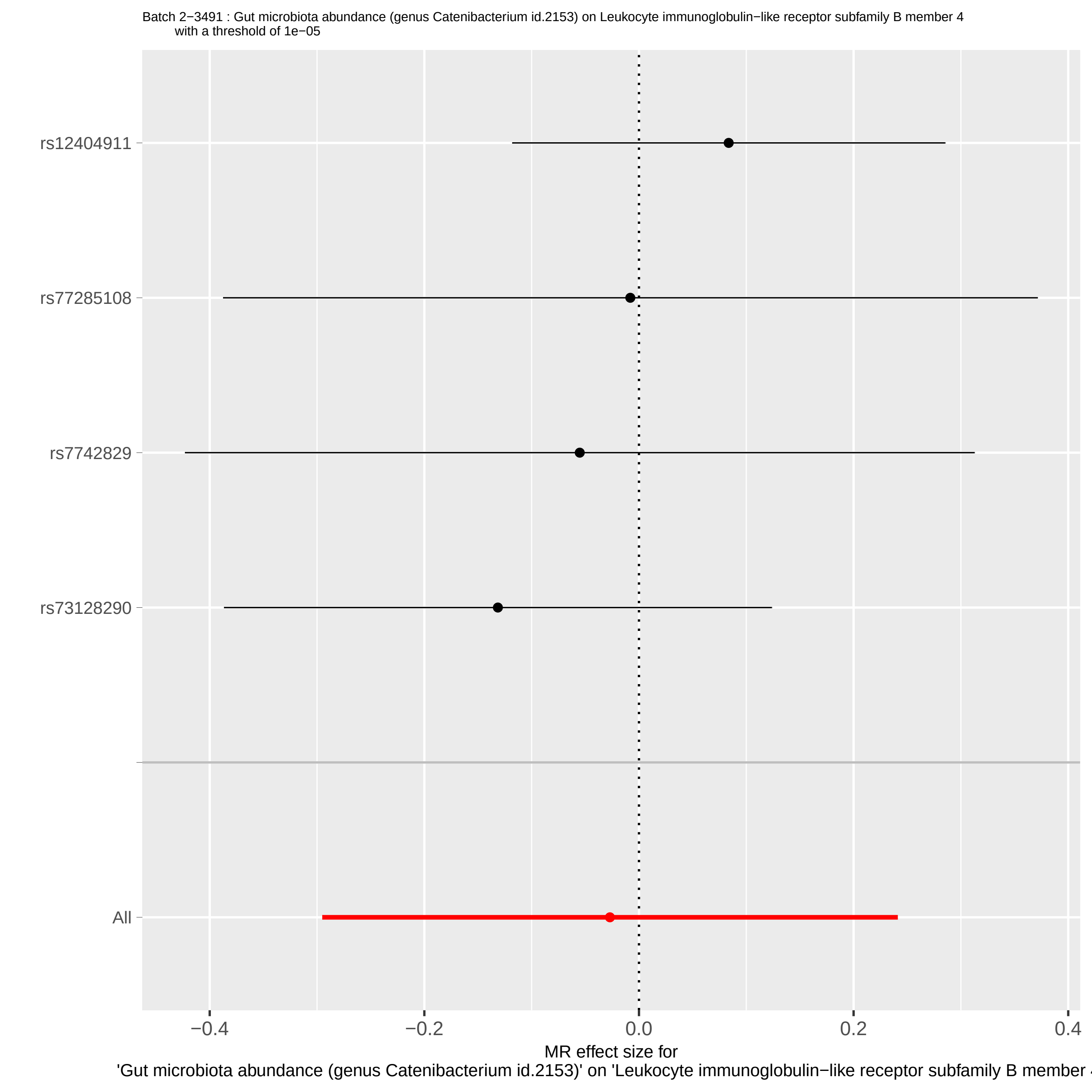
Batch 2−3491 : Gut microbiota abundance (genus Catenibacterium id.2153) on Leukocyte immunoglobulin−like receptor subfamily B member 4
with a threshold of 1e−05
rs12404911
rs77285108
rs7742829
rs73128290
All
−0.4
−0.2
0.0
0.2
0.4
MR effect size for
'Gut microbiota abundance (genus Catenibacterium id.2153)' on 'Leukocyte immunoglobulin−like receptor subfamily B member

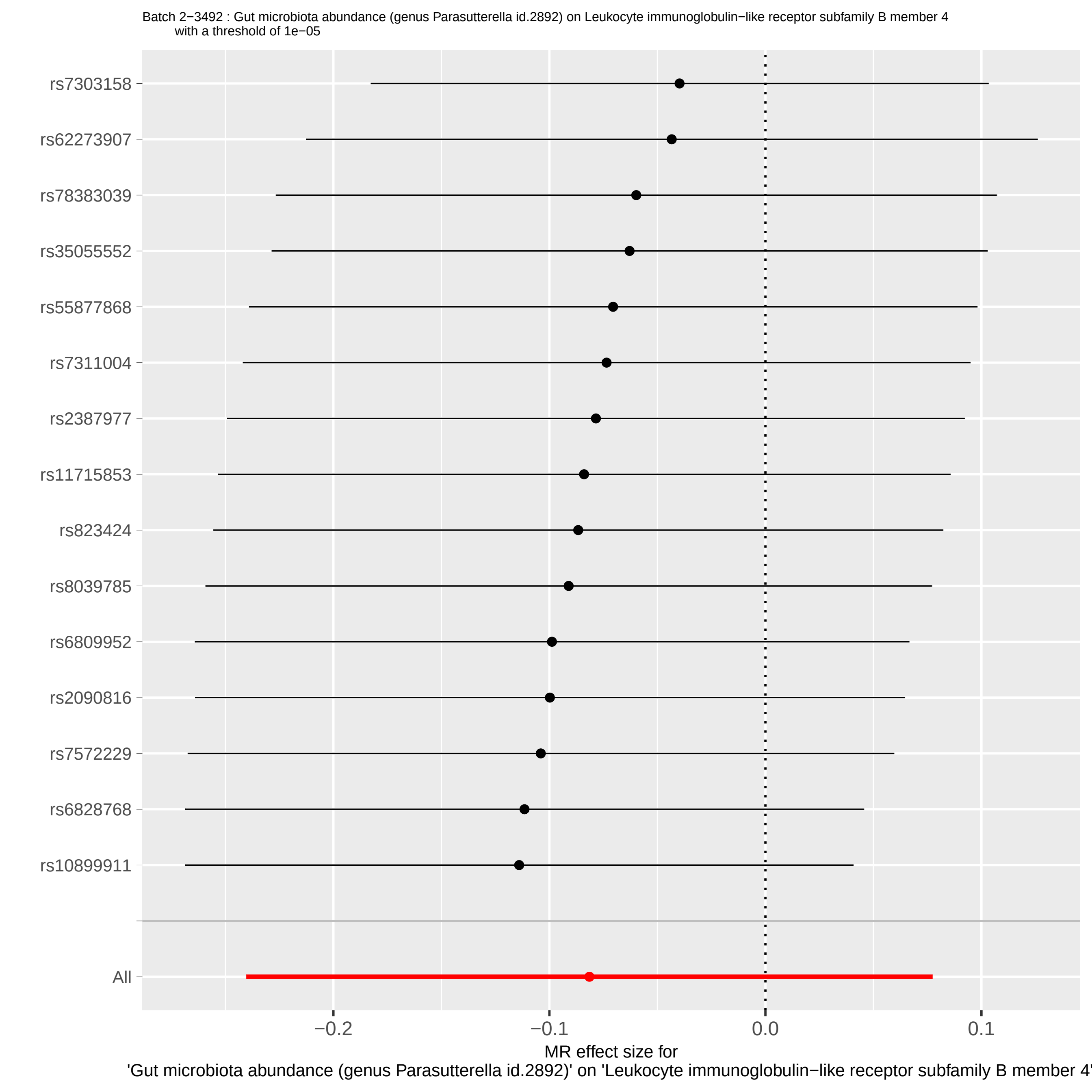
Batch 2−3492 : Gut microbiota abundance (genus Parasutterella id.2892) on Leukocyte immunoglobulin−like receptor subfamily B member 4
with a threshold of 1e−05
rs7303158
rs62273907
rs78383039
rs35055552
rs55877868
rs7311004
rs2387977
rs11715853
rs823424
rs8039785
rs6809952
rs2090816
rs7572229
rs6828768
rs10899911
All
−0.2
−0.1
0.0
0.1
MR effect size for
'Gut microbiota abundance (genus Parasutterella id.2892)' on 'Leukocyte immunoglobulin−like receptor subfamily B member 4'

Batch 2−3493 : Gut microbiota abundance (genus Catenibacterium id.2153) on Leukocyte immunoglobulin−like receptor subfamily B member 5
with a threshold of 1e−05

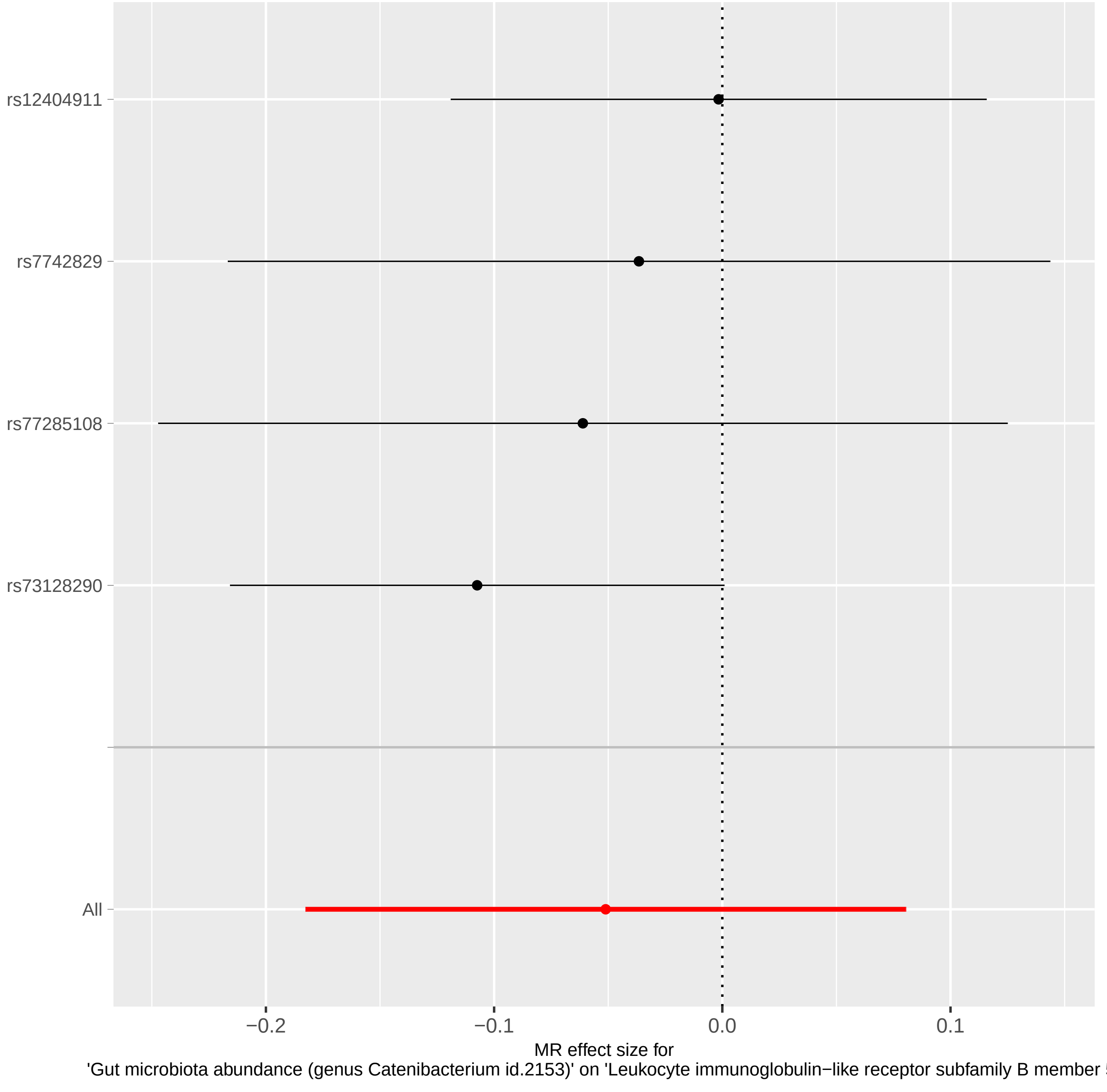

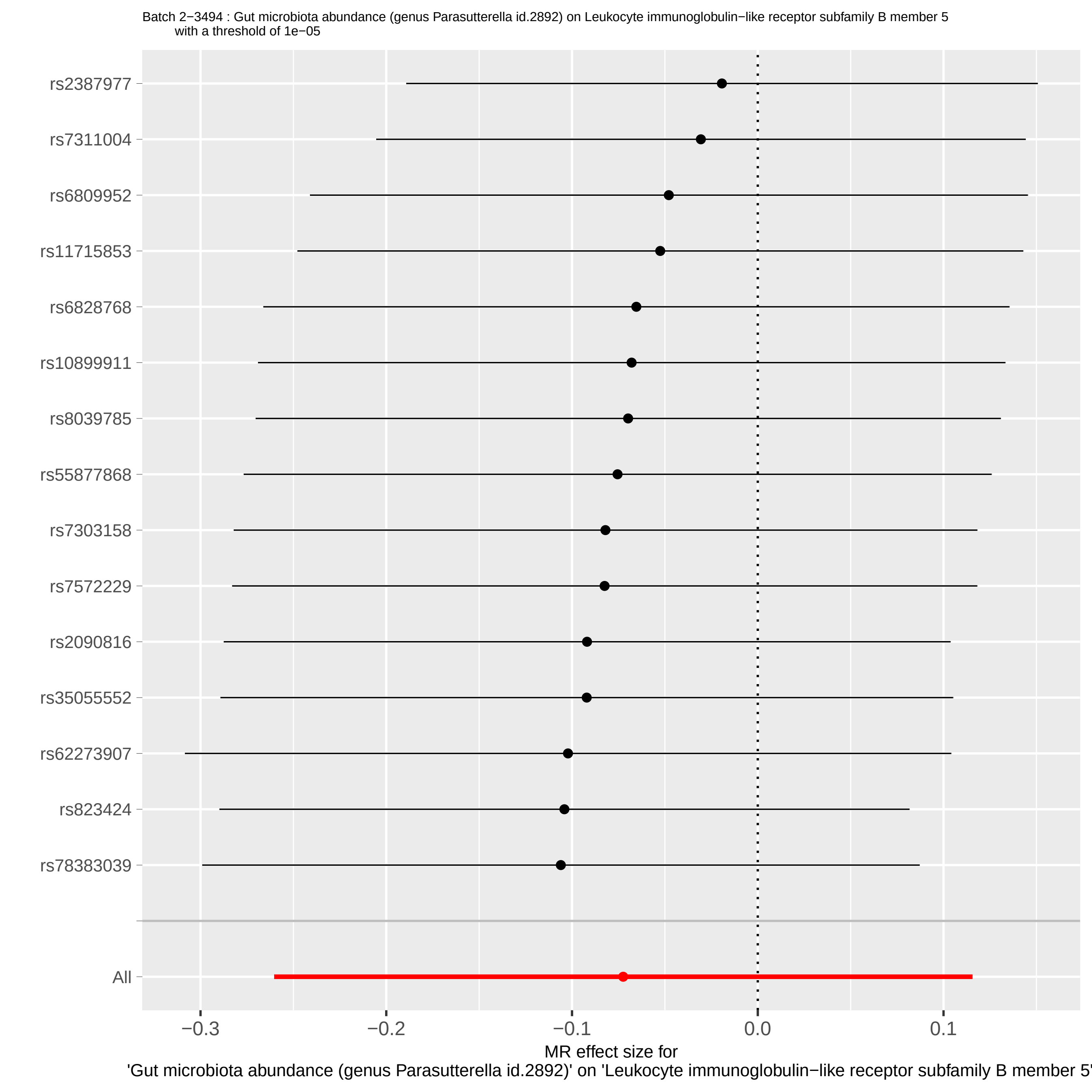
Batch 2−3494 : Gut microbiota abundance (genus Parasutterella id.2892) on Leukocyte immunoglobulin−like receptor subfamily B member 5
with a threshold of 1e−05
rs2387977
rs7311004
rs6809952
rs11715853
rs6828768
rs10899911
rs8039785
rs55877868
rs7303158
rs7572229
rs2090816
rs35055552
rs62273907
rs823424
rs78383039
All
−0.3
−0.2
−0.1
0.0
0.1
MR effect size for
'Gut microbiota abundance (genus Parasutterella id.2892)' on 'Leukocyte immunoglobulin−like receptor subfamily B member 5'

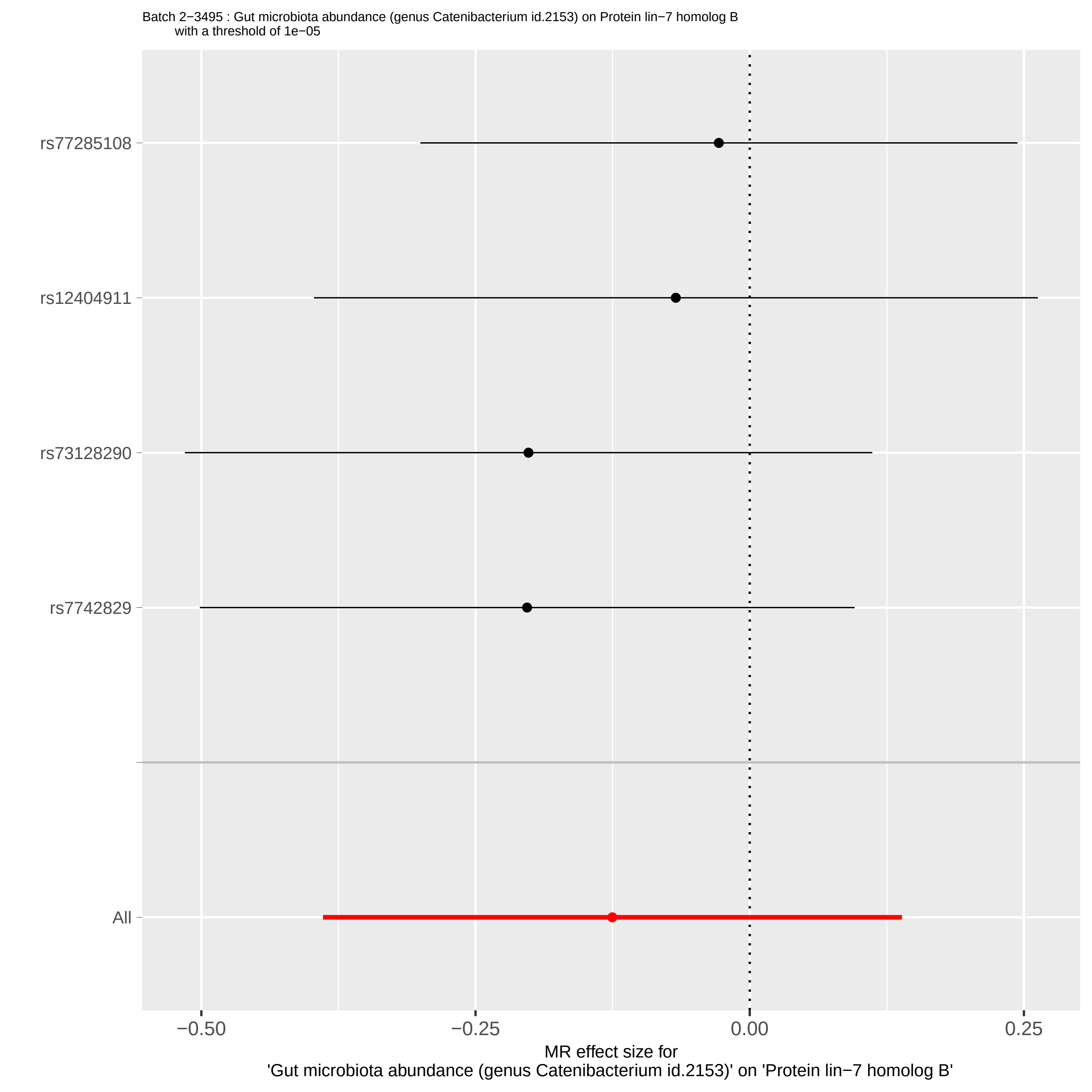
Batch 2−3495 : Gut microbiota abundance (genus Catenibacterium id.2153) on Protein lin−7 homolog B
with a threshold of 1e−05
rs77285108
rs12404911
rs73128290
rs7742829
All
−0.50
−0.25
0.00
0.25
MR effect size for
'Gut microbiota abundance (genus Catenibacterium id.2153)' on 'Protein lin−7 homolog B'

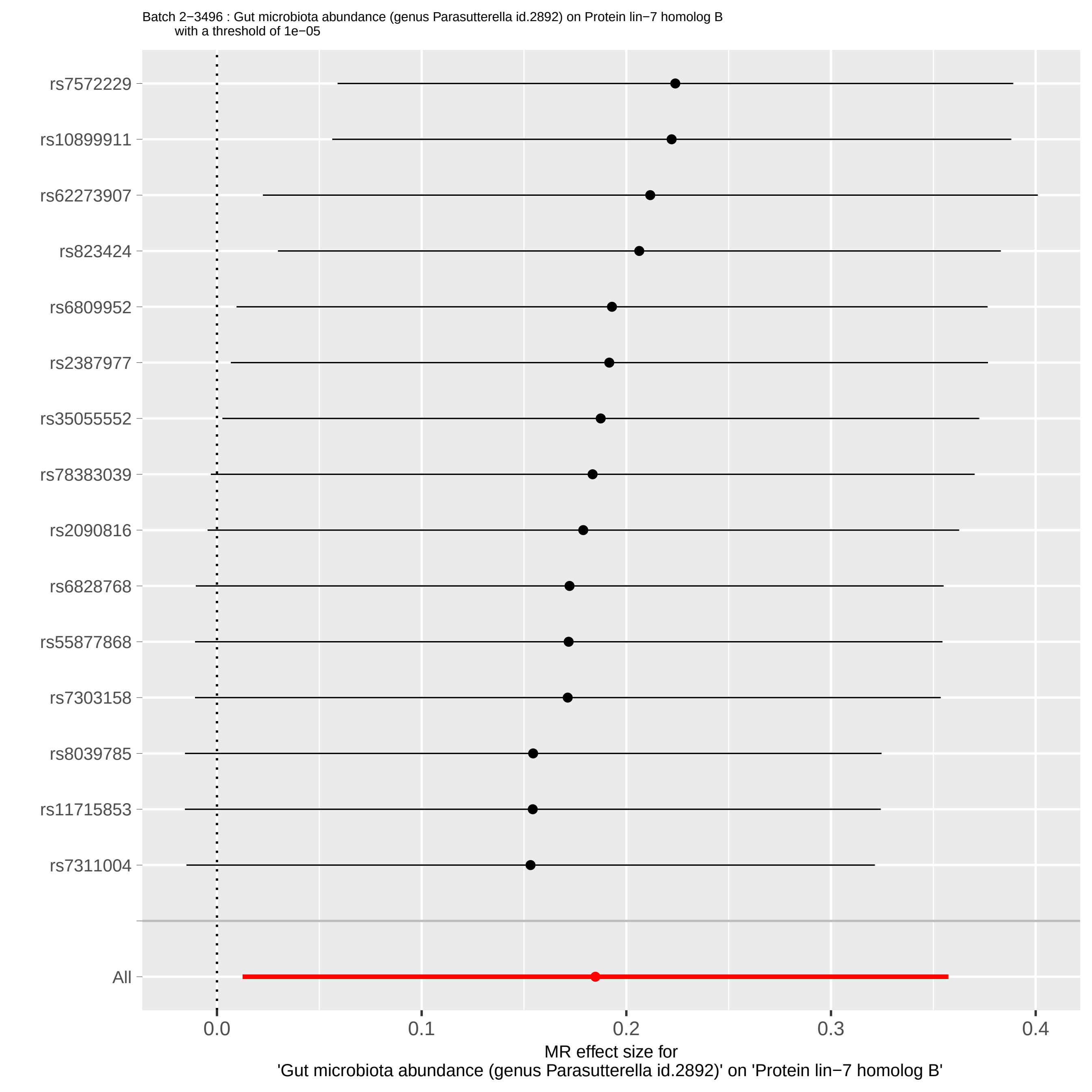

Batch 2−3496 : Gut microbiota abundance (genus Parasutterella id.2892) on Protein lin−7 homolog B
with a threshold of 1e−05
rs7572229
rs10899911
rs62273907
rs823424
rs6809952
rs2387977
rs35055552
rs78383039
rs2090816
rs6828768
rs55877868
rs7303158
rs8039785
rs11715853
rs7311004
All
0.0
0.1
0.2
0.3
0.4
MR effect size for
'Gut microbiota abundance (genus Parasutterella id.2892)' on 'Protein lin−7 homolog B'

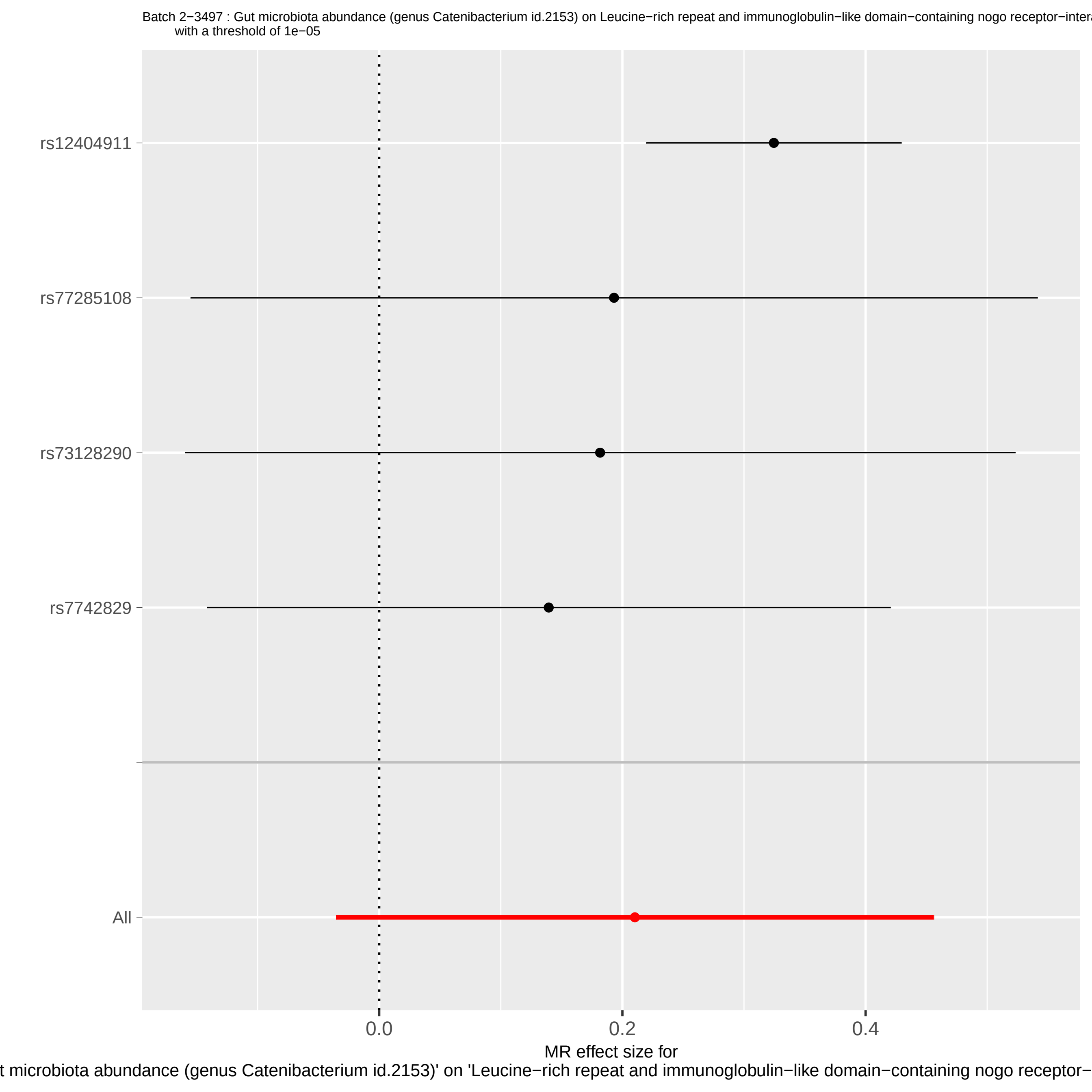
Batch 2−3497 : Gut microbiota abundance (genus Catenibacterium id.2153) on Leucine−rich repeat and immunoglobulin−like domain−containing nogo receptor−intera
with a threshold of 1e−05
rs12404911
rs77285108
rs73128290
rs7742829
All
0.0
0.2
0.4
MR effect size for
t microbiota abundance (genus Catenibacterium id.2153)' on 'Leucine−rich repeat and immunoglobulin−like domain−containing nogo receptor−

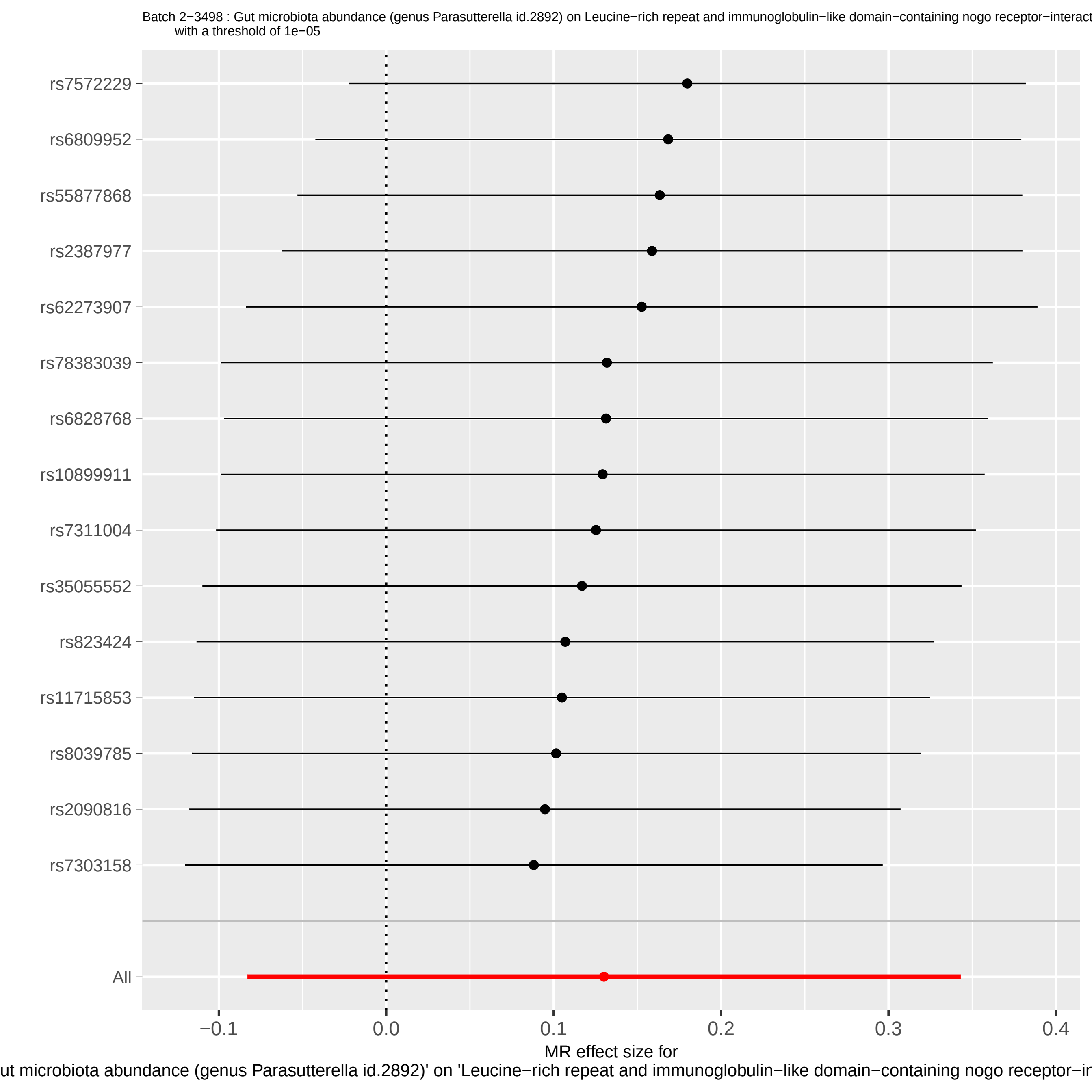
Batch 2−3498 : Gut microbiota abundance (genus Parasutterella id.2892) on Leucine−rich repeat and immunoglobulin−like domain−containing nogo receptor−interact
with a threshold of 1e−05
rs7572229
rs6809952
rs55877868
rs2387977
rs62273907
rs78383039
rs6828768
rs10899911
rs7311004
rs35055552
rs823424
rs11715853
rs8039785
rs2090816
rs7303158
All
−0.1
0.0
0.1
0.2
0.3
0.4
MR effect size for
ut microbiota abundance (genus Parasutterella id.2892)' on 'Leucine−rich repeat and immunoglobulin−like domain−containing nogo receptor−in

Batch 2−3499 : Gut microbiota abundance (genus Catenibacterium id.2153) on Leucine−rich repeat and immunoglobulin−like domain−containing nogo receptor−intera
with a threshold of 1e−05

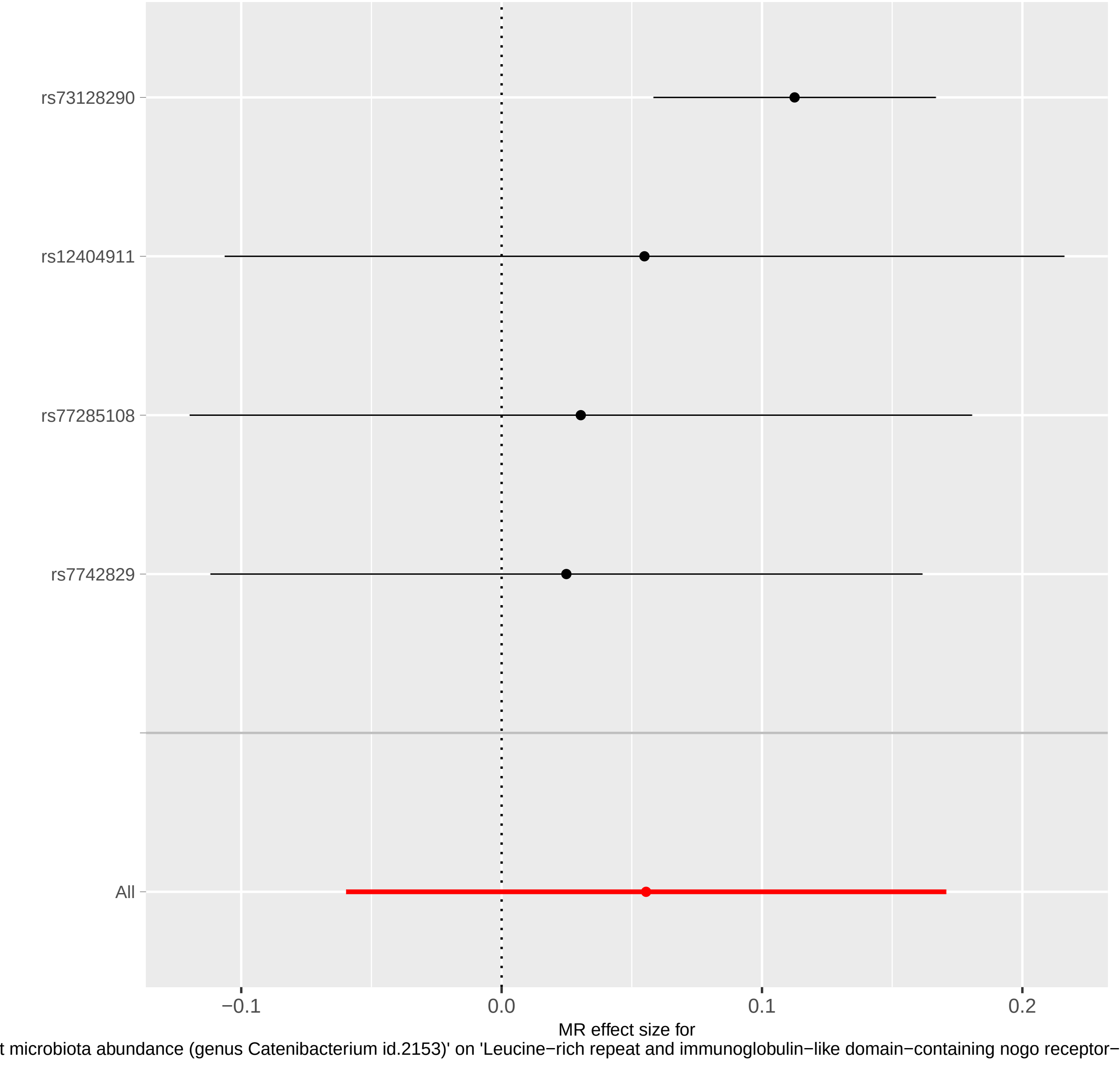

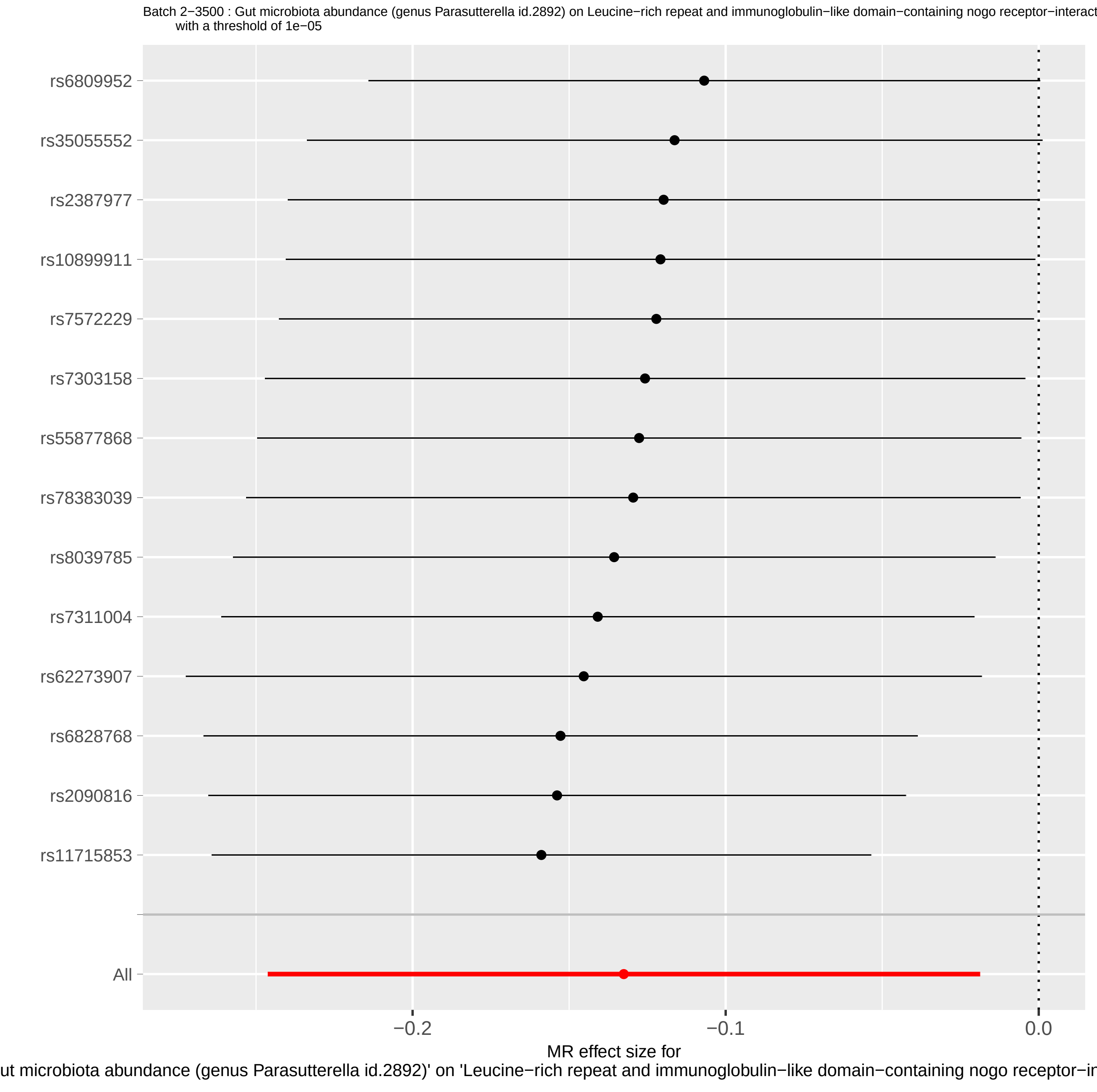

Batch 2−3500 : Gut microbiota abundance (genus Parasutterella id.2892) on Leucine−rich repeat and immunoglobulin−like domain−containing nogo receptor−interact
with a threshold of 1e−05
rs6809952
rs35055552
rs2387977
rs10899911
rs7572229
rs7303158
rs55877868
rs78383039
rs8039785
rs7311004
rs62273907
rs6828768
rs2090816
rs11715853
All
−0.2
−0.1
0.0
MR effect size for
ut microbiota abundance (genus Parasutterella id.2892)' on 'Leucine−rich repeat and immunoglobulin−like domain−containing nogo receptor−in

Batch 2−3501 : Gut microbiota abundance (genus Catenibacterium id.2153) on Gastric triacylglycerol lipase
with a threshold of 1e−05

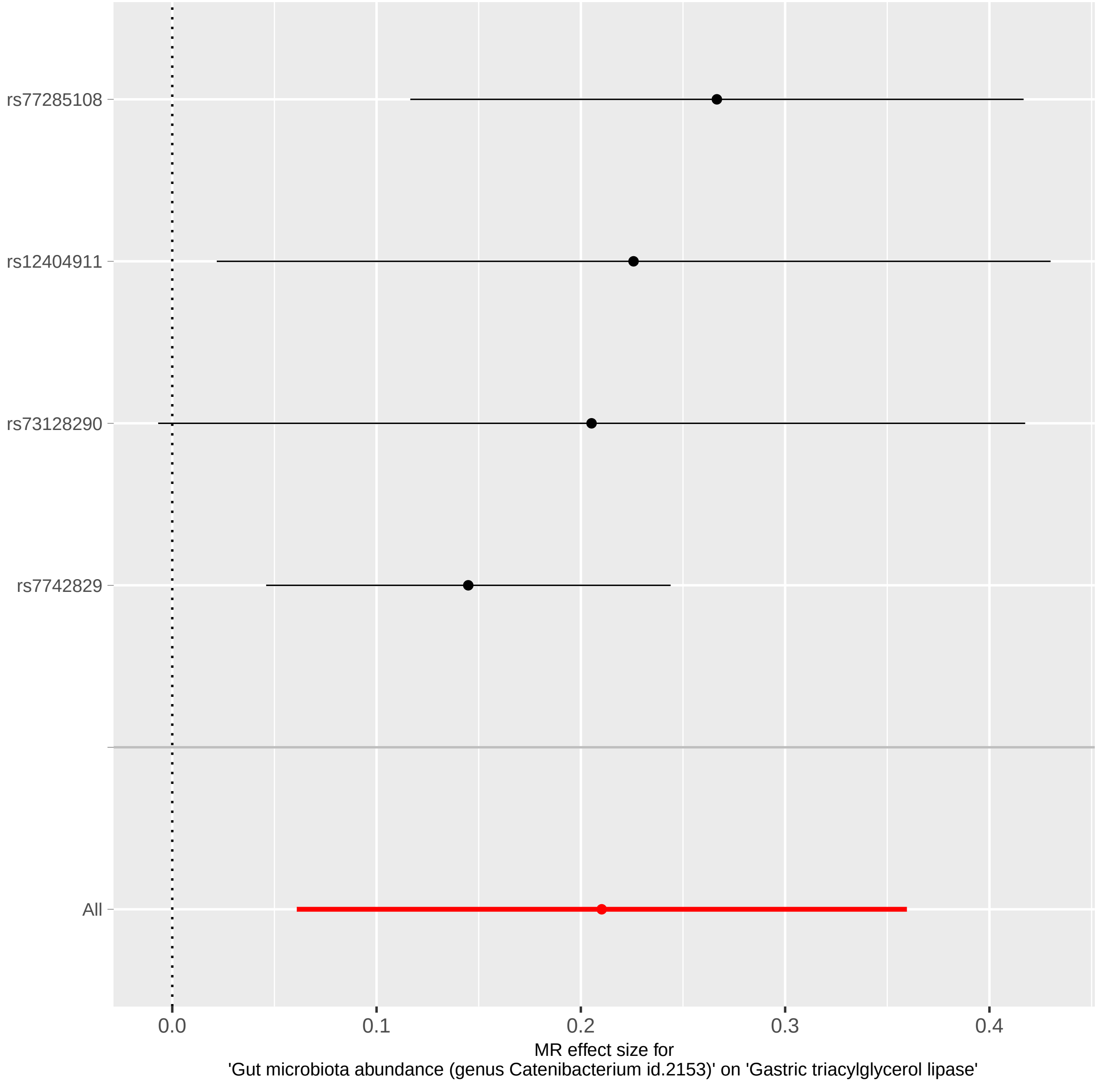

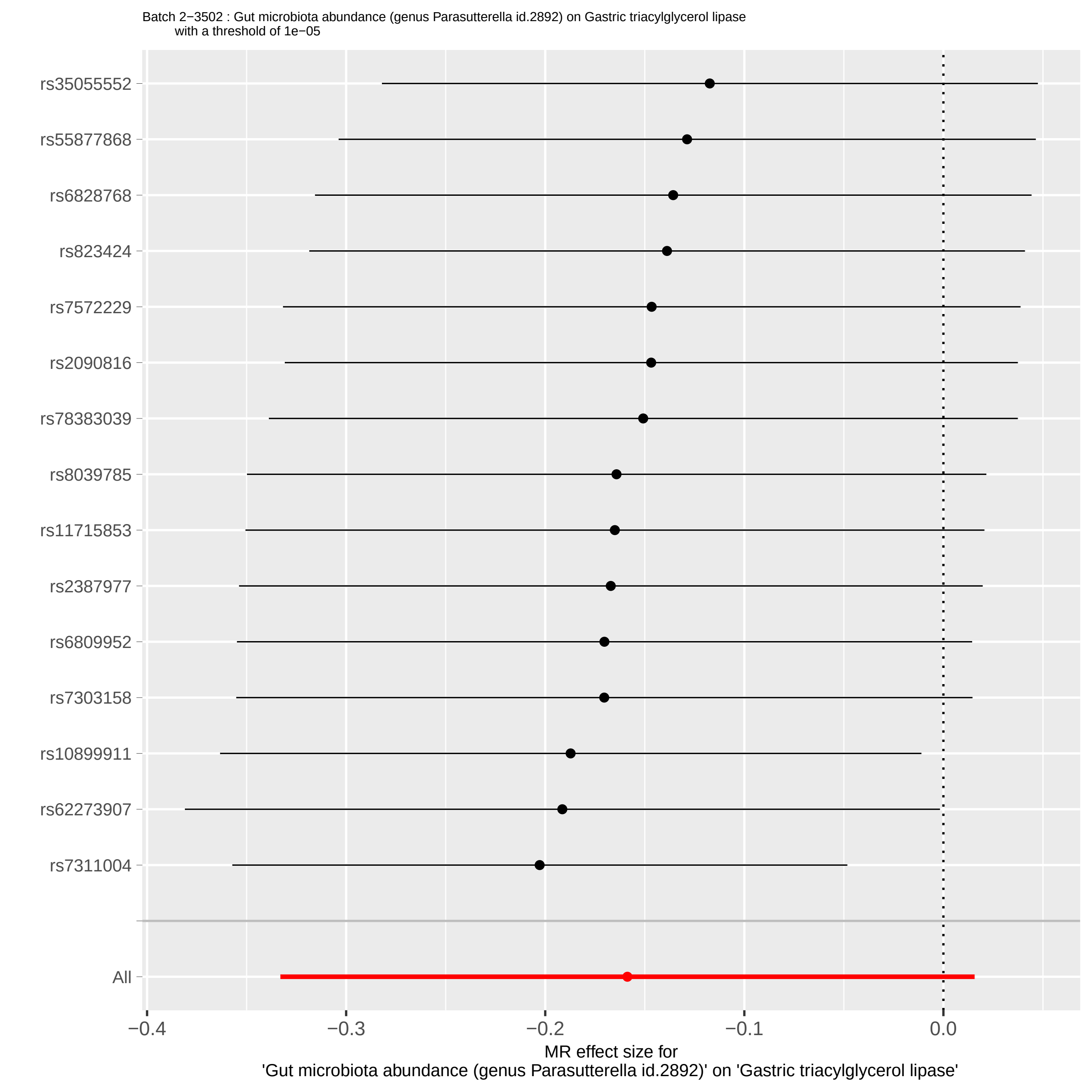

Batch 2−3502 : Gut microbiota abundance (genus Parasutterella id.2892) on Gastric triacylglycerol lipase
with a threshold of 1e−05
rs35055552
rs55877868
rs6828768
rs823424
rs7572229
rs2090816
rs78383039
rs8039785
rs11715853
rs2387977
rs6809952
rs7303158
rs10899911
rs62273907
rs7311004
All
−0.4
−0.3
−0.2
−0.1
0.0
MR effect size for
'Gut microbiota abundance (genus Parasutterella id.2892)' on 'Gastric triacylglycerol lipase'

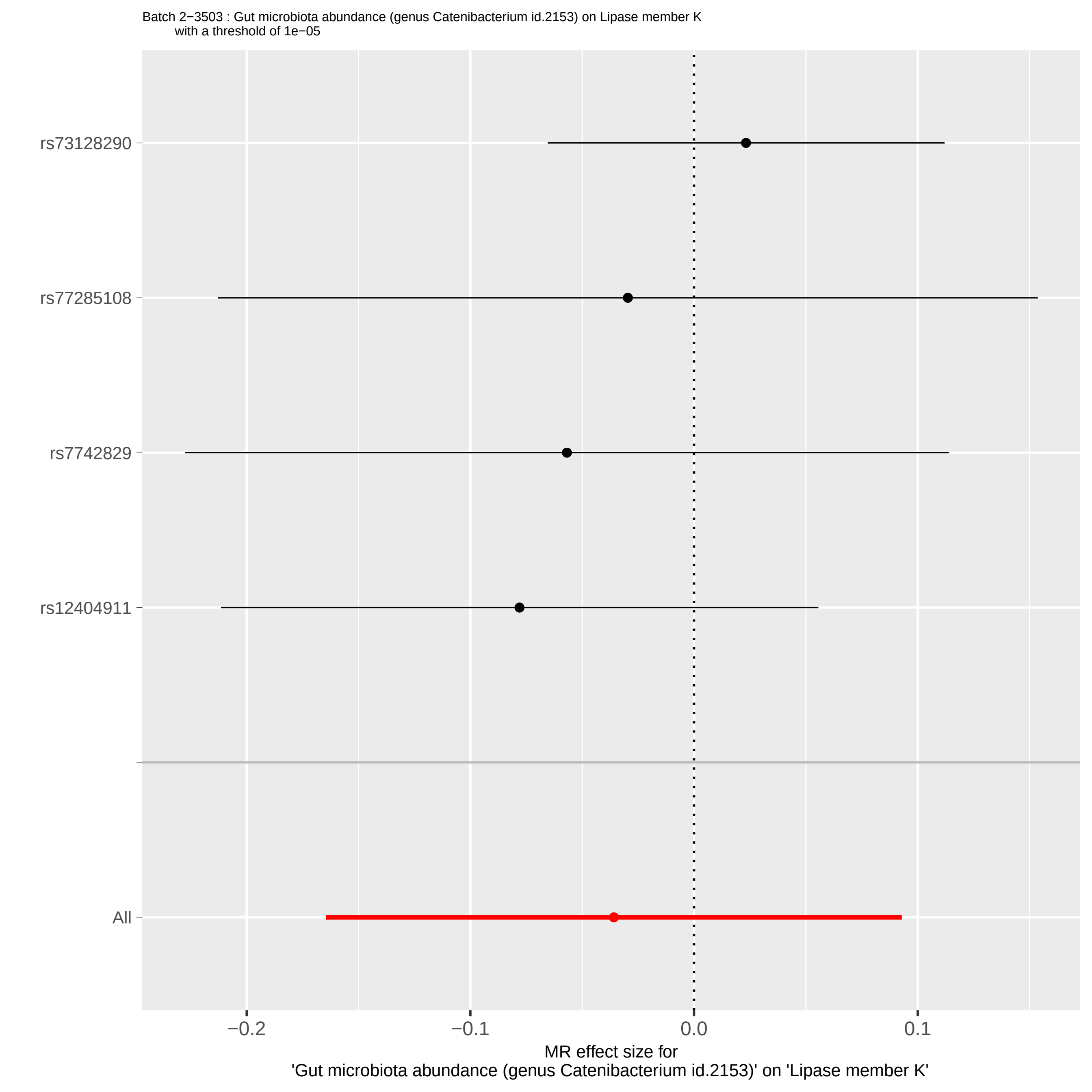
Batch 2−3503 : Gut microbiota abundance (genus Catenibacterium id.2153) on Lipase member K
with a threshold of 1e−05
rs73128290
rs77285108
rs7742829
rs12404911
All
−0.2
−0.1
0.0
0.1
MR effect size for
'Gut microbiota abundance (genus Catenibacterium id.2153)' on 'Lipase member K'

Batch 2−3504 : Gut microbiota abundance (genus Parasutterella id.2892) on Lipase member K
with a threshold of 1e−05

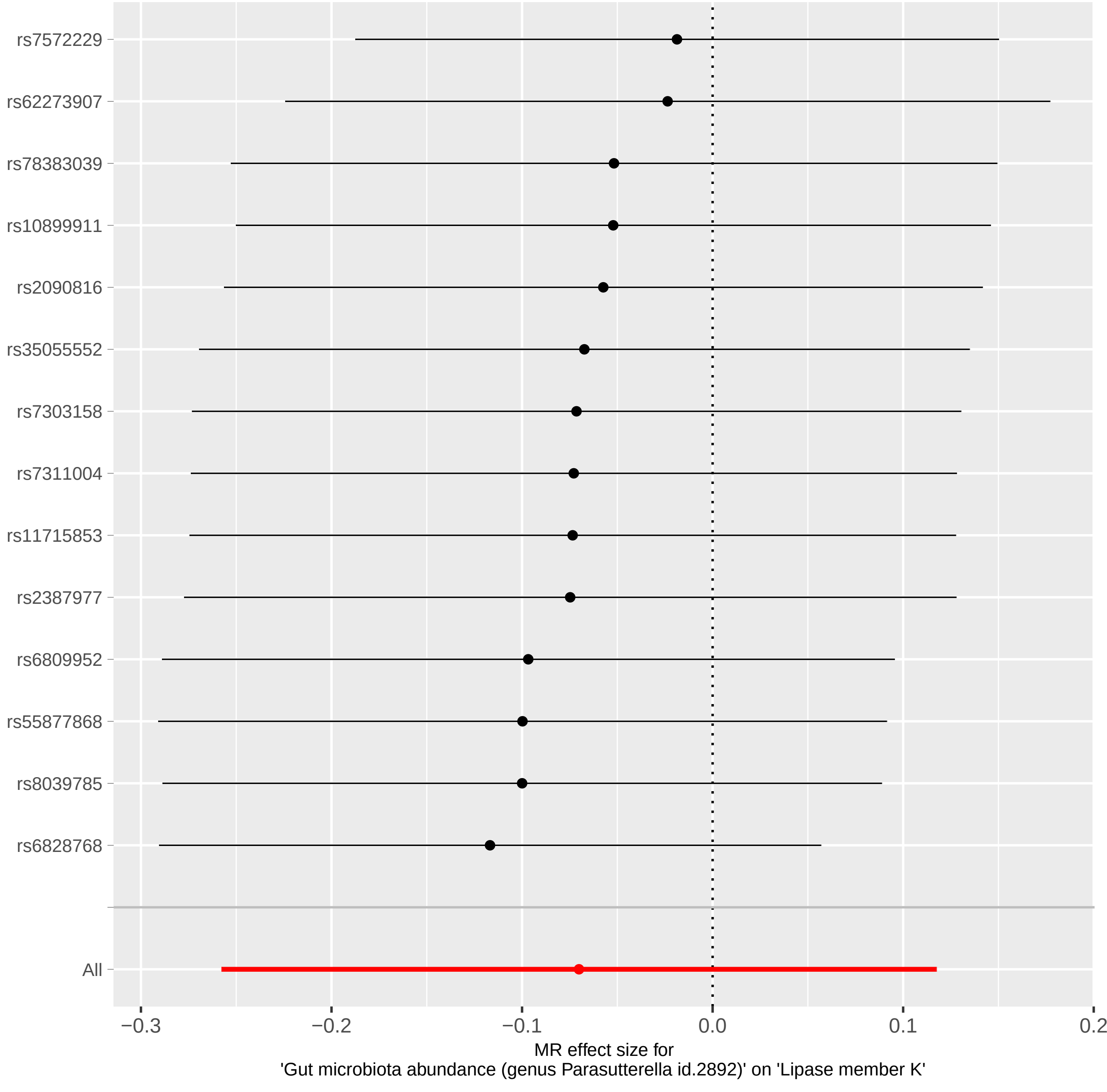

Batch 2−3505 : Gut microbiota abundance (genus Catenibacterium id.2153) on Lipase member N
with a threshold of 1e−05

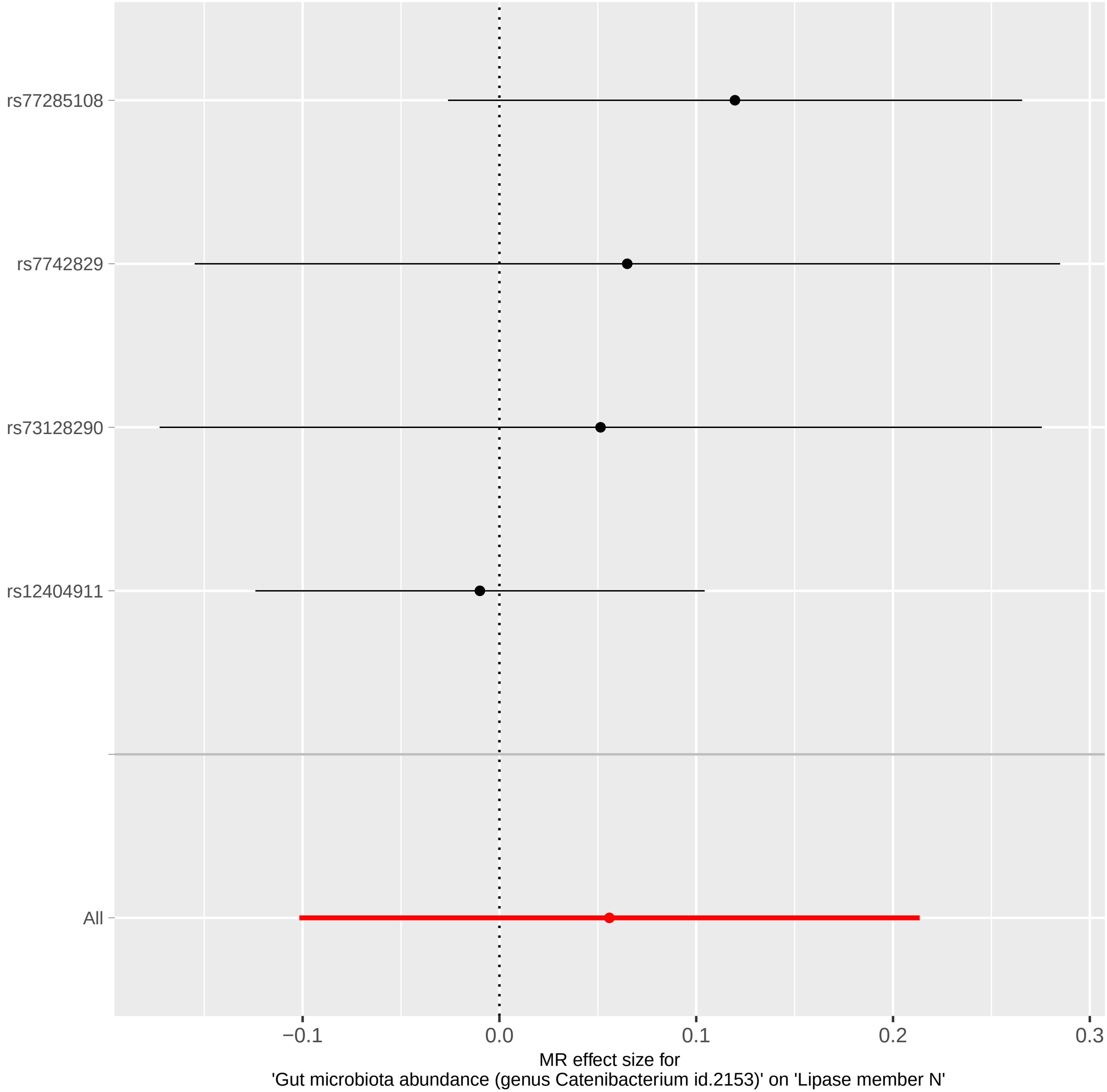

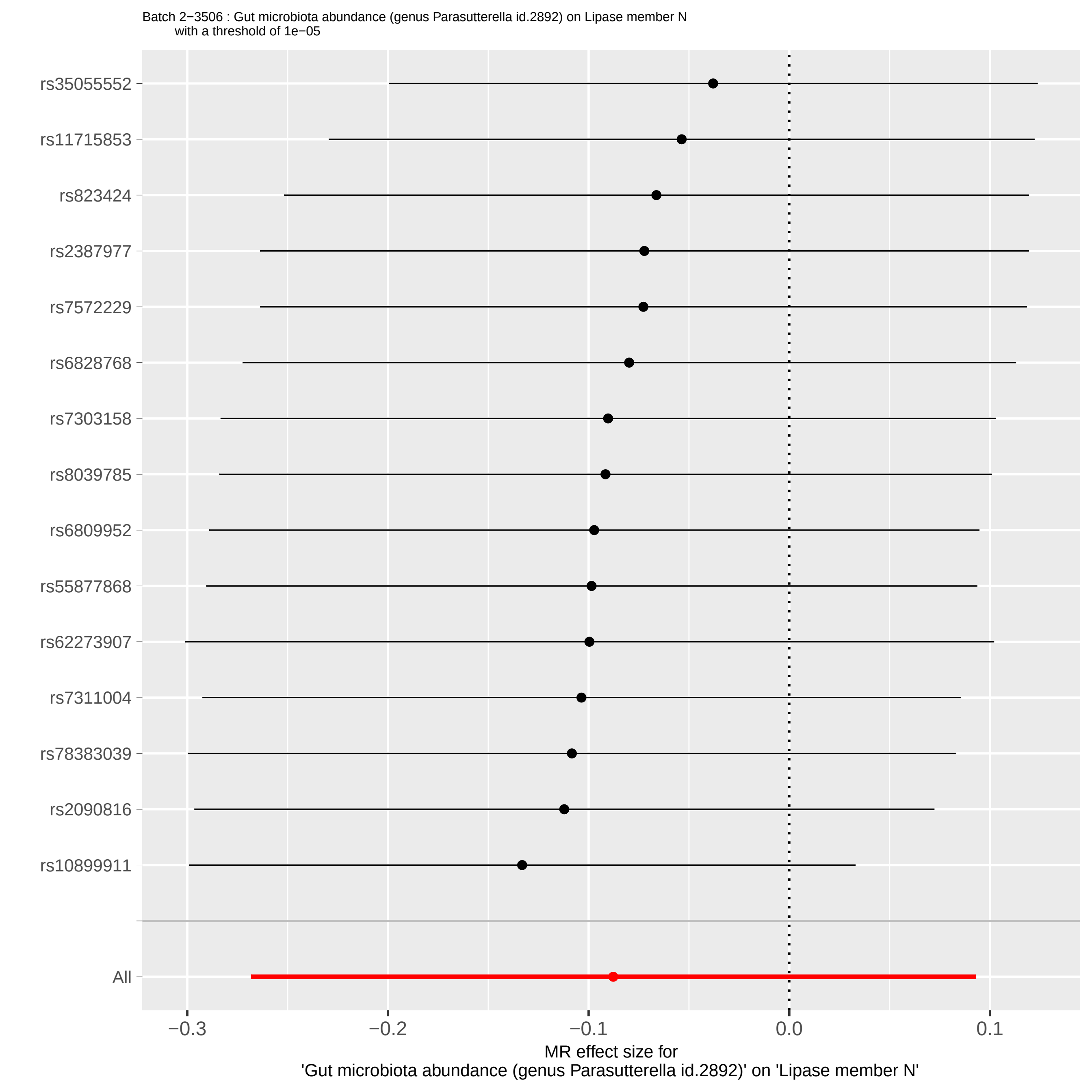
Batch 2−3506 : Gut microbiota abundance (genus Parasutterella id.2892) on Lipase member N
with a threshold of 1e−05
rs35055552
rs11715853
rs823424
rs2387977
rs7572229
rs6828768
rs7303158
rs8039785
rs6809952
rs55877868
rs62273907
rs7311004
rs78383039
rs2090816
rs10899911
All
−0.3
−0.2
−0.1
0.0
0.1
MR effect size for
'Gut microbiota abundance (genus Parasutterella id.2892)' on 'Lipase member N'

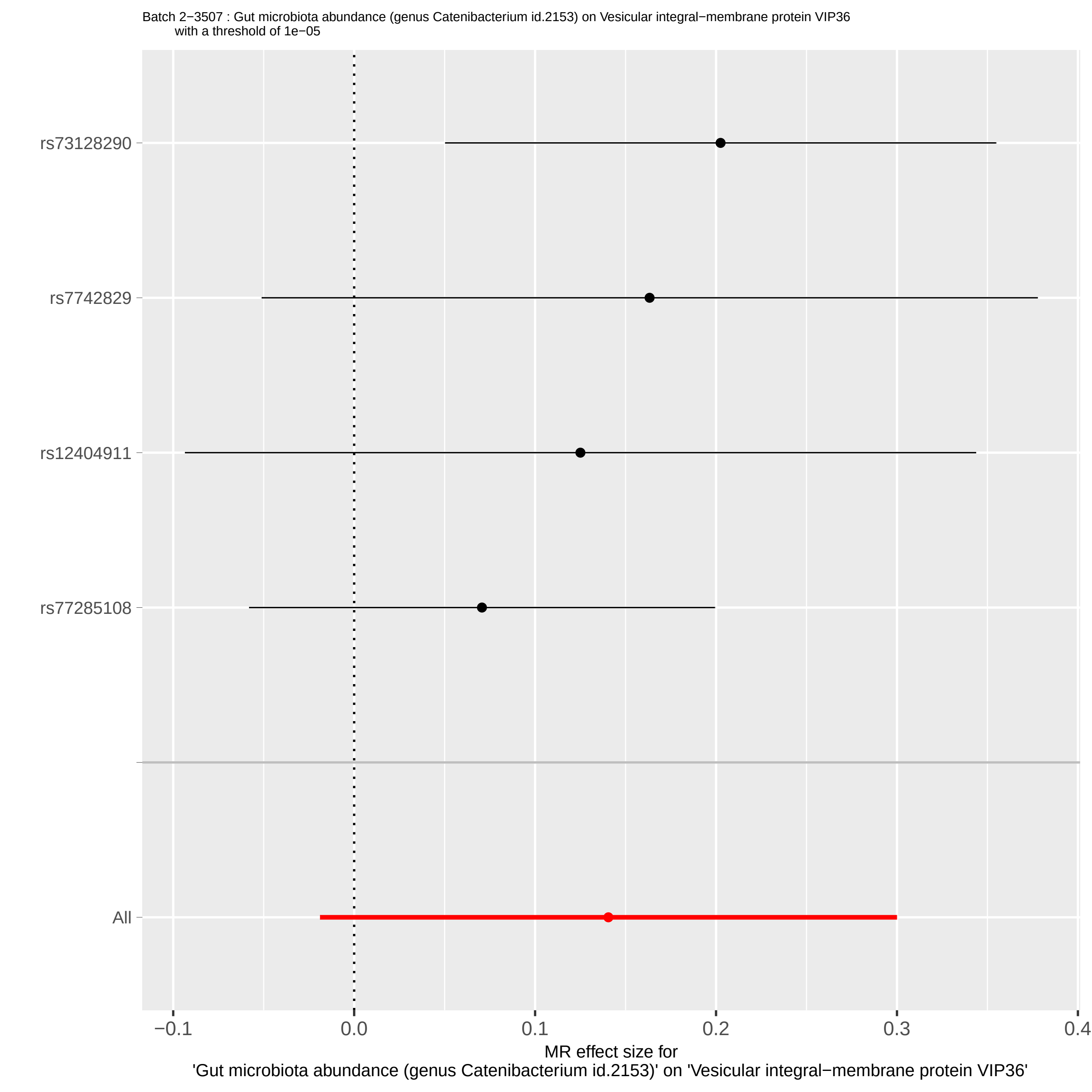

Batch 2−3507 : Gut microbiota abundance (genus Catenibacterium id.2153) on Vesicular integral−membrane protein VIP36
with a threshold of 1e−05
rs73128290
rs7742829
rs12404911
rs77285108
All
−0.1
0.0
0.1
0.2
0.3
0.4
MR effect size for
'Gut microbiota abundance (genus Catenibacterium id.2153)' on 'Vesicular integral−membrane protein VIP36'

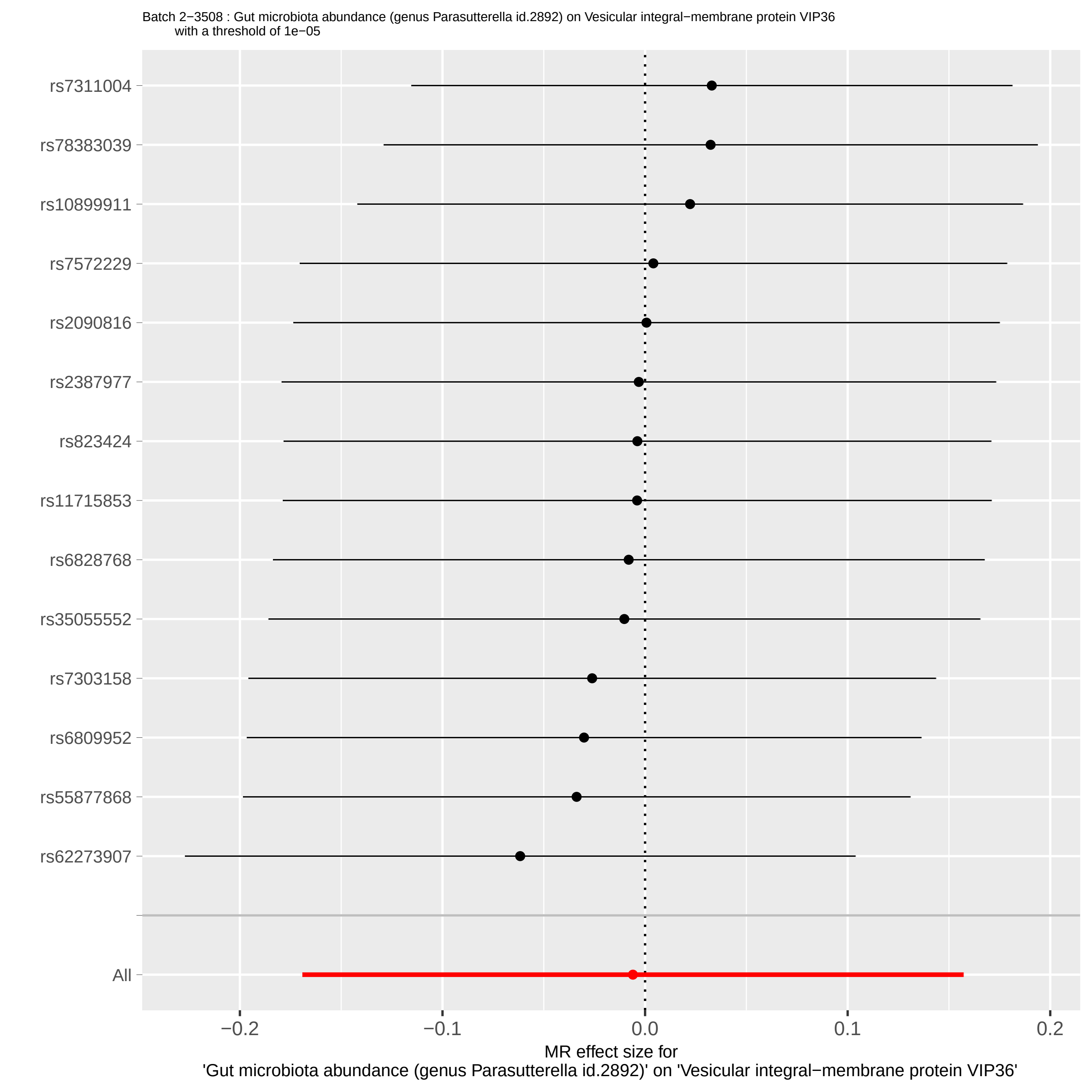

Batch 2−3508 : Gut microbiota abundance (genus Parasutterella id.2892) on Vesicular integral−membrane protein VIP36
with a threshold of 1e−05
rs7311004
rs78383039
rs10899911
rs7572229
rs2090816
rs2387977
rs823424
rs11715853
rs6828768
rs35055552
rs7303158
rs6809952
rs55877868
rs62273907
All
−0.2
−0.1
0.0
0.1
0.2
MR effect size for
'Gut microbiota abundance (genus Parasutterella id.2892)' on 'Vesicular integral−membrane protein VIP36'

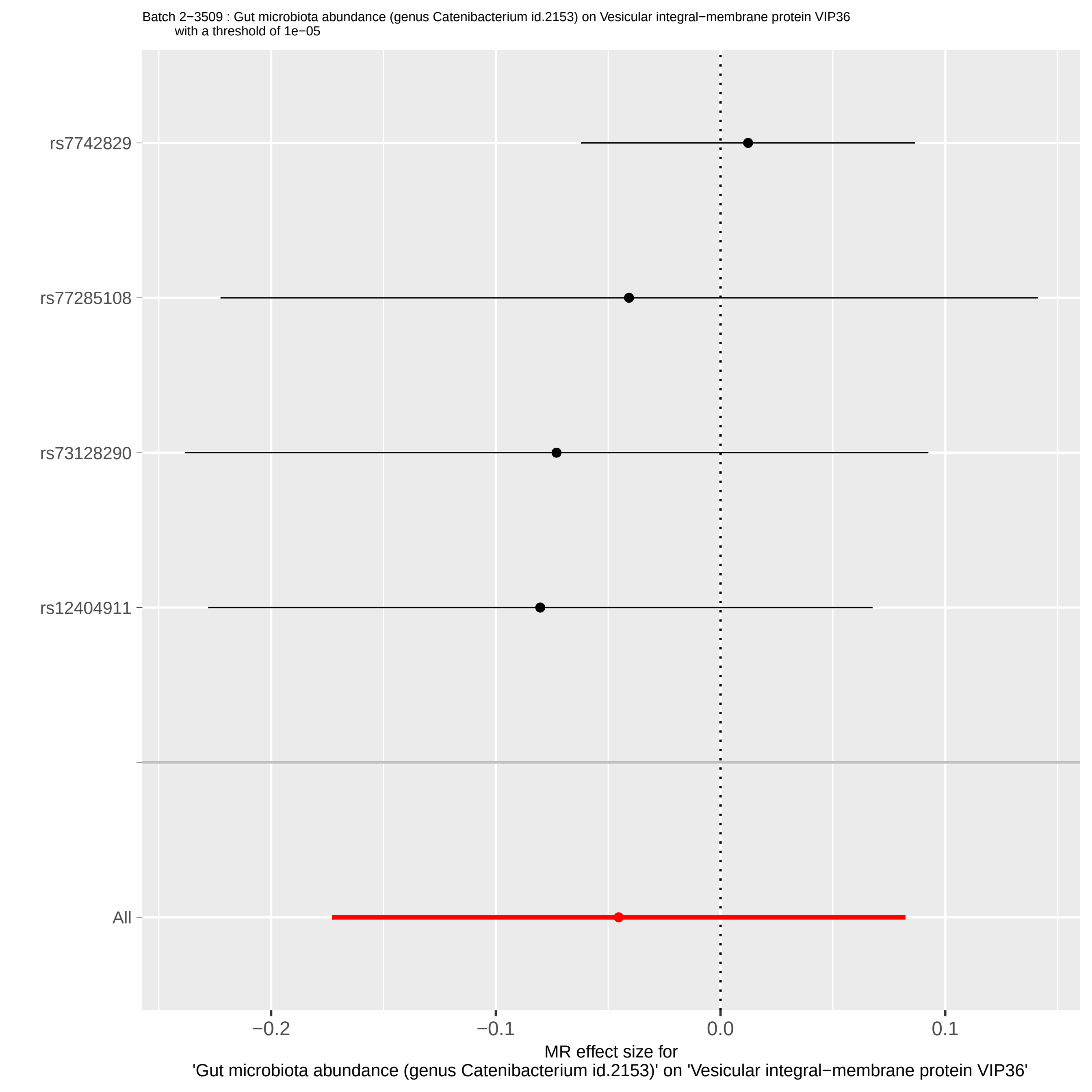
Batch 2−3509 : Gut microbiota abundance (genus Catenibacterium id.2153) on Vesicular integral−membrane protein VIP36
with a threshold of 1e−05
rs7742829
rs77285108
rs73128290
rs12404911
All
−0.2
−0.1
0.0
0.1
MR effect size for
'Gut microbiota abundance (genus Catenibacterium id.2153)' on 'Vesicular integral−membrane protein VIP36'

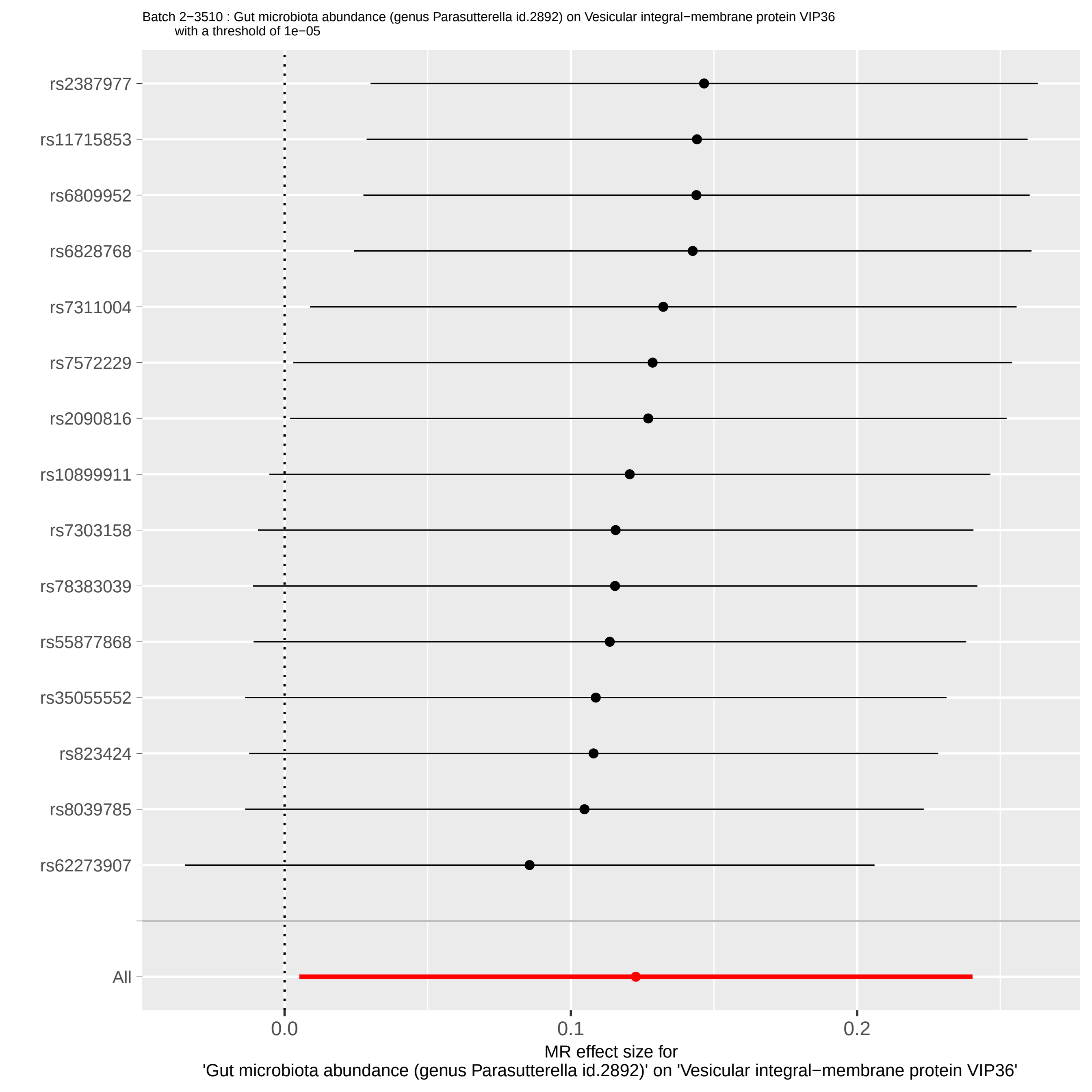
Batch 2−3510 : Gut microbiota abundance (genus Parasutterella id.2892) on Vesicular integral−membrane protein VIP36
with a threshold of 1e−05
rs2387977
rs11715853
rs6809952
rs6828768
rs7311004
rs7572229
rs2090816
rs10899911
rs7303158
rs78383039
rs55877868
rs35055552
rs823424
rs8039785
rs62273907
All
0.0
0.1
0.2
MR effect size for
'Gut microbiota abundance (genus Parasutterella id.2892)' on 'Vesicular integral−membrane protein VIP36'

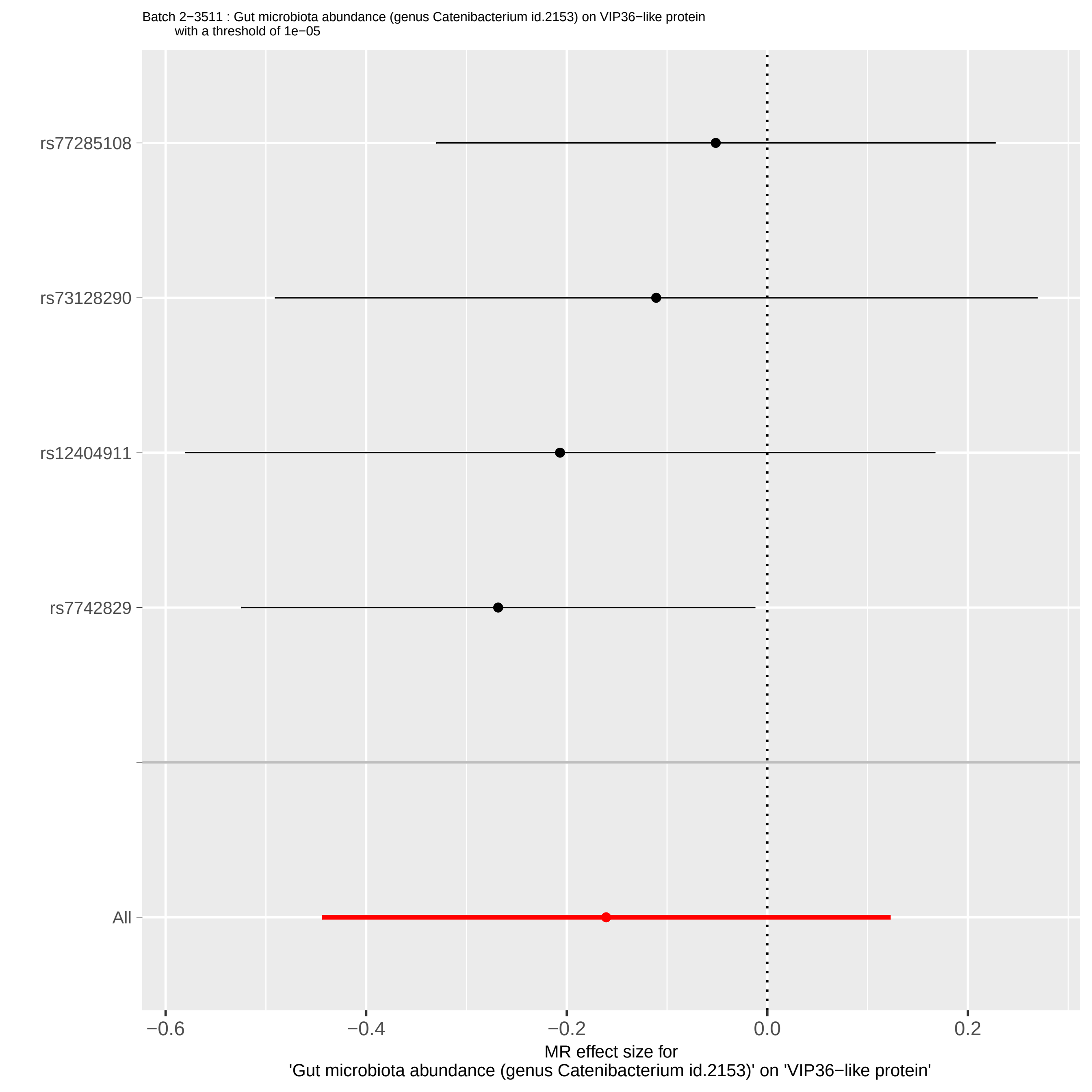

Batch 2−3511 : Gut microbiota abundance (genus Catenibacterium id.2153) on VIP36−like protein
with a threshold of 1e−05
rs77285108
rs73128290
rs12404911
rs7742829
All
−0.6
−0.4
−0.2
0.0
0.2
MR effect size for
'Gut microbiota abundance (genus Catenibacterium id.2153)' on 'VIP36−like protein'

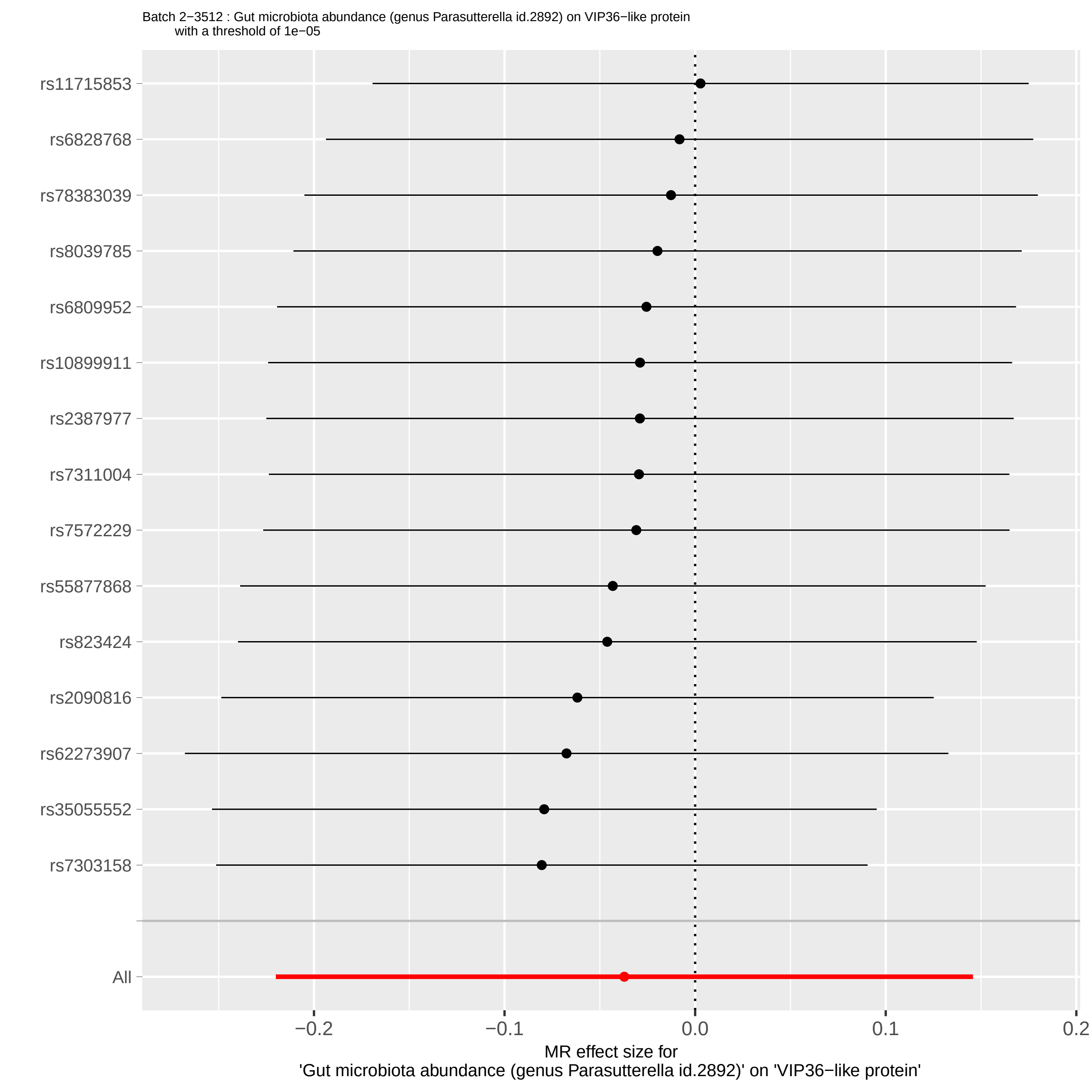

Batch 2−3512 : Gut microbiota abundance (genus Parasutterella id.2892) on VIP36−like protein
with a threshold of 1e−05
rs11715853
rs6828768
rs78383039
rs8039785
rs6809952
rs10899911
rs2387977
rs7311004
rs7572229
rs55877868
rs823424
rs2090816
rs62273907
rs35055552
rs7303158
All
−0.2
−0.1
0.0
0.1
0.2
MR effect size for
'Gut microbiota abundance (genus Parasutterella id.2892)' on 'VIP36−like protein'

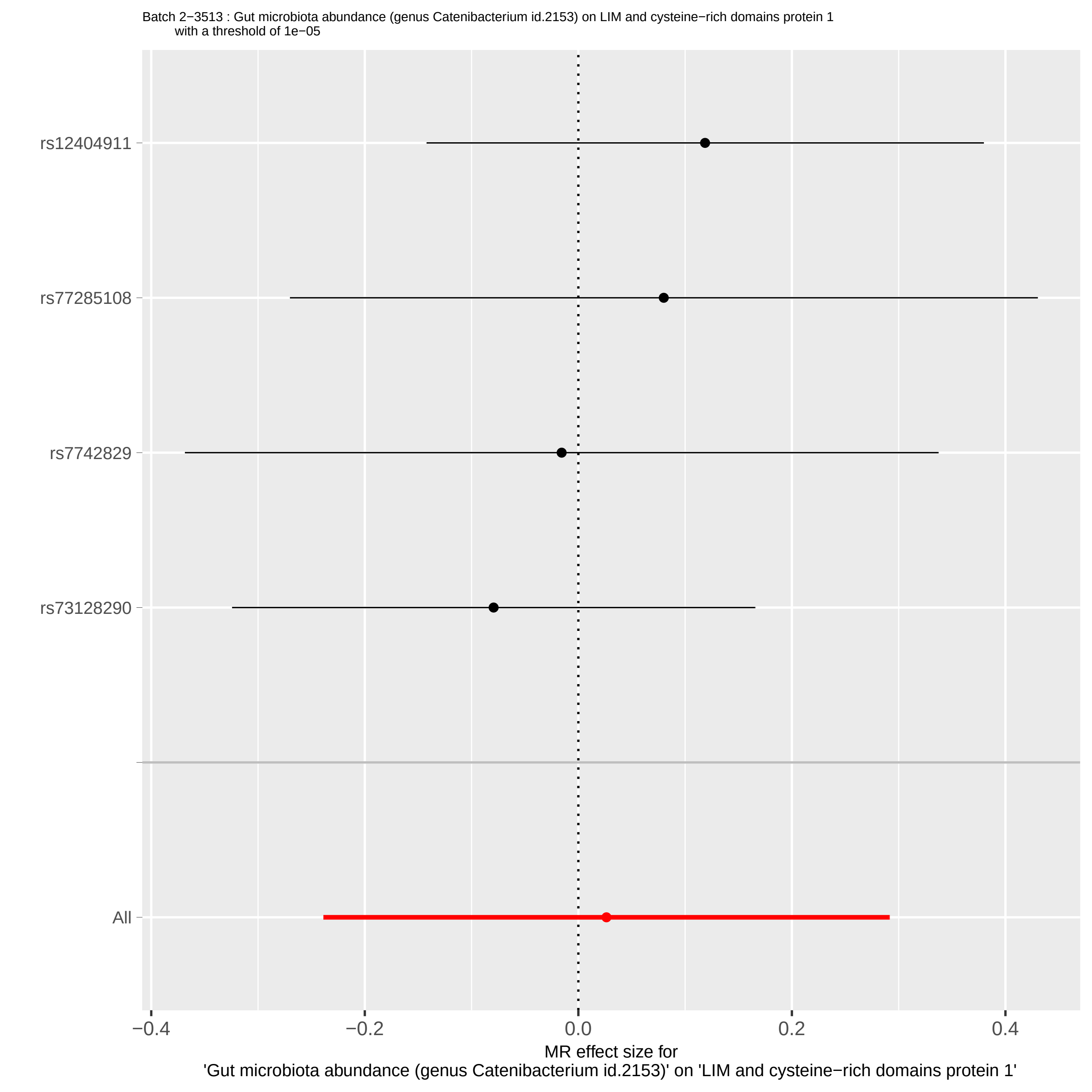

Batch 2−3513 : Gut microbiota abundance (genus Catenibacterium id.2153) on LIM and cysteine−rich domains protein 1
with a threshold of 1e−05
rs12404911
rs77285108
rs7742829
rs73128290
All
−0.4
−0.2
0.0
0.2
0.4
MR effect size for
'Gut microbiota abundance (genus Catenibacterium id.2153)' on 'LIM and cysteine−rich domains protein 1'

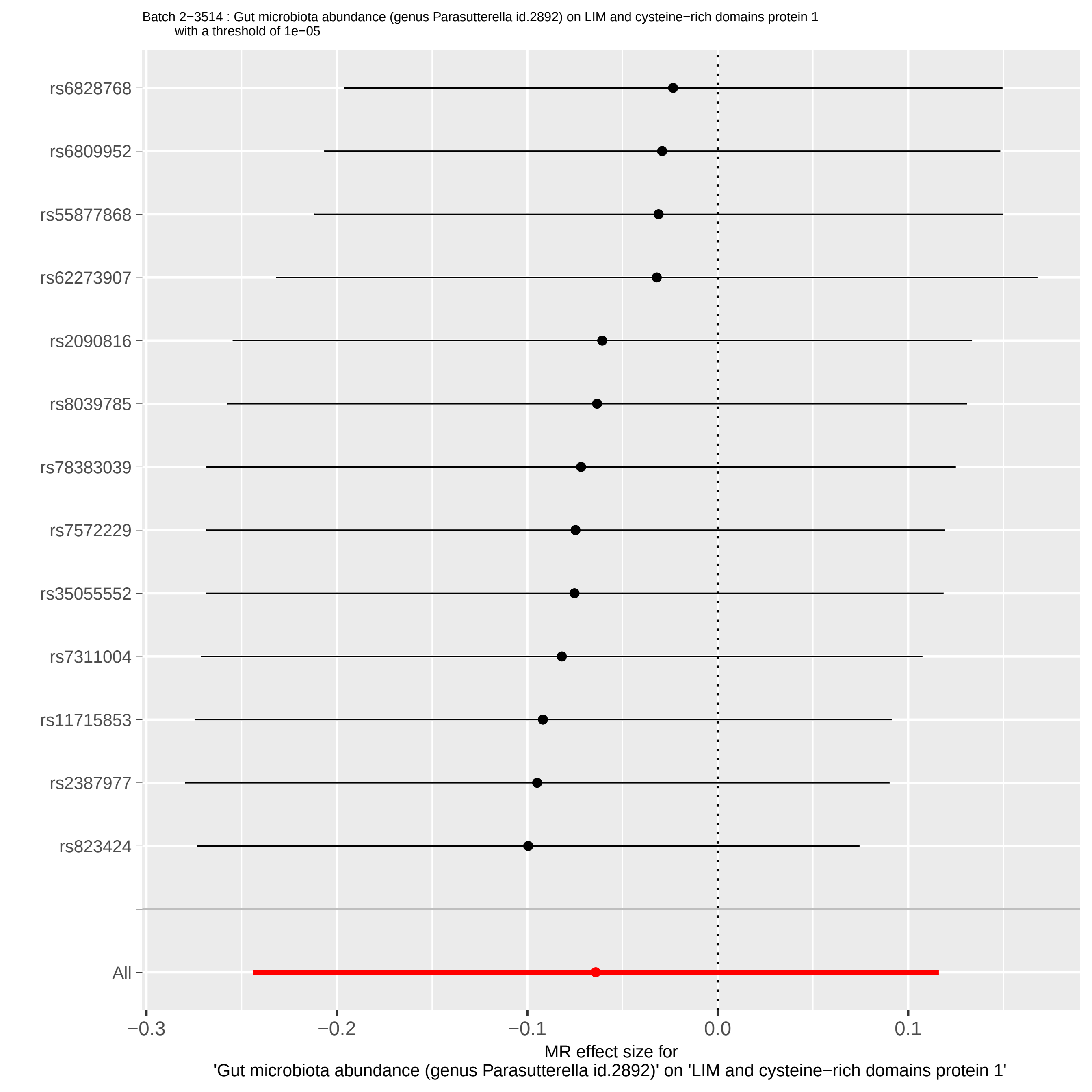
Batch 2−3514 : Gut microbiota abundance (genus Parasutterella id.2892) on LIM and cysteine−rich domains protein 1
with a threshold of 1e−05
rs6828768
rs6809952
rs55877868
rs62273907
rs2090816
rs8039785
rs78383039
rs7572229
rs35055552
rs7311004
rs11715853
rs2387977
rs823424
All
−0.3
−0.2
−0.1
0.0
0.1
MR effect size for
'Gut microbiota abundance (genus Parasutterella id.2892)' on 'LIM and cysteine−rich domains protein 1'

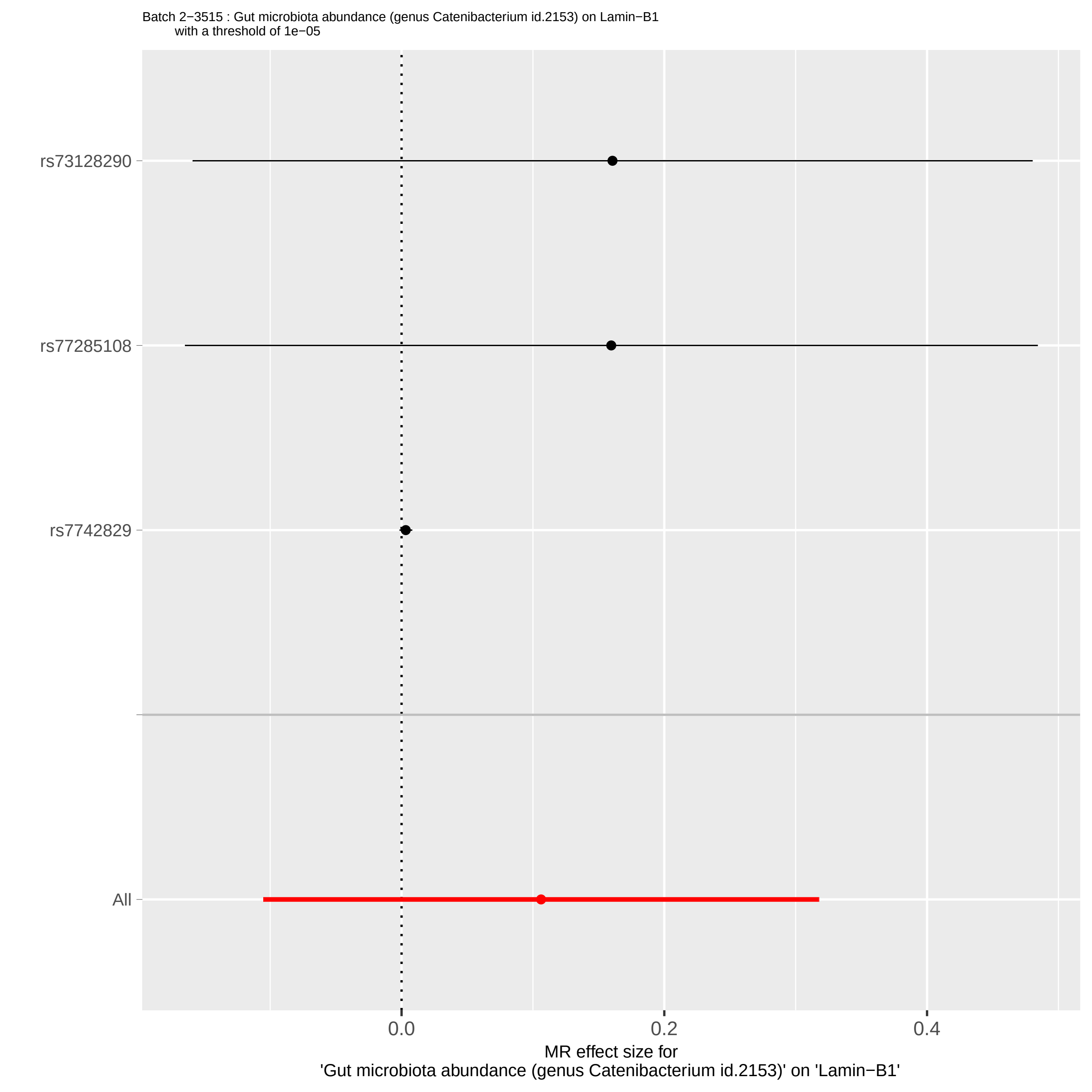

Batch 2−3515 : Gut microbiota abundance (genus Catenibacterium id.2153) on Lamin−B1
with a threshold of 1e−05
rs73128290
rs77285108
rs7742829
All
0.0
0.2
0.4
MR effect size for
'Gut microbiota abundance (genus Catenibacterium id.2153)' on 'Lamin−B1'

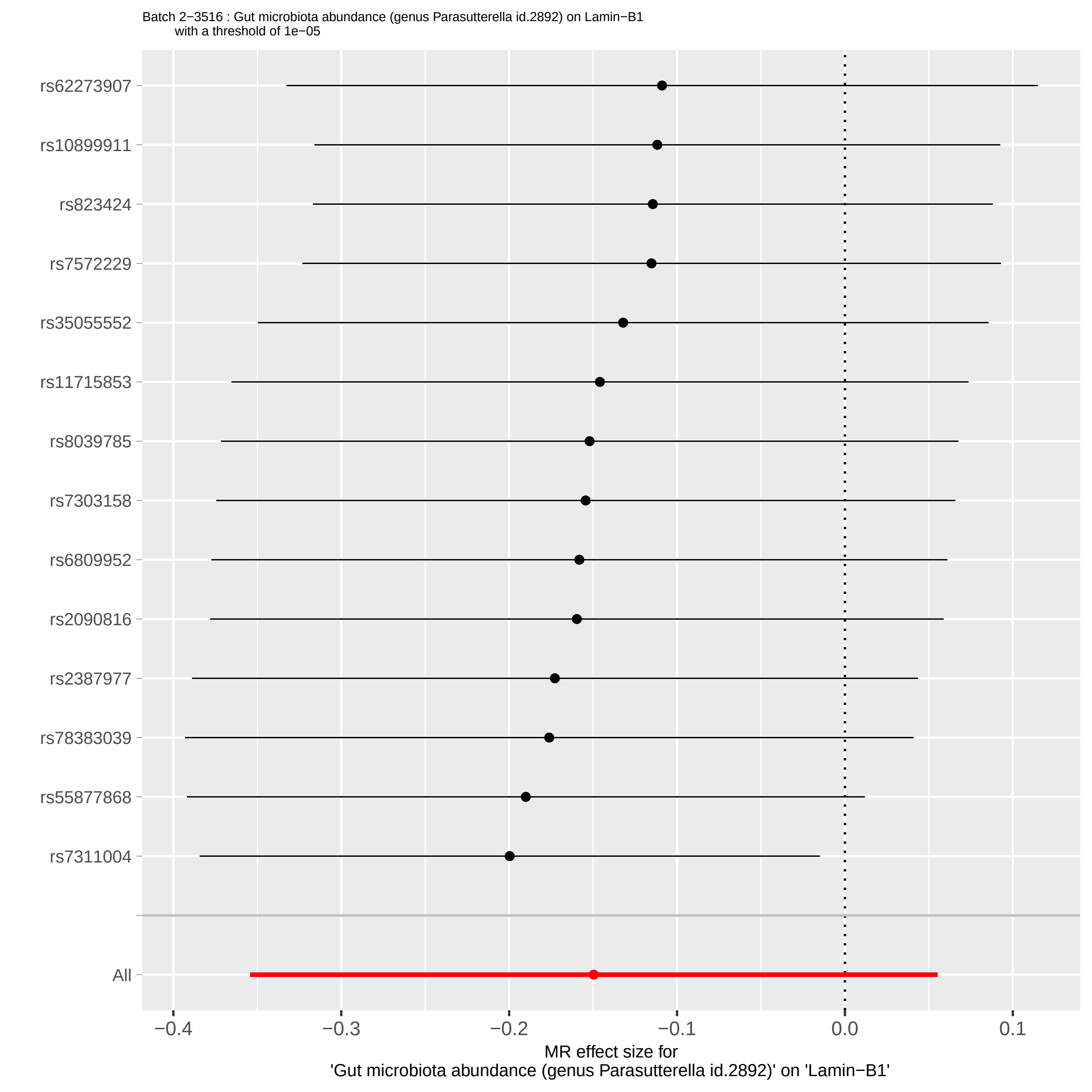

Batch 2−3516 : Gut microbiota abundance (genus Parasutterella id.2892) on Lamin−B1
with a threshold of 1e−05
rs62273907
rs10899911
rs823424
rs7572229
rs35055552
rs11715853
rs8039785
rs7303158
rs6809952
rs2090816
rs2387977
rs78383039
rs55877868
rs7311004
All
−0.4
−0.3
−0.2
−0.1
0.0
0.1
MR effect size for
'Gut microbiota abundance (genus Parasutterella id.2892)' on 'Lamin−B1'

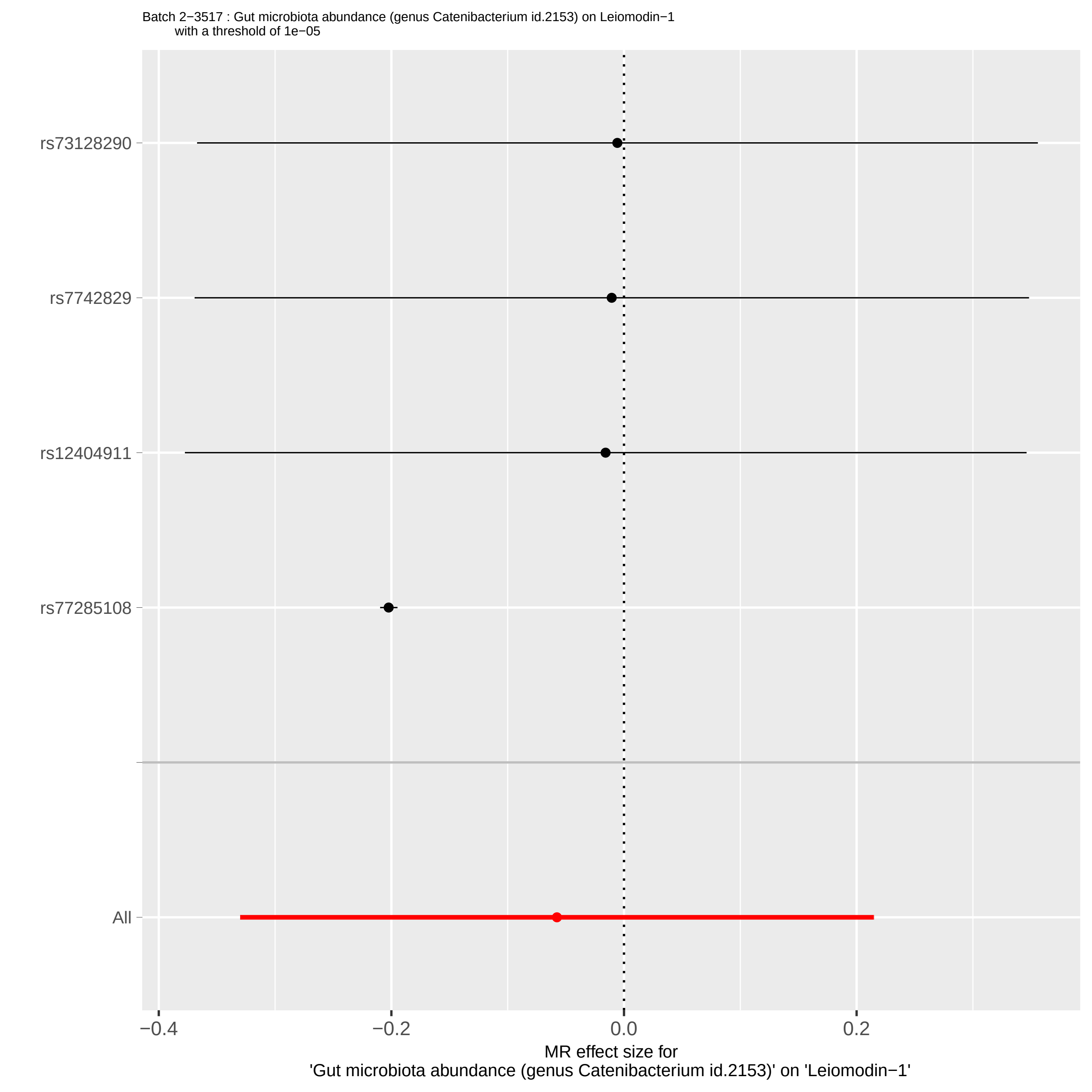
Batch 2−3517 : Gut microbiota abundance (genus Catenibacterium id.2153) on Leiomodin−1
with a threshold of 1e−05
rs73128290
rs7742829
rs12404911
rs77285108
All
−0.4
−0.2
0.0
0.2
MR effect size for
'Gut microbiota abundance (genus Catenibacterium id.2153)' on 'Leiomodin−1'

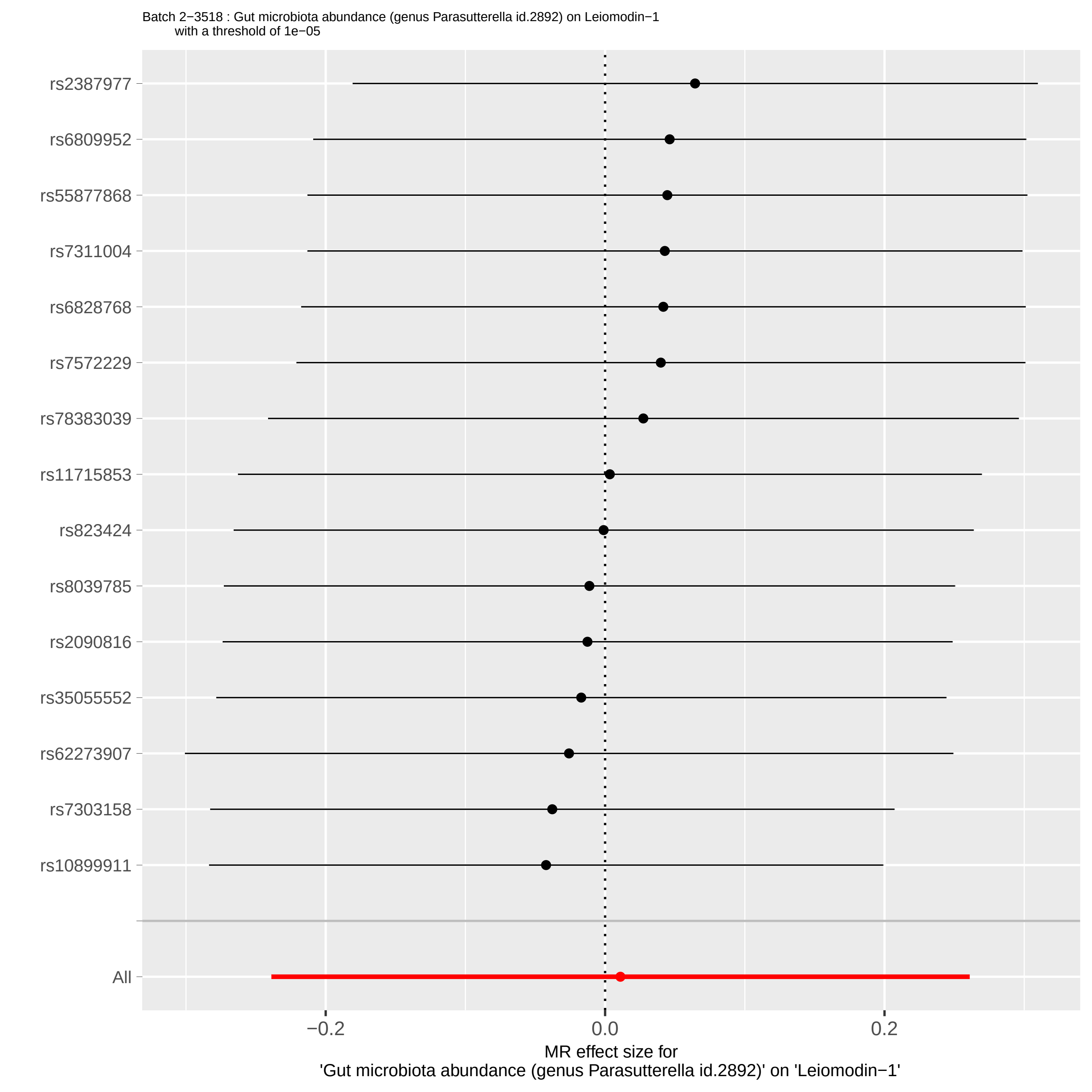
Batch 2−3518 : Gut microbiota abundance (genus Parasutterella id.2892) on Leiomodin−1
with a threshold of 1e−05
rs2387977
rs6809952
rs55877868
rs7311004
rs6828768
rs7572229
rs78383039
rs11715853
rs823424
rs8039785
rs2090816
rs35055552
rs62273907
rs7303158
rs10899911
All
−0.2
0.0
0.2
MR effect size for
'Gut microbiota abundance (genus Parasutterella id.2892)' on 'Leiomodin−1'

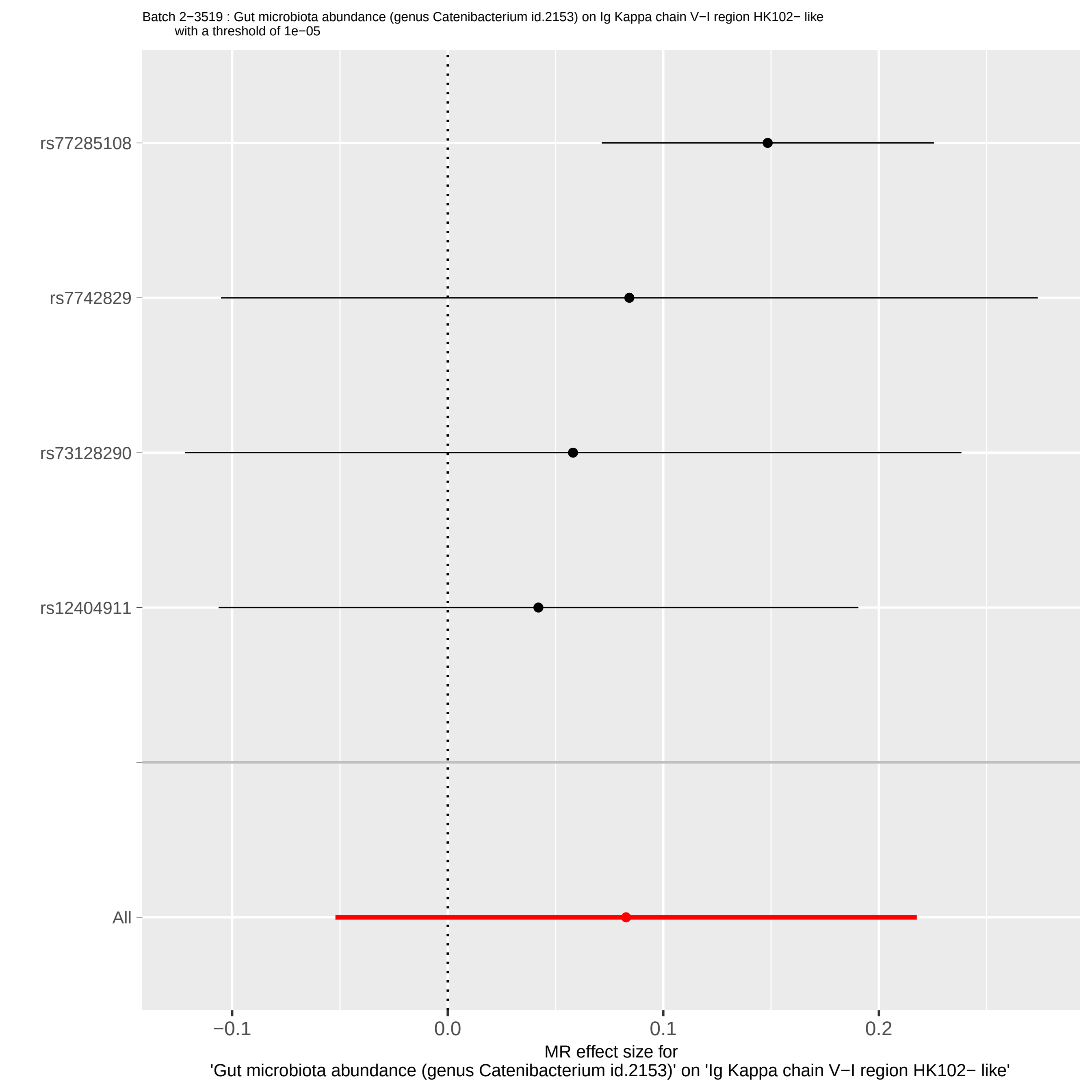

Batch 2−3519 : Gut microbiota abundance (genus Catenibacterium id.2153) on Ig Kappa chain V−I region HK102− like
with a threshold of 1e−05
rs77285108
rs7742829
rs73128290
rs12404911
All
−0.1
0.0
0.1
0.2
MR effect size for
'Gut microbiota abundance (genus Catenibacterium id.2153)' on 'Ig Kappa chain V−I region HK102− like'

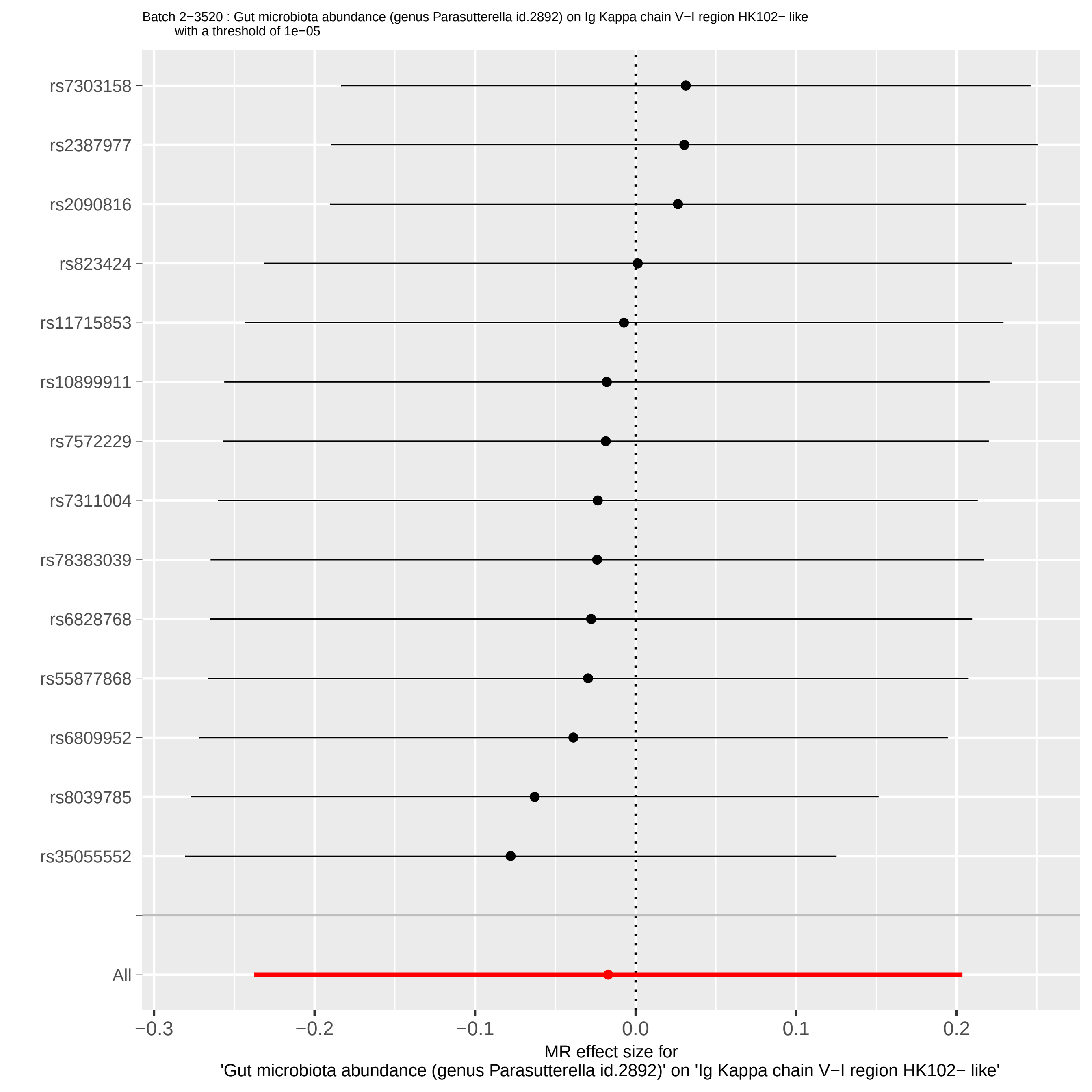

Batch 2−3520 : Gut microbiota abundance (genus Parasutterella id.2892) on Ig Kappa chain V−I region HK102− like
with a threshold of 1e−05
rs7303158
rs2387977
rs2090816
rs823424
rs11715853
rs10899911
rs7572229
rs7311004
rs78383039
rs6828768
rs55877868
rs6809952
rs8039785
rs35055552
All
−0.3
−0.2
−0.1
0.0
0.1
0.2
MR effect size for
'Gut microbiota abundance (genus Parasutterella id.2892)' on 'Ig Kappa chain V−I region HK102− like'

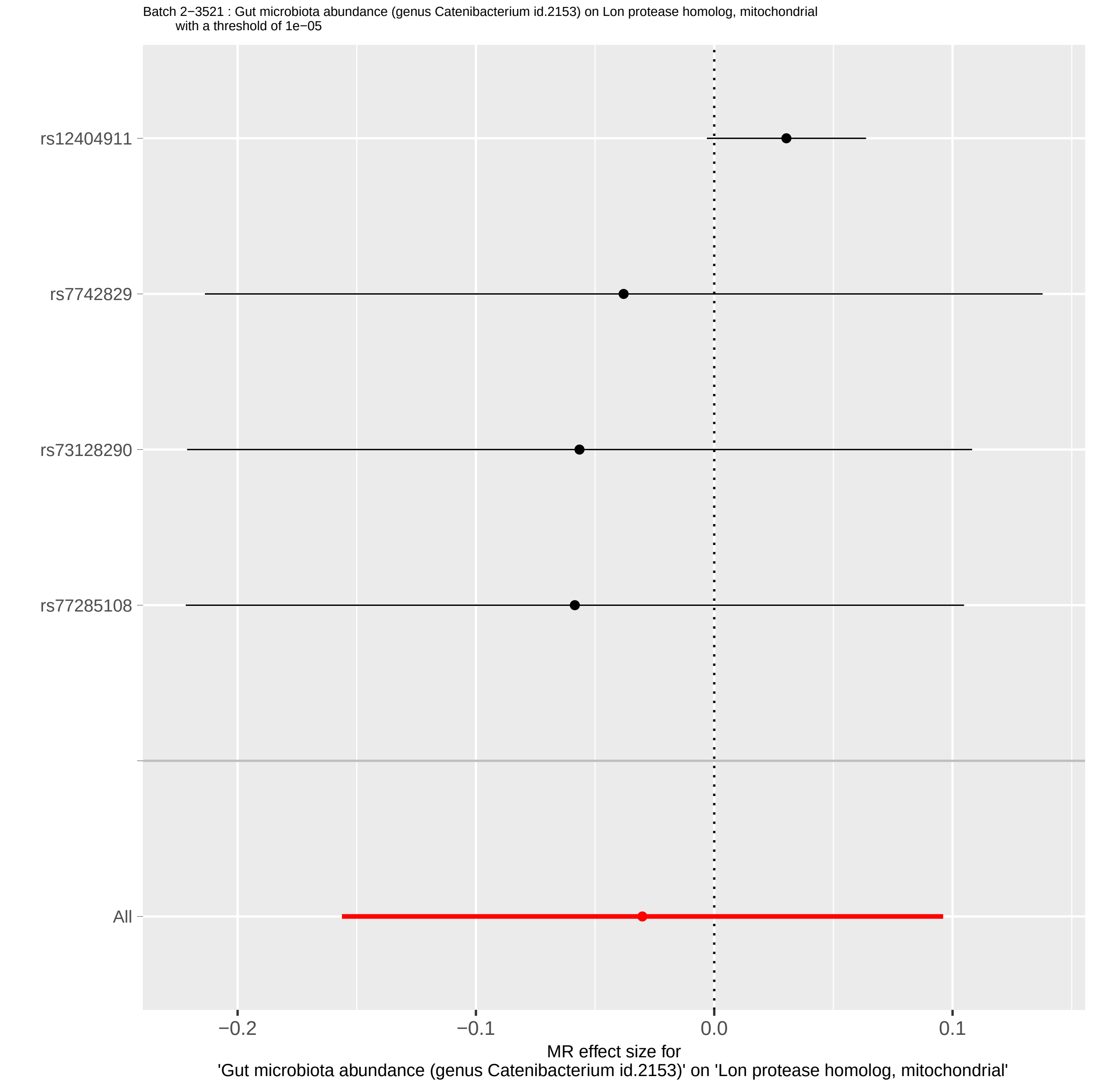

Batch 2−3521 : Gut microbiota abundance (genus Catenibacterium id.2153) on Lon protease homolog, mitochondrial
with a threshold of 1e−05
rs12404911
rs7742829
rs73128290
rs77285108
All
−0.2
−0.1
0.0
0.1
MR effect size for
'Gut microbiota abundance (genus Catenibacterium id.2153)' on 'Lon protease homolog, mitochondrial'

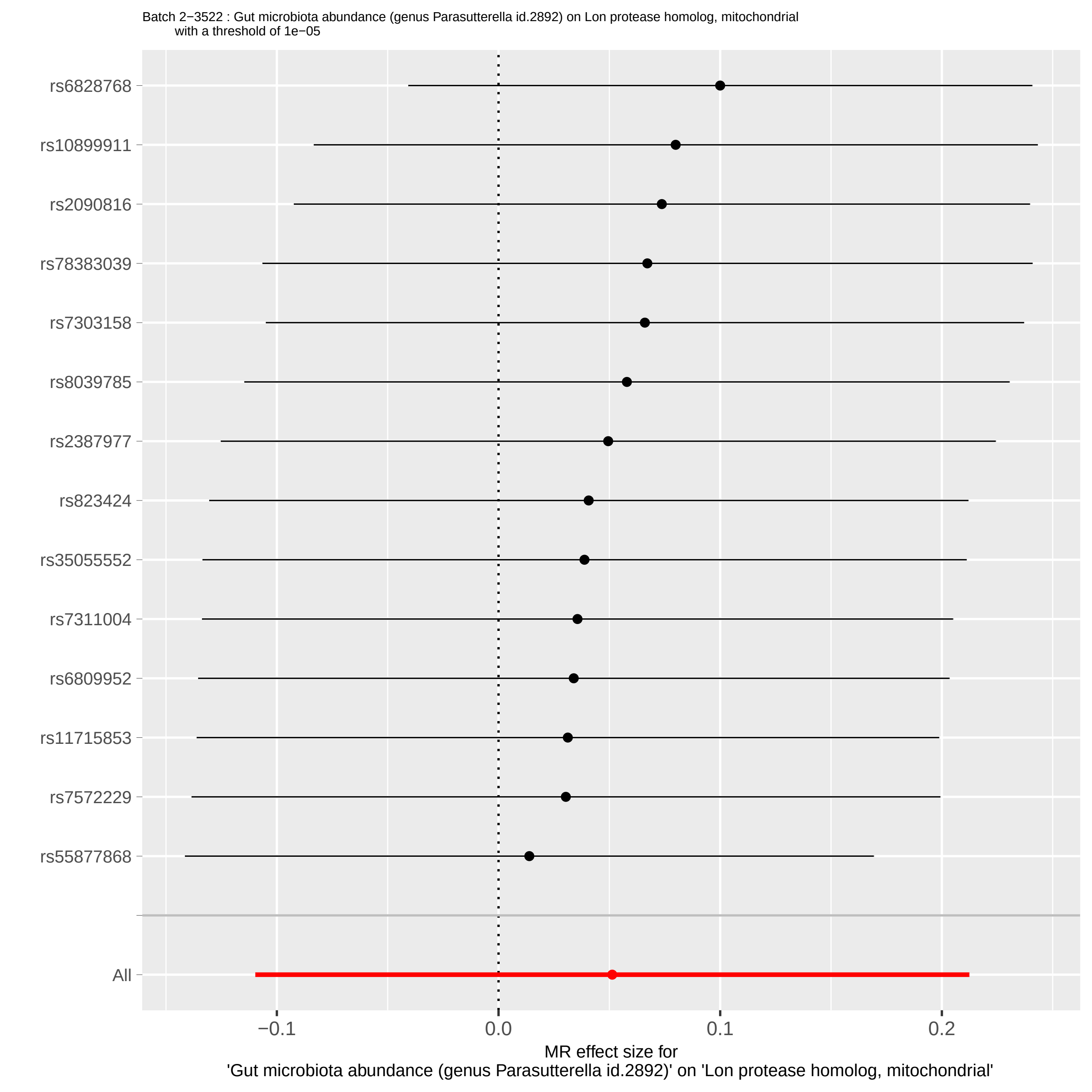
Batch 2−3522 : Gut microbiota abundance (genus Parasutterella id.2892) on Lon protease homolog, mitochondrial
with a threshold of 1e−05
rs6828768
rs10899911
rs2090816
rs78383039
rs7303158
rs8039785
rs2387977
rs823424
rs35055552
rs7311004
rs6809952
rs11715853
rs7572229
rs55877868
All
−0.1
0.0
0.1
0.2
MR effect size for
'Gut microbiota abundance (genus Parasutterella id.2892)' on 'Lon protease homolog, mitochondrial'

Batch 2−3523 : Gut microbiota abundance (genus Catenibacterium id.2153) on Lysyl oxidase homolog 2
with a threshold of 1e−05

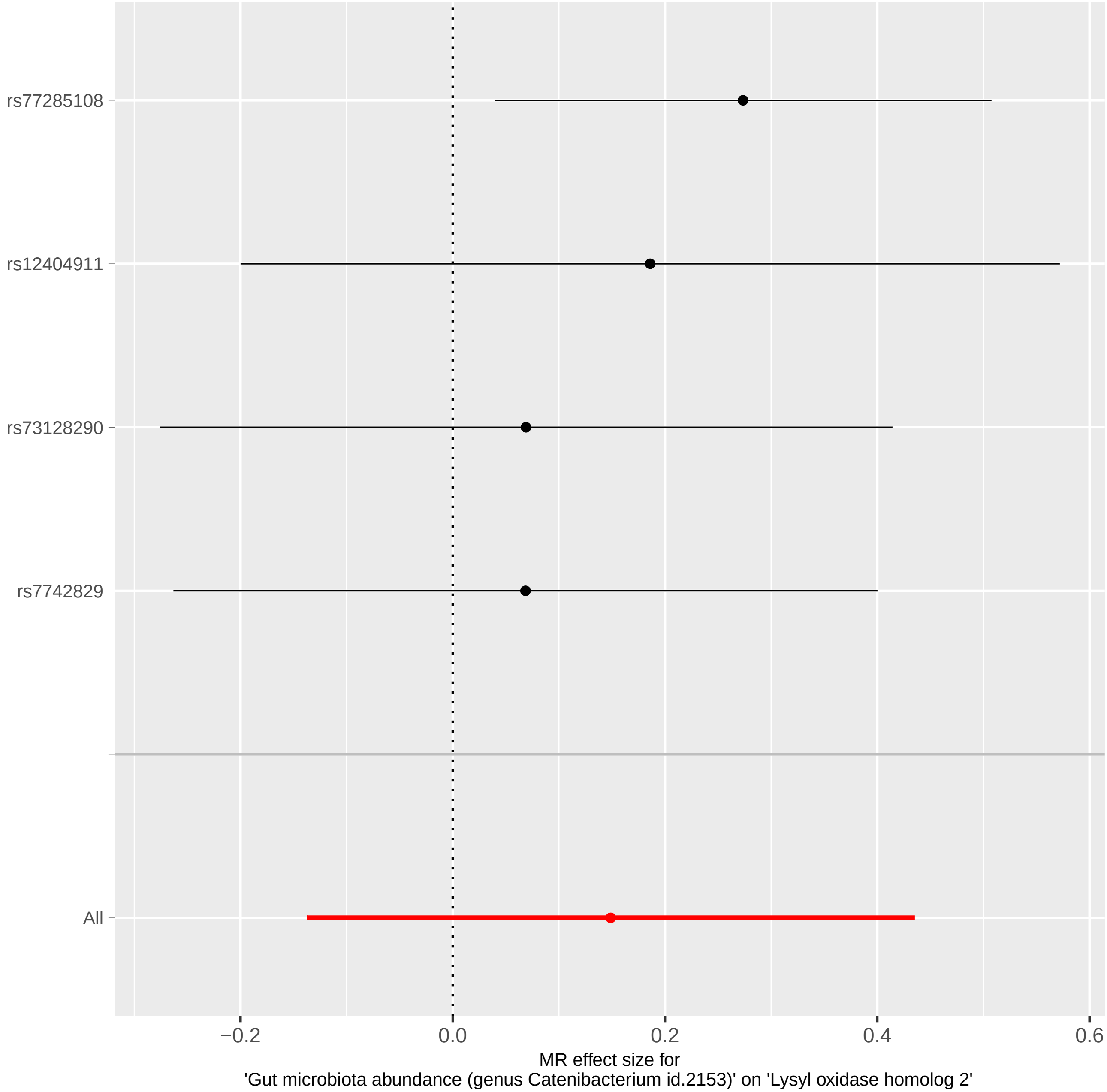

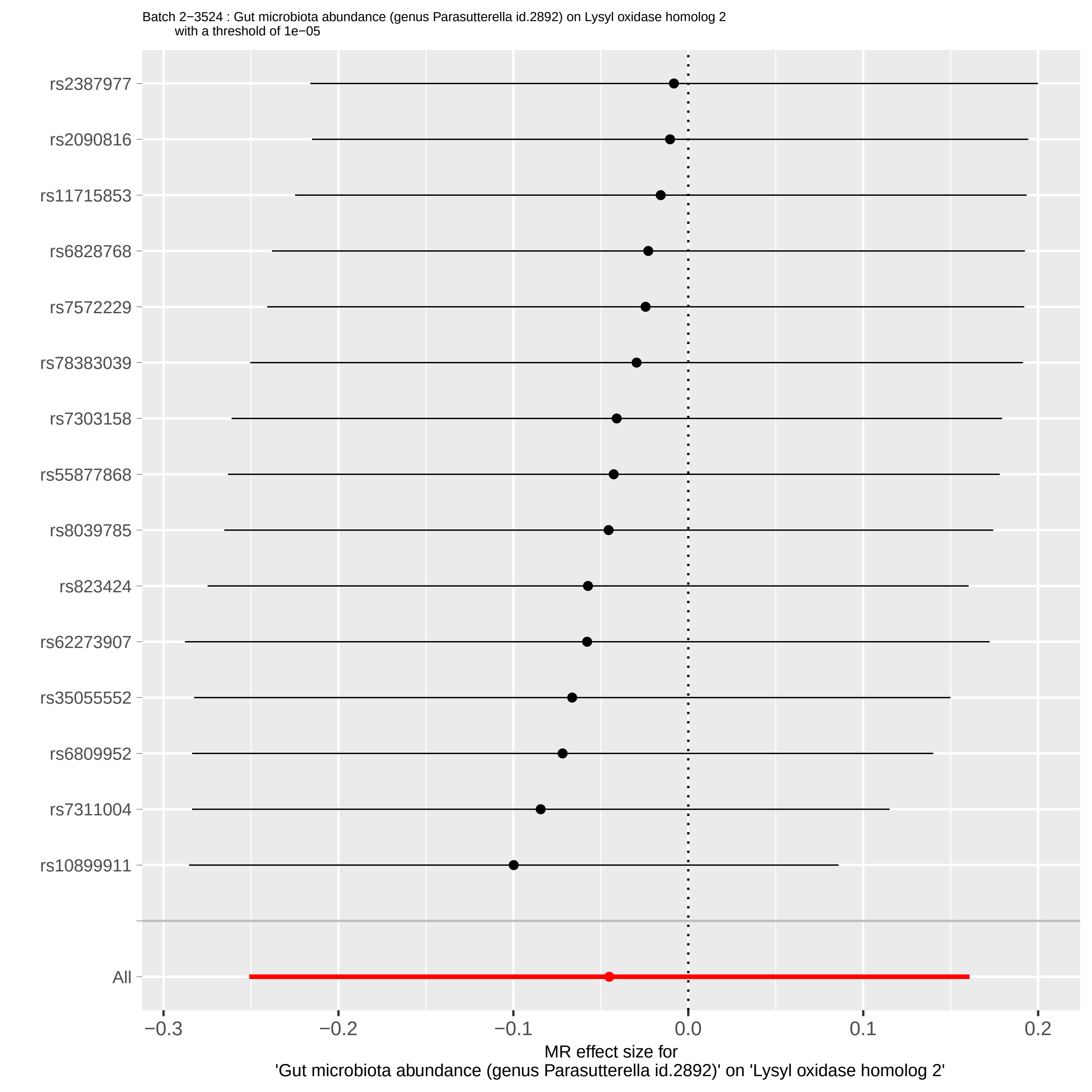
Batch 2−3524 : Gut microbiota abundance (genus Parasutterella id.2892) on Lysyl oxidase homolog 2
with a threshold of 1e−05
rs2387977
rs2090816
rs11715853
rs6828768
rs7572229
rs78383039
rs7303158
rs55877868
rs8039785
rs823424
rs62273907
rs35055552
rs6809952
rs7311004
rs10899911
All
−0.3
−0.2
−0.1
0.0
0.1
0.2
MR effect size for
'Gut microbiota abundance (genus Parasutterella id.2892)' on 'Lysyl oxidase homolog 2'

Batch 2−3525 : Gut microbiota abundance (genus Catenibacterium id.2153) on Putative apolipoprotein(a)−like protein 2
with a threshold of 1e−05

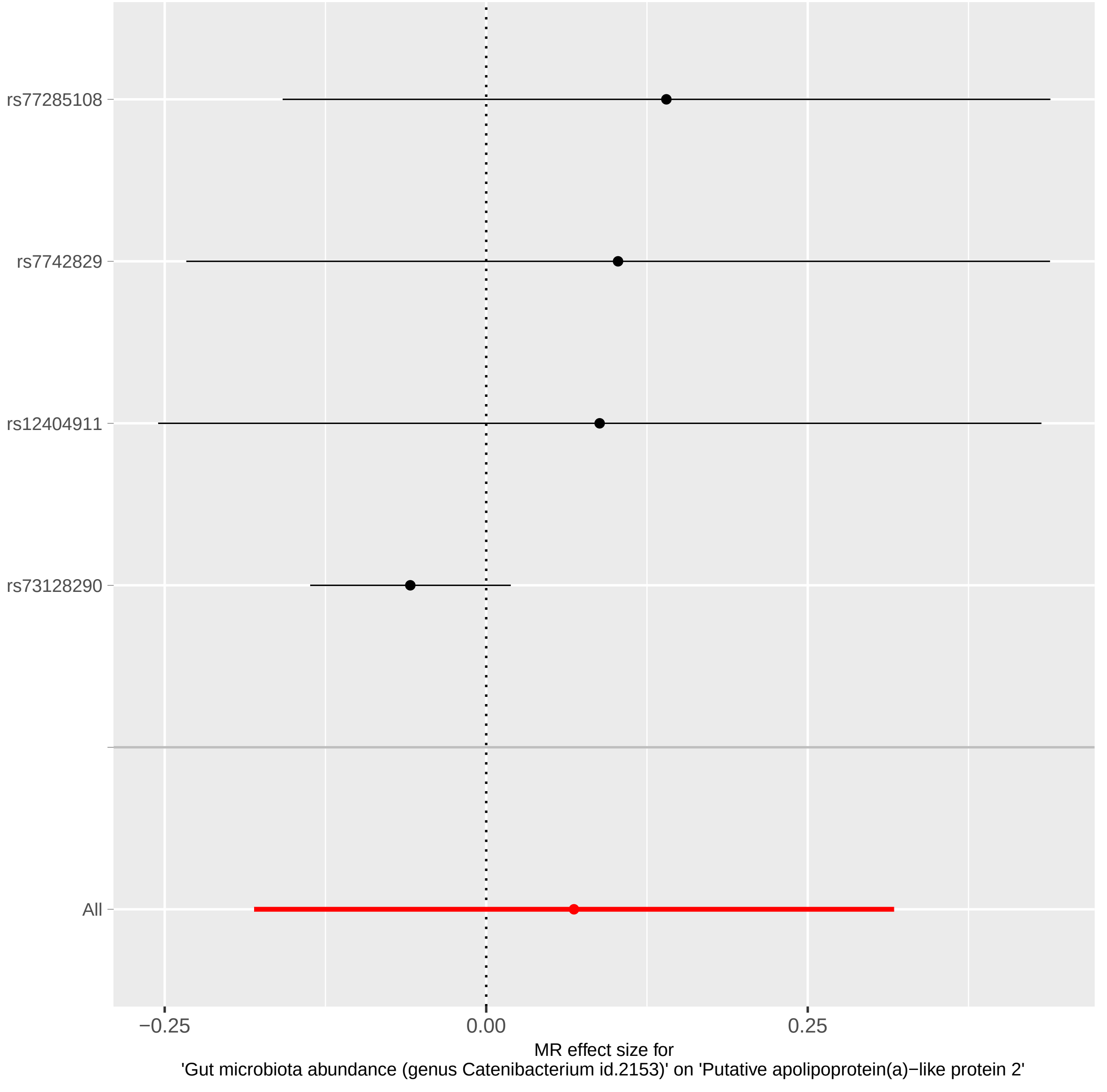

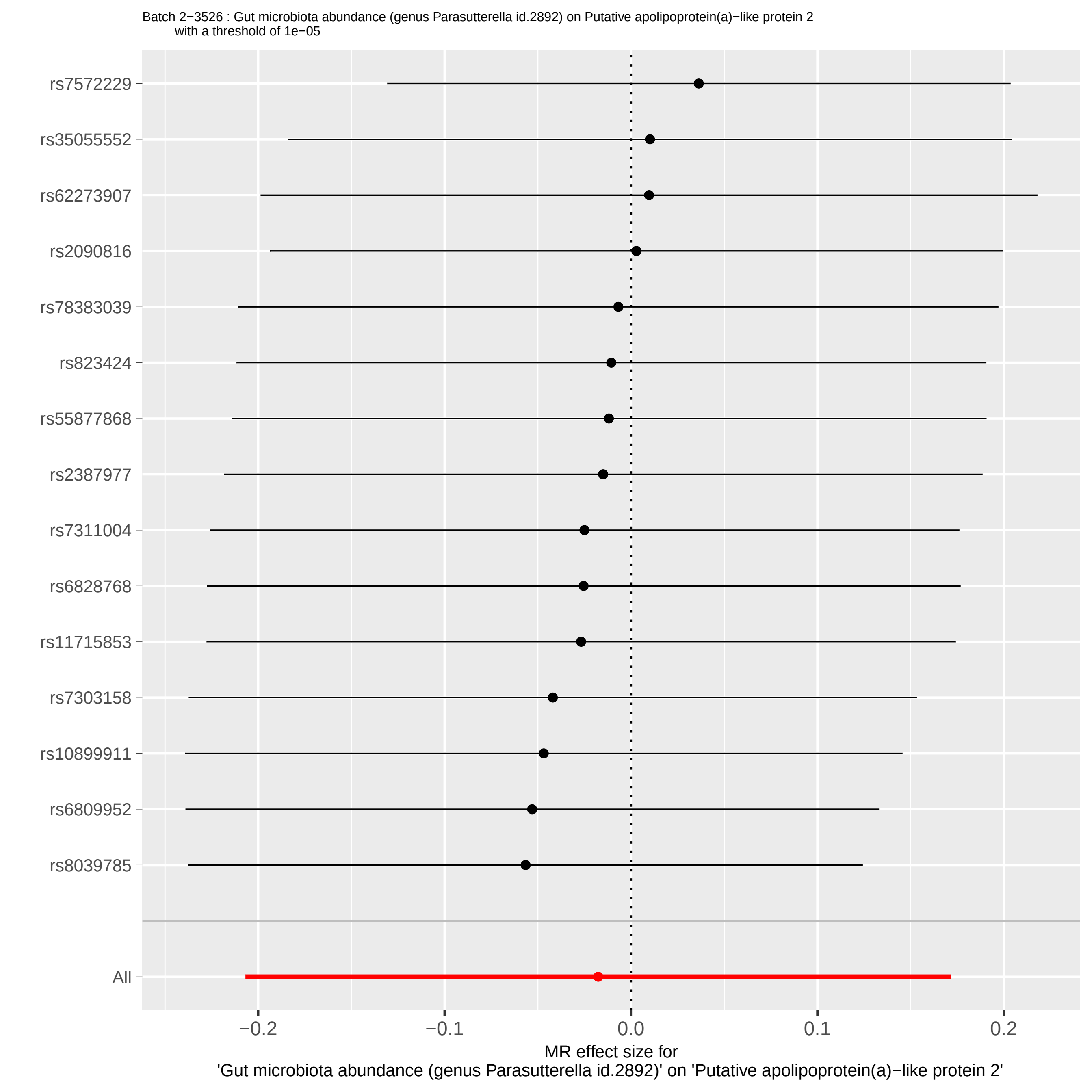
Batch 2−3526 : Gut microbiota abundance (genus Parasutterella id.2892) on Putative apolipoprotein(a)−like protein 2
with a threshold of 1e−05
rs7572229
rs35055552
rs62273907
rs2090816
rs78383039
rs823424
rs55877868
rs2387977
rs7311004
rs6828768
rs11715853
rs7303158
rs10899911
rs6809952
rs8039785
All
−0.2
−0.1
0.0
0.1
0.2
MR effect size for
'Gut microbiota abundance (genus Parasutterella id.2892)' on 'Putative apolipoprotein(a)−like protein 2'

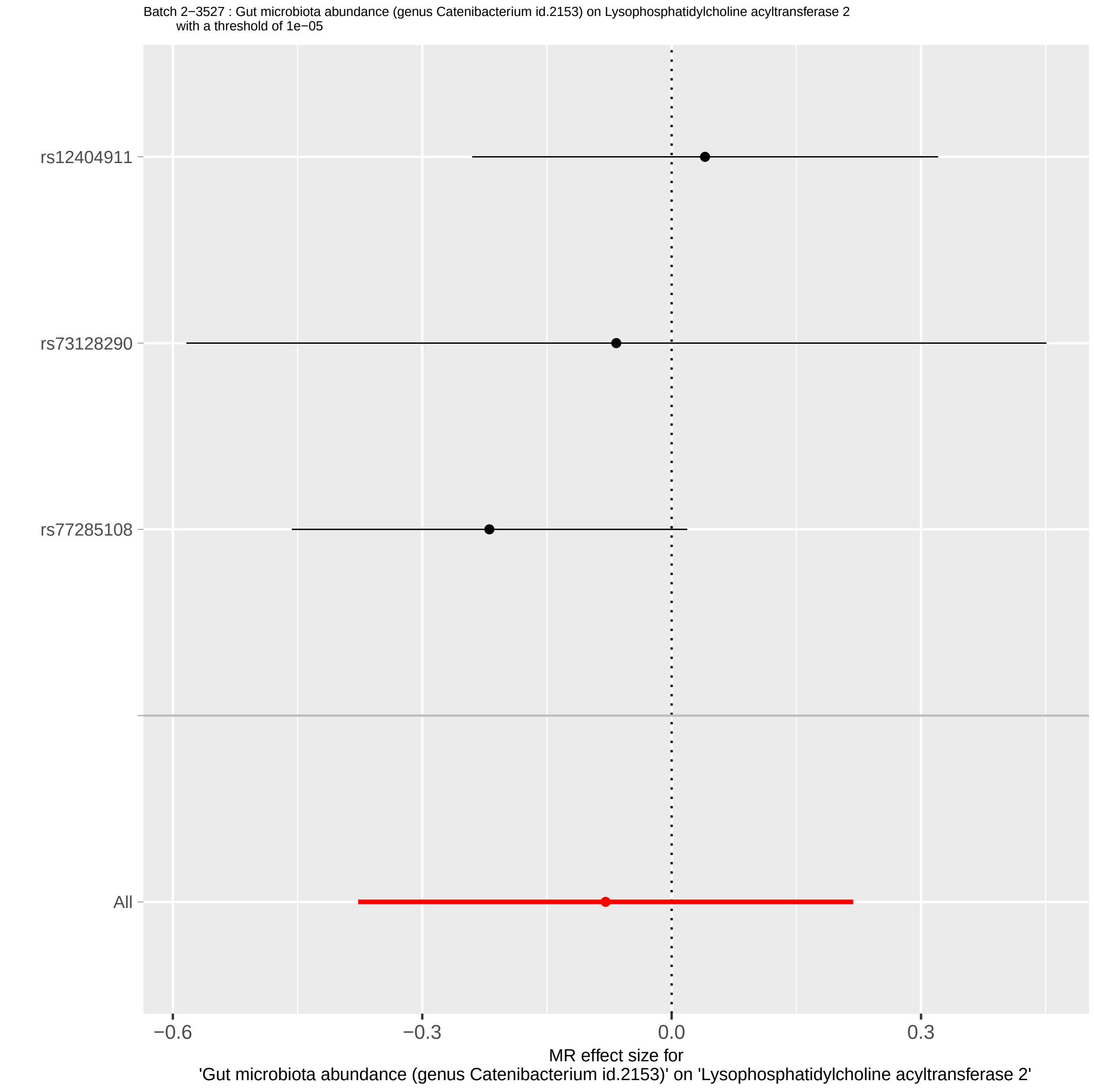
Batch 2−3527 : Gut microbiota abundance (genus Catenibacterium id.2153) on Lysophosphatidylcholine acyltransferase 2
with a threshold of 1e−05
rs12404911
rs73128290
rs77285108
All
−0.6
−0.3
0.0
0.3
MR effect size for
'Gut microbiota abundance (genus Catenibacterium id.2153)' on 'Lysophosphatidylcholine acyltransferase 2'

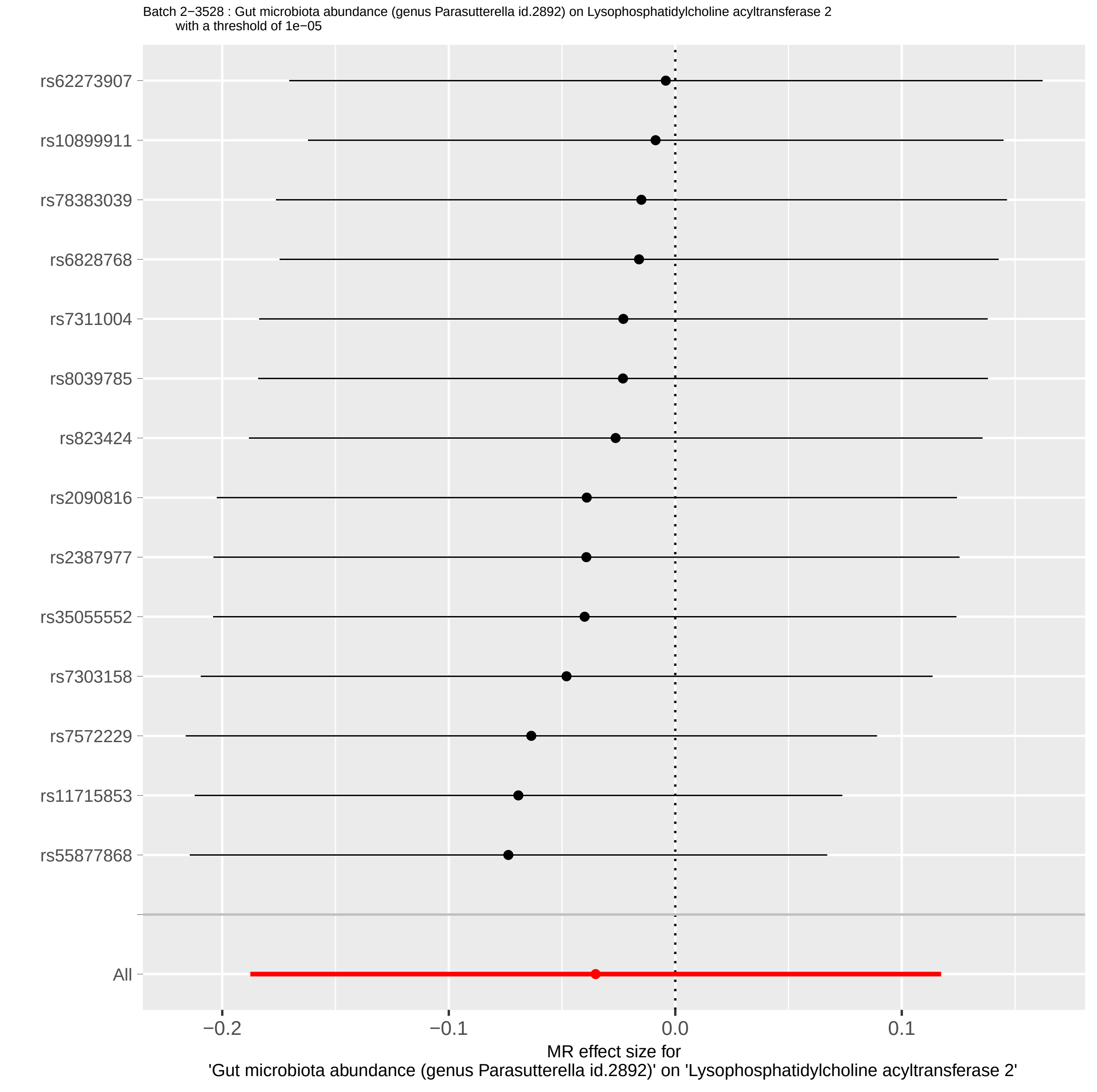

Batch 2−3528 : Gut microbiota abundance (genus Parasutterella id.2892) on Lysophosphatidylcholine acyltransferase 2
with a threshold of 1e−05
rs62273907
rs10899911
rs78383039
rs6828768
rs7311004
rs8039785
rs823424
rs2090816
rs2387977
rs35055552
rs7303158
rs7572229
rs11715853
rs55877868
All
−0.2
−0.1
0.0
0.1
MR effect size for
'Gut microbiota abundance (genus Parasutterella id.2892)' on 'Lysophosphatidylcholine acyltransferase 2'

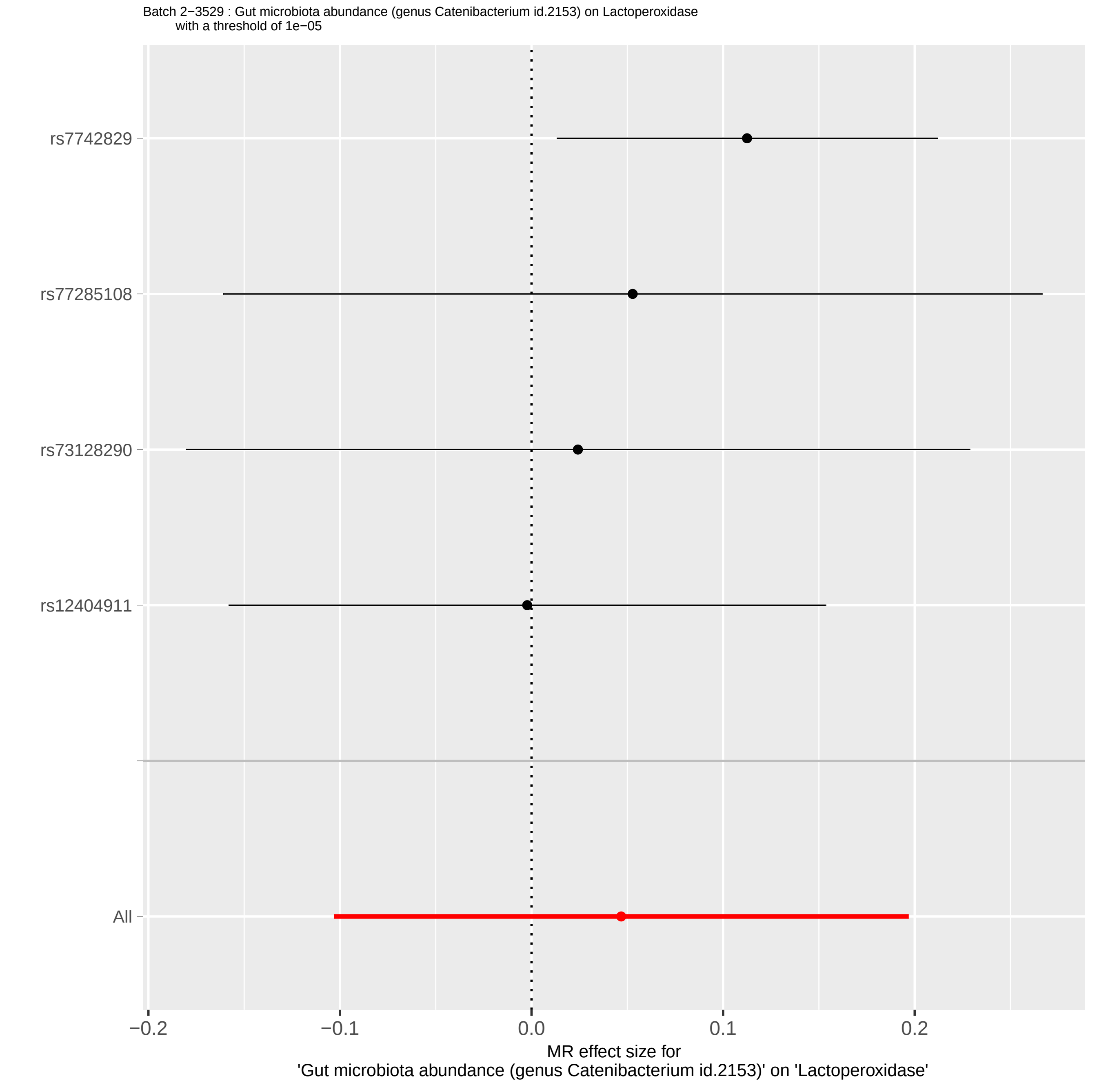
Batch 2−3529 : Gut microbiota abundance (genus Catenibacterium id.2153) on Lactoperoxidase
with a threshold of 1e−05
rs7742829
rs77285108
rs73128290
rs12404911
All
−0.2
−0.1
0.0
0.1
0.2
MR effect size for
'Gut microbiota abundance (genus Catenibacterium id.2153)' on 'Lactoperoxidase'

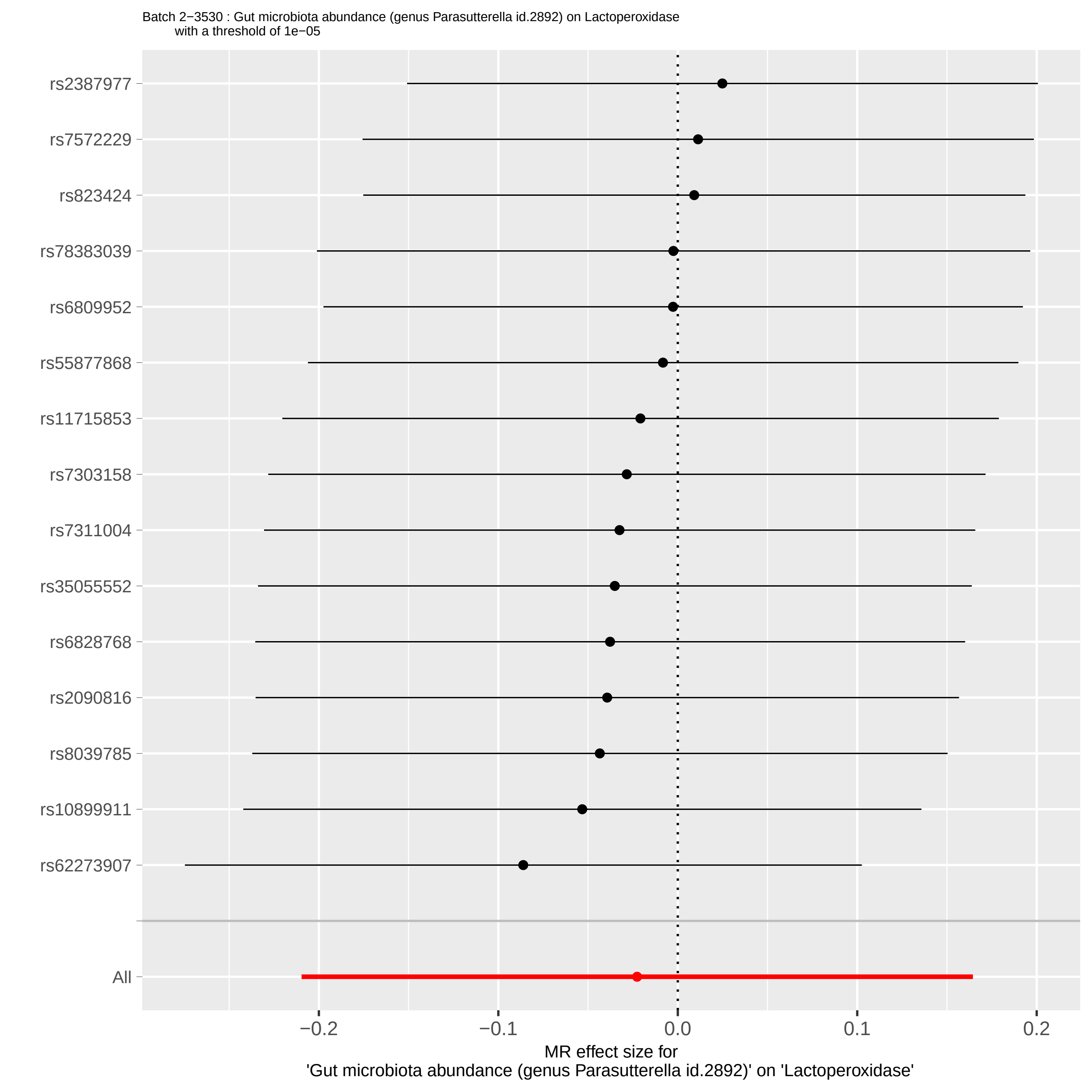

Batch 2−3530 : Gut microbiota abundance (genus Parasutterella id.2892) on Lactoperoxidase
with a threshold of 1e−05
rs2387977
rs7572229
rs823424
rs78383039
rs6809952
rs55877868
rs11715853
rs7303158
rs7311004
rs35055552
rs6828768
rs2090816
rs8039785
rs10899911
rs62273907
All
−0.2
−0.1
0.0
0.1
0.2
MR effect size for
'Gut microbiota abundance (genus Parasutterella id.2892)' on 'Lactoperoxidase'

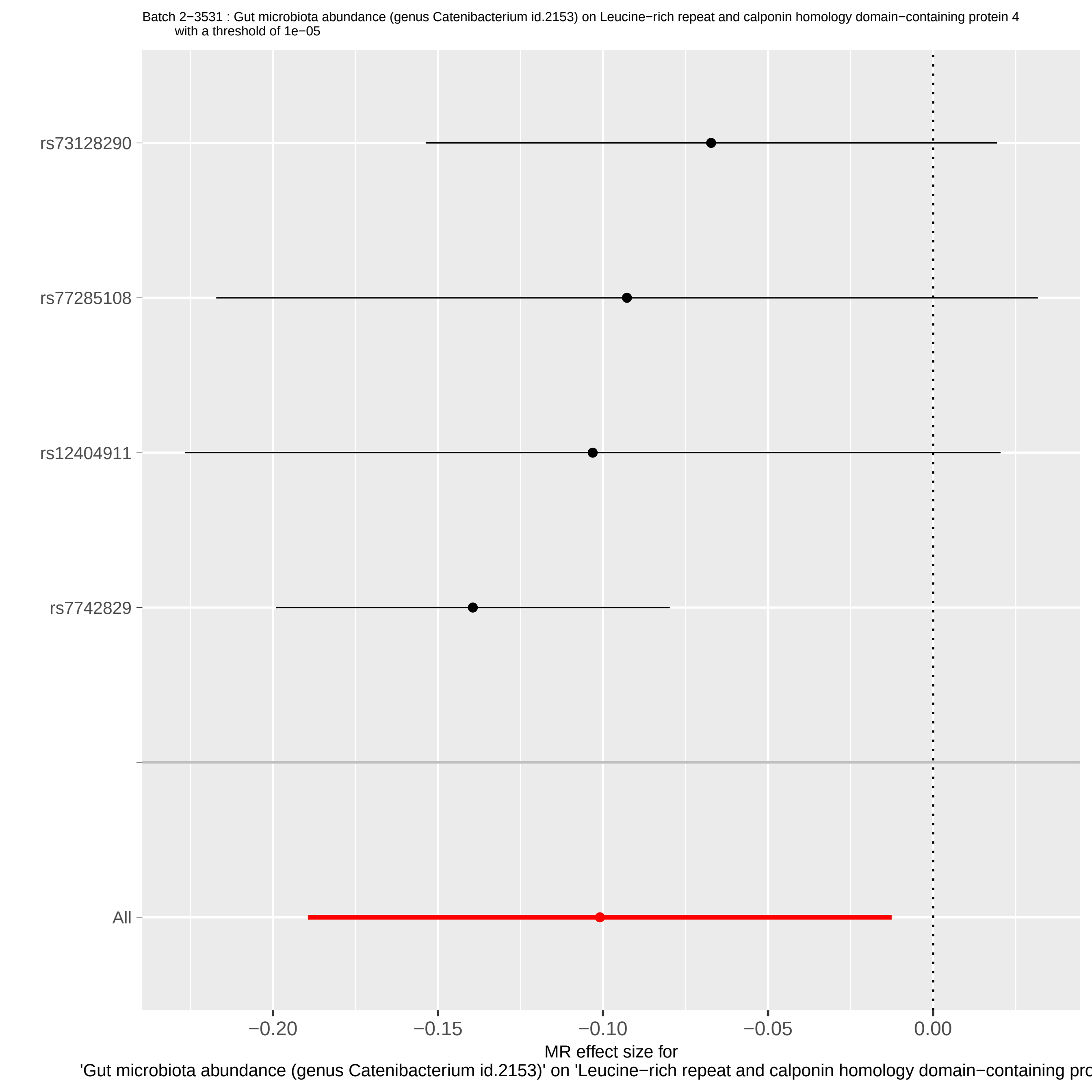

Batch 2−3531 : Gut microbiota abundance (genus Catenibacterium id.2153) on Leucine−rich repeat and calponin homology domain−containing protein 4
with a threshold of 1e−05
rs73128290
rs77285108
rs12404911
rs7742829
All
−0.20
−0.15
−0.10
−0.05
0.00
MR effect size for
'Gut microbiota abundance (genus Catenibacterium id.2153)' on 'Leucine−rich repeat and calponin homology domain−containing pro

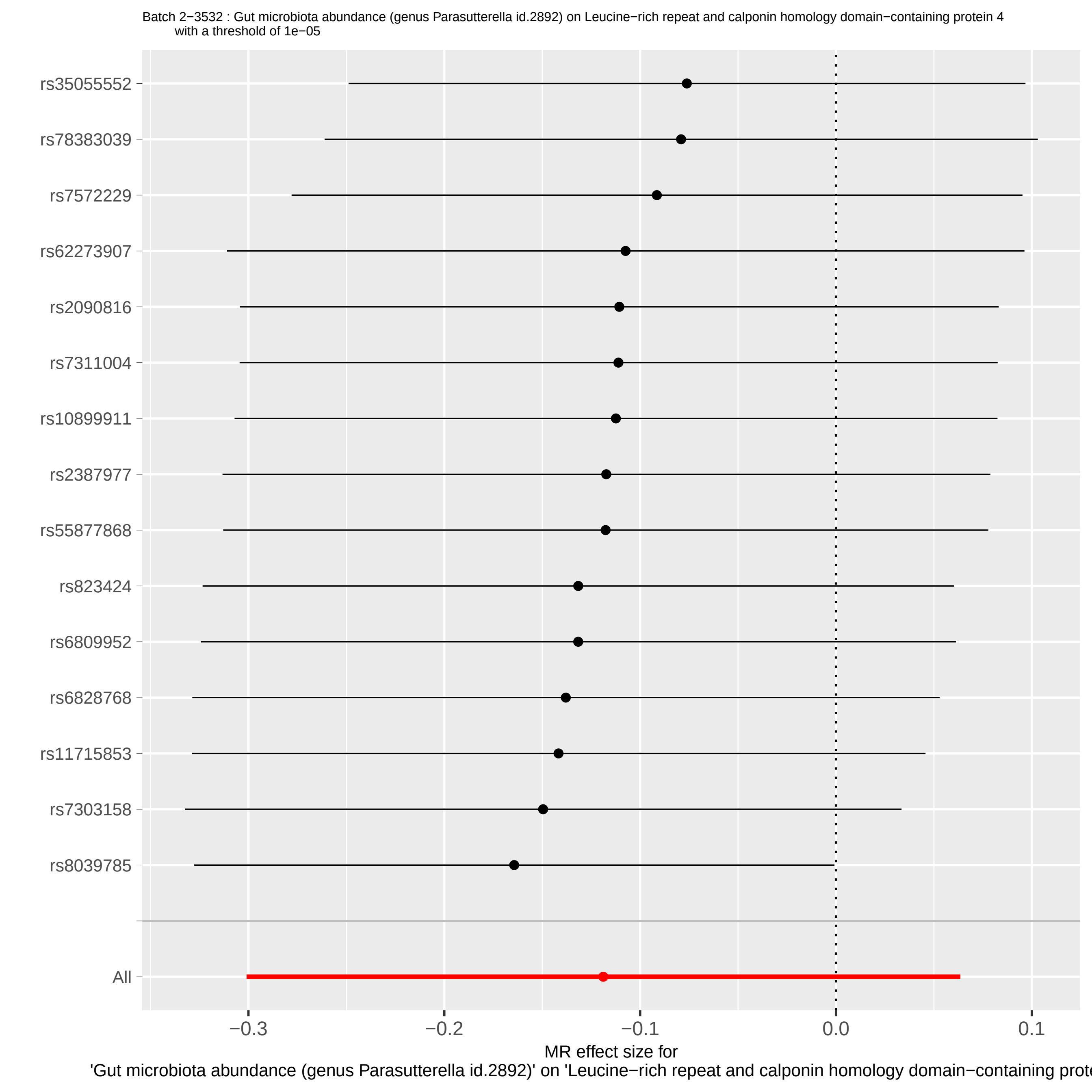
Batch 2−3532 : Gut microbiota abundance (genus Parasutterella id.2892) on Leucine−rich repeat and calponin homology domain−containing protein 4
with a threshold of 1e−05
rs35055552
rs78383039
rs7572229
rs62273907
rs2090816
rs7311004
rs10899911
rs2387977
rs55877868
rs823424
rs6809952
rs6828768
rs11715853
rs7303158
rs8039785
All
−0.3
−0.2
−0.1
0.0
0.1
MR effect size for
'Gut microbiota abundance (genus Parasutterella id.2892)' on 'Leucine−rich repeat and calponin homology domain−containing prote

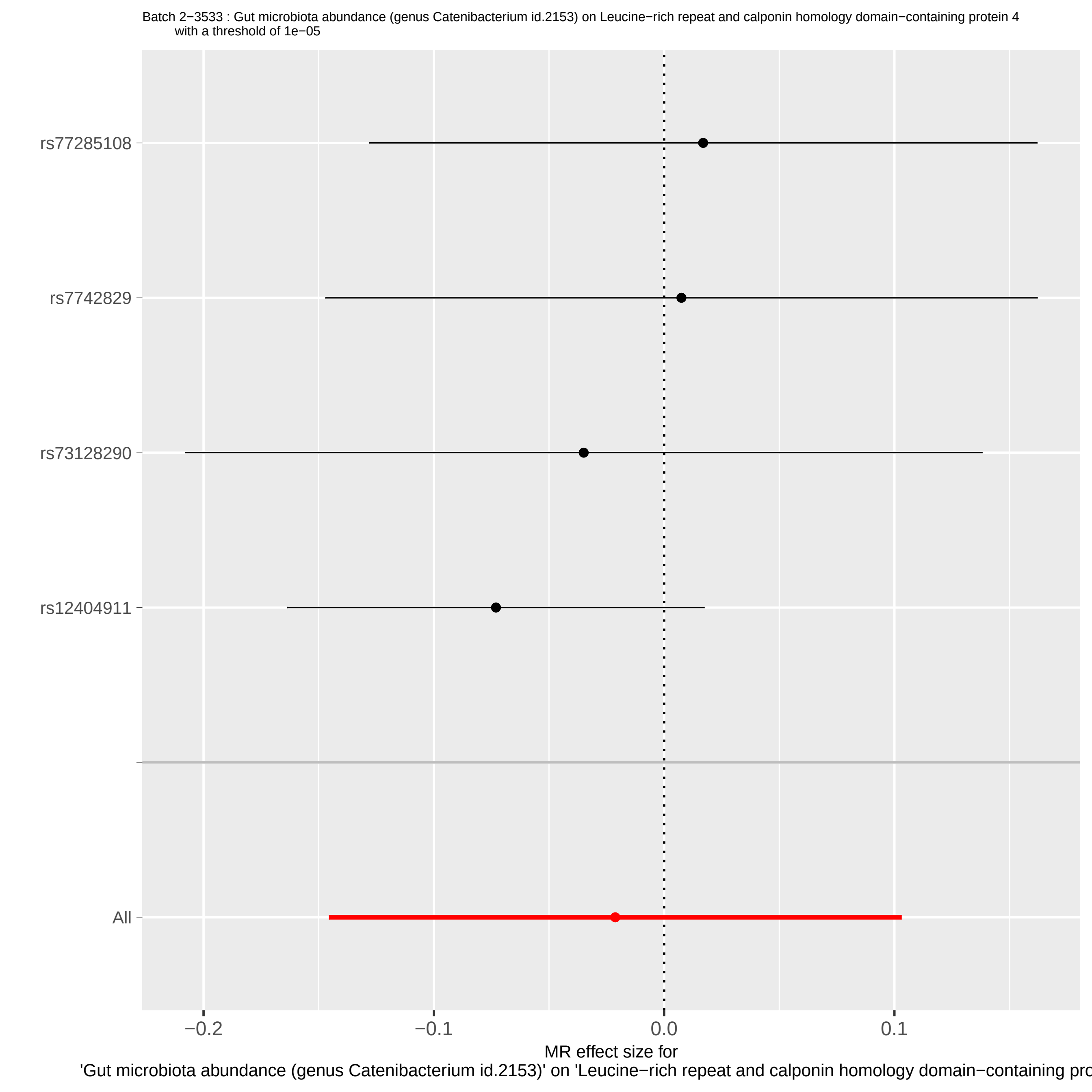

Batch 2−3533 : Gut microbiota abundance (genus Catenibacterium id.2153) on Leucine−rich repeat and calponin homology domain−containing protein 4
with a threshold of 1e−05
rs77285108
rs7742829
rs73128290
rs12404911
All
−0.2
−0.1
0.0
0.1
MR effect size for
'Gut microbiota abundance (genus Catenibacterium id.2153)' on 'Leucine−rich repeat and calponin homology domain−containing pro

Batch 2−3534 : Gut microbiota abundance (genus Parasutterella id.2892) on Leucine−rich repeat and calponin homology domain−containing protein 4 with a threshold of 1e−05

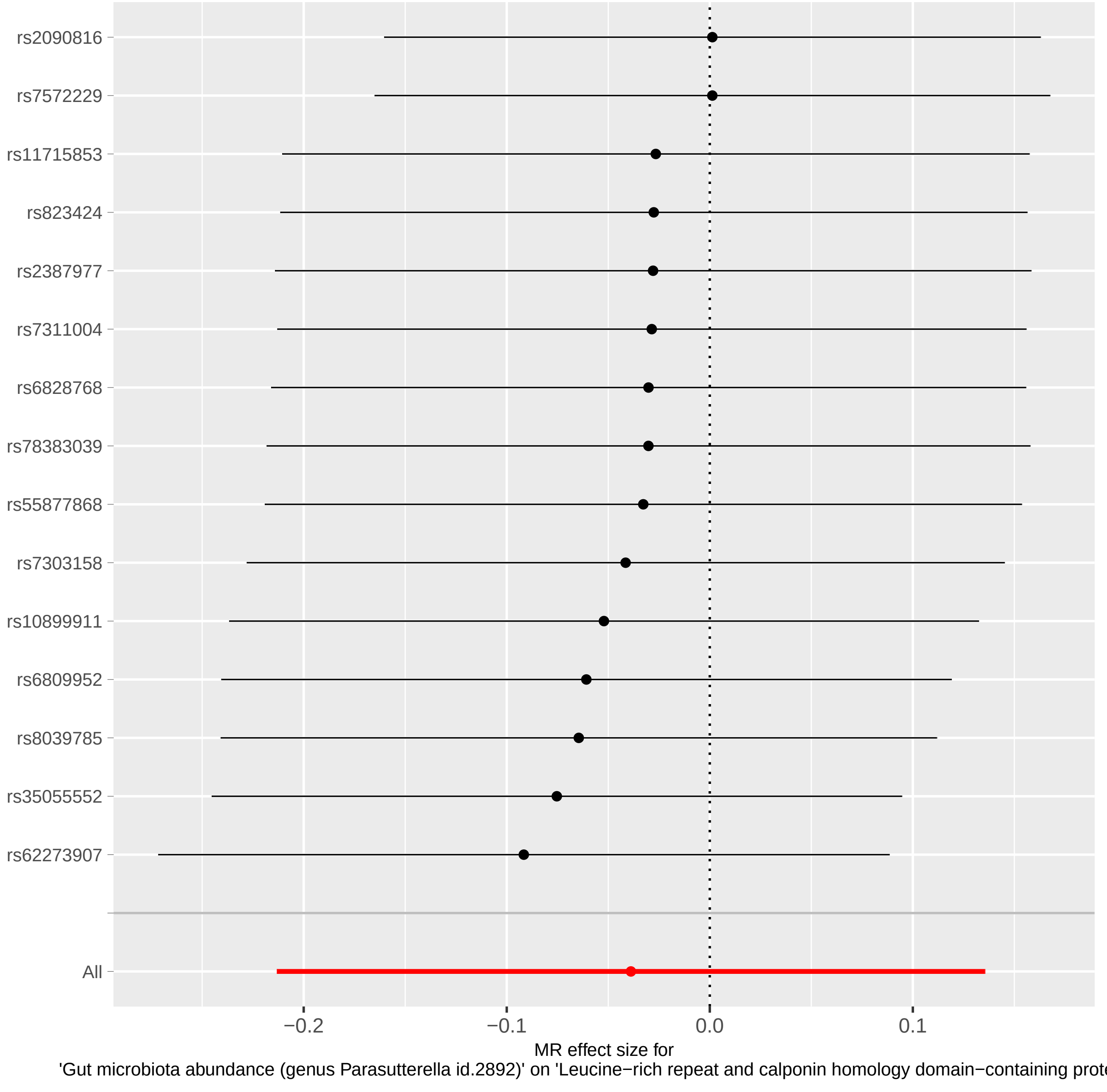

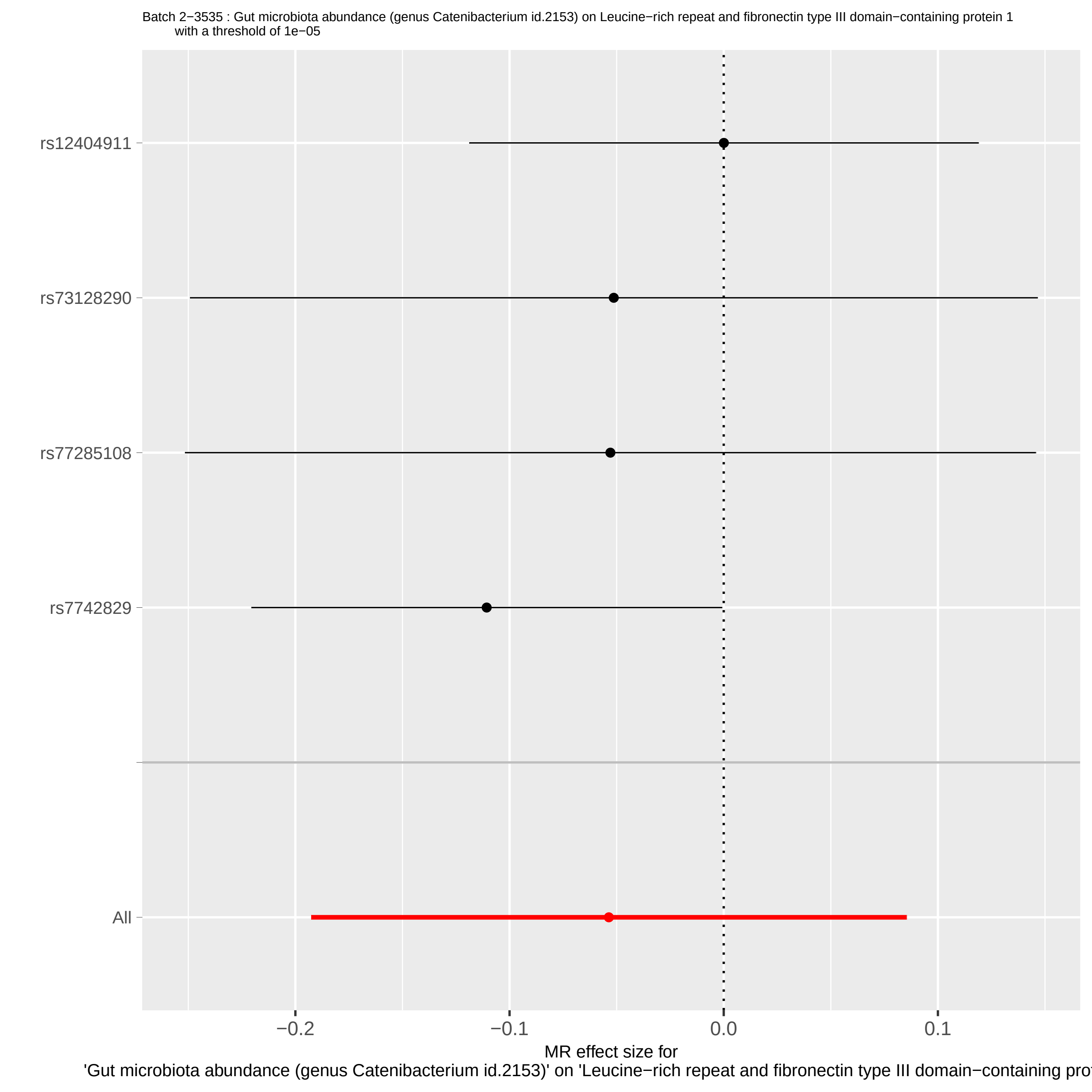

Batch 2−3535 : Gut microbiota abundance (genus Catenibacterium id.2153) on Leucine−rich repeat and fibronectin type III domain−containing protein 1 with a threshold of 1e−05
rs12404911
rs73128290
rs77285108
rs7742829
All
−0.2
−0.1
0.0
0.1
MR effect size for
'Gut microbiota abundance (genus Catenibacterium id.2153)' on 'Leucine−rich repeat and fibronectin type III domain−containing pro

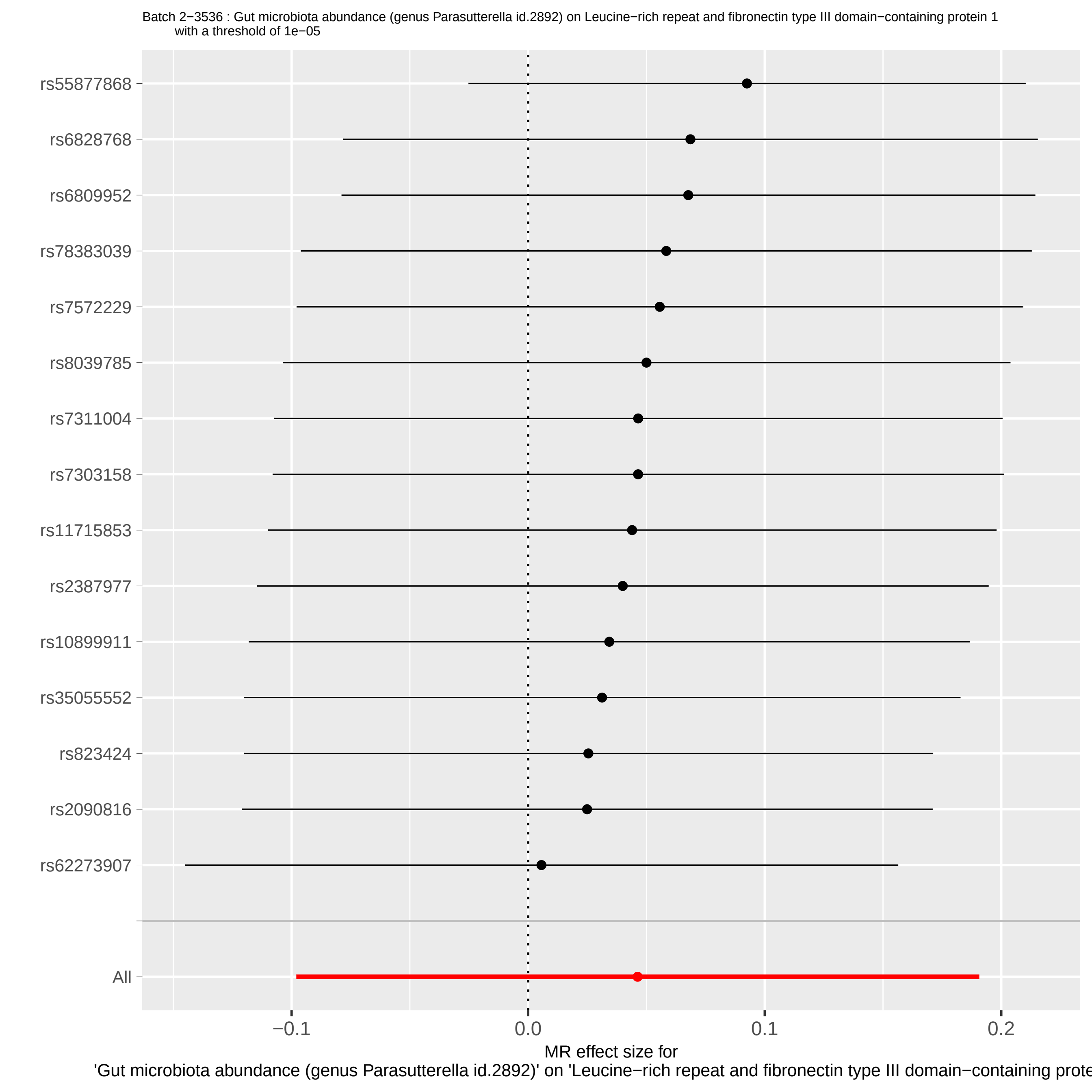

Batch 2−3536 : Gut microbiota abundance (genus Parasutterella id.2892) on Leucine−rich repeat and fibronectin type III domain−containing protein 1
with a threshold of 1e−05
rs55877868
rs6828768
rs6809952
rs78383039
rs7572229
rs8039785
rs7311004
rs7303158
rs11715853
rs2387977
rs10899911
rs35055552
rs823424
rs2090816
rs62273907
All
−0.1
0.0
0.1
0.2
MR effect size for
'Gut microbiota abundance (genus Parasutterella id.2892)' on 'Leucine−rich repeat and fibronectin type III domain−containing prote

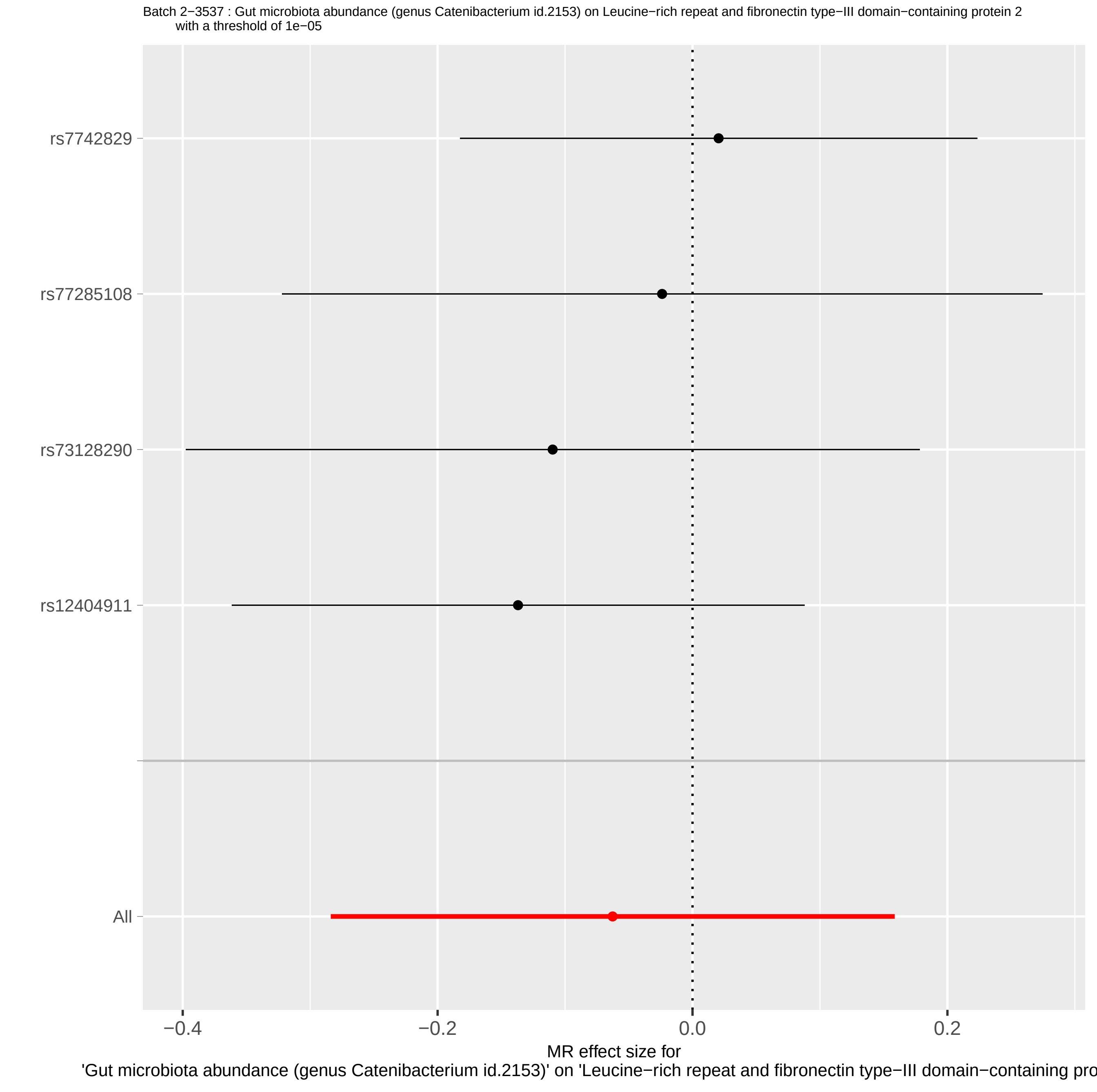

Batch 2−3537 : Gut microbiota abundance (genus Catenibacterium id.2153) on Leucine−rich repeat and fibronectin type−III domain−containing protein 2
with a threshold of 1e−05
rs7742829
rs77285108
rs73128290
rs12404911
All
−0.4
−0.2
0.0
0.2
MR effect size for
'Gut microbiota abundance (genus Catenibacterium id.2153)' on 'Leucine−rich repeat and fibronectin type−III domain−containing pro

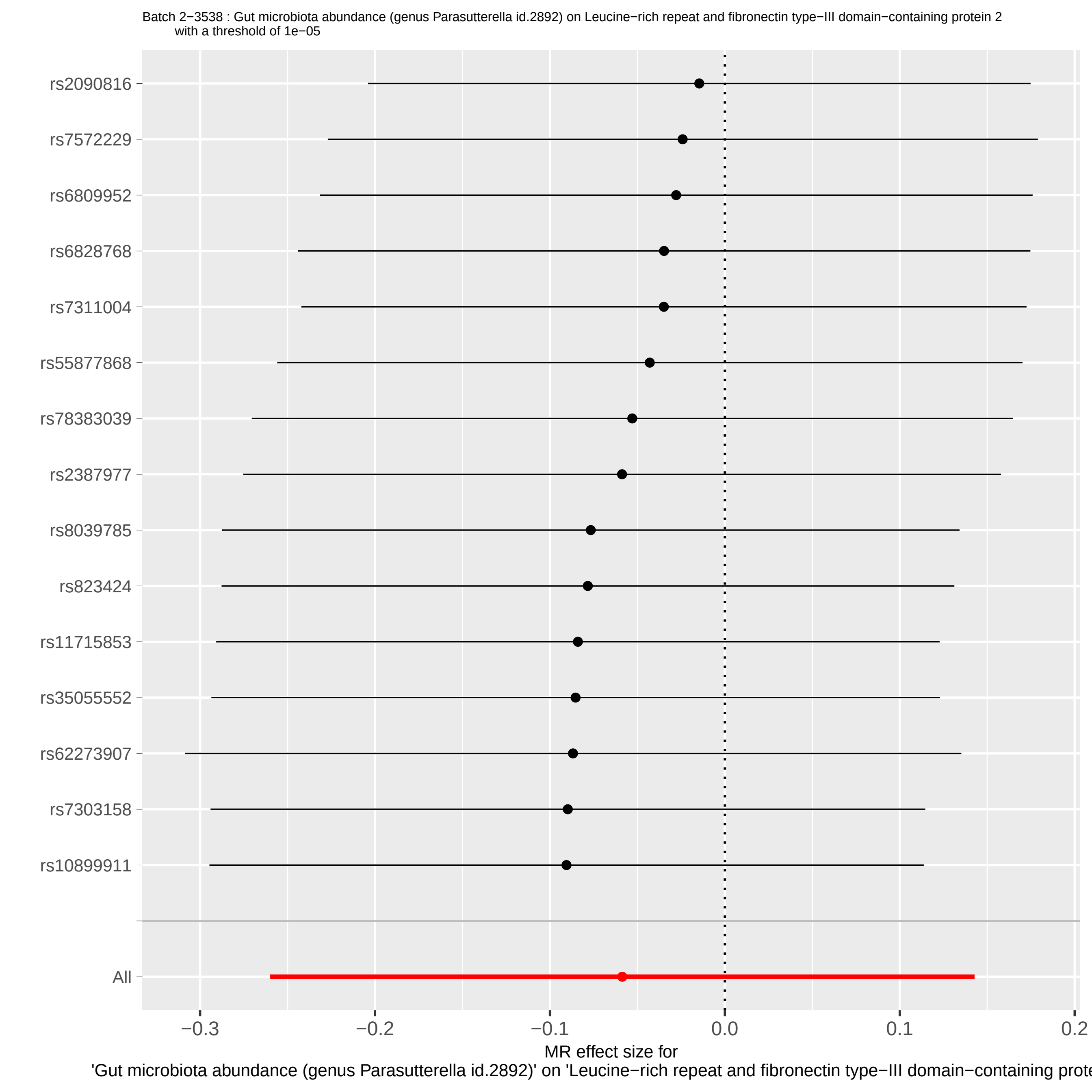

Batch 2−3538 : Gut microbiota abundance (genus Parasutterella id.2892) on Leucine−rich repeat and fibronectin type−III domain−containing protein 2
with a threshold of 1e−05
rs2090816
rs7572229
rs6809952
rs6828768
rs7311004
rs55877868
rs78383039
rs2387977
rs8039785
rs823424
rs11715853
rs35055552
rs62273907
rs7303158
rs10899911
All
−0.3
−0.2
−0.1
0.0
0.1
0.2
MR effect size for
'Gut microbiota abundance (genus Parasutterella id.2892)' on 'Leucine−rich repeat and fibronectin type−III domain−containing prote

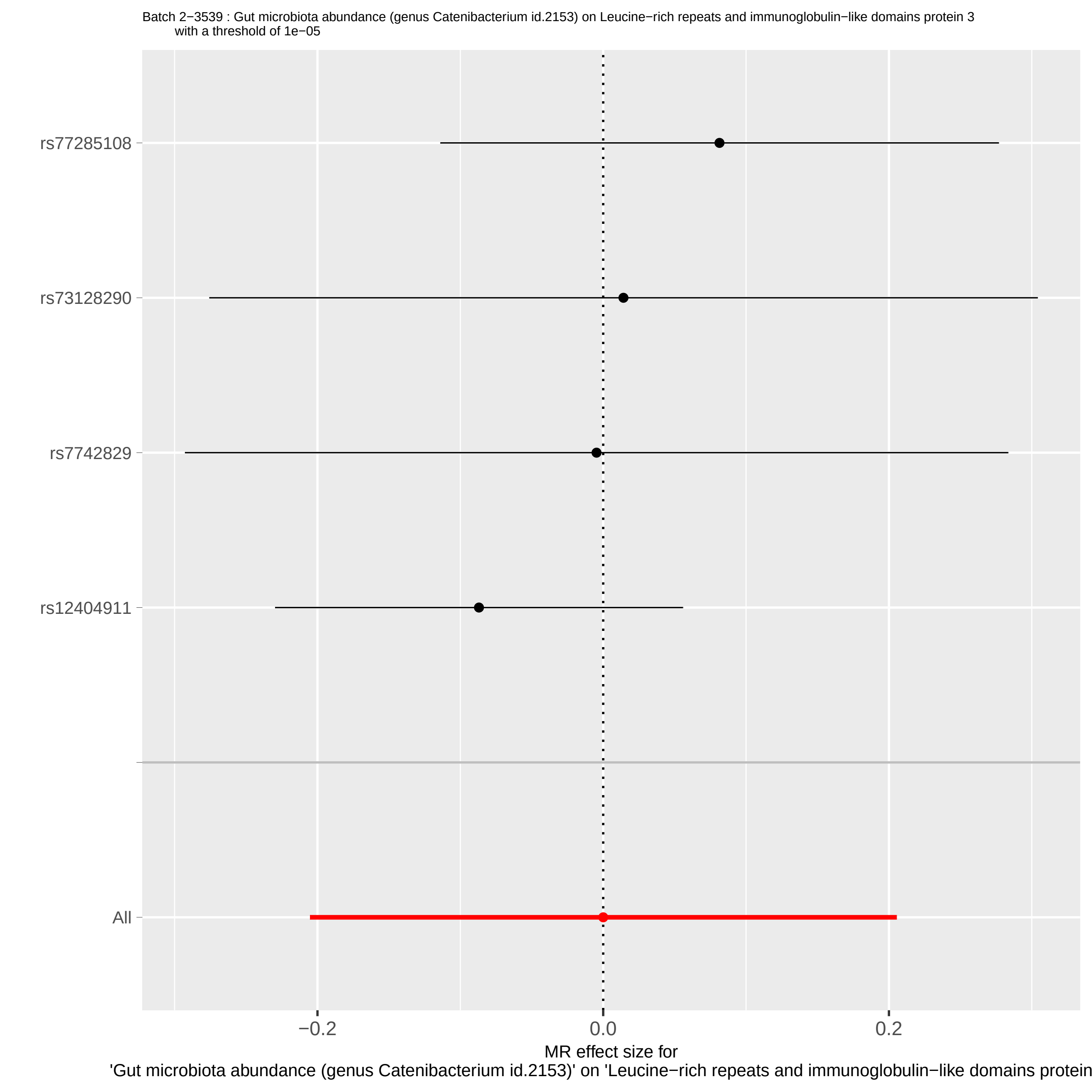

Batch 2−3539 : Gut microbiota abundance (genus Catenibacterium id.2153) on Leucine−rich repeats and immunoglobulin−like domains protein 3
with a threshold of 1e−05
rs77285108
rs73128290
rs7742829
rs12404911
All
−0.2
0.0
0.2
MR effect size for
'Gut microbiota abundance (genus Catenibacterium id.2153)' on 'Leucine−rich repeats and immunoglobulin−like domains protein

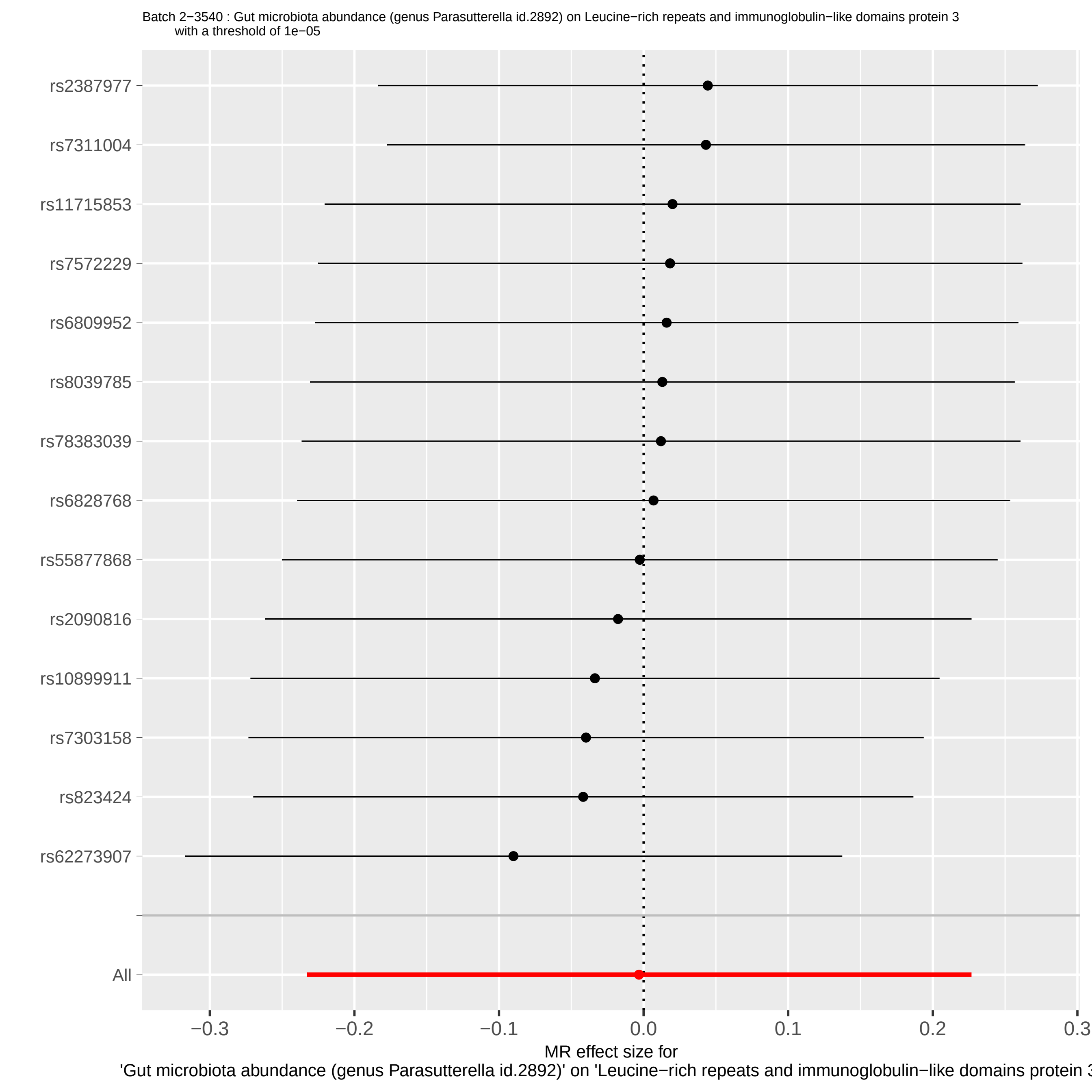
Batch 2−3540 : Gut microbiota abundance (genus Parasutterella id.2892) on Leucine−rich repeats and immunoglobulin−like domains protein 3 with a threshold of 1e−05
rs2387977
rs7311004
rs11715853
rs7572229
rs6809952
rs8039785
rs78383039
rs6828768
rs55877868
rs2090816
rs10899911
rs7303158
rs823424
rs62273907
All
−0.3
−0.2
−0.1
0.0
0.1
0.2
0.3
MR effect size for
'Gut microbiota abundance (genus Parasutterella id.2892)' on 'Leucine−rich repeats and immunoglobulin−like domains protein 3

Batch 2−3541 : Gut microbiota abundance (genus Catenibacterium id.2153) on Leucine−rich repeat, immunoglobulin−like domain and transmembrane domain−contai
with a threshold of 1e−05

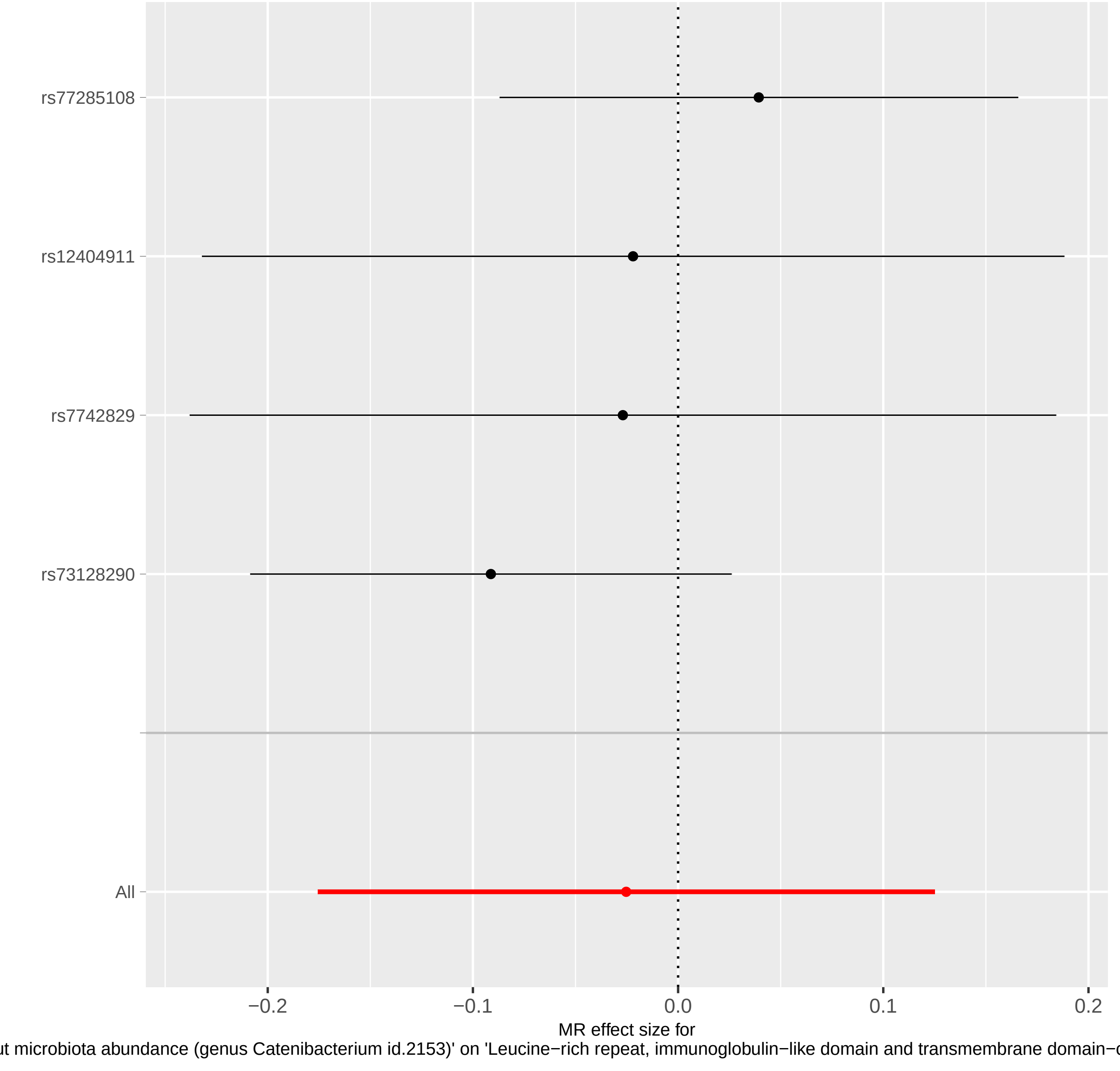

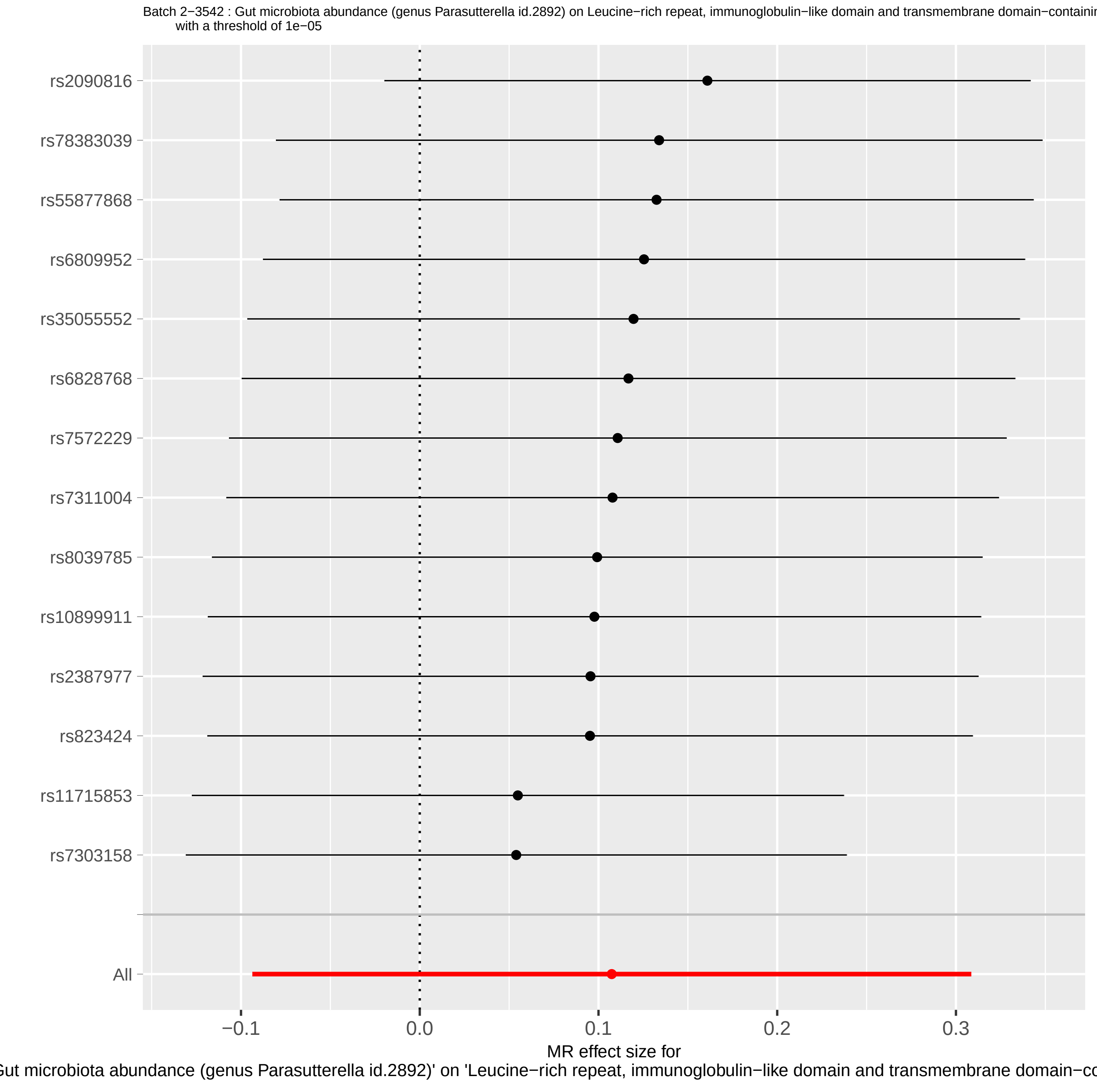

Batch 2−3542 : Gut microbiota abundance (genus Parasutterella id.2892) on Leucine−rich repeat, immunoglobulin−like domain and transmembrane domain−containin
with a threshold of 1e−05
rs2090816
rs78383039
rs55877868
rs6809952
rs35055552
rs6828768
rs7572229
rs7311004
rs8039785
rs10899911
rs2387977
rs823424
rs11715853
rs7303158
All
−0.1
0.0
0.1
0.2
0.3
MR effect size for
Gut microbiota abundance (genus Parasutterella id.2892)' on 'Leucine−rich repeat, immunoglobulin−like domain and transmembrane domain−co

Batch 2−3543 : Gut microbiota abundance (genus Catenibacterium id.2153) on Leucine−rich repeat, immunoglobulin−like domain and transmembrane domain−contai
with a threshold of 1e−05

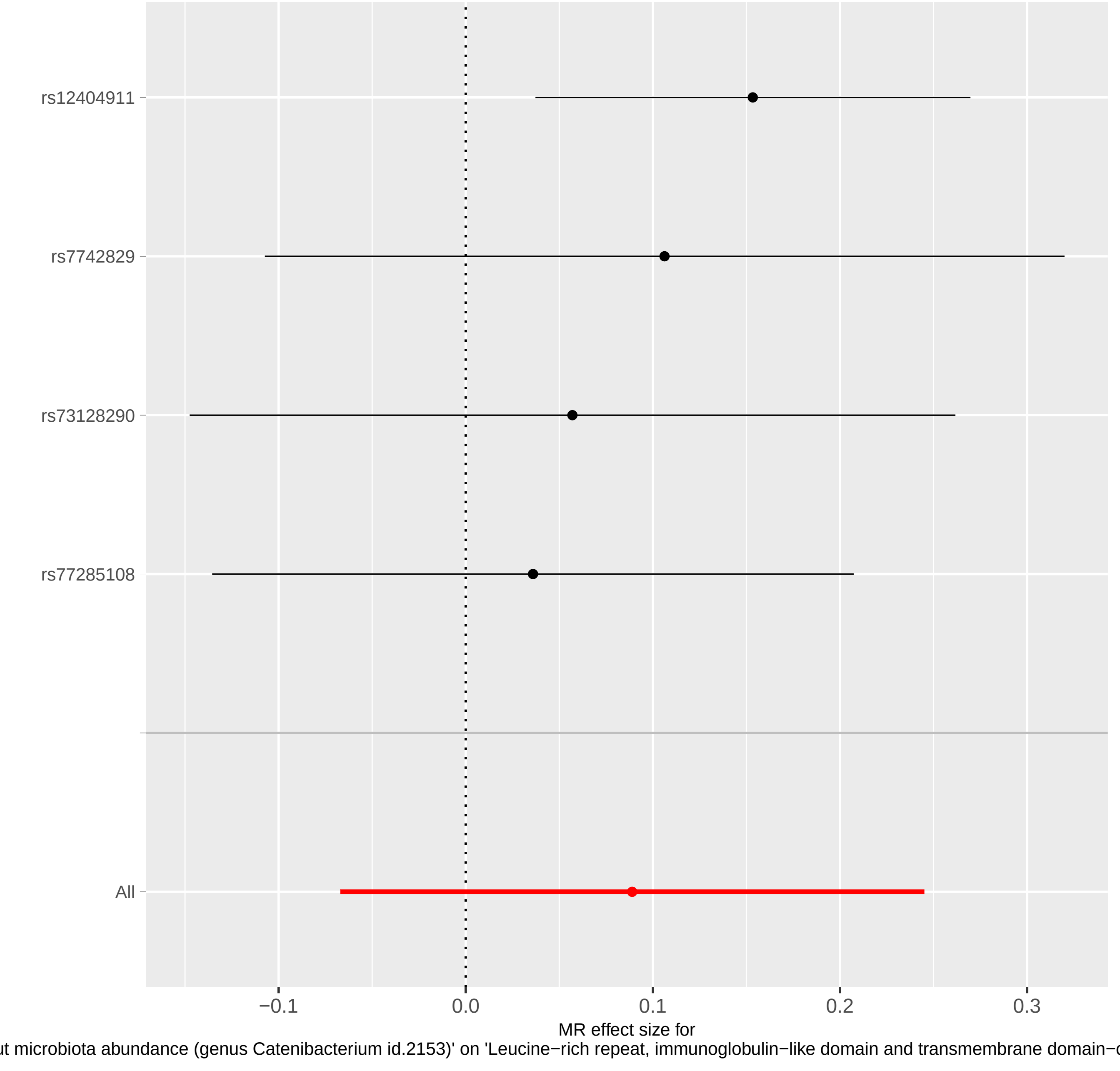

Batch 2−3544 : Gut microbiota abundance (genus Parasutterella id.2892) on Leucine−rich repeat, immunoglobulin−like domain and transmembrane domain−containi
with a threshold of 1e−05

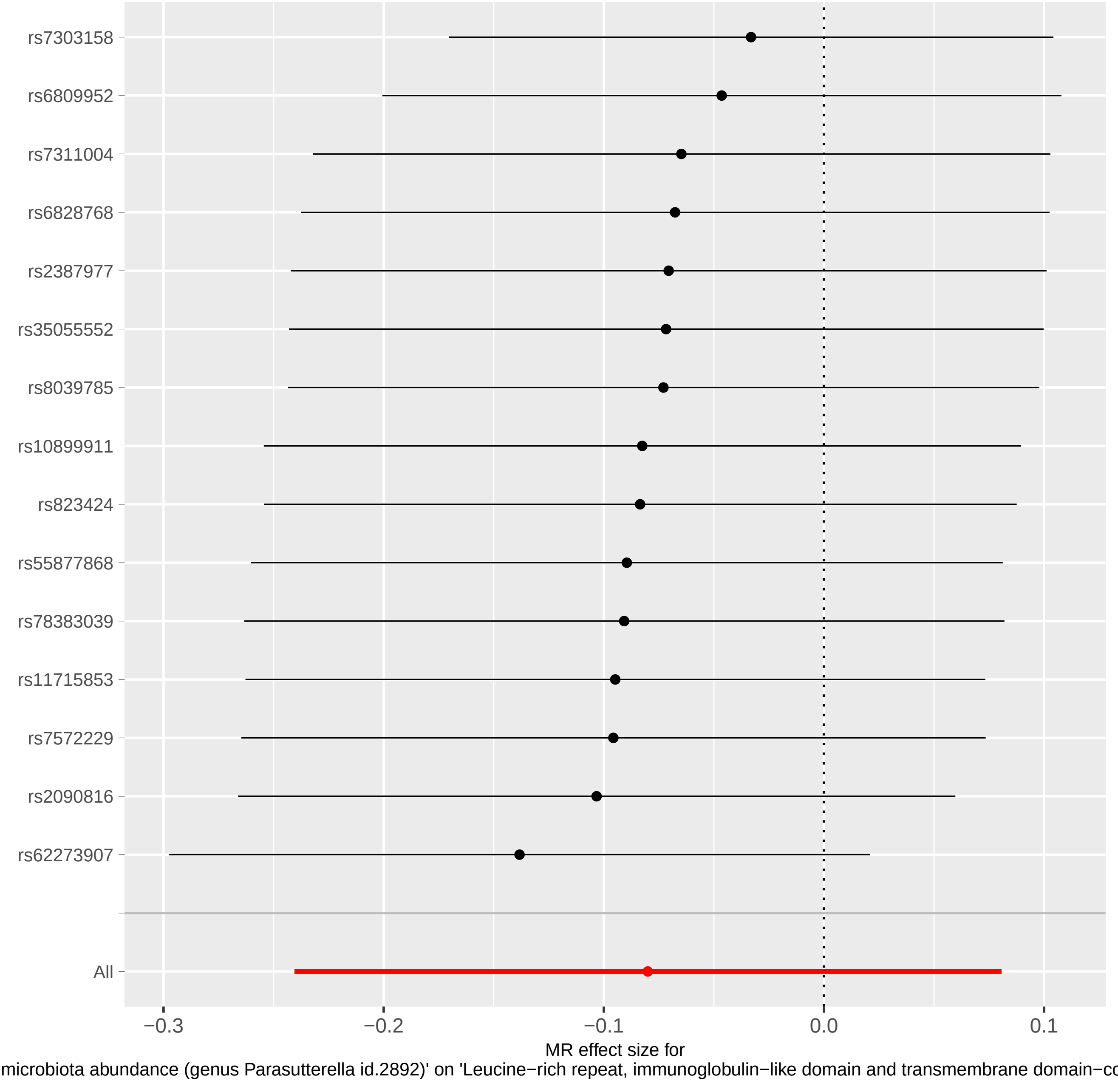

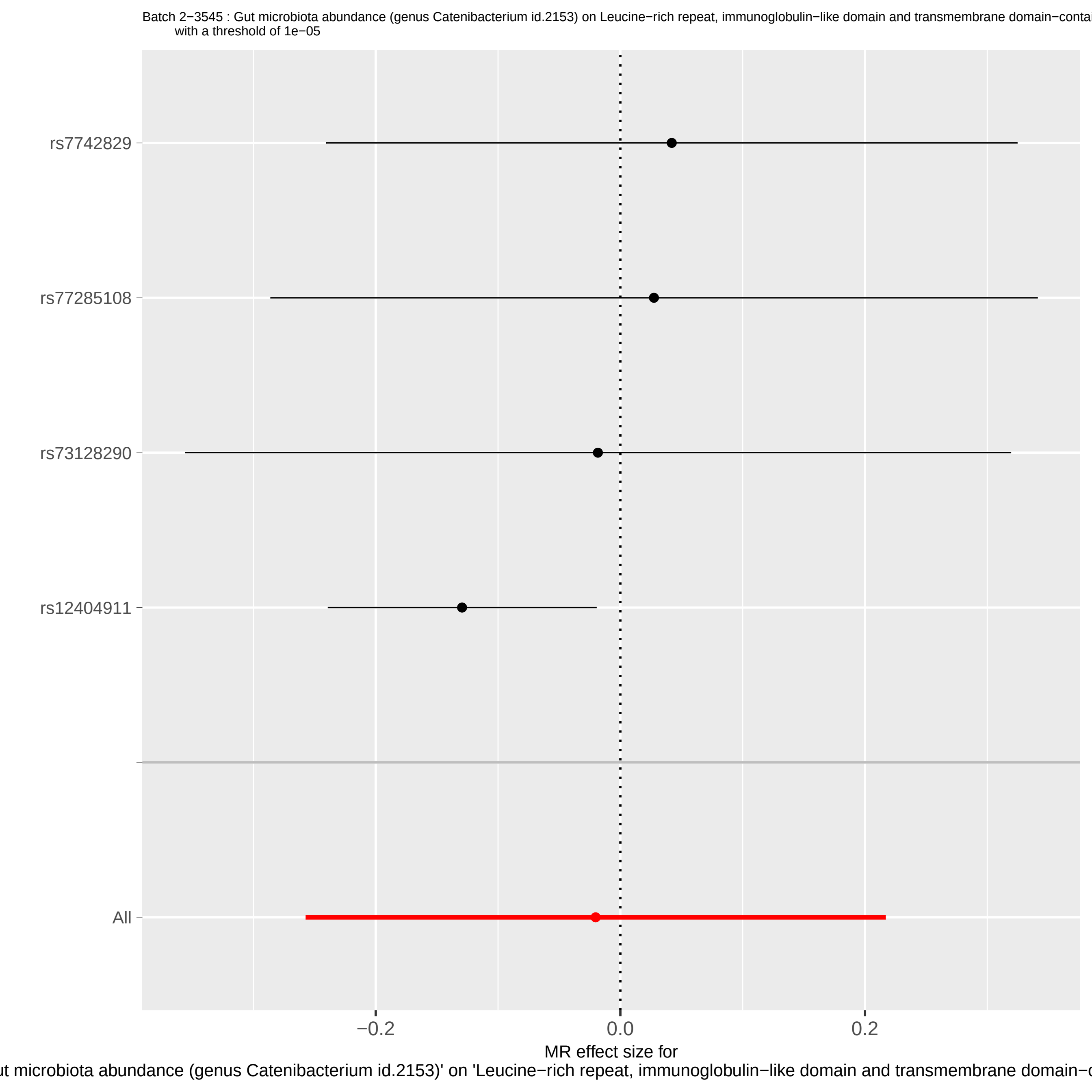

Batch 2−3545 : Gut microbiota abundance (genus Catenibacterium id.2153) on Leucine−rich repeat, immunoglobulin−like domain and transmembrane domain−conta
with a threshold of 1e−05
rs7742829
rs77285108
rs73128290
rs12404911
All
−0.2
0.0
0.2
MR effect size for
ut microbiota abundance (genus Catenibacterium id.2153)' on 'Leucine−rich repeat, immunoglobulin−like domain and transmembrane domain−c

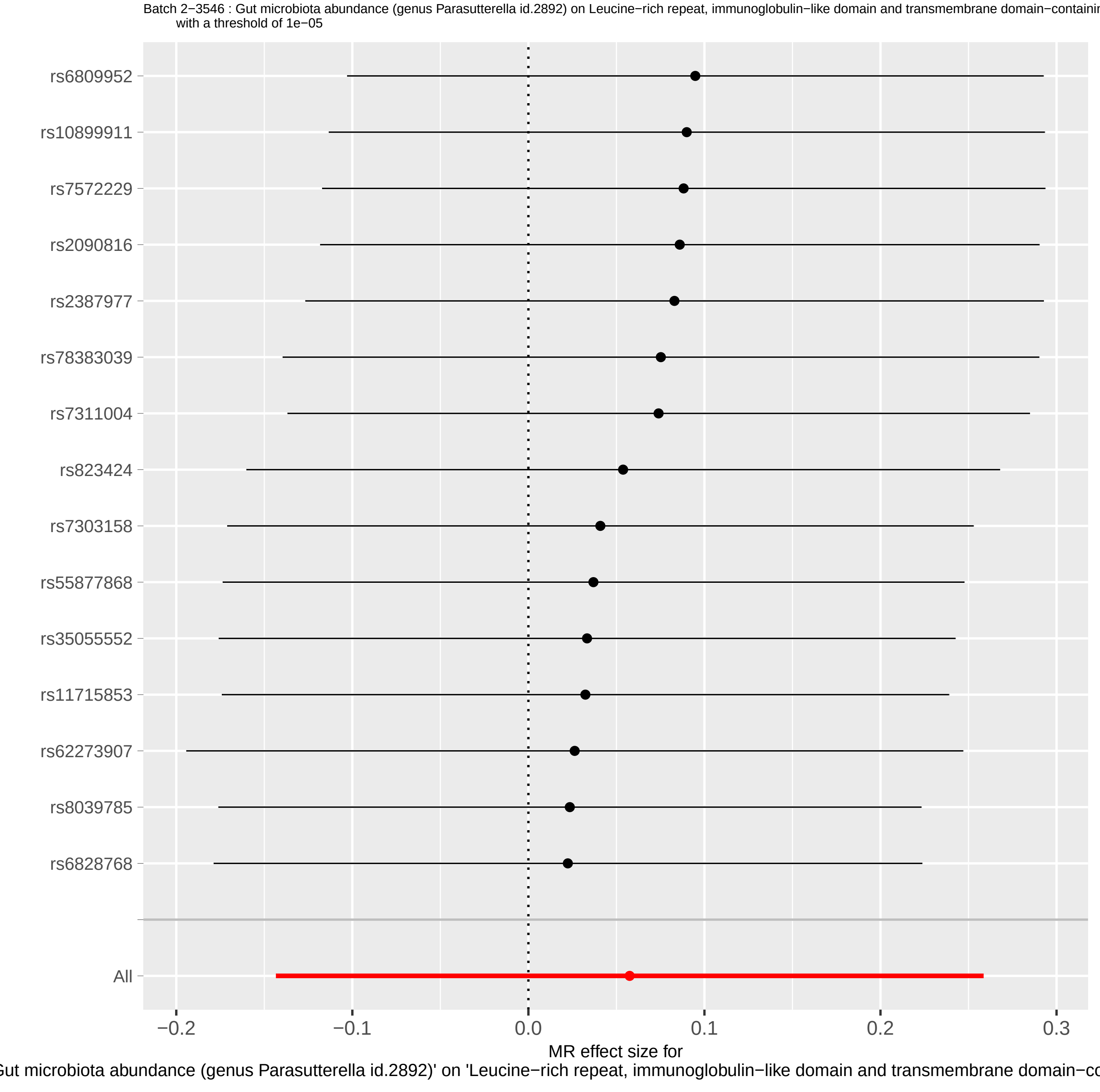

Batch 2−3546 : Gut microbiota abundance (genus Parasutterella id.2892) on Leucine−rich repeat, immunoglobulin−like domain and transmembrane domain−containin
with a threshold of 1e−05
rs6809952
rs10899911
rs7572229
rs2090816
rs2387977
rs78383039
rs7311004
rs823424
rs7303158
rs55877868
rs35055552
rs11715853
rs62273907
rs8039785
rs6828768
All
−0.2
−0.1
0.0
0.1
0.2
0.3
MR effect size for
Gut microbiota abundance (genus Parasutterella id.2892)' on 'Leucine−rich repeat, immunoglobulin−like domain and transmembrane domain−co

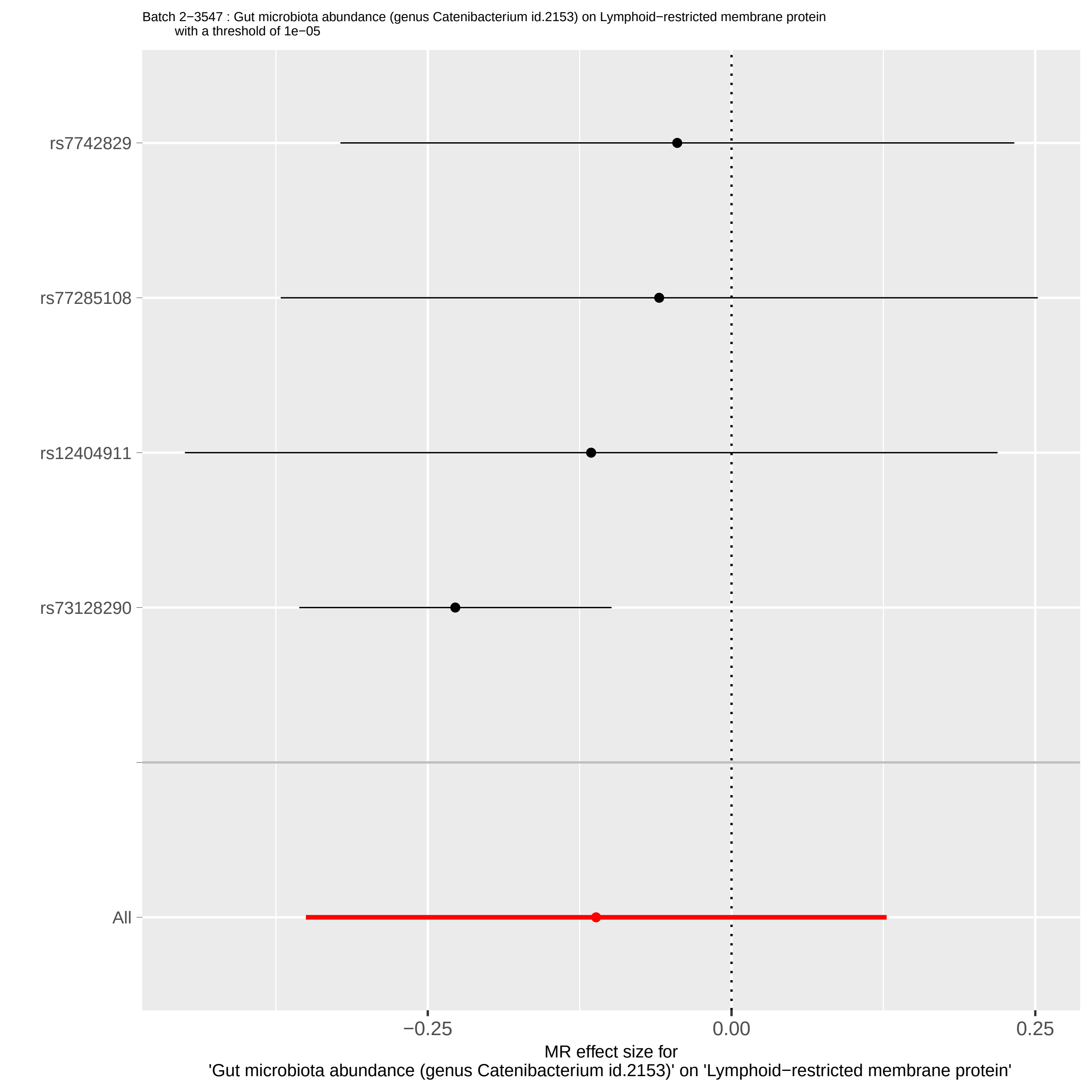

Batch 2−3547 : Gut microbiota abundance (genus Catenibacterium id.2153) on Lymphoid−restricted membrane protein
with a threshold of 1e−05
rs7742829
rs77285108
rs12404911
rs73128290
All
−0.25
0.00
0.25
MR effect size for
'Gut microbiota abundance (genus Catenibacterium id.2153)' on 'Lymphoid−restricted membrane protein'

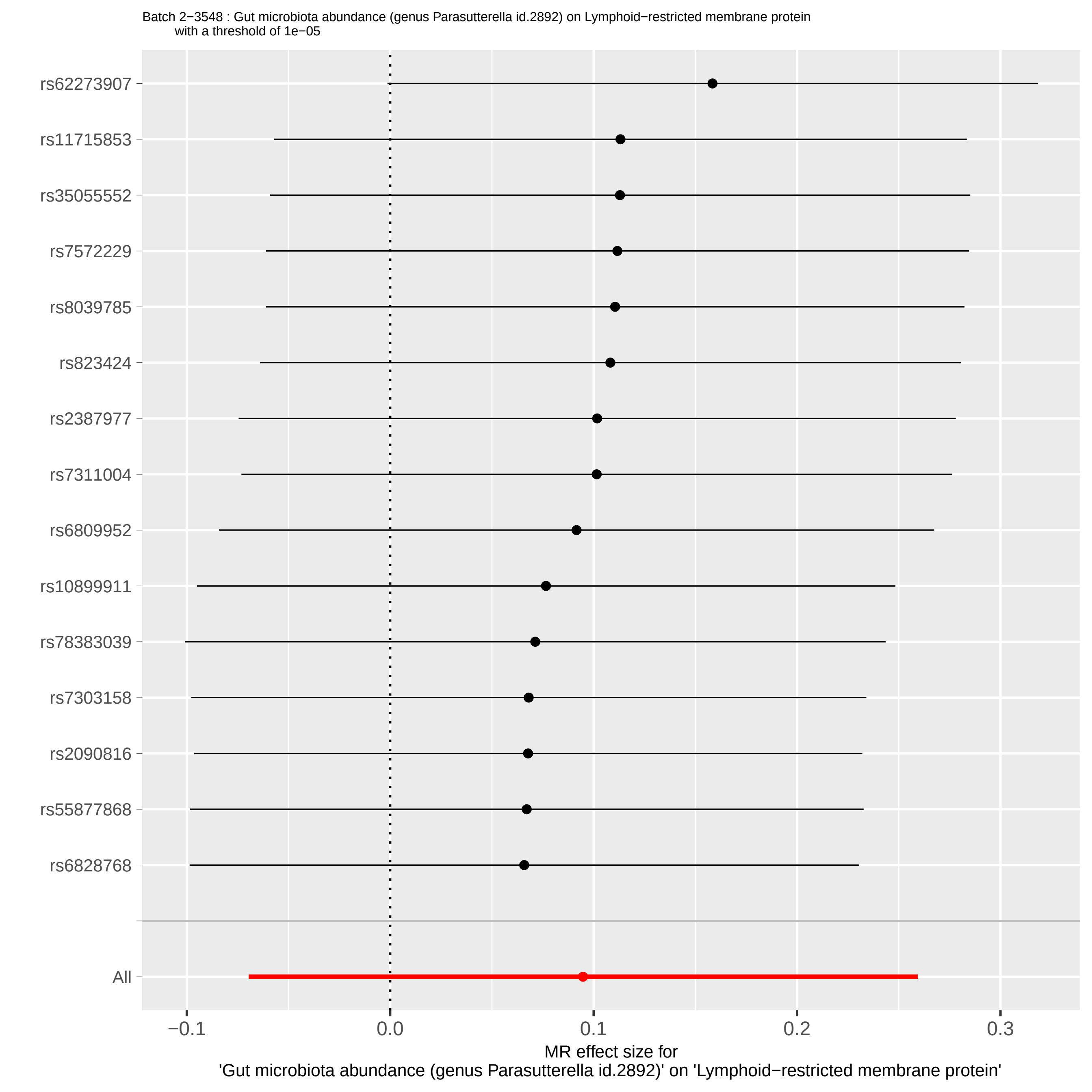

Batch 2−3548 : Gut microbiota abundance (genus Parasutterella id.2892) on Lymphoid−restricted membrane protein
with a threshold of 1e−05
rs62273907
rs11715853
rs35055552
rs7572229
rs8039785
rs823424
rs2387977
rs7311004
rs6809952
rs10899911
rs78383039
rs7303158
rs2090816
rs55877868
rs6828768
All
−0.1
0.0
0.1
0.2
0.3
MR effect size for
'Gut microbiota abundance (genus Parasutterella id.2892)' on 'Lymphoid−restricted membrane protein'

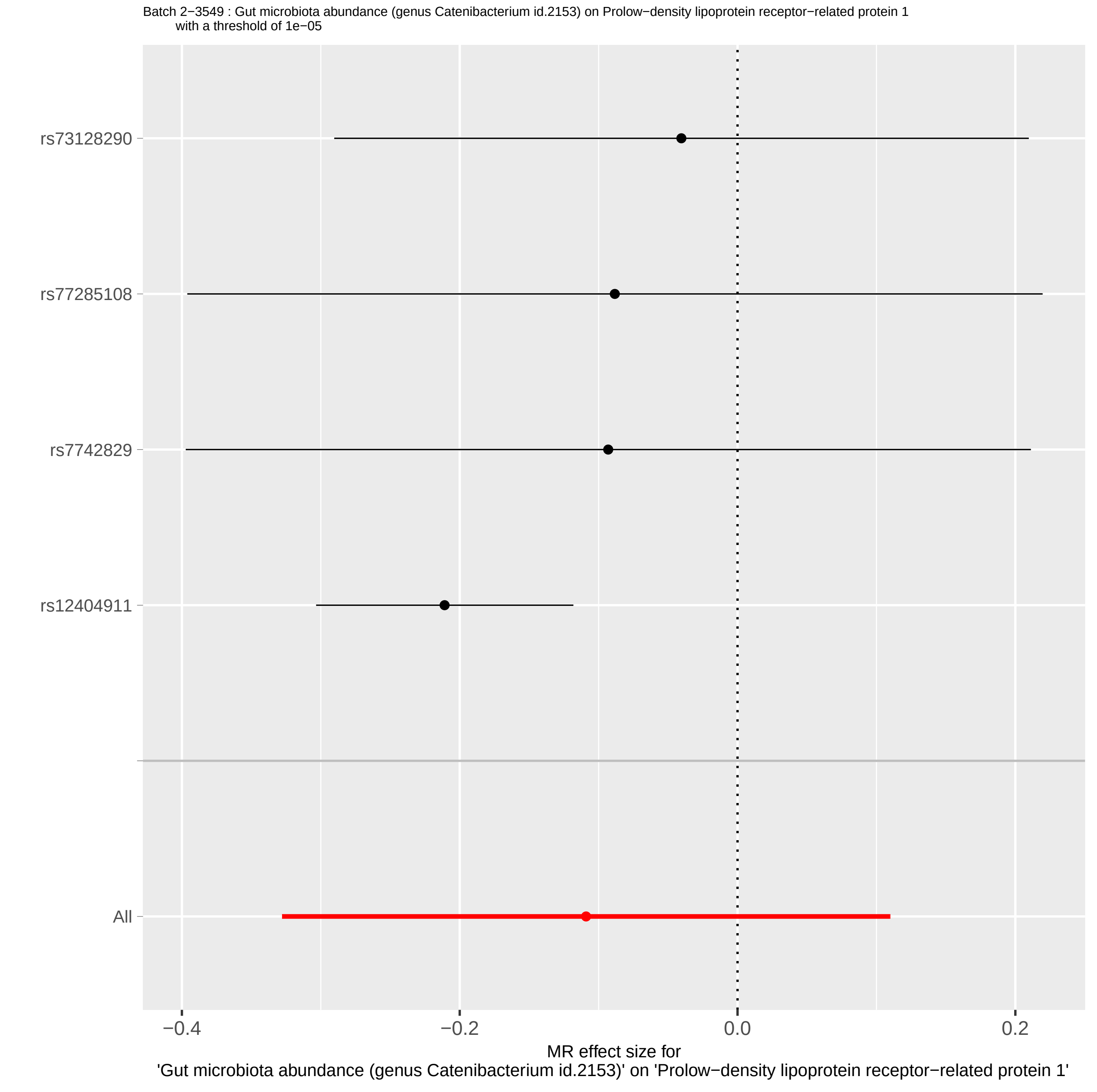
Batch 2−3549 : Gut microbiota abundance (genus Catenibacterium id.2153) on Prolow−density lipoprotein receptor−related protein 1
with a threshold of 1e−05
rs73128290
rs77285108
rs7742829
rs12404911
All
−0.4
−0.2
0.0
0.2
MR effect size for
'Gut microbiota abundance (genus Catenibacterium id.2153)' on 'Prolow−density lipoprotein receptor−related protein 1'

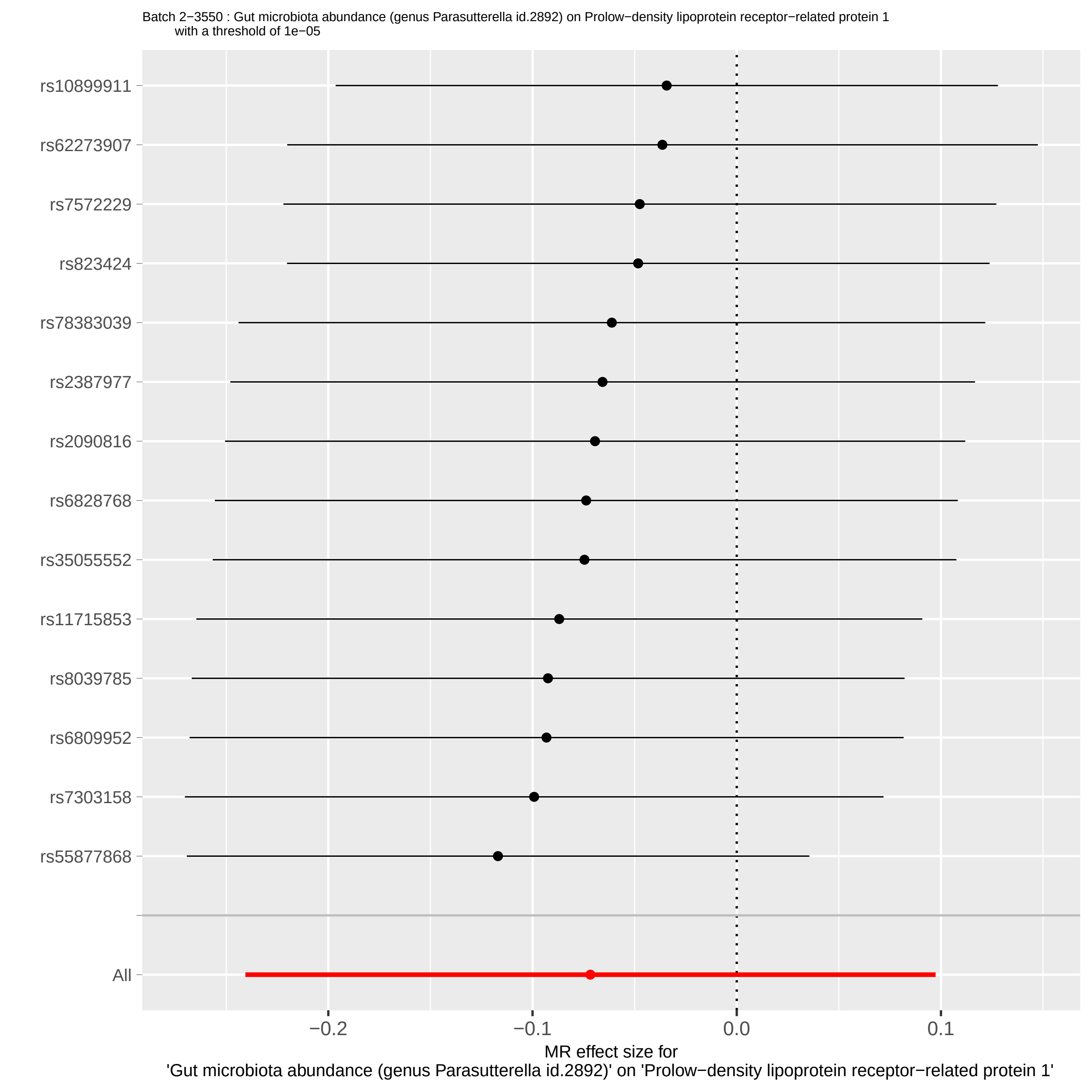

Batch 2−3550 : Gut microbiota abundance (genus Parasutterella id.2892) on Prolow−density lipoprotein receptor−related protein 1
with a threshold of 1e−05
rs10899911
rs62273907
rs7572229
rs823424
rs78383039
rs2387977
rs2090816
rs6828768
rs35055552
rs11715853
rs8039785
rs6809952
rs7303158
rs55877868
All
−0.2
−0.1
0.0
0.1
MR effect size for
'Gut microbiota abundance (genus Parasutterella id.2892)' on 'Prolow−density lipoprotein receptor−related protein 1'

Batch 2−3551 : Gut microbiota abundance (genus Catenibacterium id.2153) on Low−density lipoprotein receptor−related protein 1, soluble
with a threshold of 1e−05

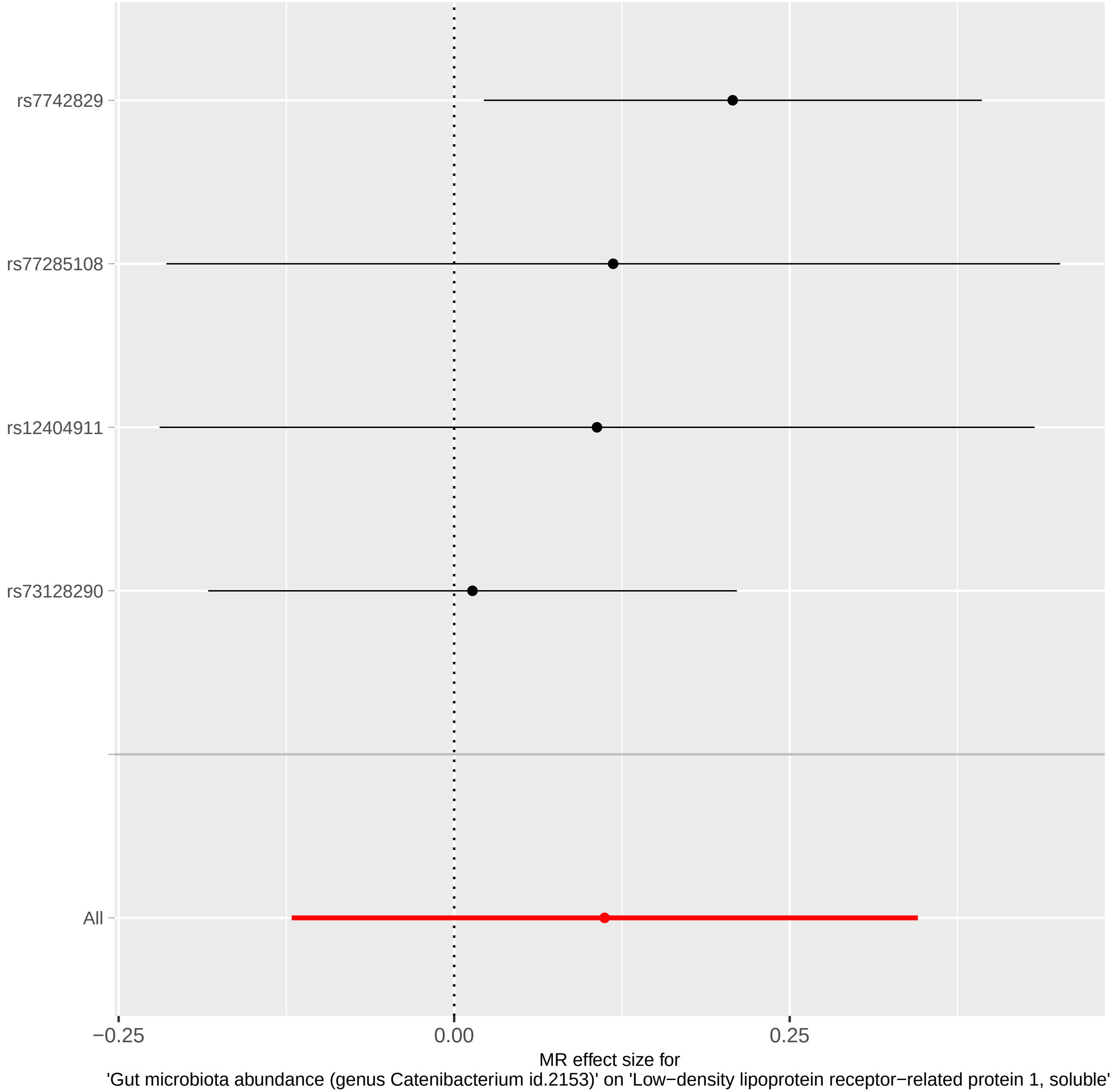

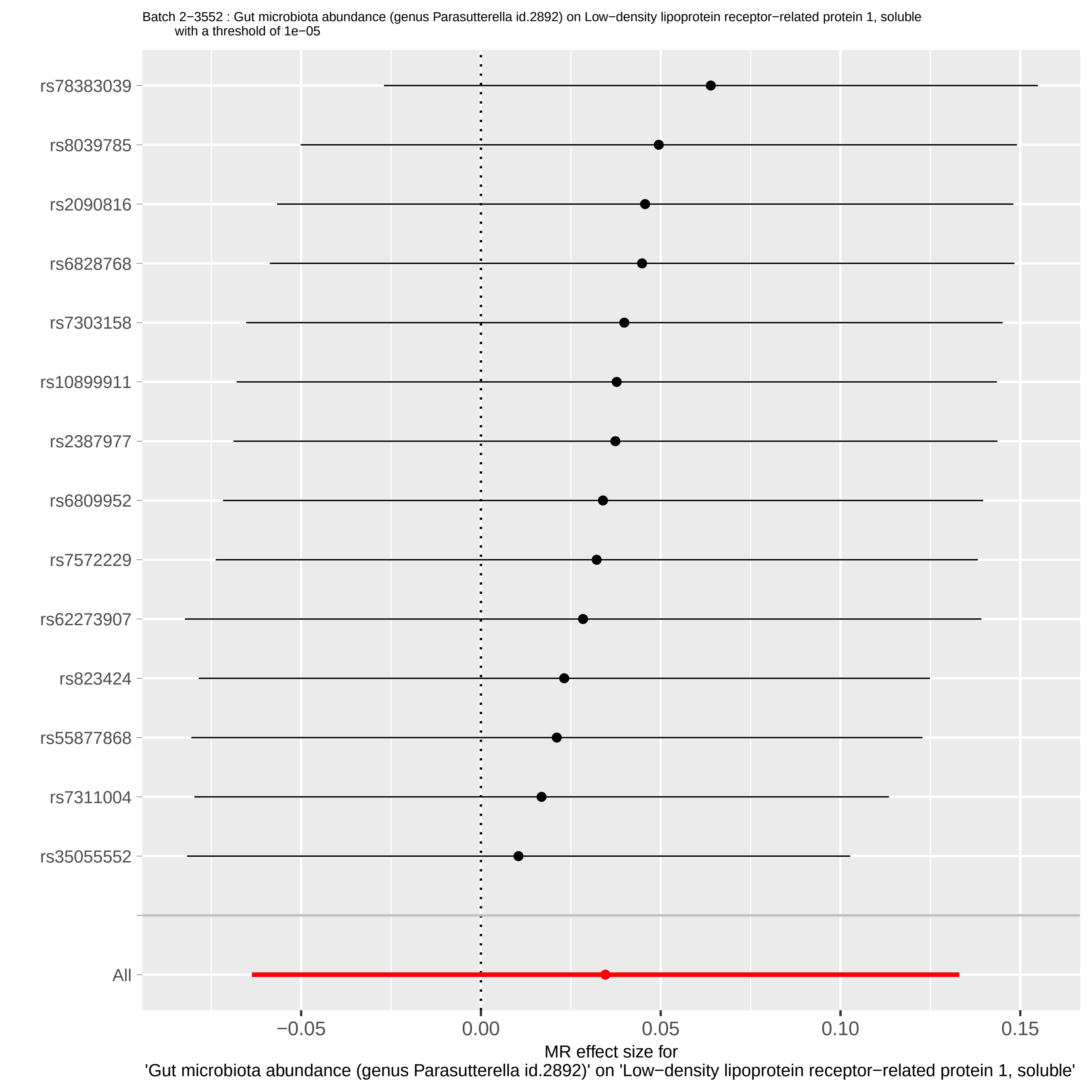
Batch 2−3552 : Gut microbiota abundance (genus Parasutterella id.2892) on Low−density lipoprotein receptor−related protein 1, soluble
with a threshold of 1e−05
rs78383039
rs8039785
rs2090816
rs6828768
rs7303158
rs10899911
rs2387977
rs6809952
rs7572229
rs62273907
rs823424
rs55877868
rs7311004
rs35055552
All
−0.05
0.00
0.05
0.10
0.15
MR effect size for
'Gut microbiota abundance (genus Parasutterella id.2892)' on 'Low−density lipoprotein receptor−related protein 1, soluble'

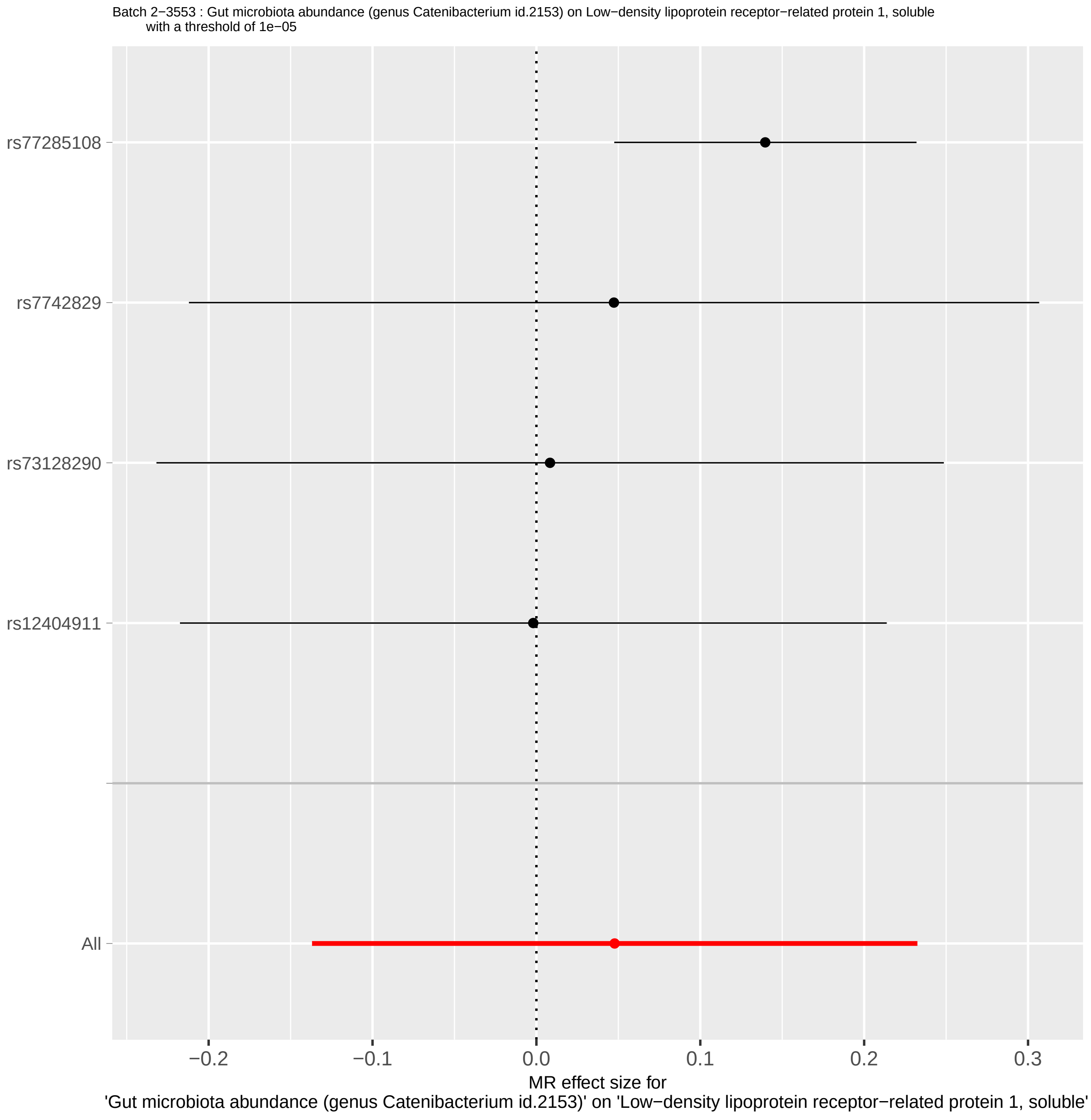
Batch 2−3553 : Gut microbiota abundance (genus Catenibacterium id.2153) on Low−density lipoprotein receptor−related protein 1, soluble
with a threshold of 1e−05
rs77285108
rs7742829
rs73128290
rs12404911
All
−0.2
−0.1
0.0
0.1
0.2
0.3
MR effect size for
'Gut microbiota abundance (genus Catenibacterium id.2153)' on 'Low−density lipoprotein receptor−related protein 1, soluble'

Batch 2−3554 : Gut microbiota abundance (genus Parasutterella id.2892) on Low−density lipoprotein receptor−related protein 1, soluble
with a threshold of 1e−05

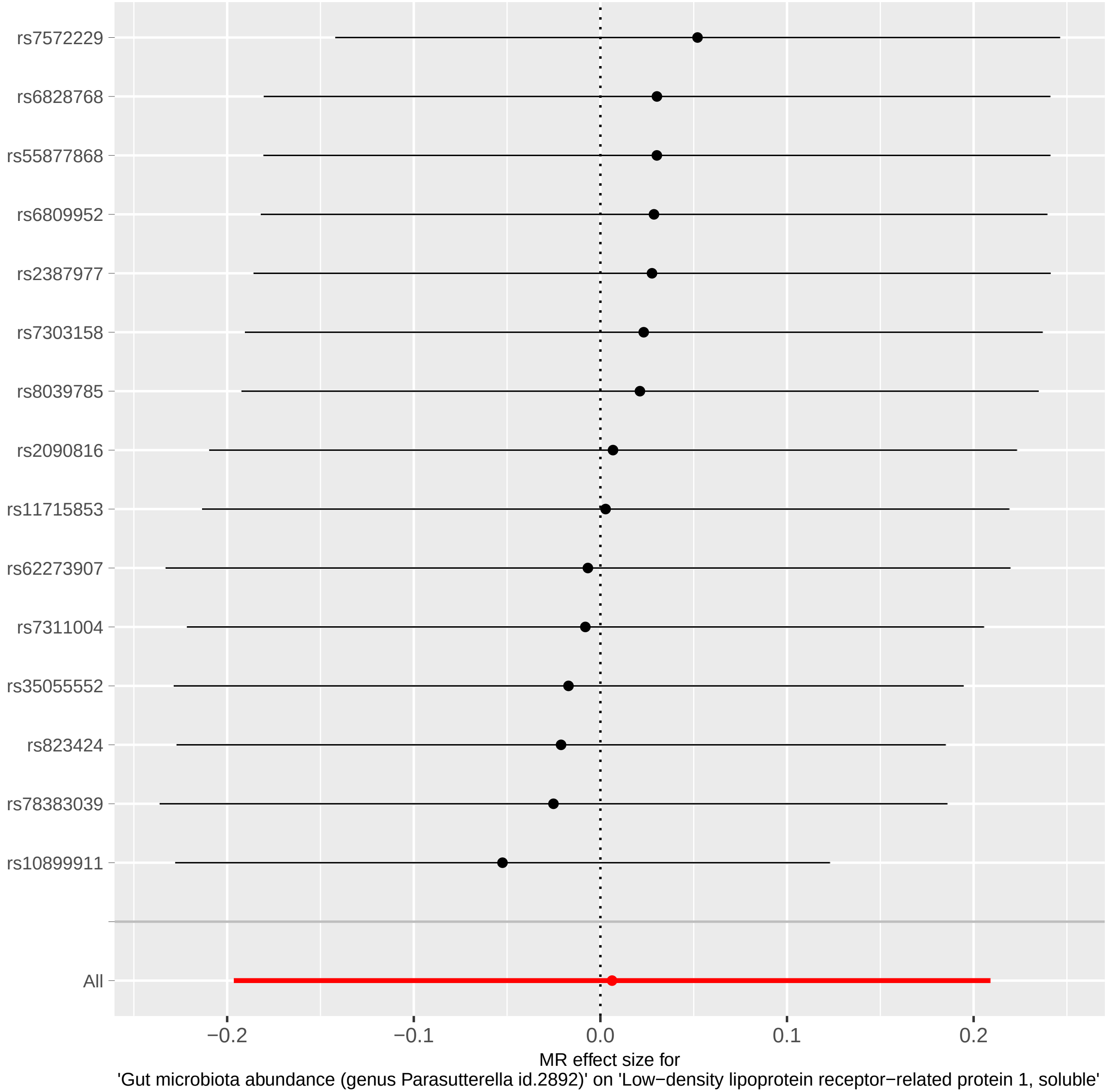

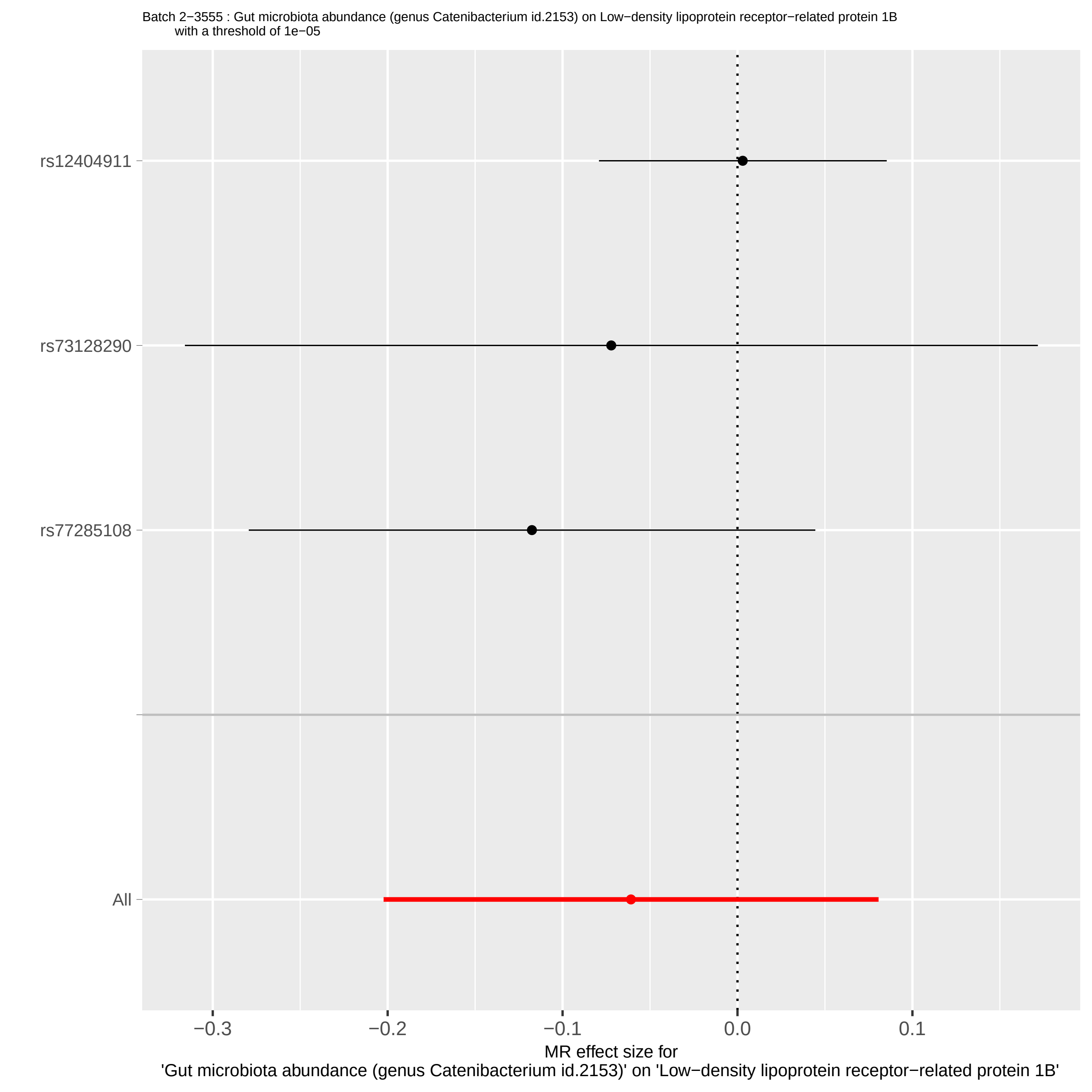

Batch 2−3555 : Gut microbiota abundance (genus Catenibacterium id.2153) on Low−density lipoprotein receptor−related protein 1B
with a threshold of 1e−05
rs12404911
rs73128290
rs77285108
All
−0.3
−0.2
−0.1
0.0
0.1
MR effect size for
'Gut microbiota abundance (genus Catenibacterium id.2153)' on 'Low−density lipoprotein receptor−related protein 1B'

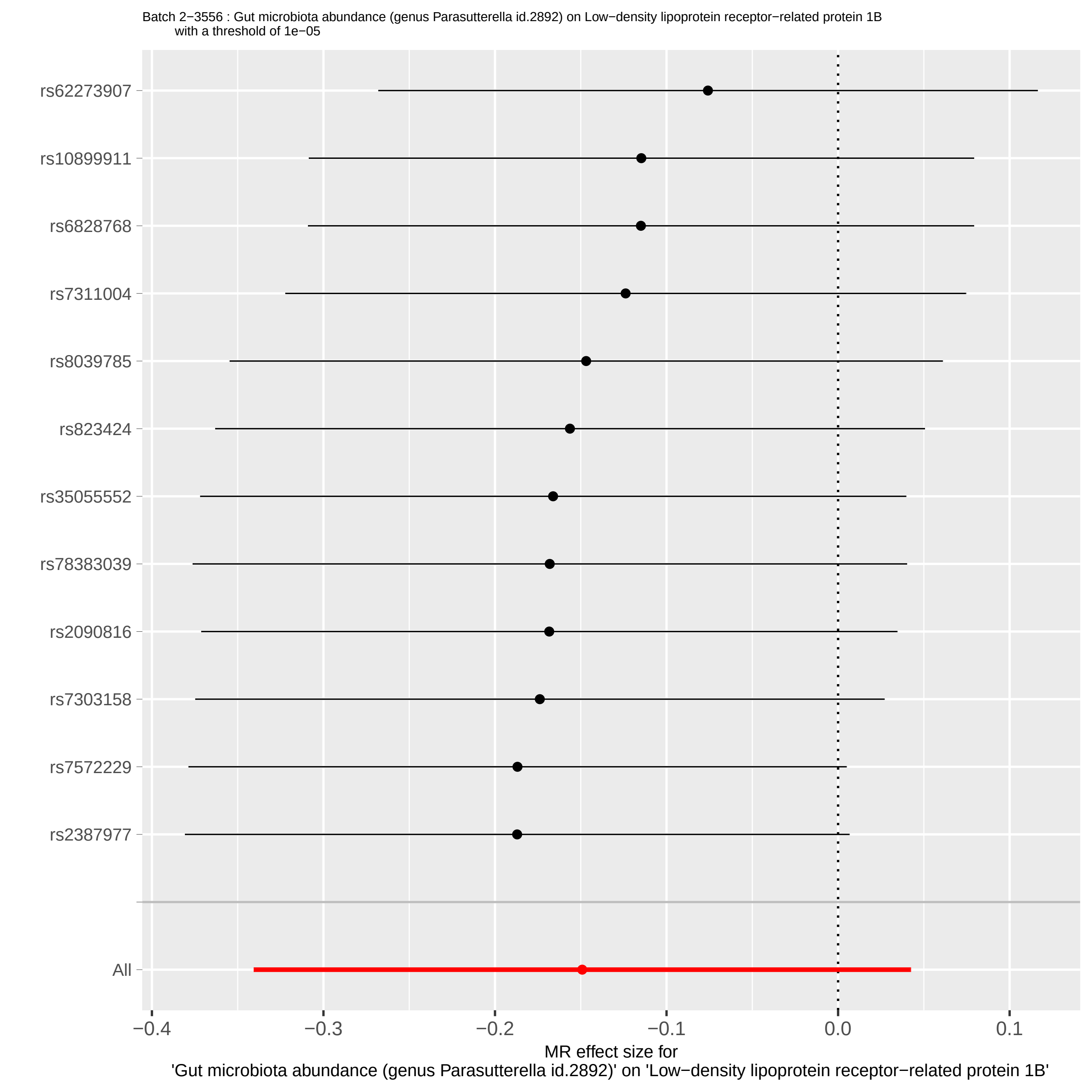
Batch 2−3556 : Gut microbiota abundance (genus Parasutterella id.2892) on Low−density lipoprotein receptor−related protein 1B
with a threshold of 1e−05
rs62273907
rs10899911
rs6828768
rs7311004
rs8039785
rs823424
rs35055552
rs78383039
rs2090816
rs7303158
rs7572229
rs2387977
All
−0.4
−0.3
−0.2
−0.1
0.0
0.1
MR effect size for
'Gut microbiota abundance (genus Parasutterella id.2892)' on 'Low−density lipoprotein receptor−related protein 1B'

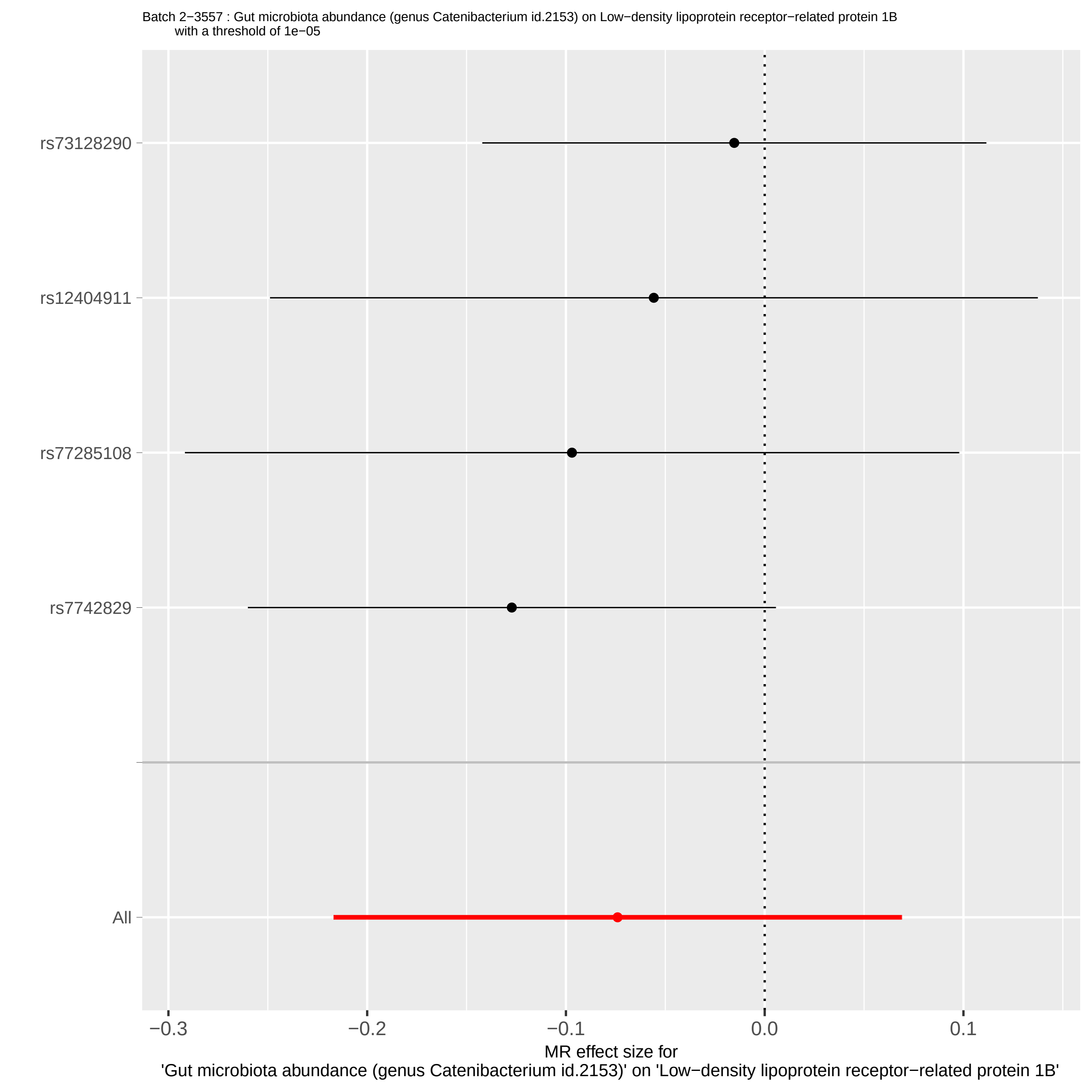

Batch 2−3557 : Gut microbiota abundance (genus Catenibacterium id.2153) on Low−density lipoprotein receptor−related protein 1B
with a threshold of 1e−05
rs73128290
rs12404911
rs77285108
rs7742829
All
−0.3
−0.2
−0.1
0.0
0.1
MR effect size for
'Gut microbiota abundance (genus Catenibacterium id.2153)' on 'Low−density lipoprotein receptor−related protein 1B'

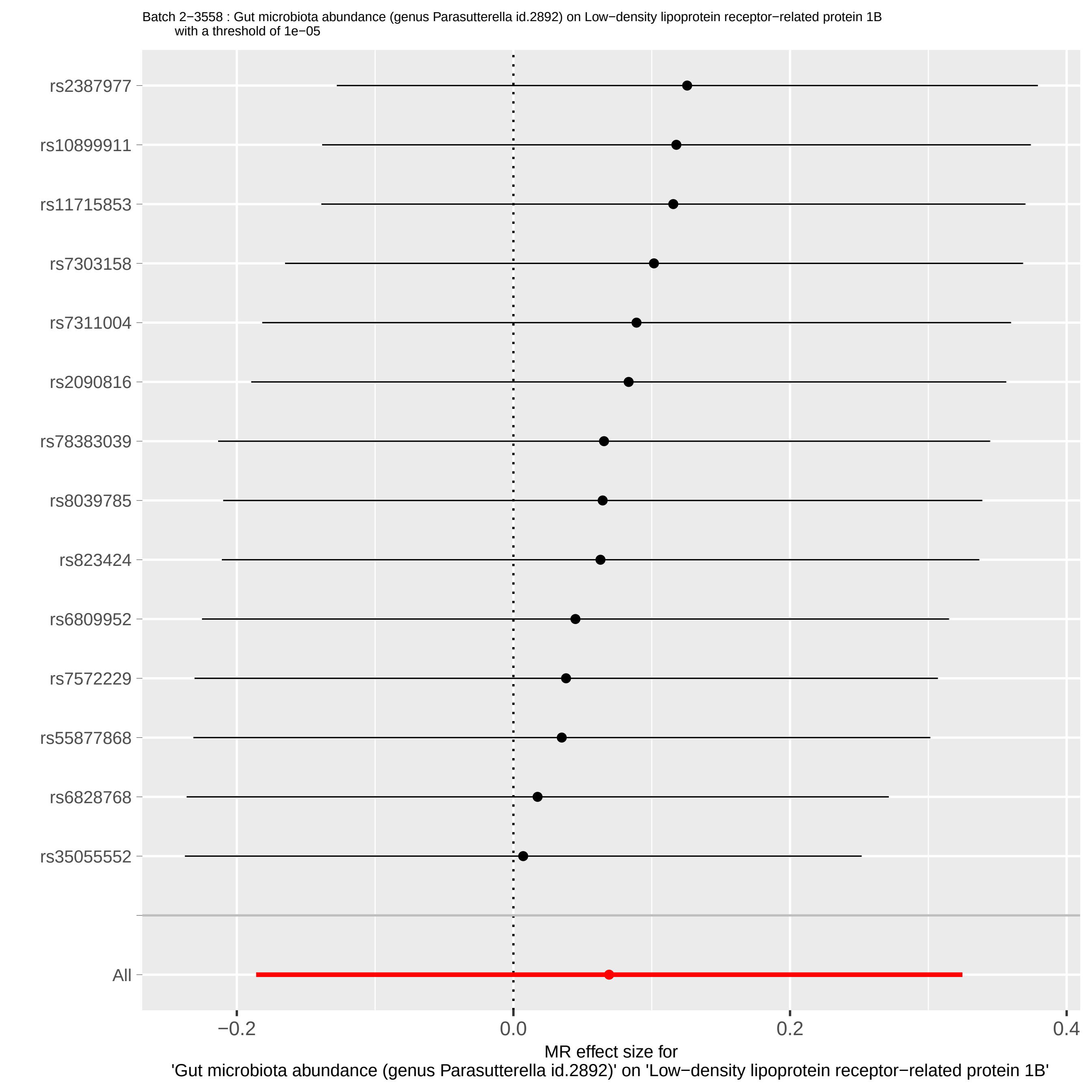

Batch 2−3558 : Gut microbiota abundance (genus Parasutterella id.2892) on Low−density lipoprotein receptor−related protein 1B
with a threshold of 1e−05
rs2387977
rs10899911
rs11715853
rs7303158
rs7311004
rs2090816
rs78383039
rs8039785
rs823424
rs6809952
rs7572229
rs55877868
rs6828768
rs35055552
All
−0.2
0.0
0.2
0.4
MR effect size for
'Gut microbiota abundance (genus Parasutterella id.2892)' on 'Low−density lipoprotein receptor−related protein 1B'

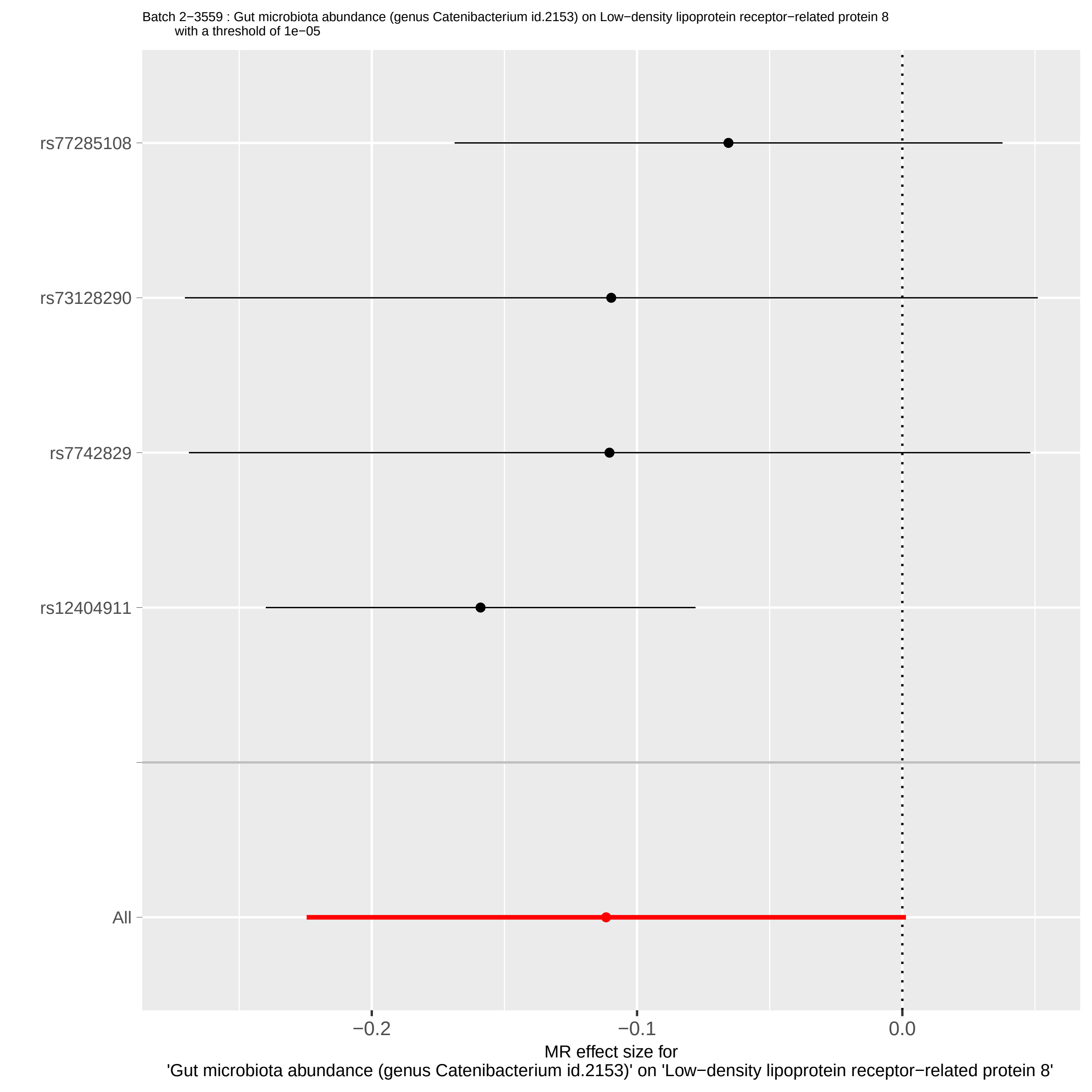

Batch 2−3559 : Gut microbiota abundance (genus Catenibacterium id.2153) on Low−density lipoprotein receptor−related protein 8
with a threshold of 1e−05
rs77285108
rs73128290
rs7742829
rs12404911
All
−0.2
−0.1
0.0
MR effect size for
'Gut microbiota abundance (genus Catenibacterium id.2153)' on 'Low−density lipoprotein receptor−related protein 8'

Batch 2−3560 : Gut microbiota abundance (genus Parasutterella id.2892) on Low−density lipoprotein receptor−related protein 8
with a threshold of 1e−05

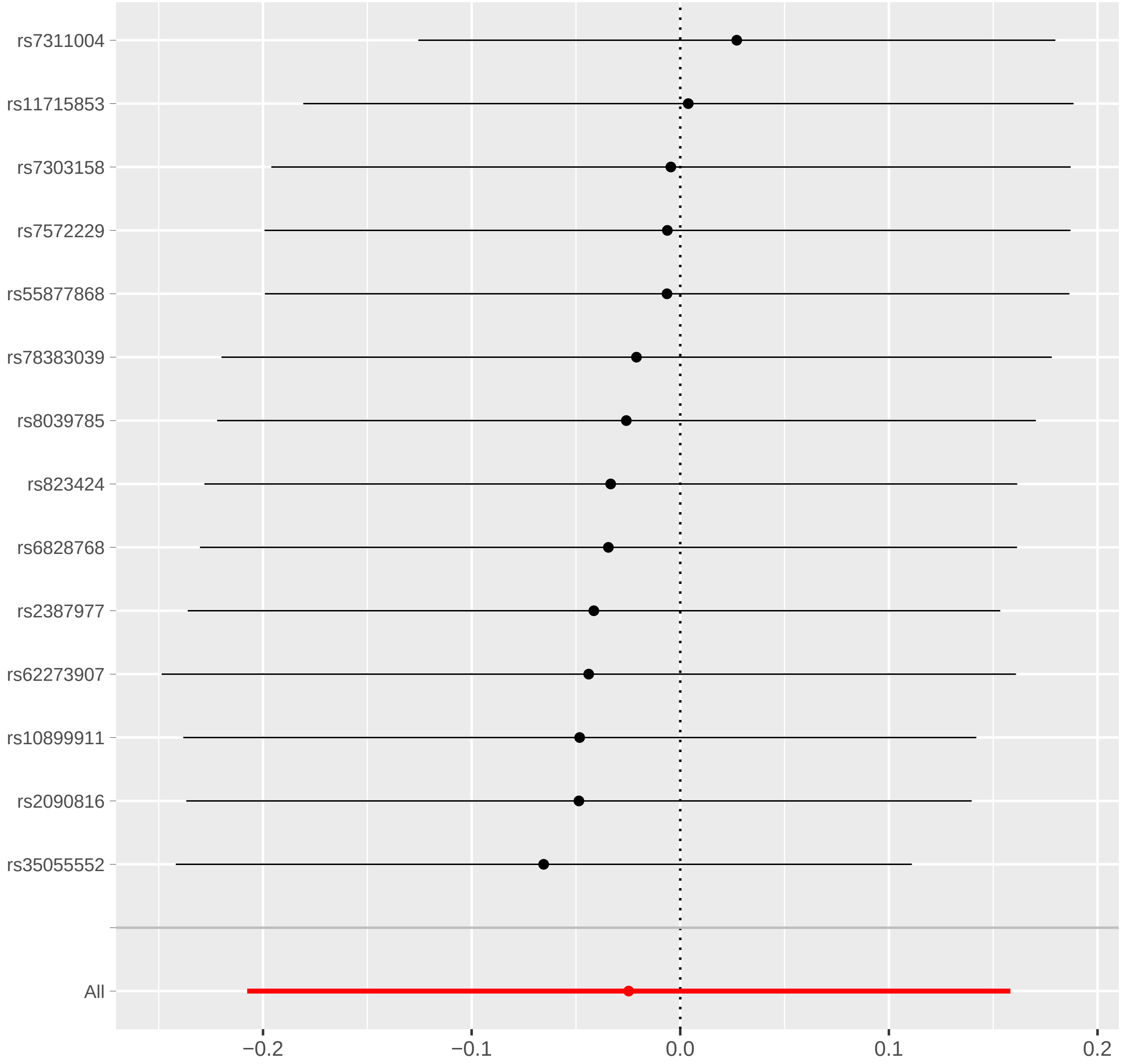

MR effect size for
'Gut microbiota abundance (genus Parasutterella id.2892)' on 'Low−density lipoprotein receptor−related protein 8'

Batch 2−3561 : Gut microbiota abundance (genus Catenibacterium id.2153) on alpha−2−macroglobulin receptor−associated protein with a threshold of 1e−05

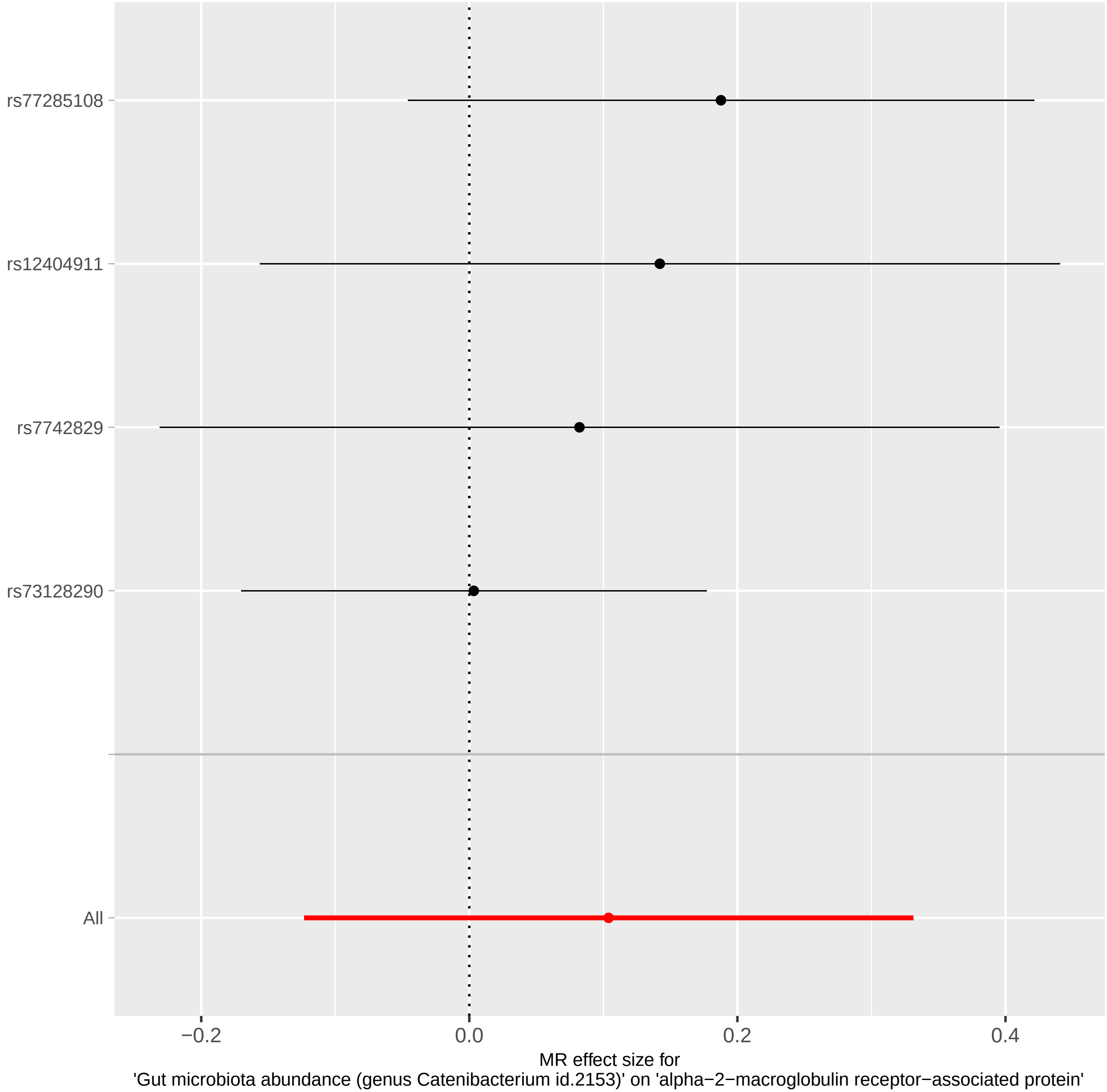

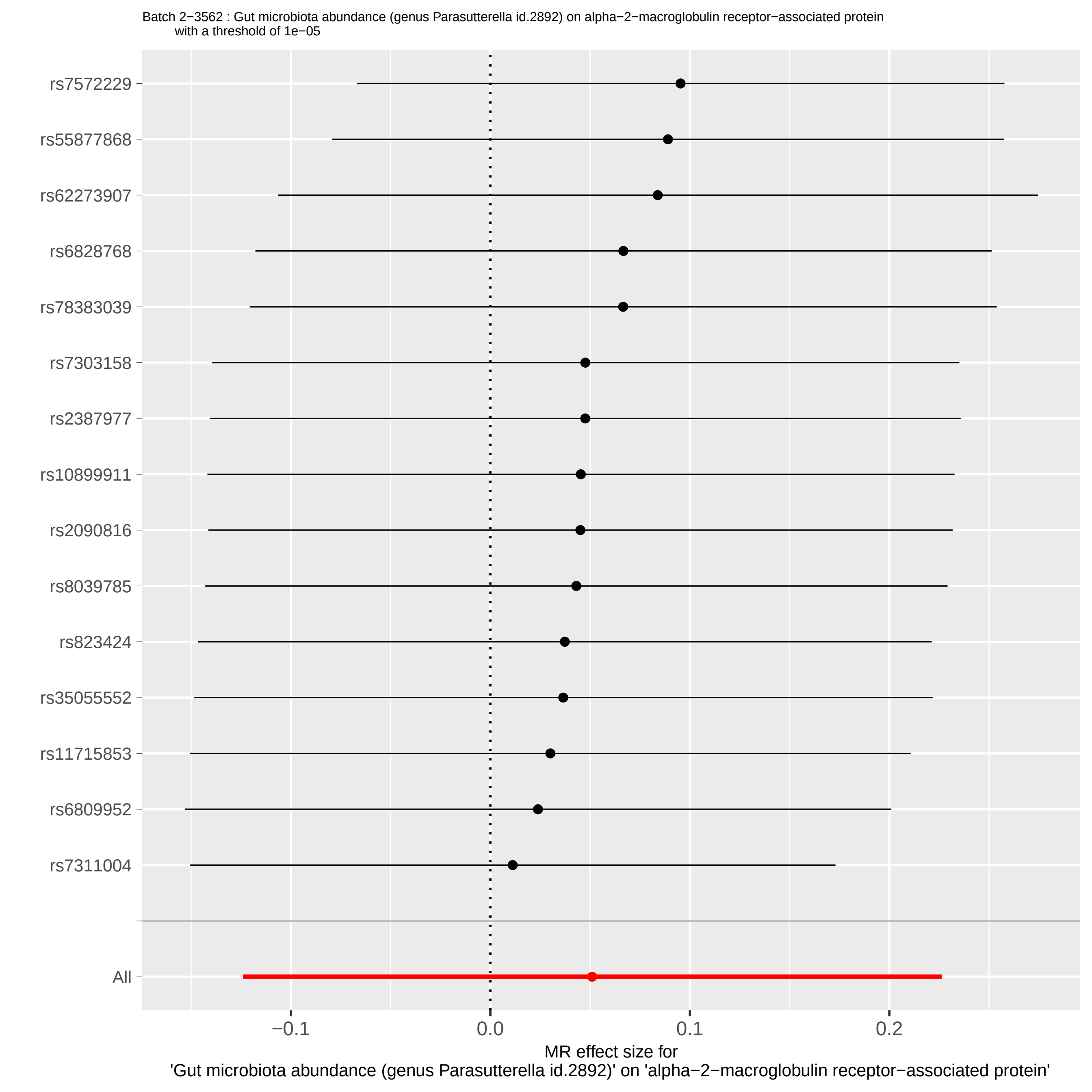

Batch 2−3562 : Gut microbiota abundance (genus Parasutterella id.2892) on alpha−2−macroglobulin receptor−associated protein with a threshold of 1e−05
rs7572229
rs55877868
rs62273907
rs6828768
rs78383039
rs7303158
rs2387977
rs10899911
rs2090816
rs8039785
rs823424
rs35055552
rs11715853
rs6809952
rs7311004
All
−0.1
0.0
0.1
0.2
MR effect size for
'Gut microbiota abundance (genus Parasutterella id.2892)' on 'alpha−2−macroglobulin receptor−associated protein'

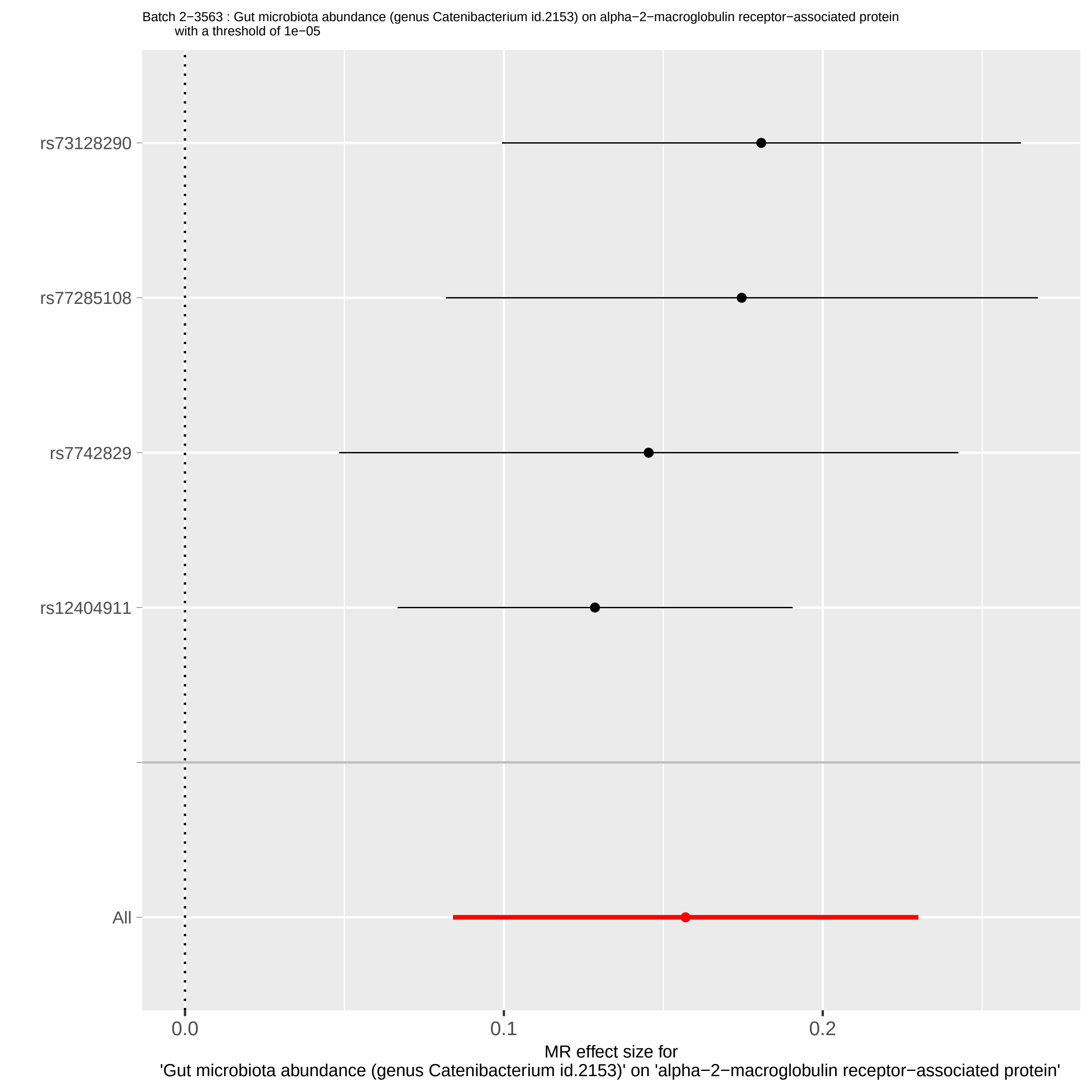
Batch 2−3563 : Gut microbiota abundance (genus Catenibacterium id.2153) on alpha−2−macroglobulin receptor−associated protein
with a threshold of 1e−05
rs73128290
rs77285108
rs7742829
rs12404911
All
0.0
0.1
0.2
MR effect size for
'Gut microbiota abundance (genus Catenibacterium id.2153)' on 'alpha−2−macroglobulin receptor−associated protein'

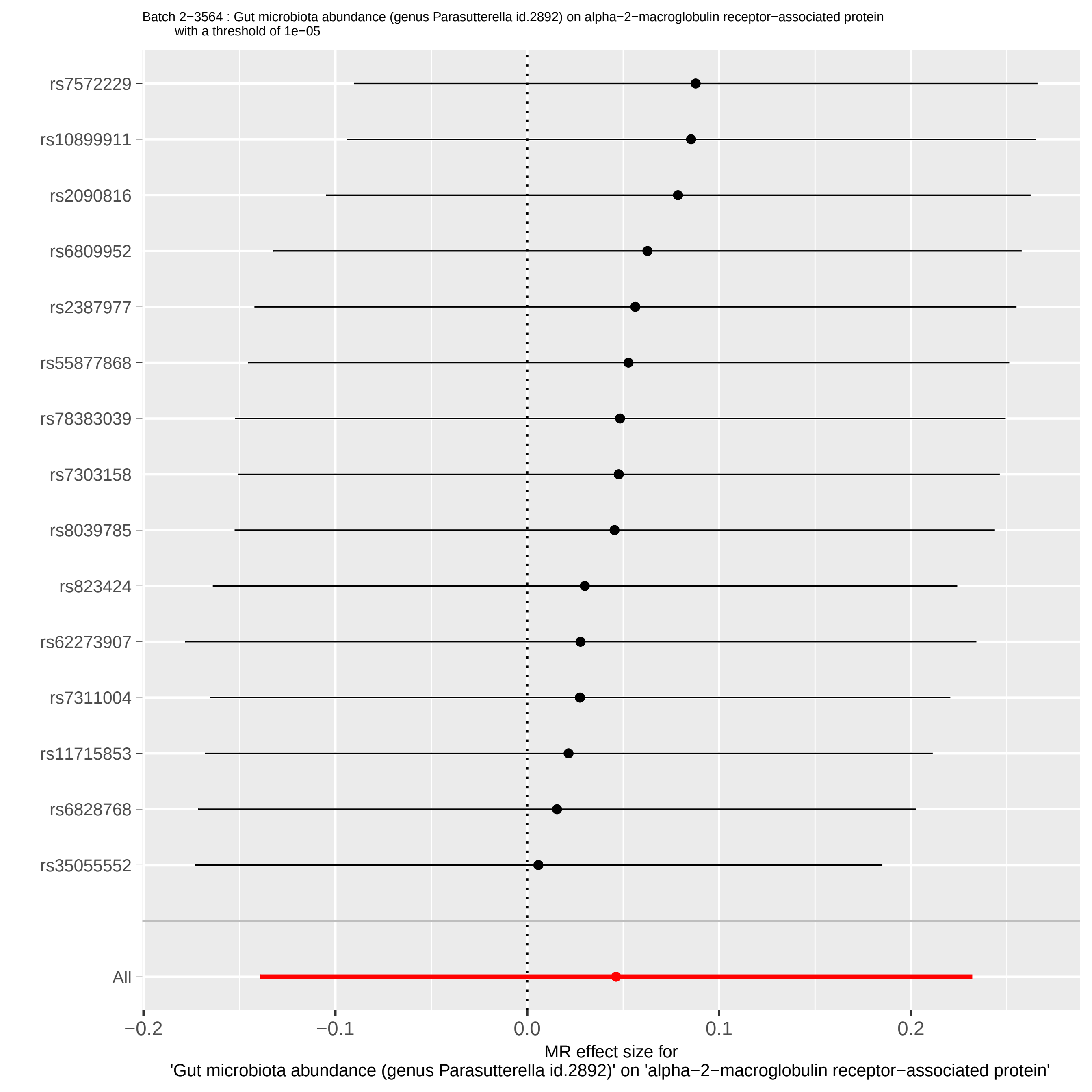
Batch 2−3564 : Gut microbiota abundance (genus Parasutterella id.2892) on alpha−2−macroglobulin receptor−associated protein with a threshold of 1e−05
rs7572229
rs10899911
rs2090816
rs6809952
rs2387977
rs55877868
rs78383039
rs7303158
rs8039785
rs823424
rs62273907
rs7311004
rs11715853
rs6828768
rs35055552
All
−0.2
−0.1
0.0
0.1
0.2
MR effect size for
'Gut microbiota abundance (genus Parasutterella id.2892)' on 'alpha−2−macroglobulin receptor−associated protein'

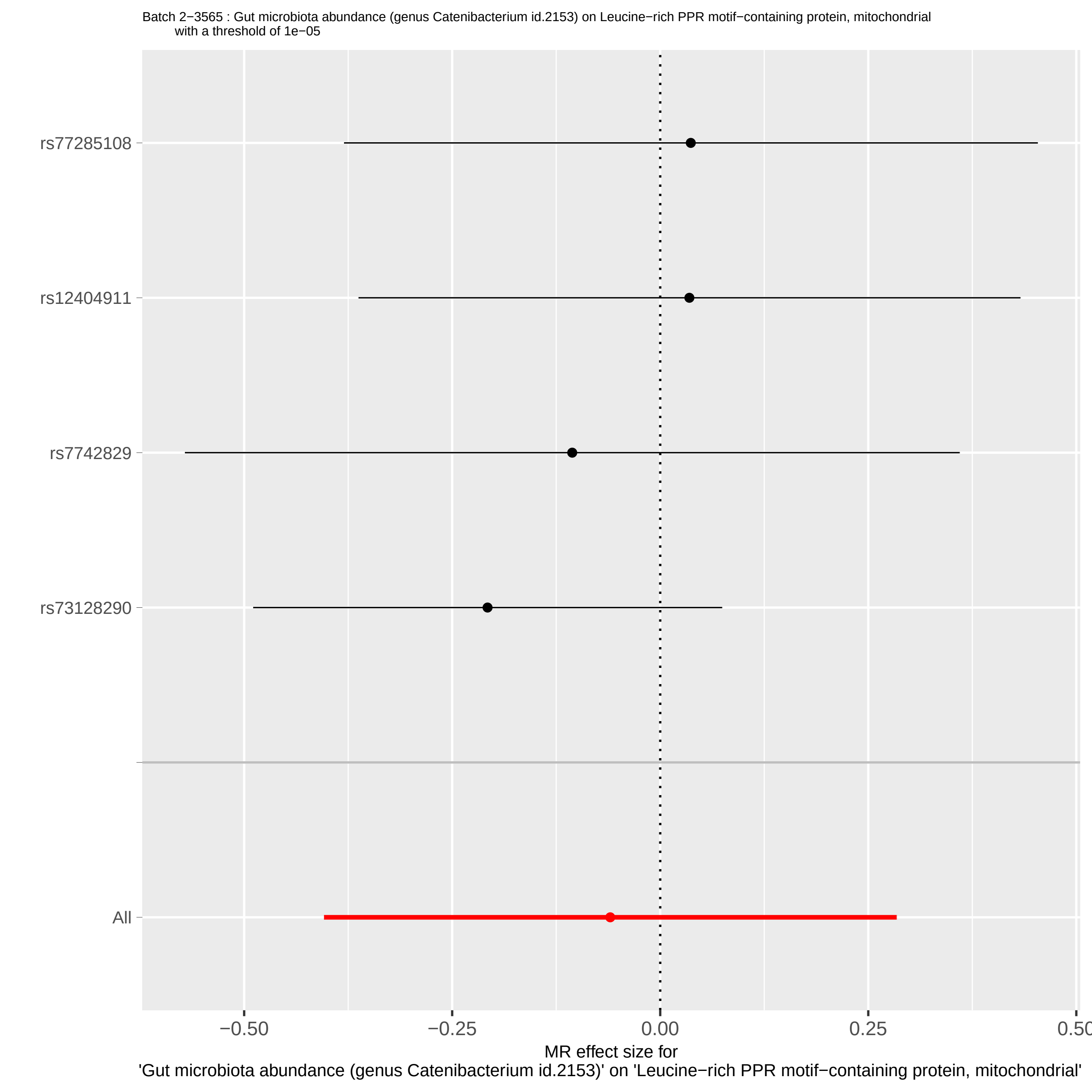
Batch 2−3565 : Gut microbiota abundance (genus Catenibacterium id.2153) on Leucine−rich PPR motif−containing protein, mitochondrial
with a threshold of 1e−05
rs77285108
rs12404911
rs7742829
rs73128290
All
−0.50
−0.25
0.00
0.25
0.50
MR effect size for
'Gut microbiota abundance (genus Catenibacterium id.2153)' on 'Leucine−rich PPR motif−containing protein, mitochondrial'

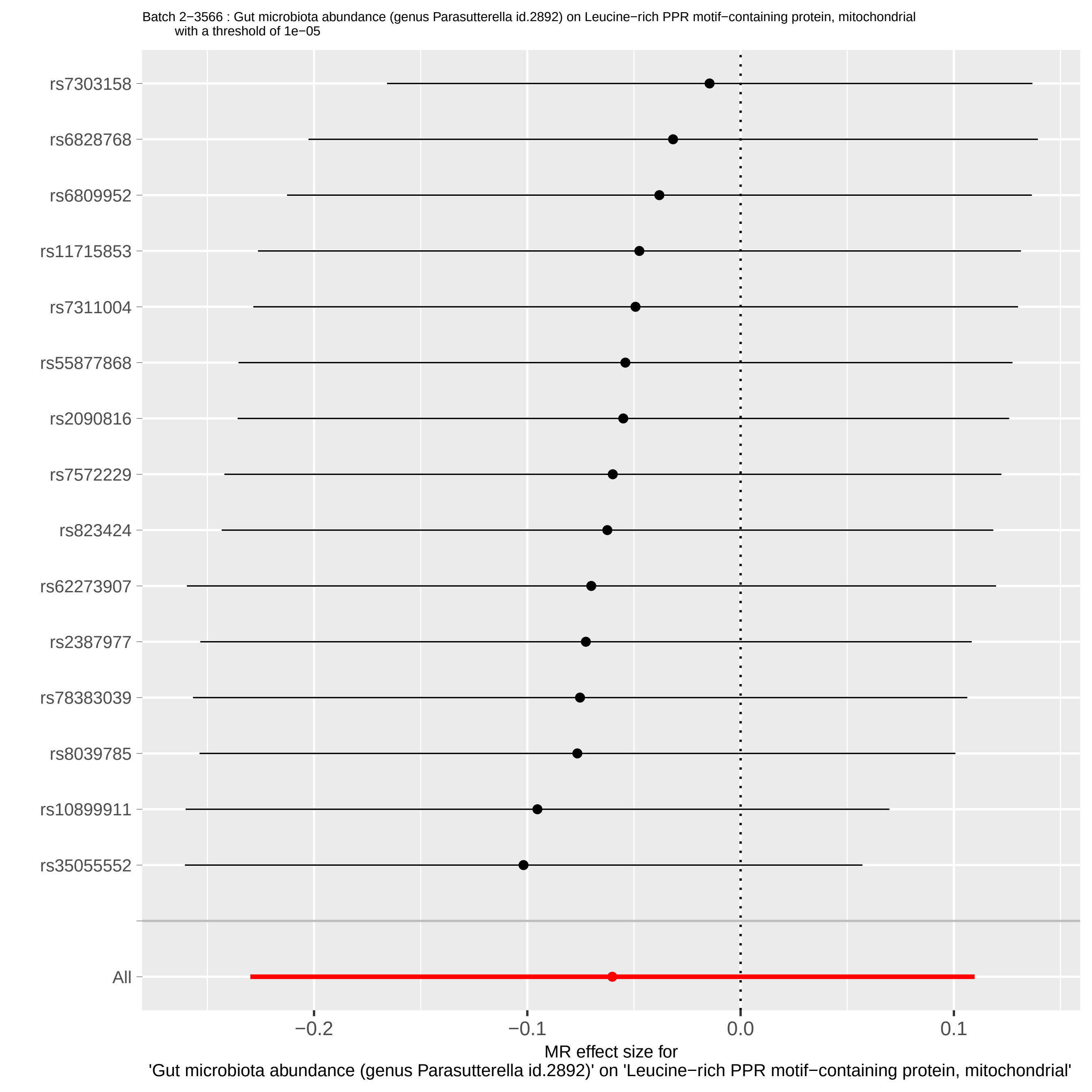

Batch 2−3566 : Gut microbiota abundance (genus Parasutterella id.2892) on Leucine−rich PPR motif−containing protein, mitochondrial
with a threshold of 1e−05
rs7303158
rs6828768
rs6809952
rs11715853
rs7311004
rs55877868
rs2090816
rs7572229
rs823424
rs62273907
rs2387977
rs78383039
rs8039785
rs10899911
rs35055552
All
−0.2
−0.1
0.0
0.1
MR effect size for
'Gut microbiota abundance (genus Parasutterella id.2892)' on 'Leucine−rich PPR motif−containing protein, mitochondrial'

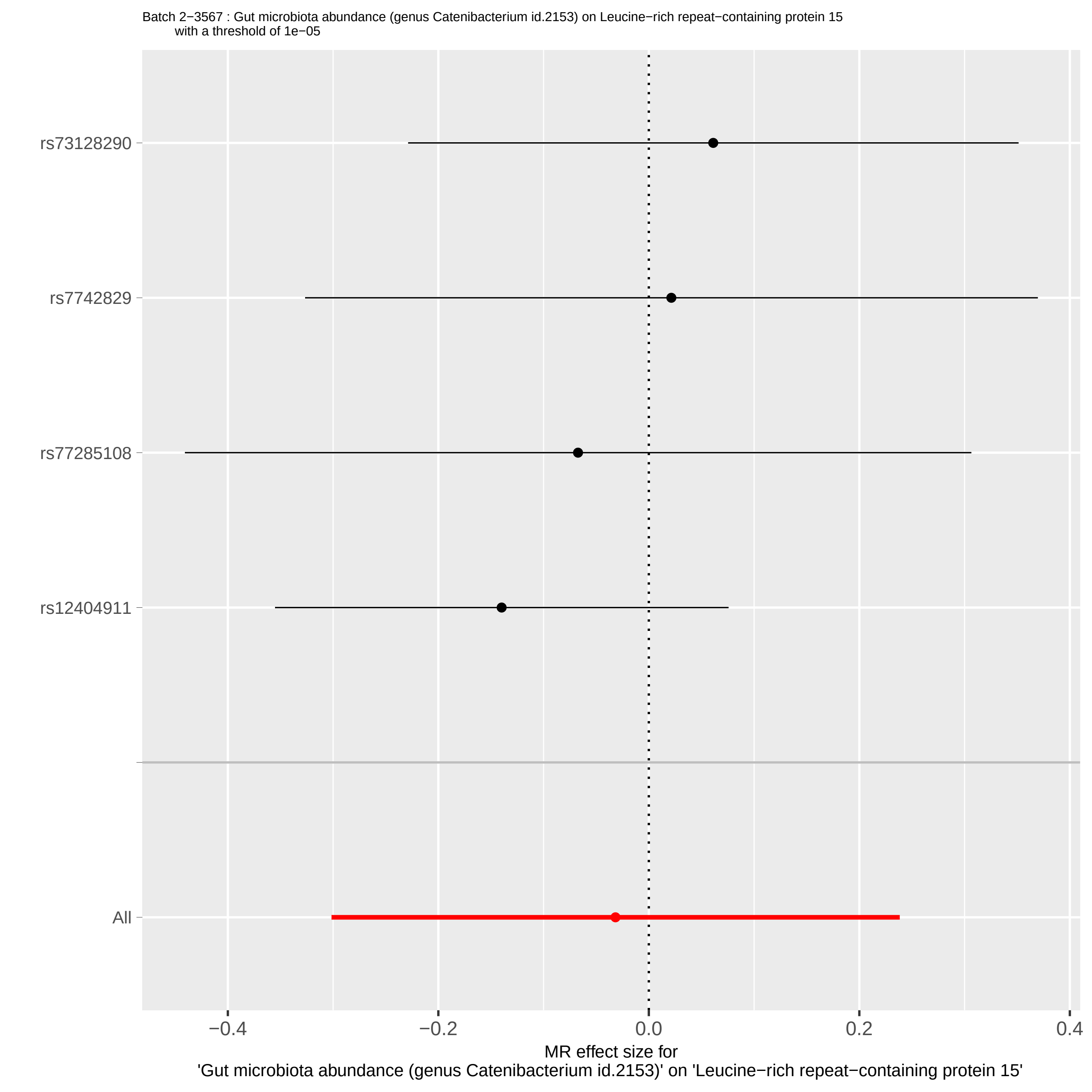
Batch 2−3567 : Gut microbiota abundance (genus Catenibacterium id.2153) on Leucine−rich repeat−containing protein 15
with a threshold of 1e−05
rs73128290
rs7742829
rs77285108
rs12404911
All
−0.4
−0.2
0.0
0.2
0.4
MR effect size for
'Gut microbiota abundance (genus Catenibacterium id.2153)' on 'Leucine−rich repeat−containing protein 15'

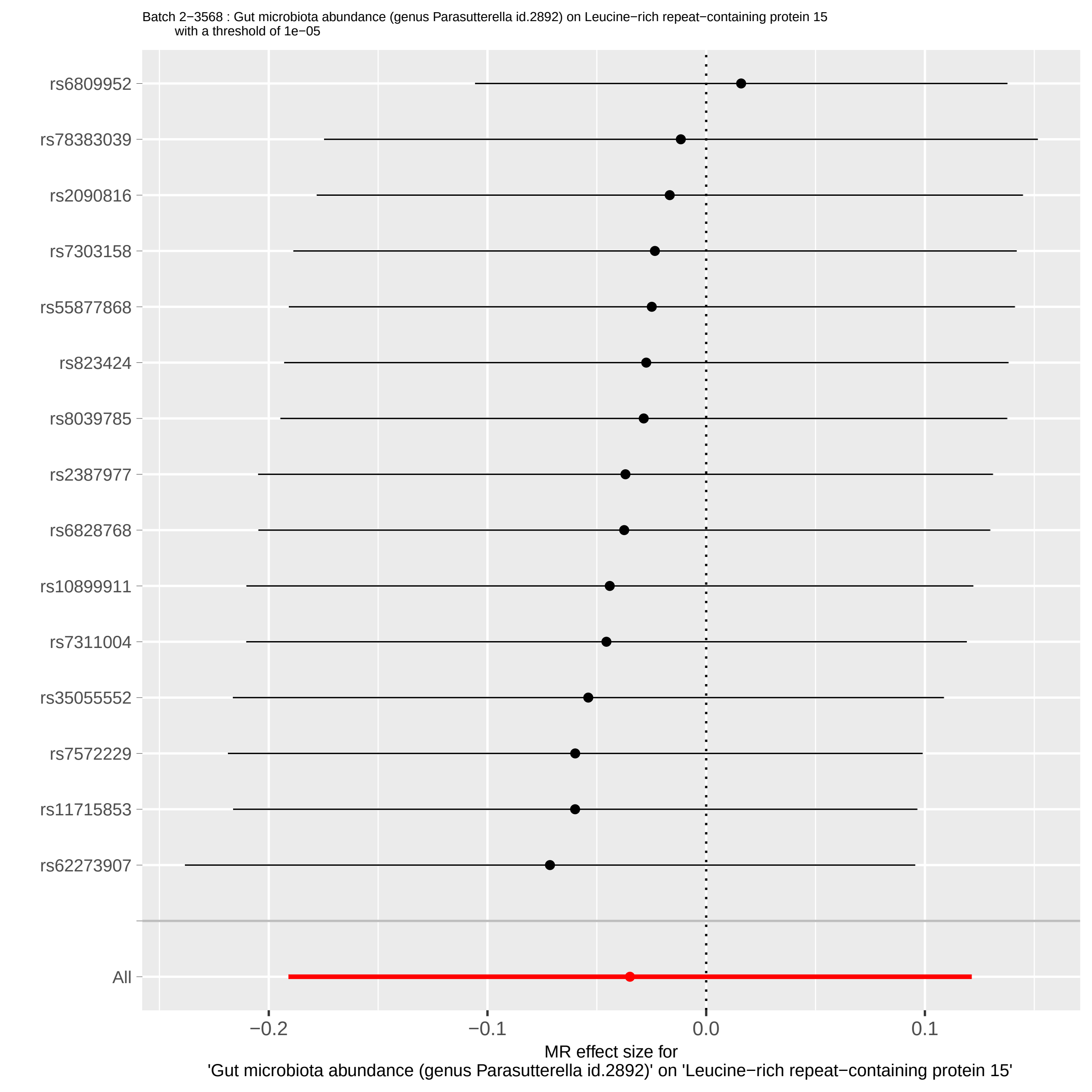

Batch 2−3568 : Gut microbiota abundance (genus Parasutterella id.2892) on Leucine−rich repeat−containing protein 15
with a threshold of 1e−05
rs6809952
rs78383039
rs2090816
rs7303158
rs55877868
rs823424
rs8039785
rs2387977
rs6828768
rs10899911
rs7311004
rs35055552
rs7572229
rs11715853
rs62273907
All
−0.2
−0.1
0.0
0.1
MR effect size for
'Gut microbiota abundance (genus Parasutterella id.2892)' on 'Leucine−rich repeat−containing protein 15'

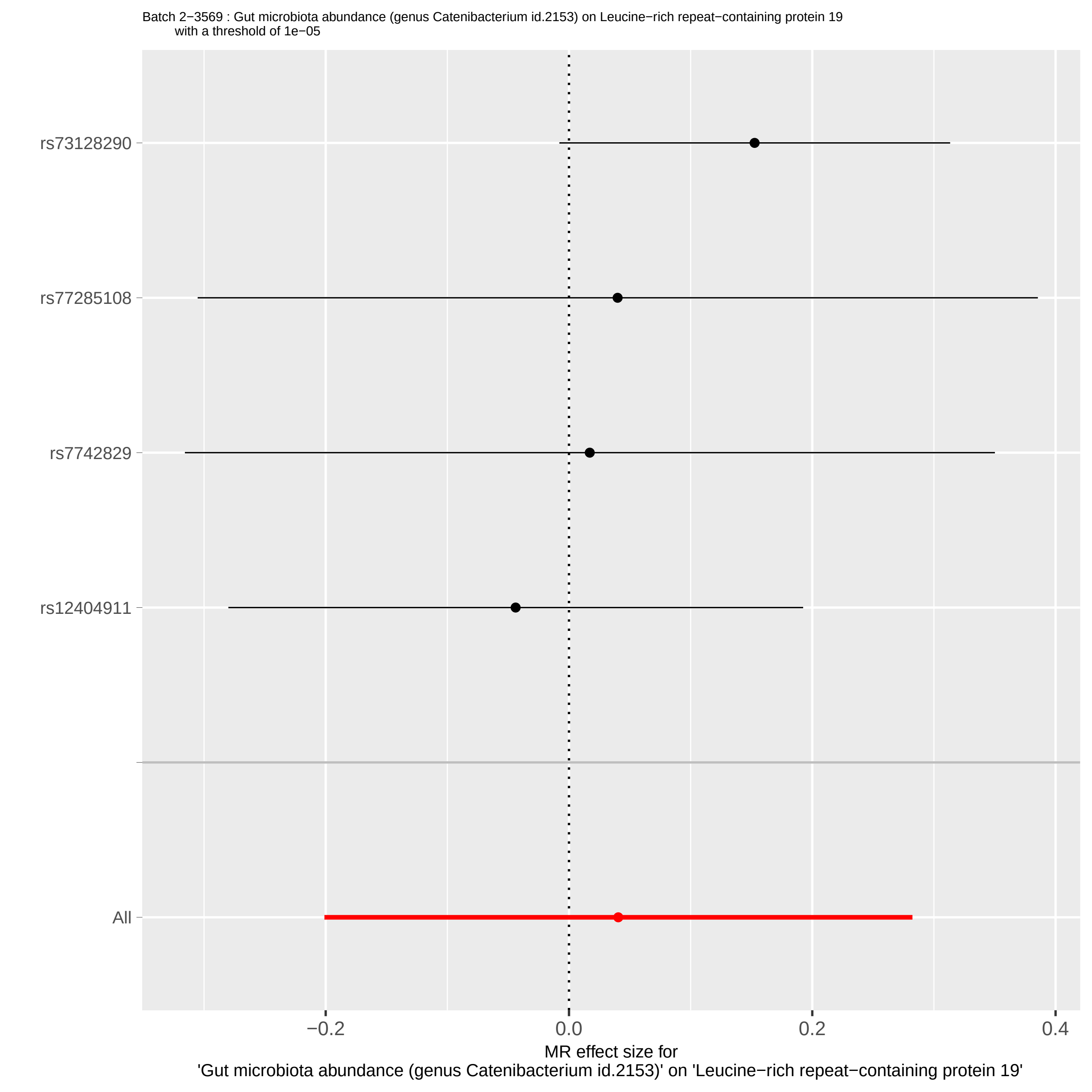
Batch 2−3569 : Gut microbiota abundance (genus Catenibacterium id.2153) on Leucine−rich repeat−containing protein 19
with a threshold of 1e−05
rs73128290
rs77285108
rs7742829
rs12404911
All
−0.2
0.0
0.2
0.4
MR effect size for
'Gut microbiota abundance (genus Catenibacterium id.2153)' on 'Leucine−rich repeat−containing protein 19'

Batch 2−3570 : Gut microbiota abundance (genus Parasutterella id.2892) on Leucine−rich repeat−containing protein 19
with a threshold of 1e−05

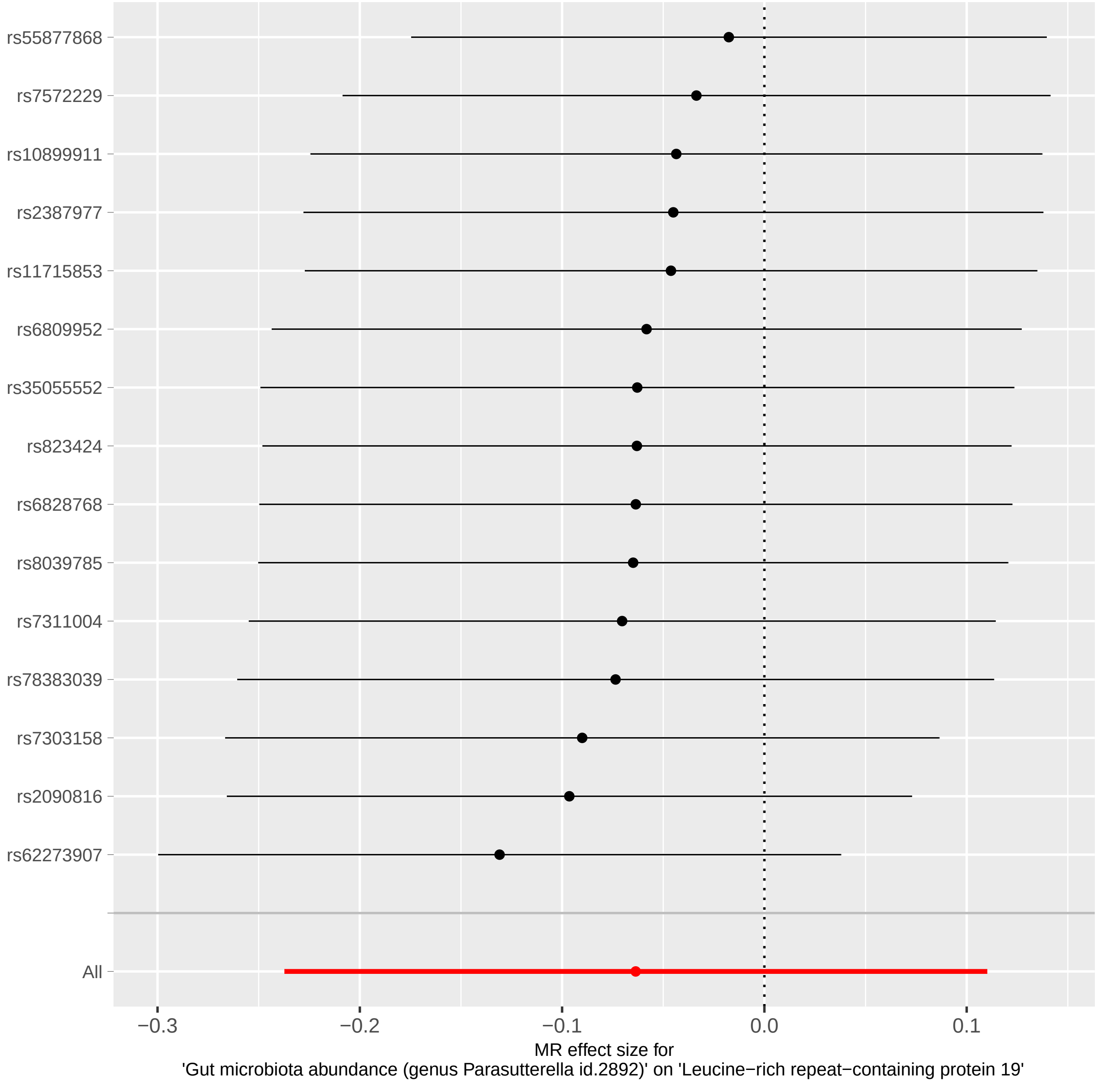

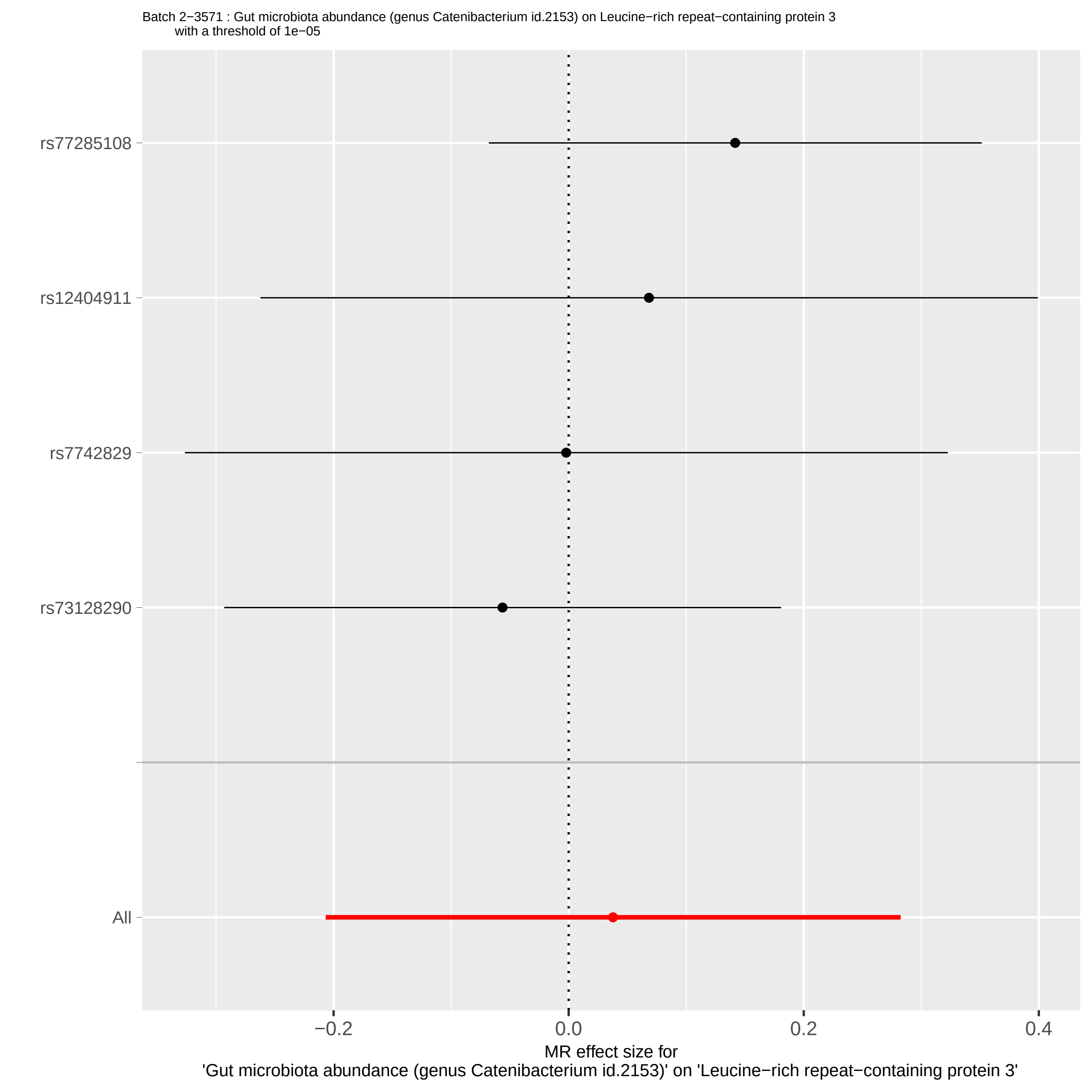
Batch 2−3571 : Gut microbiota abundance (genus Catenibacterium id.2153) on Leucine−rich repeat−containing protein 3
with a threshold of 1e−05
rs77285108
rs12404911
rs7742829
rs73128290
All
−0.2
0.0
0.2
0.4
MR effect size for
'Gut microbiota abundance (genus Catenibacterium id.2153)' on 'Leucine−rich repeat−containing protein 3'

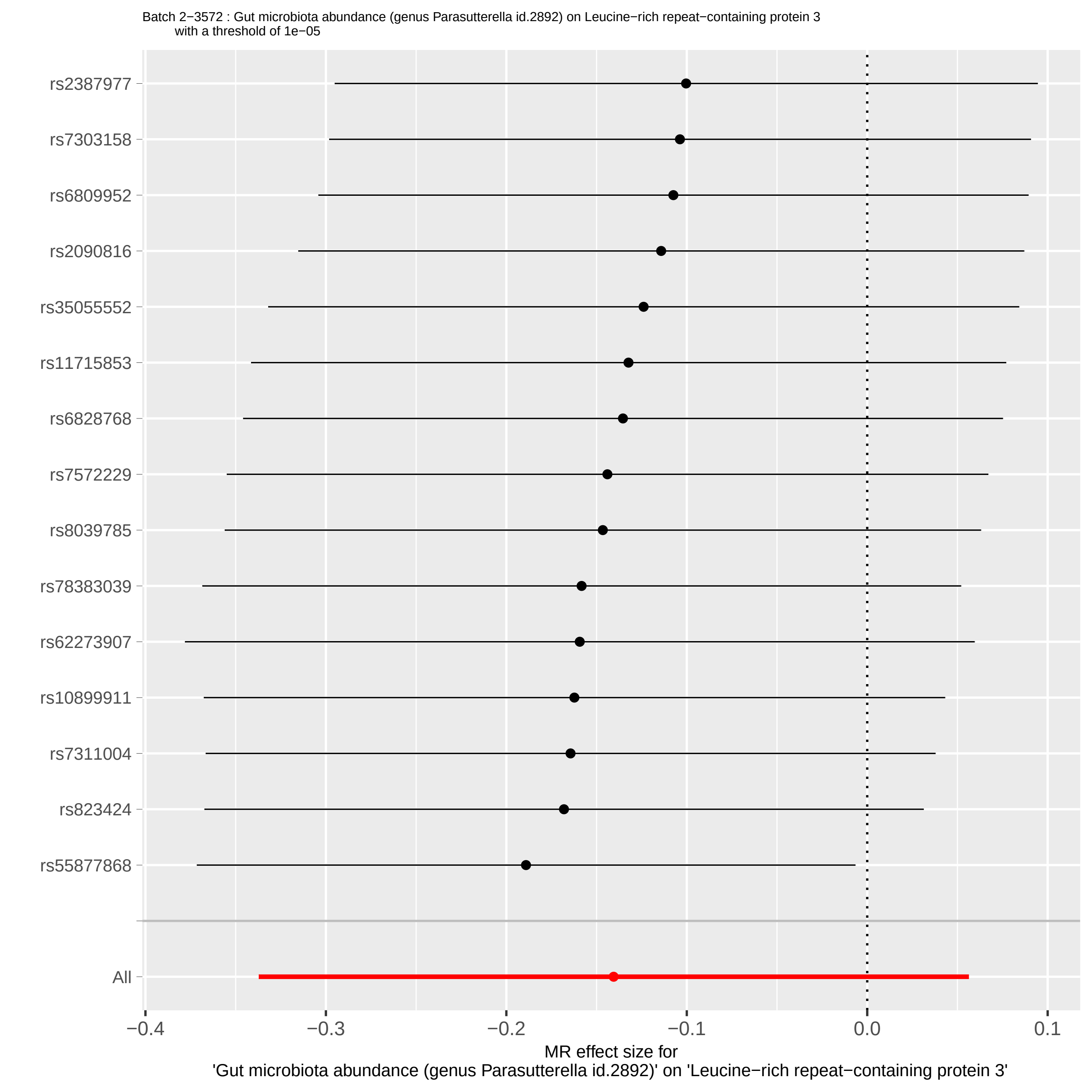

Batch 2−3572 : Gut microbiota abundance (genus Parasutterella id.2892) on Leucine−rich repeat−containing protein 3
with a threshold of 1e−05
rs2387977
rs7303158
rs6809952
rs2090816
rs35055552
rs11715853
rs6828768
rs7572229
rs8039785
rs78383039
rs62273907
rs10899911
rs7311004
rs823424
rs55877868
All
−0.4
−0.3
−0.2
−0.1
0.0
0.1
MR effect size for
'Gut microbiota abundance (genus Parasutterella id.2892)' on 'Leucine−rich repeat−containing protein 3'

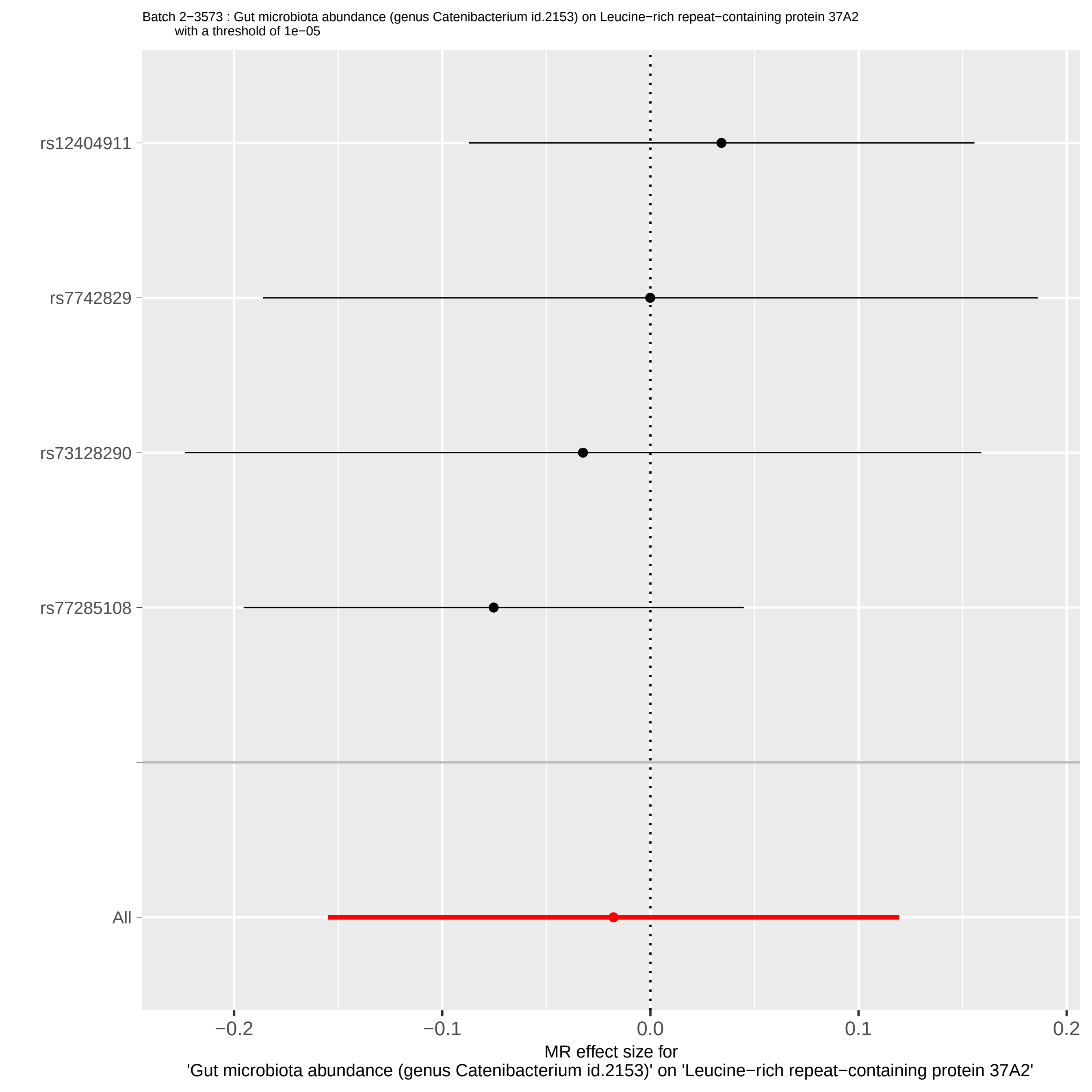
Batch 2−3573 : Gut microbiota abundance (genus Catenibacterium id.2153) on Leucine−rich repeat−containing protein 37A2
with a threshold of 1e−05
rs12404911
rs7742829
rs73128290
rs77285108
All
−0.2
−0.1
0.0
0.1
0.2
MR effect size for
'Gut microbiota abundance (genus Catenibacterium id.2153)' on 'Leucine−rich repeat−containing protein 37A2'

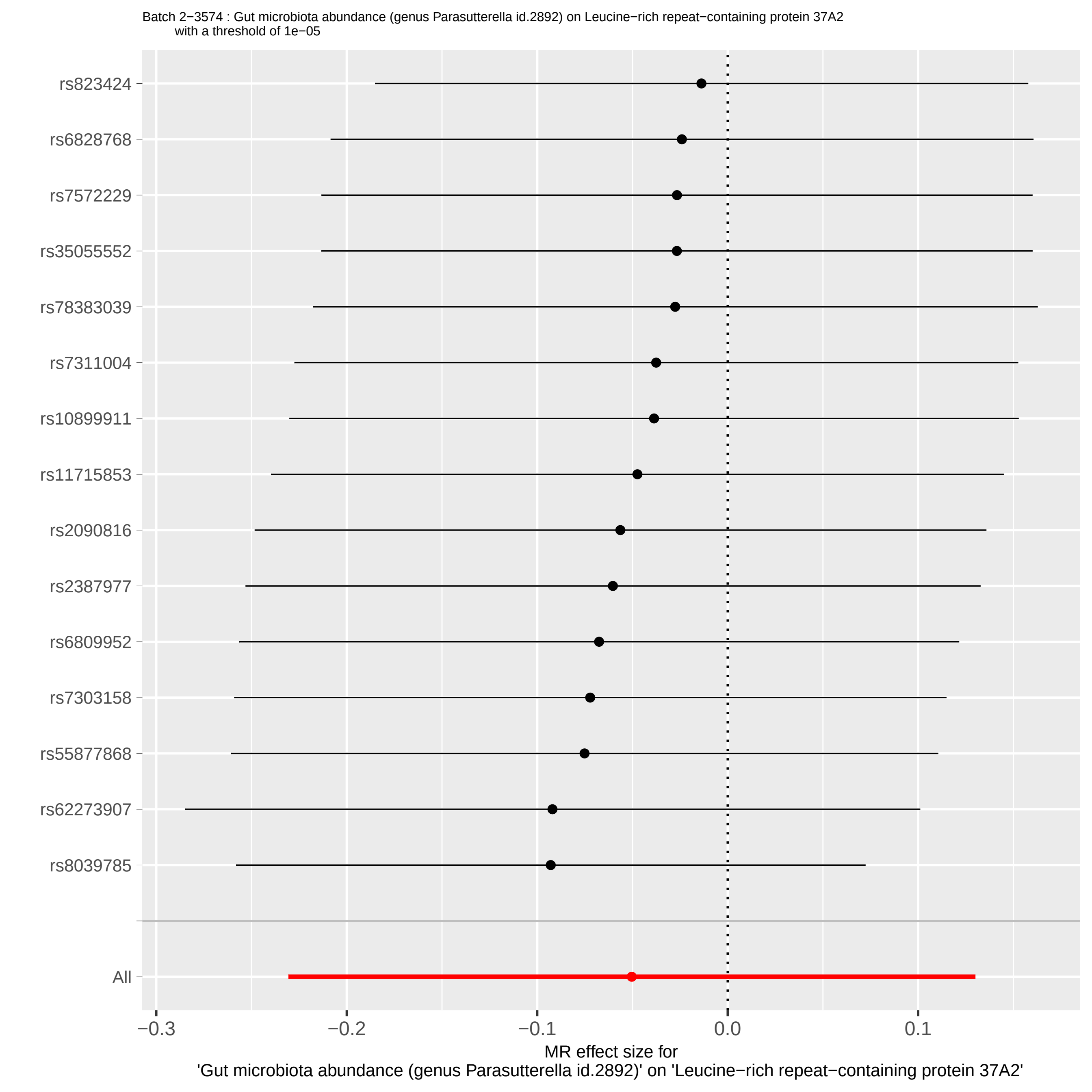

Batch 2−3574 : Gut microbiota abundance (genus Parasutterella id.2892) on Leucine−rich repeat−containing protein 37A2
with a threshold of 1e−05
rs823424
rs6828768
rs7572229
rs35055552
rs78383039
rs7311004
rs10899911
rs11715853
rs2090816
rs2387977
rs6809952
rs7303158
rs55877868
rs62273907
rs8039785
All
−0.3
−0.2
−0.1
0.0
0.1
MR effect size for
'Gut microbiota abundance (genus Parasutterella id.2892)' on 'Leucine−rich repeat−containing protein 37A2'

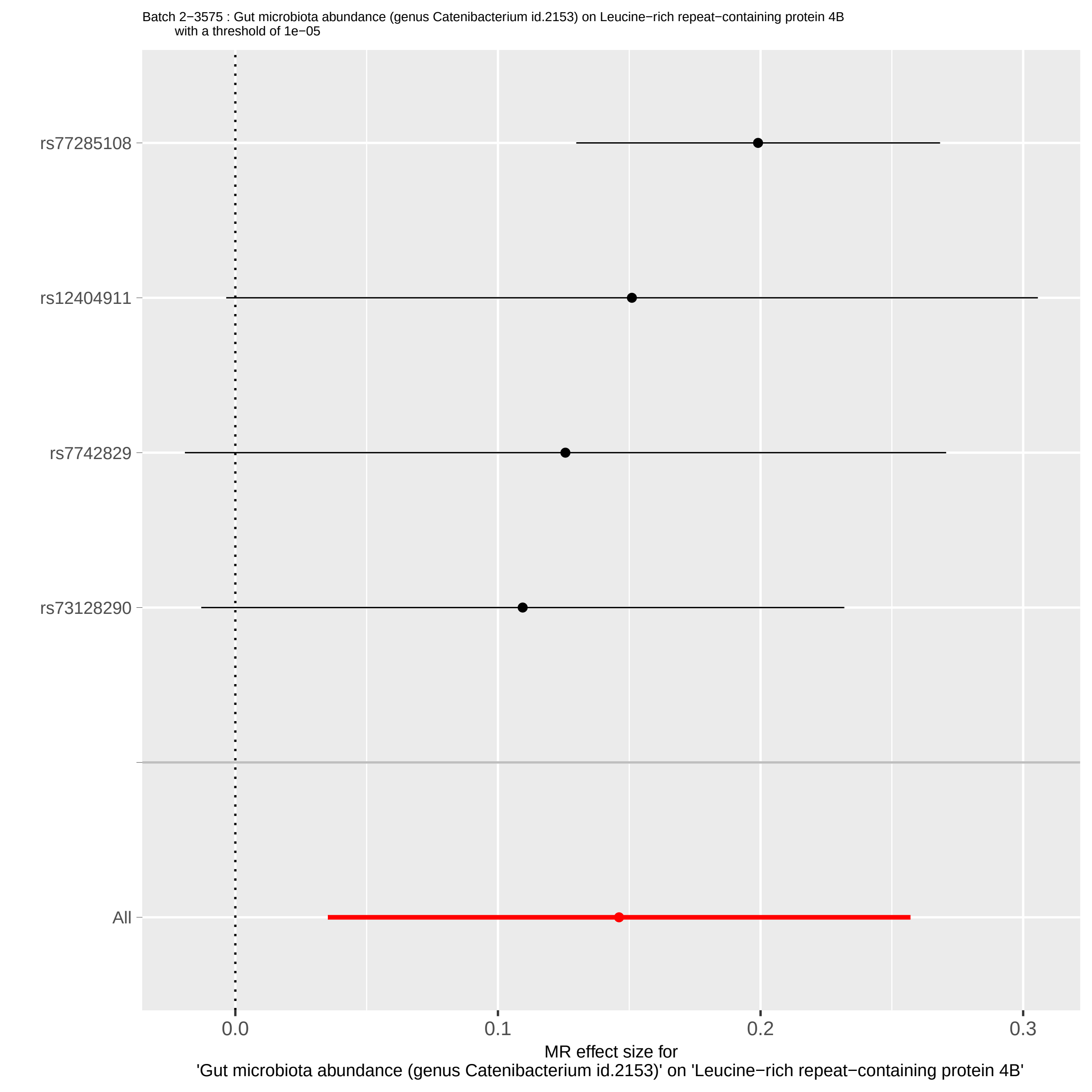
Batch 2−3575 : Gut microbiota abundance (genus Catenibacterium id.2153) on Leucine−rich repeat−containing protein 4B
with a threshold of 1e−05
rs77285108
rs12404911
rs7742829
rs73128290
All
0.0
0.1
0.2
0.3
MR effect size for
'Gut microbiota abundance (genus Catenibacterium id.2153)' on 'Leucine−rich repeat−containing protein 4B'

Batch 2−3576 : Gut microbiota abundance (genus Parasutterella id.2892) on Leucine−rich repeat−containing protein 4B
with a threshold of 1e−05

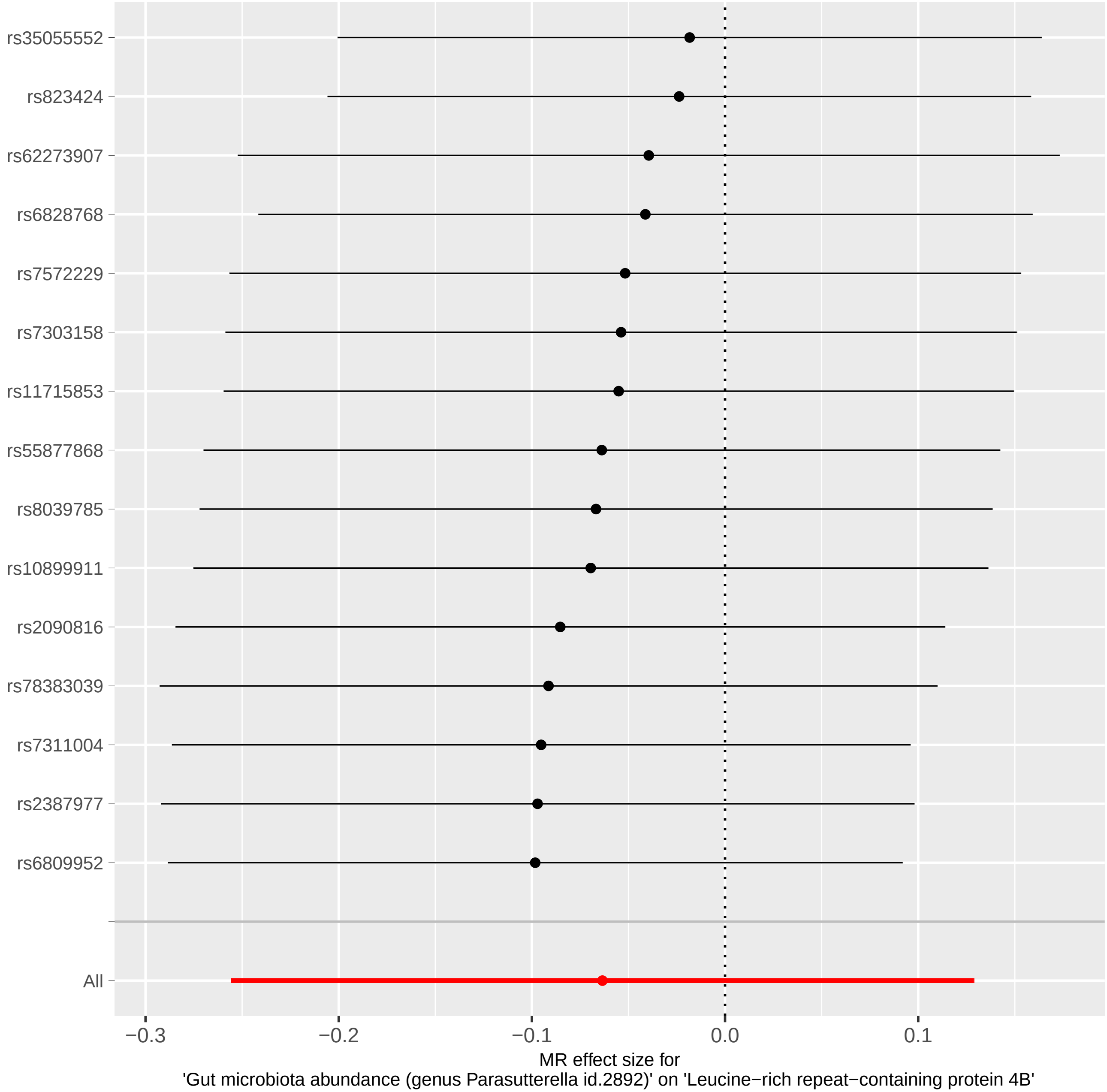

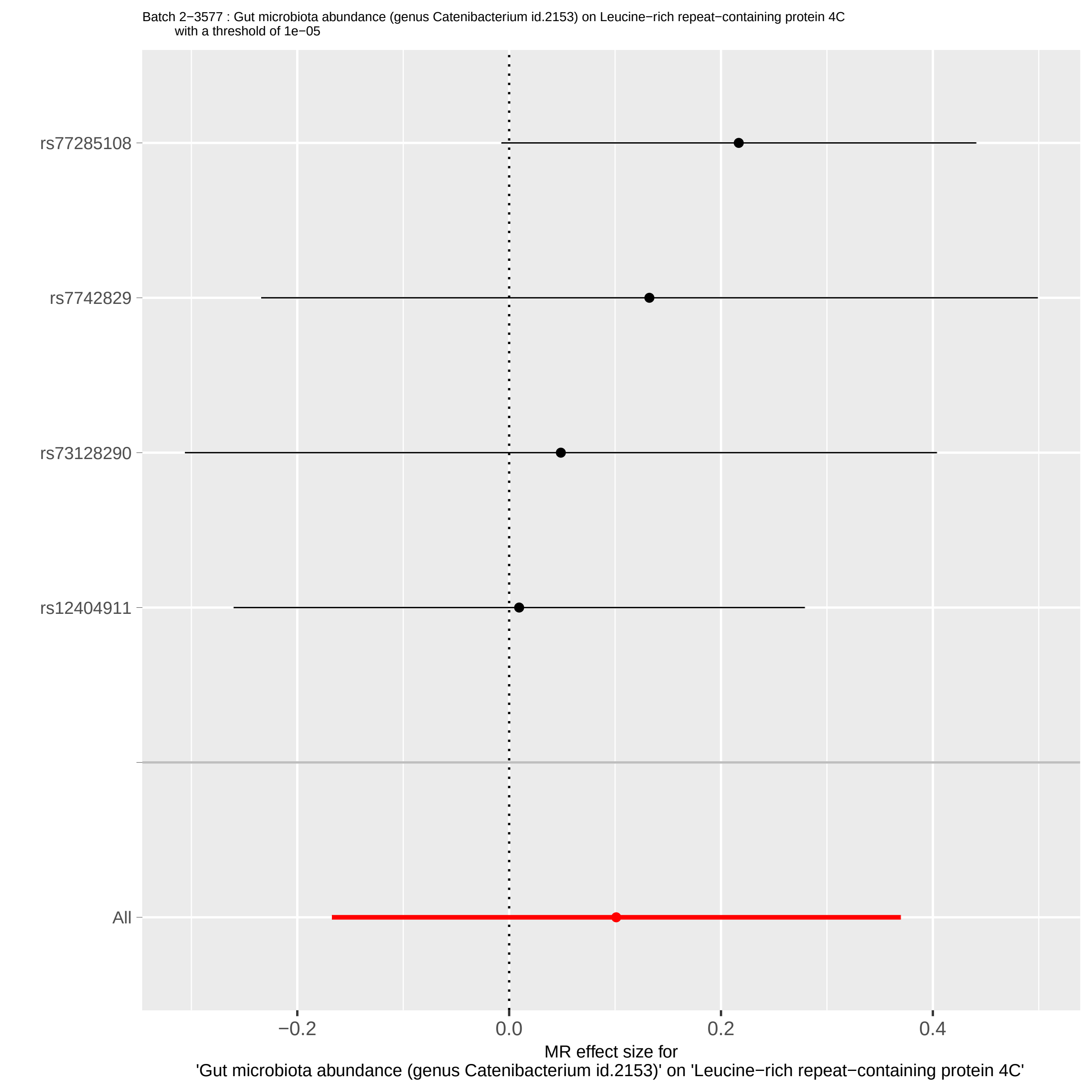

Batch 2−3577 : Gut microbiota abundance (genus Catenibacterium id.2153) on Leucine−rich repeat−containing protein 4C
with a threshold of 1e−05
rs77285108
rs7742829
rs73128290
rs12404911
All
−0.2
0.0
0.2
0.4
MR effect size for
'Gut microbiota abundance (genus Catenibacterium id.2153)' on 'Leucine−rich repeat−containing protein 4C'

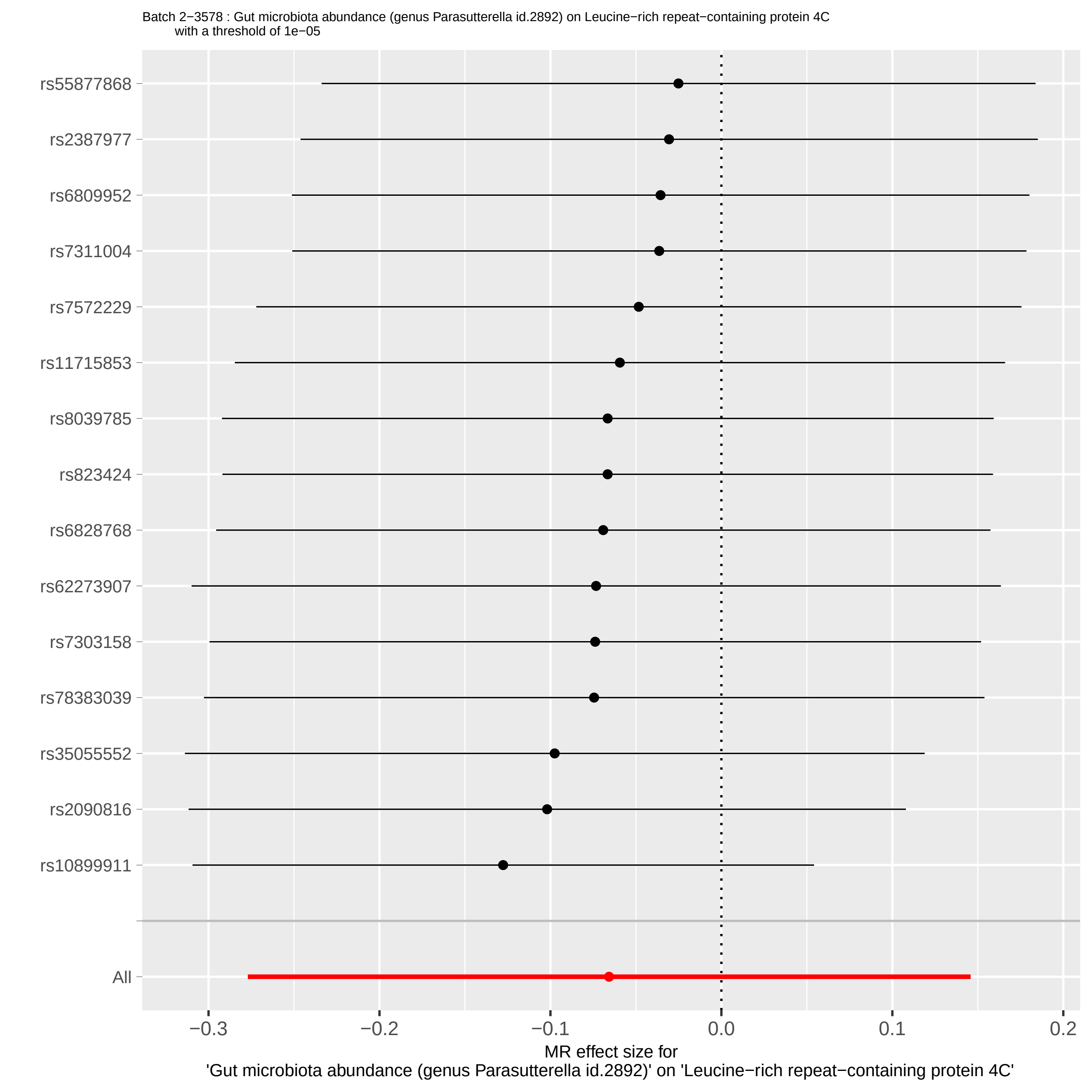
Batch 2−3578 : Gut microbiota abundance (genus Parasutterella id.2892) on Leucine−rich repeat−containing protein 4C
with a threshold of 1e−05
rs55877868
rs2387977
rs6809952
rs7311004
rs7572229
rs11715853
rs8039785
rs823424
rs6828768
rs62273907
rs7303158
rs78383039
rs35055552
rs2090816
rs10899911
All
−0.3
−0.2
−0.1
0.0
0.1
0.2
MR effect size for
'Gut microbiota abundance (genus Parasutterella id.2892)' on 'Leucine−rich repeat−containing protein 4C'

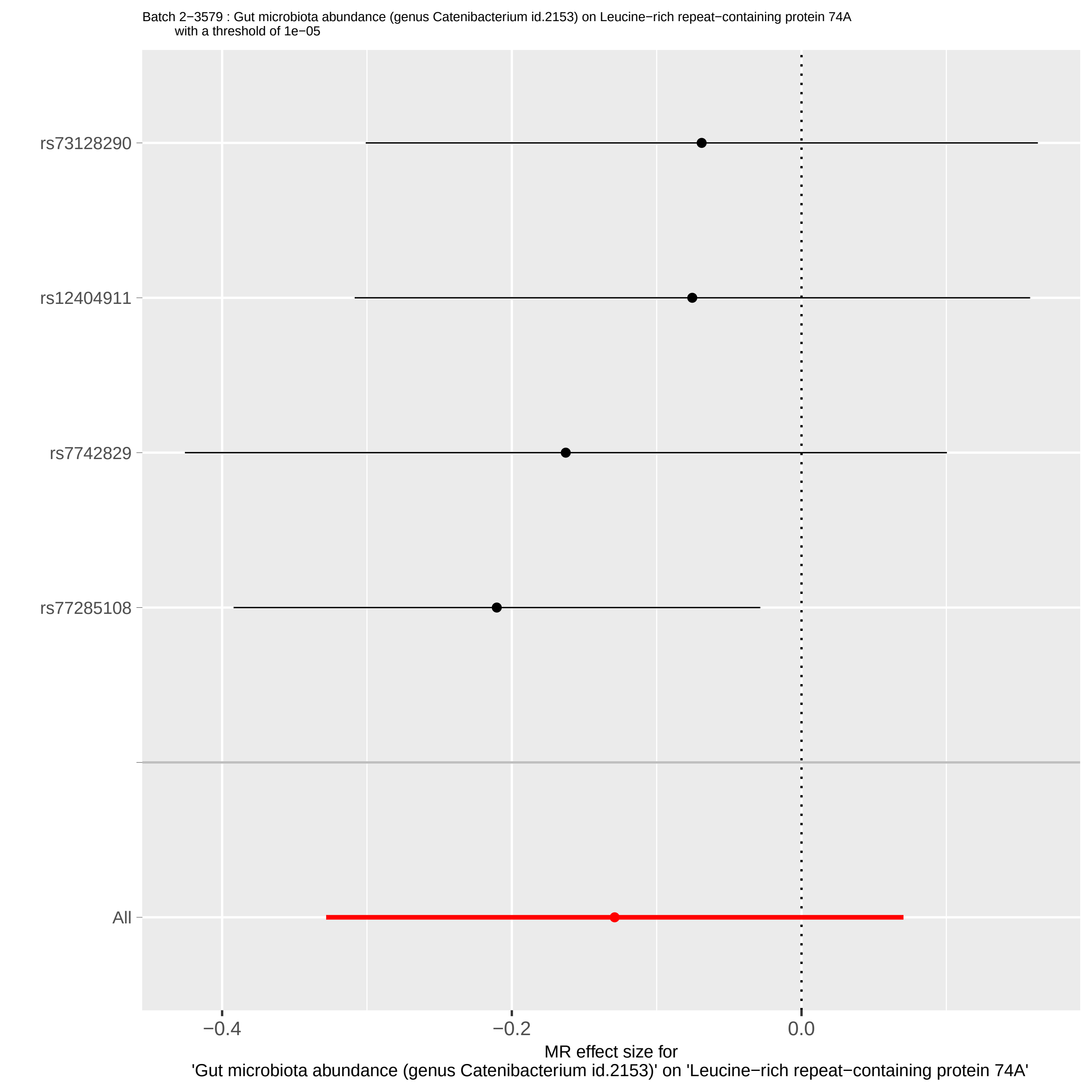

Batch 2−3579 : Gut microbiota abundance (genus Catenibacterium id.2153) on Leucine−rich repeat−containing protein 74A
with a threshold of 1e−05
rs73128290
rs12404911
rs7742829
rs77285108
All
−0.4
−0.2
0.0
MR effect size for
'Gut microbiota abundance (genus Catenibacterium id.2153)' on 'Leucine−rich repeat−containing protein 74A'

Batch 2−3580 : Gut microbiota abundance (genus Parasutterella id.2892) on Leucine−rich repeat−containing protein 74A
with a threshold of 1e−05

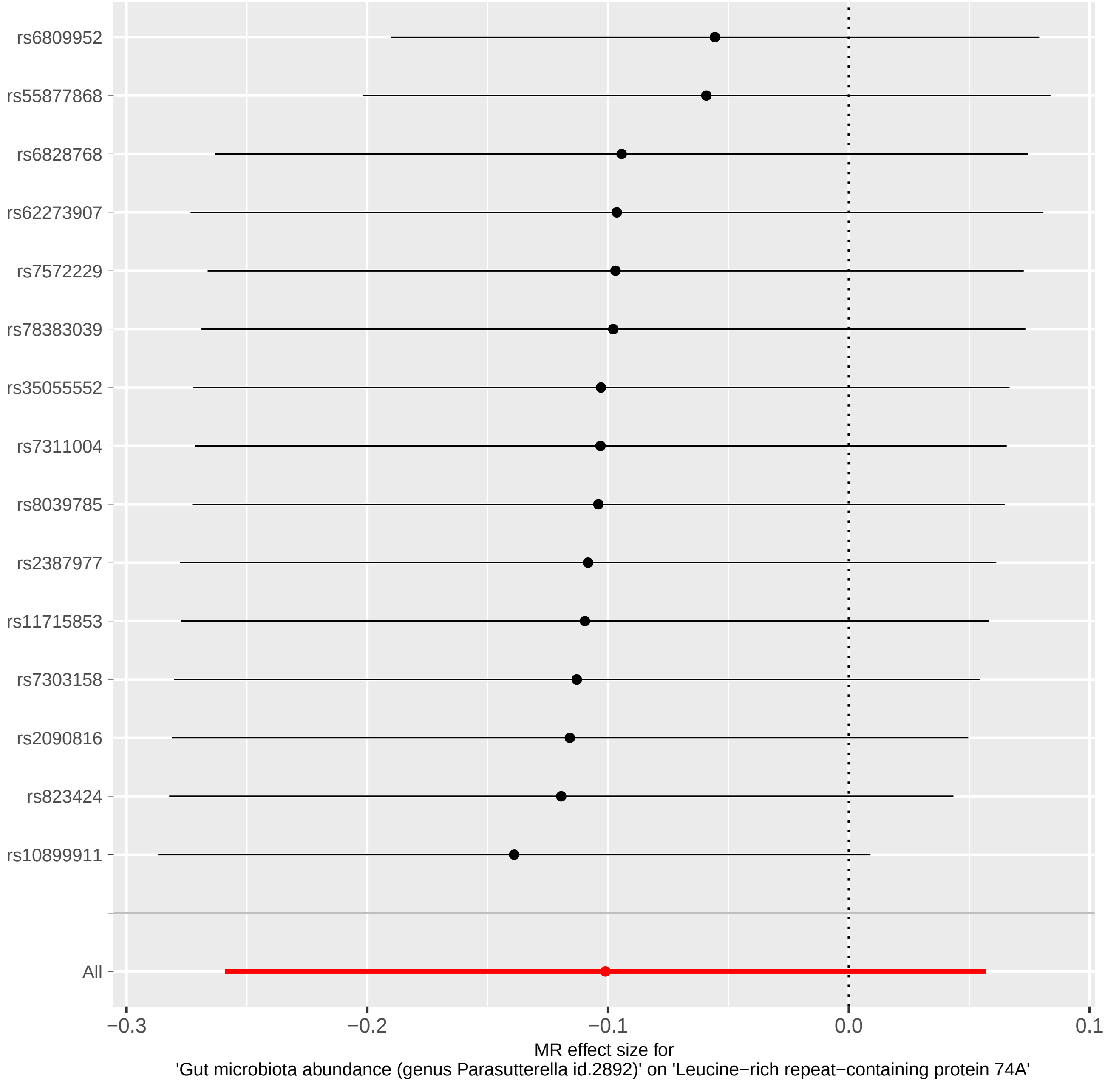

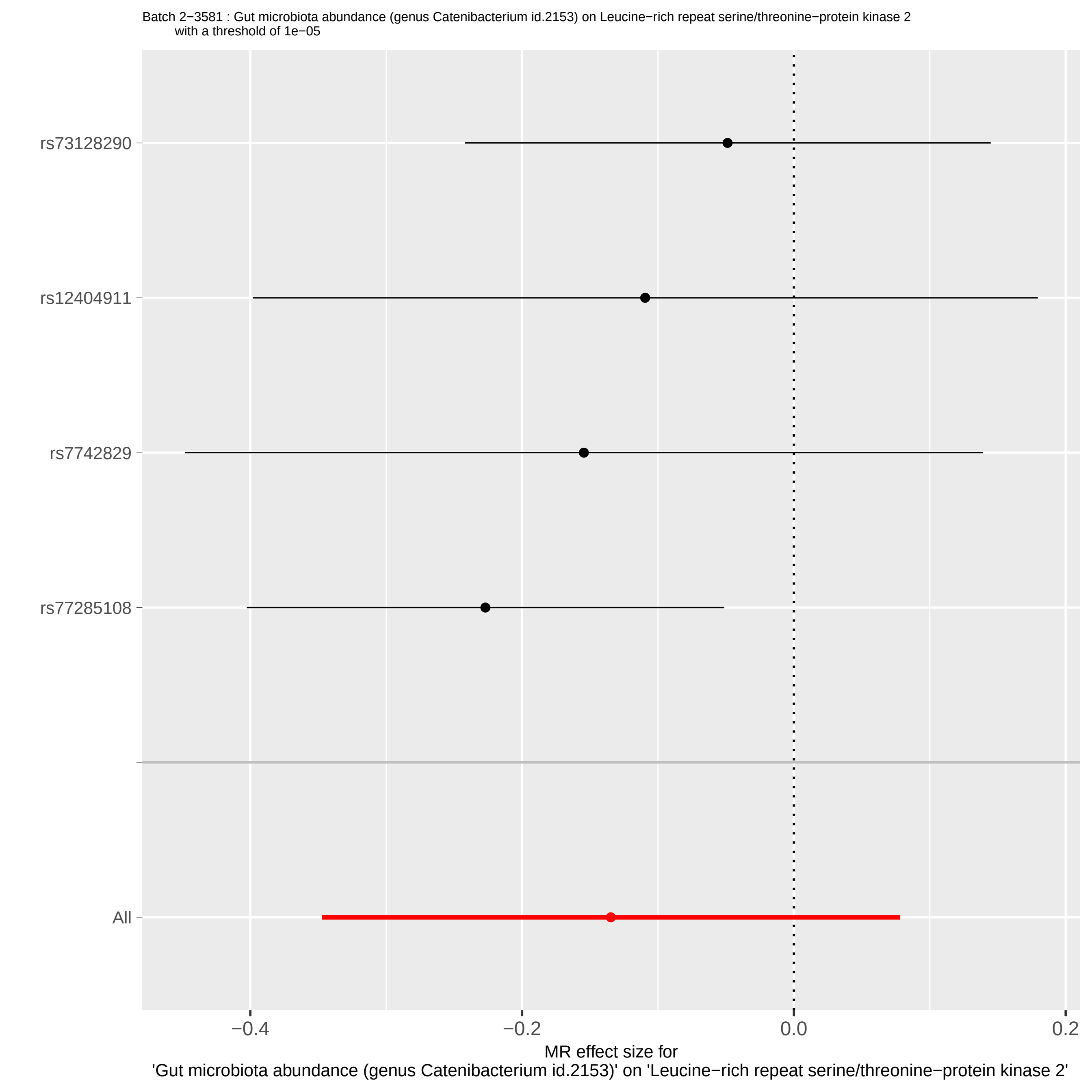
Batch 2−3581 : Gut microbiota abundance (genus Catenibacterium id.2153) on Leucine−rich repeat serine/threonine−protein kinase 2
with a threshold of 1e−05
rs73128290
rs12404911
rs7742829
rs77285108
All
−0.4
−0.2
0.0
0.2
MR effect size for
'Gut microbiota abundance (genus Catenibacterium id.2153)' on 'Leucine−rich repeat serine/threonine−protein kinase 2'

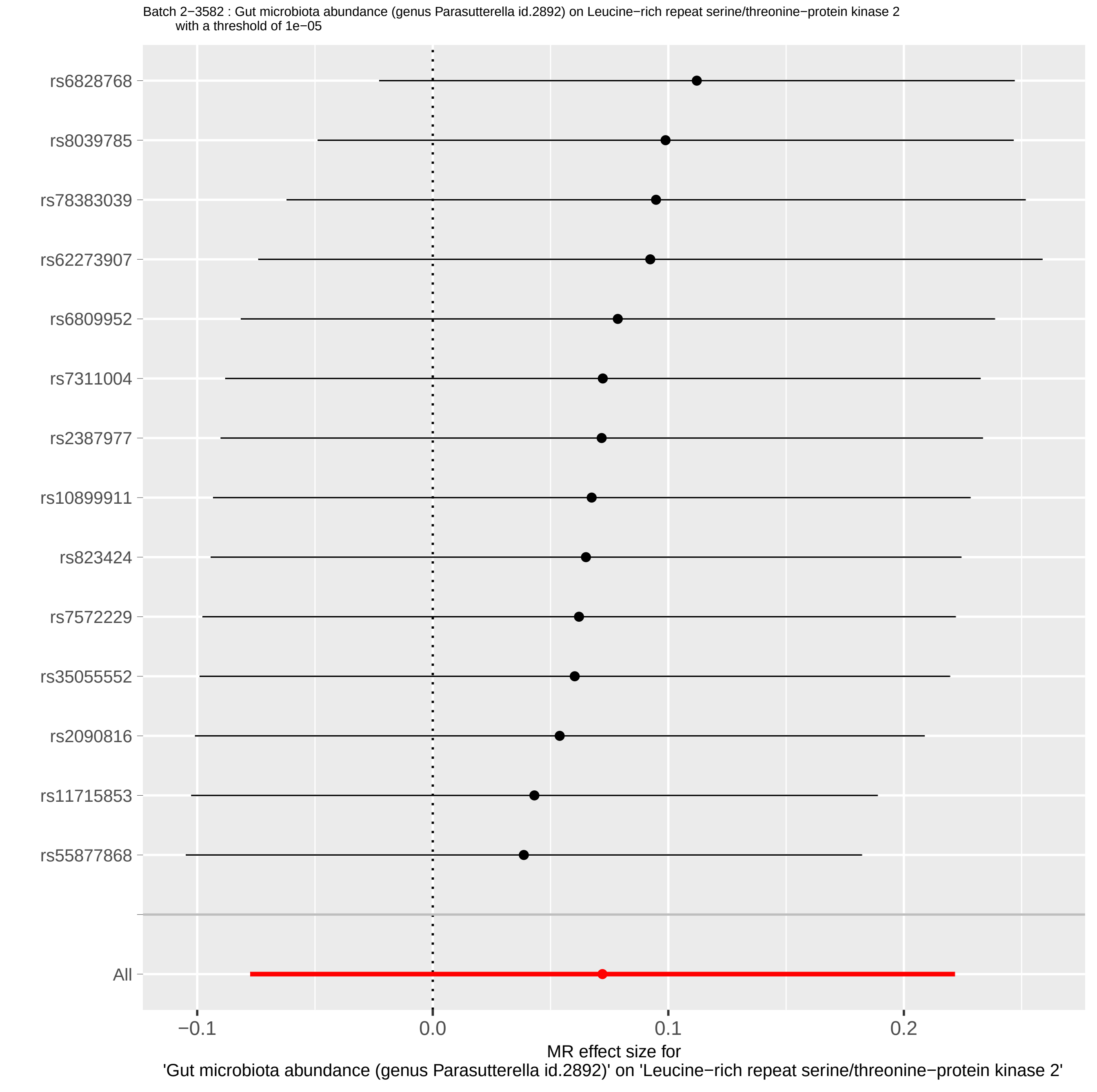
Batch 2−3582 : Gut microbiota abundance (genus Parasutterella id.2892) on Leucine−rich repeat serine/threonine−protein kinase 2
with a threshold of 1e−05
rs6828768
rs8039785
rs78383039
rs62273907
rs6809952
rs7311004
rs2387977
rs10899911
rs823424
rs7572229
rs35055552
rs2090816
rs11715853
rs55877868
All
−0.1
0.0
0.1
0.2
MR effect size for
'Gut microbiota abundance (genus Parasutterella id.2892)' on 'Leucine−rich repeat serine/threonine−protein kinase 2'

Batch 2−3583 : Gut microbiota abundance (genus Catenibacterium id.2153) on Leucine−rich repeat neuronal protein 1
with a threshold of 1e−05

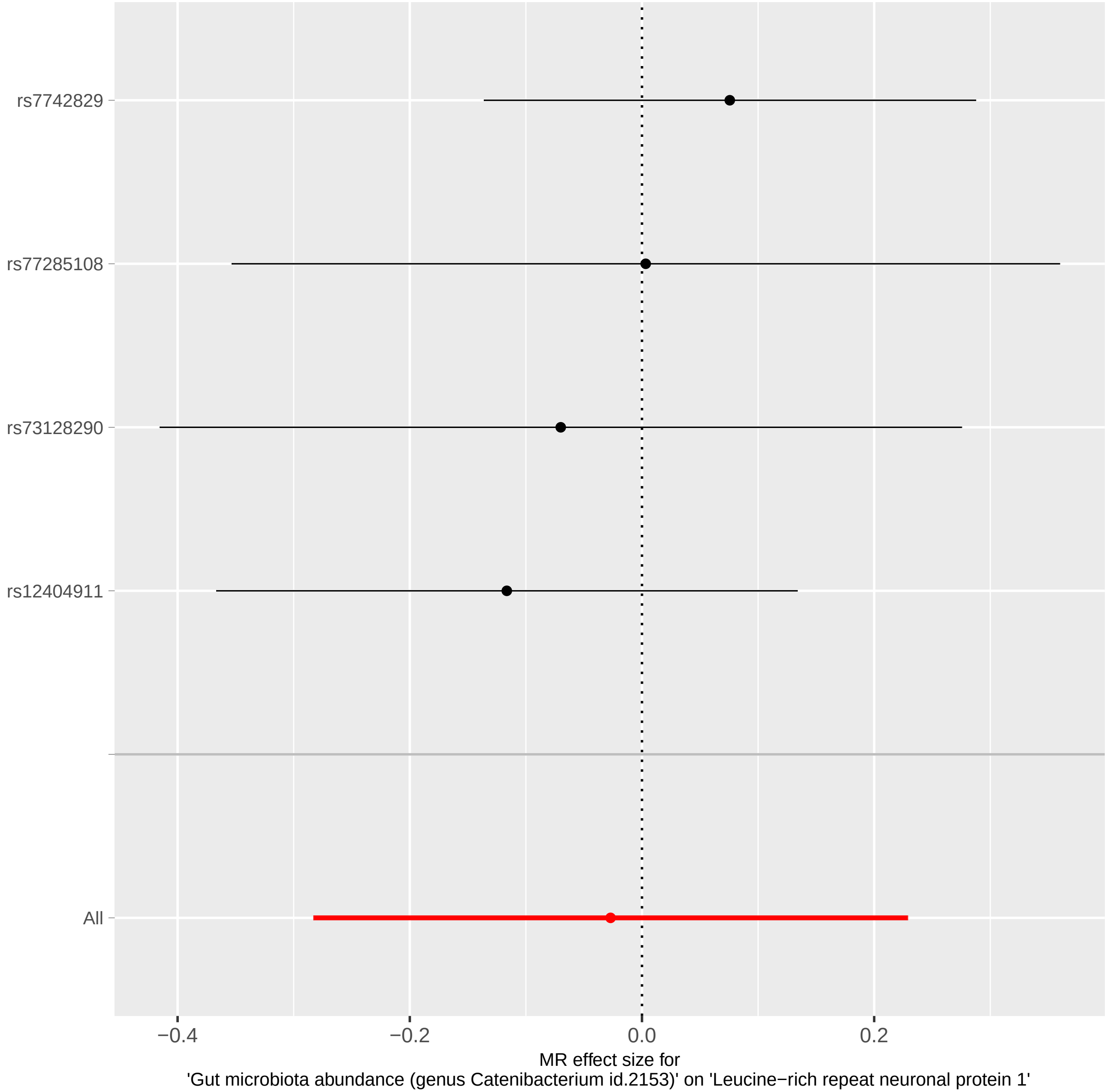

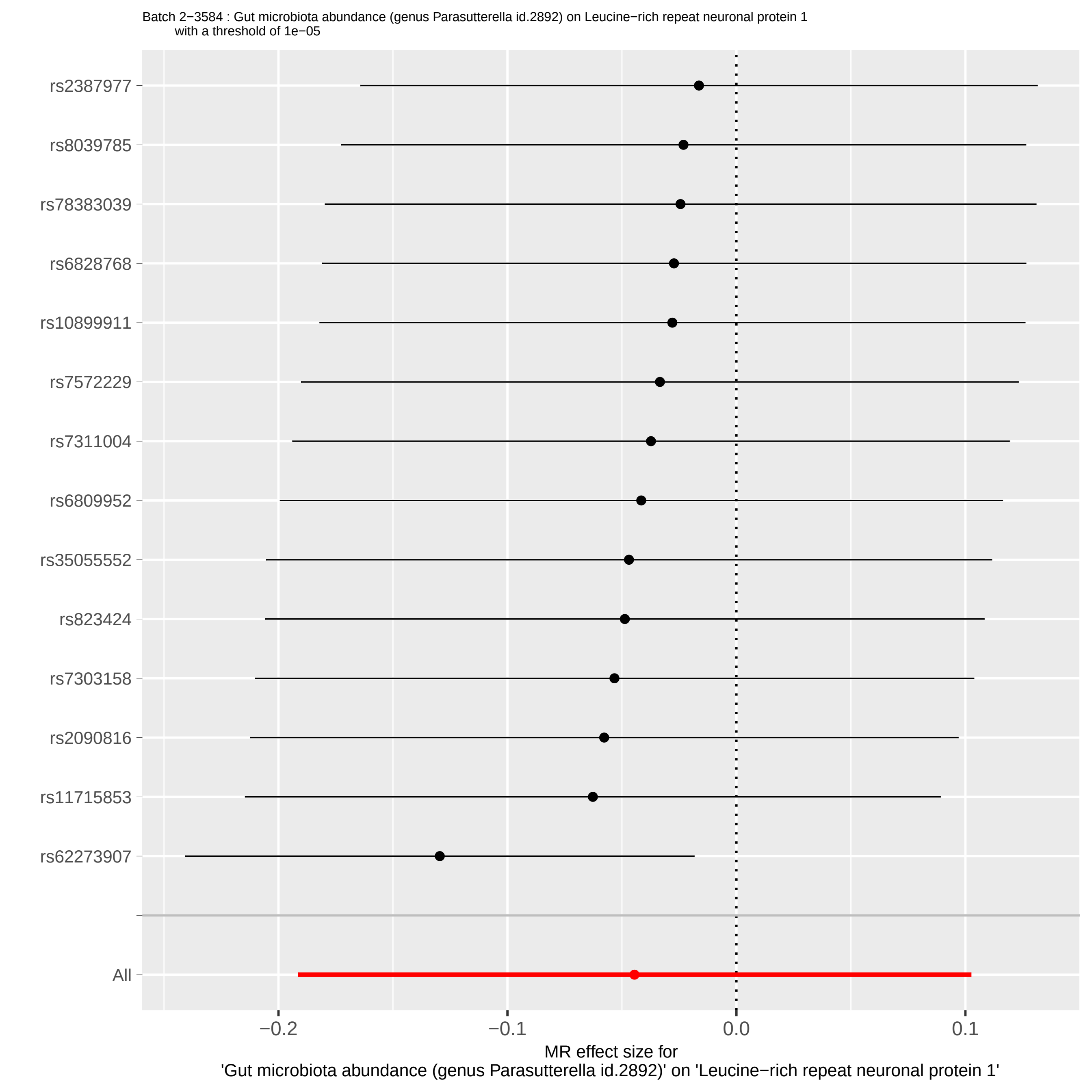
Batch 2−3584 : Gut microbiota abundance (genus Parasutterella id.2892) on Leucine−rich repeat neuronal protein 1
with a threshold of 1e−05
rs2387977
rs8039785
rs78383039
rs6828768
rs10899911
rs7572229
rs7311004
rs6809952
rs35055552
rs823424
rs7303158
rs2090816
rs11715853
rs62273907
All
−0.2
−0.1
0.0
0.1
MR effect size for
'Gut microbiota abundance (genus Parasutterella id.2892)' on 'Leucine−rich repeat neuronal protein 1'

Batch 2−3585 : Gut microbiota abundance (genus Catenibacterium id.2153) on Leucine−rich repeat neuronal protein 1
with a threshold of 1e−05

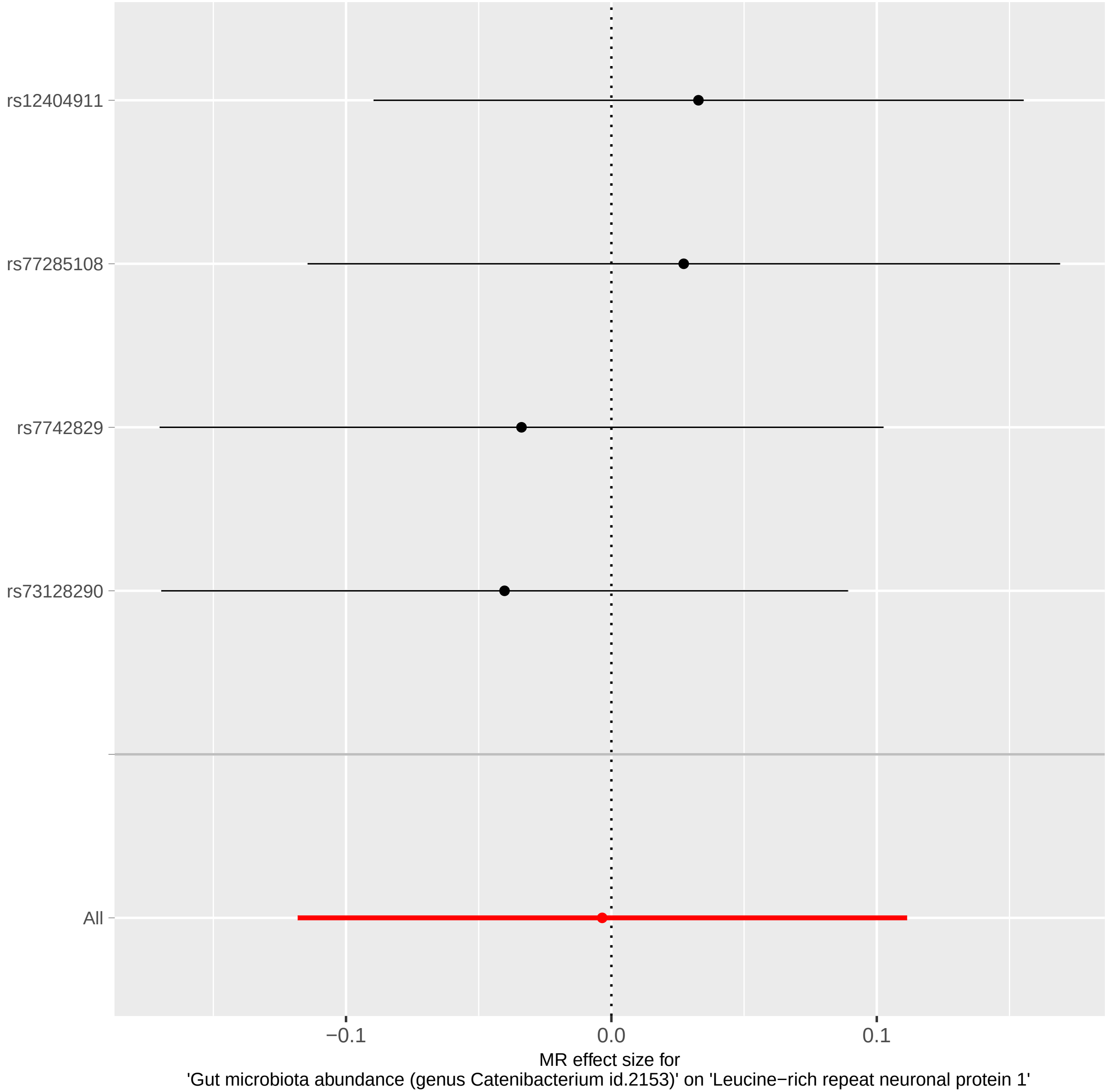

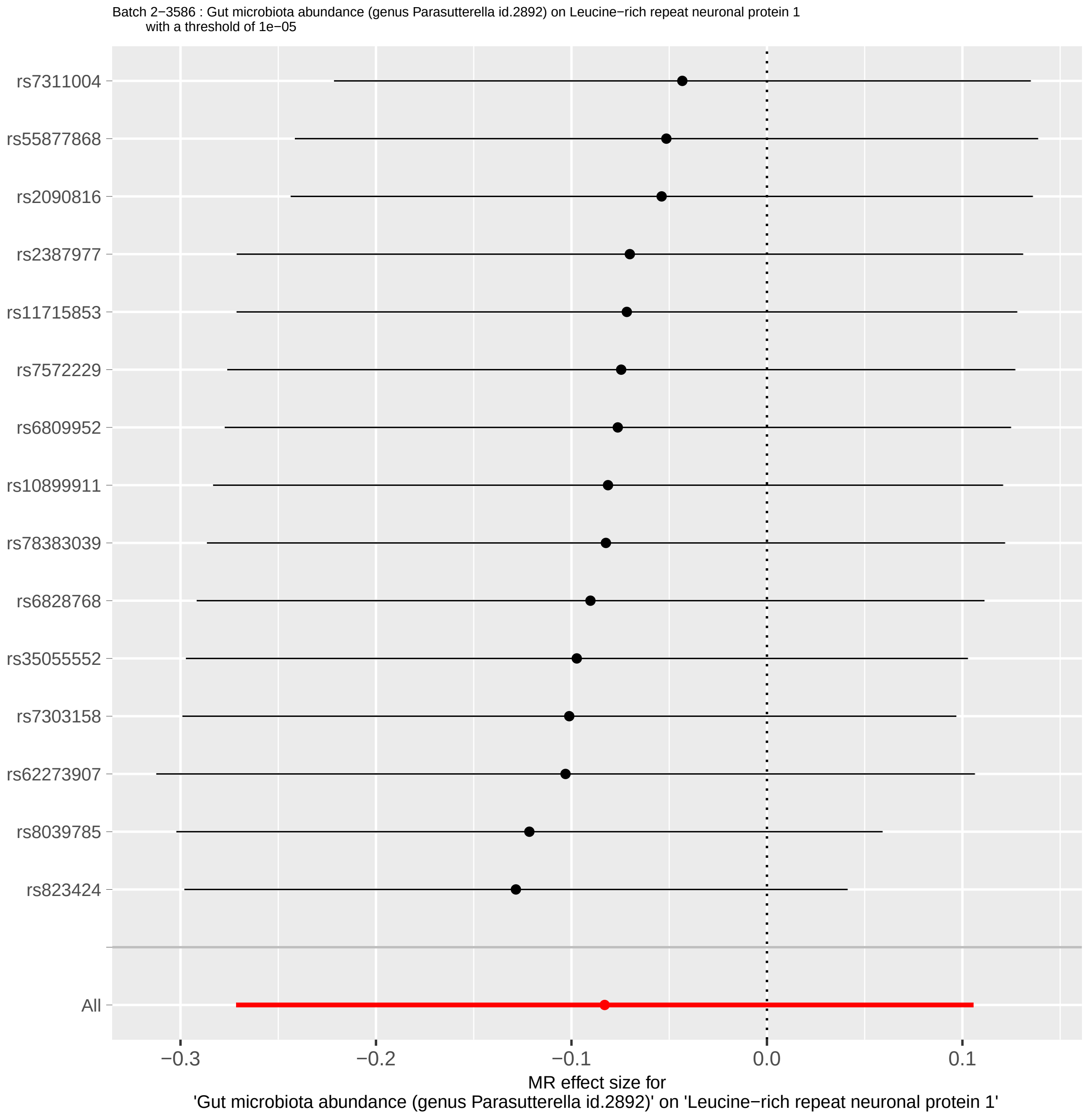
Batch 2−3586 : Gut microbiota abundance (genus Parasutterella id.2892) on Leucine−rich repeat neuronal protein 1
with a threshold of 1e−05
rs7311004
rs55877868
rs2090816
rs2387977
rs11715853
rs7572229
rs6809952
rs10899911
rs78383039
rs6828768
rs35055552
rs7303158
rs62273907
rs8039785
rs823424
All
−0.3
−0.2
−0.1
0.0
0.1
MR effect size for
'Gut microbiota abundance (genus Parasutterella id.2892)' on 'Leucine−rich repeat neuronal protein 1'

Batch 2−3587 : Gut microbiota abundance (genus Catenibacterium id.2153) on Leucine−rich repeat neuronal protein 3
with a threshold of 1e−05

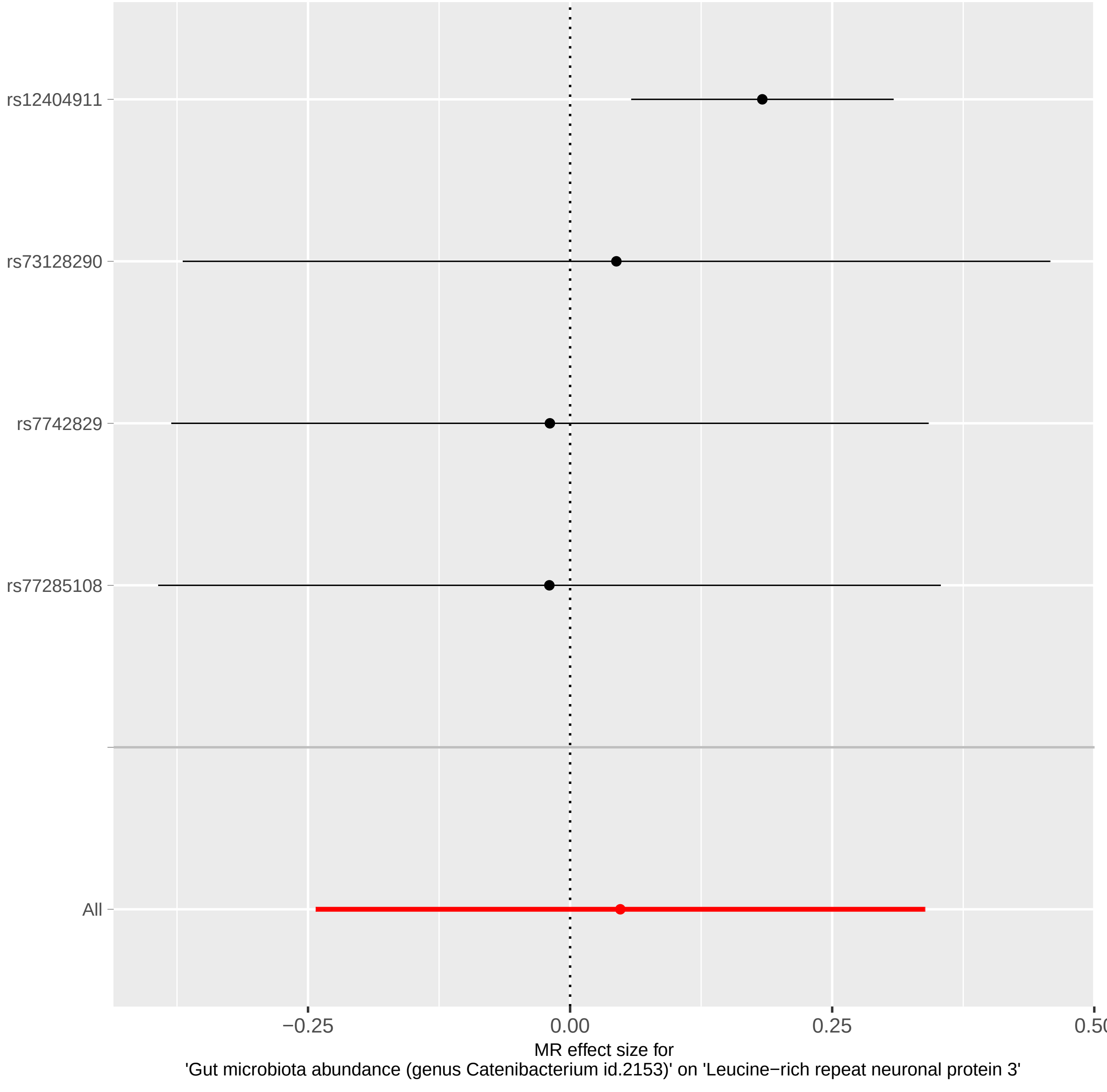

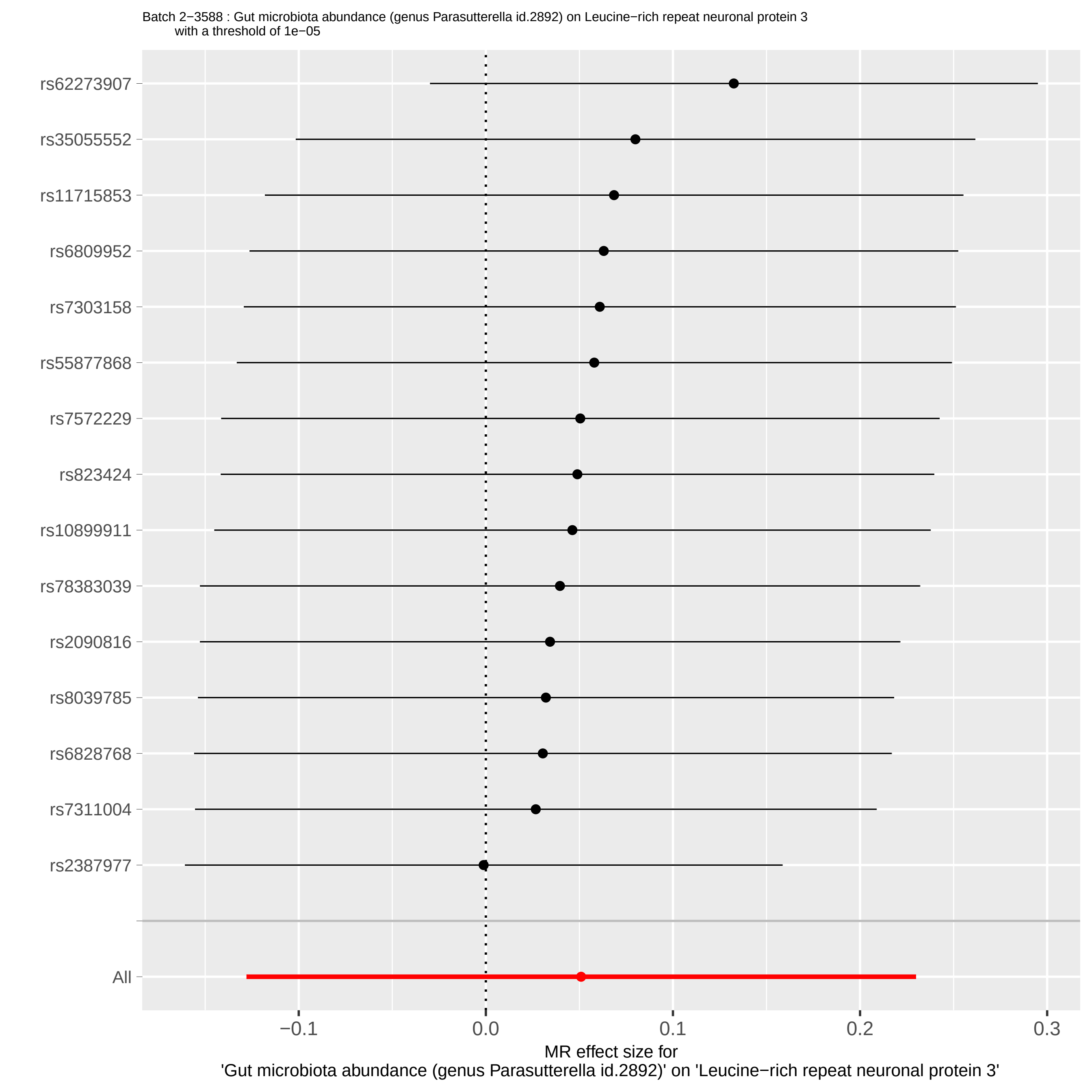

Batch 2−3588 : Gut microbiota abundance (genus Parasutterella id.2892) on Leucine−rich repeat neuronal protein 3
with a threshold of 1e−05
rs62273907
rs35055552
rs11715853
rs6809952
rs7303158
rs55877868
rs7572229
rs823424
rs10899911
rs78383039
rs2090816
rs8039785
rs6828768
rs7311004
rs2387977
All
−0.1
0.0
0.1
0.2
0.3
MR effect size for
'Gut microbiota abundance (genus Parasutterella id.2892)' on 'Leucine−rich repeat neuronal protein 3'

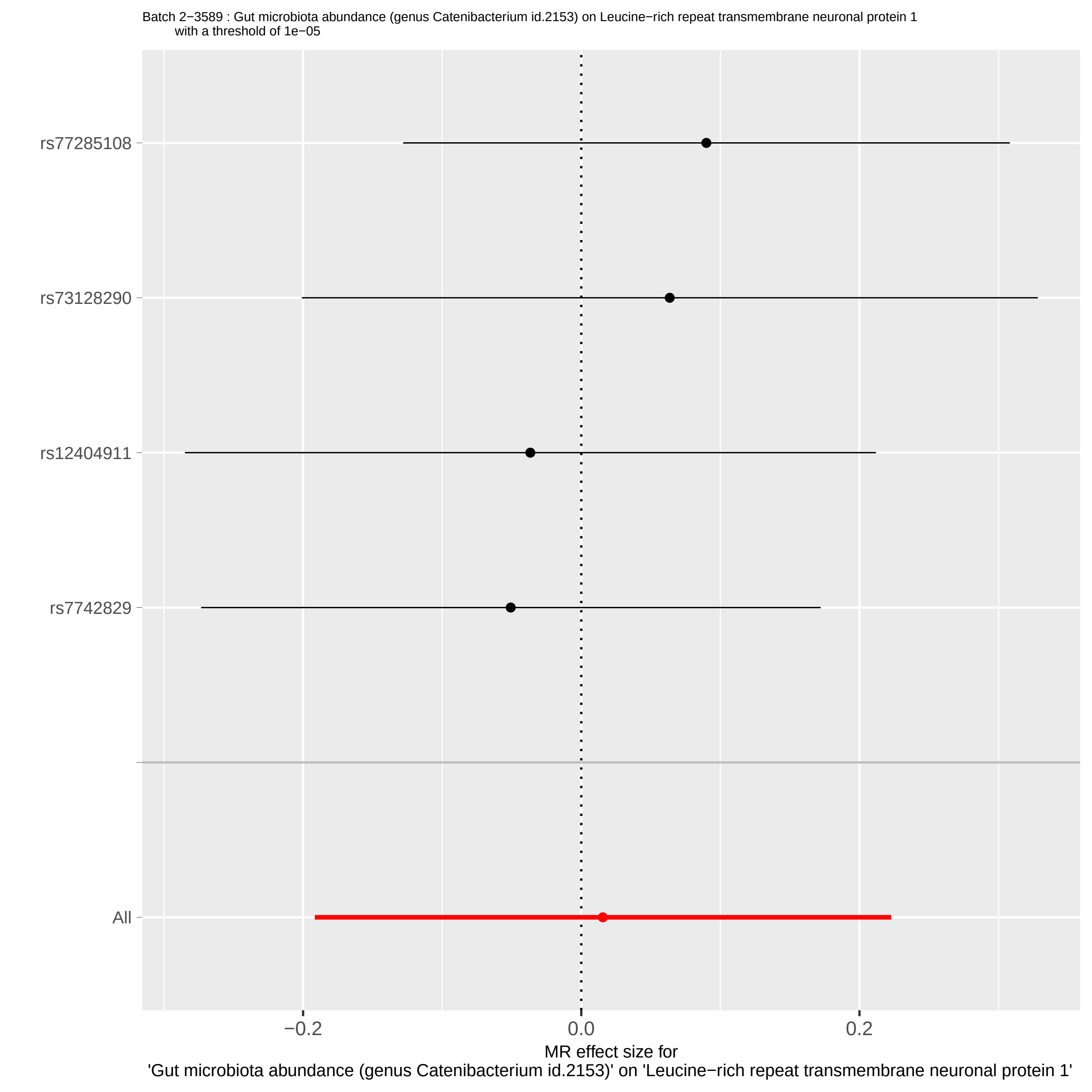
Batch 2−3589 : Gut microbiota abundance (genus Catenibacterium id.2153) on Leucine−rich repeat transmembrane neuronal protein 1
with a threshold of 1e−05
rs77285108
rs73128290
rs12404911
rs7742829
All
−0.2
0.0
0.2
MR effect size for
'Gut microbiota abundance (genus Catenibacterium id.2153)' on 'Leucine−rich repeat transmembrane neuronal protein 1'

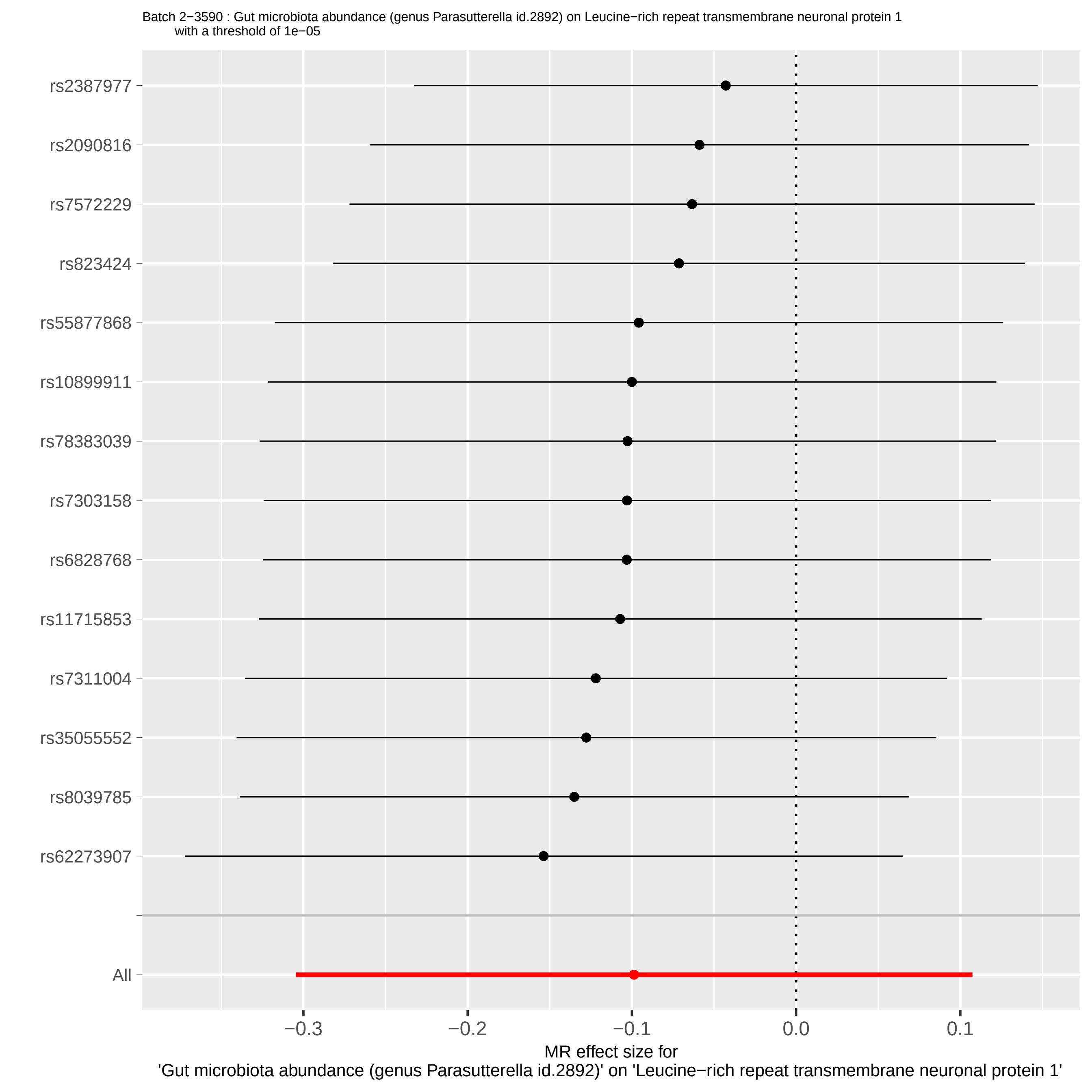
Batch 2−3590 : Gut microbiota abundance (genus Parasutterella id.2892) on Leucine−rich repeat transmembrane neuronal protein 1
with a threshold of 1e−05
rs2387977
rs2090816
rs7572229
rs823424
rs55877868
rs10899911
rs78383039
rs7303158
rs6828768
rs11715853
rs7311004
rs35055552
rs8039785
rs62273907
All
−0.3
−0.2
−0.1
0.0
0.1
MR effect size for
'Gut microbiota abundance (genus Parasutterella id.2892)' on 'Leucine−rich repeat transmembrane neuronal protein 1'

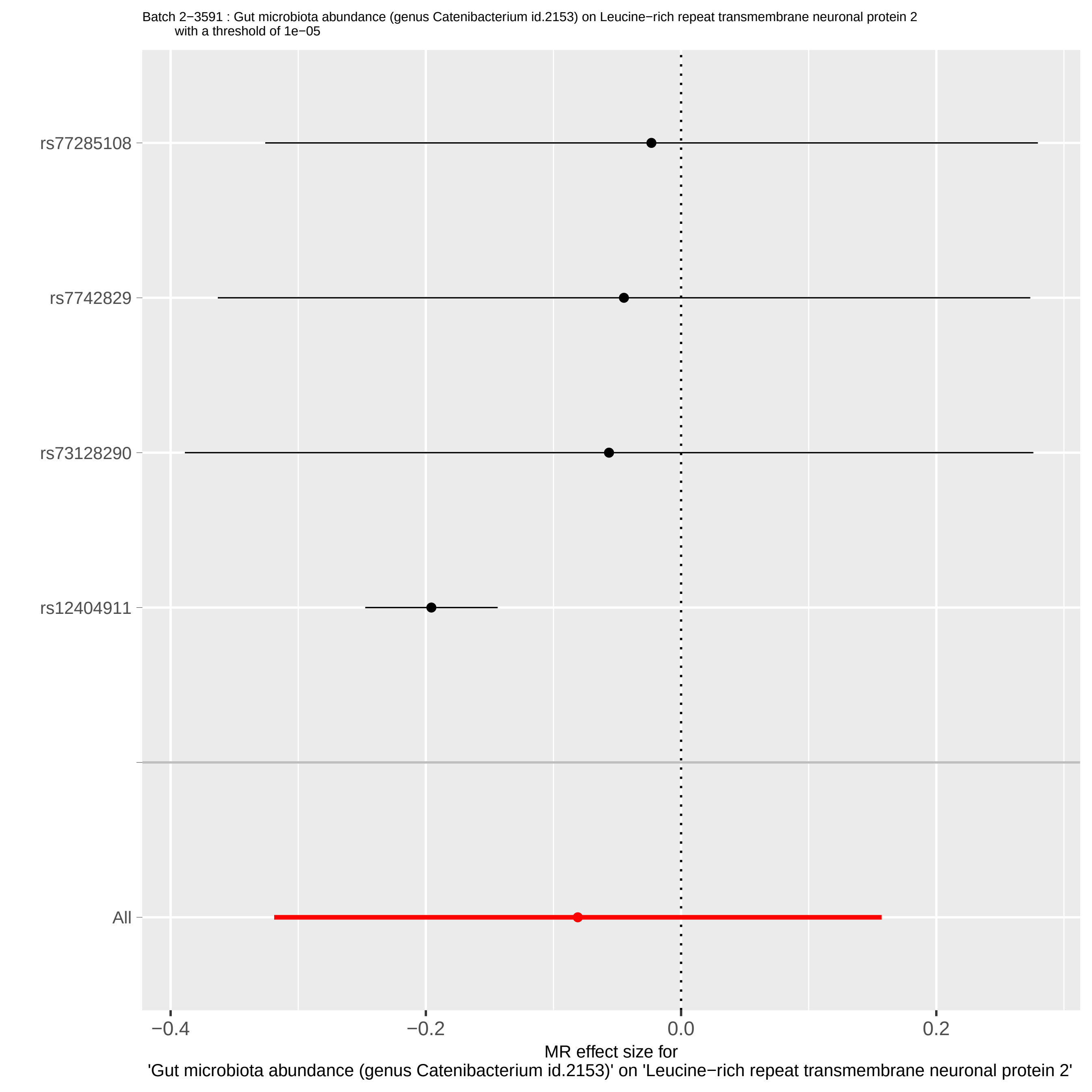

Batch 2−3591 : Gut microbiota abundance (genus Catenibacterium id.2153) on Leucine−rich repeat transmembrane neuronal protein 2
with a threshold of 1e−05
rs77285108
rs7742829
rs73128290
rs12404911
All
−0.4
−0.2
0.0
0.2
MR effect size for
'Gut microbiota abundance (genus Catenibacterium id.2153)' on 'Leucine−rich repeat transmembrane neuronal protein 2'

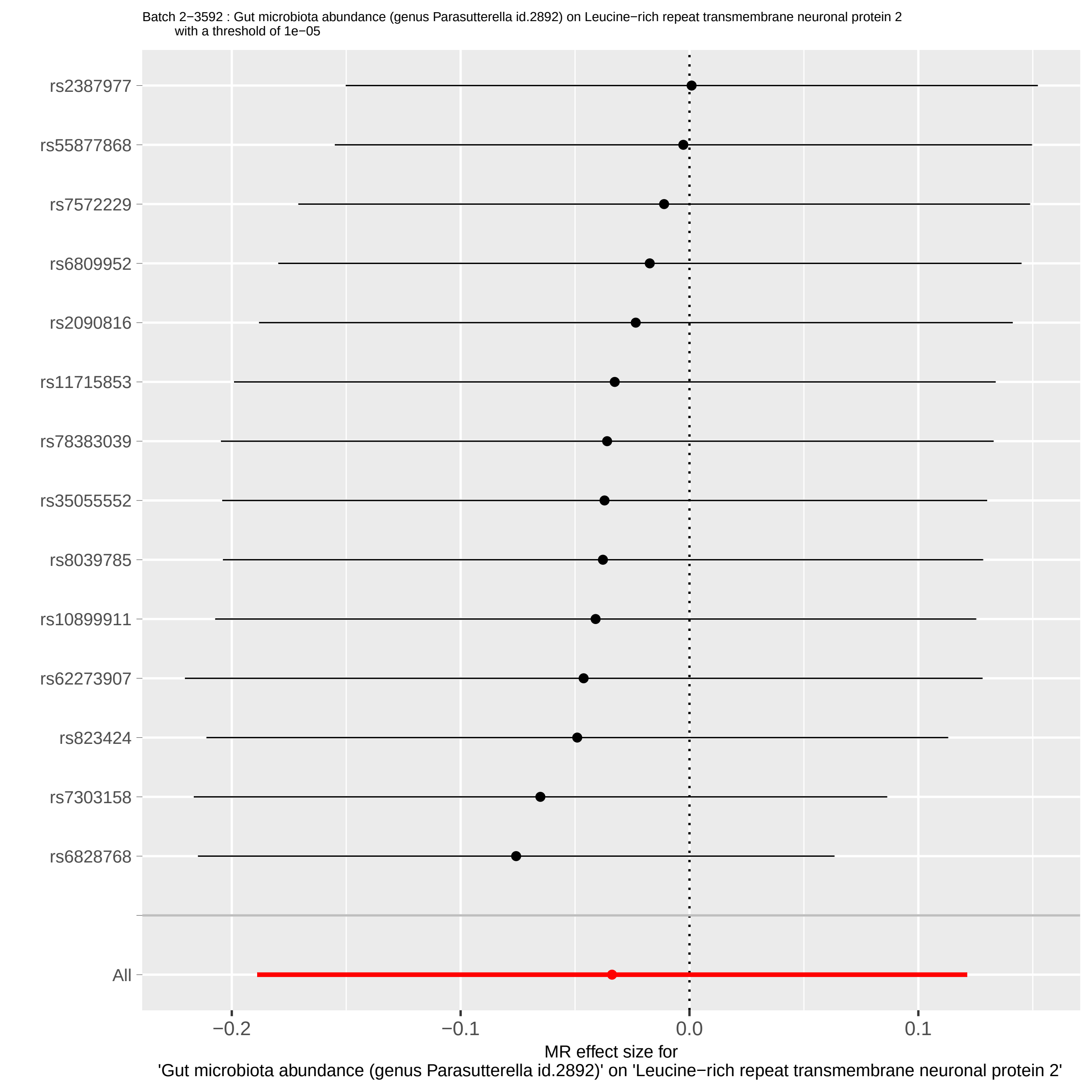

Batch 2−3592 : Gut microbiota abundance (genus Parasutterella id.2892) on Leucine−rich repeat transmembrane neuronal protein 2
with a threshold of 1e−05
rs2387977
rs55877868
rs7572229
rs6809952
rs2090816
rs11715853
rs78383039
rs35055552
rs8039785
rs10899911
rs62273907
rs823424
rs7303158
rs6828768
All
−0.2
−0.1
0.0
0.1
MR effect size for
'Gut microbiota abundance (genus Parasutterella id.2892)' on 'Leucine−rich repeat transmembrane neuronal protein 2'

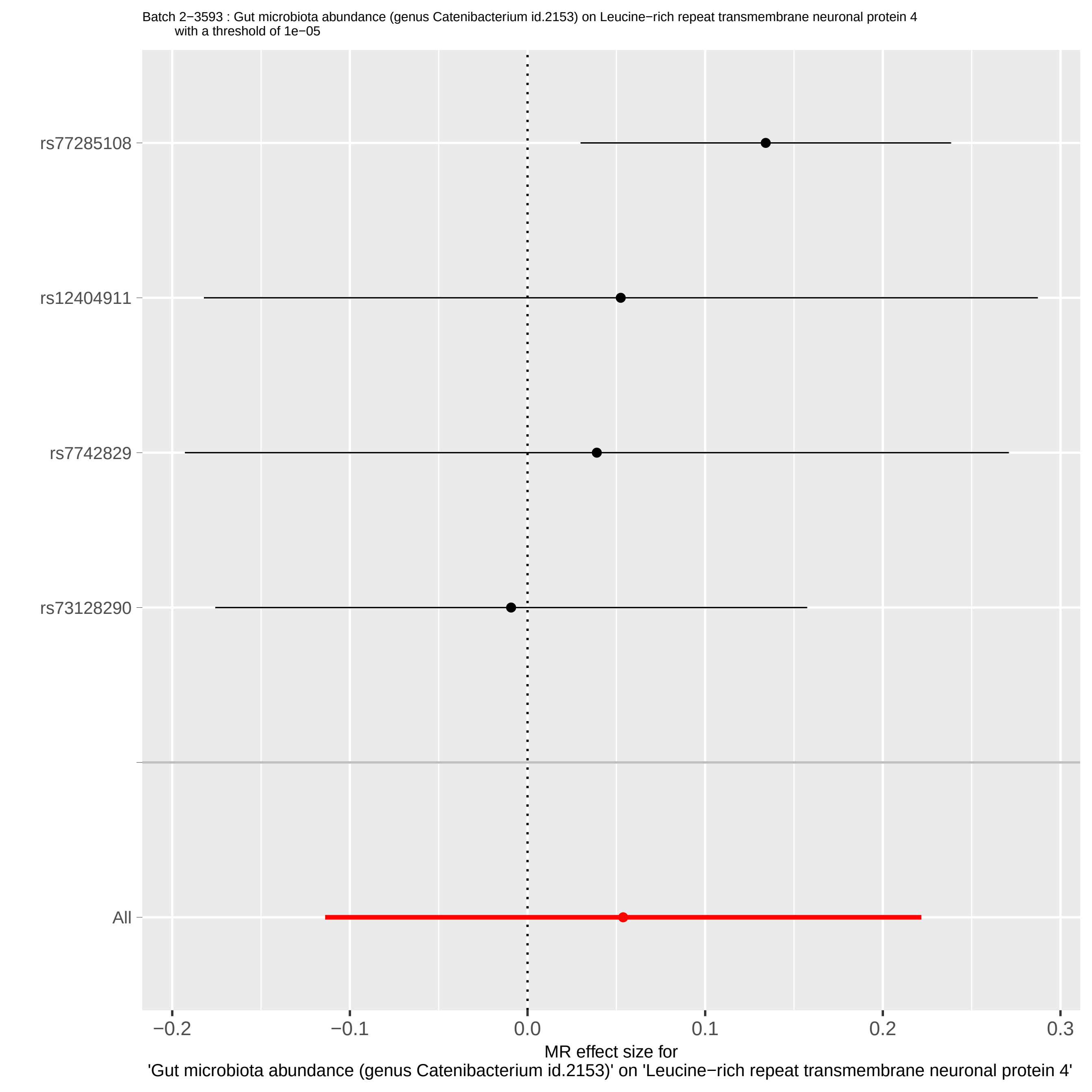

Batch 2−3593 : Gut microbiota abundance (genus Catenibacterium id.2153) on Leucine−rich repeat transmembrane neuronal protein 4
with a threshold of 1e−05
rs77285108
rs12404911
rs7742829
rs73128290
All
−0.2
−0.1
0.0
0.1
0.2
0.3
MR effect size for
'Gut microbiota abundance (genus Catenibacterium id.2153)' on 'Leucine−rich repeat transmembrane neuronal protein 4'

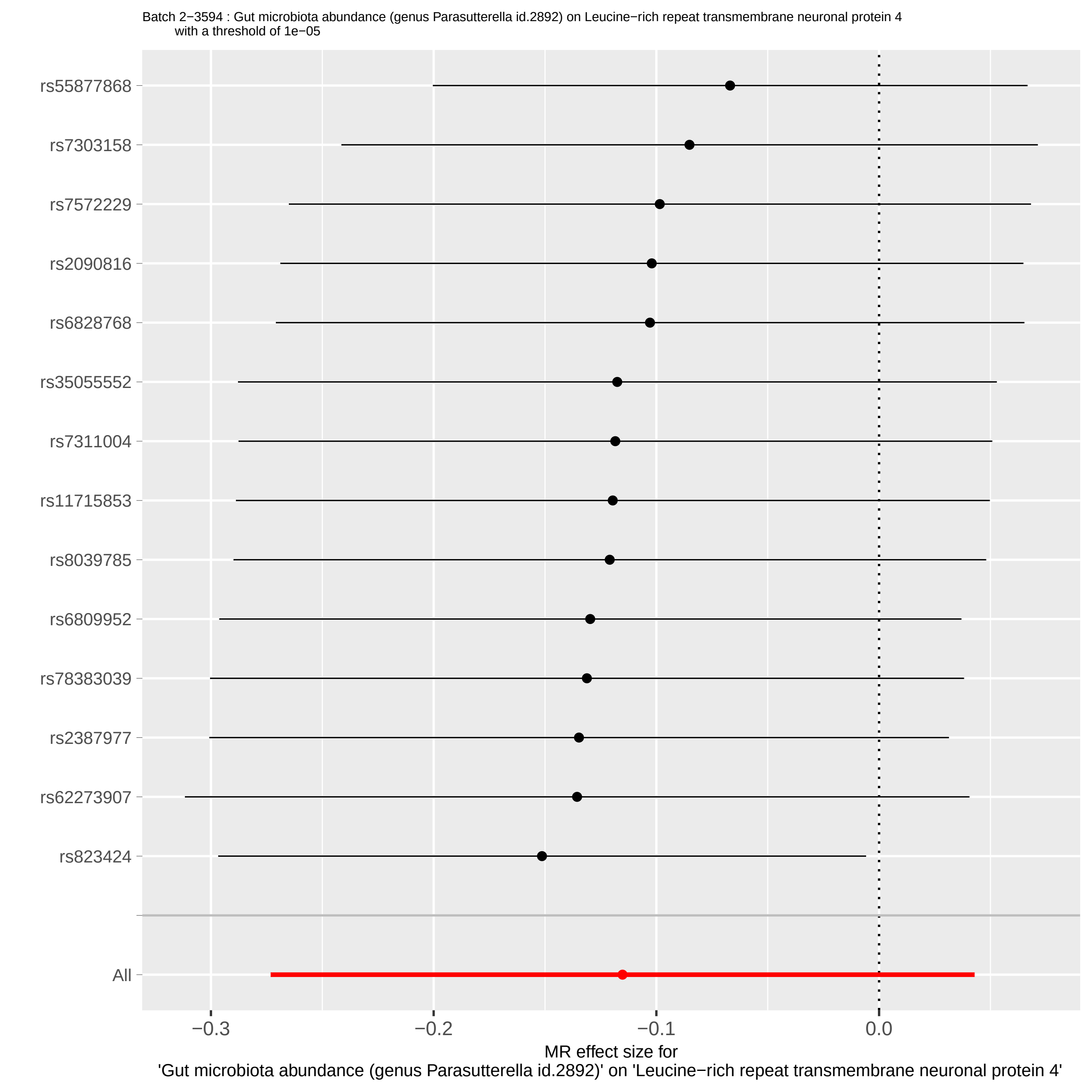
Batch 2−3594 : Gut microbiota abundance (genus Parasutterella id.2892) on Leucine−rich repeat transmembrane neuronal protein 4
with a threshold of 1e−05
rs55877868
rs7303158
rs7572229
rs2090816
rs6828768
rs35055552
rs7311004
rs11715853
rs8039785
rs6809952
rs78383039
rs2387977
rs62273907
rs823424
All
−0.3
−0.2
−0.1
0.0
MR effect size for
'Gut microbiota abundance (genus Parasutterella id.2892)' on 'Leucine−rich repeat transmembrane neuronal protein 4'

Batch 2−3595 : Gut microbiota abundance (genus Catenibacterium id.2153) on Leucine−rich repeat transmembrane neuronal protein 4
with a threshold of 1e−05

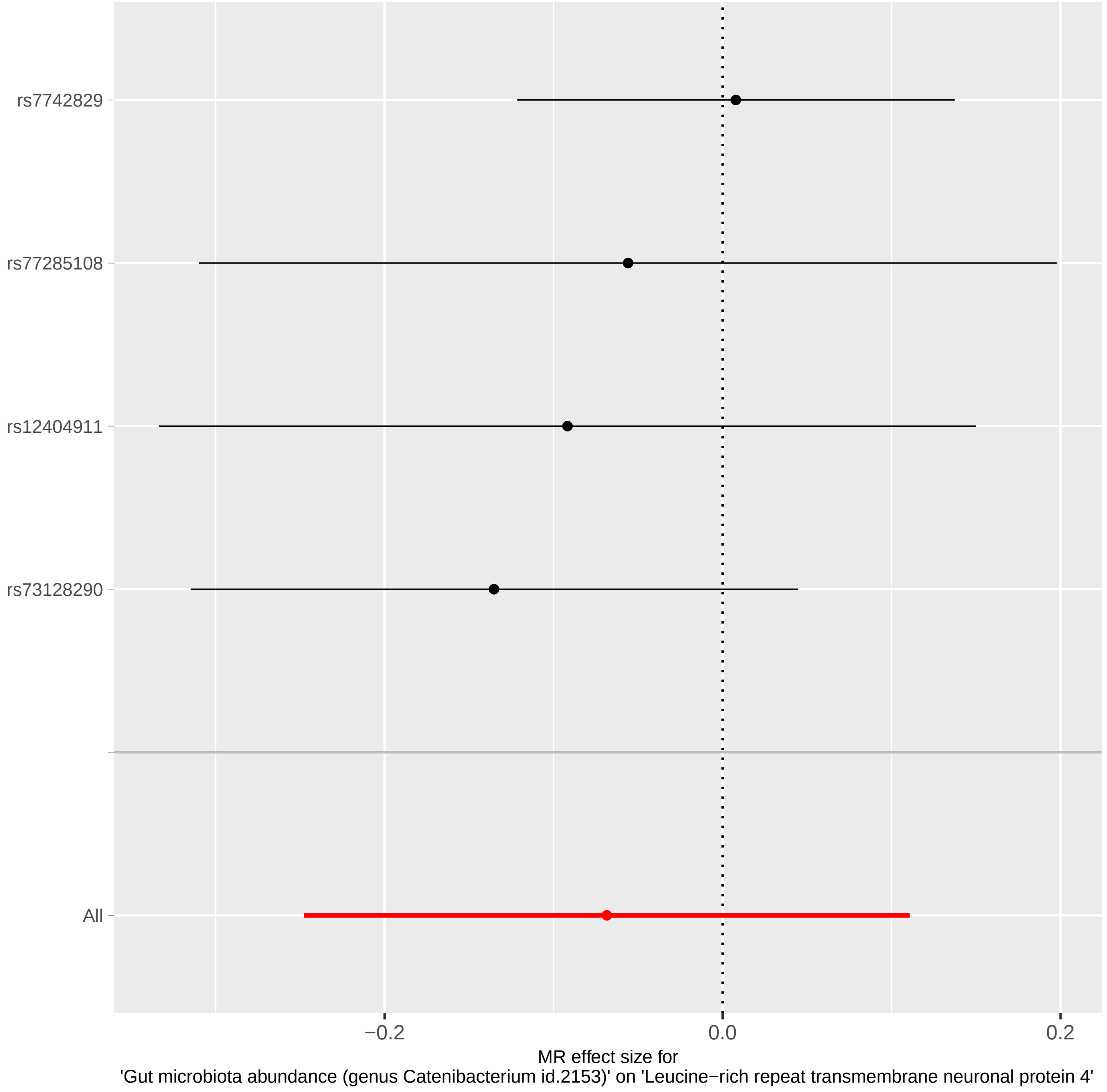

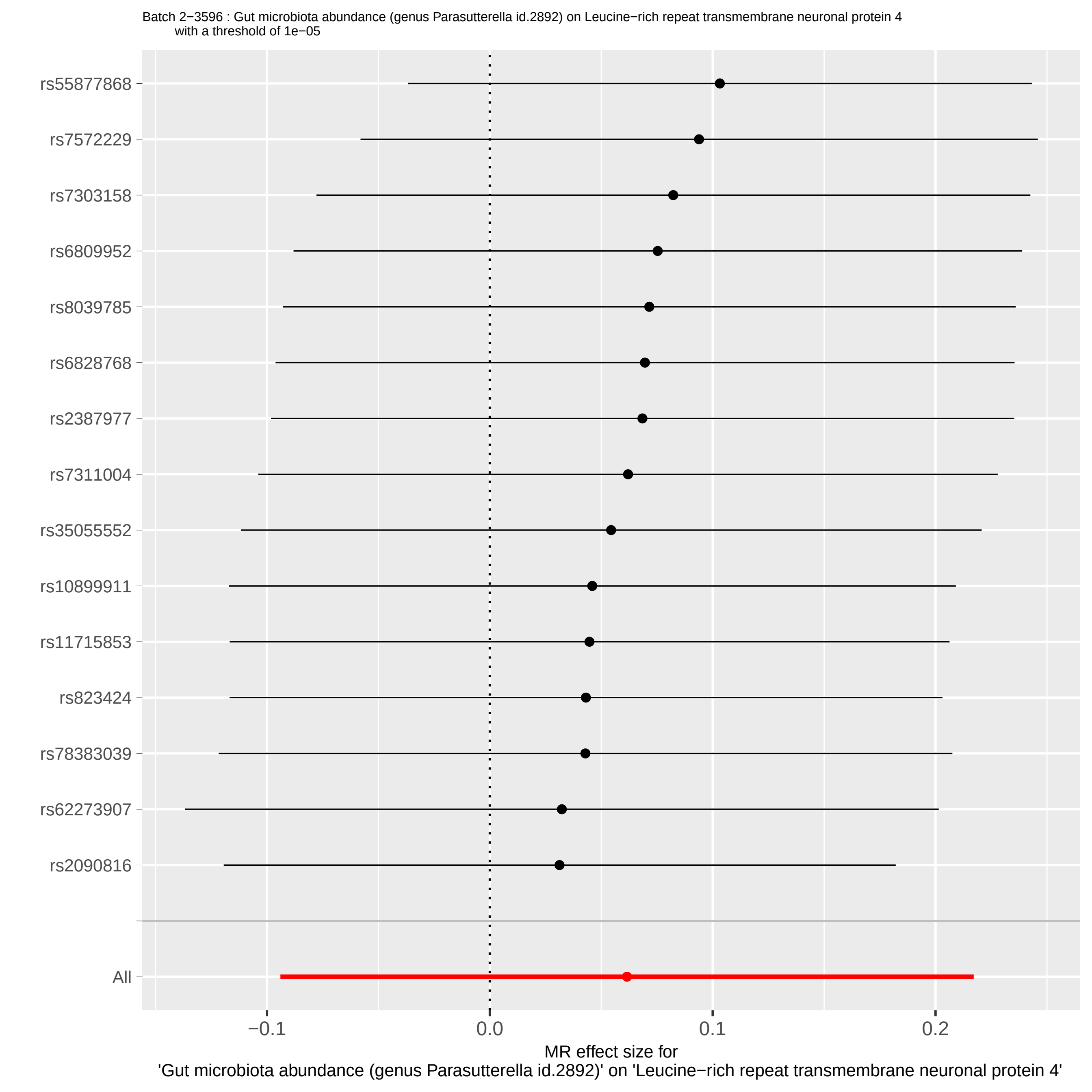

Batch 2−3596 : Gut microbiota abundance (genus Parasutterella id.2892) on Leucine−rich repeat transmembrane neuronal protein 4
with a threshold of 1e−05
rs55877868
rs7572229
rs7303158
rs6809952
rs8039785
rs6828768
rs2387977
rs7311004
rs35055552
rs10899911
rs11715853
rs823424
rs78383039
rs62273907
rs2090816
All
−0.1
0.0
0.1
0.2
MR effect size for
'Gut microbiota abundance (genus Parasutterella id.2892)' on 'Leucine−rich repeat transmembrane neuronal protein 4'

Batch 2−3597 : Gut microbiota abundance (genus Catenibacterium id.2153) on Leucine−rich repeat and transmembrane domain−containing protein 1
with a threshold of 1e−05

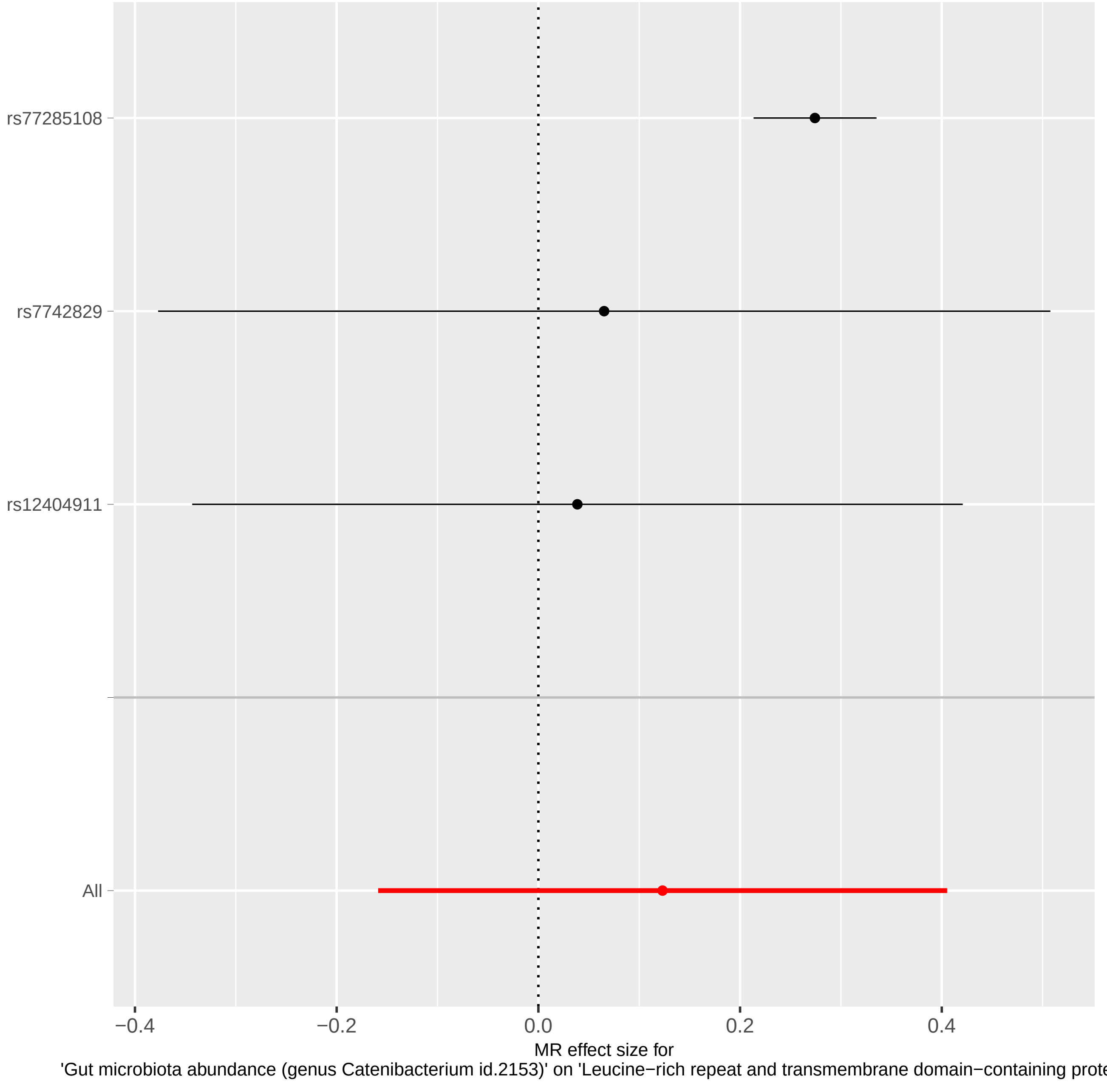

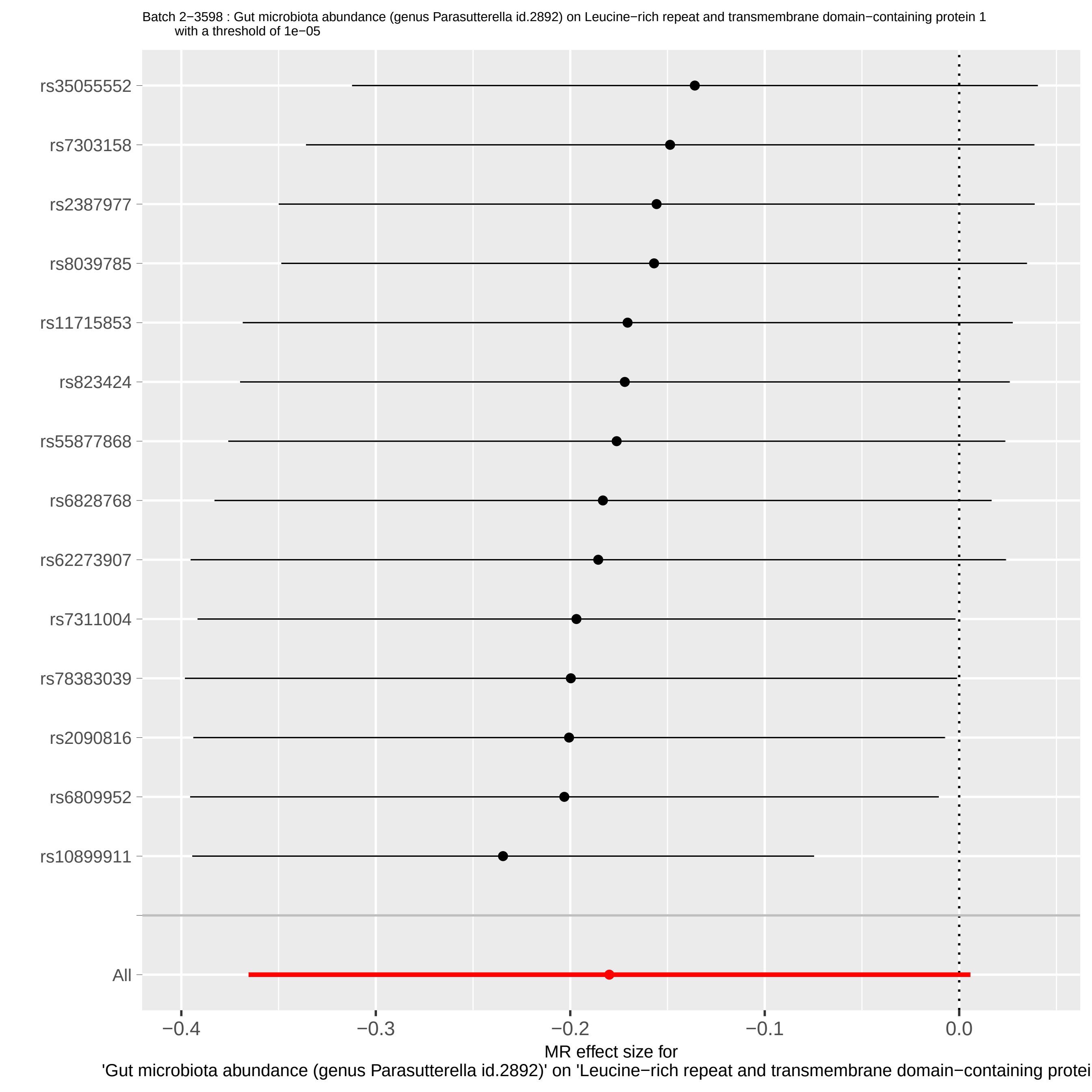

Batch 2−3598 : Gut microbiota abundance (genus Parasutterella id.2892) on Leucine−rich repeat and transmembrane domain−containing protein 1 with a threshold of 1e−05
rs35055552
rs7303158
rs2387977
rs8039785
rs11715853
rs823424
rs55877868
rs6828768
rs62273907
rs7311004
rs78383039
rs2090816
rs6809952
rs10899911
All
−0.4
−0.3
−0.2
−0.1
0.0
MR effect size for
'Gut microbiota abundance (genus Parasutterella id.2892)' on 'Leucine−rich repeat and transmembrane domain−containing protei

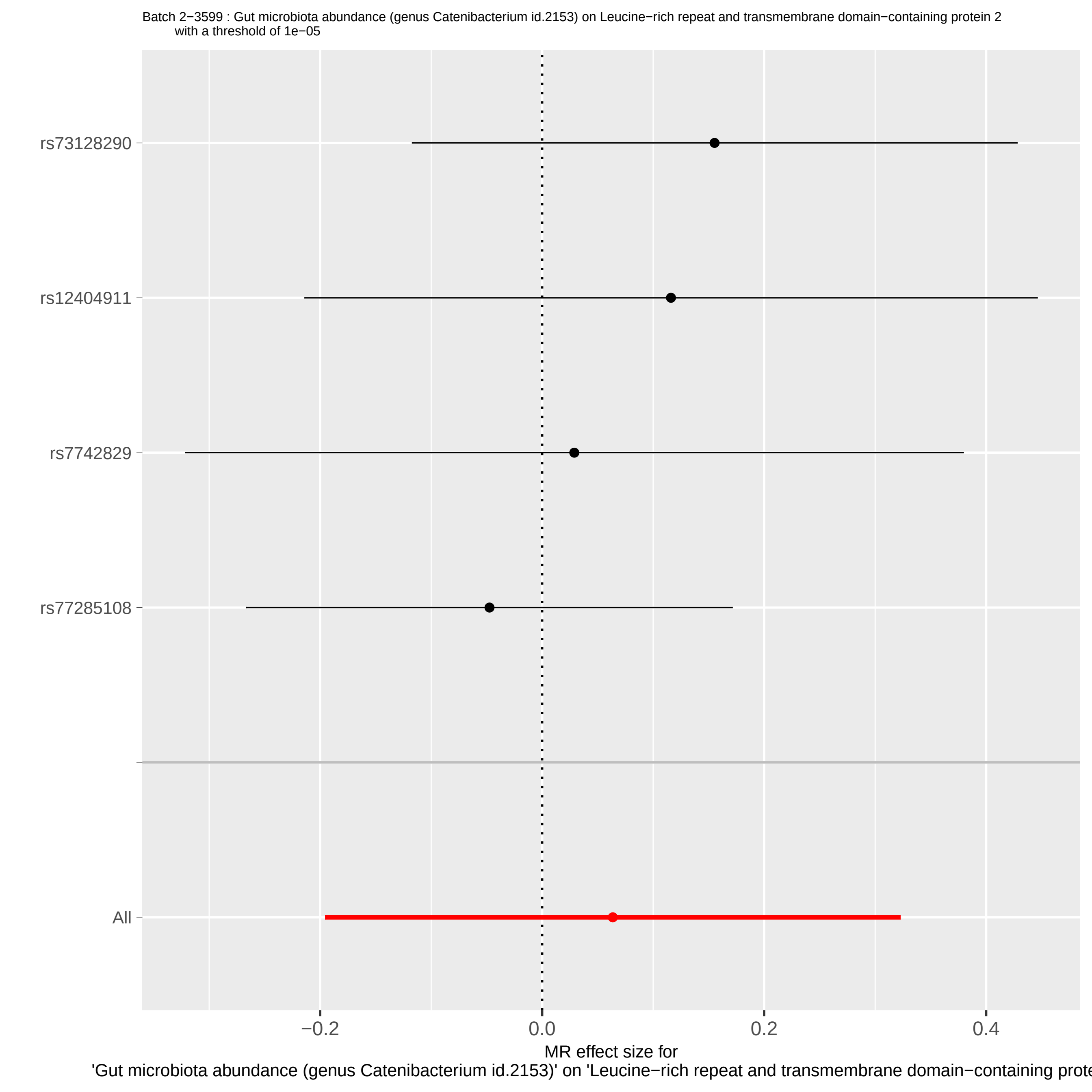
Batch 2−3599 : Gut microbiota abundance (genus Catenibacterium id.2153) on Leucine−rich repeat and transmembrane domain−containing protein 2
with a threshold of 1e−05
rs73128290
rs12404911
rs7742829
rs77285108
All
−0.2
0.0
0.2
0.4
MR effect size for
'Gut microbiota abundance (genus Catenibacterium id.2153)' on 'Leucine−rich repeat and transmembrane domain−containing prote

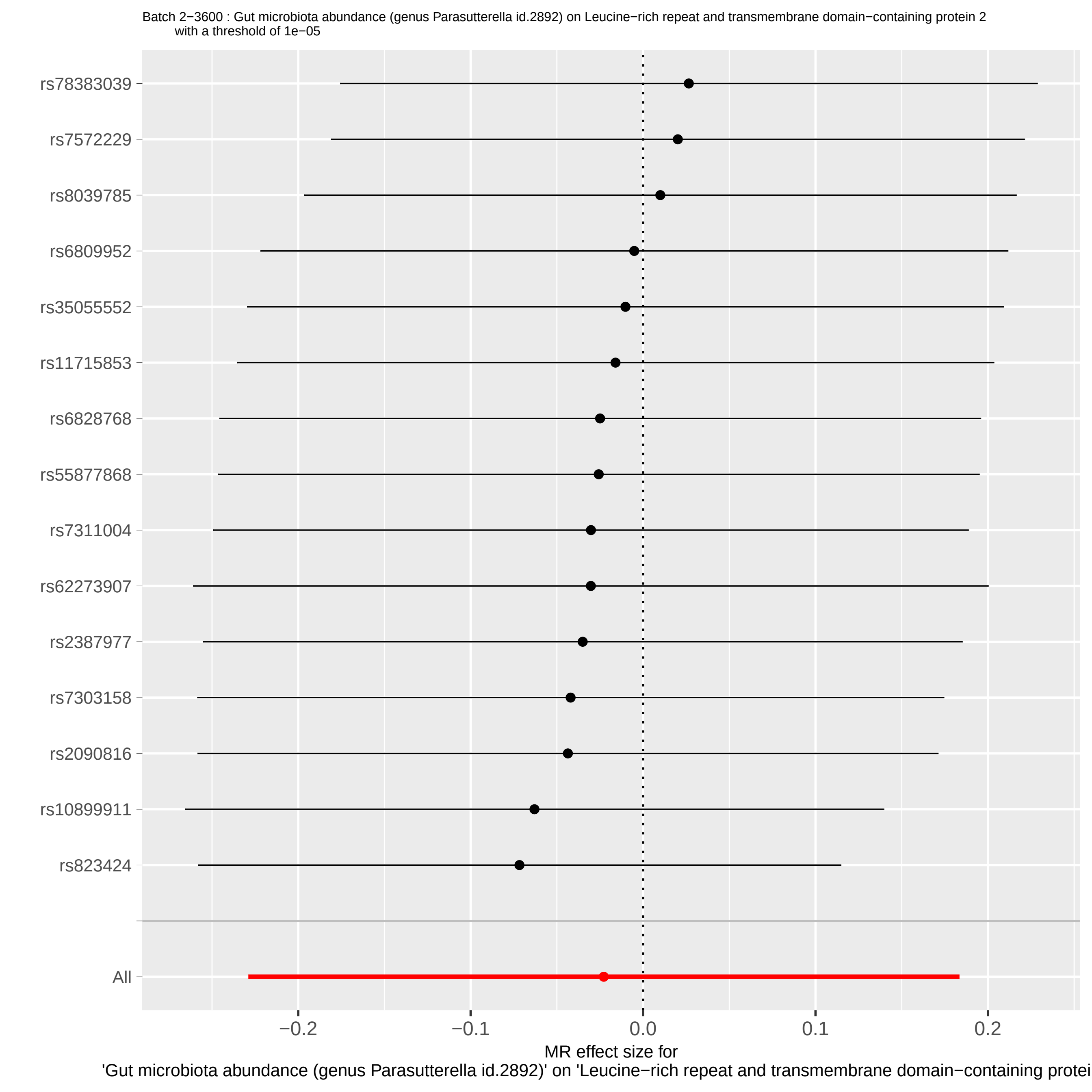

Batch 2−3600 : Gut microbiota abundance (genus Parasutterella id.2892) on Leucine−rich repeat and transmembrane domain−containing protein 2
with a threshold of 1e−05
rs78383039
rs7572229
rs8039785
rs6809952
rs35055552
rs11715853
rs6828768
rs55877868
rs7311004
rs62273907
rs2387977
rs7303158
rs2090816
rs10899911
rs823424
All
−0.2
−0.1
0.0
0.1
0.2
MR effect size for
'Gut microbiota abundance (genus Parasutterella id.2892)' on 'Leucine−rich repeat and transmembrane domain−containing protei

Batch 2−3601 : Gut microbiota abundance (genus Catenibacterium id.2153) on Limbic system−associated membrane protein
with a threshold of 1e−05

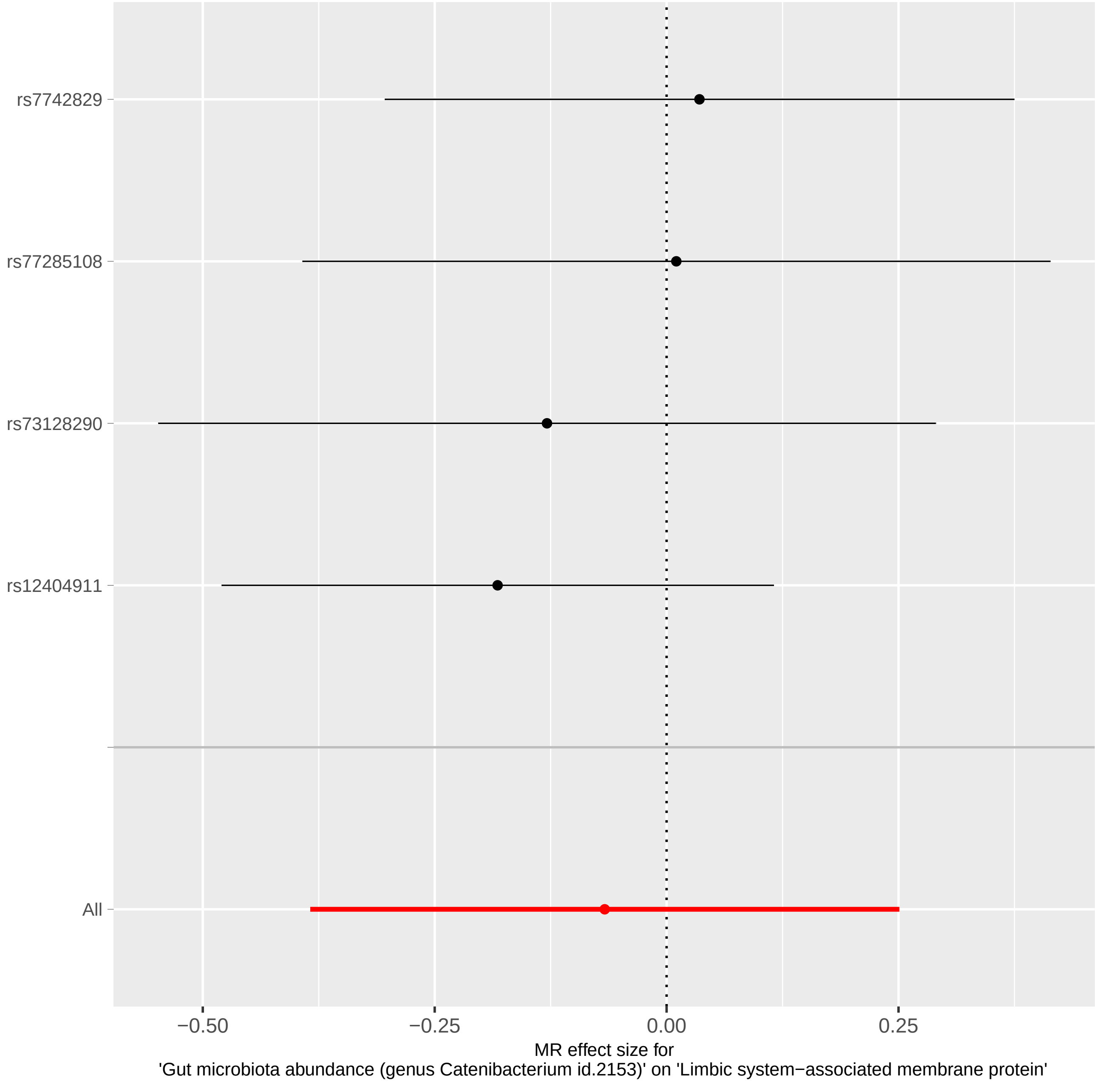

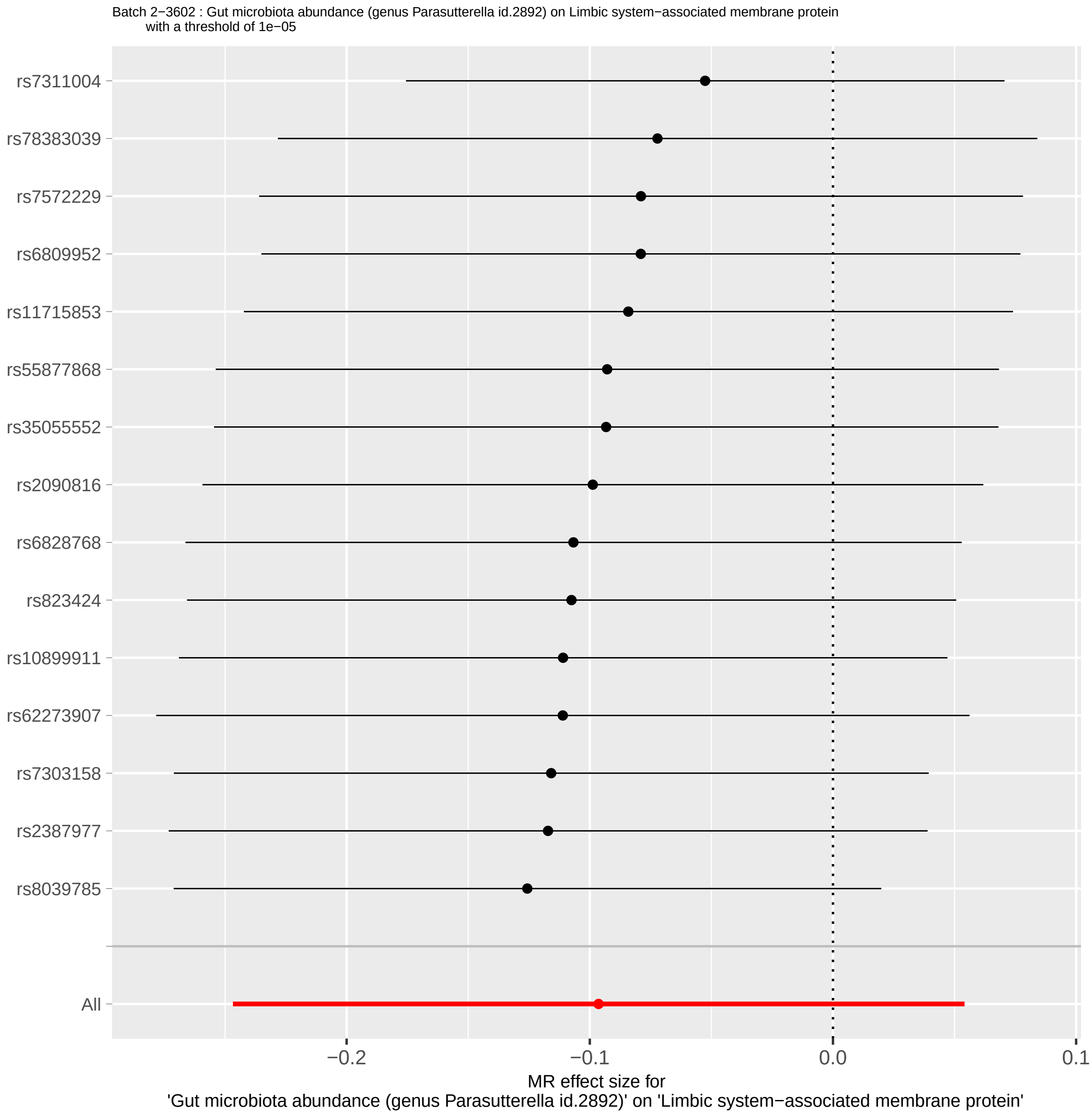

Batch 2−3602 : Gut microbiota abundance (genus Parasutterella id.2892) on Limbic system−associated membrane protein
with a threshold of 1e−05
rs7311004
rs78383039
rs7572229
rs6809952
rs11715853
rs55877868
rs35055552
rs2090816
rs6828768
rs823424
rs10899911
rs62273907
rs7303158
rs2387977
rs8039785
All
−0.2
−0.1
0.0
0.1
MR effect size for
'Gut microbiota abundance (genus Parasutterella id.2892)' on 'Limbic system−associated membrane protein'

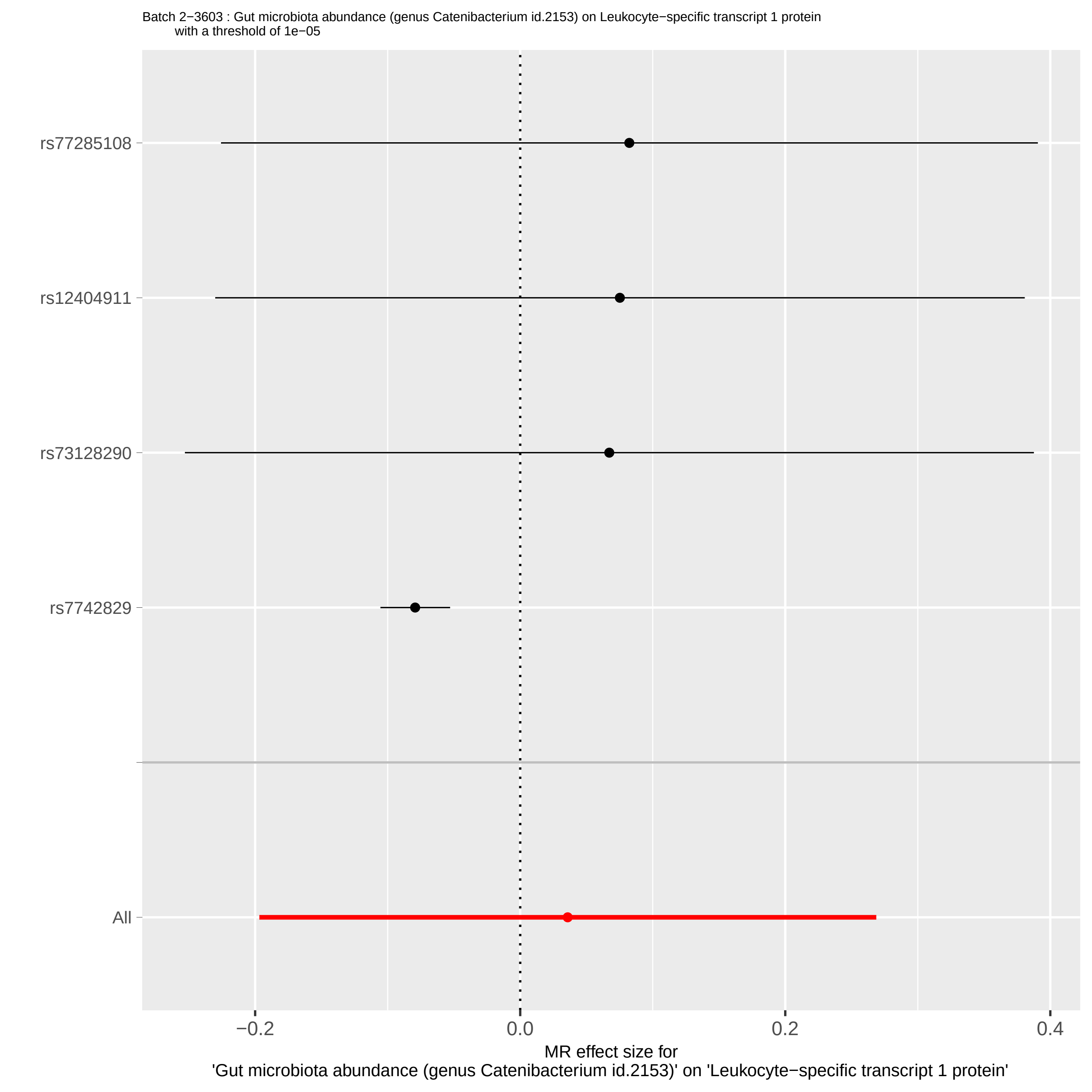

Batch 2−3603 : Gut microbiota abundance (genus Catenibacterium id.2153) on Leukocyte−specific transcript 1 protein
with a threshold of 1e−05
rs77285108
rs12404911
rs73128290
rs7742829
All
−0.2
0.0
0.2
0.4
MR effect size for
'Gut microbiota abundance (genus Catenibacterium id.2153)' on 'Leukocyte−specific transcript 1 protein'

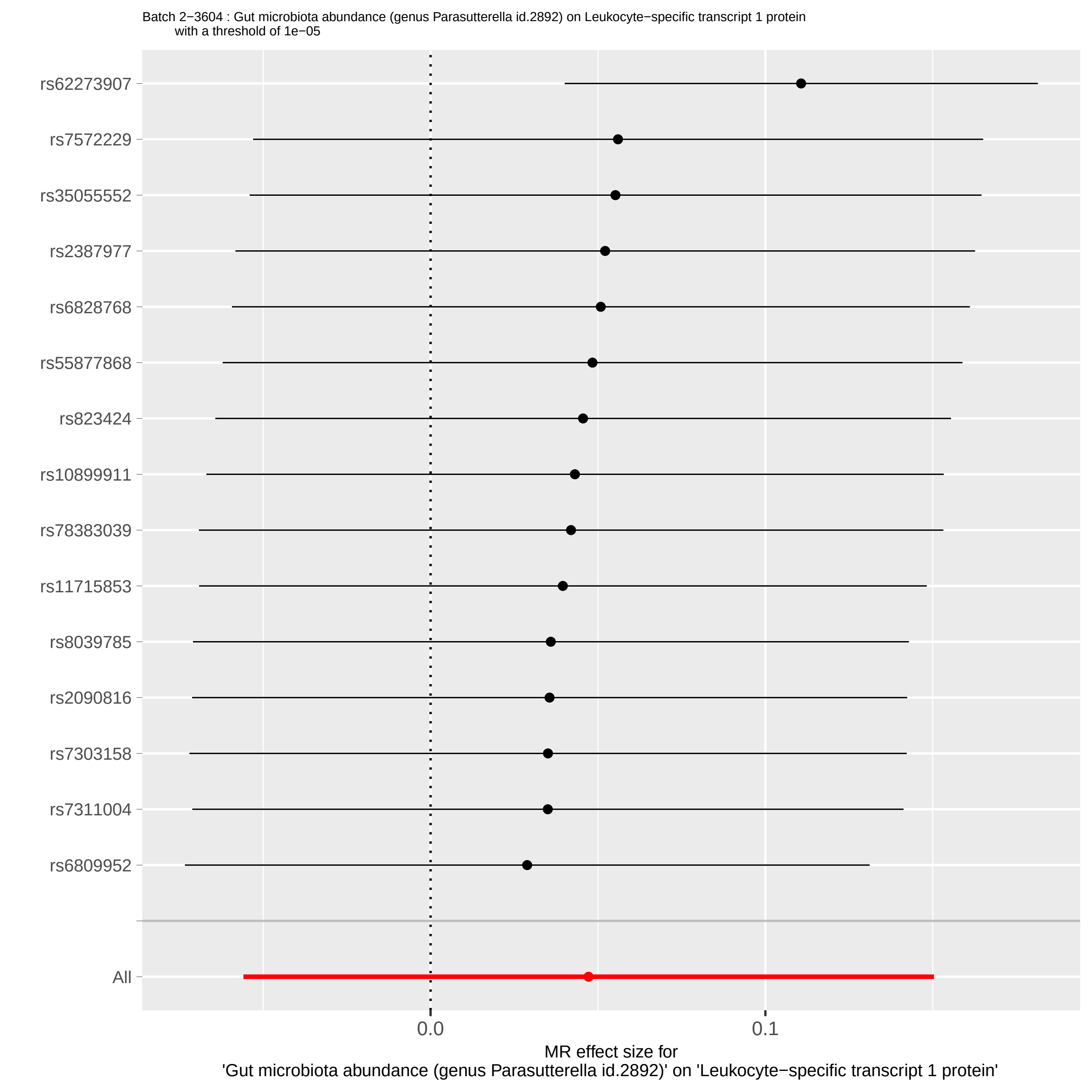
Batch 2−3604 : Gut microbiota abundance (genus Parasutterella id.2892) on Leukocyte−specific transcript 1 protein
with a threshold of 1e−05
rs62273907
rs7572229
rs35055552
rs2387977
rs6828768
rs55877868
rs823424
rs10899911
rs78383039
rs11715853
rs8039785
rs2090816
rs7303158
rs7311004
rs6809952
All
0.0
0.1
MR effect size for
'Gut microbiota abundance (genus Parasutterella id.2892)' on 'Leukocyte−specific transcript 1 protein'

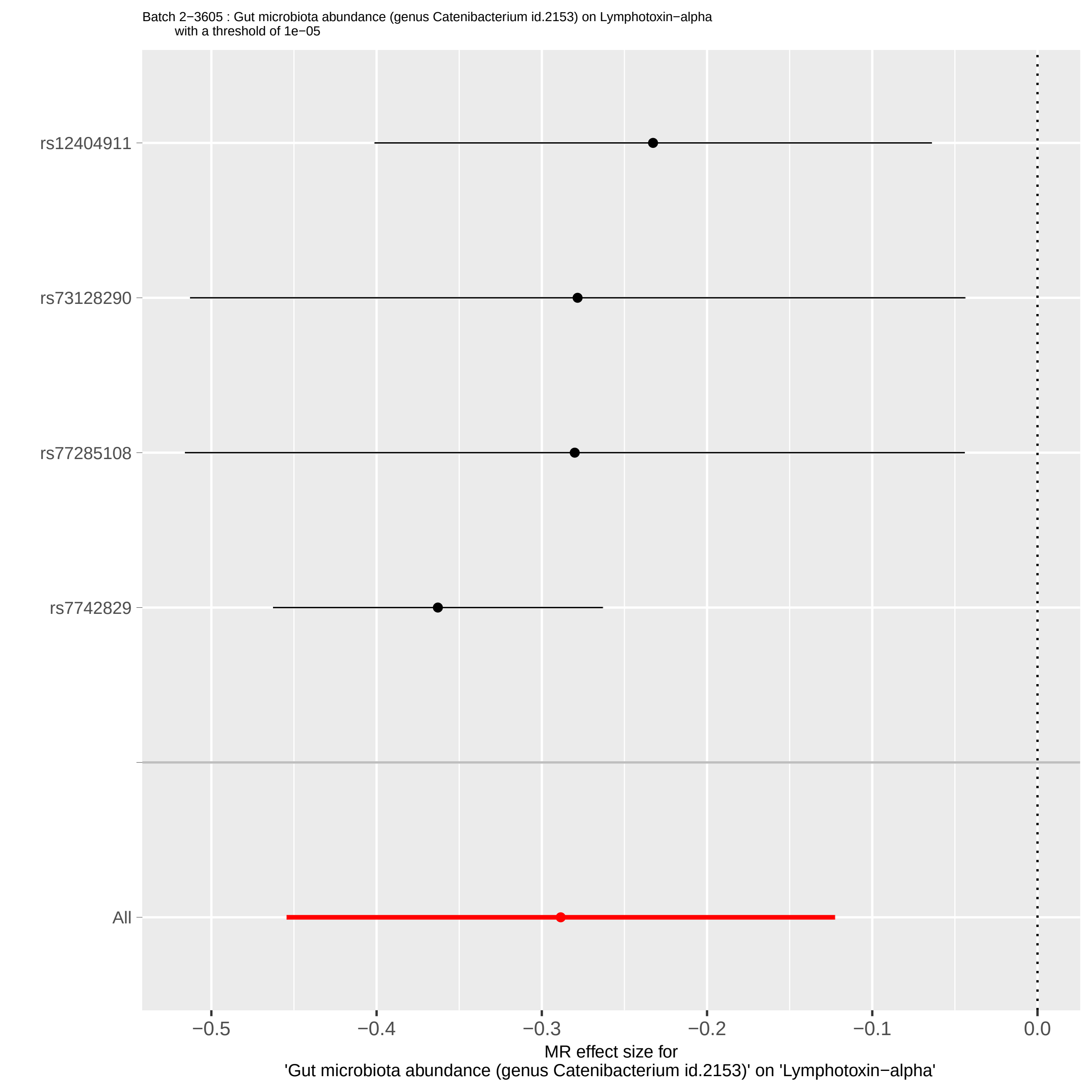
Batch 2−3605 : Gut microbiota abundance (genus Catenibacterium id.2153) on Lymphotoxin−alpha
with a threshold of 1e−05
rs12404911
rs73128290
rs77285108
rs7742829
All
−0.5
−0.4
−0.3
−0.2
−0.1
0.0
MR effect size for
'Gut microbiota abundance (genus Catenibacterium id.2153)' on 'Lymphotoxin−alpha'

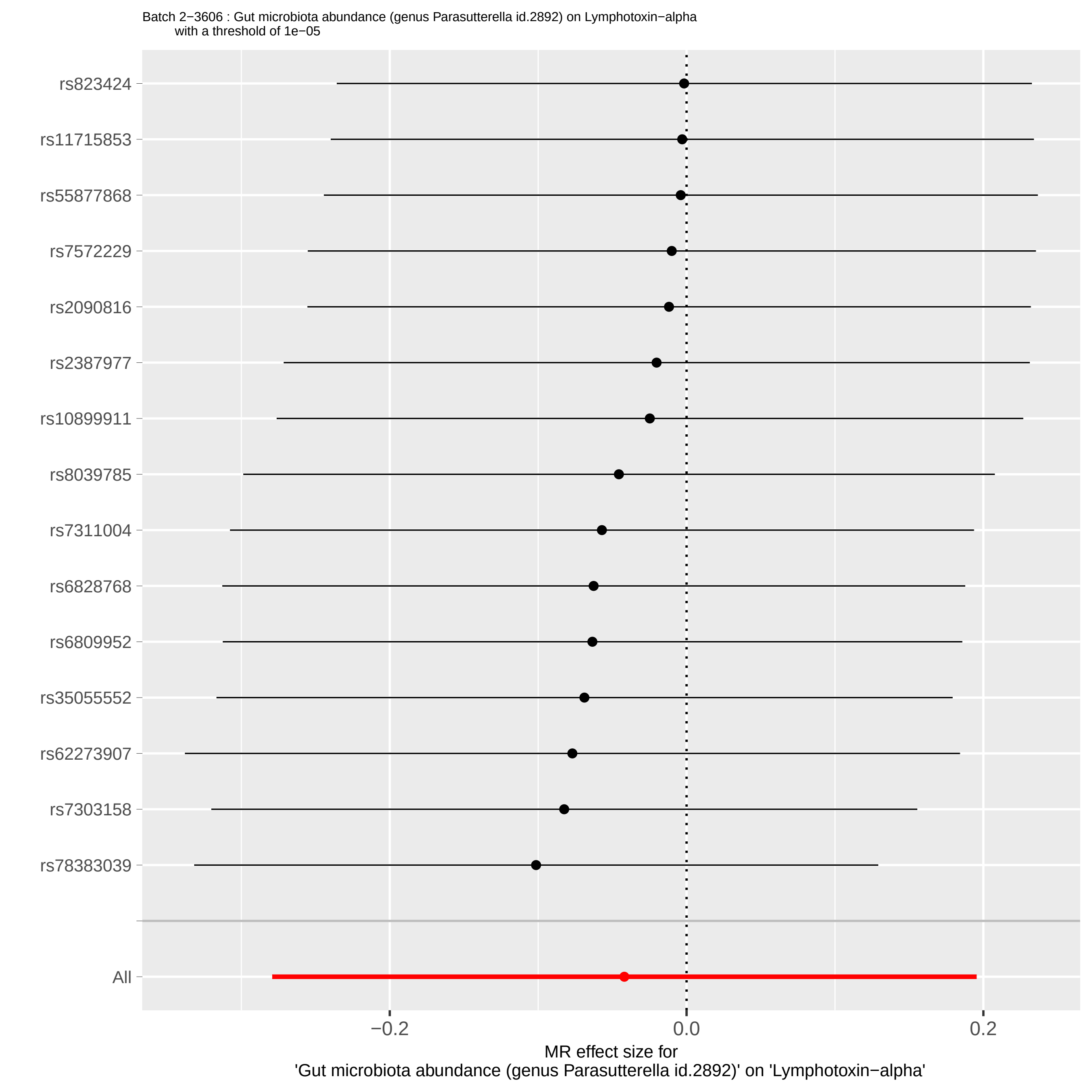
Batch 2−3606 : Gut microbiota abundance (genus Parasutterella id.2892) on Lymphotoxin−alpha
with a threshold of 1e−05
rs823424
rs11715853
rs55877868
rs7572229
rs2090816
rs2387977
rs10899911
rs8039785
rs7311004
rs6828768
rs6809952
rs35055552
rs62273907
rs7303158
rs78383039
All
−0.2
0.0
0.2
MR effect size for
'Gut microbiota abundance (genus Parasutterella id.2892)' on 'Lymphotoxin−alpha'

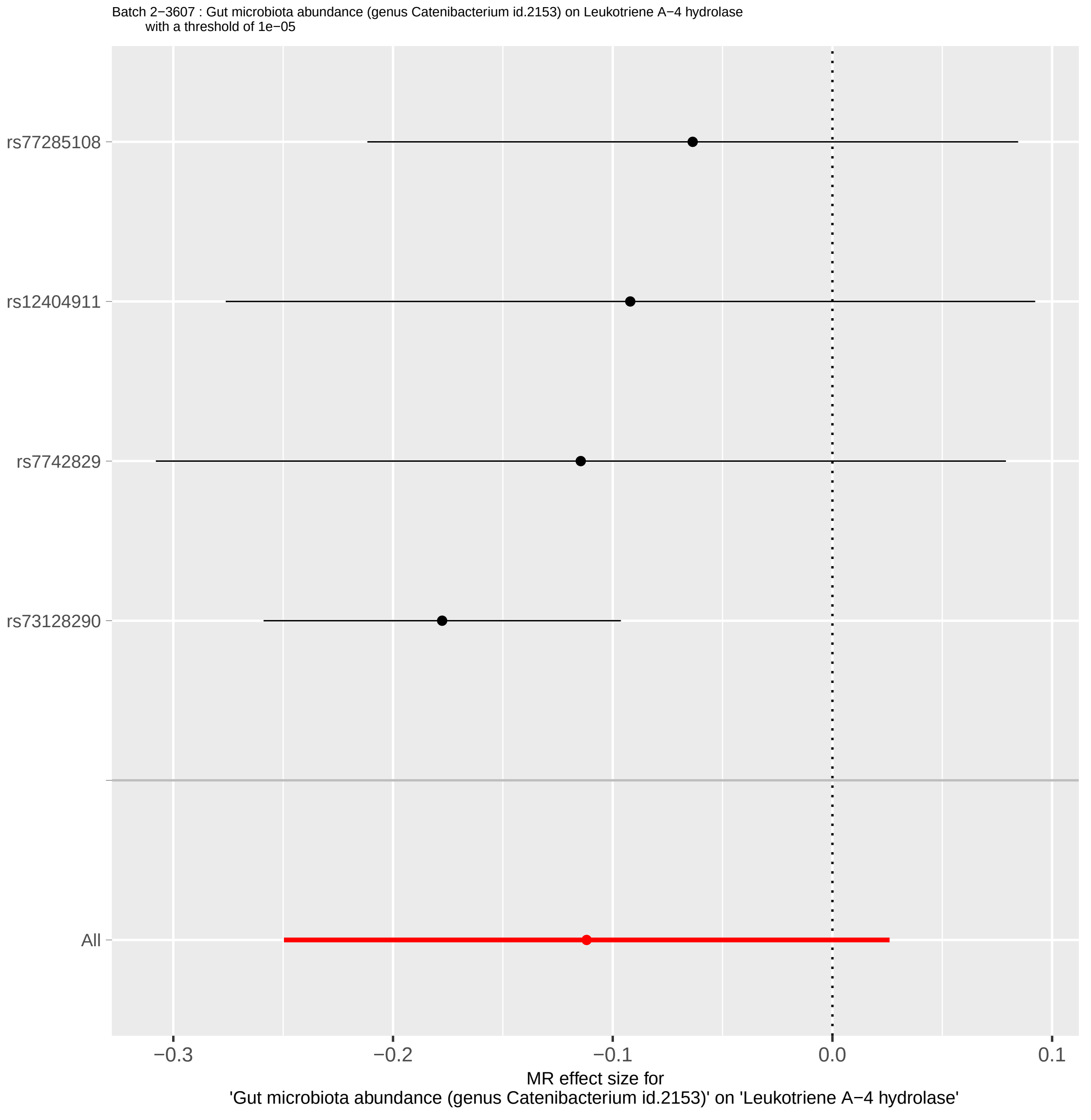

Batch 2−3607 : Gut microbiota abundance (genus Catenibacterium id.2153) on Leukotriene A−4 hydrolase
with a threshold of 1e−05
rs77285108
rs12404911
rs7742829
rs73128290
All
−0.3
−0.2
−0.1
0.0
0.1
MR effect size for
'Gut microbiota abundance (genus Catenibacterium id.2153)' on 'Leukotriene A−4 hydrolase'

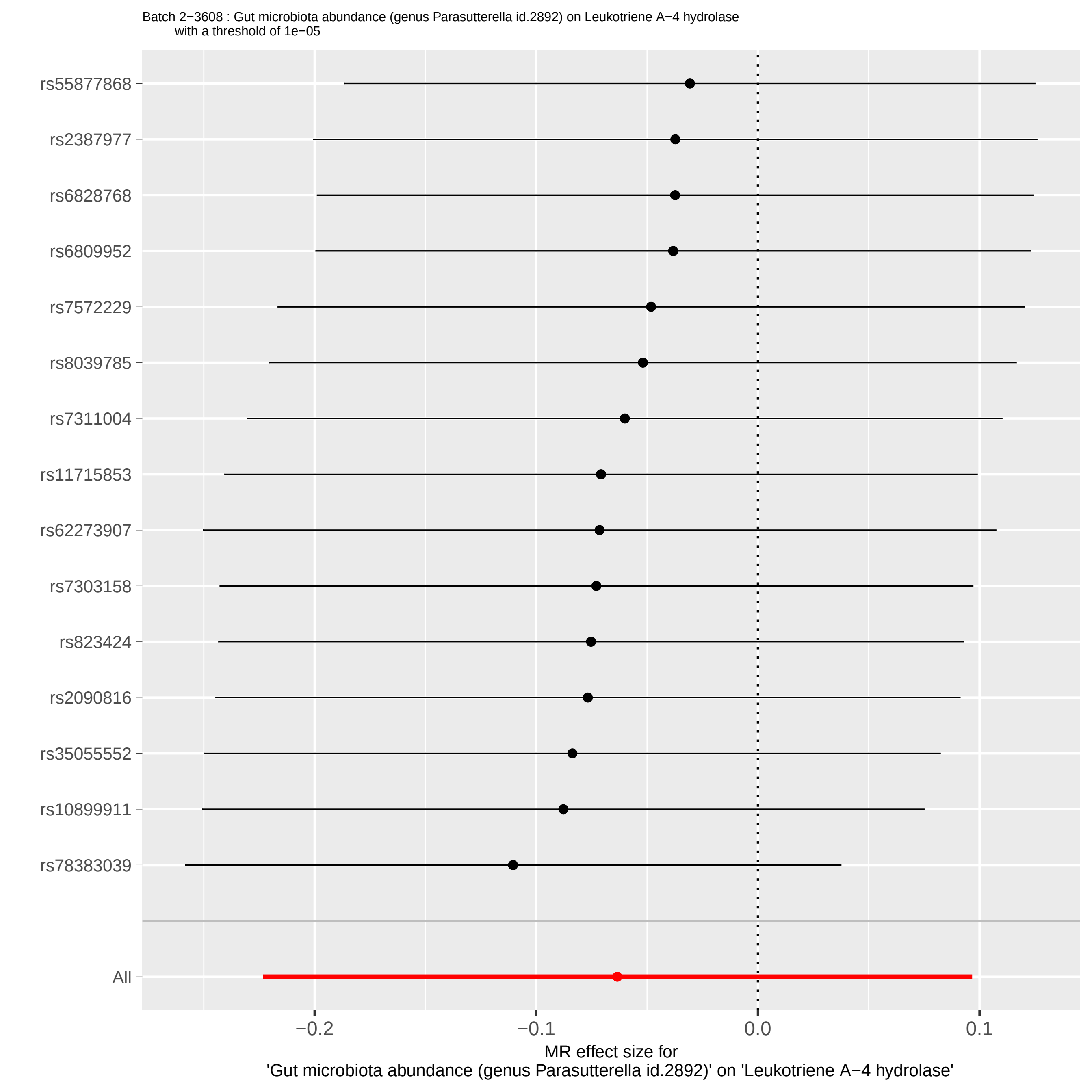
Batch 2−3608 : Gut microbiota abundance (genus Parasutterella id.2892) on Leukotriene A−4 hydrolase
with a threshold of 1e−05
rs55877868
rs2387977
rs6828768
rs6809952
rs7572229
rs8039785
rs7311004
rs11715853
rs62273907
rs7303158
rs823424
rs2090816
rs35055552
rs10899911
rs78383039
All
−0.2
−0.1
0.0
0.1
MR effect size for
'Gut microbiota abundance (genus Parasutterella id.2892)' on 'Leukotriene A−4 hydrolase'

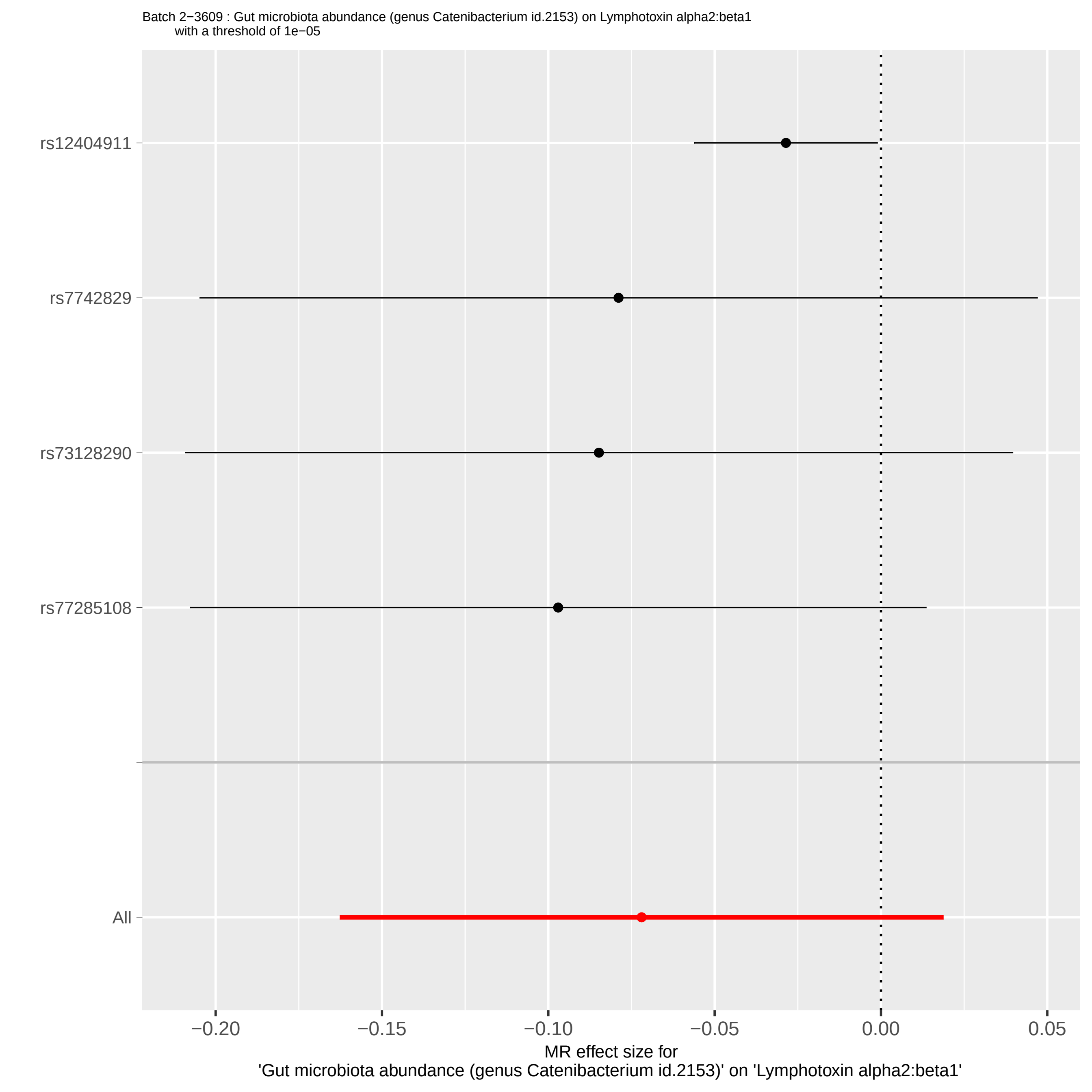
Batch 2−3609 : Gut microbiota abundance (genus Catenibacterium id.2153) on Lymphotoxin alpha2:beta1
with a threshold of 1e−05
rs12404911
rs7742829
rs73128290
rs77285108
All
−0.20
−0.15
−0.10
−0.05
0.00
0.05
MR effect size for
'Gut microbiota abundance (genus Catenibacterium id.2153)' on 'Lymphotoxin alpha2:beta1'

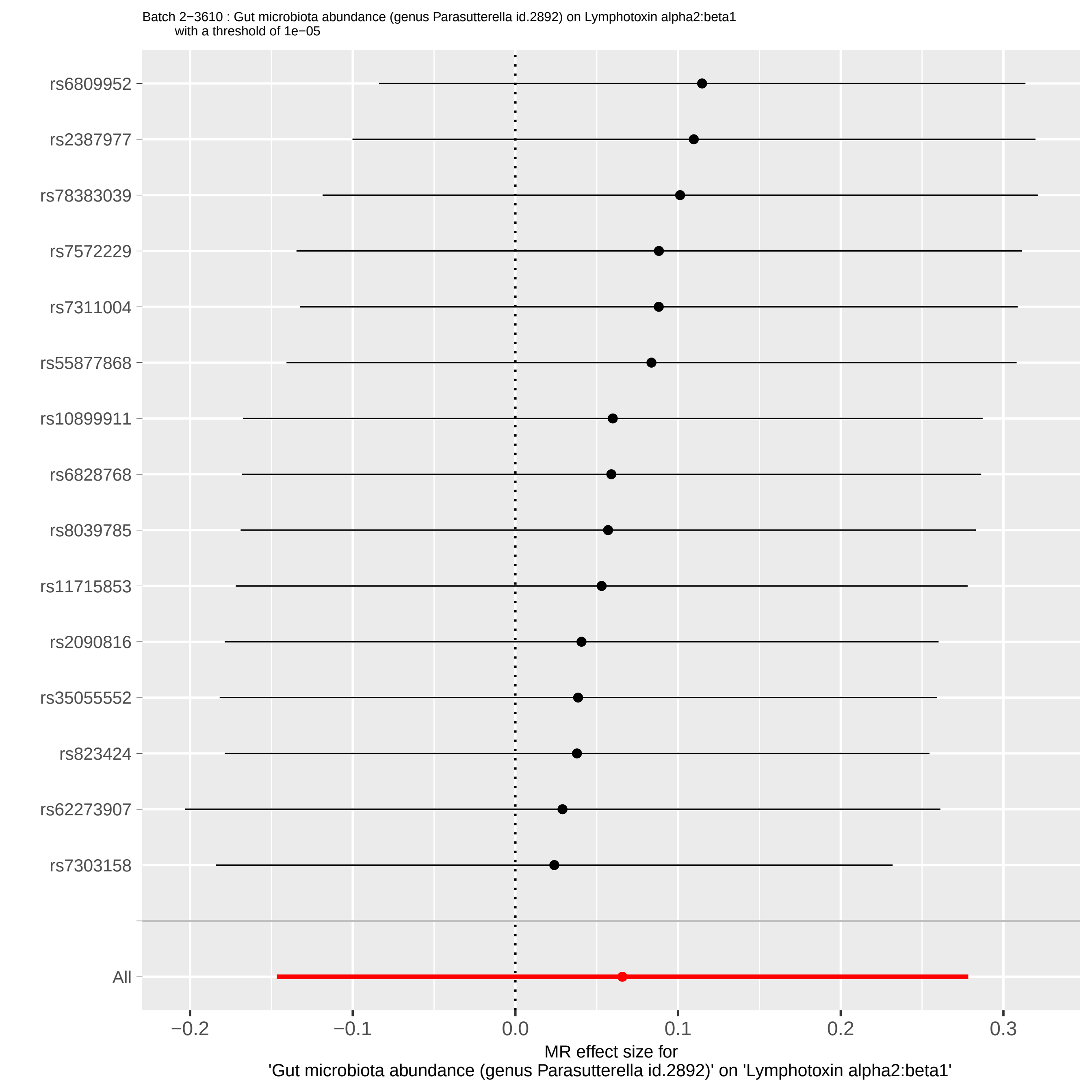

Batch 2−3610 : Gut microbiota abundance (genus Parasutterella id.2892) on Lymphotoxin alpha2:beta1
with a threshold of 1e−05
rs6809952
rs2387977
rs78383039
rs7572229
rs7311004
rs55877868
rs10899911
rs6828768
rs8039785
rs11715853
rs2090816
rs35055552
rs823424
rs62273907
rs7303158
All
−0.2
−0.1
0.0
0.1
0.2
0.3
MR effect size for
'Gut microbiota abundance (genus Parasutterella id.2892)' on 'Lymphotoxin alpha2:beta1'

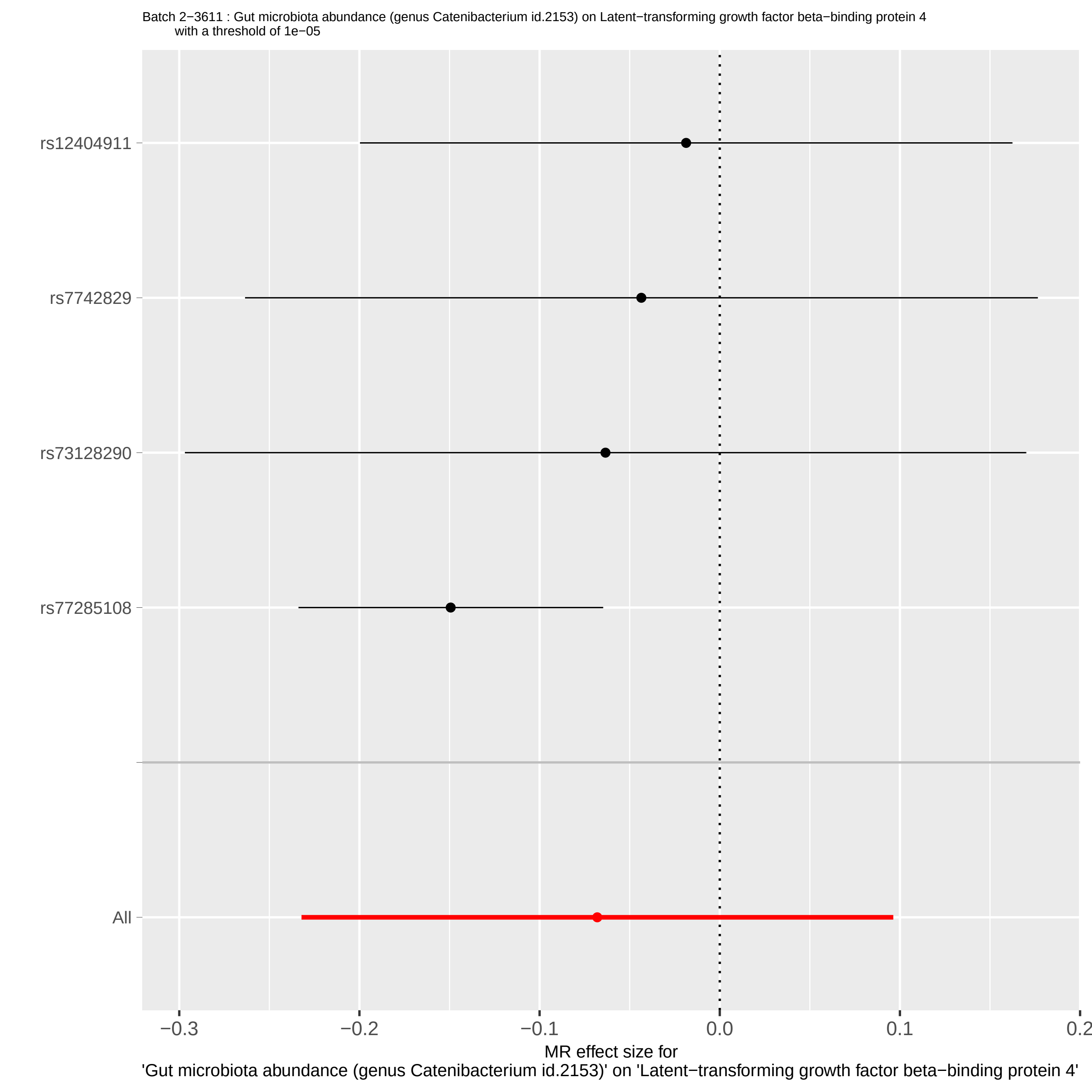
Batch 2−3611 : Gut microbiota abundance (genus Catenibacterium id.2153) on Latent−transforming growth factor beta−binding protein 4
with a threshold of 1e−05
rs12404911
rs7742829
rs73128290
rs77285108
All
−0.3
−0.2
−0.1
0.0
0.1
0.2
MR effect size for
'Gut microbiota abundance (genus Catenibacterium id.2153)' on 'Latent−transforming growth factor beta−binding protein 4'

Batch 2−3612 : Gut microbiota abundance (genus Parasutterella id.2892) on Latent−transforming growth factor beta−binding protein 4
with a threshold of 1e−05

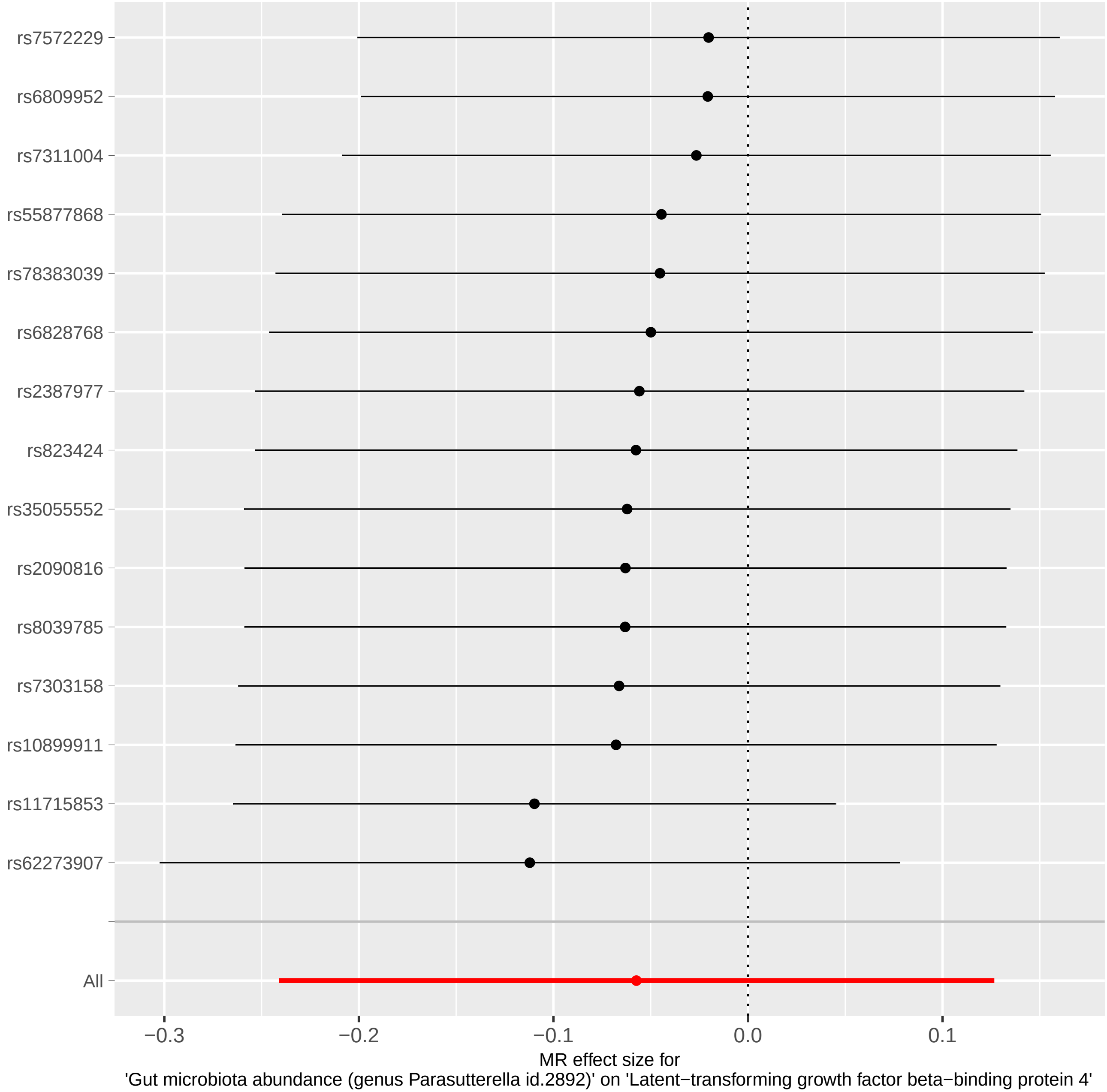

Batch 2−3613 : Gut microbiota abundance (genus Catenibacterium id.2153) on Tumor necrosis factor receptor superfamily member 3
with a threshold of 1e−05

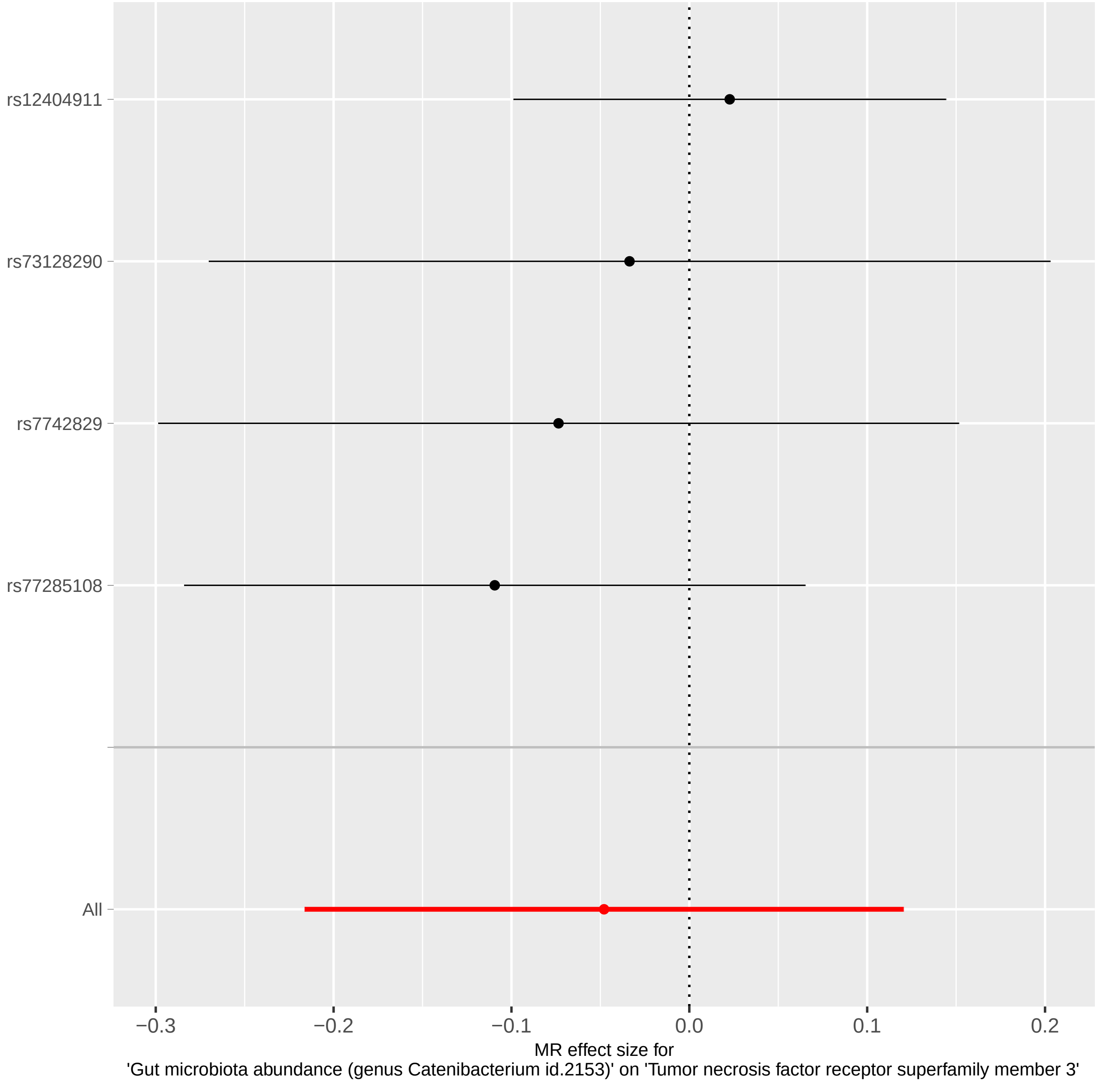

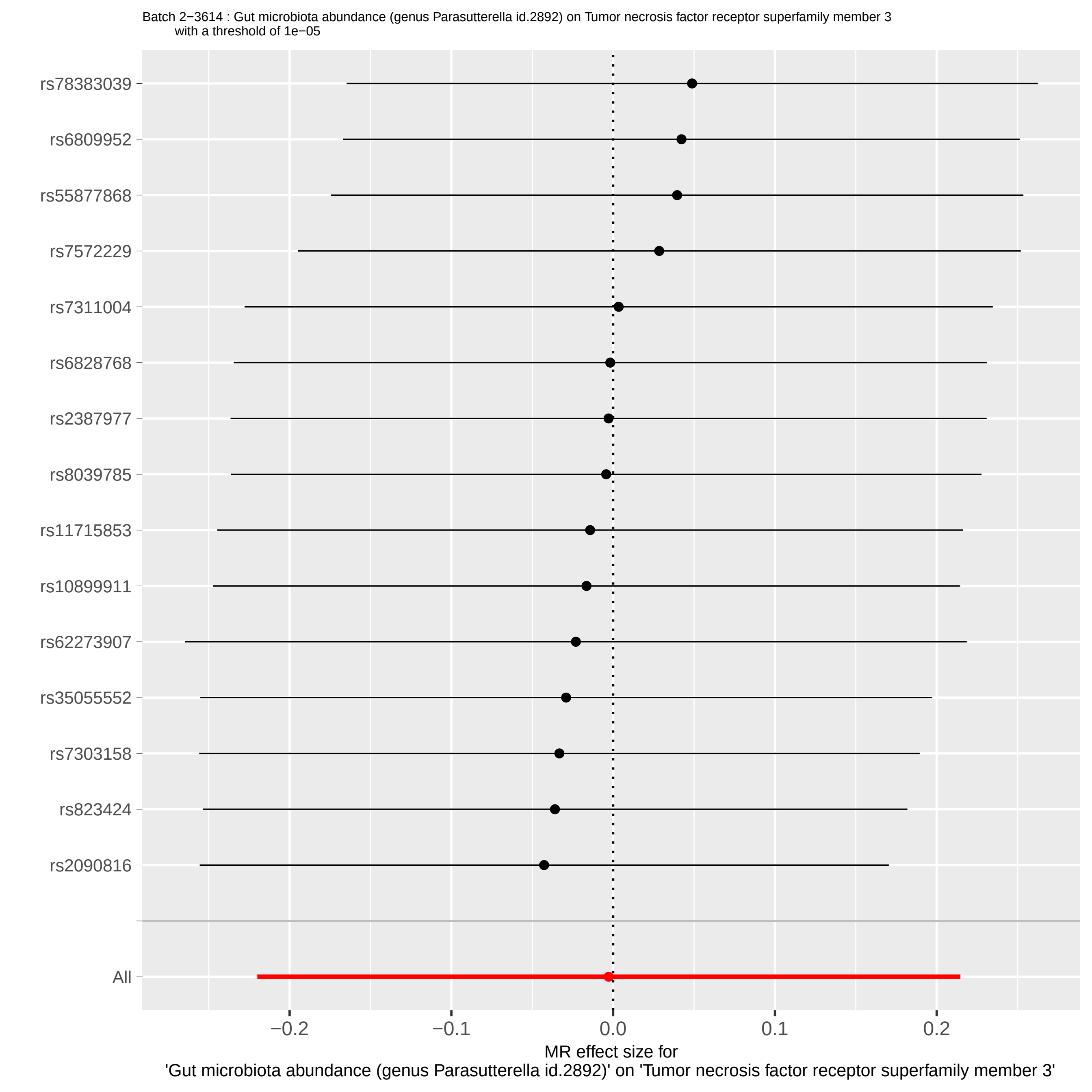
Batch 2−3614 : Gut microbiota abundance (genus Parasutterella id.2892) on Tumor necrosis factor receptor superfamily member 3
with a threshold of 1e−05
rs78383039
rs6809952
rs55877868
rs7572229
rs7311004
rs6828768
rs2387977
rs8039785
rs11715853
rs10899911
rs62273907
rs35055552
rs7303158
rs823424
rs2090816
All
−0.2
−0.1
0.0
0.1
0.2
MR effect size for
'Gut microbiota abundance (genus Parasutterella id.2892)' on 'Tumor necrosis factor receptor superfamily member 3'

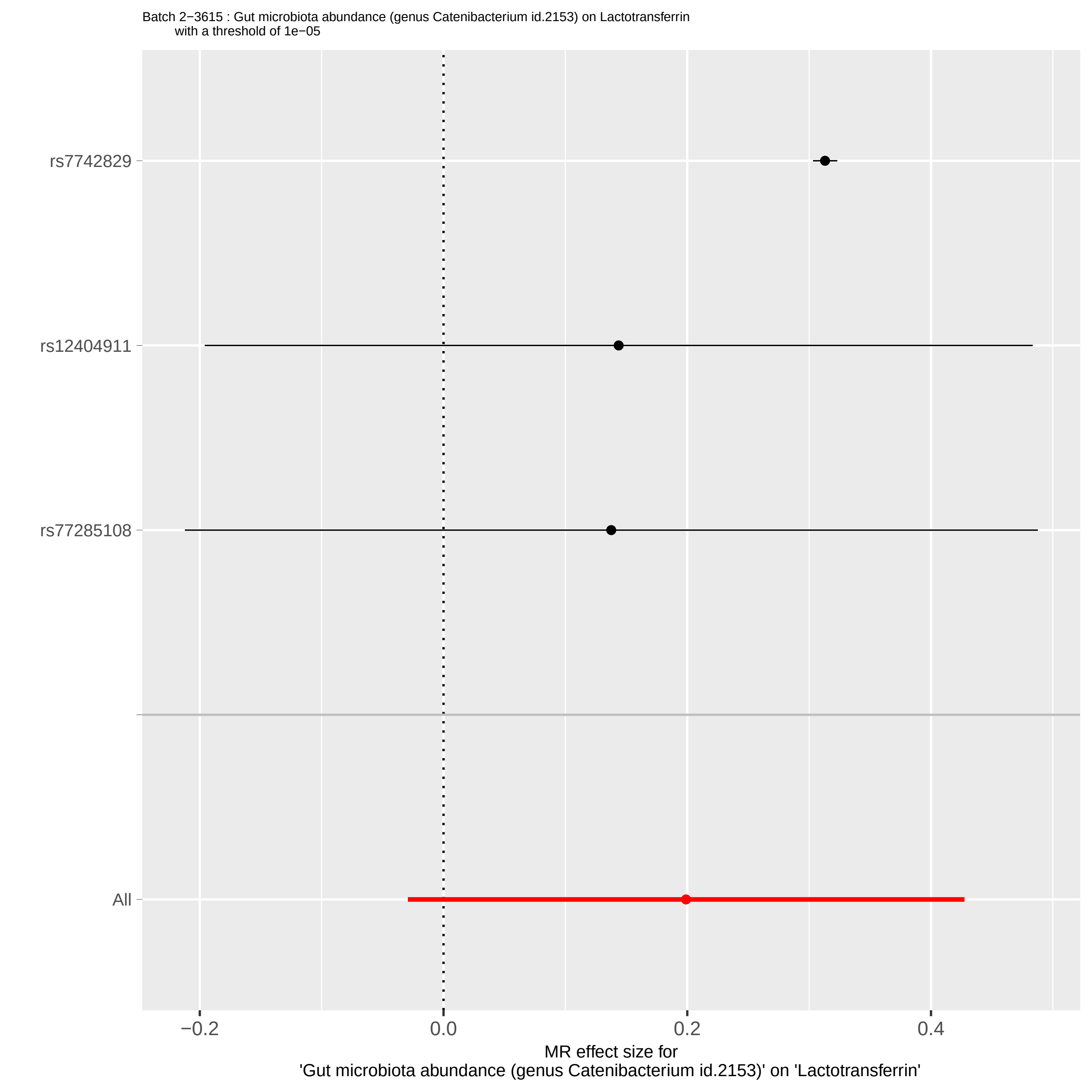
Batch 2−3615 : Gut microbiota abundance (genus Catenibacterium id.2153) on Lactotransferrin
with a threshold of 1e−05
rs7742829
rs12404911
rs77285108
All
−0.2
0.0
0.2
0.4
MR effect size for
'Gut microbiota abundance (genus Catenibacterium id.2153)' on 'Lactotransferrin'

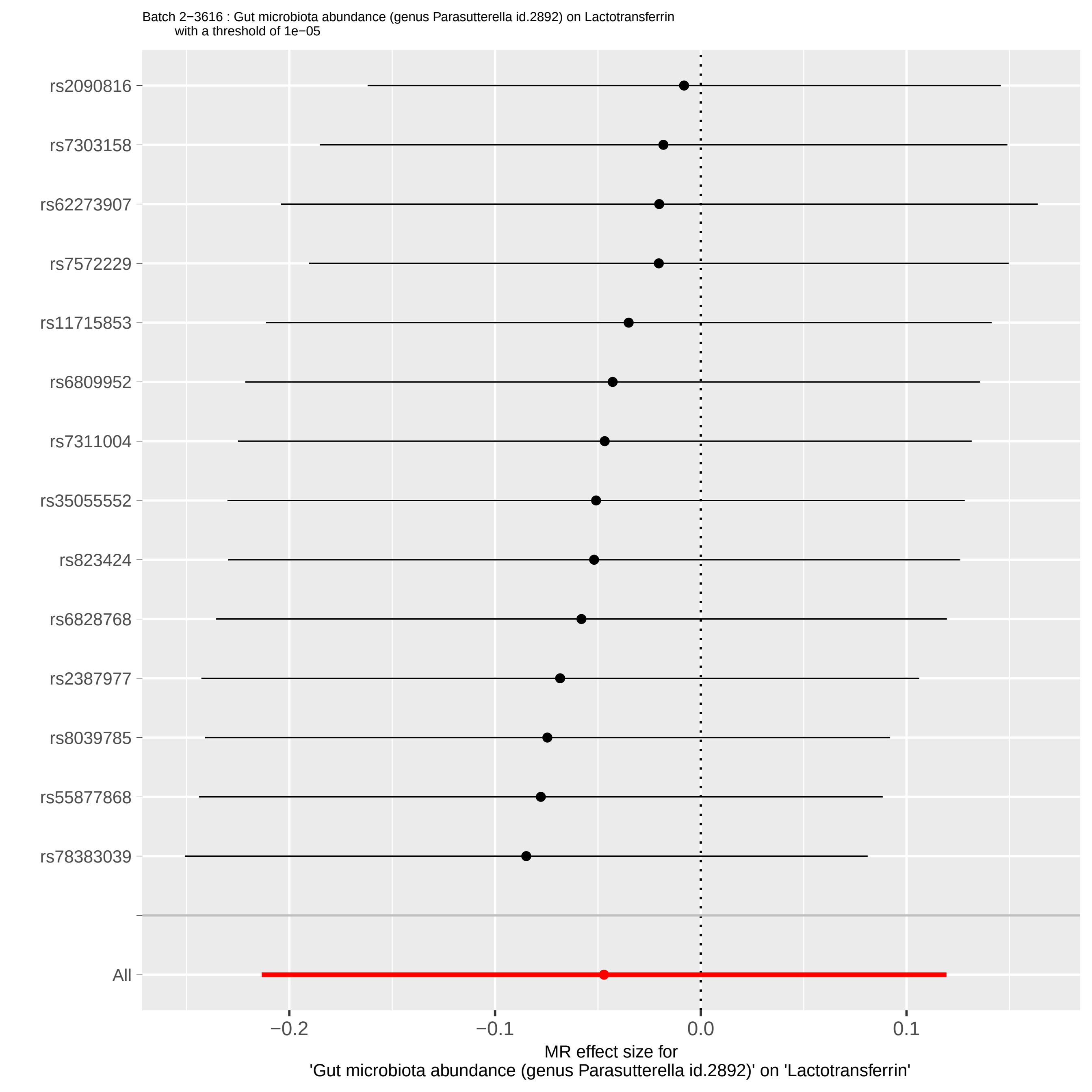

Batch 2−3616 : Gut microbiota abundance (genus Parasutterella id.2892) on Lactotransferrin
with a threshold of 1e−05
rs2090816
rs7303158
rs62273907
rs7572229
rs11715853
rs6809952
rs7311004
rs35055552
rs823424
rs6828768
rs2387977
rs8039785
rs55877868
rs78383039
All
−0.2
−0.1
0.0
0.1
MR effect size for
'Gut microbiota abundance (genus Parasutterella id.2892)' on 'Lactotransferrin'

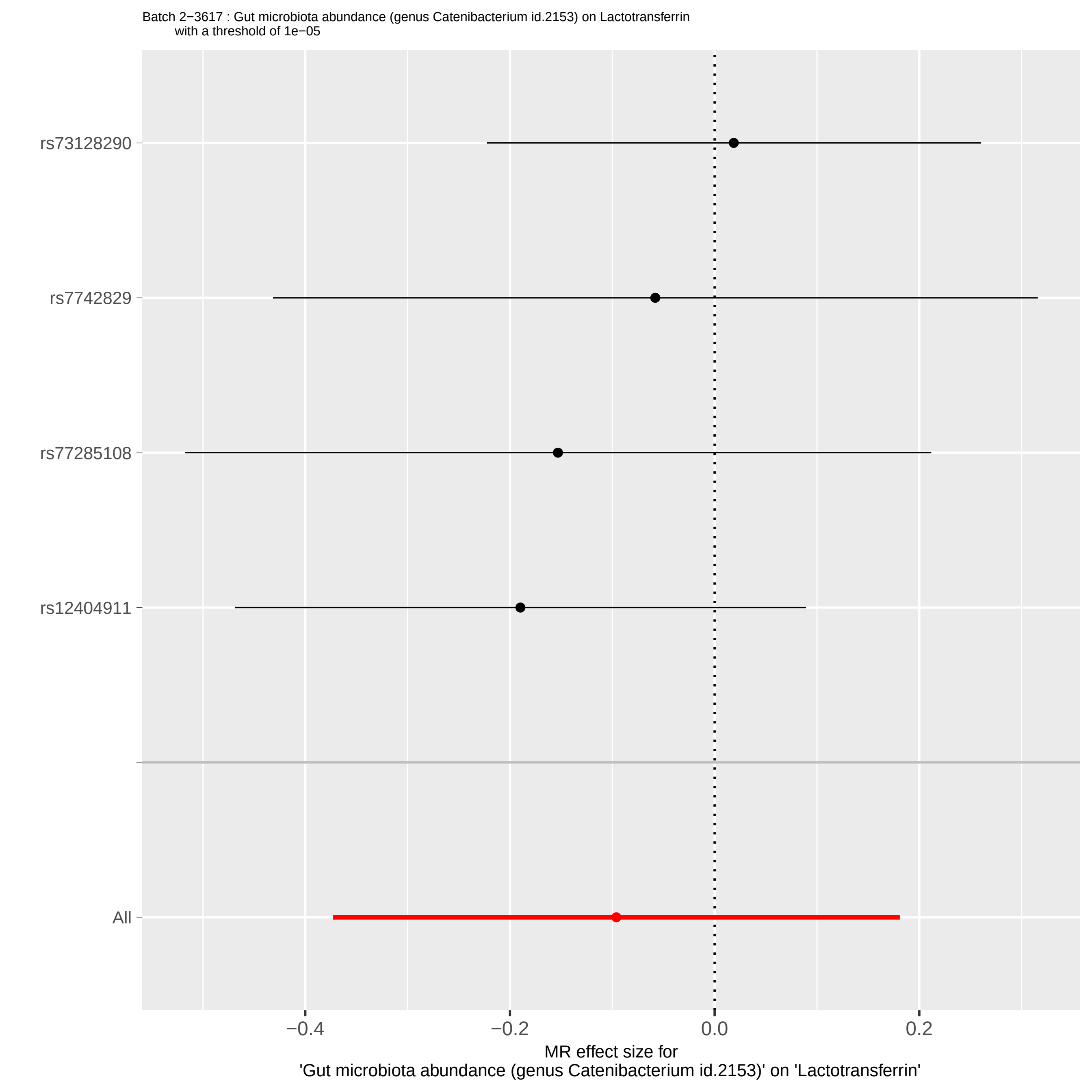

Batch 2−3617 : Gut microbiota abundance (genus Catenibacterium id.2153) on Lactotransferrin
with a threshold of 1e−05
rs73128290
rs7742829
rs77285108
rs12404911
All
−0.4
−0.2
0.0
0.2
MR effect size for
'Gut microbiota abundance (genus Catenibacterium id.2153)' on 'Lactotransferrin'

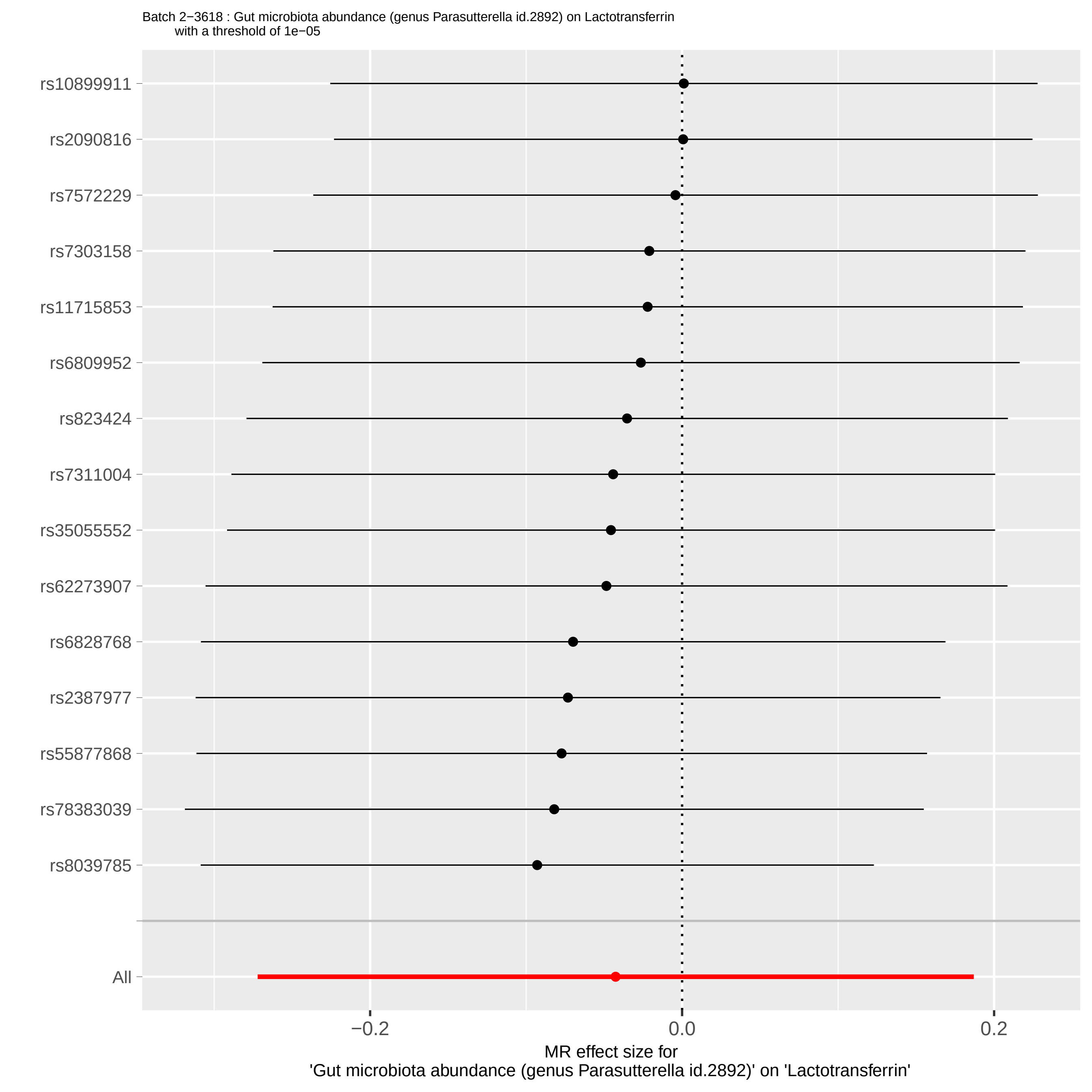
Batch 2−3618 : Gut microbiota abundance (genus Parasutterella id.2892) on Lactotransferrin
with a threshold of 1e−05
rs10899911
rs2090816
rs7572229
rs7303158
rs11715853
rs6809952
rs823424
rs7311004
rs35055552
rs62273907
rs6828768
rs2387977
rs55877868
rs78383039
rs8039785
All
−0.2
0.0
0.2
MR effect size for
'Gut microbiota abundance (genus Parasutterella id.2892)' on 'Lactotransferrin'

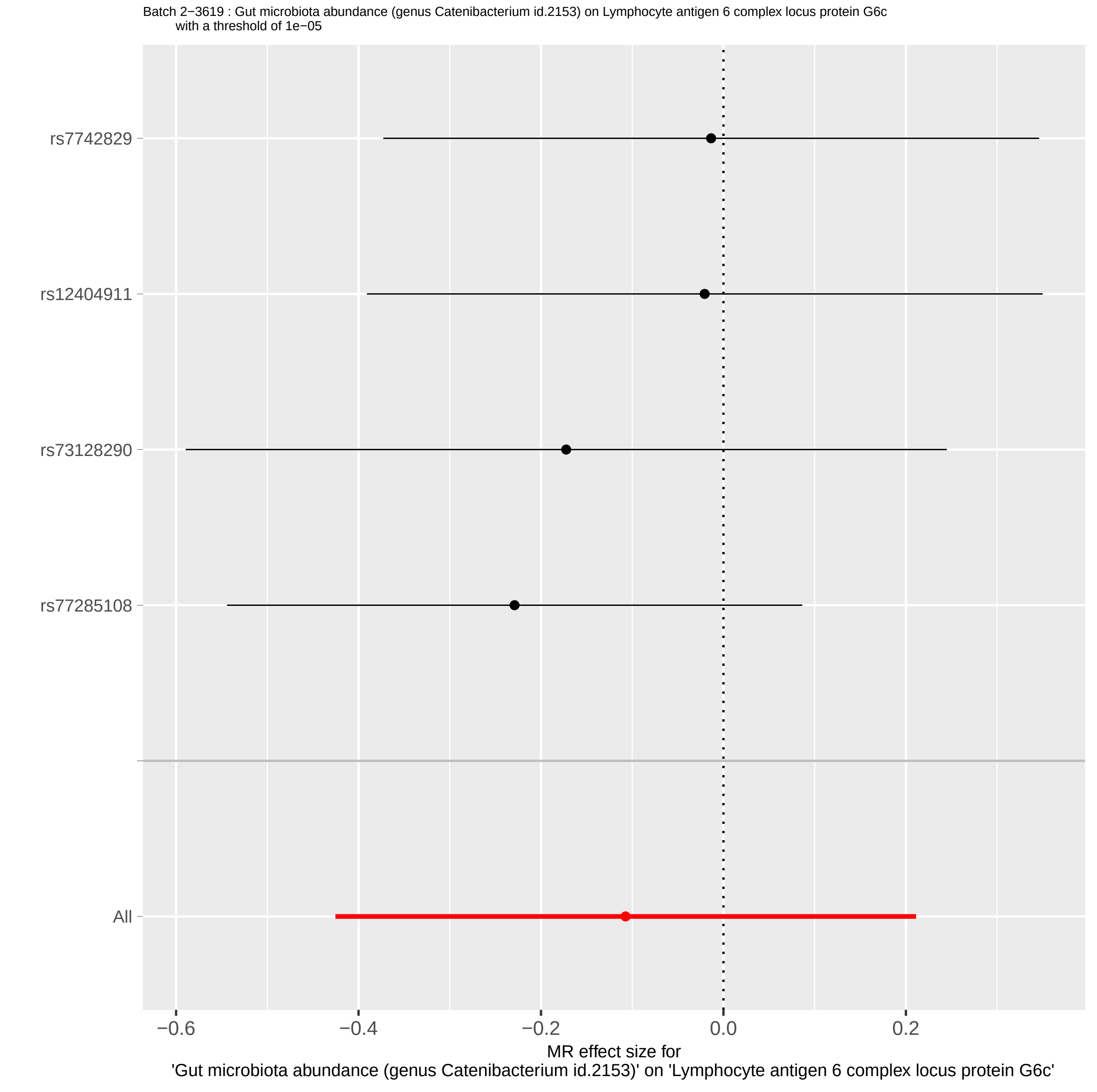

Batch 2−3619 : Gut microbiota abundance (genus Catenibacterium id.2153) on Lymphocyte antigen 6 complex locus protein G6c
with a threshold of 1e−05
rs7742829
rs12404911
rs73128290
rs77285108
All
−0.6
−0.4
−0.2
0.0
0.2
MR effect size for
'Gut microbiota abundance (genus Catenibacterium id.2153)' on 'Lymphocyte antigen 6 complex locus protein G6c'

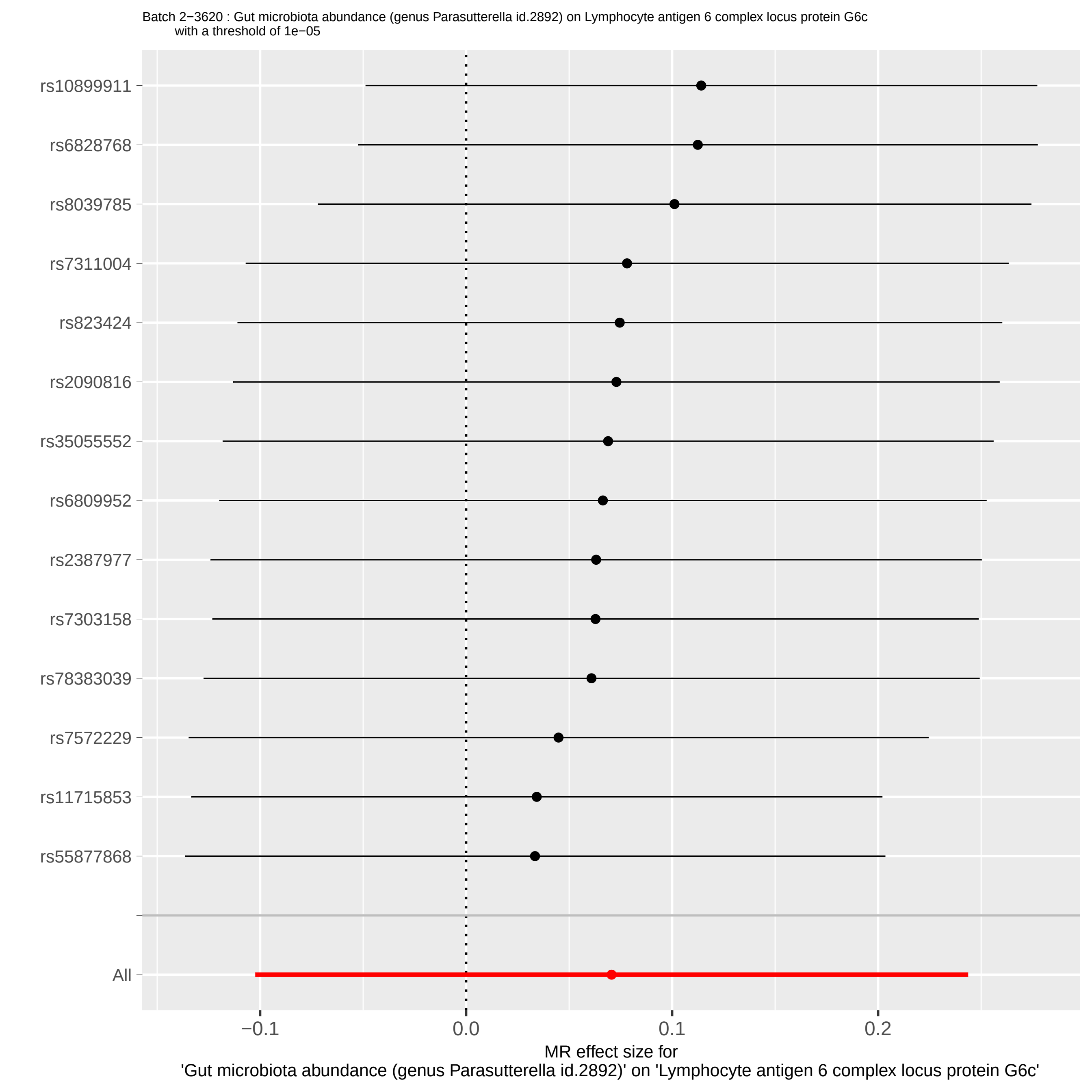
Batch 2−3620 : Gut microbiota abundance (genus Parasutterella id.2892) on Lymphocyte antigen 6 complex locus protein G6c
with a threshold of 1e−05
rs10899911
rs6828768
rs8039785
rs7311004
rs823424
rs2090816
rs35055552
rs6809952
rs2387977
rs7303158
rs78383039
rs7572229
rs11715853
rs55877868
All
−0.1
0.0
0.1
0.2
MR effect size for
'Gut microbiota abundance (genus Parasutterella id.2892)' on 'Lymphocyte antigen 6 complex locus protein G6c'

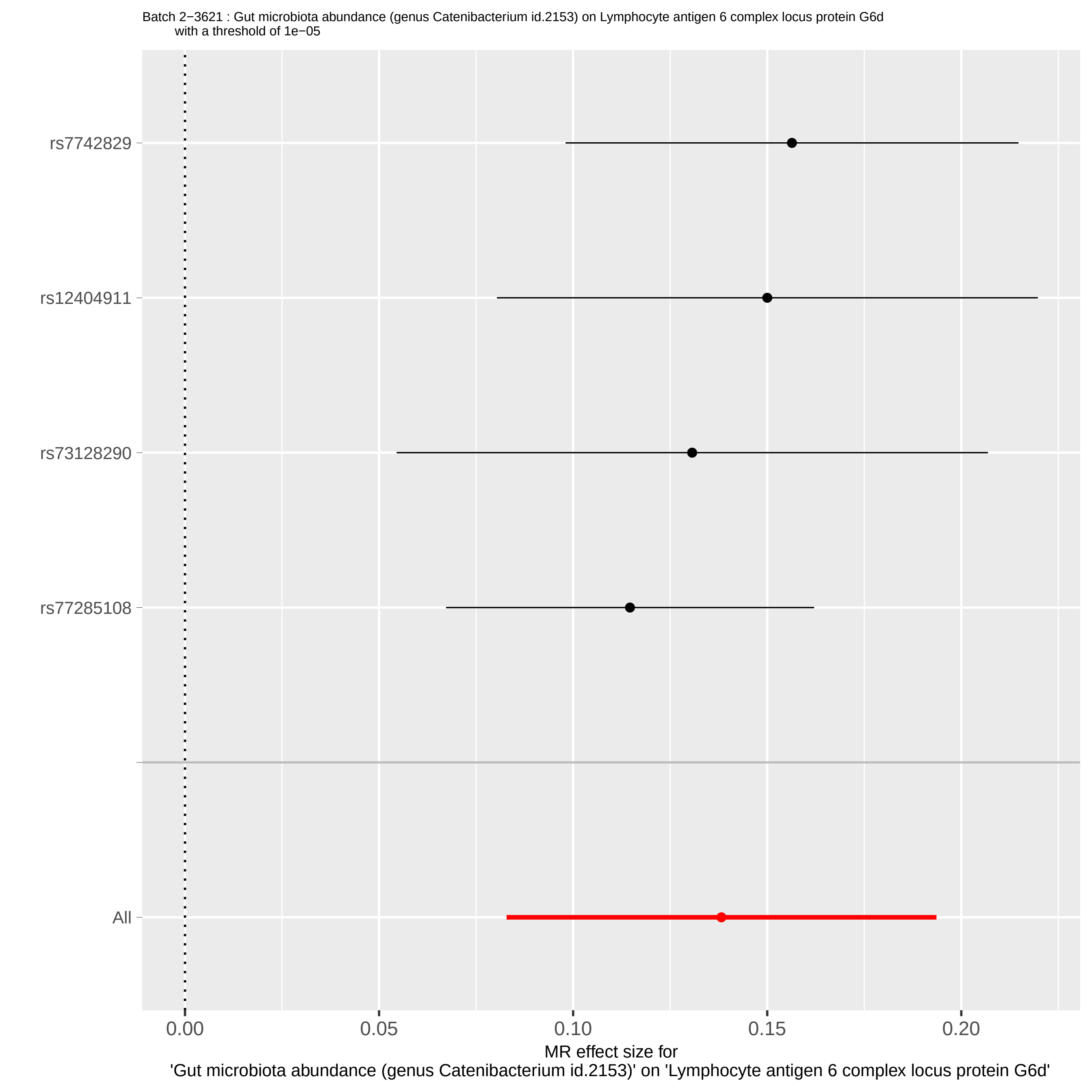
Batch 2−3621 : Gut microbiota abundance (genus Catenibacterium id.2153) on Lymphocyte antigen 6 complex locus protein G6d
with a threshold of 1e−05
rs7742829
rs12404911
rs73128290
rs77285108
All
0.00
0.05
0.10
0.15
0.20
MR effect size for
'Gut microbiota abundance (genus Catenibacterium id.2153)' on 'Lymphocyte antigen 6 complex locus protein G6d'

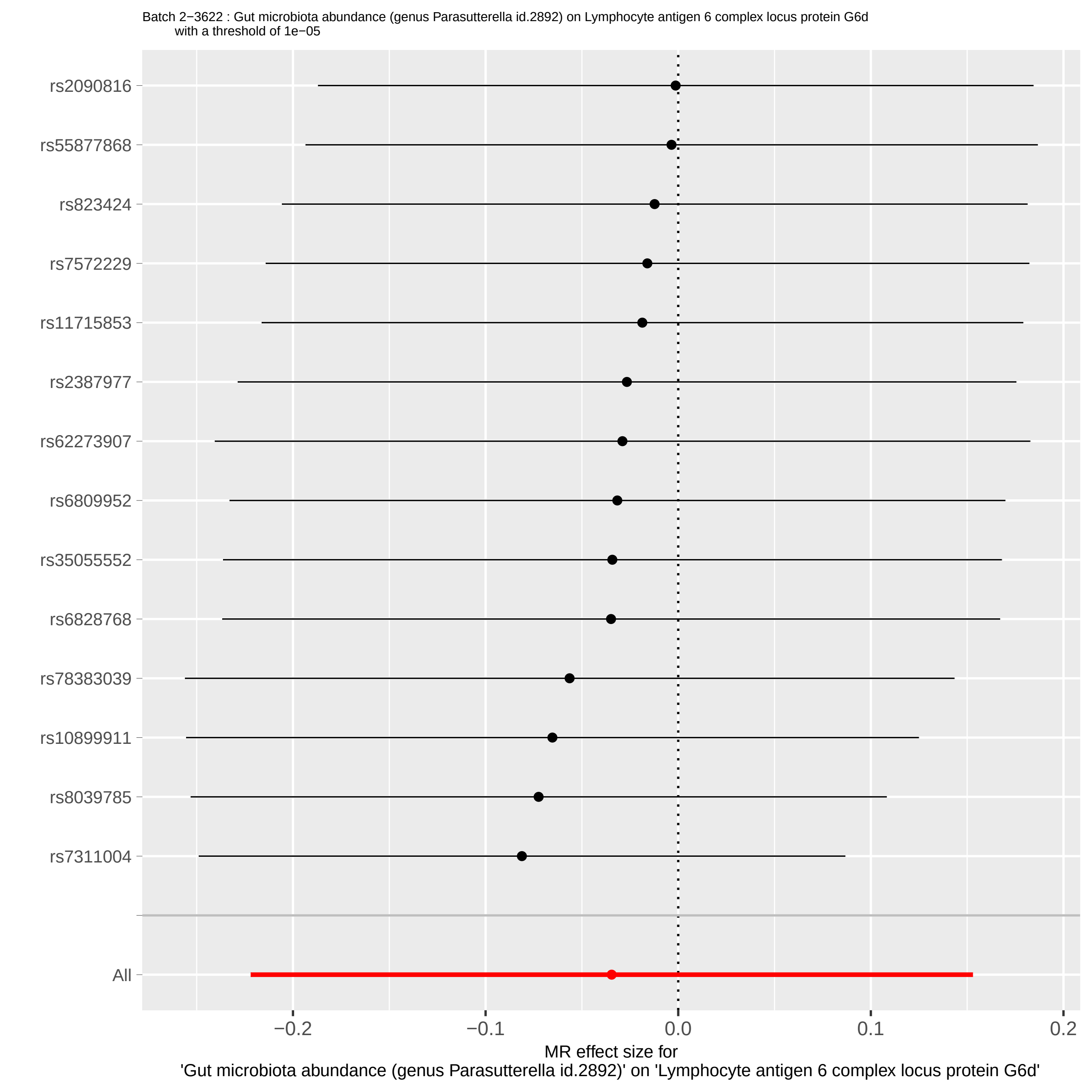
Batch 2−3622 : Gut microbiota abundance (genus Parasutterella id.2892) on Lymphocyte antigen 6 complex locus protein G6d
with a threshold of 1e−05
rs2090816
rs55877868
rs823424
rs7572229
rs11715853
rs2387977
rs62273907
rs6809952
rs35055552
rs6828768
rs78383039
rs10899911
rs8039785
rs7311004
All
−0.2
−0.1
0.0
0.1
0.2
MR effect size for
'Gut microbiota abundance (genus Parasutterella id.2892)' on 'Lymphocyte antigen 6 complex locus protein G6d'

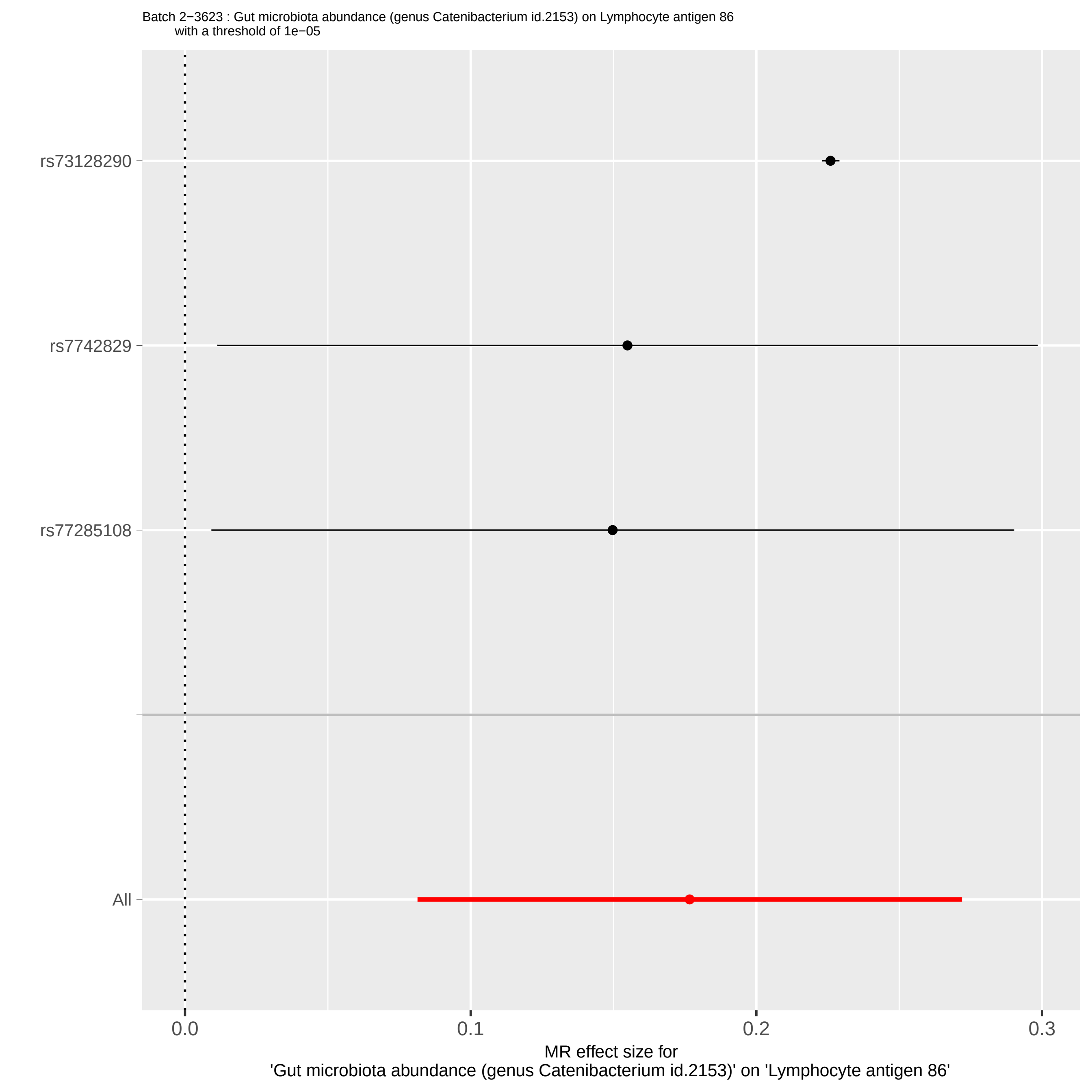

Batch 2−3623 : Gut microbiota abundance (genus Catenibacterium id.2153) on Lymphocyte antigen 86
with a threshold of 1e−05
rs73128290
rs7742829
rs77285108
All
0.0
0.1
0.2
0.3
MR effect size for
'Gut microbiota abundance (genus Catenibacterium id.2153)' on 'Lymphocyte antigen 86'

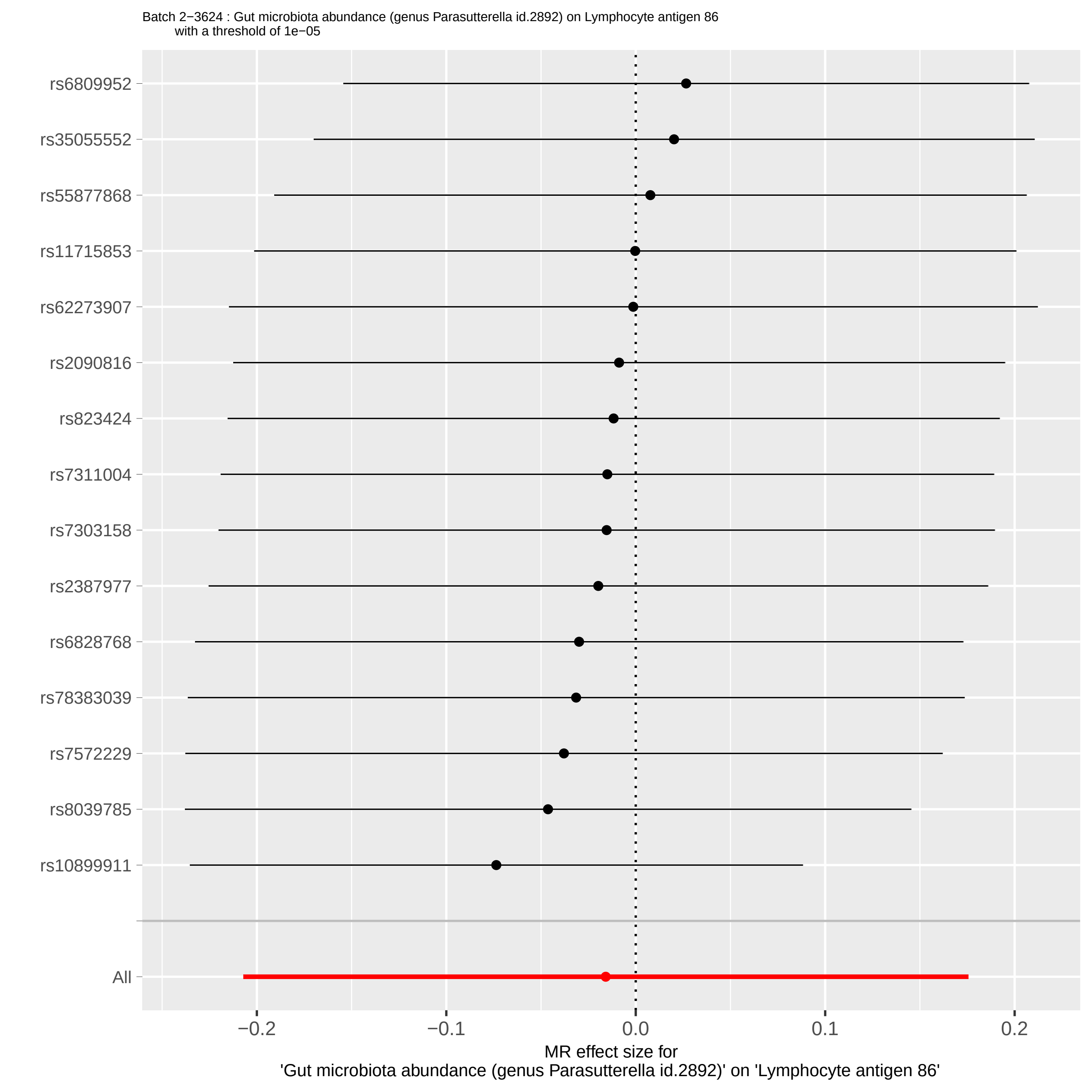

Batch 2−3624 : Gut microbiota abundance (genus Parasutterella id.2892) on Lymphocyte antigen 86
with a threshold of 1e−05
rs6809952
rs35055552
rs55877868
rs11715853
rs62273907
rs2090816
rs823424
rs7311004
rs7303158
rs2387977
rs6828768
rs78383039
rs7572229
rs8039785
rs10899911
All
−0.2
−0.1
0.0
0.1
0.2
MR effect size for
'Gut microbiota abundance (genus Parasutterella id.2892)' on 'Lymphocyte antigen 86'

Batch 2−3625 : Gut microbiota abundance (genus Catenibacterium id.2153) on T−lymphocyte surface antigen Ly−9
with a threshold of 1e−05

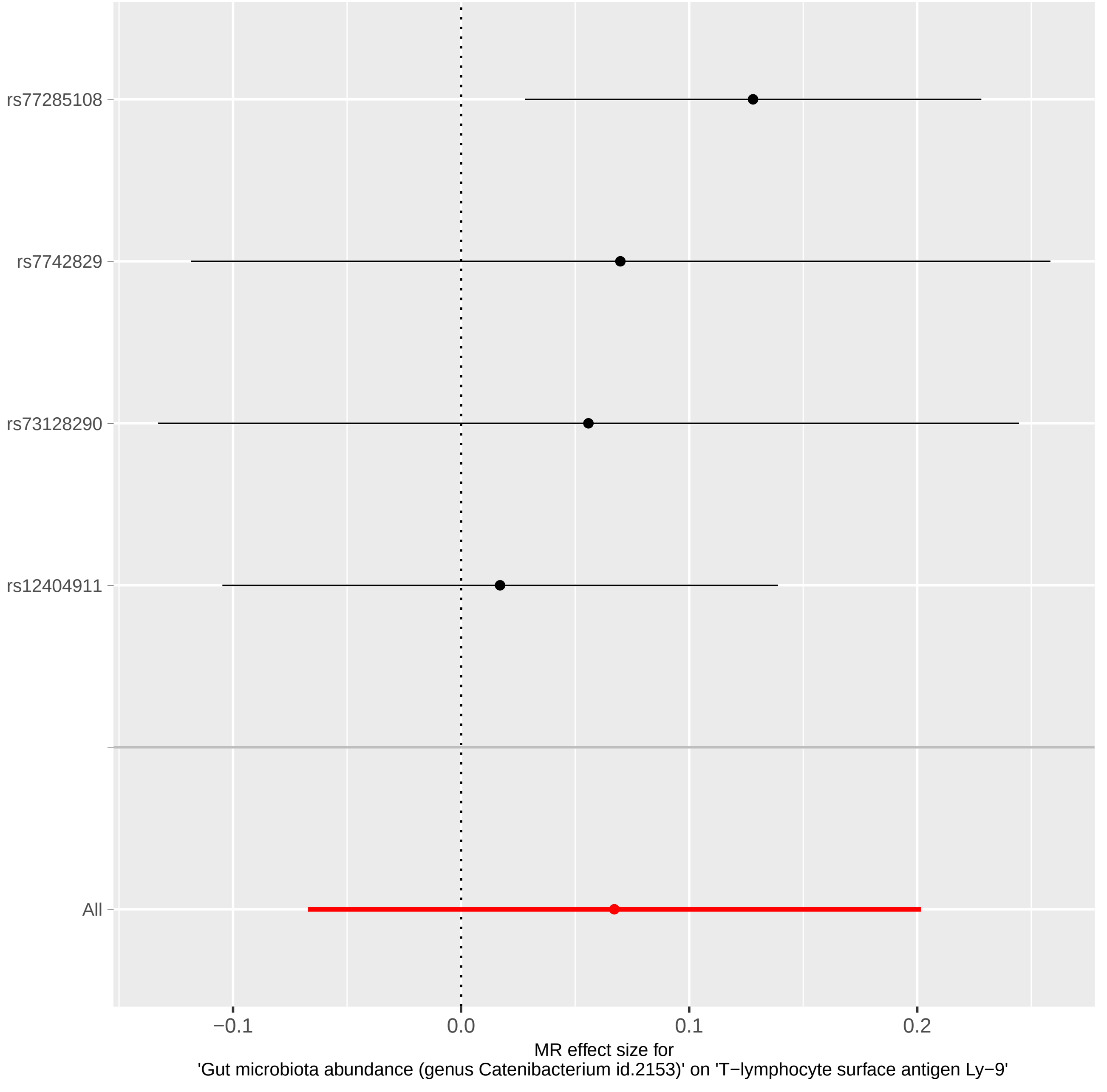

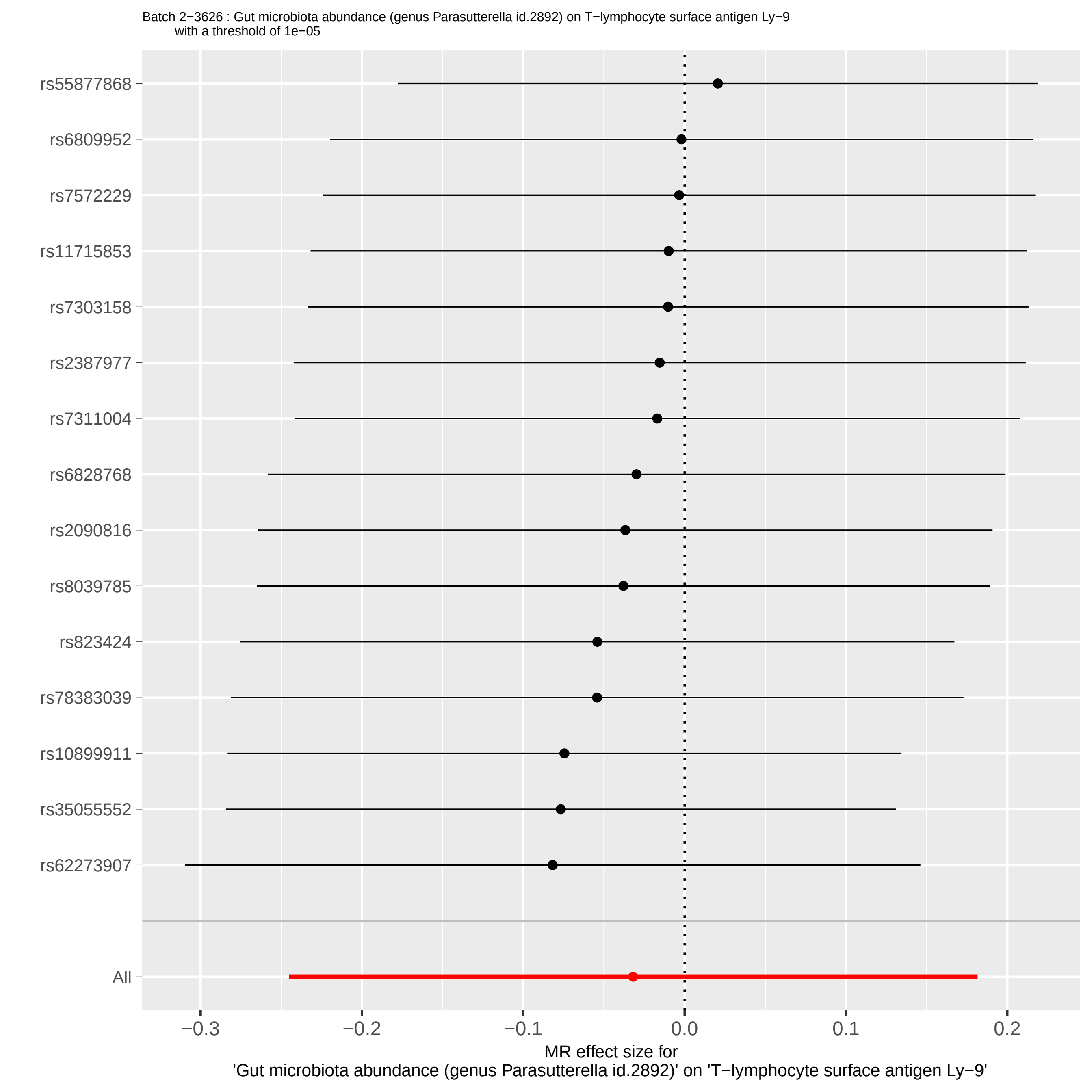

Batch 2−3626 : Gut microbiota abundance (genus Parasutterella id.2892) on T−lymphocyte surface antigen Ly−9
with a threshold of 1e−05
rs55877868
rs6809952
rs7572229
rs11715853
rs7303158
rs2387977
rs7311004
rs6828768
rs2090816
rs8039785
rs823424
rs78383039
rs10899911
rs35055552
rs62273907
All
−0.3
−0.2
−0.1
0.0
0.1
0.2
MR effect size for
'Gut microbiota abundance (genus Parasutterella id.2892)' on 'T−lymphocyte surface antigen Ly−9'

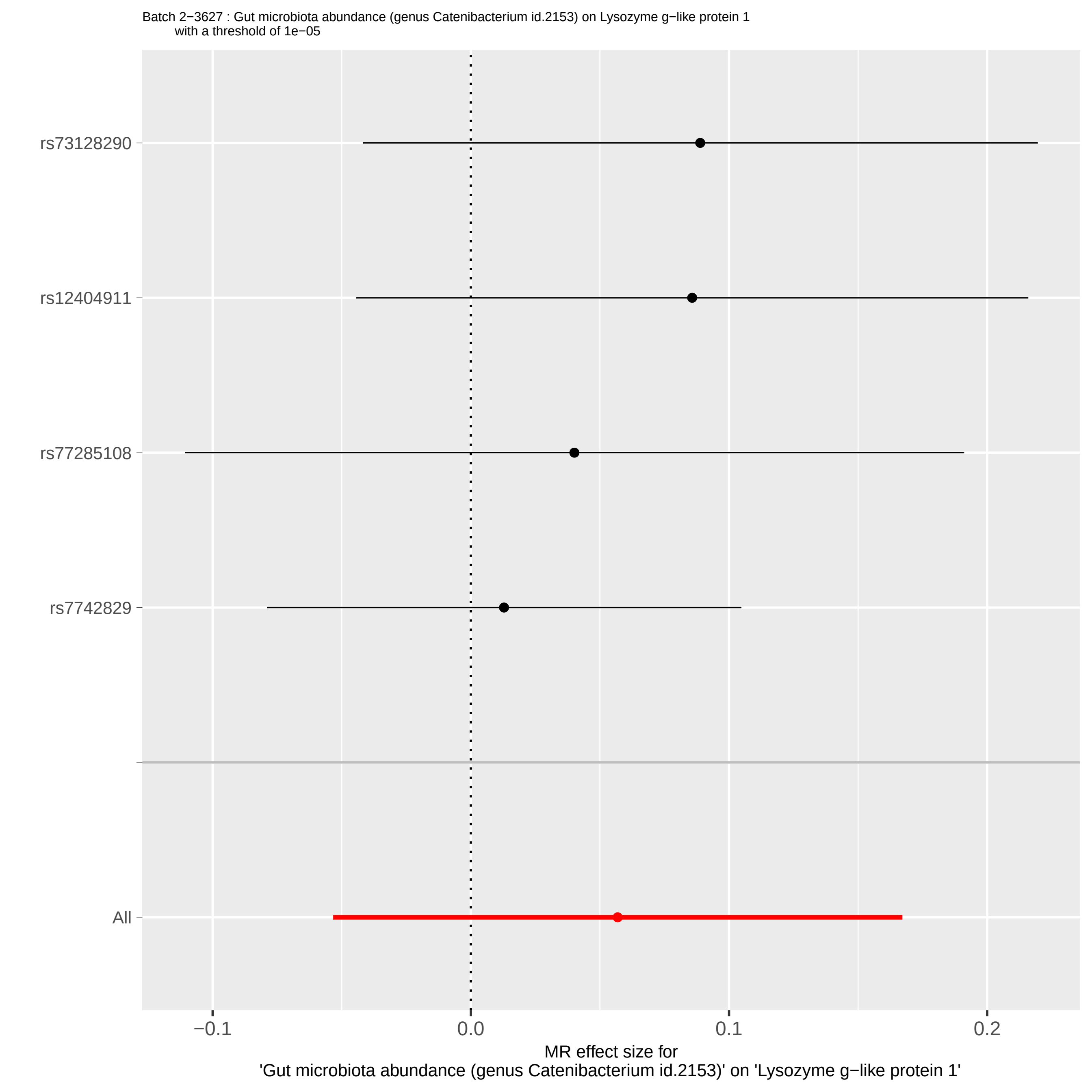
Batch 2−3627 : Gut microbiota abundance (genus Catenibacterium id.2153) on Lysozyme g−like protein 1
with a threshold of 1e−05
rs73128290
rs12404911
rs77285108
rs7742829
All
−0.1
0.0
0.1
0.2
MR effect size for
'Gut microbiota abundance (genus Catenibacterium id.2153)' on 'Lysozyme g−like protein 1'

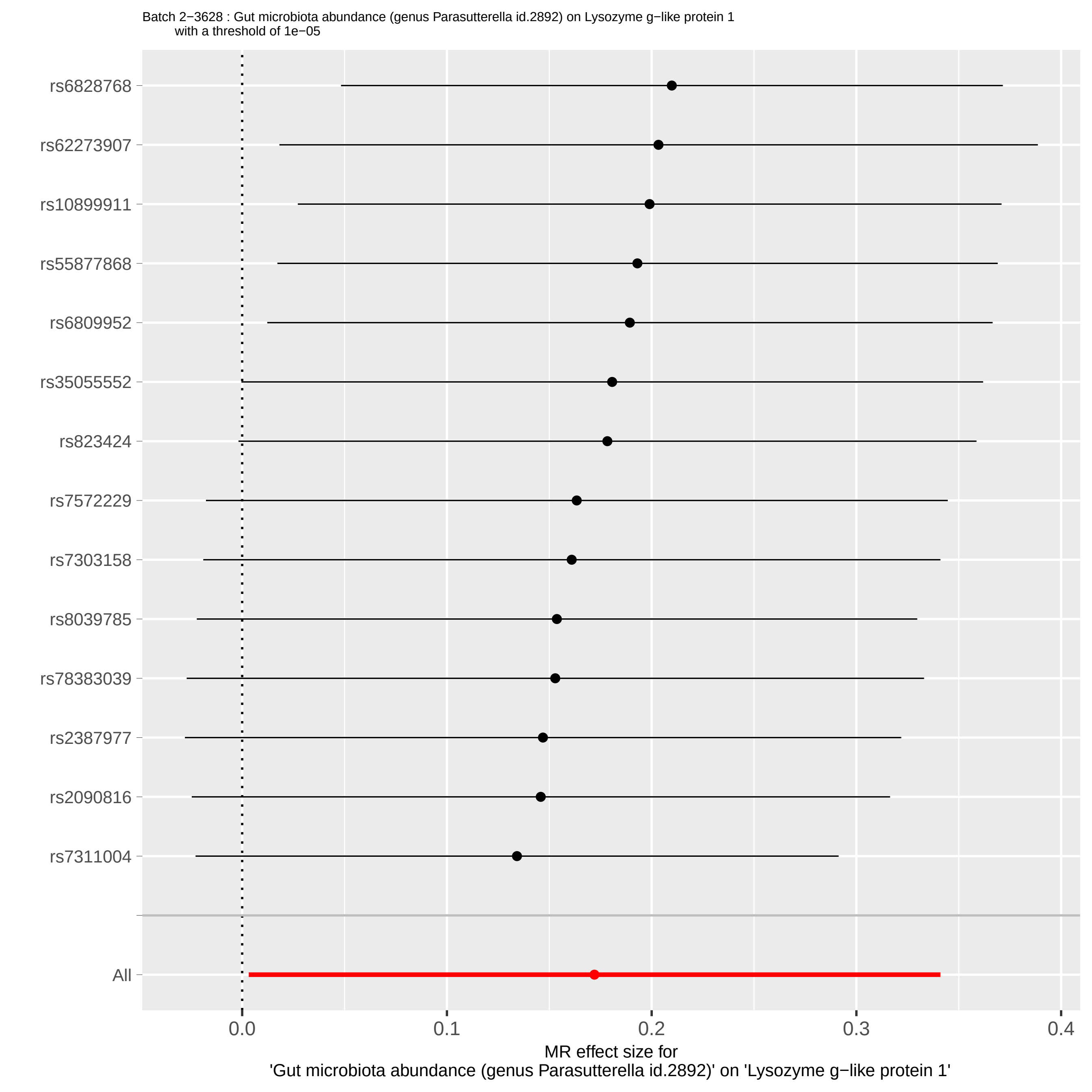
Batch 2−3628 : Gut microbiota abundance (genus Parasutterella id.2892) on Lysozyme g−like protein 1
with a threshold of 1e−05
rs6828768
rs62273907
rs10899911
rs55877868
rs6809952
rs35055552
rs823424
rs7572229
rs7303158
rs8039785
rs78383039
rs2387977
rs2090816
rs7311004
All
0.0
0.1
0.2
0.3
0.4
MR effect size for
'Gut microbiota abundance (genus Parasutterella id.2892)' on 'Lysozyme g−like protein 1'

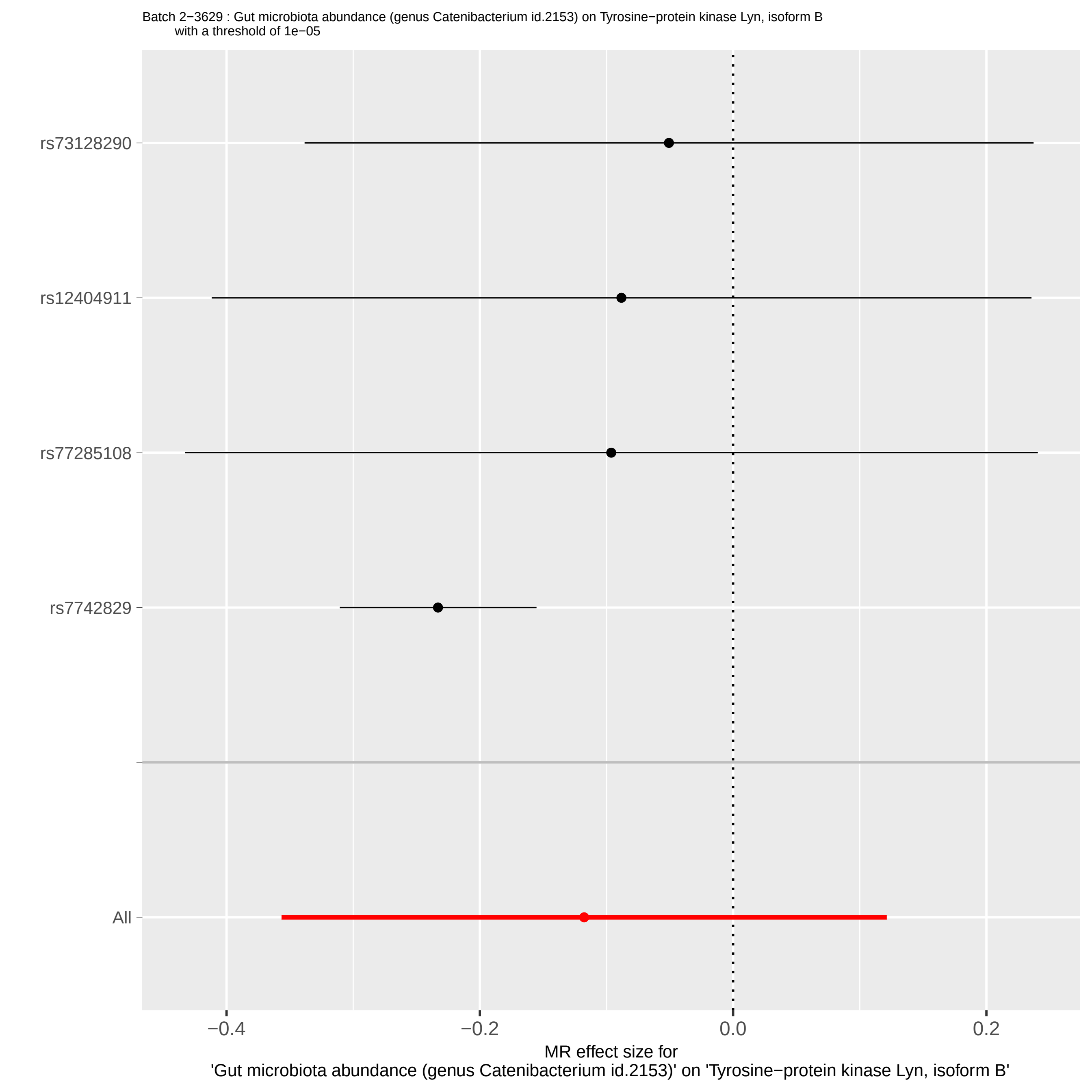
Batch 2−3629 : Gut microbiota abundance (genus Catenibacterium id.2153) on Tyrosine−protein kinase Lyn, isoform B
with a threshold of 1e−05
rs73128290
rs12404911
rs77285108
rs7742829
All
−0.4
−0.2
0.0
0.2
MR effect size for
'Gut microbiota abundance (genus Catenibacterium id.2153)' on 'Tyrosine−protein kinase Lyn, isoform B'

Batch 2−3630 : Gut microbiota abundance (genus Parasutterella id.2892) on Tyrosine−protein kinase Lyn, isoform B
with a threshold of 1e−05

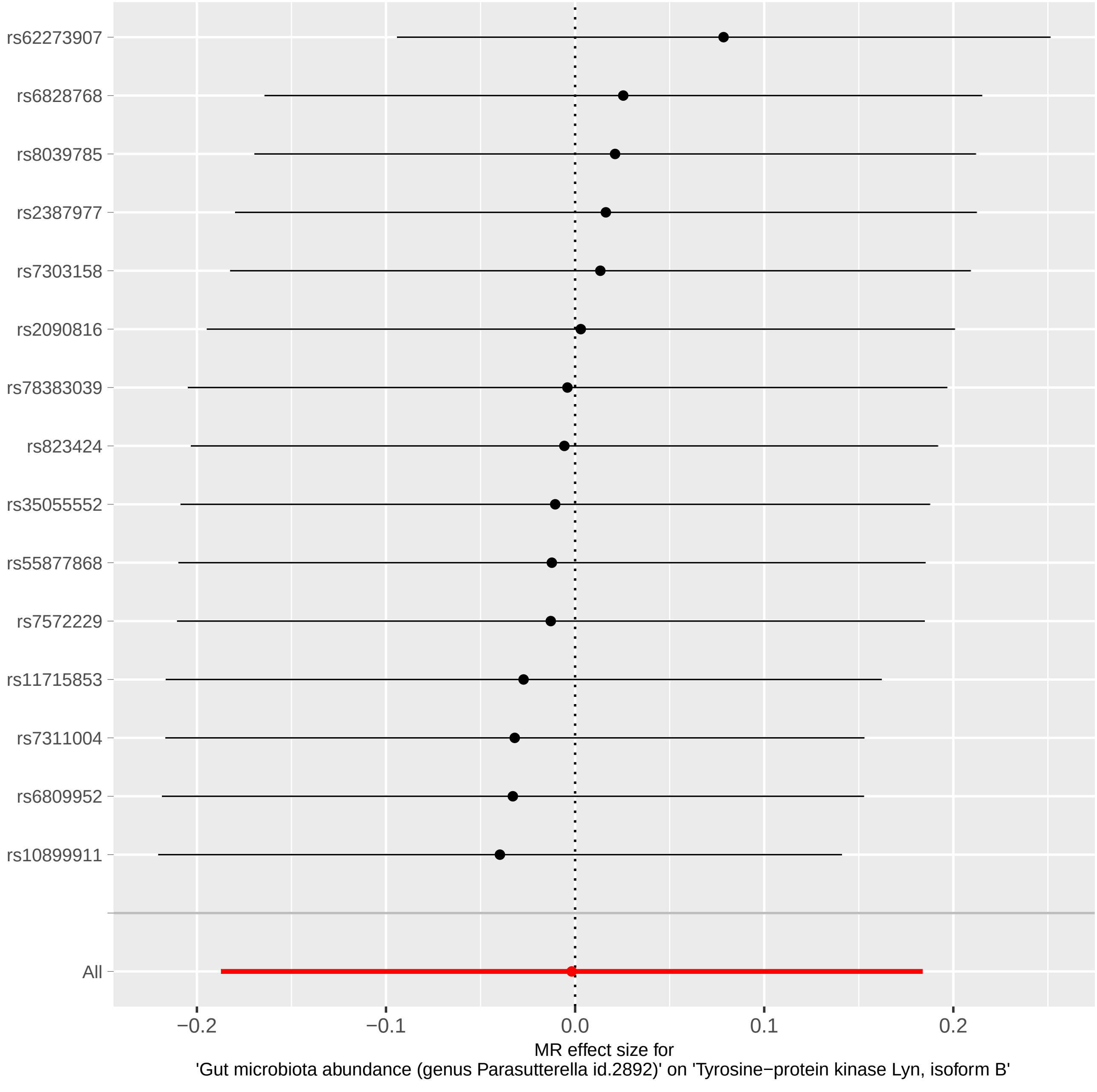

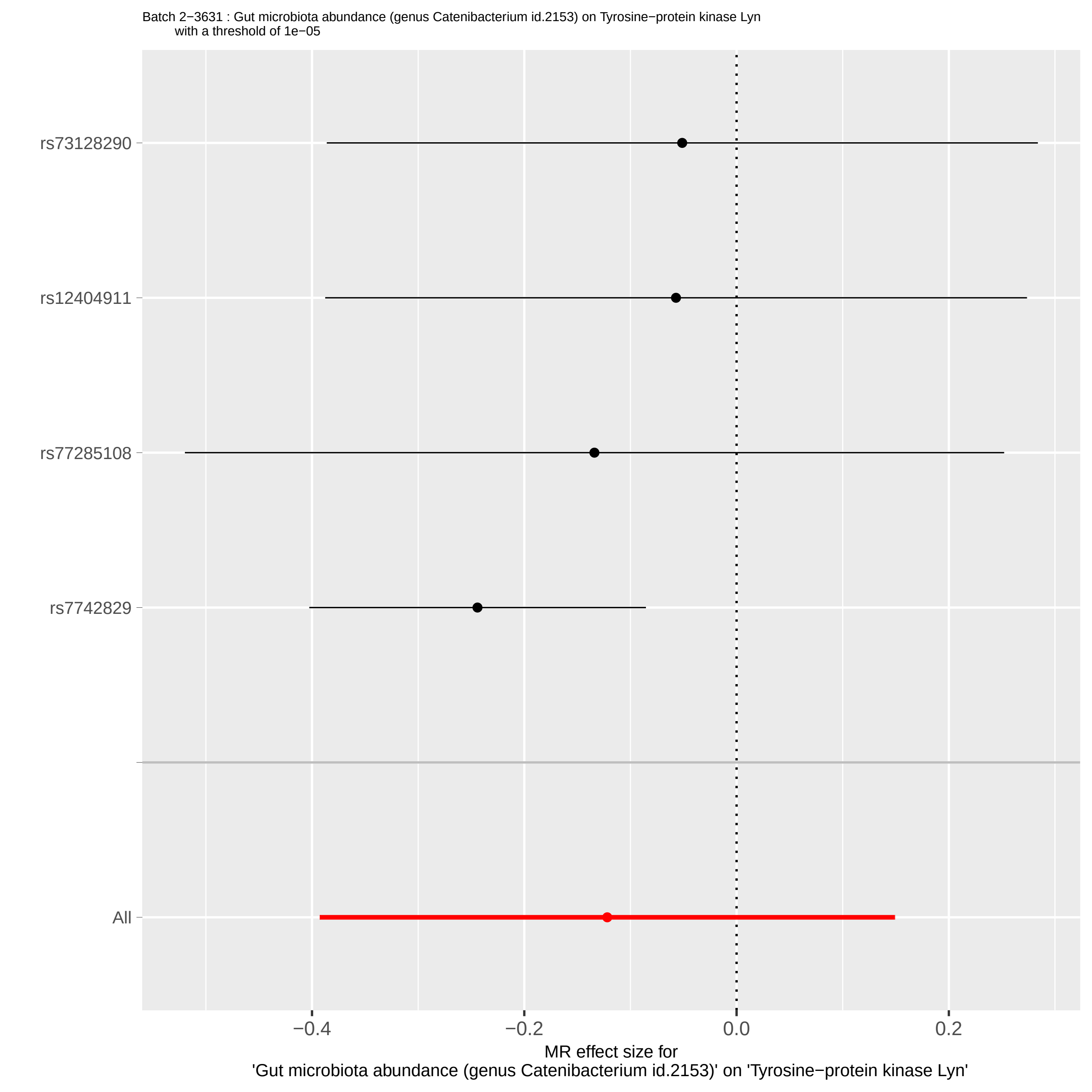
Batch 2−3631 : Gut microbiota abundance (genus Catenibacterium id.2153) on Tyrosine−protein kinase Lyn
with a threshold of 1e−05
rs73128290
rs12404911
rs77285108
rs7742829
All
−0.4
−0.2
0.0
0.2
MR effect size for
'Gut microbiota abundance (genus Catenibacterium id.2153)' on 'Tyrosine−protein kinase Lyn'

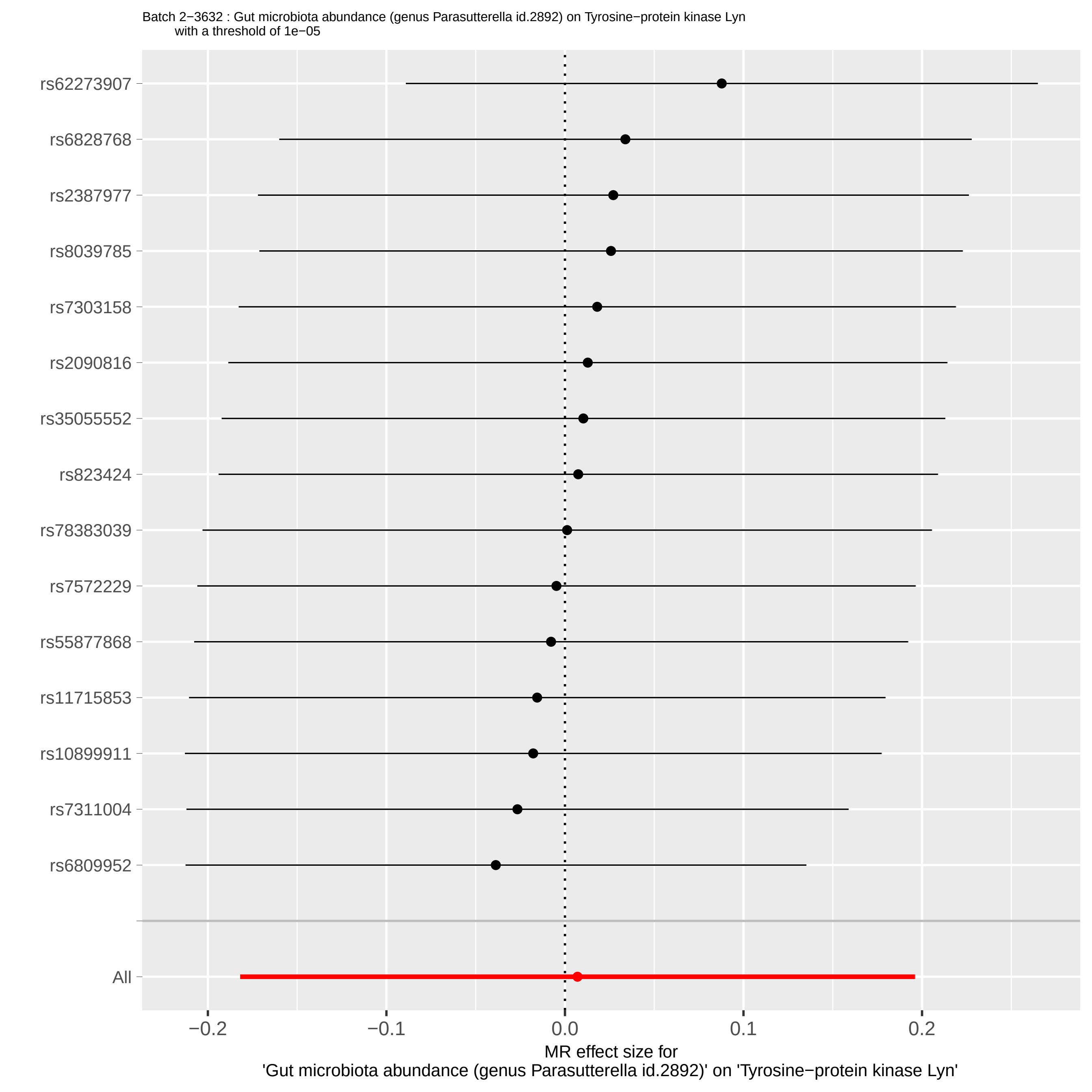

Batch 2−3632 : Gut microbiota abundance (genus Parasutterella id.2892) on Tyrosine−protein kinase Lyn
with a threshold of 1e−05
rs62273907
rs6828768
rs2387977
rs8039785
rs7303158
rs2090816
rs35055552
rs823424
rs78383039
rs7572229
rs55877868
rs11715853
rs10899911
rs7311004
rs6809952
All
−0.2
−0.1
0.0
0.1
0.2
MR effect size for
'Gut microbiota abundance (genus Parasutterella id.2892)' on 'Tyrosine−protein kinase Lyn'

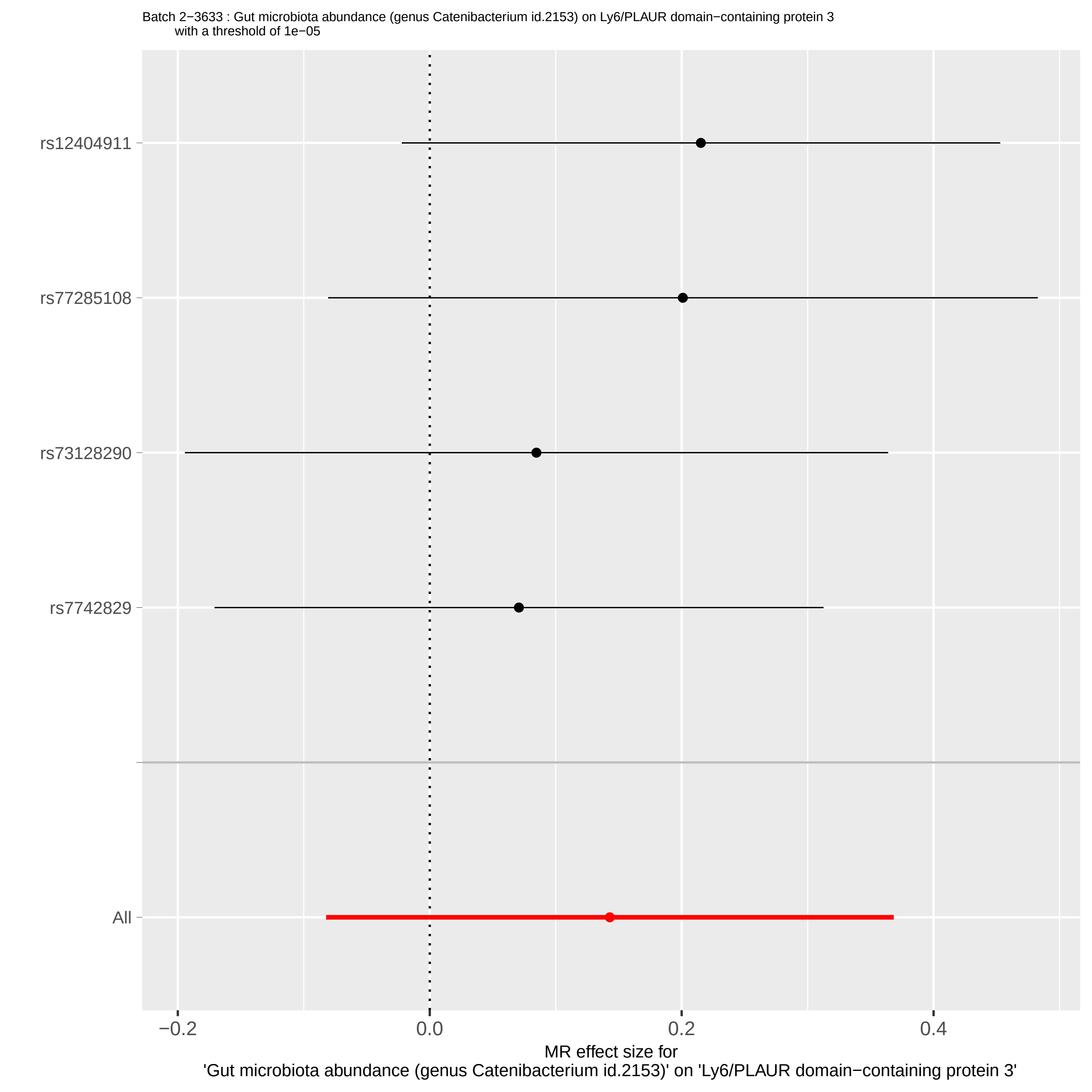

Batch 2−3633 : Gut microbiota abundance (genus Catenibacterium id.2153) on Ly6/PLAUR domain−containing protein 3
with a threshold of 1e−05
rs12404911
rs77285108
rs73128290
rs7742829
All
−0.2
0.0
0.2
0.4
MR effect size for
'Gut microbiota abundance (genus Catenibacterium id.2153)' on 'Ly6/PLAUR domain−containing protein 3'

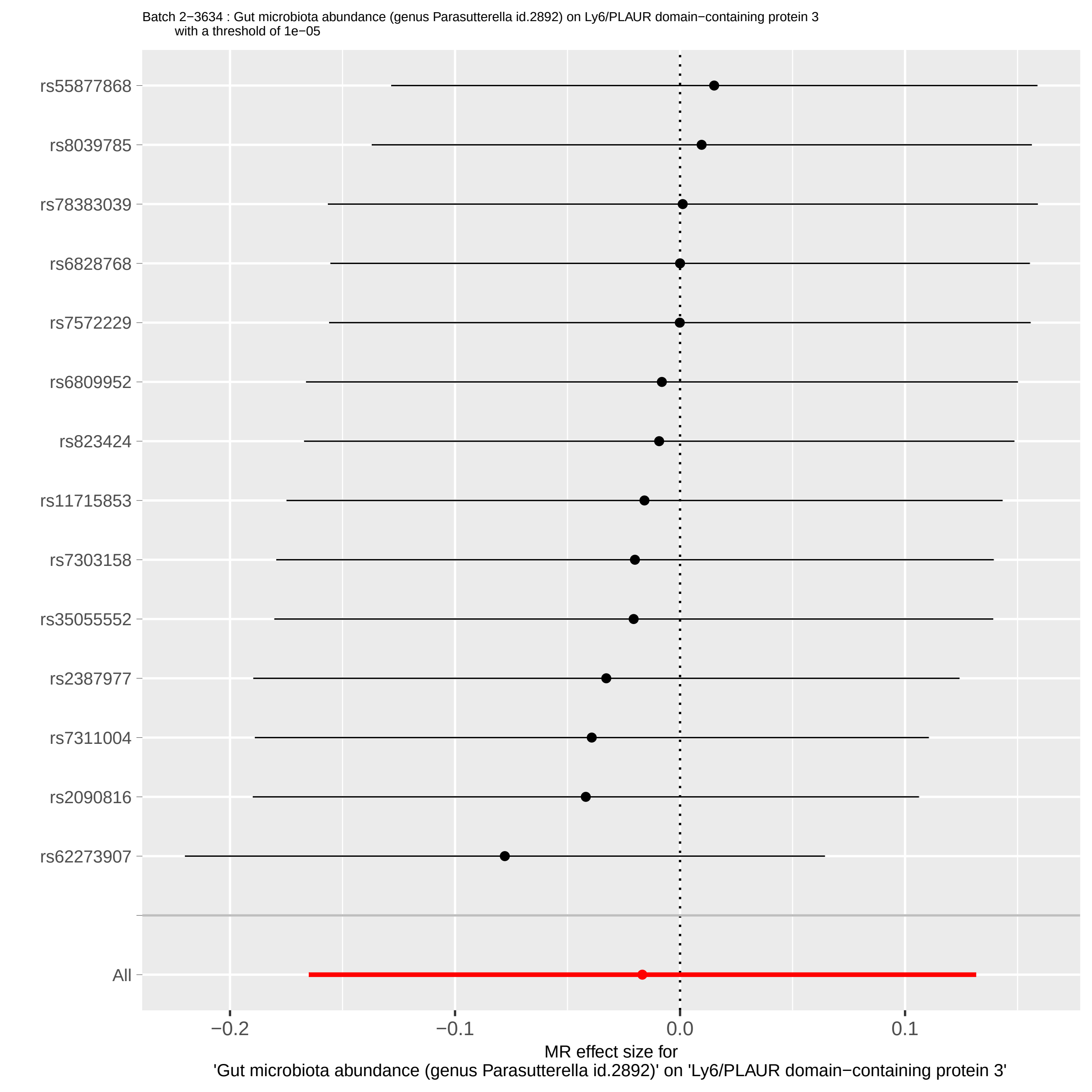

Batch 2−3634 : Gut microbiota abundance (genus Parasutterella id.2892) on Ly6/PLAUR domain−containing protein 3
with a threshold of 1e−05
rs55877868
rs8039785
rs78383039
rs6828768
rs7572229
rs6809952
rs823424
rs11715853
rs7303158
rs35055552
rs2387977
rs7311004
rs2090816
rs62273907
All
−0.2
−0.1
0.0
0.1
MR effect size for
'Gut microbiota abundance (genus Parasutterella id.2892)' on 'Ly6/PLAUR domain−containing protein 3'

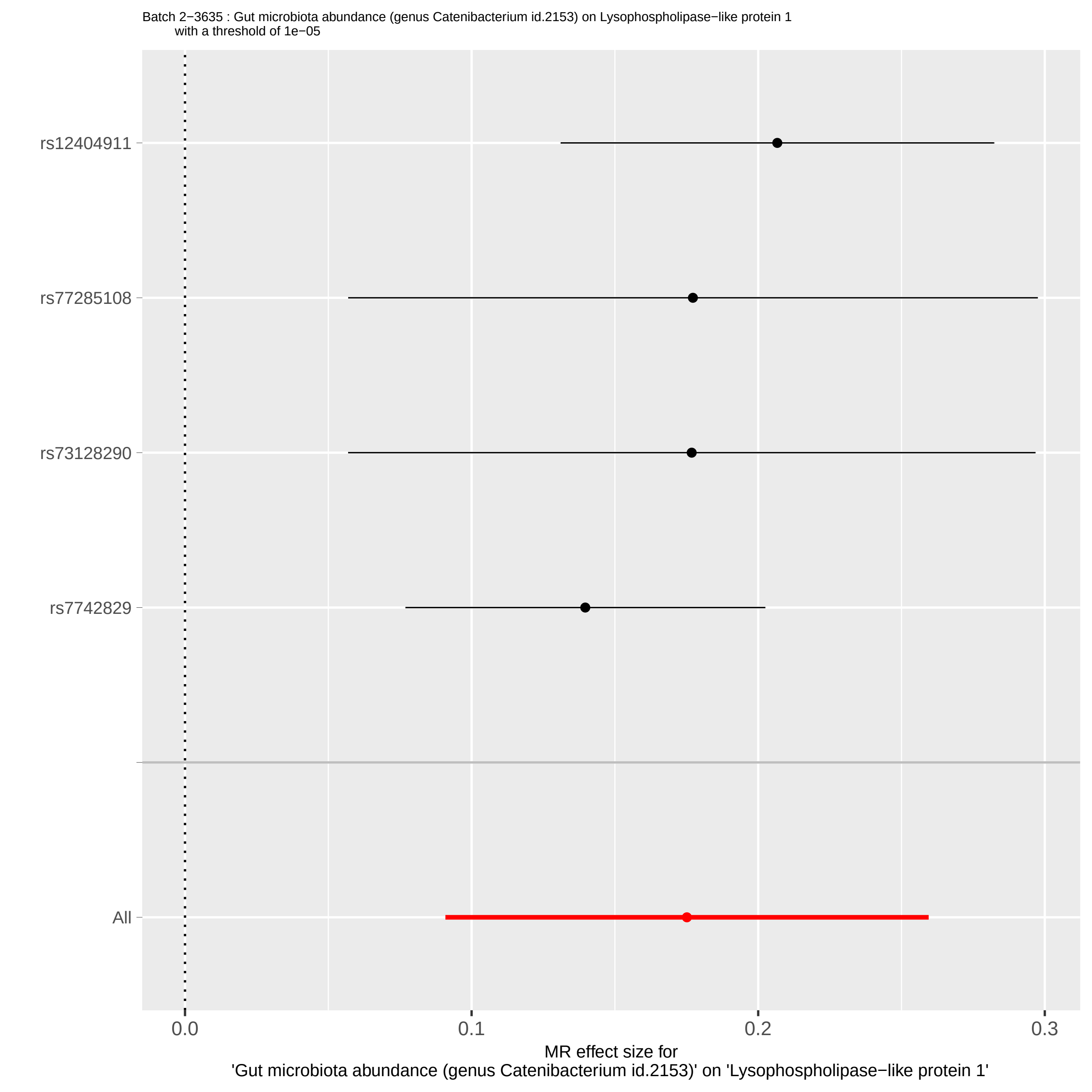
Batch 2−3635 : Gut microbiota abundance (genus Catenibacterium id.2153) on Lysophospholipase−like protein 1
with a threshold of 1e−05
rs12404911
rs77285108
rs73128290
rs7742829
All
0.0
0.1
0.2
0.3
MR effect size for
'Gut microbiota abundance (genus Catenibacterium id.2153)' on 'Lysophospholipase−like protein 1'

Batch 2−3636 : Gut microbiota abundance (genus Parasutterella id.2892) on Lysophospholipase−like protein 1
with a threshold of 1e−05

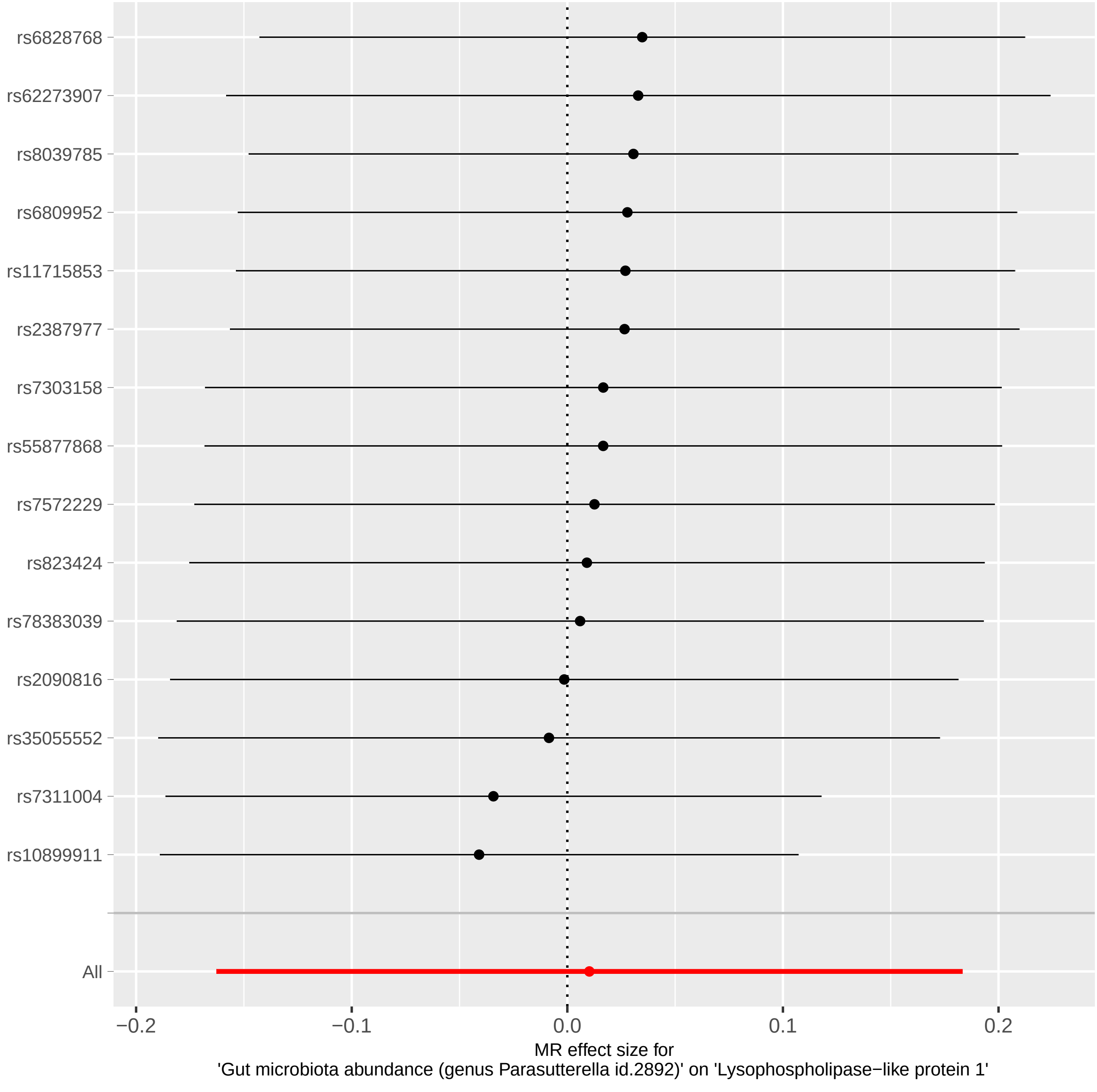

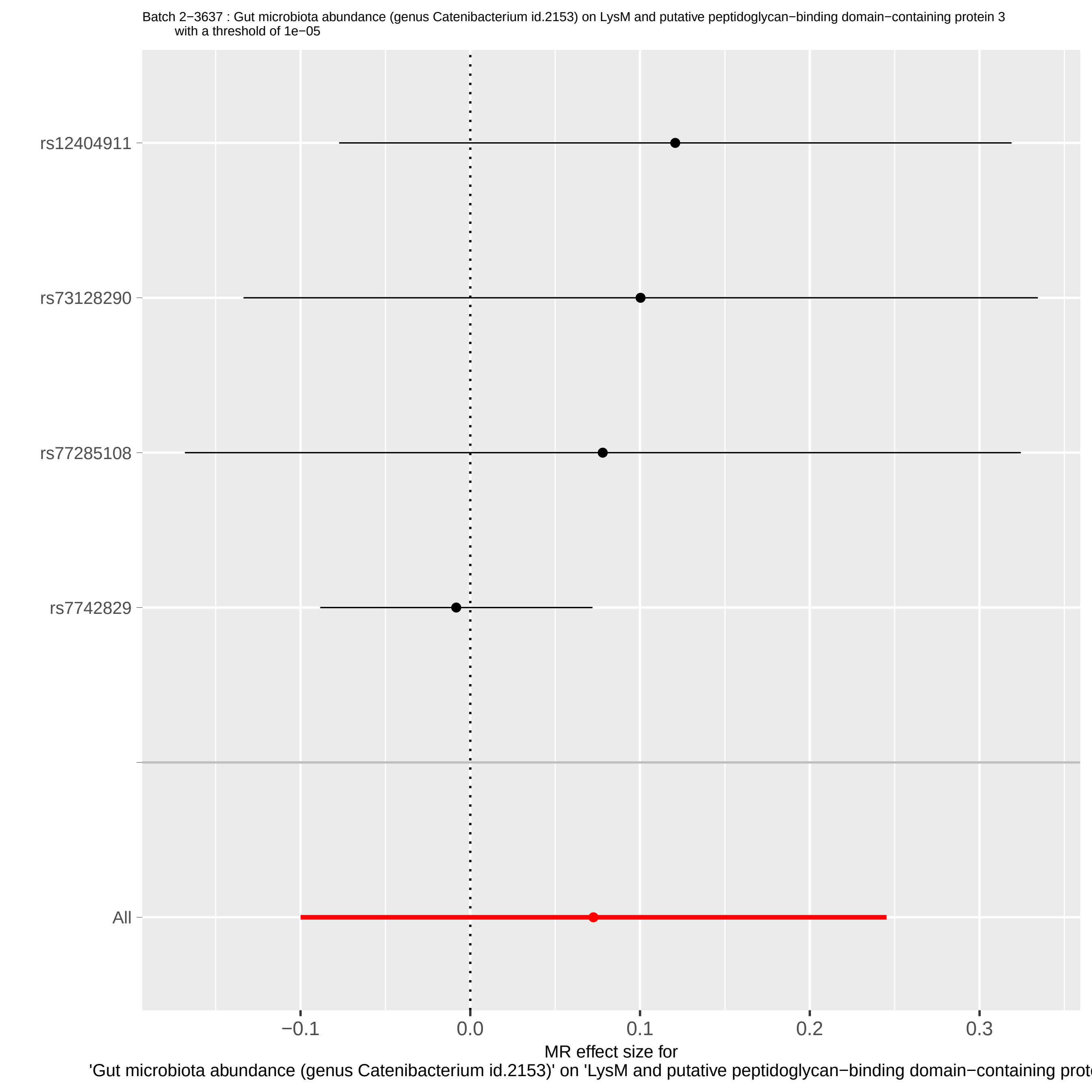
Batch 2−3637 : Gut microbiota abundance (genus Catenibacterium id.2153) on LysM and putative peptidoglycan−binding domain−containing protein 3
with a threshold of 1e−05
rs12404911
rs73128290
rs77285108
rs7742829
All
−0.1
0.0
0.1
0.2
0.3
MR effect size for
'Gut microbiota abundance (genus Catenibacterium id.2153)' on 'LysM and putative peptidoglycan−binding domain−containing prot

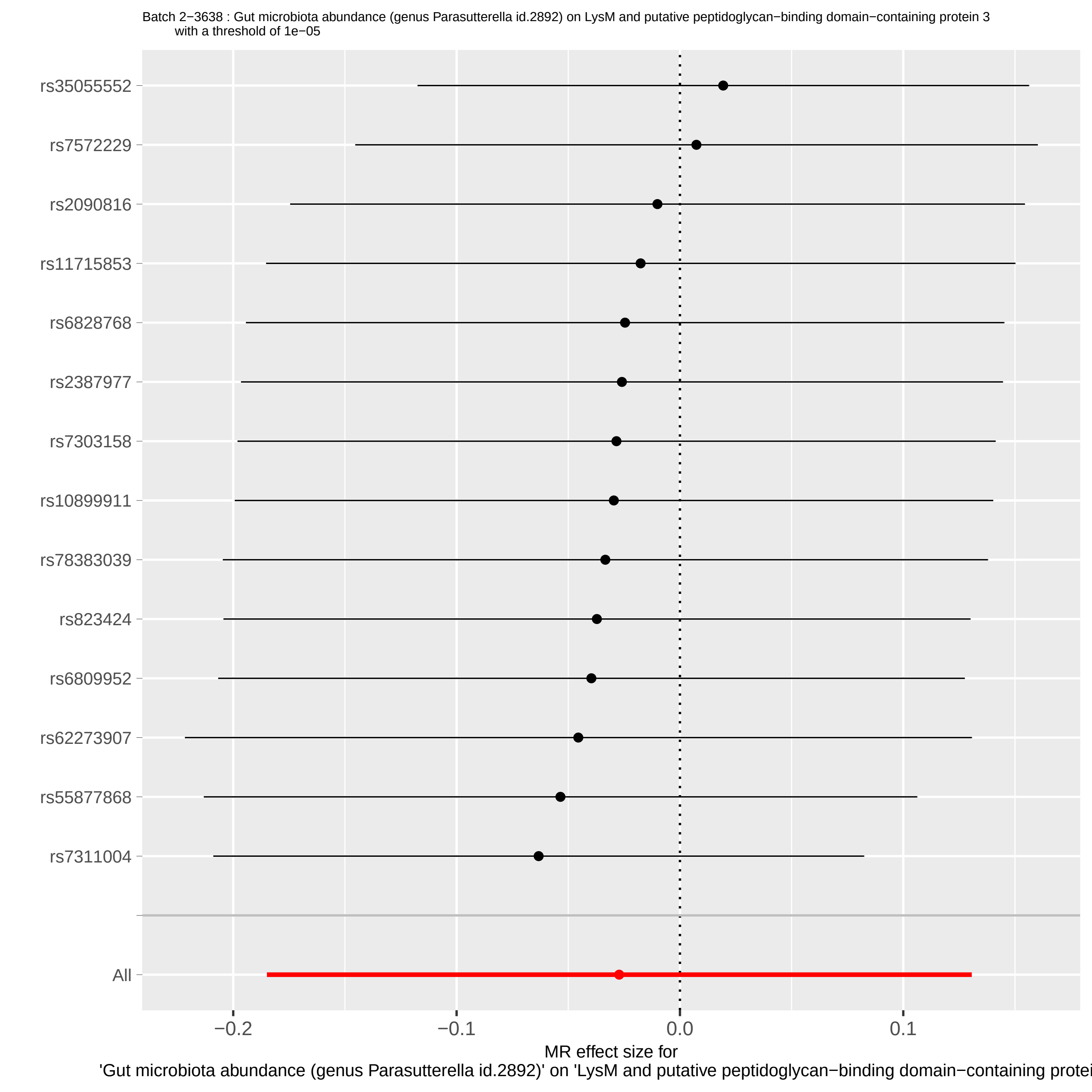

Batch 2−3638 : Gut microbiota abundance (genus Parasutterella id.2892) on LysM and putative peptidoglycan−binding domain−containing protein 3 with a threshold of 1e−05
rs35055552
rs7572229
rs2090816
rs11715853
rs6828768
rs2387977
rs7303158
rs10899911
rs78383039
rs823424
rs6809952
rs62273907
rs55877868
rs7311004
All
−0.2
−0.1
0.0
0.1
MR effect size for
'Gut microbiota abundance (genus Parasutterella id.2892)' on 'LysM and putative peptidoglycan−binding domain−containing protei

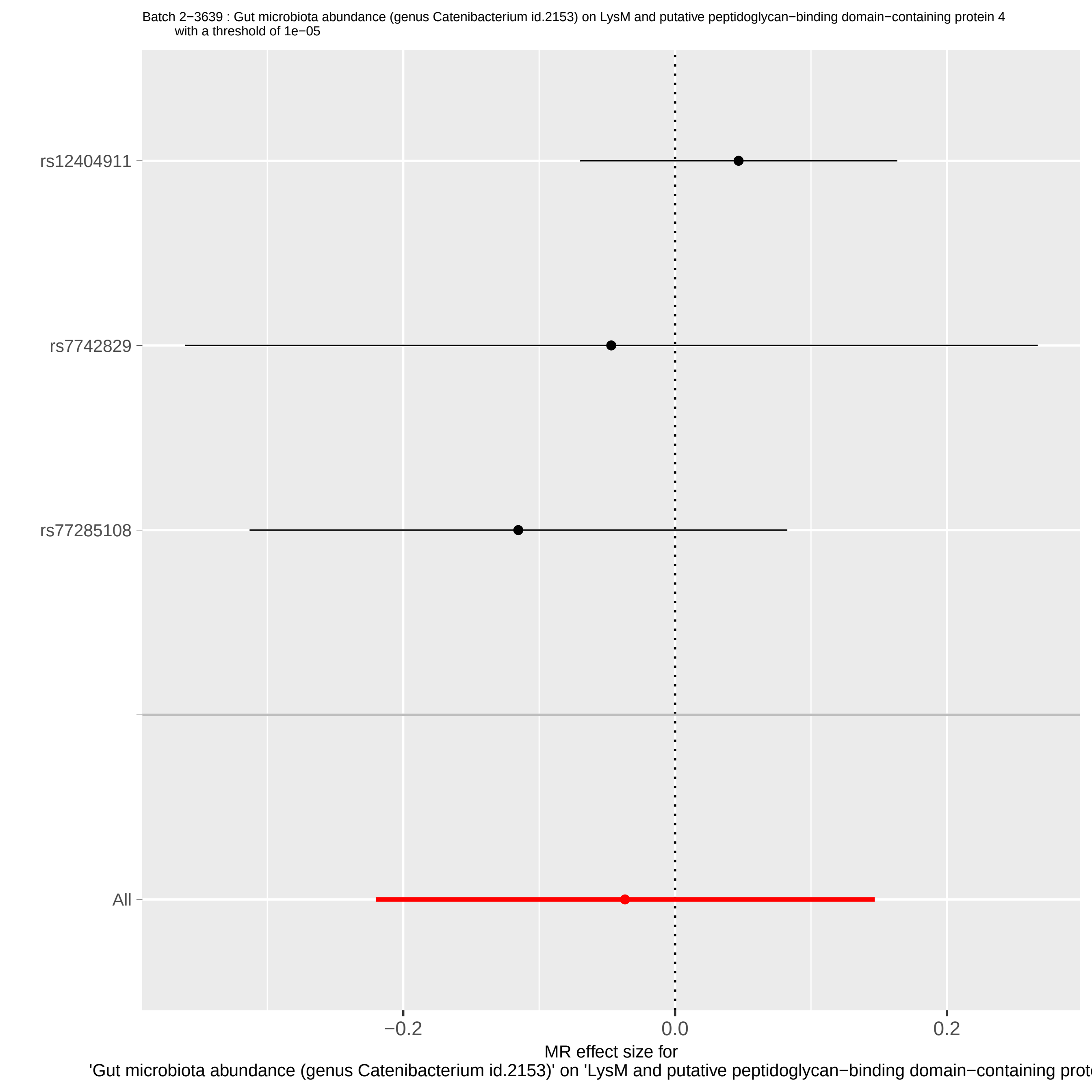
Batch 2−3639 : Gut microbiota abundance (genus Catenibacterium id.2153) on LysM and putative peptidoglycan−binding domain−containing protein 4
with a threshold of 1e−05
rs12404911
rs7742829
rs77285108
All
−0.2
0.0
0.2
MR effect size for
'Gut microbiota abundance (genus Catenibacterium id.2153)' on 'LysM and putative peptidoglycan−binding domain−containing prot

Batch 2−3640 : Gut microbiota abundance (genus Parasutterella id.2892) on LysM and putative peptidoglycan−binding domain−containing protein 4 with a threshold of 1e−05

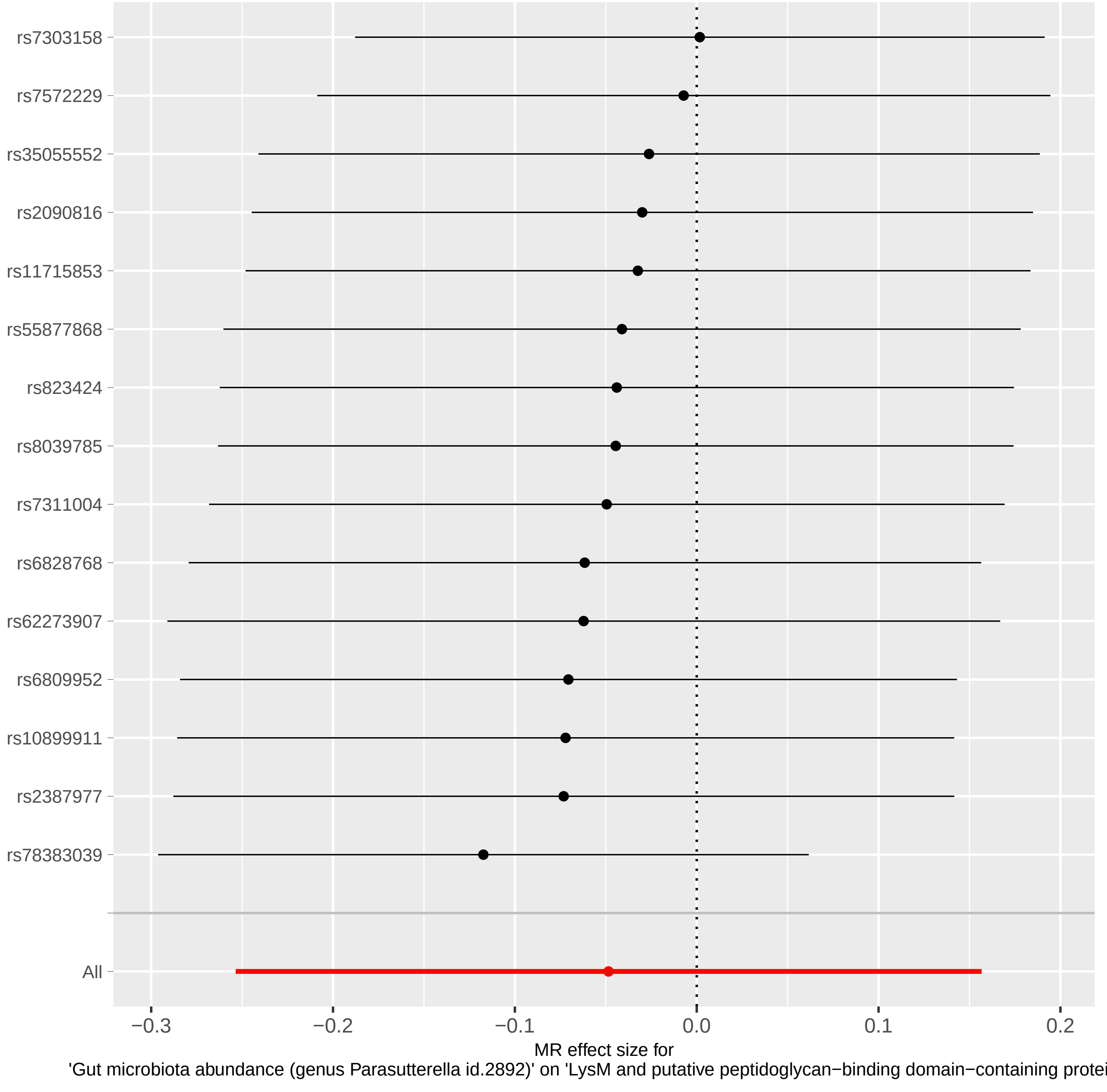

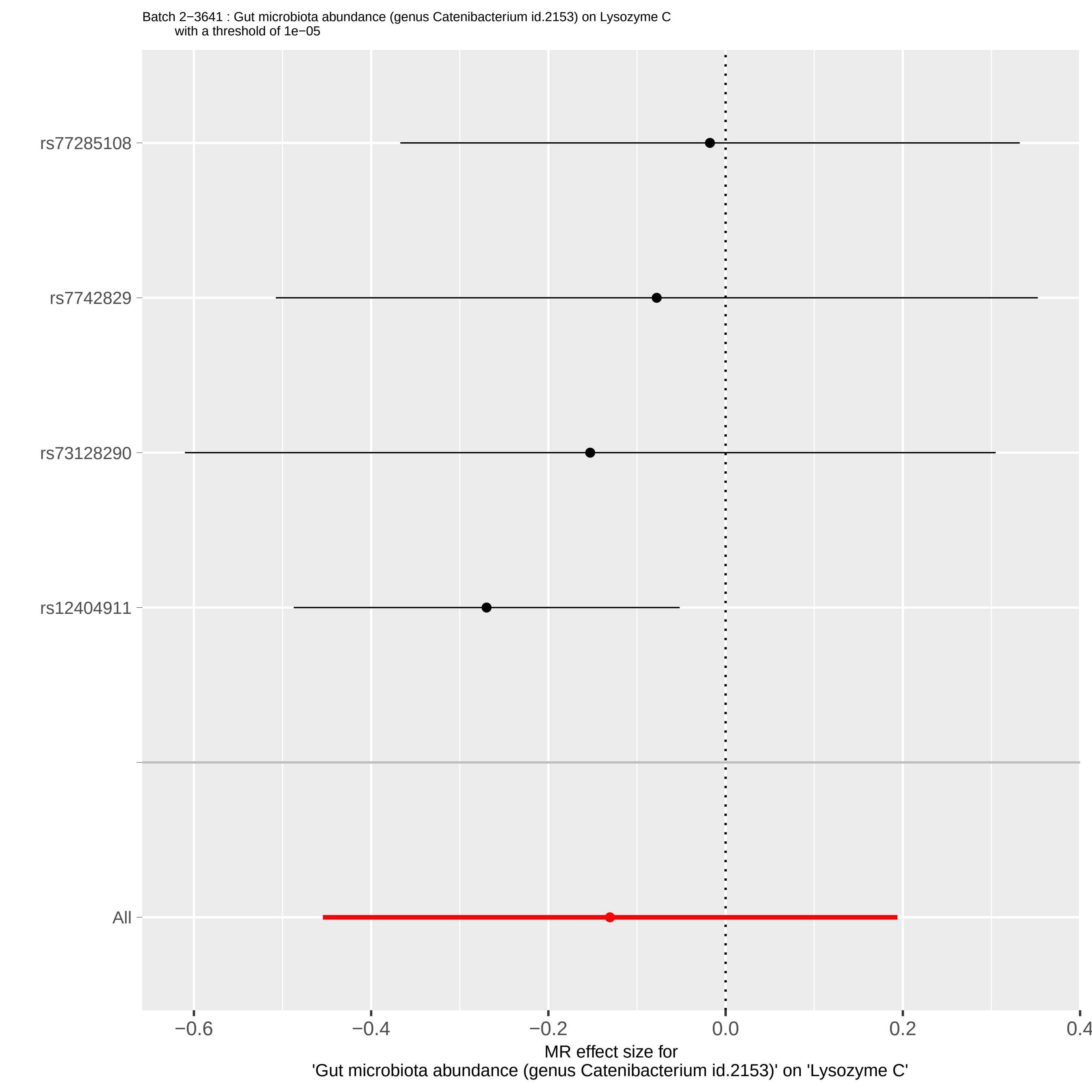

Batch 2−3641 : Gut microbiota abundance (genus Catenibacterium id.2153) on Lysozyme C
with a threshold of 1e−05
rs77285108
rs7742829
rs73128290
rs12404911
All
−0.6
−0.4
−0.2
0.0
0.2
0.4
MR effect size for
'Gut microbiota abundance (genus Catenibacterium id.2153)' on 'Lysozyme C'

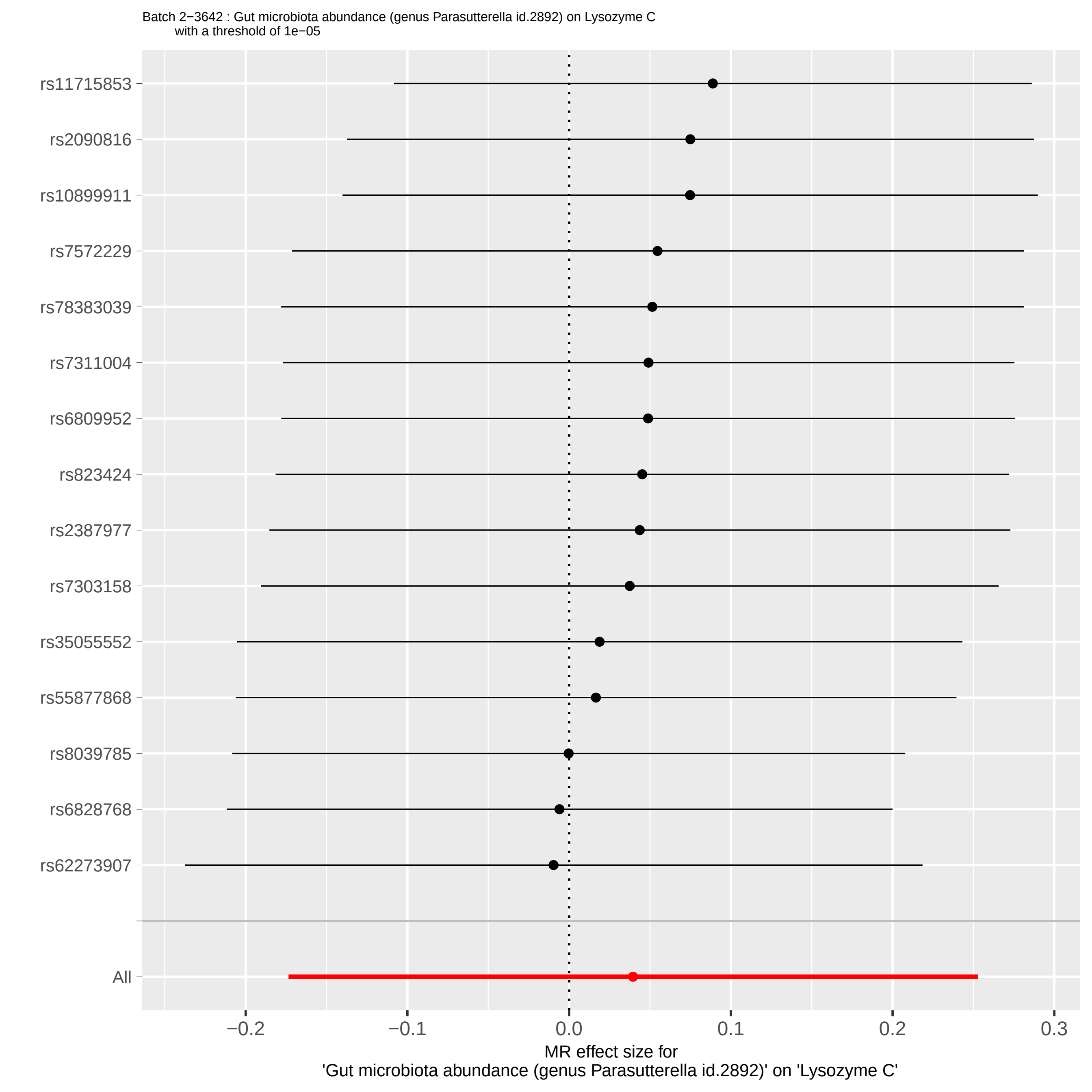
Batch 2−3642 : Gut microbiota abundance (genus Parasutterella id.2892) on Lysozyme C
with a threshold of 1e−05
rs11715853
rs2090816
rs10899911
rs7572229
rs78383039
rs7311004
rs6809952
rs823424
rs2387977
rs7303158
rs35055552
rs55877868
rs8039785
rs6828768
rs62273907
All
−0.2
−0.1
0.0
0.1
0.2
0.3
MR effect size for
'Gut microbiota abundance (genus Parasutterella id.2892)' on 'Lysozyme C'

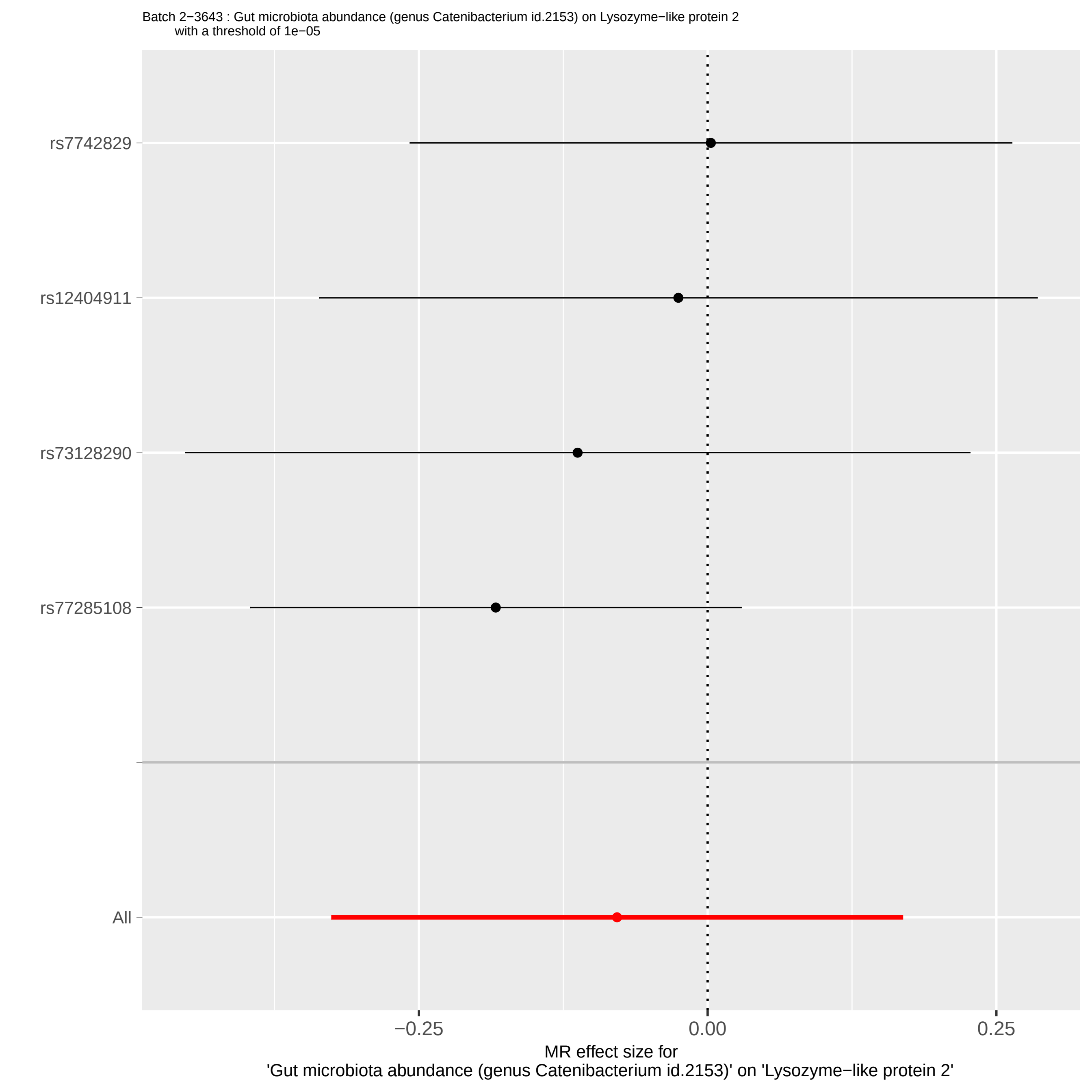
Batch 2−3643 : Gut microbiota abundance (genus Catenibacterium id.2153) on Lysozyme−like protein 2
with a threshold of 1e−05
rs7742829
rs12404911
rs73128290
rs77285108
All
−0.25
0.00
0.25
MR effect size for
'Gut microbiota abundance (genus Catenibacterium id.2153)' on 'Lysozyme−like protein 2'

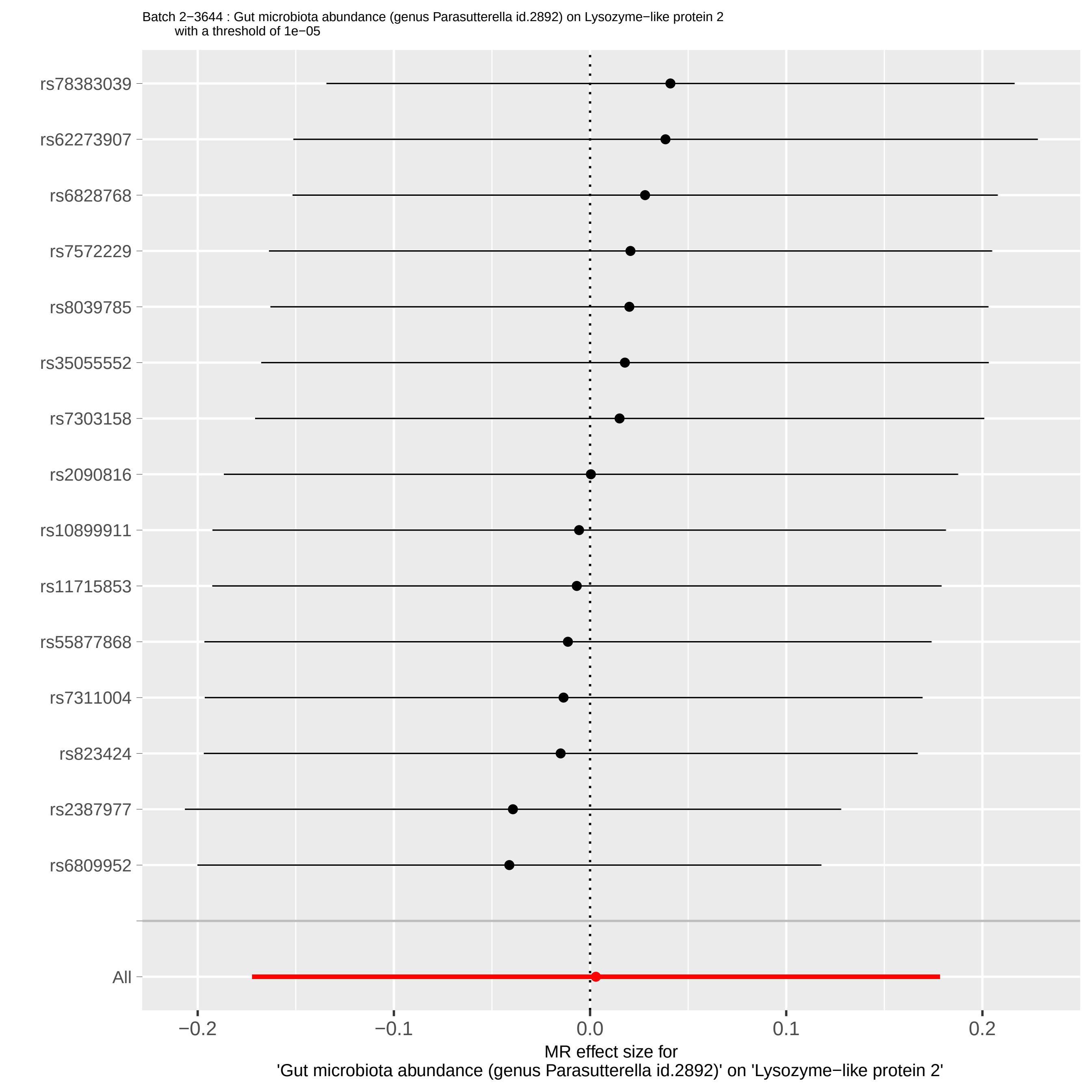

Batch 2−3644 : Gut microbiota abundance (genus Parasutterella id.2892) on Lysozyme−like protein 2
with a threshold of 1e−05
rs78383039
rs62273907
rs6828768
rs7572229
rs8039785
rs35055552
rs7303158
rs2090816
rs10899911
rs11715853
rs55877868
rs7311004
rs823424
rs2387977
rs6809952
All
−0.2
−0.1
0.0
0.1
0.2
MR effect size for
'Gut microbiota abundance (genus Parasutterella id.2892)' on 'Lysozyme−like protein 2'

Batch 2−3645 : Gut microbiota abundance (genus Catenibacterium id.2153) on Cation−dependent mannose−6−phosphate receptor
with a threshold of 1e−05

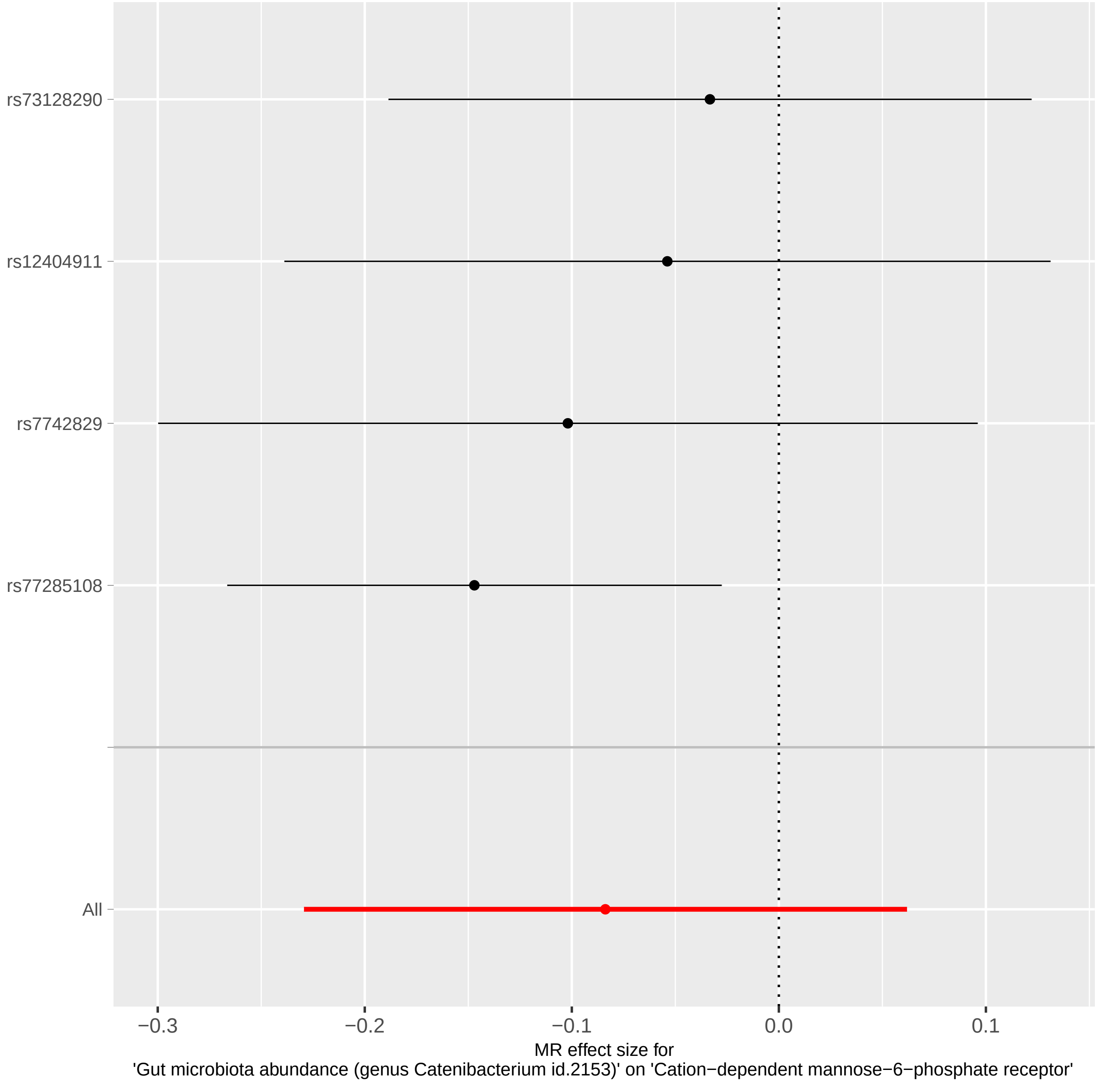

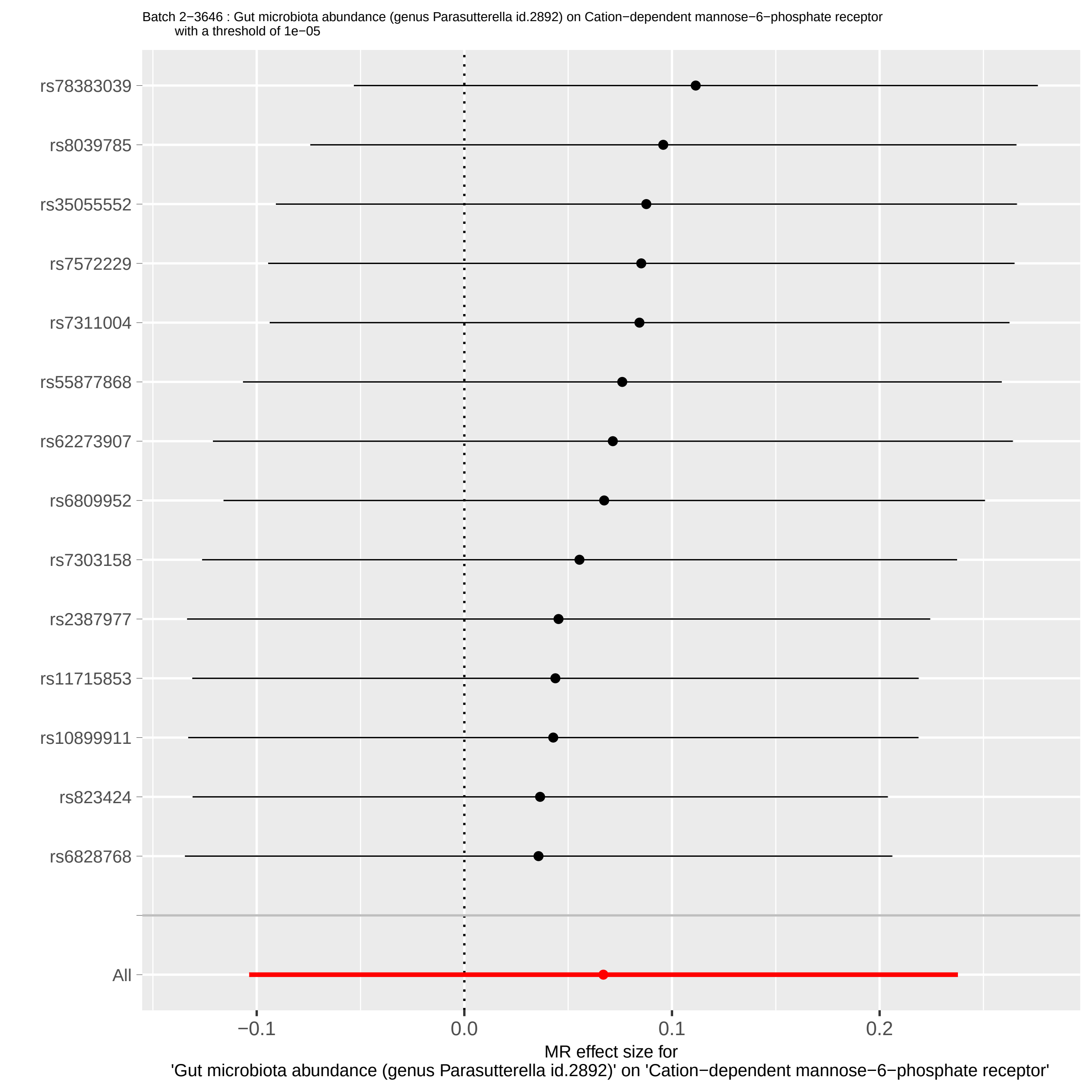

Batch 2−3646 : Gut microbiota abundance (genus Parasutterella id.2892) on Cation−dependent mannose−6−phosphate receptor
with a threshold of 1e−05
rs78383039
rs8039785
rs35055552
rs7572229
rs7311004
rs55877868
rs62273907
rs6809952
rs7303158
rs2387977
rs11715853
rs10899911
rs823424
rs6828768
All
−0.1
0.0
0.1
0.2
MR effect size for
'Gut microbiota abundance (genus Parasutterella id.2892)' on 'Cation−dependent mannose−6−phosphate receptor'

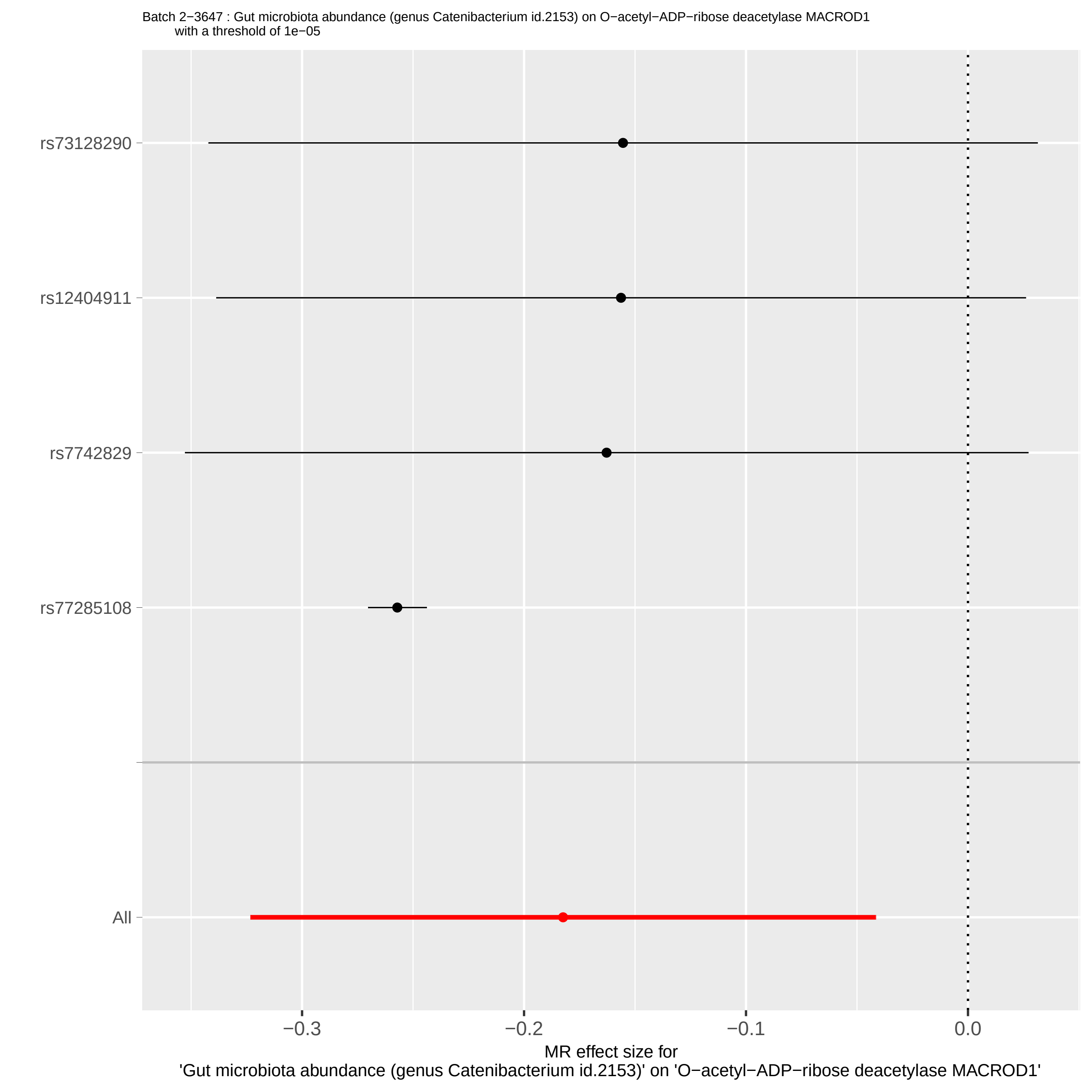
Batch 2−3647 : Gut microbiota abundance (genus Catenibacterium id.2153) on O−acetyl−ADP−ribose deacetylase MACROD1
with a threshold of 1e−05
rs73128290
rs12404911
rs7742829
rs77285108
All
−0.3
−0.2
−0.1
0.0
MR effect size for
'Gut microbiota abundance (genus Catenibacterium id.2153)' on 'O−acetyl−ADP−ribose deacetylase MACROD1'

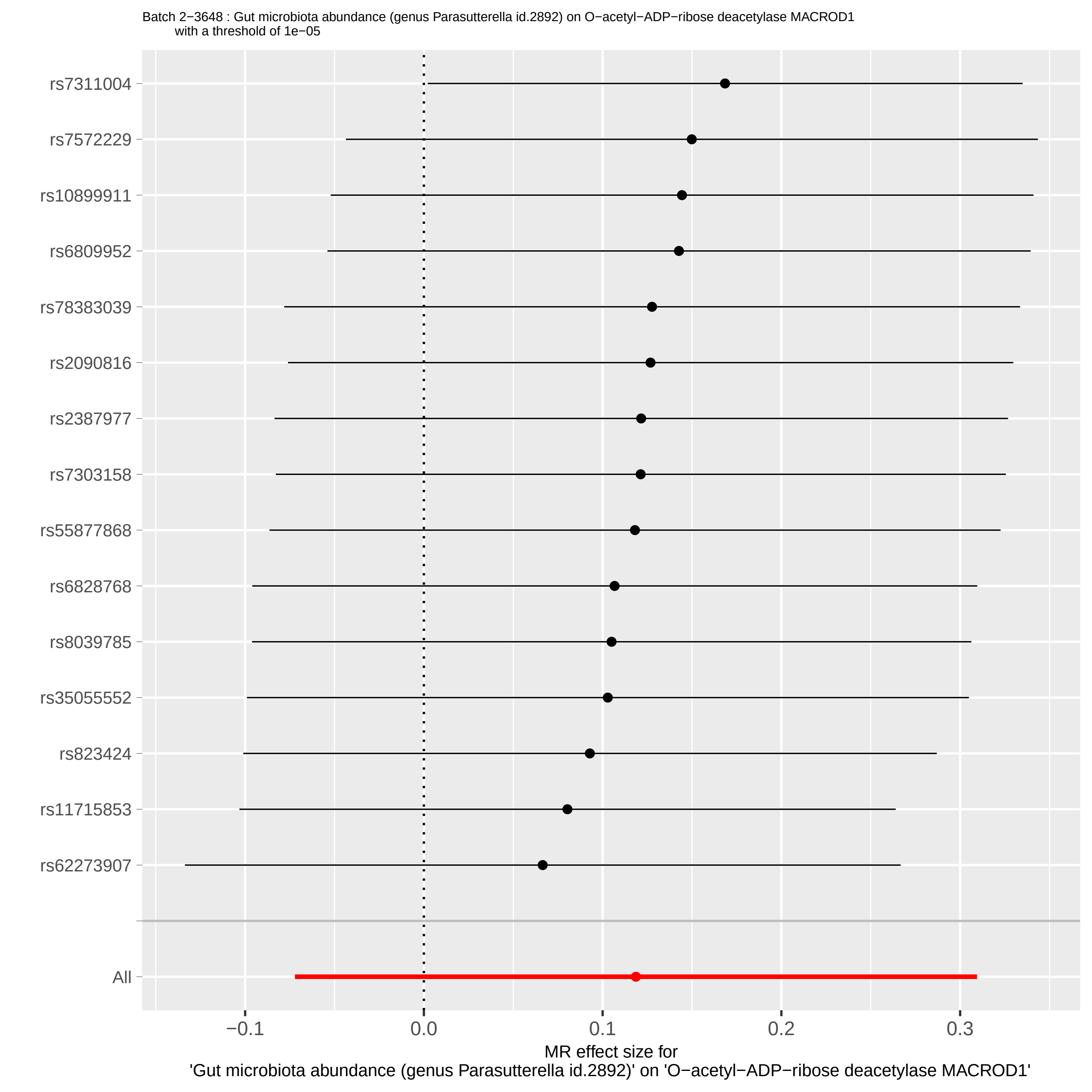
Batch 2−3648 : Gut microbiota abundance (genus Parasutterella id.2892) on O−acetyl−ADP−ribose deacetylase MACROD1
with a threshold of 1e−05
rs7311004
rs7572229
rs10899911
rs6809952
rs78383039
rs2090816
rs2387977
rs7303158
rs55877868
rs6828768
rs8039785
rs35055552
rs823424
rs11715853
rs62273907
All
−0.1
0.0
0.1
0.2
0.3
MR effect size for
'Gut microbiota abundance (genus Parasutterella id.2892)' on 'O−acetyl−ADP−ribose deacetylase MACROD1'

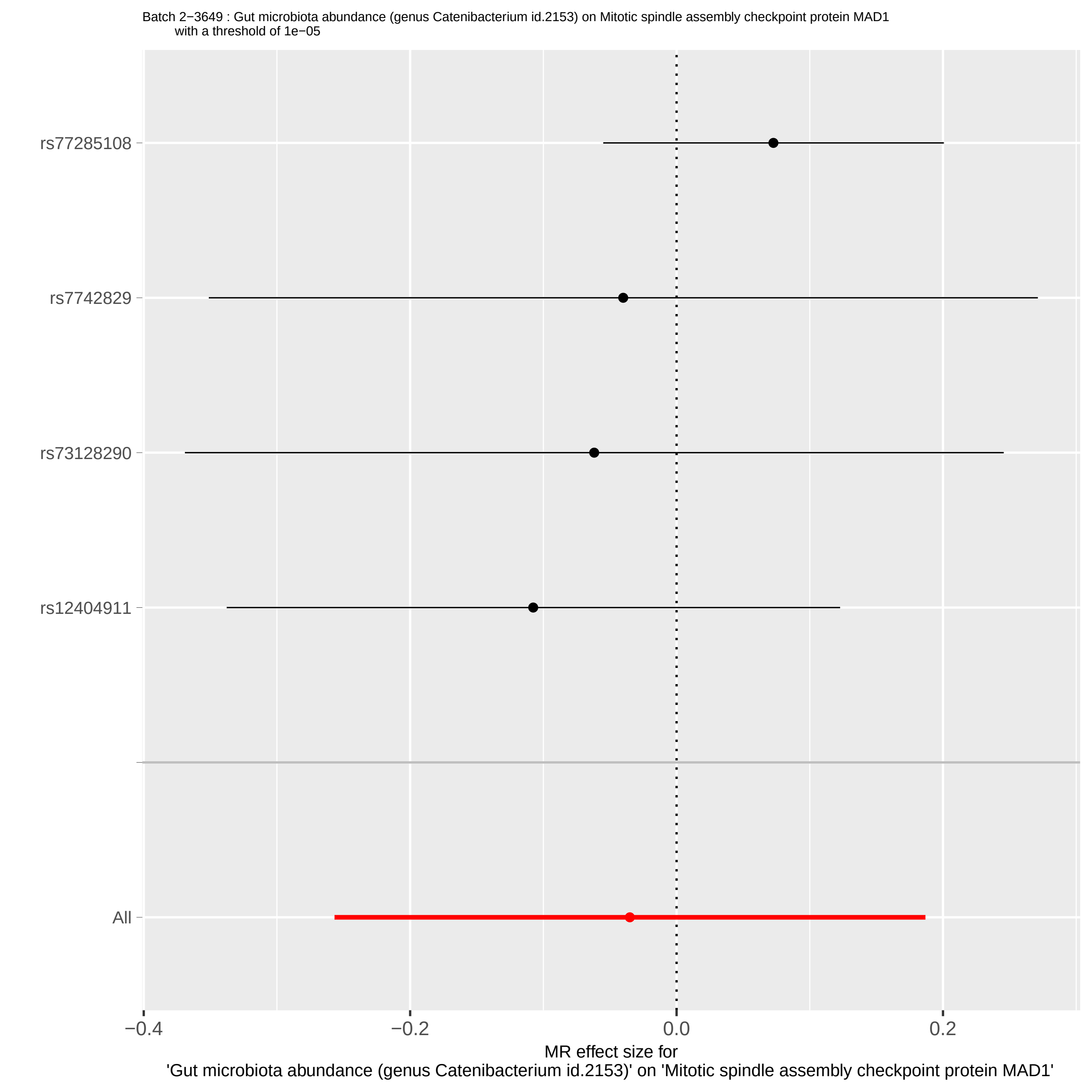
Batch 2−3649 : Gut microbiota abundance (genus Catenibacterium id.2153) on Mitotic spindle assembly checkpoint protein MAD1
with a threshold of 1e−05
rs77285108
rs7742829
rs73128290
rs12404911
All
−0.4
−0.2
0.0
0.2
MR effect size for
'Gut microbiota abundance (genus Catenibacterium id.2153)' on 'Mitotic spindle assembly checkpoint protein MAD1'

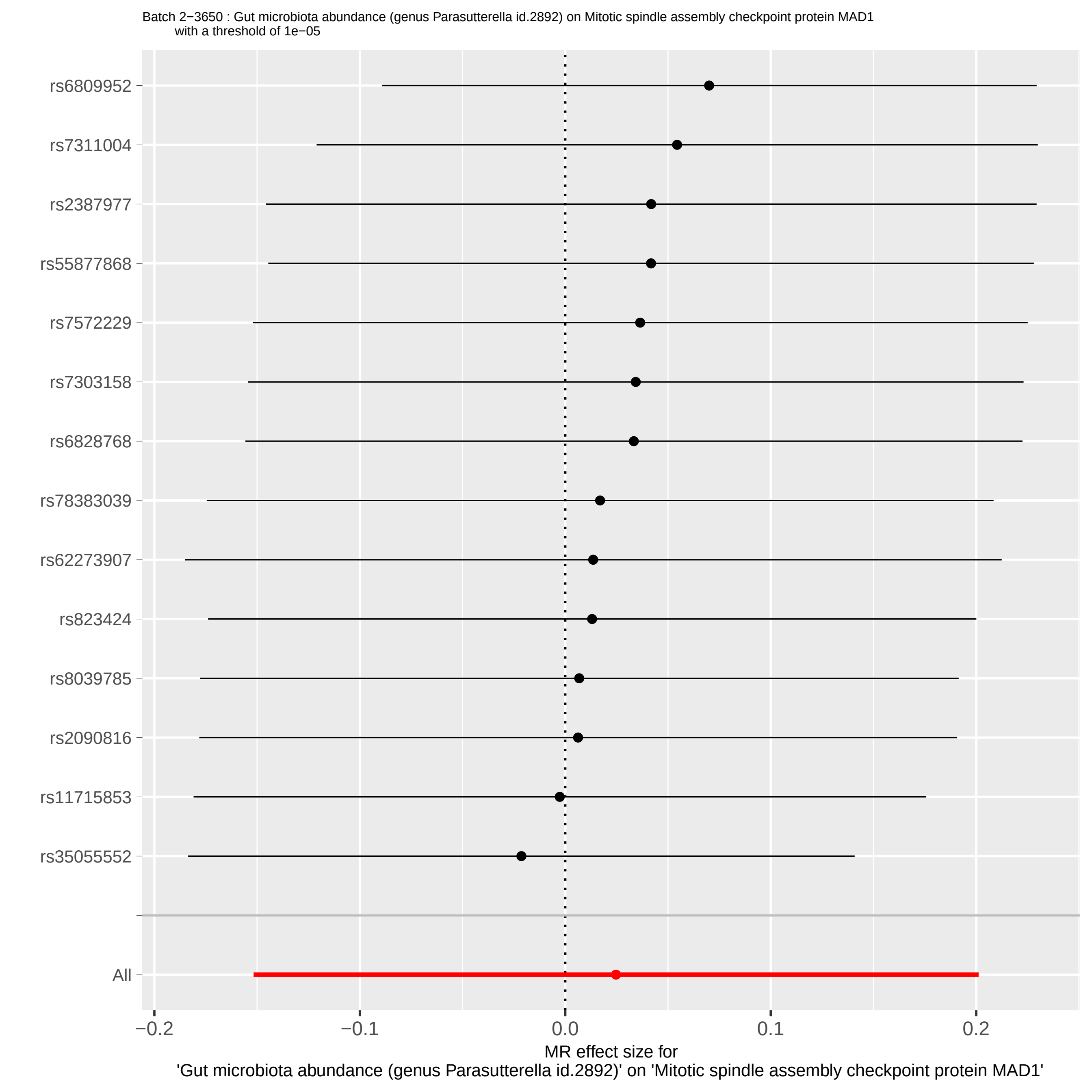
Batch 2−3650 : Gut microbiota abundance (genus Parasutterella id.2892) on Mitotic spindle assembly checkpoint protein MAD1
with a threshold of 1e−05
rs6809952
rs7311004
rs2387977
rs55877868
rs7572229
rs7303158
rs6828768
rs78383039
rs62273907
rs823424
rs8039785
rs2090816
rs11715853
rs35055552
All
−0.2
−0.1
0.0
0.1
0.2
MR effect size for
'Gut microbiota abundance (genus Parasutterella id.2892)' on 'Mitotic spindle assembly checkpoint protein MAD1'

Batch 2−3651 : Gut microbiota abundance (genus Catenibacterium id.2153) on Mucosal addressin cell adhesion molecule 1
with a threshold of 1e−05

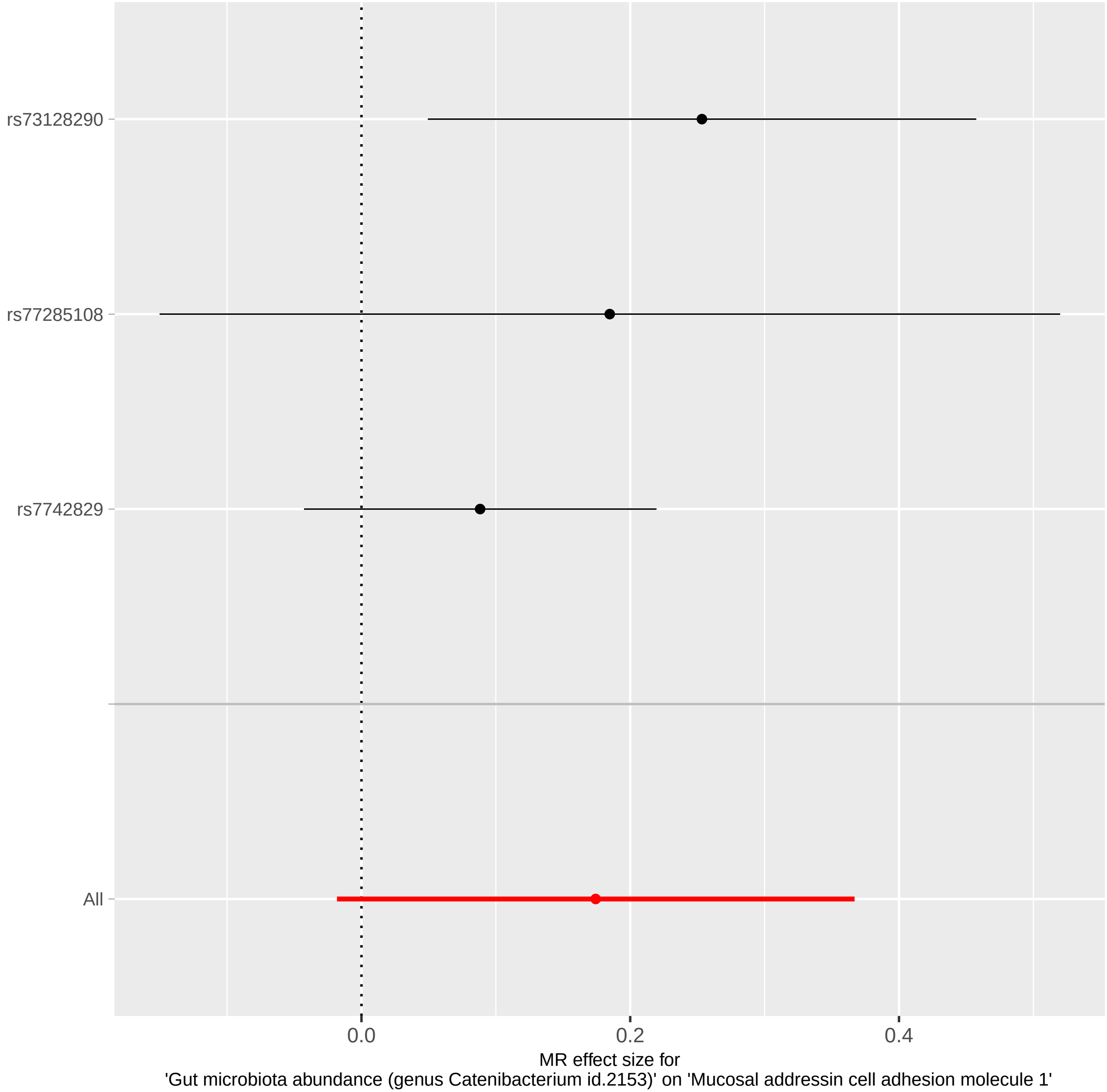

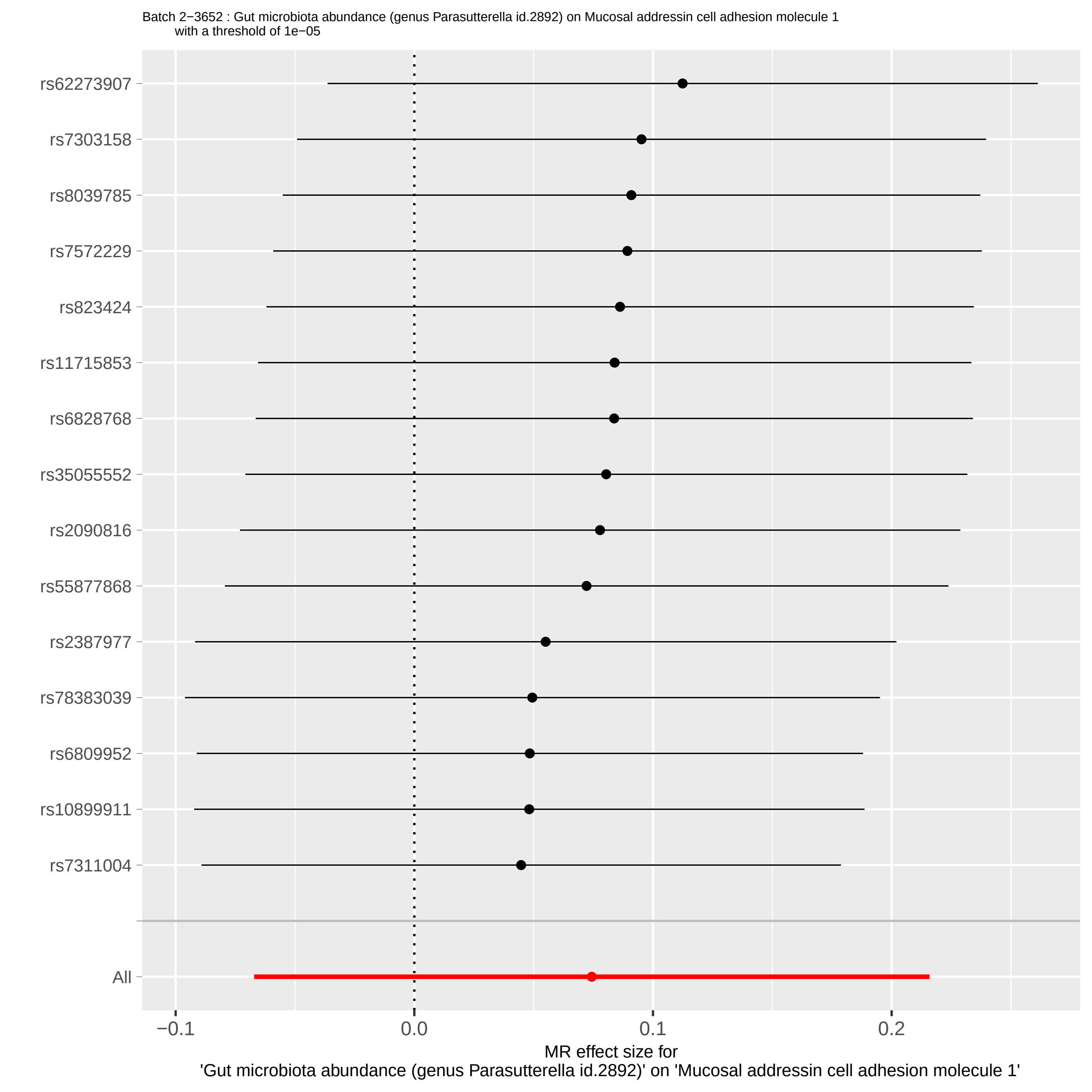
Batch 2−3652 : Gut microbiota abundance (genus Parasutterella id.2892) on Mucosal addressin cell adhesion molecule 1
with a threshold of 1e−05
rs62273907
rs7303158
rs8039785
rs7572229
rs823424
rs11715853
rs6828768
rs35055552
rs2090816
rs55877868
rs2387977
rs78383039
rs6809952
rs10899911
rs7311004
All
−0.1
0.0
0.1
0.2
MR effect size for
'Gut microbiota abundance (genus Parasutterella id.2892)' on 'Mucosal addressin cell adhesion molecule 1'

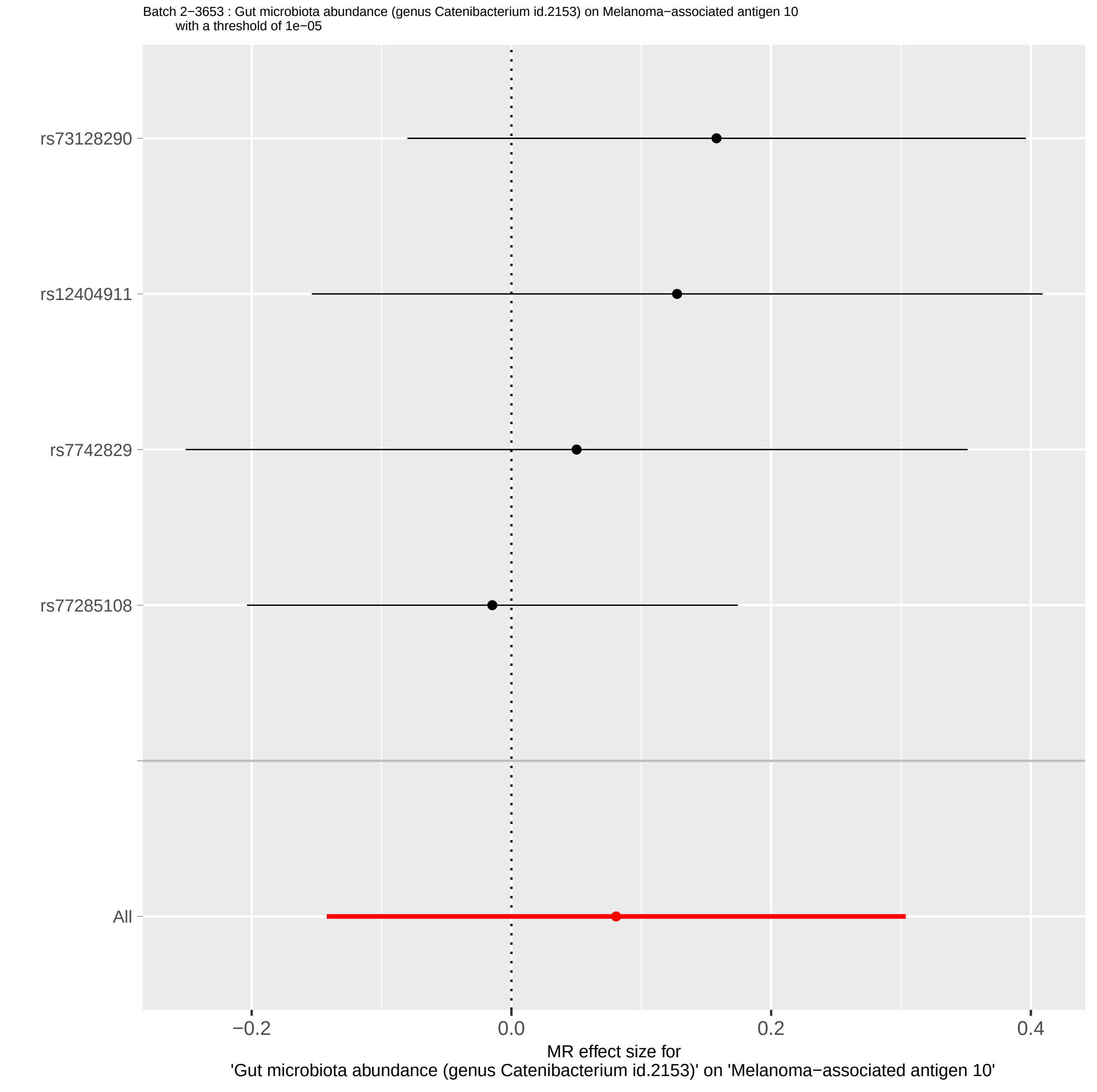

Batch 2−3653 : Gut microbiota abundance (genus Catenibacterium id.2153) on Melanoma−associated antigen 10
with a threshold of 1e−05
rs73128290
rs12404911
rs7742829
rs77285108
All
−0.2
0.0
0.2
0.4
MR effect size for
'Gut microbiota abundance (genus Catenibacterium id.2153)' on 'Melanoma−associated antigen 10'

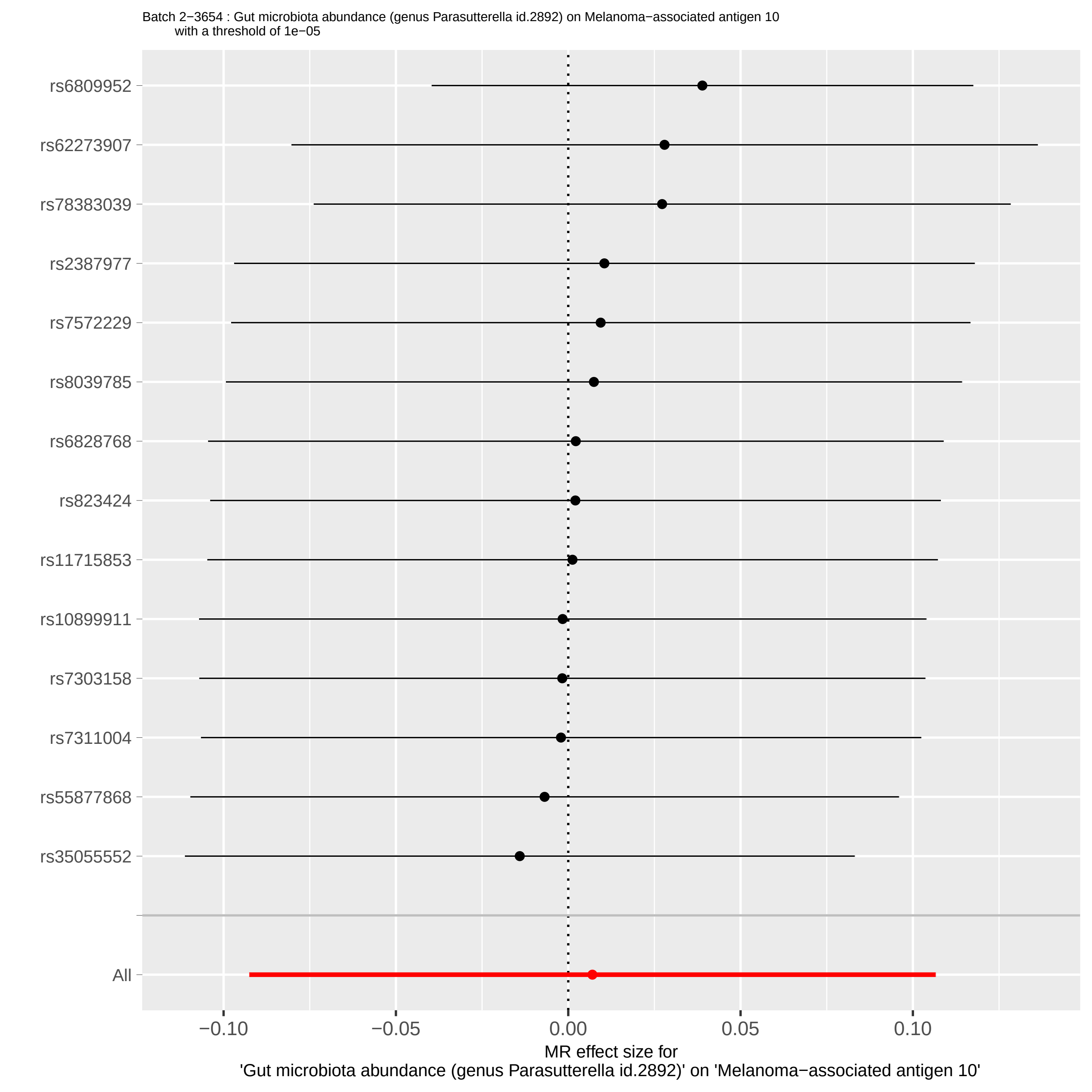

Batch 2−3654 : Gut microbiota abundance (genus Parasutterella id.2892) on Melanoma−associated antigen 10
with a threshold of 1e−05
rs6809952
rs62273907
rs78383039
rs2387977
rs7572229
rs8039785
rs6828768
rs823424
rs11715853
rs10899911
rs7303158
rs7311004
rs55877868
rs35055552
All
−0.10
−0.05
0.00
0.05
0.10
MR effect size for
'Gut microbiota abundance (genus Parasutterella id.2892)' on 'Melanoma−associated antigen 10'

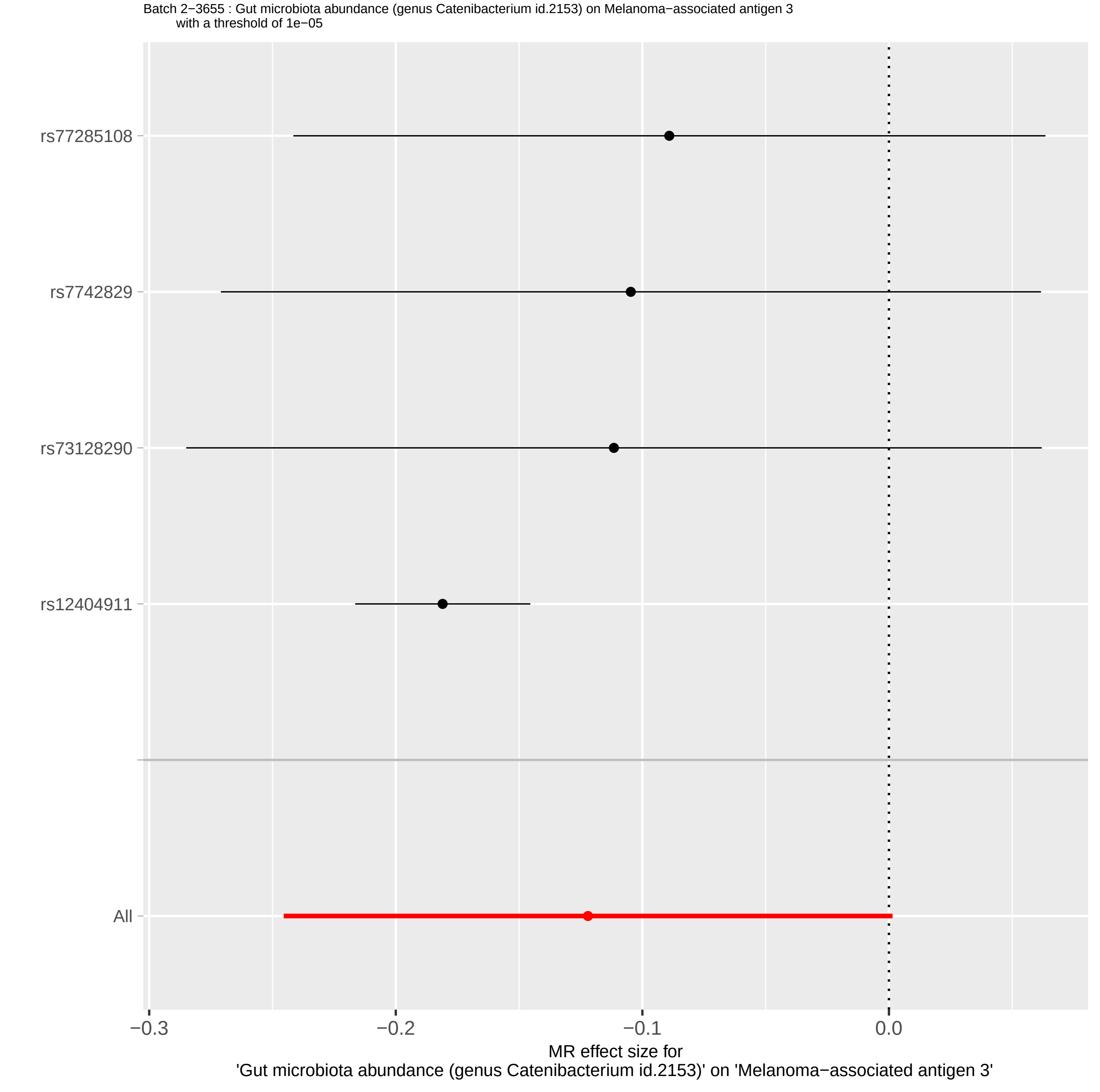

Batch 2−3655 : Gut microbiota abundance (genus Catenibacterium id.2153) on Melanoma−associated antigen 3
with a threshold of 1e−05
rs77285108
rs7742829
rs73128290
rs12404911
All
−0.3
−0.2
−0.1
0.0
MR effect size for
'Gut microbiota abundance (genus Catenibacterium id.2153)' on 'Melanoma−associated antigen 3'

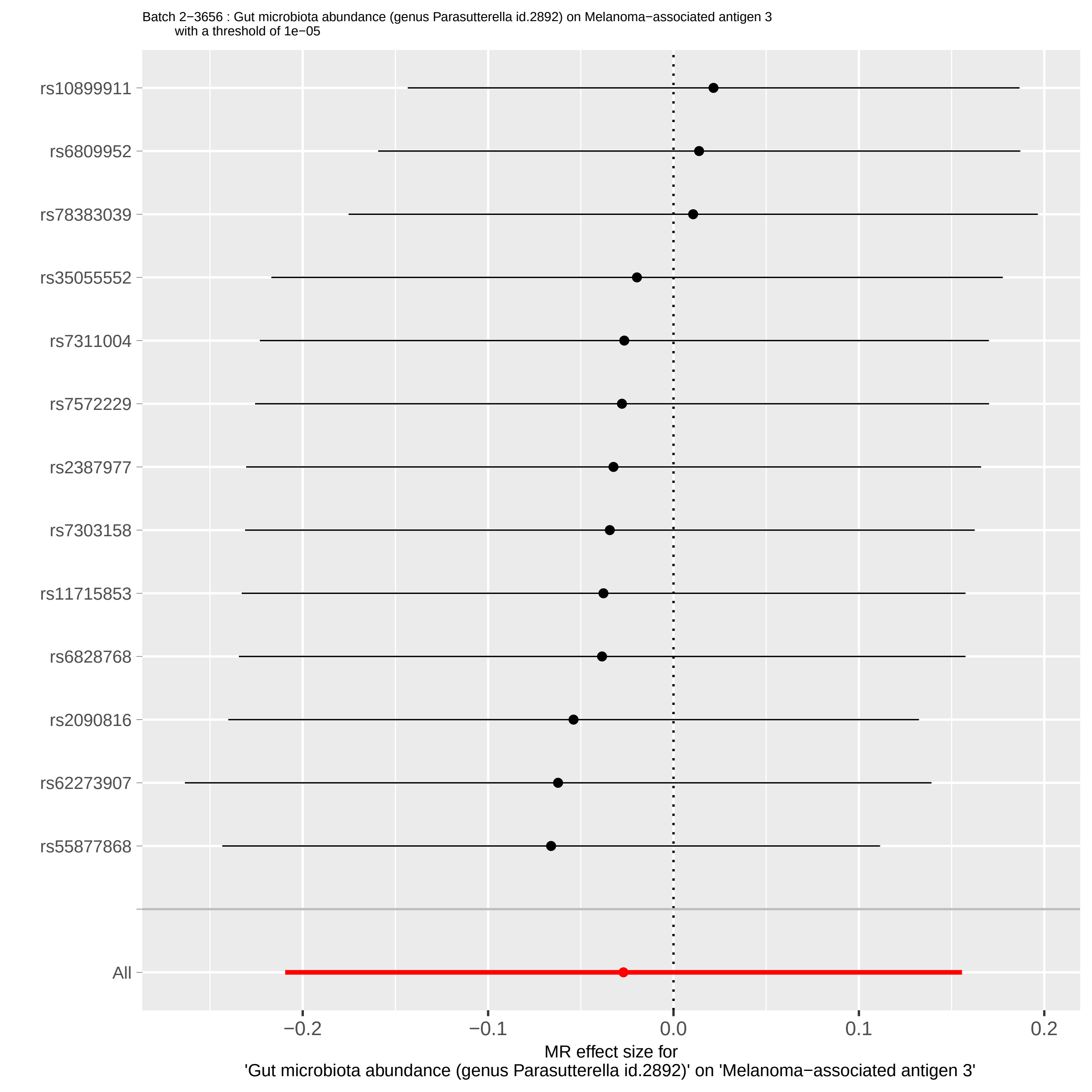

Batch 2−3656 : Gut microbiota abundance (genus Parasutterella id.2892) on Melanoma−associated antigen 3
with a threshold of 1e−05
rs10899911
rs6809952
rs78383039
rs35055552
rs7311004
rs7572229
rs2387977
rs7303158
rs11715853
rs6828768
rs2090816
rs62273907
rs55877868
All
−0.2
−0.1
0.0
0.1
0.2
MR effect size for
'Gut microbiota abundance (genus Parasutterella id.2892)' on 'Melanoma−associated antigen 3'

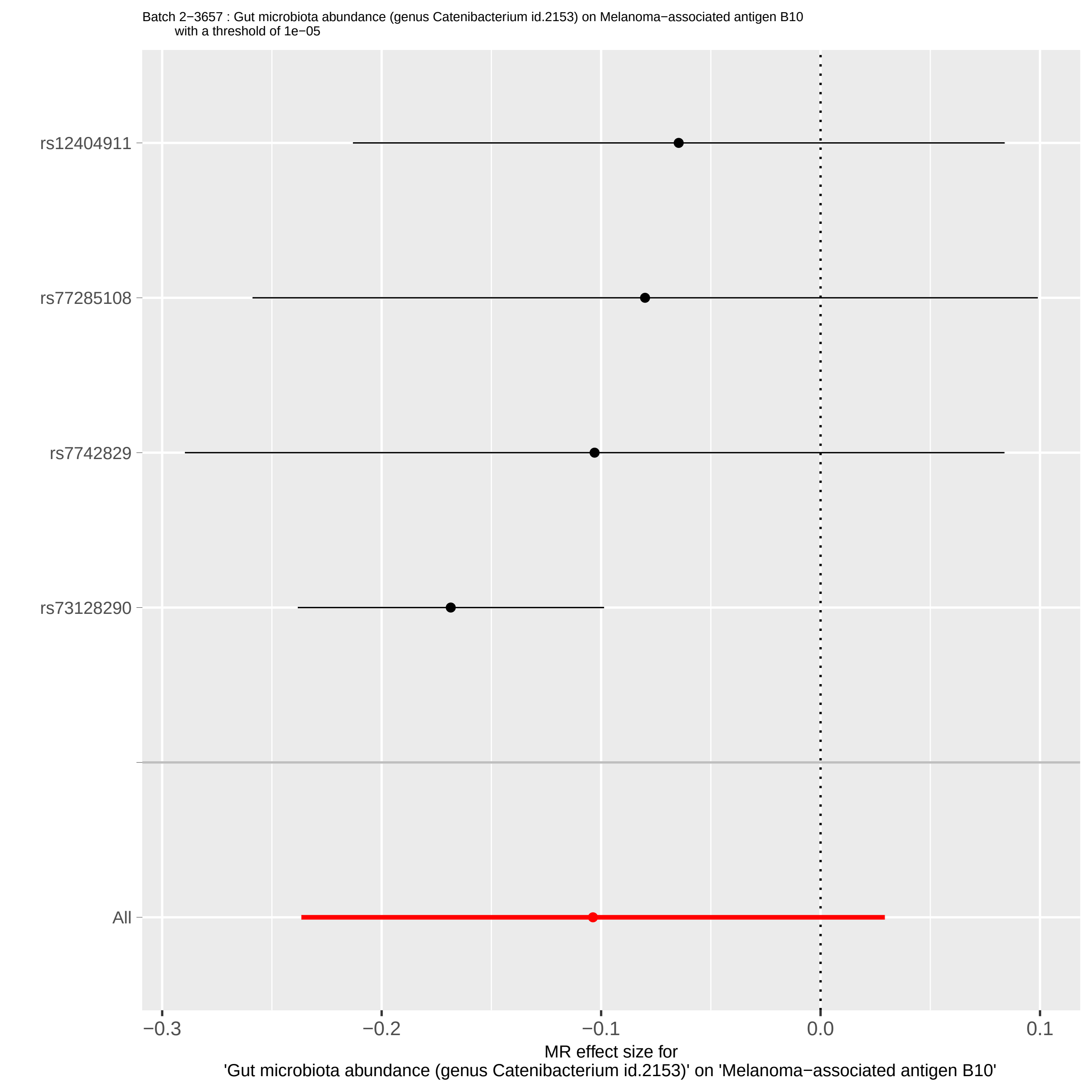
Batch 2−3657 : Gut microbiota abundance (genus Catenibacterium id.2153) on Melanoma−associated antigen B10
with a threshold of 1e−05
rs12404911
rs77285108
rs7742829
rs73128290
All
−0.3
−0.2
−0.1
0.0
0.1
MR effect size for
'Gut microbiota abundance (genus Catenibacterium id.2153)' on 'Melanoma−associated antigen B10'

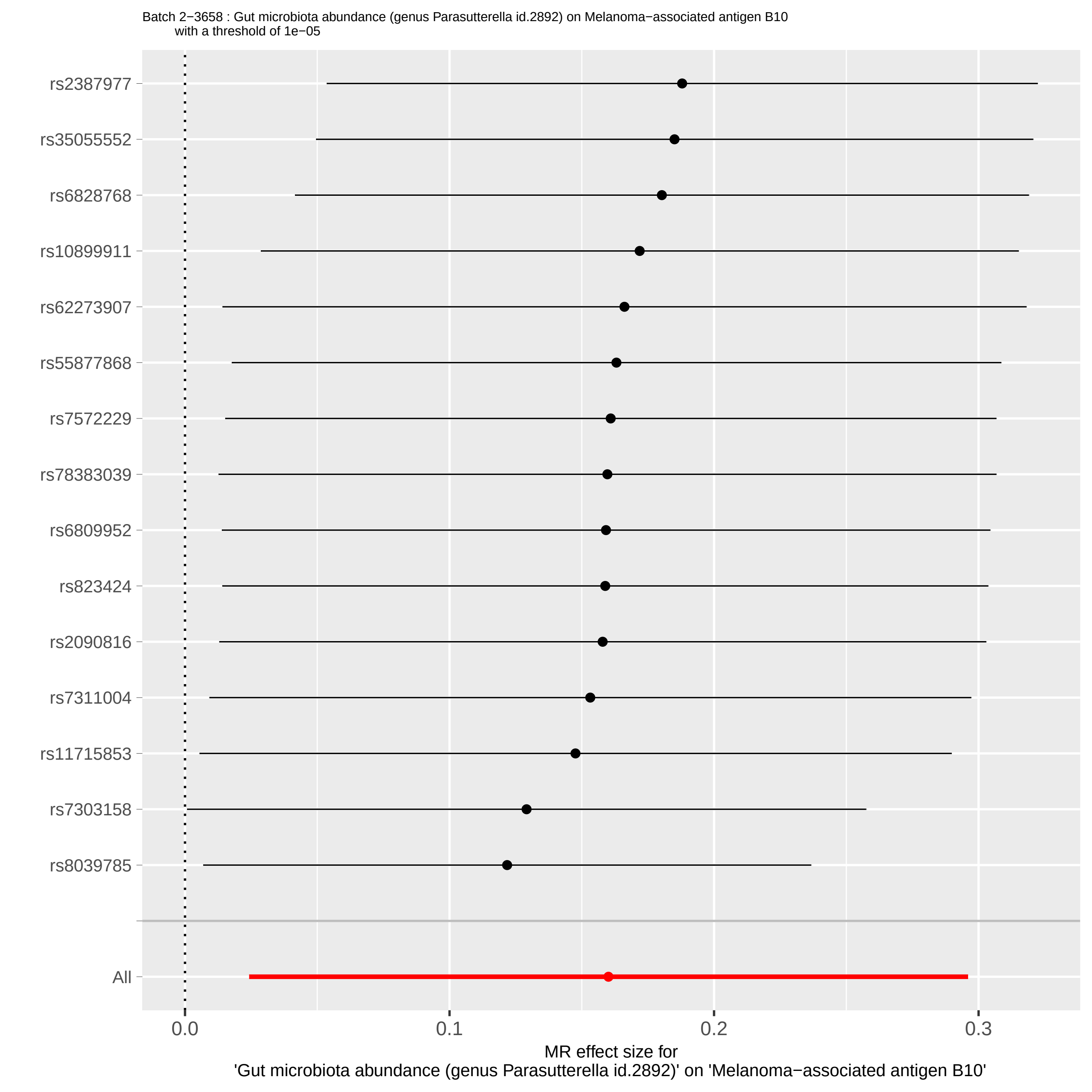

Batch 2−3658 : Gut microbiota abundance (genus Parasutterella id.2892) on Melanoma−associated antigen B10
with a threshold of 1e−05
rs2387977
rs35055552
rs6828768
rs10899911
rs62273907
rs55877868
rs7572229
rs78383039
rs6809952
rs823424
rs2090816
rs7311004
rs11715853
rs7303158
rs8039785
All
0.0
0.1
0.2
0.3
MR effect size for
'Gut microbiota abundance (genus Parasutterella id.2892)' on 'Melanoma−associated antigen B10'

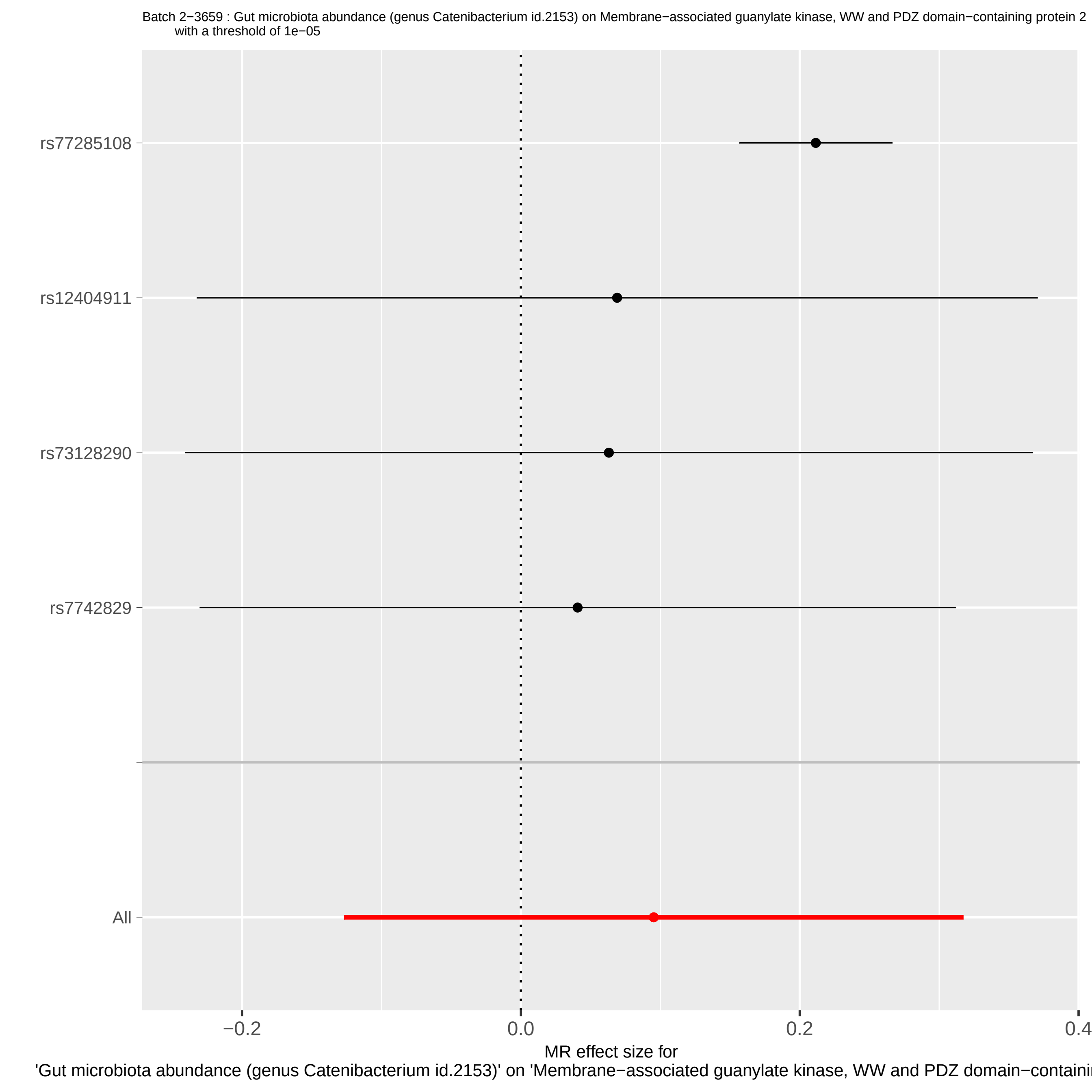

Batch 2−3659 : Gut microbiota abundance (genus Catenibacterium id.2153) on Membrane−associated guanylate kinase, WW and PDZ domain−containing protein 2
with a threshold of 1e−05
rs77285108
rs12404911
rs73128290
rs7742829
All
−0.2
0.0
0.2
0.4
MR effect size for
'Gut microbiota abundance (genus Catenibacterium id.2153)' on 'Membrane−associated guanylate kinase, WW and PDZ domain−containi

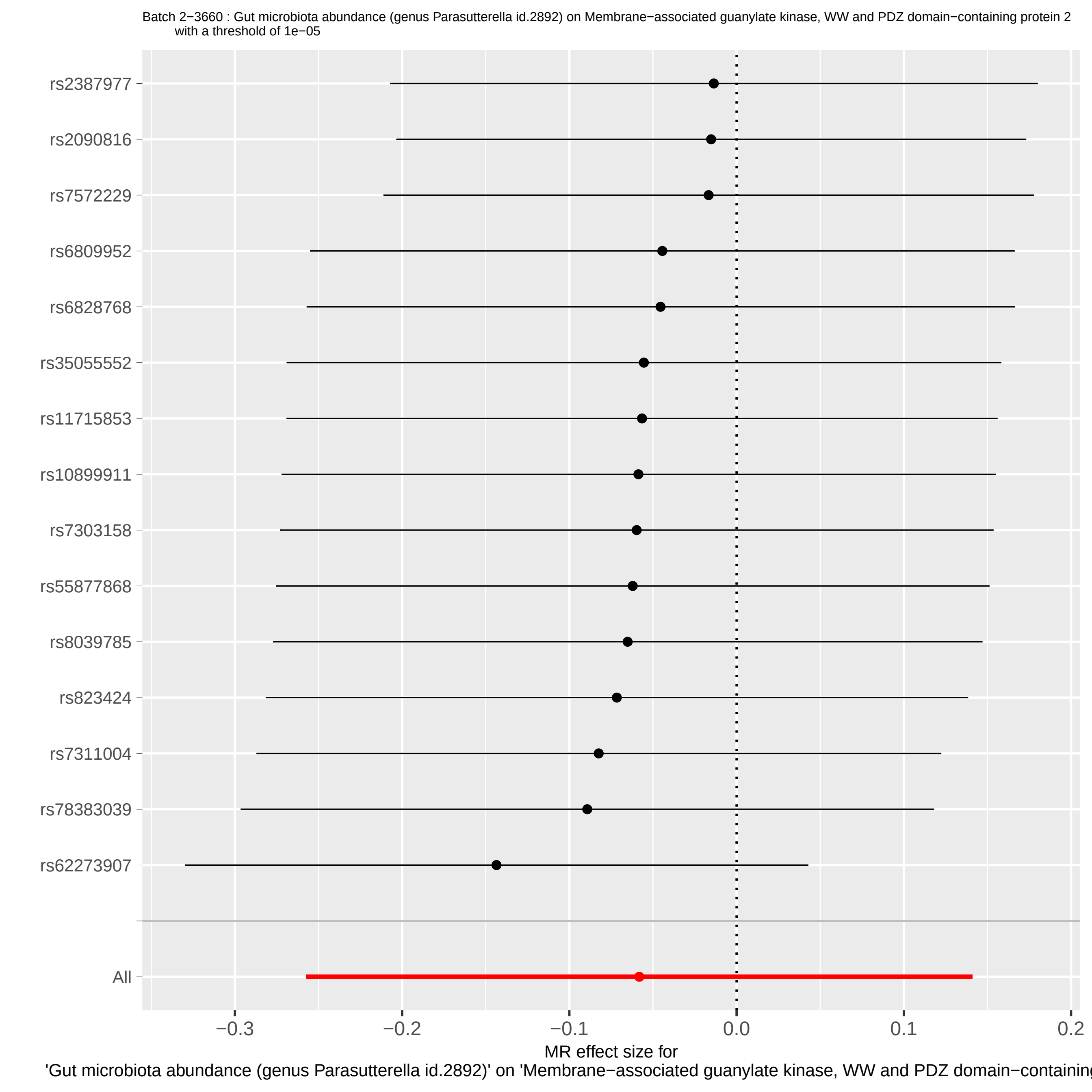
Batch 2−3660 : Gut microbiota abundance (genus Parasutterella id.2892) on Membrane−associated guanylate kinase, WW and PDZ domain−containing protein 2 with a threshold of 1e−05
rs2387977
rs2090816
rs7572229
rs6809952
rs6828768
rs35055552
rs11715853
rs10899911
rs7303158
rs55877868
rs8039785
rs823424
rs7311004
rs78383039
rs62273907
All
−0.3
−0.2
−0.1
0.0
0.1
0.2
MR effect size for
'Gut microbiota abundance (genus Parasutterella id.2892)' on 'Membrane−associated guanylate kinase, WW and PDZ domain−containin

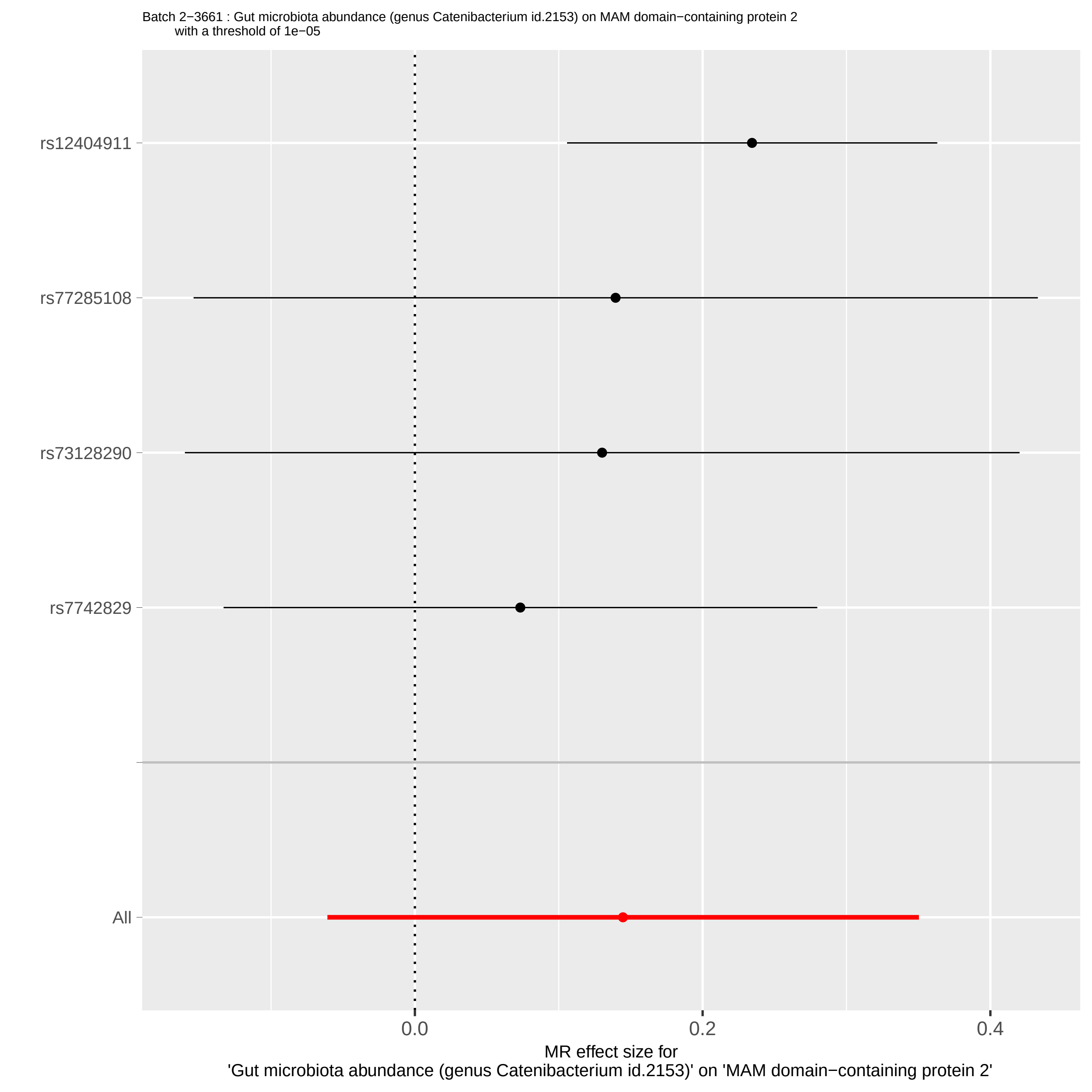
Batch 2−3661 : Gut microbiota abundance (genus Catenibacterium id.2153) on MAM domain−containing protein 2
with a threshold of 1e−05
rs12404911
rs77285108
rs73128290
rs7742829
All
0.0
0.2
0.4
MR effect size for
'Gut microbiota abundance (genus Catenibacterium id.2153)' on 'MAM domain−containing protein 2'

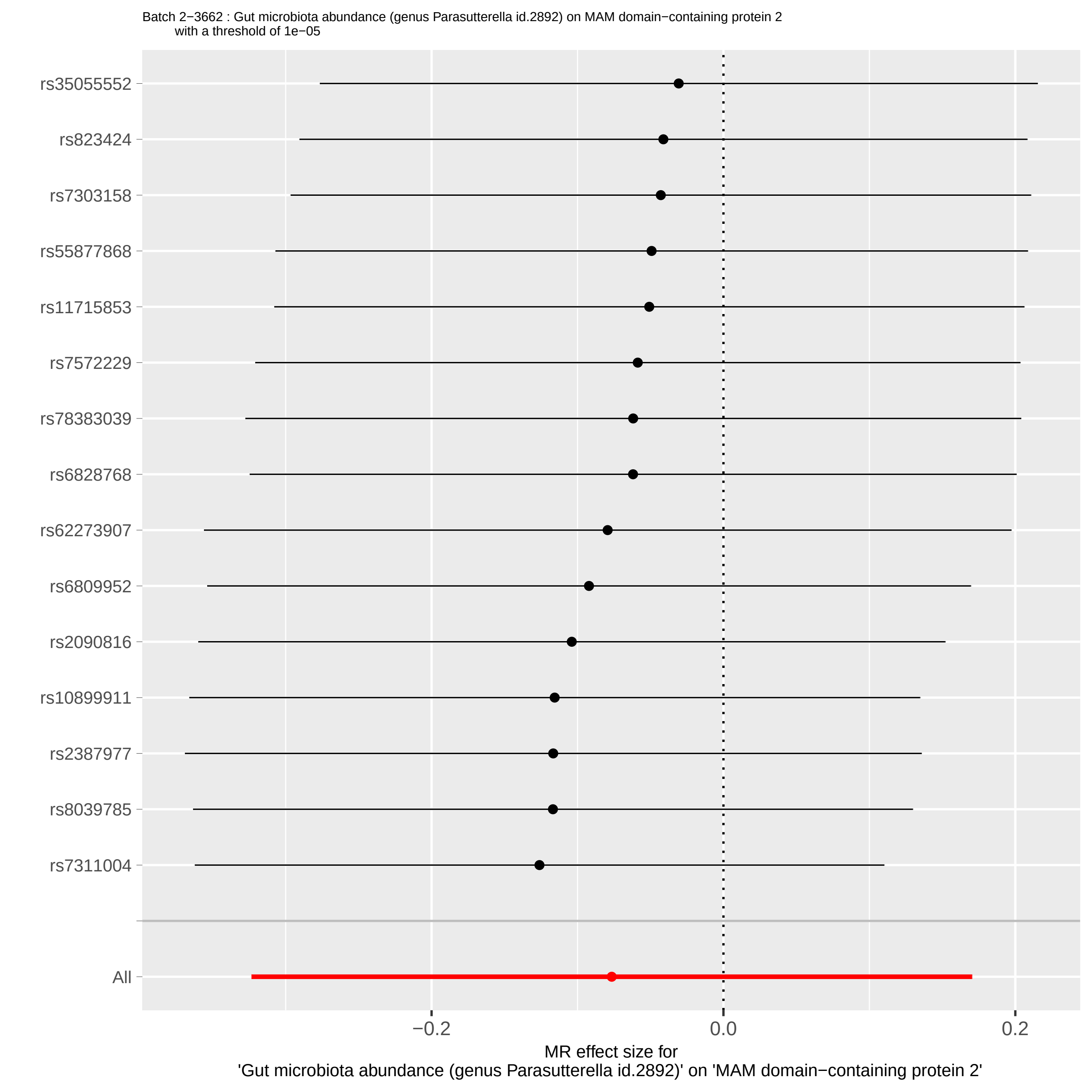
Batch 2−3662 : Gut microbiota abundance (genus Parasutterella id.2892) on MAM domain−containing protein 2
with a threshold of 1e−05
rs35055552
rs823424
rs7303158
rs55877868
rs11715853
rs7572229
rs78383039
rs6828768
rs62273907
rs6809952
rs2090816
rs10899911
rs2387977
rs8039785
rs7311004
All
−0.2
0.0
0.2
MR effect size for
'Gut microbiota abundance (genus Parasutterella id.2892)' on 'MAM domain−containing protein 2'

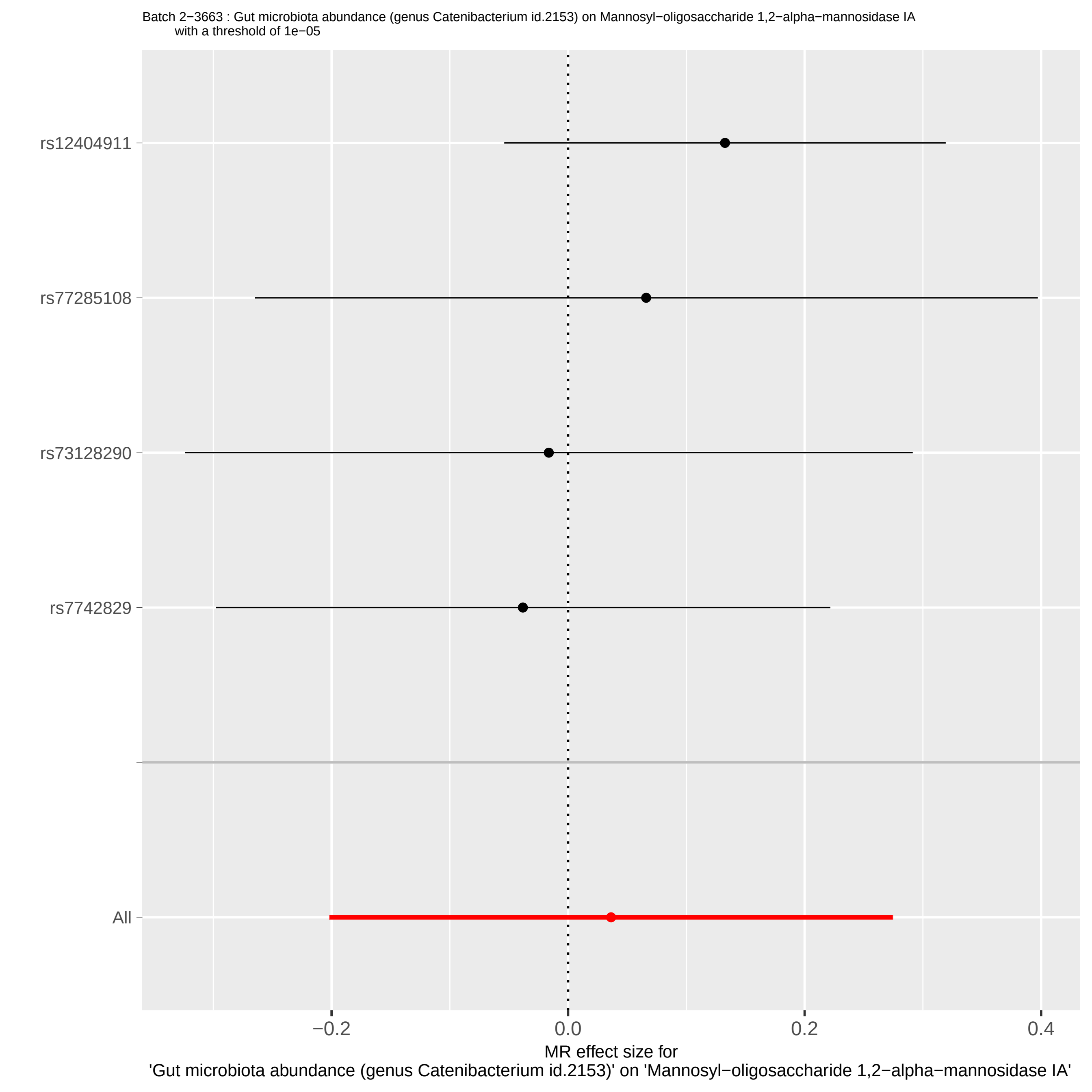

Batch 2−3663 : Gut microbiota abundance (genus Catenibacterium id.2153) on Mannosyl−oligosaccharide 1,2−alpha−mannosidase IA
with a threshold of 1e−05
rs12404911
rs77285108
rs73128290
rs7742829
All
−0.2
0.0
0.2
0.4
MR effect size for
'Gut microbiota abundance (genus Catenibacterium id.2153)' on 'Mannosyl−oligosaccharide 1,2−alpha−mannosidase IA'

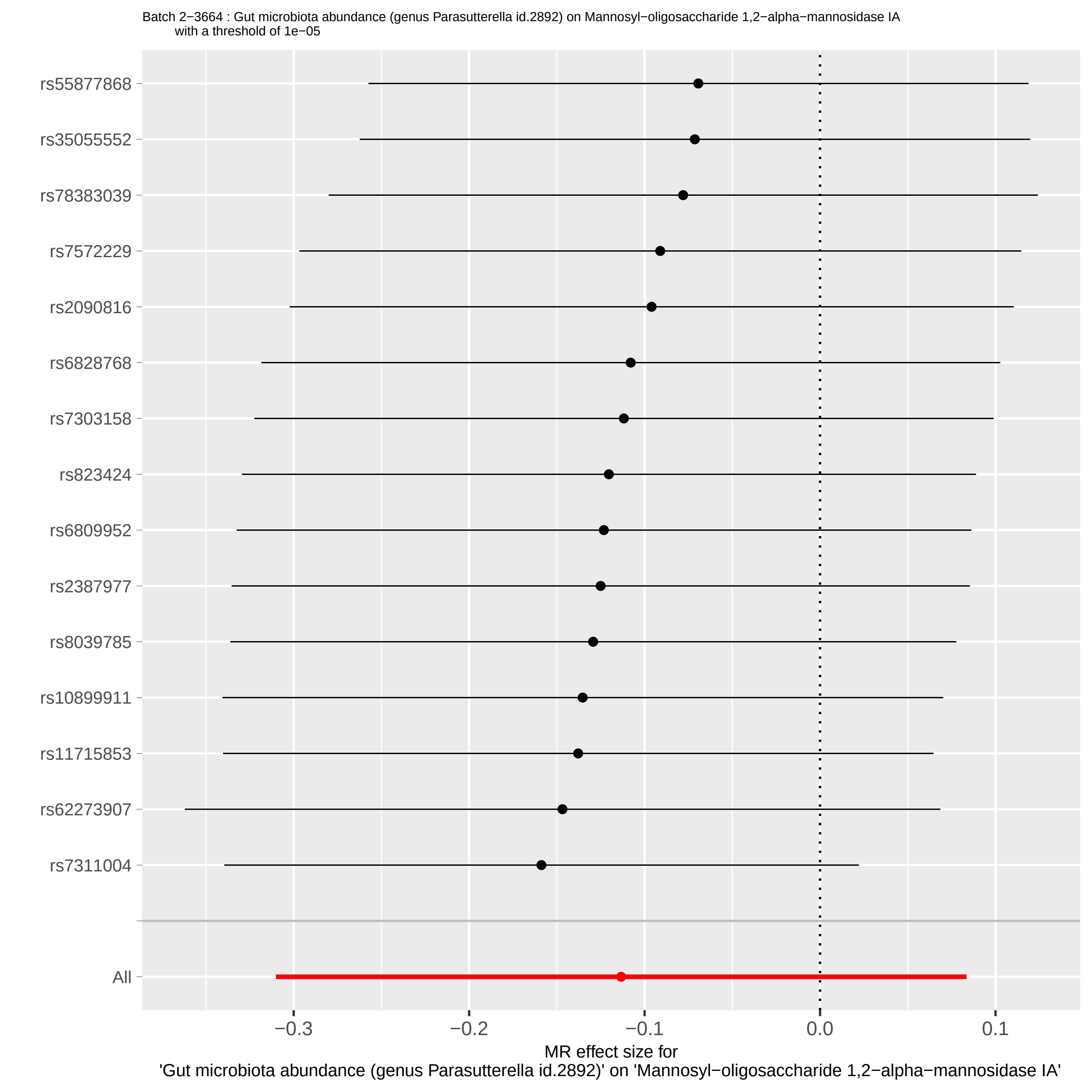
Batch 2−3664 : Gut microbiota abundance (genus Parasutterella id.2892) on Mannosyl−oligosaccharide 1,2−alpha−mannosidase IA
with a threshold of 1e−05
rs55877868
rs35055552
rs78383039
rs7572229
rs2090816
rs6828768
rs7303158
rs823424
rs6809952
rs2387977
rs8039785
rs10899911
rs11715853
rs62273907
rs7311004
All
−0.3
−0.2
−0.1
0.0
0.1
MR effect size for
'Gut microbiota abundance (genus Parasutterella id.2892)' on 'Mannosyl−oligosaccharide 1,2−alpha−mannosidase IA'

Batch 2−3665 : Gut microbiota abundance (genus Catenibacterium id.2153) on Mannosyl−oligosaccharide 1,2−alpha−mannosidase IB with a threshold of 1e−05

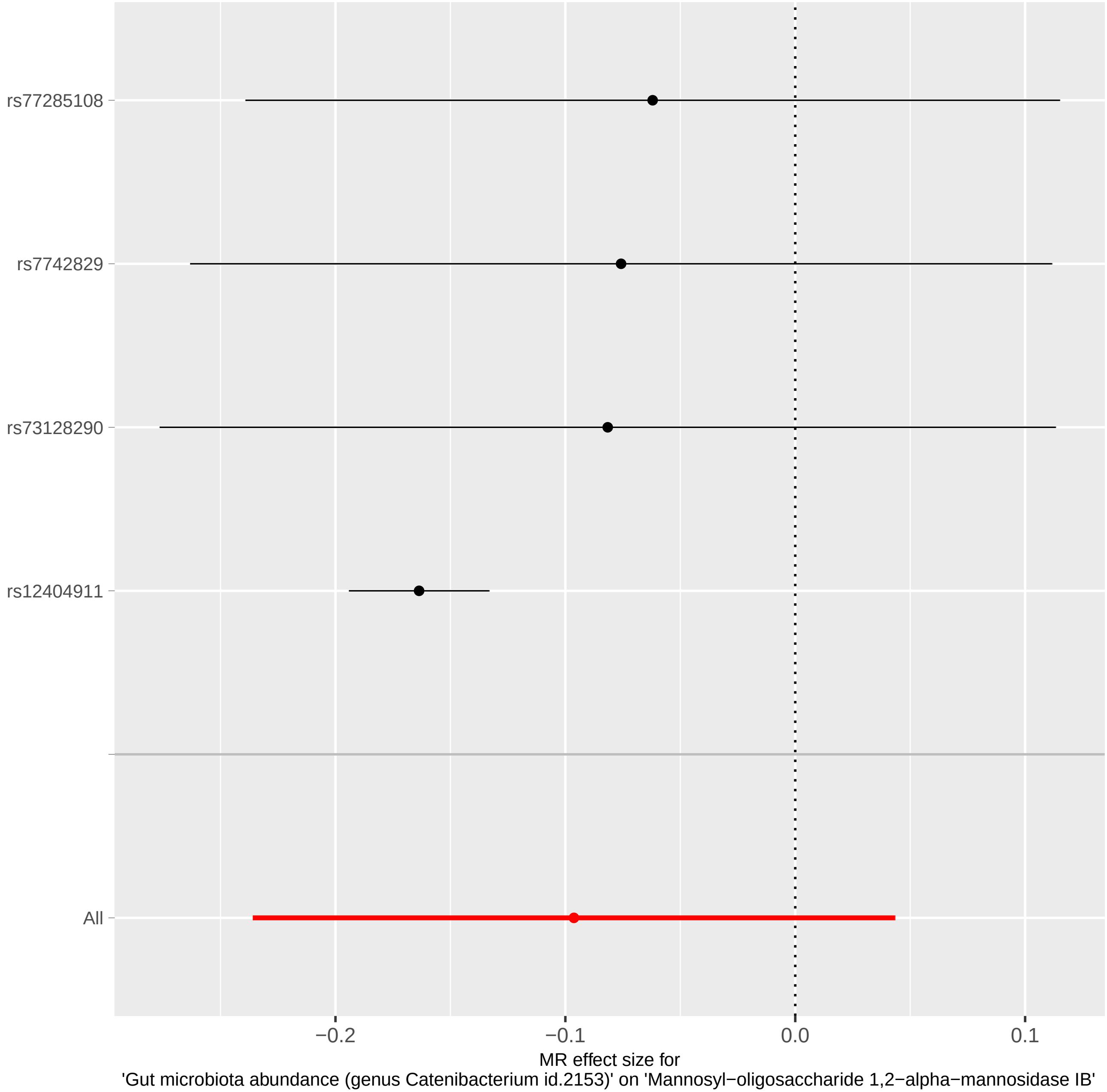

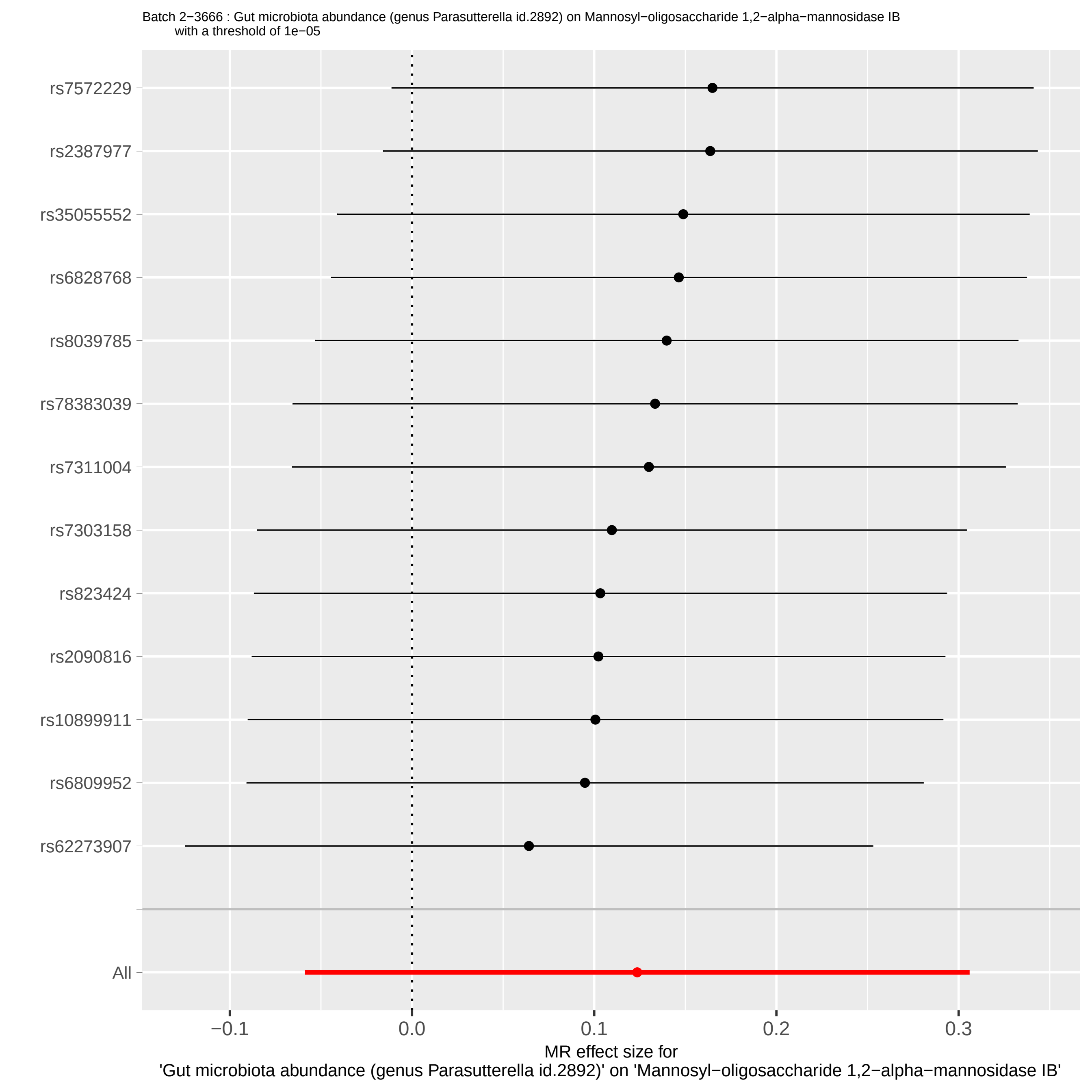

Batch 2−3666 : Gut microbiota abundance (genus Parasutterella id.2892) on Mannosyl−oligosaccharide 1,2−alpha−mannosidase IB
with a threshold of 1e−05
rs7572229
rs2387977
rs35055552
rs6828768
rs8039785
rs78383039
rs7311004
rs7303158
rs823424
rs2090816
rs10899911
rs6809952
rs62273907
All
−0.1
0.0
0.1
0.2
0.3
MR effect size for
'Gut microbiota abundance (genus Parasutterella id.2892)' on 'Mannosyl−oligosaccharide 1,2−alpha−mannosidase IB'

Batch 2−3667 : Gut microbiota abundance (genus Catenibacterium id.2153) on Endoplasmic reticulum mannosyl−oligosaccharide 1,2−alpha−mannosidase with a threshold of 1e−05

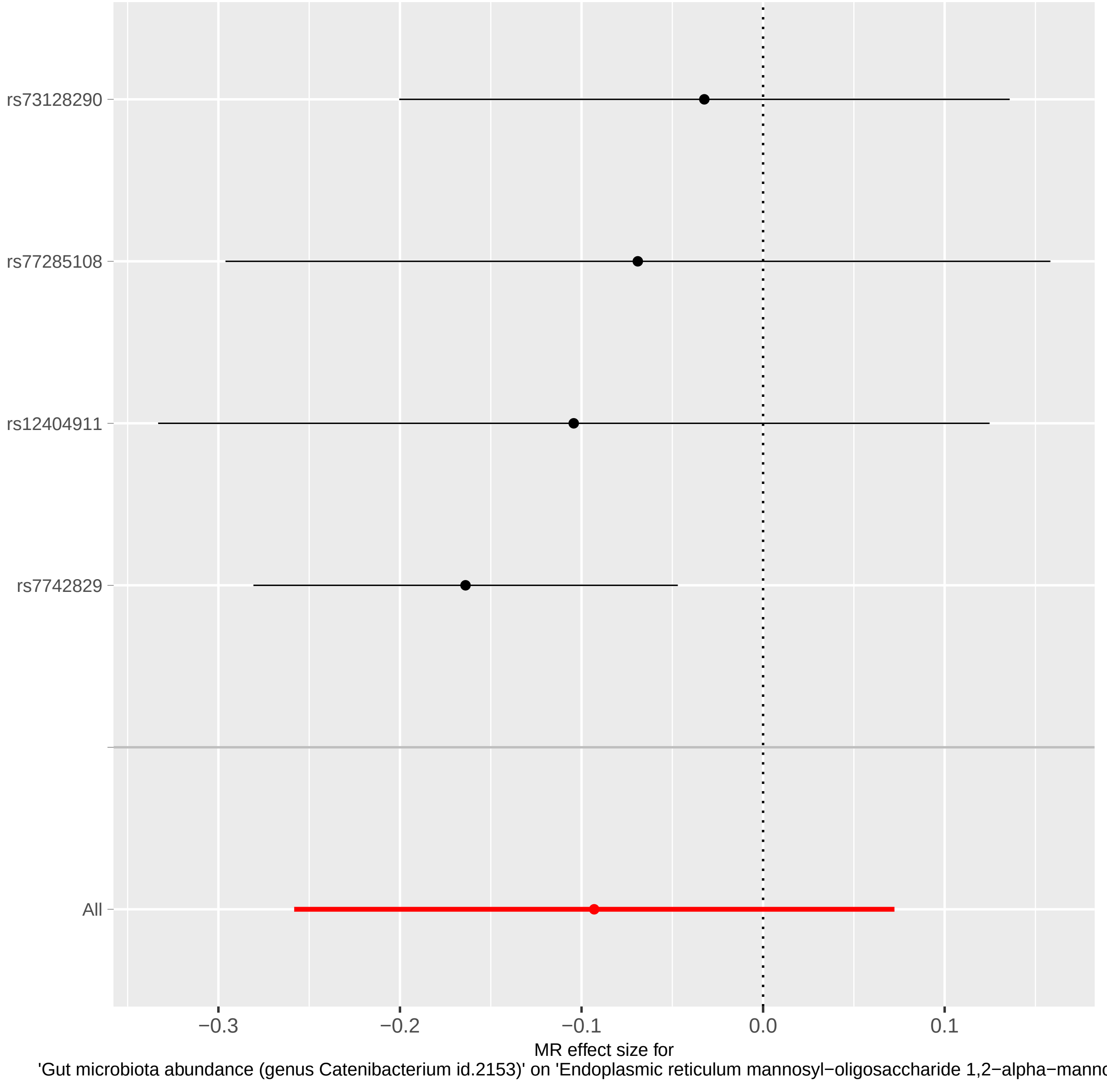

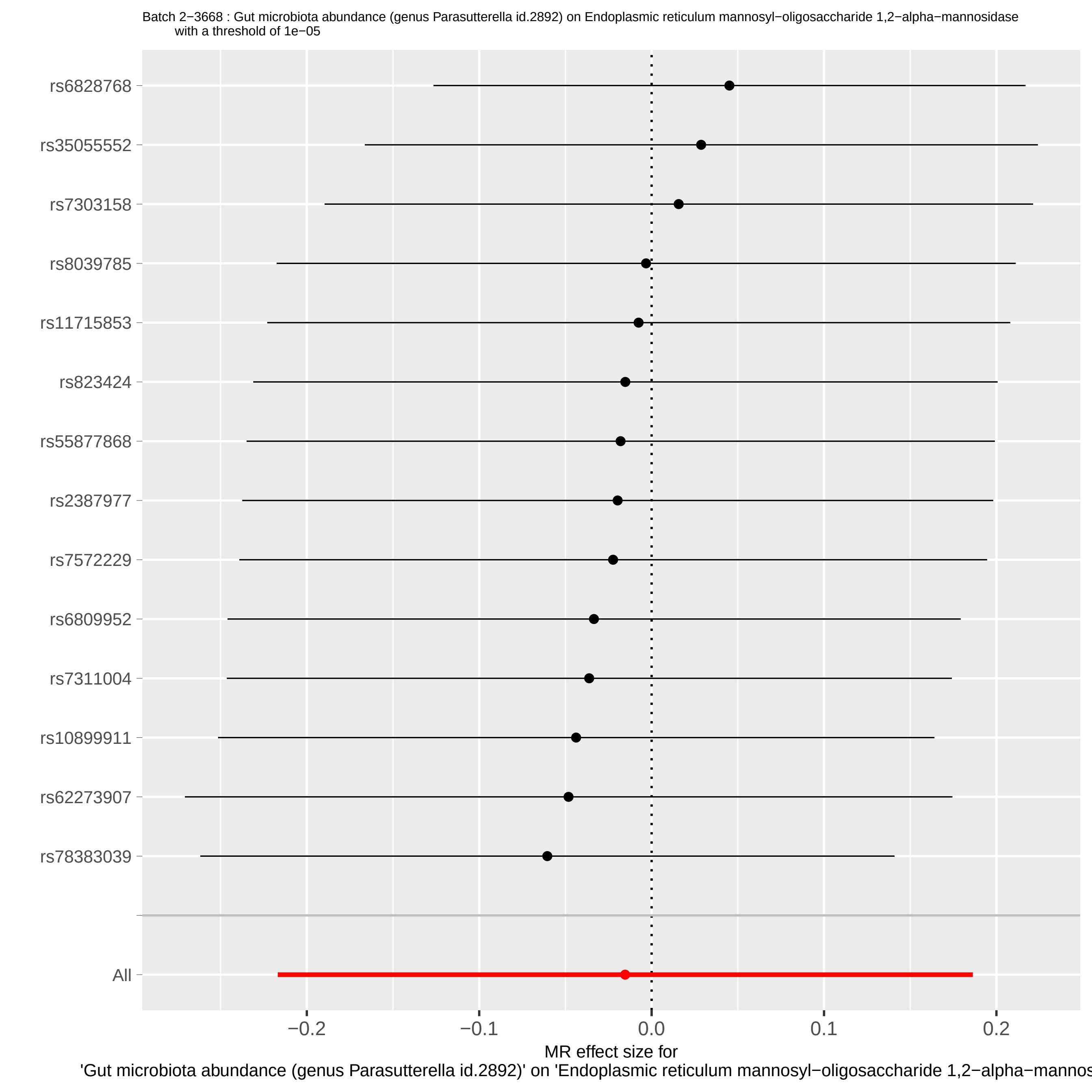
Batch 2−3668 : Gut microbiota abundance (genus Parasutterella id.2892) on Endoplasmic reticulum mannosyl−oligosaccharide 1,2−alpha−mannosidase with a threshold of 1e−05
rs6828768
rs35055552
rs7303158
rs8039785
rs11715853
rs823424
rs55877868
rs2387977
rs7572229
rs6809952
rs7311004
rs10899911
rs62273907
rs78383039
All
−0.2
−0.1
0.0
0.1
0.2
MR effect size for
'Gut microbiota abundance (genus Parasutterella id.2892)' on 'Endoplasmic reticulum mannosyl−oligosaccharide 1,2−alpha−mannos

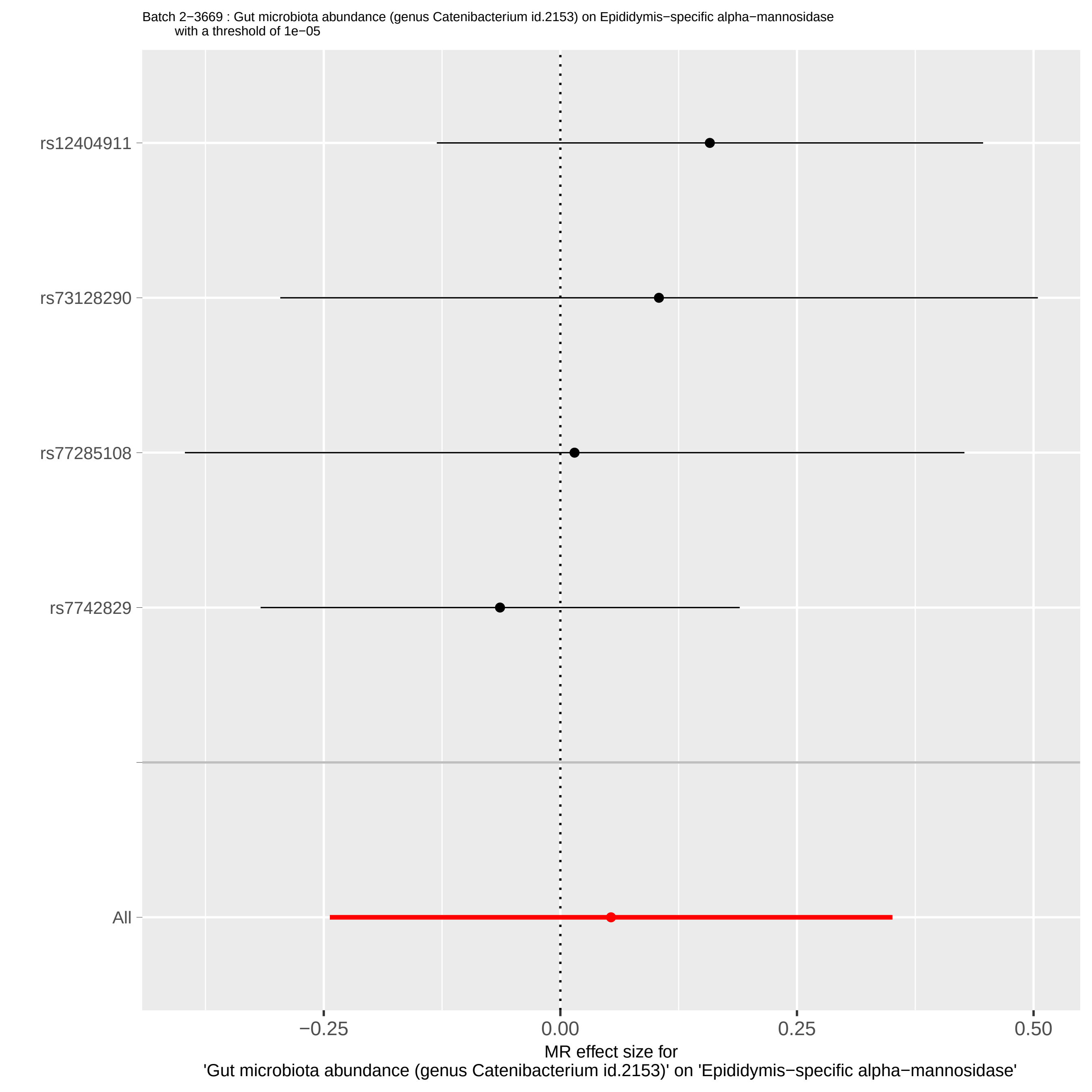
Batch 2−3669 : Gut microbiota abundance (genus Catenibacterium id.2153) on Epididymis−specific alpha−mannosidase
with a threshold of 1e−05
rs12404911
rs73128290
rs77285108
rs7742829
All
−0.25
0.00
0.25
0.50
MR effect size for
'Gut microbiota abundance (genus Catenibacterium id.2153)' on 'Epididymis−specific alpha−mannosidase'

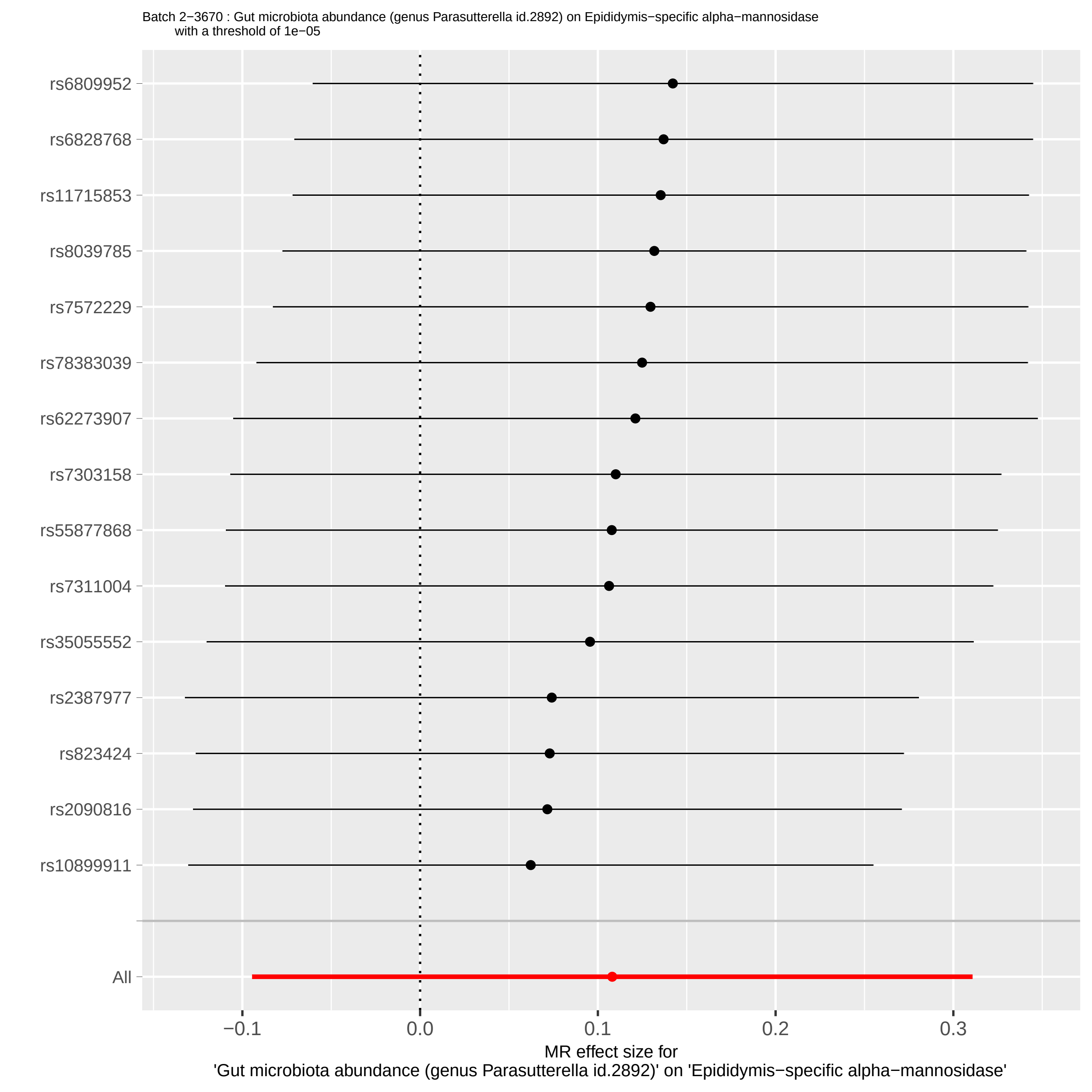
Batch 2−3670 : Gut microbiota abundance (genus Parasutterella id.2892) on Epididymis−specific alpha−mannosidase
with a threshold of 1e−05
rs6809952
rs6828768
rs11715853
rs8039785
rs7572229
rs78383039
rs62273907
rs7303158
rs55877868
rs7311004
rs35055552
rs2387977
rs823424
rs2090816
rs10899911
All
−0.1
0.0
0.1
0.2
0.3
MR effect size for
'Gut microbiota abundance (genus Parasutterella id.2892)' on 'Epididymis−specific alpha−mannosidase'

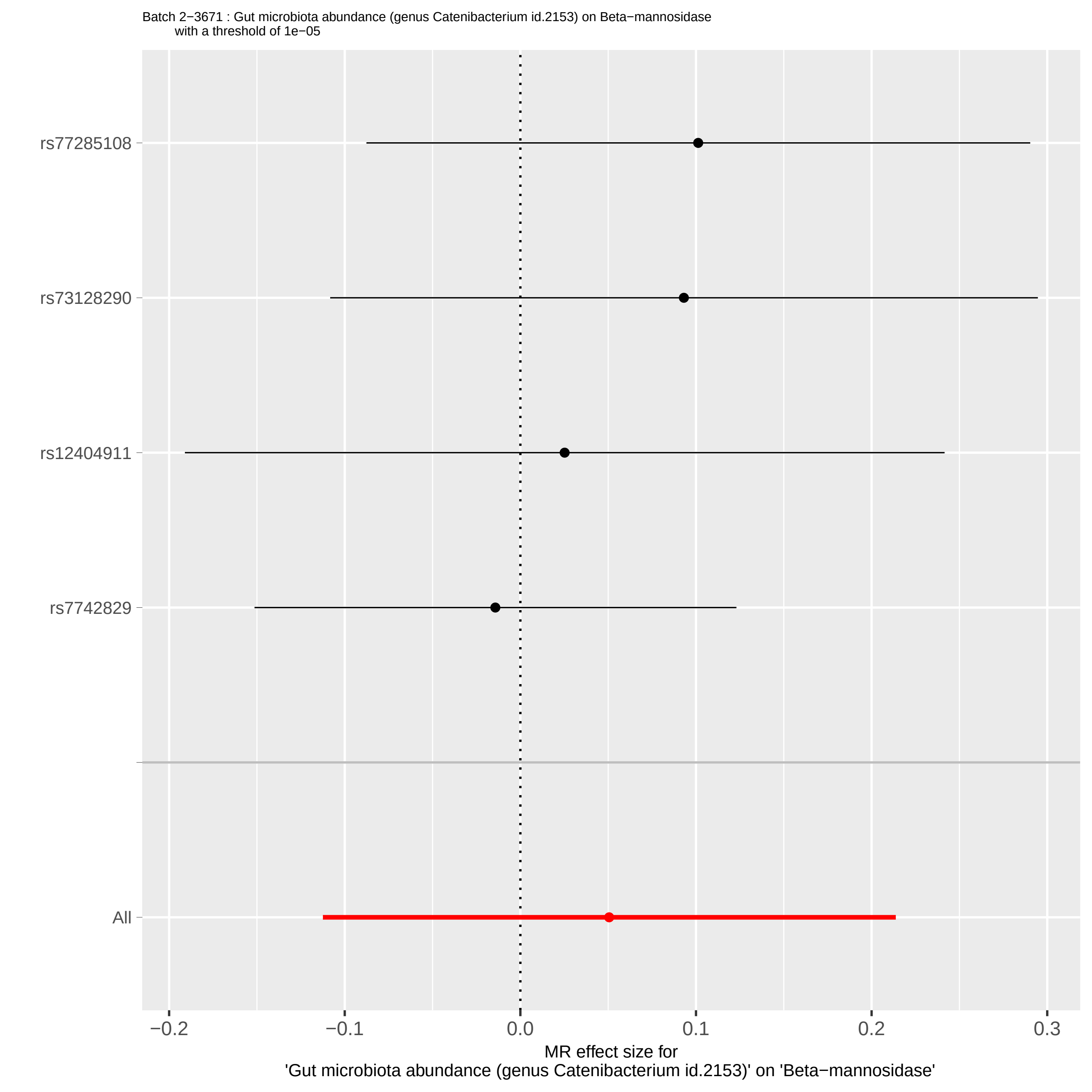

Batch 2−3671 : Gut microbiota abundance (genus Catenibacterium id.2153) on Beta−mannosidase
with a threshold of 1e−05
rs77285108
rs73128290
rs12404911
rs7742829
All
−0.2
−0.1
0.0
0.1
0.2
0.3
MR effect size for
'Gut microbiota abundance (genus Catenibacterium id.2153)' on 'Beta−mannosidase'

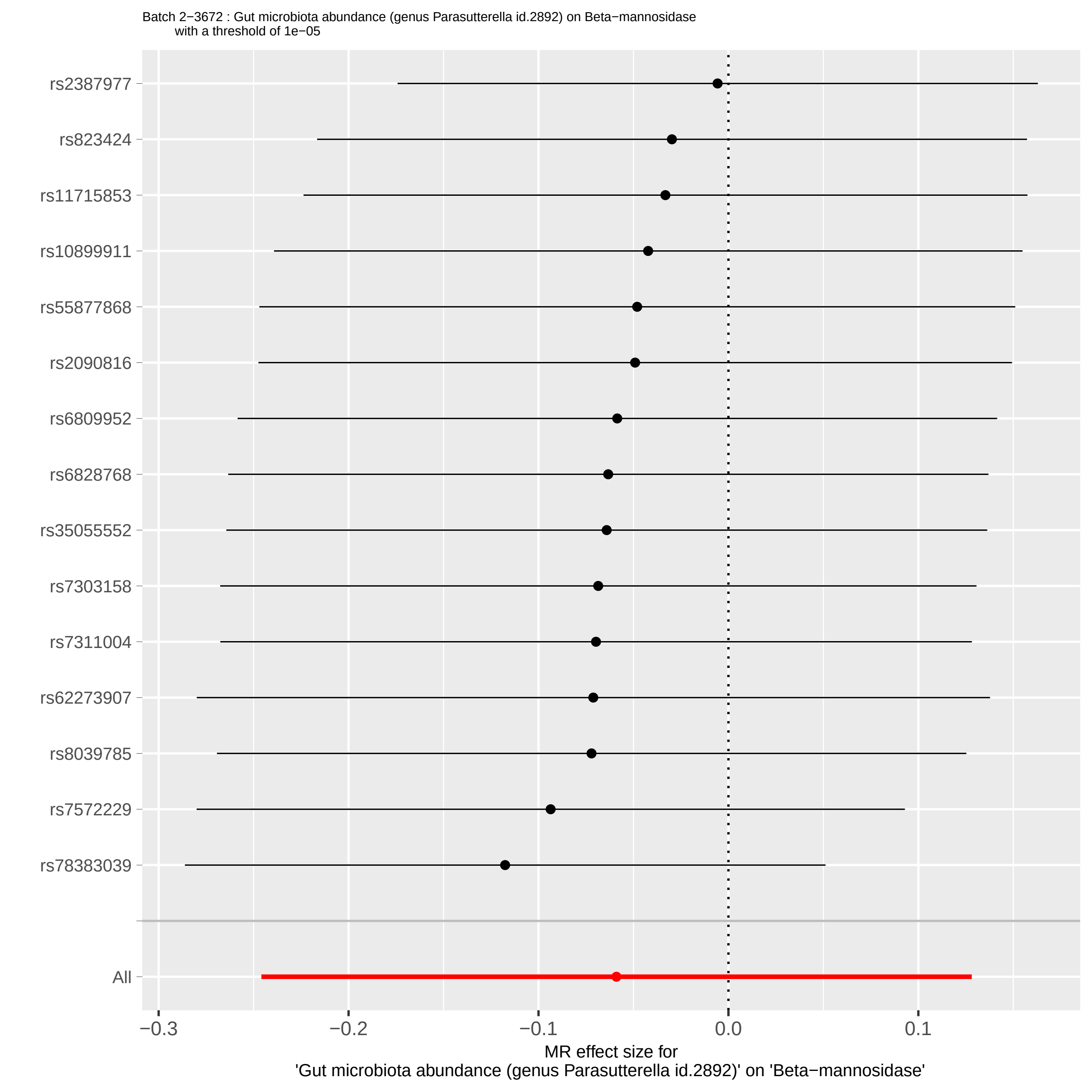

Batch 2−3672 : Gut microbiota abundance (genus Parasutterella id.2892) on Beta−mannosidase
with a threshold of 1e−05
rs2387977
rs823424
rs11715853
rs10899911
rs55877868
rs2090816
rs6809952
rs6828768
rs35055552
rs7303158
rs7311004
rs62273907
rs8039785
rs7572229
rs78383039
All
−0.3
−0.2
−0.1
0.0
0.1
MR effect size for
'Gut microbiota abundance (genus Parasutterella id.2892)' on 'Beta−mannosidase'

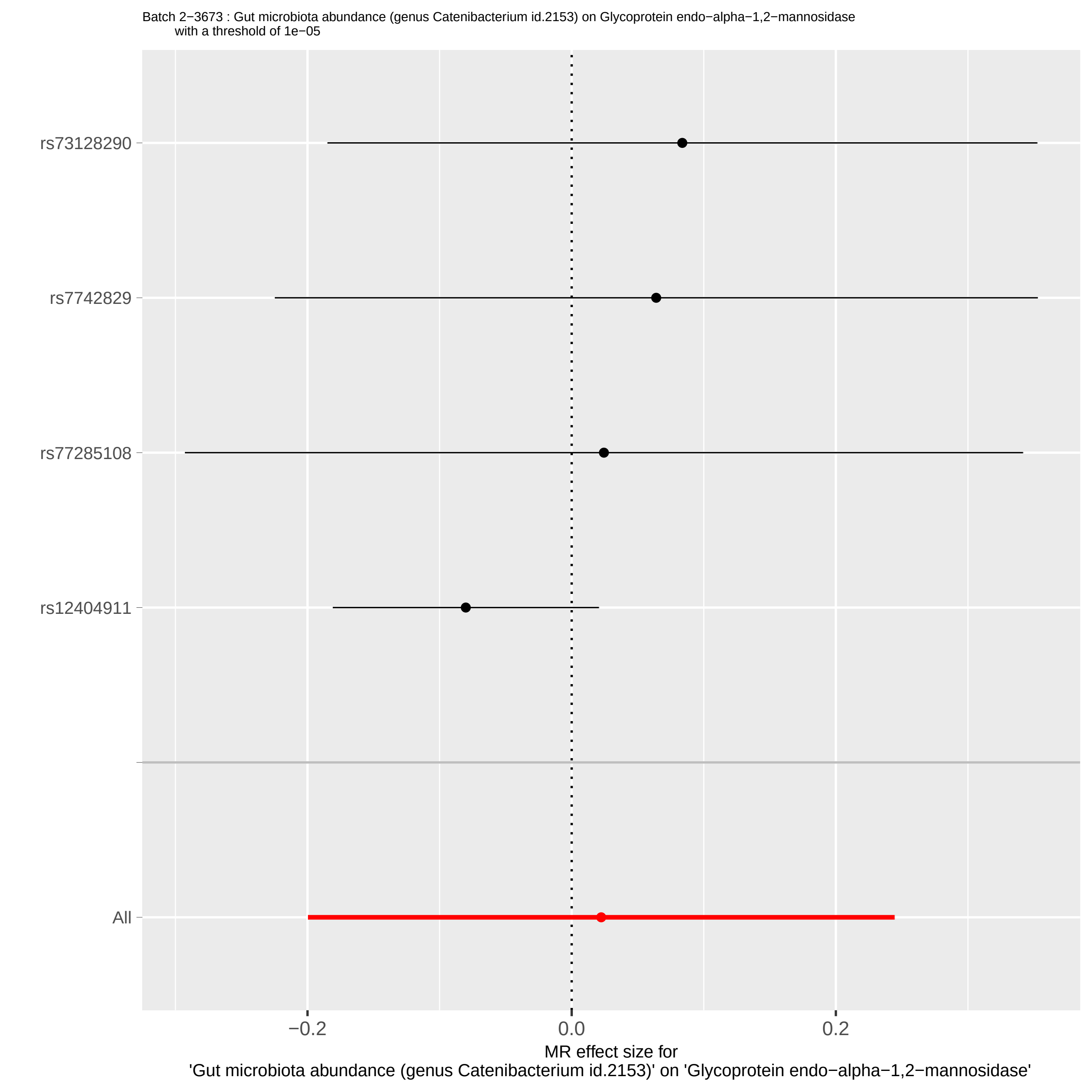

Batch 2−3673 : Gut microbiota abundance (genus Catenibacterium id.2153) on Glycoprotein endo−alpha−1,2−mannosidase
with a threshold of 1e−05
rs73128290
rs7742829
rs77285108
rs12404911
All
−0.2
0.0
0.2
MR effect size for
'Gut microbiota abundance (genus Catenibacterium id.2153)' on 'Glycoprotein endo−alpha−1,2−mannosidase'

Batch 2−3674 : Gut microbiota abundance (genus Parasutterella id.2892) on Glycoprotein endo−alpha−1,2−mannosidase with a threshold of 1e−05

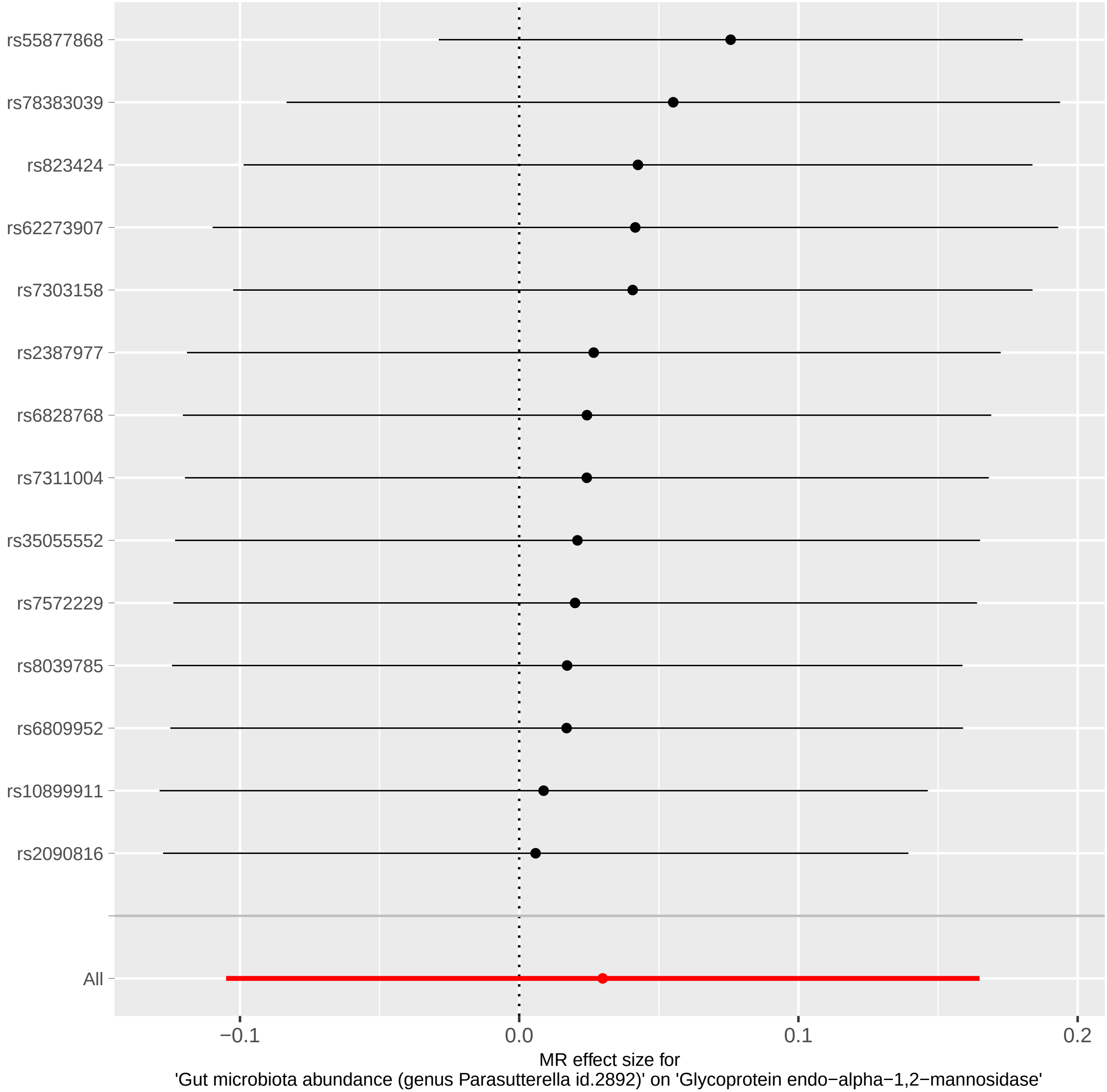

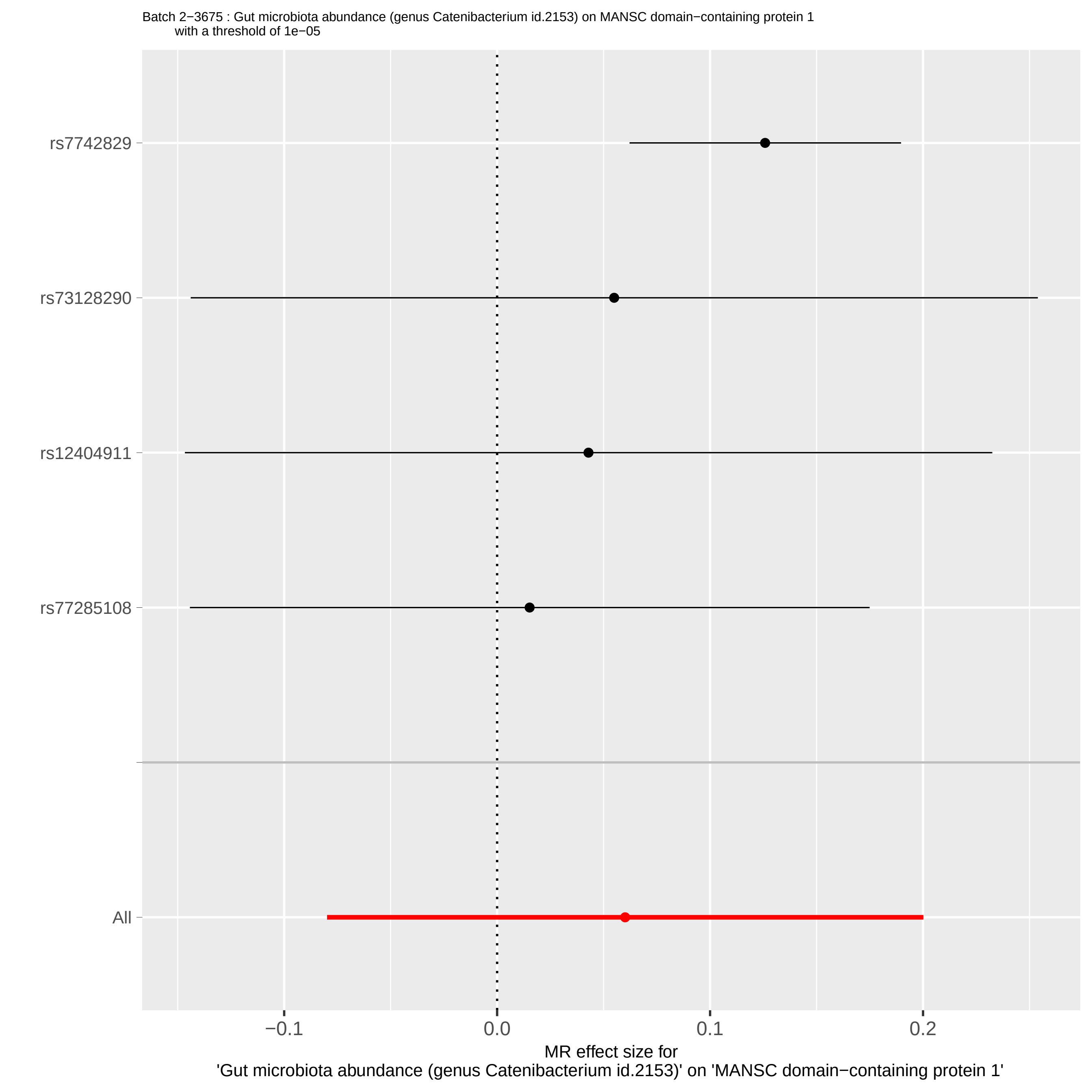

Batch 2−3675 : Gut microbiota abundance (genus Catenibacterium id.2153) on MANSC domain−containing protein 1
with a threshold of 1e−05
rs7742829
rs73128290
rs12404911
rs77285108
All
−0.1
0.0
0.1
0.2
MR effect size for
'Gut microbiota abundance (genus Catenibacterium id.2153)' on 'MANSC domain−containing protein 1'

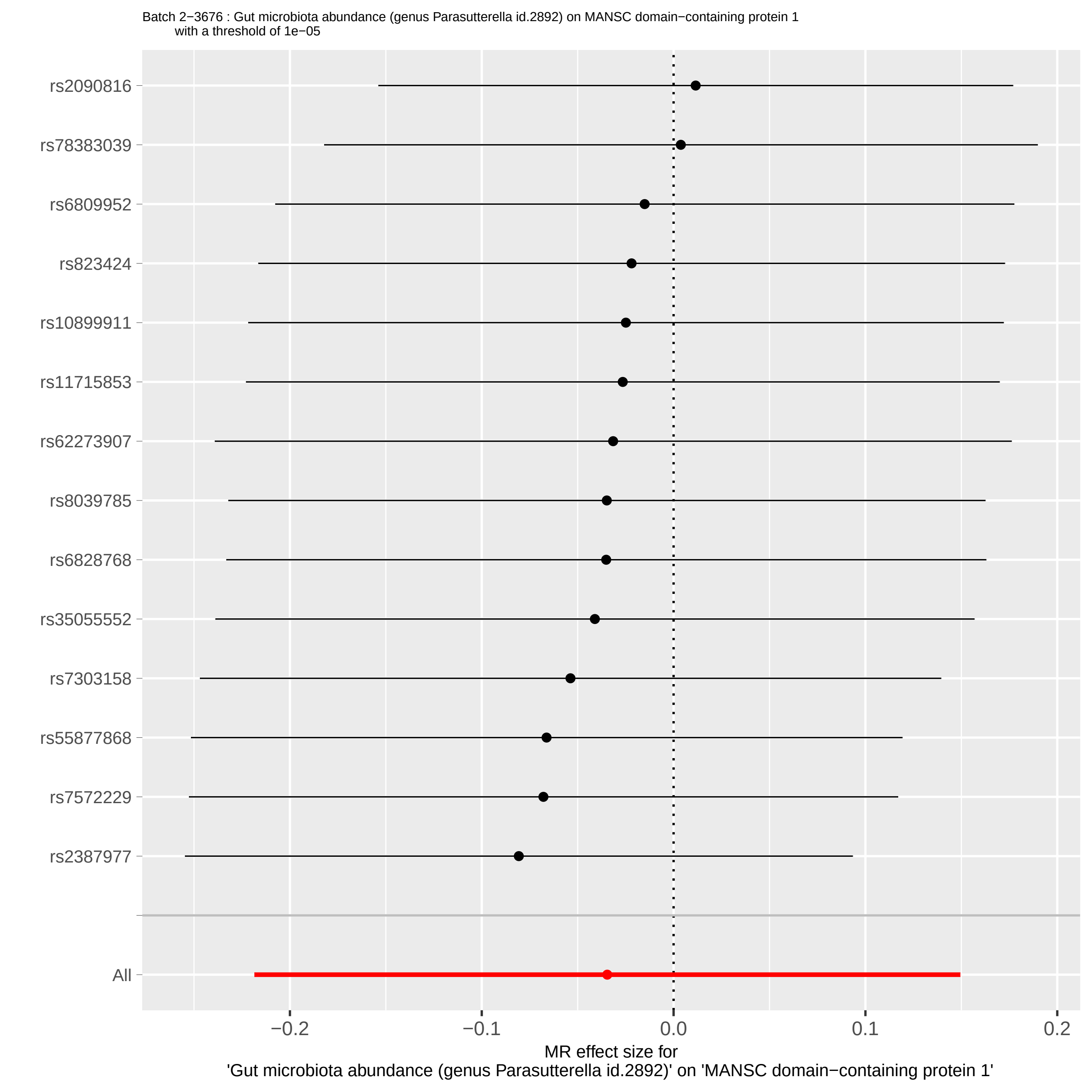

Batch 2−3676 : Gut microbiota abundance (genus Parasutterella id.2892) on MANSC domain−containing protein 1
with a threshold of 1e−05
rs2090816
rs78383039
rs6809952
rs823424
rs10899911
rs11715853
rs62273907
rs8039785
rs6828768
rs35055552
rs7303158
rs55877868
rs7572229
rs2387977
All
−0.2
−0.1
0.0
0.1
0.2
MR effect size for
'Gut microbiota abundance (genus Parasutterella id.2892)' on 'MANSC domain−containing protein 1'

Batch 2−3677 : Gut microbiota abundance (genus Catenibacterium id.2153) on MANSC domain−containing protein 4
with a threshold of 1e−05

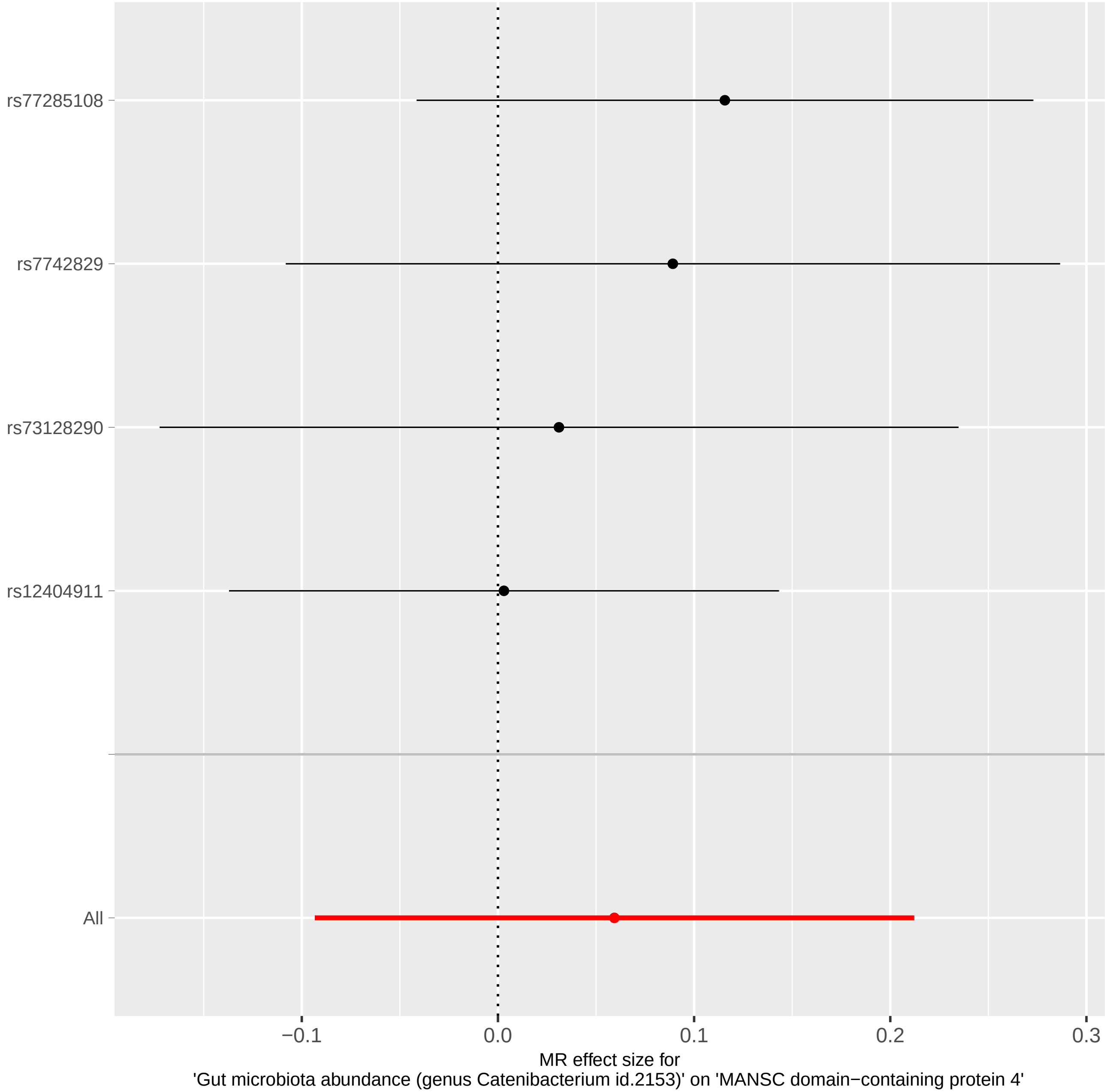

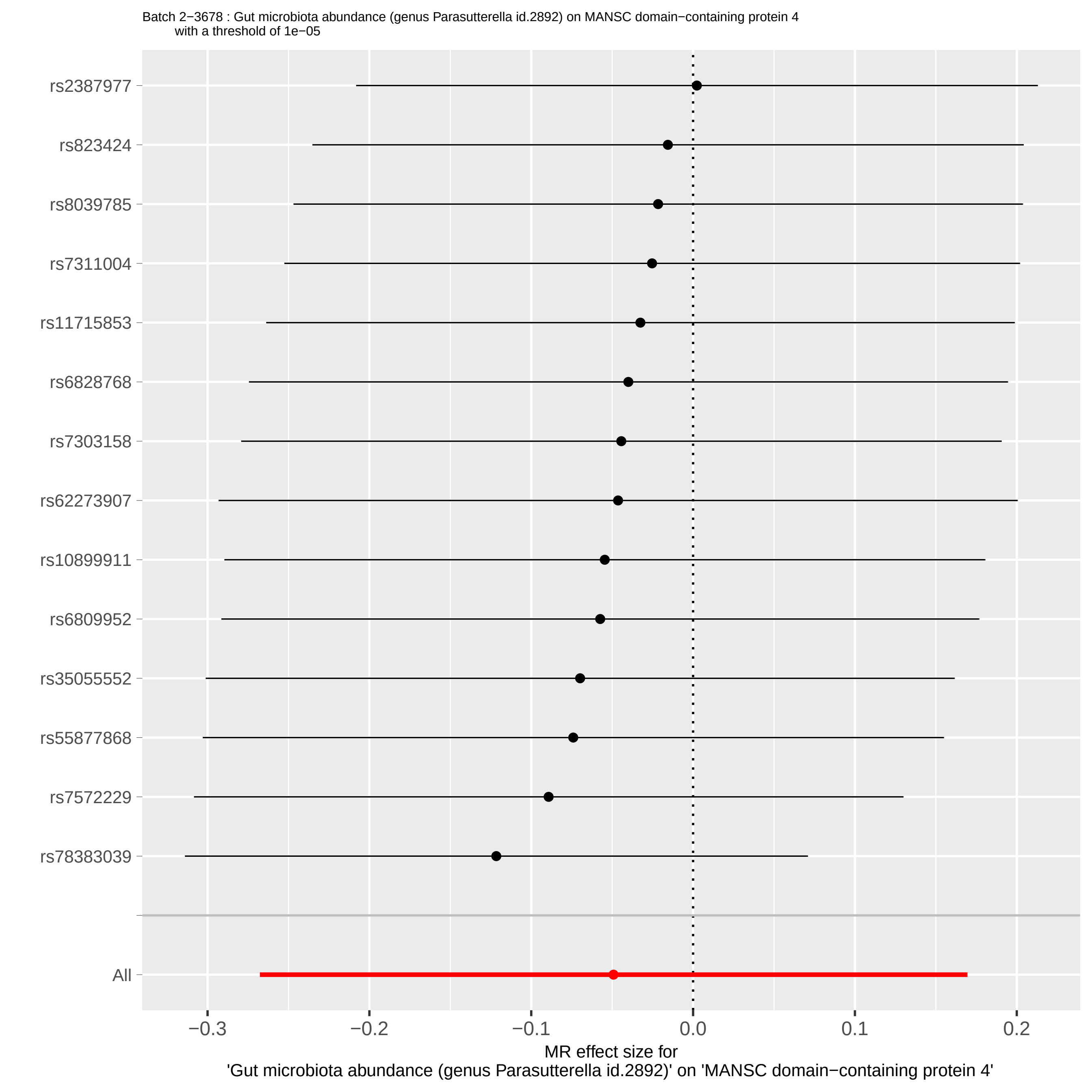

Batch 2−3678 : Gut microbiota abundance (genus Parasutterella id.2892) on MANSC domain−containing protein 4
with a threshold of 1e−05
rs2387977
rs823424
rs8039785
rs7311004
rs11715853
rs6828768
rs7303158
rs62273907
rs10899911
rs6809952
rs35055552
rs55877868
rs7572229
rs78383039
All
−0.3
−0.2
−0.1
0.0
0.1
0.2
MR effect size for
'Gut microbiota abundance (genus Parasutterella id.2892)' on 'MANSC domain−containing protein 4'

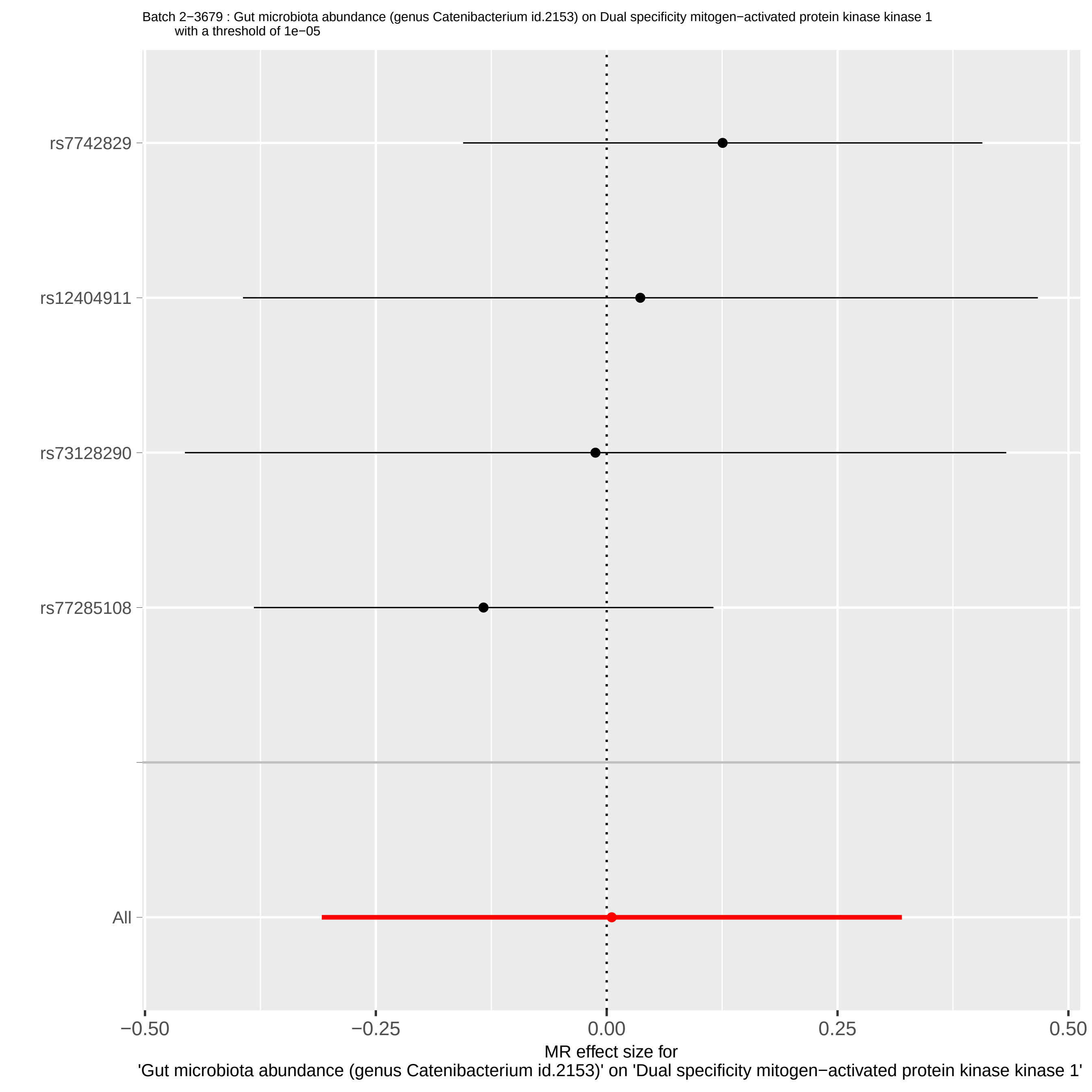

Batch 2−3679 : Gut microbiota abundance (genus Catenibacterium id.2153) on Dual specificity mitogen−activated protein kinase kinase 1
with a threshold of 1e−05
rs7742829
rs12404911
rs73128290
rs77285108
All
−0.50
−0.25
0.00
0.25
0.50
MR effect size for
'Gut microbiota abundance (genus Catenibacterium id.2153)' on 'Dual specificity mitogen−activated protein kinase kinase 1'

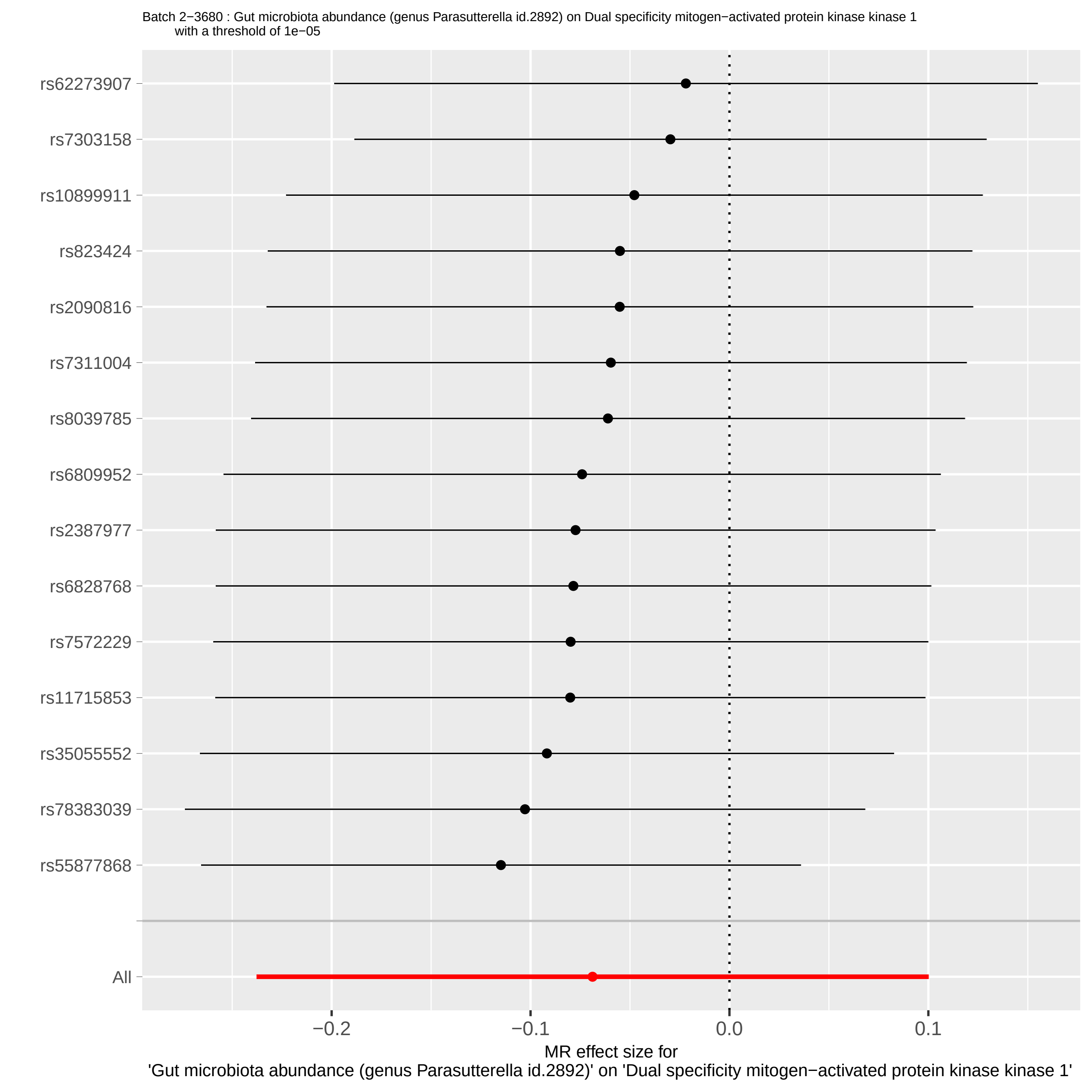
Batch 2−3680 : Gut microbiota abundance (genus Parasutterella id.2892) on Dual specificity mitogen−activated protein kinase kinase 1
with a threshold of 1e−05
rs62273907
rs7303158
rs10899911
rs823424
rs2090816
rs7311004
rs8039785
rs6809952
rs2387977
rs6828768
rs7572229
rs11715853
rs35055552
rs78383039
rs55877868
All
−0.2
−0.1
0.0
0.1
MR effect size for
'Gut microbiota abundance (genus Parasutterella id.2892)' on 'Dual specificity mitogen−activated protein kinase kinase 1'

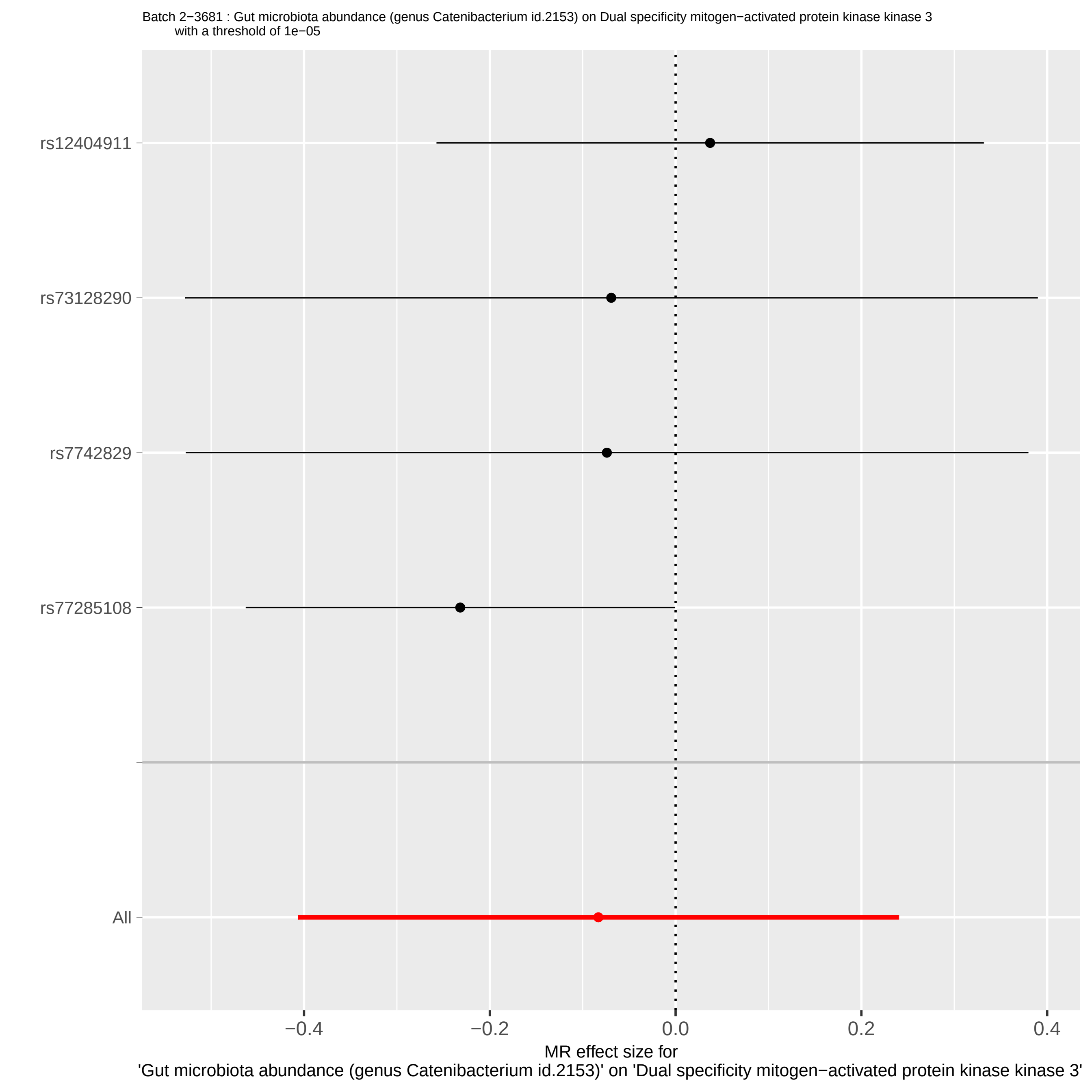
Batch 2−3681 : Gut microbiota abundance (genus Catenibacterium id.2153) on Dual specificity mitogen−activated protein kinase kinase 3
with a threshold of 1e−05
rs12404911
rs73128290
rs7742829
rs77285108
All
−0.4
−0.2
0.0
0.2
0.4
MR effect size for
'Gut microbiota abundance (genus Catenibacterium id.2153)' on 'Dual specificity mitogen−activated protein kinase kinase 3'

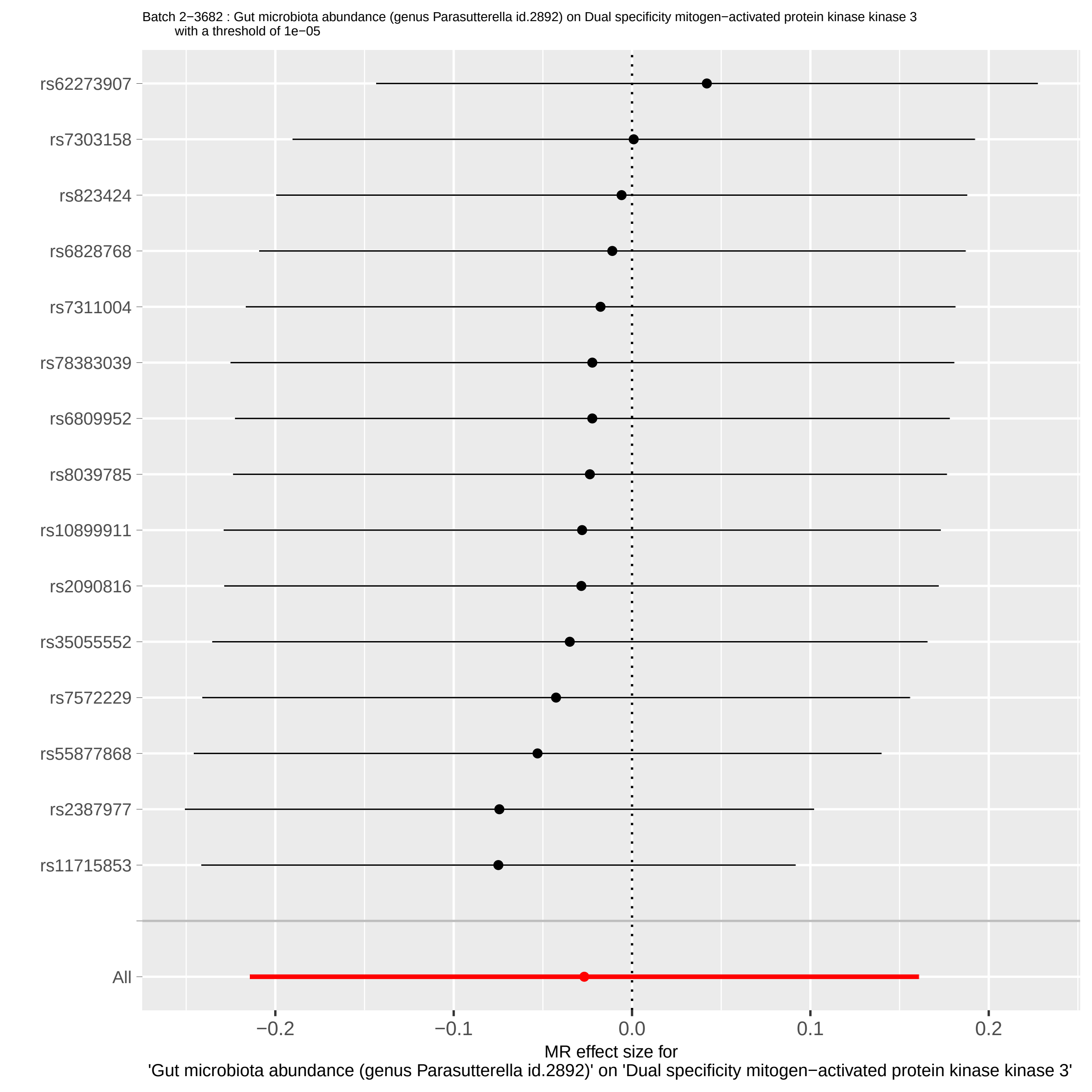
Batch 2−3682 : Gut microbiota abundance (genus Parasutterella id.2892) on Dual specificity mitogen−activated protein kinase kinase 3
with a threshold of 1e−05
rs62273907
rs7303158
rs823424
rs6828768
rs7311004
rs78383039
rs6809952
rs8039785
rs10899911
rs2090816
rs35055552
rs7572229
rs55877868
rs2387977
rs11715853
All
−0.2
−0.1
0.0
0.1
0.2
MR effect size for
'Gut microbiota abundance (genus Parasutterella id.2892)' on 'Dual specificity mitogen−activated protein kinase kinase 3'

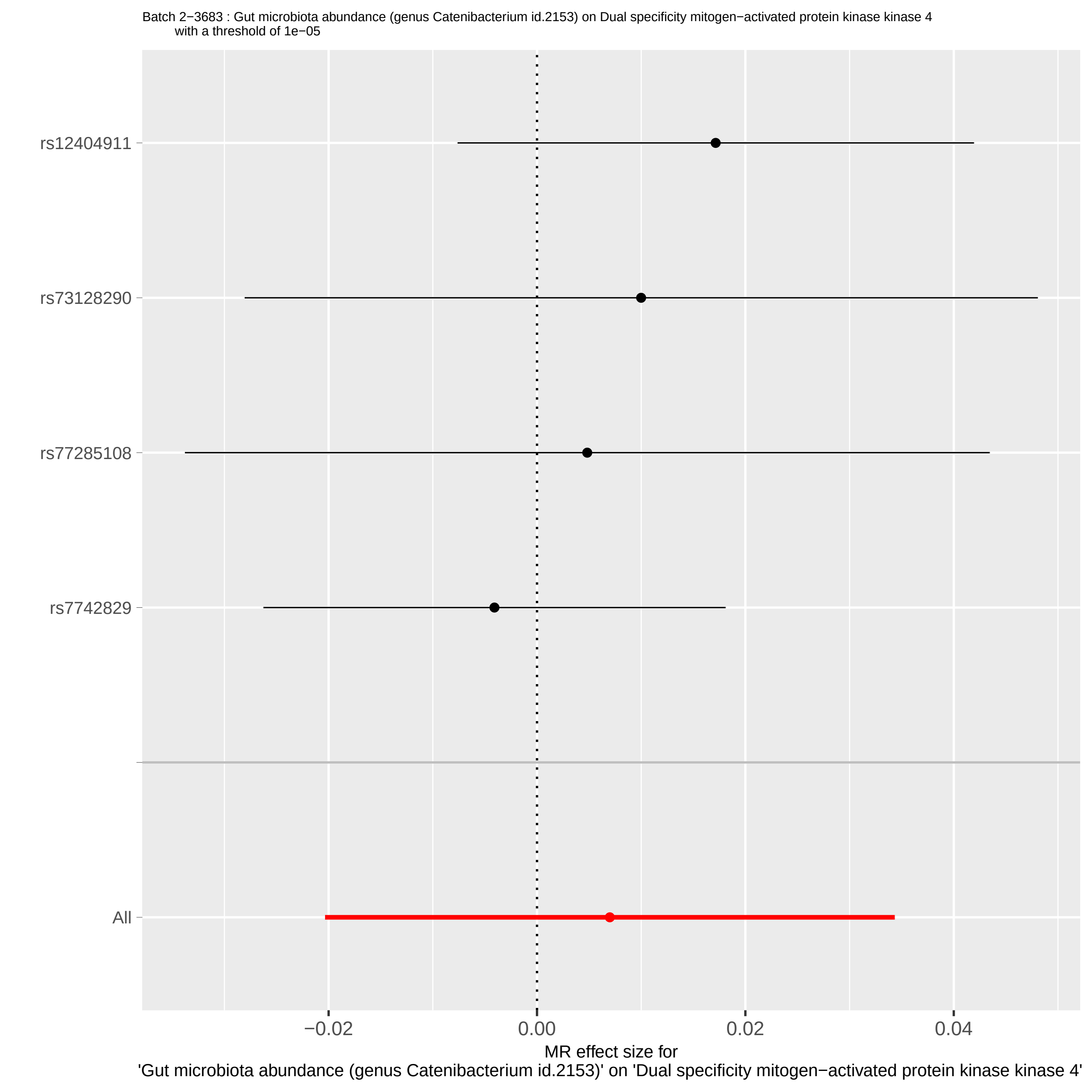
Batch 2−3683 : Gut microbiota abundance (genus Catenibacterium id.2153) on Dual specificity mitogen−activated protein kinase kinase 4
with a threshold of 1e−05
rs12404911
rs73128290
rs77285108
rs7742829
All
−0.02
0.00
0.02
0.04
MR effect size for
'Gut microbiota abundance (genus Catenibacterium id.2153)' on 'Dual specificity mitogen−activated protein kinase kinase 4'

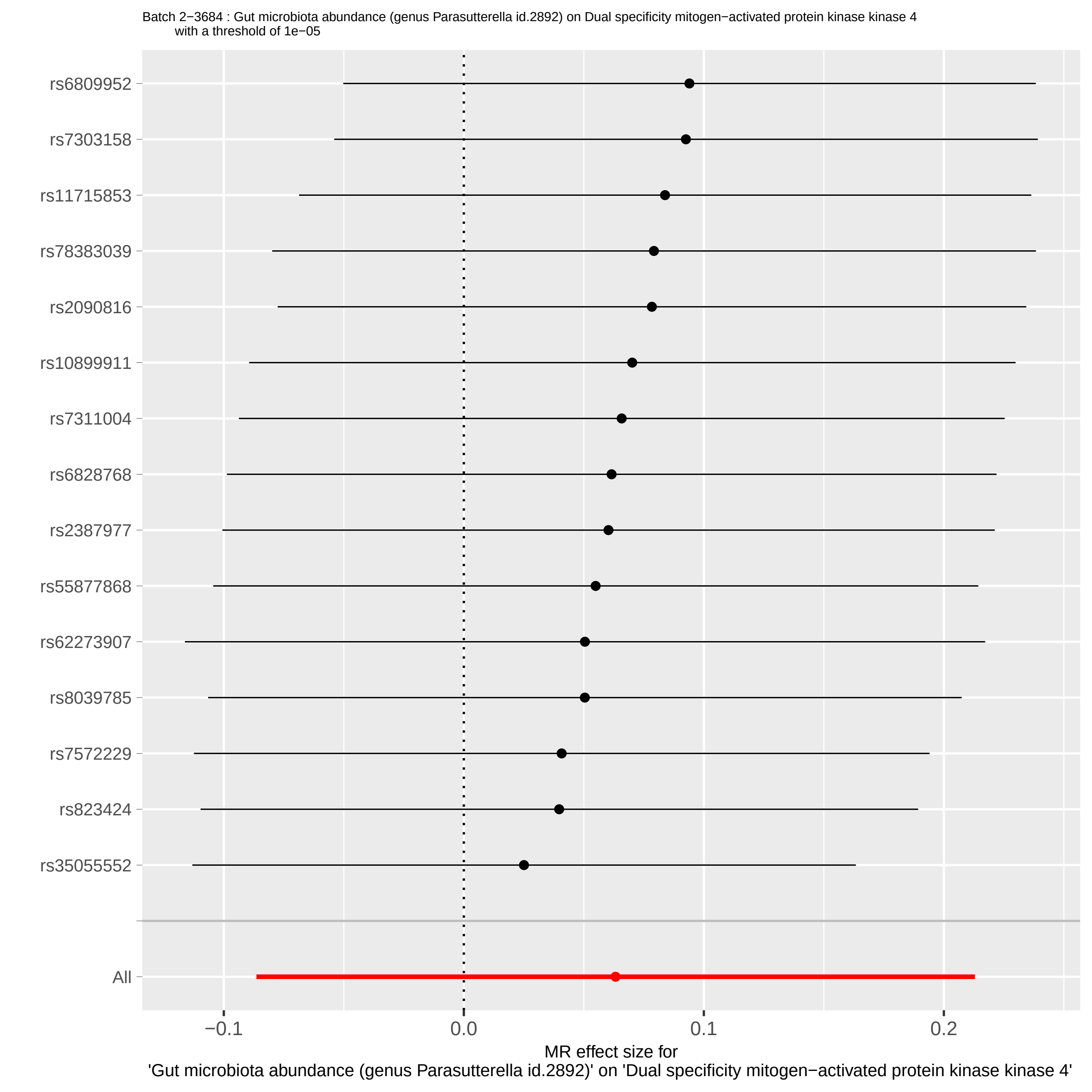

Batch 2−3684 : Gut microbiota abundance (genus Parasutterella id.2892) on Dual specificity mitogen−activated protein kinase kinase 4
with a threshold of 1e−05
rs6809952
rs7303158
rs11715853
rs78383039
rs2090816
rs10899911
rs7311004
rs6828768
rs2387977
rs55877868
rs62273907
rs8039785
rs7572229
rs823424
rs35055552
All
−0.1
0.0
0.1
0.2
MR effect size for
'Gut microbiota abundance (genus Parasutterella id.2892)' on 'Dual specificity mitogen−activated protein kinase kinase 4'

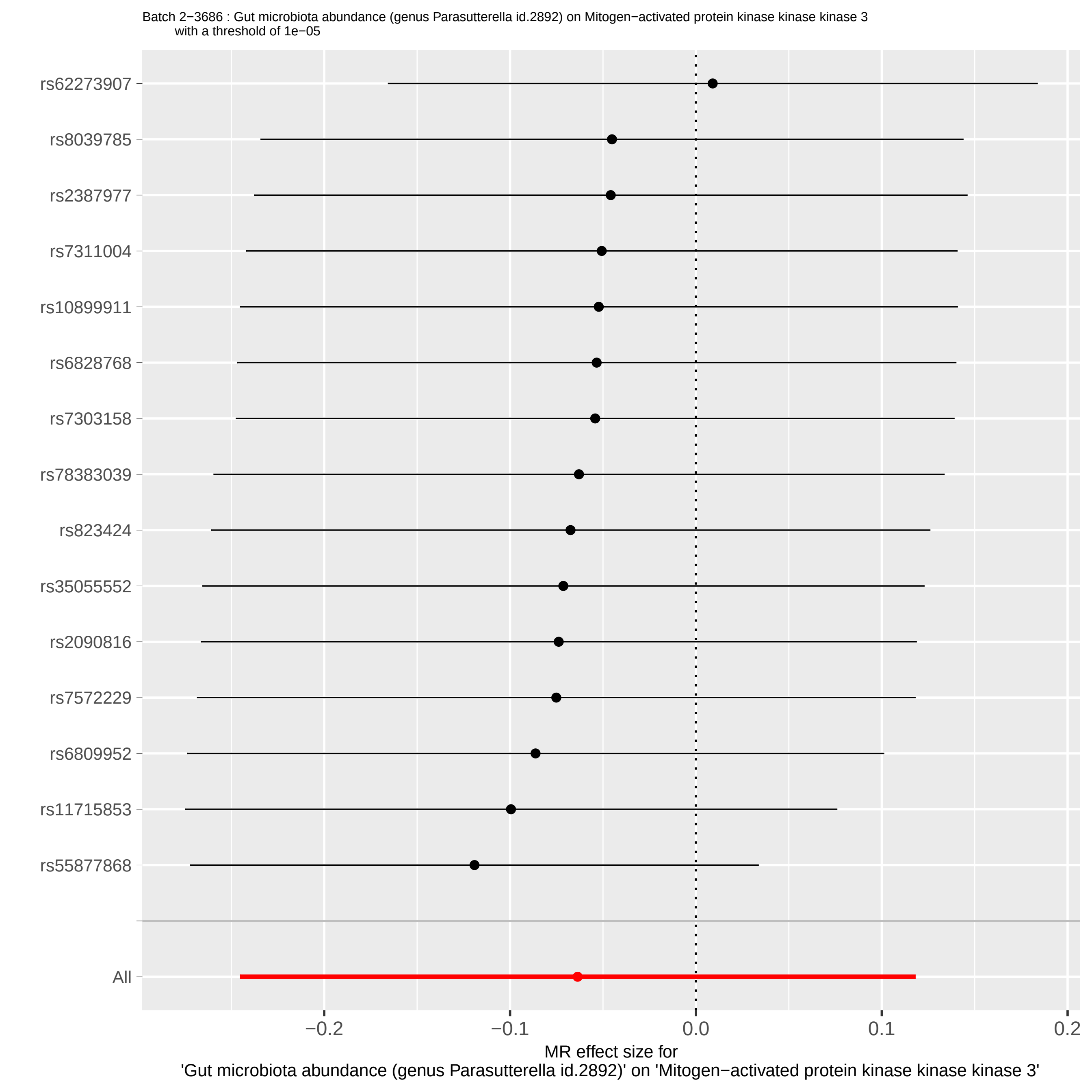

Batch 2−3686 : Gut microbiota abundance (genus Parasutterella id.2892) on Mitogen−activated protein kinase kinase kinase 3
with a threshold of 1e−05
rs62273907
rs8039785
rs2387977
rs7311004
rs10899911
rs6828768
rs7303158
rs78383039
rs823424
rs35055552
rs2090816
rs7572229
rs6809952
rs11715853
rs55877868
All
−0.2
−0.1
0.0
0.1
0.2
MR effect size for
'Gut microbiota abundance (genus Parasutterella id.2892)' on 'Mitogen−activated protein kinase kinase kinase 3'

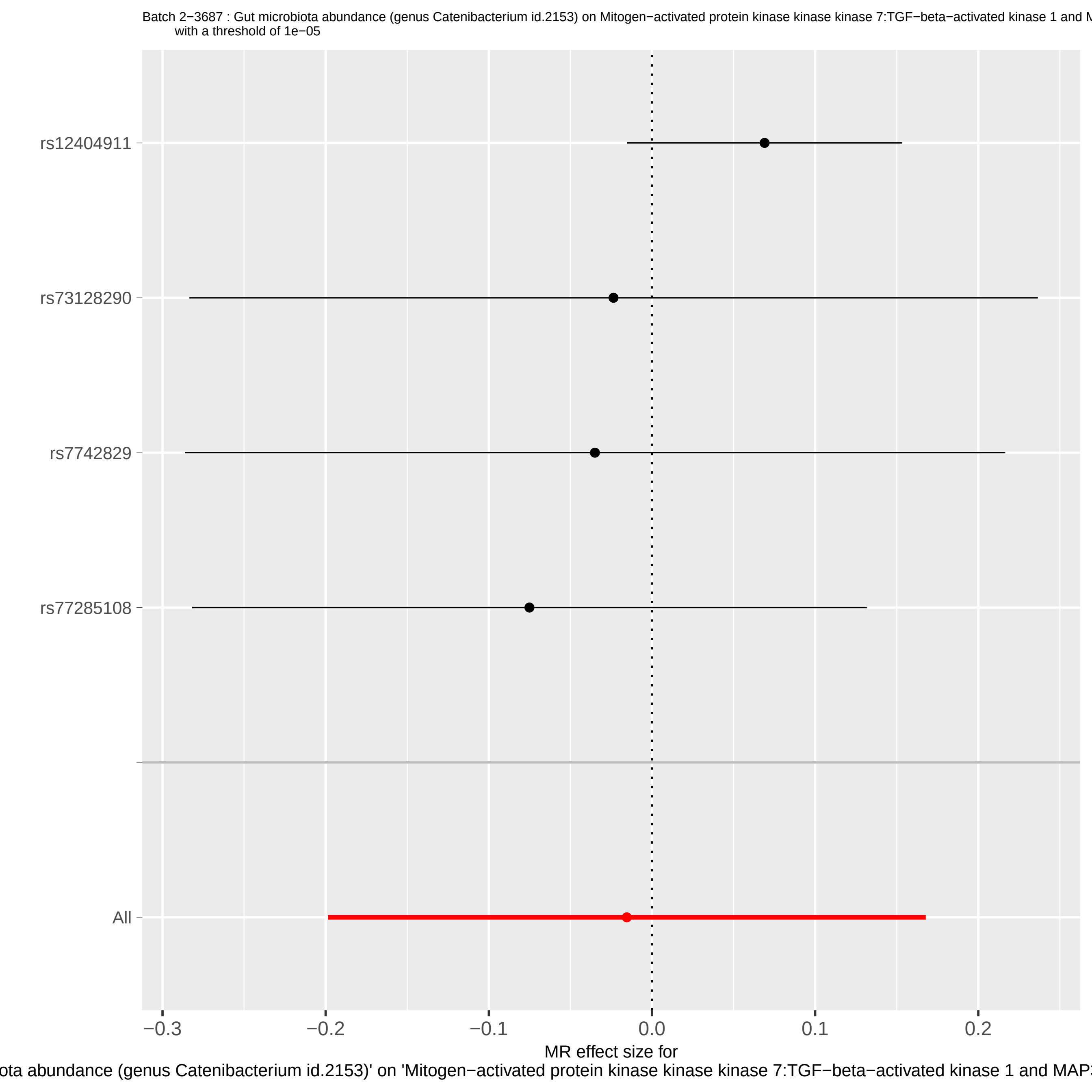
Batch 2−3687 : Gut microbiota abundance (genus Catenibacterium id.2153) on Mitogen−activated protein kinase kinase kinase 7:TGF−beta−activated kinase 1 and M
with a threshold of 1e−05
rs12404911
rs73128290
rs7742829
rs77285108
All
−0.3
−0.2
−0.1
0.0
0.1
0.2
MR effect size for
ota abundance (genus Catenibacterium id.2153)' on 'Mitogen−activated protein kinase kinase kinase 7:TGF−beta−activated kinase 1 and MAP

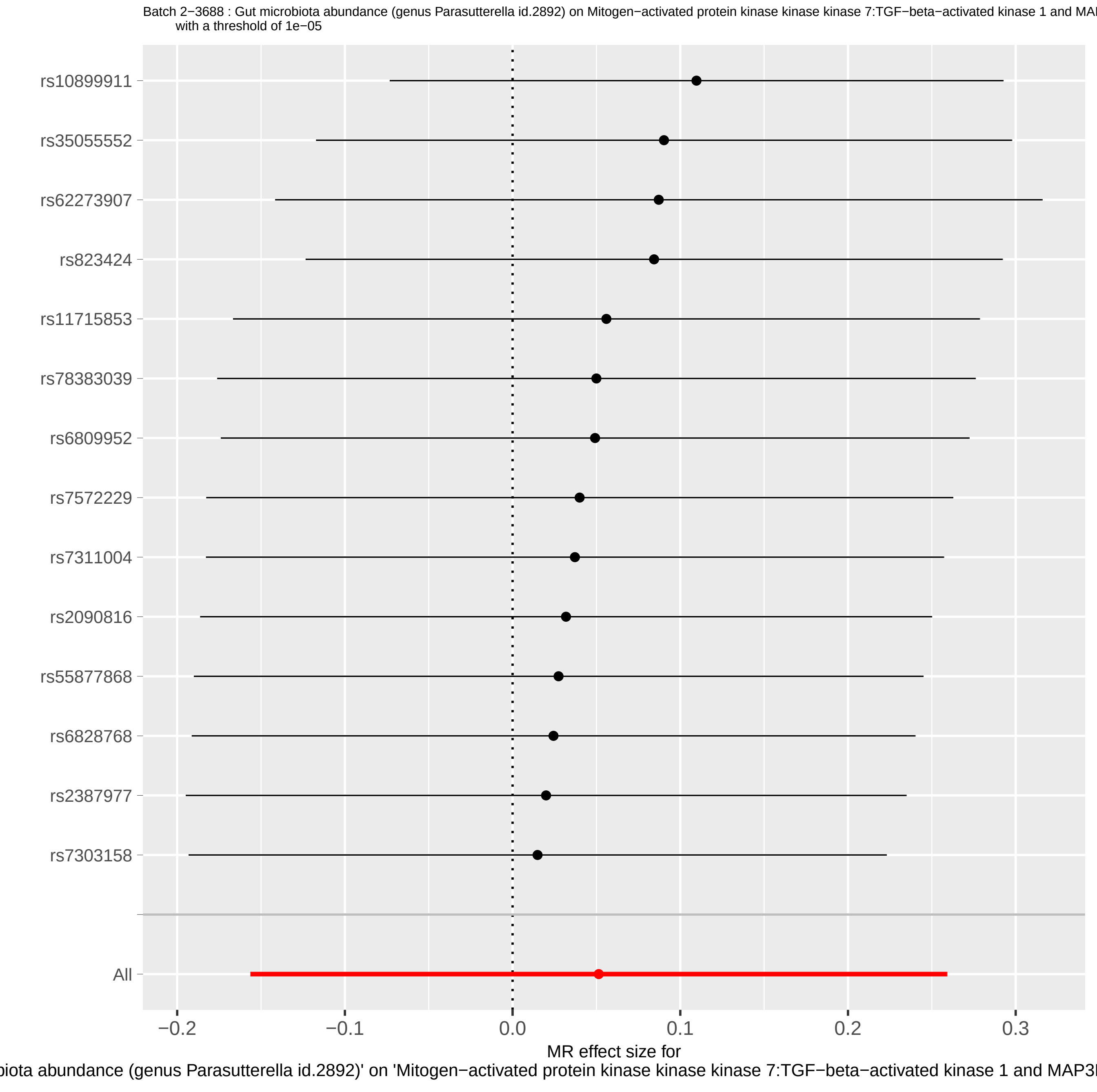

Batch 2−3688 : Gut microbiota abundance (genus Parasutterella id.2892) on Mitogen−activated protein kinase kinase kinase 7:TGF−beta−activated kinase 1 and MA
with a threshold of 1e−05
rs10899911
rs35055552
rs62273907
rs823424
rs11715853
rs78383039
rs6809952
rs7572229
rs7311004
rs2090816
rs55877868
rs6828768
rs2387977
rs7303158
All
−0.2
−0.1
0.0
0.1
0.2
0.3
MR effect size for
biota abundance (genus Parasutterella id.2892)' on 'Mitogen−activated protein kinase kinase kinase 7:TGF−beta−activated kinase 1 and MAP3

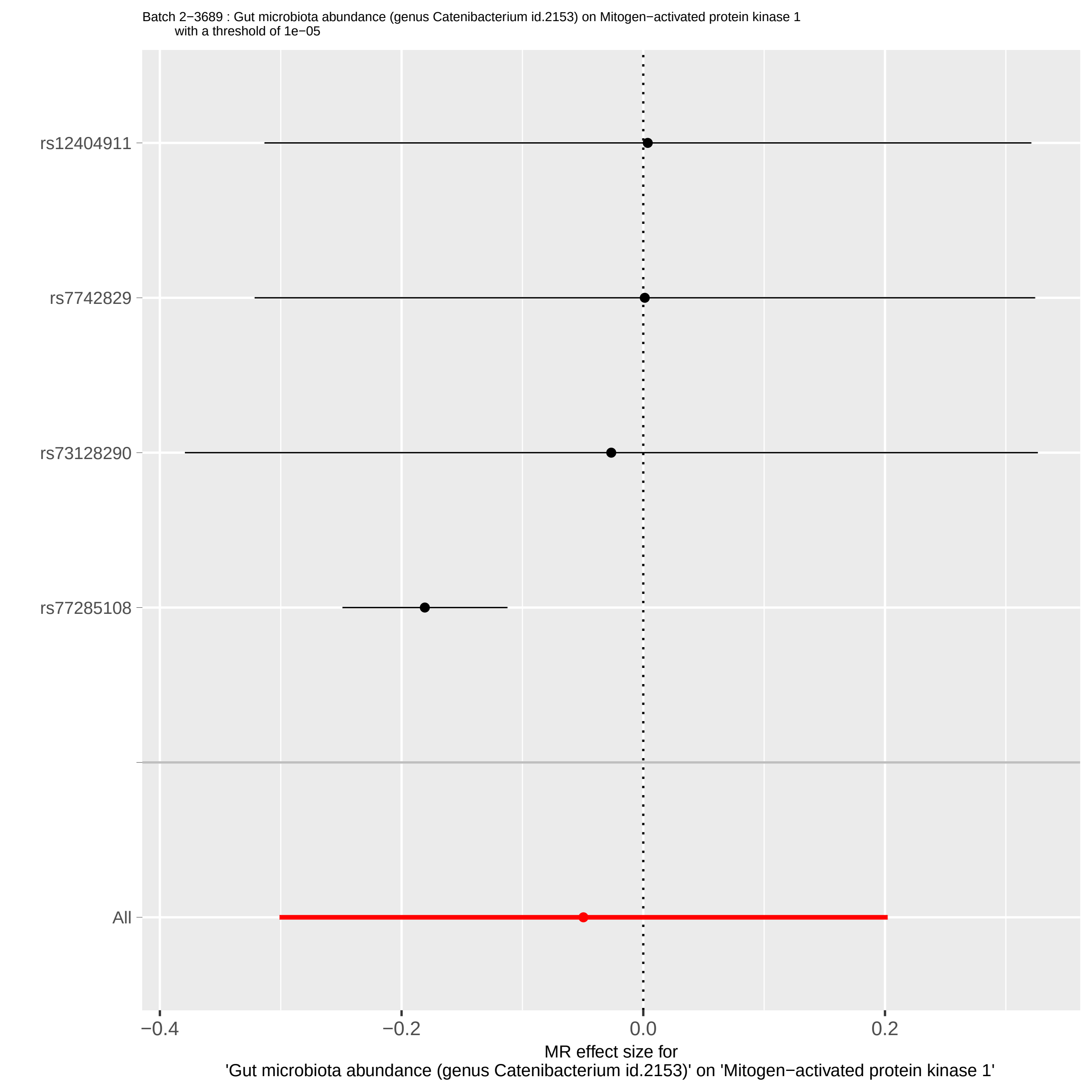
Batch 2−3689 : Gut microbiota abundance (genus Catenibacterium id.2153) on Mitogen−activated protein kinase 1
with a threshold of 1e−05
rs12404911
rs7742829
rs73128290
rs77285108
All
−0.4
−0.2
0.0
0.2
MR effect size for
'Gut microbiota abundance (genus Catenibacterium id.2153)' on 'Mitogen−activated protein kinase 1'

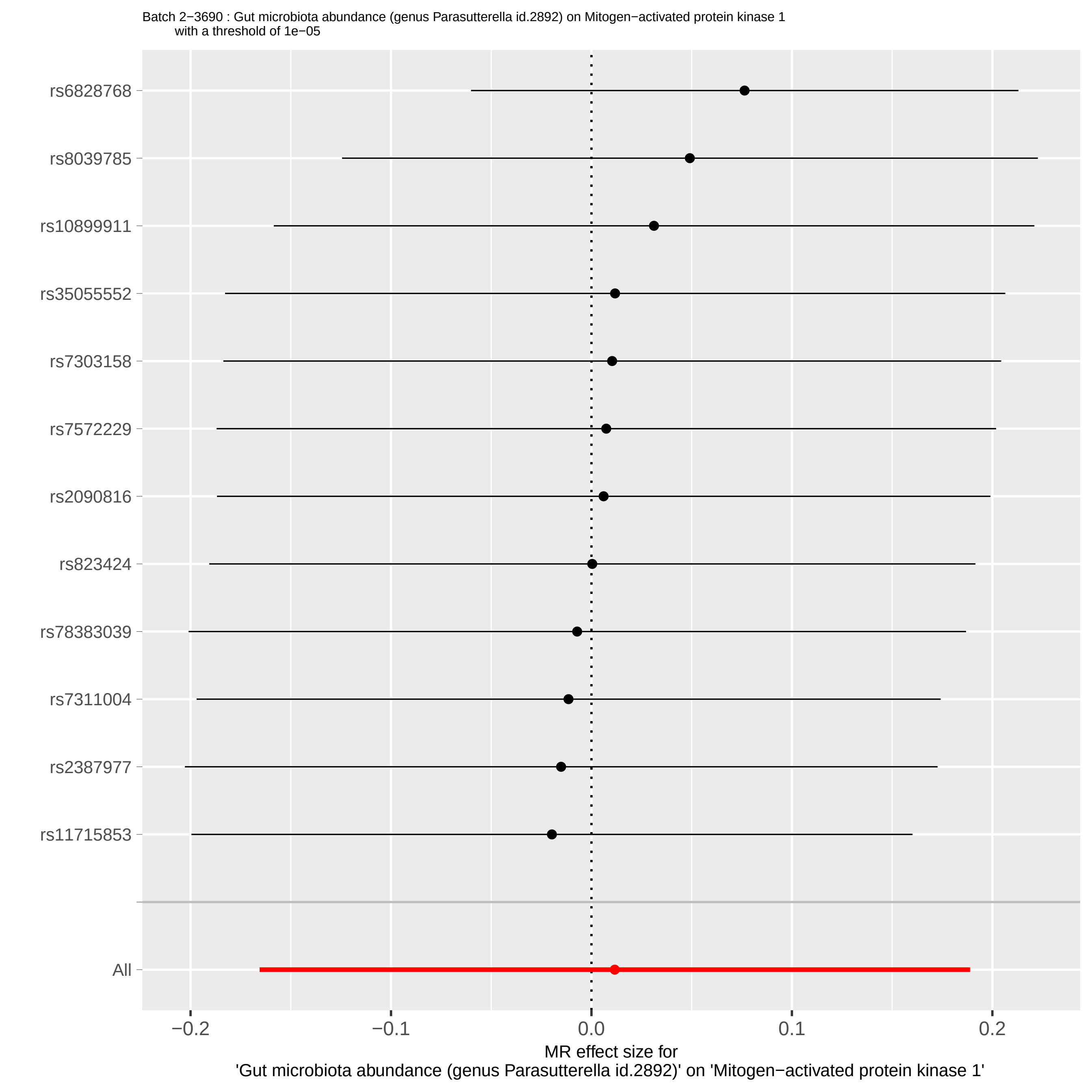
Batch 2−3690 : Gut microbiota abundance (genus Parasutterella id.2892) on Mitogen−activated protein kinase 1
with a threshold of 1e−05
rs6828768
rs8039785
rs10899911
rs35055552
rs7303158
rs7572229
rs2090816
rs823424
rs78383039
rs7311004
rs2387977
rs11715853
All
−0.2
−0.1
0.0
0.1
0.2
MR effect size for
'Gut microbiota abundance (genus Parasutterella id.2892)' on 'Mitogen−activated protein kinase 1'

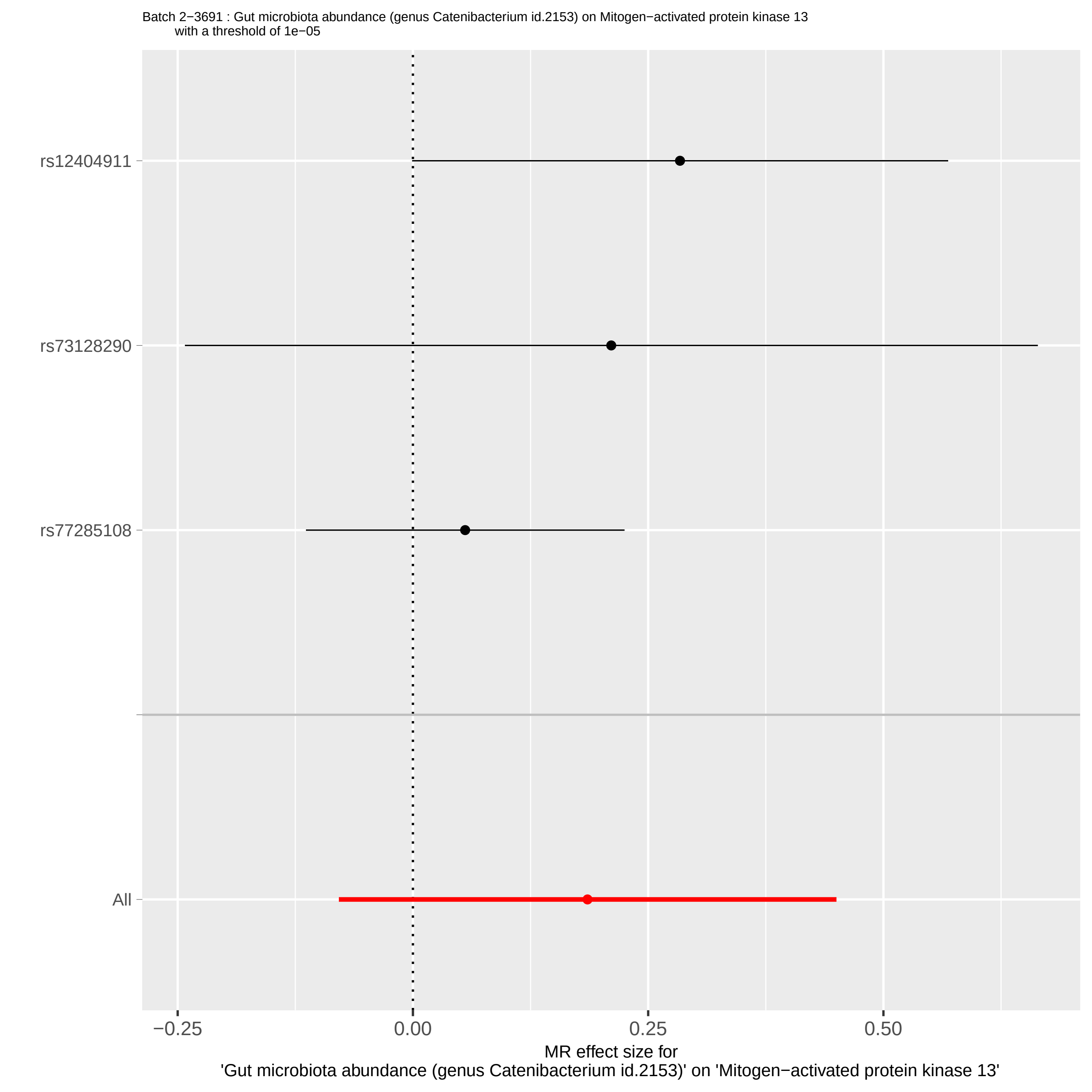

Batch 2−3691 : Gut microbiota abundance (genus Catenibacterium id.2153) on Mitogen−activated protein kinase 13
with a threshold of 1e−05
rs12404911
rs73128290
rs77285108
All
−0.25
0.00
0.25
0.50
MR effect size for
'Gut microbiota abundance (genus Catenibacterium id.2153)' on 'Mitogen−activated protein kinase 13'

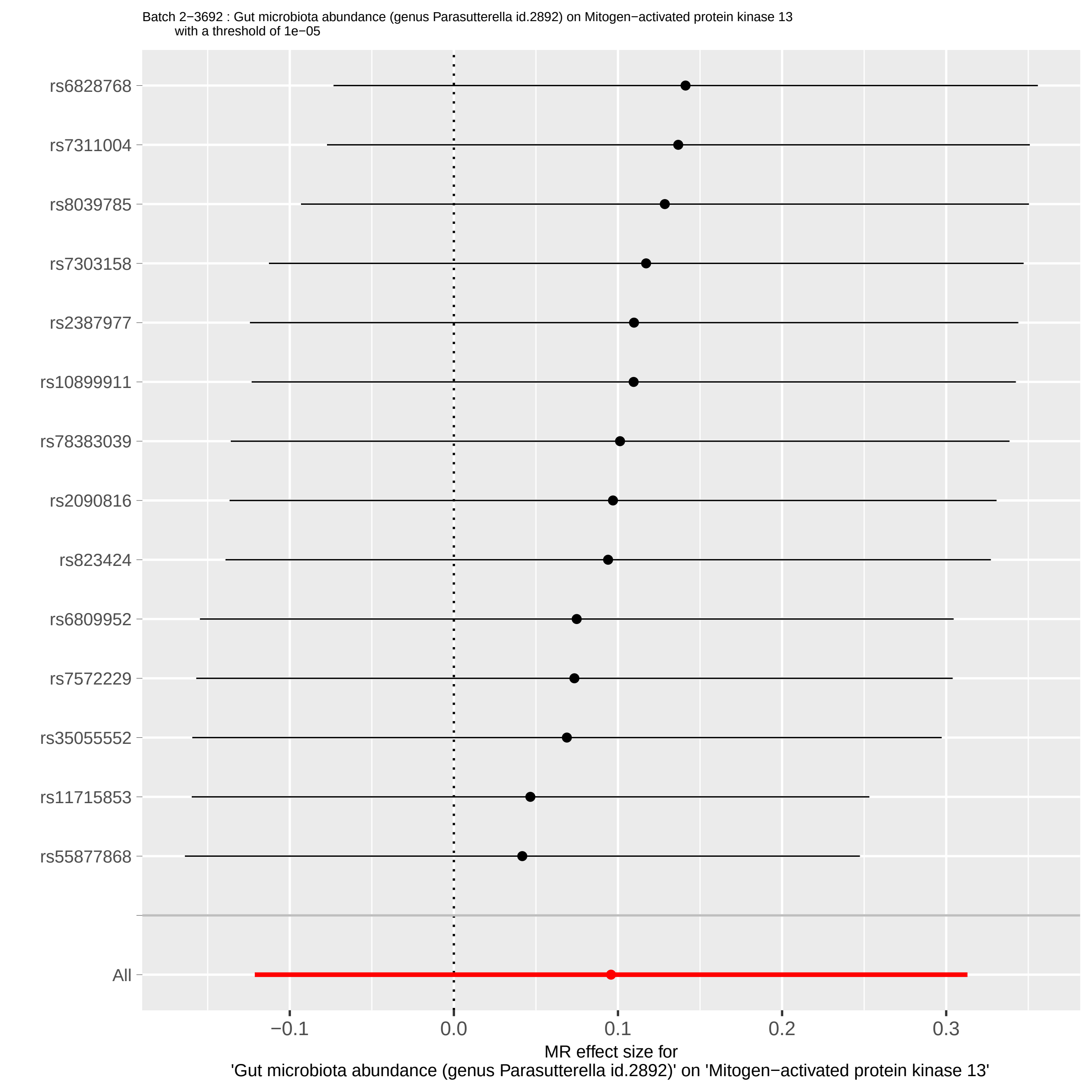
Batch 2−3692 : Gut microbiota abundance (genus Parasutterella id.2892) on Mitogen−activated protein kinase 13
with a threshold of 1e−05
rs6828768
rs7311004
rs8039785
rs7303158
rs2387977
rs10899911
rs78383039
rs2090816
rs823424
rs6809952
rs7572229
rs35055552
rs11715853
rs55877868
All
−0.1
0.0
0.1
0.2
0.3
MR effect size for
'Gut microbiota abundance (genus Parasutterella id.2892)' on 'Mitogen−activated protein kinase 13'

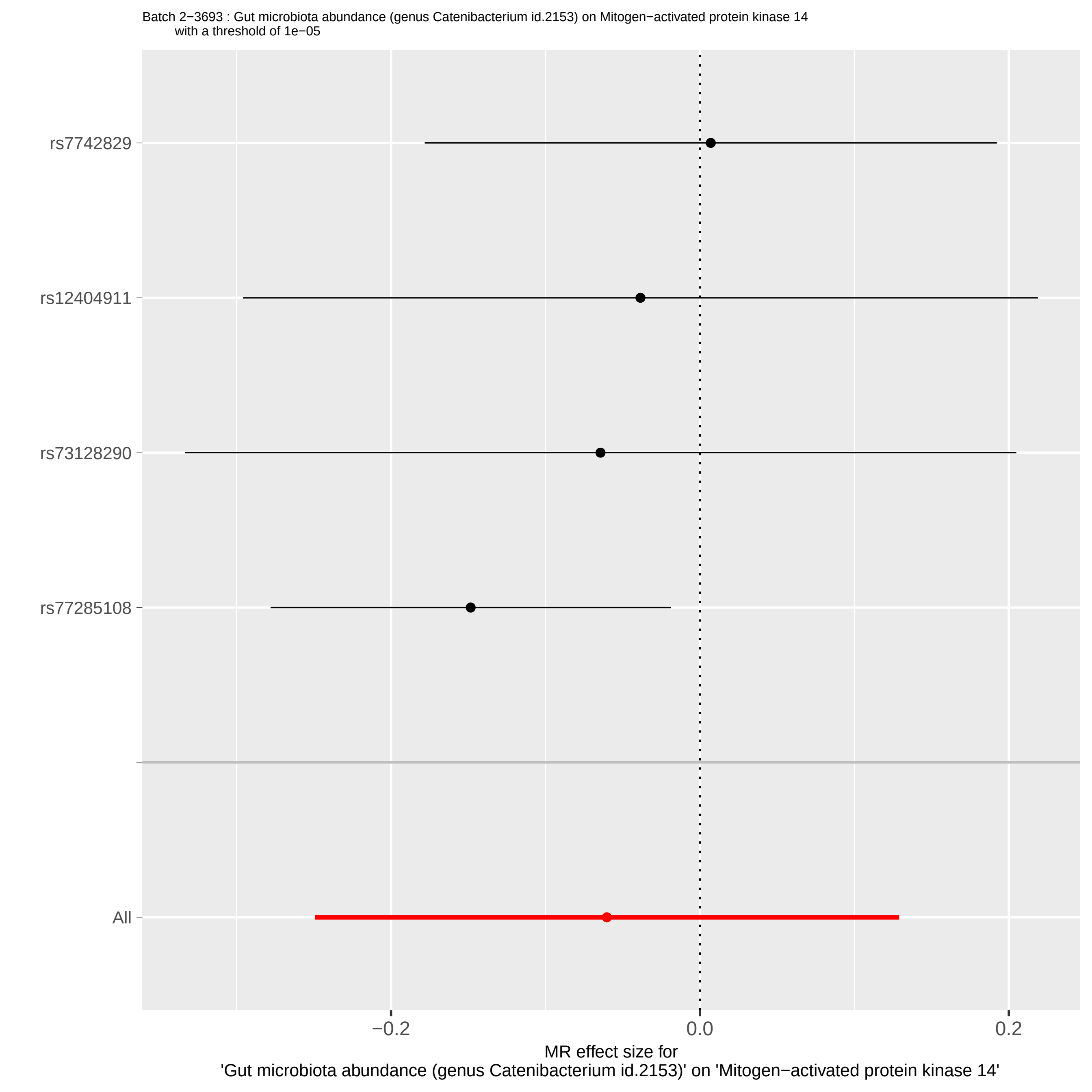
Batch 2−3693 : Gut microbiota abundance (genus Catenibacterium id.2153) on Mitogen−activated protein kinase 14
with a threshold of 1e−05
rs7742829
rs12404911
rs73128290
rs77285108
All
−0.2
0.0
0.2
MR effect size for
'Gut microbiota abundance (genus Catenibacterium id.2153)' on 'Mitogen−activated protein kinase 14'

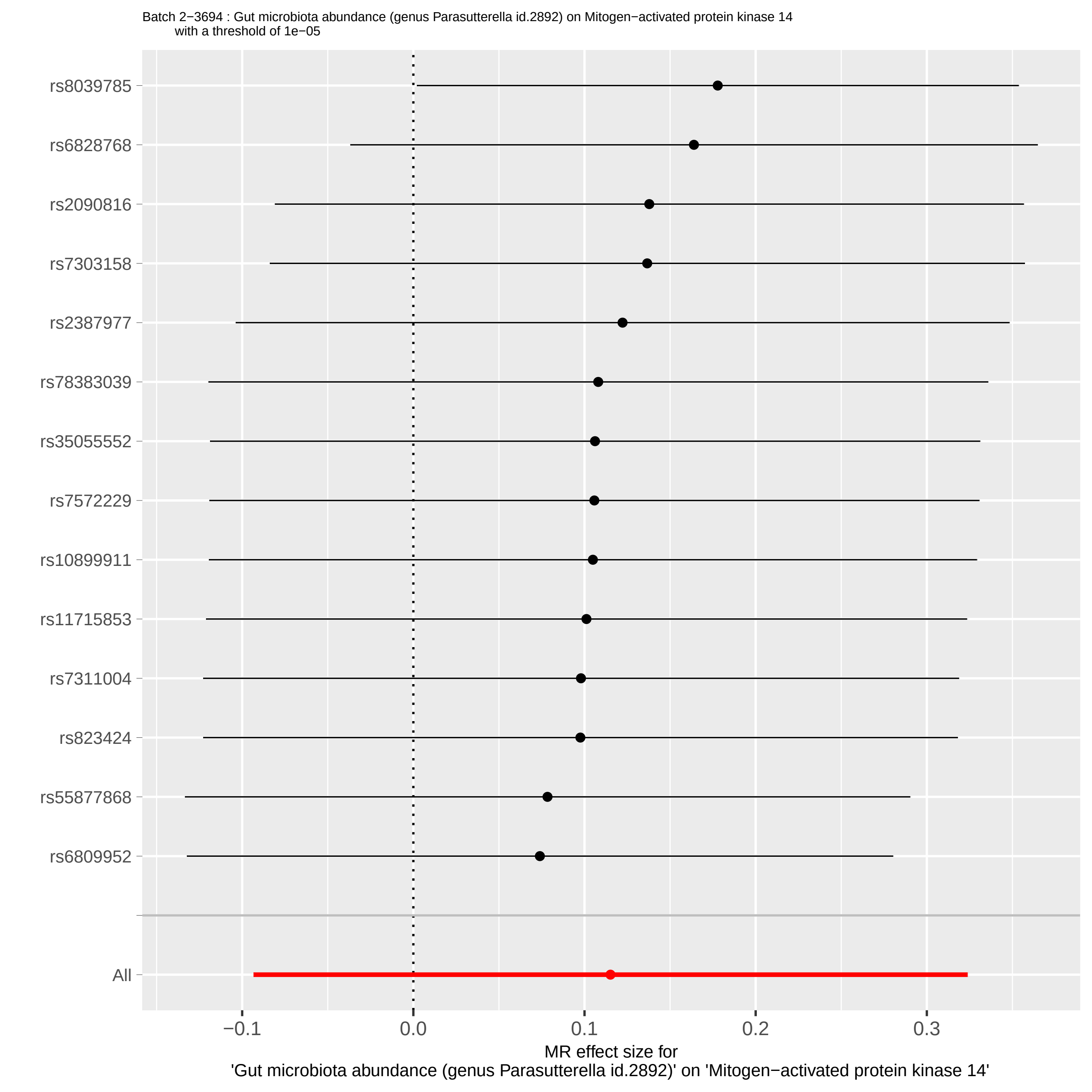

Batch 2−3694 : Gut microbiota abundance (genus Parasutterella id.2892) on Mitogen−activated protein kinase 14
with a threshold of 1e−05
rs8039785
rs6828768
rs2090816
rs7303158
rs2387977
rs78383039
rs35055552
rs7572229
rs10899911
rs11715853
rs7311004
rs823424
rs55877868
rs6809952
All
−0.1
0.0
0.1
0.2
0.3
MR effect size for
'Gut microbiota abundance (genus Parasutterella id.2892)' on 'Mitogen−activated protein kinase 14'

Batch 2−3695 : Gut microbiota abundance (genus Catenibacterium id.2153) on Mitogen−activated protein kinase 3
with a threshold of 1e−05

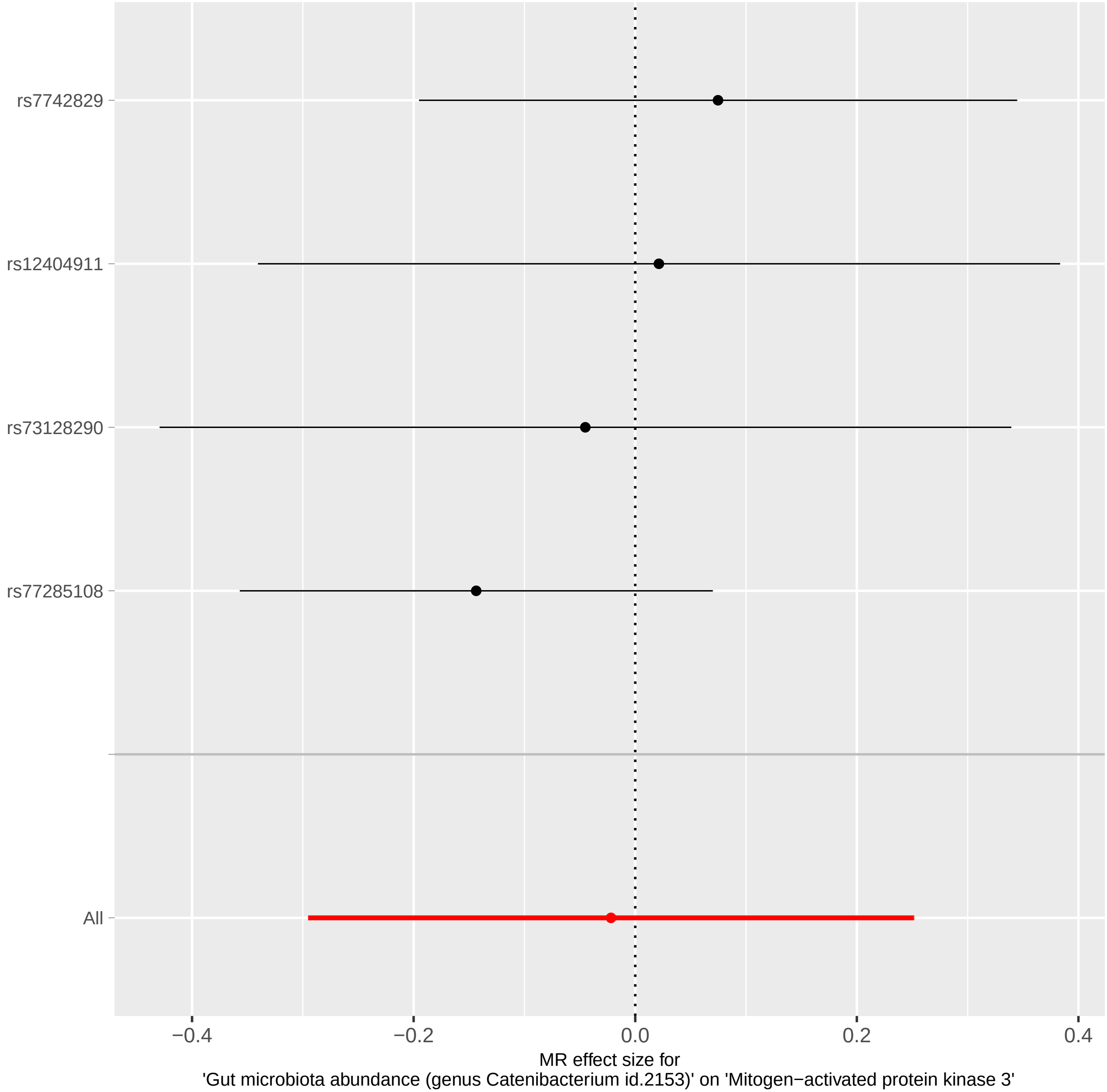

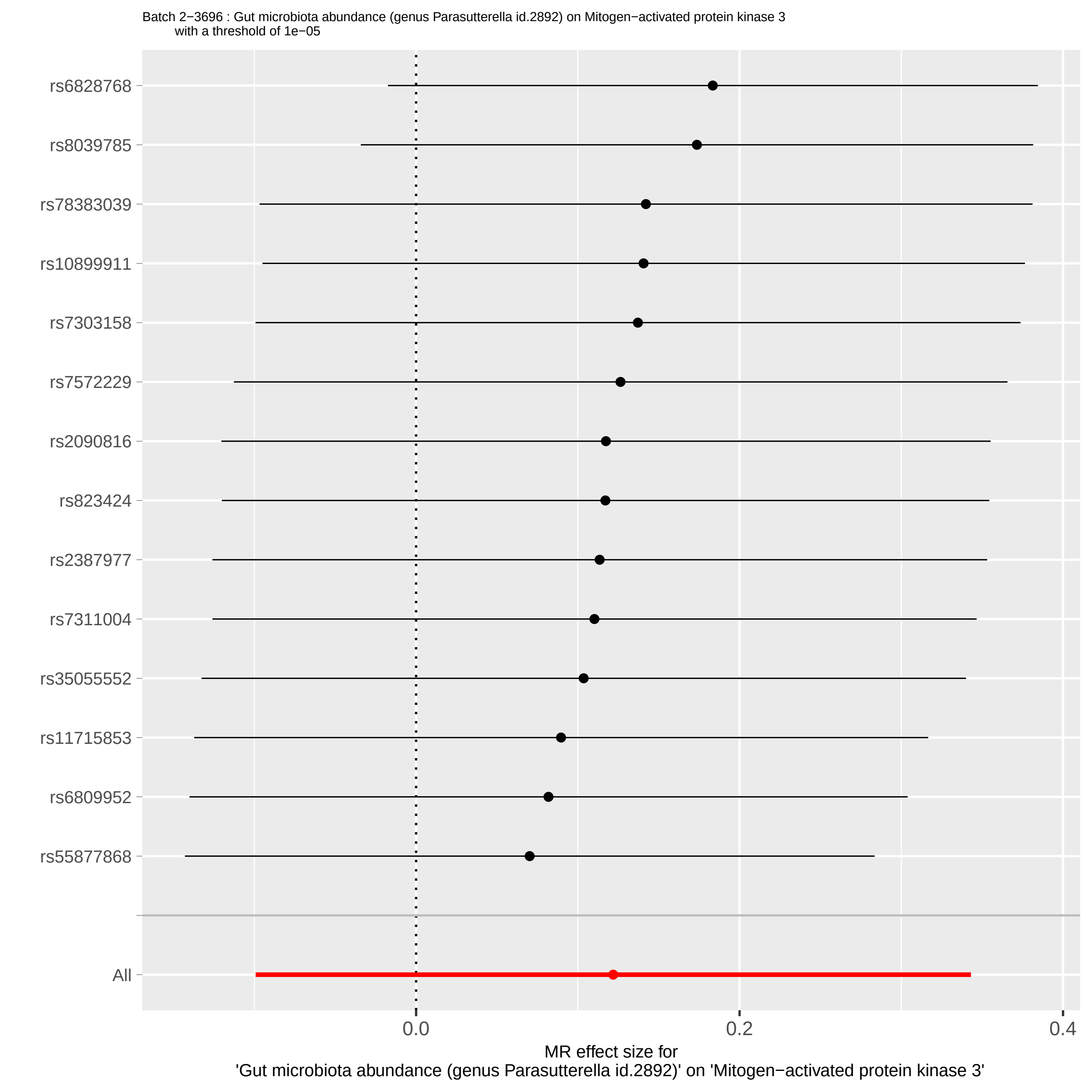

Batch 2−3696 : Gut microbiota abundance (genus Parasutterella id.2892) on Mitogen−activated protein kinase 3
with a threshold of 1e−05
rs6828768
rs8039785
rs78383039
rs10899911
rs7303158
rs7572229
rs2090816
rs823424
rs2387977
rs7311004
rs35055552
rs11715853
rs6809952
rs55877868
All
0.0
0.2
0.4
MR effect size for
'Gut microbiota abundance (genus Parasutterella id.2892)' on 'Mitogen−activated protein kinase 3'

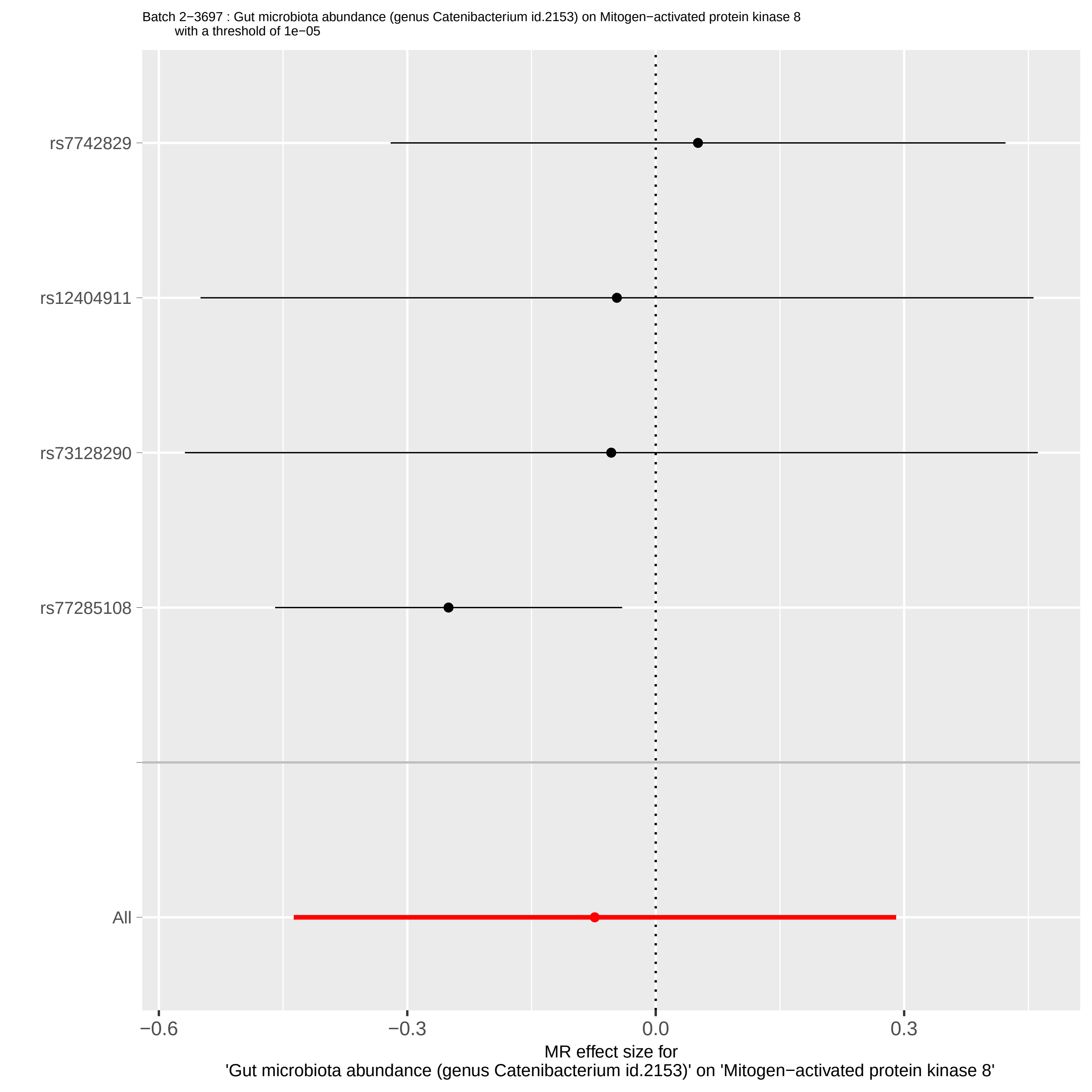
Batch 2−3697 : Gut microbiota abundance (genus Catenibacterium id.2153) on Mitogen−activated protein kinase 8
with a threshold of 1e−05
rs7742829
rs12404911
rs73128290
rs77285108
All
−0.6
−0.3
0.0
0.3
MR effect size for
'Gut microbiota abundance (genus Catenibacterium id.2153)' on 'Mitogen−activated protein kinase 8'

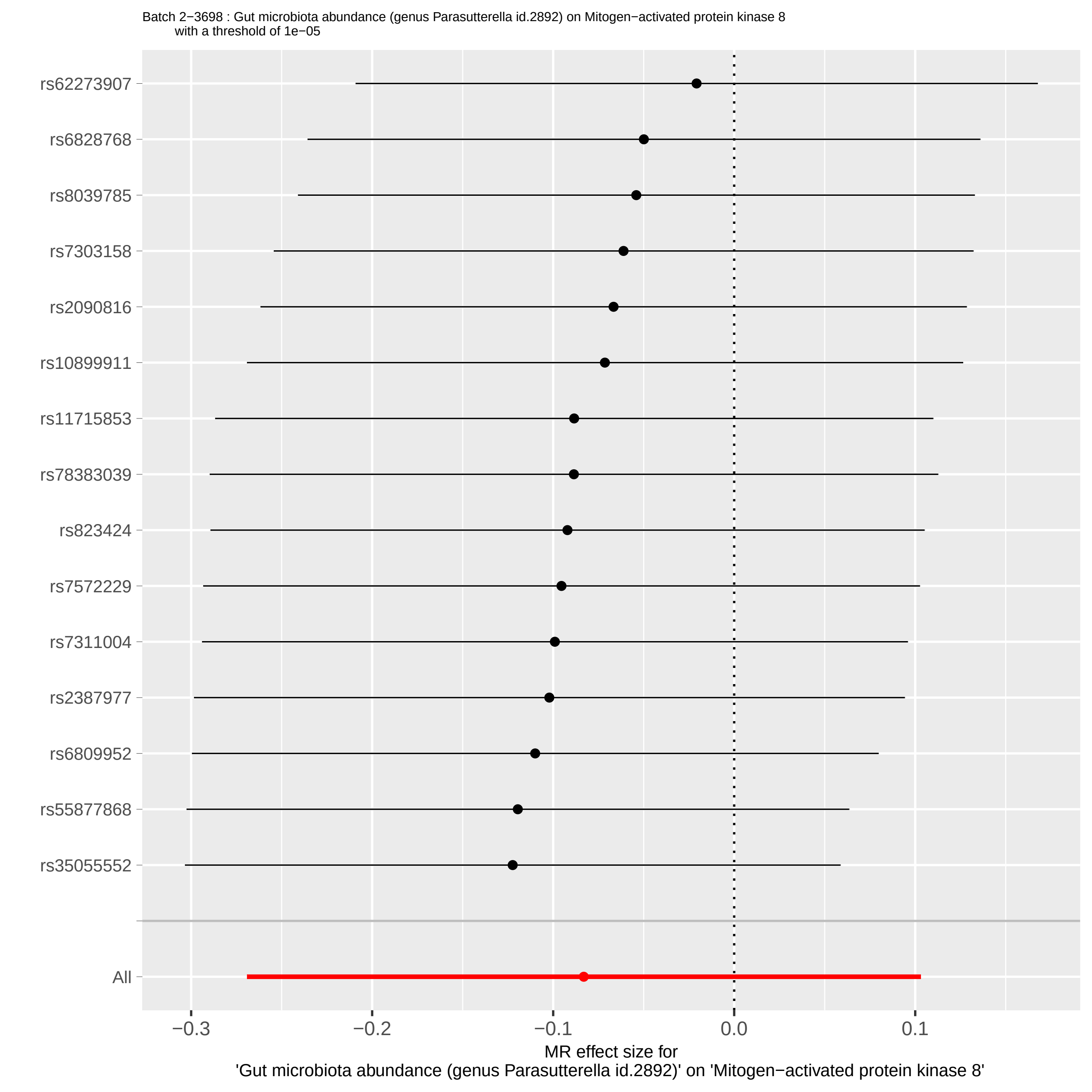
Batch 2−3698 : Gut microbiota abundance (genus Parasutterella id.2892) on Mitogen−activated protein kinase 8
with a threshold of 1e−05
rs62273907
rs6828768
rs8039785
rs7303158
rs2090816
rs10899911
rs11715853
rs78383039
rs823424
rs7572229
rs7311004
rs2387977
rs6809952
rs55877868
rs35055552
All
−0.3
−0.2
−0.1
0.0
0.1
MR effect size for
'Gut microbiota abundance (genus Parasutterella id.2892)' on 'Mitogen−activated protein kinase 8'

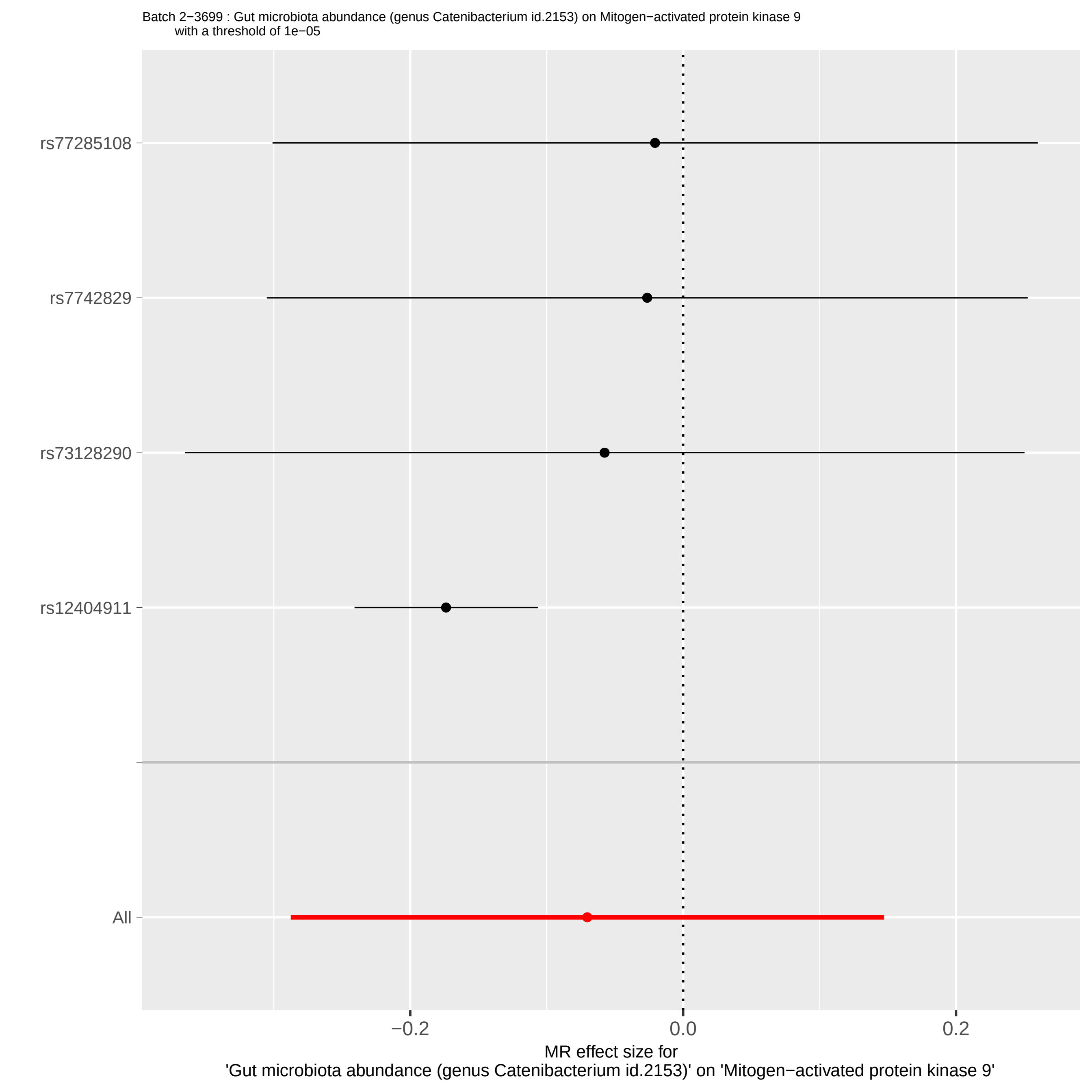
Batch 2−3699 : Gut microbiota abundance (genus Catenibacterium id.2153) on Mitogen−activated protein kinase 9
with a threshold of 1e−05
rs77285108
rs7742829
rs73128290
rs12404911
All
−0.2
0.0
0.2
MR effect size for
'Gut microbiota abundance (genus Catenibacterium id.2153)' on 'Mitogen−activated protein kinase 9'

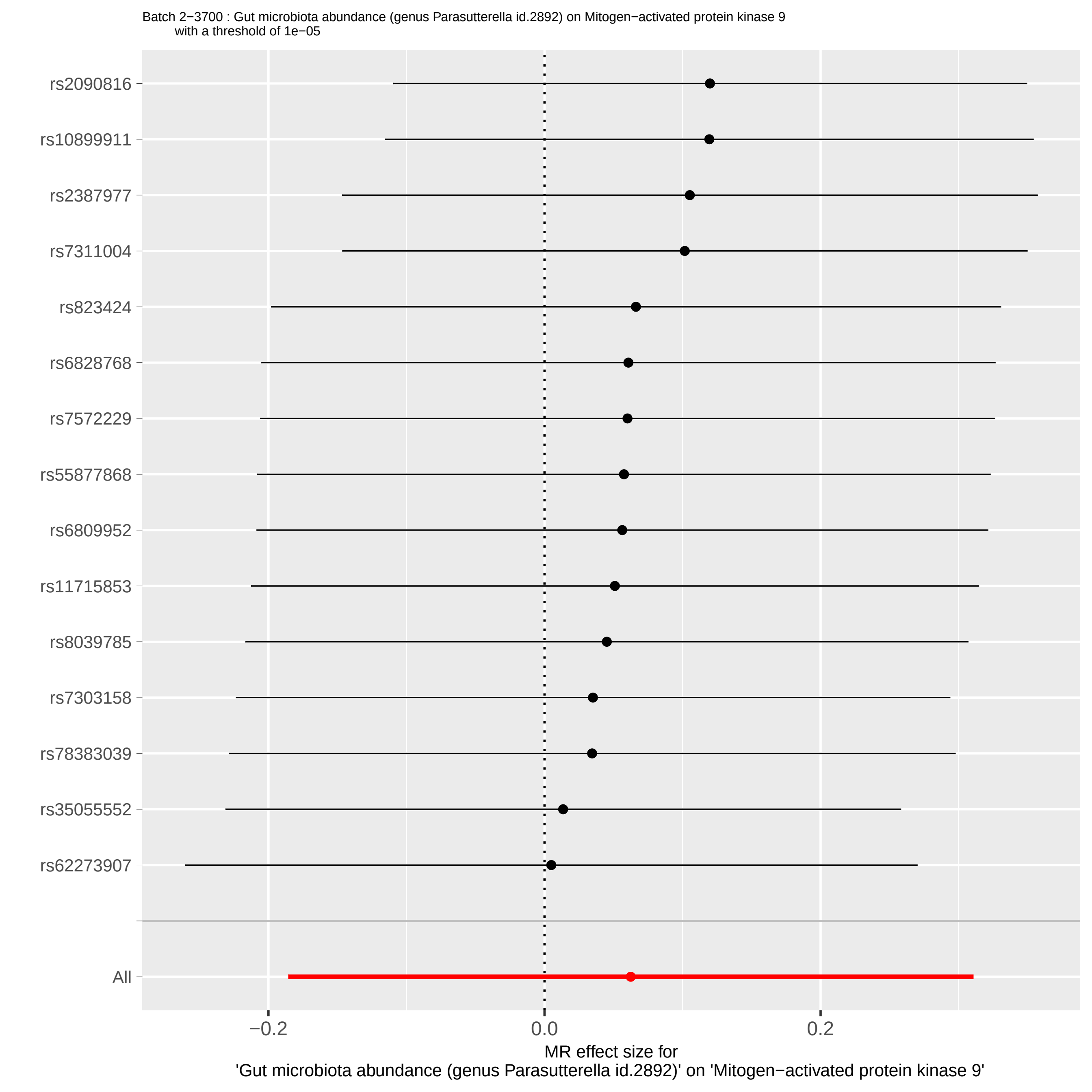
Batch 2−3700 : Gut microbiota abundance (genus Parasutterella id.2892) on Mitogen−activated protein kinase 9
with a threshold of 1e−05
rs2090816
rs10899911
rs2387977
rs7311004
rs823424
rs6828768
rs7572229
rs55877868
rs6809952
rs11715853
rs8039785
rs7303158
rs78383039
rs35055552
rs62273907
All
−0.2
0.0
0.2
MR effect size for
'Gut microbiota abundance (genus Parasutterella id.2892)' on 'Mitogen−activated protein kinase 9'

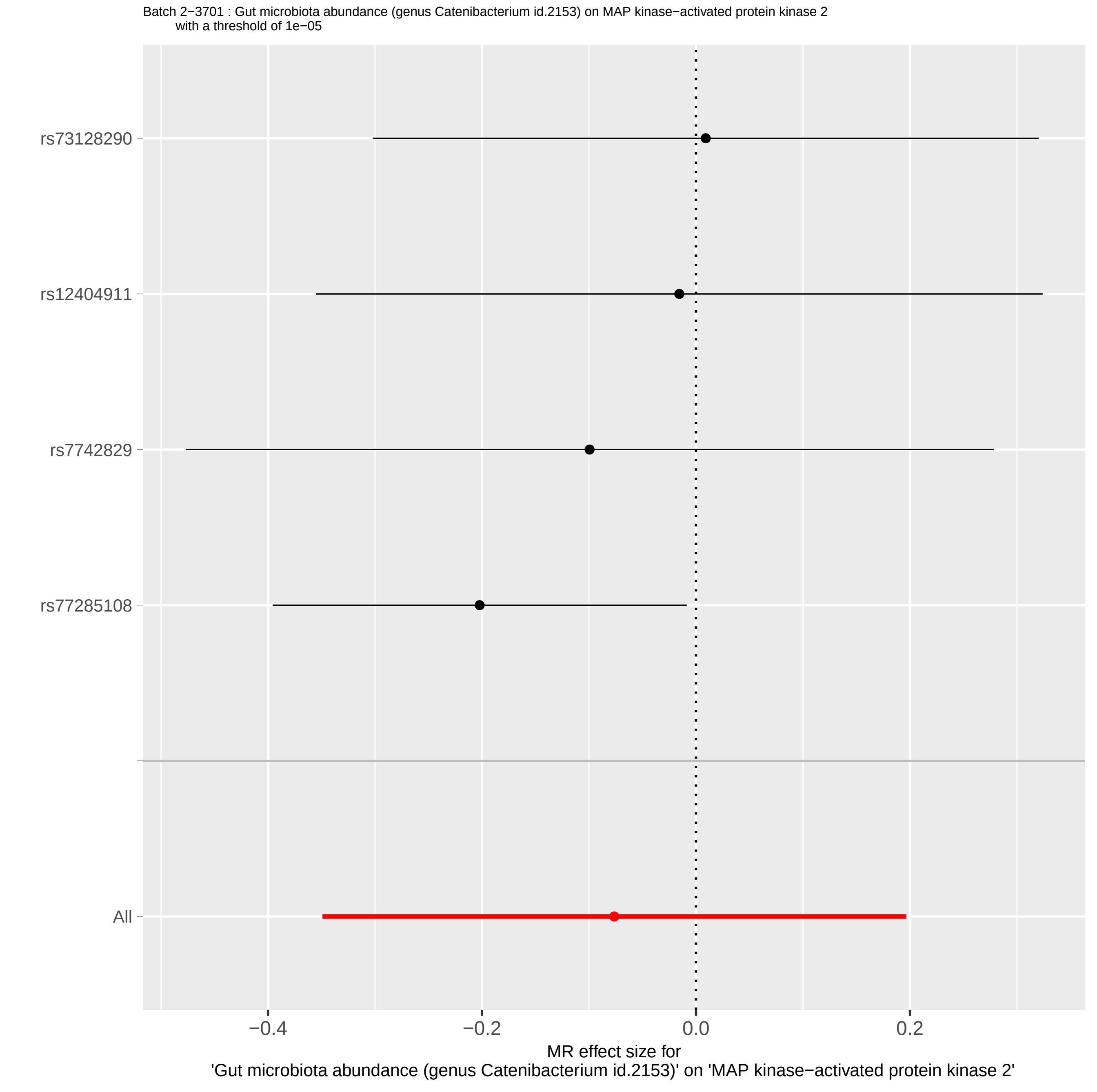
Batch 2−3701 : Gut microbiota abundance (genus Catenibacterium id.2153) on MAP kinase−activated protein kinase 2
with a threshold of 1e−05
rs73128290
rs12404911
rs7742829
rs77285108
All
−0.4
−0.2
0.0
0.2
MR effect size for
'Gut microbiota abundance (genus Catenibacterium id.2153)' on 'MAP kinase−activated protein kinase 2'

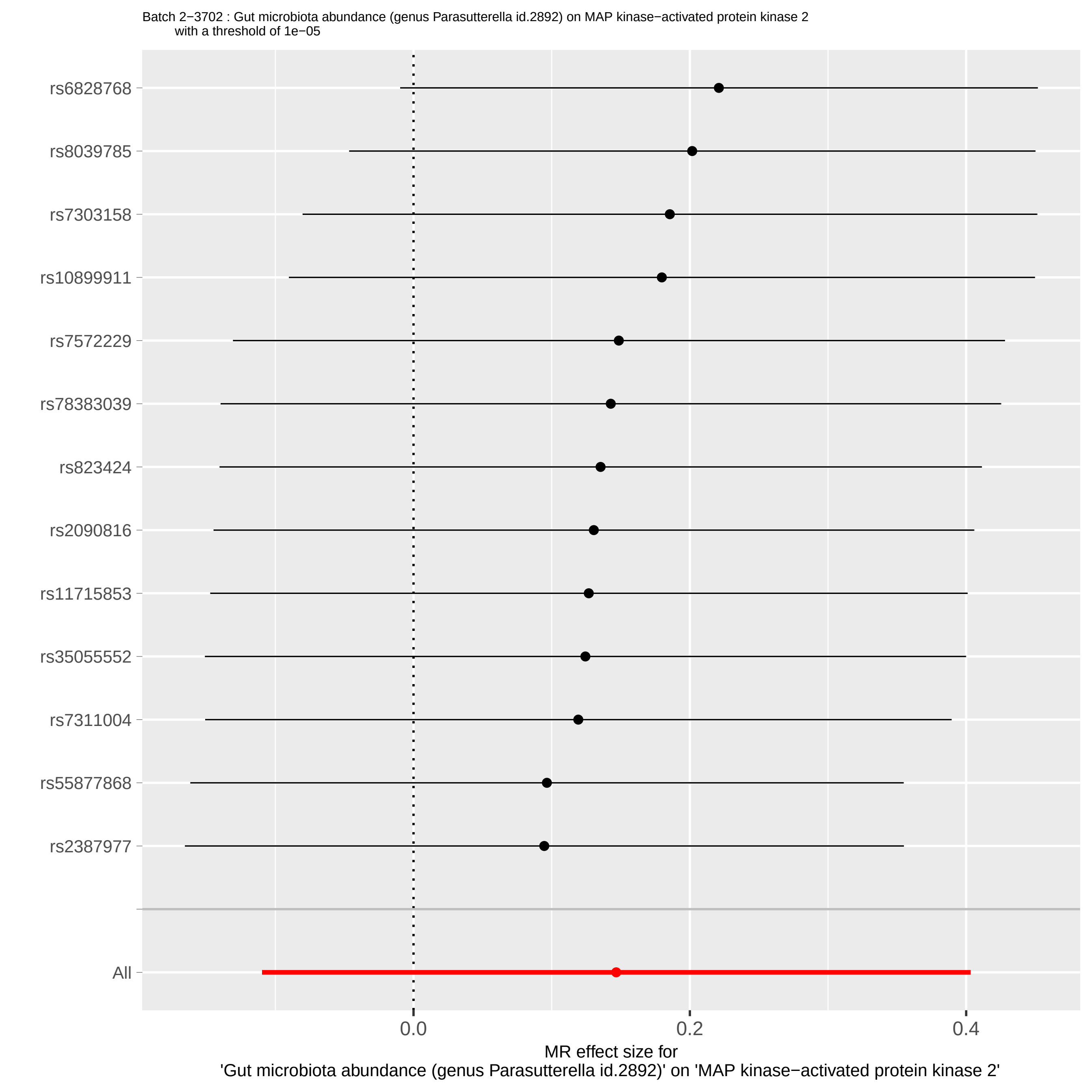
Batch 2−3702 : Gut microbiota abundance (genus Parasutterella id.2892) on MAP kinase−activated protein kinase 2
with a threshold of 1e−05
rs6828768
rs8039785
rs7303158
rs10899911
rs7572229
rs78383039
rs823424
rs2090816
rs11715853
rs35055552
rs7311004
rs55877868
rs2387977
All
0.0
0.2
0.4
MR effect size for
'Gut microbiota abundance (genus Parasutterella id.2892)' on 'MAP kinase−activated protein kinase 2'

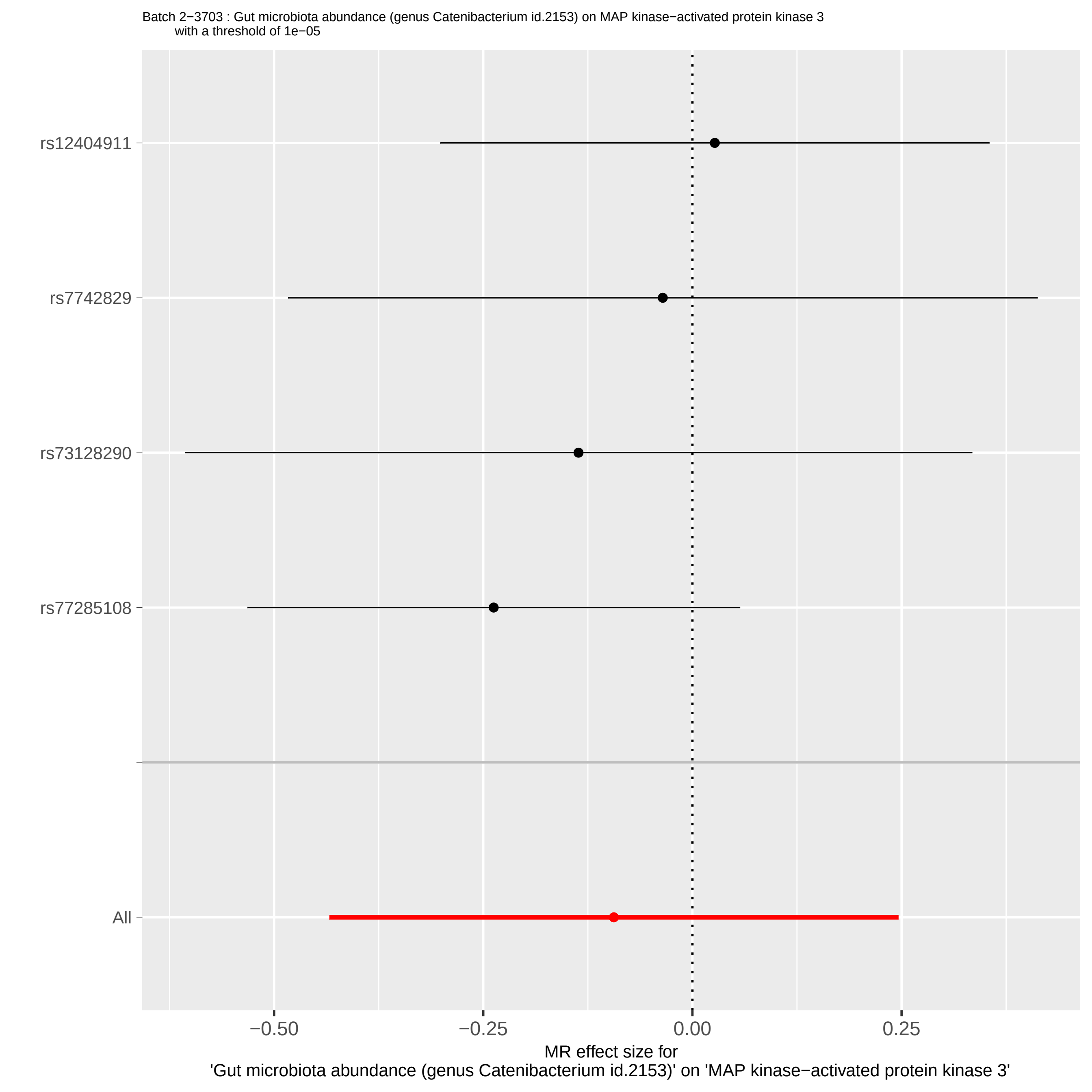
Batch 2−3703 : Gut microbiota abundance (genus Catenibacterium id.2153) on MAP kinase−activated protein kinase 3
with a threshold of 1e−05
rs12404911
rs7742829
rs73128290
rs77285108
All
−0.50
−0.25
0.00
0.25
MR effect size for
'Gut microbiota abundance (genus Catenibacterium id.2153)' on 'MAP kinase−activated protein kinase 3'

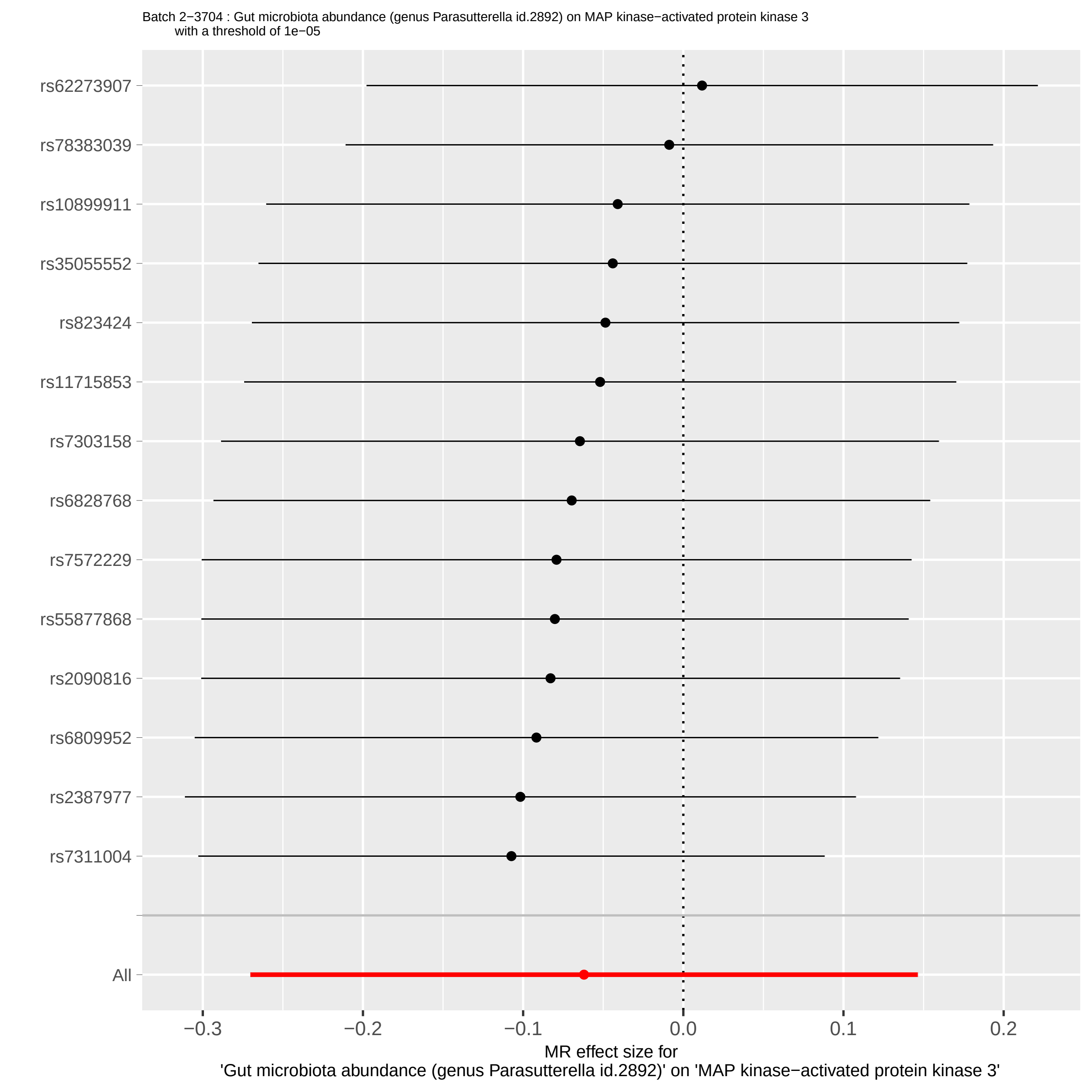

Batch 2−3704 : Gut microbiota abundance (genus Parasutterella id.2892) on MAP kinase−activated protein kinase 3
with a threshold of 1e−05
rs62273907
rs78383039
rs10899911
rs35055552
rs823424
rs11715853
rs7303158
rs6828768
rs7572229
rs55877868
rs2090816
rs6809952
rs2387977
rs7311004
All
−0.3
−0.2
−0.1
0.0
0.1
0.2
MR effect size for
'Gut microbiota abundance (genus Parasutterella id.2892)' on 'MAP kinase−activated protein kinase 3'

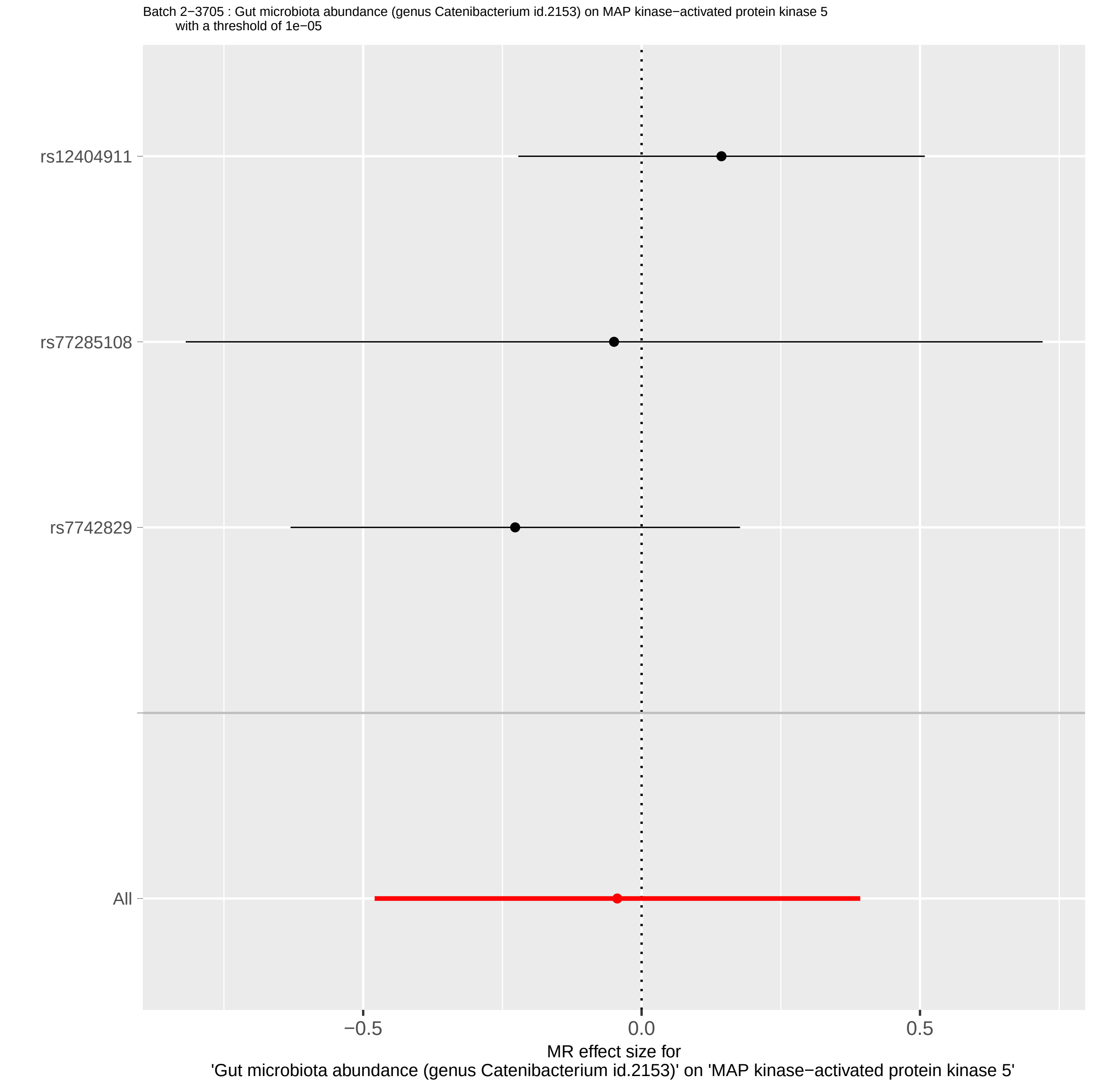

Batch 2−3705 : Gut microbiota abundance (genus Catenibacterium id.2153) on MAP kinase−activated protein kinase 5
with a threshold of 1e−05
rs12404911
rs77285108
rs7742829
All
−0.5
0.0
0.5
MR effect size for
'Gut microbiota abundance (genus Catenibacterium id.2153)' on 'MAP kinase−activated protein kinase 5'

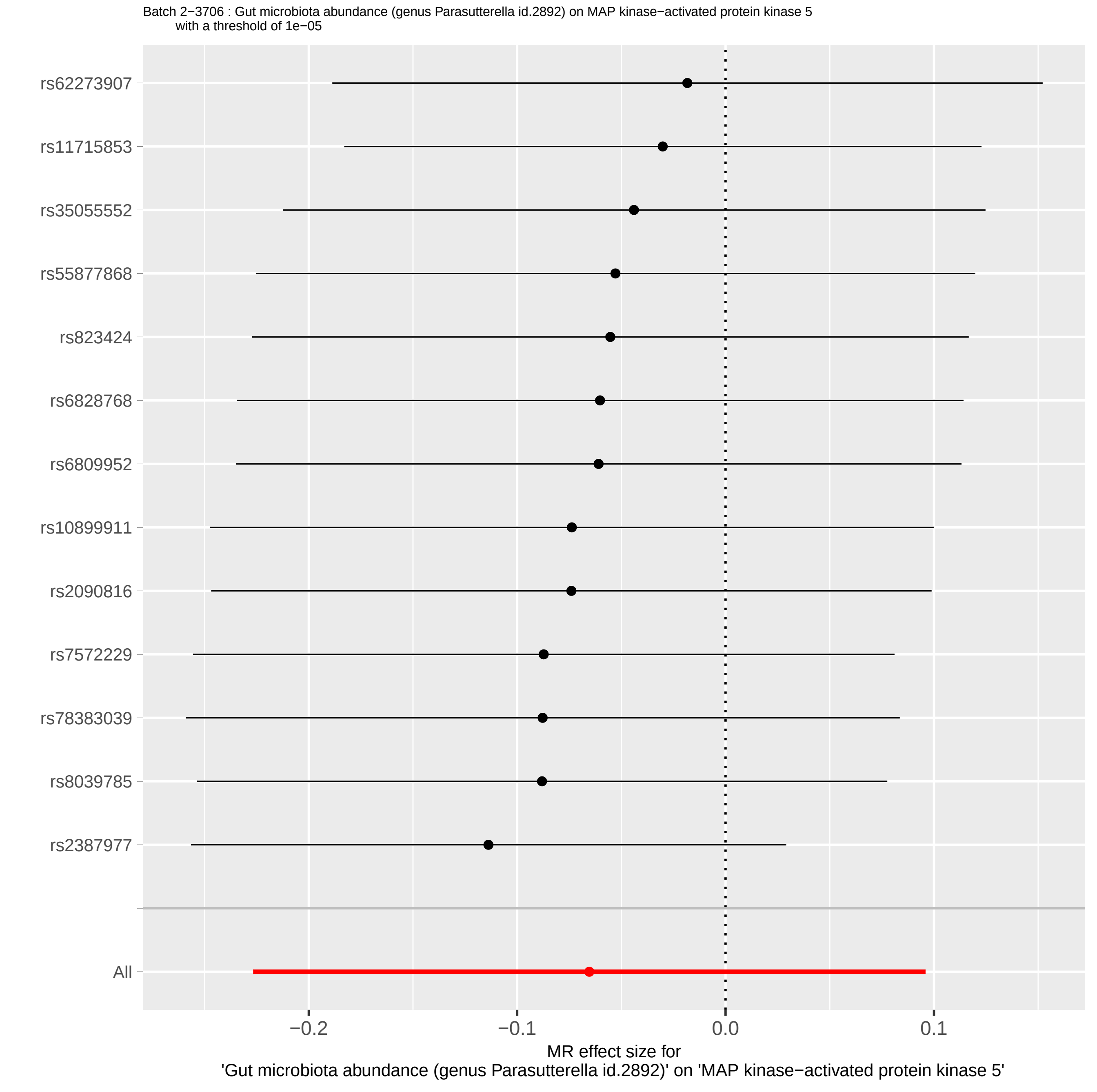

Batch 2−3706 : Gut microbiota abundance (genus Parasutterella id.2892) on MAP kinase−activated protein kinase 5
with a threshold of 1e−05
rs62273907
rs11715853
rs35055552
rs55877868
rs823424
rs6828768
rs6809952
rs10899911
rs2090816
rs7572229
rs78383039
rs8039785
rs2387977
All
−0.2
−0.1
0.0
0.1
MR effect size for
'Gut microbiota abundance (genus Parasutterella id.2892)' on 'MAP kinase−activated protein kinase 5'

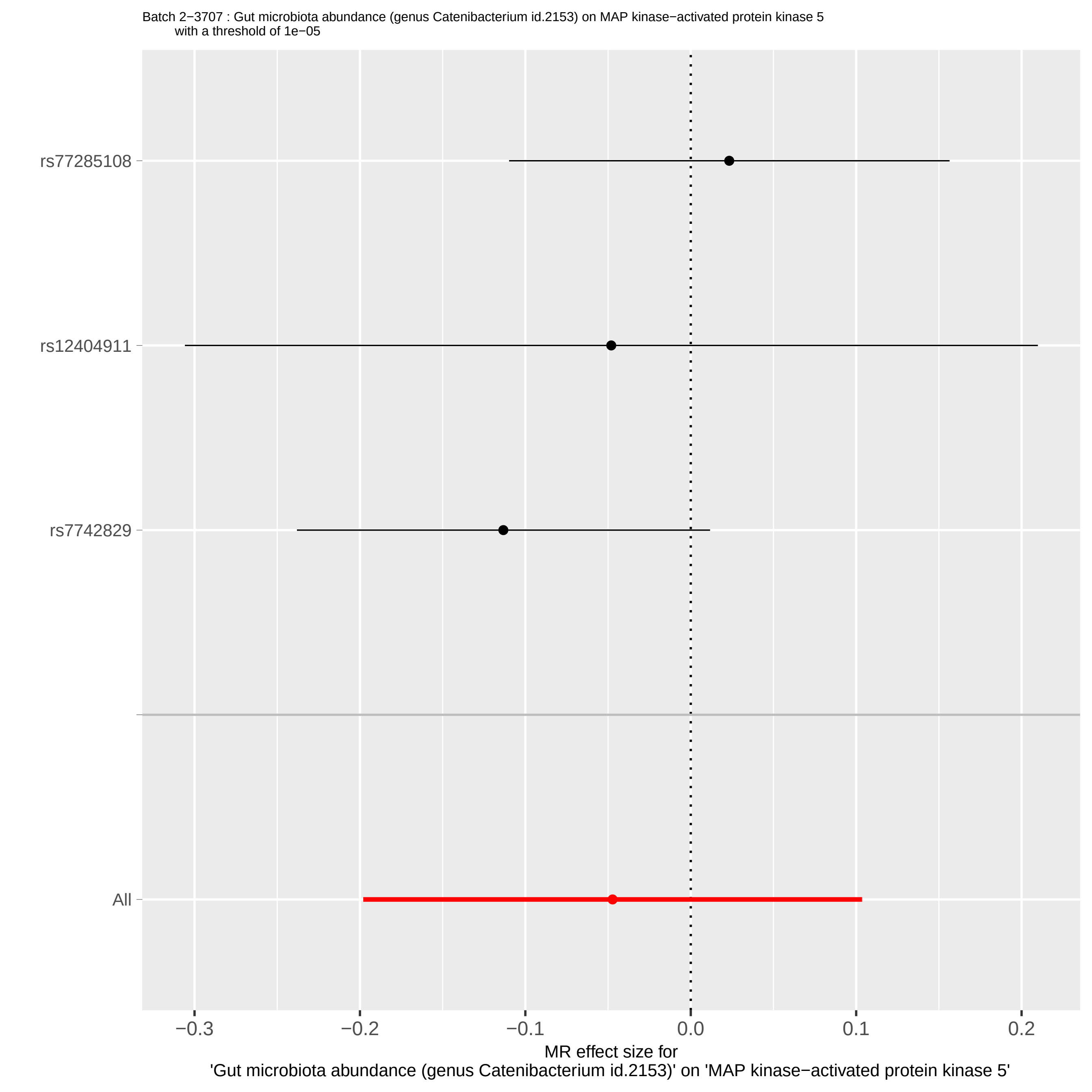
Batch 2−3707 : Gut microbiota abundance (genus Catenibacterium id.2153) on MAP kinase−activated protein kinase 5
with a threshold of 1e−05
rs77285108
rs12404911
rs7742829
All
−0.3
−0.2
−0.1
0.0
0.1
0.2
MR effect size for
'Gut microbiota abundance (genus Catenibacterium id.2153)' on 'MAP kinase−activated protein kinase 5'

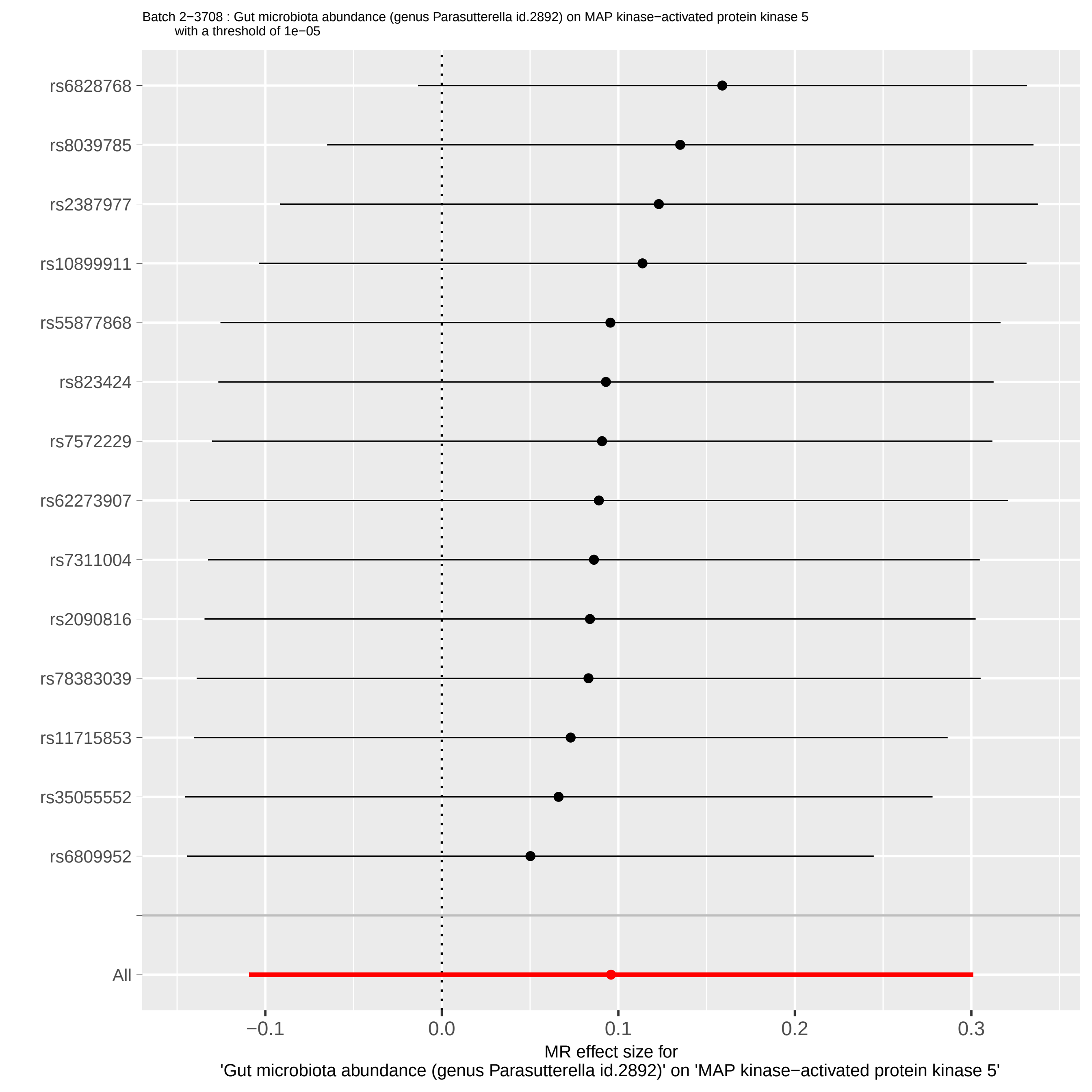
Batch 2−3708 : Gut microbiota abundance (genus Parasutterella id.2892) on MAP kinase−activated protein kinase 5
with a threshold of 1e−05
rs6828768
rs8039785
rs2387977
rs10899911
rs55877868
rs823424
rs7572229
rs62273907
rs7311004
rs2090816
rs78383039
rs11715853
rs35055552
rs6809952
All
−0.1
0.0
0.1
0.2
0.3
MR effect size for
'Gut microbiota abundance (genus Parasutterella id.2892)' on 'MAP kinase−activated protein kinase 5'

Batch 2−3709 : Gut microbiota abundance (genus Catenibacterium id.2153) on Mannan−binding lectin serine protease 1
with a threshold of 1e−05

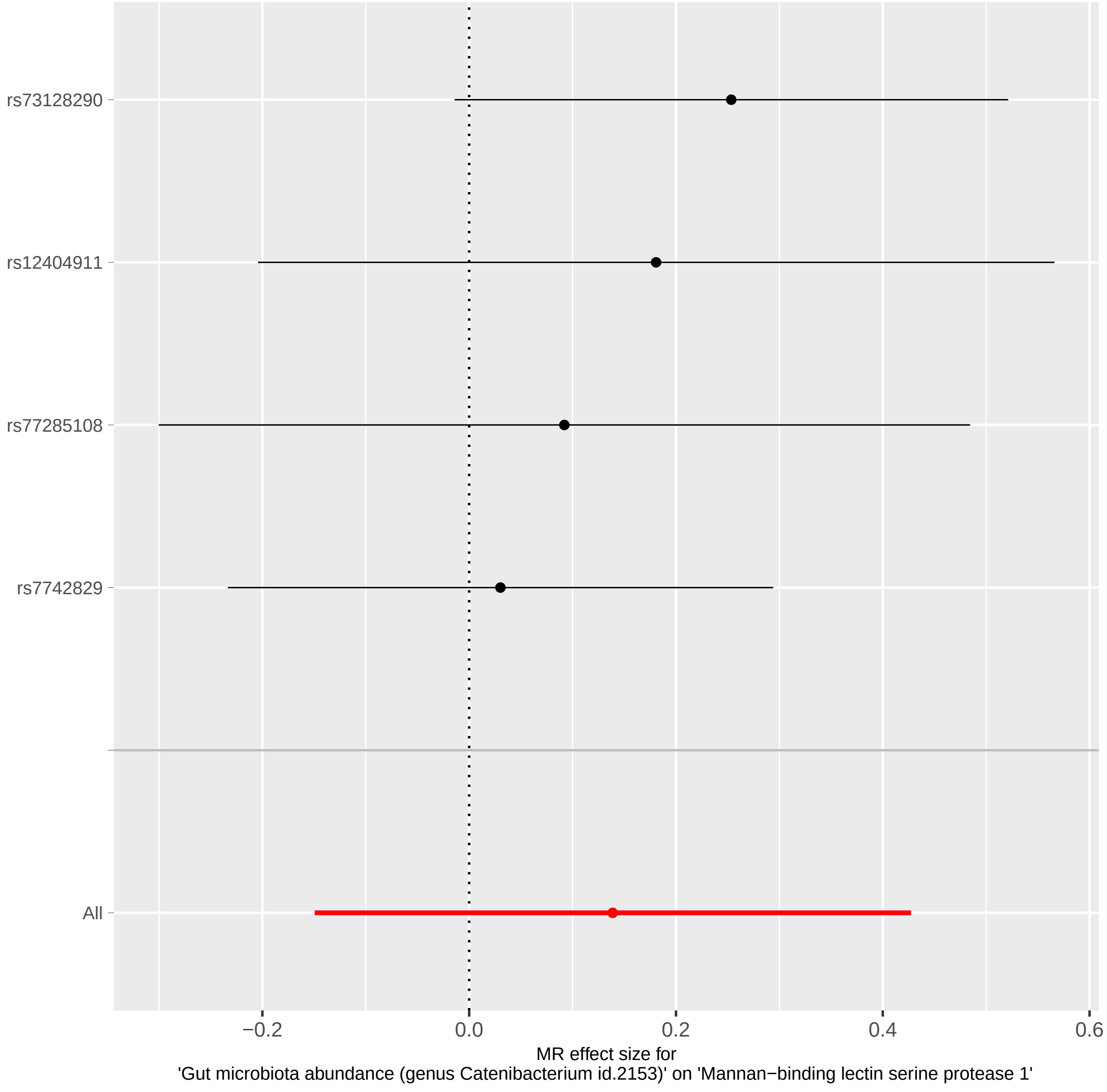

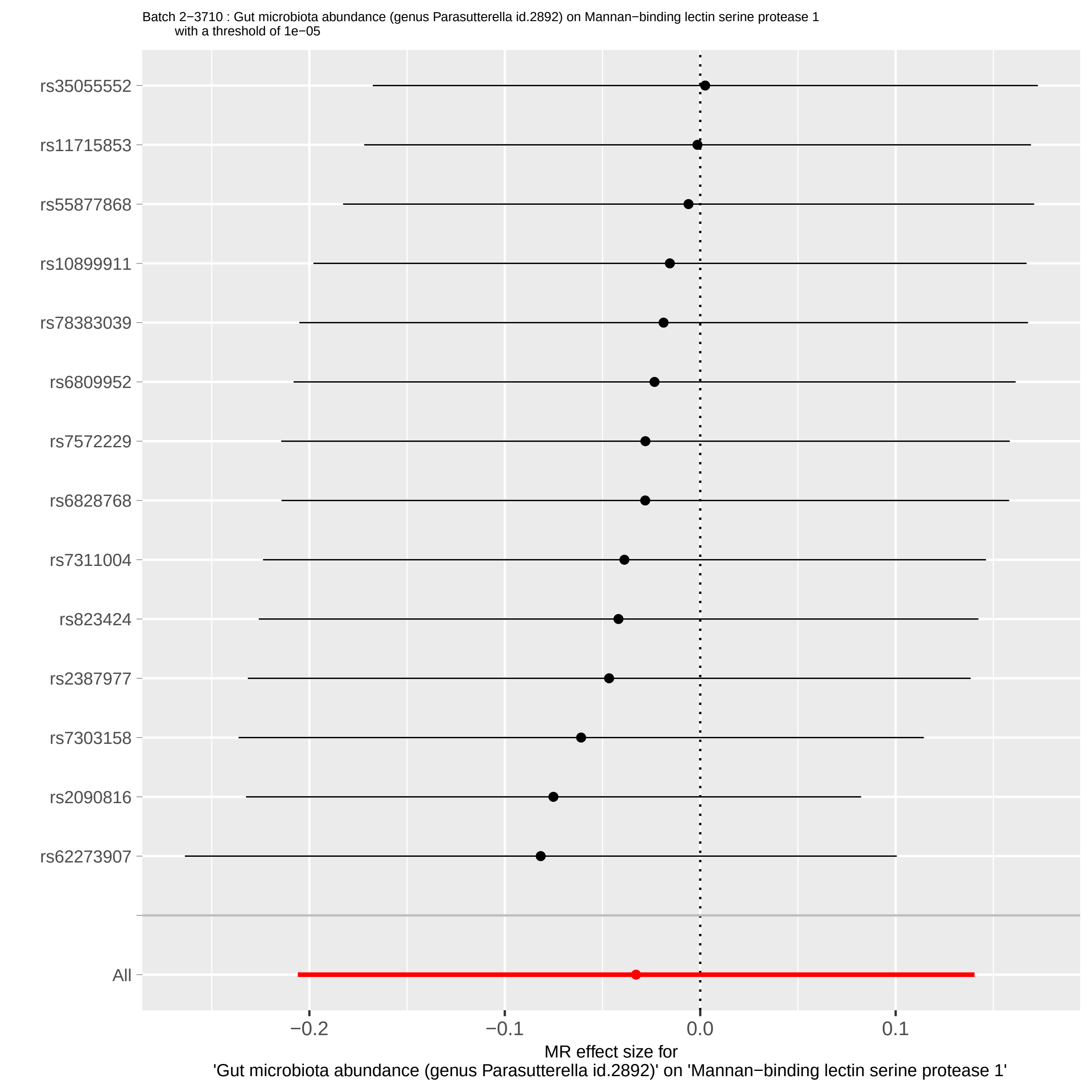

Batch 2−3710 : Gut microbiota abundance (genus Parasutterella id.2892) on Mannan−binding lectin serine protease 1
with a threshold of 1e−05
rs35055552
rs11715853
rs55877868
rs10899911
rs78383039
rs6809952
rs7572229
rs6828768
rs7311004
rs823424
rs2387977
rs7303158
rs2090816
rs62273907
All
−0.2
−0.1
0.0
0.1
MR effect size for
'Gut microbiota abundance (genus Parasutterella id.2892)' on 'Mannan−binding lectin serine protease 1'

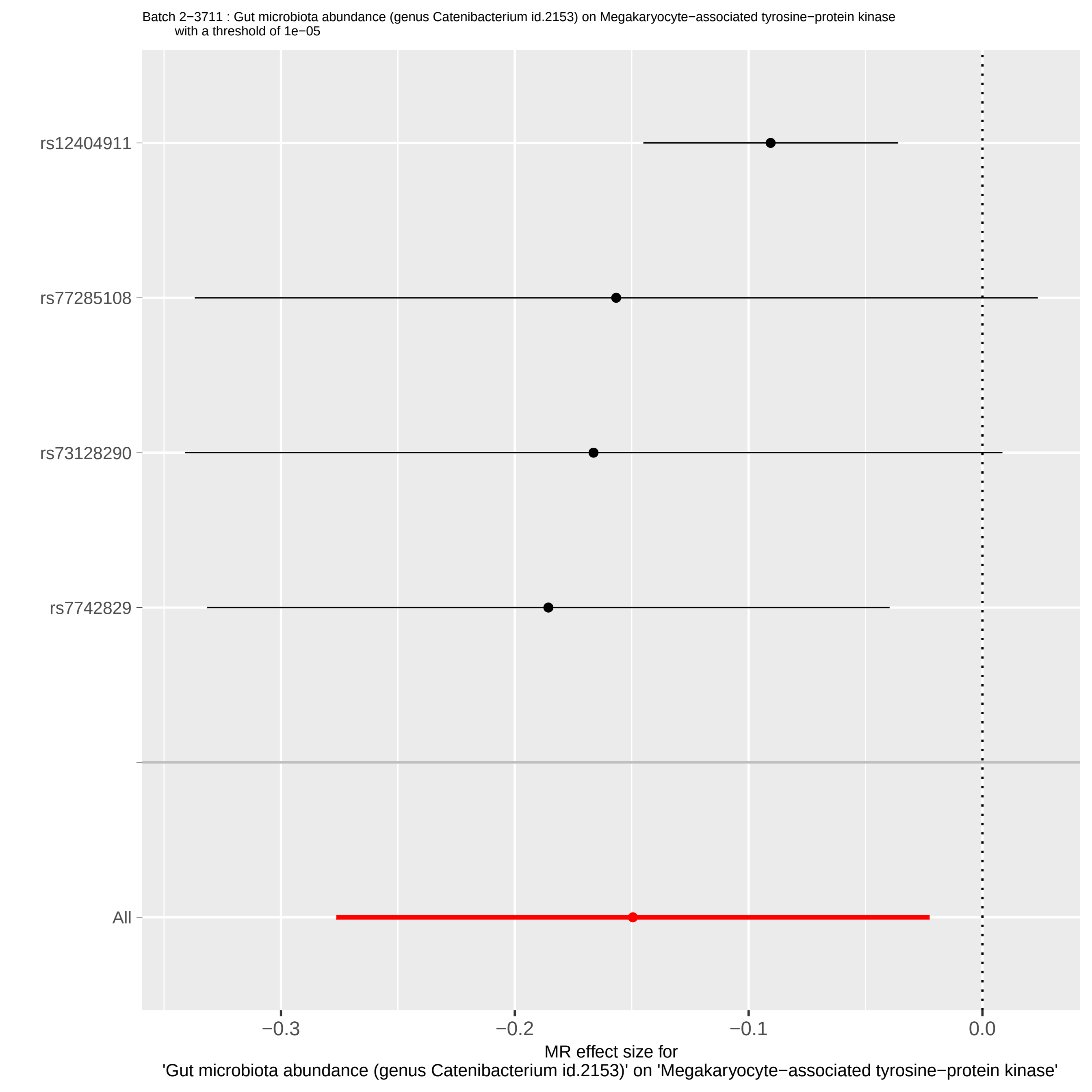
Batch 2−3711 : Gut microbiota abundance (genus Catenibacterium id.2153) on Megakaryocyte−associated tyrosine−protein kinase
with a threshold of 1e−05
rs12404911
rs77285108
rs73128290
rs7742829
All
−0.3
−0.2
−0.1
0.0
MR effect size for
'Gut microbiota abundance (genus Catenibacterium id.2153)' on 'Megakaryocyte−associated tyrosine−protein kinase'

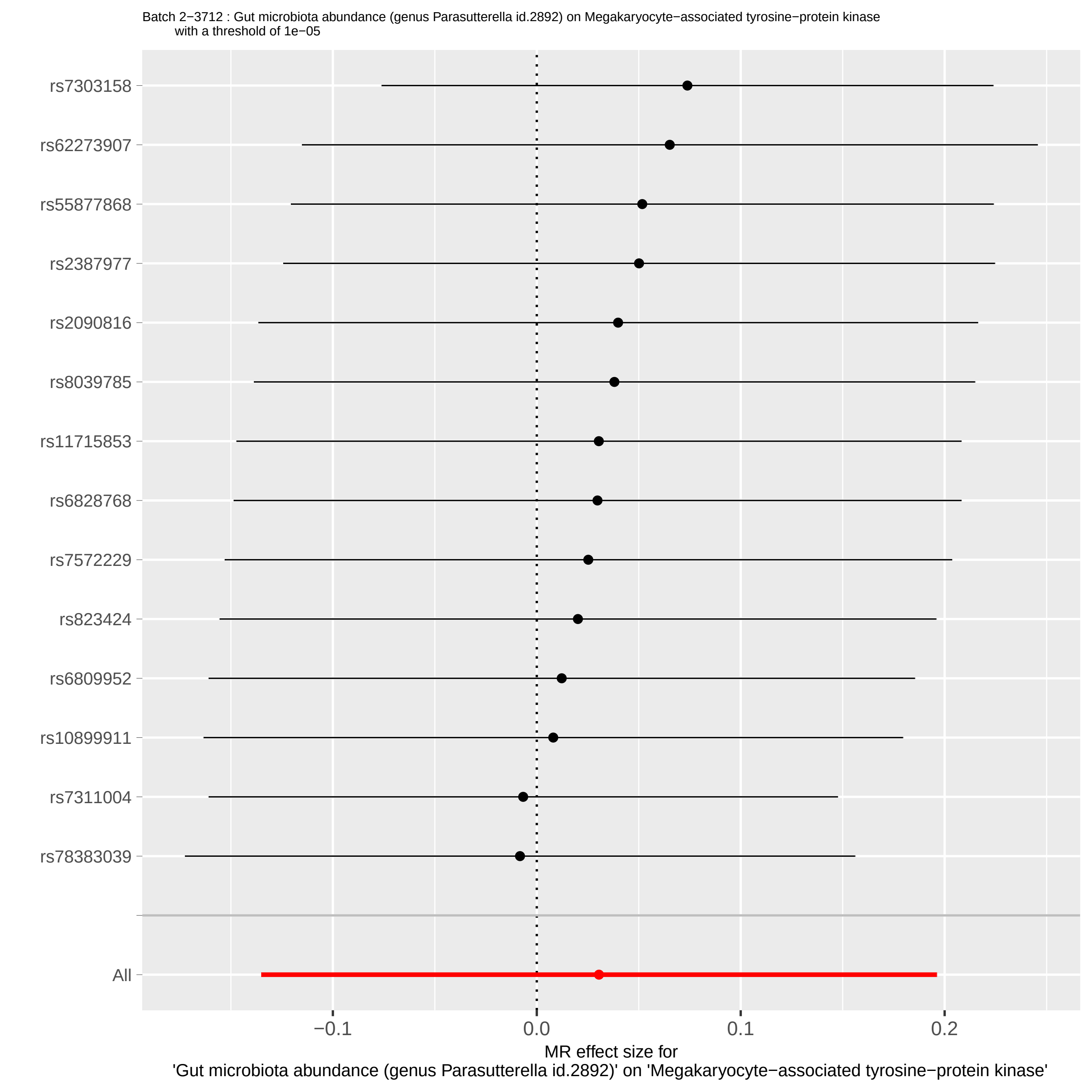
Batch 2−3712 : Gut microbiota abundance (genus Parasutterella id.2892) on Megakaryocyte−associated tyrosine−protein kinase
with a threshold of 1e−05
rs7303158
rs62273907
rs55877868
rs2387977
rs2090816
rs8039785
rs11715853
rs6828768
rs7572229
rs823424
rs6809952
rs10899911
rs7311004
rs78383039
All
−0.1
0.0
0.1
0.2
MR effect size for
'Gut microbiota abundance (genus Parasutterella id.2892)' on 'Megakaryocyte−associated tyrosine−protein kinase'

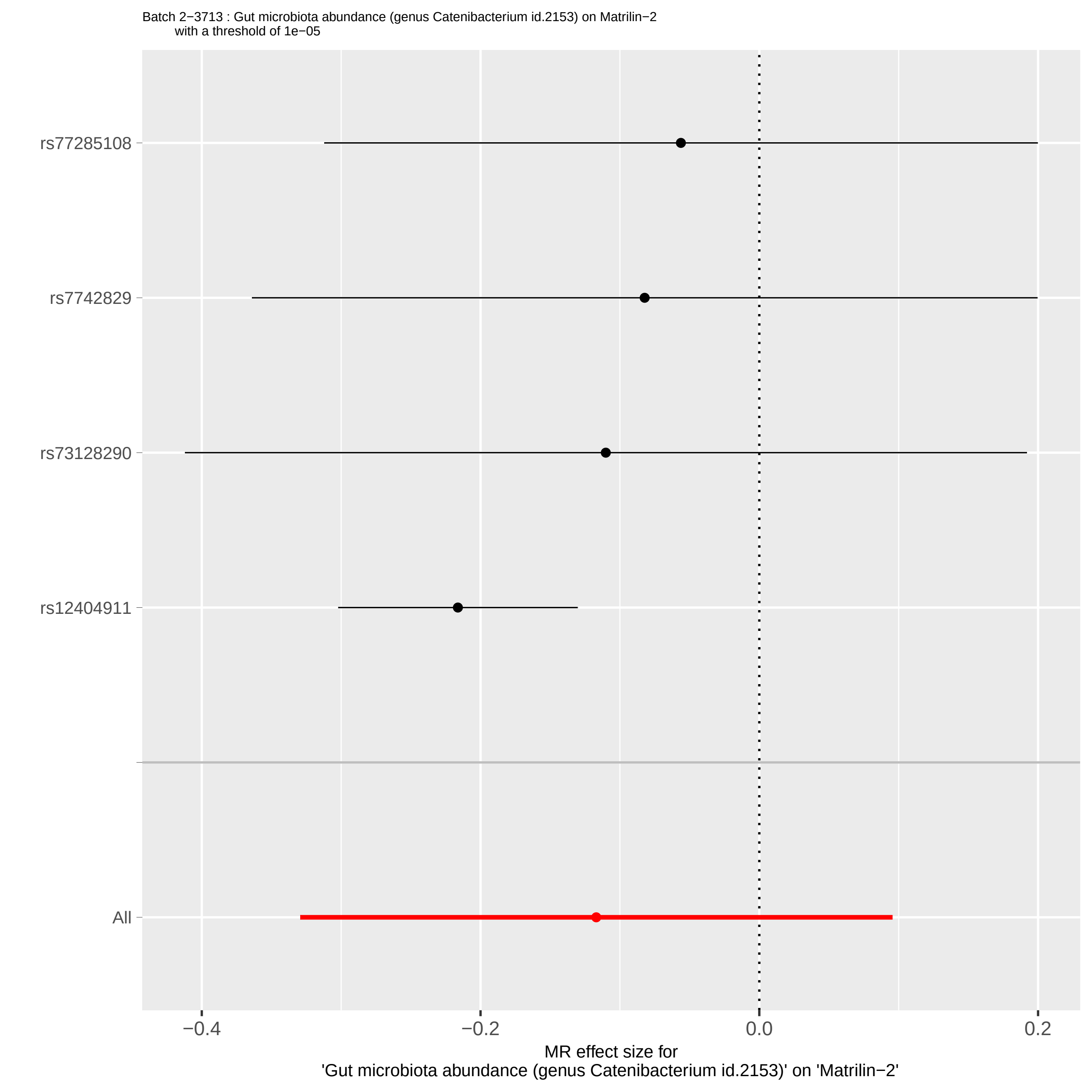

Batch 2−3713 : Gut microbiota abundance (genus Catenibacterium id.2153) on Matrilin−2
with a threshold of 1e−05
rs77285108
rs7742829
rs73128290
rs12404911
All
−0.4
−0.2
0.0
0.2
MR effect size for
'Gut microbiota abundance (genus Catenibacterium id.2153)' on 'Matrilin−2'

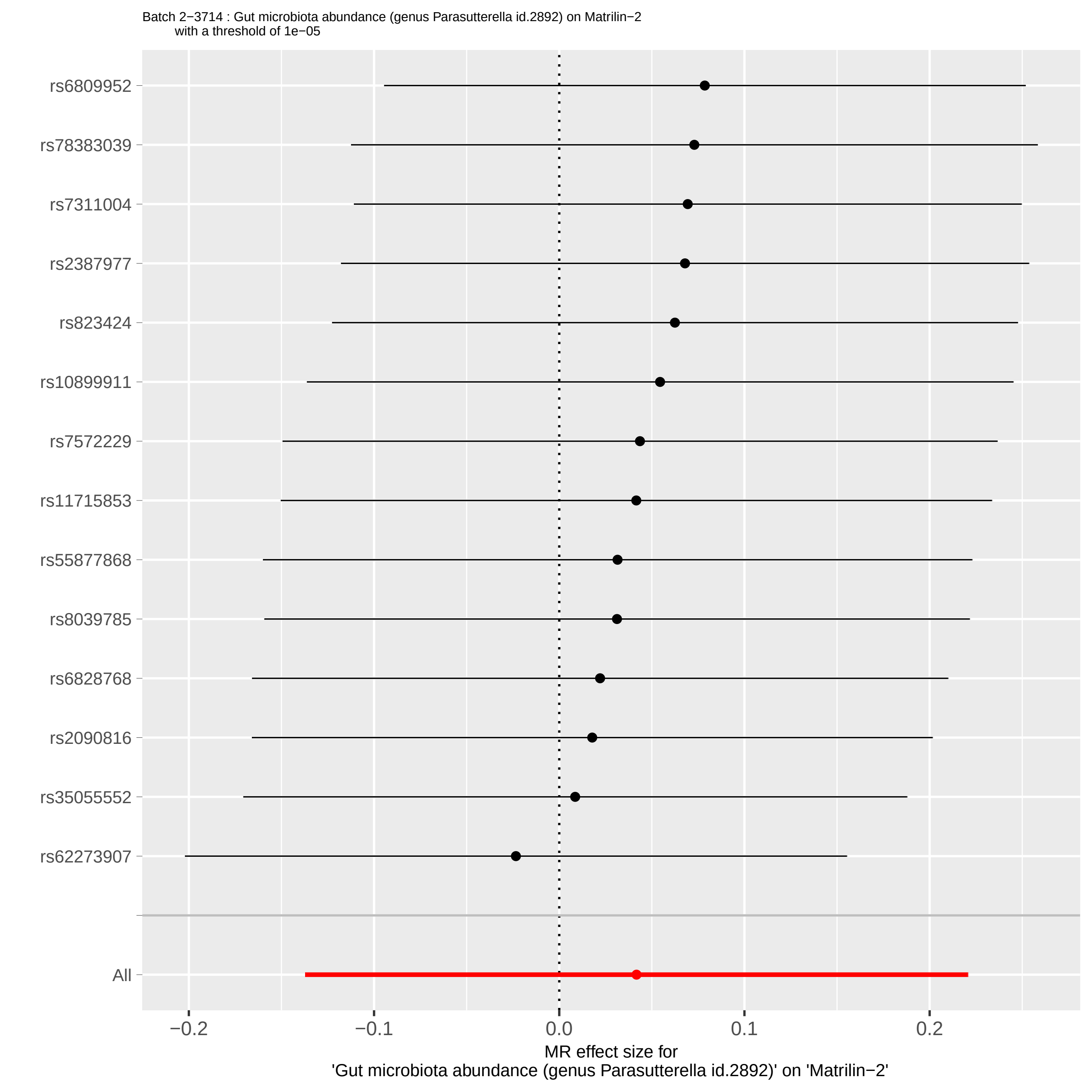
Batch 2−3714 : Gut microbiota abundance (genus Parasutterella id.2892) on Matrilin−2
with a threshold of 1e−05
rs6809952
rs78383039
rs7311004
rs2387977
rs823424
rs10899911
rs7572229
rs11715853
rs55877868
rs8039785
rs6828768
rs2090816
rs35055552
rs62273907
All
−0.2
−0.1
0.0
0.1
0.2
MR effect size for
'Gut microbiota abundance (genus Parasutterella id.2892)' on 'Matrilin−2'

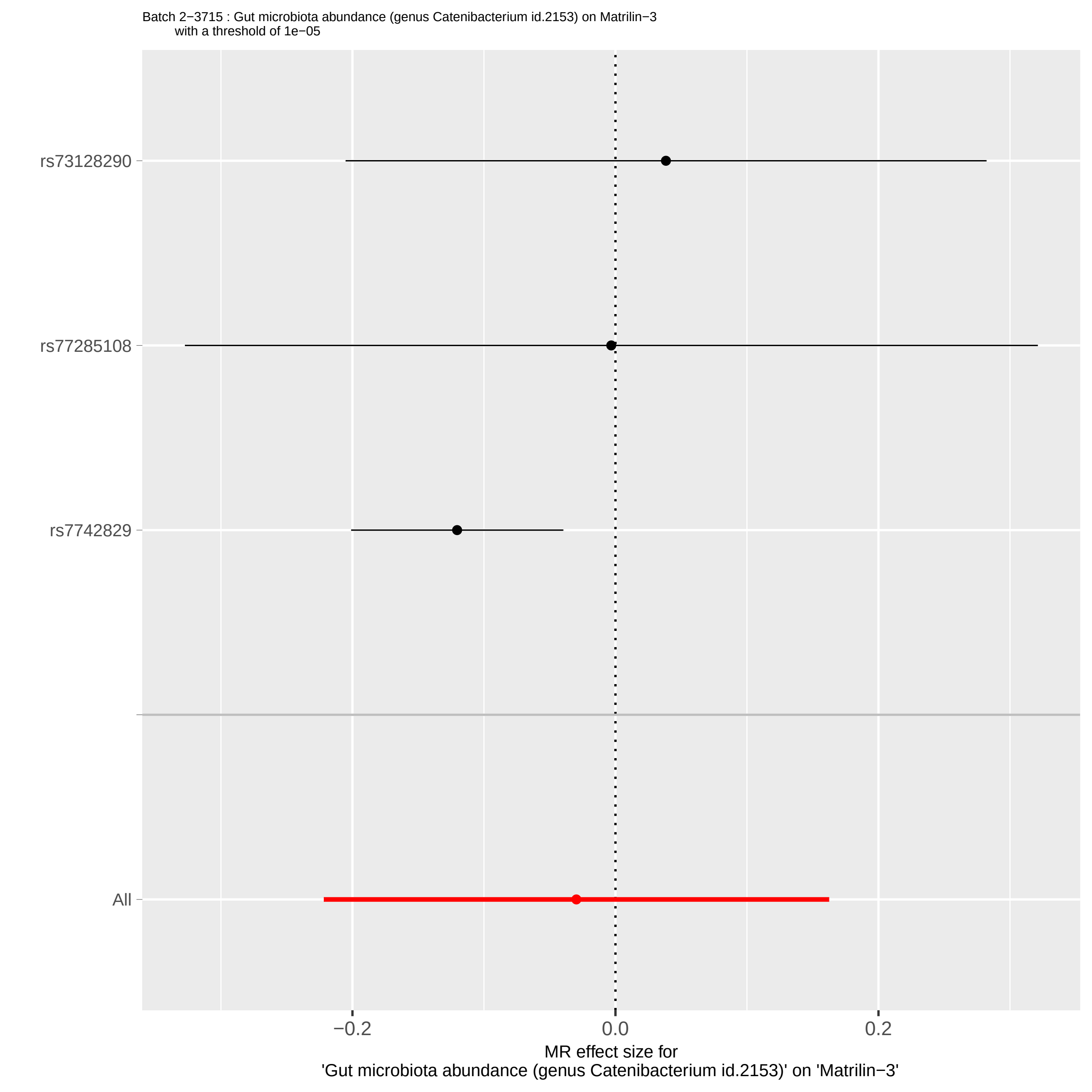

Batch 2−3715 : Gut microbiota abundance (genus Catenibacterium id.2153) on Matrilin−3
with a threshold of 1e−05
rs73128290
rs77285108
rs7742829
All
−0.2
0.0
0.2
MR effect size for
'Gut microbiota abundance (genus Catenibacterium id.2153)' on 'Matrilin−3'

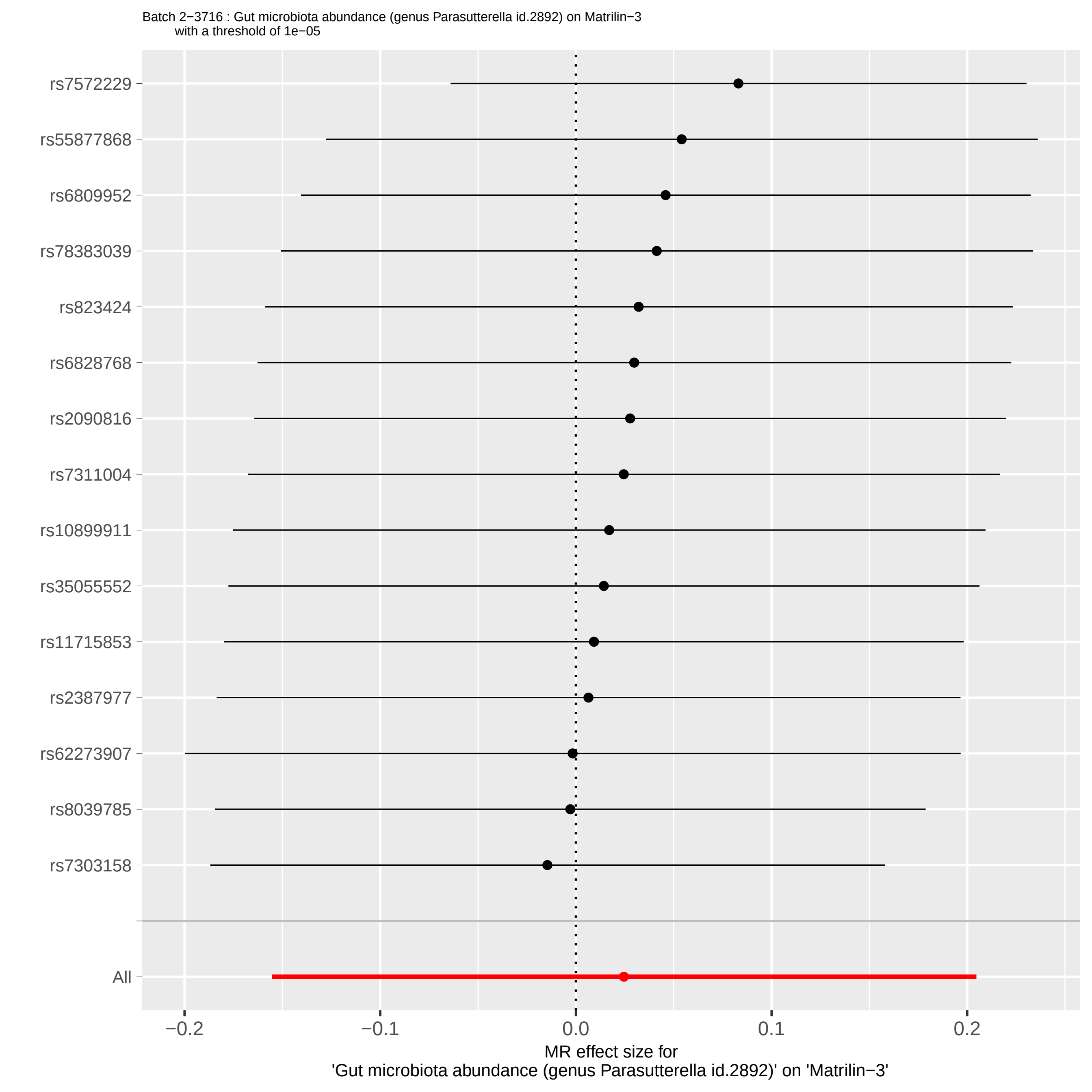
Batch 2−3716 : Gut microbiota abundance (genus Parasutterella id.2892) on Matrilin−3
with a threshold of 1e−05
rs7572229
rs55877868
rs6809952
rs78383039
rs823424
rs6828768
rs2090816
rs7311004
rs10899911
rs35055552
rs11715853
rs2387977
rs62273907
rs8039785
rs7303158
All
−0.2
−0.1
0.0
0.1
0.2
MR effect size for
'Gut microbiota abundance (genus Parasutterella id.2892)' on 'Matrilin−3'

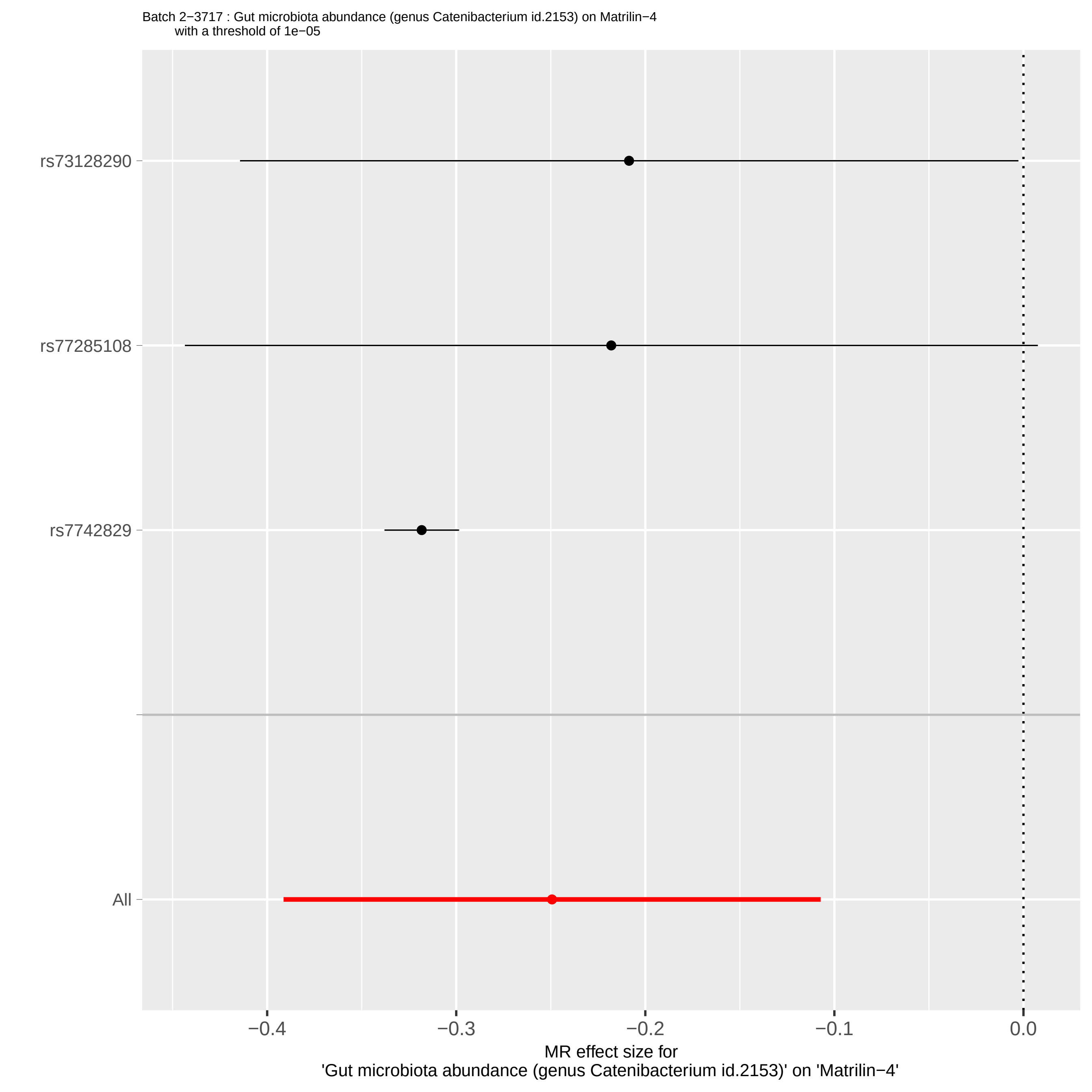
Batch 2−3717 : Gut microbiota abundance (genus Catenibacterium id.2153) on Matrilin−4
with a threshold of 1e−05
rs73128290
rs77285108
rs7742829
All
−0.4
−0.3
−0.2
−0.1
0.0
MR effect size for
'Gut microbiota abundance (genus Catenibacterium id.2153)' on 'Matrilin−4'

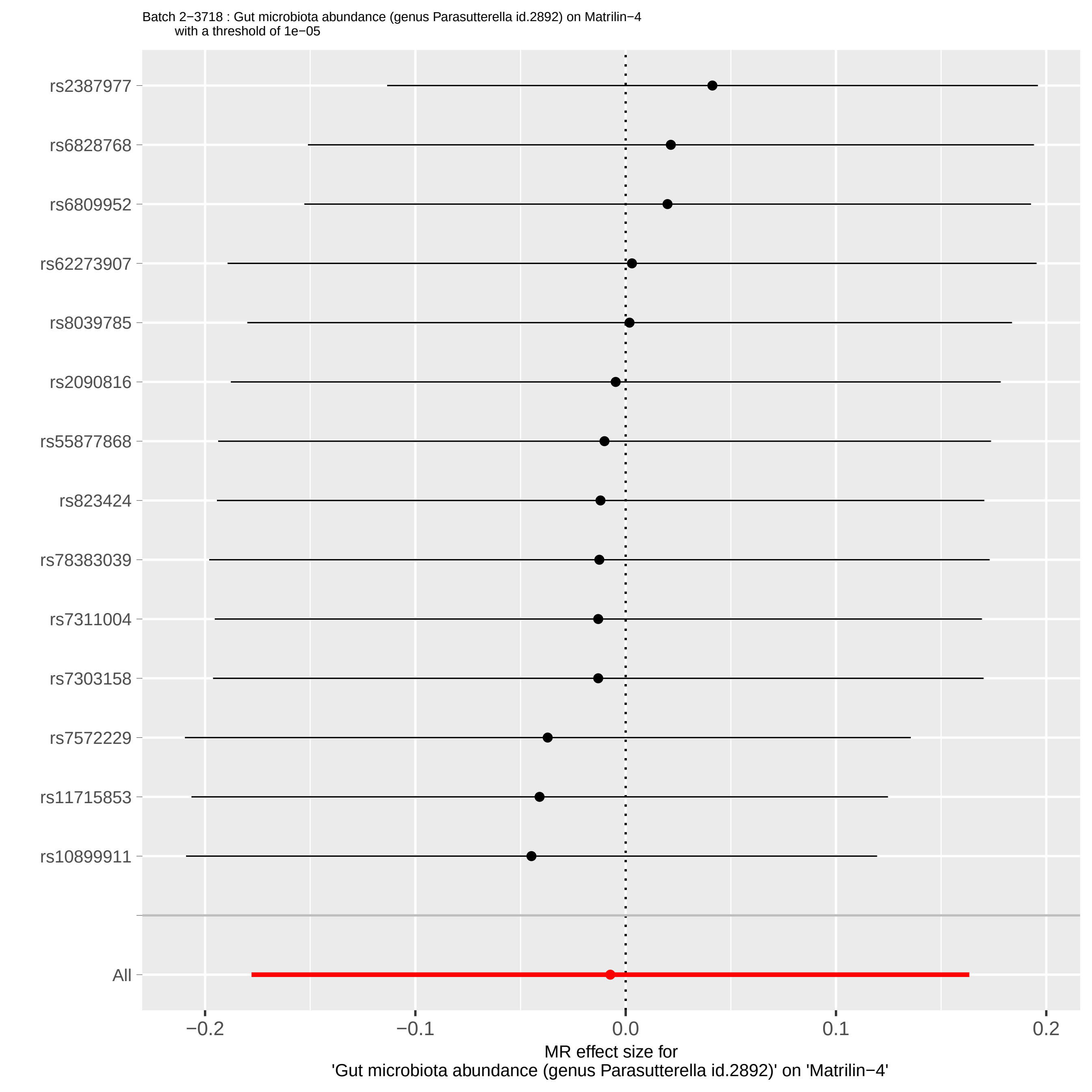

Batch 2−3718 : Gut microbiota abundance (genus Parasutterella id.2892) on Matrilin−4
with a threshold of 1e−05
rs2387977
rs6828768
rs6809952
rs62273907
rs8039785
rs2090816
rs55877868
rs823424
rs78383039
rs7311004
rs7303158
rs7572229
rs11715853
rs10899911
All
−0.2
−0.1
0.0
0.1
0.2
MR effect size for
'Gut microbiota abundance (genus Parasutterella id.2892)' on 'Matrilin−4'

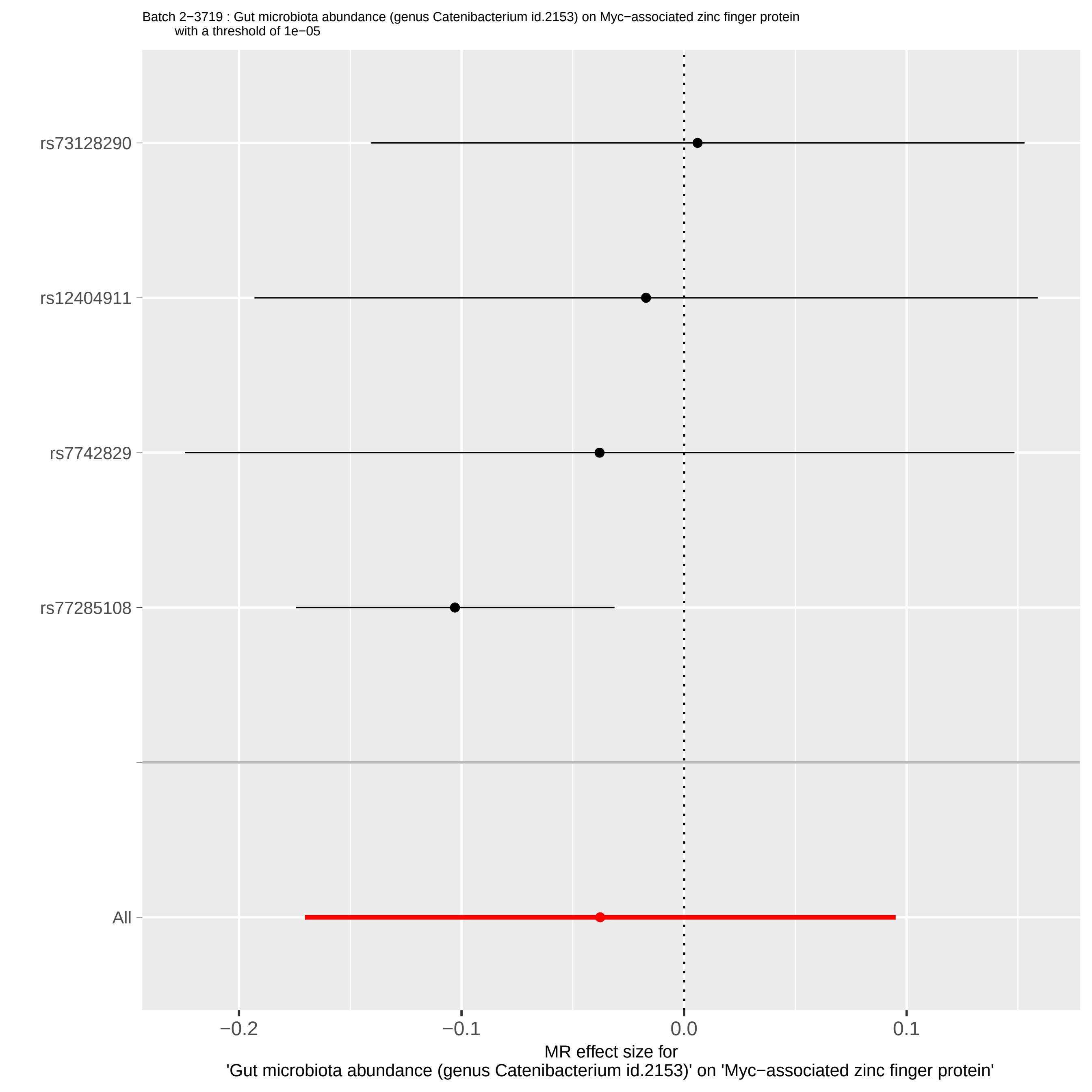

Batch 2−3719 : Gut microbiota abundance (genus Catenibacterium id.2153) on Myc−associated zinc finger protein
with a threshold of 1e−05
rs73128290
rs12404911
rs7742829
rs77285108
All
−0.2
−0.1
0.0
0.1
MR effect size for
'Gut microbiota abundance (genus Catenibacterium id.2153)' on 'Myc−associated zinc finger protein'

Batch 2−3720 : Gut microbiota abundance (genus Parasutterella id.2892) on Myc−associated zinc finger protein
with a threshold of 1e−05

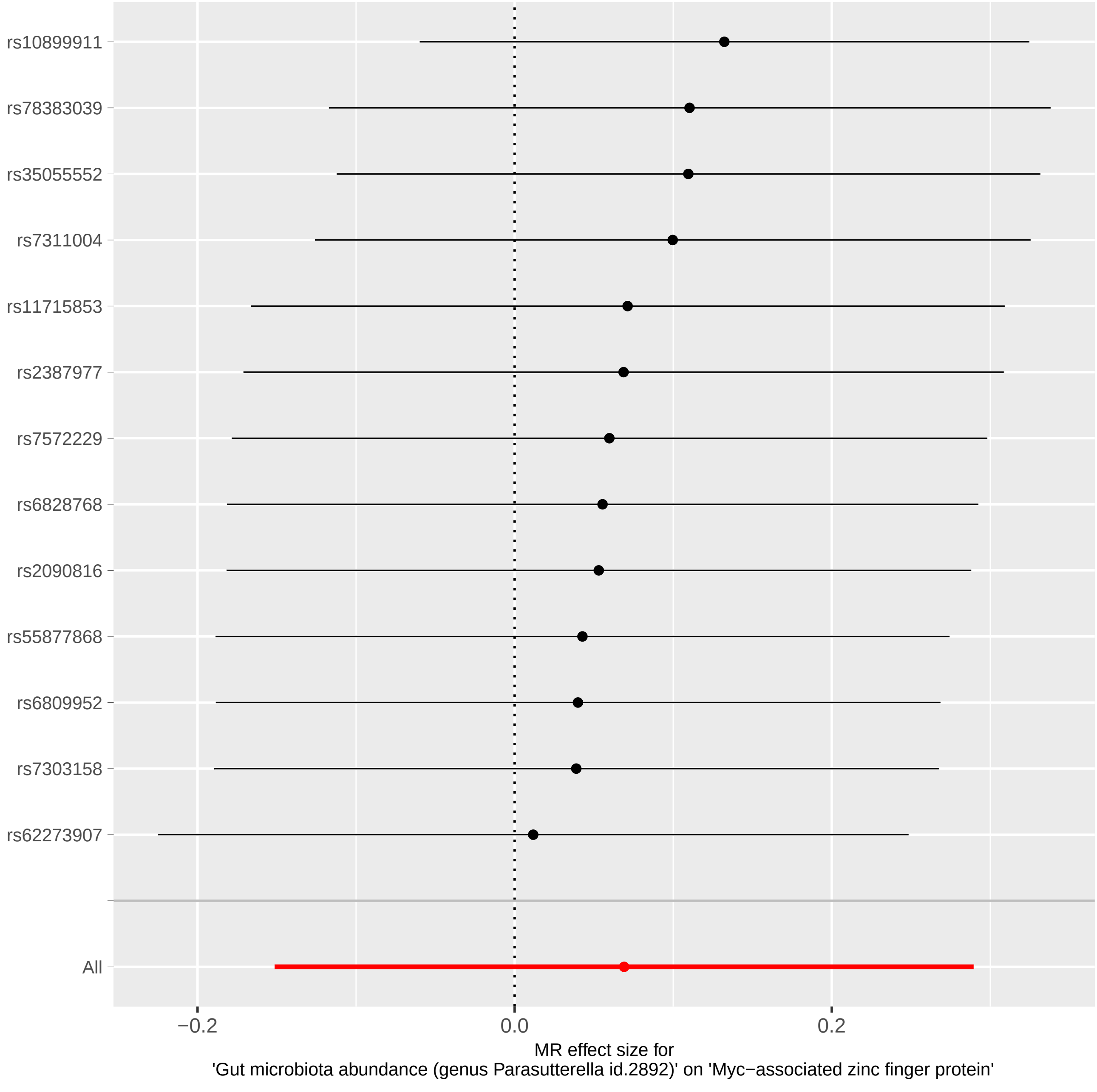

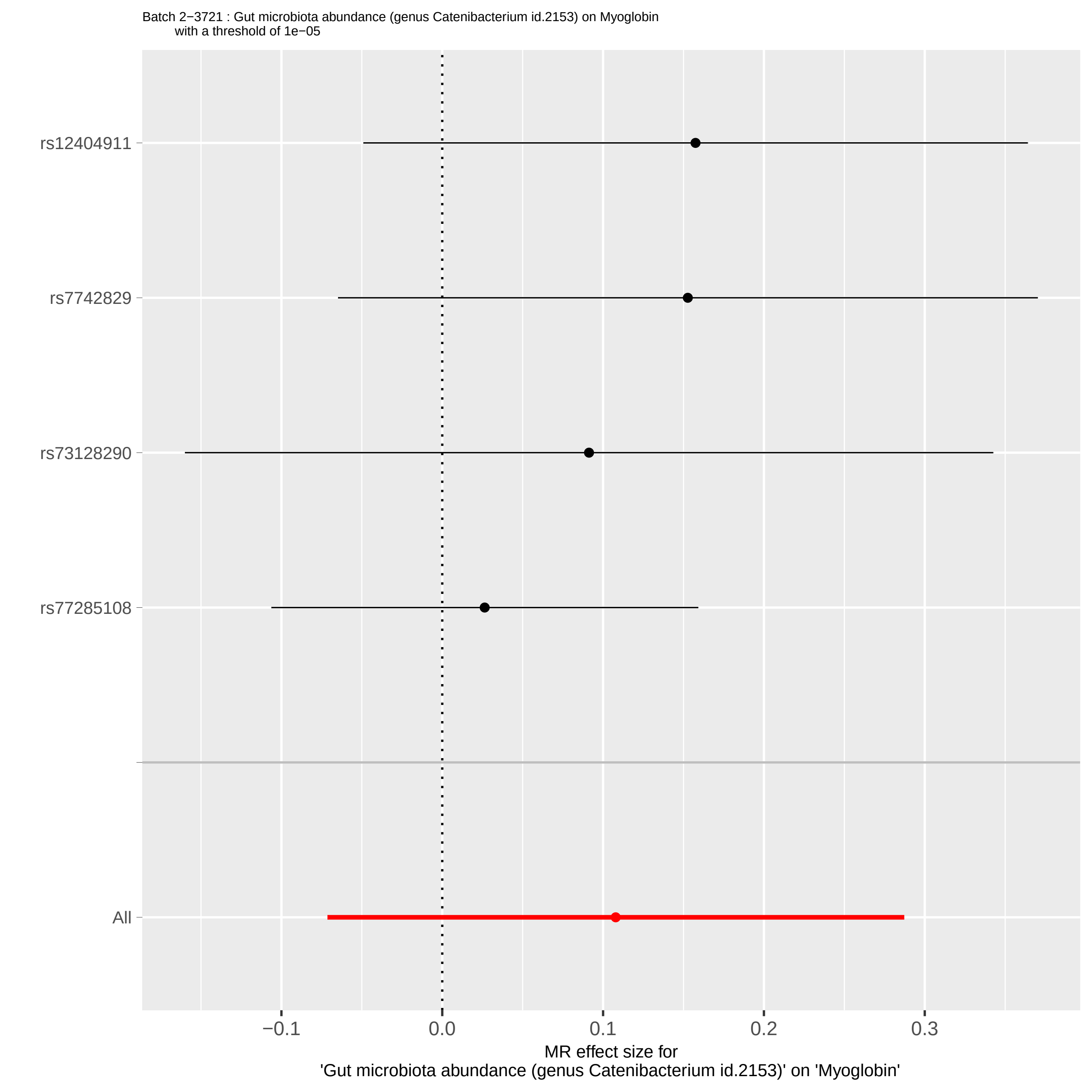
Batch 2−3721 : Gut microbiota abundance (genus Catenibacterium id.2153) on Myoglobin
with a threshold of 1e−05
rs12404911
rs7742829
rs73128290
rs77285108
All
−0.1
0.0
0.1
0.2
0.3
MR effect size for
'Gut microbiota abundance (genus Catenibacterium id.2153)' on 'Myoglobin'

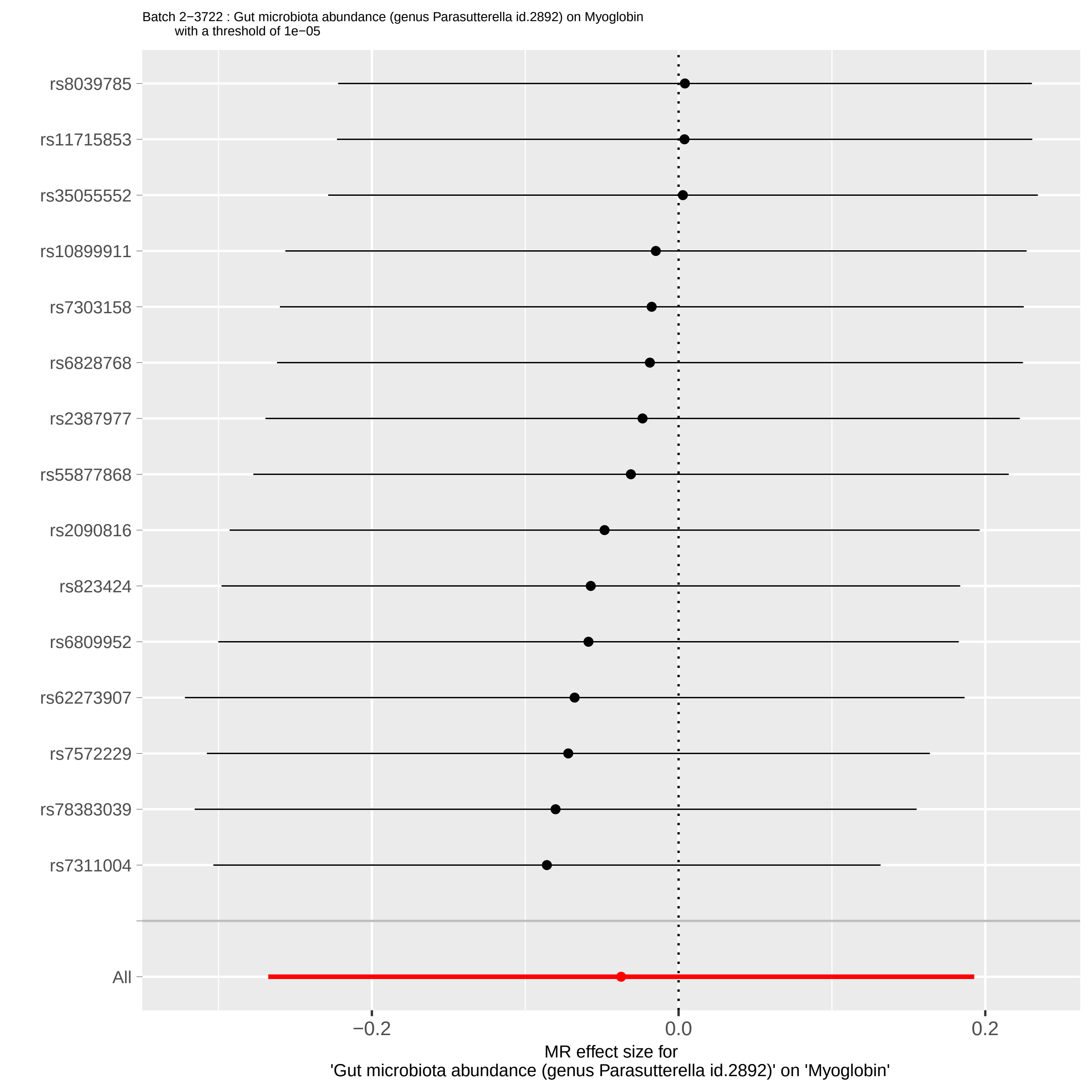
Batch 2−3722 : Gut microbiota abundance (genus Parasutterella id.2892) on Myoglobin
with a threshold of 1e−05
rs8039785
rs11715853
rs35055552
rs10899911
rs7303158
rs6828768
rs2387977
rs55877868
rs2090816
rs823424
rs6809952
rs62273907
rs7572229
rs78383039
rs7311004
All
−0.2
0.0
0.2
MR effect size for
'Gut microbiota abundance (genus Parasutterella id.2892)' on 'Myoglobin'

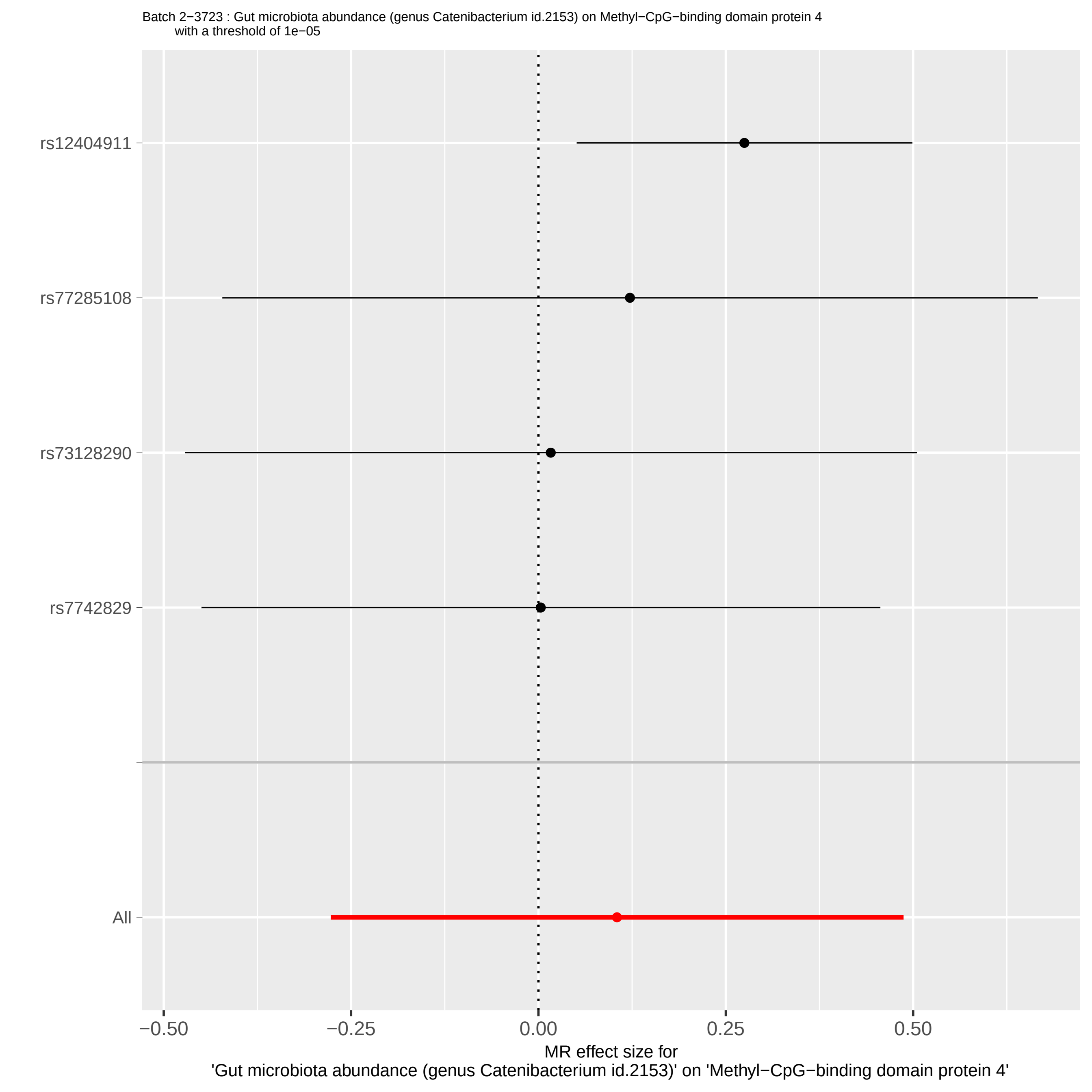

Batch 2−3723 : Gut microbiota abundance (genus Catenibacterium id.2153) on Methyl−CpG−binding domain protein 4
with a threshold of 1e−05
rs12404911
rs77285108
rs73128290
rs7742829
All
−0.50
−0.25
0.00
0.25
0.50
MR effect size for
'Gut microbiota abundance (genus Catenibacterium id.2153)' on 'Methyl−CpG−binding domain protein 4'

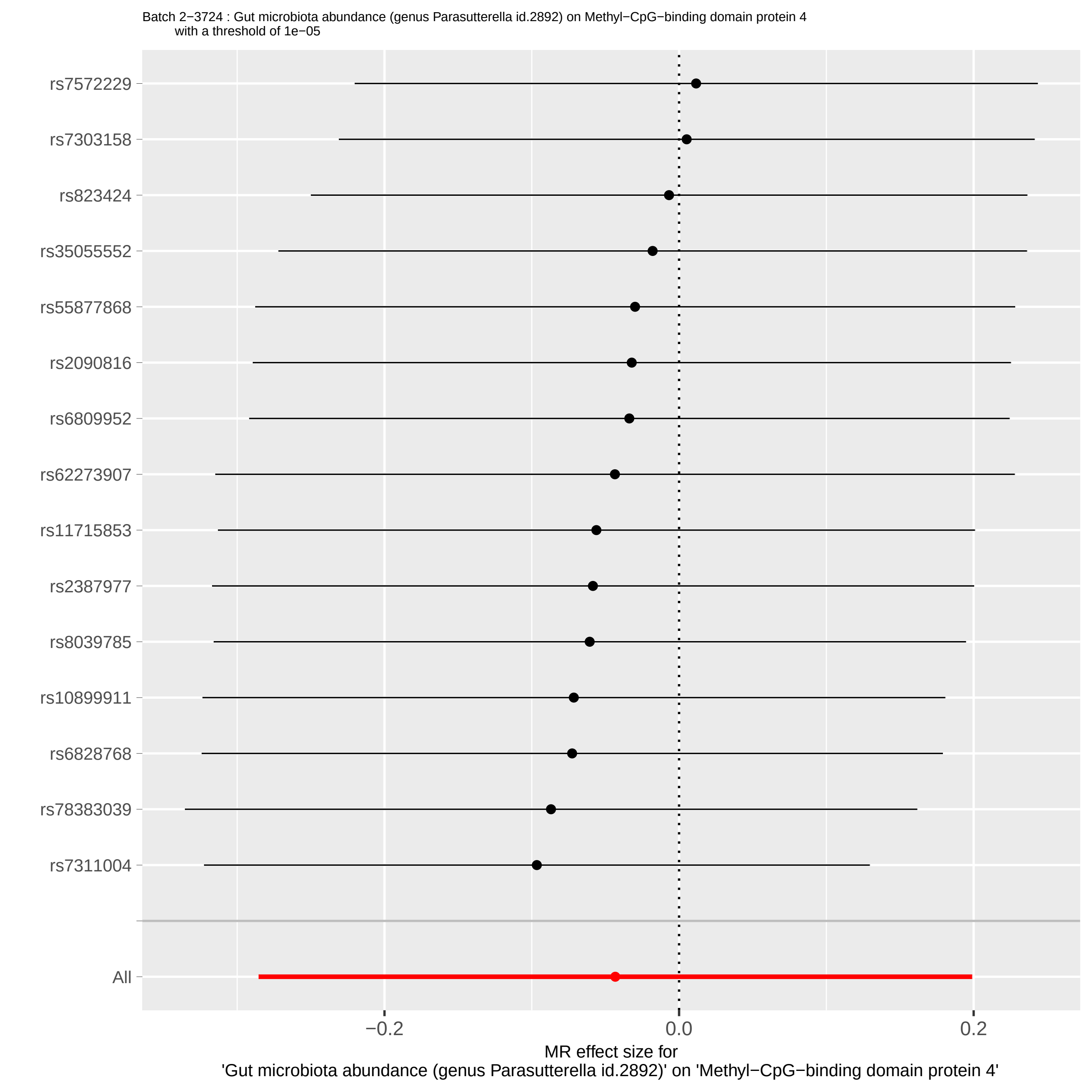

Batch 2−3724 : Gut microbiota abundance (genus Parasutterella id.2892) on Methyl−CpG−binding domain protein 4
with a threshold of 1e−05
rs7572229
rs7303158
rs823424
rs35055552
rs55877868
rs2090816
rs6809952
rs62273907
rs11715853
rs2387977
rs8039785
rs10899911
rs6828768
rs78383039
rs7311004
All
−0.2
0.0
0.2
MR effect size for
'Gut microbiota abundance (genus Parasutterella id.2892)' on 'Methyl−CpG−binding domain protein 4'

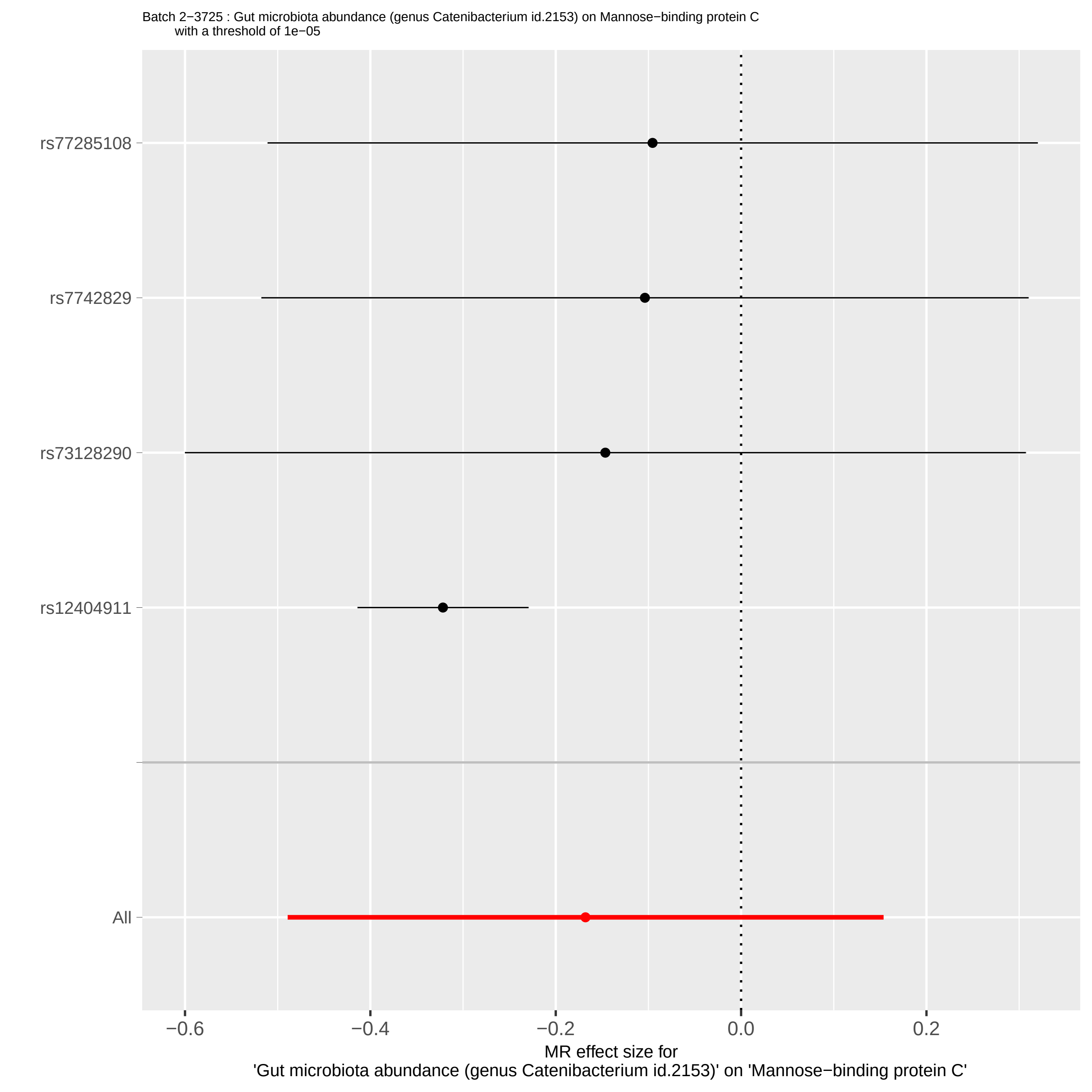

Batch 2−3725 : Gut microbiota abundance (genus Catenibacterium id.2153) on Mannose−binding protein C
with a threshold of 1e−05
rs77285108
rs7742829
rs73128290
rs12404911
All
−0.6
−0.4
−0.2
0.0
0.2
MR effect size for
'Gut microbiota abundance (genus Catenibacterium id.2153)' on 'Mannose−binding protein C'

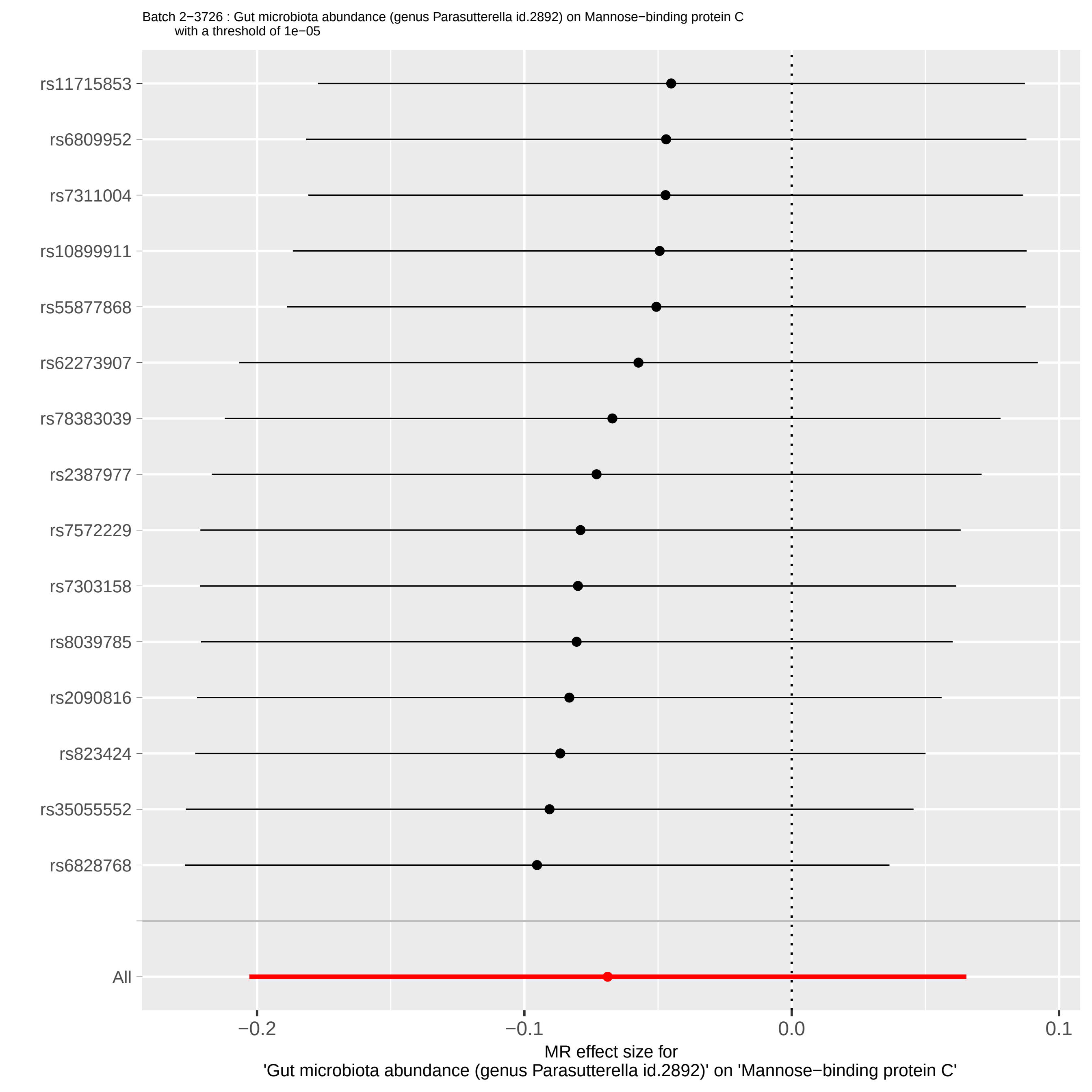

Batch 2−3726 : Gut microbiota abundance (genus Parasutterella id.2892) on Mannose−binding protein C
with a threshold of 1e−05
rs11715853
rs6809952
rs7311004
rs10899911
rs55877868
rs62273907
rs78383039
rs2387977
rs7572229
rs7303158
rs8039785
rs2090816
rs823424
rs35055552
rs6828768
All
−0.2
−0.1
0.0
0.1
MR effect size for
'Gut microbiota abundance (genus Parasutterella id.2892)' on 'Mannose−binding protein C'

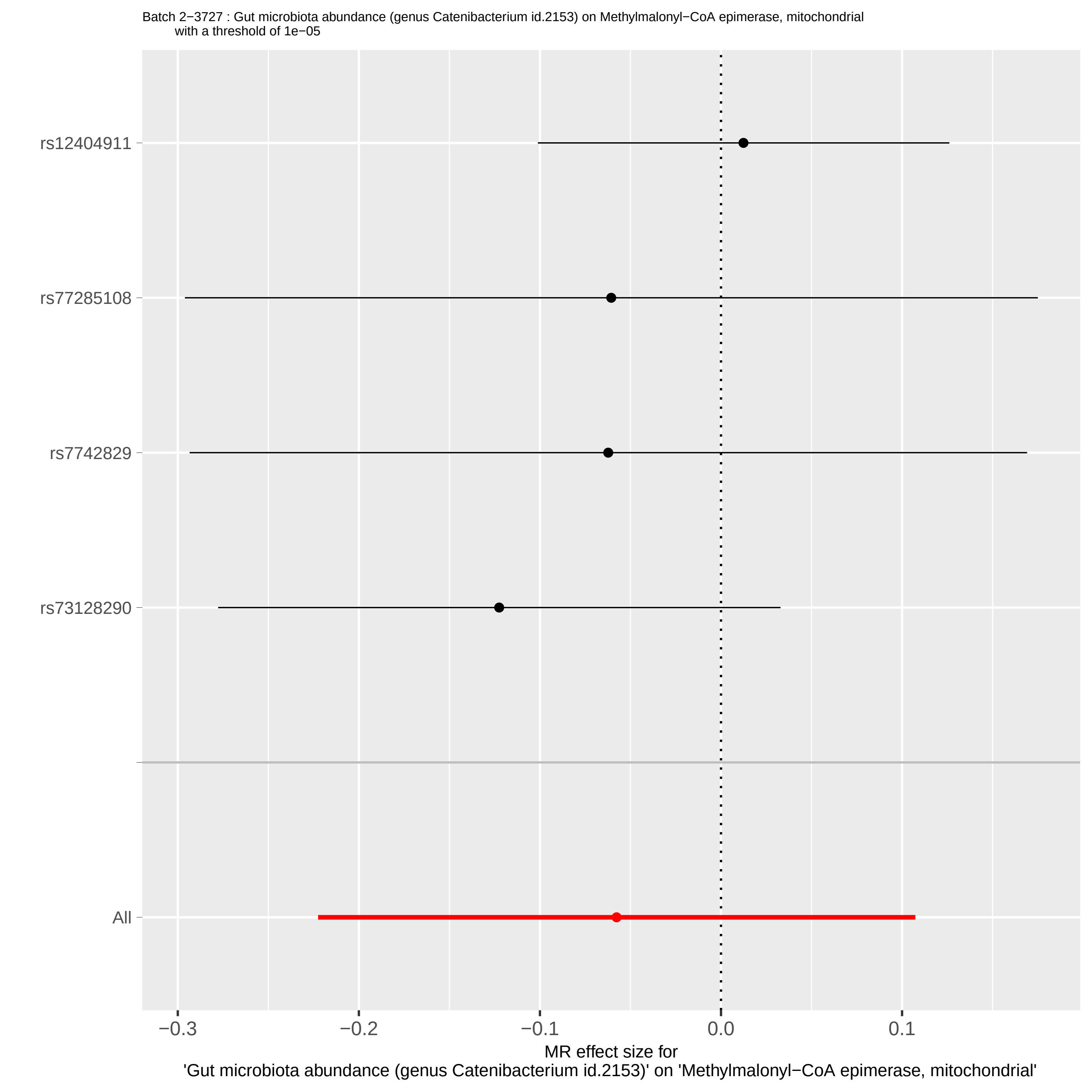

Batch 2−3727 : Gut microbiota abundance (genus Catenibacterium id.2153) on Methylmalonyl−CoA epimerase, mitochondrial
with a threshold of 1e−05
rs12404911
rs77285108
rs7742829
rs73128290
All
−0.3
−0.2
−0.1
0.0
0.1
MR effect size for
'Gut microbiota abundance (genus Catenibacterium id.2153)' on 'Methylmalonyl−CoA epimerase, mitochondrial'

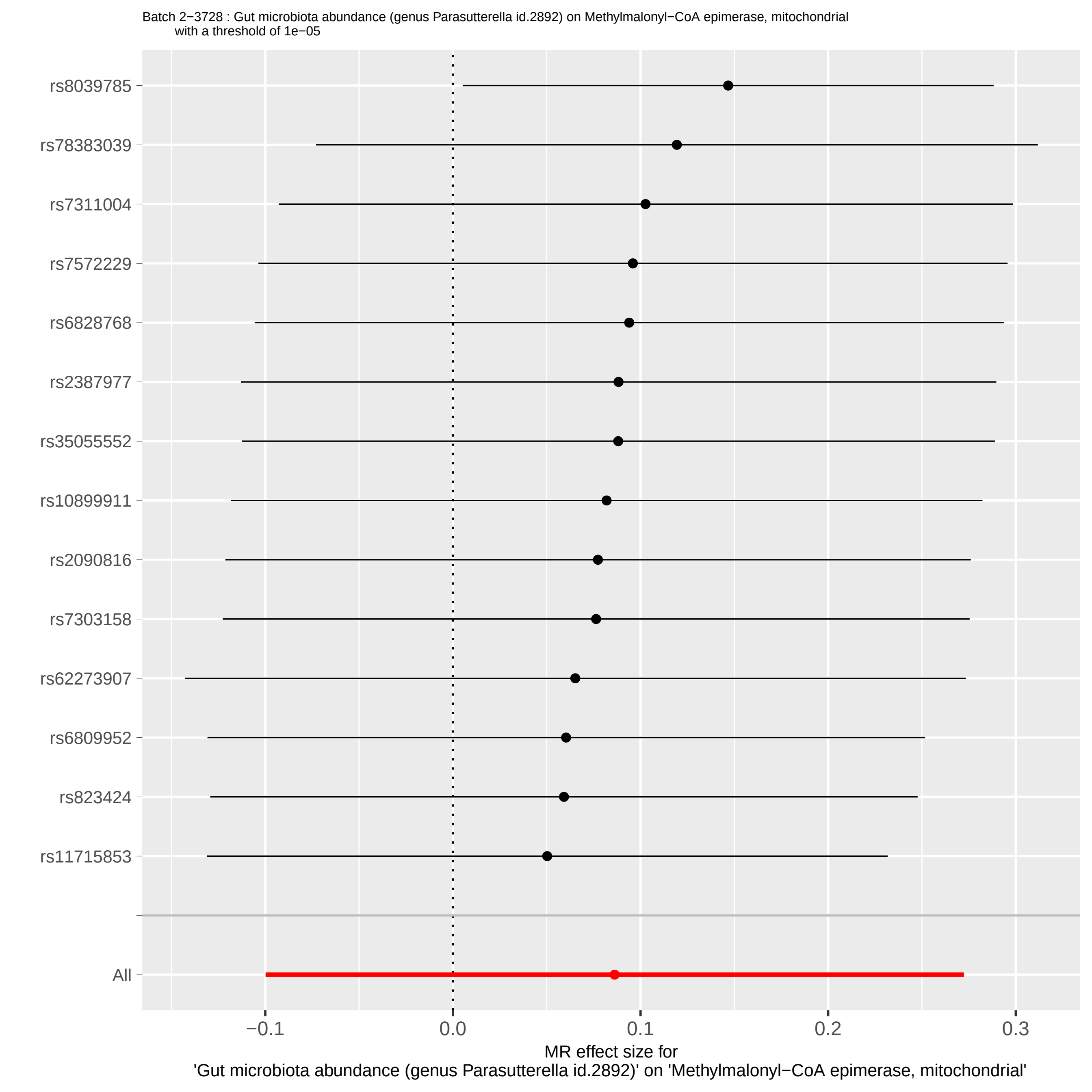

Batch 2−3728 : Gut microbiota abundance (genus Parasutterella id.2892) on Methylmalonyl−CoA epimerase, mitochondrial
with a threshold of 1e−05
rs8039785
rs78383039
rs7311004
rs7572229
rs6828768
rs2387977
rs35055552
rs10899911
rs2090816
rs7303158
rs62273907
rs6809952
rs823424
rs11715853
All
−0.1
0.0
0.1
0.2
0.3
MR effect size for
'Gut microbiota abundance (genus Parasutterella id.2892)' on 'Methylmalonyl−CoA epimerase, mitochondrial'

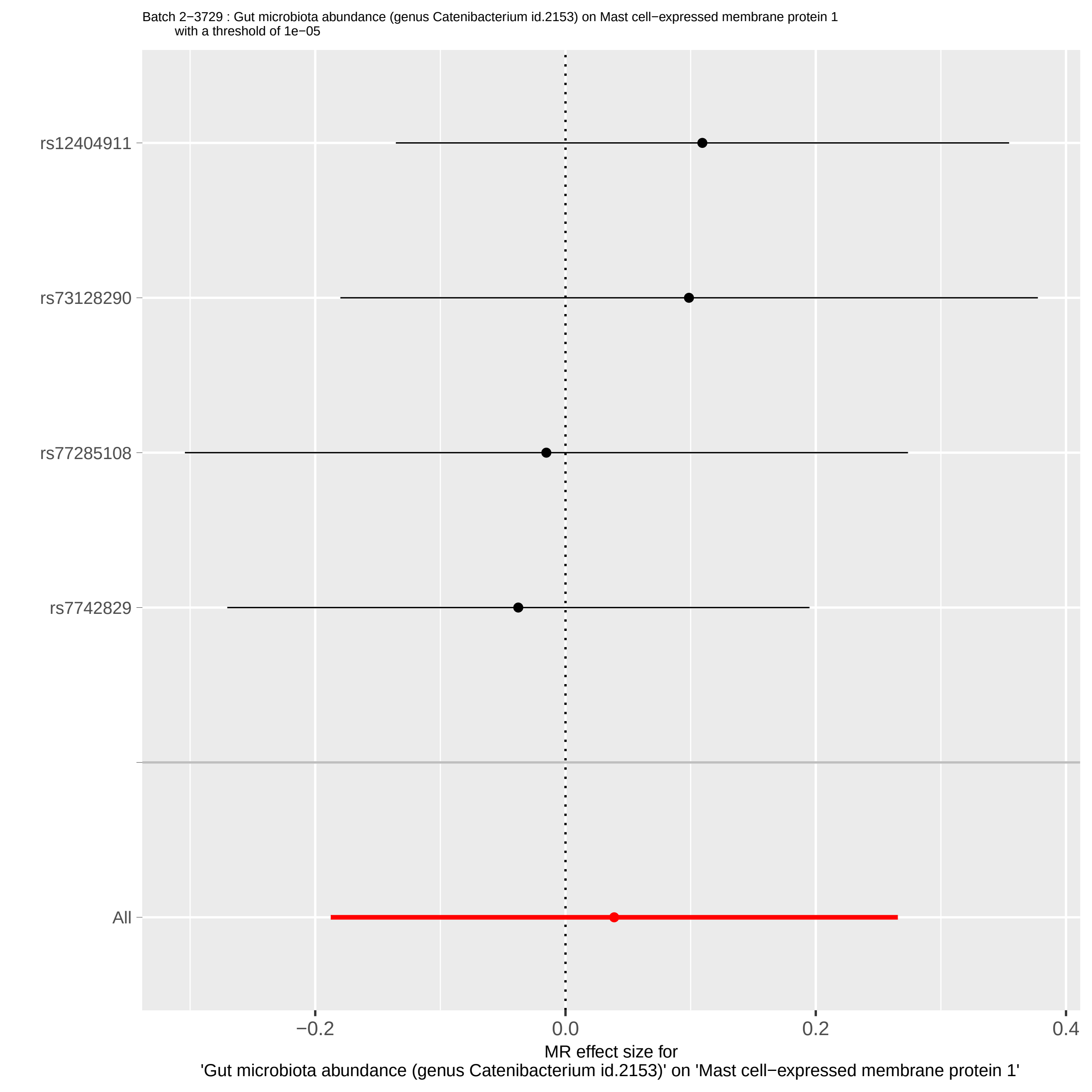

Batch 2−3729 : Gut microbiota abundance (genus Catenibacterium id.2153) on Mast cell−expressed membrane protein 1
with a threshold of 1e−05
rs12404911
rs73128290
rs77285108
rs7742829
All
−0.2
0.0
0.2
0.4
MR effect size for
'Gut microbiota abundance (genus Catenibacterium id.2153)' on 'Mast cell−expressed membrane protein 1'

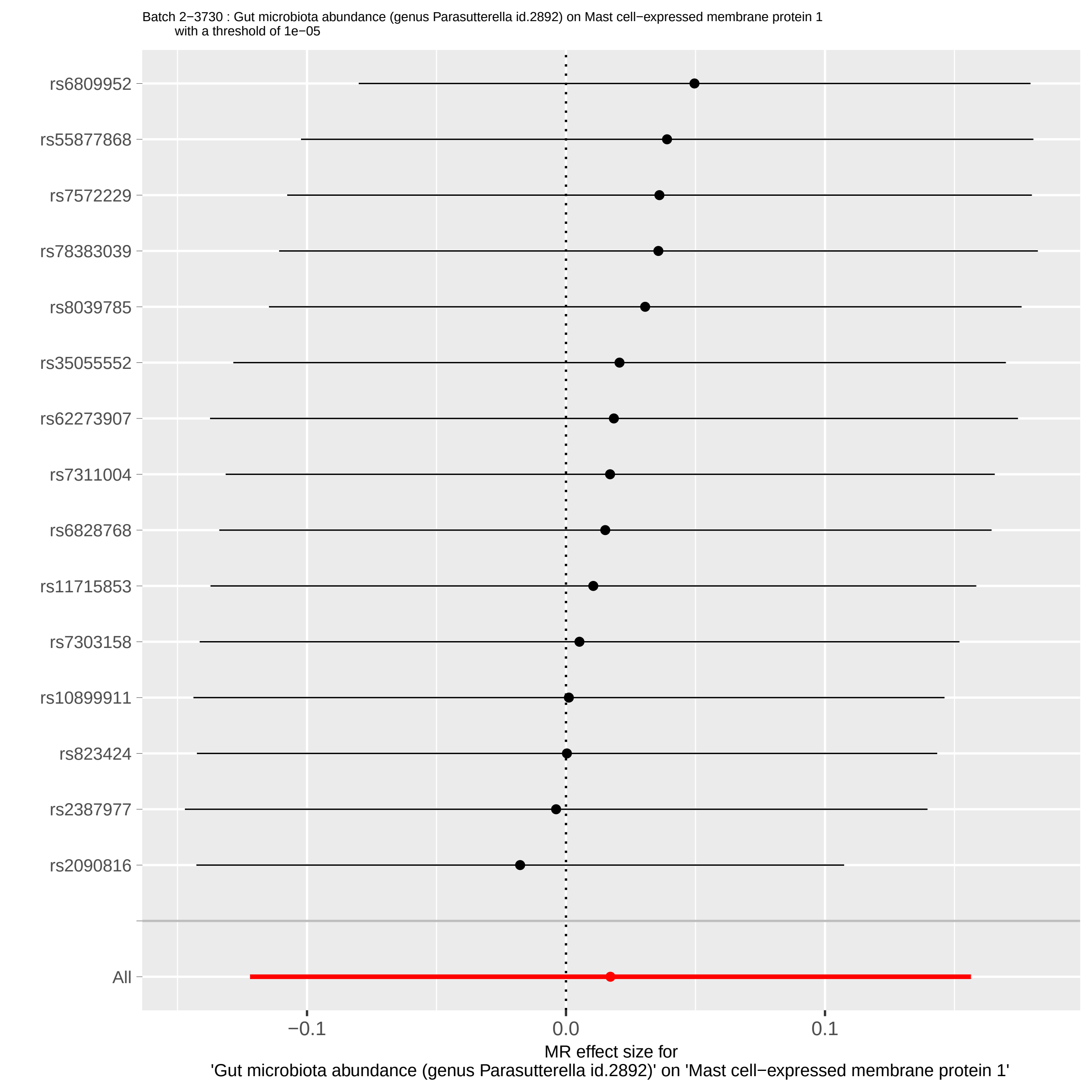
Batch 2−3730 : Gut microbiota abundance (genus Parasutterella id.2892) on Mast cell−expressed membrane protein 1
with a threshold of 1e−05
rs6809952
rs55877868
rs7572229
rs78383039
rs8039785
rs35055552
rs62273907
rs7311004
rs6828768
rs11715853
rs7303158
rs10899911
rs823424
rs2387977
rs2090816
All
−0.1
0.0
0.1
MR effect size for
'Gut microbiota abundance (genus Parasutterella id.2892)' on 'Mast cell−expressed membrane protein 1'

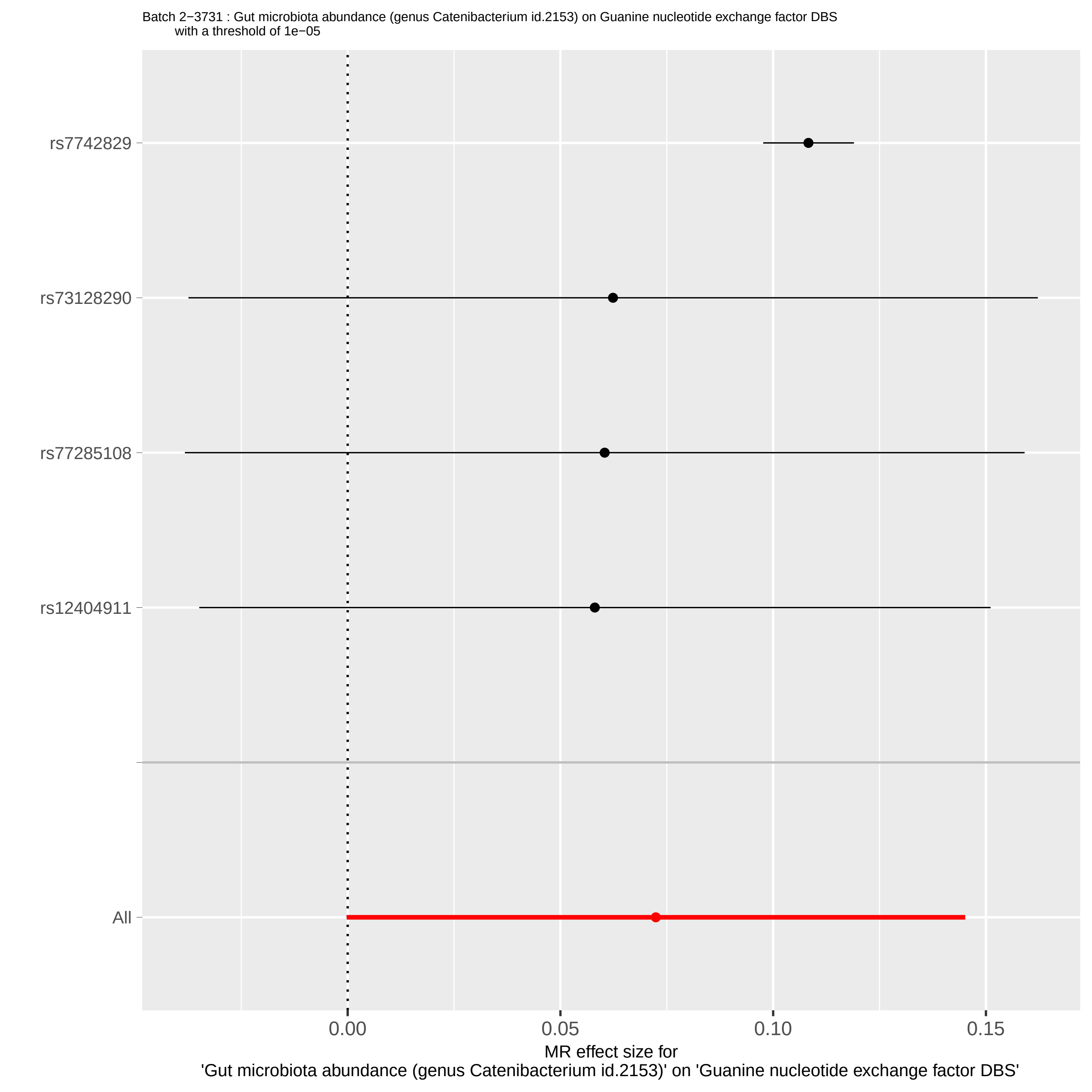
Batch 2−3731 : Gut microbiota abundance (genus Catenibacterium id.2153) on Guanine nucleotide exchange factor DBS
with a threshold of 1e−05
rs7742829
rs73128290
rs77285108
rs12404911
All
0.00
0.05
0.10
0.15
MR effect size for
'Gut microbiota abundance (genus Catenibacterium id.2153)' on 'Guanine nucleotide exchange factor DBS'

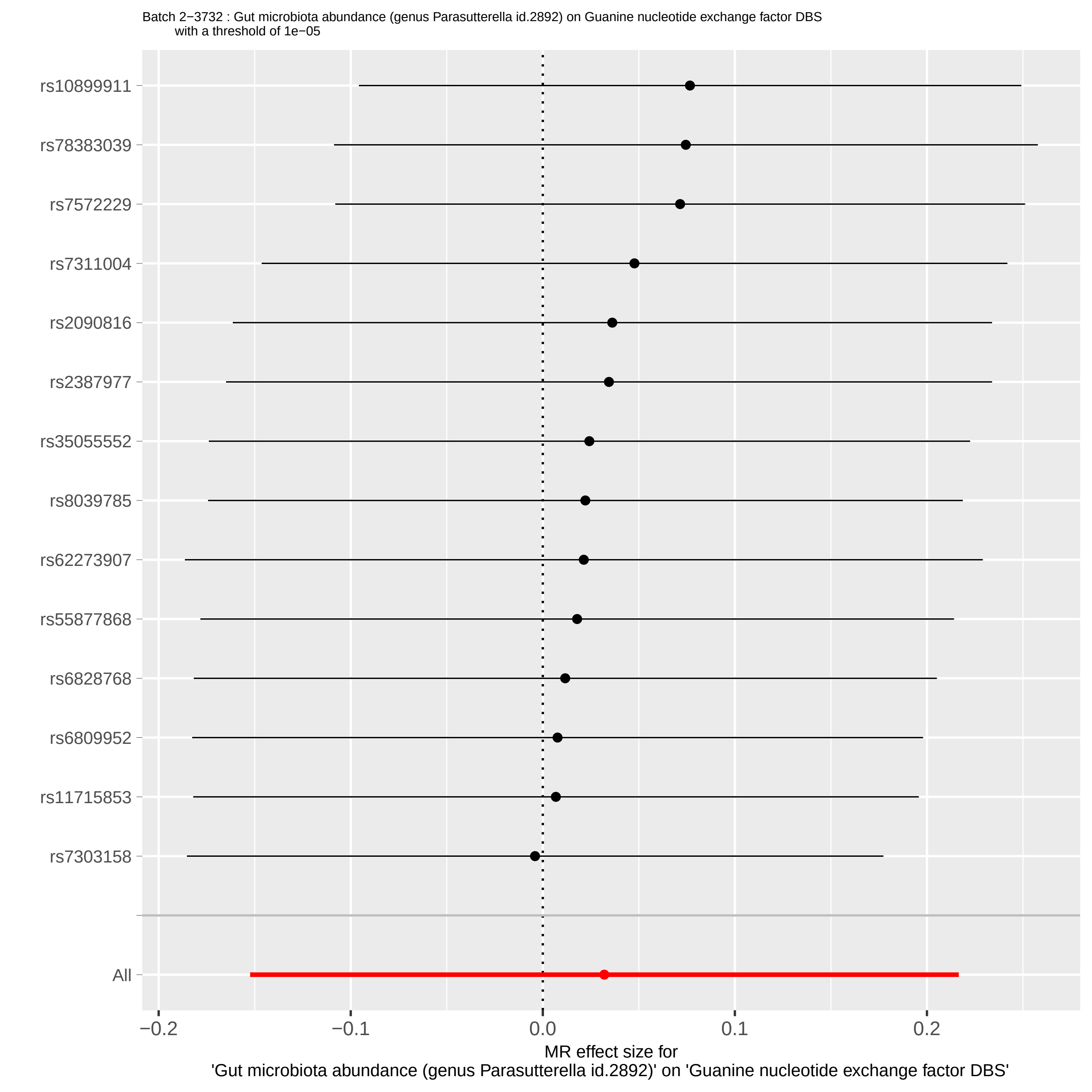

Batch 2−3732 : Gut microbiota abundance (genus Parasutterella id.2892) on Guanine nucleotide exchange factor DBS
with a threshold of 1e−05
rs10899911
rs78383039
rs7572229
rs7311004
rs2090816
rs2387977
rs35055552
rs8039785
rs62273907
rs55877868
rs6828768
rs6809952
rs11715853
rs7303158
All
−0.2
−0.1
0.0
0.1
0.2
MR effect size for
'Gut microbiota abundance (genus Parasutterella id.2892)' on 'Guanine nucleotide exchange factor DBS'

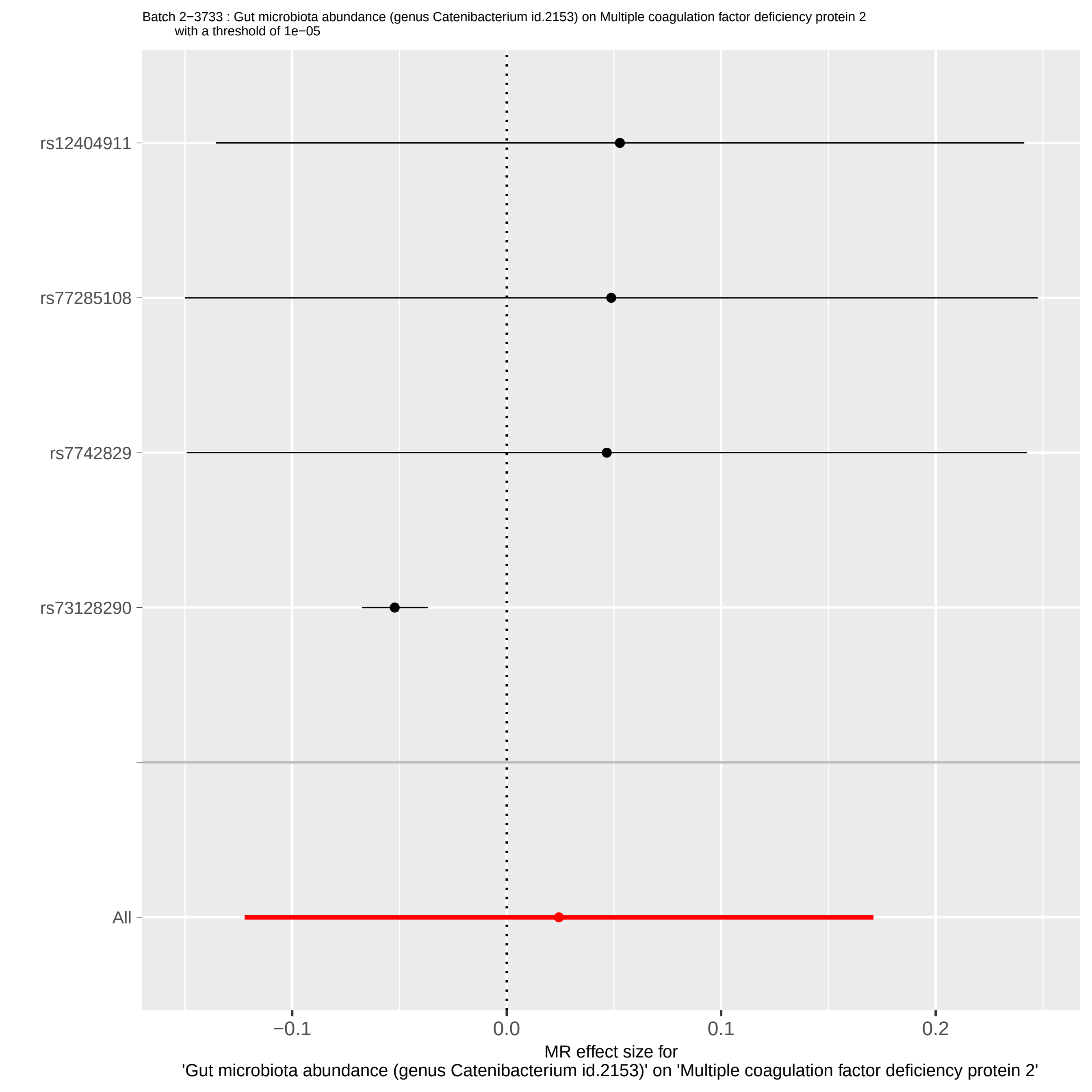

Batch 2−3733 : Gut microbiota abundance (genus Catenibacterium id.2153) on Multiple coagulation factor deficiency protein 2
with a threshold of 1e−05
rs12404911
rs77285108
rs7742829
rs73128290
All
−0.1
0.0
0.1
0.2
MR effect size for
'Gut microbiota abundance (genus Catenibacterium id.2153)' on 'Multiple coagulation factor deficiency protein 2'

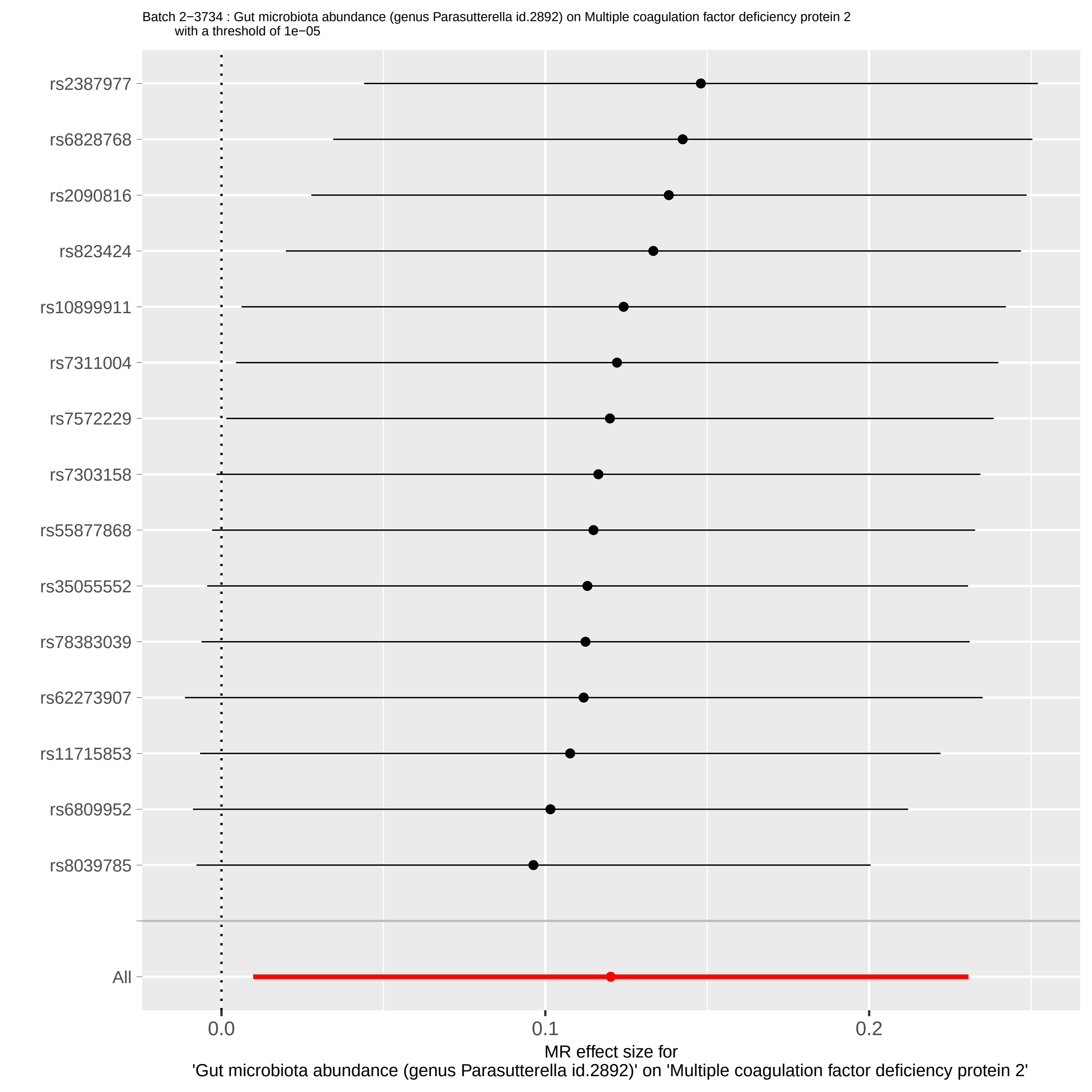
Batch 2−3734 : Gut microbiota abundance (genus Parasutterella id.2892) on Multiple coagulation factor deficiency protein 2
with a threshold of 1e−05
rs2387977
rs6828768
rs2090816
rs823424
rs10899911
rs7311004
rs7572229
rs7303158
rs55877868
rs35055552
rs78383039
rs62273907
rs11715853
rs6809952
rs8039785
All
0.0
0.1
0.2
MR effect size for
'Gut microbiota abundance (genus Parasutterella id.2892)' on 'Multiple coagulation factor deficiency protein 2'

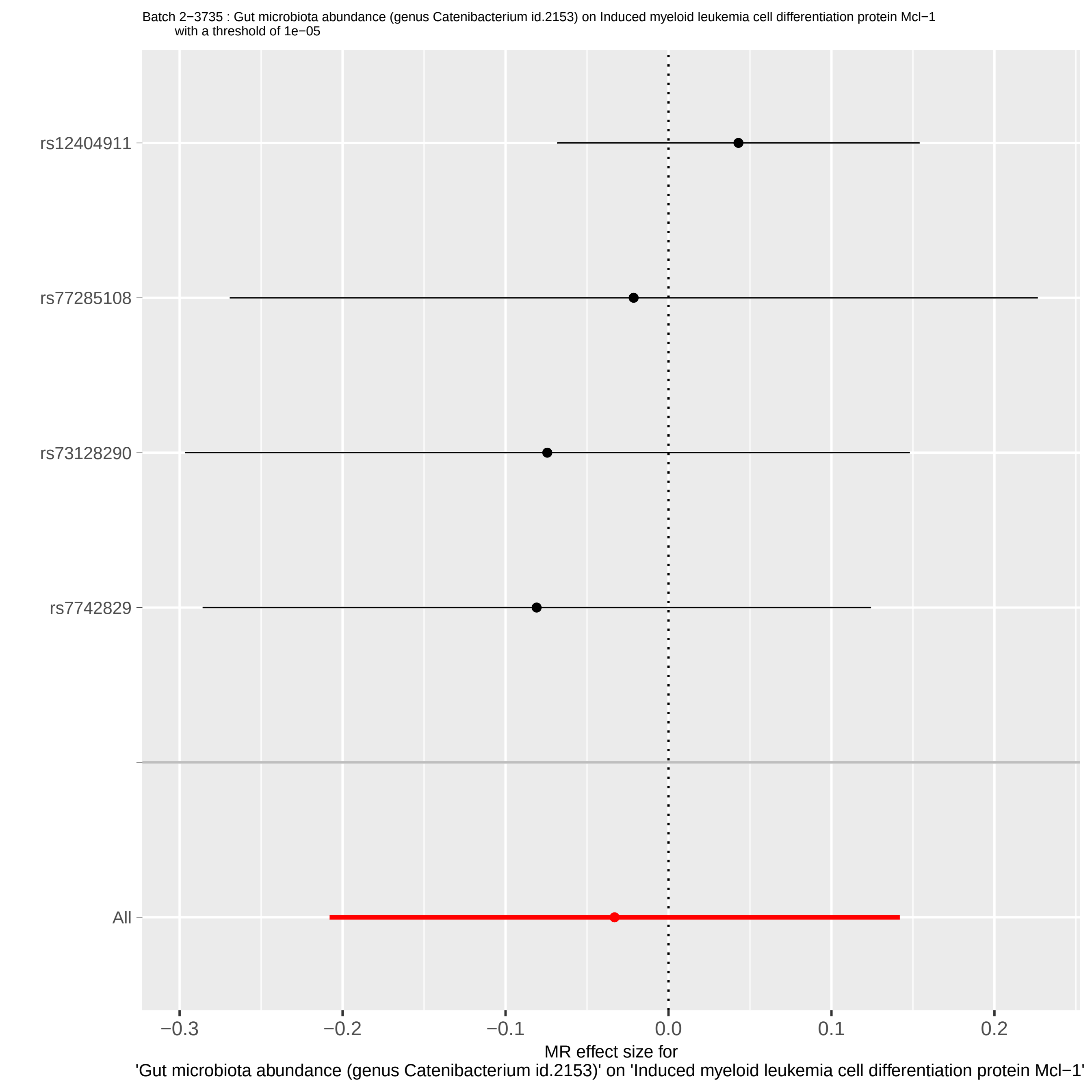
Batch 2−3735 : Gut microbiota abundance (genus Catenibacterium id.2153) on Induced myeloid leukemia cell differentiation protein Mcl−1
with a threshold of 1e−05
rs12404911
rs77285108
rs73128290
rs7742829
All
−0.3
−0.2
−0.1
0.0
0.1
0.2
MR effect size for
'Gut microbiota abundance (genus Catenibacterium id.2153)' on 'Induced myeloid leukemia cell differentiation protein Mcl−1'

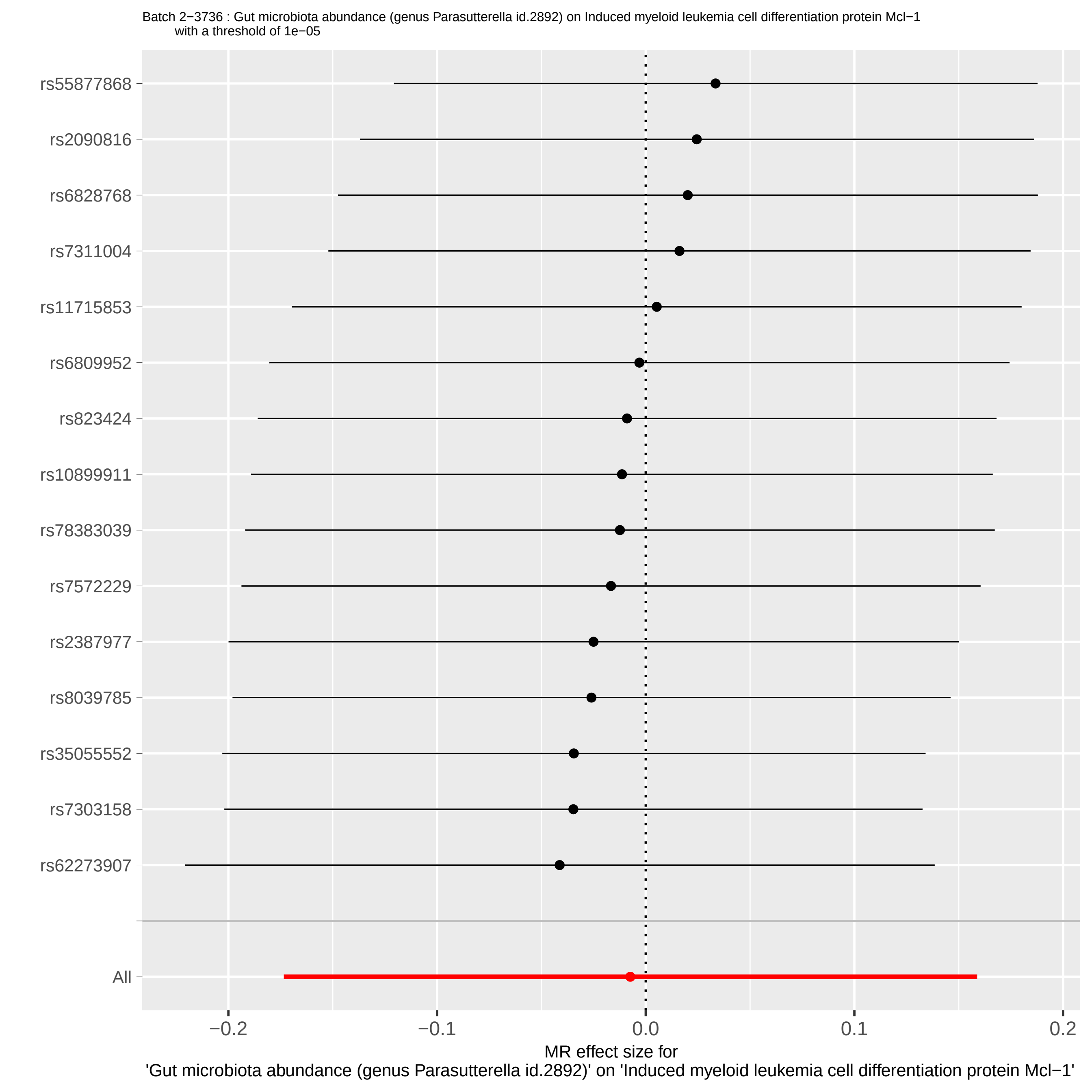

Batch 2−3736 : Gut microbiota abundance (genus Parasutterella id.2892) on Induced myeloid leukemia cell differentiation protein Mcl−1
with a threshold of 1e−05
rs55877868
rs2090816
rs6828768
rs7311004
rs11715853
rs6809952
rs823424
rs10899911
rs78383039
rs7572229
rs2387977
rs8039785
rs35055552
rs7303158
rs62273907
All
−0.2
−0.1
0.0
0.1
0.2
MR effect size for
'Gut microbiota abundance (genus Parasutterella id.2892)' on 'Induced myeloid leukemia cell differentiation protein Mcl−1'

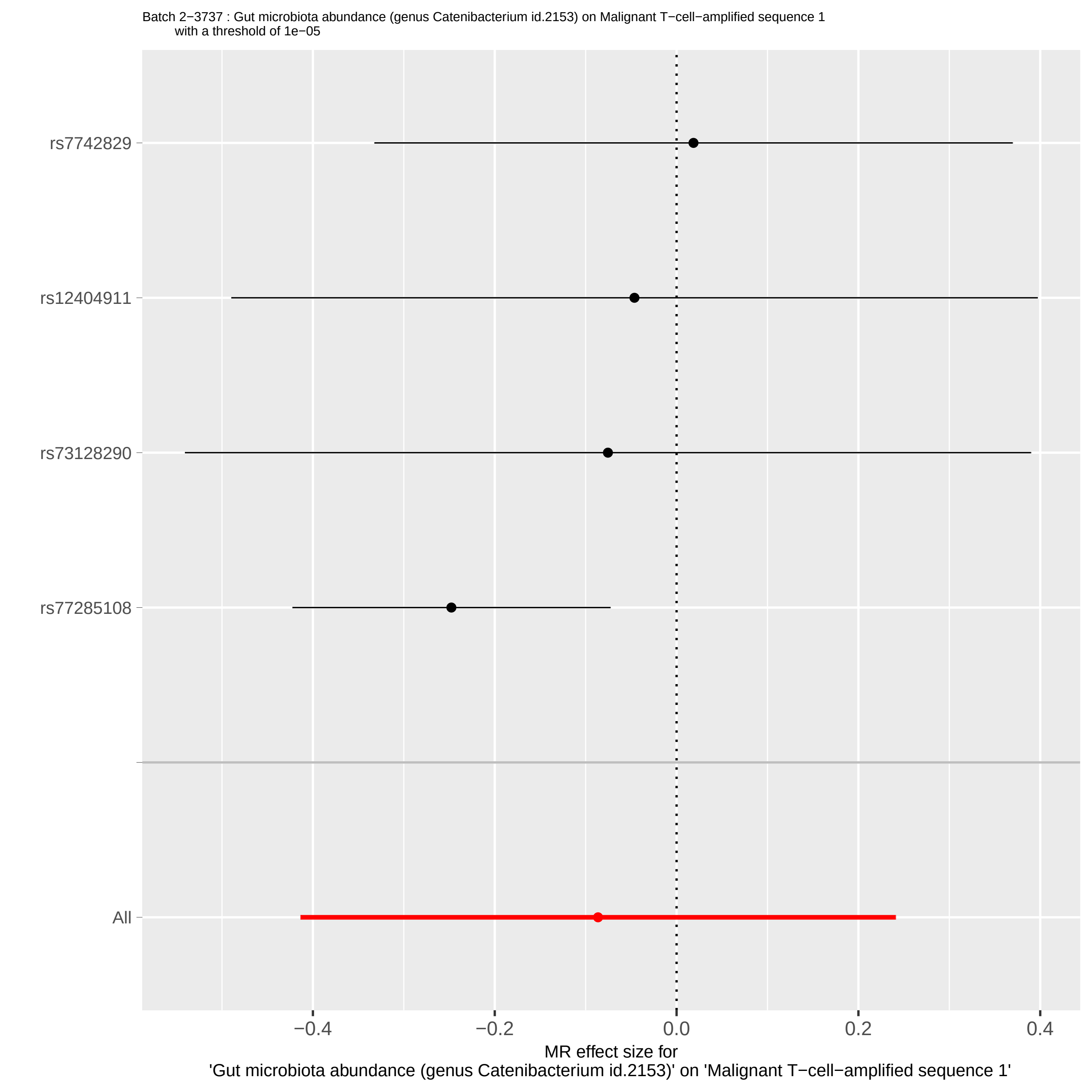

Batch 2−3737 : Gut microbiota abundance (genus Catenibacterium id.2153) on Malignant T−cell−amplified sequence 1
with a threshold of 1e−05
rs7742829
rs12404911
rs73128290
rs77285108
All
−0.4
−0.2
0.0
0.2
0.4
MR effect size for
'Gut microbiota abundance (genus Catenibacterium id.2153)' on 'Malignant T−cell−amplified sequence 1'

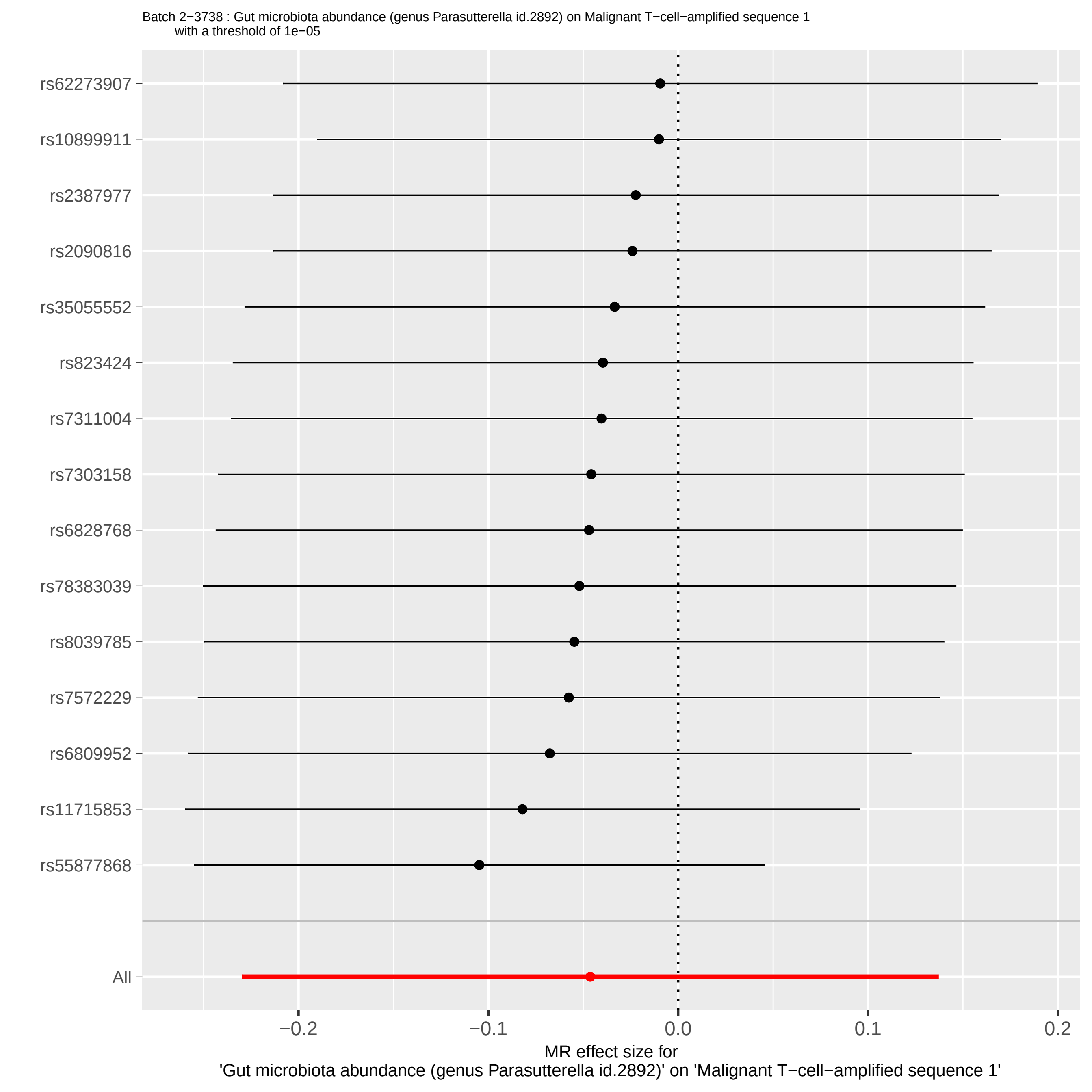

Batch 2−3738 : Gut microbiota abundance (genus Parasutterella id.2892) on Malignant T−cell−amplified sequence 1
with a threshold of 1e−05
rs62273907
rs10899911
rs2387977
rs2090816
rs35055552
rs823424
rs7311004
rs7303158
rs6828768
rs78383039
rs8039785
rs7572229
rs6809952
rs11715853
rs55877868
All
−0.2
−0.1
0.0
0.1
0.2
MR effect size for
'Gut microbiota abundance (genus Parasutterella id.2892)' on 'Malignant T−cell−amplified sequence 1'

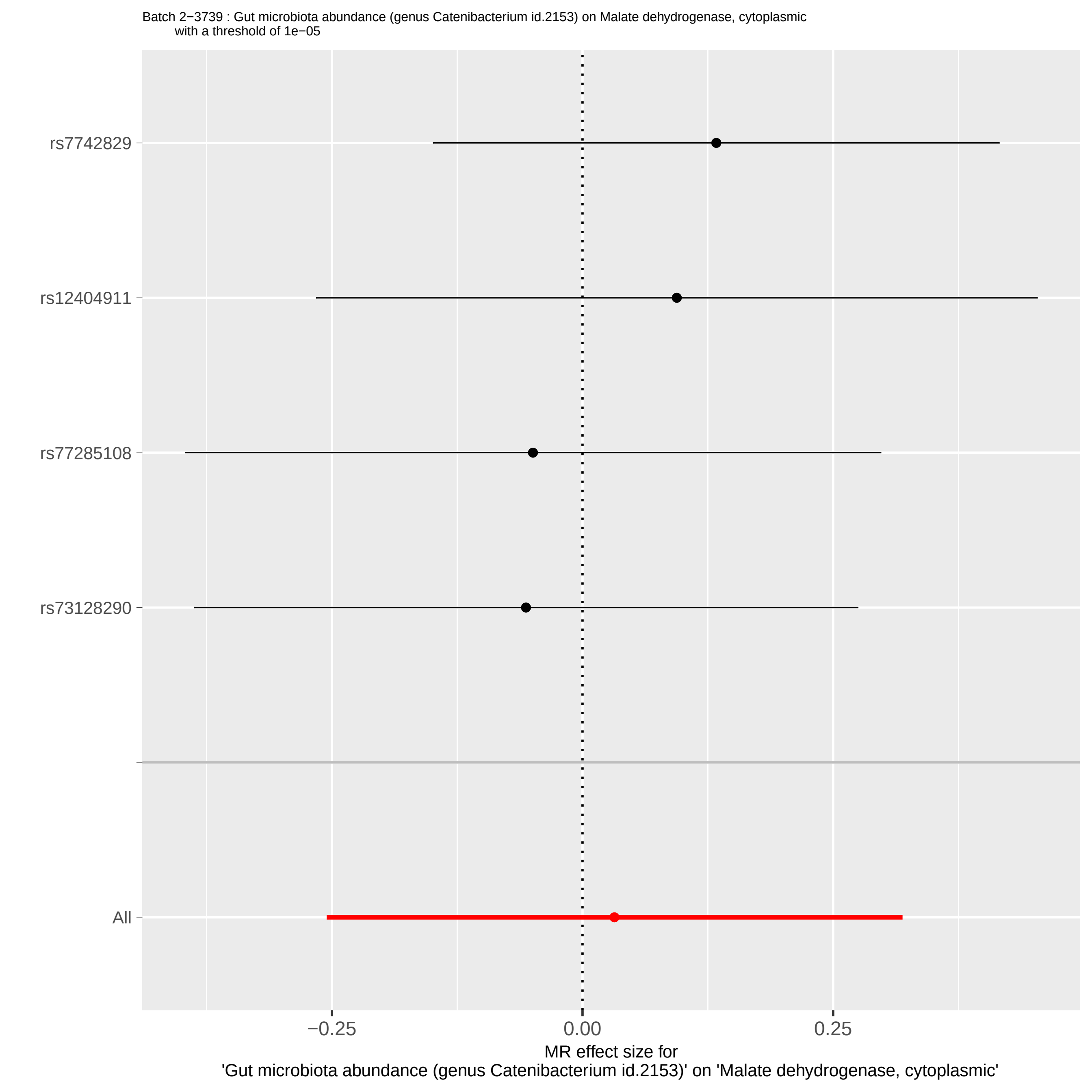

Batch 2−3739 : Gut microbiota abundance (genus Catenibacterium id.2153) on Malate dehydrogenase, cytoplasmic
with a threshold of 1e−05
rs7742829
rs12404911
rs77285108
rs73128290
All
−0.25
0.00
0.25
MR effect size for
'Gut microbiota abundance (genus Catenibacterium id.2153)' on 'Malate dehydrogenase, cytoplasmic'

Batch 2−3740 : Gut microbiota abundance (genus Parasutterella id.2892) on Malate dehydrogenase, cytoplasmic
with a threshold of 1e−05

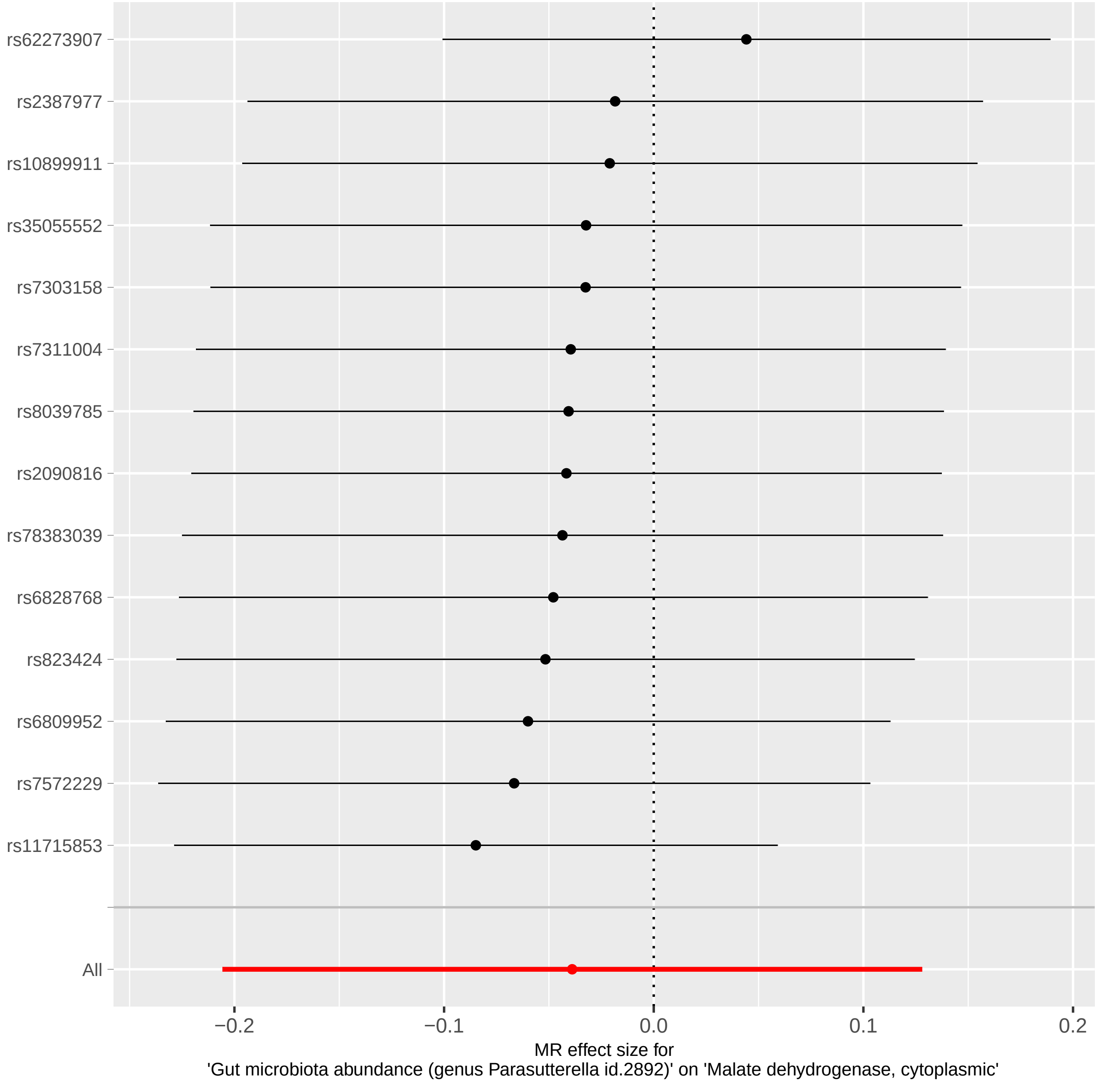

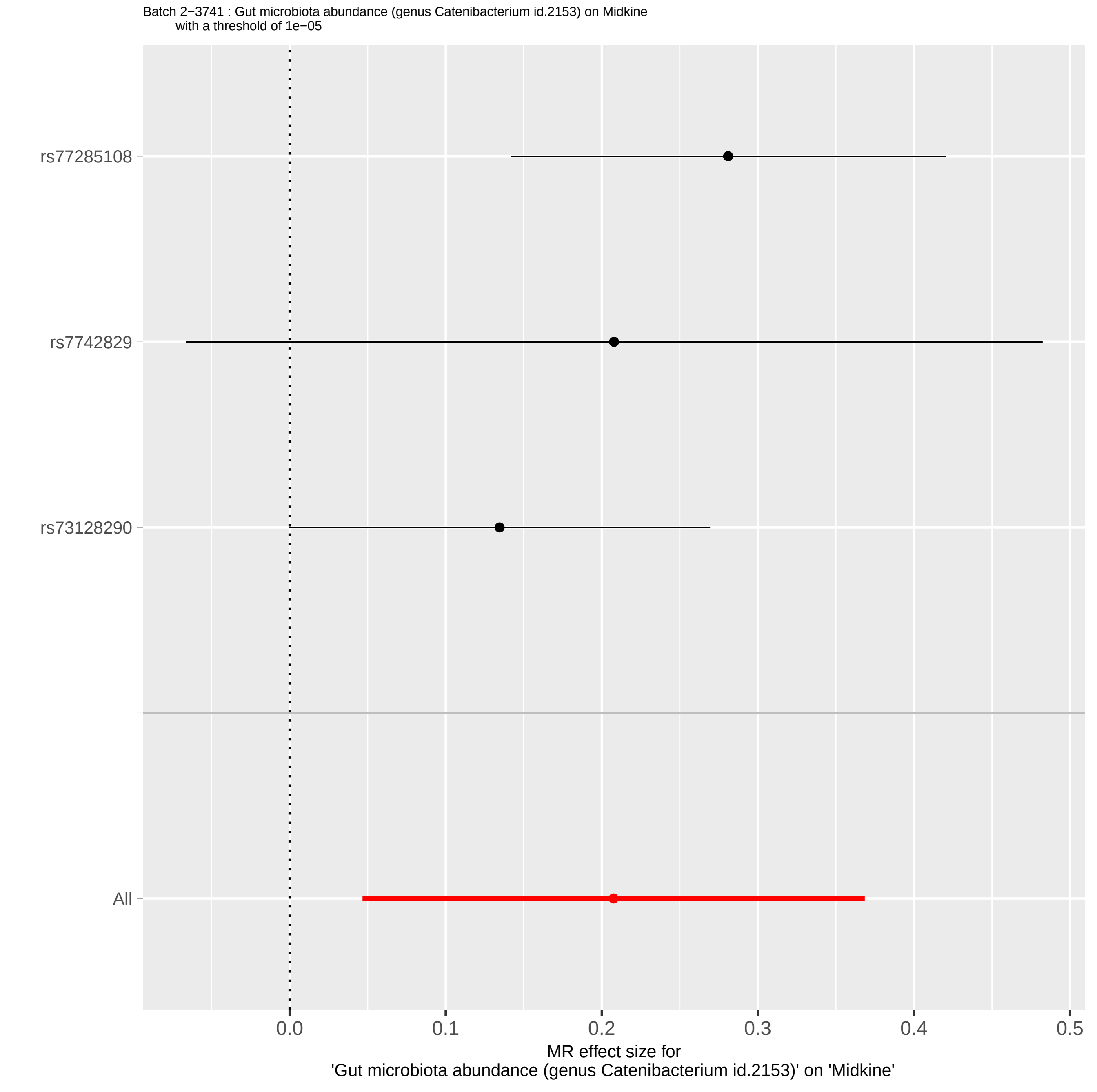
Batch 2−3741 : Gut microbiota abundance (genus Catenibacterium id.2153) on Midkine
with a threshold of 1e−05
rs77285108
rs7742829
rs73128290
All
0.0
0.1
0.2
0.3
0.4
0.5
MR effect size for
'Gut microbiota abundance (genus Catenibacterium id.2153)' on 'Midkine'

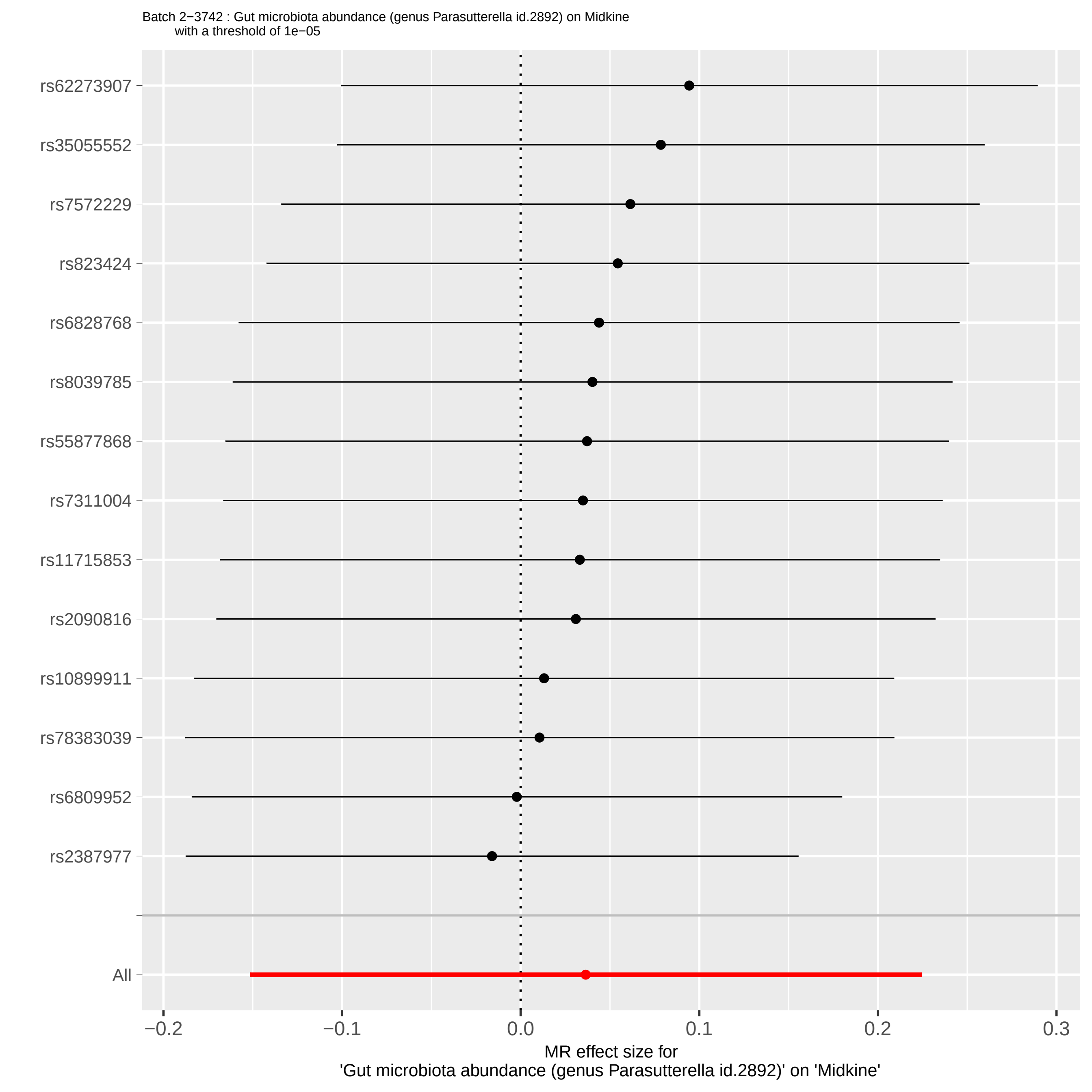
Batch 2−3742 : Gut microbiota abundance (genus Parasutterella id.2892) on Midkine
with a threshold of 1e−05
rs62273907
rs35055552
rs7572229
rs823424
rs6828768
rs8039785
rs55877868
rs7311004
rs11715853
rs2090816
rs10899911
rs78383039
rs6809952
rs2387977
All
−0.2
−0.1
0.0
0.1
0.2
0.3
MR effect size for
'Gut microbiota abundance (genus Parasutterella id.2892)' on 'Midkine'

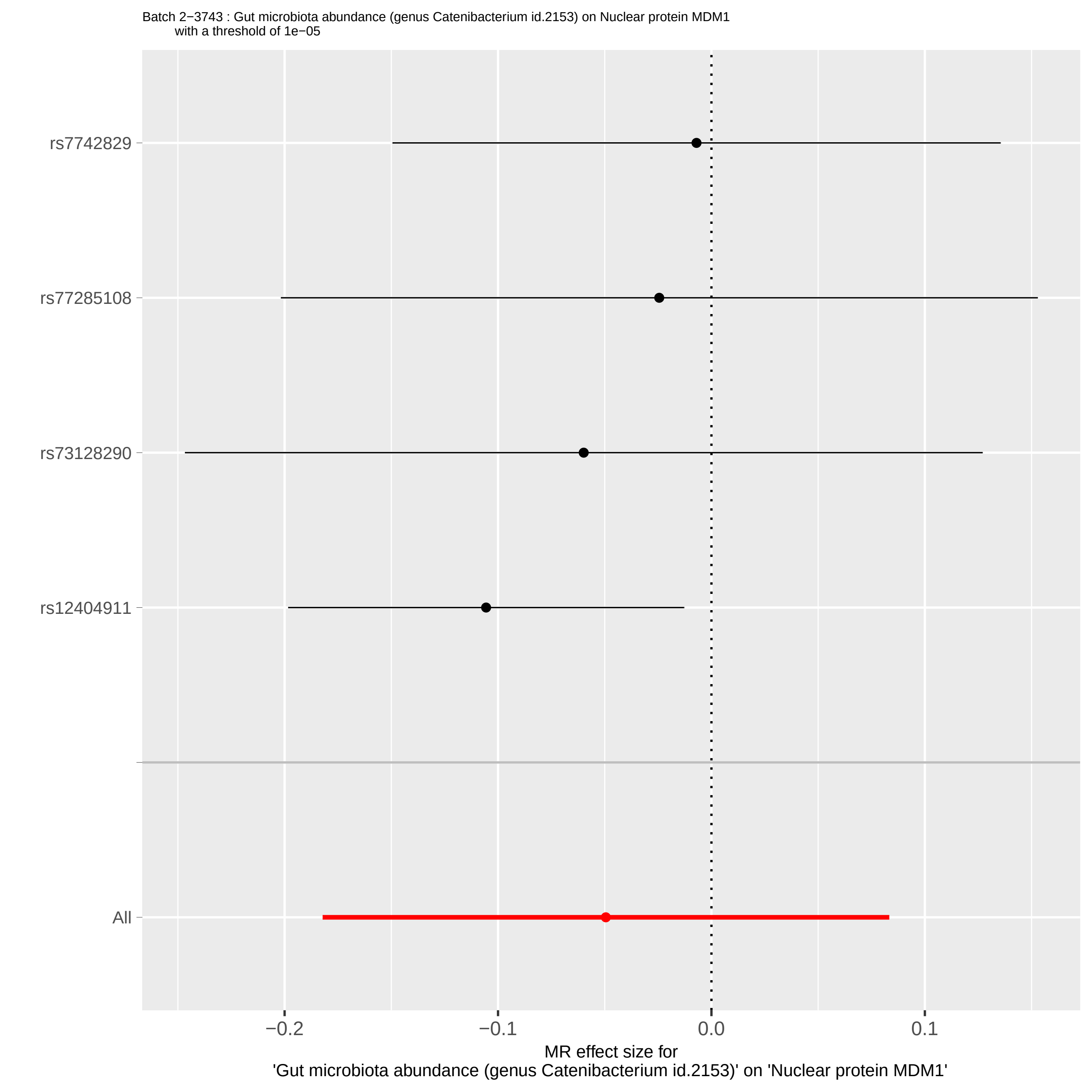
Batch 2−3743 : Gut microbiota abundance (genus Catenibacterium id.2153) on Nuclear protein MDM1
with a threshold of 1e−05
rs7742829
rs77285108
rs73128290
rs12404911
All
−0.2
−0.1
0.0
0.1
MR effect size for
'Gut microbiota abundance (genus Catenibacterium id.2153)' on 'Nuclear protein MDM1'

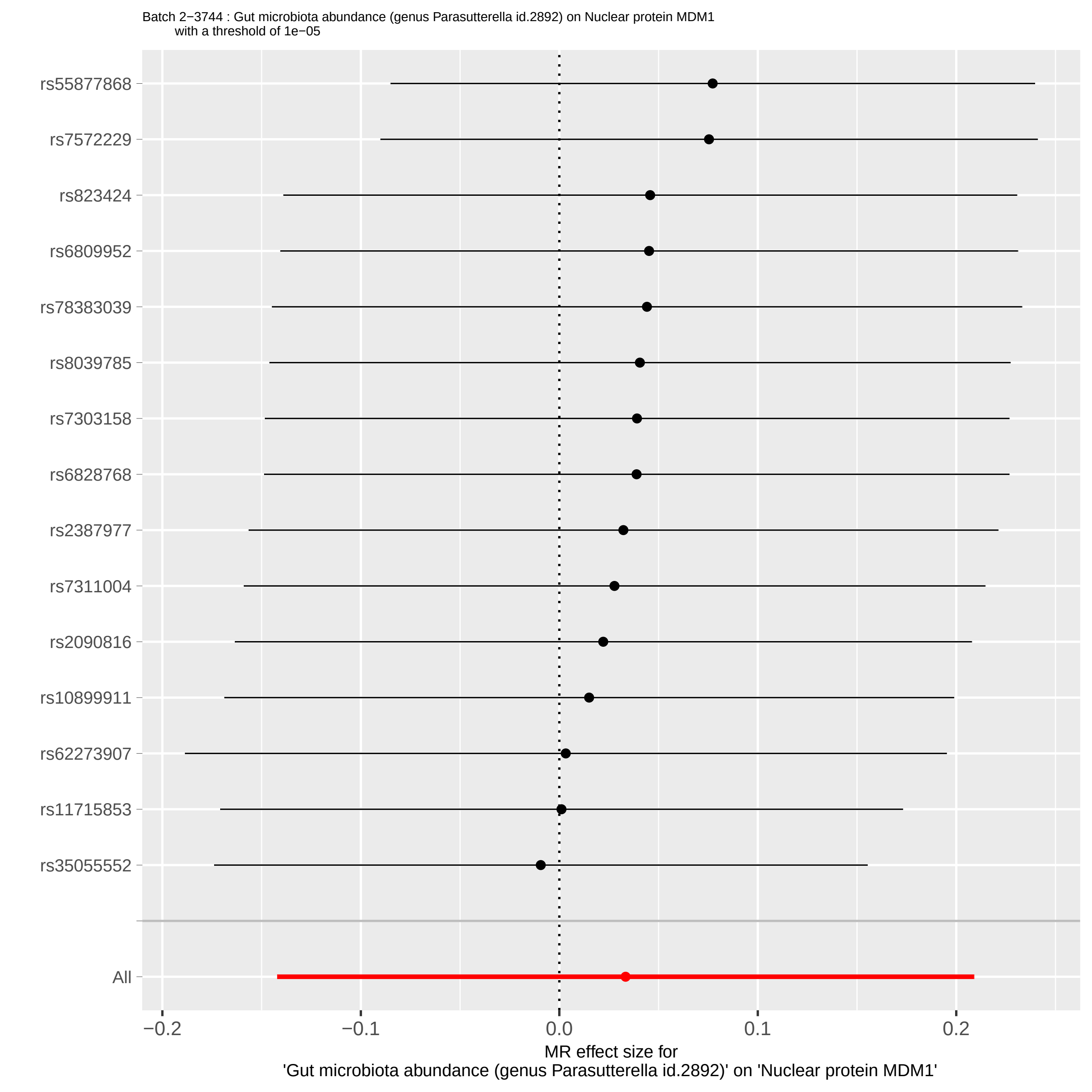

Batch 2−3744 : Gut microbiota abundance (genus Parasutterella id.2892) on Nuclear protein MDM1
with a threshold of 1e−05
rs55877868
rs7572229
rs823424
rs6809952
rs78383039
rs8039785
rs7303158
rs6828768
rs2387977
rs7311004
rs2090816
rs10899911
rs62273907
rs11715853
rs35055552
All
−0.2
−0.1
0.0
0.1
0.2
MR effect size for
'Gut microbiota abundance (genus Parasutterella id.2892)' on 'Nuclear protein MDM1'

Batch 2−3745 : Gut microbiota abundance (genus Catenibacterium id.2153) on E3 ubiquitin−protein ligase Mdm2
with a threshold of 1e−05

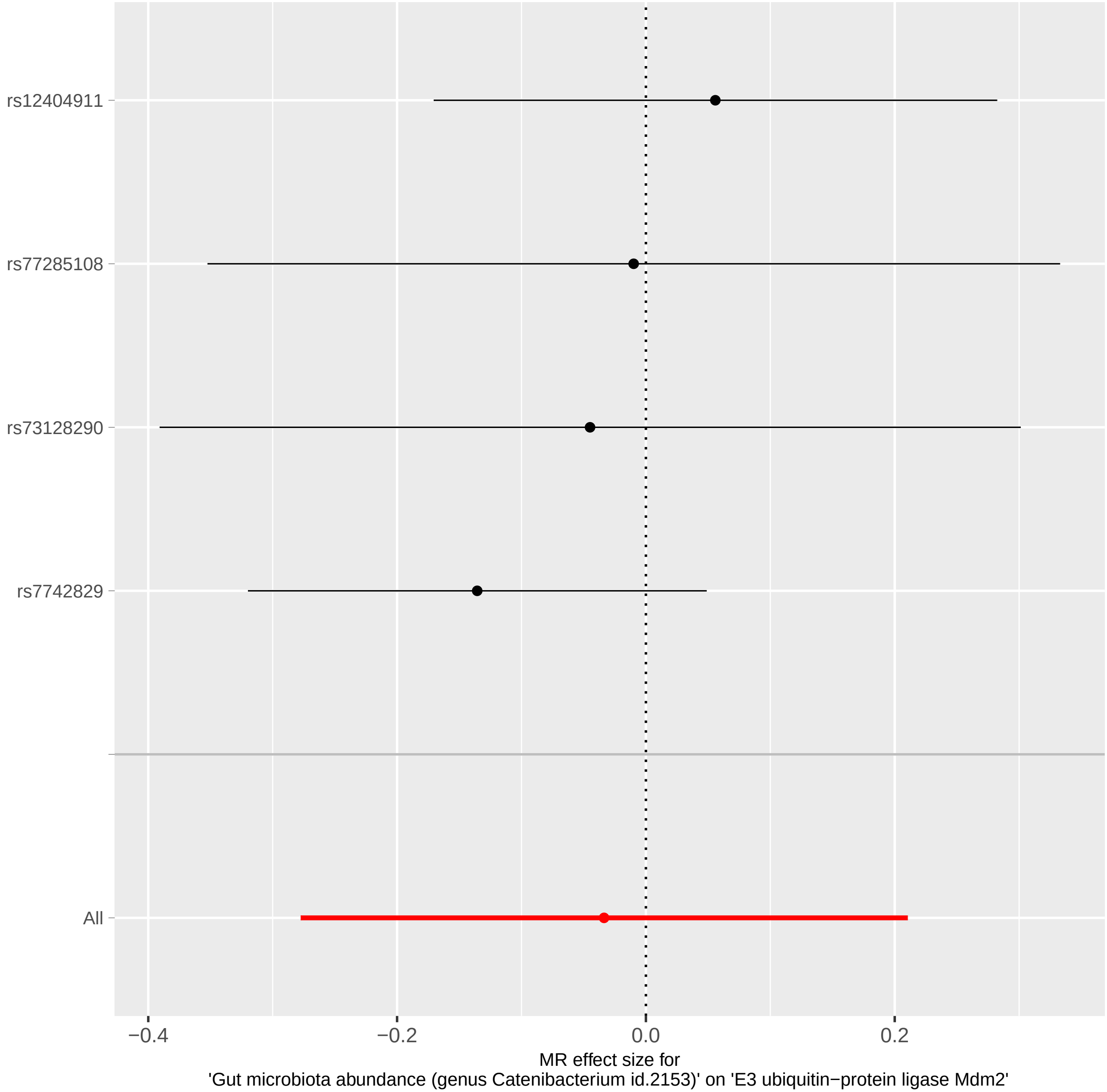

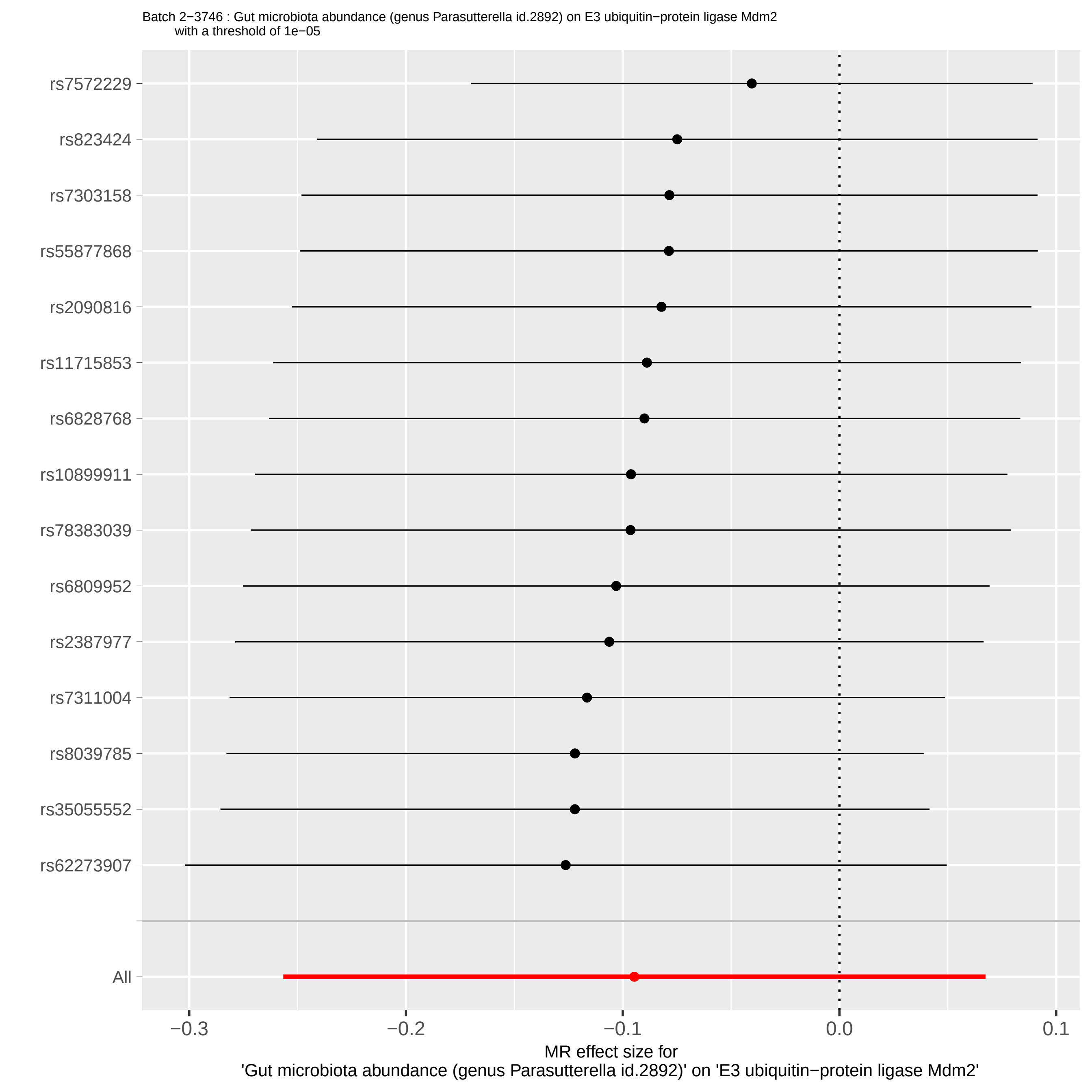

Batch 2−3746 : Gut microbiota abundance (genus Parasutterella id.2892) on E3 ubiquitin−protein ligase Mdm2
with a threshold of 1e−05
rs7572229
rs823424
rs7303158
rs55877868
rs2090816
rs11715853
rs6828768
rs10899911
rs78383039
rs6809952
rs2387977
rs7311004
rs8039785
rs35055552
rs62273907
All
−0.3
−0.2
−0.1
0.0
0.1
MR effect size for
'Gut microbiota abundance (genus Parasutterella id.2892)' on 'E3 ubiquitin−protein ligase Mdm2'

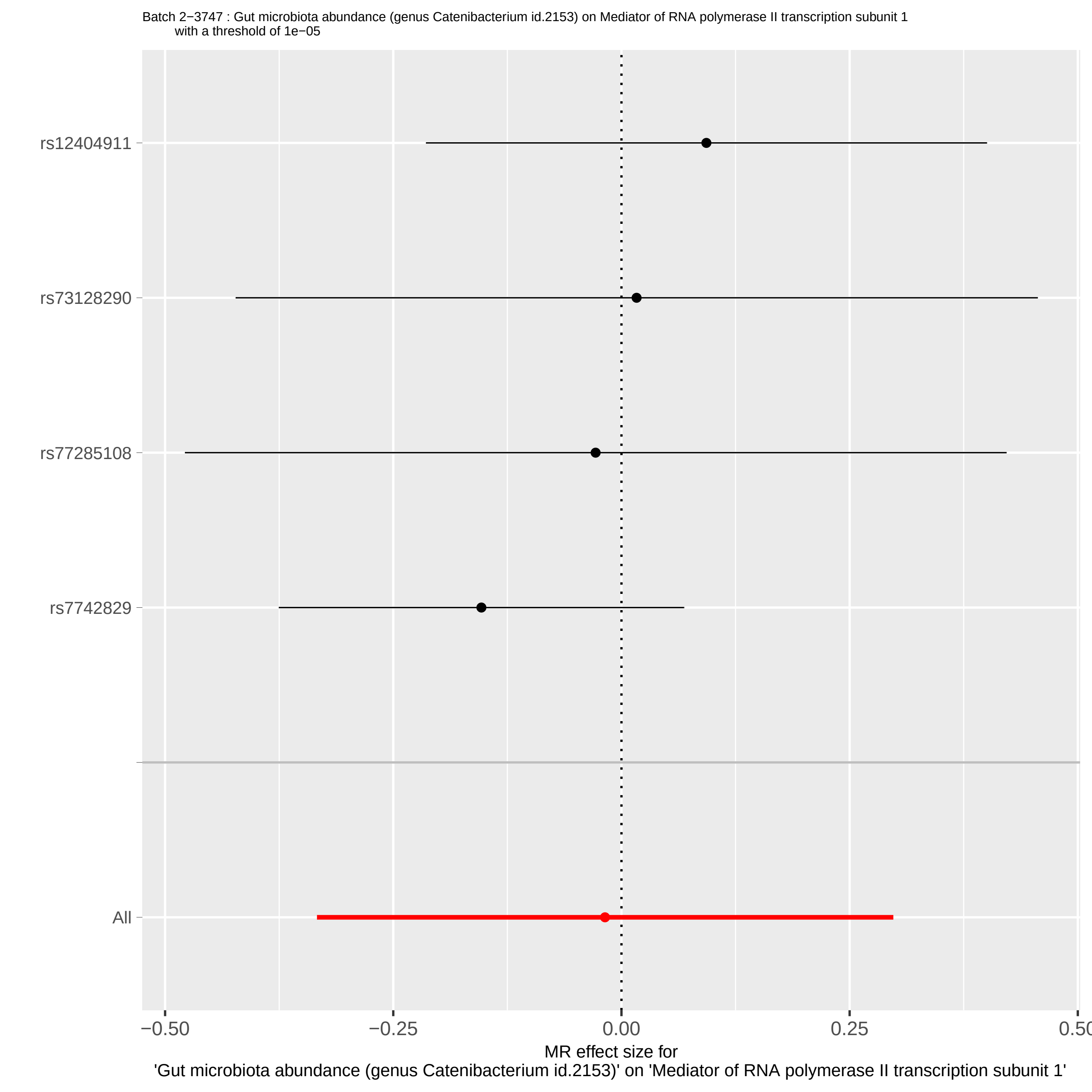

Batch 2−3747 : Gut microbiota abundance (genus Catenibacterium id.2153) on Mediator of RNA polymerase II transcription subunit 1 with a threshold of 1e−05
rs12404911
rs73128290
rs77285108
rs7742829
All
−0.50
−0.25
0.00
0.25
0.50
MR effect size for
'Gut microbiota abundance (genus Catenibacterium id.2153)' on 'Mediator of RNA polymerase II transcription subunit 1'

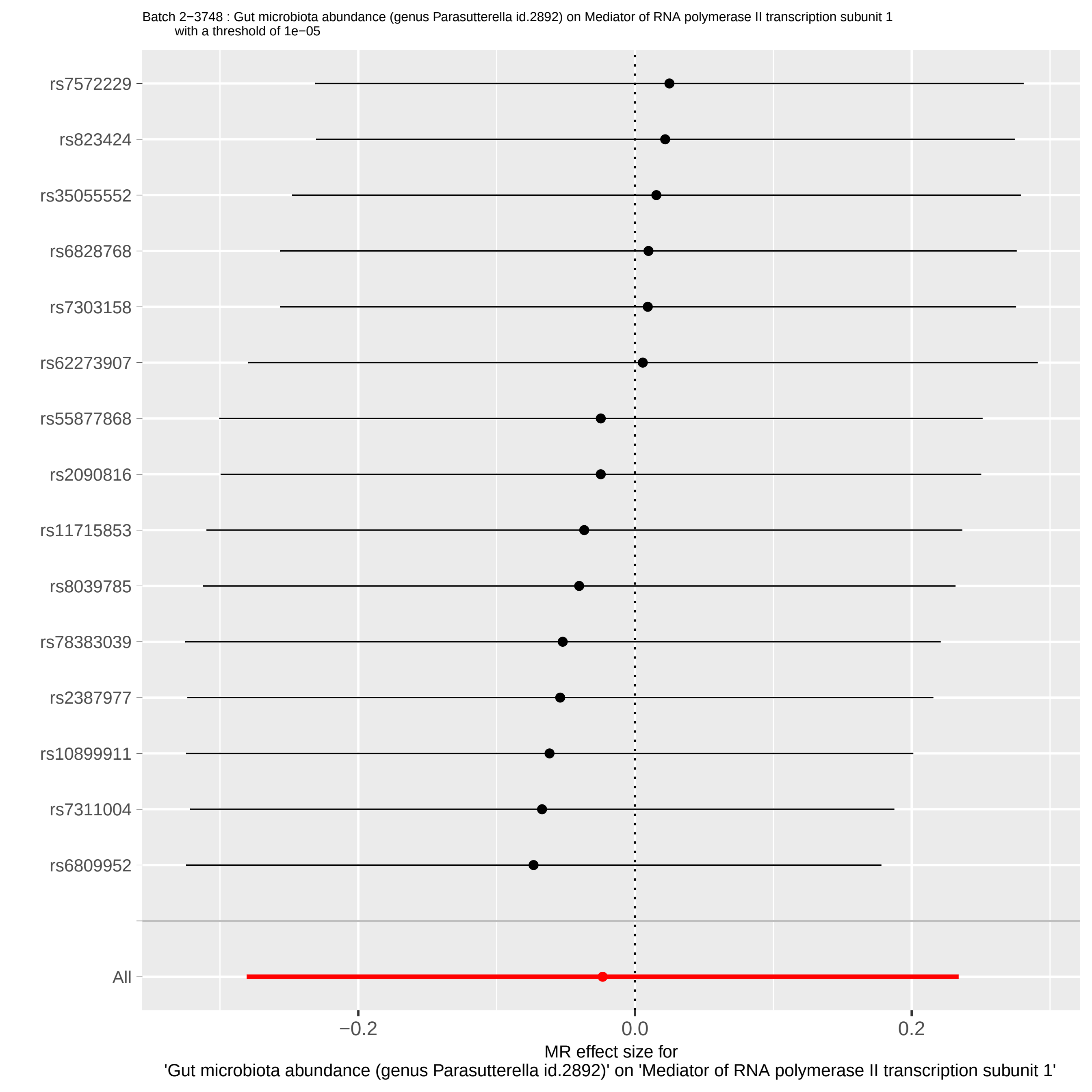

Batch 2−3748 : Gut microbiota abundance (genus Parasutterella id.2892) on Mediator of RNA polymerase II transcription subunit 1
with a threshold of 1e−05
rs7572229
rs823424
rs35055552
rs6828768
rs7303158
rs62273907
rs55877868
rs2090816
rs11715853
rs8039785
rs78383039
rs2387977
rs10899911
rs7311004
rs6809952
All
−0.2
0.0
0.2
MR effect size for
'Gut microbiota abundance (genus Parasutterella id.2892)' on 'Mediator of RNA polymerase II transcription subunit 1'

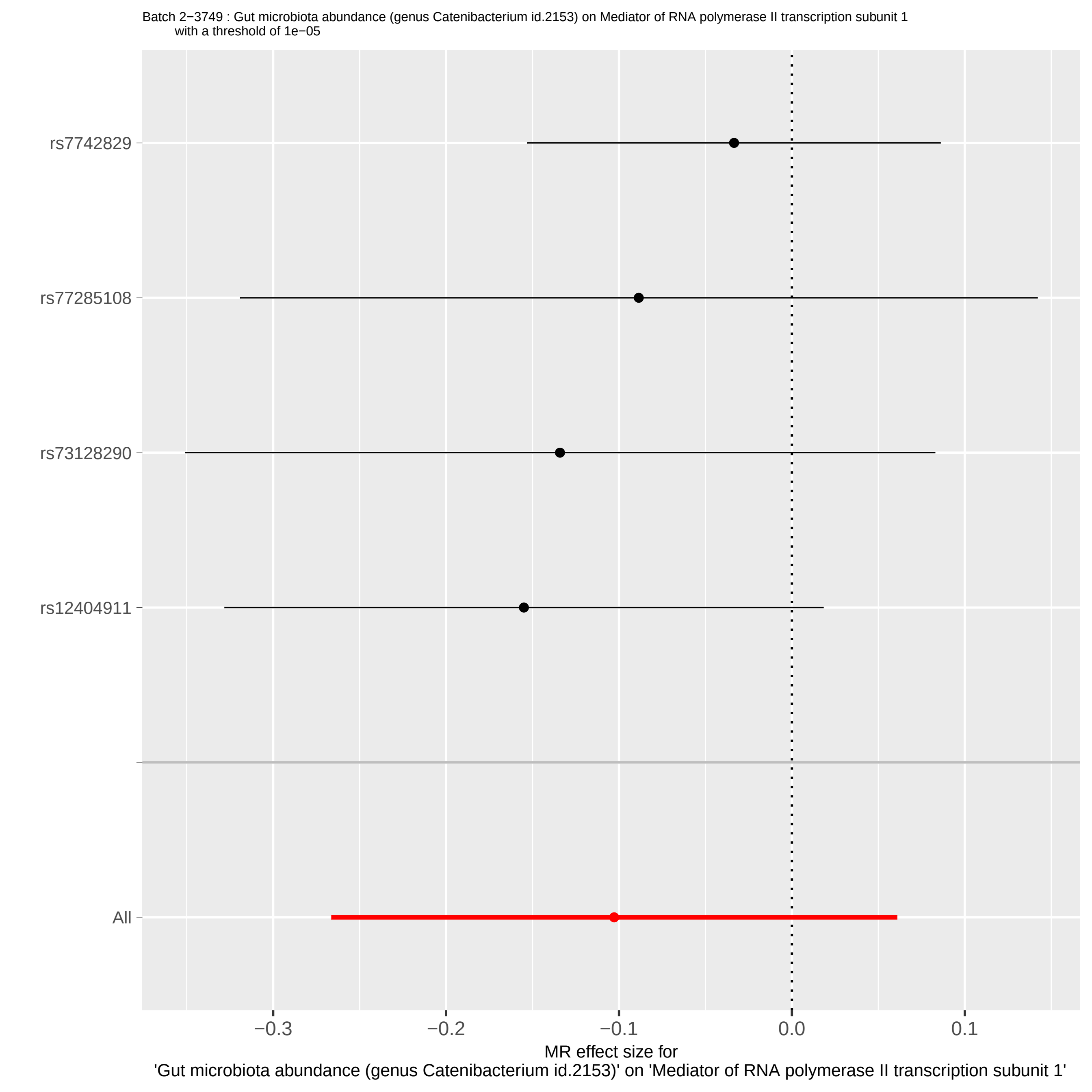
Batch 2−3749 : Gut microbiota abundance (genus Catenibacterium id.2153) on Mediator of RNA polymerase II transcription subunit 1
with a threshold of 1e−05
rs7742829
rs77285108
rs73128290
rs12404911
All
−0.3
−0.2
−0.1
0.0
0.1
MR effect size for
'Gut microbiota abundance (genus Catenibacterium id.2153)' on 'Mediator of RNA polymerase II transcription subunit 1'

Batch 2−3750 : Gut microbiota abundance (genus Parasutterella id.2892) on Mediator of RNA polymerase II transcription subunit 1 with a threshold of 1e−05

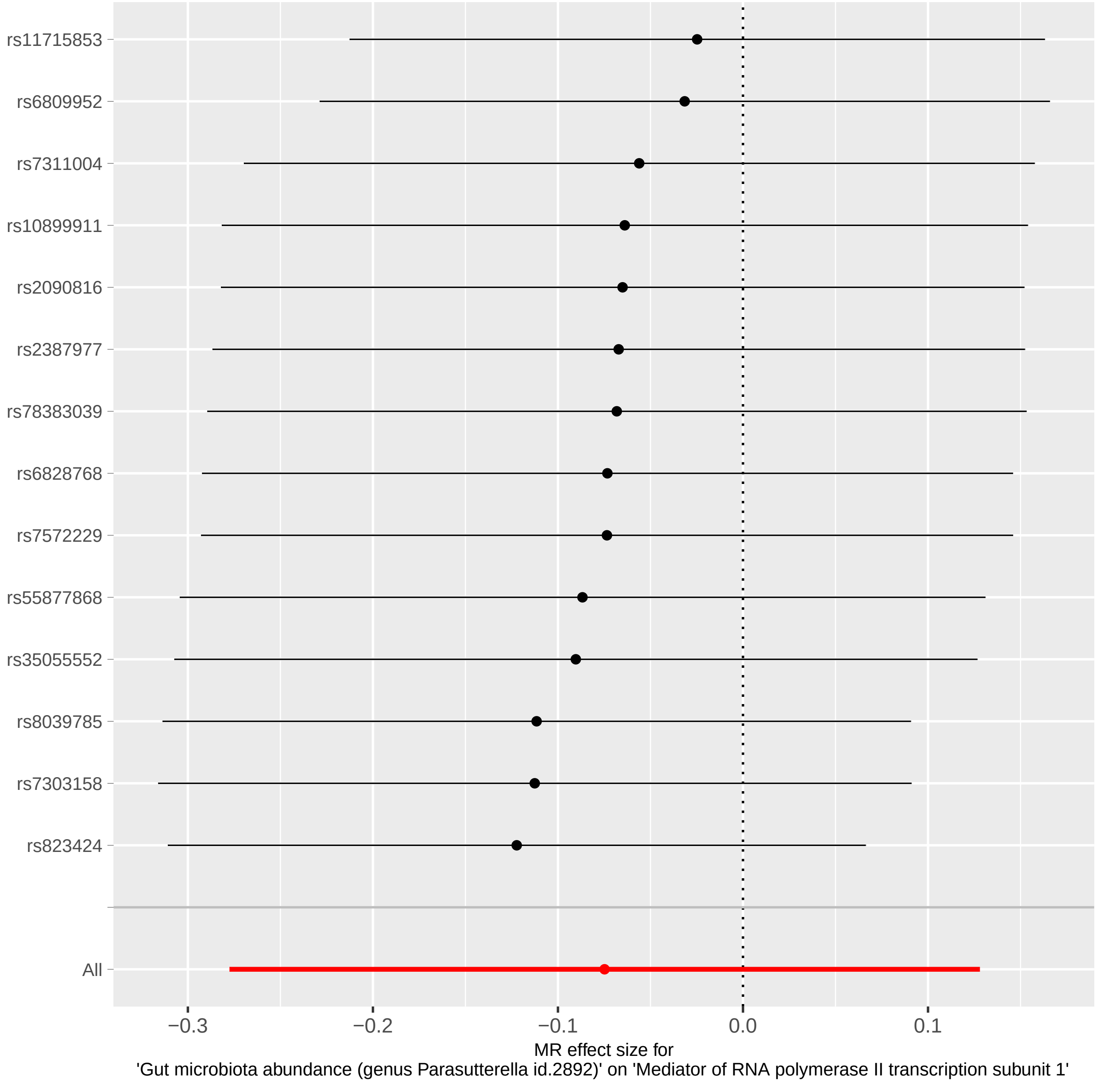

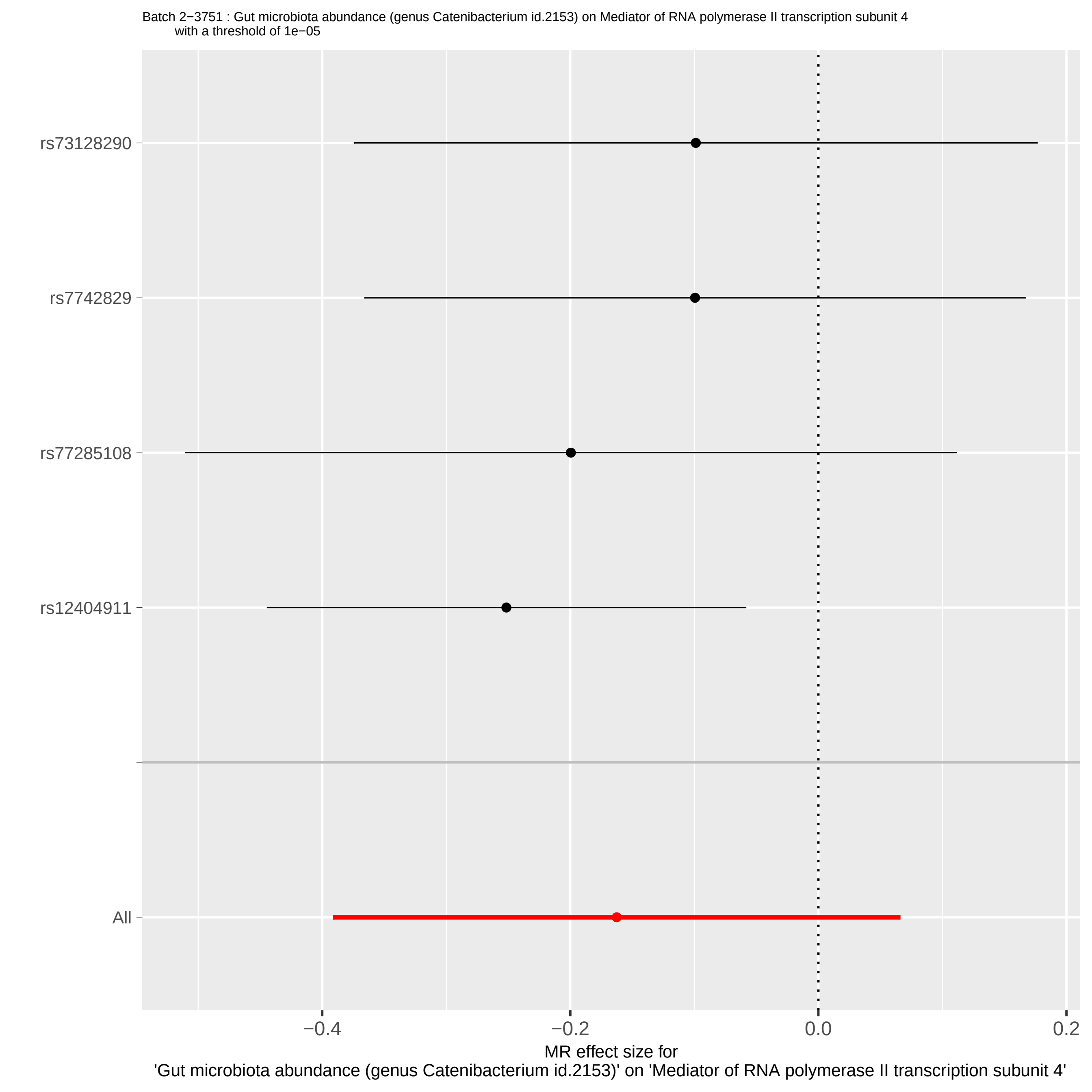
Batch 2−3751 : Gut microbiota abundance (genus Catenibacterium id.2153) on Mediator of RNA polymerase II transcription subunit 4
with a threshold of 1e−05
rs73128290
rs7742829
rs77285108
rs12404911
All
−0.4
−0.2
0.0
0.2
MR effect size for
'Gut microbiota abundance (genus Catenibacterium id.2153)' on 'Mediator of RNA polymerase II transcription subunit 4'

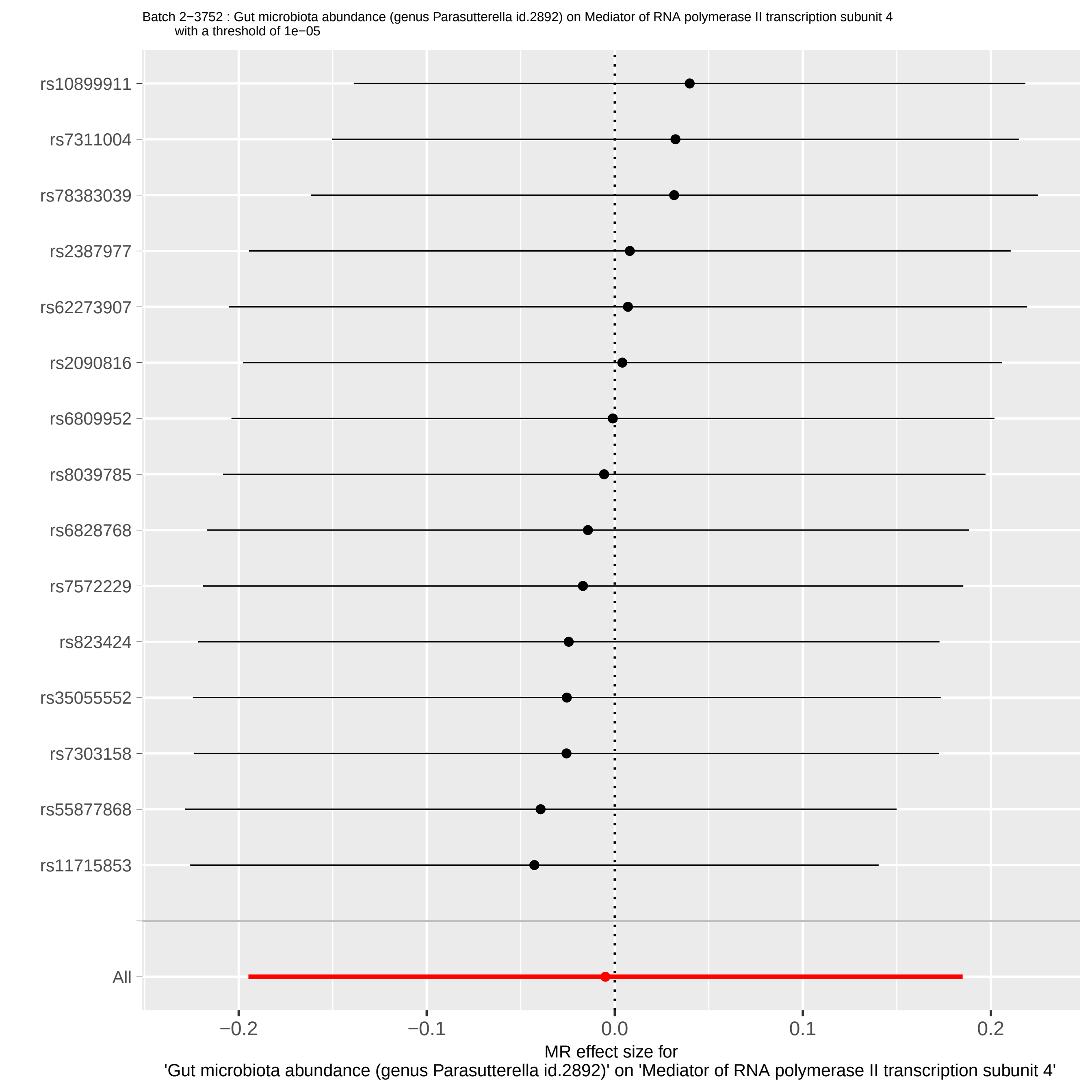
Batch 2−3752 : Gut microbiota abundance (genus Parasutterella id.2892) on Mediator of RNA polymerase II transcription subunit 4
with a threshold of 1e−05
rs10899911
rs7311004
rs78383039
rs2387977
rs62273907
rs2090816
rs6809952
rs8039785
rs6828768
rs7572229
rs823424
rs35055552
rs7303158
rs55877868
rs11715853
All
−0.2
−0.1
0.0
0.1
0.2
MR effect size for
'Gut microbiota abundance (genus Parasutterella id.2892)' on 'Mediator of RNA polymerase II transcription subunit 4'

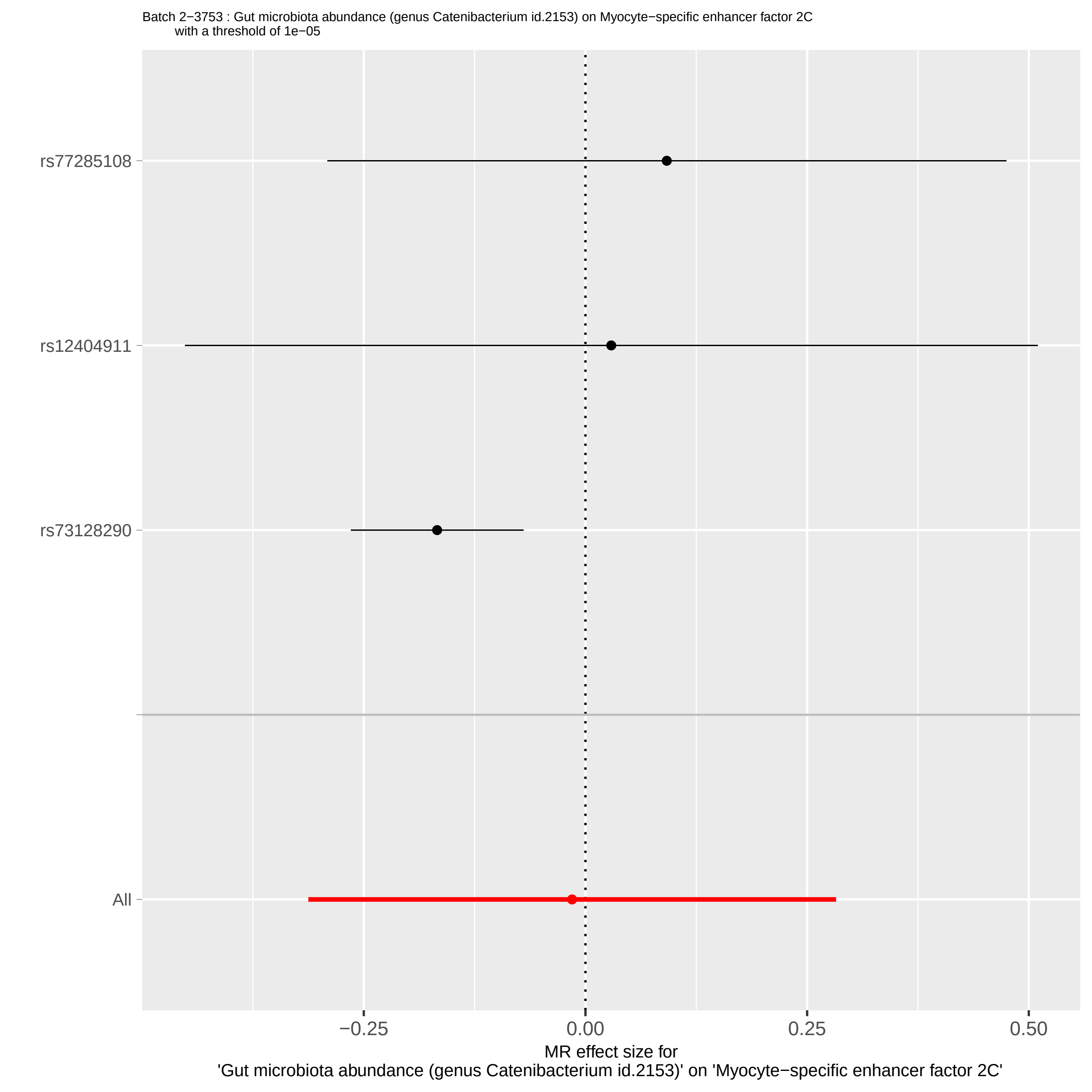

Batch 2−3753 : Gut microbiota abundance (genus Catenibacterium id.2153) on Myocyte−specific enhancer factor 2C
with a threshold of 1e−05
rs77285108
rs12404911
rs73128290
All
−0.25
0.00
0.25
0.50
MR effect size for
'Gut microbiota abundance (genus Catenibacterium id.2153)' on 'Myocyte−specific enhancer factor 2C'

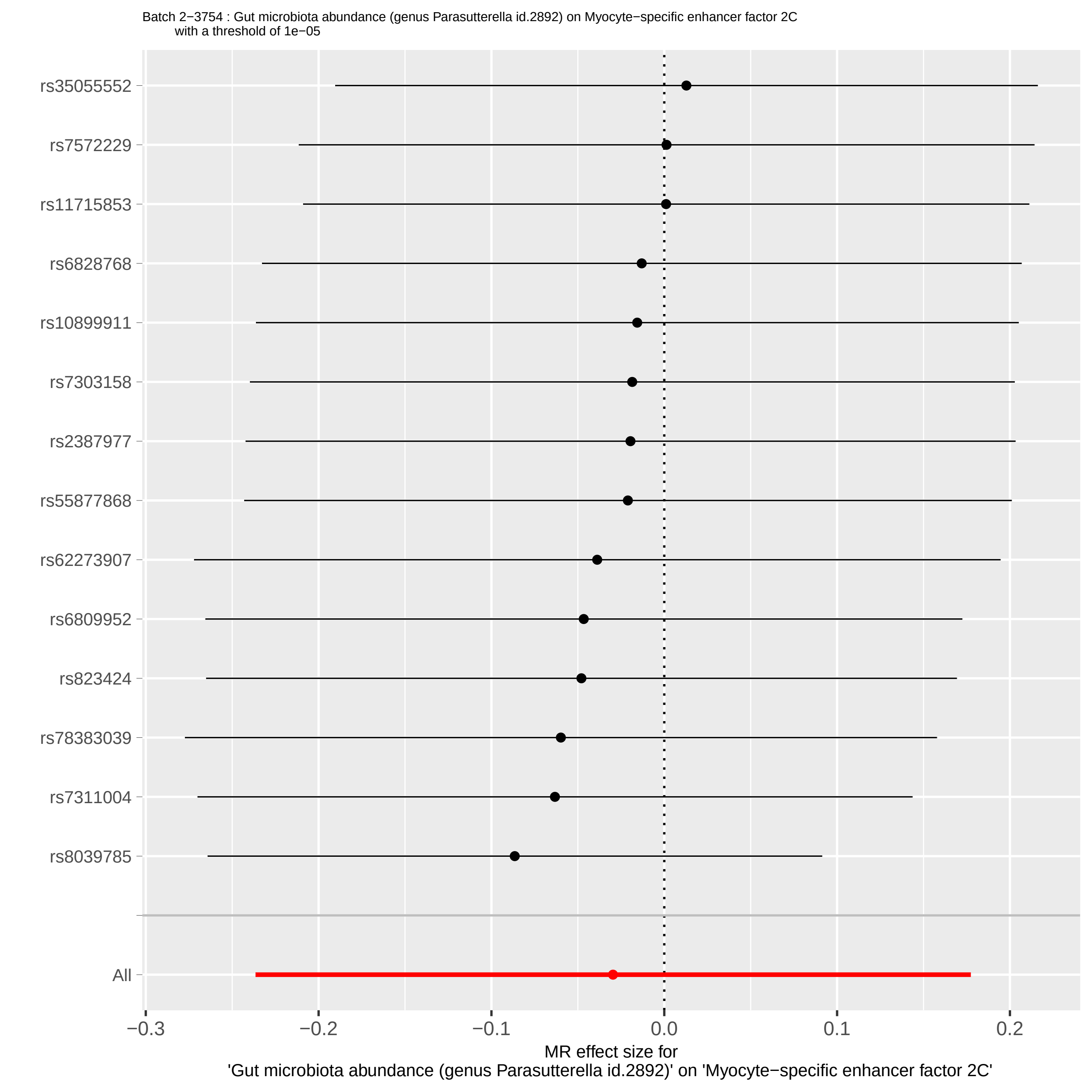
Batch 2−3754 : Gut microbiota abundance (genus Parasutterella id.2892) on Myocyte−specific enhancer factor 2C
with a threshold of 1e−05
rs35055552
rs7572229
rs11715853
rs6828768
rs10899911
rs7303158
rs2387977
rs55877868
rs62273907
rs6809952
rs823424
rs78383039
rs7311004
rs8039785
All
−0.3
−0.2
−0.1
0.0
0.1
0.2
MR effect size for
'Gut microbiota abundance (genus Parasutterella id.2892)' on 'Myocyte−specific enhancer factor 2C'

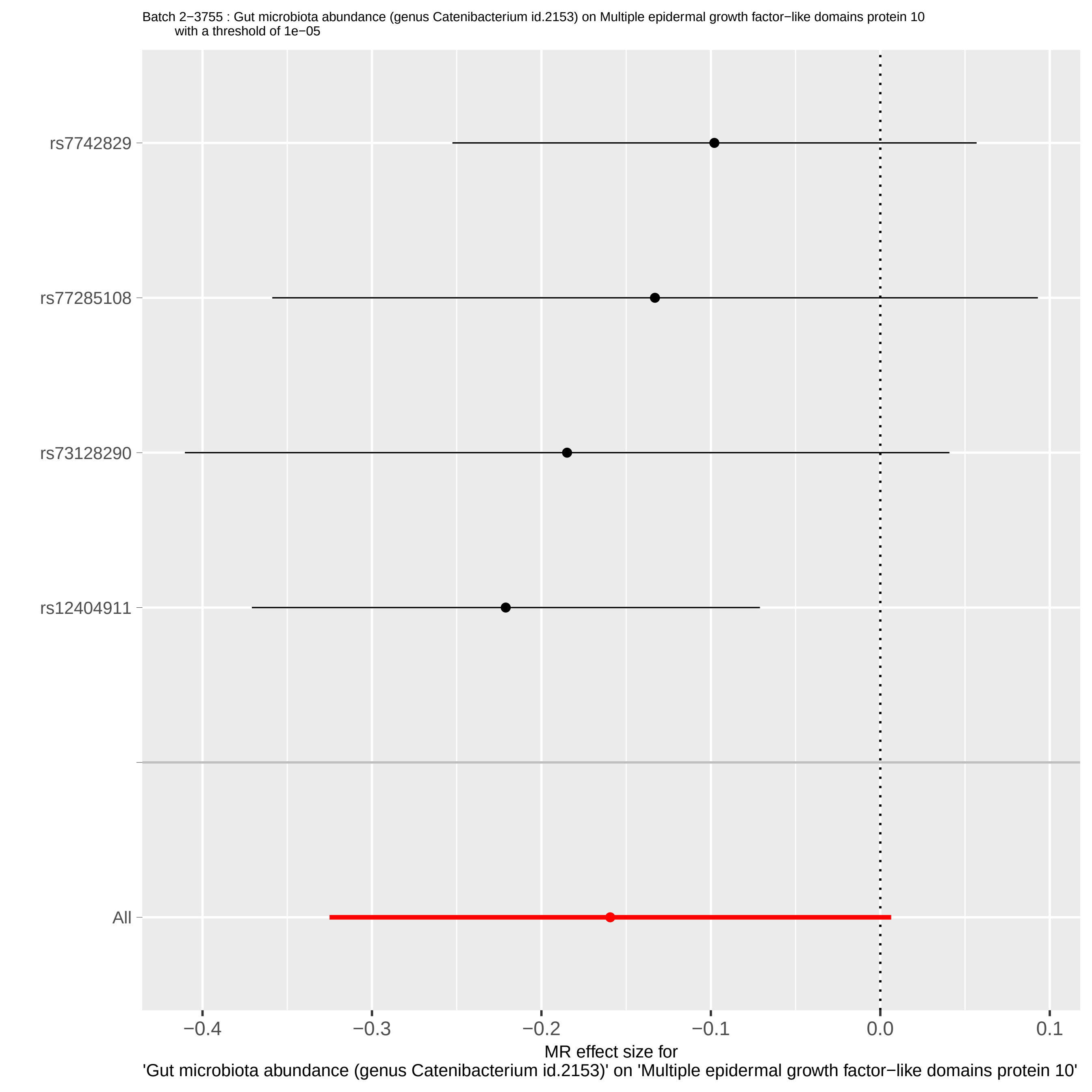
Batch 2−3755 : Gut microbiota abundance (genus Catenibacterium id.2153) on Multiple epidermal growth factor−like domains protein 10
with a threshold of 1e−05
rs7742829
rs77285108
rs73128290
rs12404911
All
−0.4
−0.3
−0.2
−0.1
0.0
0.1
MR effect size for
'Gut microbiota abundance (genus Catenibacterium id.2153)' on 'Multiple epidermal growth factor−like domains protein 10'

Batch 2−3756 : Gut microbiota abundance (genus Parasutterella id.2892) on Multiple epidermal growth factor−like domains protein 10
with a threshold of 1e−05

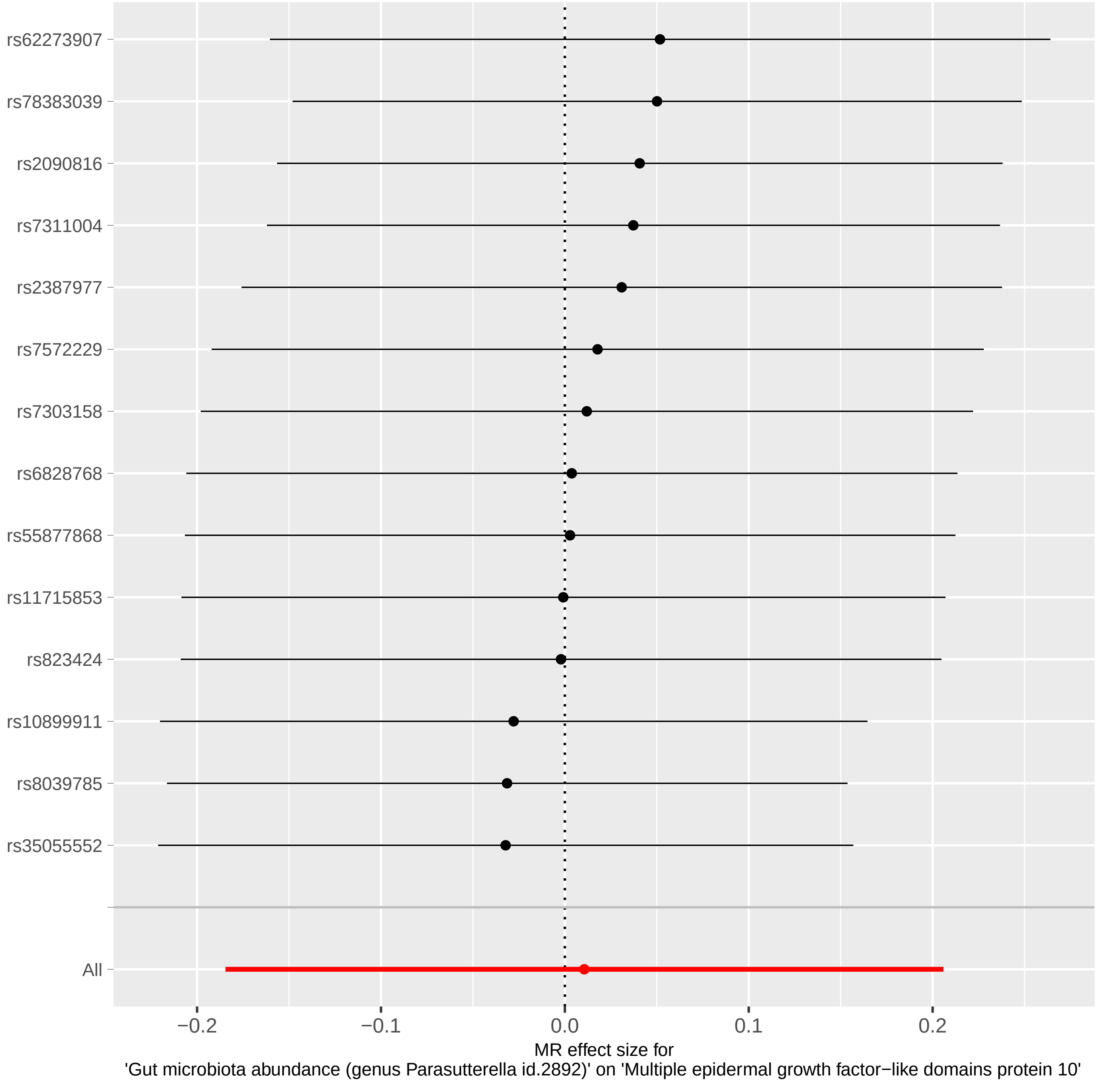

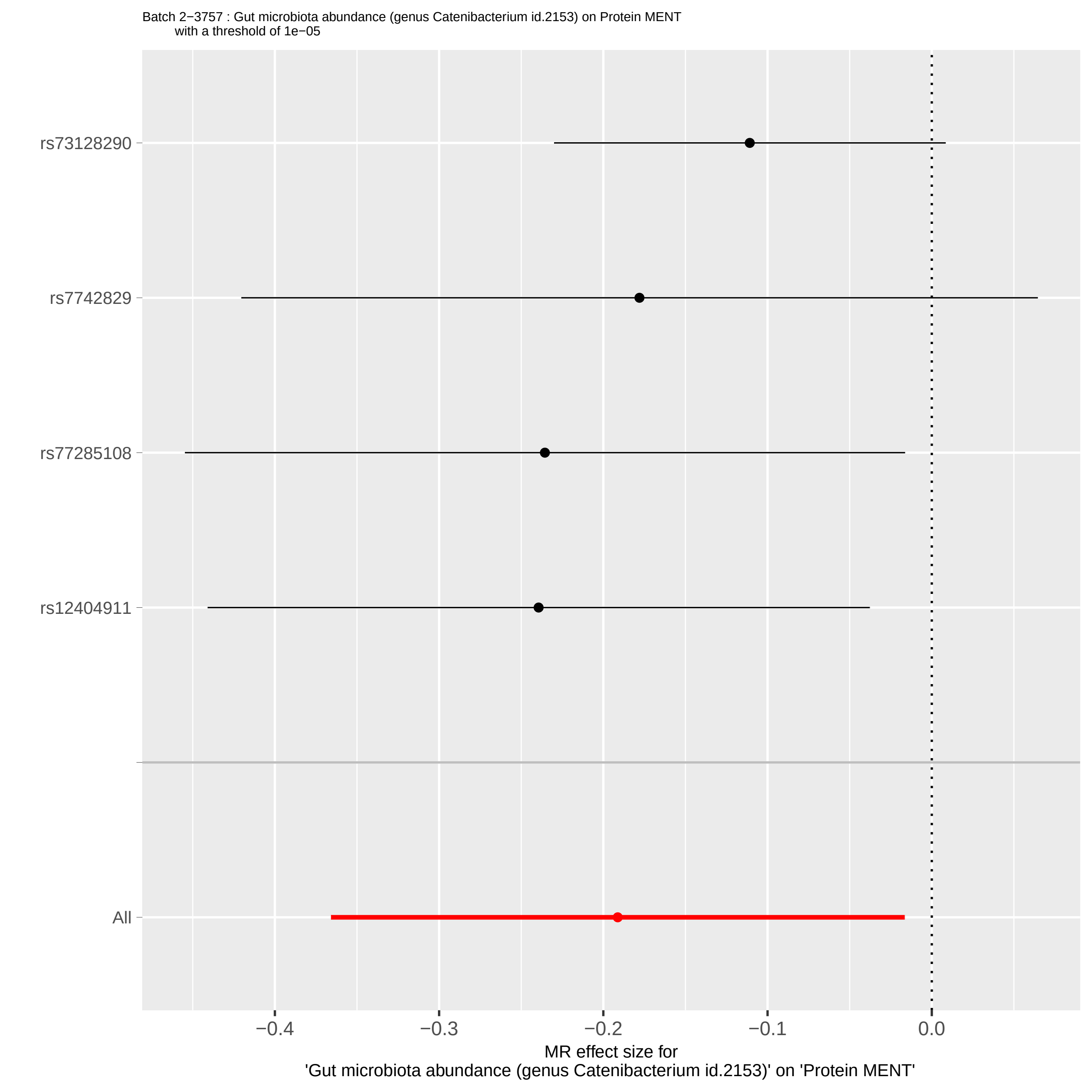
Batch 2−3757 : Gut microbiota abundance (genus Catenibacterium id.2153) on Protein MENT
with a threshold of 1e−05
rs73128290
rs7742829
rs77285108
rs12404911
All
−0.4
−0.3
−0.2
−0.1
0.0
MR effect size for
'Gut microbiota abundance (genus Catenibacterium id.2153)' on 'Protein MENT'

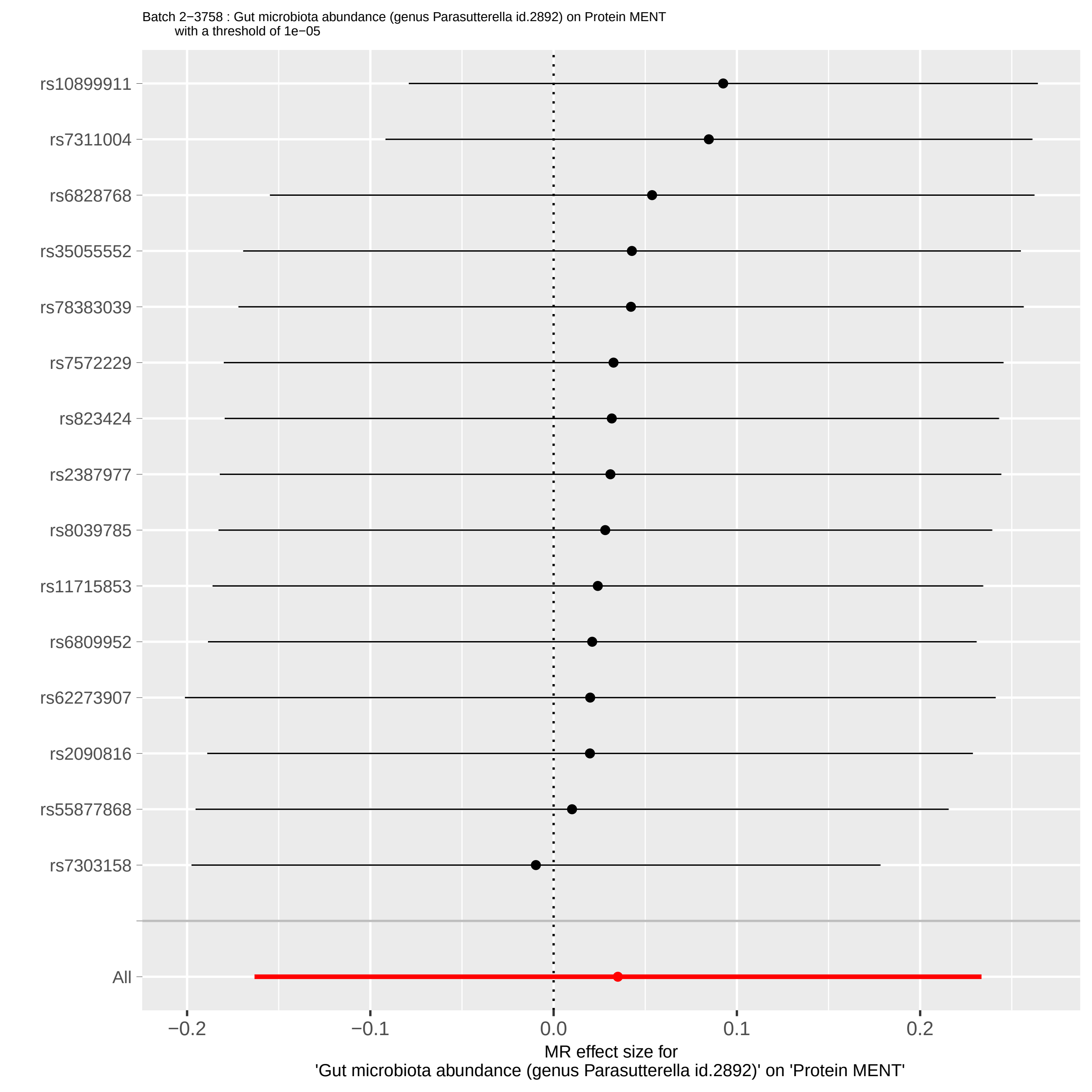
Batch 2−3758 : Gut microbiota abundance (genus Parasutterella id.2892) on Protein MENT
with a threshold of 1e−05
rs10899911
rs7311004
rs6828768
rs35055552
rs78383039
rs7572229
rs823424
rs2387977
rs8039785
rs11715853
rs6809952
rs62273907
rs2090816
rs55877868
rs7303158
All
−0.2
−0.1
0.0
0.1
0.2
MR effect size for
'Gut microbiota abundance (genus Parasutterella id.2892)' on 'Protein MENT'

Batch 2−3759 : Gut microbiota abundance (genus Catenibacterium id.2153) on Matrix extracellular phosphoglycoprotein
with a threshold of 1e−05

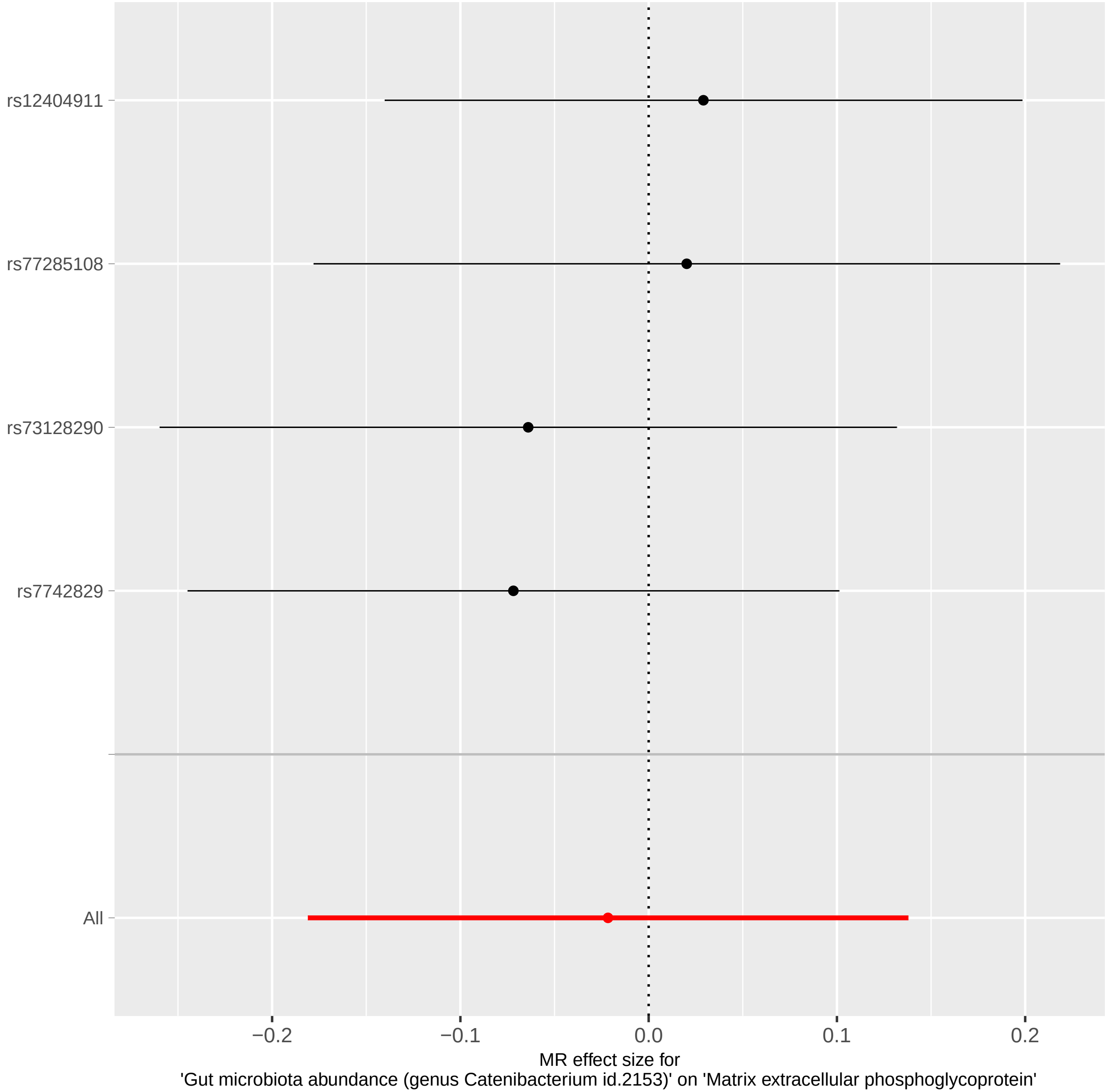

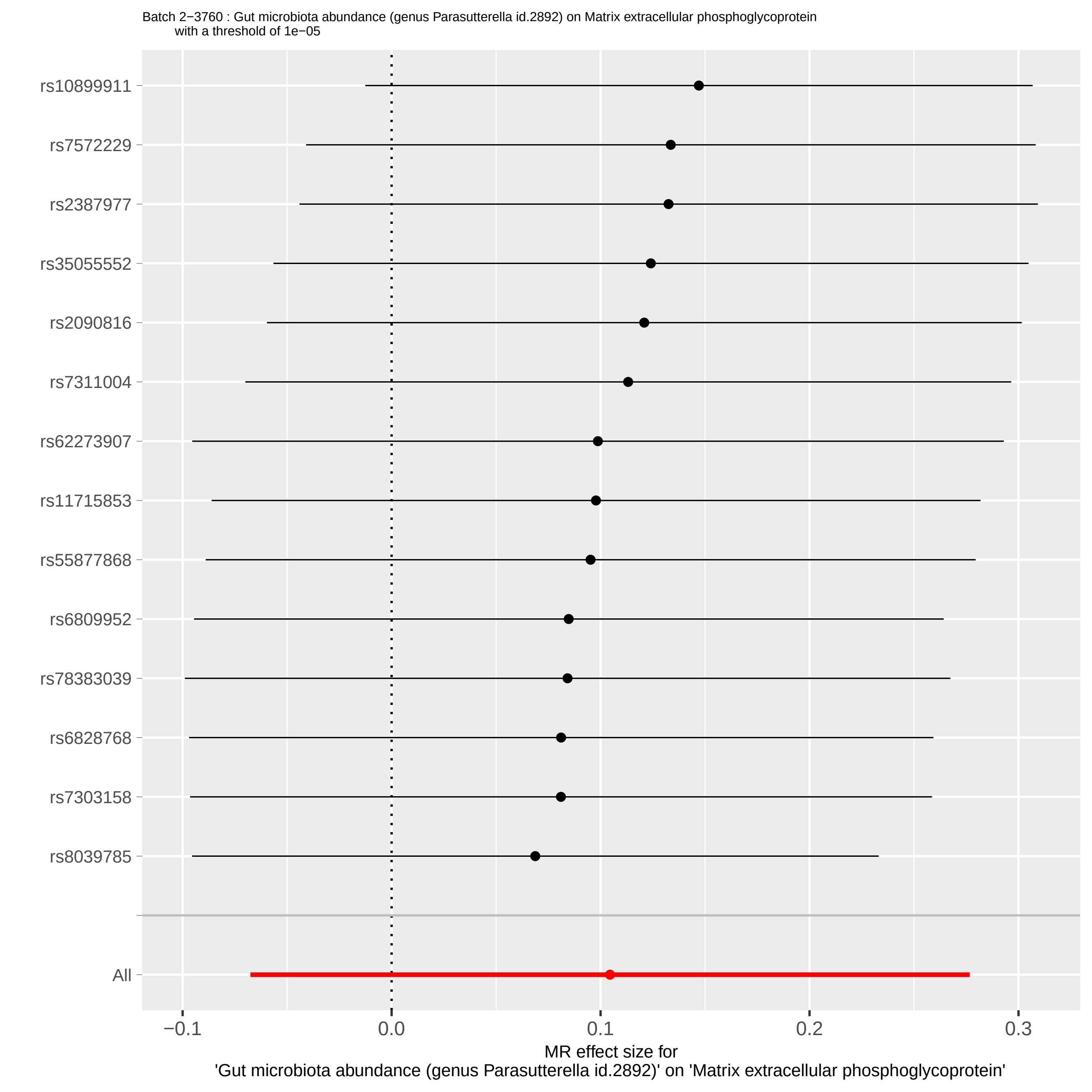

Batch 2−3760 : Gut microbiota abundance (genus Parasutterella id.2892) on Matrix extracellular phosphoglycoprotein
with a threshold of 1e−05
rs10899911
rs7572229
rs2387977
rs35055552
rs2090816
rs7311004
rs62273907
rs11715853
rs55877868
rs6809952
rs78383039
rs6828768
rs7303158
rs8039785
All
−0.1
0.0
0.1
0.2
0.3
MR effect size for
'Gut microbiota abundance (genus Parasutterella id.2892)' on 'Matrix extracellular phosphoglycoprotein'

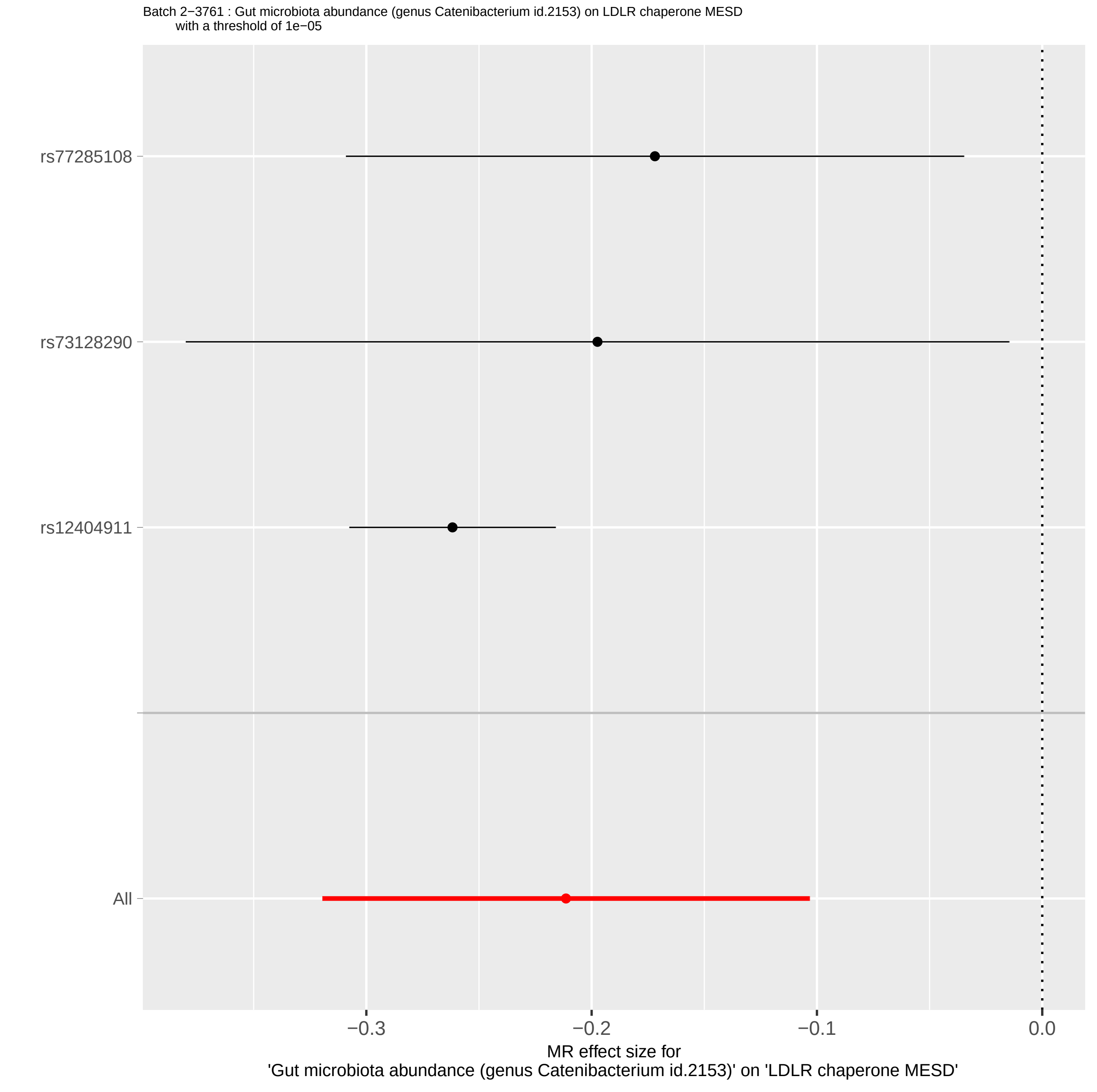

Batch 2−3761 : Gut microbiota abundance (genus Catenibacterium id.2153) on LDLR chaperone MESD
with a threshold of 1e−05
rs77285108
rs73128290
rs12404911
All
−0.3
−0.2
−0.1
0.0
MR effect size for
'Gut microbiota abundance (genus Catenibacterium id.2153)' on 'LDLR chaperone MESD'

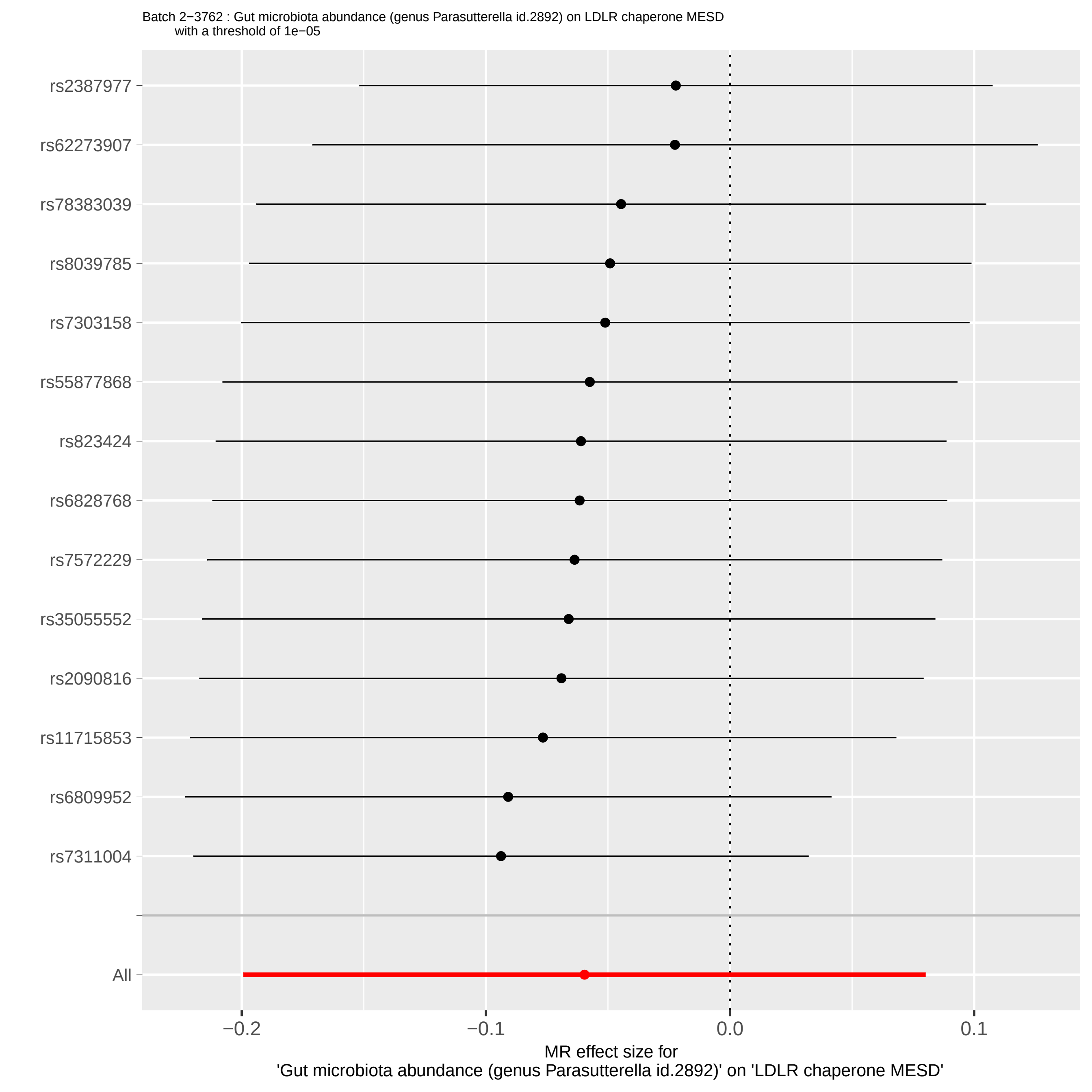
Batch 2−3762 : Gut microbiota abundance (genus Parasutterella id.2892) on LDLR chaperone MESD
with a threshold of 1e−05
rs2387977
rs62273907
rs78383039
rs8039785
rs7303158
rs55877868
rs823424
rs6828768
rs7572229
rs35055552
rs2090816
rs11715853
rs6809952
rs7311004
All
−0.2
−0.1
0.0
0.1
MR effect size for
'Gut microbiota abundance (genus Parasutterella id.2892)' on 'LDLR chaperone MESD'

Batch 2−3763 : Gut microbiota abundance (genus Catenibacterium id.2153) on Hepatocyte growth factor receptor
with a threshold of 1e−05

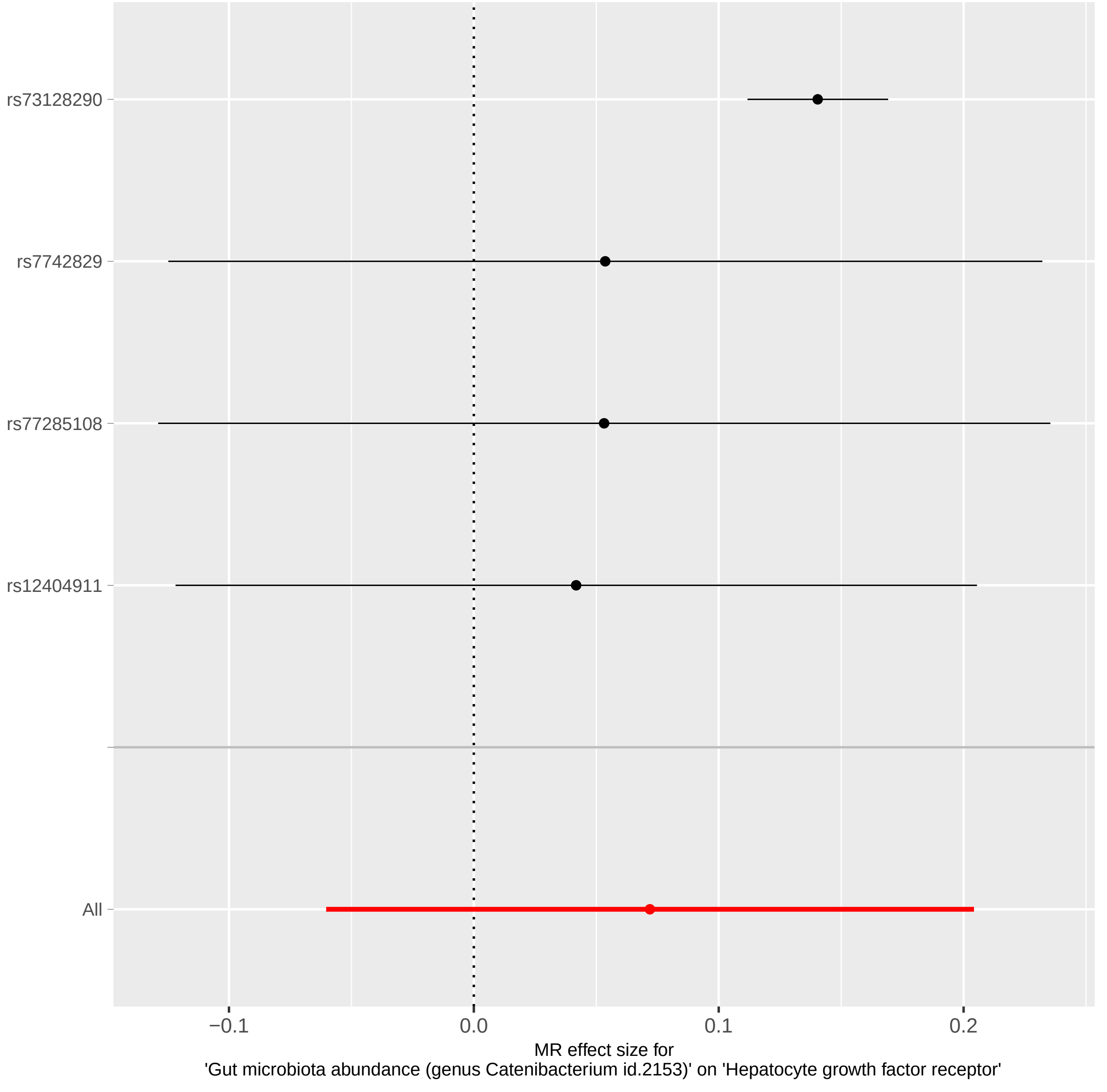

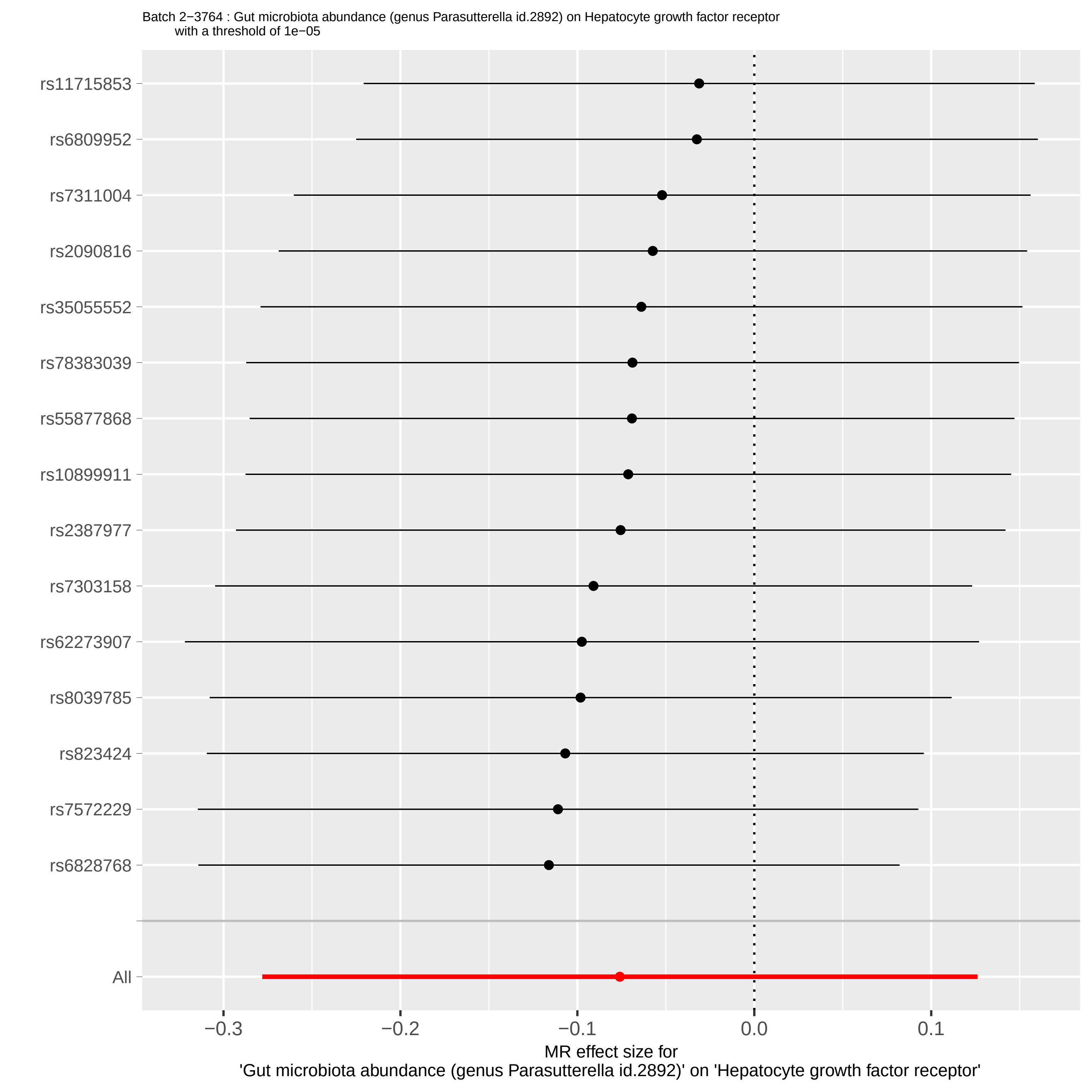

Batch 2−3764 : Gut microbiota abundance (genus Parasutterella id.2892) on Hepatocyte growth factor receptor
with a threshold of 1e−05
rs11715853
rs6809952
rs7311004
rs2090816
rs35055552
rs78383039
rs55877868
rs10899911
rs2387977
rs7303158
rs62273907
rs8039785
rs823424
rs7572229
rs6828768
All
−0.3
−0.2
−0.1
0.0
0.1
MR effect size for
'Gut microbiota abundance (genus Parasutterella id.2892)' on 'Hepatocyte growth factor receptor'

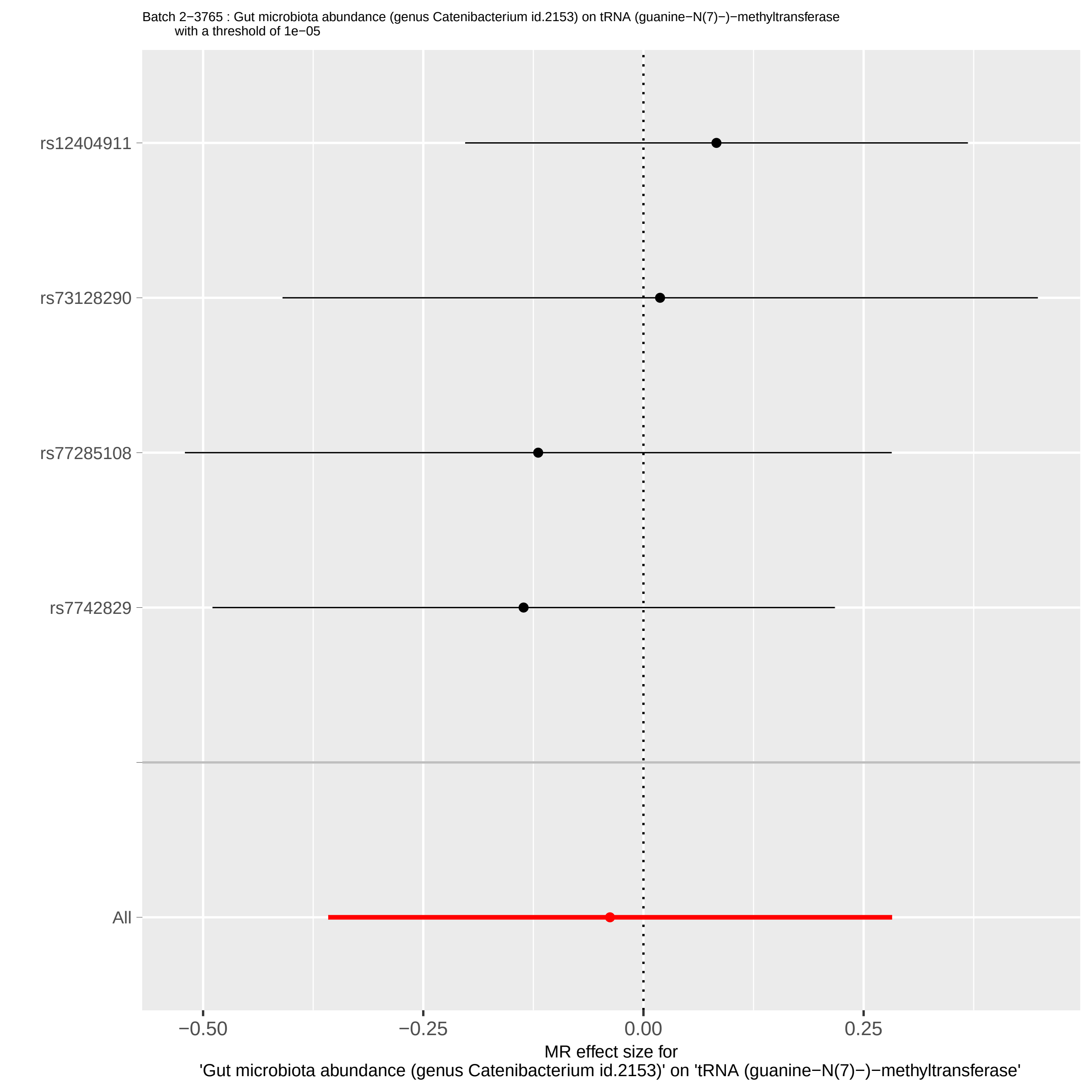

Batch 2−3765 : Gut microbiota abundance (genus Catenibacterium id.2153) on tRNA (guanine−N(7)−)−methyltransferase
with a threshold of 1e−05
rs12404911
rs73128290
rs77285108
rs7742829
All
−0.50
−0.25
0.00
0.25
MR effect size for
'Gut microbiota abundance (genus Catenibacterium id.2153)' on 'tRNA (guanine−N(7)−)−methyltransferase'

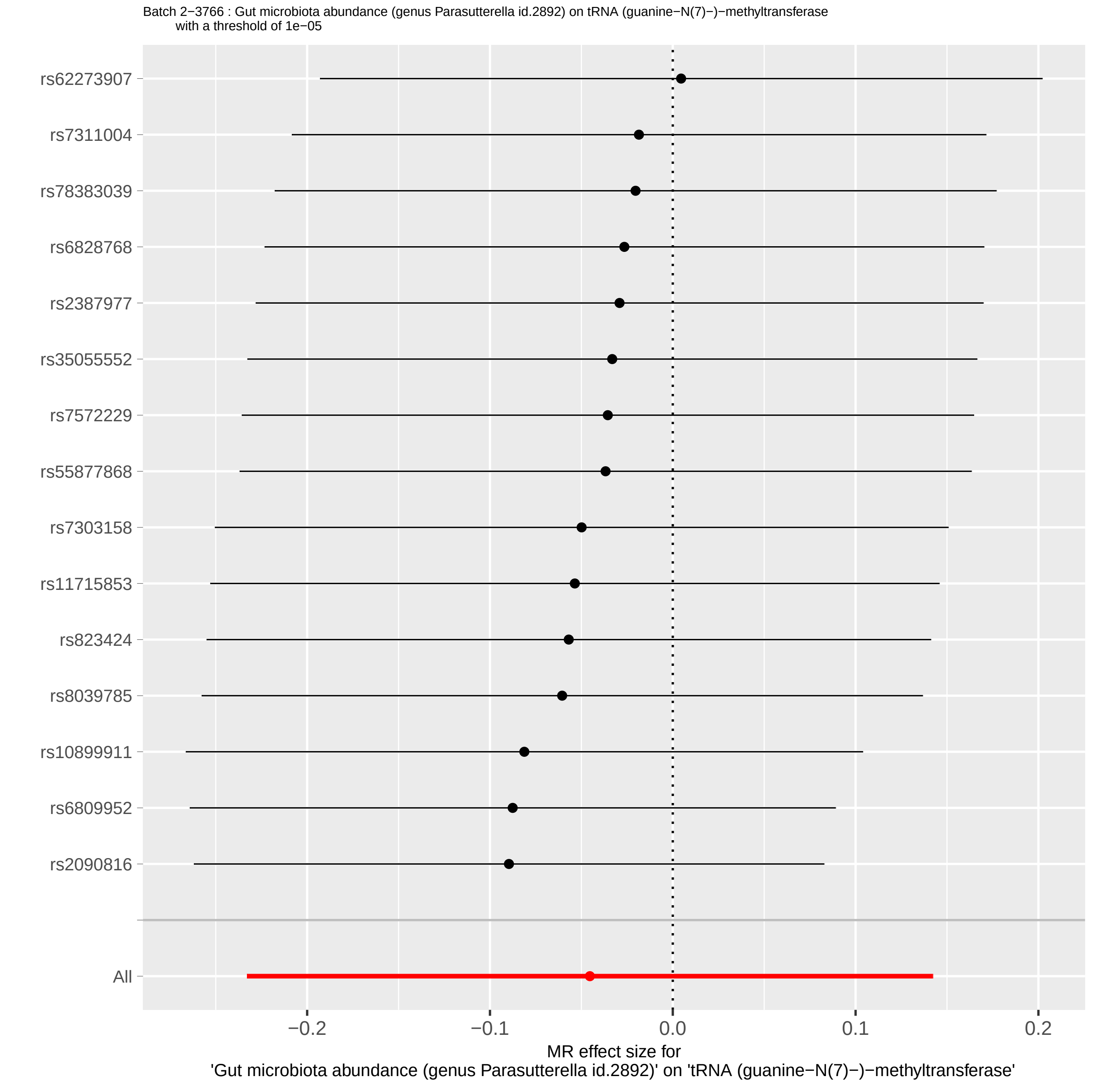

Batch 2−3766 : Gut microbiota abundance (genus Parasutterella id.2892) on tRNA (guanine−N(7)−)−methyltransferase
with a threshold of 1e−05
rs62273907
rs7311004
rs78383039
rs6828768
rs2387977
rs35055552
rs7572229
rs55877868
rs7303158
rs11715853
rs823424
rs8039785
rs10899911
rs6809952
rs2090816
All
−0.2
−0.1
0.0
0.1
0.2
MR effect size for
'Gut microbiota abundance (genus Parasutterella id.2892)' on 'tRNA (guanine−N(7)−)−methyltransferase'

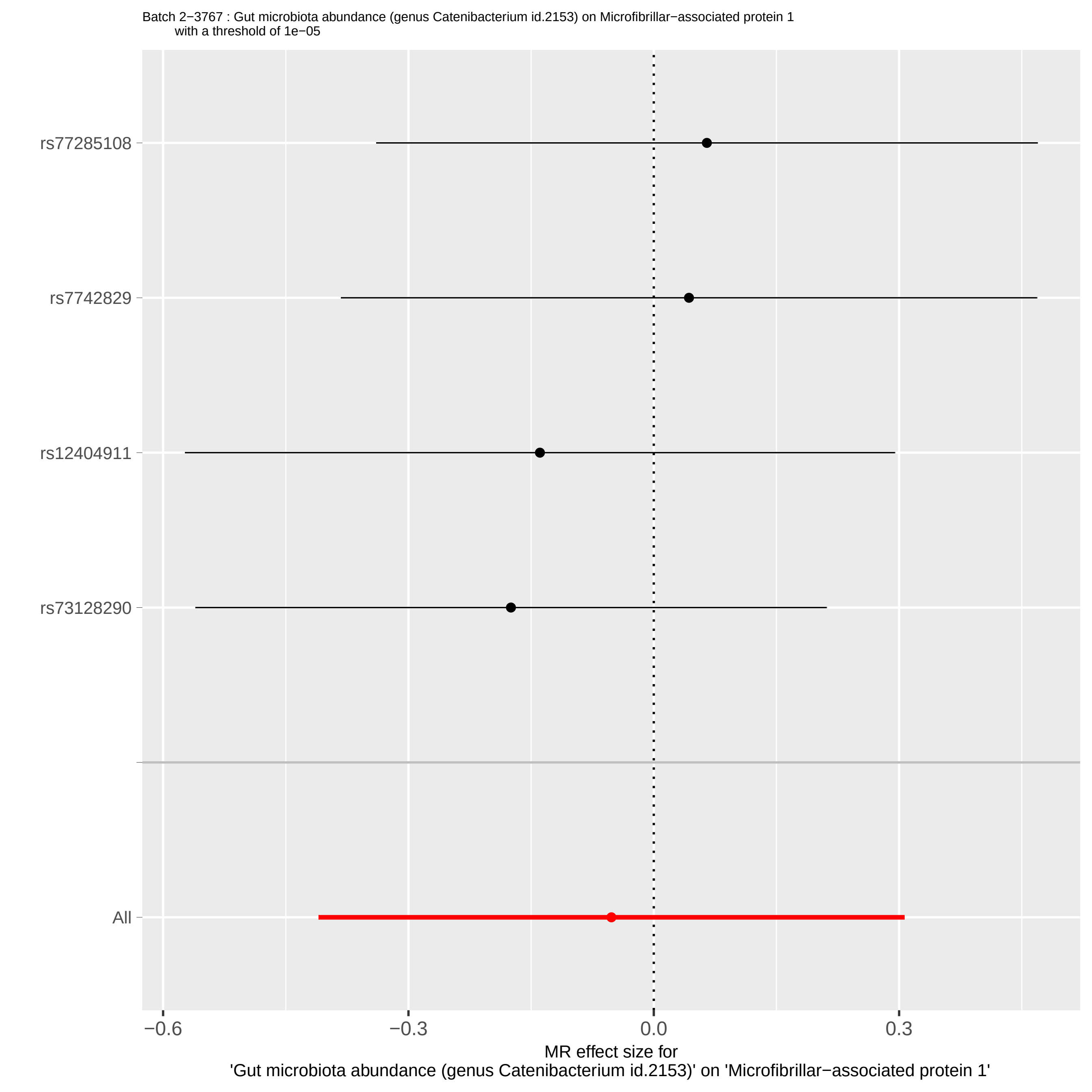
Batch 2−3767 : Gut microbiota abundance (genus Catenibacterium id.2153) on Microfibrillar−associated protein 1
with a threshold of 1e−05
rs77285108
rs7742829
rs12404911
rs73128290
All
−0.6
−0.3
0.0
0.3
MR effect size for
'Gut microbiota abundance (genus Catenibacterium id.2153)' on 'Microfibrillar−associated protein 1'

Batch 2−3768 : Gut microbiota abundance (genus Parasutterella id.2892) on Microfibrillar−associated protein 1
with a threshold of 1e−05

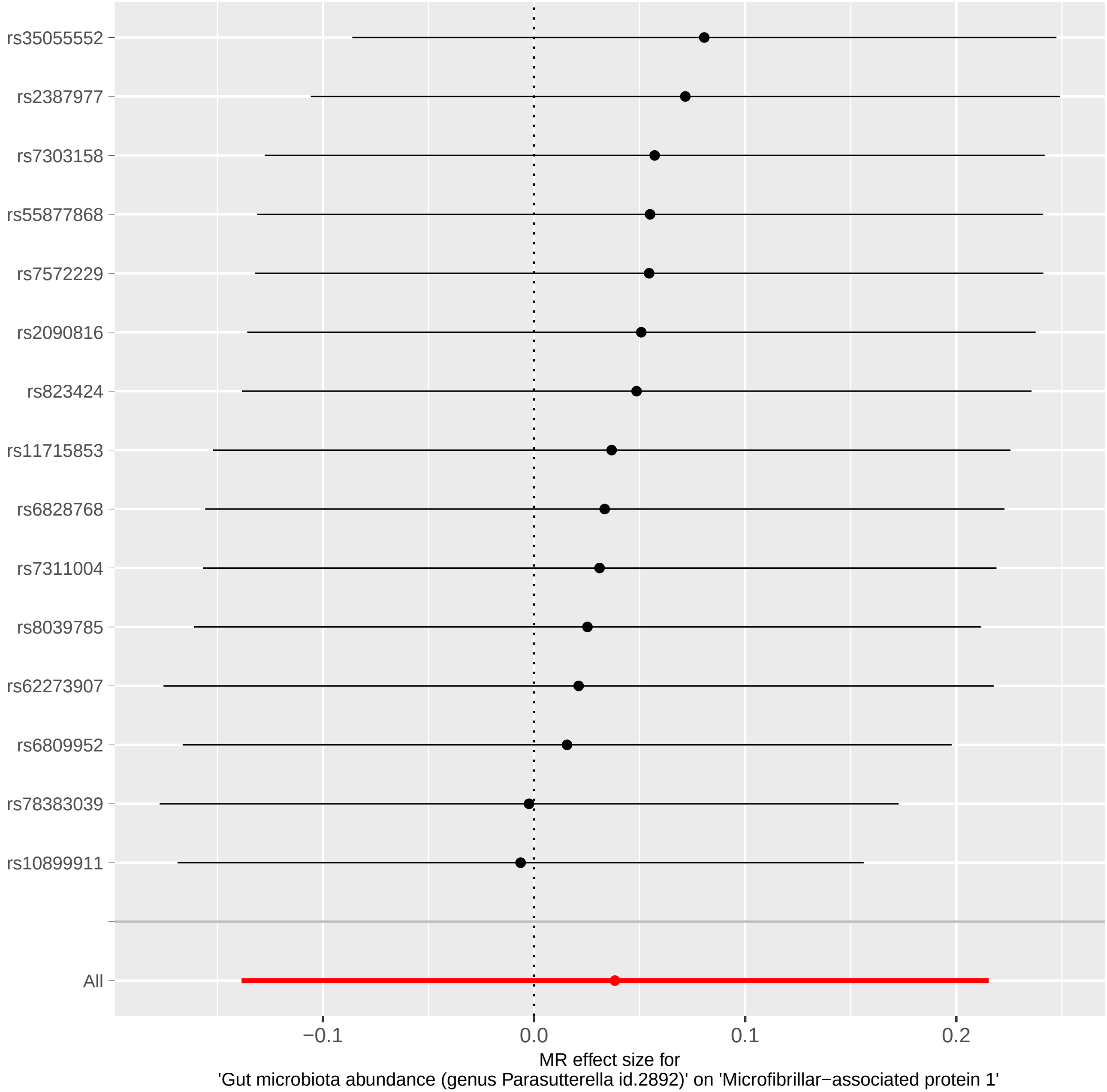

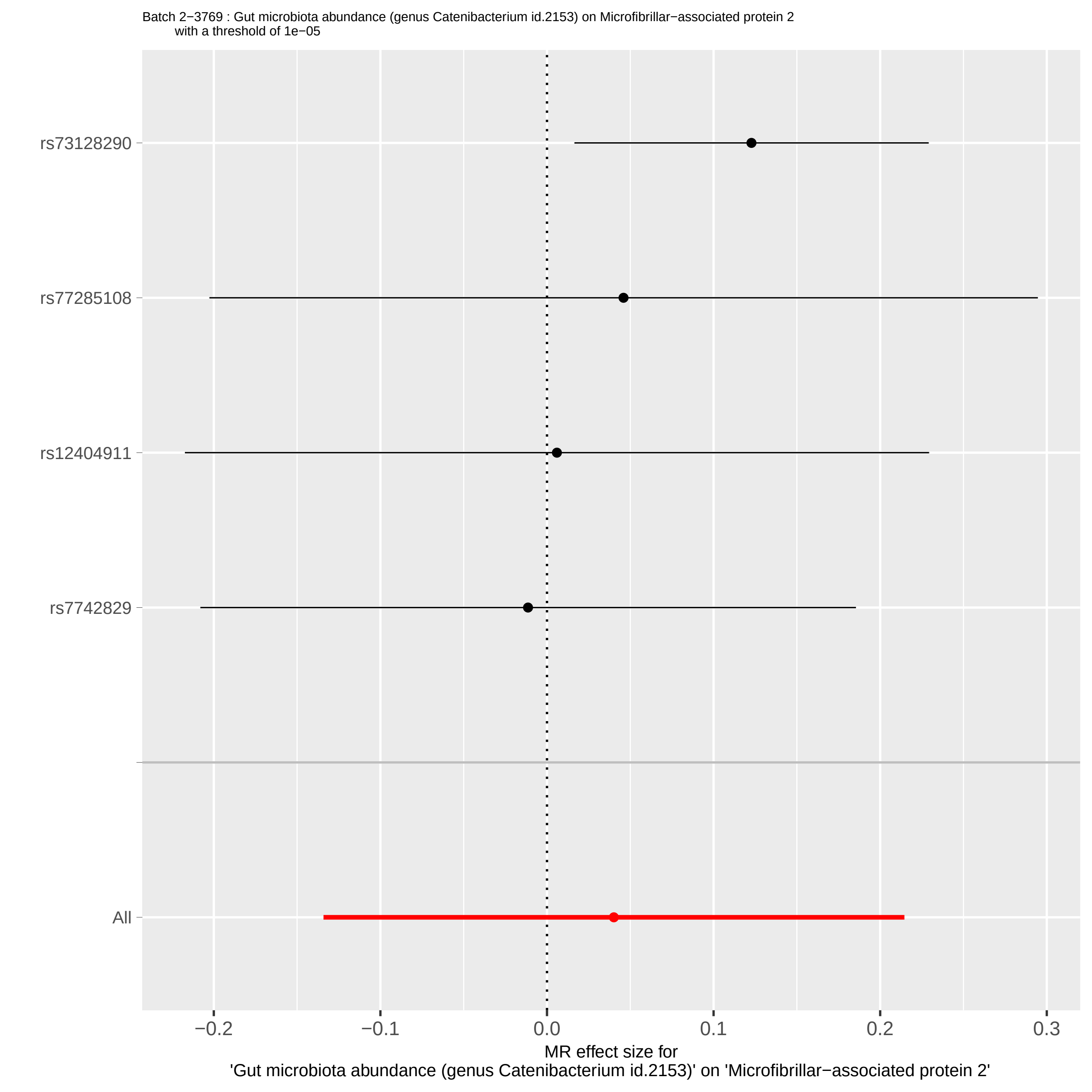

Batch 2−3769 : Gut microbiota abundance (genus Catenibacterium id.2153) on Microfibrillar−associated protein 2
with a threshold of 1e−05
rs73128290
rs77285108
rs12404911
rs7742829
All
−0.2
−0.1
0.0
0.1
0.2
0.3
MR effect size for
'Gut microbiota abundance (genus Catenibacterium id.2153)' on 'Microfibrillar−associated protein 2'

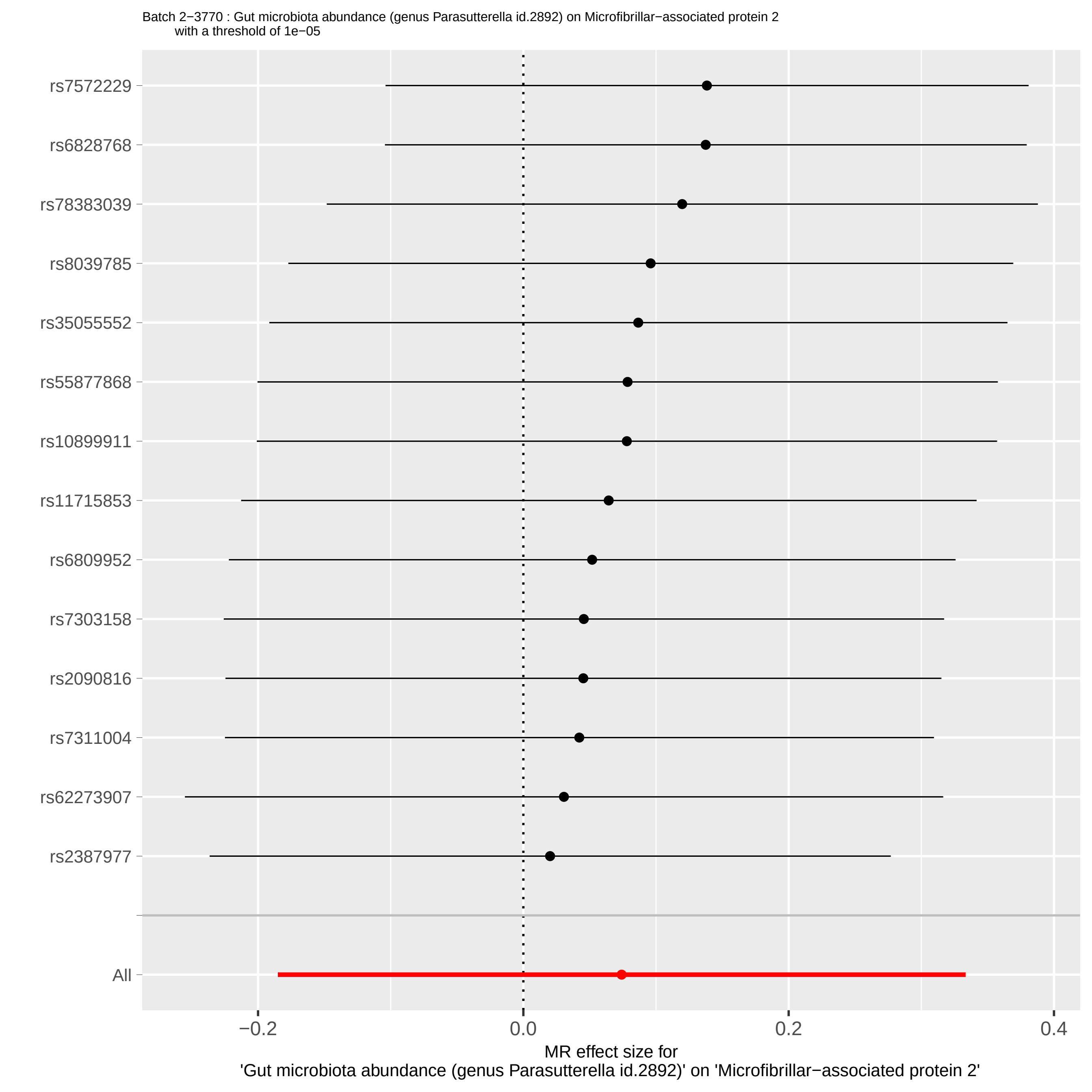
Batch 2−3770 : Gut microbiota abundance (genus Parasutterella id.2892) on Microfibrillar−associated protein 2
with a threshold of 1e−05
rs7572229
rs6828768
rs78383039
rs8039785
rs35055552
rs55877868
rs10899911
rs11715853
rs6809952
rs7303158
rs2090816
rs7311004
rs62273907
rs2387977
All
−0.2
0.0
0.2
0.4
MR effect size for
'Gut microbiota abundance (genus Parasutterella id.2892)' on 'Microfibrillar−associated protein 2'

Batch 2−3771 : Gut microbiota abundance (genus Catenibacterium id.2153) on Microfibril−associated glycoprotein 4
with a threshold of 1e−05

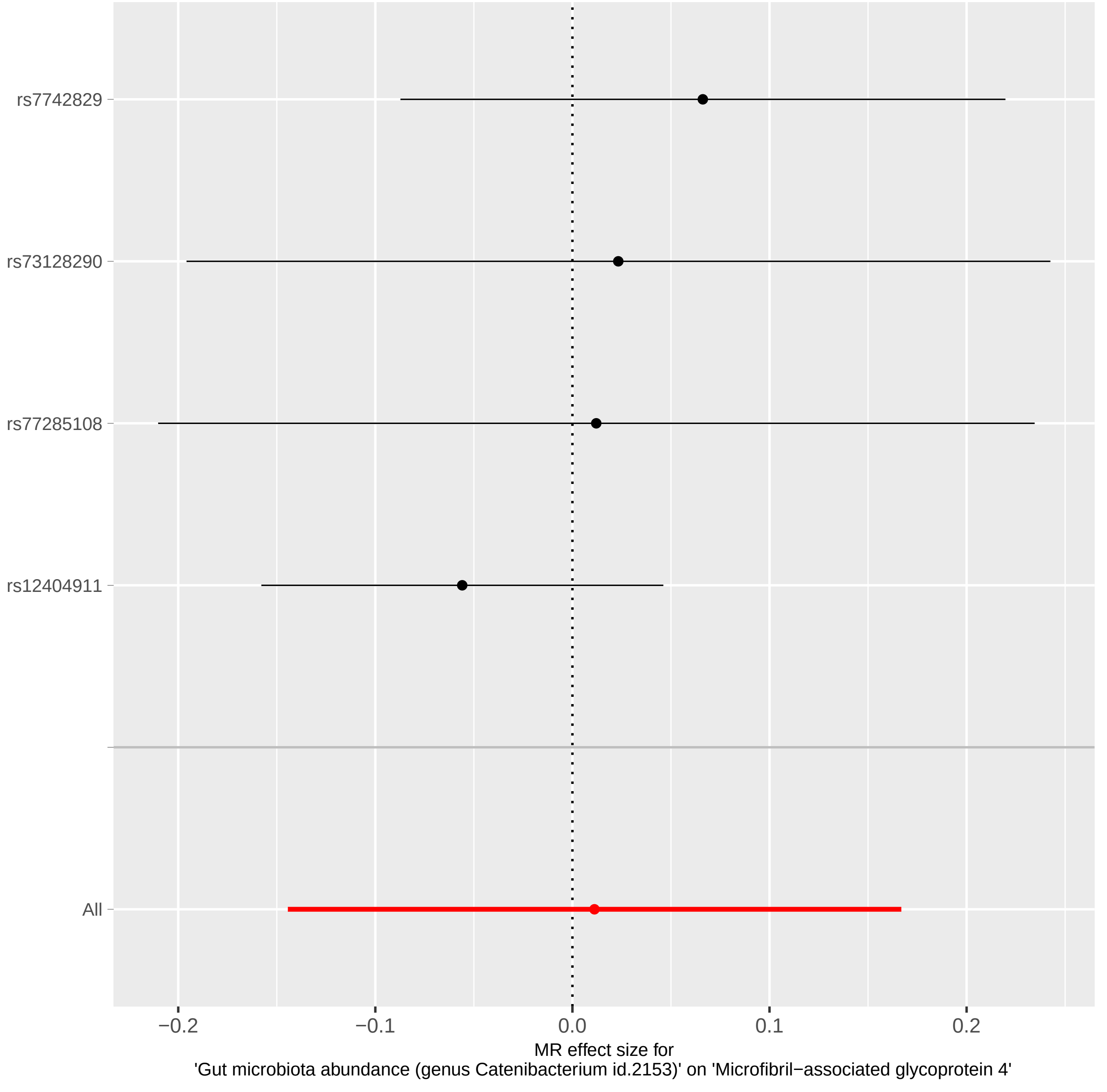

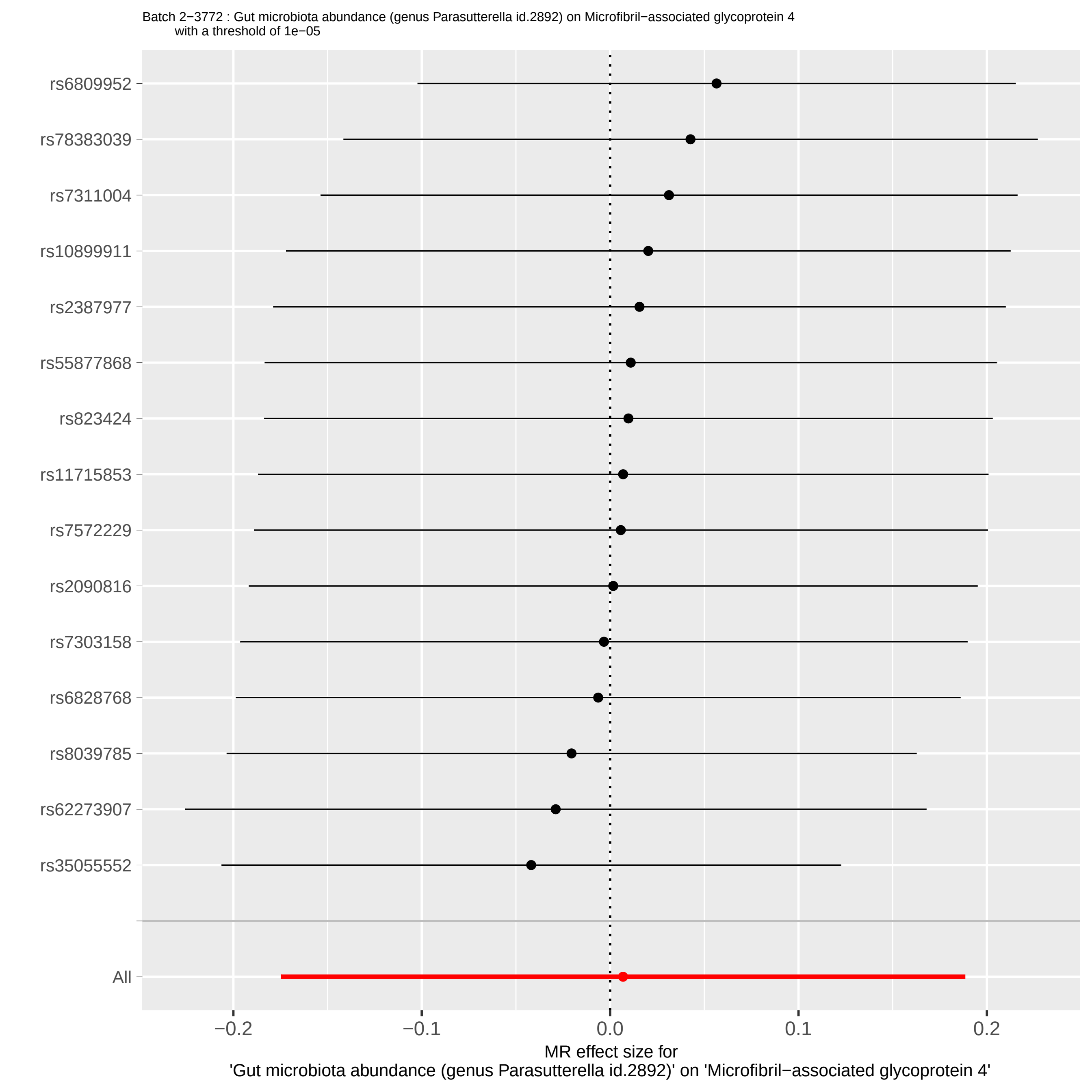
Batch 2−3772 : Gut microbiota abundance (genus Parasutterella id.2892) on Microfibril−associated glycoprotein 4
with a threshold of 1e−05
rs6809952
rs78383039
rs7311004
rs10899911
rs2387977
rs55877868
rs823424
rs11715853
rs7572229
rs2090816
rs7303158
rs6828768
rs8039785
rs62273907
rs35055552
All
−0.2
−0.1
0.0
0.1
0.2
MR effect size for
'Gut microbiota abundance (genus Parasutterella id.2892)' on 'Microfibril−associated glycoprotein 4'

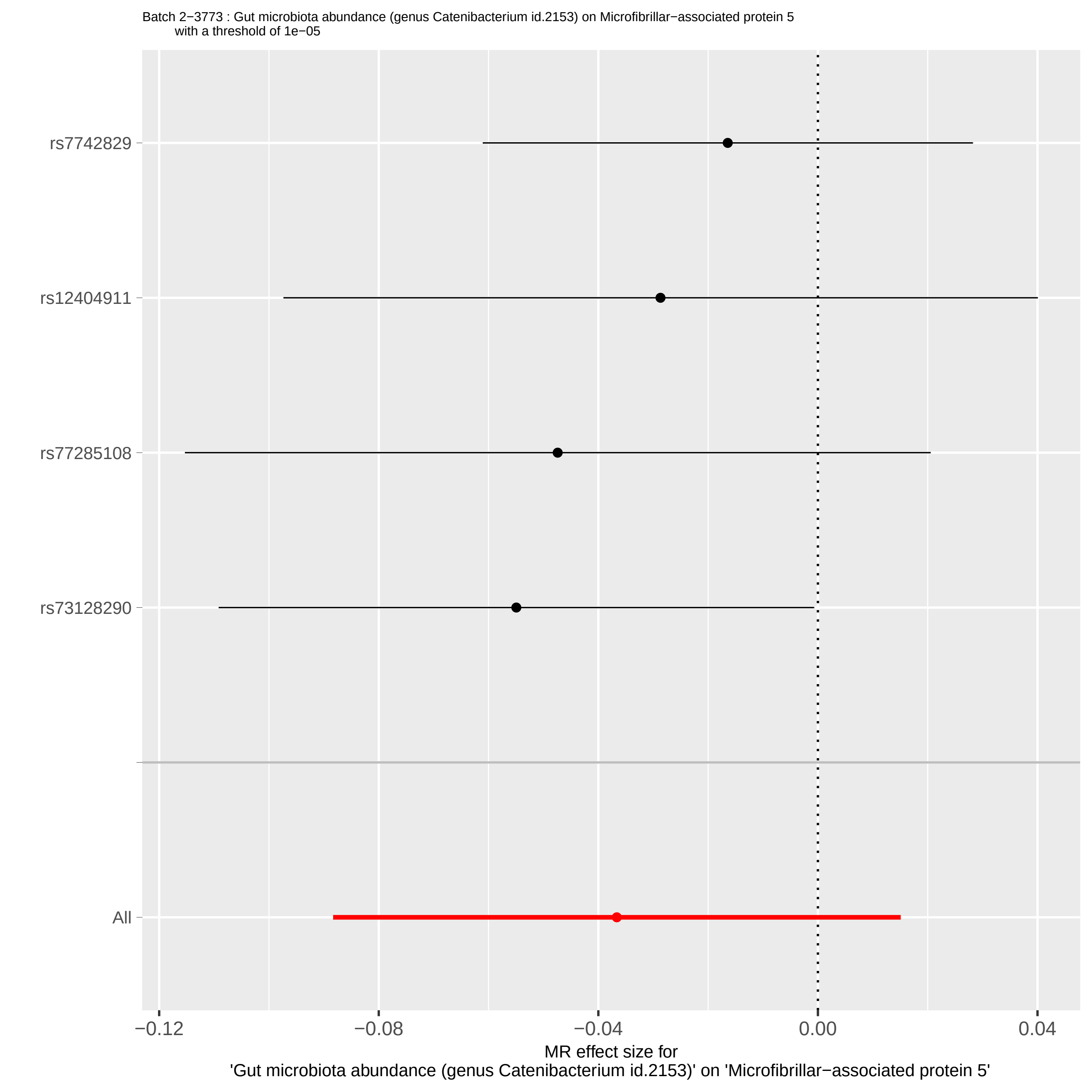

Batch 2−3773 : Gut microbiota abundance (genus Catenibacterium id.2153) on Microfibrillar−associated protein 5
with a threshold of 1e−05
rs7742829
rs12404911
rs77285108
rs73128290
All
−0.12
−0.08
−0.04
0.00
0.04
MR effect size for
'Gut microbiota abundance (genus Catenibacterium id.2153)' on 'Microfibrillar−associated protein 5'

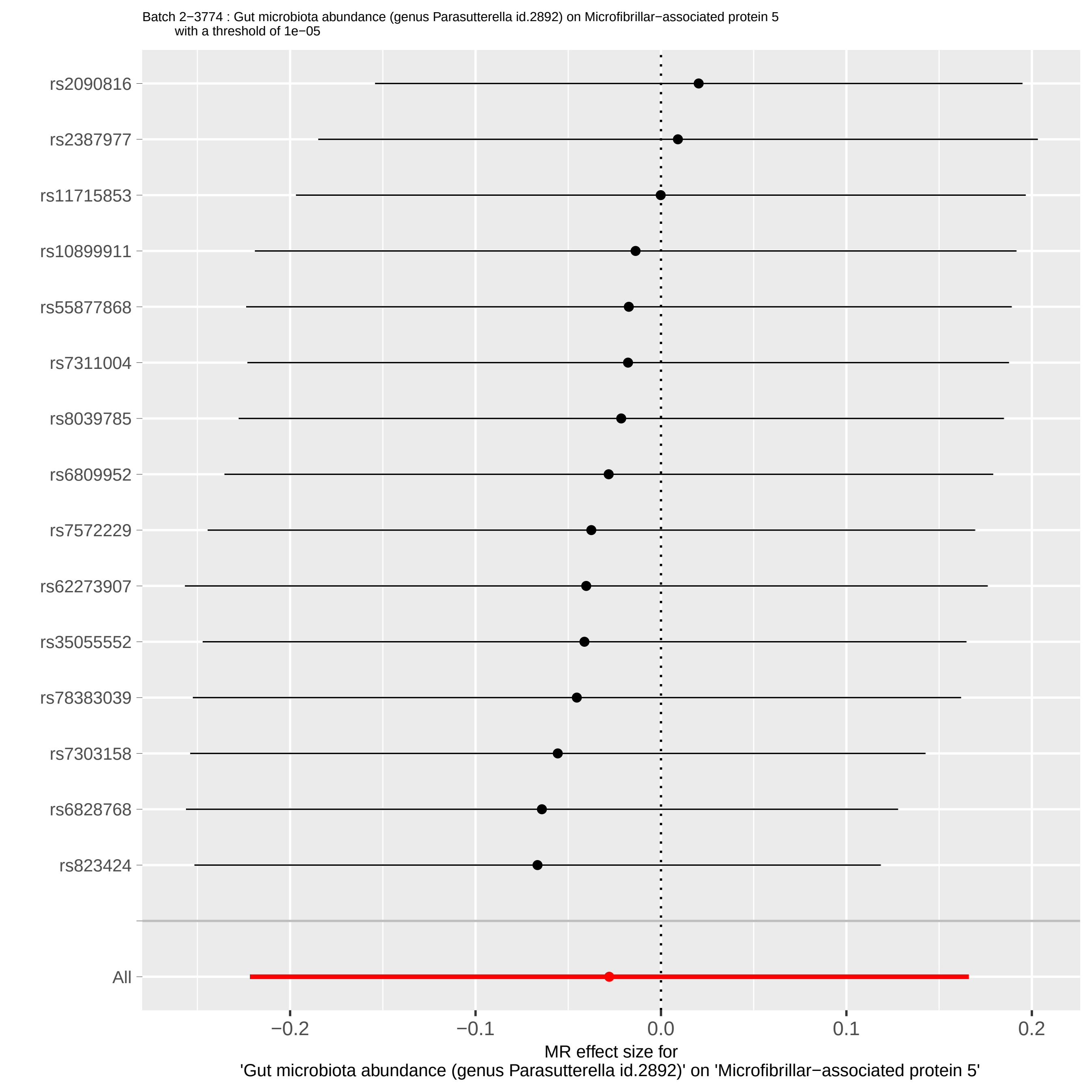
Batch 2−3774 : Gut microbiota abundance (genus Parasutterella id.2892) on Microfibrillar−associated protein 5
with a threshold of 1e−05
rs2090816
rs2387977
rs11715853
rs10899911
rs55877868
rs7311004
rs8039785
rs6809952
rs7572229
rs62273907
rs35055552
rs78383039
rs7303158
rs6828768
rs823424
All
−0.2
−0.1
0.0
0.1
0.2
MR effect size for
'Gut microbiota abundance (genus Parasutterella id.2892)' on 'Microfibrillar−associated protein 5'

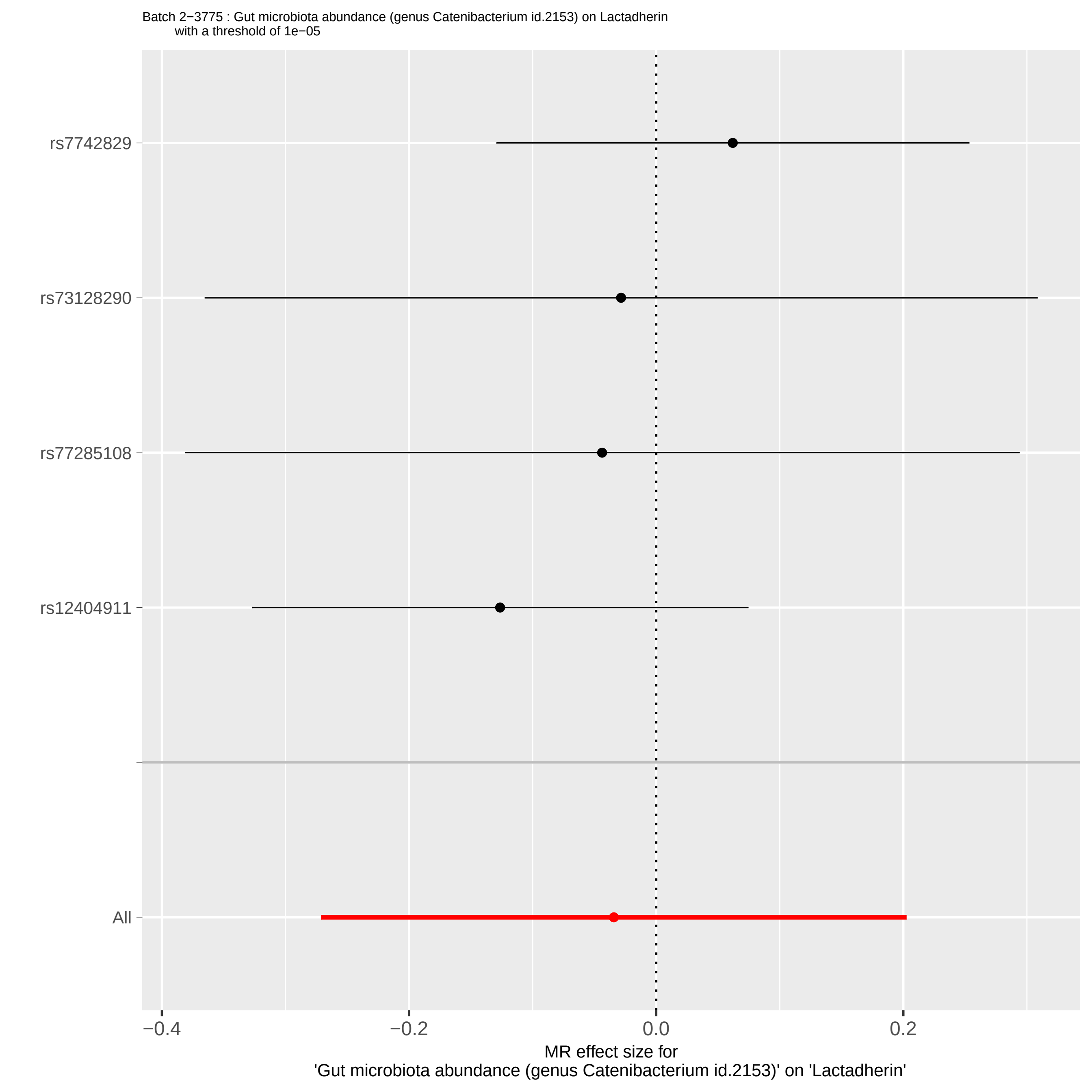

Batch 2−3775 : Gut microbiota abundance (genus Catenibacterium id.2153) on Lactadherin
with a threshold of 1e−05
rs7742829
rs73128290
rs77285108
rs12404911
All
−0.4
−0.2
0.0
0.2
MR effect size for
'Gut microbiota abundance (genus Catenibacterium id.2153)' on 'Lactadherin'

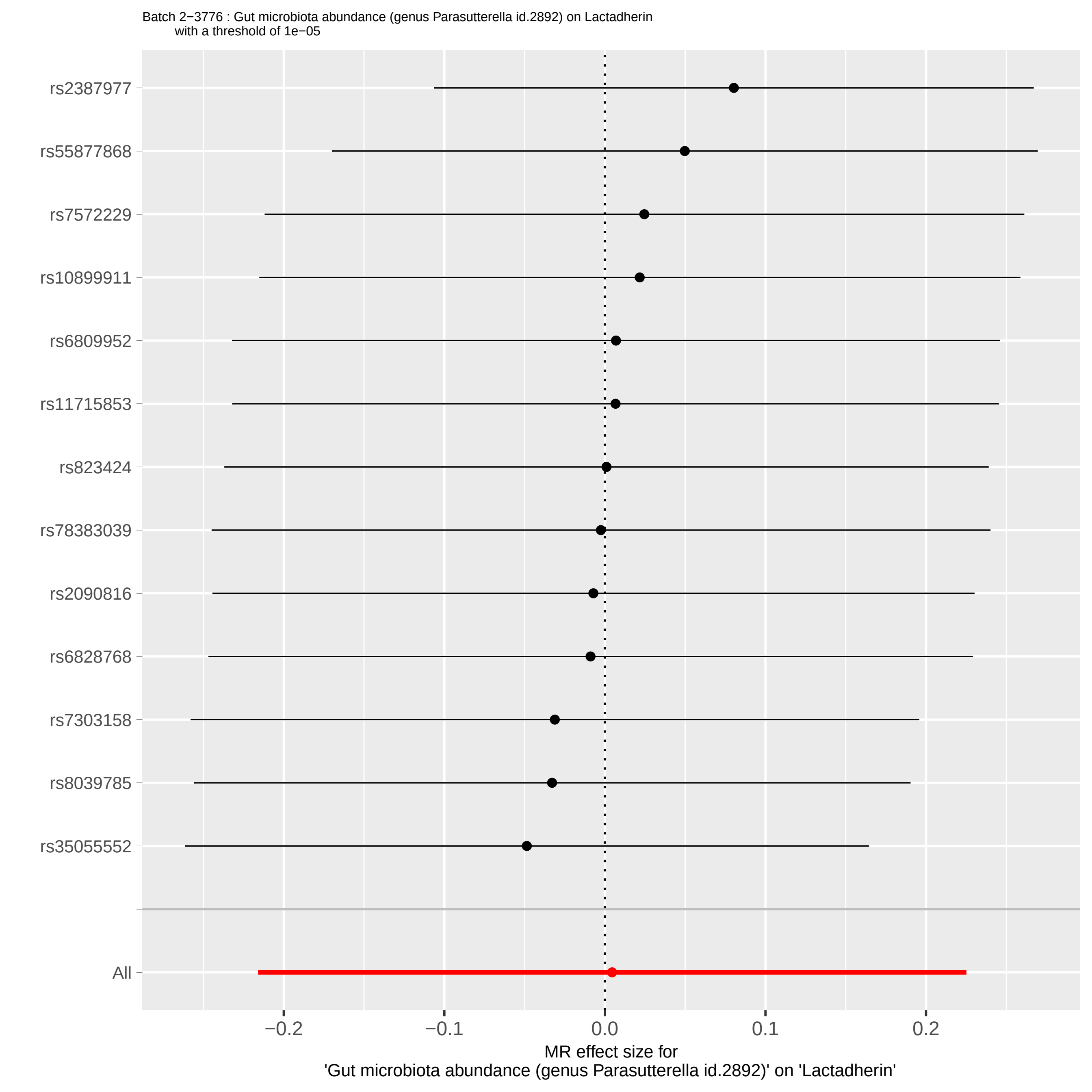

Batch 2−3776 : Gut microbiota abundance (genus Parasutterella id.2892) on Lactadherin
with a threshold of 1e−05
rs2387977
rs55877868
rs7572229
rs10899911
rs6809952
rs11715853
rs823424
rs78383039
rs2090816
rs6828768
rs7303158
rs8039785
rs35055552
All
−0.2
−0.1
0.0
0.1
0.2
MR effect size for
'Gut microbiota abundance (genus Parasutterella id.2892)' on 'Lactadherin'

Batch 2−3777 : Gut microbiota abundance (genus Catenibacterium id.2153) on Mitofusin−1
with a threshold of 1e−05

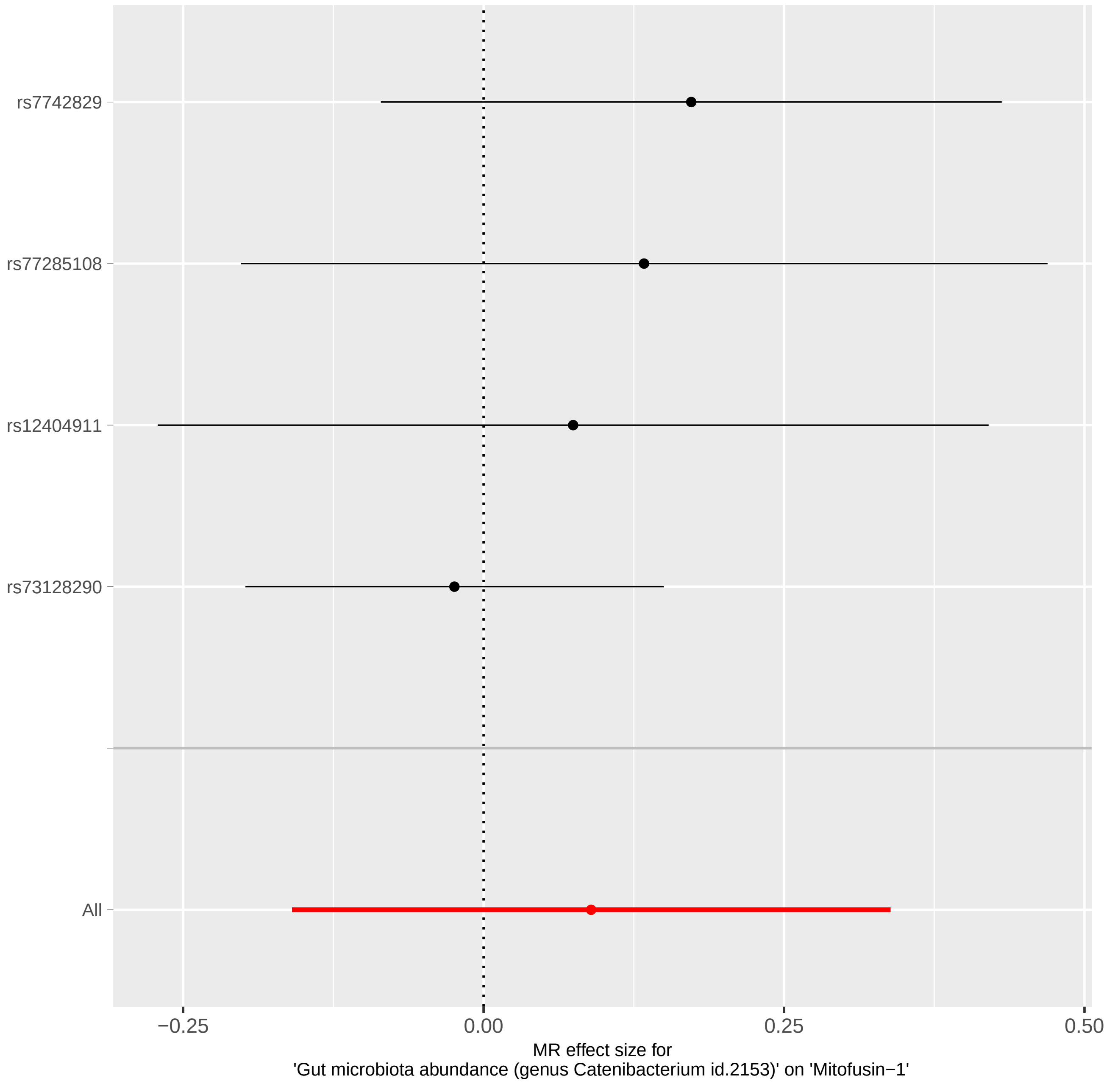

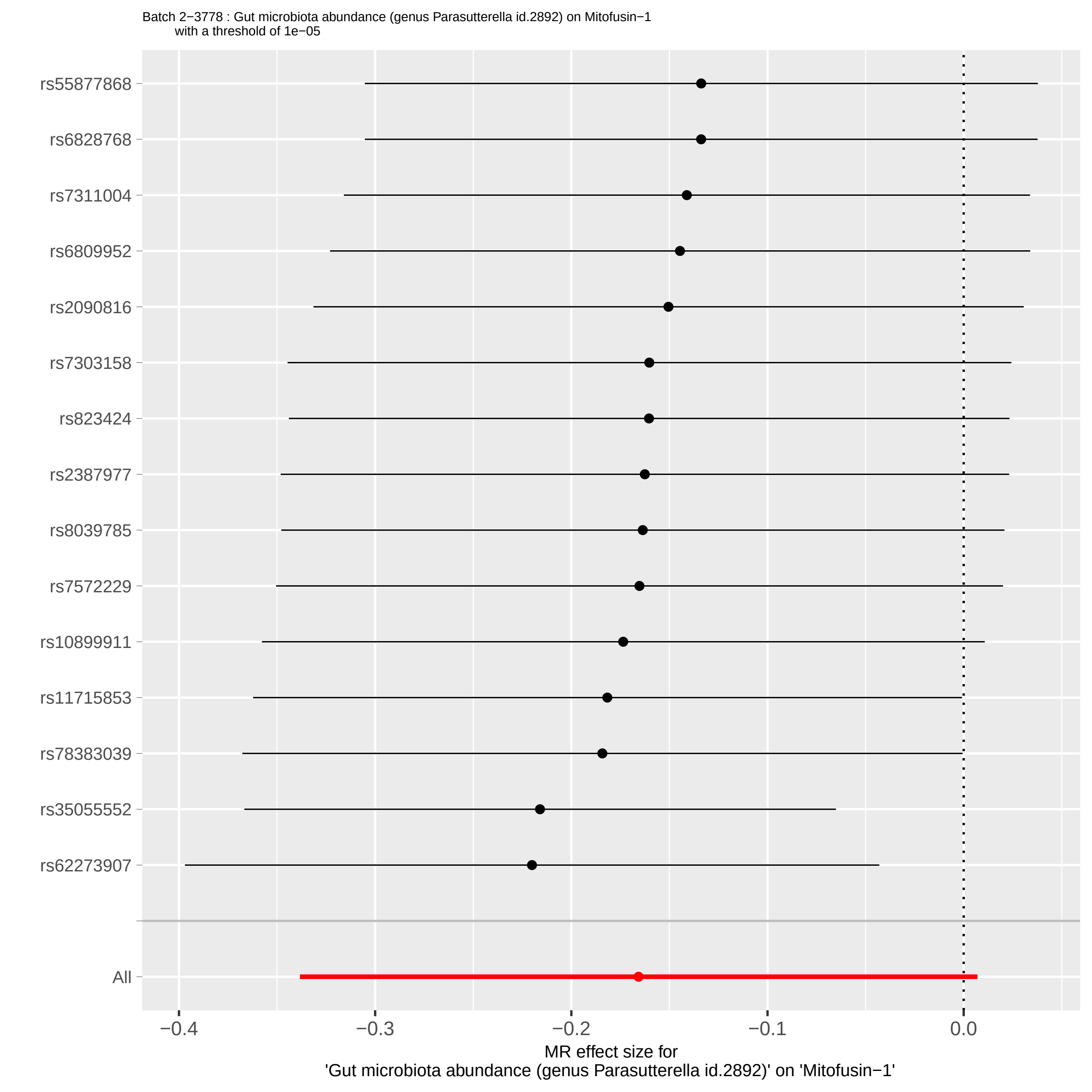
Batch 2−3778 : Gut microbiota abundance (genus Parasutterella id.2892) on Mitofusin−1
with a threshold of 1e−05
rs55877868
rs6828768
rs7311004
rs6809952
rs2090816
rs7303158
rs823424
rs2387977
rs8039785
rs7572229
rs10899911
rs11715853
rs78383039
rs35055552
rs62273907
All
−0.4
−0.3
−0.2
−0.1
0.0
MR effect size for
'Gut microbiota abundance (genus Parasutterella id.2892)' on 'Mitofusin−1'

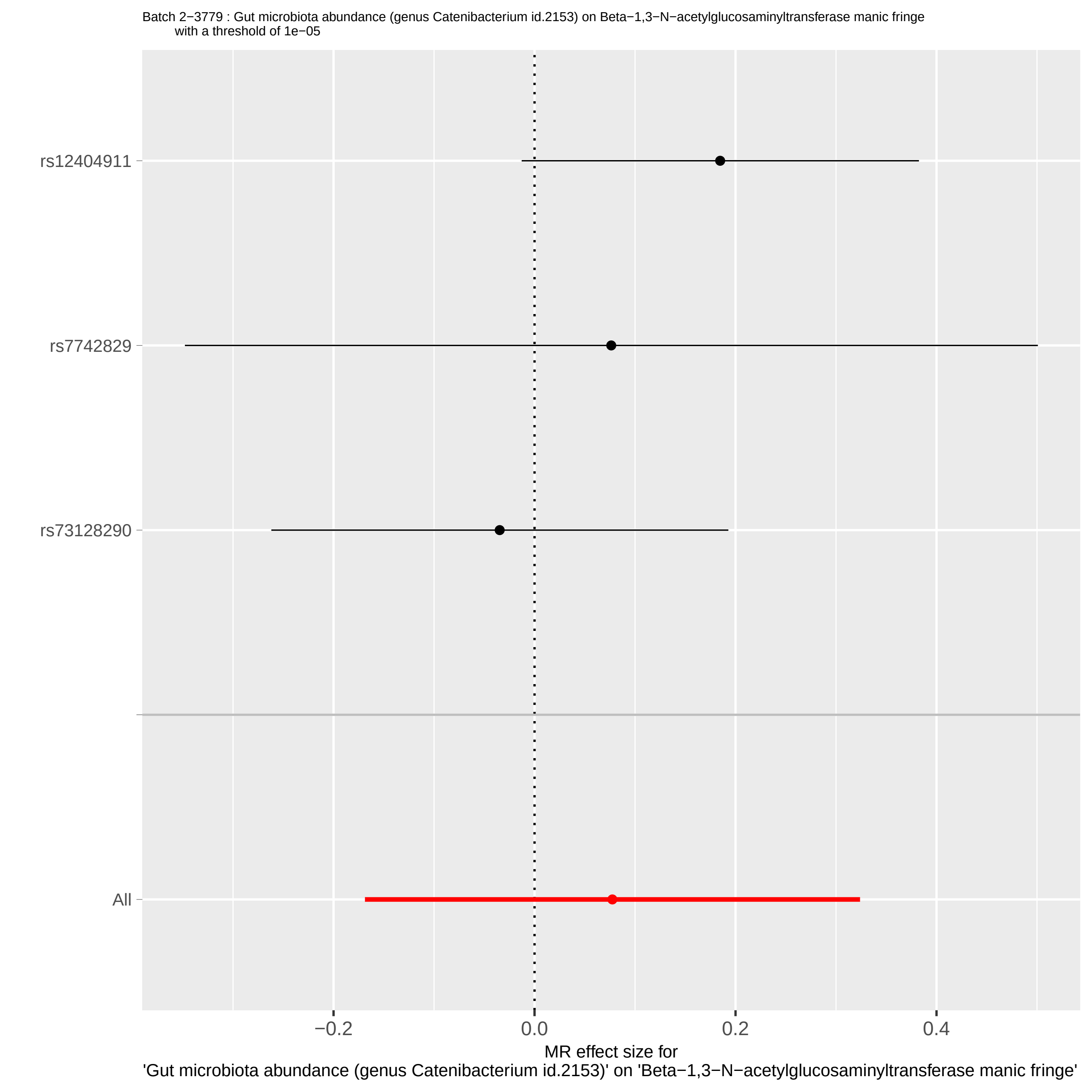
Batch 2−3779 : Gut microbiota abundance (genus Catenibacterium id.2153) on Beta−1,3−N−acetylglucosaminyltransferase manic fringe
with a threshold of 1e−05
rs12404911
rs7742829
rs73128290
All
−0.2
0.0
0.2
0.4
MR effect size for
'Gut microbiota abundance (genus Catenibacterium id.2153)' on 'Beta−1,3−N−acetylglucosaminyltransferase manic fringe'

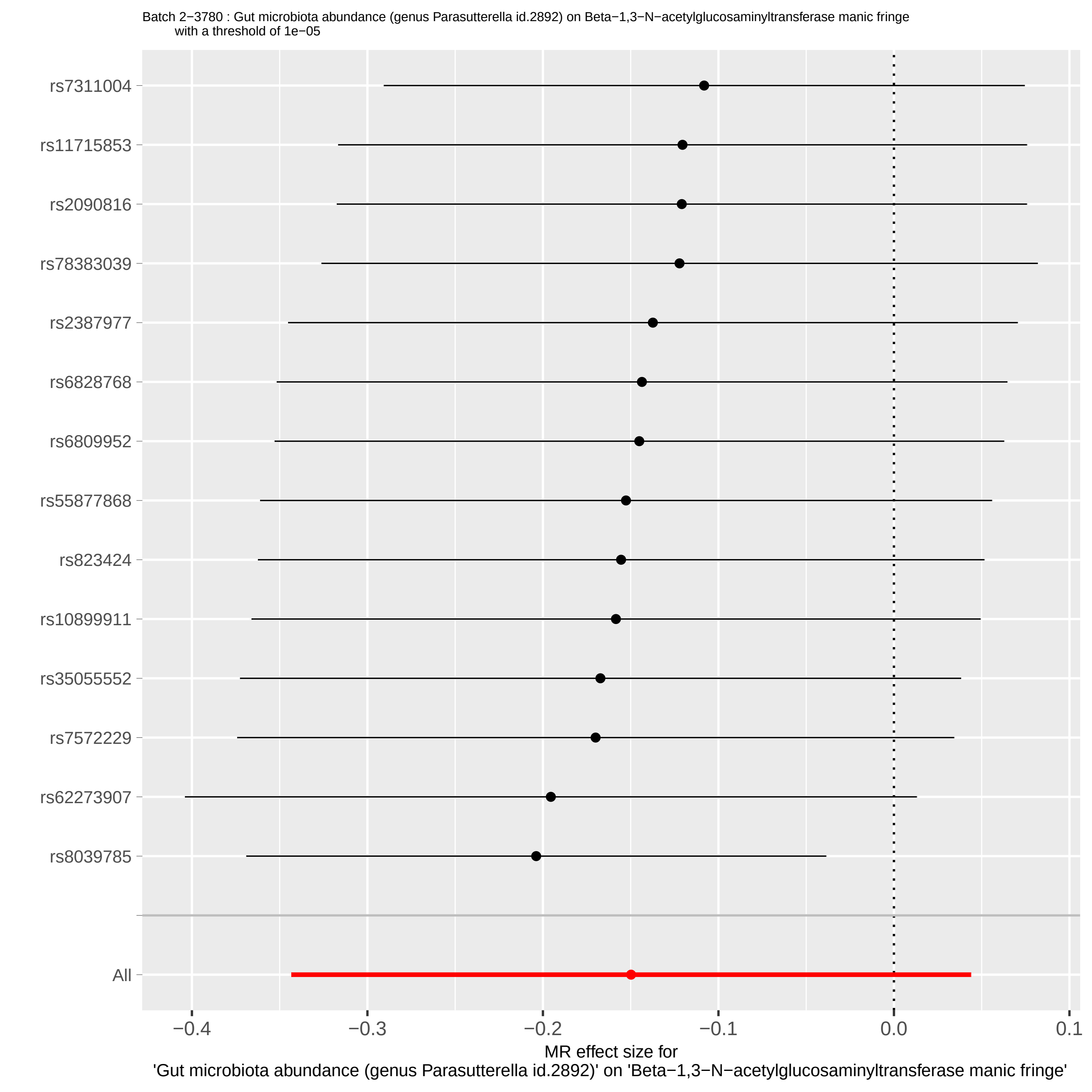

Batch 2−3780 : Gut microbiota abundance (genus Parasutterella id.2892) on Beta−1,3−N−acetylglucosaminyltransferase manic fringe
with a threshold of 1e−05
rs7311004
rs11715853
rs2090816
rs78383039
rs2387977
rs6828768
rs6809952
rs55877868
rs823424
rs10899911
rs35055552
rs7572229
rs62273907
rs8039785
All
−0.4
−0.3
−0.2
−0.1
0.0
0.1
MR effect size for
'Gut microbiota abundance (genus Parasutterella id.2892)' on 'Beta−1,3−N−acetylglucosaminyltransferase manic fringe'

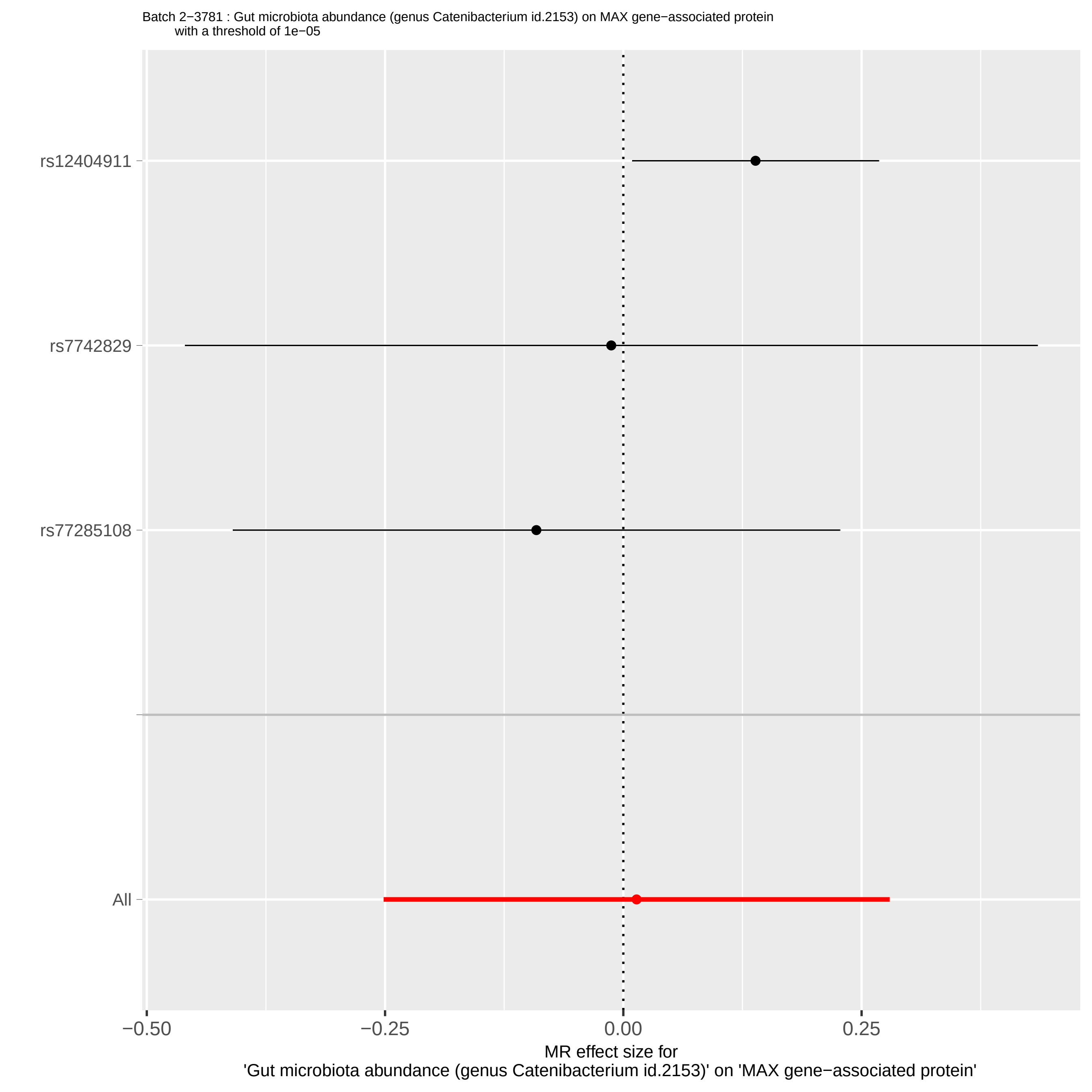

Batch 2−3781 : Gut microbiota abundance (genus Catenibacterium id.2153) on MAX gene−associated protein
with a threshold of 1e−05
rs12404911
rs7742829
rs77285108
All
−0.50
−0.25
0.00
0.25
MR effect size for
'Gut microbiota abundance (genus Catenibacterium id.2153)' on 'MAX gene−associated protein'

Batch 2−3782 : Gut microbiota abundance (genus Parasutterella id.2892) on MAX gene−associated protein
with a threshold of 1e−05

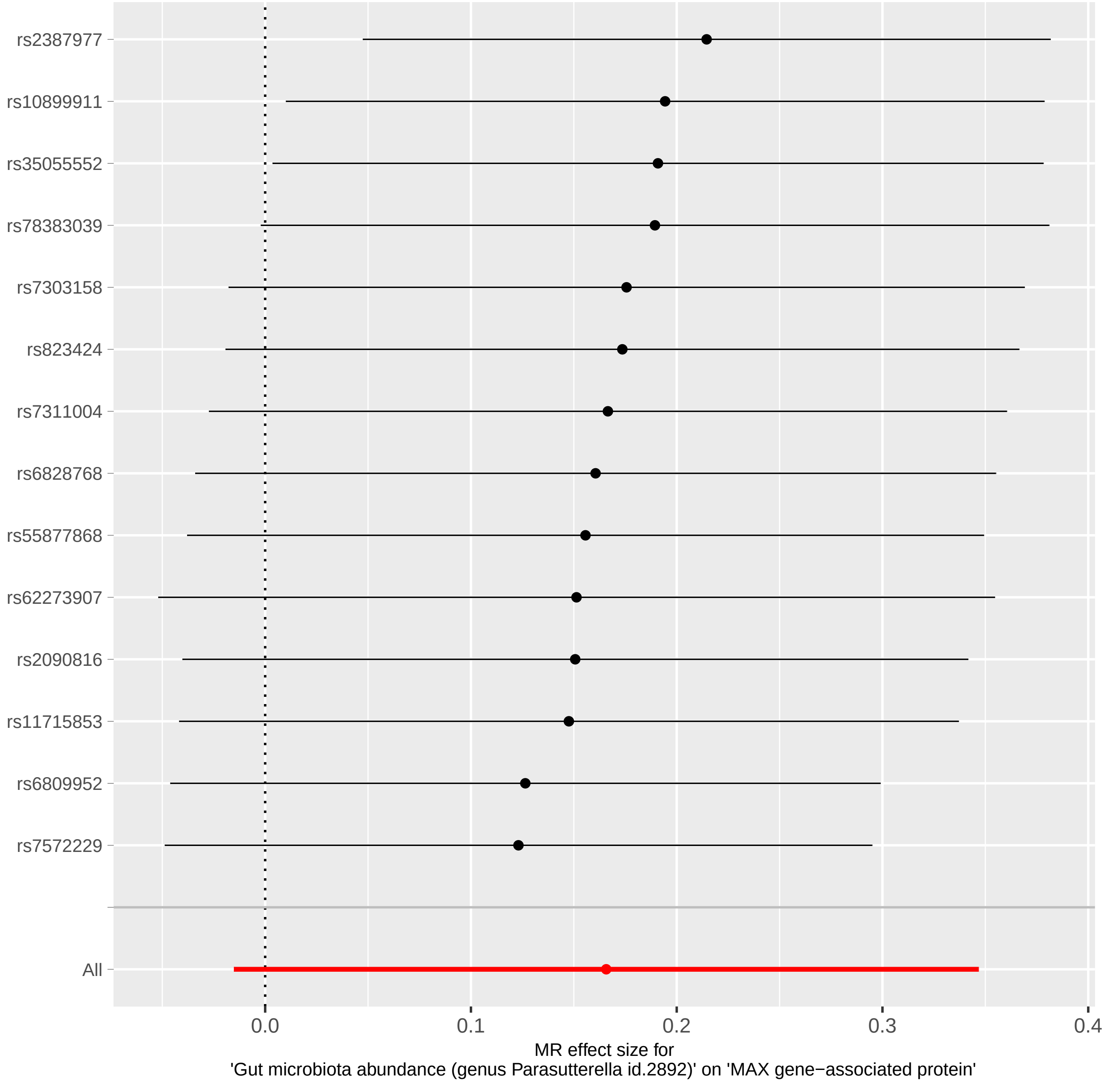

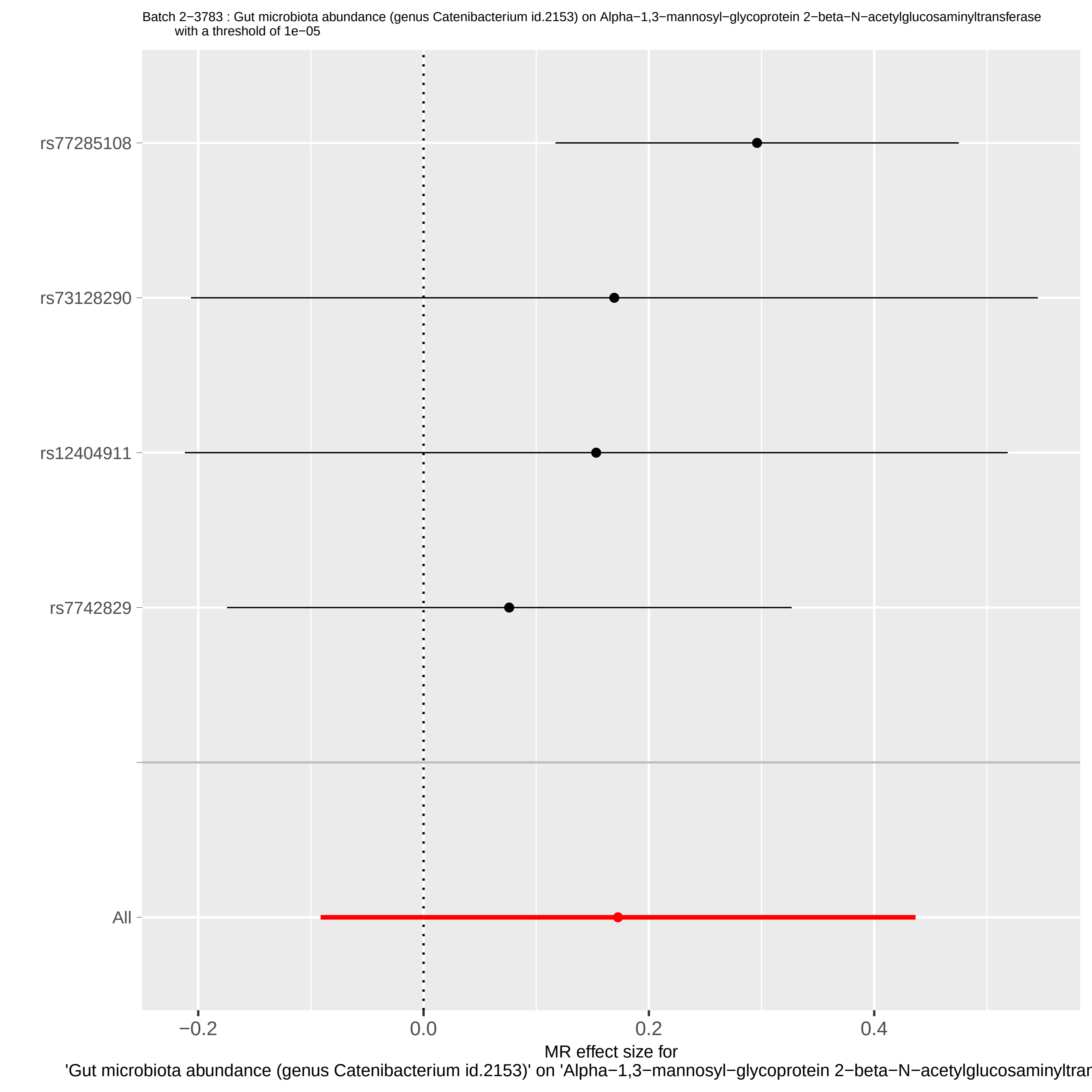
Batch 2−3783 : Gut microbiota abundance (genus Catenibacterium id.2153) on Alpha−1,3−mannosyl−glycoprotein 2−beta−N−acetylglucosaminyltransferase
with a threshold of 1e−05
rs77285108
rs73128290
rs12404911
rs7742829
All
−0.2
0.0
0.2
0.4
MR effect size for
'Gut microbiota abundance (genus Catenibacterium id.2153)' on 'Alpha−1,3−mannosyl−glycoprotein 2−beta−N−acetylglucosaminyltran

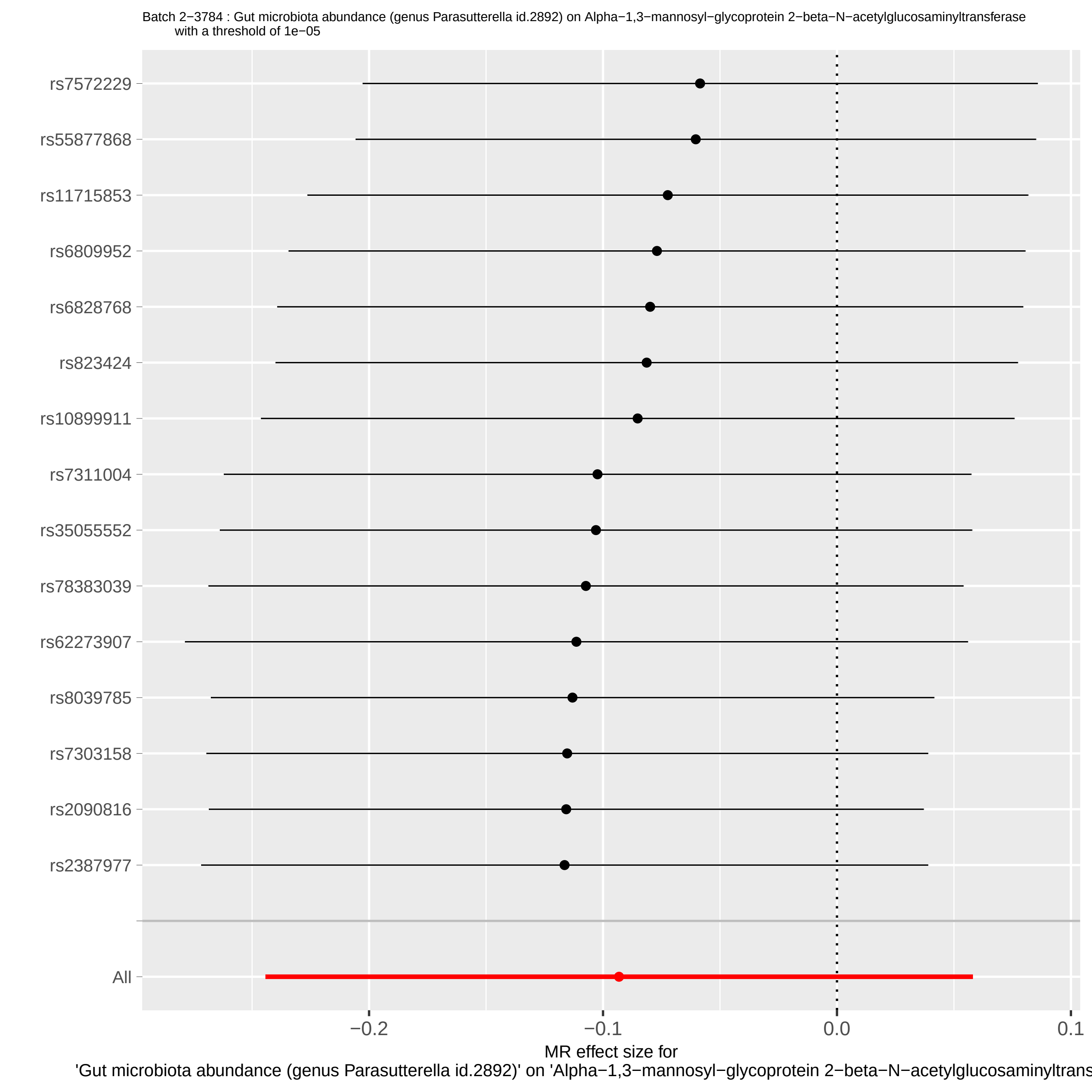
Batch 2−3784 : Gut microbiota abundance (genus Parasutterella id.2892) on Alpha−1,3−mannosyl−glycoprotein 2−beta−N−acetylglucosaminyltransferase
with a threshold of 1e−05
rs7572229
rs55877868
rs11715853
rs6809952
rs6828768
rs823424
rs10899911
rs7311004
rs35055552
rs78383039
rs62273907
rs8039785
rs7303158
rs2090816
rs2387977
All
−0.2
−0.1
0.0
0.1
MR effect size for
'Gut microbiota abundance (genus Parasutterella id.2892)' on 'Alpha−1,3−mannosyl−glycoprotein 2−beta−N−acetylglucosaminyltrans

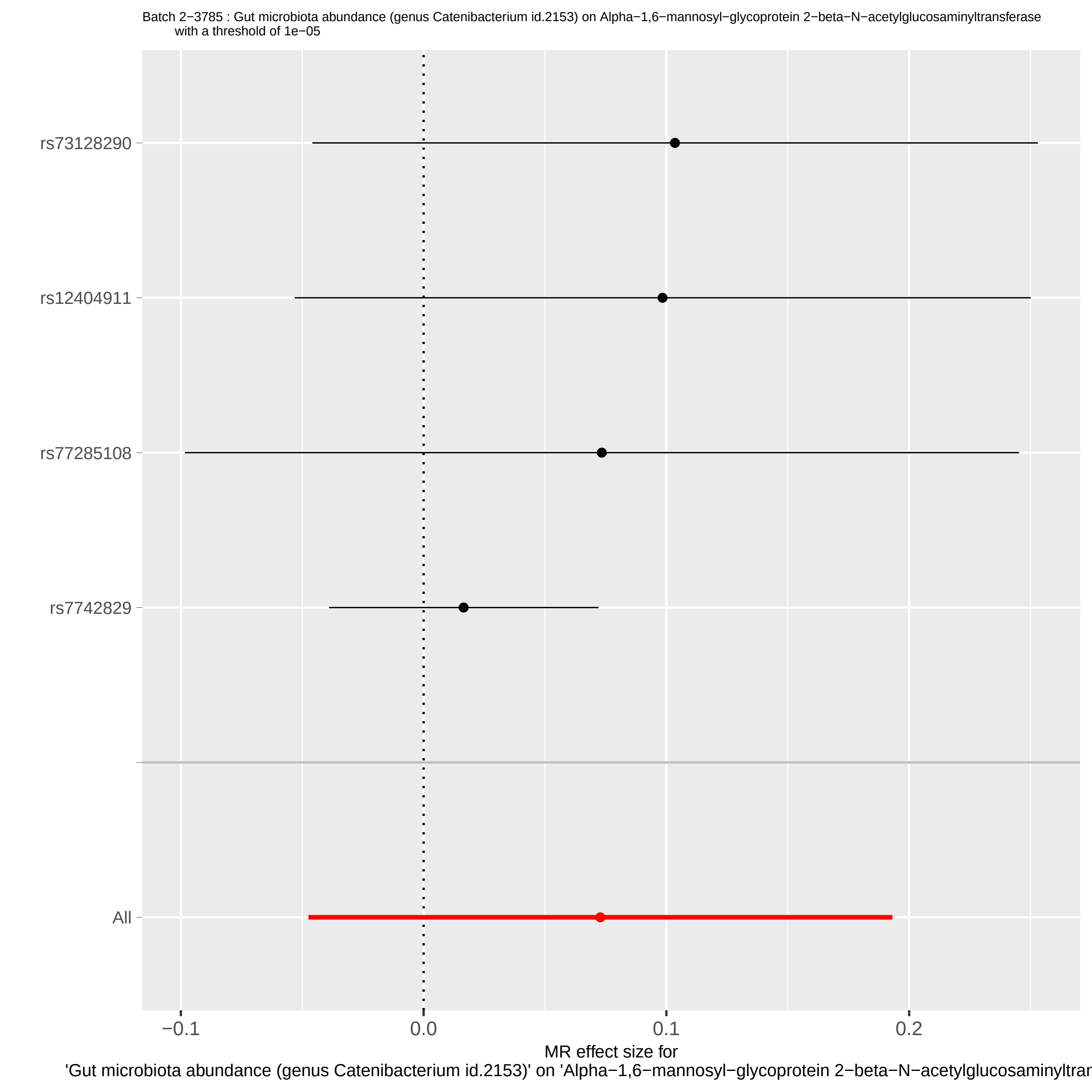
Batch 2−3785 : Gut microbiota abundance (genus Catenibacterium id.2153) on Alpha−1,6−mannosyl−glycoprotein 2−beta−N−acetylglucosaminyltransferase
with a threshold of 1e−05
rs73128290
rs12404911
rs77285108
rs7742829
All
−0.1
0.0
0.1
0.2
MR effect size for
'Gut microbiota abundance (genus Catenibacterium id.2153)' on 'Alpha−1,6−mannosyl−glycoprotein 2−beta−N−acetylglucosaminyltran

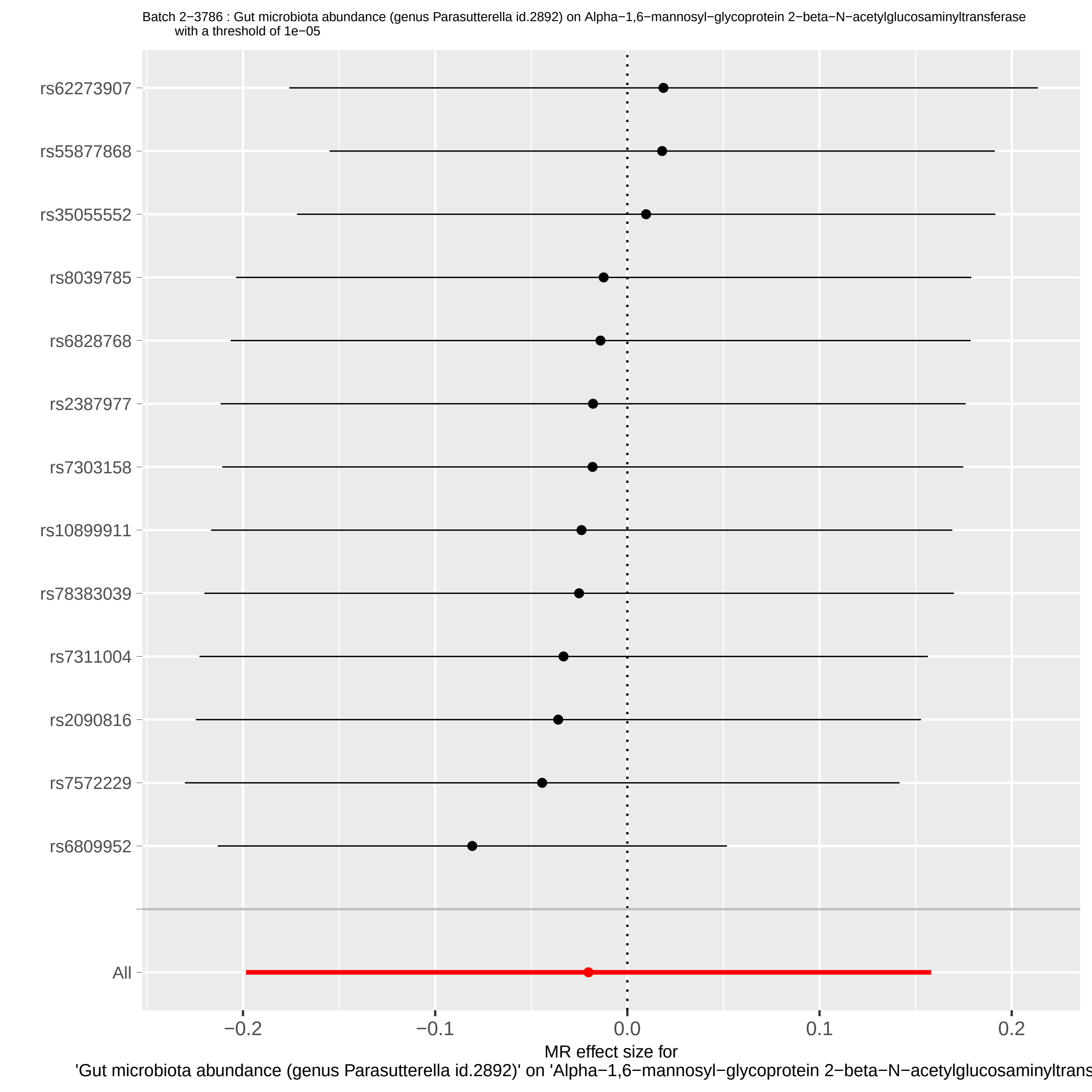

Batch 2−3786 : Gut microbiota abundance (genus Parasutterella id.2892) on Alpha−1,6−mannosyl−glycoprotein 2−beta−N−acetylglucosaminyltransferase
with a threshold of 1e−05
rs62273907
rs55877868
rs35055552
rs8039785
rs6828768
rs2387977
rs7303158
rs10899911
rs78383039
rs7311004
rs2090816
rs7572229
rs6809952
All
−0.2
−0.1
0.0
0.1
0.2
MR effect size for
'Gut microbiota abundance (genus Parasutterella id.2892)' on 'Alpha−1,6−mannosyl−glycoprotein 2−beta−N−acetylglucosaminyltrans

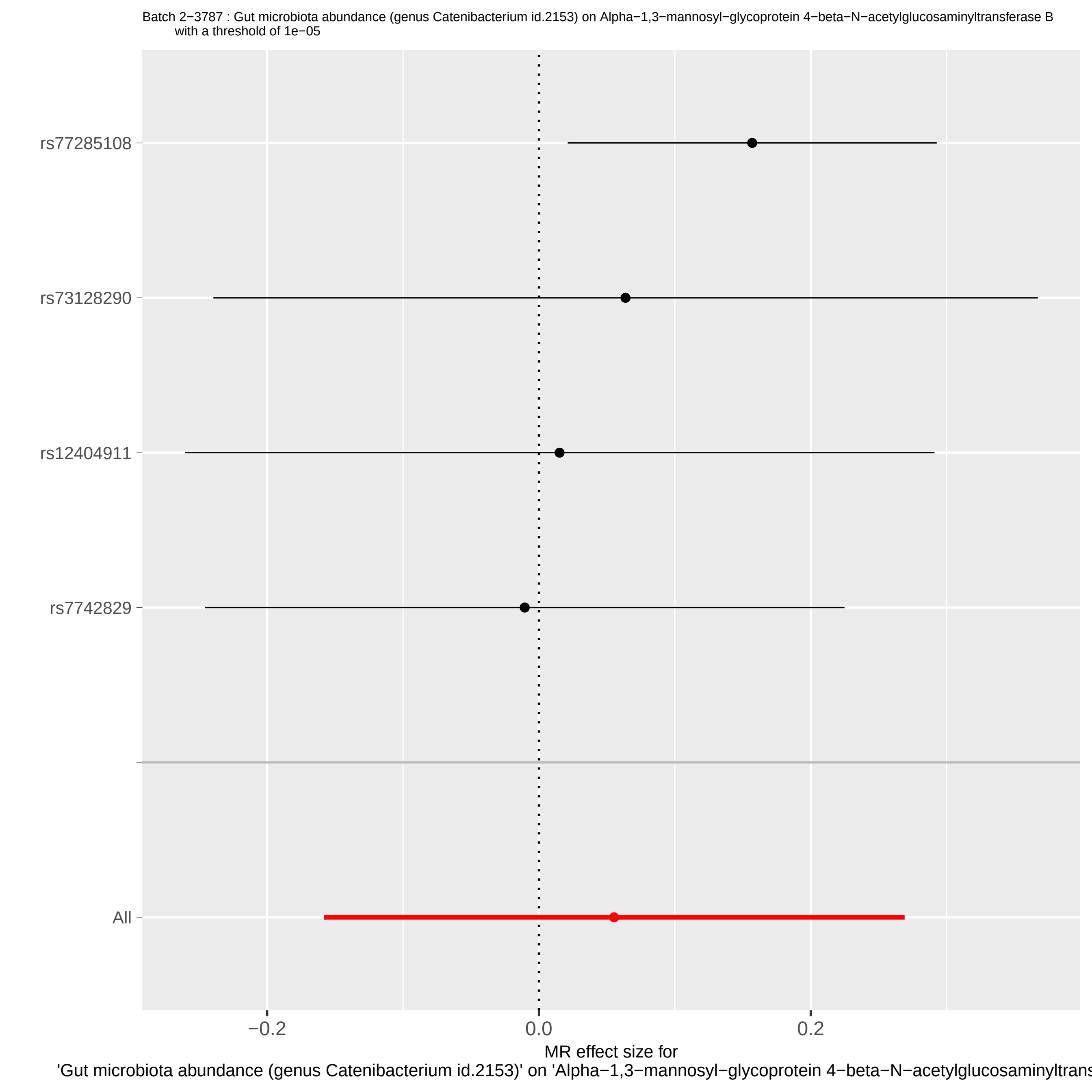
Batch 2−3787 : Gut microbiota abundance (genus Catenibacterium id.2153) on Alpha−1,3−mannosyl−glycoprotein 4−beta−N−acetylglucosaminyltransferase B with a threshold of 1e−05
rs77285108
rs73128290
rs12404911
rs7742829
All
−0.2
0.0
0.2
MR effect size for
'Gut microbiota abundance (genus Catenibacterium id.2153)' on 'Alpha−1,3−mannosyl−glycoprotein 4−beta−N−acetylglucosaminyltrans

Batch 2−3788 : Gut microbiota abundance (genus Parasutterella id.2892) on Alpha−1,3−mannosyl−glycoprotein 4−beta−N−acetylglucosaminyltransferase B
with a threshold of 1e−05

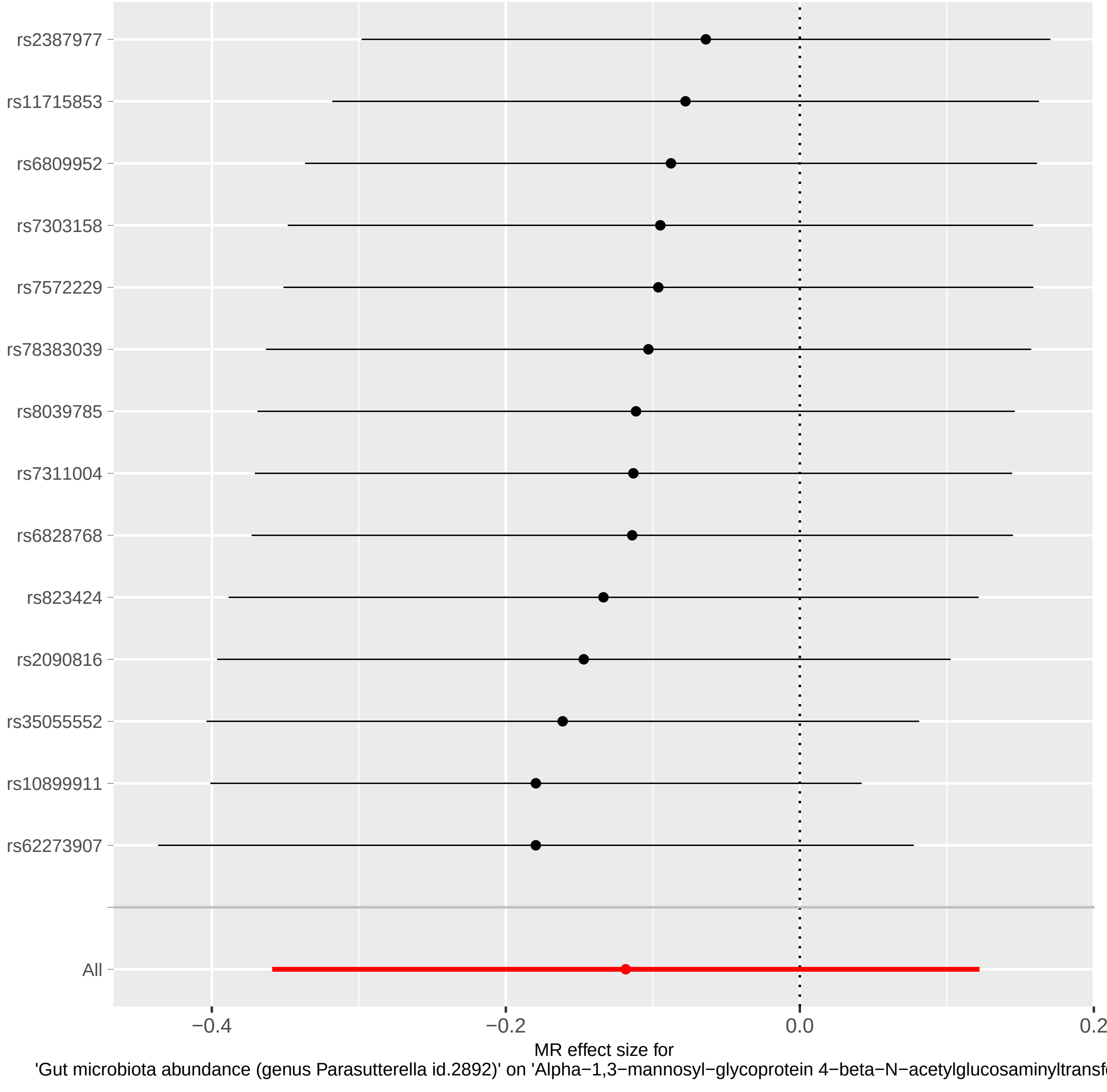

Batch 2−3789 : Gut microbiota abundance (genus Catenibacterium id.2153) on Alpha−1,3−mannosyl−glycoprotein 4−beta−N−acetylglucosaminyltransferase C
with a threshold of 1e−05

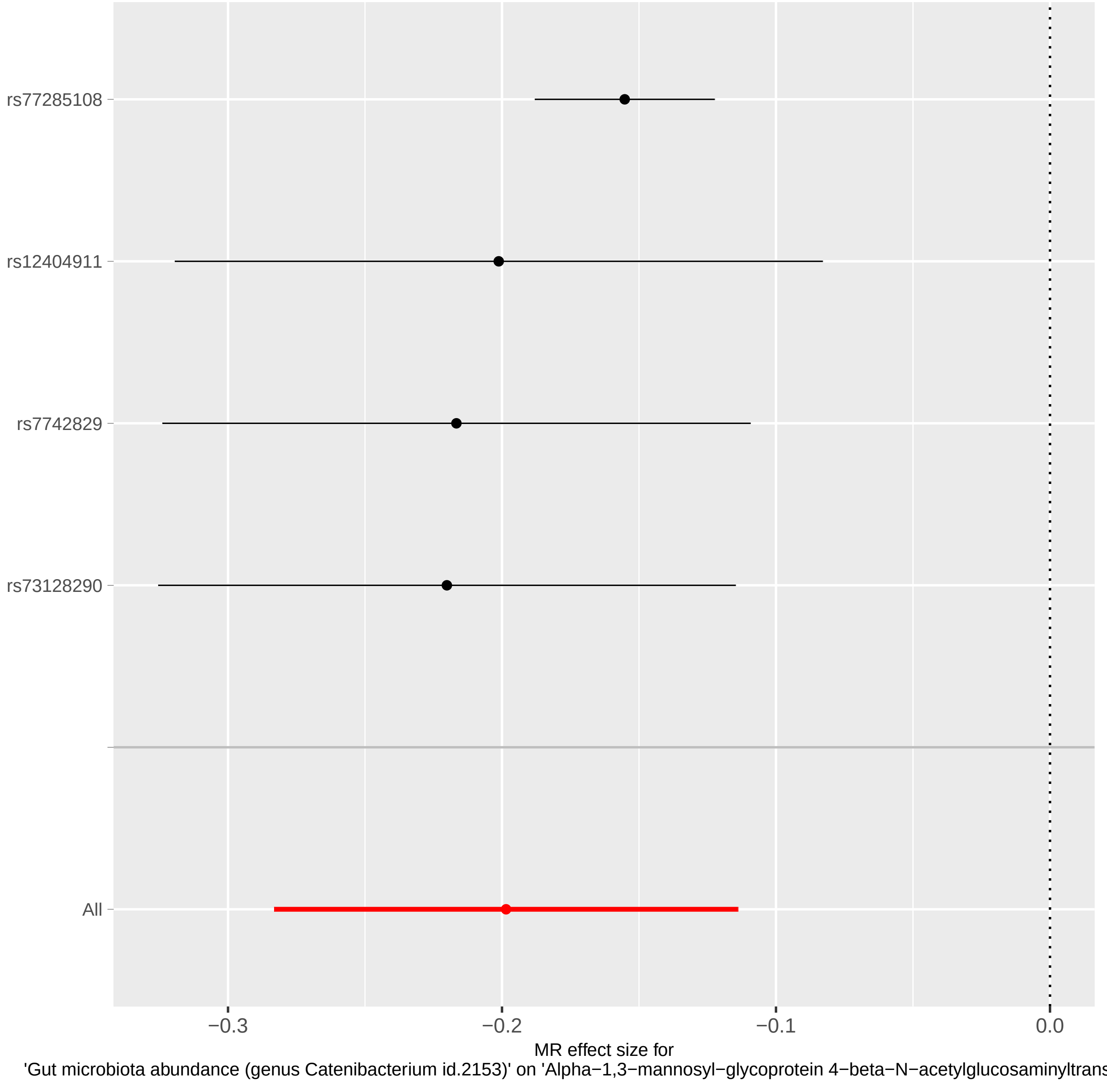

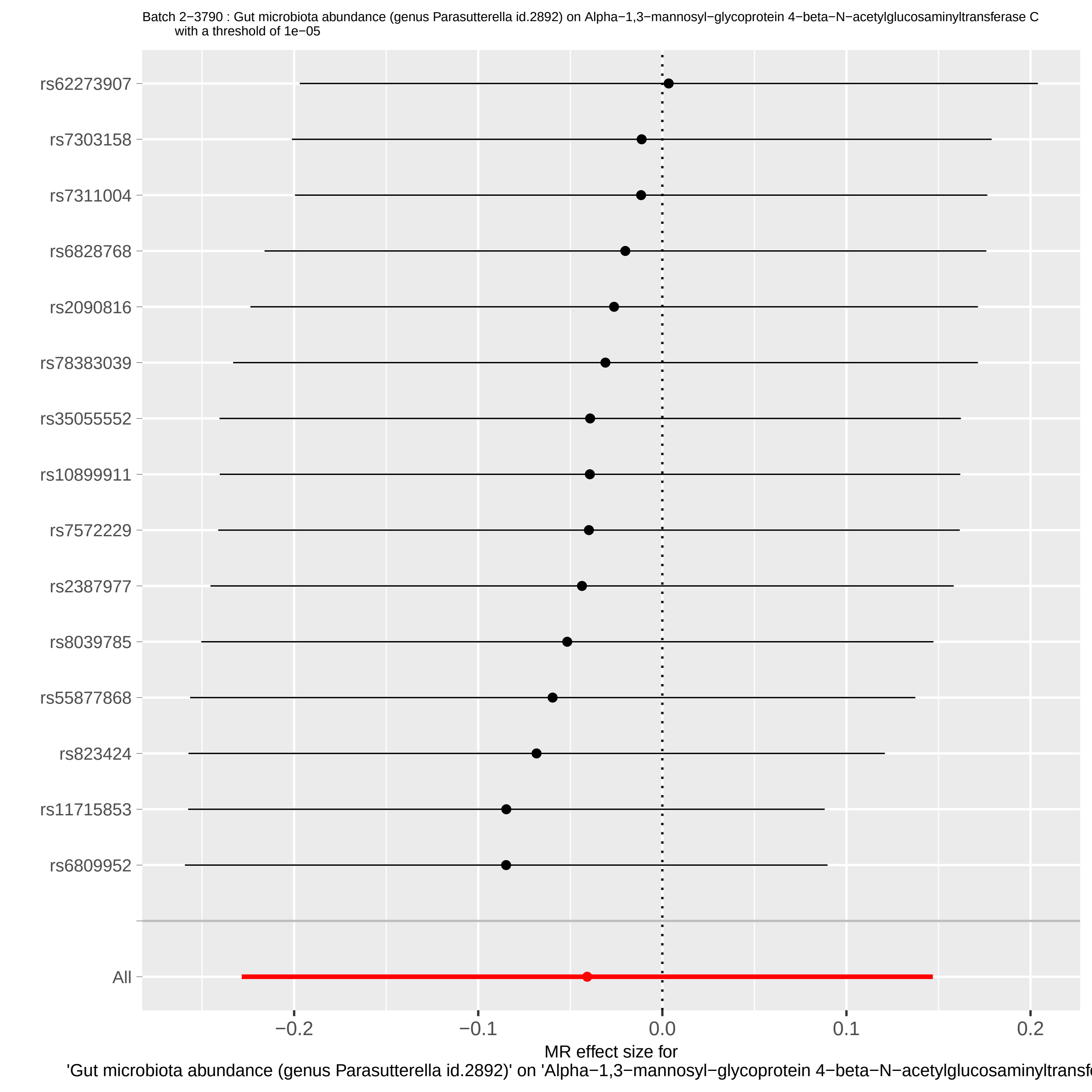

Batch 2−3790 : Gut microbiota abundance (genus Parasutterella id.2892) on Alpha−1,3−mannosyl−glycoprotein 4−beta−N−acetylglucosaminyltransferase C with a threshold of 1e−05
rs62273907
rs7303158
rs7311004
rs6828768
rs2090816
rs78383039
rs35055552
rs10899911
rs7572229
rs2387977
rs8039785
rs55877868
rs823424
rs11715853
rs6809952
All
−0.2
−0.1
0.0
0.1
0.2
MR effect size for
'Gut microbiota abundance (genus Parasutterella id.2892)' on 'Alpha−1,3−mannosyl−glycoprotein 4−beta−N−acetylglucosaminyltransf

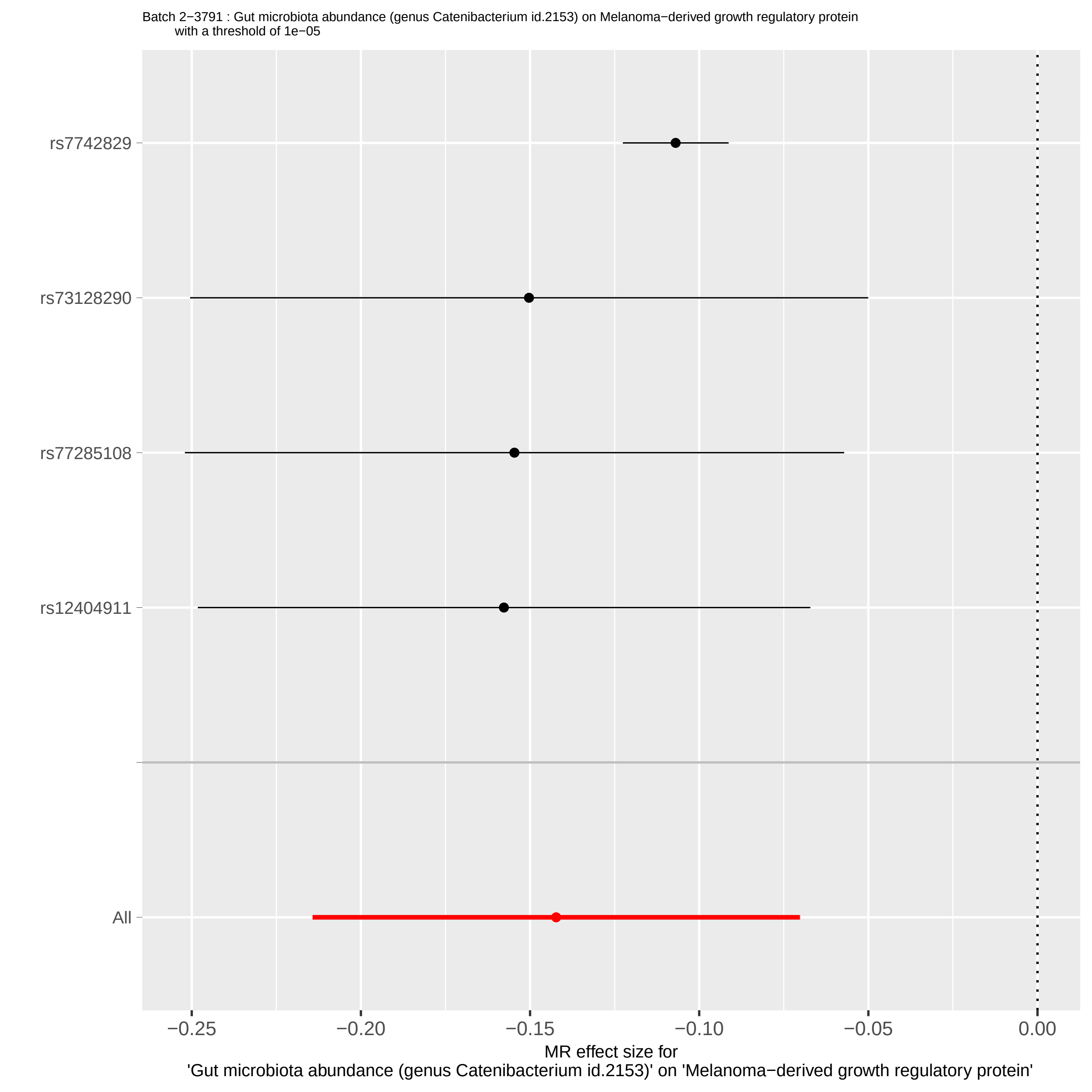

Batch 2−3791 : Gut microbiota abundance (genus Catenibacterium id.2153) on Melanoma−derived growth regulatory protein
with a threshold of 1e−05
rs7742829
rs73128290
rs77285108
rs12404911
All
−0.25
−0.20
−0.15
−0.10
−0.05
0.00
MR effect size for
'Gut microbiota abundance (genus Catenibacterium id.2153)' on 'Melanoma−derived growth regulatory protein'

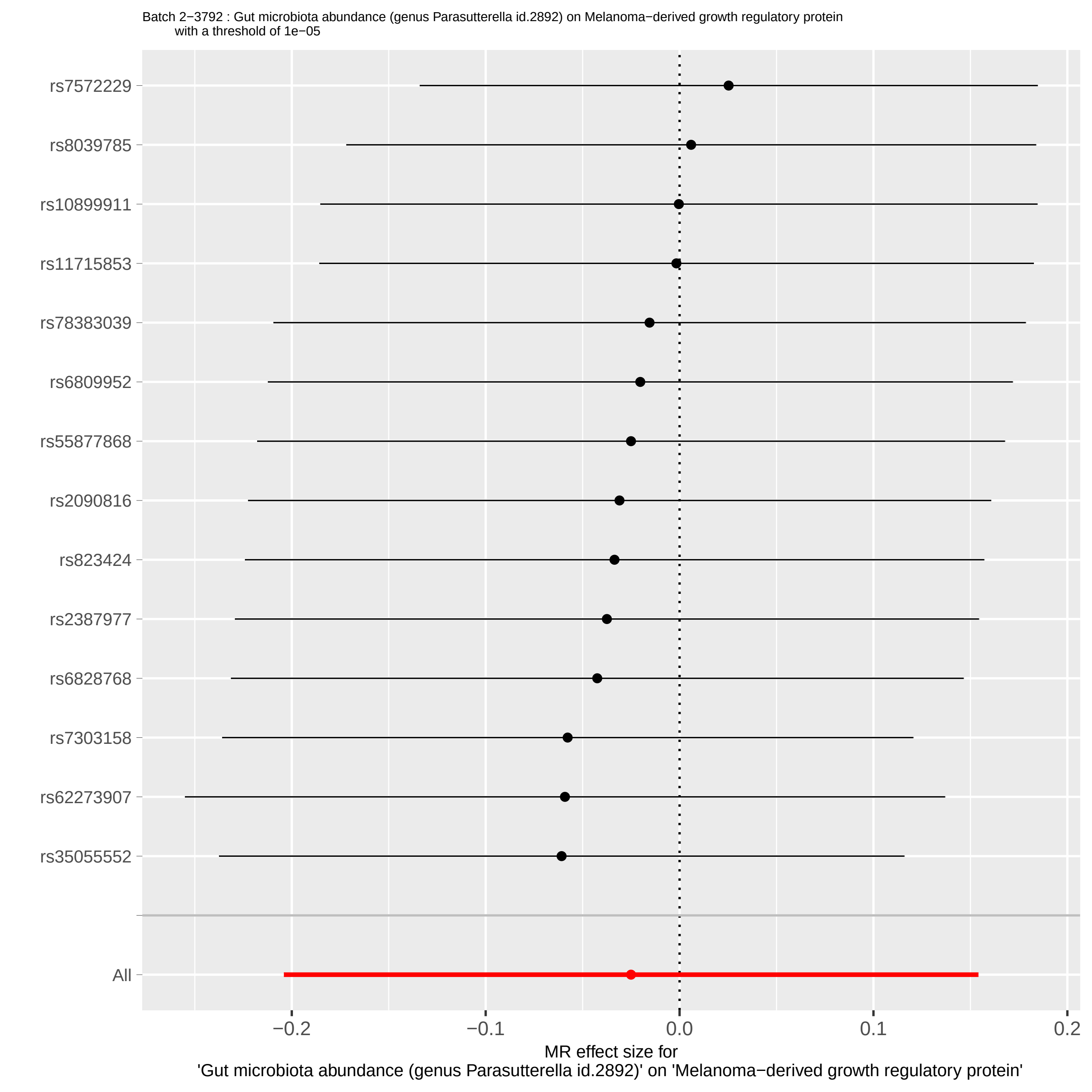

Batch 2−3792 : Gut microbiota abundance (genus Parasutterella id.2892) on Melanoma−derived growth regulatory protein
with a threshold of 1e−05
rs7572229
rs8039785
rs10899911
rs11715853
rs78383039
rs6809952
rs55877868
rs2090816
rs823424
rs2387977
rs6828768
rs7303158
rs62273907
rs35055552
All
−0.2
−0.1
0.0
0.1
0.2
MR effect size for
'Gut microbiota abundance (genus Parasutterella id.2892)' on 'Melanoma−derived growth regulatory protein'

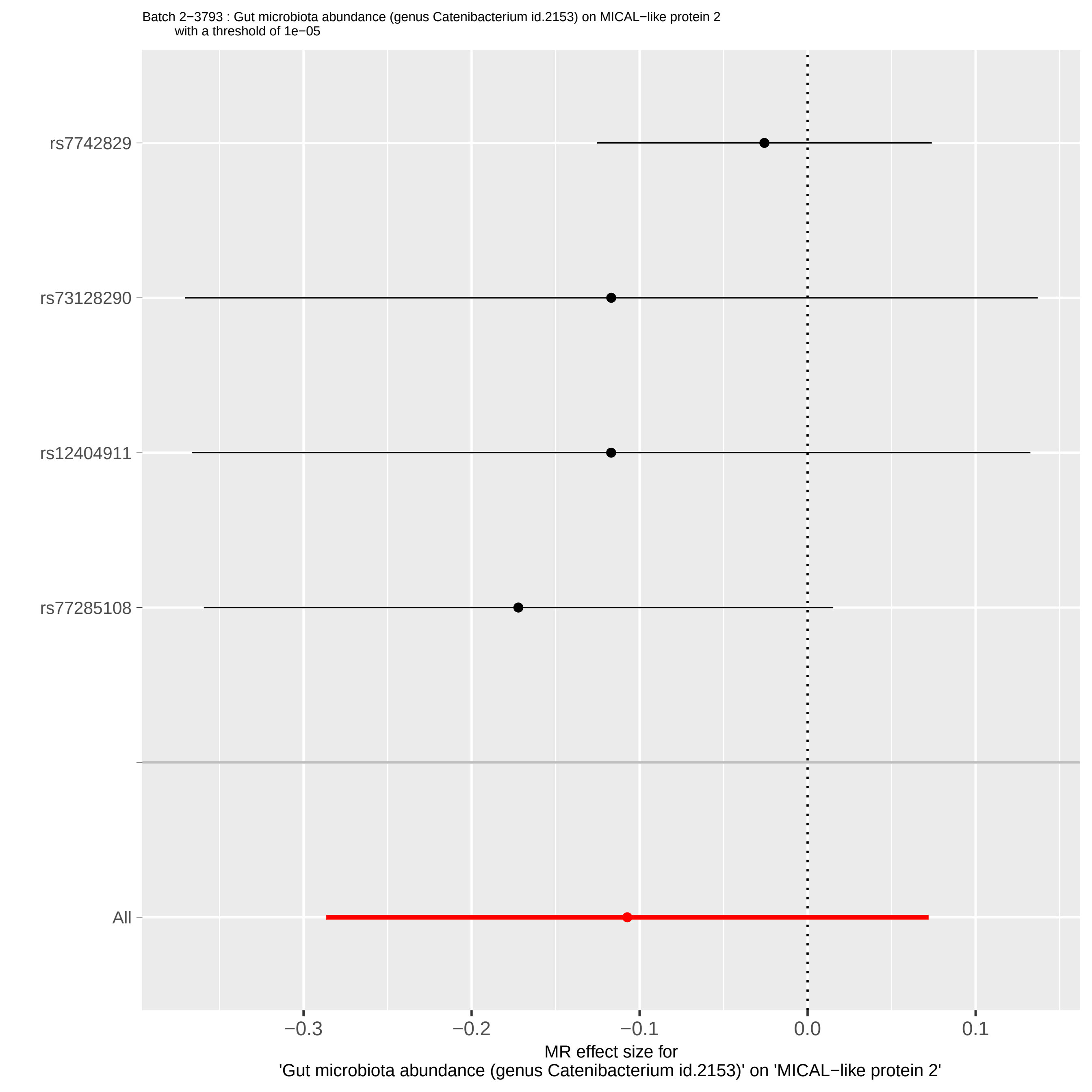

Batch 2−3793 : Gut microbiota abundance (genus Catenibacterium id.2153) on MICAL−like protein 2
with a threshold of 1e−05
rs7742829
rs73128290
rs12404911
rs77285108
All
−0.3
−0.2
−0.1
0.0
0.1
MR effect size for
'Gut microbiota abundance (genus Catenibacterium id.2153)' on 'MICAL−like protein 2'

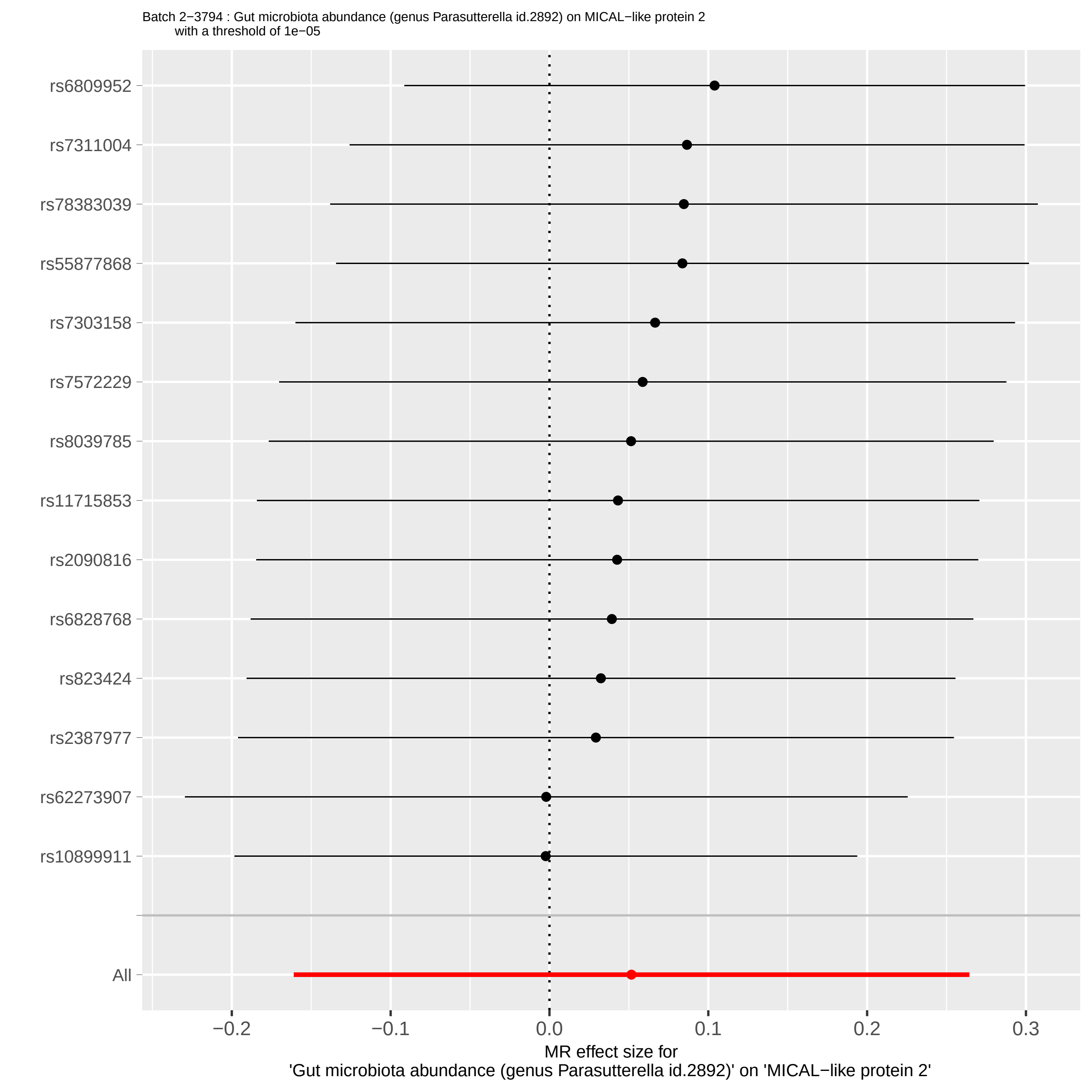

Batch 2−3794 : Gut microbiota abundance (genus Parasutterella id.2892) on MICAL−like protein 2
with a threshold of 1e−05
rs6809952
rs7311004
rs78383039
rs55877868
rs7303158
rs7572229
rs8039785
rs11715853
rs2090816
rs6828768
rs823424
rs2387977
rs62273907
rs10899911
All
−0.2
−0.1
0.0
0.1
0.2
0.3
MR effect size for
'Gut microbiota abundance (genus Parasutterella id.2892)' on 'MICAL−like protein 2'

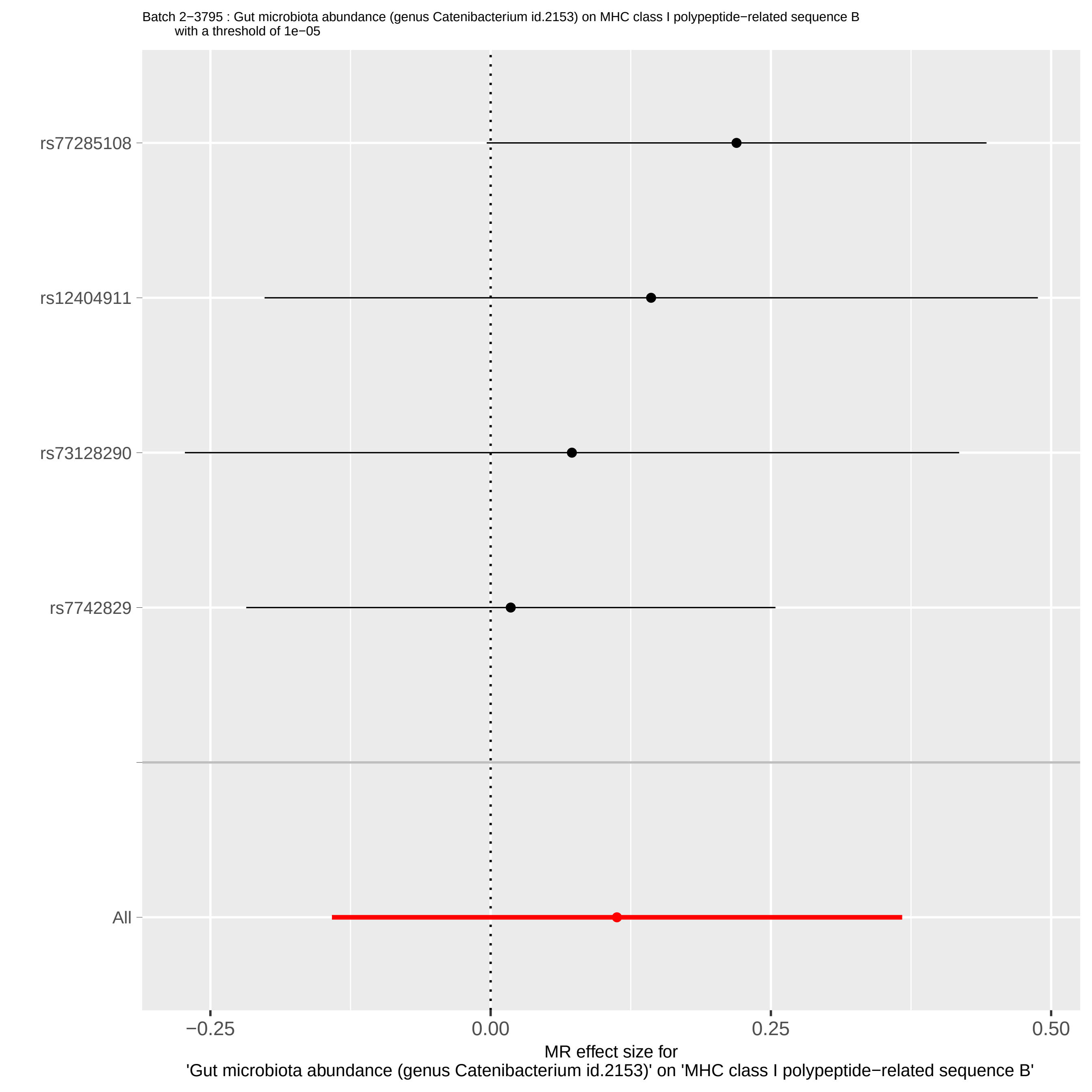
Batch 2−3795 : Gut microbiota abundance (genus Catenibacterium id.2153) on MHC class I polypeptide−related sequence B
with a threshold of 1e−05
rs77285108
rs12404911
rs73128290
rs7742829
All
−0.25
0.00
0.25
0.50
MR effect size for
'Gut microbiota abundance (genus Catenibacterium id.2153)' on 'MHC class I polypeptide−related sequence B'

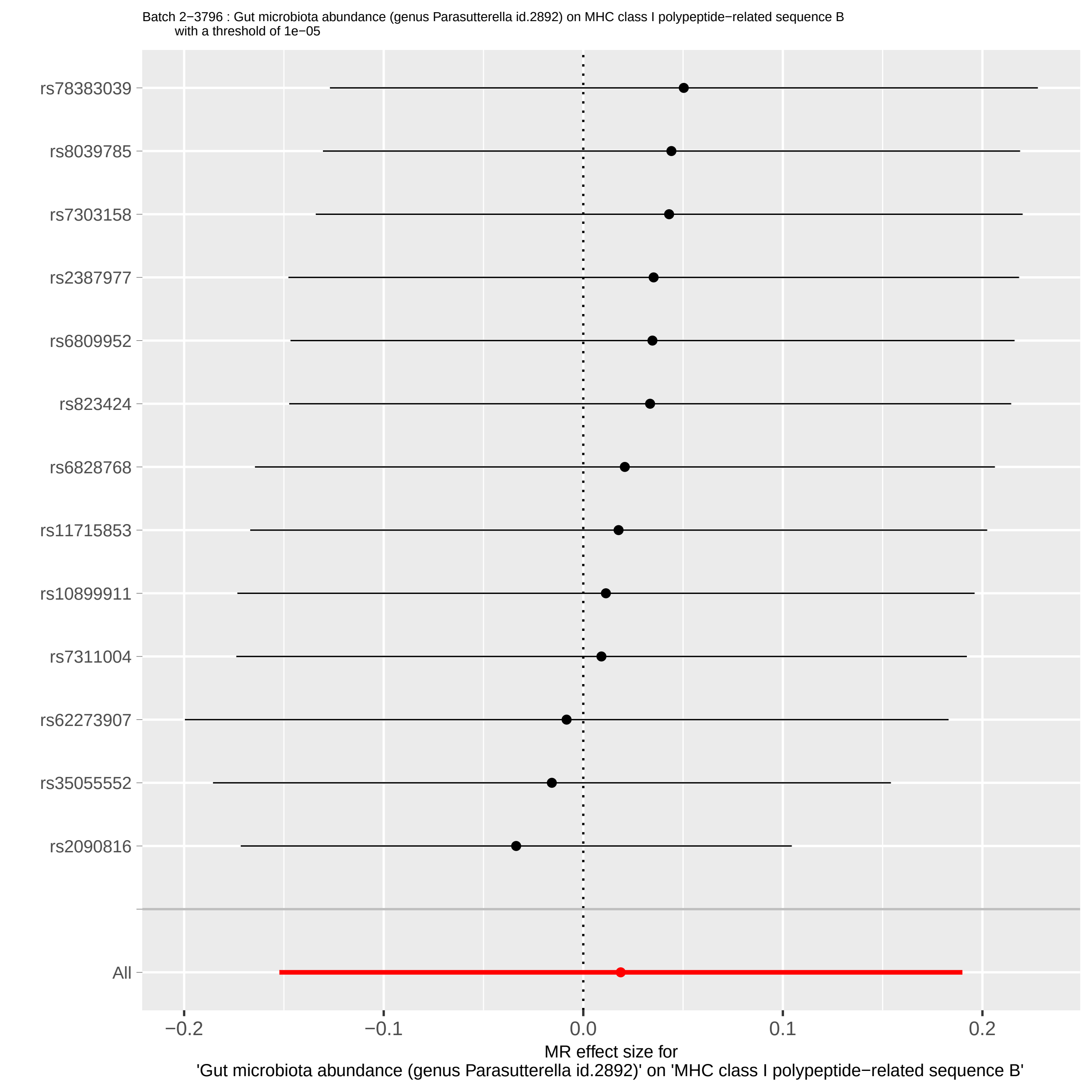
Batch 2−3796 : Gut microbiota abundance (genus Parasutterella id.2892) on MHC class I polypeptide−related sequence B
with a threshold of 1e−05
rs78383039
rs8039785
rs7303158
rs2387977
rs6809952
rs823424
rs6828768
rs11715853
rs10899911
rs7311004
rs62273907
rs35055552
rs2090816
All
−0.2
−0.1
0.0
0.1
0.2
MR effect size for
'Gut microbiota abundance (genus Parasutterella id.2892)' on 'MHC class I polypeptide−related sequence B'

Batch 2−3797 : Gut microbiota abundance (genus Catenibacterium id.2153) on Probable E3 ubiquitin−protein ligase MID2
with a threshold of 1e−05

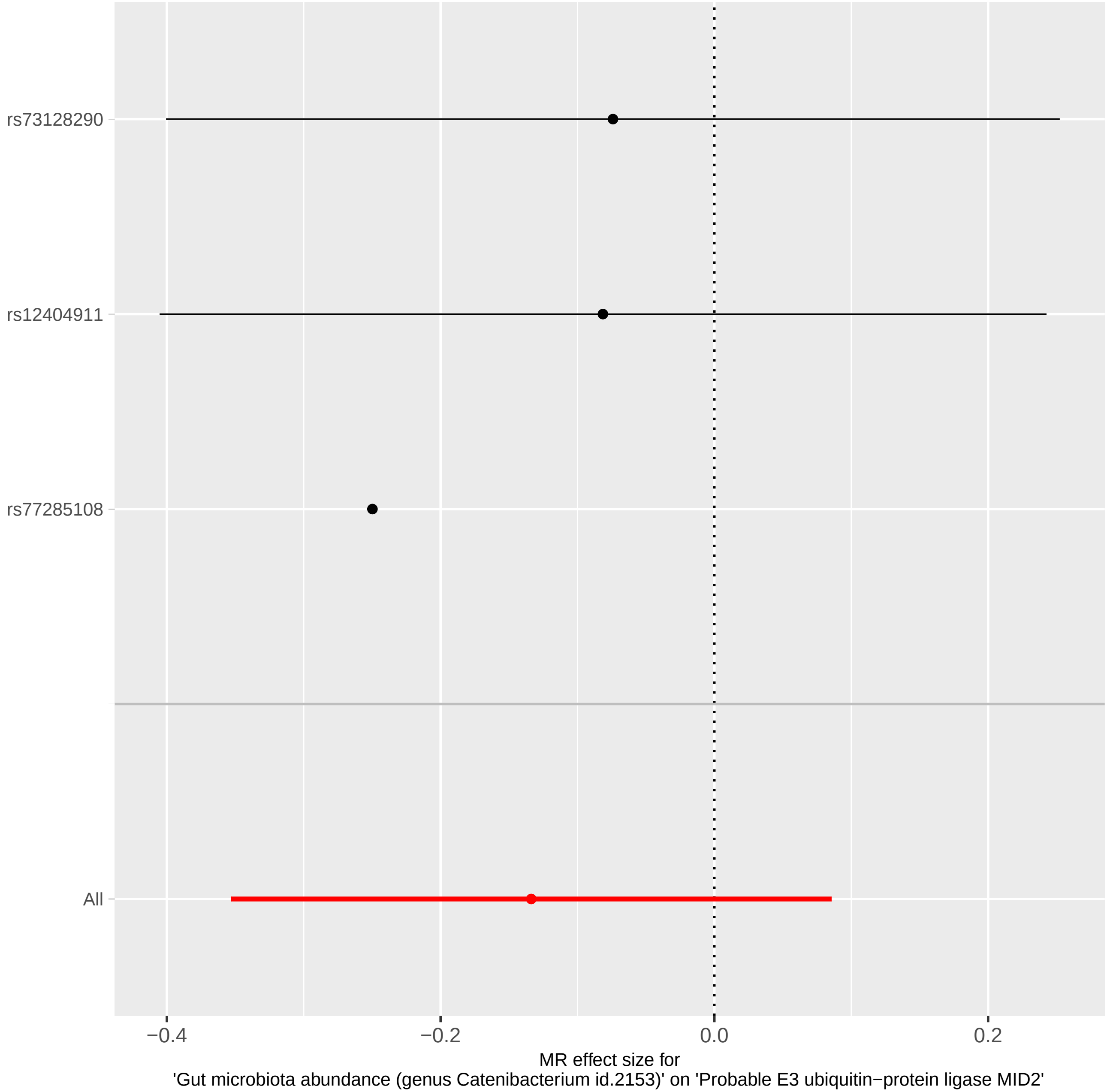

Batch 2−3798 : Gut microbiota abundance (genus Parasutterella id.2892) on Probable E3 ubiquitin−protein ligase MID2
with a threshold of 1e−05

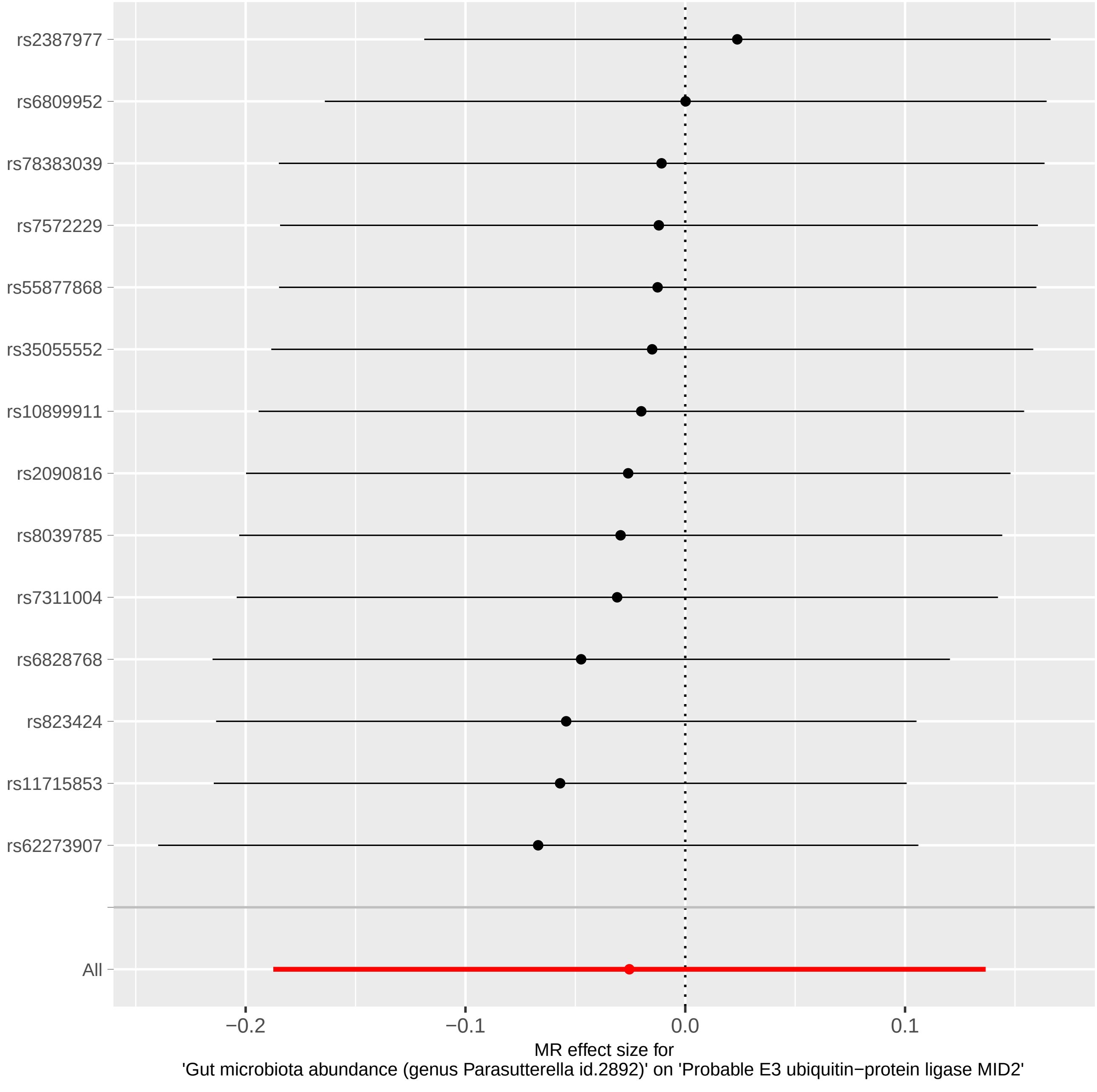

Batch 2−3799 : Gut microbiota abundance (genus Catenibacterium id.2153) on Macrophage migration inhibitory factor
with a threshold of 1e−05

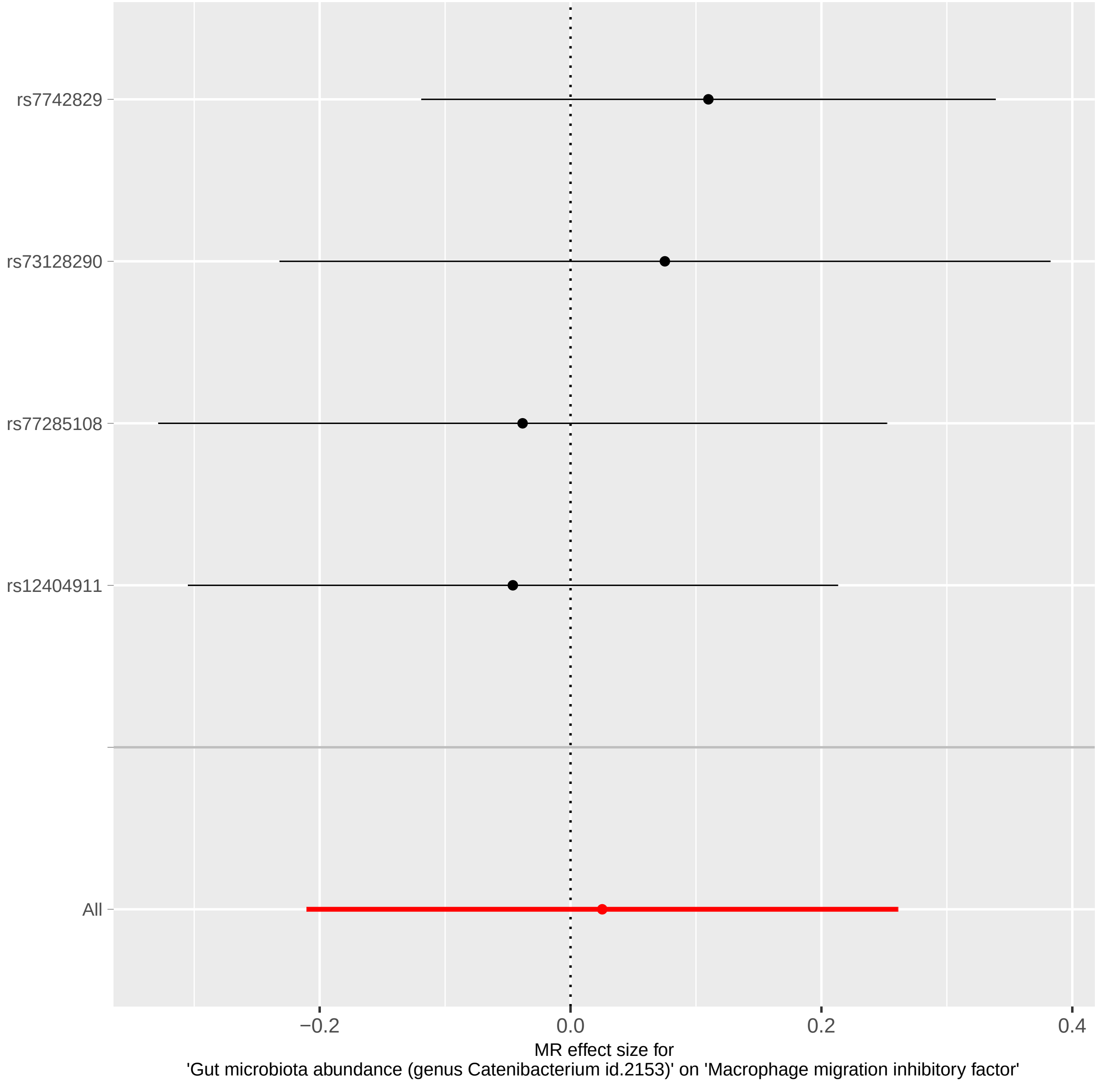

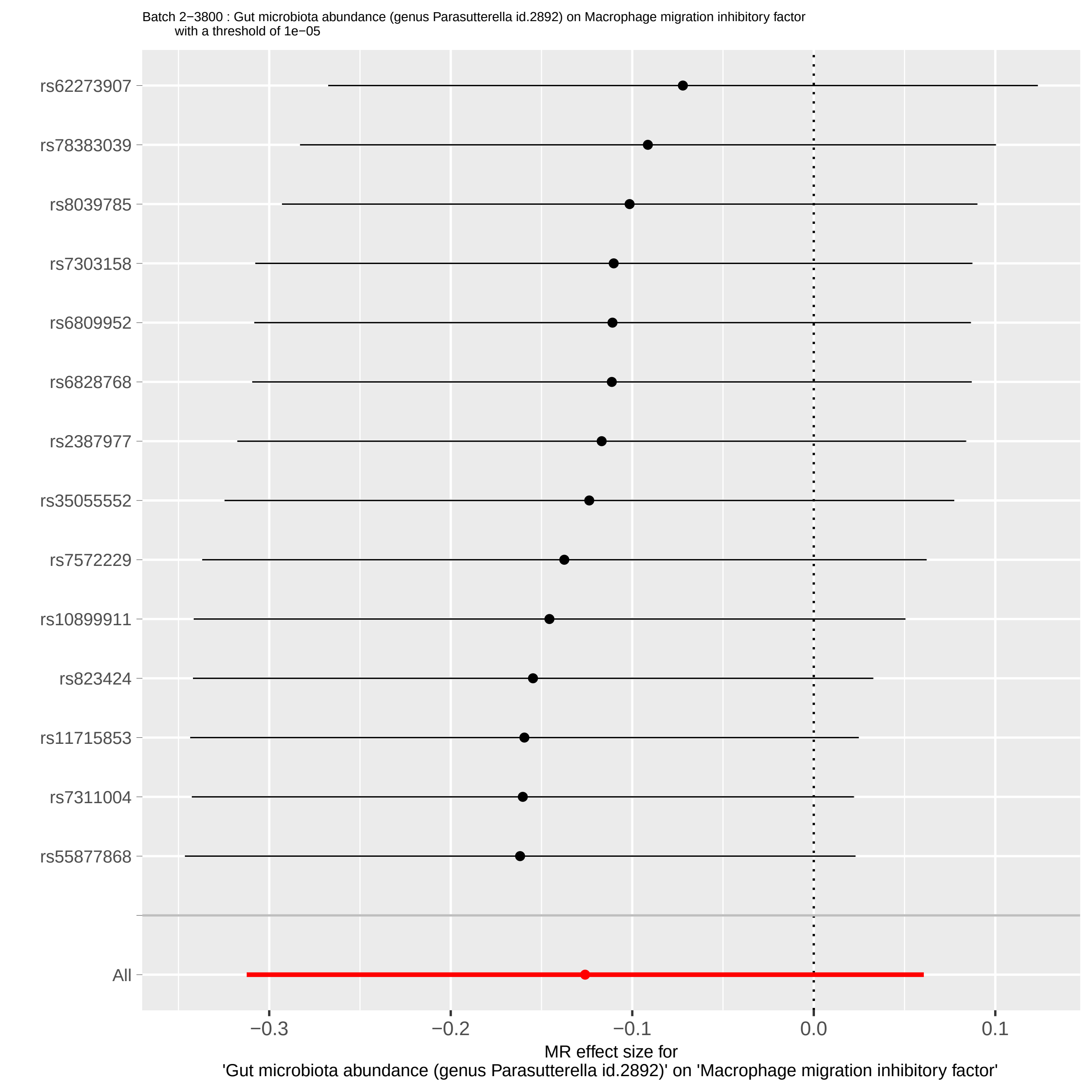
Batch 2−3800 : Gut microbiota abundance (genus Parasutterella id.2892) on Macrophage migration inhibitory factor
with a threshold of 1e−05
rs62273907
rs78383039
rs8039785
rs7303158
rs6809952
rs6828768
rs2387977
rs35055552
rs7572229
rs10899911
rs823424
rs11715853
rs7311004
rs55877868
All
−0.3
−0.2
−0.1
0.0
0.1
MR effect size for
'Gut microbiota abundance (genus Parasutterella id.2892)' on 'Macrophage migration inhibitory factor'

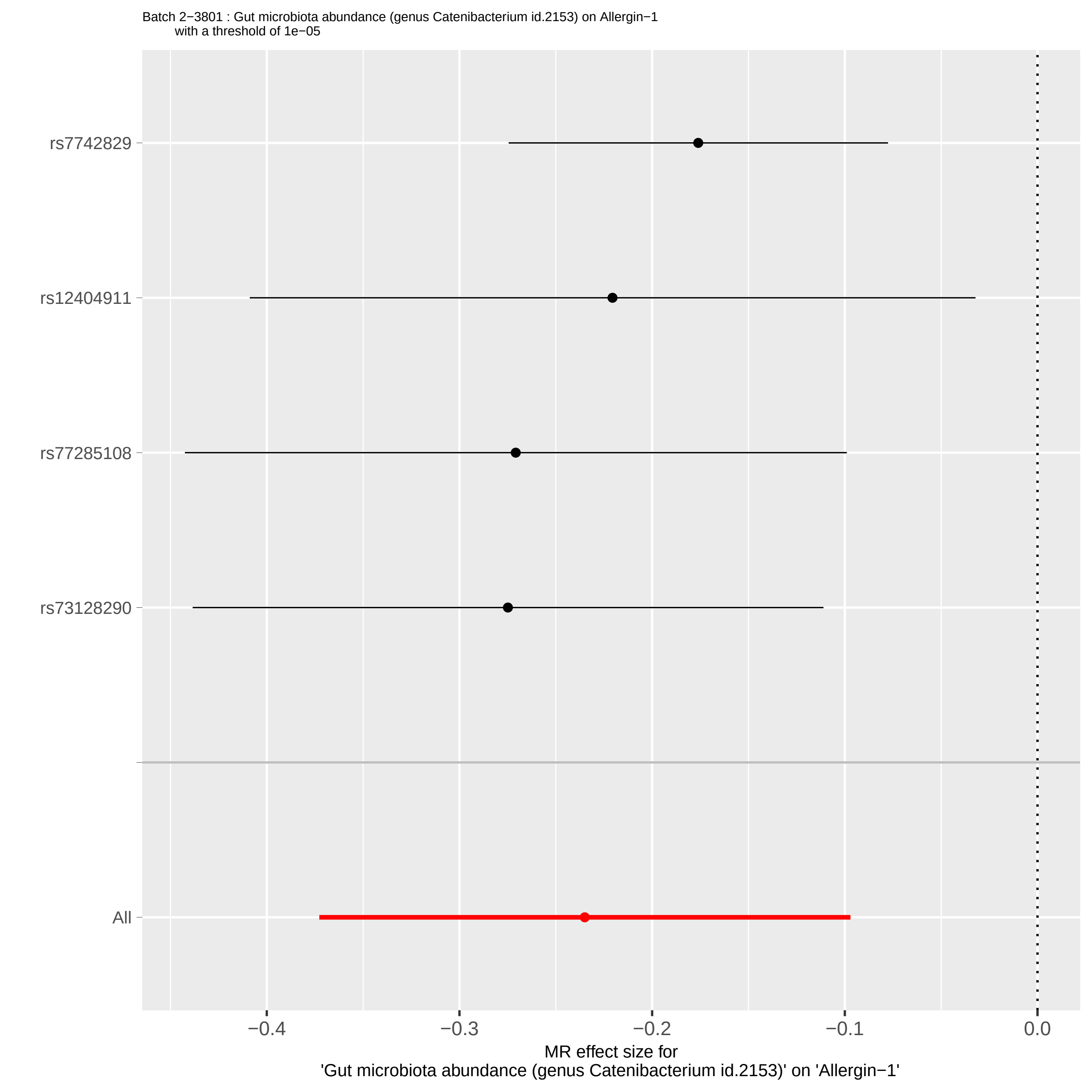
Batch 2−3801 : Gut microbiota abundance (genus Catenibacterium id.2153) on Allergin−1
with a threshold of 1e−05
rs7742829
rs12404911
rs77285108
rs73128290
All
−0.4
−0.3
−0.2
−0.1
0.0
MR effect size for
'Gut microbiota abundance (genus Catenibacterium id.2153)' on 'Allergin−1'

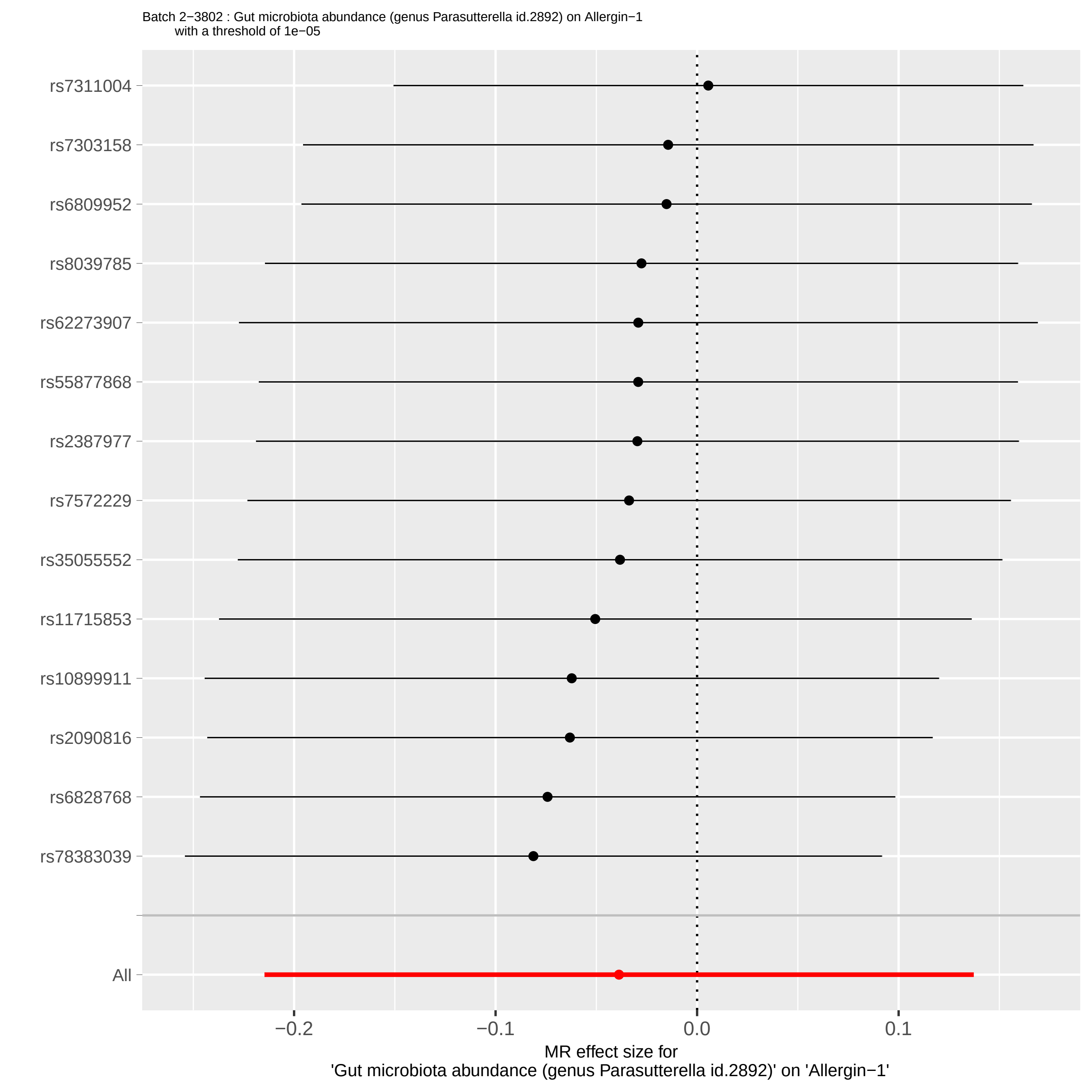
Batch 2−3802 : Gut microbiota abundance (genus Parasutterella id.2892) on Allergin−1
with a threshold of 1e−05
rs7311004
rs7303158
rs6809952
rs8039785
rs62273907
rs55877868
rs2387977
rs7572229
rs35055552
rs11715853
rs10899911
rs2090816
rs6828768
rs78383039
All
−0.2
−0.1
0.0
0.1
MR effect size for
'Gut microbiota abundance (genus Parasutterella id.2892)' on 'Allergin−1'

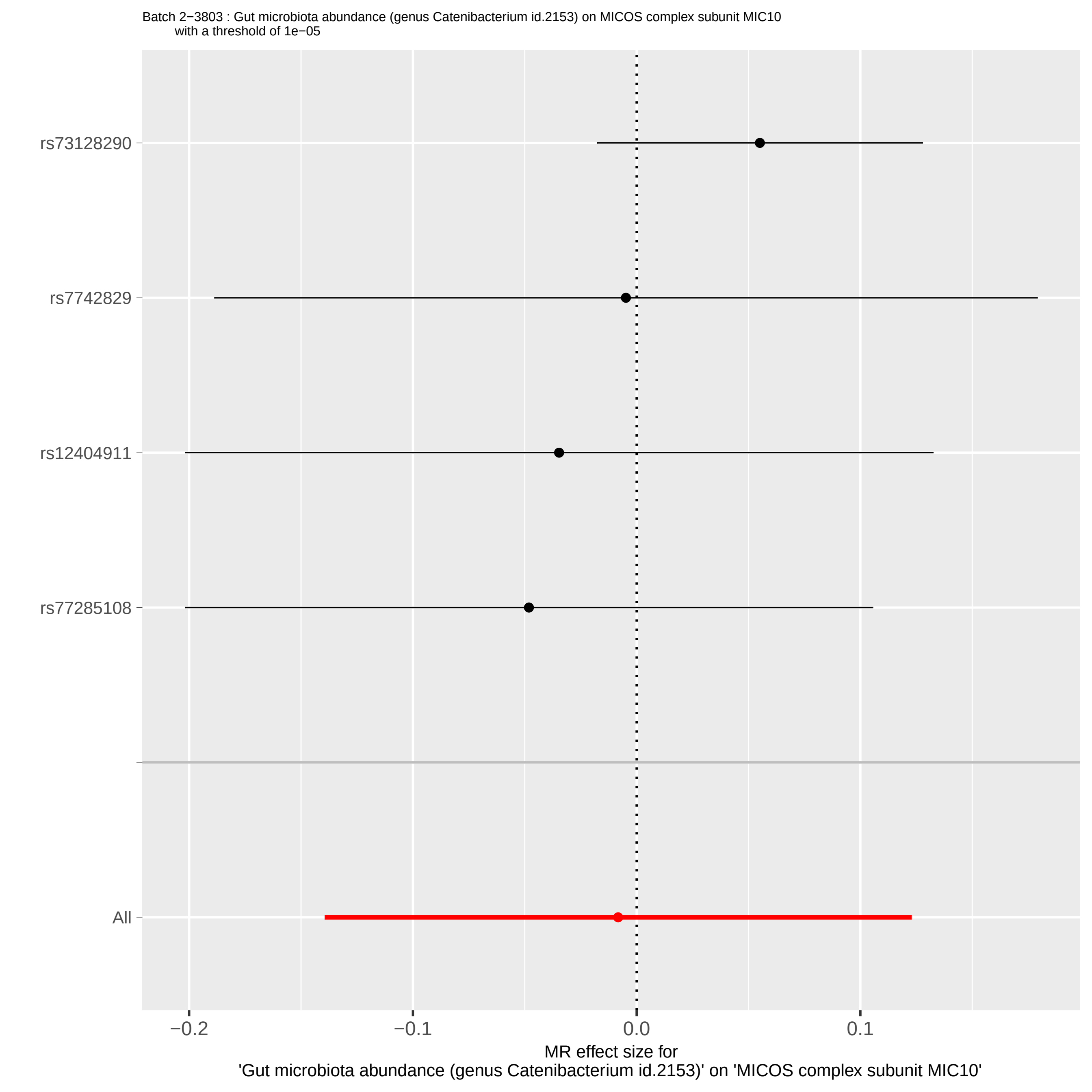

Batch 2−3803 : Gut microbiota abundance (genus Catenibacterium id.2153) on MICOS complex subunit MIC10
with a threshold of 1e−05
rs73128290
rs7742829
rs12404911
rs77285108
All
−0.2
−0.1
0.0
0.1
MR effect size for
'Gut microbiota abundance (genus Catenibacterium id.2153)' on 'MICOS complex subunit MIC10'

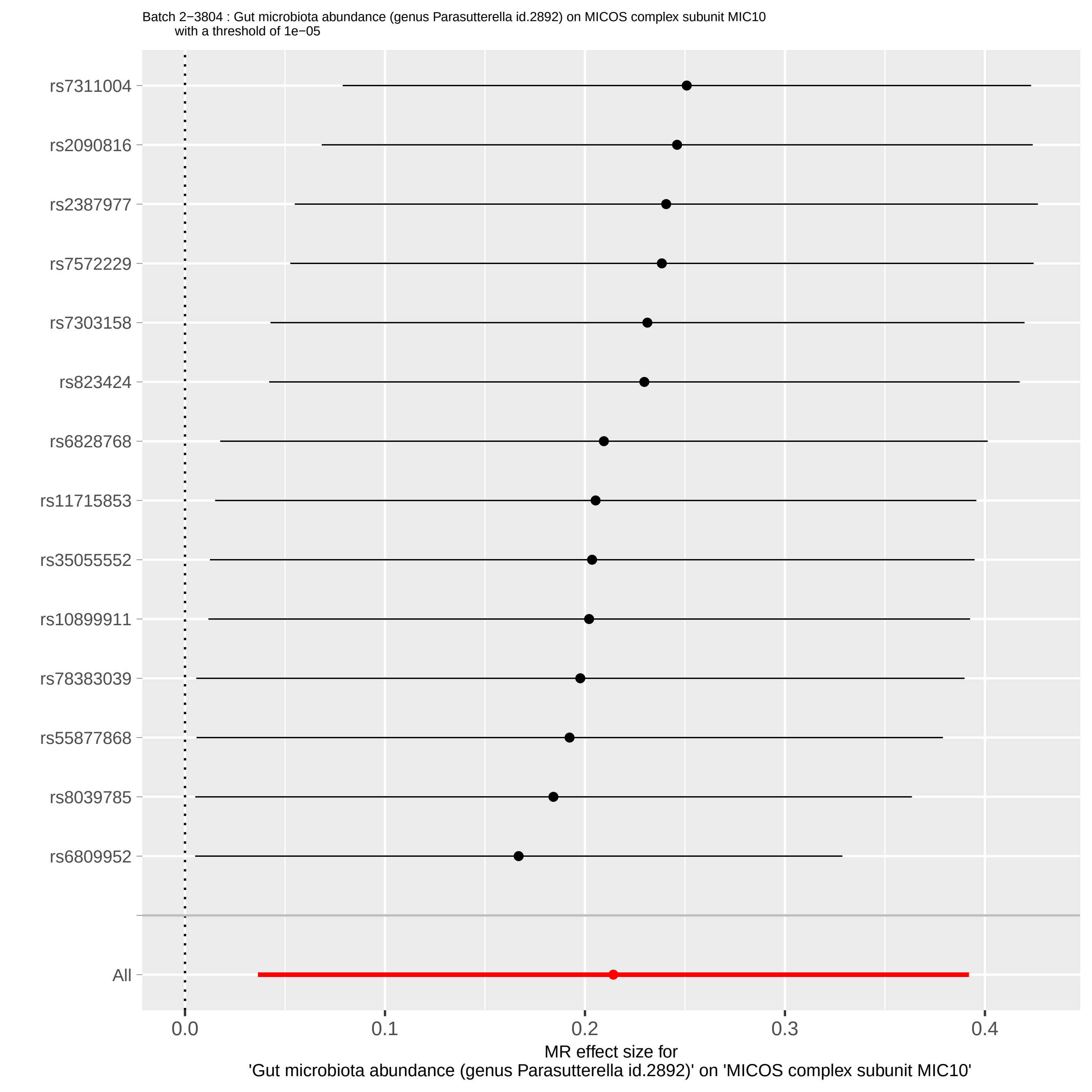
Batch 2−3804 : Gut microbiota abundance (genus Parasutterella id.2892) on MICOS complex subunit MIC10
with a threshold of 1e−05
rs7311004
rs2090816
rs2387977
rs7572229
rs7303158
rs823424
rs6828768
rs11715853
rs35055552
rs10899911
rs78383039
rs55877868
rs8039785
rs6809952
All
0.0
0.1
0.2
0.3
0.4
MR effect size for
'Gut microbiota abundance (genus Parasutterella id.2892)' on 'MICOS complex subunit MIC10'

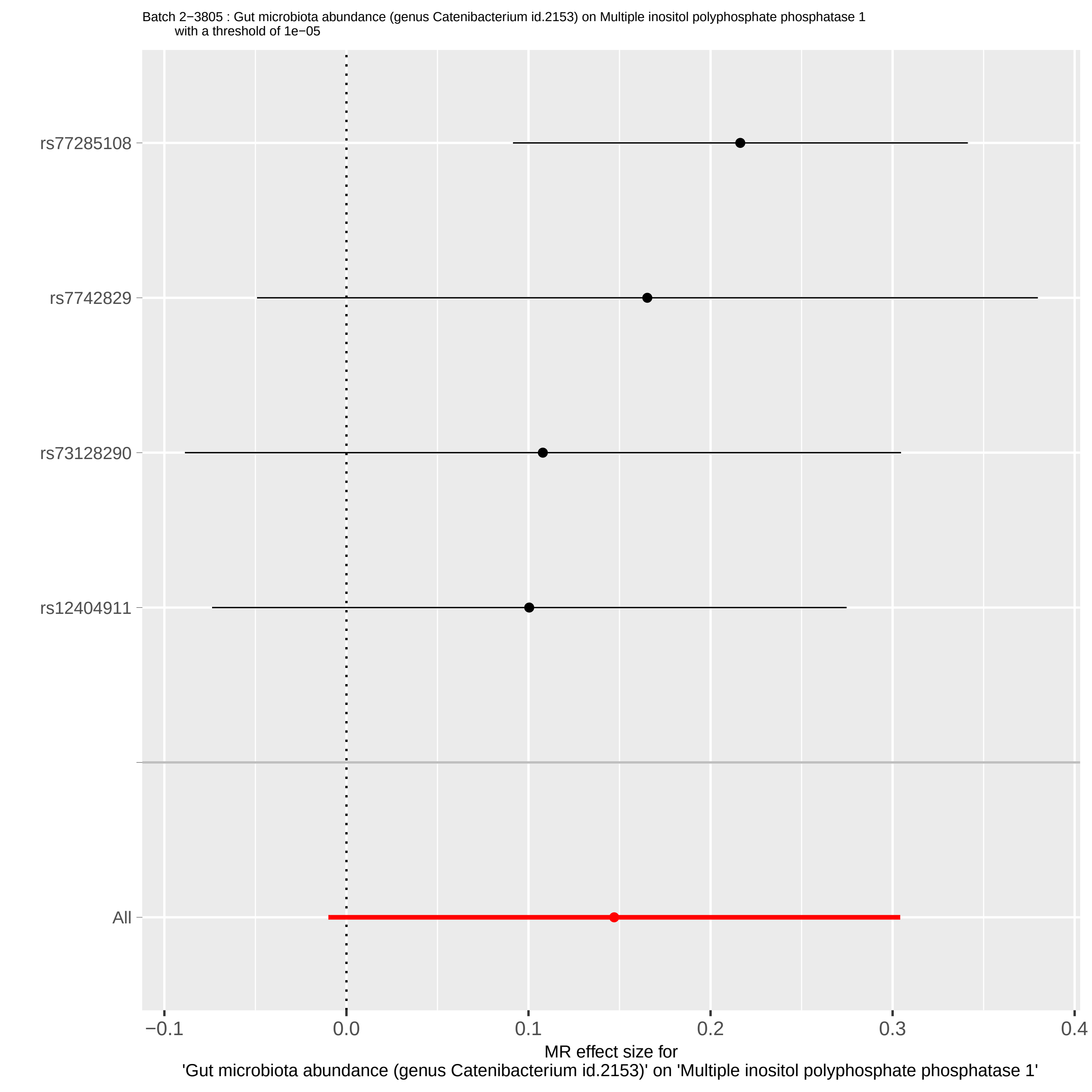
Batch 2−3805 : Gut microbiota abundance (genus Catenibacterium id.2153) on Multiple inositol polyphosphate phosphatase 1
with a threshold of 1e−05
rs77285108
rs7742829
rs73128290
rs12404911
All
−0.1
0.0
0.1
0.2
0.3
0.4
MR effect size for
'Gut microbiota abundance (genus Catenibacterium id.2153)' on 'Multiple inositol polyphosphate phosphatase 1'

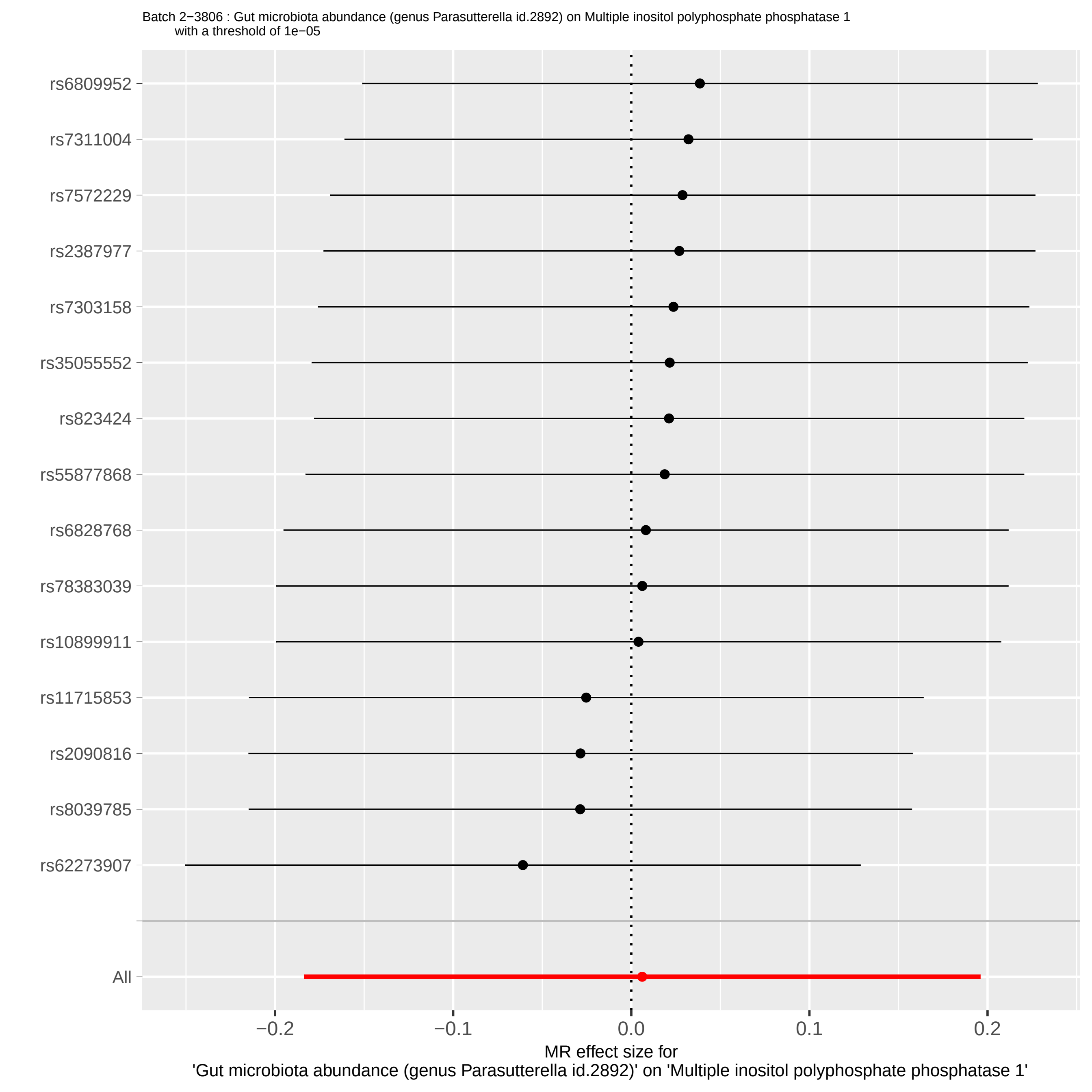

Batch 2−3806 : Gut microbiota abundance (genus Parasutterella id.2892) on Multiple inositol polyphosphate phosphatase 1
with a threshold of 1e−05
rs6809952
rs7311004
rs7572229
rs2387977
rs7303158
rs35055552
rs823424
rs55877868
rs6828768
rs78383039
rs10899911
rs11715853
rs2090816
rs8039785
rs62273907
All
−0.2
−0.1
0.0
0.1
0.2
MR effect size for
'Gut microbiota abundance (genus Parasutterella id.2892)' on 'Multiple inositol polyphosphate phosphatase 1'

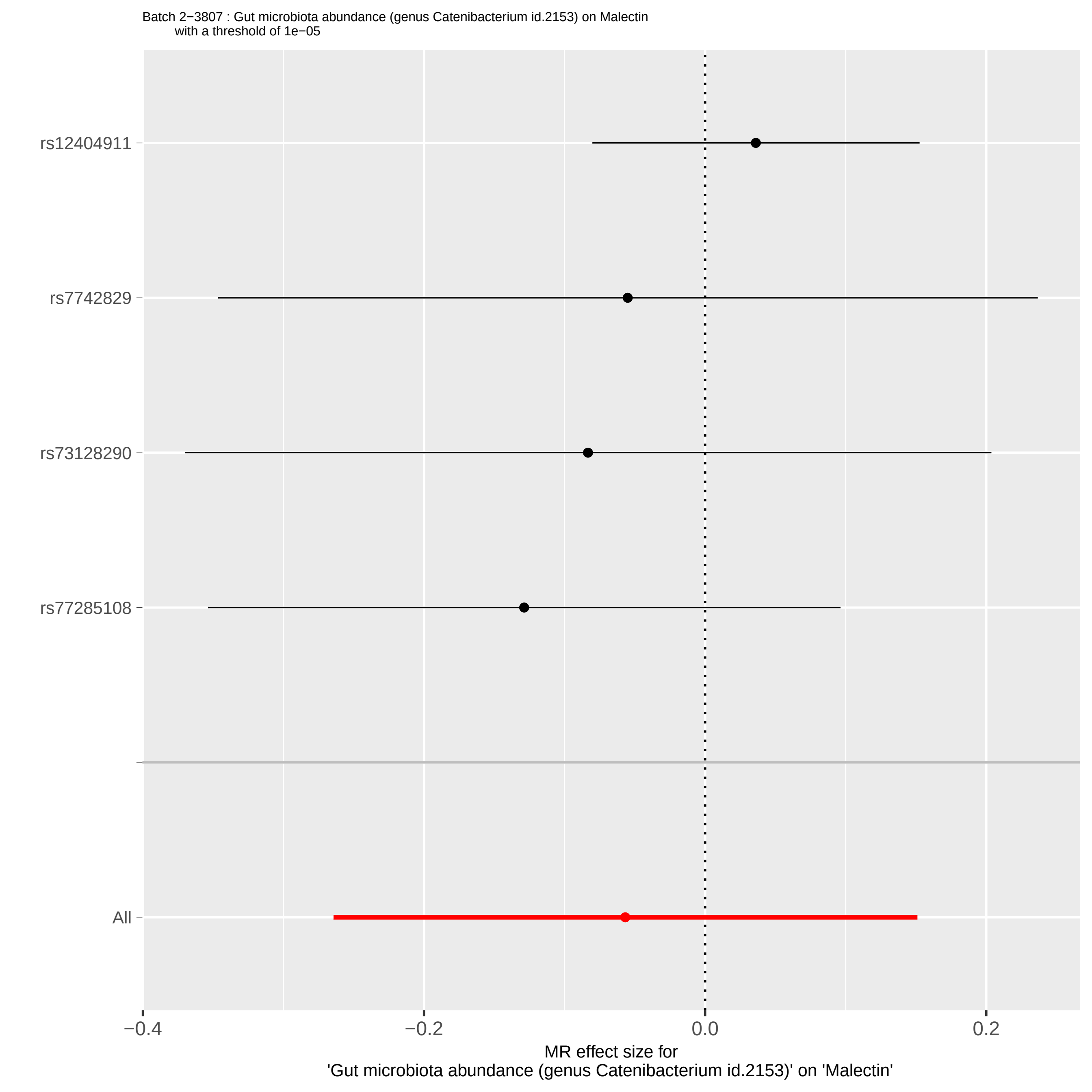
Batch 2−3807 : Gut microbiota abundance (genus Catenibacterium id.2153) on Malectin
with a threshold of 1e−05
rs12404911
rs7742829
rs73128290
rs77285108
All
−0.4
−0.2
0.0
0.2
MR effect size for
'Gut microbiota abundance (genus Catenibacterium id.2153)' on 'Malectin'

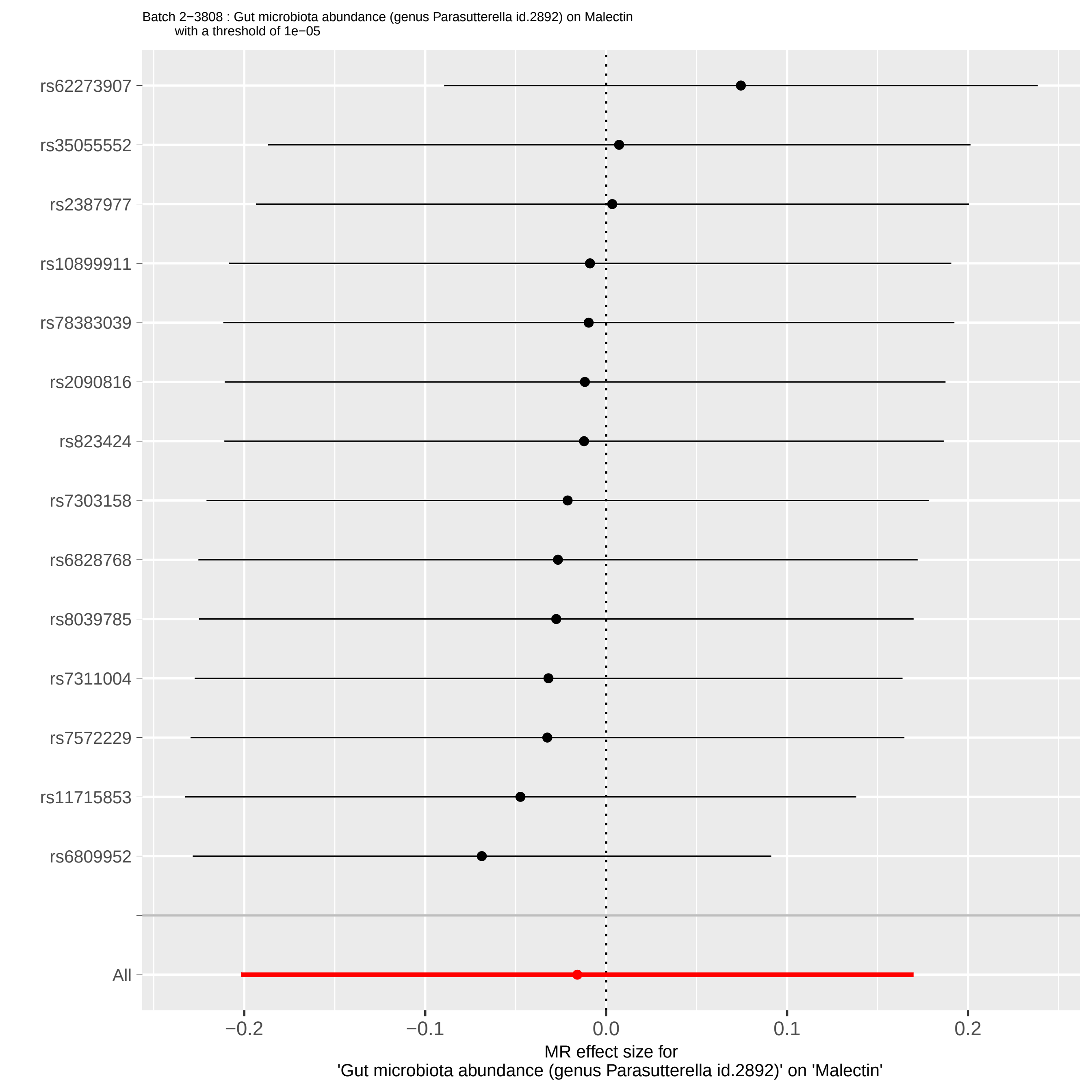

Batch 2−3808 : Gut microbiota abundance (genus Parasutterella id.2892) on Malectin
with a threshold of 1e−05
rs62273907
rs35055552
rs2387977
rs10899911
rs78383039
rs2090816
rs823424
rs7303158
rs6828768
rs8039785
rs7311004
rs7572229
rs11715853
rs6809952
All
−0.2
−0.1
0.0
0.1
0.2
MR effect size for
'Gut microbiota abundance (genus Parasutterella id.2892)' on 'Malectin'

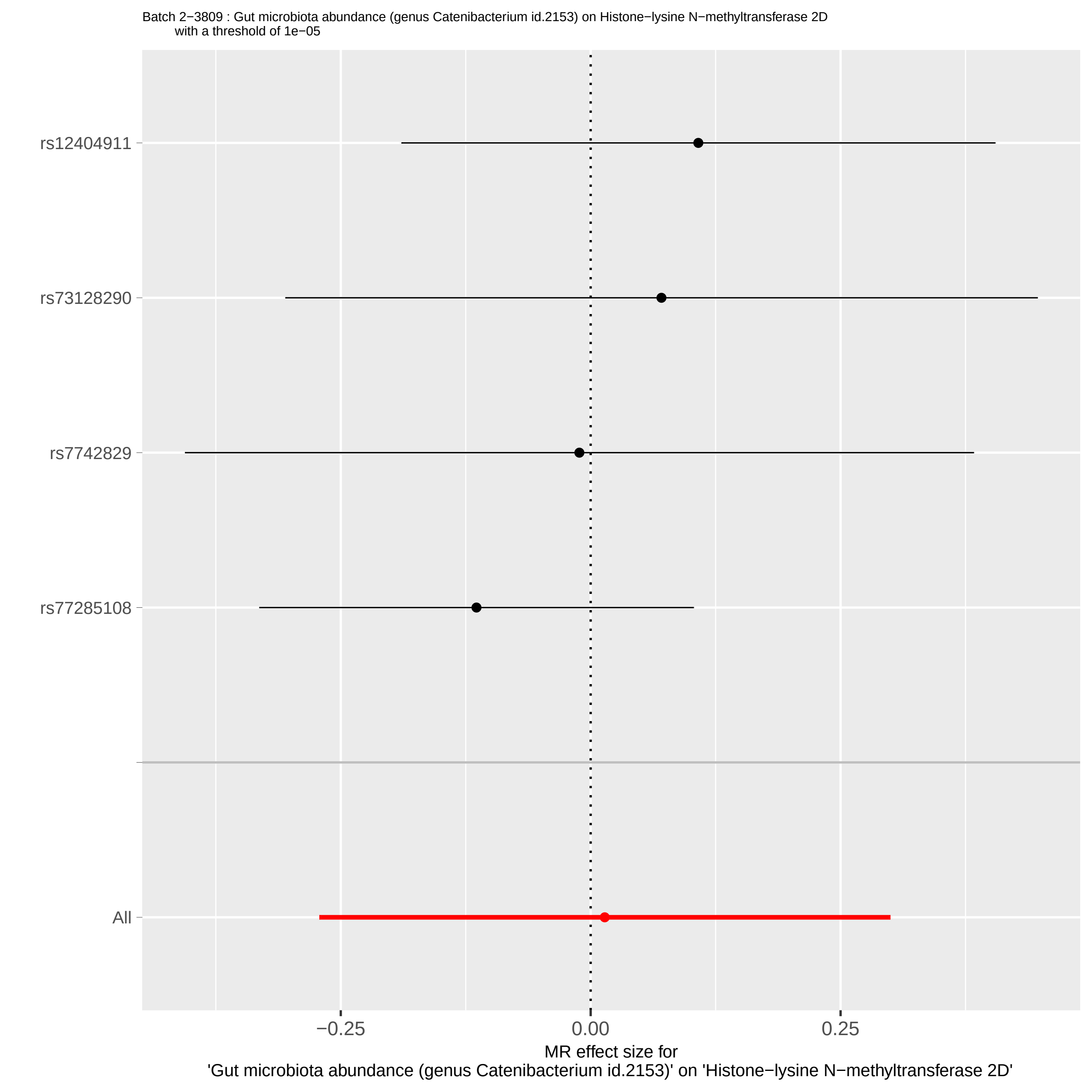
Batch 2−3809 : Gut microbiota abundance (genus Catenibacterium id.2153) on Histone−lysine N−methyltransferase 2D
with a threshold of 1e−05
rs12404911
rs73128290
rs7742829
rs77285108
All
−0.25
0.00
0.25
MR effect size for
'Gut microbiota abundance (genus Catenibacterium id.2153)' on 'Histone−lysine N−methyltransferase 2D'

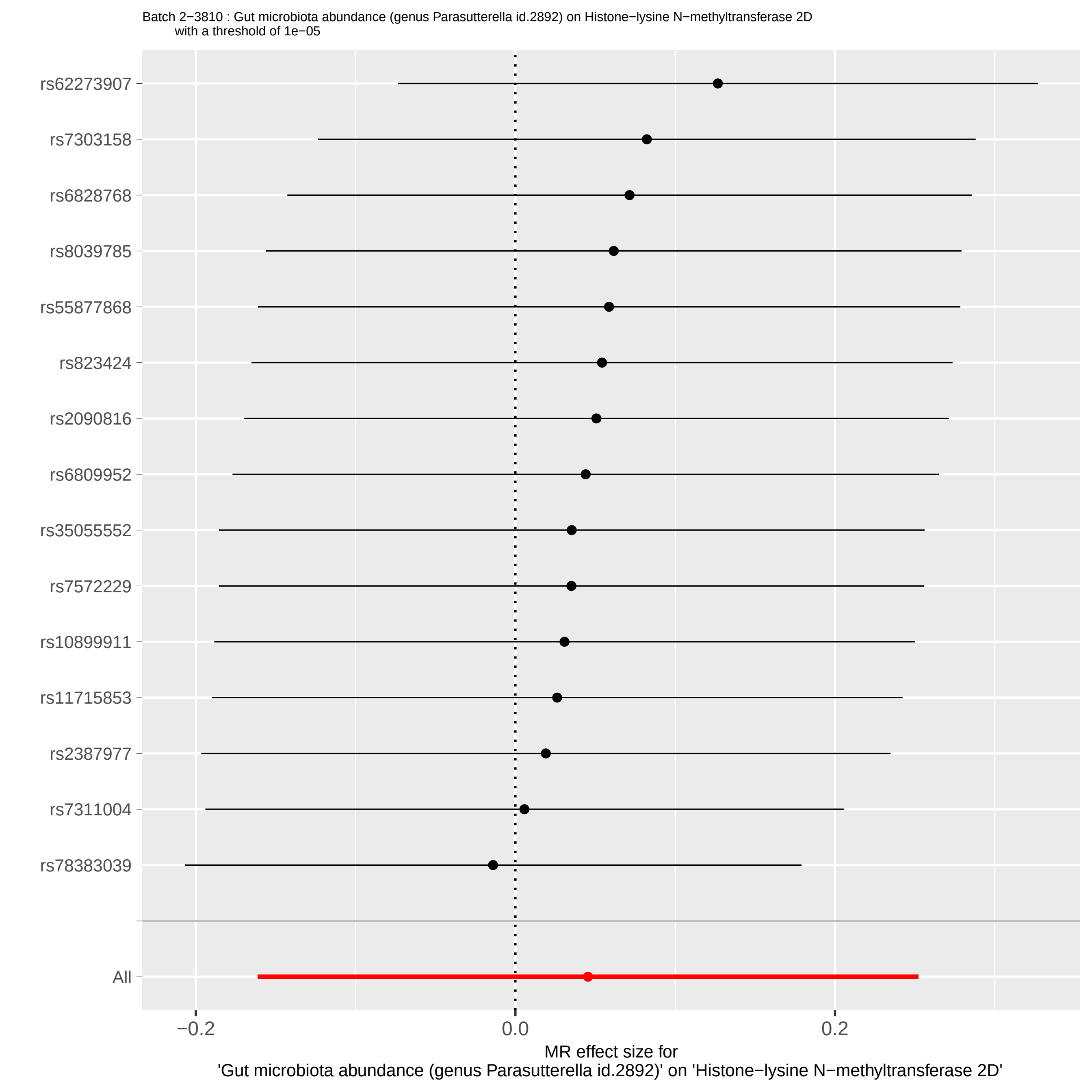
Batch 2−3810 : Gut microbiota abundance (genus Parasutterella id.2892) on Histone−lysine N−methyltransferase 2D
with a threshold of 1e−05
rs62273907
rs7303158
rs6828768
rs8039785
rs55877868
rs823424
rs2090816
rs6809952
rs35055552
rs7572229
rs10899911
rs11715853
rs2387977
rs7311004
rs78383039
All
−0.2
0.0
0.2
MR effect size for
'Gut microbiota abundance (genus Parasutterella id.2892)' on 'Histone−lysine N−methyltransferase 2D'

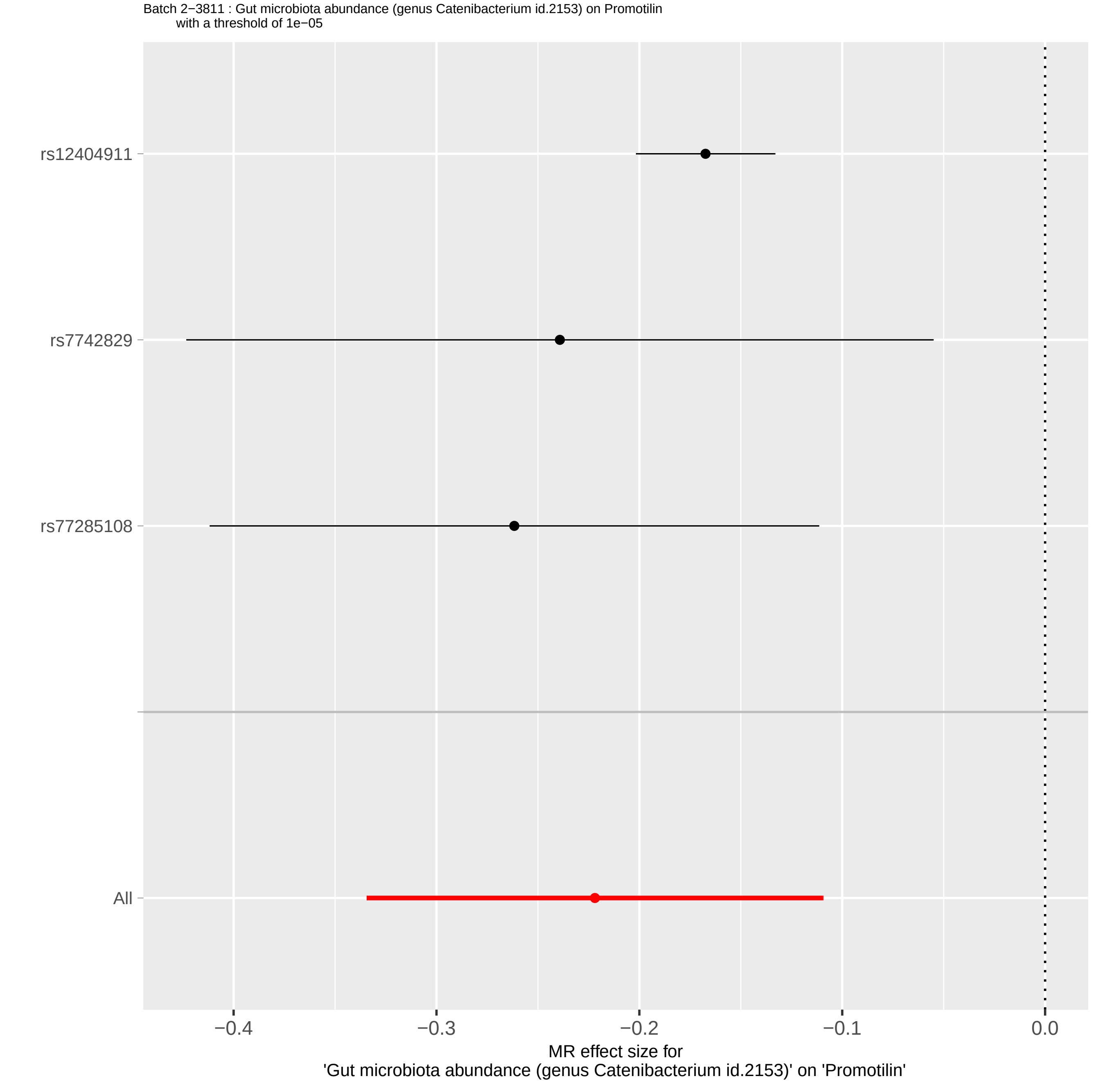
Batch 2−3811 : Gut microbiota abundance (genus Catenibacterium id.2153) on Promotilin
with a threshold of 1e−05
rs12404911
rs7742829
rs77285108
All
−0.4
−0.3
−0.2
−0.1
0.0
MR effect size for
'Gut microbiota abundance (genus Catenibacterium id.2153)' on 'Promotilin'

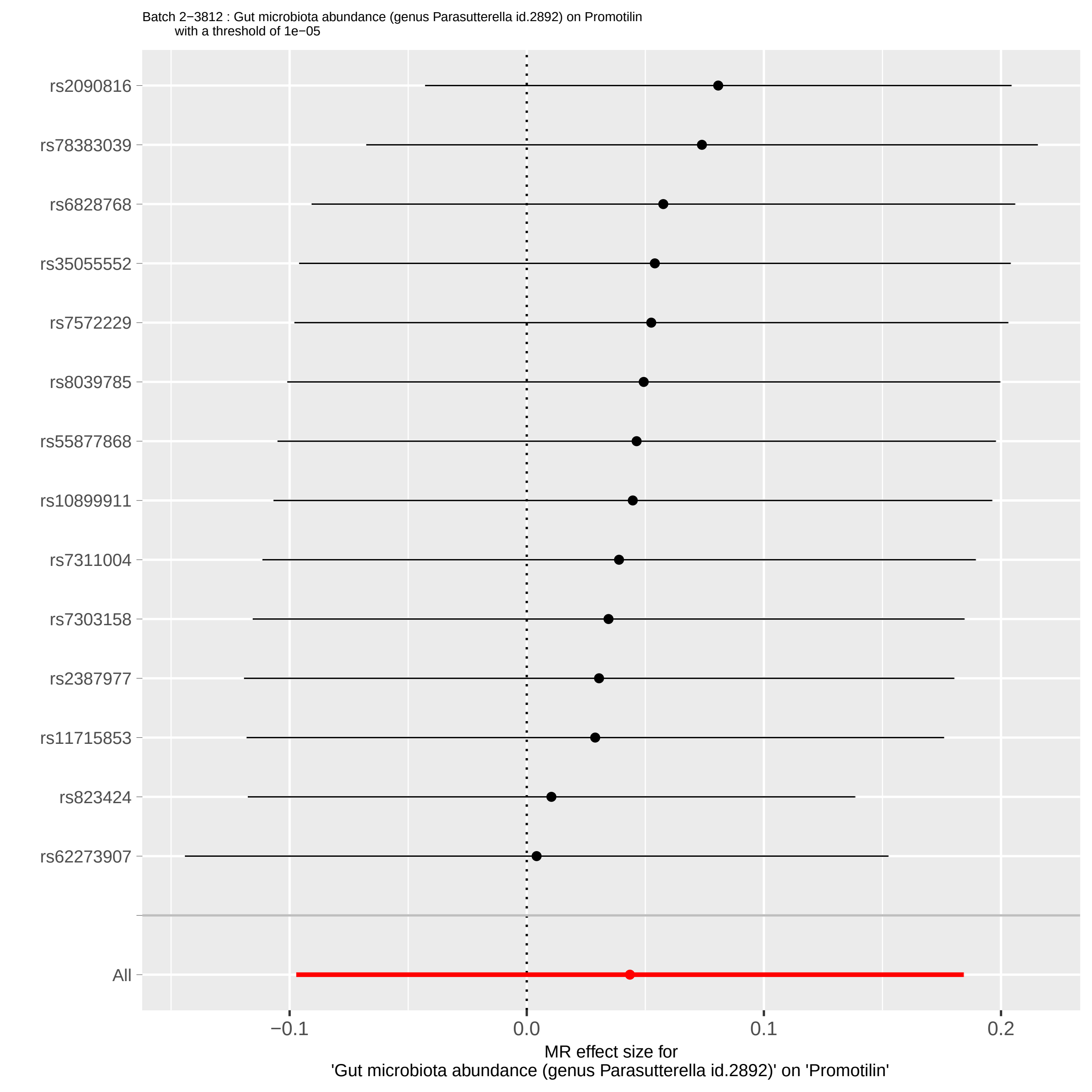

Batch 2−3812 : Gut microbiota abundance (genus Parasutterella id.2892) on Promotilin
with a threshold of 1e−05
rs2090816
rs78383039
rs6828768
rs35055552
rs7572229
rs8039785
rs55877868
rs10899911
rs7311004
rs7303158
rs2387977
rs11715853
rs823424
rs62273907
All
−0.1
0.0
0.1
0.2
MR effect size for
'Gut microbiota abundance (genus Parasutterella id.2892)' on 'Promotilin'

Batch 2−3813 : Gut microbiota abundance (genus Catenibacterium id.2153) on Malonyl−CoA decarboxylase, mitochondrial with a threshold of 1e−05

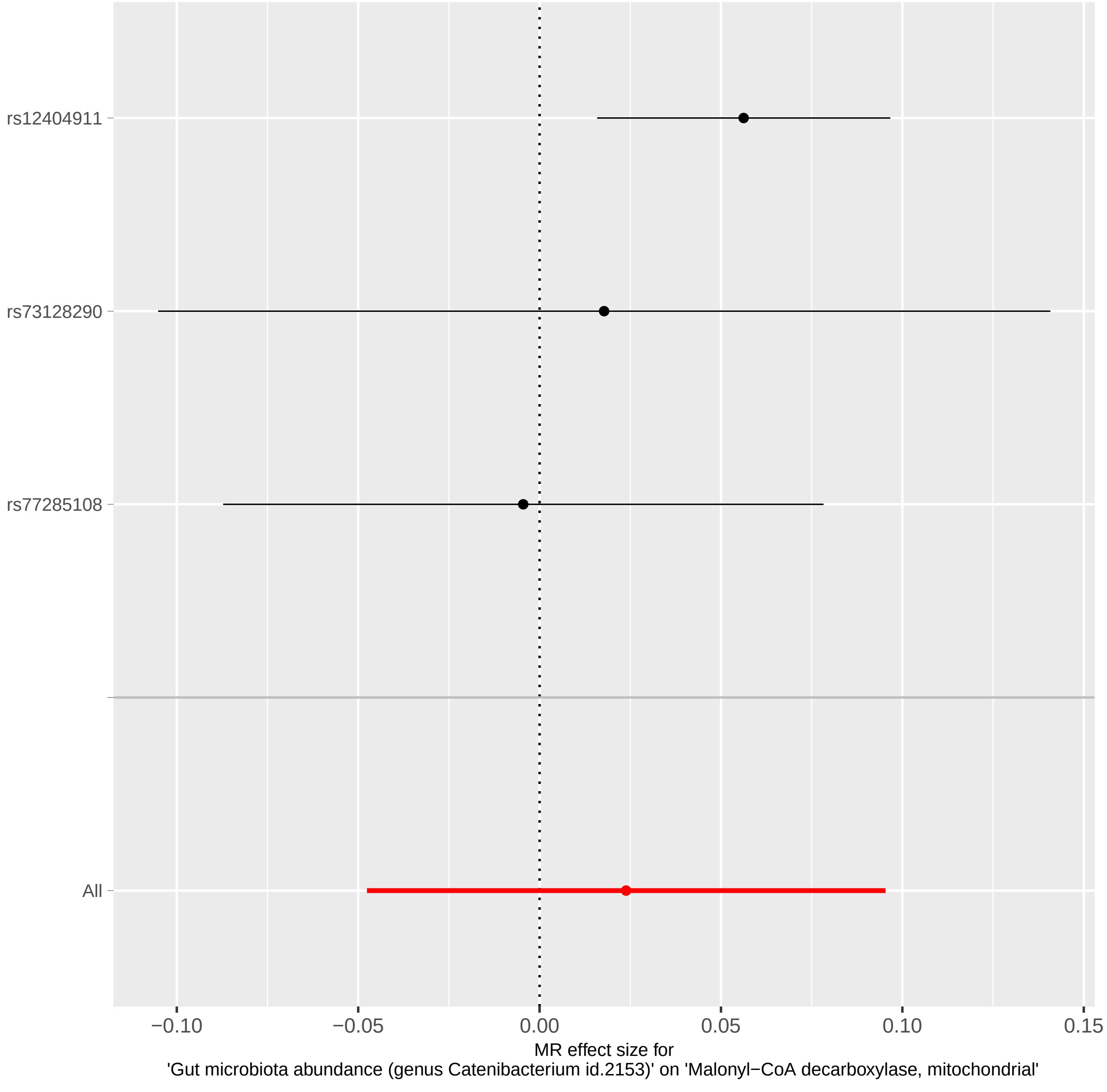

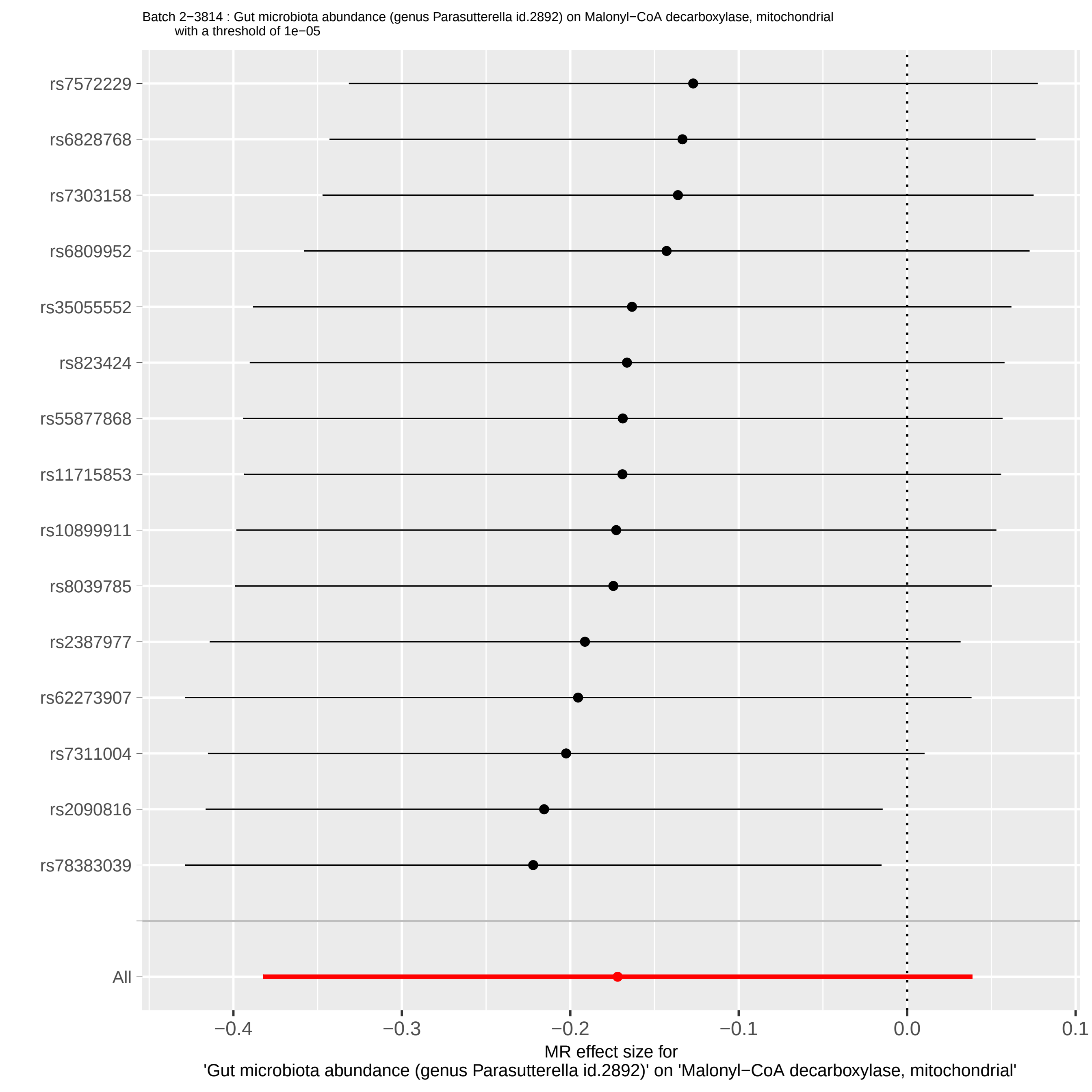
Batch 2−3814 : Gut microbiota abundance (genus Parasutterella id.2892) on Malonyl−CoA decarboxylase, mitochondrial
with a threshold of 1e−05
rs7572229
rs6828768
rs7303158
rs6809952
rs35055552
rs823424
rs55877868
rs11715853
rs10899911
rs8039785
rs2387977
rs62273907
rs7311004
rs2090816
rs78383039
All
−0.4
−0.3
−0.2
−0.1
0.0
0.1
MR effect size for
'Gut microbiota abundance (genus Parasutterella id.2892)' on 'Malonyl−CoA decarboxylase, mitochondrial'

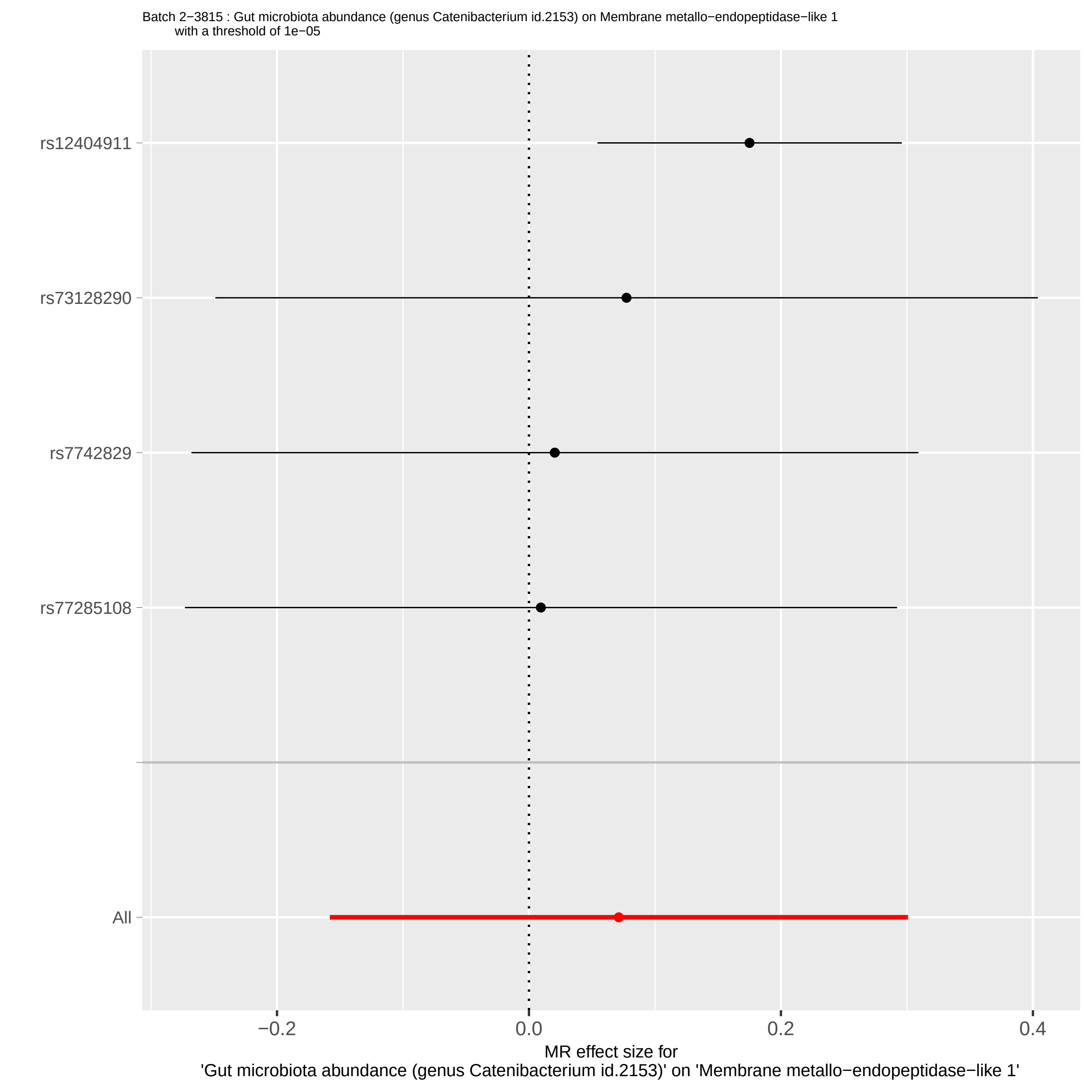
Batch 2−3815 : Gut microbiota abundance (genus Catenibacterium id.2153) on Membrane metallo−endopeptidase−like 1
with a threshold of 1e−05
rs12404911
rs73128290
rs7742829
rs77285108
All
−0.2
0.0
0.2
0.4
MR effect size for
'Gut microbiota abundance (genus Catenibacterium id.2153)' on 'Membrane metallo−endopeptidase−like 1'

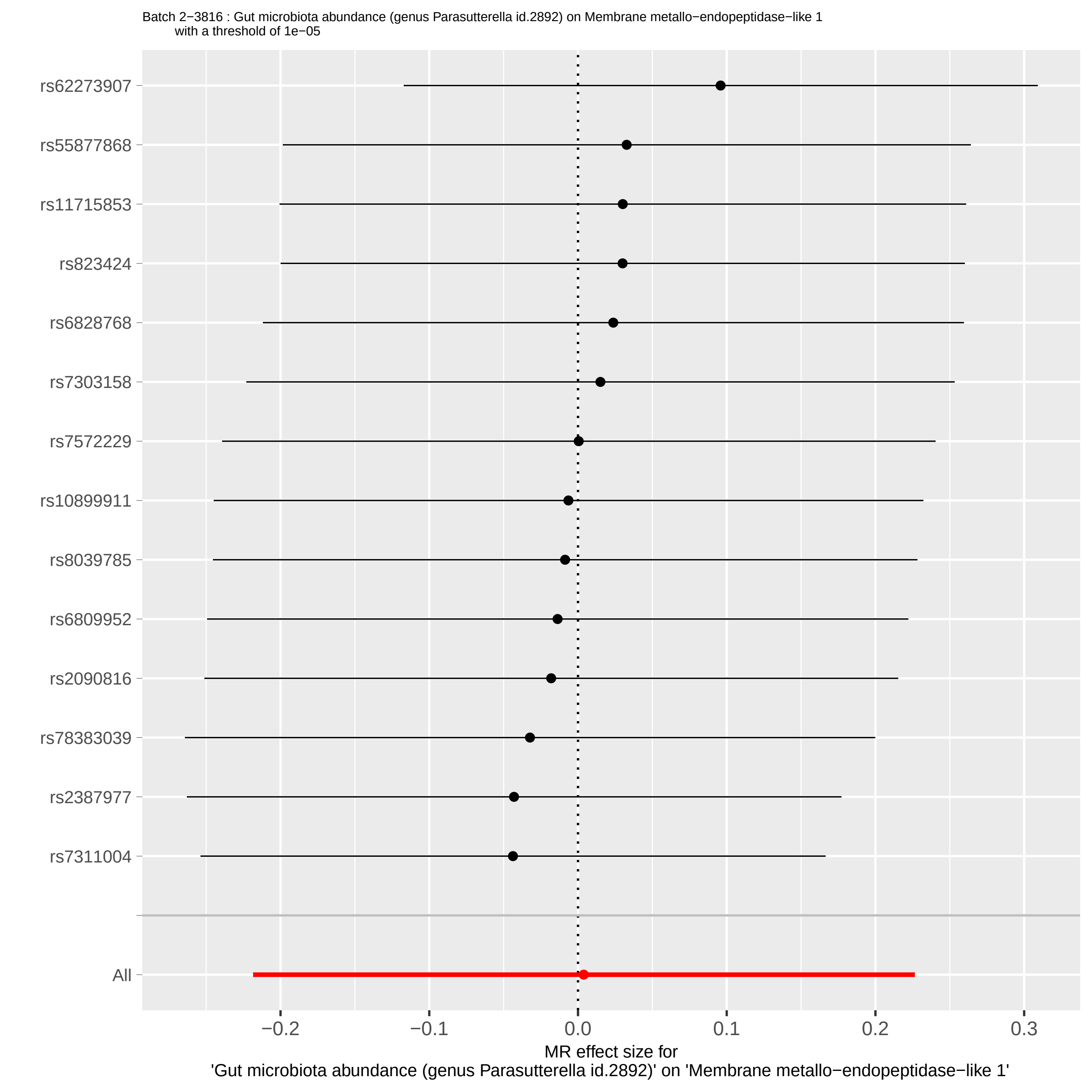
Batch 2−3816 : Gut microbiota abundance (genus Parasutterella id.2892) on Membrane metallo−endopeptidase−like 1
with a threshold of 1e−05
rs62273907
rs55877868
rs11715853
rs823424
rs6828768
rs7303158
rs7572229
rs10899911
rs8039785
rs6809952
rs2090816
rs78383039
rs2387977
rs7311004
All
−0.2
−0.1
0.0
0.1
0.2
0.3
MR effect size for
'Gut microbiota abundance (genus Parasutterella id.2892)' on 'Membrane metallo−endopeptidase−like 1'

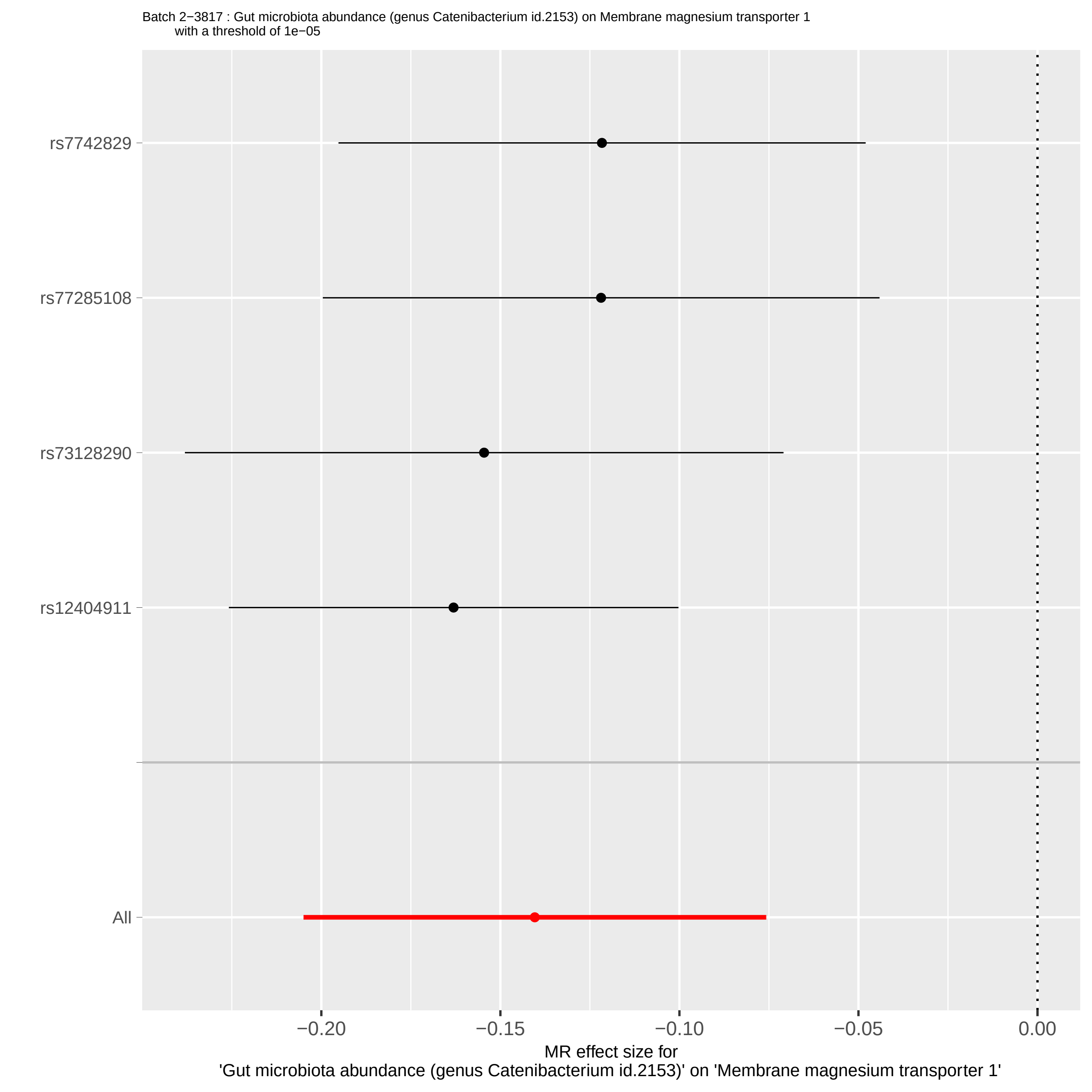
Batch 2−3817 : Gut microbiota abundance (genus Catenibacterium id.2153) on Membrane magnesium transporter 1
with a threshold of 1e−05
rs7742829
rs77285108
rs73128290
rs12404911
All
−0.20
−0.15
−0.10
−0.05
0.00
MR effect size for
'Gut microbiota abundance (genus Catenibacterium id.2153)' on 'Membrane magnesium transporter 1'

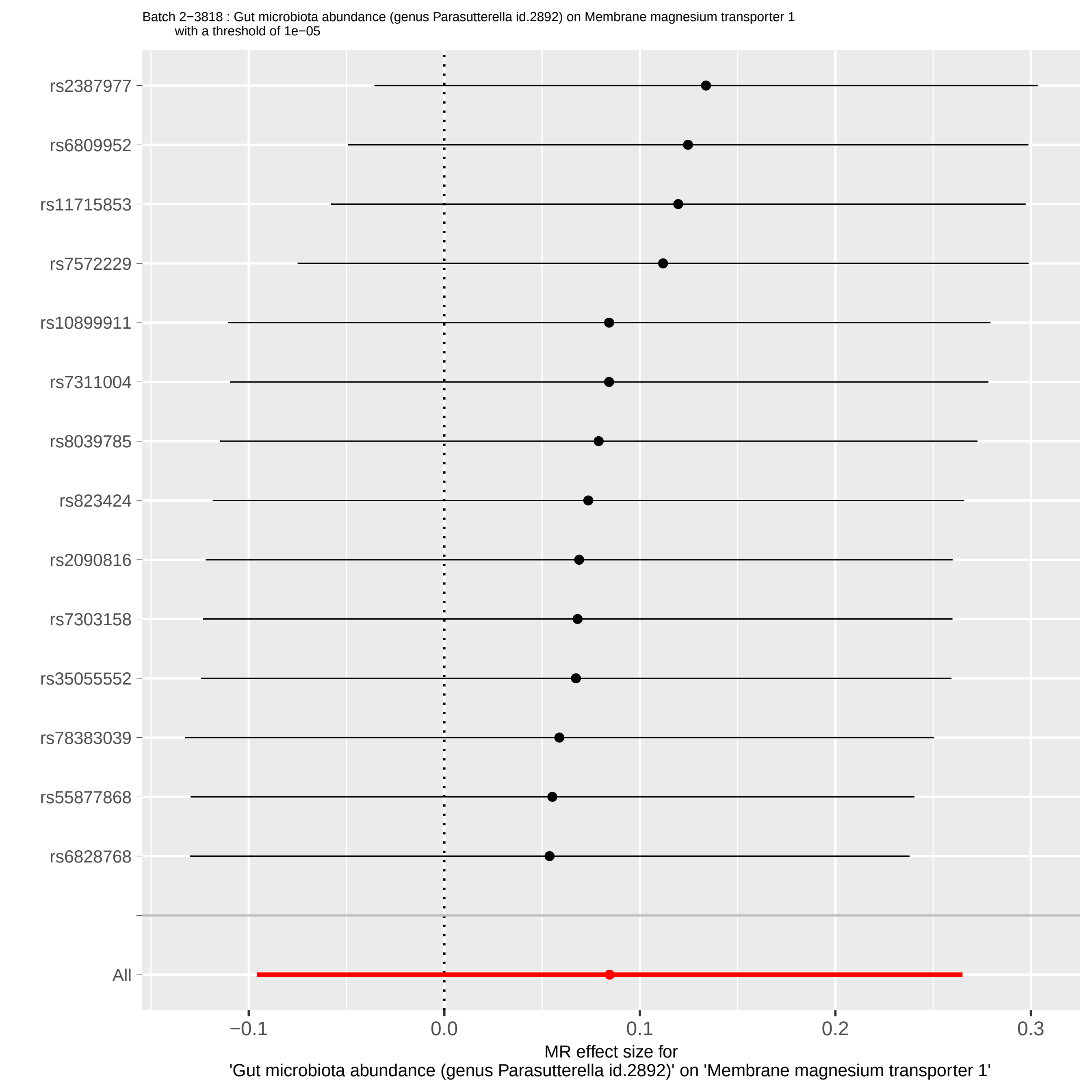
Batch 2−3818 : Gut microbiota abundance (genus Parasutterella id.2892) on Membrane magnesium transporter 1
with a threshold of 1e−05
rs2387977
rs6809952
rs11715853
rs7572229
rs10899911
rs7311004
rs8039785
rs823424
rs2090816
rs7303158
rs35055552
rs78383039
rs55877868
rs6828768
All
−0.1
0.0
0.1
0.2
0.3
MR effect size for
'Gut microbiota abundance (genus Parasutterella id.2892)' on 'Membrane magnesium transporter 1'

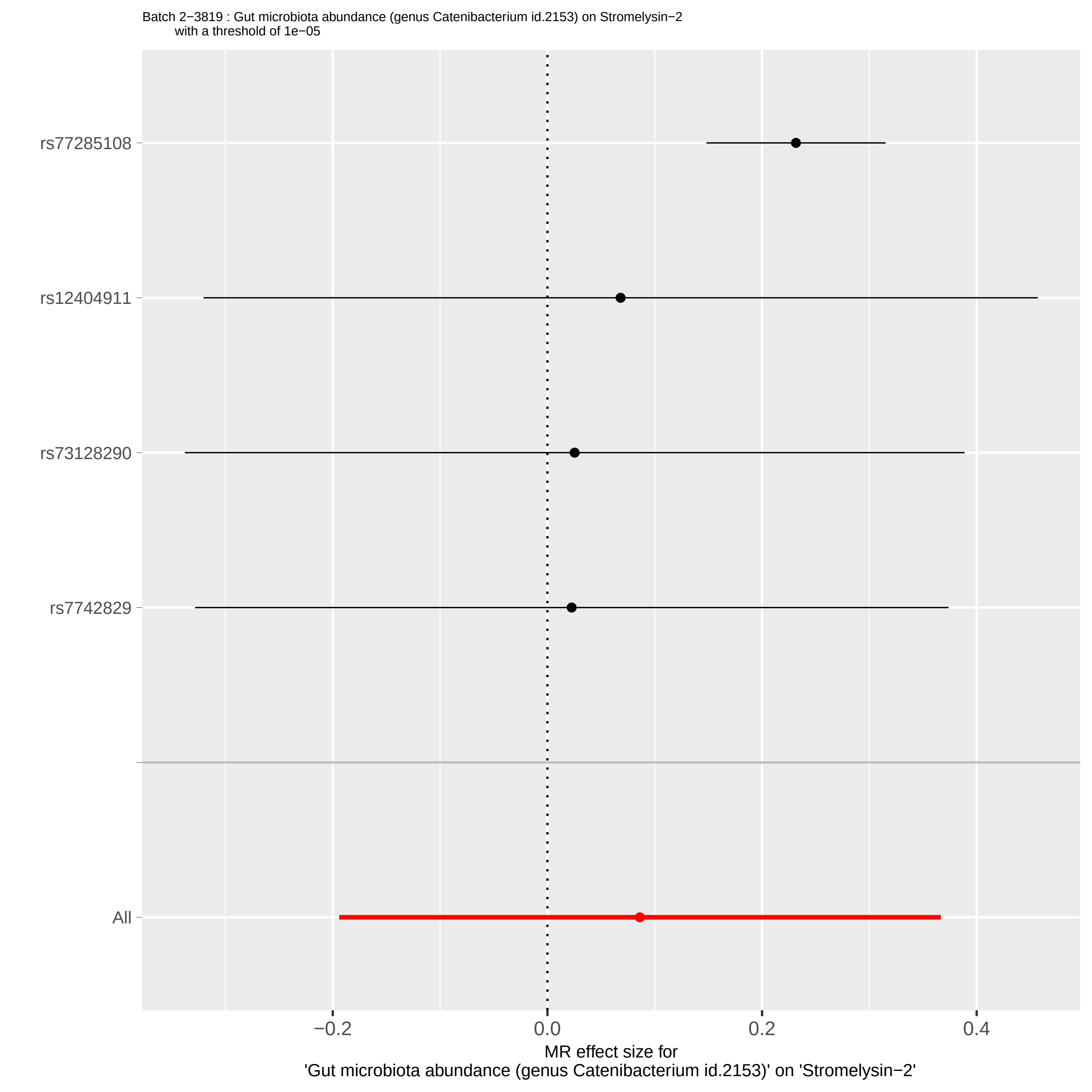

Batch 2−3819 : Gut microbiota abundance (genus Catenibacterium id.2153) on Stromelysin−2
with a threshold of 1e−05
rs77285108
rs12404911
rs73128290
rs7742829
All
−0.2
0.0
0.2
0.4
MR effect size for
'Gut microbiota abundance (genus Catenibacterium id.2153)' on 'Stromelysin−2'

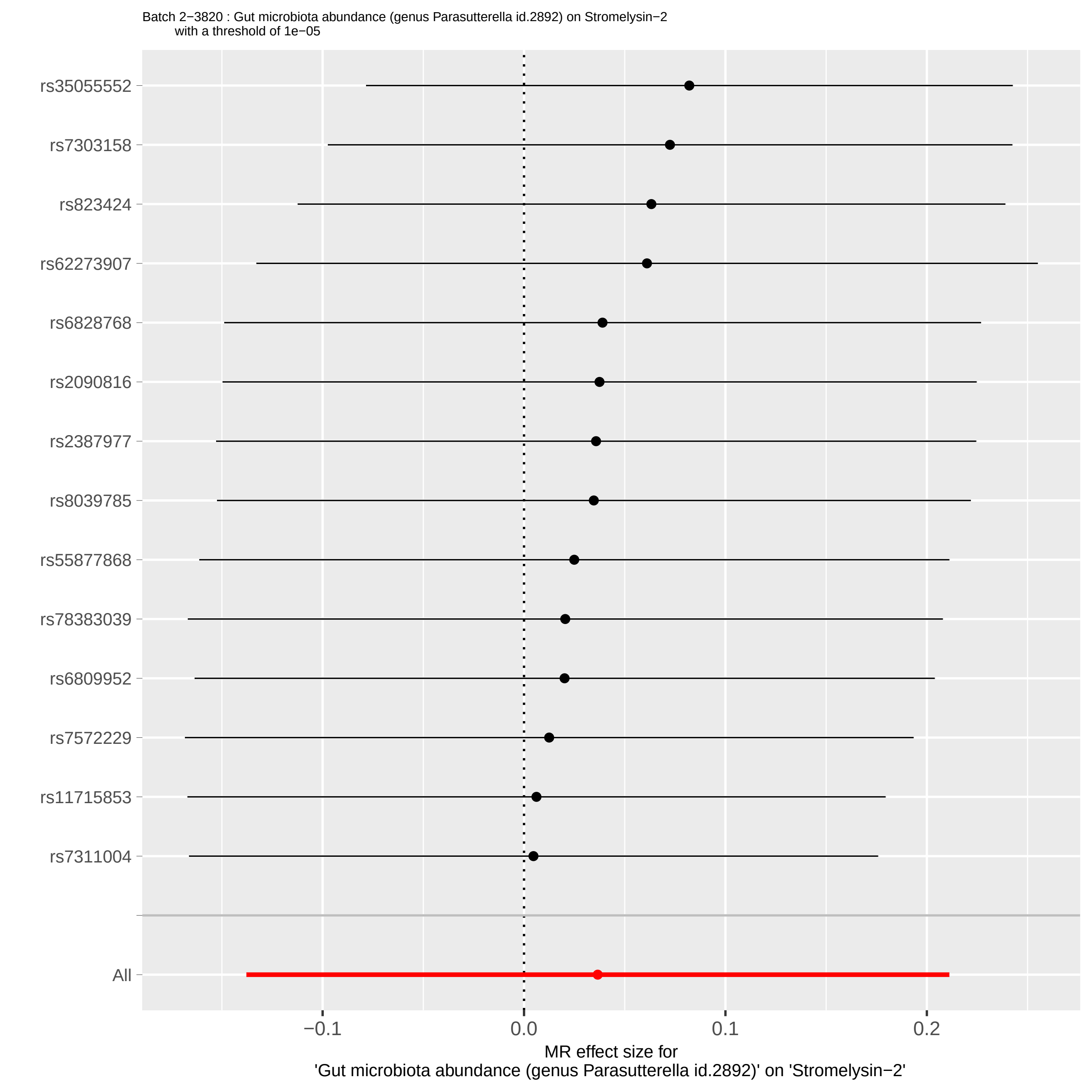

Batch 2−3820 : Gut microbiota abundance (genus Parasutterella id.2892) on Stromelysin−2
with a threshold of 1e−05
rs35055552
rs7303158
rs823424
rs62273907
rs6828768
rs2090816
rs2387977
rs8039785
rs55877868
rs78383039
rs6809952
rs7572229
rs11715853
rs7311004
All
−0.1
0.0
0.1
0.2
MR effect size for
'Gut microbiota abundance (genus Parasutterella id.2892)' on 'Stromelysin−2'

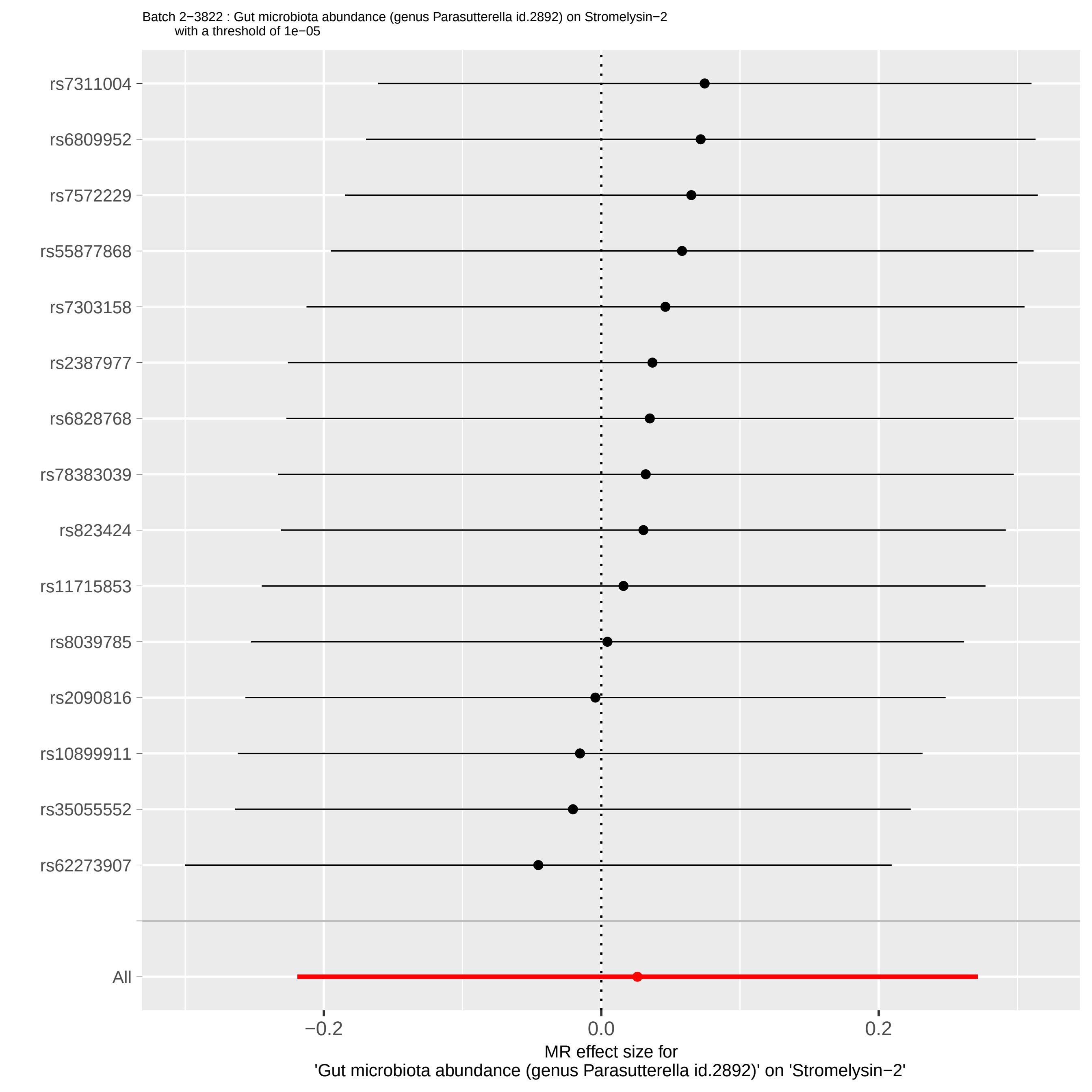

Batch 2−3822 : Gut microbiota abundance (genus Parasutterella id.2892) on Stromelysin−2
with a threshold of 1e−05
rs7311004
rs6809952
rs7572229
rs55877868
rs7303158
rs2387977
rs6828768
rs78383039
rs823424
rs11715853
rs8039785
rs2090816
rs10899911
rs35055552
rs62273907
All
−0.2
0.0
0.2
MR effect size for
'Gut microbiota abundance (genus Parasutterella id.2892)' on 'Stromelysin−2'

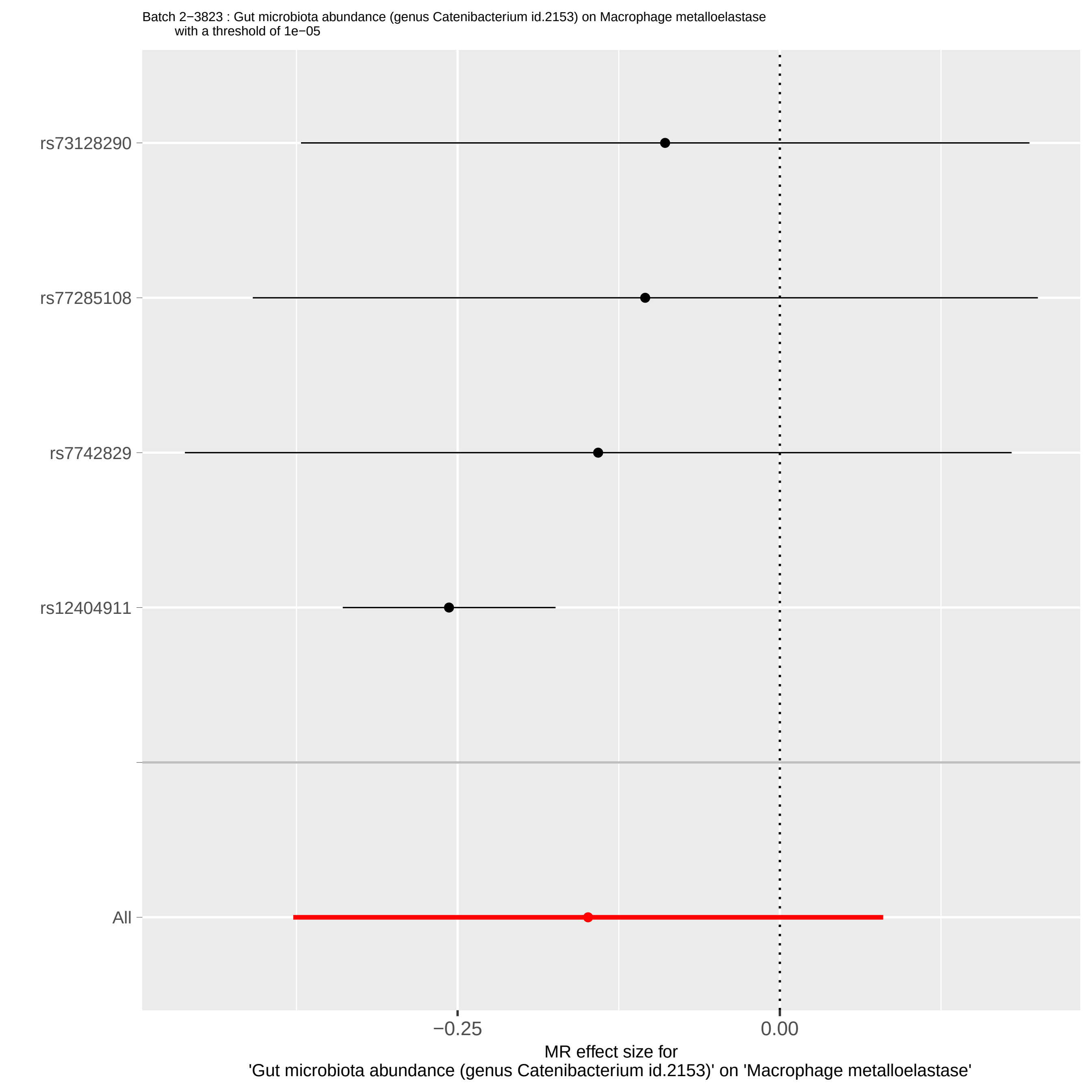
Batch 2−3823 : Gut microbiota abundance (genus Catenibacterium id.2153) on Macrophage metalloelastase
with a threshold of 1e−05
rs73128290
rs77285108
rs7742829
rs12404911
All
−0.25
0.00
MR effect size for
'Gut microbiota abundance (genus Catenibacterium id.2153)' on 'Macrophage metalloelastase'

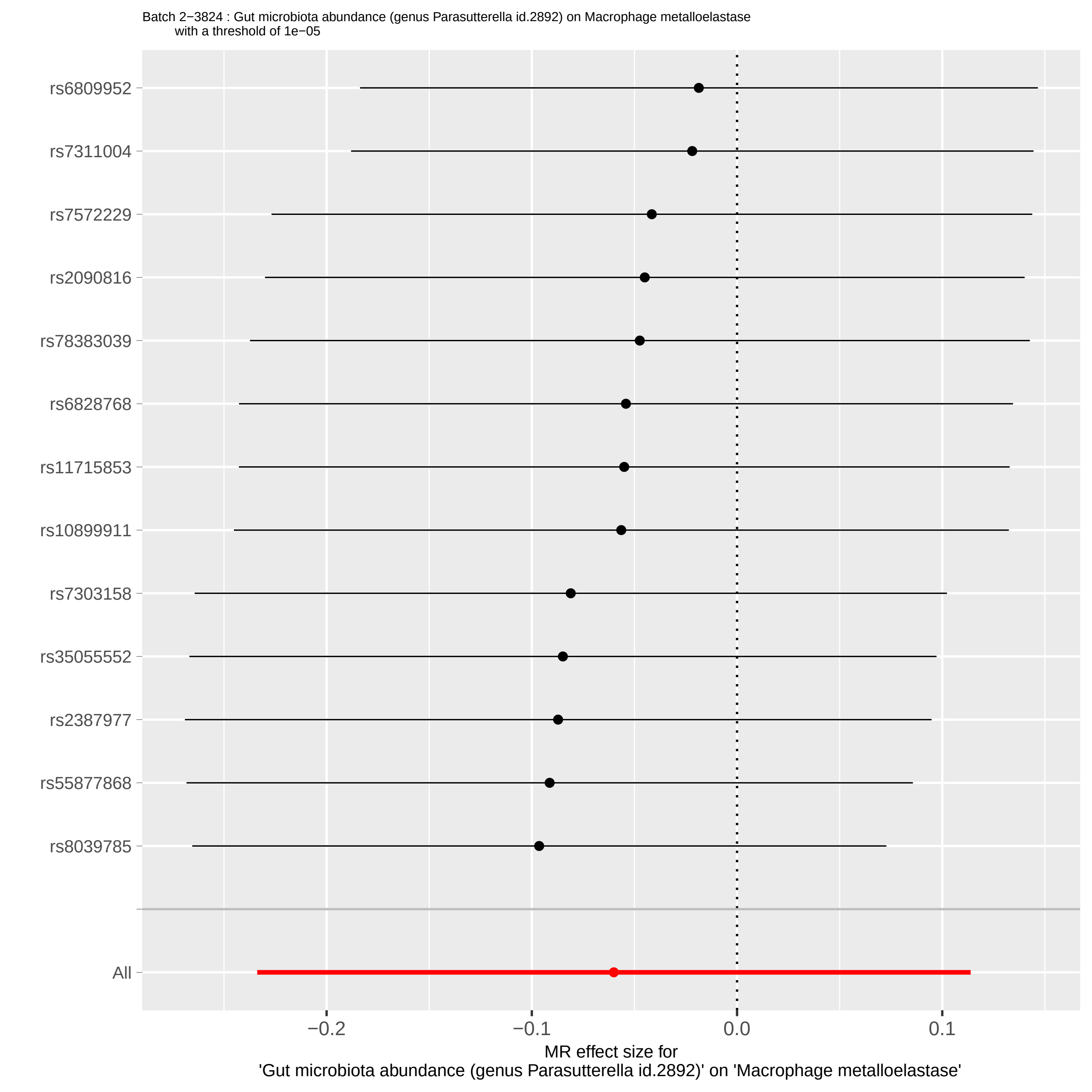
Batch 2−3824 : Gut microbiota abundance (genus Parasutterella id.2892) on Macrophage metalloelastase
with a threshold of 1e−05
rs6809952
rs7311004
rs7572229
rs2090816
rs78383039
rs6828768
rs11715853
rs10899911
rs7303158
rs35055552
rs2387977
rs55877868
rs8039785
All
−0.2
−0.1
0.0
0.1
MR effect size for
'Gut microbiota abundance (genus Parasutterella id.2892)' on 'Macrophage metalloelastase'

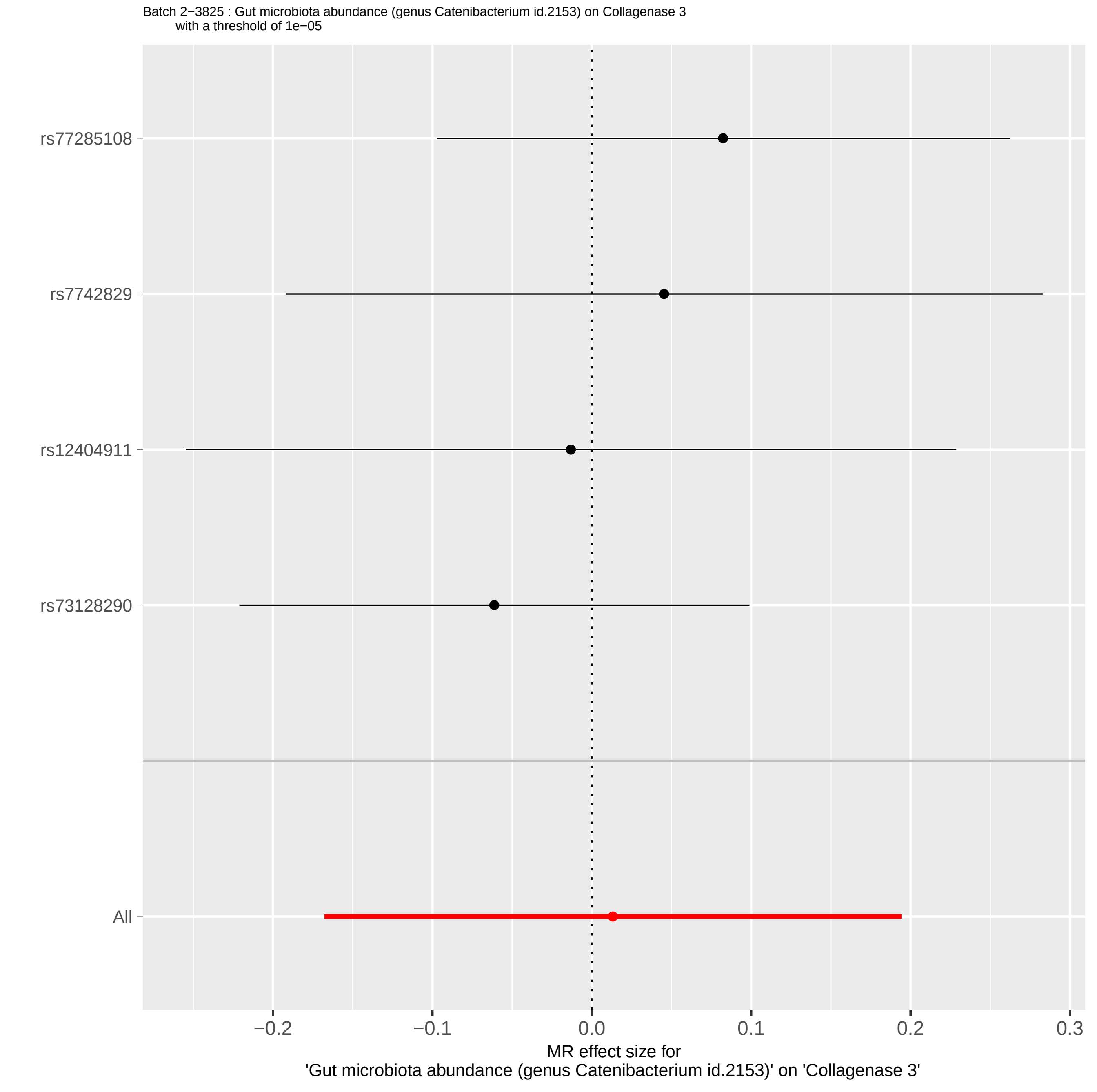
Batch 2−3825 : Gut microbiota abundance (genus Catenibacterium id.2153) on Collagenase 3
with a threshold of 1e−05
rs77285108
rs7742829
rs12404911
rs73128290
All
−0.2
−0.1
0.0
0.1
0.2
0.3
MR effect size for
'Gut microbiota abundance (genus Catenibacterium id.2153)' on 'Collagenase 3'

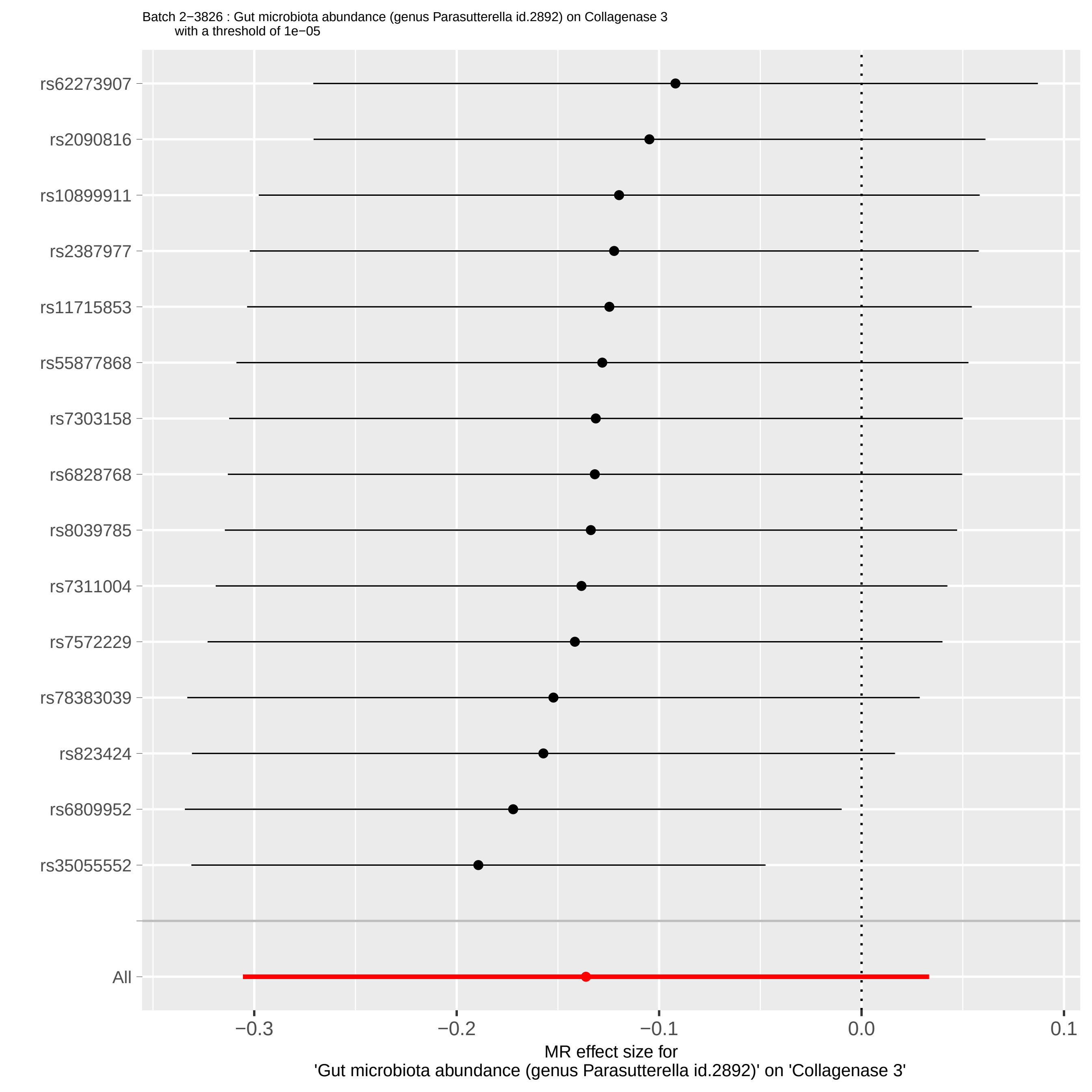

Batch 2−3826 : Gut microbiota abundance (genus Parasutterella id.2892) on Collagenase 3
with a threshold of 1e−05
rs62273907
rs2090816
rs10899911
rs2387977
rs11715853
rs55877868
rs7303158
rs6828768
rs8039785
rs7311004
rs7572229
rs78383039
rs823424
rs6809952
rs35055552
All
−0.3
−0.2
−0.1
0.0
0.1
MR effect size for
'Gut microbiota abundance (genus Parasutterella id.2892)' on 'Collagenase 3'

Batch 2−3827 : Gut microbiota abundance (genus Catenibacterium id.2153) on Matrix metalloproteinase−14
with a threshold of 1e−05

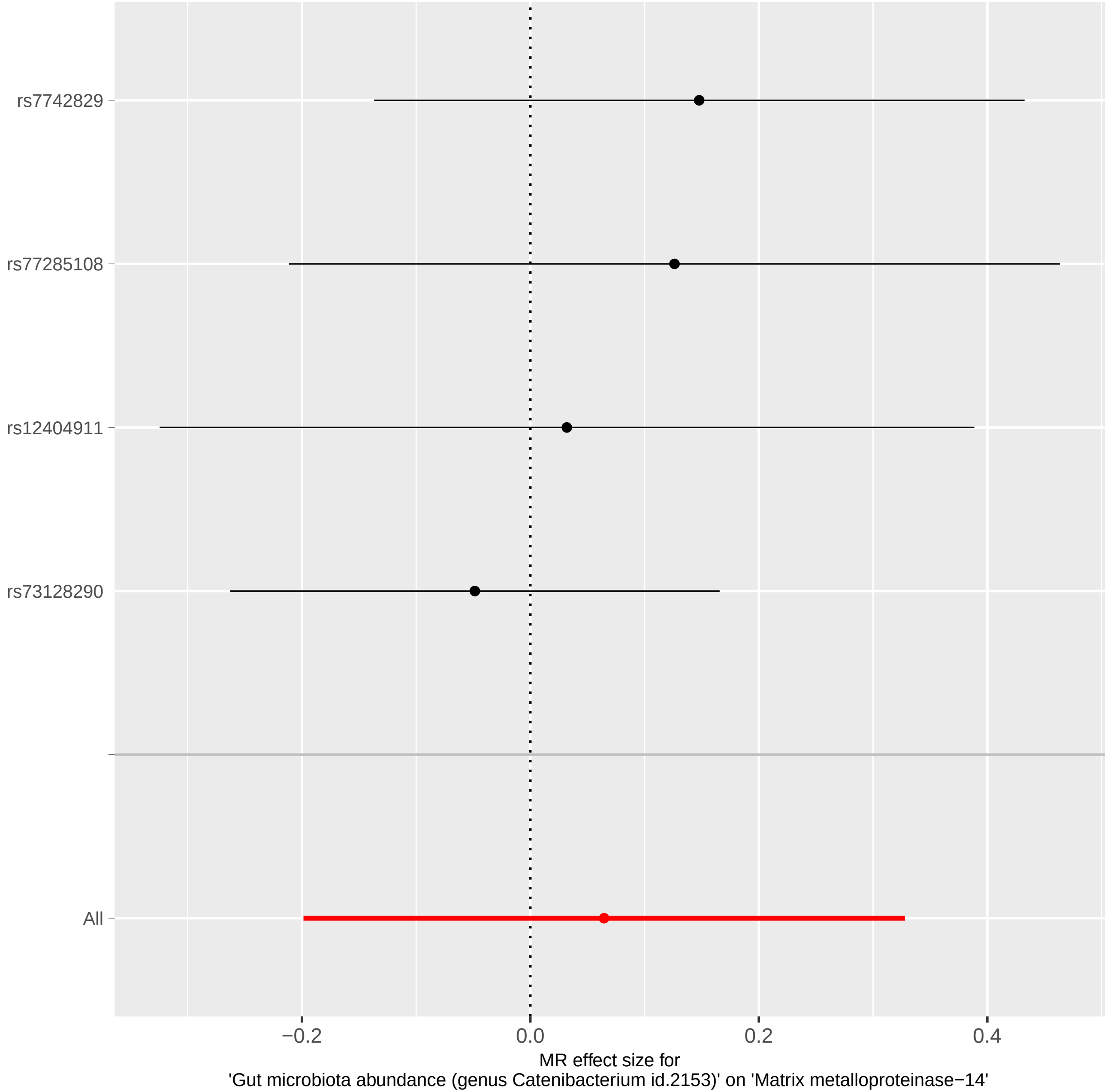

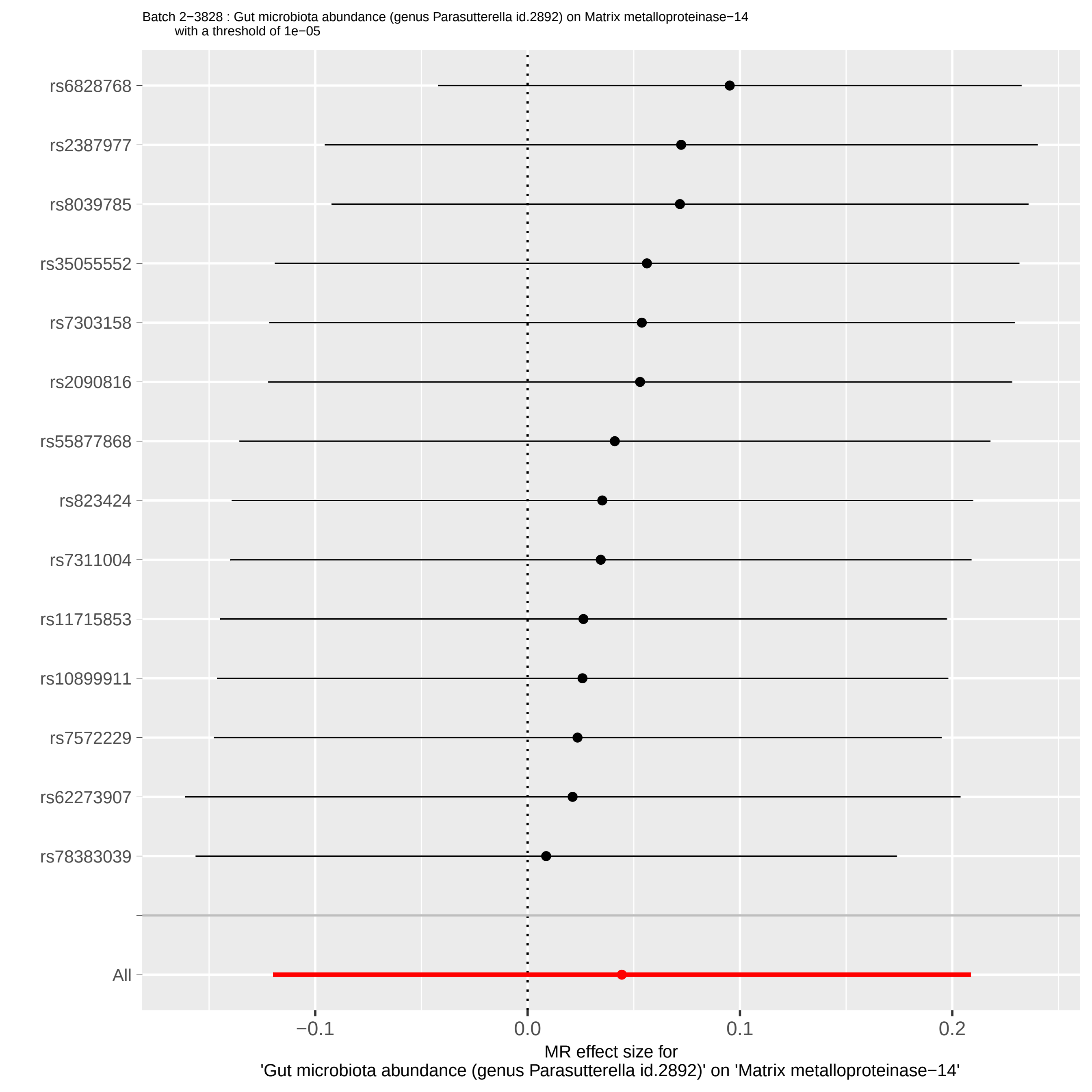
Batch 2−3828 : Gut microbiota abundance (genus Parasutterella id.2892) on Matrix metalloproteinase−14
with a threshold of 1e−05
rs6828768
rs2387977
rs8039785
rs35055552
rs7303158
rs2090816
rs55877868
rs823424
rs7311004
rs11715853
rs10899911
rs7572229
rs62273907
rs78383039
All
−0.1
0.0
0.1
0.2
MR effect size for
'Gut microbiota abundance (genus Parasutterella id.2892)' on 'Matrix metalloproteinase−14'

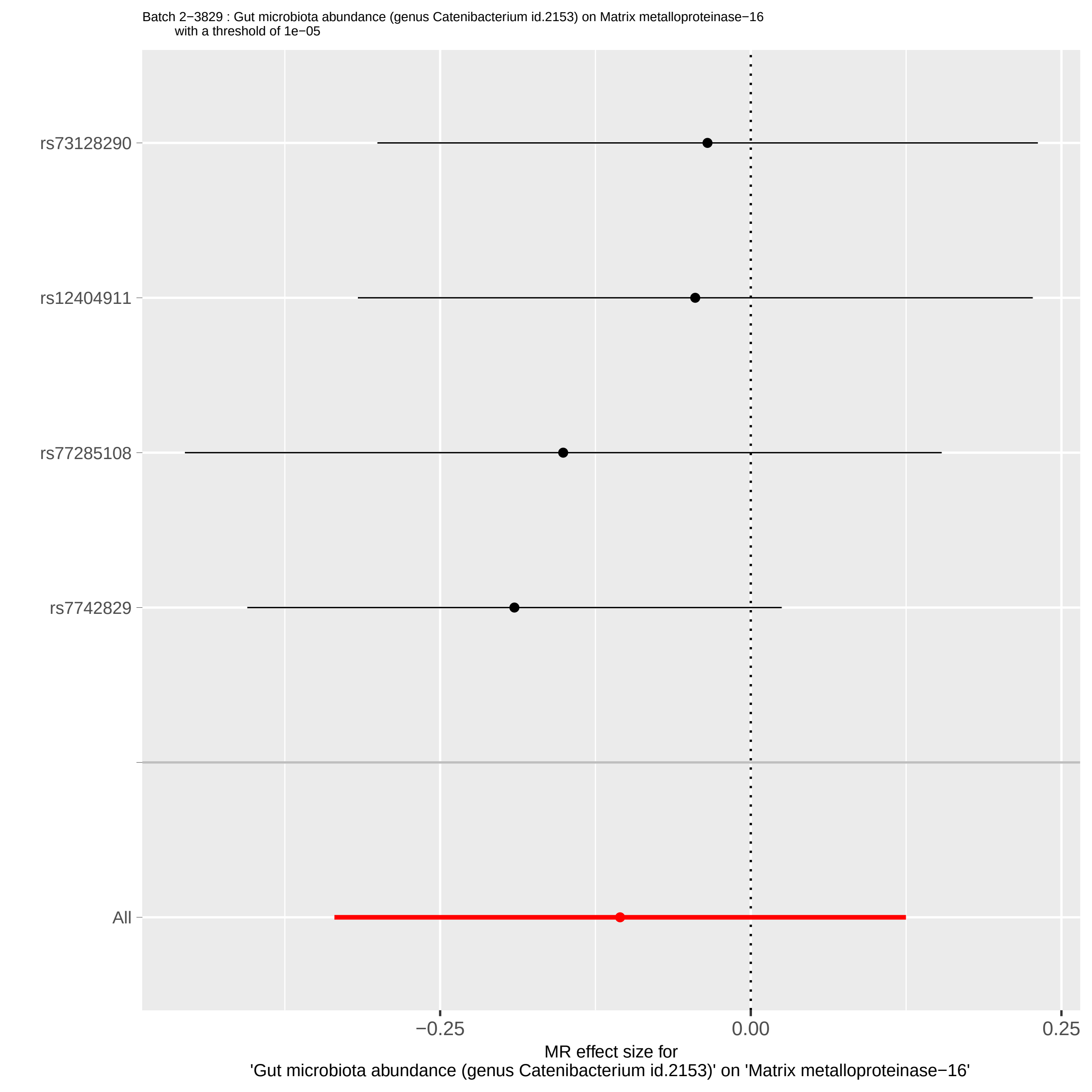
Batch 2−3829 : Gut microbiota abundance (genus Catenibacterium id.2153) on Matrix metalloproteinase−16
with a threshold of 1e−05
rs73128290
rs12404911
rs77285108
rs7742829
All
−0.25
0.00
0.25
MR effect size for
'Gut microbiota abundance (genus Catenibacterium id.2153)' on 'Matrix metalloproteinase−16'

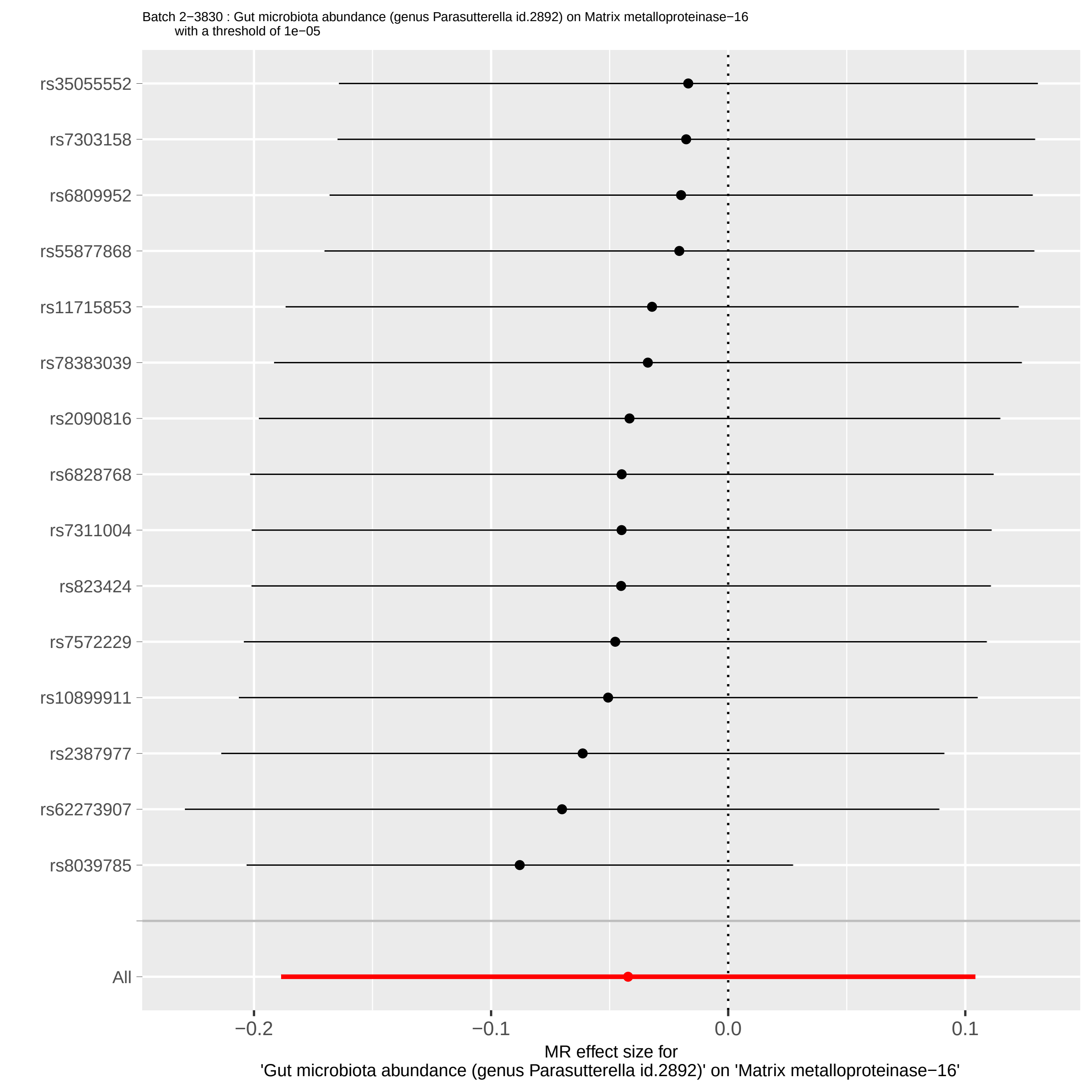
Batch 2−3830 : Gut microbiota abundance (genus Parasutterella id.2892) on Matrix metalloproteinase−16
with a threshold of 1e−05
rs35055552
rs7303158
rs6809952
rs55877868
rs11715853
rs78383039
rs2090816
rs6828768
rs7311004
rs823424
rs7572229
rs10899911
rs2387977
rs62273907
rs8039785
All
−0.2
−0.1
0.0
0.1
MR effect size for
'Gut microbiota abundance (genus Parasutterella id.2892)' on 'Matrix metalloproteinase−16'

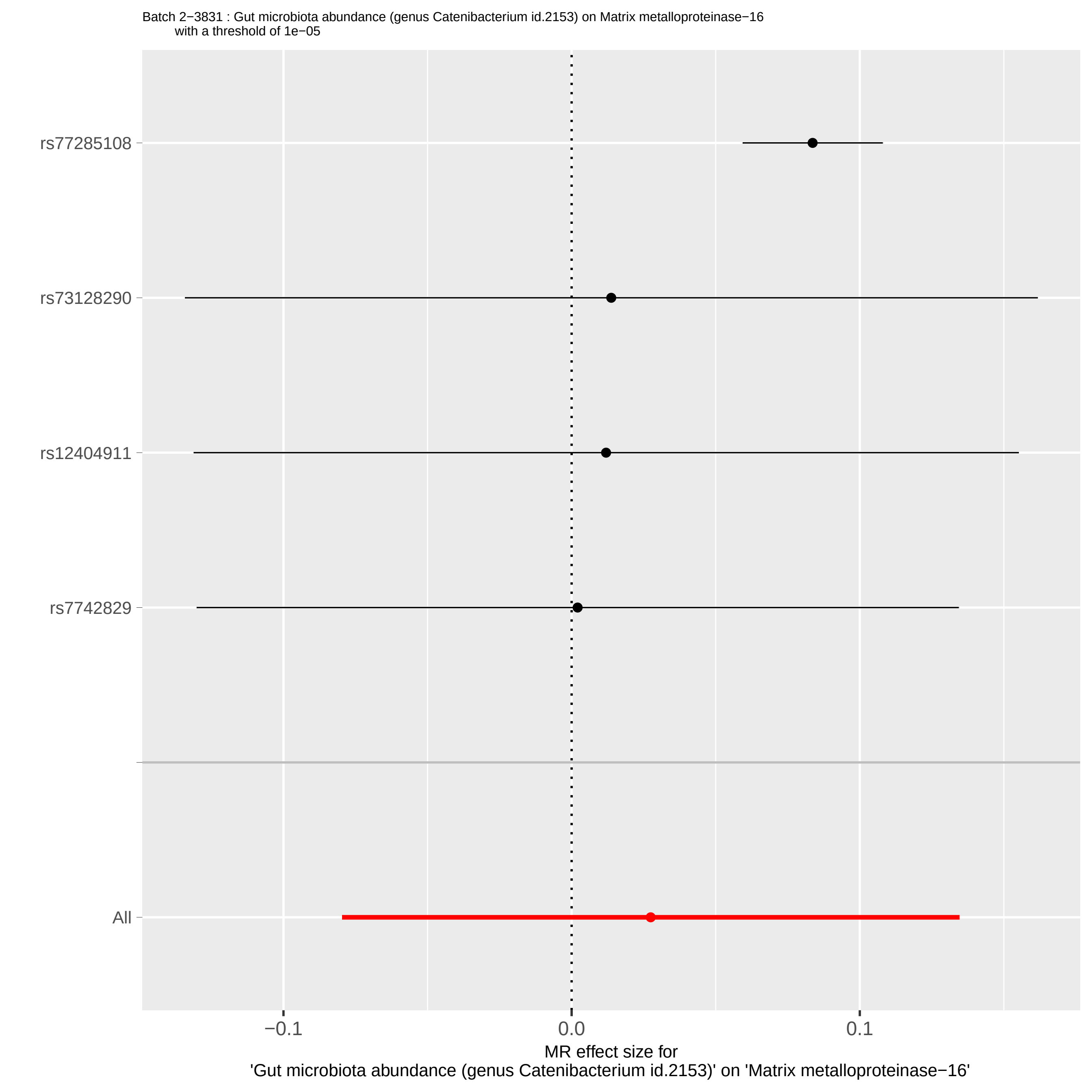

Batch 2−3831 : Gut microbiota abundance (genus Catenibacterium id.2153) on Matrix metalloproteinase−16
with a threshold of 1e−05
rs77285108
rs73128290
rs12404911
rs7742829
All
−0.1
0.0
0.1
MR effect size for
'Gut microbiota abundance (genus Catenibacterium id.2153)' on 'Matrix metalloproteinase−16'

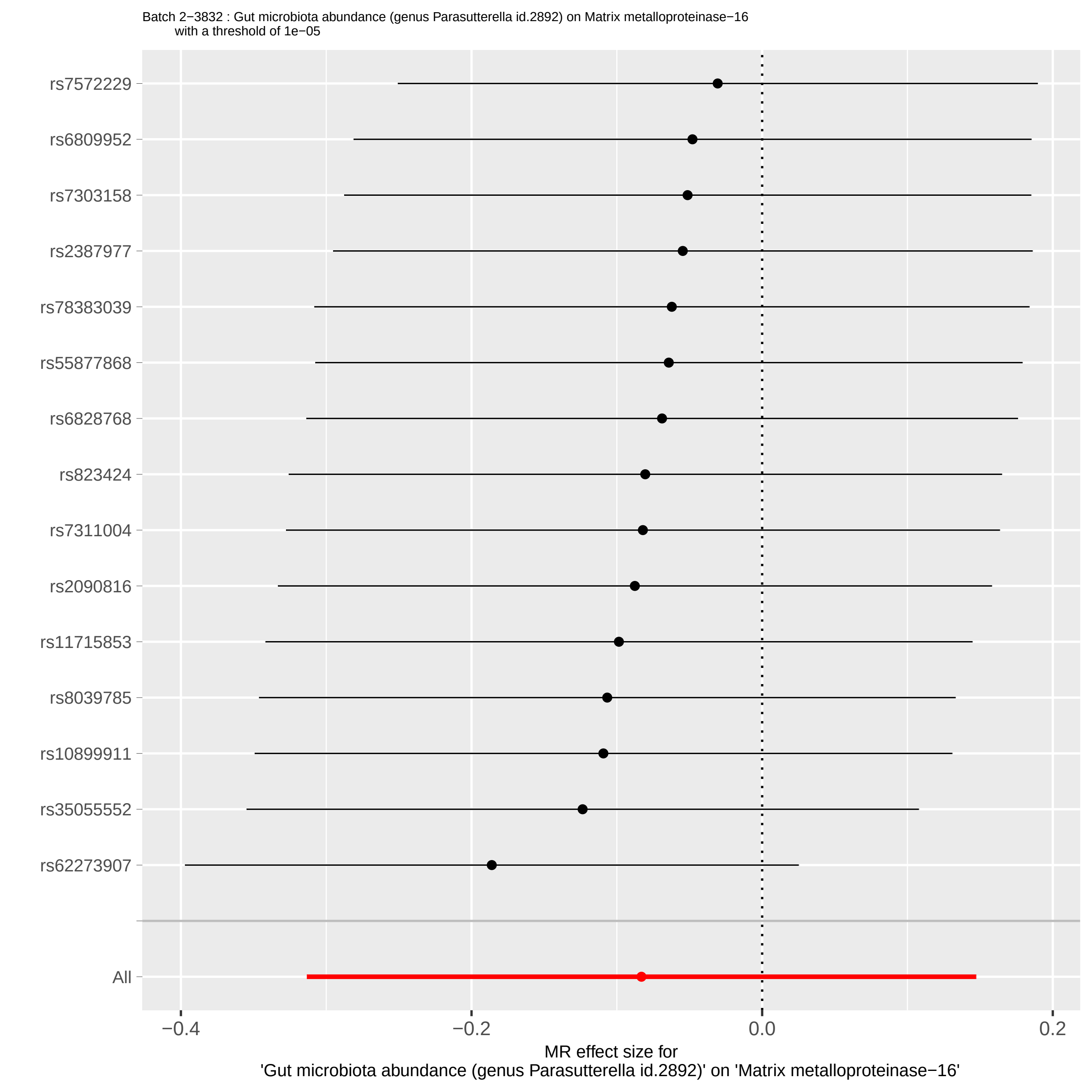

Batch 2−3832 : Gut microbiota abundance (genus Parasutterella id.2892) on Matrix metalloproteinase−16
with a threshold of 1e−05
rs7572229
rs6809952
rs7303158
rs2387977
rs78383039
rs55877868
rs6828768
rs823424
rs7311004
rs2090816
rs11715853
rs8039785
rs10899911
rs35055552
rs62273907
All
−0.4
−0.2
0.0
0.2
MR effect size for
'Gut microbiota abundance (genus Parasutterella id.2892)' on 'Matrix metalloproteinase−16'

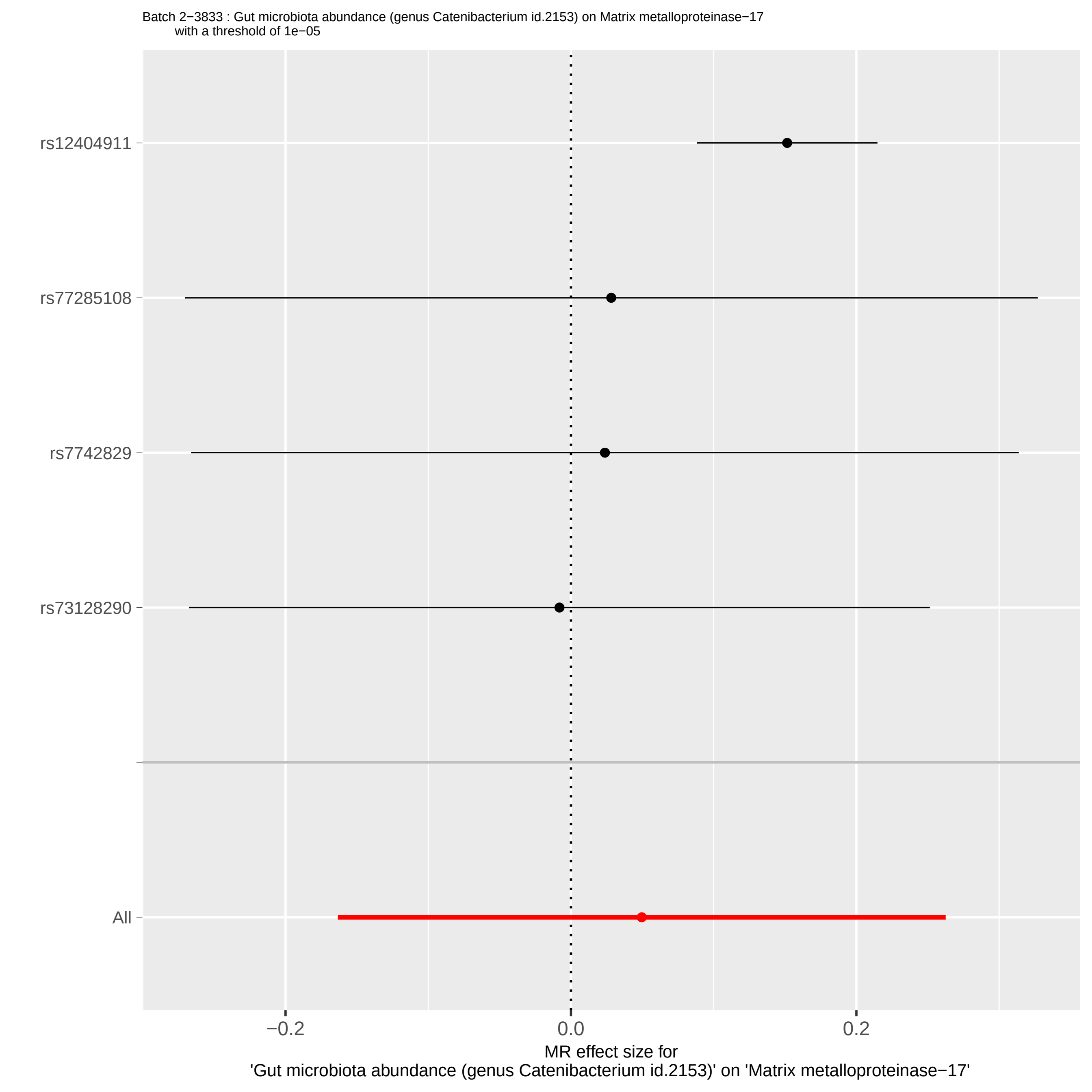
Batch 2−3833 : Gut microbiota abundance (genus Catenibacterium id.2153) on Matrix metalloproteinase−17
with a threshold of 1e−05
rs12404911
rs77285108
rs7742829
rs73128290
All
−0.2
0.0
0.2
MR effect size for
'Gut microbiota abundance (genus Catenibacterium id.2153)' on 'Matrix metalloproteinase−17'

Batch 2−3834 : Gut microbiota abundance (genus Parasutterella id.2892) on Matrix metalloproteinase−17
with a threshold of 1e−05

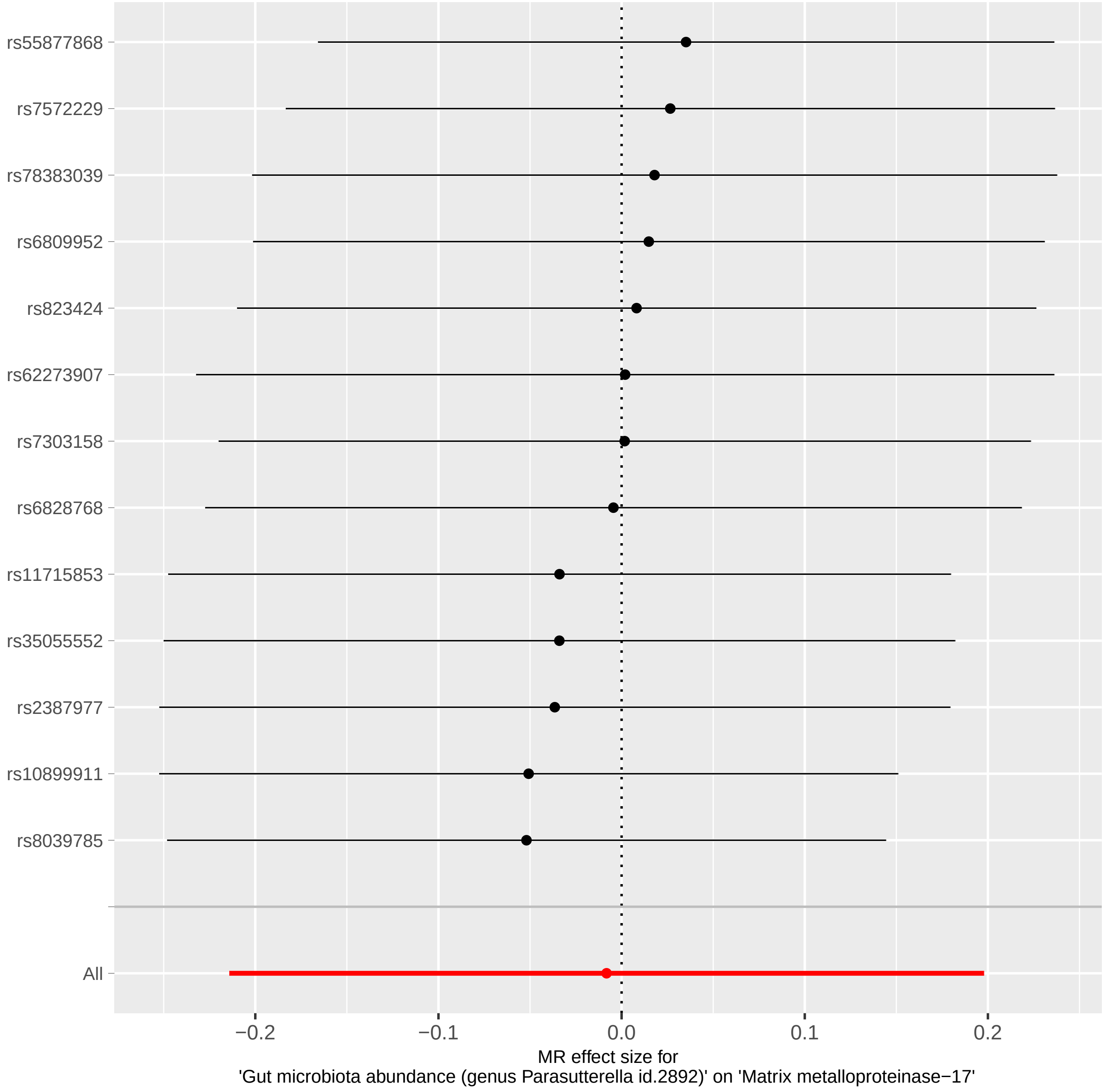

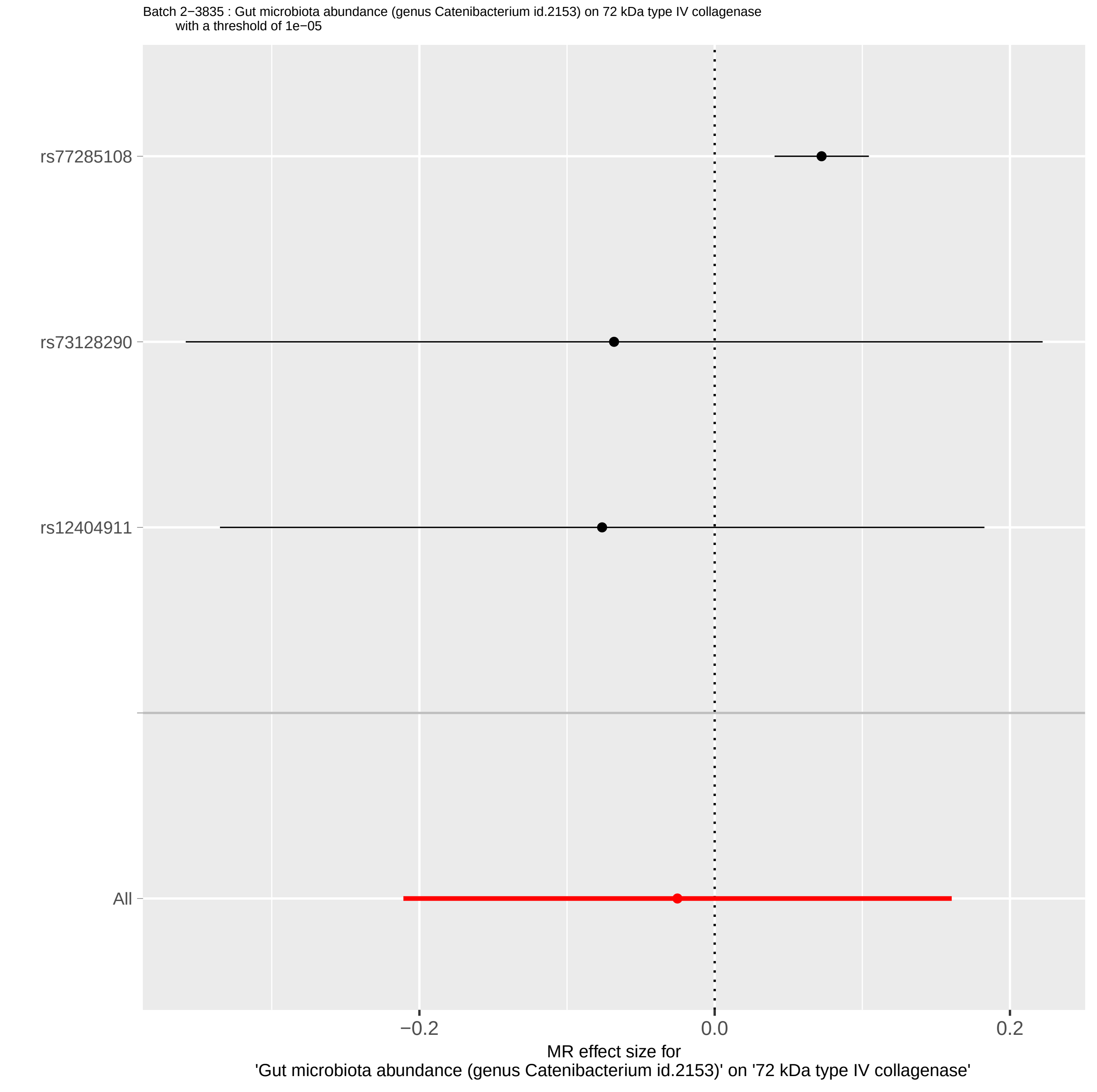
Batch 2−3835 : Gut microbiota abundance (genus Catenibacterium id.2153) on 72 kDa type IV collagenase
with a threshold of 1e−05
rs77285108
rs73128290
rs12404911
All
−0.2
0.0
0.2
MR effect size for
'Gut microbiota abundance (genus Catenibacterium id.2153)' on '72 kDa type IV collagenase'

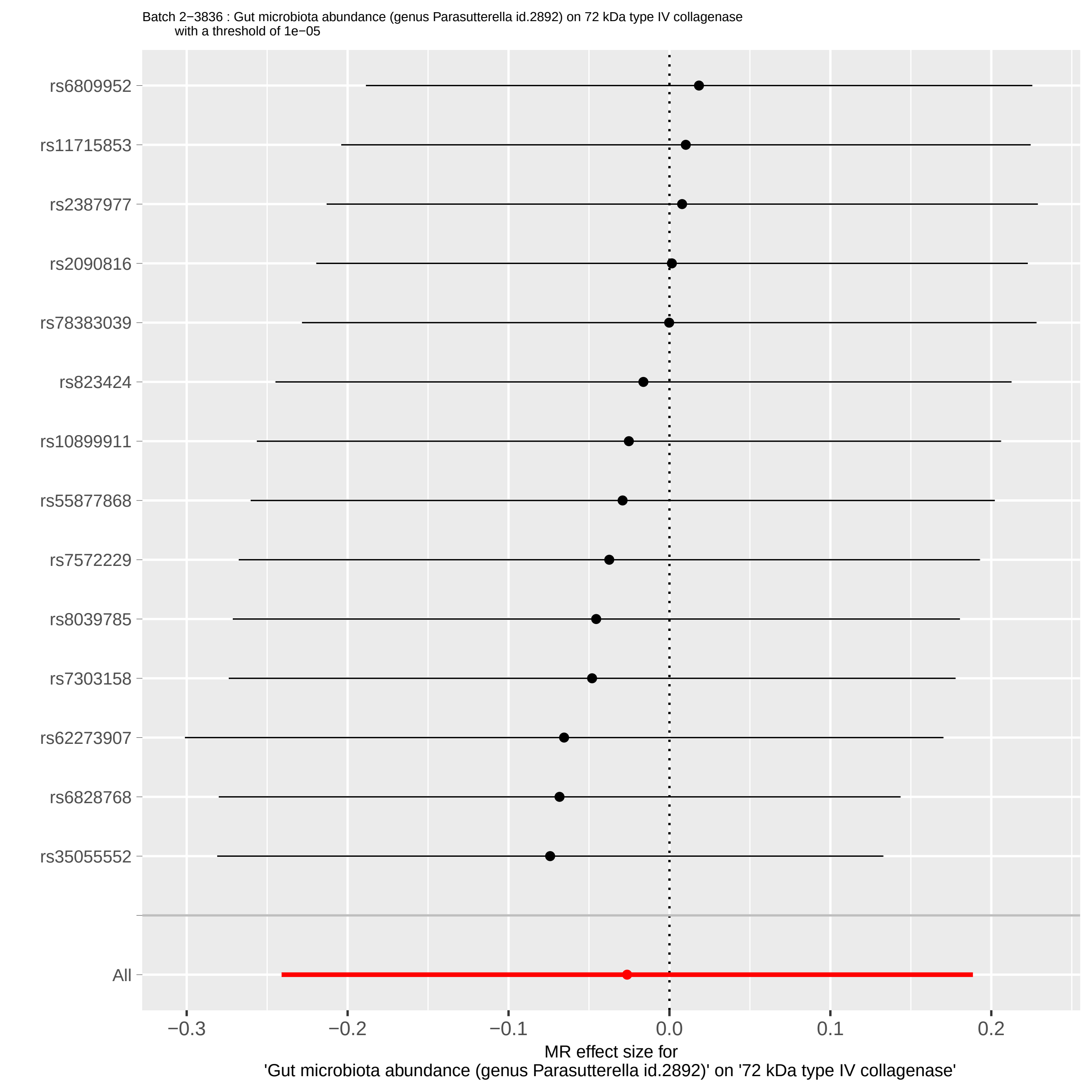

Batch 2−3836 : Gut microbiota abundance (genus Parasutterella id.2892) on 72 kDa type IV collagenase
with a threshold of 1e−05
rs6809952
rs11715853
rs2387977
rs2090816
rs78383039
rs823424
rs10899911
rs55877868
rs7572229
rs8039785
rs7303158
rs62273907
rs6828768
rs35055552
All
−0.3
−0.2
−0.1
0.0
0.1
0.2
MR effect size for
'Gut microbiota abundance (genus Parasutterella id.2892)' on '72 kDa type IV collagenase'

Batch 2−3837 : Gut microbiota abundance (genus Catenibacterium id.2153) on Matrilysin
with a threshold of 1e−05

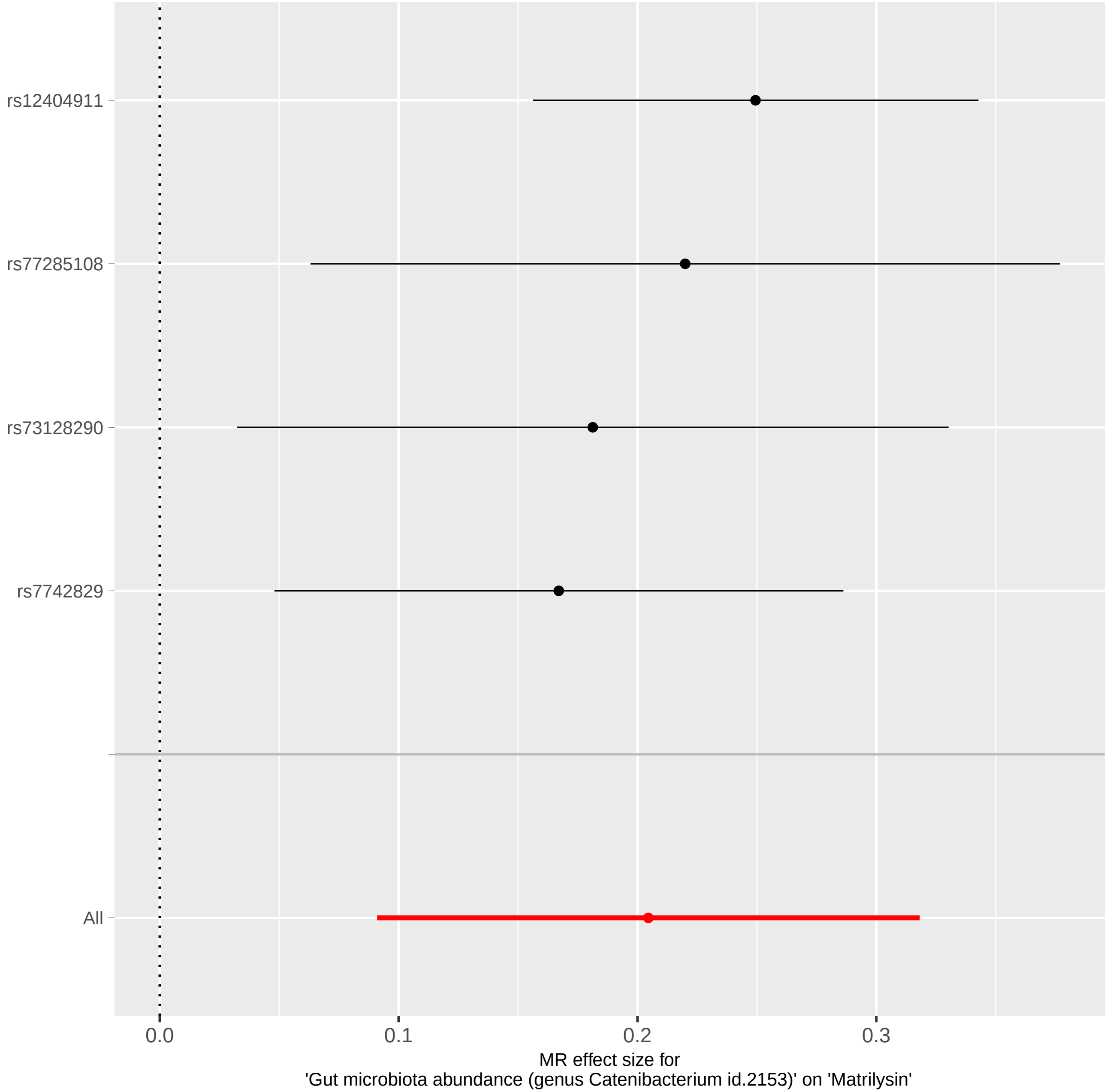

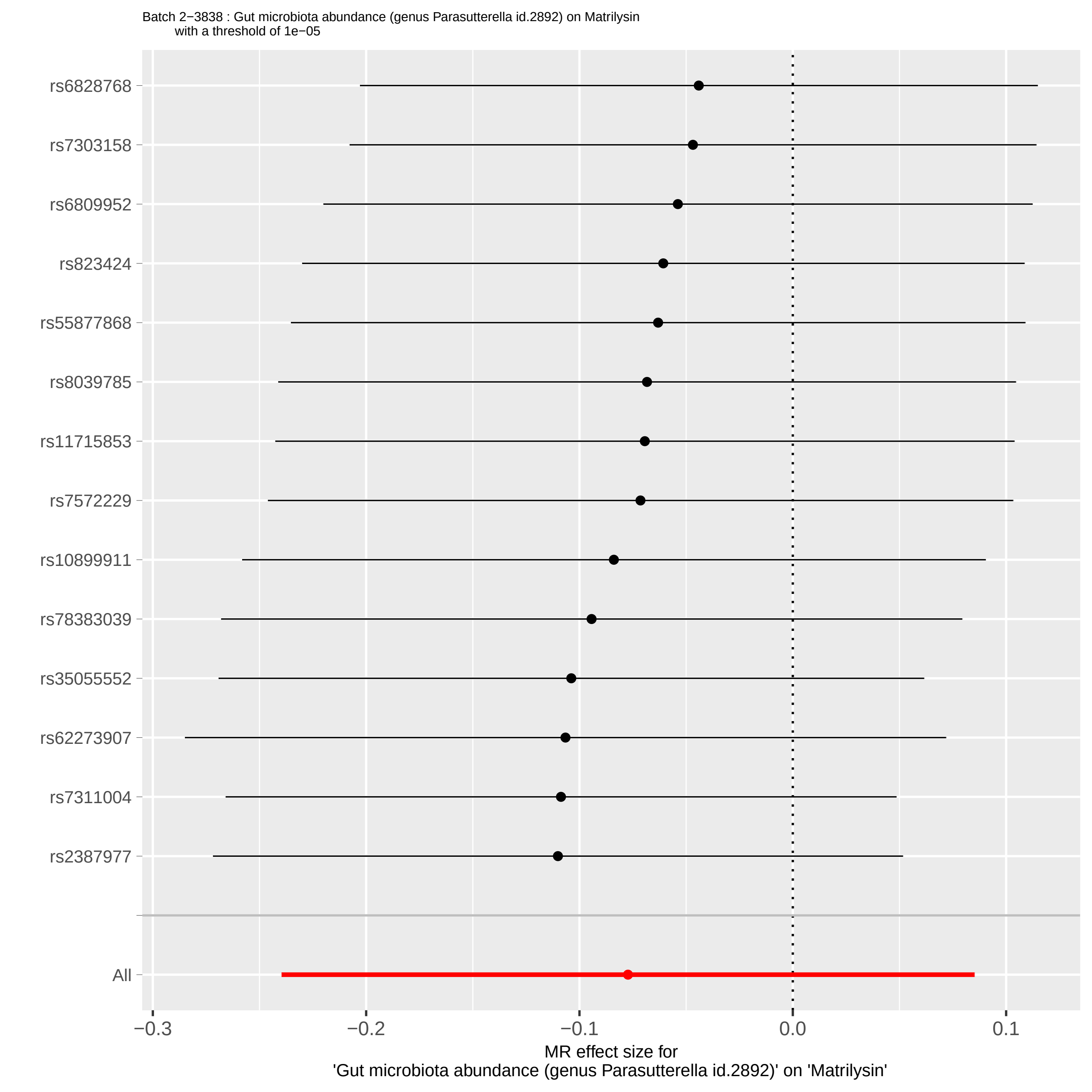
Batch 2−3838 : Gut microbiota abundance (genus Parasutterella id.2892) on Matrilysin
with a threshold of 1e−05
rs6828768
rs7303158
rs6809952
rs823424
rs55877868
rs8039785
rs11715853
rs7572229
rs10899911
rs78383039
rs35055552
rs62273907
rs7311004
rs2387977
All
−0.3
−0.2
−0.1
0.0
0.1
MR effect size for
'Gut microbiota abundance (genus Parasutterella id.2892)' on 'Matrilysin'

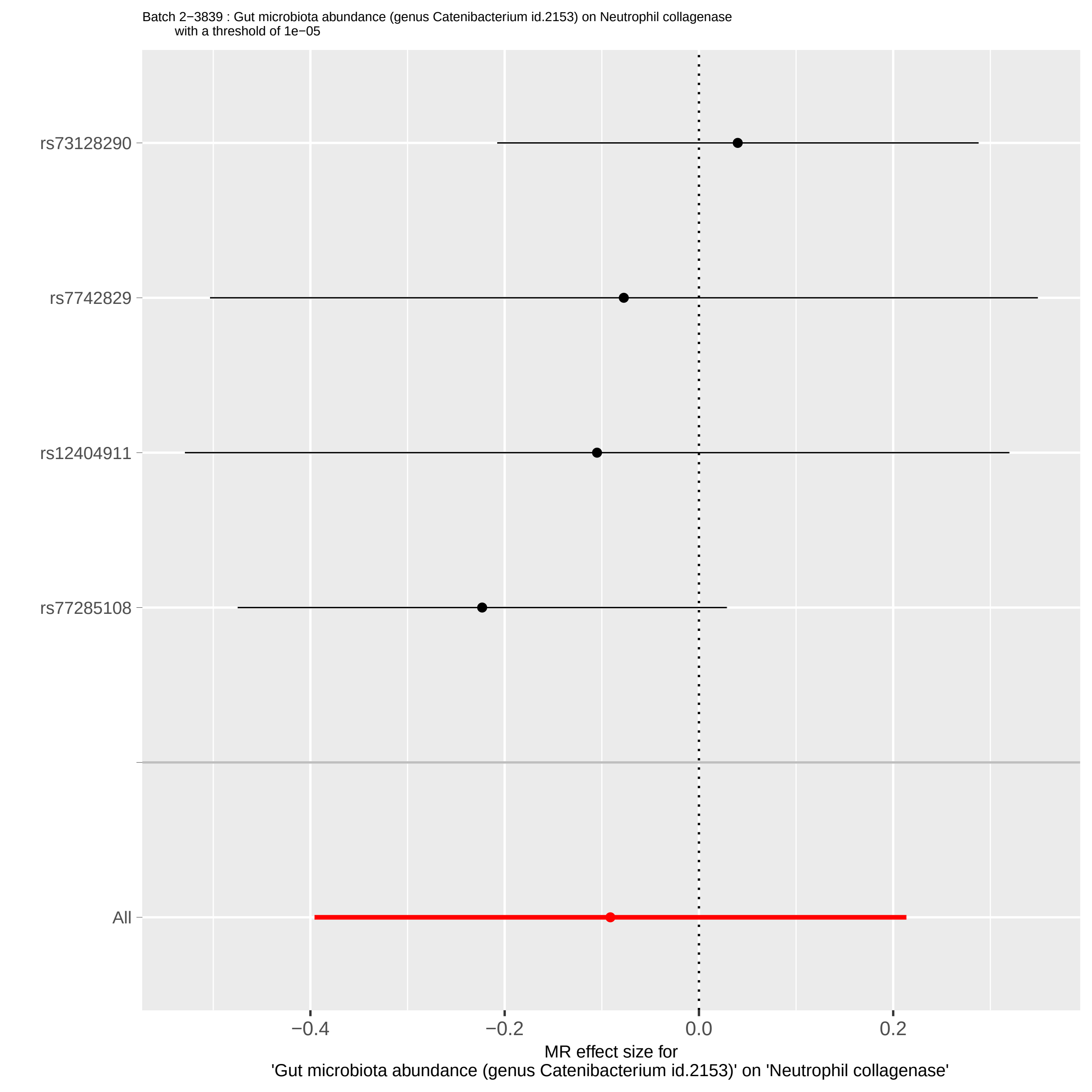

Batch 2−3839 : Gut microbiota abundance (genus Catenibacterium id.2153) on Neutrophil collagenase
with a threshold of 1e−05
rs73128290
rs7742829
rs12404911
rs77285108
All
−0.4
−0.2
0.0
0.2
MR effect size for
'Gut microbiota abundance (genus Catenibacterium id.2153)' on 'Neutrophil collagenase'

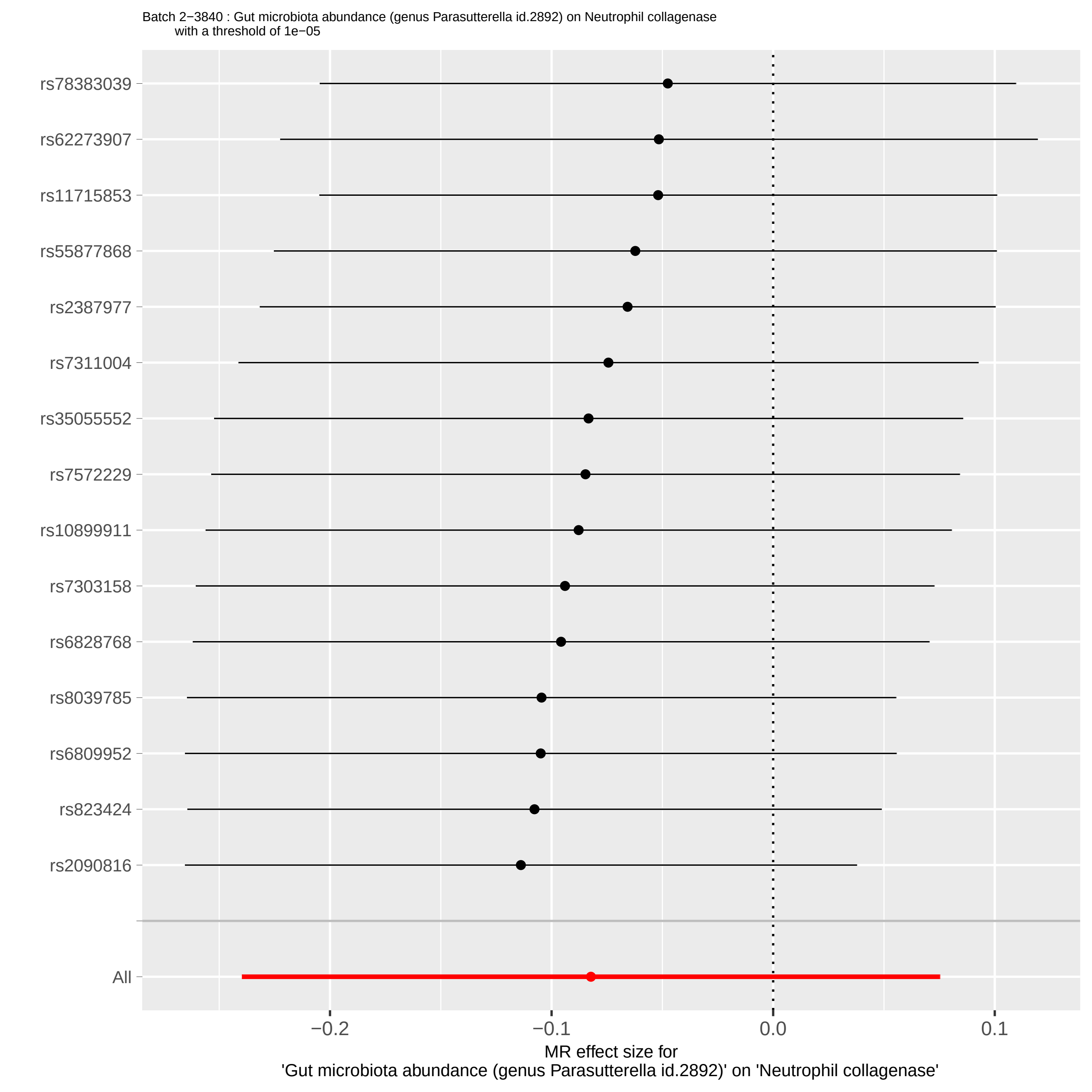

Batch 2−3840 : Gut microbiota abundance (genus Parasutterella id.2892) on Neutrophil collagenase
with a threshold of 1e−05
rs78383039
rs62273907
rs11715853
rs55877868
rs2387977
rs7311004
rs35055552
rs7572229
rs10899911
rs7303158
rs6828768
rs8039785
rs6809952
rs823424
rs2090816
All
−0.2
−0.1
0.0
0.1
MR effect size for
'Gut microbiota abundance (genus Parasutterella id.2892)' on 'Neutrophil collagenase'

Batch 2−3841 : Gut microbiota abundance (genus Catenibacterium id.2153) on Matrix metalloproteinase−9
with a threshold of 1e−05

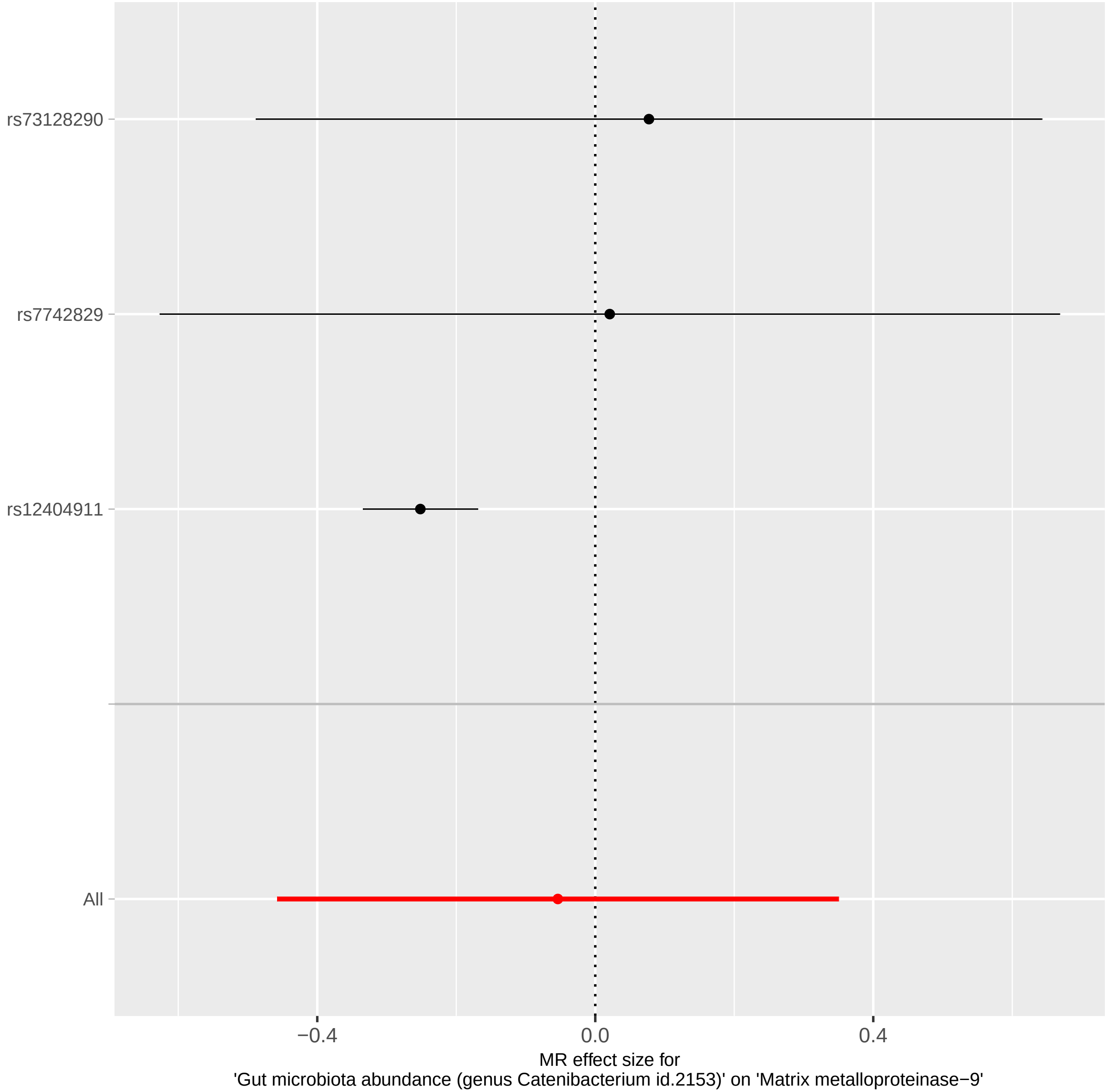

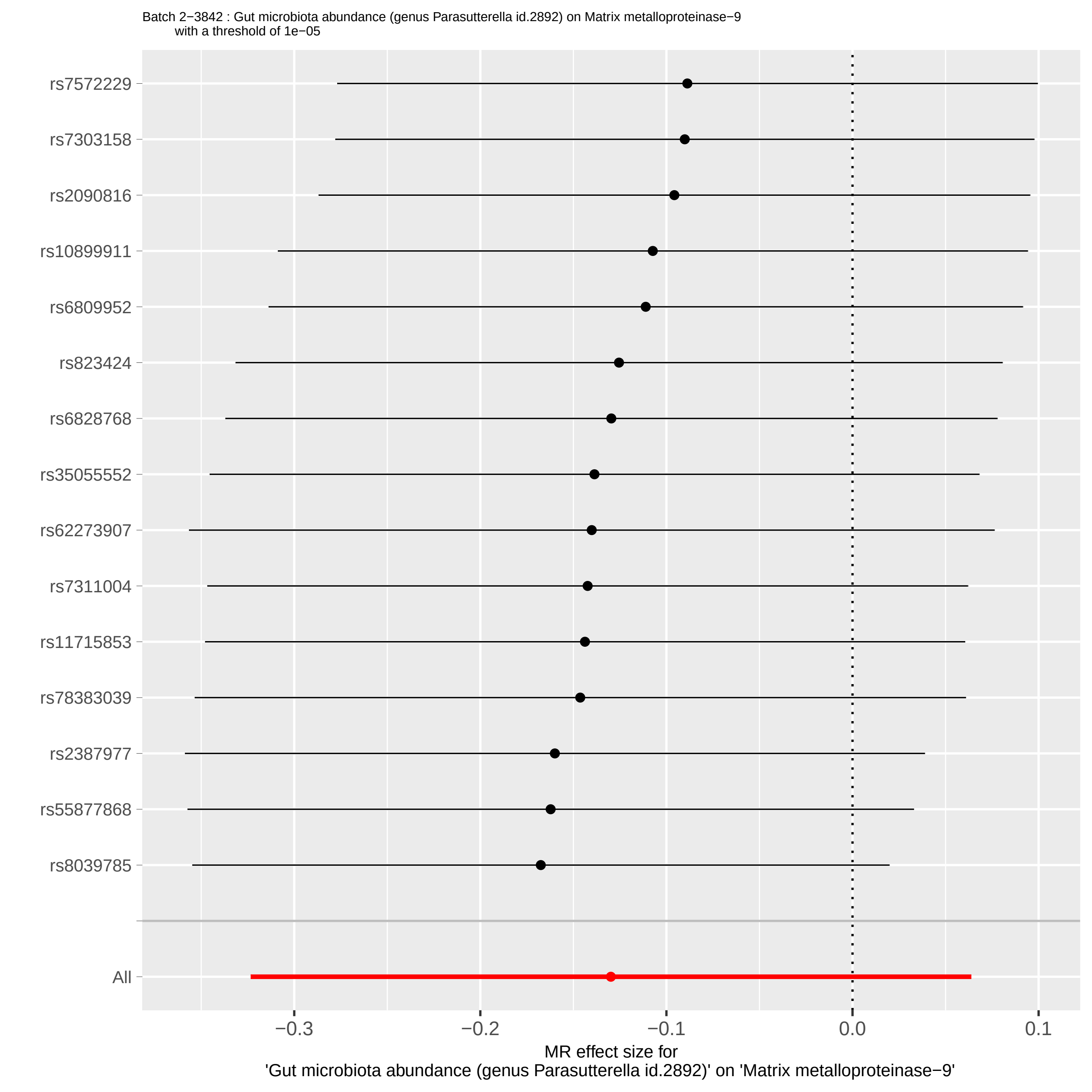

Batch 2−3842 : Gut microbiota abundance (genus Parasutterella id.2892) on Matrix metalloproteinase−9
with a threshold of 1e−05
rs7572229
rs7303158
rs2090816
rs10899911
rs6809952
rs823424
rs6828768
rs35055552
rs62273907
rs7311004
rs11715853
rs78383039
rs2387977
rs55877868
rs8039785
All
−0.3
−0.2
−0.1
0.0
0.1
MR effect size for
'Gut microbiota abundance (genus Parasutterella id.2892)' on 'Matrix metalloproteinase−9'

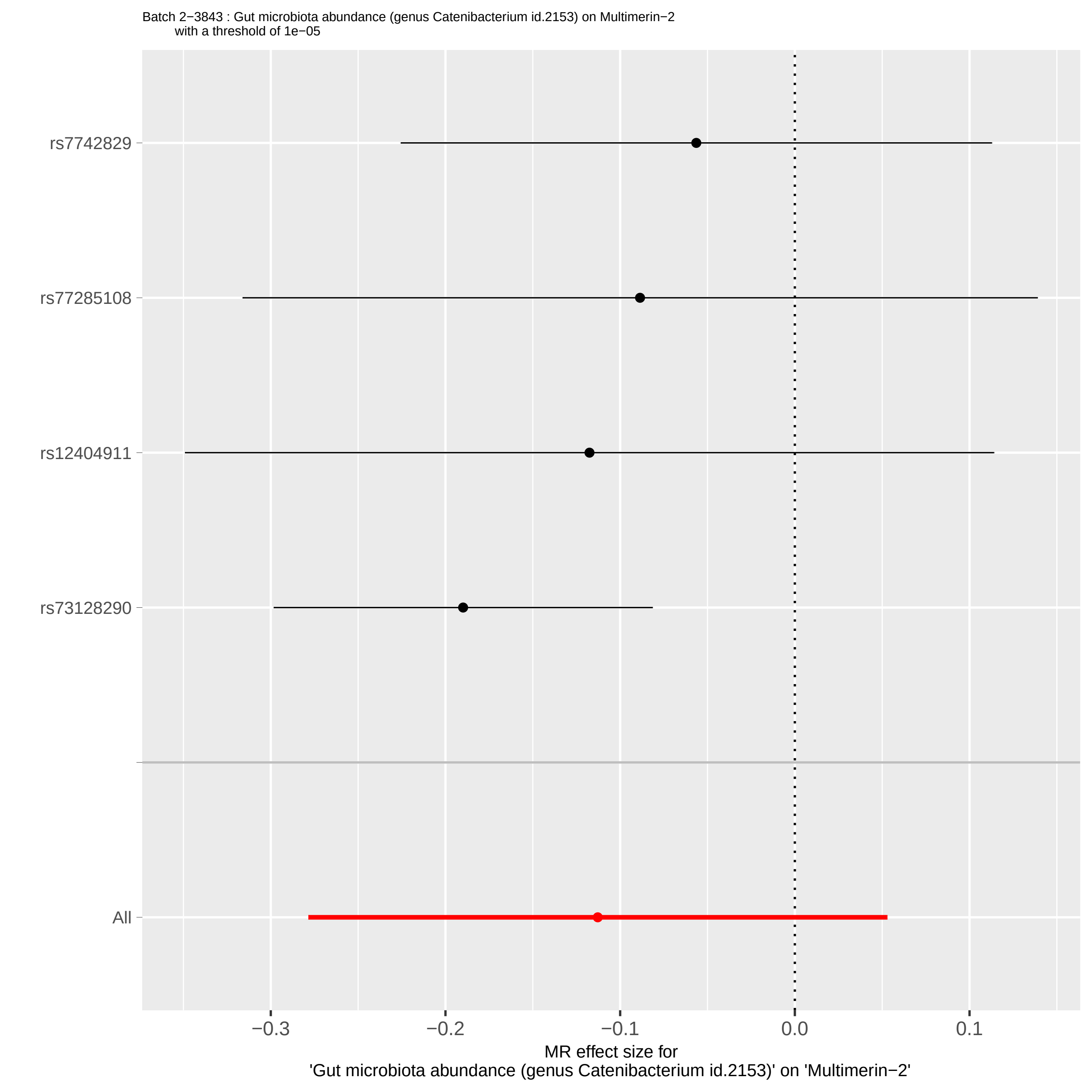

Batch 2−3843 : Gut microbiota abundance (genus Catenibacterium id.2153) on Multimerin−2
with a threshold of 1e−05
rs7742829
rs77285108
rs12404911
rs73128290
All
−0.3
−0.2
−0.1
0.0
0.1
MR effect size for
'Gut microbiota abundance (genus Catenibacterium id.2153)' on 'Multimerin−2'

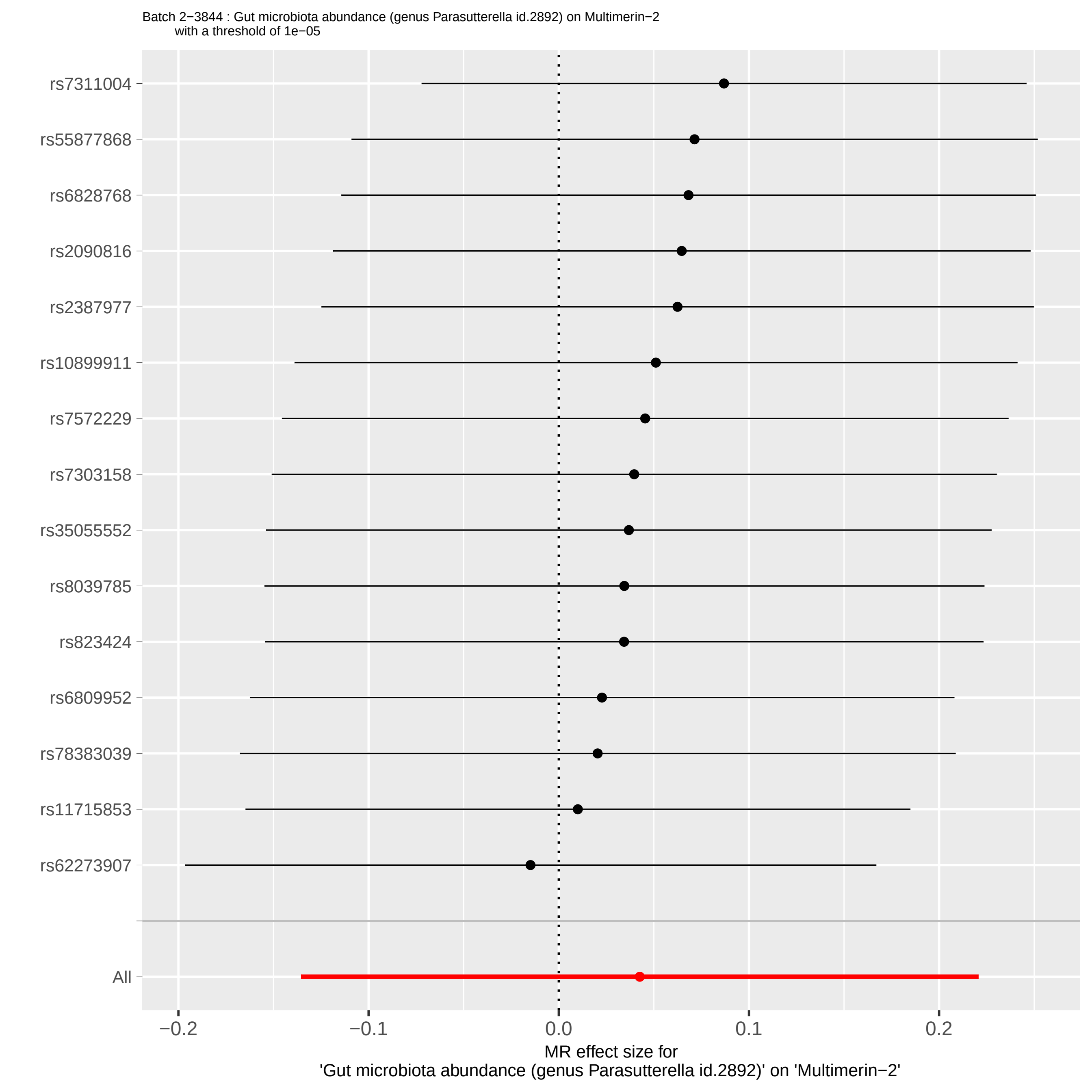
Batch 2−3844 : Gut microbiota abundance (genus Parasutterella id.2892) on Multimerin−2
with a threshold of 1e−05
rs7311004
rs55877868
rs6828768
rs2090816
rs2387977
rs10899911
rs7572229
rs7303158
rs35055552
rs8039785
rs823424
rs6809952
rs78383039
rs11715853
rs62273907
All
−0.2
−0.1
0.0
0.1
0.2
MR effect size for
'Gut microbiota abundance (genus Parasutterella id.2892)' on 'Multimerin−2'

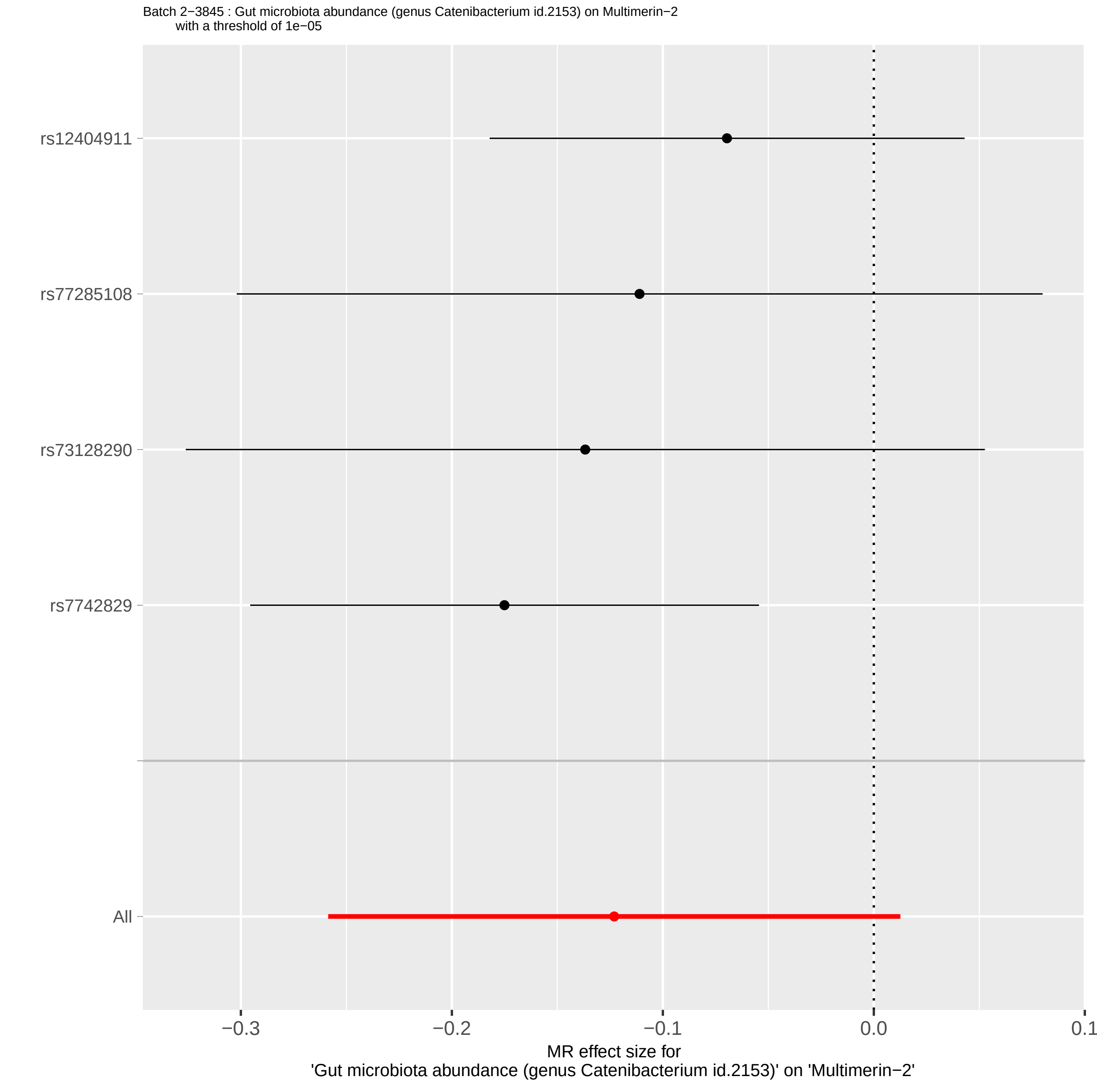
Batch 2−3845 : Gut microbiota abundance (genus Catenibacterium id.2153) on Multimerin−2
with a threshold of 1e−05
rs12404911
rs77285108
rs73128290
rs7742829
All
−0.3
−0.2
−0.1
0.0
0.1
MR effect size for
'Gut microbiota abundance (genus Catenibacterium id.2153)' on 'Multimerin−2'

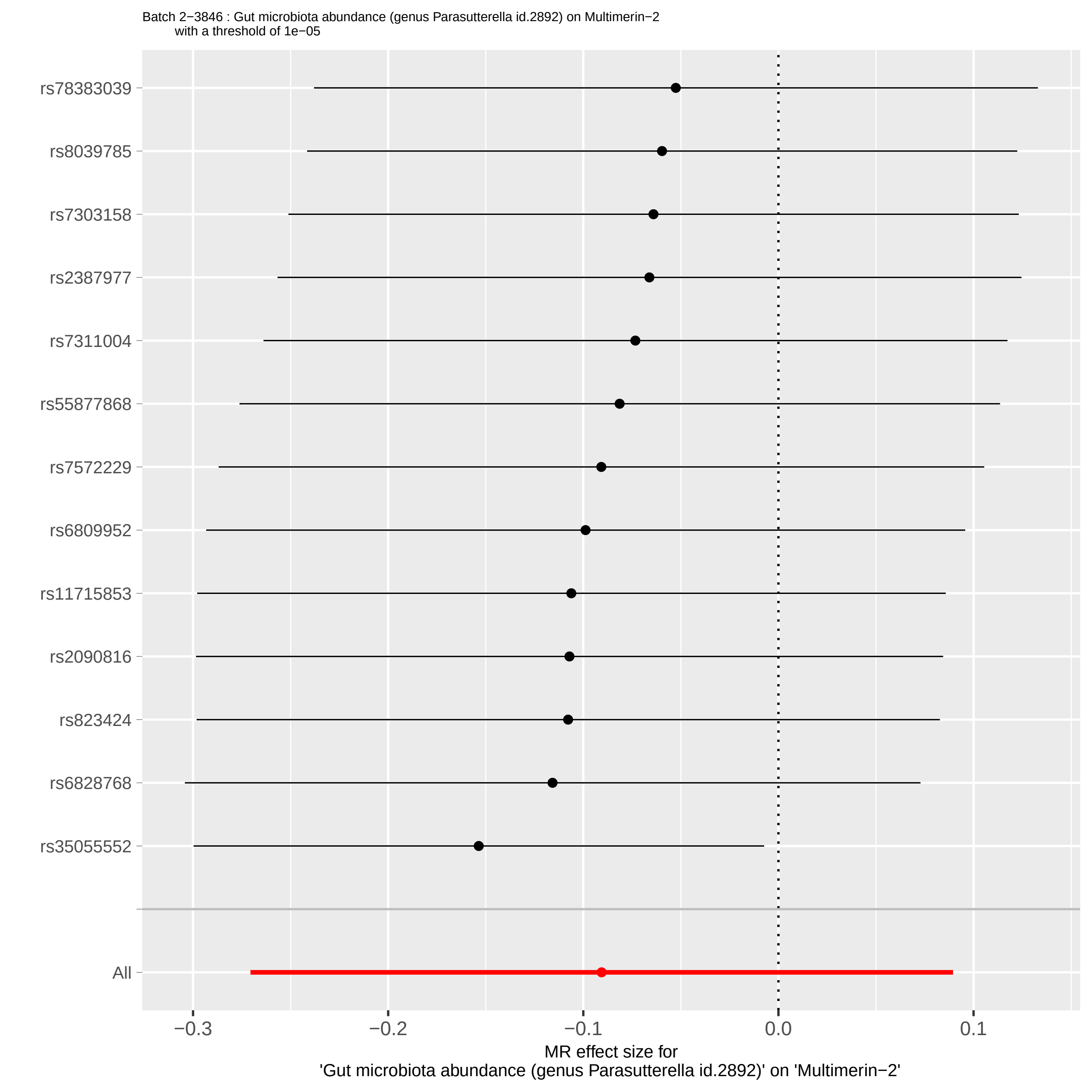

Batch 2−3846 : Gut microbiota abundance (genus Parasutterella id.2892) on Multimerin−2
with a threshold of 1e−05
rs78383039
rs8039785
rs7303158
rs2387977
rs7311004
rs55877868
rs7572229
rs6809952
rs11715853
rs2090816
rs823424
rs6828768
rs35055552
All
−0.3
−0.2
−0.1
0.0
0.1
MR effect size for
'Gut microbiota abundance (genus Parasutterella id.2892)' on 'Multimerin−2'

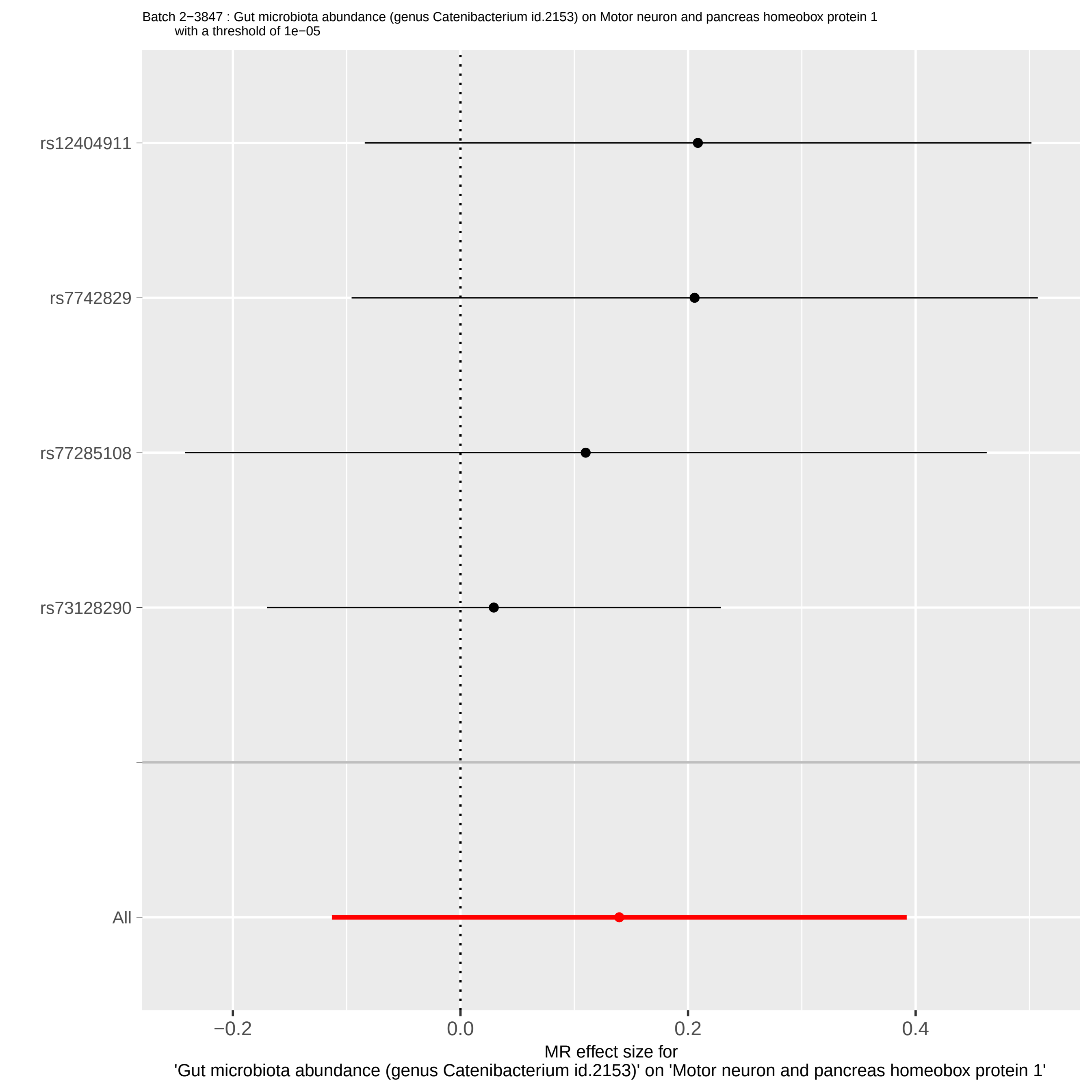

Batch 2−3847 : Gut microbiota abundance (genus Catenibacterium id.2153) on Motor neuron and pancreas homeobox protein 1
with a threshold of 1e−05
rs12404911
rs7742829
rs77285108
rs73128290
All
−0.2
0.0
0.2
0.4
MR effect size for
'Gut microbiota abundance (genus Catenibacterium id.2153)' on 'Motor neuron and pancreas homeobox protein 1'

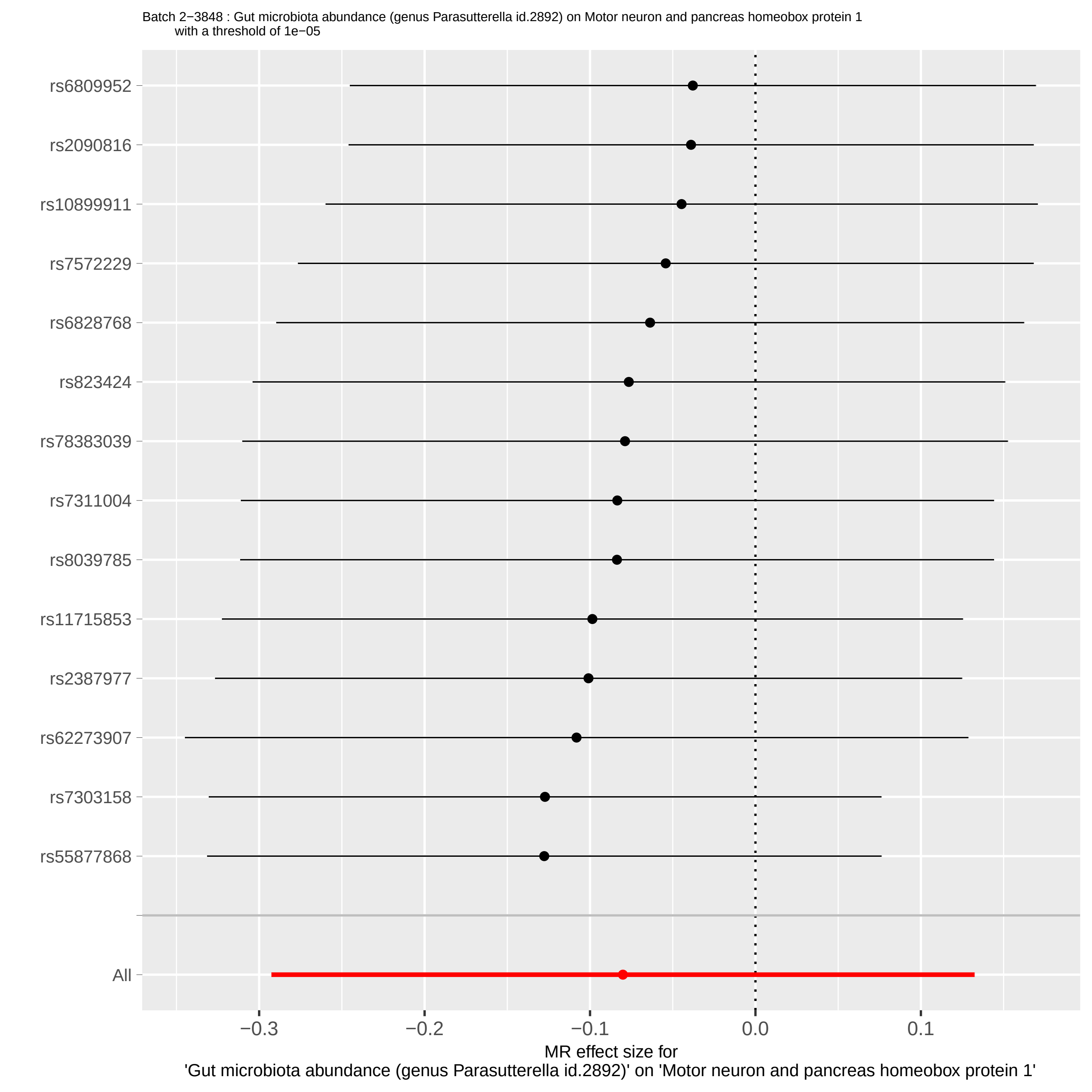
Batch 2−3848 : Gut microbiota abundance (genus Parasutterella id.2892) on Motor neuron and pancreas homeobox protein 1
with a threshold of 1e−05
rs6809952
rs2090816
rs10899911
rs7572229
rs6828768
rs823424
rs78383039
rs7311004
rs8039785
rs11715853
rs2387977
rs62273907
rs7303158
rs55877868
All
−0.3
−0.2
−0.1
0.0
0.1
MR effect size for
'Gut microbiota abundance (genus Parasutterella id.2892)' on 'Motor neuron and pancreas homeobox protein 1'

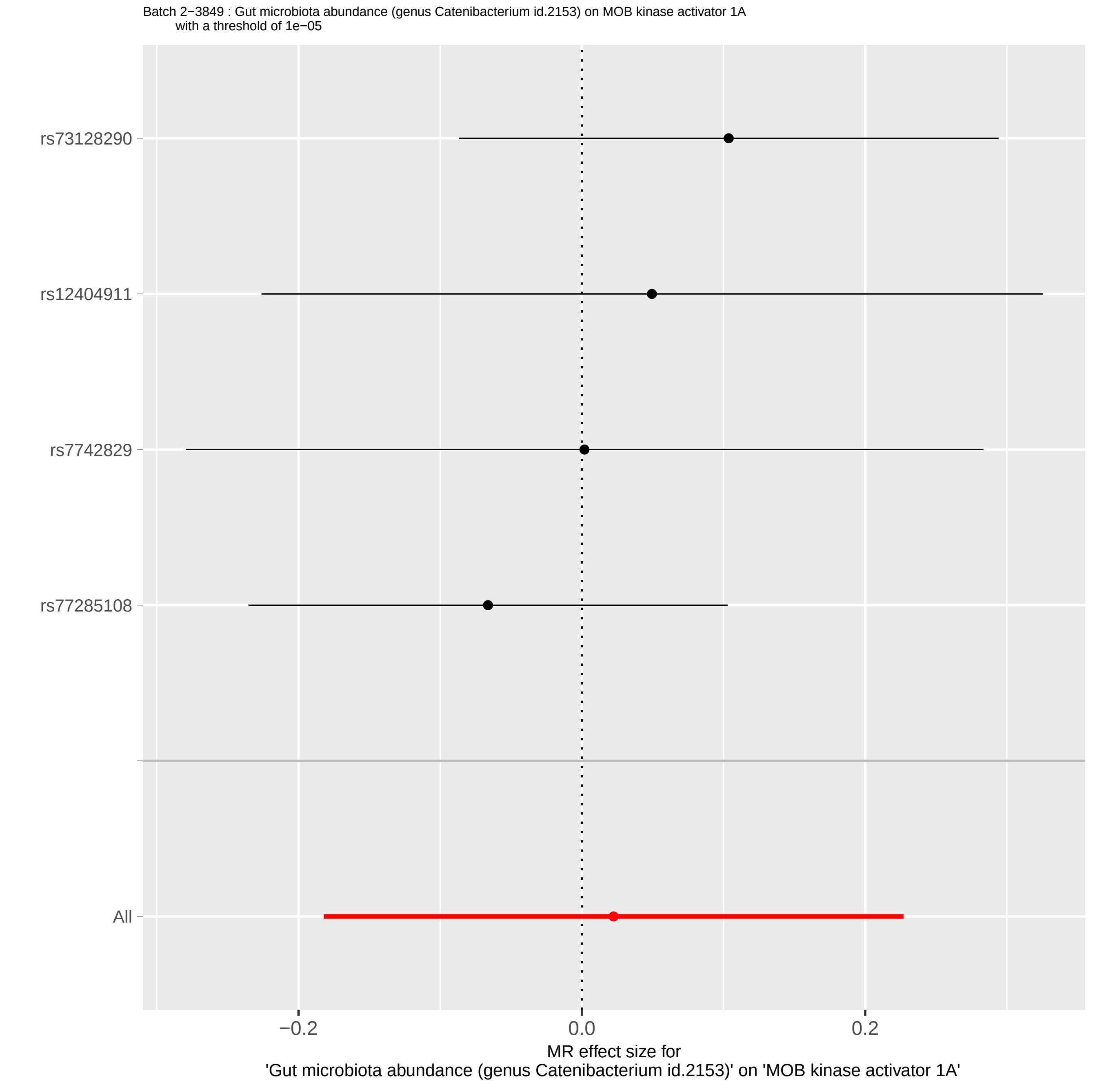
Batch 2−3849 : Gut microbiota abundance (genus Catenibacterium id.2153) on MOB kinase activator 1A
with a threshold of 1e−05
rs73128290
rs12404911
rs7742829
rs77285108
All
−0.2
0.0
0.2
MR effect size for
'Gut microbiota abundance (genus Catenibacterium id.2153)' on 'MOB kinase activator 1A'

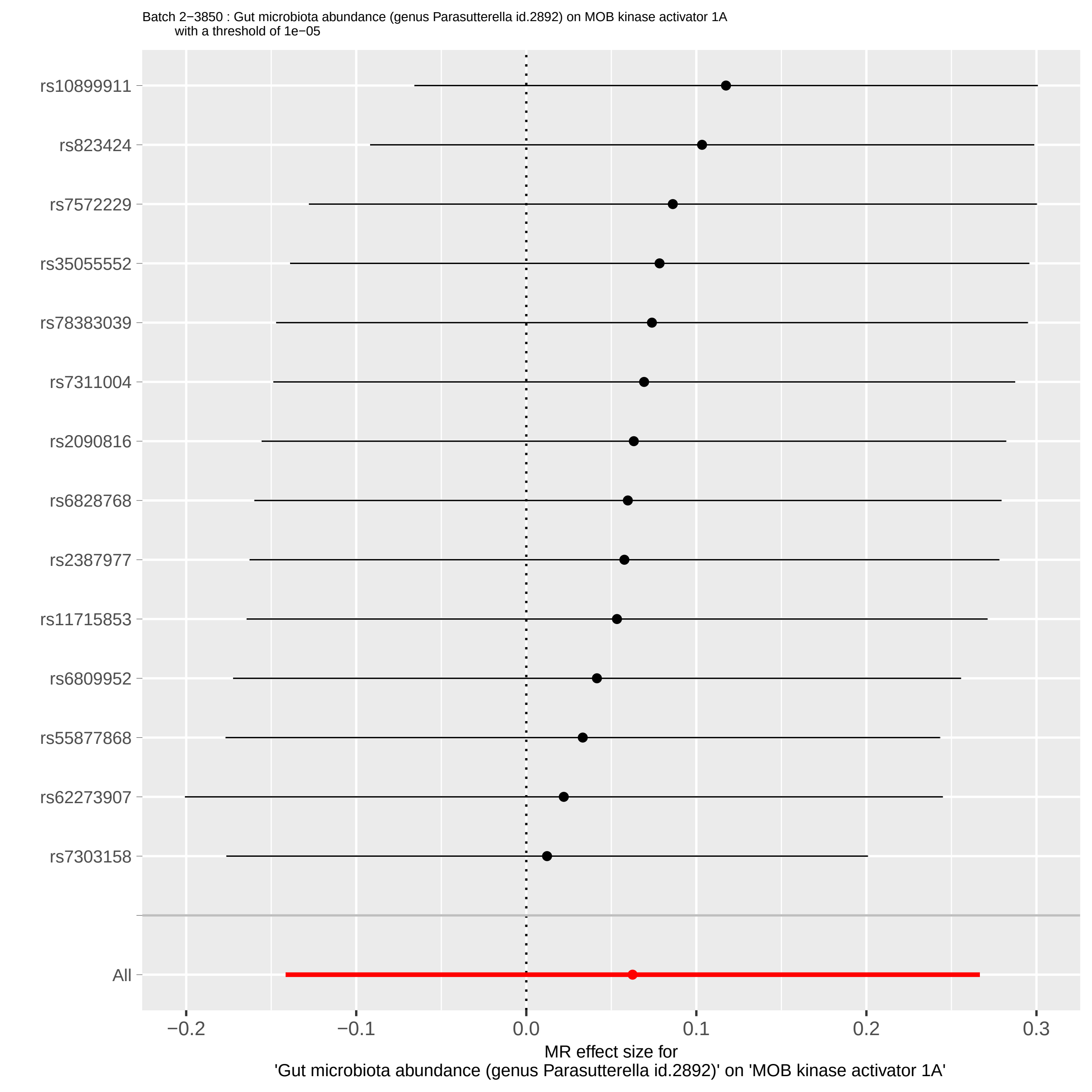

Batch 2−3850 : Gut microbiota abundance (genus Parasutterella id.2892) on MOB kinase activator 1A
with a threshold of 1e−05
rs10899911
rs823424
rs7572229
rs35055552
rs78383039
rs7311004
rs2090816
rs6828768
rs2387977
rs11715853
rs6809952
rs55877868
rs62273907
rs7303158
All
−0.2
−0.1
0.0
0.1
0.2
0.3
MR effect size for
'Gut microbiota abundance (genus Parasutterella id.2892)' on 'MOB kinase activator 1A'

Batch 2−3851 : Gut microbiota abundance (genus Catenibacterium id.2153) on Adenylyltransferase and sulfurtransferase MOCS3
with a threshold of 1e−05

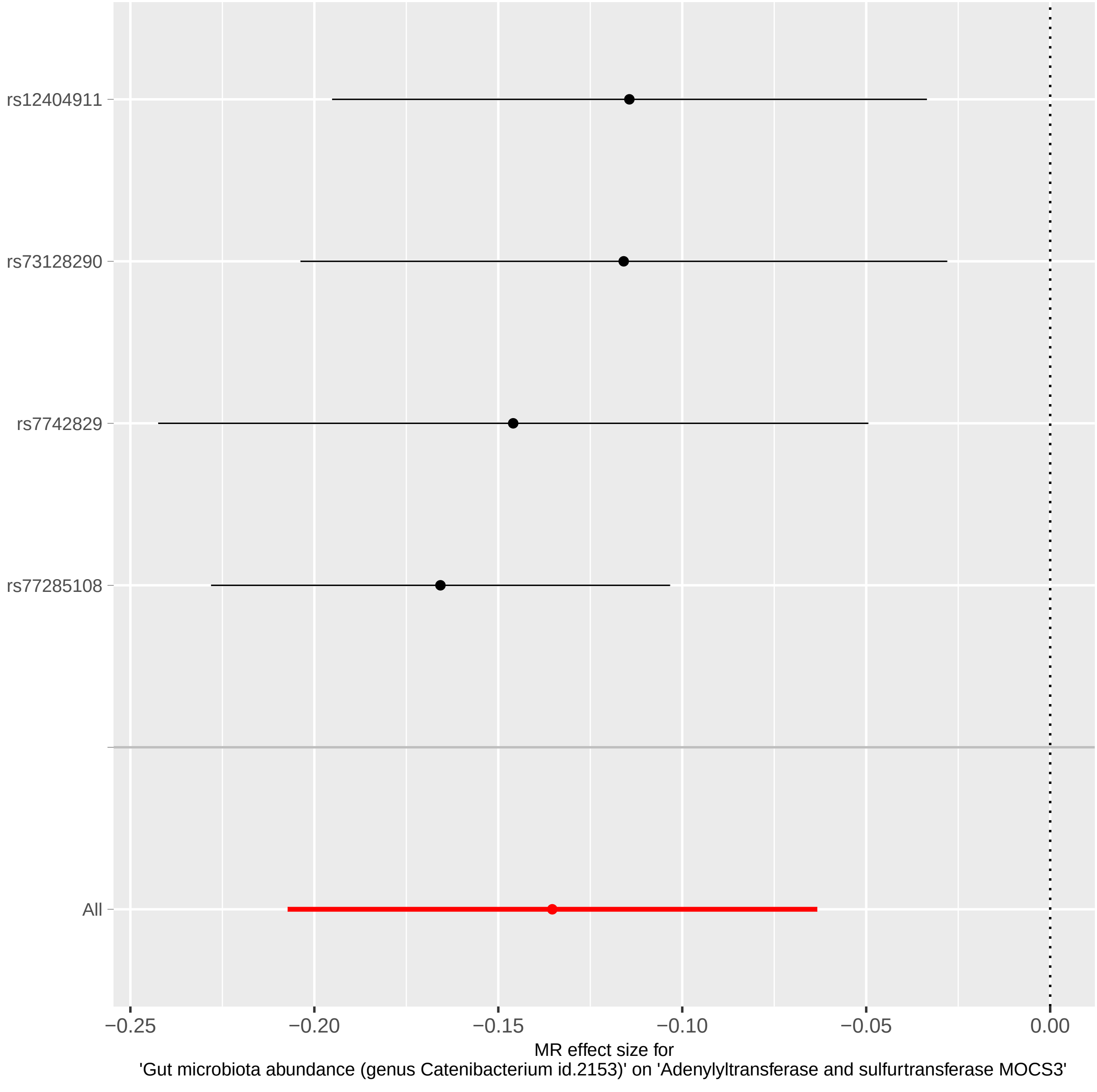

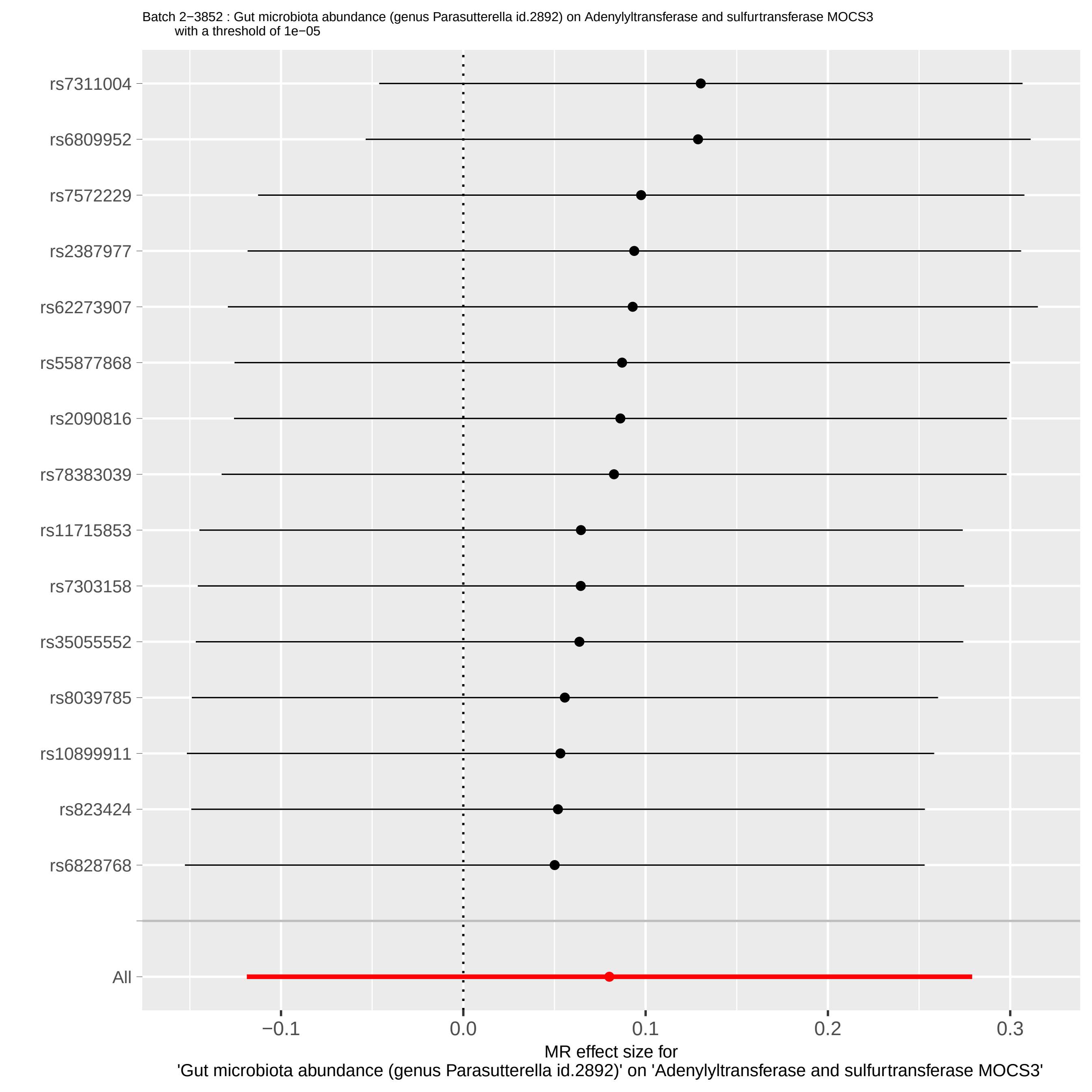
Batch 2−3852 : Gut microbiota abundance (genus Parasutterella id.2892) on Adenylyltransferase and sulfurtransferase MOCS3
with a threshold of 1e−05
rs7311004
rs6809952
rs7572229
rs2387977
rs62273907
rs55877868
rs2090816
rs78383039
rs11715853
rs7303158
rs35055552
rs8039785
rs10899911
rs823424
rs6828768
All
−0.1
0.0
0.1
0.2
0.3
MR effect size for
'Gut microbiota abundance (genus Parasutterella id.2892)' on 'Adenylyltransferase and sulfurtransferase MOCS3'

Batch 2−3853 : Gut microbiota abundance (genus Catenibacterium id.2153) on Multiple PDZ domain protein
with a threshold of 1e−05

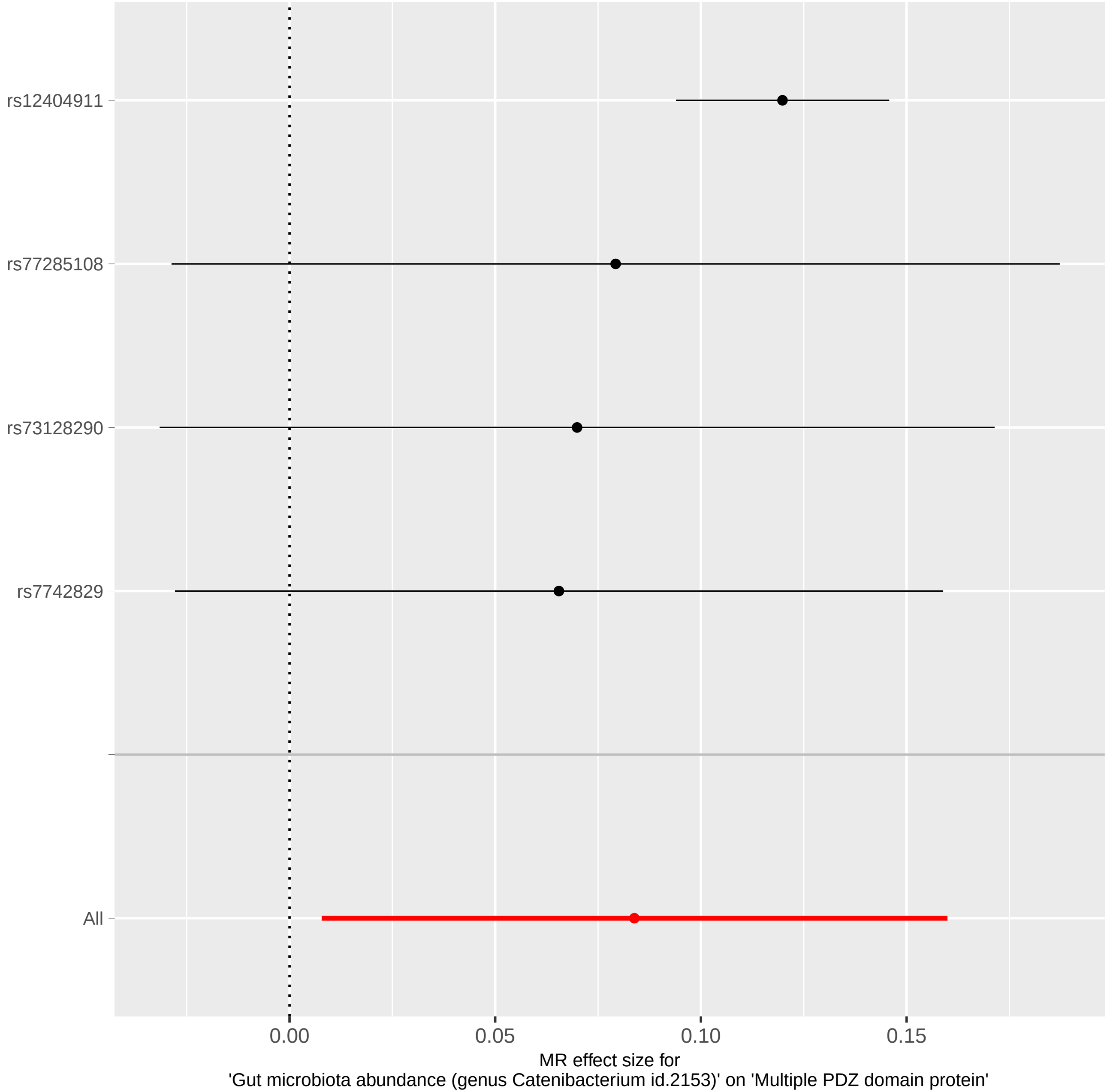

Batch 2−3854 : Gut microbiota abundance (genus Parasutterella id.2892) on Multiple PDZ domain protein
with a threshold of 1e−05

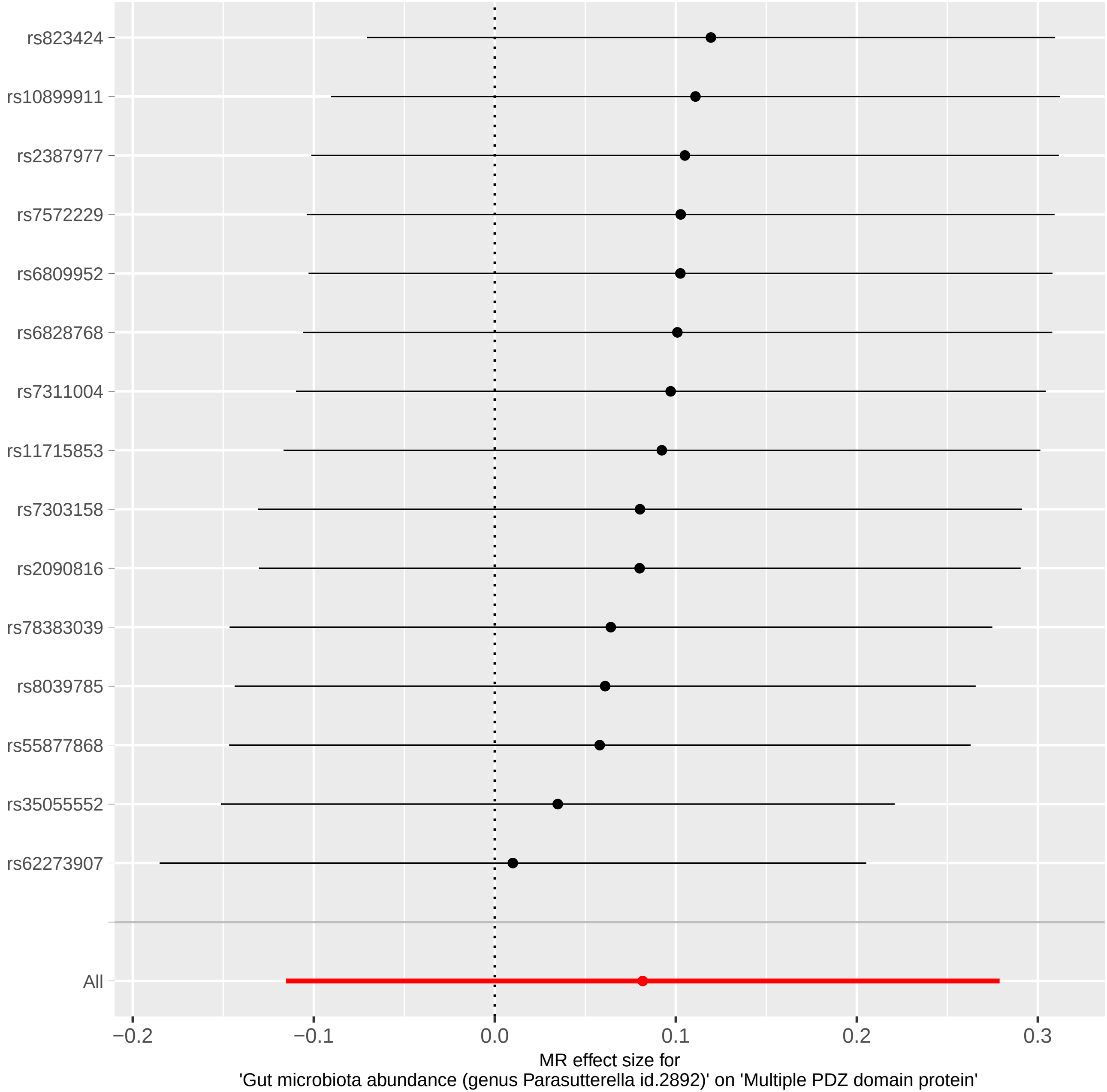

Batch 2−3855 : Gut microbiota abundance (genus Catenibacterium id.2153) on DNA−3−methyladenine glycosylase
with a threshold of 1e−05

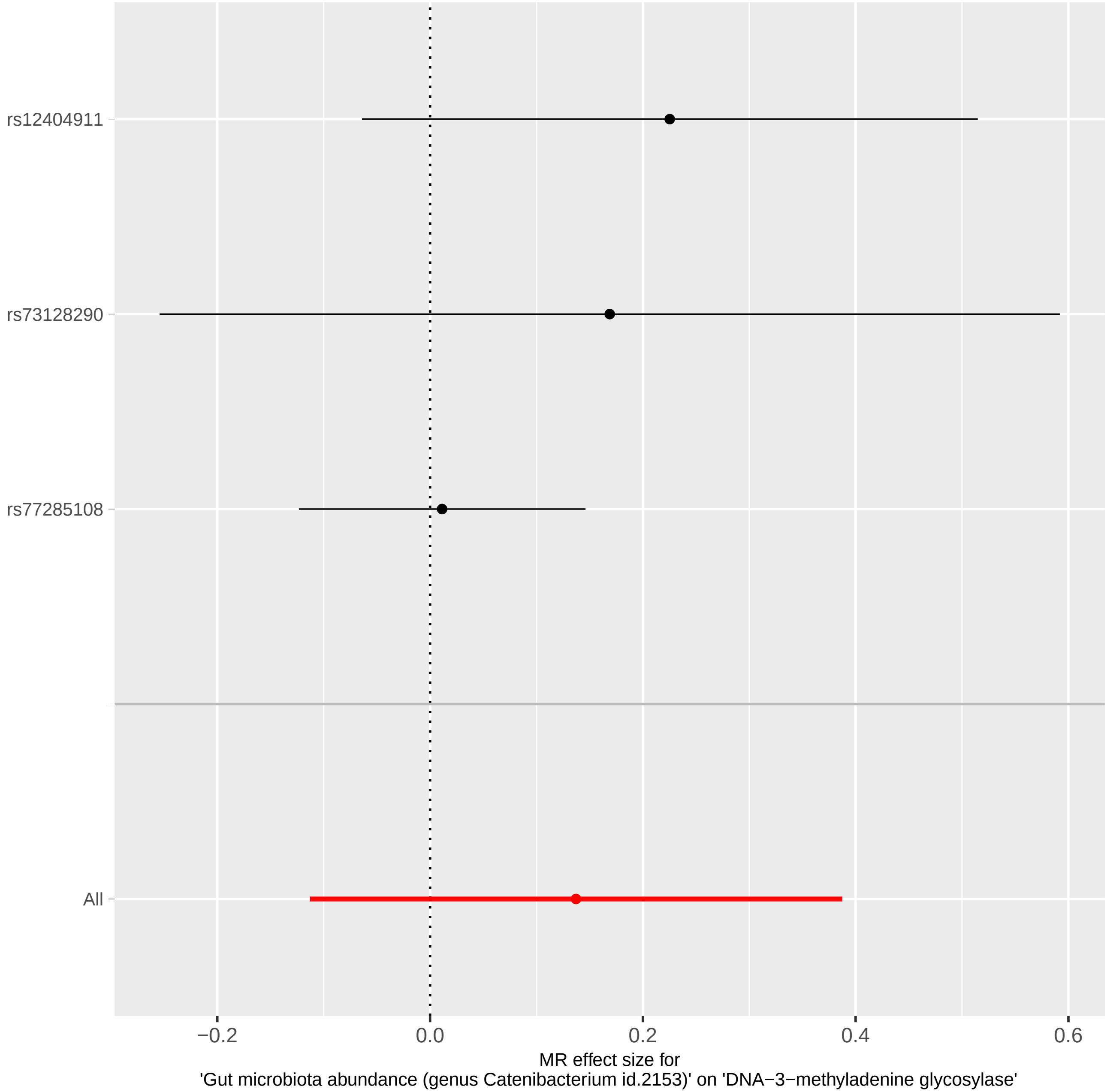

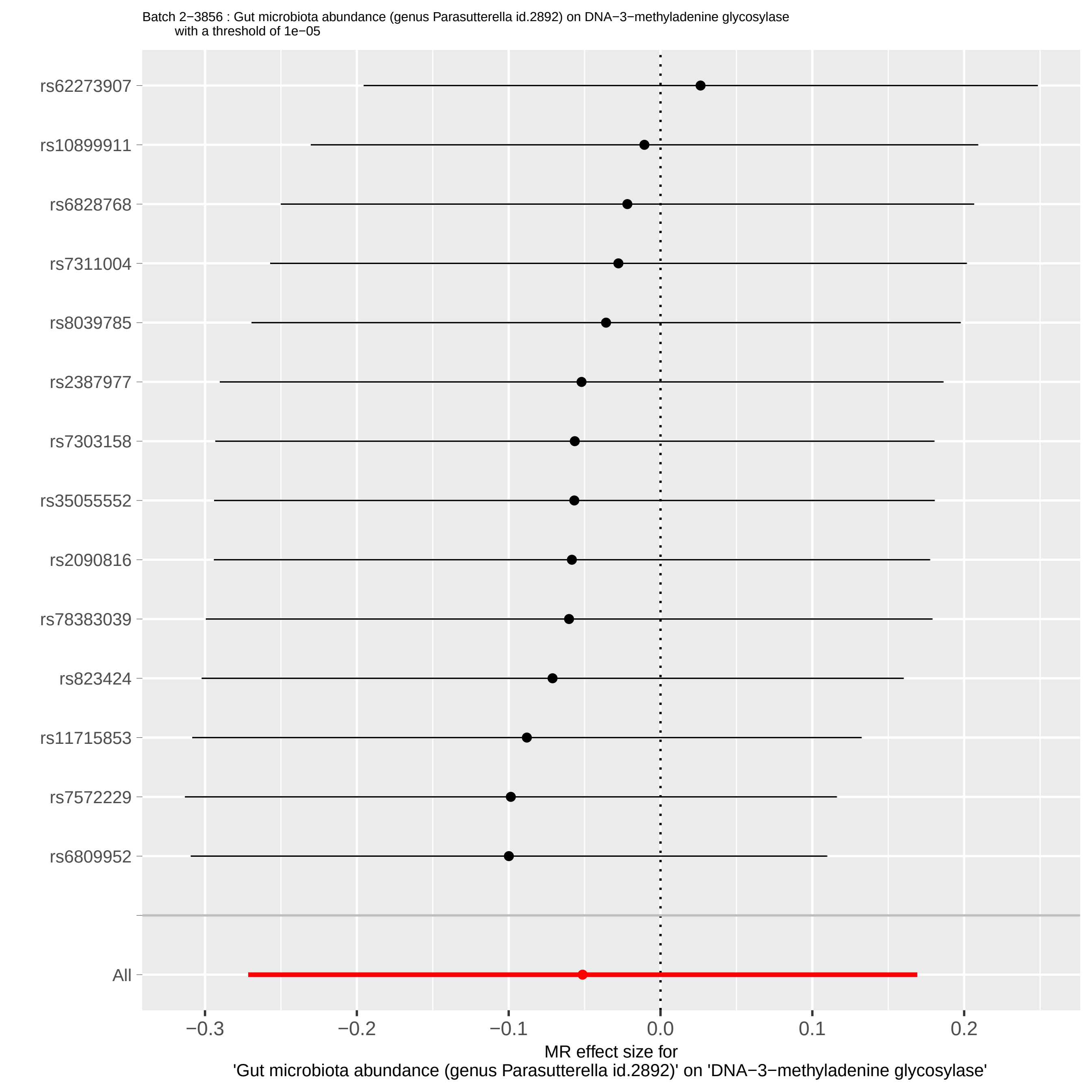

Batch 2−3856 : Gut microbiota abundance (genus Parasutterella id.2892) on DNA−3−methyladenine glycosylase
with a threshold of 1e−05
rs62273907
rs10899911
rs6828768
rs7311004
rs8039785
rs2387977
rs7303158
rs35055552
rs2090816
rs78383039
rs823424
rs11715853
rs7572229
rs6809952
All
−0.3
−0.2
−0.1
0.0
0.1
0.2
MR effect size for
'Gut microbiota abundance (genus Parasutterella id.2892)' on 'DNA−3−methyladenine glycosylase'

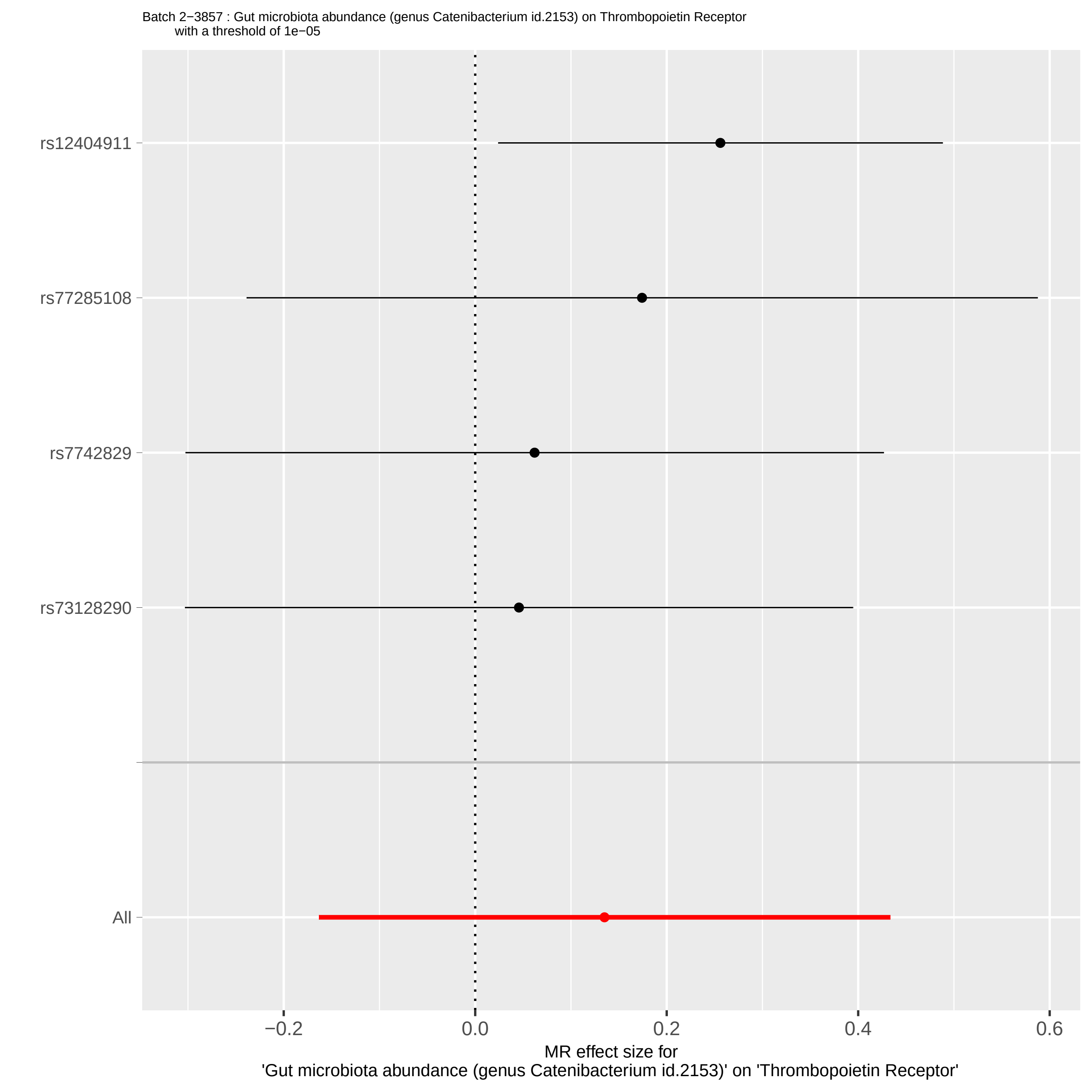
Batch 2−3857 : Gut microbiota abundance (genus Catenibacterium id.2153) on Thrombopoietin Receptor
with a threshold of 1e−05
rs12404911
rs77285108
rs7742829
rs73128290
All
−0.2
0.0
0.2
0.4
0.6
MR effect size for
'Gut microbiota abundance (genus Catenibacterium id.2153)' on 'Thrombopoietin Receptor'

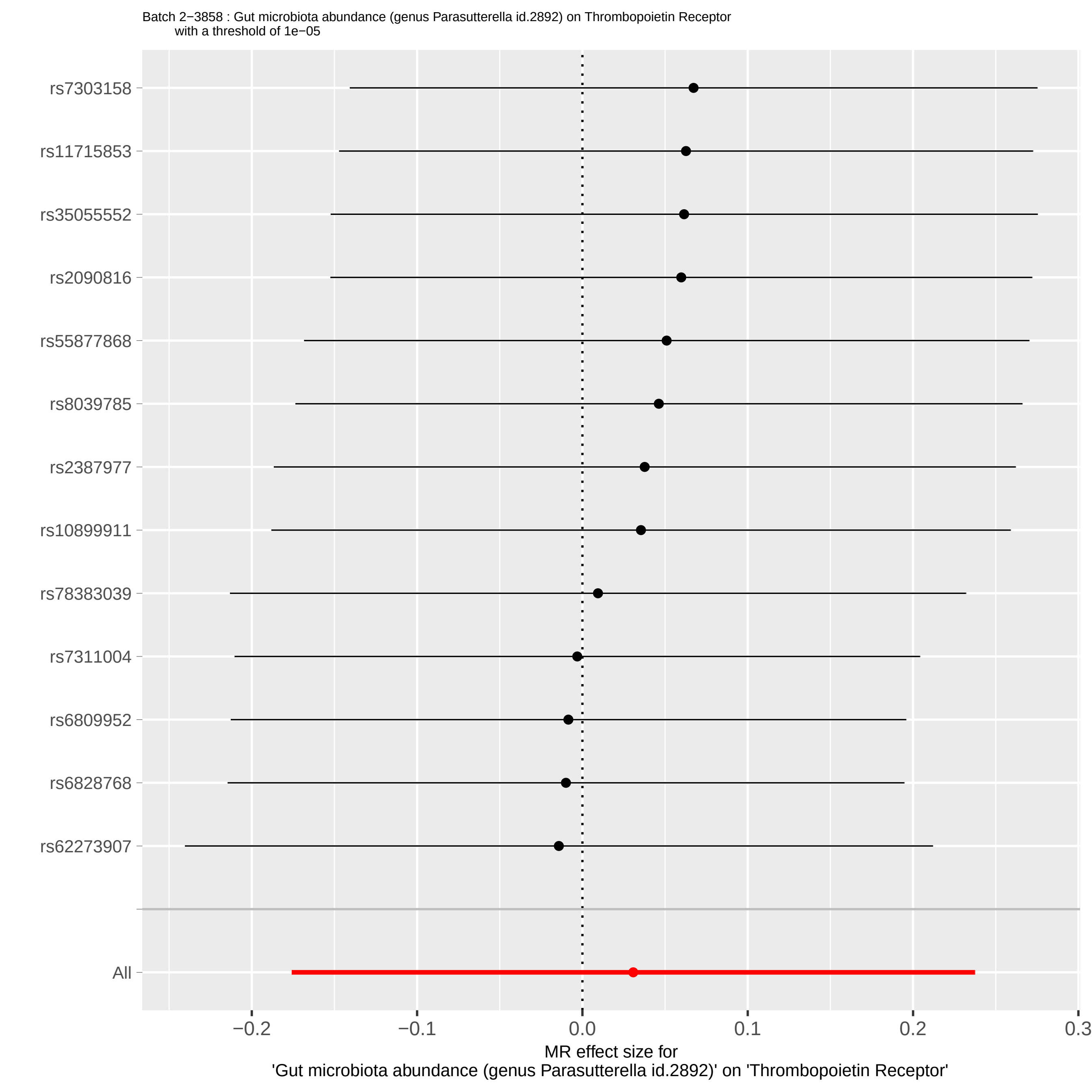

Batch 2−3858 : Gut microbiota abundance (genus Parasutterella id.2892) on Thrombopoietin Receptor
with a threshold of 1e−05
rs7303158
rs11715853
rs35055552
rs2090816
rs55877868
rs8039785
rs2387977
rs10899911
rs78383039
rs7311004
rs6809952
rs6828768
rs62273907
All
−0.2
−0.1
0.0
0.1
0.2
0.3
MR effect size for
'Gut microbiota abundance (genus Parasutterella id.2892)' on 'Thrombopoietin Receptor'

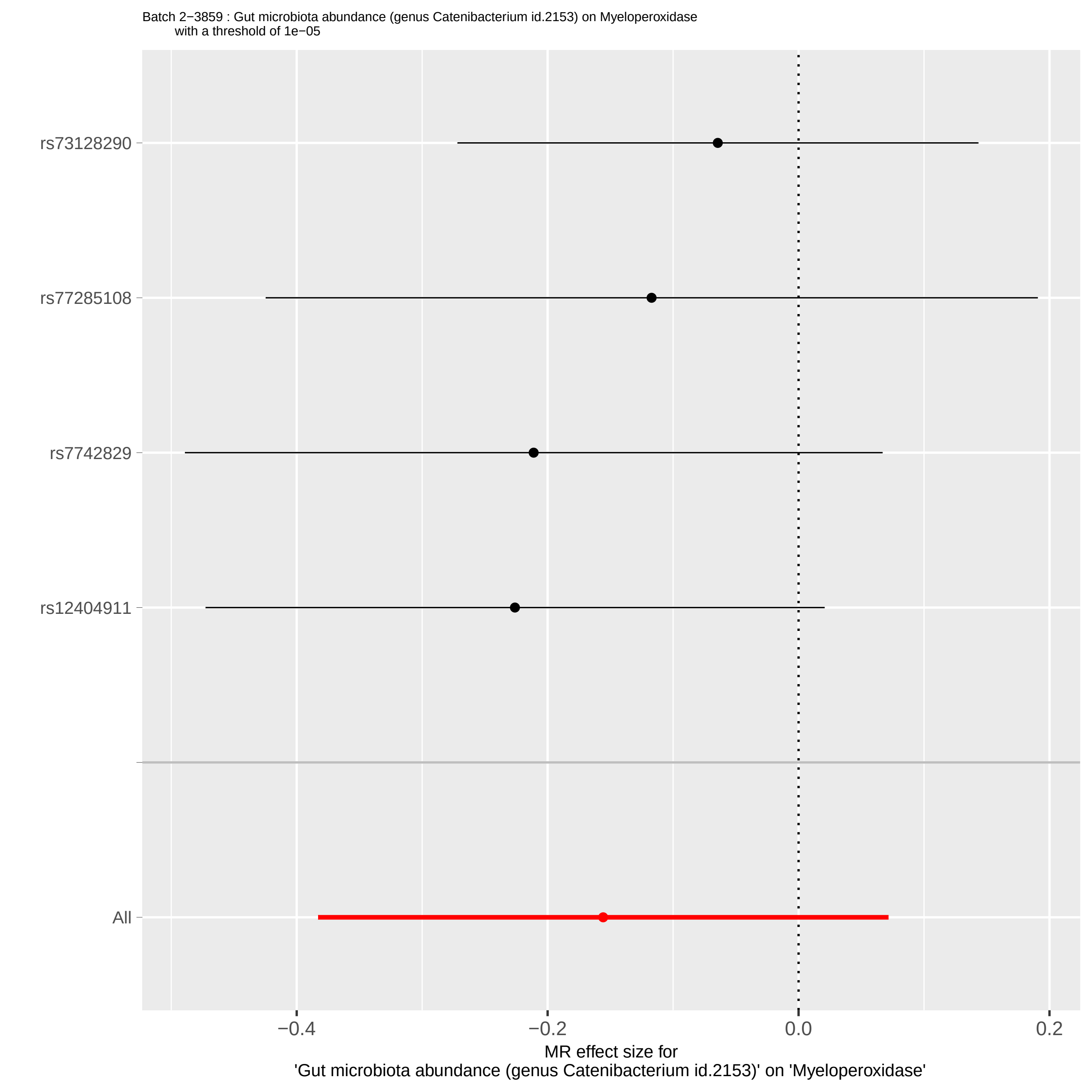
Batch 2−3859 : Gut microbiota abundance (genus Catenibacterium id.2153) on Myeloperoxidase
with a threshold of 1e−05
rs73128290
rs77285108
rs7742829
rs12404911
All
−0.4
−0.2
0.0
0.2
MR effect size for
'Gut microbiota abundance (genus Catenibacterium id.2153)' on 'Myeloperoxidase'

Batch 2−3860 : Gut microbiota abundance (genus Parasutterella id.2892) on Myeloperoxidase
with a threshold of 1e−05

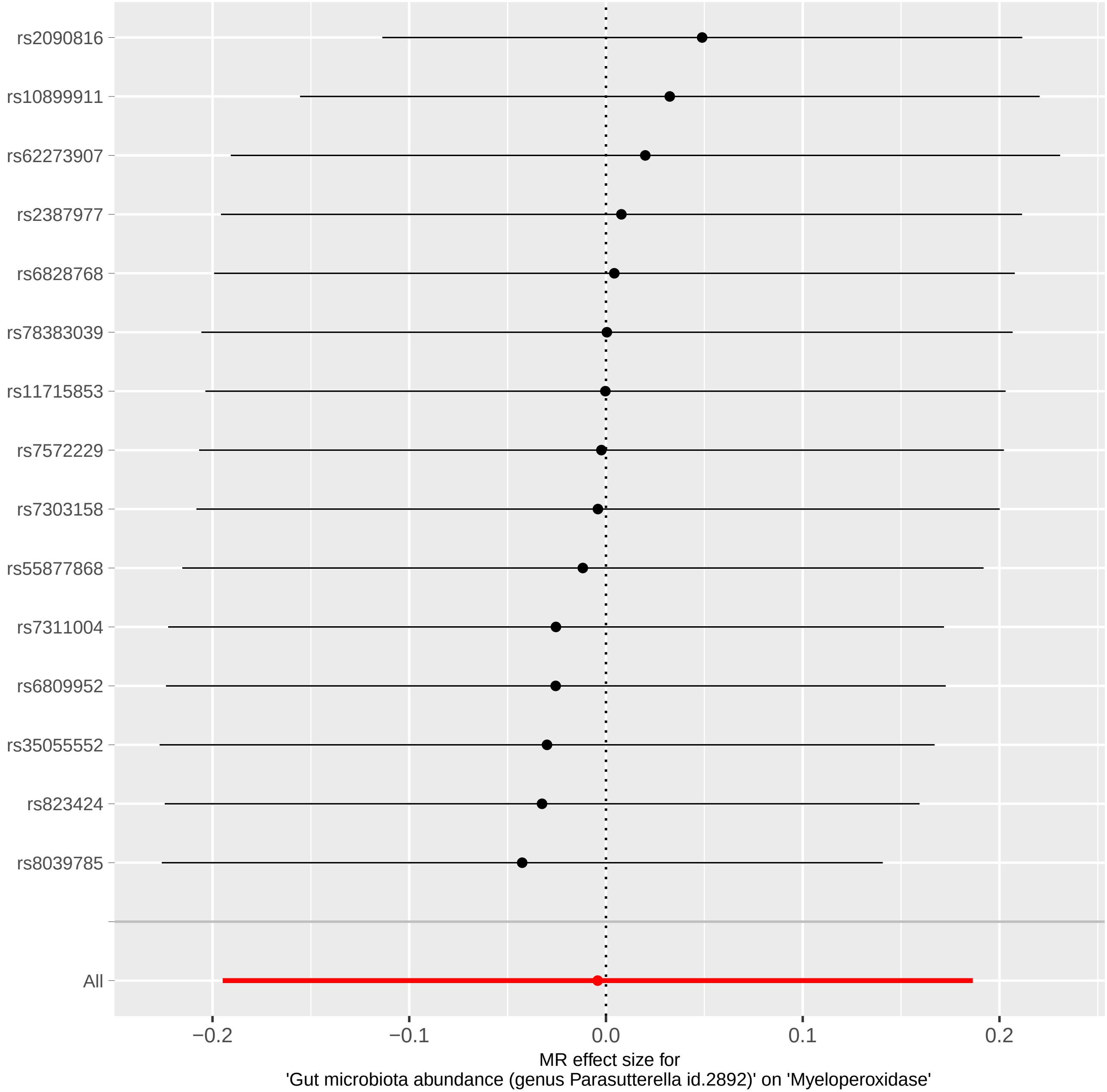

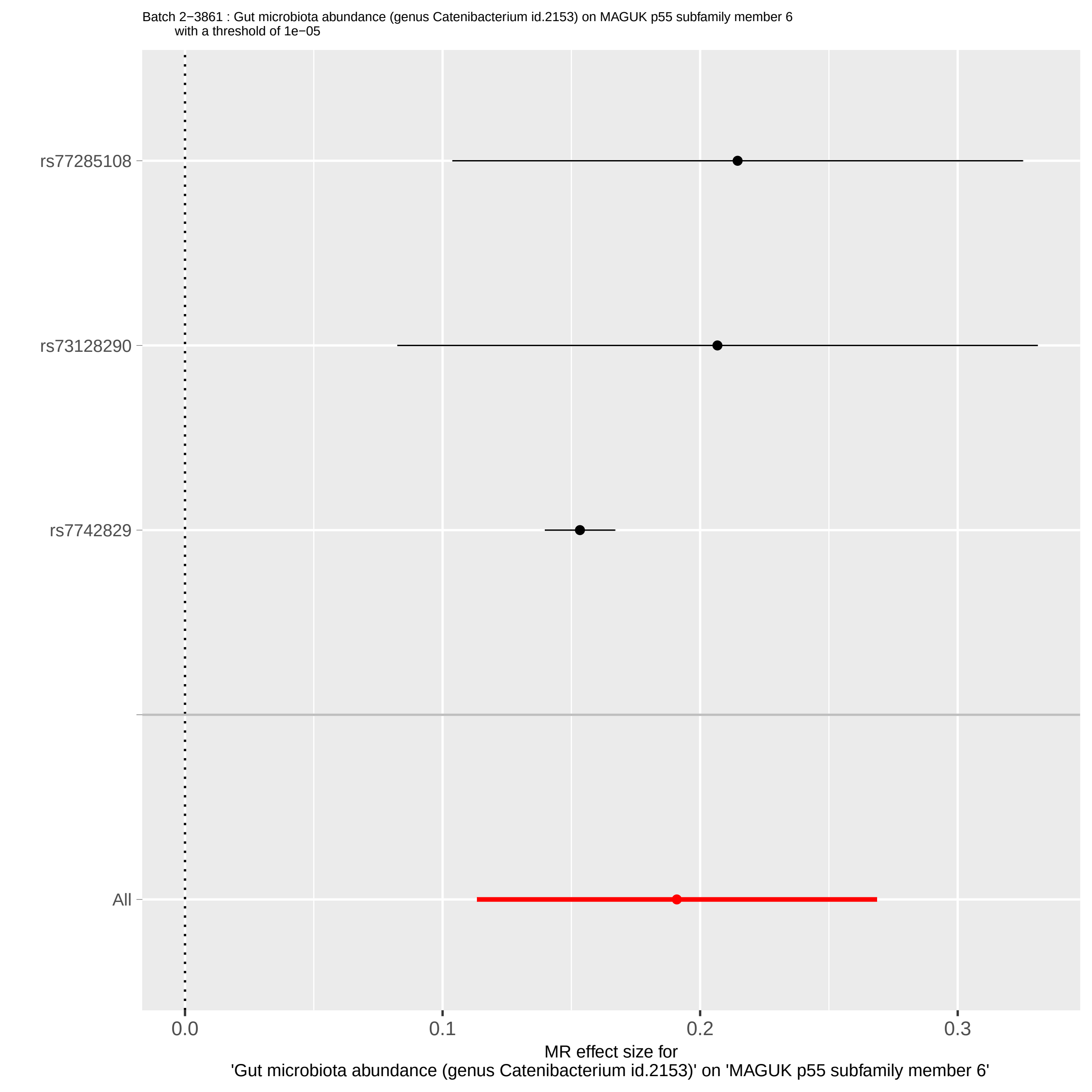

Batch 2−3861 : Gut microbiota abundance (genus Catenibacterium id.2153) on MAGUK p55 subfamily member 6
with a threshold of 1e−05
rs77285108
rs73128290
rs7742829
All
0.0
0.1
0.2
0.3
MR effect size for
'Gut microbiota abundance (genus Catenibacterium id.2153)' on 'MAGUK p55 subfamily member 6'

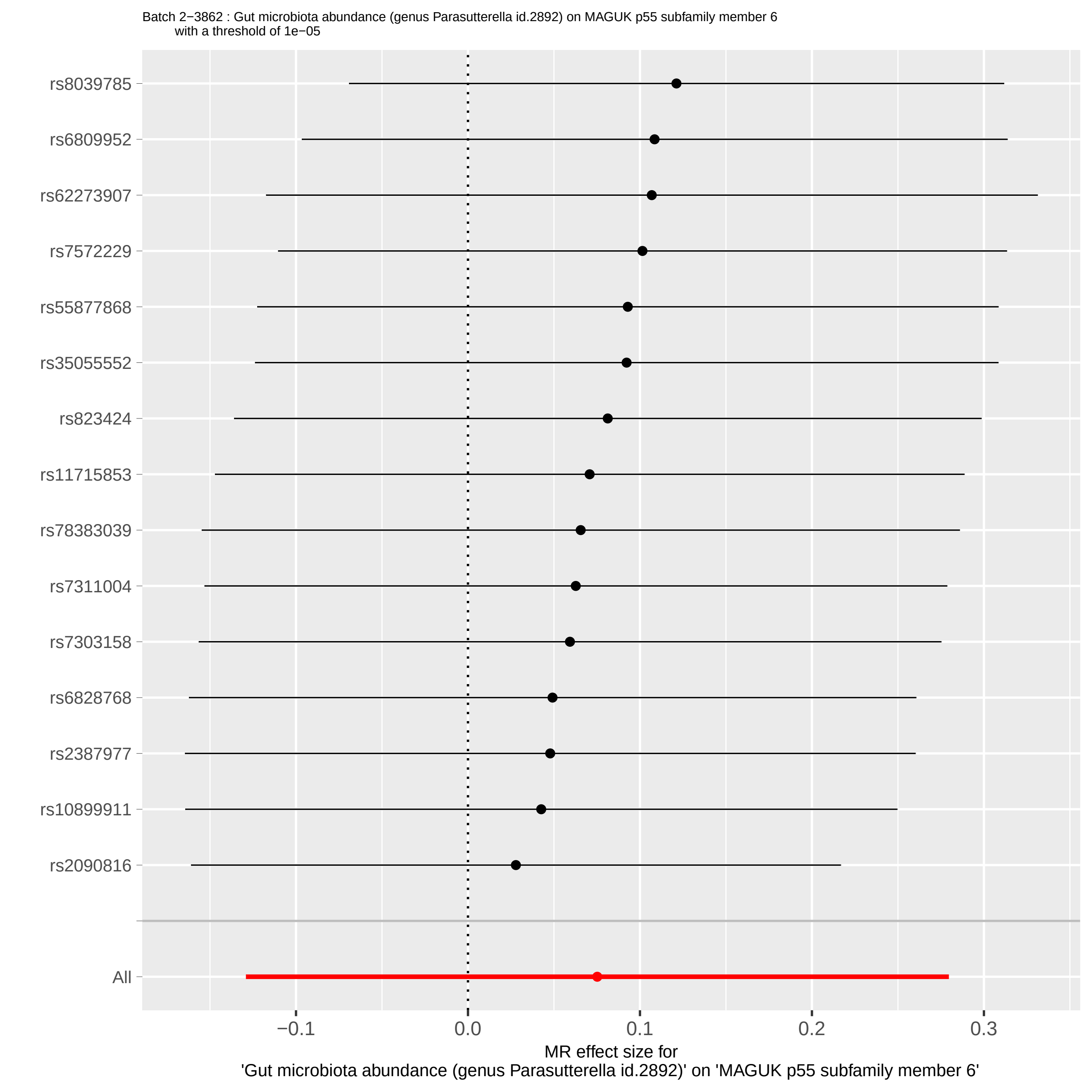
Batch 2−3862 : Gut microbiota abundance (genus Parasutterella id.2892) on MAGUK p55 subfamily member 6
with a threshold of 1e−05
rs8039785
rs6809952
rs62273907
rs7572229
rs55877868
rs35055552
rs823424
rs11715853
rs78383039
rs7311004
rs7303158
rs6828768
rs2387977
rs10899911
rs2090816
All
−0.1
0.0
0.1
0.2
0.3
MR effect size for
'Gut microbiota abundance (genus Parasutterella id.2892)' on 'MAGUK p55 subfamily member 6'

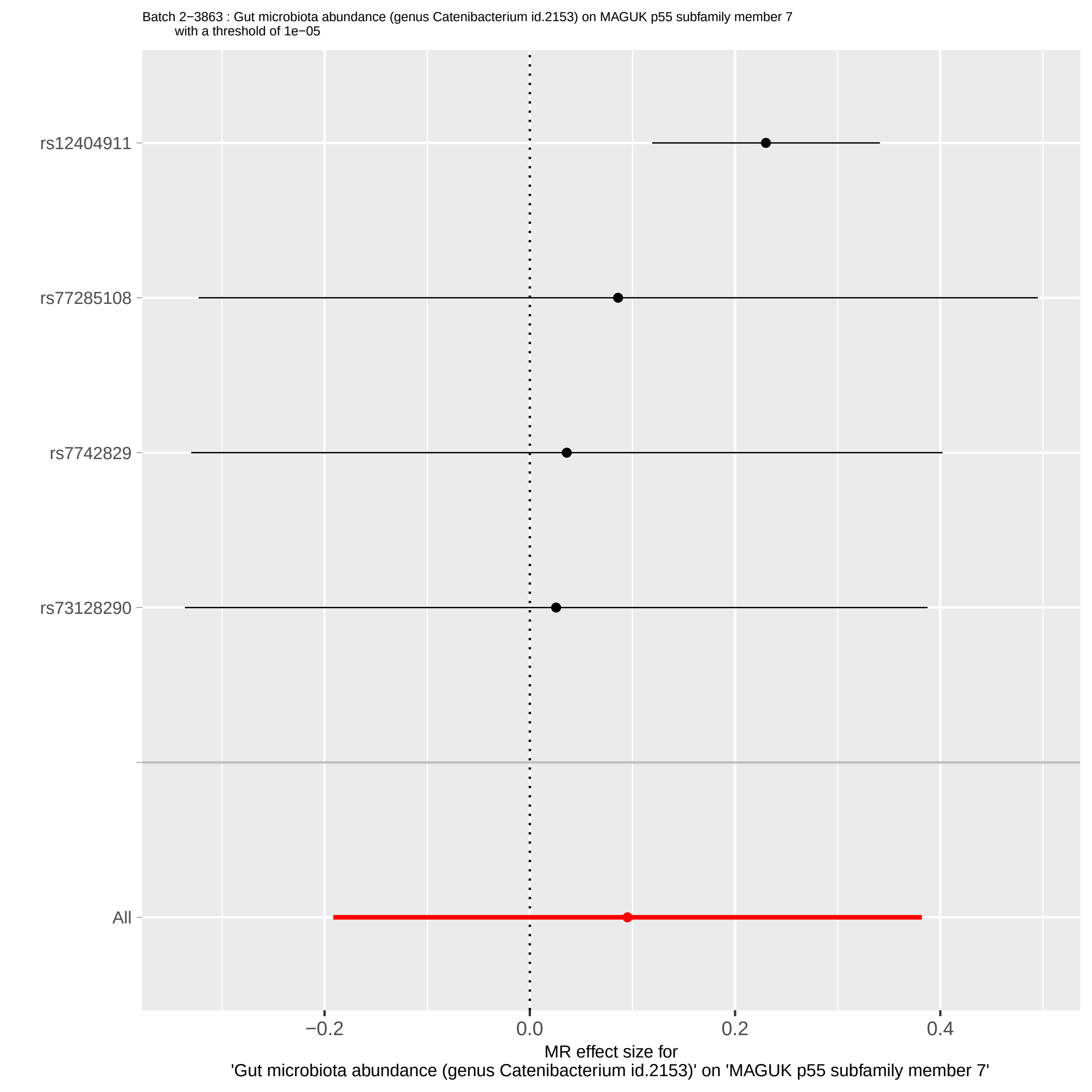

Batch 2−3863 : Gut microbiota abundance (genus Catenibacterium id.2153) on MAGUK p55 subfamily member 7
with a threshold of 1e−05
rs12404911
rs77285108
rs7742829
rs73128290
All
−0.2
0.0
0.2
0.4
MR effect size for
'Gut microbiota abundance (genus Catenibacterium id.2153)' on 'MAGUK p55 subfamily member 7'

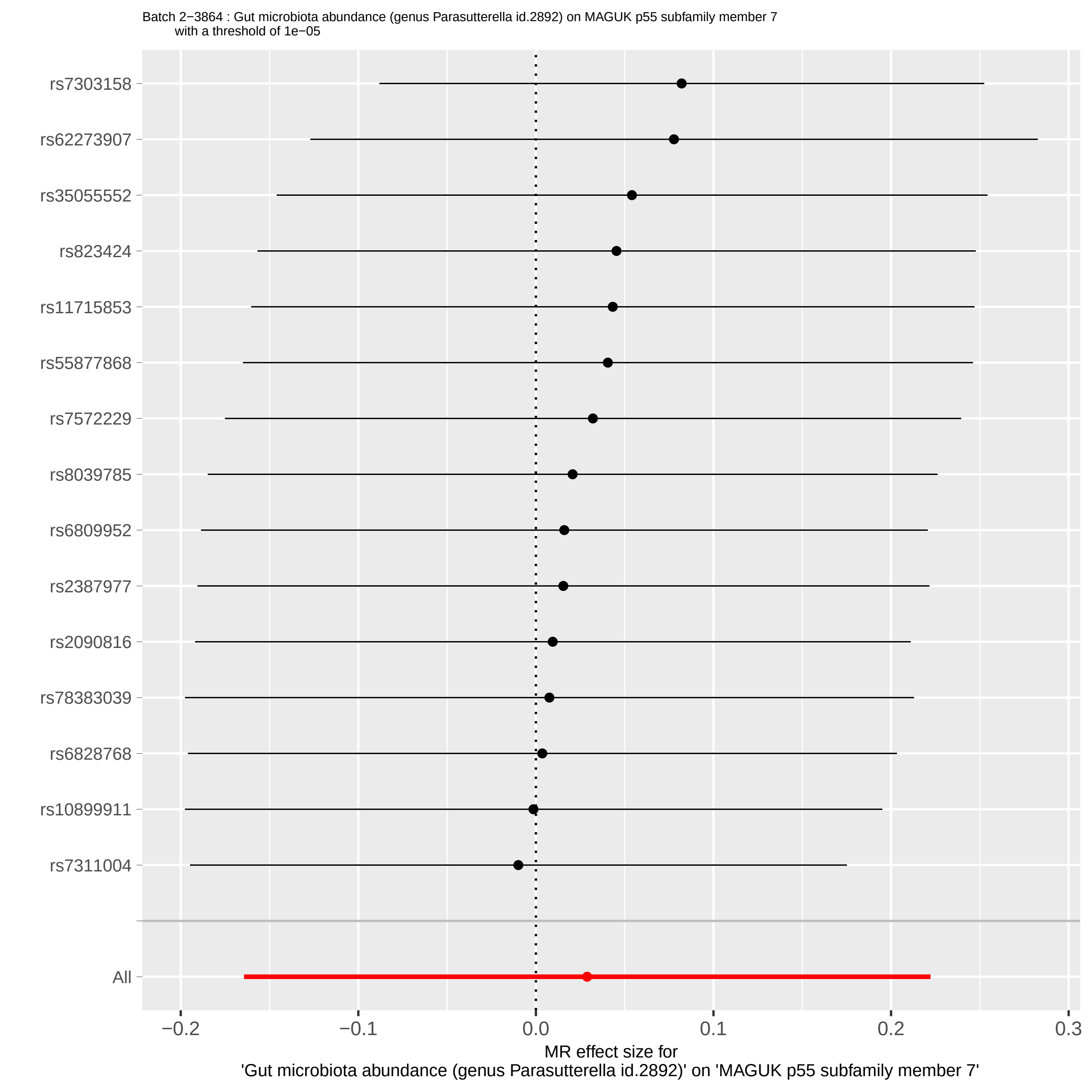
Batch 2−3864 : Gut microbiota abundance (genus Parasutterella id.2892) on MAGUK p55 subfamily member 7
with a threshold of 1e−05
rs7303158
rs62273907
rs35055552
rs823424
rs11715853
rs55877868
rs7572229
rs8039785
rs6809952
rs2387977
rs2090816
rs78383039
rs6828768
rs10899911
rs7311004
All
−0.2
−0.1
0.0
0.1
0.2
0.3
MR effect size for
'Gut microbiota abundance (genus Parasutterella id.2892)' on 'MAGUK p55 subfamily member 7'

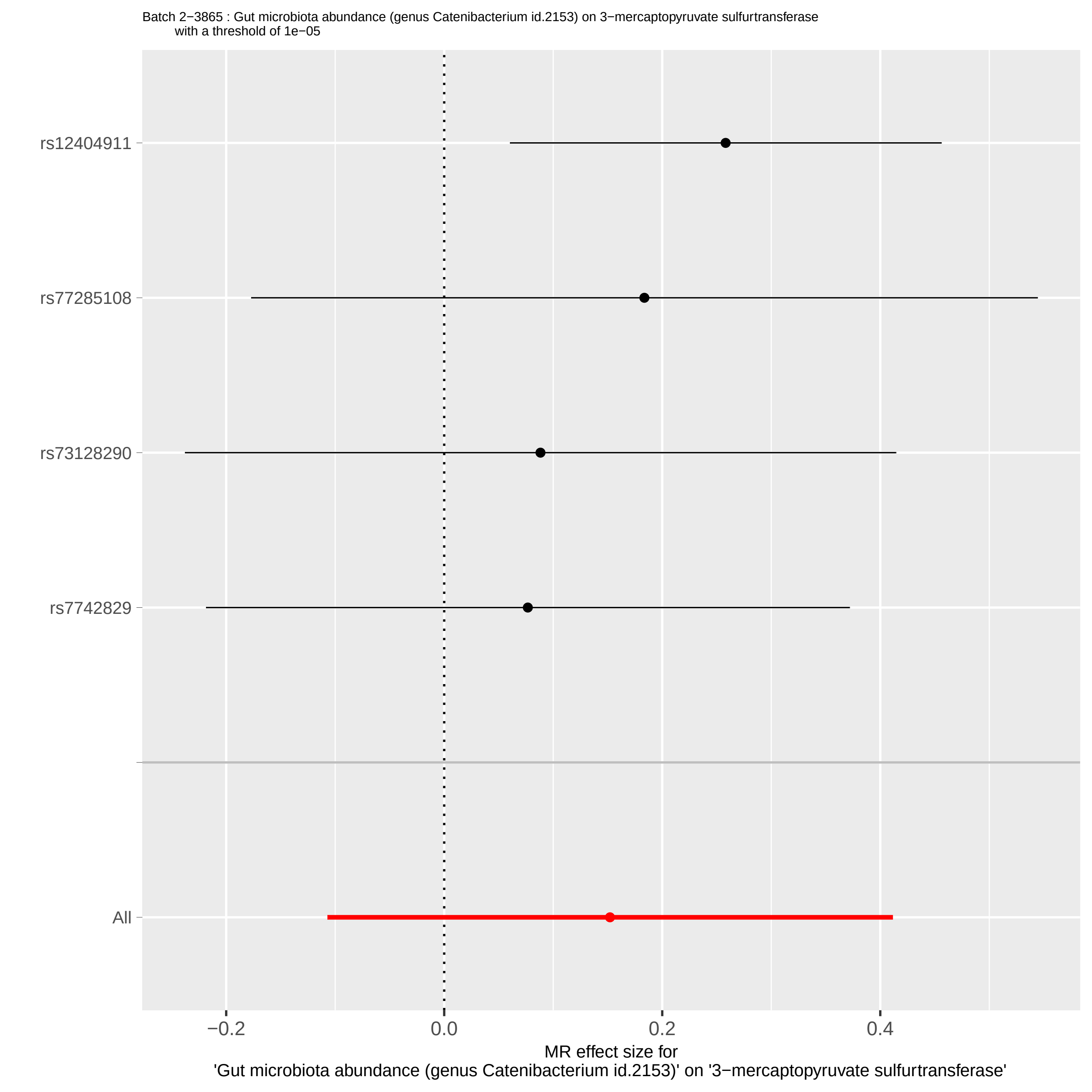

Batch 2−3865 : Gut microbiota abundance (genus Catenibacterium id.2153) on 3−mercaptopyruvate sulfurtransferase
with a threshold of 1e−05
rs12404911
rs77285108
rs73128290
rs7742829
All
−0.2
0.0
0.2
0.4
MR effect size for
'Gut microbiota abundance (genus Catenibacterium id.2153)' on '3−mercaptopyruvate sulfurtransferase'

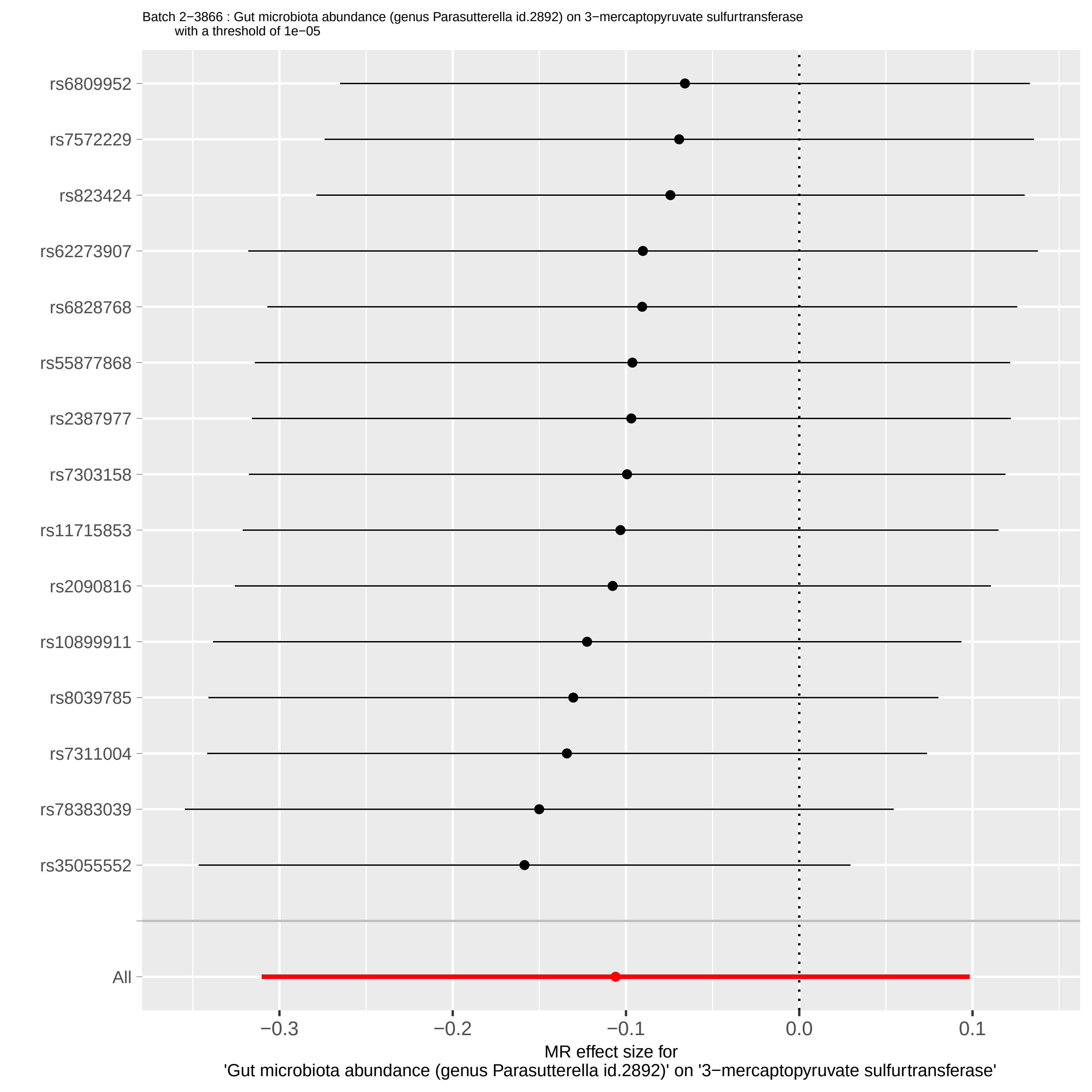

Batch 2−3866 : Gut microbiota abundance (genus Parasutterella id.2892) on 3−mercaptopyruvate sulfurtransferase
with a threshold of 1e−05
rs6809952
rs7572229
rs823424
rs62273907
rs6828768
rs55877868
rs2387977
rs7303158
rs11715853
rs2090816
rs10899911
rs8039785
rs7311004
rs78383039
rs35055552
All
−0.3
−0.2
−0.1
0.0
0.1
MR effect size for
'Gut microbiota abundance (genus Parasutterella id.2892)' on '3−mercaptopyruvate sulfurtransferase'

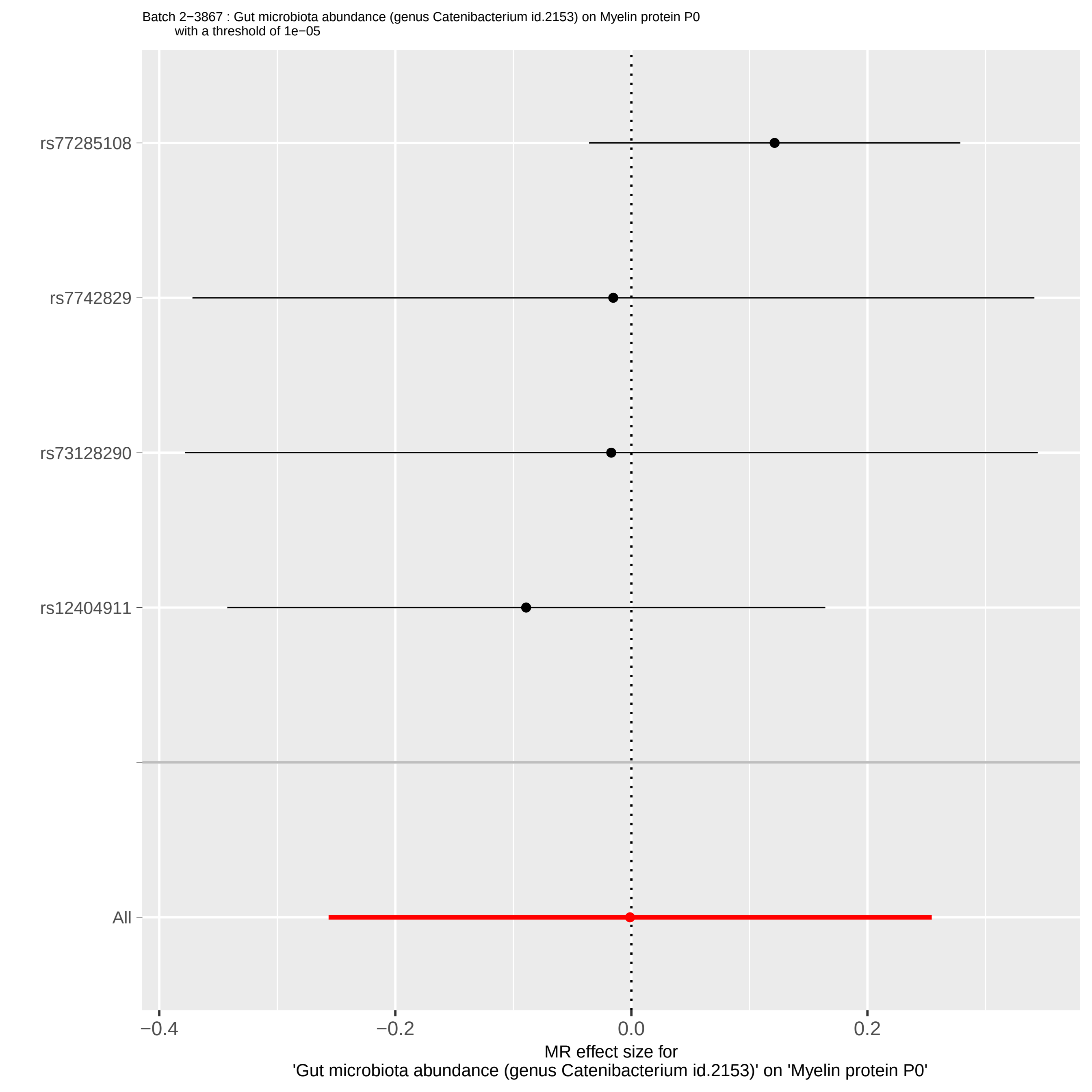

Batch 2−3867 : Gut microbiota abundance (genus Catenibacterium id.2153) on Myelin protein P0
with a threshold of 1e−05
rs77285108
rs7742829
rs73128290
rs12404911
All
−0.4
−0.2
0.0
0.2
MR effect size for
'Gut microbiota abundance (genus Catenibacterium id.2153)' on 'Myelin protein P0'

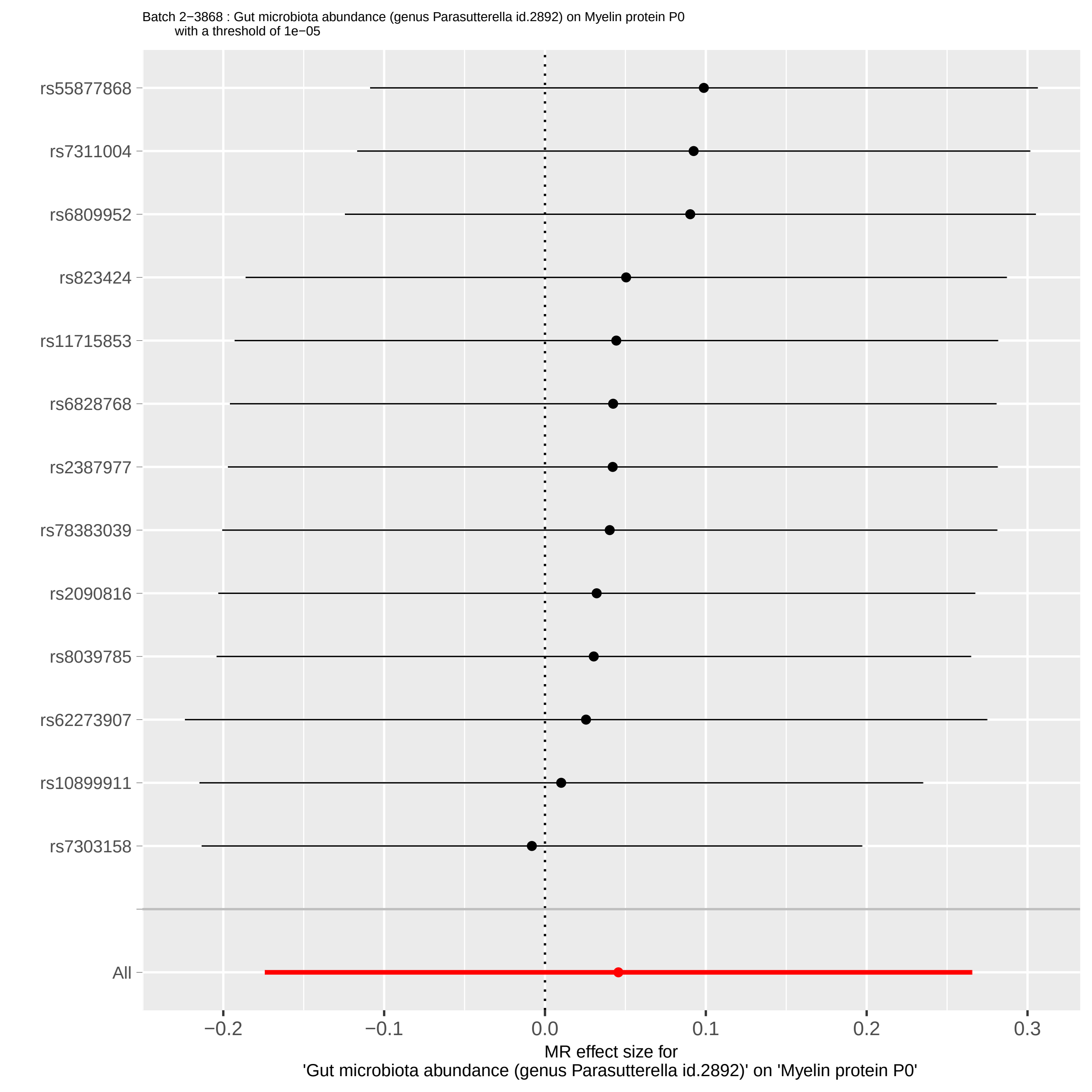
Batch 2−3868 : Gut microbiota abundance (genus Parasutterella id.2892) on Myelin protein P0
with a threshold of 1e−05
rs55877868
rs7311004
rs6809952
rs823424
rs11715853
rs6828768
rs2387977
rs78383039
rs2090816
rs8039785
rs62273907
rs10899911
rs7303158
All
−0.2
−0.1
0.0
0.1
0.2
0.3
MR effect size for
'Gut microbiota abundance (genus Parasutterella id.2892)' on 'Myelin protein P0'

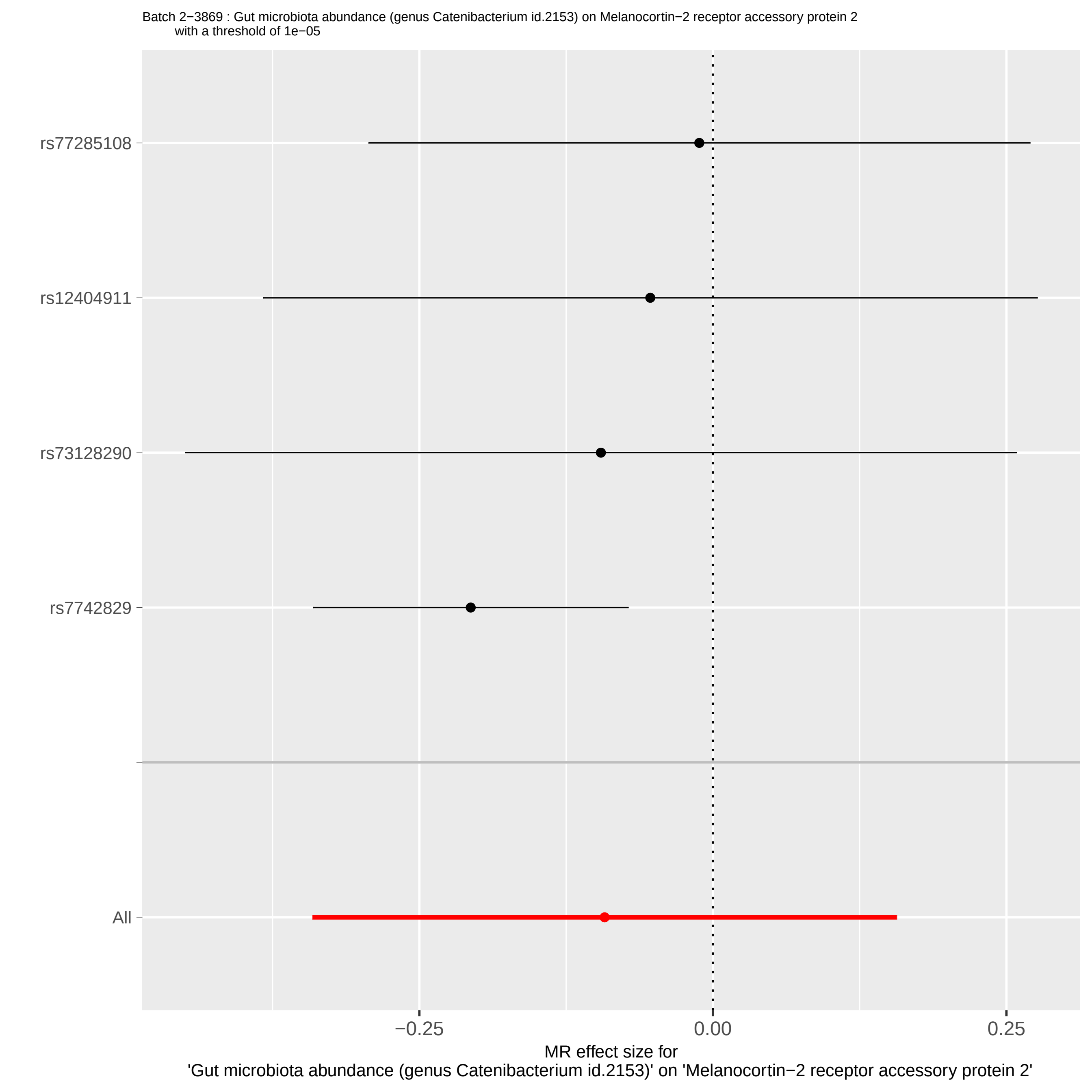

Batch 2−3869 : Gut microbiota abundance (genus Catenibacterium id.2153) on Melanocortin−2 receptor accessory protein 2
with a threshold of 1e−05
rs77285108
rs12404911
rs73128290
rs7742829
All
−0.25
0.00
0.25
MR effect size for
'Gut microbiota abundance (genus Catenibacterium id.2153)' on 'Melanocortin−2 receptor accessory protein 2'

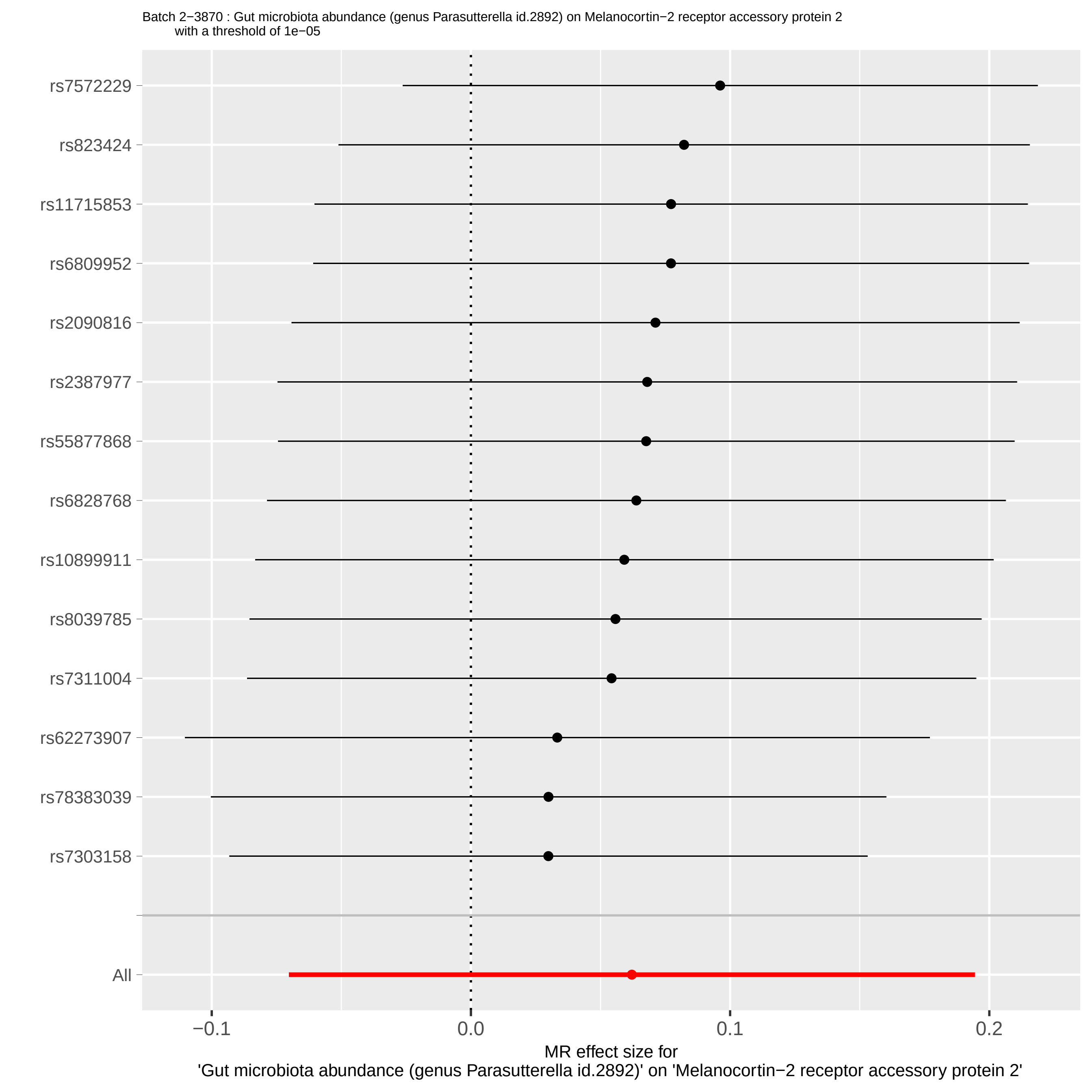

Batch 2−3870 : Gut microbiota abundance (genus Parasutterella id.2892) on Melanocortin−2 receptor accessory protein 2
with a threshold of 1e−05
rs7572229
rs823424
rs11715853
rs6809952
rs2090816
rs2387977
rs55877868
rs6828768
rs10899911
rs8039785
rs7311004
rs62273907
rs78383039
rs7303158
All
−0.1
0.0
0.1
0.2
MR effect size for
'Gut microbiota abundance (genus Parasutterella id.2892)' on 'Melanocortin−2 receptor accessory protein 2'

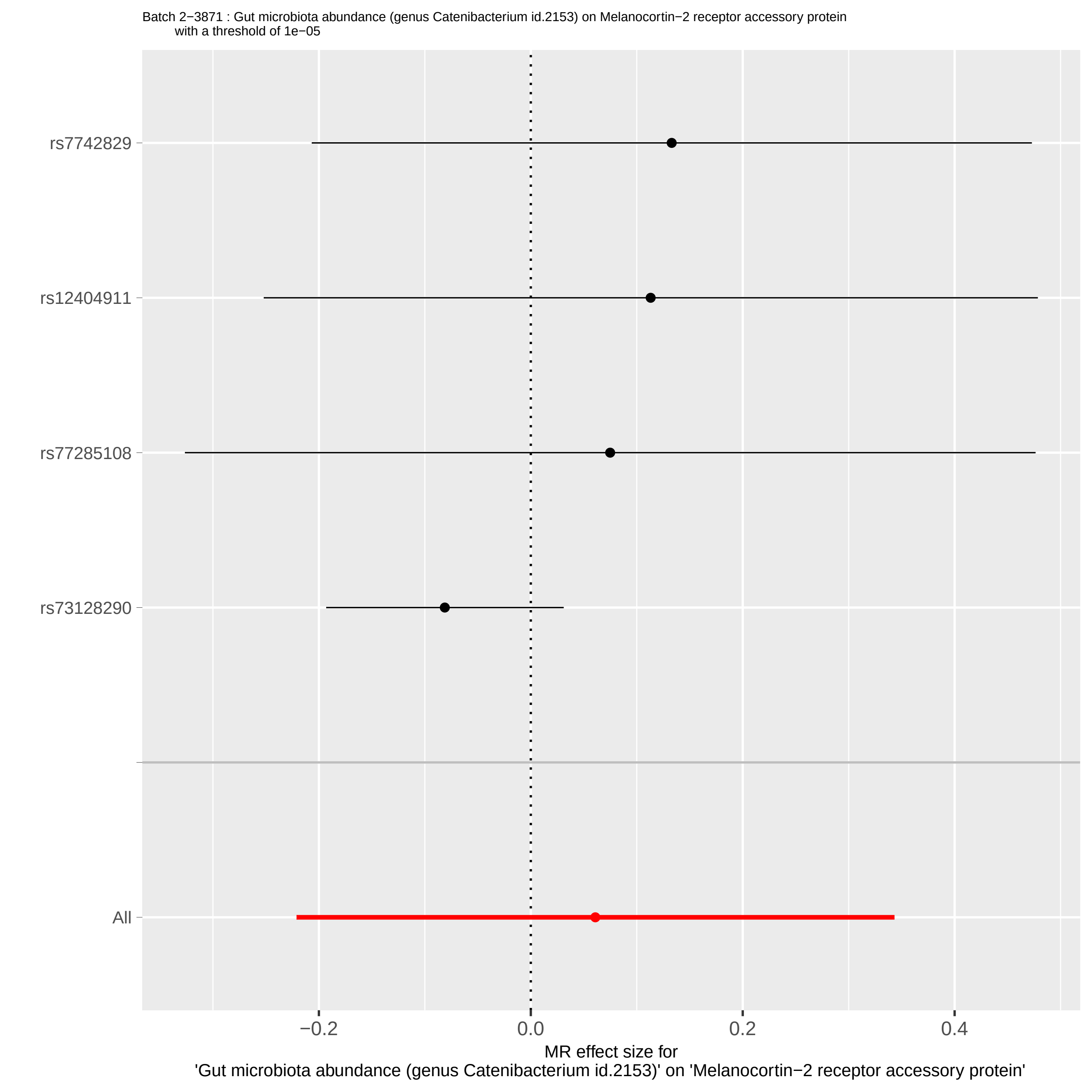
Batch 2−3871 : Gut microbiota abundance (genus Catenibacterium id.2153) on Melanocortin−2 receptor accessory protein
with a threshold of 1e−05
rs7742829
rs12404911
rs77285108
rs73128290
All
−0.2
0.0
0.2
0.4
MR effect size for
'Gut microbiota abundance (genus Catenibacterium id.2153)' on 'Melanocortin−2 receptor accessory protein'

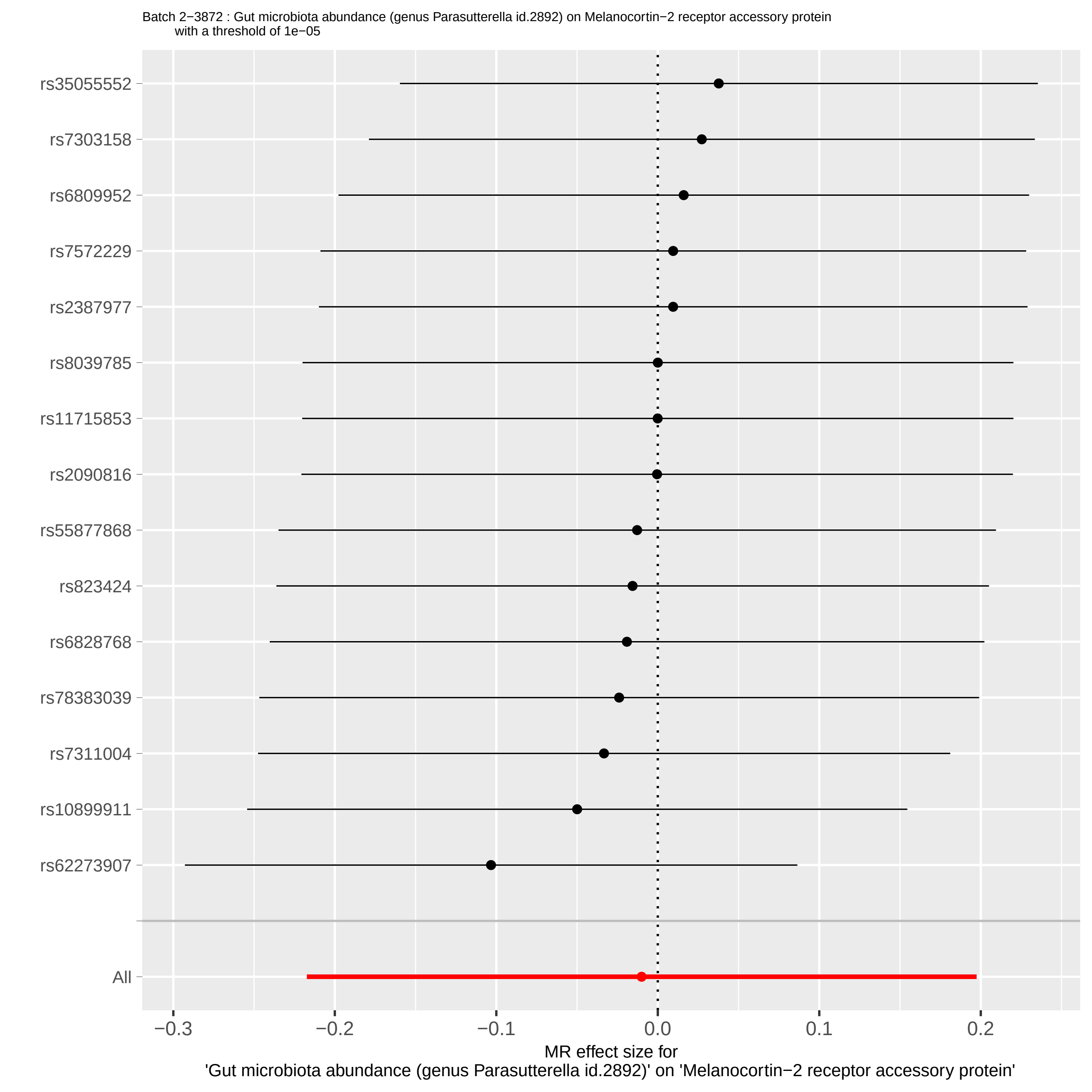

Batch 2−3872 : Gut microbiota abundance (genus Parasutterella id.2892) on Melanocortin−2 receptor accessory protein
with a threshold of 1e−05
rs35055552
rs7303158
rs6809952
rs7572229
rs2387977
rs8039785
rs11715853
rs2090816
rs55877868
rs823424
rs6828768
rs78383039
rs7311004
rs10899911
rs62273907
All
−0.3
−0.2
−0.1
0.0
0.1
0.2
MR effect size for
'Gut microbiota abundance (genus Parasutterella id.2892)' on 'Melanocortin−2 receptor accessory protein'

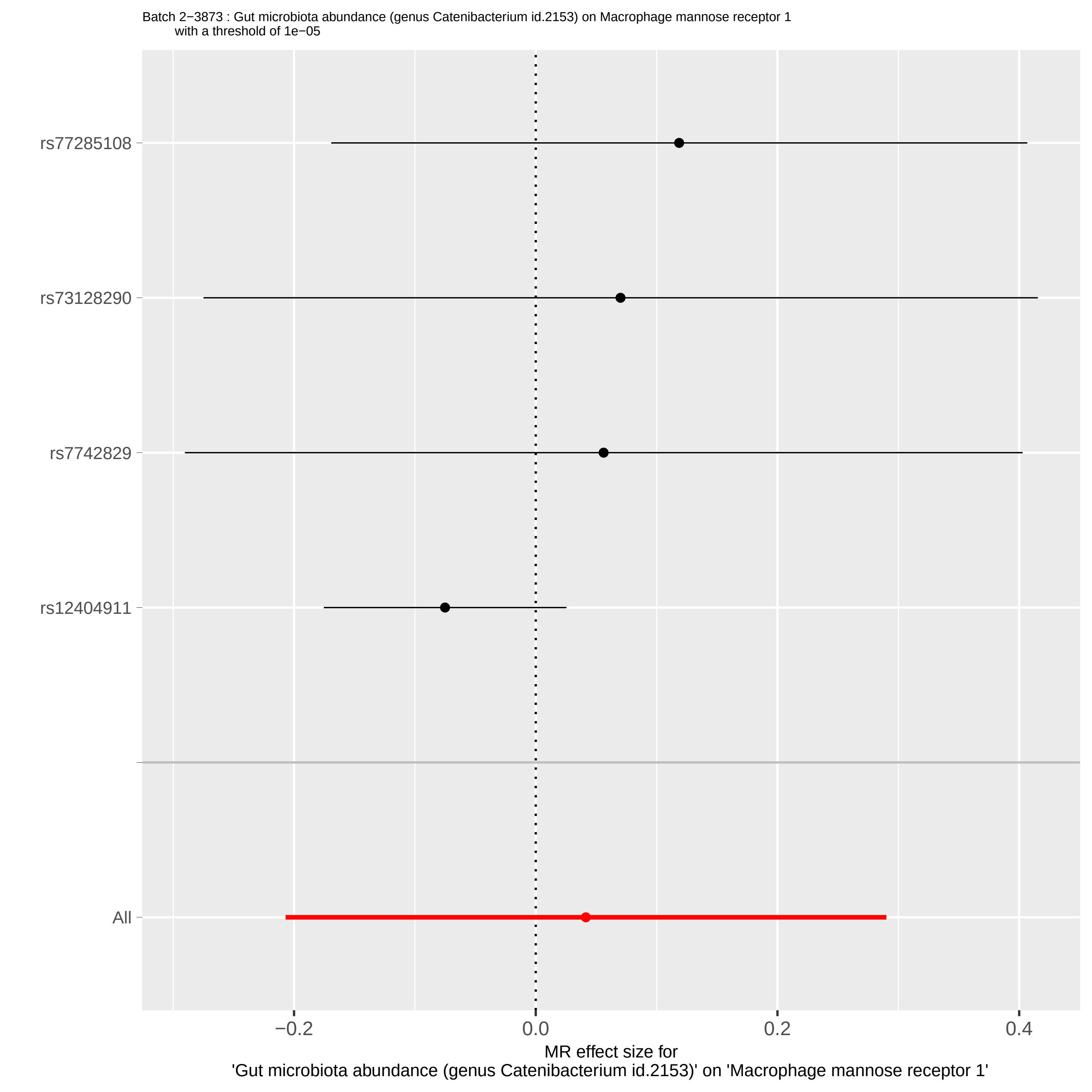

Batch 2−3873 : Gut microbiota abundance (genus Catenibacterium id.2153) on Macrophage mannose receptor 1
with a threshold of 1e−05
rs77285108
rs73128290
rs7742829
rs12404911
All
−0.2
0.0
0.2
0.4
MR effect size for
'Gut microbiota abundance (genus Catenibacterium id.2153)' on 'Macrophage mannose receptor 1'

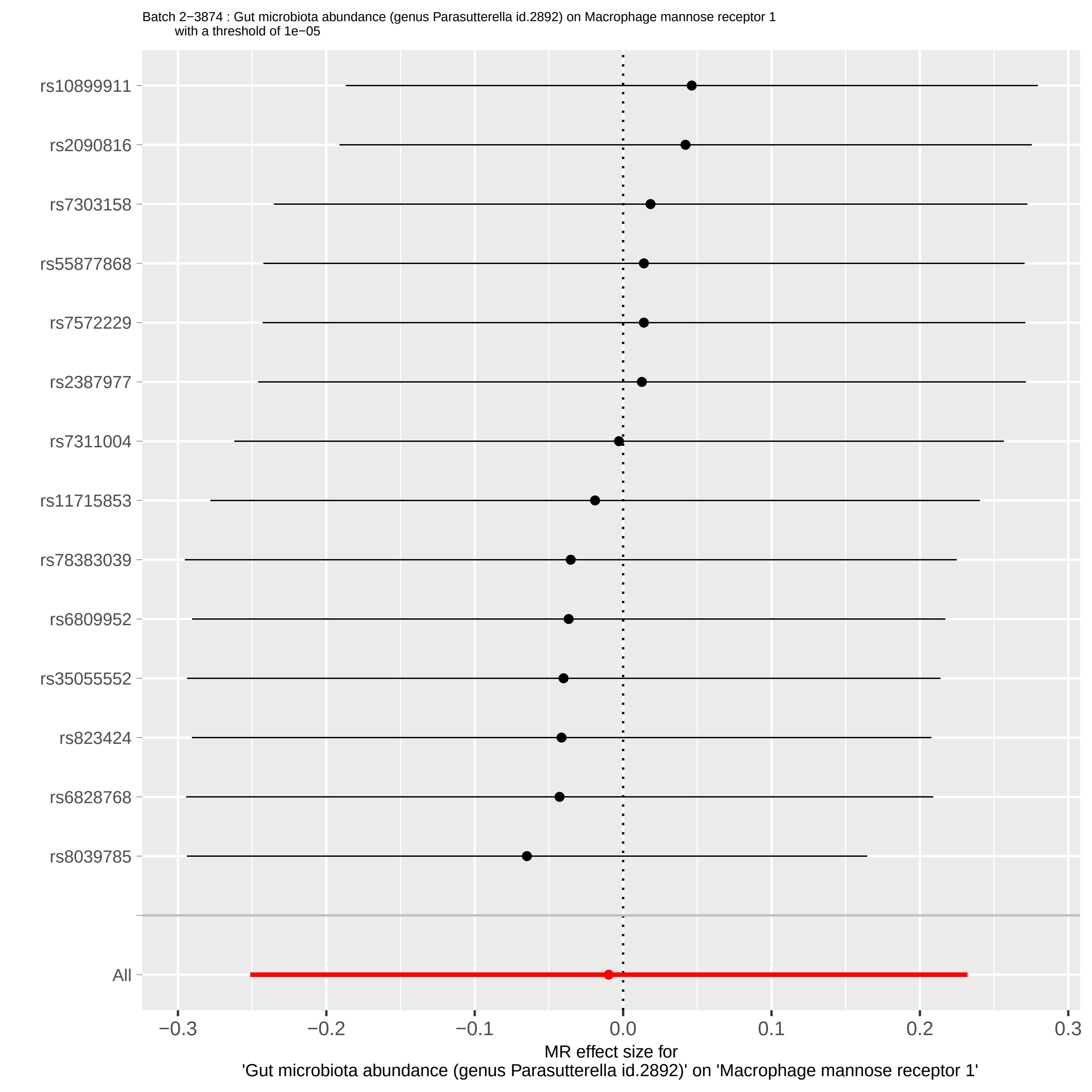
Batch 2−3874 : Gut microbiota abundance (genus Parasutterella id.2892) on Macrophage mannose receptor 1
with a threshold of 1e−05
rs10899911
rs2090816
rs7303158
rs55877868
rs7572229
rs2387977
rs7311004
rs11715853
rs78383039
rs6809952
rs35055552
rs823424
rs6828768
rs8039785
All
−0.3
−0.2
−0.1
0.0
0.1
0.2
0.3
MR effect size for
'Gut microbiota abundance (genus Parasutterella id.2892)' on 'Macrophage mannose receptor 1'

Batch 2−3875 : Gut microbiota abundance (genus Catenibacterium id.2153) on C−type mannose receptor 2
with a threshold of 1e−05

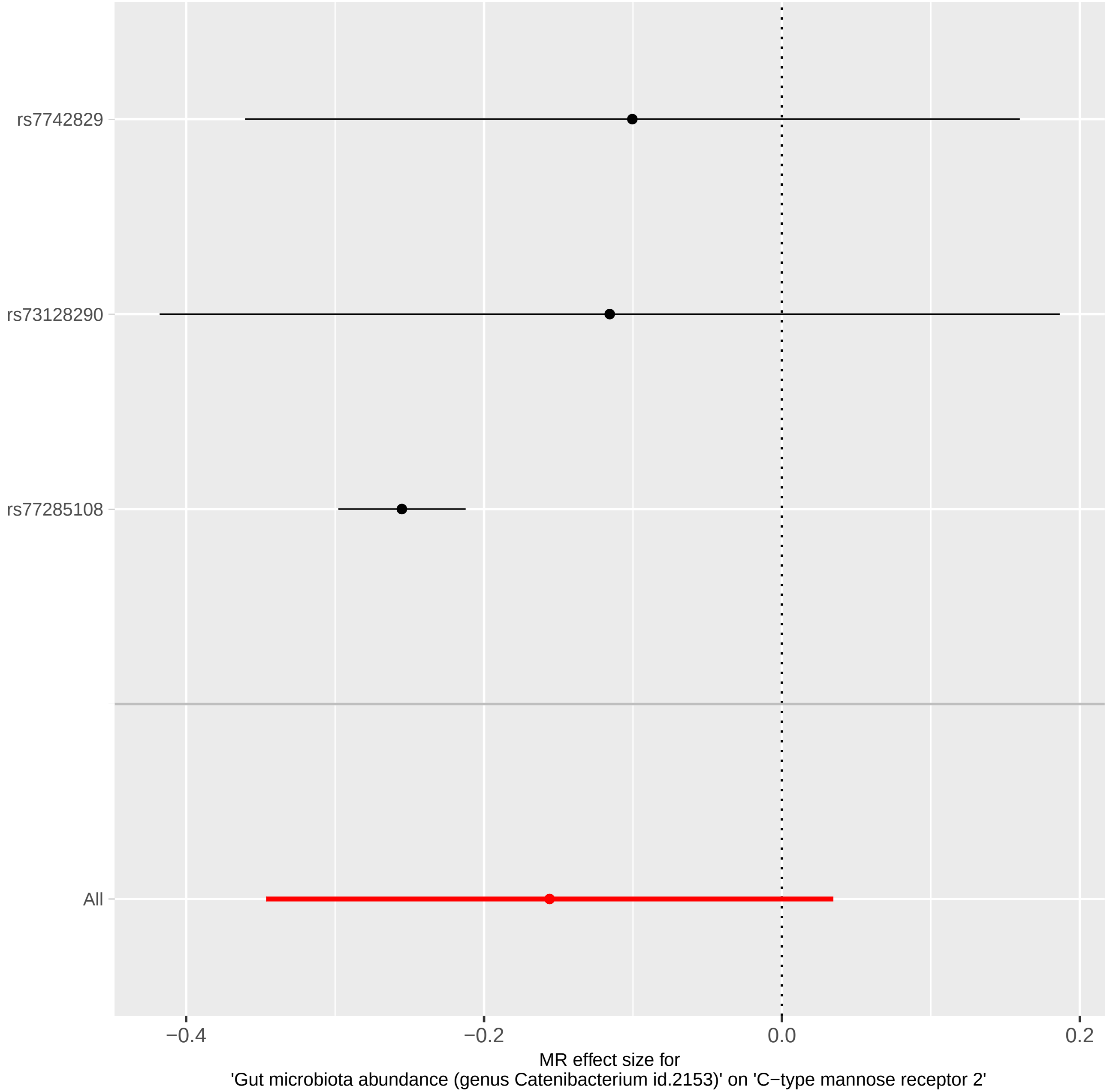

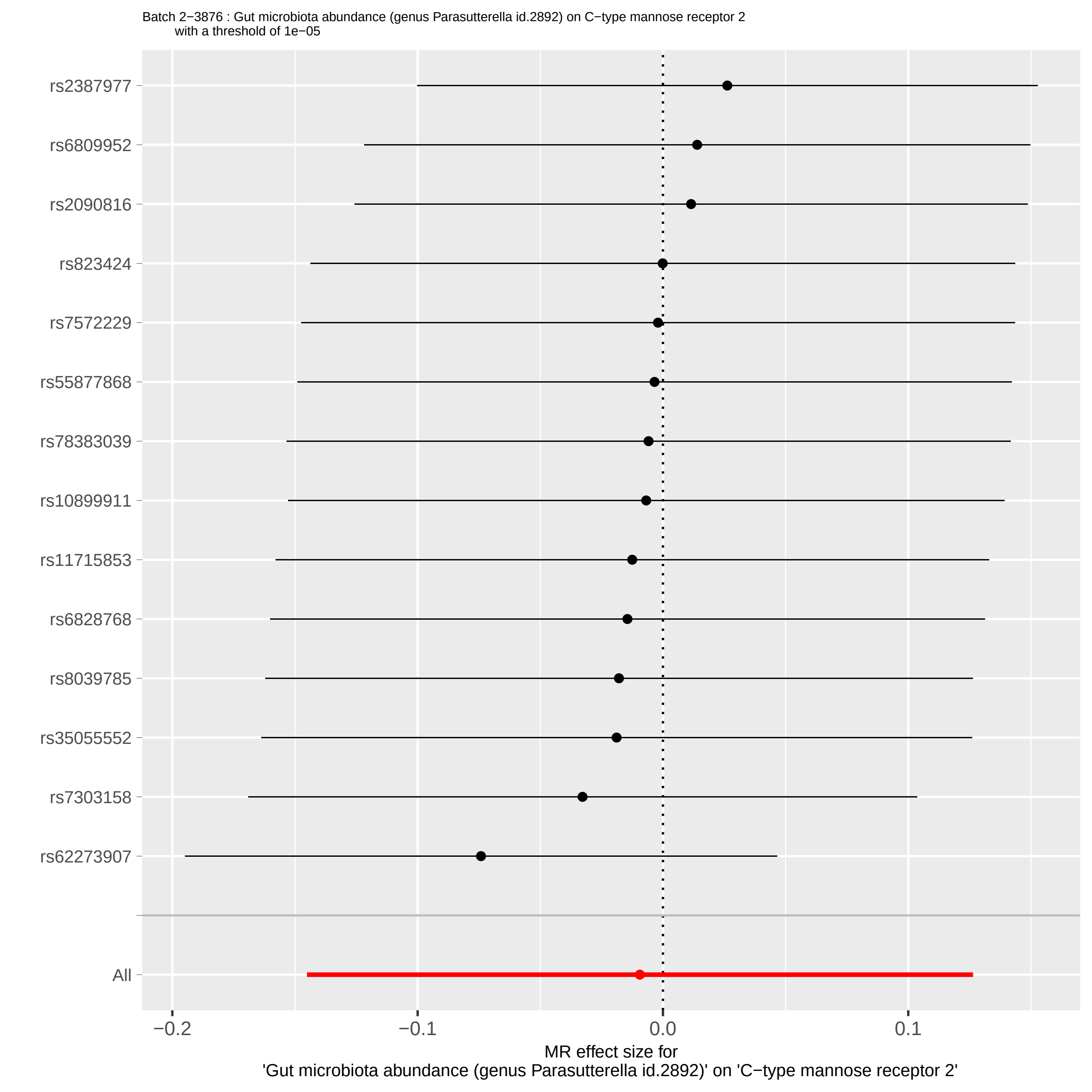
Batch 2−3876 : Gut microbiota abundance (genus Parasutterella id.2892) on C−type mannose receptor 2
with a threshold of 1e−05
rs2387977
rs6809952
rs2090816
rs823424
rs7572229
rs55877868
rs78383039
rs10899911
rs11715853
rs6828768
rs8039785
rs35055552
rs7303158
rs62273907
All
−0.2
−0.1
0.0
0.1
MR effect size for
'Gut microbiota abundance (genus Parasutterella id.2892)' on 'C−type mannose receptor 2'

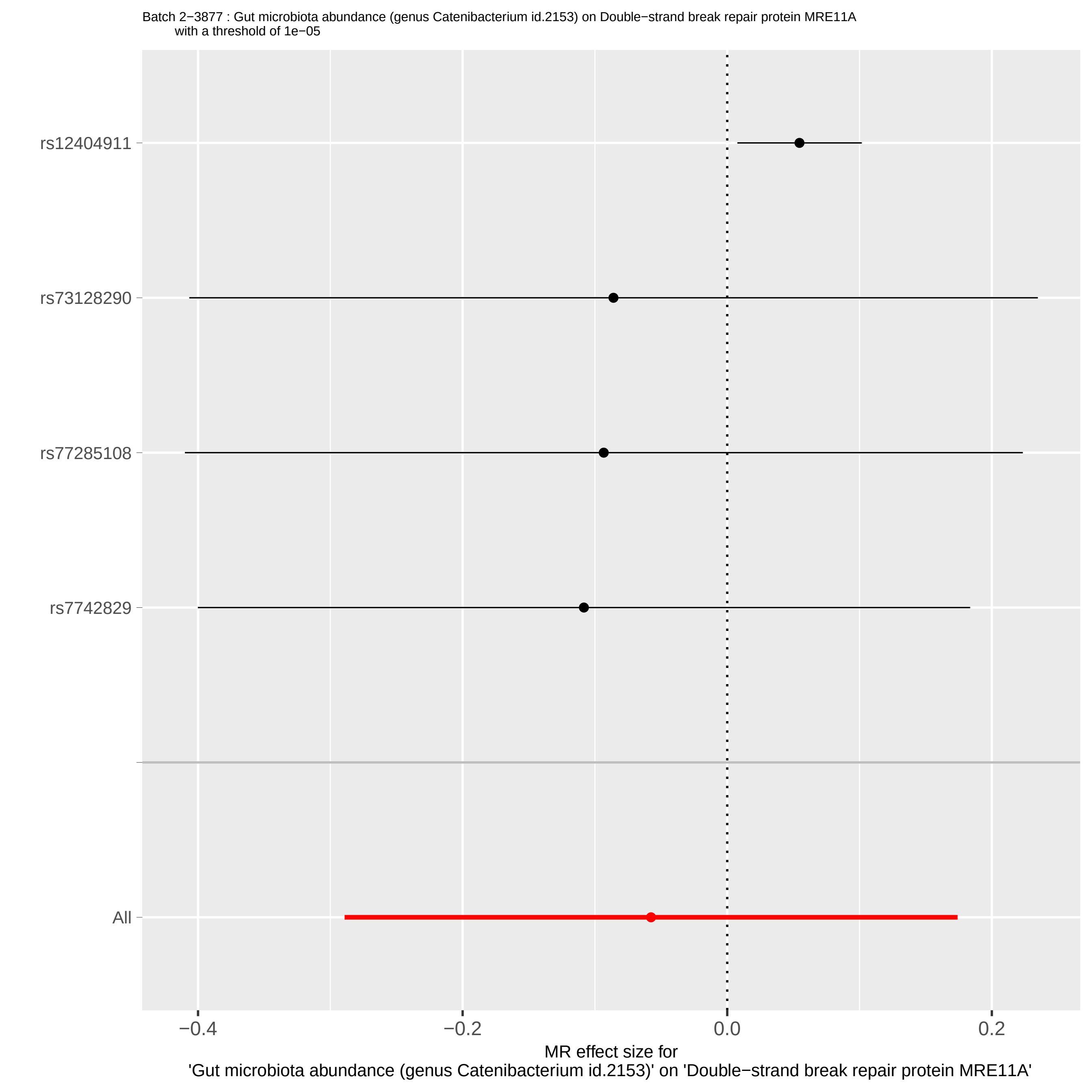
Batch 2−3877 : Gut microbiota abundance (genus Catenibacterium id.2153) on Double−strand break repair protein MRE11A
with a threshold of 1e−05
rs12404911
rs73128290
rs77285108
rs7742829
All
−0.4
−0.2
0.0
0.2
MR effect size for
'Gut microbiota abundance (genus Catenibacterium id.2153)' on 'Double−strand break repair protein MRE11A'

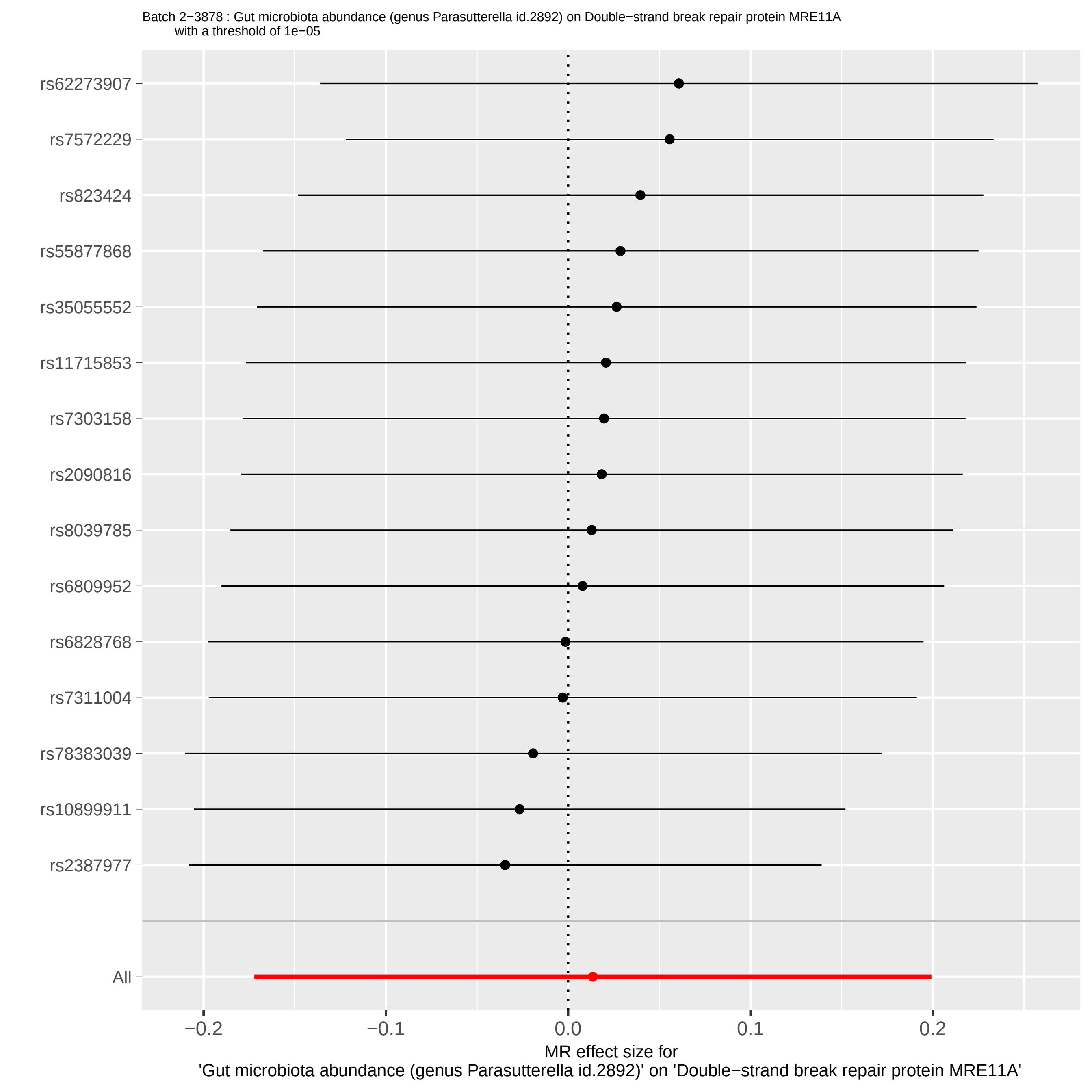

Batch 2−3878 : Gut microbiota abundance (genus Parasutterella id.2892) on Double−strand break repair protein MRE11A
with a threshold of 1e−05
rs62273907
rs7572229
rs823424
rs55877868
rs35055552
rs11715853
rs7303158
rs2090816
rs8039785
rs6809952
rs6828768
rs7311004
rs78383039
rs10899911
rs2387977
All
−0.2
−0.1
0.0
0.1
0.2
MR effect size for
'Gut microbiota abundance (genus Parasutterella id.2892)' on 'Double−strand break repair protein MRE11A'

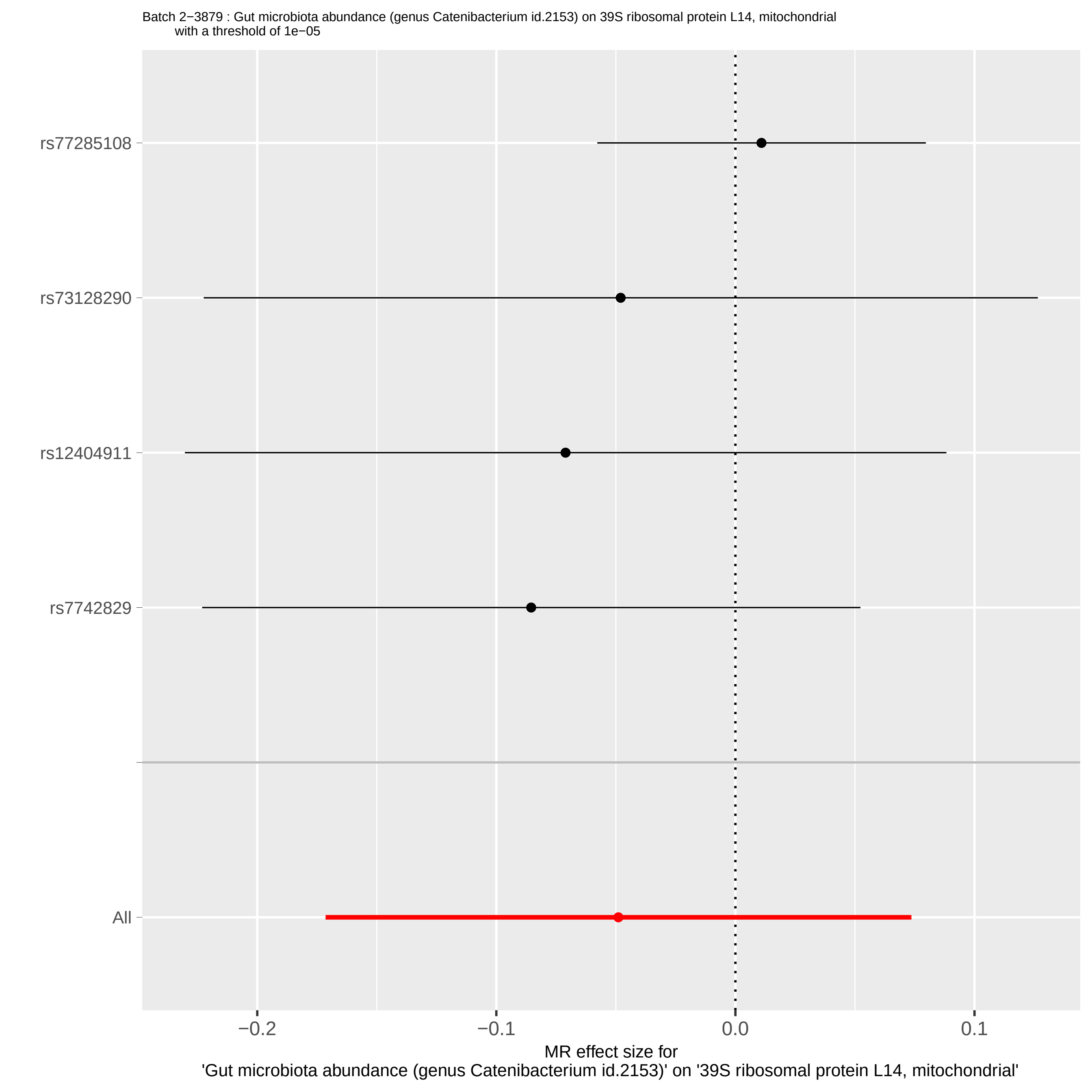

Batch 2−3879 : Gut microbiota abundance (genus Catenibacterium id.2153) on 39S ribosomal protein L14, mitochondrial
with a threshold of 1e−05
rs77285108
rs73128290
rs12404911
rs7742829
All
−0.2
−0.1
0.0
0.1
MR effect size for
'Gut microbiota abundance (genus Catenibacterium id.2153)' on '39S ribosomal protein L14, mitochondrial'

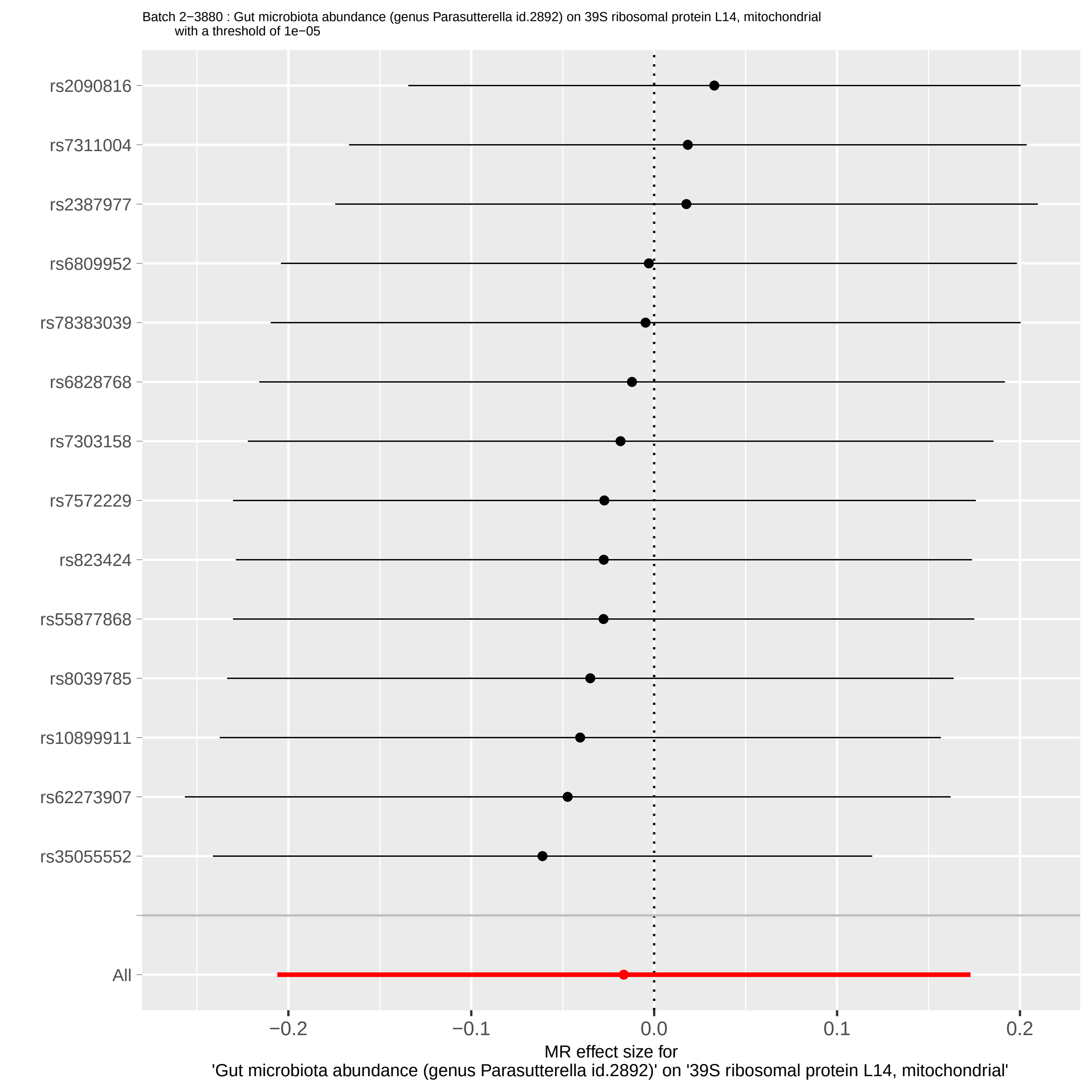
Batch 2−3880 : Gut microbiota abundance (genus Parasutterella id.2892) on 39S ribosomal protein L14, mitochondrial
with a threshold of 1e−05
rs2090816
rs7311004
rs2387977
rs6809952
rs78383039
rs6828768
rs7303158
rs7572229
rs823424
rs55877868
rs8039785
rs10899911
rs62273907
rs35055552
All
−0.2
−0.1
0.0
0.1
0.2
MR effect size for
'Gut microbiota abundance (genus Parasutterella id.2892)' on '39S ribosomal protein L14, mitochondrial'

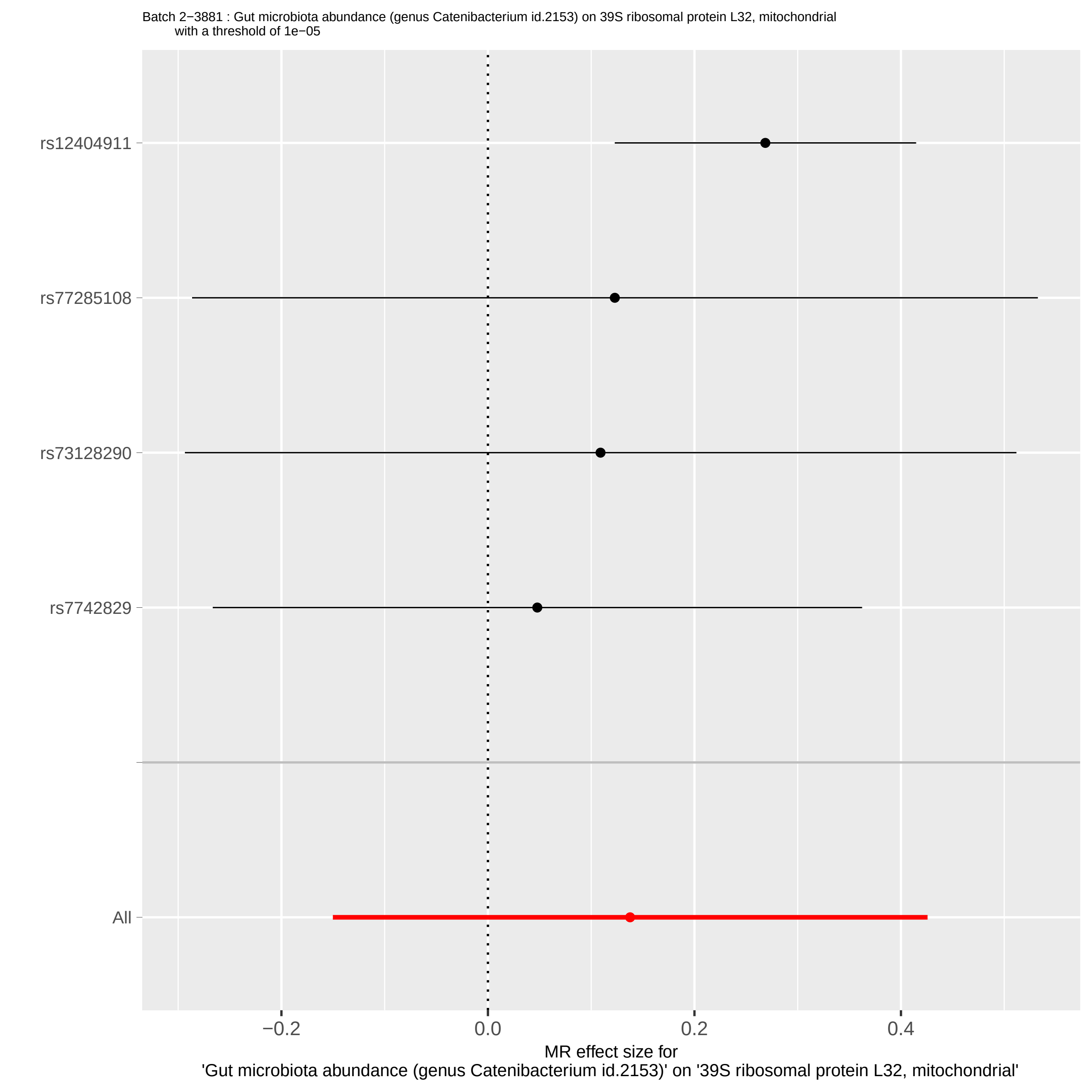
Batch 2−3881 : Gut microbiota abundance (genus Catenibacterium id.2153) on 39S ribosomal protein L32, mitochondrial
with a threshold of 1e−05
rs12404911
rs77285108
rs73128290
rs7742829
All
−0.2
0.0
0.2
0.4
MR effect size for
'Gut microbiota abundance (genus Catenibacterium id.2153)' on '39S ribosomal protein L32, mitochondrial'

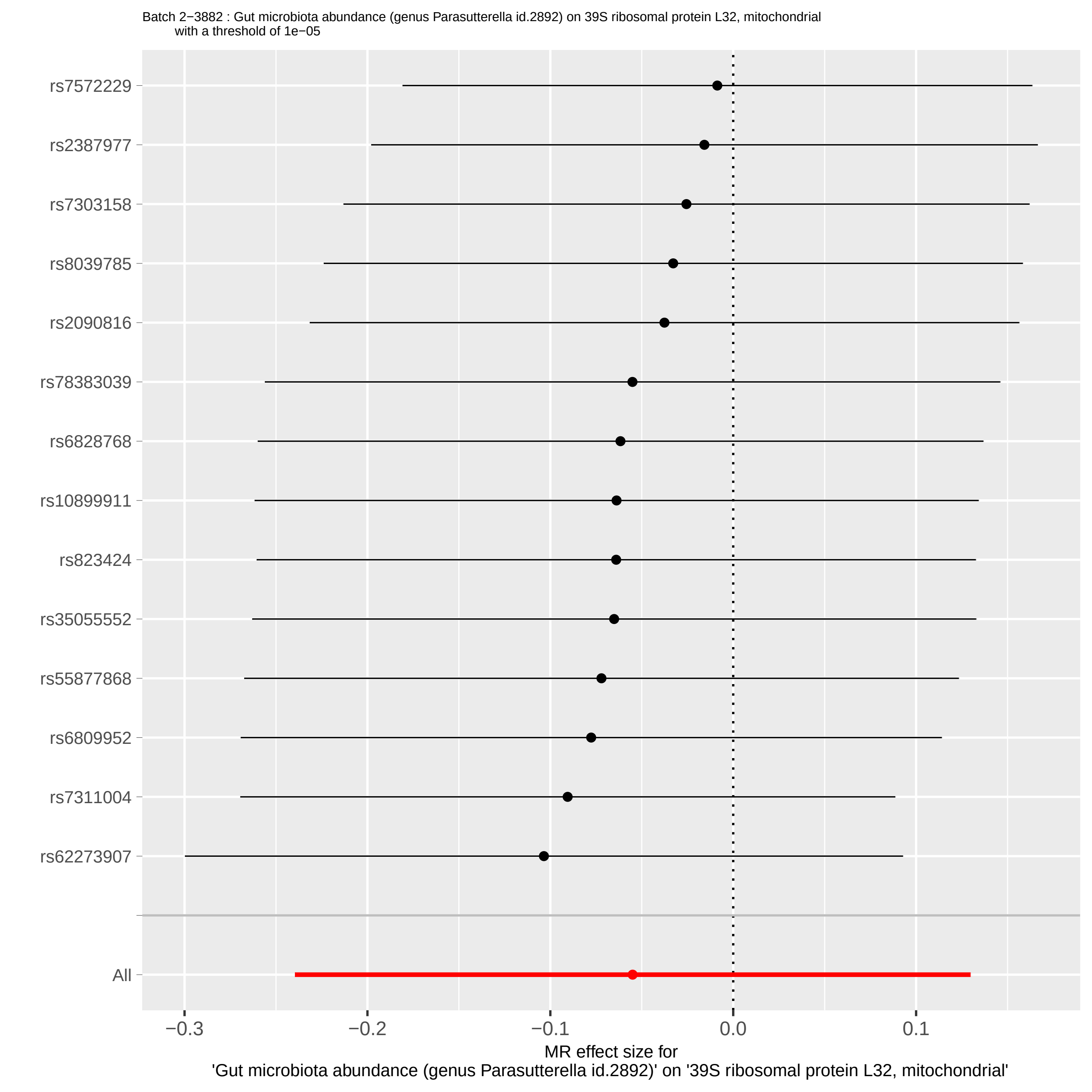
Batch 2−3882 : Gut microbiota abundance (genus Parasutterella id.2892) on 39S ribosomal protein L32, mitochondrial
with a threshold of 1e−05
rs7572229
rs2387977
rs7303158
rs8039785
rs2090816
rs78383039
rs6828768
rs10899911
rs823424
rs35055552
rs55877868
rs6809952
rs7311004
rs62273907
All
−0.3
−0.2
−0.1
0.0
0.1
MR effect size for
'Gut microbiota abundance (genus Parasutterella id.2892)' on '39S ribosomal protein L32, mitochondrial'

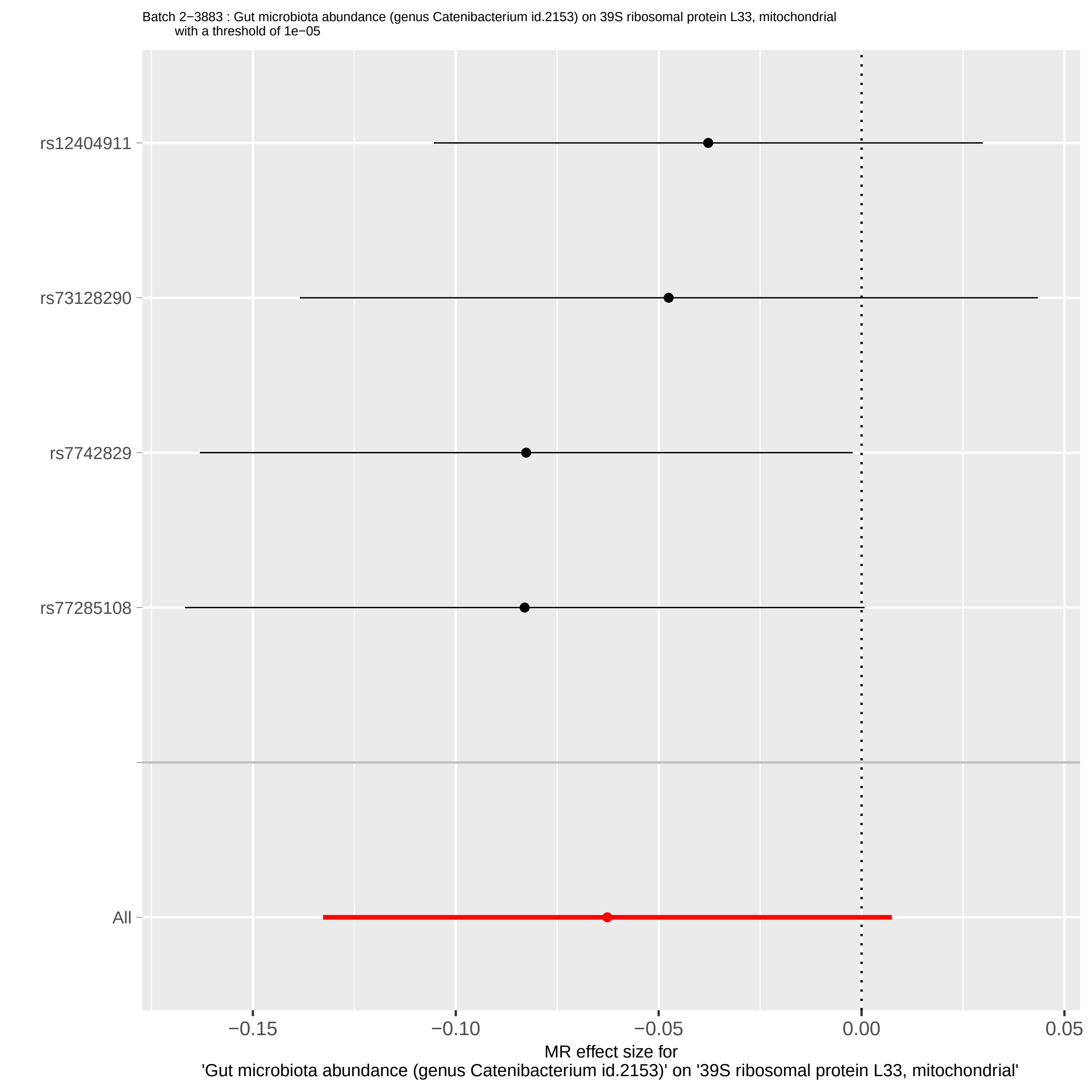
Batch 2−3883 : Gut microbiota abundance (genus Catenibacterium id.2153) on 39S ribosomal protein L33, mitochondrial
with a threshold of 1e−05
rs12404911
rs73128290
rs7742829
rs77285108
All
−0.15
−0.10
−0.05
0.00
0.05
MR effect size for
'Gut microbiota abundance (genus Catenibacterium id.2153)' on '39S ribosomal protein L33, mitochondrial'

Batch 2−3884 : Gut microbiota abundance (genus Parasutterella id.2892) on 39S ribosomal protein L33, mitochondrial
with a threshold of 1e−05

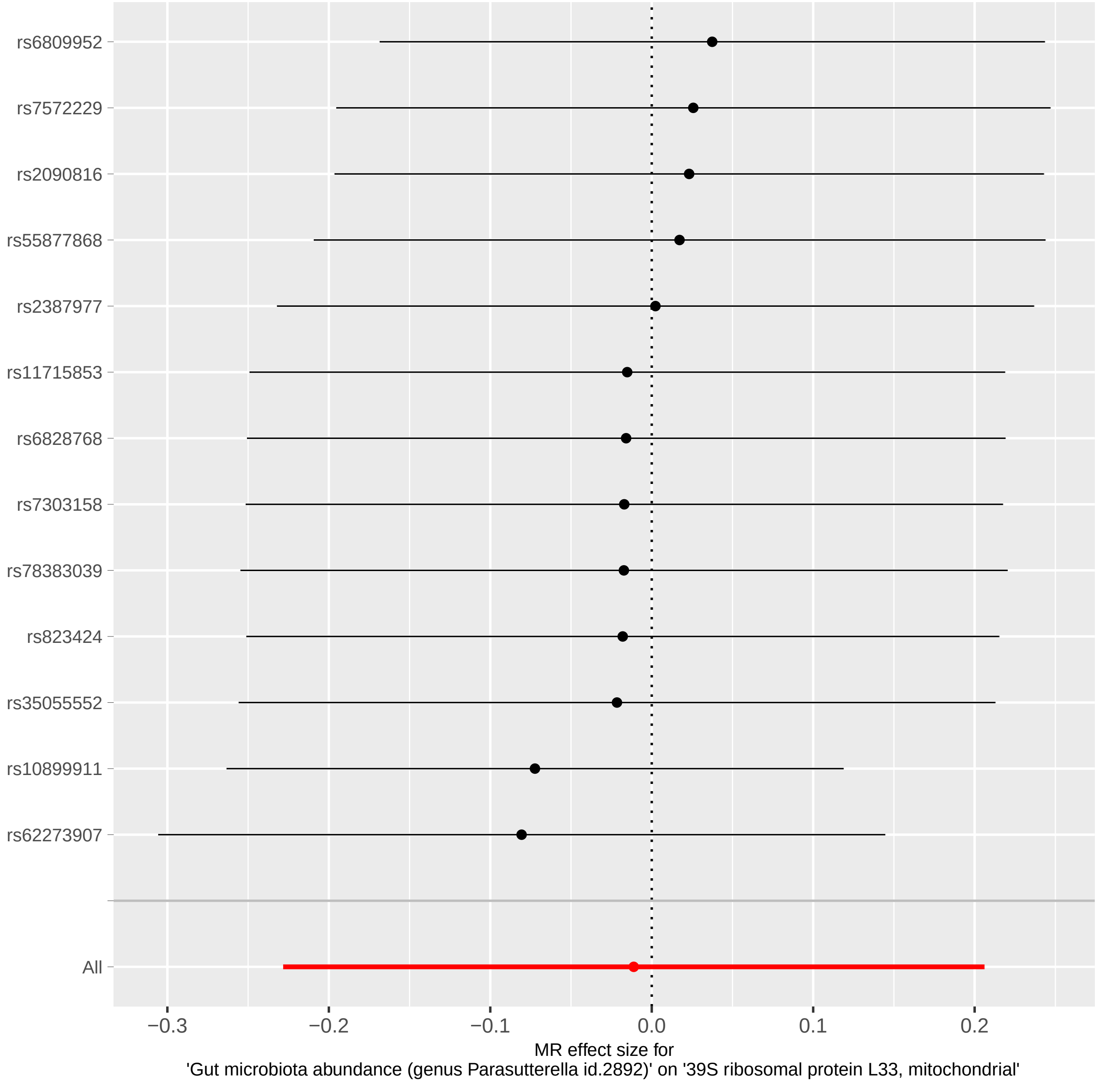

Batch 2−3885 : Gut microbiota abundance (genus Catenibacterium id.2153) on 39S ribosomal protein L34, mitochondrial
with a threshold of 1e−05

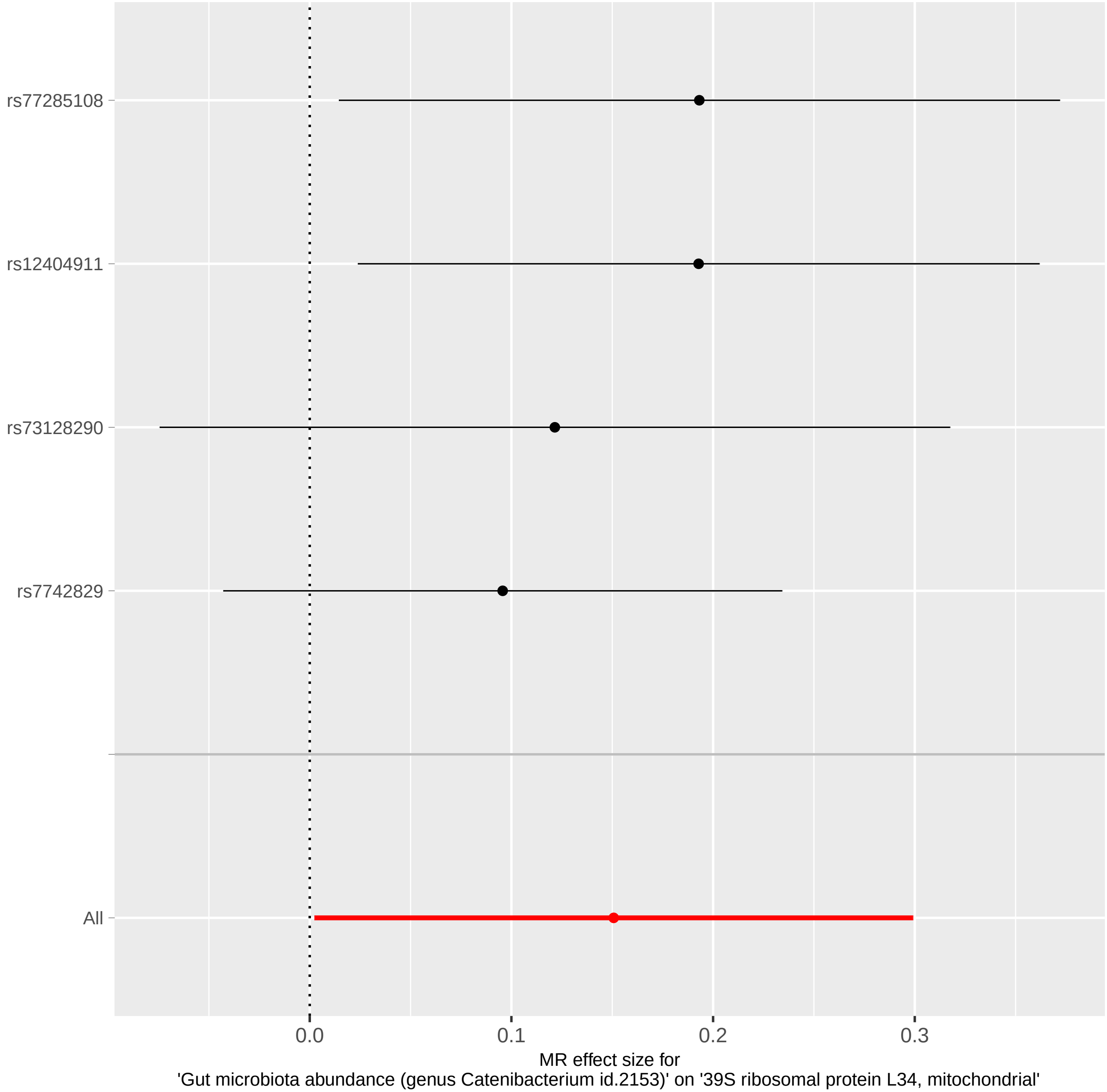

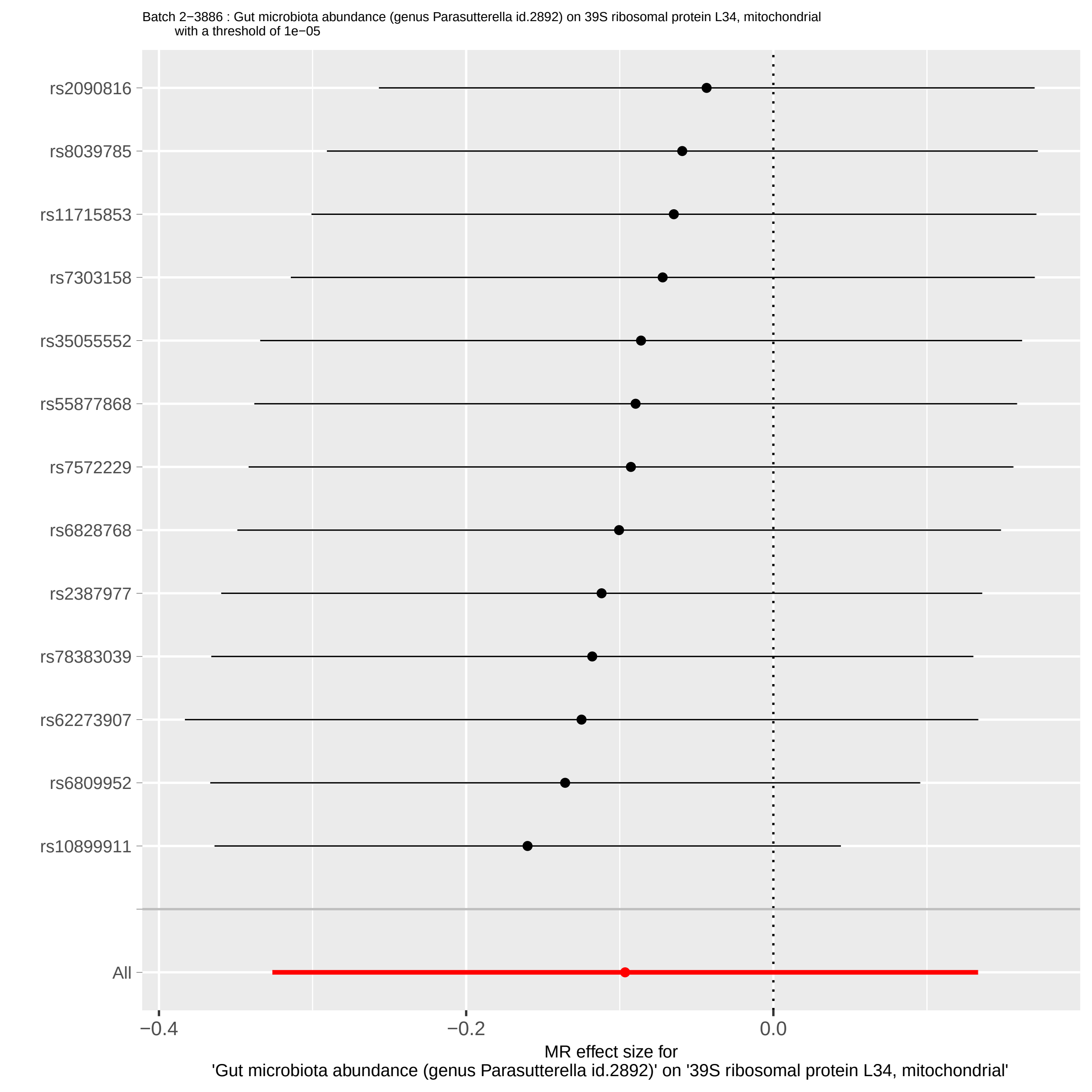

Batch 2−3886 : Gut microbiota abundance (genus Parasutterella id.2892) on 39S ribosomal protein L34, mitochondrial
with a threshold of 1e−05
rs2090816
rs8039785
rs11715853
rs7303158
rs35055552
rs55877868
rs7572229
rs6828768
rs2387977
rs78383039
rs62273907
rs6809952
rs10899911
All
−0.4
−0.2
0.0
MR effect size for
'Gut microbiota abundance (genus Parasutterella id.2892)' on '39S ribosomal protein L34, mitochondrial'

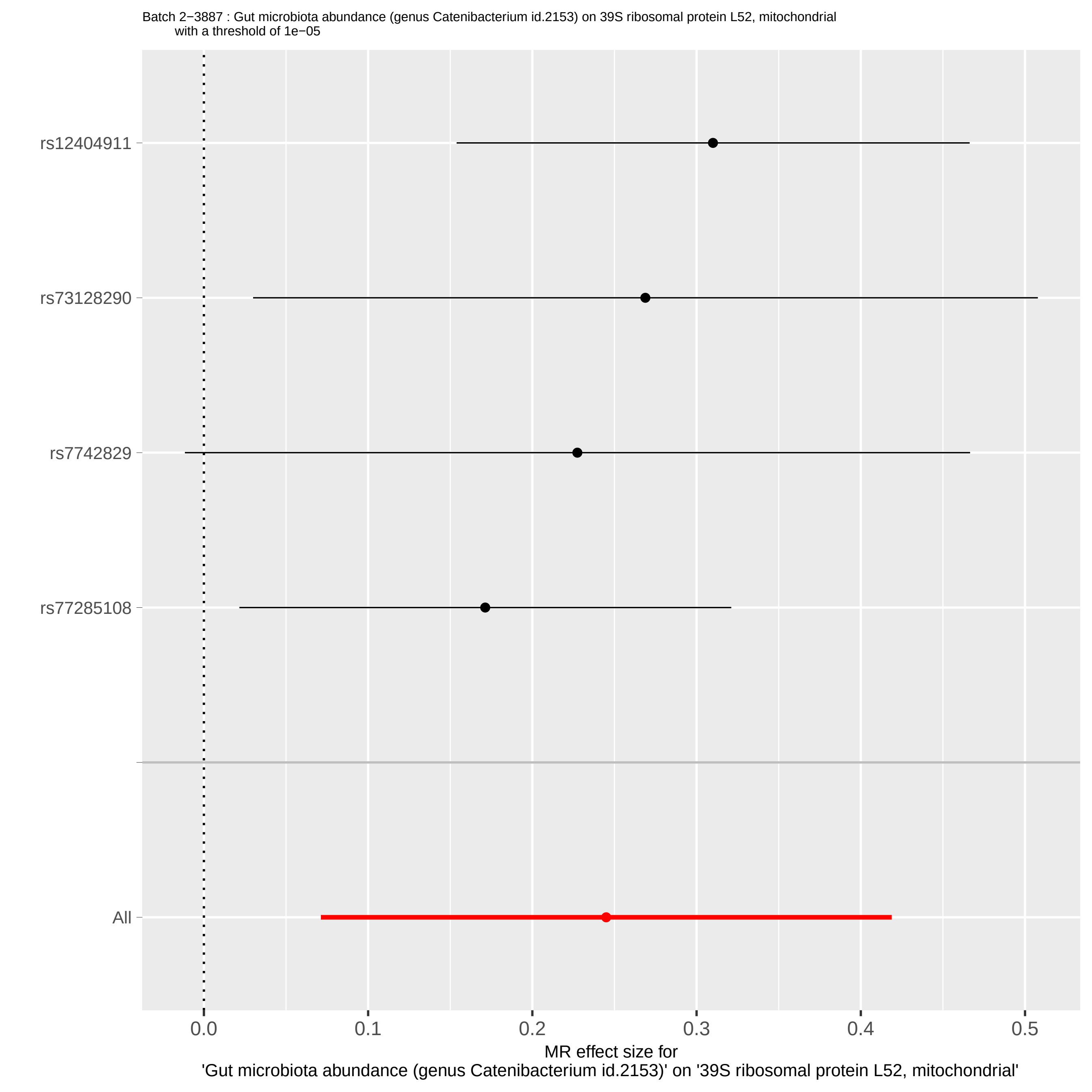
Batch 2−3887 : Gut microbiota abundance (genus Catenibacterium id.2153) on 39S ribosomal protein L52, mitochondrial
with a threshold of 1e−05
rs12404911
rs73128290
rs7742829
rs77285108
All
0.0
0.1
0.2
0.3
0.4
0.5
MR effect size for
'Gut microbiota abundance (genus Catenibacterium id.2153)' on '39S ribosomal protein L52, mitochondrial'

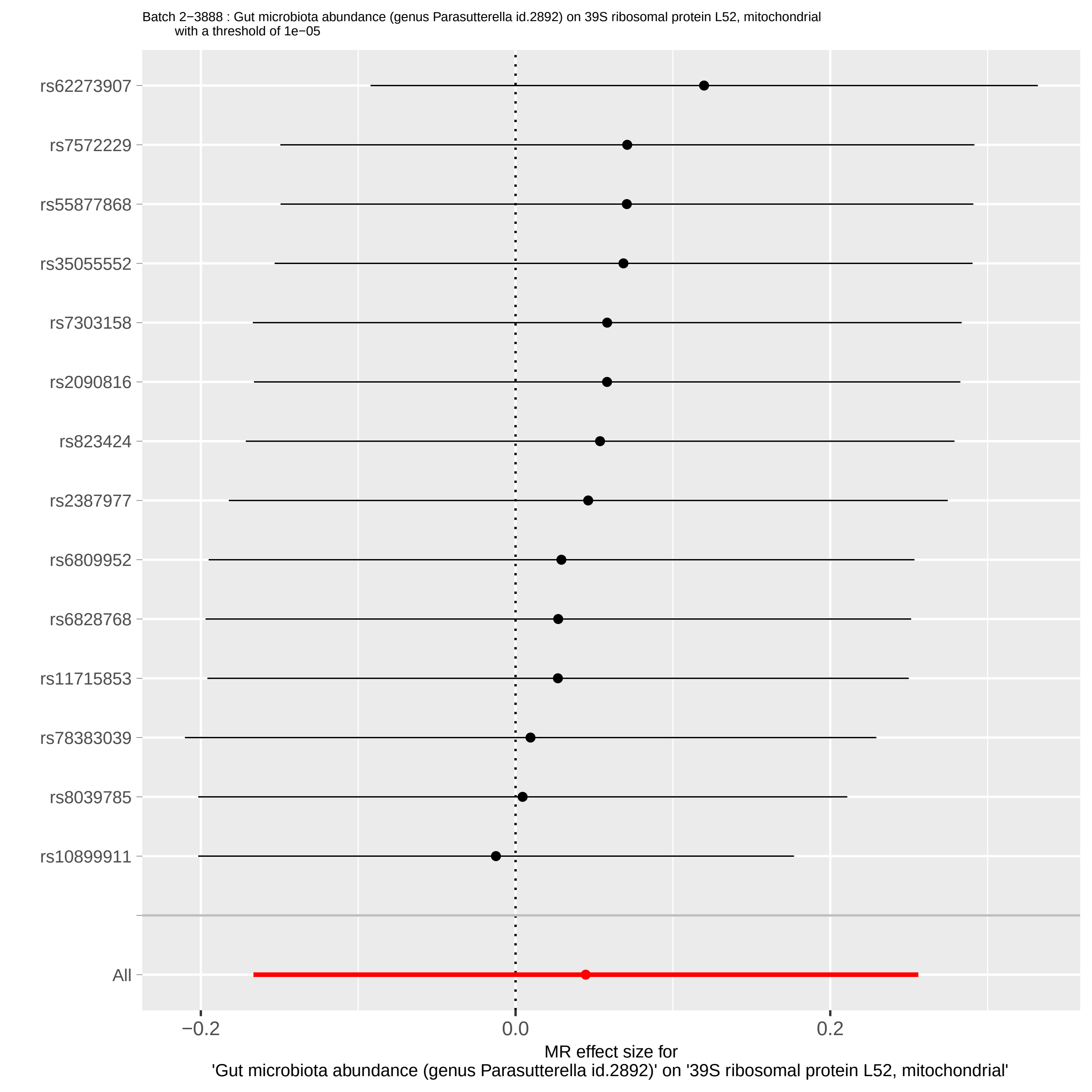

Batch 2−3888 : Gut microbiota abundance (genus Parasutterella id.2892) on 39S ribosomal protein L52, mitochondrial
with a threshold of 1e−05
rs62273907
rs7572229
rs55877868
rs35055552
rs7303158
rs2090816
rs823424
rs2387977
rs6809952
rs6828768
rs11715853
rs78383039
rs8039785
rs10899911
All
−0.2
0.0
0.2
MR effect size for
'Gut microbiota abundance (genus Parasutterella id.2892)' on '39S ribosomal protein L52, mitochondrial'

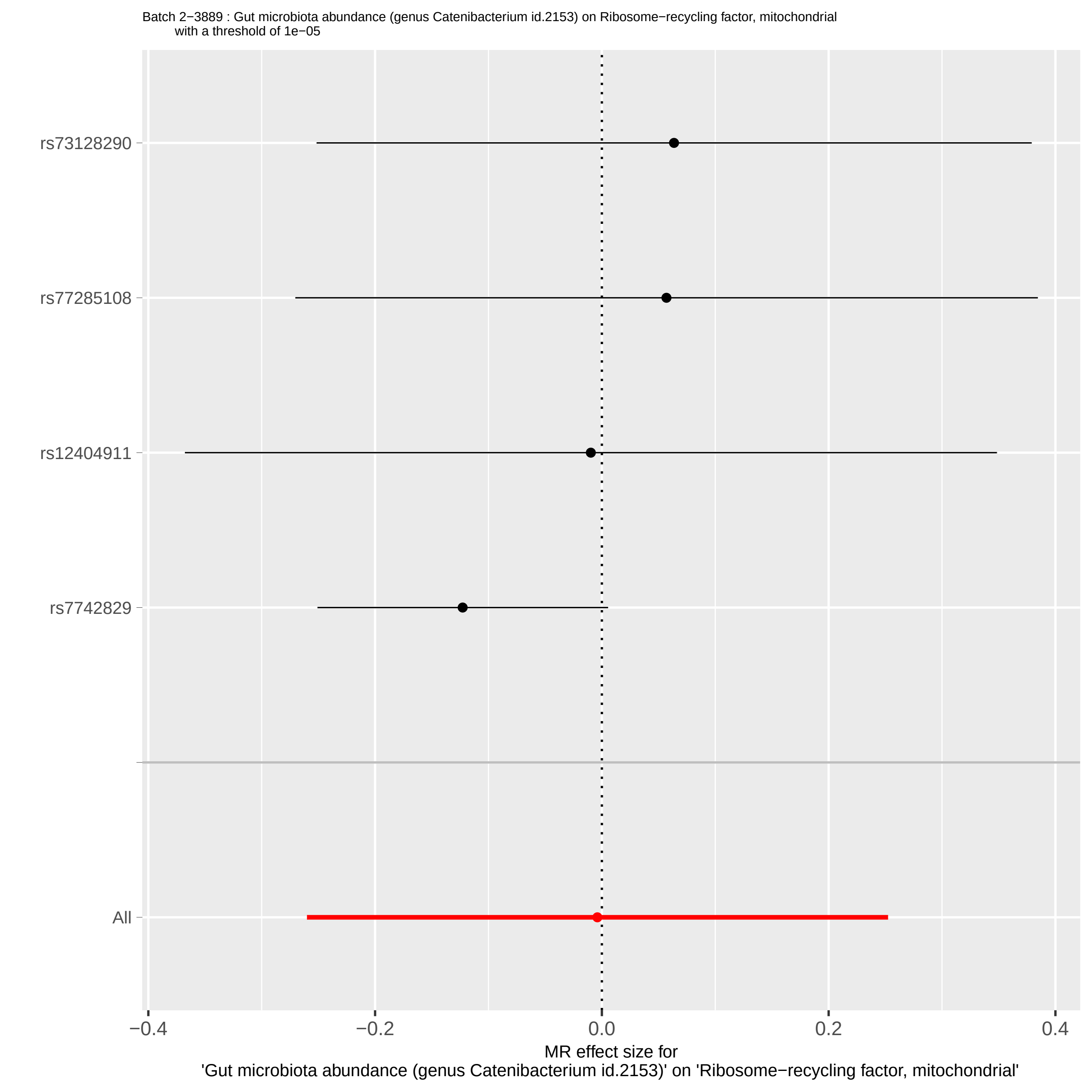
Batch 2−3889 : Gut microbiota abundance (genus Catenibacterium id.2153) on Ribosome−recycling factor, mitochondrial
with a threshold of 1e−05
rs73128290
rs77285108
rs12404911
rs7742829
All
−0.4
−0.2
0.0
0.2
0.4
MR effect size for
'Gut microbiota abundance (genus Catenibacterium id.2153)' on 'Ribosome−recycling factor, mitochondrial'

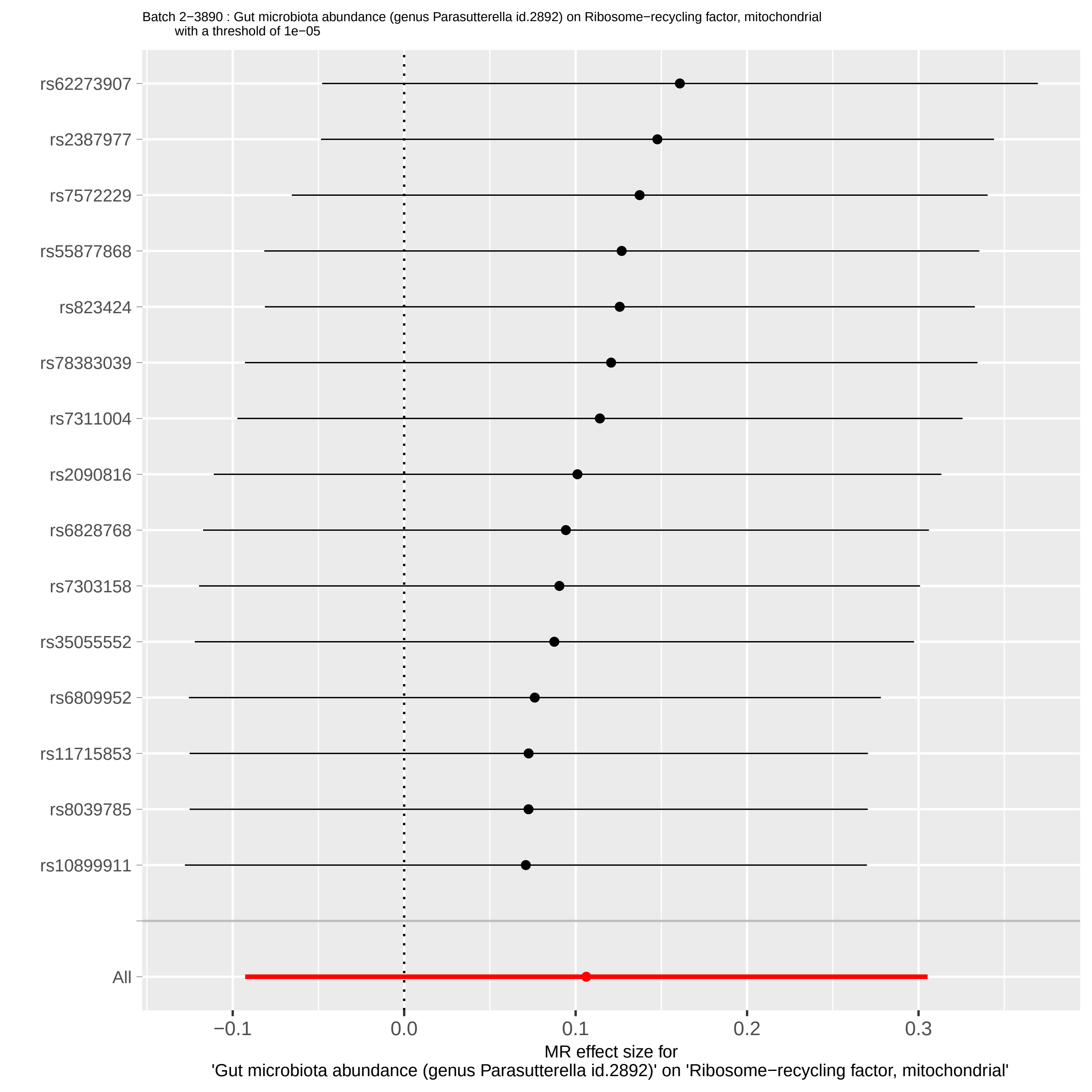

Batch 2−3890 : Gut microbiota abundance (genus Parasutterella id.2892) on Ribosome−recycling factor, mitochondrial
with a threshold of 1e−05
rs62273907
rs2387977
rs7572229
rs55877868
rs823424
rs78383039
rs7311004
rs2090816
rs6828768
rs7303158
rs35055552
rs6809952
rs11715853
rs8039785
rs10899911
All
−0.1
0.0
0.1
0.2
0.3
MR effect size for
'Gut microbiota abundance (genus Parasutterella id.2892)' on 'Ribosome−recycling factor, mitochondrial'

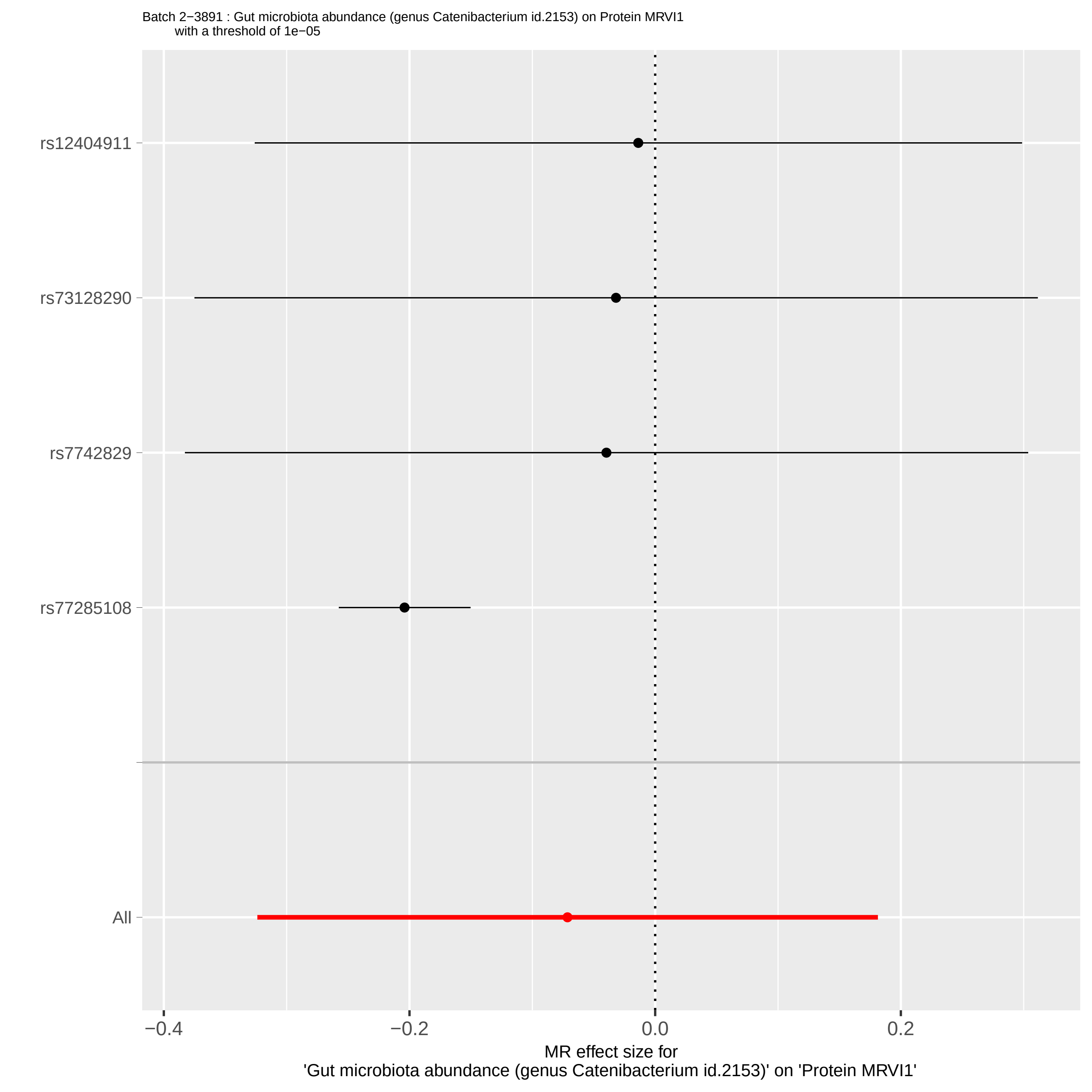

Batch 2−3891 : Gut microbiota abundance (genus Catenibacterium id.2153) on Protein MRVI1
with a threshold of 1e−05
rs12404911
rs73128290
rs7742829
rs77285108
All
−0.4
−0.2
0.0
0.2
MR effect size for
'Gut microbiota abundance (genus Catenibacterium id.2153)' on 'Protein MRVI1'

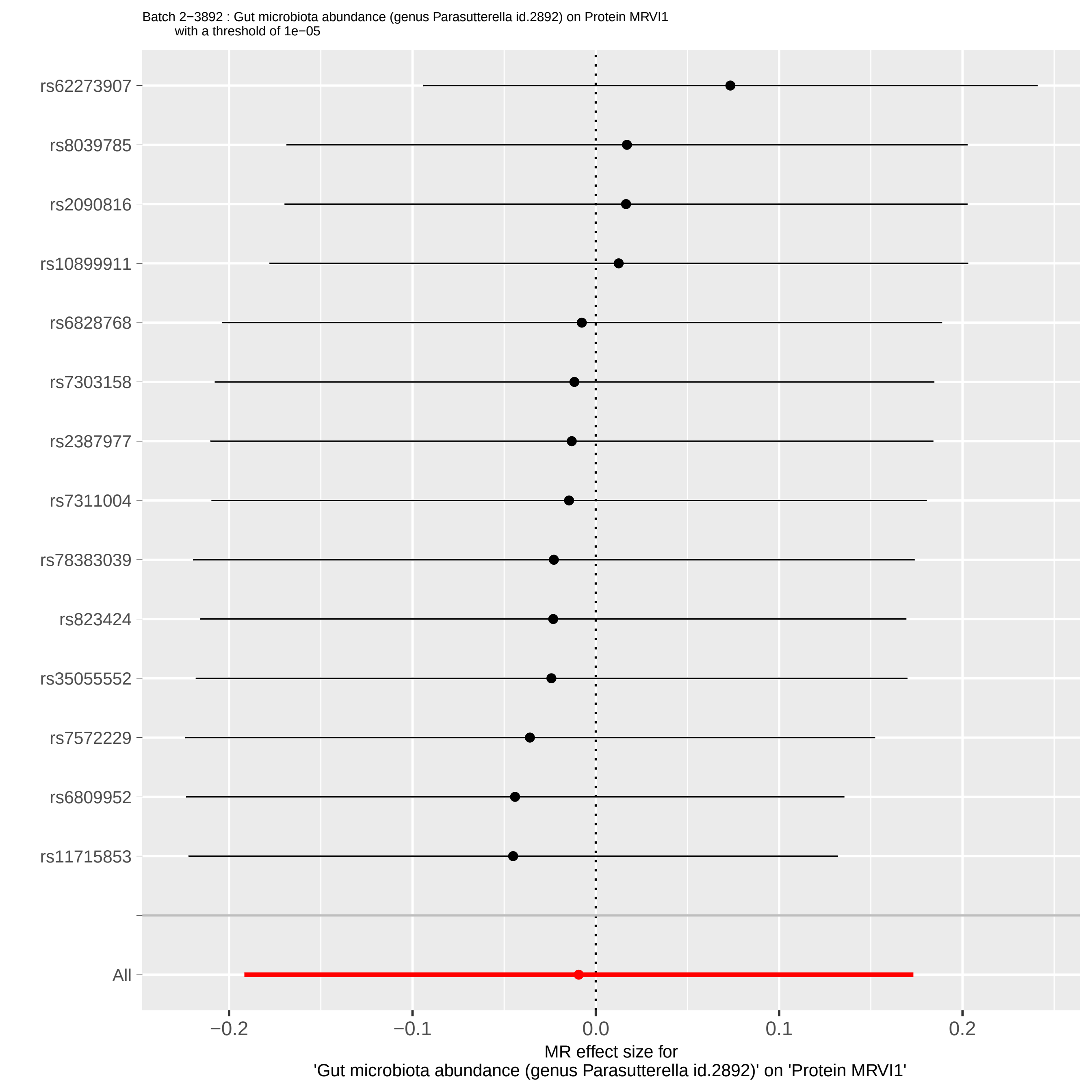
Batch 2−3892 : Gut microbiota abundance (genus Parasutterella id.2892) on Protein MRVI1
with a threshold of 1e−05
rs62273907
rs8039785
rs2090816
rs10899911
rs6828768
rs7303158
rs2387977
rs7311004
rs78383039
rs823424
rs35055552
rs7572229
rs6809952
rs11715853
All
−0.2
−0.1
0.0
0.1
0.2
MR effect size for
'Gut microbiota abundance (genus Parasutterella id.2892)' on 'Protein MRVI1'

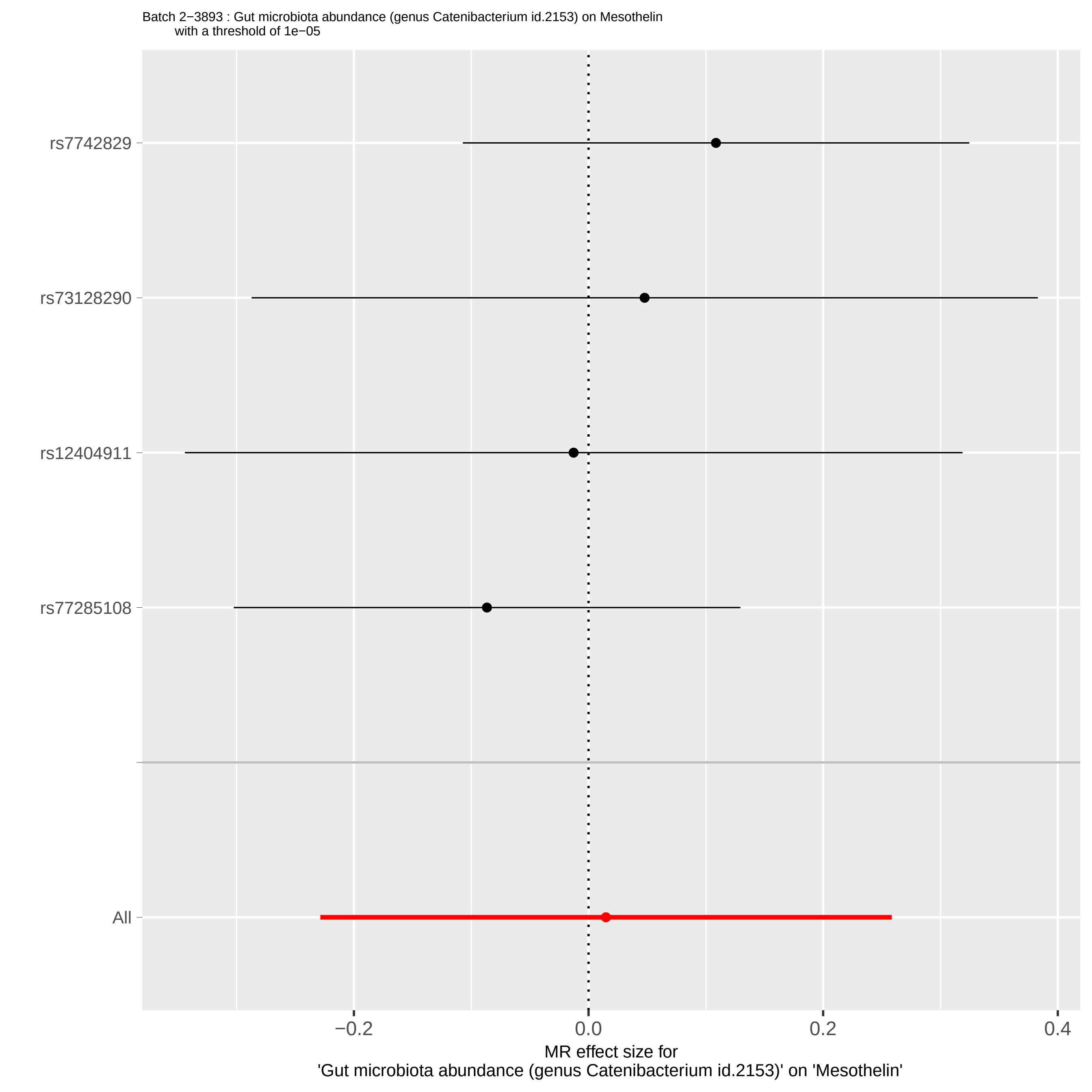

Batch 2−3893 : Gut microbiota abundance (genus Catenibacterium id.2153) on Mesothelin
with a threshold of 1e−05
rs7742829
rs73128290
rs12404911
rs77285108
All
−0.2
0.0
0.2
0.4
MR effect size for
'Gut microbiota abundance (genus Catenibacterium id.2153)' on 'Mesothelin'

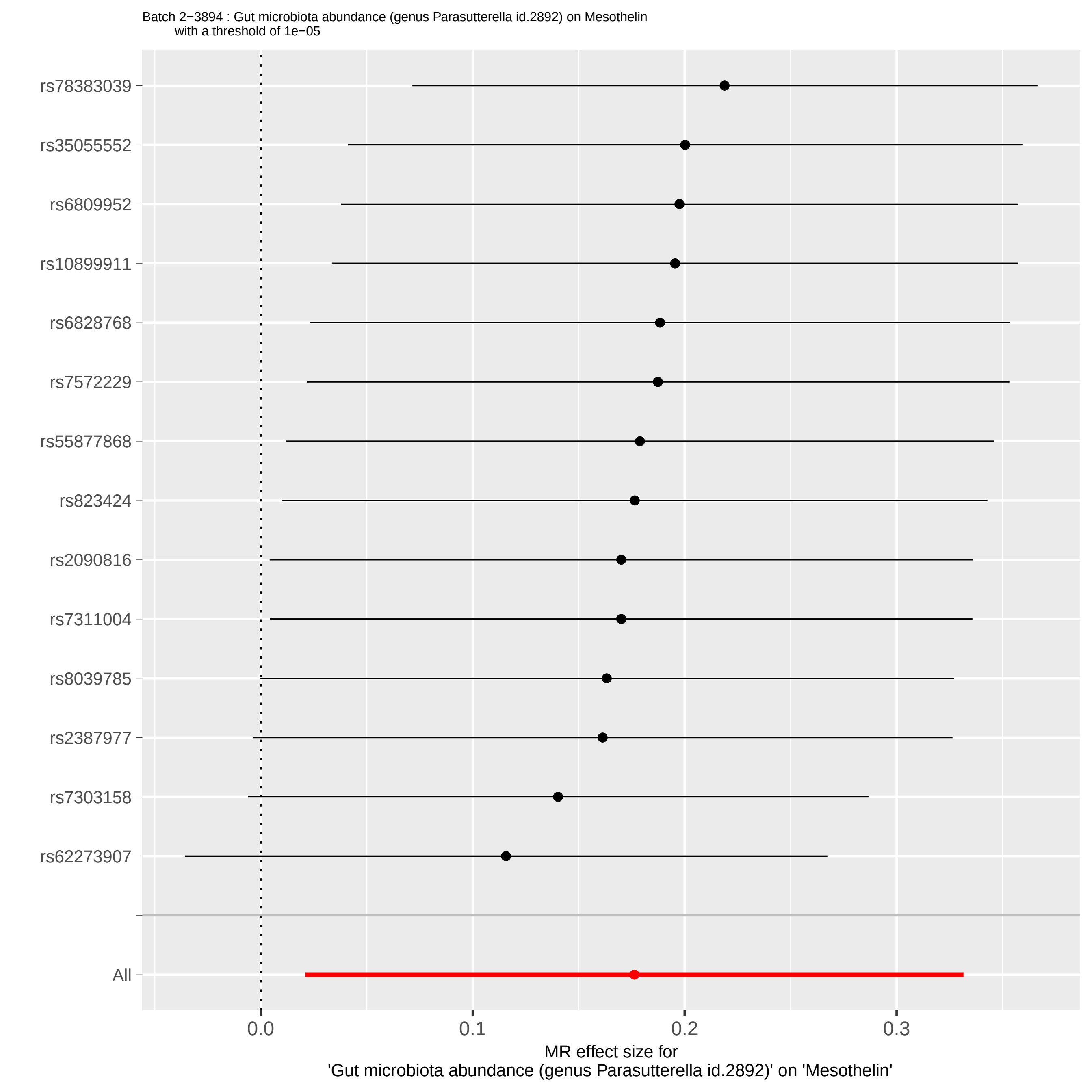
Batch 2−3894 : Gut microbiota abundance (genus Parasutterella id.2892) on Mesothelin
with a threshold of 1e−05
rs78383039
rs35055552
rs6809952
rs10899911
rs6828768
rs7572229
rs55877868
rs823424
rs2090816
rs7311004
rs8039785
rs2387977
rs7303158
rs62273907
All
0.0
0.1
0.2
0.3
MR effect size for
'Gut microbiota abundance (genus Parasutterella id.2892)' on 'Mesothelin'

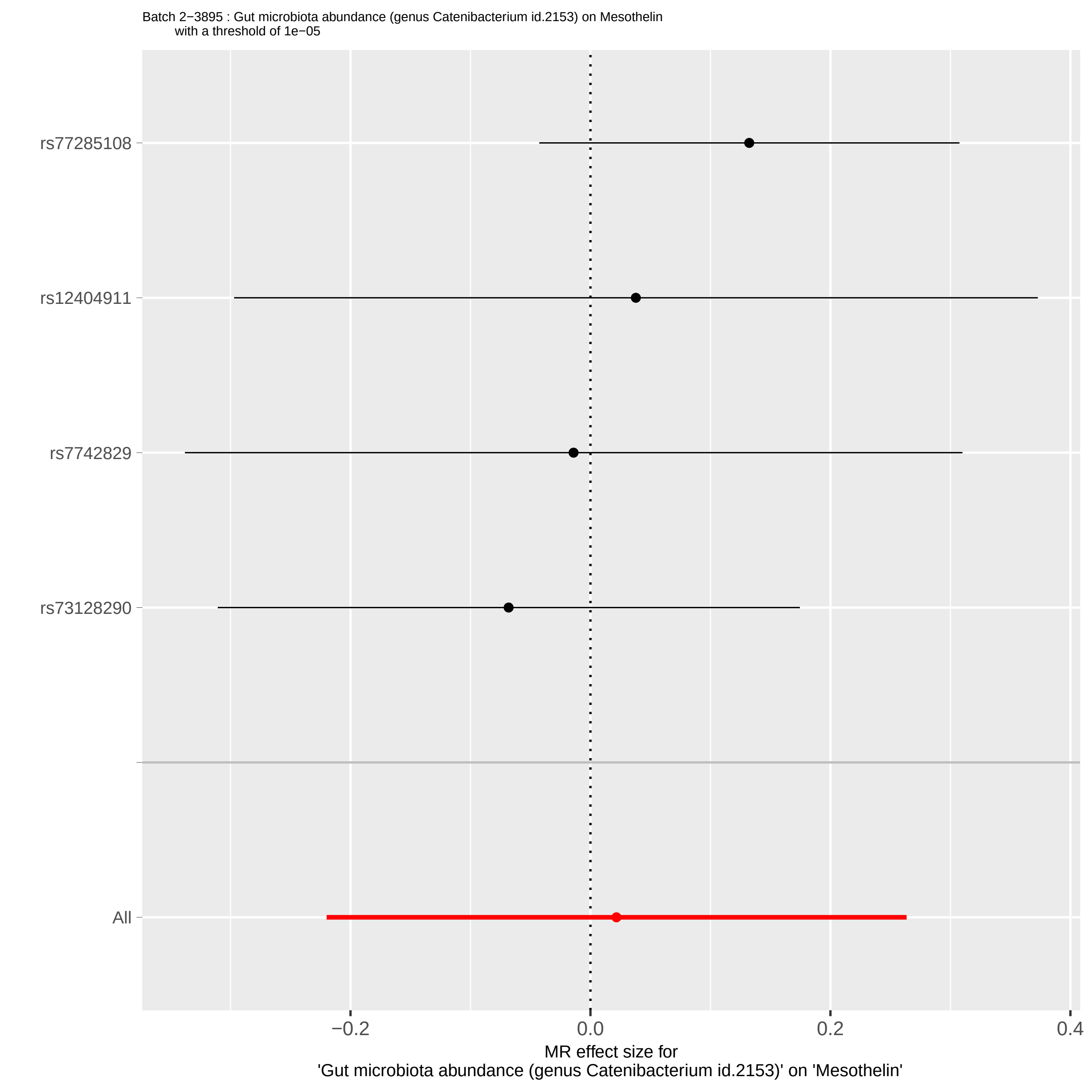

Batch 2−3895 : Gut microbiota abundance (genus Catenibacterium id.2153) on Mesothelin
with a threshold of 1e−05
rs77285108
rs12404911
rs7742829
rs73128290
All
−0.2
0.0
0.2
0.4
MR effect size for
'Gut microbiota abundance (genus Catenibacterium id.2153)' on 'Mesothelin'

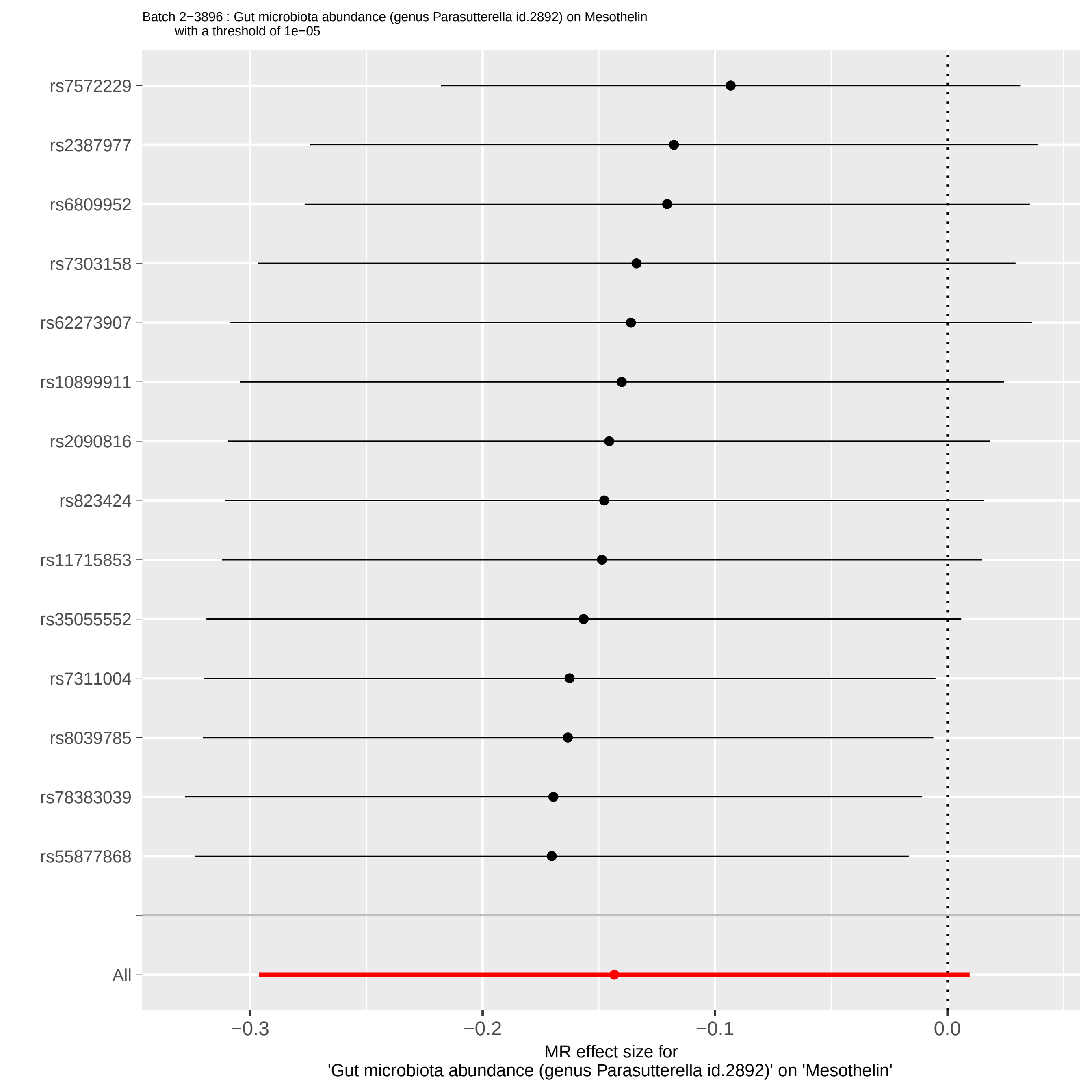
Batch 2−3896 : Gut microbiota abundance (genus Parasutterella id.2892) on Mesothelin
with a threshold of 1e−05
rs7572229
rs2387977
rs6809952
rs7303158
rs62273907
rs10899911
rs2090816
rs823424
rs11715853
rs35055552
rs7311004
rs8039785
rs78383039
rs55877868
All
−0.3
−0.2
−0.1
0.0
MR effect size for
'Gut microbiota abundance (genus Parasutterella id.2892)' on 'Mesothelin'

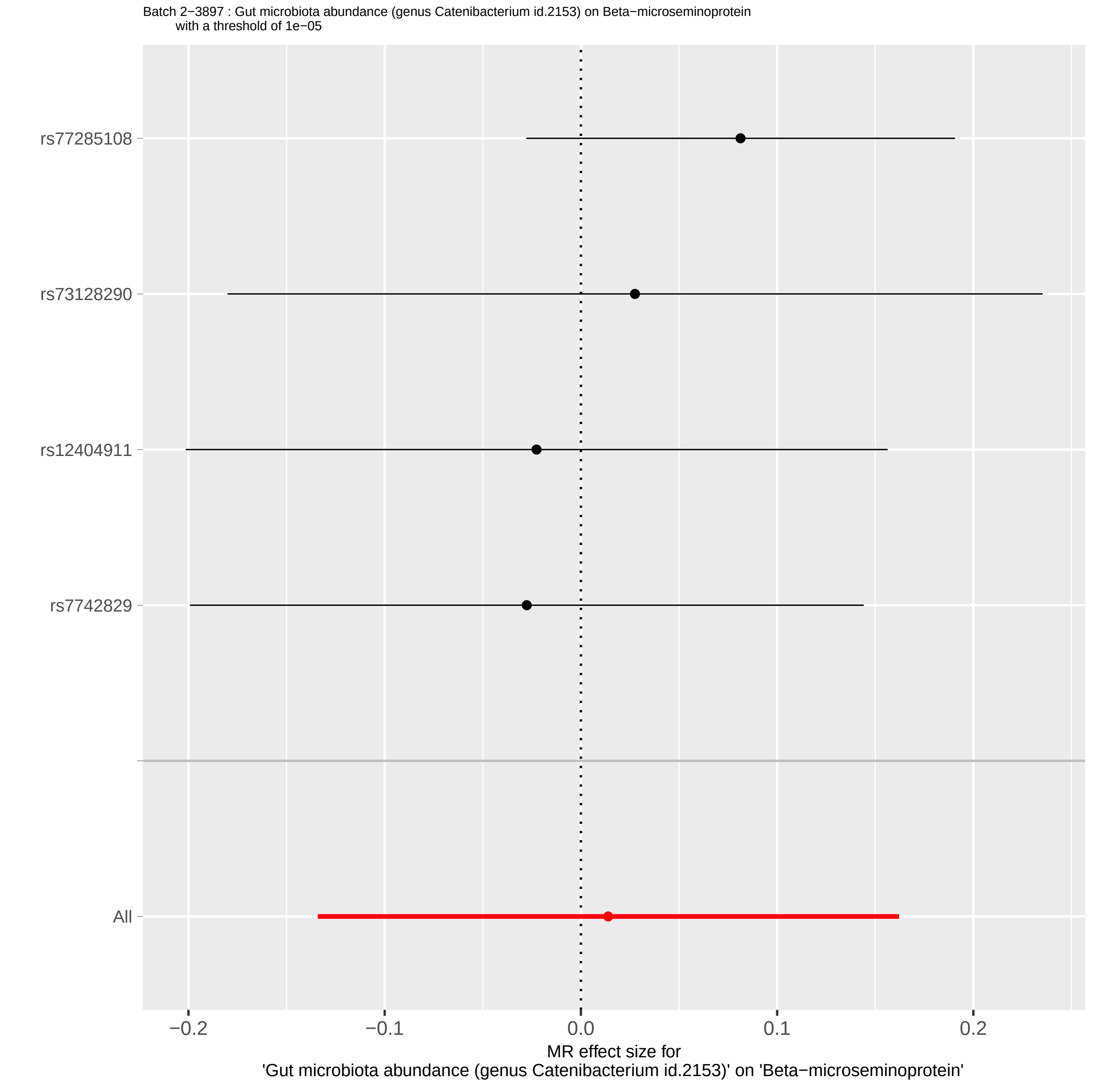

Batch 2−3897 : Gut microbiota abundance (genus Catenibacterium id.2153) on Beta−microseminoprotein
with a threshold of 1e−05
rs77285108
rs73128290
rs12404911
rs7742829
All
−0.2
−0.1
0.0
0.1
0.2
MR effect size for
'Gut microbiota abundance (genus Catenibacterium id.2153)' on 'Beta−microseminoprotein'

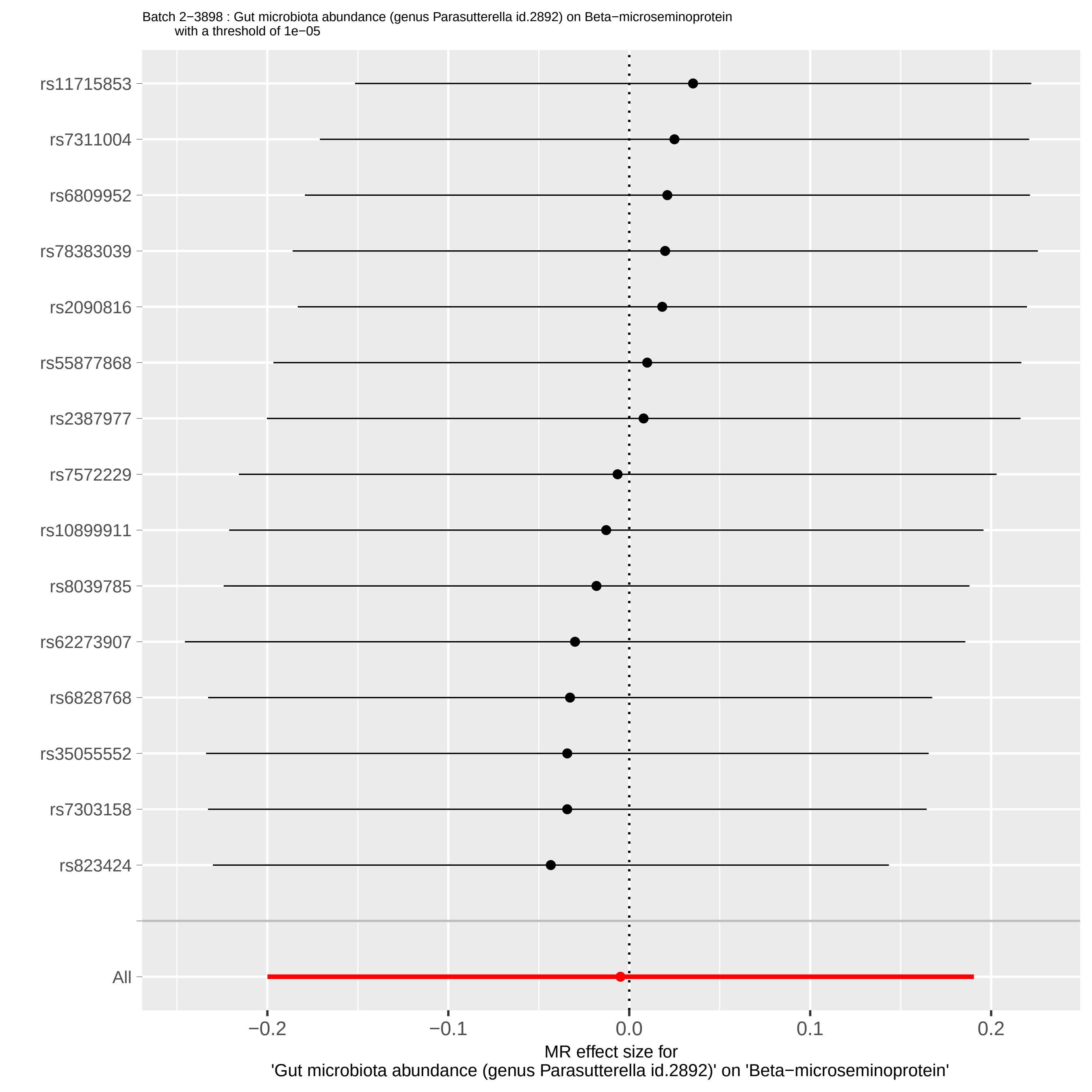
Batch 2−3898 : Gut microbiota abundance (genus Parasutterella id.2892) on Beta−microseminoprotein
with a threshold of 1e−05
rs11715853
rs7311004
rs6809952
rs78383039
rs2090816
rs55877868
rs2387977
rs7572229
rs10899911
rs8039785
rs62273907
rs6828768
rs35055552
rs7303158
rs823424
All
−0.2
−0.1
0.0
0.1
0.2
MR effect size for
'Gut microbiota abundance (genus Parasutterella id.2892)' on 'Beta−microseminoprotein'

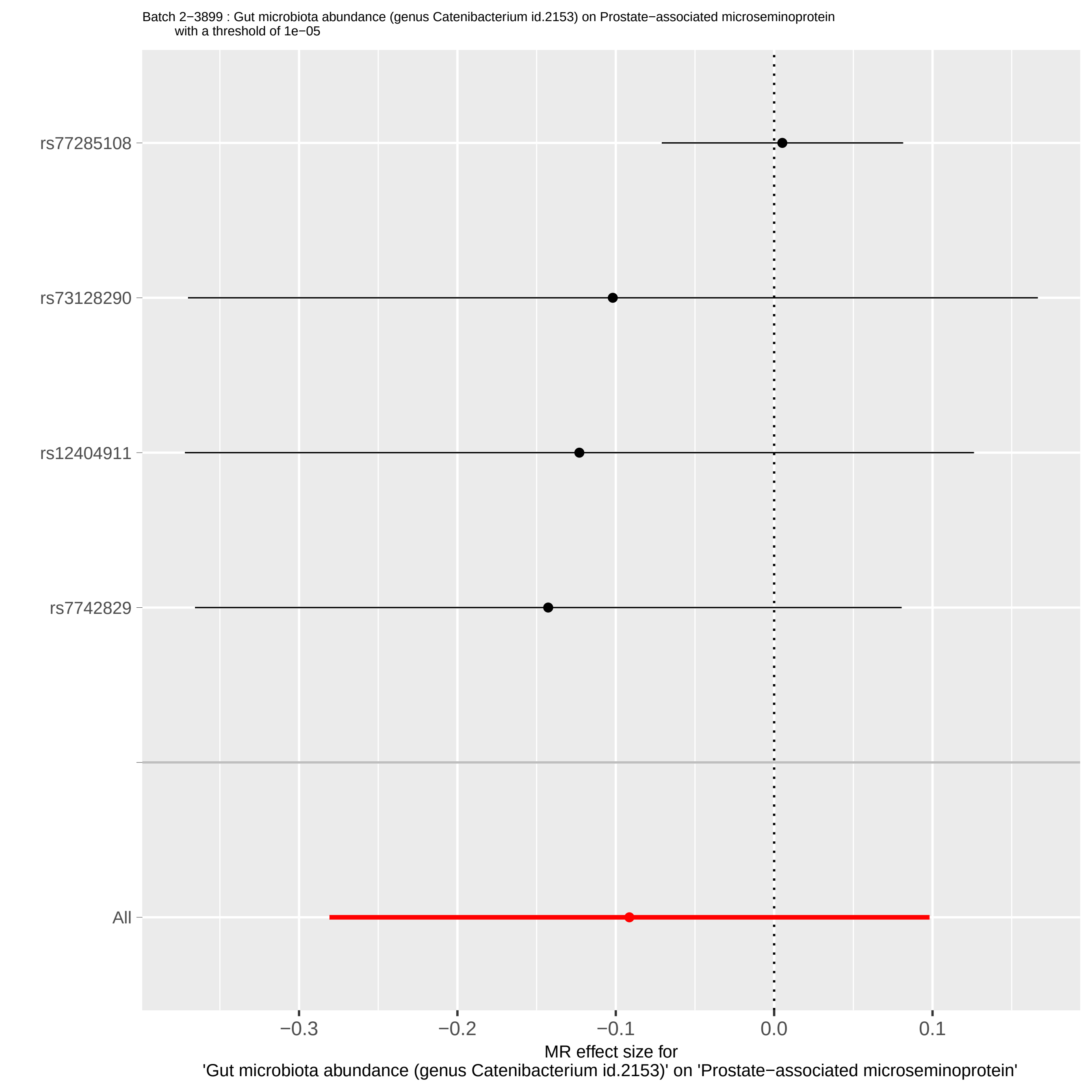
Batch 2−3899 : Gut microbiota abundance (genus Catenibacterium id.2153) on Prostate−associated microseminoprotein
with a threshold of 1e−05
rs77285108
rs73128290
rs12404911
rs7742829
All
−0.3
−0.2
−0.1
0.0
0.1
MR effect size for
'Gut microbiota abundance (genus Catenibacterium id.2153)' on 'Prostate−associated microseminoprotein'

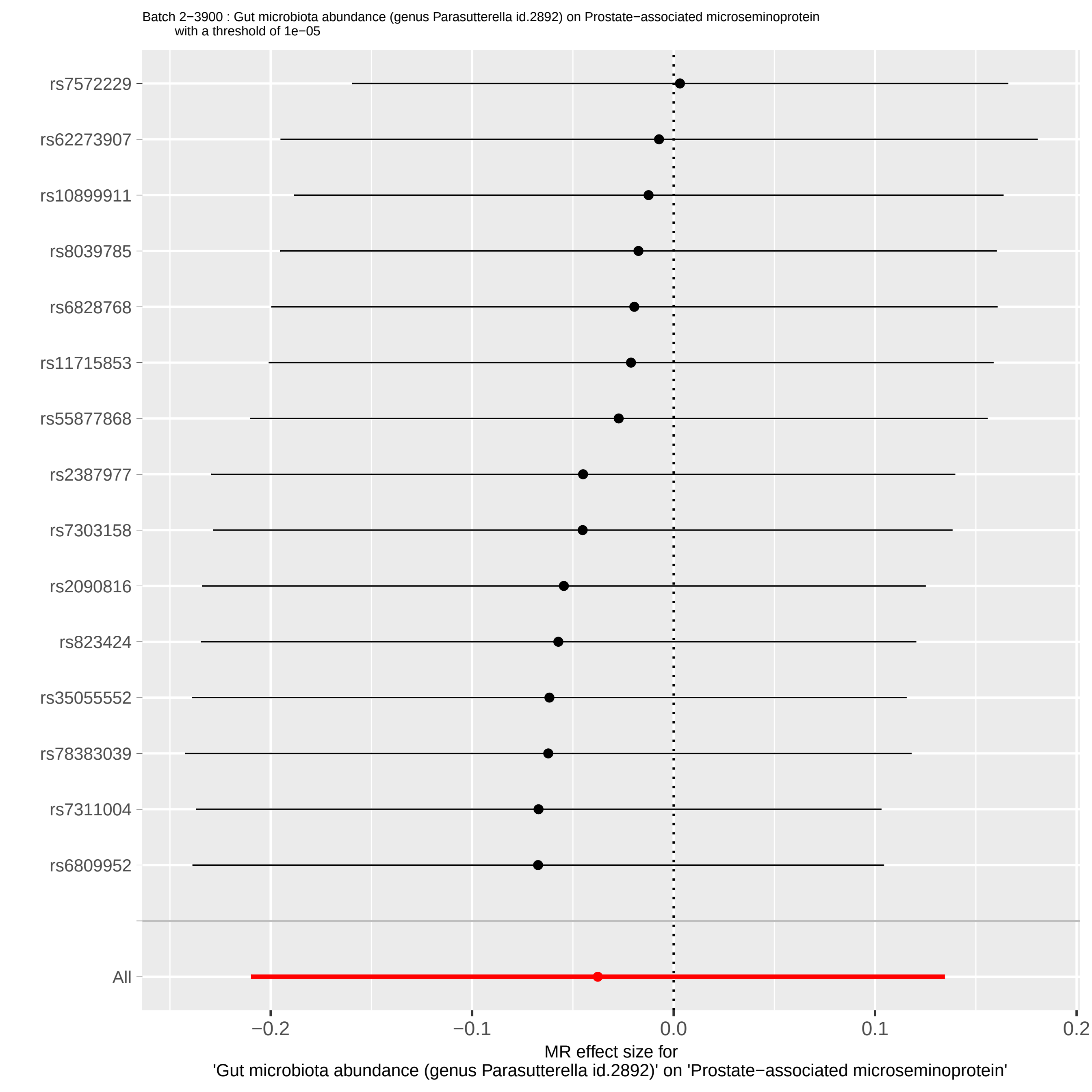

Batch 2−3900 : Gut microbiota abundance (genus Parasutterella id.2892) on Prostate−associated microseminoprotein
with a threshold of 1e−05
rs7572229
rs62273907
rs10899911
rs8039785
rs6828768
rs11715853
rs55877868
rs2387977
rs7303158
rs2090816
rs823424
rs35055552
rs78383039
rs7311004
rs6809952
All
−0.2
−0.1
0.0
0.1
0.2
MR effect size for
'Gut microbiota abundance (genus Parasutterella id.2892)' on 'Prostate−associated microseminoprotein'

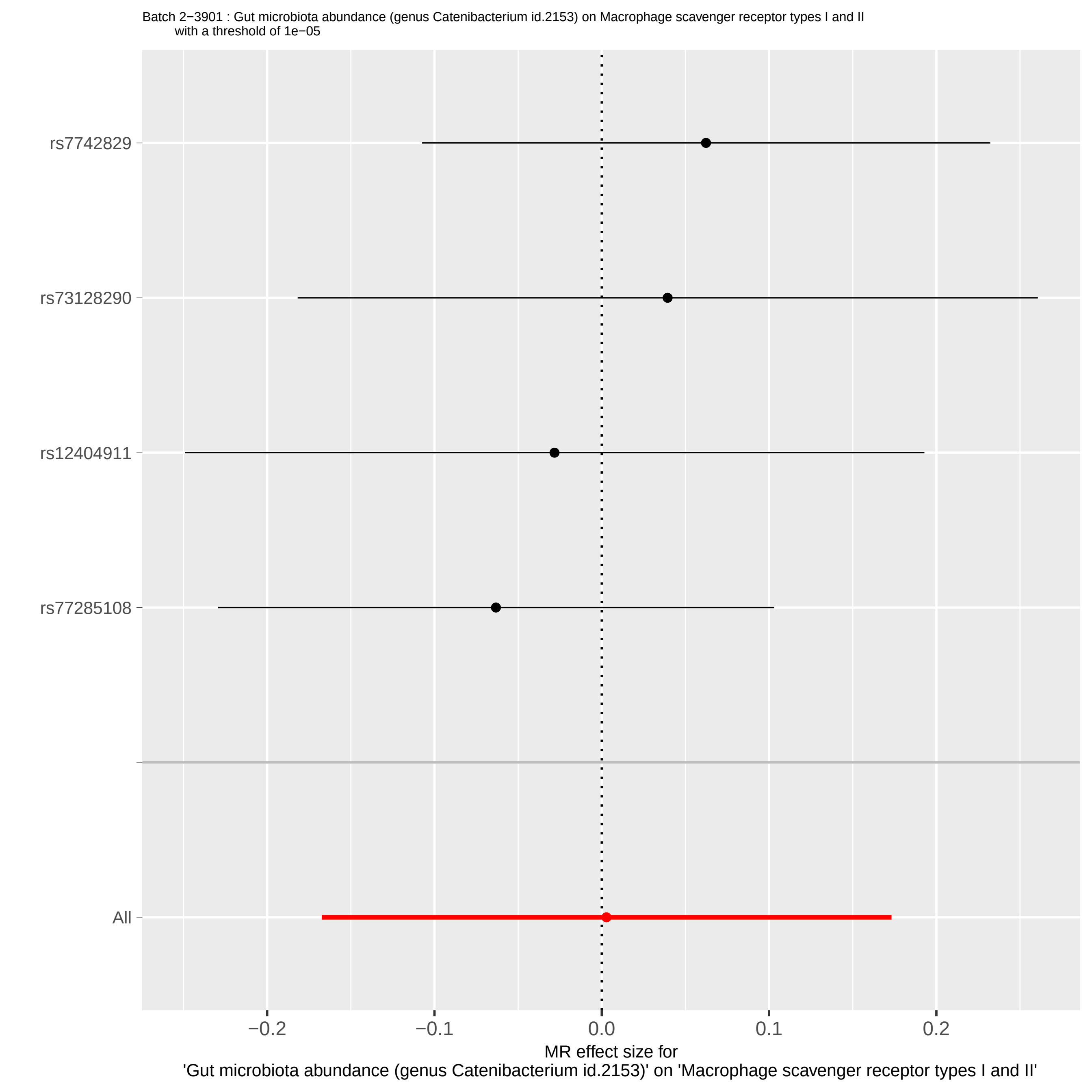
Batch 2−3901 : Gut microbiota abundance (genus Catenibacterium id.2153) on Macrophage scavenger receptor types I and II
with a threshold of 1e−05
rs7742829
rs73128290
rs12404911
rs77285108
All
−0.2
−0.1
0.0
0.1
0.2
MR effect size for
'Gut microbiota abundance (genus Catenibacterium id.2153)' on 'Macrophage scavenger receptor types I and II'

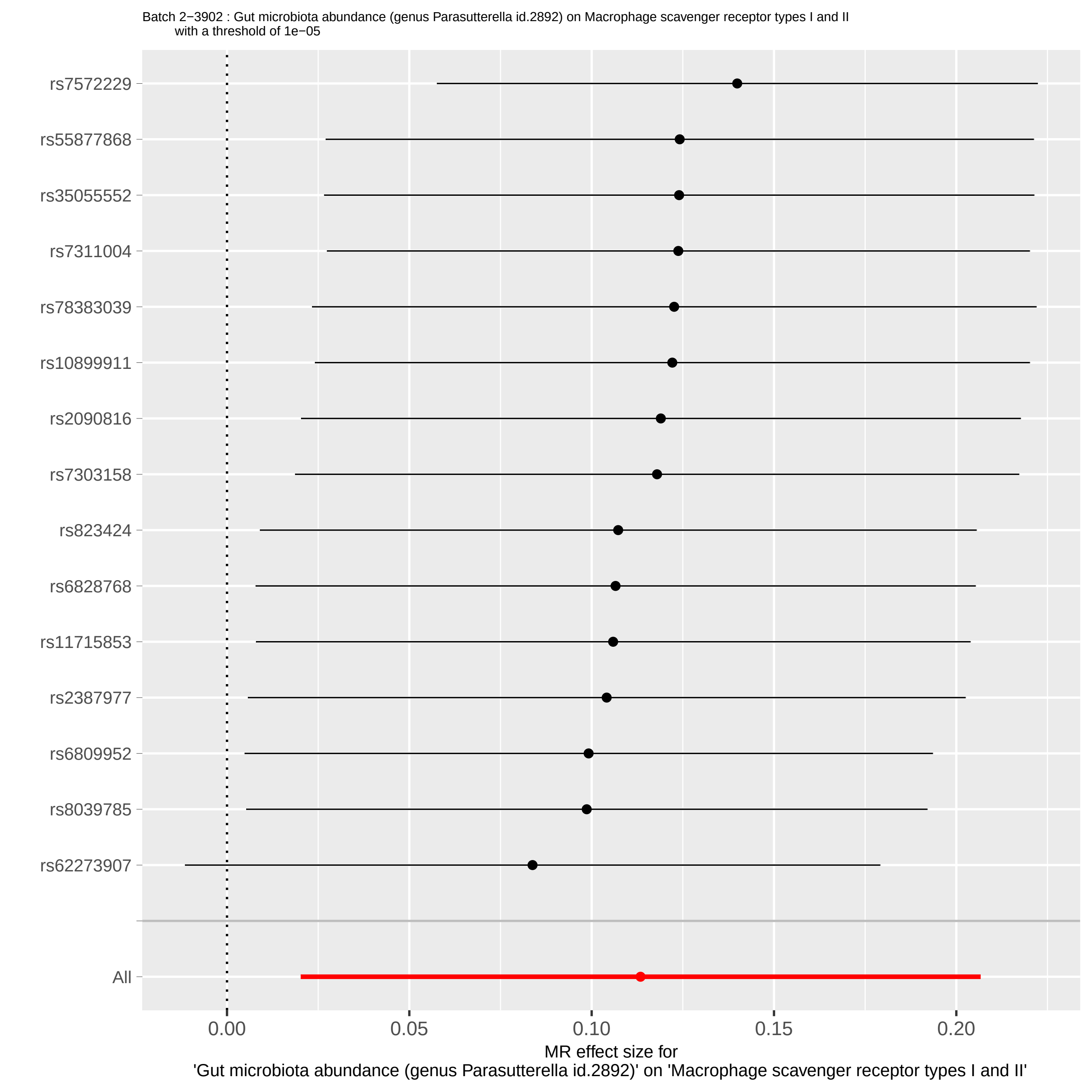

Batch 2−3902 : Gut microbiota abundance (genus Parasutterella id.2892) on Macrophage scavenger receptor types I and II
with a threshold of 1e−05
rs7572229
rs55877868
rs35055552
rs7311004
rs78383039
rs10899911
rs2090816
rs7303158
rs823424
rs6828768
rs11715853
rs2387977
rs6809952
rs8039785
rs62273907
All
0.00
0.05
0.10
0.15
0.20
MR effect size for
'Gut microbiota abundance (genus Parasutterella id.2892)' on 'Macrophage scavenger receptor types I and II'

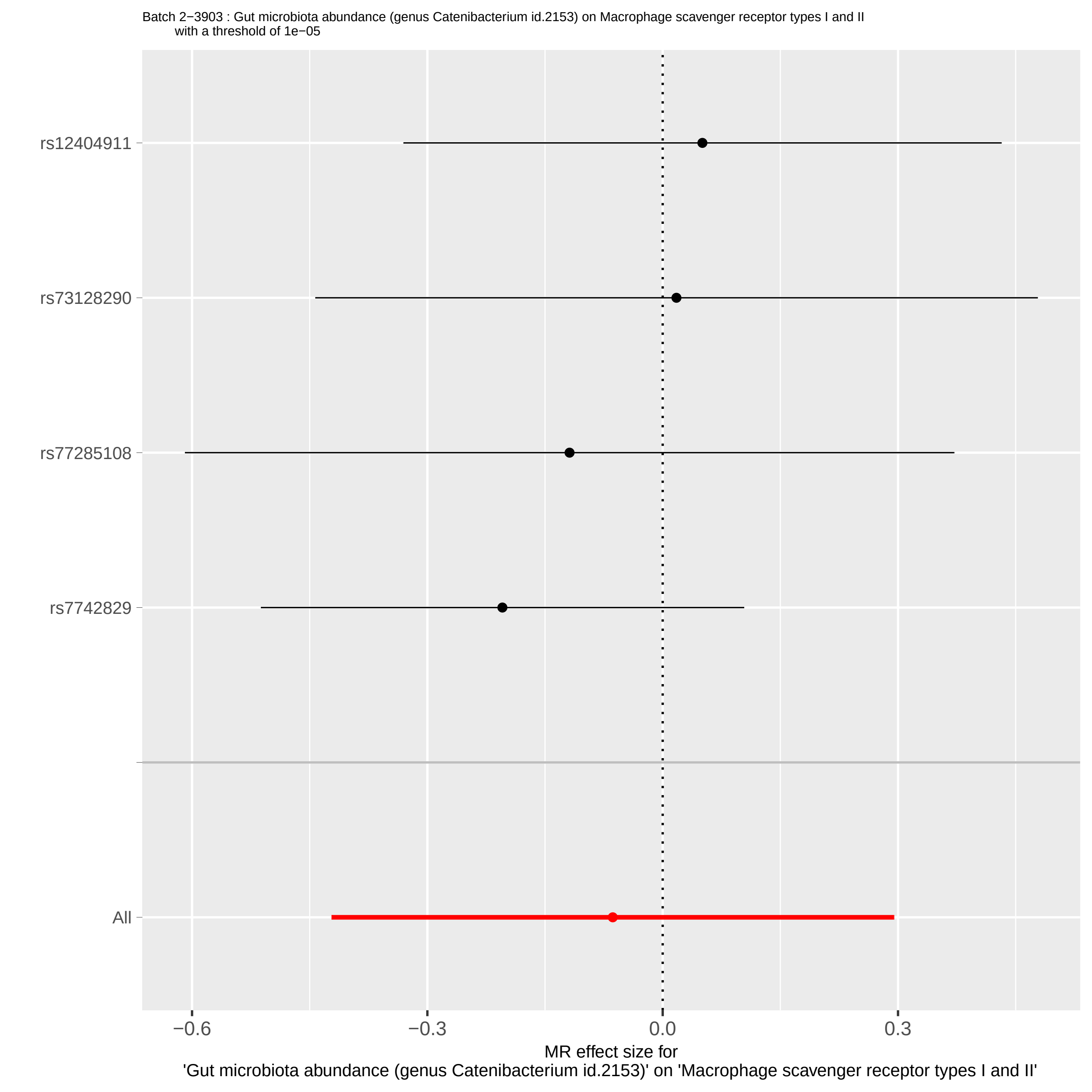
Batch 2−3903 : Gut microbiota abundance (genus Catenibacterium id.2153) on Macrophage scavenger receptor types I and II
with a threshold of 1e−05
rs12404911
rs73128290
rs77285108
rs7742829
All
−0.6
−0.3
0.0
0.3
MR effect size for
'Gut microbiota abundance (genus Catenibacterium id.2153)' on 'Macrophage scavenger receptor types I and II'

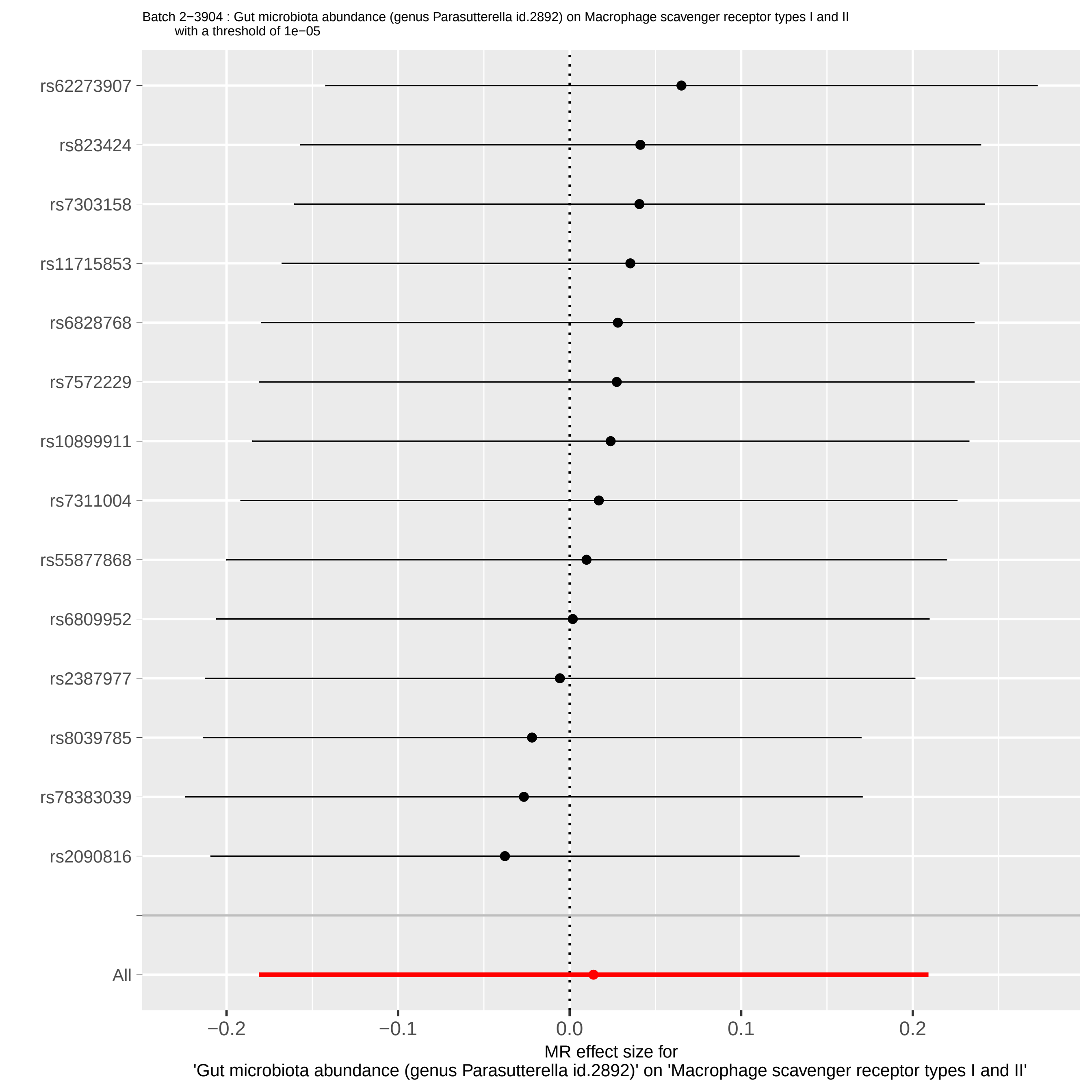

Batch 2−3904 : Gut microbiota abundance (genus Parasutterella id.2892) on Macrophage scavenger receptor types I and II
with a threshold of 1e−05
rs62273907
rs823424
rs7303158
rs11715853
rs6828768
rs7572229
rs10899911
rs7311004
rs55877868
rs6809952
rs2387977
rs8039785
rs78383039
rs2090816
All
−0.2
−0.1
0.0
0.1
0.2
MR effect size for
'Gut microbiota abundance (genus Parasutterella id.2892)' on 'Macrophage scavenger receptor types I and II'

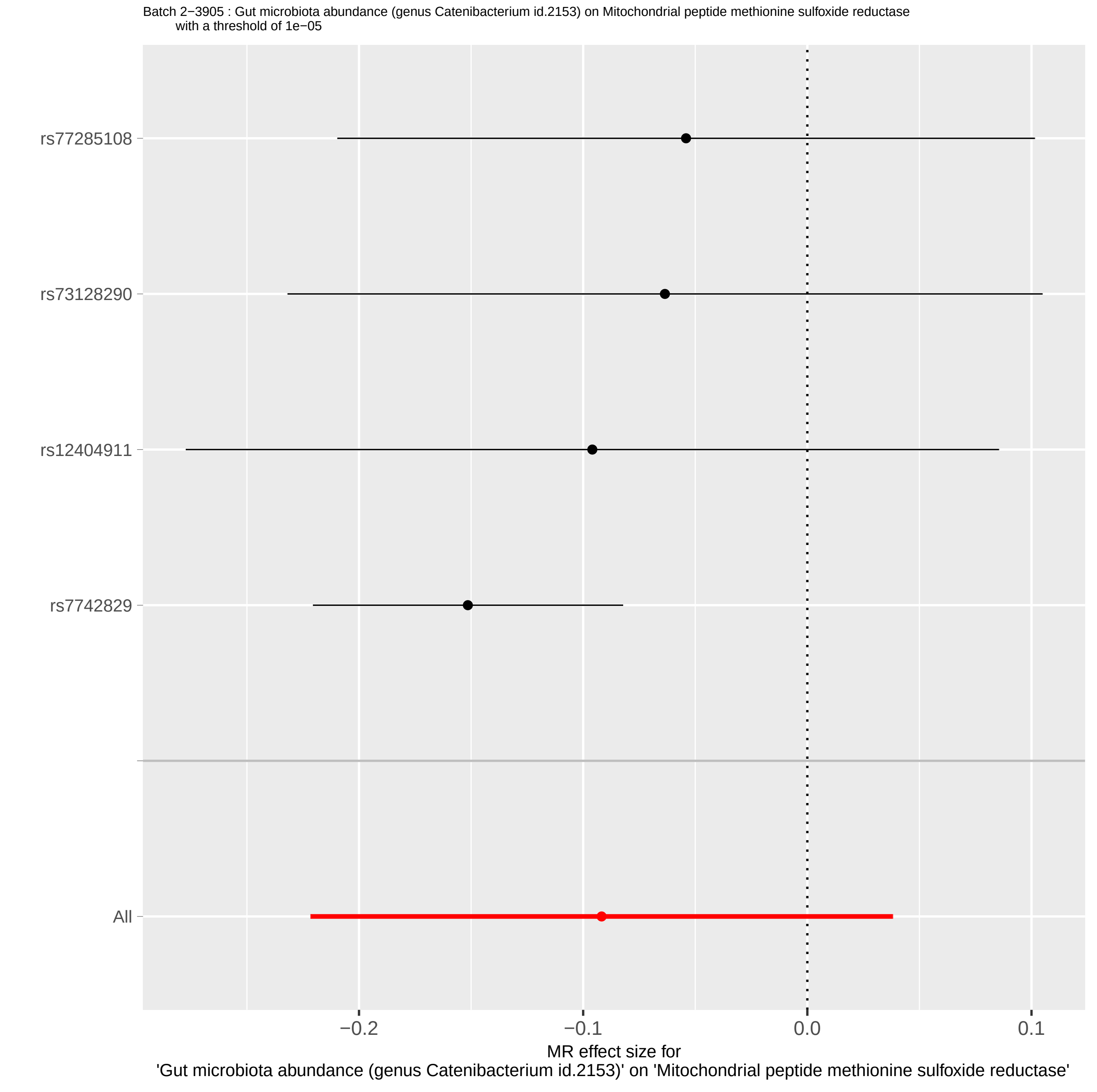

Batch 2−3905 : Gut microbiota abundance (genus Catenibacterium id.2153) on Mitochondrial peptide methionine sulfoxide reductase
with a threshold of 1e−05
rs77285108
rs73128290
rs12404911
rs7742829
All
−0.2
−0.1
0.0
0.1
MR effect size for
'Gut microbiota abundance (genus Catenibacterium id.2153)' on 'Mitochondrial peptide methionine sulfoxide reductase'

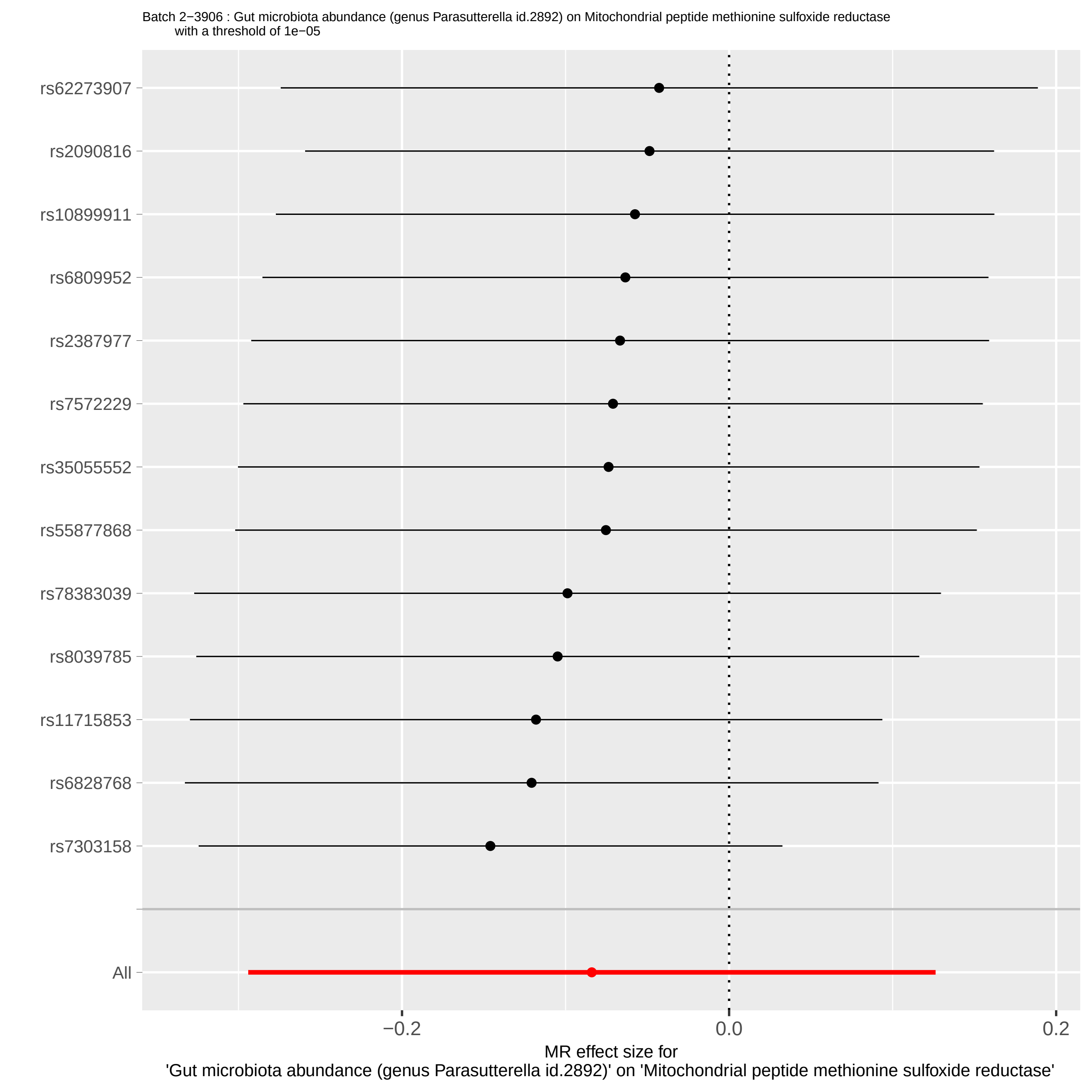

Batch 2−3906 : Gut microbiota abundance (genus Parasutterella id.2892) on Mitochondrial peptide methionine sulfoxide reductase
with a threshold of 1e−05
rs62273907
rs2090816
rs10899911
rs6809952
rs2387977
rs7572229
rs35055552
rs55877868
rs78383039
rs8039785
rs11715853
rs6828768
rs7303158
All
−0.2
0.0
0.2
MR effect size for
'Gut microbiota abundance (genus Parasutterella id.2892)' on 'Mitochondrial peptide methionine sulfoxide reductase'

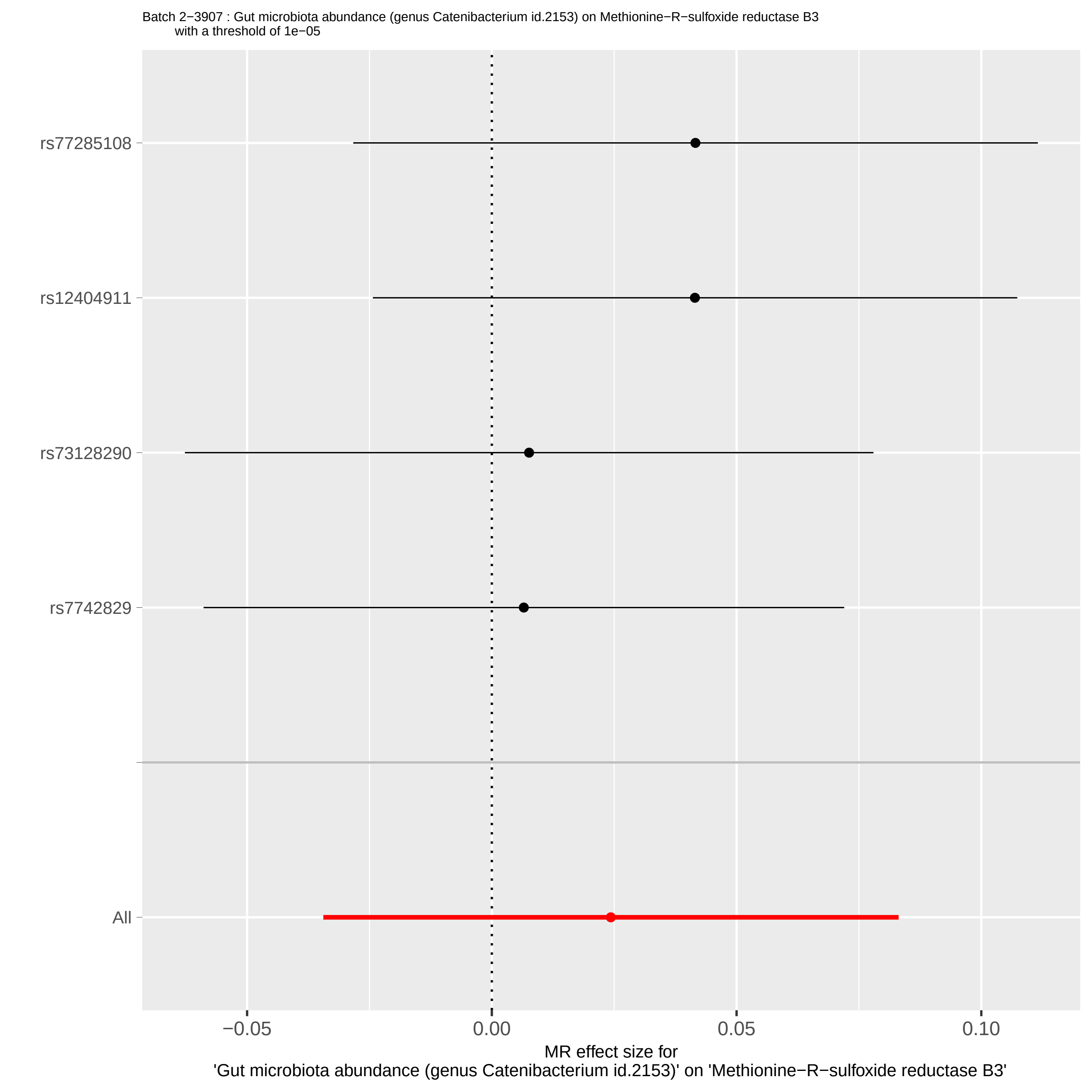
Batch 2−3907 : Gut microbiota abundance (genus Catenibacterium id.2153) on Methionine−R−sulfoxide reductase B3
with a threshold of 1e−05
rs77285108
rs12404911
rs73128290
rs7742829
All
−0.05
0.00
0.05
0.10
MR effect size for
'Gut microbiota abundance (genus Catenibacterium id.2153)' on 'Methionine−R−sulfoxide reductase B3'

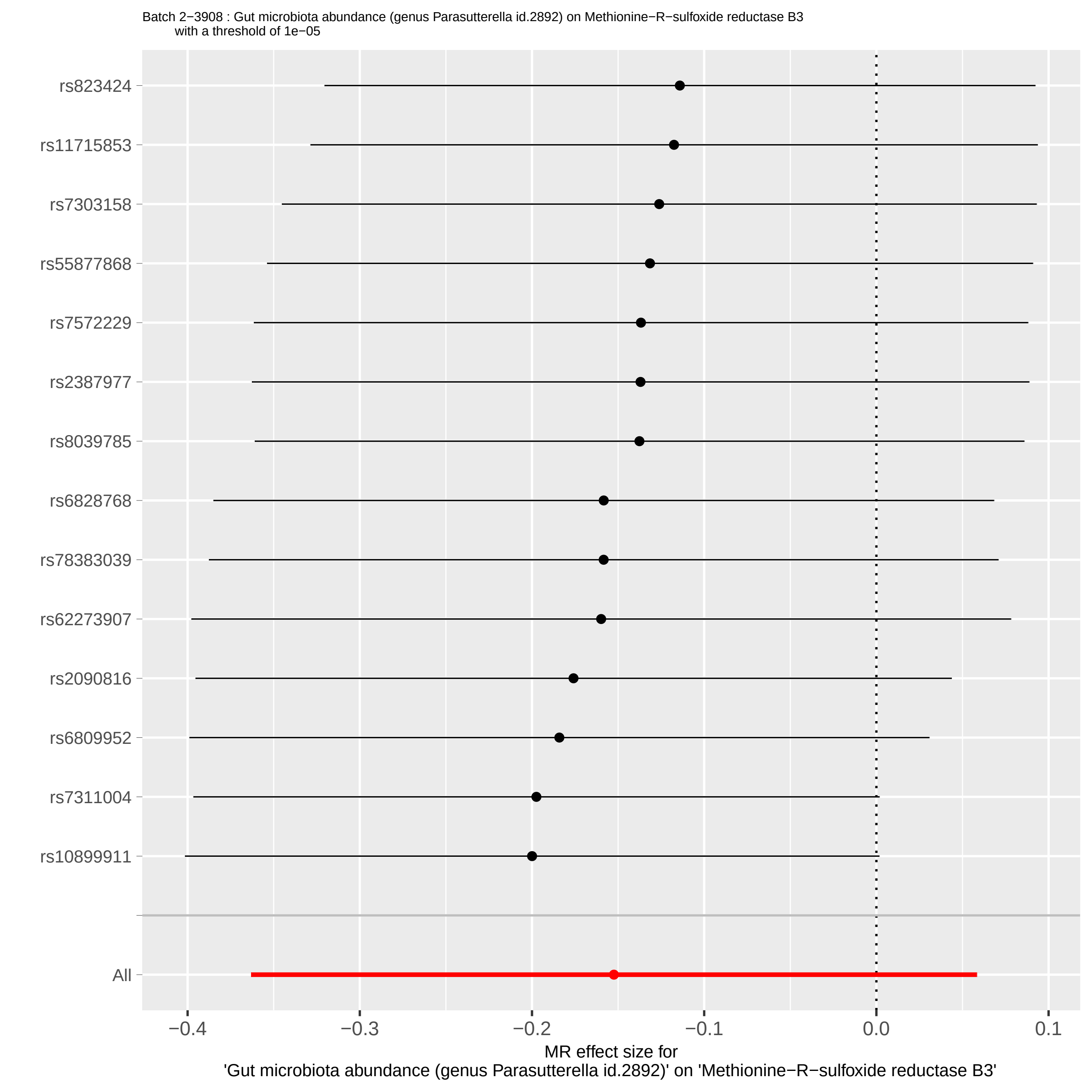
Batch 2−3908 : Gut microbiota abundance (genus Parasutterella id.2892) on Methionine−R−sulfoxide reductase B3
with a threshold of 1e−05
rs823424
rs11715853
rs7303158
rs55877868
rs7572229
rs2387977
rs8039785
rs6828768
rs78383039
rs62273907
rs2090816
rs6809952
rs7311004
rs10899911
All
−0.4
−0.3
−0.2
−0.1
0.0
0.1
MR effect size for
'Gut microbiota abundance (genus Parasutterella id.2892)' on 'Methionine−R−sulfoxide reductase B3'

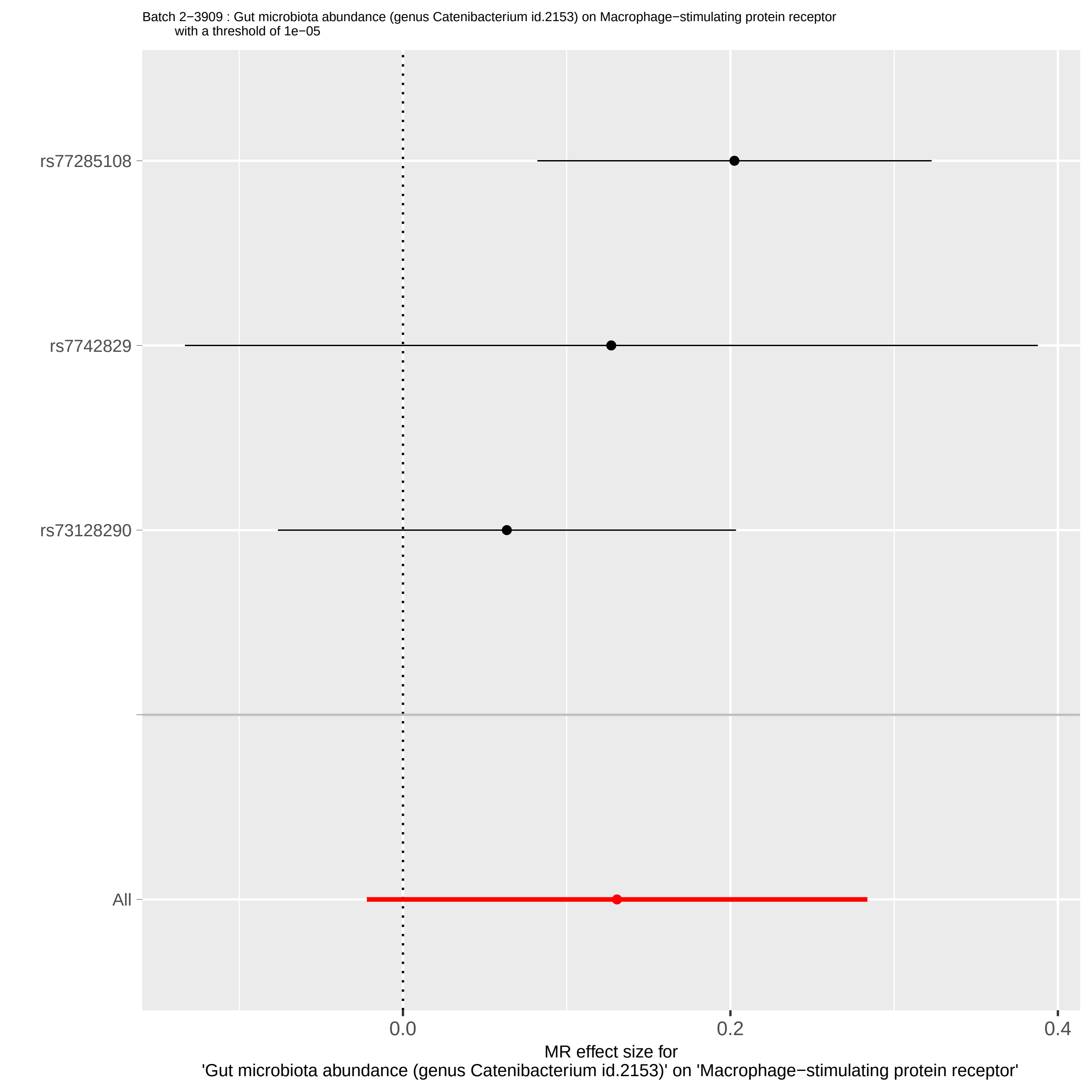
Batch 2−3909 : Gut microbiota abundance (genus Catenibacterium id.2153) on Macrophage−stimulating protein receptor
with a threshold of 1e−05
rs77285108
rs7742829
rs73128290
All
0.0
0.2
0.4
MR effect size for
'Gut microbiota abundance (genus Catenibacterium id.2153)' on 'Macrophage−stimulating protein receptor'

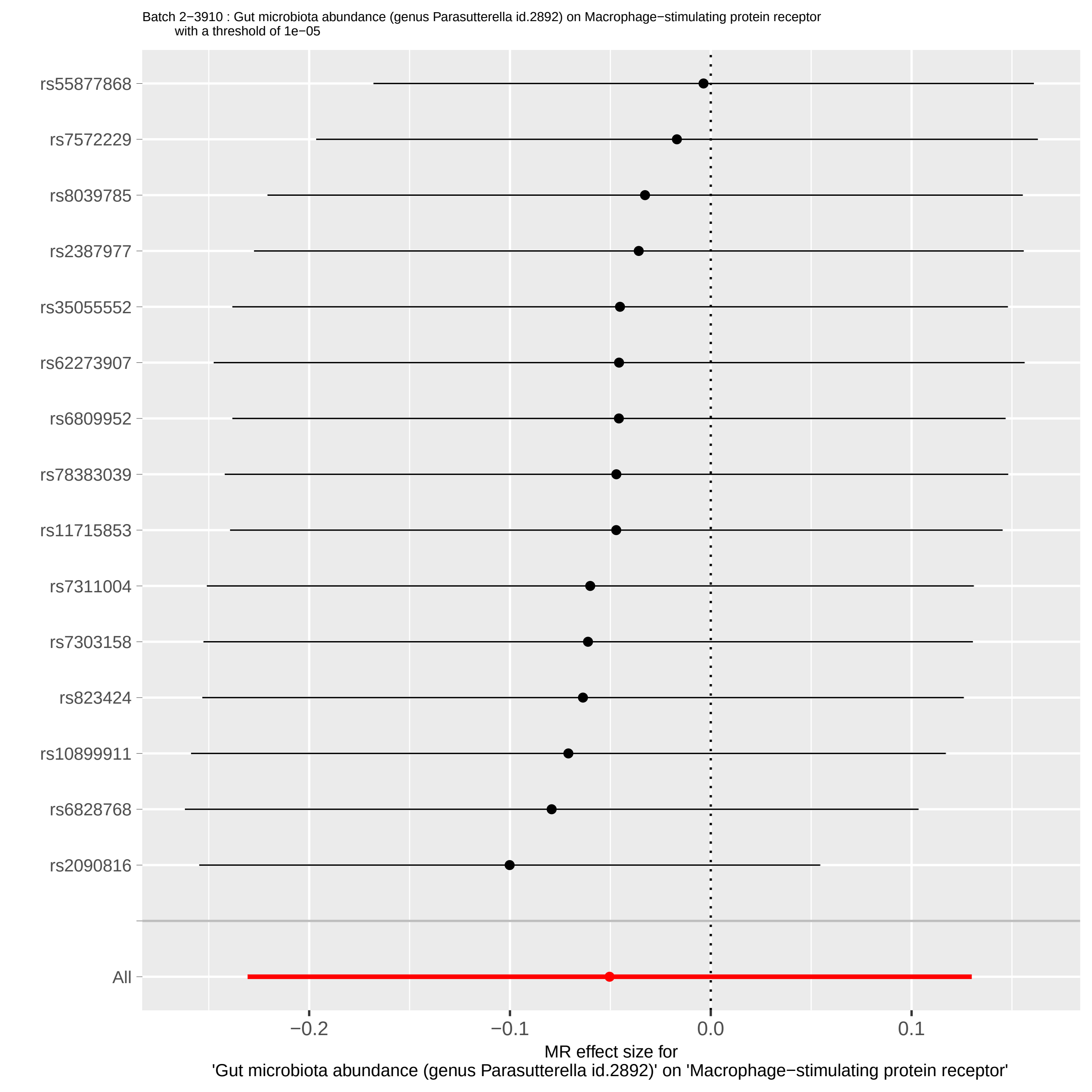

Batch 2−3910 : Gut microbiota abundance (genus Parasutterella id.2892) on Macrophage−stimulating protein receptor
with a threshold of 1e−05
rs55877868
rs7572229
rs8039785
rs2387977
rs35055552
rs62273907
rs6809952
rs78383039
rs11715853
rs7311004
rs7303158
rs823424
rs10899911
rs6828768
rs2090816
All
−0.2
−0.1
0.0
0.1
MR effect size for
'Gut microbiota abundance (genus Parasutterella id.2892)' on 'Macrophage−stimulating protein receptor'

Batch 2−3911 : Gut microbiota abundance (genus Catenibacterium id.2153) on Growth/differentiation factor 8
with a threshold of 1e−05

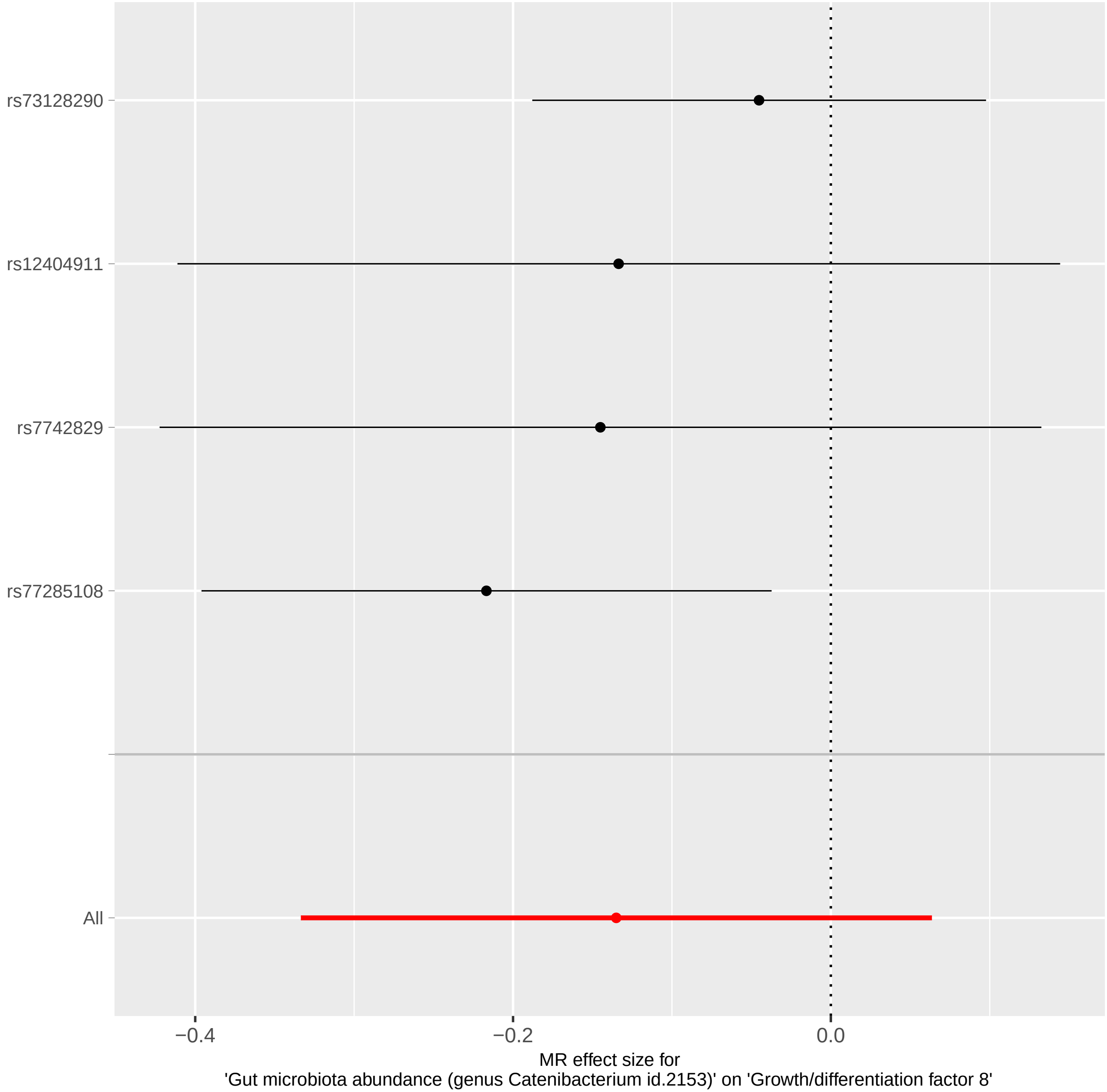

Batch 2−3912 : Gut microbiota abundance (genus Parasutterella id.2892) on Growth/differentiation factor 8
with a threshold of 1e−05

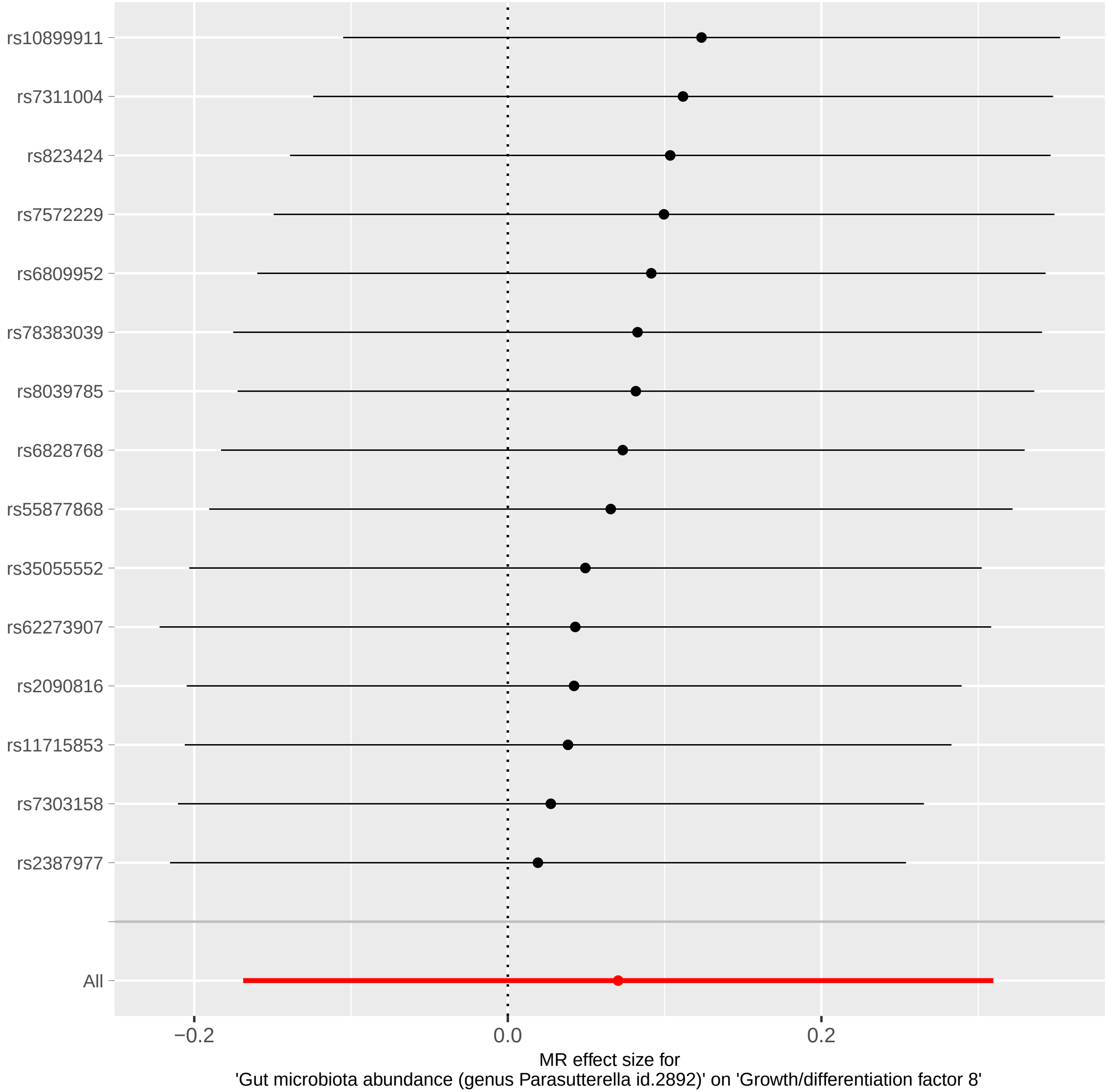

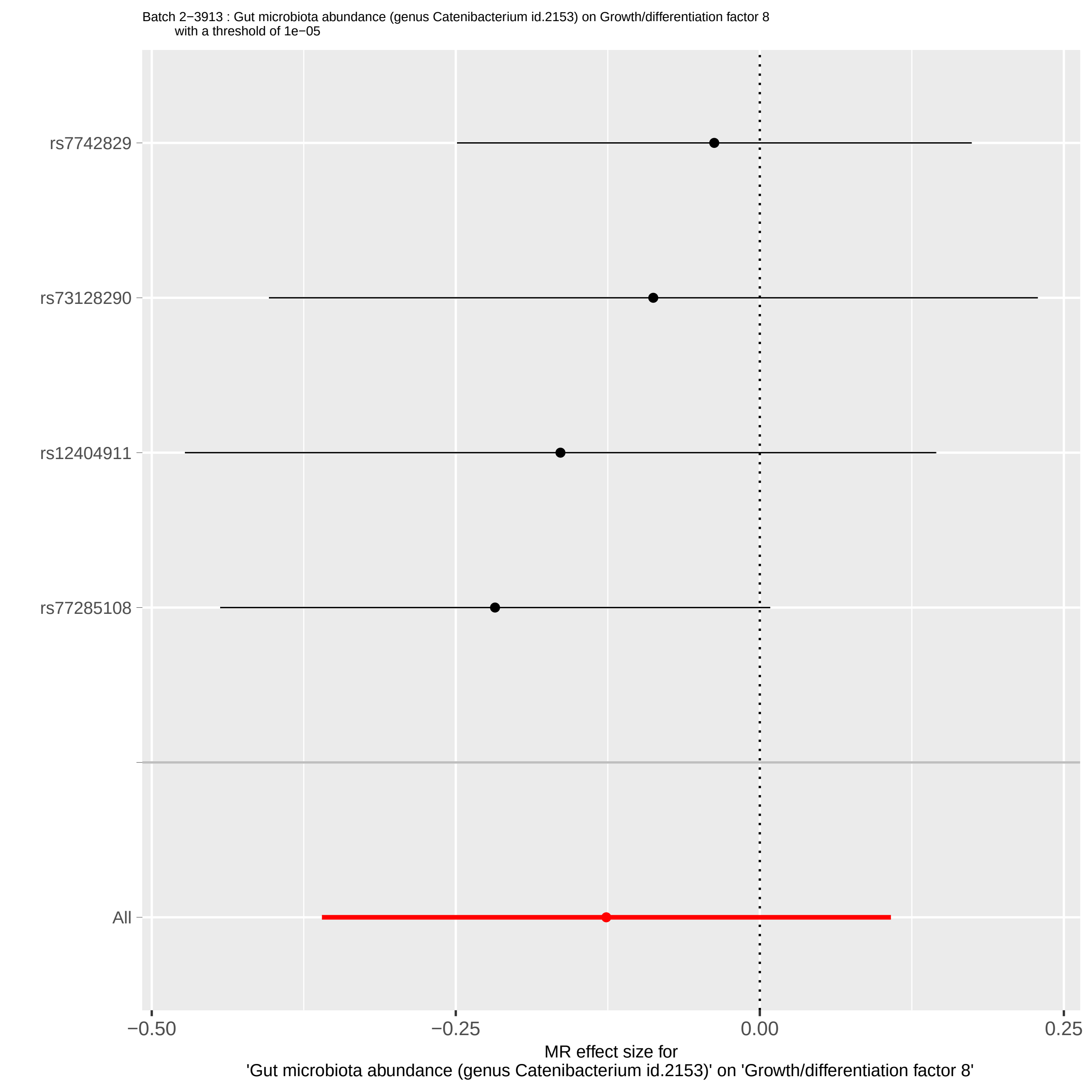

Batch 2−3913 : Gut microbiota abundance (genus Catenibacterium id.2153) on Growth/differentiation factor 8
with a threshold of 1e−05
rs7742829
rs73128290
rs12404911
rs77285108
All
−0.50
−0.25
0.00
0.25
MR effect size for
'Gut microbiota abundance (genus Catenibacterium id.2153)' on 'Growth/differentiation factor 8'

Batch 2−3914 : Gut microbiota abundance (genus Parasutterella id.2892) on Growth/differentiation factor 8
with a threshold of 1e−05

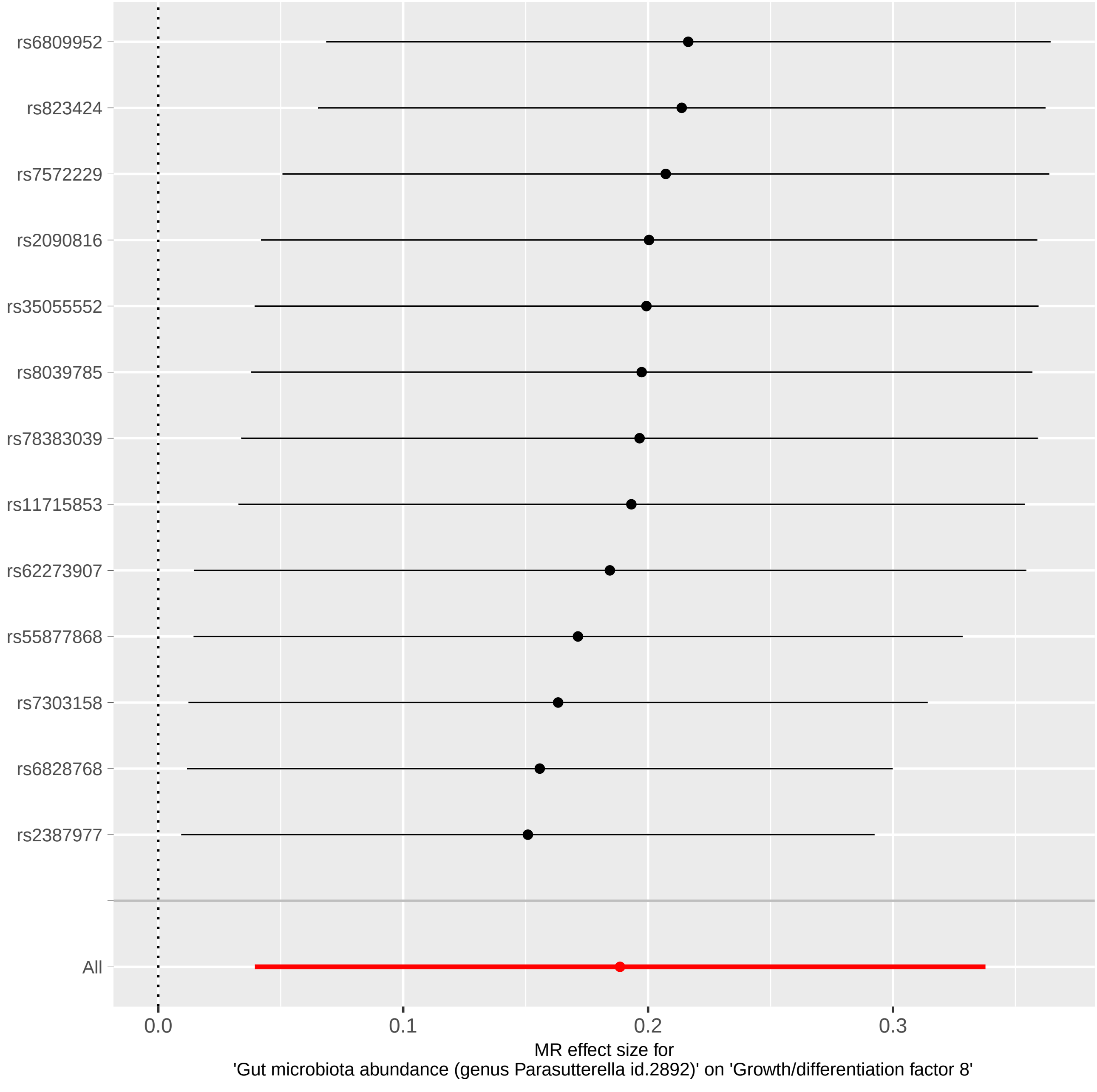

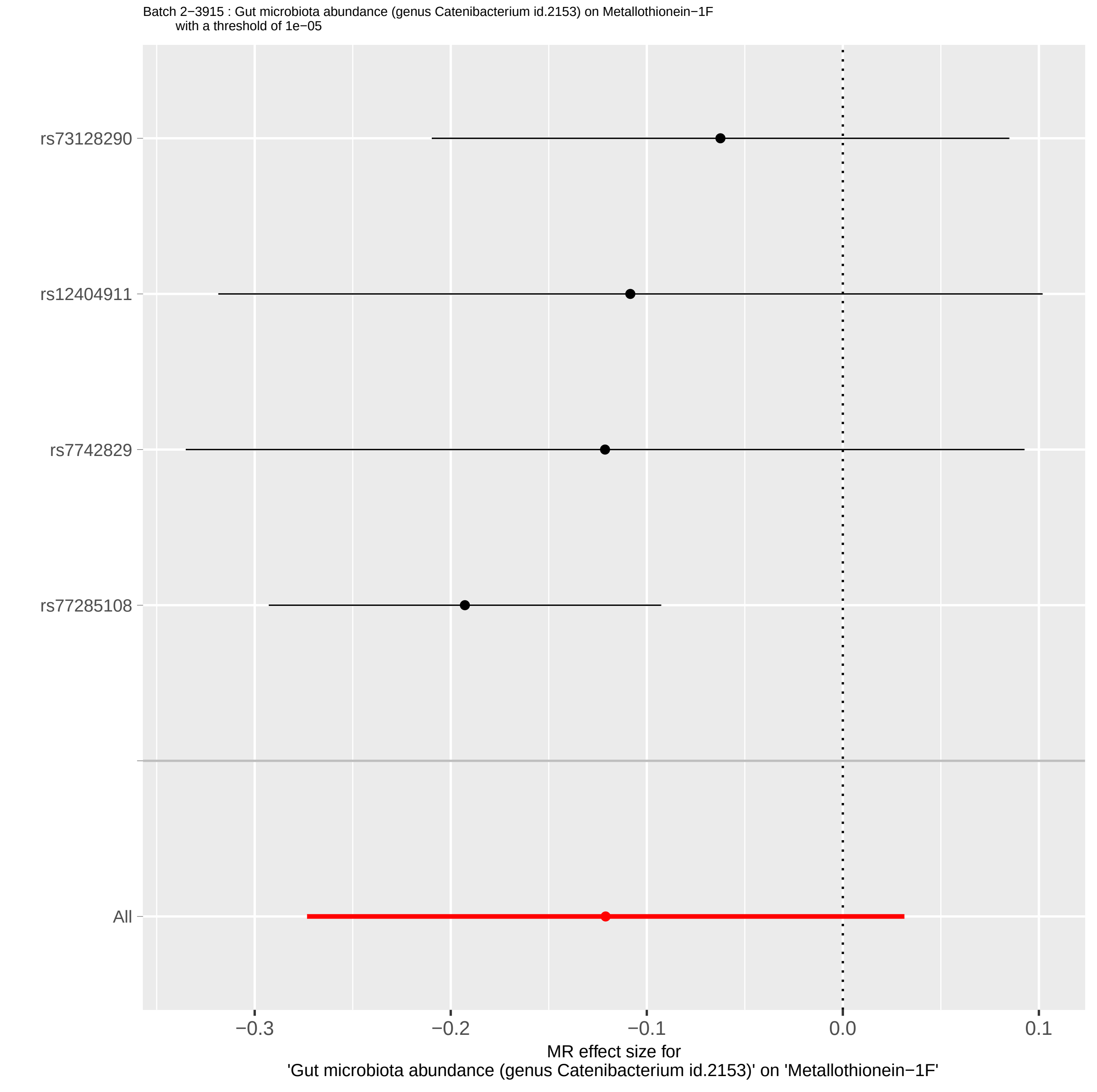

Batch 2−3915 : Gut microbiota abundance (genus Catenibacterium id.2153) on Metallothionein−1F
with a threshold of 1e−05
rs73128290
rs12404911
rs7742829
rs77285108
All
−0.3
−0.2
−0.1
0.0
0.1
MR effect size for
'Gut microbiota abundance (genus Catenibacterium id.2153)' on 'Metallothionein−1F'

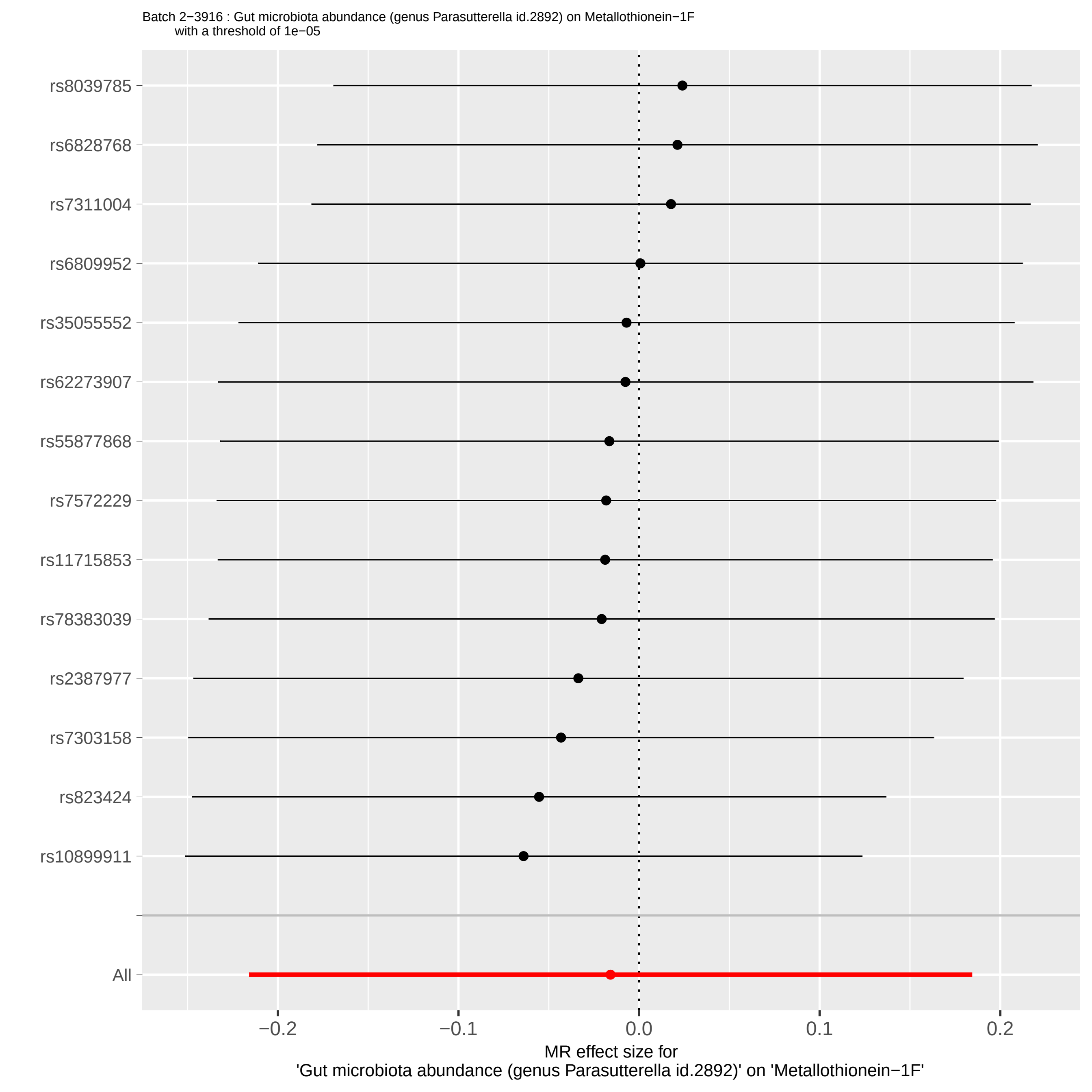
Batch 2−3916 : Gut microbiota abundance (genus Parasutterella id.2892) on Metallothionein−1F
with a threshold of 1e−05
rs8039785
rs6828768
rs7311004
rs6809952
rs35055552
rs62273907
rs55877868
rs7572229
rs11715853
rs78383039
rs2387977
rs7303158
rs823424
rs10899911
All
−0.2
−0.1
0.0
0.1
0.2
MR effect size for
'Gut microbiota abundance (genus Parasutterella id.2892)' on 'Metallothionein−1F'

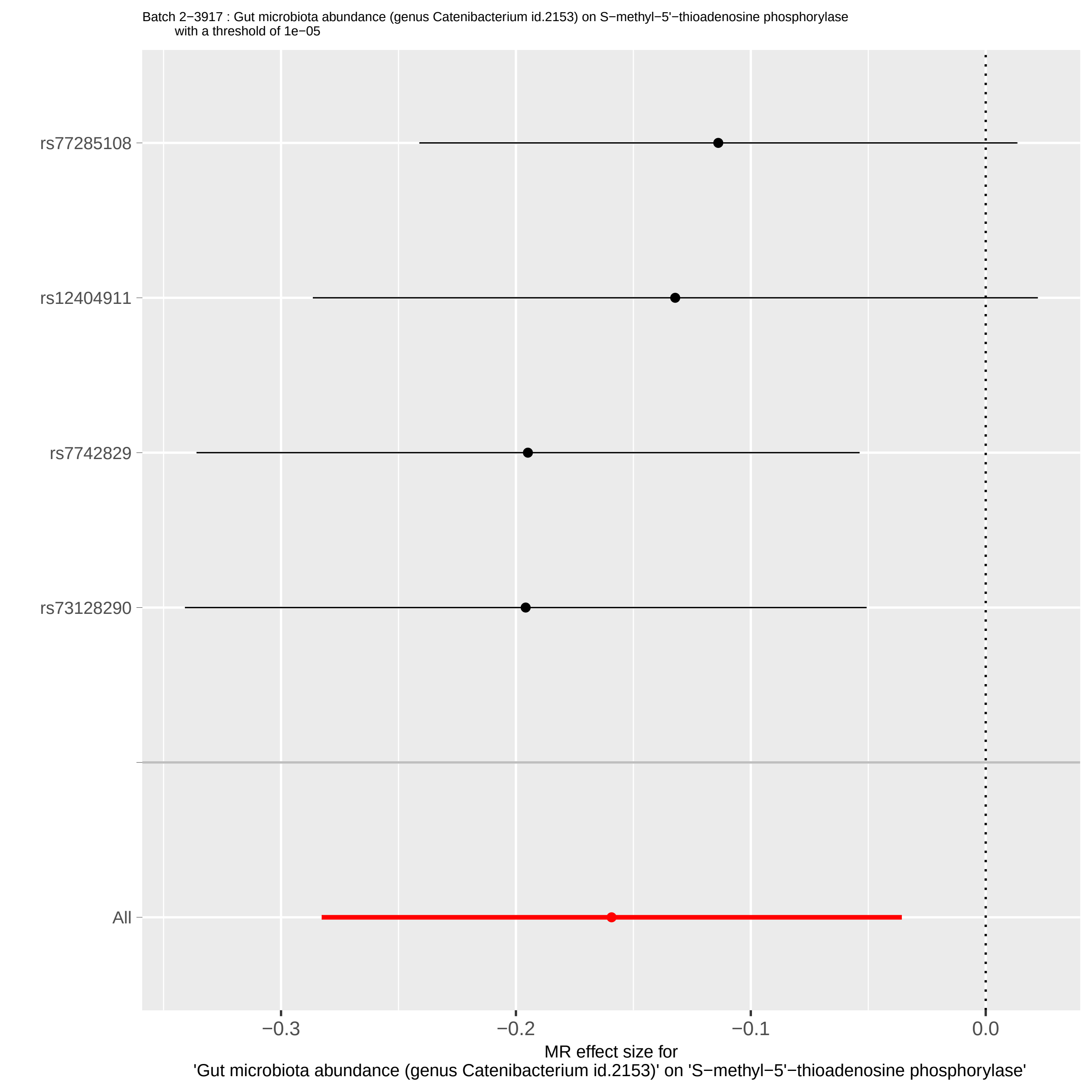

Batch 2−3917 : Gut microbiota abundance (genus Catenibacterium id.2153) on S−methyl−5'−thioadenosine phosphorylase
with a threshold of 1e−05
rs77285108
rs12404911
rs7742829
rs73128290
All
−0.3
−0.2
−0.1
0.0
MR effect size for
'Gut microbiota abundance (genus Catenibacterium id.2153)' on 'S−methyl−5'−thioadenosine phosphorylase'

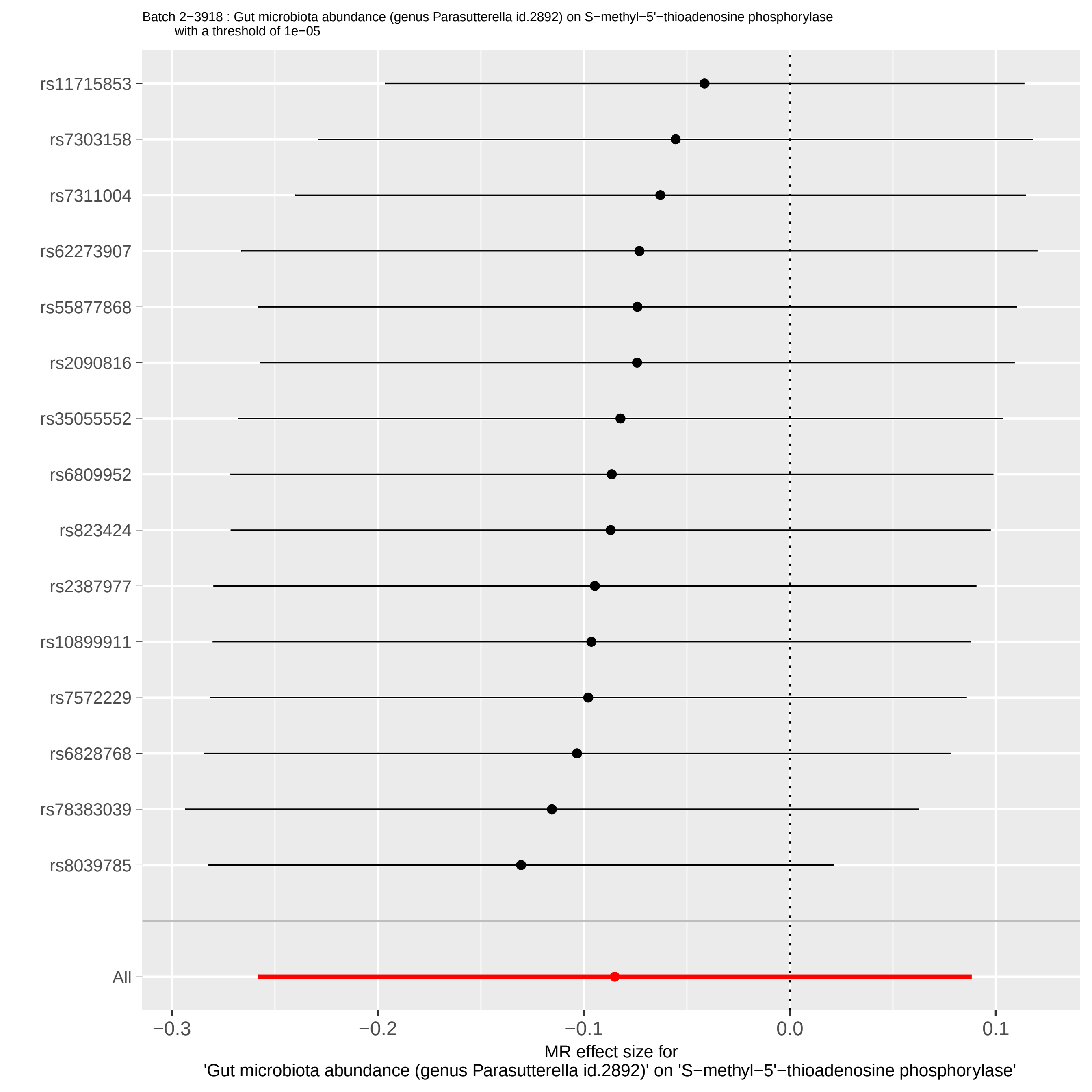

Batch 2−3918 : Gut microbiota abundance (genus Parasutterella id.2892) on S−methyl−5'−thioadenosine phosphorylase
with a threshold of 1e−05
rs11715853
rs7303158
rs7311004
rs62273907
rs55877868
rs2090816
rs35055552
rs6809952
rs823424
rs2387977
rs10899911
rs7572229
rs6828768
rs78383039
rs8039785
All
−0.3
−0.2
−0.1
0.0
0.1
MR effect size for
'Gut microbiota abundance (genus Parasutterella id.2892)' on 'S−methyl−5'−thioadenosine phosphorylase'

Batch 2−3919 : Gut microbiota abundance (genus Catenibacterium id.2153) on Protein p13 MTCP−1
with a threshold of 1e−05

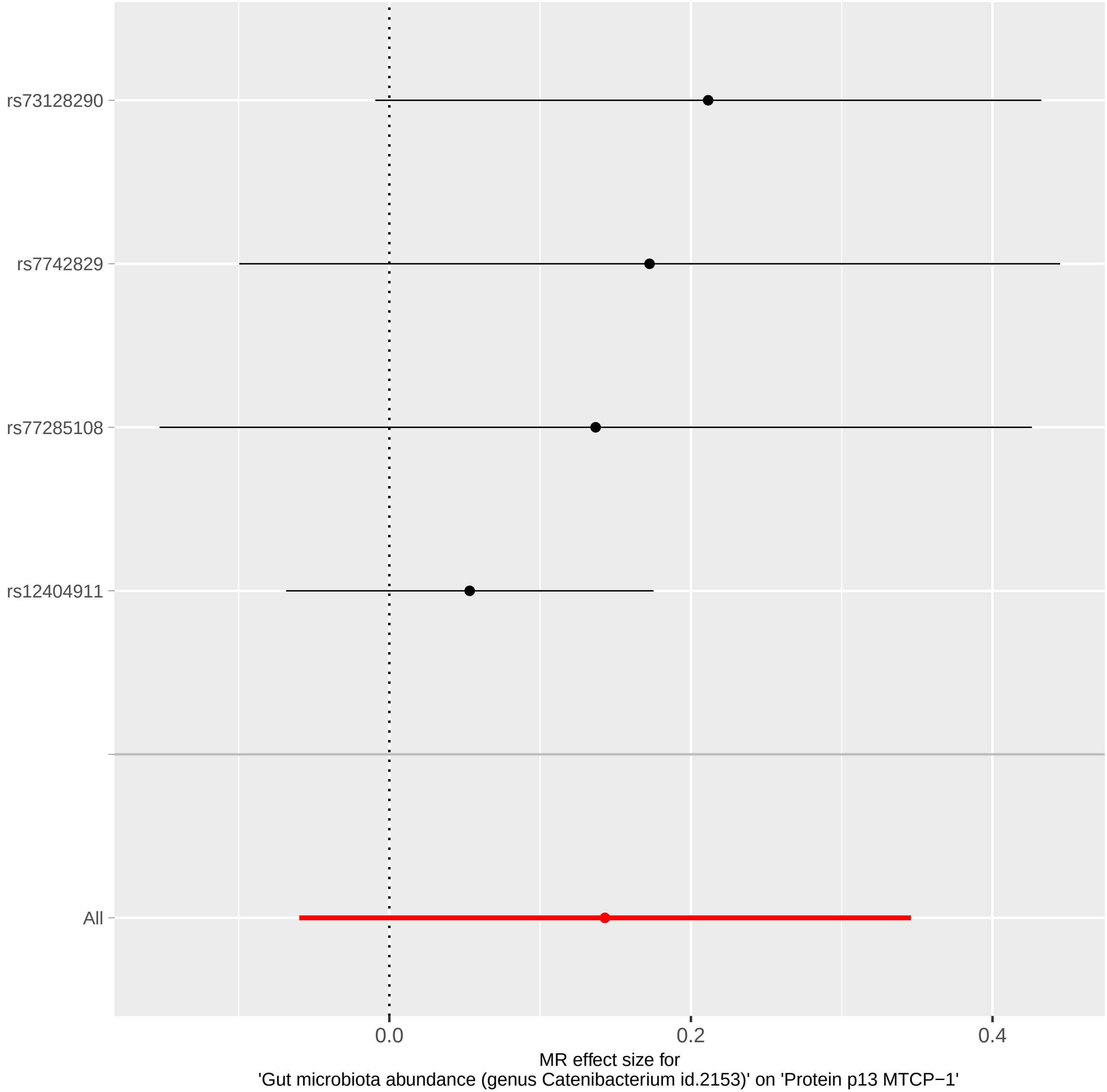

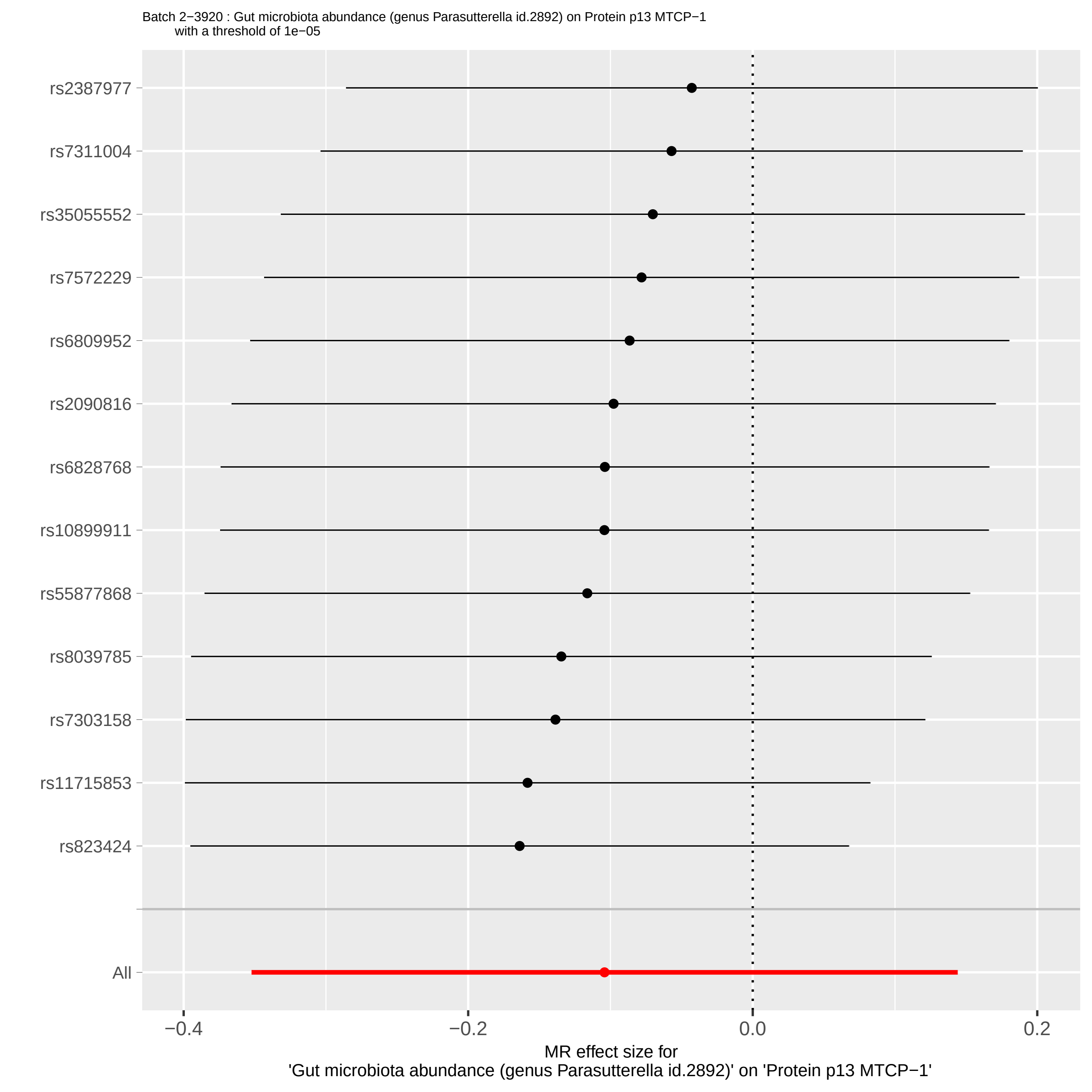
Batch 2−3920 : Gut microbiota abundance (genus Parasutterella id.2892) on Protein p13 MTCP−1
with a threshold of 1e−05
rs2387977
rs7311004
rs35055552
rs7572229
rs6809952
rs2090816
rs6828768
rs10899911
rs55877868
rs8039785
rs7303158
rs11715853
rs823424
All
−0.4
−0.2
0.0
0.2
MR effect size for
'Gut microbiota abundance (genus Parasutterella id.2892)' on 'Protein p13 MTCP−1'

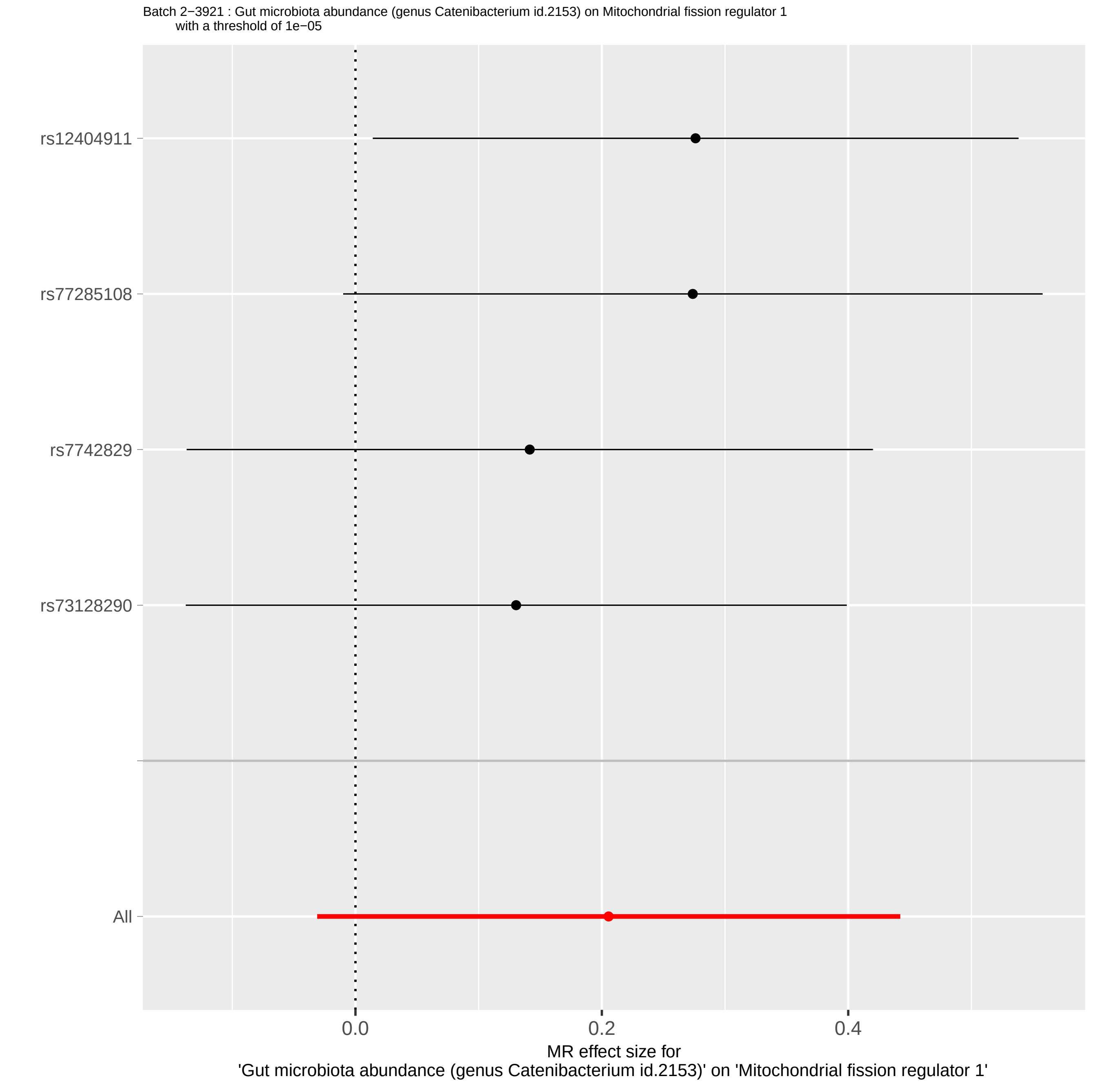

Batch 2−3921 : Gut microbiota abundance (genus Catenibacterium id.2153) on Mitochondrial fission regulator 1
with a threshold of 1e−05
rs12404911
rs77285108
rs7742829
rs73128290
All
0.0
0.2
0.4
MR effect size for
'Gut microbiota abundance (genus Catenibacterium id.2153)' on 'Mitochondrial fission regulator 1'

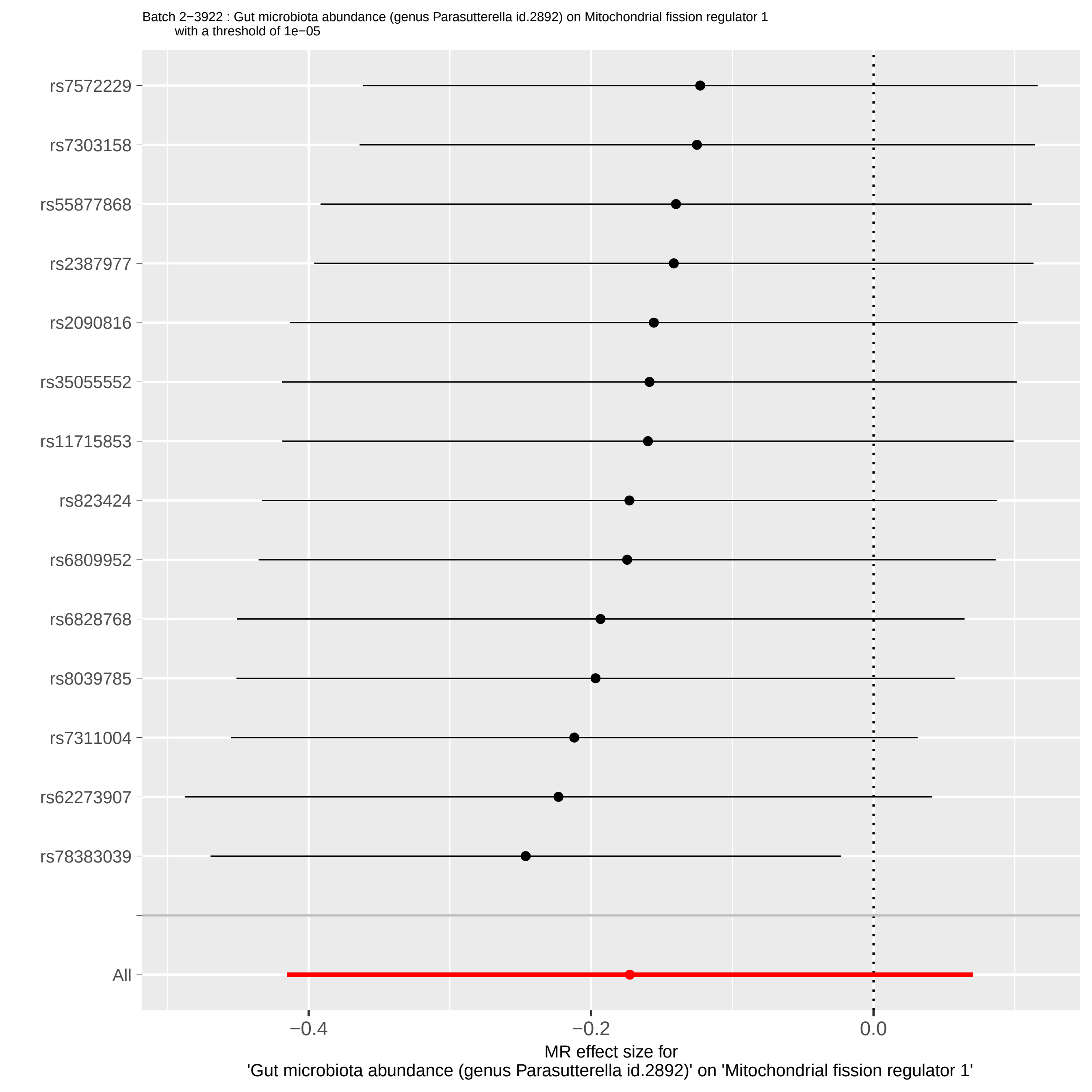

Batch 2−3922 : Gut microbiota abundance (genus Parasutterella id.2892) on Mitochondrial fission regulator 1
with a threshold of 1e−05
rs7572229
rs7303158
rs55877868
rs2387977
rs2090816
rs35055552
rs11715853
rs823424
rs6809952
rs6828768
rs8039785
rs7311004
rs62273907
rs78383039
All
−0.4
−0.2
0.0
MR effect size for
'Gut microbiota abundance (genus Parasutterella id.2892)' on 'Mitochondrial fission regulator 1'

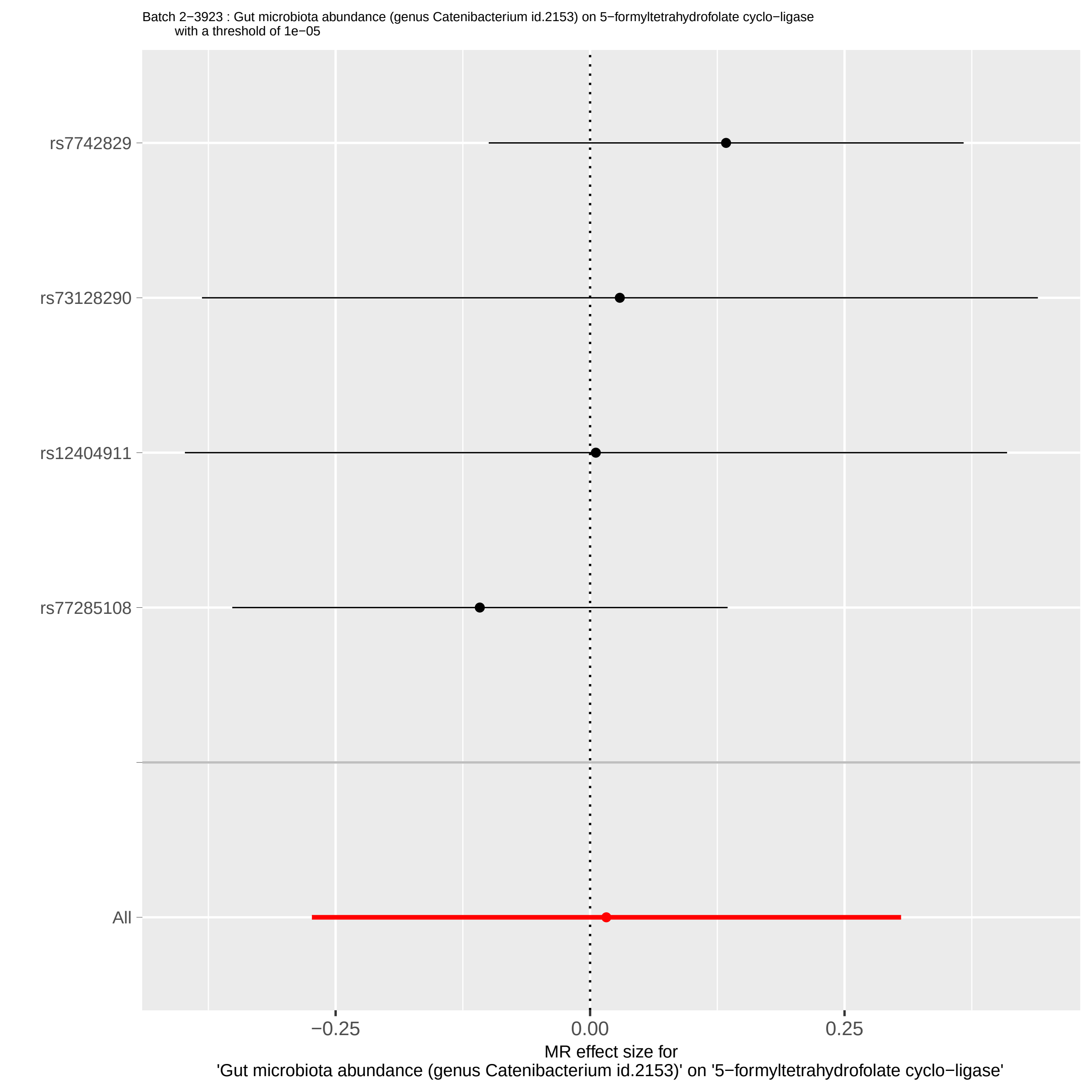
Batch 2−3923 : Gut microbiota abundance (genus Catenibacterium id.2153) on 5−formyltetrahydrofolate cyclo−ligase
with a threshold of 1e−05
rs7742829
rs73128290
rs12404911
rs77285108
All
−0.25
0.00
0.25
MR effect size for
'Gut microbiota abundance (genus Catenibacterium id.2153)' on '5−formyltetrahydrofolate cyclo−ligase'

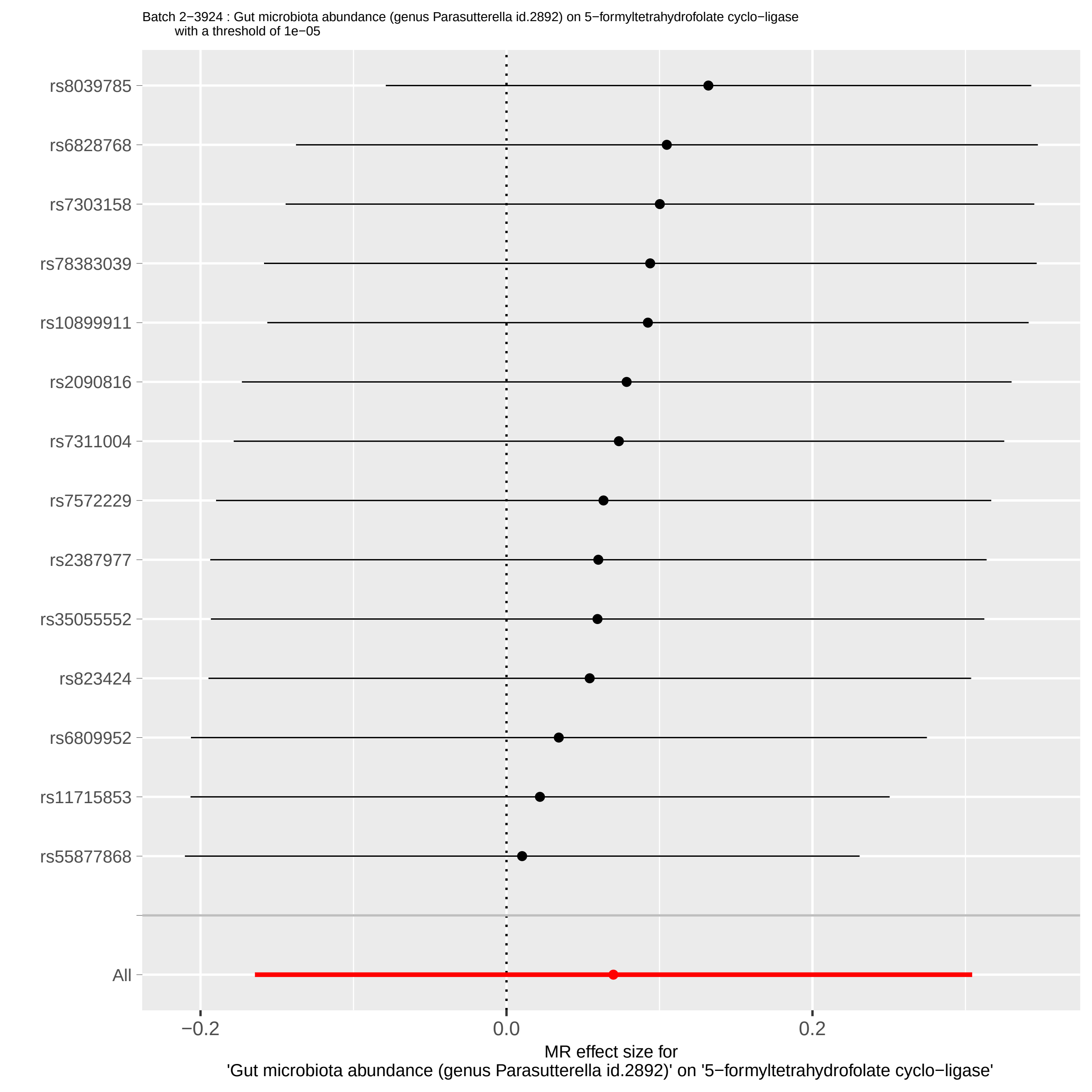
Batch 2−3924 : Gut microbiota abundance (genus Parasutterella id.2892) on 5−formyltetrahydrofolate cyclo−ligase
with a threshold of 1e−05
rs8039785
rs6828768
rs7303158
rs78383039
rs10899911
rs2090816
rs7311004
rs7572229
rs2387977
rs35055552
rs823424
rs6809952
rs11715853
rs55877868
All
−0.2
0.0
0.2
MR effect size for
'Gut microbiota abundance (genus Parasutterella id.2892)' on '5−formyltetrahydrofolate cyclo−ligase'

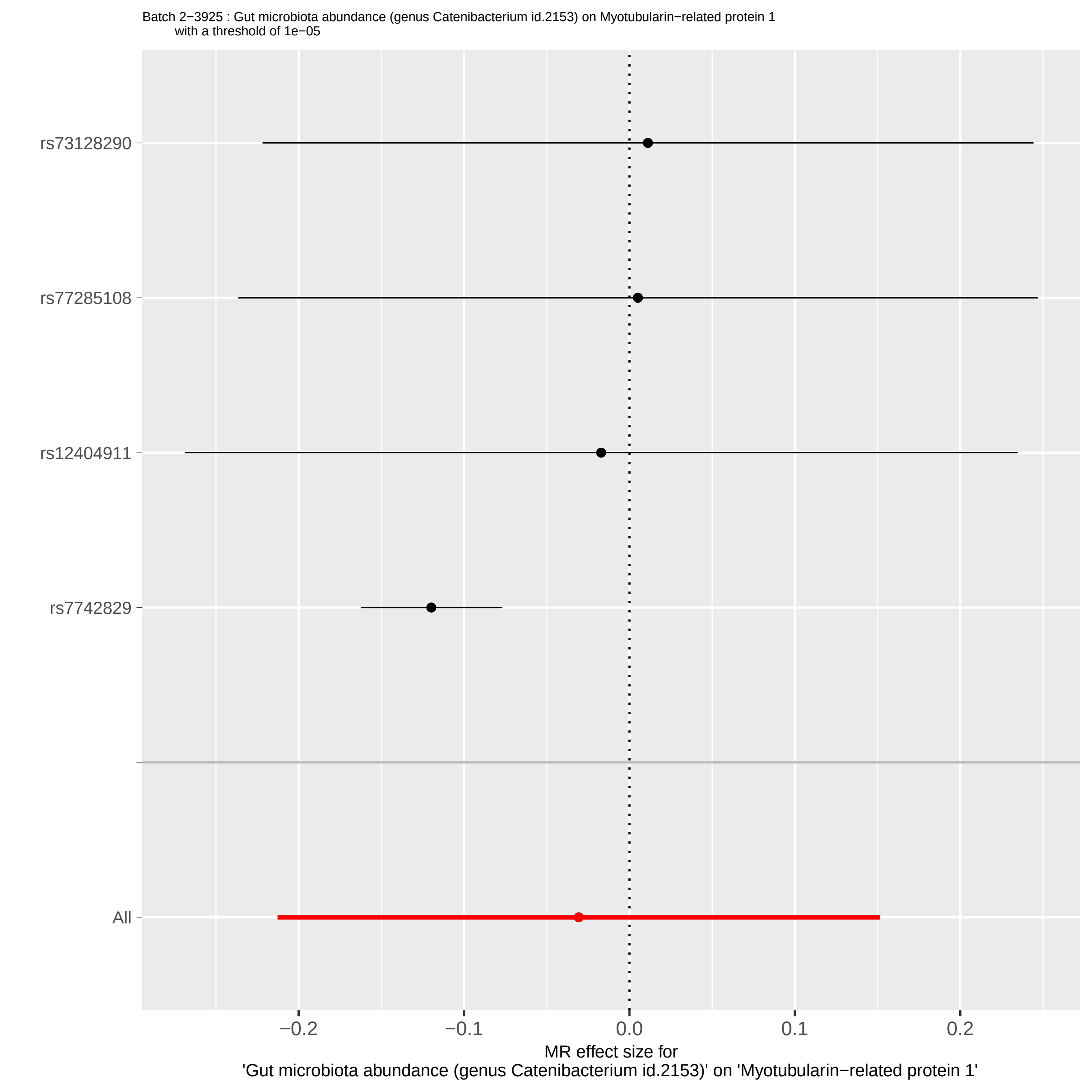

Batch 2−3925 : Gut microbiota abundance (genus Catenibacterium id.2153) on Myotubularin−related protein 1
with a threshold of 1e−05
rs73128290
rs77285108
rs12404911
rs7742829
All
−0.2
−0.1
0.0
0.1
0.2
MR effect size for
'Gut microbiota abundance (genus Catenibacterium id.2153)' on 'Myotubularin−related protein 1'

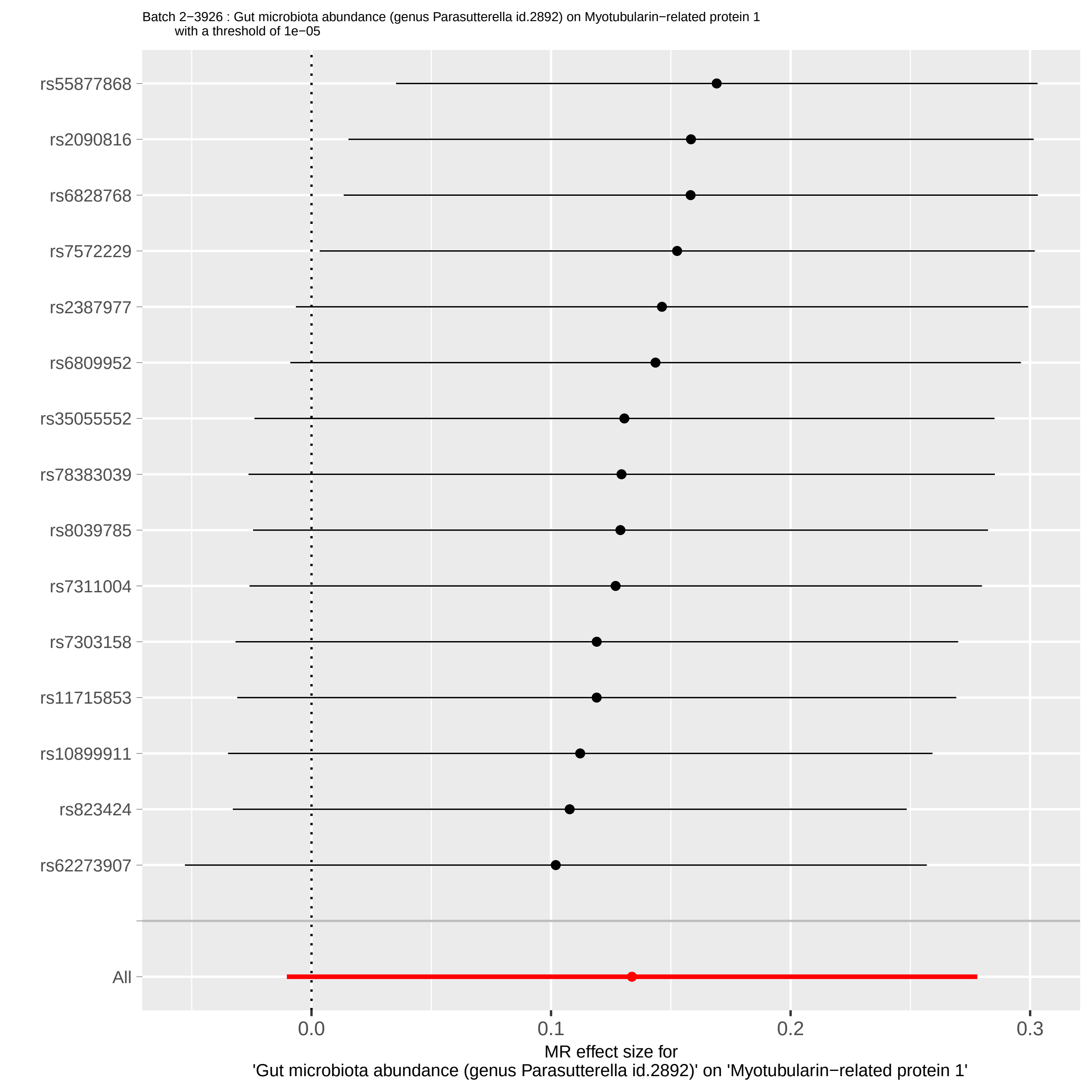
Batch 2−3926 : Gut microbiota abundance (genus Parasutterella id.2892) on Myotubularin−related protein 1
with a threshold of 1e−05
rs55877868
rs2090816
rs6828768
rs7572229
rs2387977
rs6809952
rs35055552
rs78383039
rs8039785
rs7311004
rs7303158
rs11715853
rs10899911
rs823424
rs62273907
All
0.0
0.1
0.2
0.3
MR effect size for
'Gut microbiota abundance (genus Parasutterella id.2892)' on 'Myotubularin−related protein 1'

Batch 2−3927 : Gut microbiota abundance (genus Catenibacterium id.2153) on Poly(A) RNA polymerase, mitochondrial
with a threshold of 1e−05

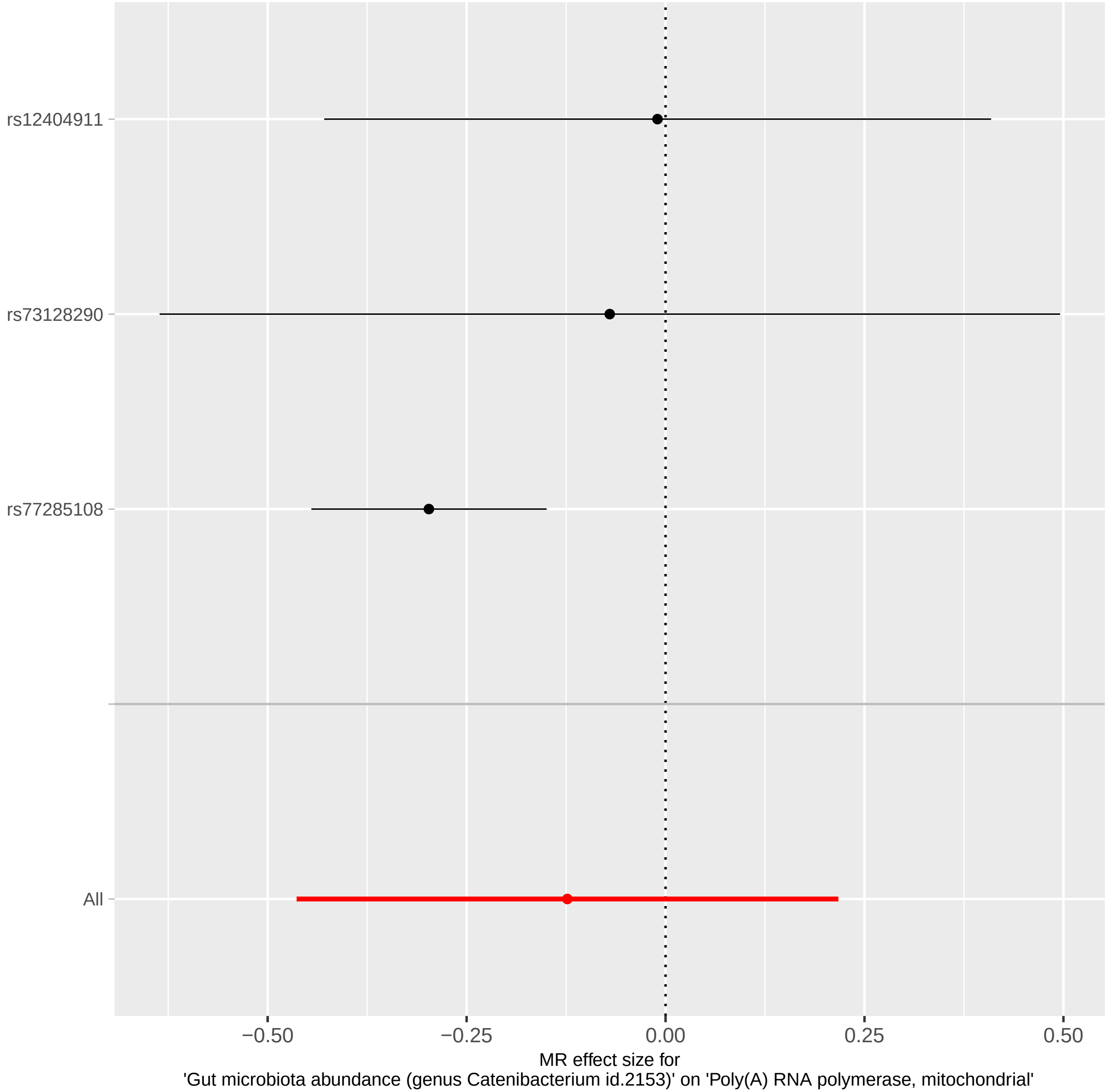

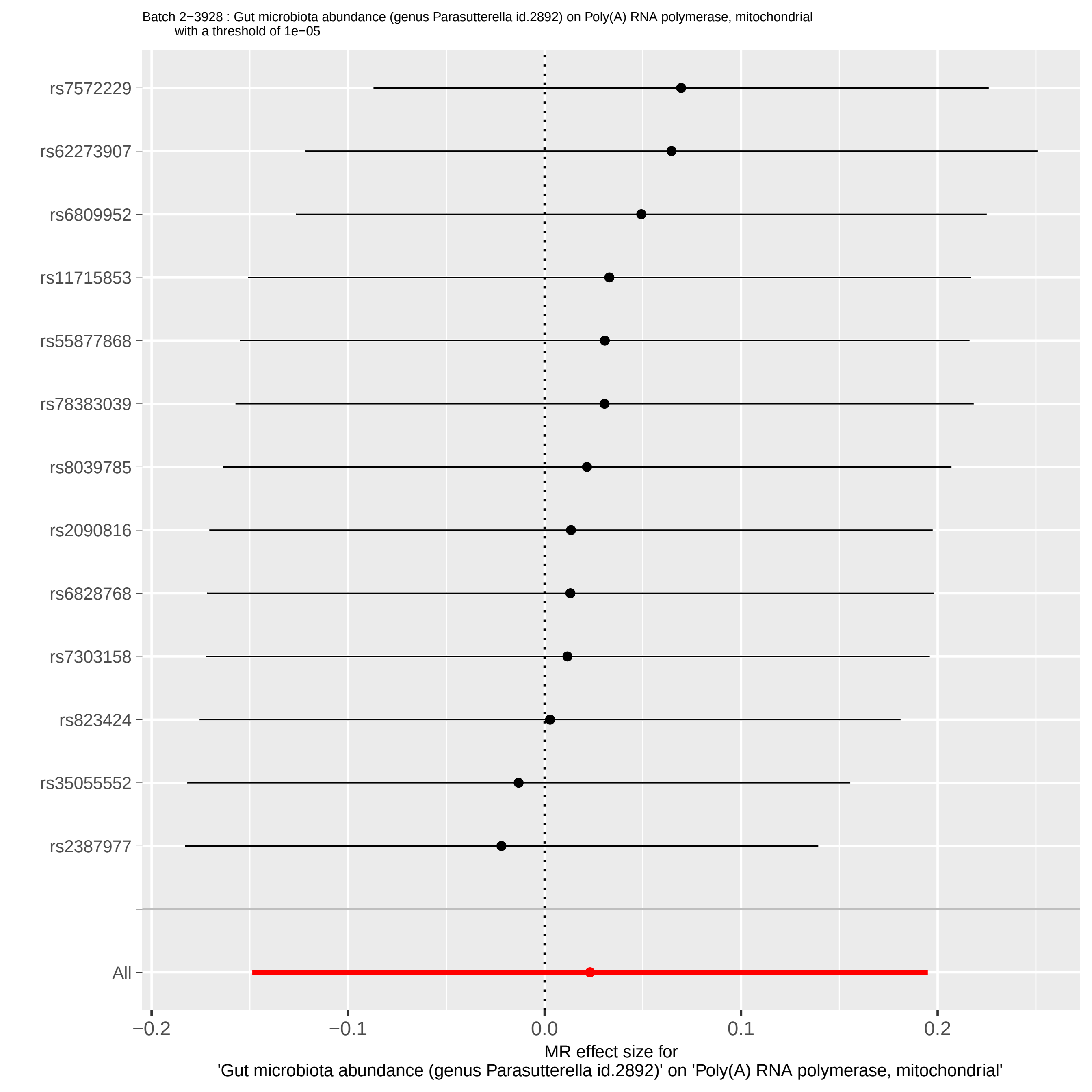

Batch 2−3928 : Gut microbiota abundance (genus Parasutterella id.2892) on Poly(A) RNA polymerase, mitochondrial
with a threshold of 1e−05
rs7572229
rs62273907
rs6809952
rs11715853
rs55877868
rs78383039
rs8039785
rs2090816
rs6828768
rs7303158
rs823424
rs35055552
rs2387977
All
−0.2
−0.1
0.0
0.1
0.2
MR effect size for
'Gut microbiota abundance (genus Parasutterella id.2892)' on 'Poly(A) RNA polymerase, mitochondrial'

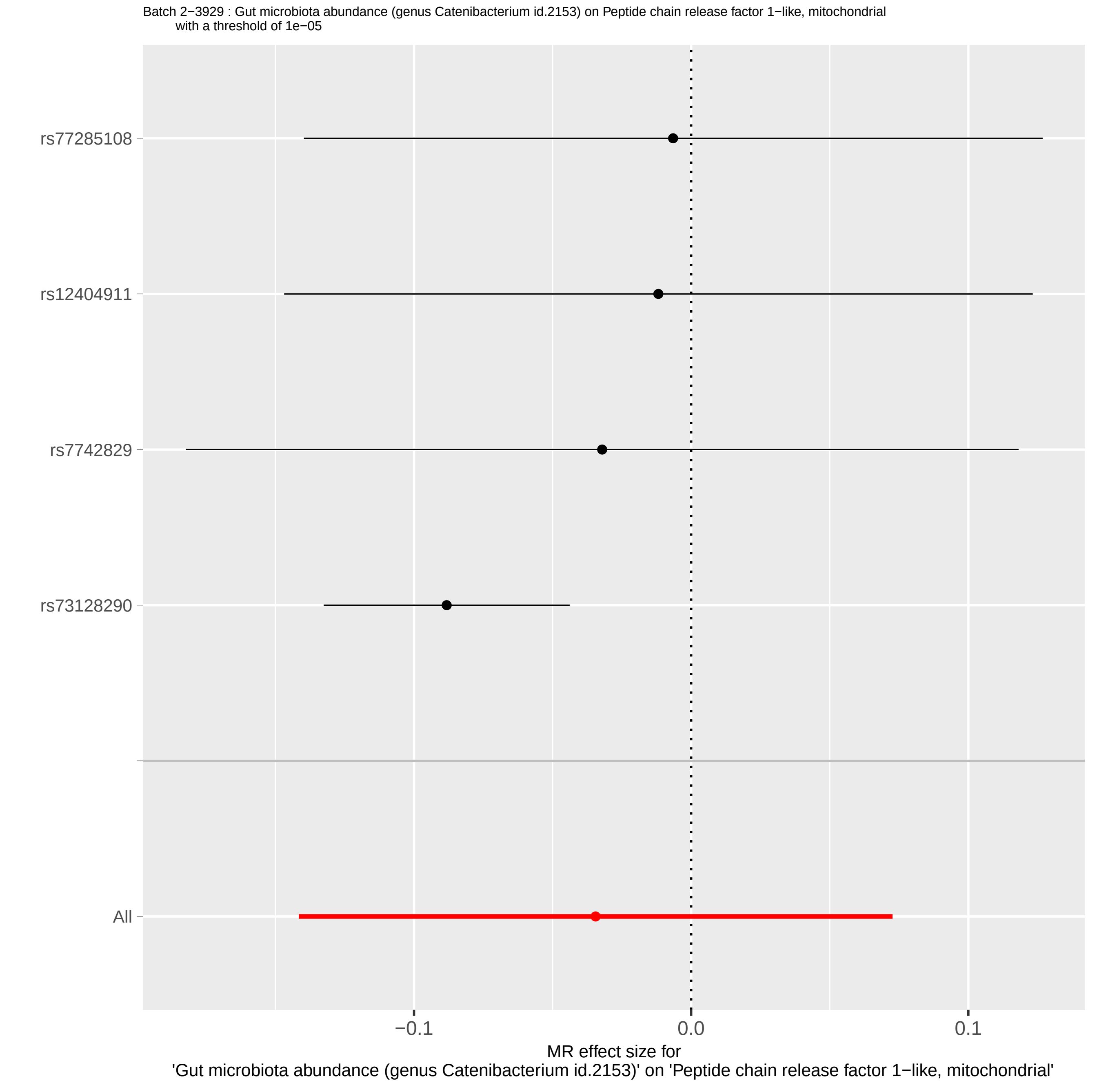
Batch 2−3929 : Gut microbiota abundance (genus Catenibacterium id.2153) on Peptide chain release factor 1−like, mitochondrial
with a threshold of 1e−05
rs77285108
rs12404911
rs7742829
rs73128290
All
−0.1
0.0
0.1
MR effect size for
'Gut microbiota abundance (genus Catenibacterium id.2153)' on 'Peptide chain release factor 1−like, mitochondrial'

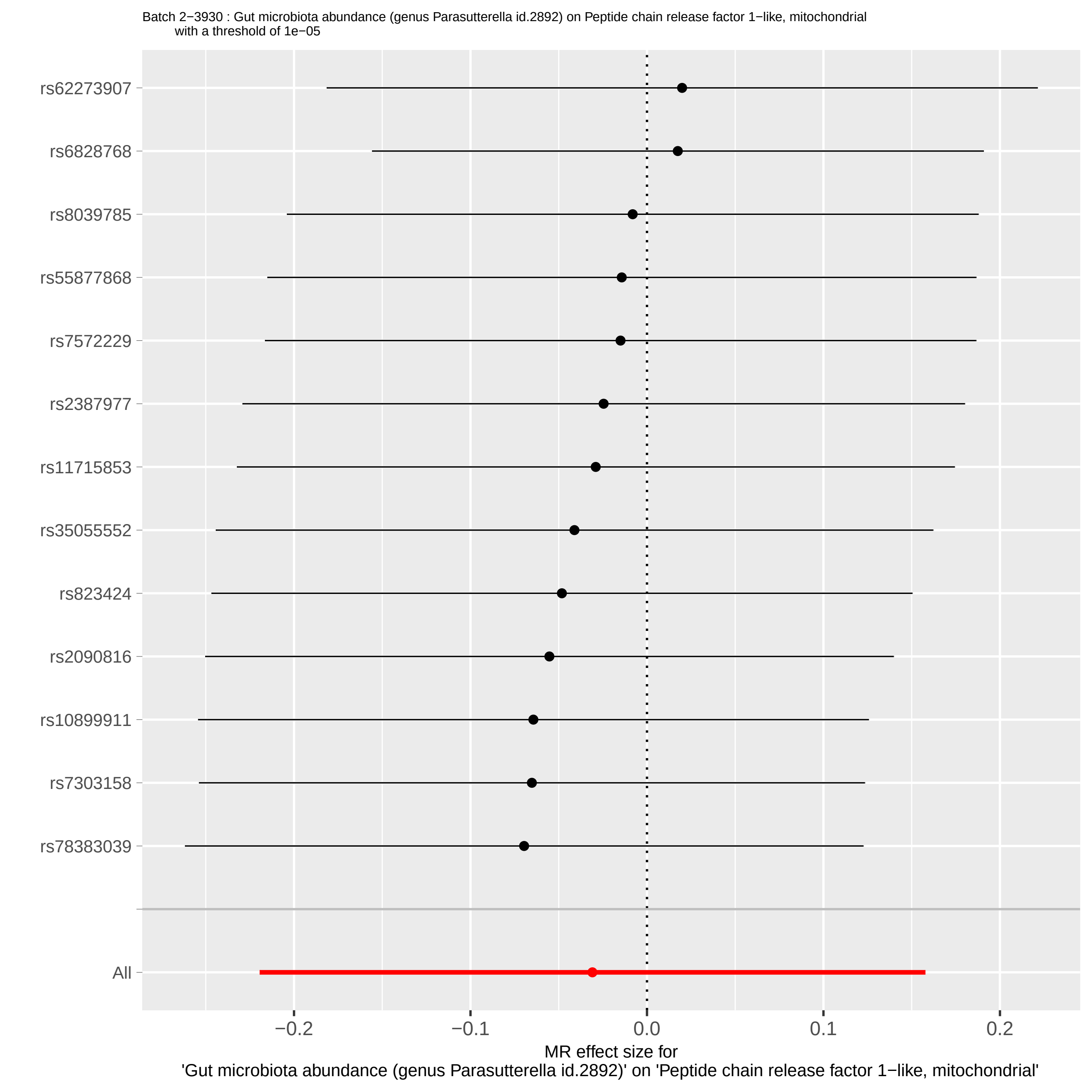

Batch 2−3930 : Gut microbiota abundance (genus Parasutterella id.2892) on Peptide chain release factor 1−like, mitochondrial
with a threshold of 1e−05
rs62273907
rs6828768
rs8039785
rs55877868
rs7572229
rs2387977
rs11715853
rs35055552
rs823424
rs2090816
rs10899911
rs7303158
rs78383039
All
−0.2
−0.1
0.0
0.1
0.2
MR effect size for
'Gut microbiota abundance (genus Parasutterella id.2892)' on 'Peptide chain release factor 1−like, mitochondrial'

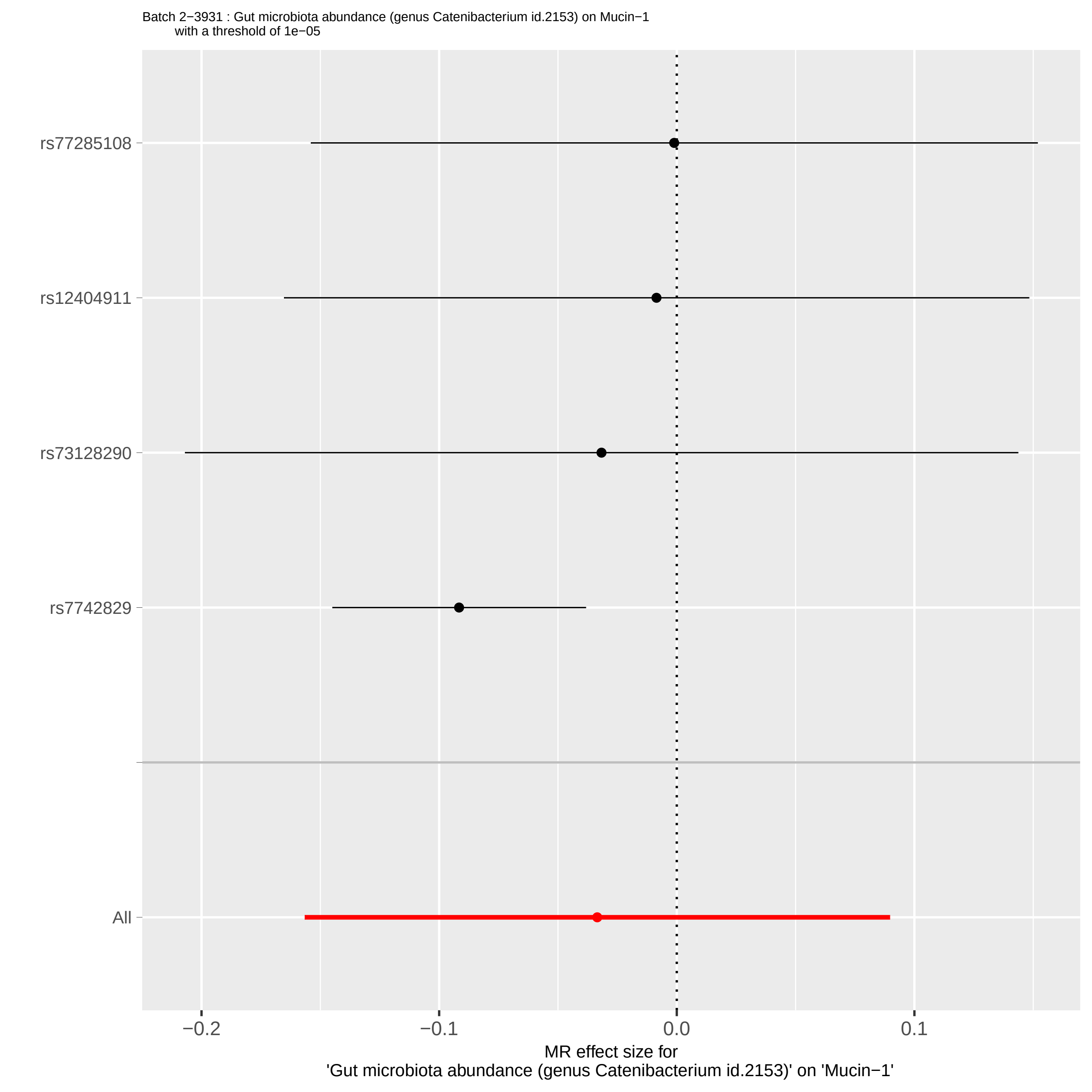
Batch 2−3931 : Gut microbiota abundance (genus Catenibacterium id.2153) on Mucin−1
with a threshold of 1e−05
rs77285108
rs12404911
rs73128290
rs7742829
All
−0.2
−0.1
0.0
0.1
MR effect size for
'Gut microbiota abundance (genus Catenibacterium id.2153)' on 'Mucin−1'

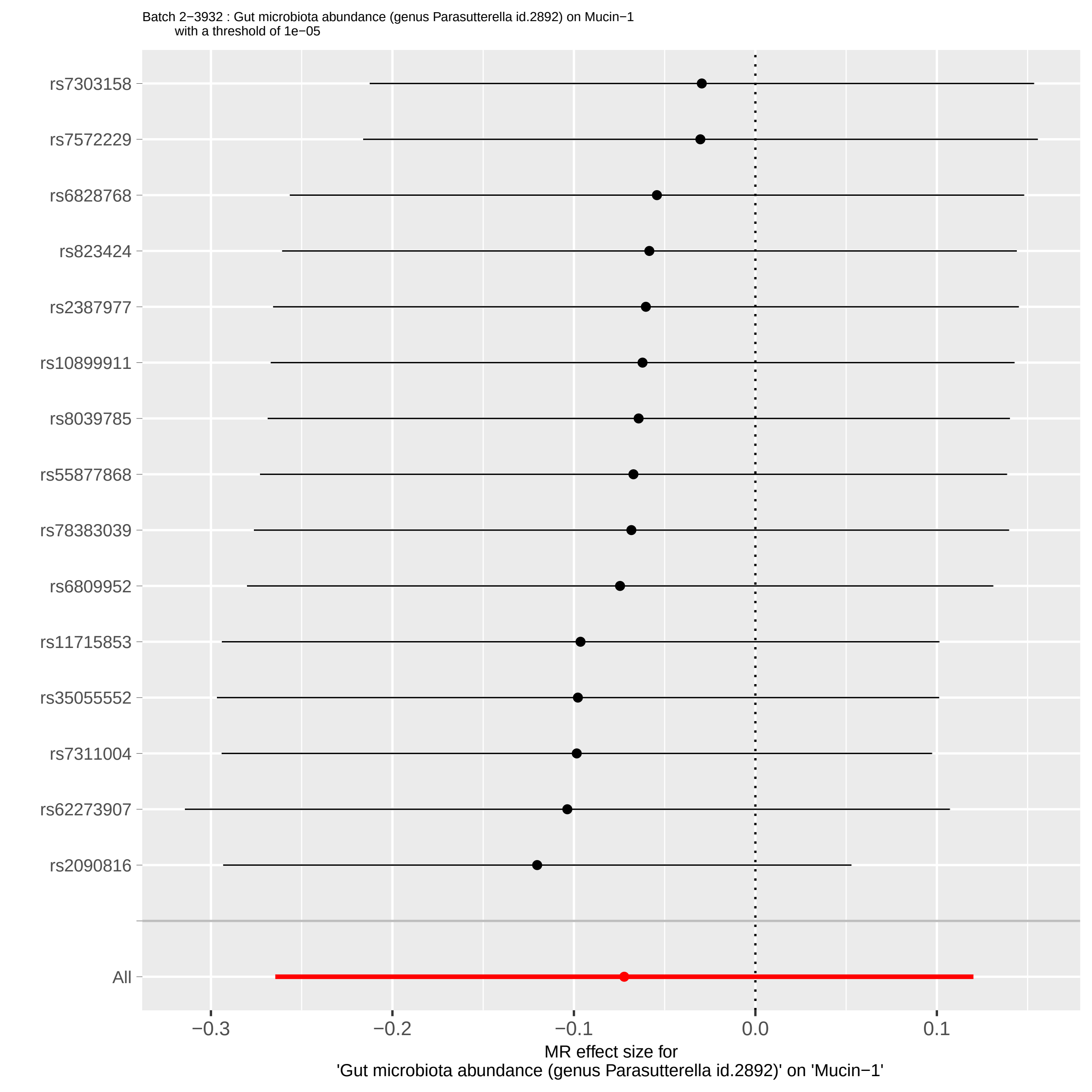

Batch 2−3932 : Gut microbiota abundance (genus Parasutterella id.2892) on Mucin−1
with a threshold of 1e−05
rs7303158
rs7572229
rs6828768
rs823424
rs2387977
rs10899911
rs8039785
rs55877868
rs78383039
rs6809952
rs11715853
rs35055552
rs7311004
rs62273907
rs2090816
All
−0.3
−0.2
−0.1
0.0
0.1
MR effect size for
'Gut microbiota abundance (genus Parasutterella id.2892)' on 'Mucin−1'

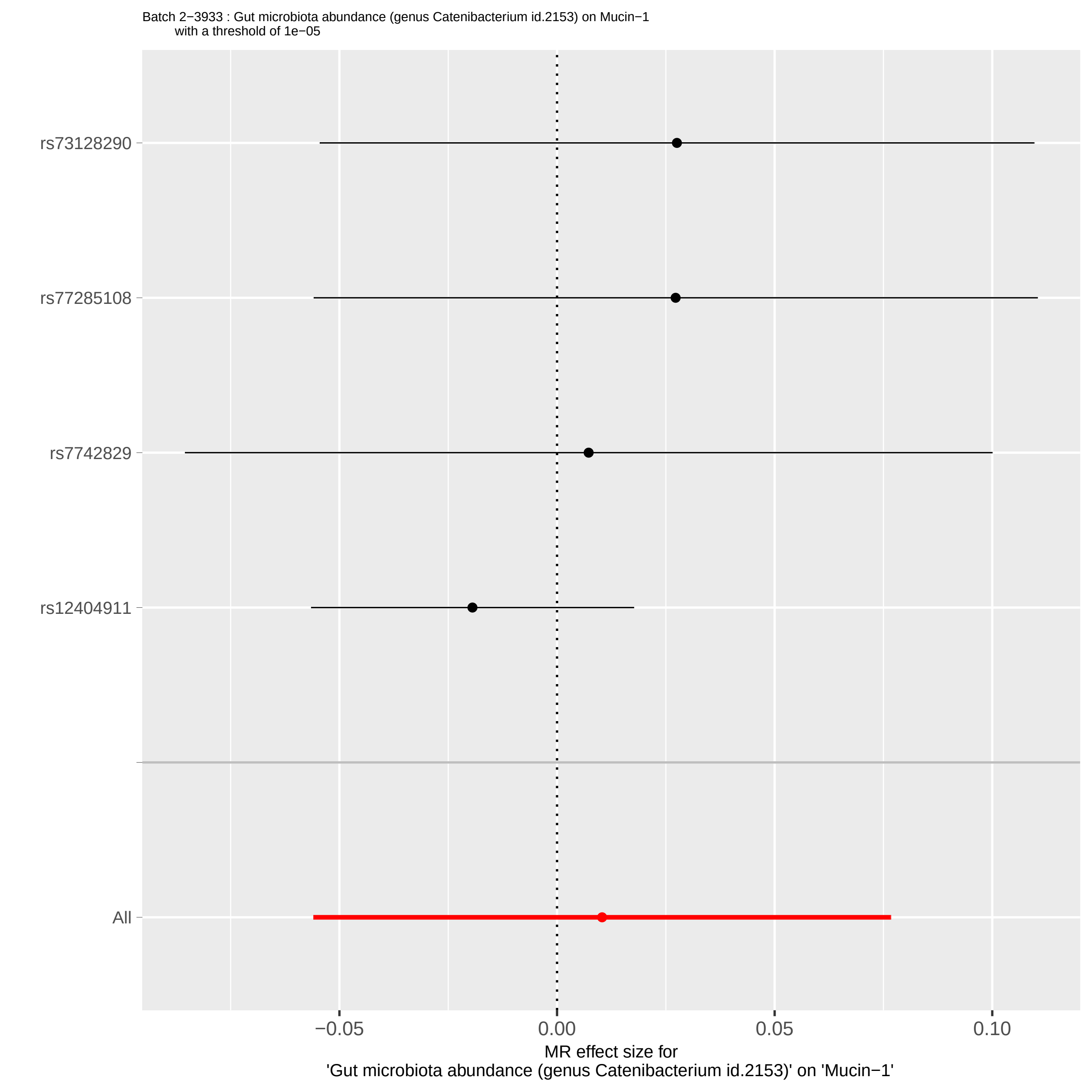
Batch 2−3933 : Gut microbiota abundance (genus Catenibacterium id.2153) on Mucin−1
with a threshold of 1e−05
rs73128290
rs77285108
rs7742829
rs12404911
All
−0.05
0.00
0.05
0.10
MR effect size for
'Gut microbiota abundance (genus Catenibacterium id.2153)' on 'Mucin−1'

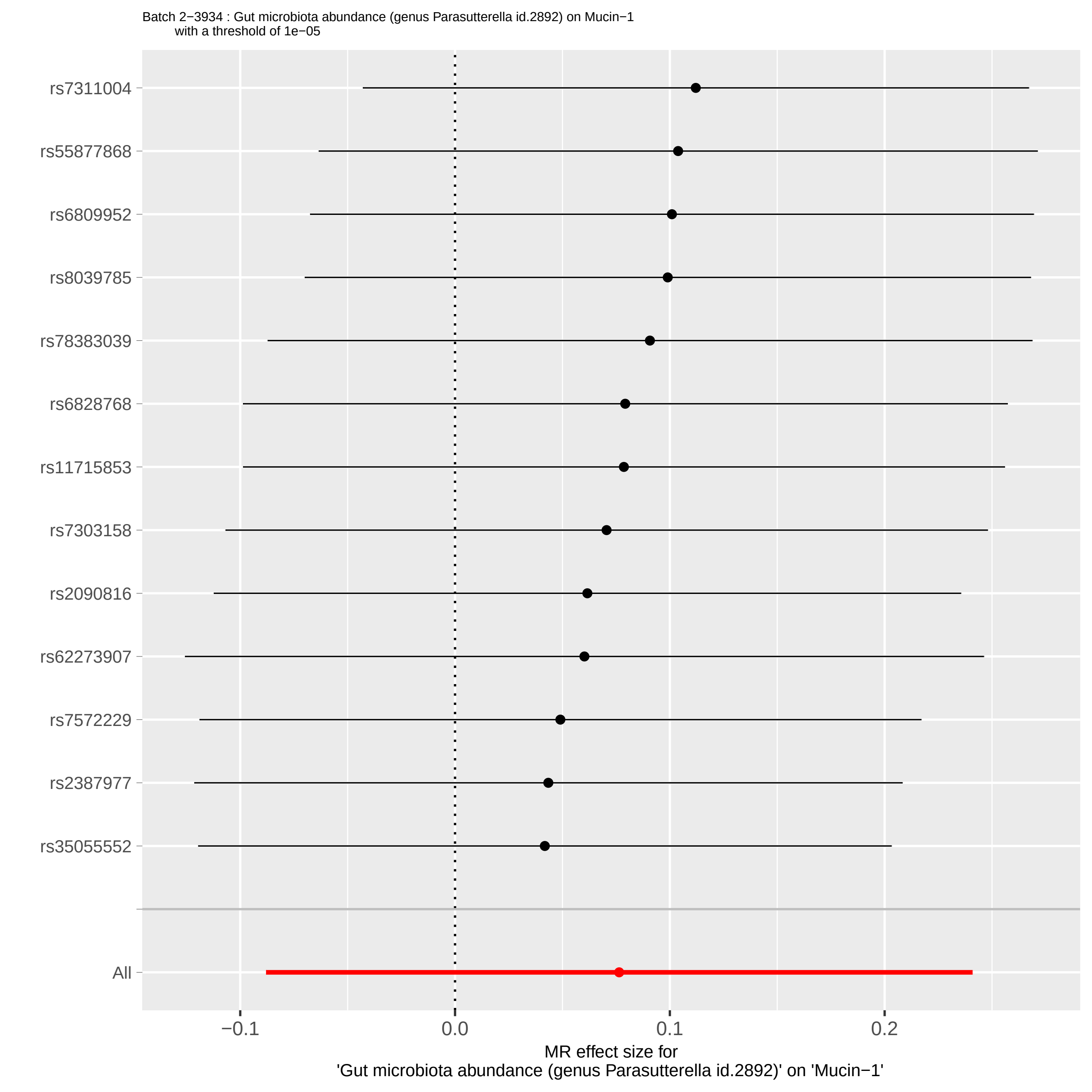
Batch 2−3934 : Gut microbiota abundance (genus Parasutterella id.2892) on Mucin−1
with a threshold of 1e−05
rs7311004
rs55877868
rs6809952
rs8039785
rs78383039
rs6828768
rs11715853
rs7303158
rs2090816
rs62273907
rs7572229
rs2387977
rs35055552
All
−0.1
0.0
0.1
0.2
MR effect size for
'Gut microbiota abundance (genus Parasutterella id.2892)' on 'Mucin−1'

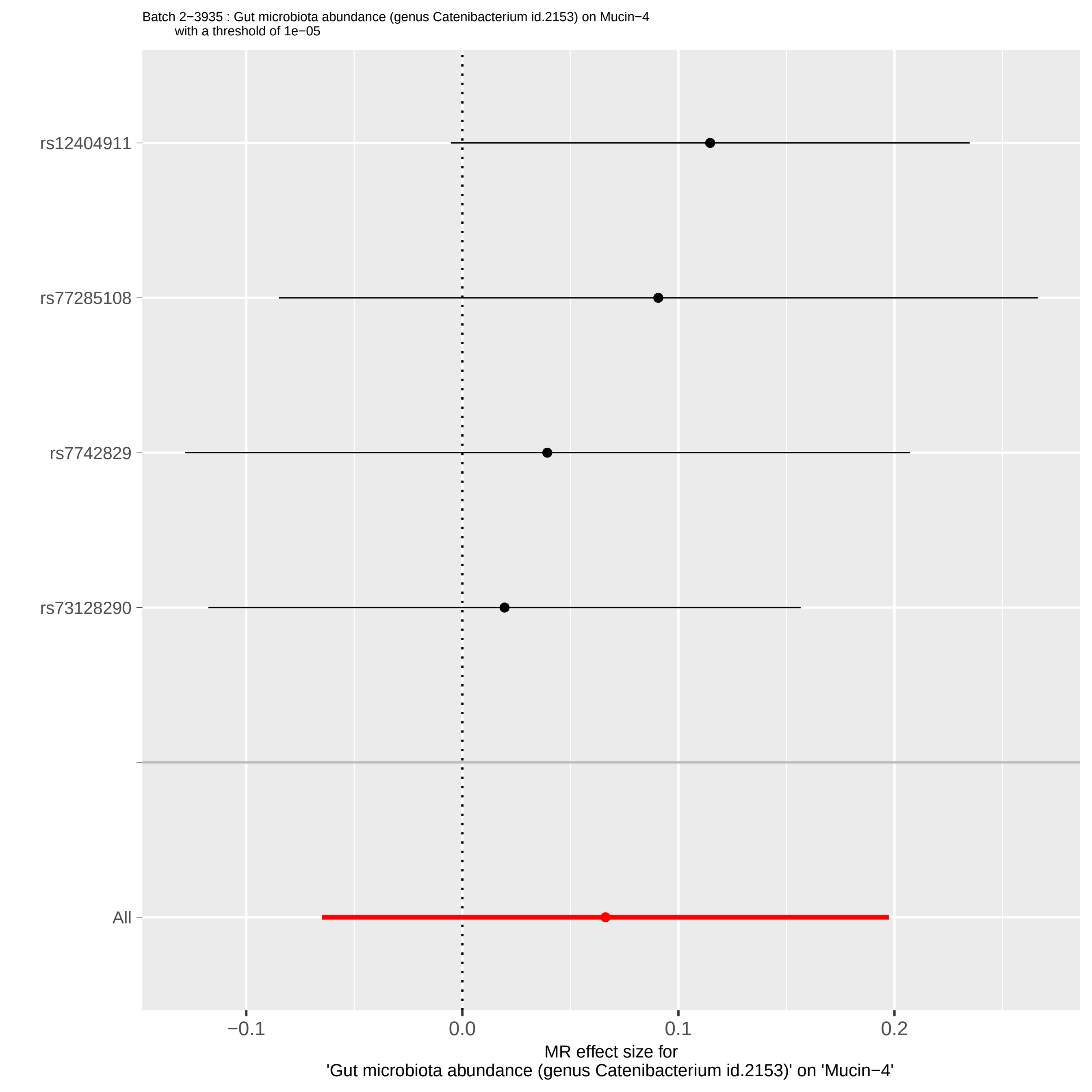
Batch 2−3935 : Gut microbiota abundance (genus Catenibacterium id.2153) on Mucin−4
with a threshold of 1e−05
rs12404911
rs77285108
rs7742829
rs73128290
All
−0.1
0.0
0.1
0.2
MR effect size for
'Gut microbiota abundance (genus Catenibacterium id.2153)' on 'Mucin−4'

Batch 2−3936 : Gut microbiota abundance (genus Parasutterella id.2892) on Mucin−4
with a threshold of 1e−05

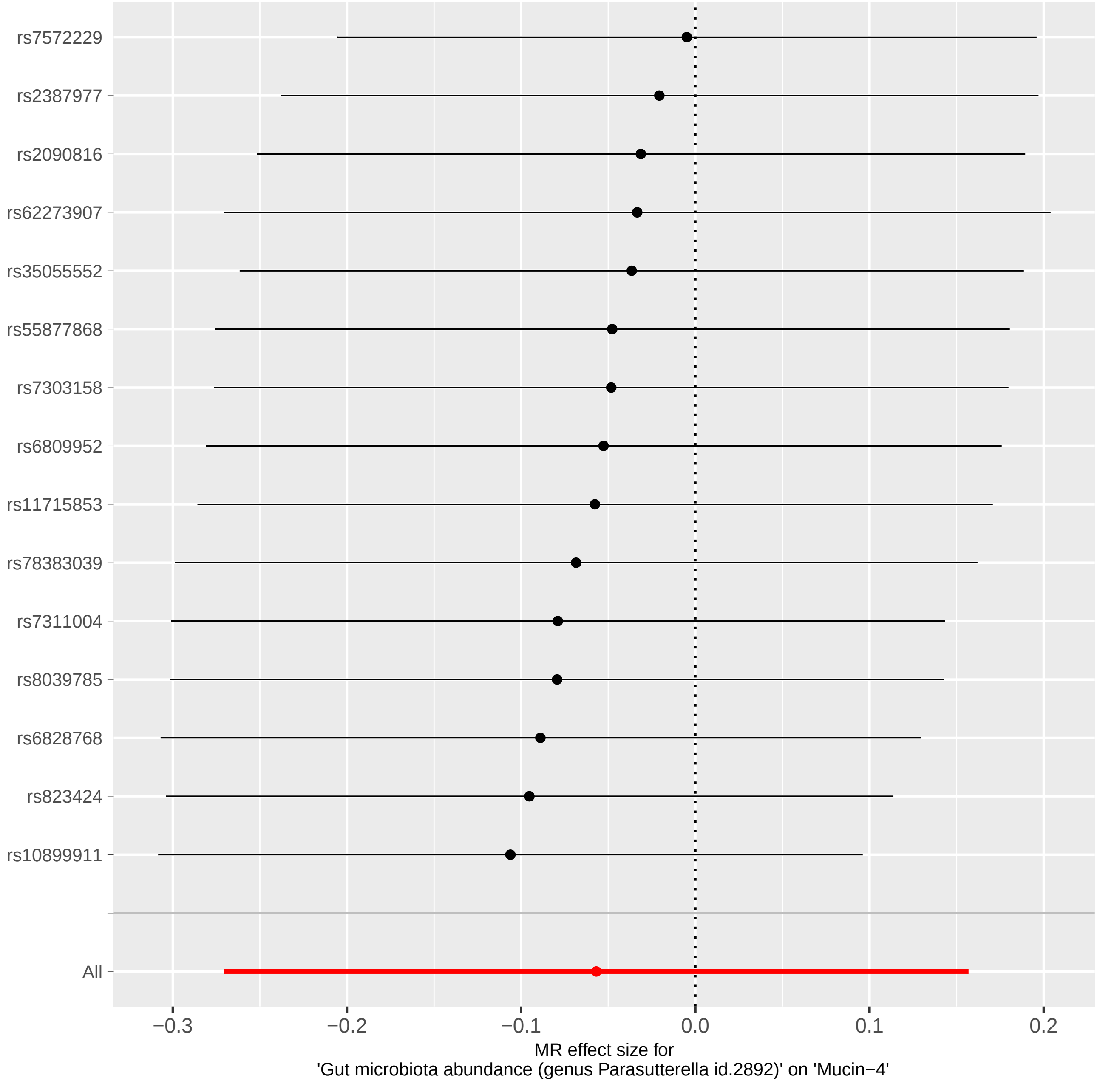

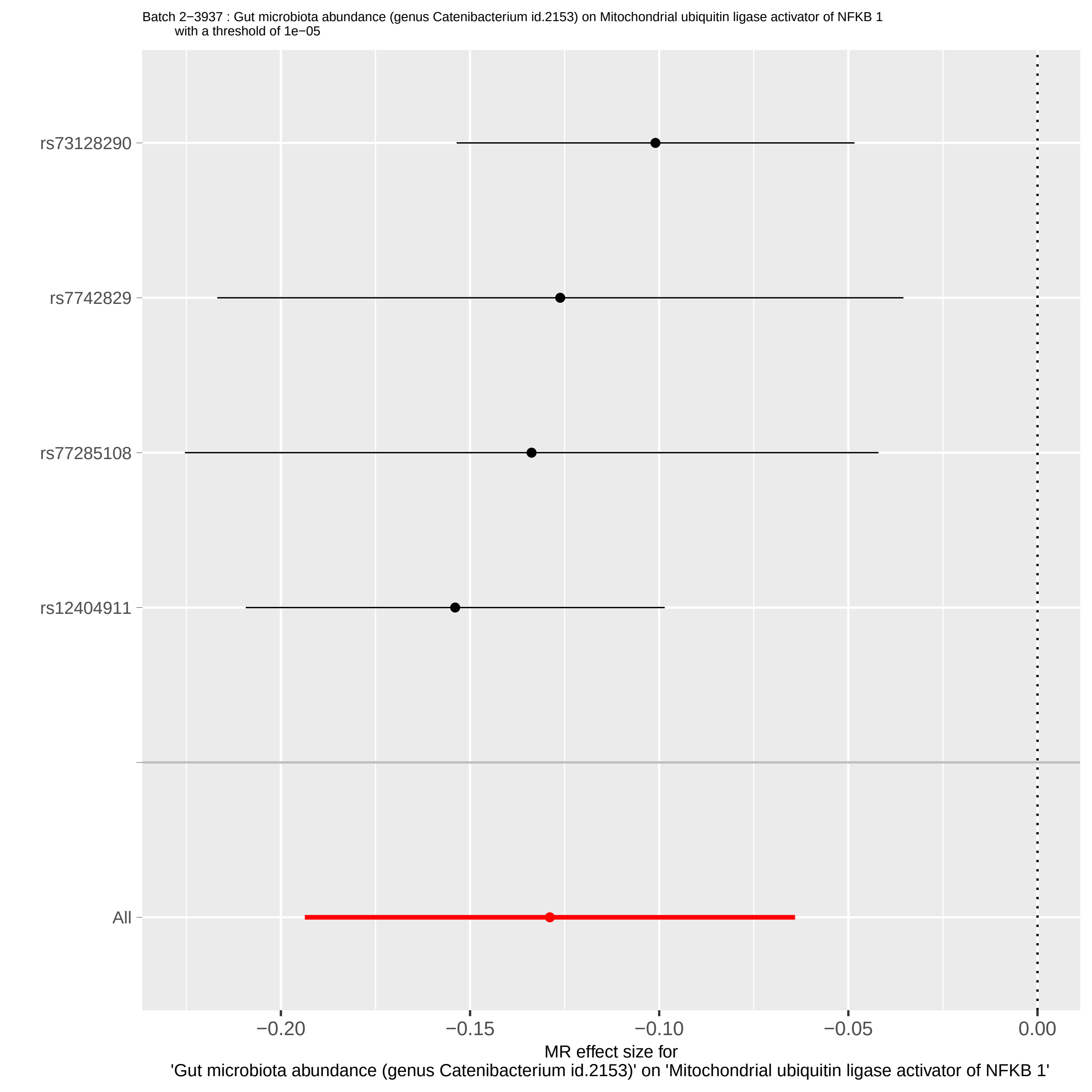

Batch 2−3937 : Gut microbiota abundance (genus Catenibacterium id.2153) on Mitochondrial ubiquitin ligase activator of NFKB 1
with a threshold of 1e−05
rs73128290
rs7742829
rs77285108
rs12404911
All
−0.20
−0.15
−0.10
−0.05
0.00
MR effect size for
'Gut microbiota abundance (genus Catenibacterium id.2153)' on 'Mitochondrial ubiquitin ligase activator of NFKB 1'

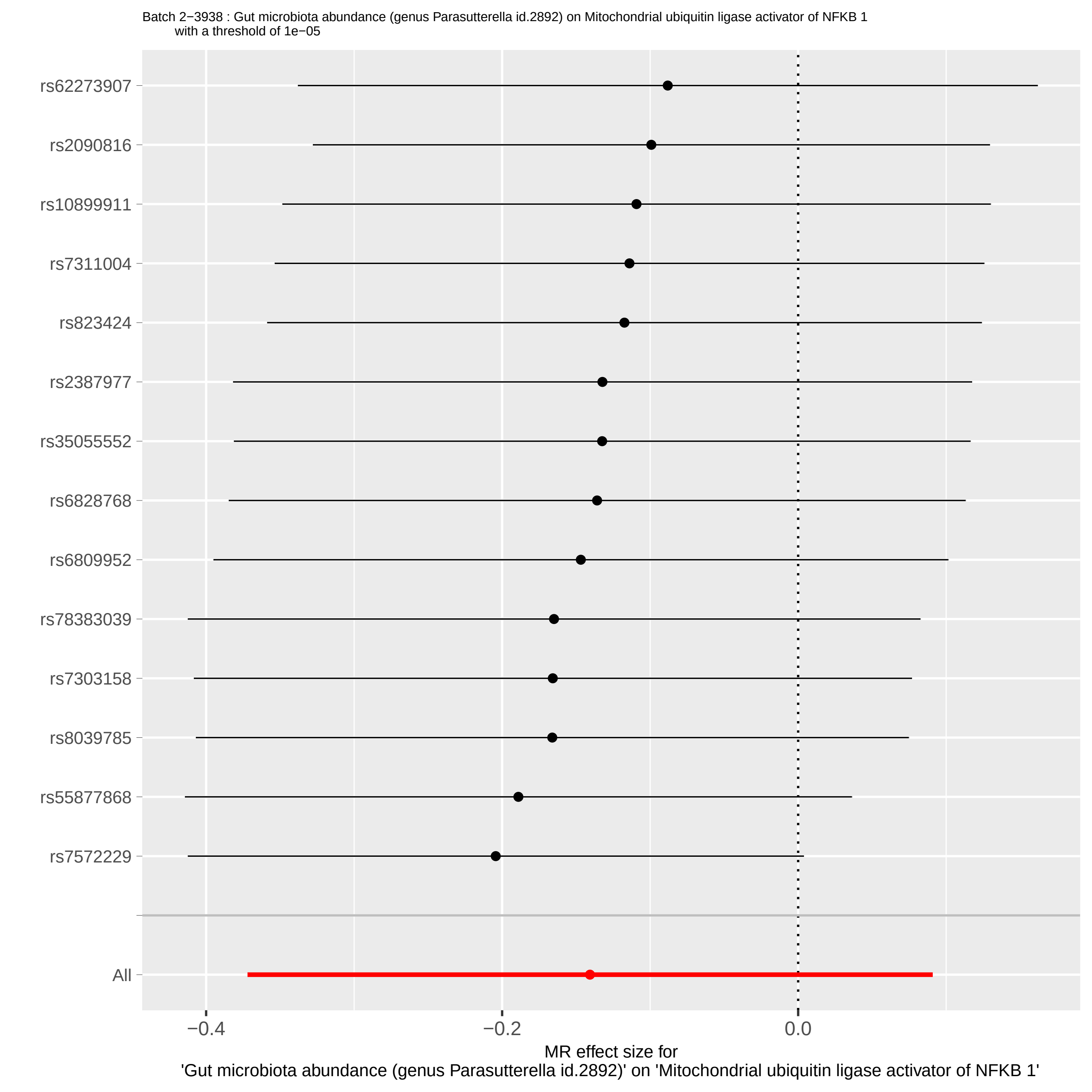
Batch 2−3938 : Gut microbiota abundance (genus Parasutterella id.2892) on Mitochondrial ubiquitin ligase activator of NFKB 1
with a threshold of 1e−05
rs62273907
rs2090816
rs10899911
rs7311004
rs823424
rs2387977
rs35055552
rs6828768
rs6809952
rs78383039
rs7303158
rs8039785
rs55877868
rs7572229
All
−0.4
−0.2
0.0
MR effect size for
'Gut microbiota abundance (genus Parasutterella id.2892)' on 'Mitochondrial ubiquitin ligase activator of NFKB 1'

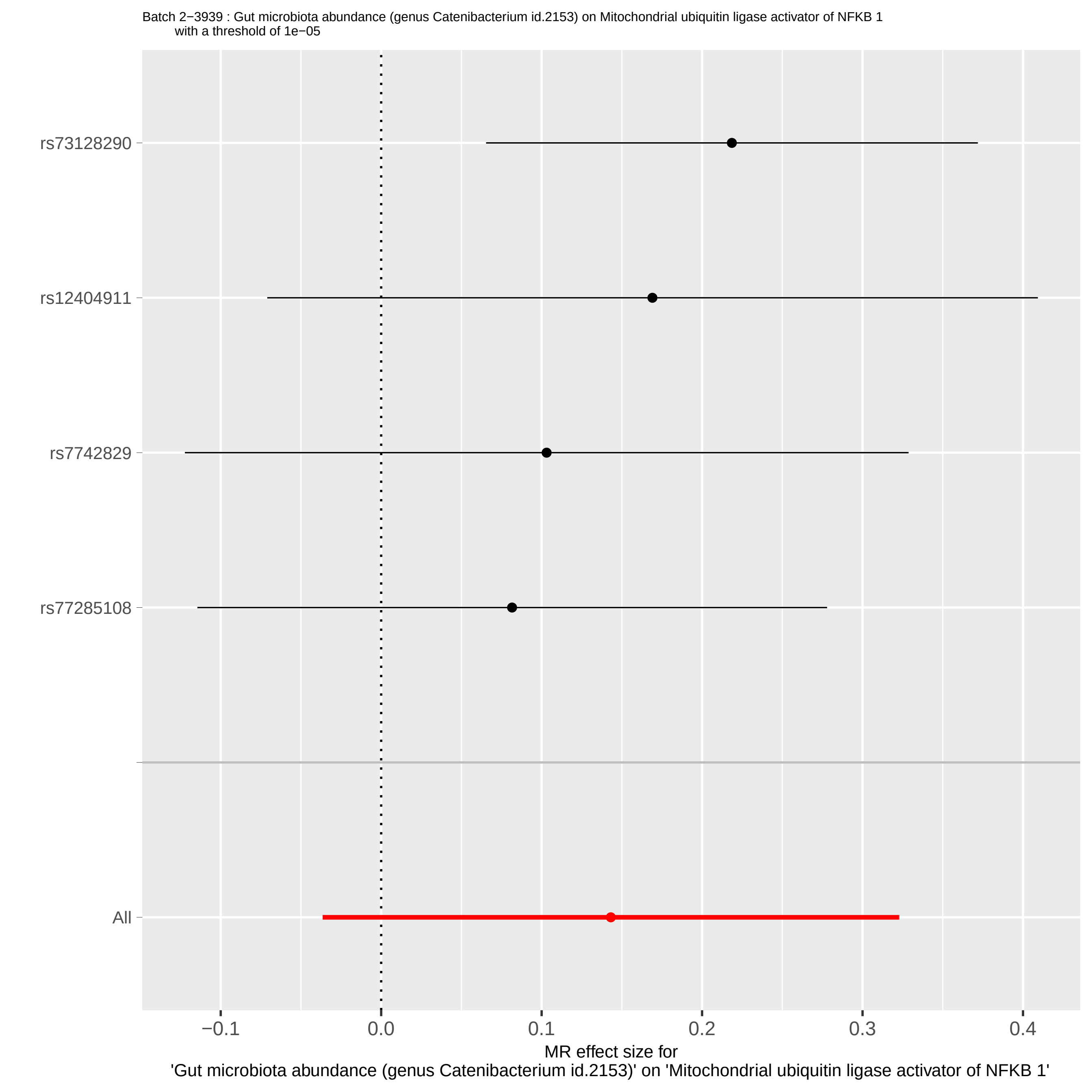
Batch 2−3939 : Gut microbiota abundance (genus Catenibacterium id.2153) on Mitochondrial ubiquitin ligase activator of NFKB 1
with a threshold of 1e−05
rs73128290
rs12404911
rs7742829
rs77285108
All
−0.1
0.0
0.1
0.2
0.3
0.4
MR effect size for
'Gut microbiota abundance (genus Catenibacterium id.2153)' on 'Mitochondrial ubiquitin ligase activator of NFKB 1'

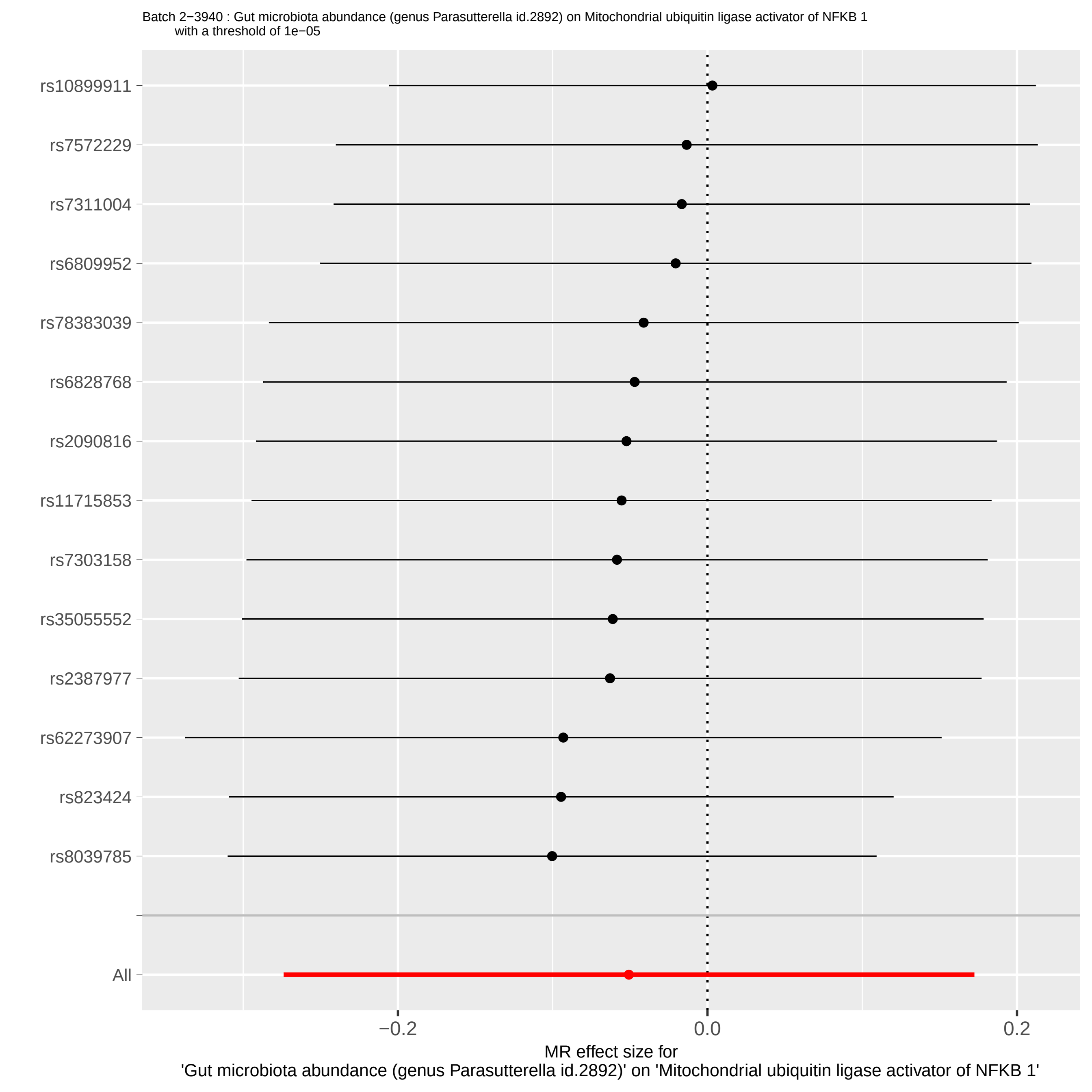

Batch 2−3940 : Gut microbiota abundance (genus Parasutterella id.2892) on Mitochondrial ubiquitin ligase activator of NFKB 1
with a threshold of 1e−05
rs10899911
rs7572229
rs7311004
rs6809952
rs78383039
rs6828768
rs2090816
rs11715853
rs7303158
rs35055552
rs2387977
rs62273907
rs823424
rs8039785
All
−0.2
0.0
0.2
MR effect size for
'Gut microbiota abundance (genus Parasutterella id.2892)' on 'Mitochondrial ubiquitin ligase activator of NFKB 1'

Batch 2−3941 : Gut microbiota abundance (genus Catenibacterium id.2153) on Muscle, skeletal receptor tyrosine−protein kinase
with a threshold of 1e−05

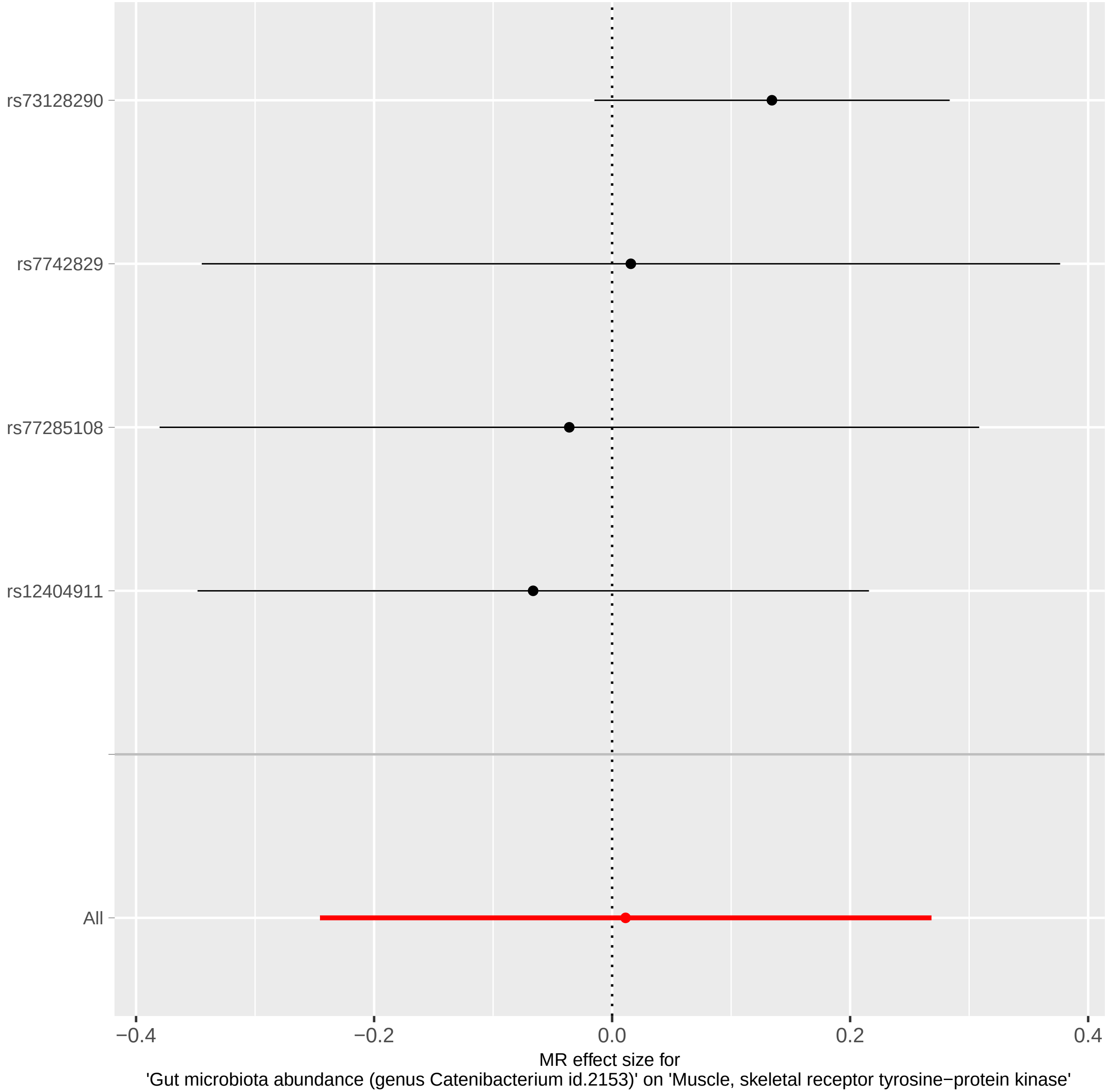

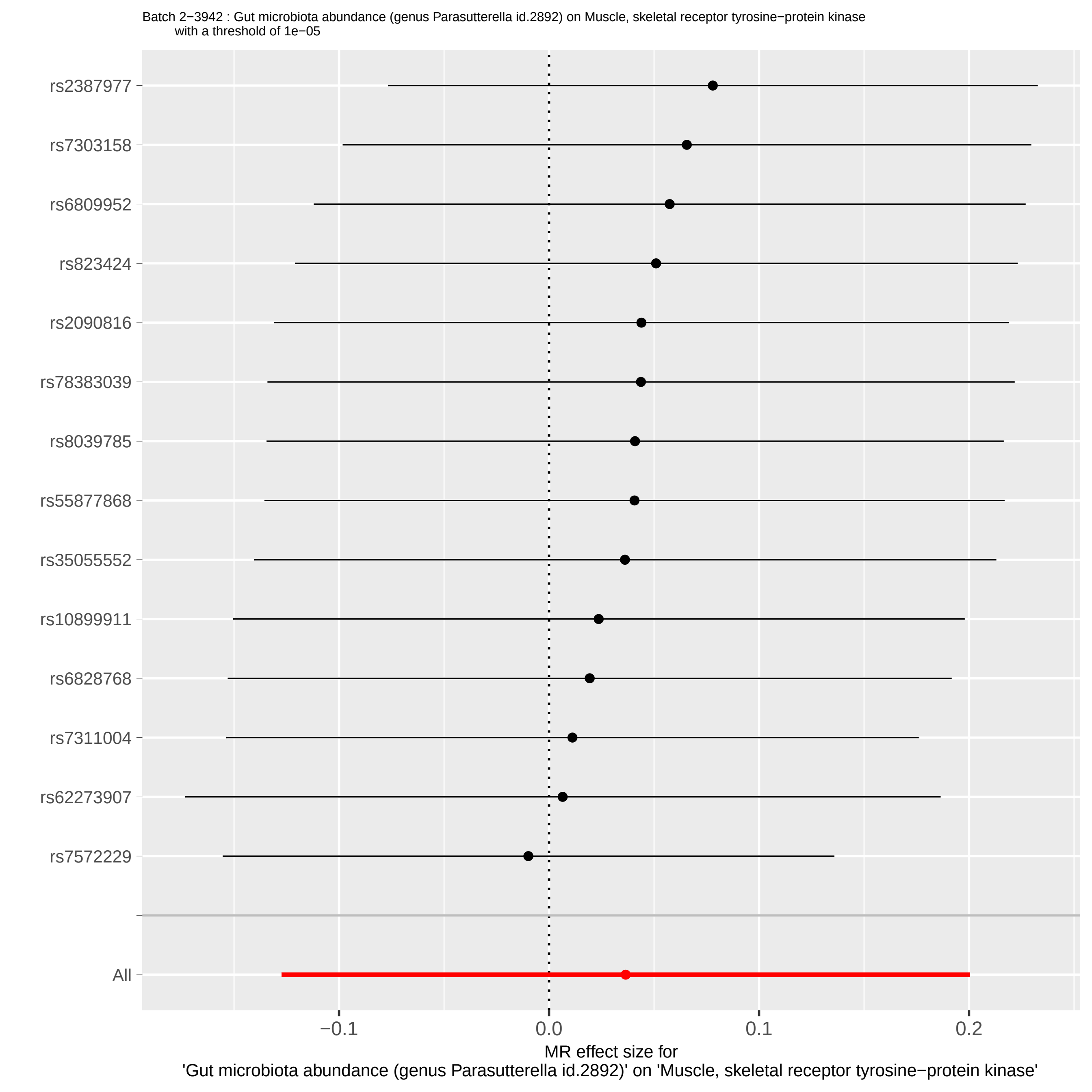

Batch 2−3942 : Gut microbiota abundance (genus Parasutterella id.2892) on Muscle, skeletal receptor tyrosine−protein kinase
with a threshold of 1e−05
rs2387977
rs7303158
rs6809952
rs823424
rs2090816
rs78383039
rs8039785
rs55877868
rs35055552
rs10899911
rs6828768
rs7311004
rs62273907
rs7572229
All
−0.1
0.0
0.1
0.2
MR effect size for
'Gut microbiota abundance (genus Parasutterella id.2892)' on 'Muscle, skeletal receptor tyrosine−protein kinase'

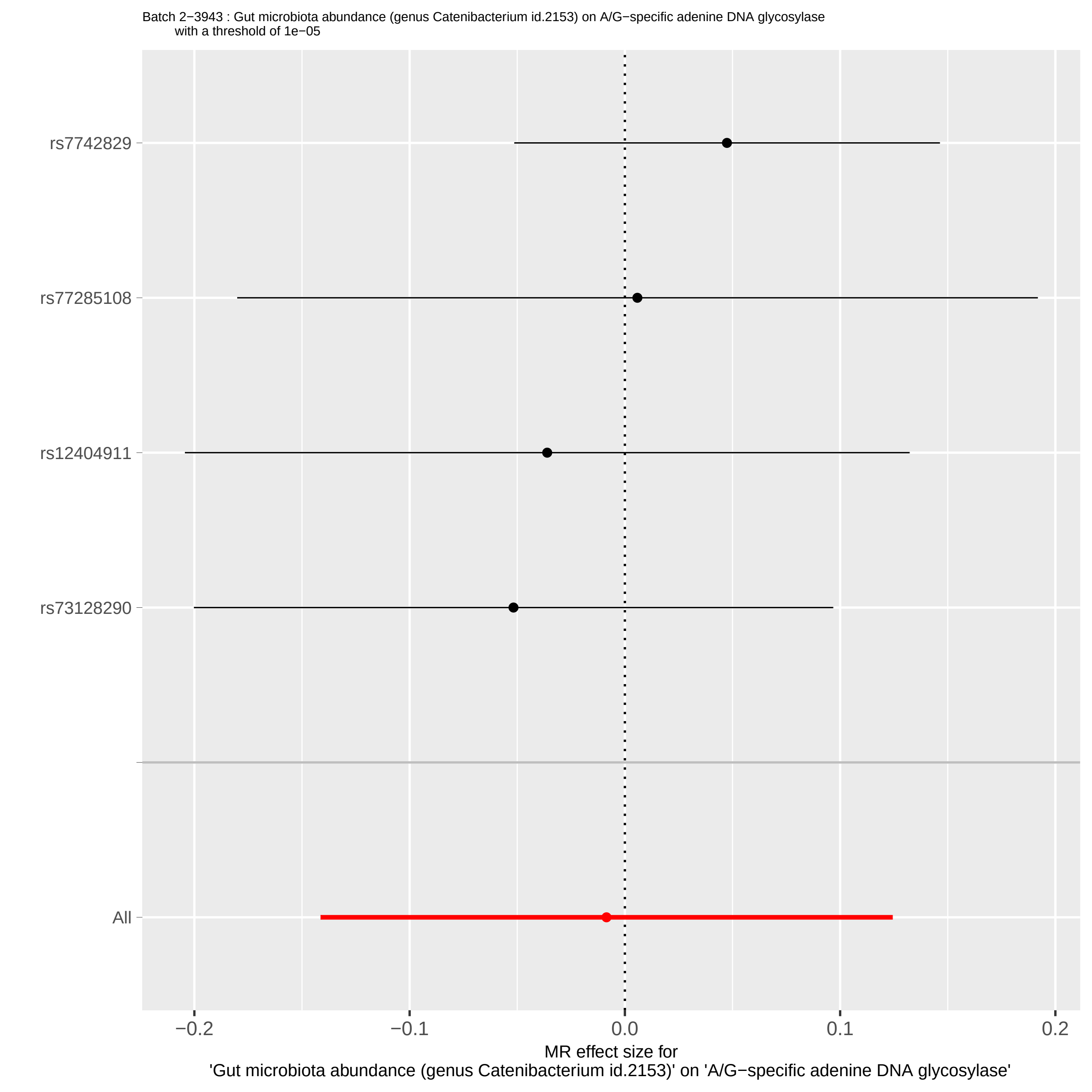
Batch 2−3943 : Gut microbiota abundance (genus Catenibacterium id.2153) on A/G−specific adenine DNA glycosylase
with a threshold of 1e−05
rs7742829
rs77285108
rs12404911
rs73128290
All
−0.2
−0.1
0.0
0.1
0.2
MR effect size for
'Gut microbiota abundance (genus Catenibacterium id.2153)' on 'A/G−specific adenine DNA glycosylase'

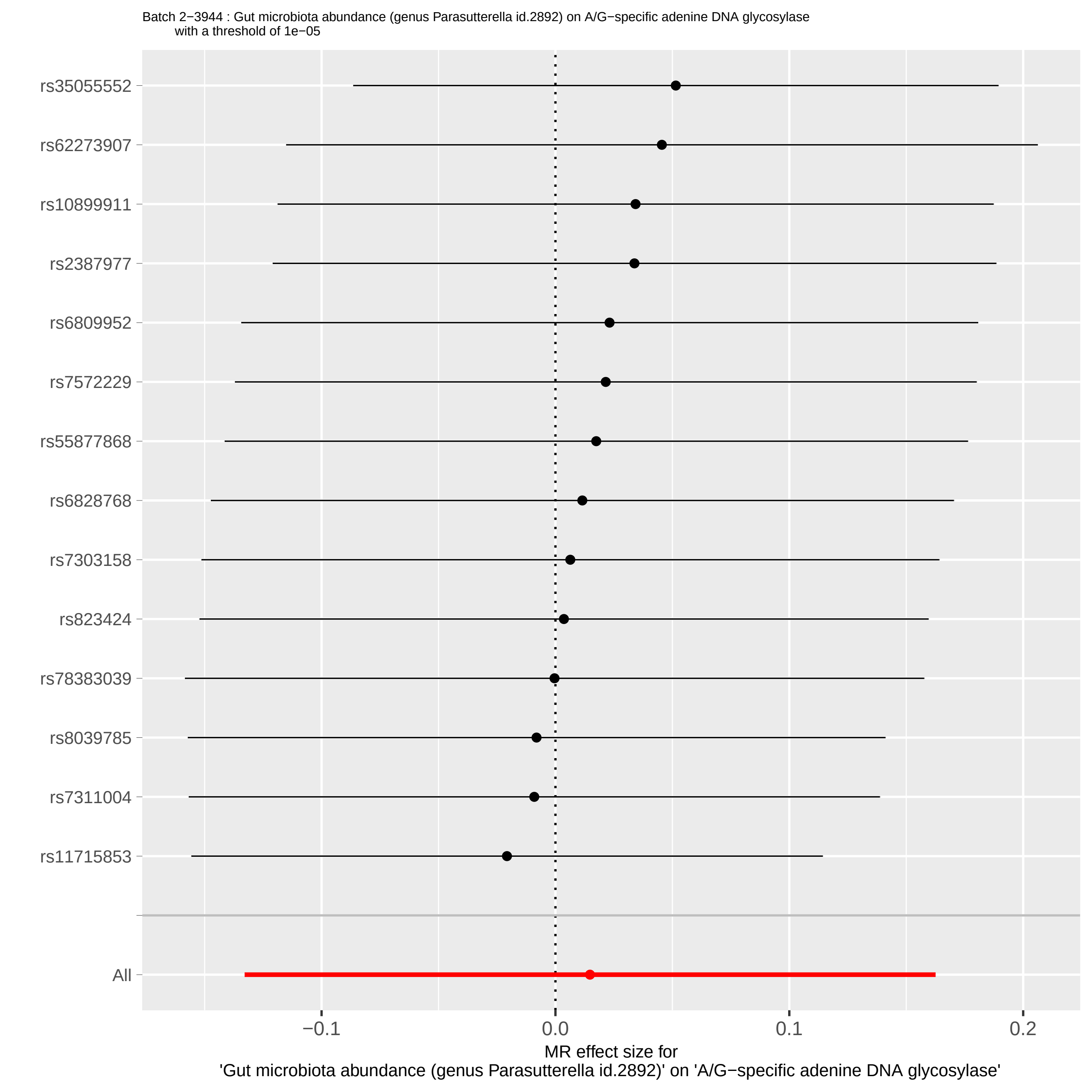

Batch 2−3944 : Gut microbiota abundance (genus Parasutterella id.2892) on A/G−specific adenine DNA glycosylase
with a threshold of 1e−05
rs35055552
rs62273907
rs10899911
rs2387977
rs6809952
rs7572229
rs55877868
rs6828768
rs7303158
rs823424
rs78383039
rs8039785
rs7311004
rs11715853
All
−0.1
0.0
0.1
0.2
MR effect size for
'Gut microbiota abundance (genus Parasutterella id.2892)' on 'A/G−specific adenine DNA glycosylase'

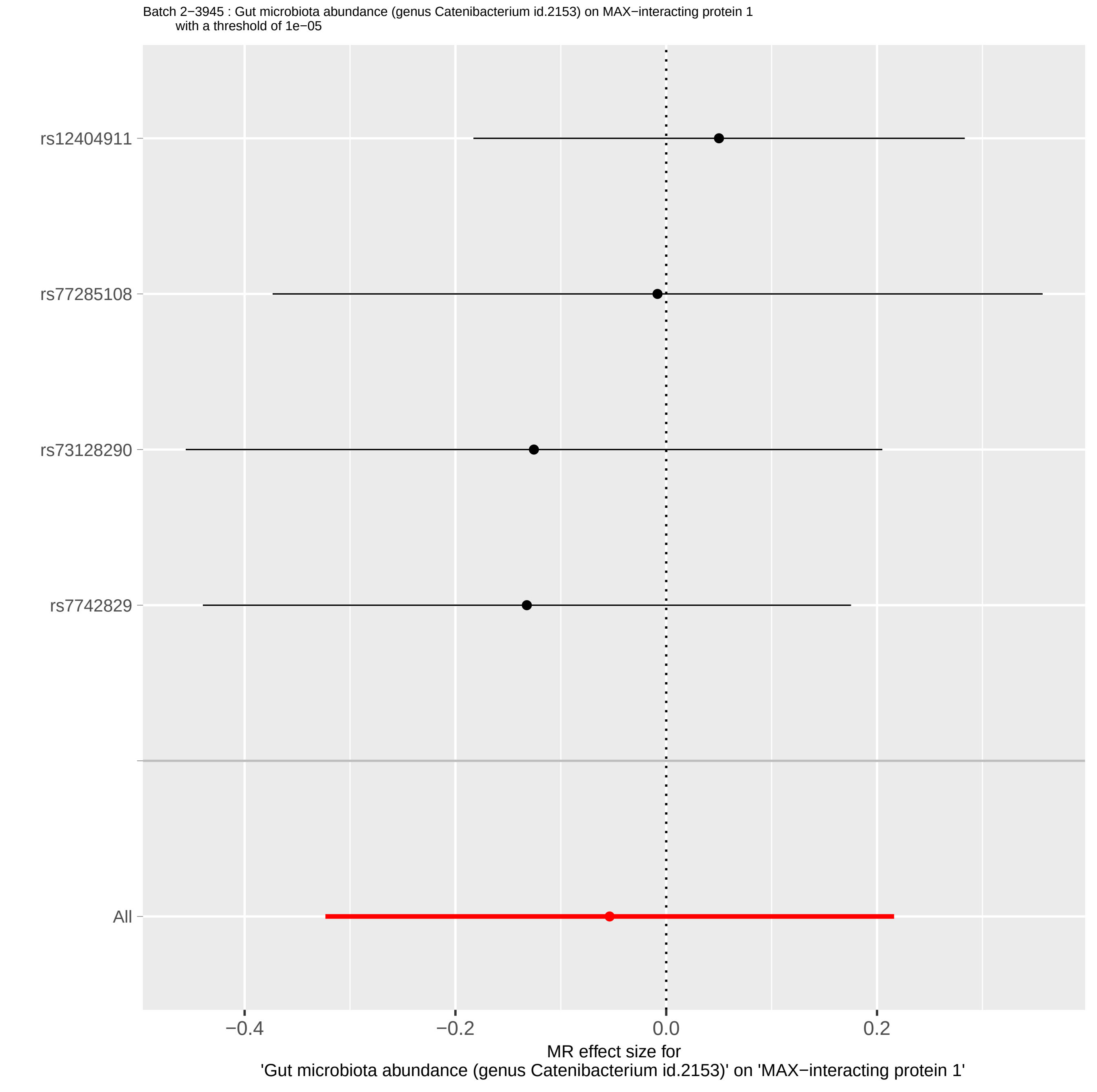

Batch 2−3945 : Gut microbiota abundance (genus Catenibacterium id.2153) on MAX−interacting protein 1
with a threshold of 1e−05
rs12404911
rs77285108
rs73128290
rs7742829
All
−0.4
−0.2
0.0
0.2
MR effect size for
'Gut microbiota abundance (genus Catenibacterium id.2153)' on 'MAX−interacting protein 1'

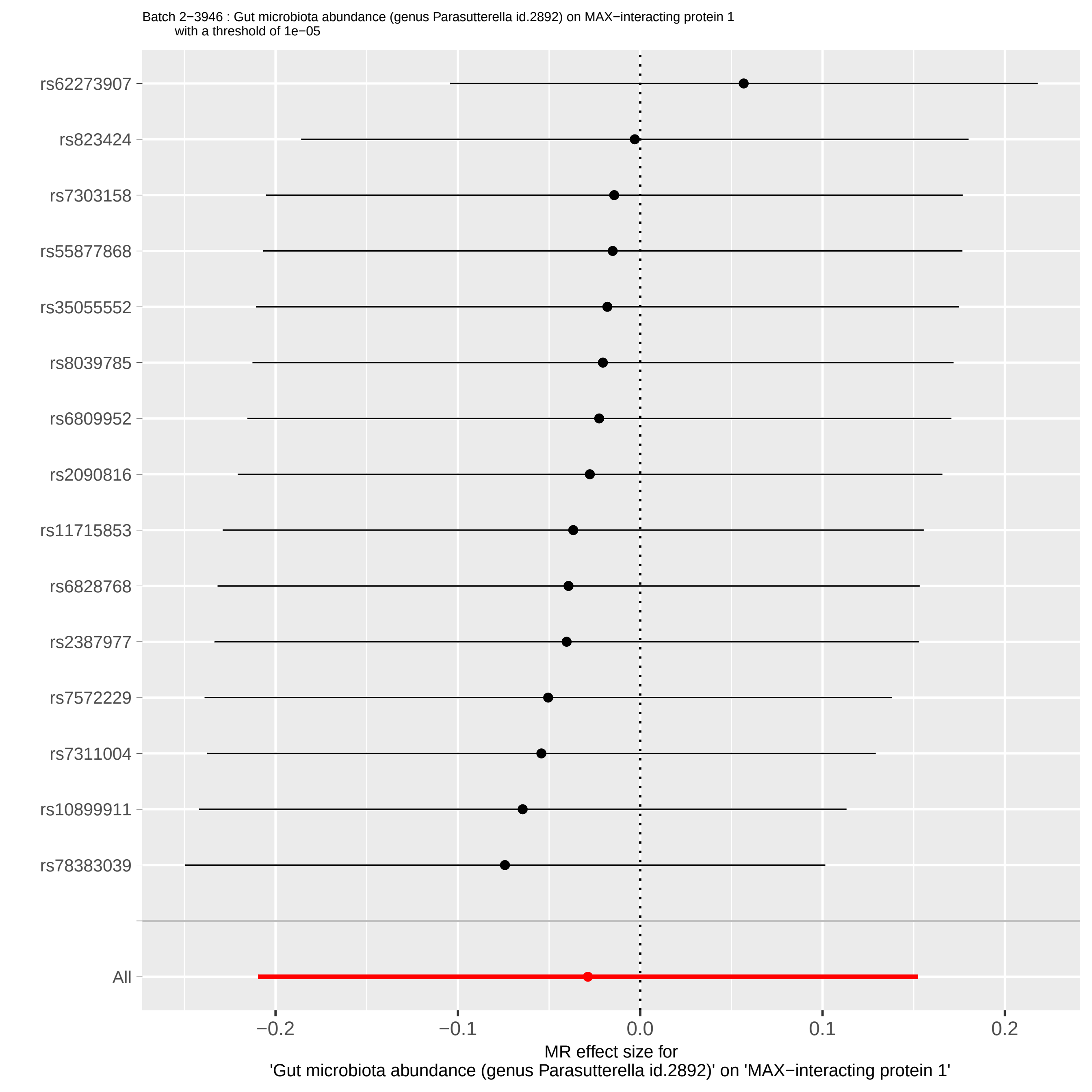

Batch 2−3946 : Gut microbiota abundance (genus Parasutterella id.2892) on MAX−interacting protein 1
with a threshold of 1e−05
rs62273907
rs823424
rs7303158
rs55877868
rs35055552
rs8039785
rs6809952
rs2090816
rs11715853
rs6828768
rs2387977
rs7572229
rs7311004
rs10899911
rs78383039
All
−0.2
−0.1
0.0
0.1
0.2
MR effect size for
'Gut microbiota abundance (genus Parasutterella id.2892)' on 'MAX−interacting protein 1'

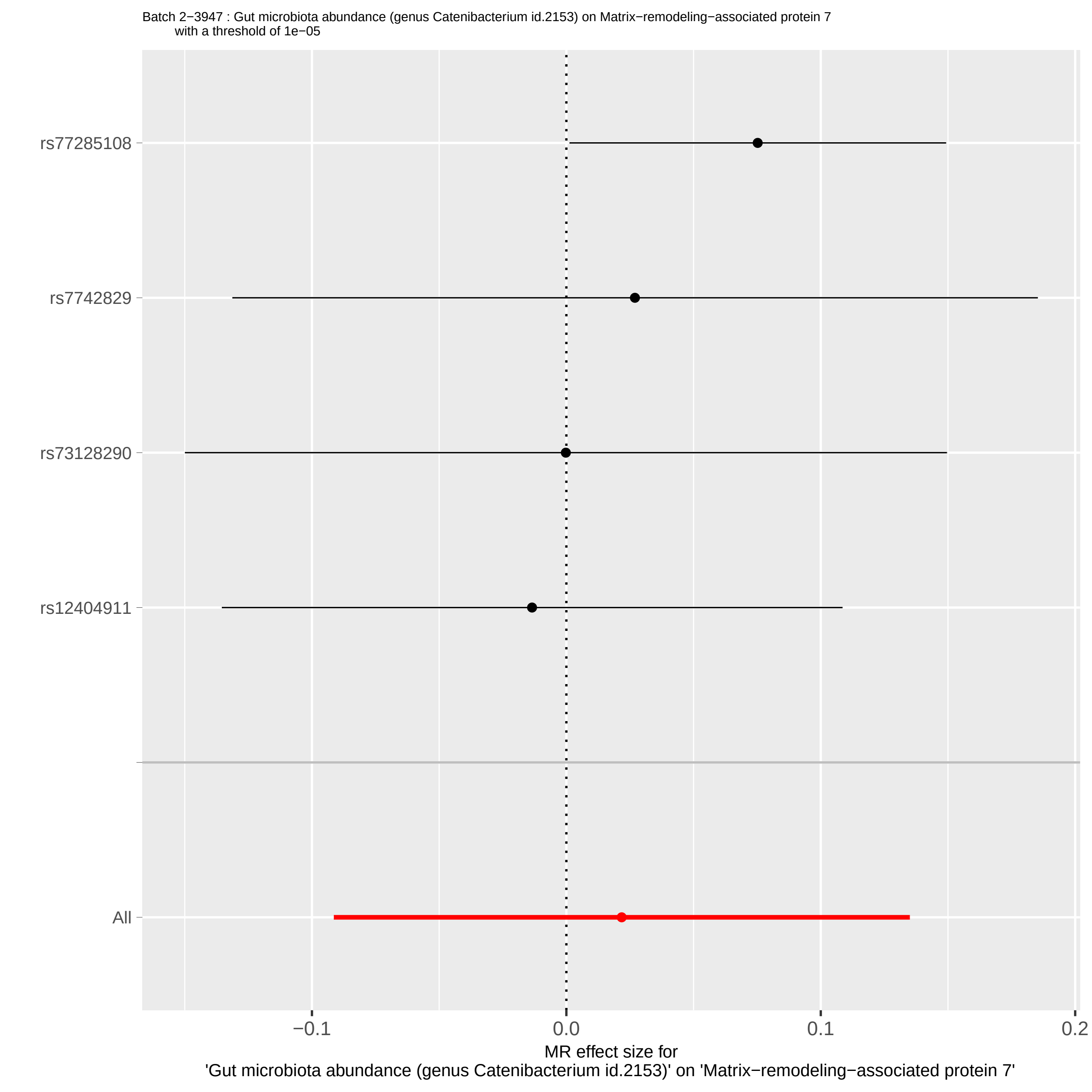
Batch 2−3947 : Gut microbiota abundance (genus Catenibacterium id.2153) on Matrix−remodeling−associated protein 7
with a threshold of 1e−05
rs77285108
rs7742829
rs73128290
rs12404911
All
−0.1
0.0
0.1
0.2
MR effect size for
'Gut microbiota abundance (genus Catenibacterium id.2153)' on 'Matrix−remodeling−associated protein 7'

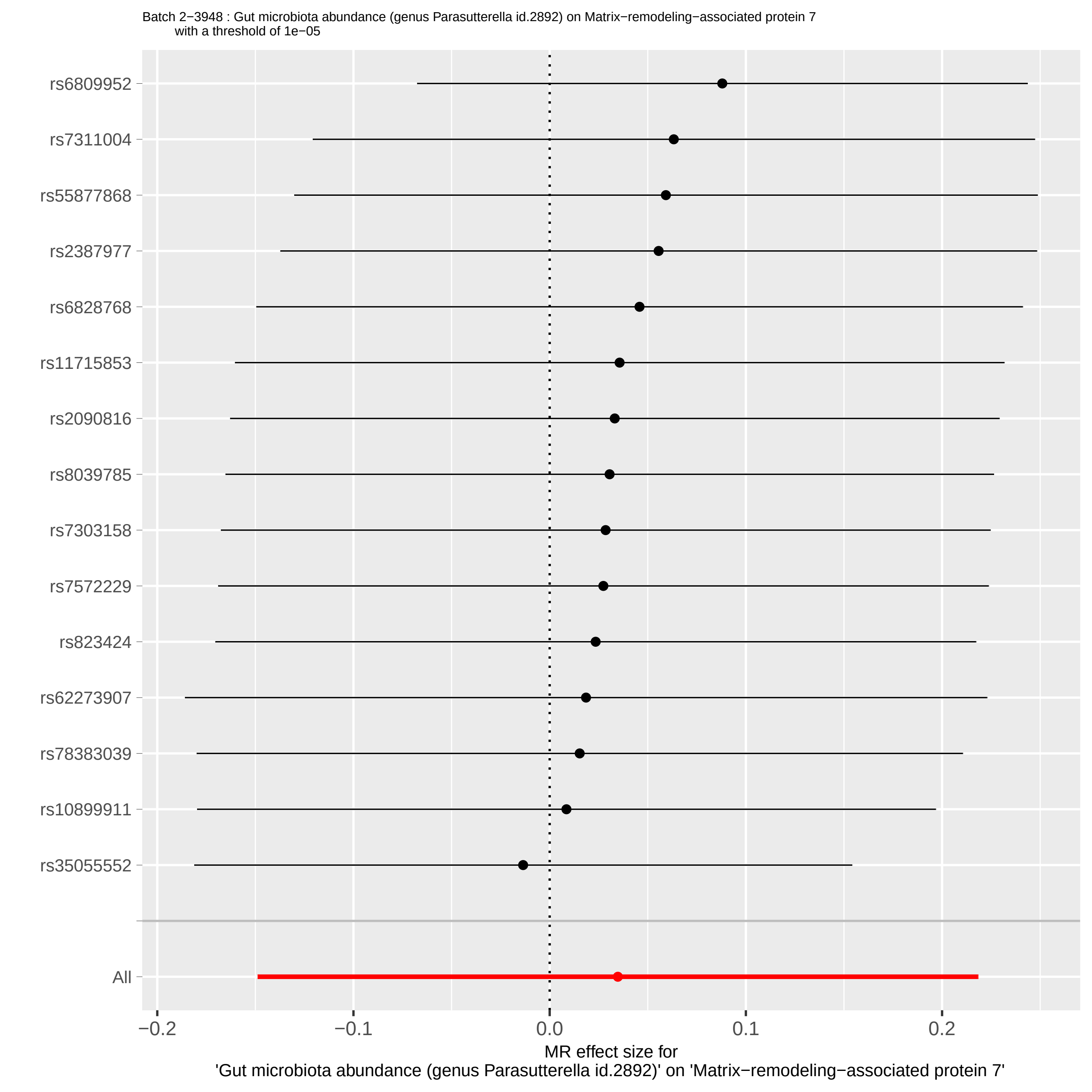

Batch 2−3948 : Gut microbiota abundance (genus Parasutterella id.2892) on Matrix−remodeling−associated protein 7
with a threshold of 1e−05
rs6809952
rs7311004
rs55877868
rs2387977
rs6828768
rs11715853
rs2090816
rs8039785
rs7303158
rs7572229
rs823424
rs62273907
rs78383039
rs10899911
rs35055552
All
−0.2
−0.1
0.0
0.1
0.2
MR effect size for
'Gut microbiota abundance (genus Parasutterella id.2892)' on 'Matrix−remodeling−associated protein 7'

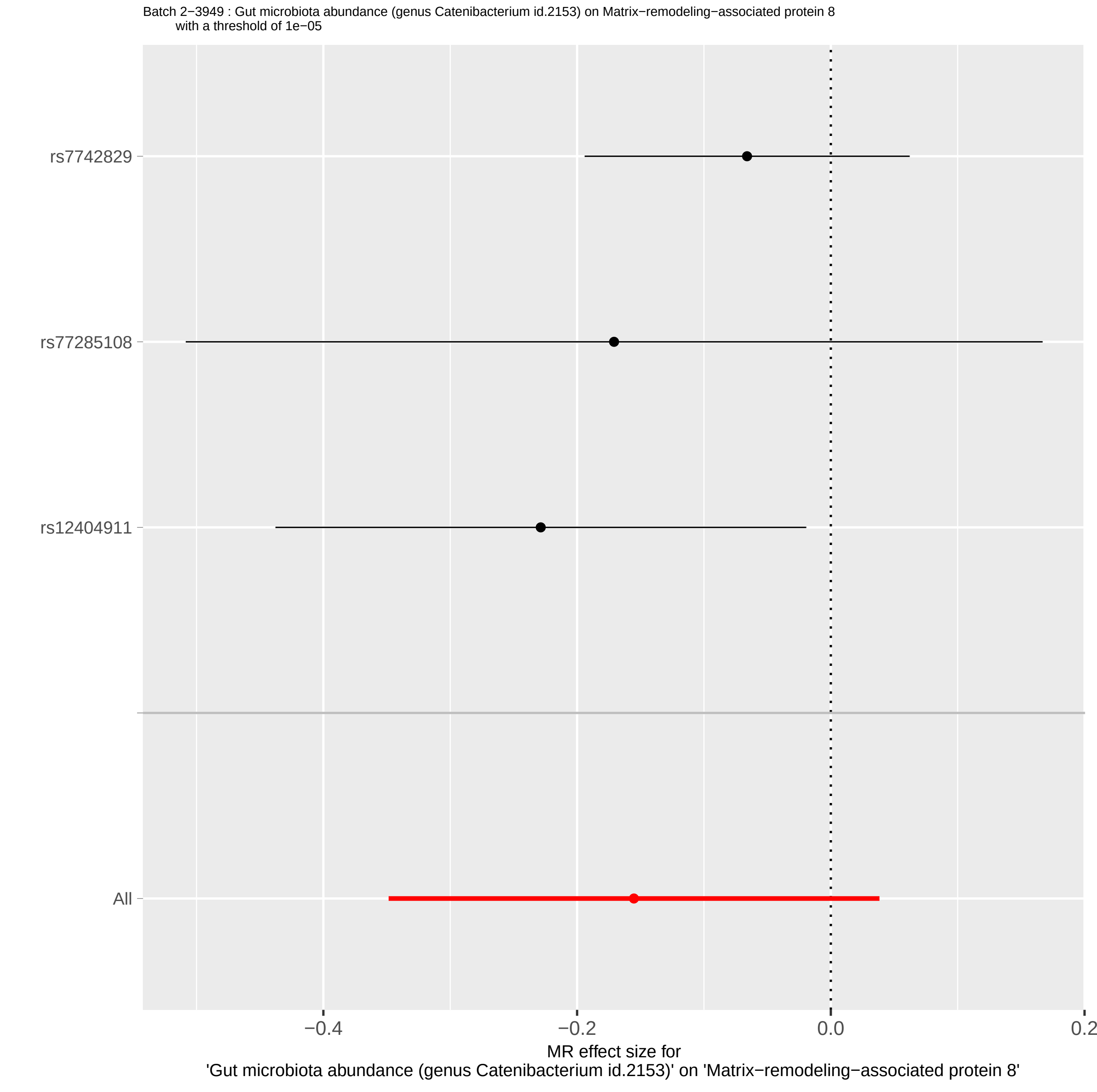
Batch 2−3949 : Gut microbiota abundance (genus Catenibacterium id.2153) on Matrix−remodeling−associated protein 8
with a threshold of 1e−05
rs7742829
rs77285108
rs12404911
All
−0.4
−0.2
0.0
0.2
MR effect size for
'Gut microbiota abundance (genus Catenibacterium id.2153)' on 'Matrix−remodeling−associated protein 8'

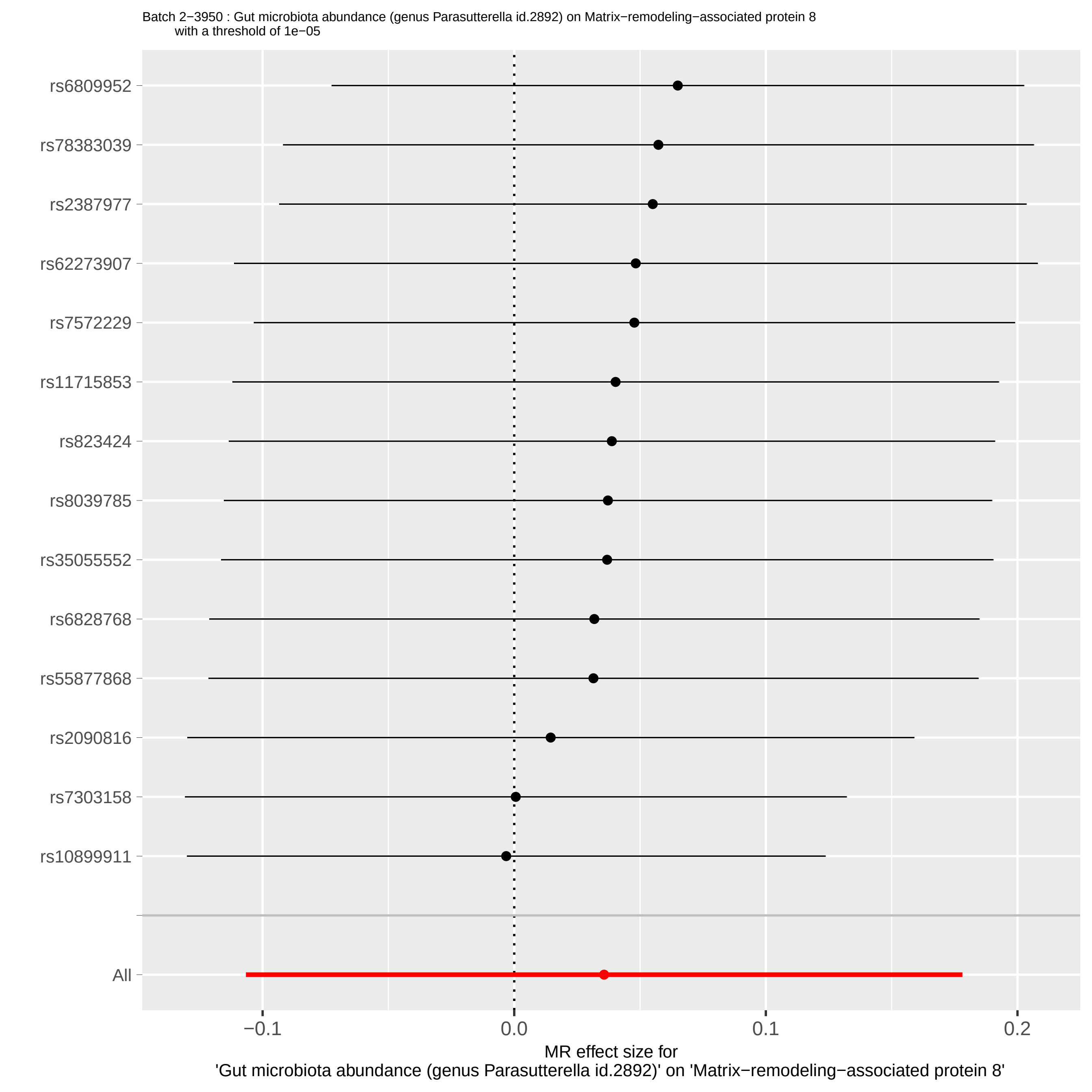
Batch 2−3950 : Gut microbiota abundance (genus Parasutterella id.2892) on Matrix−remodeling−associated protein 8
with a threshold of 1e−05
rs6809952
rs78383039
rs2387977
rs62273907
rs7572229
rs11715853
rs823424
rs8039785
rs35055552
rs6828768
rs55877868
rs2090816
rs7303158
rs10899911
All
−0.1
0.0
0.1
0.2
MR effect size for
'Gut microbiota abundance (genus Parasutterella id.2892)' on 'Matrix−remodeling−associated protein 8'

Batch 2−3951 : Gut microbiota abundance (genus Catenibacterium id.2153) on Matrix−remodeling−associated protein 8
with a threshold of 1e−05

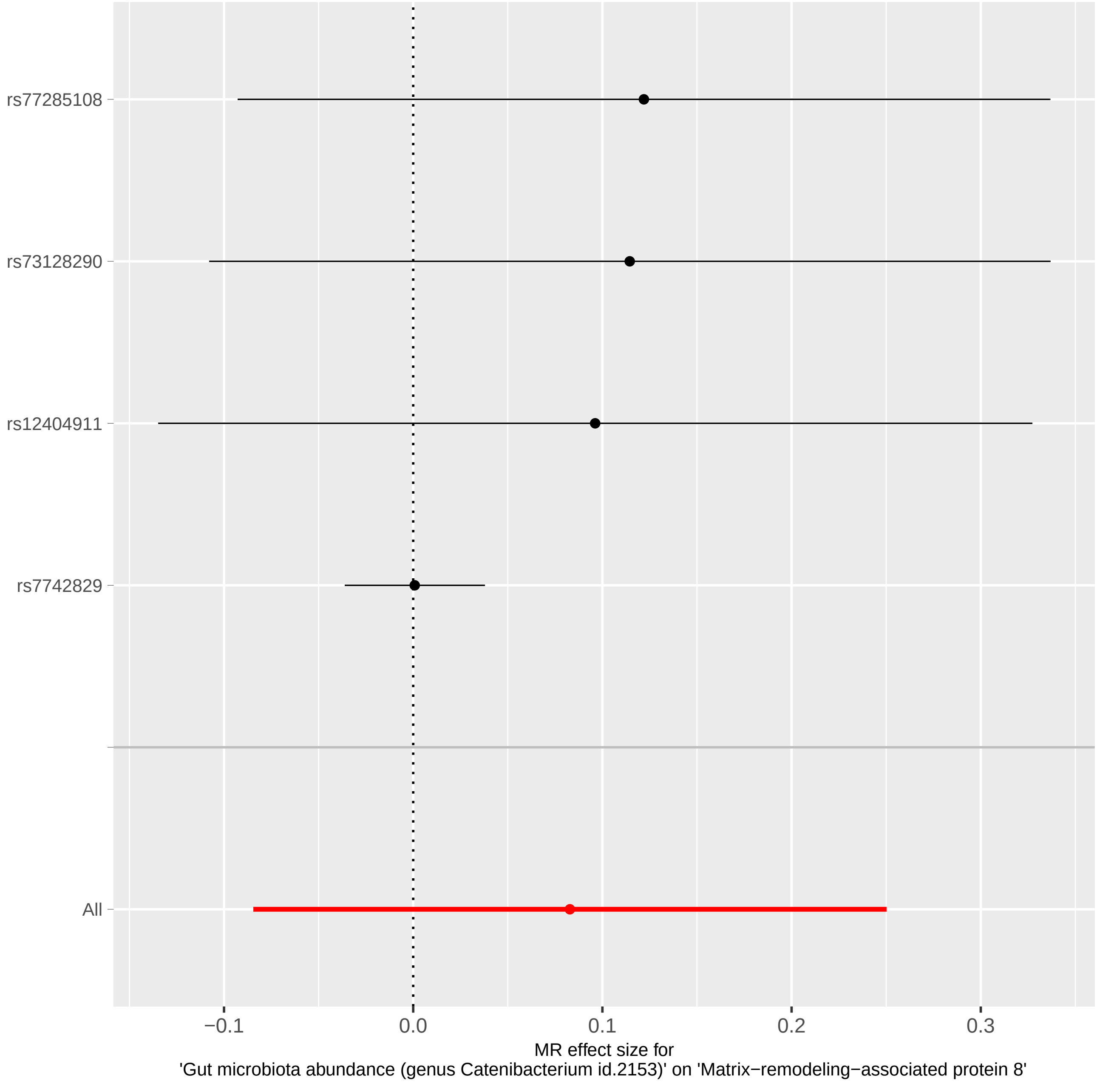

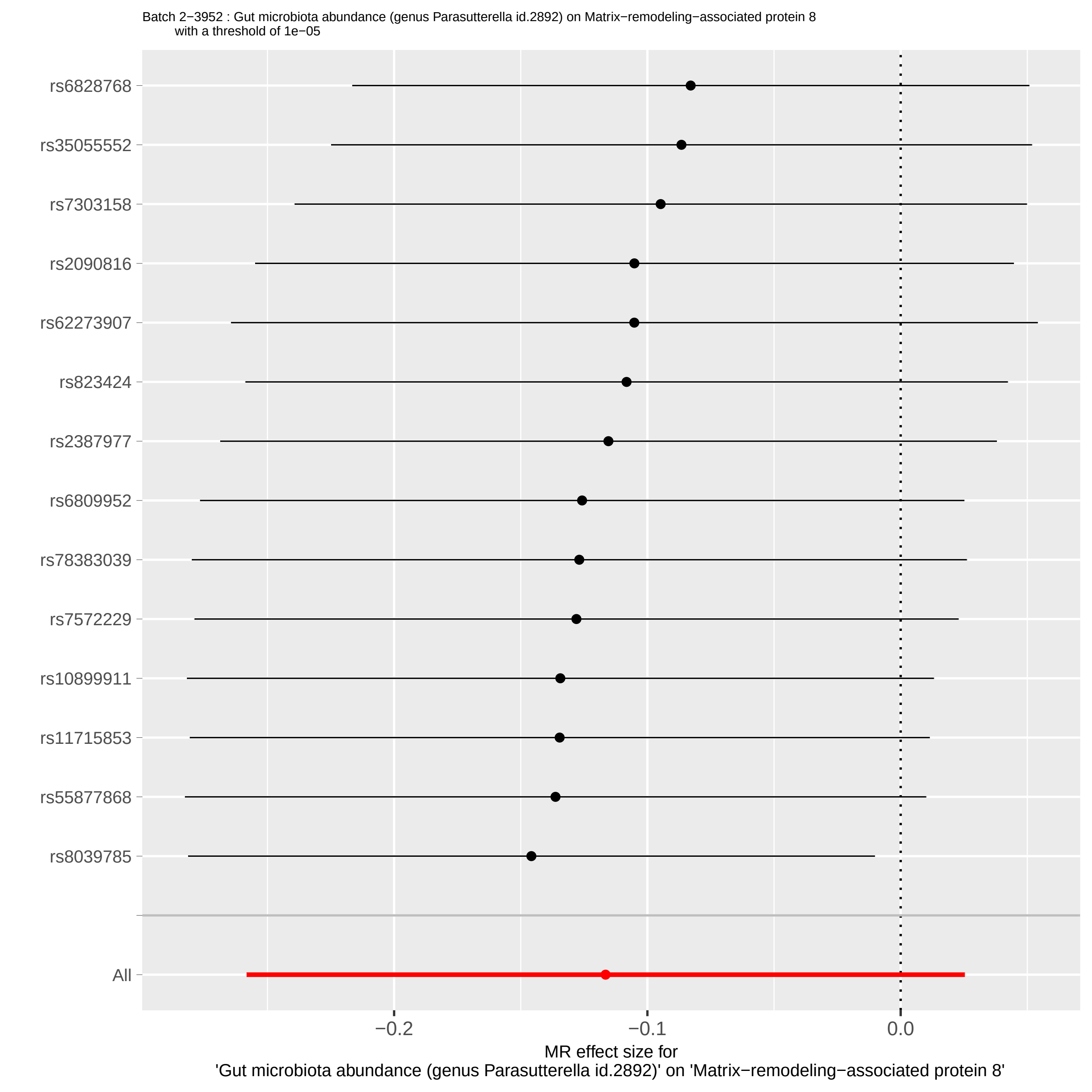

Batch 2−3952 : Gut microbiota abundance (genus Parasutterella id.2892) on Matrix−remodeling−associated protein 8
with a threshold of 1e−05
rs6828768
rs35055552
rs7303158
rs2090816
rs62273907
rs823424
rs2387977
rs6809952
rs78383039
rs7572229
rs10899911
rs11715853
rs55877868
rs8039785
All
−0.2
−0.1
0.0
MR effect size for
'Gut microbiota abundance (genus Parasutterella id.2892)' on 'Matrix−remodeling−associated protein 8'

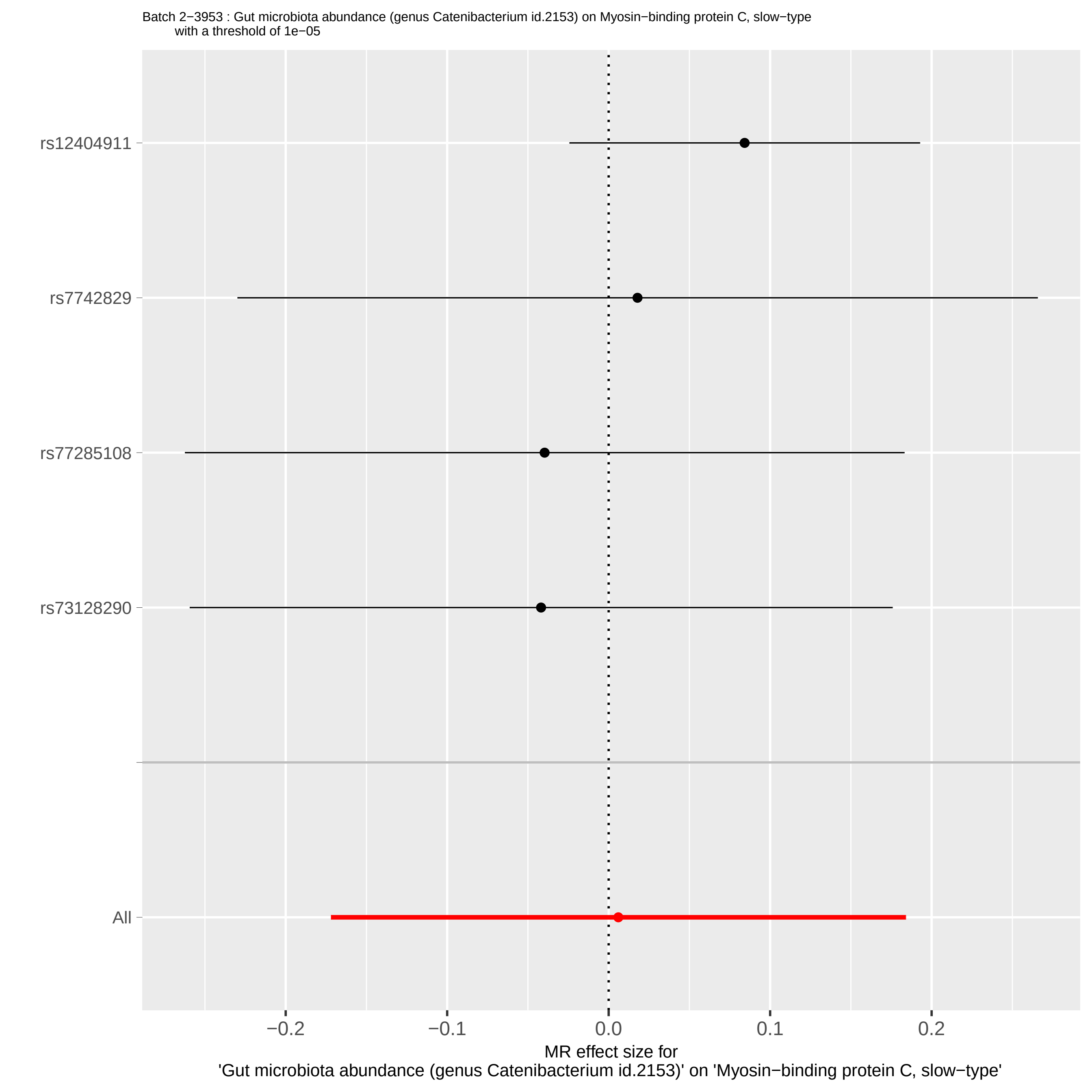

Batch 2−3953 : Gut microbiota abundance (genus Catenibacterium id.2153) on Myosin−binding protein C, slow−type
with a threshold of 1e−05
rs12404911
rs7742829
rs77285108
rs73128290
All
−0.2
−0.1
0.0
0.1
0.2
MR effect size for
'Gut microbiota abundance (genus Catenibacterium id.2153)' on 'Myosin−binding protein C, slow−type'

Batch 2−3954 : Gut microbiota abundance (genus Parasutterella id.2892) on Myosin−binding protein C, slow−type
with a threshold of 1e−05

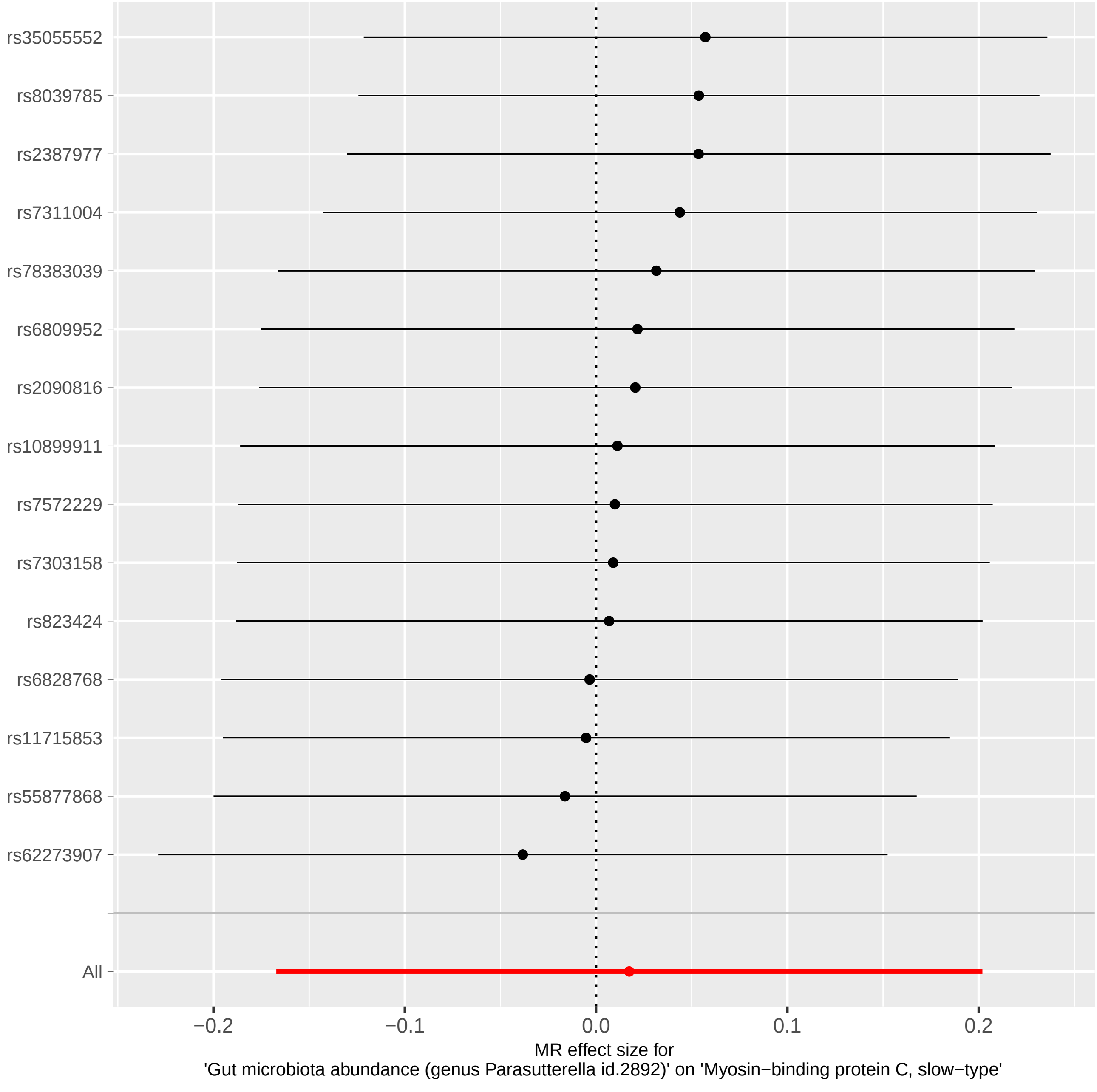

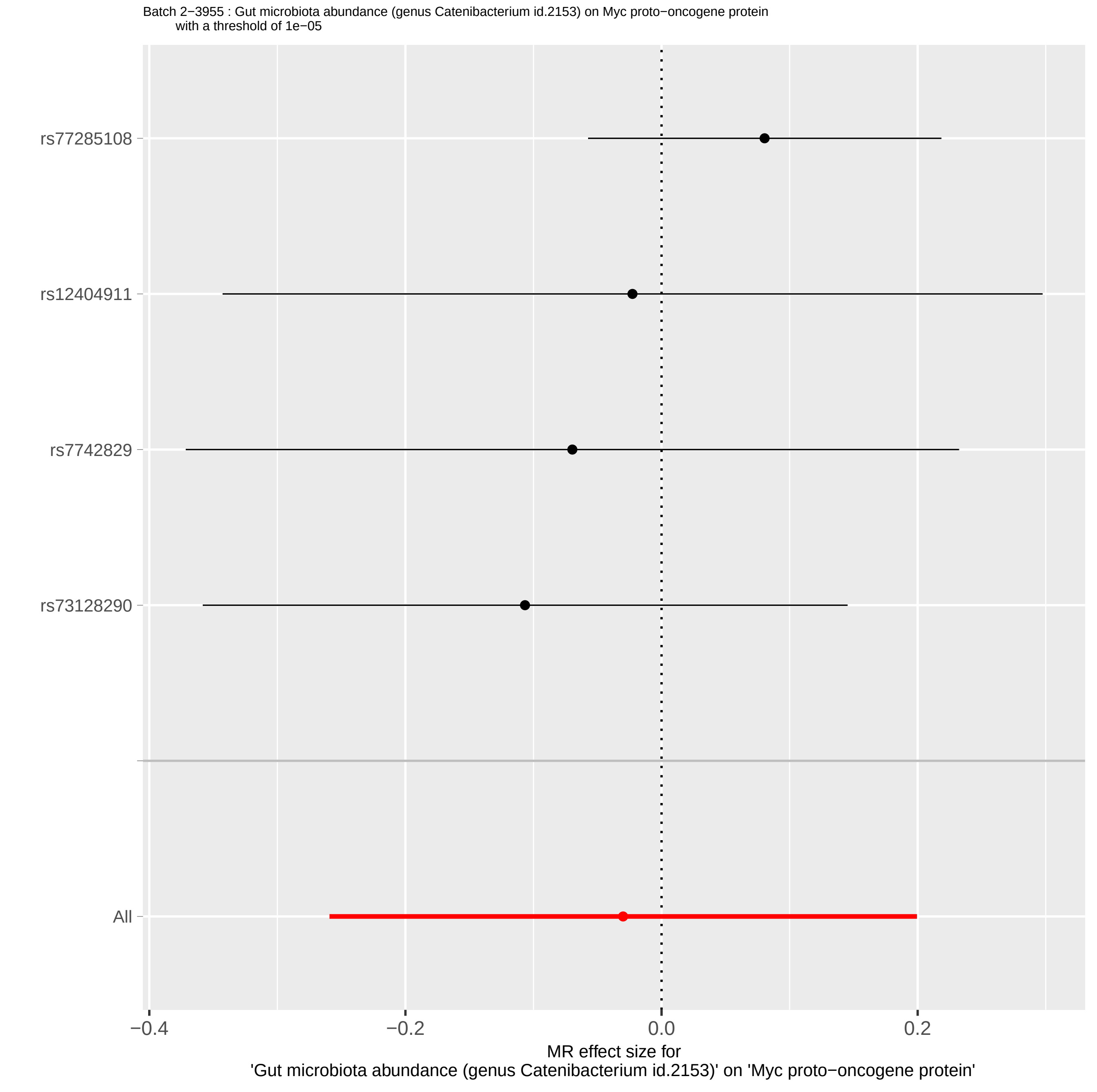
Batch 2−3955 : Gut microbiota abundance (genus Catenibacterium id.2153) on Myc proto−oncogene protein
with a threshold of 1e−05
rs77285108
rs12404911
rs7742829
rs73128290
All
−0.4
−0.2
0.0
0.2
MR effect size for
'Gut microbiota abundance (genus Catenibacterium id.2153)' on 'Myc proto−oncogene protein'

Batch 2−3956 : Gut microbiota abundance (genus Parasutterella id.2892) on Myc proto−oncogene protein
with a threshold of 1e−05

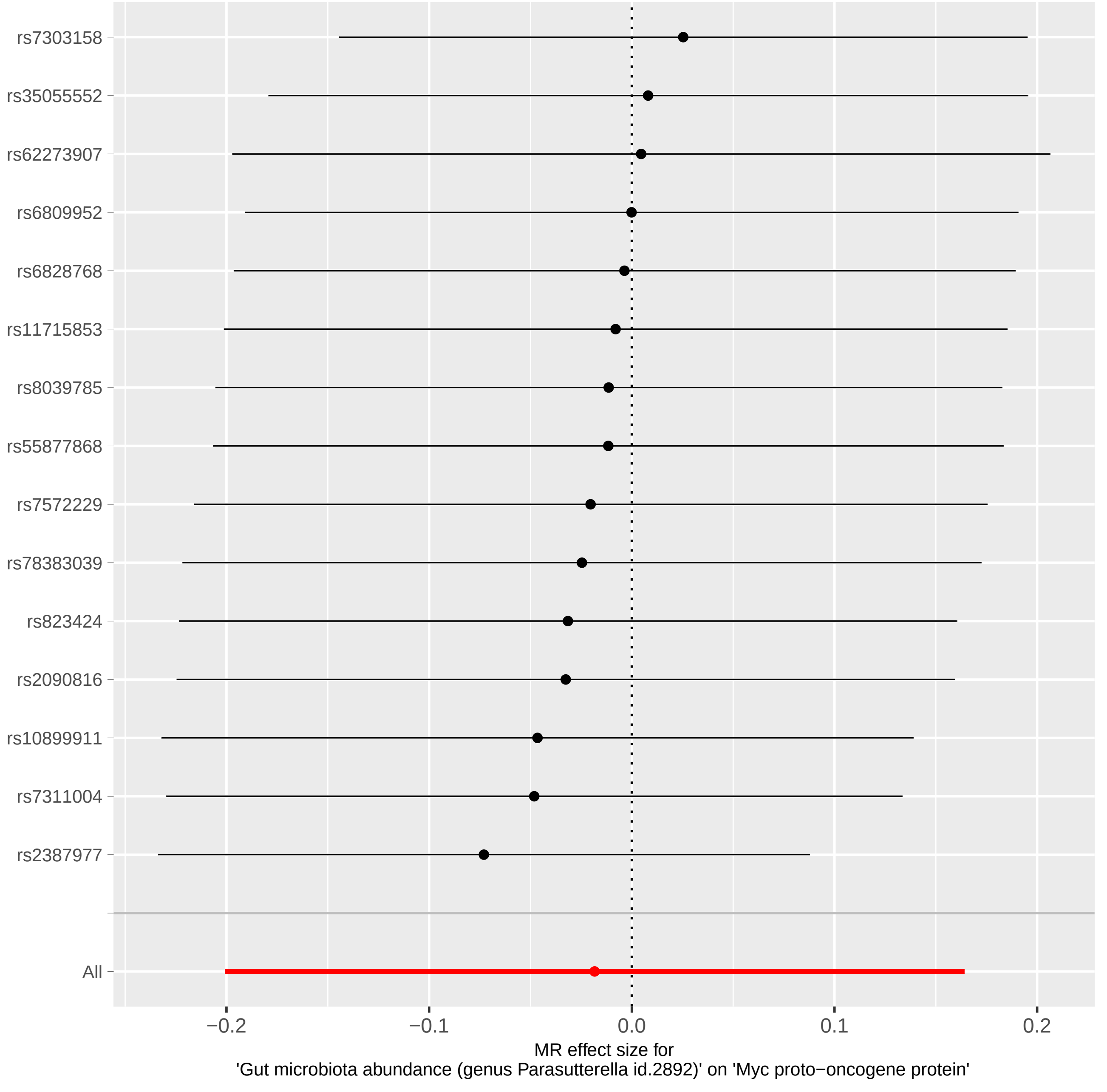

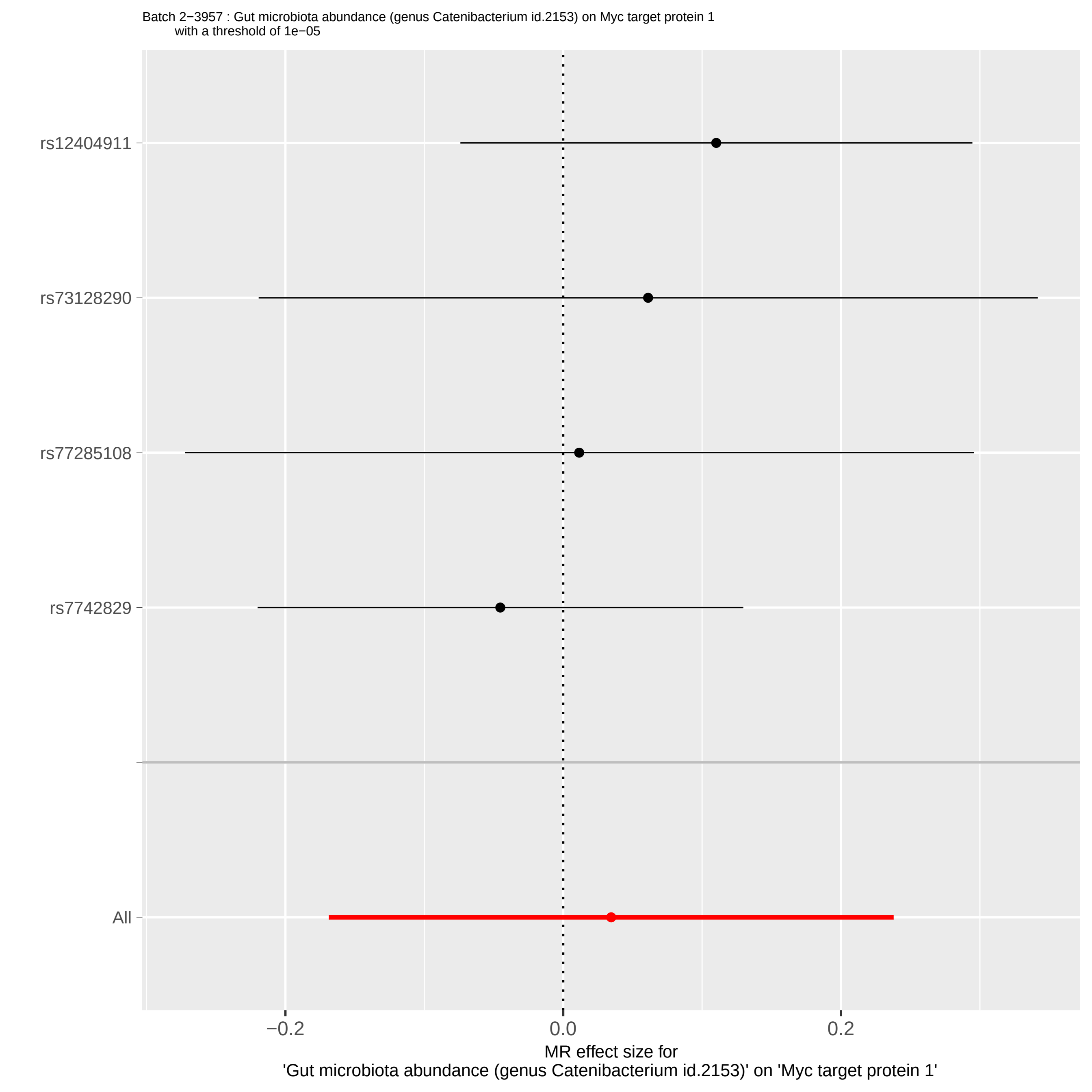

Batch 2−3957 : Gut microbiota abundance (genus Catenibacterium id.2153) on Myc target protein 1
with a threshold of 1e−05
rs12404911
rs73128290
rs77285108
rs7742829
All
−0.2
0.0
0.2
MR effect size for
'Gut microbiota abundance (genus Catenibacterium id.2153)' on 'Myc target protein 1'

Batch 2−3958 : Gut microbiota abundance (genus Parasutterella id.2892) on Myc target protein 1
with a threshold of 1e−05

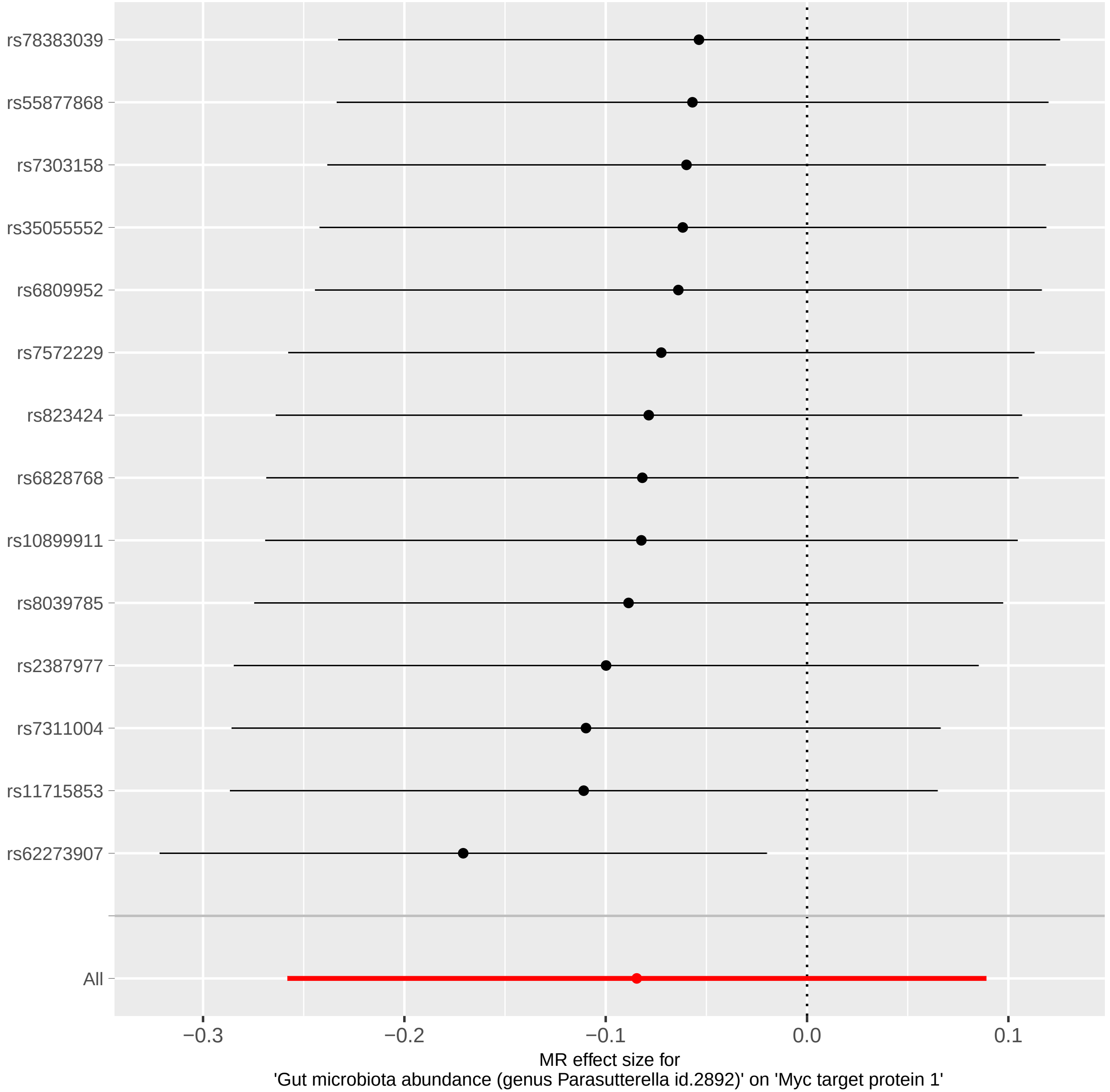

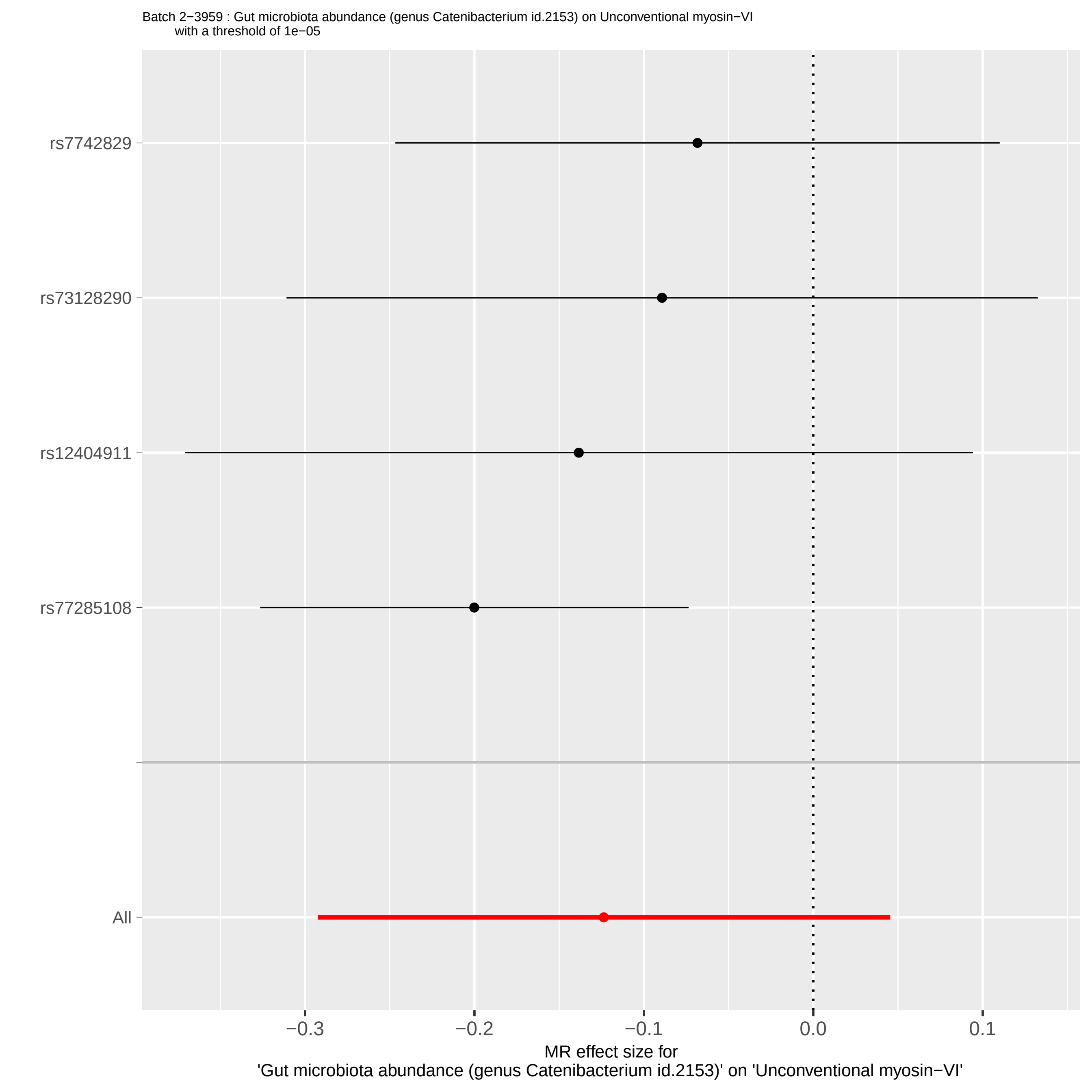
Batch 2−3959 : Gut microbiota abundance (genus Catenibacterium id.2153) on Unconventional myosin−VI
with a threshold of 1e−05
rs7742829
rs73128290
rs12404911
rs77285108
All
−0.3
−0.2
−0.1
0.0
0.1
MR effect size for
'Gut microbiota abundance (genus Catenibacterium id.2153)' on 'Unconventional myosin−VI'

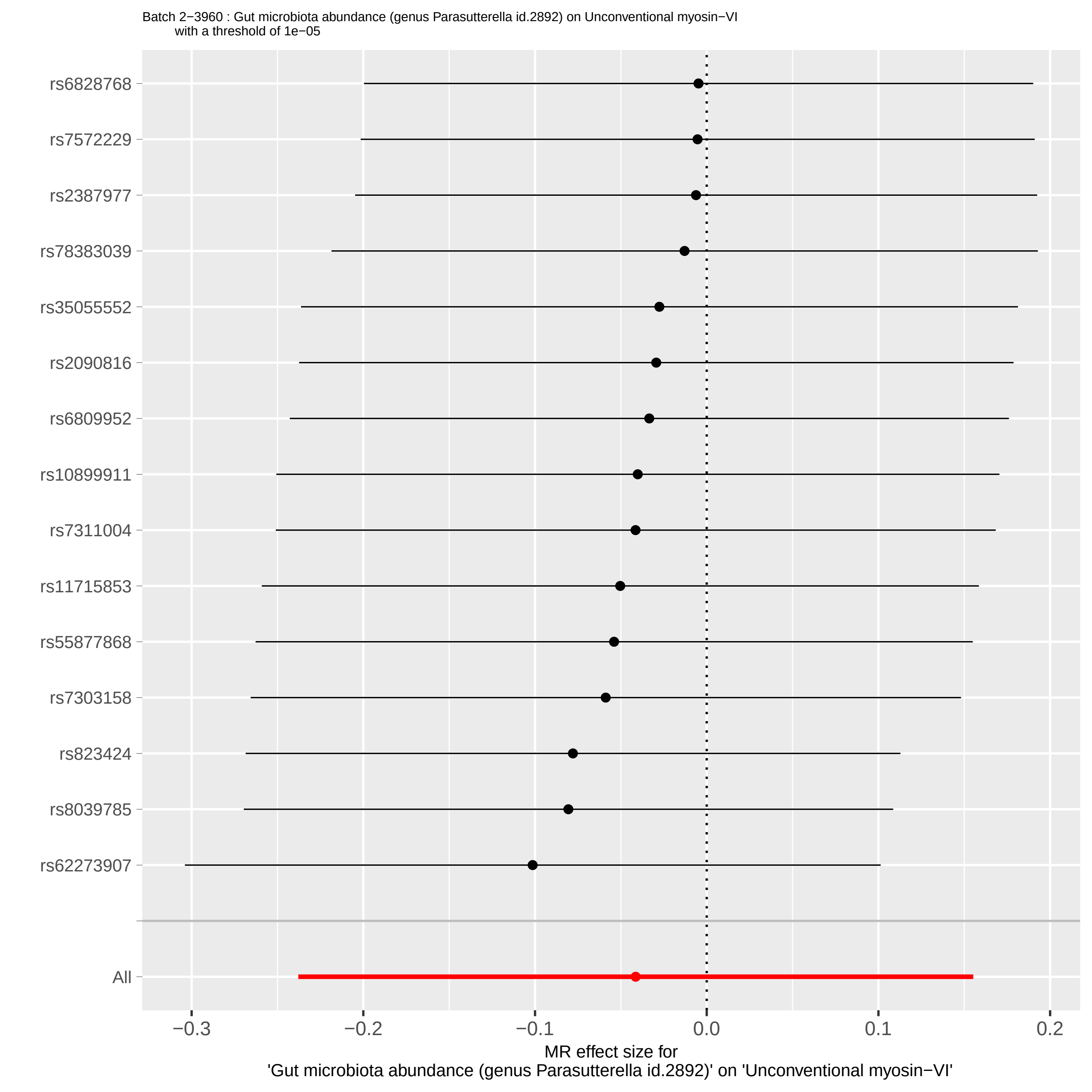

Batch 2−3960 : Gut microbiota abundance (genus Parasutterella id.2892) on Unconventional myosin−VI
with a threshold of 1e−05
rs6828768
rs7572229
rs2387977
rs78383039
rs35055552
rs2090816
rs6809952
rs10899911
rs7311004
rs11715853
rs55877868
rs7303158
rs823424
rs8039785
rs62273907
All
−0.3
−0.2
−0.1
0.0
0.1
0.2
MR effect size for
'Gut microbiota abundance (genus Parasutterella id.2892)' on 'Unconventional myosin−VI'

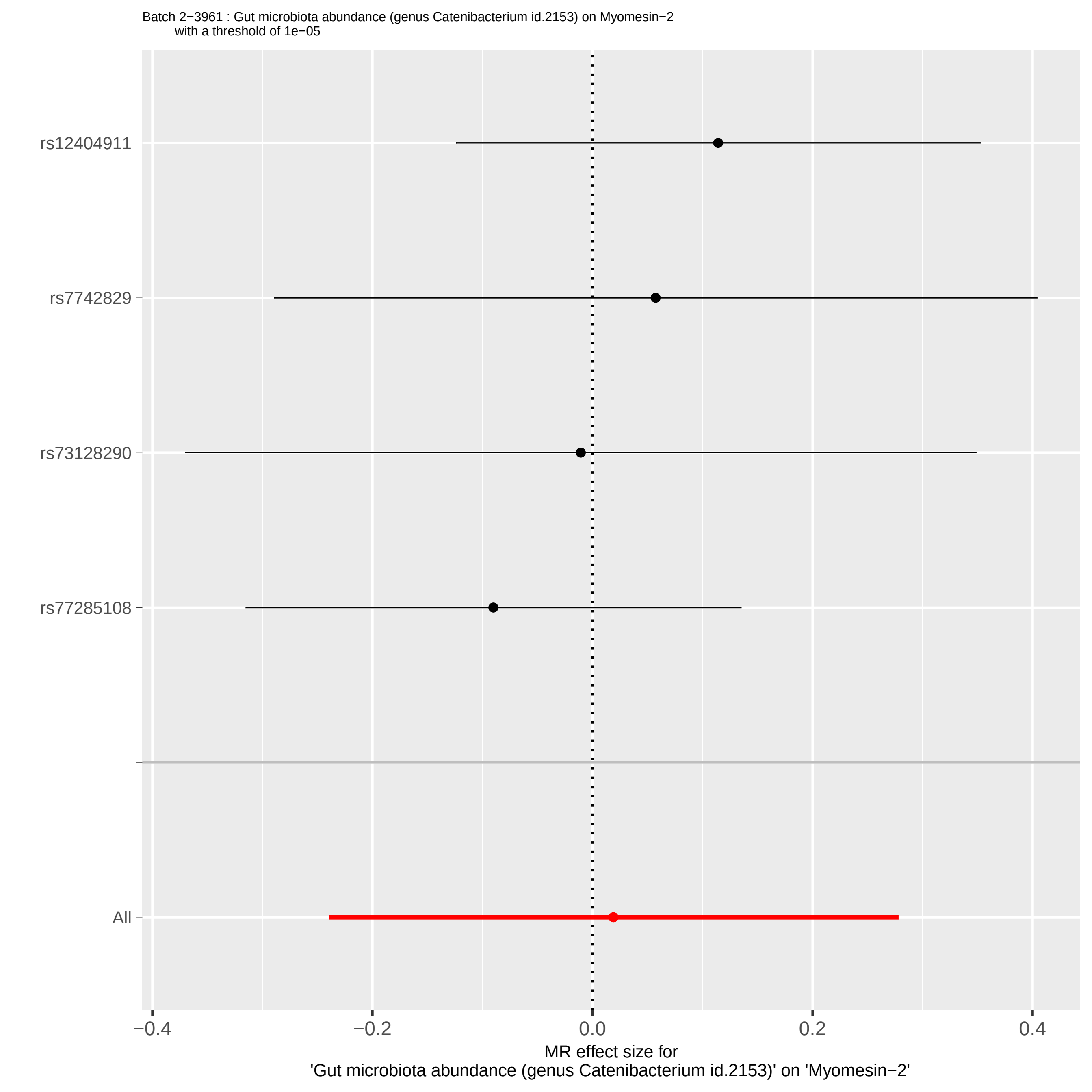
Batch 2−3961 : Gut microbiota abundance (genus Catenibacterium id.2153) on Myomesin−2
with a threshold of 1e−05
rs12404911
rs7742829
rs73128290
rs77285108
All
−0.4
−0.2
0.0
0.2
0.4
MR effect size for
'Gut microbiota abundance (genus Catenibacterium id.2153)' on 'Myomesin−2'

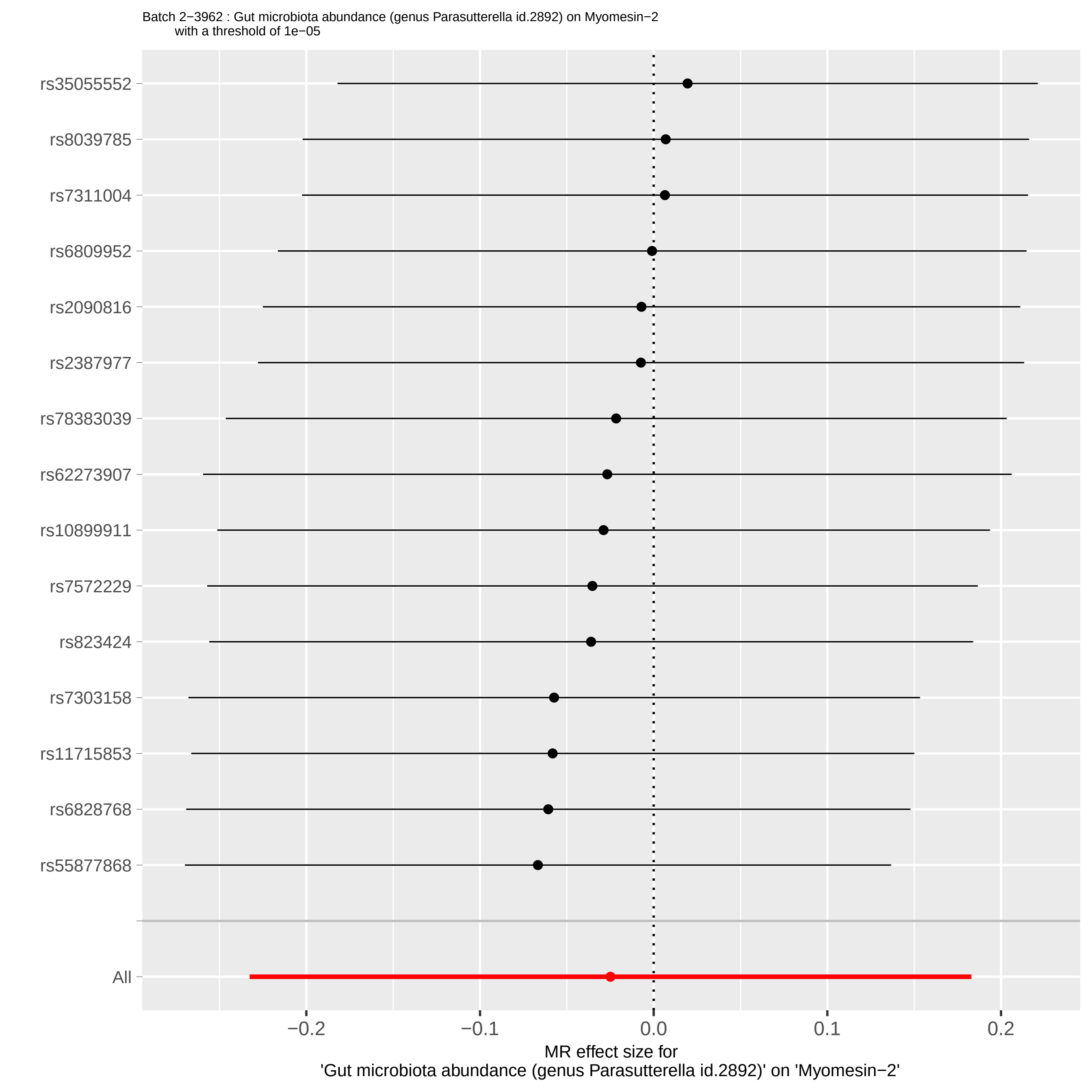
Batch 2−3962 : Gut microbiota abundance (genus Parasutterella id.2892) on Myomesin−2
with a threshold of 1e−05
rs35055552
rs8039785
rs7311004
rs6809952
rs2090816
rs2387977
rs78383039
rs62273907
rs10899911
rs7572229
rs823424
rs7303158
rs11715853
rs6828768
rs55877868
All
−0.2
−0.1
0.0
0.1
0.2
MR effect size for
'Gut microbiota abundance (genus Parasutterella id.2892)' on 'Myomesin−2'

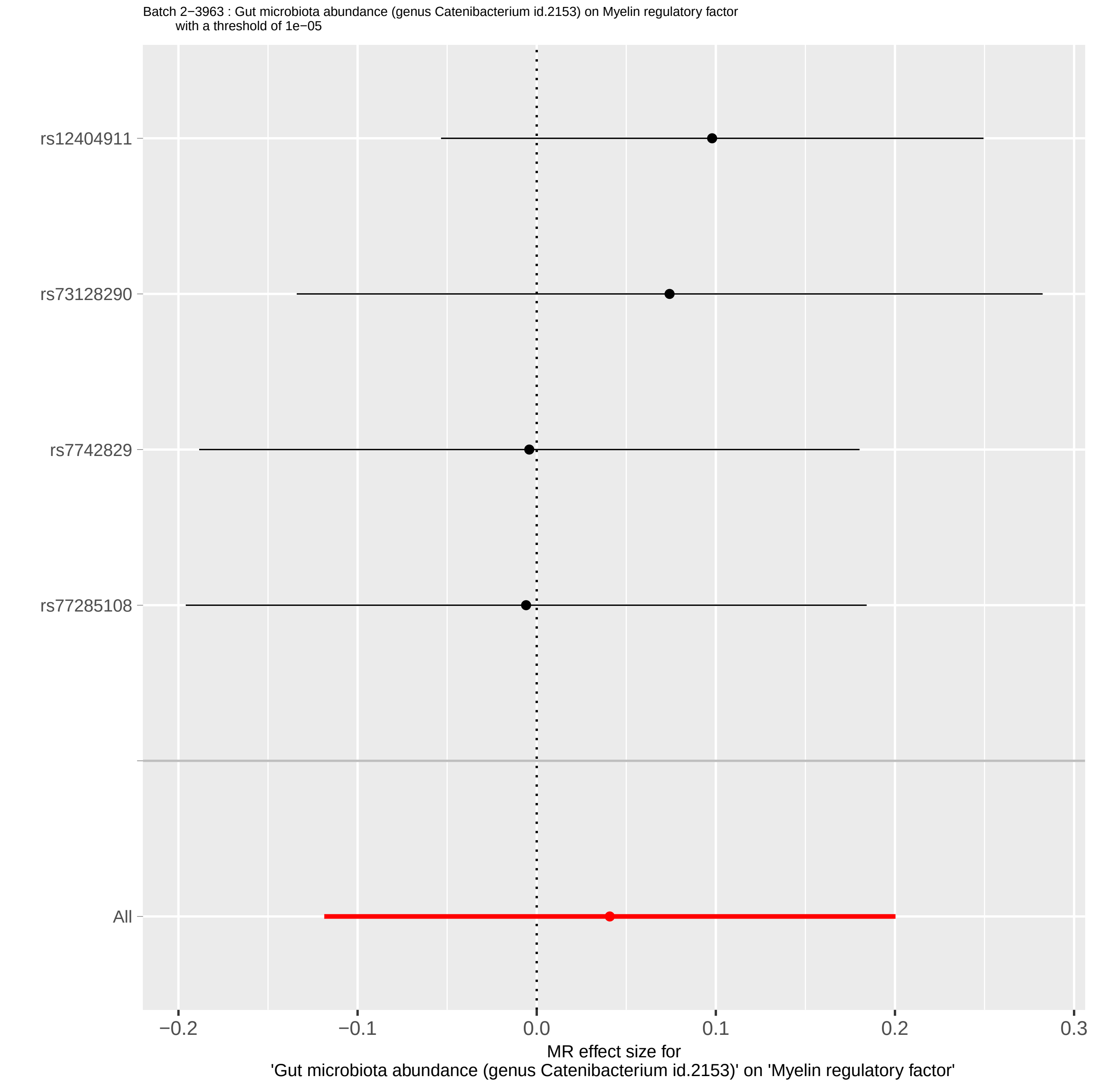

Batch 2−3963 : Gut microbiota abundance (genus Catenibacterium id.2153) on Myelin regulatory factor
with a threshold of 1e−05
rs12404911
rs73128290
rs7742829
rs77285108
All
−0.2
−0.1
0.0
0.1
0.2
0.3
MR effect size for
'Gut microbiota abundance (genus Catenibacterium id.2153)' on 'Myelin regulatory factor'

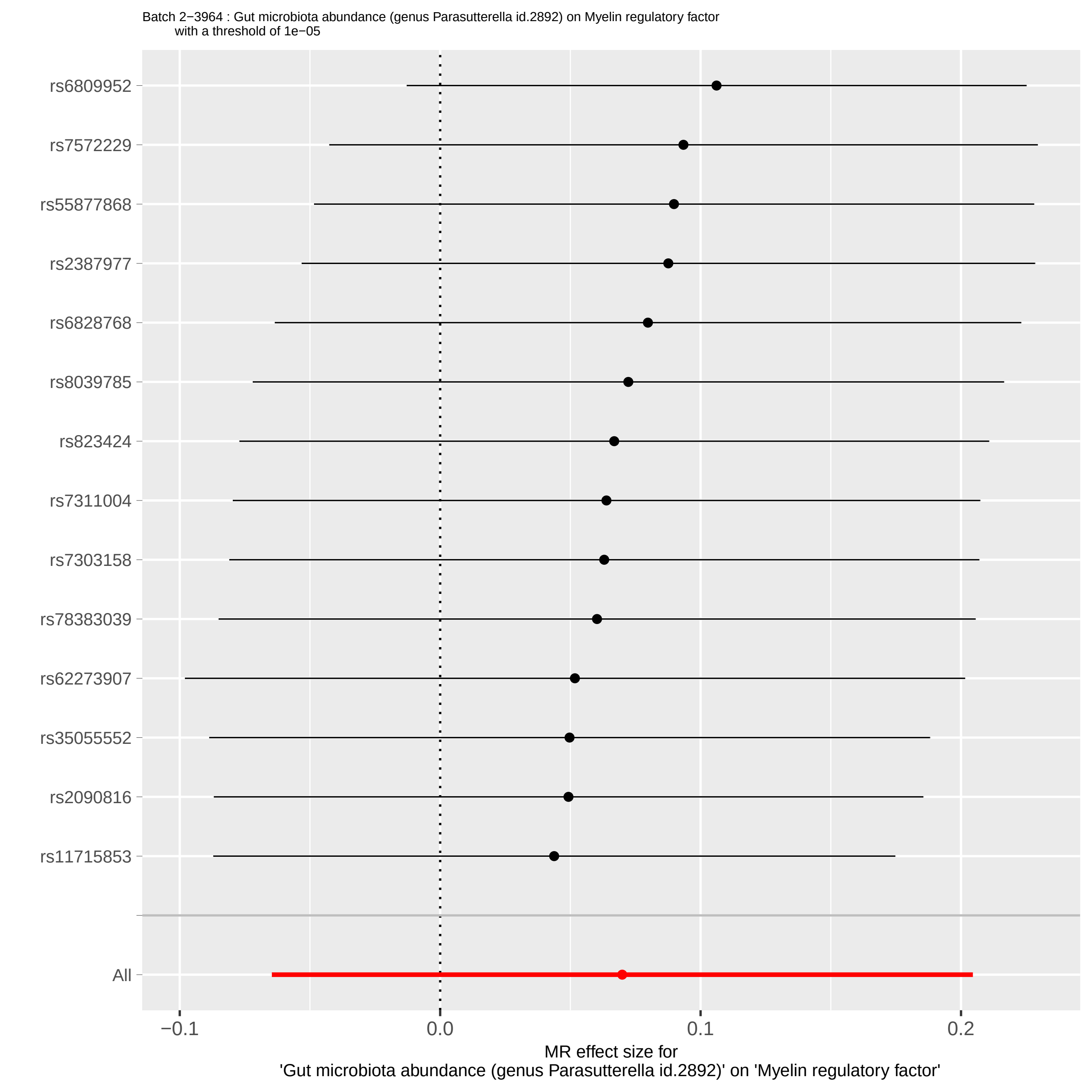
Batch 2−3964 : Gut microbiota abundance (genus Parasutterella id.2892) on Myelin regulatory factor
with a threshold of 1e−05
rs6809952
rs7572229
rs55877868
rs2387977
rs6828768
rs8039785
rs823424
rs7311004
rs7303158
rs78383039
rs62273907
rs35055552
rs2090816
rs11715853
All
−0.1
0.0
0.1
0.2
MR effect size for
'Gut microbiota abundance (genus Parasutterella id.2892)' on 'Myelin regulatory factor'

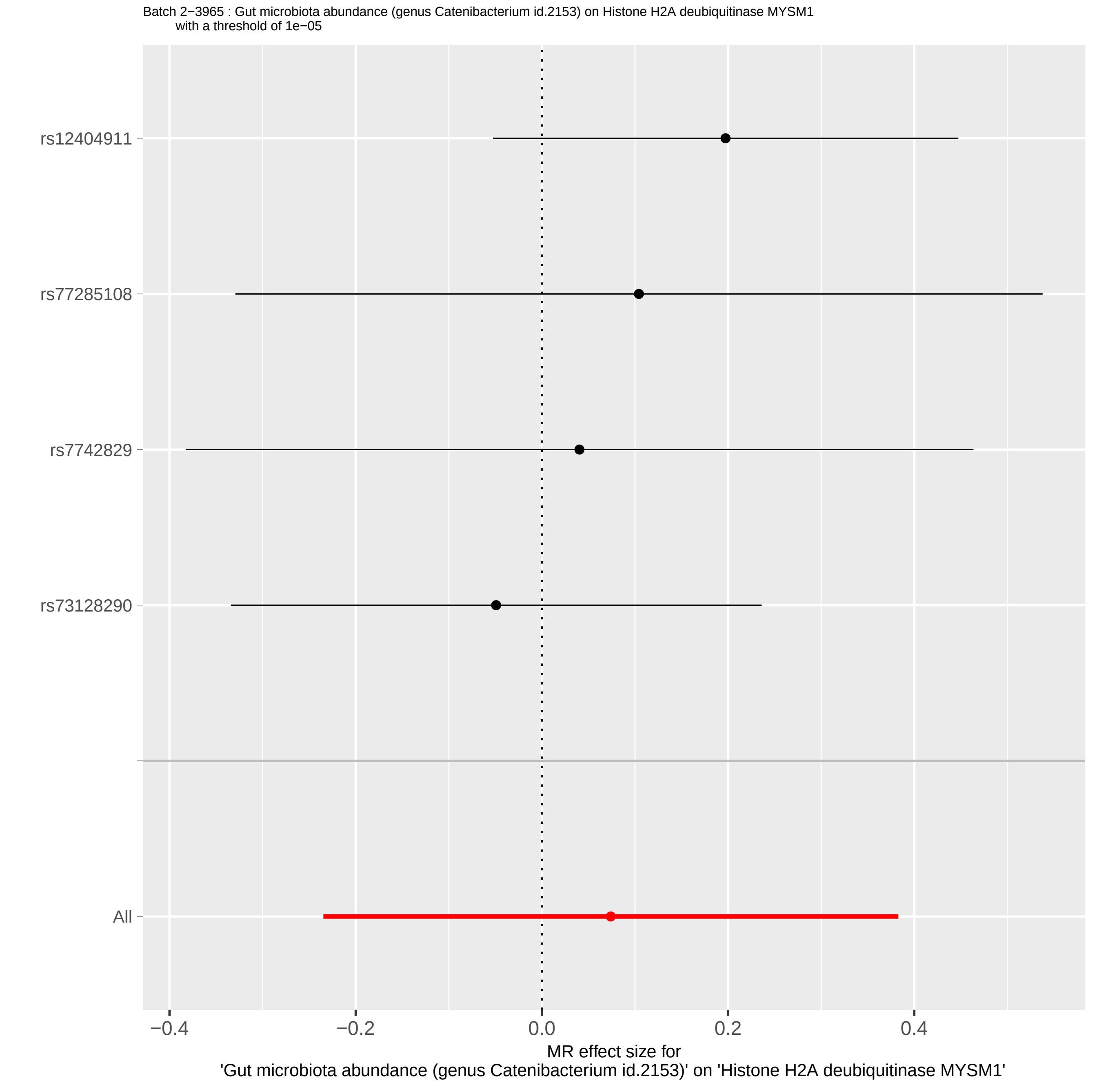

Batch 2−3965 : Gut microbiota abundance (genus Catenibacterium id.2153) on Histone H2A deubiquitinase MYSM1
with a threshold of 1e−05
rs12404911
rs77285108
rs7742829
rs73128290
All
−0.4
−0.2
0.0
0.2
0.4
MR effect size for
'Gut microbiota abundance (genus Catenibacterium id.2153)' on 'Histone H2A deubiquitinase MYSM1'

Batch 2−3966 : Gut microbiota abundance (genus Parasutterella id.2892) on Histone H2A deubiquitinase MYSM1
with a threshold of 1e−05

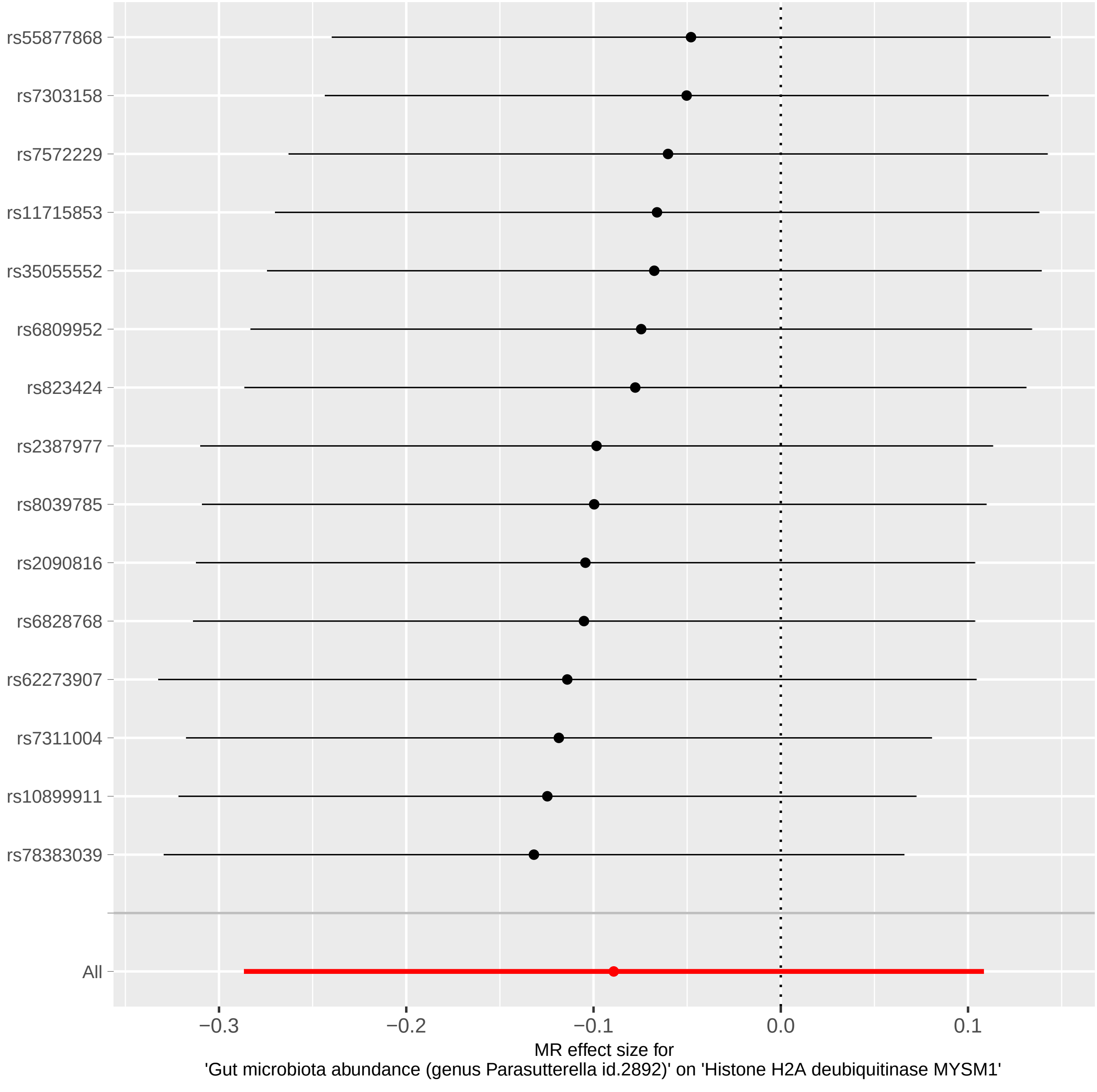

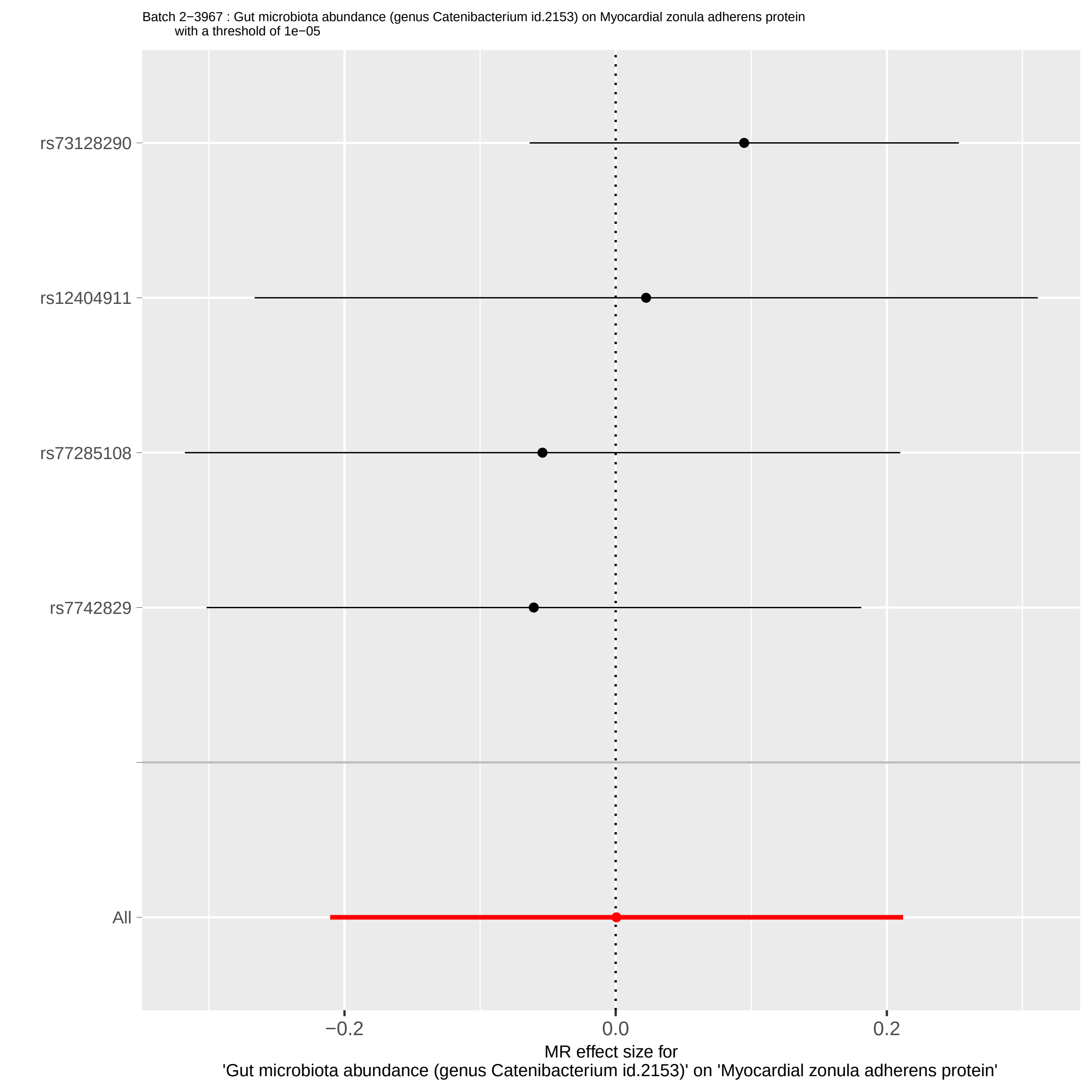
Batch 2−3967 : Gut microbiota abundance (genus Catenibacterium id.2153) on Myocardial zonula adherens protein
with a threshold of 1e−05
rs73128290
rs12404911
rs77285108
rs7742829
All
−0.2
0.0
0.2
MR effect size for
'Gut microbiota abundance (genus Catenibacterium id.2153)' on 'Myocardial zonula adherens protein'

Batch 2−3968 : Gut microbiota abundance (genus Parasutterella id.2892) on Myocardial zonula adherens protein
with a threshold of 1e−05

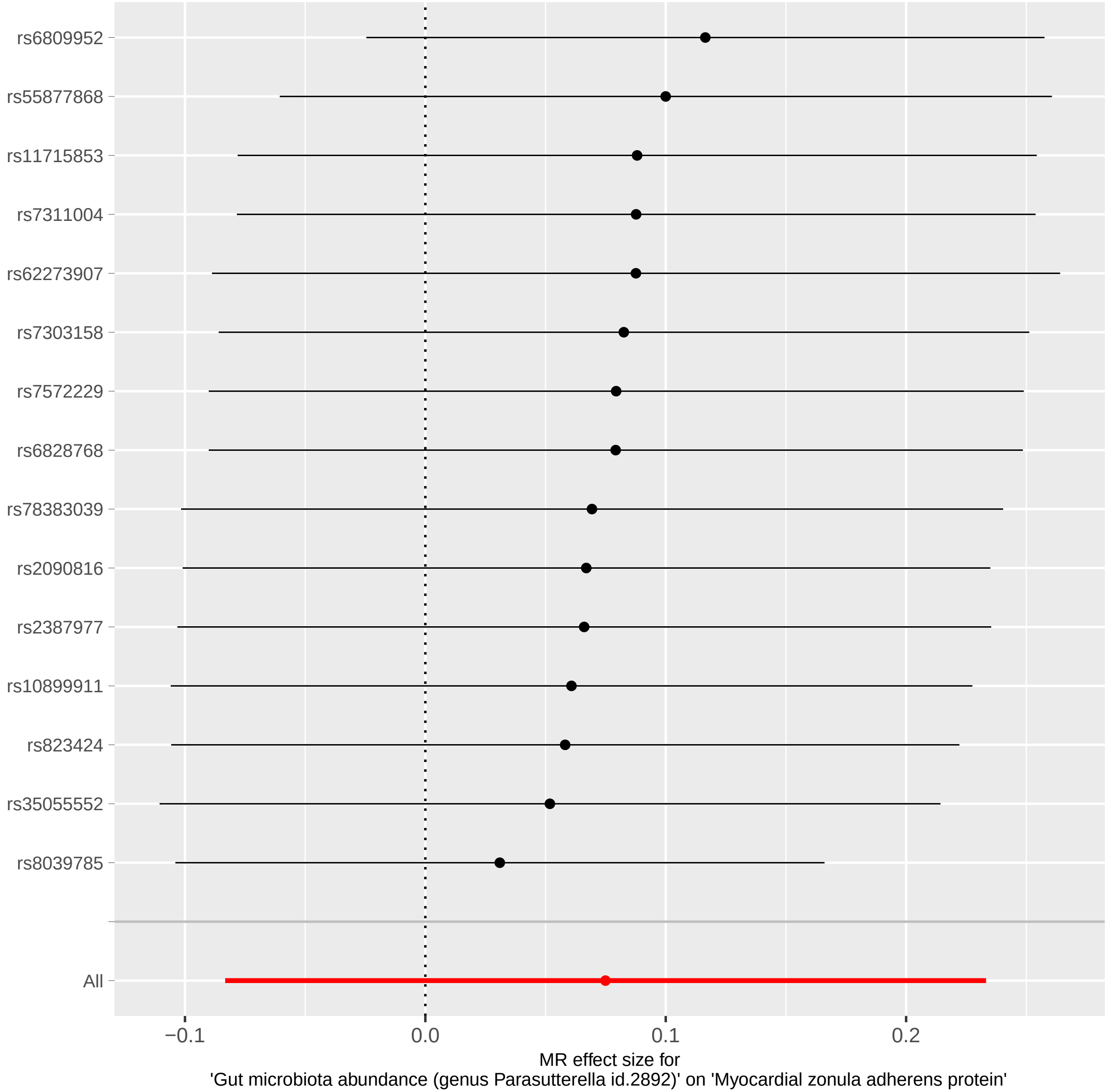

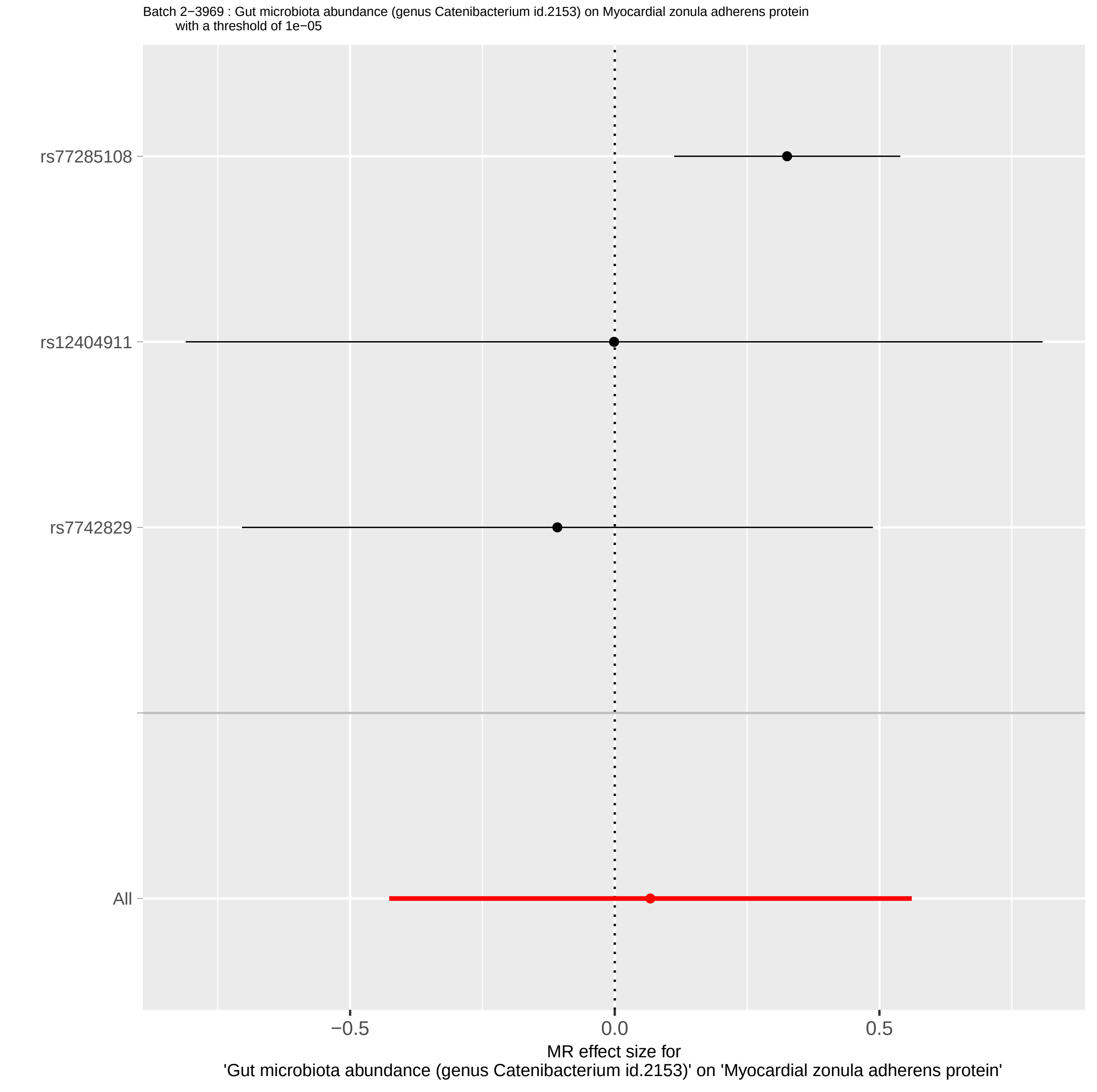

Batch 2−3969 : Gut microbiota abundance (genus Catenibacterium id.2153) on Myocardial zonula adherens protein
with a threshold of 1e−05
rs77285108
rs12404911
rs7742829
All
−0.5
0.0
0.5
MR effect size for
'Gut microbiota abundance (genus Catenibacterium id.2153)' on 'Myocardial zonula adherens protein'

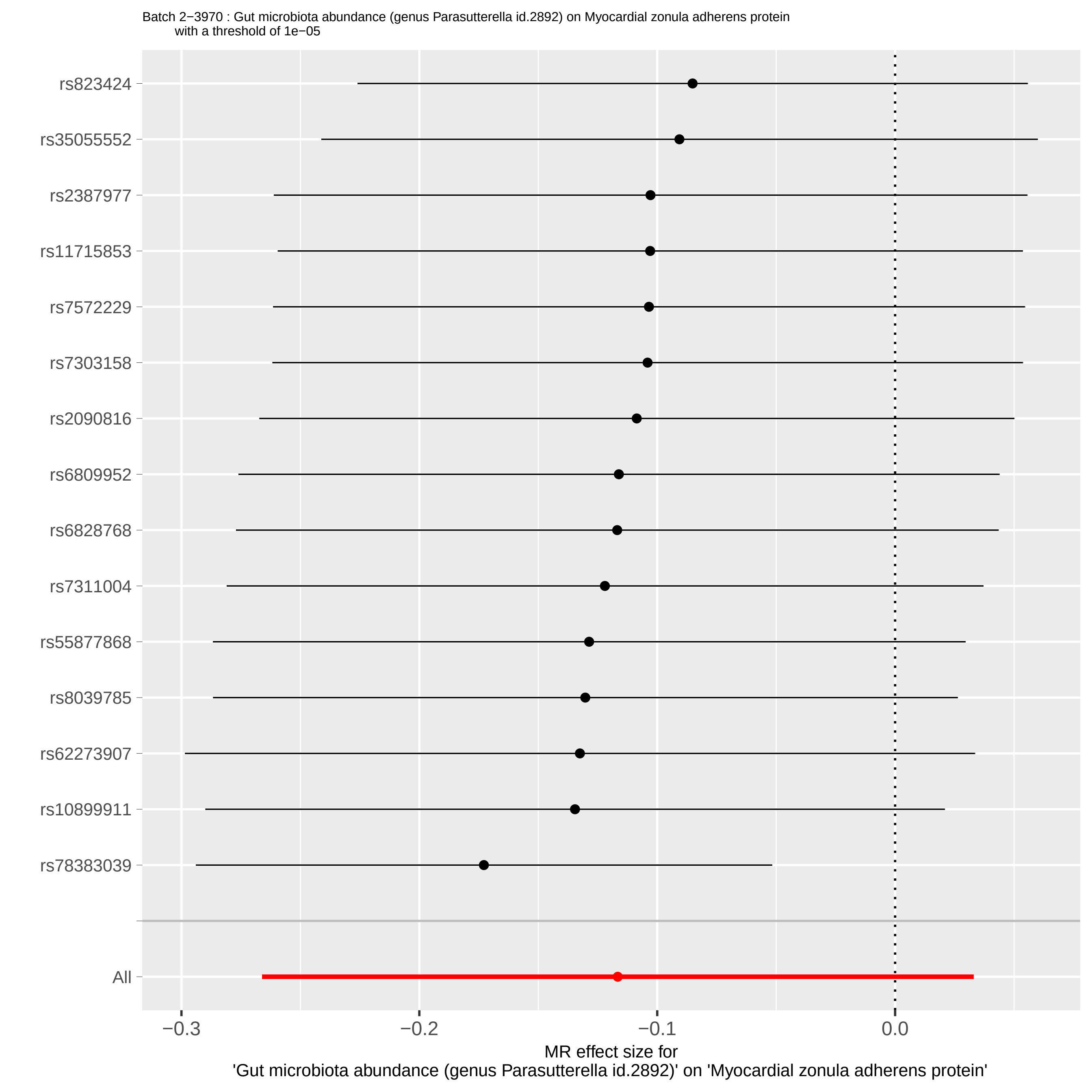

Batch 2−3970 : Gut microbiota abundance (genus Parasutterella id.2892) on Myocardial zonula adherens protein
with a threshold of 1e−05
rs823424
rs35055552
rs2387977
rs11715853
rs7572229
rs7303158
rs2090816
rs6809952
rs6828768
rs7311004
rs55877868
rs8039785
rs62273907
rs10899911
rs78383039
All
−0.3
−0.2
−0.1
0.0
MR effect size for
'Gut microbiota abundance (genus Parasutterella id.2892)' on 'Myocardial zonula adherens protein'

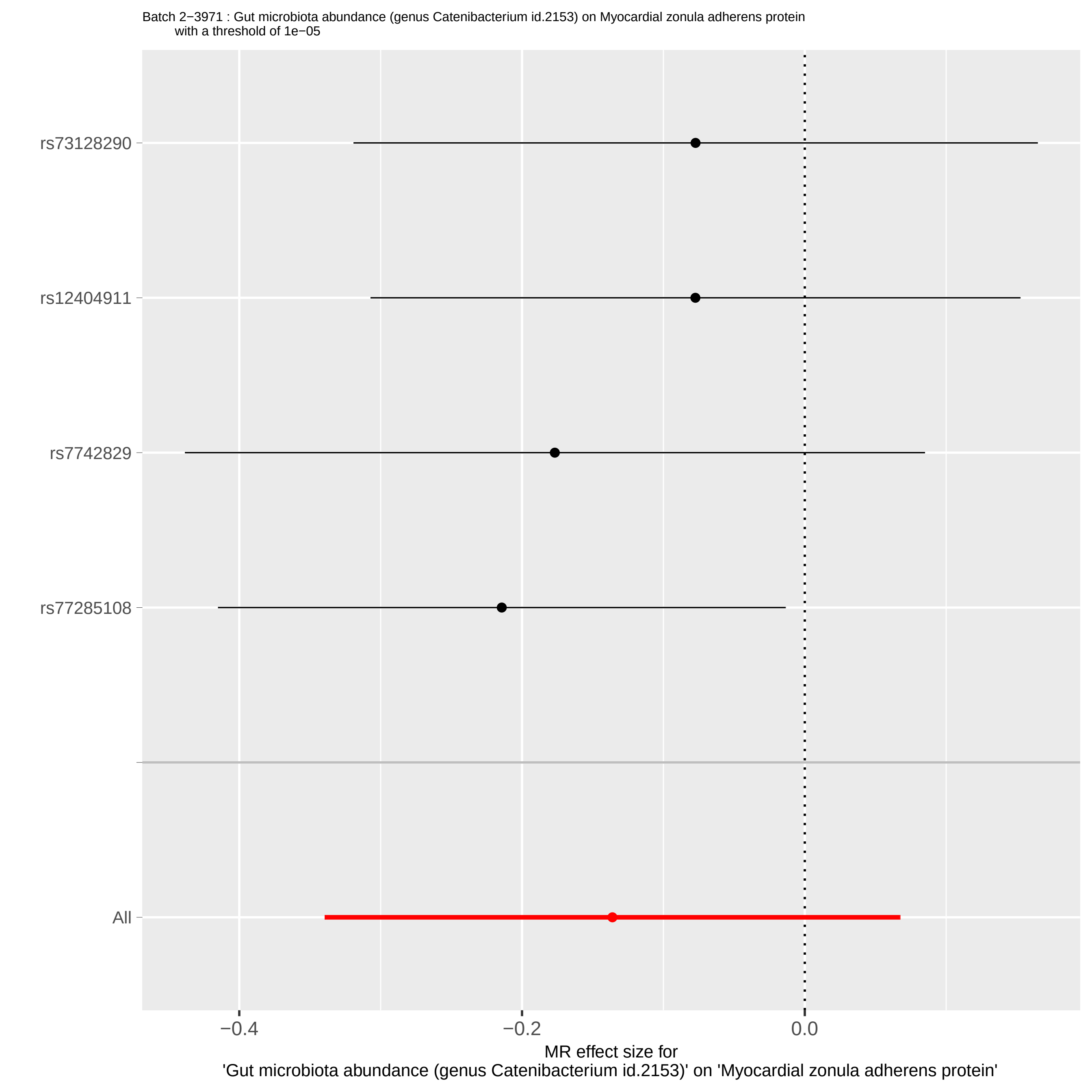

Batch 2−3971 : Gut microbiota abundance (genus Catenibacterium id.2153) on Myocardial zonula adherens protein
with a threshold of 1e−05
rs73128290
rs12404911
rs7742829
rs77285108
All
−0.4
−0.2
0.0
MR effect size for
'Gut microbiota abundance (genus Catenibacterium id.2153)' on 'Myocardial zonula adherens protein'

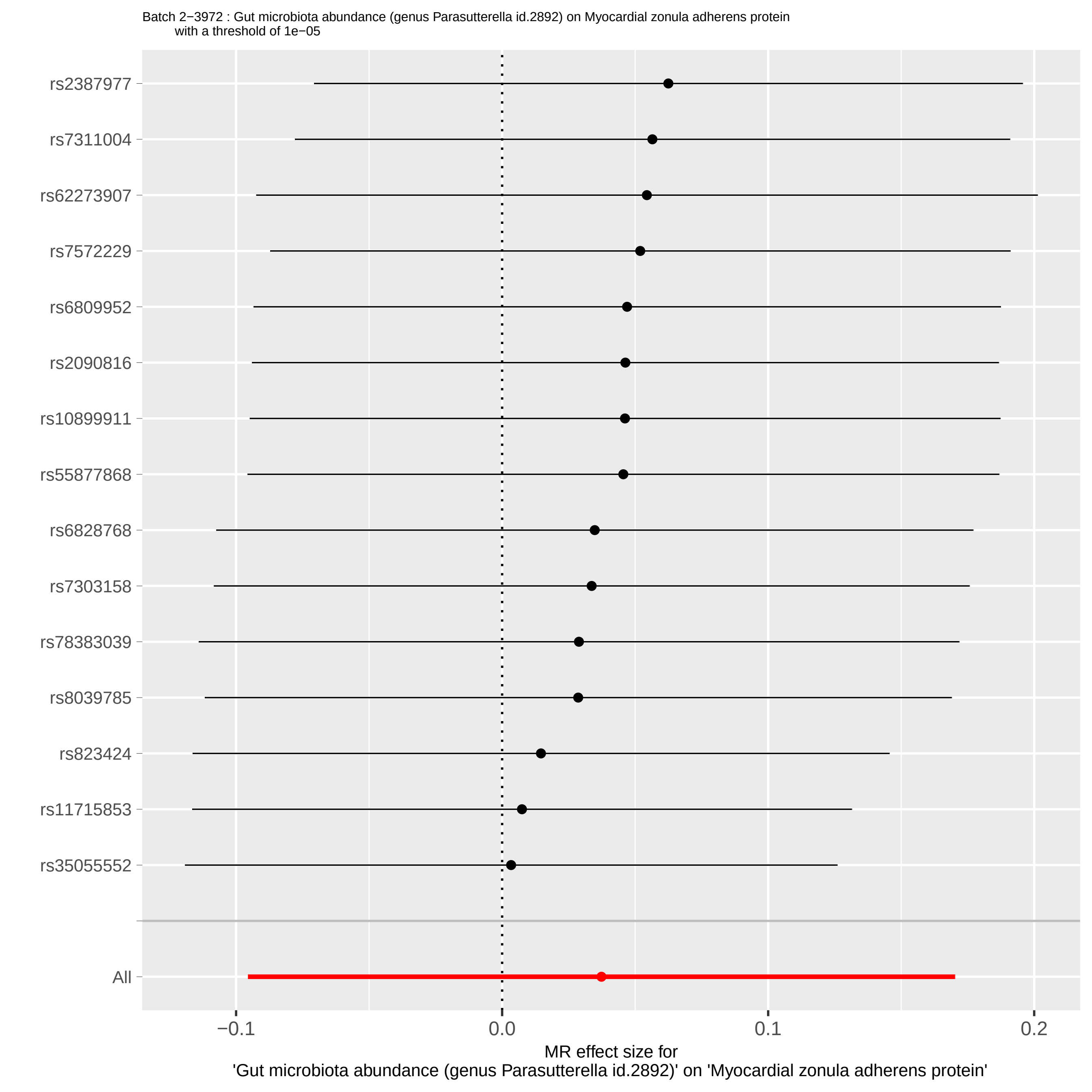
Batch 2−3972 : Gut microbiota abundance (genus Parasutterella id.2892) on Myocardial zonula adherens protein
with a threshold of 1e−05
rs2387977
rs7311004
rs62273907
rs7572229
rs6809952
rs2090816
rs10899911
rs55877868
rs6828768
rs7303158
rs78383039
rs8039785
rs823424
rs11715853
rs35055552
All
−0.1
0.0
0.1
0.2
MR effect size for
'Gut microbiota abundance (genus Parasutterella id.2892)' on 'Myocardial zonula adherens protein'

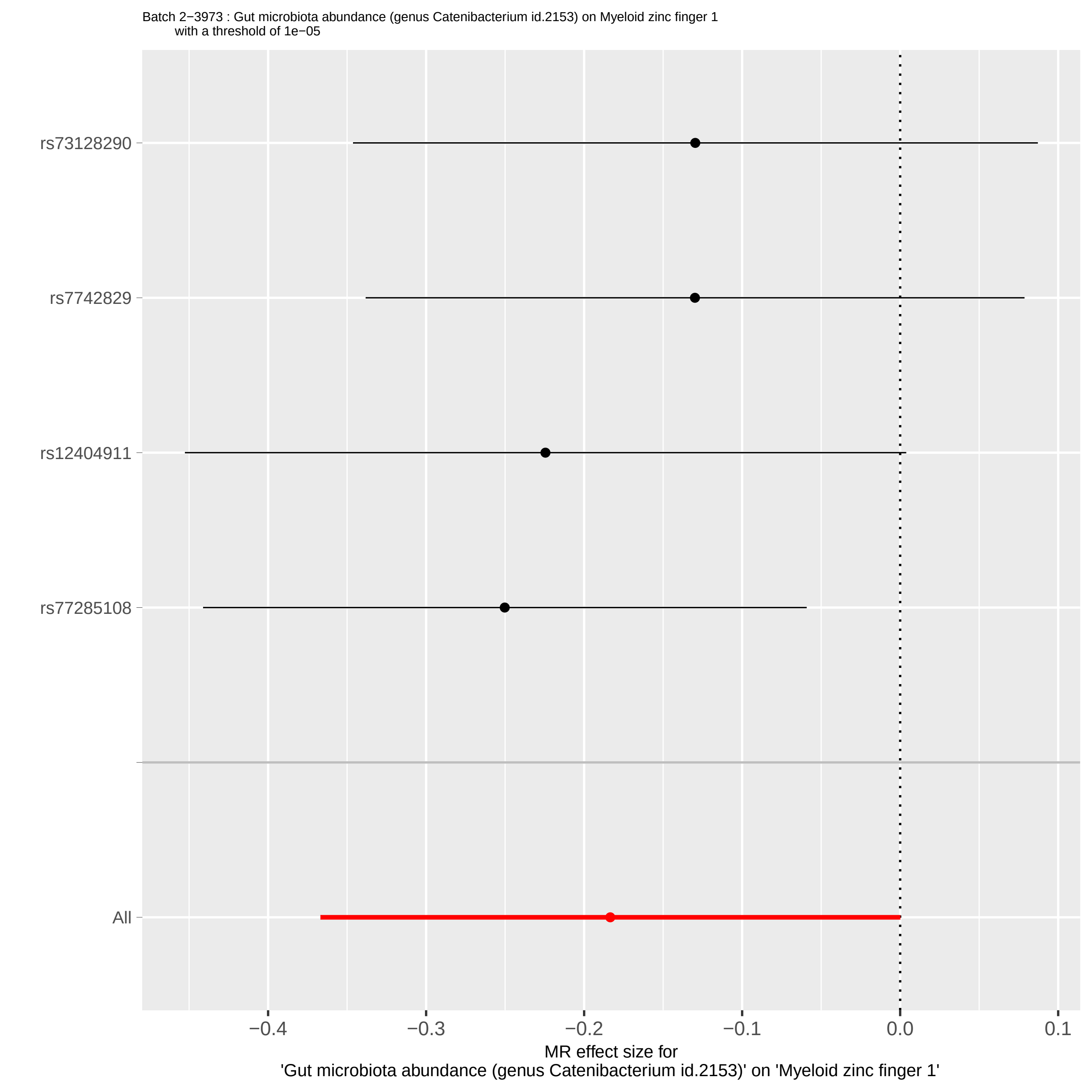

Batch 2−3973 : Gut microbiota abundance (genus Catenibacterium id.2153) on Myeloid zinc finger 1
with a threshold of 1e−05
rs73128290
rs7742829
rs12404911
rs77285108
All
−0.4
−0.3
−0.2
−0.1
0.0
0.1
MR effect size for
'Gut microbiota abundance (genus Catenibacterium id.2153)' on 'Myeloid zinc finger 1'

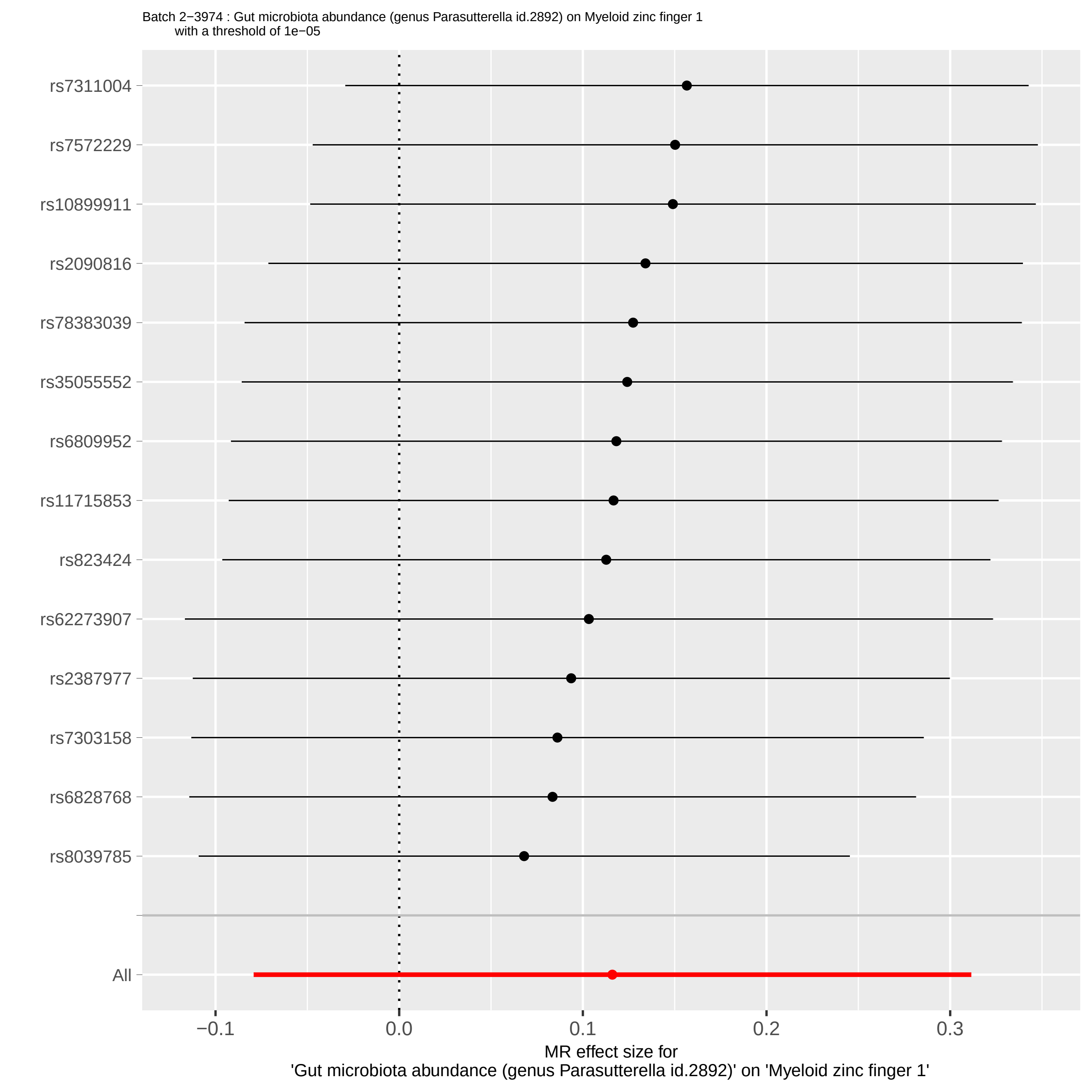
Batch 2−3974 : Gut microbiota abundance (genus Parasutterella id.2892) on Myeloid zinc finger 1
with a threshold of 1e−05
rs7311004
rs7572229
rs10899911
rs2090816
rs78383039
rs35055552
rs6809952
rs11715853
rs823424
rs62273907
rs2387977
rs7303158
rs6828768
rs8039785
All
−0.1
0.0
0.1
0.2
0.3
MR effect size for
'Gut microbiota abundance (genus Parasutterella id.2892)' on 'Myeloid zinc finger 1'

Batch 2−3975 : Gut microbiota abundance (genus Catenibacterium id.2153) on Mitotic−spindle organizing protein 1
with a threshold of 1e−05

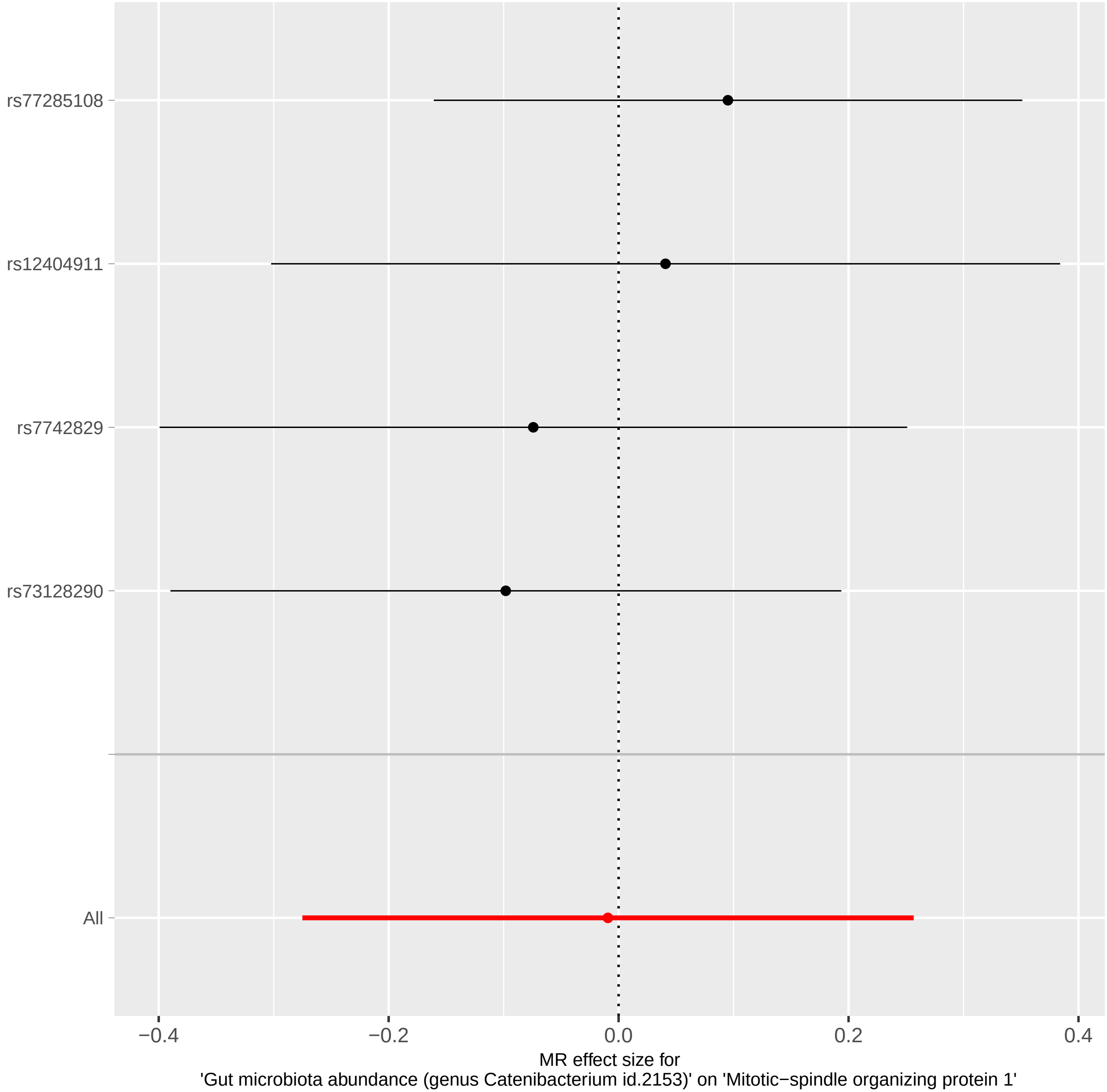

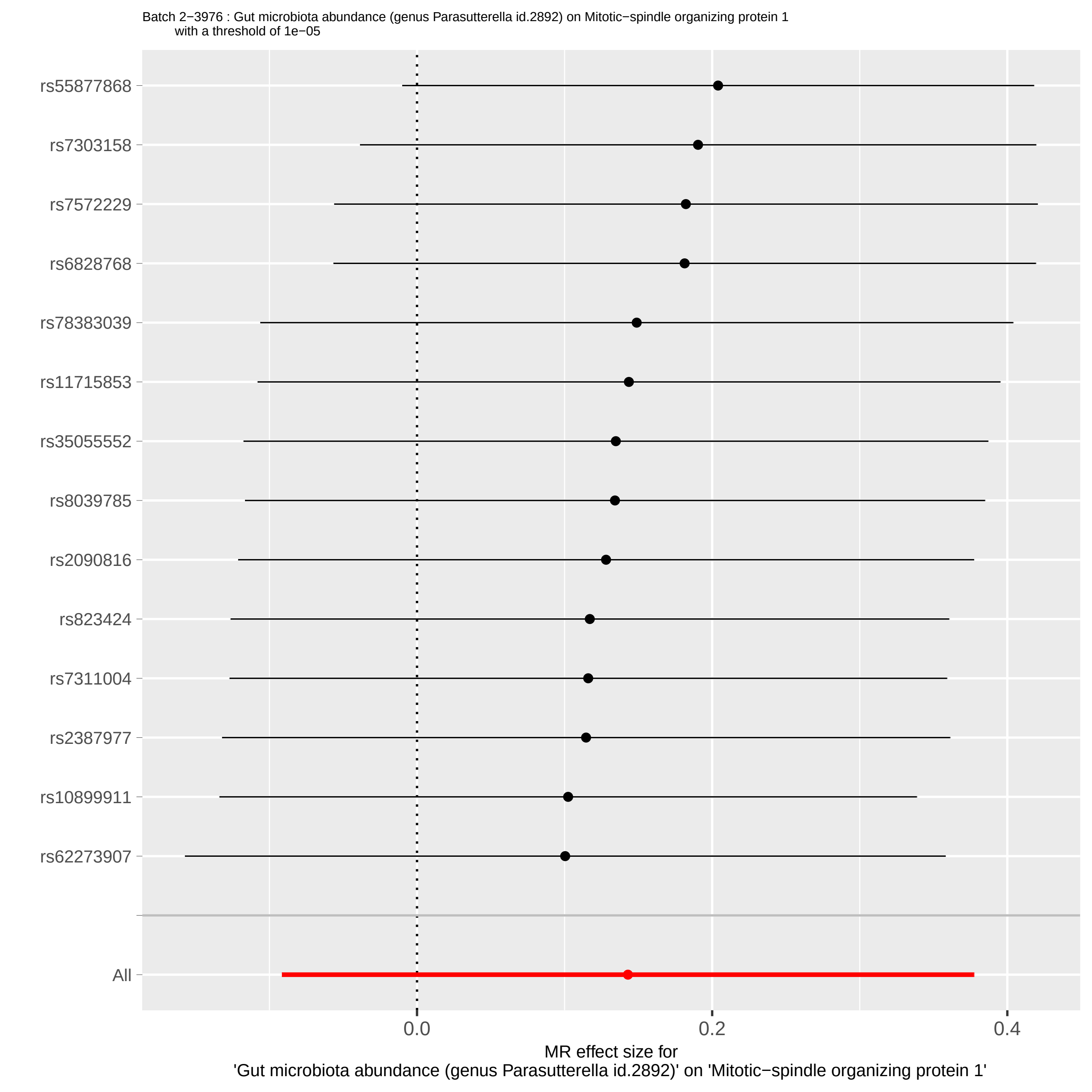
Batch 2−3976 : Gut microbiota abundance (genus Parasutterella id.2892) on Mitotic−spindle organizing protein 1
with a threshold of 1e−05
rs55877868
rs7303158
rs7572229
rs6828768
rs78383039
rs11715853
rs35055552
rs8039785
rs2090816
rs823424
rs7311004
rs2387977
rs10899911
rs62273907
All
0.0
0.2
0.4
MR effect size for
'Gut microbiota abundance (genus Parasutterella id.2892)' on 'Mitotic−spindle organizing protein 1'

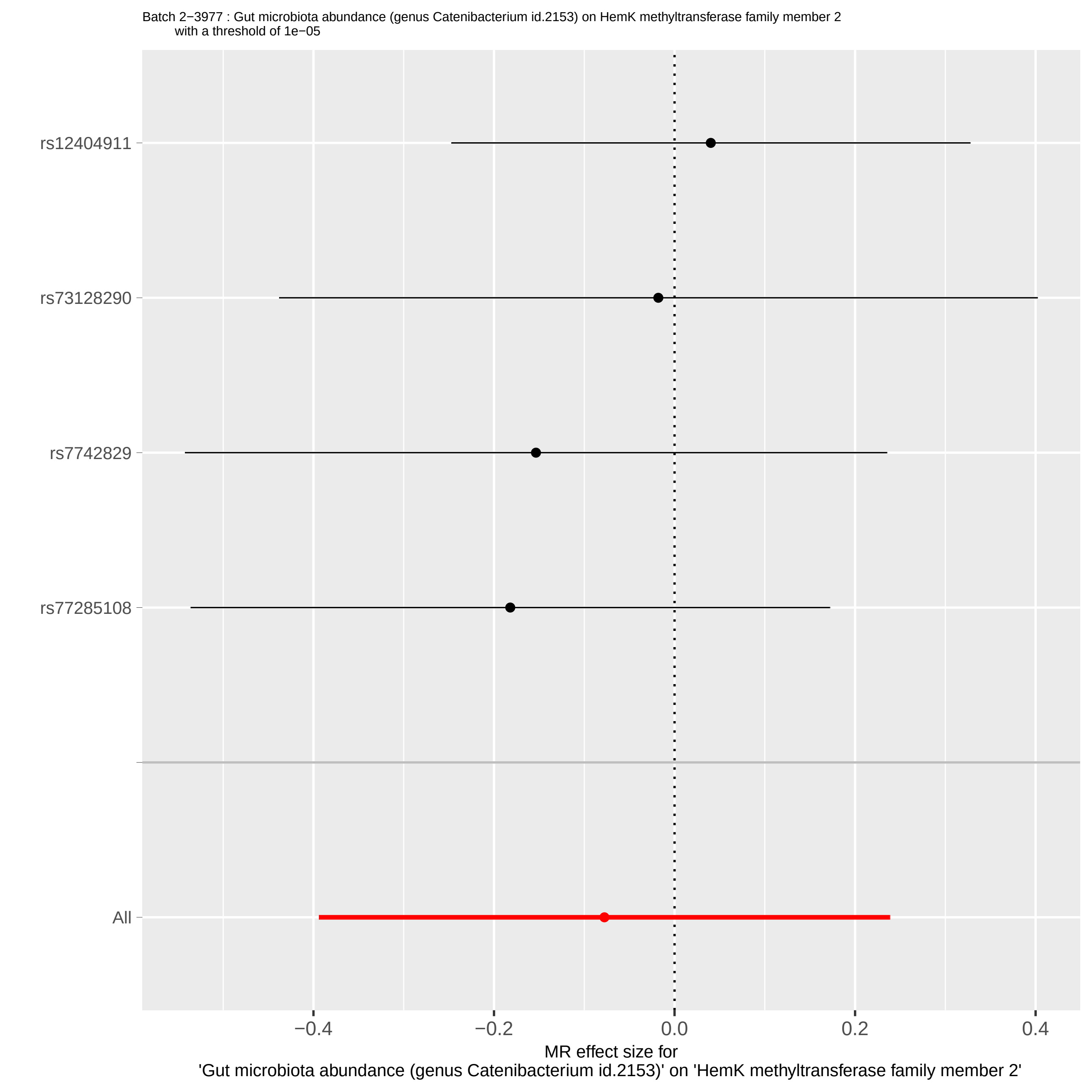
Batch 2−3977 : Gut microbiota abundance (genus Catenibacterium id.2153) on HemK methyltransferase family member 2
with a threshold of 1e−05
rs12404911
rs73128290
rs7742829
rs77285108
All
−0.4
−0.2
0.0
0.2
0.4
MR effect size for
'Gut microbiota abundance (genus Catenibacterium id.2153)' on 'HemK methyltransferase family member 2'

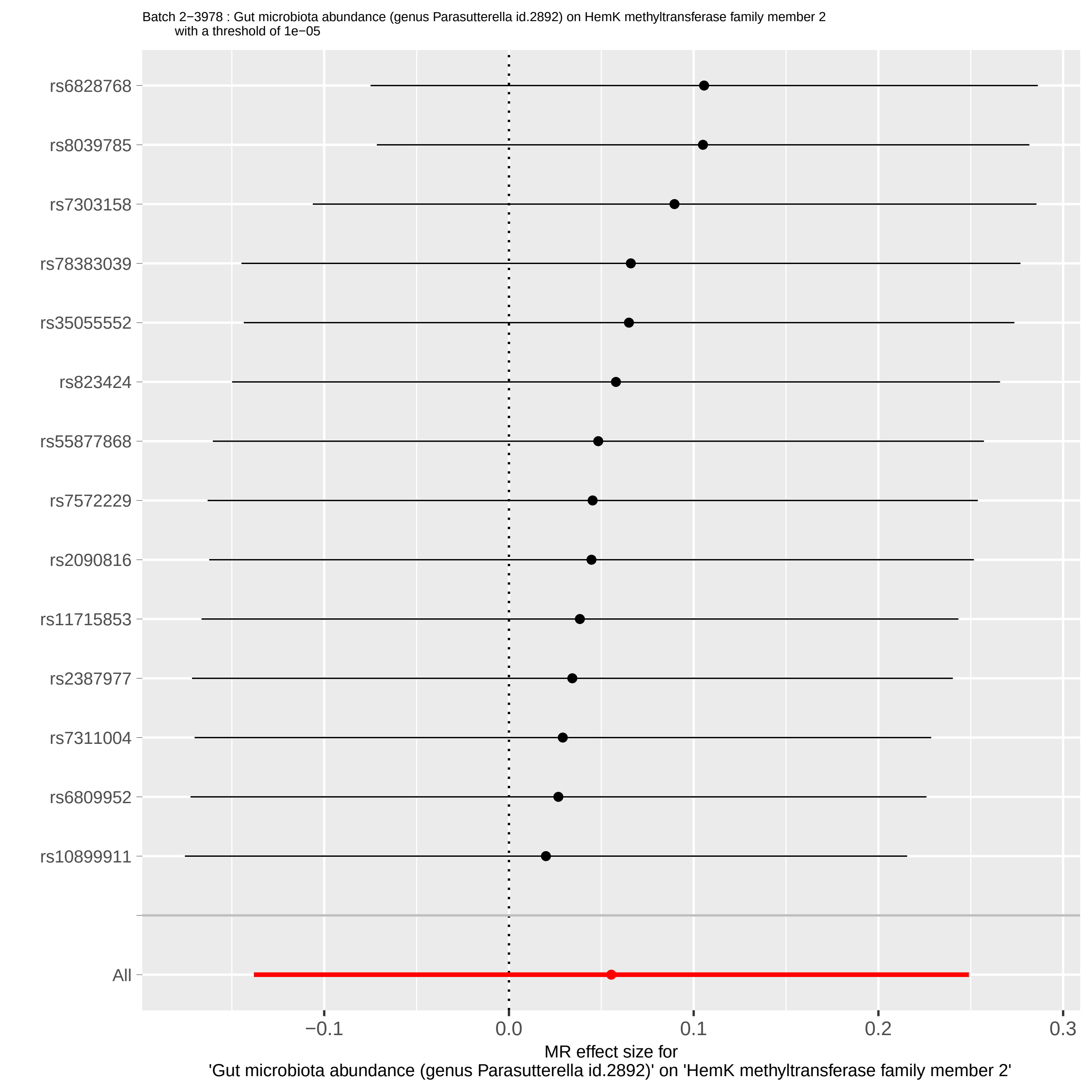
Batch 2−3978 : Gut microbiota abundance (genus Parasutterella id.2892) on HemK methyltransferase family member 2
with a threshold of 1e−05
rs6828768
rs8039785
rs7303158
rs78383039
rs35055552
rs823424
rs55877868
rs7572229
rs2090816
rs11715853
rs2387977
rs7311004
rs6809952
rs10899911
All
−0.1
0.0
0.1
0.2
0.3
MR effect size for
'Gut microbiota abundance (genus Parasutterella id.2892)' on 'HemK methyltransferase family member 2'

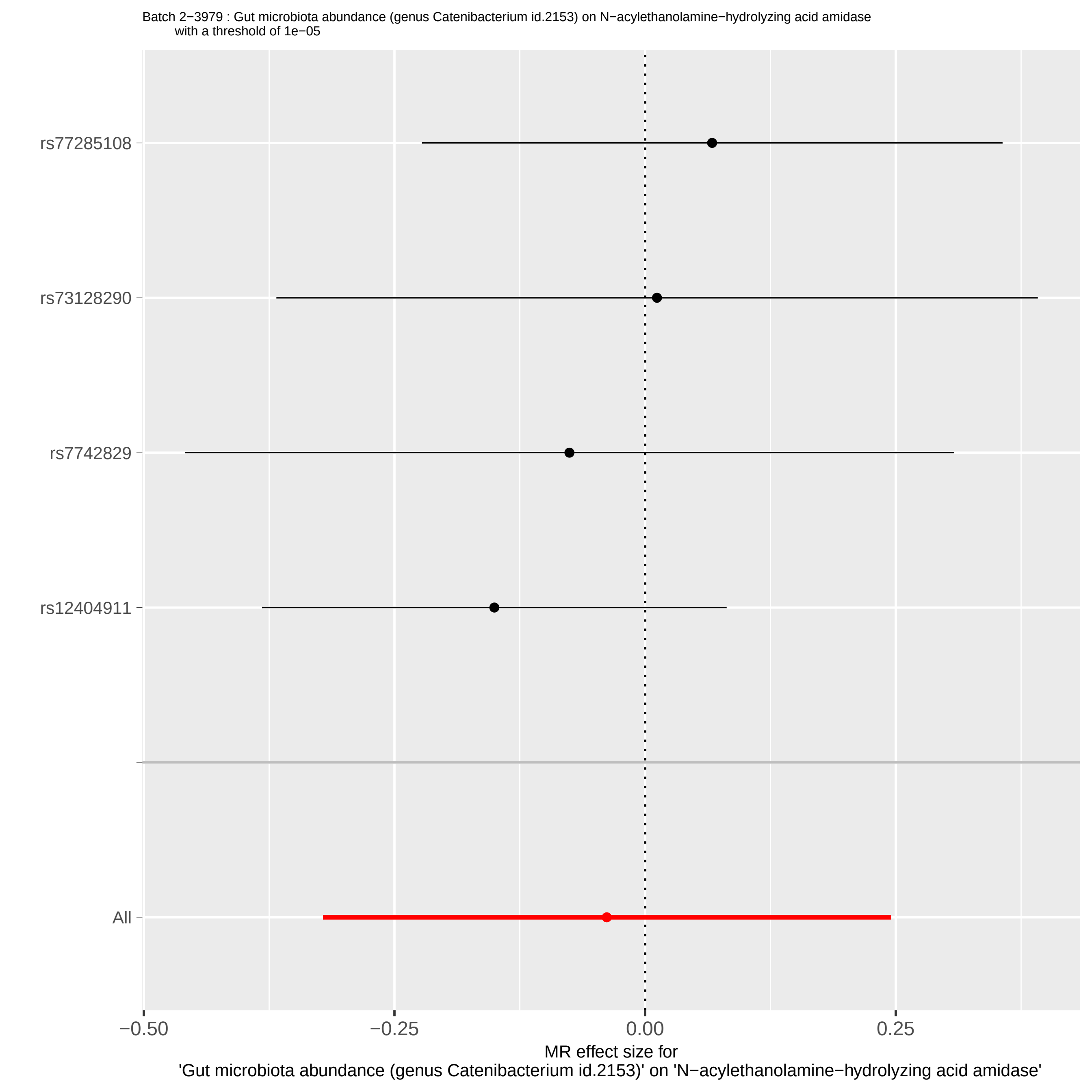
Batch 2−3979 : Gut microbiota abundance (genus Catenibacterium id.2153) on N−acylethanolamine−hydrolyzing acid amidase
with a threshold of 1e−05
rs77285108
rs73128290
rs7742829
rs12404911
All
−0.50
−0.25
0.00
0.25
MR effect size for
'Gut microbiota abundance (genus Catenibacterium id.2153)' on 'N−acylethanolamine−hydrolyzing acid amidase'

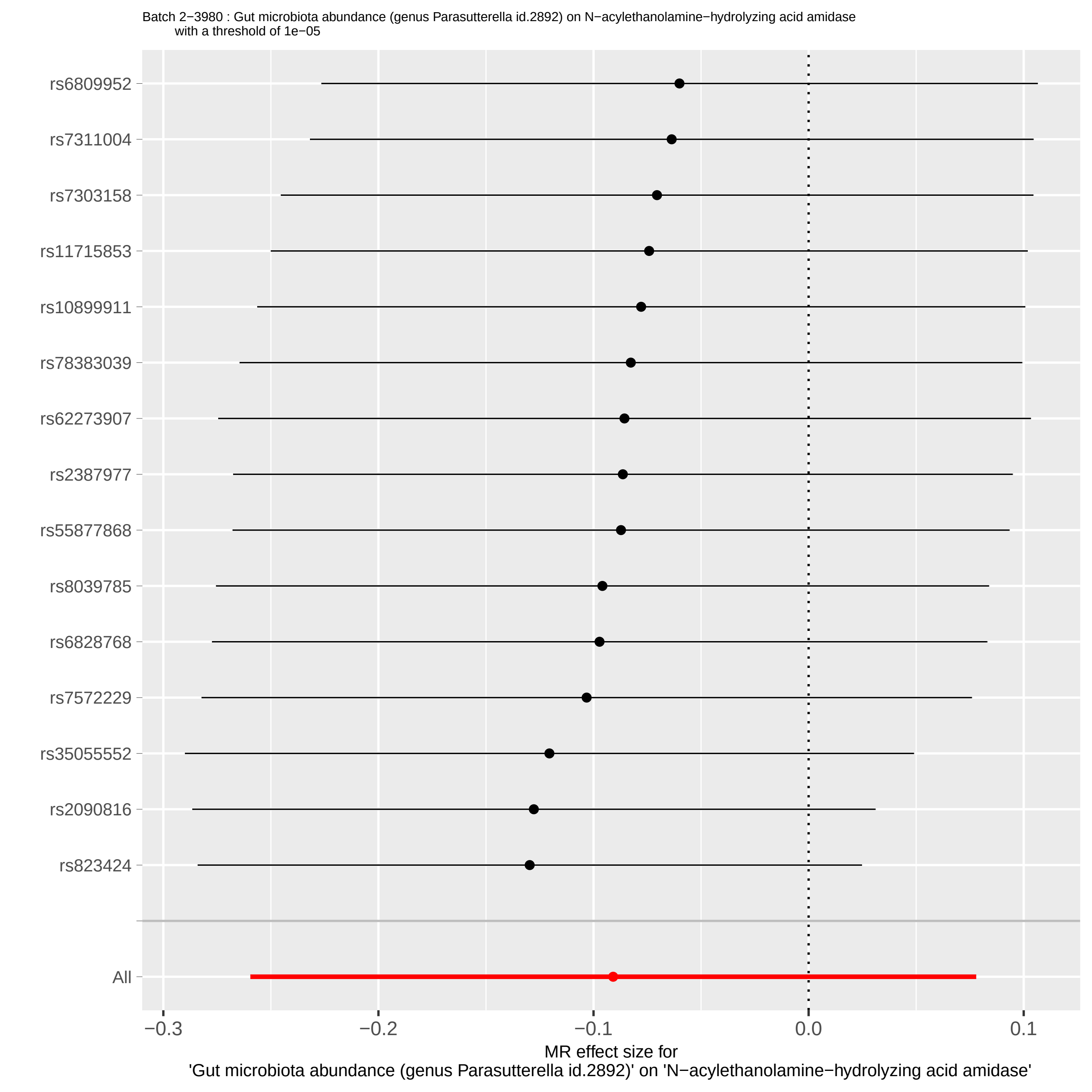

Batch 2−3980 : Gut microbiota abundance (genus Parasutterella id.2892) on N−acylethanolamine−hydrolyzing acid amidase
with a threshold of 1e−05
rs6809952
rs7311004
rs7303158
rs11715853
rs10899911
rs78383039
rs62273907
rs2387977
rs55877868
rs8039785
rs6828768
rs7572229
rs35055552
rs2090816
rs823424
All
−0.3
−0.2
−0.1
0.0
0.1
MR effect size for
'Gut microbiota abundance (genus Parasutterella id.2892)' on 'N−acylethanolamine−hydrolyzing acid amidase'

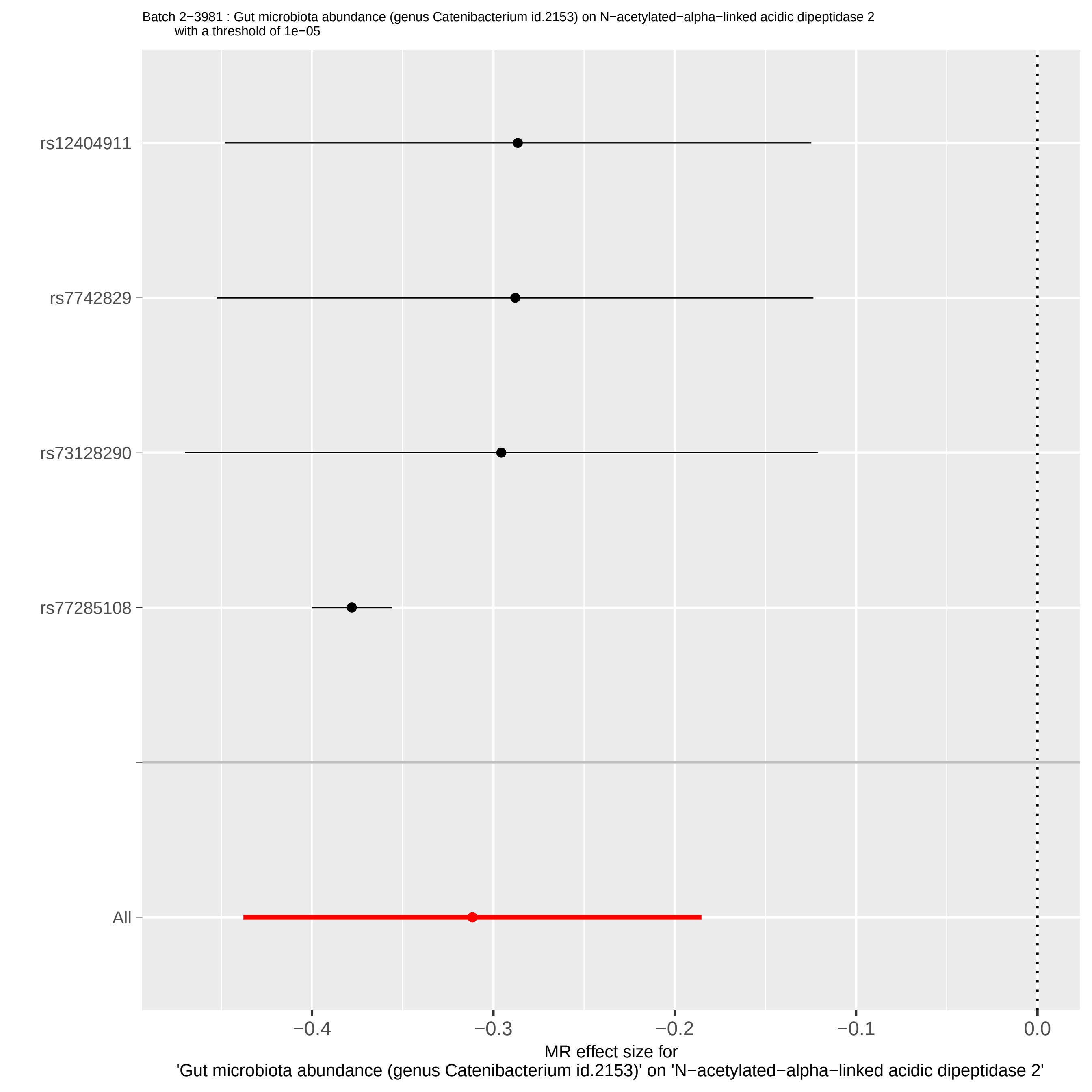
Batch 2−3981 : Gut microbiota abundance (genus Catenibacterium id.2153) on N−acetylated−alpha−linked acidic dipeptidase 2
with a threshold of 1e−05
rs12404911
rs7742829
rs73128290
rs77285108
All
−0.4
−0.3
−0.2
−0.1
0.0
MR effect size for
'Gut microbiota abundance (genus Catenibacterium id.2153)' on 'N−acetylated−alpha−linked acidic dipeptidase 2'

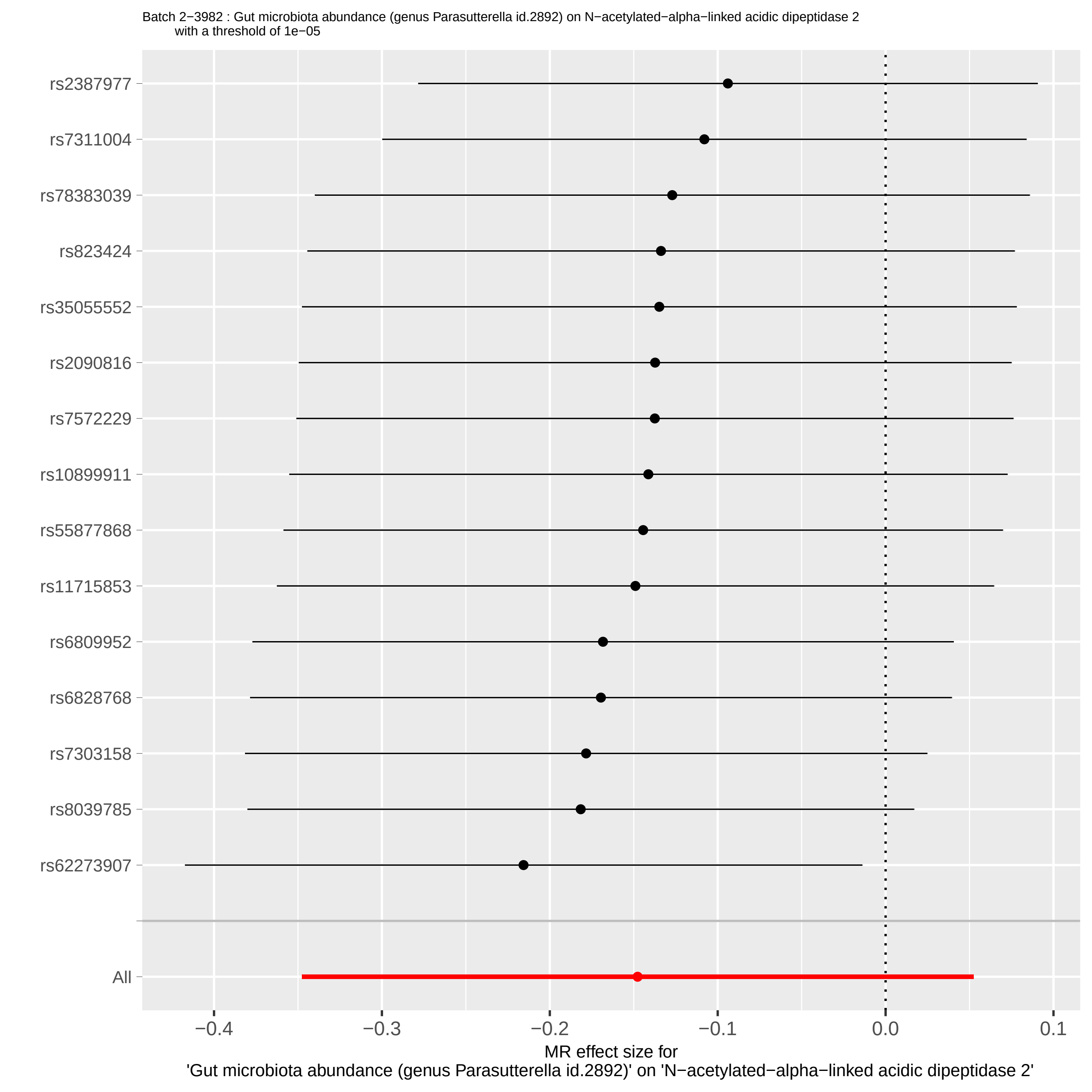

Batch 2−3982 : Gut microbiota abundance (genus Parasutterella id.2892) on N−acetylated−alpha−linked acidic dipeptidase 2
with a threshold of 1e−05
rs2387977
rs7311004
rs78383039
rs823424
rs35055552
rs2090816
rs7572229
rs10899911
rs55877868
rs11715853
rs6809952
rs6828768
rs7303158
rs8039785
rs62273907
All
−0.4
−0.3
−0.2
−0.1
0.0
0.1
MR effect size for
'Gut microbiota abundance (genus Parasutterella id.2892)' on 'N−acetylated−alpha−linked acidic dipeptidase 2'

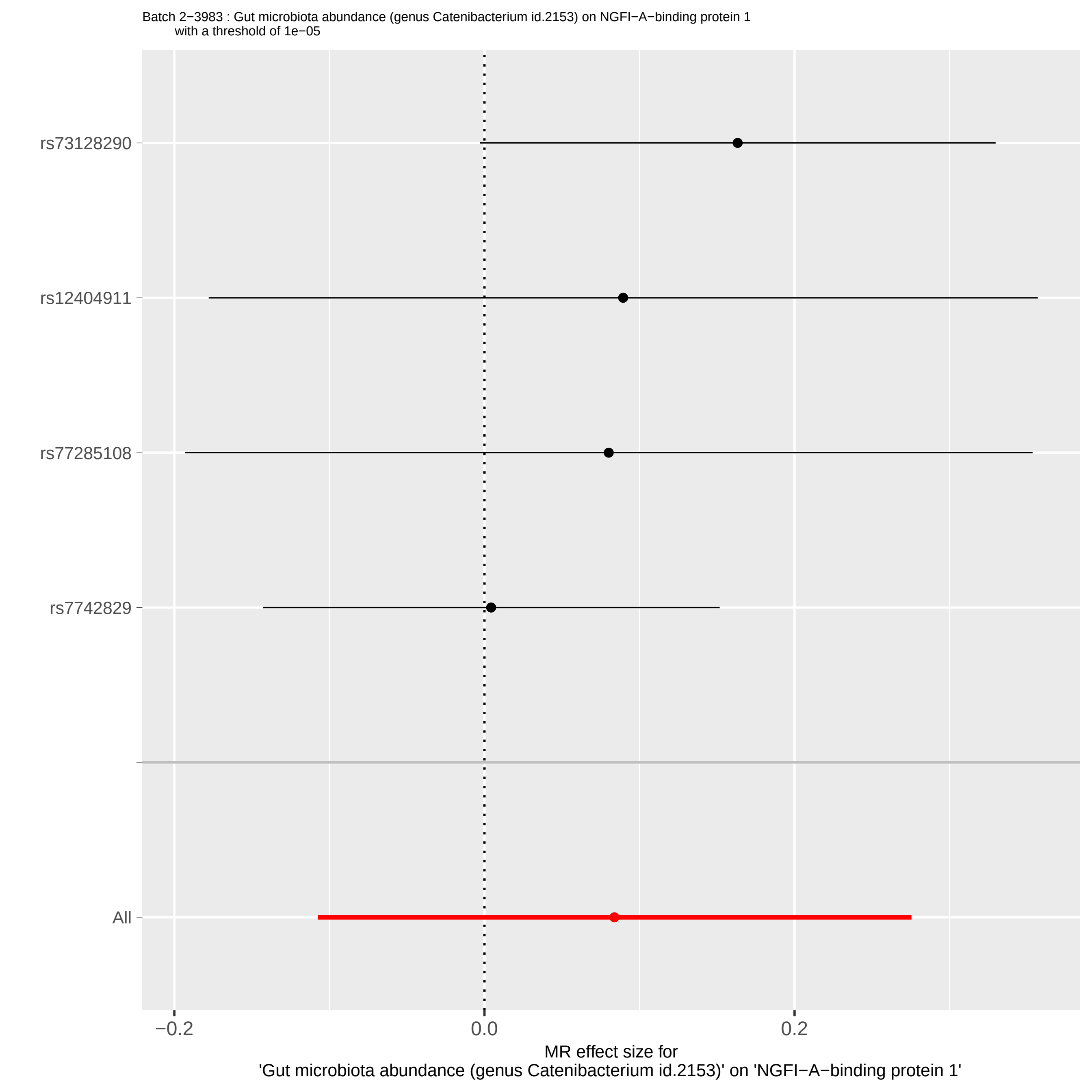
Batch 2−3983 : Gut microbiota abundance (genus Catenibacterium id.2153) on NGFI−A−binding protein 1
with a threshold of 1e−05
rs73128290
rs12404911
rs77285108
rs7742829
All
−0.2
0.0
0.2
MR effect size for
'Gut microbiota abundance (genus Catenibacterium id.2153)' on 'NGFI−A−binding protein 1'

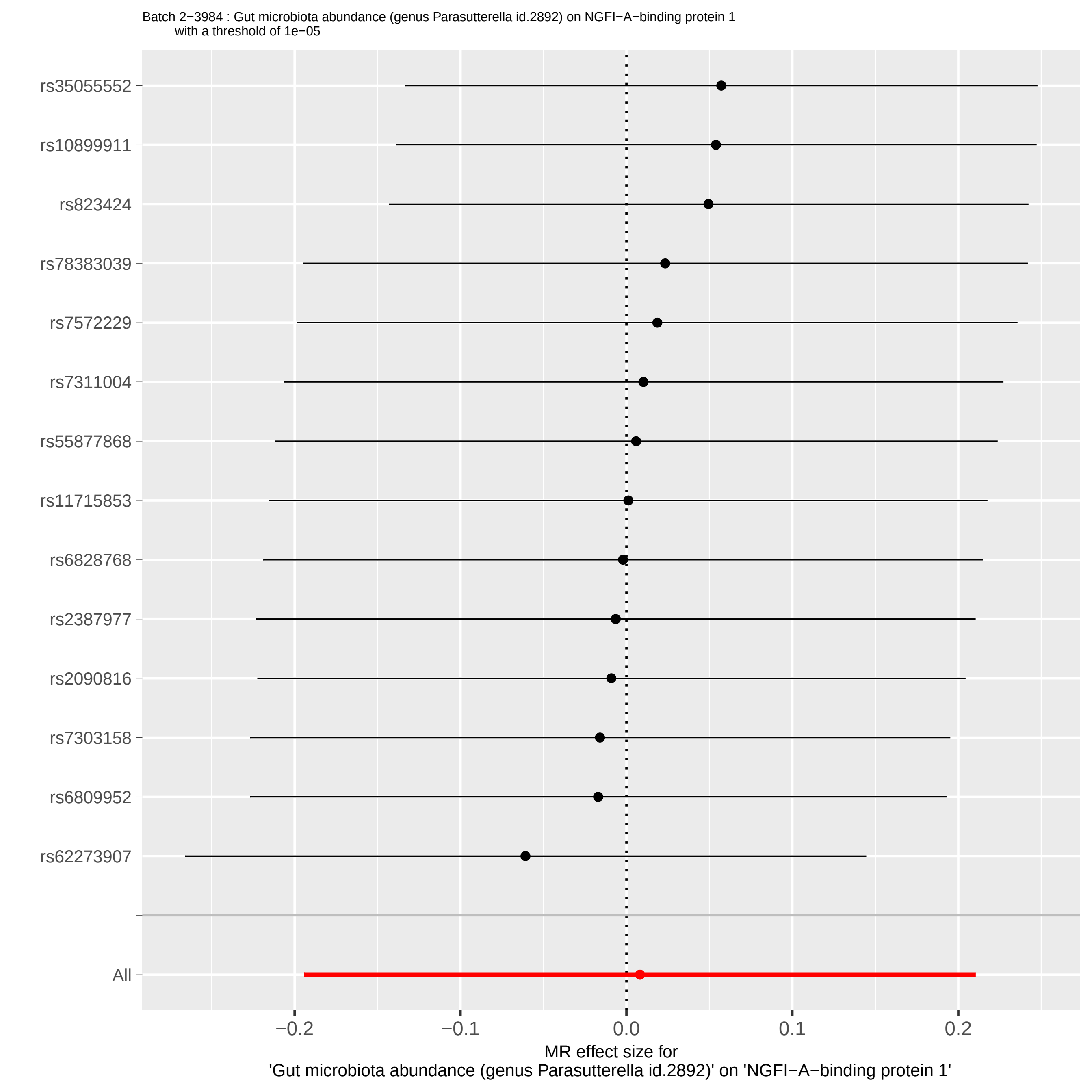
Batch 2−3984 : Gut microbiota abundance (genus Parasutterella id.2892) on NGFI−A−binding protein 1
with a threshold of 1e−05
rs35055552
rs10899911
rs823424
rs78383039
rs7572229
rs7311004
rs55877868
rs11715853
rs6828768
rs2387977
rs2090816
rs7303158
rs6809952
rs62273907
All
−0.2
−0.1
0.0
0.1
0.2
MR effect size for
'Gut microbiota abundance (genus Parasutterella id.2892)' on 'NGFI−A−binding protein 1'

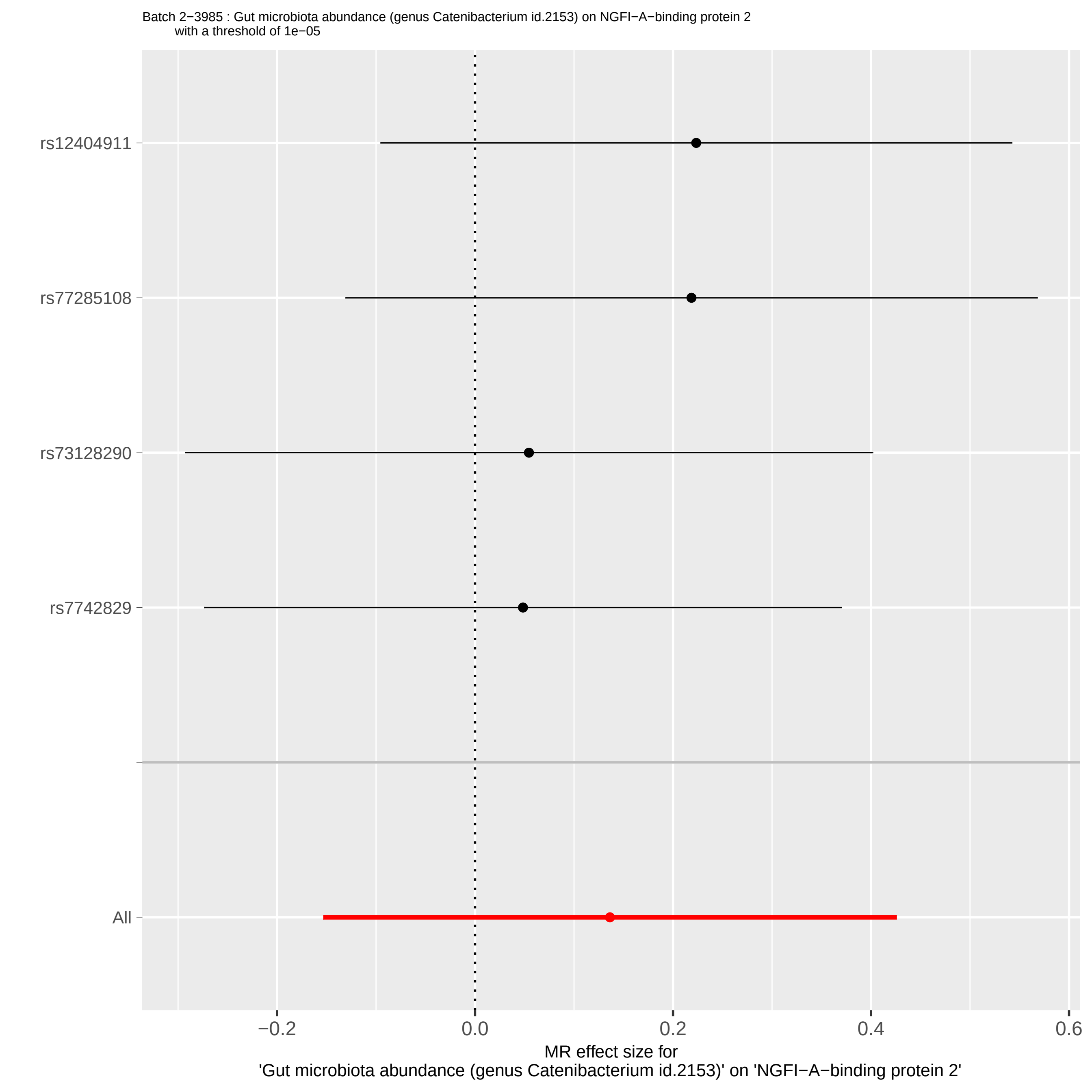

Batch 2−3985 : Gut microbiota abundance (genus Catenibacterium id.2153) on NGFI−A−binding protein 2
with a threshold of 1e−05
rs12404911
rs77285108
rs73128290
rs7742829
All
−0.2
0.0
0.2
0.4
0.6
MR effect size for
'Gut microbiota abundance (genus Catenibacterium id.2153)' on 'NGFI−A−binding protein 2'

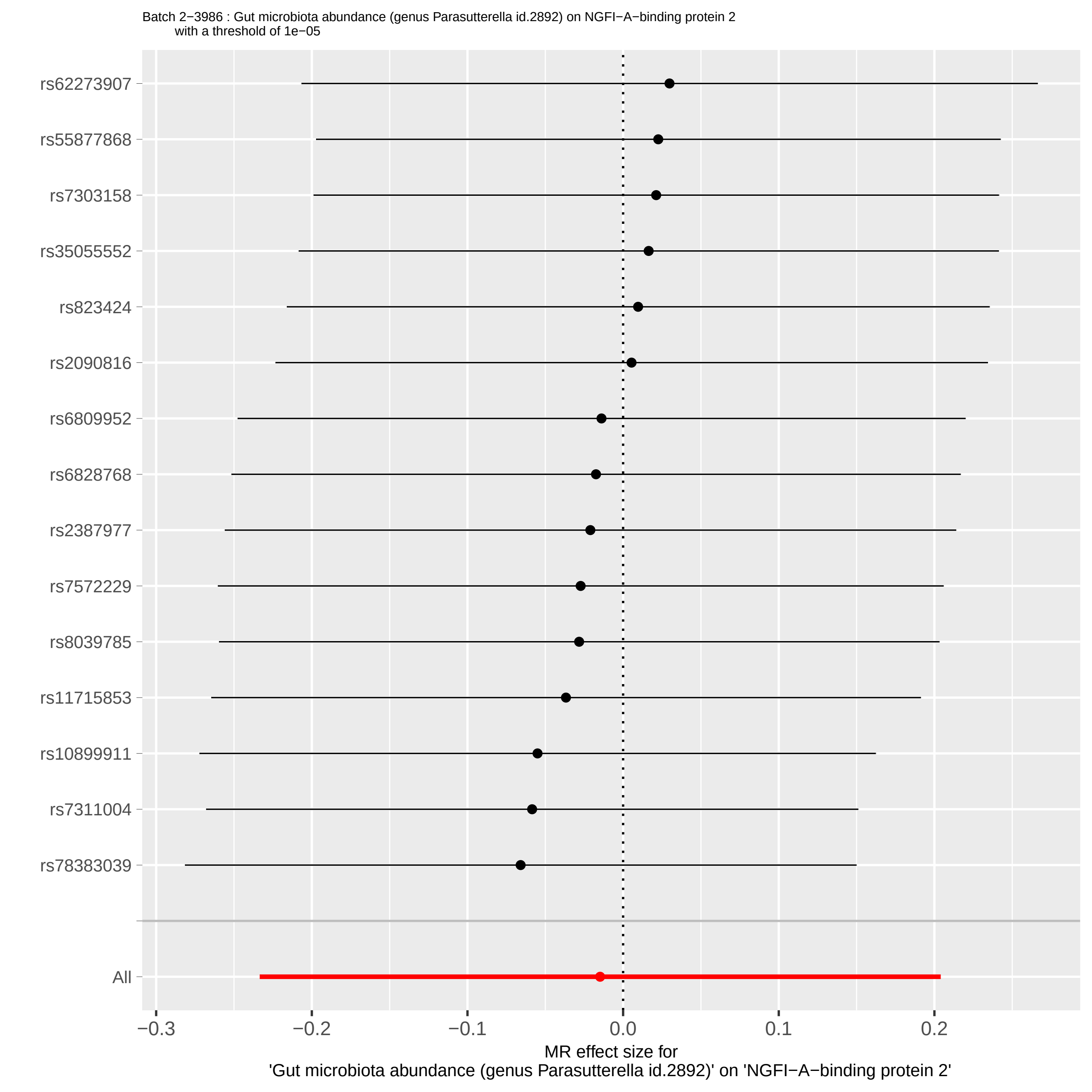
Batch 2−3986 : Gut microbiota abundance (genus Parasutterella id.2892) on NGFI−A−binding protein 2
with a threshold of 1e−05
rs62273907
rs55877868
rs7303158
rs35055552
rs823424
rs2090816
rs6809952
rs6828768
rs2387977
rs7572229
rs8039785
rs11715853
rs10899911
rs7311004
rs78383039
All
−0.3
−0.2
−0.1
0.0
0.1
0.2
MR effect size for
'Gut microbiota abundance (genus Parasutterella id.2892)' on 'NGFI−A−binding protein 2'

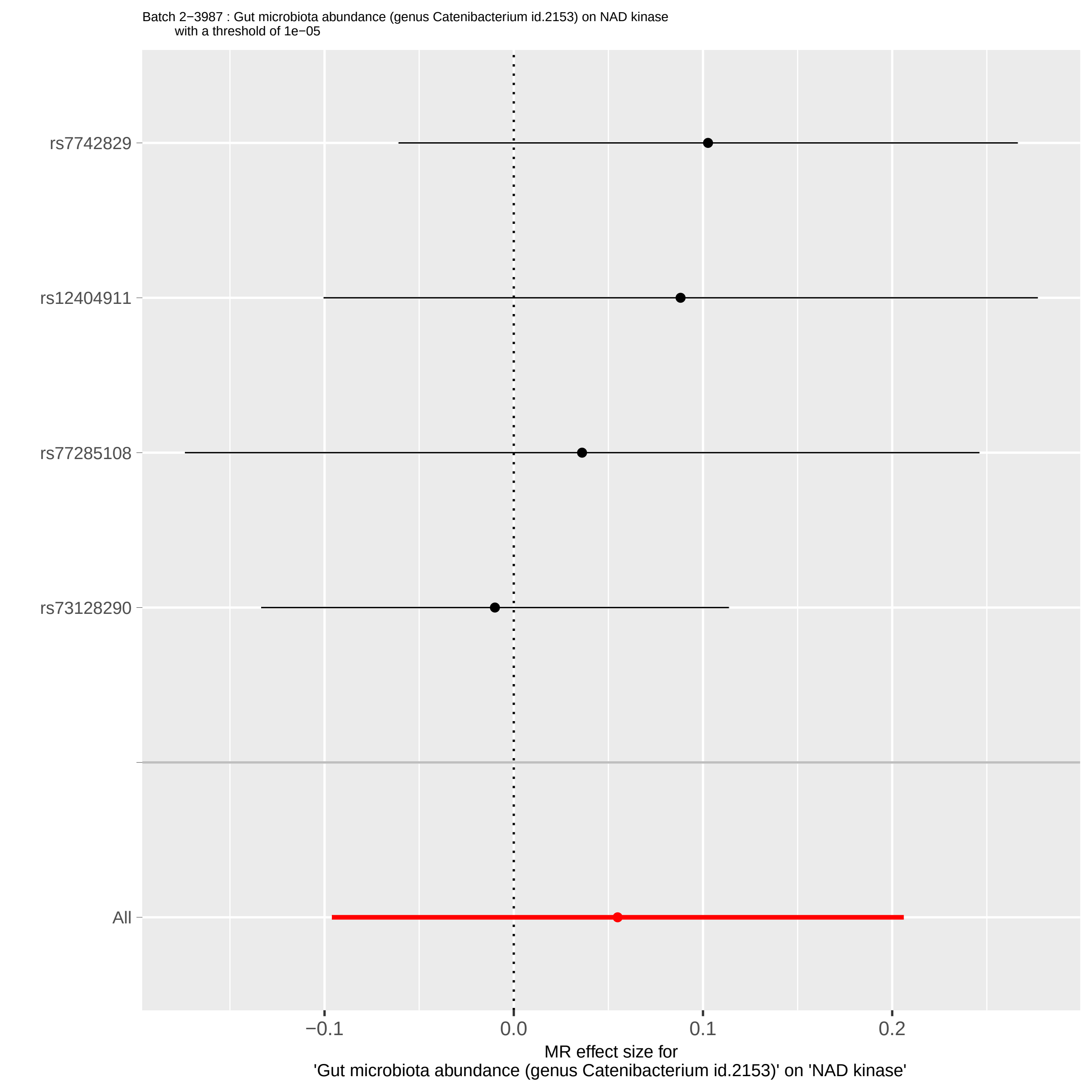
Batch 2−3987 : Gut microbiota abundance (genus Catenibacterium id.2153) on NAD kinase
with a threshold of 1e−05
rs7742829
rs12404911
rs77285108
rs73128290
All
−0.1
0.0
0.1
0.2
MR effect size for
'Gut microbiota abundance (genus Catenibacterium id.2153)' on 'NAD kinase'

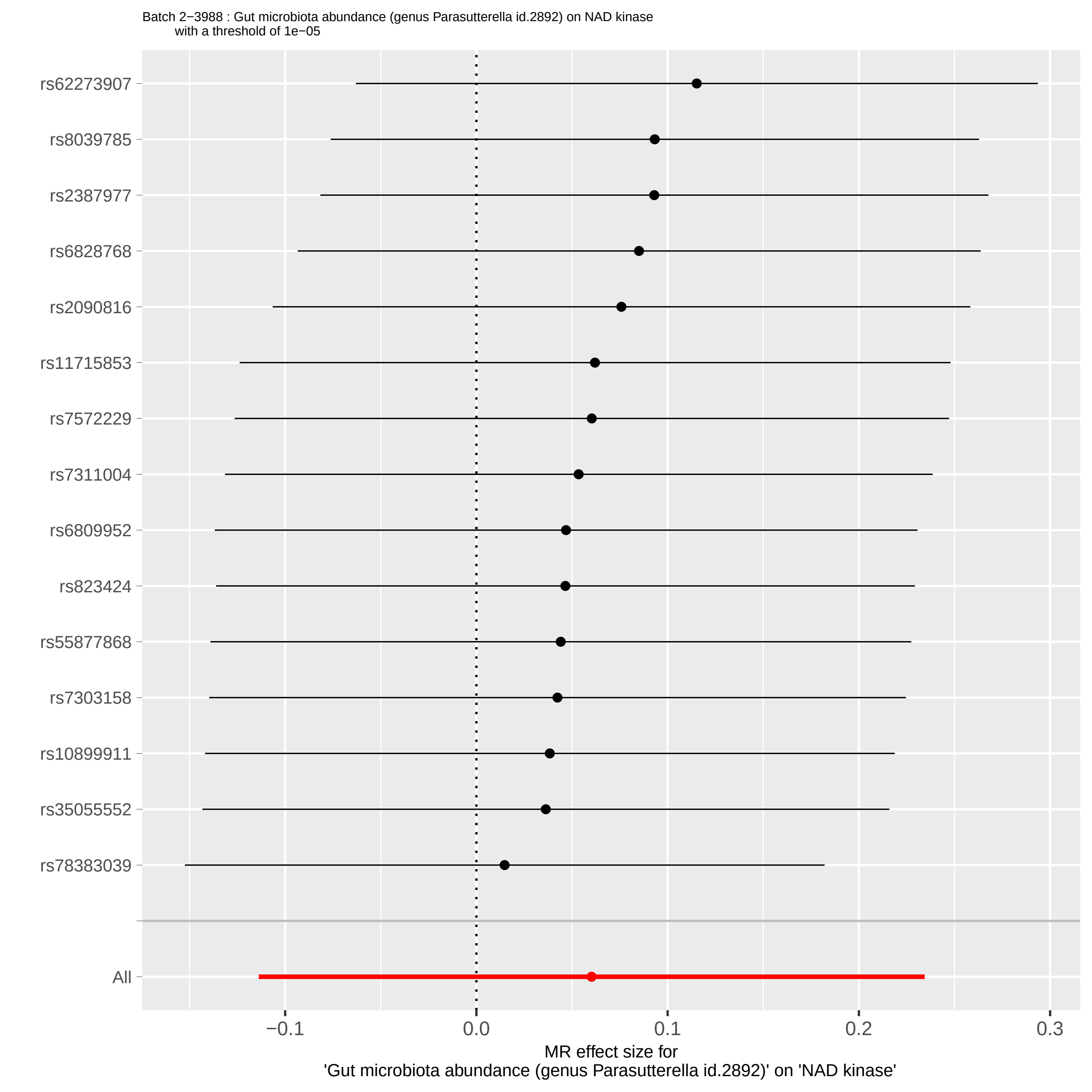
Batch 2−3988 : Gut microbiota abundance (genus Parasutterella id.2892) on NAD kinase
with a threshold of 1e−05
rs62273907
rs8039785
rs2387977
rs6828768
rs2090816
rs11715853
rs7572229
rs7311004
rs6809952
rs823424
rs55877868
rs7303158
rs10899911
rs35055552
rs78383039
All
−0.1
0.0
0.1
0.2
0.3
MR effect size for
'Gut microbiota abundance (genus Parasutterella id.2892)' on 'NAD kinase'

Batch 2−3989 : Gut microbiota abundance (genus Catenibacterium id.2153) on N−acetyl−D−glucosamine kinase
with a threshold of 1e−05

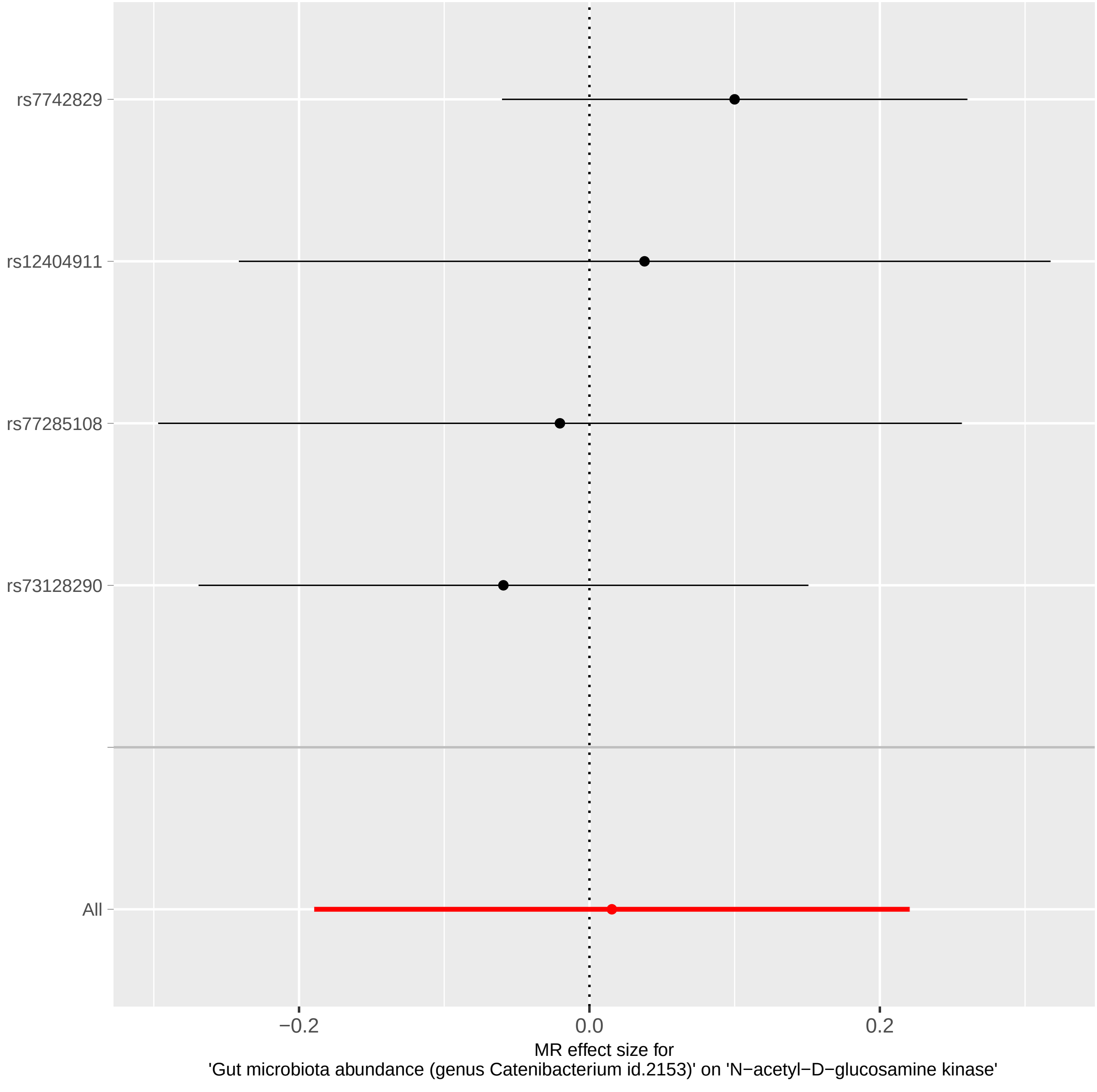

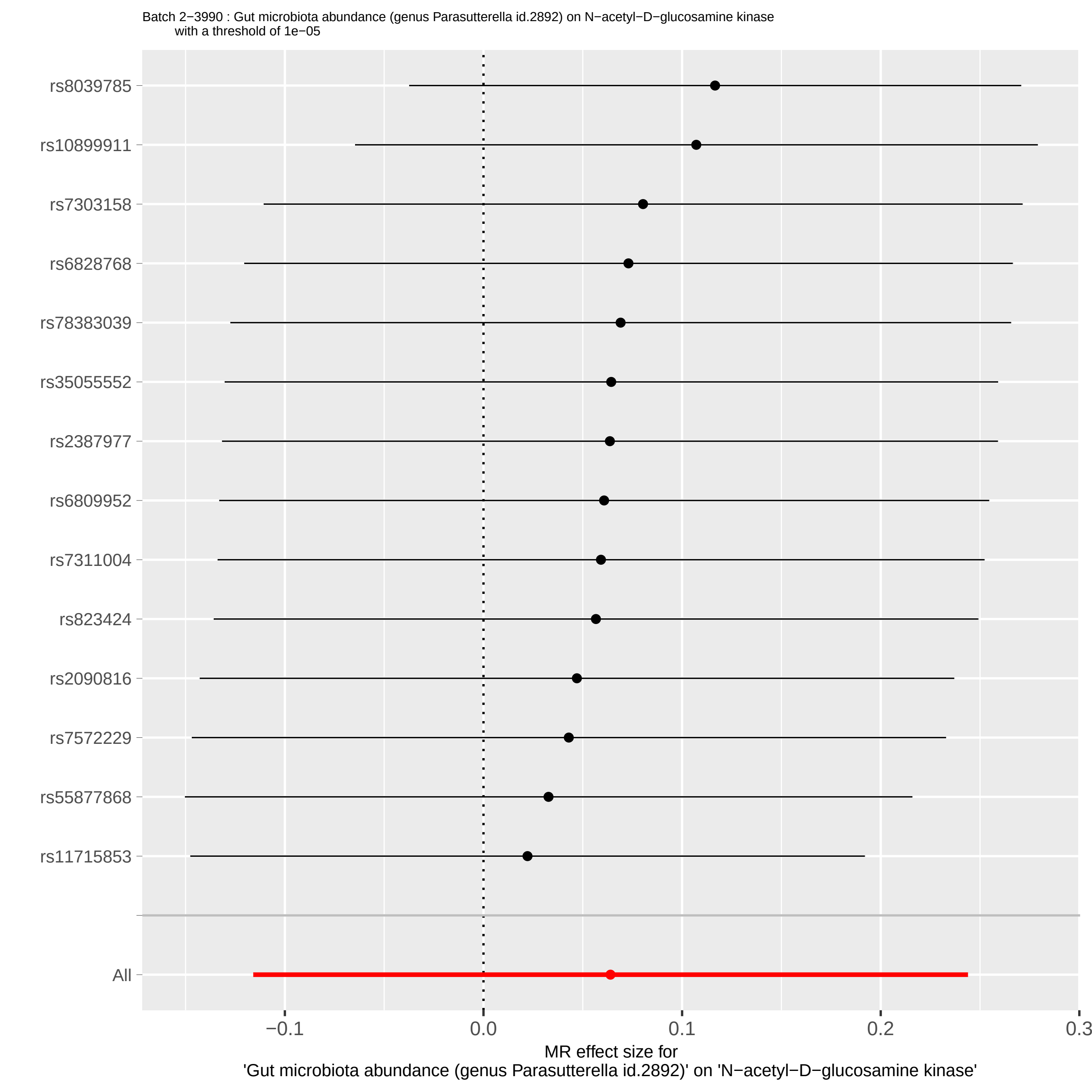

Batch 2−3990 : Gut microbiota abundance (genus Parasutterella id.2892) on N−acetyl−D−glucosamine kinase
with a threshold of 1e−05
rs8039785
rs10899911
rs7303158
rs6828768
rs78383039
rs35055552
rs2387977
rs6809952
rs7311004
rs823424
rs2090816
rs7572229
rs55877868
rs11715853
All
−0.1
0.0
0.1
0.2
0.3
MR effect size for
'Gut microbiota abundance (genus Parasutterella id.2892)' on 'N−acetyl−D−glucosamine kinase'

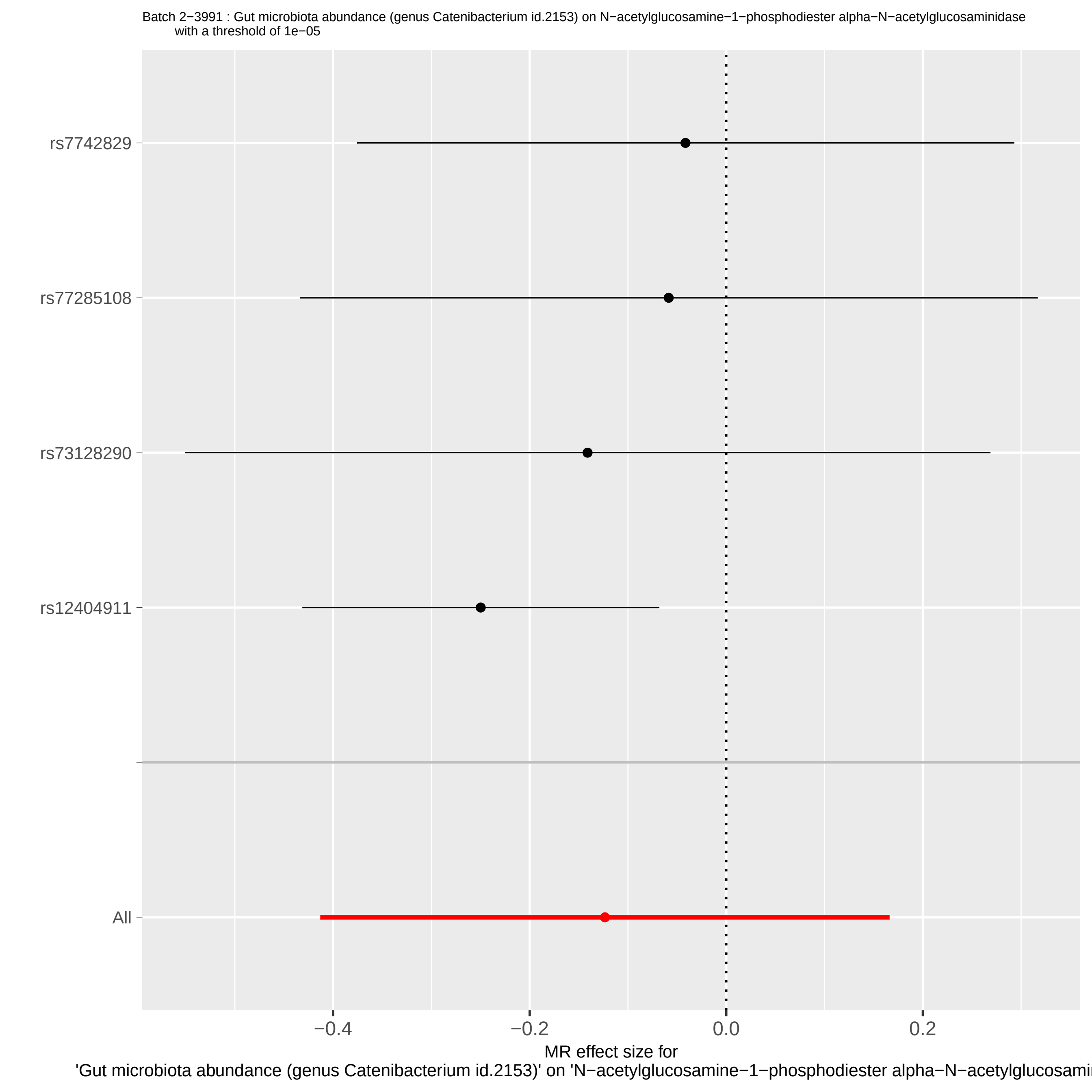
Batch 2−3991 : Gut microbiota abundance (genus Catenibacterium id.2153) on N−acetylglucosamine−1−phosphodiester alpha−N−acetylglucosaminidase
with a threshold of 1e−05
rs7742829
rs77285108
rs73128290
rs12404911
All
−0.4
−0.2
0.0
0.2
MR effect size for
'Gut microbiota abundance (genus Catenibacterium id.2153)' on 'N−acetylglucosamine−1−phosphodiester alpha−N−acetylglucosami

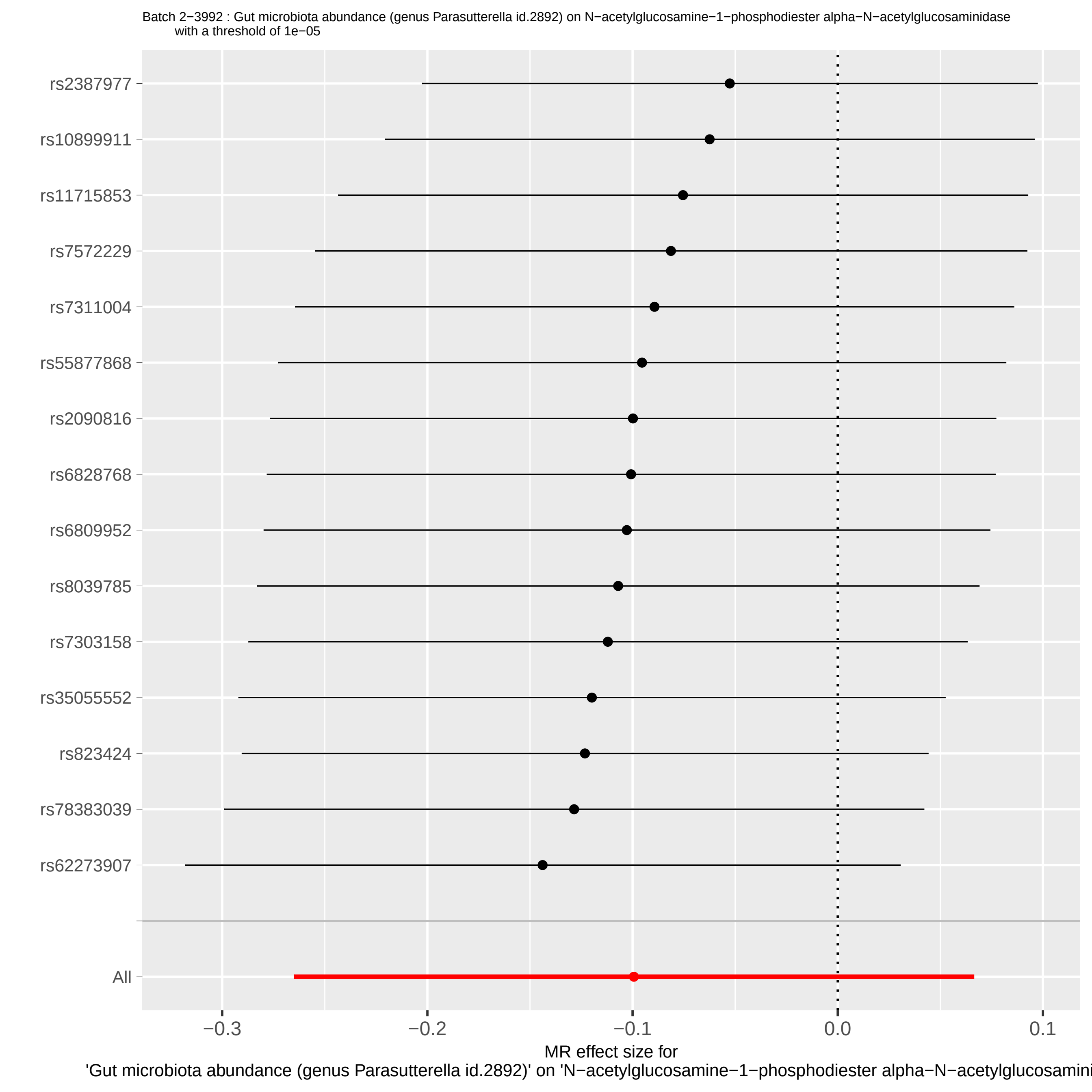
Batch 2−3992 : Gut microbiota abundance (genus Parasutterella id.2892) on N−acetylglucosamine−1−phosphodiester alpha−N−acetylglucosaminidase with a threshold of 1e−05
rs2387977
rs10899911
rs11715853
rs7572229
rs7311004
rs55877868
rs2090816
rs6828768
rs6809952
rs8039785
rs7303158
rs35055552
rs823424
rs78383039
rs62273907
All
−0.3
−0.2
−0.1
0.0
0.1
MR effect size for
'Gut microbiota abundance (genus Parasutterella id.2892)' on 'N−acetylglucosamine−1−phosphodiester alpha−N−acetylglucosamini

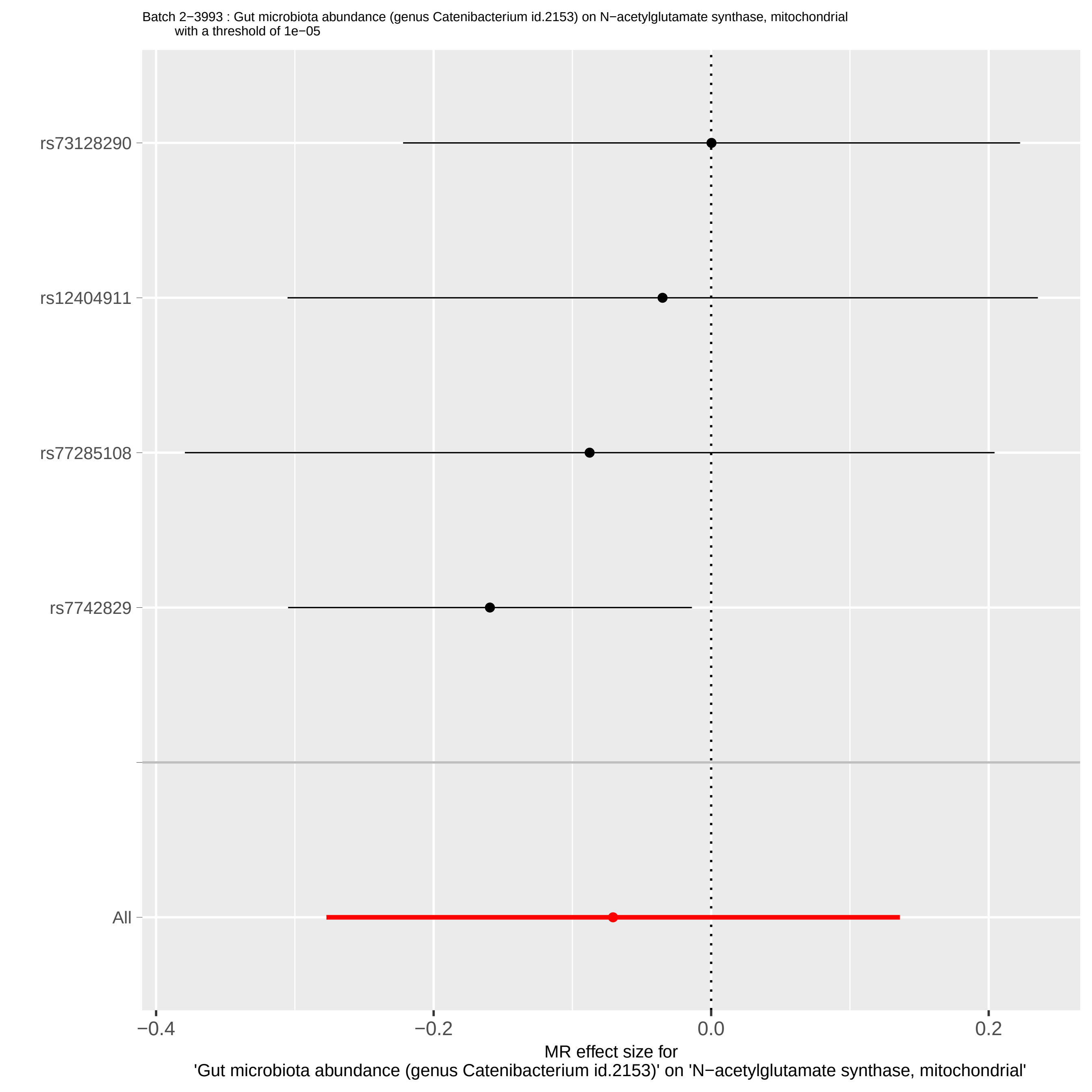
Batch 2−3993 : Gut microbiota abundance (genus Catenibacterium id.2153) on N−acetylglutamate synthase, mitochondrial
with a threshold of 1e−05
rs73128290
rs12404911
rs77285108
rs7742829
All
−0.4
−0.2
0.0
0.2
MR effect size for
'Gut microbiota abundance (genus Catenibacterium id.2153)' on 'N−acetylglutamate synthase, mitochondrial'

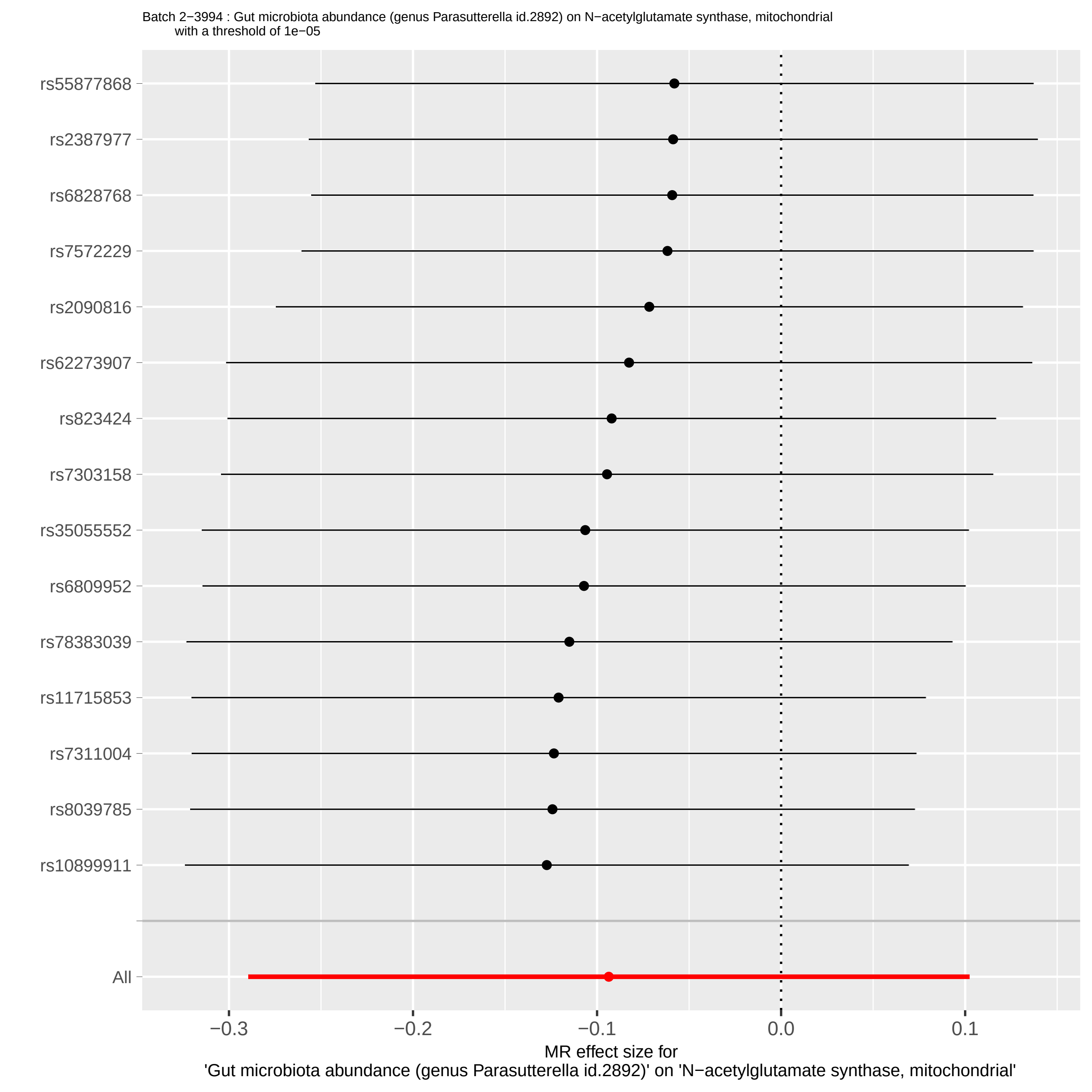

Batch 2−3994 : Gut microbiota abundance (genus Parasutterella id.2892) on N−acetylglutamate synthase, mitochondrial
with a threshold of 1e−05
rs55877868
rs2387977
rs6828768
rs7572229
rs2090816
rs62273907
rs823424
rs7303158
rs35055552
rs6809952
rs78383039
rs11715853
rs7311004
rs8039785
rs10899911
All
−0.3
−0.2
−0.1
0.0
0.1
MR effect size for
'Gut microbiota abundance (genus Parasutterella id.2892)' on 'N−acetylglutamate synthase, mitochondrial'

Batch 2−3995 : Gut microbiota abundance (genus Catenibacterium id.2153) on Nicotinamide phosphoribosyltransferase
with a threshold of 1e−05

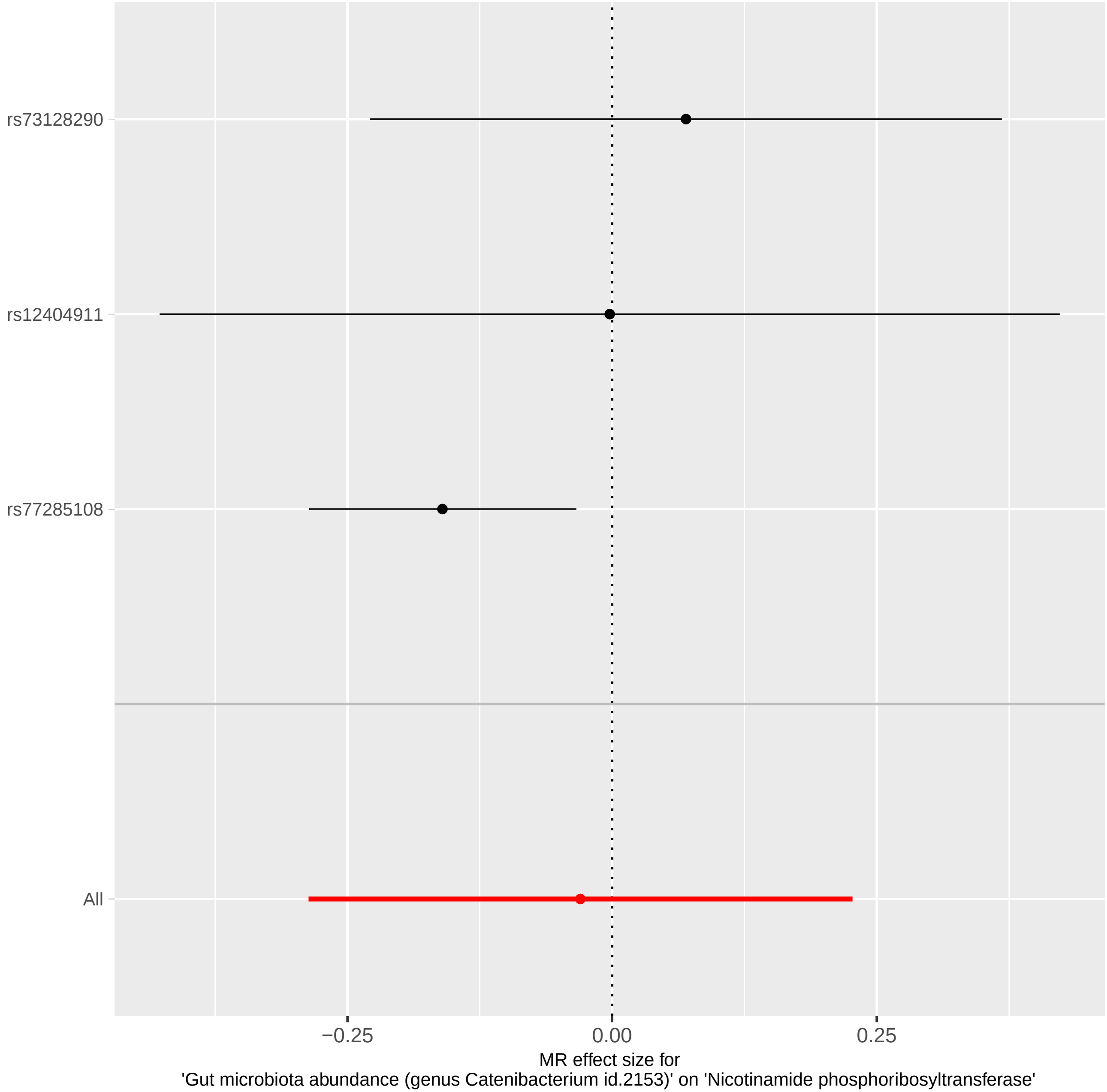

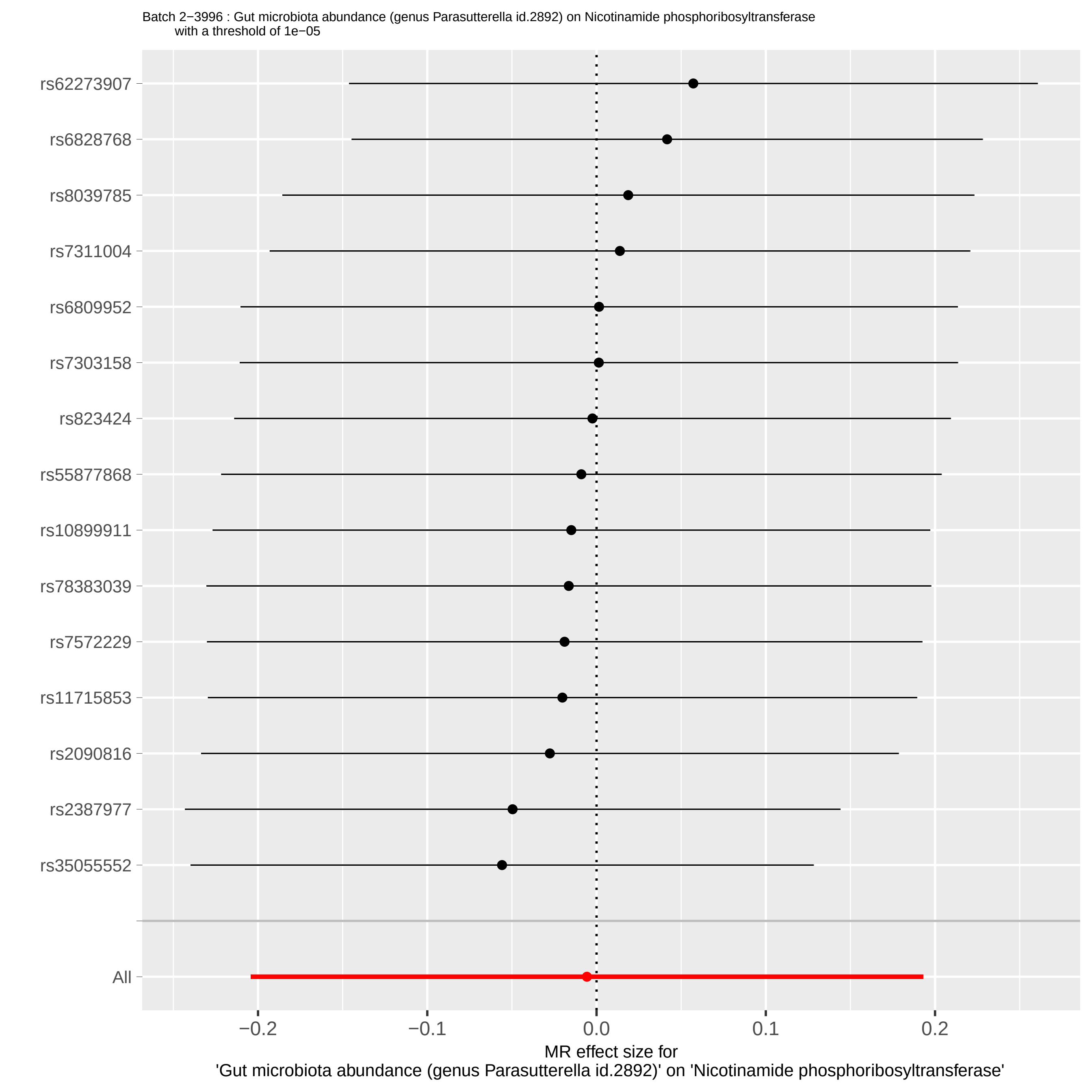

Batch 2−3996 : Gut microbiota abundance (genus Parasutterella id.2892) on Nicotinamide phosphoribosyltransferase
with a threshold of 1e−05
rs62273907
rs6828768
rs8039785
rs7311004
rs6809952
rs7303158
rs823424
rs55877868
rs10899911
rs78383039
rs7572229
rs11715853
rs2090816
rs2387977
rs35055552
All
−0.2
−0.1
0.0
0.1
0.2
MR effect size for
'Gut microbiota abundance (genus Parasutterella id.2892)' on 'Nicotinamide phosphoribosyltransferase'

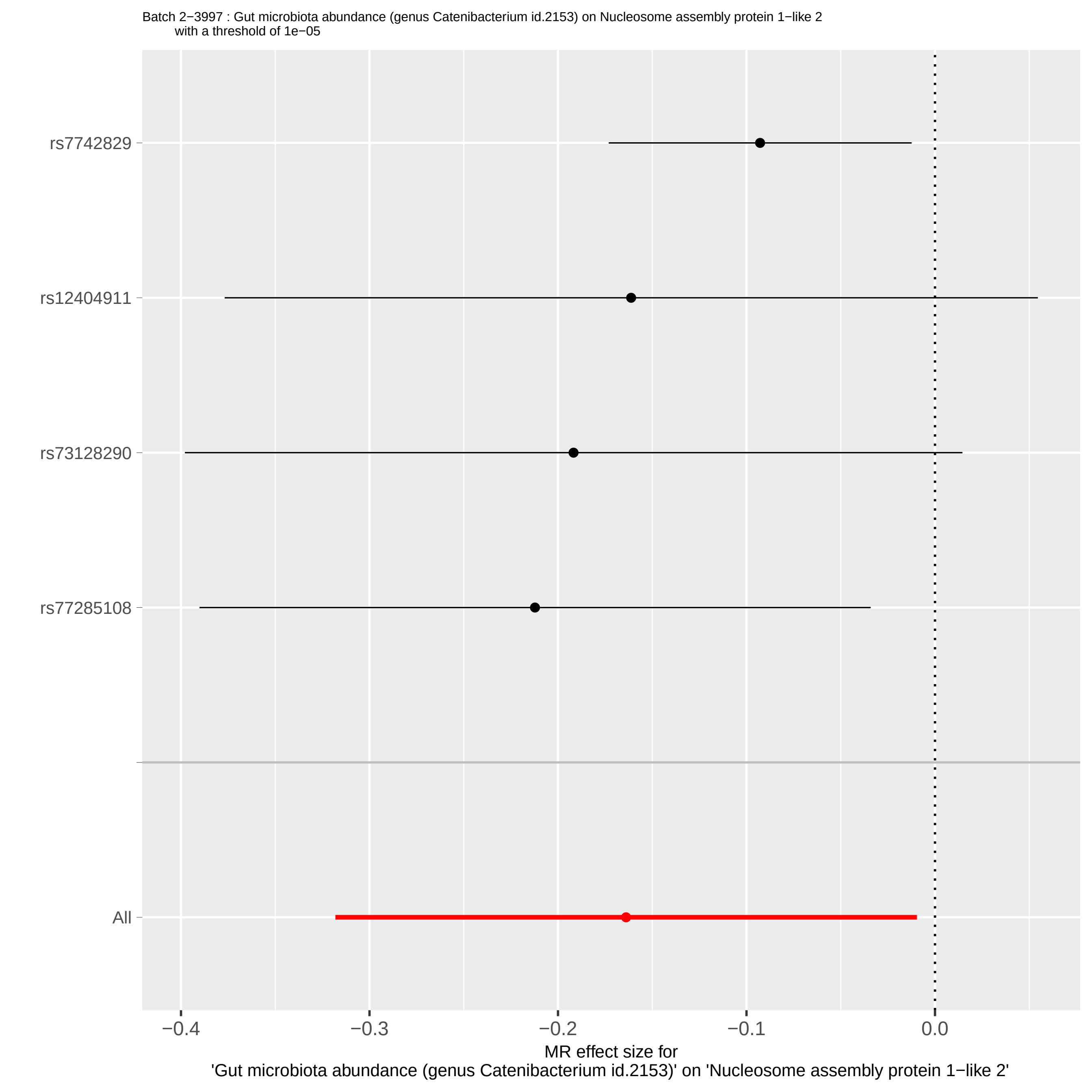

Batch 2−3997 : Gut microbiota abundance (genus Catenibacterium id.2153) on Nucleosome assembly protein 1−like 2
with a threshold of 1e−05
rs7742829
rs12404911
rs73128290
rs77285108
All
−0.4
−0.3
−0.2
−0.1
0.0
MR effect size for
'Gut microbiota abundance (genus Catenibacterium id.2153)' on 'Nucleosome assembly protein 1−like 2'

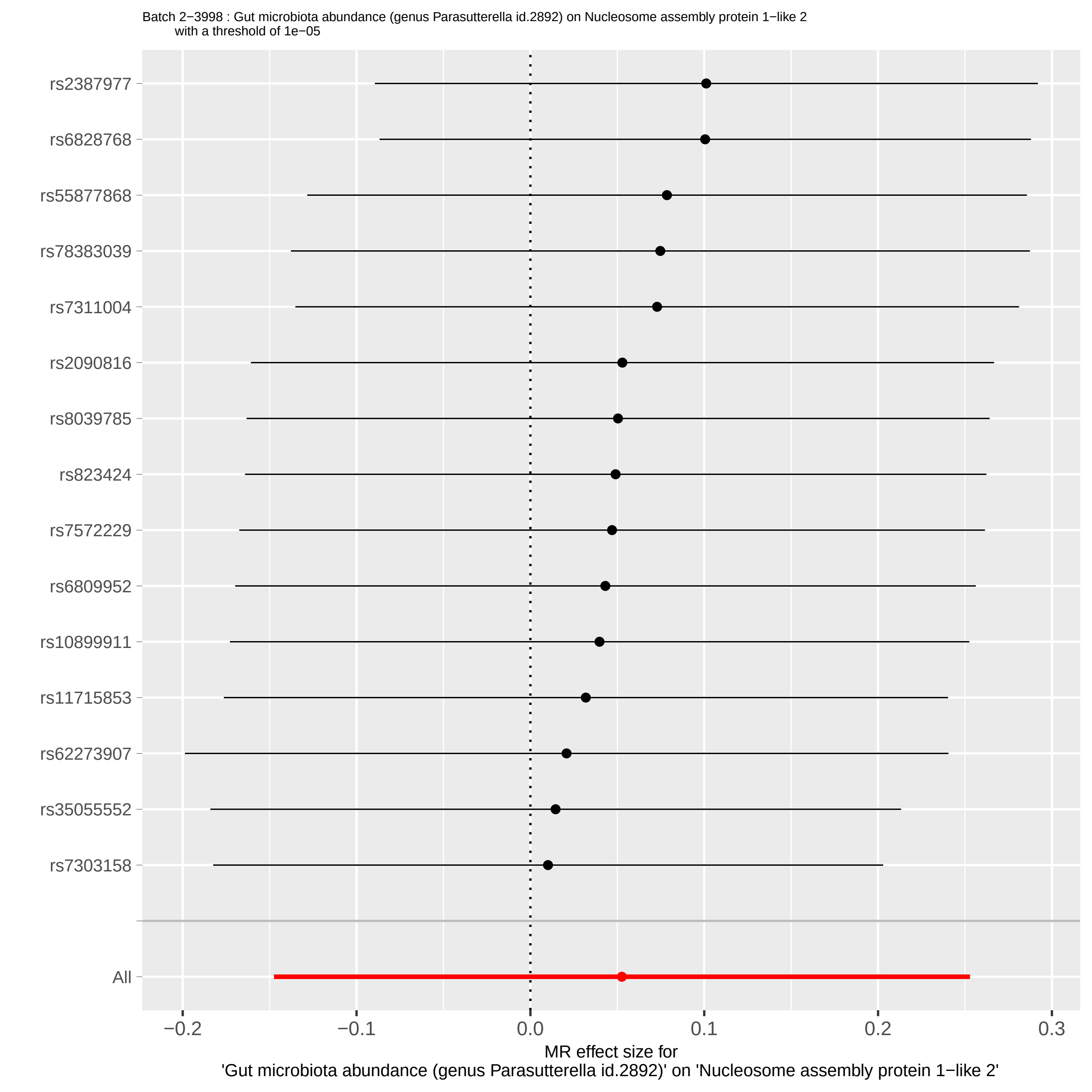

Batch 2−3998 : Gut microbiota abundance (genus Parasutterella id.2892) on Nucleosome assembly protein 1−like 2
with a threshold of 1e−05
rs2387977
rs6828768
rs55877868
rs78383039
rs7311004
rs2090816
rs8039785
rs823424
rs7572229
rs6809952
rs10899911
rs11715853
rs62273907
rs35055552
rs7303158
All
−0.2
−0.1
0.0
0.1
0.2
0.3
MR effect size for
'Gut microbiota abundance (genus Parasutterella id.2892)' on 'Nucleosome assembly protein 1−like 2'

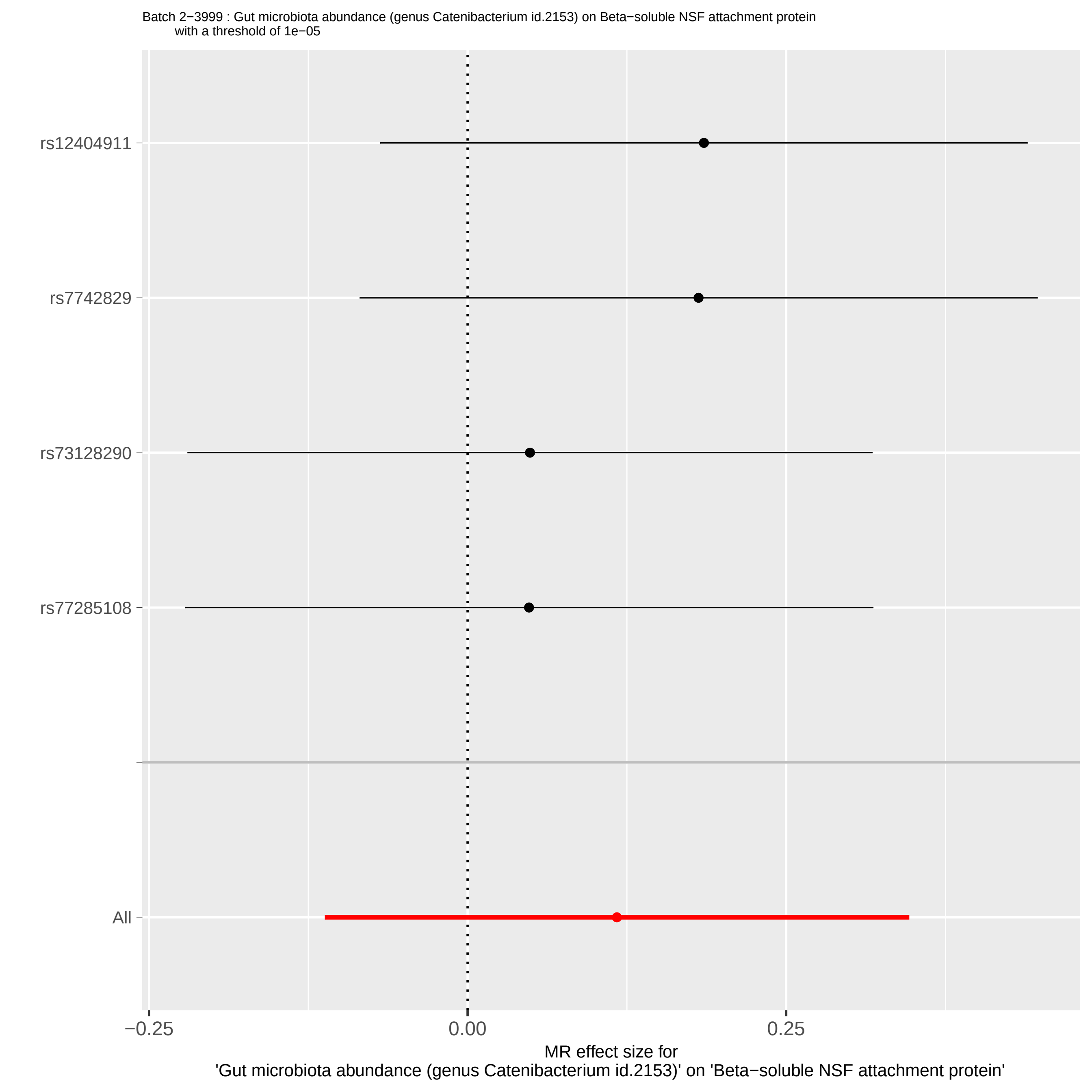
Batch 2−3999 : Gut microbiota abundance (genus Catenibacterium id.2153) on Beta−soluble NSF attachment protein
with a threshold of 1e−05
rs12404911
rs7742829
rs73128290
rs77285108
All
−0.25
0.00
0.25
MR effect size for
'Gut microbiota abundance (genus Catenibacterium id.2153)' on 'Beta−soluble NSF attachment protein'

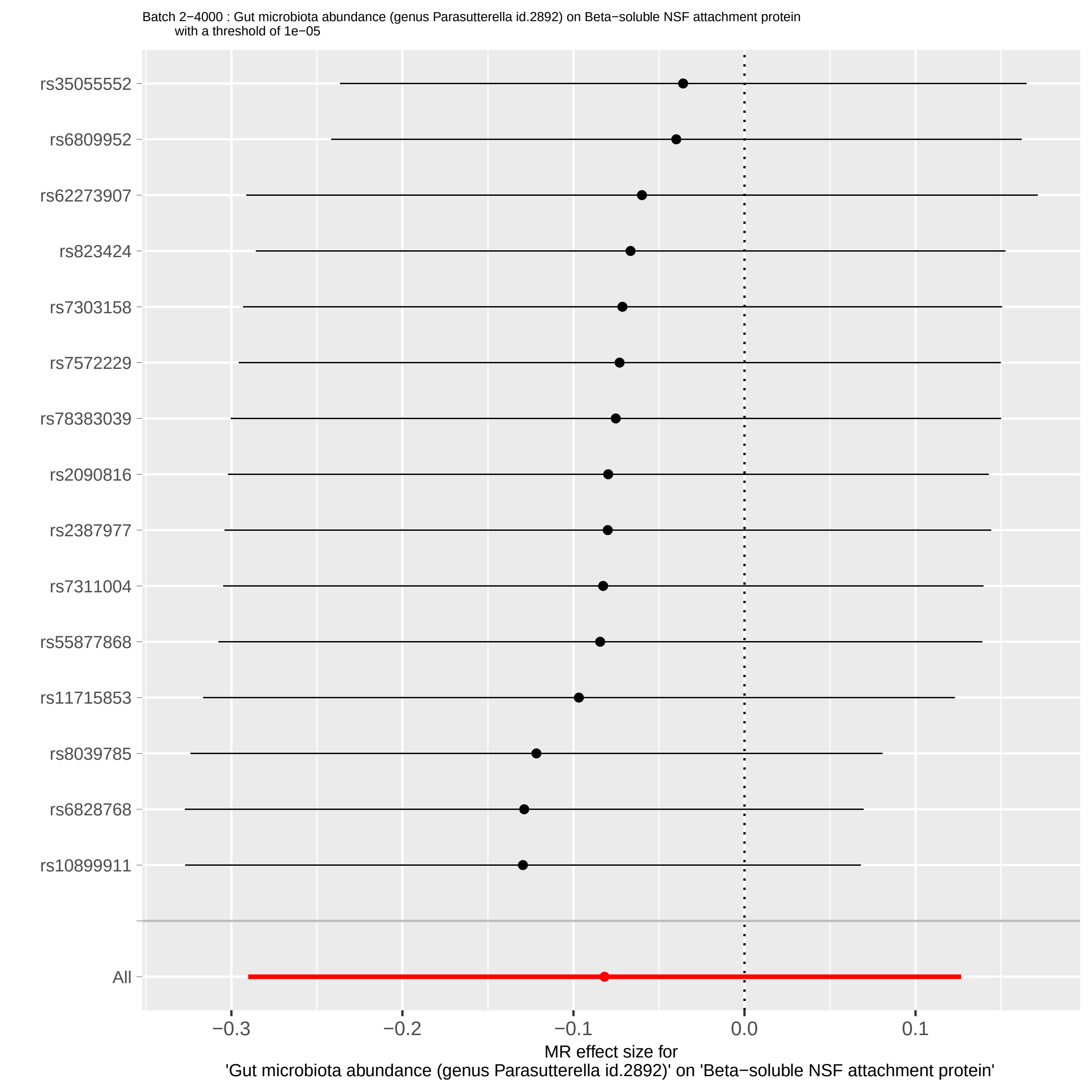
Batch 2−4000 : Gut microbiota abundance (genus Parasutterella id.2892) on Beta−soluble NSF attachment protein
with a threshold of 1e−05
rs35055552
rs6809952
rs62273907
rs823424
rs7303158
rs7572229
rs78383039
rs2090816
rs2387977
rs7311004
rs55877868
rs11715853
rs8039785
rs6828768
rs10899911
All
−0.3
−0.2
−0.1
0.0
0.1
MR effect size for
'Gut microbiota abundance (genus Parasutterella id.2892)' on 'Beta−soluble NSF attachment protein'

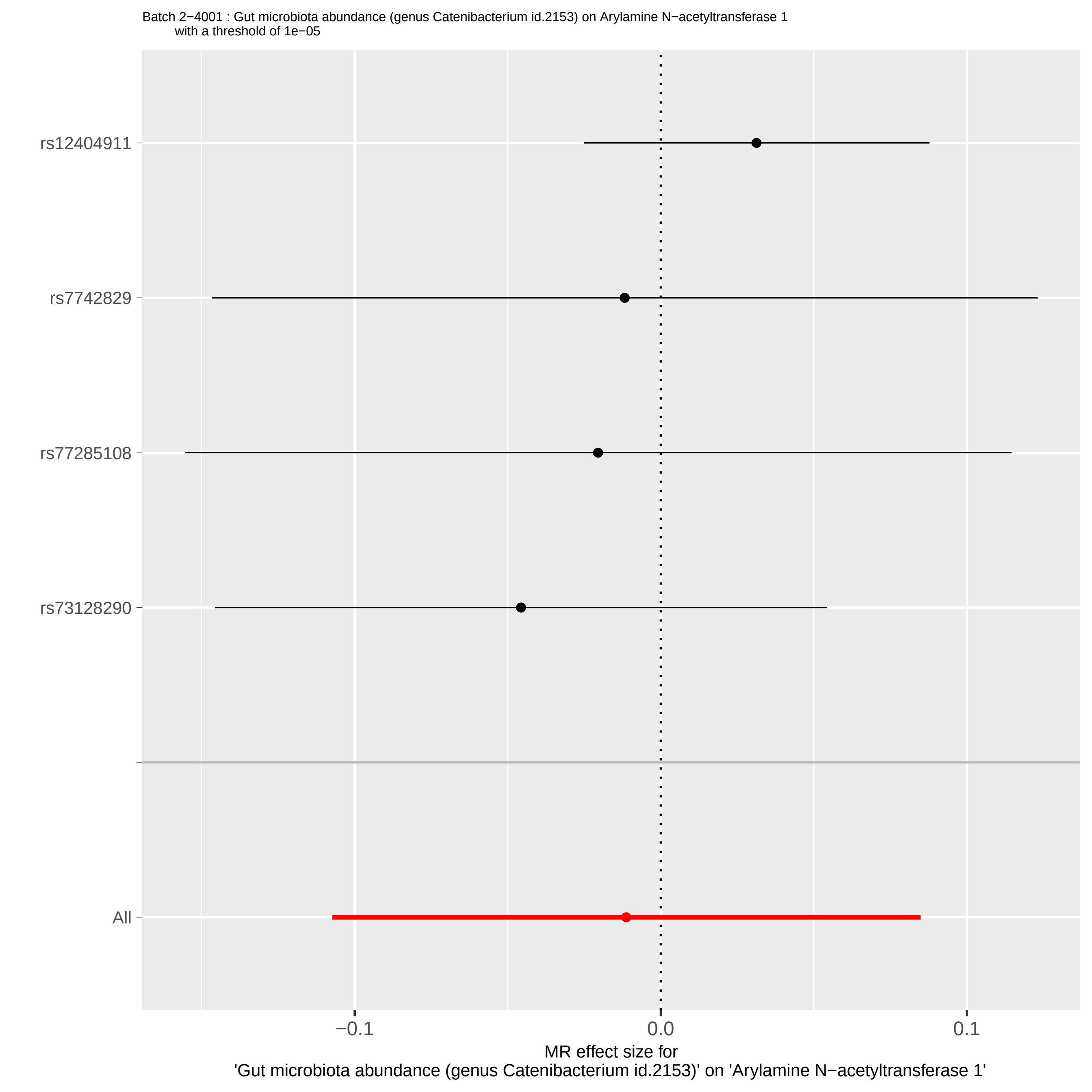
Batch 2−4001 : Gut microbiota abundance (genus Catenibacterium id.2153) on Arylamine N−acetyltransferase 1
with a threshold of 1e−05
rs12404911
rs7742829
rs77285108
rs73128290
All
−0.1
0.0
0.1
MR effect size for
'Gut microbiota abundance (genus Catenibacterium id.2153)' on 'Arylamine N−acetyltransferase 1'

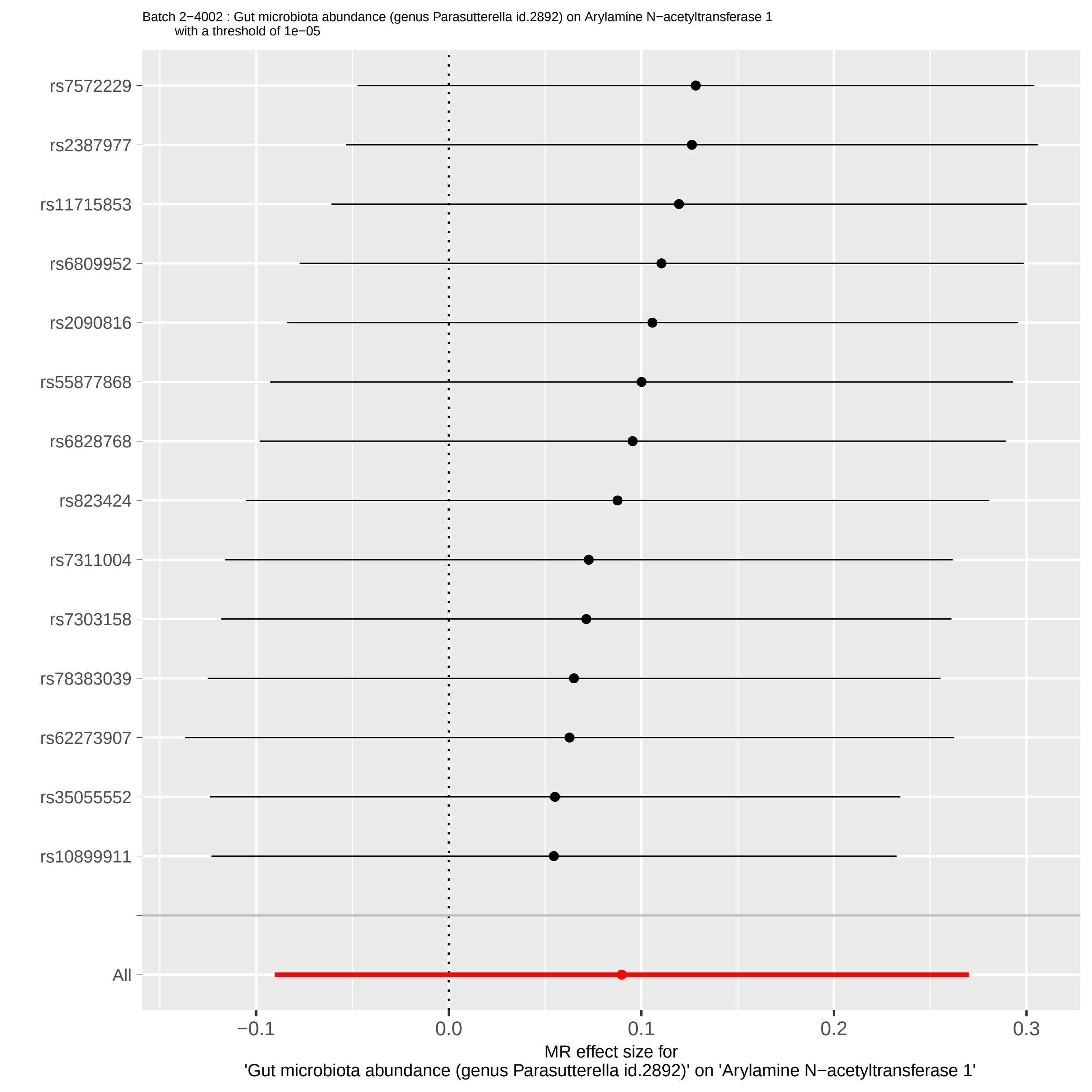

Batch 2−4002 : Gut microbiota abundance (genus Parasutterella id.2892) on Arylamine N−acetyltransferase 1
with a threshold of 1e−05
rs7572229
rs2387977
rs11715853
rs6809952
rs2090816
rs55877868
rs6828768
rs823424
rs7311004
rs7303158
rs78383039
rs62273907
rs35055552
rs10899911
All
−0.1
0.0
0.1
0.2
0.3
MR effect size for
'Gut microbiota abundance (genus Parasutterella id.2892)' on 'Arylamine N−acetyltransferase 1'

Batch 2−4003 : Gut microbiota abundance (genus Catenibacterium id.2153) on N−acetyltransferase 14
with a threshold of 1e−05

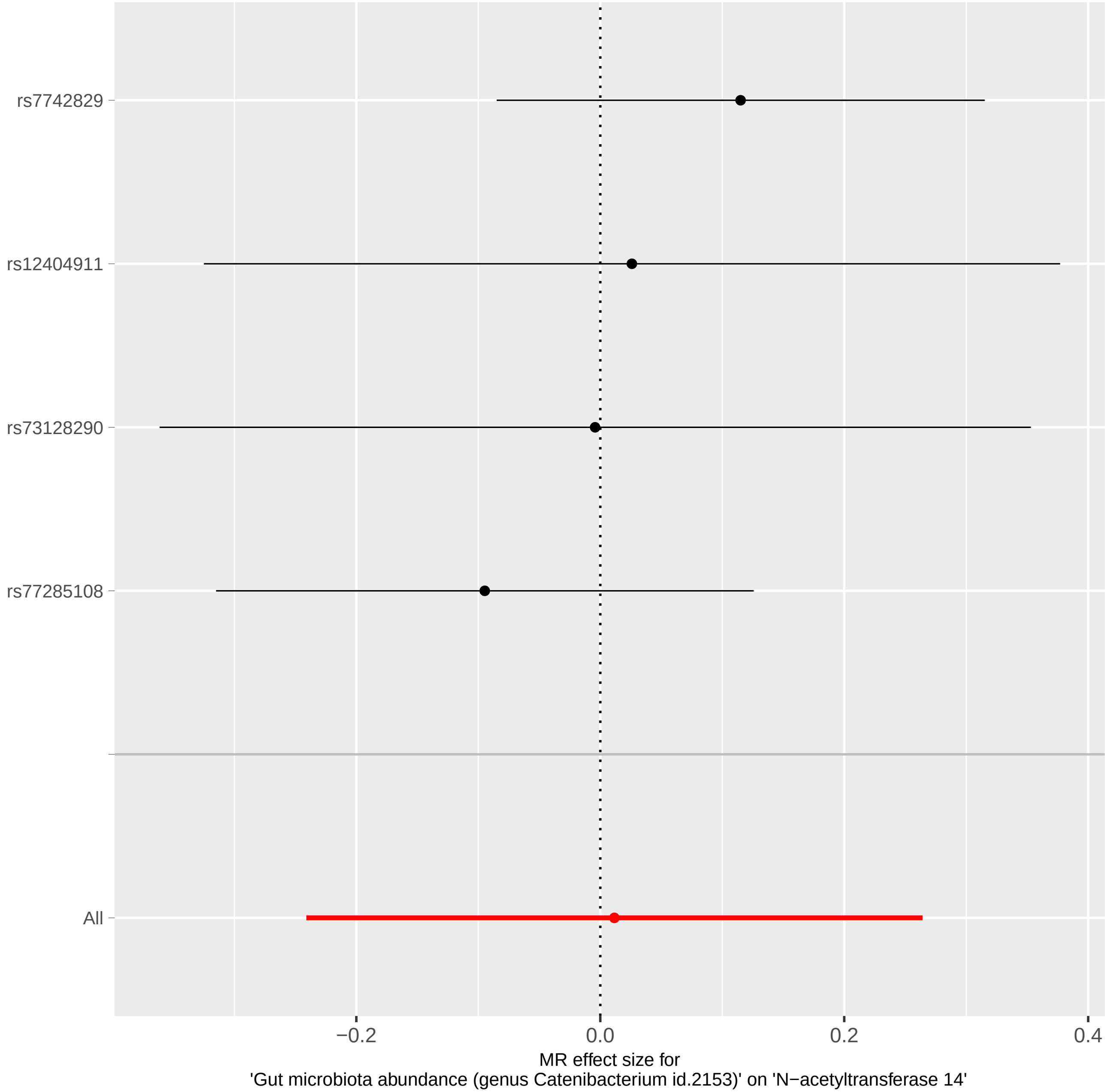

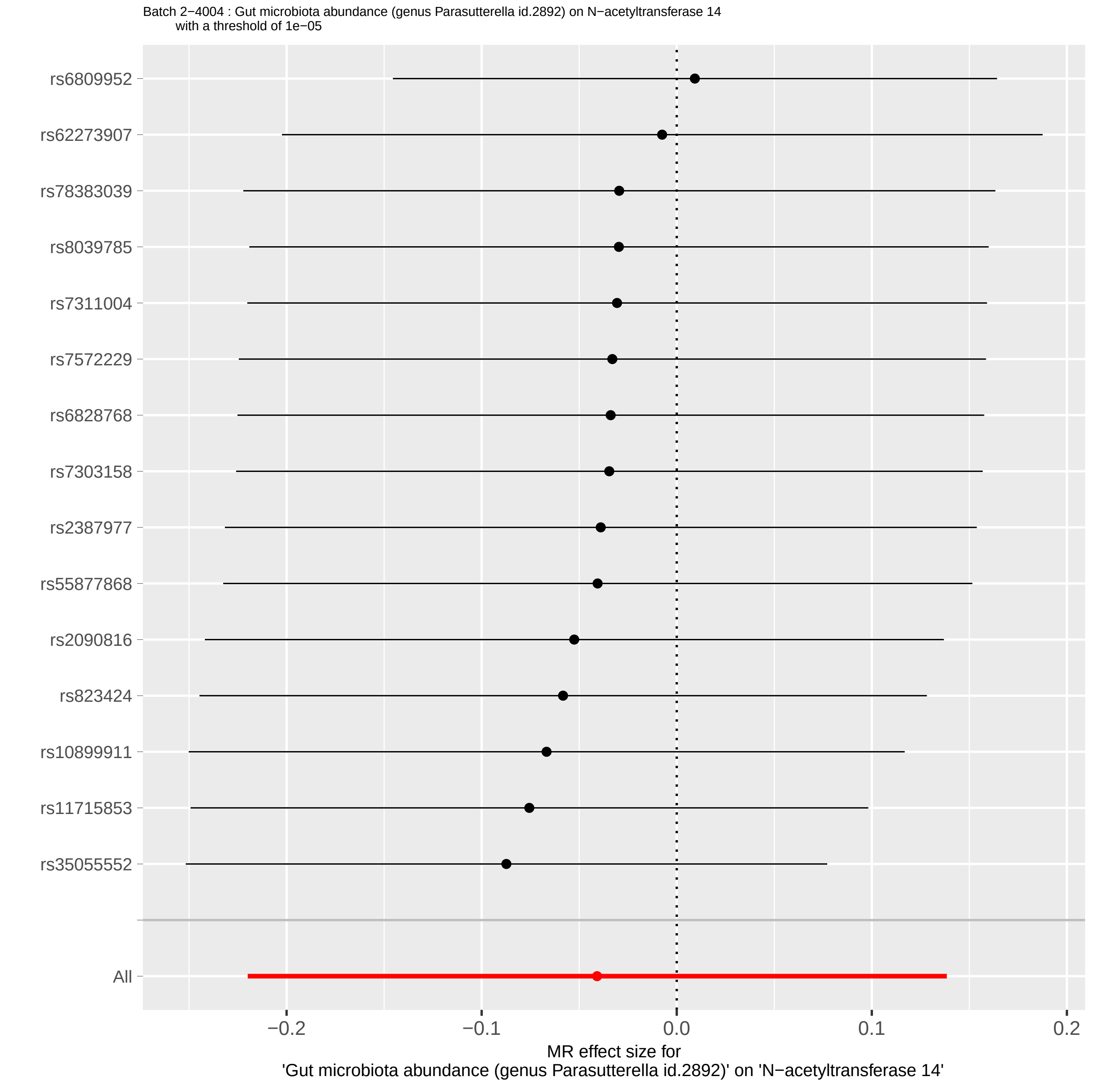

Batch 2−4004 : Gut microbiota abundance (genus Parasutterella id.2892) on N−acetyltransferase 14
with a threshold of 1e−05
rs6809952
rs62273907
rs78383039
rs8039785
rs7311004
rs7572229
rs6828768
rs7303158
rs2387977
rs55877868
rs2090816
rs823424
rs10899911
rs11715853
rs35055552
All
−0.2
−0.1
0.0
0.1
0.2
MR effect size for
'Gut microbiota abundance (genus Parasutterella id.2892)' on 'N−acetyltransferase 14'

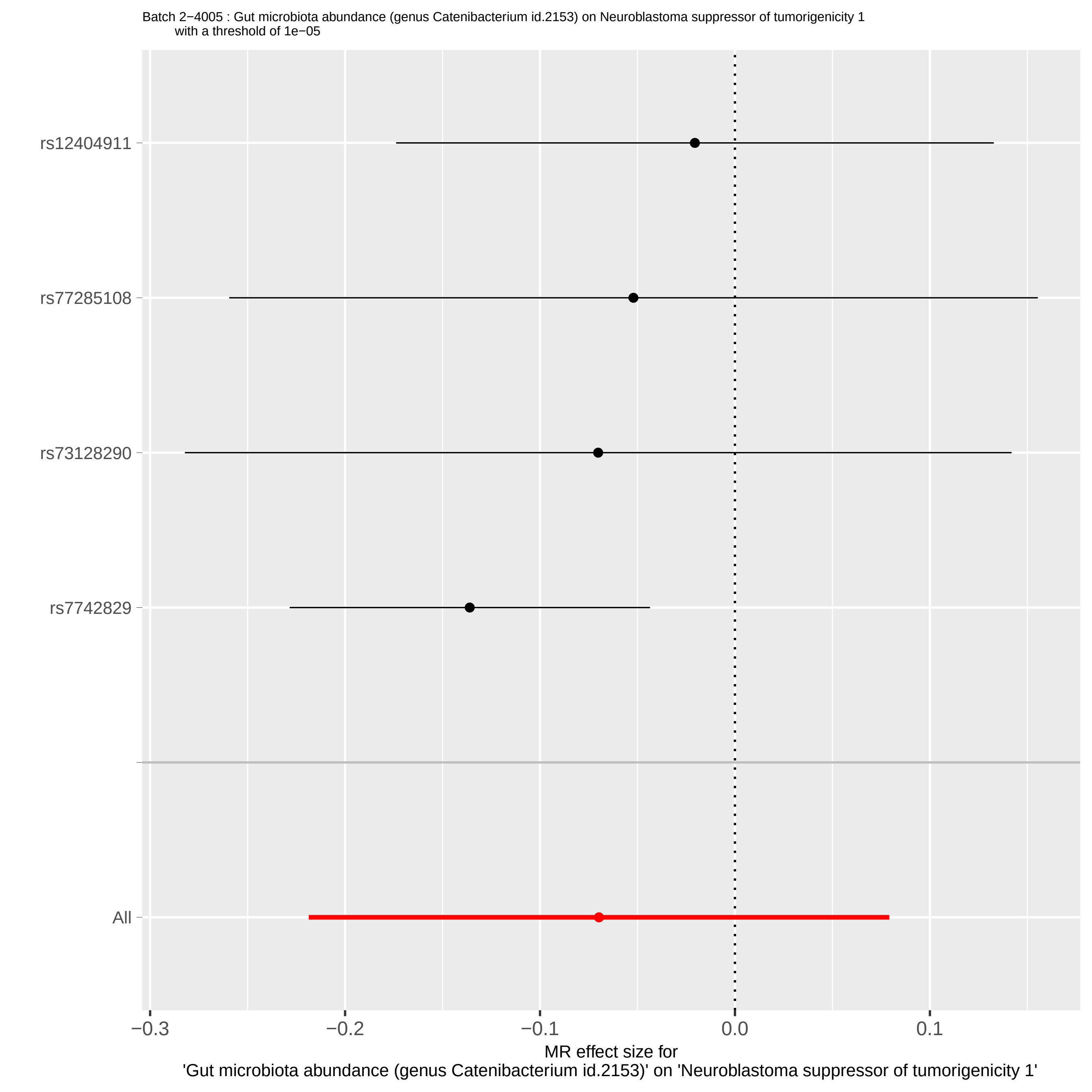
Batch 2−4005 : Gut microbiota abundance (genus Catenibacterium id.2153) on Neuroblastoma suppressor of tumorigenicity 1
with a threshold of 1e−05
rs12404911
rs77285108
rs73128290
rs7742829
All
−0.3
−0.2
−0.1
0.0
0.1
MR effect size for
'Gut microbiota abundance (genus Catenibacterium id.2153)' on 'Neuroblastoma suppressor of tumorigenicity 1'

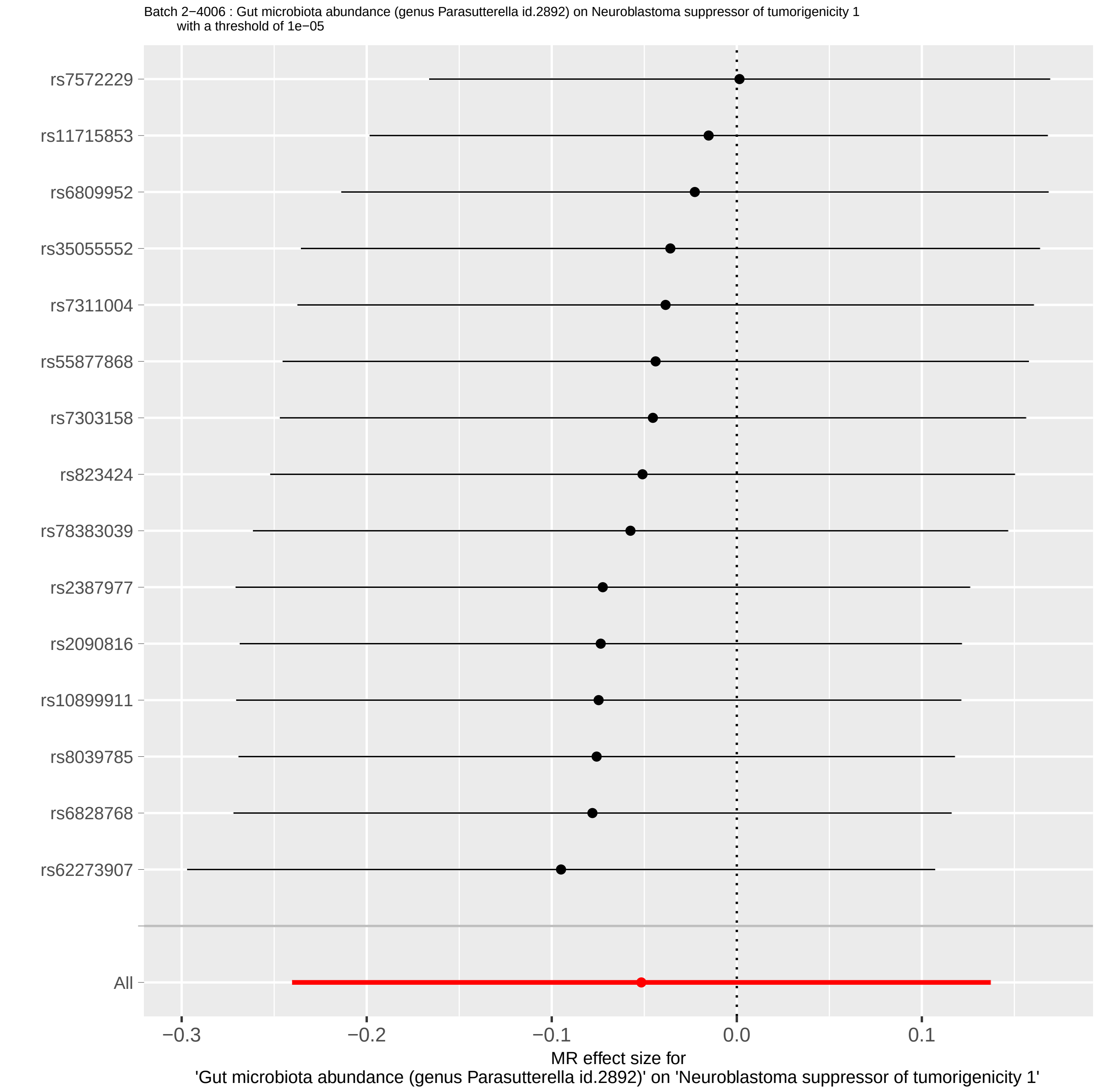
Batch 2−4006 : Gut microbiota abundance (genus Parasutterella id.2892) on Neuroblastoma suppressor of tumorigenicity 1
with a threshold of 1e−05
rs7572229
rs11715853
rs6809952
rs35055552
rs7311004
rs55877868
rs7303158
rs823424
rs78383039
rs2387977
rs2090816
rs10899911
rs8039785
rs6828768
rs62273907
All
−0.3
−0.2
−0.1
0.0
0.1
MR effect size for
'Gut microbiota abundance (genus Parasutterella id.2892)' on 'Neuroblastoma suppressor of tumorigenicity 1'

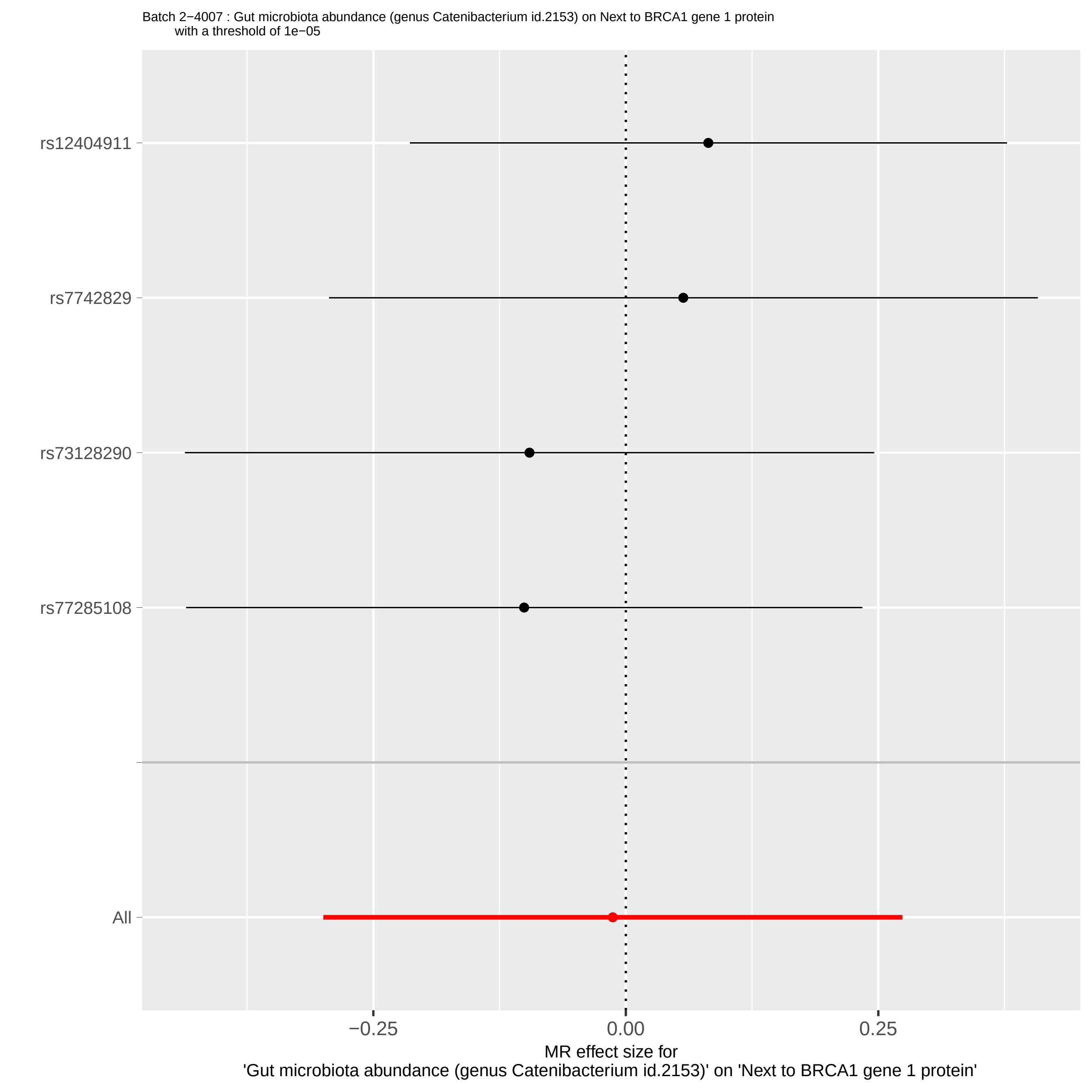
Batch 2−4007 : Gut microbiota abundance (genus Catenibacterium id.2153) on Next to BRCA1 gene 1 protein
with a threshold of 1e−05
rs12404911
rs7742829
rs73128290
rs77285108
All
−0.25
0.00
0.25
MR effect size for
'Gut microbiota abundance (genus Catenibacterium id.2153)' on 'Next to BRCA1 gene 1 protein'

Batch 2−4008 : Gut microbiota abundance (genus Parasutterella id.2892) on Next to BRCA1 gene 1 protein
with a threshold of 1e−05

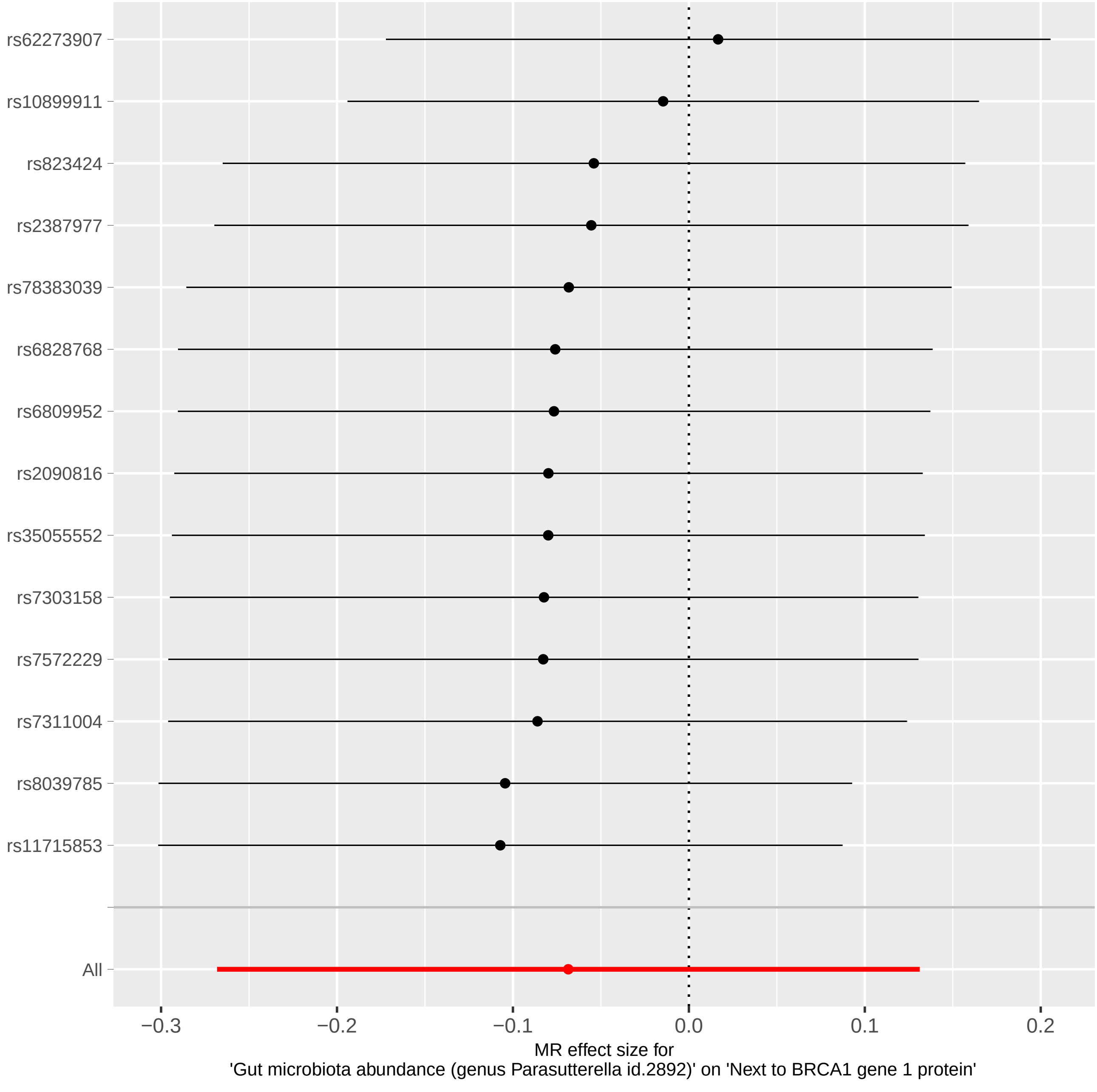

Batch 2−4009 : Gut microbiota abundance (genus Catenibacterium id.2153) on Next to BRCA1 gene 1 protein
with a threshold of 1e−05

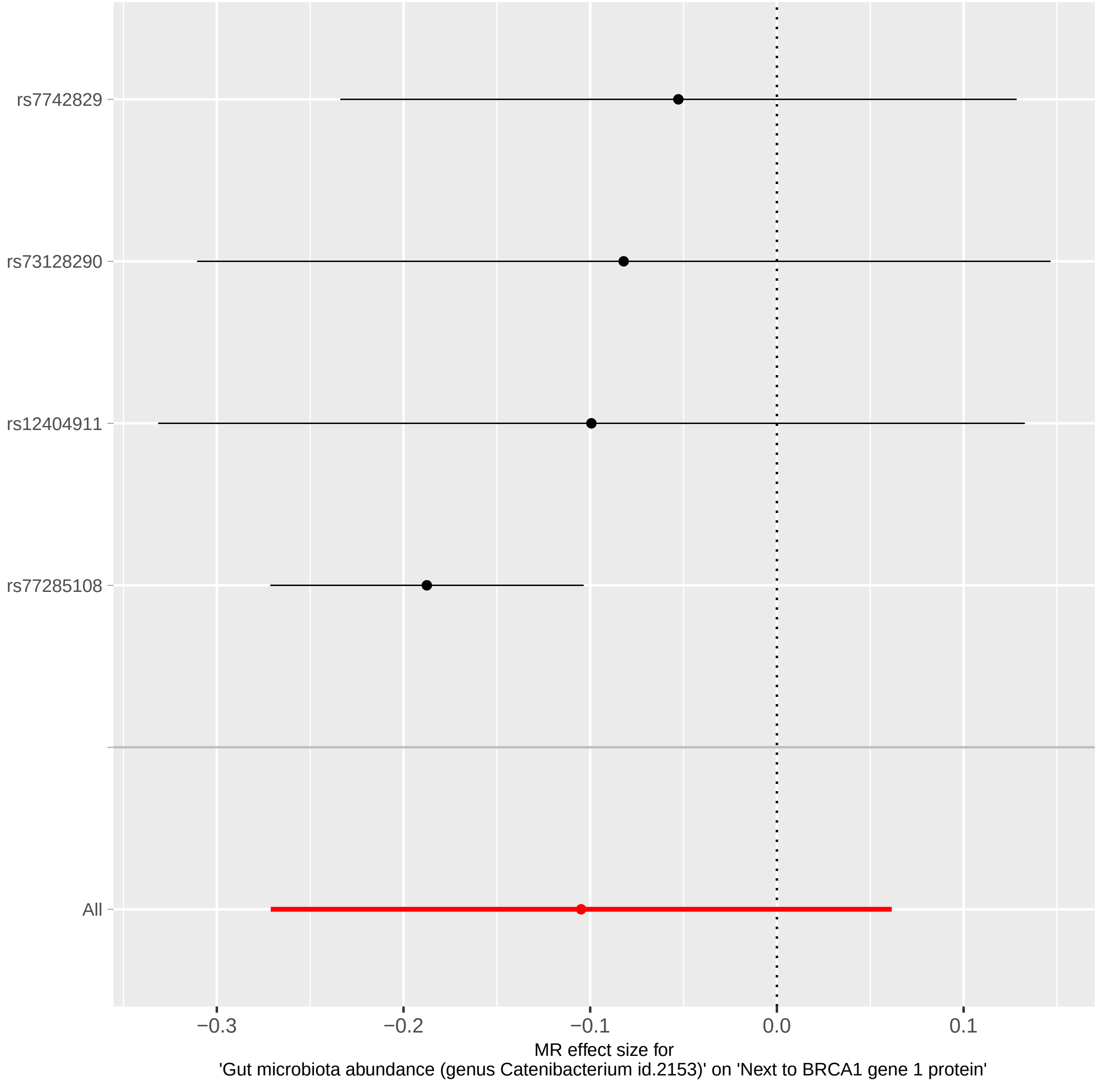

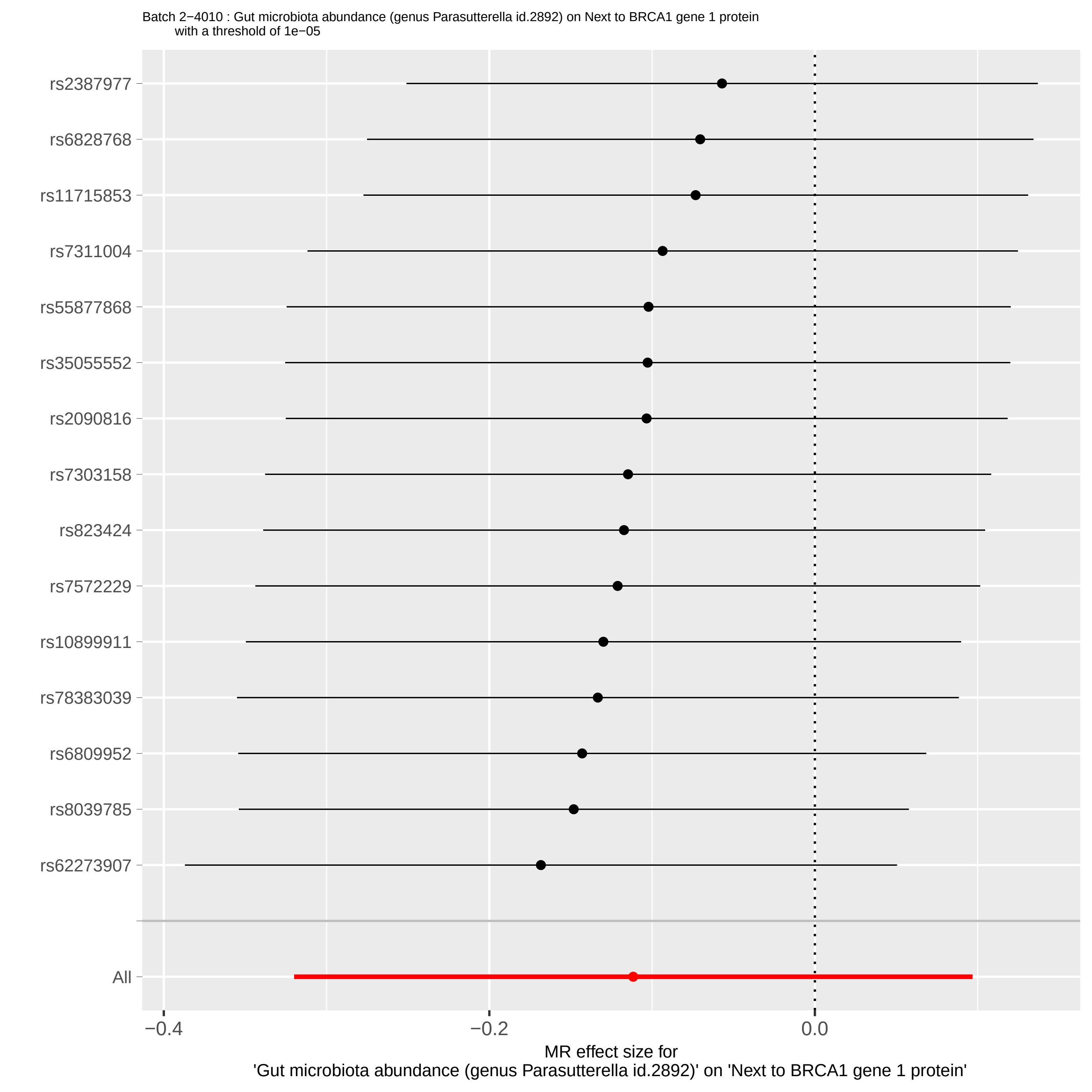

Batch 2−4010 : Gut microbiota abundance (genus Parasutterella id.2892) on Next to BRCA1 gene 1 protein
with a threshold of 1e−05
rs2387977
rs6828768
rs11715853
rs7311004
rs55877868
rs35055552
rs2090816
rs7303158
rs823424
rs7572229
rs10899911
rs78383039
rs6809952
rs8039785
rs62273907
All
−0.4
−0.2
0.0
MR effect size for
'Gut microbiota abundance (genus Parasutterella id.2892)' on 'Next to BRCA1 gene 1 protein'

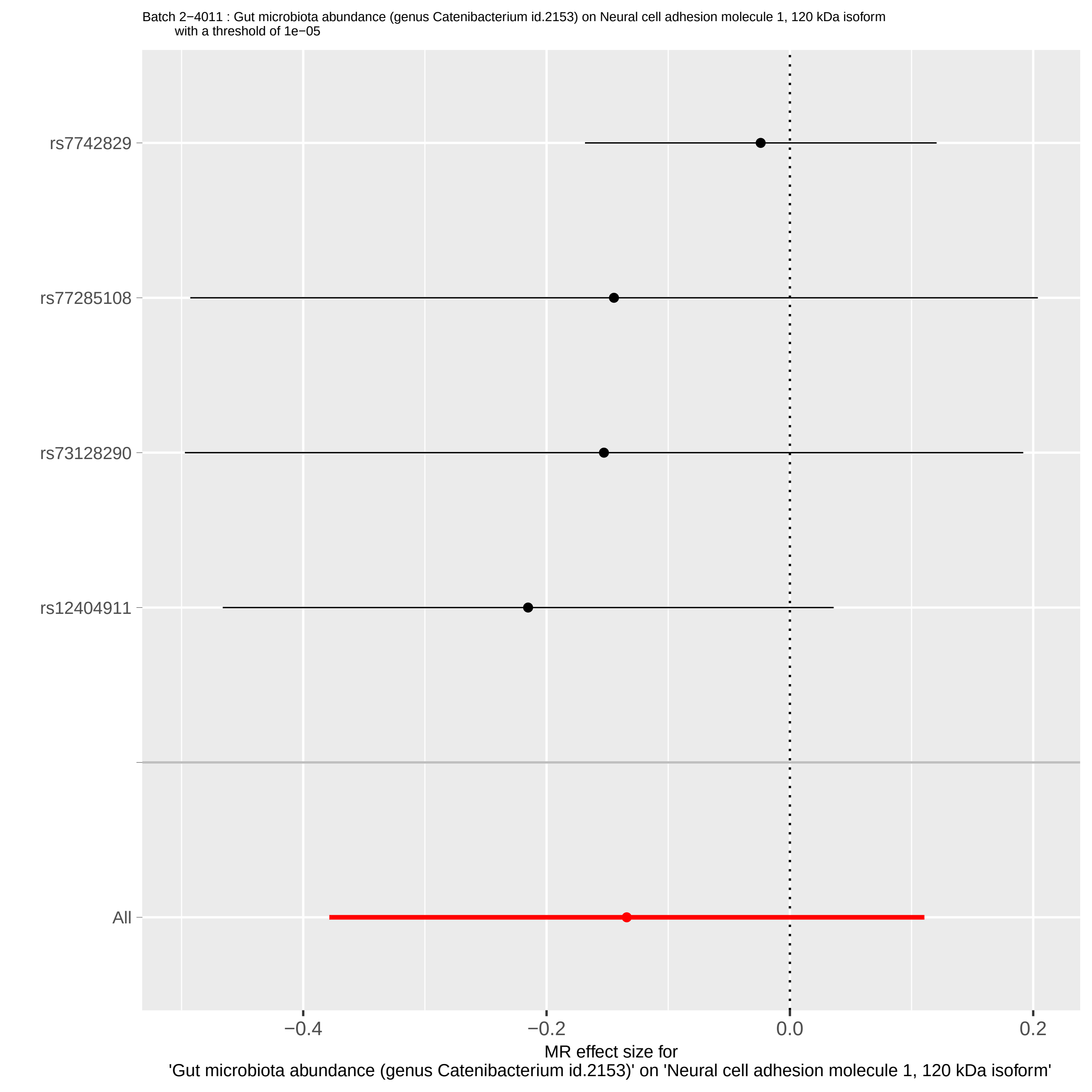

Batch 2−4011 : Gut microbiota abundance (genus Catenibacterium id.2153) on Neural cell adhesion molecule 1, 120 kDa isoform
with a threshold of 1e−05
rs7742829
rs77285108
rs73128290
rs12404911
All
−0.4
−0.2
0.0
0.2
MR effect size for
'Gut microbiota abundance (genus Catenibacterium id.2153)' on 'Neural cell adhesion molecule 1, 120 kDa isoform'

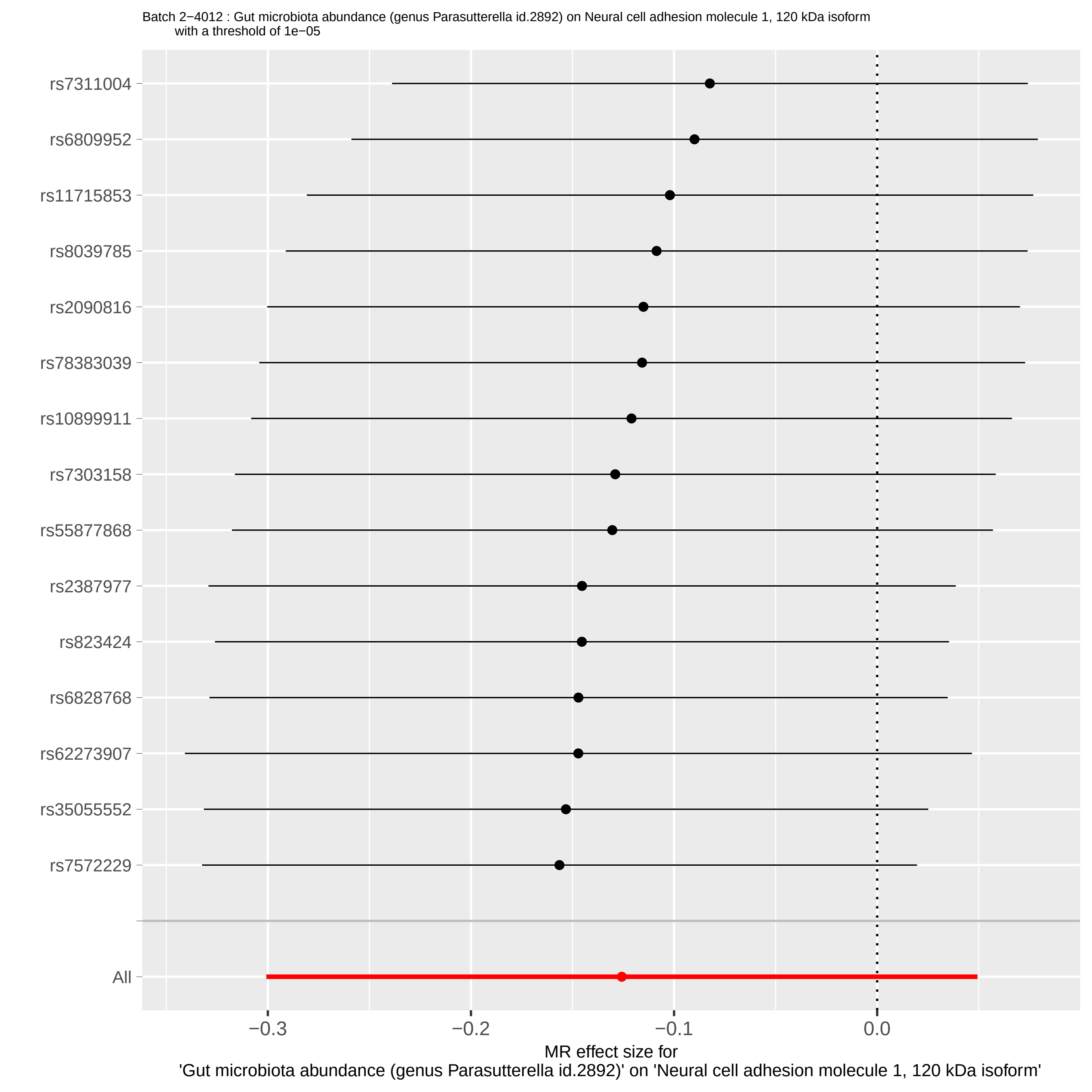

Batch 2−4012 : Gut microbiota abundance (genus Parasutterella id.2892) on Neural cell adhesion molecule 1, 120 kDa isoform
with a threshold of 1e−05
rs7311004
rs6809952
rs11715853
rs8039785
rs2090816
rs78383039
rs10899911
rs7303158
rs55877868
rs2387977
rs823424
rs6828768
rs62273907
rs35055552
rs7572229
All
−0.3
−0.2
−0.1
0.0
MR effect size for
'Gut microbiota abundance (genus Parasutterella id.2892)' on 'Neural cell adhesion molecule 1, 120 kDa isoform'

Batch 2−4013 : Gut microbiota abundance (genus Catenibacterium id.2153) on Neural cell adhesion molecule 1
with a threshold of 1e−05

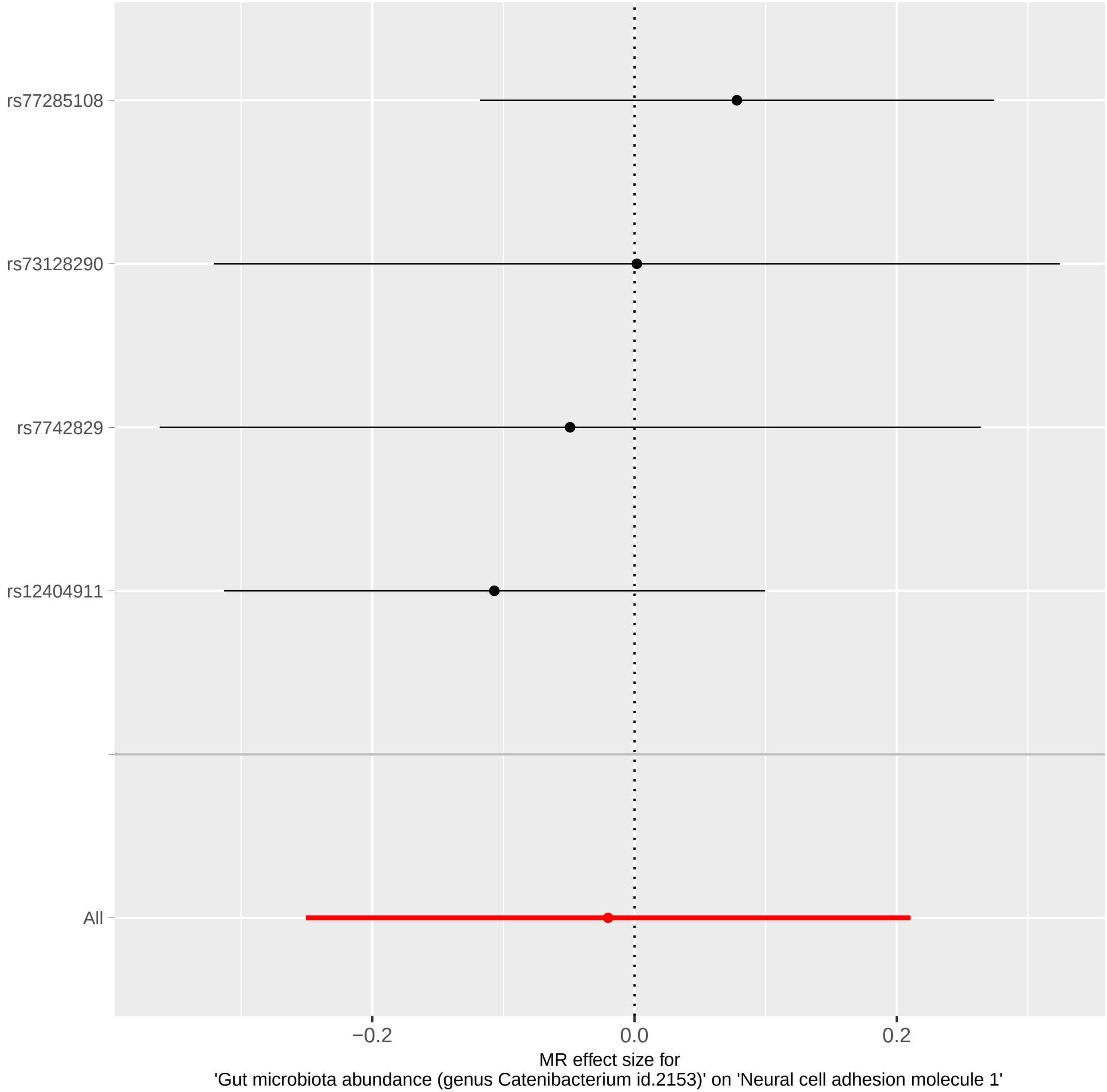

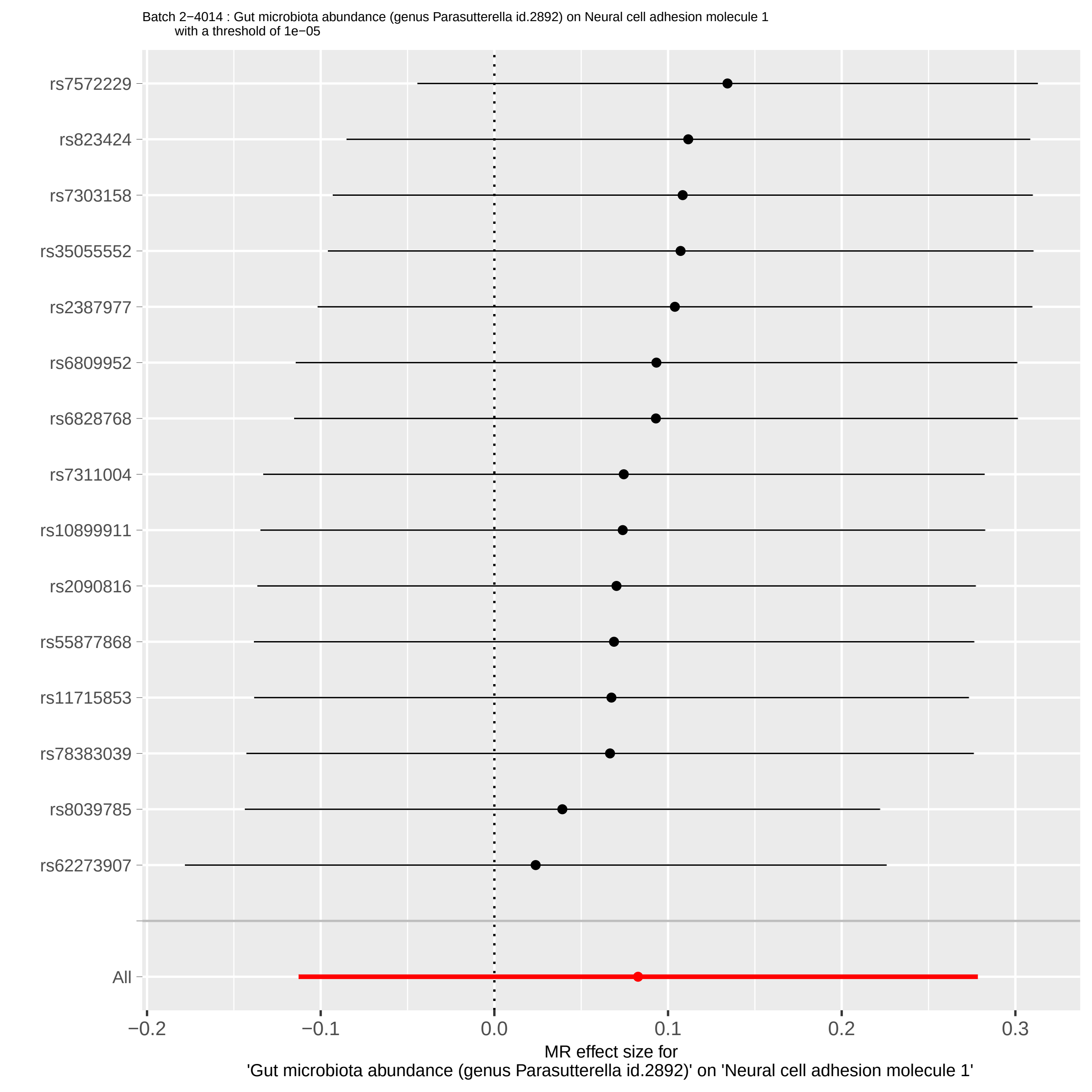
Batch 2−4014 : Gut microbiota abundance (genus Parasutterella id.2892) on Neural cell adhesion molecule 1
with a threshold of 1e−05
rs7572229
rs823424
rs7303158
rs35055552
rs2387977
rs6809952
rs6828768
rs7311004
rs10899911
rs2090816
rs55877868
rs11715853
rs78383039
rs8039785
rs62273907
All
−0.2
−0.1
0.0
0.1
0.2
0.3
MR effect size for
'Gut microbiota abundance (genus Parasutterella id.2892)' on 'Neural cell adhesion molecule 1'

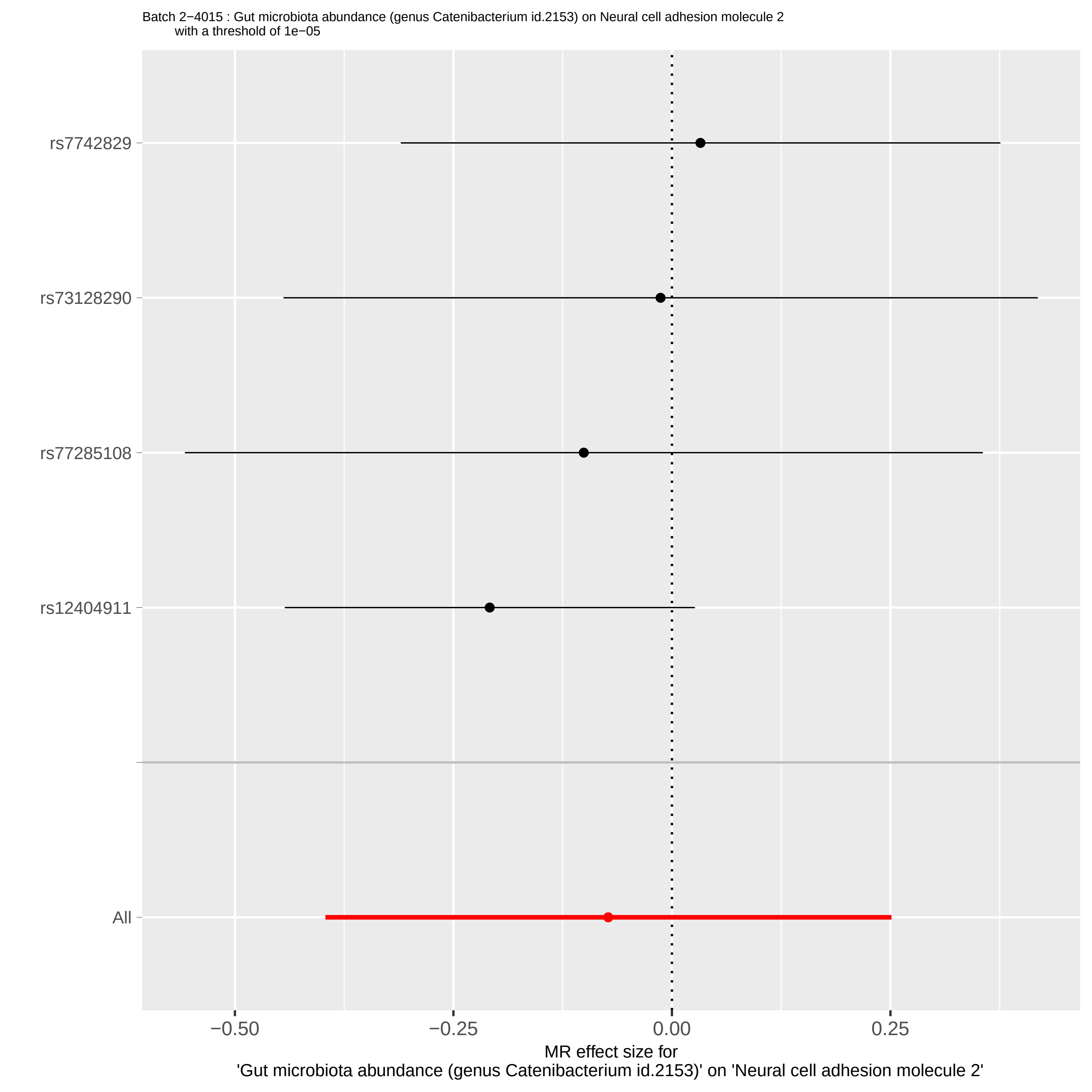

Batch 2−4015 : Gut microbiota abundance (genus Catenibacterium id.2153) on Neural cell adhesion molecule 2
with a threshold of 1e−05
rs7742829
rs73128290
rs77285108
rs12404911
All
−0.50
−0.25
0.00
0.25
MR effect size for
'Gut microbiota abundance (genus Catenibacterium id.2153)' on 'Neural cell adhesion molecule 2'

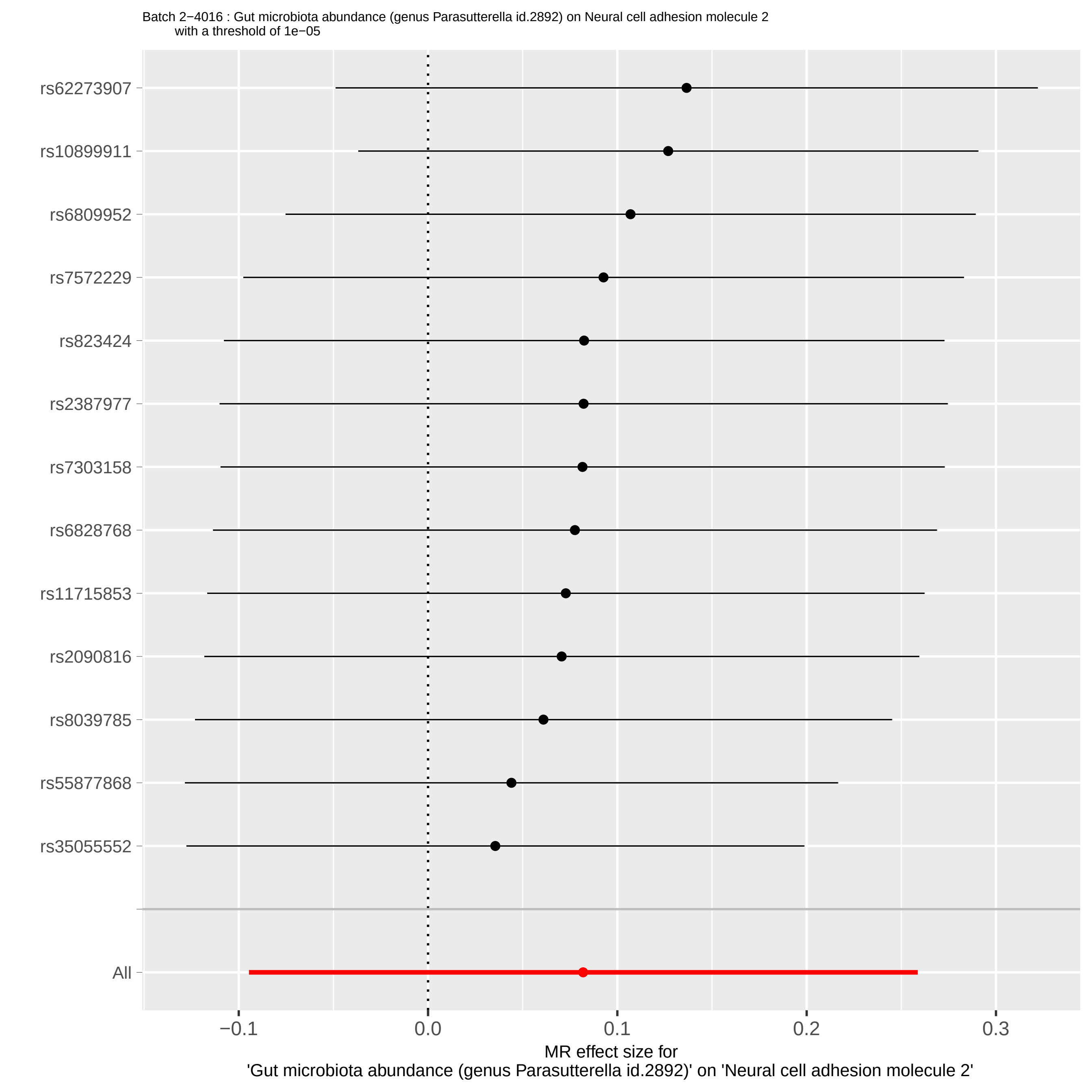

Batch 2−4016 : Gut microbiota abundance (genus Parasutterella id.2892) on Neural cell adhesion molecule 2
with a threshold of 1e−05
rs62273907
rs10899911
rs6809952
rs7572229
rs823424
rs2387977
rs7303158
rs6828768
rs11715853
rs2090816
rs8039785
rs55877868
rs35055552
All
−0.1
0.0
0.1
0.2
0.3
MR effect size for
'Gut microbiota abundance (genus Parasutterella id.2892)' on 'Neural cell adhesion molecule 2'

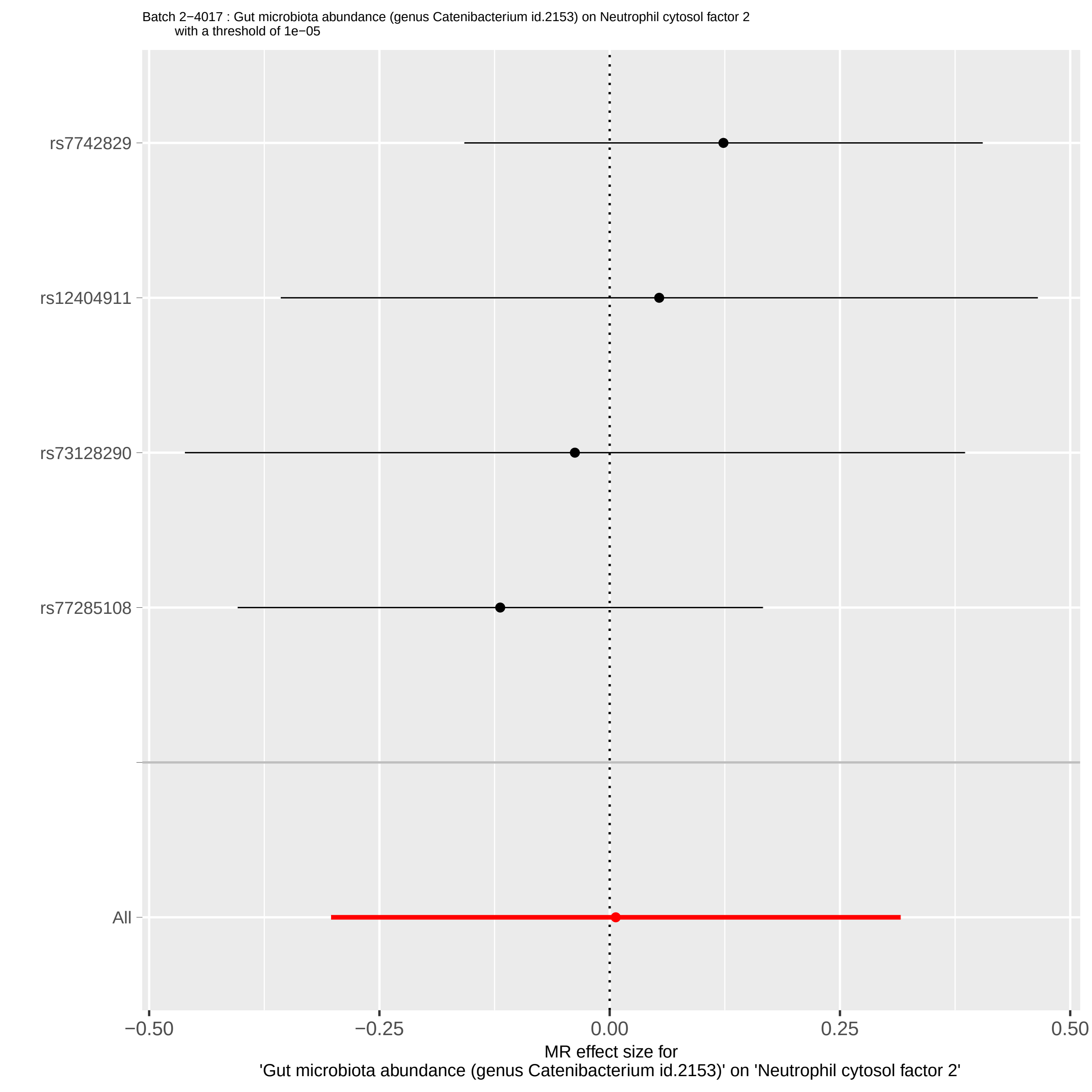
Batch 2−4017 : Gut microbiota abundance (genus Catenibacterium id.2153) on Neutrophil cytosol factor 2
with a threshold of 1e−05
rs7742829
rs12404911
rs73128290
rs77285108
All
−0.50
−0.25
0.00
0.25
0.50
MR effect size for
'Gut microbiota abundance (genus Catenibacterium id.2153)' on 'Neutrophil cytosol factor 2'

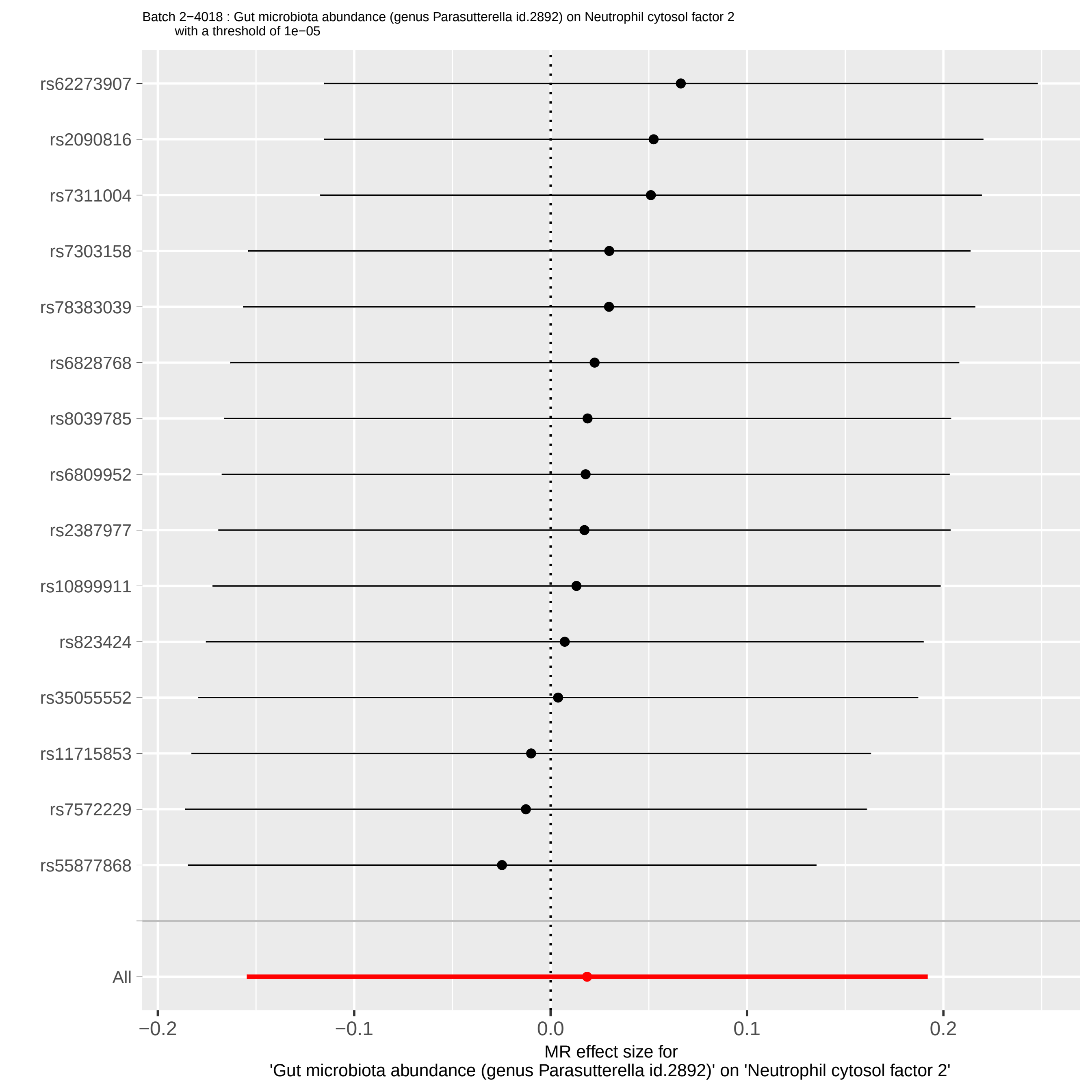
Batch 2−4018 : Gut microbiota abundance (genus Parasutterella id.2892) on Neutrophil cytosol factor 2
with a threshold of 1e−05
rs62273907
rs2090816
rs7311004
rs7303158
rs78383039
rs6828768
rs8039785
rs6809952
rs2387977
rs10899911
rs823424
rs35055552
rs11715853
rs7572229
rs55877868
All
−0.2
−0.1
0.0
0.1
0.2
MR effect size for
'Gut microbiota abundance (genus Parasutterella id.2892)' on 'Neutrophil cytosol factor 2'

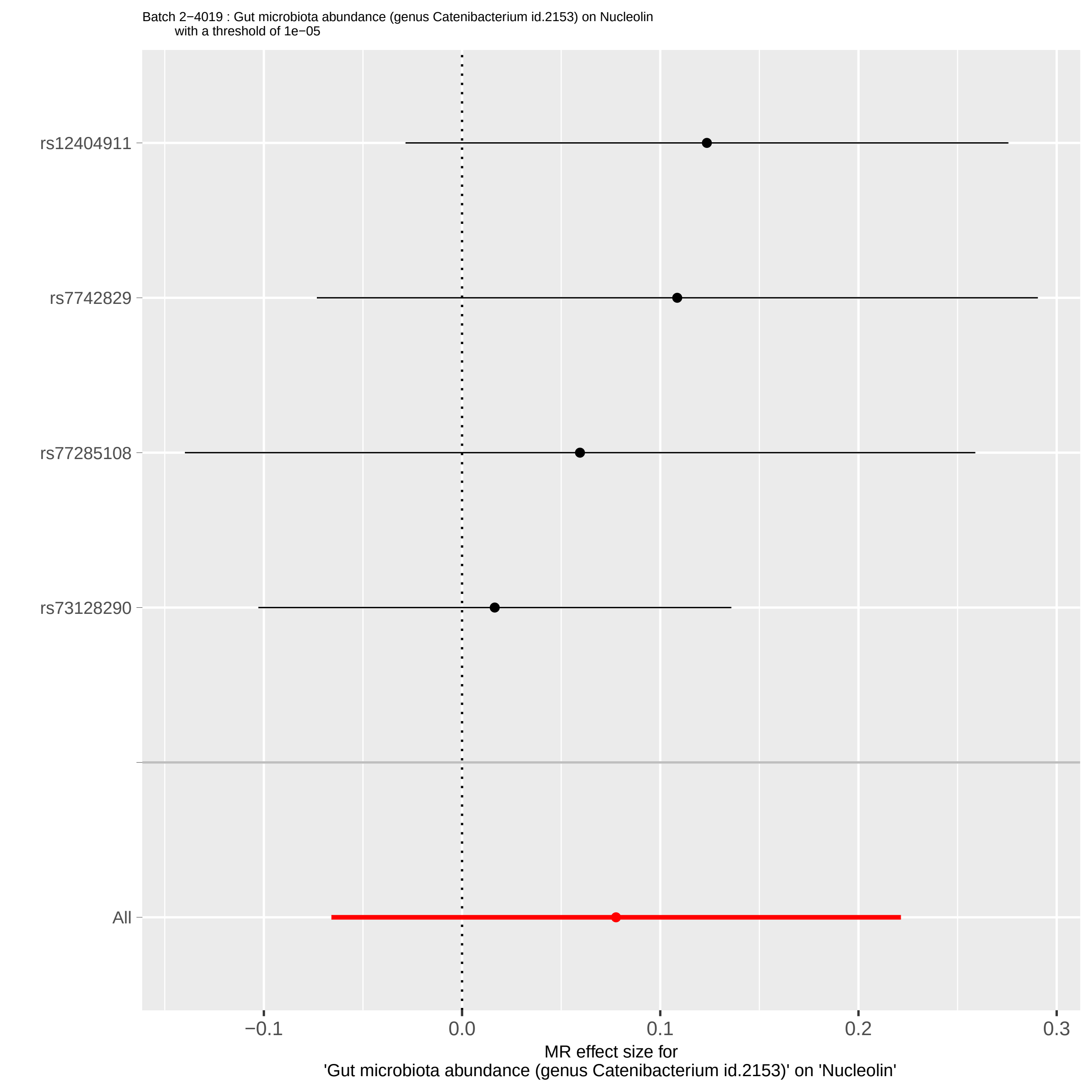
Batch 2−4019 : Gut microbiota abundance (genus Catenibacterium id.2153) on Nucleolin
with a threshold of 1e−05
rs12404911
rs7742829
rs77285108
rs73128290
All
−0.1
0.0
0.1
0.2
0.3
MR effect size for
'Gut microbiota abundance (genus Catenibacterium id.2153)' on 'Nucleolin'

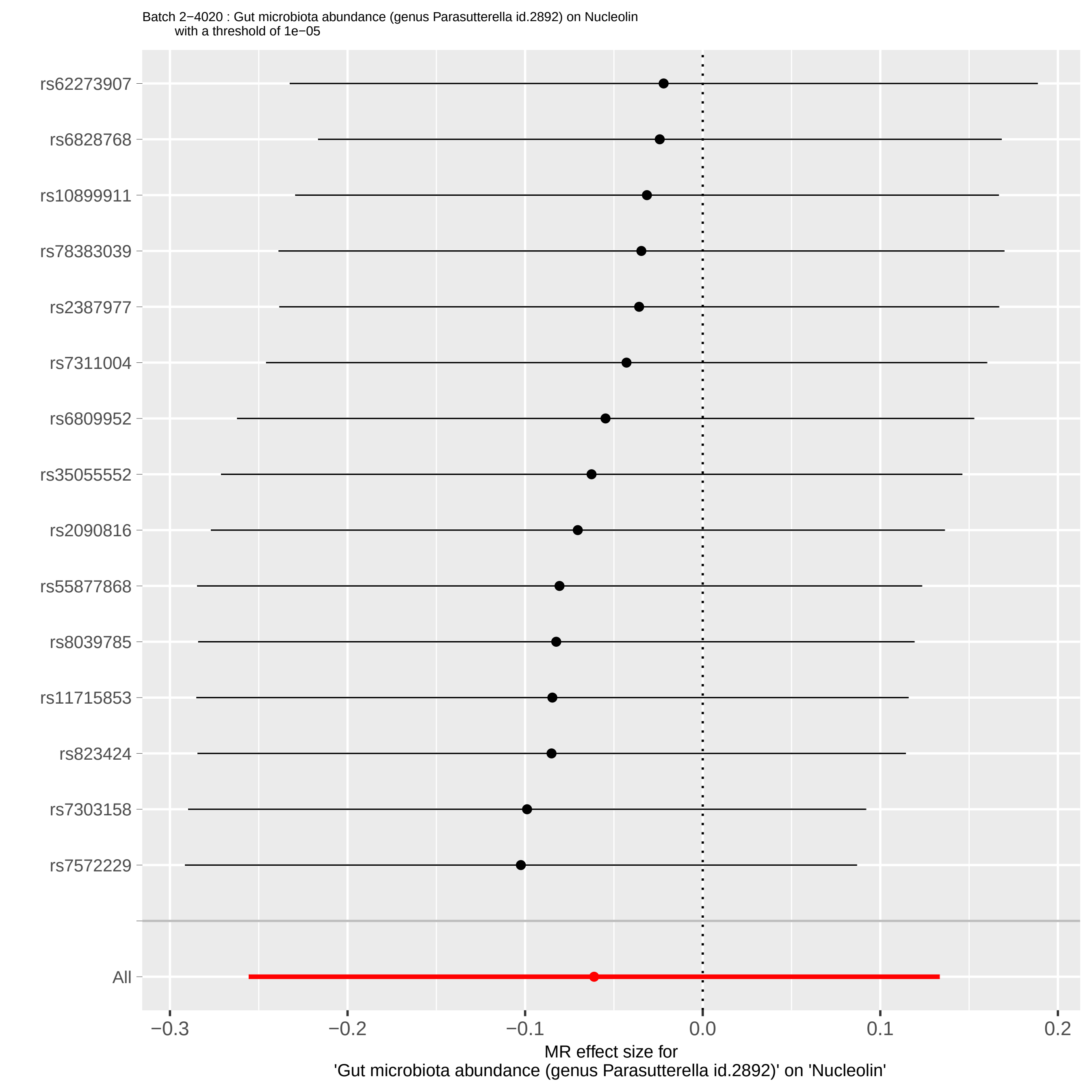

Batch 2−4020 : Gut microbiota abundance (genus Parasutterella id.2892) on Nucleolin
with a threshold of 1e−05
rs62273907
rs6828768
rs10899911
rs78383039
rs2387977
rs7311004
rs6809952
rs35055552
rs2090816
rs55877868
rs8039785
rs11715853
rs823424
rs7303158
rs7572229
All
−0.3
−0.2
−0.1
0.0
0.1
0.2
MR effect size for
'Gut microbiota abundance (genus Parasutterella id.2892)' on 'Nucleolin'

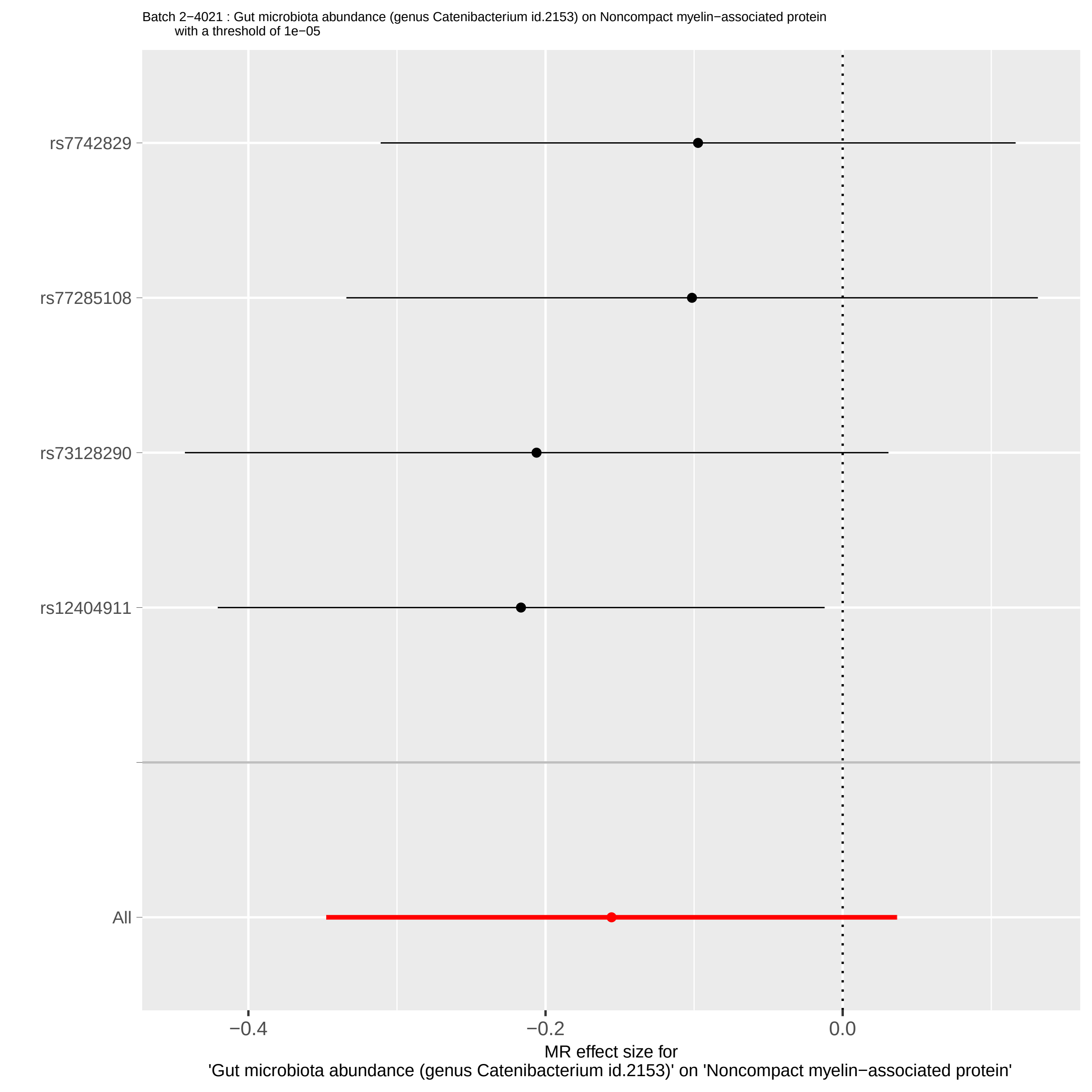
Batch 2−4021 : Gut microbiota abundance (genus Catenibacterium id.2153) on Noncompact myelin−associated protein
with a threshold of 1e−05
rs7742829
rs77285108
rs73128290
rs12404911
All
−0.4
−0.2
0.0
MR effect size for
'Gut microbiota abundance (genus Catenibacterium id.2153)' on 'Noncompact myelin−associated protein'

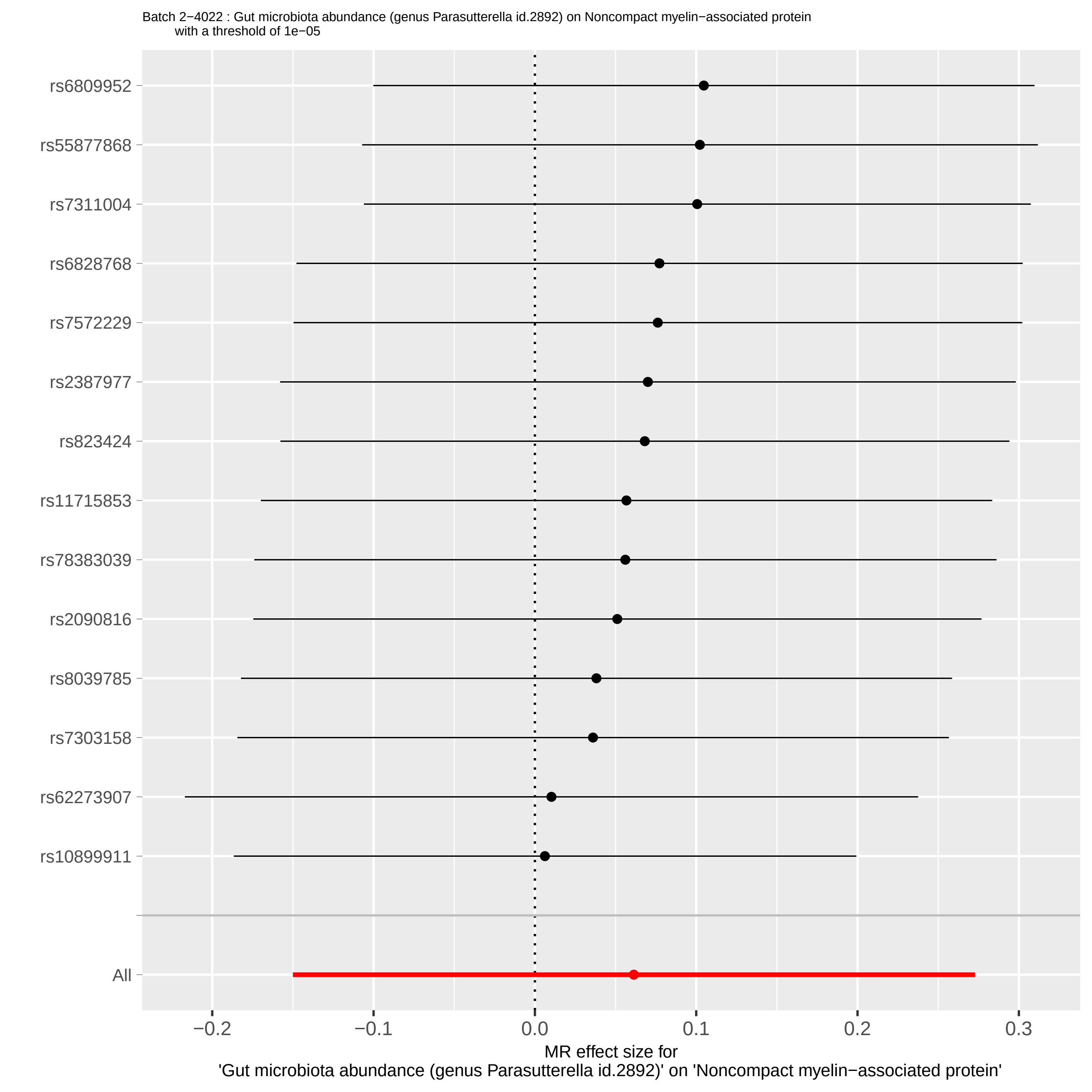
Batch 2−4022 : Gut microbiota abundance (genus Parasutterella id.2892) on Noncompact myelin−associated protein
with a threshold of 1e−05
rs6809952
rs55877868
rs7311004
rs6828768
rs7572229
rs2387977
rs823424
rs11715853
rs78383039
rs2090816
rs8039785
rs7303158
rs62273907
rs10899911
All
−0.2
−0.1
0.0
0.1
0.2
0.3
MR effect size for
'Gut microbiota abundance (genus Parasutterella id.2892)' on 'Noncompact myelin−associated protein'

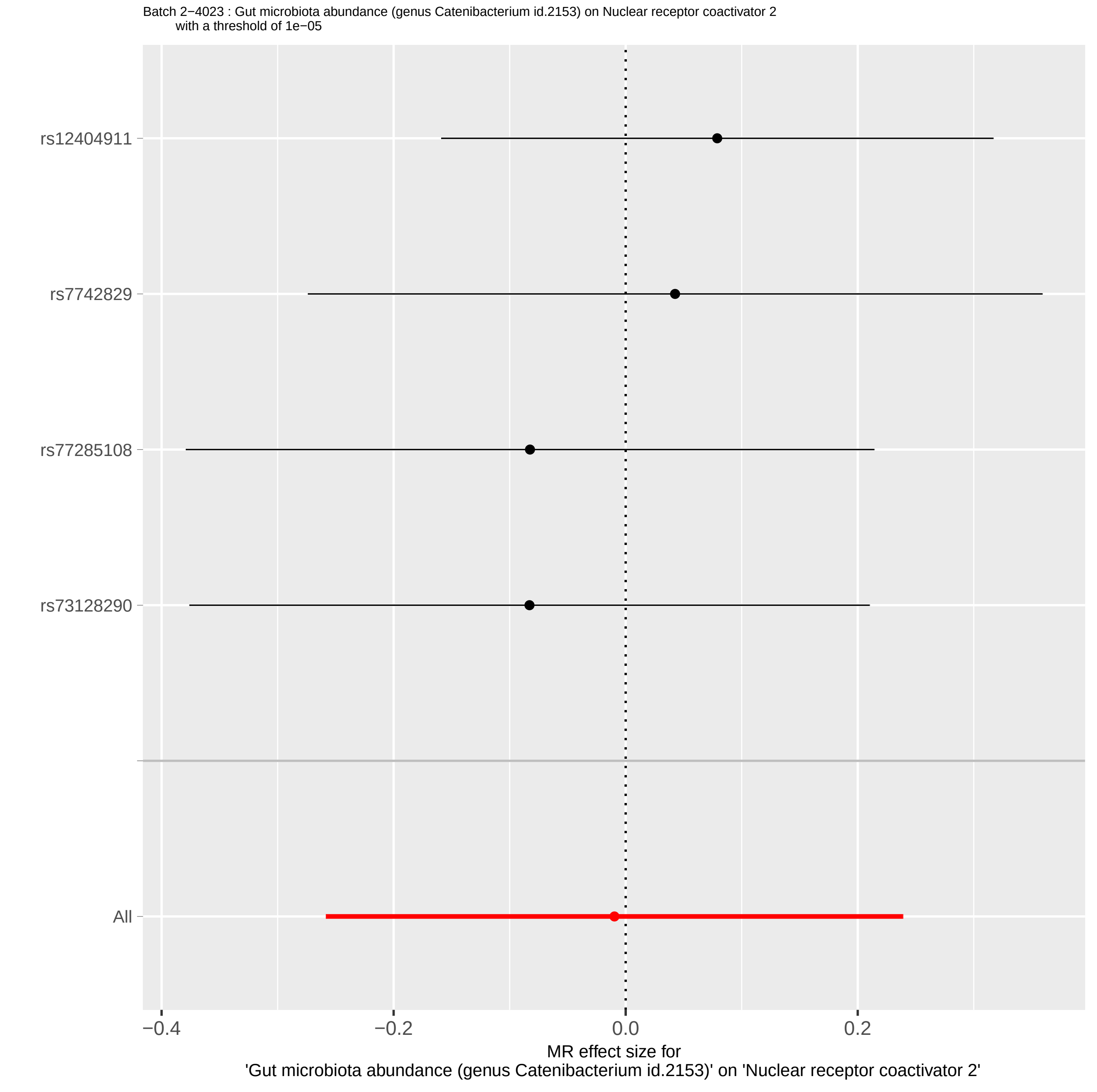
Batch 2−4023 : Gut microbiota abundance (genus Catenibacterium id.2153) on Nuclear receptor coactivator 2
with a threshold of 1e−05
rs12404911
rs7742829
rs77285108
rs73128290
All
−0.4
−0.2
0.0
0.2
MR effect size for
'Gut microbiota abundance (genus Catenibacterium id.2153)' on 'Nuclear receptor coactivator 2'

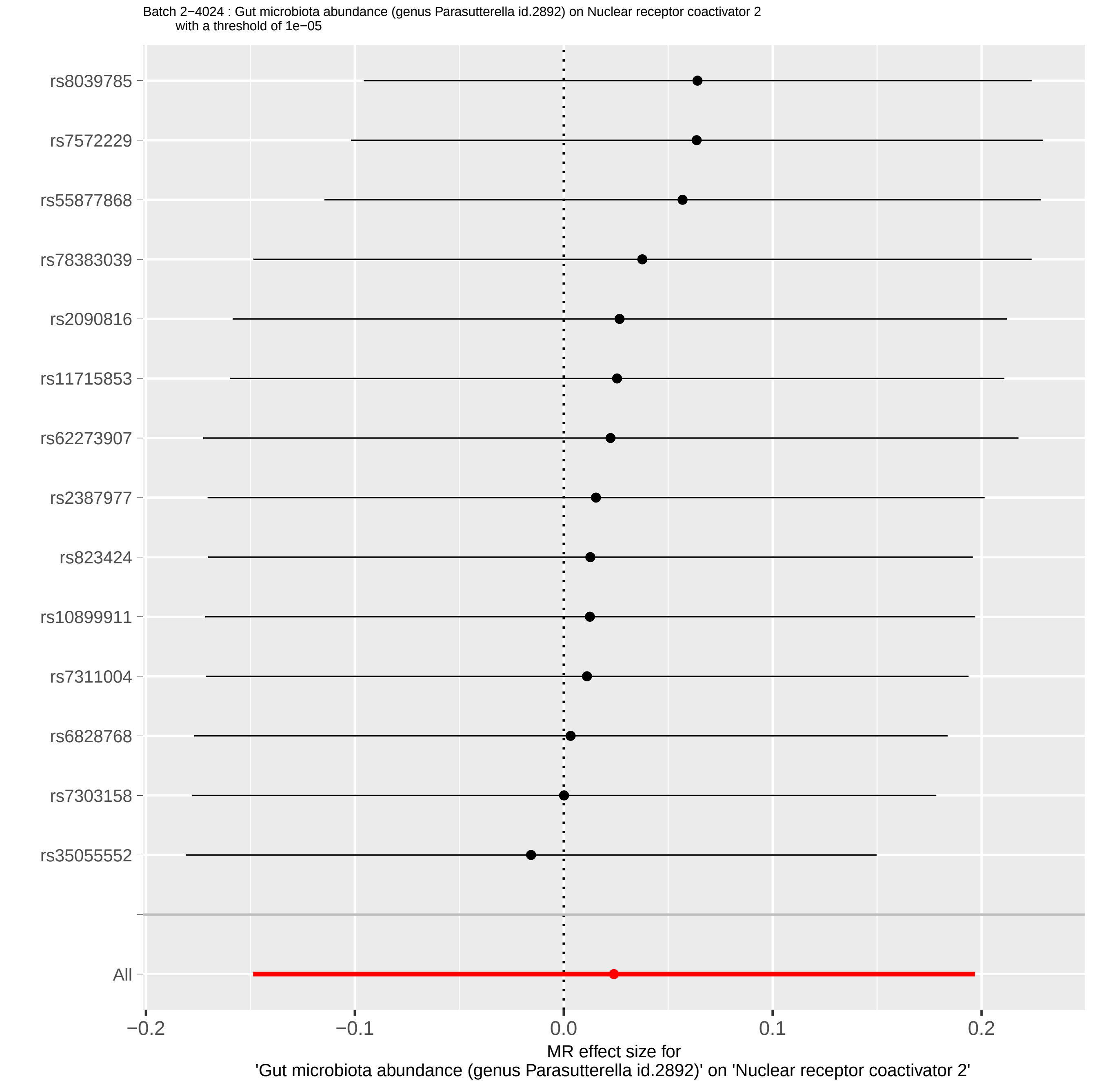
Batch 2−4024 : Gut microbiota abundance (genus Parasutterella id.2892) on Nuclear receptor coactivator 2
with a threshold of 1e−05
rs8039785
rs7572229
rs55877868
rs78383039
rs2090816
rs11715853
rs62273907
rs2387977
rs823424
rs10899911
rs7311004
rs6828768
rs7303158
rs35055552
All
−0.2
−0.1
0.0
0.1
0.2
MR effect size for
'Gut microbiota abundance (genus Parasutterella id.2892)' on 'Nuclear receptor coactivator 2'

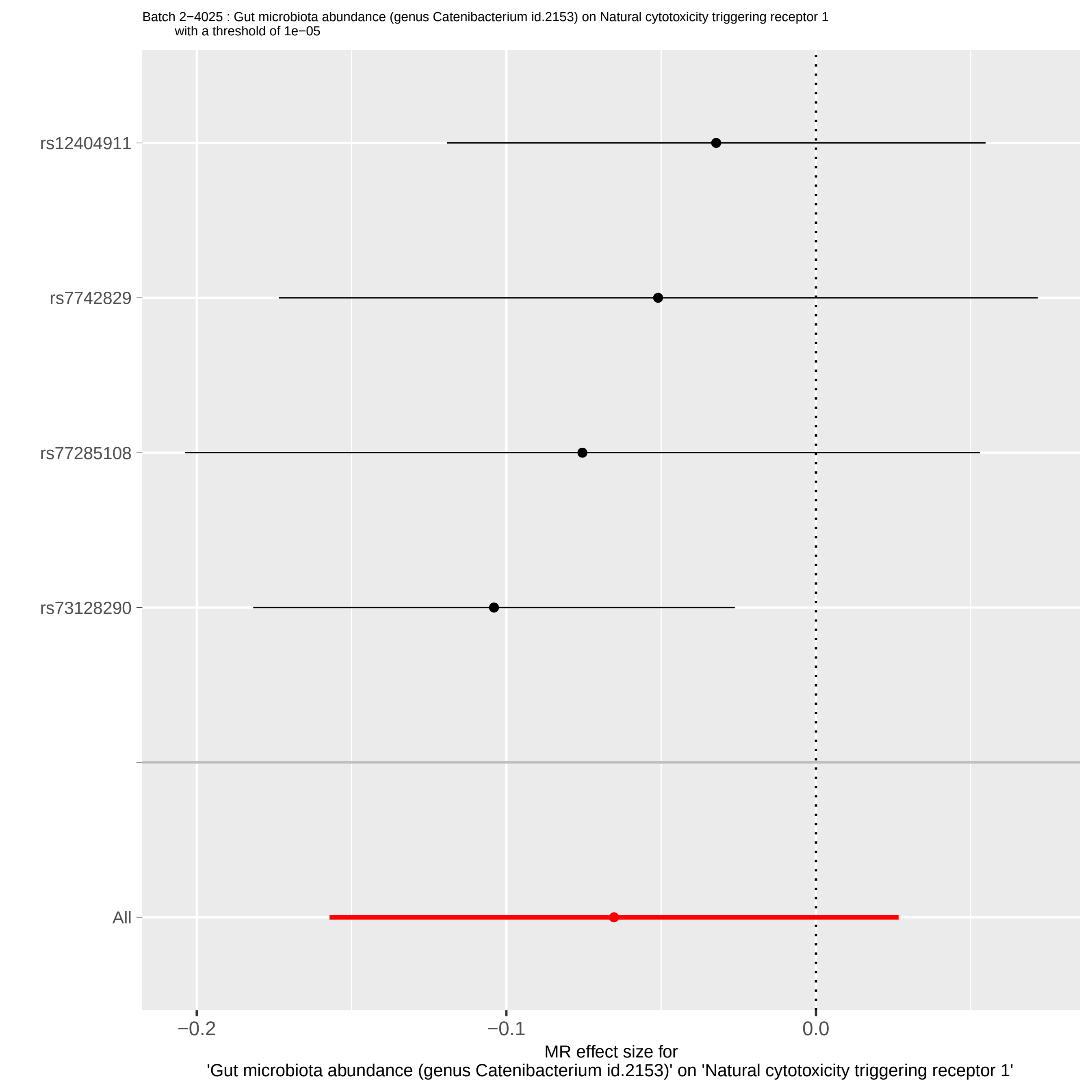
Batch 2−4025 : Gut microbiota abundance (genus Catenibacterium id.2153) on Natural cytotoxicity triggering receptor 1
with a threshold of 1e−05
rs12404911
rs7742829
rs77285108
rs73128290
All
−0.2
−0.1
0.0
MR effect size for
'Gut microbiota abundance (genus Catenibacterium id.2153)' on 'Natural cytotoxicity triggering receptor 1'

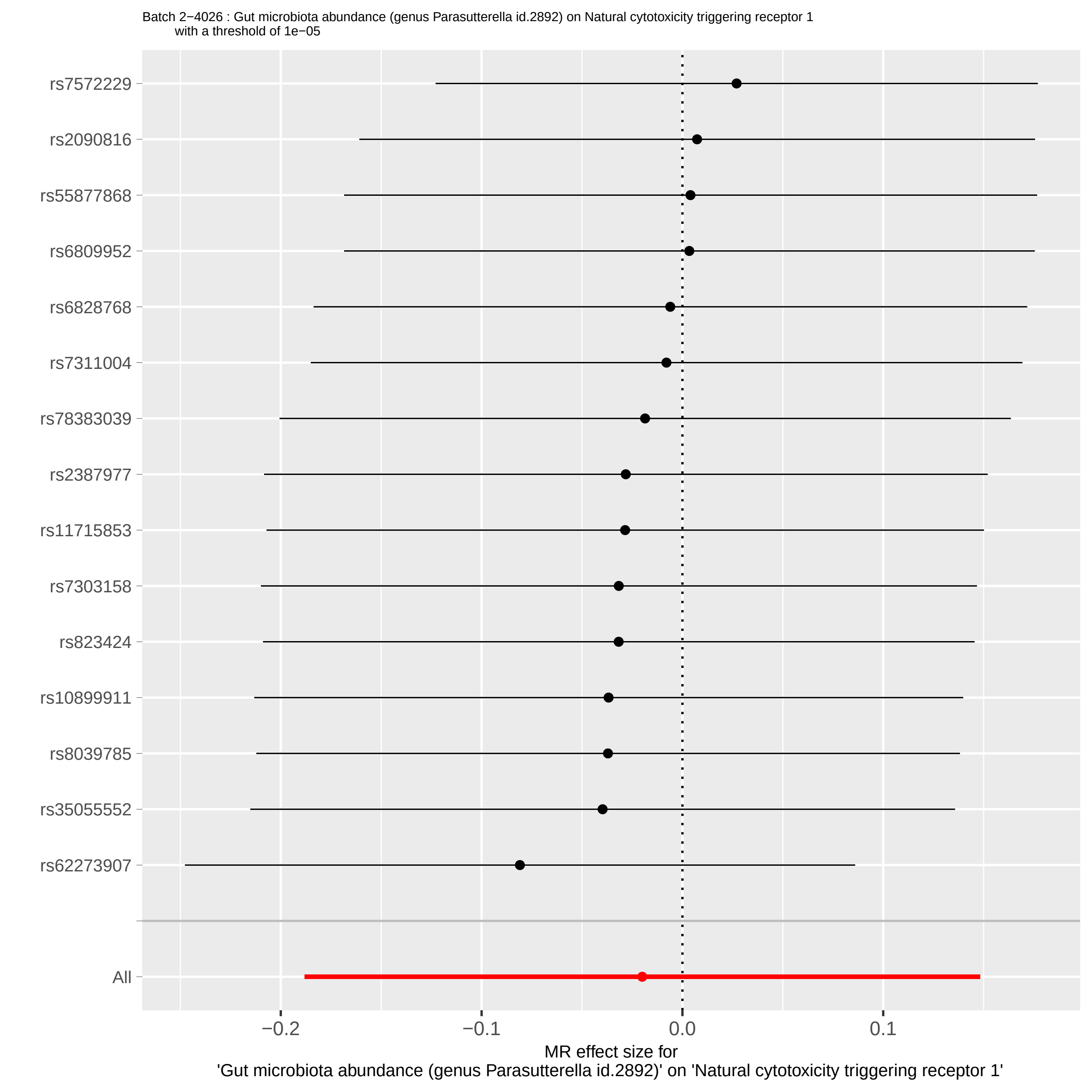
Batch 2−4026 : Gut microbiota abundance (genus Parasutterella id.2892) on Natural cytotoxicity triggering receptor 1
with a threshold of 1e−05
rs7572229
rs2090816
rs55877868
rs6809952
rs6828768
rs7311004
rs78383039
rs2387977
rs11715853
rs7303158
rs823424
rs10899911
rs8039785
rs35055552
rs62273907
All
−0.2
−0.1
0.0
0.1
MR effect size for
'Gut microbiota abundance (genus Parasutterella id.2892)' on 'Natural cytotoxicity triggering receptor 1'

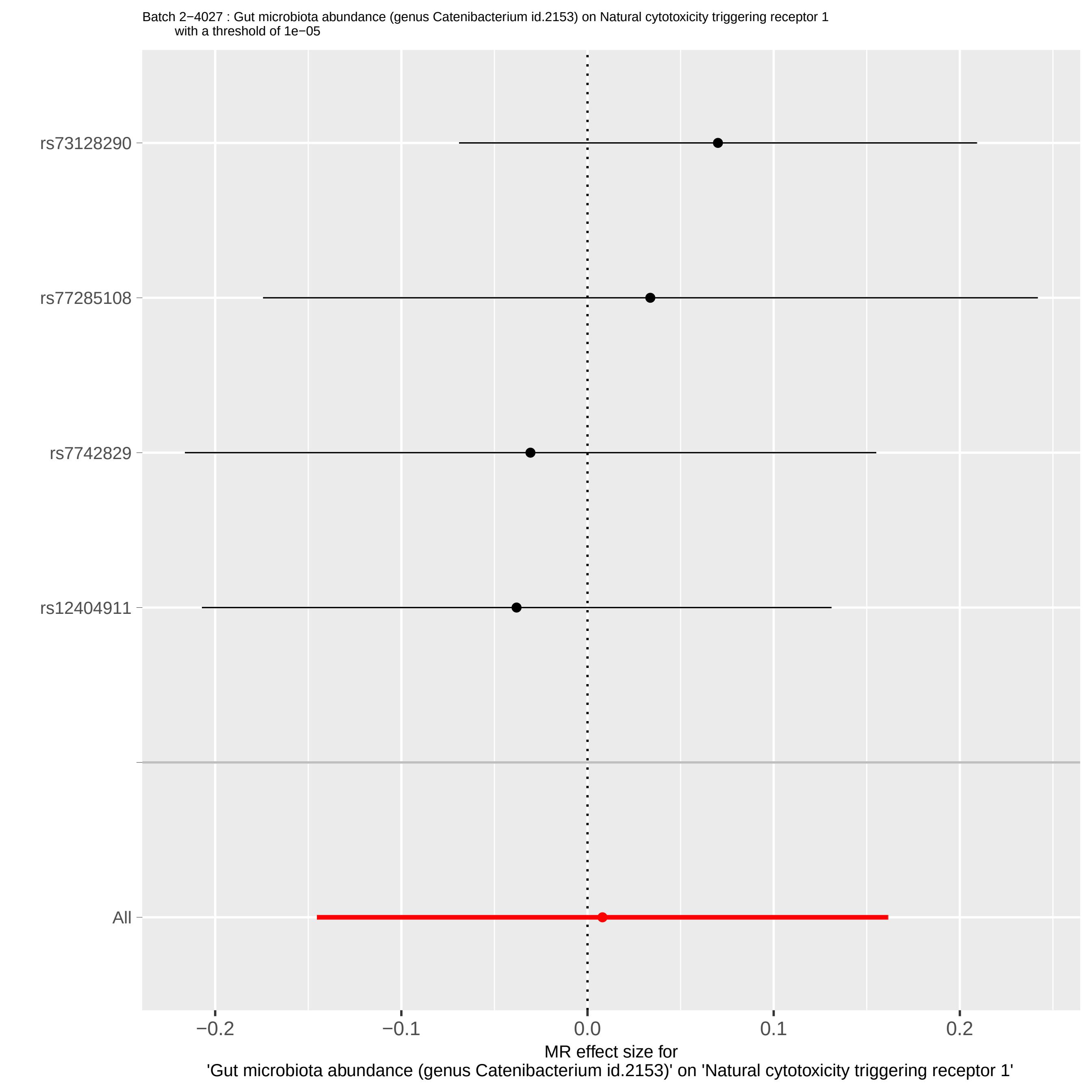
Batch 2−4027 : Gut microbiota abundance (genus Catenibacterium id.2153) on Natural cytotoxicity triggering receptor 1
with a threshold of 1e−05
rs73128290
rs77285108
rs7742829
rs12404911
All
−0.2
−0.1
0.0
0.1
0.2
MR effect size for
'Gut microbiota abundance (genus Catenibacterium id.2153)' on 'Natural cytotoxicity triggering receptor 1'

Batch 2−4028 : Gut microbiota abundance (genus Parasutterella id.2892) on Natural cytotoxicity triggering receptor 1
with a threshold of 1e−05

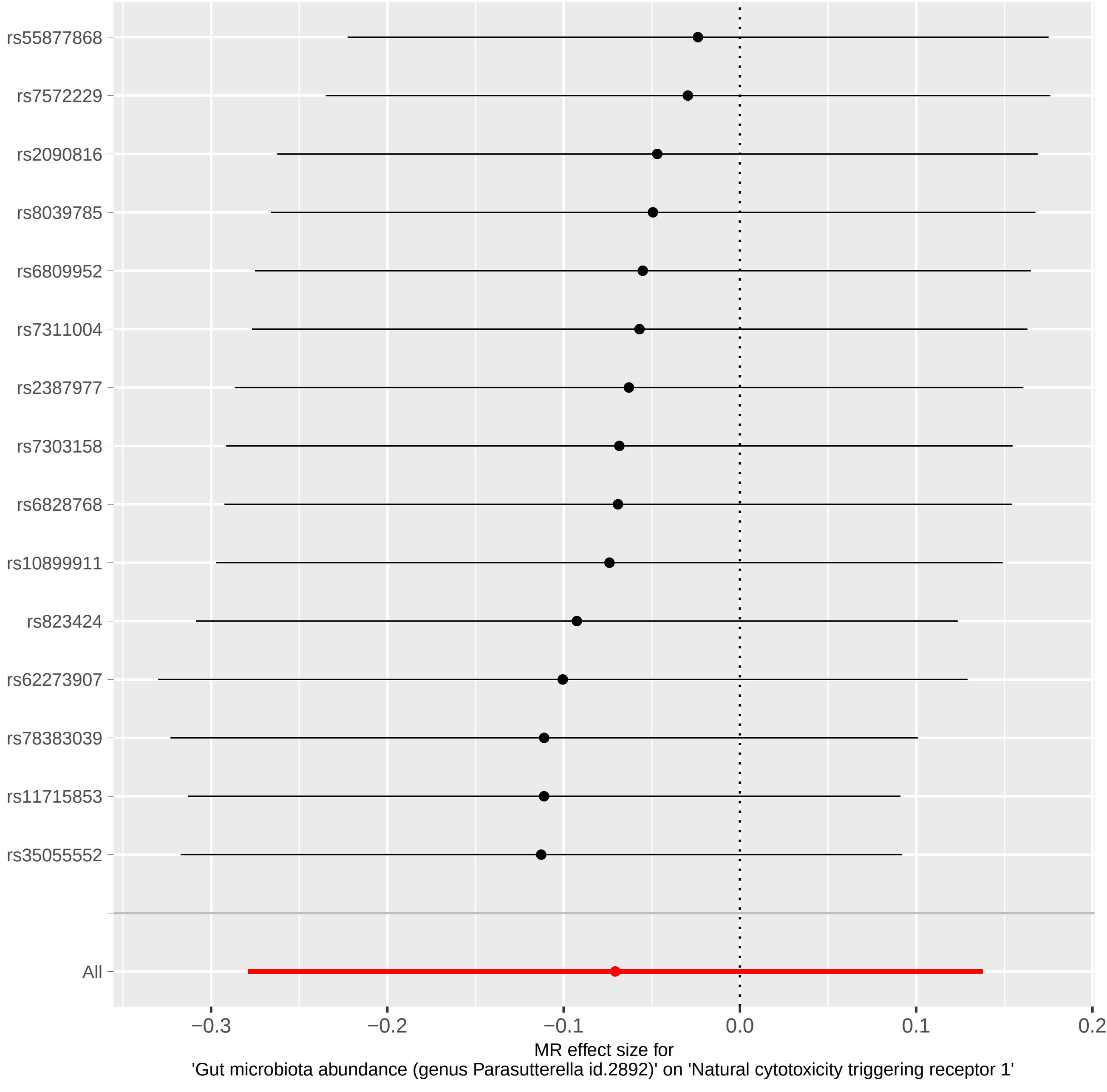

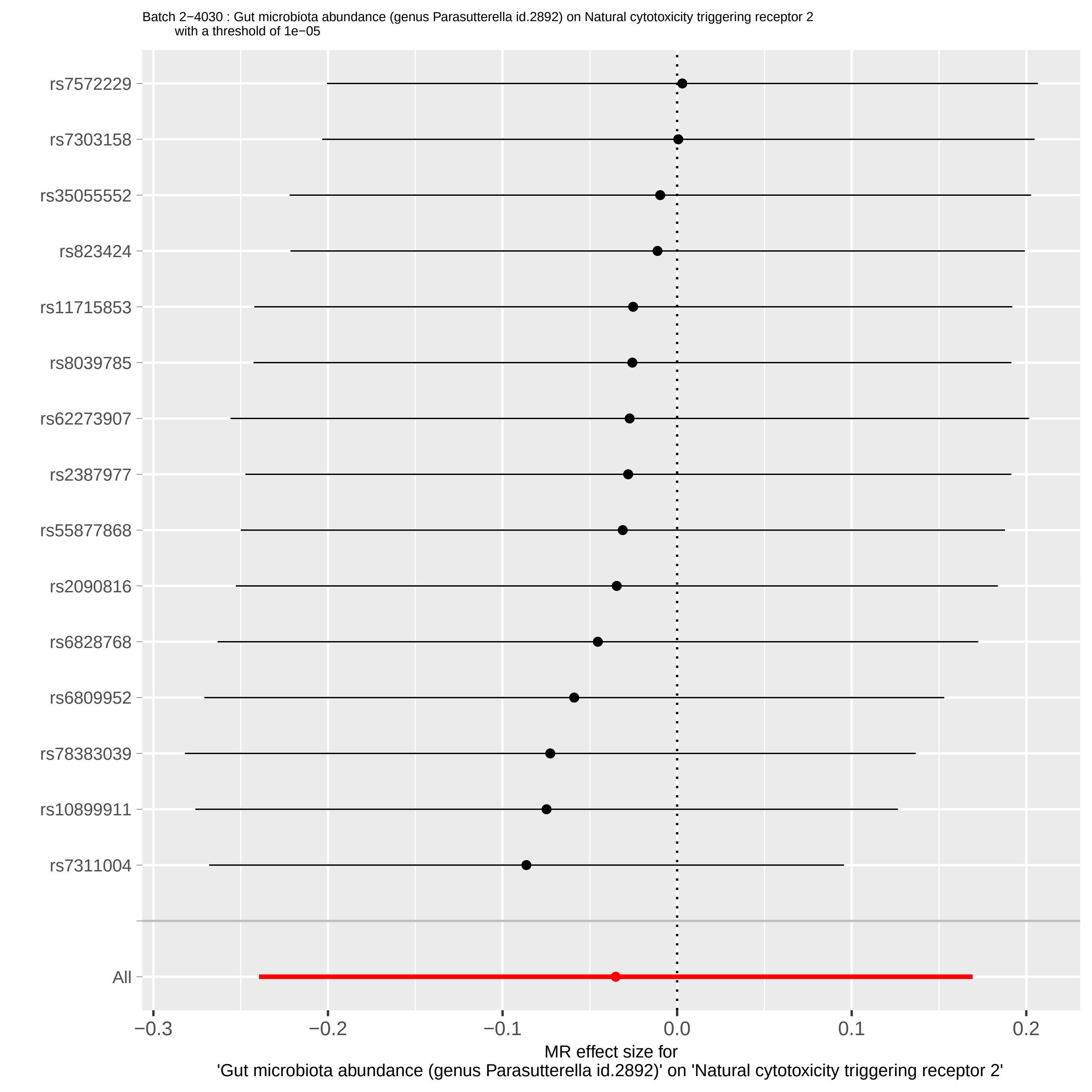
Batch 2−4030 : Gut microbiota abundance (genus Parasutterella id.2892) on Natural cytotoxicity triggering receptor 2
with a threshold of 1e−05
rs7572229
rs7303158
rs35055552
rs823424
rs11715853
rs8039785
rs62273907
rs2387977
rs55877868
rs2090816
rs6828768
rs6809952
rs78383039
rs10899911
rs7311004
All
−0.3
−0.2
−0.1
0.0
0.1
0.2
MR effect size for
'Gut microbiota abundance (genus Parasutterella id.2892)' on 'Natural cytotoxicity triggering receptor 2'

Batch 2−4031 : Gut microbiota abundance (genus Catenibacterium id.2153) on Natural cytotoxicity triggering receptor 3
with a threshold of 1e−05

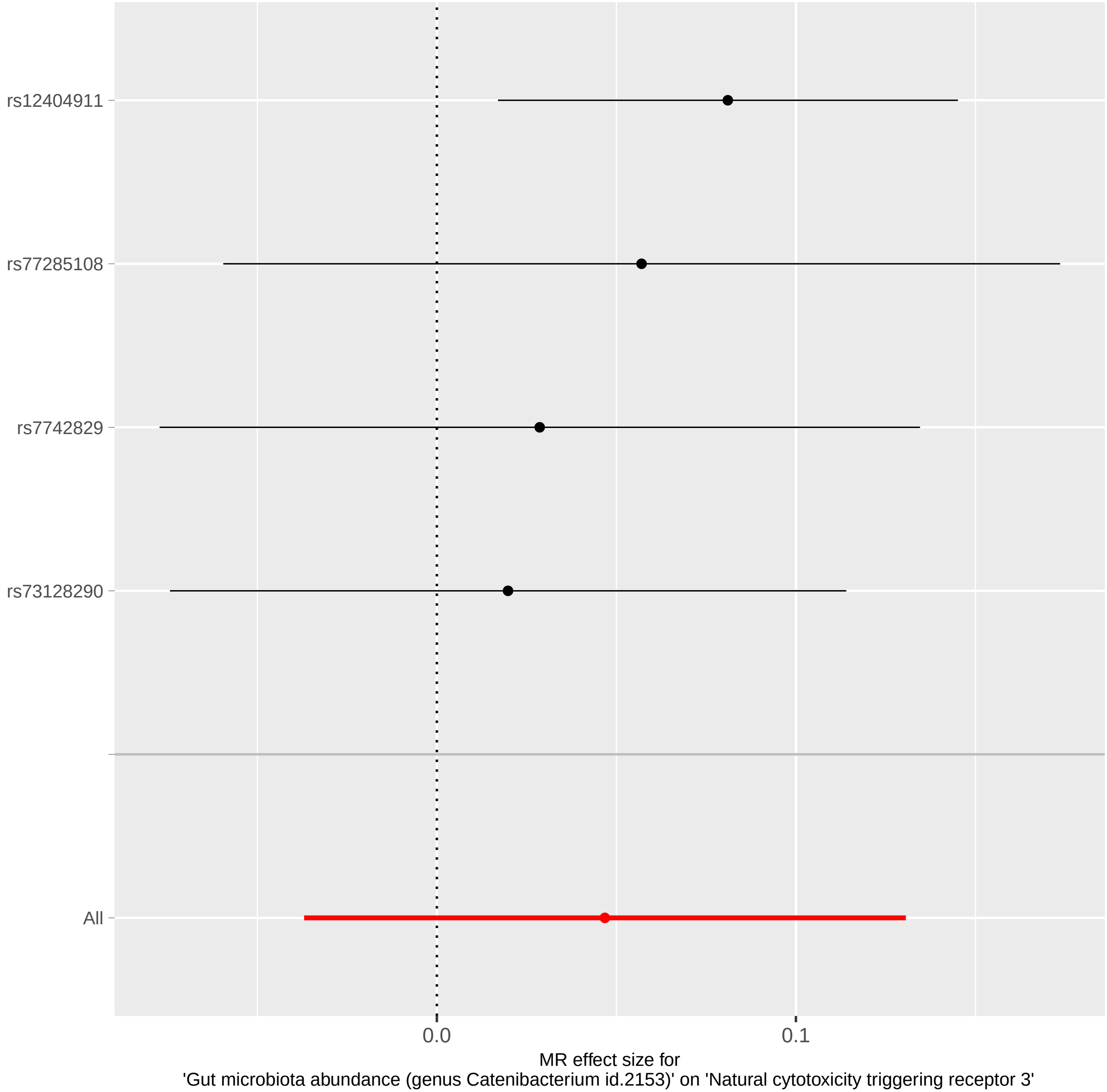

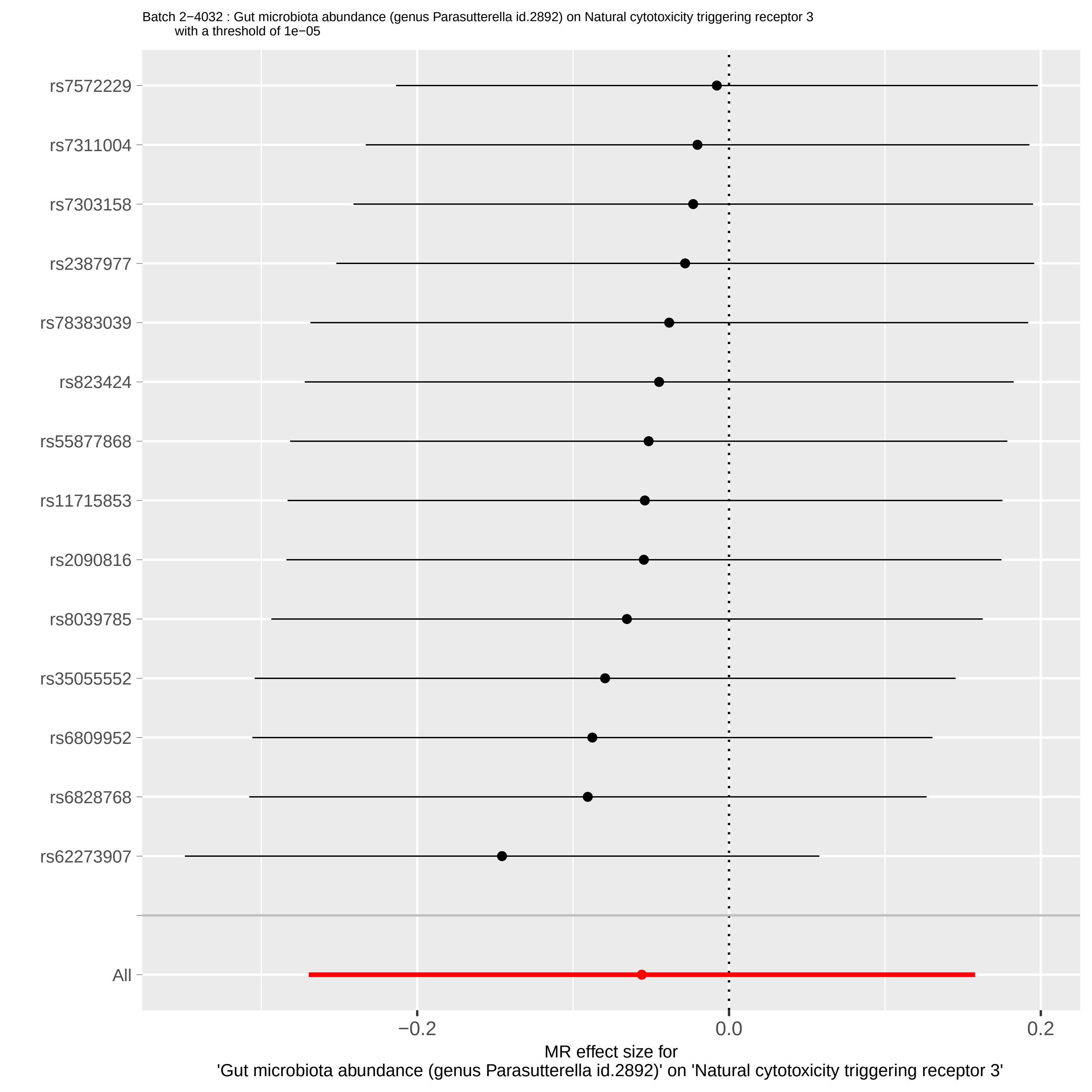

Batch 2−4032 : Gut microbiota abundance (genus Parasutterella id.2892) on Natural cytotoxicity triggering receptor 3
with a threshold of 1e−05
rs7572229
rs7311004
rs7303158
rs2387977
rs78383039
rs823424
rs55877868
rs11715853
rs2090816
rs8039785
rs35055552
rs6809952
rs6828768
rs62273907
All
−0.2
0.0
0.2
MR effect size for
'Gut microbiota abundance (genus Parasutterella id.2892)' on 'Natural cytotoxicity triggering receptor 3'

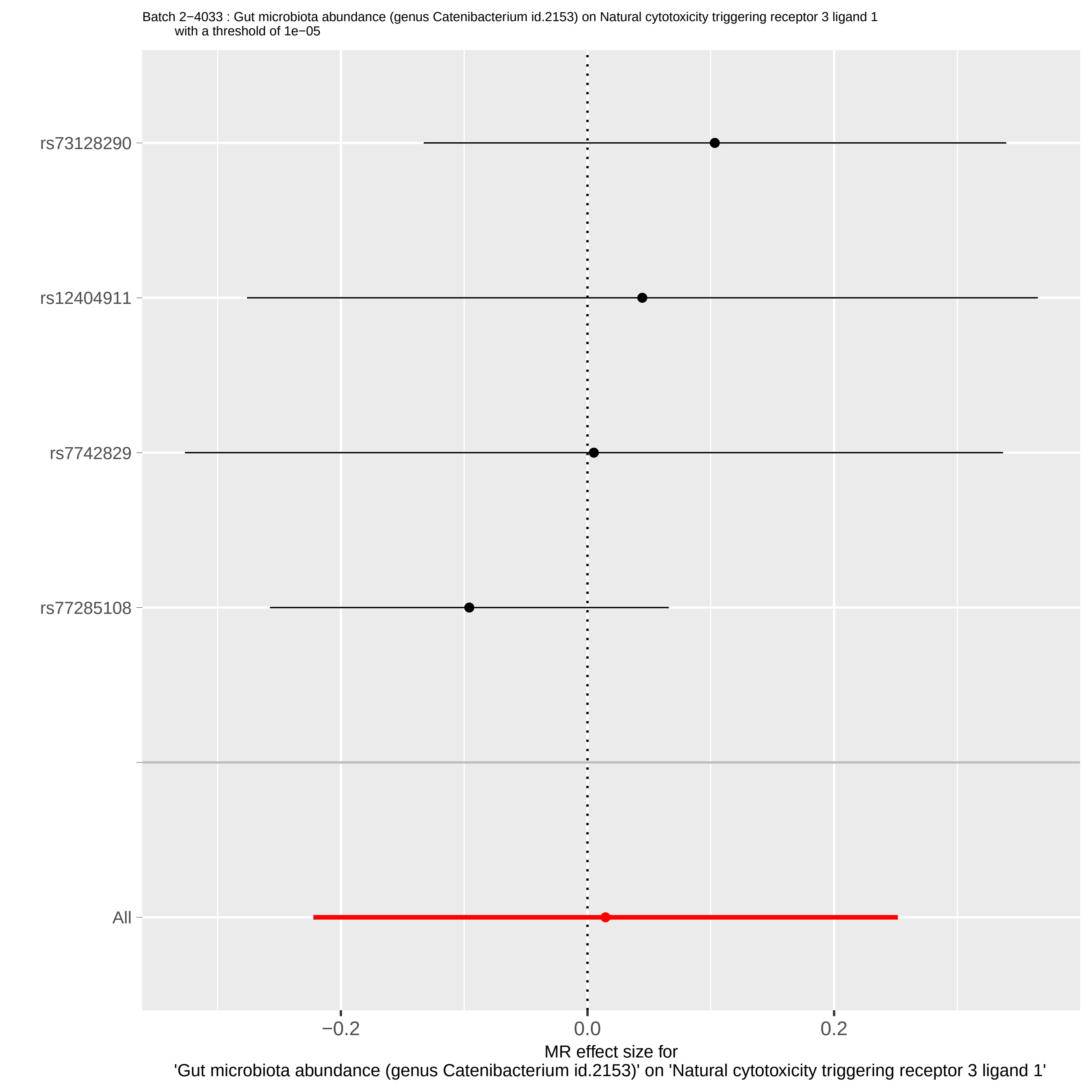

Batch 2−4033 : Gut microbiota abundance (genus Catenibacterium id.2153) on Natural cytotoxicity triggering receptor 3 ligand 1
with a threshold of 1e−05
rs73128290
rs12404911
rs7742829
rs77285108
All
−0.2
0.0
0.2
MR effect size for
'Gut microbiota abundance (genus Catenibacterium id.2153)' on 'Natural cytotoxicity triggering receptor 3 ligand 1'

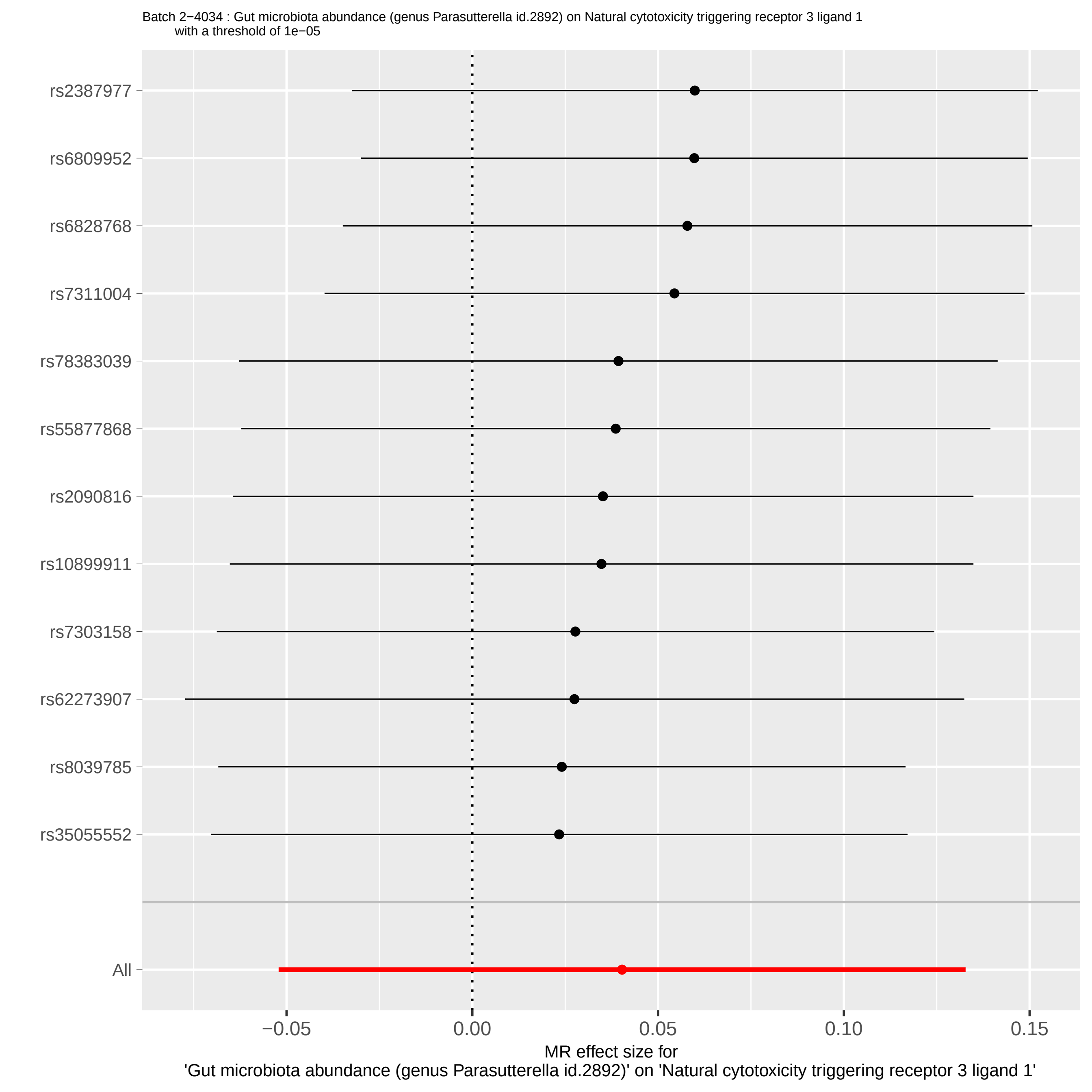
Batch 2−4034 : Gut microbiota abundance (genus Parasutterella id.2892) on Natural cytotoxicity triggering receptor 3 ligand 1
with a threshold of 1e−05
rs2387977
rs6809952
rs6828768
rs7311004
rs78383039
rs55877868
rs2090816
rs10899911
rs7303158
rs62273907
rs8039785
rs35055552
All
−0.05
0.00
0.05
0.10
0.15
MR effect size for
'Gut microbiota abundance (genus Parasutterella id.2892)' on 'Natural cytotoxicity triggering receptor 3 ligand 1'

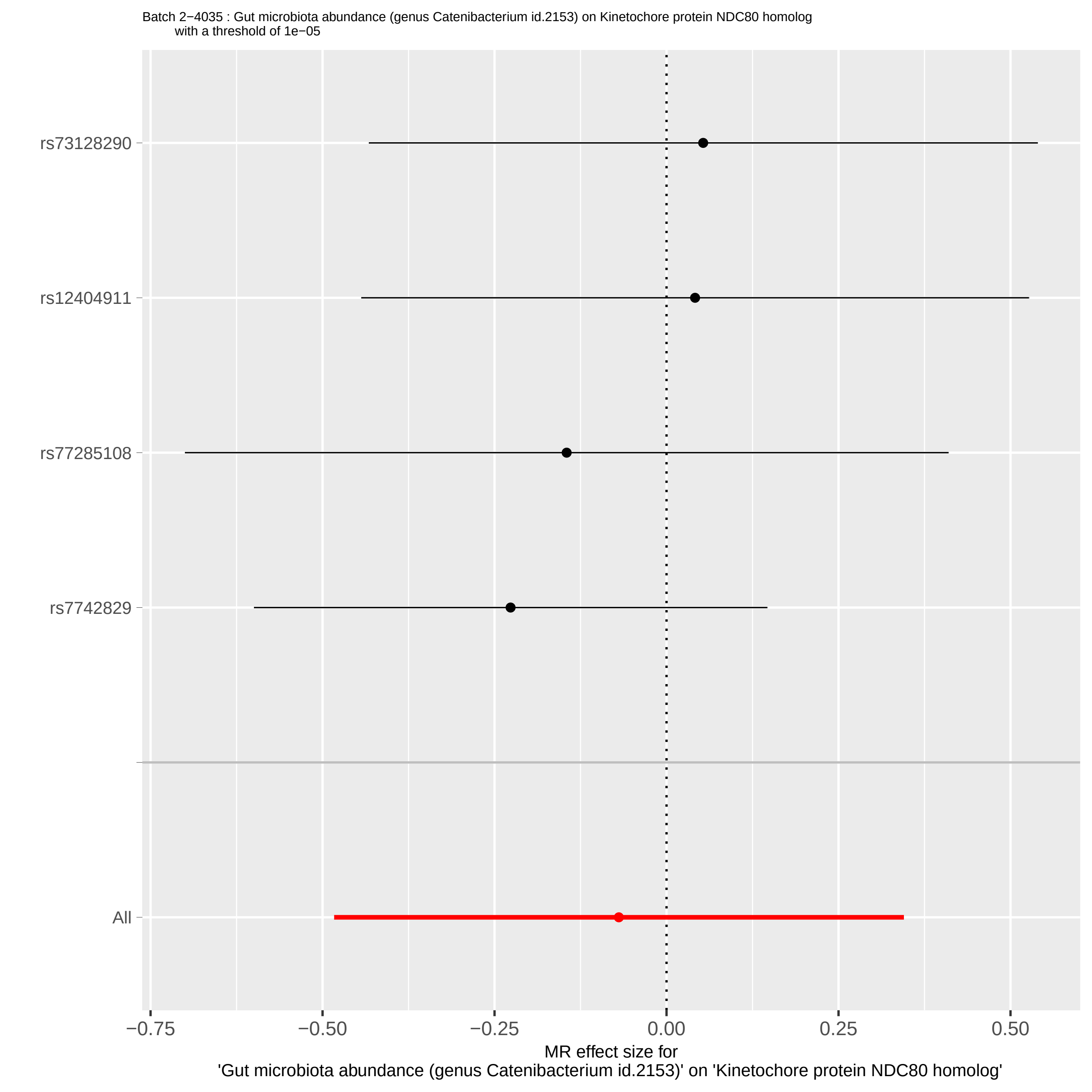
Batch 2−4035 : Gut microbiota abundance (genus Catenibacterium id.2153) on Kinetochore protein NDC80 homolog
with a threshold of 1e−05
rs73128290
rs12404911
rs77285108
rs7742829
All
−0.75
−0.50
−0.25
0.00
0.25
0.50
MR effect size for
'Gut microbiota abundance (genus Catenibacterium id.2153)' on 'Kinetochore protein NDC80 homolog'

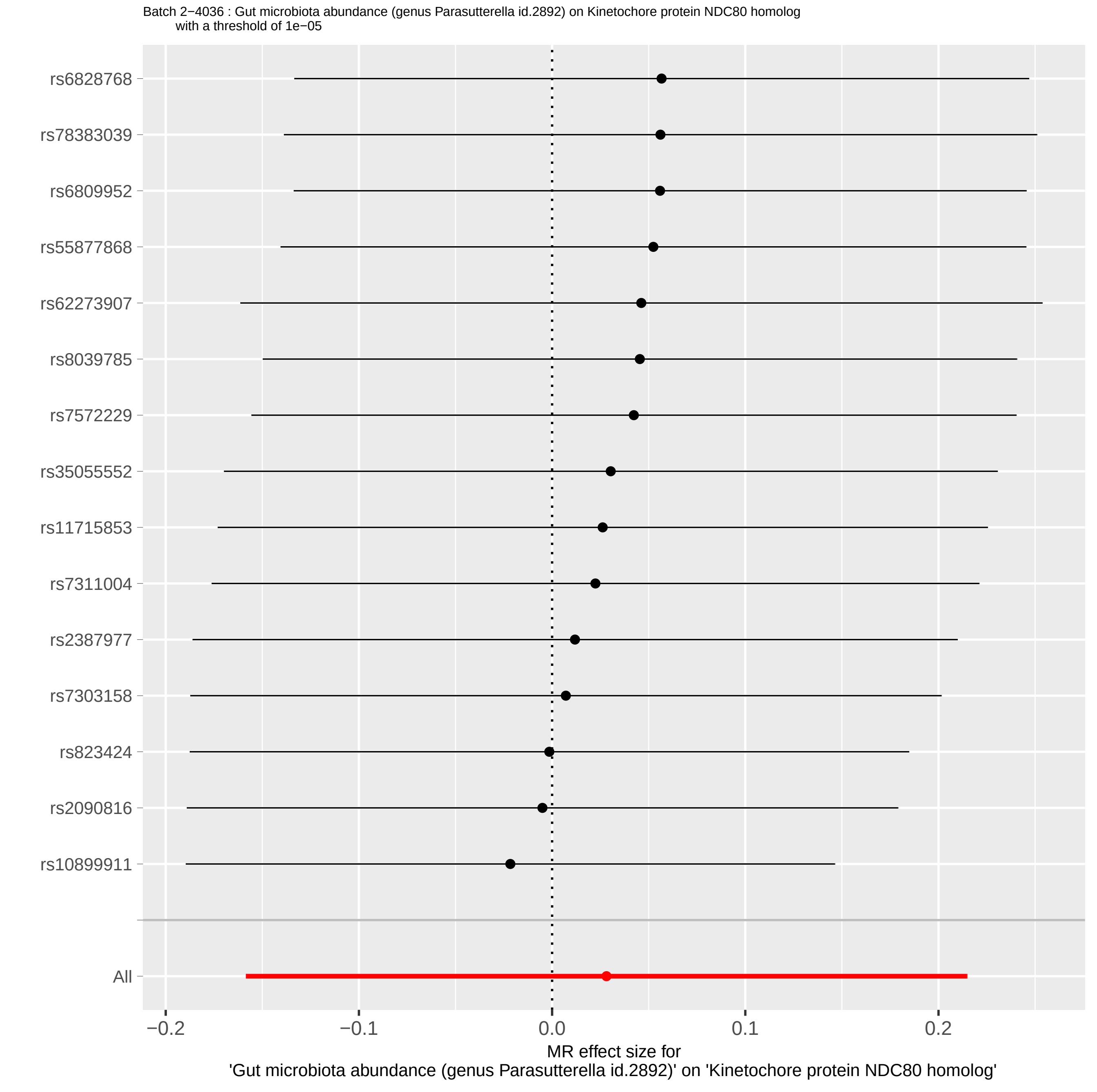

Batch 2−4036 : Gut microbiota abundance (genus Parasutterella id.2892) on Kinetochore protein NDC80 homolog
with a threshold of 1e−05
rs6828768
rs78383039
rs6809952
rs55877868
rs62273907
rs8039785
rs7572229
rs35055552
rs11715853
rs7311004
rs2387977
rs7303158
rs823424
rs2090816
rs10899911
All
−0.2
−0.1
0.0
0.1
0.2
MR effect size for
'Gut microbiota abundance (genus Parasutterella id.2892)' on 'Kinetochore protein NDC80 homolog'

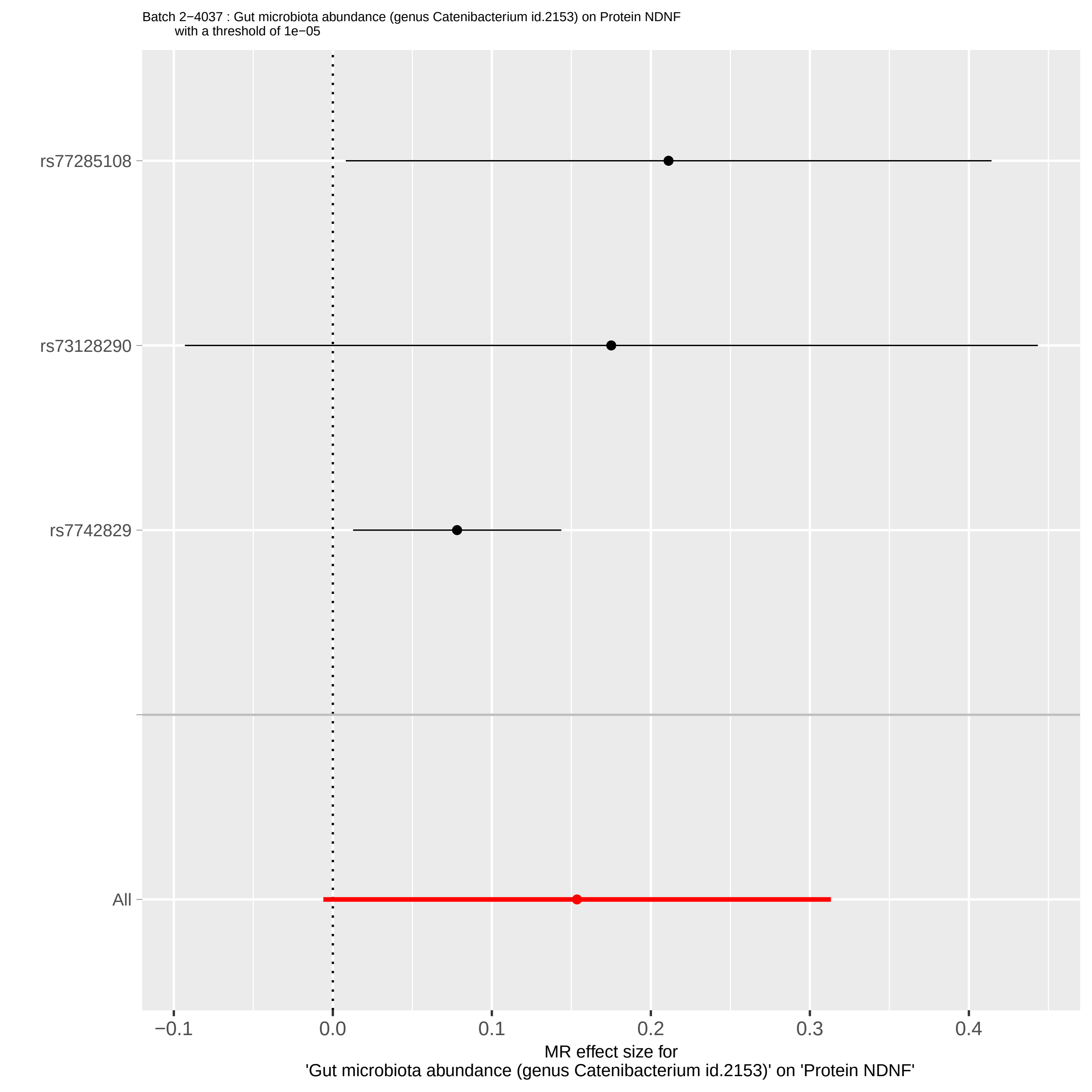

Batch 2−4037 : Gut microbiota abundance (genus Catenibacterium id.2153) on Protein NDNF
with a threshold of 1e−05
rs77285108
rs73128290
rs7742829
All
−0.1
0.0
0.1
0.2
0.3
0.4
MR effect size for
'Gut microbiota abundance (genus Catenibacterium id.2153)' on 'Protein NDNF'

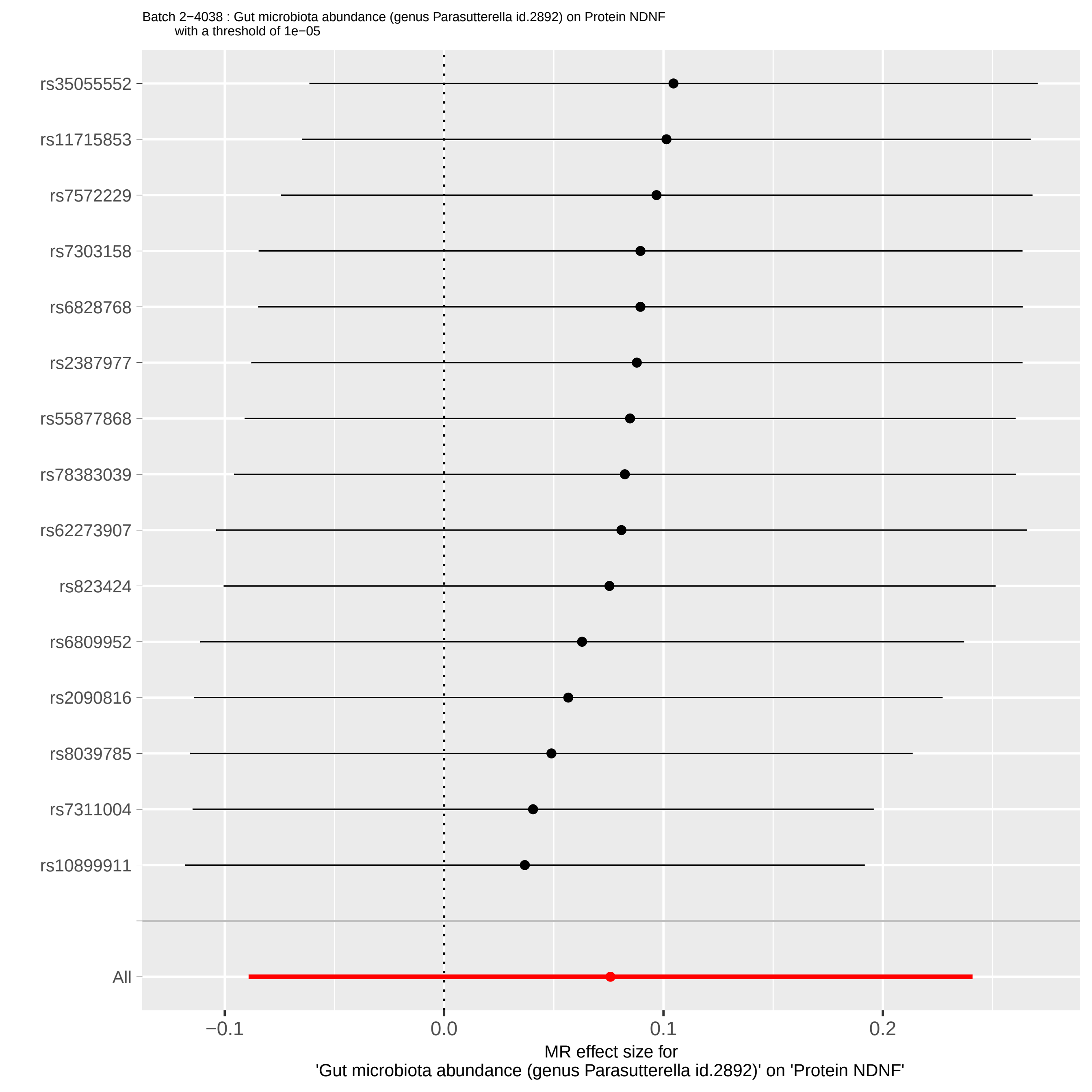
Batch 2−4038 : Gut microbiota abundance (genus Parasutterella id.2892) on Protein NDNF
with a threshold of 1e−05
rs35055552
rs11715853
rs7572229
rs7303158
rs6828768
rs2387977
rs55877868
rs78383039
rs62273907
rs823424
rs6809952
rs2090816
rs8039785
rs7311004
rs10899911
All
−0.1
0.0
0.1
0.2
MR effect size for
'Gut microbiota abundance (genus Parasutterella id.2892)' on 'Protein NDNF'

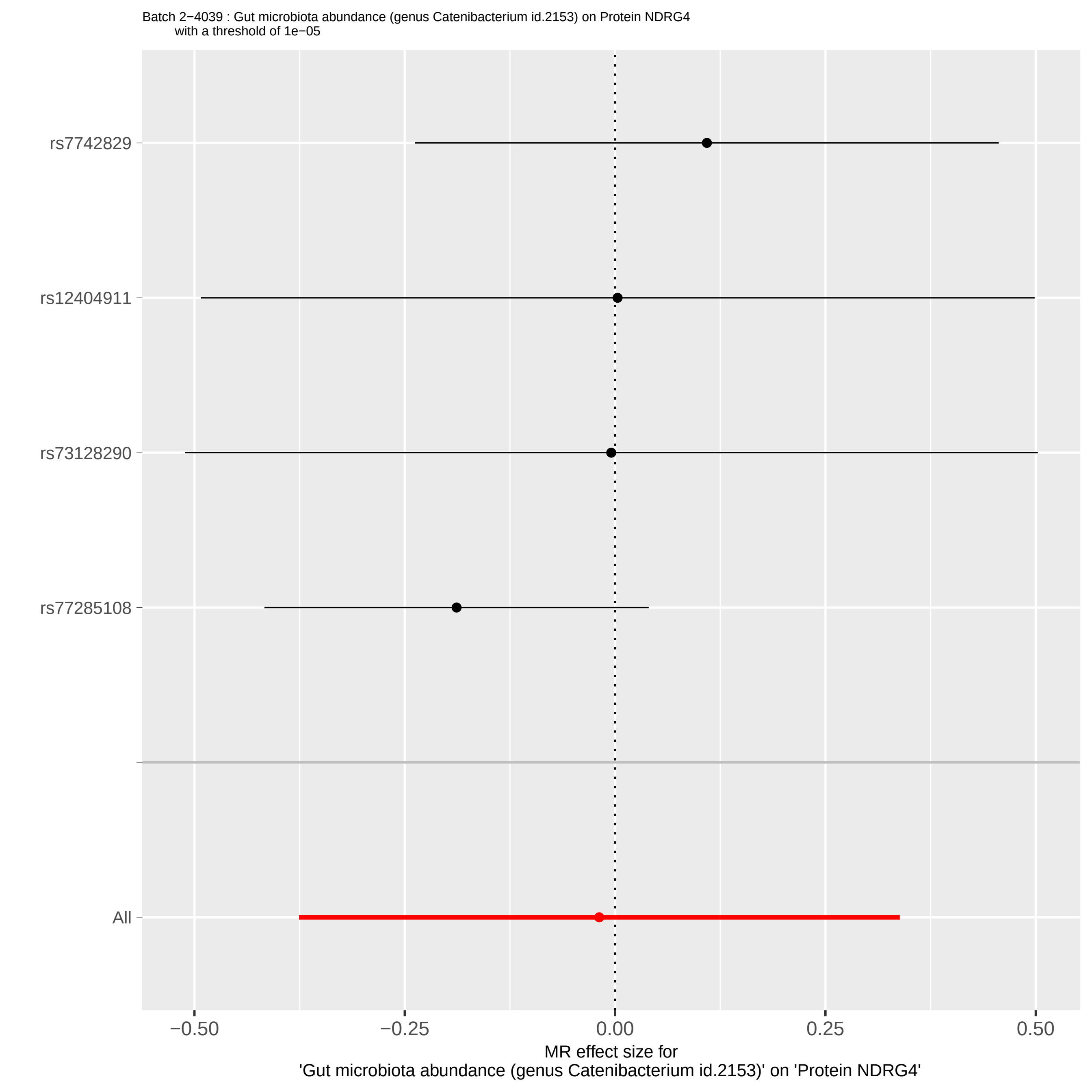

Batch 2−4039 : Gut microbiota abundance (genus Catenibacterium id.2153) on Protein NDRG4
with a threshold of 1e−05
rs7742829
rs12404911
rs73128290
rs77285108
All
−0.50
−0.25
0.00
0.25
0.50
MR effect size for
'Gut microbiota abundance (genus Catenibacterium id.2153)' on 'Protein NDRG4'

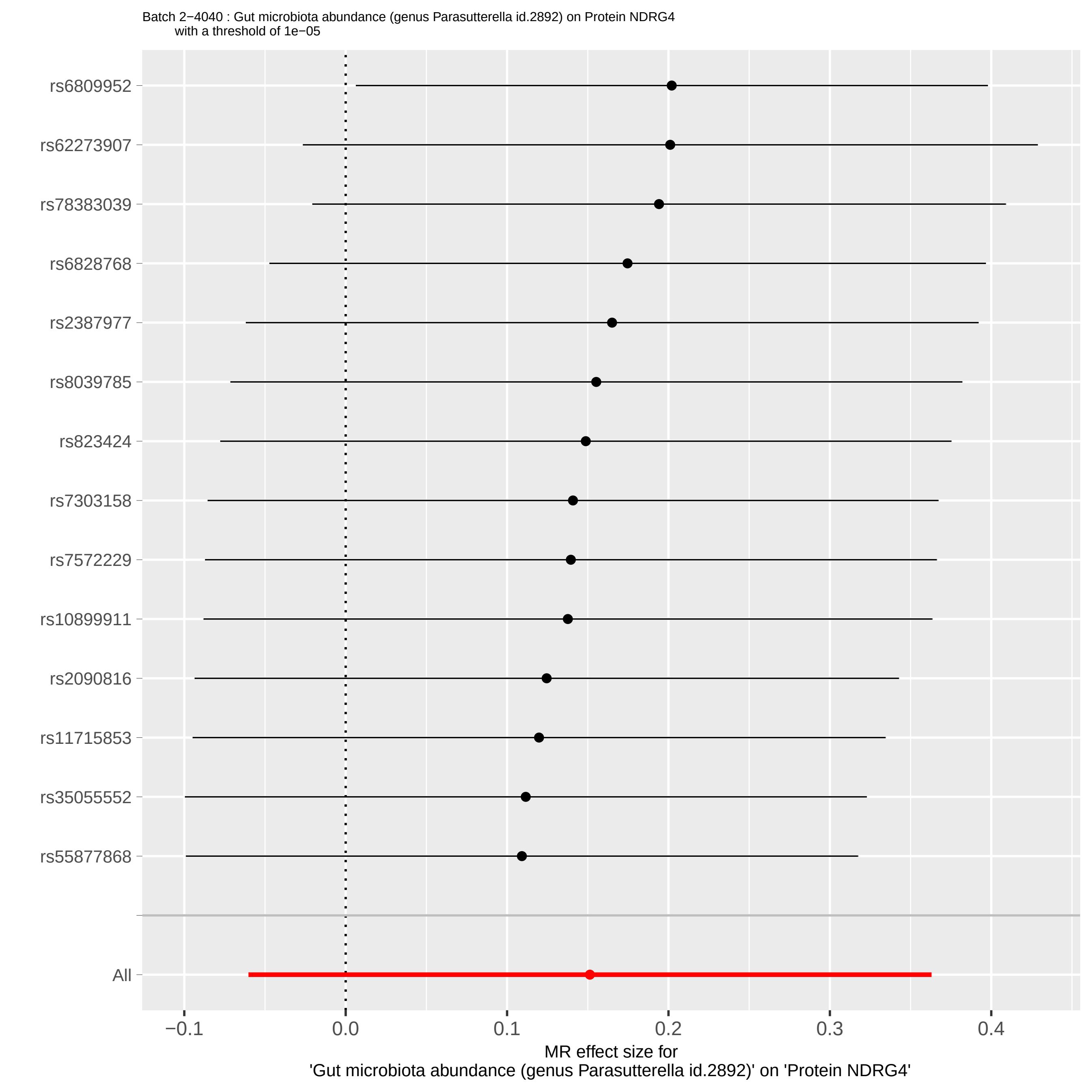
Batch 2−4040 : Gut microbiota abundance (genus Parasutterella id.2892) on Protein NDRG4
with a threshold of 1e−05
rs6809952
rs62273907
rs78383039
rs6828768
rs2387977
rs8039785
rs823424
rs7303158
rs7572229
rs10899911
rs2090816
rs11715853
rs35055552
rs55877868
All
−0.1
0.0
0.1
0.2
0.3
0.4
MR effect size for
'Gut microbiota abundance (genus Parasutterella id.2892)' on 'Protein NDRG4'

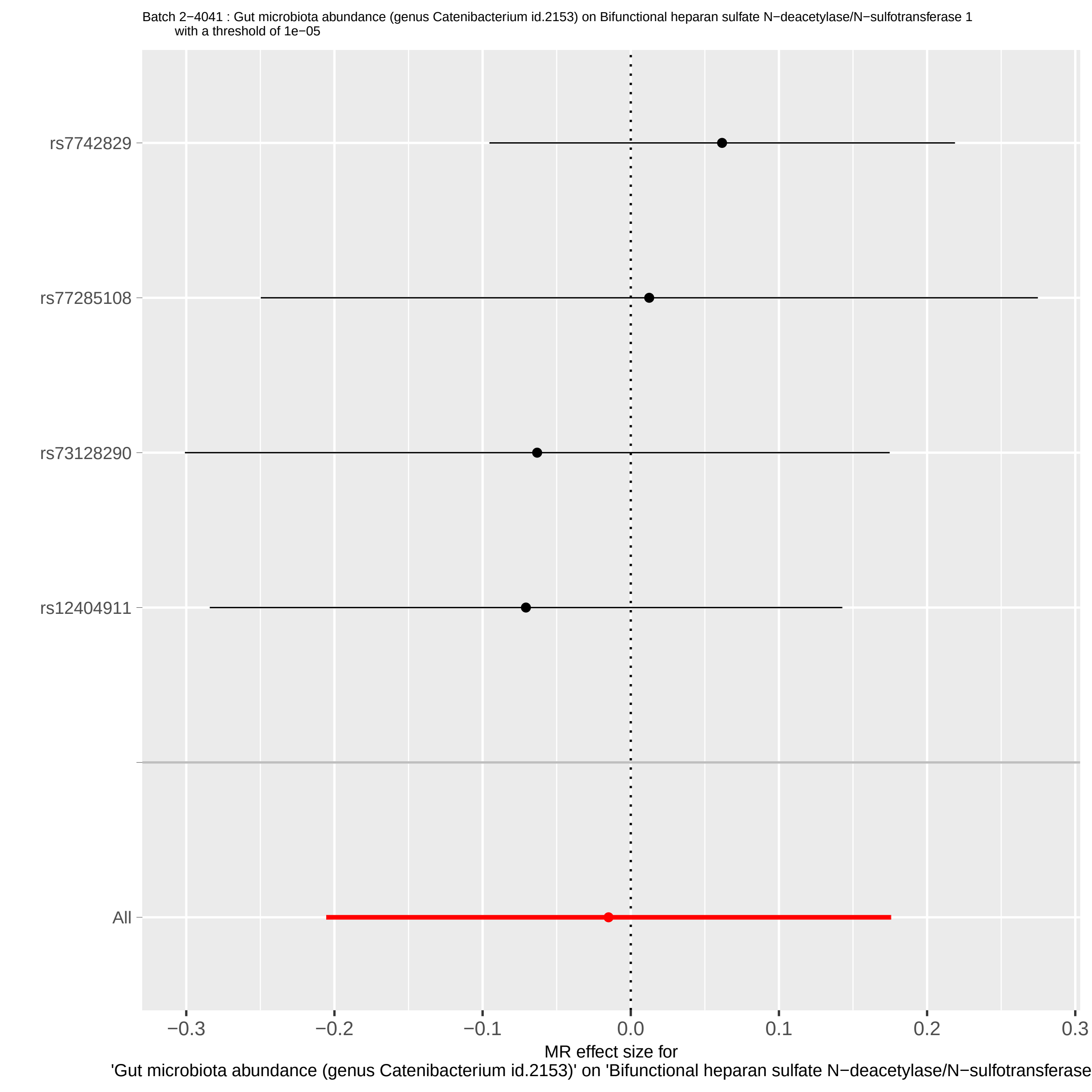

Batch 2−4041 : Gut microbiota abundance (genus Catenibacterium id.2153) on Bifunctional heparan sulfate N−deacetylase/N−sulfotransferase 1
with a threshold of 1e−05
rs7742829
rs77285108
rs73128290
rs12404911
All
−0.3
−0.2
−0.1
0.0
0.1
0.2
0.3
MR effect size for
'Gut microbiota abundance (genus Catenibacterium id.2153)' on 'Bifunctional heparan sulfate N−deacetylase/N−sulfotransferase

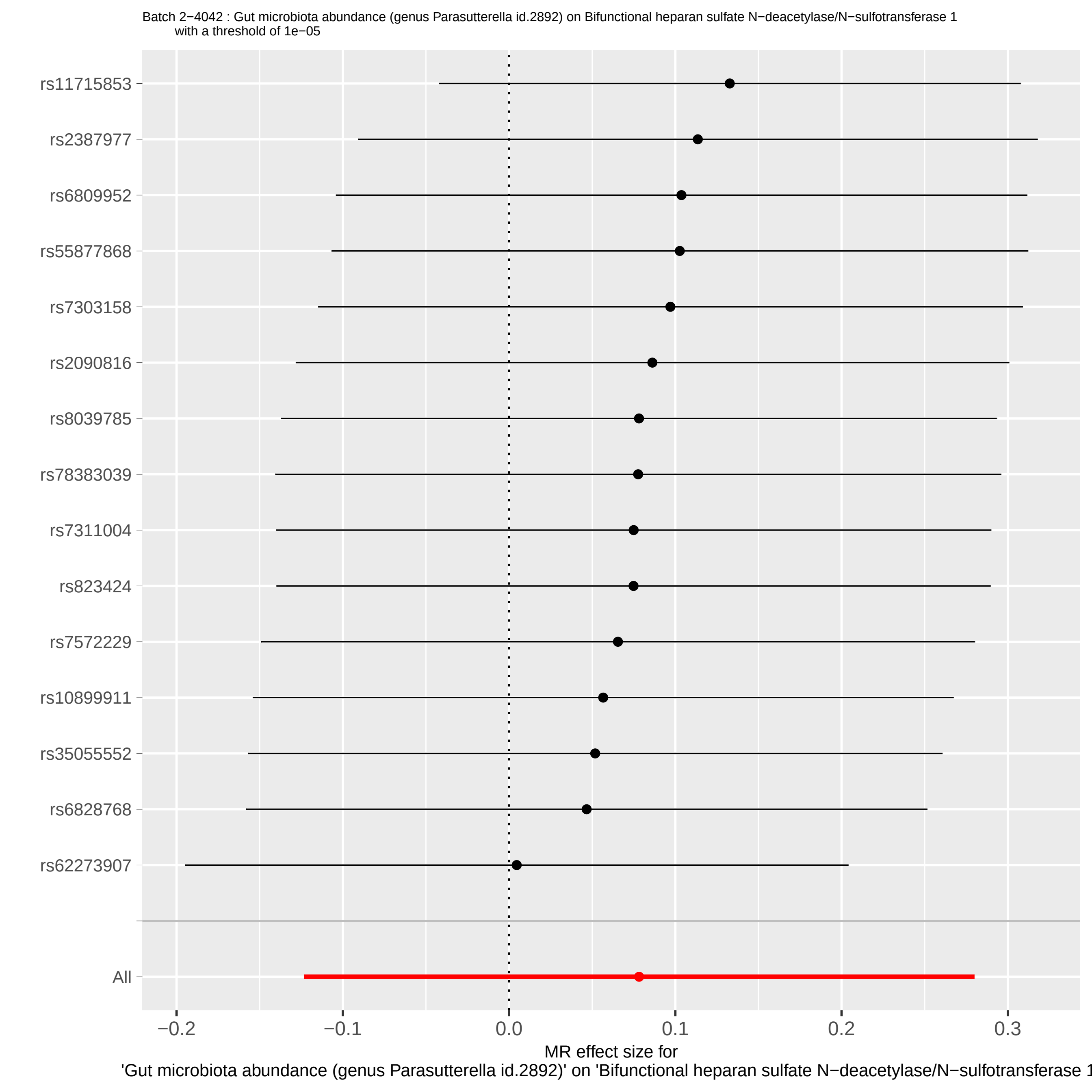

Batch 2−4042 : Gut microbiota abundance (genus Parasutterella id.2892) on Bifunctional heparan sulfate N−deacetylase/N−sulfotransferase 1
with a threshold of 1e−05
rs11715853
rs2387977
rs6809952
rs55877868
rs7303158
rs2090816
rs8039785
rs78383039
rs7311004
rs823424
rs7572229
rs10899911
rs35055552
rs6828768
rs62273907
All
−0.2
−0.1
0.0
0.1
0.2
0.3
MR effect size for
'Gut microbiota abundance (genus Parasutterella id.2892)' on 'Bifunctional heparan sulfate N−deacetylase/N−sulfotransferase 1

Batch 2−4043 : Gut microbiota abundance (genus Catenibacterium id.2153) on NADH dehydrogenase [ubiquinone] 1 beta subcomplex subunit 11, mitochondrial with a threshold of 1e−05

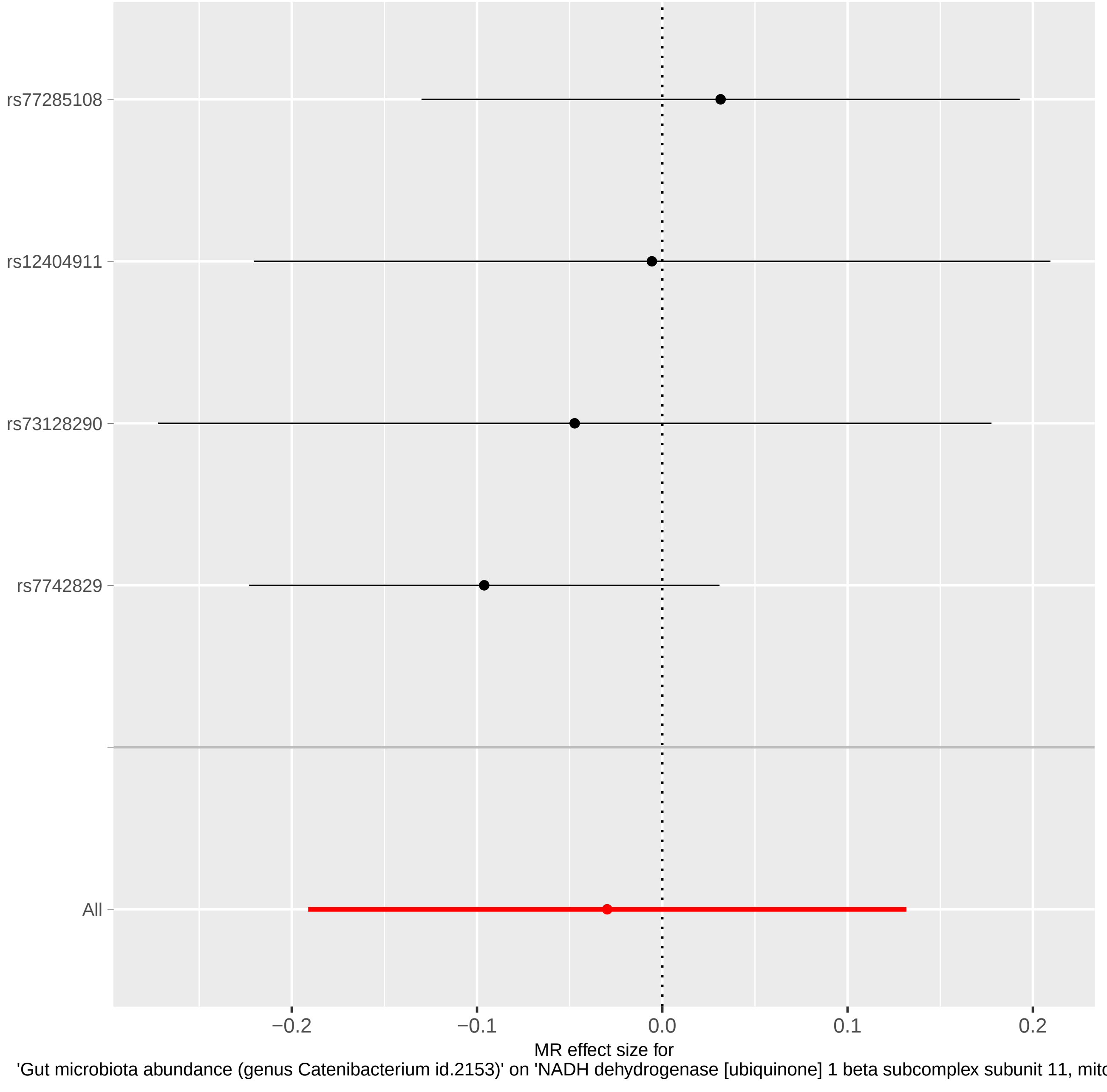

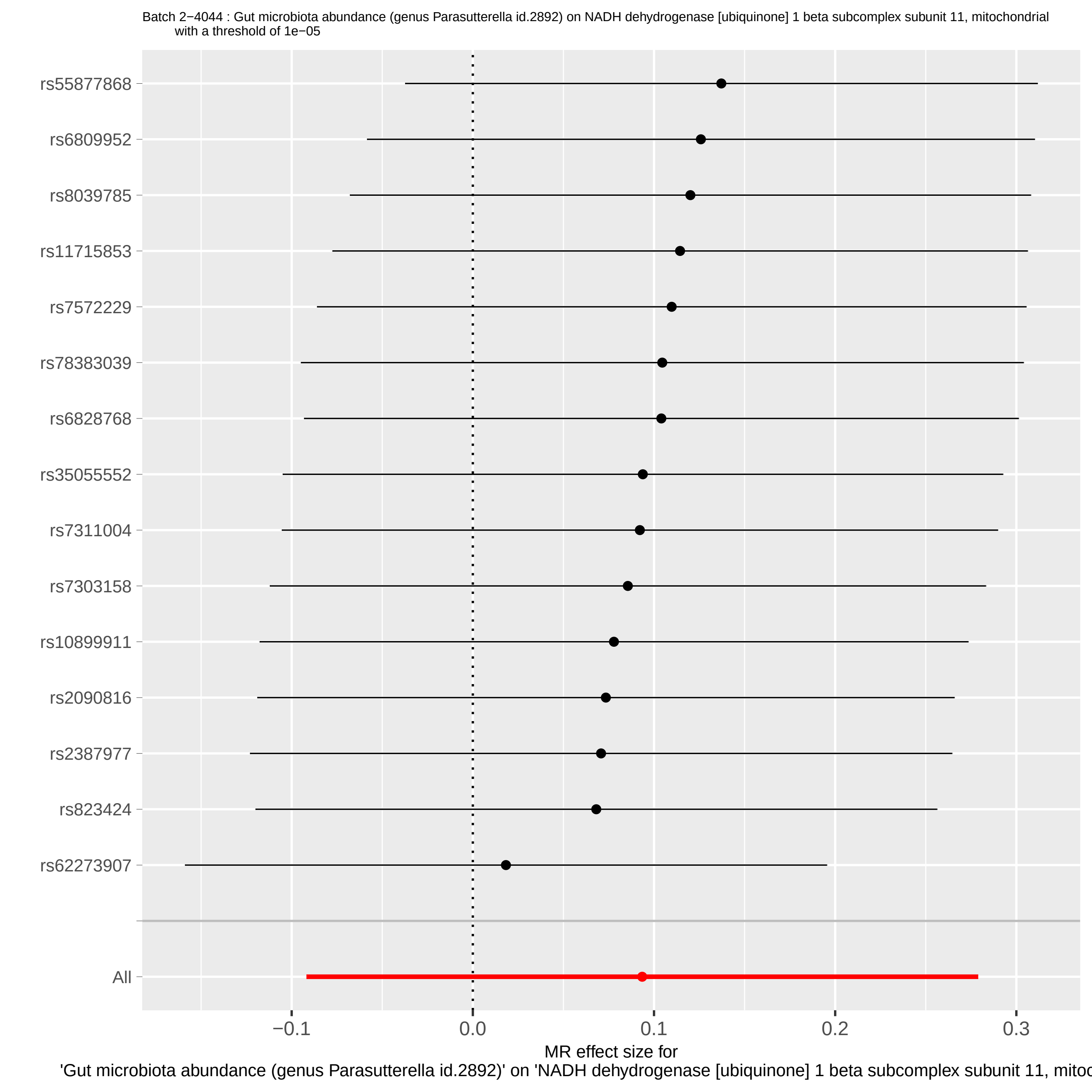

Batch 2−4044 : Gut microbiota abundance (genus Parasutterella id.2892) on NADH dehydrogenase [ubiquinone] 1 beta subcomplex subunit 11, mitochondrial with a threshold of 1e−05
rs55877868
rs6809952
rs8039785
rs11715853
rs7572229
rs78383039
rs6828768
rs35055552
rs7311004
rs7303158
rs10899911
rs2090816
rs2387977
rs823424
rs62273907
All
−0.1
0.0
0.1
0.2
0.3
MR effect size for
'Gut microbiota abundance (genus Parasutterella id.2892)' on 'NADH dehydrogenase [ubiquinone] 1 beta subcomplex subunit 11, mitoc

Batch 2−4045 : Gut microbiota abundance (genus Catenibacterium id.2153) on NADH dehydrogenase [ubiquinone] 1 beta subcomplex subunit 4
with a threshold of 1e−05

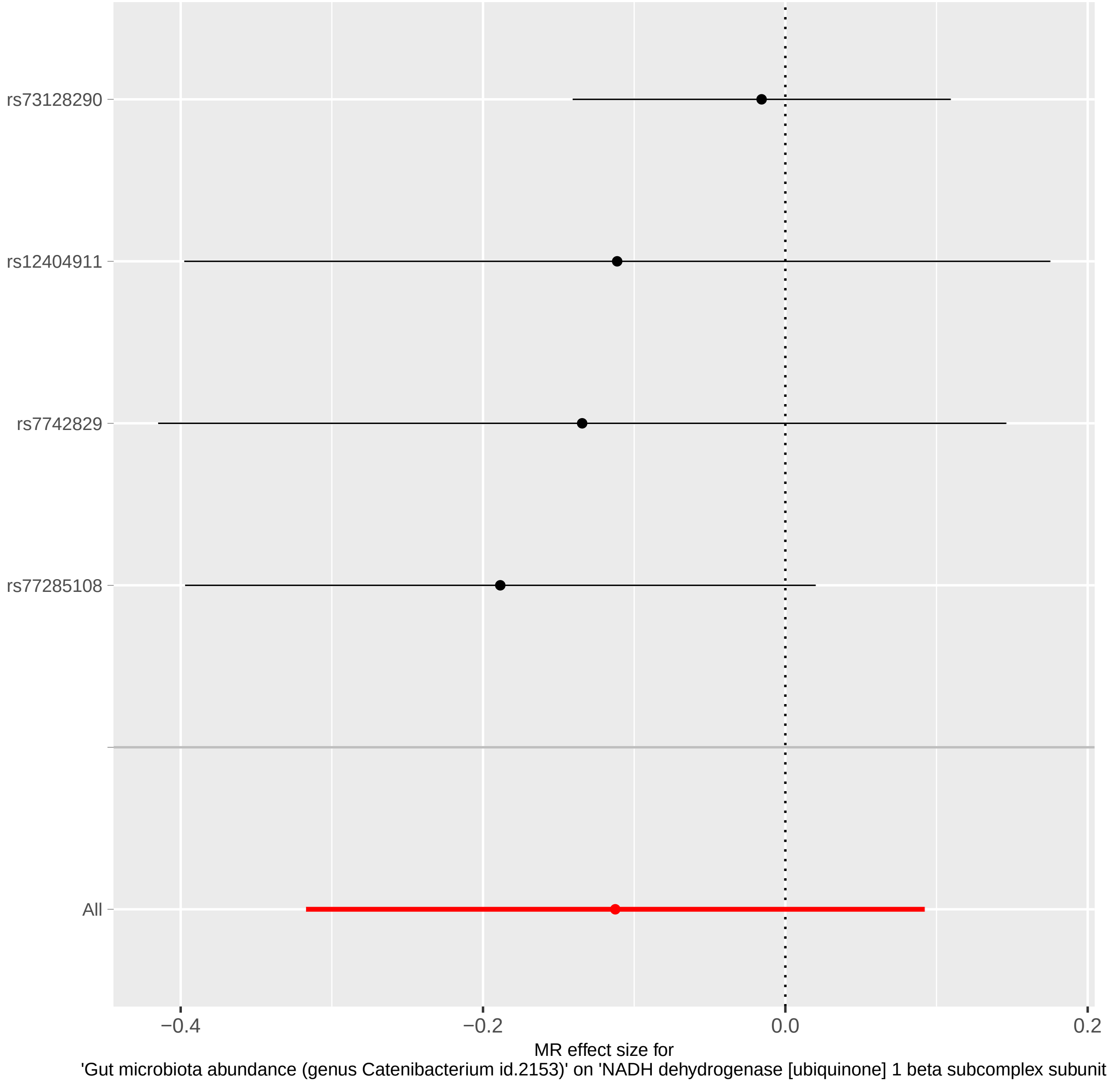

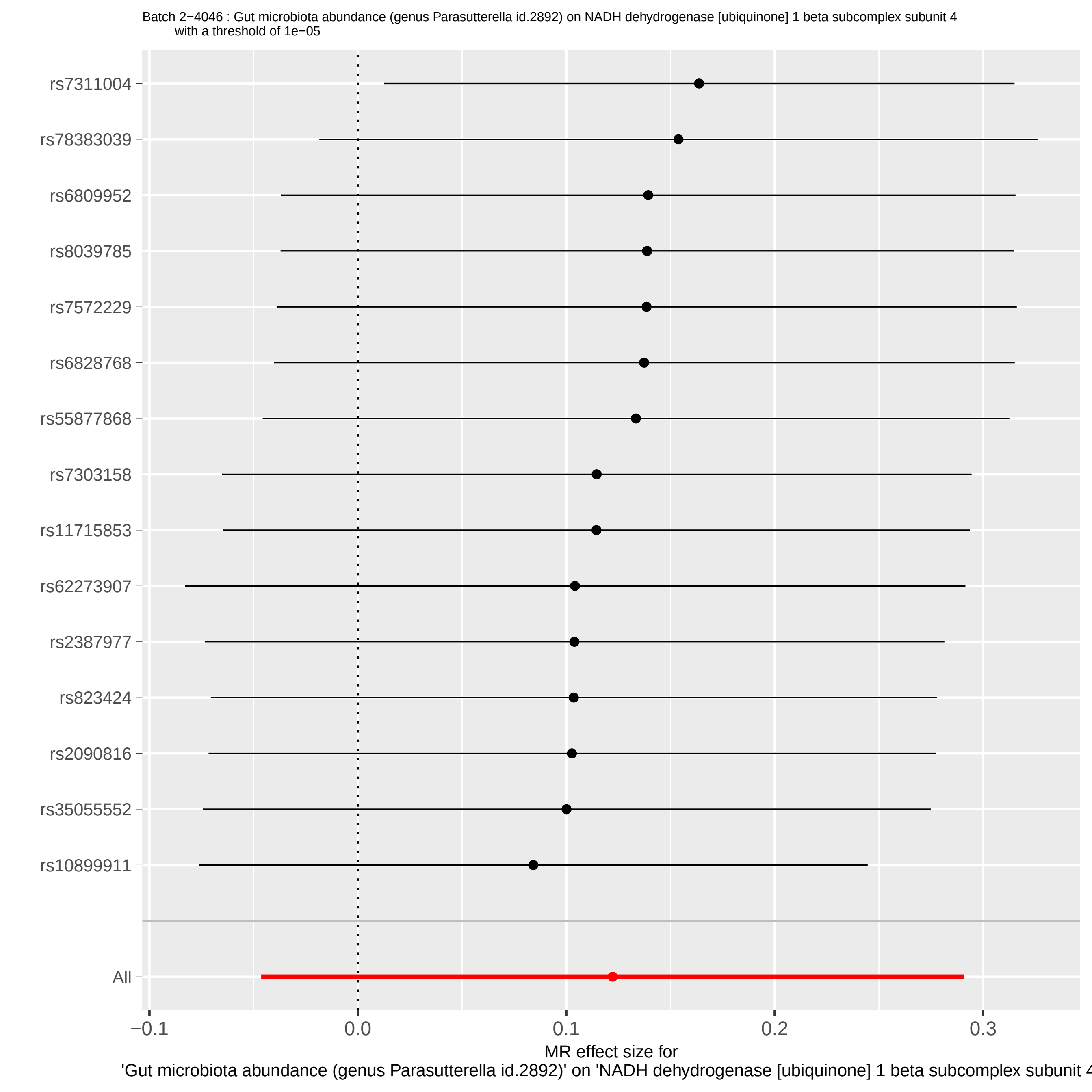

Batch 2−4046 : Gut microbiota abundance (genus Parasutterella id.2892) on NADH dehydrogenase [ubiquinone] 1 beta subcomplex subunit 4 with a threshold of 1e−05
rs7311004
rs78383039
rs6809952
rs8039785
rs7572229
rs6828768
rs55877868
rs7303158
rs11715853
rs62273907
rs2387977
rs823424
rs2090816
rs35055552
rs10899911
All
−0.1
0.0
0.1
0.2
0.3
MR effect size for
'Gut microbiota abundance (genus Parasutterella id.2892)' on 'NADH dehydrogenase [ubiquinone] 1 beta subcomplex subunit 4

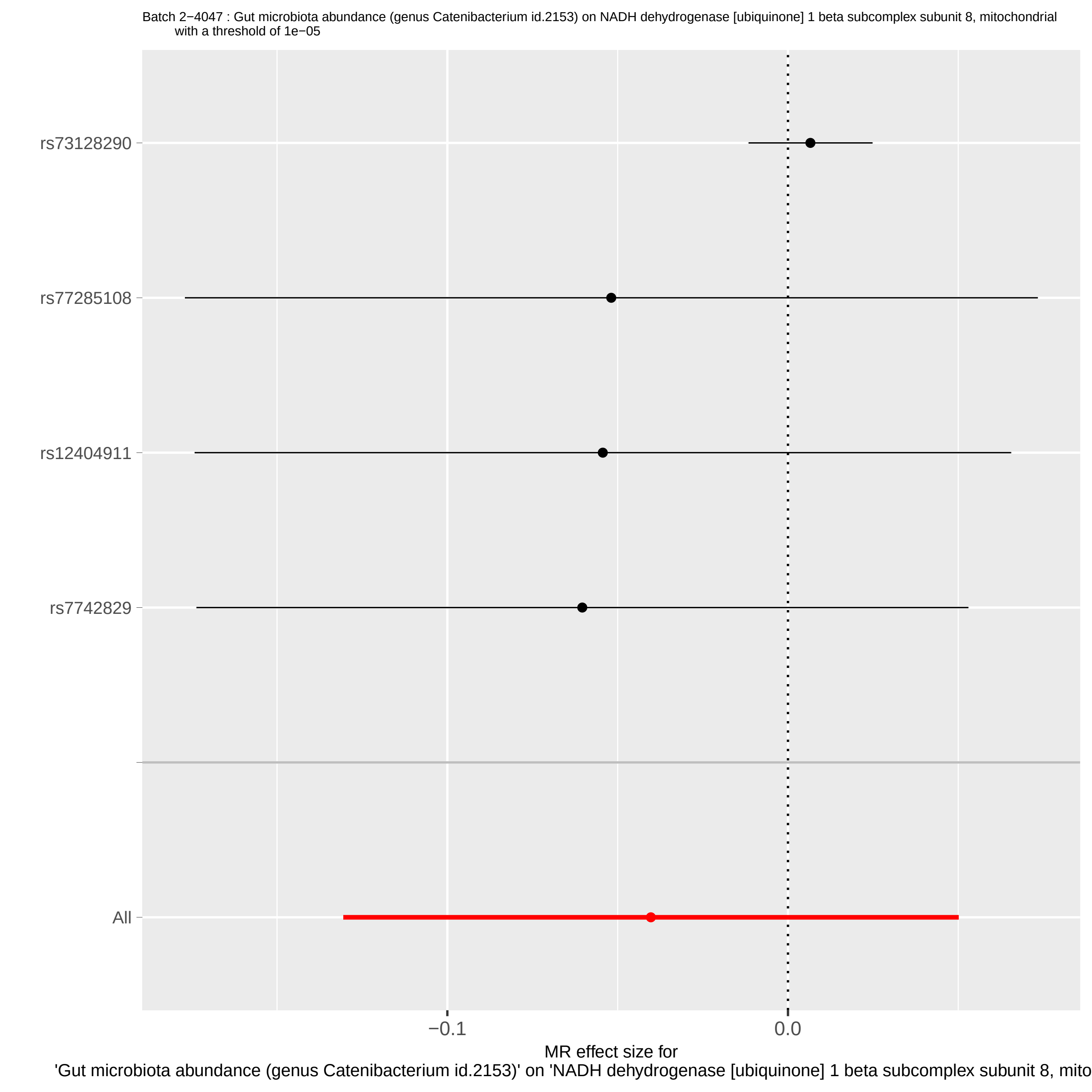

Batch 2−4047 : Gut microbiota abundance (genus Catenibacterium id.2153) on NADH dehydrogenase [ubiquinone] 1 beta subcomplex subunit 8, mitochondrial
with a threshold of 1e−05
rs73128290
rs77285108
rs12404911
rs7742829
All
−0.1
0.0
MR effect size for
'Gut microbiota abundance (genus Catenibacterium id.2153)' on 'NADH dehydrogenase [ubiquinone] 1 beta subcomplex subunit 8, mito

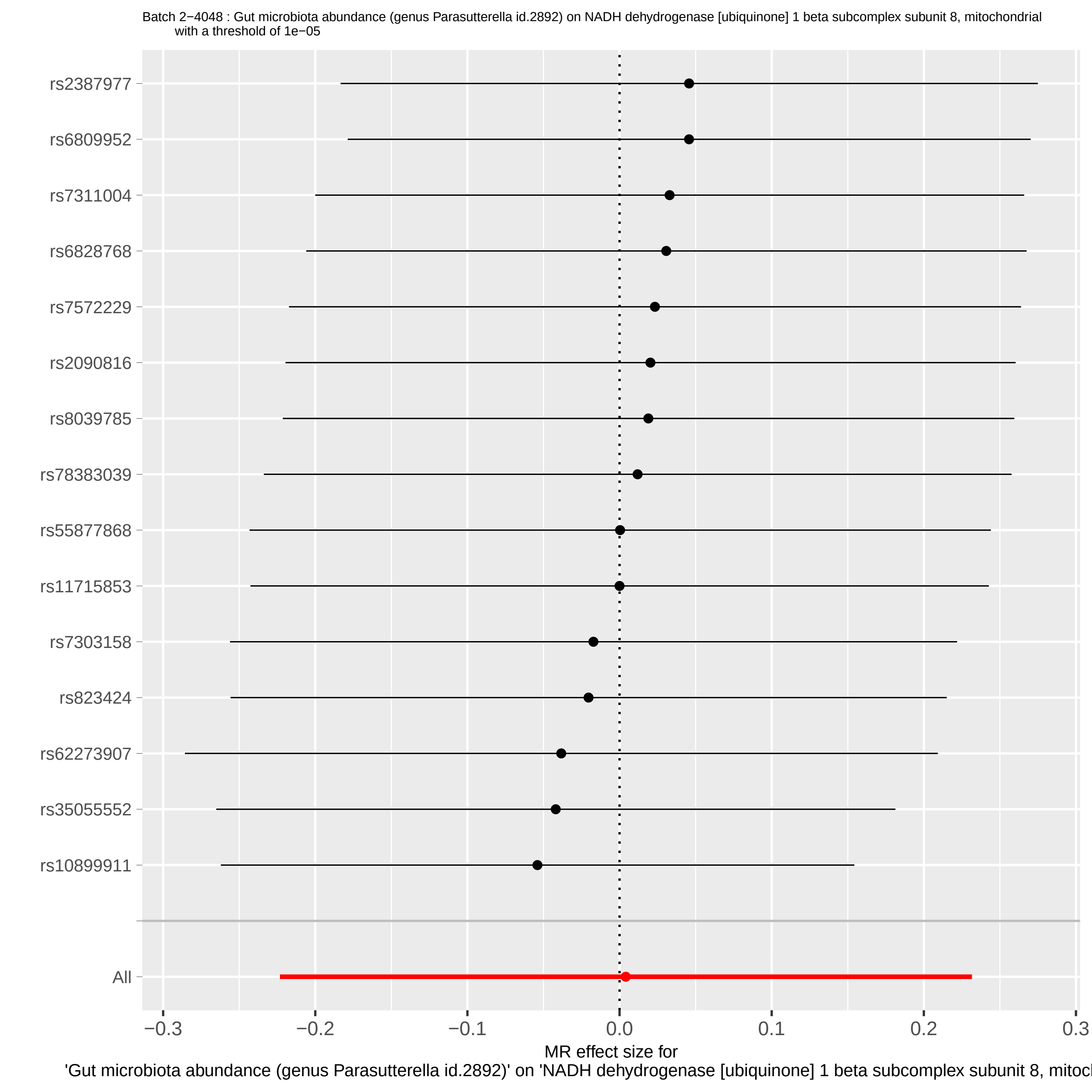

Batch 2−4048 : Gut microbiota abundance (genus Parasutterella id.2892) on NADH dehydrogenase [ubiquinone] 1 beta subcomplex subunit 8, mitochondrial with a threshold of 1e−05
rs2387977
rs6809952
rs7311004
rs6828768
rs7572229
rs2090816
rs8039785
rs78383039
rs55877868
rs11715853
rs7303158
rs823424
rs62273907
rs35055552
rs10899911
All
−0.3
−0.2
−0.1
0.0
0.1
0.2
0.3
MR effect size for
'Gut microbiota abundance (genus Parasutterella id.2892)' on 'NADH dehydrogenase [ubiquinone] 1 beta subcomplex subunit 8, mitoc

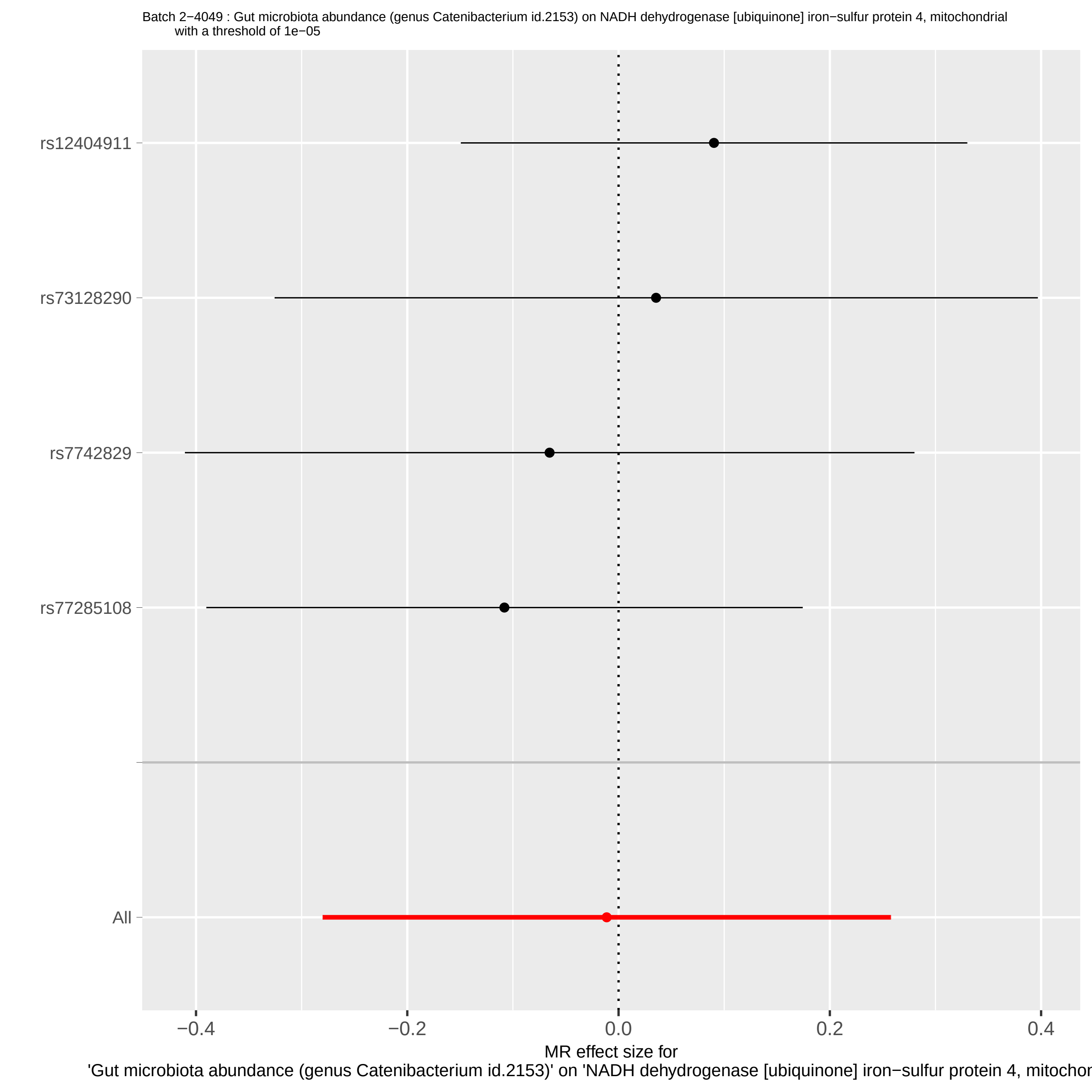

Batch 2−4049 : Gut microbiota abundance (genus Catenibacterium id.2153) on NADH dehydrogenase [ubiquinone] iron−sulfur protein 4, mitochondrial
with a threshold of 1e−05
rs12404911
rs73128290
rs7742829
rs77285108
All
−0.4
−0.2
0.0
0.2
0.4
MR effect size for
'Gut microbiota abundance (genus Catenibacterium id.2153)' on 'NADH dehydrogenase [ubiquinone] iron−sulfur protein 4, mitochon

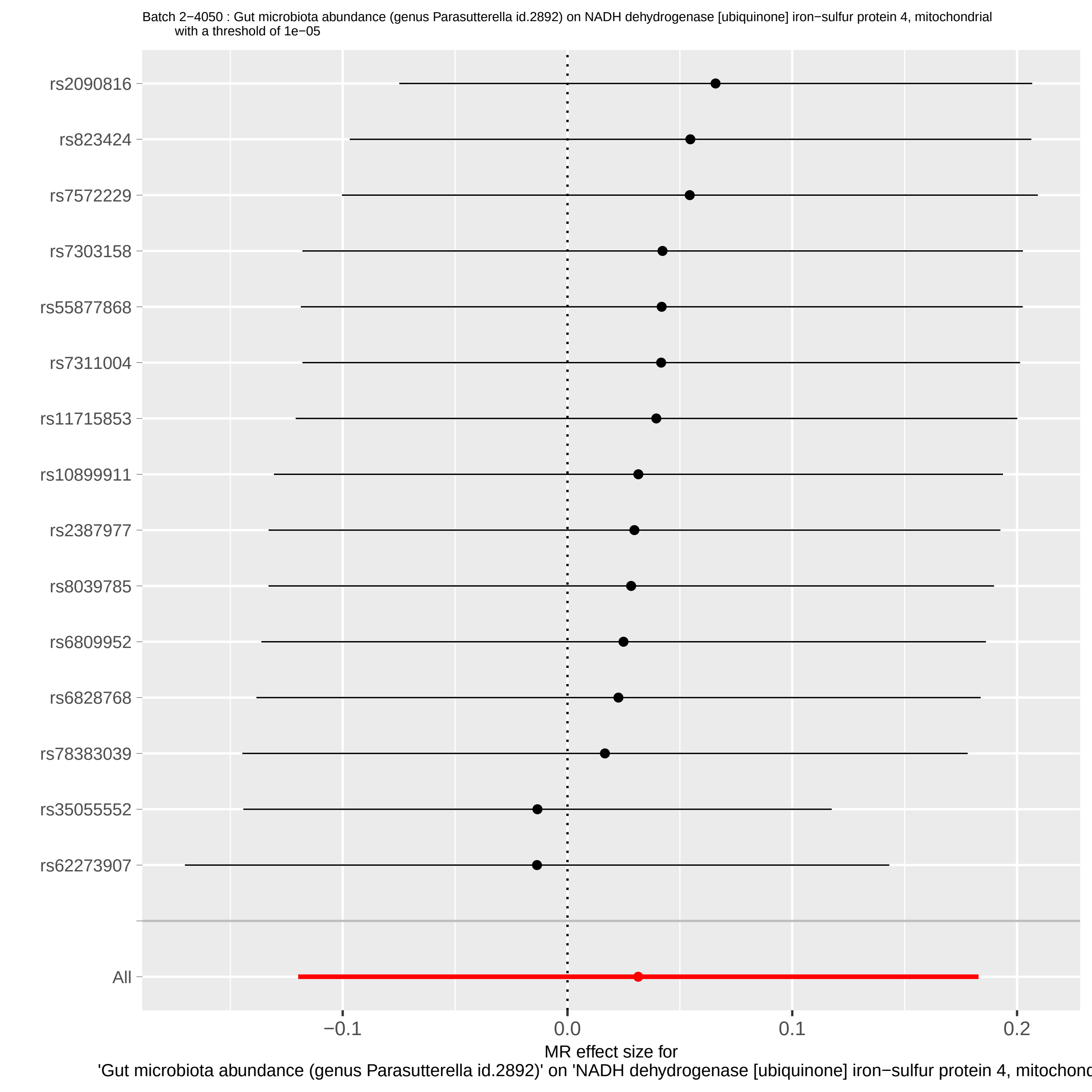
Batch 2−4050 : Gut microbiota abundance (genus Parasutterella id.2892) on NADH dehydrogenase [ubiquinone] iron−sulfur protein 4, mitochondrial
with a threshold of 1e−05
rs2090816
rs823424
rs7572229
rs7303158
rs55877868
rs7311004
rs11715853
rs10899911
rs2387977
rs8039785
rs6809952
rs6828768
rs78383039
rs35055552
rs62273907
All
−0.1
0.0
0.1
0.2
MR effect size for
'Gut microbiota abundance (genus Parasutterella id.2892)' on 'NADH dehydrogenase [ubiquinone] iron−sulfur protein 4, mitochond

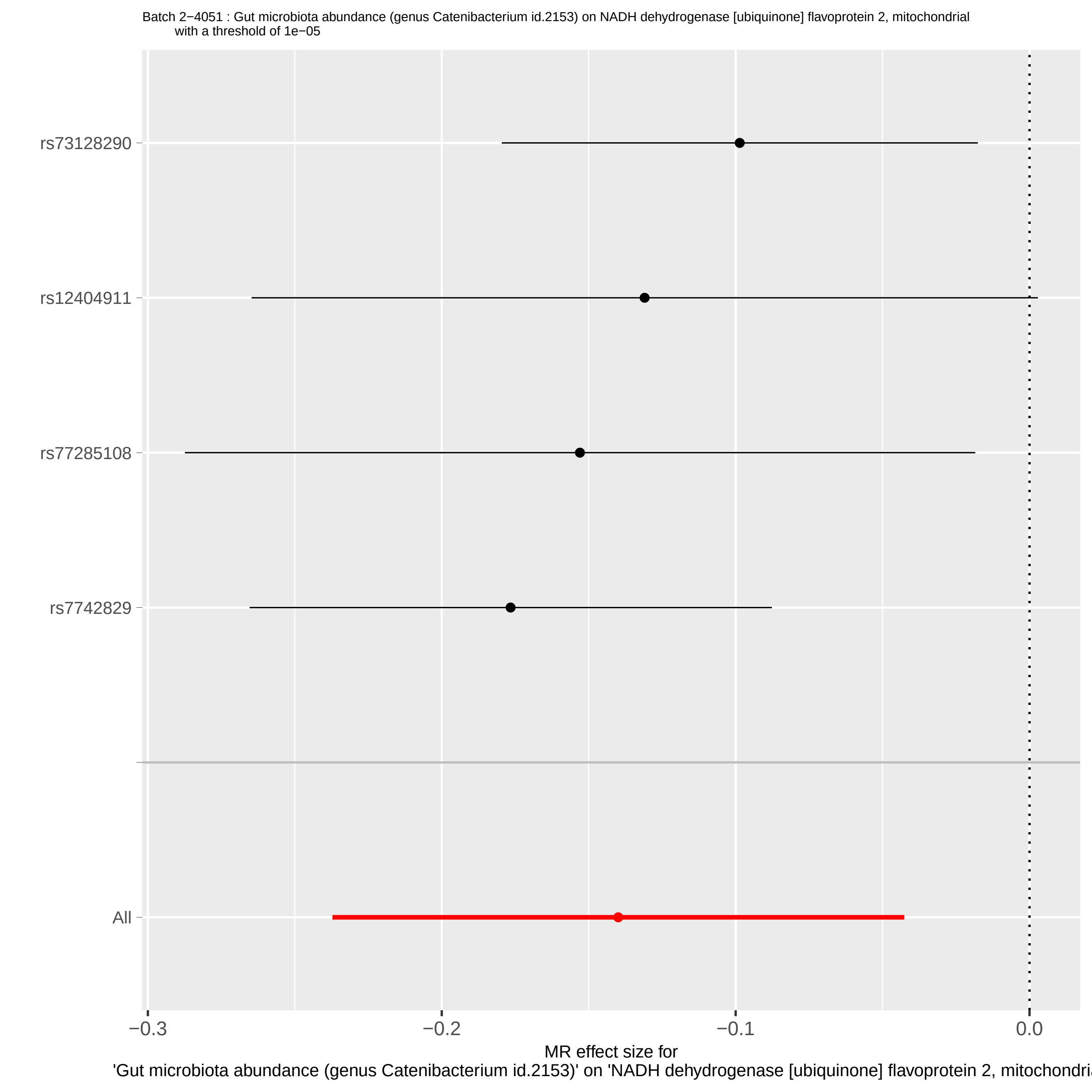
Batch 2−4051 : Gut microbiota abundance (genus Catenibacterium id.2153) on NADH dehydrogenase [ubiquinone] flavoprotein 2, mitochondrial
with a threshold of 1e−05
rs73128290
rs12404911
rs77285108
rs7742829
All
−0.3
−0.2
−0.1
0.0
MR effect size for
'Gut microbiota abundance (genus Catenibacterium id.2153)' on 'NADH dehydrogenase [ubiquinone] flavoprotein 2, mitochondri

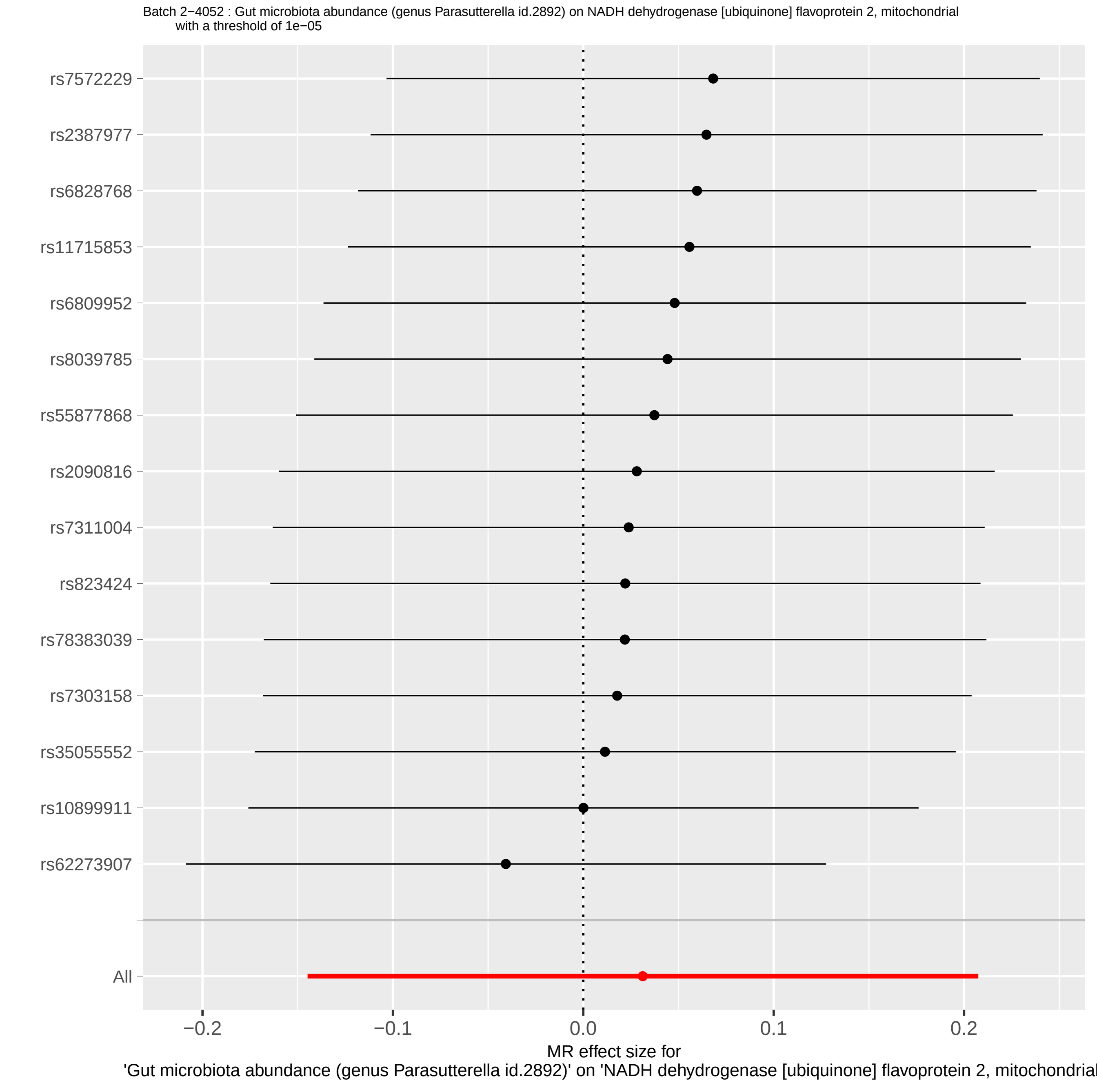
Batch 2−4052 : Gut microbiota abundance (genus Parasutterella id.2892) on NADH dehydrogenase [ubiquinone] flavoprotein 2, mitochondrial
with a threshold of 1e−05
rs7572229
rs2387977
rs6828768
rs11715853
rs6809952
rs8039785
rs55877868
rs2090816
rs7311004
rs823424
rs78383039
rs7303158
rs35055552
rs10899911
rs62273907
All
−0.2
−0.1
0.0
0.1
0.2
MR effect size for
'Gut microbiota abundance (genus Parasutterella id.2892)' on 'NADH dehydrogenase [ubiquinone] flavoprotein 2, mitochondrial

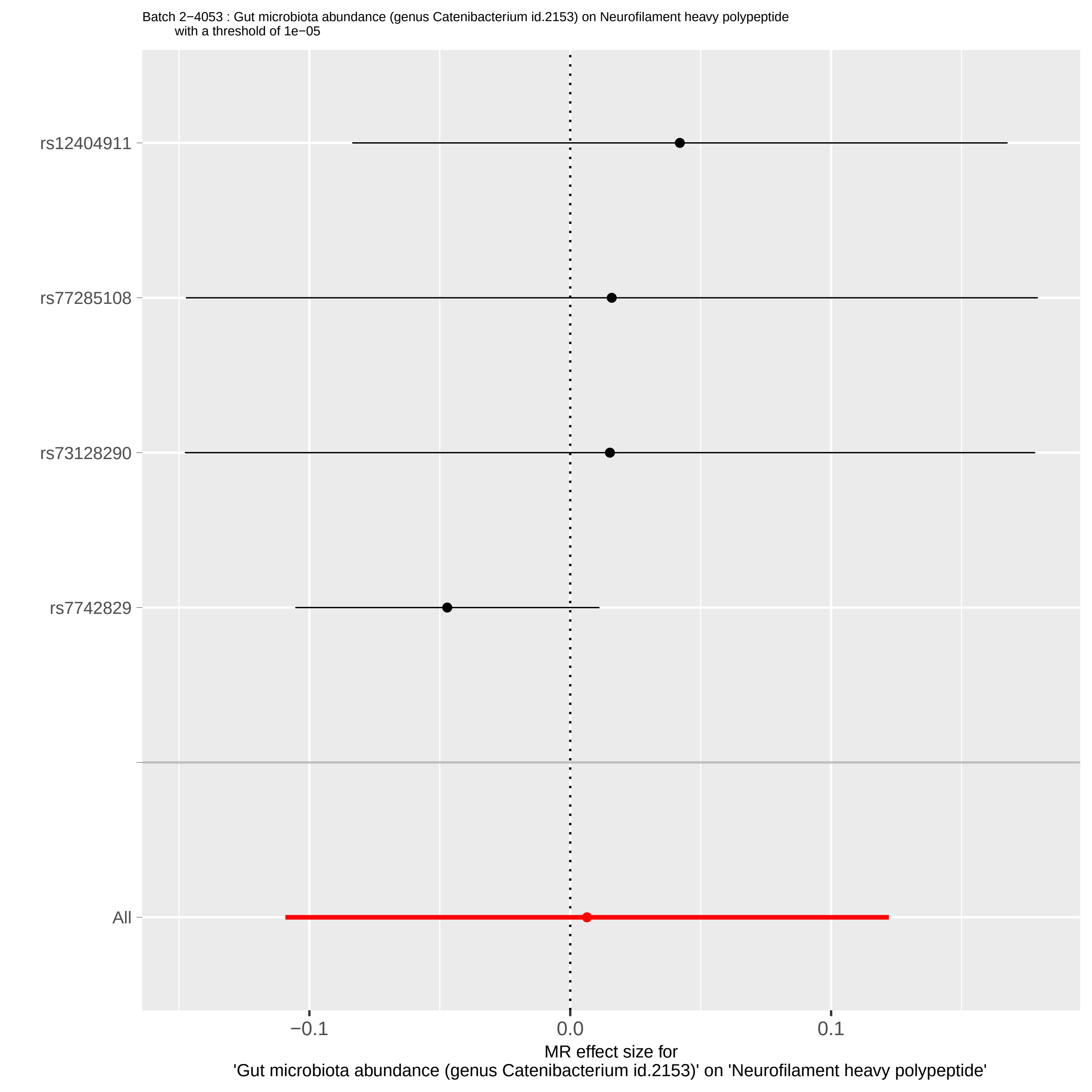
Batch 2−4053 : Gut microbiota abundance (genus Catenibacterium id.2153) on Neurofilament heavy polypeptide
with a threshold of 1e−05
rs12404911
rs77285108
rs73128290
rs7742829
All
−0.1
0.0
0.1
MR effect size for
'Gut microbiota abundance (genus Catenibacterium id.2153)' on 'Neurofilament heavy polypeptide'

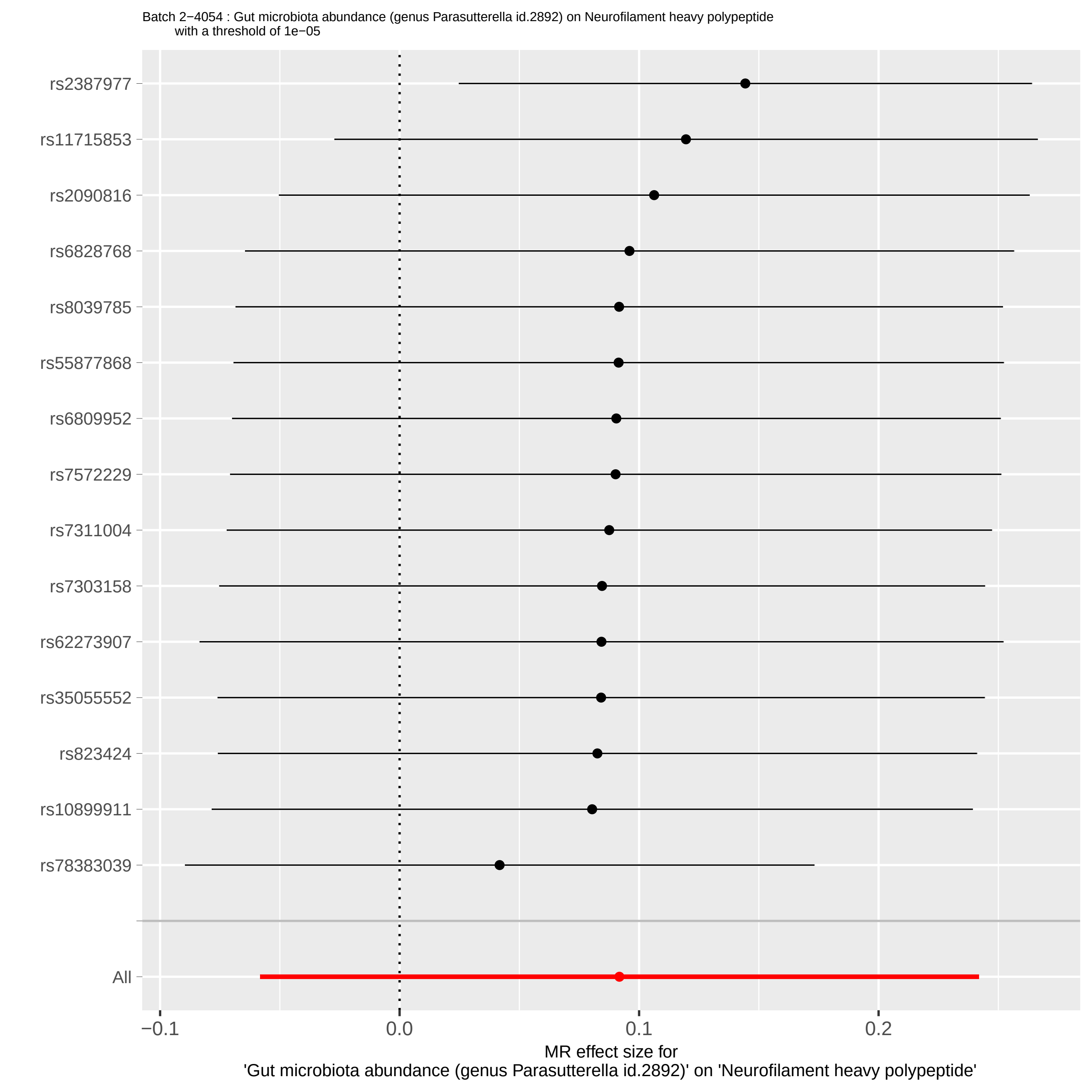

Batch 2−4054 : Gut microbiota abundance (genus Parasutterella id.2892) on Neurofilament heavy polypeptide
with a threshold of 1e−05
rs2387977
rs11715853
rs2090816
rs6828768
rs8039785
rs55877868
rs6809952
rs7572229
rs7311004
rs7303158
rs62273907
rs35055552
rs823424
rs10899911
rs78383039
All
−0.1
0.0
0.1
0.2
MR effect size for
'Gut microbiota abundance (genus Parasutterella id.2892)' on 'Neurofilament heavy polypeptide'

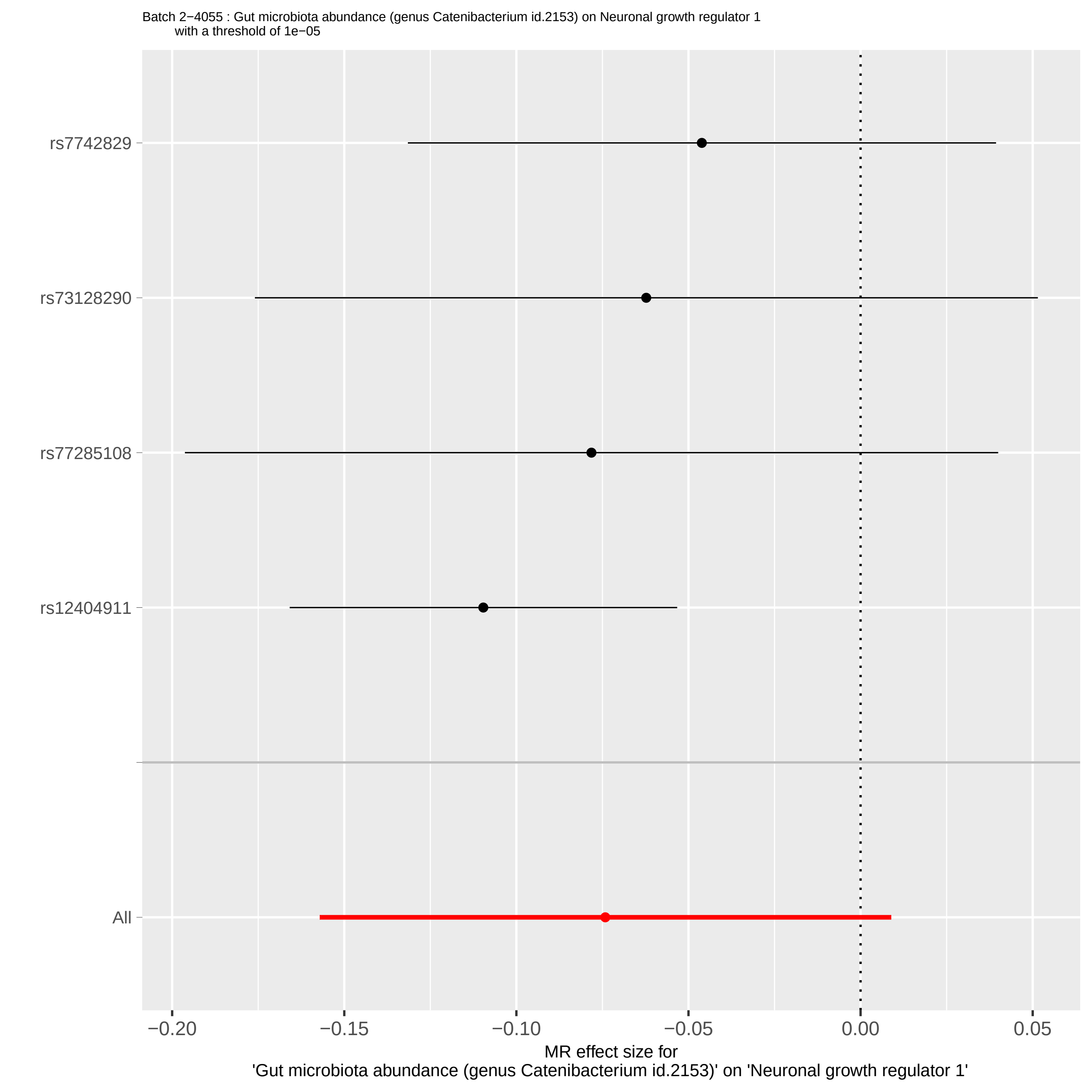
Batch 2−4055 : Gut microbiota abundance (genus Catenibacterium id.2153) on Neuronal growth regulator 1
with a threshold of 1e−05
rs7742829
rs73128290
rs77285108
rs12404911
All
−0.20
−0.15
−0.10
−0.05
0.00
0.05
MR effect size for
'Gut microbiota abundance (genus Catenibacterium id.2153)' on 'Neuronal growth regulator 1'

Batch 2−4056 : Gut microbiota abundance (genus Parasutterella id.2892) on Neuronal growth regulator 1
with a threshold of 1e−05

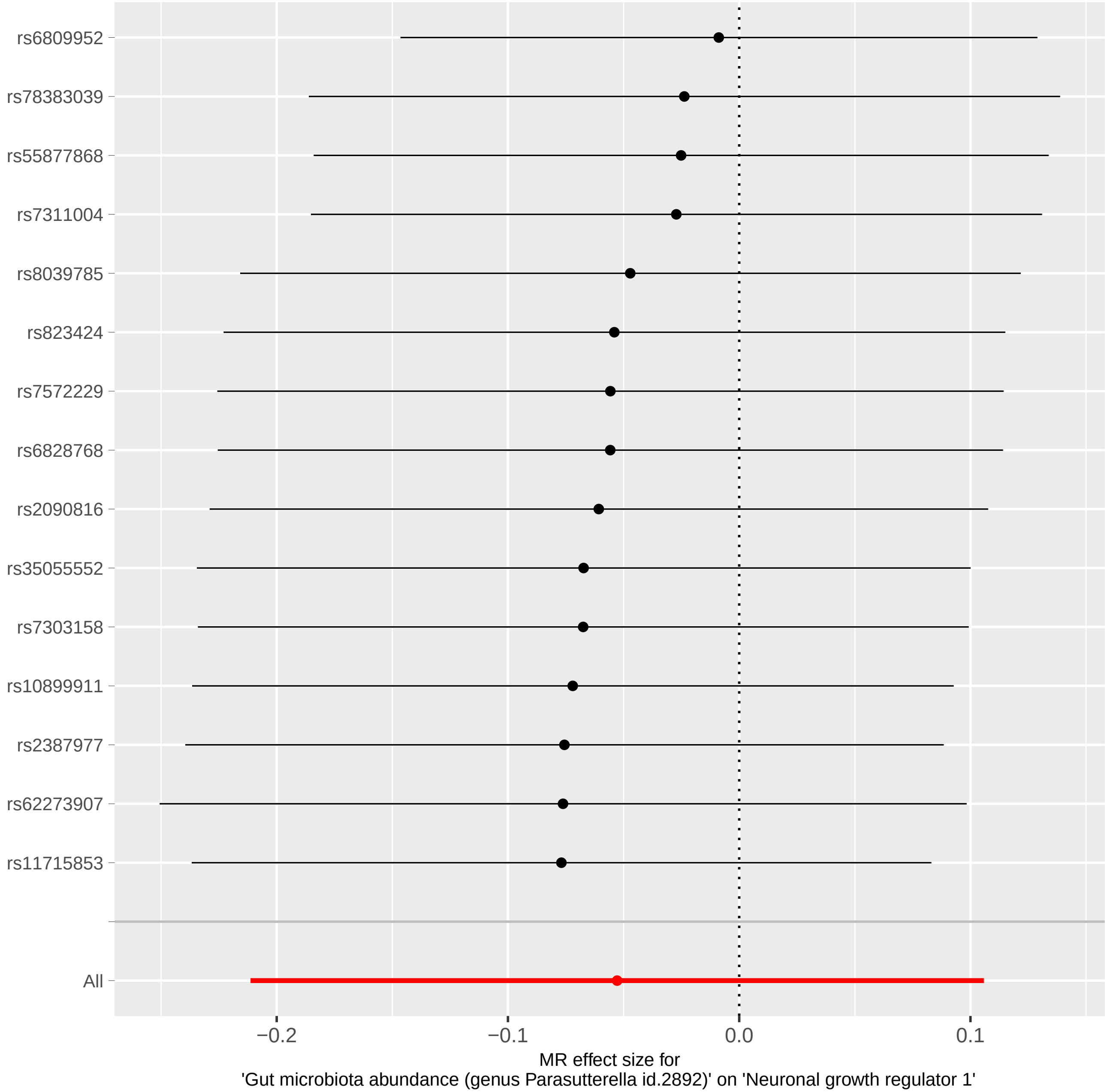

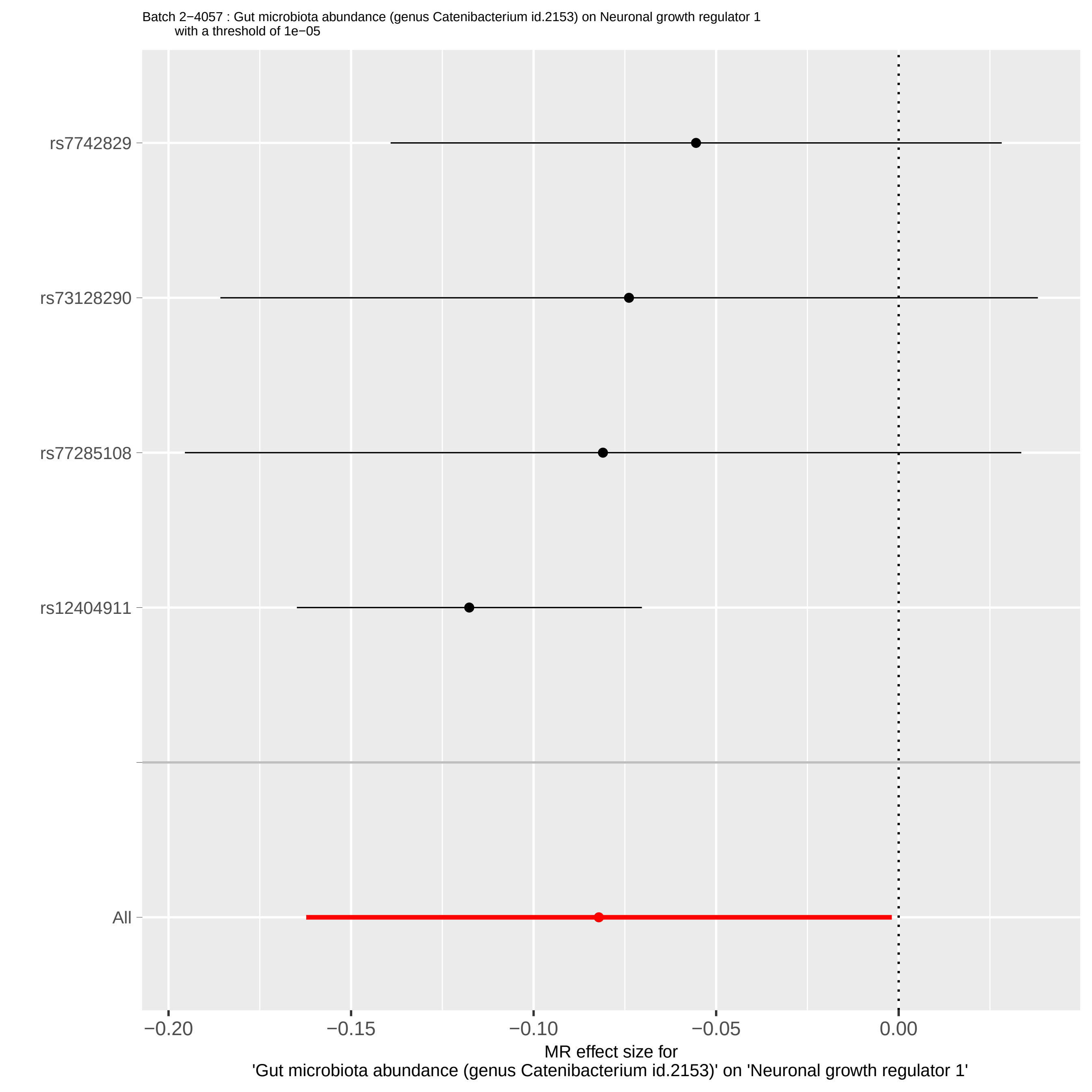
Batch 2−4057 : Gut microbiota abundance (genus Catenibacterium id.2153) on Neuronal growth regulator 1
with a threshold of 1e−05
rs7742829
rs73128290
rs77285108
rs12404911
All
−0.20
−0.15
−0.10
−0.05
0.00
MR effect size for
'Gut microbiota abundance (genus Catenibacterium id.2153)' on 'Neuronal growth regulator 1'

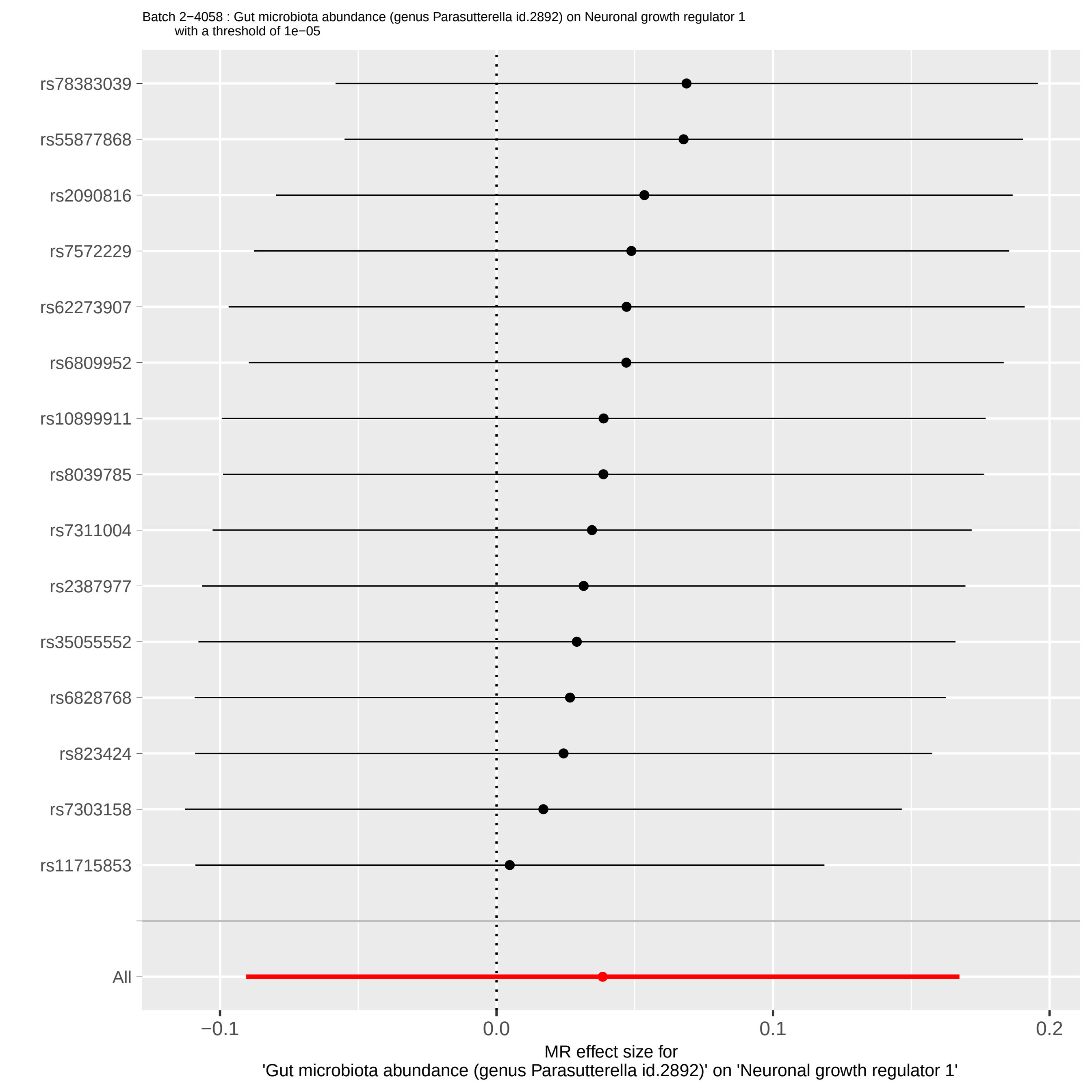

Batch 2−4058 : Gut microbiota abundance (genus Parasutterella id.2892) on Neuronal growth regulator 1
with a threshold of 1e−05
rs78383039
rs55877868
rs2090816
rs7572229
rs62273907
rs6809952
rs10899911
rs8039785
rs7311004
rs2387977
rs35055552
rs6828768
rs823424
rs7303158
rs11715853
All
−0.1
0.0
0.1
0.2
MR effect size for
'Gut microbiota abundance (genus Parasutterella id.2892)' on 'Neuronal growth regulator 1'

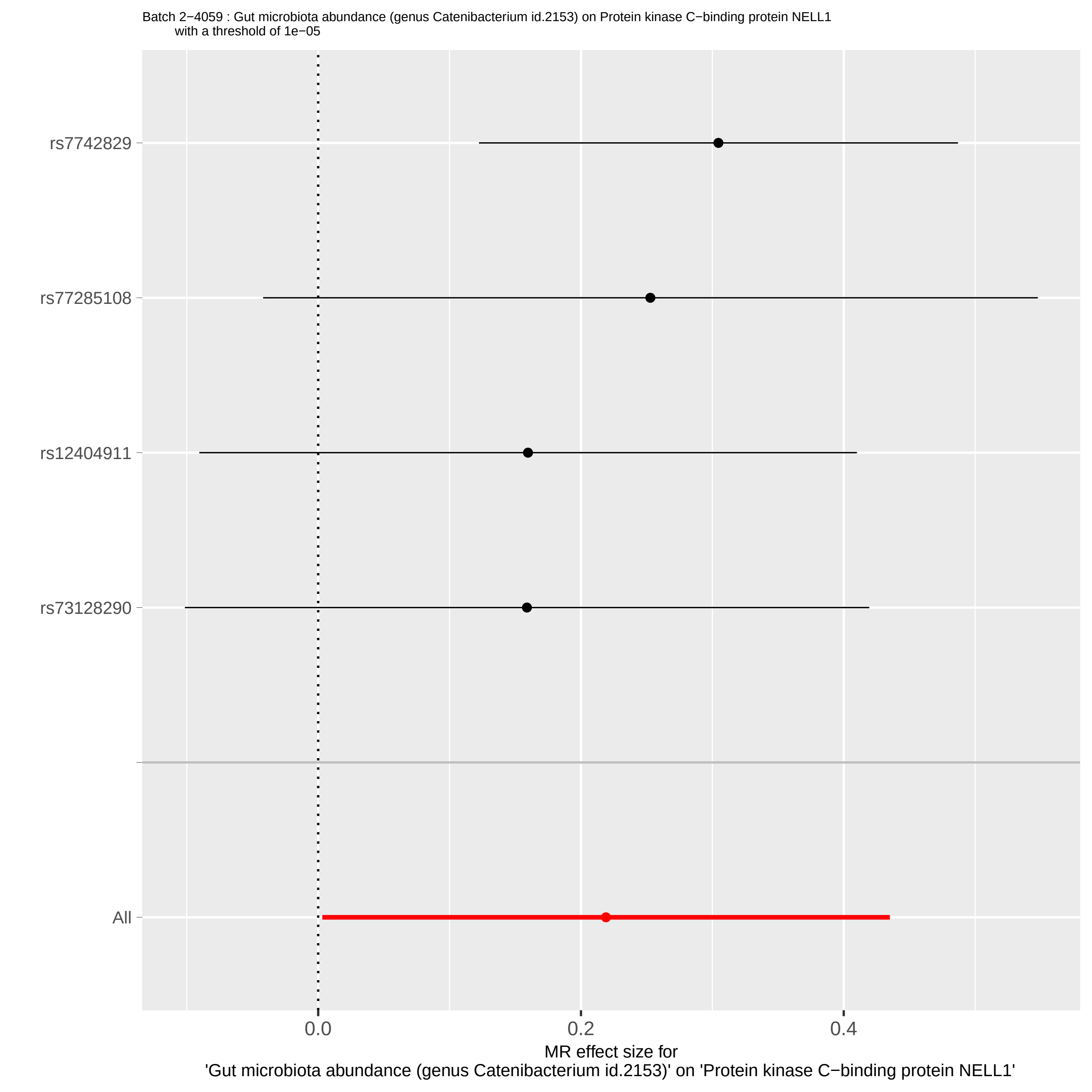
Batch 2−4059 : Gut microbiota abundance (genus Catenibacterium id.2153) on Protein kinase C−binding protein NELL1
with a threshold of 1e−05
rs7742829
rs77285108
rs12404911
rs73128290
All
0.0
0.2
0.4
MR effect size for
'Gut microbiota abundance (genus Catenibacterium id.2153)' on 'Protein kinase C−binding protein NELL1'

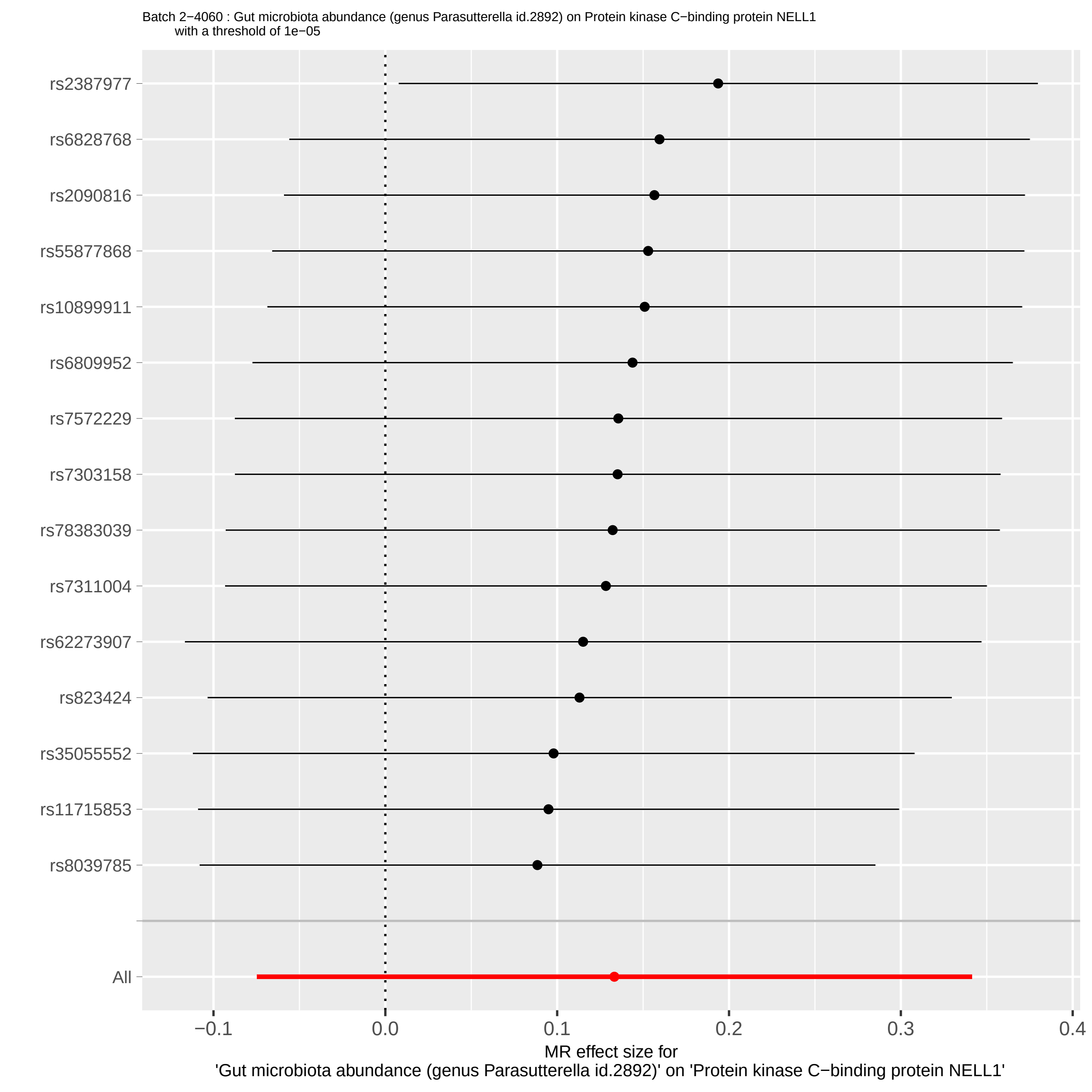

Batch 2−4060 : Gut microbiota abundance (genus Parasutterella id.2892) on Protein kinase C−binding protein NELL1
with a threshold of 1e−05
rs2387977
rs6828768
rs2090816
rs55877868
rs10899911
rs6809952
rs7572229
rs7303158
rs78383039
rs7311004
rs62273907
rs823424
rs35055552
rs11715853
rs8039785
All
−0.1
0.0
0.1
0.2
0.3
0.4
MR effect size for
'Gut microbiota abundance (genus Parasutterella id.2892)' on 'Protein kinase C−binding protein NELL1'

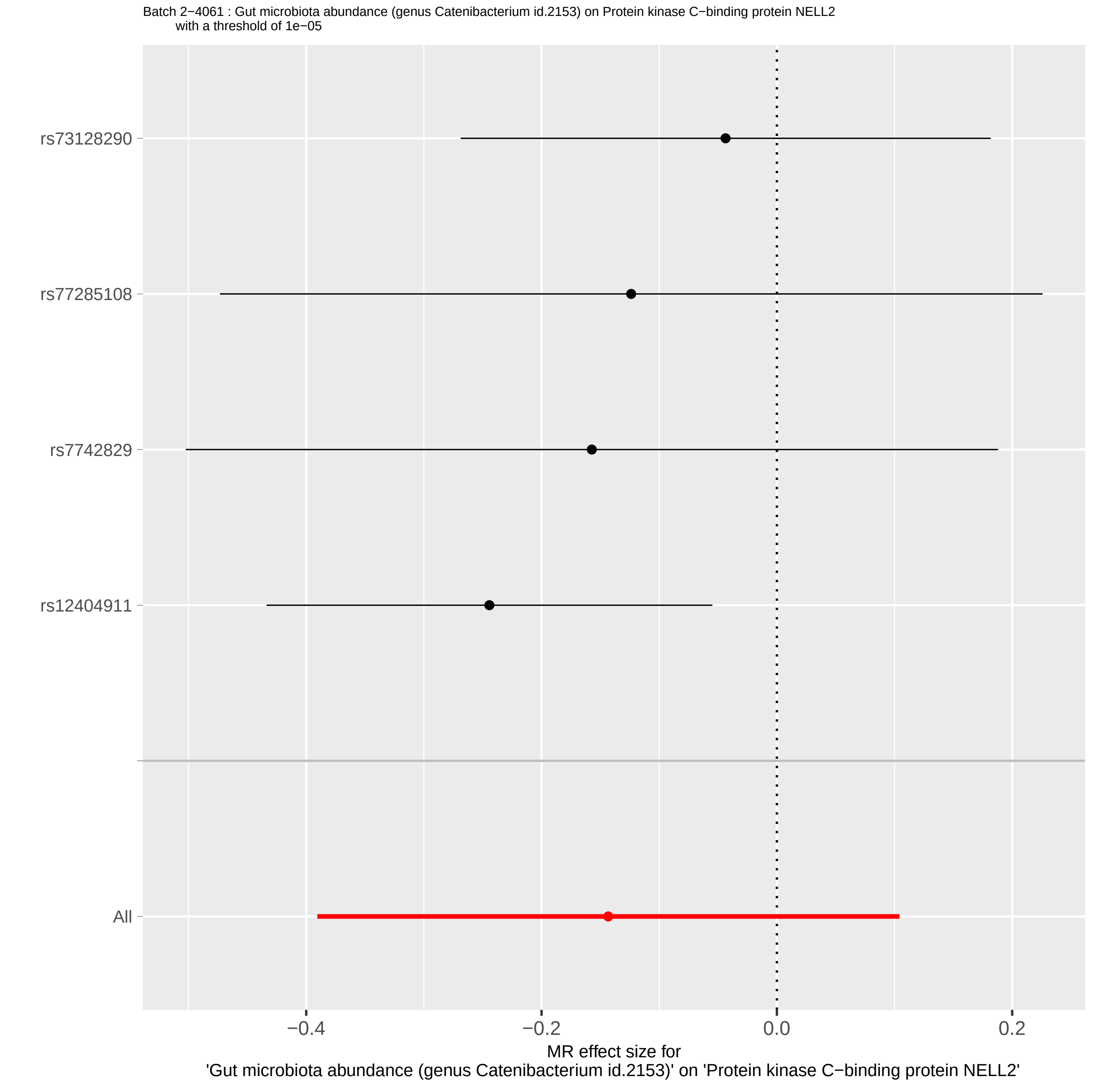

Batch 2−4061 : Gut microbiota abundance (genus Catenibacterium id.2153) on Protein kinase C−binding protein NELL2
with a threshold of 1e−05
rs73128290
rs77285108
rs7742829
rs12404911
All
−0.4
−0.2
0.0
0.2
MR effect size for
'Gut microbiota abundance (genus Catenibacterium id.2153)' on 'Protein kinase C−binding protein NELL2'

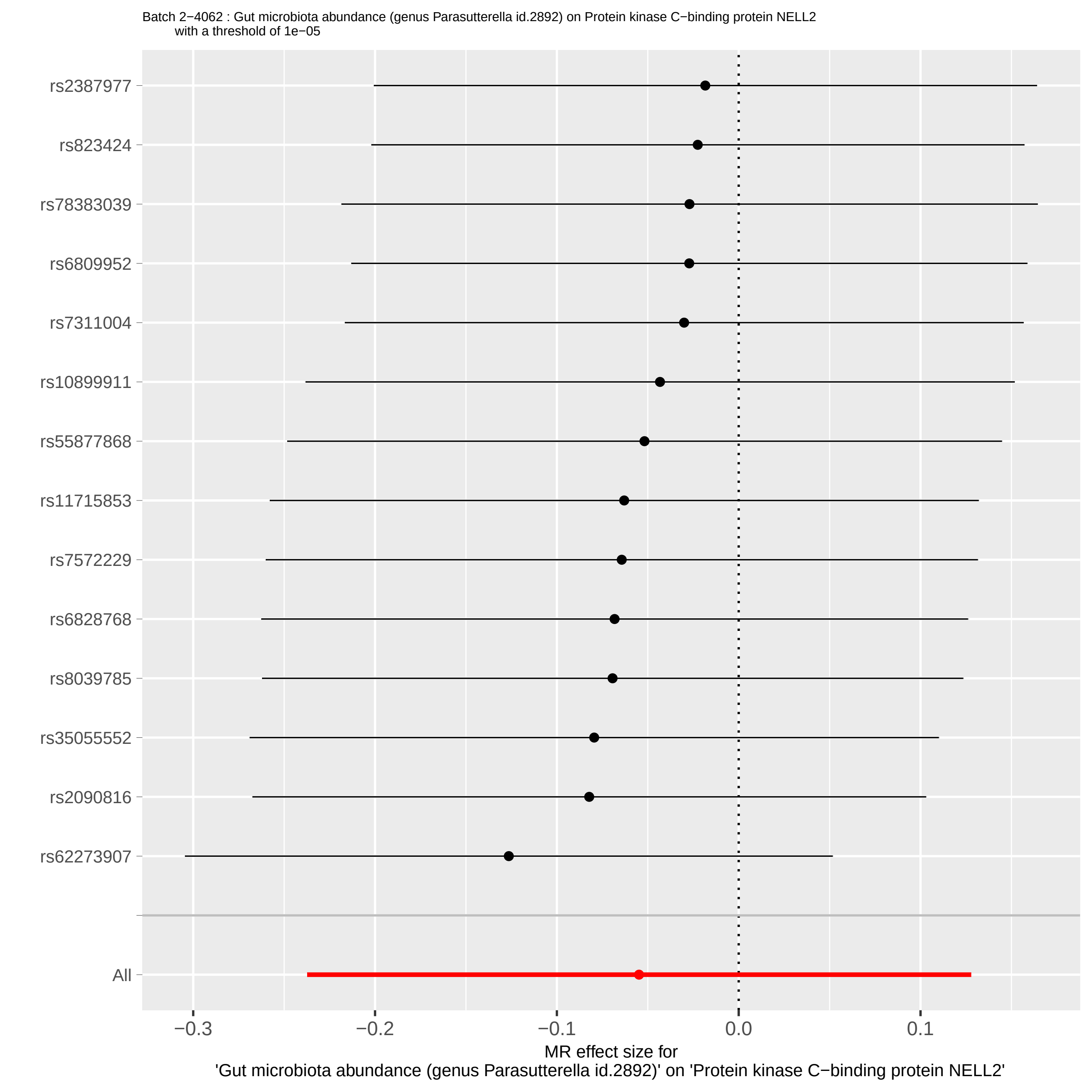

Batch 2−4062 : Gut microbiota abundance (genus Parasutterella id.2892) on Protein kinase C−binding protein NELL2
with a threshold of 1e−05
rs2387977
rs823424
rs78383039
rs6809952
rs7311004
rs10899911
rs55877868
rs11715853
rs7572229
rs6828768
rs8039785
rs35055552
rs2090816
rs62273907
All
−0.3
−0.2
−0.1
0.0
0.1
MR effect size for
'Gut microbiota abundance (genus Parasutterella id.2892)' on 'Protein kinase C−binding protein NELL2'

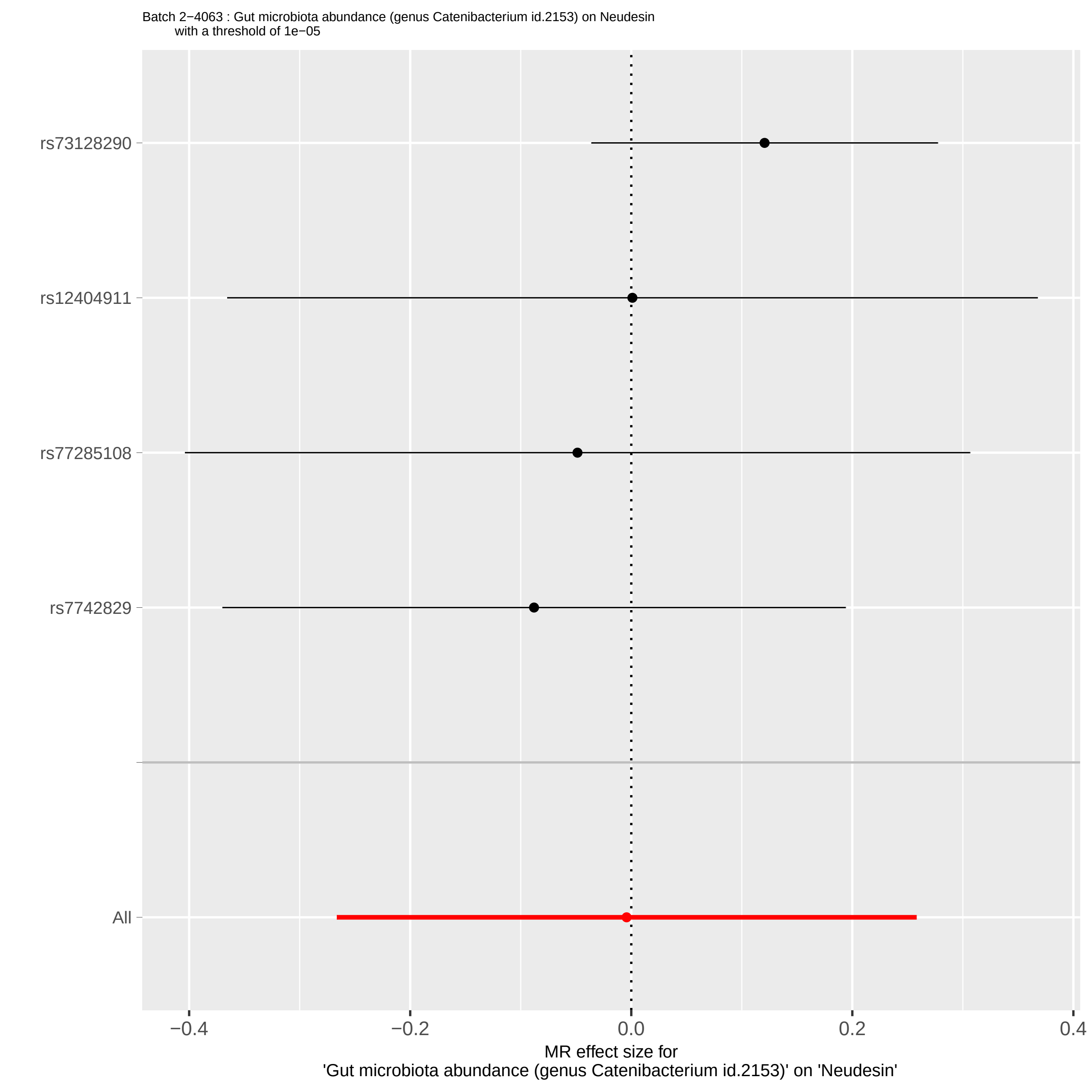
Batch 2−4063 : Gut microbiota abundance (genus Catenibacterium id.2153) on Neudesin
with a threshold of 1e−05
rs73128290
rs12404911
rs77285108
rs7742829
All
−0.4
−0.2
0.0
0.2
0.4
MR effect size for
'Gut microbiota abundance (genus Catenibacterium id.2153)' on 'Neudesin'

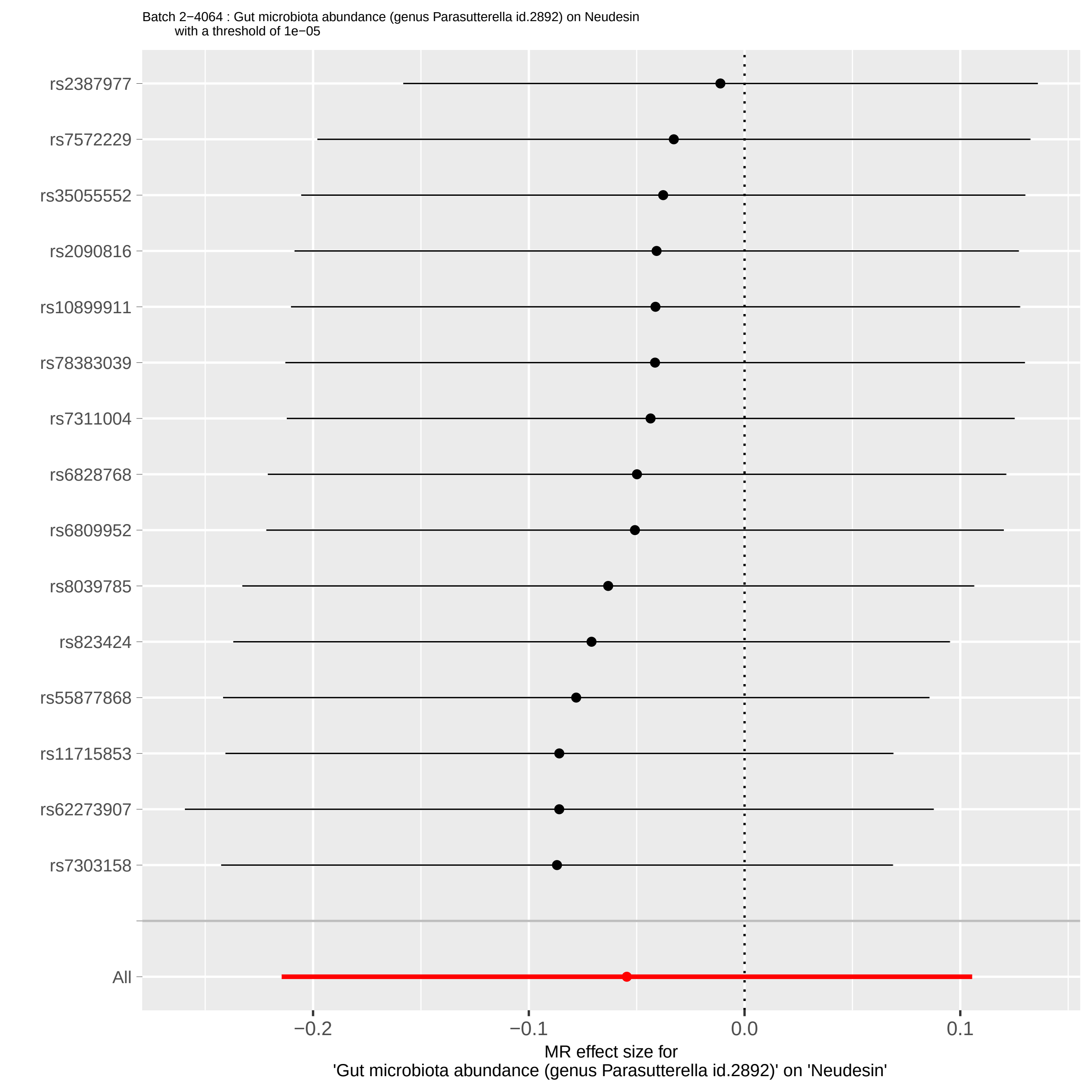

Batch 2−4064 : Gut microbiota abundance (genus Parasutterella id.2892) on Neudesin
with a threshold of 1e−05
rs2387977
rs7572229
rs35055552
rs2090816
rs10899911
rs78383039
rs7311004
rs6828768
rs6809952
rs8039785
rs823424
rs55877868
rs11715853
rs62273907
rs7303158
All
−0.2
−0.1
0.0
0.1
MR effect size for
'Gut microbiota abundance (genus Parasutterella id.2892)' on 'Neudesin'

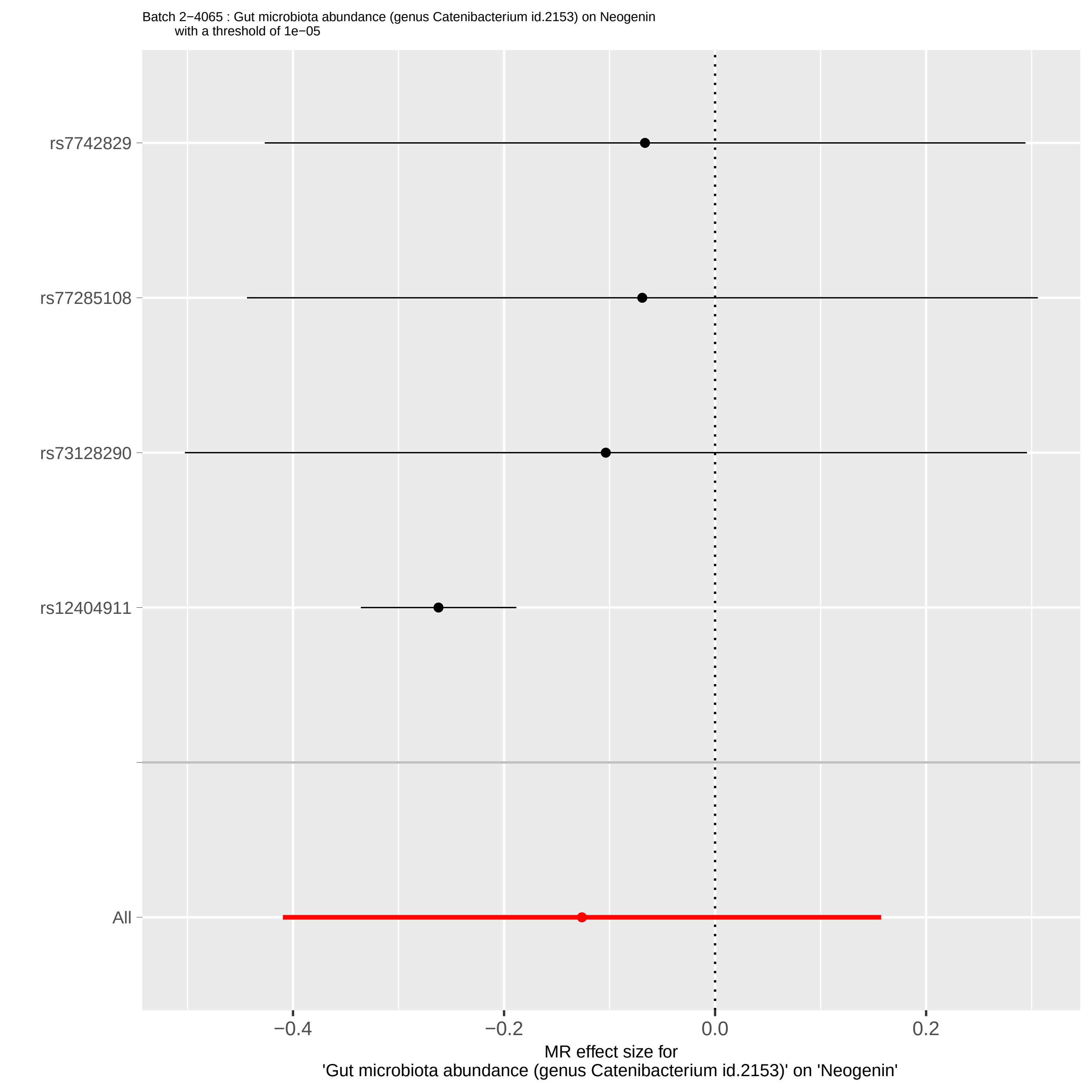

Batch 2−4065 : Gut microbiota abundance (genus Catenibacterium id.2153) on Neogenin
with a threshold of 1e−05
rs7742829
rs77285108
rs73128290
rs12404911
All
−0.4
−0.2
0.0
0.2
MR effect size for
'Gut microbiota abundance (genus Catenibacterium id.2153)' on 'Neogenin'

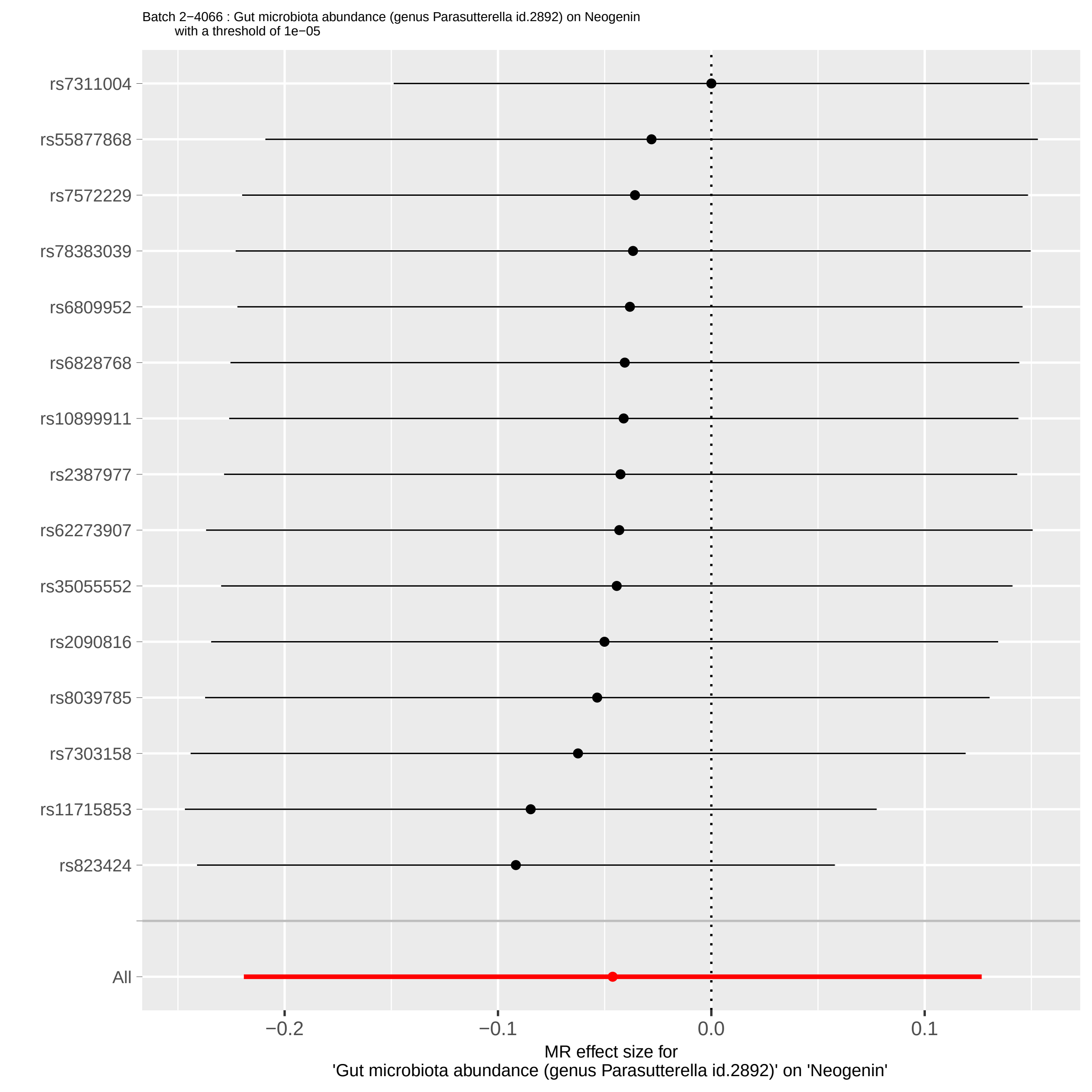

Batch 2−4066 : Gut microbiota abundance (genus Parasutterella id.2892) on Neogenin
with a threshold of 1e−05
rs7311004
rs55877868
rs7572229
rs78383039
rs6809952
rs6828768
rs10899911
rs2387977
rs62273907
rs35055552
rs2090816
rs8039785
rs7303158
rs11715853
rs823424
All
−0.2
−0.1
0.0
0.1
MR effect size for
'Gut microbiota abundance (genus Parasutterella id.2892)' on 'Neogenin'

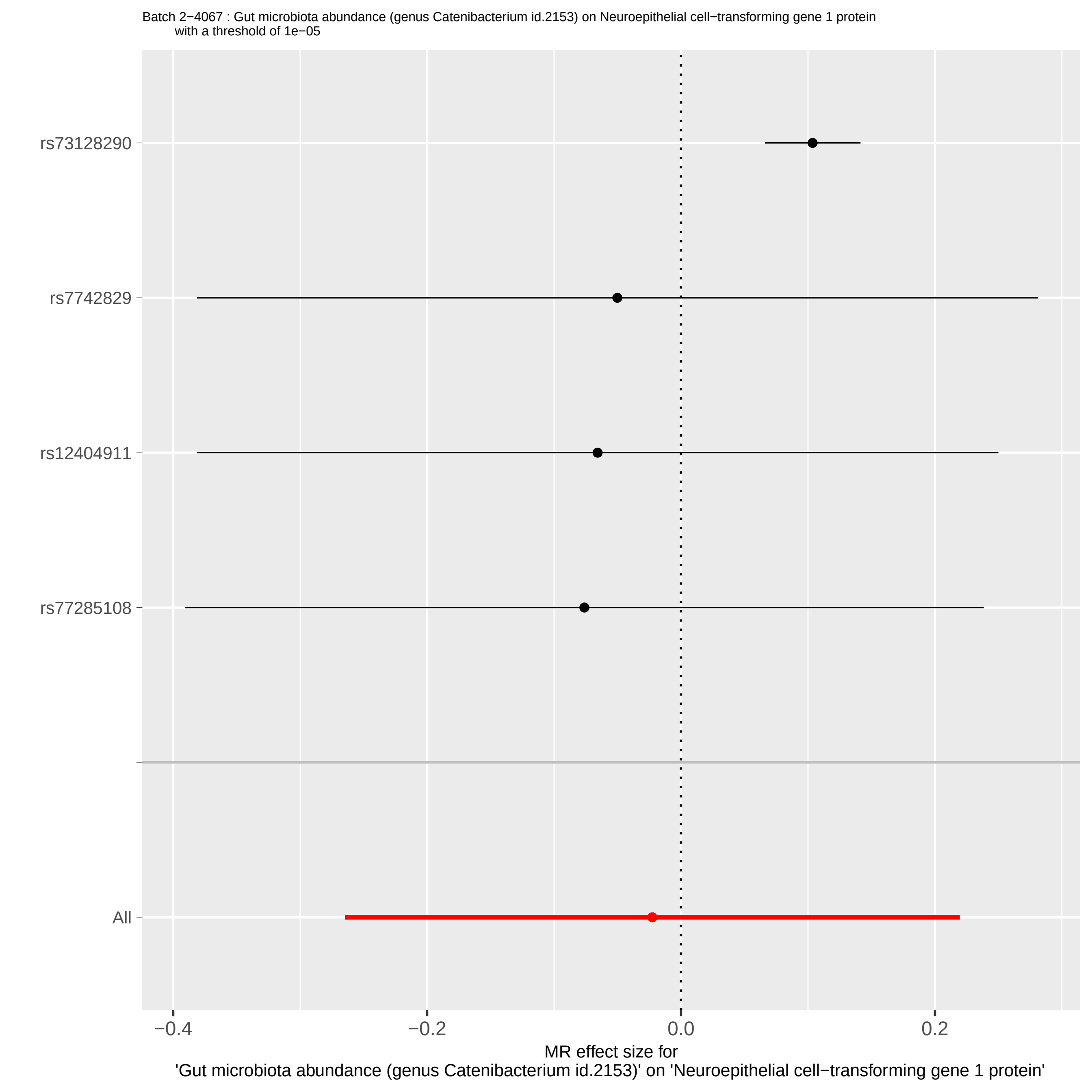

Batch 2−4067 : Gut microbiota abundance (genus Catenibacterium id.2153) on Neuroepithelial cell−transforming gene 1 protein
with a threshold of 1e−05
rs73128290
rs7742829
rs12404911
rs77285108
All
−0.4
−0.2
0.0
0.2
MR effect size for
'Gut microbiota abundance (genus Catenibacterium id.2153)' on 'Neuroepithelial cell−transforming gene 1 protein'

Batch 2−4068 : Gut microbiota abundance (genus Parasutterella id.2892) on Neuroepithelial cell−transforming gene 1 protein
with a threshold of 1e−05

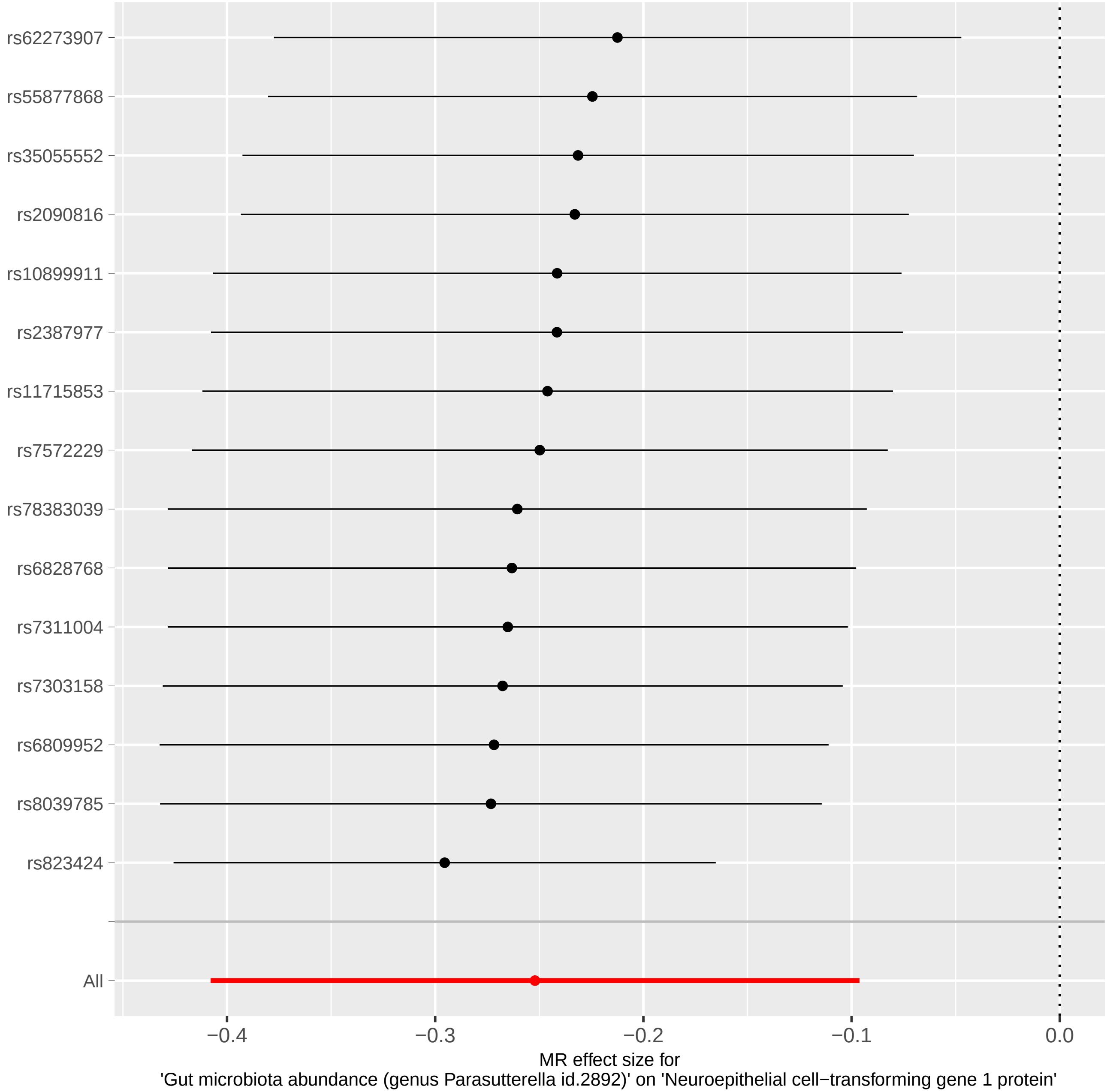

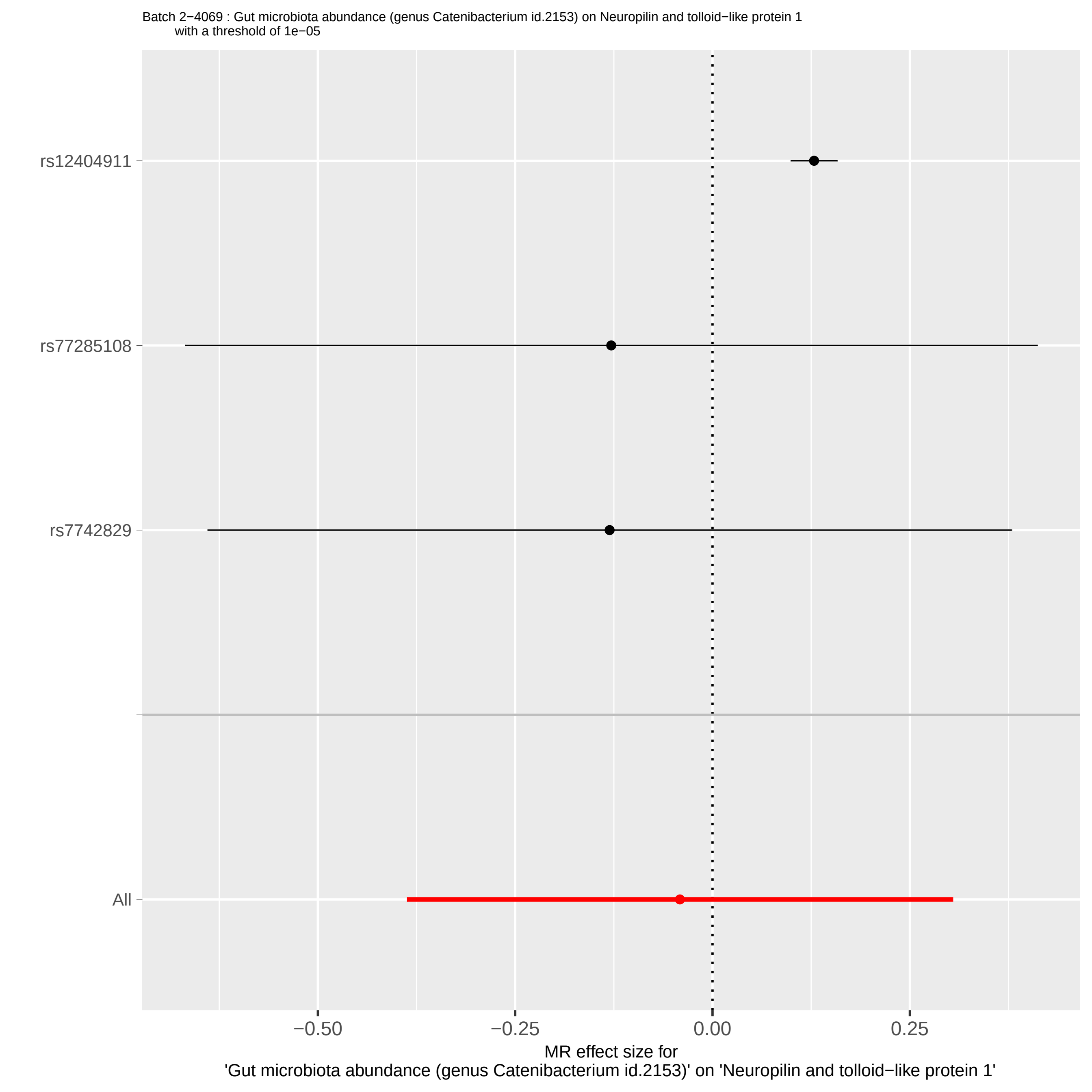
Batch 2−4069 : Gut microbiota abundance (genus Catenibacterium id.2153) on Neuropilin and tolloid−like protein 1
with a threshold of 1e−05
rs12404911
rs77285108
rs7742829
All
−0.50
−0.25
0.00
0.25
MR effect size for
'Gut microbiota abundance (genus Catenibacterium id.2153)' on 'Neuropilin and tolloid−like protein 1'

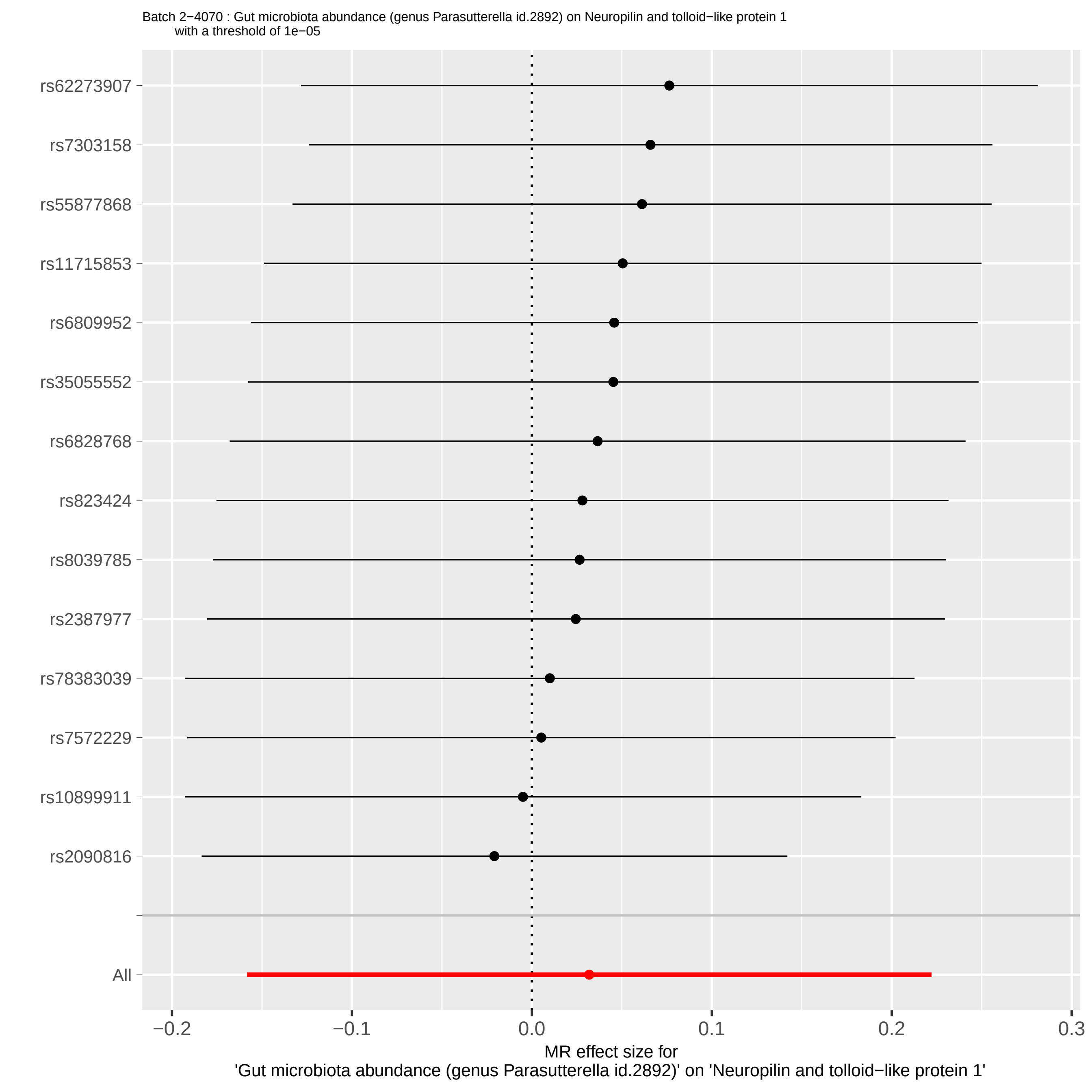
Batch 2−4070 : Gut microbiota abundance (genus Parasutterella id.2892) on Neuropilin and tolloid−like protein 1
with a threshold of 1e−05
rs62273907
rs7303158
rs55877868
rs11715853
rs6809952
rs35055552
rs6828768
rs823424
rs8039785
rs2387977
rs78383039
rs7572229
rs10899911
rs2090816
All
−0.2
−0.1
0.0
0.1
0.2
0.3
MR effect size for
'Gut microbiota abundance (genus Parasutterella id.2892)' on 'Neuropilin and tolloid−like protein 1'

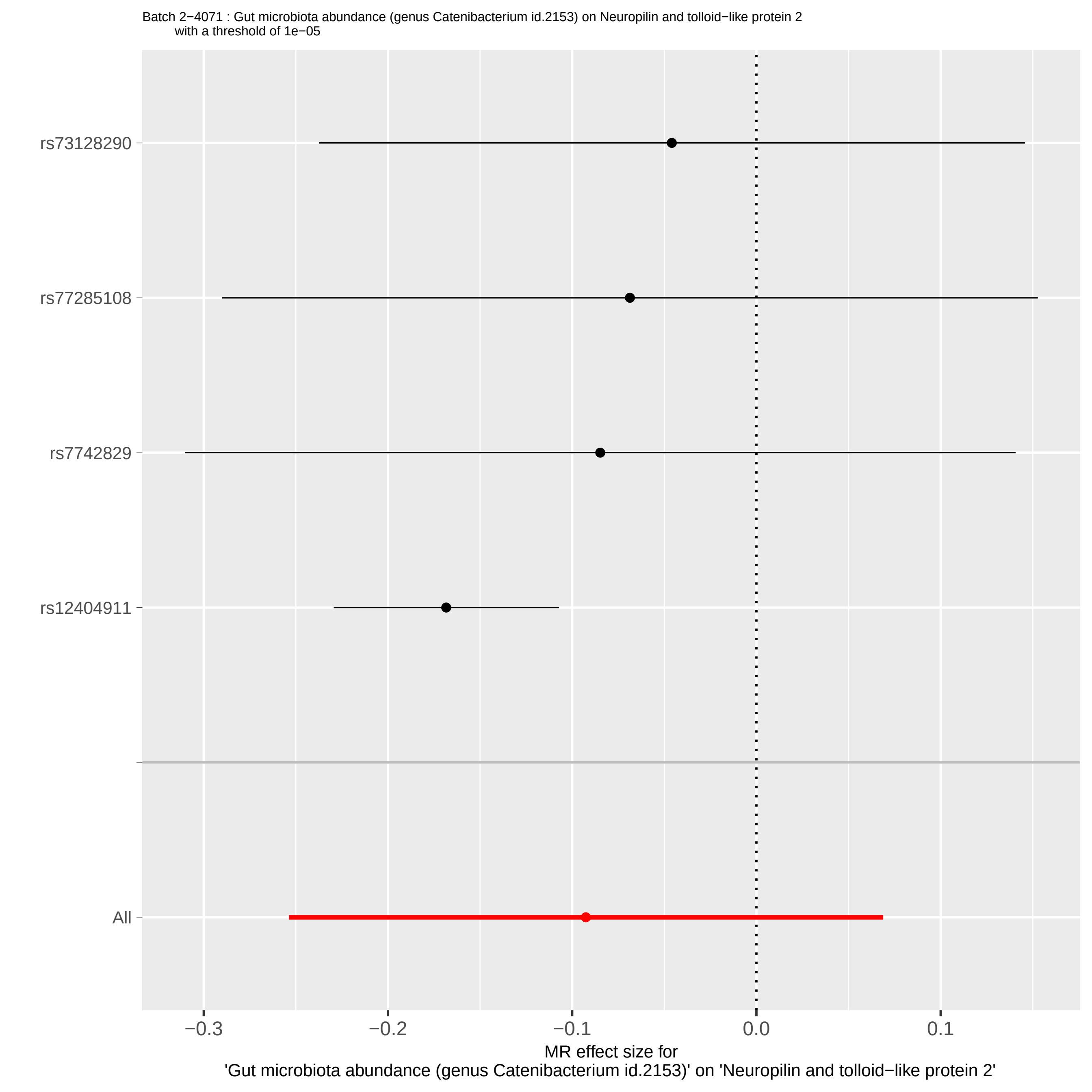
Batch 2−4071 : Gut microbiota abundance (genus Catenibacterium id.2153) on Neuropilin and tolloid−like protein 2
with a threshold of 1e−05
rs73128290
rs77285108
rs7742829
rs12404911
All
−0.3
−0.2
−0.1
0.0
0.1
MR effect size for
'Gut microbiota abundance (genus Catenibacterium id.2153)' on 'Neuropilin and tolloid−like protein 2'

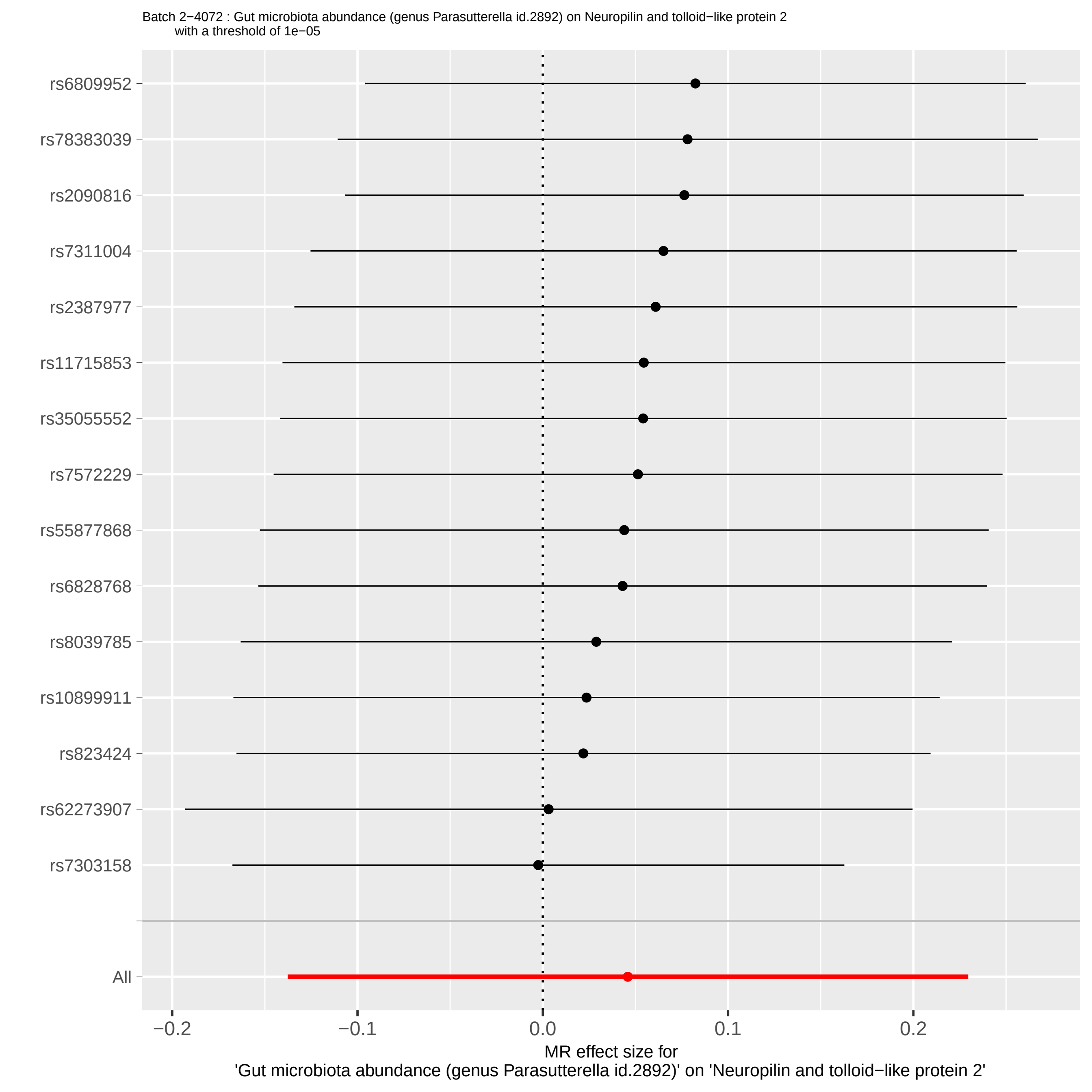

Batch 2−4072 : Gut microbiota abundance (genus Parasutterella id.2892) on Neuropilin and tolloid−like protein 2
with a threshold of 1e−05
rs6809952
rs78383039
rs2090816
rs7311004
rs2387977
rs11715853
rs35055552
rs7572229
rs55877868
rs6828768
rs8039785
rs10899911
rs823424
rs62273907
rs7303158
All
−0.2
−0.1
0.0
0.1
0.2
MR effect size for
'Gut microbiota abundance (genus Parasutterella id.2892)' on 'Neuropilin and tolloid−like protein 2'

Batch 2−4073 : Gut microbiota abundance (genus Catenibacterium id.2153) on E3 ubiquitin−protein ligase NEURL1
with a threshold of 1e−05

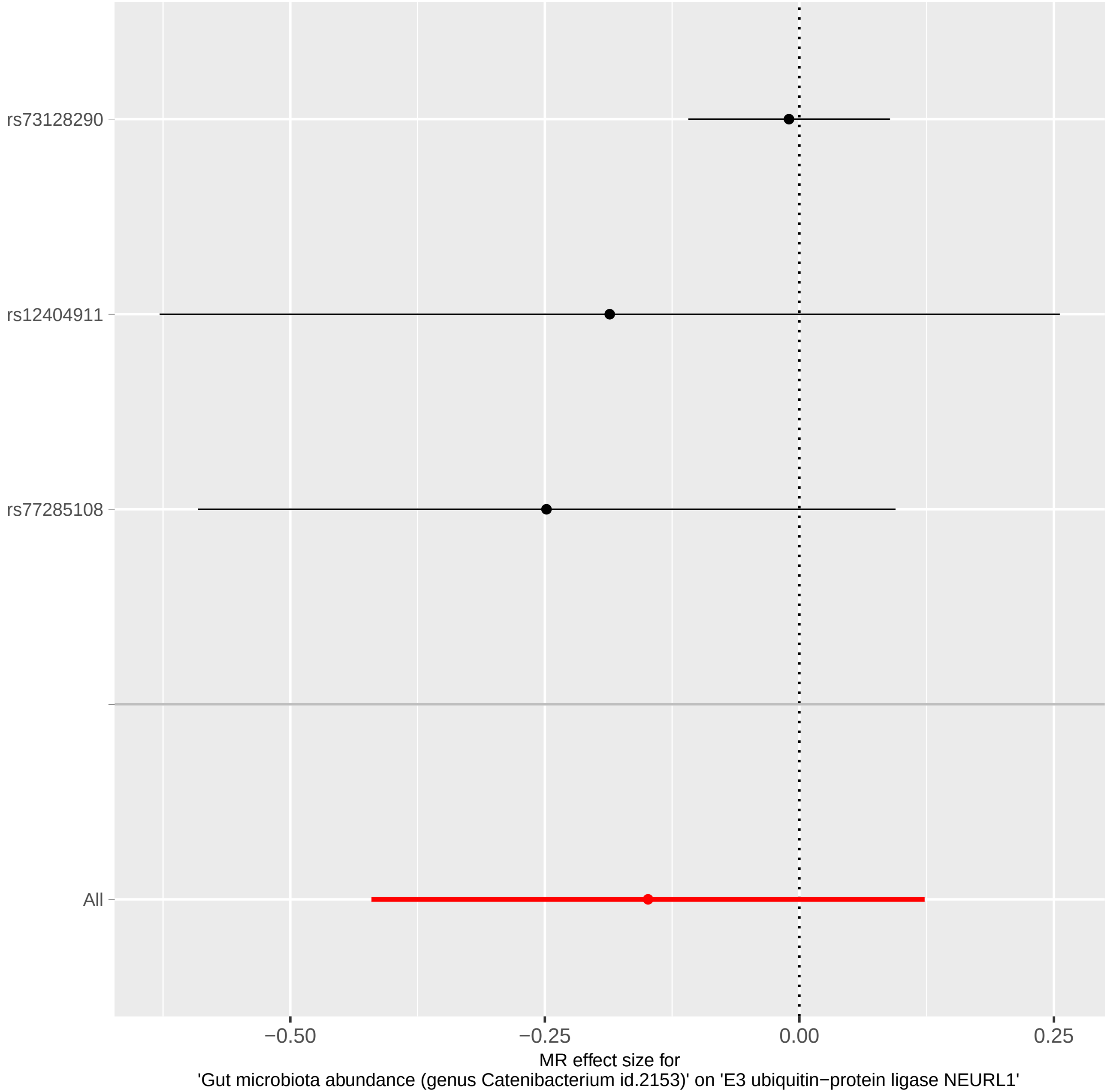

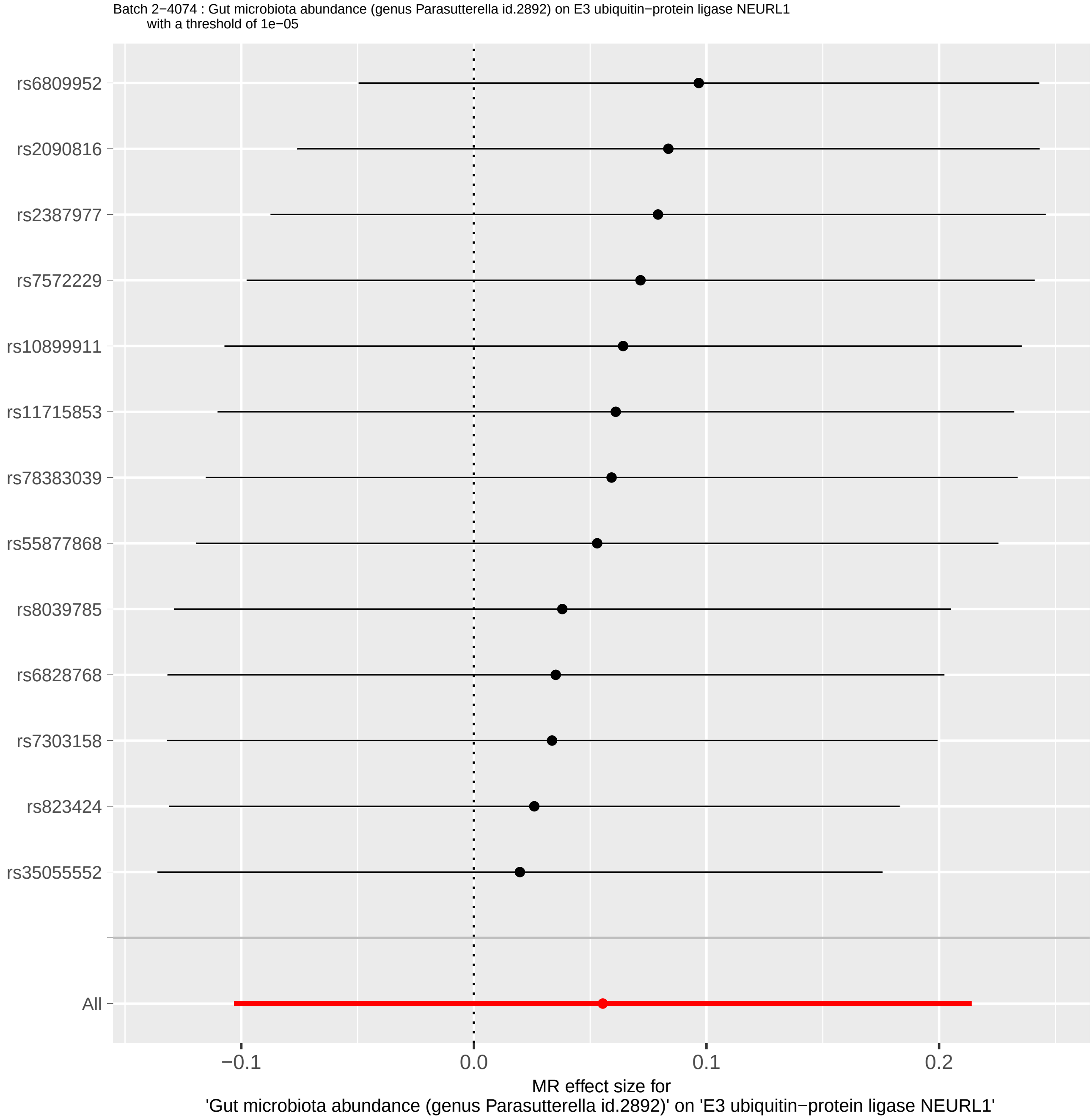
Batch 2−4074 : Gut microbiota abundance (genus Parasutterella id.2892) on E3 ubiquitin−protein ligase NEURL1
with a threshold of 1e−05
rs6809952
rs2090816
rs2387977
rs7572229
rs10899911
rs11715853
rs78383039
rs55877868
rs8039785
rs6828768
rs7303158
rs823424
rs35055552
All
−0.1
0.0
0.1
0.2
MR effect size for
'Gut microbiota abundance (genus Parasutterella id.2892)' on 'E3 ubiquitin−protein ligase NEURL1'

Batch 2−4075 : Gut microbiota abundance (genus Catenibacterium id.2153) on Neuralized−like protein 4
with a threshold of 1e−05

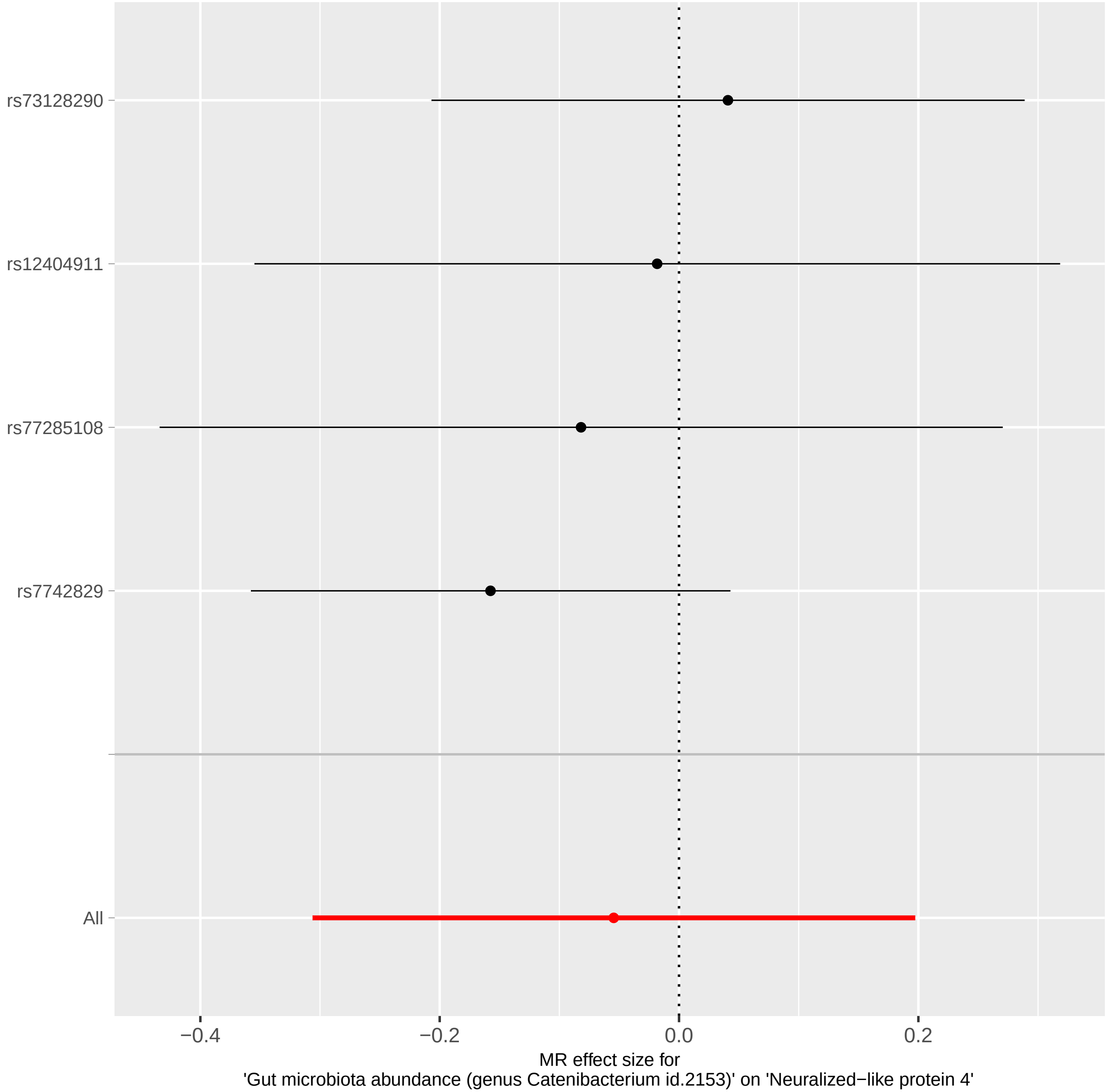

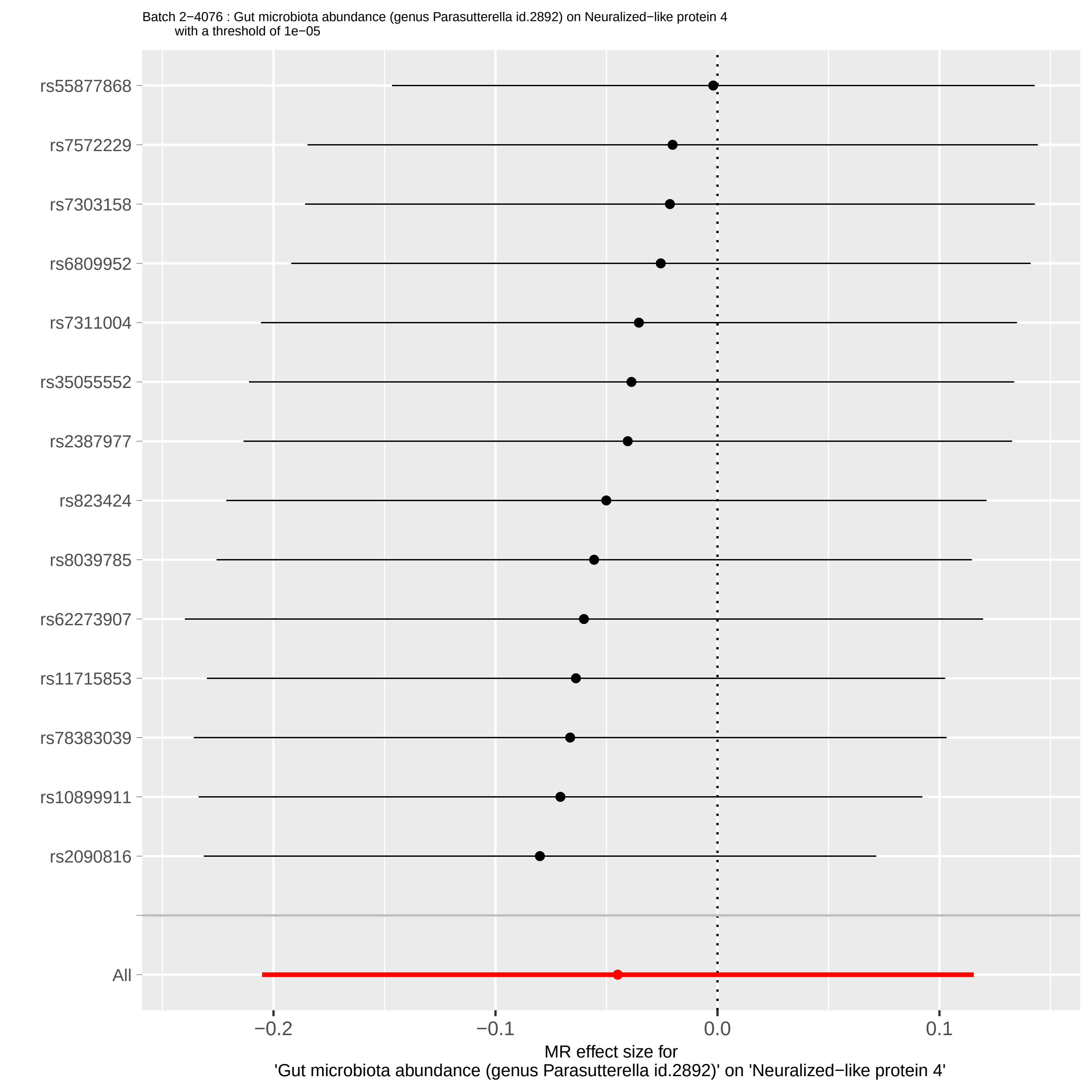

Batch 2−4076 : Gut microbiota abundance (genus Parasutterella id.2892) on Neuralized−like protein 4
with a threshold of 1e−05
rs55877868
rs7572229
rs7303158
rs6809952
rs7311004
rs35055552
rs2387977
rs823424
rs8039785
rs62273907
rs11715853
rs78383039
rs10899911
rs2090816
All
−0.2
−0.1
0.0
0.1
MR effect size for
'Gut microbiota abundance (genus Parasutterella id.2892)' on 'Neuralized−like protein 4'

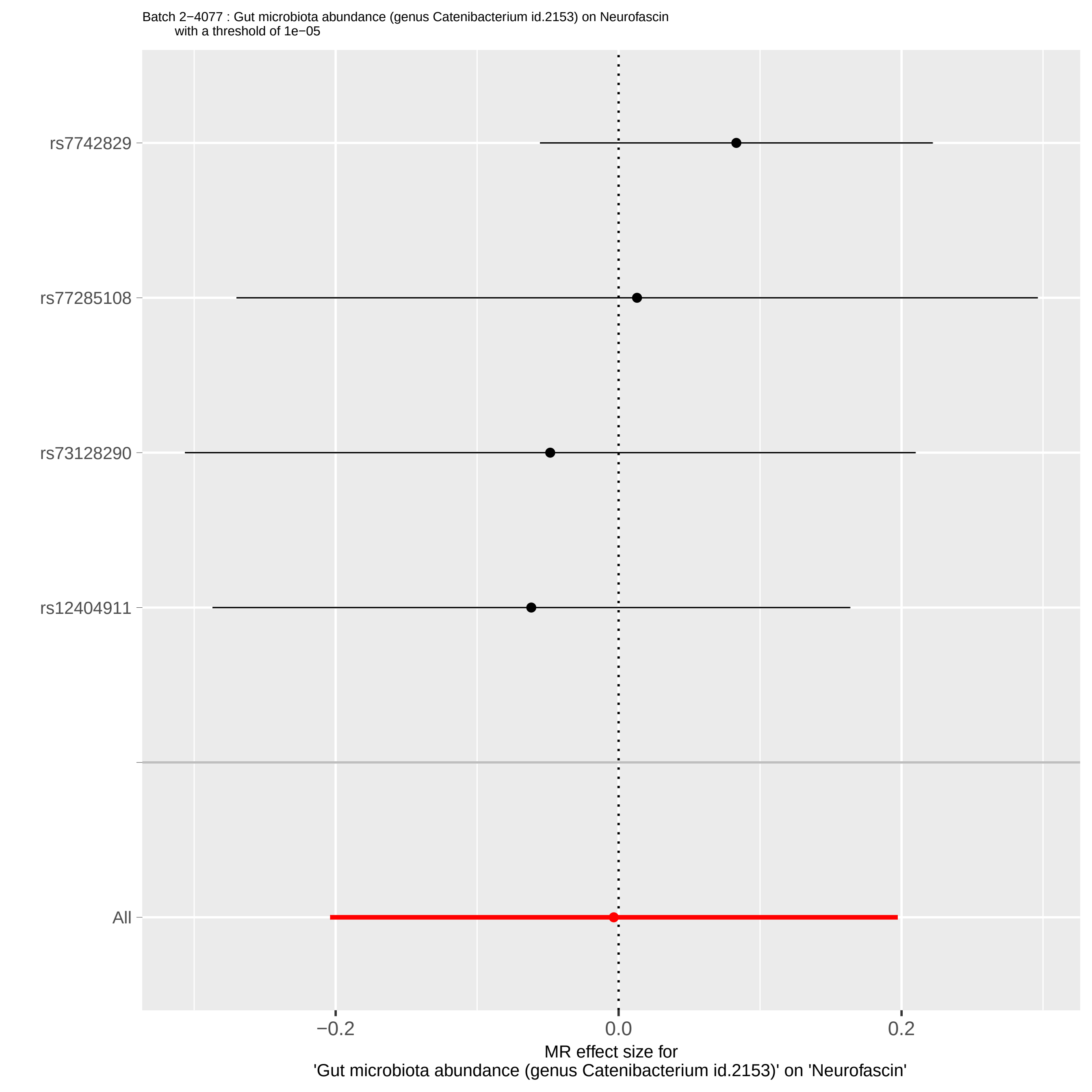

Batch 2−4077 : Gut microbiota abundance (genus Catenibacterium id.2153) on Neurofascin
with a threshold of 1e−05
rs7742829
rs77285108
rs73128290
rs12404911
All
−0.2
0.0
0.2
MR effect size for
'Gut microbiota abundance (genus Catenibacterium id.2153)' on 'Neurofascin'

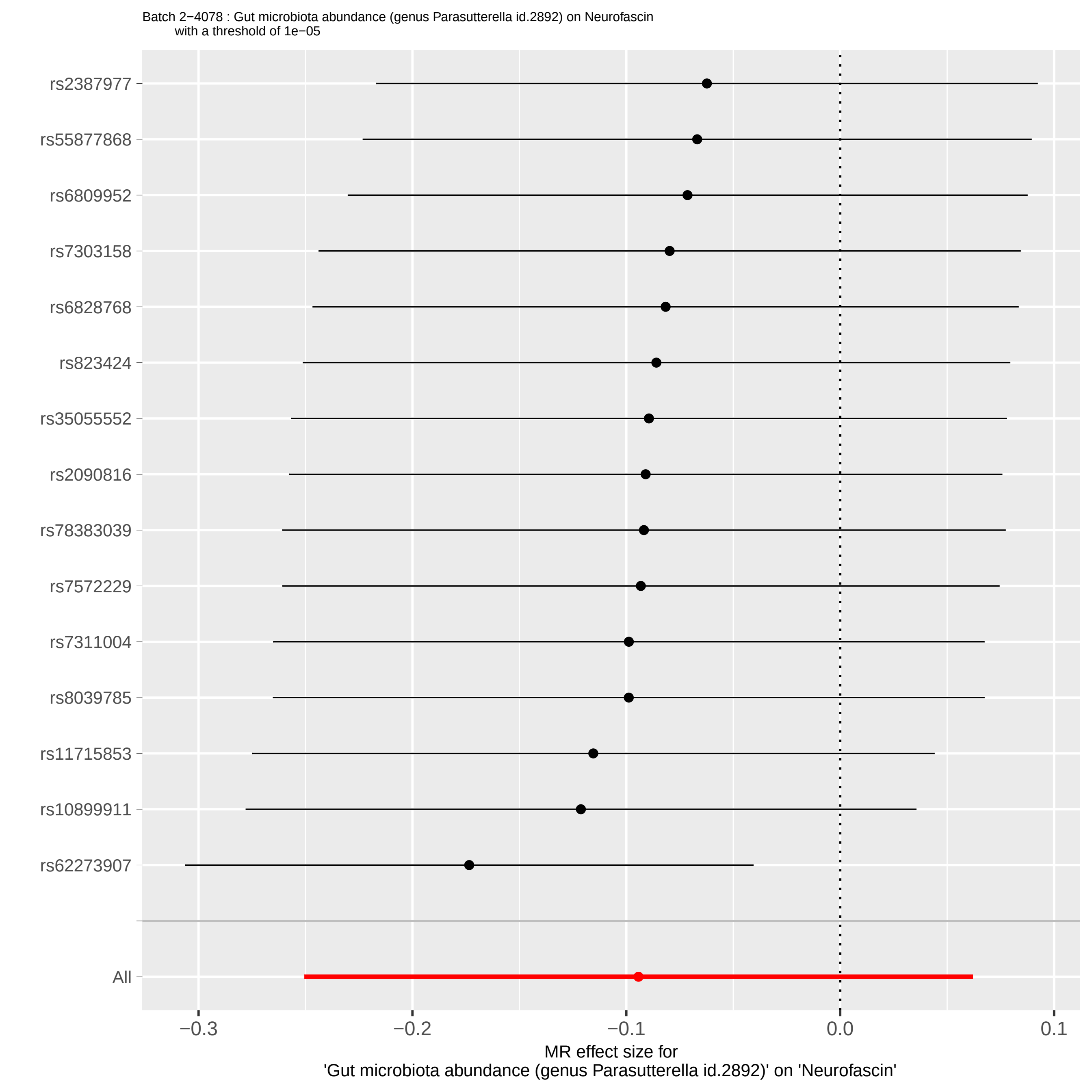
Batch 2−4078 : Gut microbiota abundance (genus Parasutterella id.2892) on Neurofascin
with a threshold of 1e−05
rs2387977
rs55877868
rs6809952
rs7303158
rs6828768
rs823424
rs35055552
rs2090816
rs78383039
rs7572229
rs7311004
rs8039785
rs11715853
rs10899911
rs62273907
All
−0.3
−0.2
−0.1
0.0
0.1
MR effect size for
'Gut microbiota abundance (genus Parasutterella id.2892)' on 'Neurofascin'

Batch 2−4079 : Gut microbiota abundance (genus Catenibacterium id.2153) on Nuclear factor erythroid 2−related factor 1
with a threshold of 1e−05

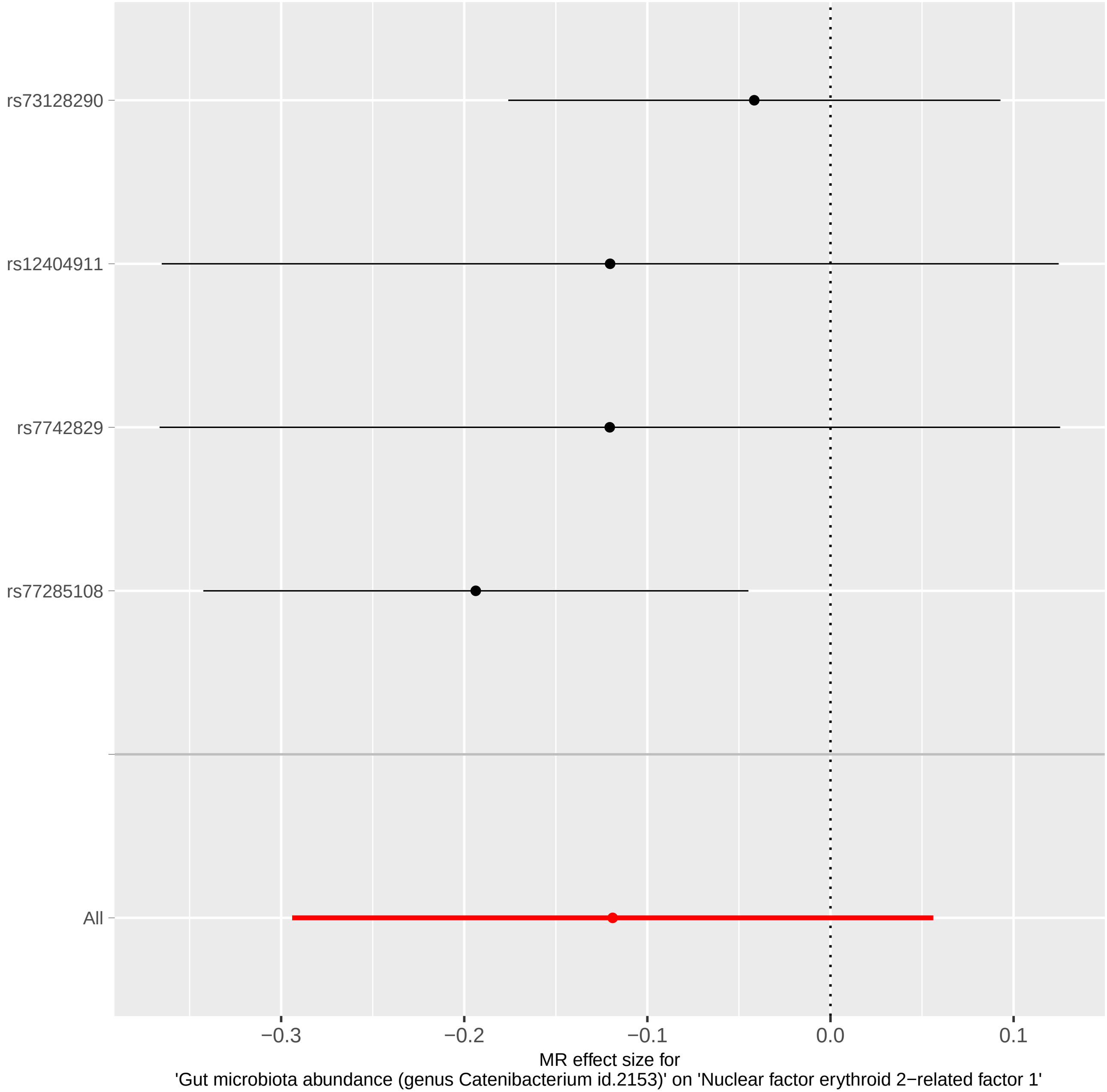

Batch 2−4080 : Gut microbiota abundance (genus Parasutterella id.2892) on Nuclear factor erythroid 2−related factor 1
with a threshold of 1e−05

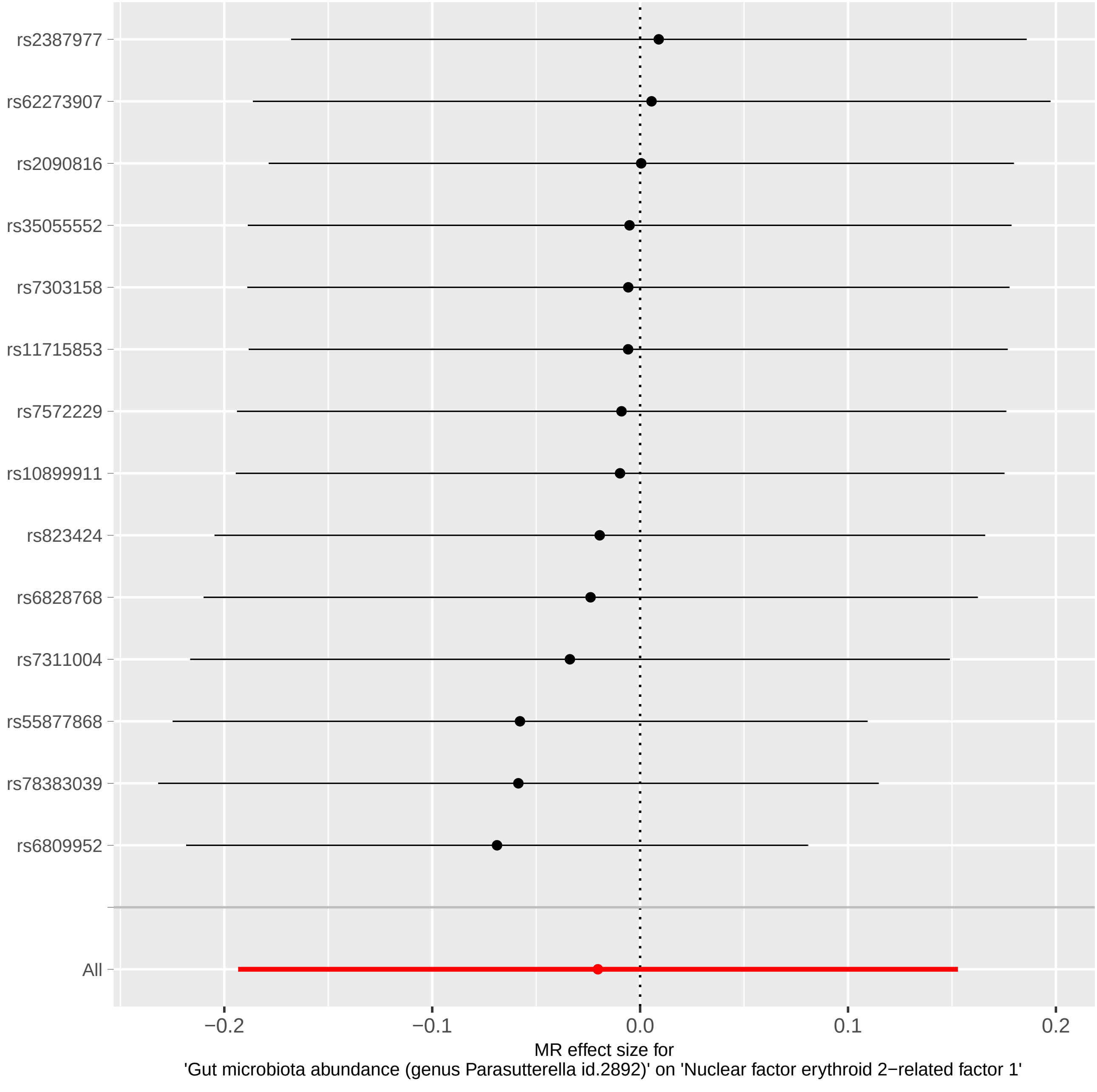

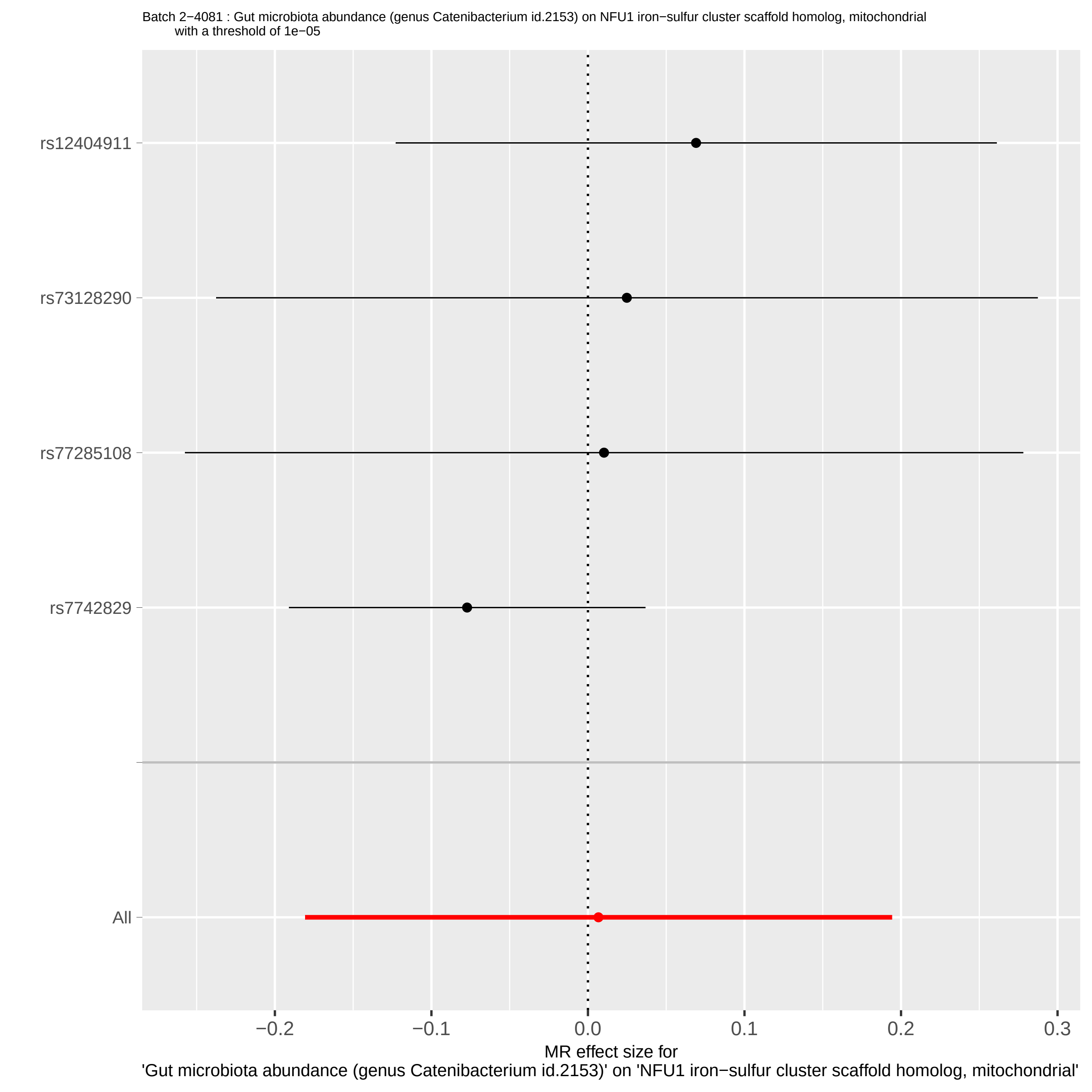

Batch 2−4081 : Gut microbiota abundance (genus Catenibacterium id.2153) on NFU1 iron−sulfur cluster scaffold homolog, mitochondrial
with a threshold of 1e−05
rs12404911
rs73128290
rs77285108
rs7742829
All
−0.2
−0.1
0.0
0.1
0.2
0.3
MR effect size for
'Gut microbiota abundance (genus Catenibacterium id.2153)' on 'NFU1 iron−sulfur cluster scaffold homolog, mitochondrial'

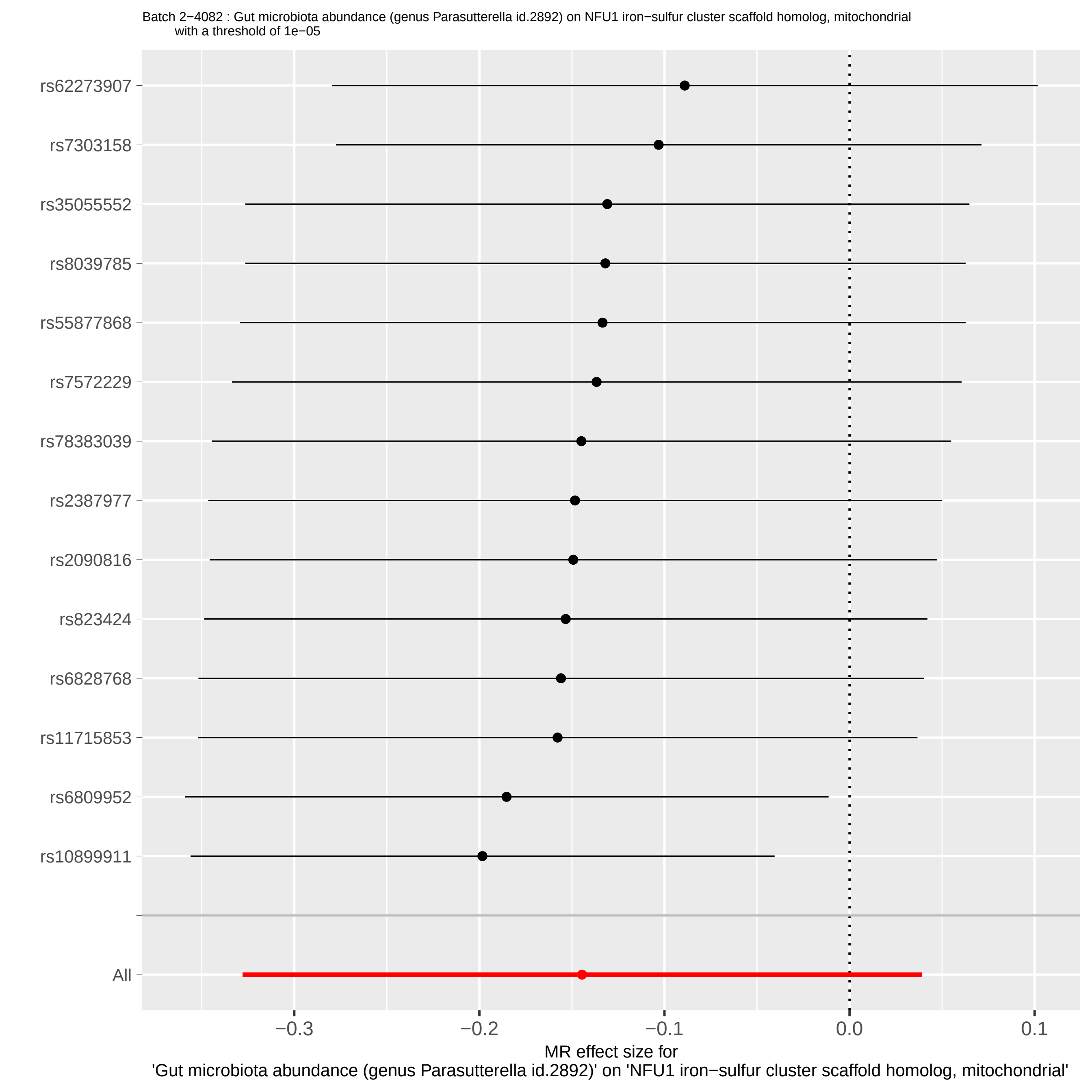

Batch 2−4082 : Gut microbiota abundance (genus Parasutterella id.2892) on NFU1 iron−sulfur cluster scaffold homolog, mitochondrial
with a threshold of 1e−05
rs62273907
rs7303158
rs35055552
rs8039785
rs55877868
rs7572229
rs78383039
rs2387977
rs2090816
rs823424
rs6828768
rs11715853
rs6809952
rs10899911
All
−0.3
−0.2
−0.1
0.0
0.1
MR effect size for
'Gut microbiota abundance (genus Parasutterella id.2892)' on 'NFU1 iron−sulfur cluster scaffold homolog, mitochondrial'

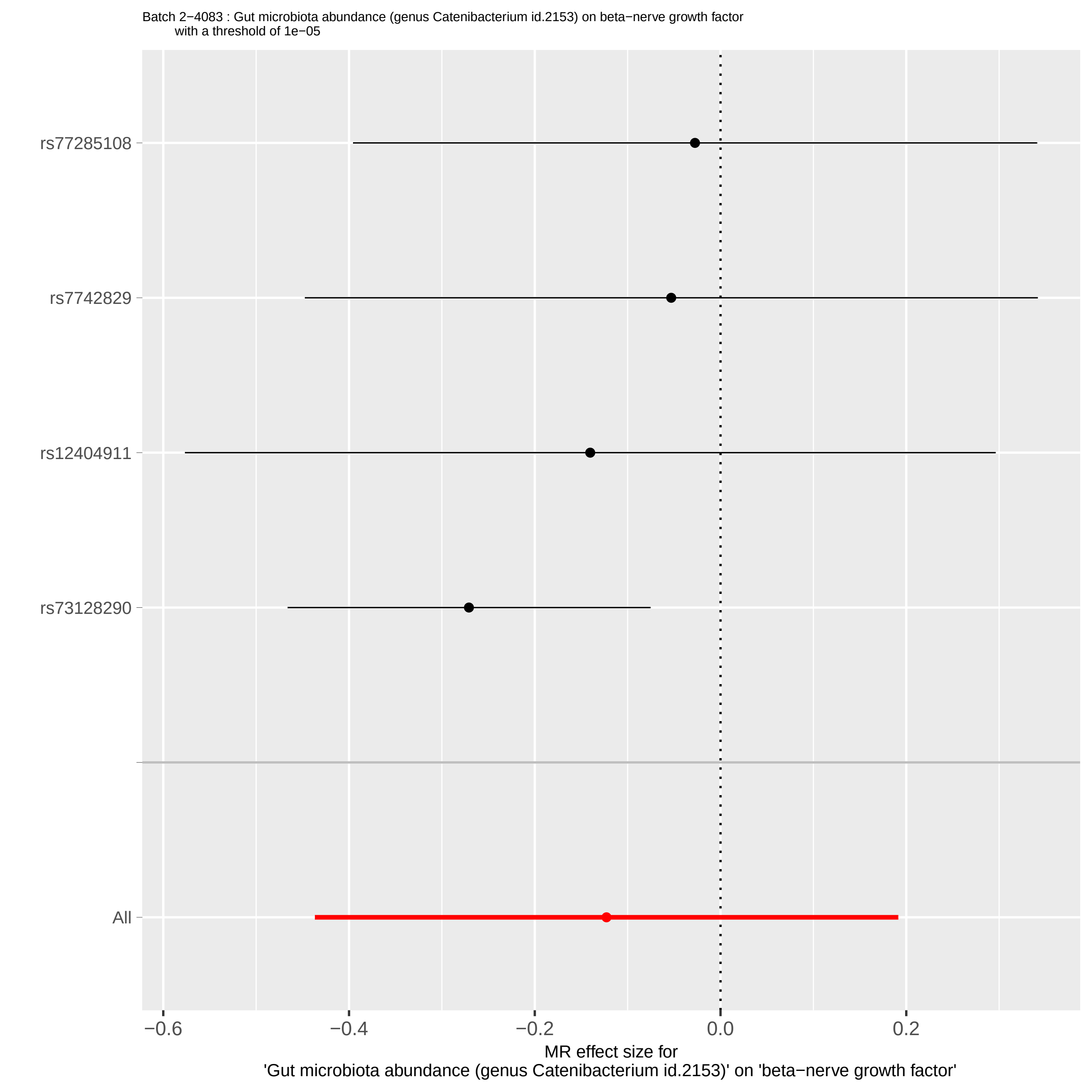

Batch 2−4083 : Gut microbiota abundance (genus Catenibacterium id.2153) on beta−nerve growth factor
with a threshold of 1e−05
rs77285108
rs7742829
rs12404911
rs73128290
All
−0.6
−0.4
−0.2
0.0
0.2
MR effect size for
'Gut microbiota abundance (genus Catenibacterium id.2153)' on 'beta−nerve growth factor'

Batch 2−4084 : Gut microbiota abundance (genus Parasutterella id.2892) on beta−nerve growth factor
with a threshold of 1e−05

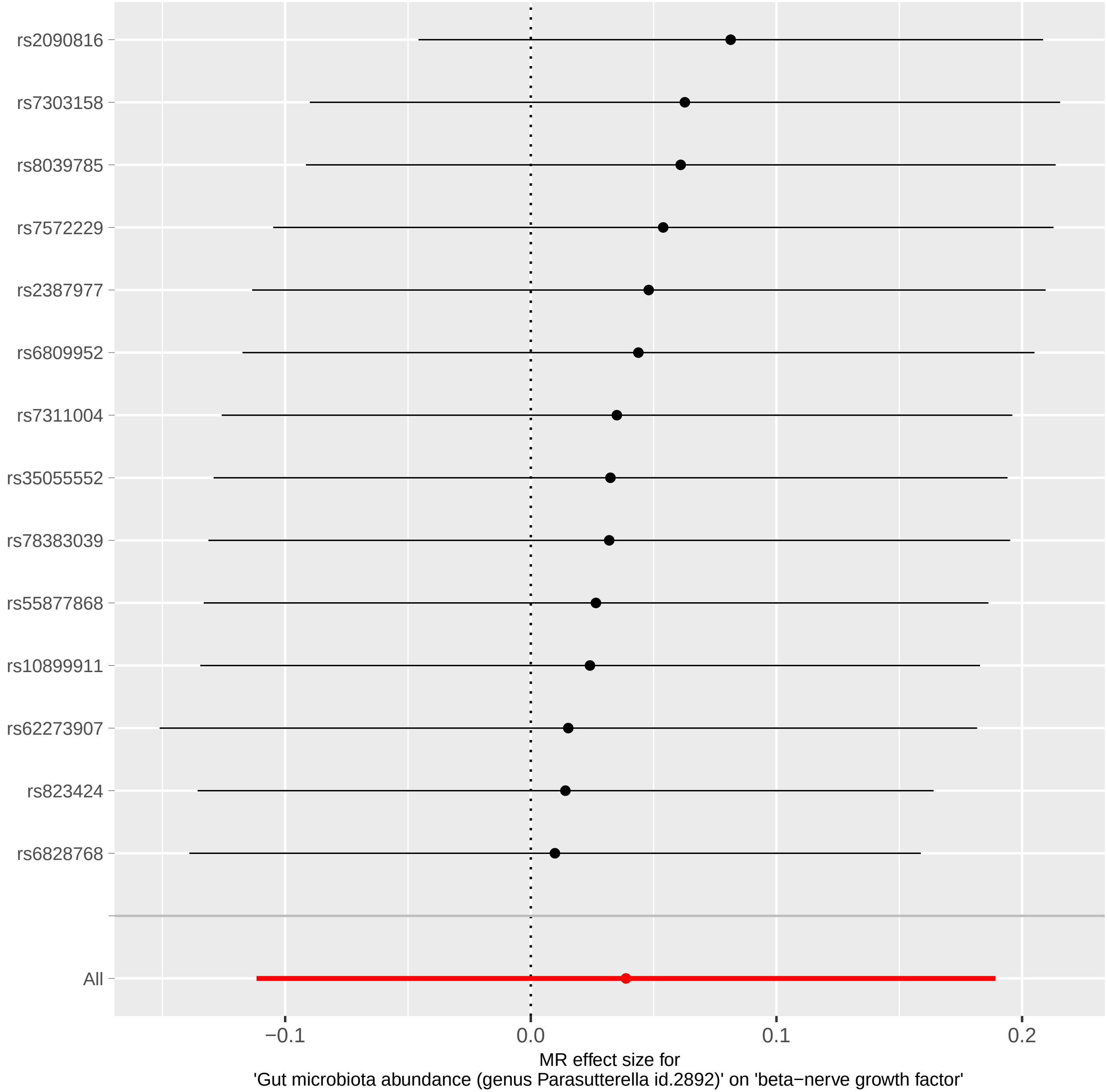

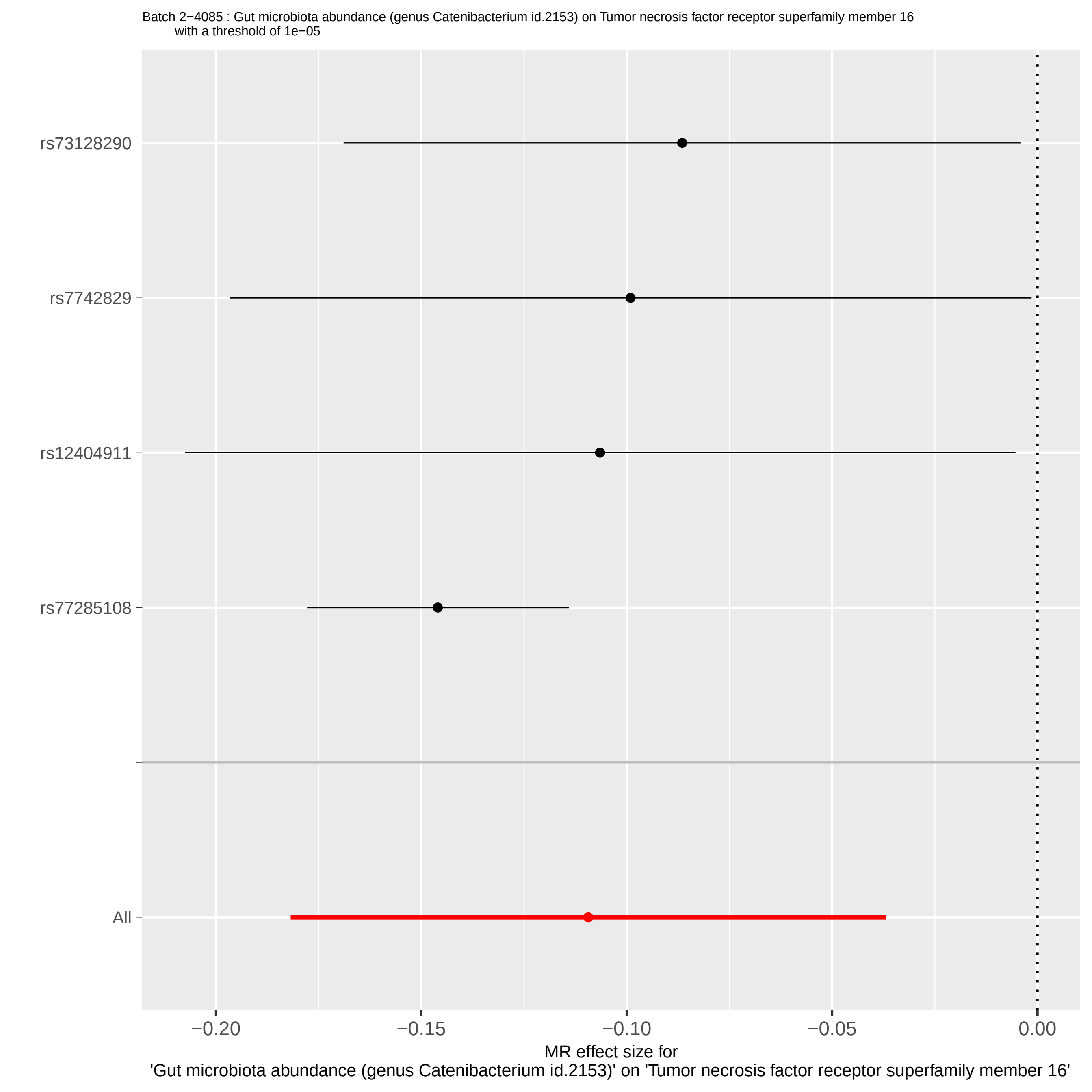
Batch 2−4085 : Gut microbiota abundance (genus Catenibacterium id.2153) on Tumor necrosis factor receptor superfamily member 16
with a threshold of 1e−05
rs73128290
rs7742829
rs12404911
rs77285108
All
−0.20
−0.15
−0.10
−0.05
0.00
MR effect size for
'Gut microbiota abundance (genus Catenibacterium id.2153)' on 'Tumor necrosis factor receptor superfamily member 16'

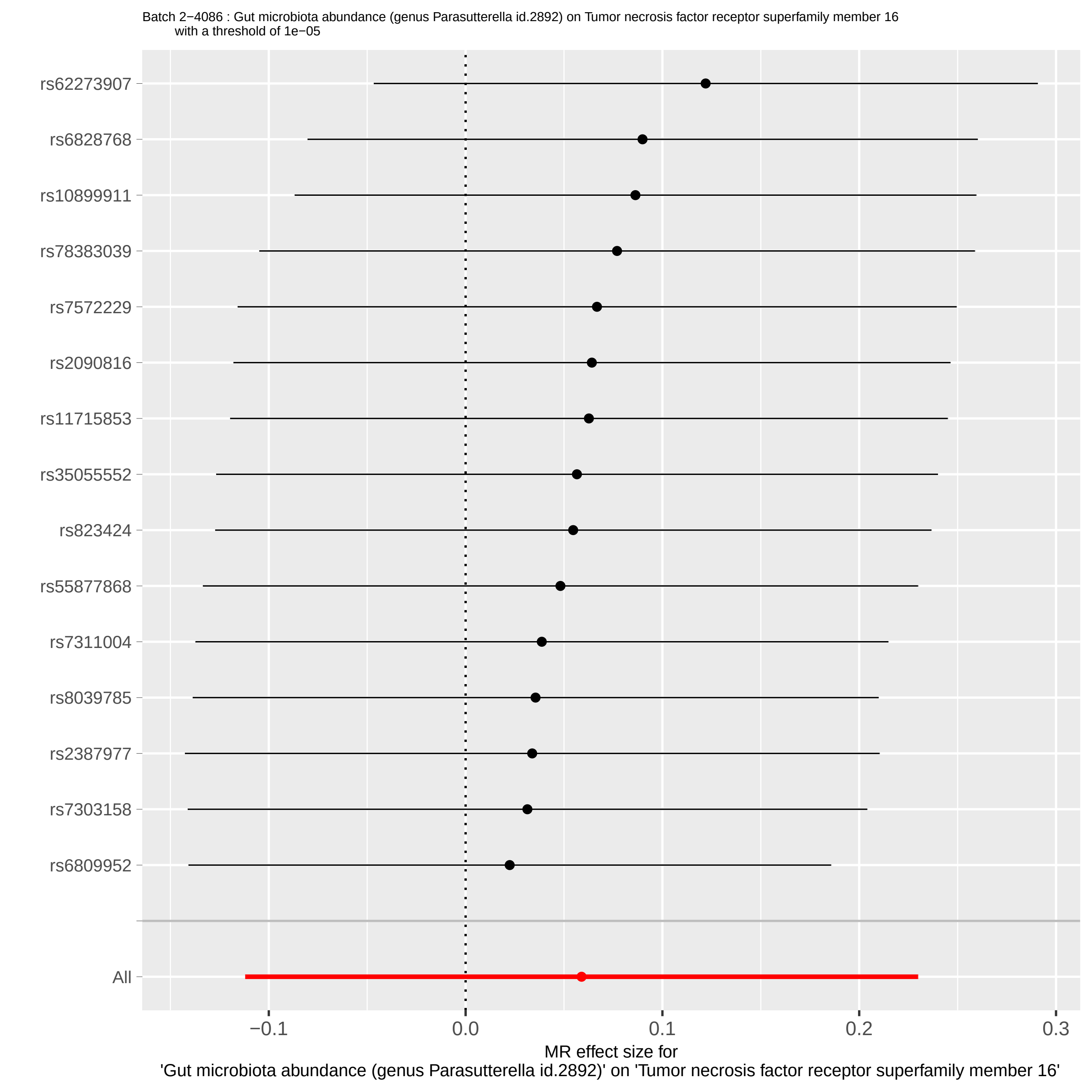
Batch 2−4086 : Gut microbiota abundance (genus Parasutterella id.2892) on Tumor necrosis factor receptor superfamily member 16
with a threshold of 1e−05
rs62273907
rs6828768
rs10899911
rs78383039
rs7572229
rs2090816
rs11715853
rs35055552
rs823424
rs55877868
rs7311004
rs8039785
rs2387977
rs7303158
rs6809952
All
−0.1
0.0
0.1
0.2
0.3
MR effect size for
'Gut microbiota abundance (genus Parasutterella id.2892)' on 'Tumor necrosis factor receptor superfamily member 16'

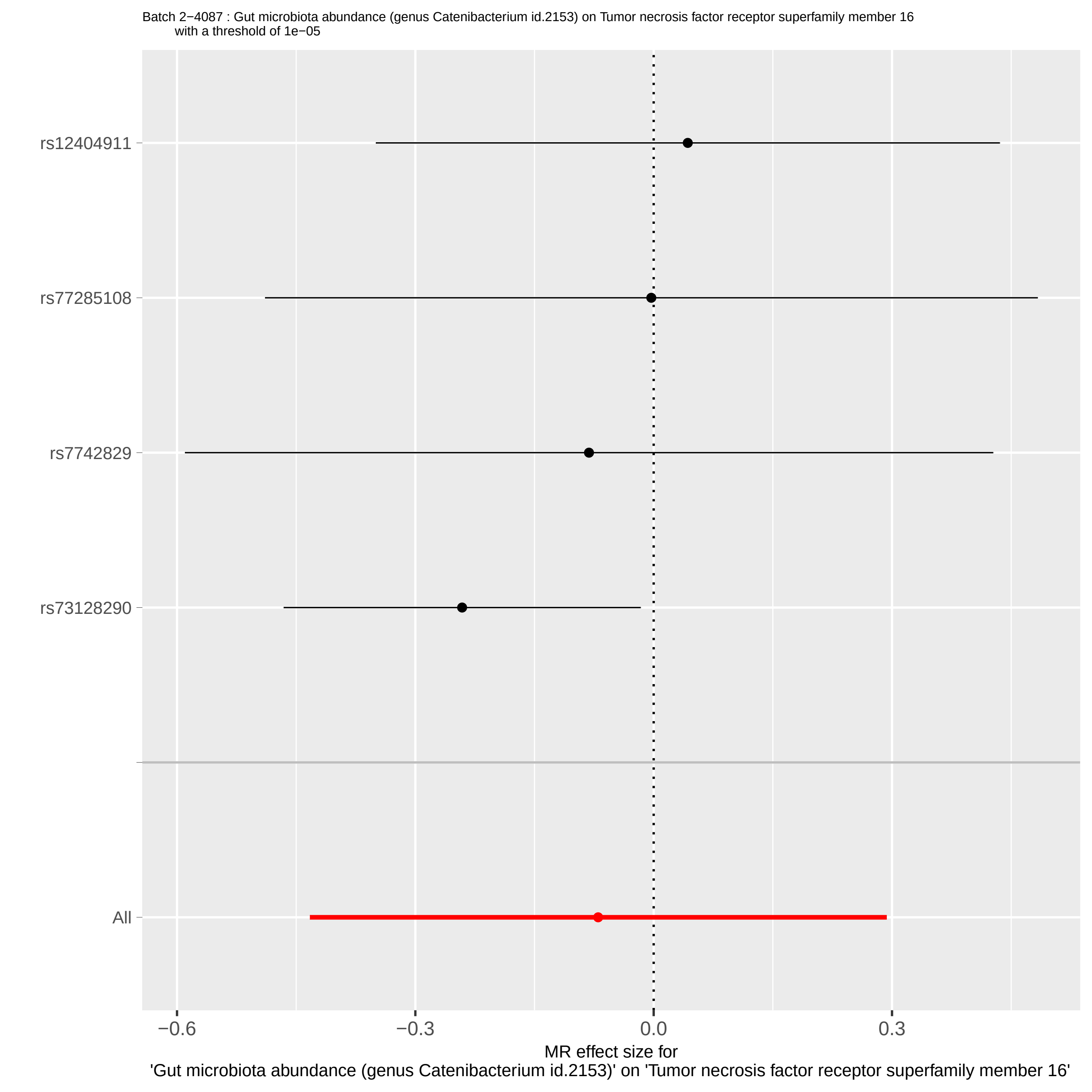
Batch 2−4087 : Gut microbiota abundance (genus Catenibacterium id.2153) on Tumor necrosis factor receptor superfamily member 16
with a threshold of 1e−05
rs12404911
rs77285108
rs7742829
rs73128290
All
−0.6
−0.3
0.0
0.3
MR effect size for
'Gut microbiota abundance (genus Catenibacterium id.2153)' on 'Tumor necrosis factor receptor superfamily member 16'

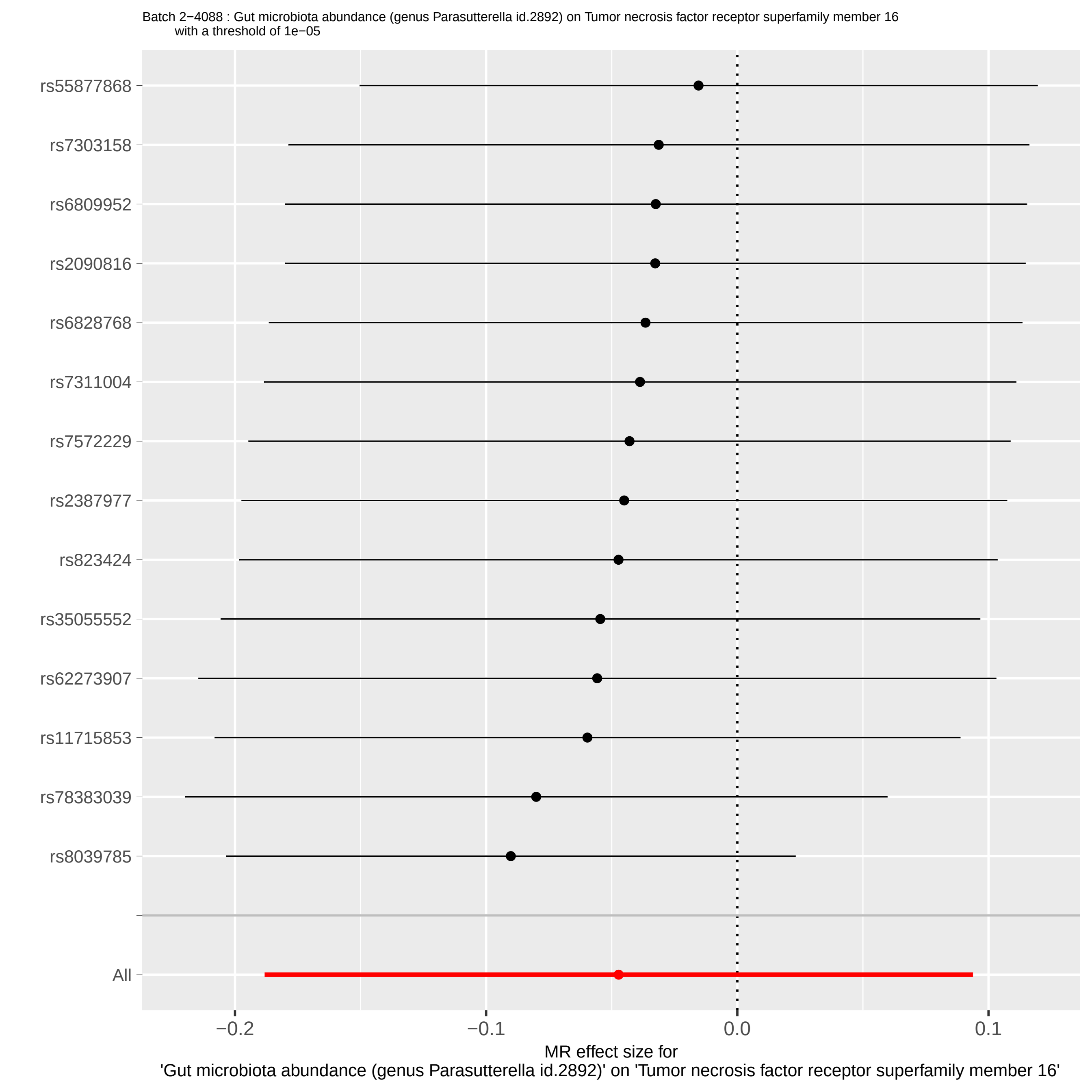
Batch 2−4088 : Gut microbiota abundance (genus Parasutterella id.2892) on Tumor necrosis factor receptor superfamily member 16
with a threshold of 1e−05
rs55877868
rs7303158
rs6809952
rs2090816
rs6828768
rs7311004
rs7572229
rs2387977
rs823424
rs35055552
rs62273907
rs11715853
rs78383039
rs8039785
All
−0.2
−0.1
0.0
0.1
MR effect size for
'Gut microbiota abundance (genus Parasutterella id.2892)' on 'Tumor necrosis factor receptor superfamily member 16'

Batch 2−4089 : Gut microbiota abundance (genus Catenibacterium id.2153) on Neugrin
with a threshold of 1e−05

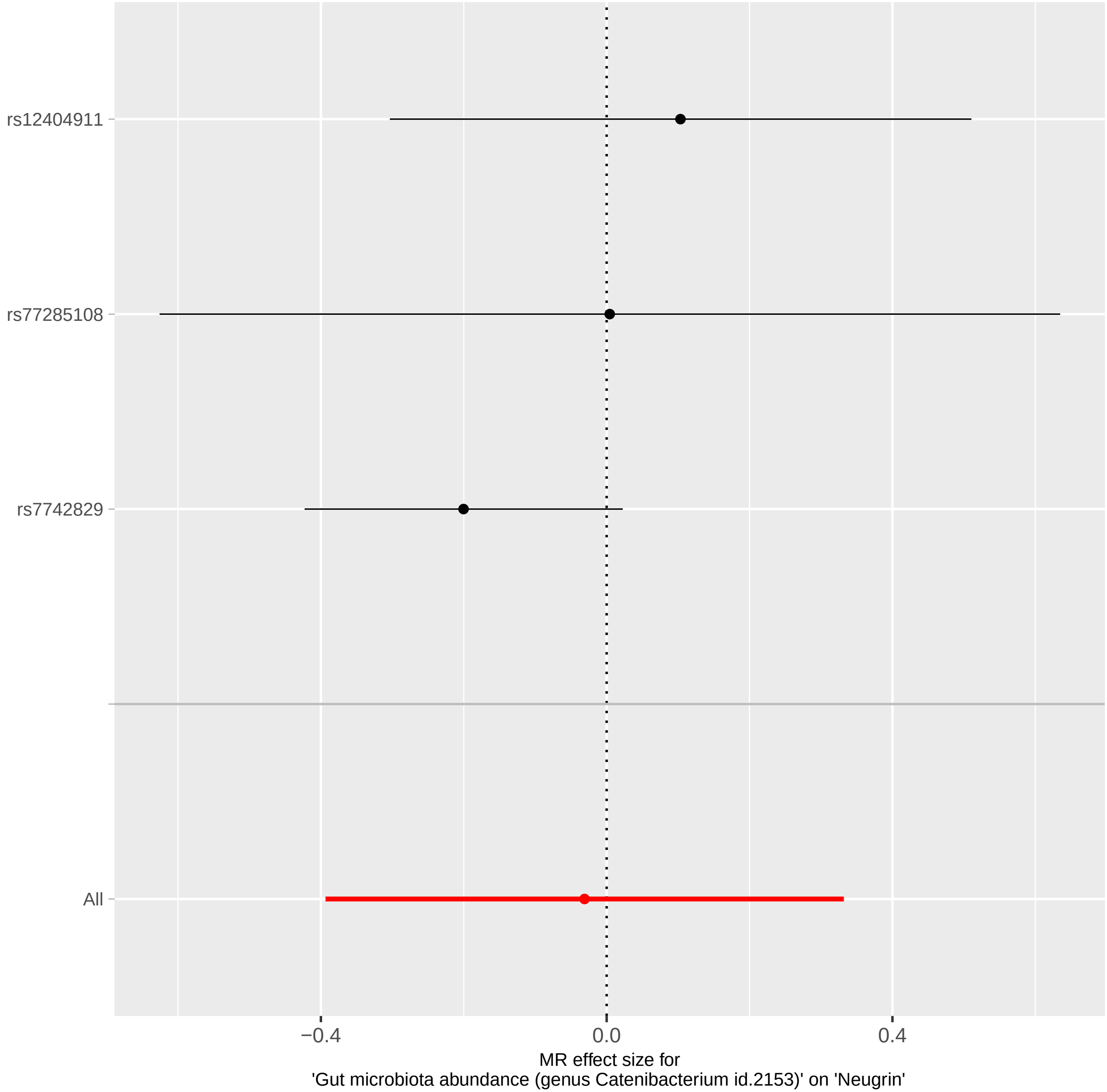

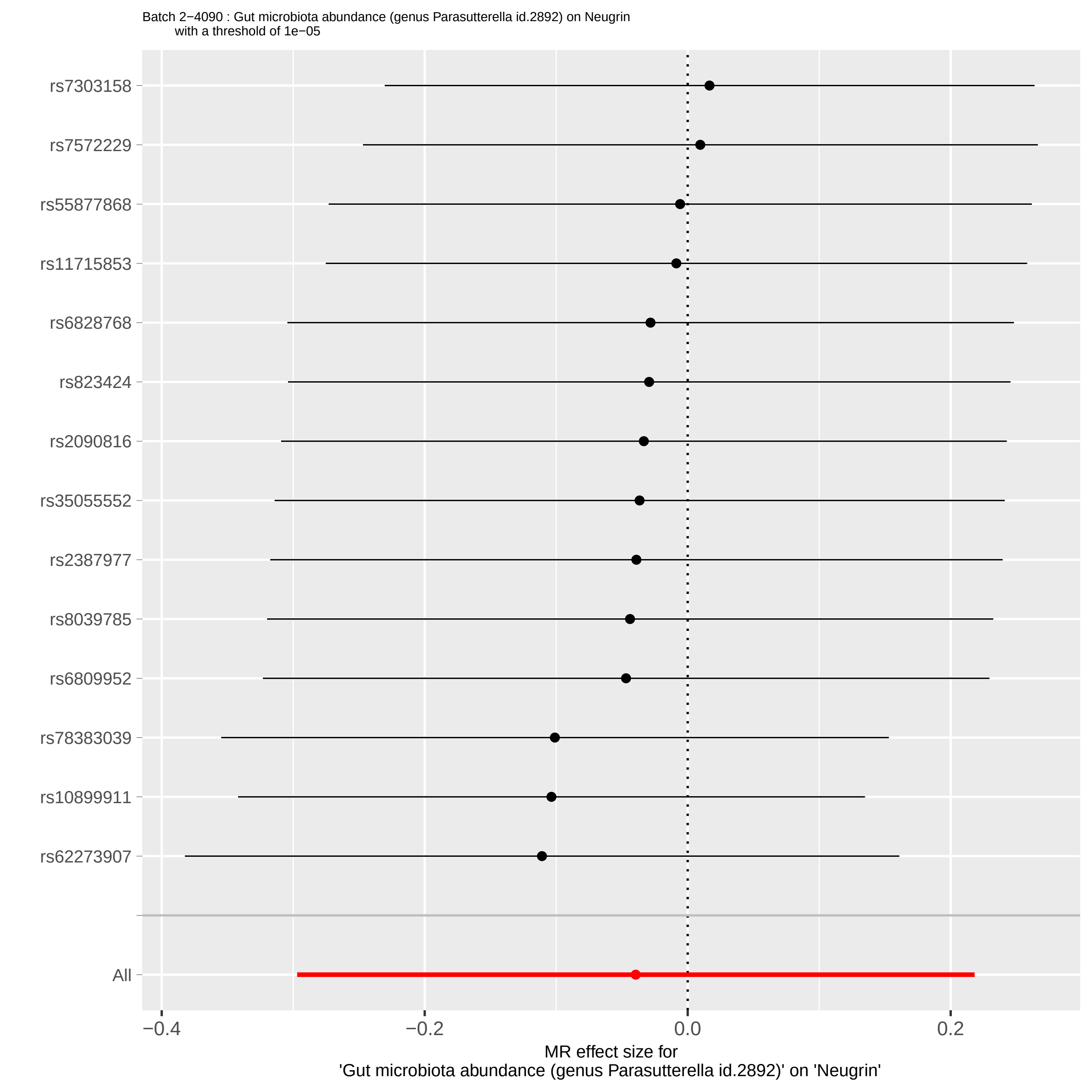
Batch 2−4090 : Gut microbiota abundance (genus Parasutterella id.2892) on Neugrin
with a threshold of 1e−05
rs7303158
rs7572229
rs55877868
rs11715853
rs6828768
rs823424
rs2090816
rs35055552
rs2387977
rs8039785
rs6809952
rs78383039
rs10899911
rs62273907
All
−0.4
−0.2
0.0
0.2
MR effect size for
'Gut microbiota abundance (genus Parasutterella id.2892)' on 'Neugrin'

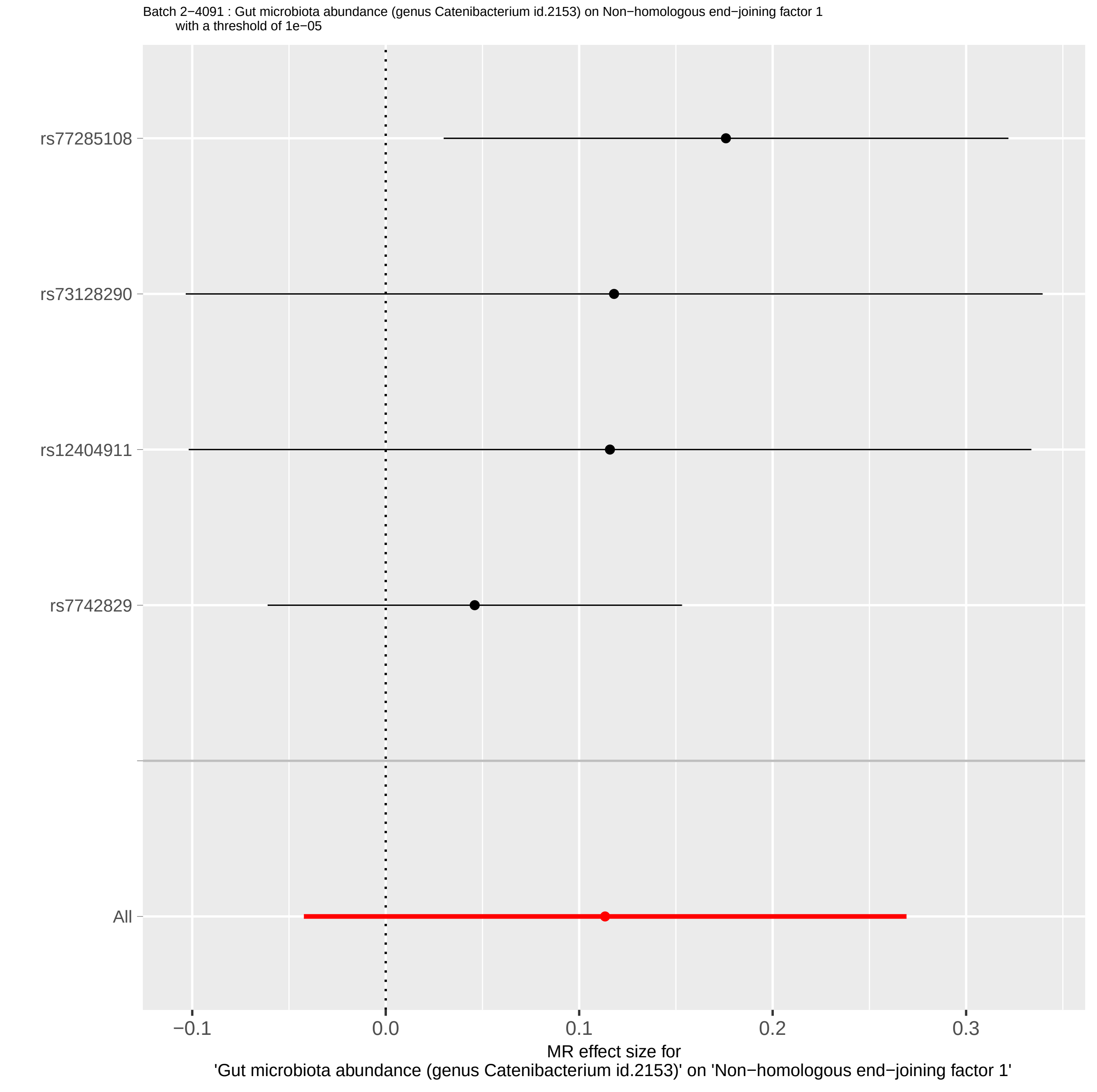

Batch 2−4091 : Gut microbiota abundance (genus Catenibacterium id.2153) on Non−homologous end−joining factor 1
with a threshold of 1e−05
rs77285108
rs73128290
rs12404911
rs7742829
All
−0.1
0.0
0.1
0.2
0.3
MR effect size for
'Gut microbiota abundance (genus Catenibacterium id.2153)' on 'Non−homologous end−joining factor 1'

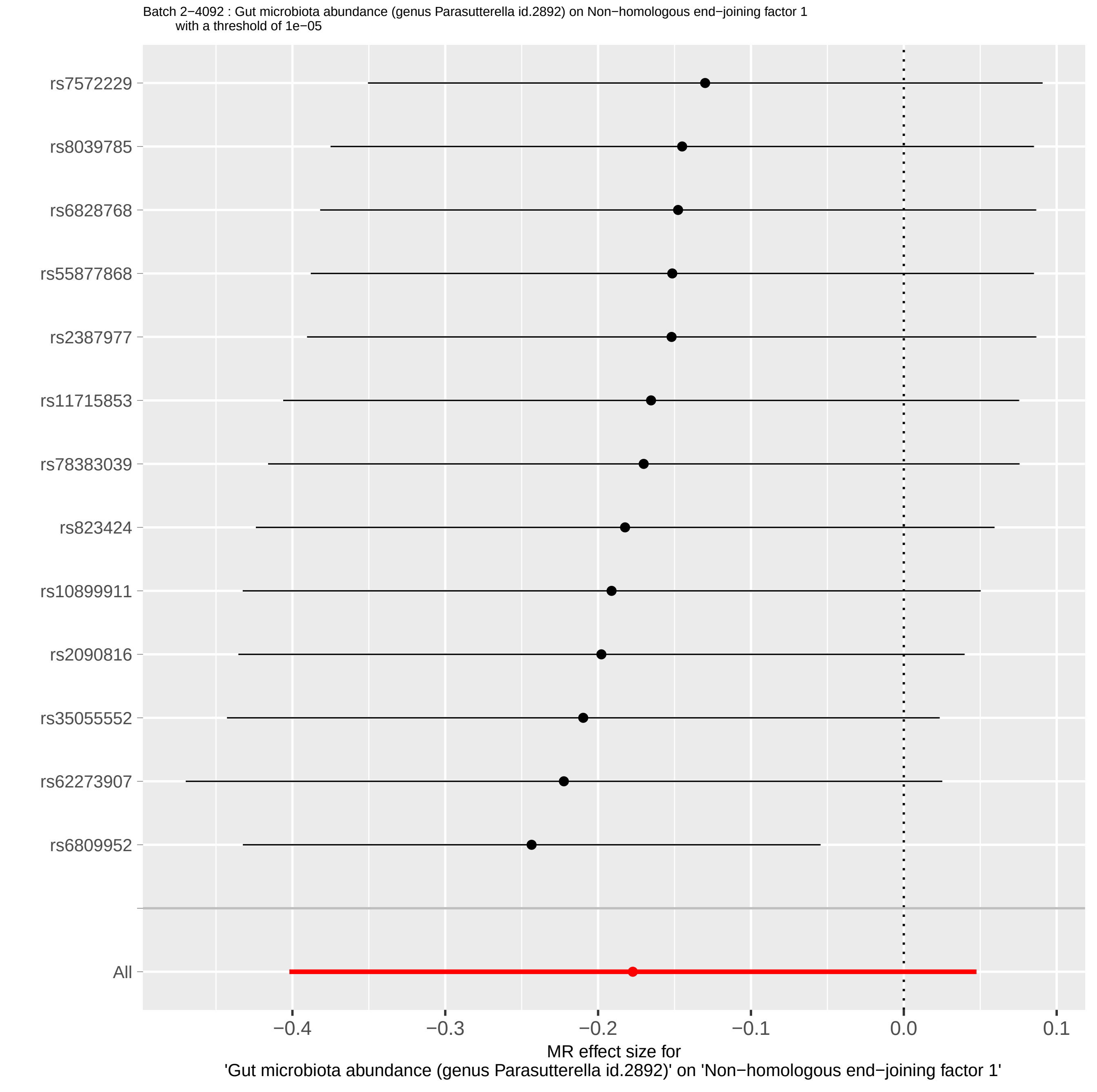

Batch 2−4092 : Gut microbiota abundance (genus Parasutterella id.2892) on Non−homologous end−joining factor 1
with a threshold of 1e−05
rs7572229
rs8039785
rs6828768
rs55877868
rs2387977
rs11715853
rs78383039
rs823424
rs10899911
rs2090816
rs35055552
rs62273907
rs6809952
All
−0.4
−0.3
−0.2
−0.1
0.0
0.1
MR effect size for
'Gut microbiota abundance (genus Parasutterella id.2892)' on 'Non−homologous end−joining factor 1'

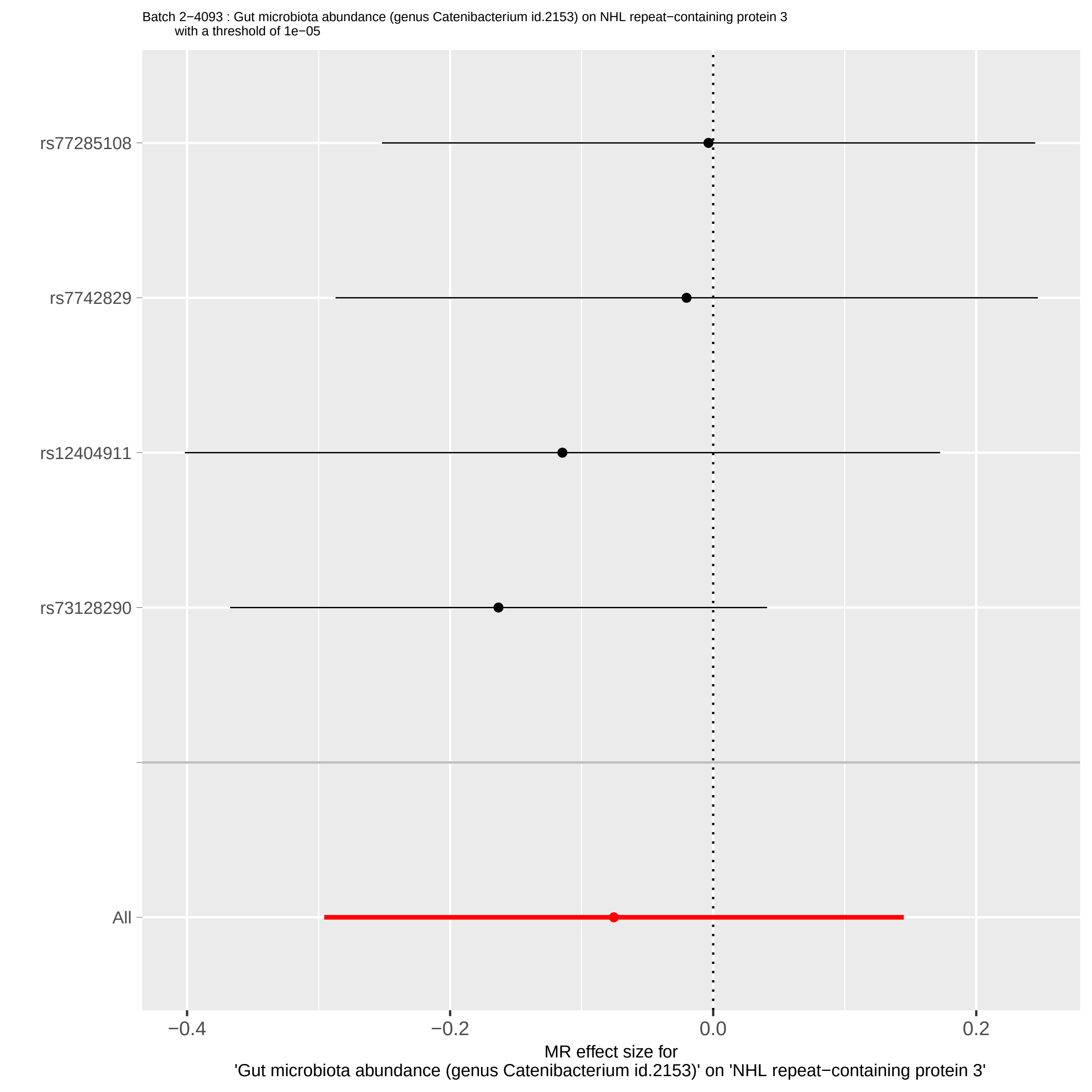
Batch 2−4093 : Gut microbiota abundance (genus Catenibacterium id.2153) on NHL repeat−containing protein 3
with a threshold of 1e−05
rs77285108
rs7742829
rs12404911
rs73128290
All
−0.4
−0.2
0.0
0.2
MR effect size for
'Gut microbiota abundance (genus Catenibacterium id.2153)' on 'NHL repeat−containing protein 3'

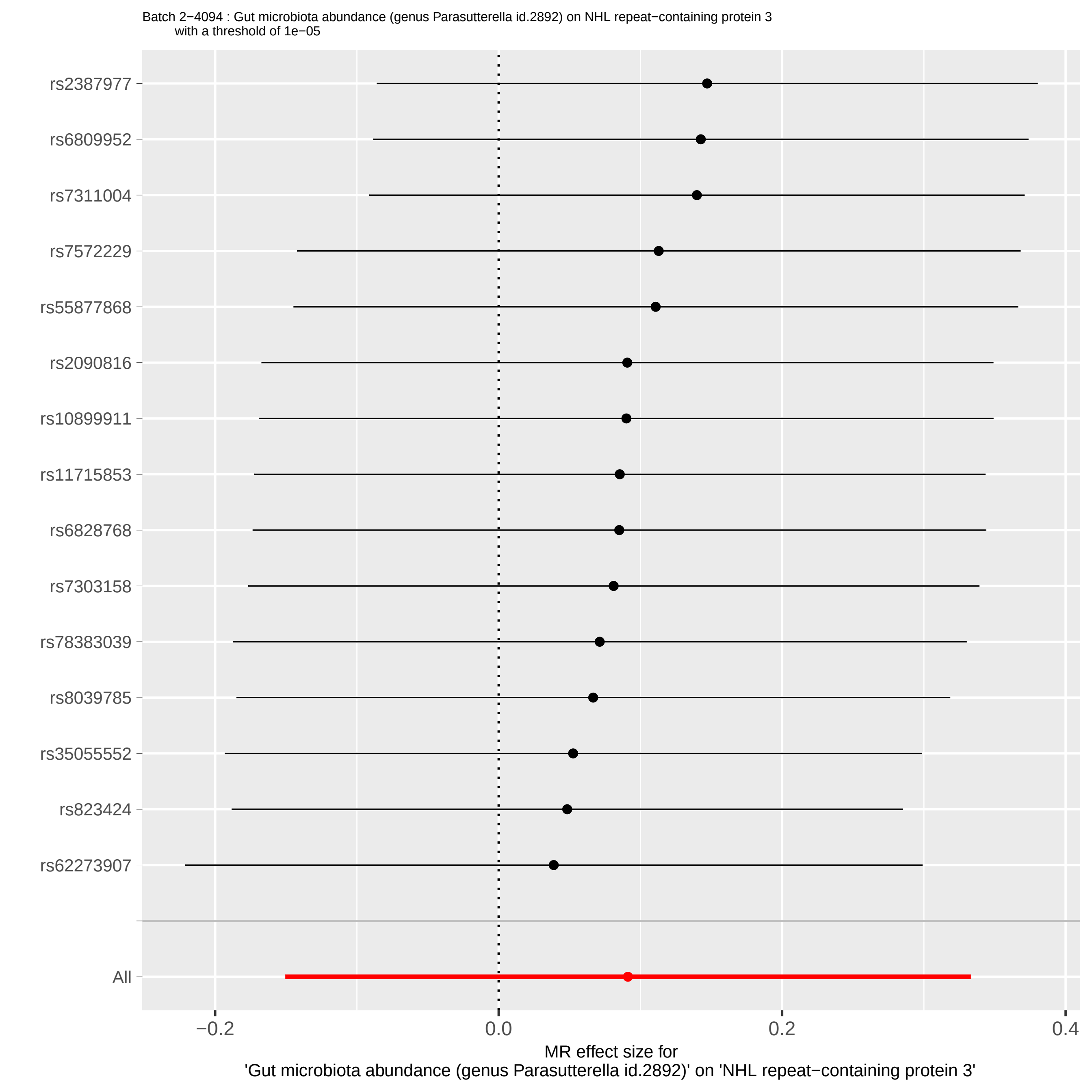
Batch 2−4094 : Gut microbiota abundance (genus Parasutterella id.2892) on NHL repeat−containing protein 3
with a threshold of 1e−05
rs2387977
rs6809952
rs7311004
rs7572229
rs55877868
rs2090816
rs10899911
rs11715853
rs6828768
rs7303158
rs78383039
rs8039785
rs35055552
rs823424
rs62273907
All
−0.2
0.0
0.2
0.4
MR effect size for
'Gut microbiota abundance (genus Parasutterella id.2892)' on 'NHL repeat−containing protein 3'

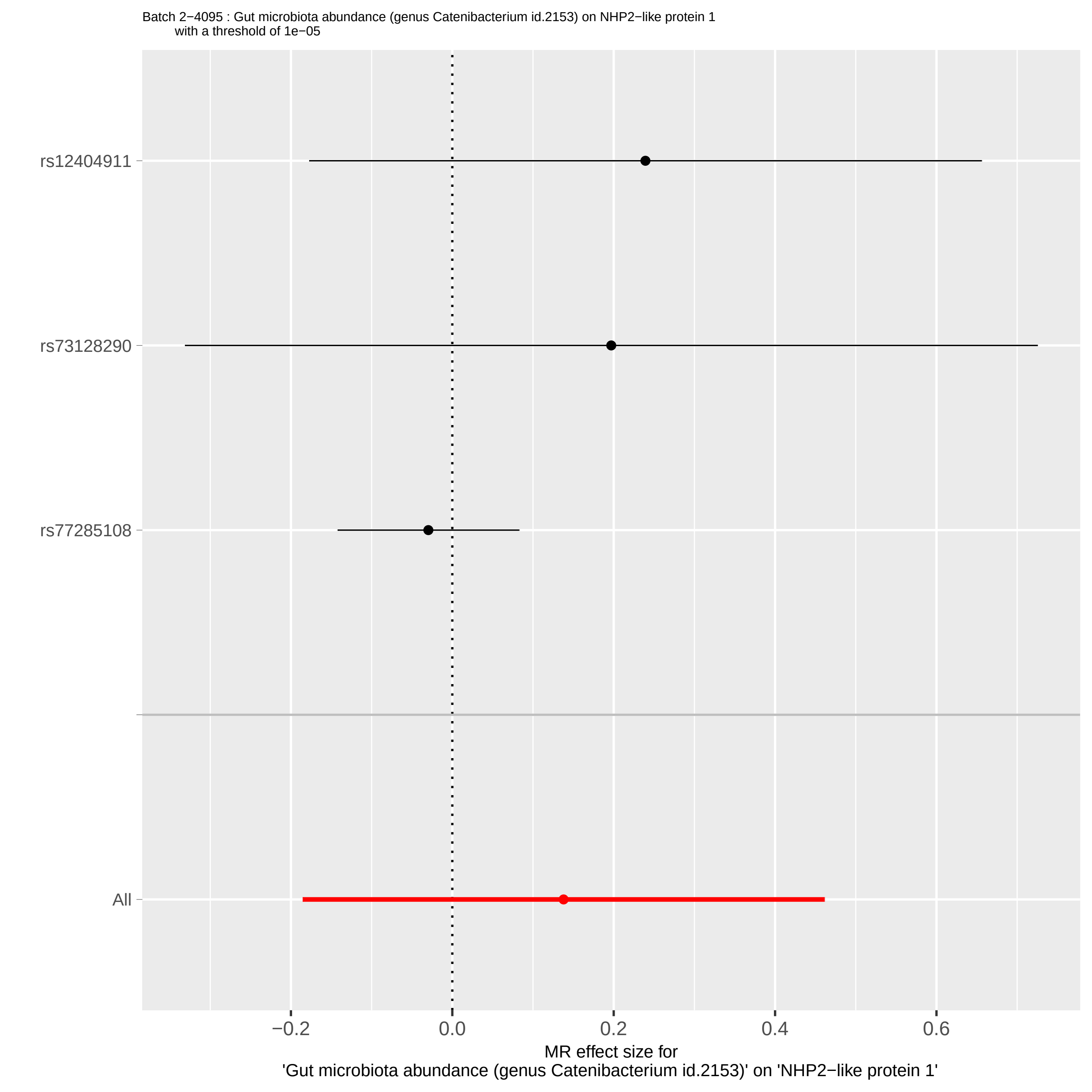

Batch 2−4095 : Gut microbiota abundance (genus Catenibacterium id.2153) on NHP2−like protein 1
with a threshold of 1e−05
rs12404911
rs73128290
rs77285108
All
−0.2
0.0
0.2
0.4
0.6
MR effect size for
'Gut microbiota abundance (genus Catenibacterium id.2153)' on 'NHP2−like protein 1'

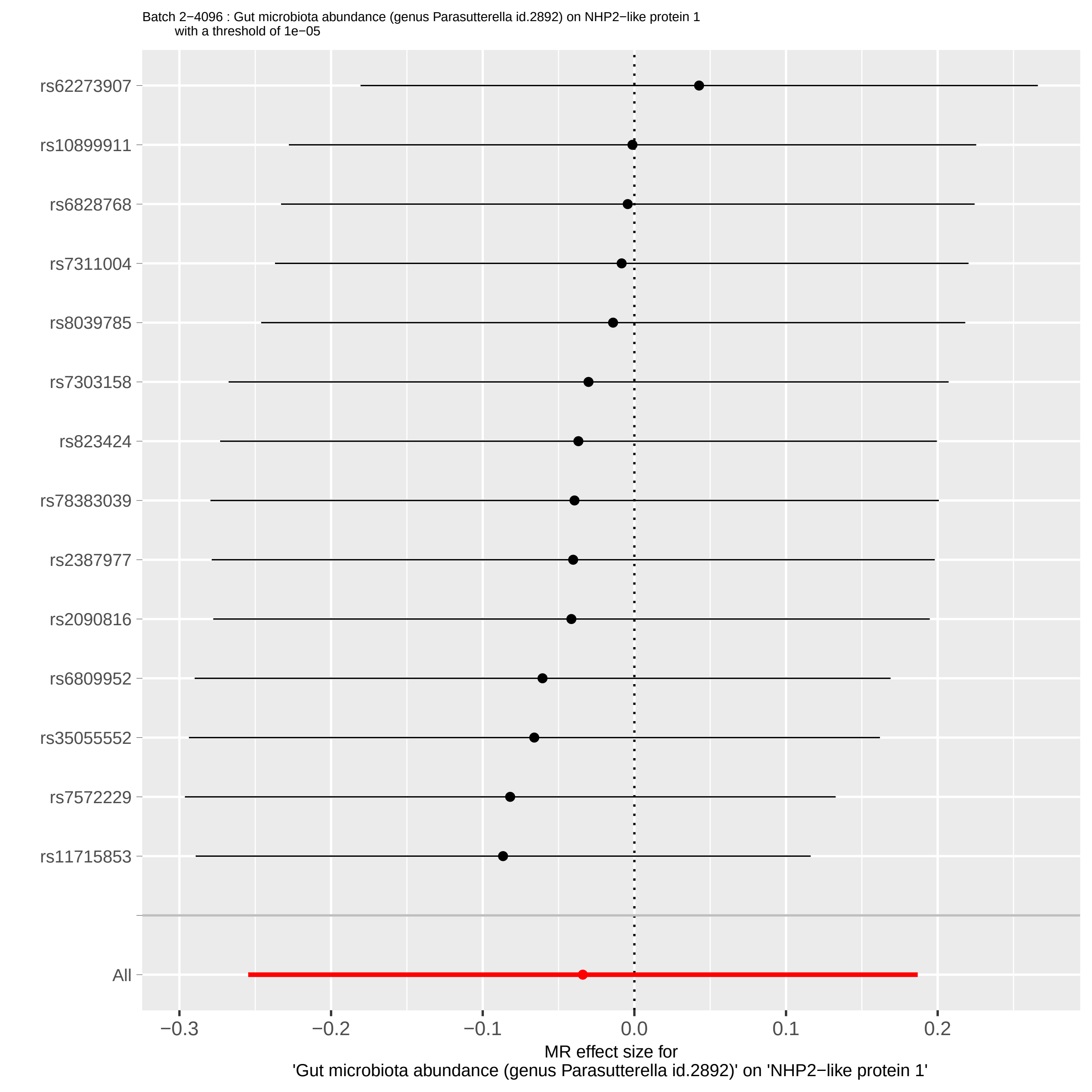

Batch 2−4096 : Gut microbiota abundance (genus Parasutterella id.2892) on NHP2−like protein 1
with a threshold of 1e−05
rs62273907
rs10899911
rs6828768
rs7311004
rs8039785
rs7303158
rs823424
rs78383039
rs2387977
rs2090816
rs6809952
rs35055552
rs7572229
rs11715853
All
−0.3
−0.2
−0.1
0.0
0.1
0.2
MR effect size for
'Gut microbiota abundance (genus Parasutterella id.2892)' on 'NHP2−like protein 1'

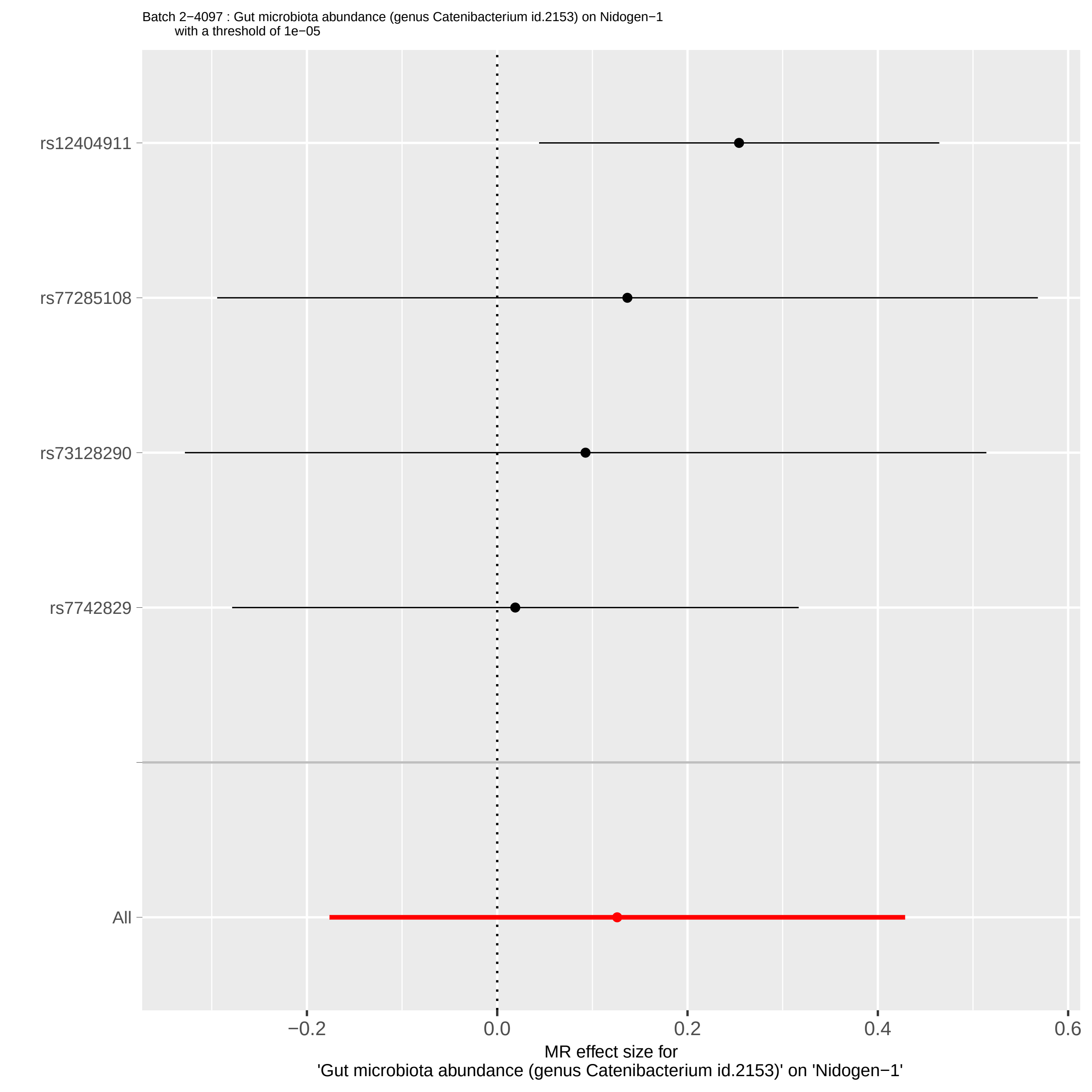
Batch 2−4097 : Gut microbiota abundance (genus Catenibacterium id.2153) on Nidogen−1
with a threshold of 1e−05
rs12404911
rs77285108
rs73128290
rs7742829
All
−0.2
0.0
0.2
0.4
0.6
MR effect size for
'Gut microbiota abundance (genus Catenibacterium id.2153)' on 'Nidogen−1'

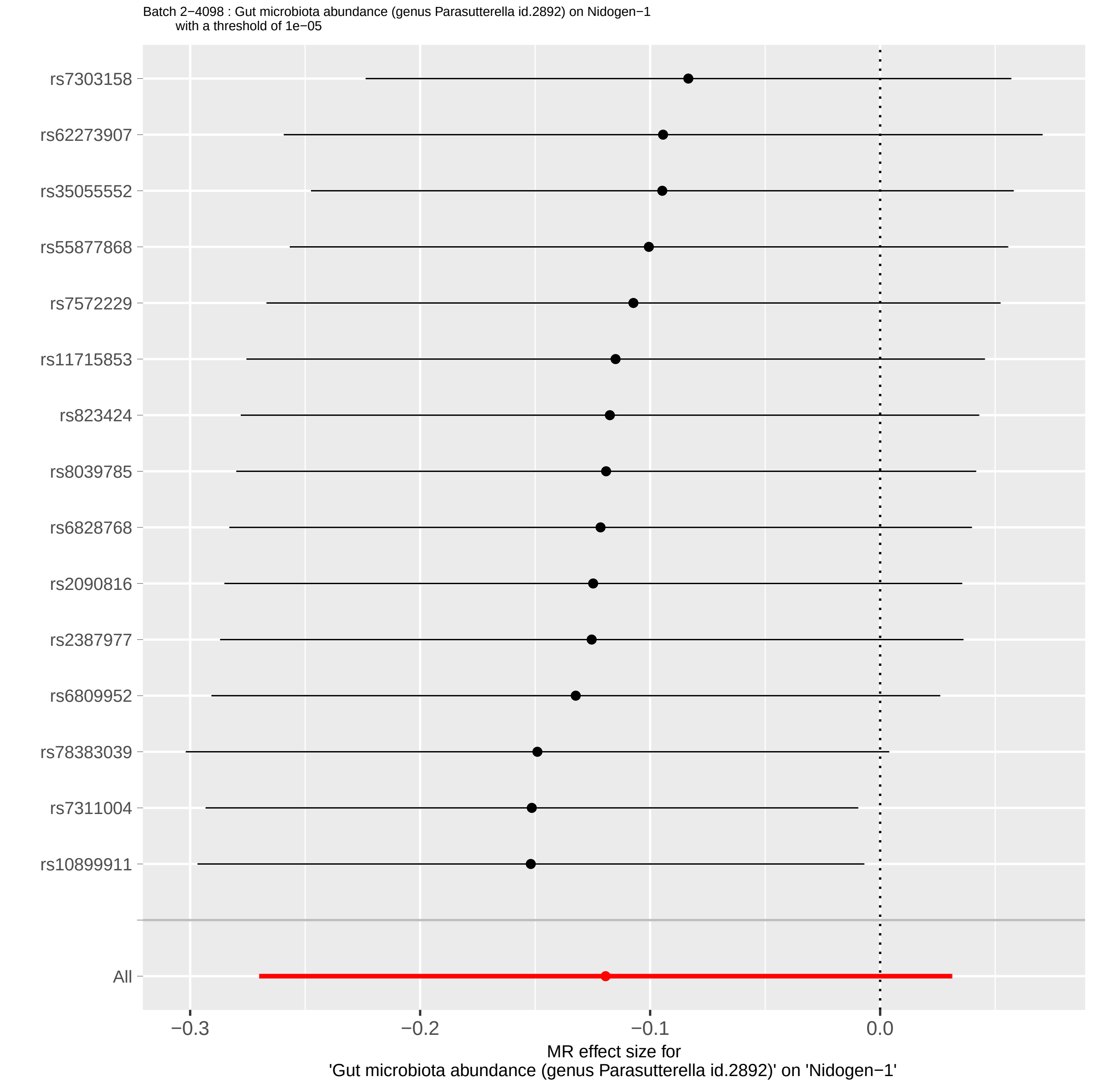

Batch 2−4098 : Gut microbiota abundance (genus Parasutterella id.2892) on Nidogen−1
with a threshold of 1e−05
rs7303158
rs62273907
rs35055552
rs55877868
rs7572229
rs11715853
rs823424
rs8039785
rs6828768
rs2090816
rs2387977
rs6809952
rs78383039
rs7311004
rs10899911
All
−0.3
−0.2
−0.1
0.0
MR effect size for
'Gut microbiota abundance (genus Parasutterella id.2892)' on 'Nidogen−1'

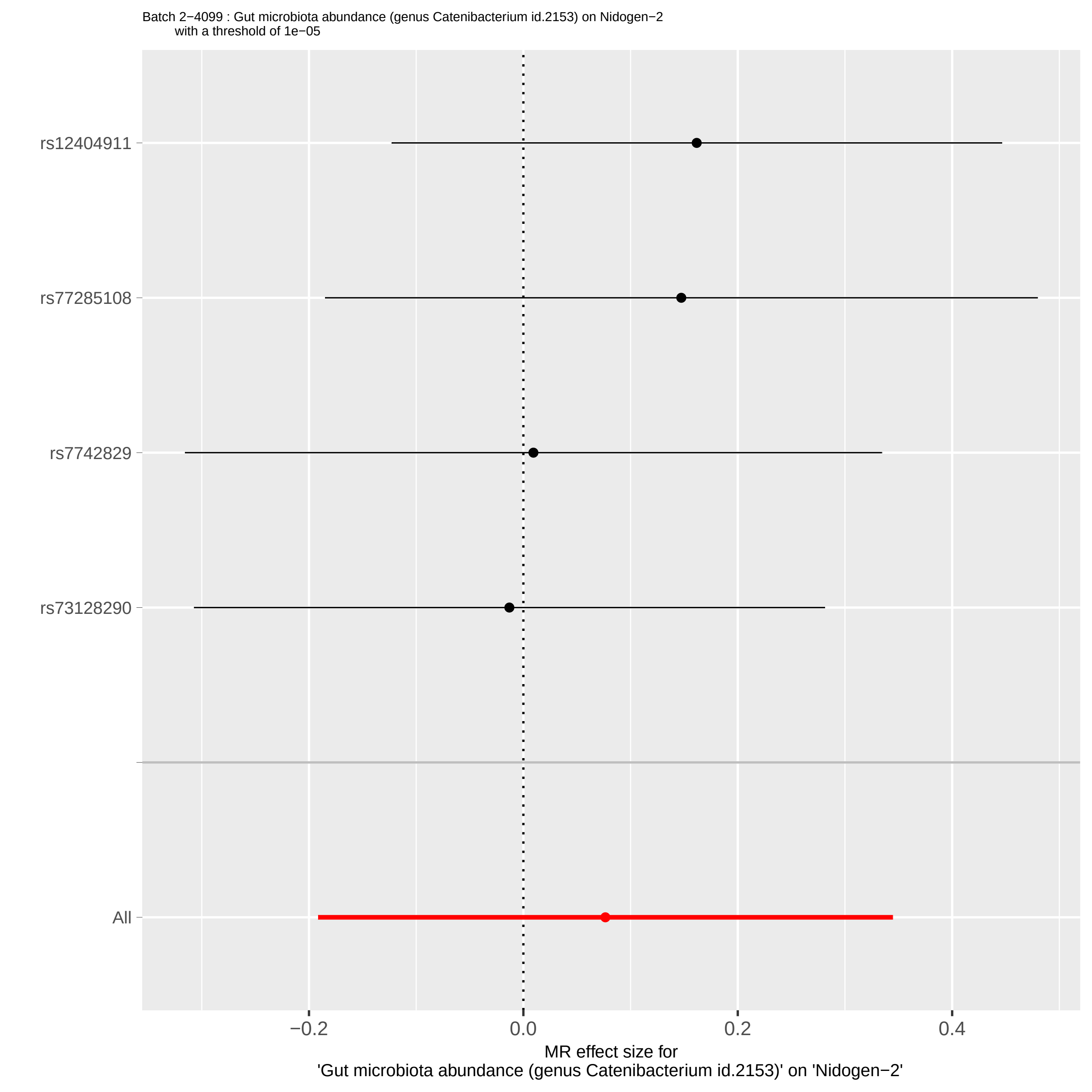

Batch 2−4099 : Gut microbiota abundance (genus Catenibacterium id.2153) on Nidogen−2
with a threshold of 1e−05
rs12404911
rs77285108
rs7742829
rs73128290
All
−0.2
0.0
0.2
0.4
MR effect size for
'Gut microbiota abundance (genus Catenibacterium id.2153)' on 'Nidogen−2'

Batch 2−4100 : Gut microbiota abundance (genus Parasutterella id.2892) on Nidogen−2
with a threshold of 1e−05

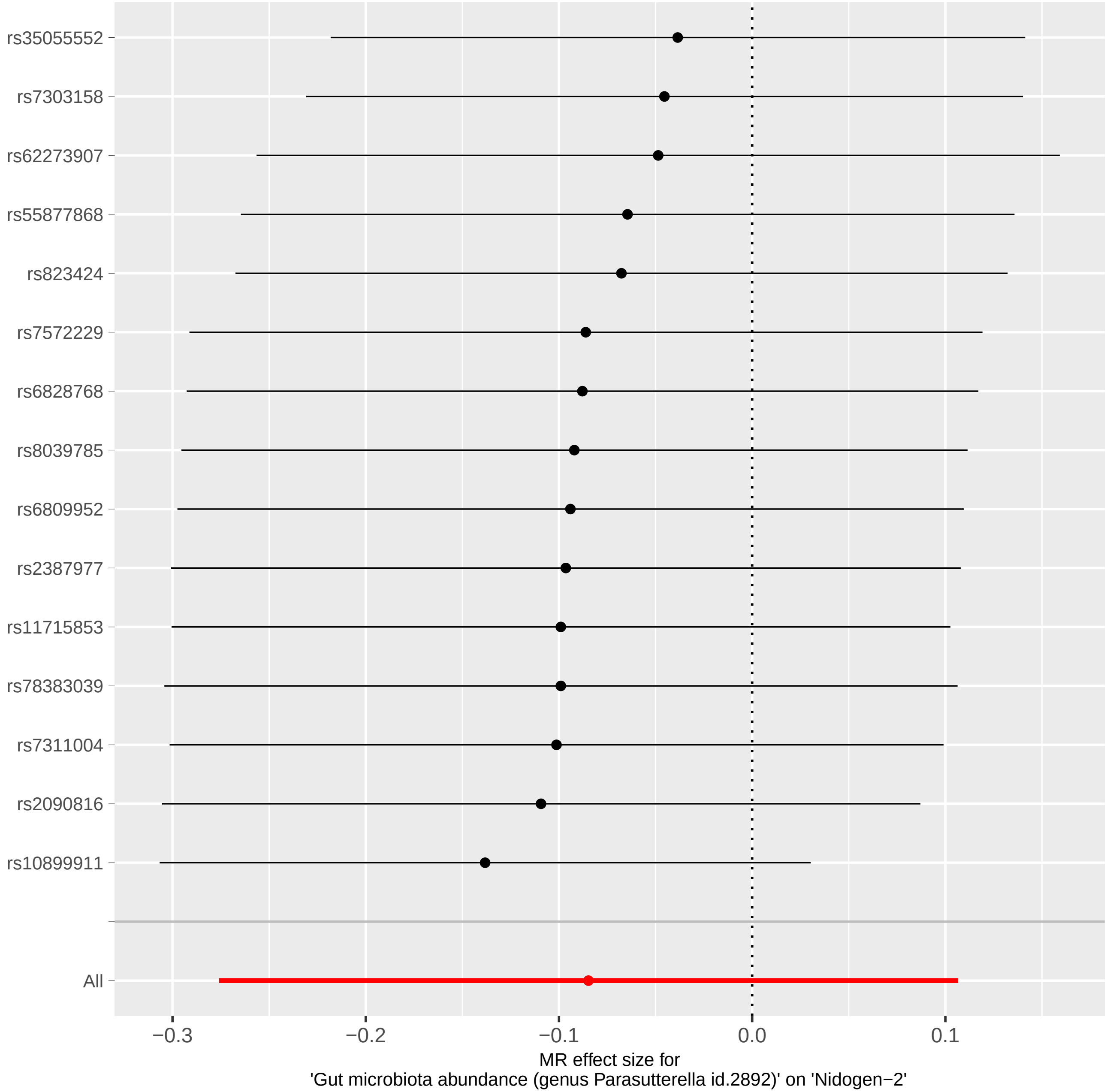

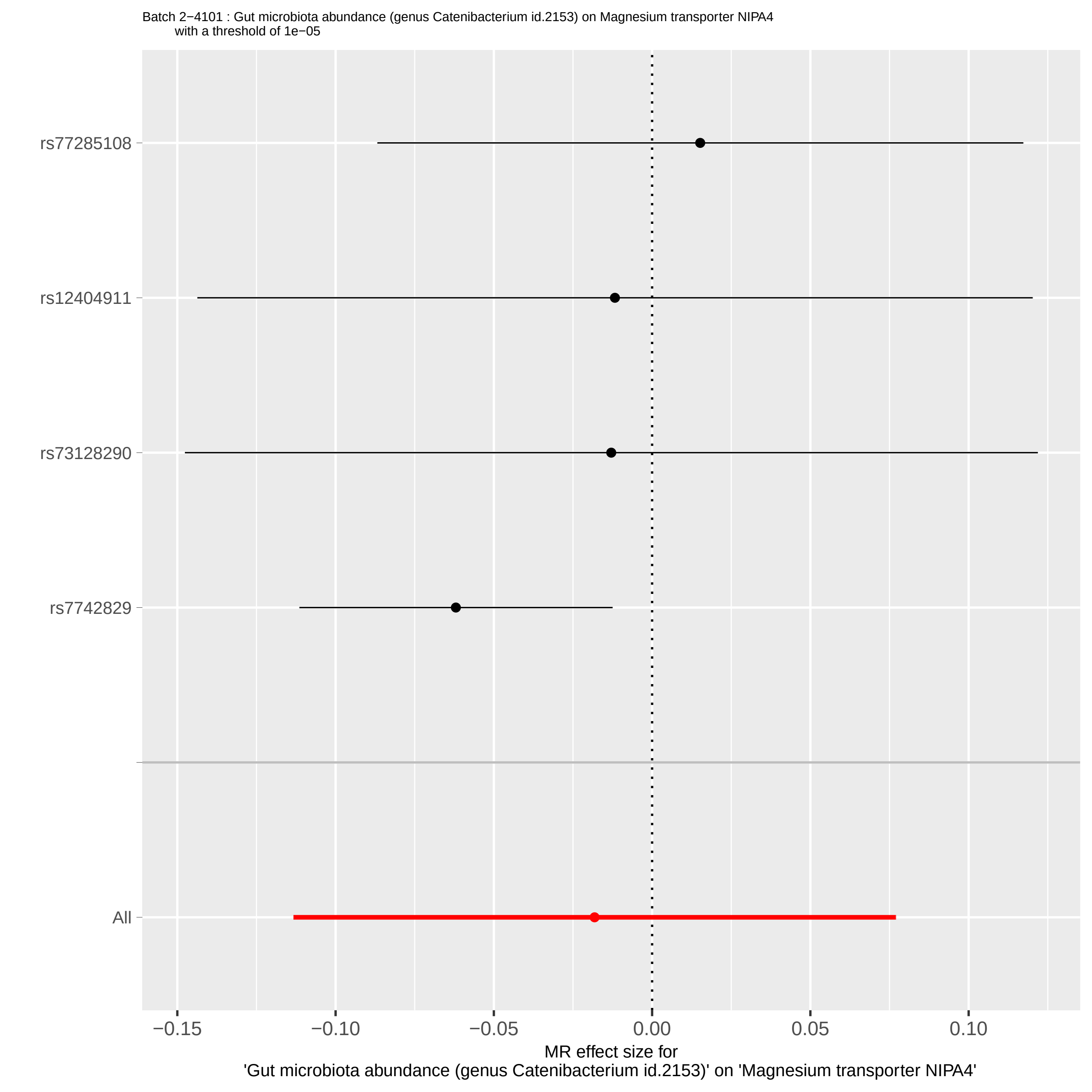

Batch 2−4101 : Gut microbiota abundance (genus Catenibacterium id.2153) on Magnesium transporter NIPA4
with a threshold of 1e−05
rs77285108
rs12404911
rs73128290
rs7742829
All
−0.15
−0.10
−0.05
0.00
0.05
0.10
MR effect size for
'Gut microbiota abundance (genus Catenibacterium id.2153)' on 'Magnesium transporter NIPA4'

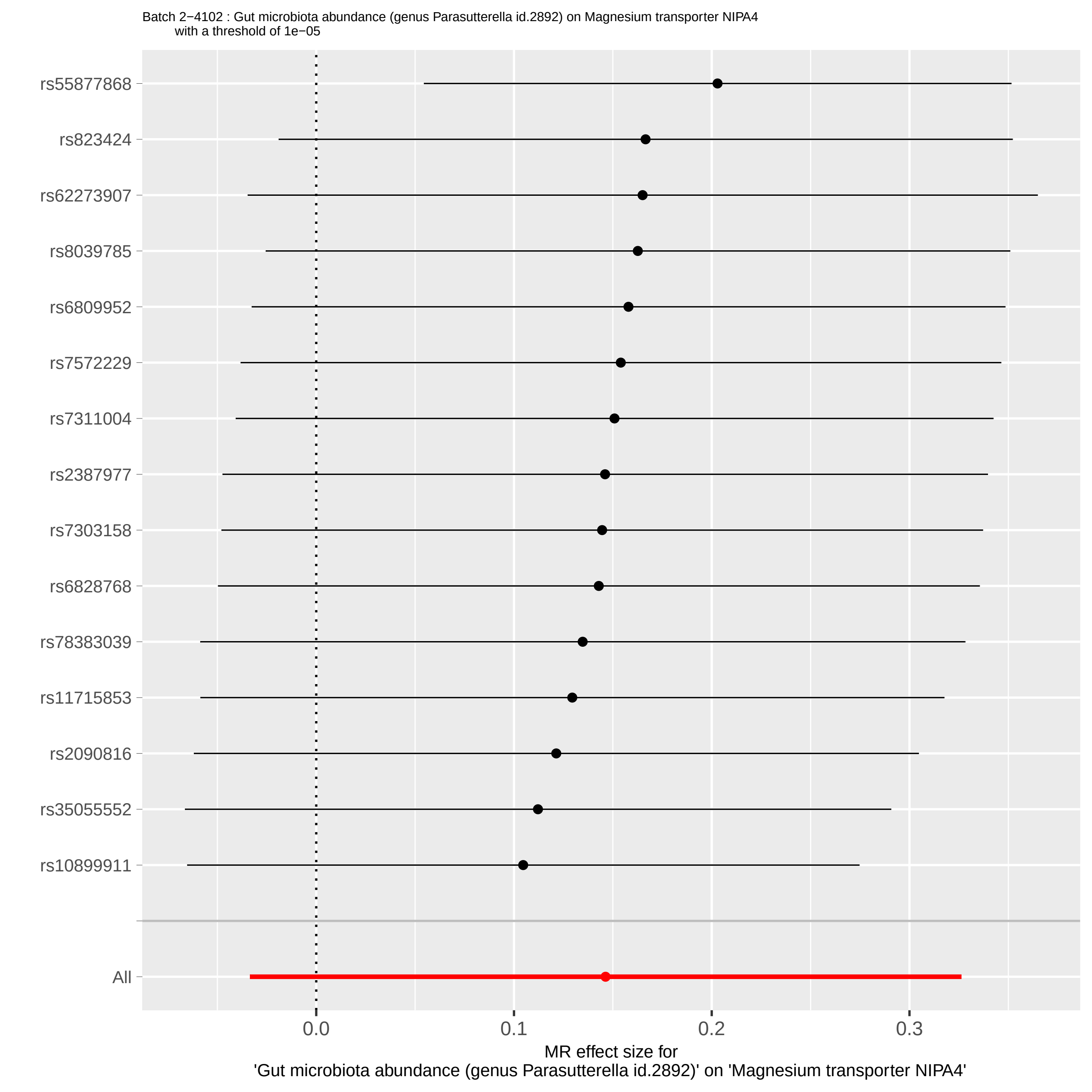

Batch 2−4102 : Gut microbiota abundance (genus Parasutterella id.2892) on Magnesium transporter NIPA4
with a threshold of 1e−05
rs55877868
rs823424
rs62273907
rs8039785
rs6809952
rs7572229
rs7311004
rs2387977
rs7303158
rs6828768
rs78383039
rs11715853
rs2090816
rs35055552
rs10899911
All
0.0
0.1
0.2
0.3
MR effect size for
'Gut microbiota abundance (genus Parasutterella id.2892)' on 'Magnesium transporter NIPA4'

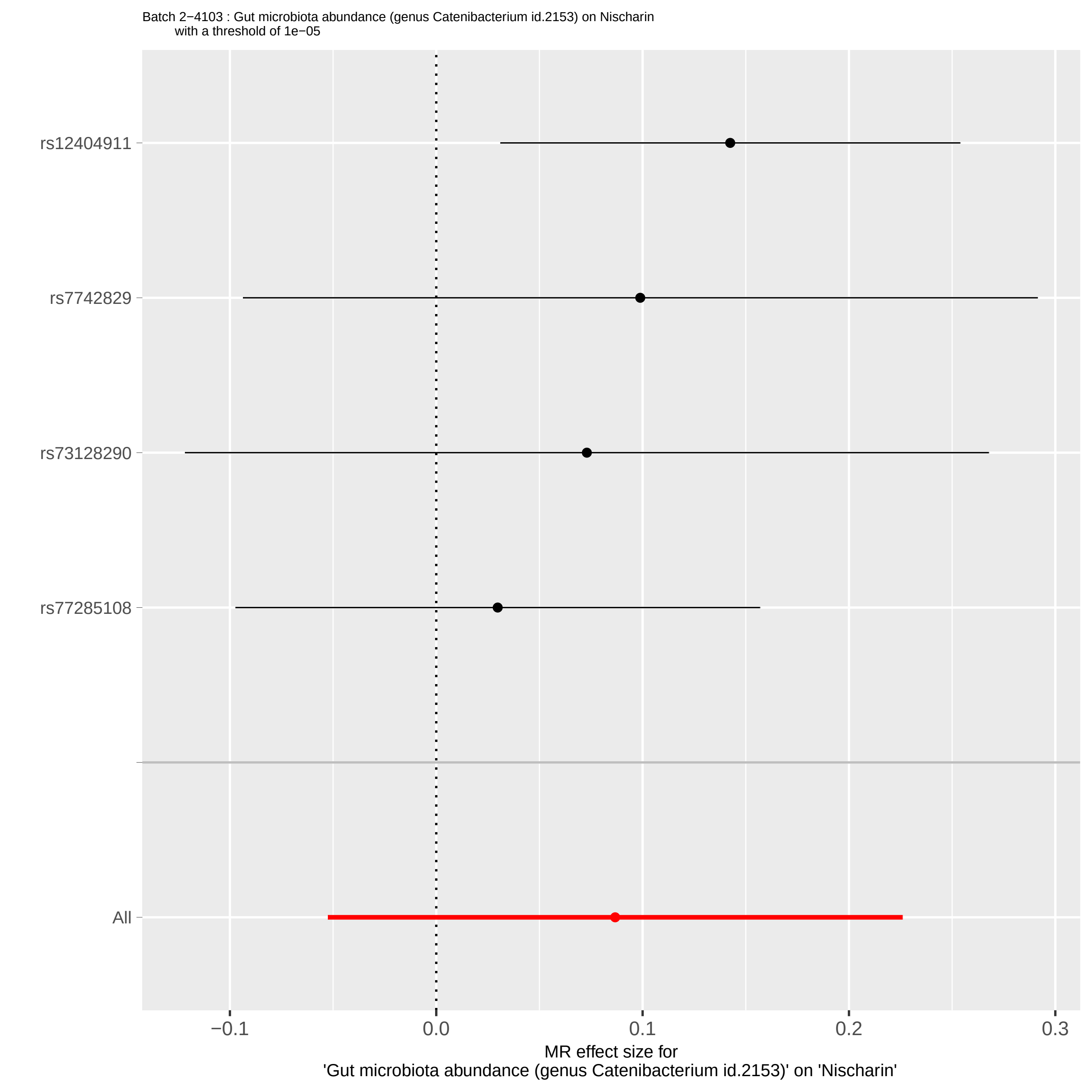
Batch 2−4103 : Gut microbiota abundance (genus Catenibacterium id.2153) on Nischarin
with a threshold of 1e−05
rs12404911
rs7742829
rs73128290
rs77285108
All
−0.1
0.0
0.1
0.2
0.3
MR effect size for
'Gut microbiota abundance (genus Catenibacterium id.2153)' on 'Nischarin'

Batch 2−4104 : Gut microbiota abundance (genus Parasutterella id.2892) on Nischarin
with a threshold of 1e−05

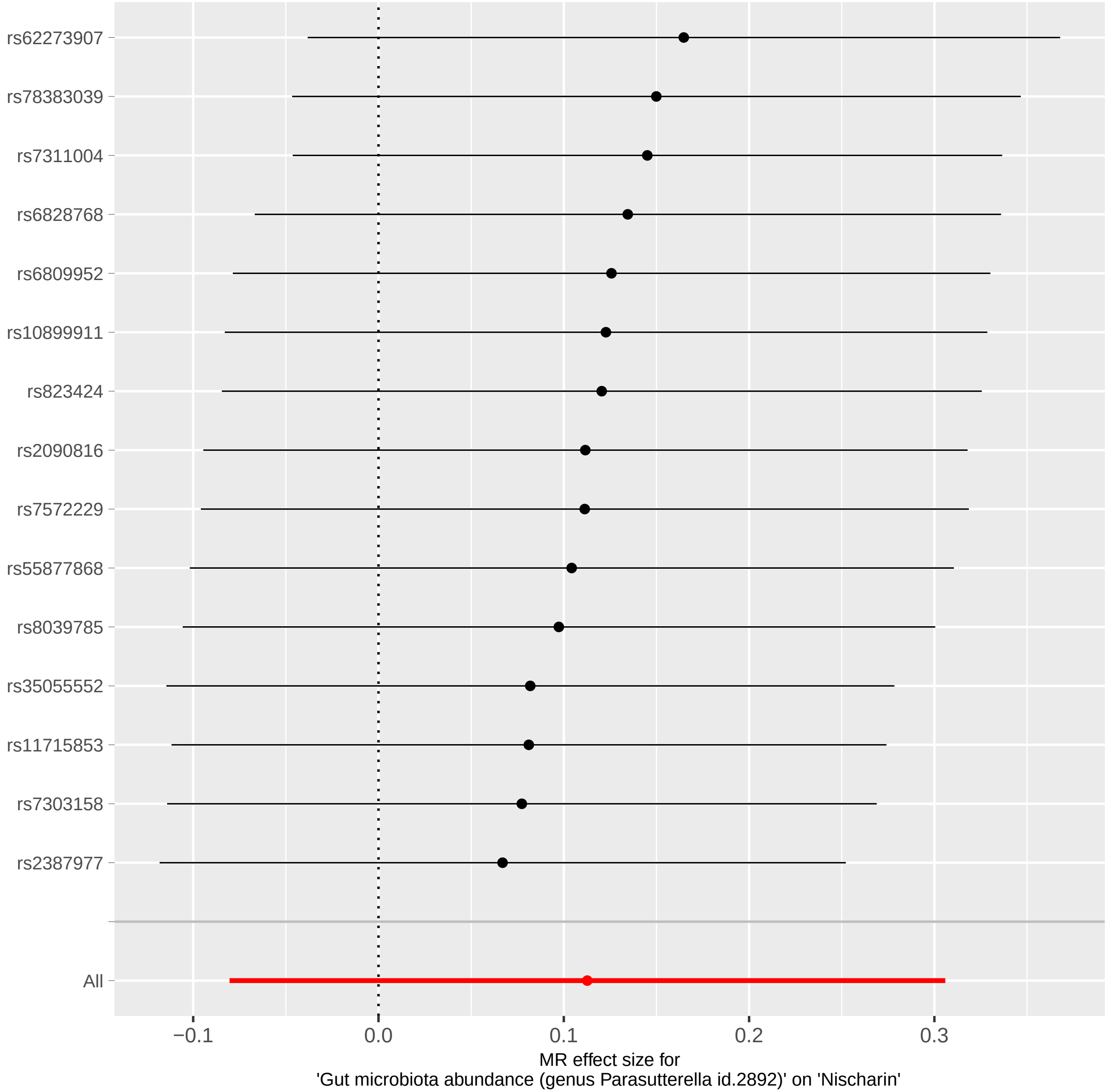

Batch 2−4105 : Gut microbiota abundance (genus Catenibacterium id.2153) on Neuroligin−1
with a threshold of 1e−05

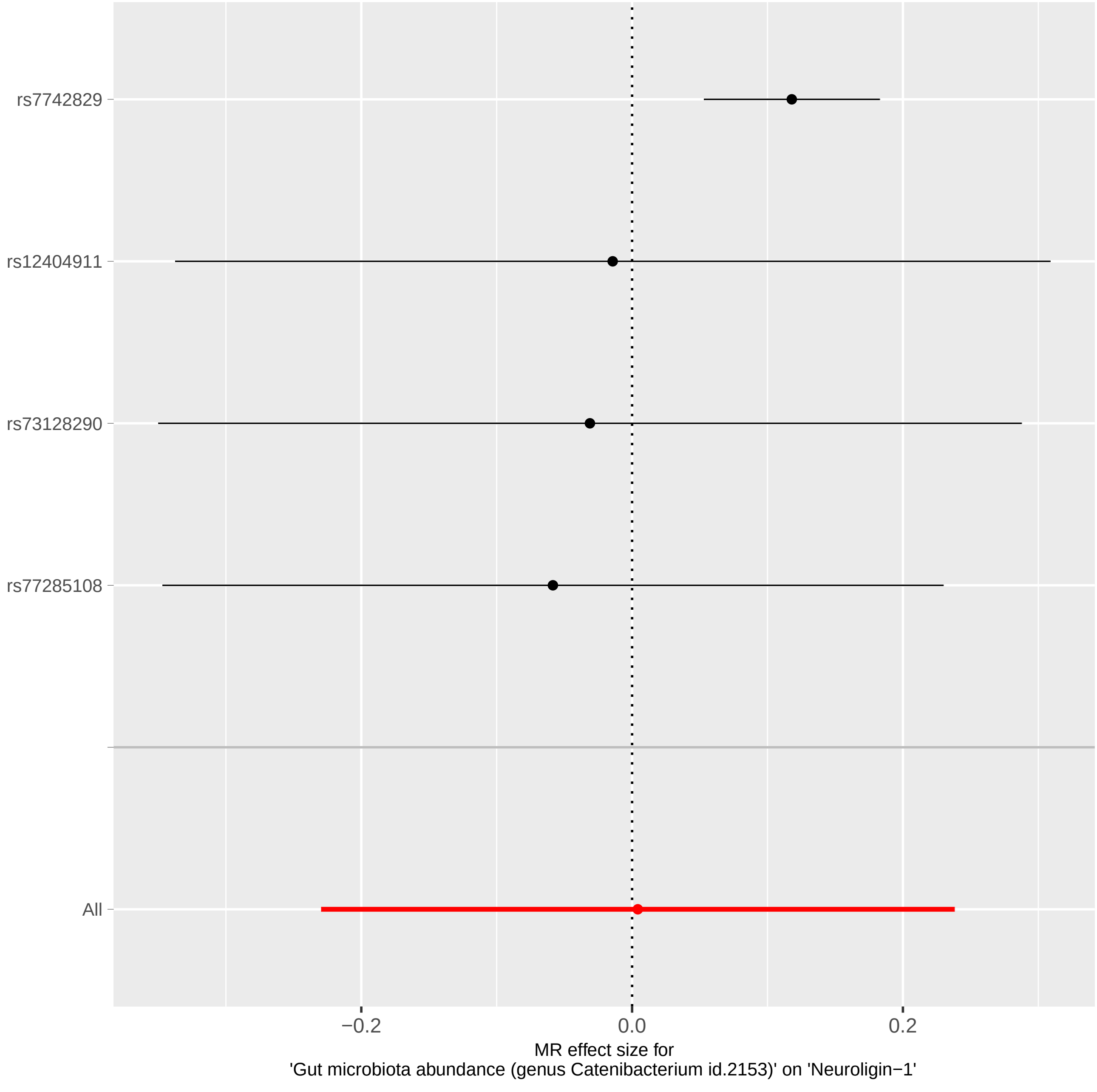

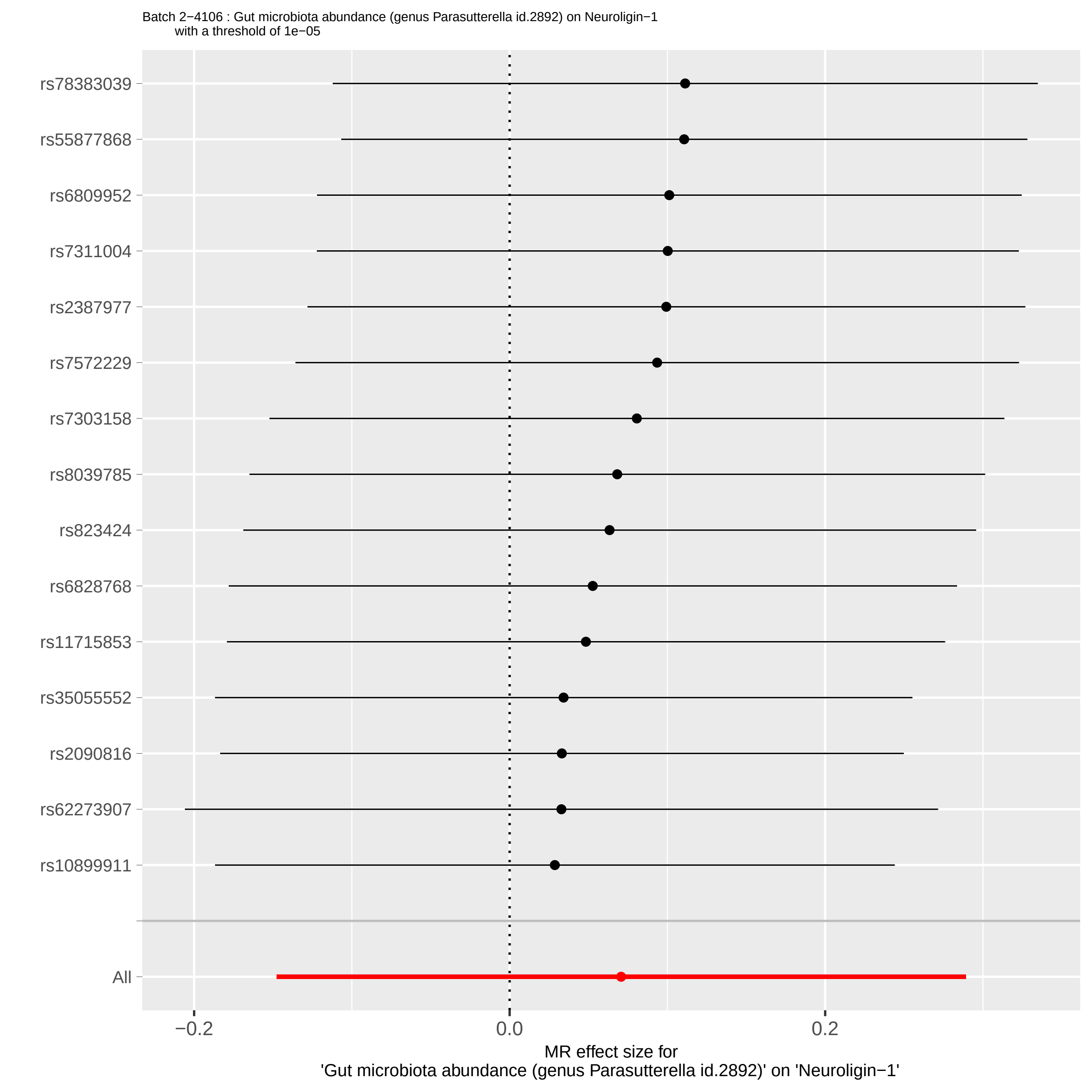
Batch 2−4106 : Gut microbiota abundance (genus Parasutterella id.2892) on Neuroligin−1
with a threshold of 1e−05
rs78383039
rs55877868
rs6809952
rs7311004
rs2387977
rs7572229
rs7303158
rs8039785
rs823424
rs6828768
rs11715853
rs35055552
rs2090816
rs62273907
rs10899911
All
−0.2
0.0
0.2
MR effect size for
'Gut microbiota abundance (genus Parasutterella id.2892)' on 'Neuroligin−1'

Batch 2−4107 : Gut microbiota abundance (genus Catenibacterium id.2153) on Neuroligin−4, X−linked
with a threshold of 1e−05

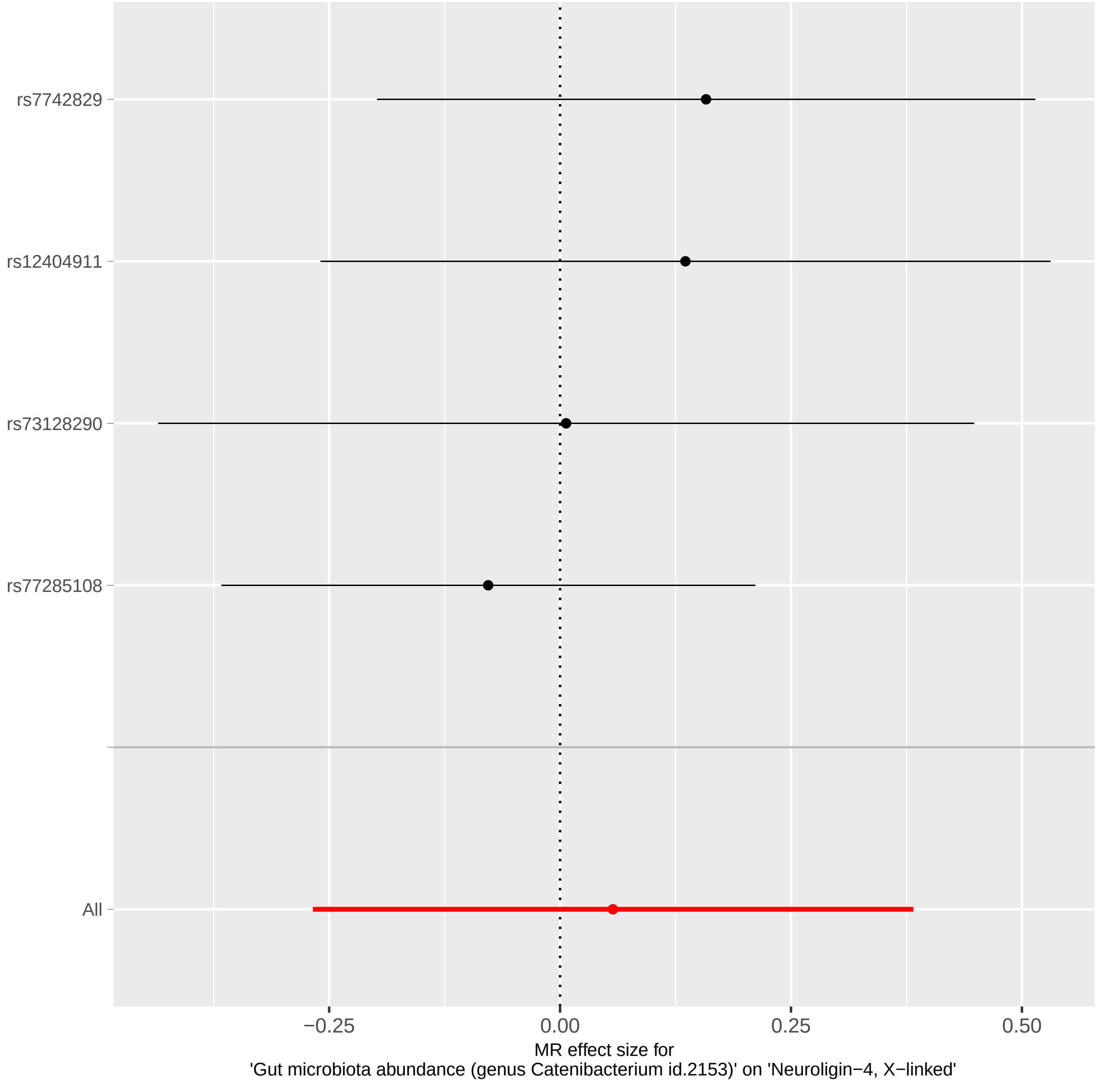

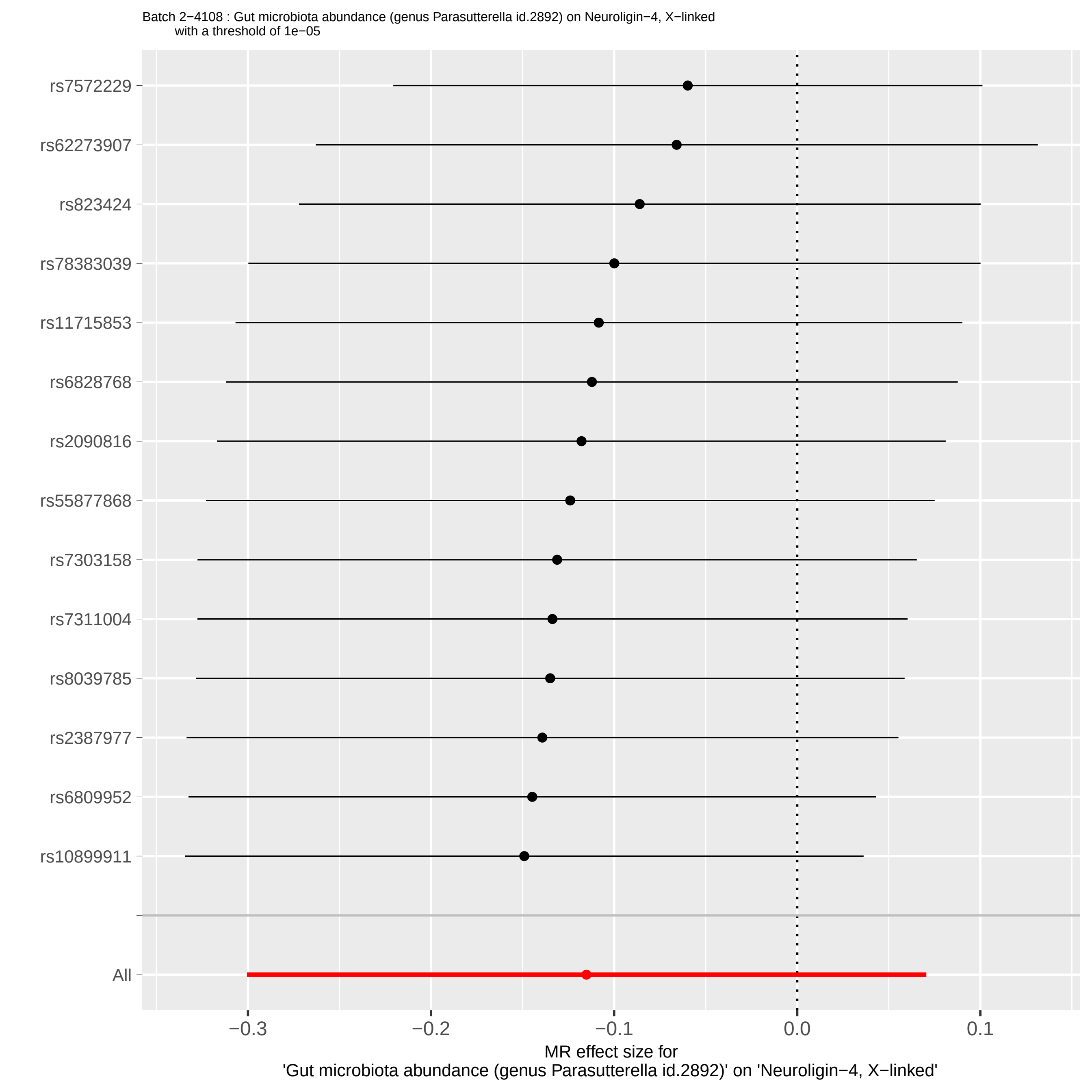
Batch 2−4108 : Gut microbiota abundance (genus Parasutterella id.2892) on Neuroligin−4, X−linked
with a threshold of 1e−05
rs7572229
rs62273907
rs823424
rs78383039
rs11715853
rs6828768
rs2090816
rs55877868
rs7303158
rs7311004
rs8039785
rs2387977
rs6809952
rs10899911
All
−0.3
−0.2
−0.1
0.0
0.1
MR effect size for
'Gut microbiota abundance (genus Parasutterella id.2892)' on 'Neuroligin−4, X−linked'

Batch 2−4109 : Gut microbiota abundance (genus Catenibacterium id.2153) on NACHT, LRR and PYD domains−containing protein 10
with a threshold of 1e−05

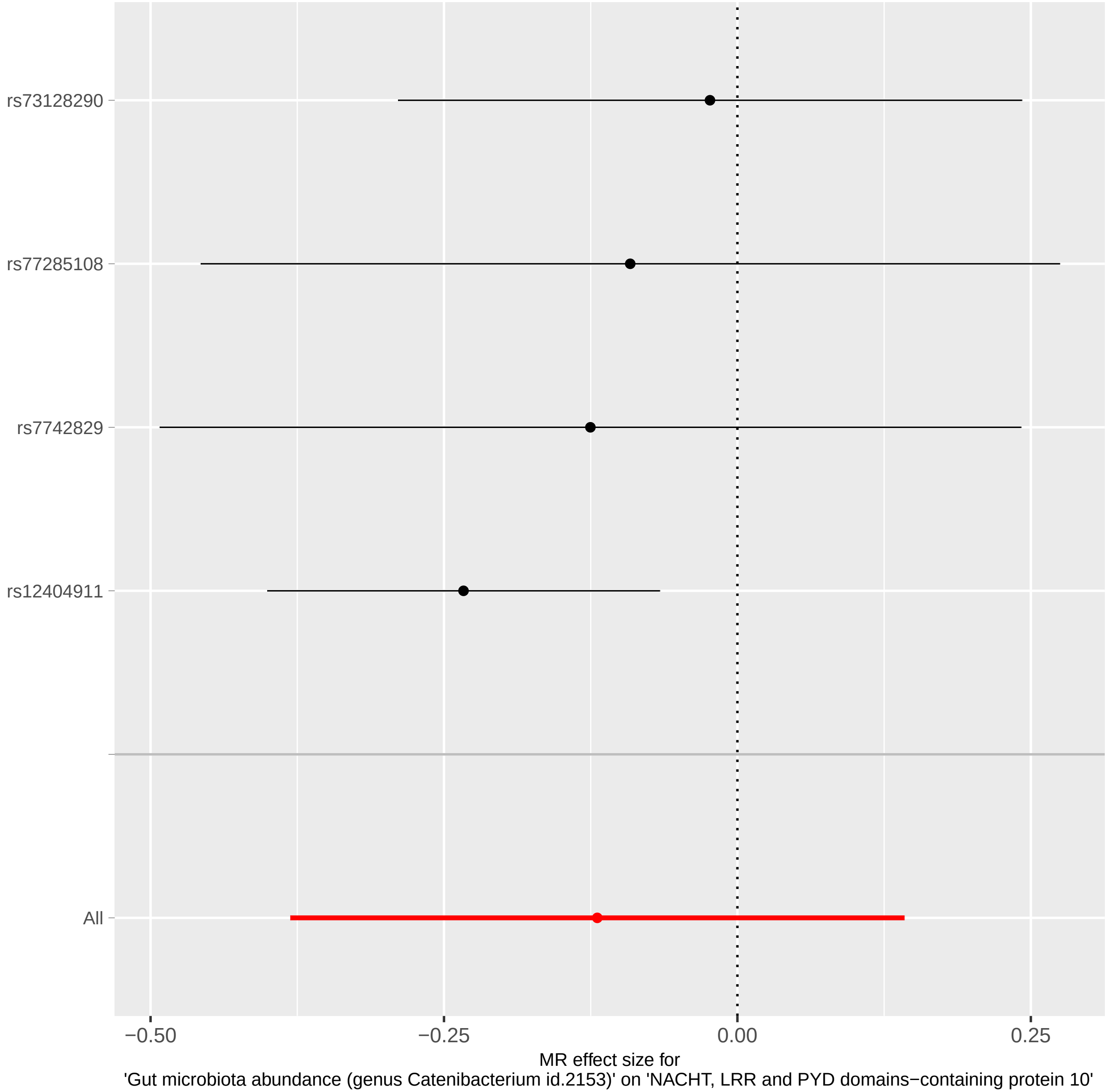

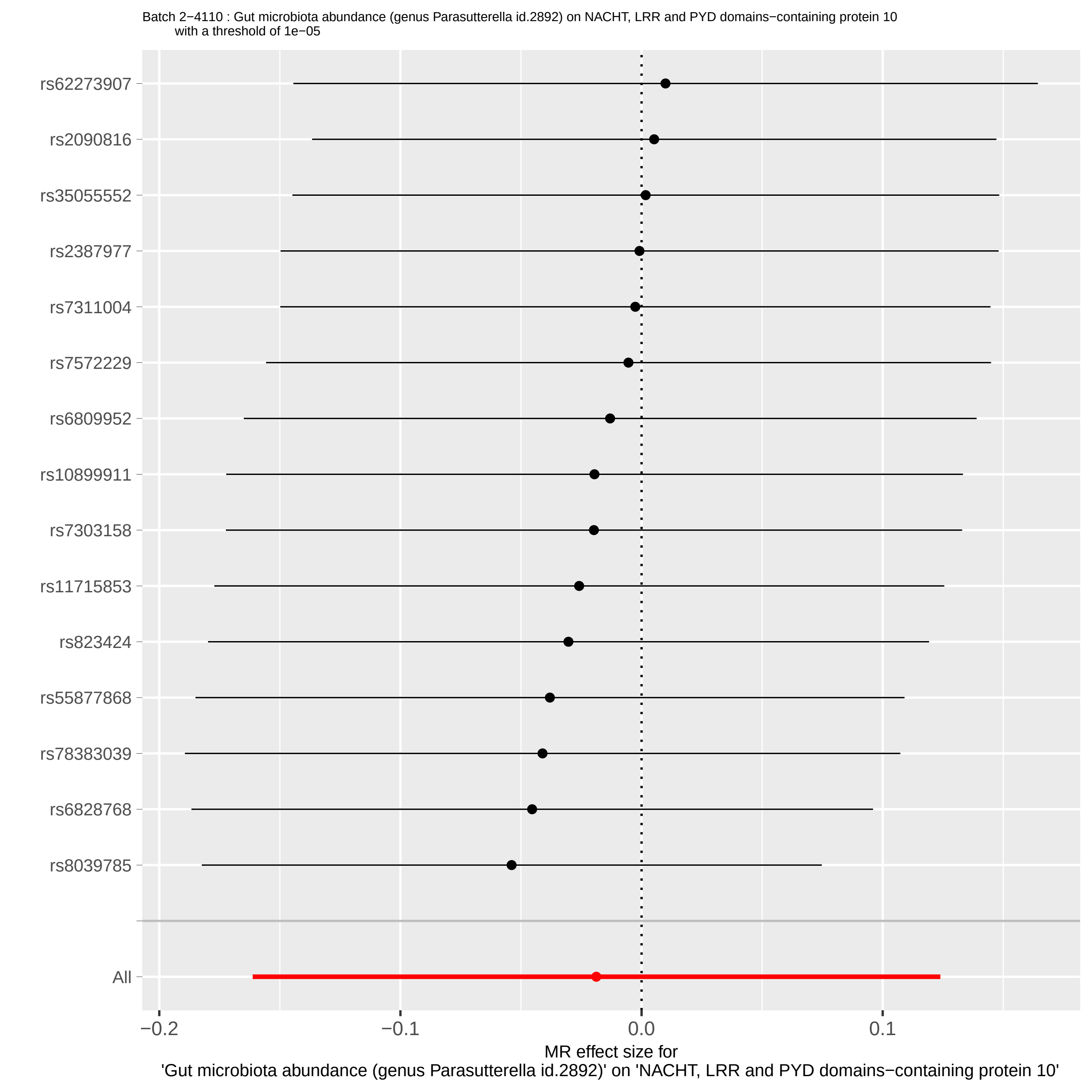

Batch 2−4110 : Gut microbiota abundance (genus Parasutterella id.2892) on NACHT, LRR and PYD domains−containing protein 10
with a threshold of 1e−05
rs62273907
rs2090816
rs35055552
rs2387977
rs7311004
rs7572229
rs6809952
rs10899911
rs7303158
rs11715853
rs823424
rs55877868
rs78383039
rs6828768
rs8039785
All
−0.2
−0.1
0.0
0.1
MR effect size for
'Gut microbiota abundance (genus Parasutterella id.2892)' on 'NACHT, LRR and PYD domains−containing protein 10'

Batch 2−4111 : Gut microbiota abundance (genus Catenibacterium id.2153) on NACHT, LRR and PYD domains−containing protein 1
with a threshold of 1e−05

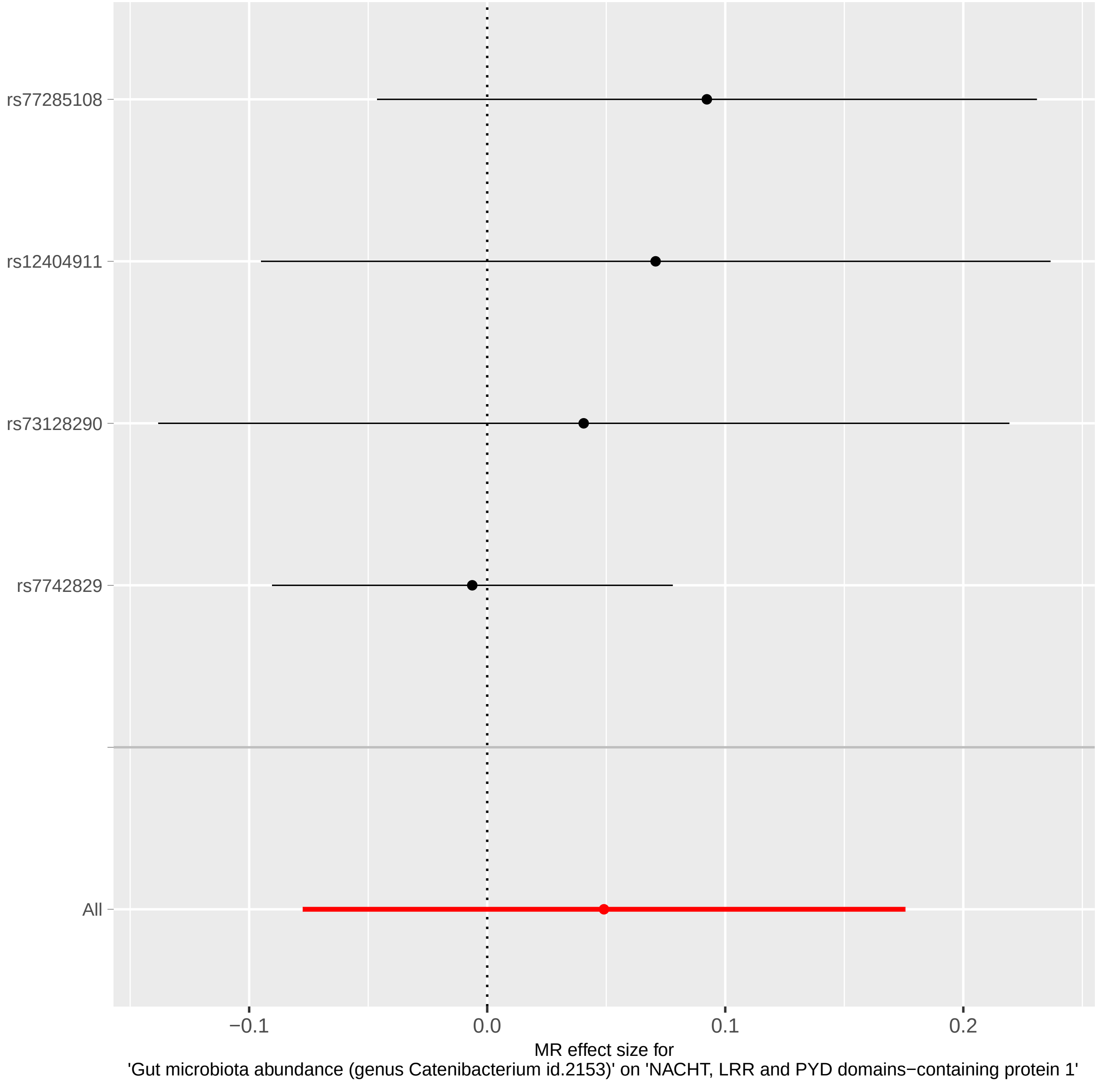

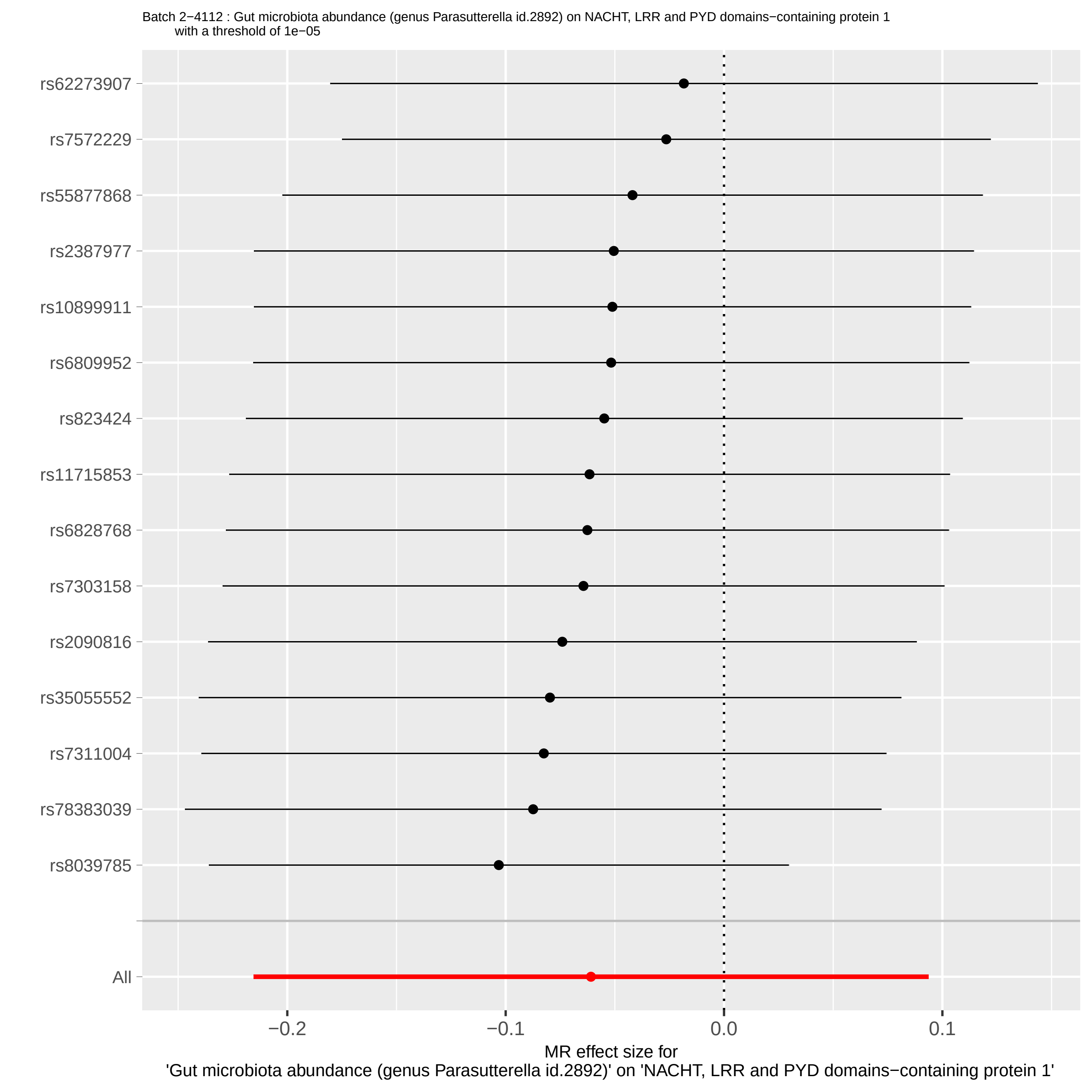
Batch 2−4112 : Gut microbiota abundance (genus Parasutterella id.2892) on NACHT, LRR and PYD domains−containing protein 1
with a threshold of 1e−05
rs62273907
rs7572229
rs55877868
rs2387977
rs10899911
rs6809952
rs823424
rs11715853
rs6828768
rs7303158
rs2090816
rs35055552
rs7311004
rs78383039
rs8039785
All
−0.2
−0.1
0.0
0.1
MR effect size for
'Gut microbiota abundance (genus Parasutterella id.2892)' on 'NACHT, LRR and PYD domains−containing protein 1'

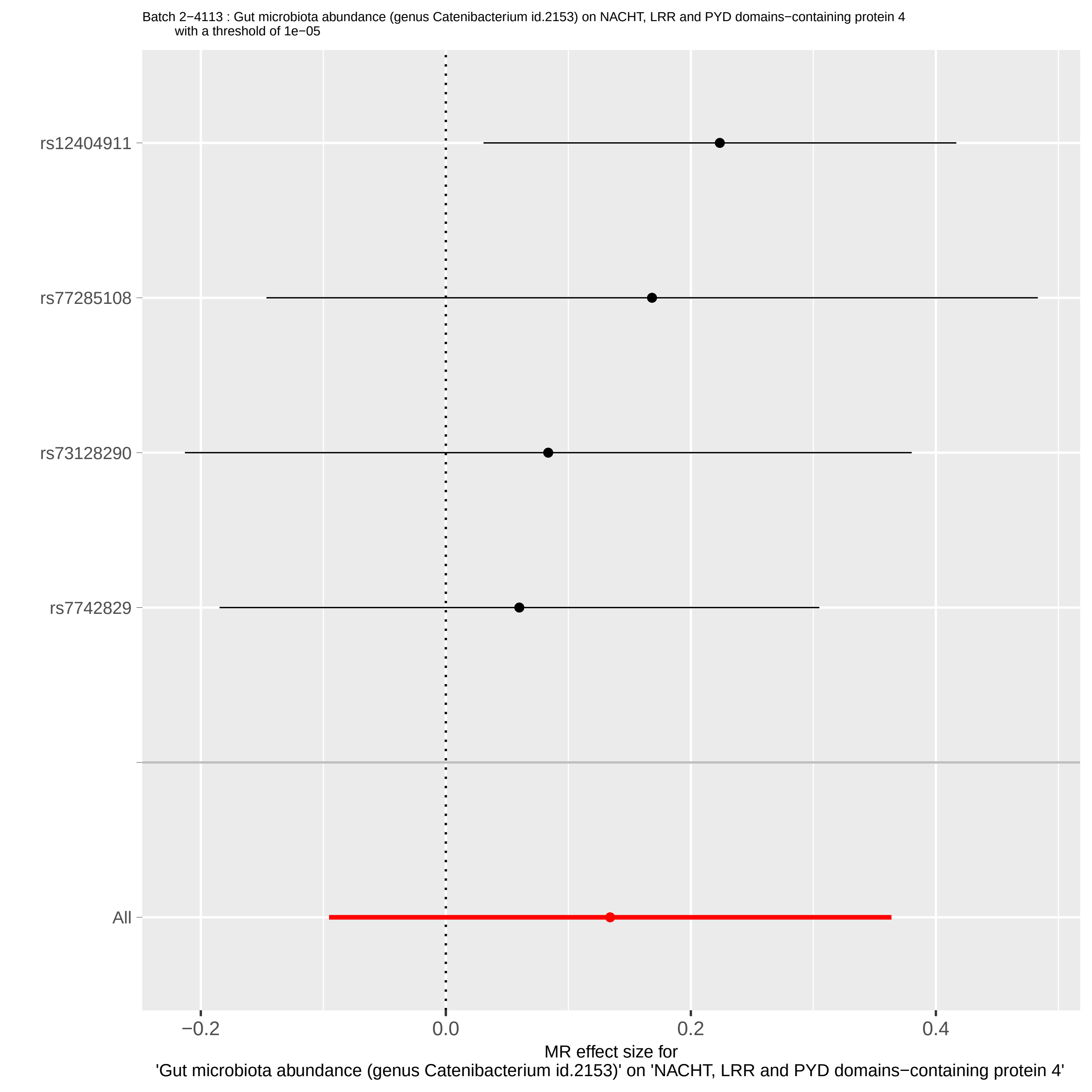

Batch 2−4113 : Gut microbiota abundance (genus Catenibacterium id.2153) on NACHT, LRR and PYD domains−containing protein 4
with a threshold of 1e−05
rs12404911
rs77285108
rs73128290
rs7742829
All
−0.2
0.0
0.2
0.4
MR effect size for
'Gut microbiota abundance (genus Catenibacterium id.2153)' on 'NACHT, LRR and PYD domains−containing protein 4'

Batch 2−4114 : Gut microbiota abundance (genus Parasutterella id.2892) on NACHT, LRR and PYD domains−containing protein 4
with a threshold of 1e−05

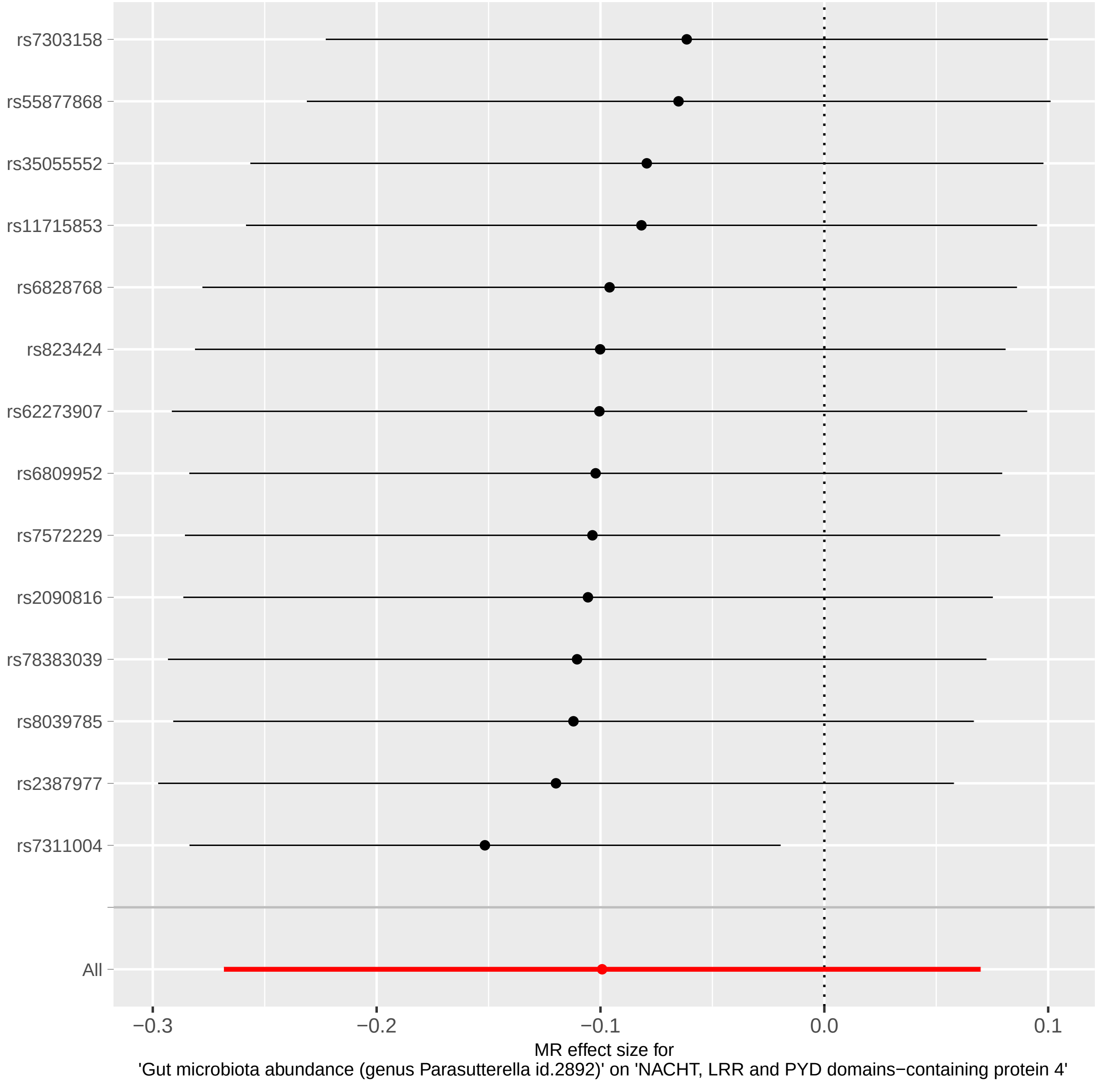

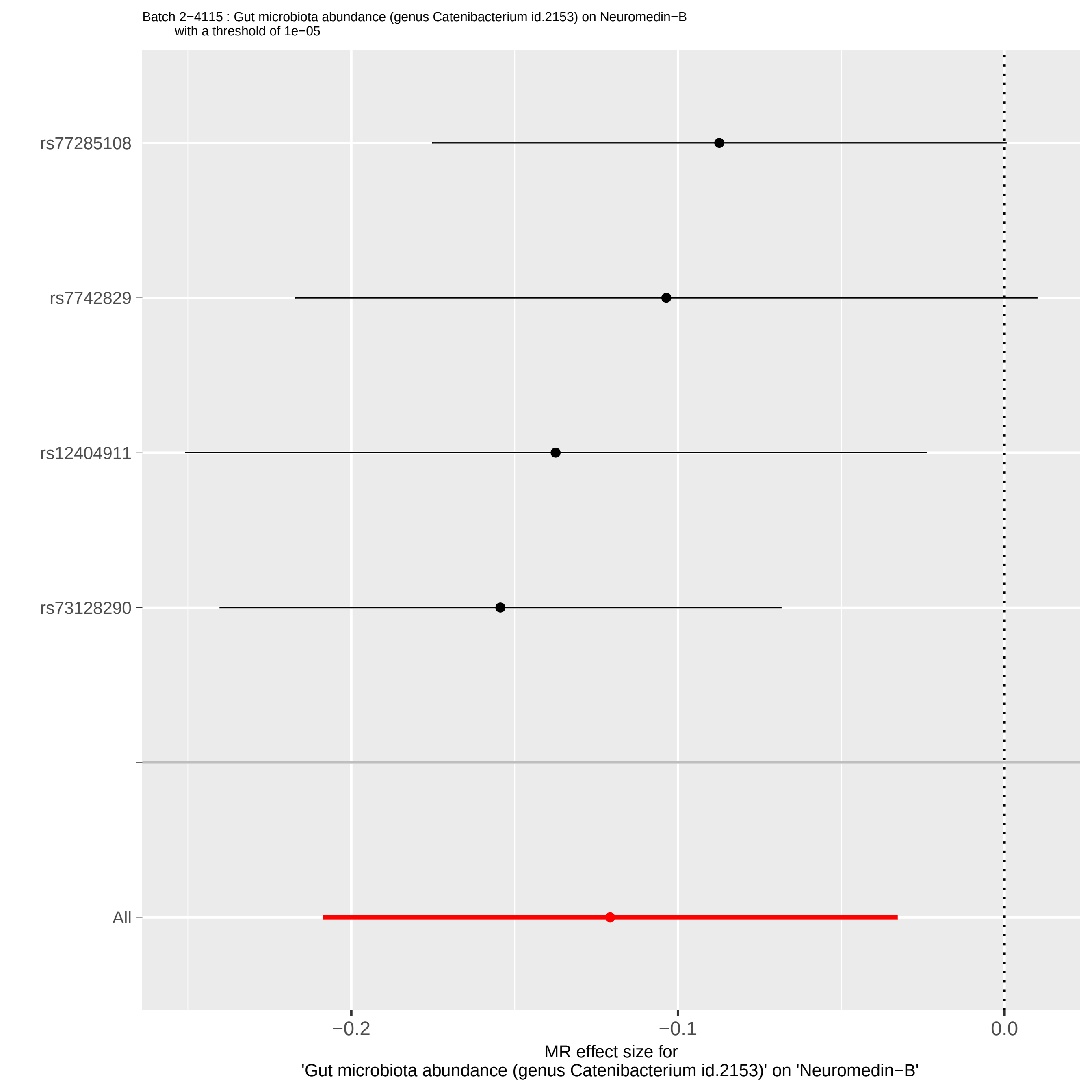
Batch 2−4115 : Gut microbiota abundance (genus Catenibacterium id.2153) on Neuromedin−B
with a threshold of 1e−05
rs77285108
rs7742829
rs12404911
rs73128290
All
−0.2
−0.1
0.0
MR effect size for
'Gut microbiota abundance (genus Catenibacterium id.2153)' on 'Neuromedin−B'

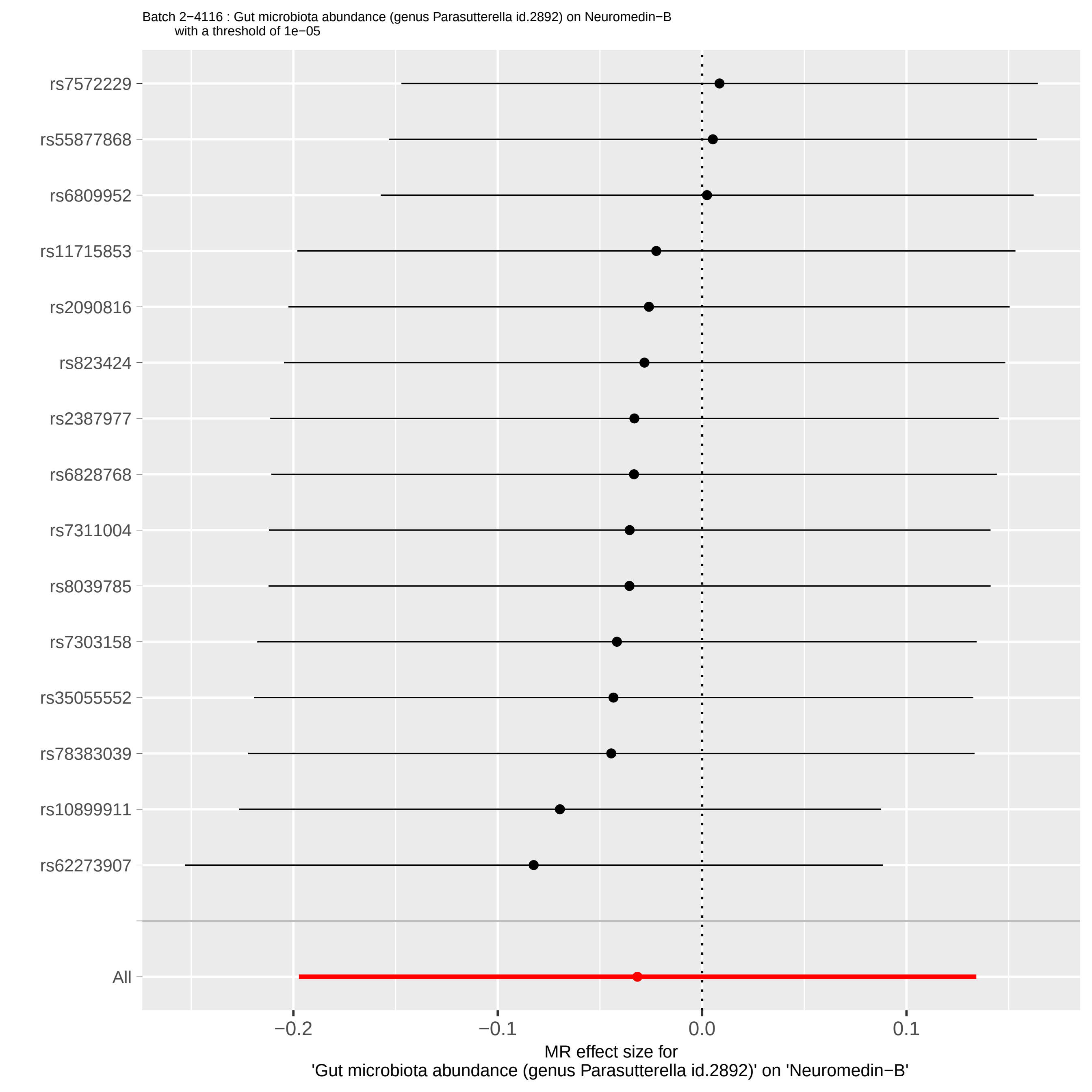

Batch 2−4116 : Gut microbiota abundance (genus Parasutterella id.2892) on Neuromedin−B
with a threshold of 1e−05
rs7572229
rs55877868
rs6809952
rs11715853
rs2090816
rs823424
rs2387977
rs6828768
rs7311004
rs8039785
rs7303158
rs35055552
rs78383039
rs10899911
rs62273907
All
−0.2
−0.1
0.0
0.1
MR effect size for
'Gut microbiota abundance (genus Parasutterella id.2892)' on 'Neuromedin−B'

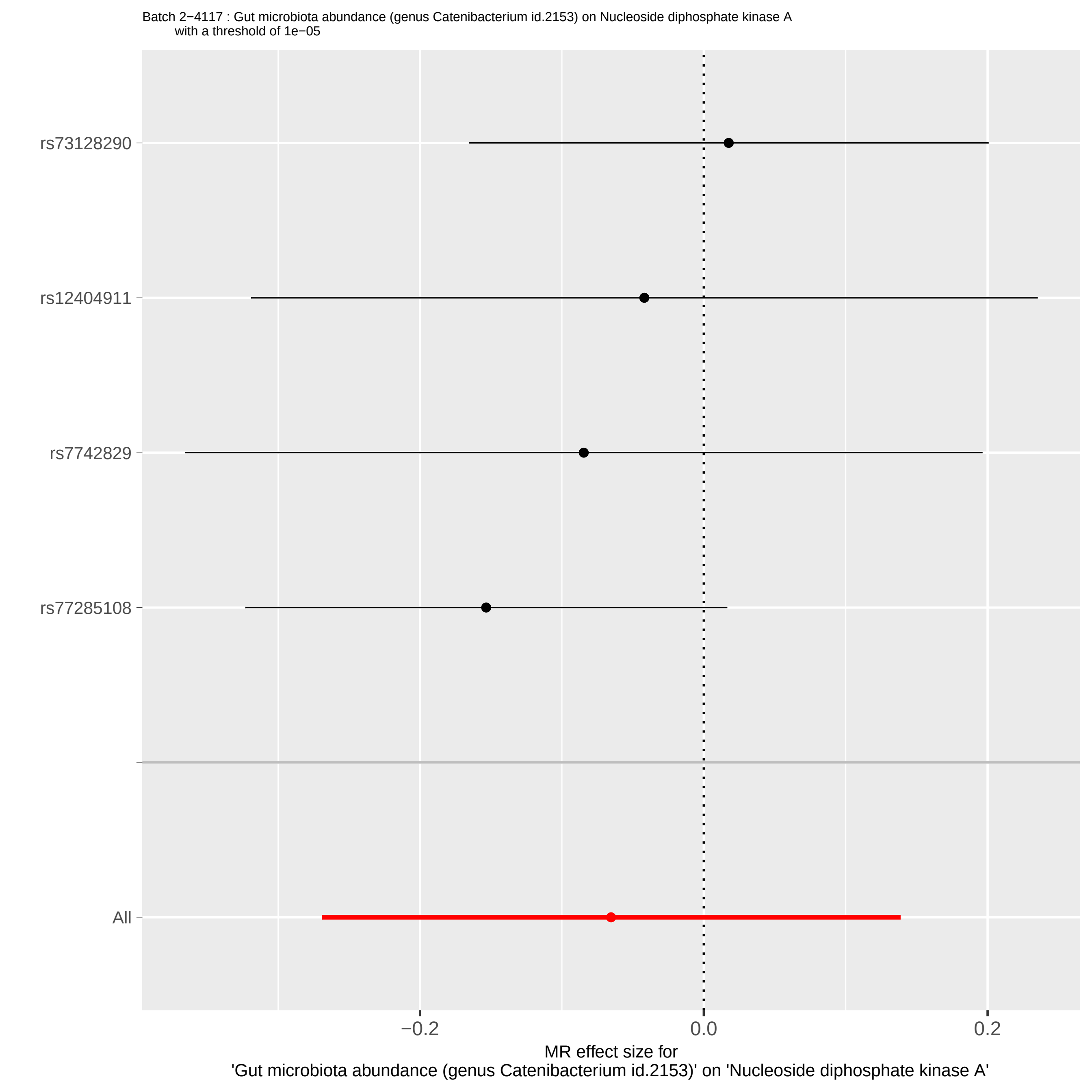
Batch 2−4117 : Gut microbiota abundance (genus Catenibacterium id.2153) on Nucleoside diphosphate kinase A
with a threshold of 1e−05
rs73128290
rs12404911
rs7742829
rs77285108
All
−0.2
0.0
0.2
MR effect size for
'Gut microbiota abundance (genus Catenibacterium id.2153)' on 'Nucleoside diphosphate kinase A'

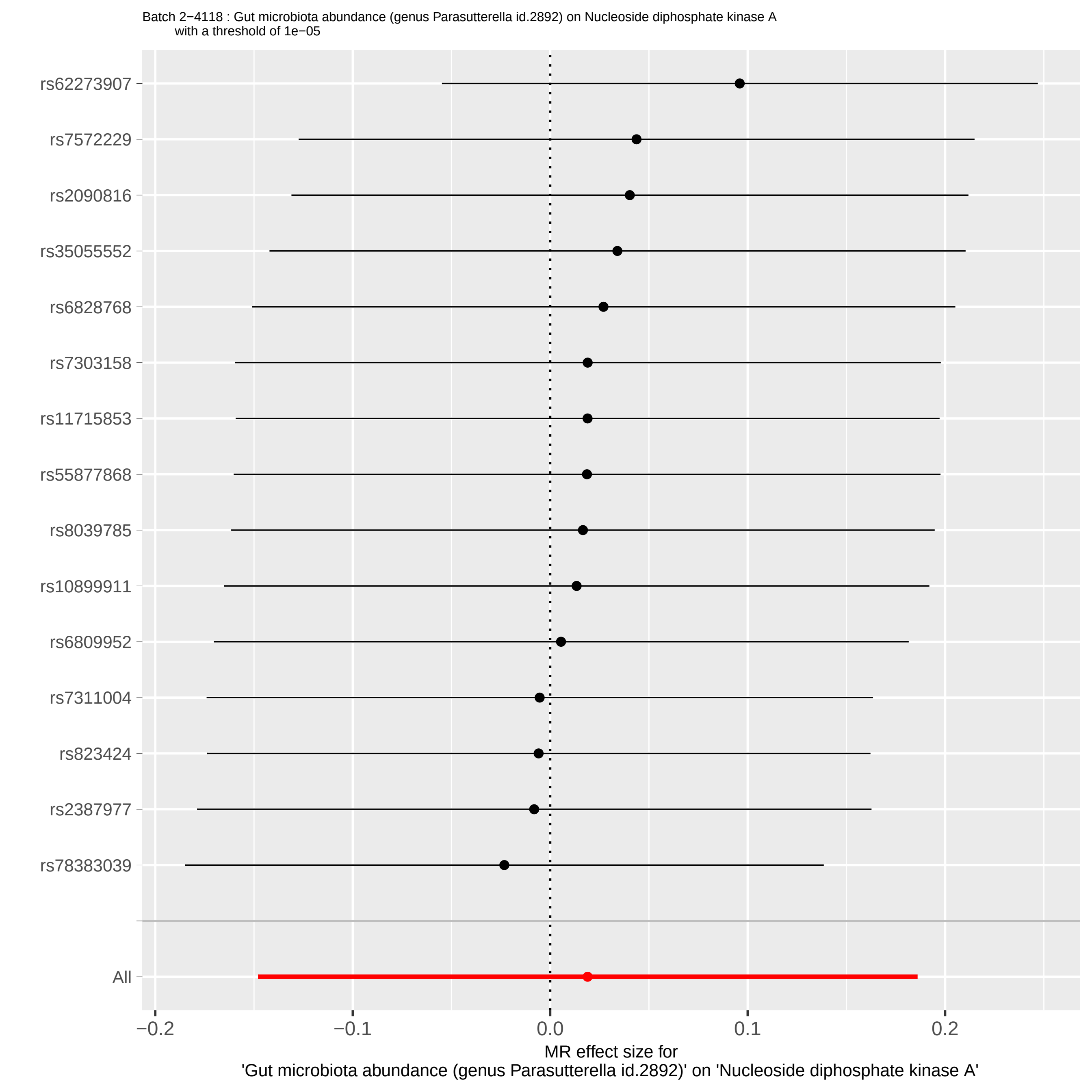
Batch 2−4118 : Gut microbiota abundance (genus Parasutterella id.2892) on Nucleoside diphosphate kinase A
with a threshold of 1e−05
rs62273907
rs7572229
rs2090816
rs35055552
rs6828768
rs7303158
rs11715853
rs55877868
rs8039785
rs10899911
rs6809952
rs7311004
rs823424
rs2387977
rs78383039
All
−0.2
−0.1
0.0
0.1
0.2
MR effect size for
'Gut microbiota abundance (genus Parasutterella id.2892)' on 'Nucleoside diphosphate kinase A'

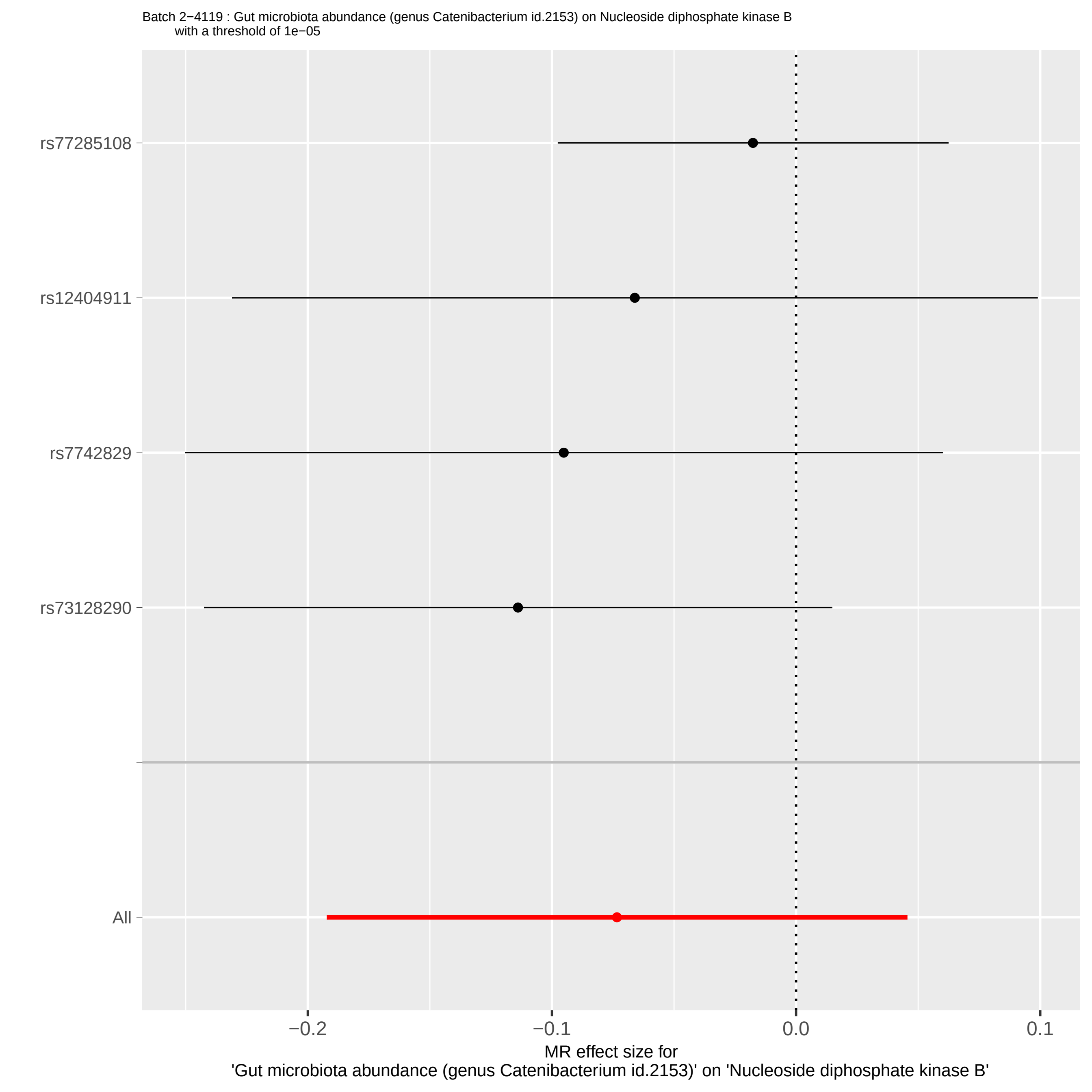
Batch 2−4119 : Gut microbiota abundance (genus Catenibacterium id.2153) on Nucleoside diphosphate kinase B
with a threshold of 1e−05
rs77285108
rs12404911
rs7742829
rs73128290
All
−0.2
−0.1
0.0
0.1
MR effect size for
'Gut microbiota abundance (genus Catenibacterium id.2153)' on 'Nucleoside diphosphate kinase B'

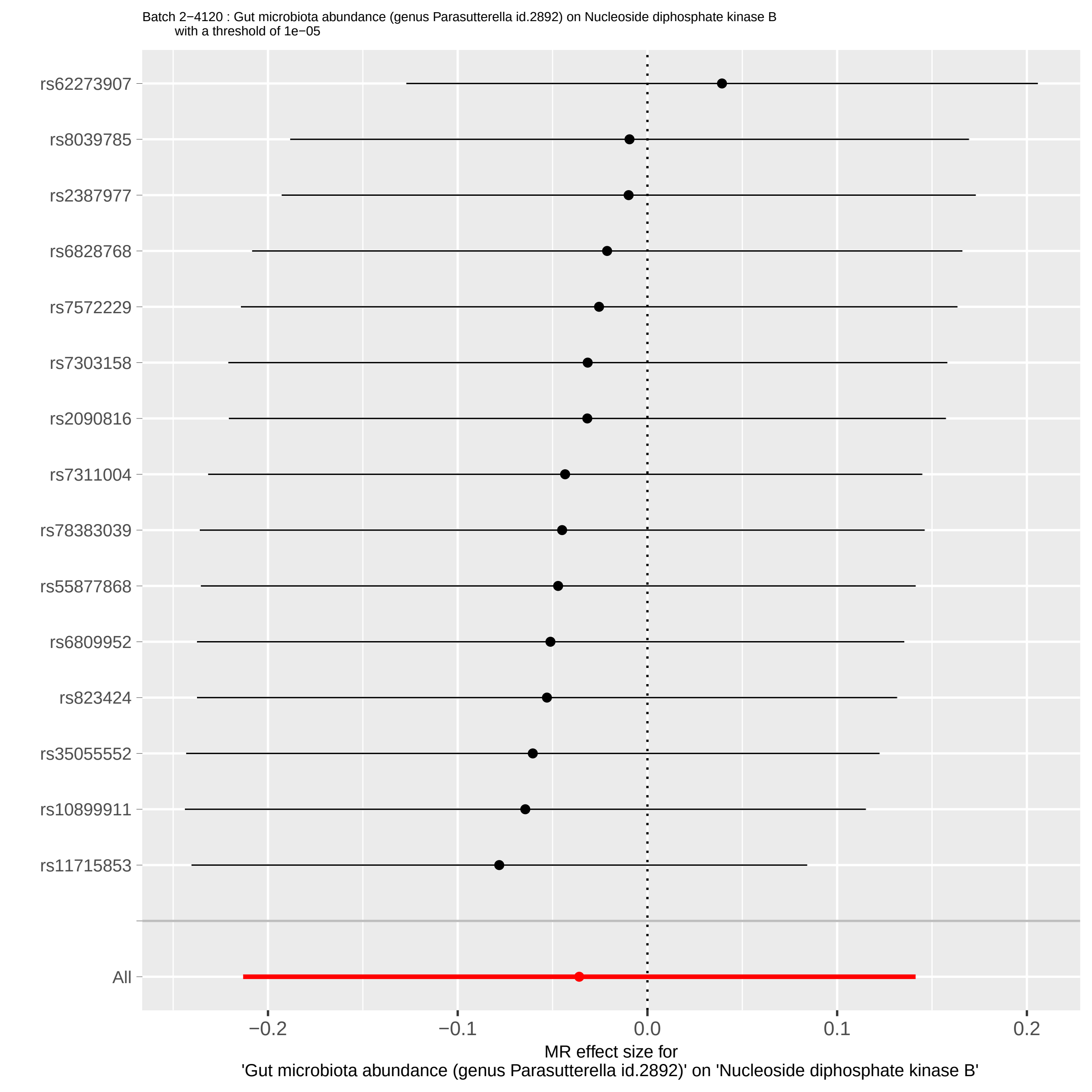
Batch 2−4120 : Gut microbiota abundance (genus Parasutterella id.2892) on Nucleoside diphosphate kinase B
with a threshold of 1e−05
rs62273907
rs8039785
rs2387977
rs6828768
rs7572229
rs7303158
rs2090816
rs7311004
rs78383039
rs55877868
rs6809952
rs823424
rs35055552
rs10899911
rs11715853
All
−0.2
−0.1
0.0
0.1
0.2
MR effect size for
'Gut microbiota abundance (genus Parasutterella id.2892)' on 'Nucleoside diphosphate kinase B'

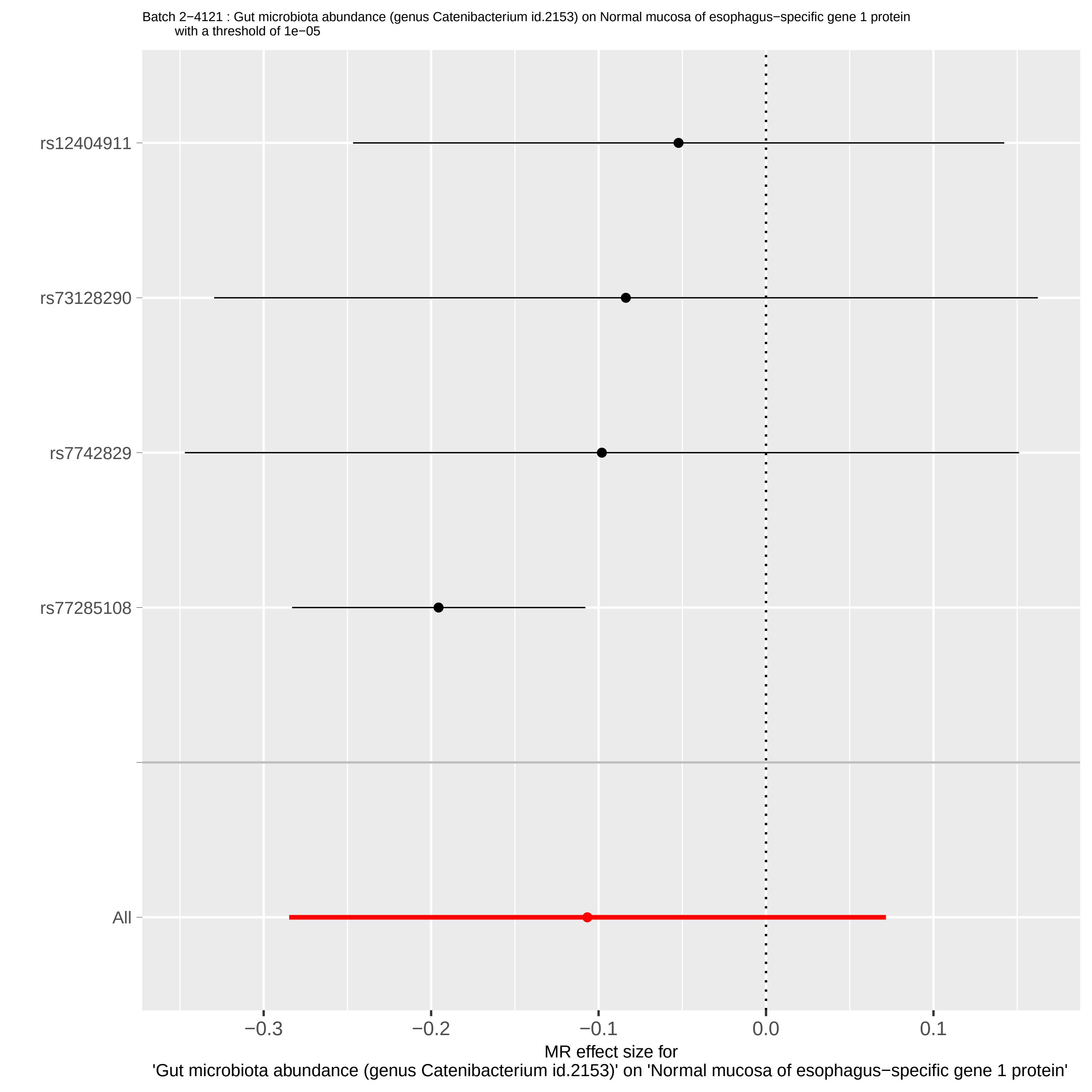
Batch 2−4121 : Gut microbiota abundance (genus Catenibacterium id.2153) on Normal mucosa of esophagus−specific gene 1 protein
with a threshold of 1e−05
rs12404911
rs73128290
rs7742829
rs77285108
All
−0.3
−0.2
−0.1
0.0
0.1
MR effect size for
'Gut microbiota abundance (genus Catenibacterium id.2153)' on 'Normal mucosa of esophagus−specific gene 1 protein'

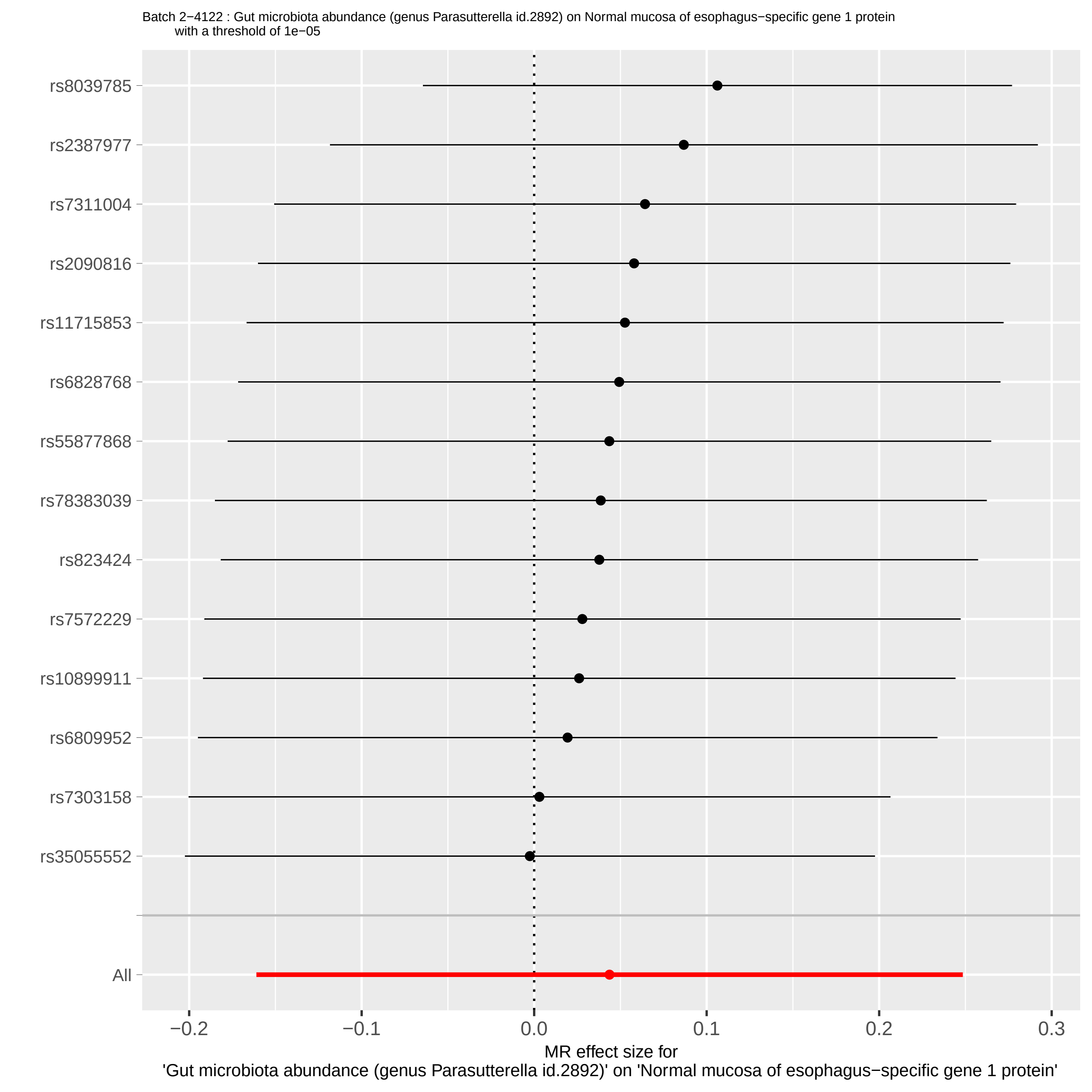

Batch 2−4122 : Gut microbiota abundance (genus Parasutterella id.2892) on Normal mucosa of esophagus−specific gene 1 protein with a threshold of 1e−05
rs8039785
rs2387977
rs7311004
rs2090816
rs11715853
rs6828768
rs55877868
rs78383039
rs823424
rs7572229
rs10899911
rs6809952
rs7303158
rs35055552
All
−0.2
−0.1
0.0
0.1
0.2
0.3
MR effect size for
'Gut microbiota abundance (genus Parasutterella id.2892)' on 'Normal mucosa of esophagus−specific gene 1 protein'

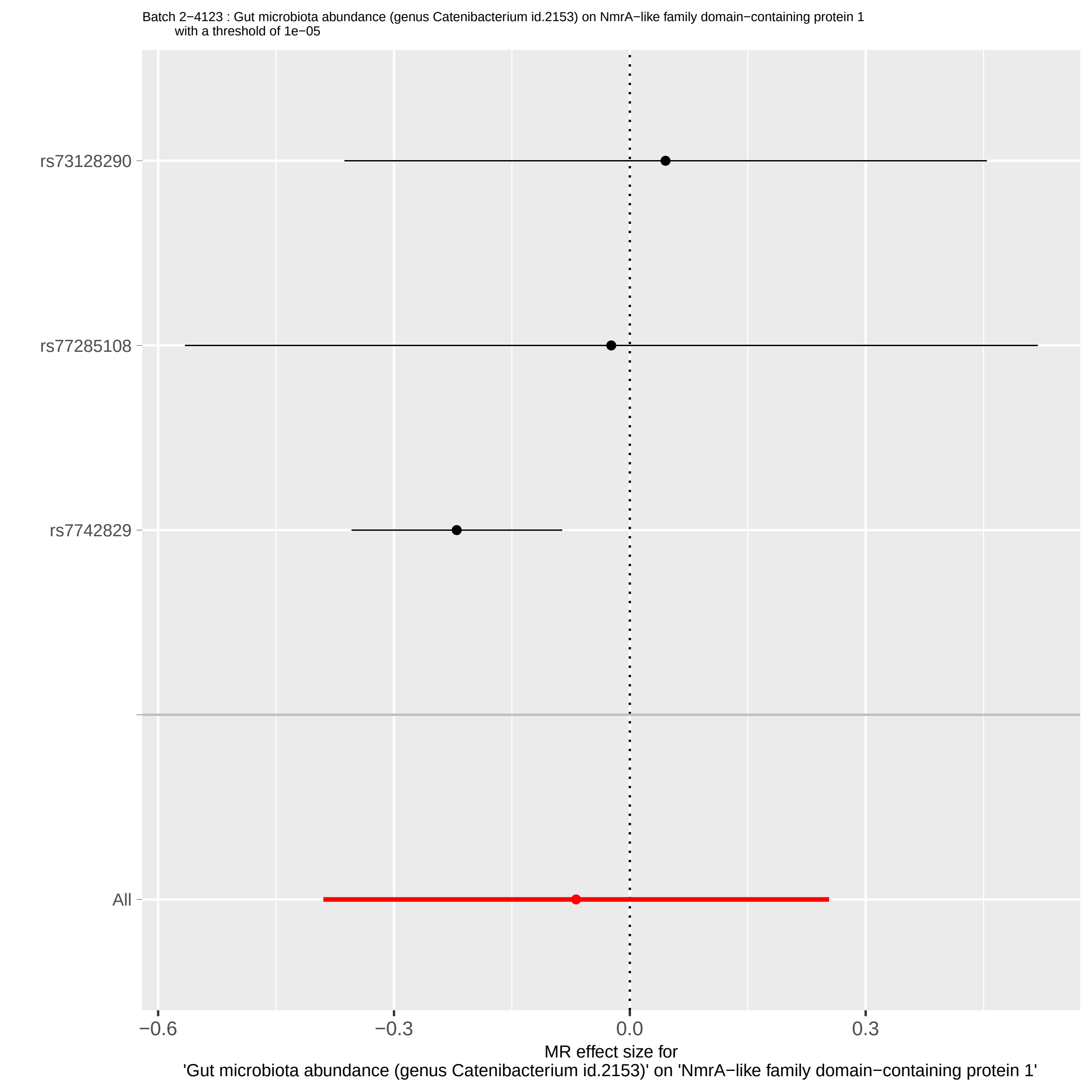

Batch 2−4123 : Gut microbiota abundance (genus Catenibacterium id.2153) on NmrA−like family domain−containing protein 1
with a threshold of 1e−05
rs73128290
rs77285108
rs7742829
All
−0.6
−0.3
0.0
0.3
MR effect size for
'Gut microbiota abundance (genus Catenibacterium id.2153)' on 'NmrA−like family domain−containing protein 1'

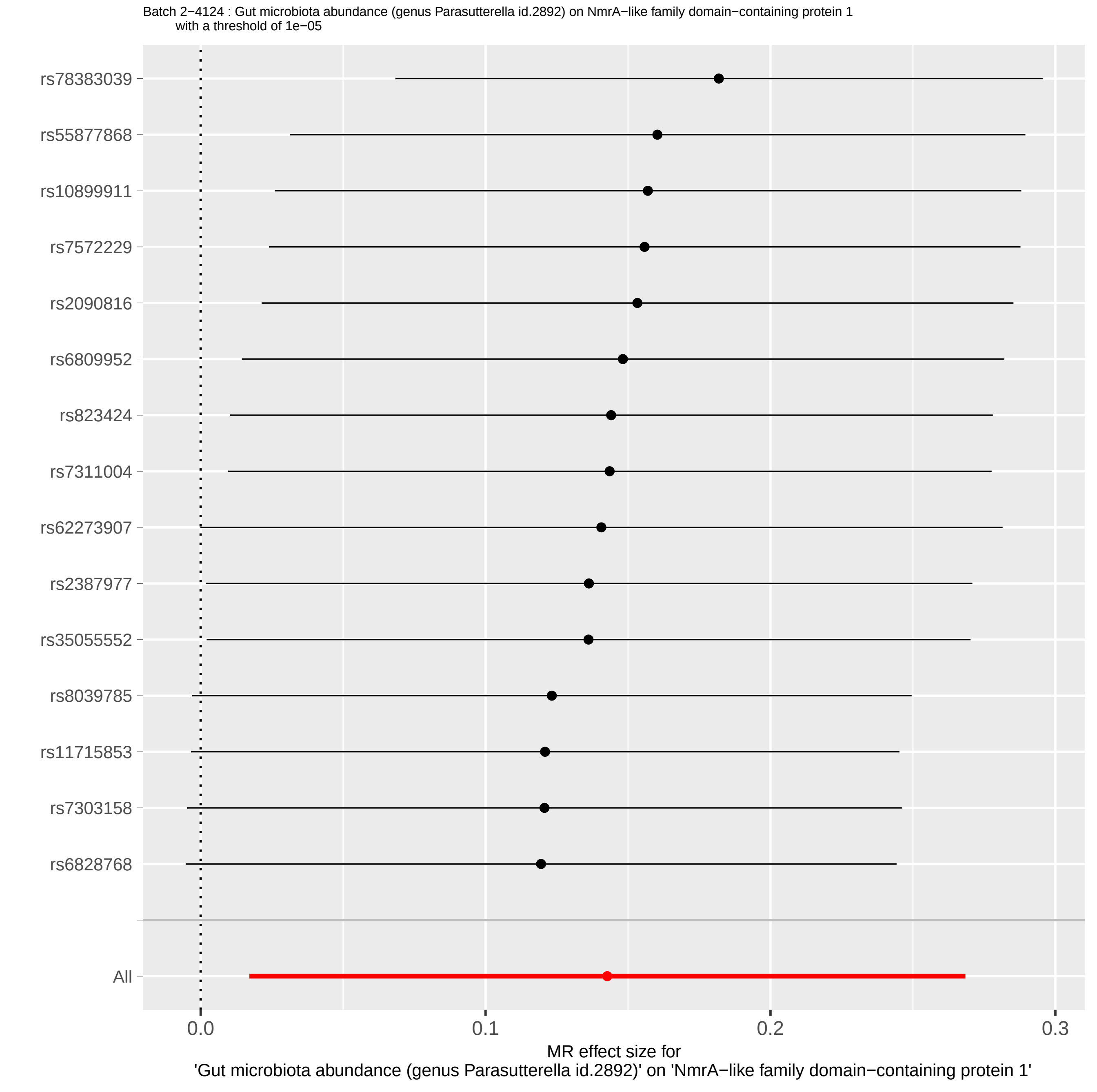
Batch 2−4124 : Gut microbiota abundance (genus Parasutterella id.2892) on NmrA−like family domain−containing protein 1
with a threshold of 1e−05
rs78383039
rs55877868
rs10899911
rs7572229
rs2090816
rs6809952
rs823424
rs7311004
rs62273907
rs2387977
rs35055552
rs8039785
rs11715853
rs7303158
rs6828768
All
0.0
0.1
0.2
0.3
MR effect size for
'Gut microbiota abundance (genus Parasutterella id.2892)' on 'NmrA−like family domain−containing protein 1'

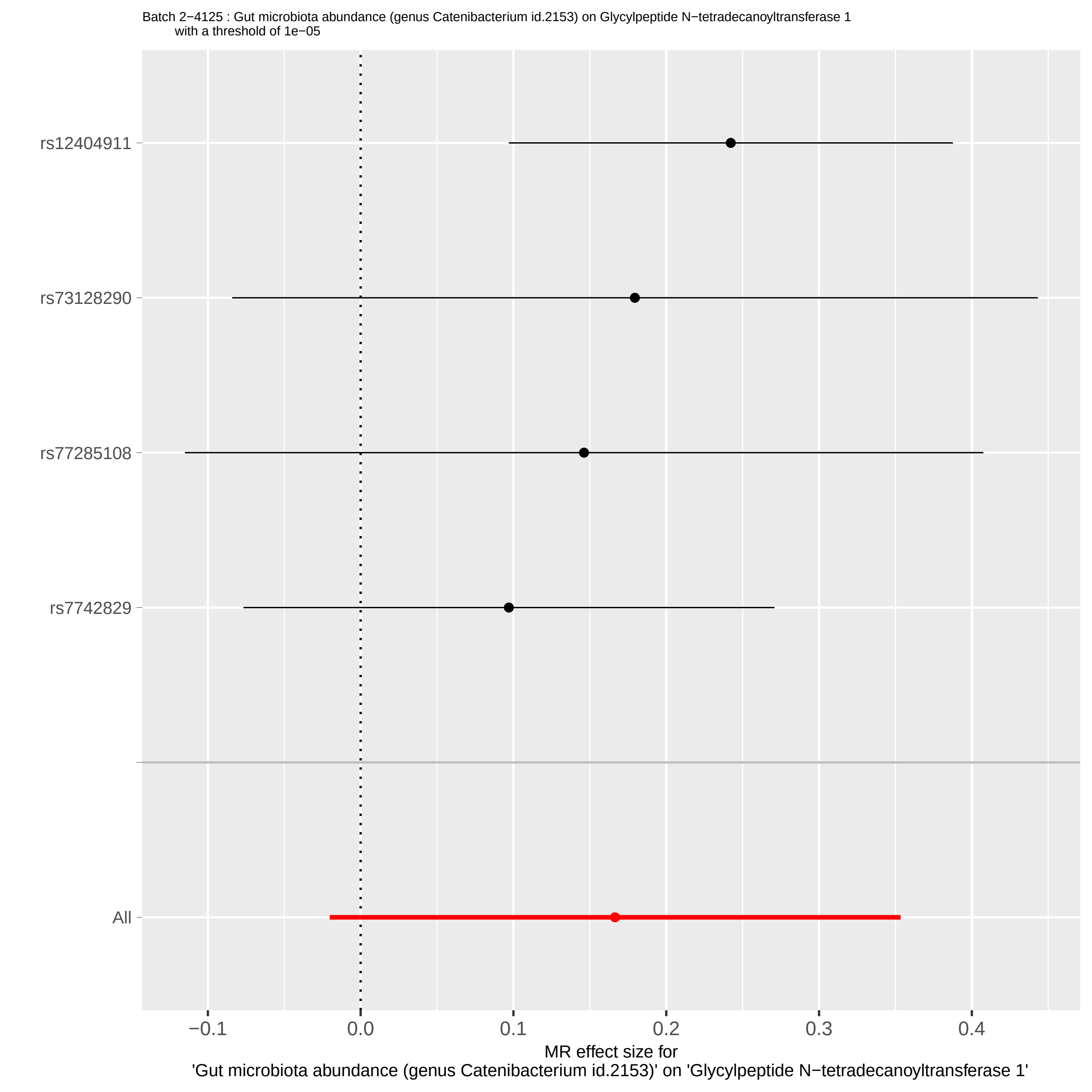

Batch 2−4125 : Gut microbiota abundance (genus Catenibacterium id.2153) on Glycylpeptide N−tetradecanoyltransferase 1
with a threshold of 1e−05
rs12404911
rs73128290
rs77285108
rs7742829
All
−0.1
0.0
0.1
0.2
0.3
0.4
MR effect size for
'Gut microbiota abundance (genus Catenibacterium id.2153)' on 'Glycylpeptide N−tetradecanoyltransferase 1'

Batch 2−4126 : Gut microbiota abundance (genus Parasutterella id.2892) on Glycylpeptide N−tetradecanoyltransferase 1
with a threshold of 1e−05

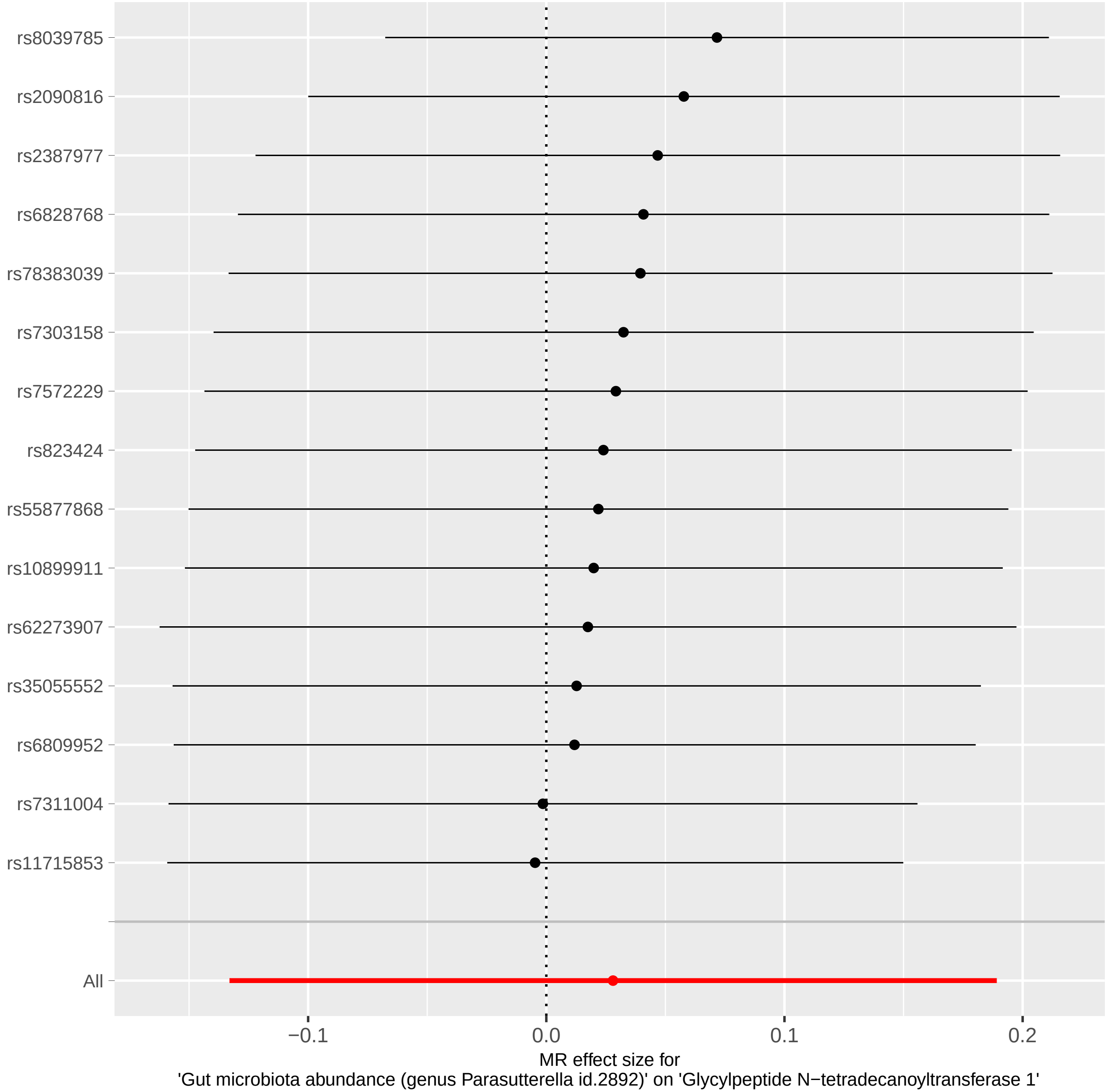

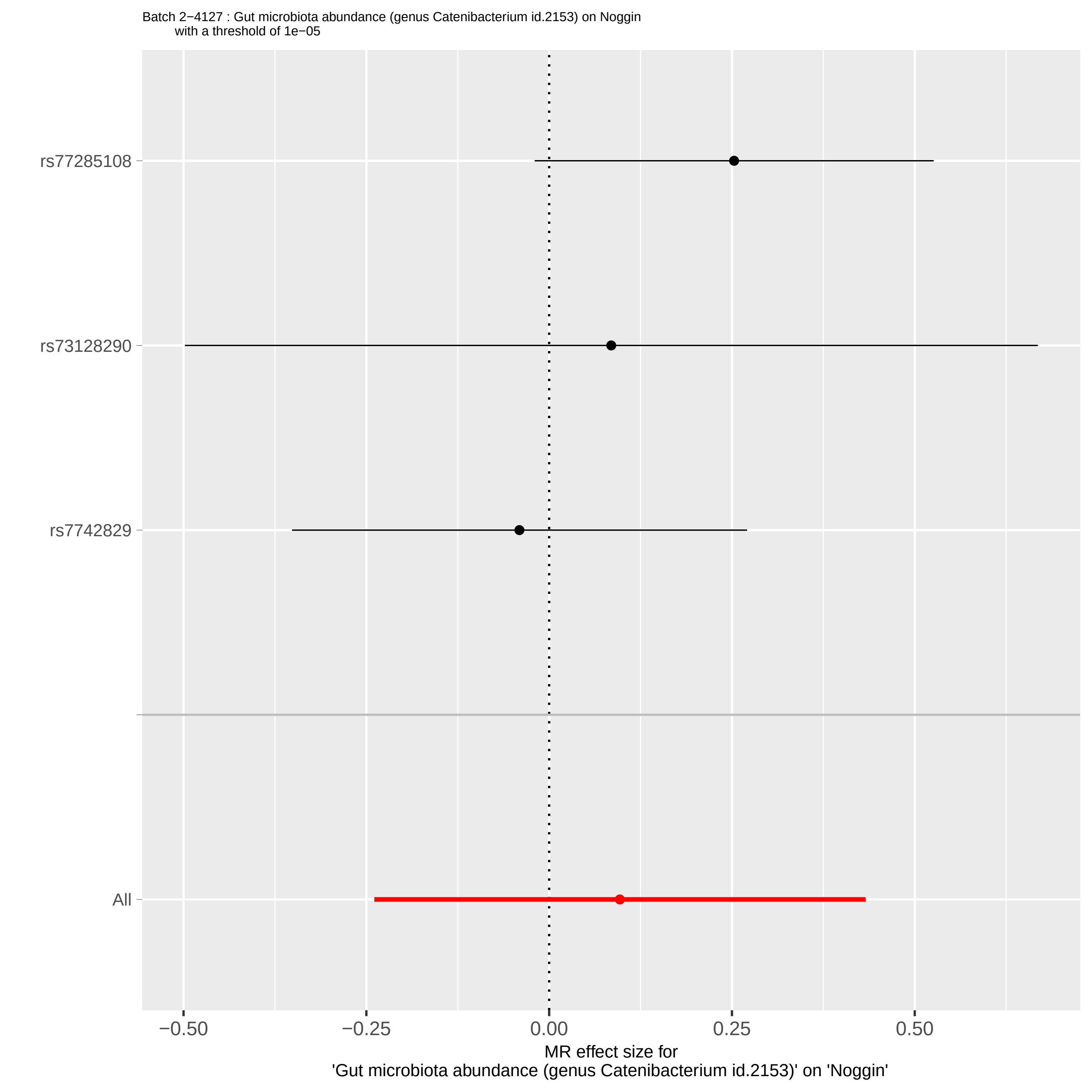

Batch 2−4127 : Gut microbiota abundance (genus Catenibacterium id.2153) on Noggin
with a threshold of 1e−05
rs77285108
rs73128290
rs7742829
All
−0.50
−0.25
0.00
0.25
0.50
MR effect size for
'Gut microbiota abundance (genus Catenibacterium id.2153)' on 'Noggin'

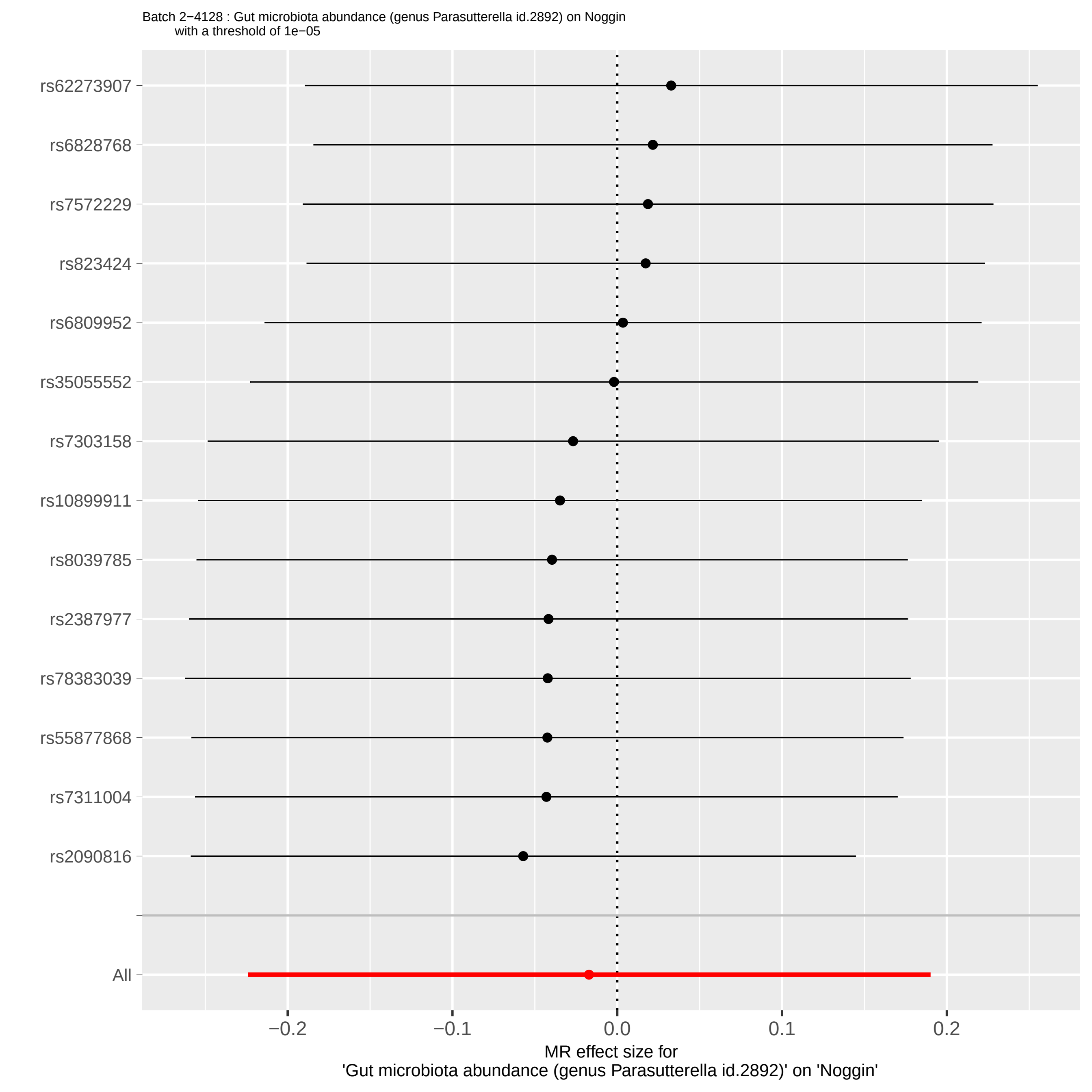
Batch 2−4128 : Gut microbiota abundance (genus Parasutterella id.2892) on Noggin
with a threshold of 1e−05
rs62273907
rs6828768
rs7572229
rs823424
rs6809952
rs35055552
rs7303158
rs10899911
rs8039785
rs2387977
rs78383039
rs55877868
rs7311004
rs2090816
All
−0.2
−0.1
0.0
0.1
0.2
MR effect size for
'Gut microbiota abundance (genus Parasutterella id.2892)' on 'Noggin'

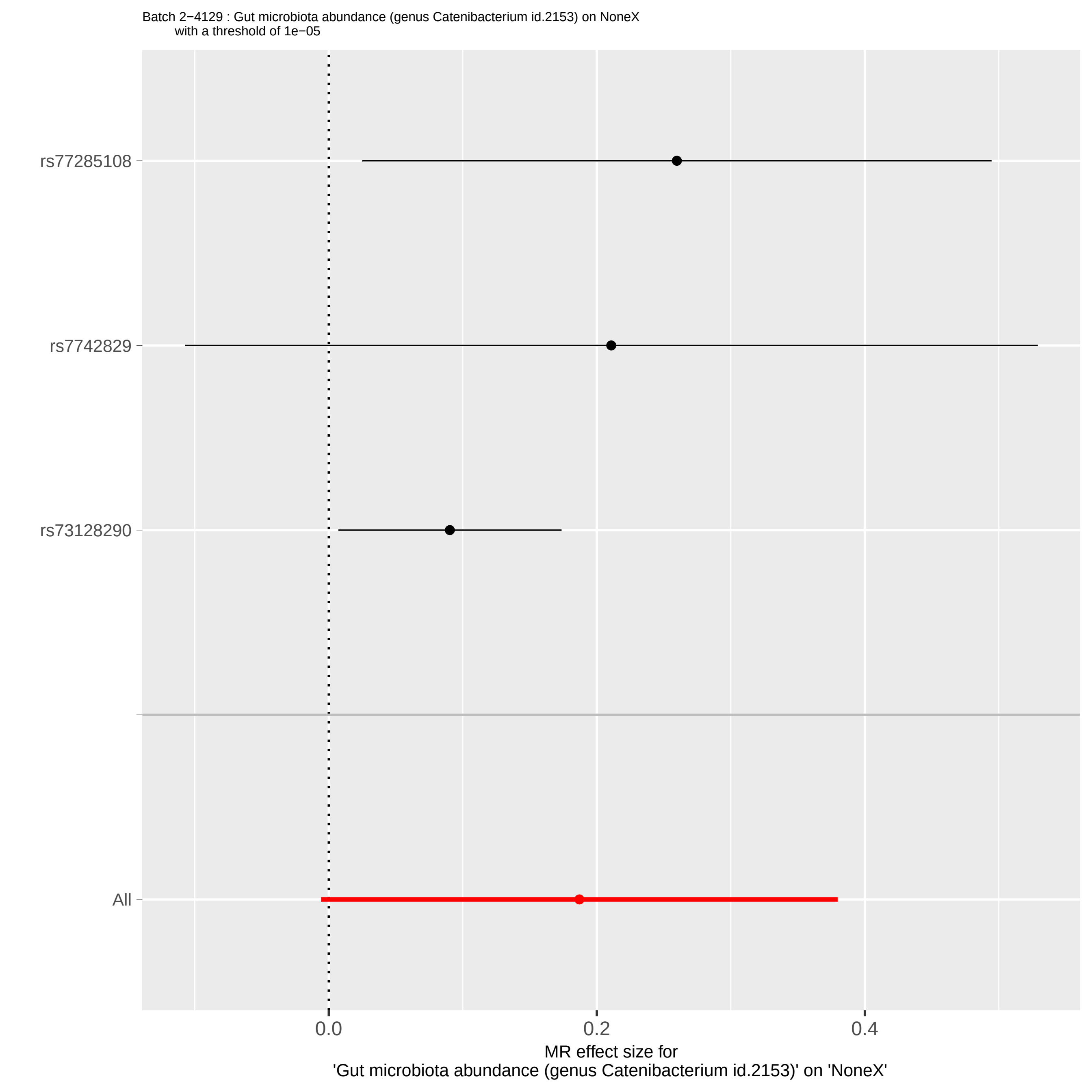
Batch 2−4129 : Gut microbiota abundance (genus Catenibacterium id.2153) on NoneX
with a threshold of 1e−05
rs77285108
rs7742829
rs73128290
All
0.0
0.2
0.4
MR effect size for
'Gut microbiota abundance (genus Catenibacterium id.2153)' on 'NoneX'

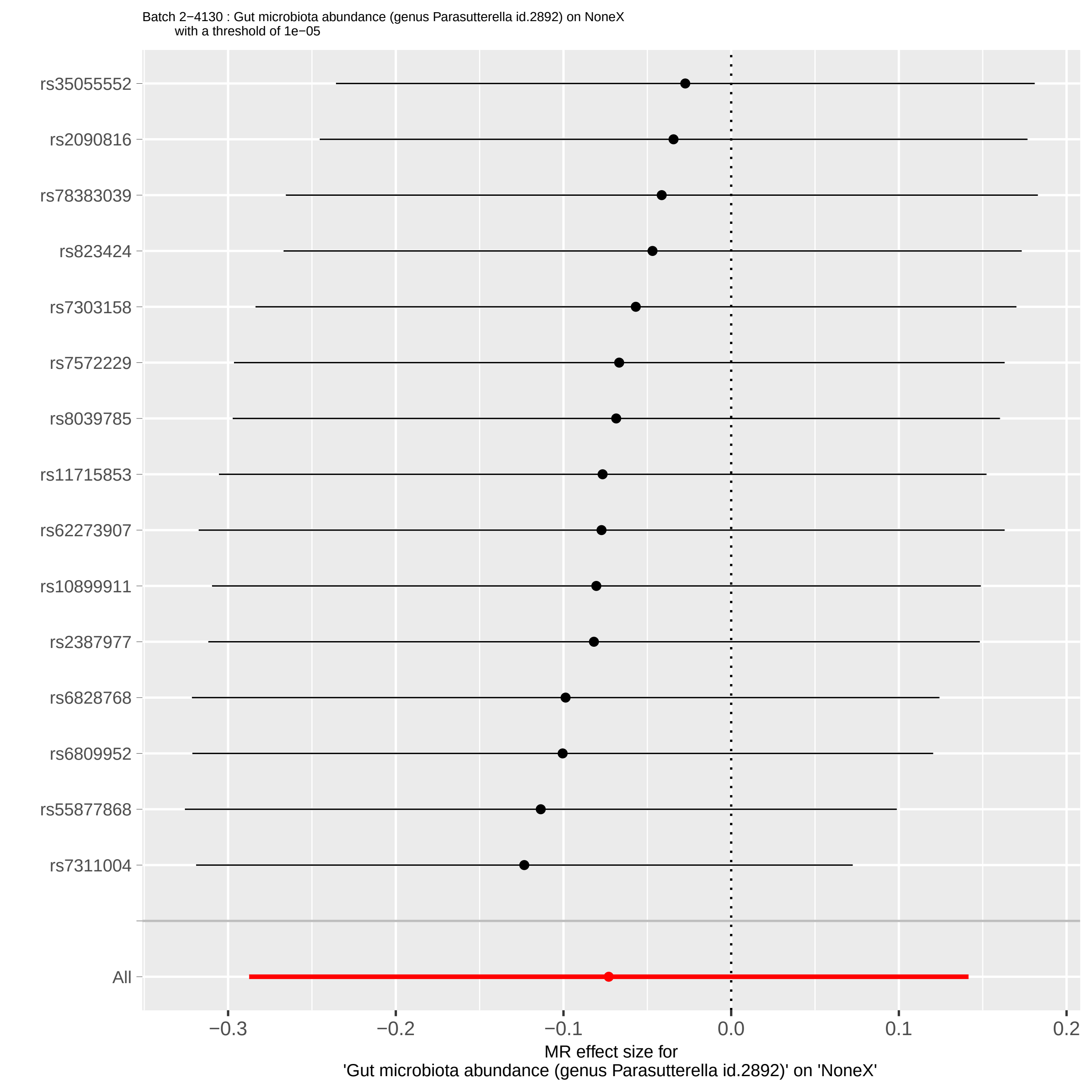
Batch 2−4130 : Gut microbiota abundance (genus Parasutterella id.2892) on NoneX
with a threshold of 1e−05
rs35055552
rs2090816
rs78383039
rs823424
rs7303158
rs7572229
rs8039785
rs11715853
rs62273907
rs10899911
rs2387977
rs6828768
rs6809952
rs55877868
rs7311004
All
−0.3
−0.2
−0.1
0.0
0.1
0.2
MR effect size for
'Gut microbiota abundance (genus Parasutterella id.2892)' on 'NoneX'

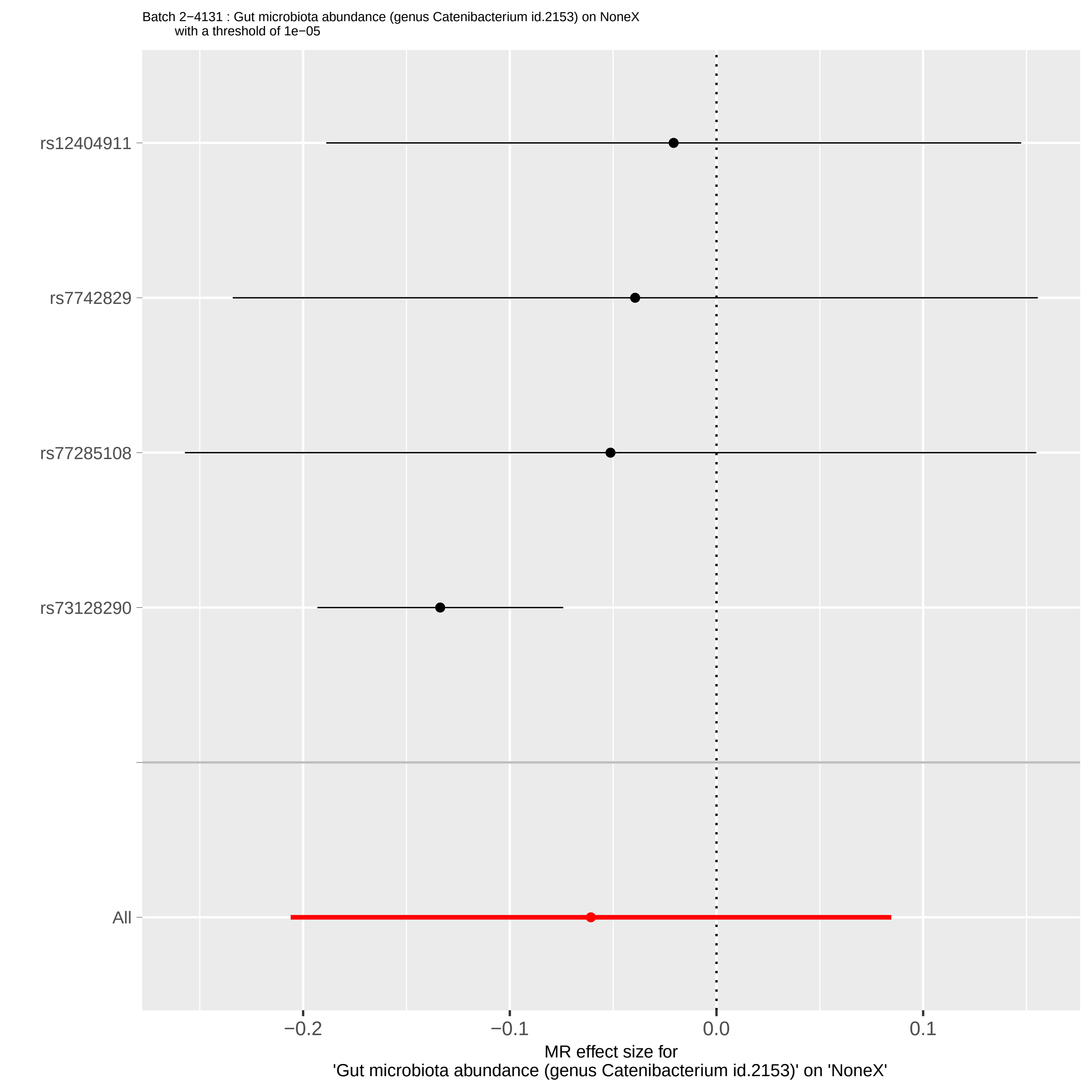
Batch 2−4131 : Gut microbiota abundance (genus Catenibacterium id.2153) on NoneX
with a threshold of 1e−05
rs12404911
rs7742829
rs77285108
rs73128290
All
−0.2
−0.1
0.0
0.1
MR effect size for
'Gut microbiota abundance (genus Catenibacterium id.2153)' on 'NoneX'

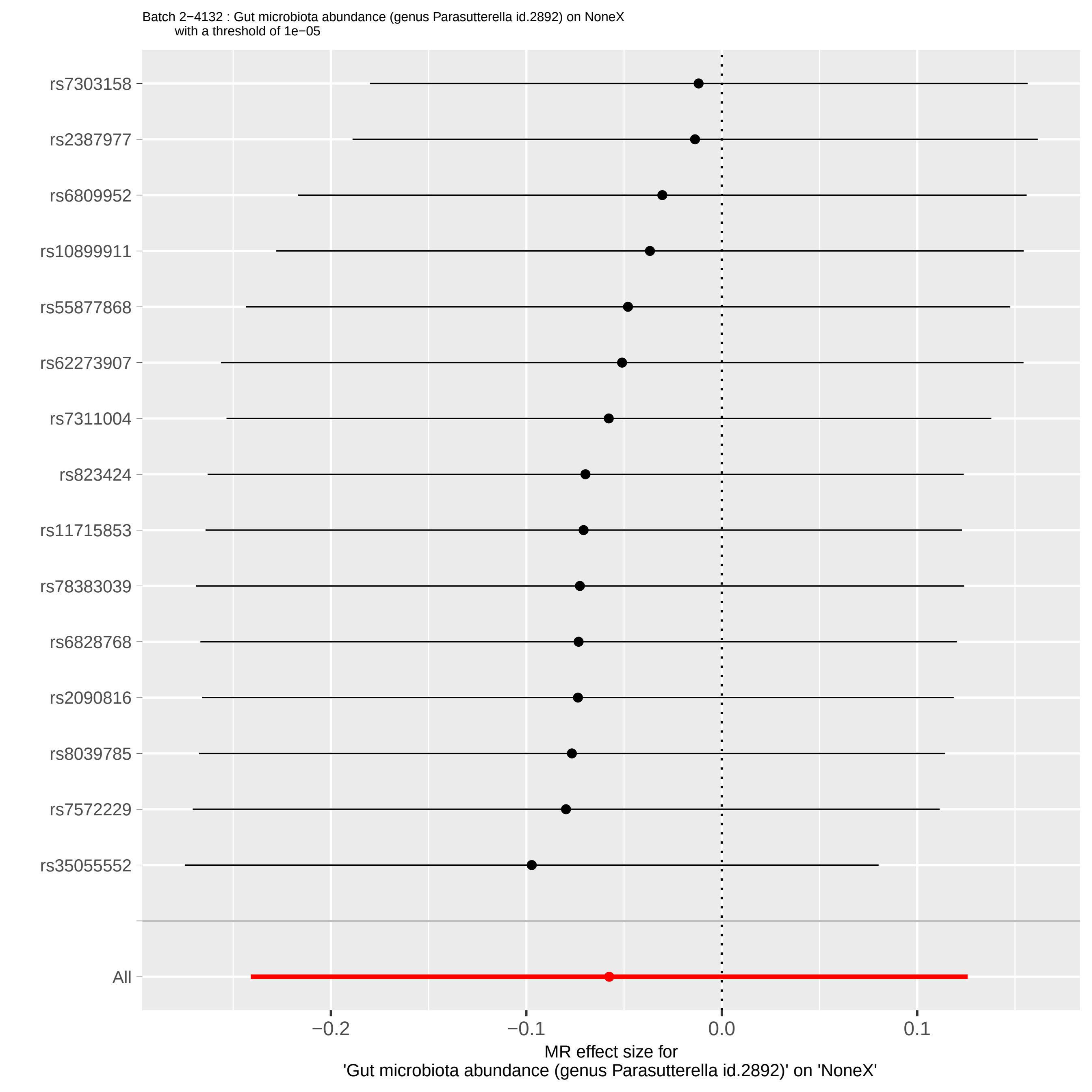
Batch 2−4132 : Gut microbiota abundance (genus Parasutterella id.2892) on NoneX
with a threshold of 1e−05
rs7303158
rs2387977
rs6809952
rs10899911
rs55877868
rs62273907
rs7311004
rs823424
rs11715853
rs78383039
rs6828768
rs2090816
rs8039785
rs7572229
rs35055552
All
−0.2
−0.1
0.0
0.1
MR effect size for
'Gut microbiota abundance (genus Parasutterella id.2892)' on 'NoneX'

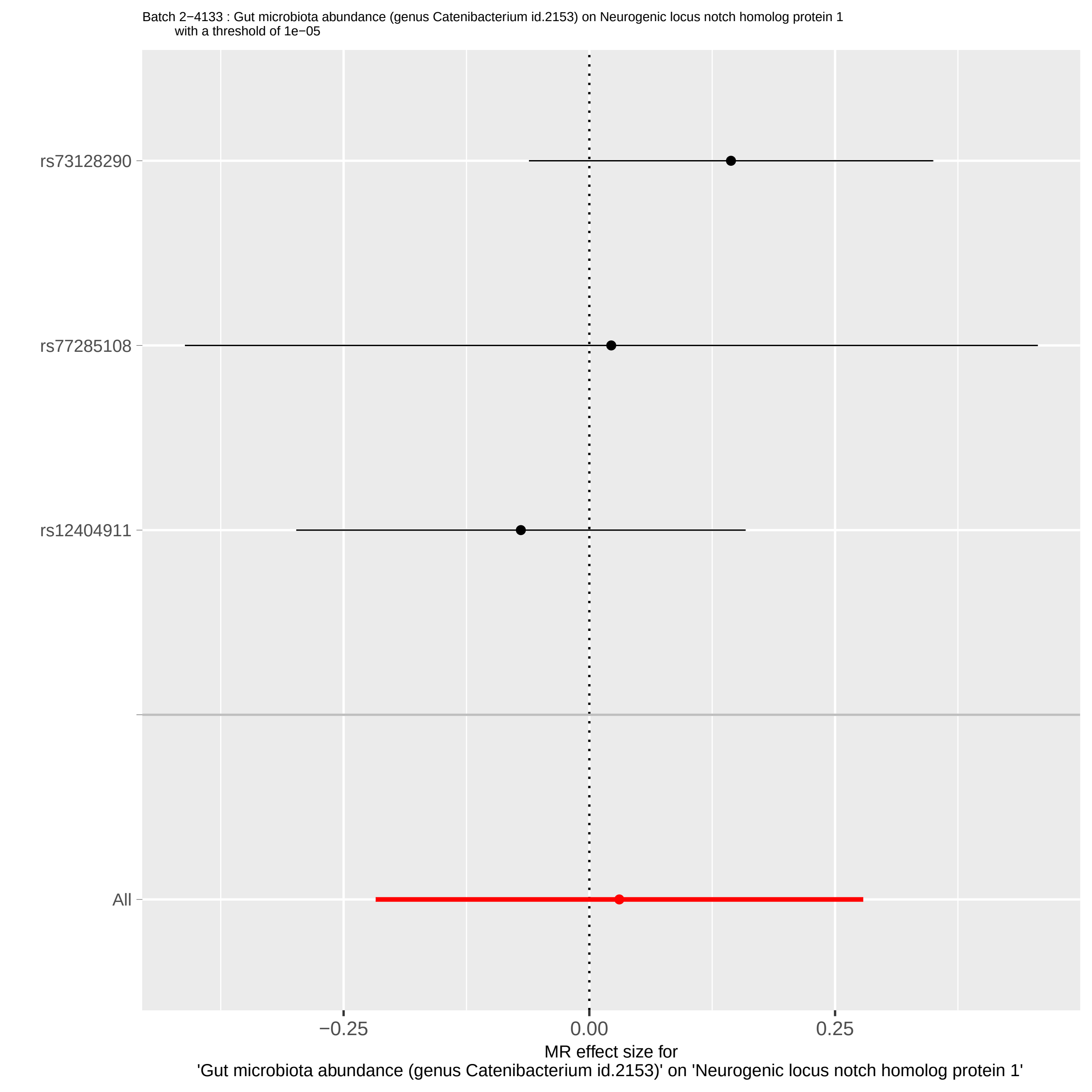

Batch 2−4133 : Gut microbiota abundance (genus Catenibacterium id.2153) on Neurogenic locus notch homolog protein 1
with a threshold of 1e−05
rs73128290
rs77285108
rs12404911
All
−0.25
0.00
0.25
MR effect size for
'Gut microbiota abundance (genus Catenibacterium id.2153)' on 'Neurogenic locus notch homolog protein 1'

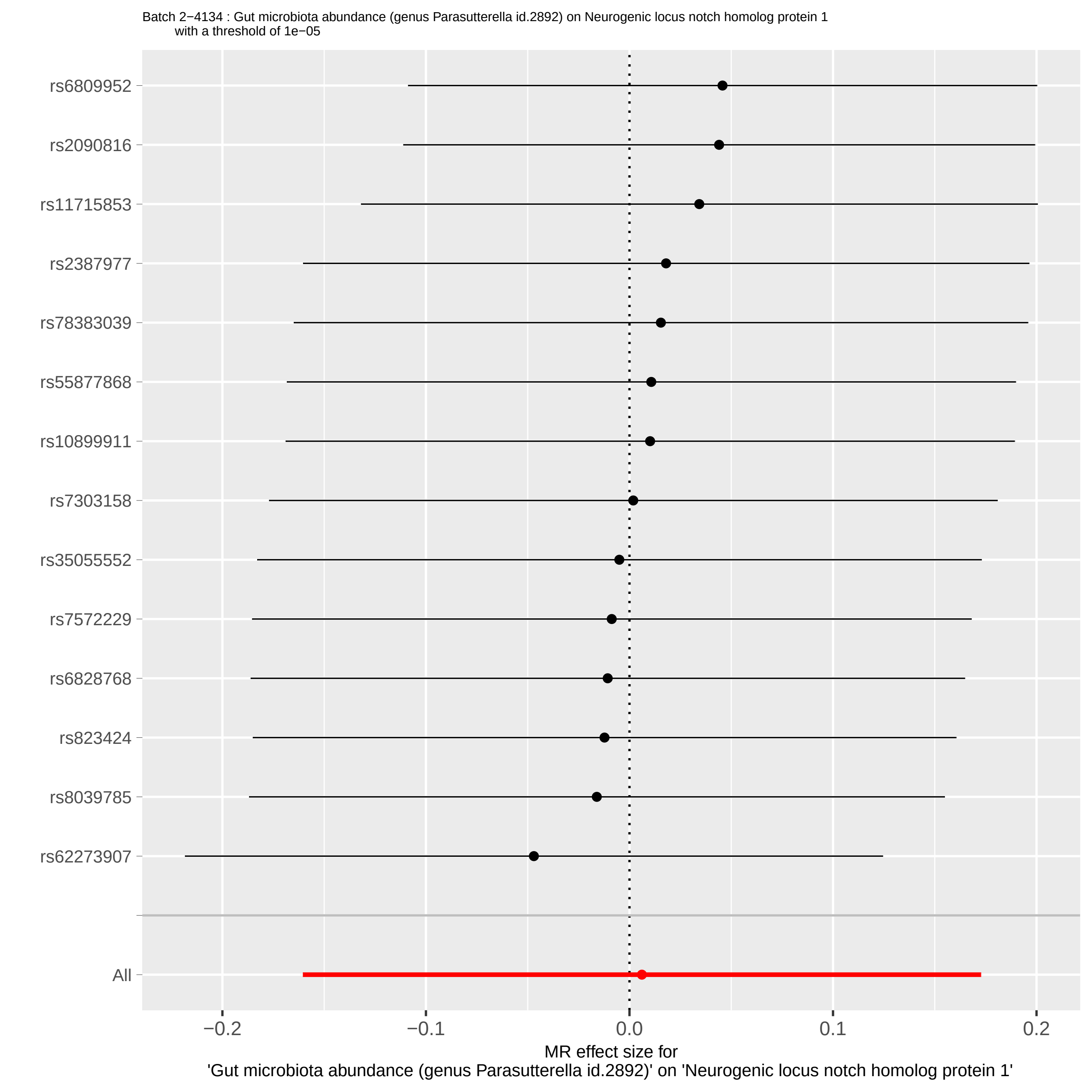
Batch 2−4134 : Gut microbiota abundance (genus Parasutterella id.2892) on Neurogenic locus notch homolog protein 1
with a threshold of 1e−05
rs6809952
rs2090816
rs11715853
rs2387977
rs78383039
rs55877868
rs10899911
rs7303158
rs35055552
rs7572229
rs6828768
rs823424
rs8039785
rs62273907
All
−0.2
−0.1
0.0
0.1
0.2
MR effect size for
'Gut microbiota abundance (genus Parasutterella id.2892)' on 'Neurogenic locus notch homolog protein 1'

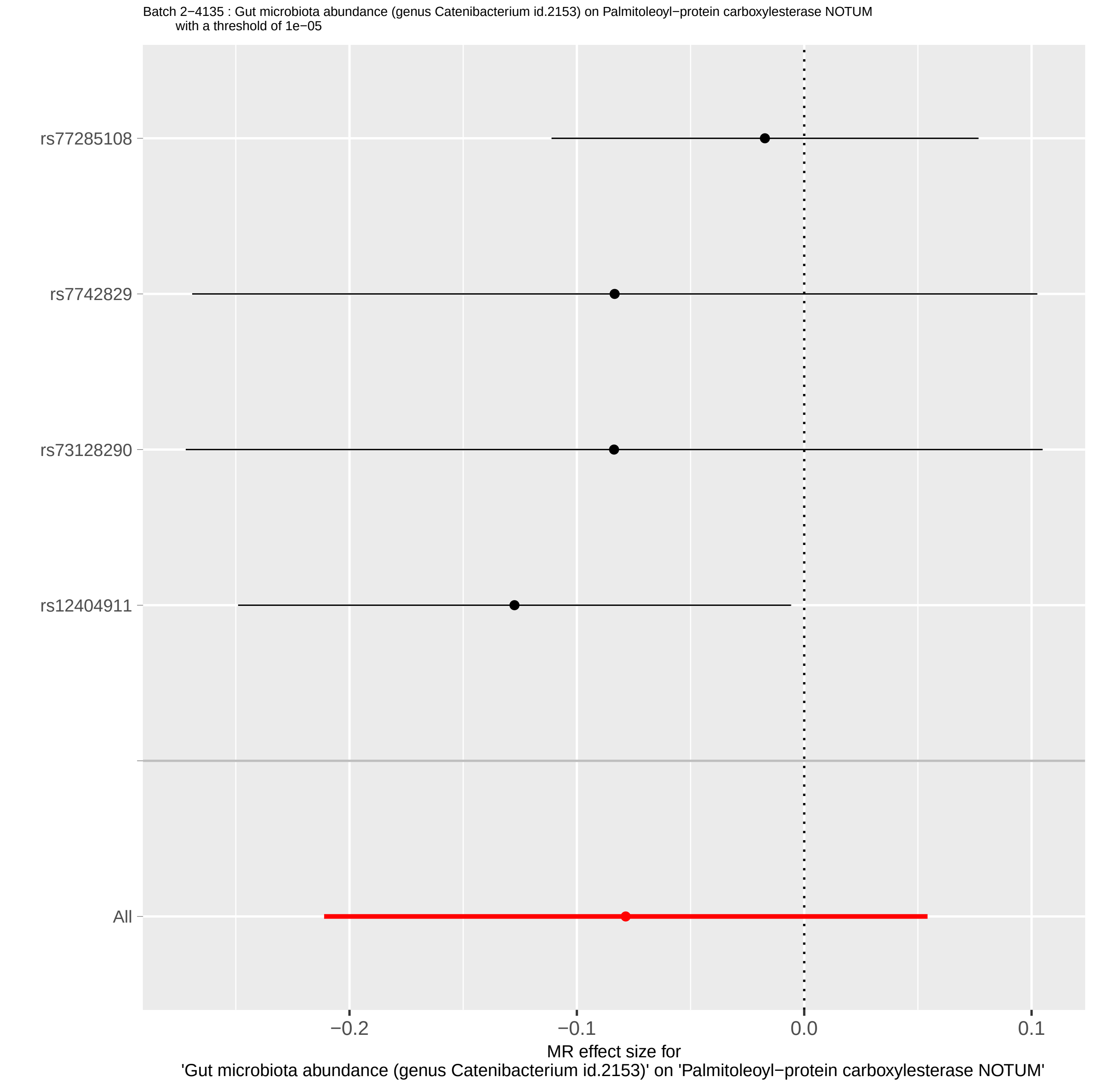

Batch 2−4135 : Gut microbiota abundance (genus Catenibacterium id.2153) on Palmitoleoyl−protein carboxylesterase NOTUM
with a threshold of 1e−05
rs77285108
rs7742829
rs73128290
rs12404911
All
−0.2
−0.1
0.0
0.1
MR effect size for
'Gut microbiota abundance (genus Catenibacterium id.2153)' on 'Palmitoleoyl−protein carboxylesterase NOTUM'

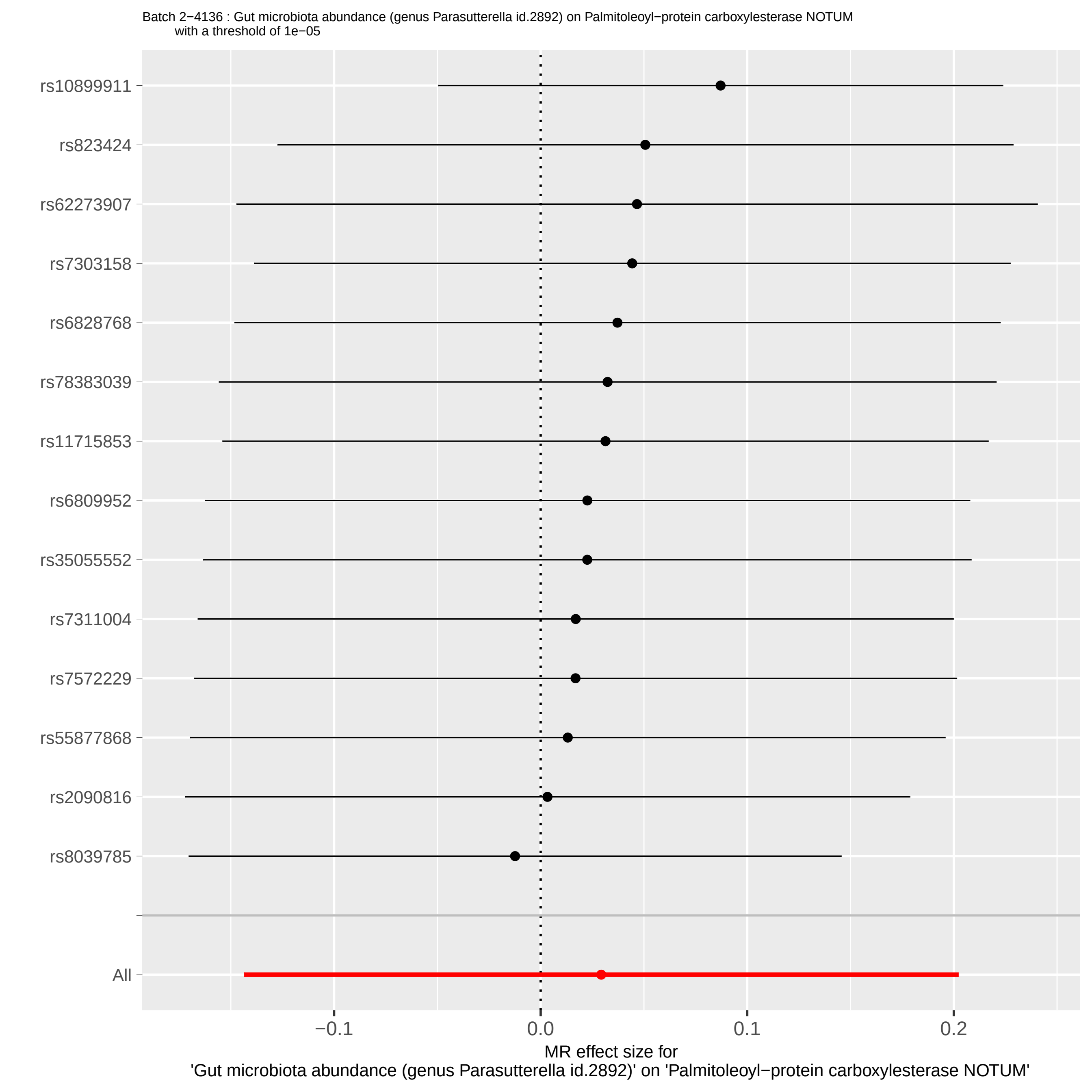

Batch 2−4136 : Gut microbiota abundance (genus Parasutterella id.2892) on Palmitoleoyl−protein carboxylesterase NOTUM
with a threshold of 1e−05
rs10899911
rs823424
rs62273907
rs7303158
rs6828768
rs78383039
rs11715853
rs6809952
rs35055552
rs7311004
rs7572229
rs55877868
rs2090816
rs8039785
All
−0.1
0.0
0.1
0.2
MR effect size for
'Gut microbiota abundance (genus Parasutterella id.2892)' on 'Palmitoleoyl−protein carboxylesterase NOTUM'

Batch 2−4137 : Gut microbiota abundance (genus Catenibacterium id.2153) on Protein NOV homolog
with a threshold of 1e−05

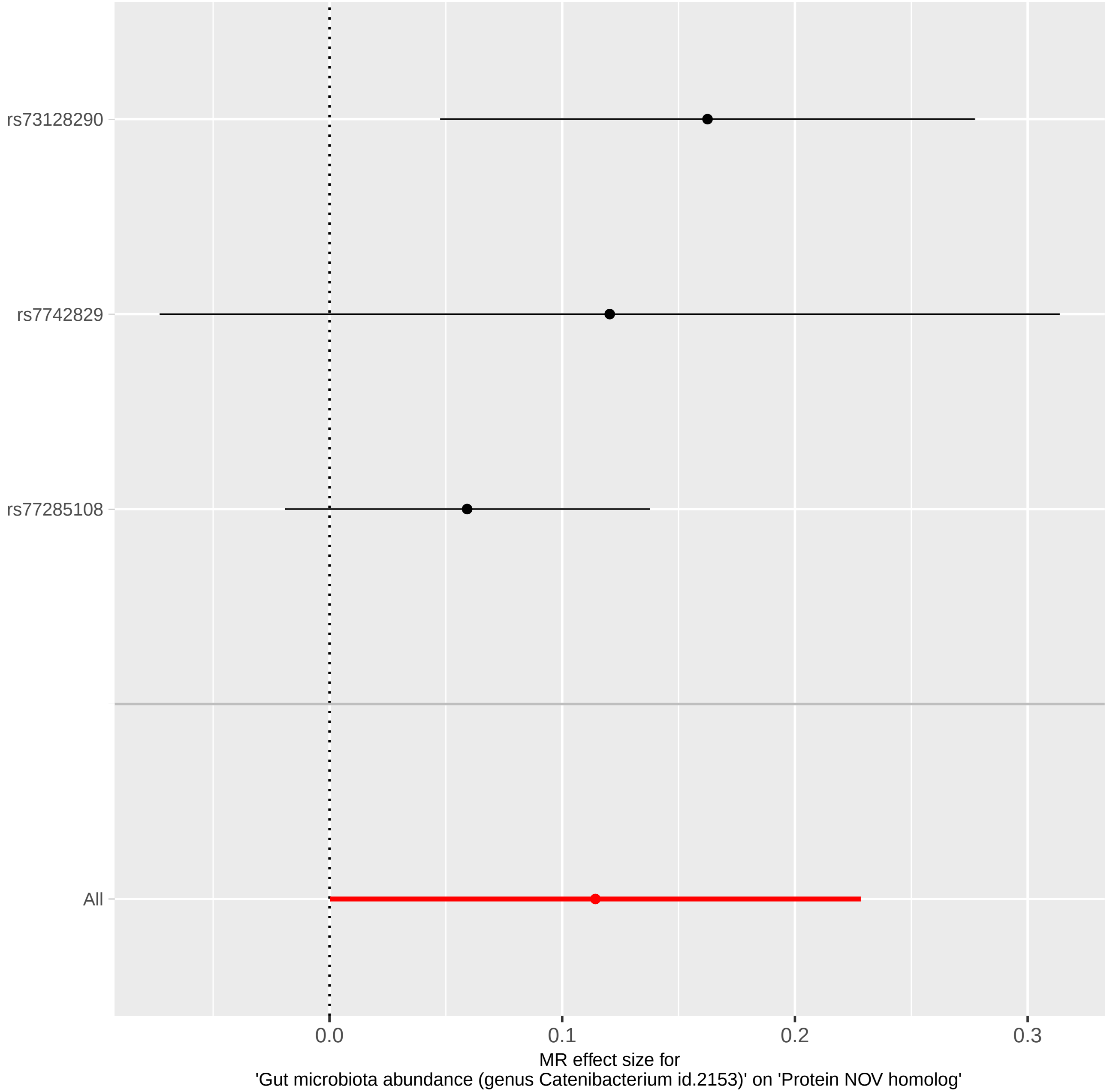

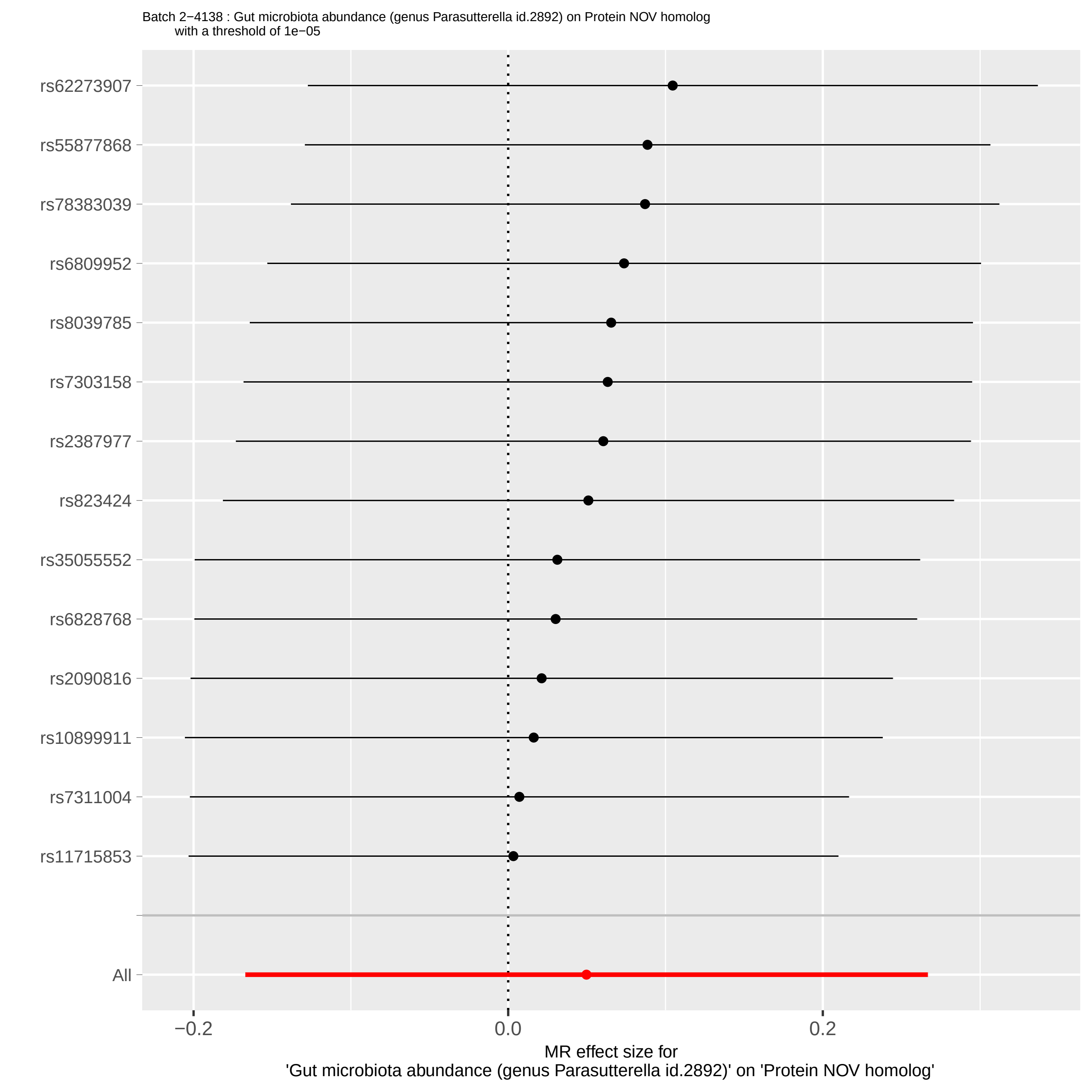

Batch 2−4138 : Gut microbiota abundance (genus Parasutterella id.2892) on Protein NOV homolog
with a threshold of 1e−05
rs62273907
rs55877868
rs78383039
rs6809952
rs8039785
rs7303158
rs2387977
rs823424
rs35055552
rs6828768
rs2090816
rs10899911
rs7311004
rs11715853
All
−0.2
0.0
0.2
MR effect size for
'Gut microbiota abundance (genus Parasutterella id.2892)' on 'Protein NOV homolog'

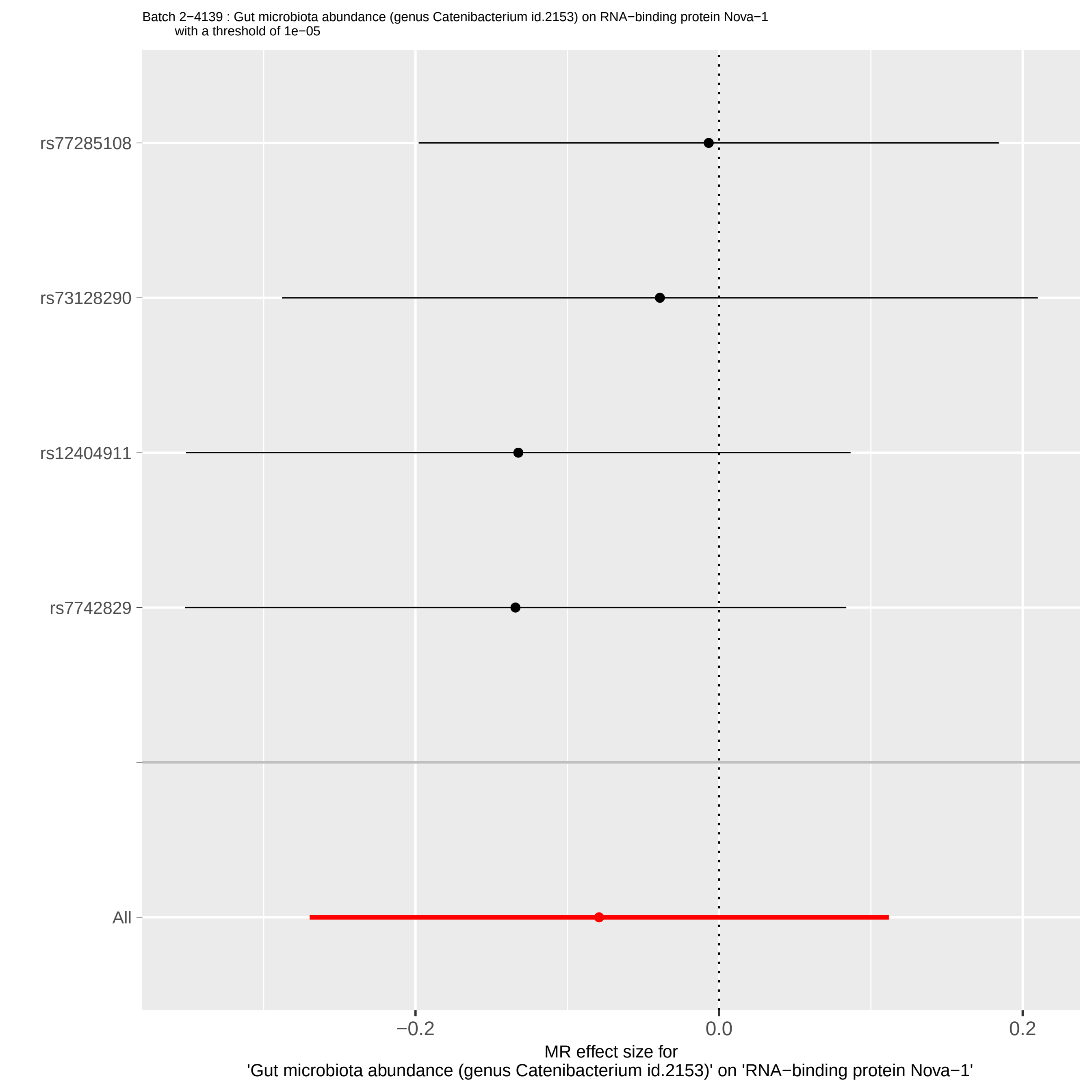

Batch 2−4139 : Gut microbiota abundance (genus Catenibacterium id.2153) on RNA−binding protein Nova−1
with a threshold of 1e−05
rs77285108
rs73128290
rs12404911
rs7742829
All
−0.2
0.0
0.2
MR effect size for
'Gut microbiota abundance (genus Catenibacterium id.2153)' on 'RNA−binding protein Nova−1'

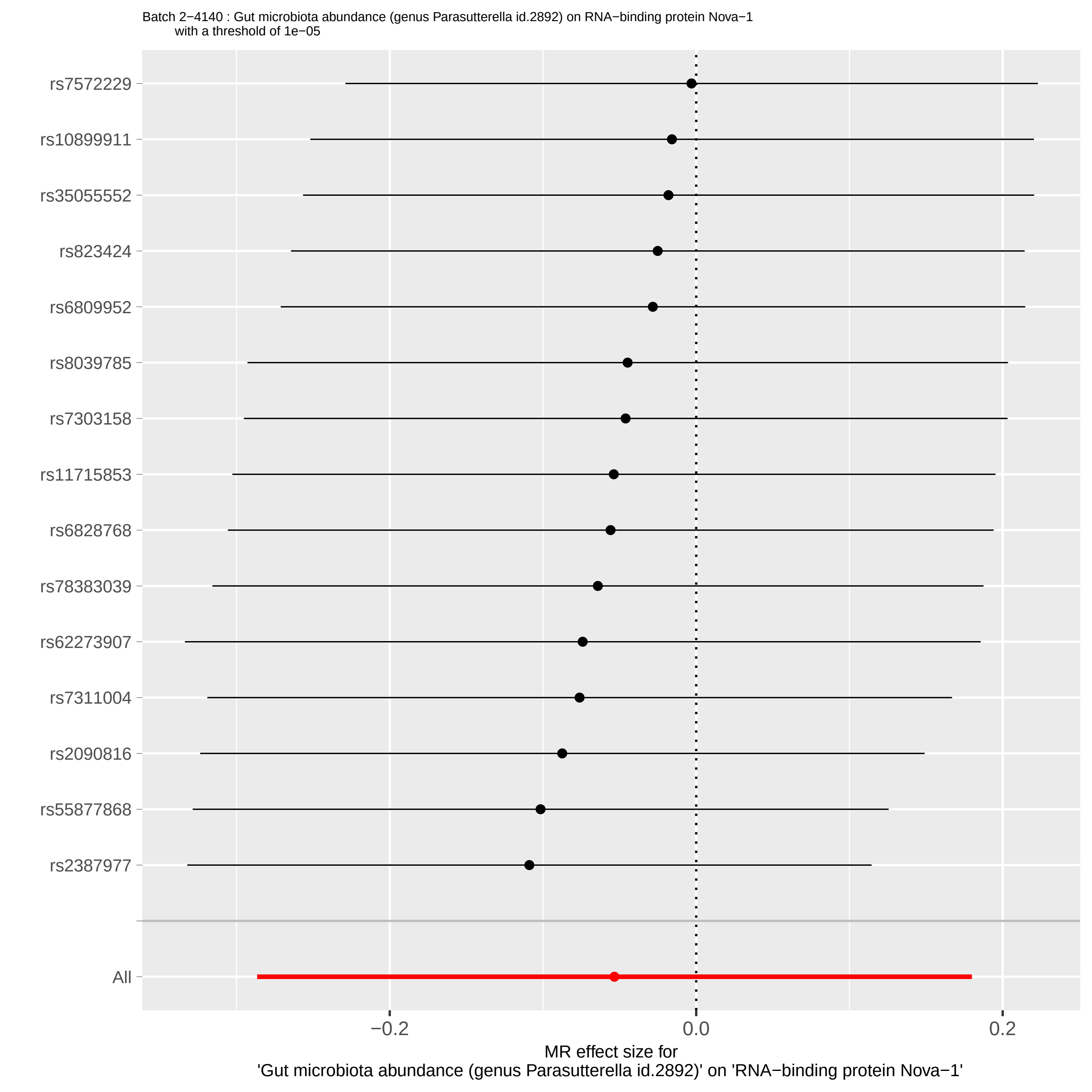

Batch 2−4140 : Gut microbiota abundance (genus Parasutterella id.2892) on RNA−binding protein Nova−1
with a threshold of 1e−05
rs7572229
rs10899911
rs35055552
rs823424
rs6809952
rs8039785
rs7303158
rs11715853
rs6828768
rs78383039
rs62273907
rs7311004
rs2090816
rs55877868
rs2387977
All
−0.2
0.0
0.2
MR effect size for
'Gut microbiota abundance (genus Parasutterella id.2892)' on 'RNA−binding protein Nova−1'

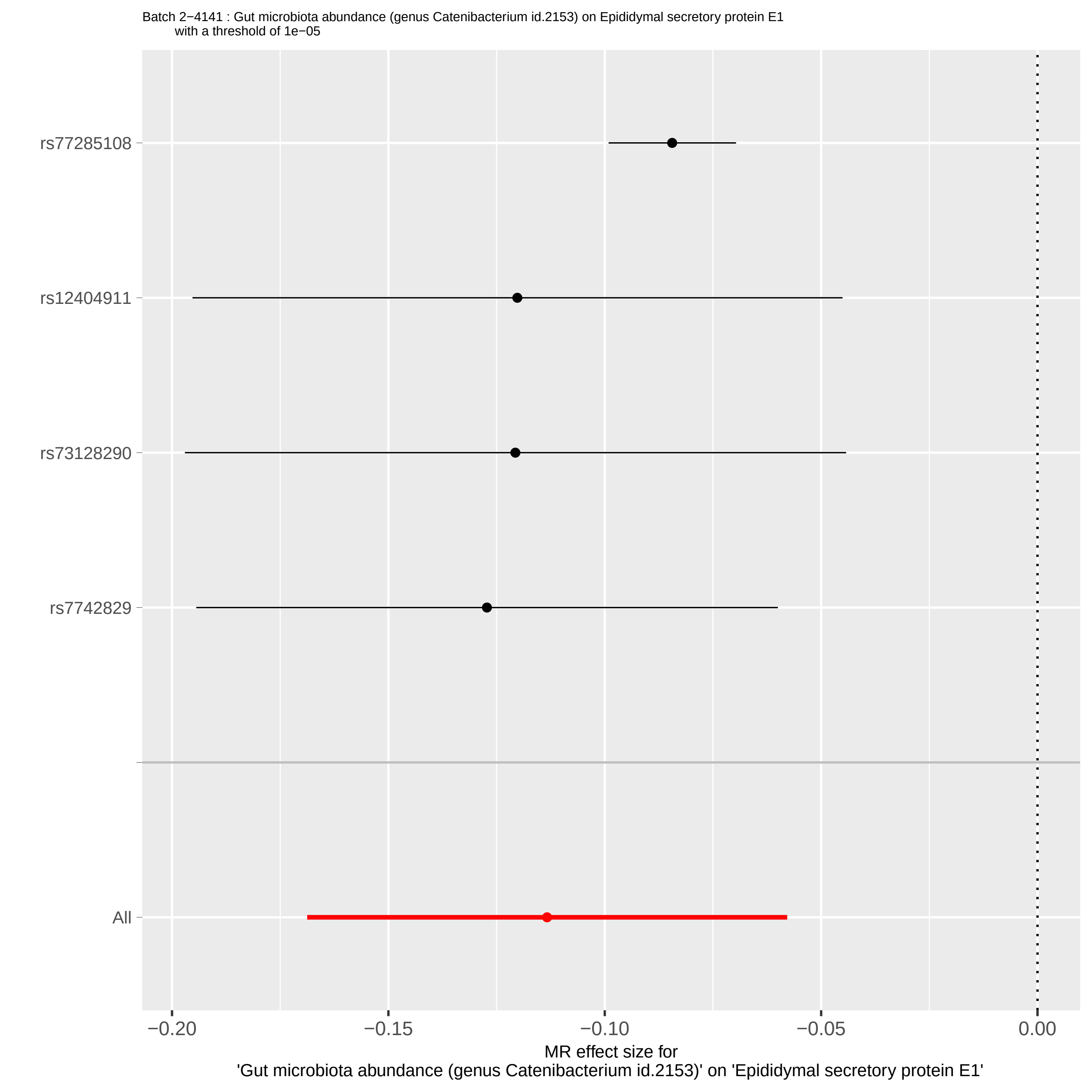

Batch 2−4141 : Gut microbiota abundance (genus Catenibacterium id.2153) on Epididymal secretory protein E1
with a threshold of 1e−05
rs77285108
rs12404911
rs73128290
rs7742829
All
−0.20
−0.15
−0.10
−0.05
0.00
MR effect size for
'Gut microbiota abundance (genus Catenibacterium id.2153)' on 'Epididymal secretory protein E1'

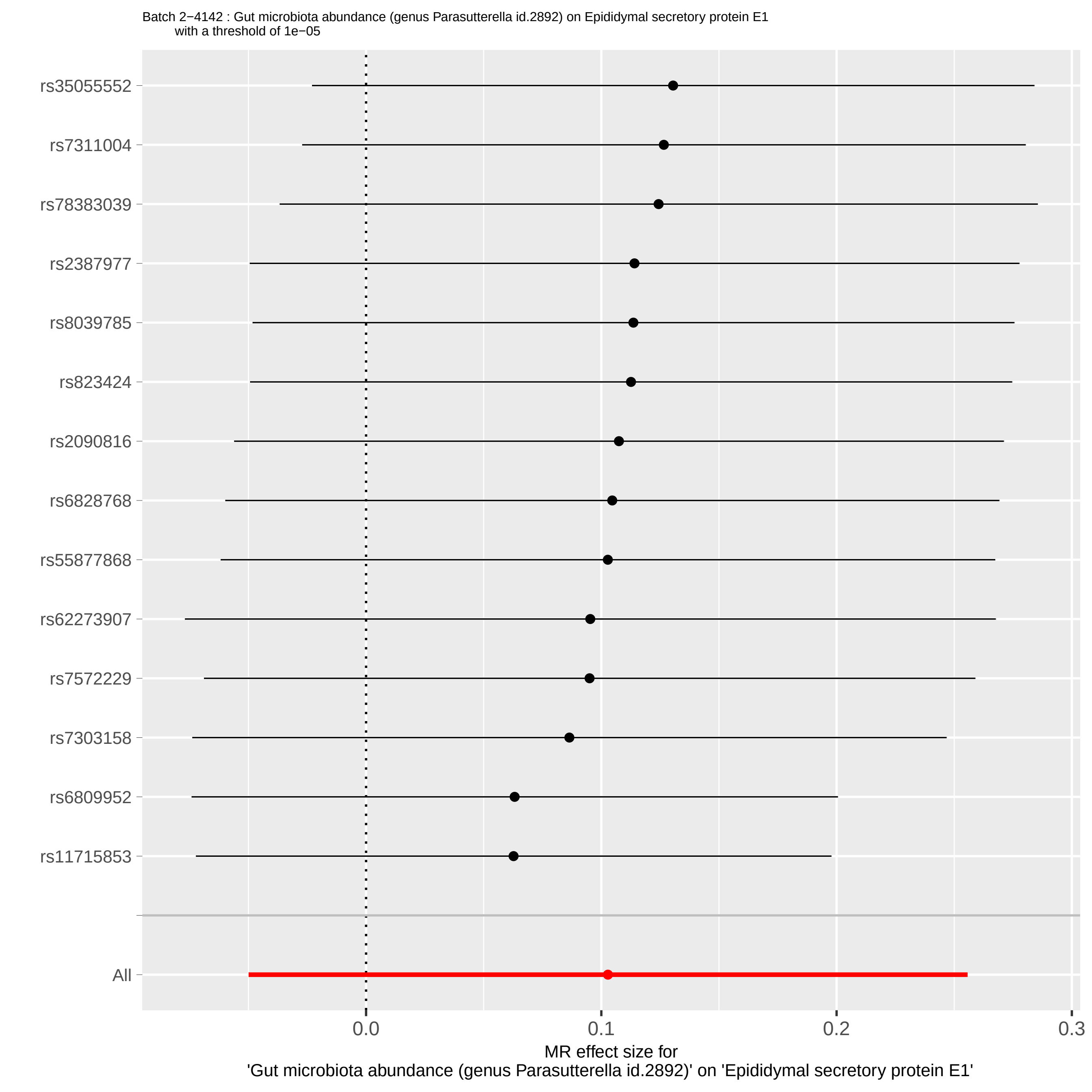
Batch 2−4142 : Gut microbiota abundance (genus Parasutterella id.2892) on Epididymal secretory protein E1
with a threshold of 1e−05
rs35055552
rs7311004
rs78383039
rs2387977
rs8039785
rs823424
rs2090816
rs6828768
rs55877868
rs62273907
rs7572229
rs7303158
rs6809952
rs11715853
All
0.0
0.1
0.2
0.3
MR effect size for
'Gut microbiota abundance (genus Parasutterella id.2892)' on 'Epididymal secretory protein E1'

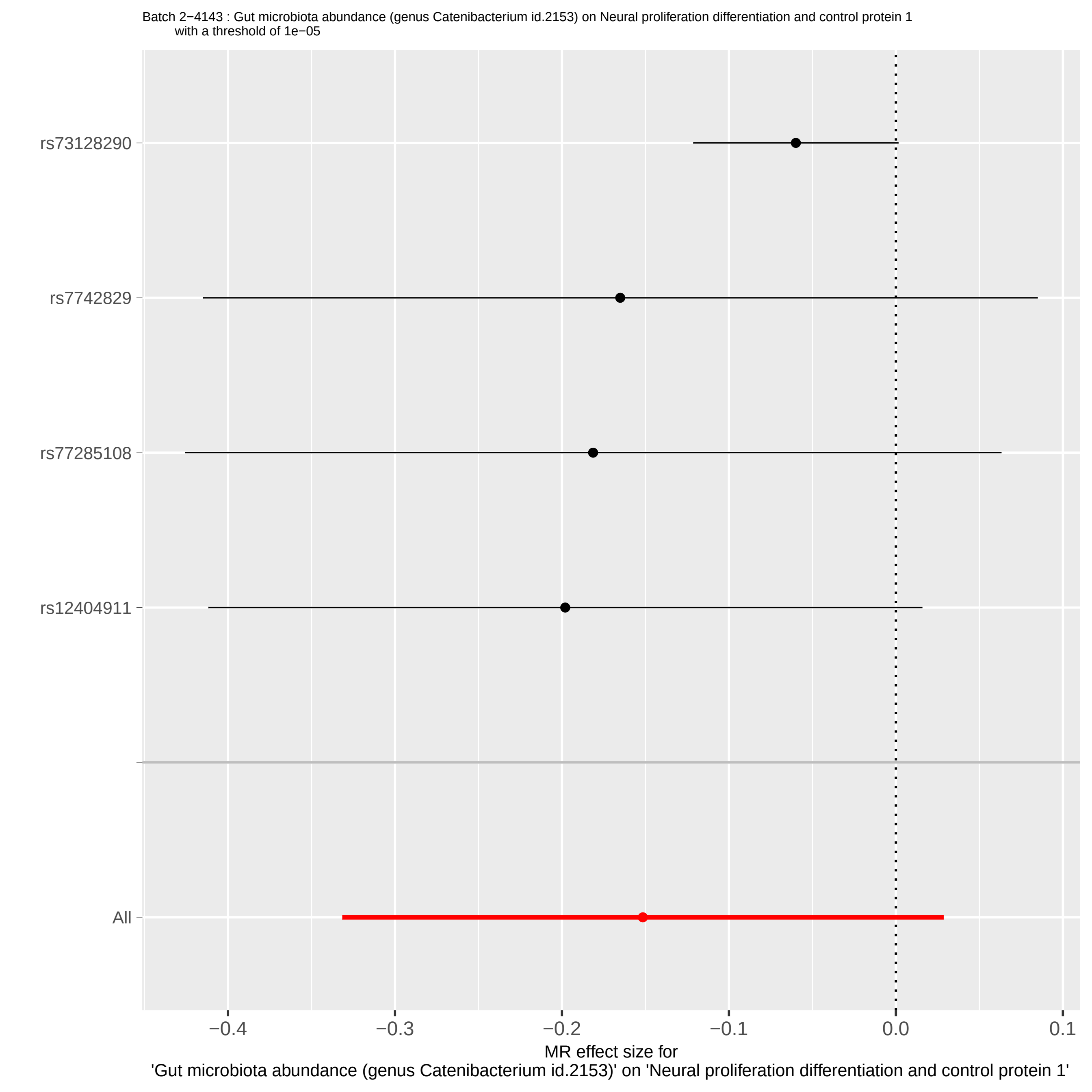

Batch 2−4143 : Gut microbiota abundance (genus Catenibacterium id.2153) on Neural proliferation differentiation and control protein 1
with a threshold of 1e−05
rs73128290
rs7742829
rs77285108
rs12404911
All
−0.4
−0.3
−0.2
−0.1
0.0
0.1
MR effect size for
'Gut microbiota abundance (genus Catenibacterium id.2153)' on 'Neural proliferation differentiation and control protein 1'

Batch 2−4144 : Gut microbiota abundance (genus Parasutterella id.2892) on Neural proliferation differentiation and control protein 1 with a threshold of 1e−05

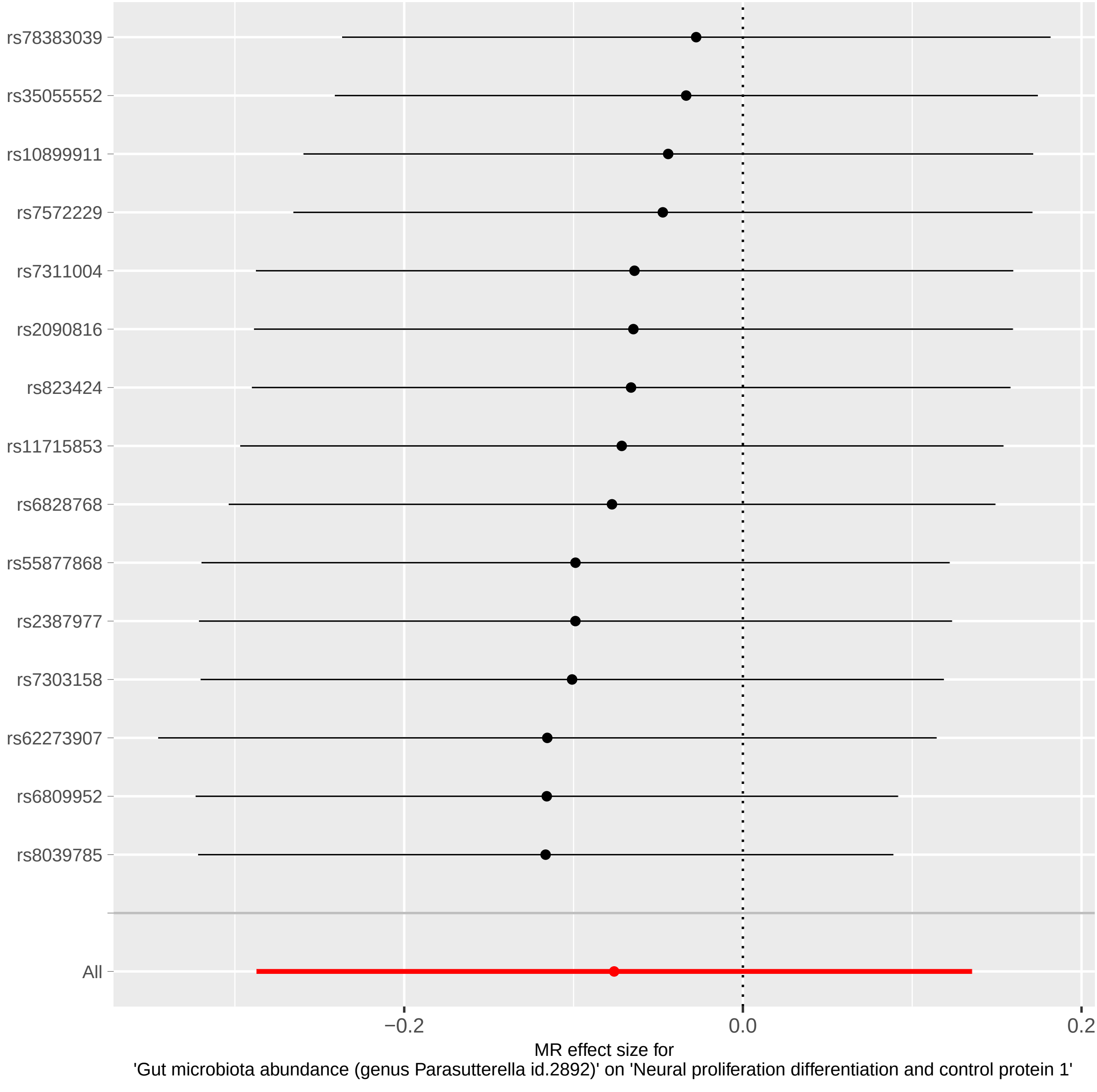

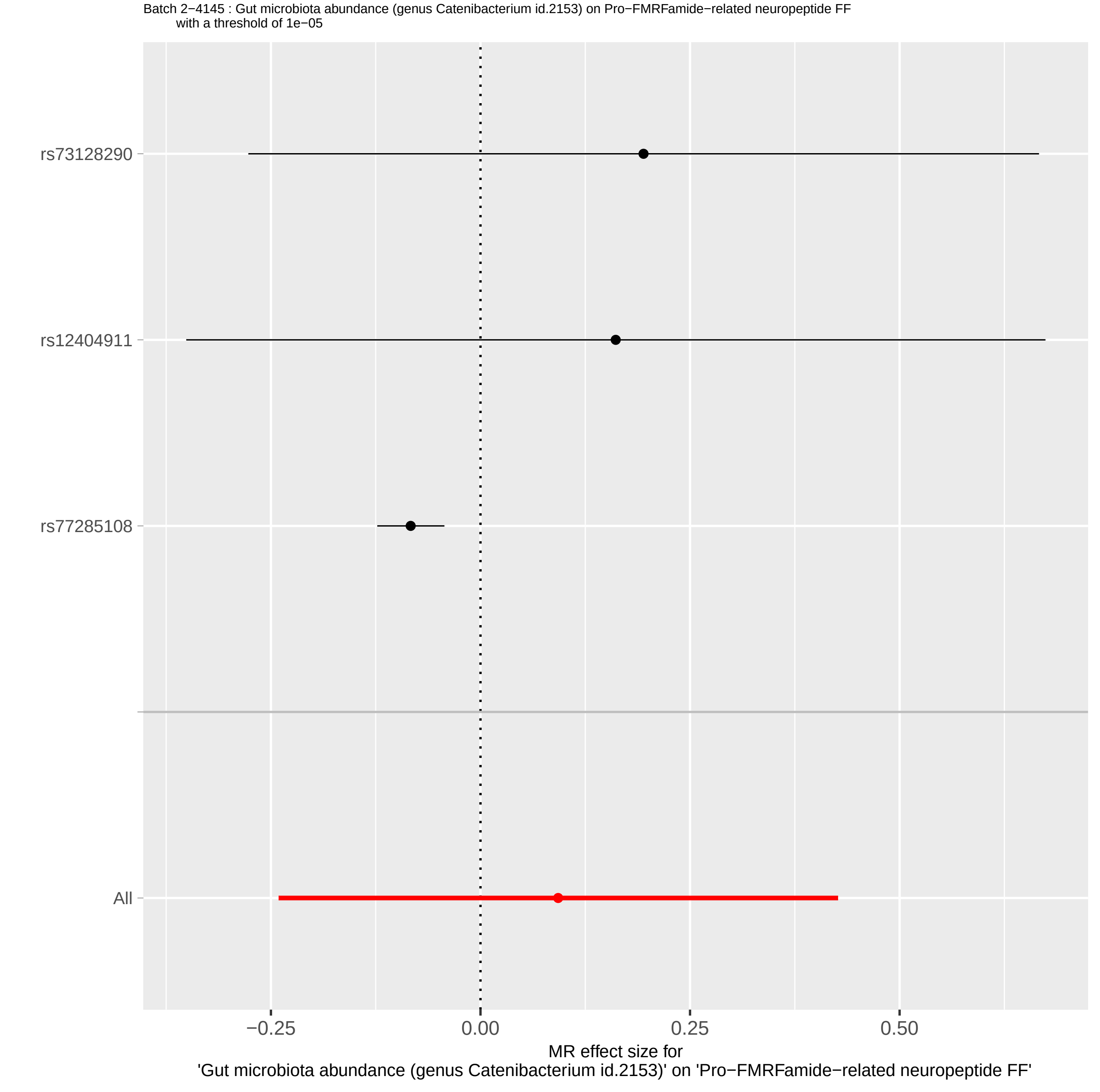
Batch 2−4145 : Gut microbiota abundance (genus Catenibacterium id.2153) on Pro−FMRFamide−related neuropeptide FF
with a threshold of 1e−05
rs73128290
rs12404911
rs77285108
All
−0.25
0.00
0.25
0.50
MR effect size for
'Gut microbiota abundance (genus Catenibacterium id.2153)' on 'Pro−FMRFamide−related neuropeptide FF'

Batch 2−4146 : Gut microbiota abundance (genus Parasutterella id.2892) on Pro−FMRFamide−related neuropeptide FF
with a threshold of 1e−05

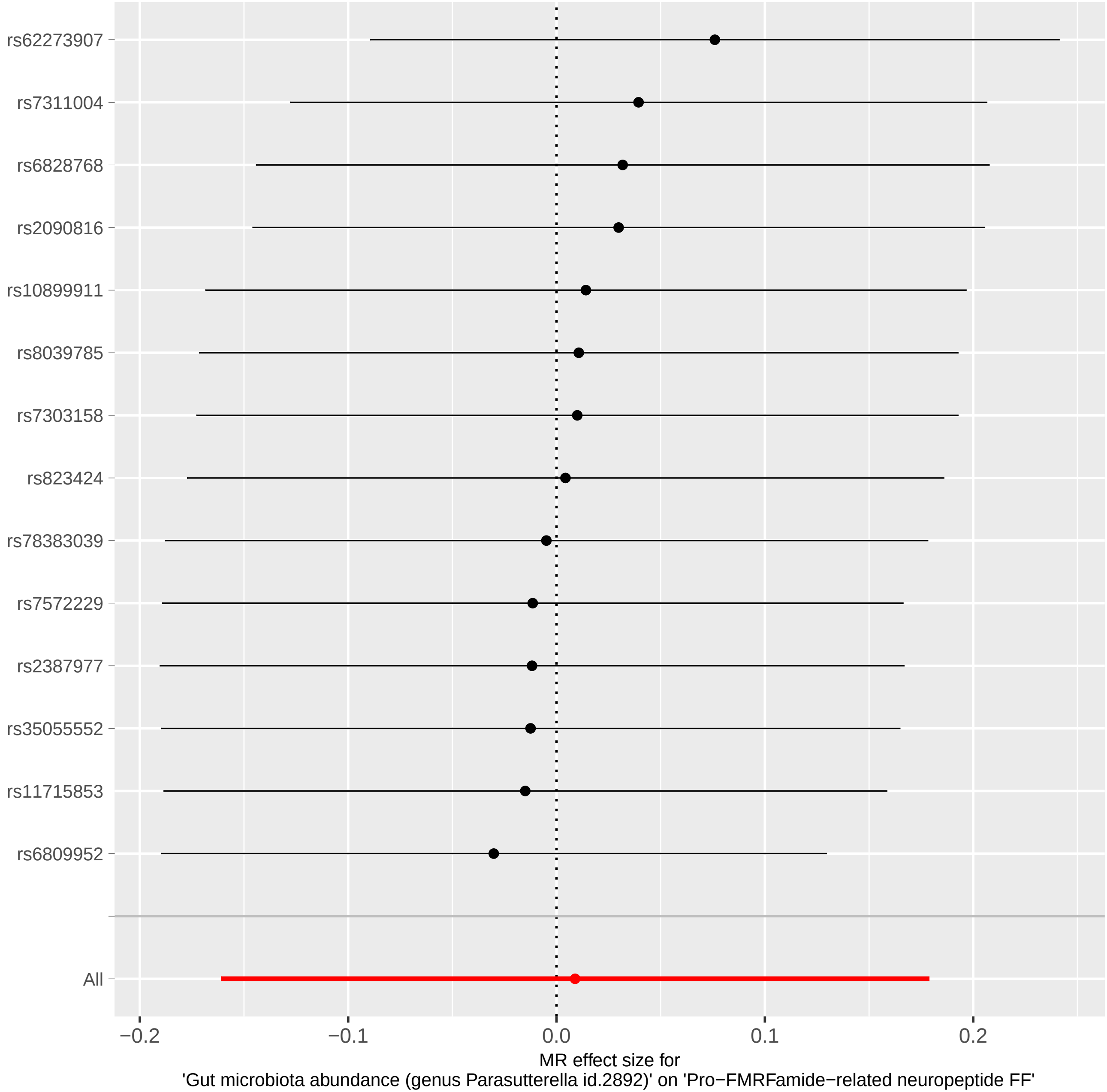

Batch 2−4147 : Gut microbiota abundance (genus Catenibacterium id.2153) on Nuclear pore complex−interacting protein family member B3
with a threshold of 1e−05

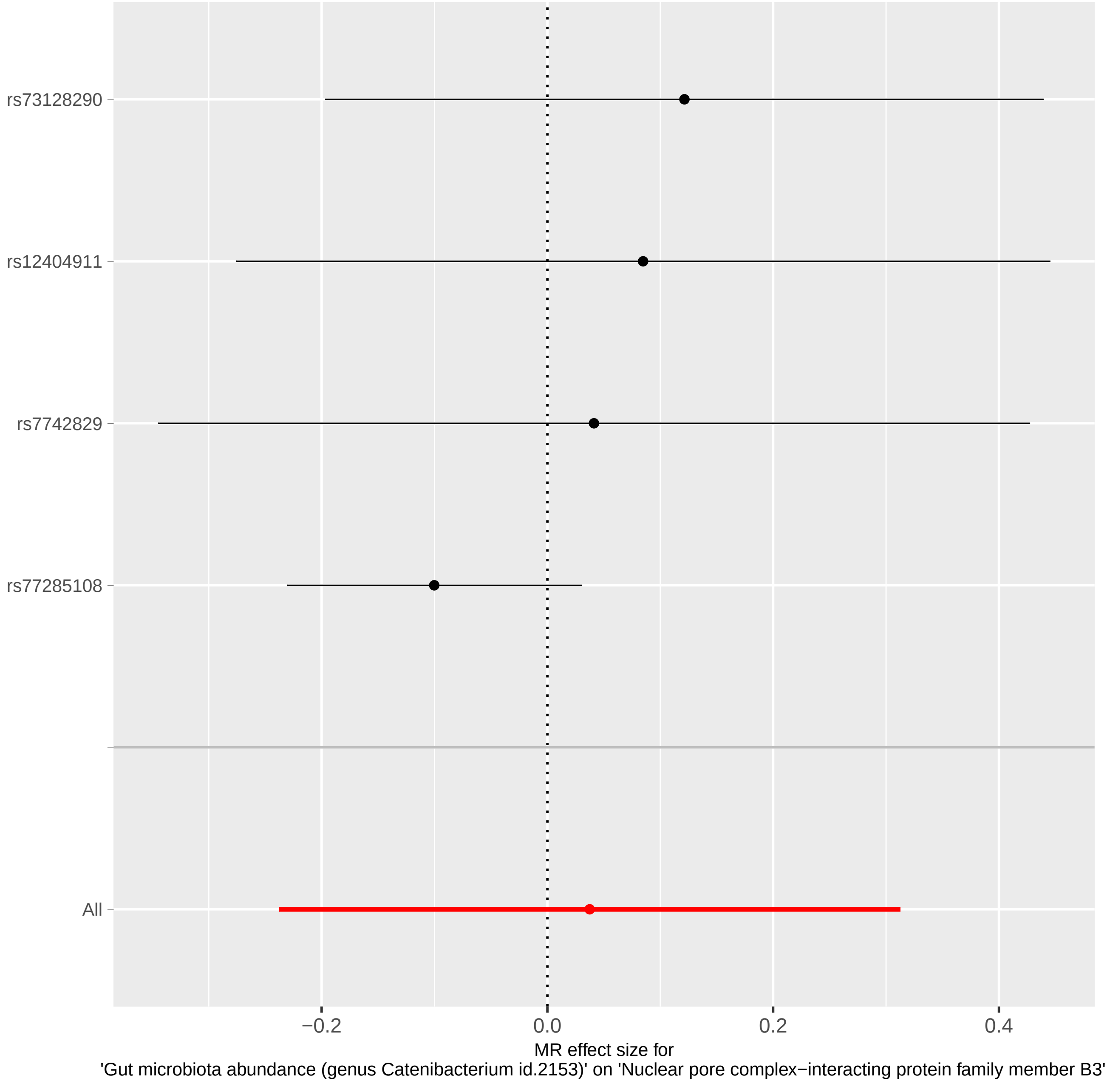

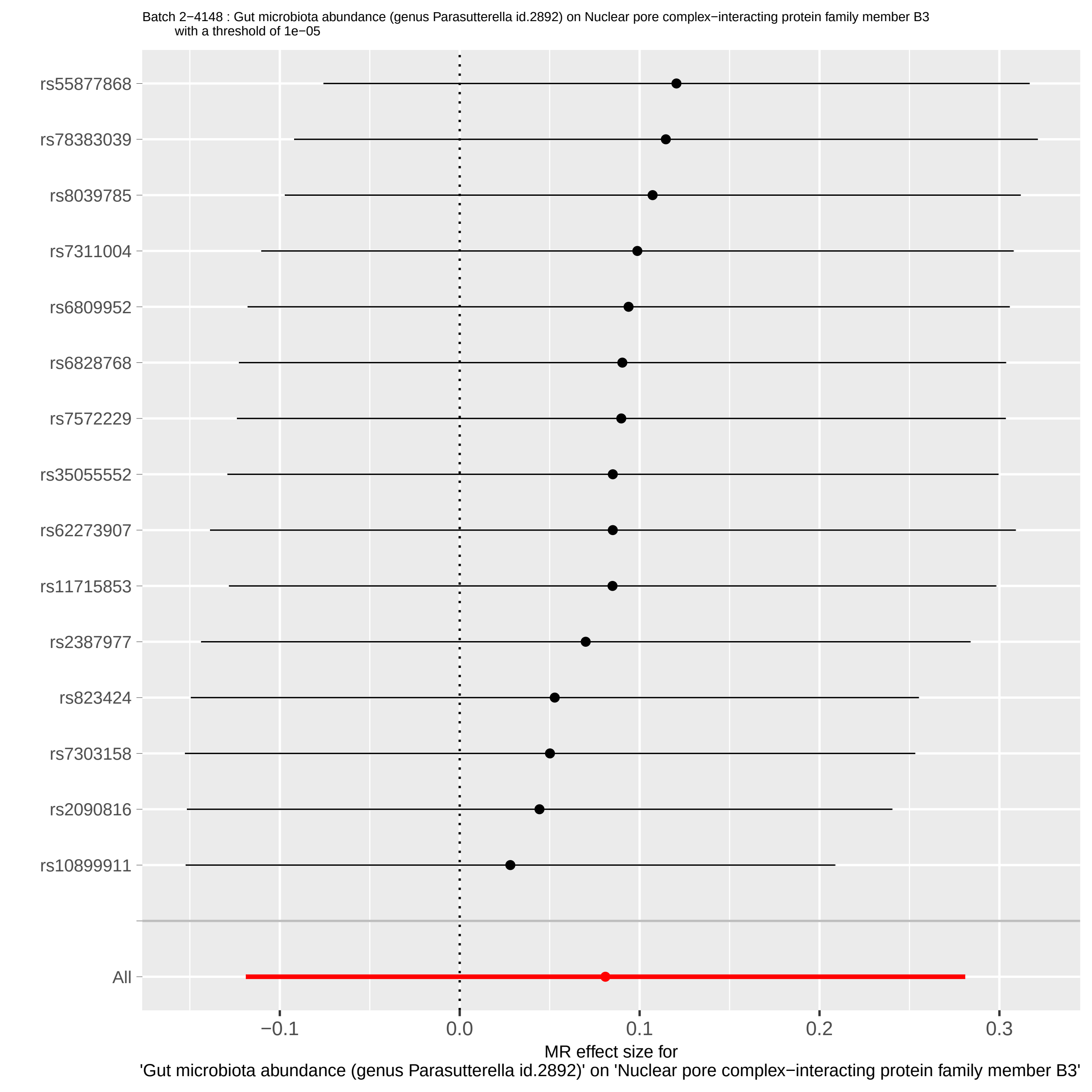

Batch 2−4148 : Gut microbiota abundance (genus Parasutterella id.2892) on Nuclear pore complex−interacting protein family member B3
with a threshold of 1e−05
rs55877868
rs78383039
rs8039785
rs7311004
rs6809952
rs6828768
rs7572229
rs35055552
rs62273907
rs11715853
rs2387977
rs823424
rs7303158
rs2090816
rs10899911
All
−0.1
0.0
0.1
0.2
0.3
MR effect size for
'Gut microbiota abundance (genus Parasutterella id.2892)' on 'Nuclear pore complex−interacting protein family member B3'

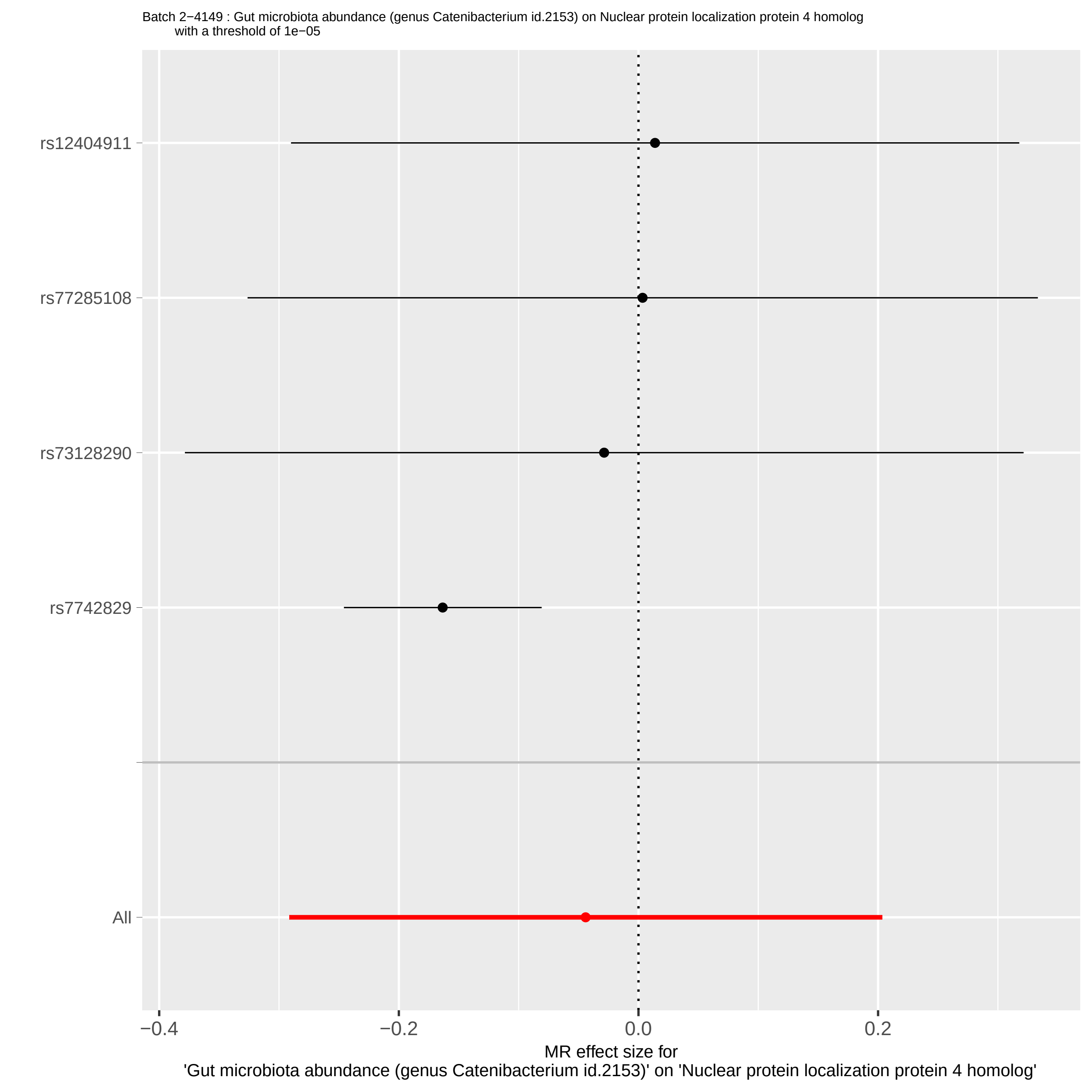

Batch 2−4149 : Gut microbiota abundance (genus Catenibacterium id.2153) on Nuclear protein localization protein 4 homolog
with a threshold of 1e−05
rs12404911
rs77285108
rs73128290
rs7742829
All
−0.4
−0.2
0.0
0.2
MR effect size for
'Gut microbiota abundance (genus Catenibacterium id.2153)' on 'Nuclear protein localization protein 4 homolog'

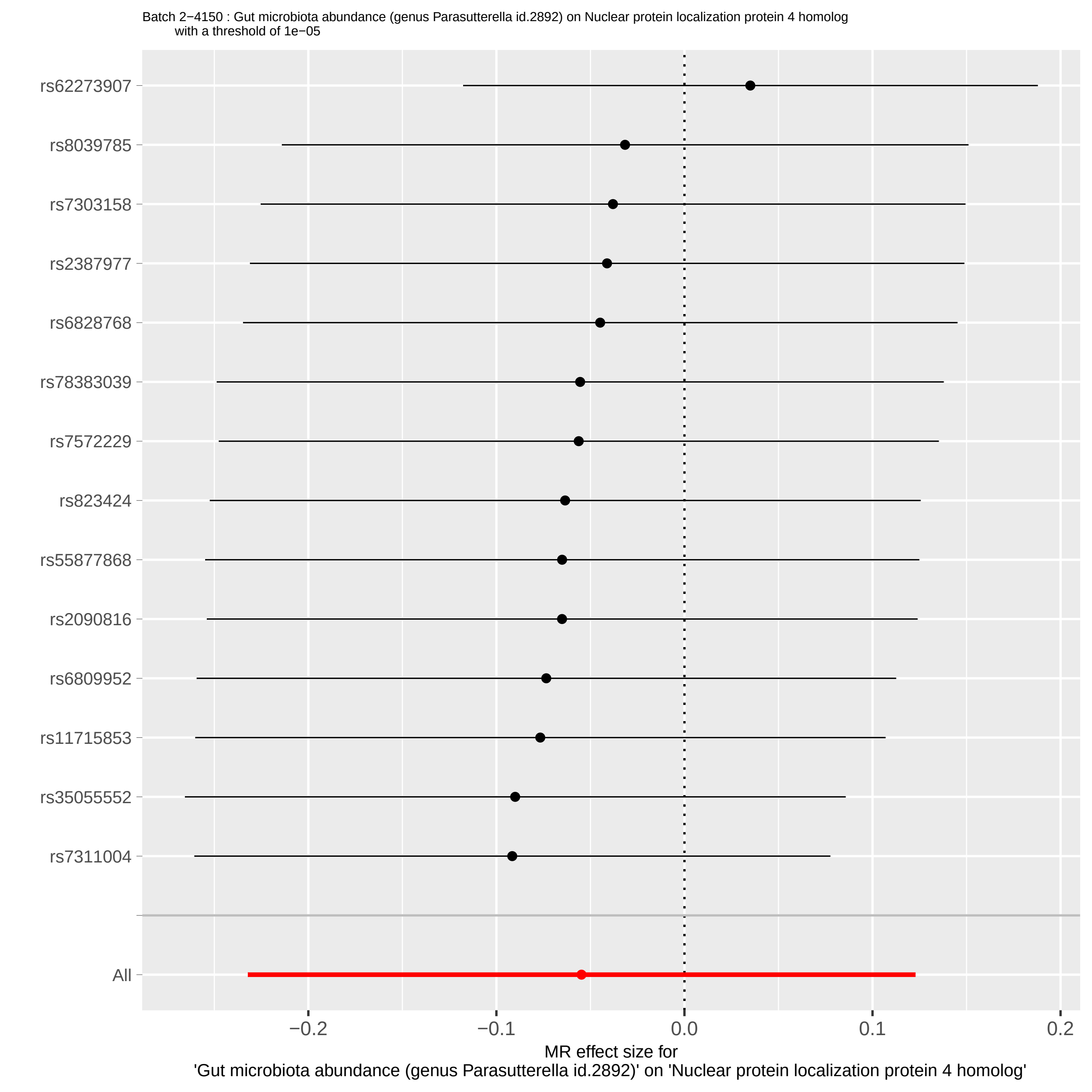
Batch 2−4150 : Gut microbiota abundance (genus Parasutterella id.2892) on Nuclear protein localization protein 4 homolog
with a threshold of 1e−05
rs62273907
rs8039785
rs7303158
rs2387977
rs6828768
rs78383039
rs7572229
rs823424
rs55877868
rs2090816
rs6809952
rs11715853
rs35055552
rs7311004
All
−0.2
−0.1
0.0
0.1
0.2
MR effect size for
'Gut microbiota abundance (genus Parasutterella id.2892)' on 'Nuclear protein localization protein 4 homolog'

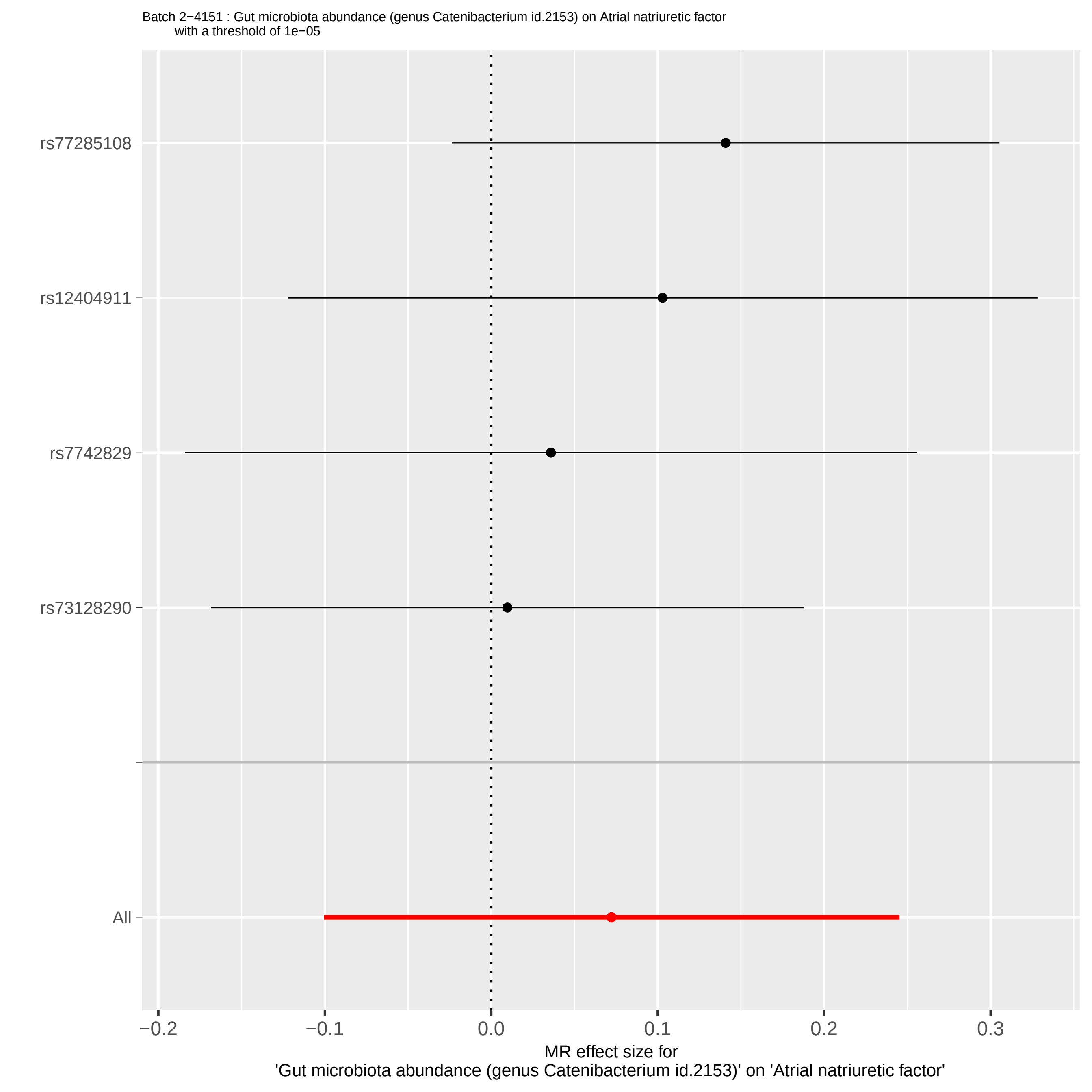

Batch 2−4151 : Gut microbiota abundance (genus Catenibacterium id.2153) on Atrial natriuretic factor
with a threshold of 1e−05
rs77285108
rs12404911
rs7742829
rs73128290
All
−0.2
−0.1
0.0
0.1
0.2
0.3
MR effect size for
'Gut microbiota abundance (genus Catenibacterium id.2153)' on 'Atrial natriuretic factor'

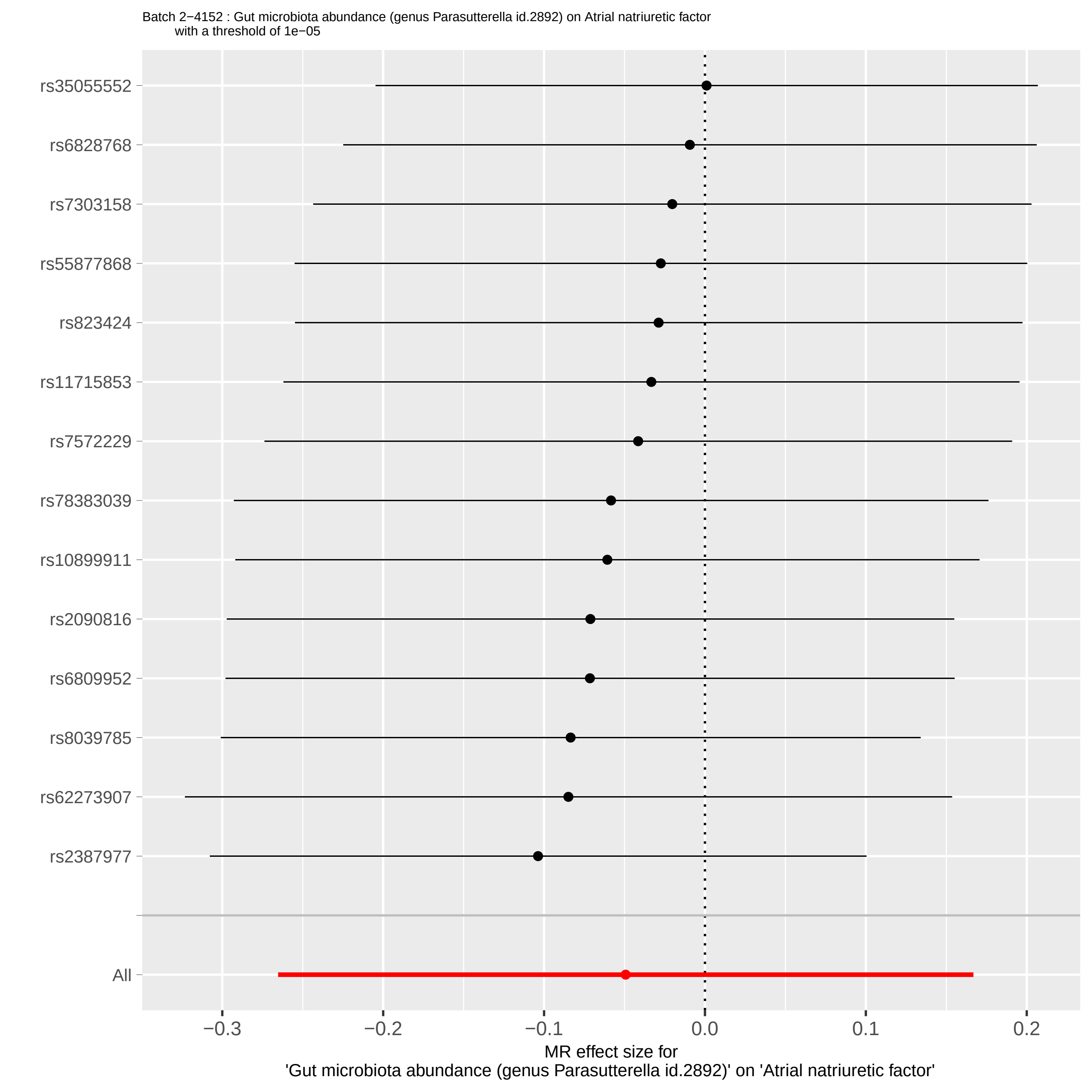
Batch 2−4152 : Gut microbiota abundance (genus Parasutterella id.2892) on Atrial natriuretic factor
with a threshold of 1e−05
rs35055552
rs6828768
rs7303158
rs55877868
rs823424
rs11715853
rs7572229
rs78383039
rs10899911
rs2090816
rs6809952
rs8039785
rs62273907
rs2387977
All
−0.3
−0.2
−0.1
0.0
0.1
0.2
MR effect size for
'Gut microbiota abundance (genus Parasutterella id.2892)' on 'Atrial natriuretic factor'

Batch 2−4153 : Gut microbiota abundance (genus Catenibacterium id.2153) on Brain natriuretic peptide 32
with a threshold of 1e−05

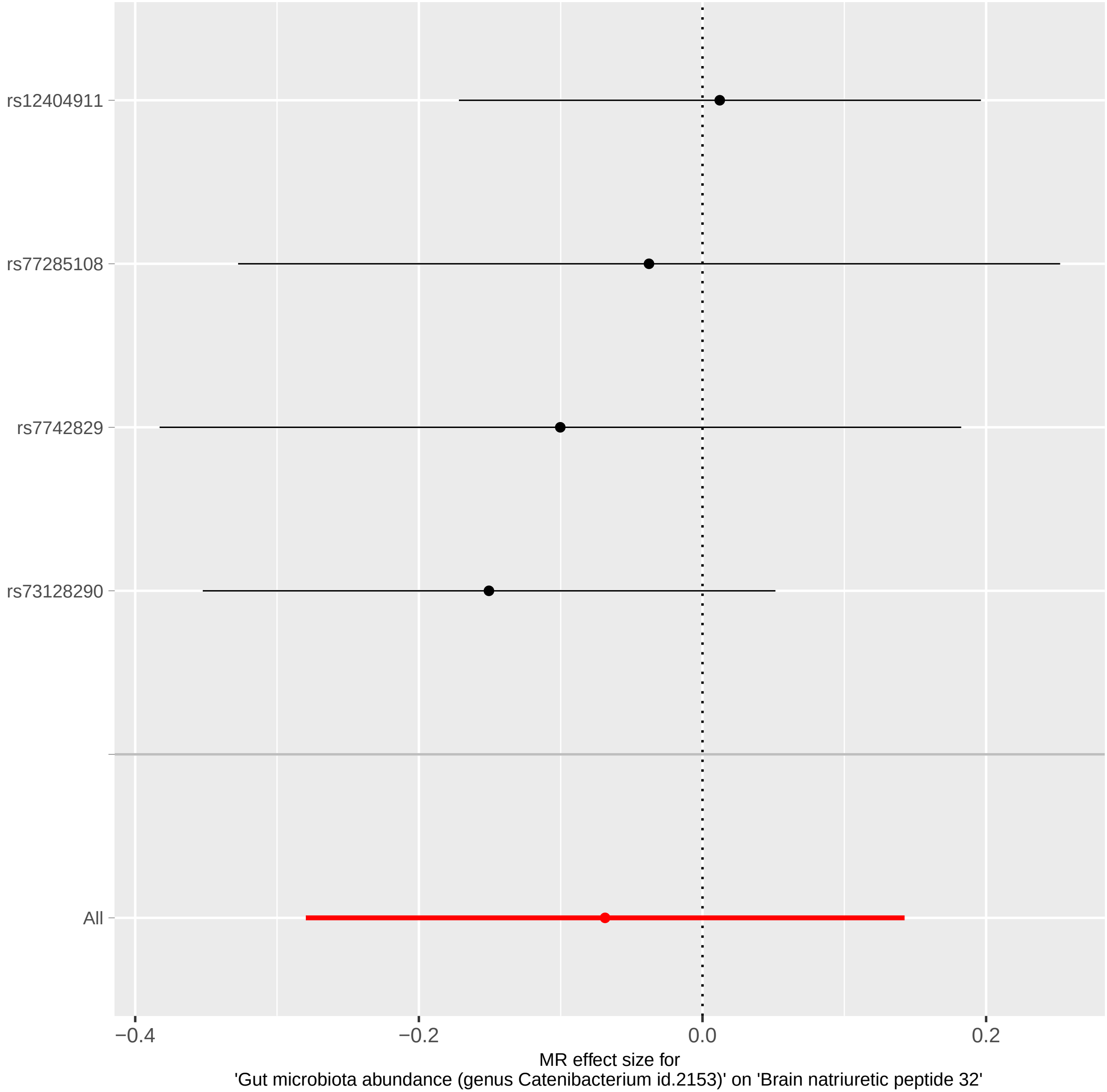

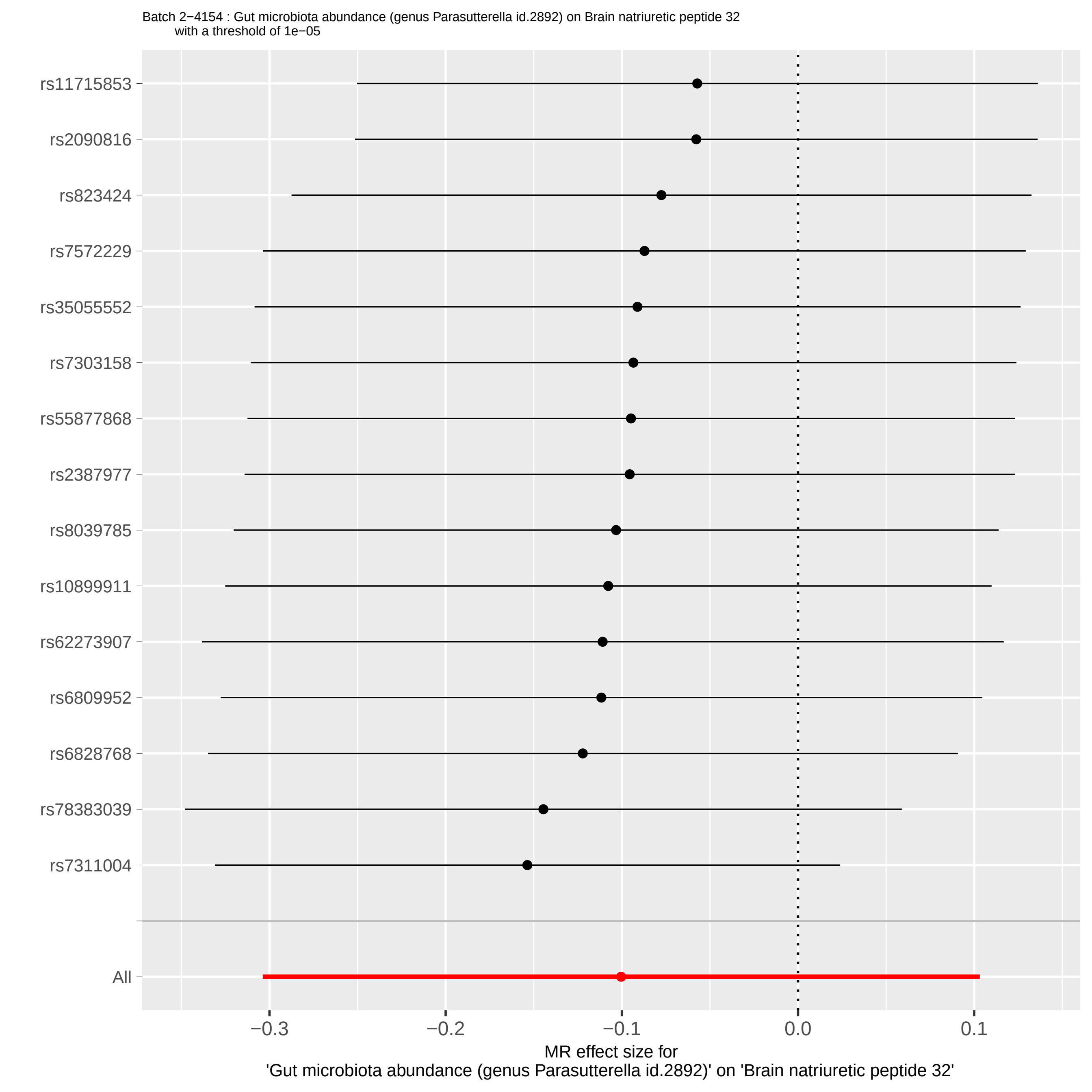
Batch 2−4154 : Gut microbiota abundance (genus Parasutterella id.2892) on Brain natriuretic peptide 32
with a threshold of 1e−05
rs11715853
rs2090816
rs823424
rs7572229
rs35055552
rs7303158
rs55877868
rs2387977
rs8039785
rs10899911
rs62273907
rs6809952
rs6828768
rs78383039
rs7311004
All
−0.3
−0.2
−0.1
0.0
0.1
MR effect size for
'Gut microbiota abundance (genus Parasutterella id.2892)' on 'Brain natriuretic peptide 32'

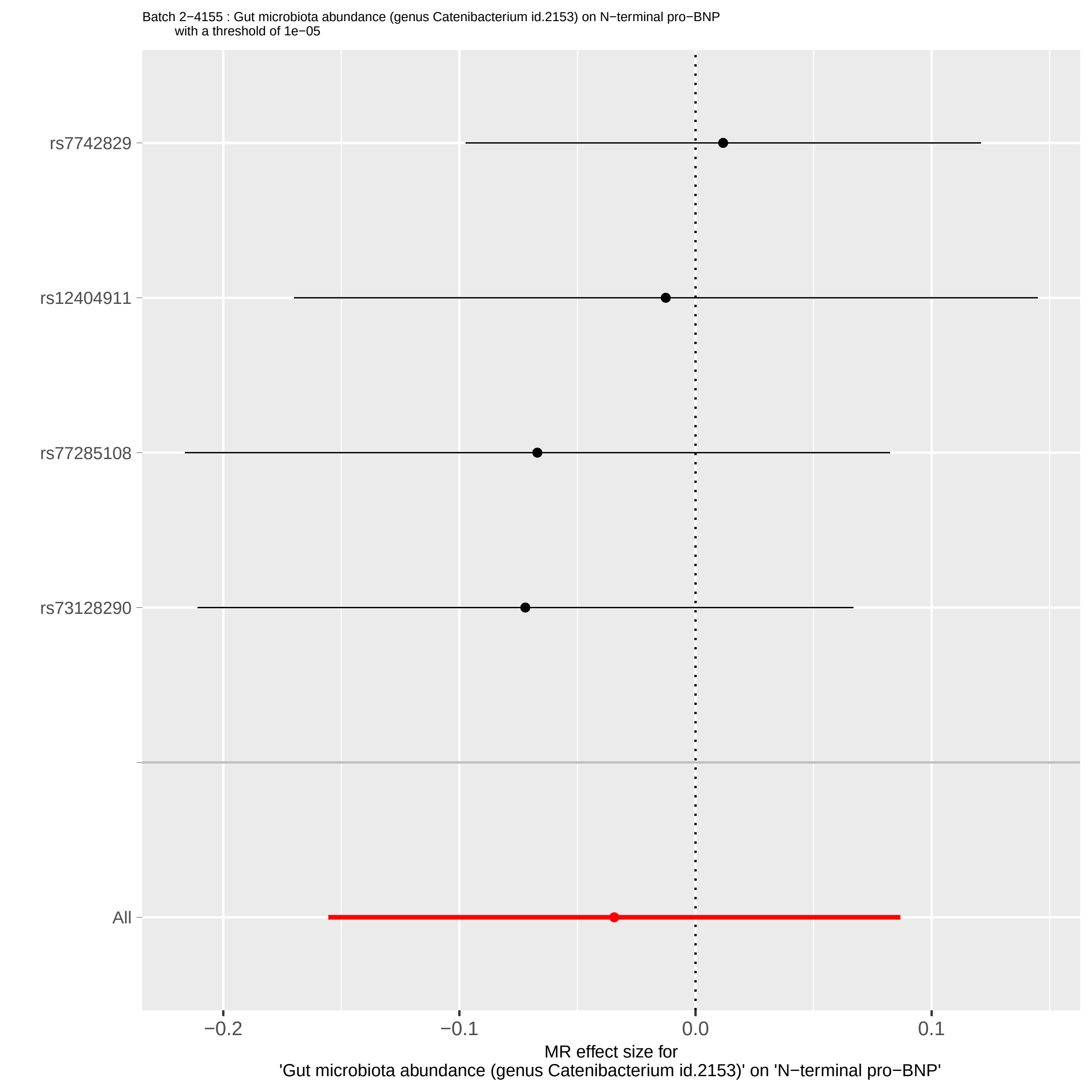

Batch 2−4155 : Gut microbiota abundance (genus Catenibacterium id.2153) on N−terminal pro−BNP
with a threshold of 1e−05
rs7742829
rs12404911
rs77285108
rs73128290
All
−0.2
−0.1
0.0
0.1
MR effect size for
'Gut microbiota abundance (genus Catenibacterium id.2153)' on 'N−terminal pro−BNP'

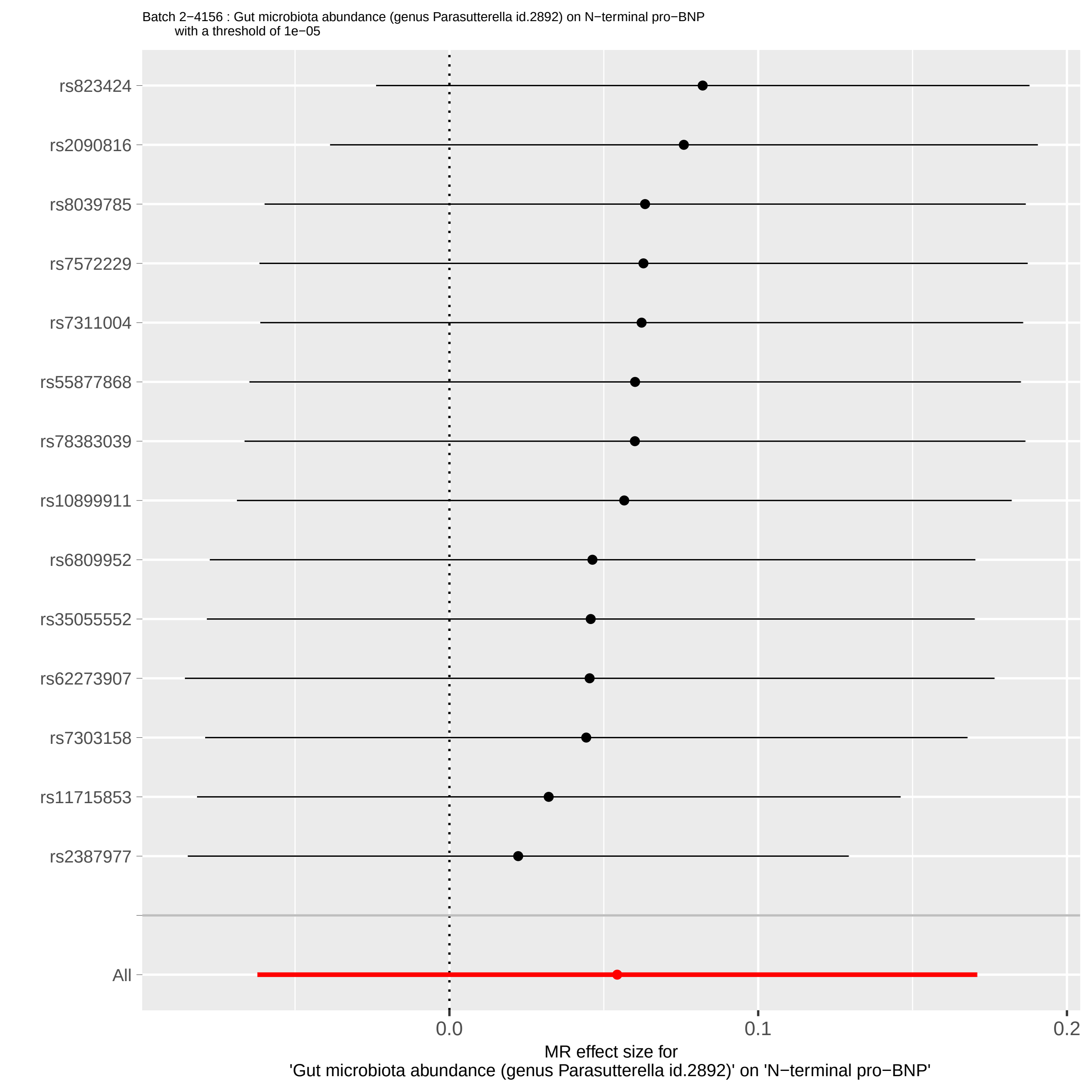
Batch 2−4156 : Gut microbiota abundance (genus Parasutterella id.2892) on N−terminal pro−BNP
with a threshold of 1e−05
rs823424
rs2090816
rs8039785
rs7572229
rs7311004
rs55877868
rs78383039
rs10899911
rs6809952
rs35055552
rs62273907
rs7303158
rs11715853
rs2387977
All
0.0
0.1
0.2
MR effect size for
'Gut microbiota abundance (genus Parasutterella id.2892)' on 'N−terminal pro−BNP'

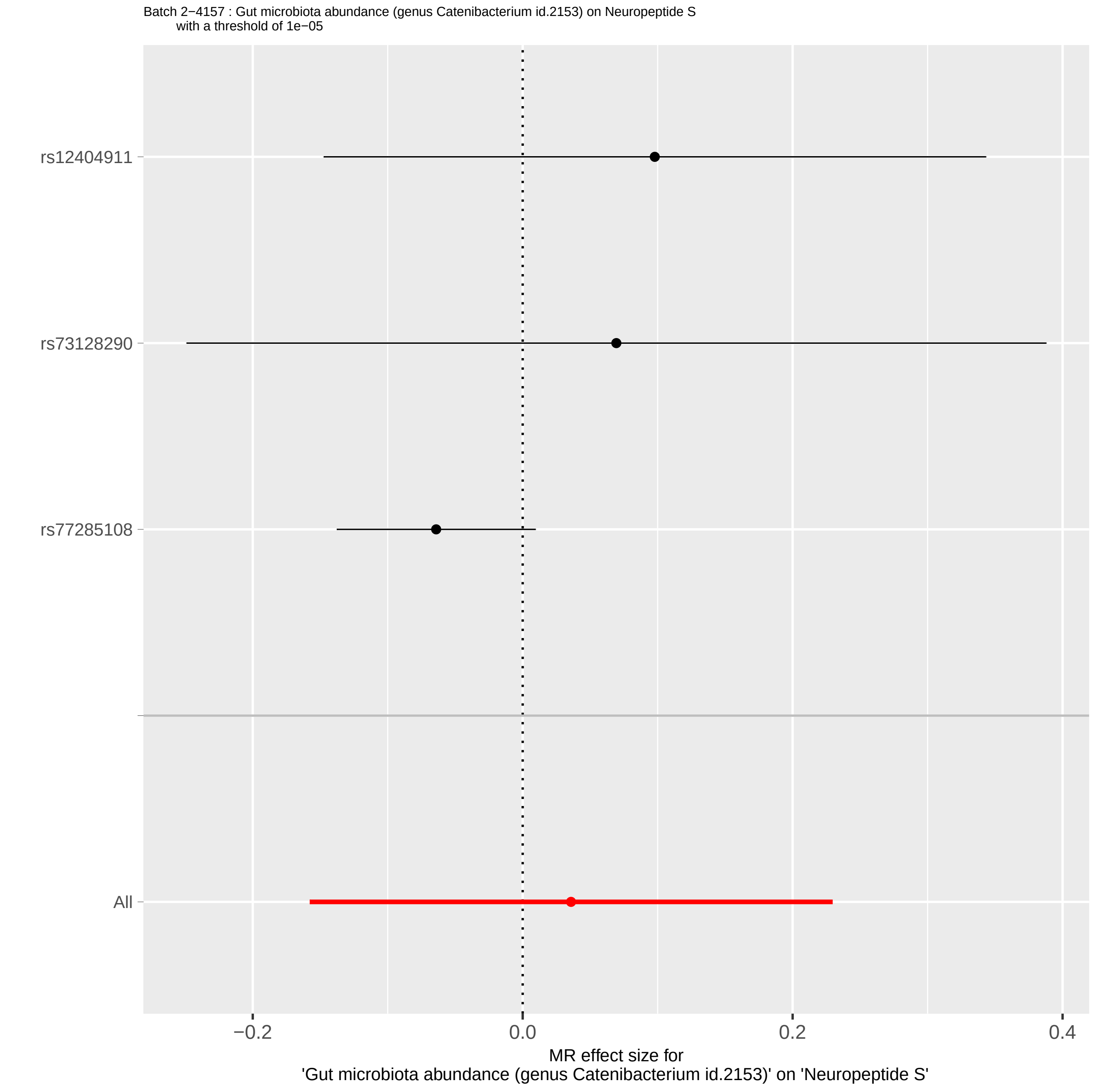

Batch 2−4157 : Gut microbiota abundance (genus Catenibacterium id.2153) on Neuropeptide S
with a threshold of 1e−05
rs12404911
rs73128290
rs77285108
All
−0.2
0.0
0.2
0.4
MR effect size for
'Gut microbiota abundance (genus Catenibacterium id.2153)' on 'Neuropeptide S'

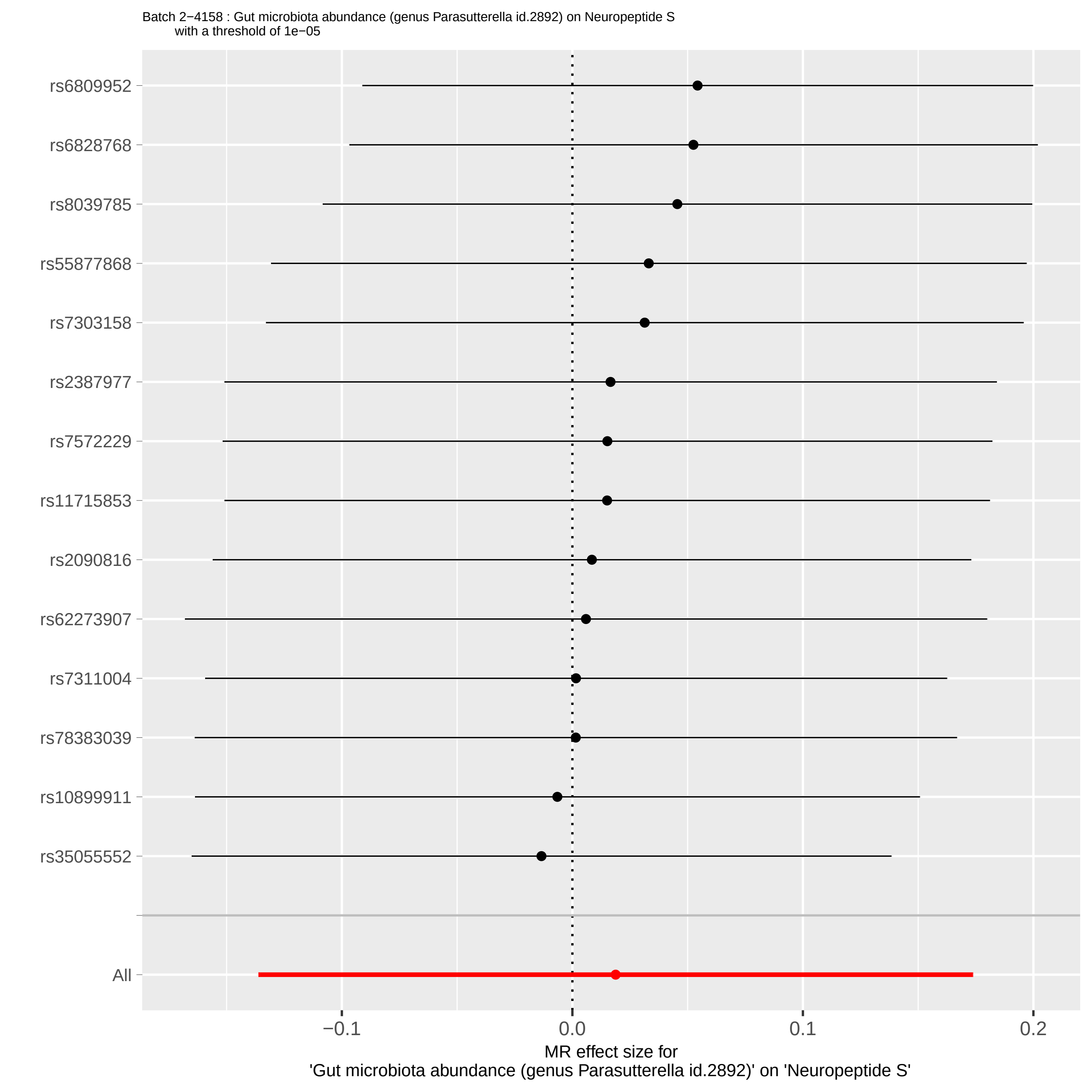
Batch 2−4158 : Gut microbiota abundance (genus Parasutterella id.2892) on Neuropeptide S
with a threshold of 1e−05
rs6809952
rs6828768
rs8039785
rs55877868
rs7303158
rs2387977
rs7572229
rs11715853
rs2090816
rs62273907
rs7311004
rs78383039
rs10899911
rs35055552
All
−0.1
0.0
0.1
0.2
MR effect size for
'Gut microbiota abundance (genus Parasutterella id.2892)' on 'Neuropeptide S'

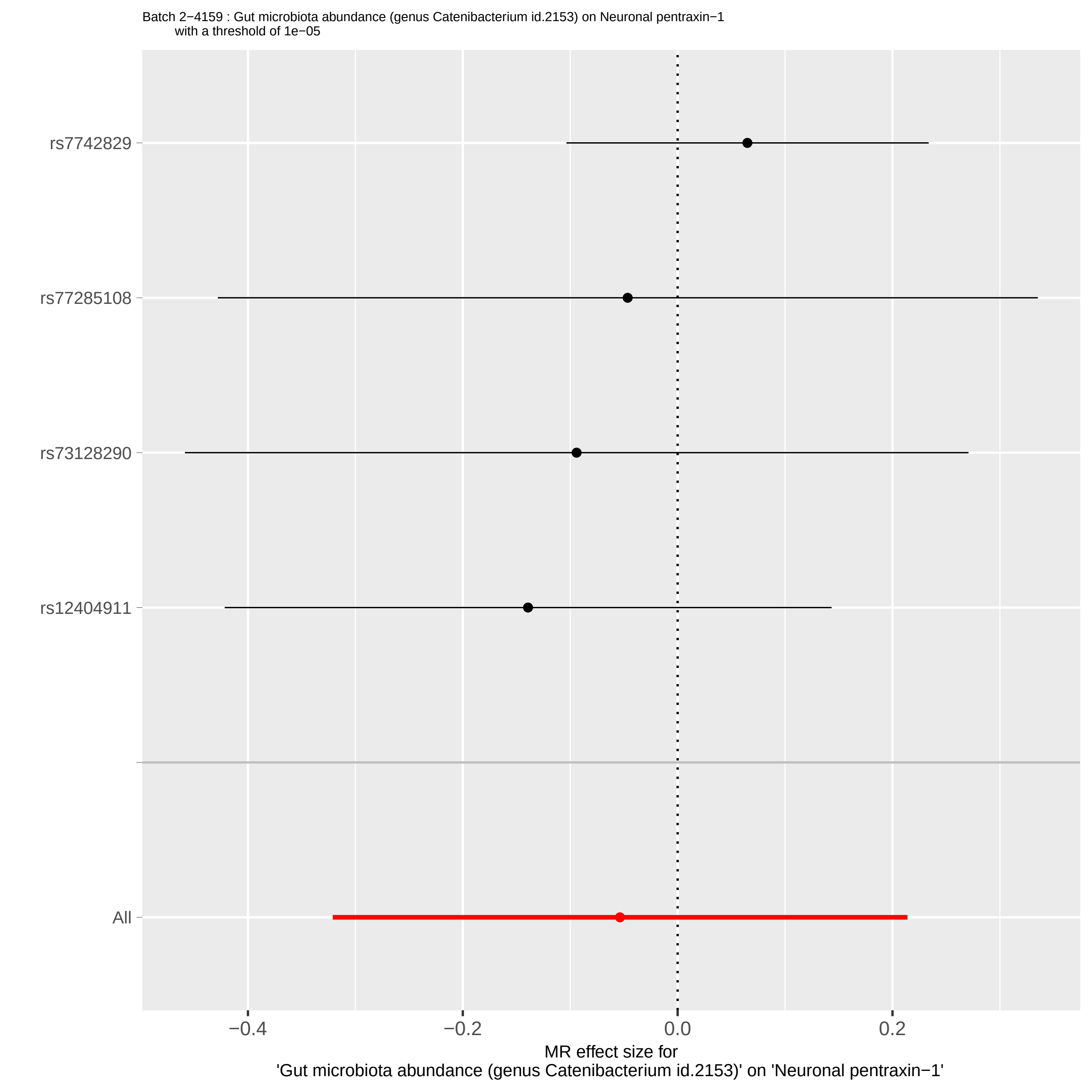
Batch 2−4159 : Gut microbiota abundance (genus Catenibacterium id.2153) on Neuronal pentraxin−1
with a threshold of 1e−05
rs7742829
rs77285108
rs73128290
rs12404911
All
−0.4
−0.2
0.0
0.2
MR effect size for
'Gut microbiota abundance (genus Catenibacterium id.2153)' on 'Neuronal pentraxin−1'

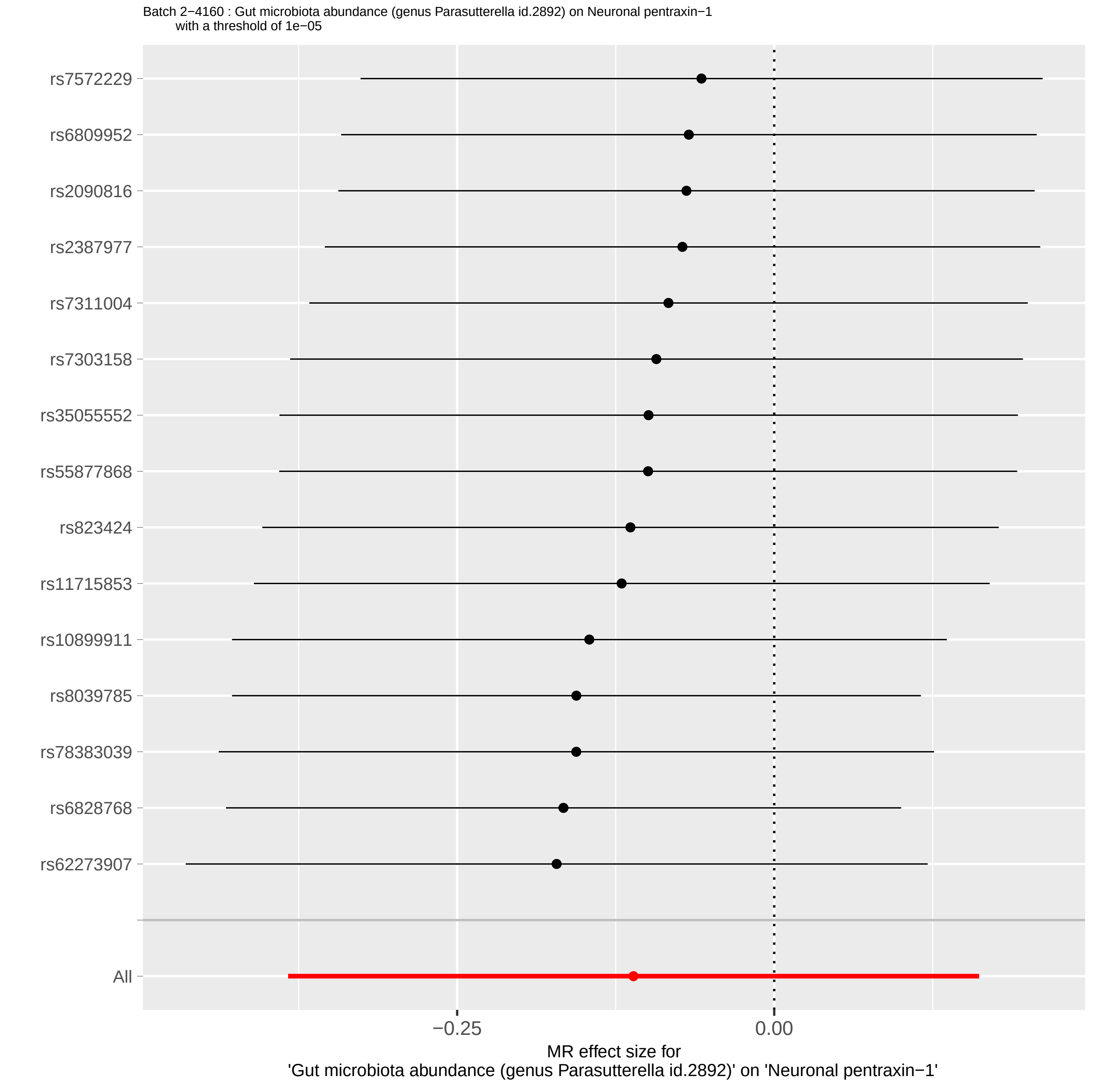
Batch 2−4160 : Gut microbiota abundance (genus Parasutterella id.2892) on Neuronal pentraxin−1
with a threshold of 1e−05
rs7572229
rs6809952
rs2090816
rs2387977
rs7311004
rs7303158
rs35055552
rs55877868
rs823424
rs11715853
rs10899911
rs8039785
rs78383039
rs6828768
rs62273907
All
−0.25
0.00
MR effect size for
'Gut microbiota abundance (genus Parasutterella id.2892)' on 'Neuronal pentraxin−1'

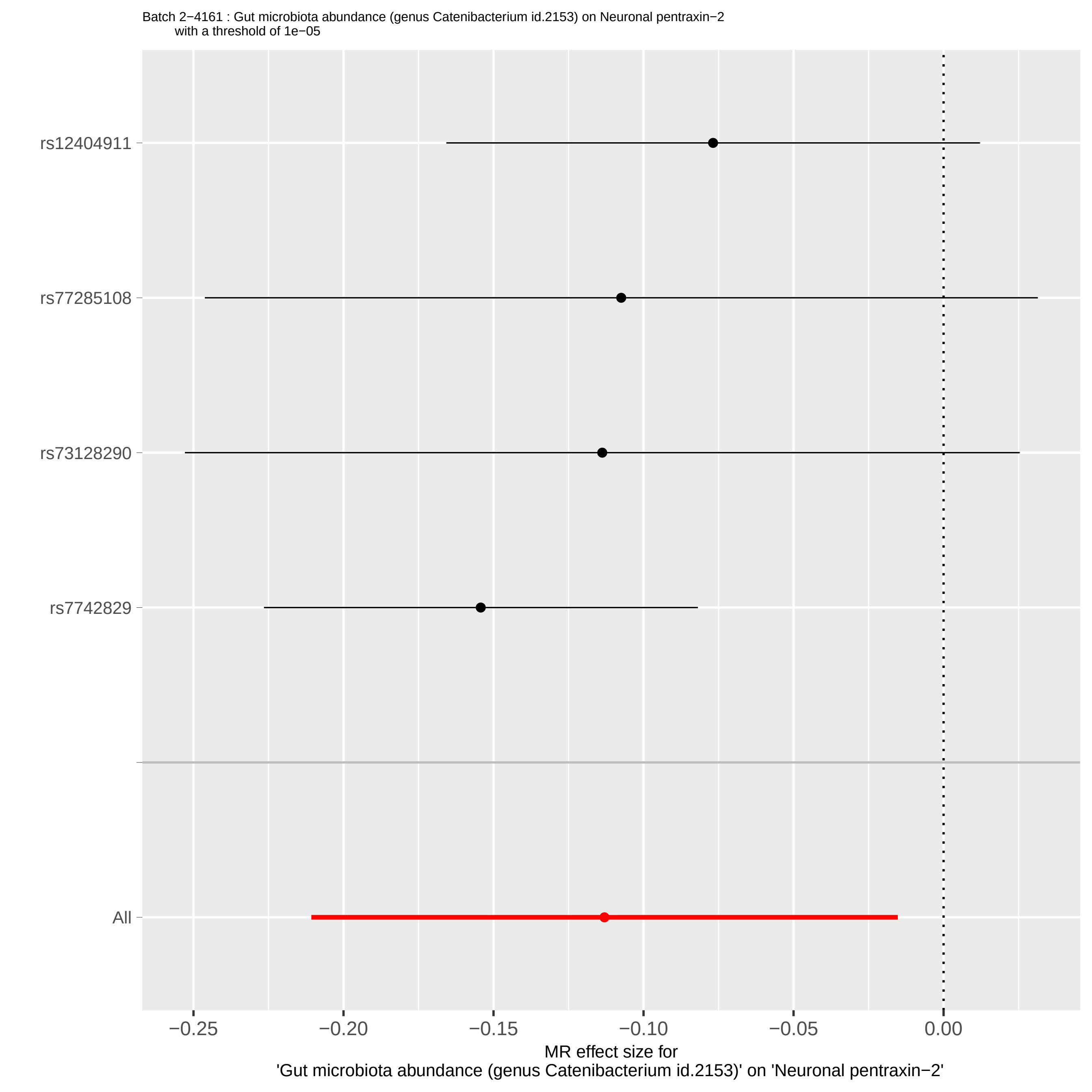

Batch 2−4161 : Gut microbiota abundance (genus Catenibacterium id.2153) on Neuronal pentraxin−2
with a threshold of 1e−05
rs12404911
rs77285108
rs73128290
rs7742829
All
−0.25
−0.20
−0.15
−0.10
−0.05
0.00
MR effect size for
'Gut microbiota abundance (genus Catenibacterium id.2153)' on 'Neuronal pentraxin−2'

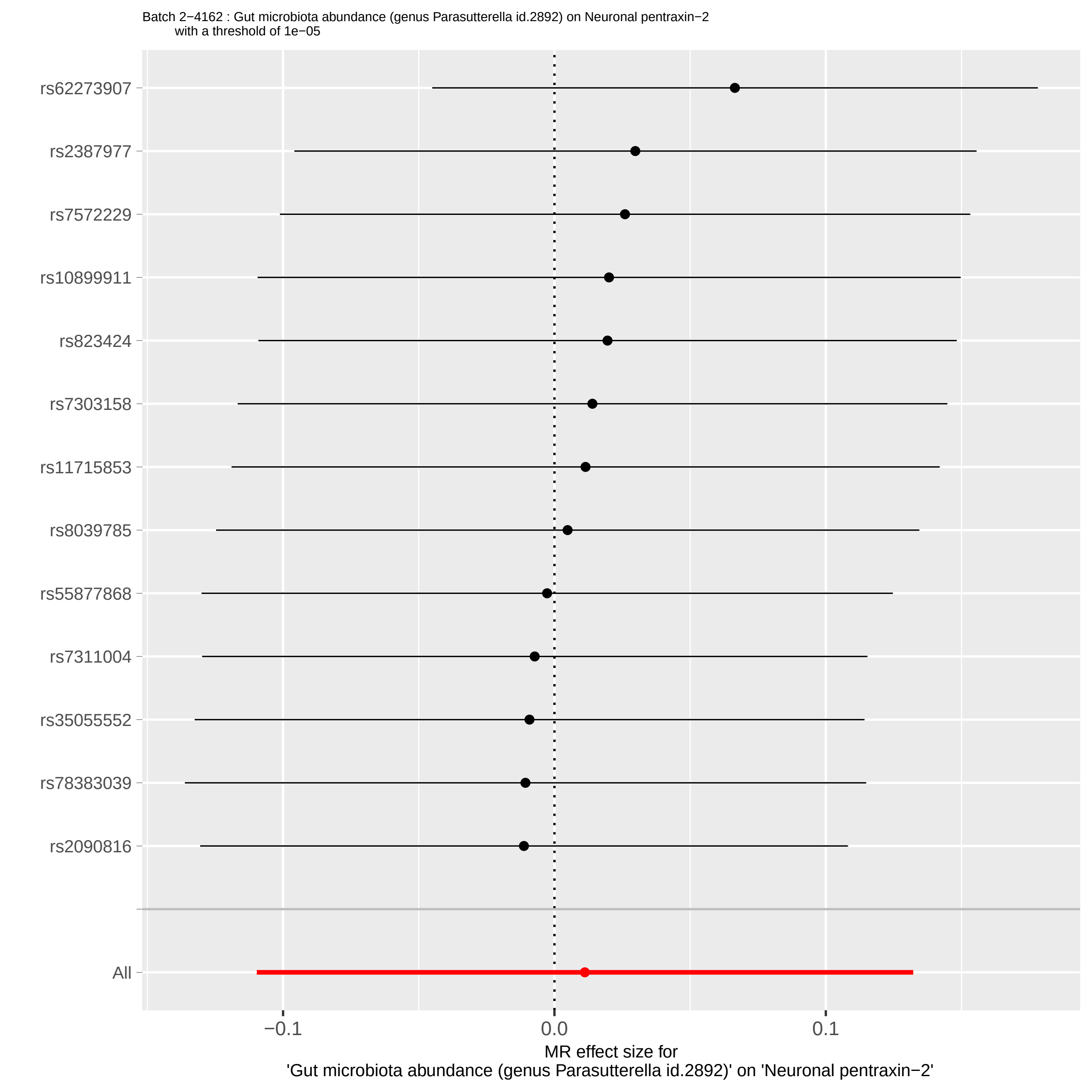
Batch 2−4162 : Gut microbiota abundance (genus Parasutterella id.2892) on Neuronal pentraxin−2
with a threshold of 1e−05
rs62273907
rs2387977
rs7572229
rs10899911
rs823424
rs7303158
rs11715853
rs8039785
rs55877868
rs7311004
rs35055552
rs78383039
rs2090816
All
−0.1
0.0
0.1
MR effect size for
'Gut microbiota abundance (genus Parasutterella id.2892)' on 'Neuronal pentraxin−2'

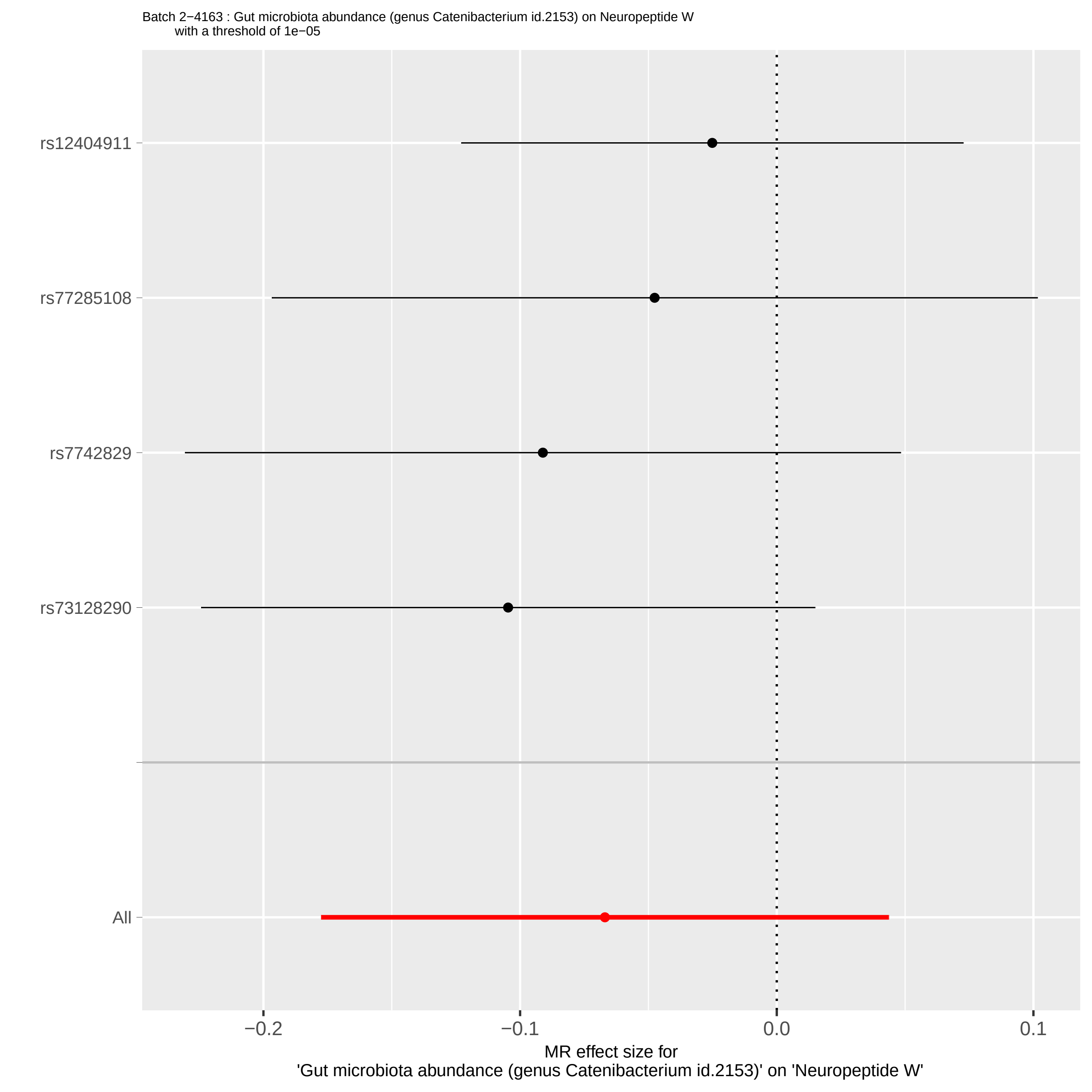
Batch 2−4163 : Gut microbiota abundance (genus Catenibacterium id.2153) on Neuropeptide W
with a threshold of 1e−05
rs12404911
rs77285108
rs7742829
rs73128290
All
−0.2
−0.1
0.0
0.1
MR effect size for
'Gut microbiota abundance (genus Catenibacterium id.2153)' on 'Neuropeptide W'

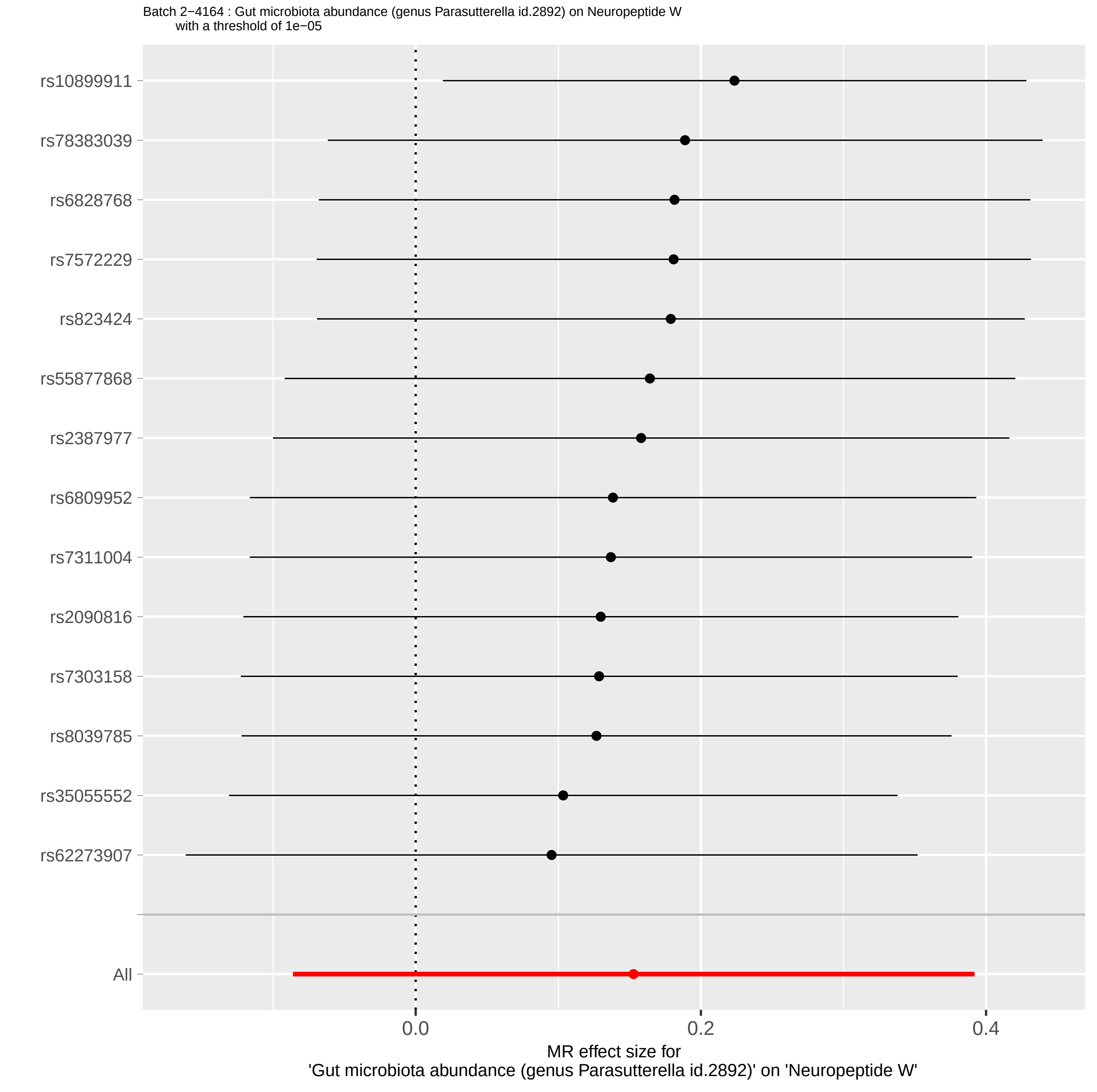

Batch 2−4164 : Gut microbiota abundance (genus Parasutterella id.2892) on Neuropeptide W
with a threshold of 1e−05
rs10899911
rs78383039
rs6828768
rs7572229
rs823424
rs55877868
rs2387977
rs6809952
rs7311004
rs2090816
rs7303158
rs8039785
rs35055552
rs62273907
All
0.0
0.2
0.4
MR effect size for
'Gut microbiota abundance (genus Parasutterella id.2892)' on 'Neuropeptide W'

Batch 2−4165 : Gut microbiota abundance (genus Catenibacterium id.2153) on NAD(P)H dehydrogenase [quinone] 1
with a threshold of 1e−05

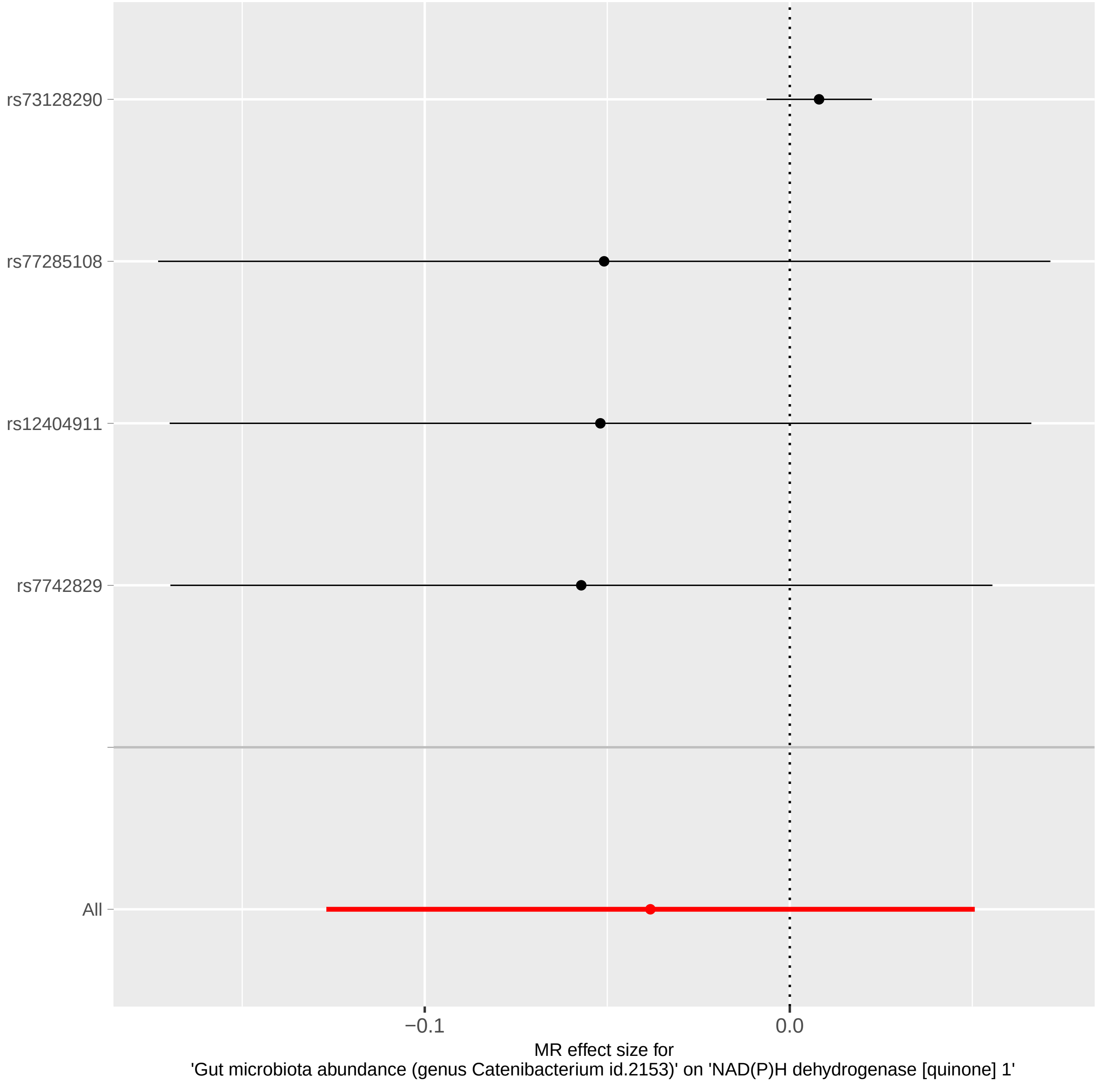

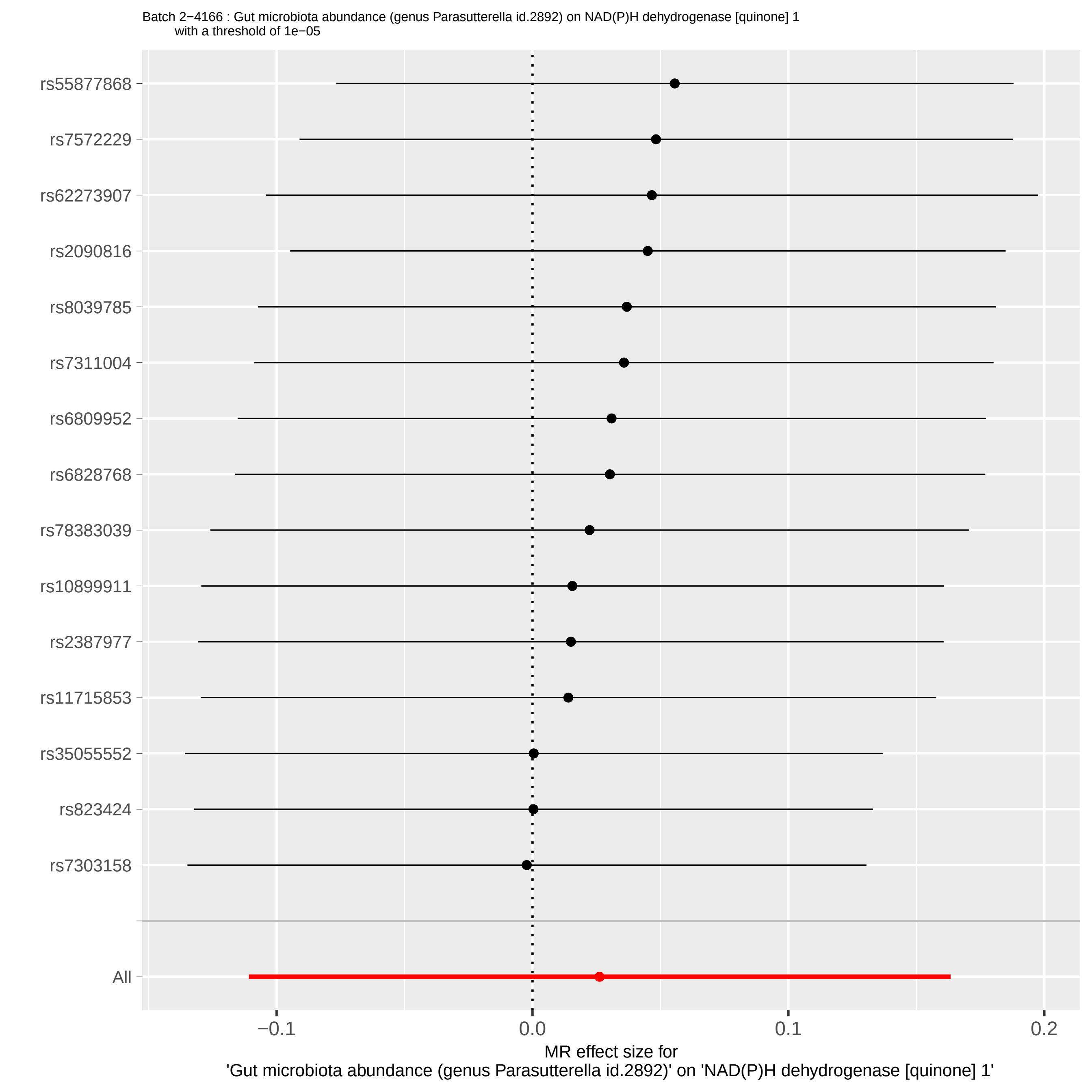
Batch 2−4166 : Gut microbiota abundance (genus Parasutterella id.2892) on NAD(P)H dehydrogenase [quinone] 1
with a threshold of 1e−05
rs55877868
rs7572229
rs62273907
rs2090816
rs8039785
rs7311004
rs6809952
rs6828768
rs78383039
rs10899911
rs2387977
rs11715853
rs35055552
rs823424
rs7303158
All
−0.1
0.0
0.1
0.2
MR effect size for
'Gut microbiota abundance (genus Parasutterella id.2892)' on 'NAD(P)H dehydrogenase [quinone] 1'

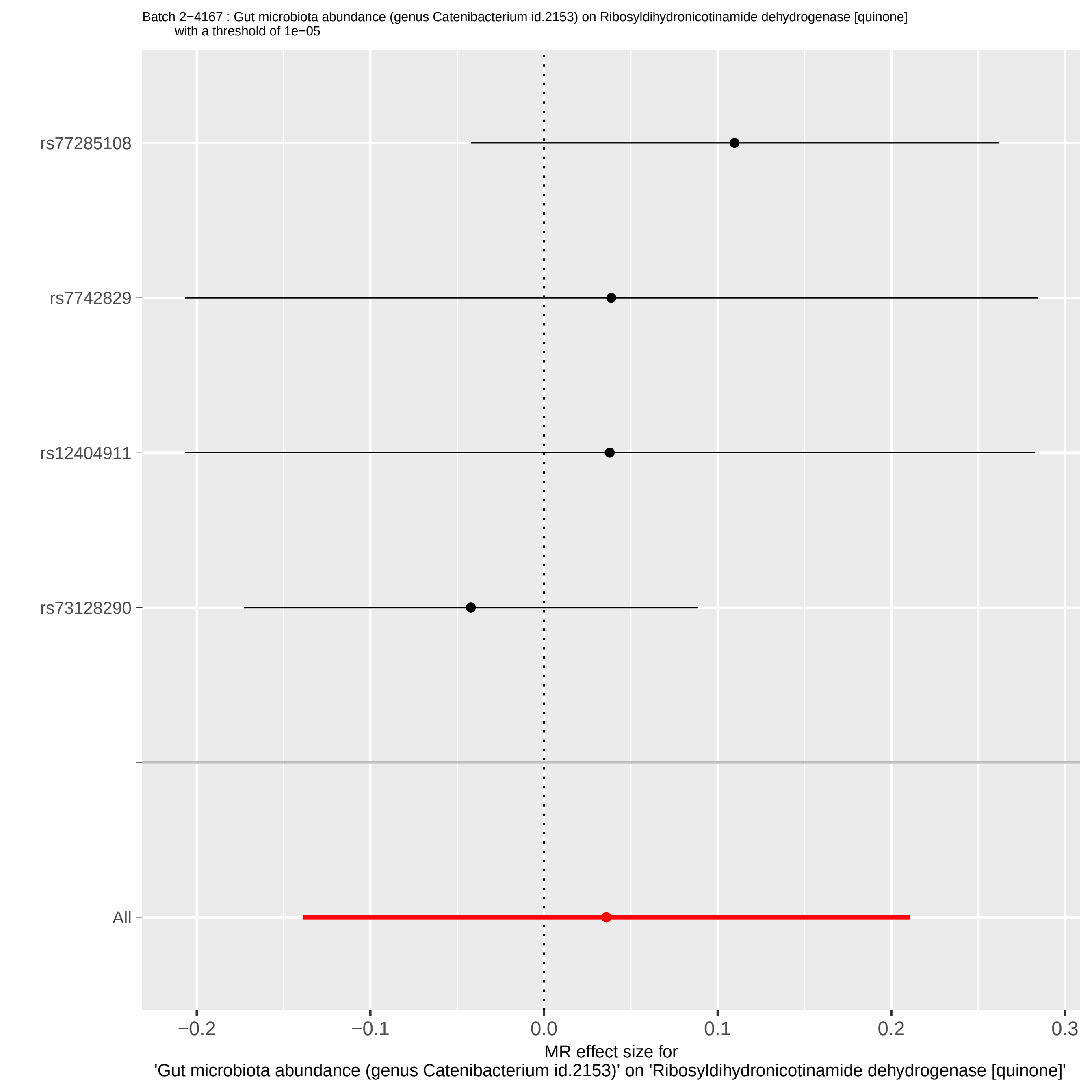
Batch 2−4167 : Gut microbiota abundance (genus Catenibacterium id.2153) on Ribosyldihydronicotinamide dehydrogenase [quinone] with a threshold of 1e−05
rs77285108
rs7742829
rs12404911
rs73128290
All
−0.2
−0.1
0.0
0.1
0.2
0.3
MR effect size for
'Gut microbiota abundance (genus Catenibacterium id.2153)' on 'Ribosyldihydronicotinamide dehydrogenase [quinone]'

Batch 2−4168 : Gut microbiota abundance (genus Parasutterella id.2892) on Ribosyldihydronicotinamide dehydrogenase [quinone] with a threshold of 1e−05

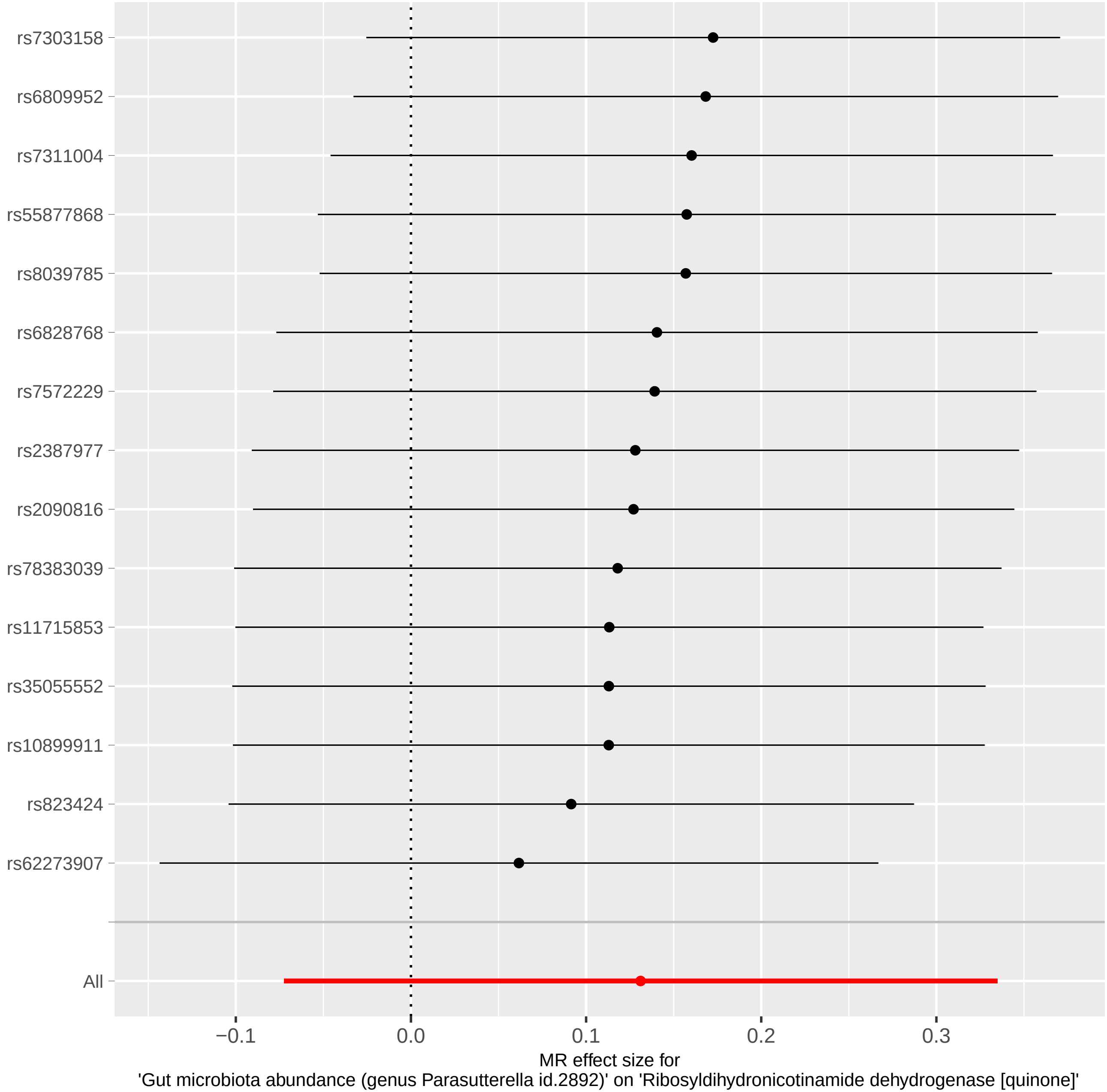

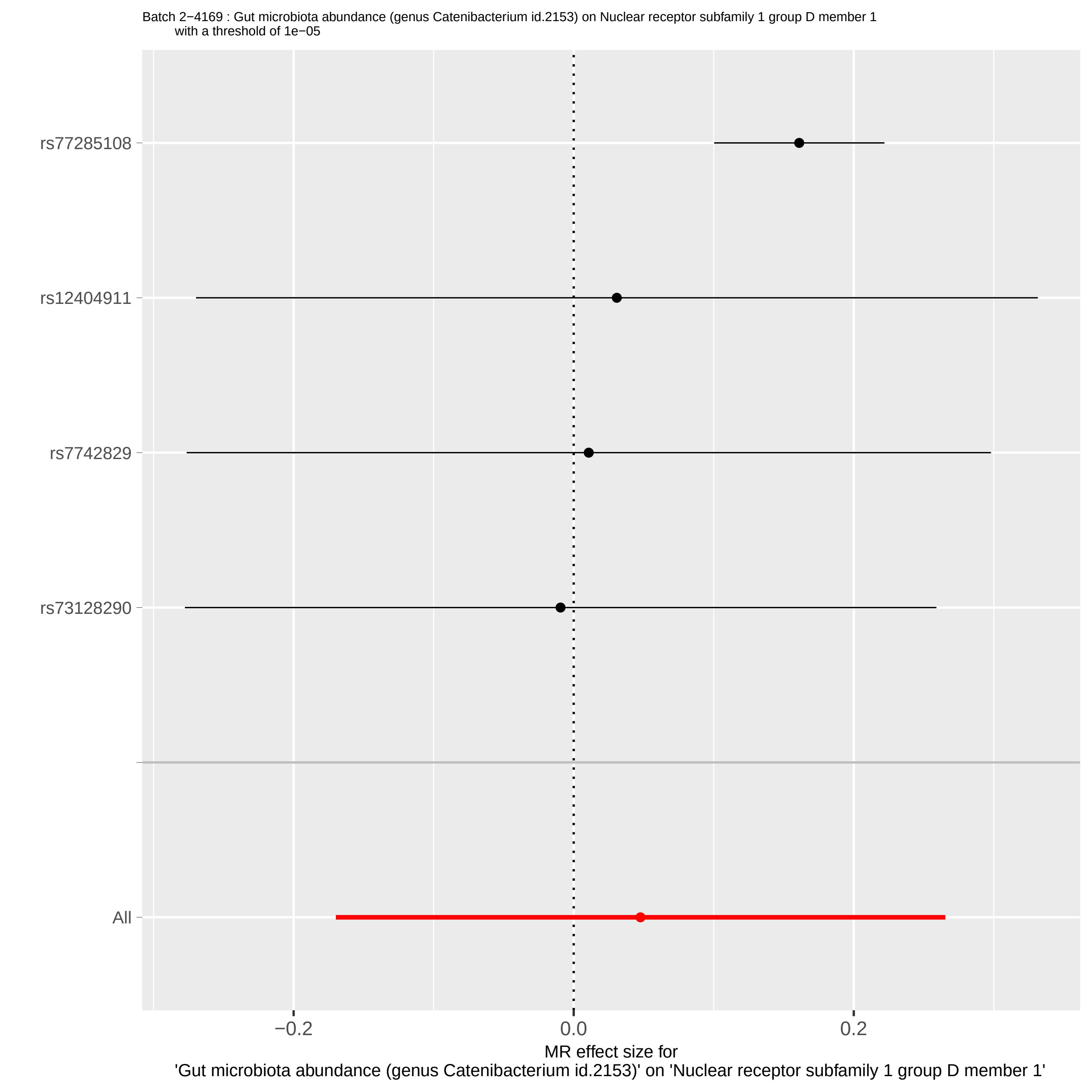

Batch 2−4169 : Gut microbiota abundance (genus Catenibacterium id.2153) on Nuclear receptor subfamily 1 group D member 1
with a threshold of 1e−05
rs77285108
rs12404911
rs7742829
rs73128290
All
−0.2
0.0
0.2
MR effect size for
'Gut microbiota abundance (genus Catenibacterium id.2153)' on 'Nuclear receptor subfamily 1 group D member 1'

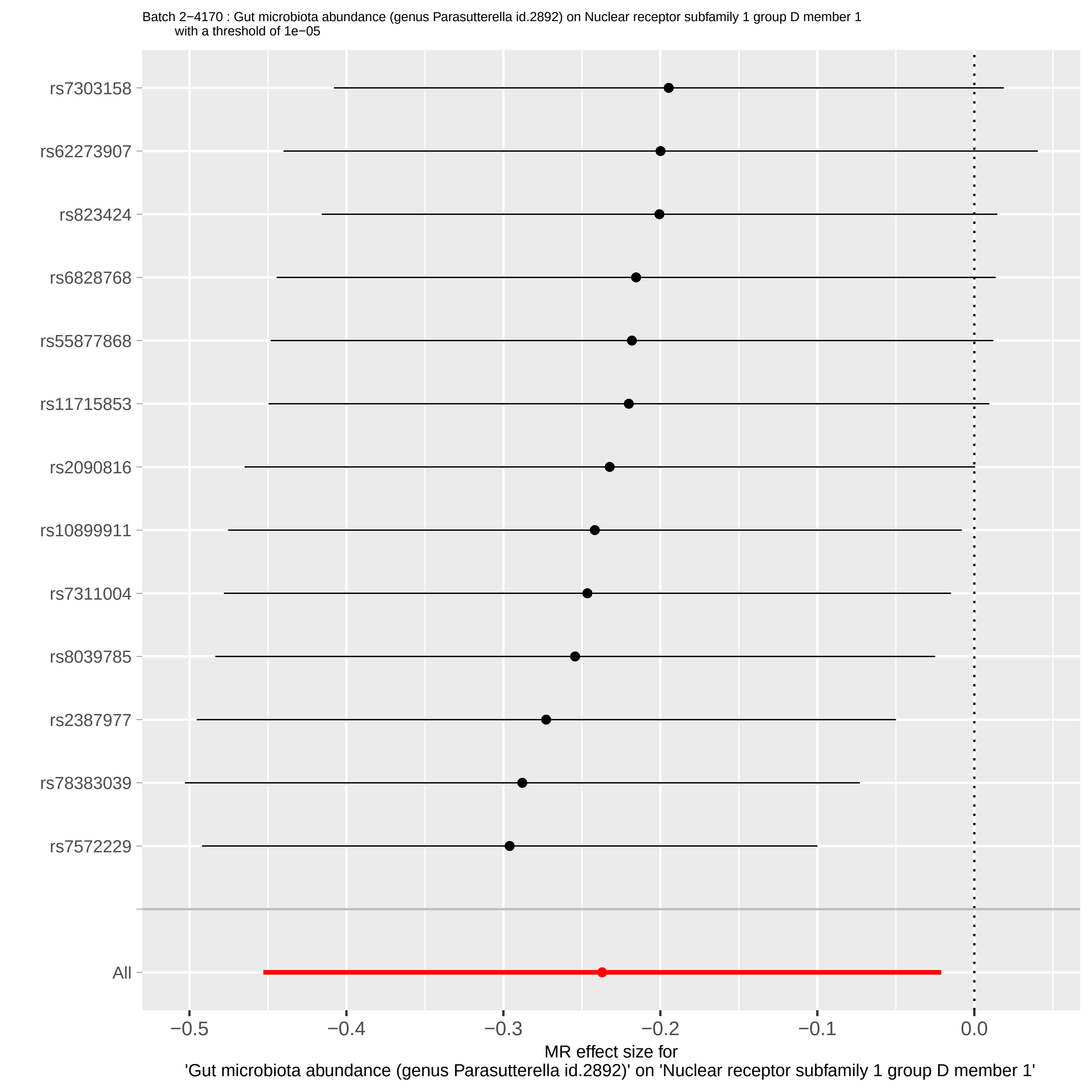
Batch 2−4170 : Gut microbiota abundance (genus Parasutterella id.2892) on Nuclear receptor subfamily 1 group D member 1
with a threshold of 1e−05
rs7303158
rs62273907
rs823424
rs6828768
rs55877868
rs11715853
rs2090816
rs10899911
rs7311004
rs8039785
rs2387977
rs78383039
rs7572229
All
−0.5
−0.4
−0.3
−0.2
−0.1
0.0
MR effect size for
'Gut microbiota abundance (genus Parasutterella id.2892)' on 'Nuclear receptor subfamily 1 group D member 1'

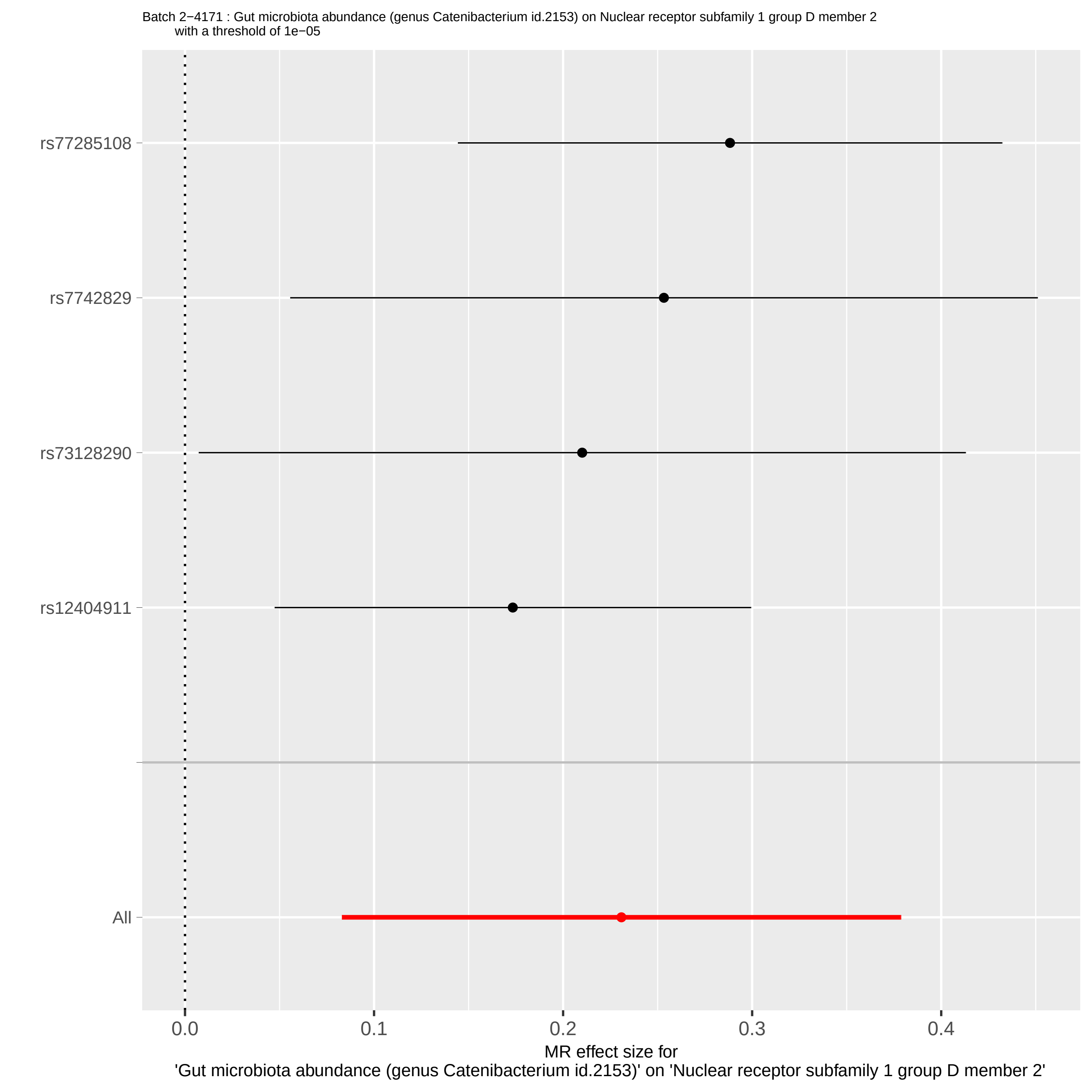

Batch 2−4171 : Gut microbiota abundance (genus Catenibacterium id.2153) on Nuclear receptor subfamily 1 group D member 2
with a threshold of 1e−05
rs77285108
rs7742829
rs73128290
rs12404911
All
0.0
0.1
0.2
0.3
0.4
MR effect size for
'Gut microbiota abundance (genus Catenibacterium id.2153)' on 'Nuclear receptor subfamily 1 group D member 2'

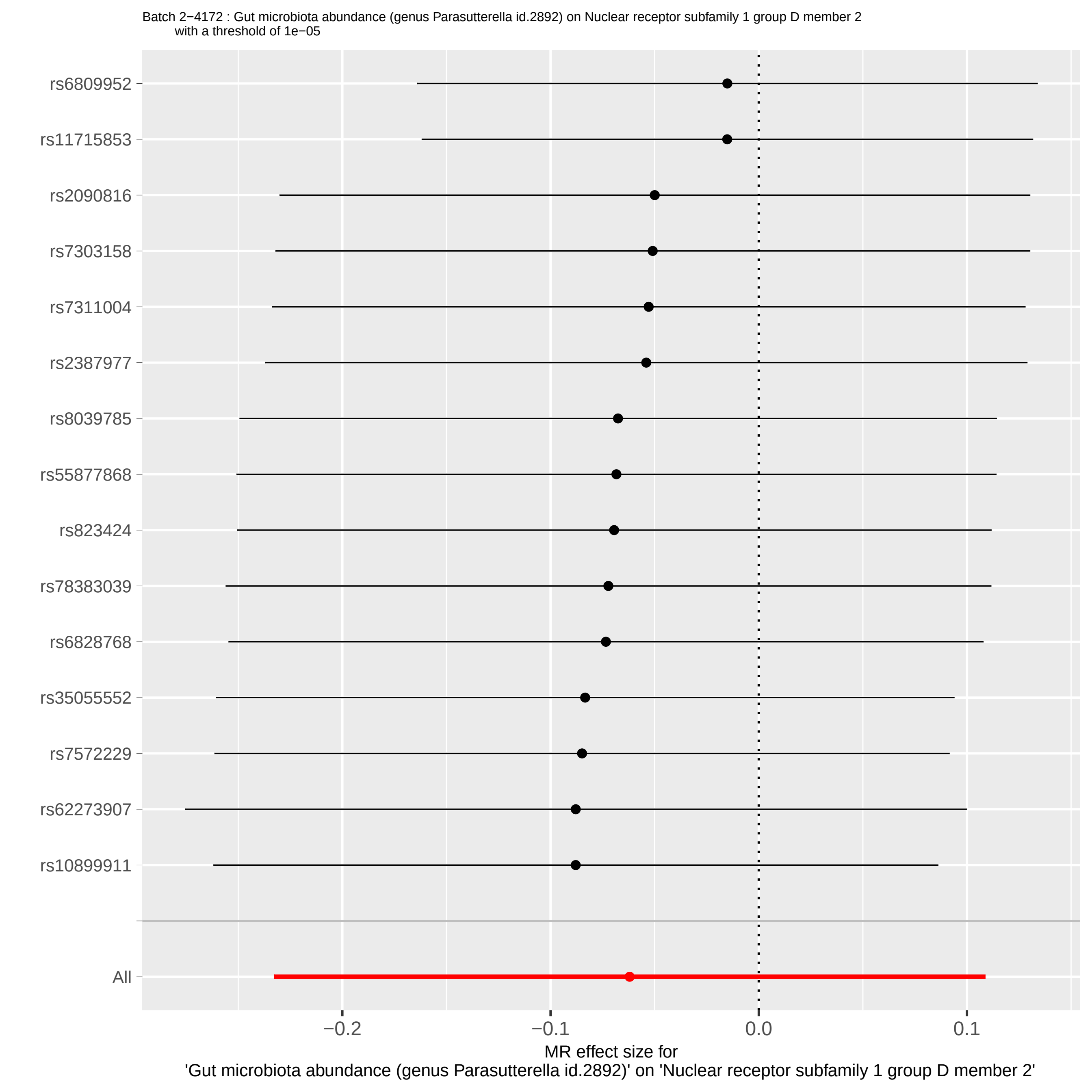

Batch 2−4172 : Gut microbiota abundance (genus Parasutterella id.2892) on Nuclear receptor subfamily 1 group D member 2
with a threshold of 1e−05
rs6809952
rs11715853
rs2090816
rs7303158
rs7311004
rs2387977
rs8039785
rs55877868
rs823424
rs78383039
rs6828768
rs35055552
rs7572229
rs62273907
rs10899911
All
−0.2
−0.1
0.0
0.1
MR effect size for
'Gut microbiota abundance (genus Parasutterella id.2892)' on 'Nuclear receptor subfamily 1 group D member 2'

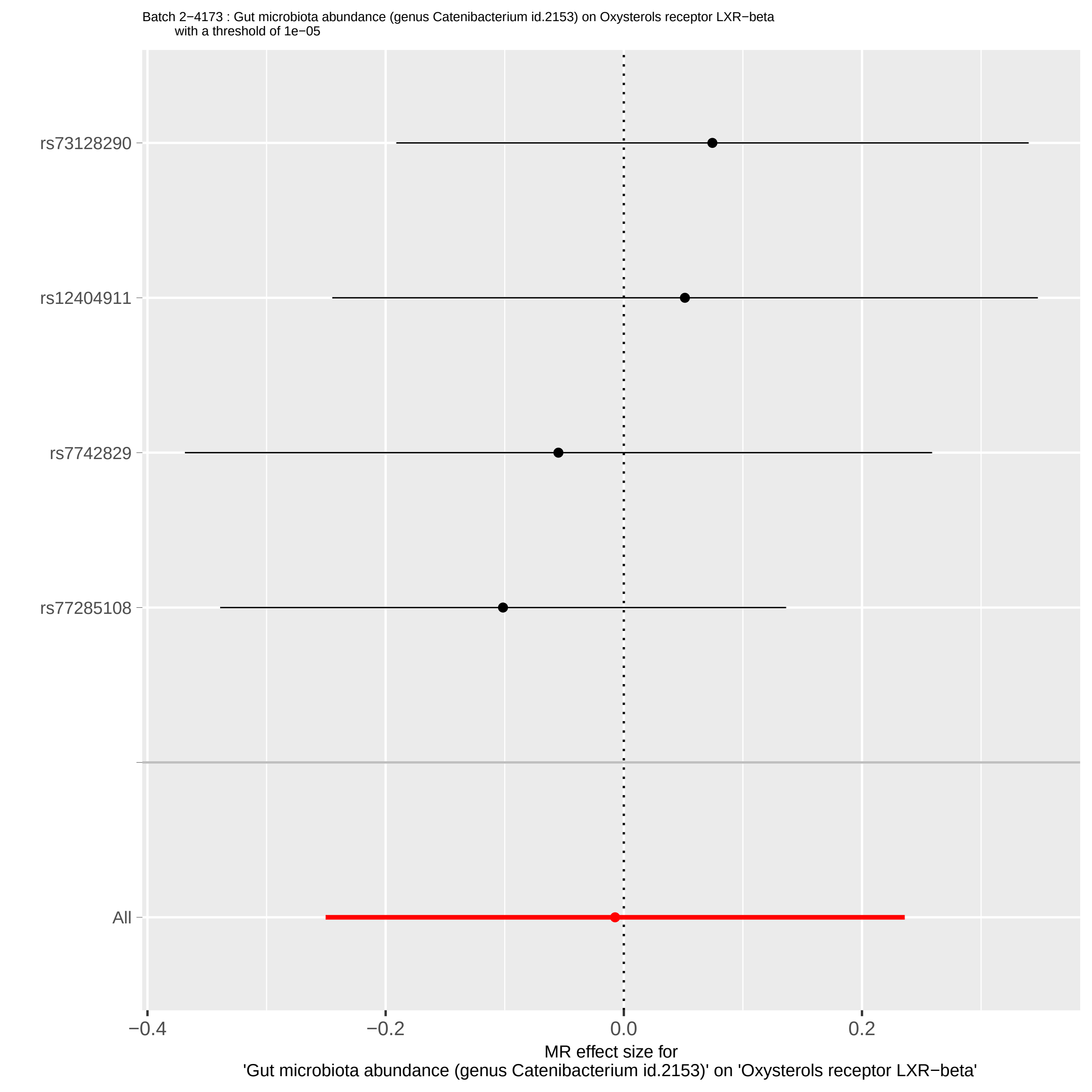
Batch 2−4173 : Gut microbiota abundance (genus Catenibacterium id.2153) on Oxysterols receptor LXR−beta
with a threshold of 1e−05
rs73128290
rs12404911
rs7742829
rs77285108
All
−0.4
−0.2
0.0
0.2
MR effect size for
'Gut microbiota abundance (genus Catenibacterium id.2153)' on 'Oxysterols receptor LXR−beta'

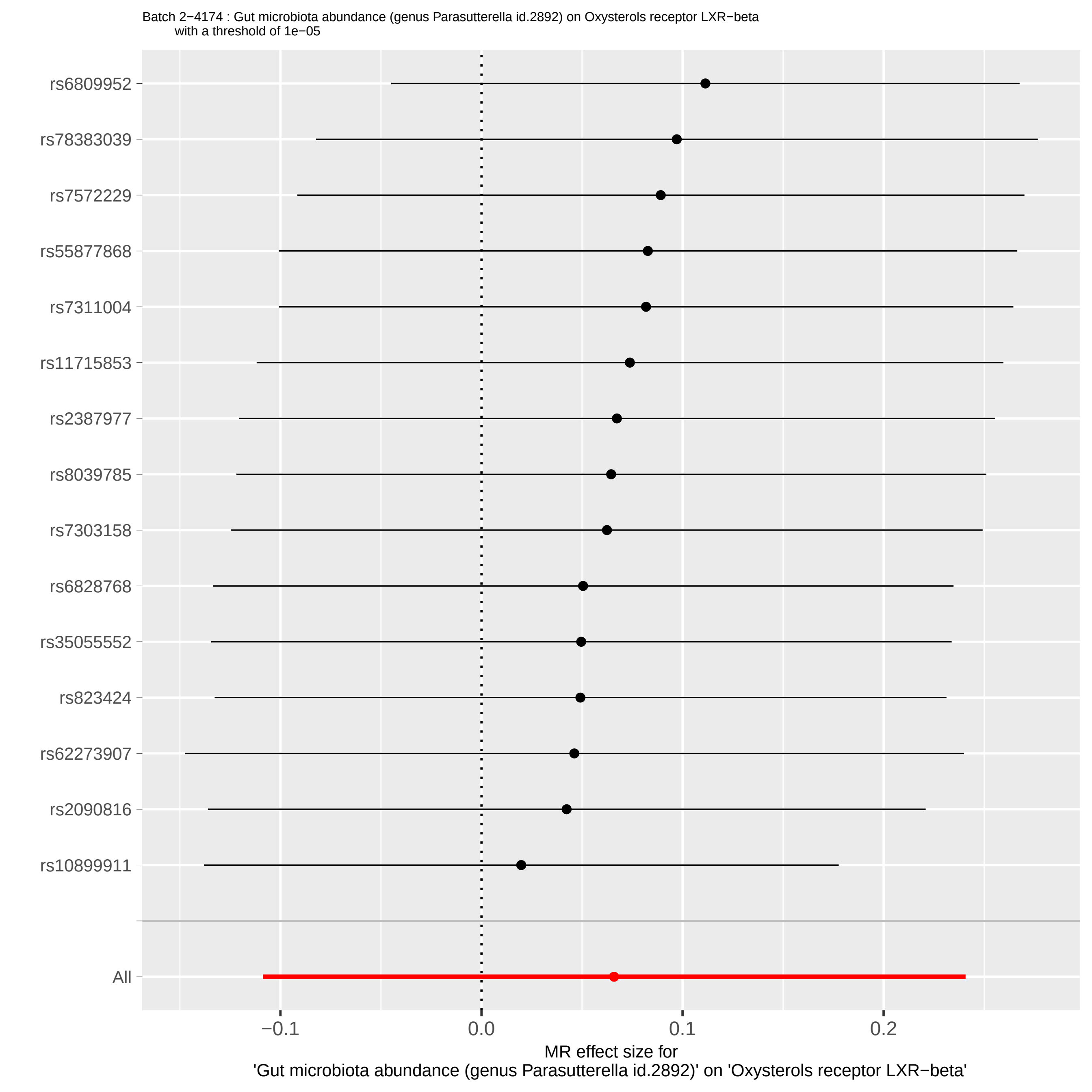
Batch 2−4174 : Gut microbiota abundance (genus Parasutterella id.2892) on Oxysterols receptor LXR−beta
with a threshold of 1e−05
rs6809952
rs78383039
rs7572229
rs55877868
rs7311004
rs11715853
rs2387977
rs8039785
rs7303158
rs6828768
rs35055552
rs823424
rs62273907
rs2090816
rs10899911
All
−0.1
0.0
0.1
0.2
MR effect size for
'Gut microbiota abundance (genus Parasutterella id.2892)' on 'Oxysterols receptor LXR−beta'

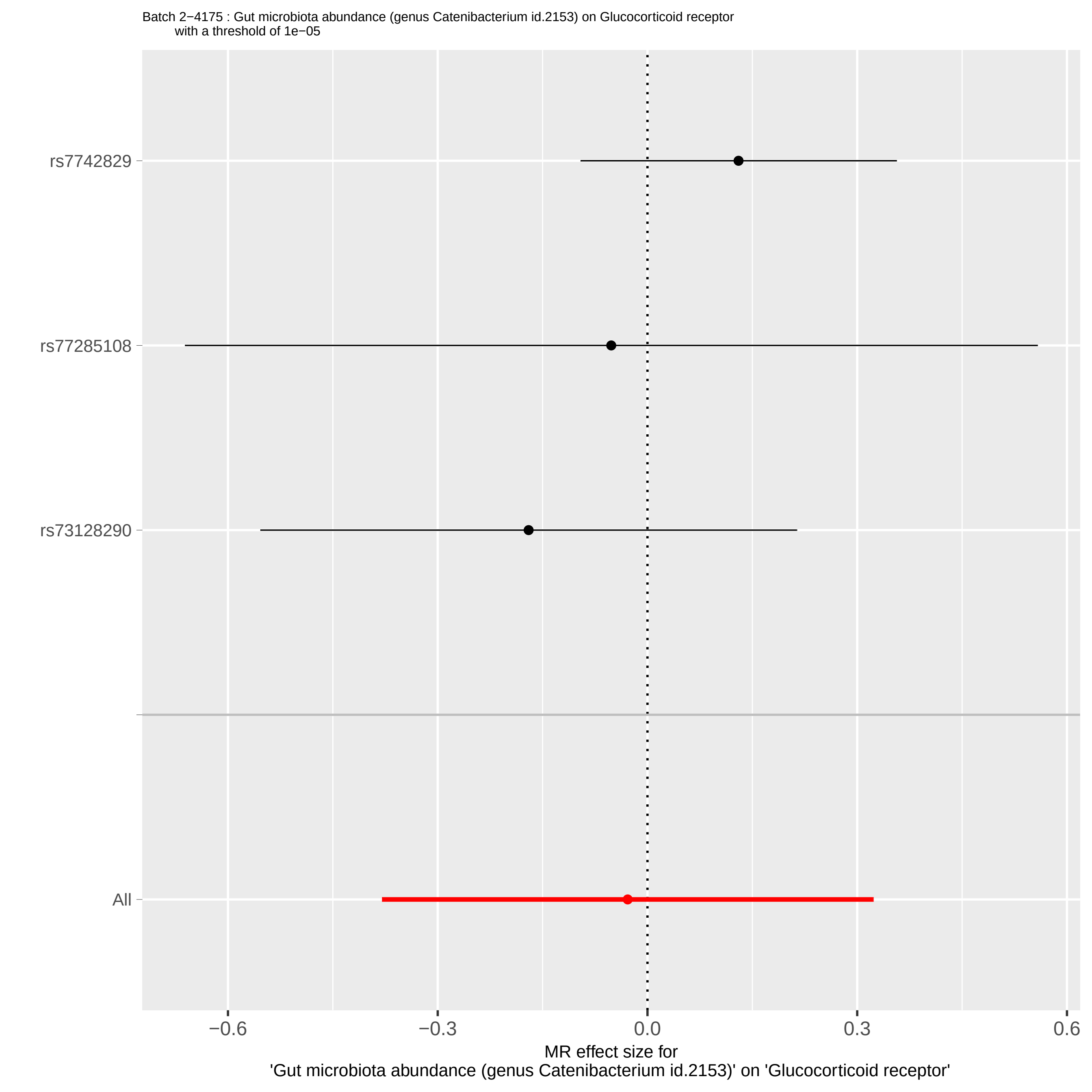
Batch 2−4175 : Gut microbiota abundance (genus Catenibacterium id.2153) on Glucocorticoid receptor
with a threshold of 1e−05
rs7742829
rs77285108
rs73128290
All
−0.6
−0.3
0.0
0.3
0.6
MR effect size for
'Gut microbiota abundance (genus Catenibacterium id.2153)' on 'Glucocorticoid receptor'

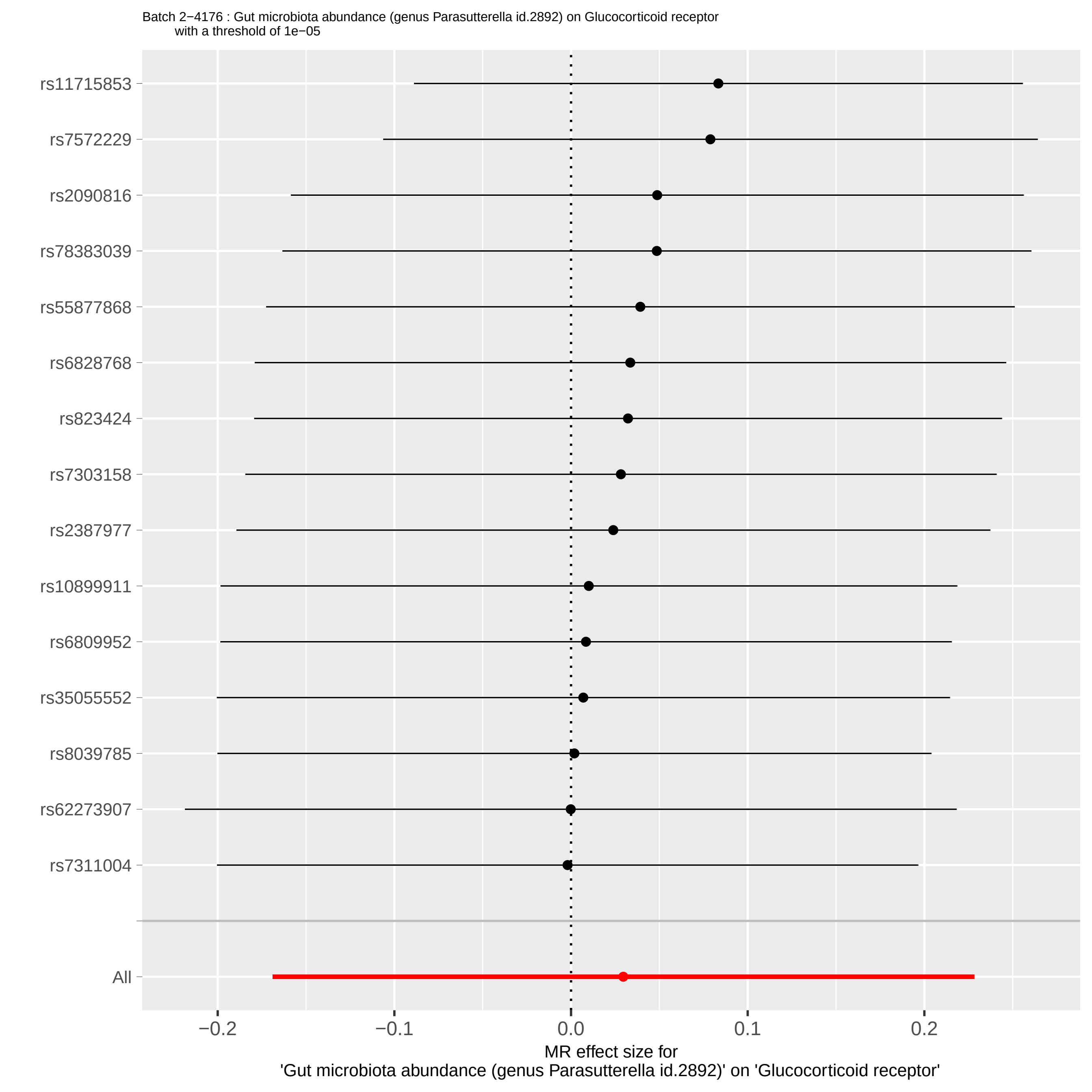
Batch 2−4176 : Gut microbiota abundance (genus Parasutterella id.2892) on Glucocorticoid receptor
with a threshold of 1e−05
rs11715853
rs7572229
rs2090816
rs78383039
rs55877868
rs6828768
rs823424
rs7303158
rs2387977
rs10899911
rs6809952
rs35055552
rs8039785
rs62273907
rs7311004
All
−0.2
−0.1
0.0
0.1
0.2
MR effect size for
'Gut microbiota abundance (genus Parasutterella id.2892)' on 'Glucocorticoid receptor'

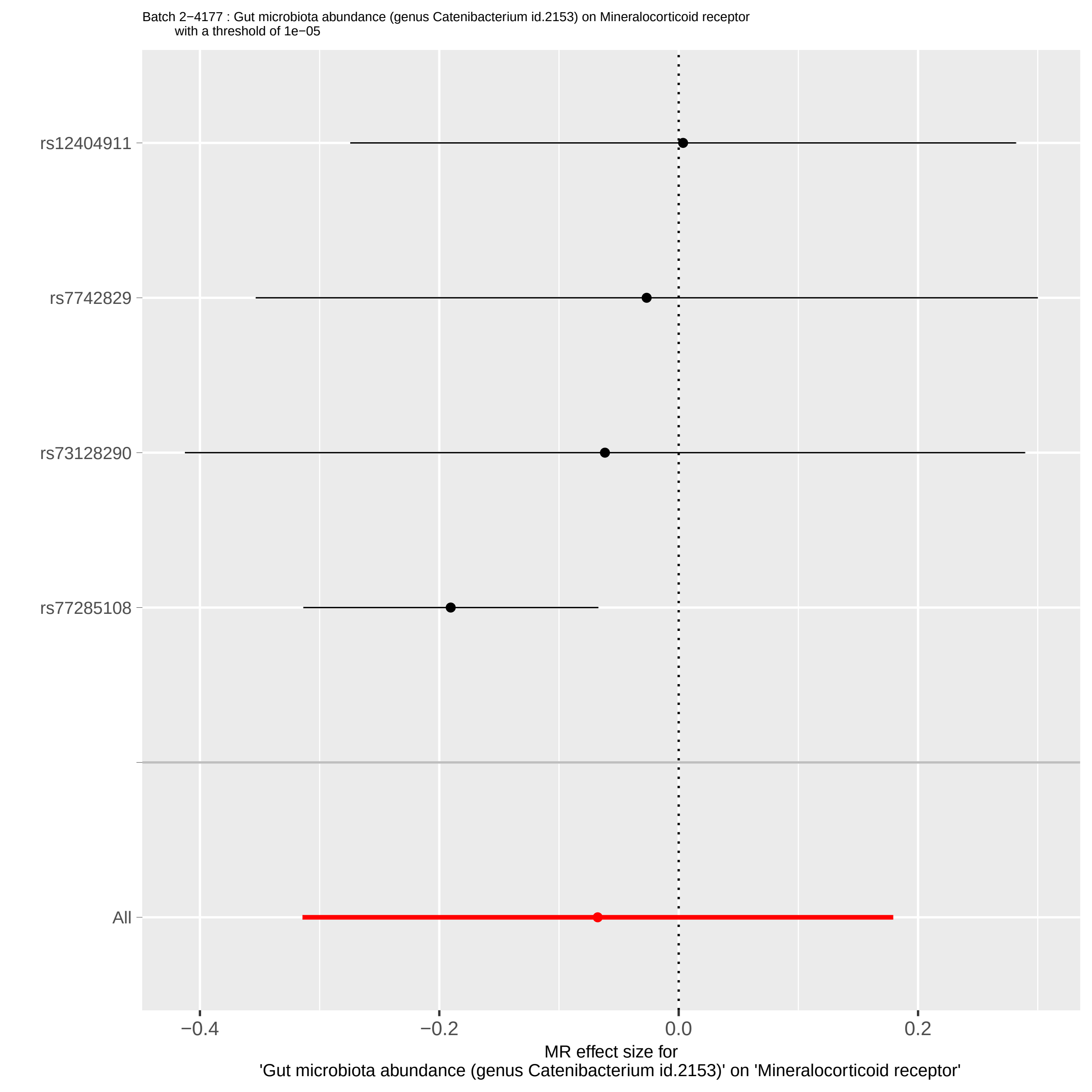
Batch 2−4177 : Gut microbiota abundance (genus Catenibacterium id.2153) on Mineralocorticoid receptor
with a threshold of 1e−05
rs12404911
rs7742829
rs73128290
rs77285108
All
−0.4
−0.2
0.0
0.2
MR effect size for
'Gut microbiota abundance (genus Catenibacterium id.2153)' on 'Mineralocorticoid receptor'

Batch 2−4178 : Gut microbiota abundance (genus Parasutterella id.2892) on Mineralocorticoid receptor
with a threshold of 1e−05

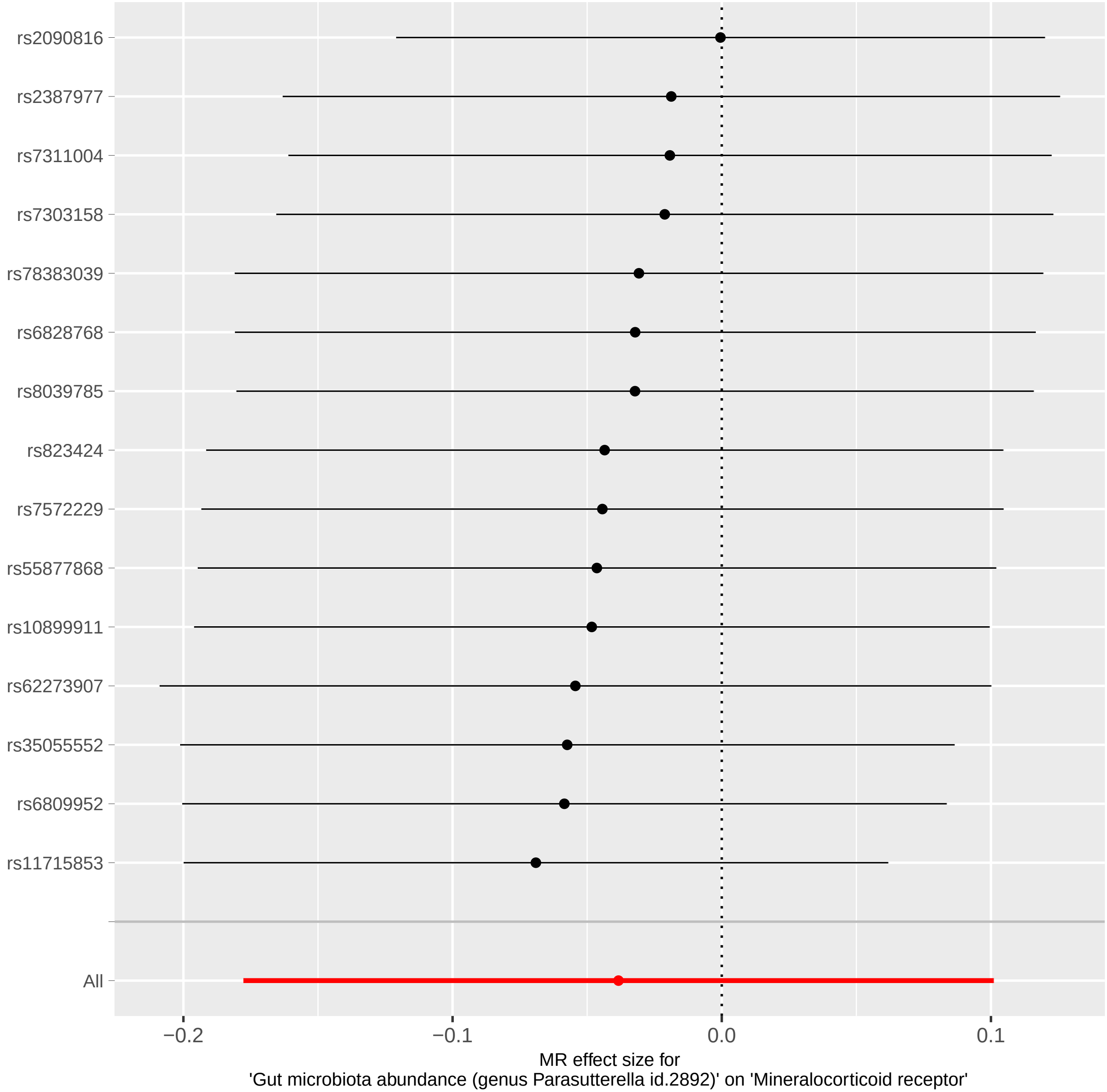

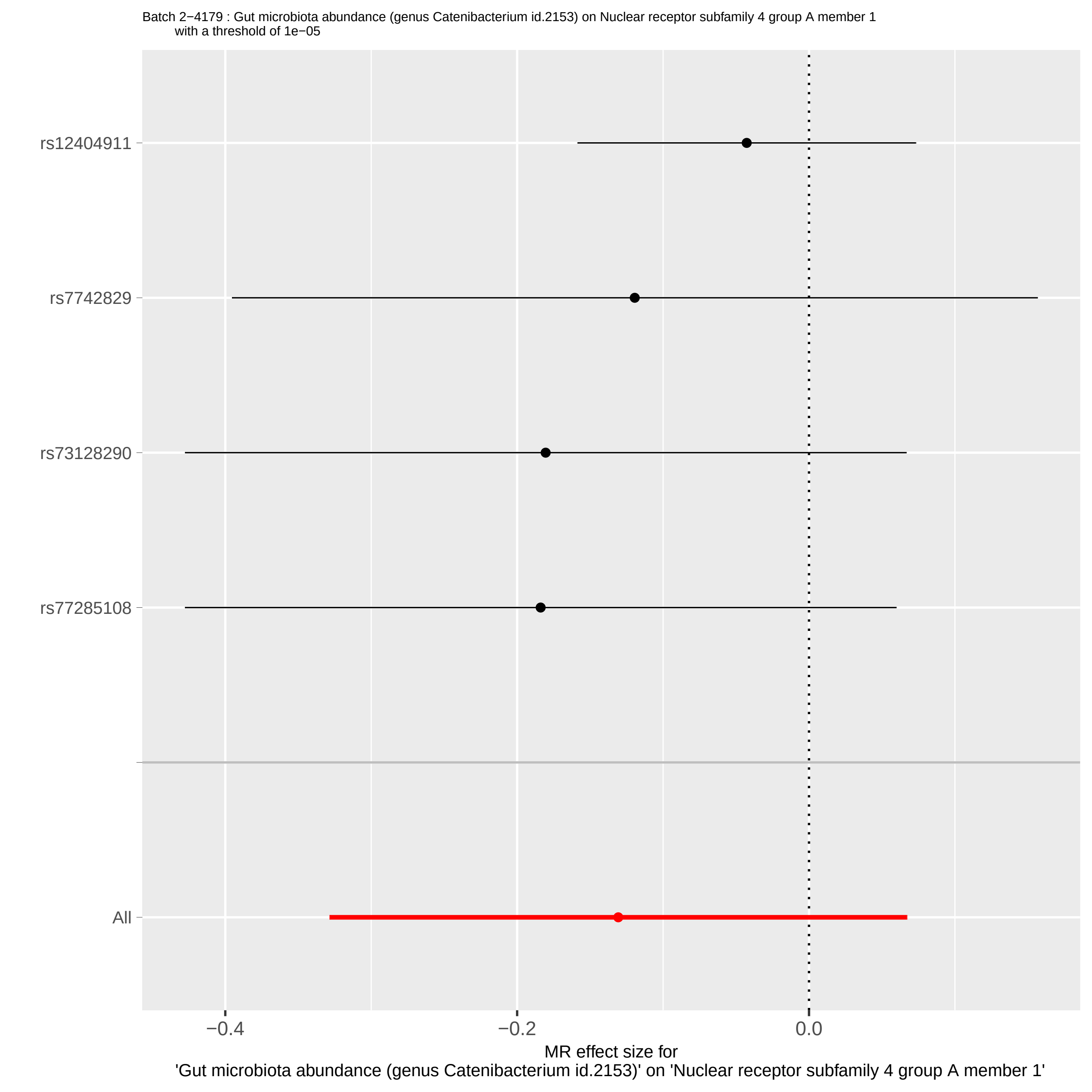

Batch 2−4179 : Gut microbiota abundance (genus Catenibacterium id.2153) on Nuclear receptor subfamily 4 group A member 1
with a threshold of 1e−05
rs12404911
rs7742829
rs73128290
rs77285108
All
−0.4
−0.2
0.0
MR effect size for
'Gut microbiota abundance (genus Catenibacterium id.2153)' on 'Nuclear receptor subfamily 4 group A member 1'

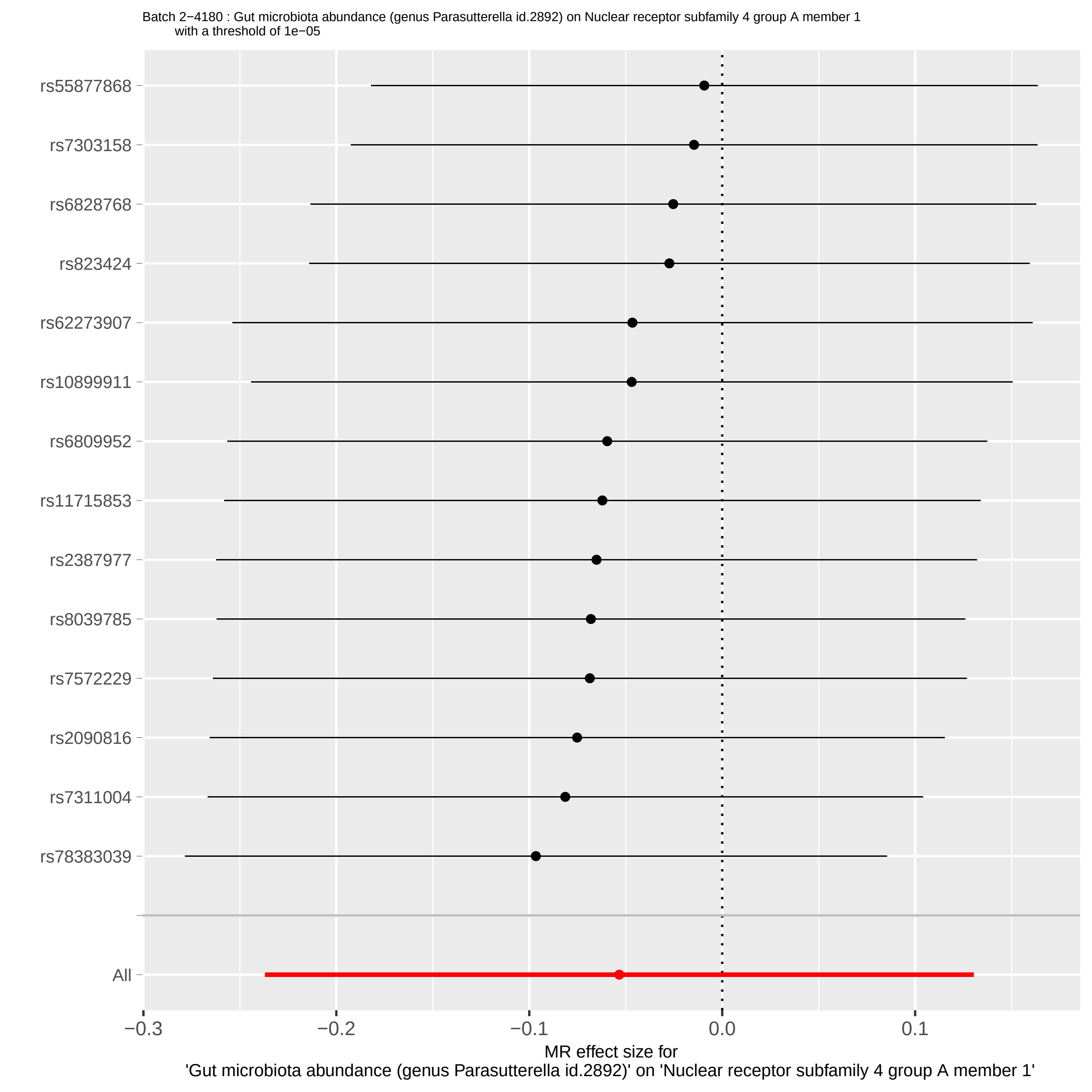
Batch 2−4180 : Gut microbiota abundance (genus Parasutterella id.2892) on Nuclear receptor subfamily 4 group A member 1
with a threshold of 1e−05
rs55877868
rs7303158
rs6828768
rs823424
rs62273907
rs10899911
rs6809952
rs11715853
rs2387977
rs8039785
rs7572229
rs2090816
rs7311004
rs78383039
All
−0.3
−0.2
−0.1
0.0
0.1
MR effect size for
'Gut microbiota abundance (genus Parasutterella id.2892)' on 'Nuclear receptor subfamily 4 group A member 1'

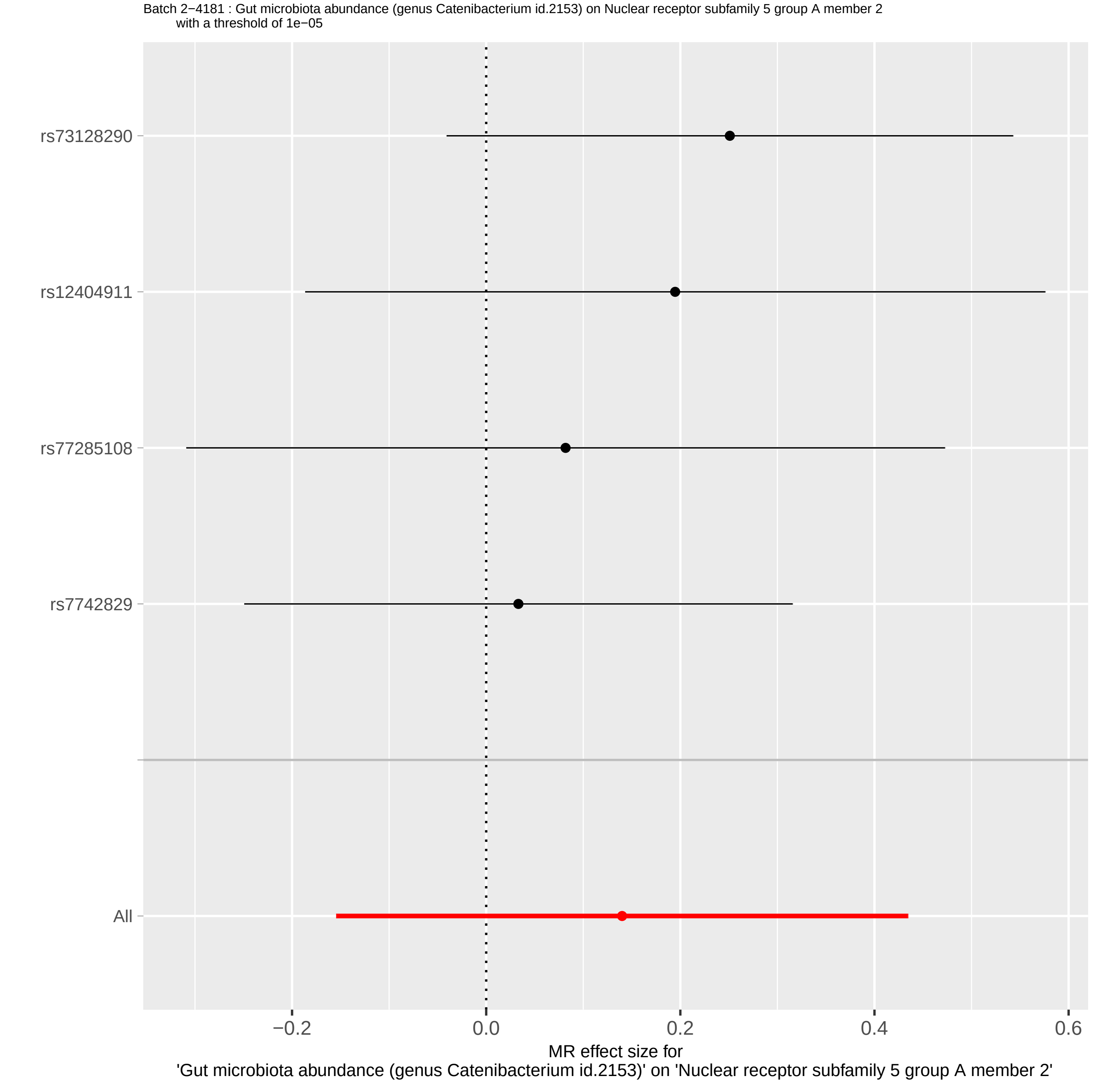

Batch 2−4181 : Gut microbiota abundance (genus Catenibacterium id.2153) on Nuclear receptor subfamily 5 group A member 2
with a threshold of 1e−05
rs73128290
rs12404911
rs77285108
rs7742829
All
−0.2
0.0
0.2
0.4
0.6
MR effect size for
'Gut microbiota abundance (genus Catenibacterium id.2153)' on 'Nuclear receptor subfamily 5 group A member 2'

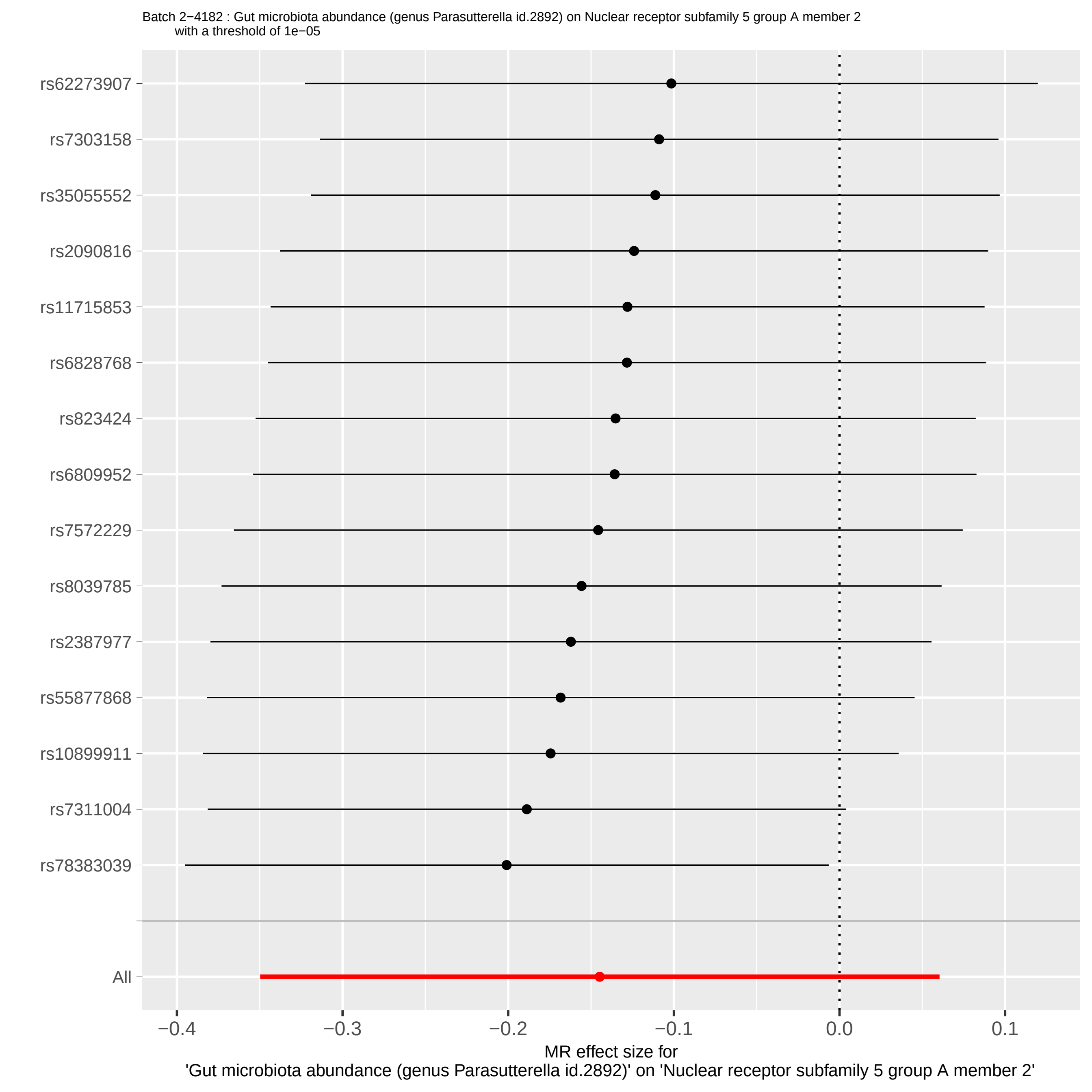
Batch 2−4182 : Gut microbiota abundance (genus Parasutterella id.2892) on Nuclear receptor subfamily 5 group A member 2
with a threshold of 1e−05
rs62273907
rs7303158
rs35055552
rs2090816
rs11715853
rs6828768
rs823424
rs6809952
rs7572229
rs8039785
rs2387977
rs55877868
rs10899911
rs7311004
rs78383039
All
−0.4
−0.3
−0.2
−0.1
0.0
0.1
MR effect size for
'Gut microbiota abundance (genus Parasutterella id.2892)' on 'Nuclear receptor subfamily 5 group A member 2'

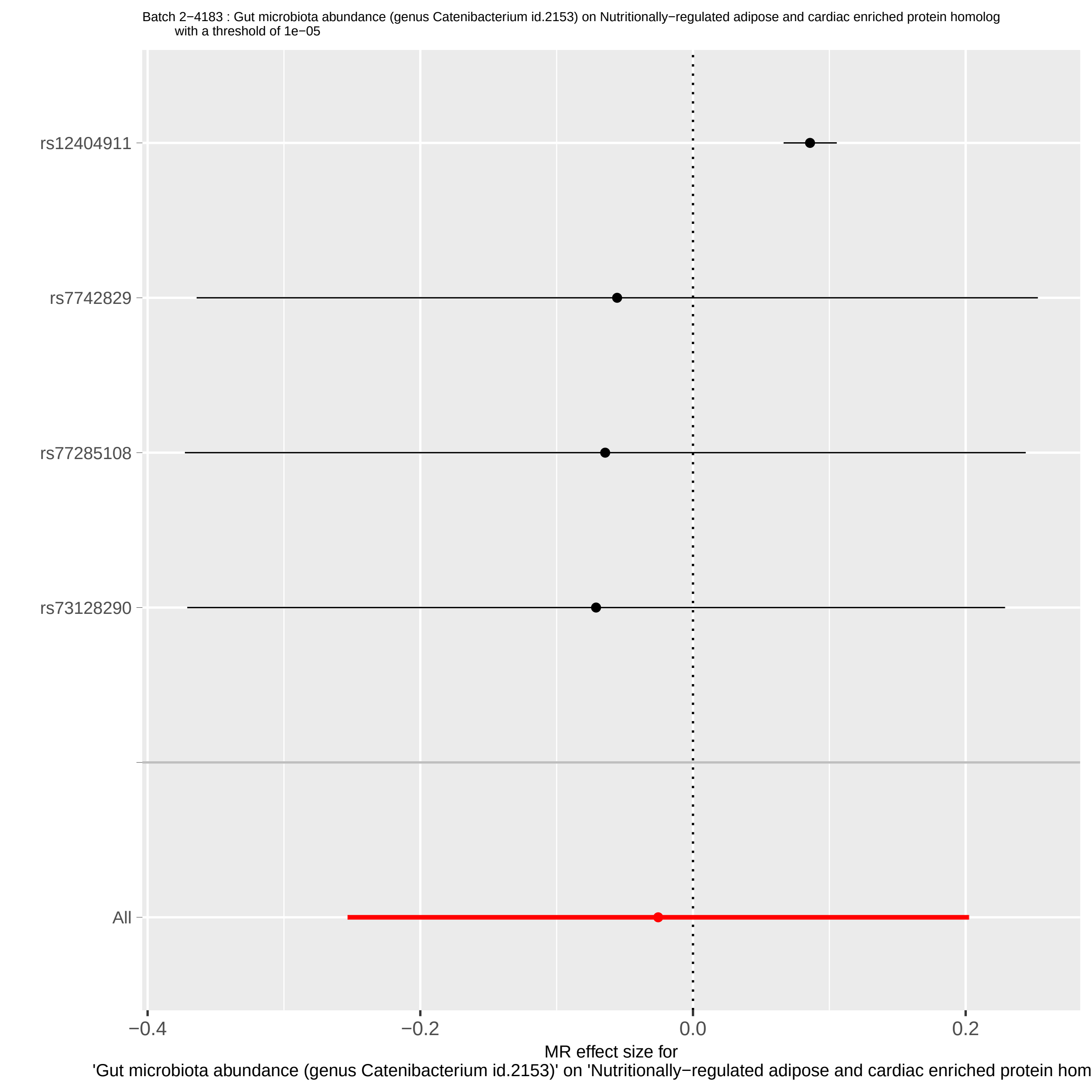

Batch 2−4183 : Gut microbiota abundance (genus Catenibacterium id.2153) on Nutritionally−regulated adipose and cardiac enriched protein homolog
with a threshold of 1e−05
rs12404911
rs7742829
rs77285108
rs73128290
All
−0.4
−0.2
0.0
0.2
MR effect size for
'Gut microbiota abundance (genus Catenibacterium id.2153)' on 'Nutritionally−regulated adipose and cardiac enriched protein hom

Batch 2−4184 : Gut microbiota abundance (genus Parasutterella id.2892) on Nutritionally−regulated adipose and cardiac enriched protein homolog with a threshold of 1e−05

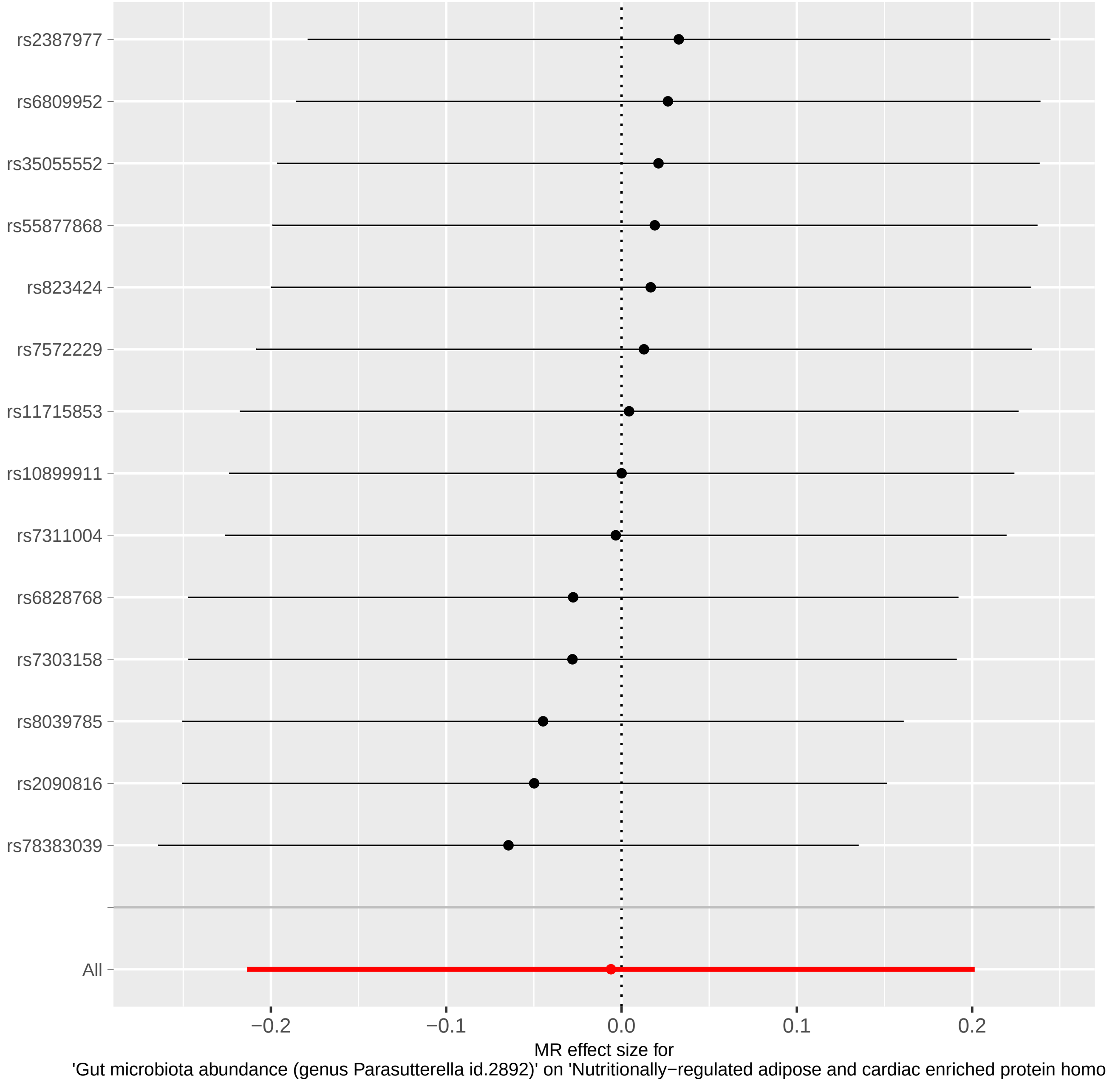

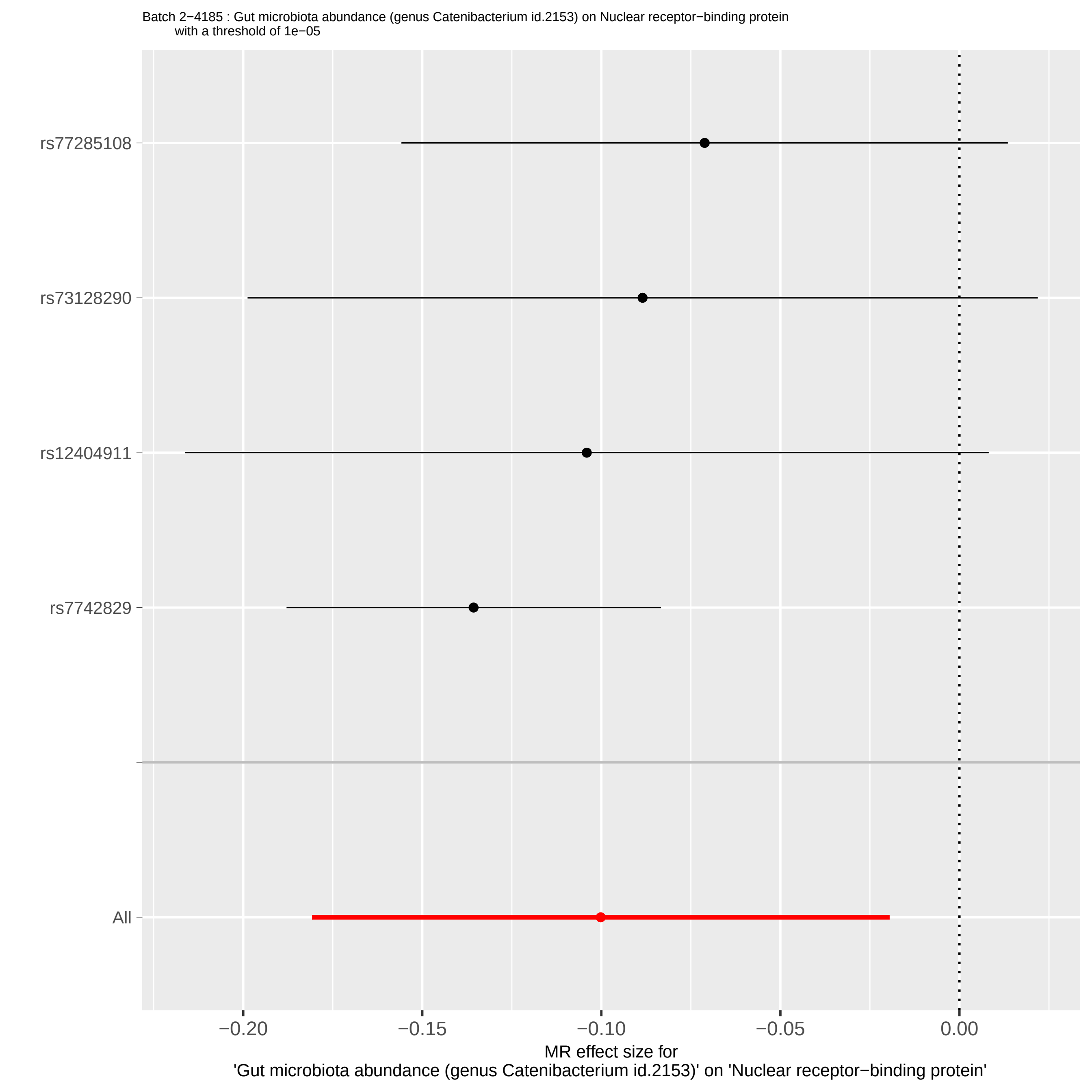

Batch 2−4185 : Gut microbiota abundance (genus Catenibacterium id.2153) on Nuclear receptor−binding protein
with a threshold of 1e−05
rs77285108
rs73128290
rs12404911
rs7742829
All
−0.20
−0.15
−0.10
−0.05
0.00
MR effect size for
'Gut microbiota abundance (genus Catenibacterium id.2153)' on 'Nuclear receptor−binding protein'

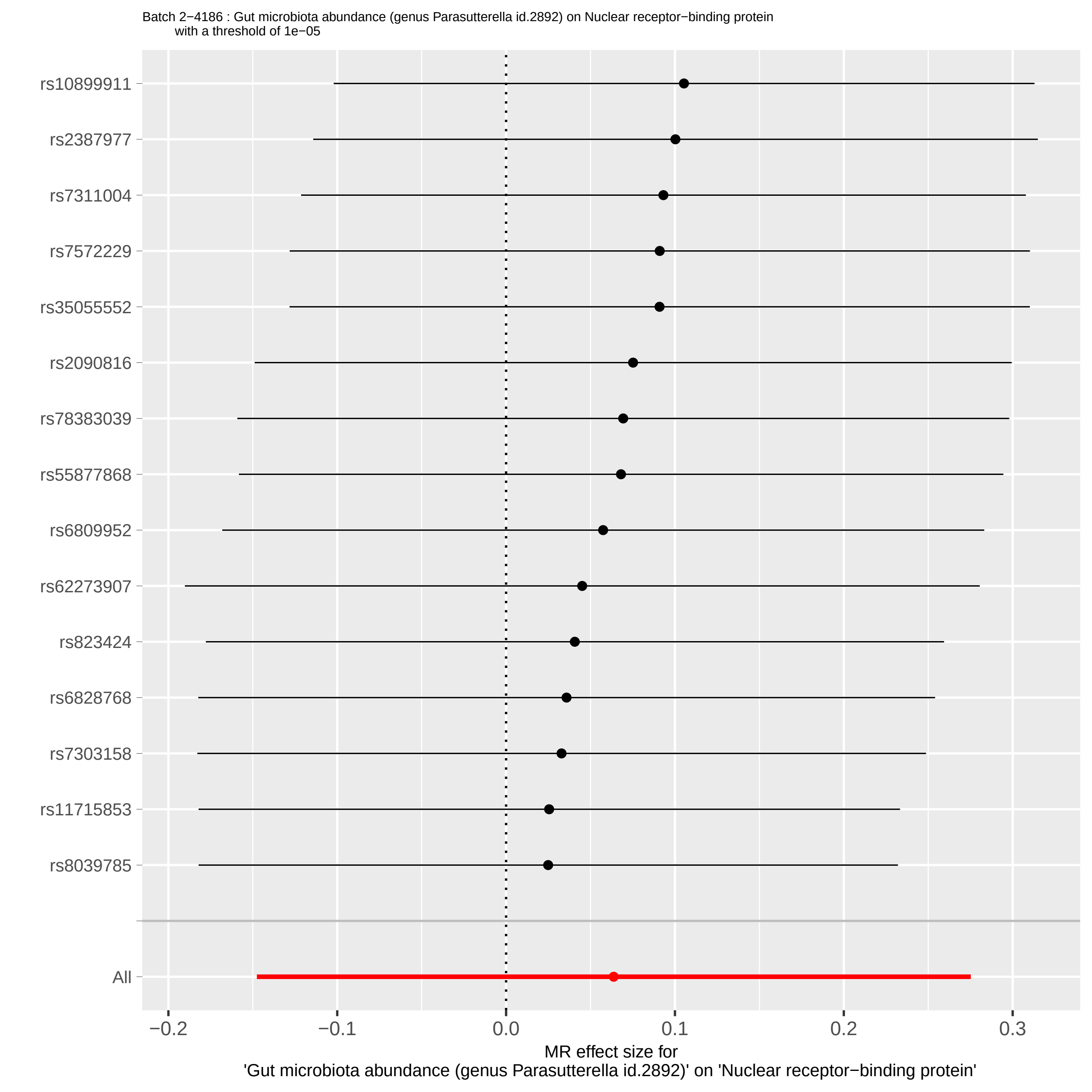
Batch 2−4186 : Gut microbiota abundance (genus Parasutterella id.2892) on Nuclear receptor−binding protein
with a threshold of 1e−05
rs10899911
rs2387977
rs7311004
rs7572229
rs35055552
rs2090816
rs78383039
rs55877868
rs6809952
rs62273907
rs823424
rs6828768
rs7303158
rs11715853
rs8039785
All
−0.2
−0.1
0.0
0.1
0.2
0.3
MR effect size for
'Gut microbiota abundance (genus Parasutterella id.2892)' on 'Nuclear receptor−binding protein'

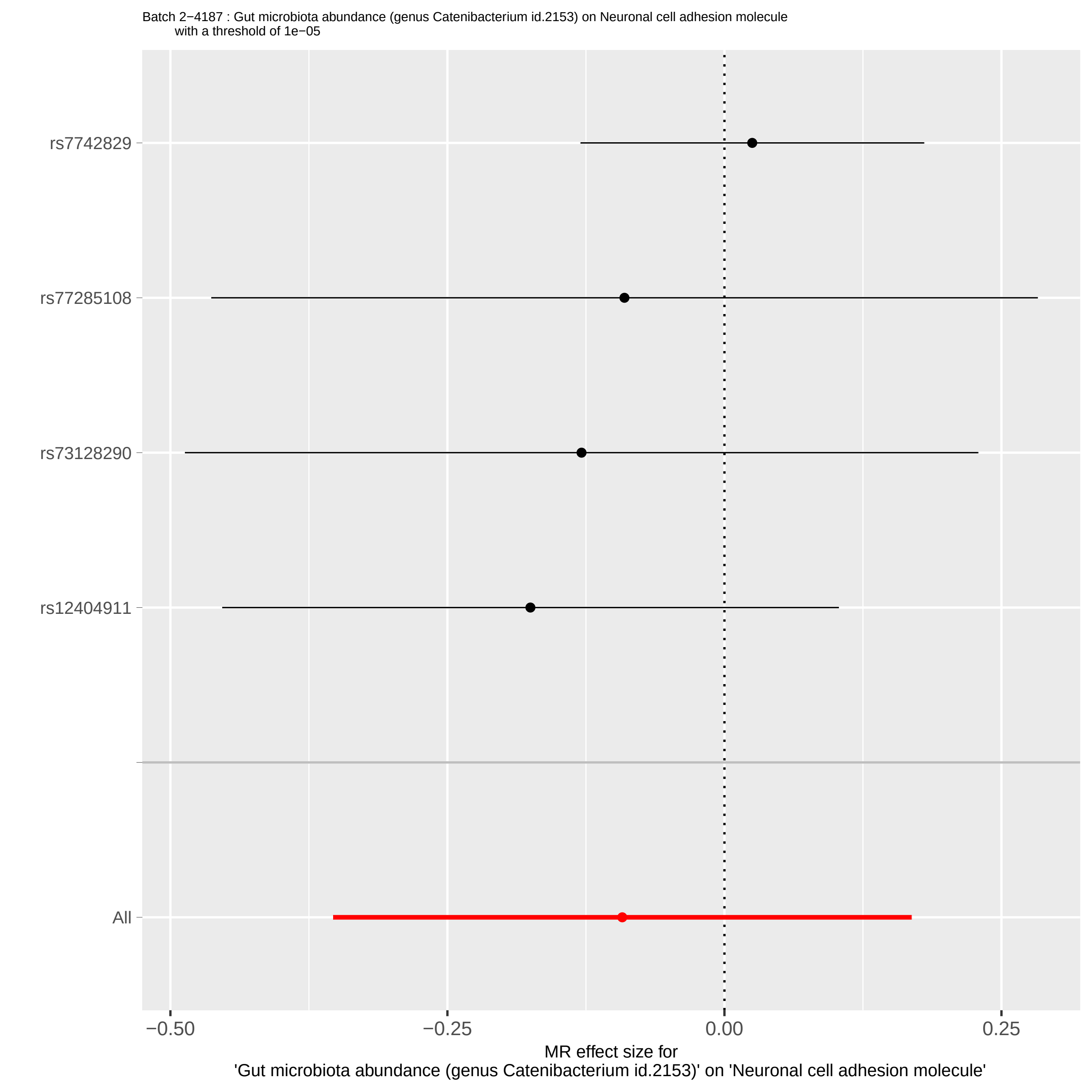
Batch 2−4187 : Gut microbiota abundance (genus Catenibacterium id.2153) on Neuronal cell adhesion molecule
with a threshold of 1e−05
rs7742829
rs77285108
rs73128290
rs12404911
All
−0.50
−0.25
0.00
0.25
MR effect size for
'Gut microbiota abundance (genus Catenibacterium id.2153)' on 'Neuronal cell adhesion molecule'

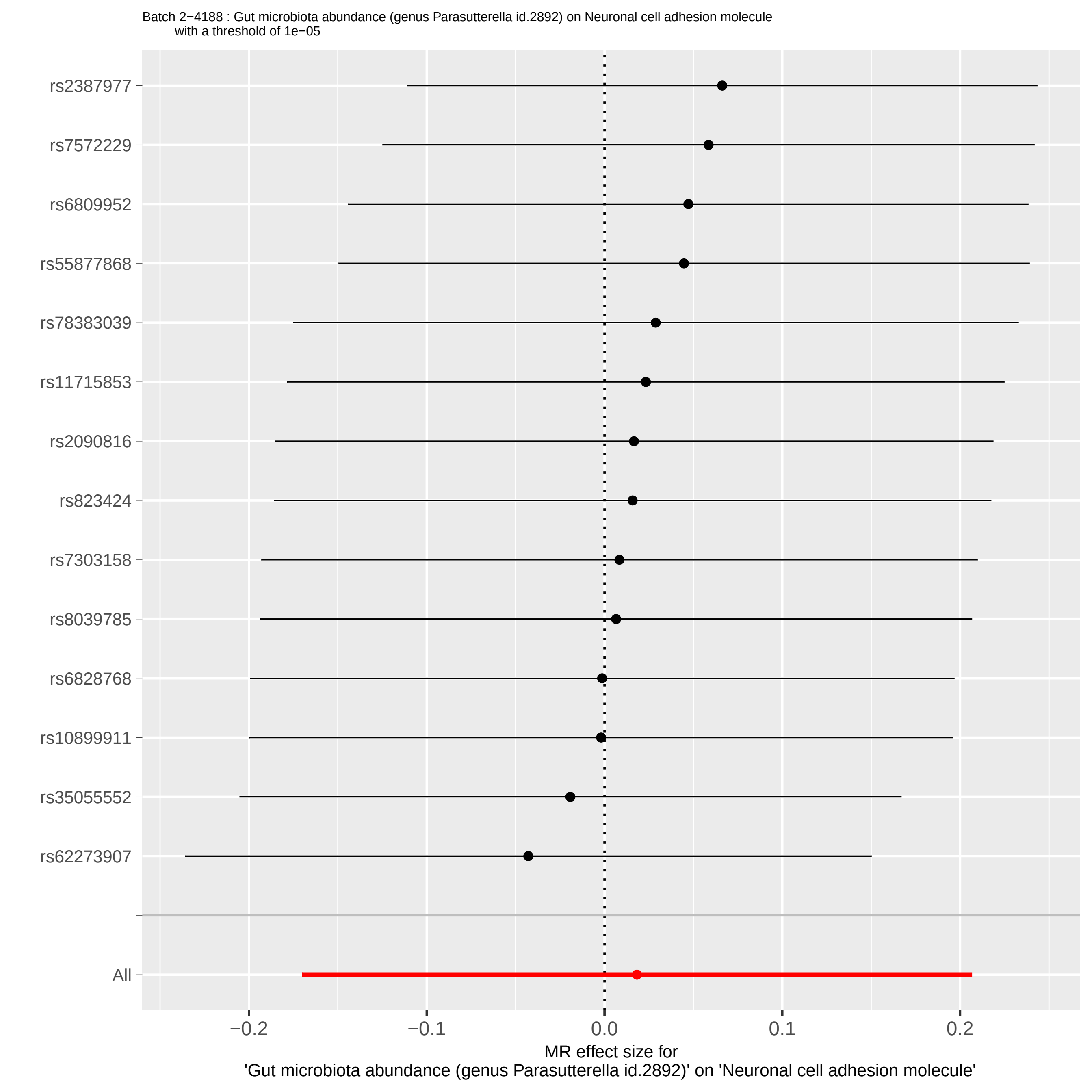
Batch 2−4188 : Gut microbiota abundance (genus Parasutterella id.2892) on Neuronal cell adhesion molecule
with a threshold of 1e−05
rs2387977
rs7572229
rs6809952
rs55877868
rs78383039
rs11715853
rs2090816
rs823424
rs7303158
rs8039785
rs6828768
rs10899911
rs35055552
rs62273907
All
−0.2
−0.1
0.0
0.1
0.2
MR effect size for
'Gut microbiota abundance (genus Parasutterella id.2892)' on 'Neuronal cell adhesion molecule'

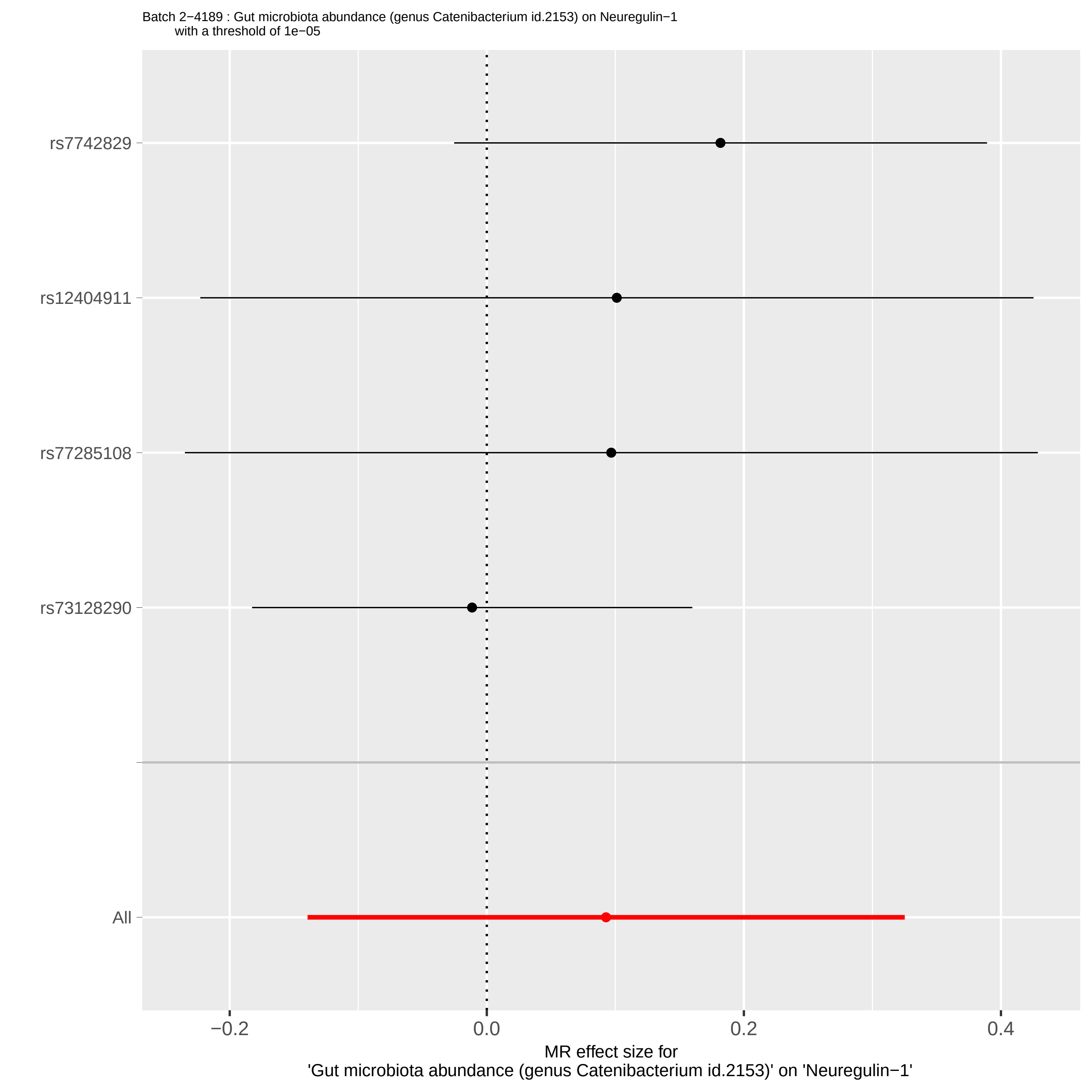

Batch 2−4189 : Gut microbiota abundance (genus Catenibacterium id.2153) on Neuregulin−1
with a threshold of 1e−05
rs7742829
rs12404911
rs77285108
rs73128290
All
−0.2
0.0
0.2
0.4
MR effect size for
'Gut microbiota abundance (genus Catenibacterium id.2153)' on 'Neuregulin−1'

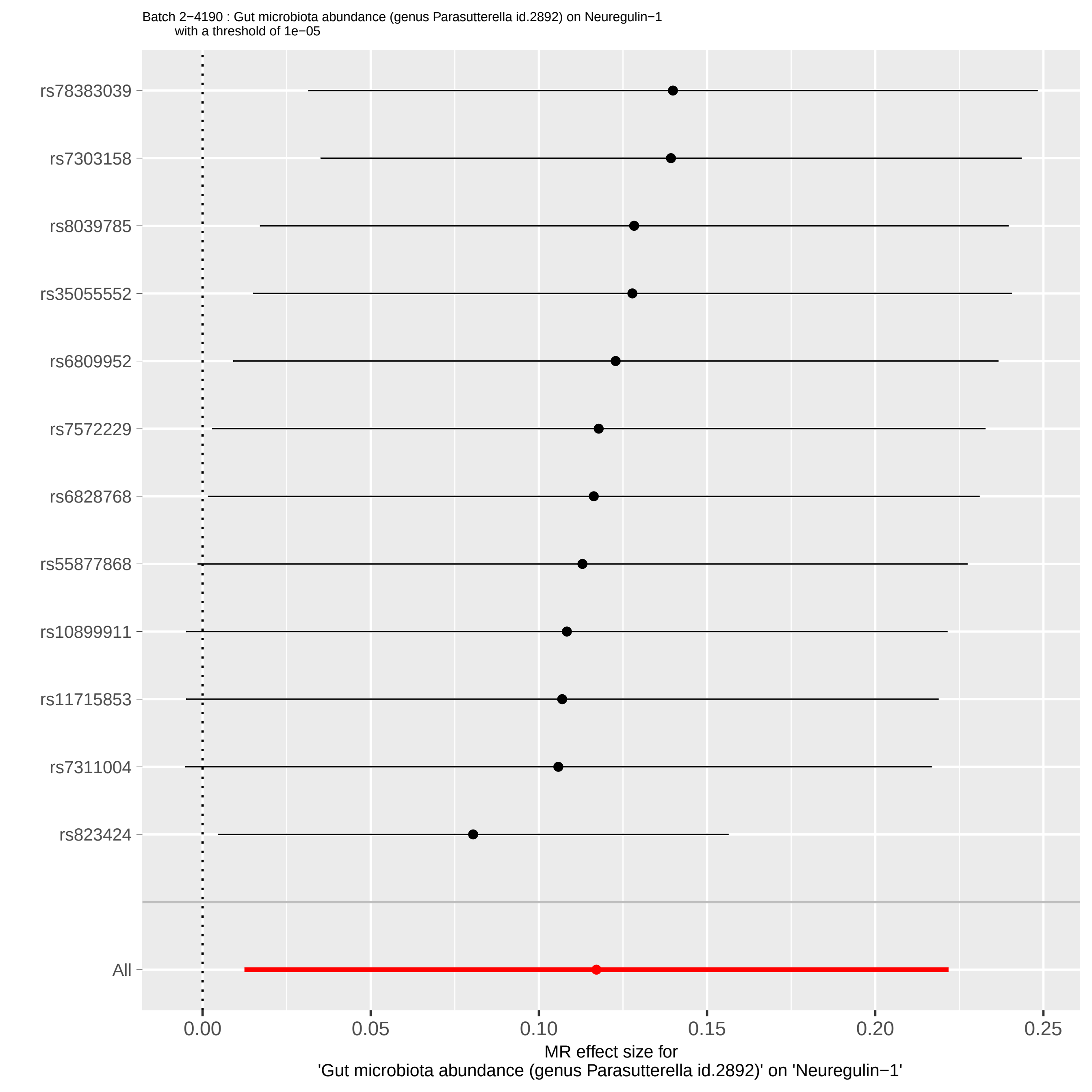
Batch 2−4190 : Gut microbiota abundance (genus Parasutterella id.2892) on Neuregulin−1
with a threshold of 1e−05
rs78383039
rs7303158
rs8039785
rs35055552
rs6809952
rs7572229
rs6828768
rs55877868
rs10899911
rs11715853
rs7311004
rs823424
All
0.00
0.05
0.10
0.15
0.20
0.25
MR effect size for
'Gut microbiota abundance (genus Parasutterella id.2892)' on 'Neuregulin−1'

Batch 2−4191 : Gut microbiota abundance (genus Catenibacterium id.2153) on Pro−neuregulin−2, membrane−bound isoform
with a threshold of 1e−05

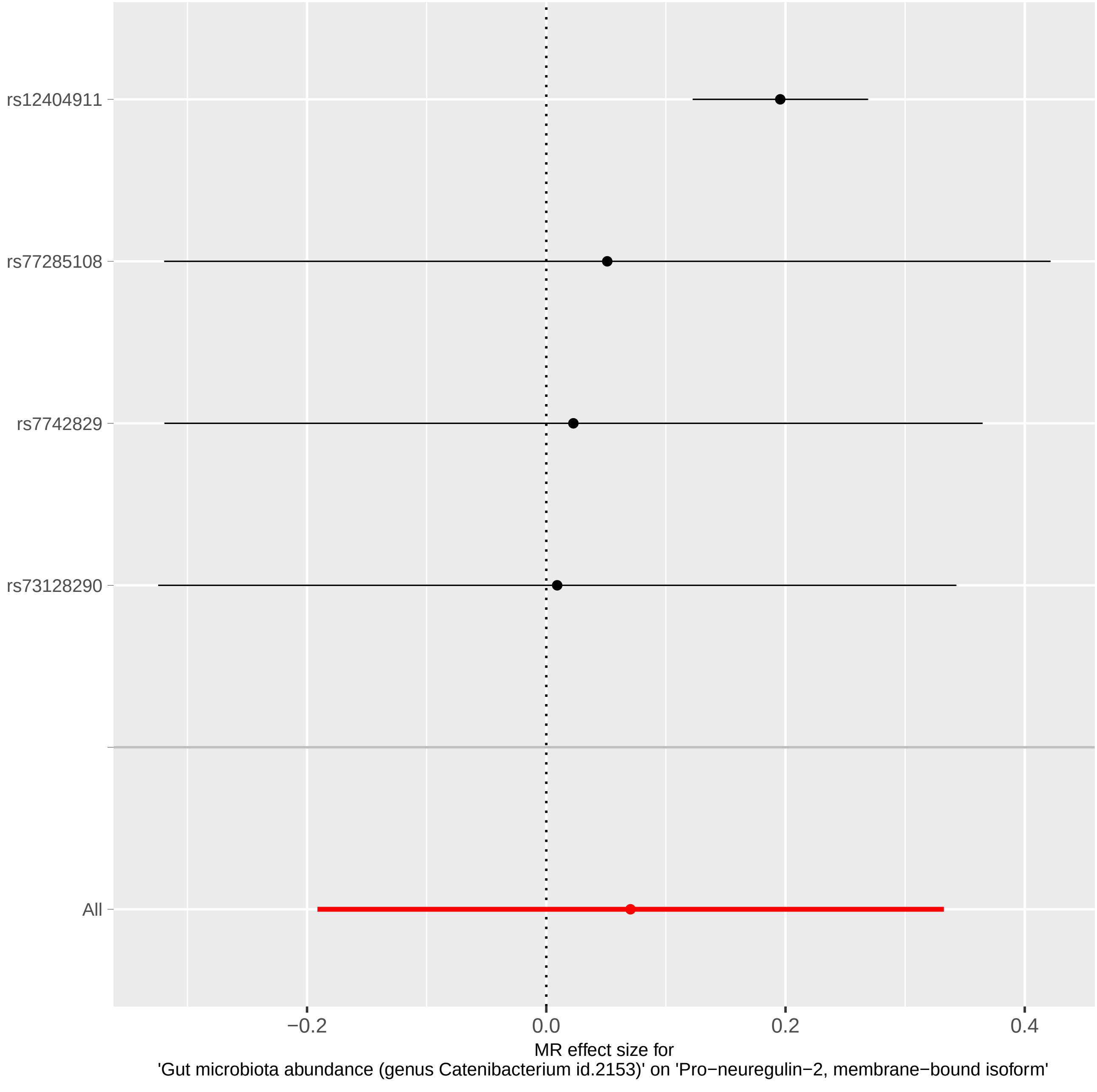

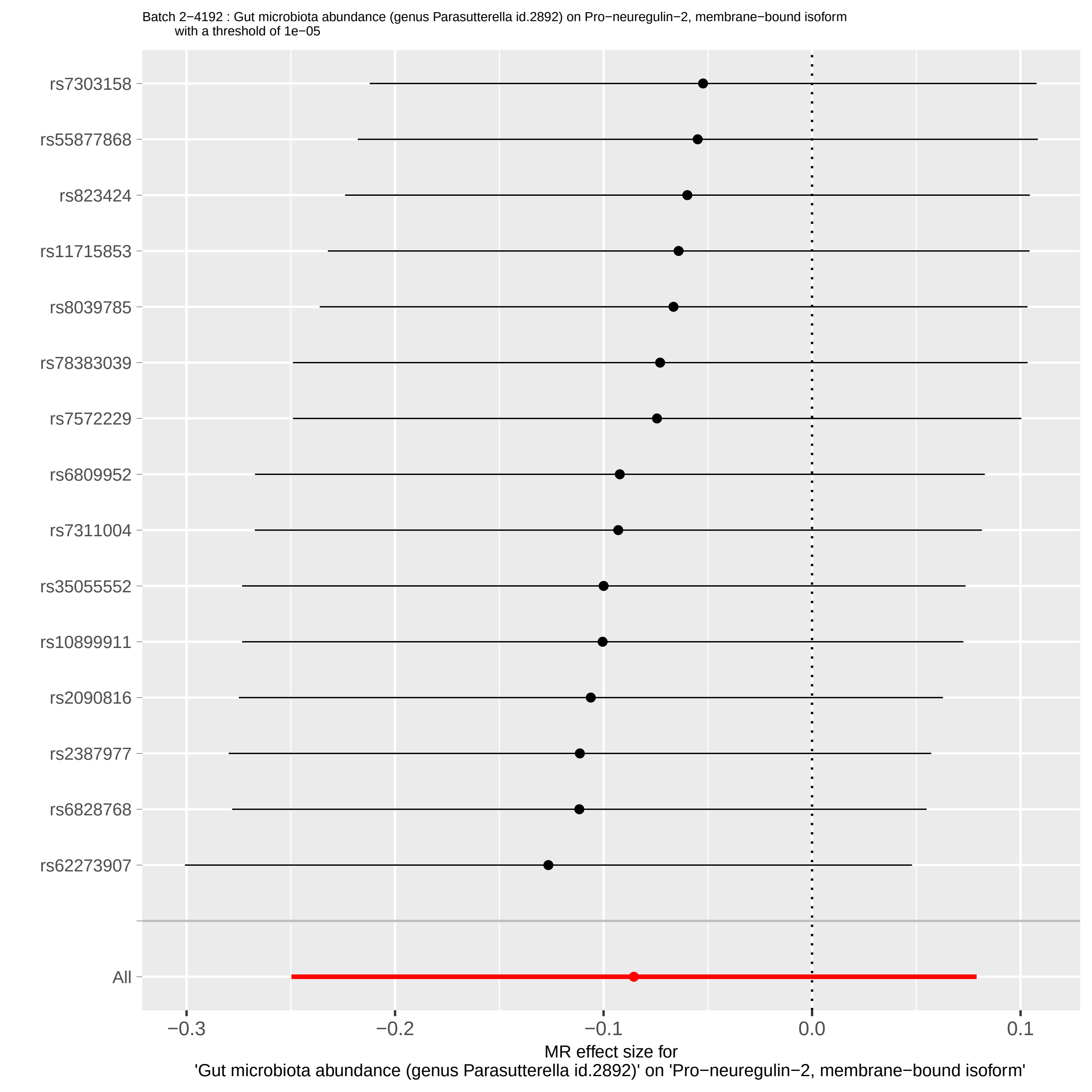
Batch 2−4192 : Gut microbiota abundance (genus Parasutterella id.2892) on Pro−neuregulin−2, membrane−bound isoform
with a threshold of 1e−05
rs7303158
rs55877868
rs823424
rs11715853
rs8039785
rs78383039
rs7572229
rs6809952
rs7311004
rs35055552
rs10899911
rs2090816
rs2387977
rs6828768
rs62273907
All
−0.3
−0.2
−0.1
0.0
0.1
MR effect size for
'Gut microbiota abundance (genus Parasutterella id.2892)' on 'Pro−neuregulin−2, membrane−bound isoform'

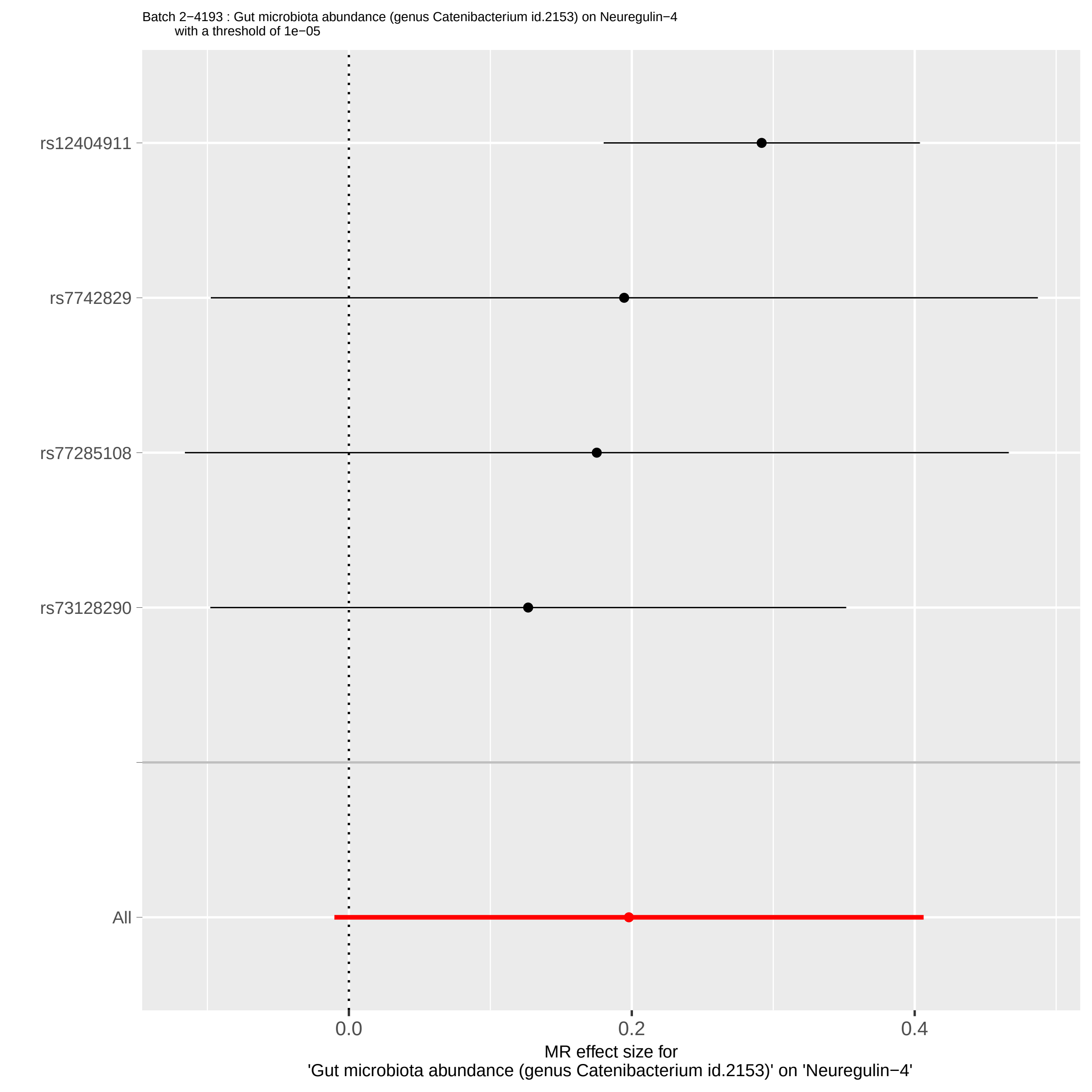

Batch 2−4193 : Gut microbiota abundance (genus Catenibacterium id.2153) on Neuregulin−4
with a threshold of 1e−05
rs12404911
rs7742829
rs77285108
rs73128290
All
0.0
0.2
0.4
MR effect size for
'Gut microbiota abundance (genus Catenibacterium id.2153)' on 'Neuregulin−4'

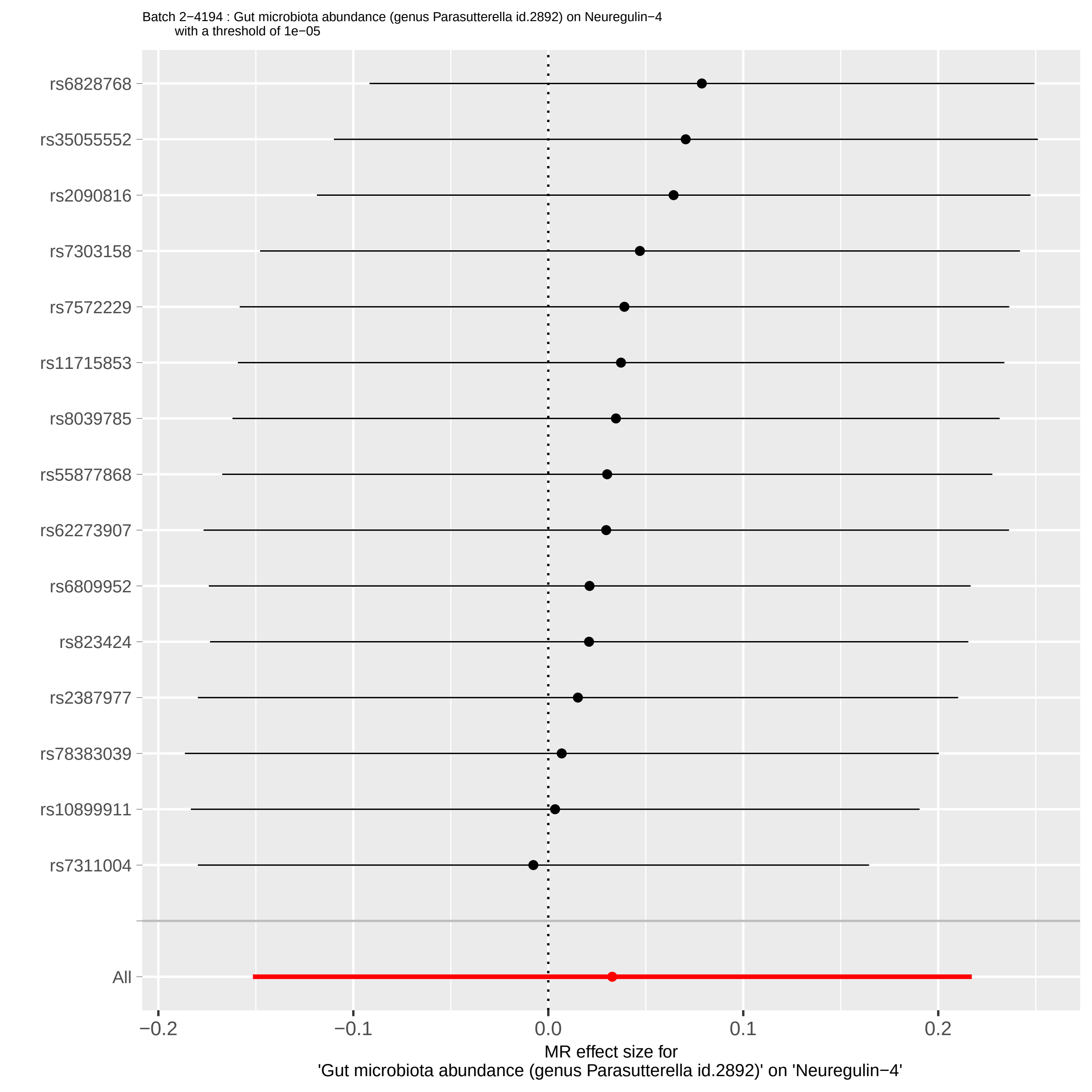
Batch 2−4194 : Gut microbiota abundance (genus Parasutterella id.2892) on Neuregulin−4
with a threshold of 1e−05
rs6828768
rs35055552
rs2090816
rs7303158
rs7572229
rs11715853
rs8039785
rs55877868
rs62273907
rs6809952
rs823424
rs2387977
rs78383039
rs10899911
rs7311004
All
−0.2
−0.1
0.0
0.1
0.2
MR effect size for
'Gut microbiota abundance (genus Parasutterella id.2892)' on 'Neuregulin−4'

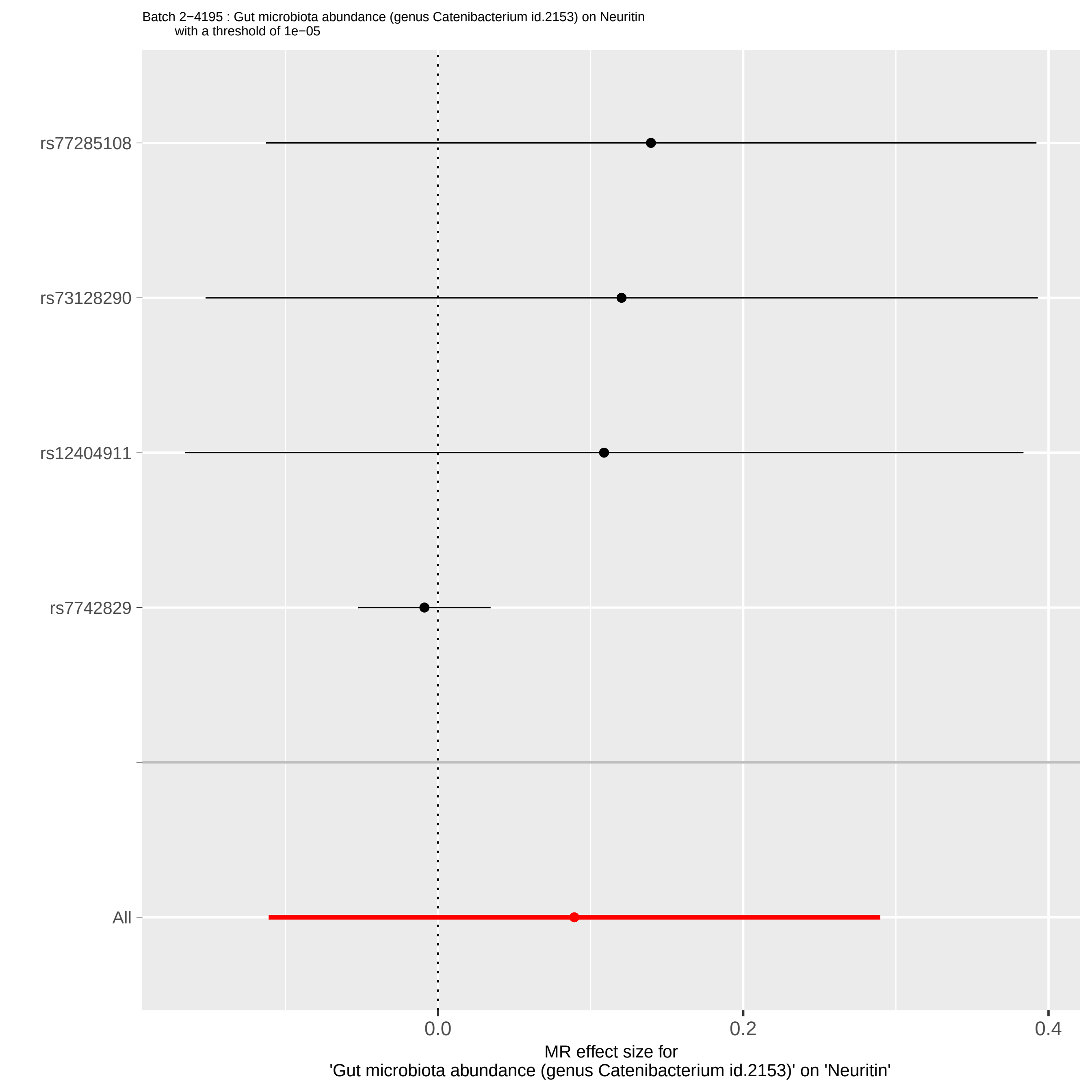

Batch 2−4195 : Gut microbiota abundance (genus Catenibacterium id.2153) on Neuritin
with a threshold of 1e−05
rs77285108
rs73128290
rs12404911
rs7742829
All
0.0
0.2
0.4
MR effect size for
'Gut microbiota abundance (genus Catenibacterium id.2153)' on 'Neuritin'

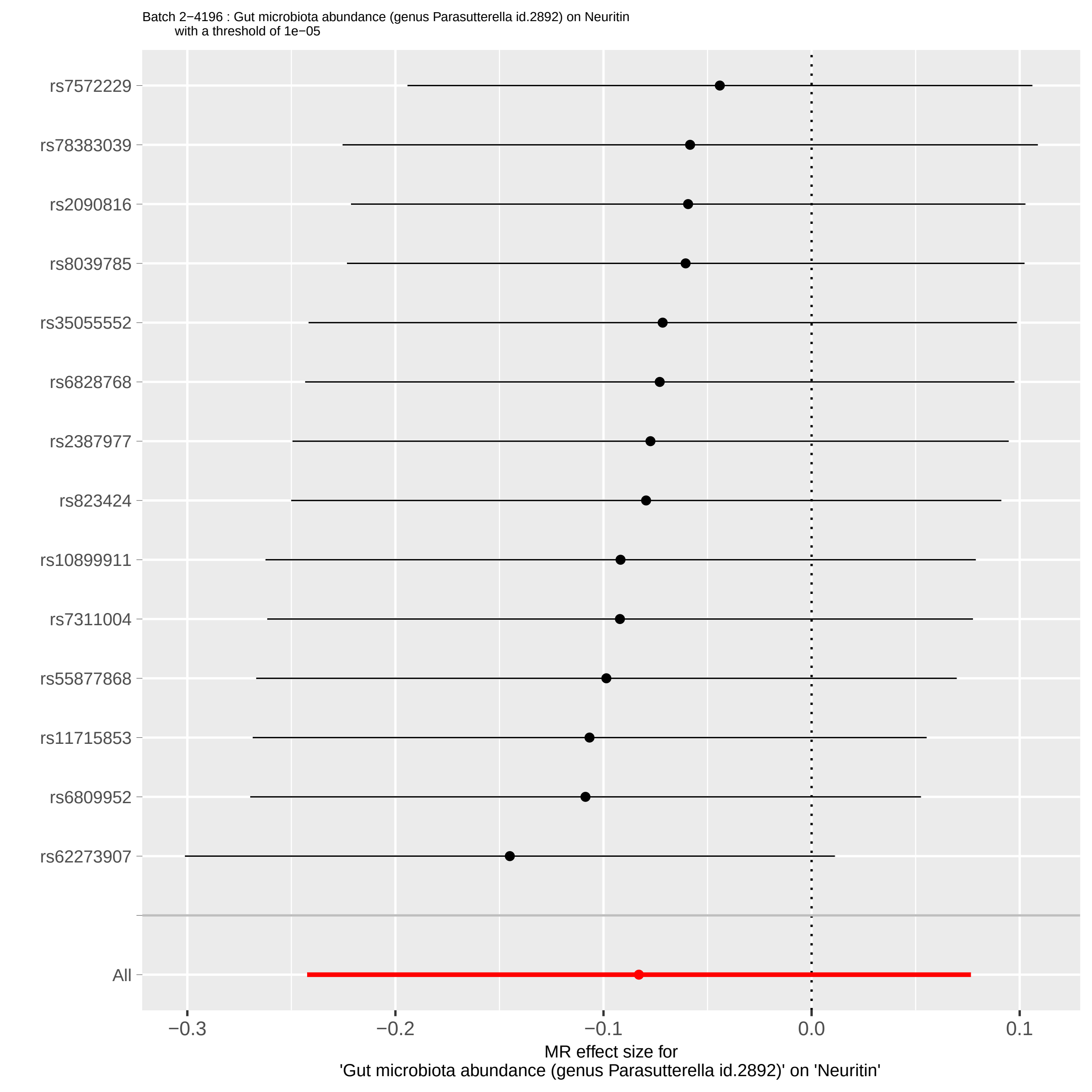
Batch 2−4196 : Gut microbiota abundance (genus Parasutterella id.2892) on Neuritin
with a threshold of 1e−05
rs7572229
rs78383039
rs2090816
rs8039785
rs35055552
rs6828768
rs2387977
rs823424
rs10899911
rs7311004
rs55877868
rs11715853
rs6809952
rs62273907
All
−0.3
−0.2
−0.1
0.0
0.1
MR effect size for
'Gut microbiota abundance (genus Parasutterella id.2892)' on 'Neuritin'

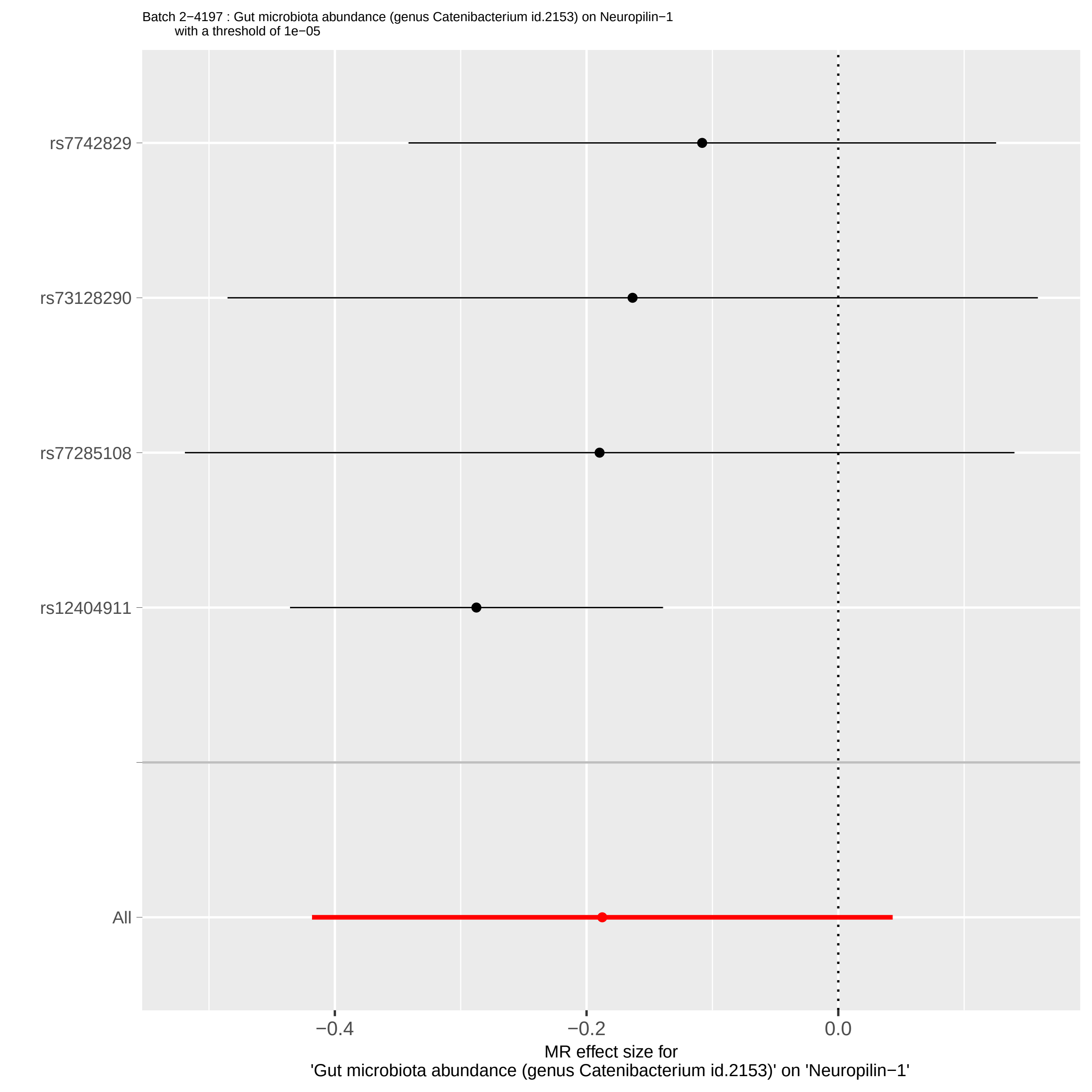

Batch 2−4197 : Gut microbiota abundance (genus Catenibacterium id.2153) on Neuropilin−1
with a threshold of 1e−05
rs7742829
rs73128290
rs77285108
rs12404911
All
−0.4
−0.2
0.0
MR effect size for
'Gut microbiota abundance (genus Catenibacterium id.2153)' on 'Neuropilin−1'

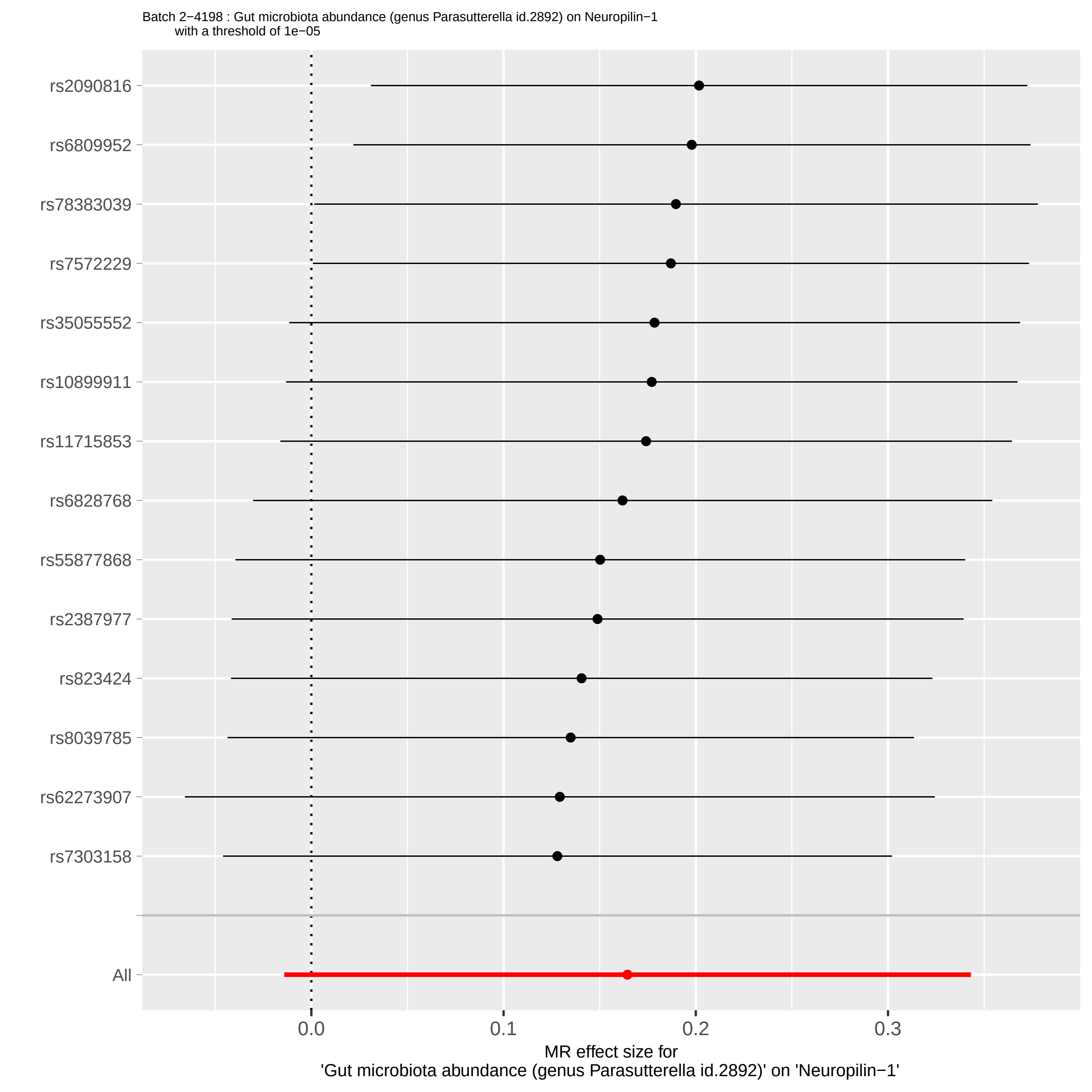
Batch 2−4198 : Gut microbiota abundance (genus Parasutterella id.2892) on Neuropilin−1
with a threshold of 1e−05
rs2090816
rs6809952
rs78383039
rs7572229
rs35055552
rs10899911
rs11715853
rs6828768
rs55877868
rs2387977
rs823424
rs8039785
rs62273907
rs7303158
All
0.0
0.1
0.2
0.3
MR effect size for
'Gut microbiota abundance (genus Parasutterella id.2892)' on 'Neuropilin−1'

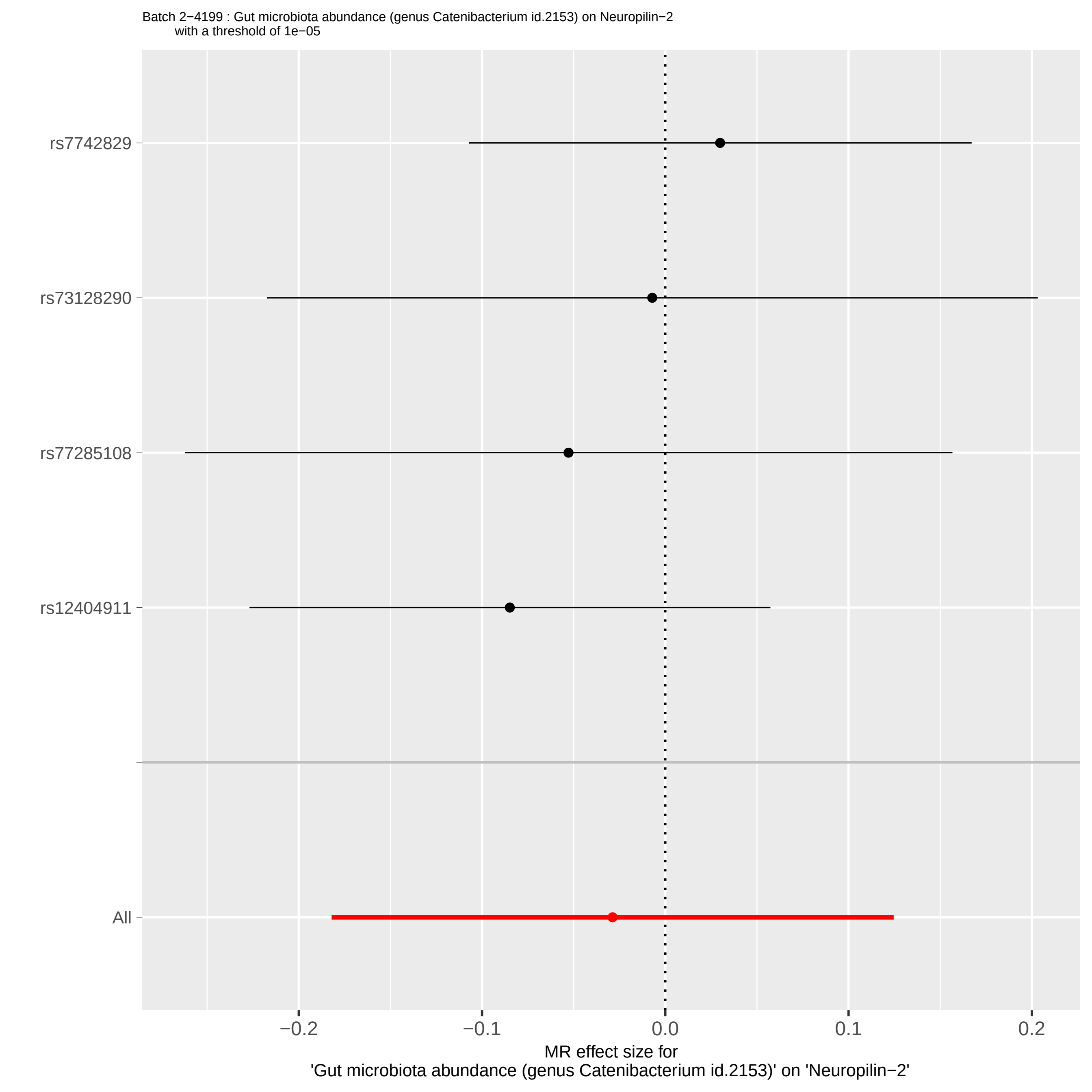
Batch 2−4199 : Gut microbiota abundance (genus Catenibacterium id.2153) on Neuropilin−2
with a threshold of 1e−05
rs7742829
rs73128290
rs77285108
rs12404911
All
−0.2
−0.1
0.0
0.1
0.2
MR effect size for
'Gut microbiota abundance (genus Catenibacterium id.2153)' on 'Neuropilin−2'

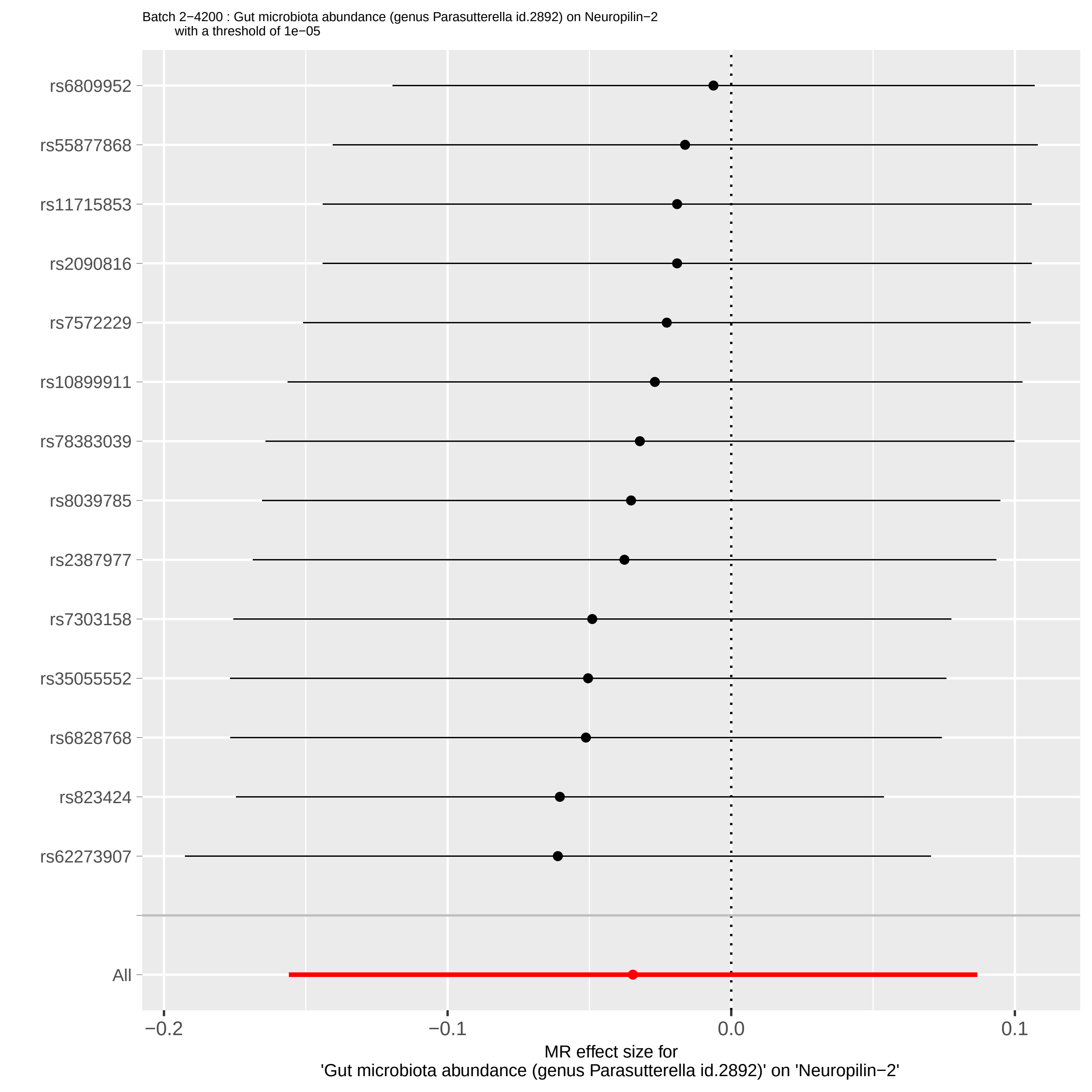
Batch 2−4200 : Gut microbiota abundance (genus Parasutterella id.2892) on Neuropilin−2
with a threshold of 1e−05
rs6809952
rs55877868
rs11715853
rs2090816
rs7572229
rs10899911
rs78383039
rs8039785
rs2387977
rs7303158
rs35055552
rs6828768
rs823424
rs62273907
All
−0.2
−0.1
0.0
0.1
MR effect size for
'Gut microbiota abundance (genus Parasutterella id.2892)' on 'Neuropilin−2'

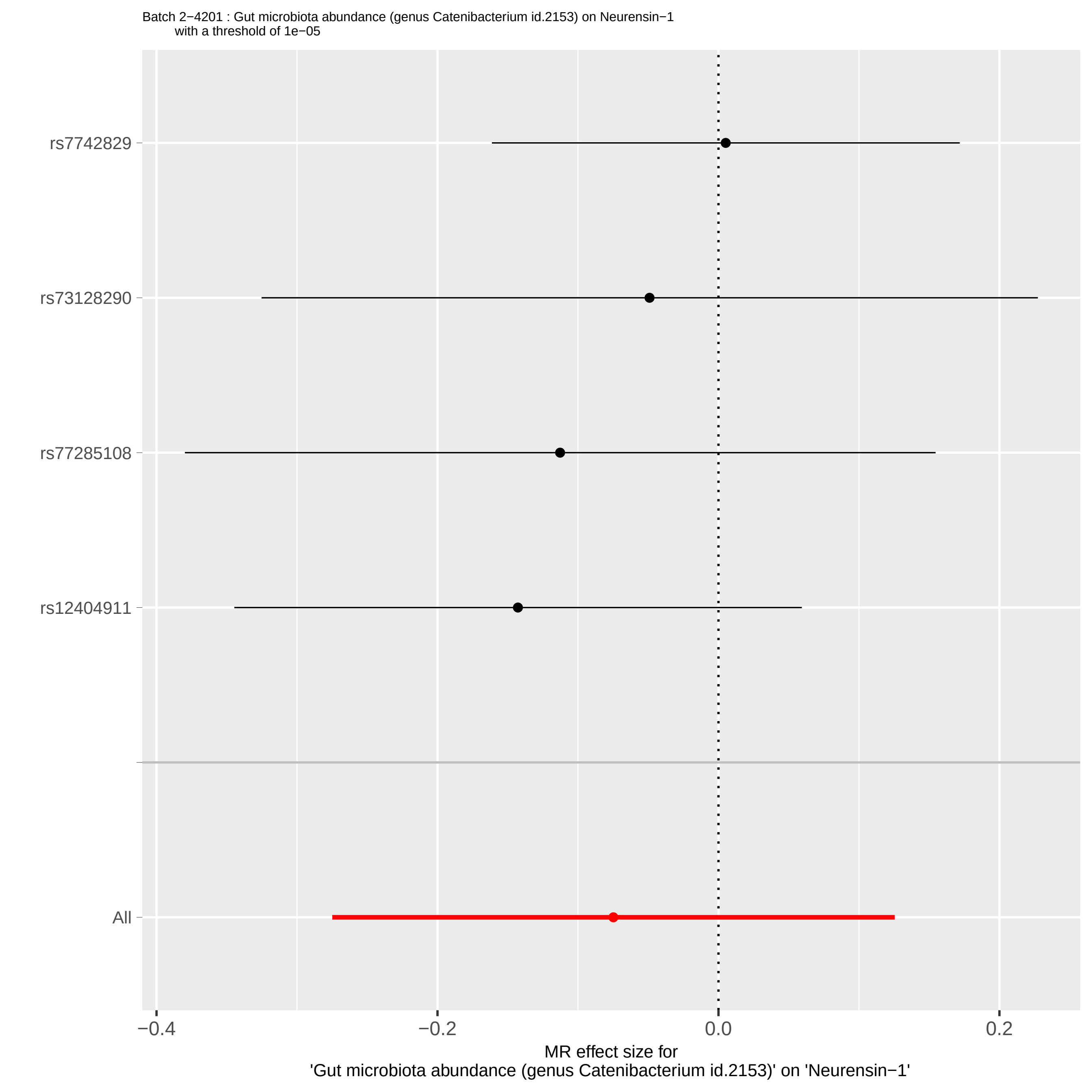

Batch 2−4201 : Gut microbiota abundance (genus Catenibacterium id.2153) on Neurensin−1
with a threshold of 1e−05
rs7742829
rs73128290
rs77285108
rs12404911
All
−0.4
−0.2
0.0
0.2
MR effect size for
'Gut microbiota abundance (genus Catenibacterium id.2153)' on 'Neurensin−1'

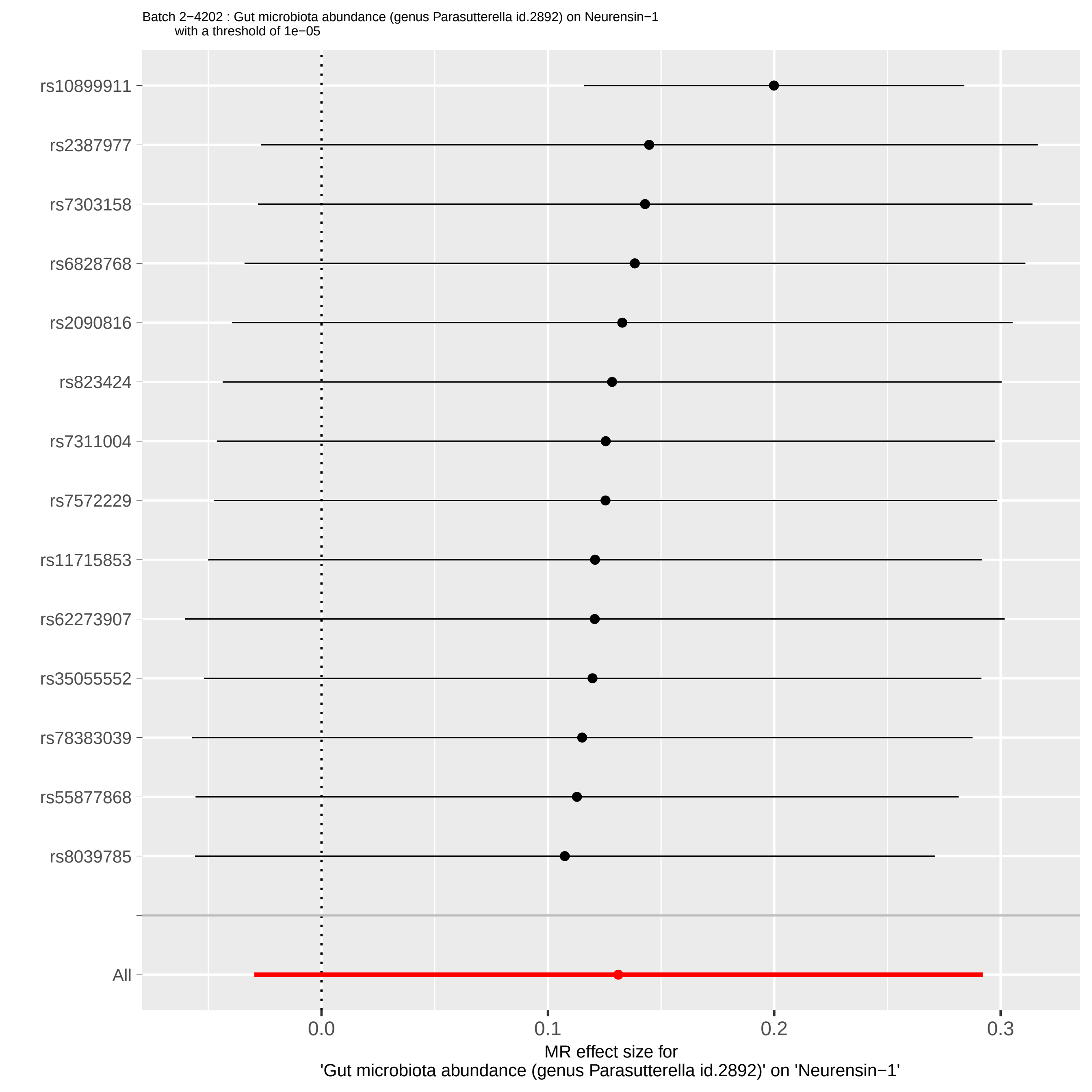
Batch 2−4202 : Gut microbiota abundance (genus Parasutterella id.2892) on Neurensin−1
with a threshold of 1e−05
rs10899911
rs2387977
rs7303158
rs6828768
rs2090816
rs823424
rs7311004
rs7572229
rs11715853
rs62273907
rs35055552
rs78383039
rs55877868
rs8039785
All
0.0
0.1
0.2
0.3
MR effect size for
'Gut microbiota abundance (genus Parasutterella id.2892)' on 'Neurensin−1'

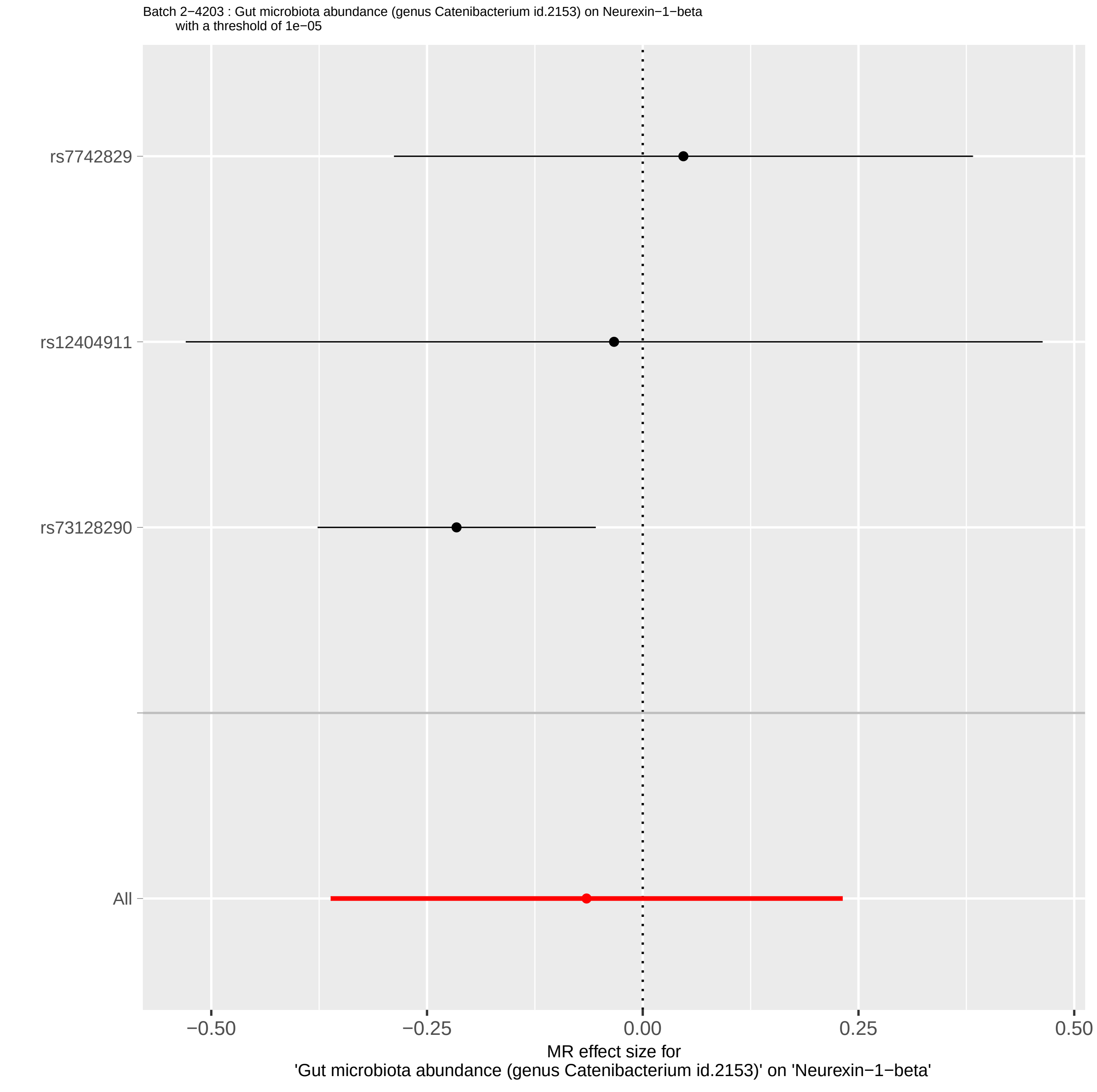
Batch 2−4203 : Gut microbiota abundance (genus Catenibacterium id.2153) on Neurexin−1−beta
with a threshold of 1e−05
rs7742829
rs12404911
rs73128290
All
−0.50
−0.25
0.00
0.25
0.50
MR effect size for
'Gut microbiota abundance (genus Catenibacterium id.2153)' on 'Neurexin−1−beta'

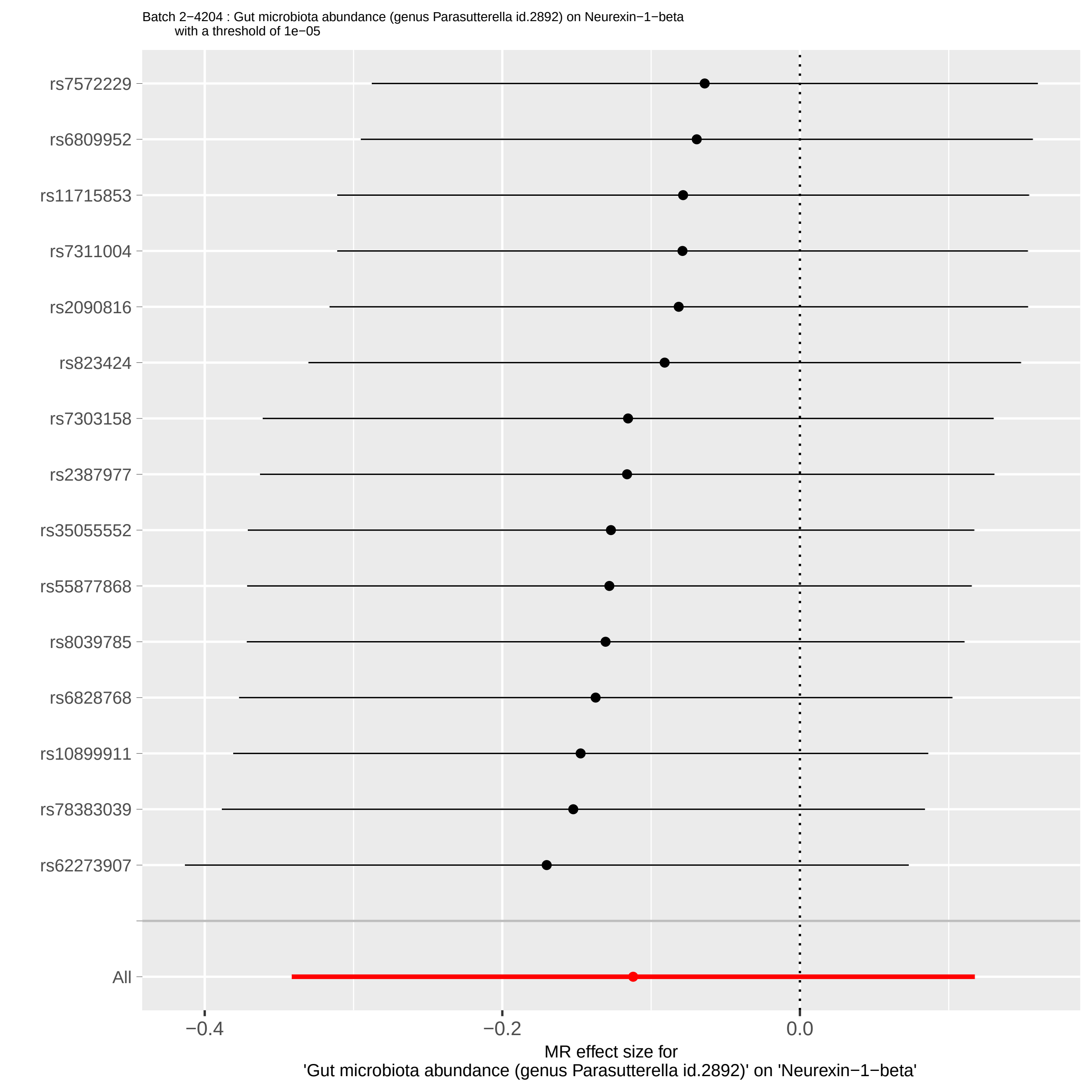
Batch 2−4204 : Gut microbiota abundance (genus Parasutterella id.2892) on Neurexin−1−beta
with a threshold of 1e−05
rs7572229
rs6809952
rs11715853
rs7311004
rs2090816
rs823424
rs7303158
rs2387977
rs35055552
rs55877868
rs8039785
rs6828768
rs10899911
rs78383039
rs62273907
All
−0.4
−0.2
0.0
MR effect size for
'Gut microbiota abundance (genus Parasutterella id.2892)' on 'Neurexin−1−beta'

Batch 2−4205 : Gut microbiota abundance (genus Catenibacterium id.2153) on Neurexin−1
with a threshold of 1e−05

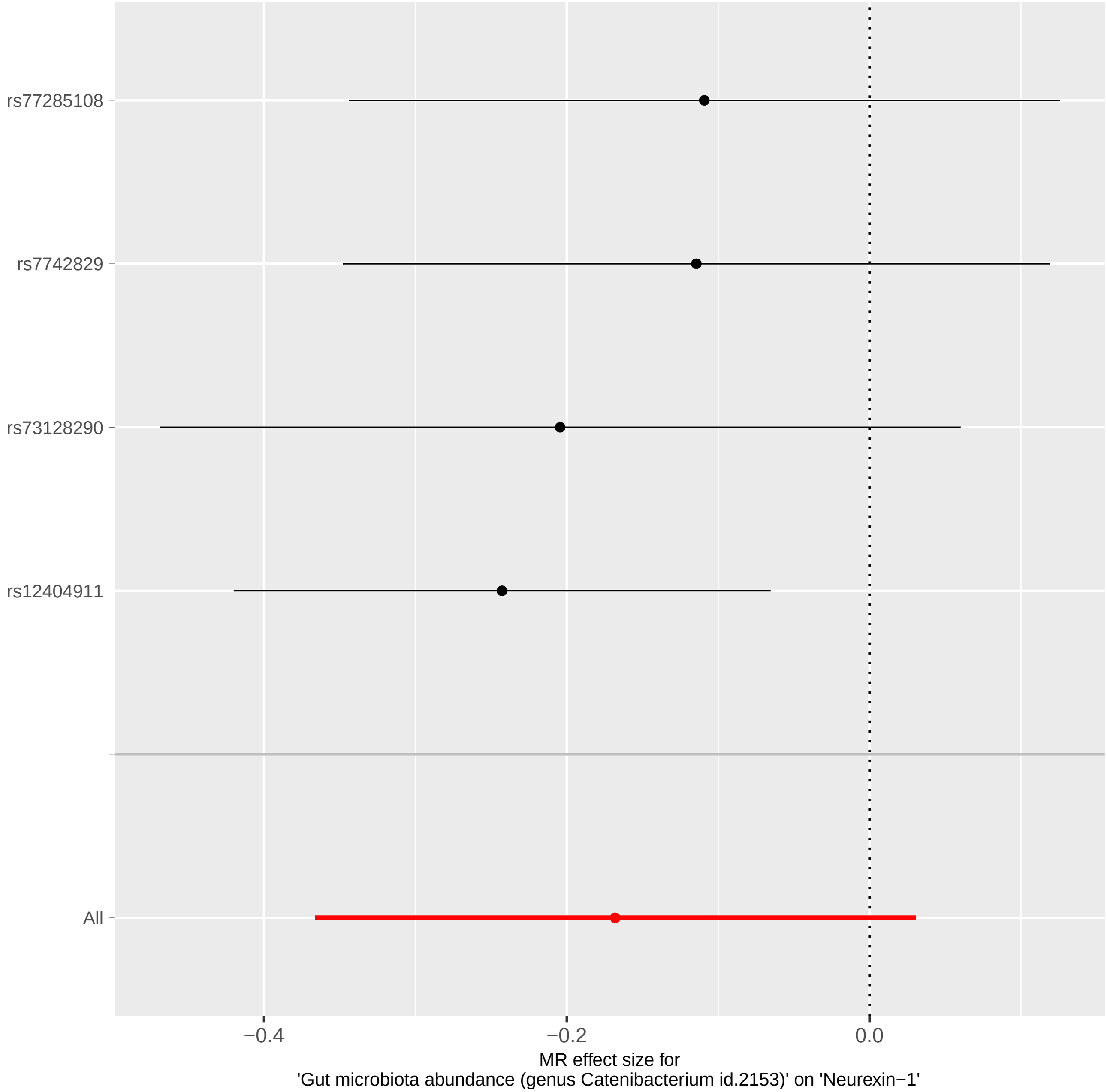

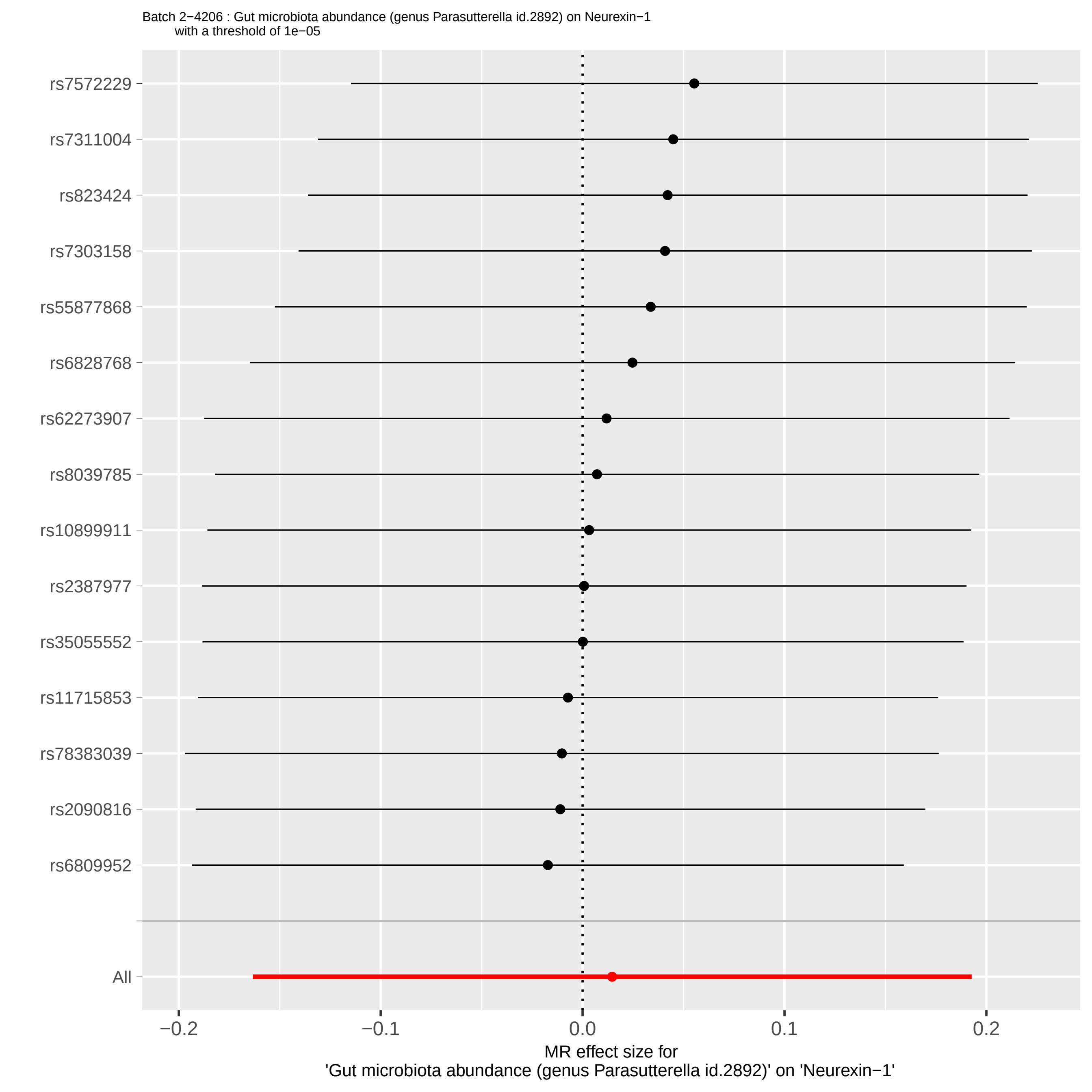

Batch 2−4206 : Gut microbiota abundance (genus Parasutterella id.2892) on Neurexin−1
with a threshold of 1e−05
rs7572229
rs7311004
rs823424
rs7303158
rs55877868
rs6828768
rs62273907
rs8039785
rs10899911
rs2387977
rs35055552
rs11715853
rs78383039
rs2090816
rs6809952
All
−0.2
−0.1
0.0
0.1
0.2
MR effect size for
'Gut microbiota abundance (genus Parasutterella id.2892)' on 'Neurexin−1'

Batch 2−4207 : Gut microbiota abundance (genus Catenibacterium id.2153) on Neurexin−2−beta
with a threshold of 1e−05

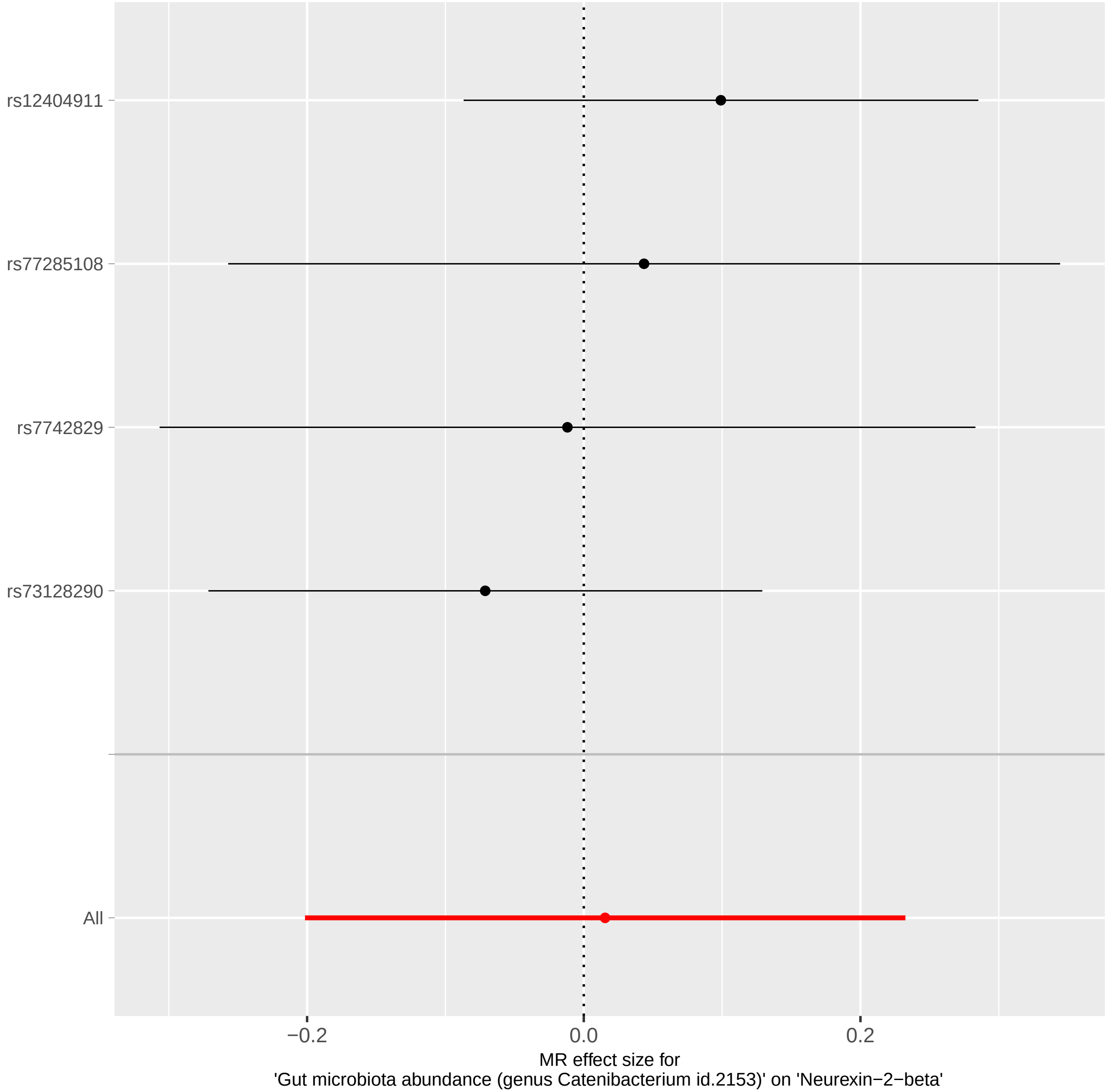

Batch 2−4208 : Gut microbiota abundance (genus Parasutterella id.2892) on Neurexin−2−beta
with a threshold of 1e−05

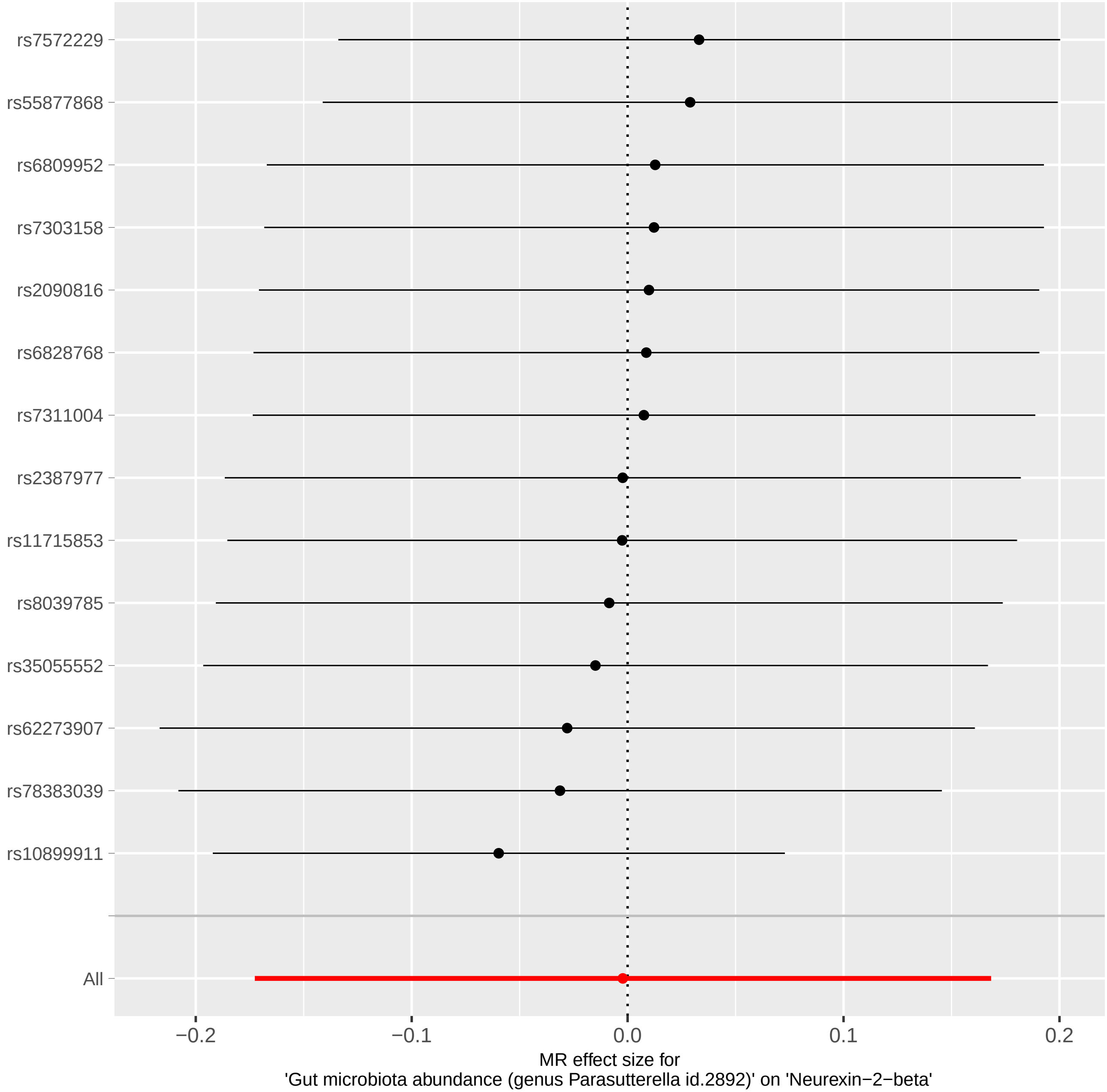

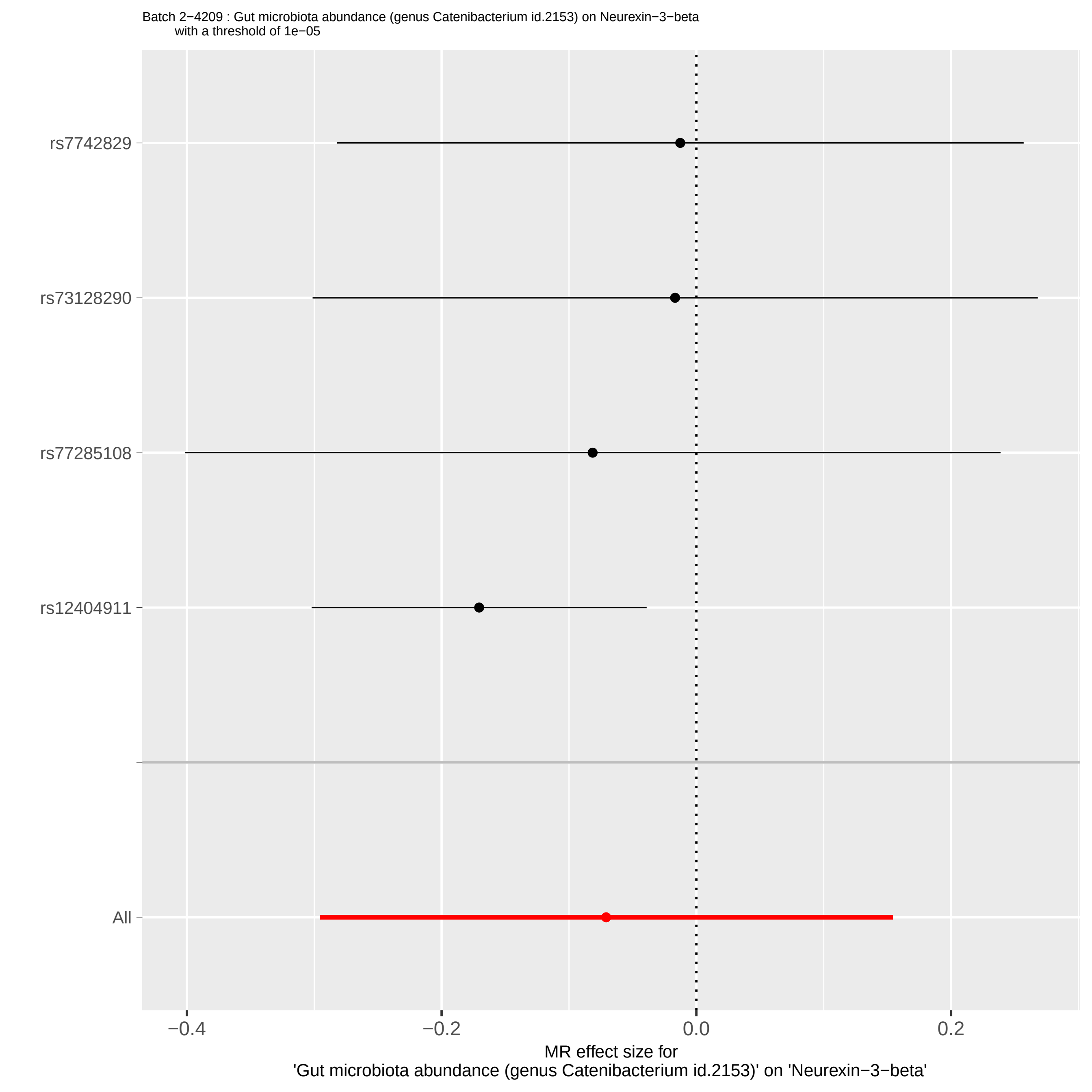

Batch 2−4209 : Gut microbiota abundance (genus Catenibacterium id.2153) on Neurexin−3−beta
with a threshold of 1e−05
rs7742829
rs73128290
rs77285108
rs12404911
All
−0.4
−0.2
0.0
0.2
MR effect size for
'Gut microbiota abundance (genus Catenibacterium id.2153)' on 'Neurexin−3−beta'

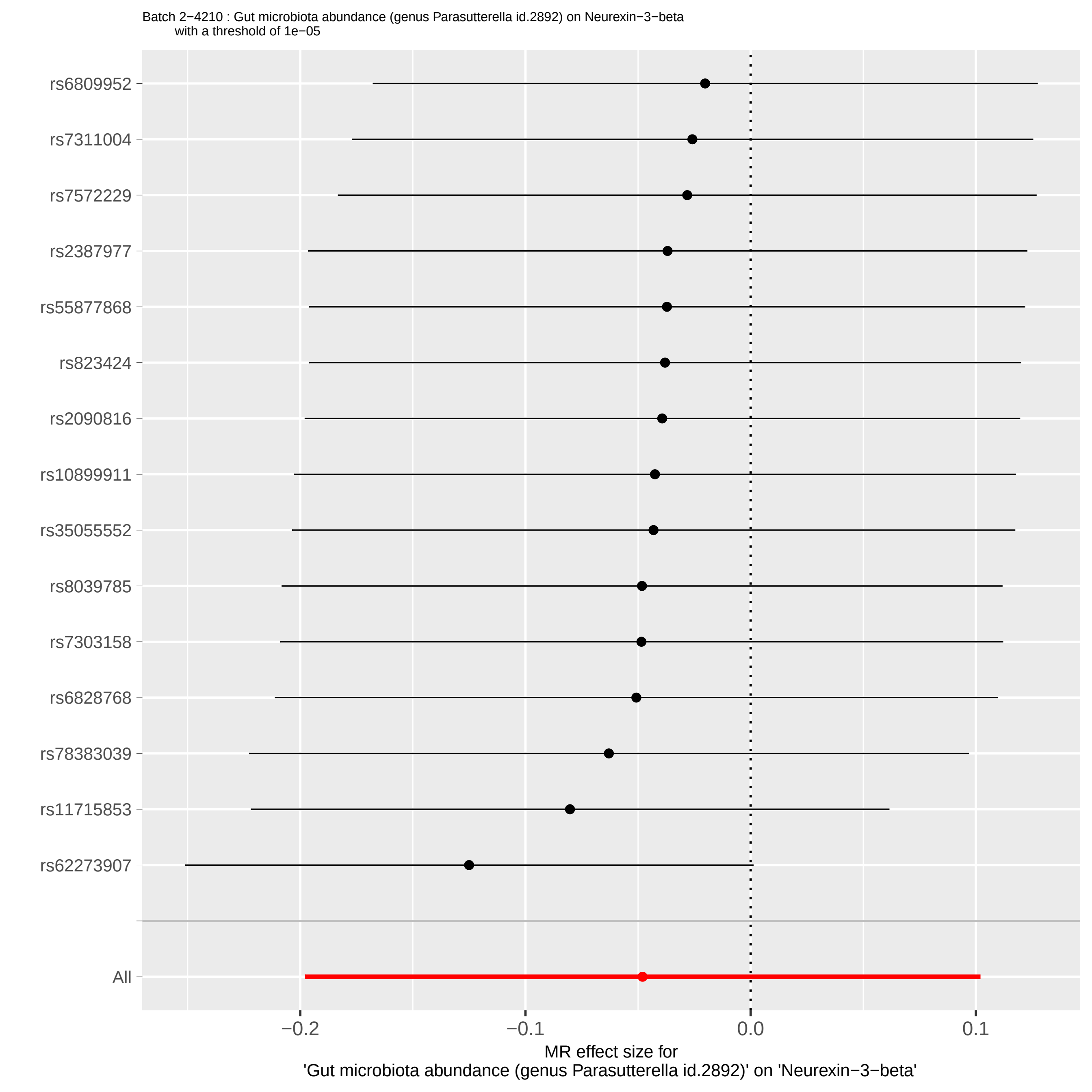
Batch 2−4210 : Gut microbiota abundance (genus Parasutterella id.2892) on Neurexin−3−beta
with a threshold of 1e−05
rs6809952
rs7311004
rs7572229
rs2387977
rs55877868
rs823424
rs2090816
rs10899911
rs35055552
rs8039785
rs7303158
rs6828768
rs78383039
rs11715853
rs62273907
All
−0.2
−0.1
0.0
0.1
MR effect size for
'Gut microbiota abundance (genus Parasutterella id.2892)' on 'Neurexin−3−beta'

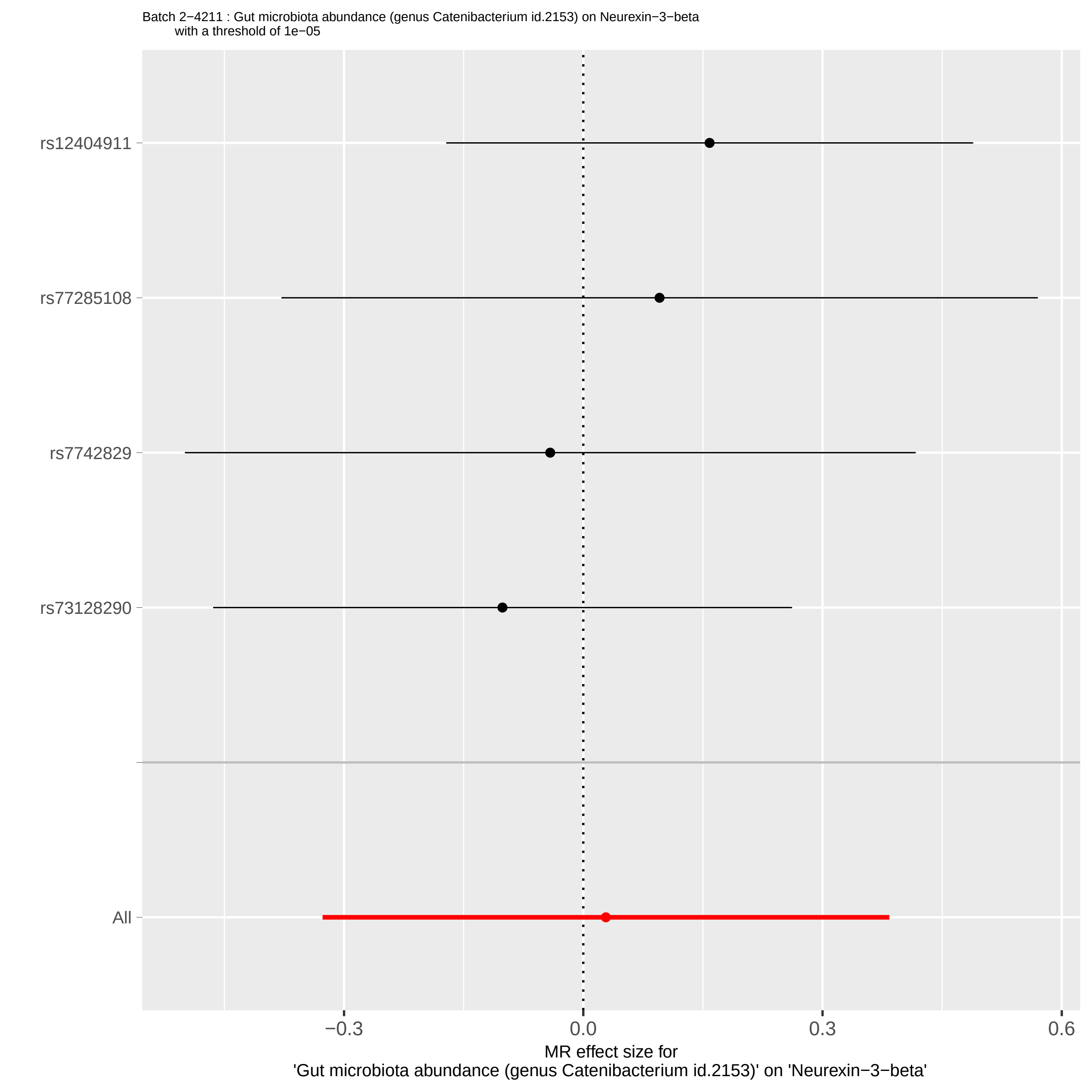
Batch 2−4211 : Gut microbiota abundance (genus Catenibacterium id.2153) on Neurexin−3−beta
with a threshold of 1e−05
rs12404911
rs77285108
rs7742829
rs73128290
All
−0.3
0.0
0.3
0.6
MR effect size for
'Gut microbiota abundance (genus Catenibacterium id.2153)' on 'Neurexin−3−beta'

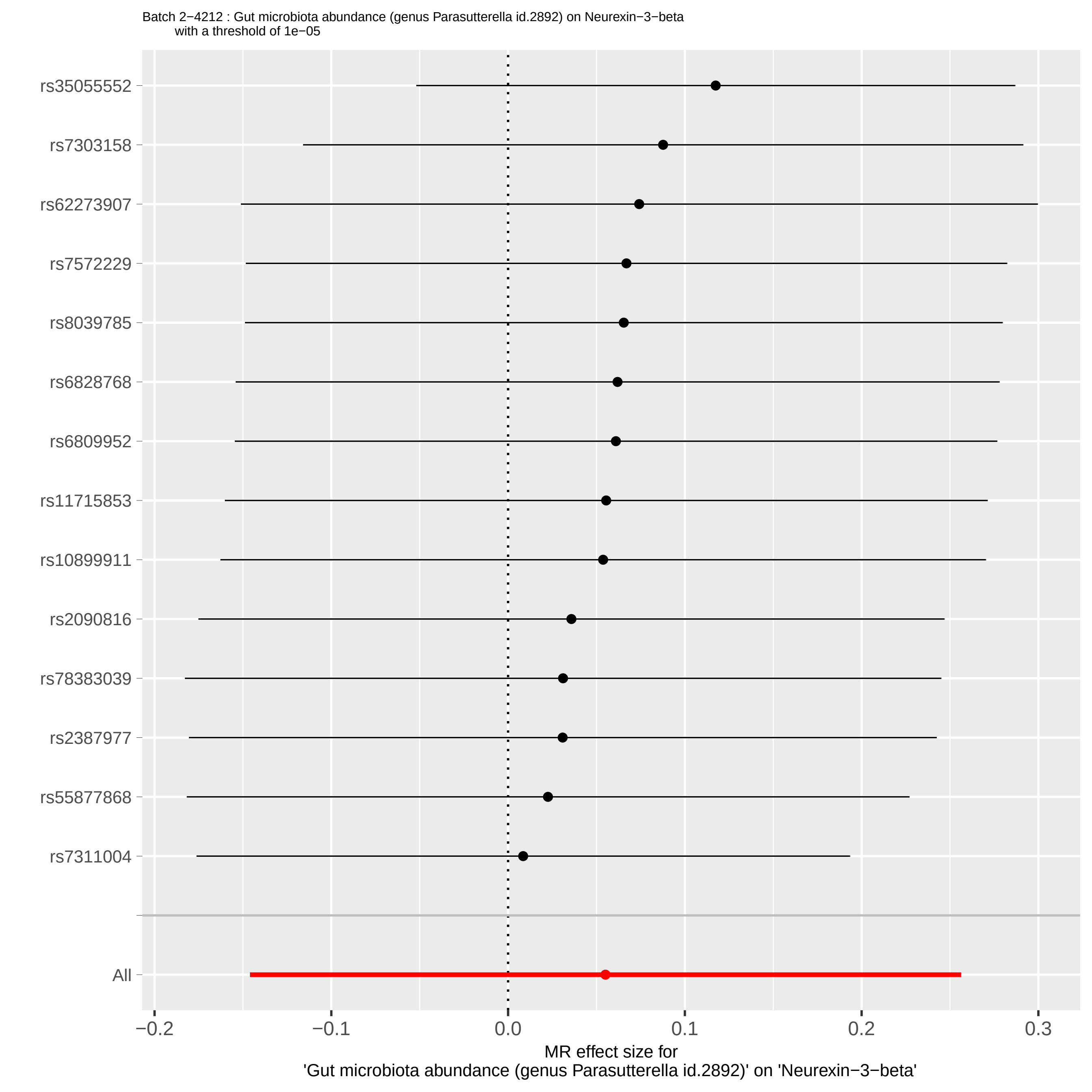

Batch 2−4212 : Gut microbiota abundance (genus Parasutterella id.2892) on Neurexin−3−beta
with a threshold of 1e−05
rs35055552
rs7303158
rs62273907
rs7572229
rs8039785
rs6828768
rs6809952
rs11715853
rs10899911
rs2090816
rs78383039
rs2387977
rs55877868
rs7311004
All
−0.2
−0.1
0.0
0.1
0.2
0.3
MR effect size for
'Gut microbiota abundance (genus Parasutterella id.2892)' on 'Neurexin−3−beta'

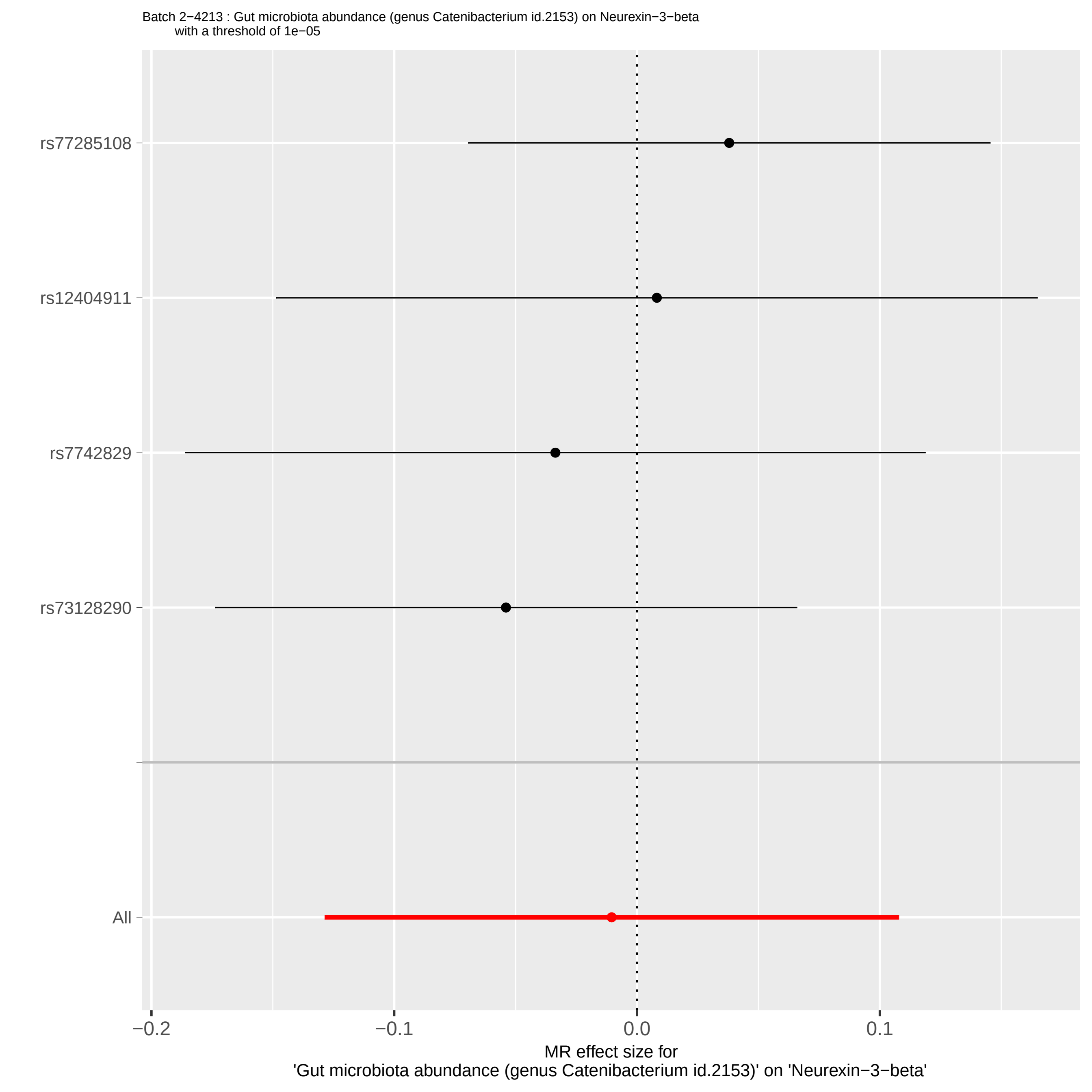

Batch 2−4213 : Gut microbiota abundance (genus Catenibacterium id.2153) on Neurexin−3−beta
with a threshold of 1e−05
rs77285108
rs12404911
rs7742829
rs73128290
All
−0.2
−0.1
0.0
0.1
MR effect size for
'Gut microbiota abundance (genus Catenibacterium id.2153)' on 'Neurexin−3−beta'

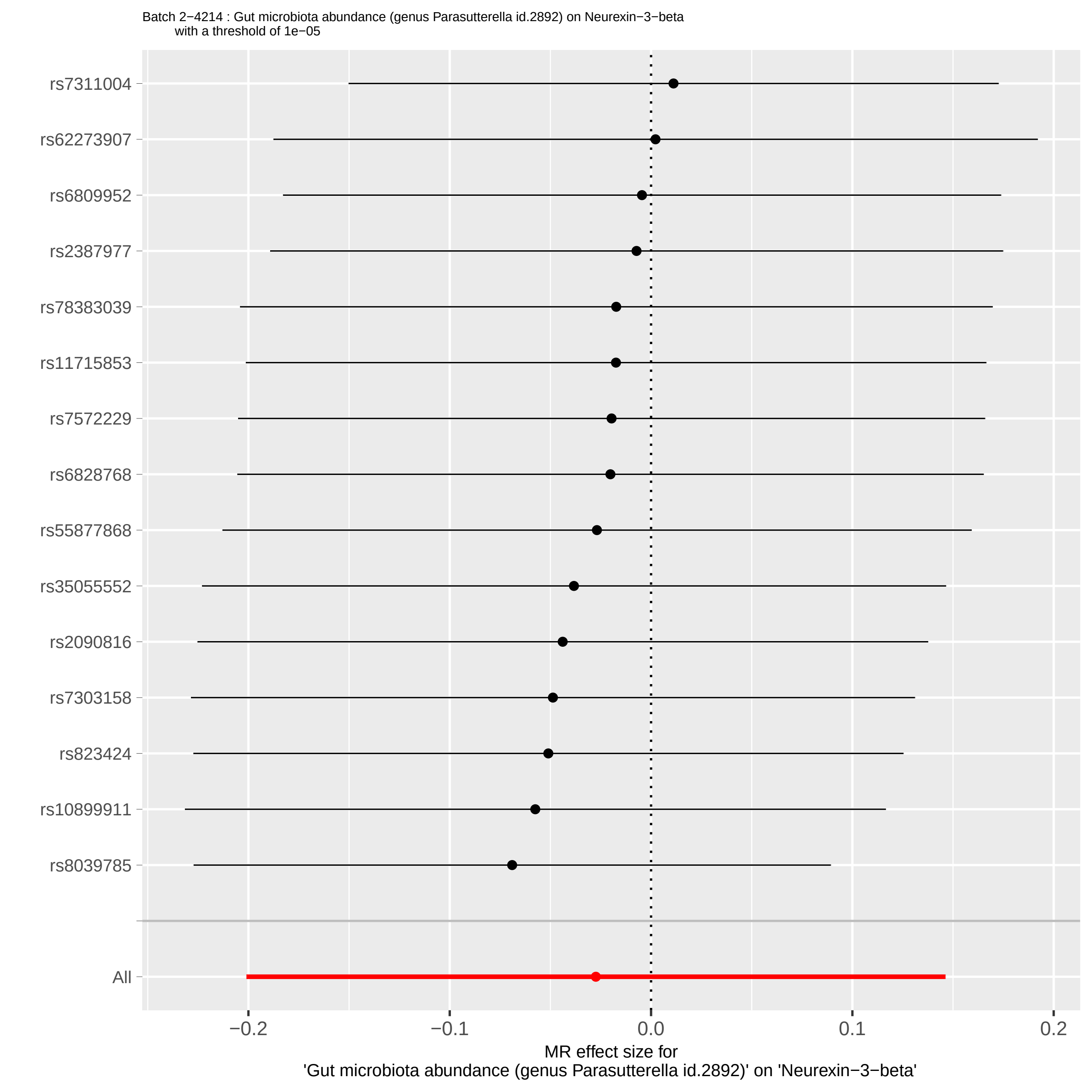

Batch 2−4214 : Gut microbiota abundance (genus Parasutterella id.2892) on Neurexin−3−beta
with a threshold of 1e−05
rs7311004
rs62273907
rs6809952
rs2387977
rs78383039
rs11715853
rs7572229
rs6828768
rs55877868
rs35055552
rs2090816
rs7303158
rs823424
rs10899911
rs8039785
All
−0.2
−0.1
0.0
0.1
0.2
MR effect size for
'Gut microbiota abundance (genus Parasutterella id.2892)' on 'Neurexin−3−beta'

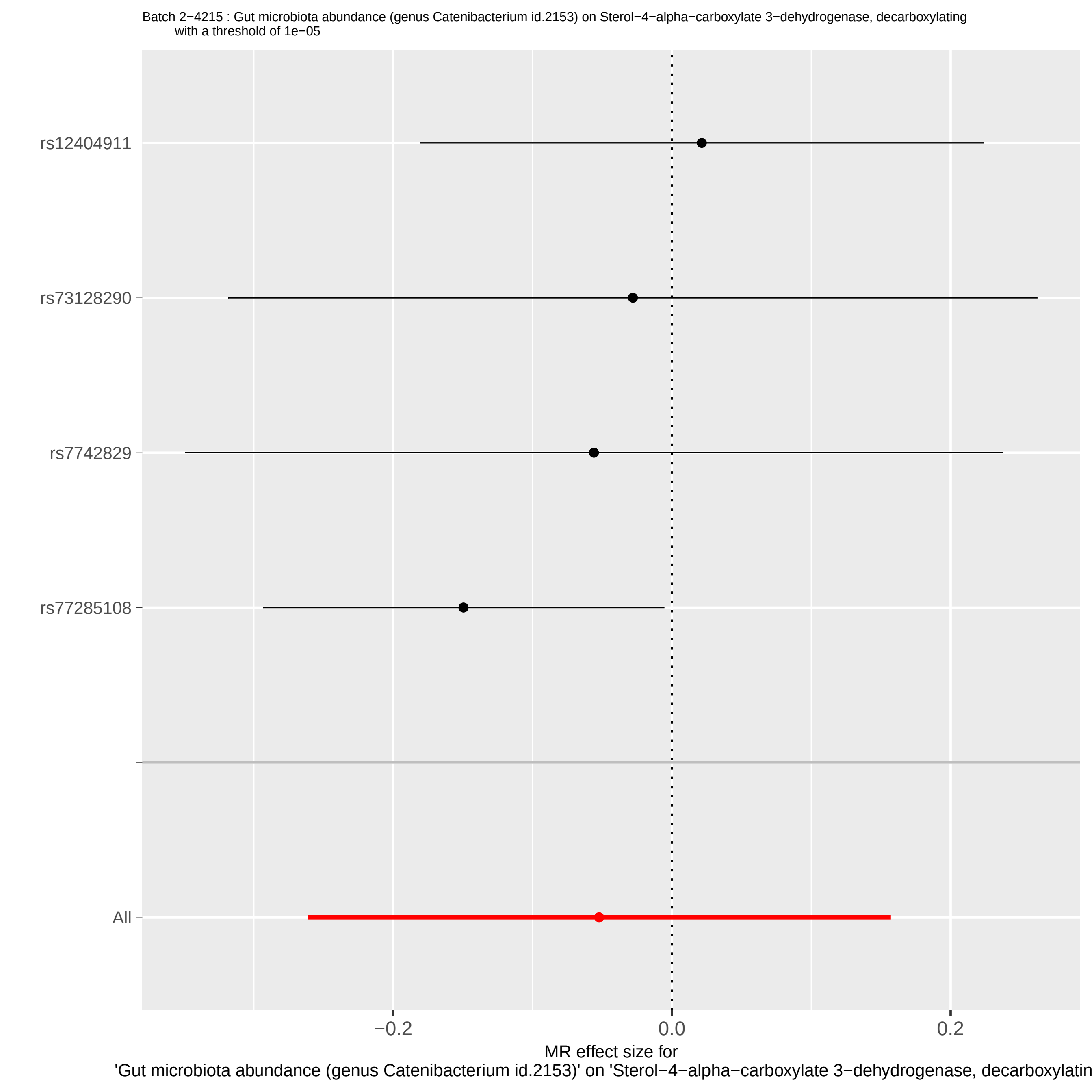
Batch 2−4215 : Gut microbiota abundance (genus Catenibacterium id.2153) on Sterol−4−alpha−carboxylate 3−dehydrogenase, decarboxylating
with a threshold of 1e−05
rs12404911
rs73128290
rs7742829
rs77285108
All
−0.2
0.0
0.2
MR effect size for
'Gut microbiota abundance (genus Catenibacterium id.2153)' on 'Sterol−4−alpha−carboxylate 3−dehydrogenase, decarboxylatin

Batch 2−4216 : Gut microbiota abundance (genus Parasutterella id.2892) on Sterol−4−alpha−carboxylate 3−dehydrogenase, decarboxylating with a threshold of 1e−05

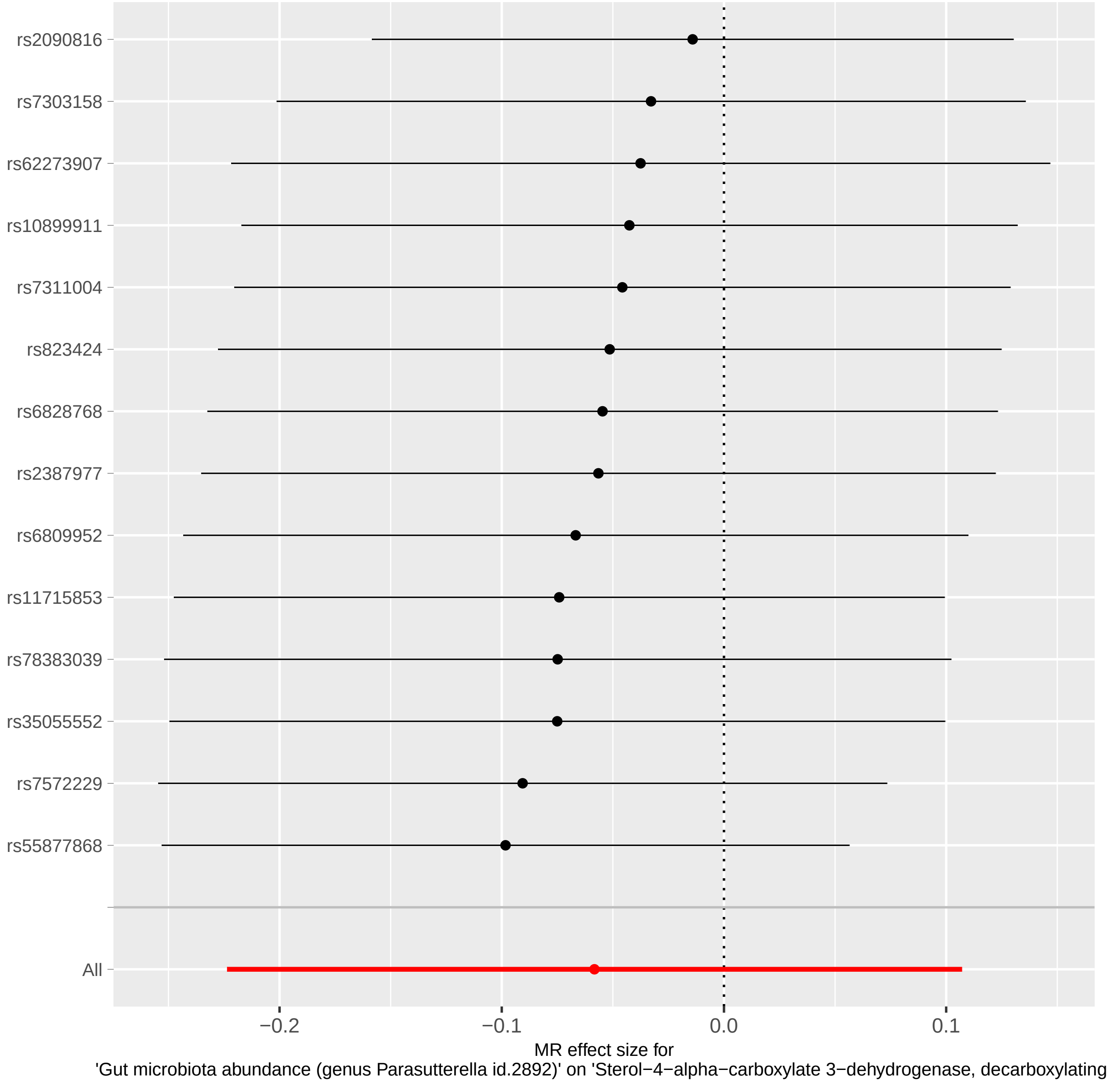

Batch 2−4217 : Gut microbiota abundance (genus Catenibacterium id.2153) on Vesicle−fusing ATPase
with a threshold of 1e−05

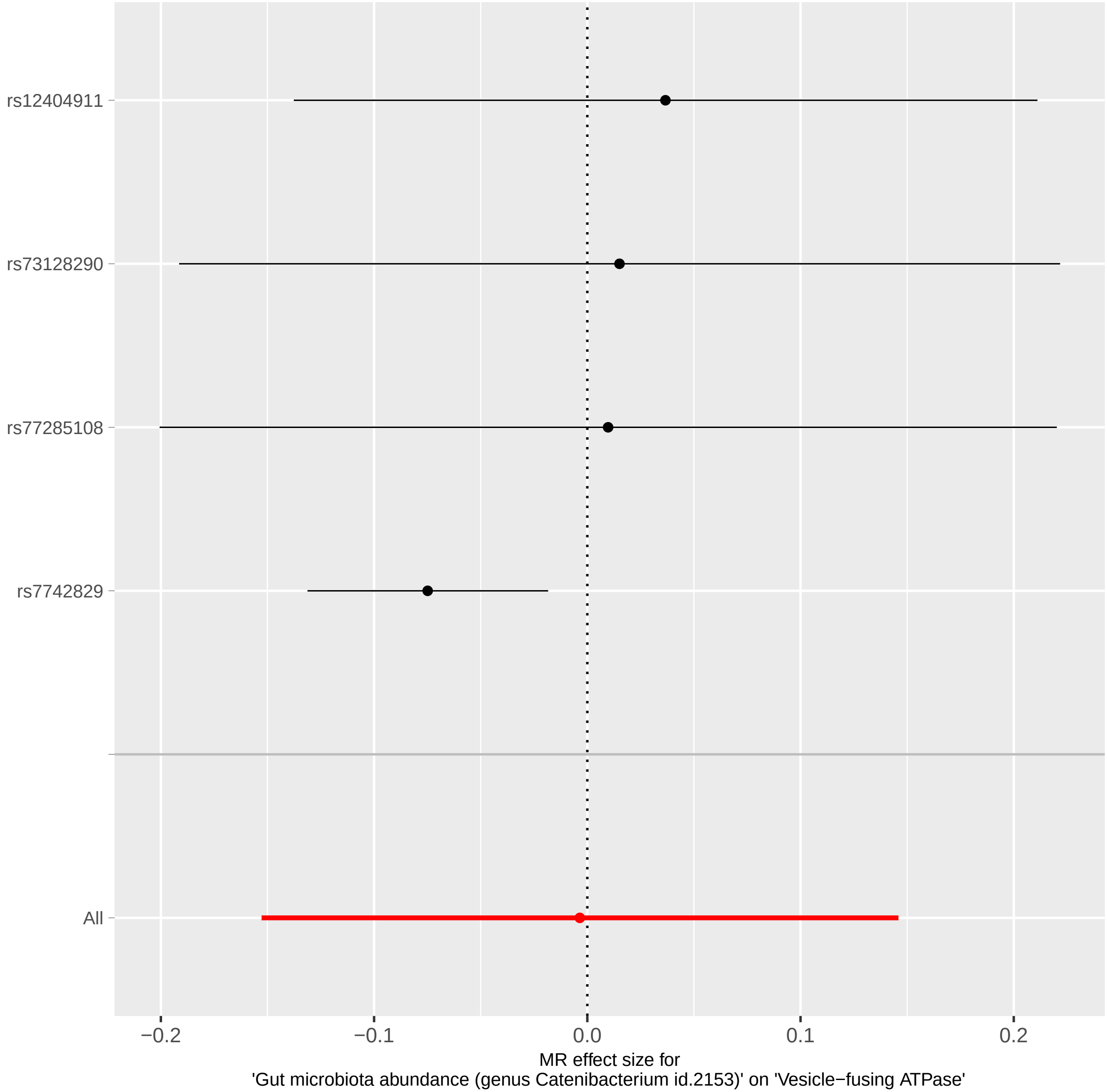

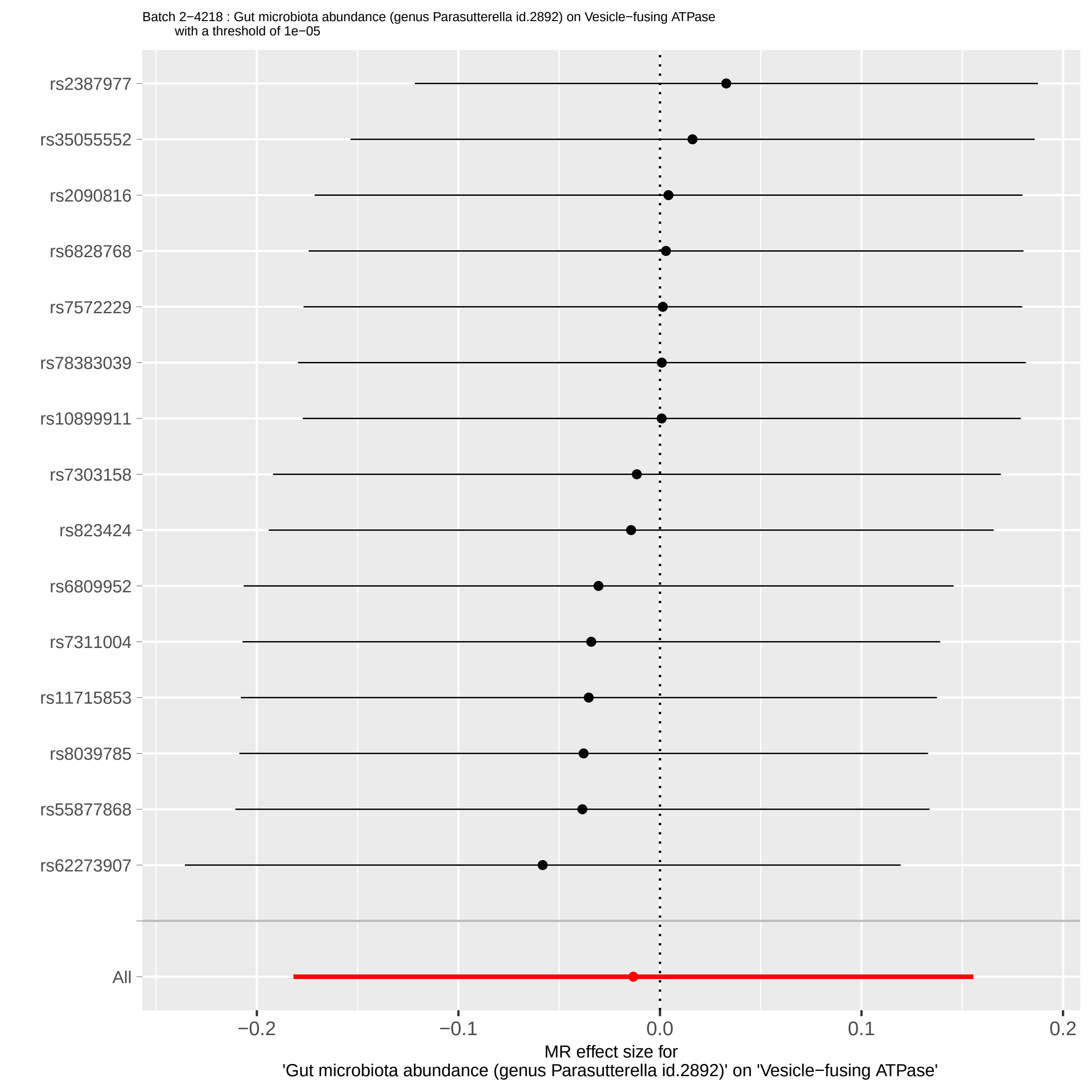

Batch 2−4218 : Gut microbiota abundance (genus Parasutterella id.2892) on Vesicle−fusing ATPase
with a threshold of 1e−05
rs2387977
rs35055552
rs2090816
rs6828768
rs7572229
rs78383039
rs10899911
rs7303158
rs823424
rs6809952
rs7311004
rs11715853
rs8039785
rs55877868
rs62273907
All
−0.2
−0.1
0.0
0.1
0.2
MR effect size for
'Gut microbiota abundance (genus Parasutterella id.2892)' on 'Vesicle−fusing ATPase'

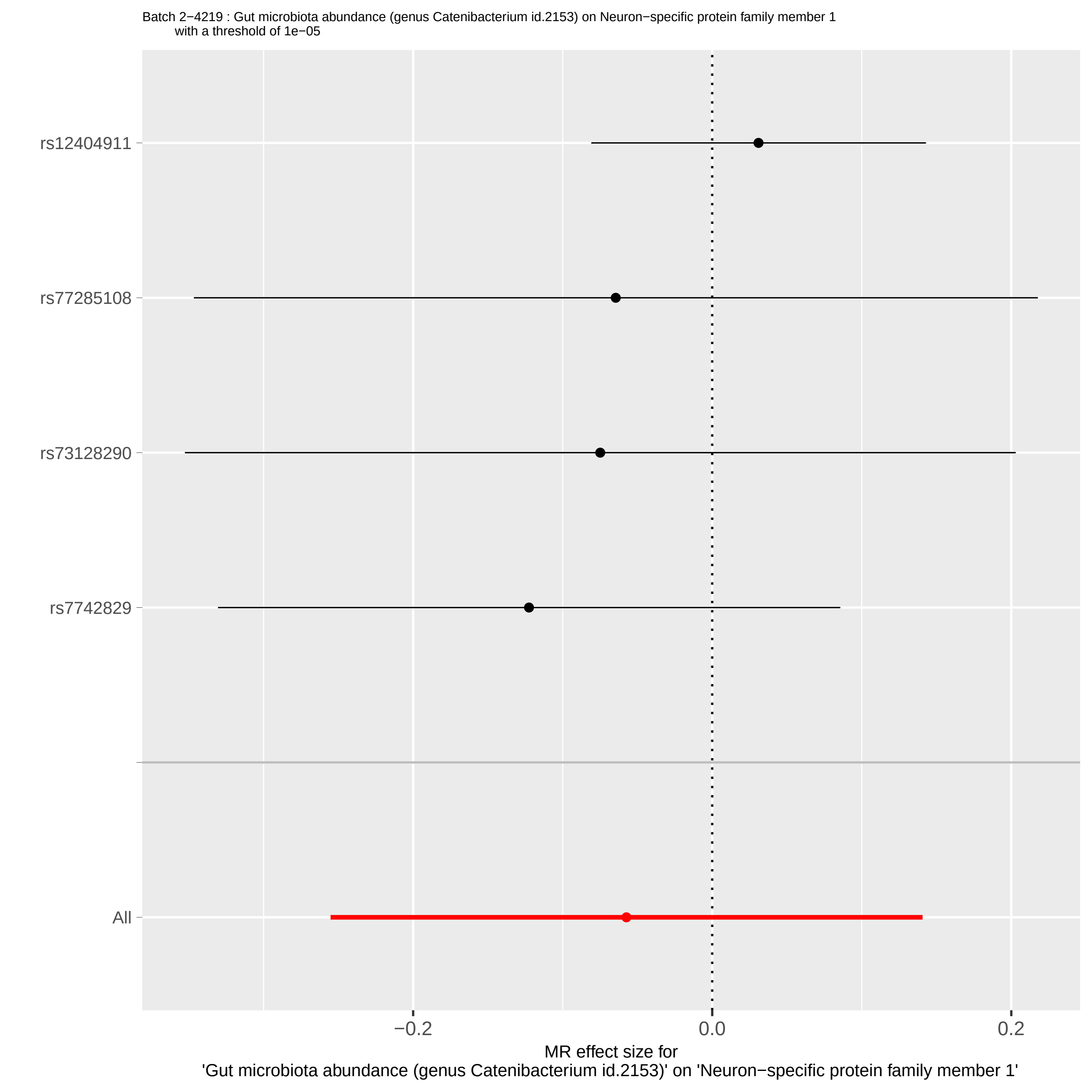

Batch 2−4219 : Gut microbiota abundance (genus Catenibacterium id.2153) on Neuron−specific protein family member 1
with a threshold of 1e−05
rs12404911
rs77285108
rs73128290
rs7742829
All
−0.2
0.0
0.2
MR effect size for
'Gut microbiota abundance (genus Catenibacterium id.2153)' on 'Neuron−specific protein family member 1'

Batch 2−4220 : Gut microbiota abundance (genus Parasutterella id.2892) on Neuron−specific protein family member 1
with a threshold of 1e−05

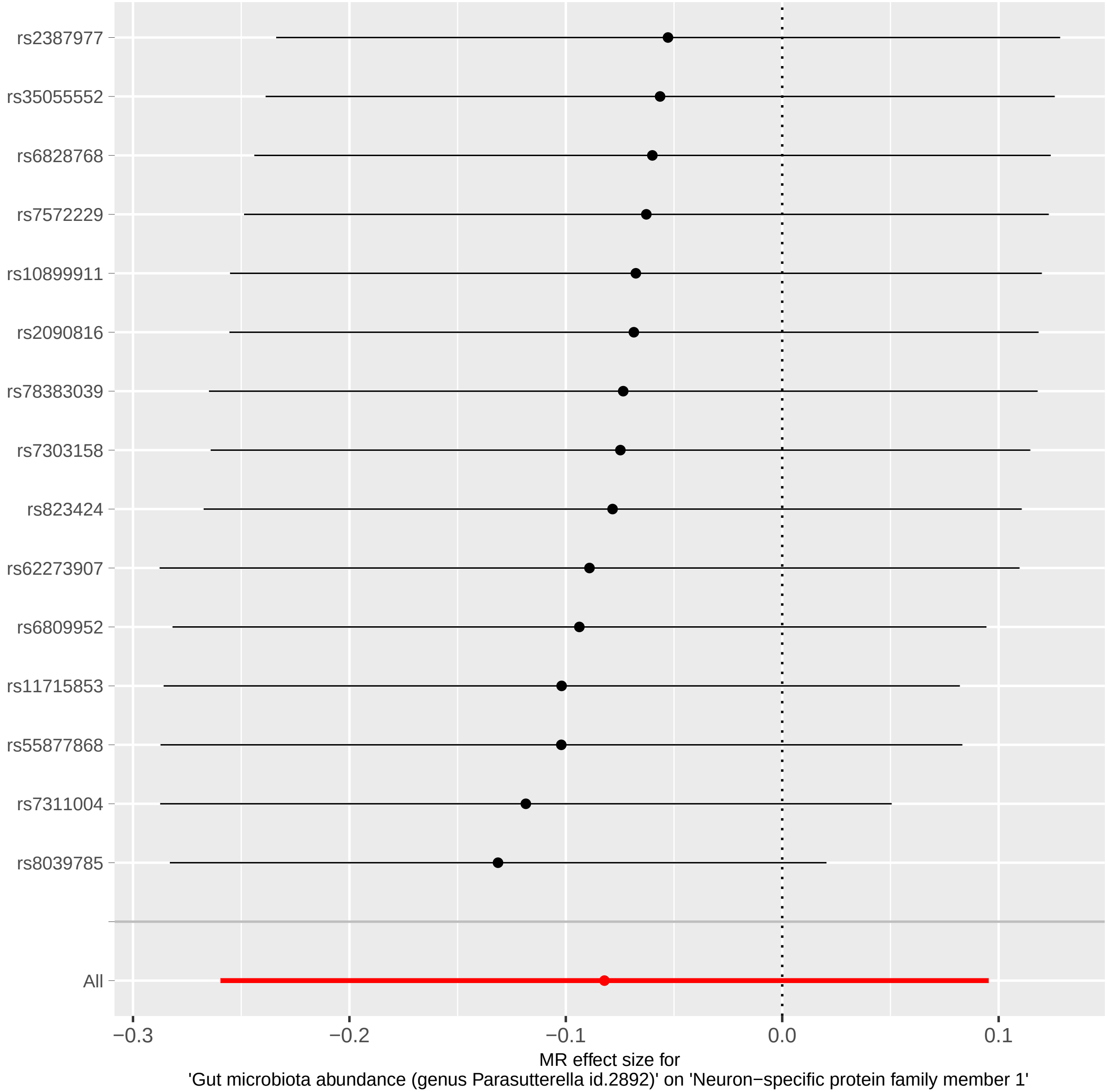

Batch 2−4221 : Gut microbiota abundance (genus Catenibacterium id.2153) on Neuron−specific protein family member 2
with a threshold of 1e−05

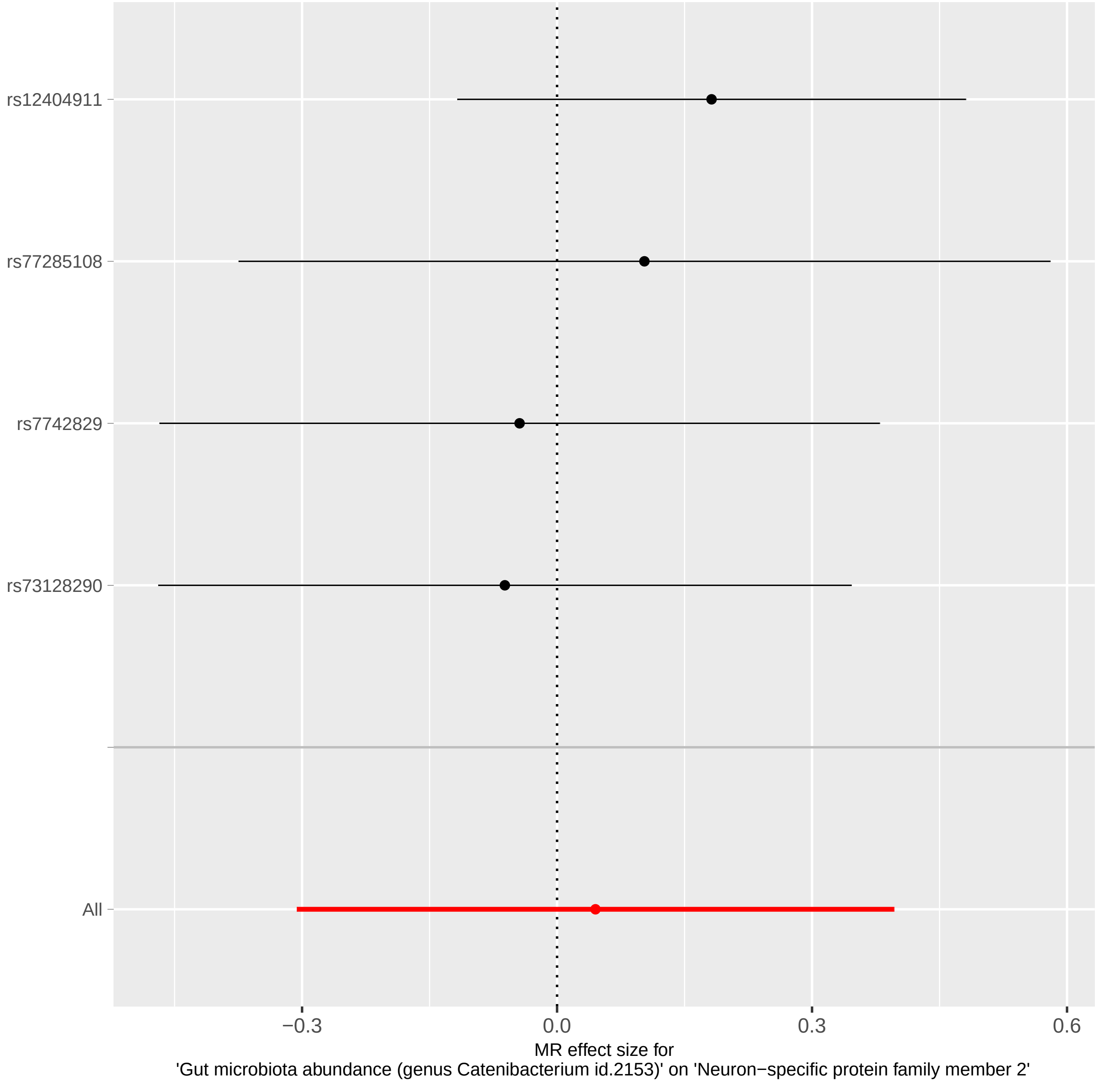

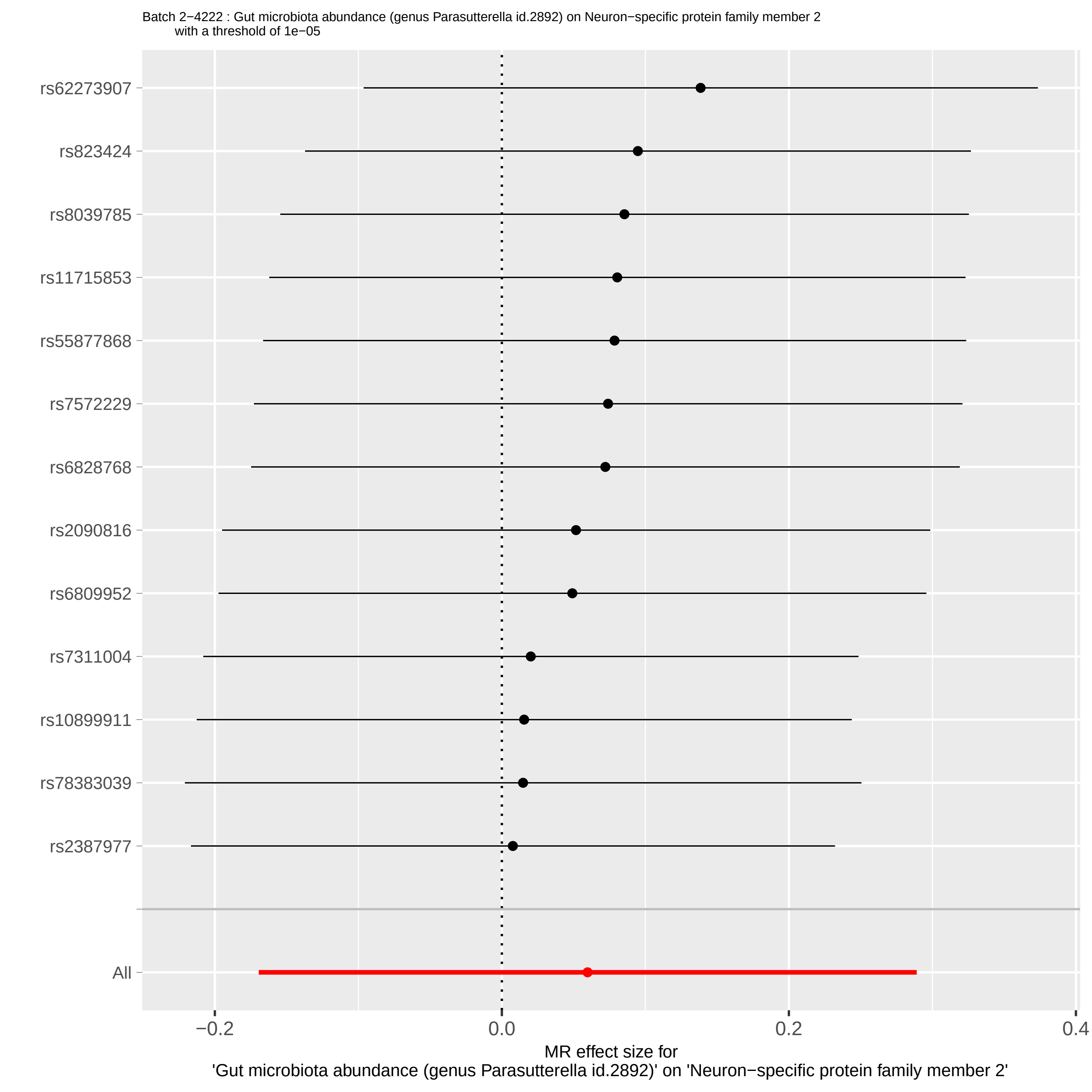

Batch 2−4222 : Gut microbiota abundance (genus Parasutterella id.2892) on Neuron−specific protein family member 2
with a threshold of 1e−05
rs62273907
rs823424
rs8039785
rs11715853
rs55877868
rs7572229
rs6828768
rs2090816
rs6809952
rs7311004
rs10899911
rs78383039
rs2387977
All
−0.2
0.0
0.2
0.4
MR effect size for
'Gut microbiota abundance (genus Parasutterella id.2892)' on 'Neuron−specific protein family member 2'

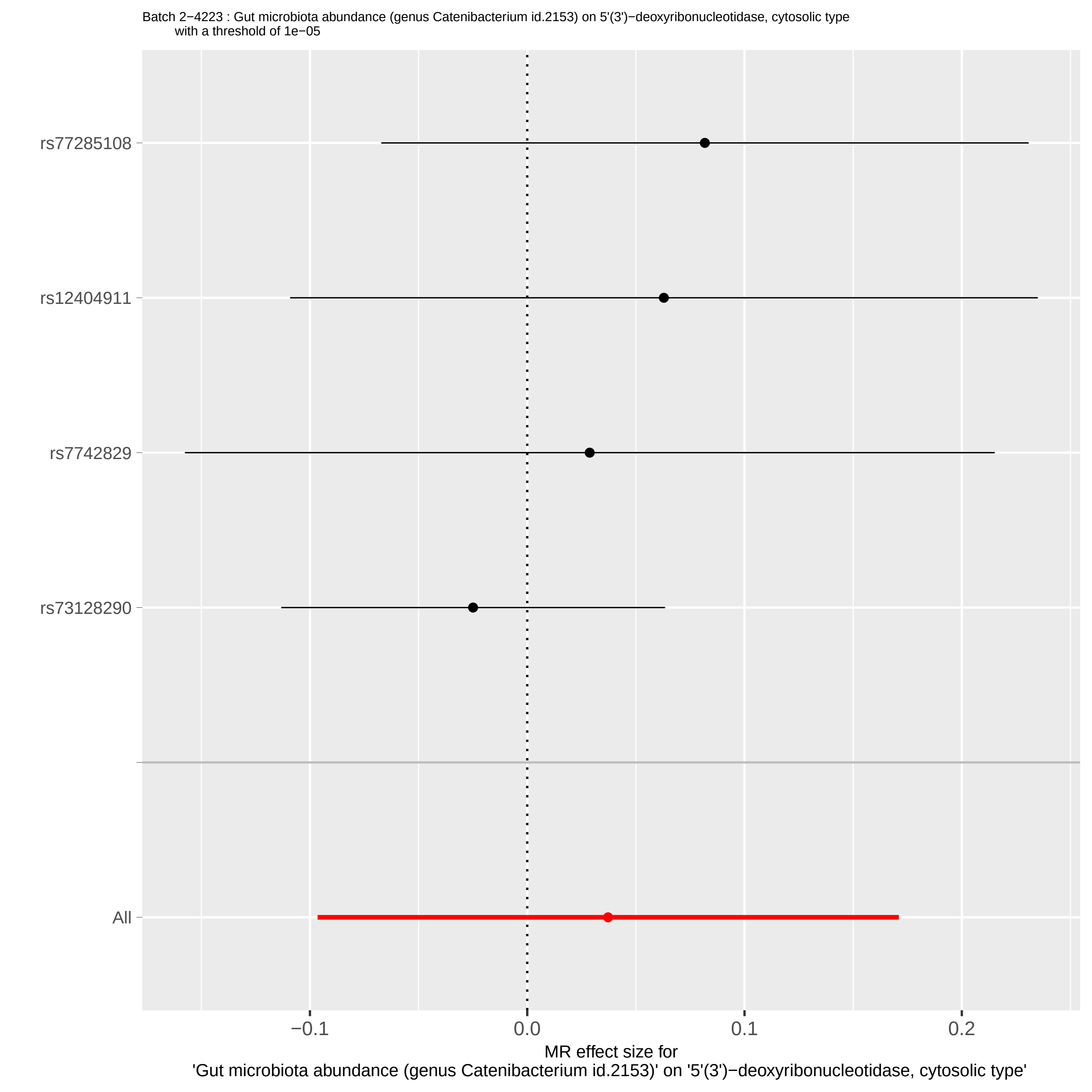
Batch 2−4223 : Gut microbiota abundance (genus Catenibacterium id.2153) on 5'(3')−deoxyribonucleotidase, cytosolic type
with a threshold of 1e−05
rs77285108
rs12404911
rs7742829
rs73128290
All
−0.1
0.0
0.1
0.2
MR effect size for
'Gut microbiota abundance (genus Catenibacterium id.2153)' on '5'(3')−deoxyribonucleotidase, cytosolic type'

Batch 2−4224 : Gut microbiota abundance (genus Parasutterella id.2892) on 5'(3')−deoxyribonucleotidase, cytosolic type with a threshold of 1e−05

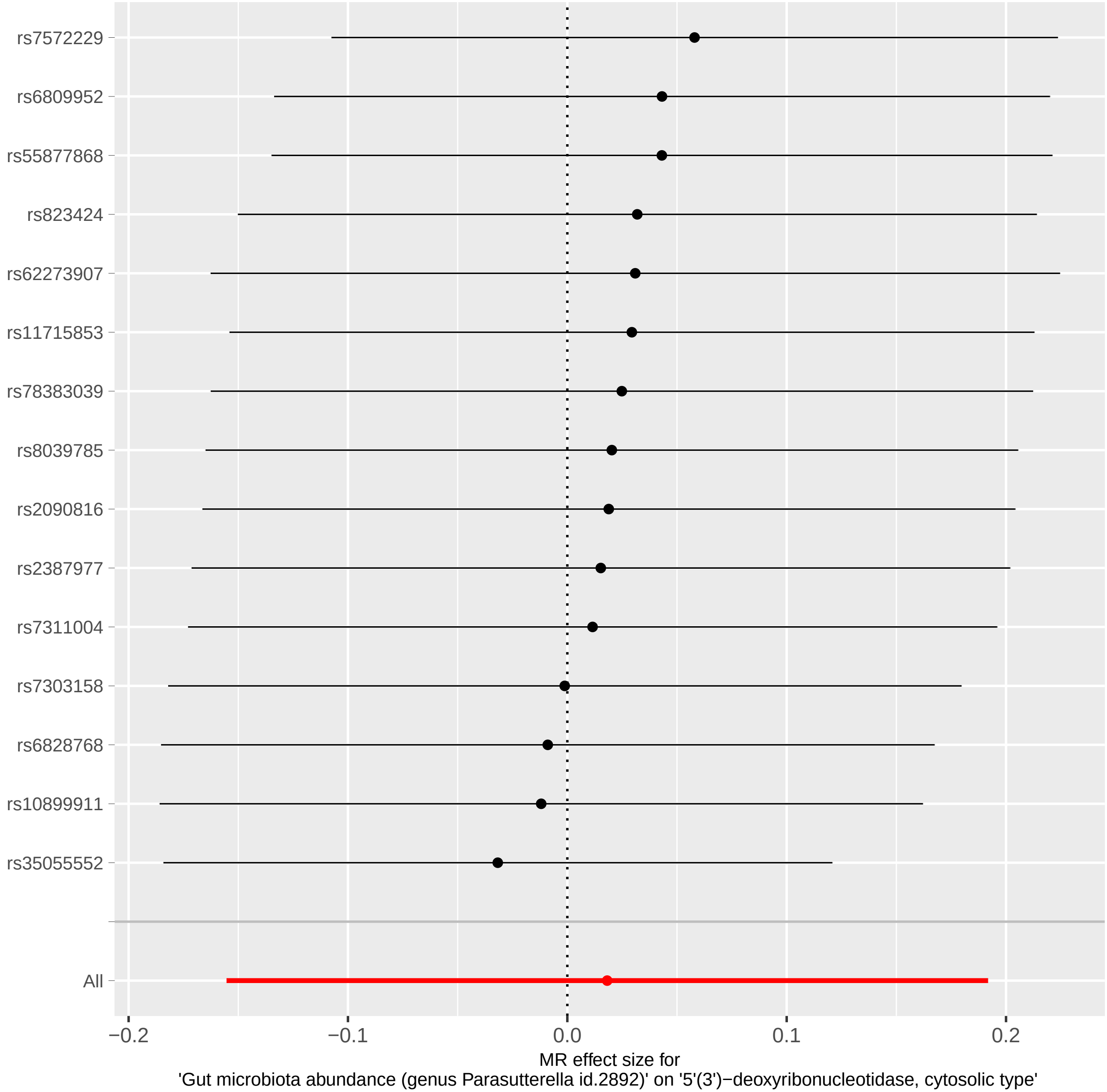

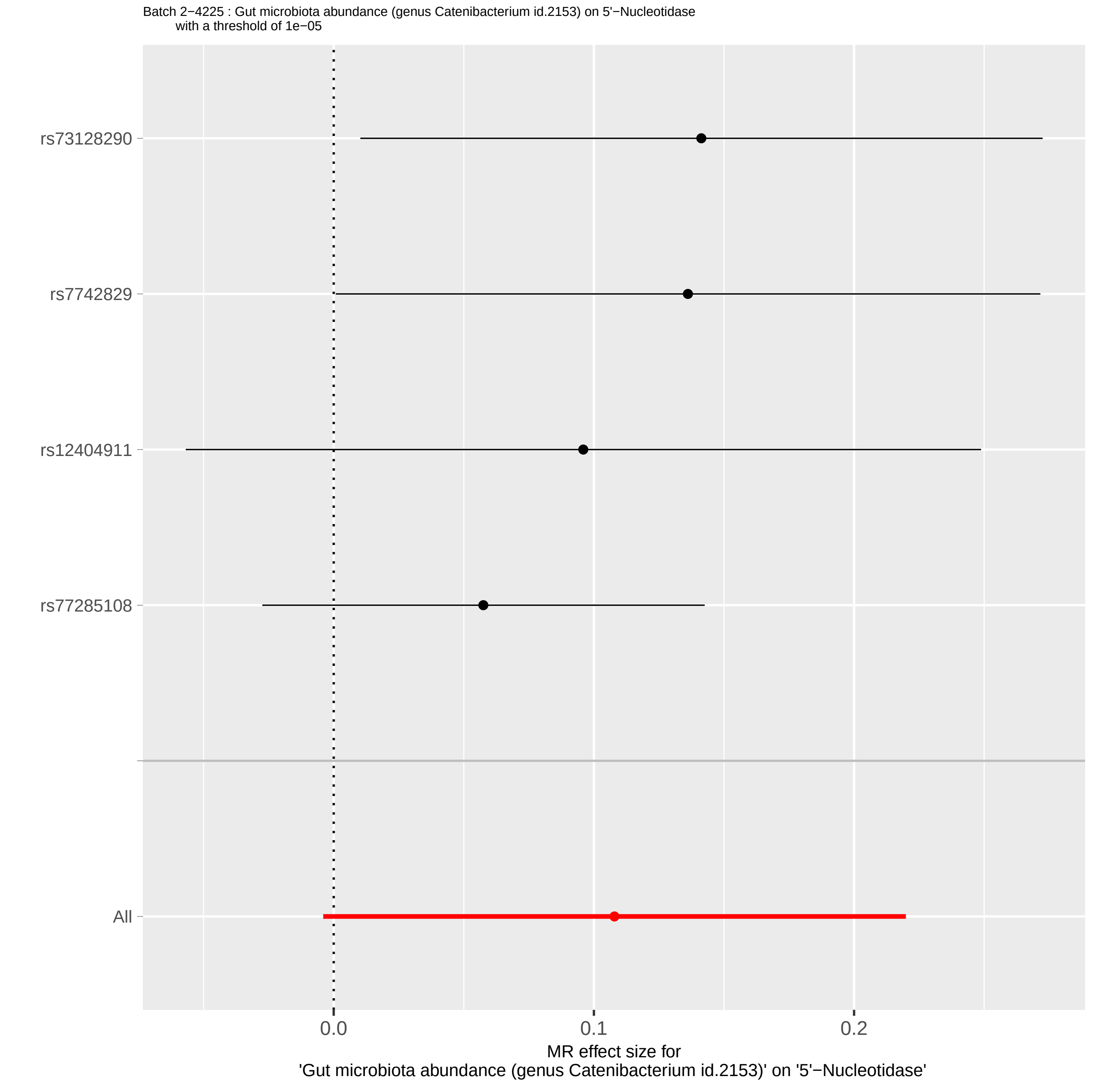
Batch 2−4225 : Gut microbiota abundance (genus Catenibacterium id.2153) on 5'−Nucleotidase
with a threshold of 1e−05
rs73128290
rs7742829
rs12404911
rs77285108
All
0.0
0.1
0.2
MR effect size for
'Gut microbiota abundance (genus Catenibacterium id.2153)' on '5'−Nucleotidase'

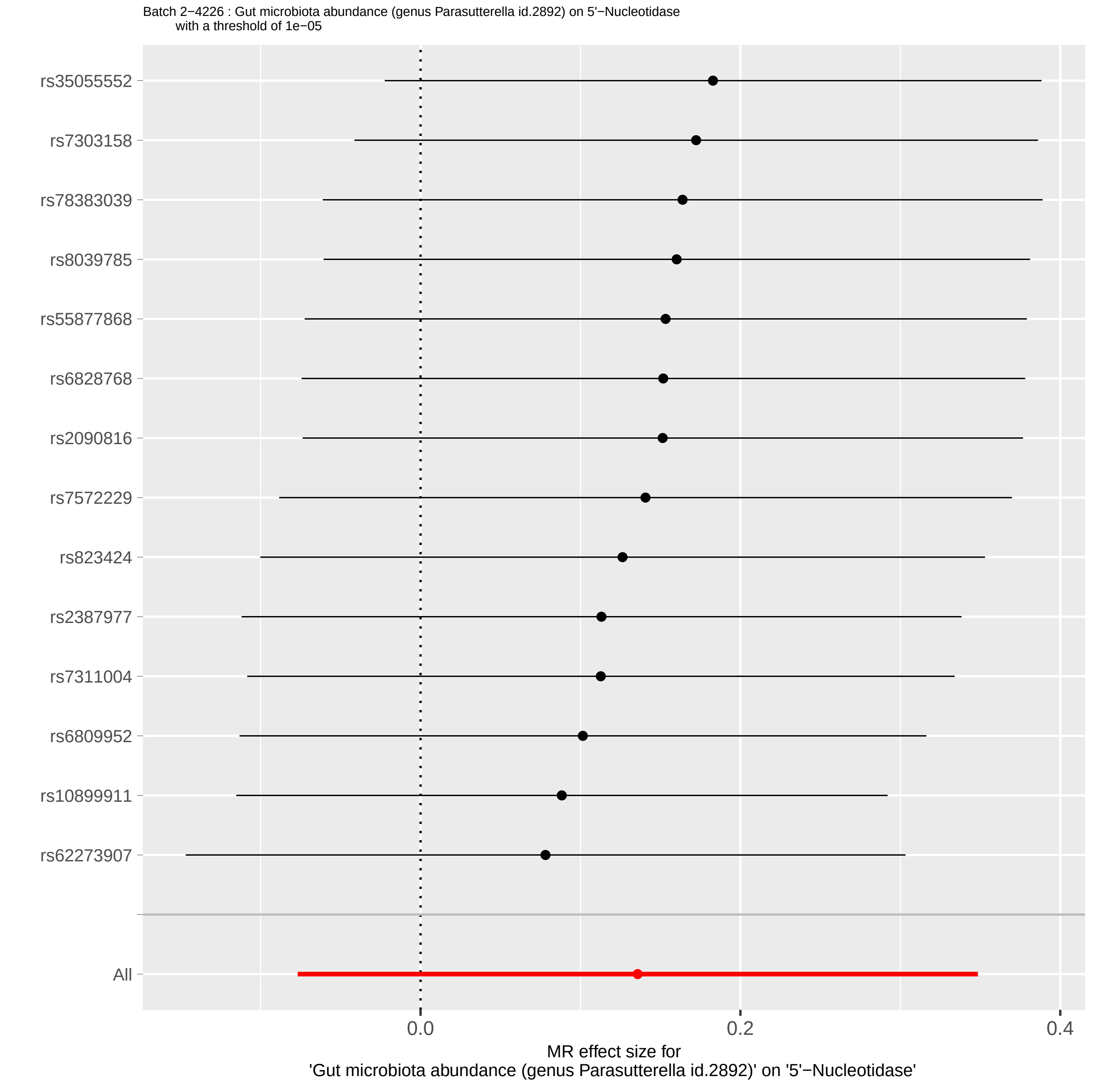
Batch 2−4226 : Gut microbiota abundance (genus Parasutterella id.2892) on 5'−Nucleotidase
with a threshold of 1e−05
rs35055552
rs7303158
rs78383039
rs8039785
rs55877868
rs6828768
rs2090816
rs7572229
rs823424
rs2387977
rs7311004
rs6809952
rs10899911
rs62273907
All
0.0
0.2
0.4
MR effect size for
'Gut microbiota abundance (genus Parasutterella id.2892)' on '5'−Nucleotidase'

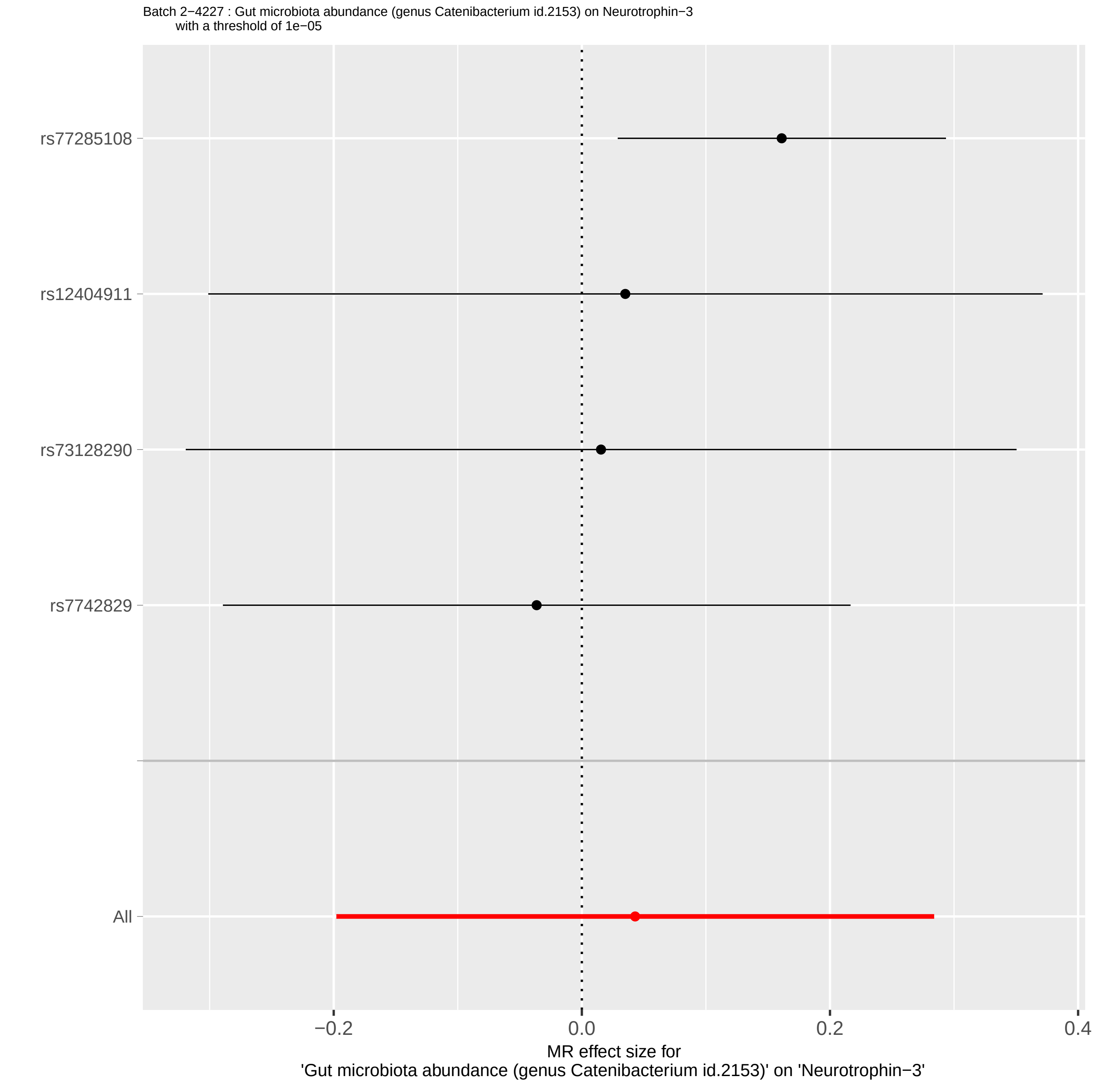
Batch 2−4227 : Gut microbiota abundance (genus Catenibacterium id.2153) on Neurotrophin−3
with a threshold of 1e−05
rs77285108
rs12404911
rs73128290
rs7742829
All
−0.2
0.0
0.2
0.4
MR effect size for
'Gut microbiota abundance (genus Catenibacterium id.2153)' on 'Neurotrophin−3'

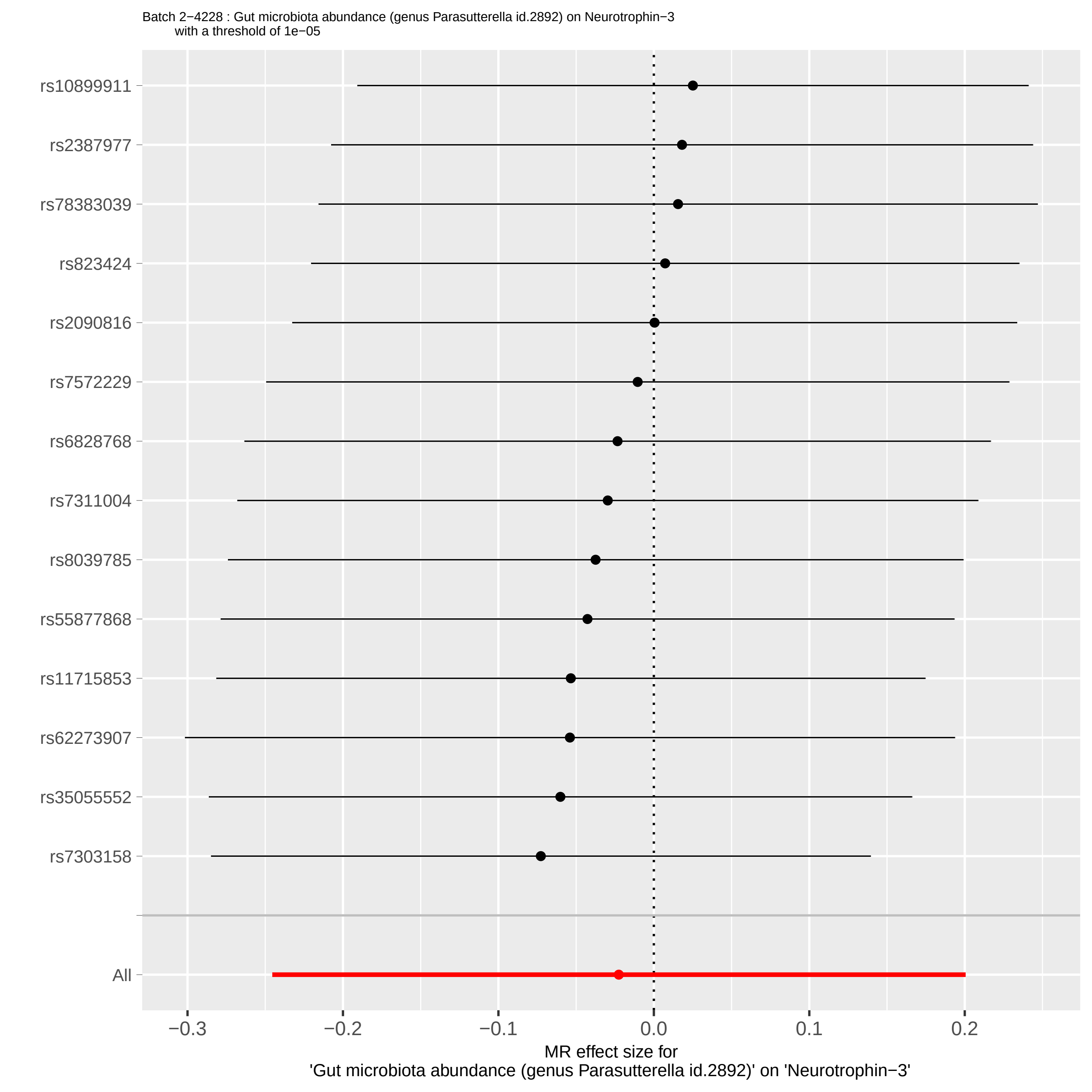
Batch 2−4228 : Gut microbiota abundance (genus Parasutterella id.2892) on Neurotrophin−3
with a threshold of 1e−05
rs10899911
rs2387977
rs78383039
rs823424
rs2090816
rs7572229
rs6828768
rs7311004
rs8039785
rs55877868
rs11715853
rs62273907
rs35055552
rs7303158
All
−0.3
−0.2
−0.1
0.0
0.1
0.2
MR effect size for
'Gut microbiota abundance (genus Parasutterella id.2892)' on 'Neurotrophin−3'

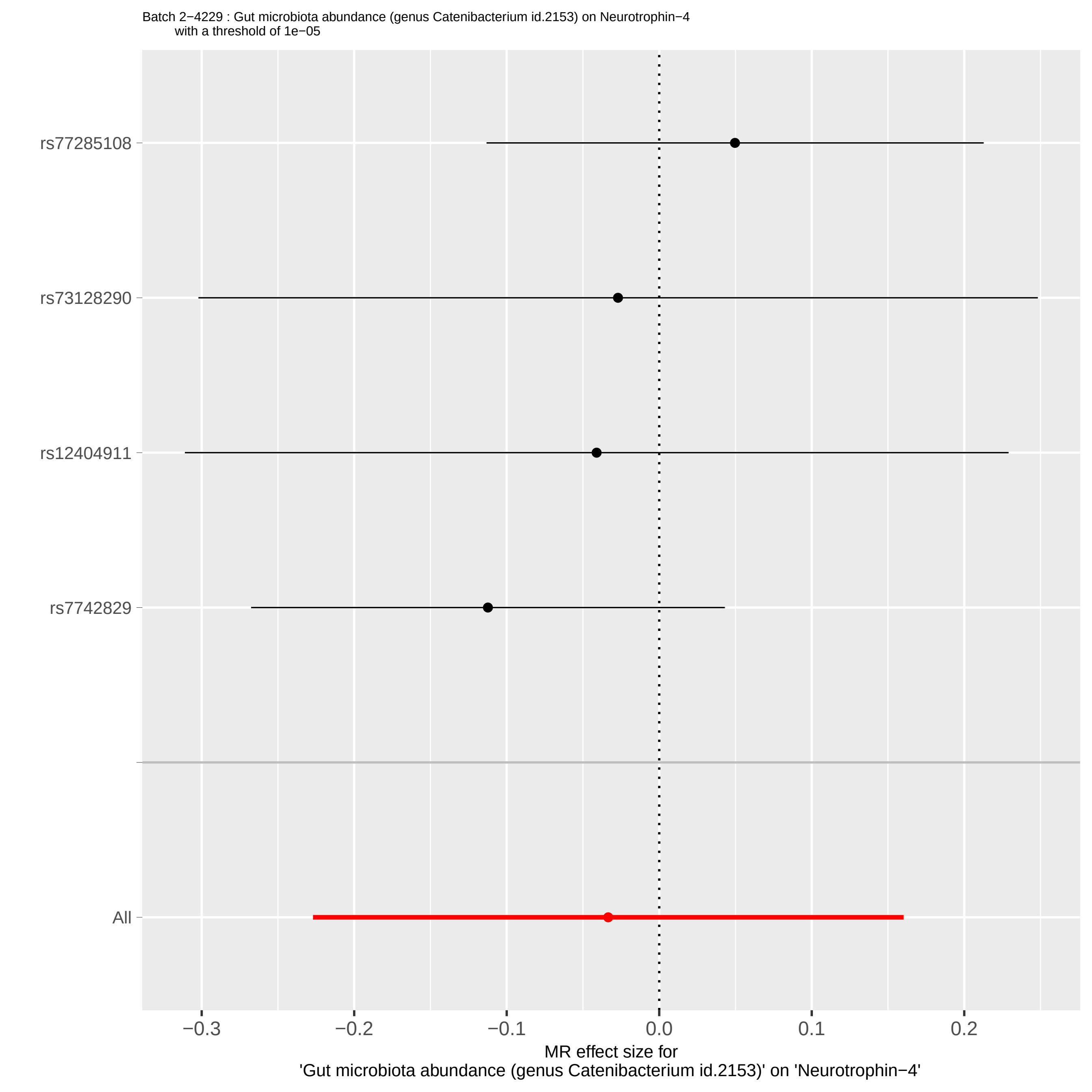
Batch 2−4229 : Gut microbiota abundance (genus Catenibacterium id.2153) on Neurotrophin−4
with a threshold of 1e−05
rs77285108
rs73128290
rs12404911
rs7742829
All
−0.3
−0.2
−0.1
0.0
0.1
0.2
MR effect size for
'Gut microbiota abundance (genus Catenibacterium id.2153)' on 'Neurotrophin−4'

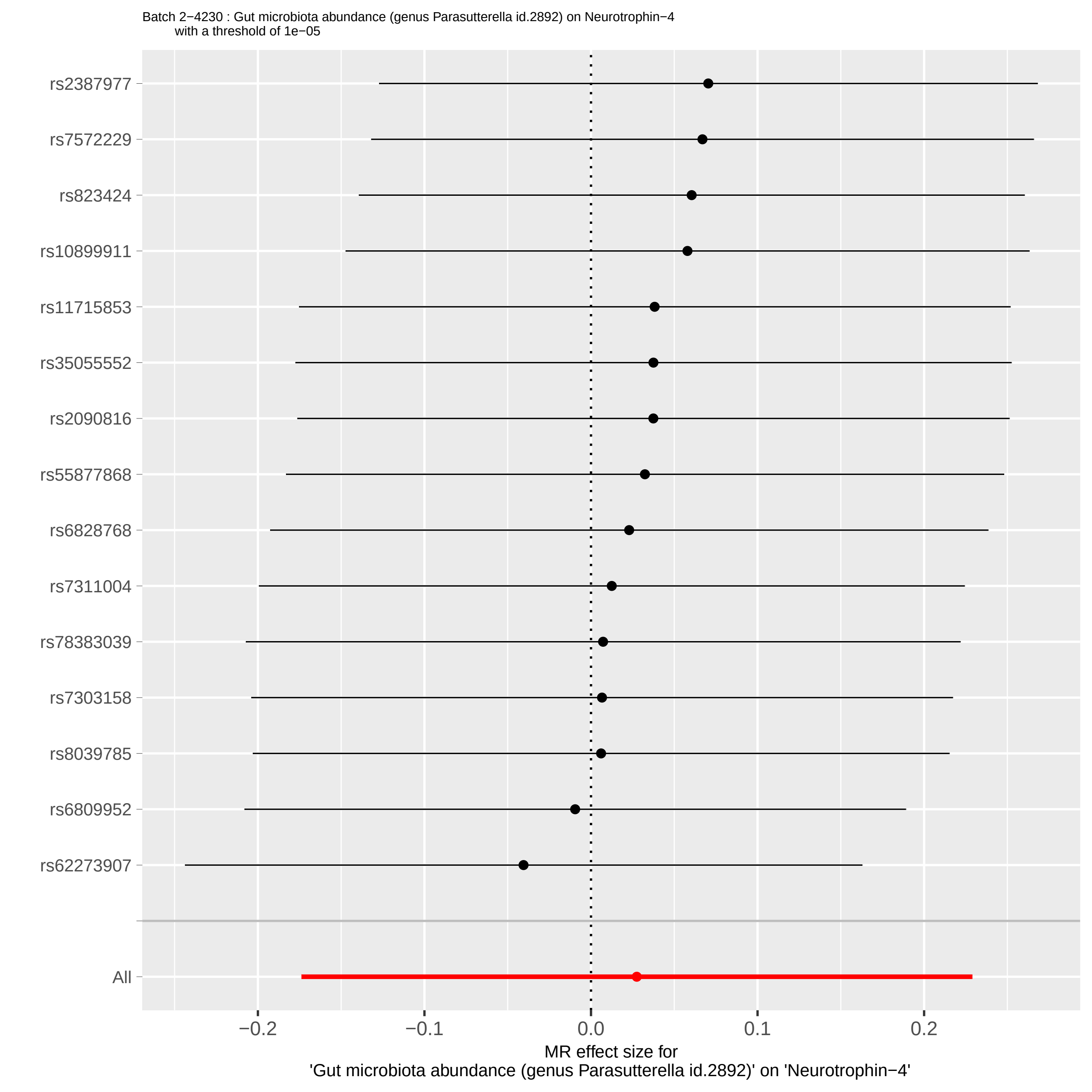
Batch 2−4230 : Gut microbiota abundance (genus Parasutterella id.2892) on Neurotrophin−4
with a threshold of 1e−05
rs2387977
rs7572229
rs823424
rs10899911
rs11715853
rs35055552
rs2090816
rs55877868
rs6828768
rs7311004
rs78383039
rs7303158
rs8039785
rs6809952
rs62273907
All
−0.2
−0.1
0.0
0.1
0.2
MR effect size for
'Gut microbiota abundance (genus Parasutterella id.2892)' on 'Neurotrophin−4'

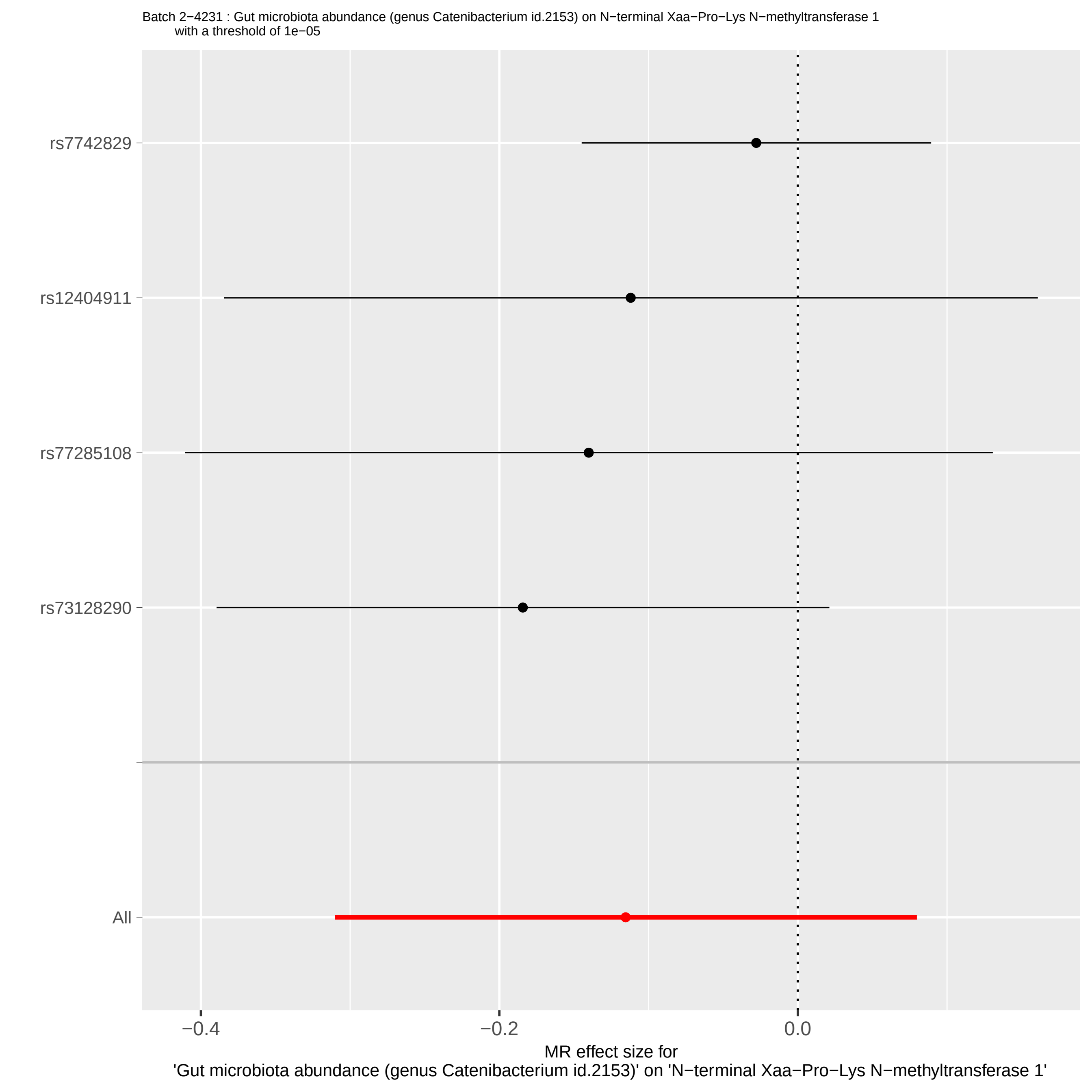

Batch 2−4231 : Gut microbiota abundance (genus Catenibacterium id.2153) on N−terminal Xaa−Pro−Lys N−methyltransferase 1
with a threshold of 1e−05
rs7742829
rs12404911
rs77285108
rs73128290
All
−0.4
−0.2
0.0
MR effect size for
'Gut microbiota abundance (genus Catenibacterium id.2153)' on 'N−terminal Xaa−Pro−Lys N−methyltransferase 1'

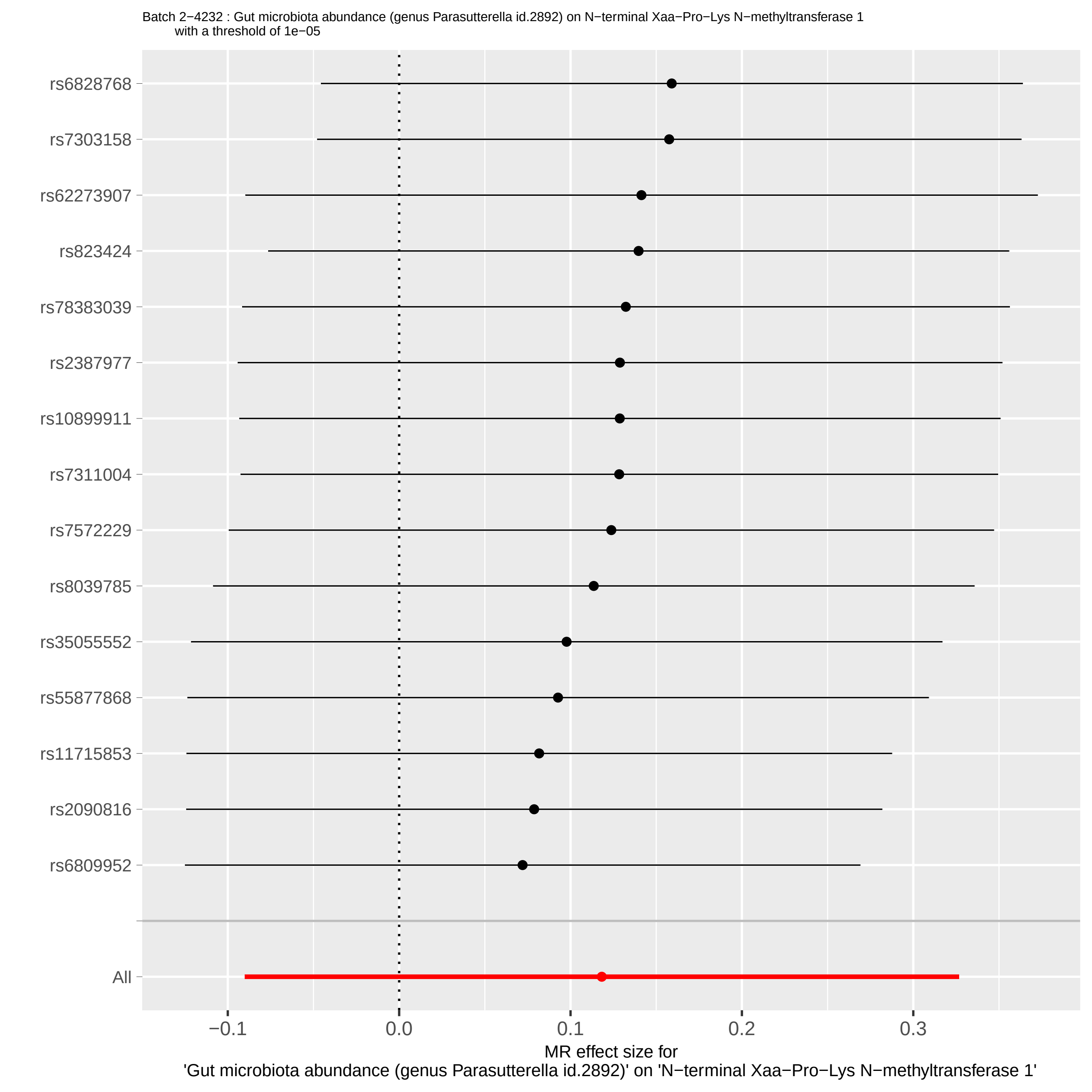
Batch 2−4232 : Gut microbiota abundance (genus Parasutterella id.2892) on N−terminal Xaa−Pro−Lys N−methyltransferase 1
with a threshold of 1e−05
rs6828768
rs7303158
rs62273907
rs823424
rs78383039
rs2387977
rs10899911
rs7311004
rs7572229
rs8039785
rs35055552
rs55877868
rs11715853
rs2090816
rs6809952
All
−0.1
0.0
0.1
0.2
0.3
MR effect size for
'Gut microbiota abundance (genus Parasutterella id.2892)' on 'N−terminal Xaa−Pro−Lys N−methyltransferase 1'

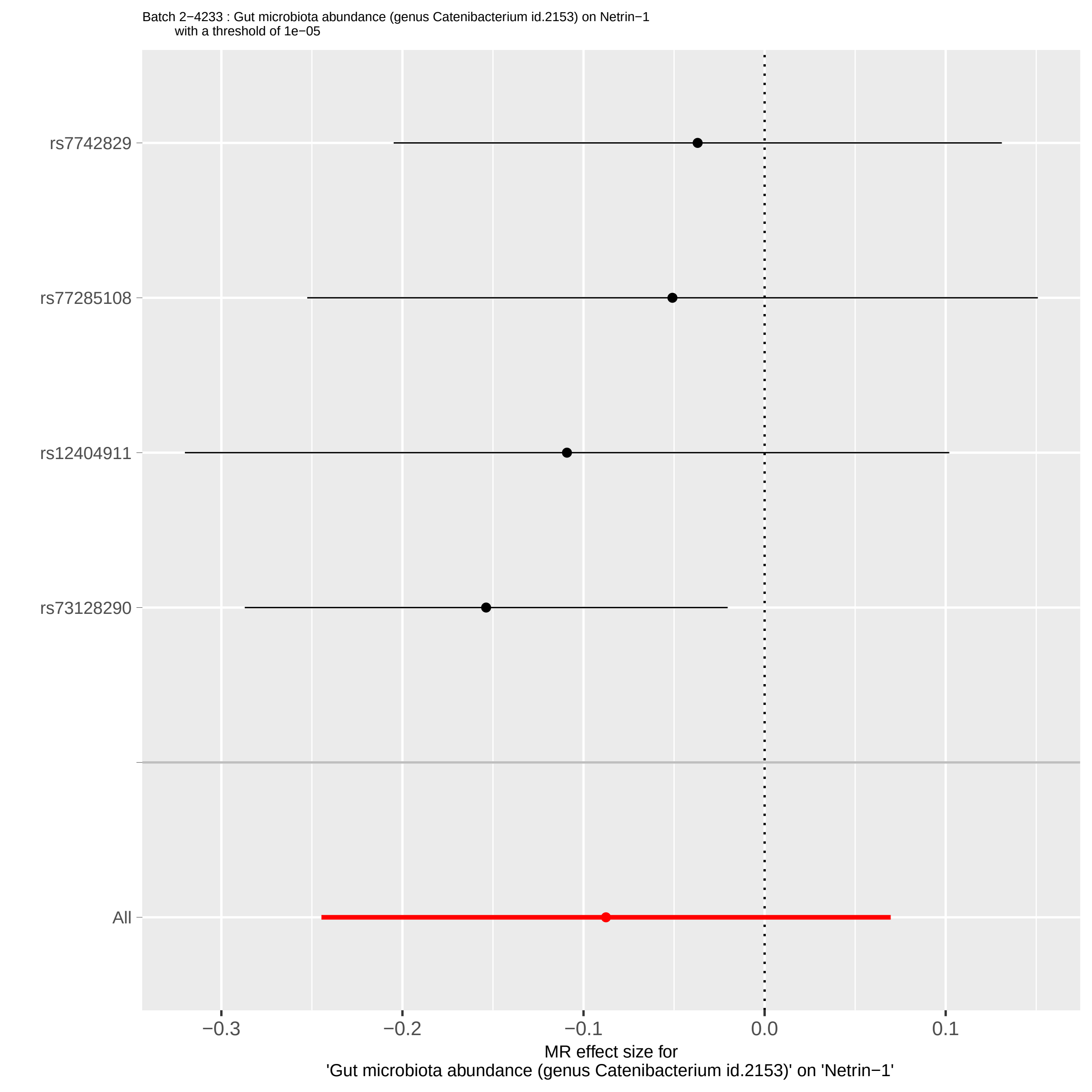

Batch 2−4233 : Gut microbiota abundance (genus Catenibacterium id.2153) on Netrin−1
with a threshold of 1e−05
rs7742829
rs77285108
rs12404911
rs73128290
All
−0.3
−0.2
−0.1
0.0
0.1
MR effect size for
'Gut microbiota abundance (genus Catenibacterium id.2153)' on 'Netrin−1'

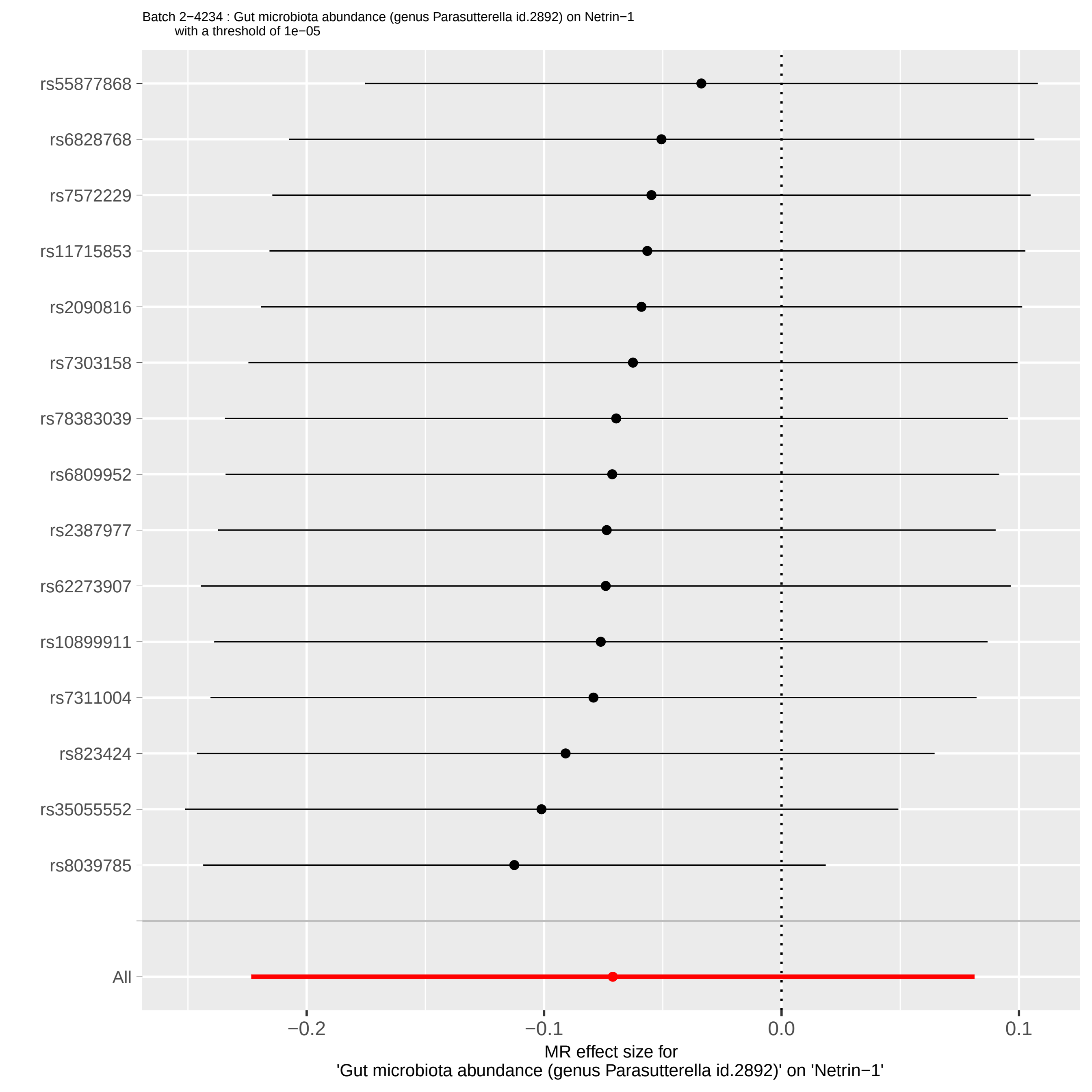

Batch 2−4234 : Gut microbiota abundance (genus Parasutterella id.2892) on Netrin−1
with a threshold of 1e−05
rs55877868
rs6828768
rs7572229
rs11715853
rs2090816
rs7303158
rs78383039
rs6809952
rs2387977
rs62273907
rs10899911
rs7311004
rs823424
rs35055552
rs8039785
All
−0.2
−0.1
0.0
0.1
MR effect size for
'Gut microbiota abundance (genus Parasutterella id.2892)' on 'Netrin−1'

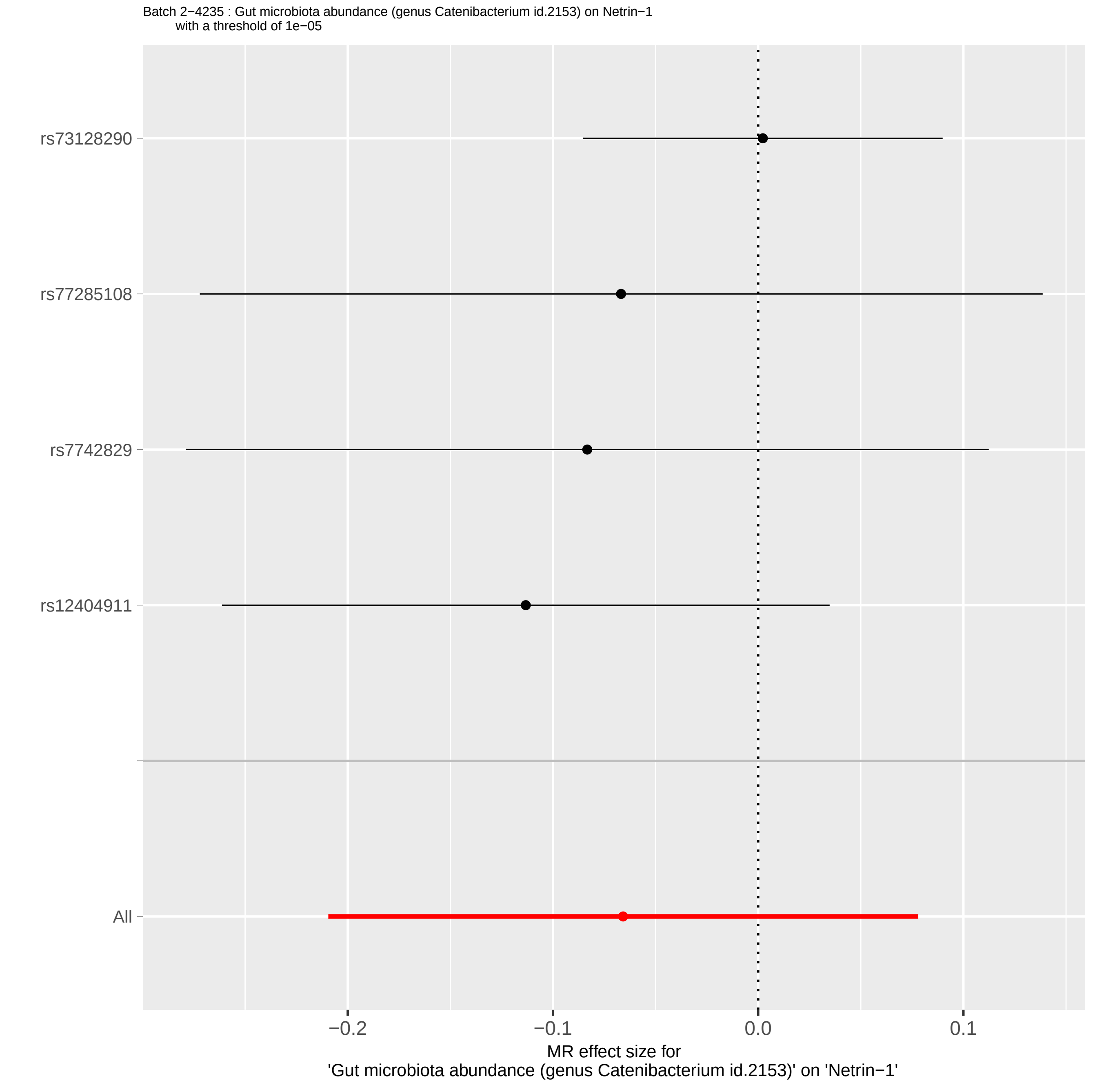

Batch 2−4235 : Gut microbiota abundance (genus Catenibacterium id.2153) on Netrin−1
with a threshold of 1e−05
rs73128290
rs77285108
rs7742829
rs12404911
All
−0.2
−0.1
0.0
0.1
MR effect size for
'Gut microbiota abundance (genus Catenibacterium id.2153)' on 'Netrin−1'

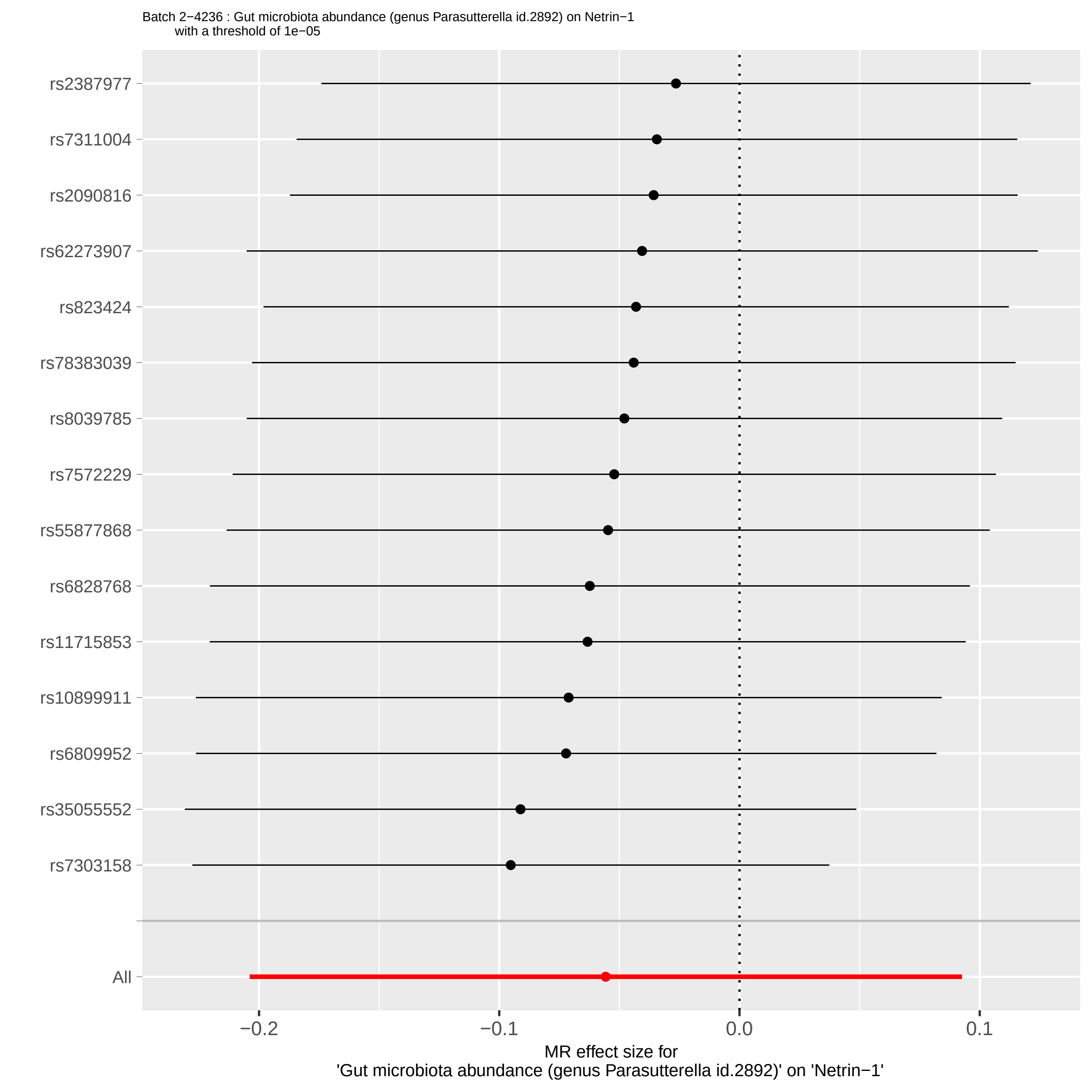
Batch 2−4236 : Gut microbiota abundance (genus Parasutterella id.2892) on Netrin−1
with a threshold of 1e−05
rs2387977
rs7311004
rs2090816
rs62273907
rs823424
rs78383039
rs8039785
rs7572229
rs55877868
rs6828768
rs11715853
rs10899911
rs6809952
rs35055552
rs7303158
All
−0.2
−0.1
0.0
0.1
MR effect size for
'Gut microbiota abundance (genus Parasutterella id.2892)' on 'Netrin−1'

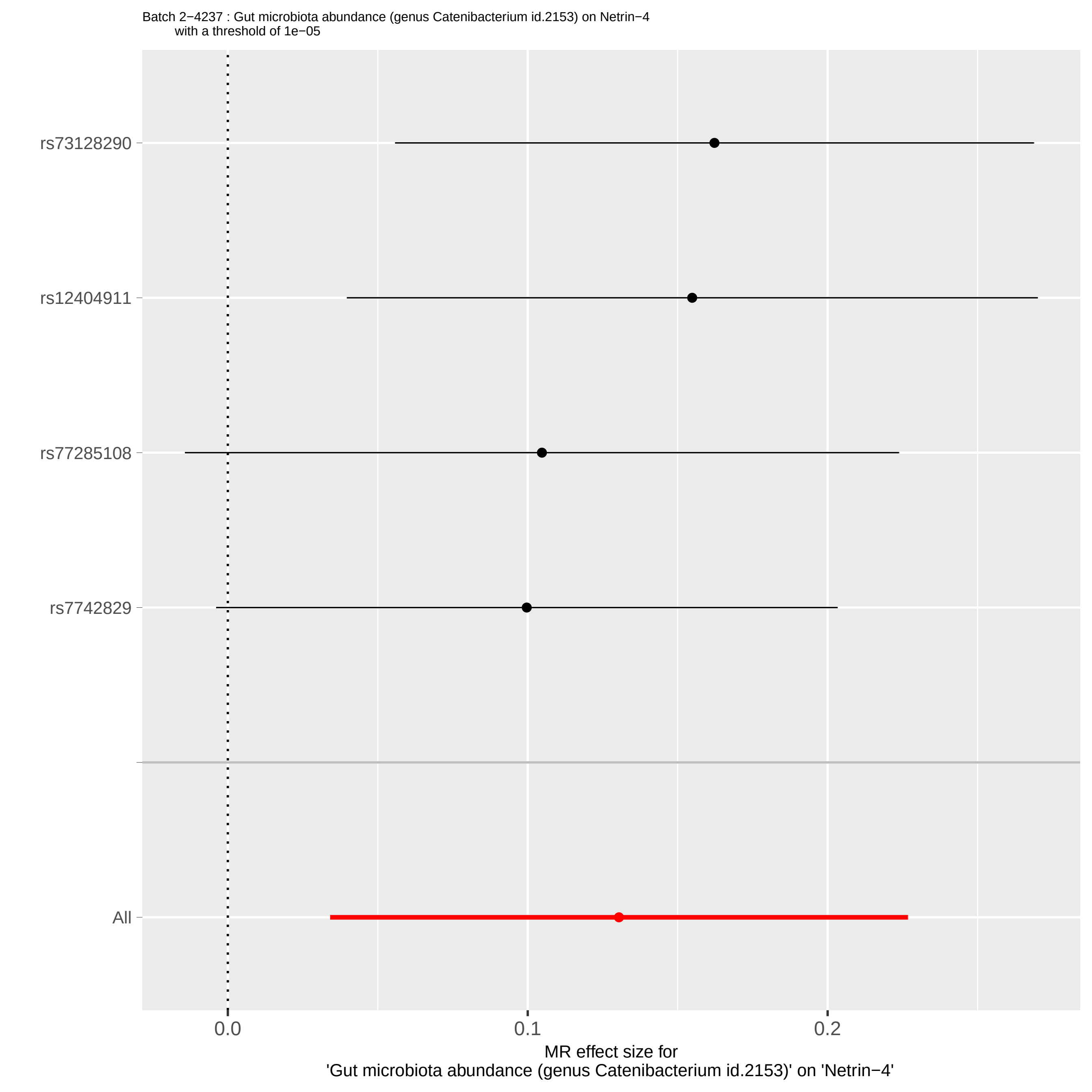

Batch 2−4237 : Gut microbiota abundance (genus Catenibacterium id.2153) on Netrin−4
with a threshold of 1e−05
rs73128290
rs12404911
rs77285108
rs7742829
All
0.0
0.1
0.2
MR effect size for
'Gut microbiota abundance (genus Catenibacterium id.2153)' on 'Netrin−4'

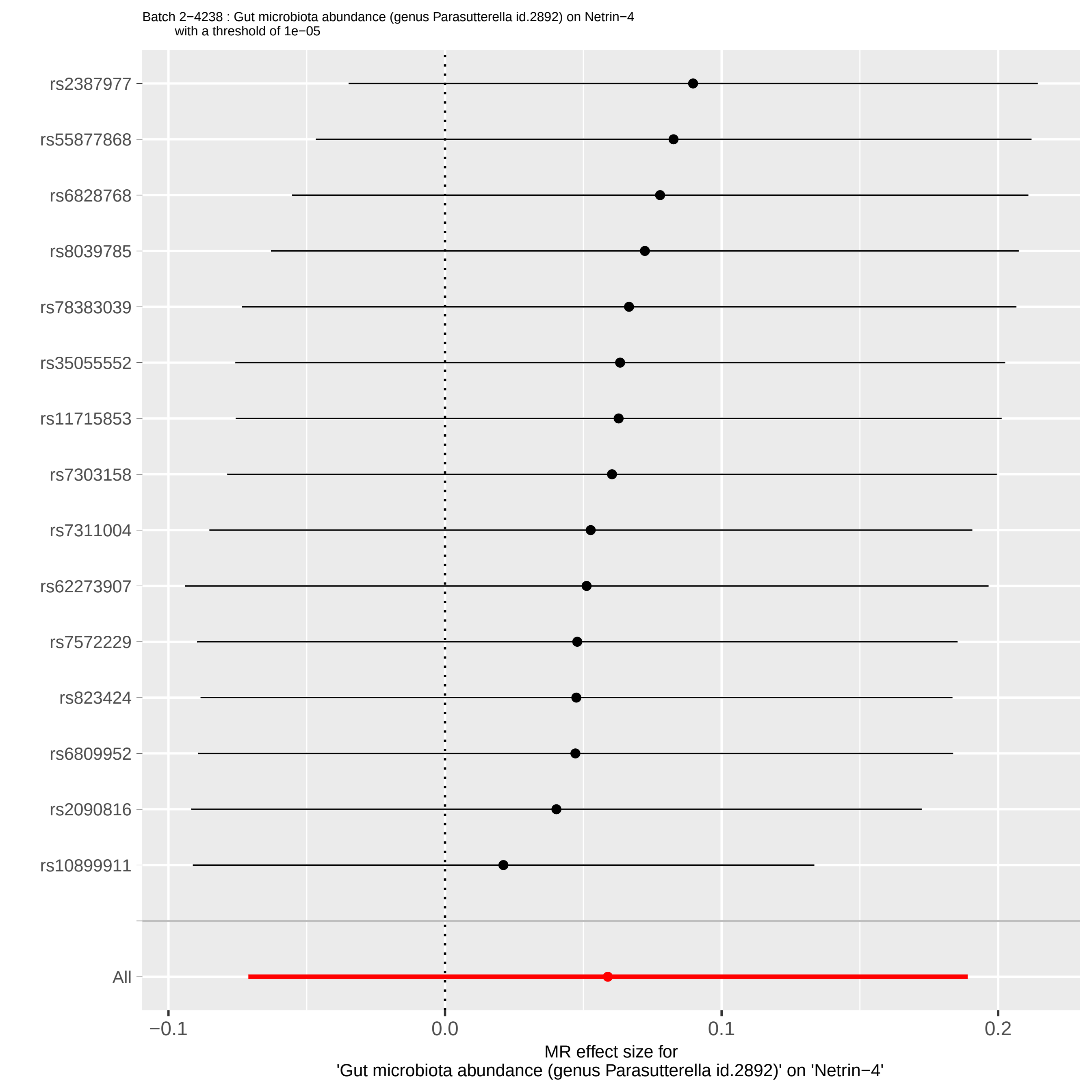

Batch 2−4238 : Gut microbiota abundance (genus Parasutterella id.2892) on Netrin−4
with a threshold of 1e−05
rs2387977
rs55877868
rs6828768
rs8039785
rs78383039
rs35055552
rs11715853
rs7303158
rs7311004
rs62273907
rs7572229
rs823424
rs6809952
rs2090816
rs10899911
All
−0.1
0.0
0.1
0.2
MR effect size for
'Gut microbiota abundance (genus Parasutterella id.2892)' on 'Netrin−4'

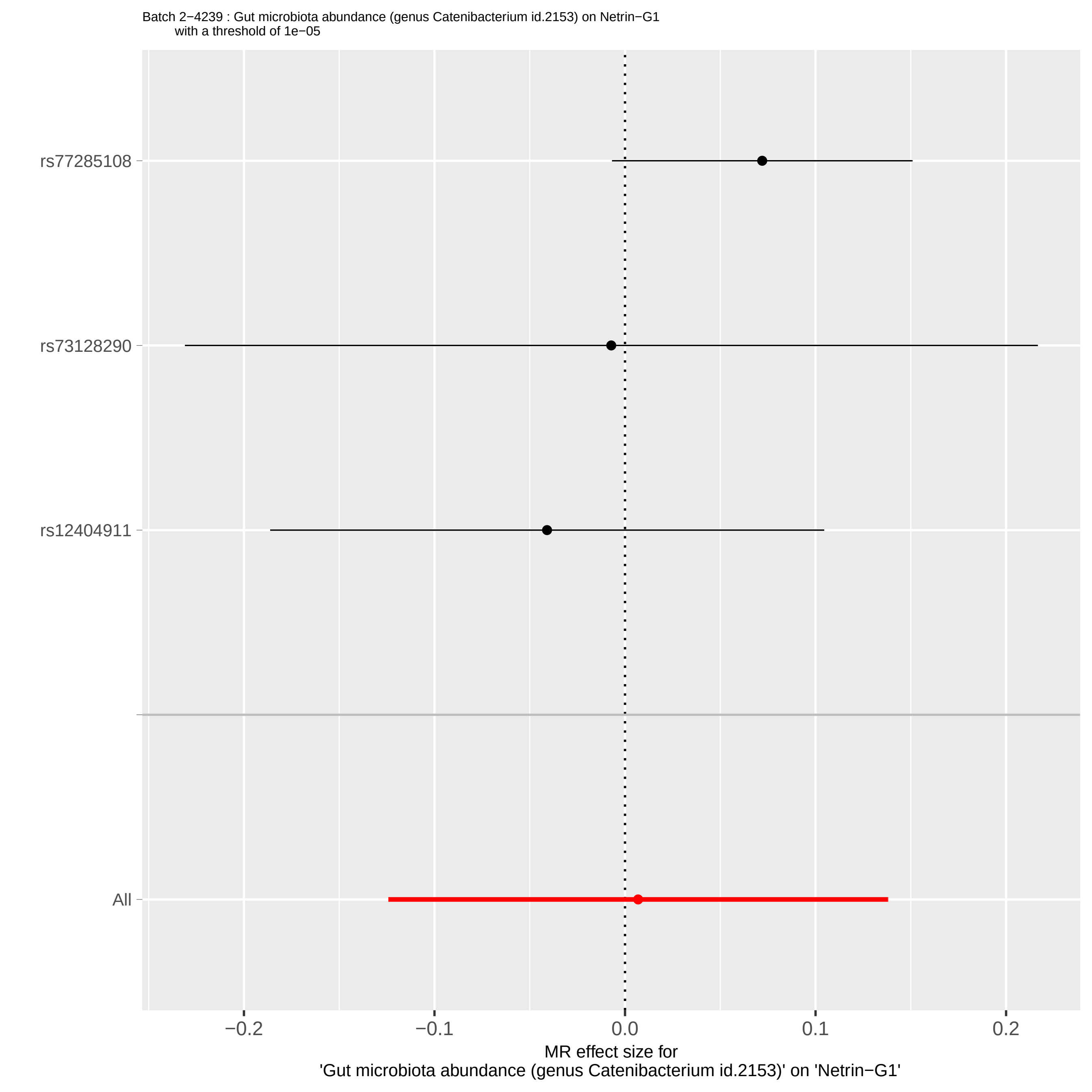

Batch 2−4239 : Gut microbiota abundance (genus Catenibacterium id.2153) on Netrin−G1
with a threshold of 1e−05
rs77285108
rs73128290
rs12404911
All
−0.2
−0.1
0.0
0.1
0.2
MR effect size for
'Gut microbiota abundance (genus Catenibacterium id.2153)' on 'Netrin−G1'

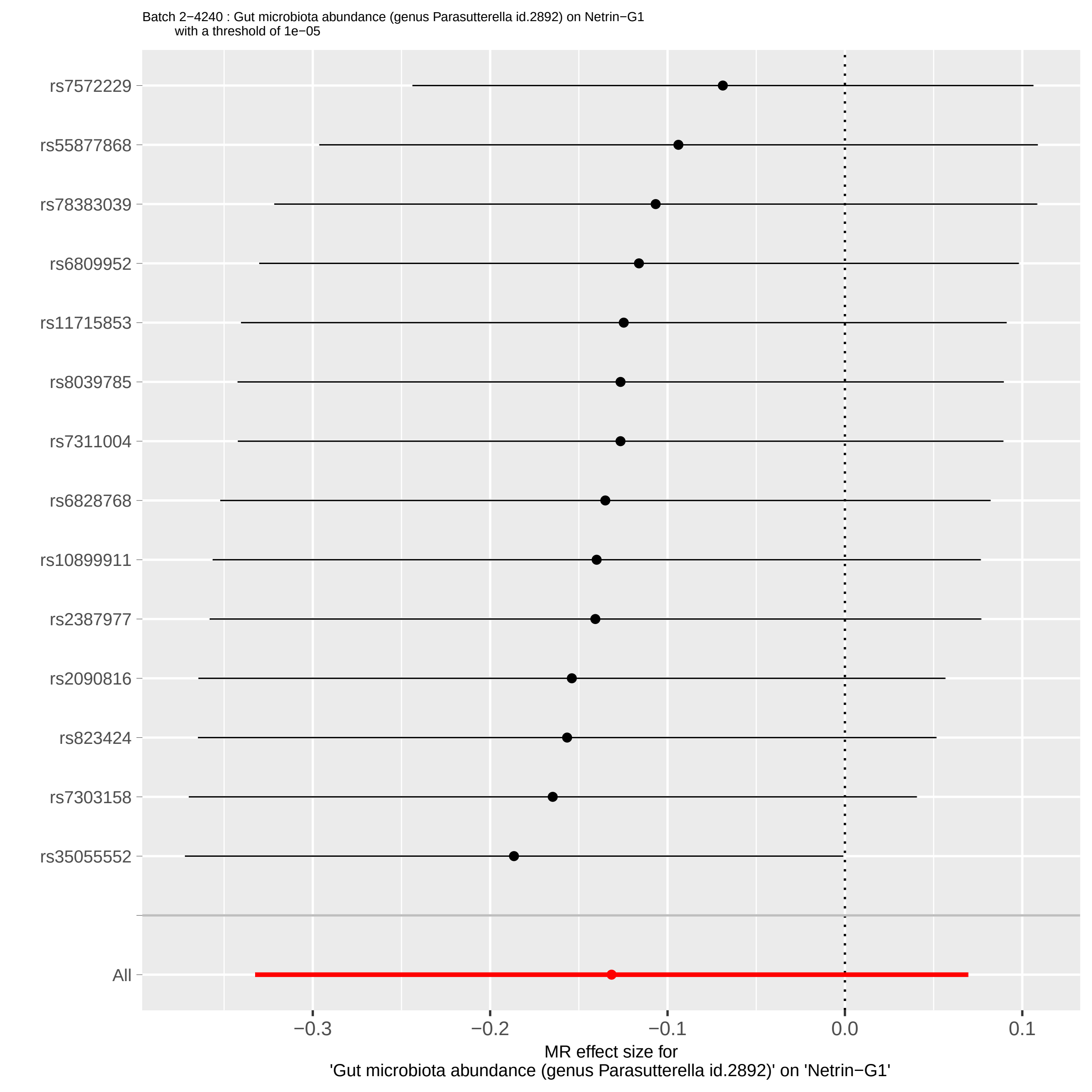

Batch 2−4240 : Gut microbiota abundance (genus Parasutterella id.2892) on Netrin−G1
with a threshold of 1e−05
rs7572229
rs55877868
rs78383039
rs6809952
rs11715853
rs8039785
rs7311004
rs6828768
rs10899911
rs2387977
rs2090816
rs823424
rs7303158
rs35055552
All
−0.3
−0.2
−0.1
0.0
0.1
MR effect size for
'Gut microbiota abundance (genus Parasutterella id.2892)' on 'Netrin−G1'

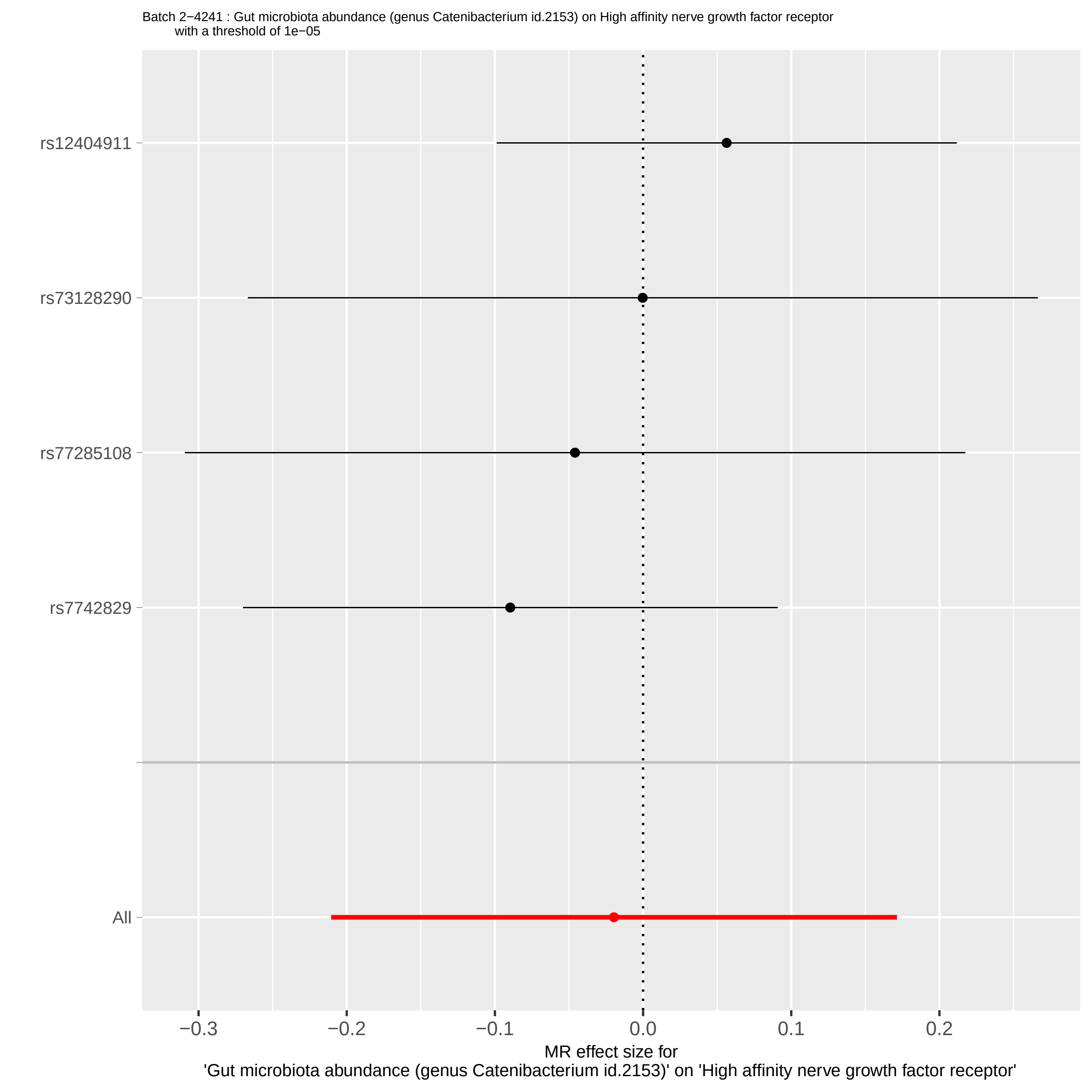
Batch 2−4241 : Gut microbiota abundance (genus Catenibacterium id.2153) on High affinity nerve growth factor receptor
with a threshold of 1e−05
rs12404911
rs73128290
rs77285108
rs7742829
All
−0.3
−0.2
−0.1
0.0
0.1
0.2
MR effect size for
'Gut microbiota abundance (genus Catenibacterium id.2153)' on 'High affinity nerve growth factor receptor'

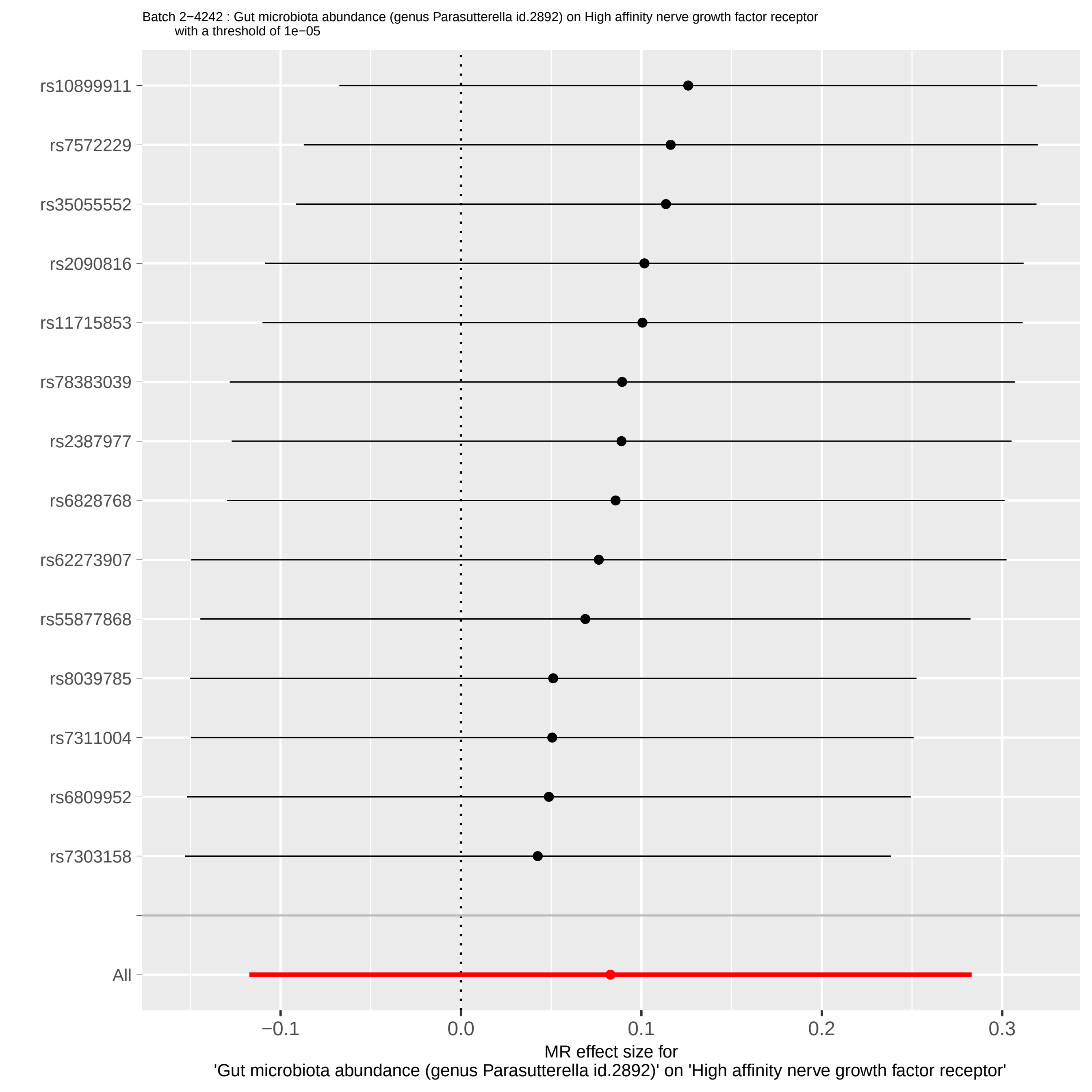
Batch 2−4242 : Gut microbiota abundance (genus Parasutterella id.2892) on High affinity nerve growth factor receptor
with a threshold of 1e−05
rs10899911
rs7572229
rs35055552
rs2090816
rs11715853
rs78383039
rs2387977
rs6828768
rs62273907
rs55877868
rs8039785
rs7311004
rs6809952
rs7303158
All
−0.1
0.0
0.1
0.2
0.3
MR effect size for
'Gut microbiota abundance (genus Parasutterella id.2892)' on 'High affinity nerve growth factor receptor'

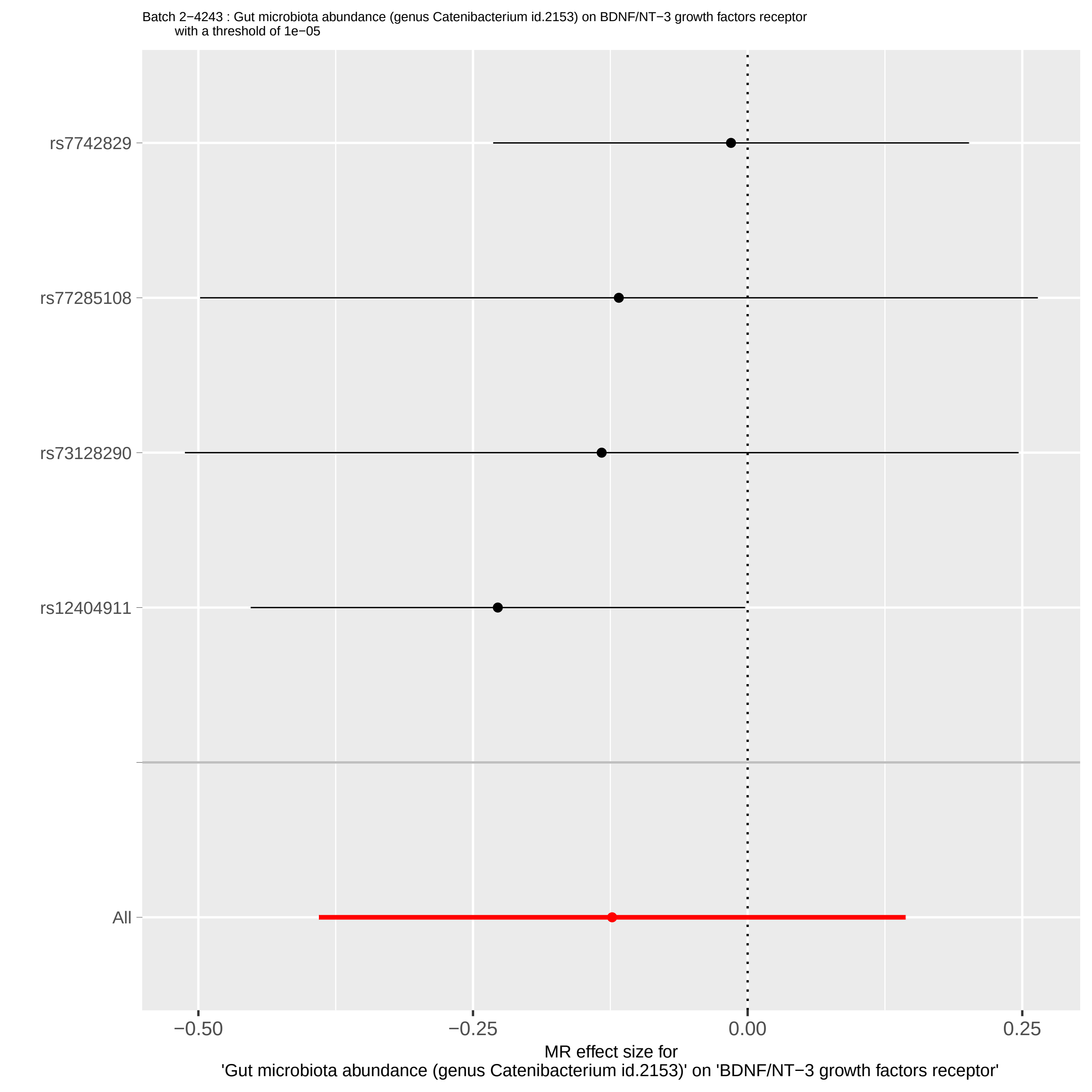

Batch 2−4243 : Gut microbiota abundance (genus Catenibacterium id.2153) on BDNF/NT−3 growth factors receptor
with a threshold of 1e−05
rs7742829
rs77285108
rs73128290
rs12404911
All
−0.50
−0.25
0.00
0.25
MR effect size for
'Gut microbiota abundance (genus Catenibacterium id.2153)' on 'BDNF/NT−3 growth factors receptor'

Batch 2−4244 : Gut microbiota abundance (genus Parasutterella id.2892) on BDNF/NT−3 growth factors receptor
with a threshold of 1e−05

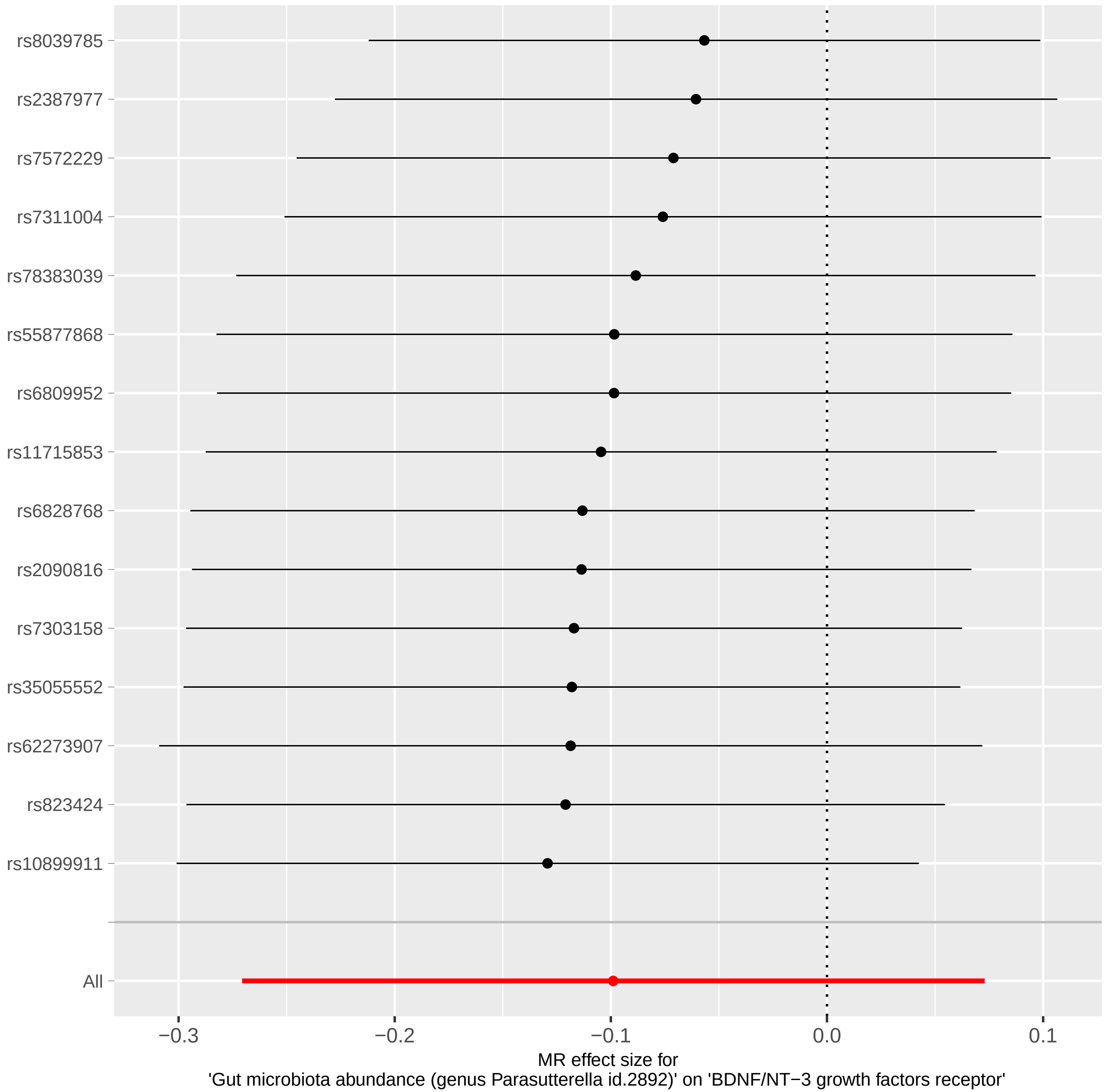

Batch 2−4245 : Gut microbiota abundance (genus Catenibacterium id.2153) on NT−3 growth factor receptor
with a threshold of 1e−05

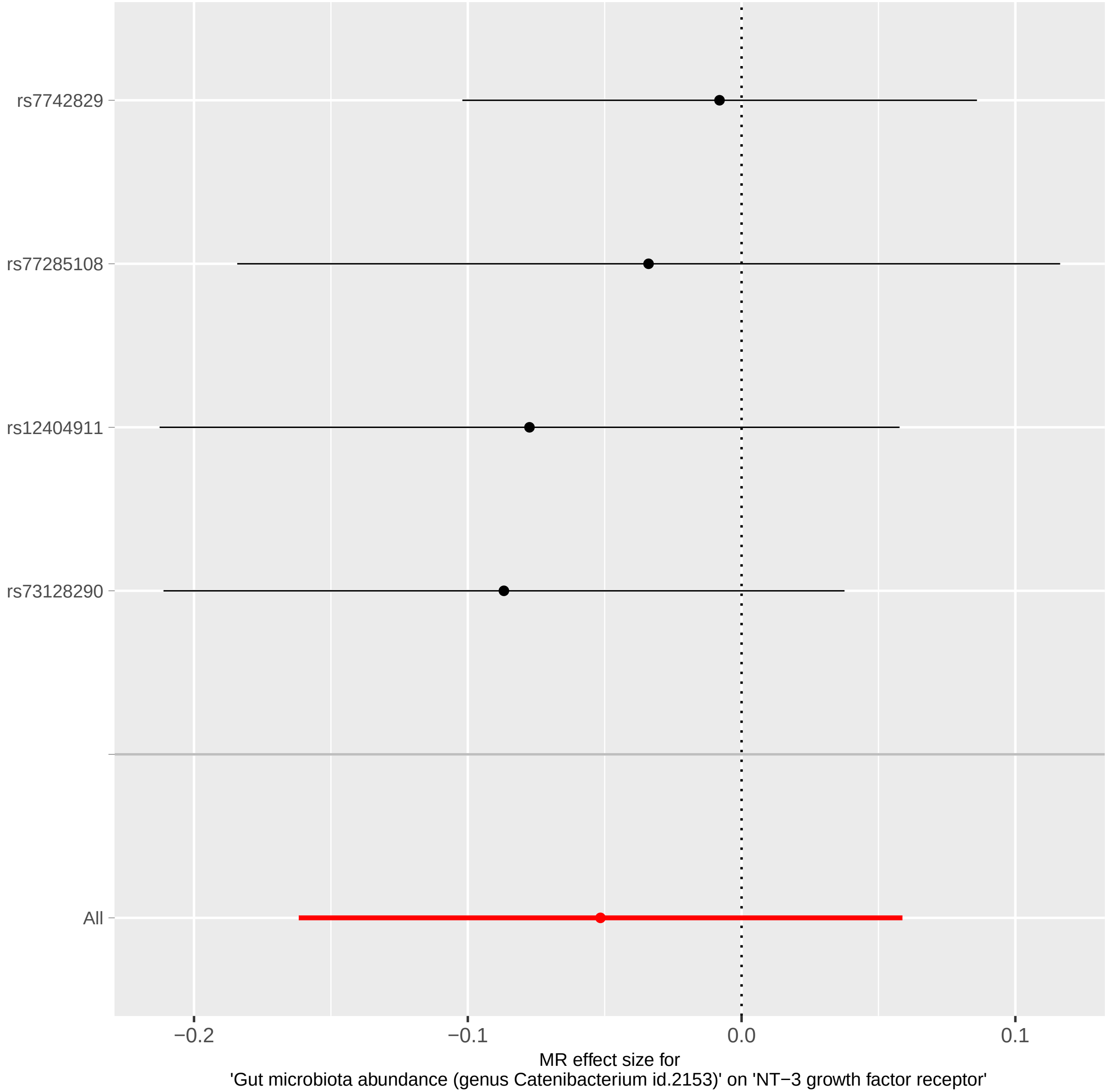

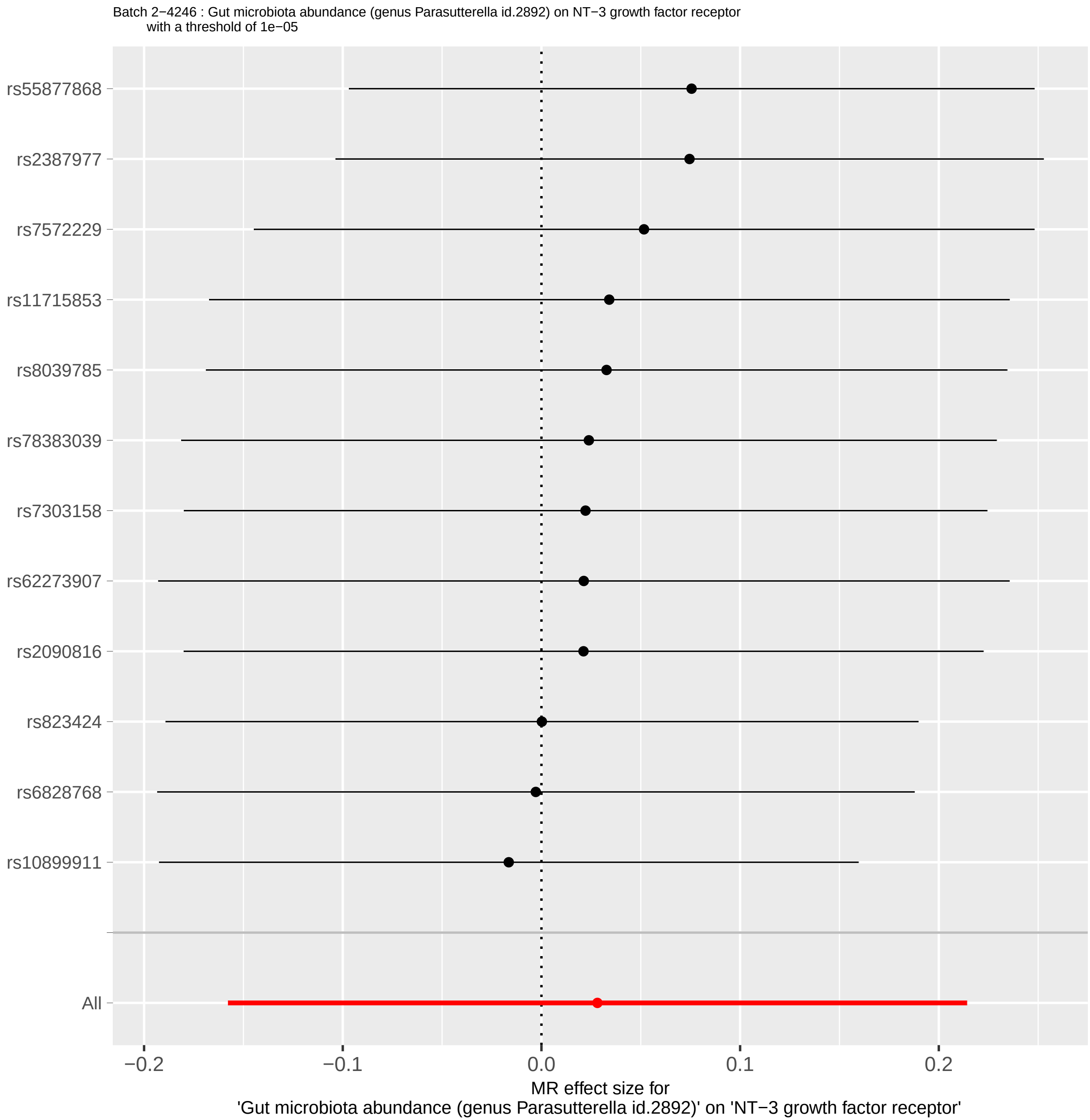
Batch 2−4246 : Gut microbiota abundance (genus Parasutterella id.2892) on NT−3 growth factor receptor
with a threshold of 1e−05
rs55877868
rs2387977
rs7572229
rs11715853
rs8039785
rs78383039
rs7303158
rs62273907
rs2090816
rs823424
rs6828768
rs10899911
All
−0.2
−0.1
0.0
0.1
0.2
MR effect size for
'Gut microbiota abundance (genus Parasutterella id.2892)' on 'NT−3 growth factor receptor'

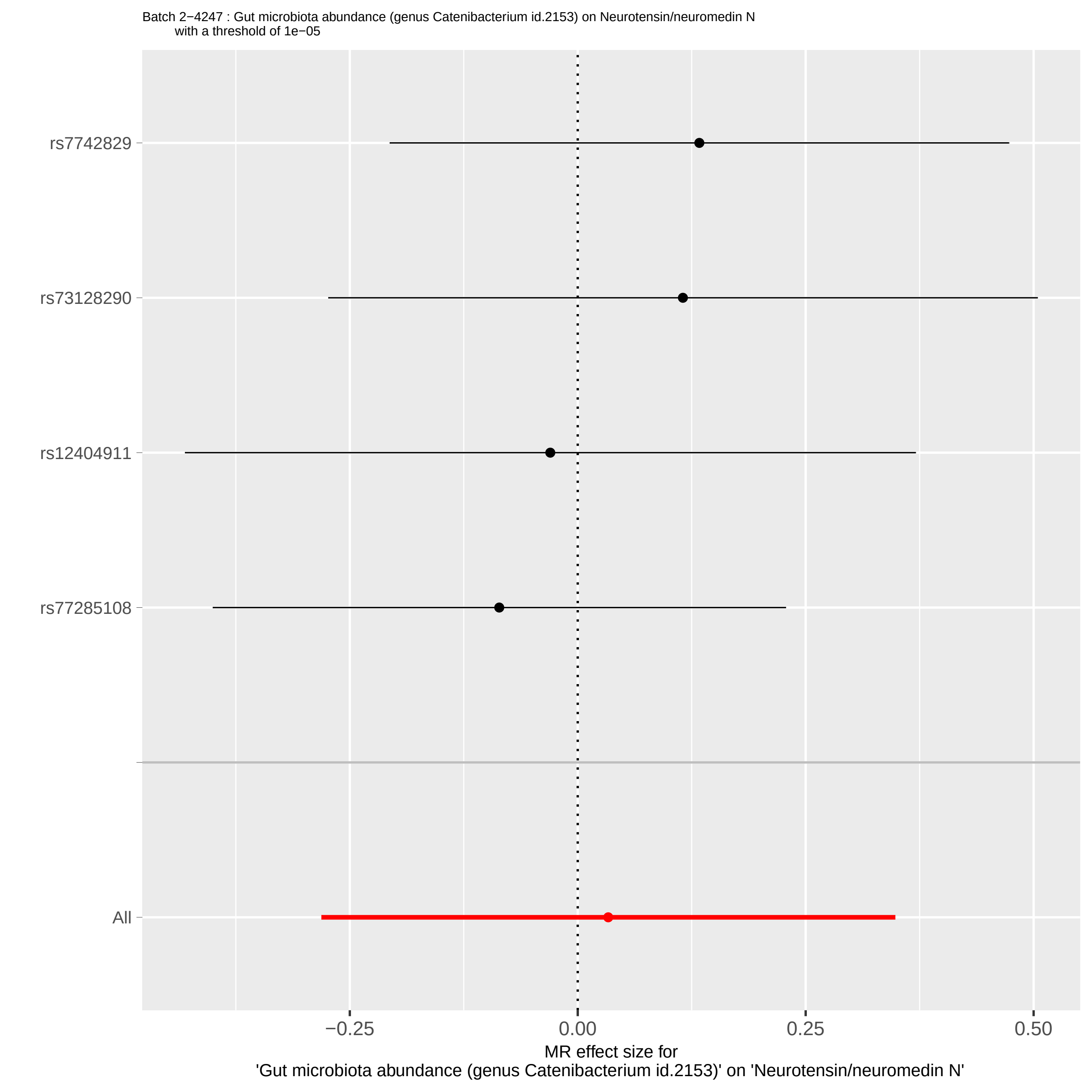

Batch 2−4247 : Gut microbiota abundance (genus Catenibacterium id.2153) on Neurotensin/neuromedin N
with a threshold of 1e−05
rs7742829
rs73128290
rs12404911
rs77285108
All
−0.25
0.00
0.25
0.50
MR effect size for
'Gut microbiota abundance (genus Catenibacterium id.2153)' on 'Neurotensin/neuromedin N'

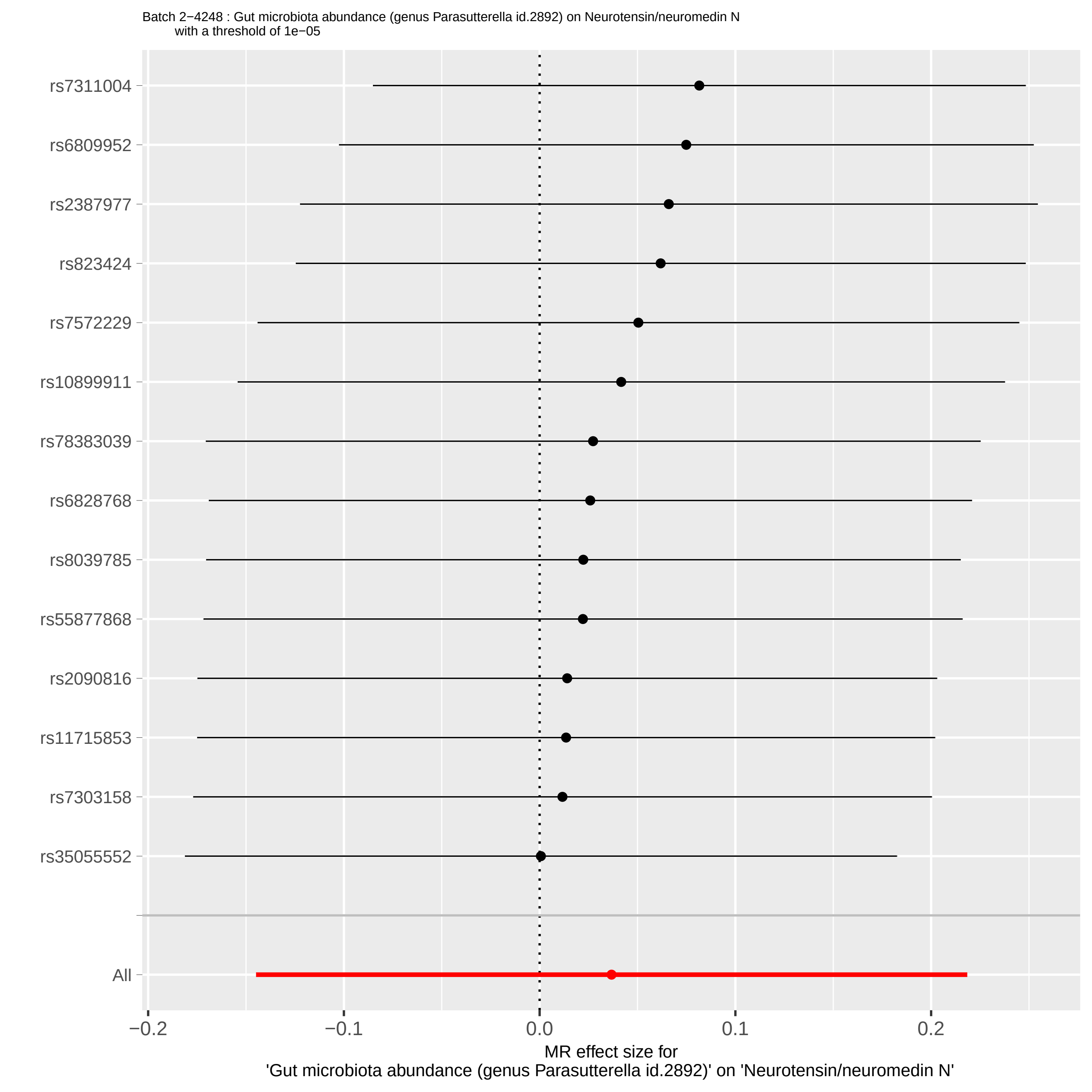
Batch 2−4248 : Gut microbiota abundance (genus Parasutterella id.2892) on Neurotensin/neuromedin N
with a threshold of 1e−05
rs7311004
rs6809952
rs2387977
rs823424
rs7572229
rs10899911
rs78383039
rs6828768
rs8039785
rs55877868
rs2090816
rs11715853
rs7303158
rs35055552
All
−0.2
−0.1
0.0
0.1
0.2
MR effect size for
'Gut microbiota abundance (genus Parasutterella id.2892)' on 'Neurotensin/neuromedin N'

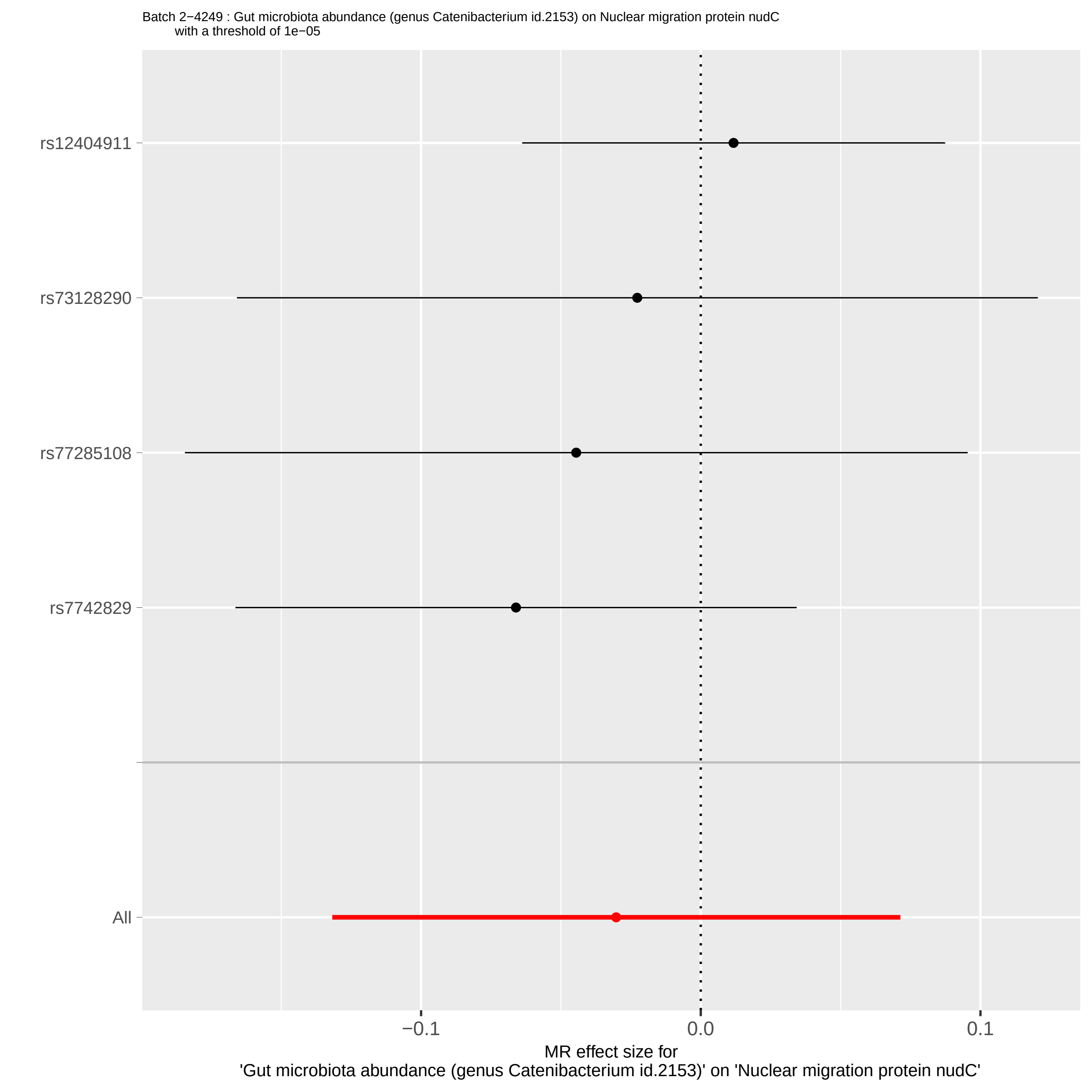

Batch 2−4249 : Gut microbiota abundance (genus Catenibacterium id.2153) on Nuclear migration protein nudC
with a threshold of 1e−05
rs12404911
rs73128290
rs77285108
rs7742829
All
−0.1
0.0
0.1
MR effect size for
'Gut microbiota abundance (genus Catenibacterium id.2153)' on 'Nuclear migration protein nudC'

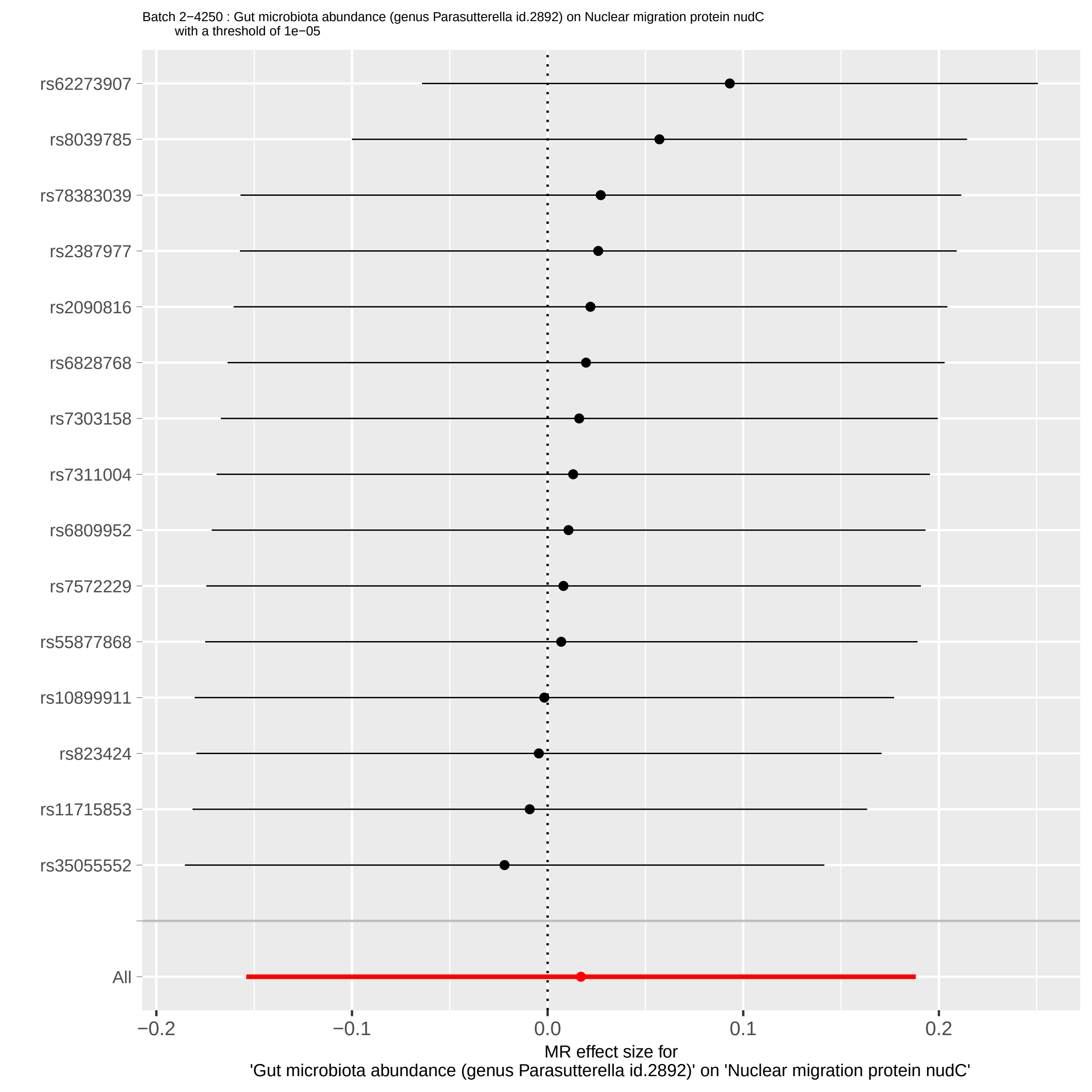

Batch 2−4250 : Gut microbiota abundance (genus Parasutterella id.2892) on Nuclear migration protein nudC
with a threshold of 1e−05
rs62273907
rs8039785
rs78383039
rs2387977
rs2090816
rs6828768
rs7303158
rs7311004
rs6809952
rs7572229
rs55877868
rs10899911
rs823424
rs11715853
rs35055552
All
−0.2
−0.1
0.0
0.1
0.2
MR effect size for
'Gut microbiota abundance (genus Parasutterella id.2892)' on 'Nuclear migration protein nudC'

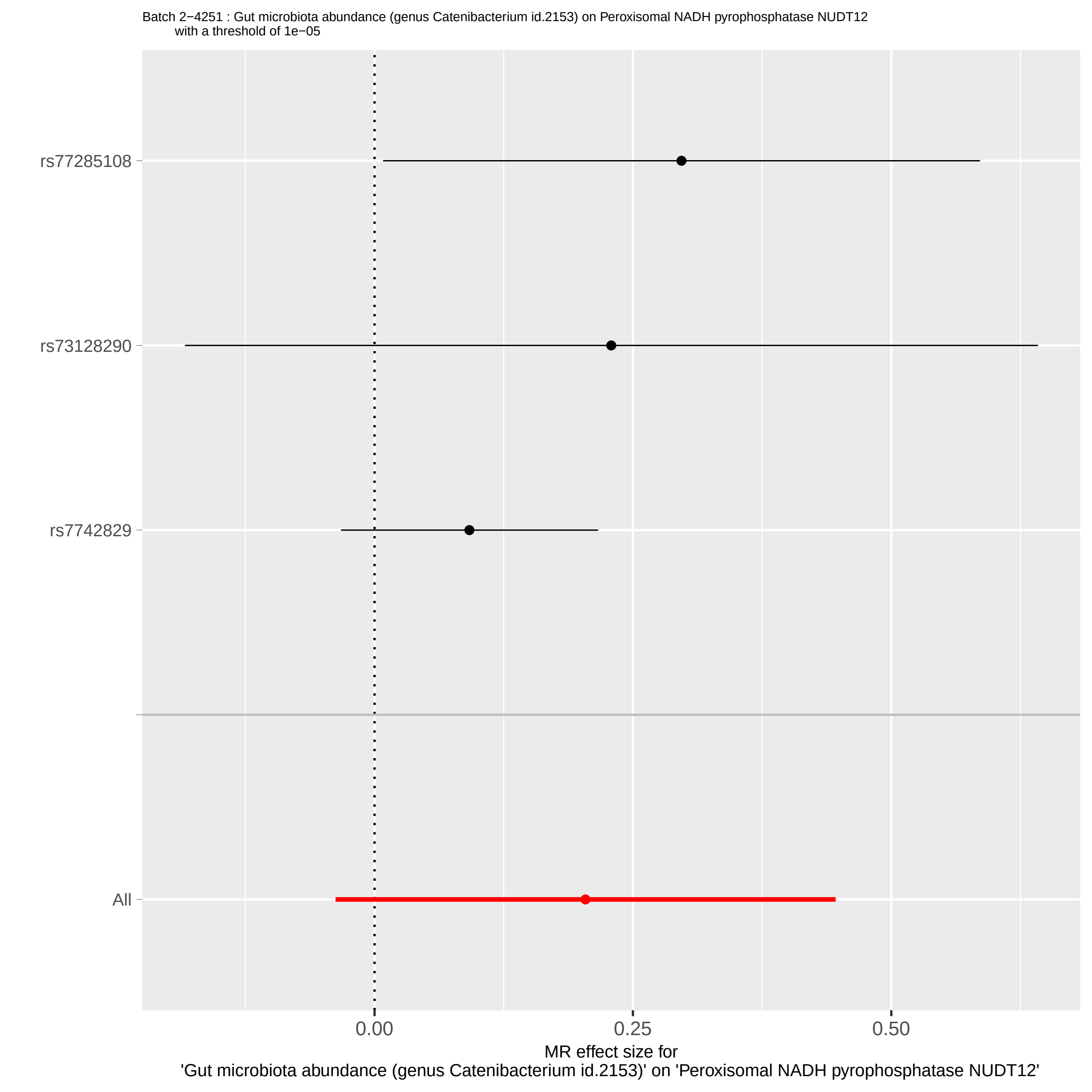

Batch 2−4251 : Gut microbiota abundance (genus Catenibacterium id.2153) on Peroxisomal NADH pyrophosphatase NUDT12
with a threshold of 1e−05
rs77285108
rs73128290
rs7742829
All
0.00
0.25
0.50
MR effect size for
'Gut microbiota abundance (genus Catenibacterium id.2153)' on 'Peroxisomal NADH pyrophosphatase NUDT12'

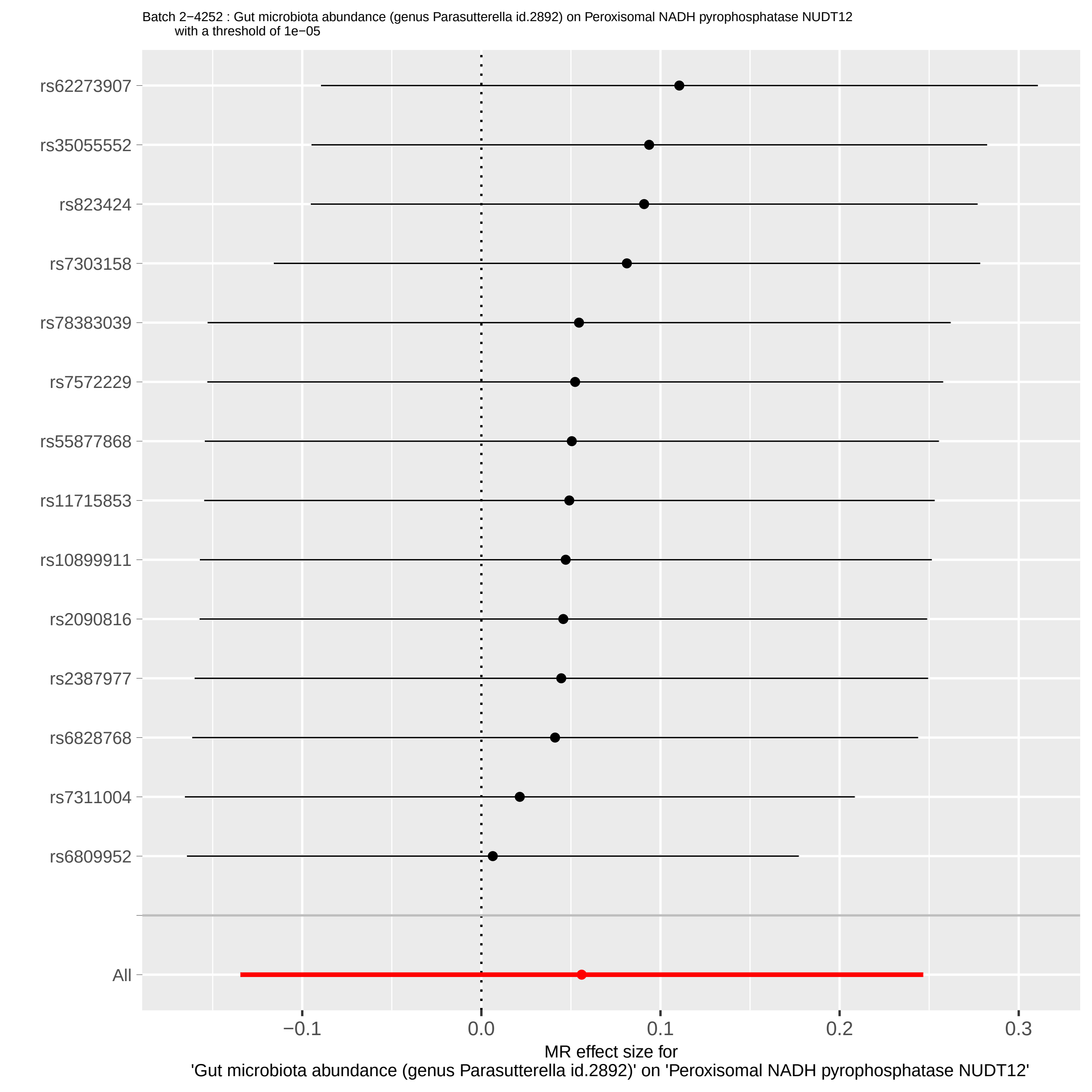

Batch 2−4252 : Gut microbiota abundance (genus Parasutterella id.2892) on Peroxisomal NADH pyrophosphatase NUDT12
with a threshold of 1e−05
rs62273907
rs35055552
rs823424
rs7303158
rs78383039
rs7572229
rs55877868
rs11715853
rs10899911
rs2090816
rs2387977
rs6828768
rs7311004
rs6809952
All
−0.1
0.0
0.1
0.2
0.3
MR effect size for
'Gut microbiota abundance (genus Parasutterella id.2892)' on 'Peroxisomal NADH pyrophosphatase NUDT12'

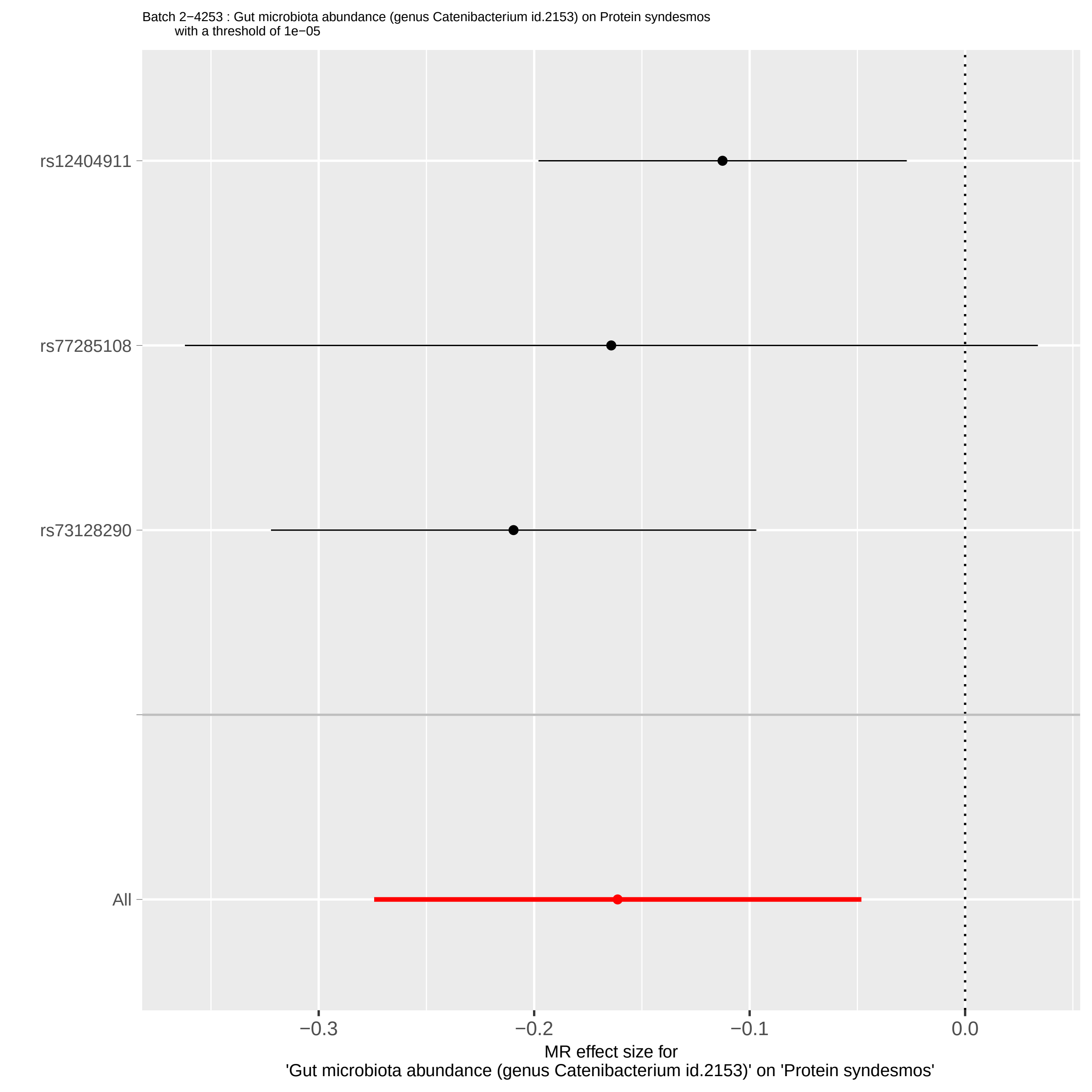
Batch 2−4253 : Gut microbiota abundance (genus Catenibacterium id.2153) on Protein syndesmos
with a threshold of 1e−05
rs12404911
rs77285108
rs73128290
All
−0.3
−0.2
−0.1
0.0
MR effect size for
'Gut microbiota abundance (genus Catenibacterium id.2153)' on 'Protein syndesmos'

Batch 2−4254 : Gut microbiota abundance (genus Parasutterella id.2892) on Protein syndesmos
with a threshold of 1e−05

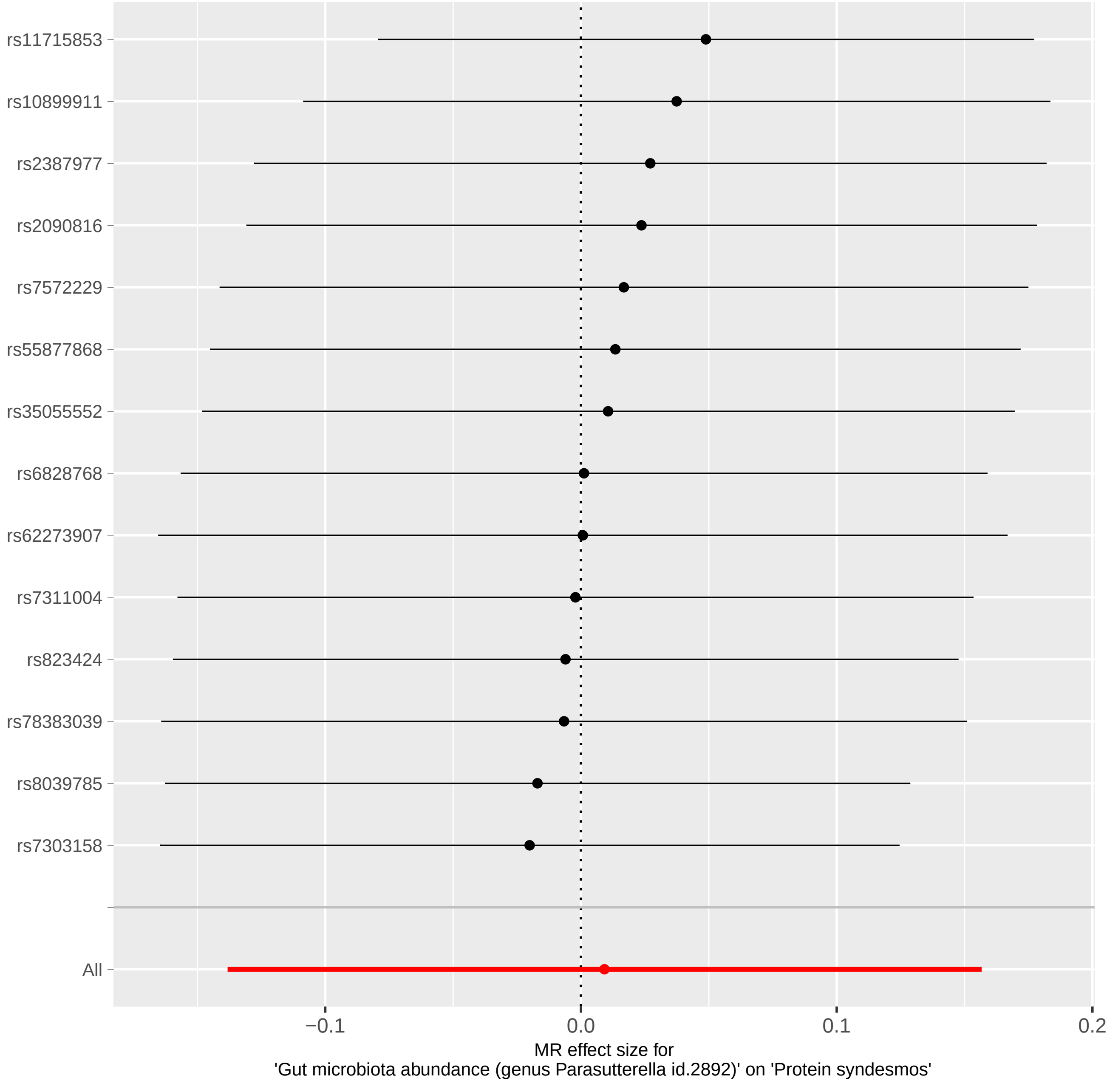

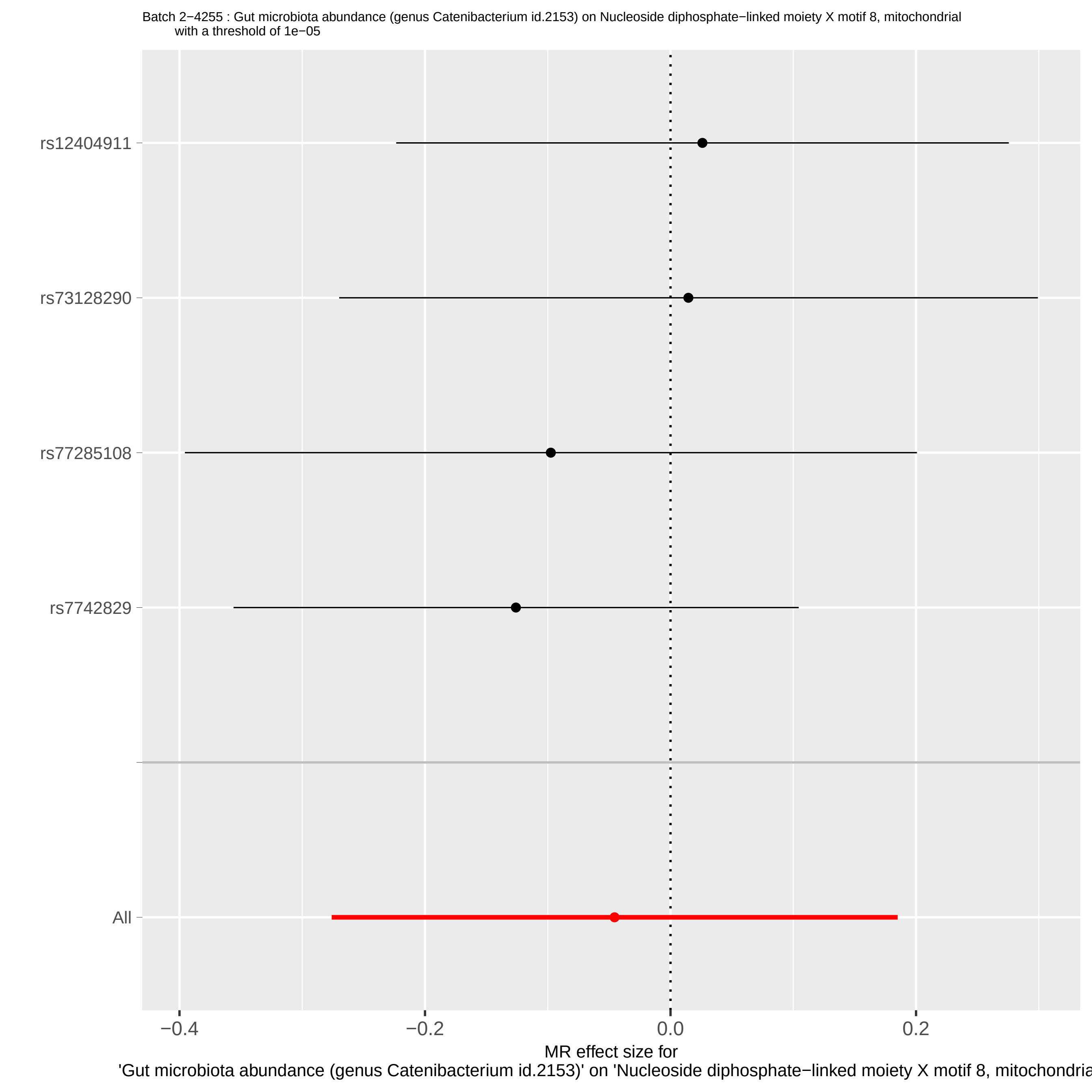
Batch 2−4255 : Gut microbiota abundance (genus Catenibacterium id.2153) on Nucleoside diphosphate−linked moiety X motif 8, mitochondrial
with a threshold of 1e−05
rs12404911
rs73128290
rs77285108
rs7742829
All
−0.4
−0.2
0.0
0.2
MR effect size for
'Gut microbiota abundance (genus Catenibacterium id.2153)' on 'Nucleoside diphosphate−linked moiety X motif 8, mitochondria

Batch 2−4256 : Gut microbiota abundance (genus Parasutterella id.2892) on Nucleoside diphosphate−linked moiety X motif 8, mitochondrial
with a threshold of 1e−05

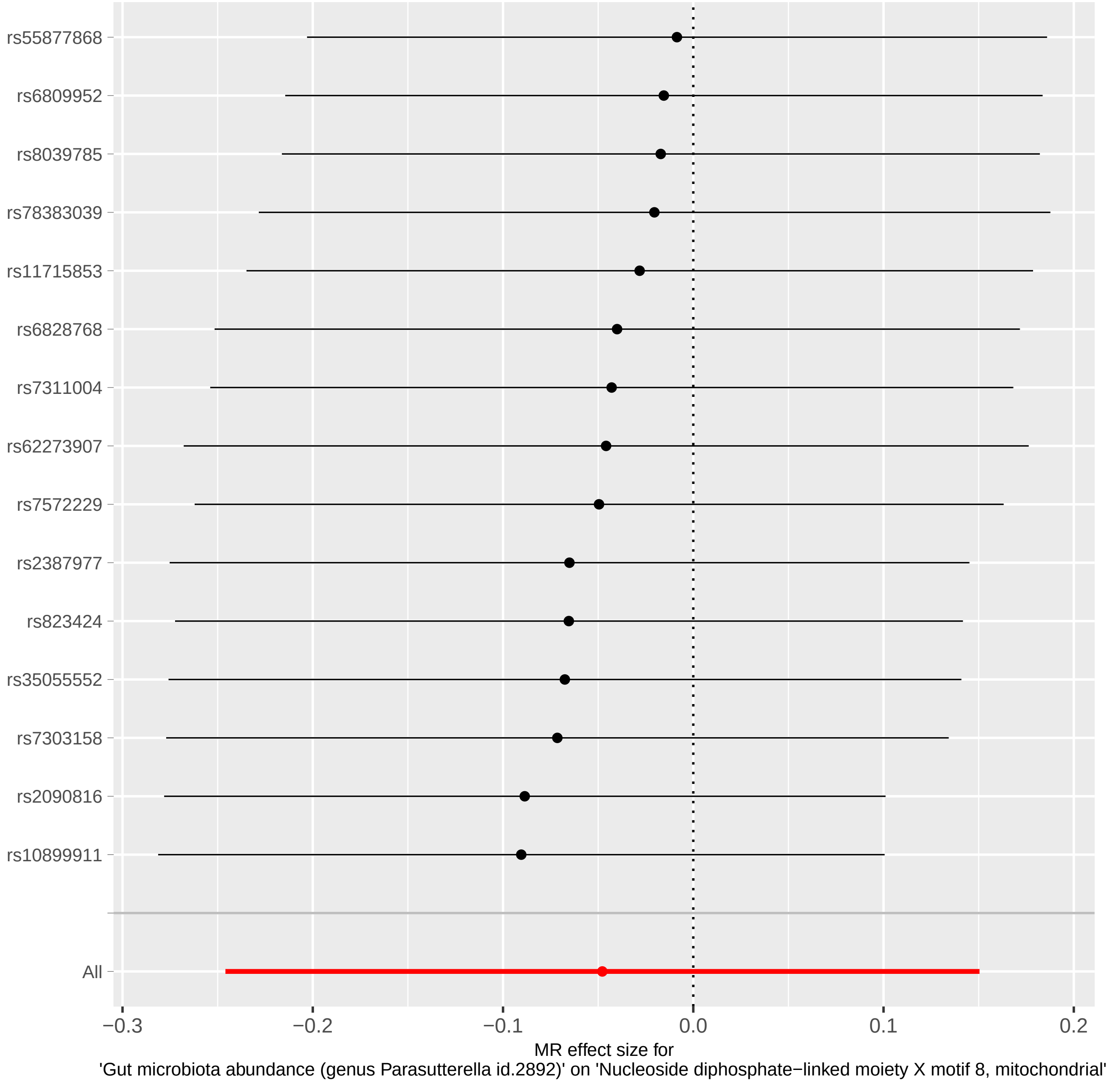

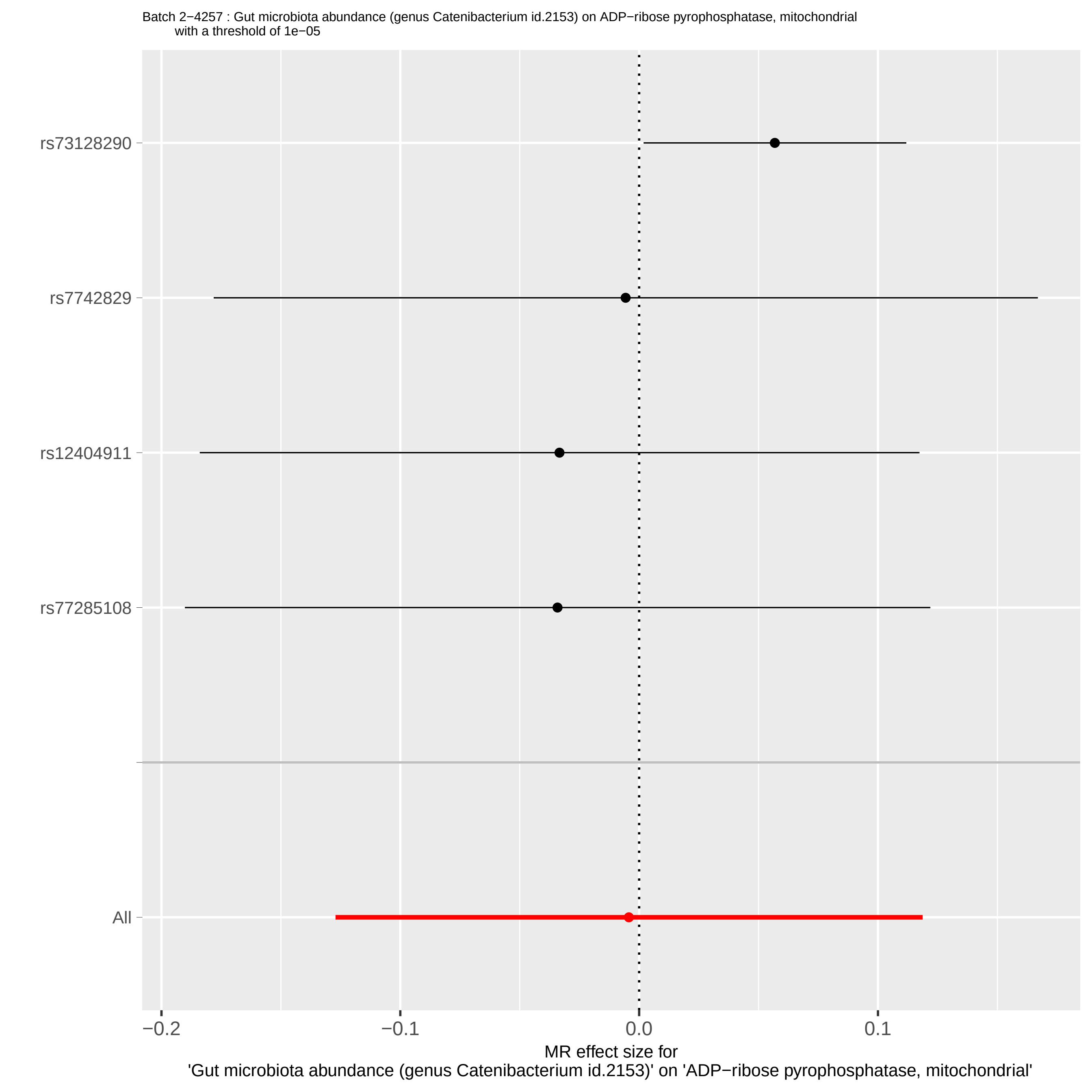

Batch 2−4257 : Gut microbiota abundance (genus Catenibacterium id.2153) on ADP−ribose pyrophosphatase, mitochondrial
with a threshold of 1e−05
rs73128290
rs7742829
rs12404911
rs77285108
All
−0.2
−0.1
0.0
0.1
MR effect size for
'Gut microbiota abundance (genus Catenibacterium id.2153)' on 'ADP−ribose pyrophosphatase, mitochondrial'

Batch 2−4258 : Gut microbiota abundance (genus Parasutterella id.2892) on ADP−ribose pyrophosphatase, mitochondrial
with a threshold of 1e−05

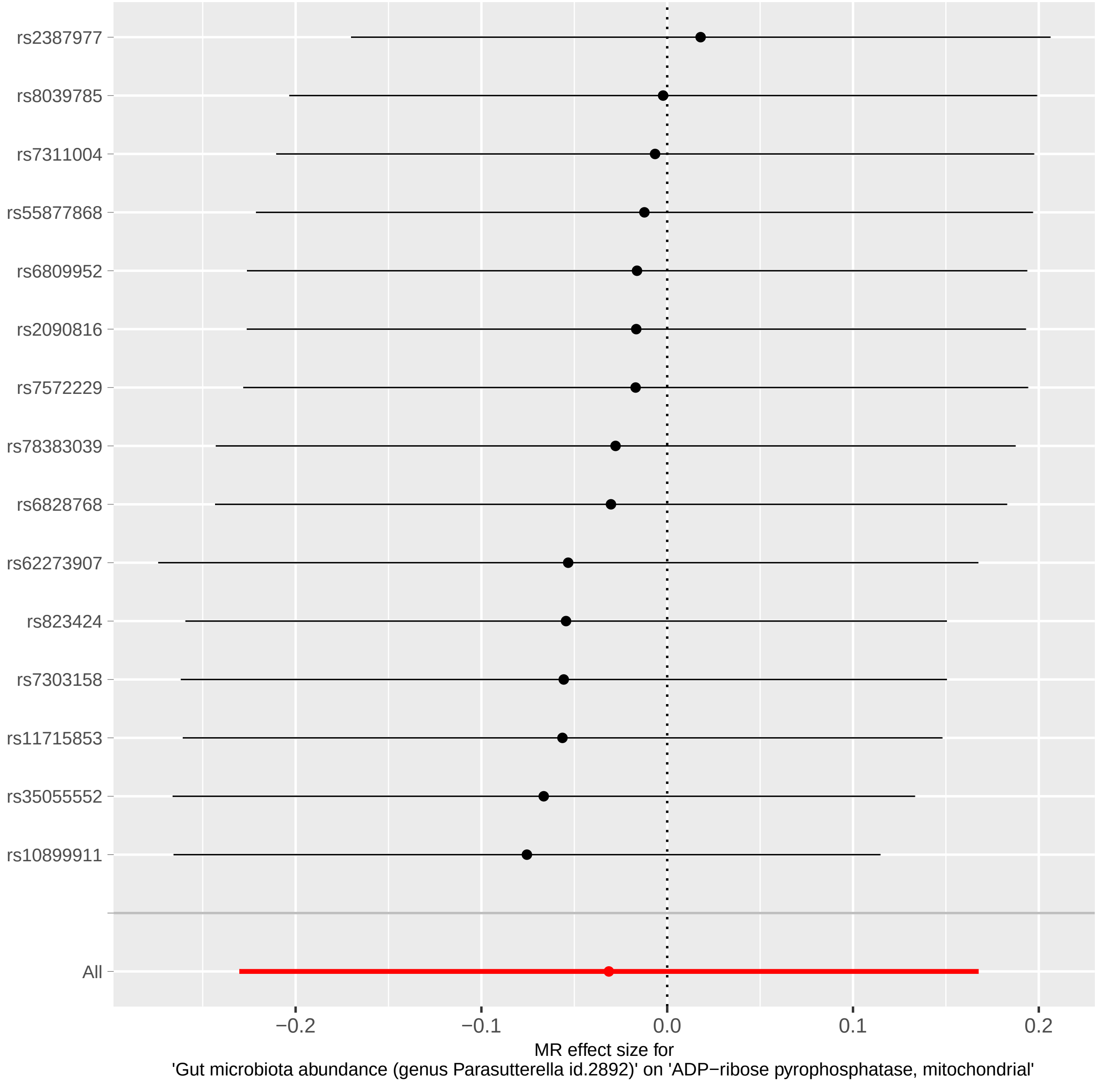

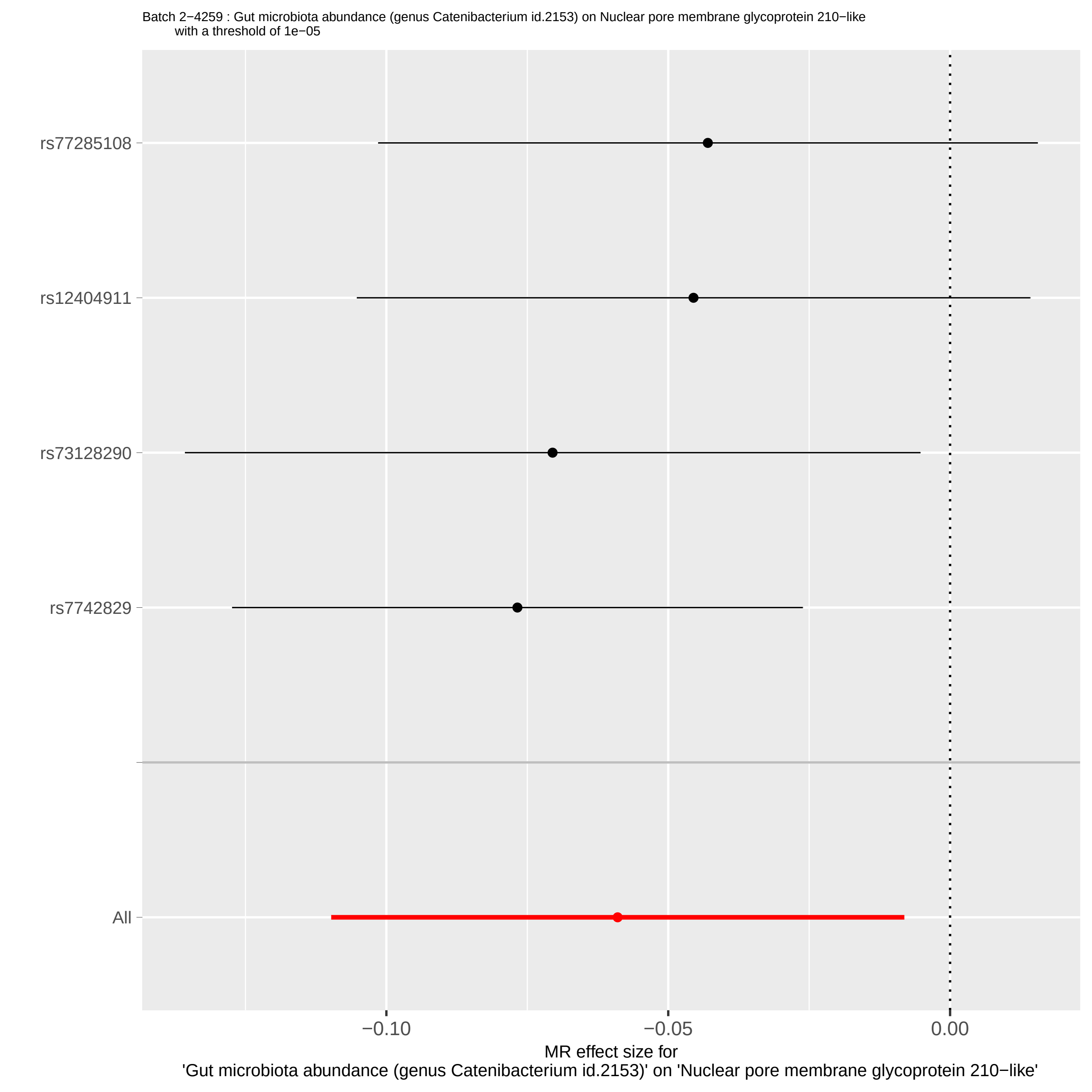

Batch 2−4259 : Gut microbiota abundance (genus Catenibacterium id.2153) on Nuclear pore membrane glycoprotein 210−like
with a threshold of 1e−05
rs77285108
rs12404911
rs73128290
rs7742829
All
−0.10
−0.05
0.00
MR effect size for
'Gut microbiota abundance (genus Catenibacterium id.2153)' on 'Nuclear pore membrane glycoprotein 210−like'

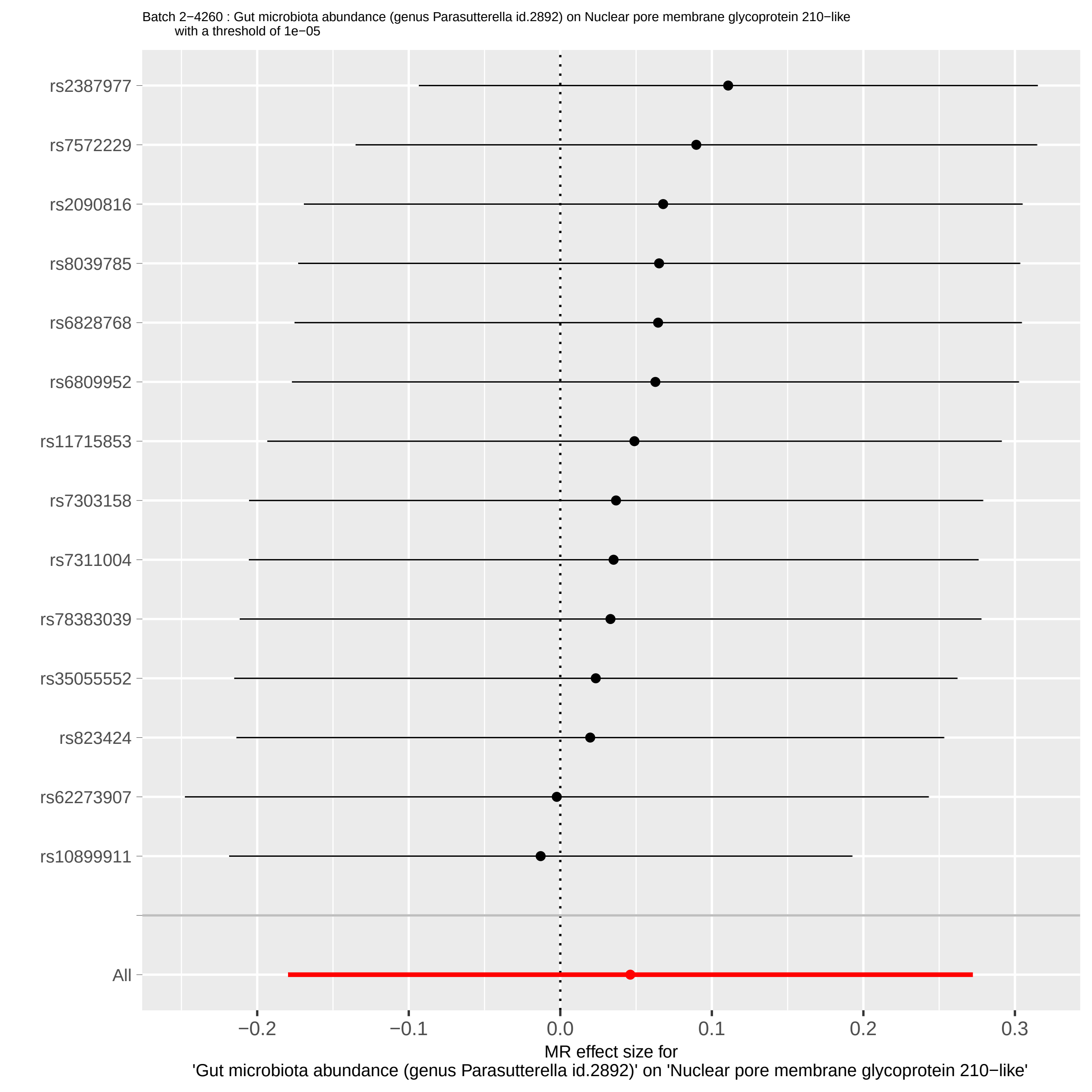
Batch 2−4260 : Gut microbiota abundance (genus Parasutterella id.2892) on Nuclear pore membrane glycoprotein 210−like
with a threshold of 1e−05
rs2387977
rs7572229
rs2090816
rs8039785
rs6828768
rs6809952
rs11715853
rs7303158
rs7311004
rs78383039
rs35055552
rs823424
rs62273907
rs10899911
All
−0.2
−0.1
0.0
0.1
0.2
0.3
MR effect size for
'Gut microbiota abundance (genus Parasutterella id.2892)' on 'Nuclear pore membrane glycoprotein 210−like'

Batch 2−4261 : Gut microbiota abundance (genus Catenibacterium id.2153) on Nuclear RNA export factor 1
with a threshold of 1e−05

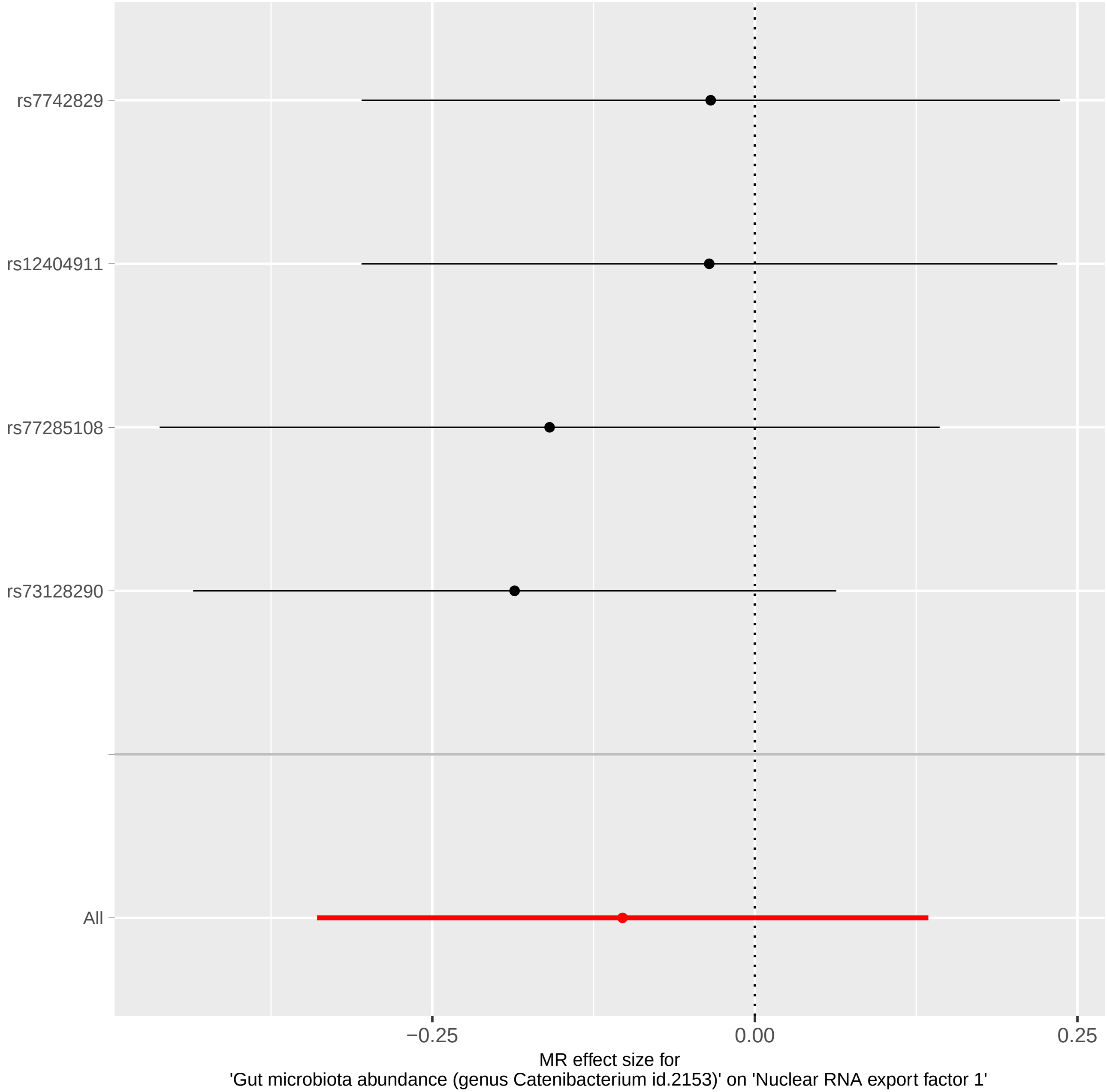

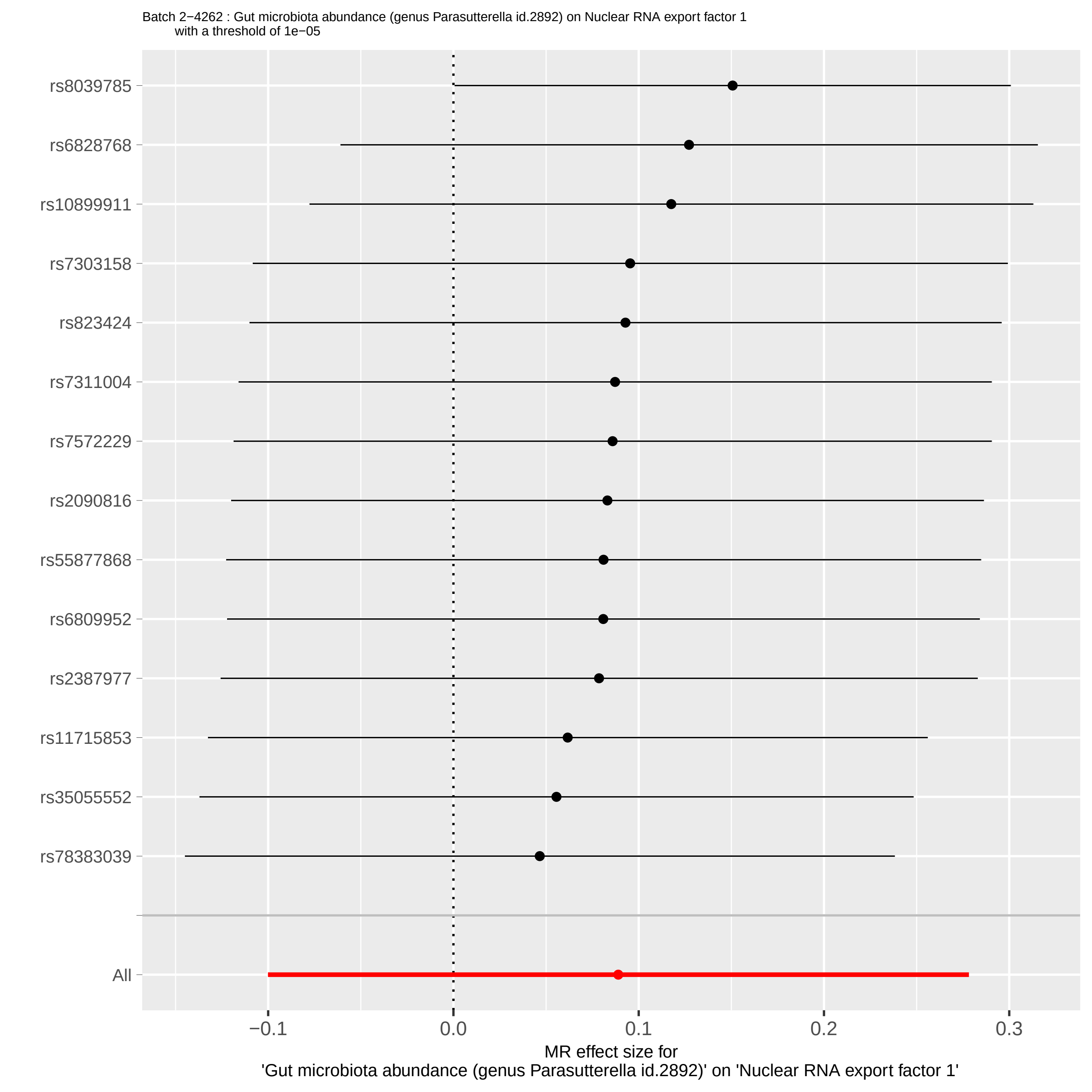

Batch 2−4262 : Gut microbiota abundance (genus Parasutterella id.2892) on Nuclear RNA export factor 1
with a threshold of 1e−05
rs8039785
rs6828768
rs10899911
rs7303158
rs823424
rs7311004
rs7572229
rs2090816
rs55877868
rs6809952
rs2387977
rs11715853
rs35055552
rs78383039
All
−0.1
0.0
0.1
0.2
0.3
MR effect size for
'Gut microbiota abundance (genus Parasutterella id.2892)' on 'Nuclear RNA export factor 1'

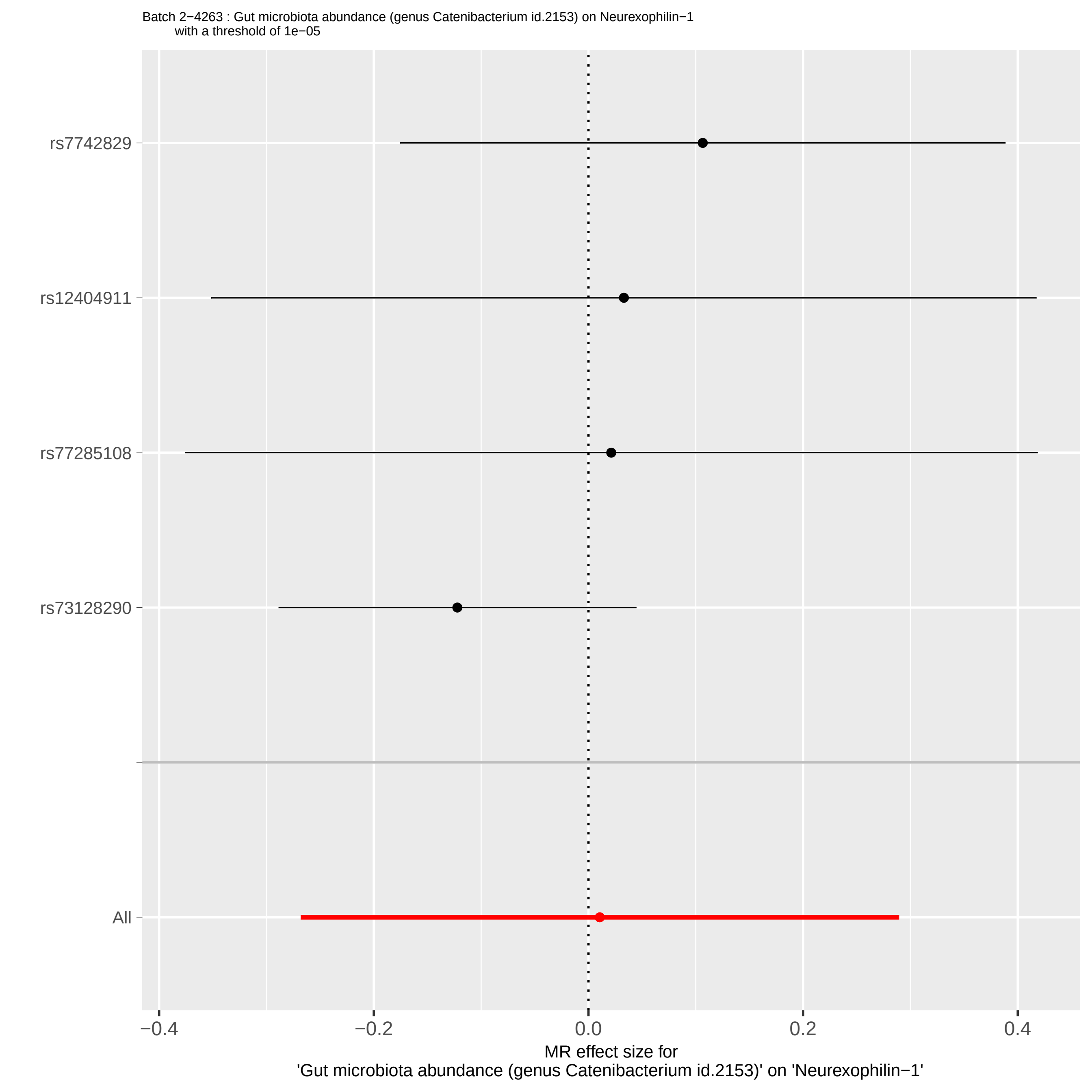

Batch 2−4263 : Gut microbiota abundance (genus Catenibacterium id.2153) on Neurexophilin−1
with a threshold of 1e−05
rs7742829
rs12404911
rs77285108
rs73128290
All
−0.4
−0.2
0.0
0.2
0.4
MR effect size for
'Gut microbiota abundance (genus Catenibacterium id.2153)' on 'Neurexophilin−1'

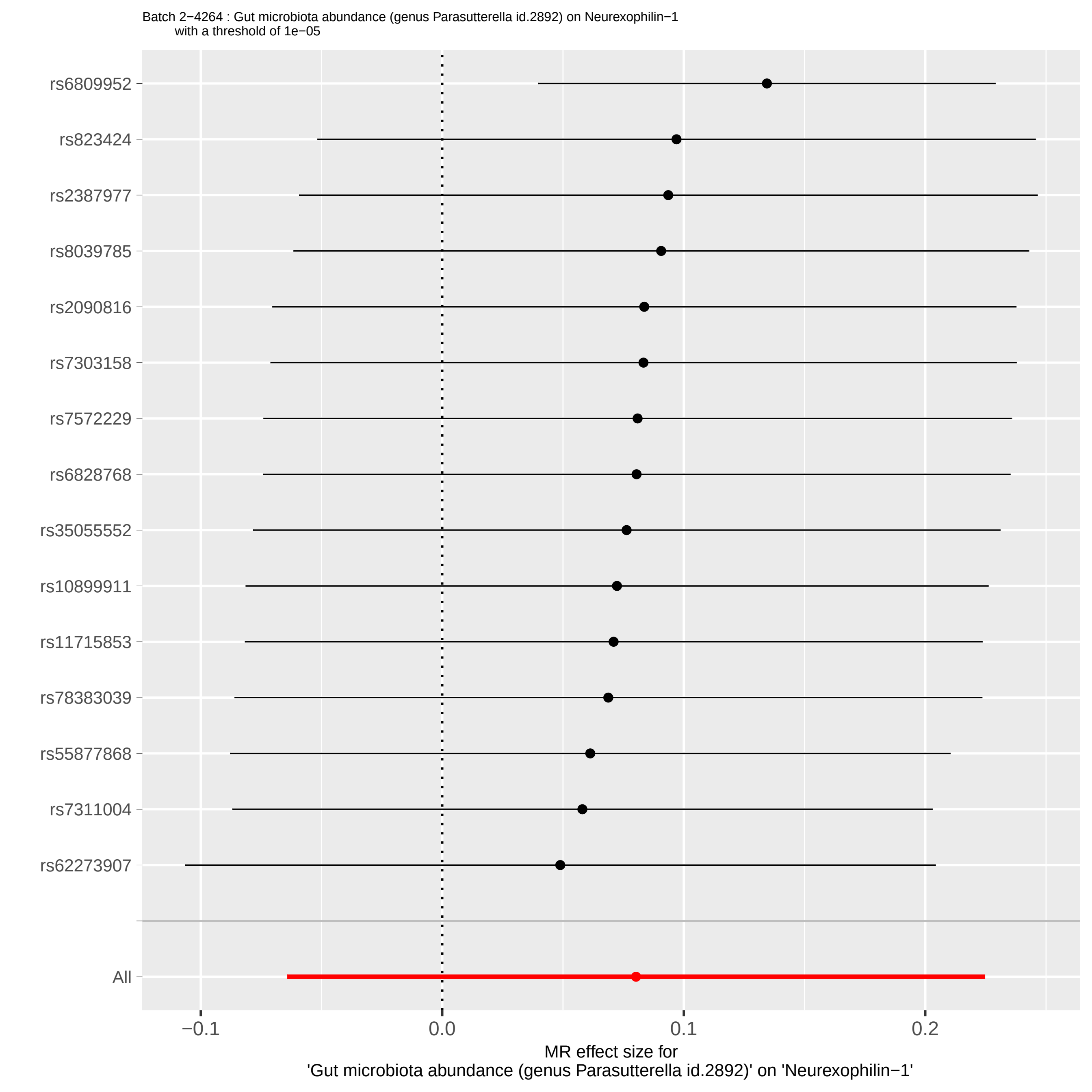

Batch 2−4264 : Gut microbiota abundance (genus Parasutterella id.2892) on Neurexophilin−1
with a threshold of 1e−05
rs6809952
rs823424
rs2387977
rs8039785
rs2090816
rs7303158
rs7572229
rs6828768
rs35055552
rs10899911
rs11715853
rs78383039
rs55877868
rs7311004
rs62273907
All
−0.1
0.0
0.1
0.2
MR effect size for
'Gut microbiota abundance (genus Parasutterella id.2892)' on 'Neurexophilin−1'

Batch 2−4265 : Gut microbiota abundance (genus Catenibacterium id.2153) on Neurexophilin−3
with a threshold of 1e−05

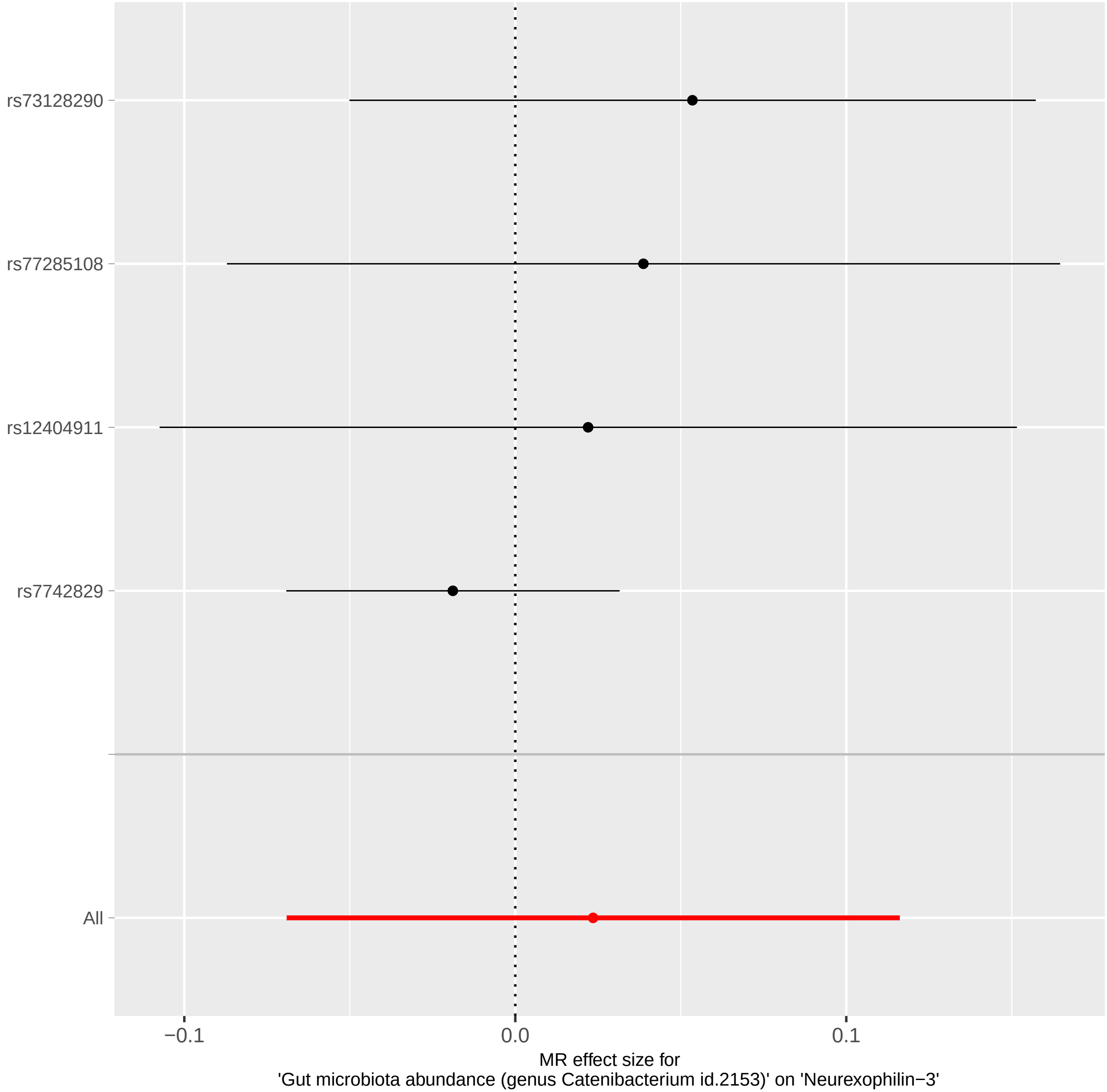

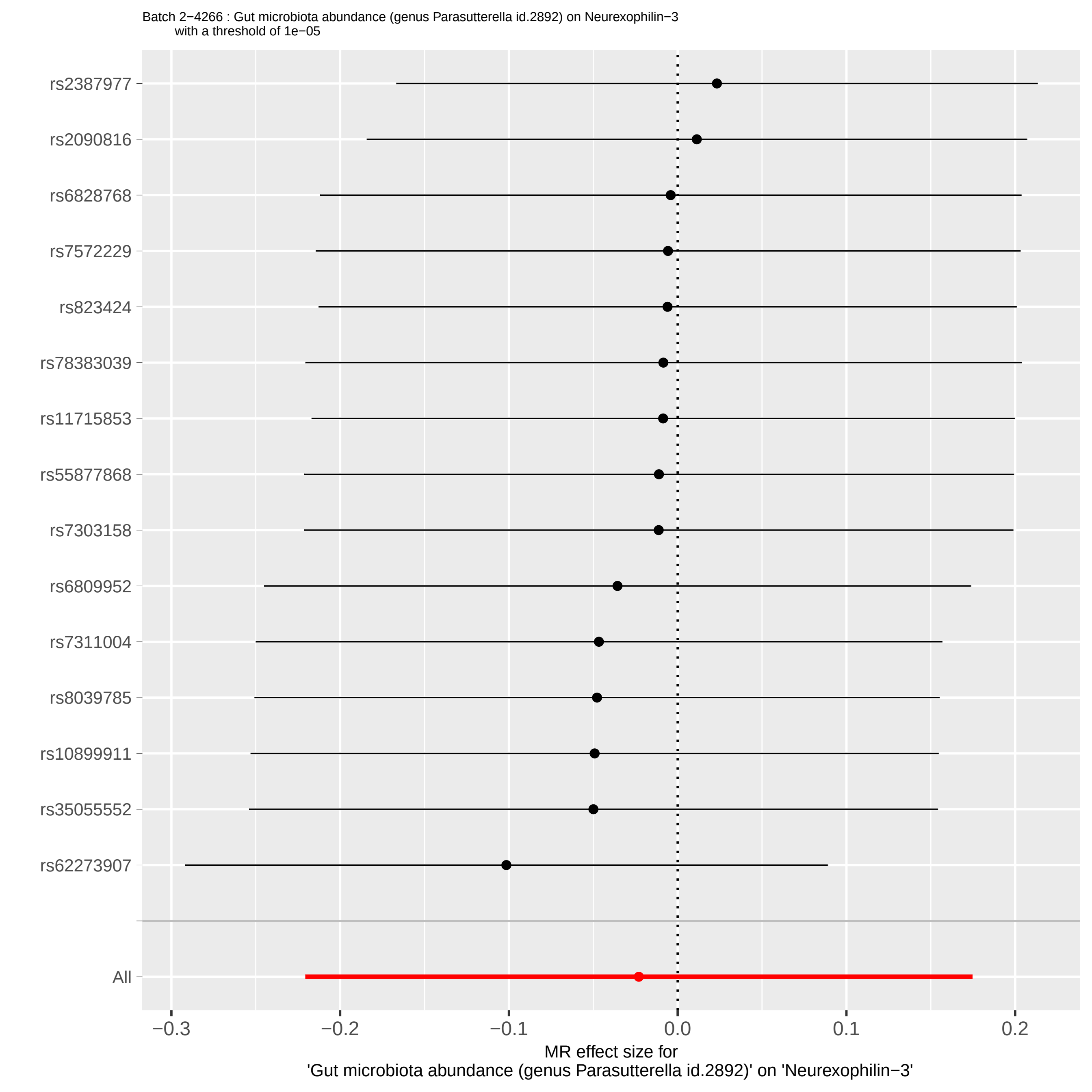
Batch 2−4266 : Gut microbiota abundance (genus Parasutterella id.2892) on Neurexophilin−3
with a threshold of 1e−05
rs2387977
rs2090816
rs6828768
rs7572229
rs823424
rs78383039
rs11715853
rs55877868
rs7303158
rs6809952
rs7311004
rs8039785
rs10899911
rs35055552
rs62273907
All
−0.3
−0.2
−0.1
0.0
0.1
0.2
MR effect size for
'Gut microbiota abundance (genus Parasutterella id.2892)' on 'Neurexophilin−3'

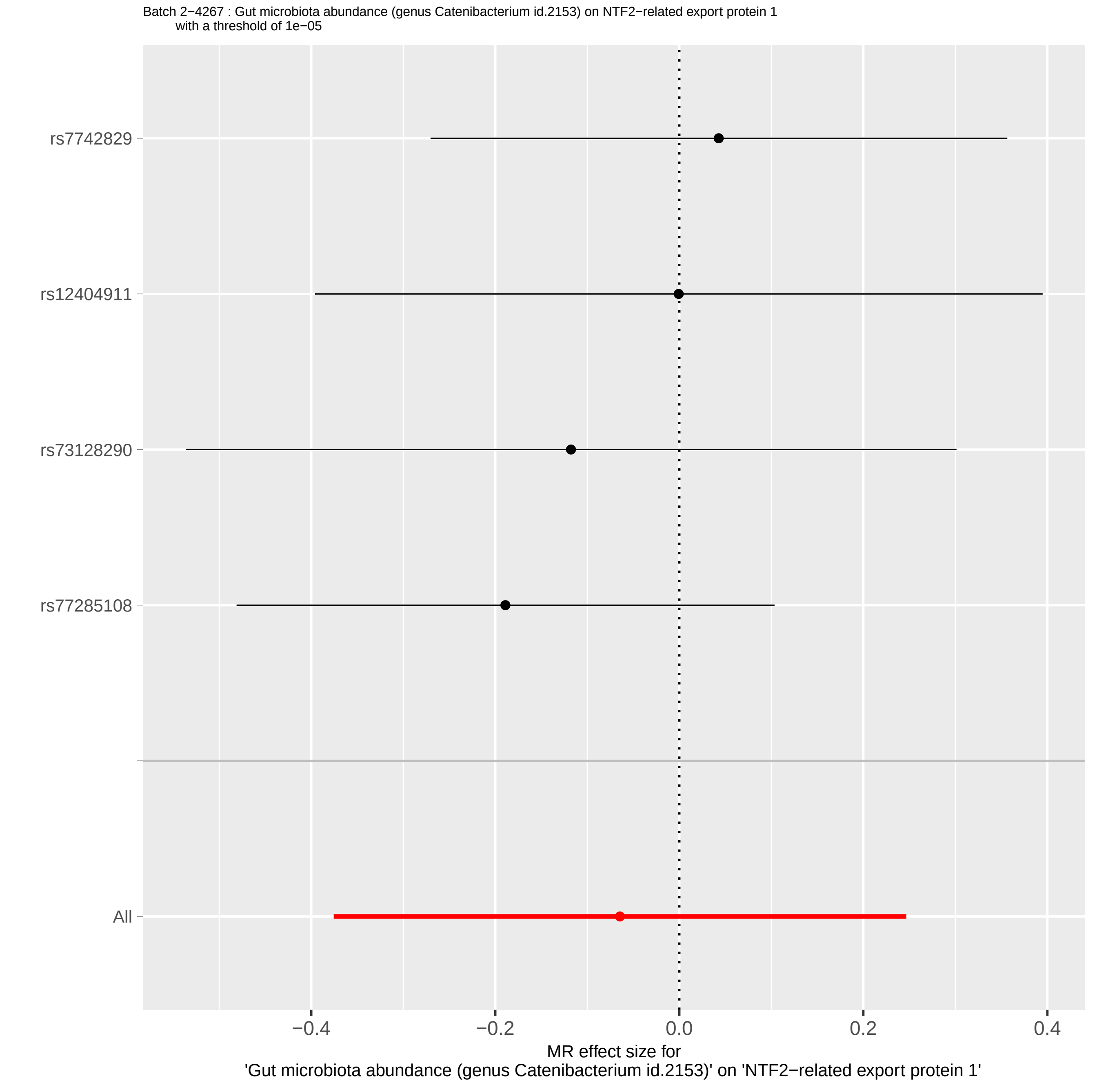
Batch 2−4267 : Gut microbiota abundance (genus Catenibacterium id.2153) on NTF2−related export protein 1
with a threshold of 1e−05
rs7742829
rs12404911
rs73128290
rs77285108
All
−0.4
−0.2
0.0
0.2
0.4
MR effect size for
'Gut microbiota abundance (genus Catenibacterium id.2153)' on 'NTF2−related export protein 1'

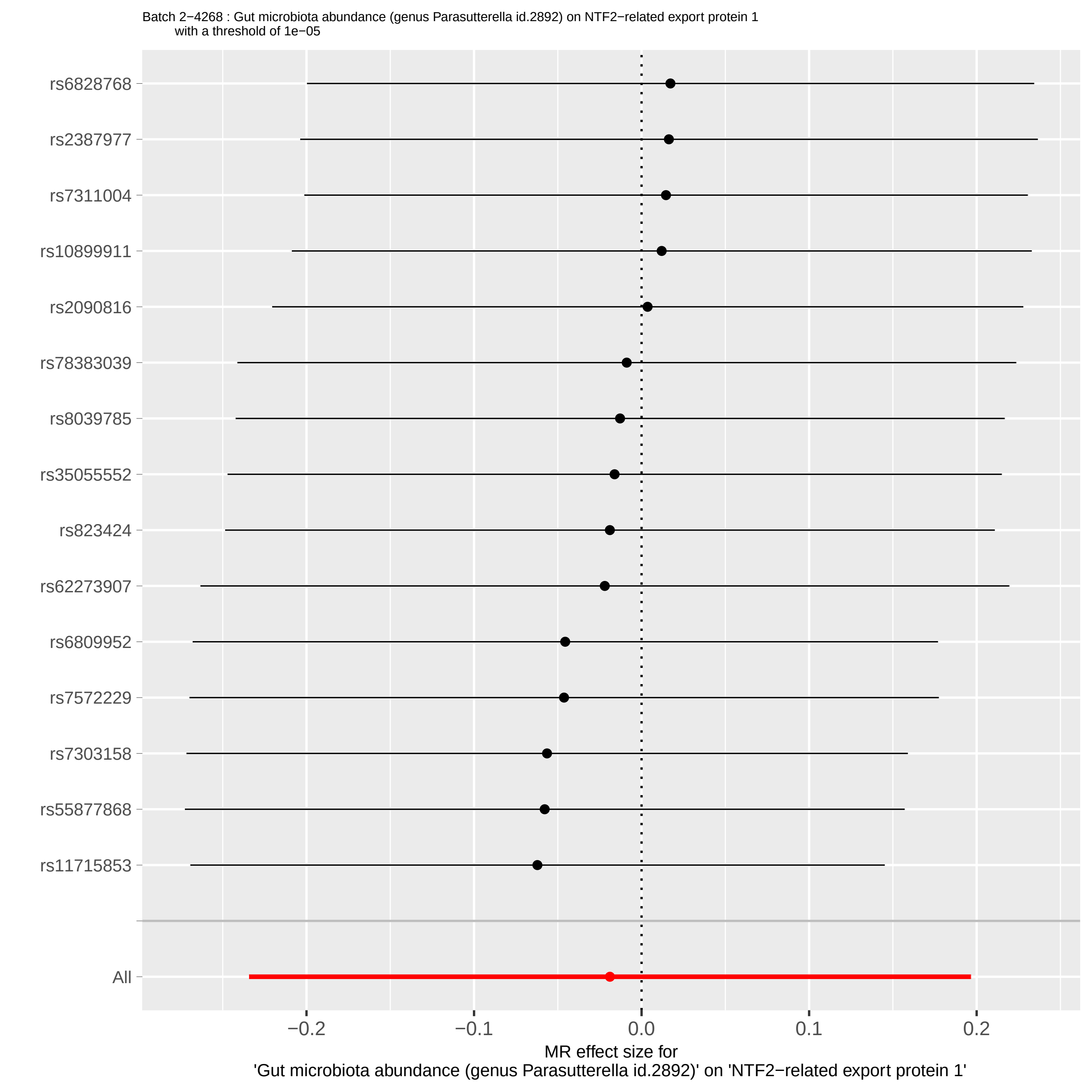
Batch 2−4268 : Gut microbiota abundance (genus Parasutterella id.2892) on NTF2−related export protein 1
with a threshold of 1e−05
rs6828768
rs2387977
rs7311004
rs10899911
rs2090816
rs78383039
rs8039785
rs35055552
rs823424
rs62273907
rs6809952
rs7572229
rs7303158
rs55877868
rs11715853
All
−0.2
−0.1
0.0
0.1
0.2
MR effect size for
'Gut microbiota abundance (genus Parasutterella id.2892)' on 'NTF2−related export protein 1'

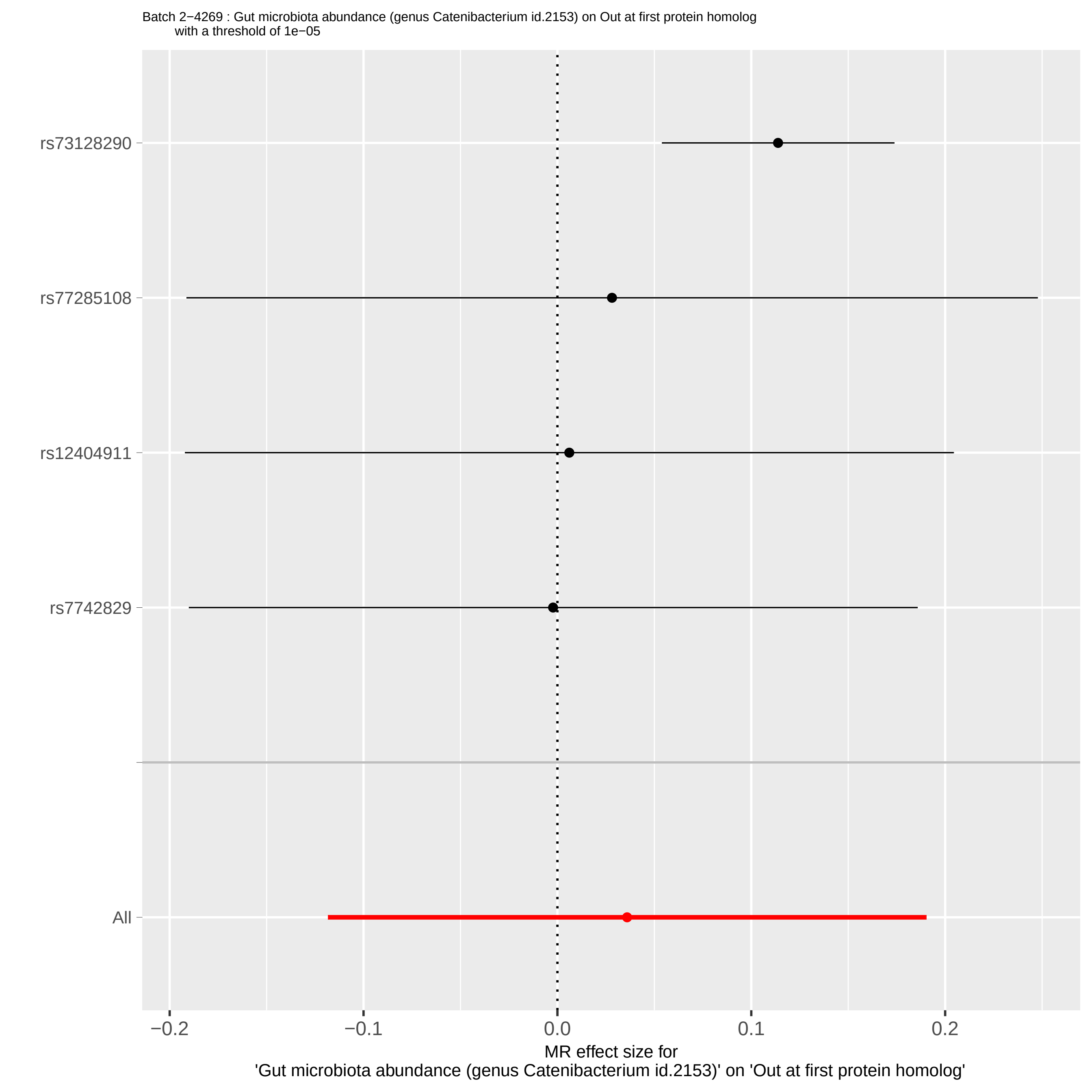
Batch 2−4269 : Gut microbiota abundance (genus Catenibacterium id.2153) on Out at first protein homolog
with a threshold of 1e−05
rs73128290
rs77285108
rs12404911
rs7742829
All
−0.2
−0.1
0.0
0.1
0.2
MR effect size for
'Gut microbiota abundance (genus Catenibacterium id.2153)' on 'Out at first protein homolog'

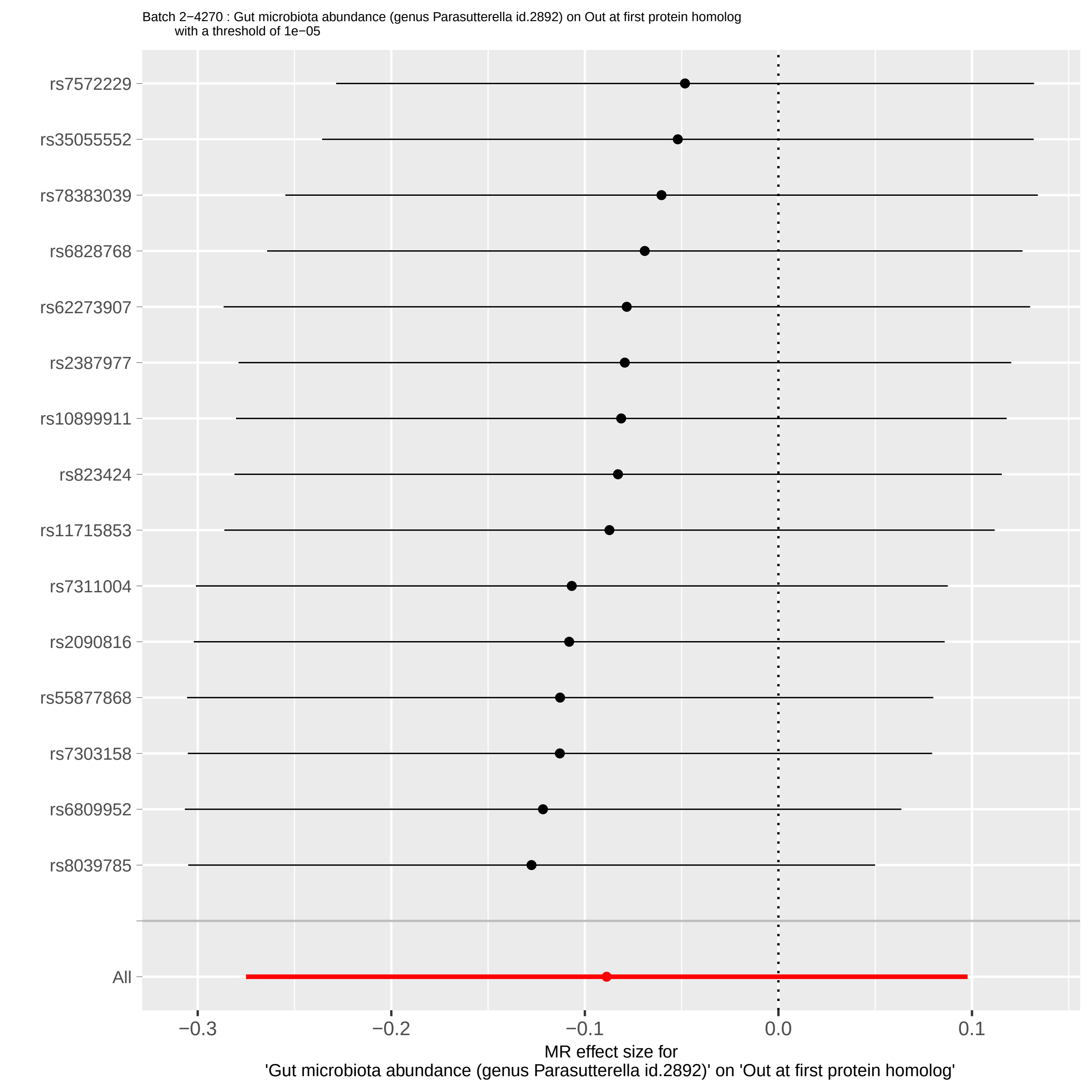

Batch 2−4270 : Gut microbiota abundance (genus Parasutterella id.2892) on Out at first protein homolog
with a threshold of 1e−05
rs7572229
rs35055552
rs78383039
rs6828768
rs62273907
rs2387977
rs10899911
rs823424
rs11715853
rs7311004
rs2090816
rs55877868
rs7303158
rs6809952
rs8039785
All
−0.3
−0.2
−0.1
0.0
0.1
MR effect size for
'Gut microbiota abundance (genus Parasutterella id.2892)' on 'Out at first protein homolog'

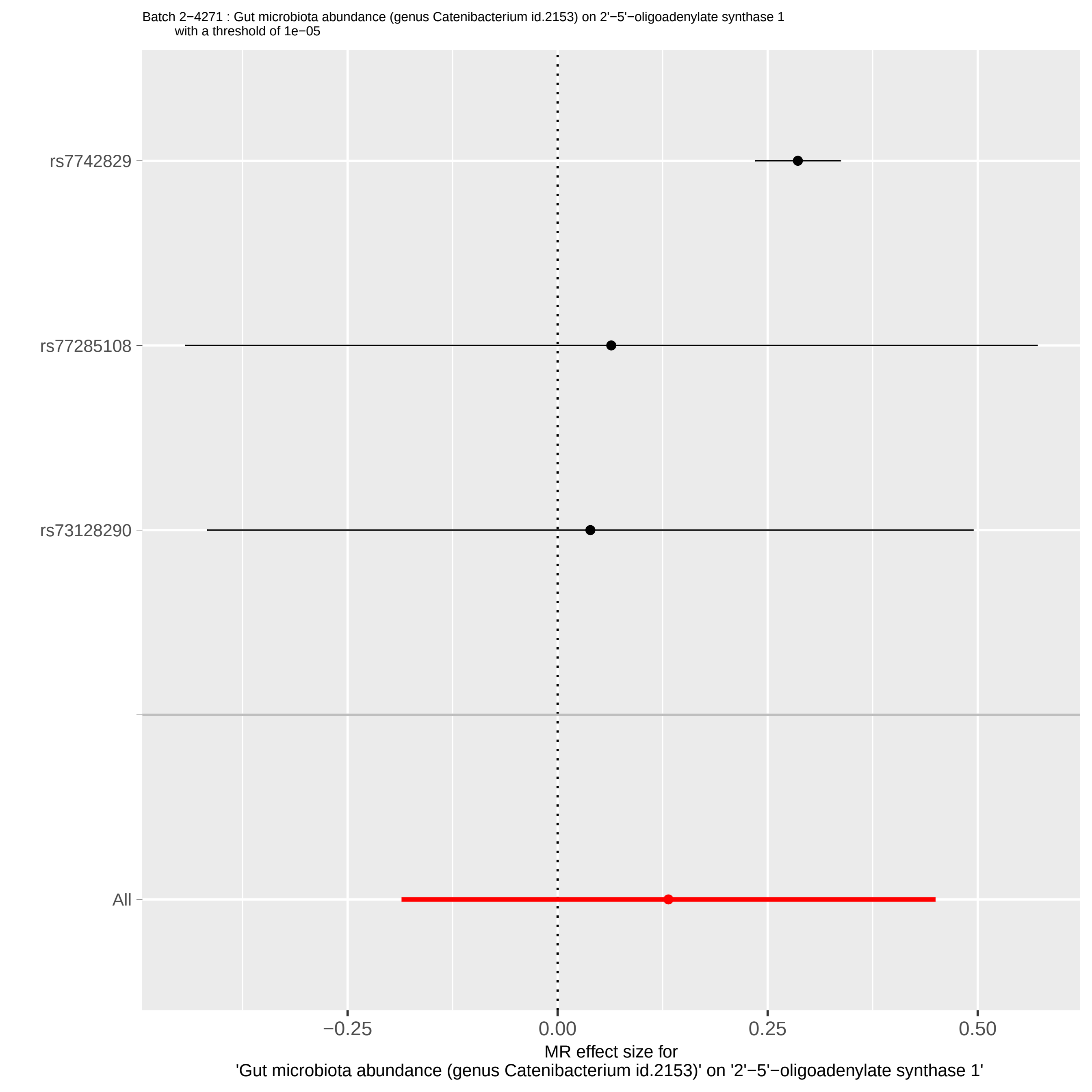

Batch 2−4271 : Gut microbiota abundance (genus Catenibacterium id.2153) on 2'−5'−oligoadenylate synthase 1
with a threshold of 1e−05
rs7742829
rs77285108
rs73128290
All
−0.25
0.00
0.25
0.50
MR effect size for
'Gut microbiota abundance (genus Catenibacterium id.2153)' on '2'−5'−oligoadenylate synthase 1'

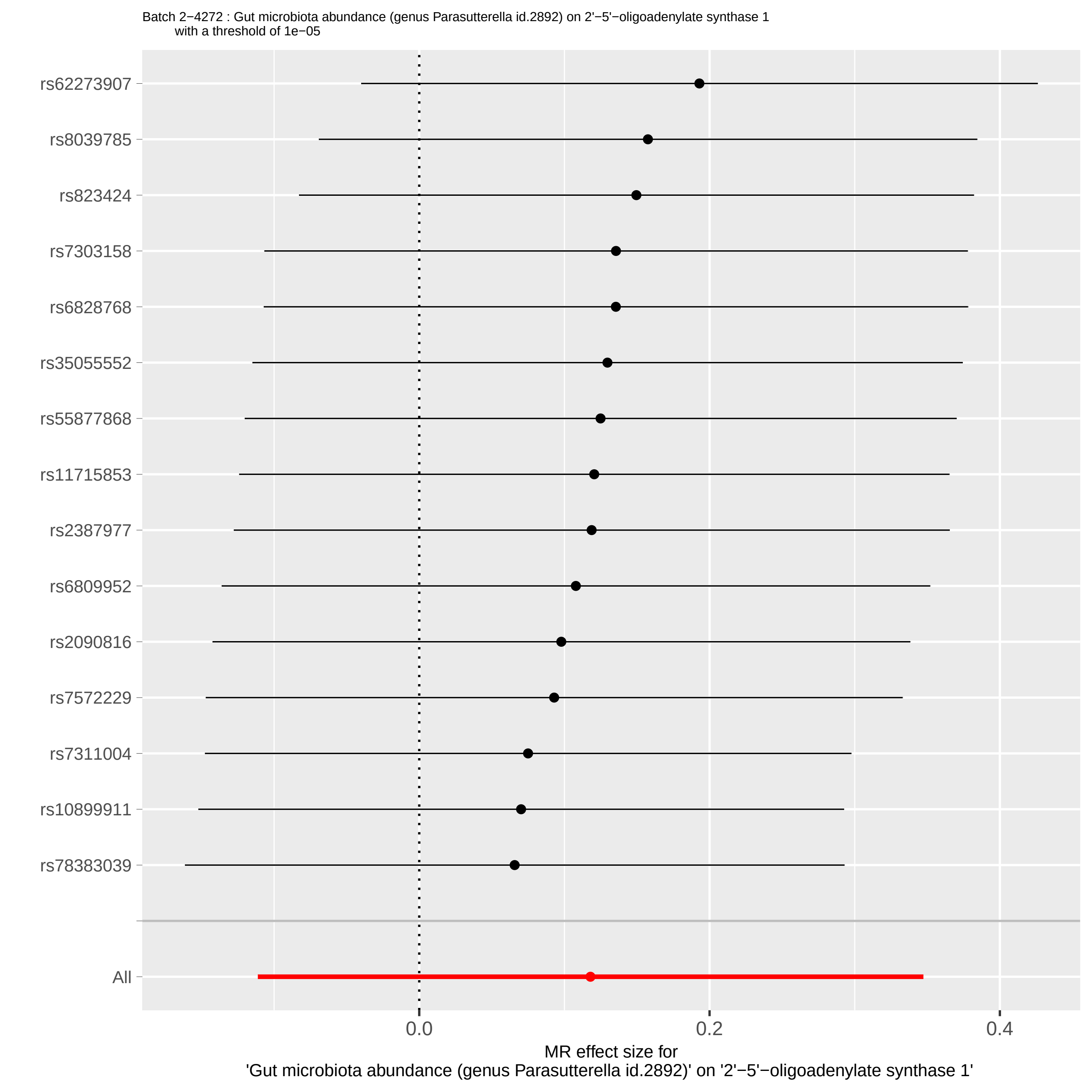
Batch 2−4272 : Gut microbiota abundance (genus Parasutterella id.2892) on 2'−5'−oligoadenylate synthase 1
with a threshold of 1e−05
rs62273907
rs8039785
rs823424
rs7303158
rs6828768
rs35055552
rs55877868
rs11715853
rs2387977
rs6809952
rs2090816
rs7572229
rs7311004
rs10899911
rs78383039
All
0.0
0.2
0.4
MR effect size for
'Gut microbiota abundance (genus Parasutterella id.2892)' on '2'−5'−oligoadenylate synthase 1'

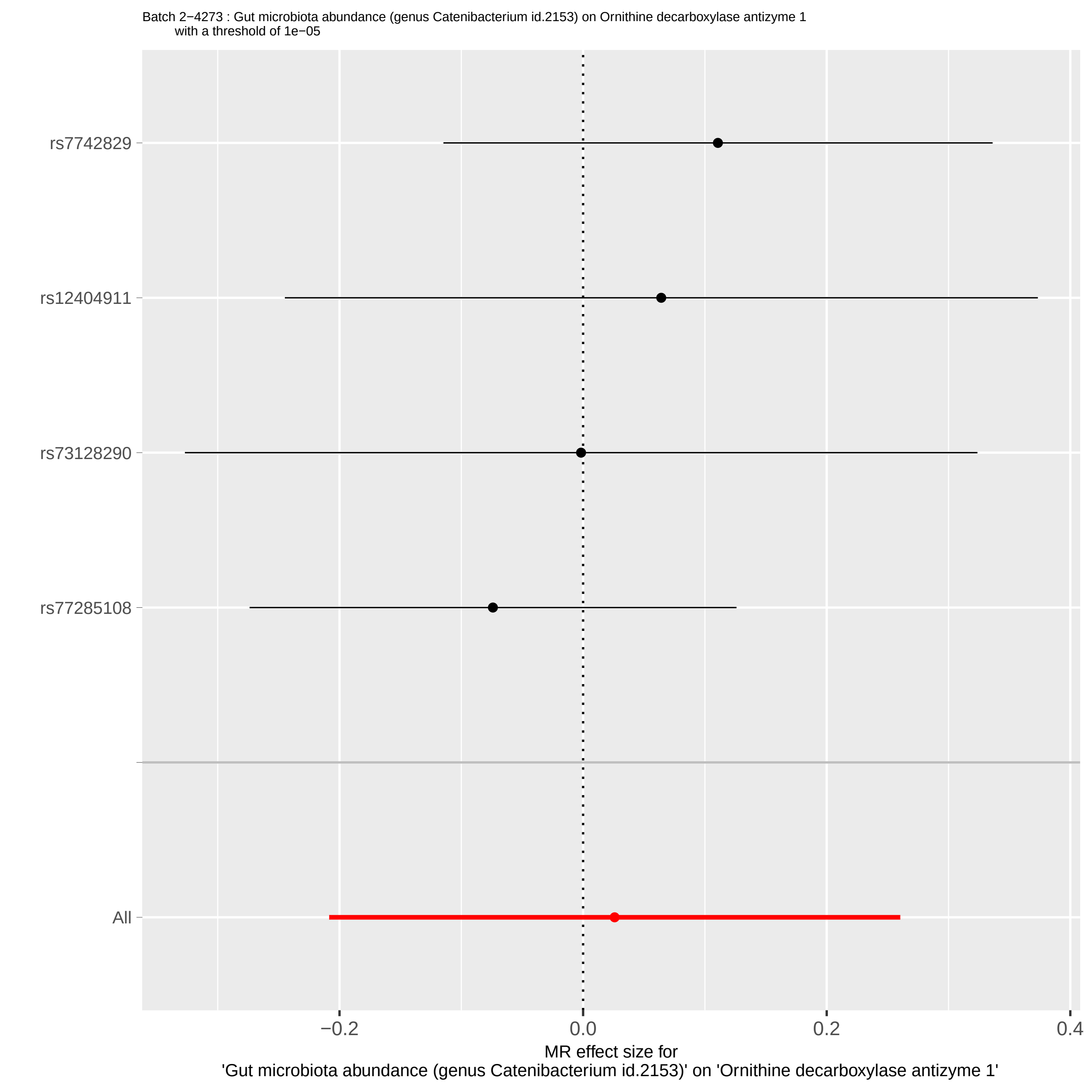
Batch 2−4273 : Gut microbiota abundance (genus Catenibacterium id.2153) on Ornithine decarboxylase antizyme 1
with a threshold of 1e−05
rs7742829
rs12404911
rs73128290
rs77285108
All
−0.2
0.0
0.2
0.4
MR effect size for
'Gut microbiota abundance (genus Catenibacterium id.2153)' on 'Ornithine decarboxylase antizyme 1'

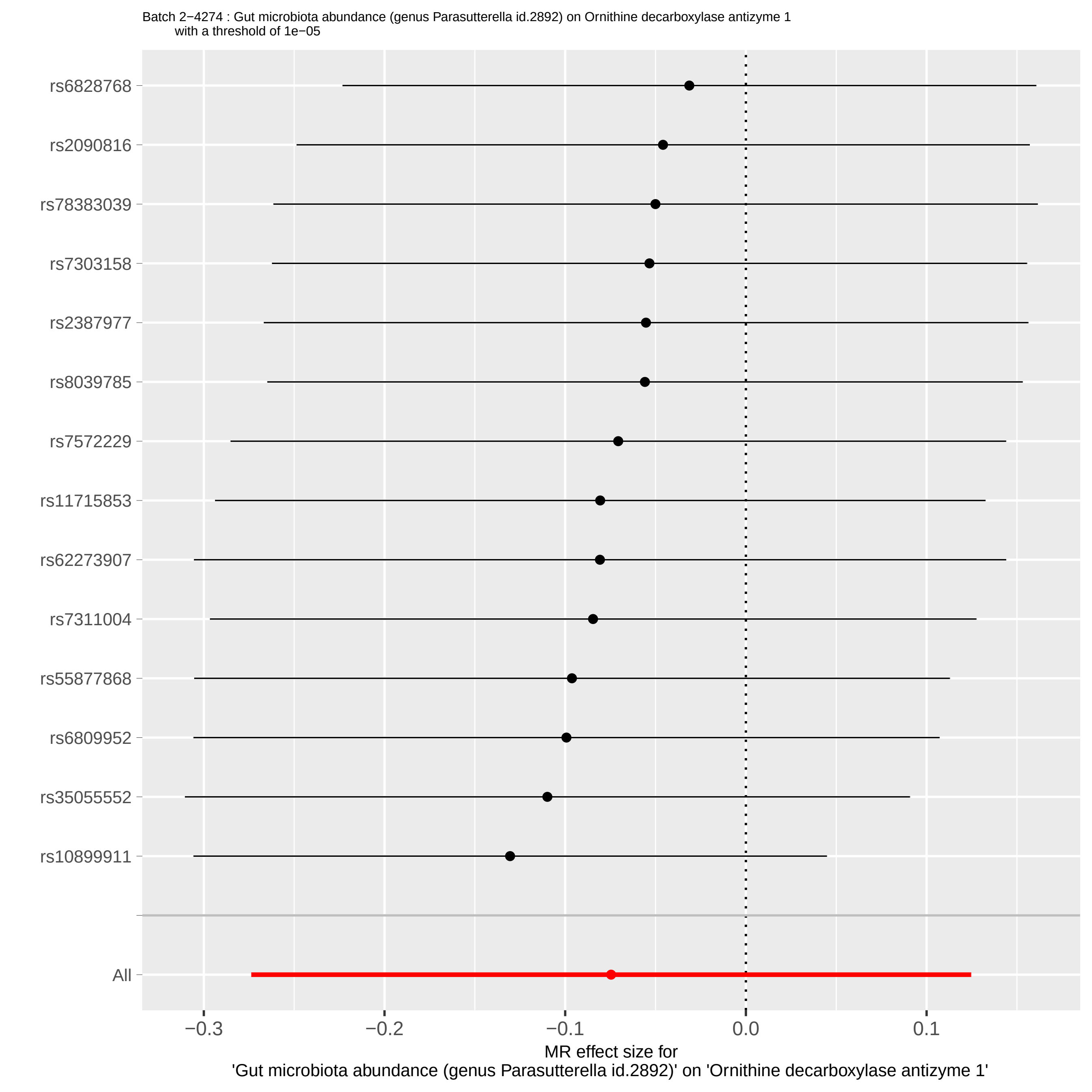
Batch 2−4274 : Gut microbiota abundance (genus Parasutterella id.2892) on Ornithine decarboxylase antizyme 1
with a threshold of 1e−05
rs6828768
rs2090816
rs78383039
rs7303158
rs2387977
rs8039785
rs7572229
rs11715853
rs62273907
rs7311004
rs55877868
rs6809952
rs35055552
rs10899911
All
−0.3
−0.2
−0.1
0.0
0.1
MR effect size for
'Gut microbiota abundance (genus Parasutterella id.2892)' on 'Ornithine decarboxylase antizyme 1'

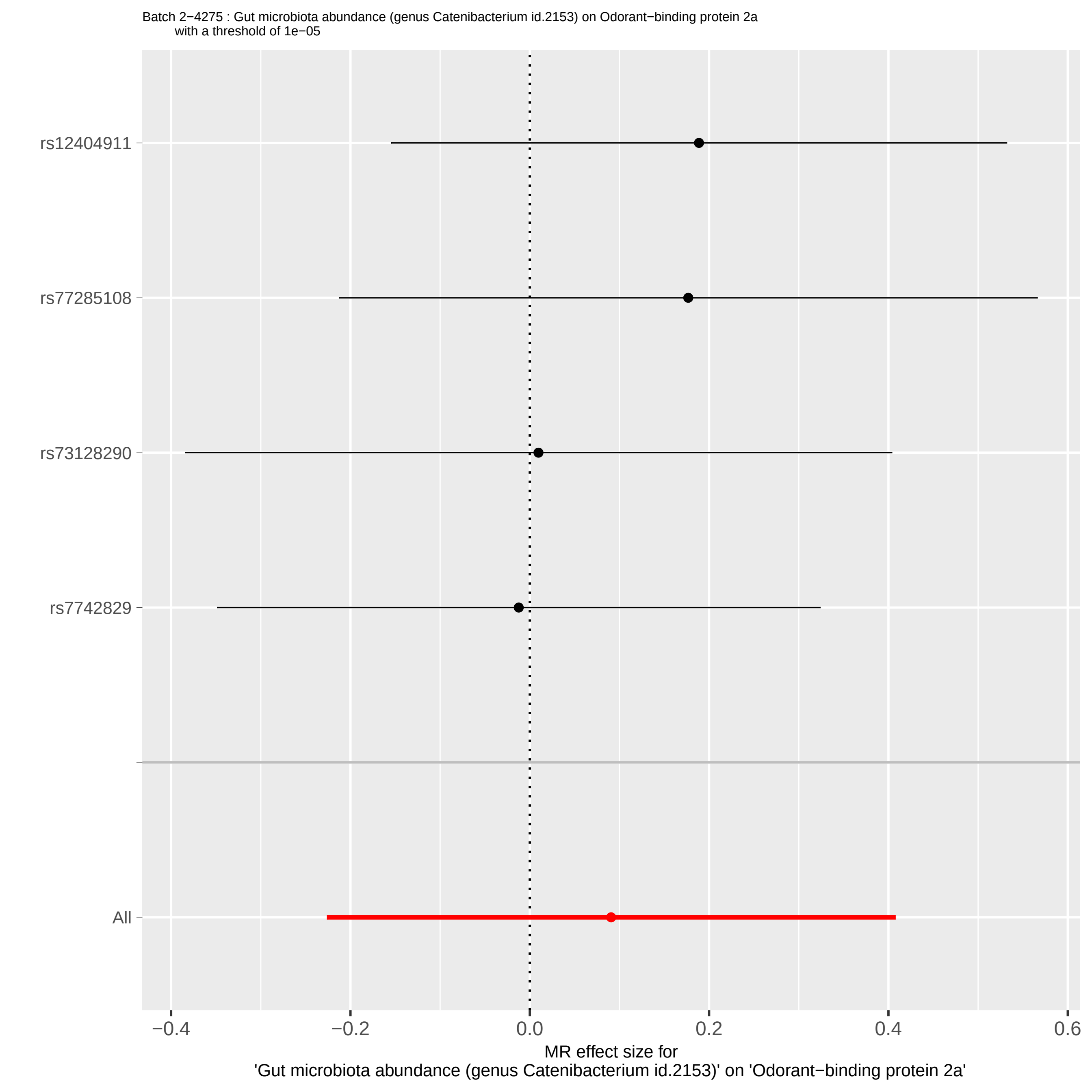

Batch 2−4275 : Gut microbiota abundance (genus Catenibacterium id.2153) on Odorant−binding protein 2a
with a threshold of 1e−05
rs12404911
rs77285108
rs73128290
rs7742829
All
−0.4
−0.2
0.0
0.2
0.4
0.6
MR effect size for
'Gut microbiota abundance (genus Catenibacterium id.2153)' on 'Odorant−binding protein 2a'

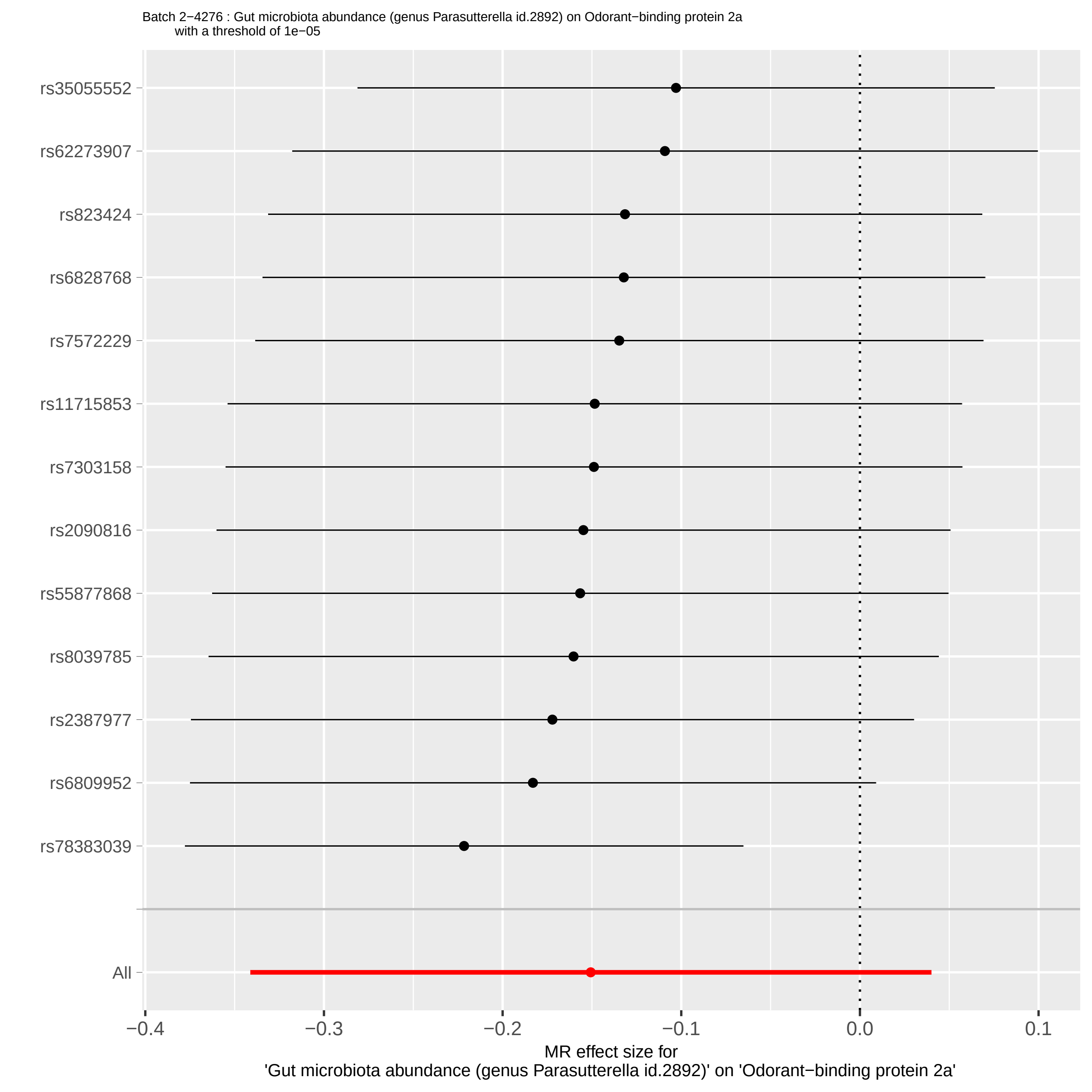
Batch 2−4276 : Gut microbiota abundance (genus Parasutterella id.2892) on Odorant−binding protein 2a
with a threshold of 1e−05
rs35055552
rs62273907
rs823424
rs6828768
rs7572229
rs11715853
rs7303158
rs2090816
rs55877868
rs8039785
rs2387977
rs6809952
rs78383039
All
−0.4
−0.3
−0.2
−0.1
0.0
0.1
MR effect size for
'Gut microbiota abundance (genus Parasutterella id.2892)' on 'Odorant−binding protein 2a'

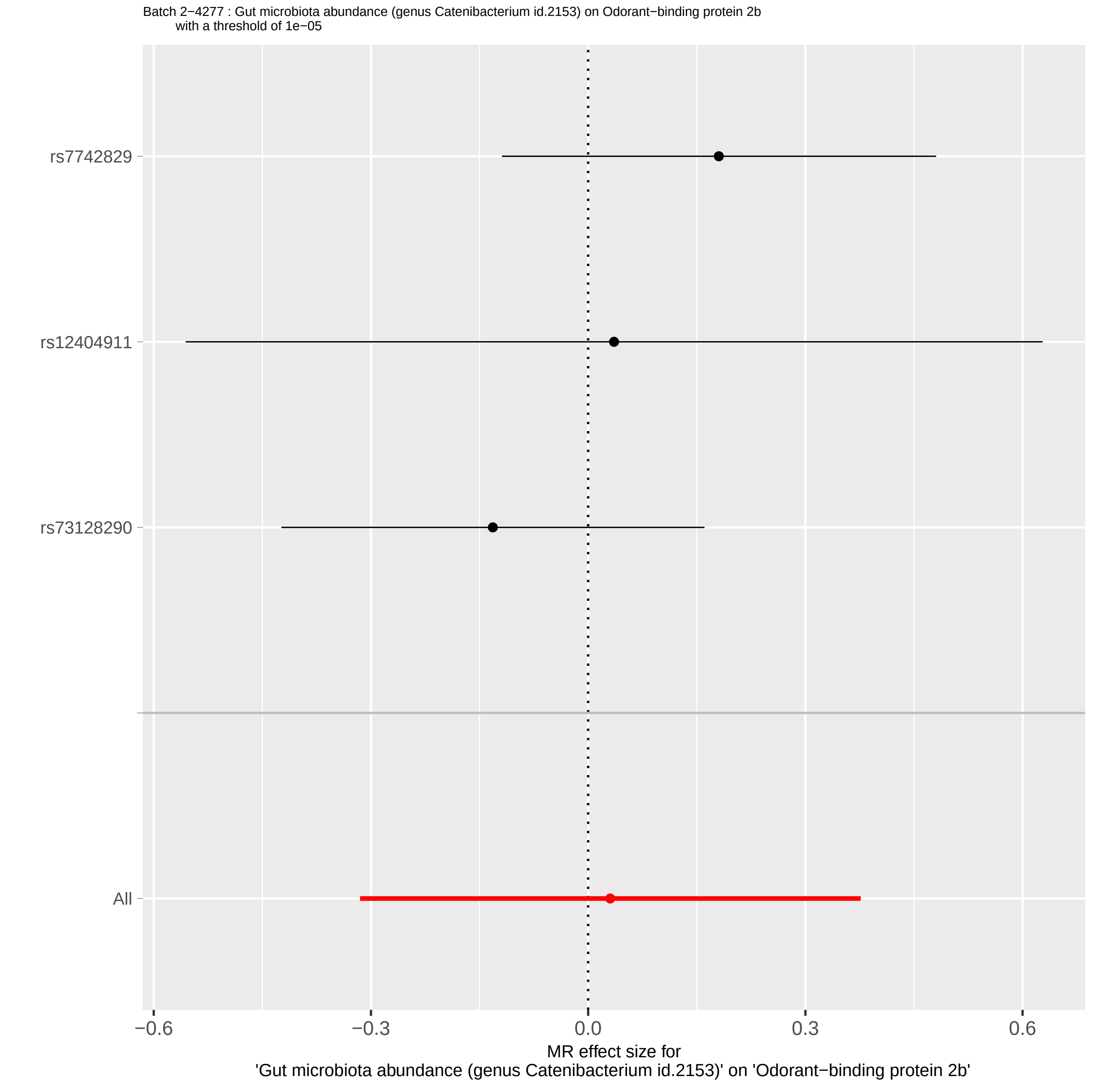

Batch 2−4277 : Gut microbiota abundance (genus Catenibacterium id.2153) on Odorant−binding protein 2b
with a threshold of 1e−05
rs7742829
rs12404911
rs73128290
All
−0.6
−0.3
0.0
0.3
0.6
MR effect size for
'Gut microbiota abundance (genus Catenibacterium id.2153)' on 'Odorant−binding protein 2b'

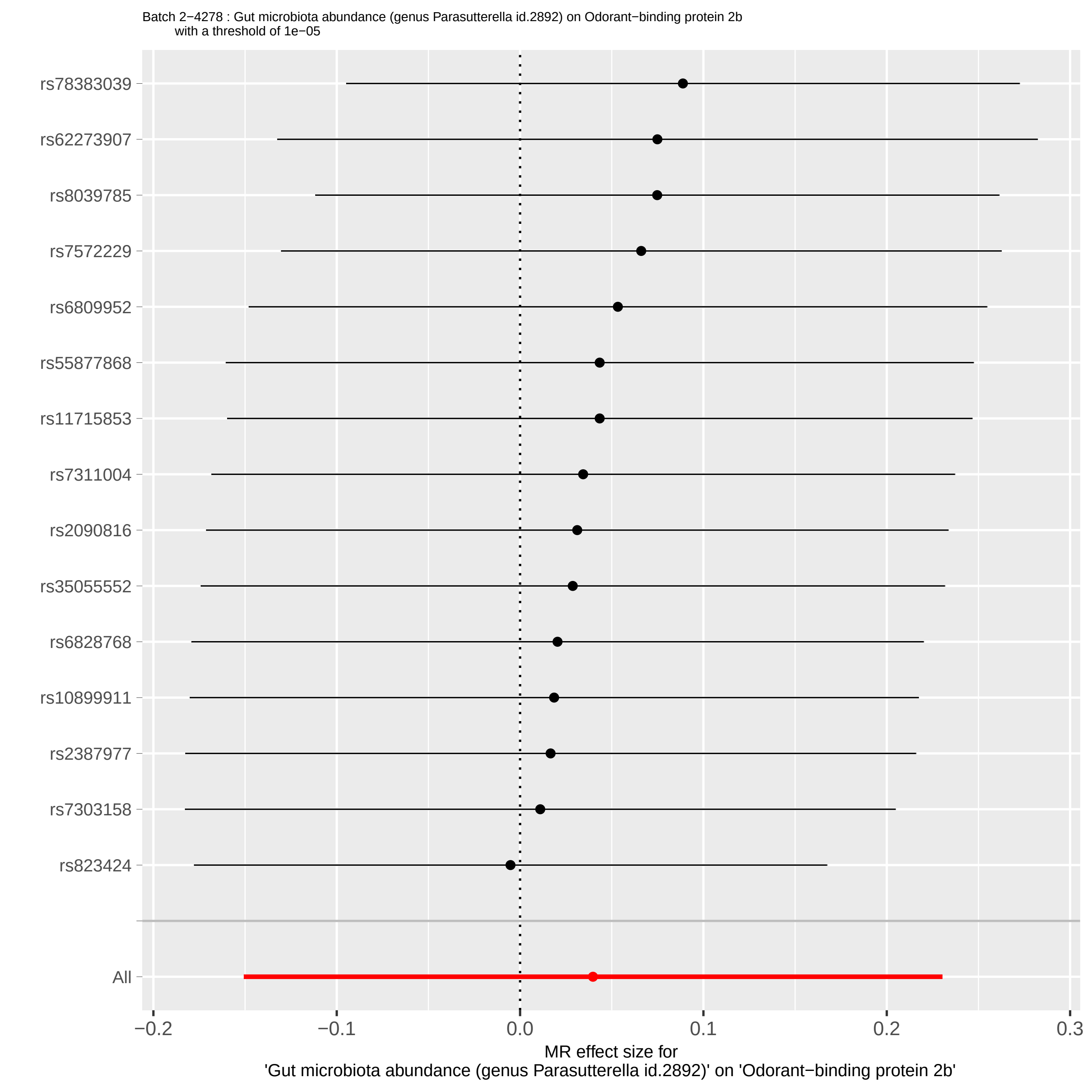

Batch 2−4278 : Gut microbiota abundance (genus Parasutterella id.2892) on Odorant−binding protein 2b
with a threshold of 1e−05
rs78383039
rs62273907
rs8039785
rs7572229
rs6809952
rs55877868
rs11715853
rs7311004
rs2090816
rs35055552
rs6828768
rs10899911
rs2387977
rs7303158
rs823424
All
−0.2
−0.1
0.0
0.1
0.2
0.3
MR effect size for
'Gut microbiota abundance (genus Parasutterella id.2892)' on 'Odorant−binding protein 2b'

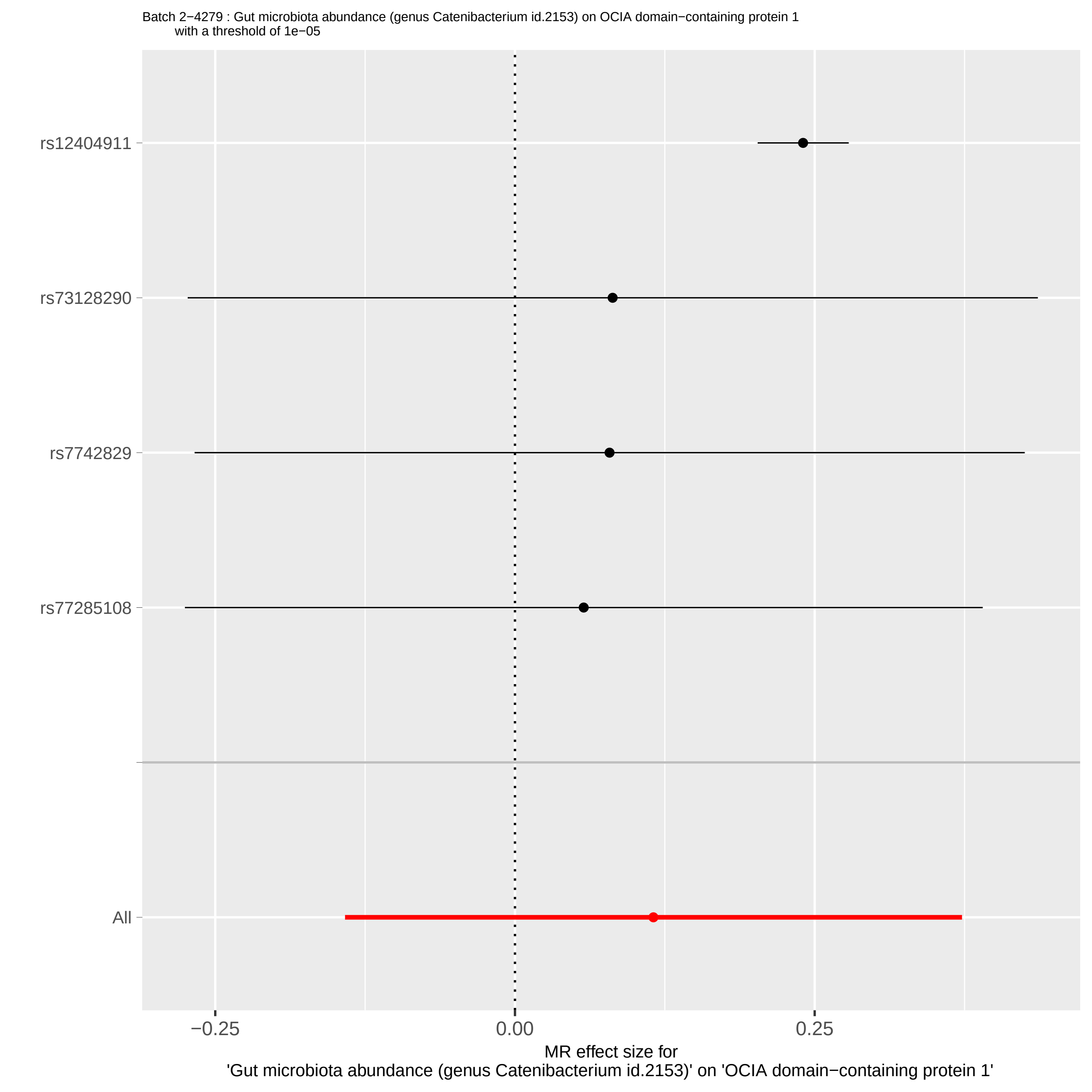

Batch 2−4279 : Gut microbiota abundance (genus Catenibacterium id.2153) on OCIA domain−containing protein 1
with a threshold of 1e−05
rs12404911
rs73128290
rs7742829
rs77285108
All
−0.25
0.00
0.25
MR effect size for
'Gut microbiota abundance (genus Catenibacterium id.2153)' on 'OCIA domain−containing protein 1'

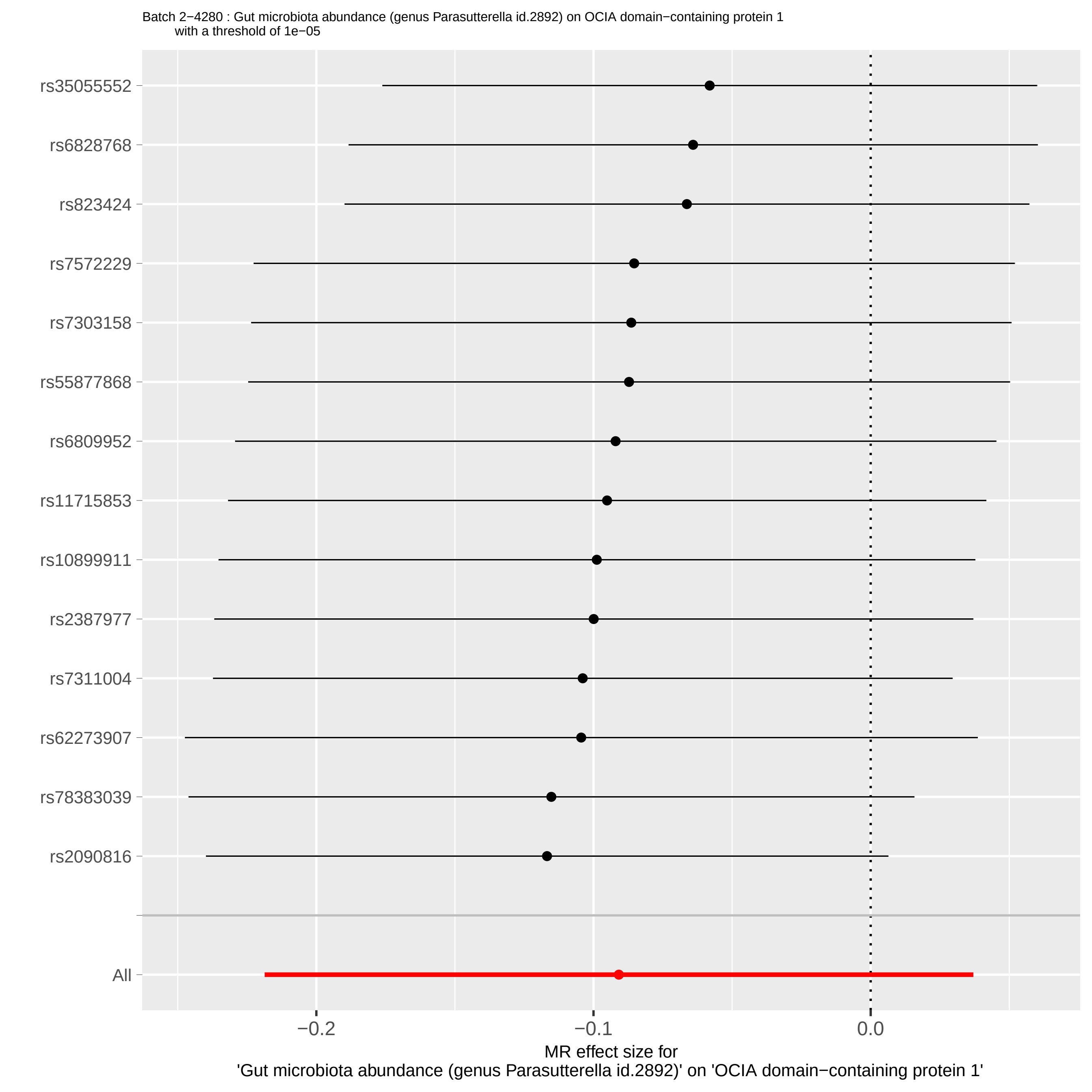

Batch 2−4280 : Gut microbiota abundance (genus Parasutterella id.2892) on OCIA domain−containing protein 1
with a threshold of 1e−05
rs35055552
rs6828768
rs823424
rs7572229
rs7303158
rs55877868
rs6809952
rs11715853
rs10899911
rs2387977
rs7311004
rs62273907
rs78383039
rs2090816
All
−0.2
−0.1
0.0
MR effect size for
'Gut microbiota abundance (genus Parasutterella id.2892)' on 'OCIA domain−containing protein 1'

Batch 2−4281 : Gut microbiota abundance (genus Catenibacterium id.2153) on Inositol polyphosphate 5−phosphatase OCRL−1
with a threshold of 1e−05

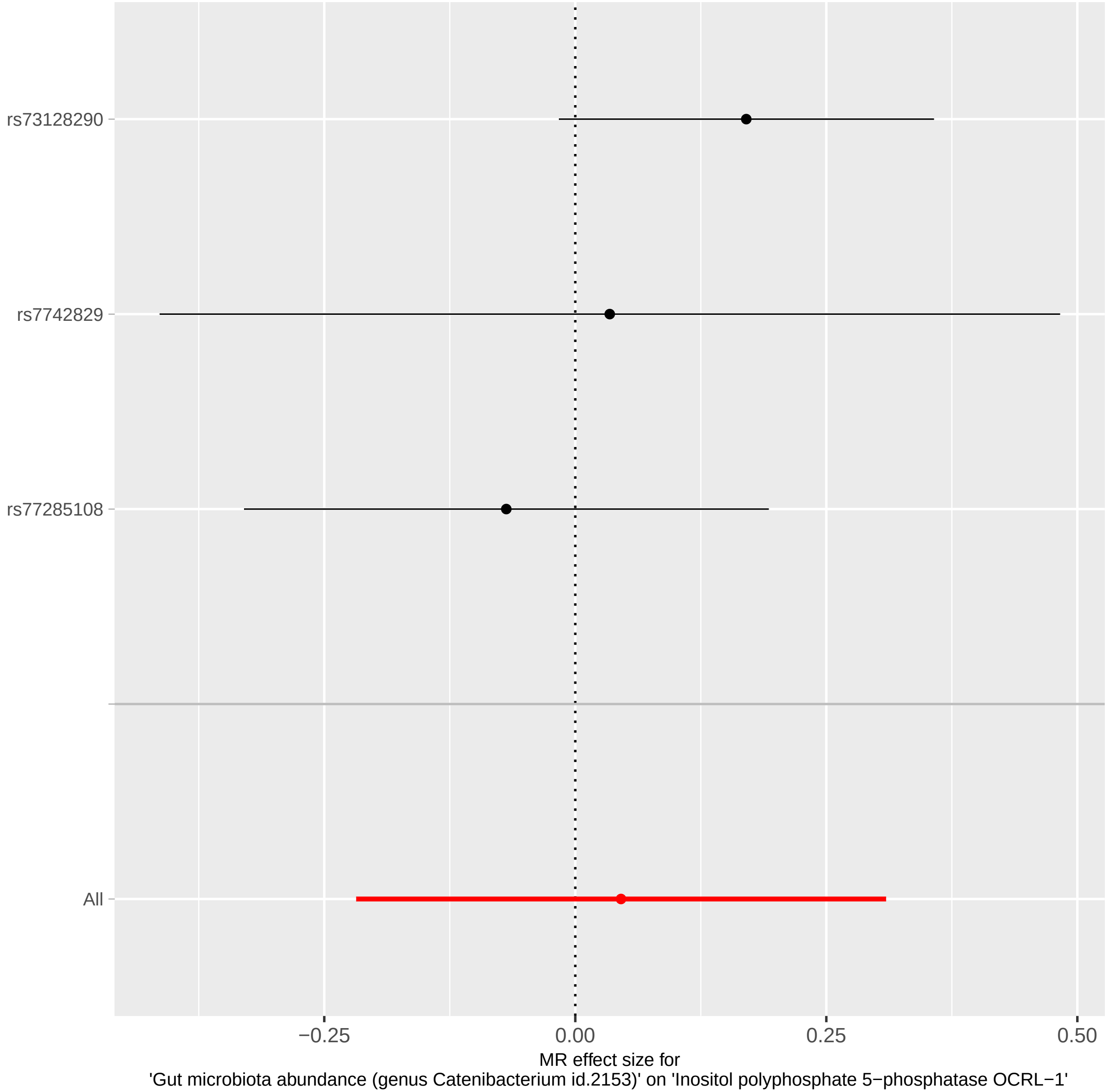

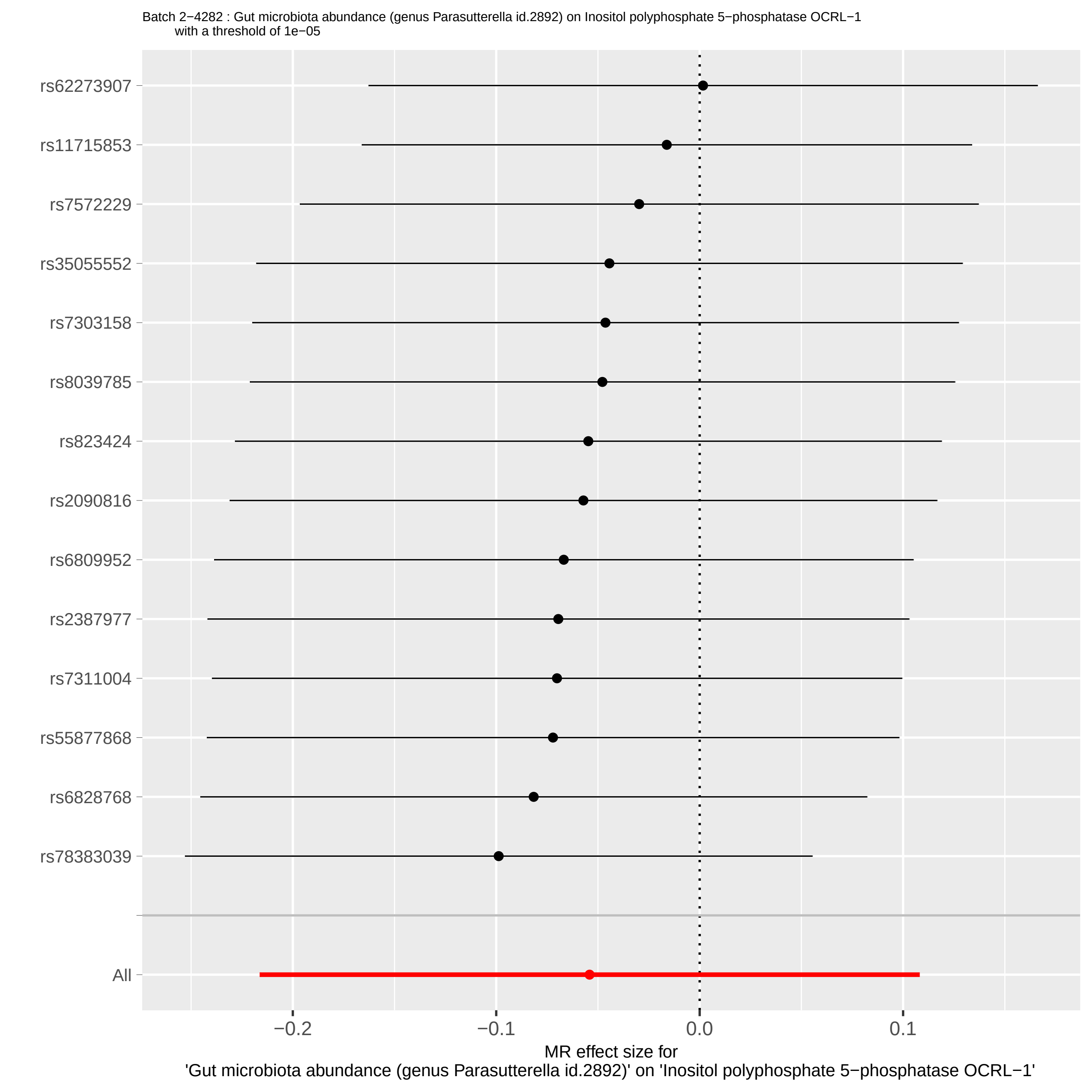
Batch 2−4282 : Gut microbiota abundance (genus Parasutterella id.2892) on Inositol polyphosphate 5−phosphatase OCRL−1
with a threshold of 1e−05
rs62273907
rs11715853
rs7572229
rs35055552
rs7303158
rs8039785
rs823424
rs2090816
rs6809952
rs2387977
rs7311004
rs55877868
rs6828768
rs78383039
All
−0.2
−0.1
0.0
0.1
MR effect size for
'Gut microbiota abundance (genus Parasutterella id.2892)' on 'Inositol polyphosphate 5−phosphatase OCRL−1'

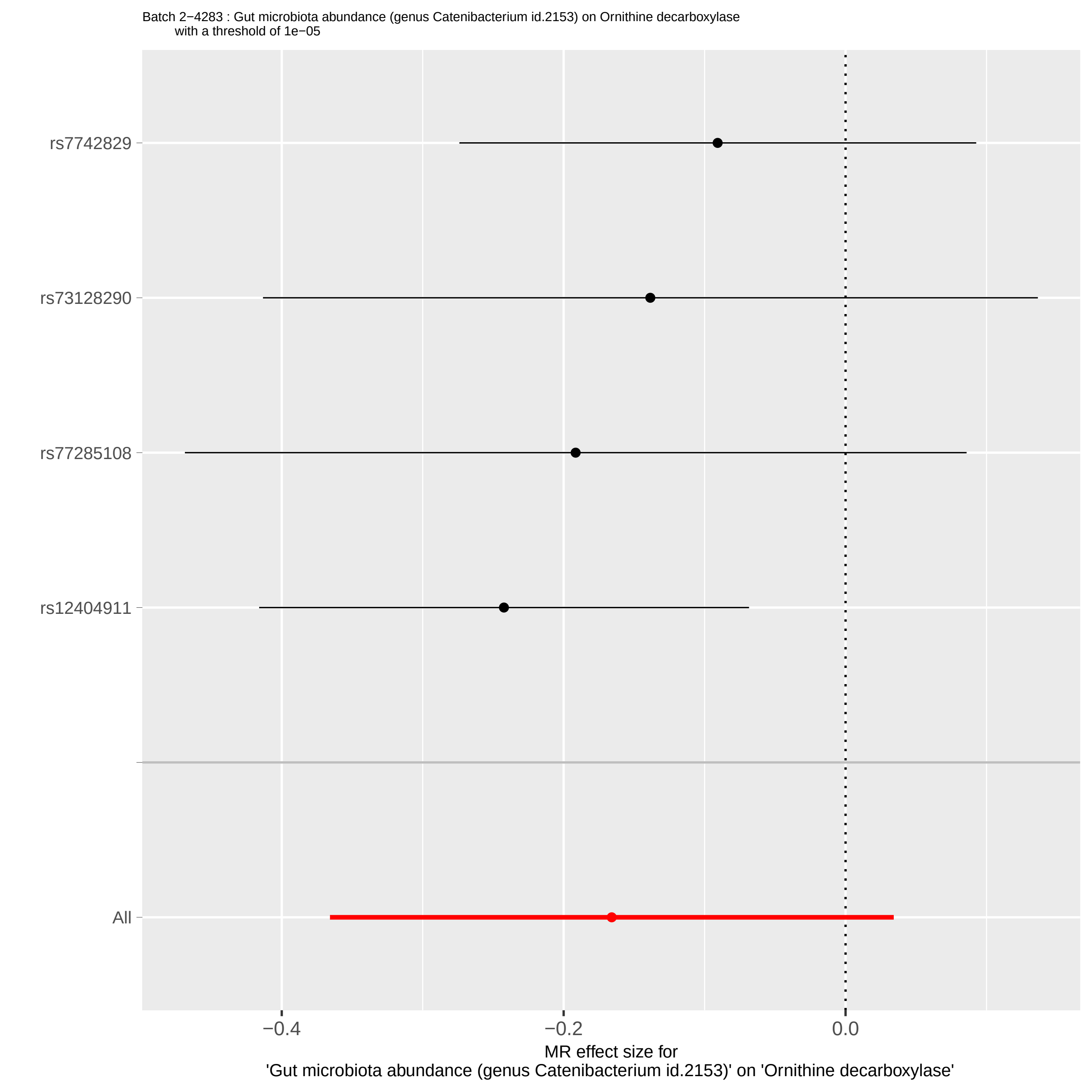

Batch 2−4283 : Gut microbiota abundance (genus Catenibacterium id.2153) on Ornithine decarboxylase
with a threshold of 1e−05
rs7742829
rs73128290
rs77285108
rs12404911
All
−0.4
−0.2
0.0
MR effect size for
'Gut microbiota abundance (genus Catenibacterium id.2153)' on 'Ornithine decarboxylase'

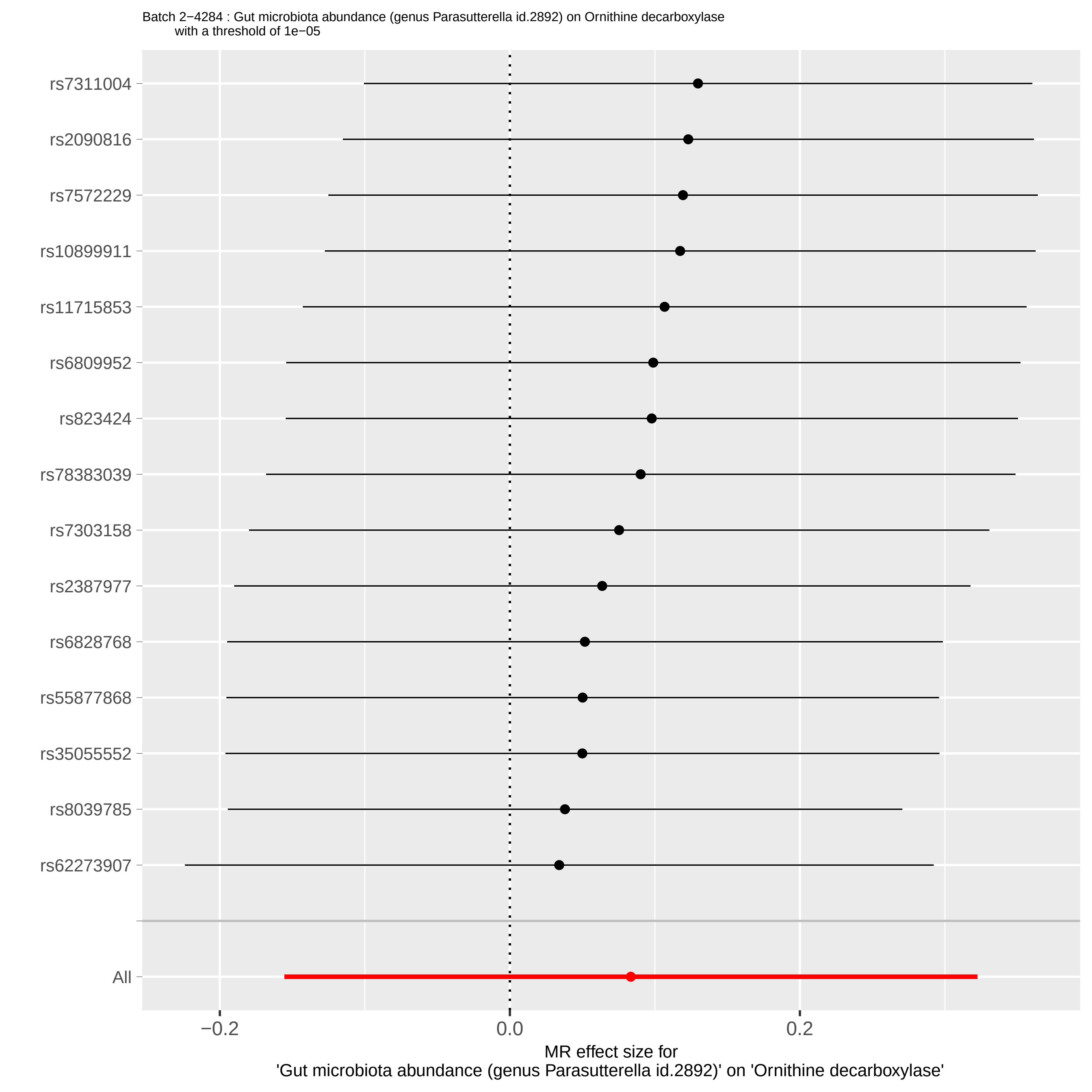
Batch 2−4284 : Gut microbiota abundance (genus Parasutterella id.2892) on Ornithine decarboxylase
with a threshold of 1e−05
rs7311004
rs2090816
rs7572229
rs10899911
rs11715853
rs6809952
rs823424
rs78383039
rs7303158
rs2387977
rs6828768
rs55877868
rs35055552
rs8039785
rs62273907
All
−0.2
0.0
0.2
MR effect size for
'Gut microbiota abundance (genus Parasutterella id.2892)' on 'Ornithine decarboxylase'

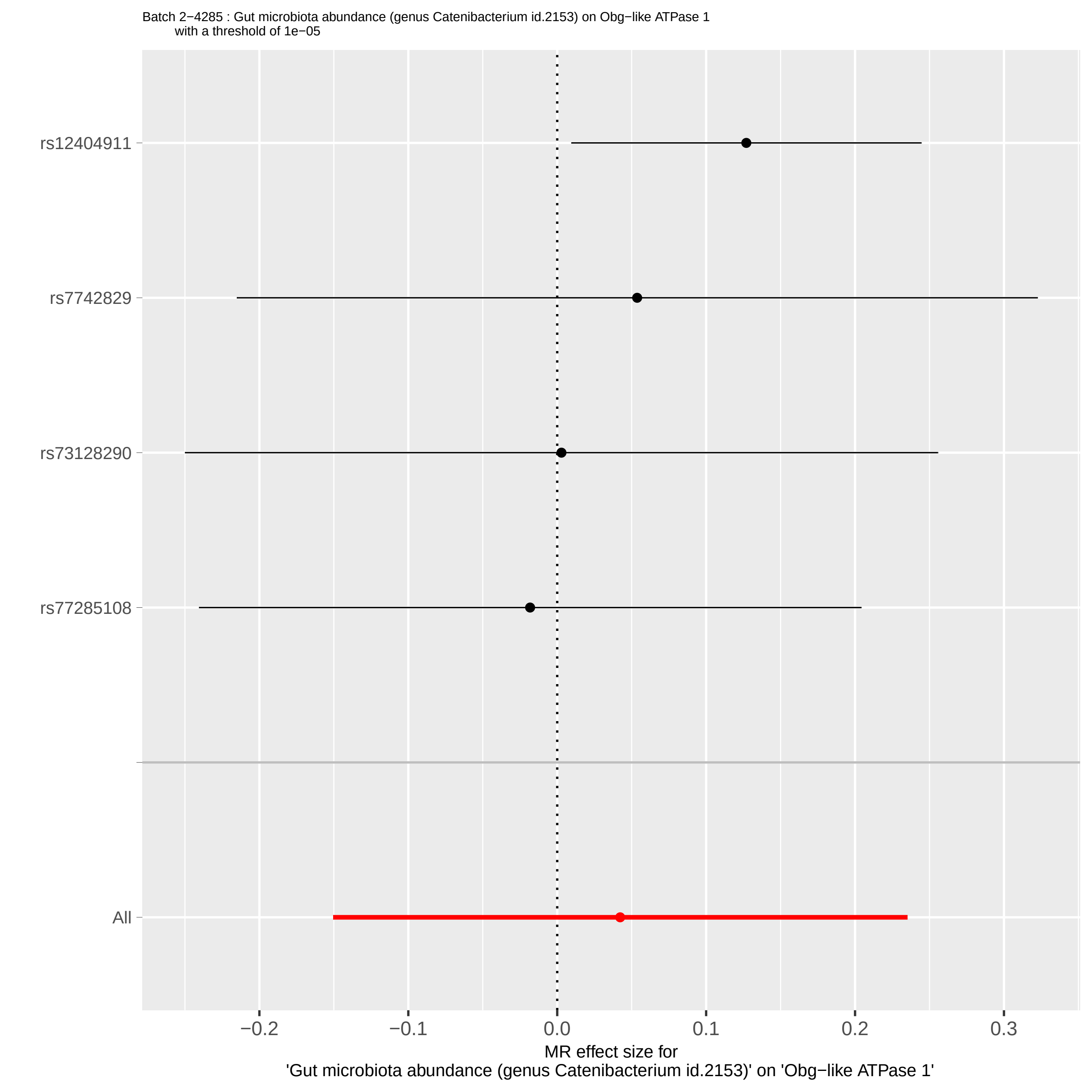

Batch 2−4285 : Gut microbiota abundance (genus Catenibacterium id.2153) on Obg−like ATPase 1
with a threshold of 1e−05
rs12404911
rs7742829
rs73128290
rs77285108
All
−0.2
−0.1
0.0
0.1
0.2
0.3
MR effect size for
'Gut microbiota abundance (genus Catenibacterium id.2153)' on 'Obg−like ATPase 1'

Batch 2−4286 : Gut microbiota abundance (genus Parasutterella id.2892) on Obg−like ATPase 1
with a threshold of 1e−05

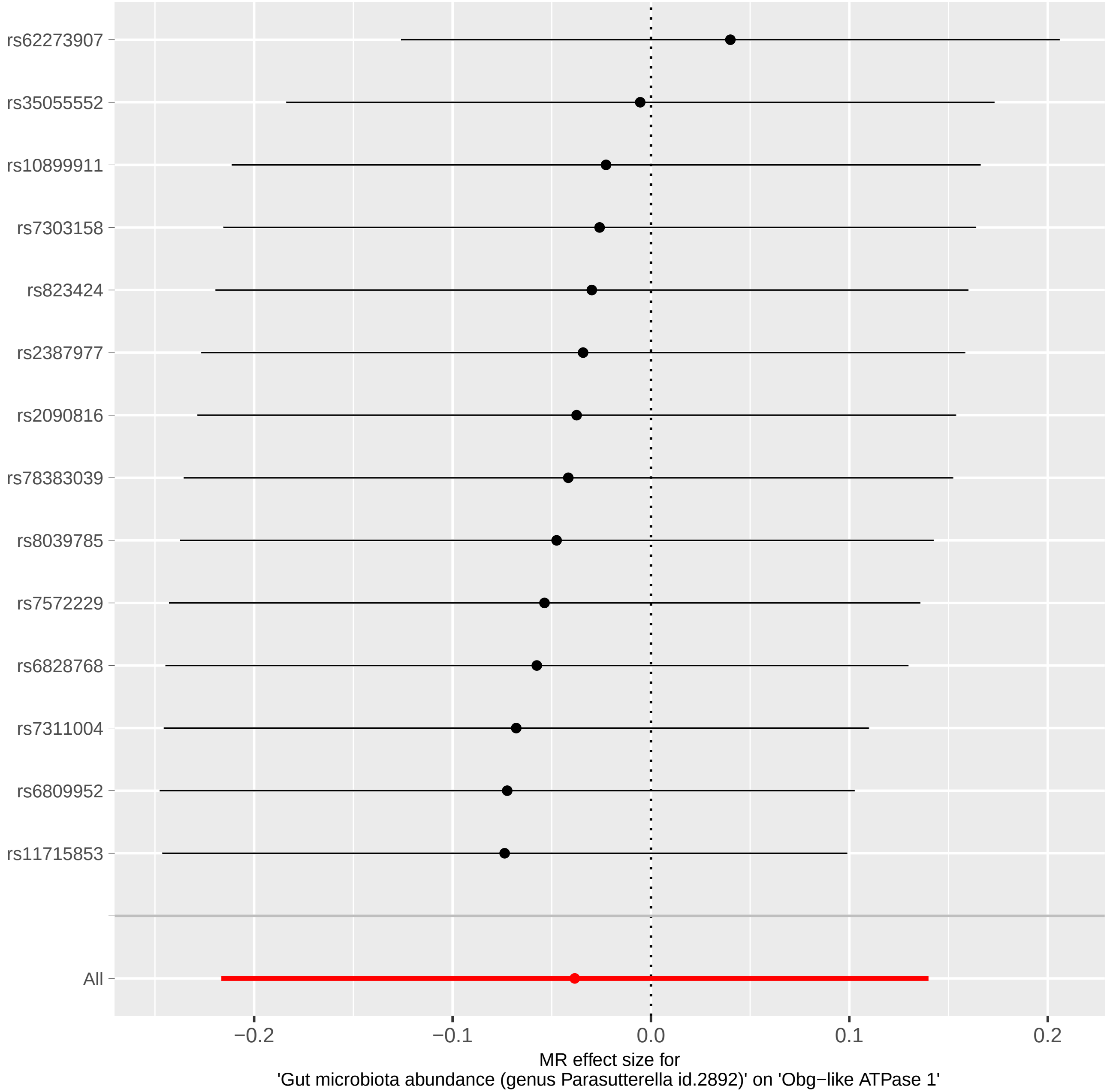

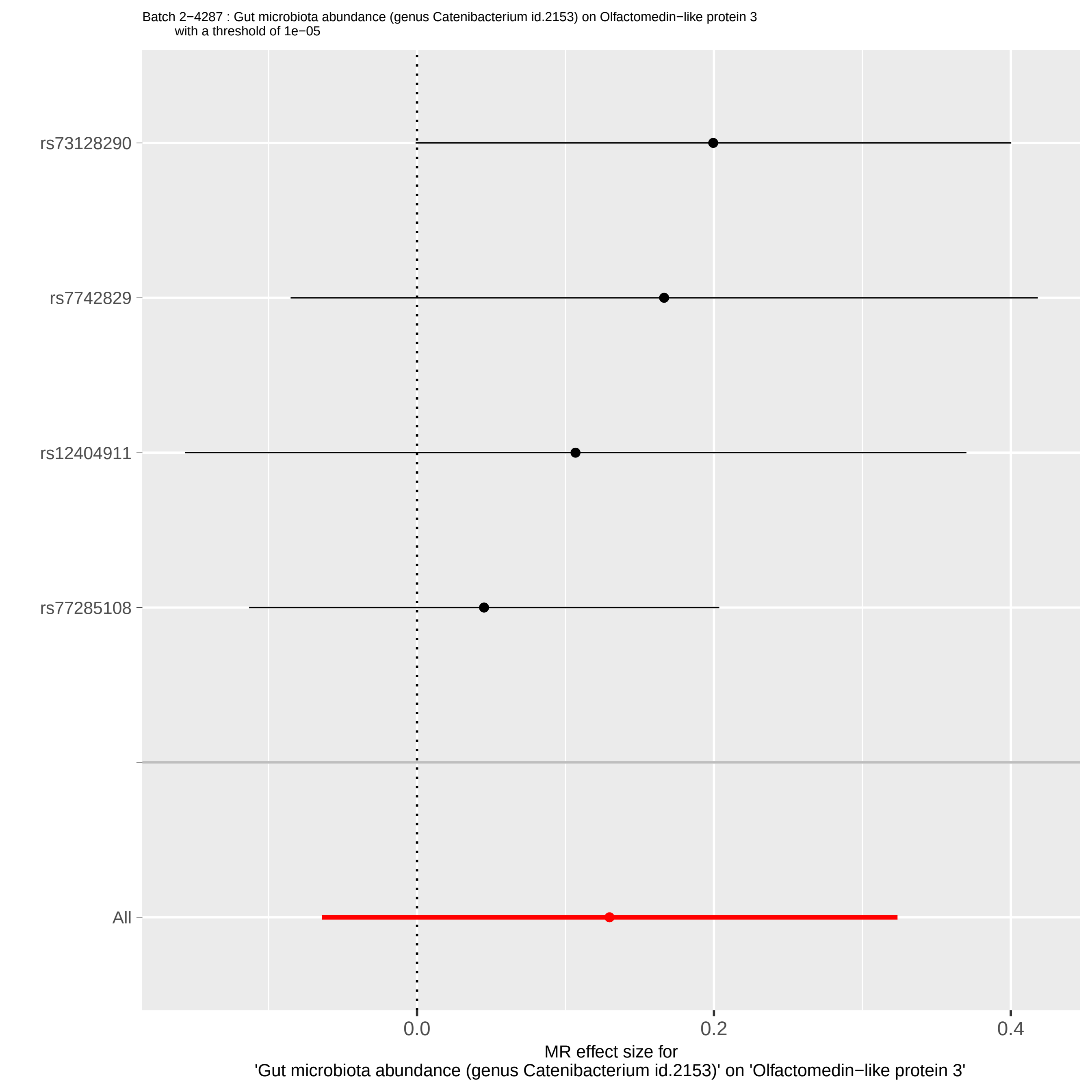
Batch 2−4287 : Gut microbiota abundance (genus Catenibacterium id.2153) on Olfactomedin−like protein 3
with a threshold of 1e−05
rs73128290
rs7742829
rs12404911
rs77285108
All
0.0
0.2
0.4
MR effect size for
'Gut microbiota abundance (genus Catenibacterium id.2153)' on 'Olfactomedin−like protein 3'

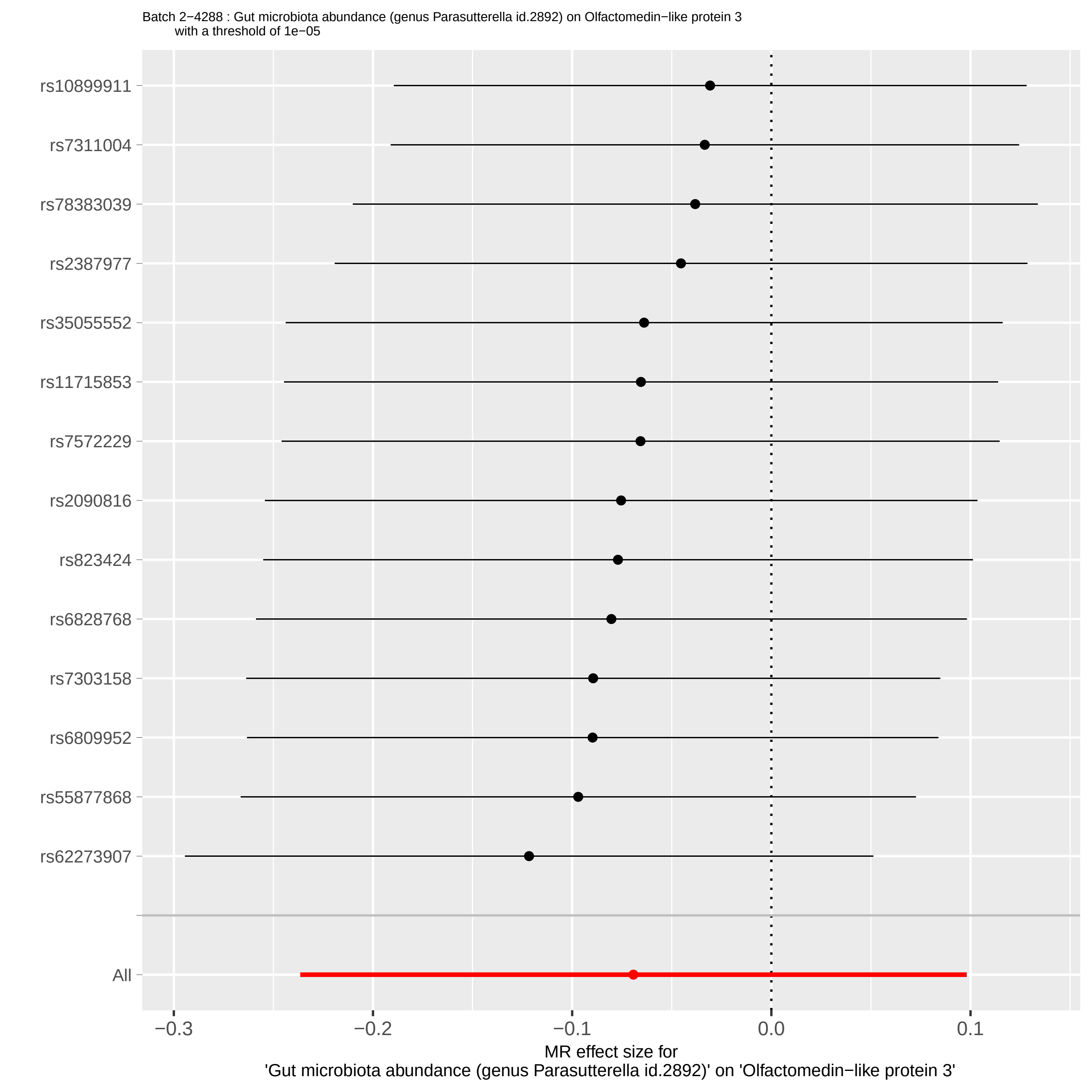
Batch 2−4288 : Gut microbiota abundance (genus Parasutterella id.2892) on Olfactomedin−like protein 3
with a threshold of 1e−05
rs10899911
rs7311004
rs78383039
rs2387977
rs35055552
rs11715853
rs7572229
rs2090816
rs823424
rs6828768
rs7303158
rs6809952
rs55877868
rs62273907
All
−0.3
−0.2
−0.1
0.0
0.1
MR effect size for
'Gut microbiota abundance (genus Parasutterella id.2892)' on 'Olfactomedin−like protein 3'

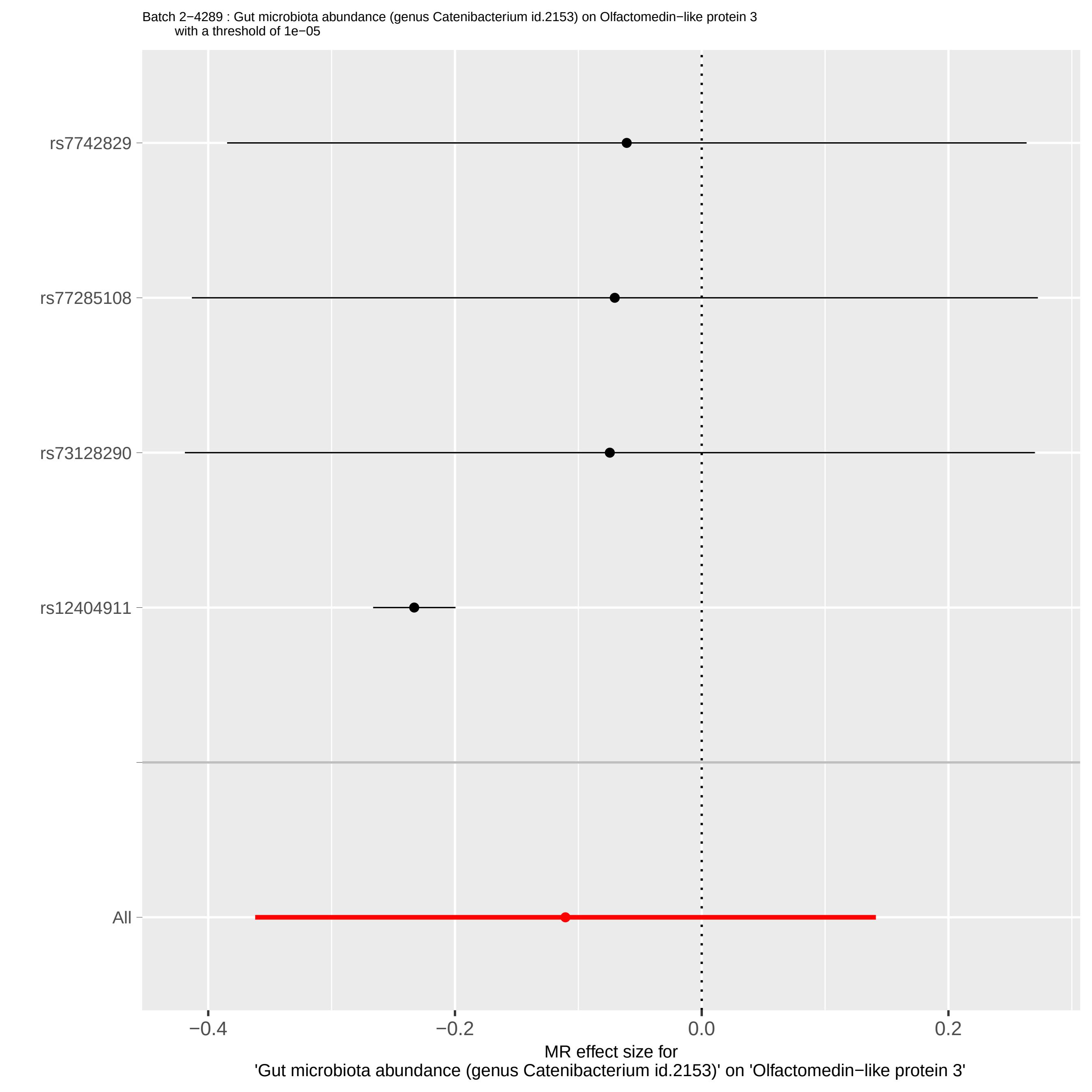
Batch 2−4289 : Gut microbiota abundance (genus Catenibacterium id.2153) on Olfactomedin−like protein 3
with a threshold of 1e−05
rs7742829
rs77285108
rs73128290
rs12404911
All
−0.4
−0.2
0.0
0.2
MR effect size for
'Gut microbiota abundance (genus Catenibacterium id.2153)' on 'Olfactomedin−like protein 3'

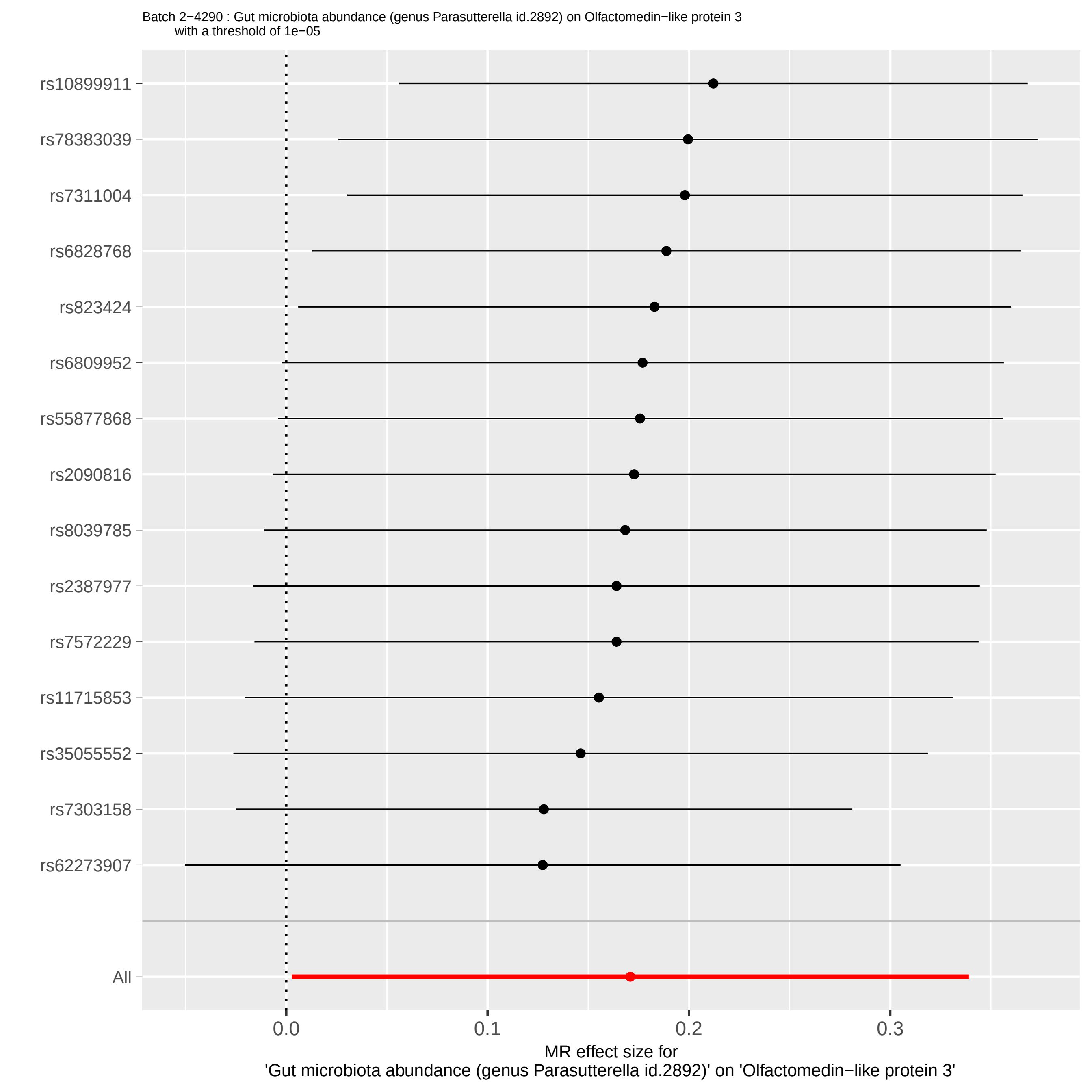
Batch 2−4290 : Gut microbiota abundance (genus Parasutterella id.2892) on Olfactomedin−like protein 3
with a threshold of 1e−05
rs10899911
rs78383039
rs7311004
rs6828768
rs823424
rs6809952
rs55877868
rs2090816
rs8039785
rs2387977
rs7572229
rs11715853
rs35055552
rs7303158
rs62273907
All
0.0
0.1
0.2
0.3
MR effect size for
'Gut microbiota abundance (genus Parasutterella id.2892)' on 'Olfactomedin−like protein 3'

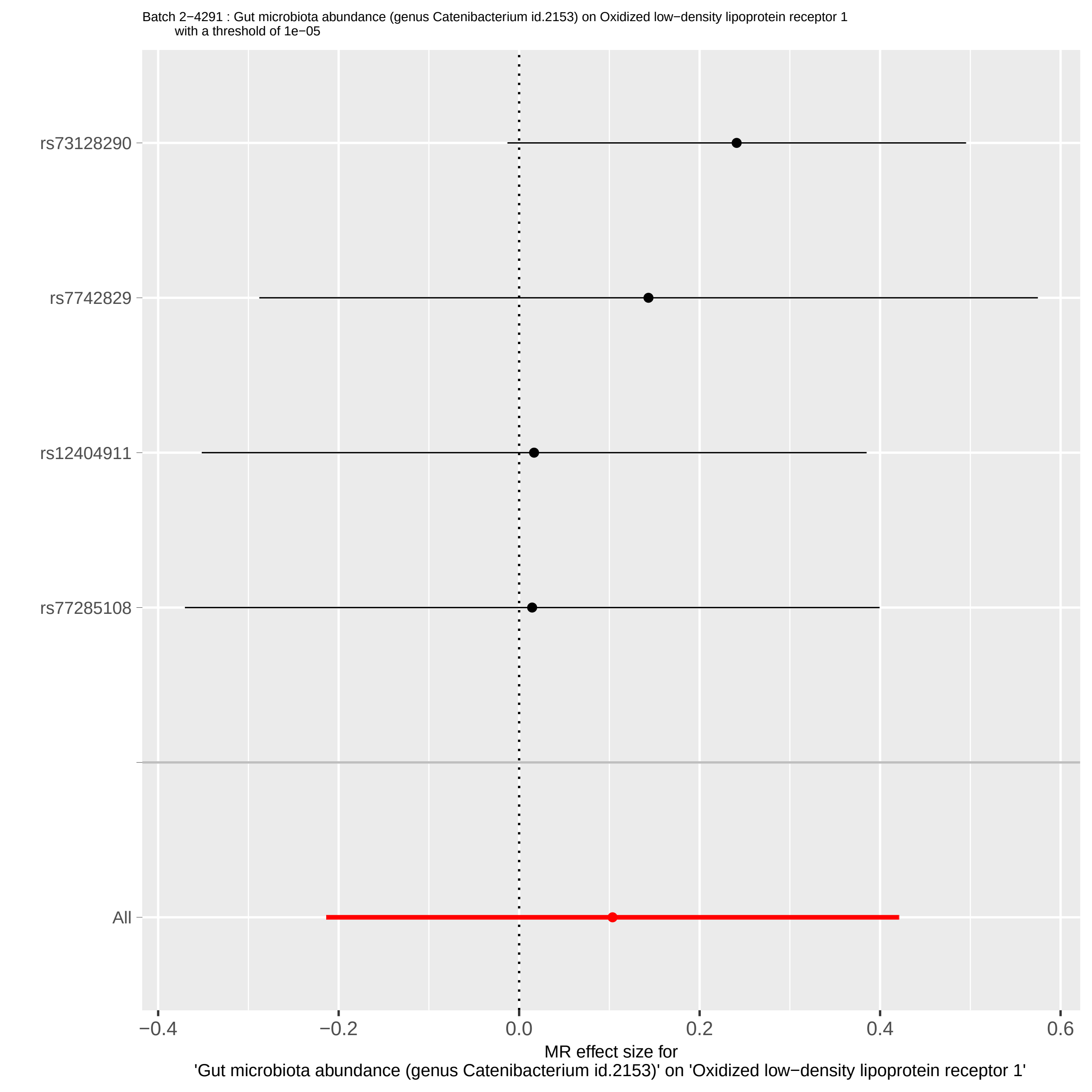

Batch 2−4291 : Gut microbiota abundance (genus Catenibacterium id.2153) on Oxidized low−density lipoprotein receptor 1
with a threshold of 1e−05
rs73128290
rs7742829
rs12404911
rs77285108
All
−0.4
−0.2
0.0
0.2
0.4
0.6
MR effect size for
'Gut microbiota abundance (genus Catenibacterium id.2153)' on 'Oxidized low−density lipoprotein receptor 1'

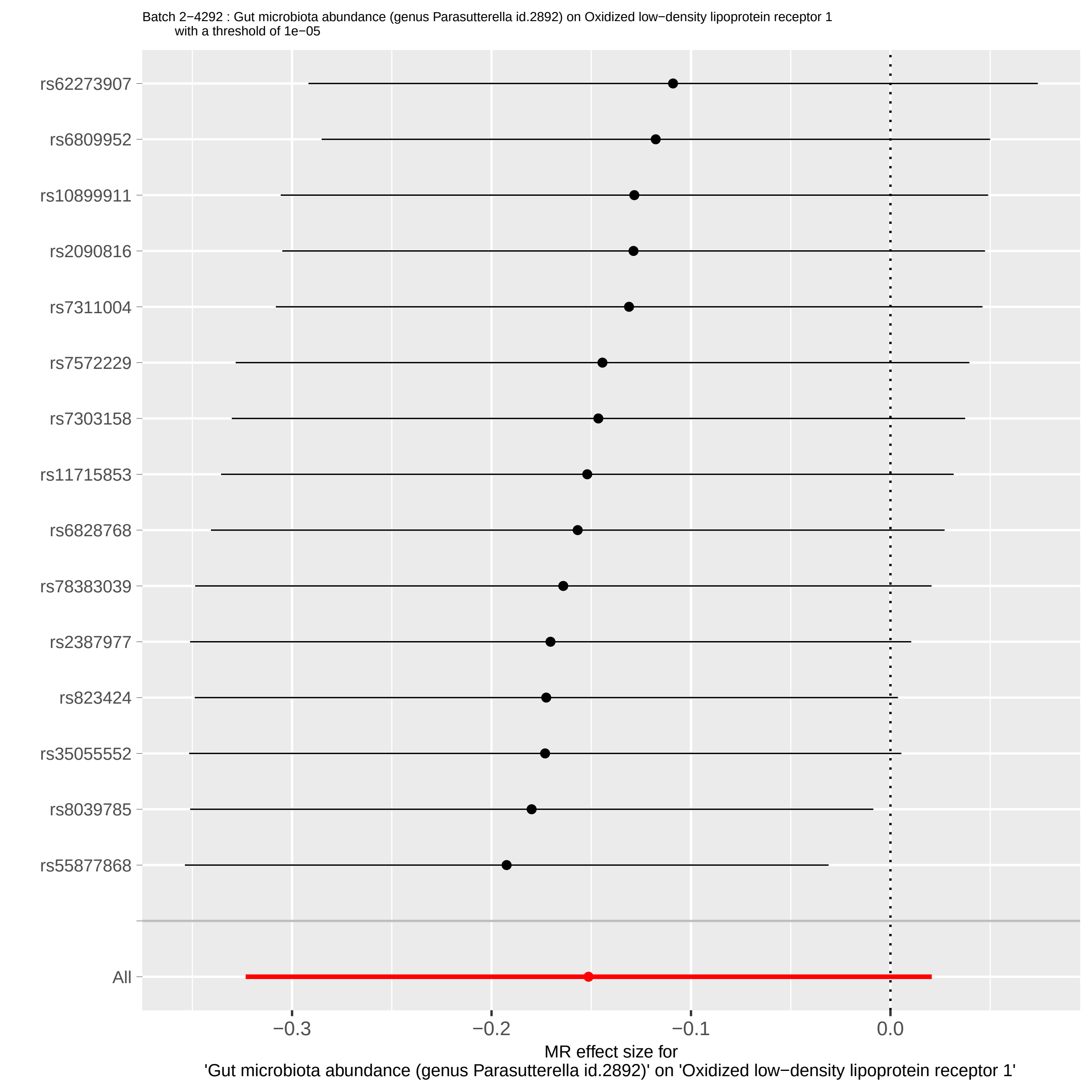

Batch 2−4292 : Gut microbiota abundance (genus Parasutterella id.2892) on Oxidized low−density lipoprotein receptor 1
with a threshold of 1e−05
rs62273907
rs6809952
rs10899911
rs2090816
rs7311004
rs7572229
rs7303158
rs11715853
rs6828768
rs78383039
rs2387977
rs823424
rs35055552
rs8039785
rs55877868
All
−0.3
−0.2
−0.1
0.0
MR effect size for
'Gut microbiota abundance (genus Parasutterella id.2892)' on 'Oxidized low−density lipoprotein receptor 1'

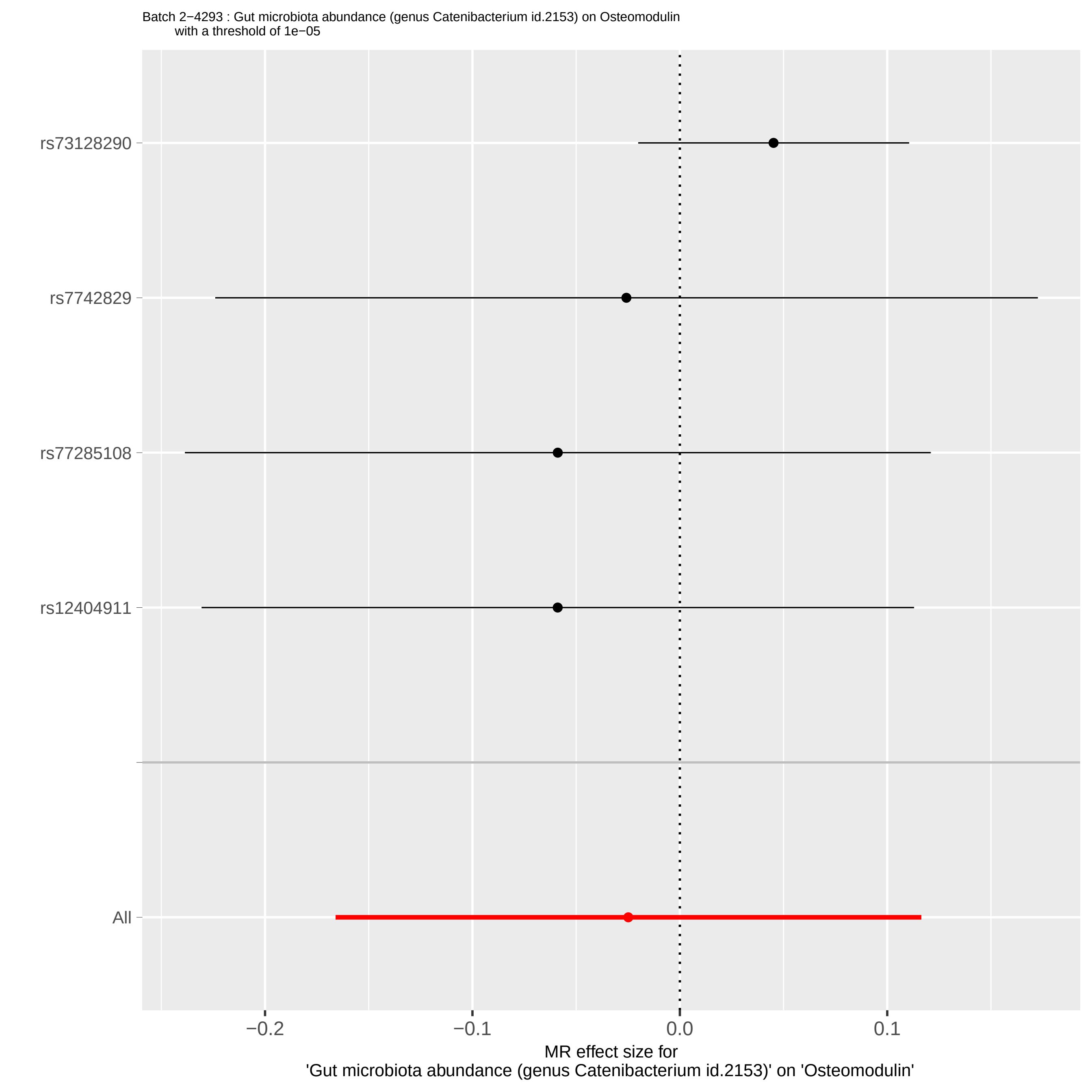

Batch 2−4293 : Gut microbiota abundance (genus Catenibacterium id.2153) on Osteomodulin
with a threshold of 1e−05
rs73128290
rs7742829
rs77285108
rs12404911
All
−0.2
−0.1
0.0
0.1
MR effect size for
'Gut microbiota abundance (genus Catenibacterium id.2153)' on 'Osteomodulin'

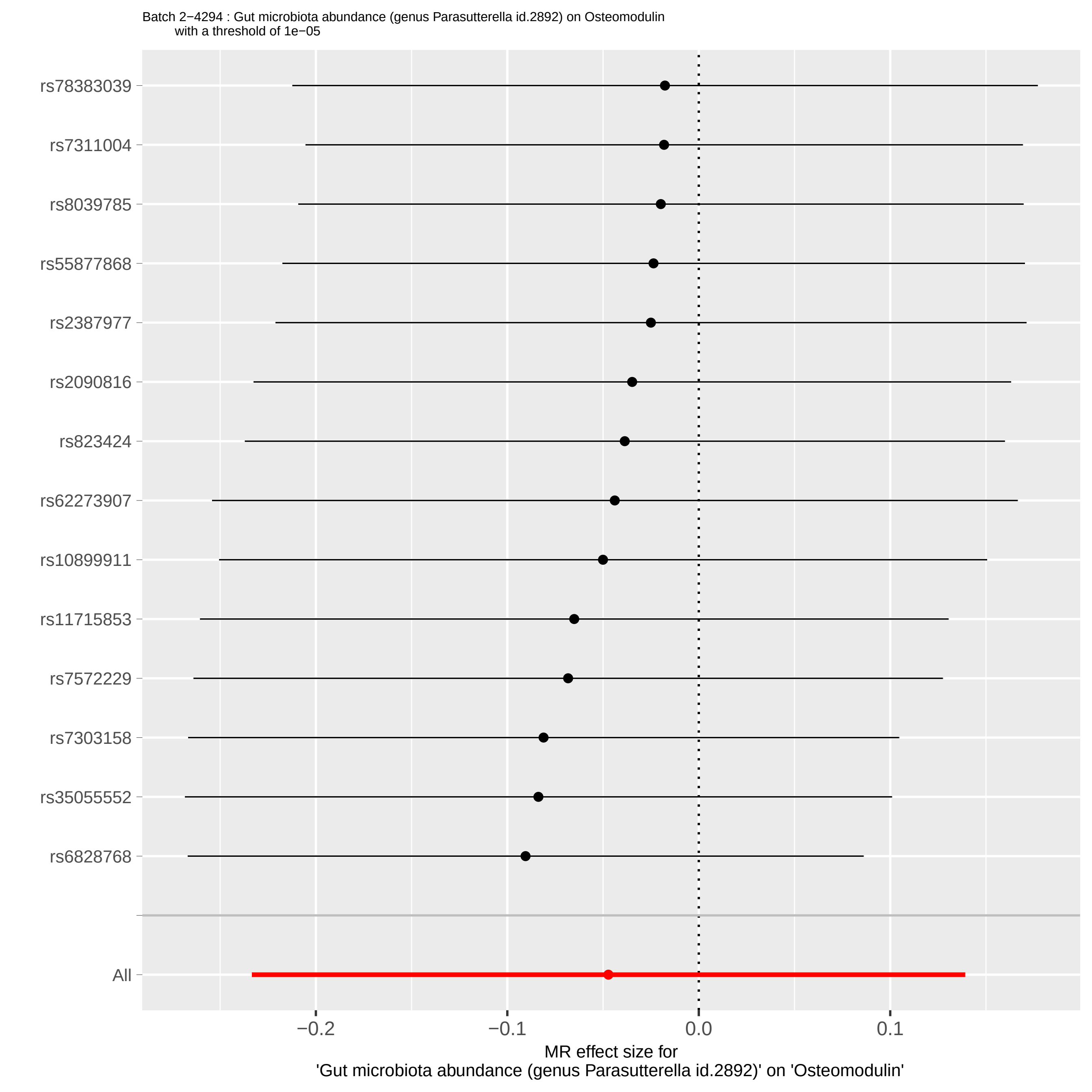
Batch 2−4294 : Gut microbiota abundance (genus Parasutterella id.2892) on Osteomodulin
with a threshold of 1e−05
rs78383039
rs7311004
rs8039785
rs55877868
rs2387977
rs2090816
rs823424
rs62273907
rs10899911
rs11715853
rs7572229
rs7303158
rs35055552
rs6828768
All
−0.2
−0.1
0.0
0.1
MR effect size for
'Gut microbiota abundance (genus Parasutterella id.2892)' on 'Osteomodulin'

Batch 2−4295 : Gut microbiota abundance (genus Catenibacterium id.2153) on Opalin
with a threshold of 1e−05

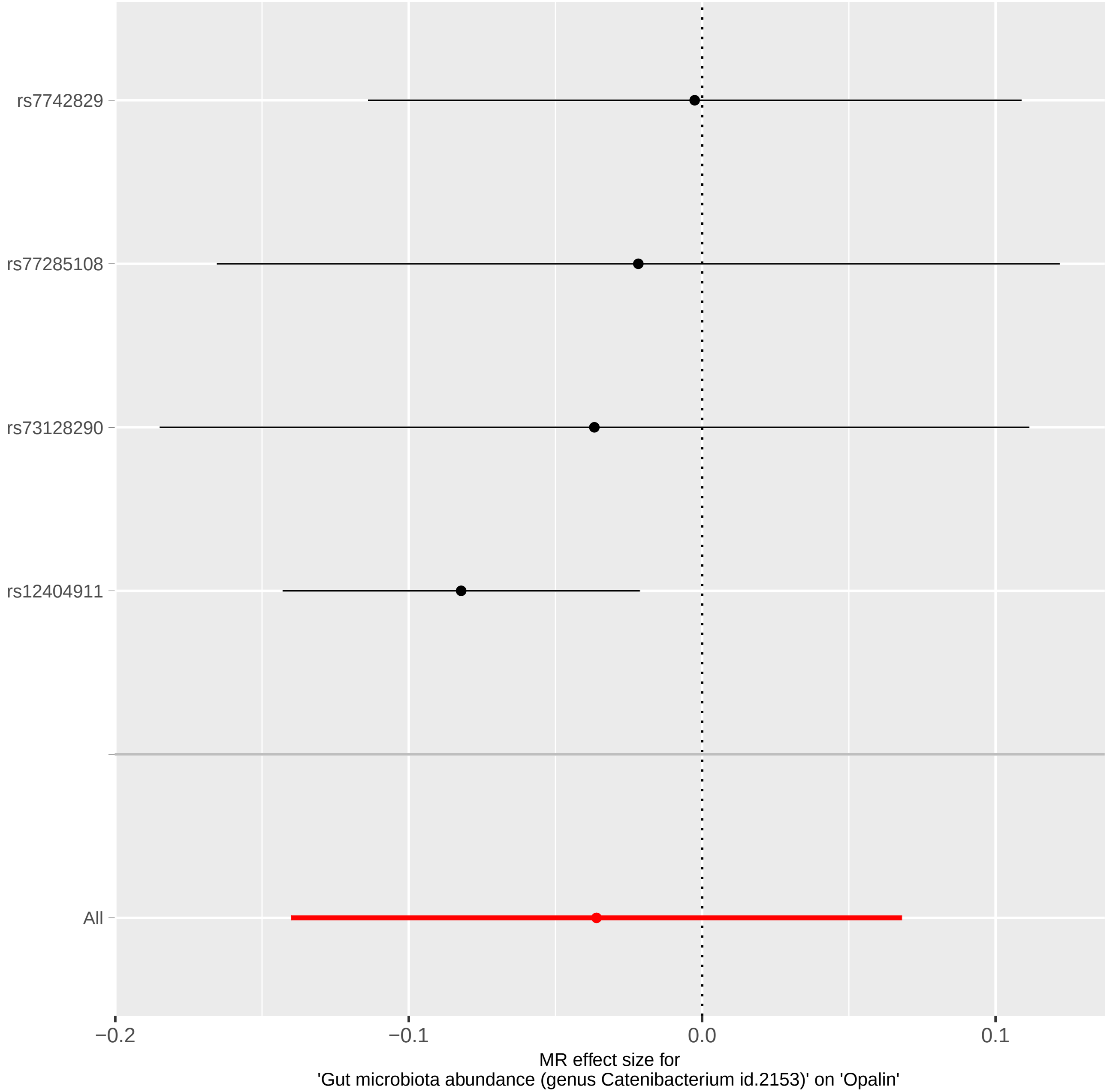

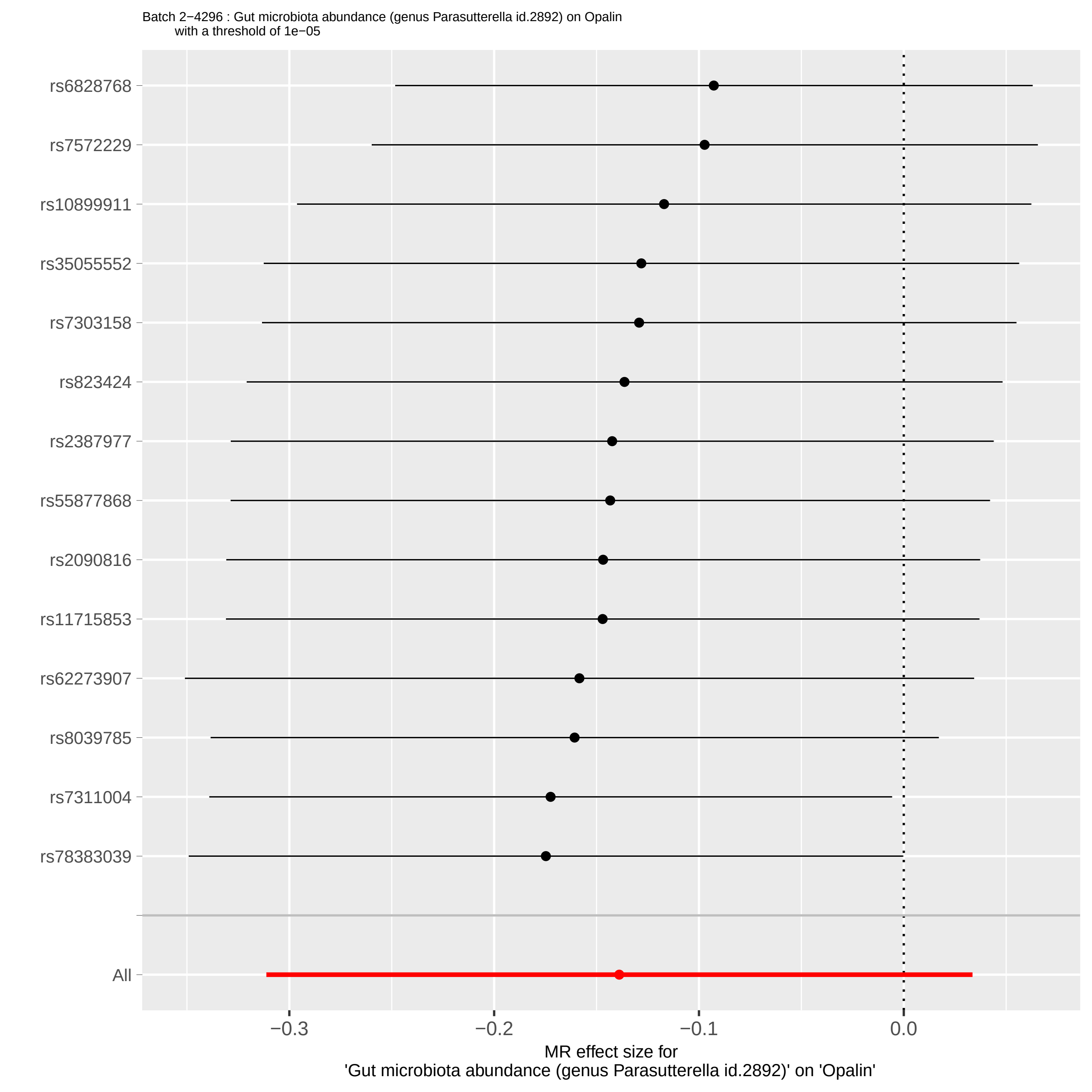
Batch 2−4296 : Gut microbiota abundance (genus Parasutterella id.2892) on Opalin
with a threshold of 1e−05
rs6828768
rs7572229
rs10899911
rs35055552
rs7303158
rs823424
rs2387977
rs55877868
rs2090816
rs11715853
rs62273907
rs8039785
rs7311004
rs78383039
All
−0.3
−0.2
−0.1
0.0
MR effect size for
'Gut microbiota abundance (genus Parasutterella id.2892)' on 'Opalin'

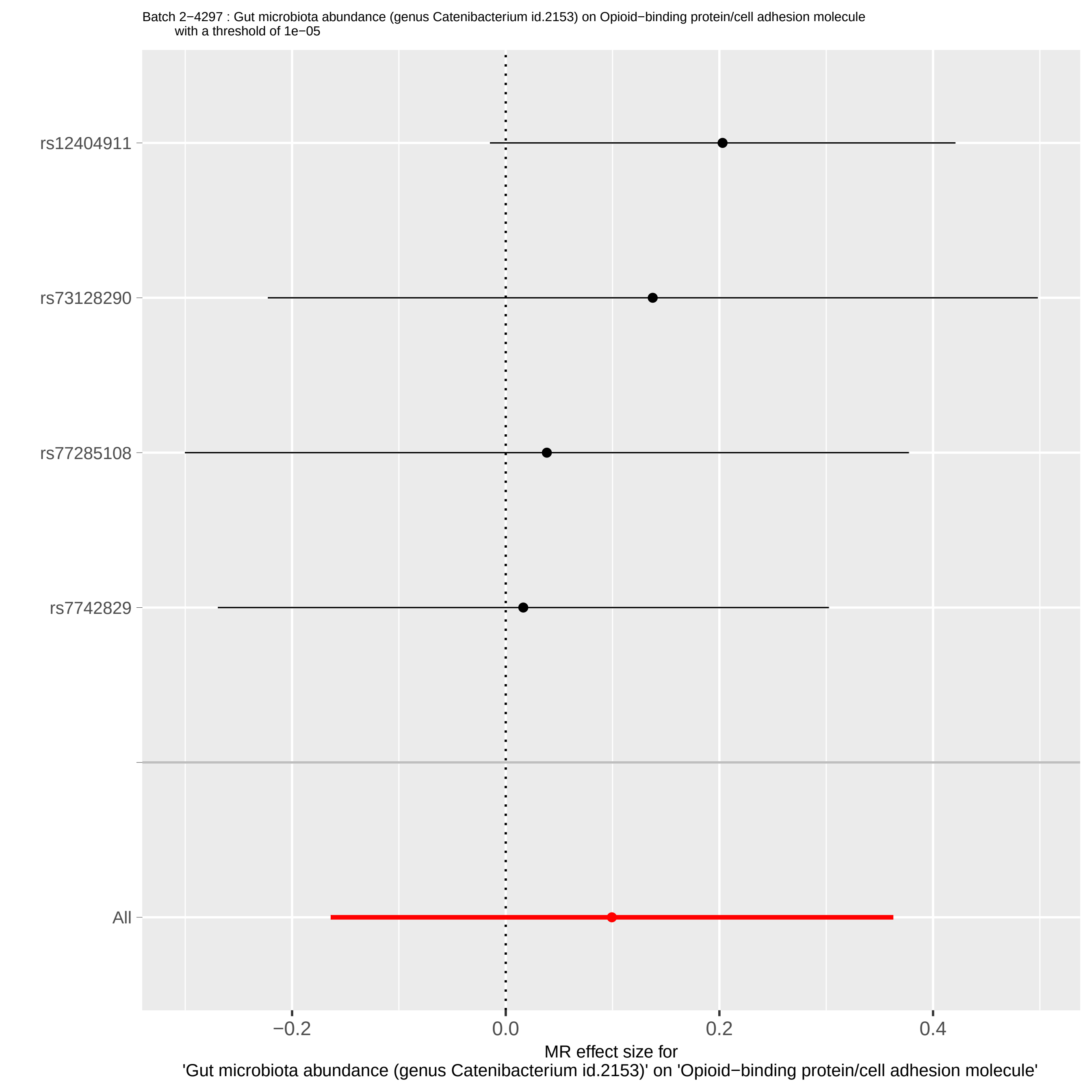

Batch 2−4297 : Gut microbiota abundance (genus Catenibacterium id.2153) on Opioid−binding protein/cell adhesion molecule
with a threshold of 1e−05
rs12404911
rs73128290
rs77285108
rs7742829
All
−0.2
0.0
0.2
0.4
MR effect size for
'Gut microbiota abundance (genus Catenibacterium id.2153)' on 'Opioid−binding protein/cell adhesion molecule'

Batch 2−4298 : Gut microbiota abundance (genus Parasutterella id.2892) on Opioid−binding protein/cell adhesion molecule with a threshold of 1e−05

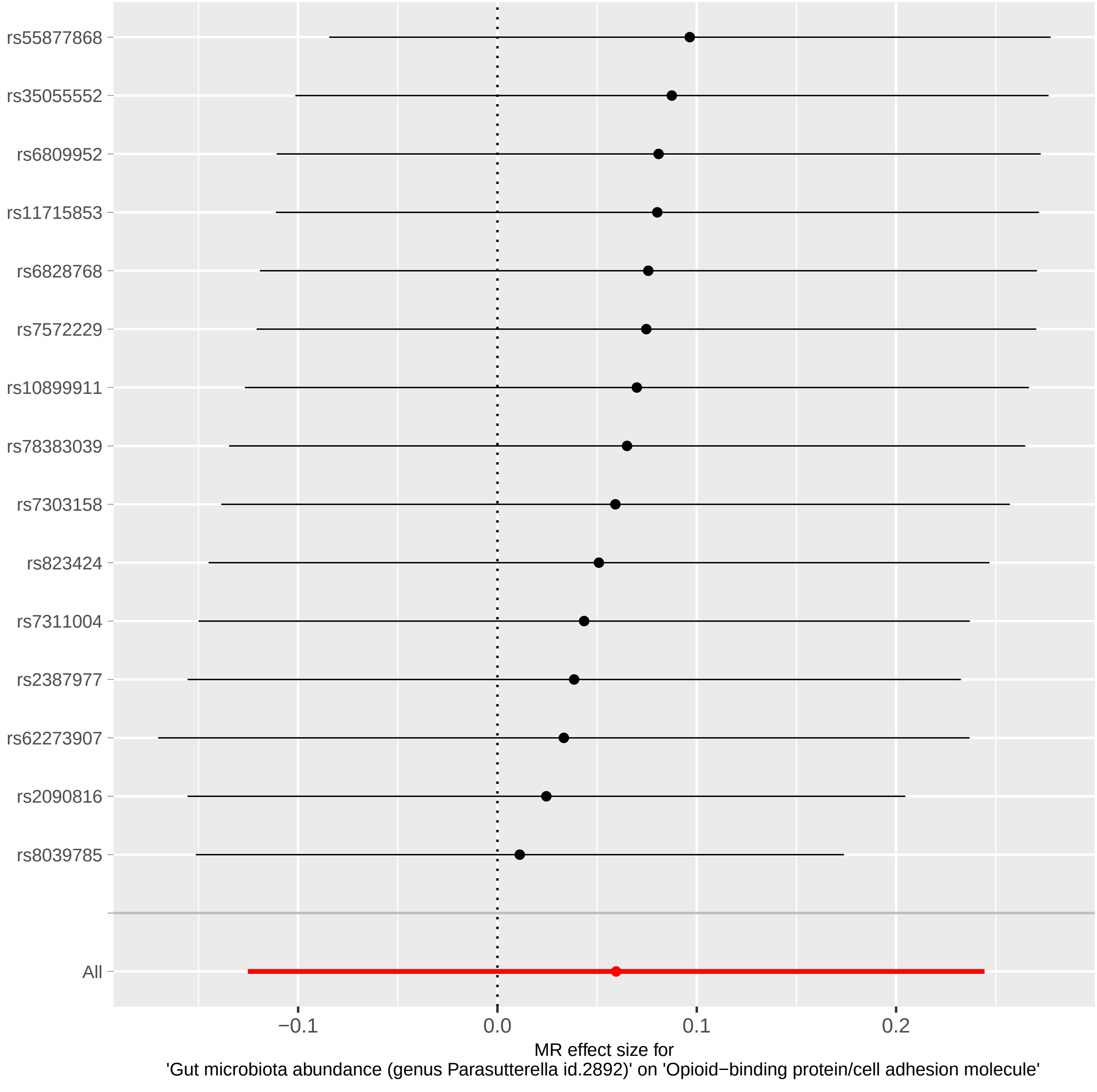

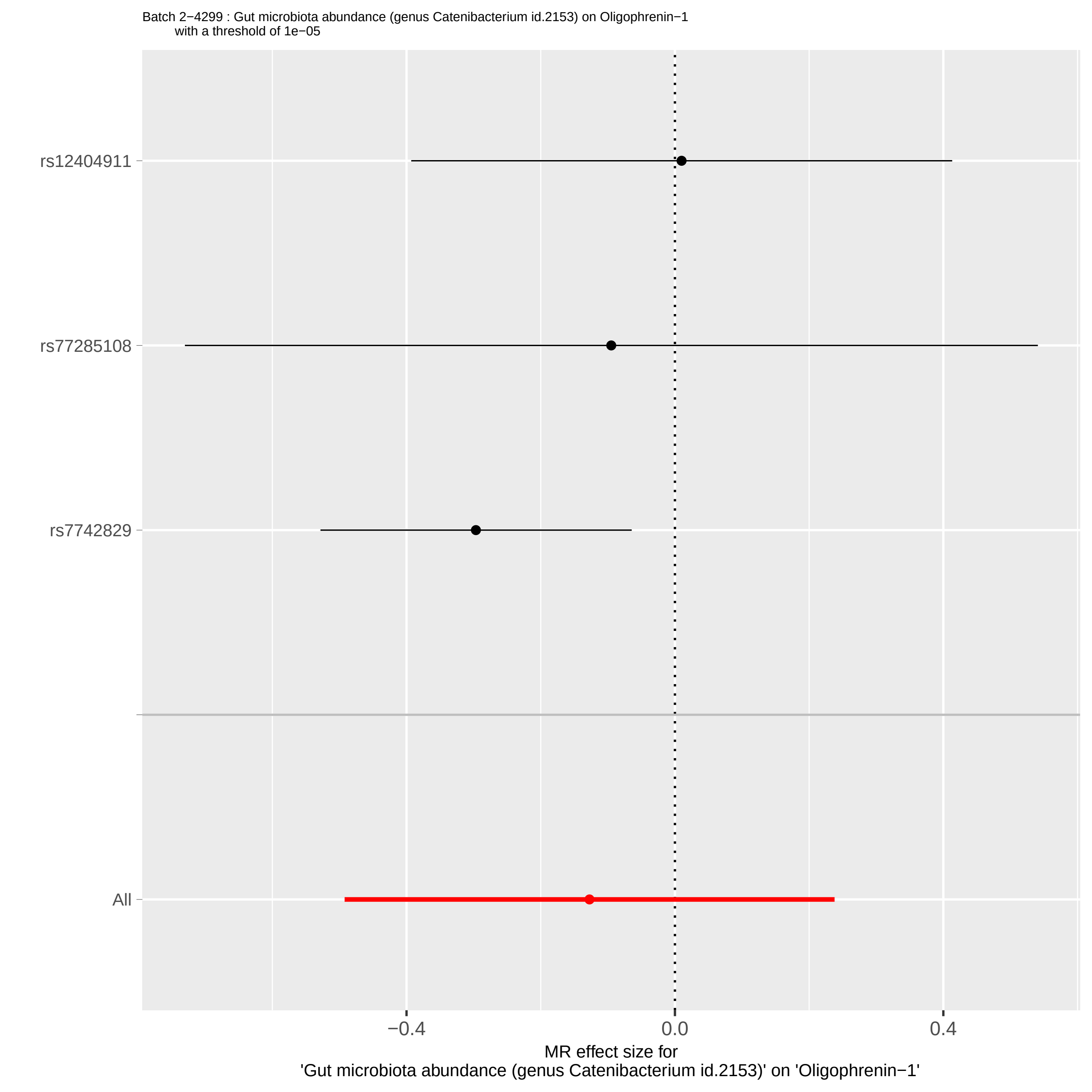
Batch 2−4299 : Gut microbiota abundance (genus Catenibacterium id.2153) on Oligophrenin−1
with a threshold of 1e−05
rs12404911
rs77285108
rs7742829
All
−0.4
0.0
0.4
MR effect size for
'Gut microbiota abundance (genus Catenibacterium id.2153)' on 'Oligophrenin−1'

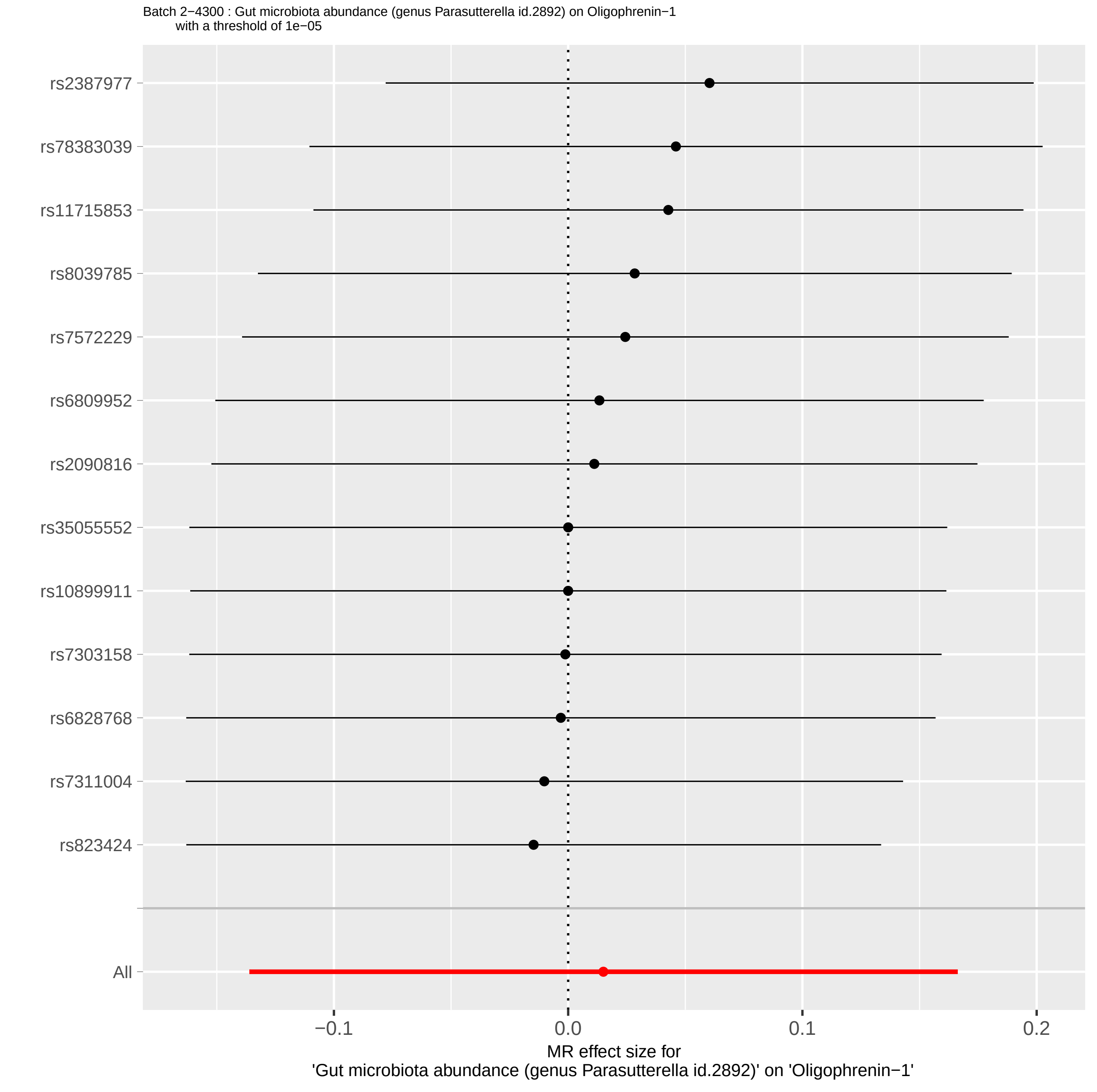
Batch 2−4300 : Gut microbiota abundance (genus Parasutterella id.2892) on Oligophrenin−1
with a threshold of 1e−05
rs2387977
rs78383039
rs11715853
rs8039785
rs7572229
rs6809952
rs2090816
rs35055552
rs10899911
rs7303158
rs6828768
rs7311004
rs823424
All
−0.1
0.0
0.1
0.2
MR effect size for
'Gut microbiota abundance (genus Parasutterella id.2892)' on 'Oligophrenin−1'

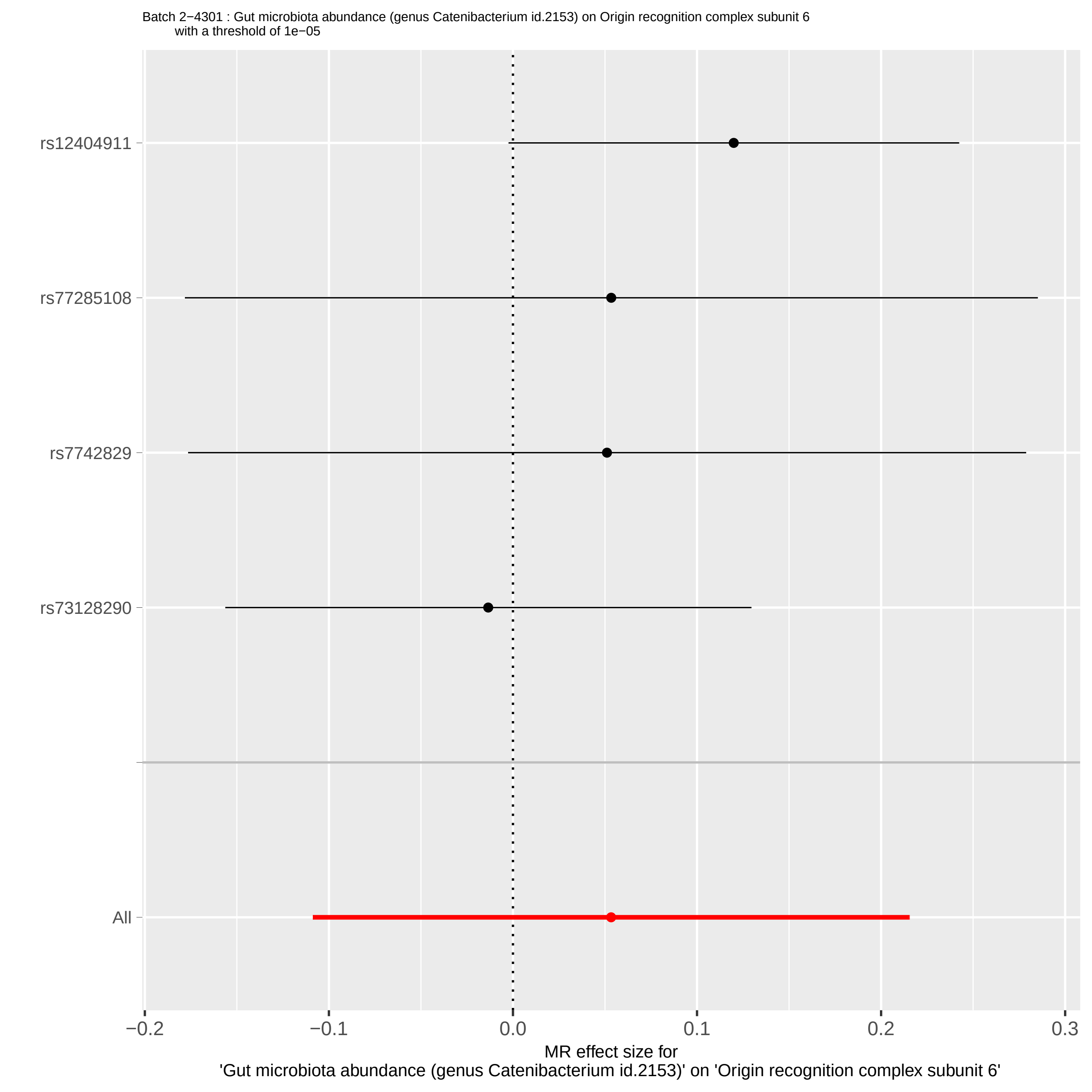

Batch 2−4301 : Gut microbiota abundance (genus Catenibacterium id.2153) on Origin recognition complex subunit 6
with a threshold of 1e−05
rs12404911
rs77285108
rs7742829
rs73128290
All
−0.2
−0.1
0.0
0.1
0.2
0.3
MR effect size for
'Gut microbiota abundance (genus Catenibacterium id.2153)' on 'Origin recognition complex subunit 6'

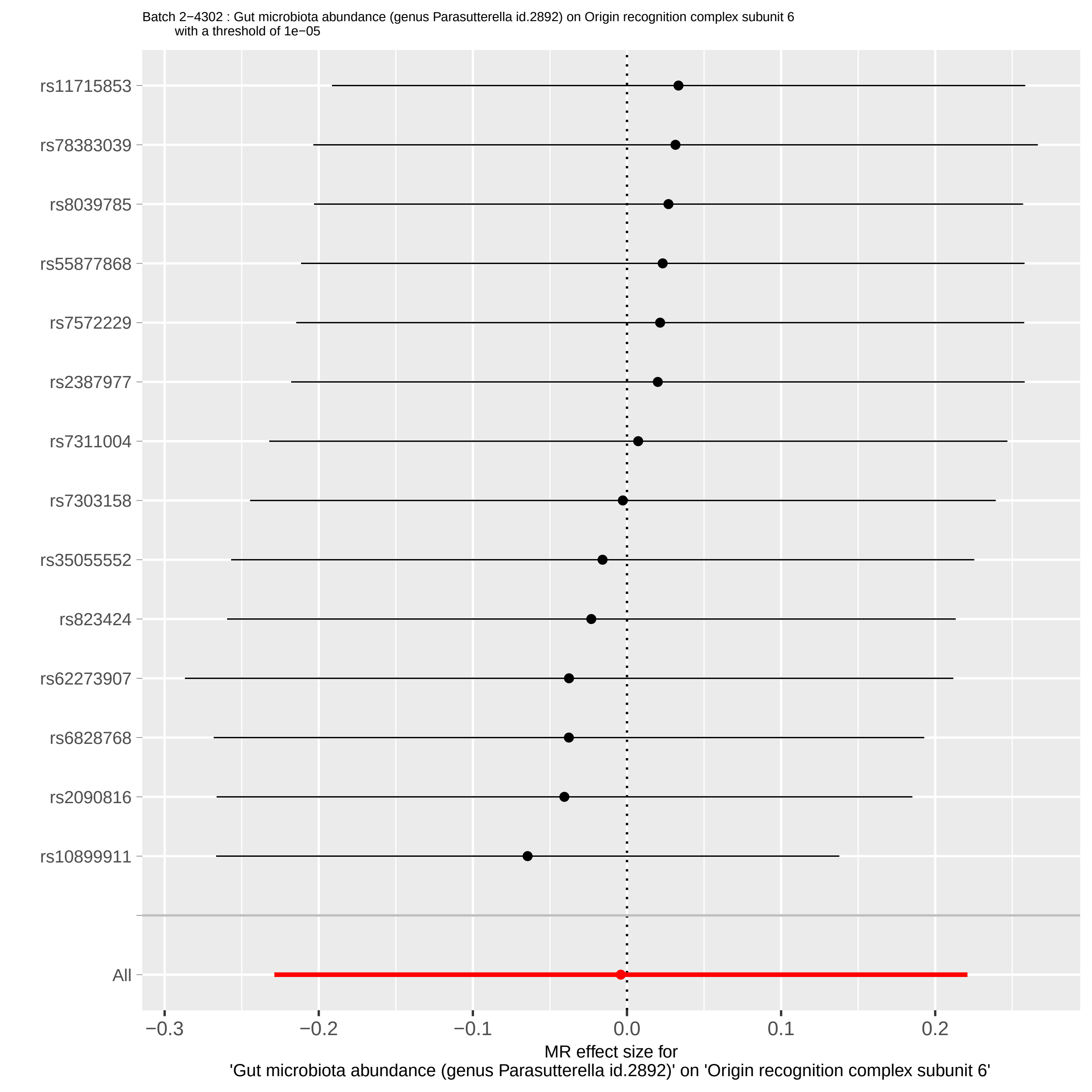

Batch 2−4302 : Gut microbiota abundance (genus Parasutterella id.2892) on Origin recognition complex subunit 6
with a threshold of 1e−05
rs11715853
rs78383039
rs8039785
rs55877868
rs7572229
rs2387977
rs7311004
rs7303158
rs35055552
rs823424
rs62273907
rs6828768
rs2090816
rs10899911
All
−0.3
−0.2
−0.1
0.0
0.1
0.2
MR effect size for
'Gut microbiota abundance (genus Parasutterella id.2892)' on 'Origin recognition complex subunit 6'

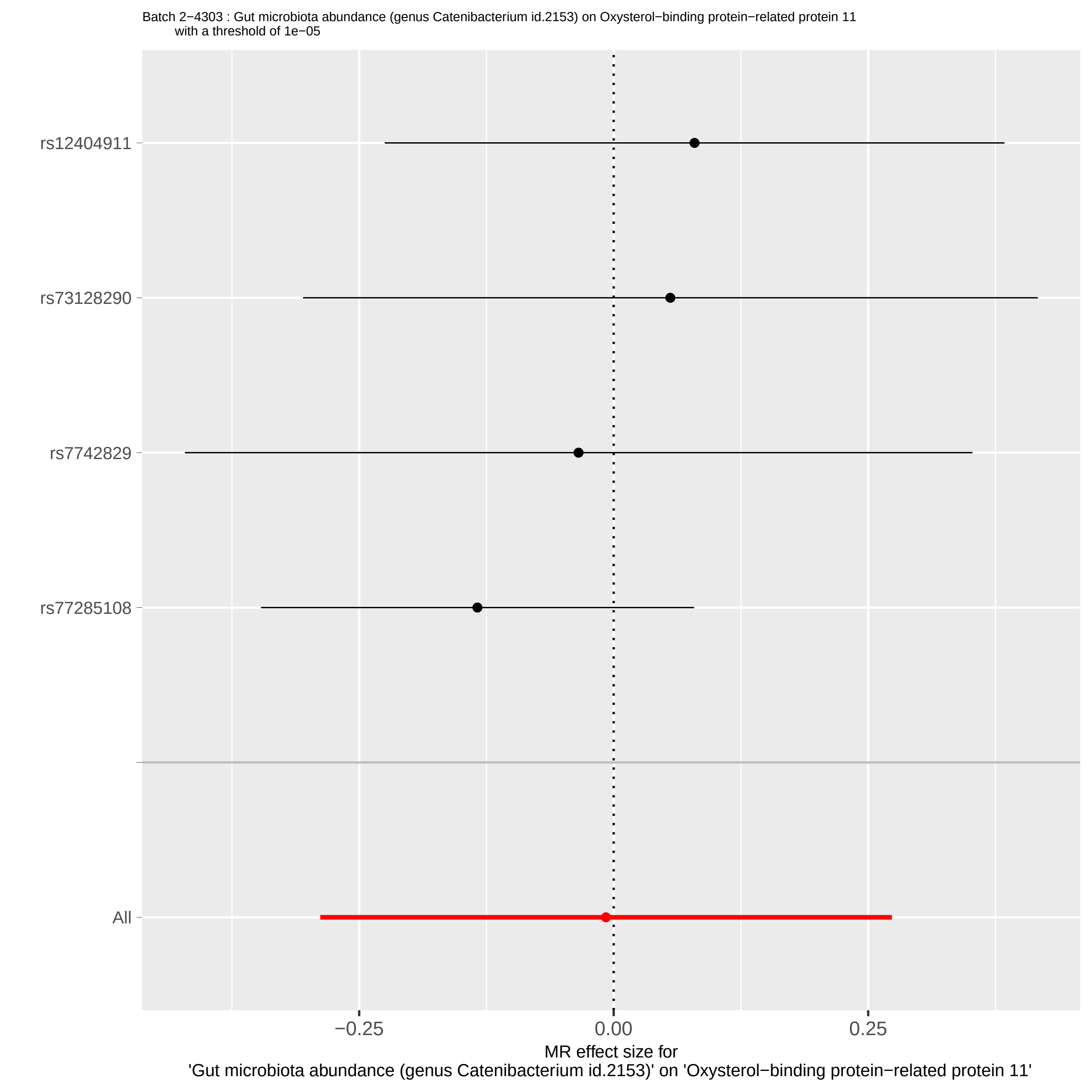

Batch 2−4303 : Gut microbiota abundance (genus Catenibacterium id.2153) on Oxysterol−binding protein−related protein 11
with a threshold of 1e−05
rs12404911
rs73128290
rs7742829
rs77285108
All
−0.25
0.00
0.25
MR effect size for
'Gut microbiota abundance (genus Catenibacterium id.2153)' on 'Oxysterol−binding protein−related protein 11'

Batch 2−4304 : Gut microbiota abundance (genus Parasutterella id.2892) on Oxysterol−binding protein−related protein 11
with a threshold of 1e−05

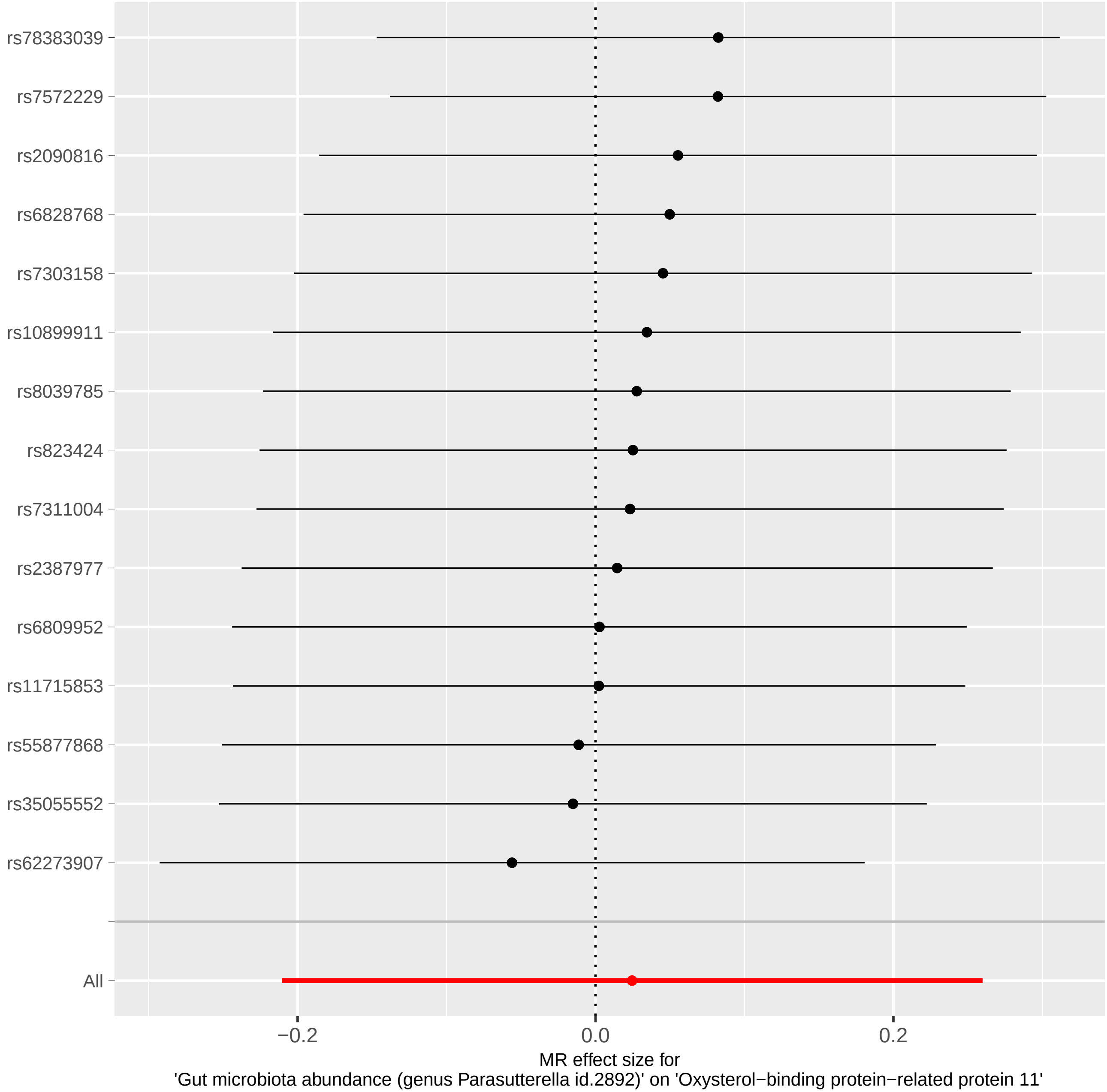

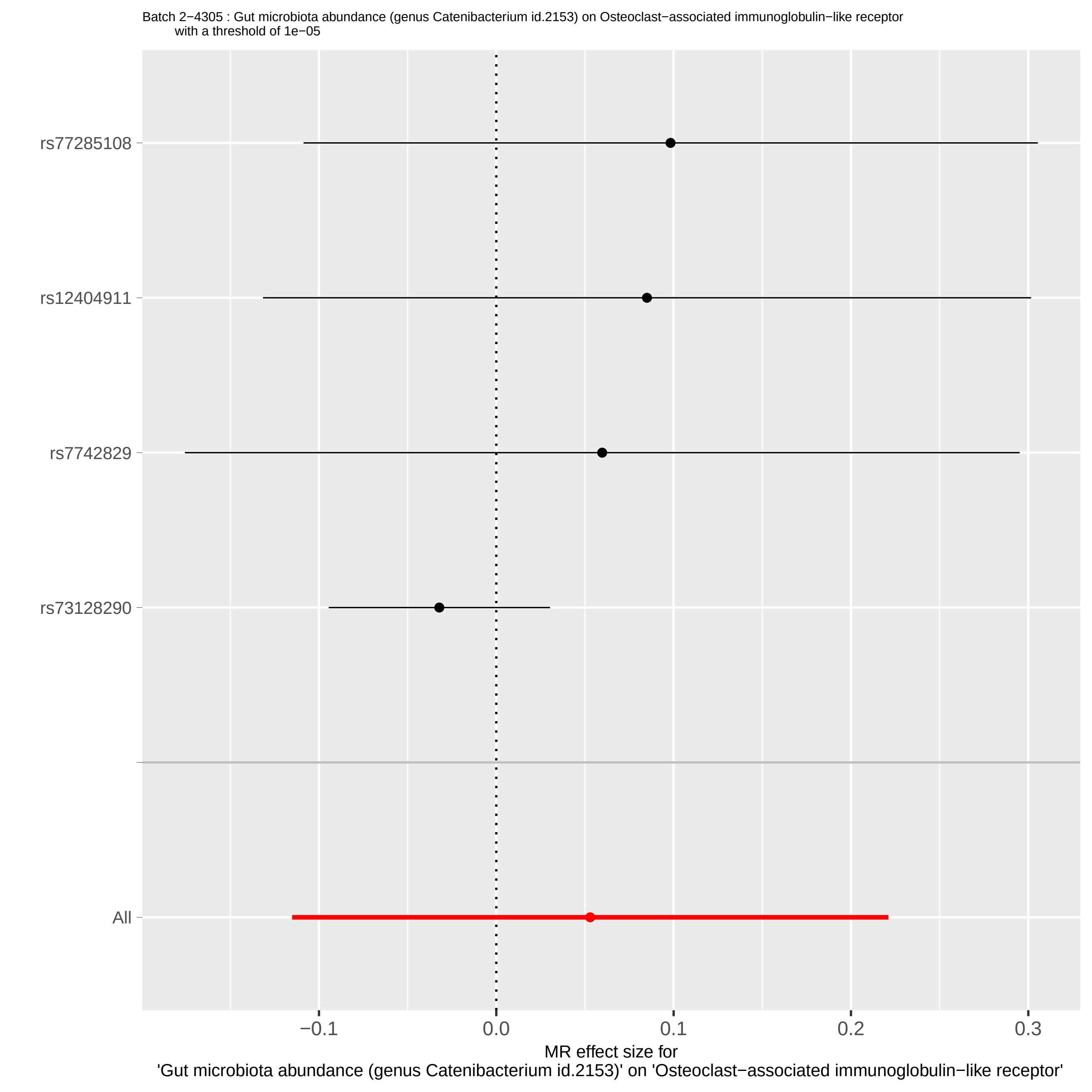

Batch 2−4305 : Gut microbiota abundance (genus Catenibacterium id.2153) on Osteoclast−associated immunoglobulin−like receptor
with a threshold of 1e−05
rs77285108
rs12404911
rs7742829
rs73128290
All
−0.1
0.0
0.1
0.2
0.3
MR effect size for
'Gut microbiota abundance (genus Catenibacterium id.2153)' on 'Osteoclast−associated immunoglobulin−like receptor'

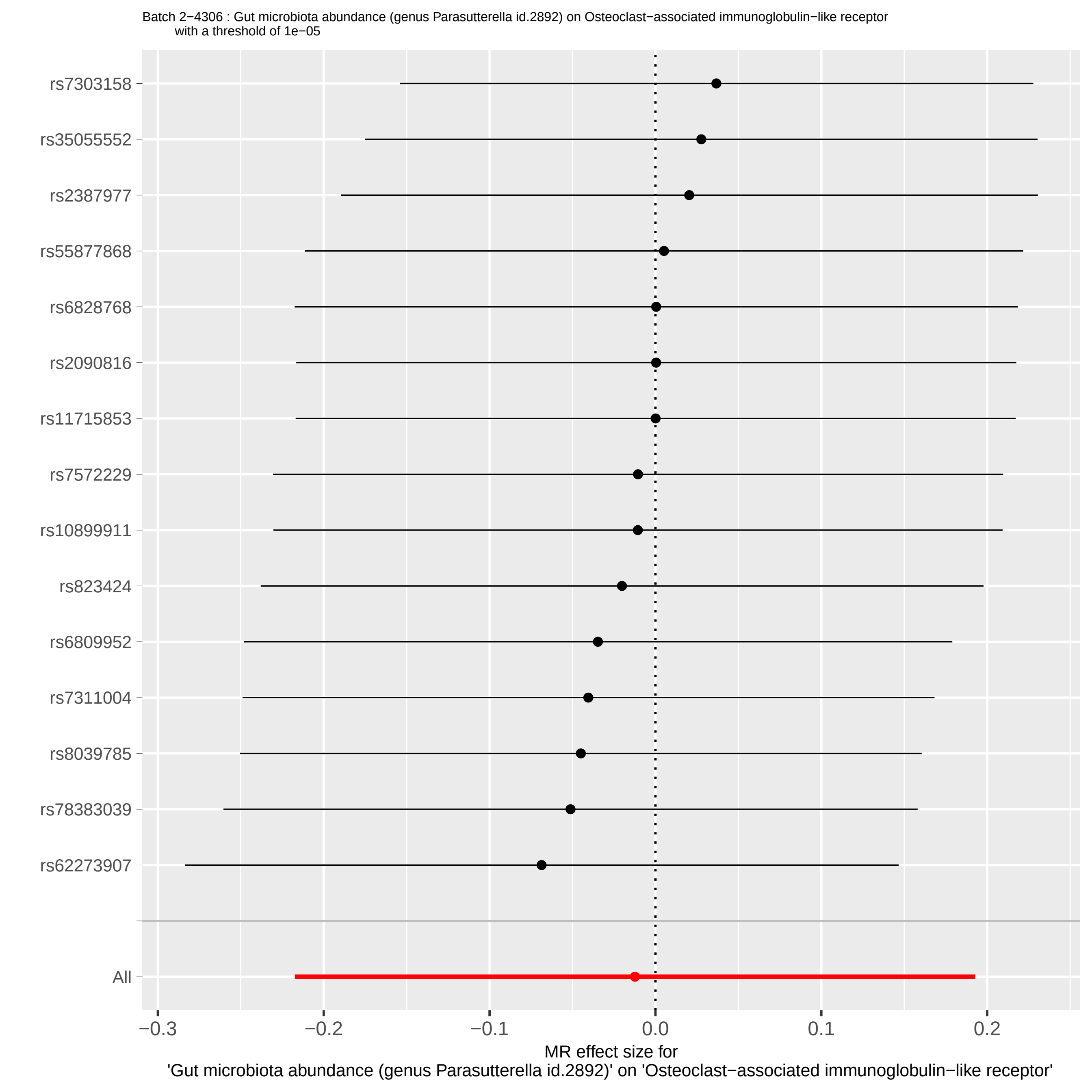
Batch 2−4306 : Gut microbiota abundance (genus Parasutterella id.2892) on Osteoclast−associated immunoglobulin−like receptor
with a threshold of 1e−05
rs7303158
rs35055552
rs2387977
rs55877868
rs6828768
rs2090816
rs11715853
rs7572229
rs10899911
rs823424
rs6809952
rs7311004
rs8039785
rs78383039
rs62273907
All
−0.3
−0.2
−0.1
0.0
0.1
0.2
MR effect size for
'Gut microbiota abundance (genus Parasutterella id.2892)' on 'Osteoclast−associated immunoglobulin−like receptor'

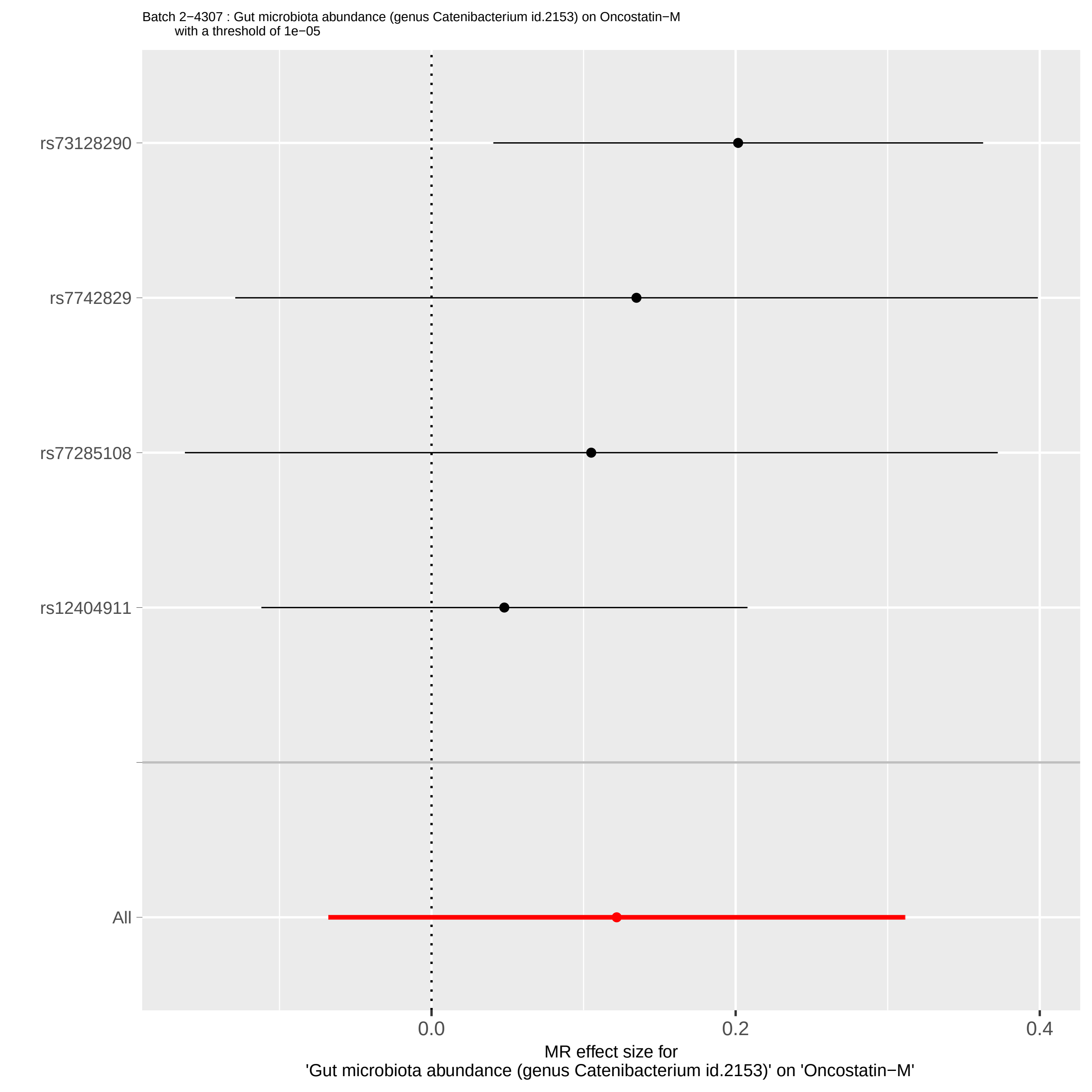
Batch 2−4307 : Gut microbiota abundance (genus Catenibacterium id.2153) on Oncostatin−M
with a threshold of 1e−05
rs73128290
rs7742829
rs77285108
rs12404911
All
0.0
0.2
0.4
MR effect size for
'Gut microbiota abundance (genus Catenibacterium id.2153)' on 'Oncostatin−M'

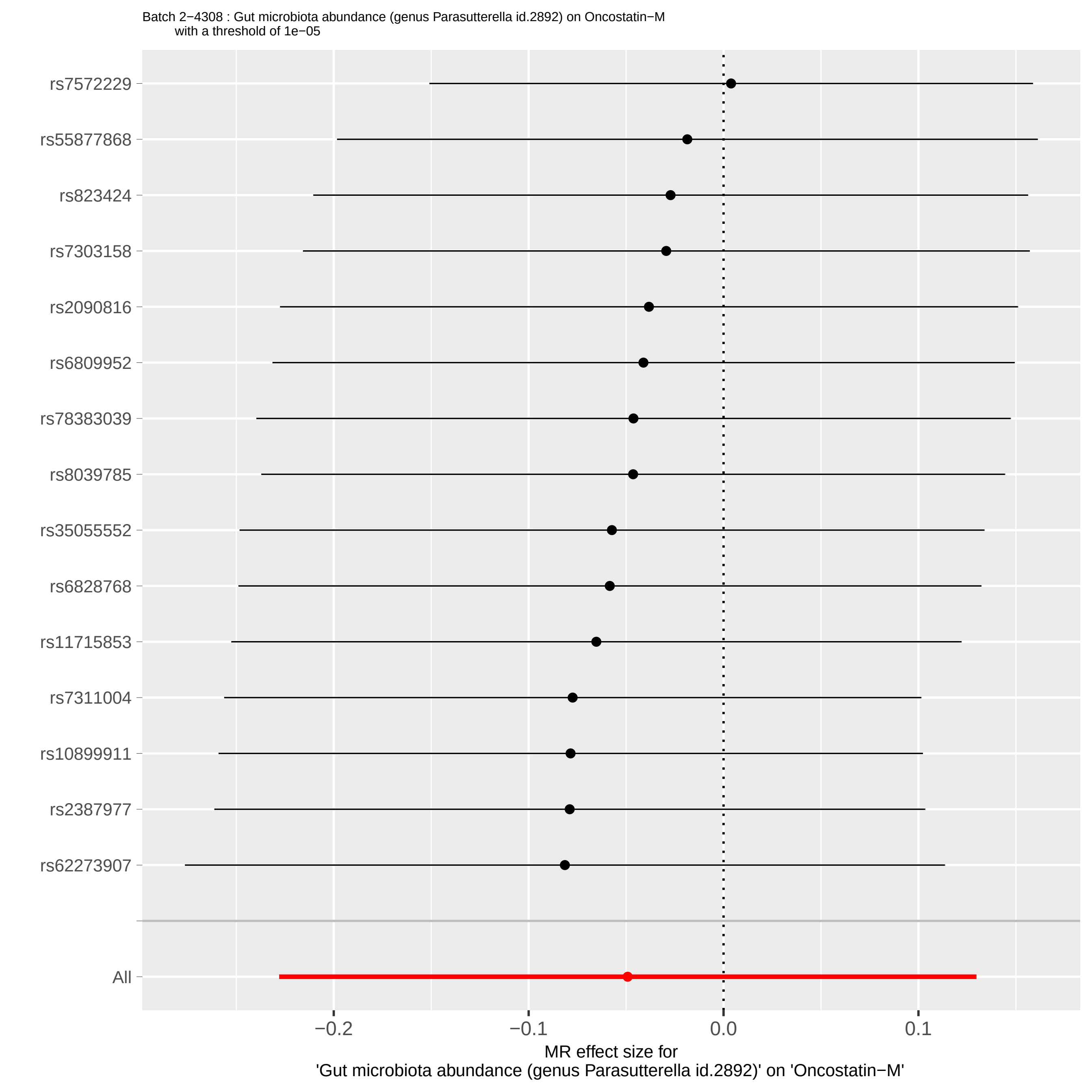

Batch 2−4308 : Gut microbiota abundance (genus Parasutterella id.2892) on Oncostatin−M
with a threshold of 1e−05
rs7572229
rs55877868
rs823424
rs7303158
rs2090816
rs6809952
rs78383039
rs8039785
rs35055552
rs6828768
rs11715853
rs7311004
rs10899911
rs2387977
rs62273907
All
−0.2
−0.1
0.0
0.1
MR effect size for
'Gut microbiota abundance (genus Parasutterella id.2892)' on 'Oncostatin−M'

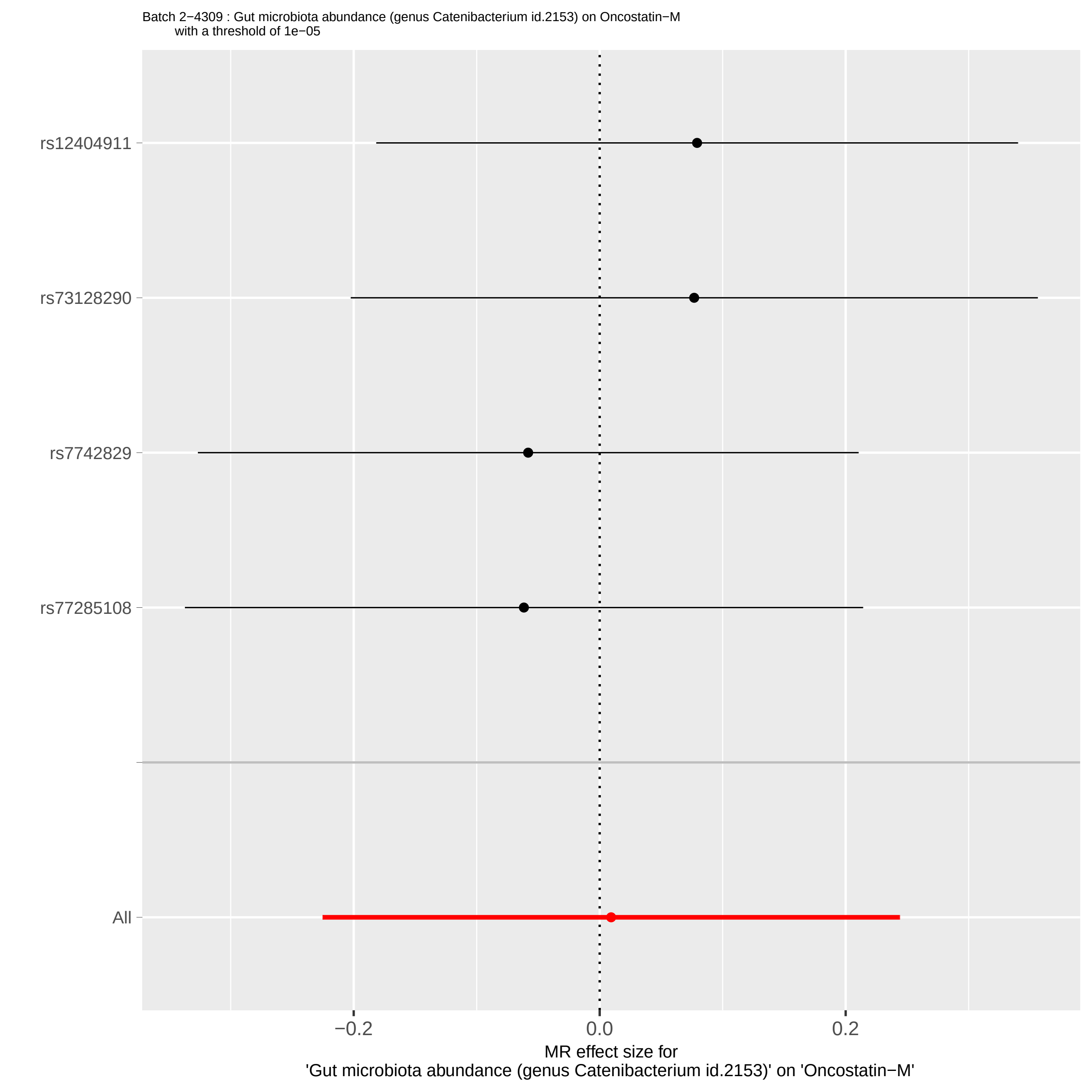
Batch 2−4309 : Gut microbiota abundance (genus Catenibacterium id.2153) on Oncostatin−M
with a threshold of 1e−05
rs12404911
rs73128290
rs7742829
rs77285108
All
−0.2
0.0
0.2
MR effect size for
'Gut microbiota abundance (genus Catenibacterium id.2153)' on 'Oncostatin−M'

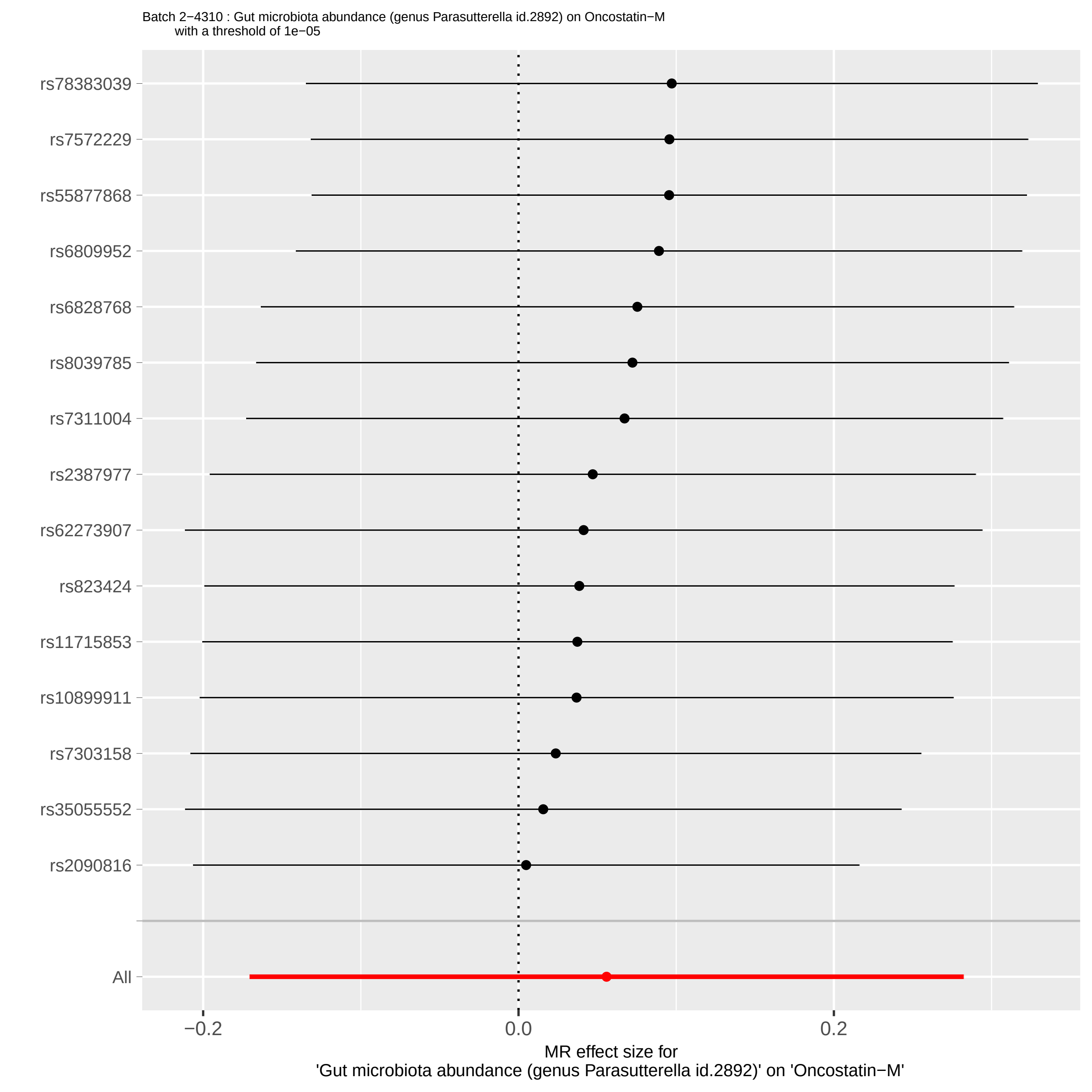

Batch 2−4310 : Gut microbiota abundance (genus Parasutterella id.2892) on Oncostatin−M
with a threshold of 1e−05
rs78383039
rs7572229
rs55877868
rs6809952
rs6828768
rs8039785
rs7311004
rs2387977
rs62273907
rs823424
rs11715853
rs10899911
rs7303158
rs35055552
rs2090816
All
−0.2
0.0
0.2
MR effect size for
'Gut microbiota abundance (genus Parasutterella id.2892)' on 'Oncostatin−M'

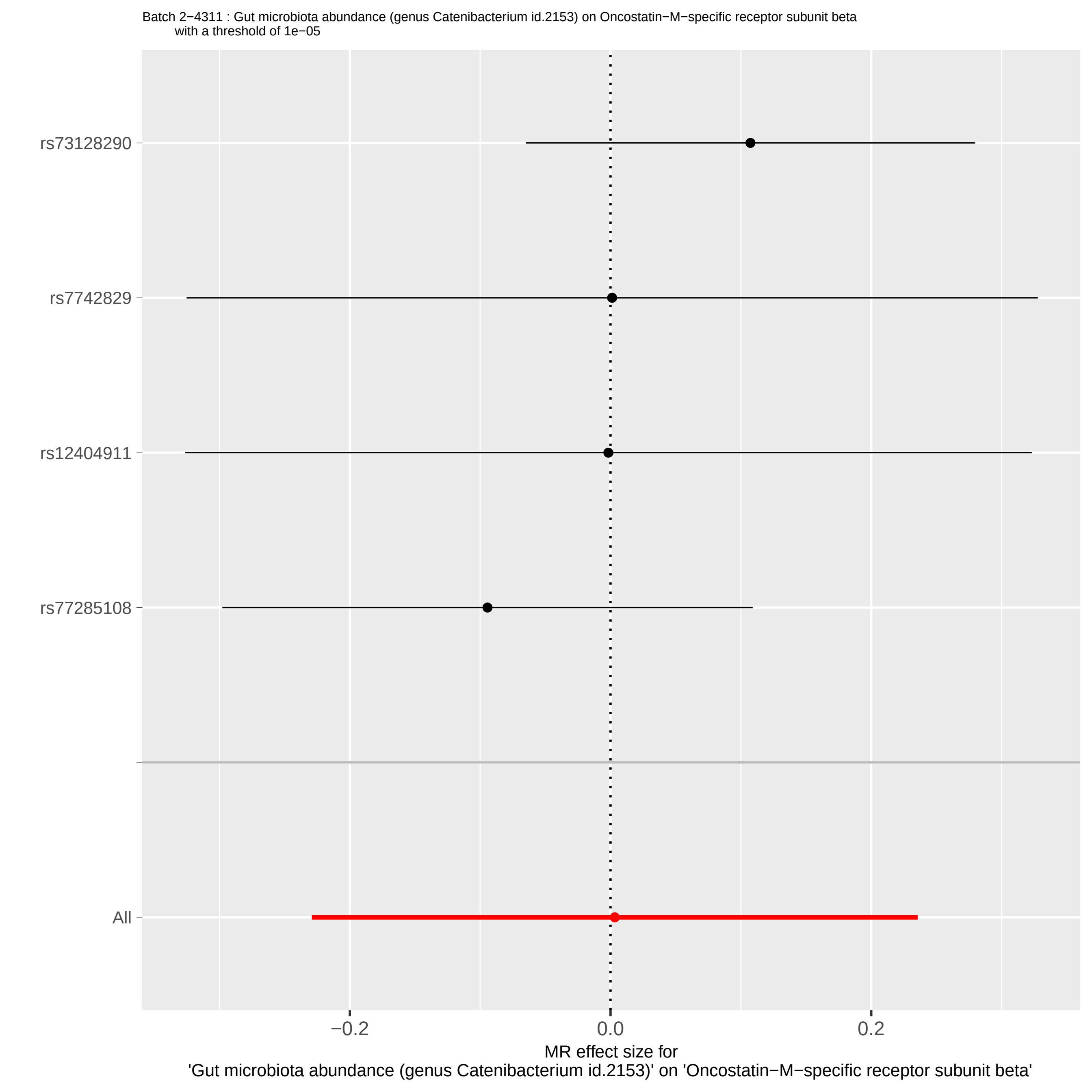
Batch 2−4311 : Gut microbiota abundance (genus Catenibacterium id.2153) on Oncostatin−M−specific receptor subunit beta
with a threshold of 1e−05
rs73128290
rs7742829
rs12404911
rs77285108
All
−0.2
0.0
0.2
MR effect size for
'Gut microbiota abundance (genus Catenibacterium id.2153)' on 'Oncostatin−M−specific receptor subunit beta'

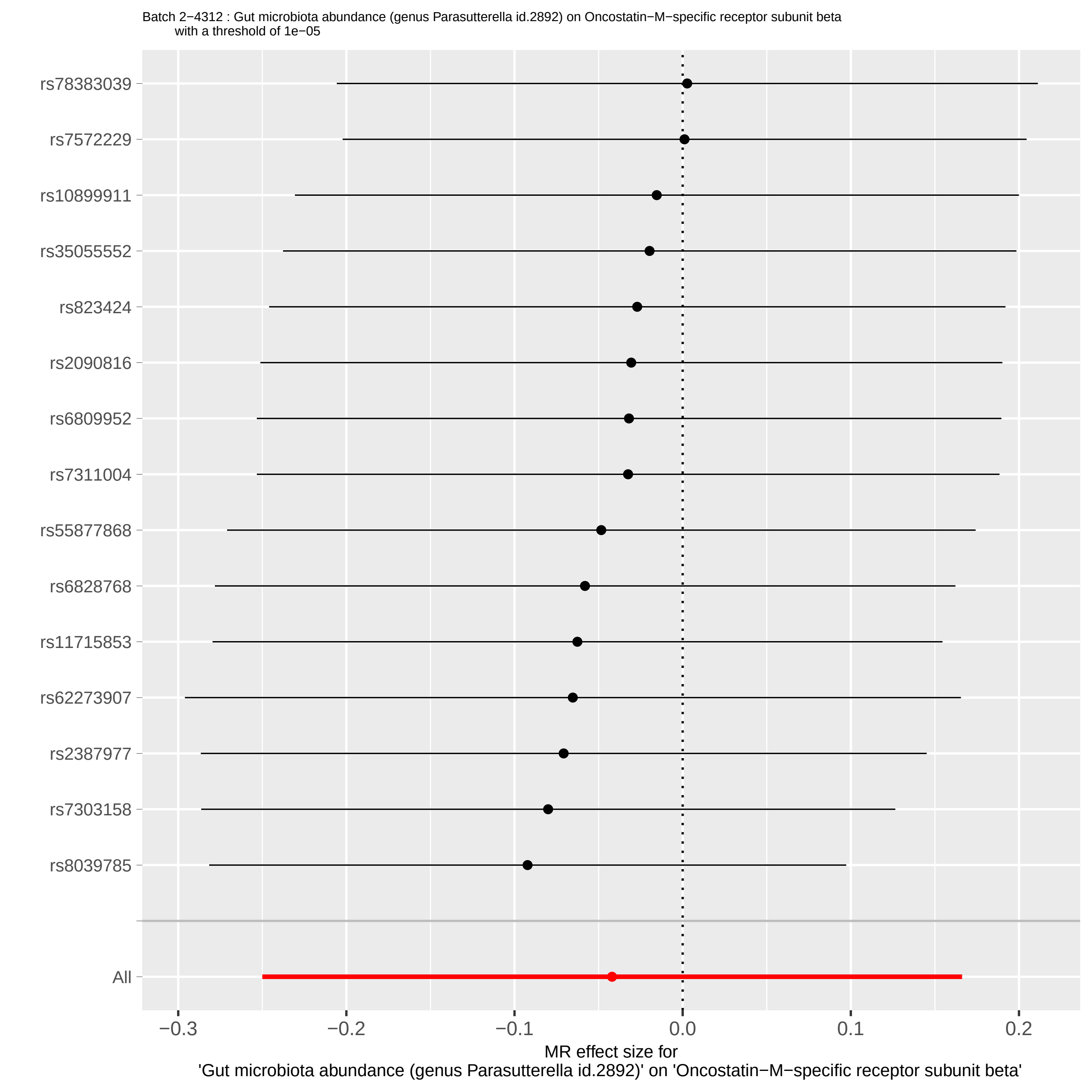
Batch 2−4312 : Gut microbiota abundance (genus Parasutterella id.2892) on Oncostatin−M−specific receptor subunit beta
with a threshold of 1e−05
rs78383039
rs7572229
rs10899911
rs35055552
rs823424
rs2090816
rs6809952
rs7311004
rs55877868
rs6828768
rs11715853
rs62273907
rs2387977
rs7303158
rs8039785
All
−0.3
−0.2
−0.1
0.0
0.1
0.2
MR effect size for
'Gut microbiota abundance (genus Parasutterella id.2892)' on 'Oncostatin−M−specific receptor subunit beta'

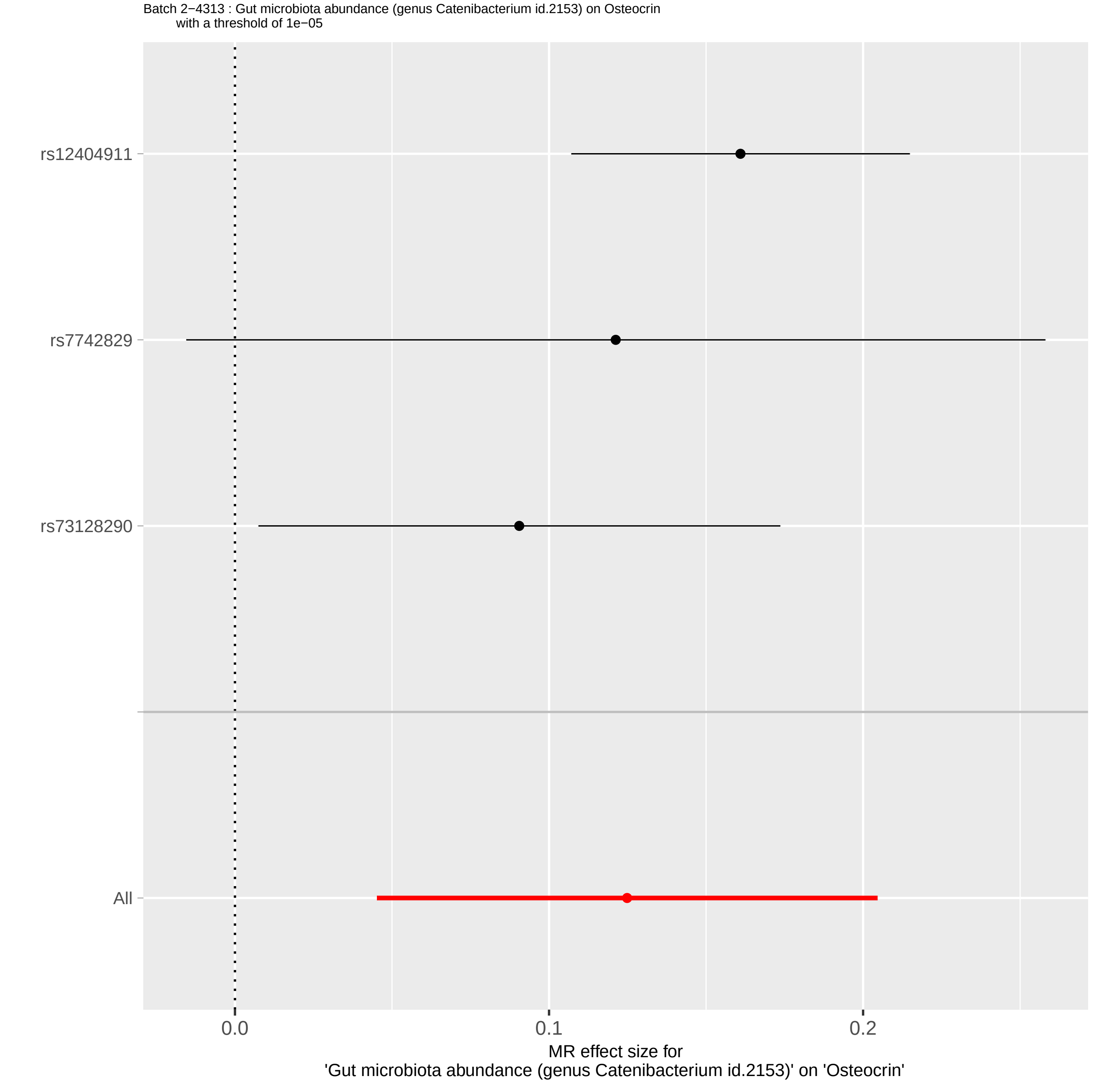
Batch 2−4313 : Gut microbiota abundance (genus Catenibacterium id.2153) on Osteocrin
with a threshold of 1e−05
rs12404911
rs7742829
rs73128290
All
0.0
0.1
0.2
MR effect size for
'Gut microbiota abundance (genus Catenibacterium id.2153)' on 'Osteocrin'

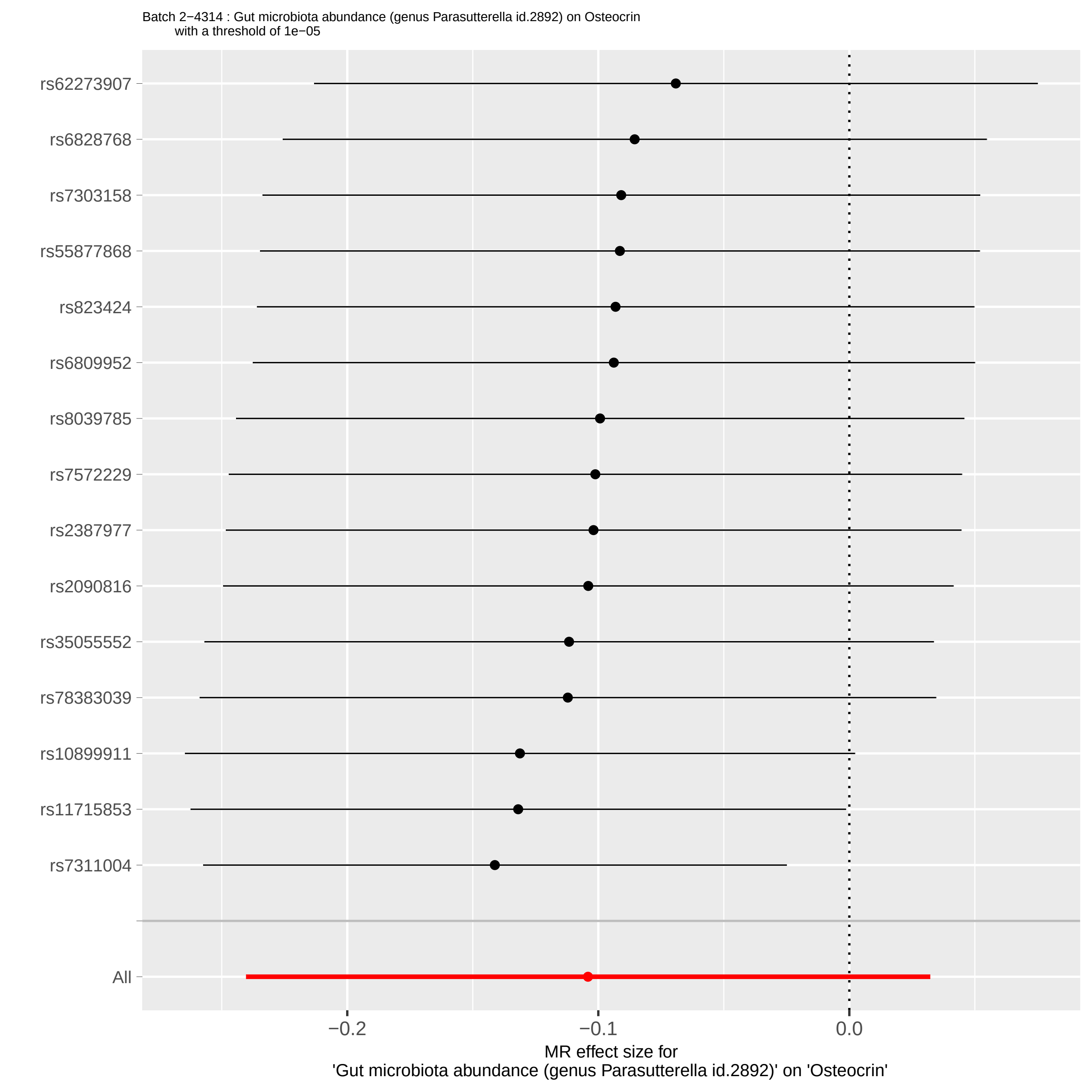

Batch 2−4314 : Gut microbiota abundance (genus Parasutterella id.2892) on Osteocrin
with a threshold of 1e−05
rs62273907
rs6828768
rs7303158
rs55877868
rs823424
rs6809952
rs8039785
rs7572229
rs2387977
rs2090816
rs35055552
rs78383039
rs10899911
rs11715853
rs7311004
All
−0.2
−0.1
0.0
MR effect size for
'Gut microbiota abundance (genus Parasutterella id.2892)' on 'Osteocrin'

Batch 2−4315 : Gut microbiota abundance (genus Catenibacterium id.2153) on Ubiquitin thioesterase OTUB2
with a threshold of 1e−05

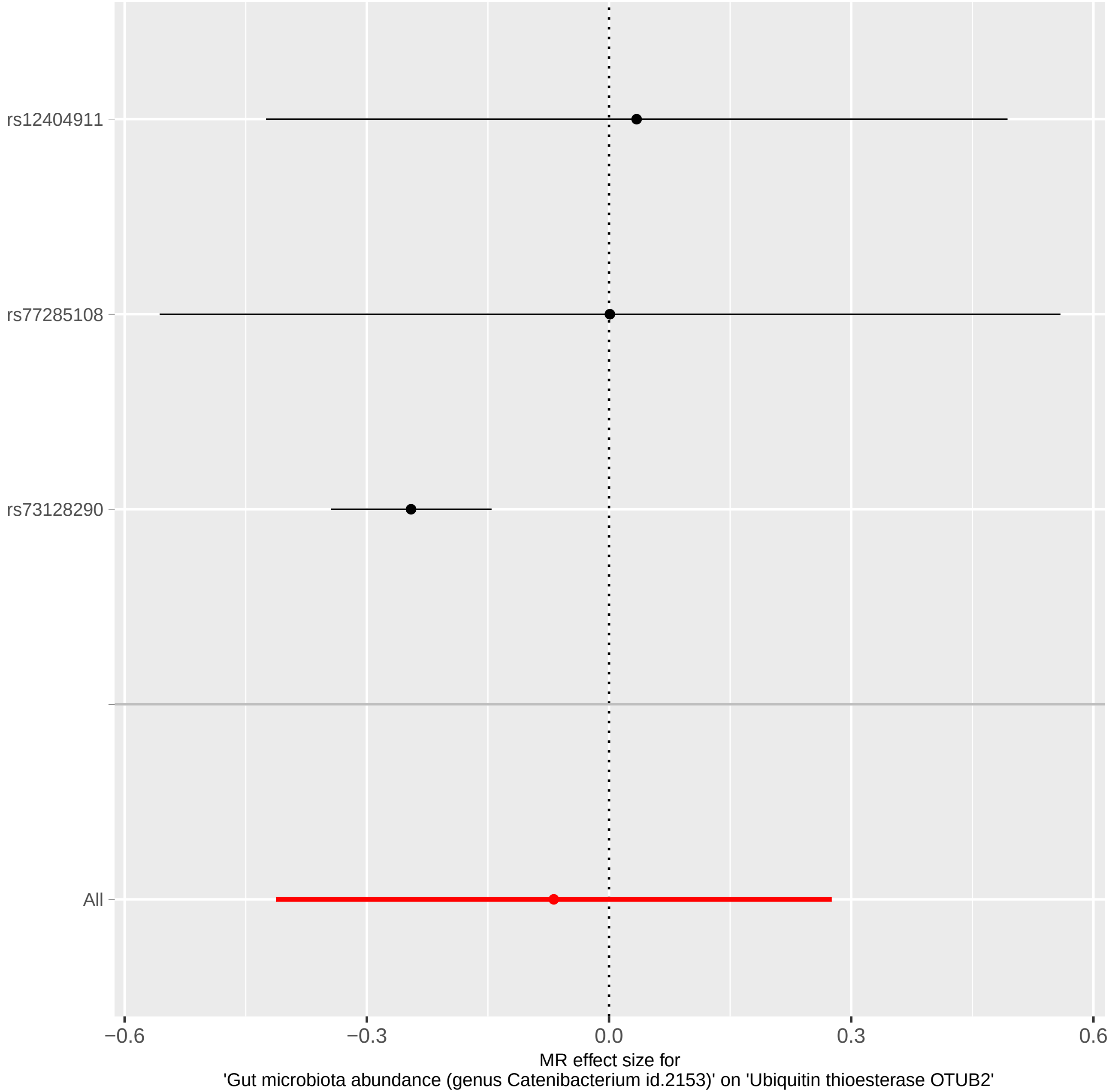

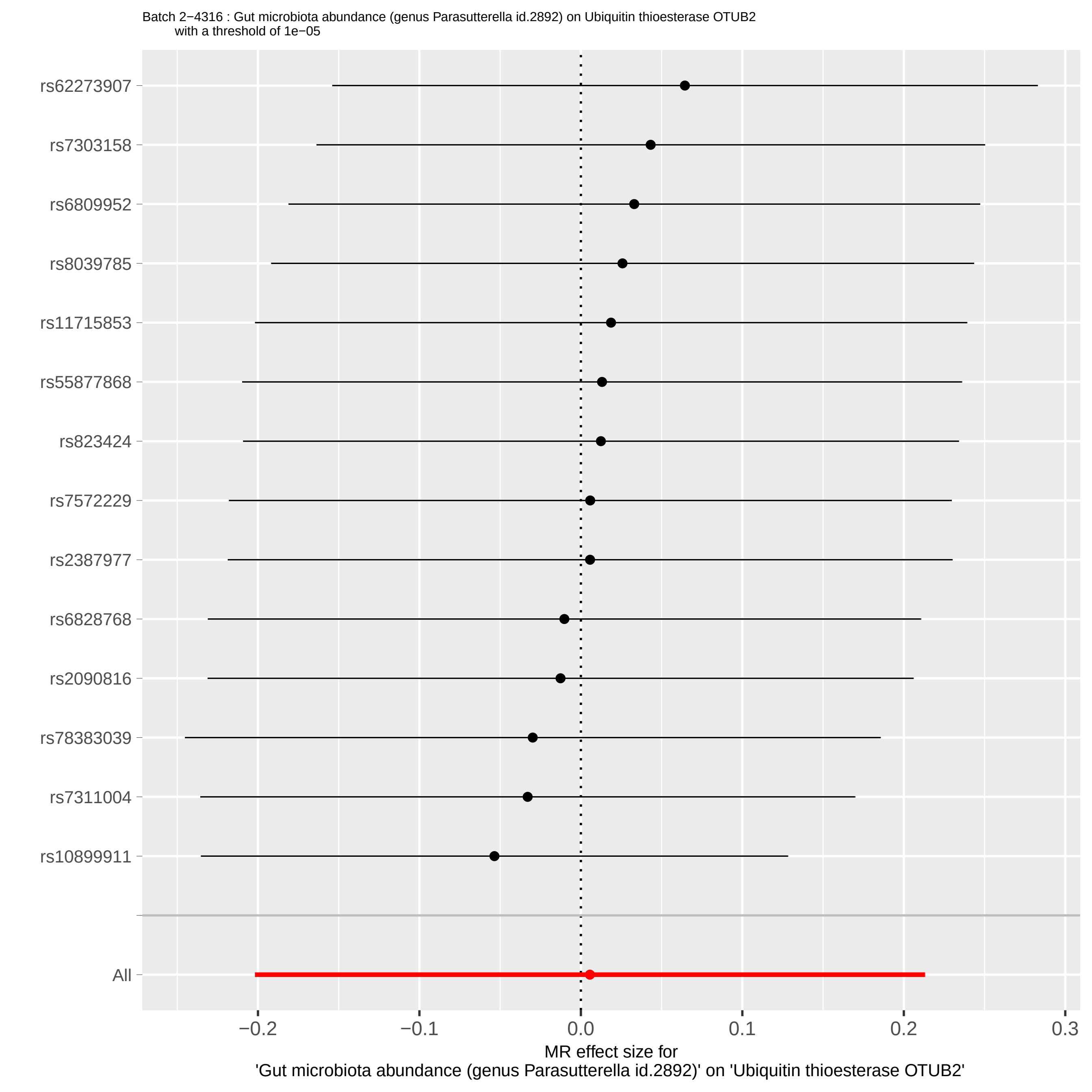

Batch 2−4316 : Gut microbiota abundance (genus Parasutterella id.2892) on Ubiquitin thioesterase OTUB2
with a threshold of 1e−05
rs62273907
rs7303158
rs6809952
rs8039785
rs11715853
rs55877868
rs823424
rs7572229
rs2387977
rs6828768
rs2090816
rs78383039
rs7311004
rs10899911
All
−0.2
−0.1
0.0
0.1
0.2
0.3
MR effect size for
'Gut microbiota abundance (genus Parasutterella id.2892)' on 'Ubiquitin thioesterase OTUB2'

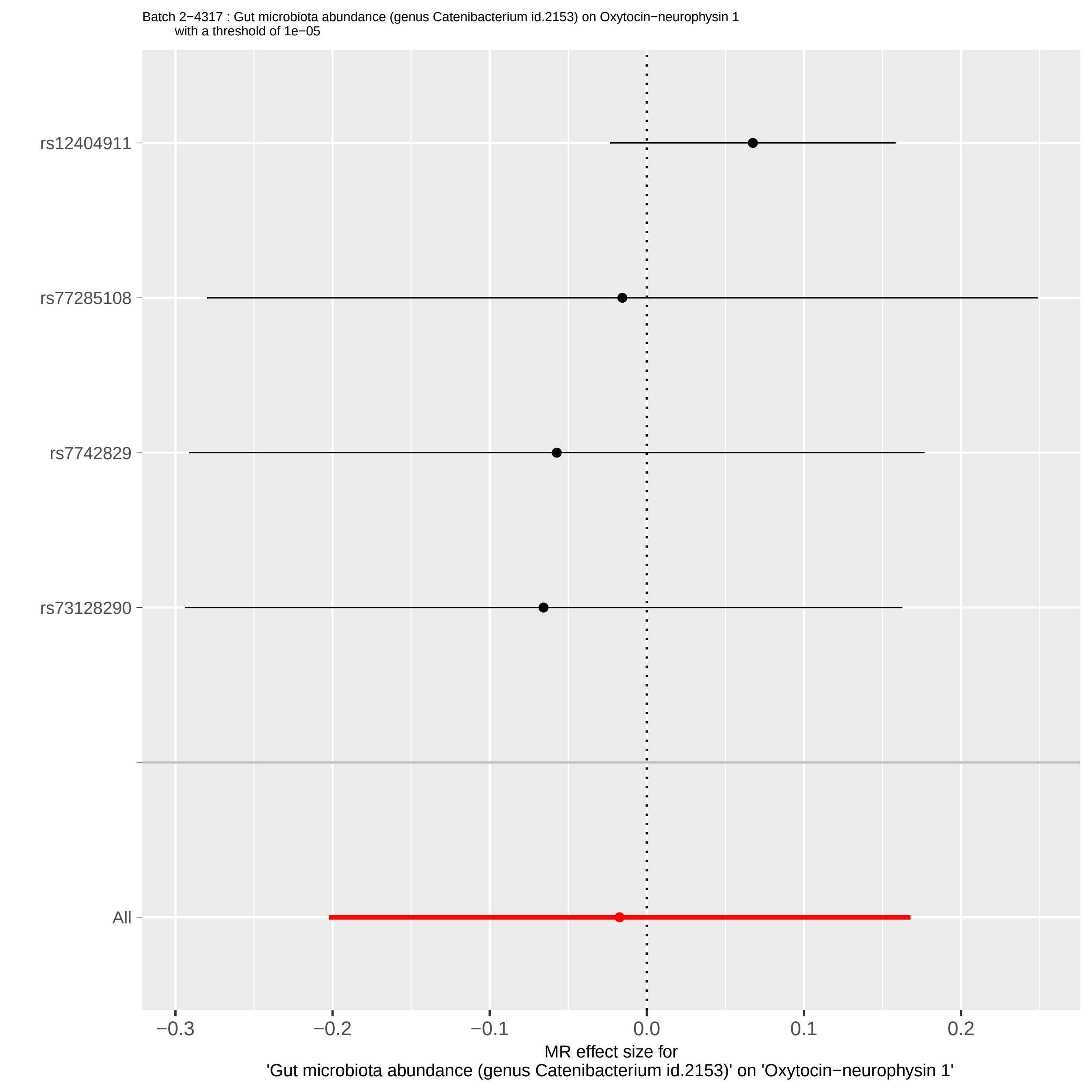
Batch 2−4317 : Gut microbiota abundance (genus Catenibacterium id.2153) on Oxytocin−neurophysin 1
with a threshold of 1e−05
rs12404911
rs77285108
rs7742829
rs73128290
All
−0.3
−0.2
−0.1
0.0
0.1
0.2
MR effect size for
'Gut microbiota abundance (genus Catenibacterium id.2153)' on 'Oxytocin−neurophysin 1'

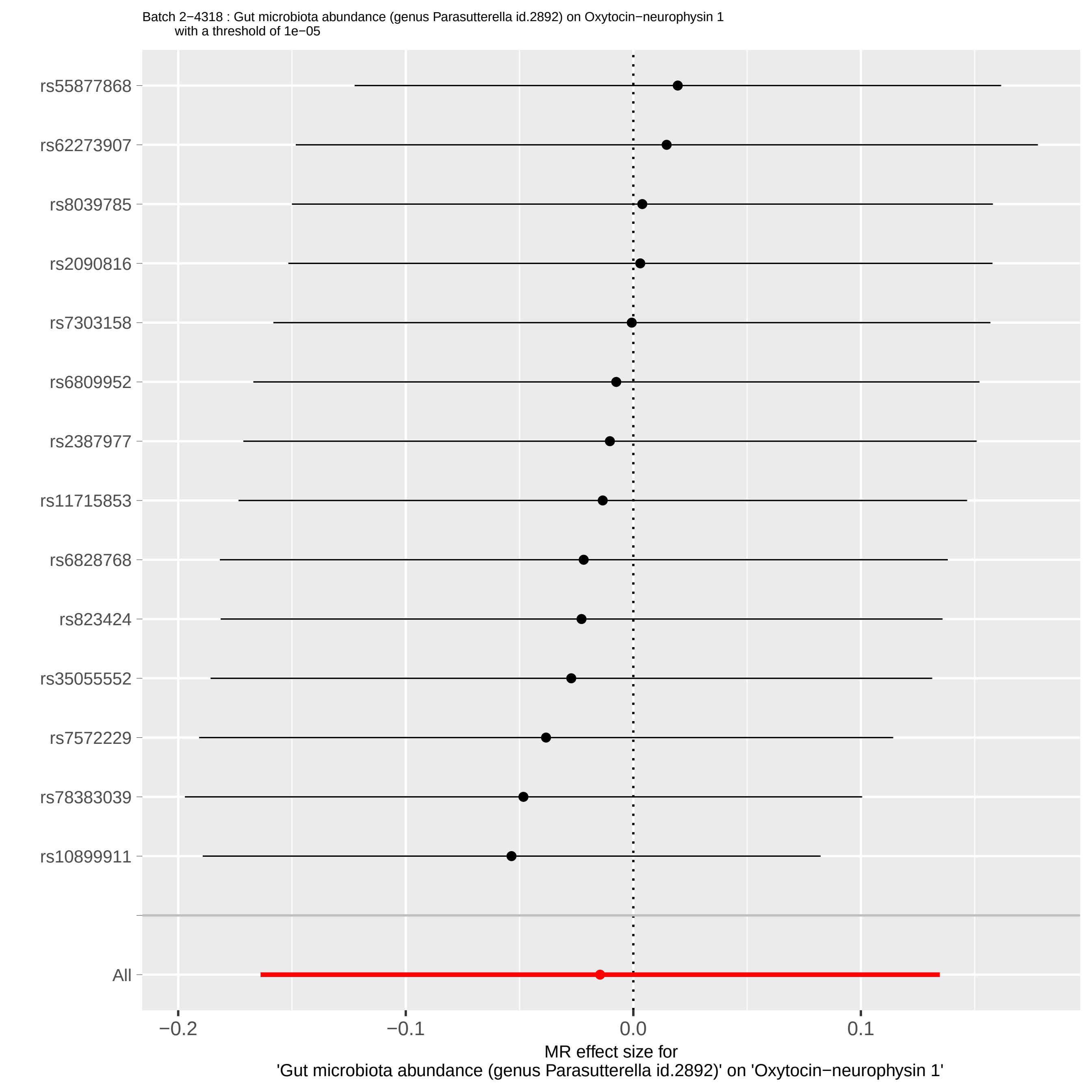
Batch 2−4318 : Gut microbiota abundance (genus Parasutterella id.2892) on Oxytocin−neurophysin 1
with a threshold of 1e−05
rs55877868
rs62273907
rs8039785
rs2090816
rs7303158
rs6809952
rs2387977
rs11715853
rs6828768
rs823424
rs35055552
rs7572229
rs78383039
rs10899911
All
−0.2
−0.1
0.0
0.1
MR effect size for
'Gut microbiota abundance (genus Parasutterella id.2892)' on 'Oxytocin−neurophysin 1'

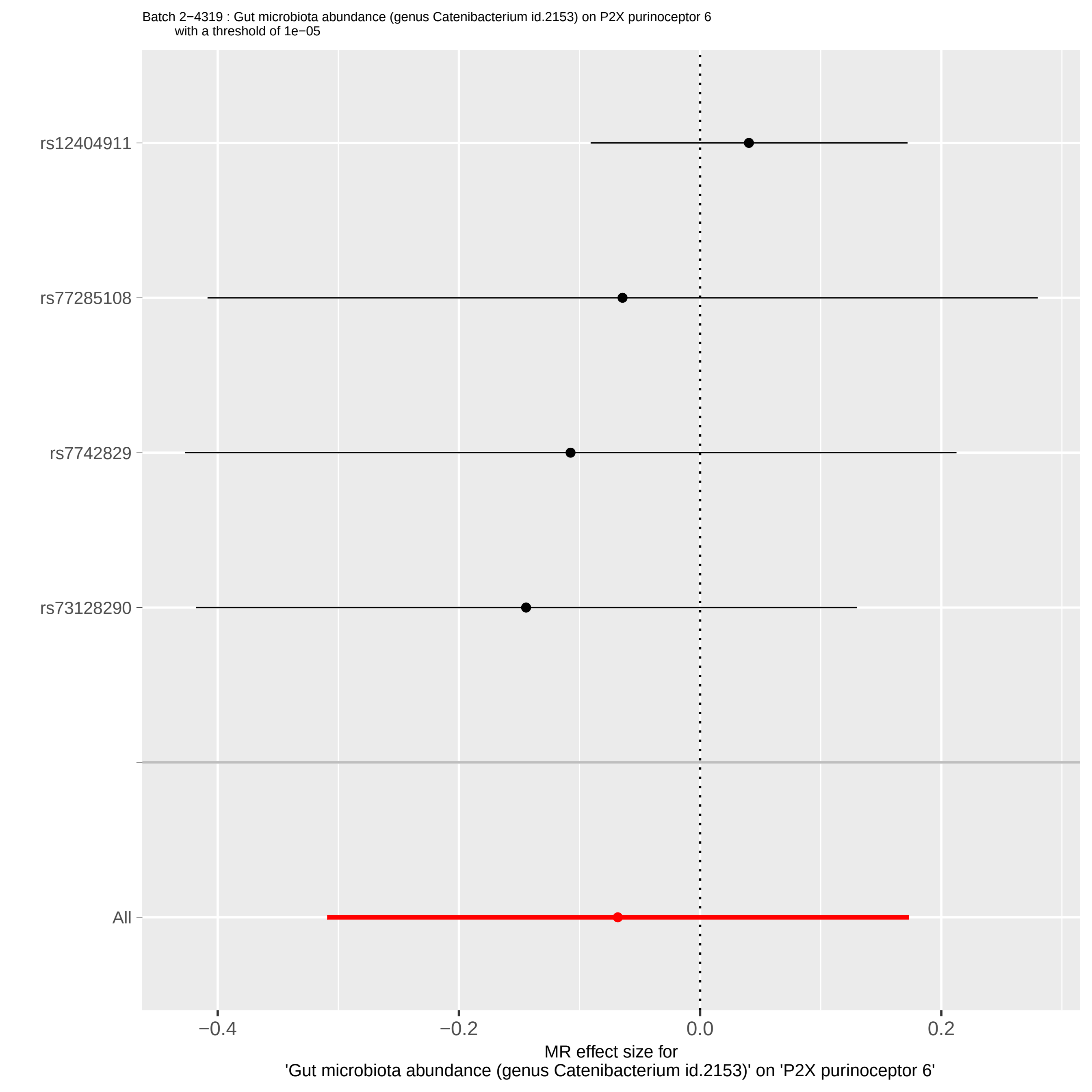
Batch 2−4319 : Gut microbiota abundance (genus Catenibacterium id.2153) on P2X purinoceptor 6
with a threshold of 1e−05
rs12404911
rs77285108
rs7742829
rs73128290
All
−0.4
−0.2
0.0
0.2
MR effect size for
'Gut microbiota abundance (genus Catenibacterium id.2153)' on 'P2X purinoceptor 6'

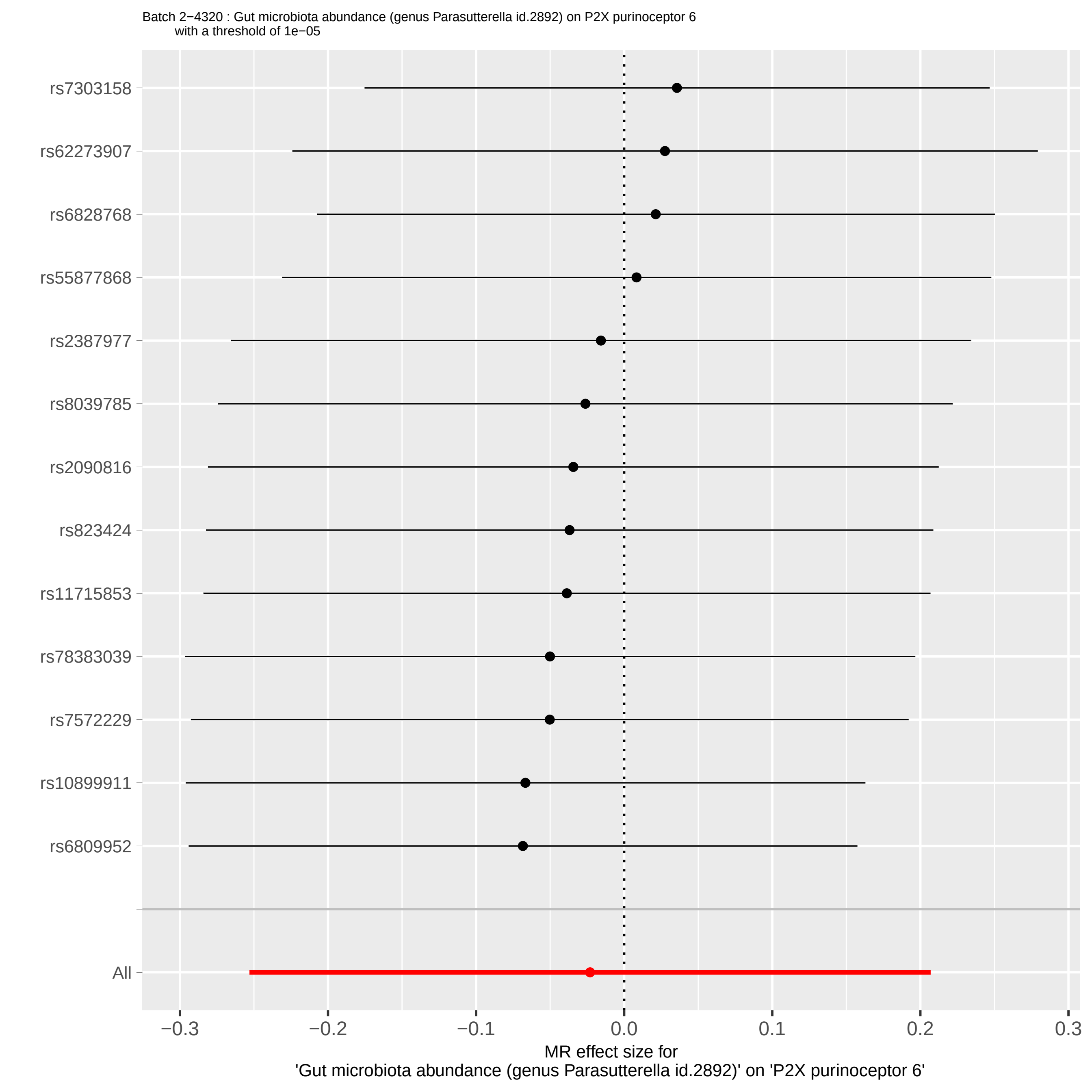
Batch 2−4320 : Gut microbiota abundance (genus Parasutterella id.2892) on P2X purinoceptor 6
with a threshold of 1e−05
rs7303158
rs62273907
rs6828768
rs55877868
rs2387977
rs8039785
rs2090816
rs823424
rs11715853
rs78383039
rs7572229
rs10899911
rs6809952
All
−0.3
−0.2
−0.1
0.0
0.1
0.2
0.3
MR effect size for
'Gut microbiota abundance (genus Parasutterella id.2892)' on 'P2X purinoceptor 6'

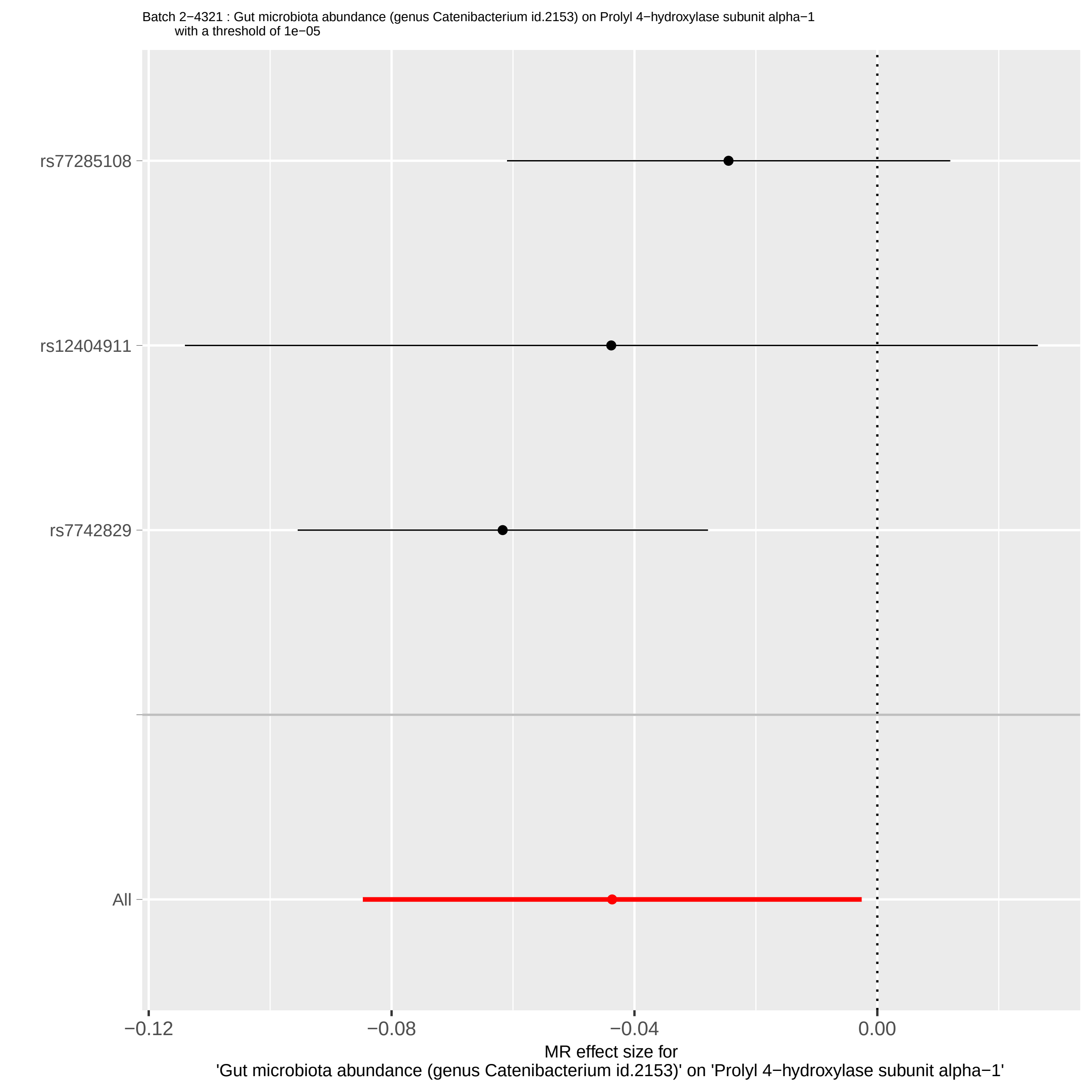
Batch 2−4321 : Gut microbiota abundance (genus Catenibacterium id.2153) on Prolyl 4−hydroxylase subunit alpha−1
with a threshold of 1e−05
rs77285108
rs12404911
rs7742829
All
−0.12
−0.08
−0.04
0.00
MR effect size for
'Gut microbiota abundance (genus Catenibacterium id.2153)' on 'Prolyl 4−hydroxylase subunit alpha−1'

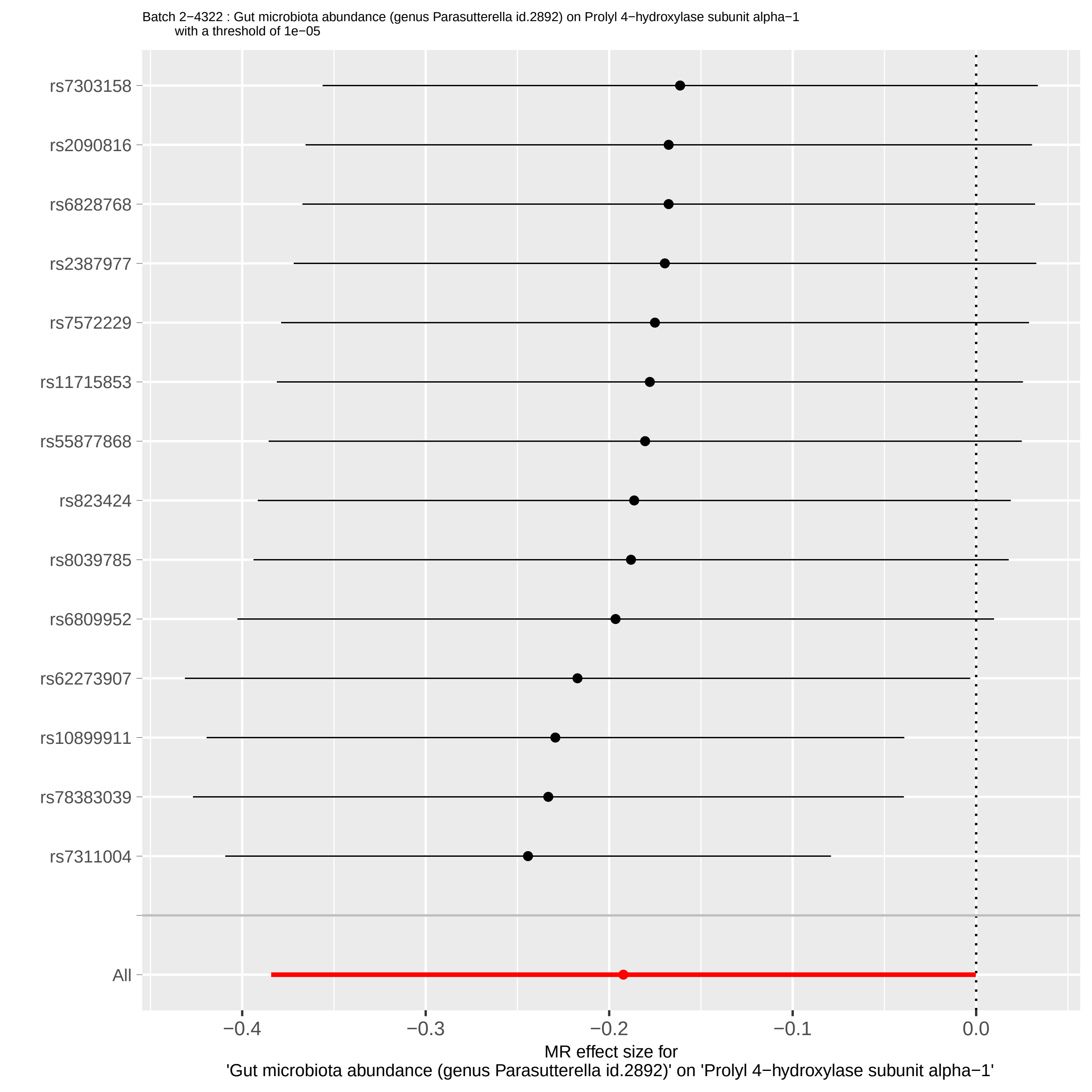

Batch 2−4322 : Gut microbiota abundance (genus Parasutterella id.2892) on Prolyl 4−hydroxylase subunit alpha−1
with a threshold of 1e−05
rs7303158
rs2090816
rs6828768
rs2387977
rs7572229
rs11715853
rs55877868
rs823424
rs8039785
rs6809952
rs62273907
rs10899911
rs78383039
rs7311004
All
−0.4
−0.3
−0.2
−0.1
0.0
MR effect size for
'Gut microbiota abundance (genus Parasutterella id.2892)' on 'Prolyl 4−hydroxylase subunit alpha−1'

Batch 2−4323 : Gut microbiota abundance (genus Catenibacterium id.2153) on Prolyl 4−hydroxylase subunit alpha−2
with a threshold of 1e−05

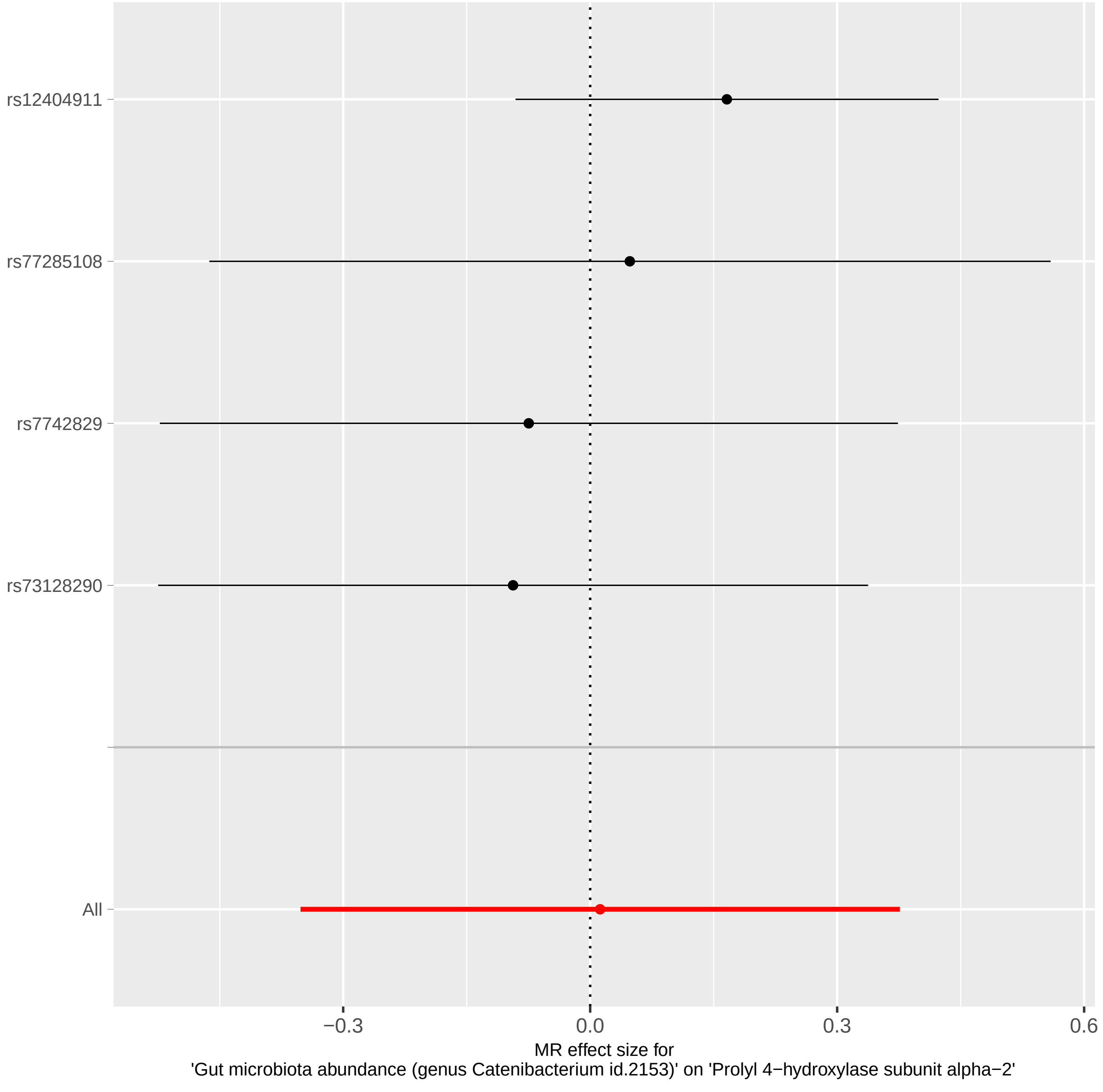

Batch 2−4324 : Gut microbiota abundance (genus Parasutterella id.2892) on Prolyl 4−hydroxylase subunit alpha−2
with a threshold of 1e−05

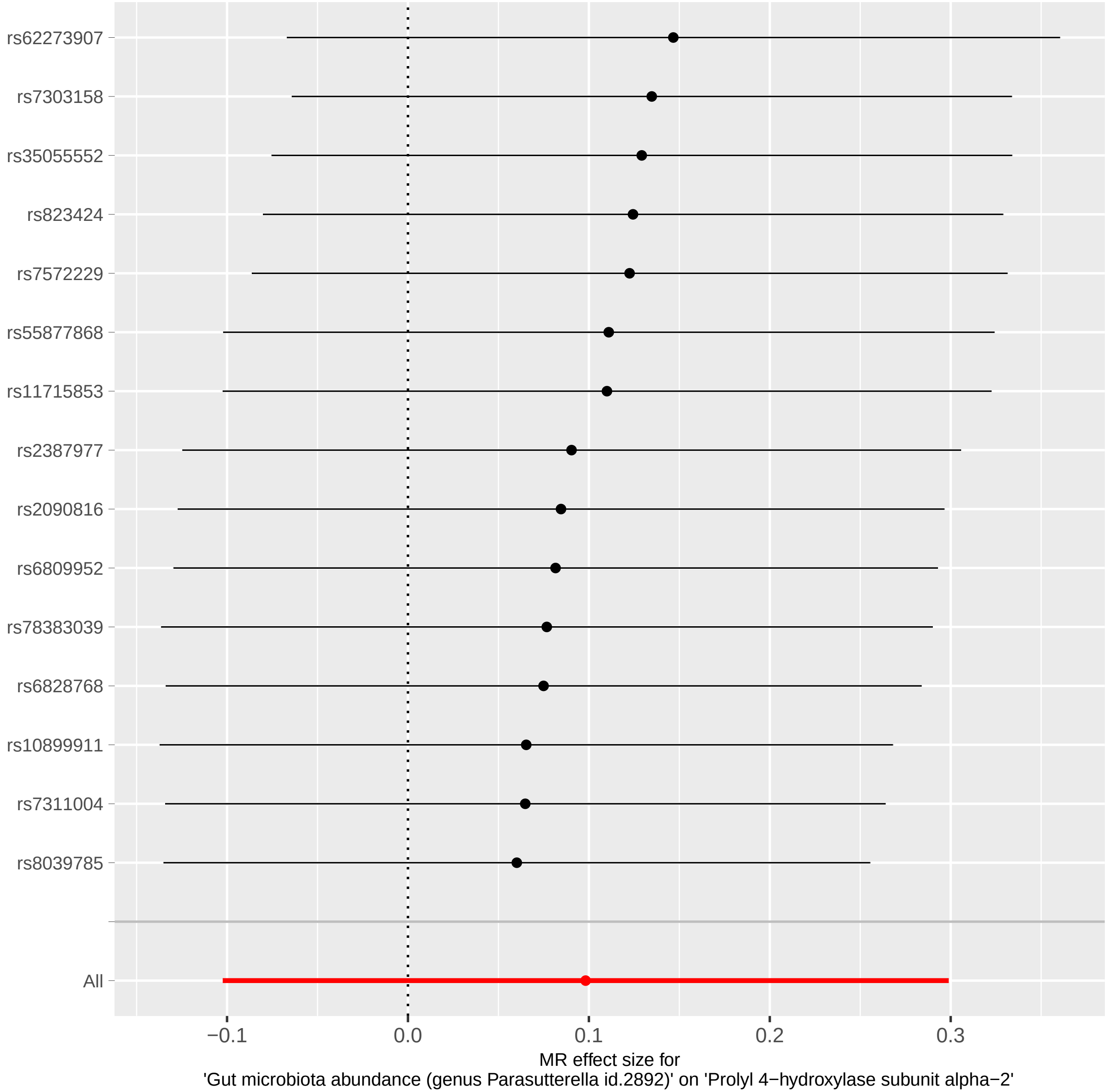

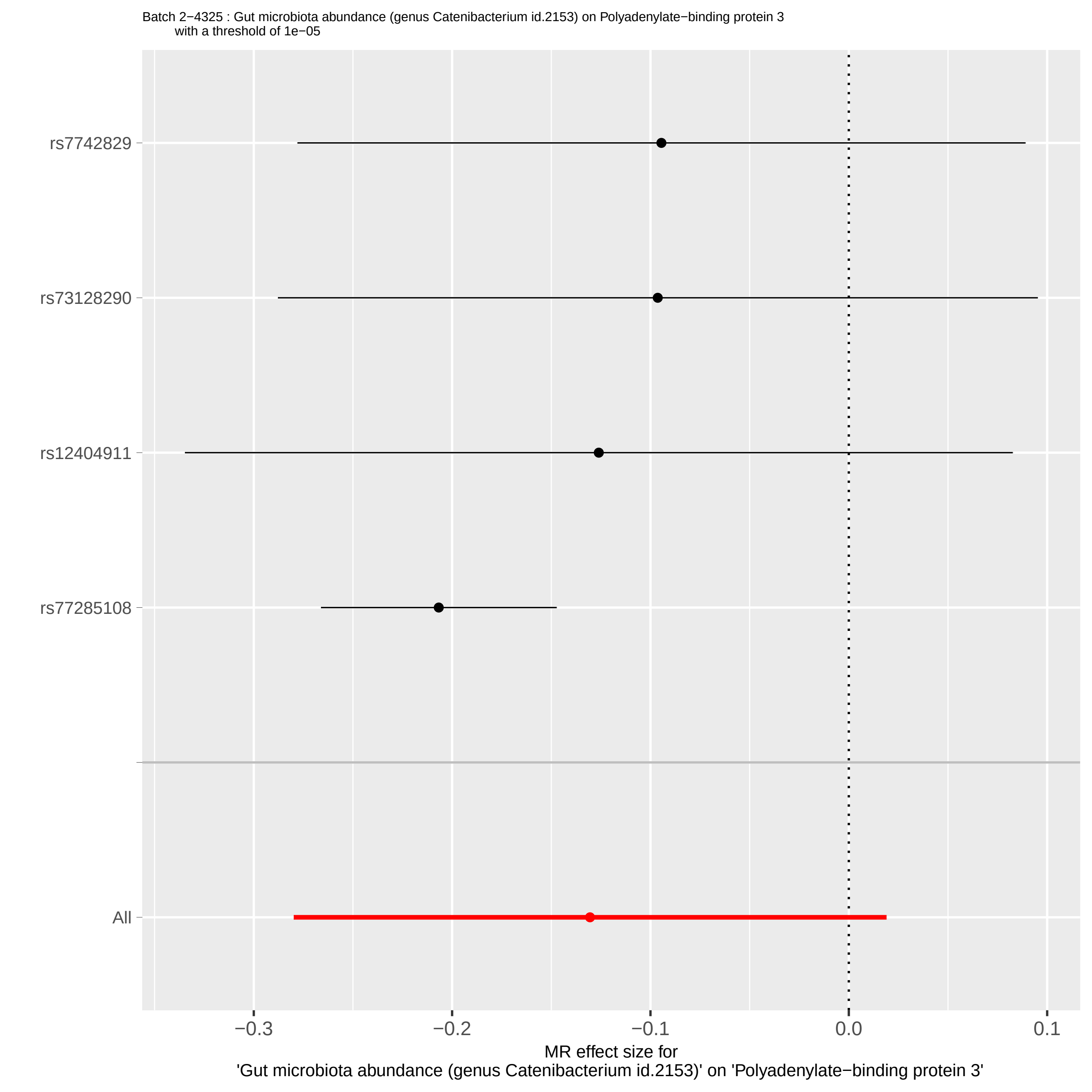
Batch 2−4325 : Gut microbiota abundance (genus Catenibacterium id.2153) on Polyadenylate−binding protein 3
with a threshold of 1e−05
rs7742829
rs73128290
rs12404911
rs77285108
All
−0.3
−0.2
−0.1
0.0
0.1
MR effect size for
'Gut microbiota abundance (genus Catenibacterium id.2153)' on 'Polyadenylate−binding protein 3'

Batch 2−4326 : Gut microbiota abundance (genus Parasutterella id.2892) on Polyadenylate−binding protein 3
with a threshold of 1e−05

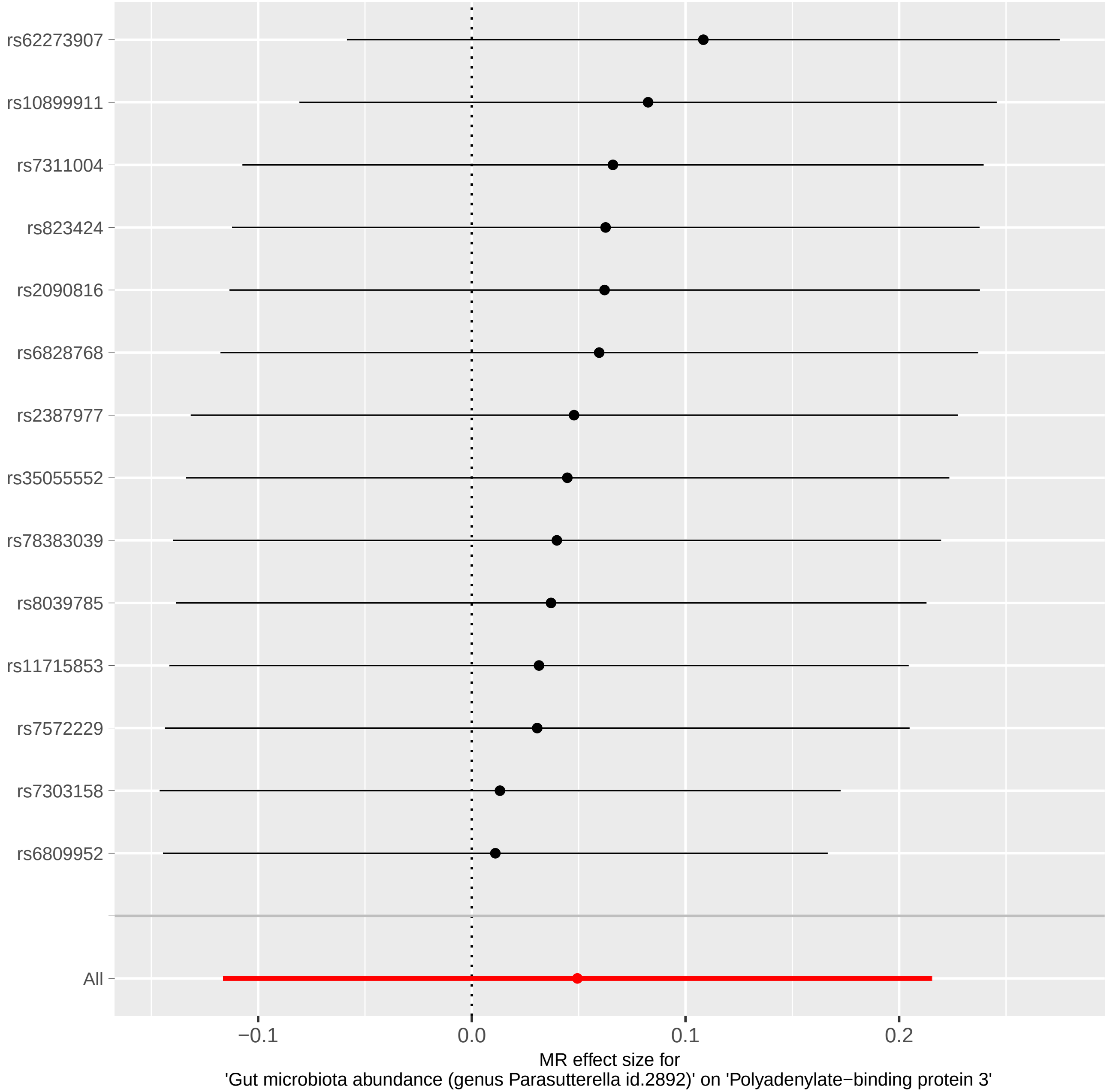

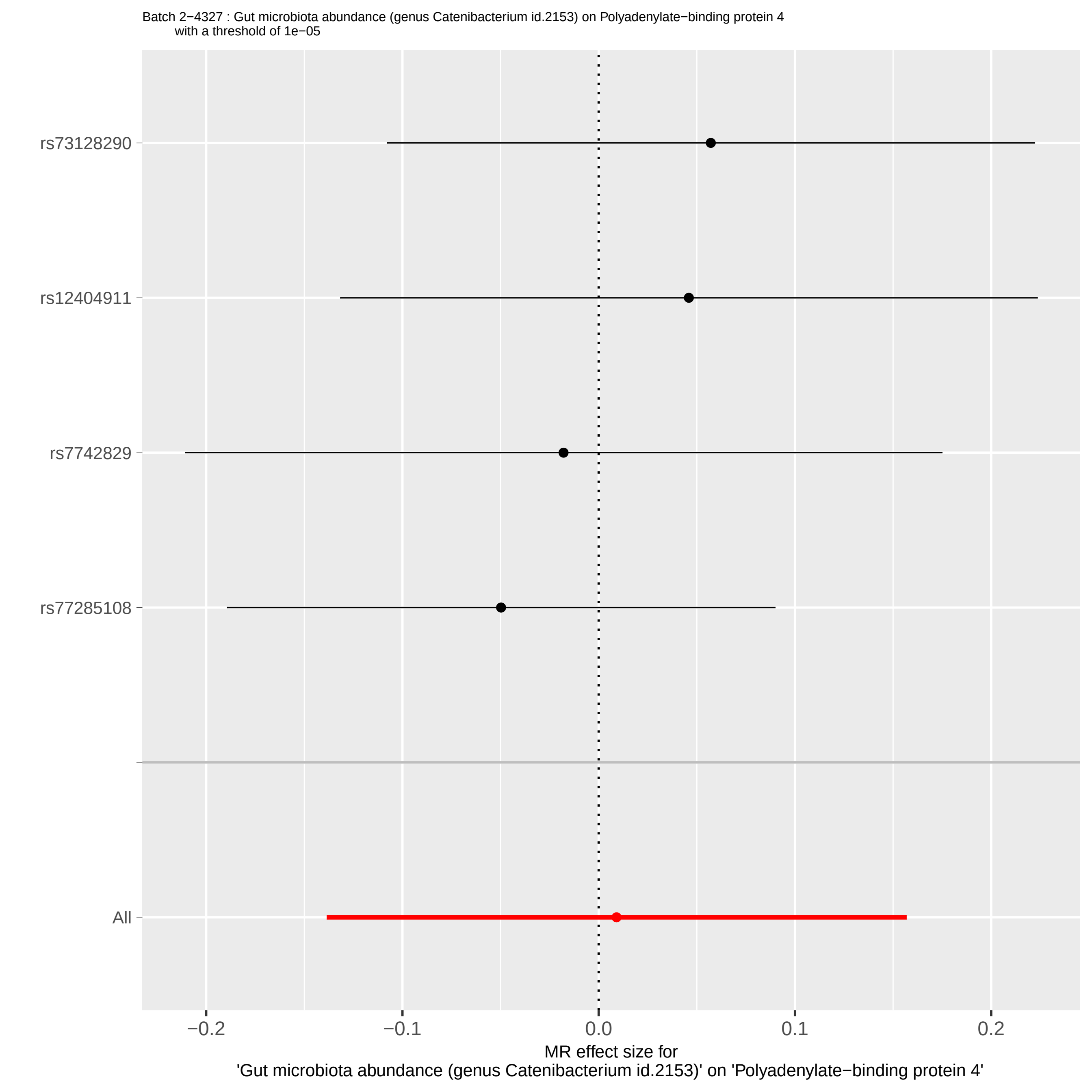
Batch 2−4327 : Gut microbiota abundance (genus Catenibacterium id.2153) on Polyadenylate−binding protein 4
with a threshold of 1e−05
rs73128290
rs12404911
rs7742829
rs77285108
All
−0.2
−0.1
0.0
0.1
0.2
MR effect size for
'Gut microbiota abundance (genus Catenibacterium id.2153)' on 'Polyadenylate−binding protein 4'

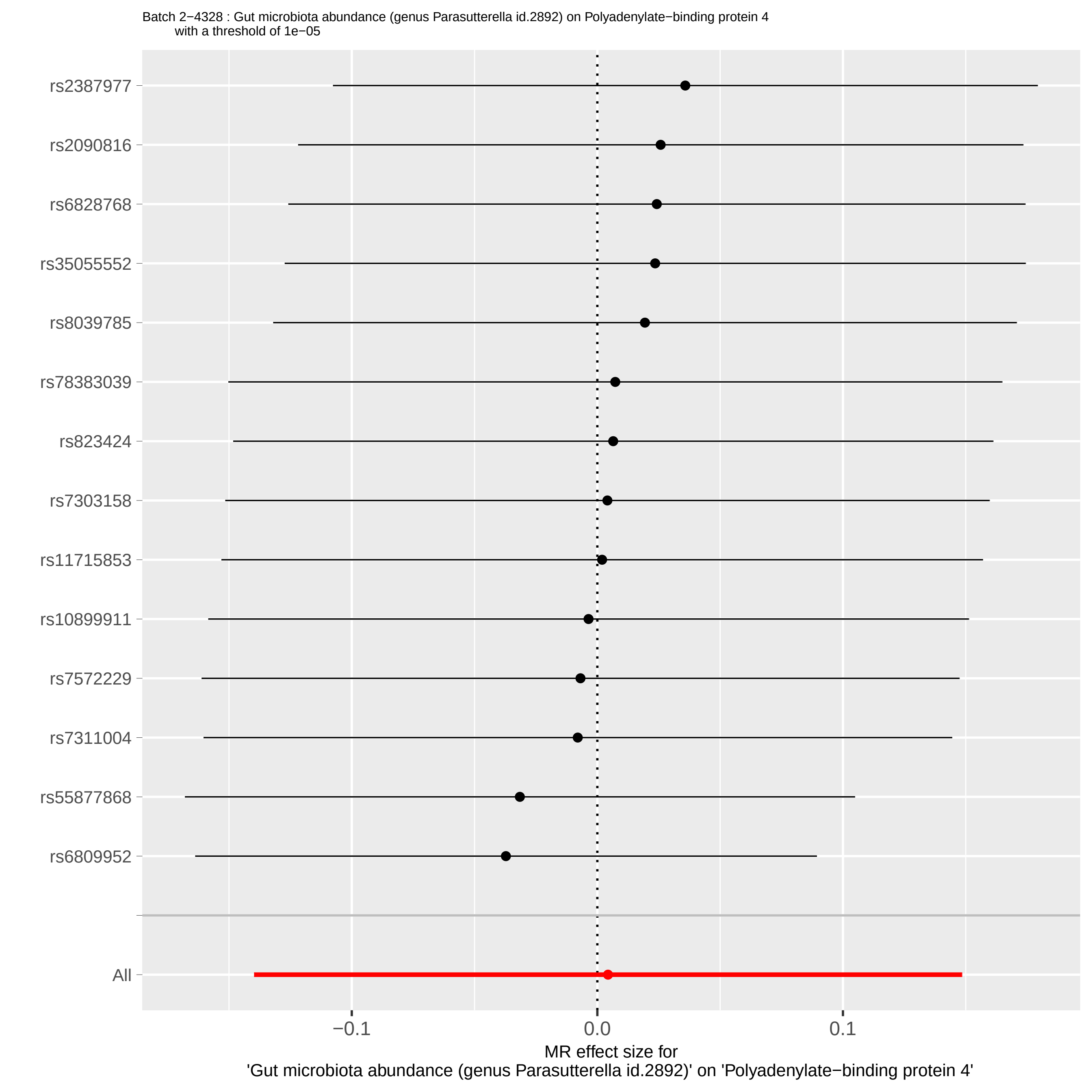
Batch 2−4328 : Gut microbiota abundance (genus Parasutterella id.2892) on Polyadenylate−binding protein 4
with a threshold of 1e−05
rs2387977
rs2090816
rs6828768
rs35055552
rs8039785
rs78383039
rs823424
rs7303158
rs11715853
rs10899911
rs7572229
rs7311004
rs55877868
rs6809952
All
−0.1
0.0
0.1
MR effect size for
'Gut microbiota abundance (genus Parasutterella id.2892)' on 'Polyadenylate−binding protein 4'

Batch 2−4329 : Gut microbiota abundance (genus Catenibacterium id.2153) on Marginal zone B− and B1−cell−specific protein
with a threshold of 1e−05

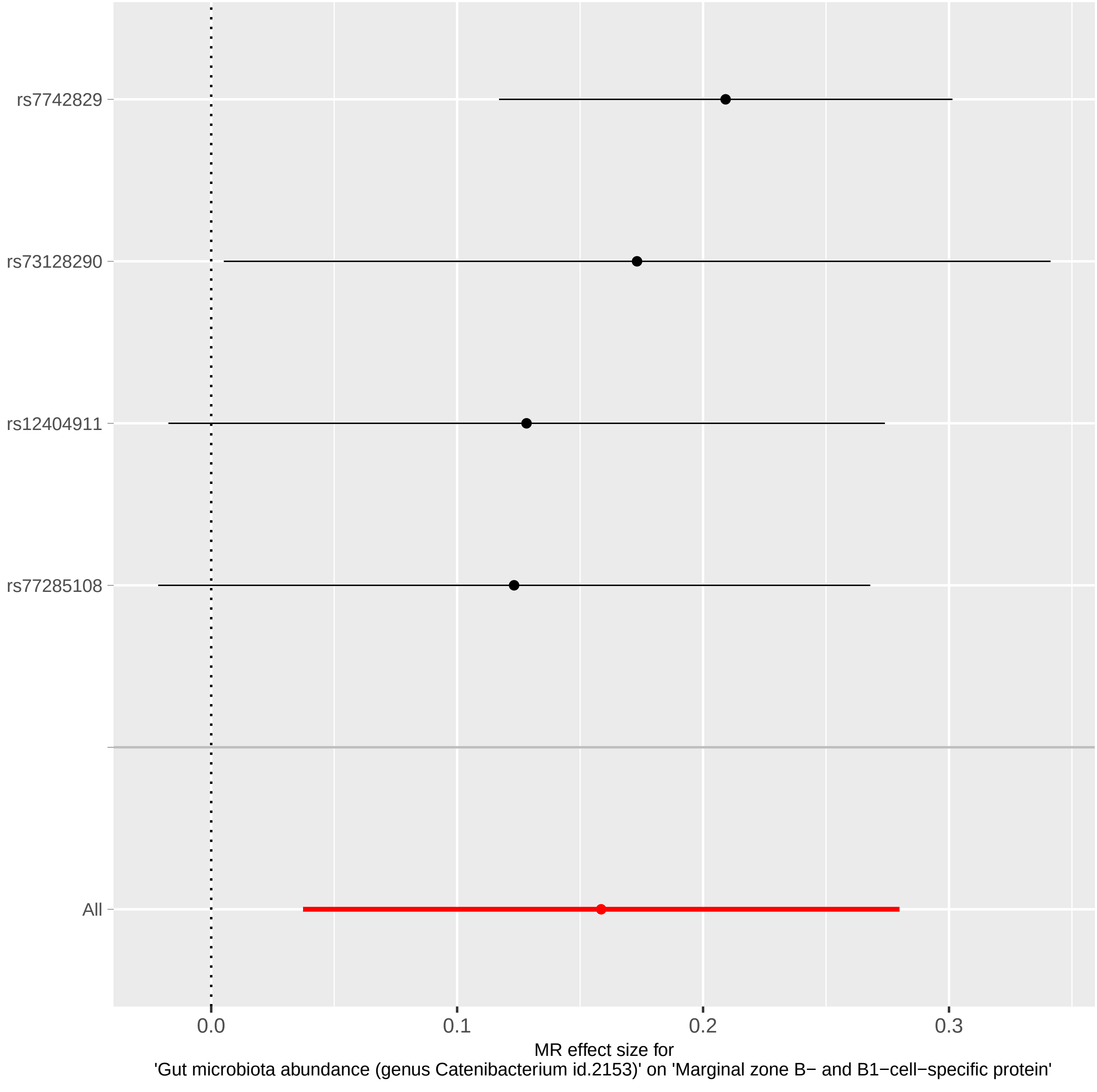

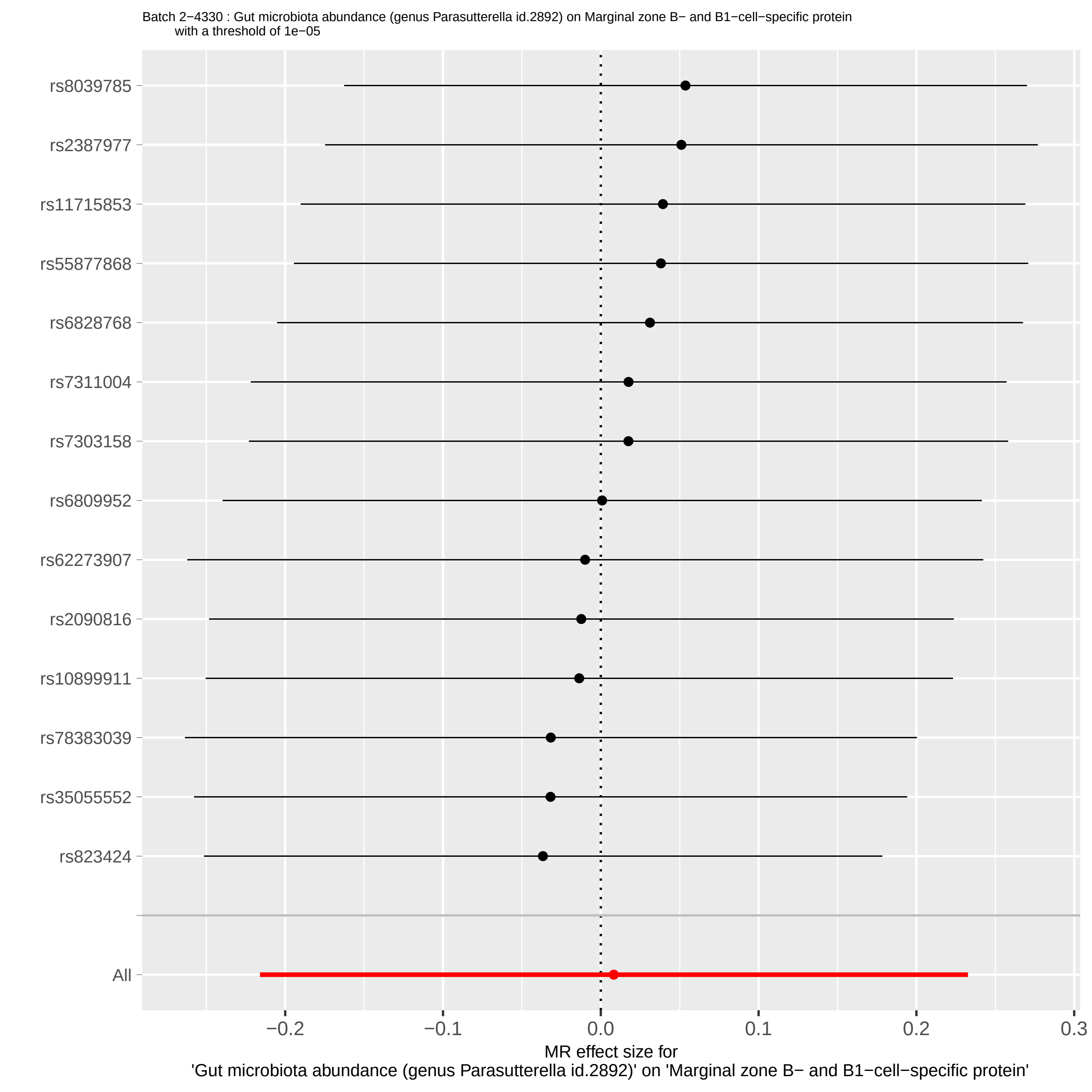

Batch 2−4330 : Gut microbiota abundance (genus Parasutterella id.2892) on Marginal zone B− and B1−cell−specific protein
with a threshold of 1e−05
rs8039785
rs2387977
rs11715853
rs55877868
rs6828768
rs7311004
rs7303158
rs6809952
rs62273907
rs2090816
rs10899911
rs78383039
rs35055552
rs823424
All
−0.2
−0.1
0.0
0.1
0.2
0.3
MR effect size for
'Gut microbiota abundance (genus Parasutterella id.2892)' on 'Marginal zone B− and B1−cell−specific protein'

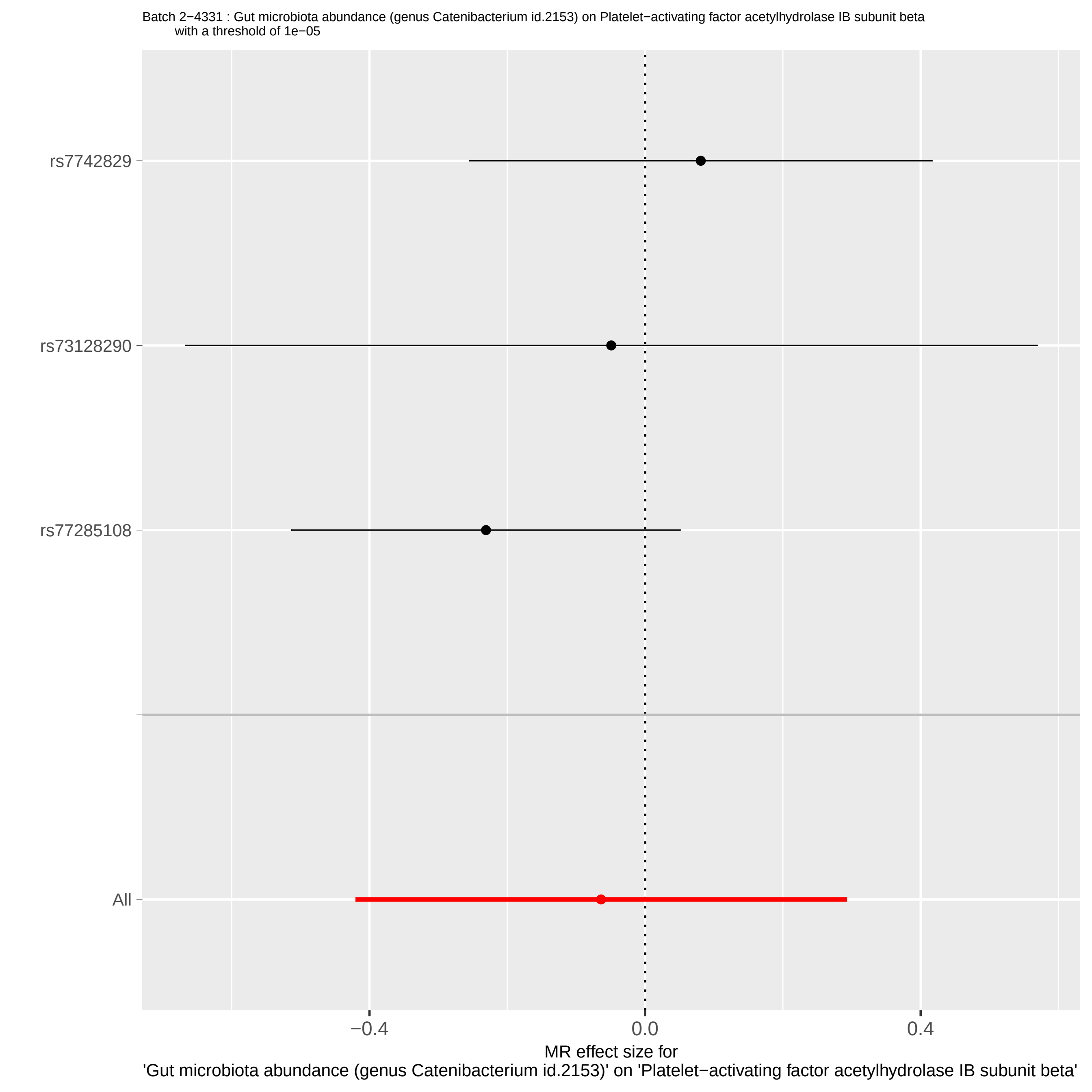
Batch 2−4331 : Gut microbiota abundance (genus Catenibacterium id.2153) on Platelet−activating factor acetylhydrolase IB subunit beta
with a threshold of 1e−05
rs7742829
rs73128290
rs77285108
All
−0.4
0.0
0.4
MR effect size for
'Gut microbiota abundance (genus Catenibacterium id.2153)' on 'Platelet−activating factor acetylhydrolase IB subunit beta'

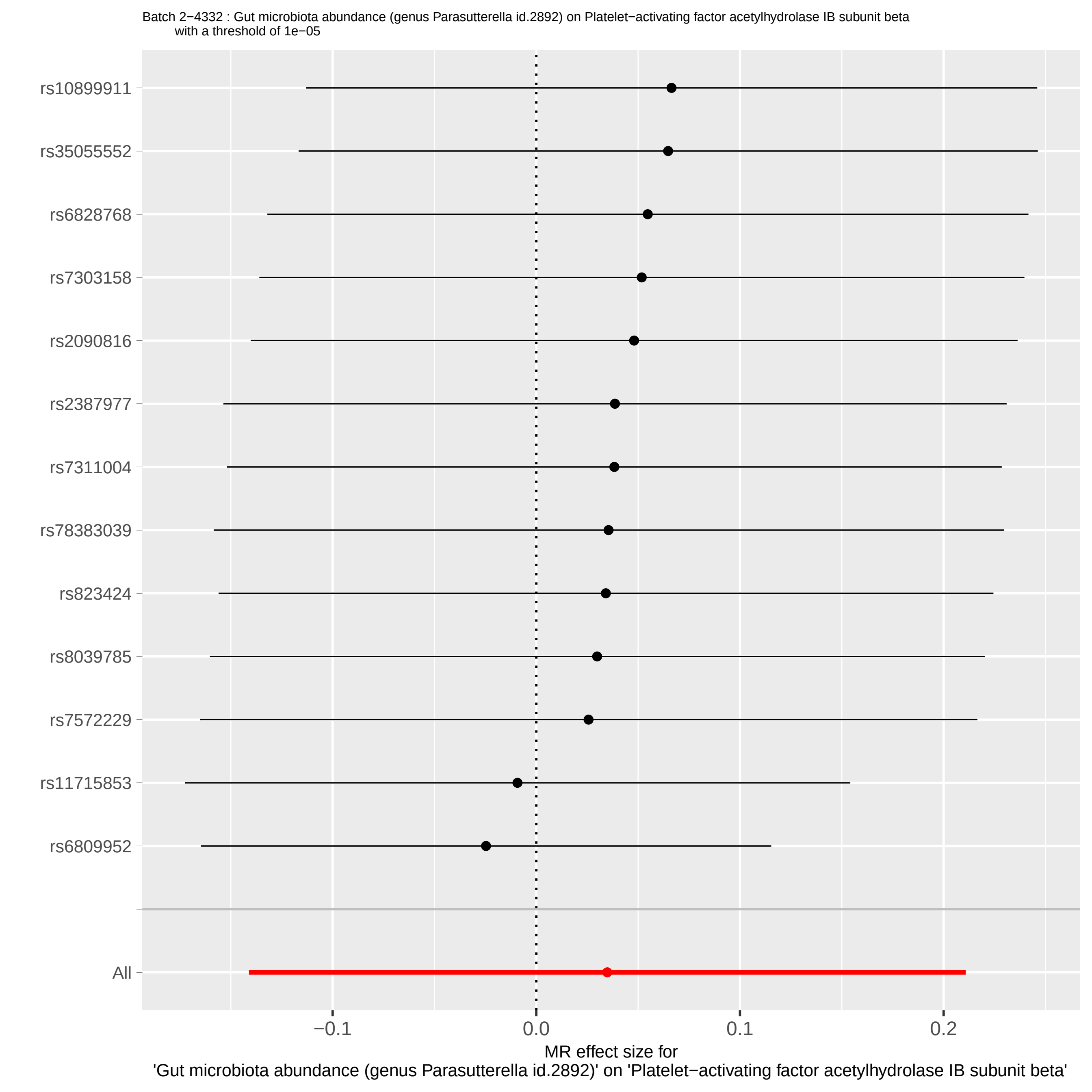

Batch 2−4332 : Gut microbiota abundance (genus Parasutterella id.2892) on Platelet−activating factor acetylhydrolase IB subunit beta
with a threshold of 1e−05
rs10899911
rs35055552
rs6828768
rs7303158
rs2090816
rs2387977
rs7311004
rs78383039
rs823424
rs8039785
rs7572229
rs11715853
rs6809952
All
−0.1
0.0
0.1
0.2
MR effect size for
'Gut microbiota abundance (genus Parasutterella id.2892)' on 'Platelet−activating factor acetylhydrolase IB subunit beta'

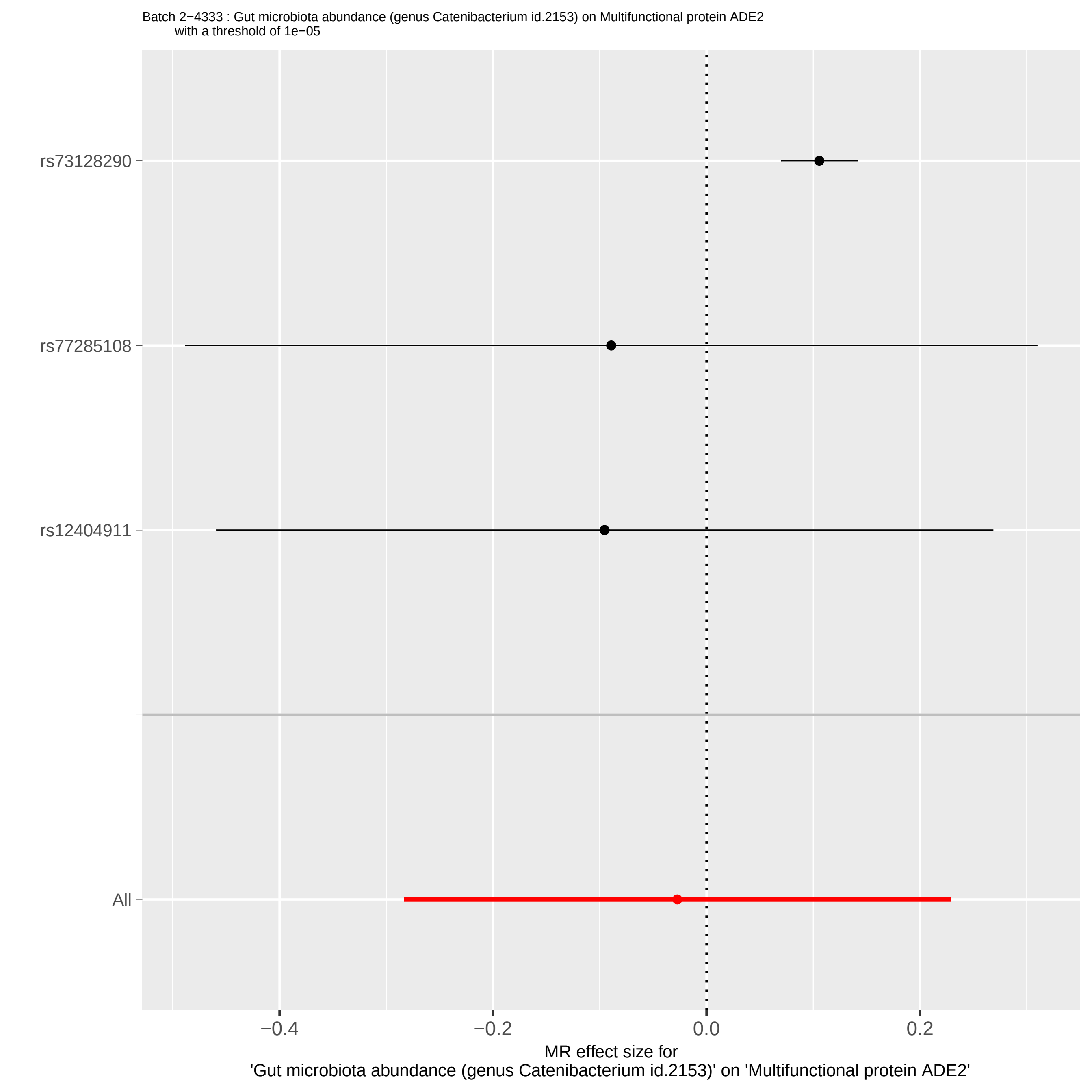
Batch 2−4333 : Gut microbiota abundance (genus Catenibacterium id.2153) on Multifunctional protein ADE2
with a threshold of 1e−05
rs73128290
rs77285108
rs12404911
All
−0.4
−0.2
0.0
0.2
MR effect size for
'Gut microbiota abundance (genus Catenibacterium id.2153)' on 'Multifunctional protein ADE2'

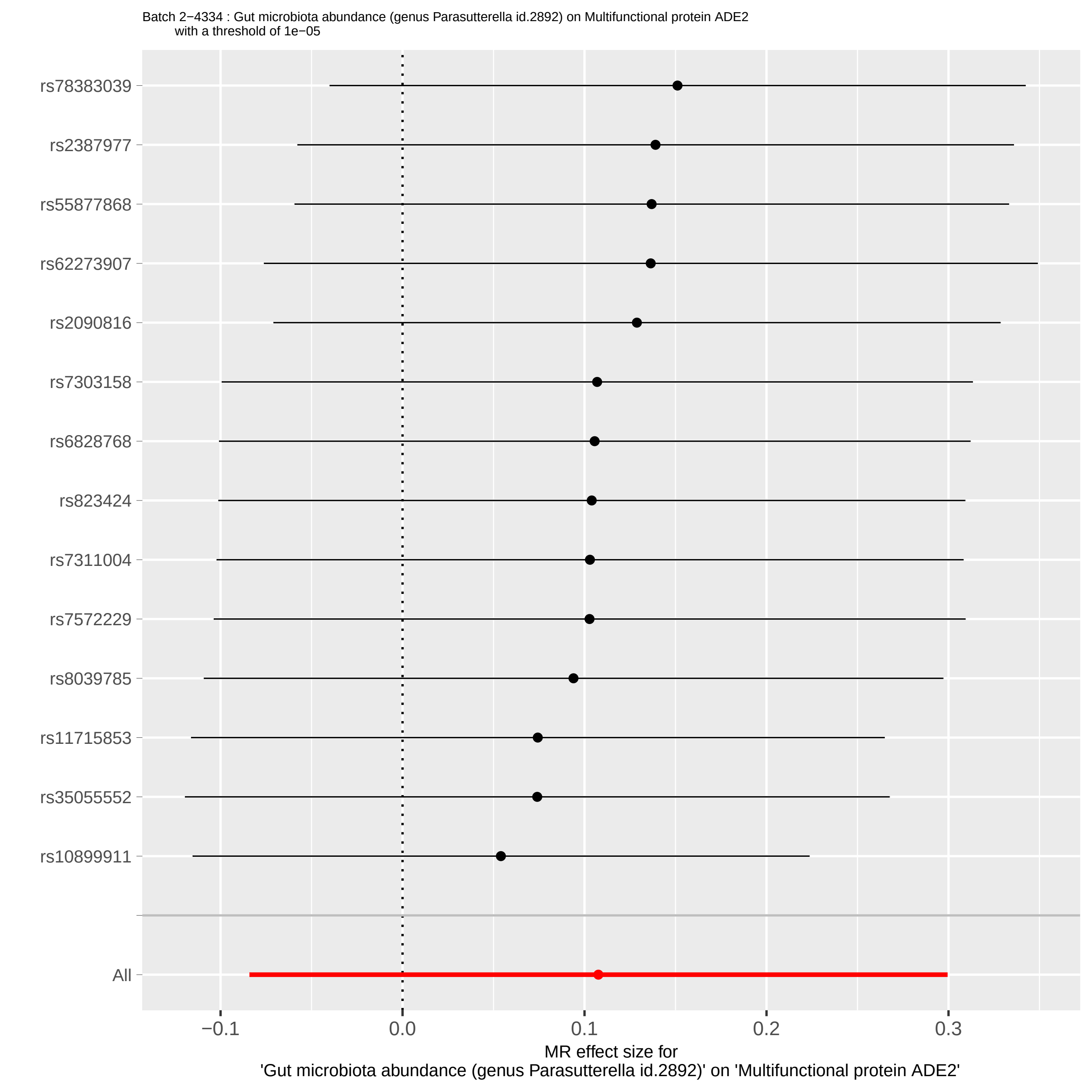

Batch 2−4334 : Gut microbiota abundance (genus Parasutterella id.2892) on Multifunctional protein ADE2
with a threshold of 1e−05
rs78383039
rs2387977
rs55877868
rs62273907
rs2090816
rs7303158
rs6828768
rs823424
rs7311004
rs7572229
rs8039785
rs11715853
rs35055552
rs10899911
All
−0.1
0.0
0.1
0.2
0.3
MR effect size for
'Gut microbiota abundance (genus Parasutterella id.2892)' on 'Multifunctional protein ADE2'

Batch 2−4335 : Gut microbiota abundance (genus Catenibacterium id.2153) on Polyadenylate−binding protein−interacting protein 1
with a threshold of 1e−05

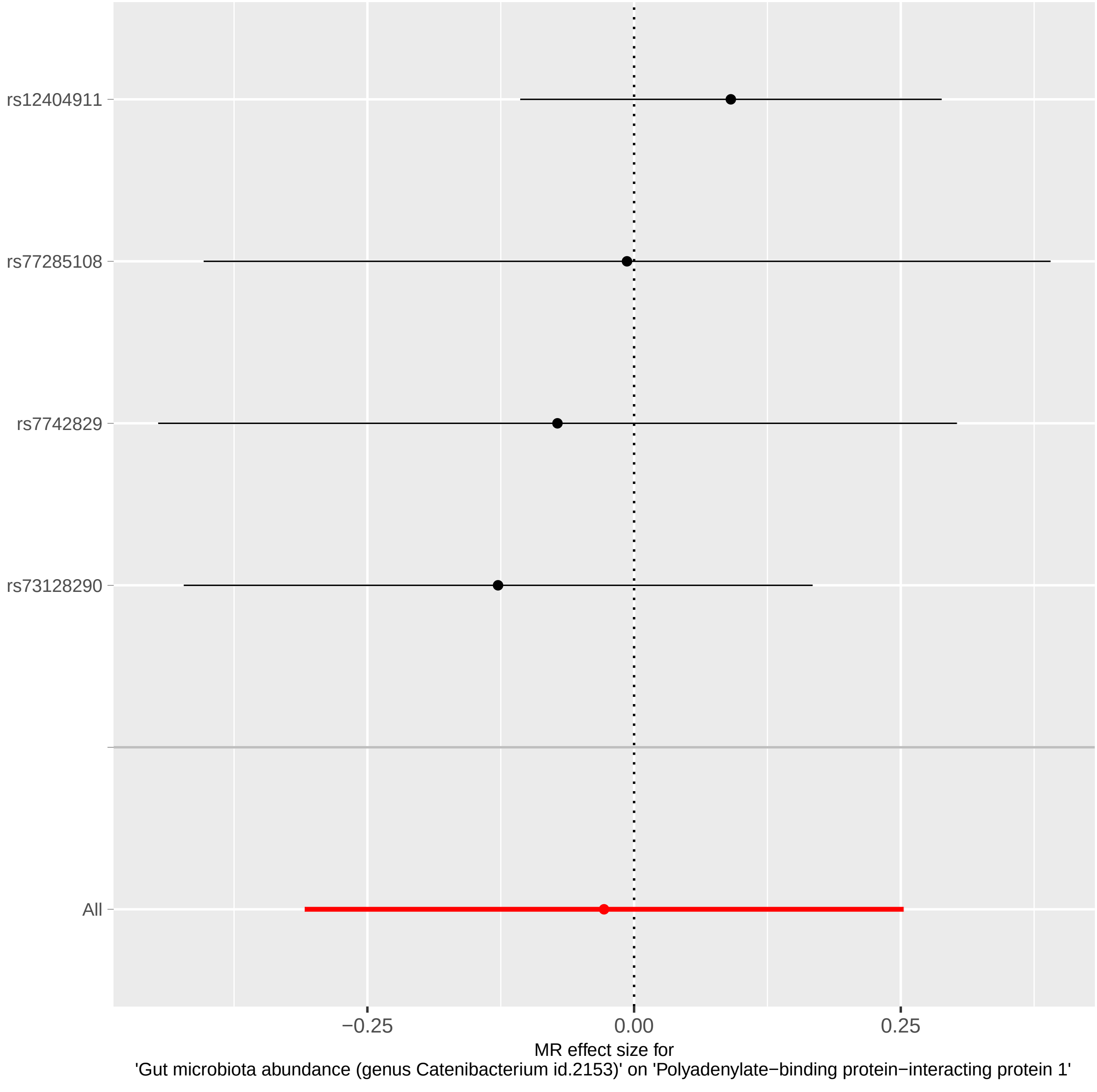

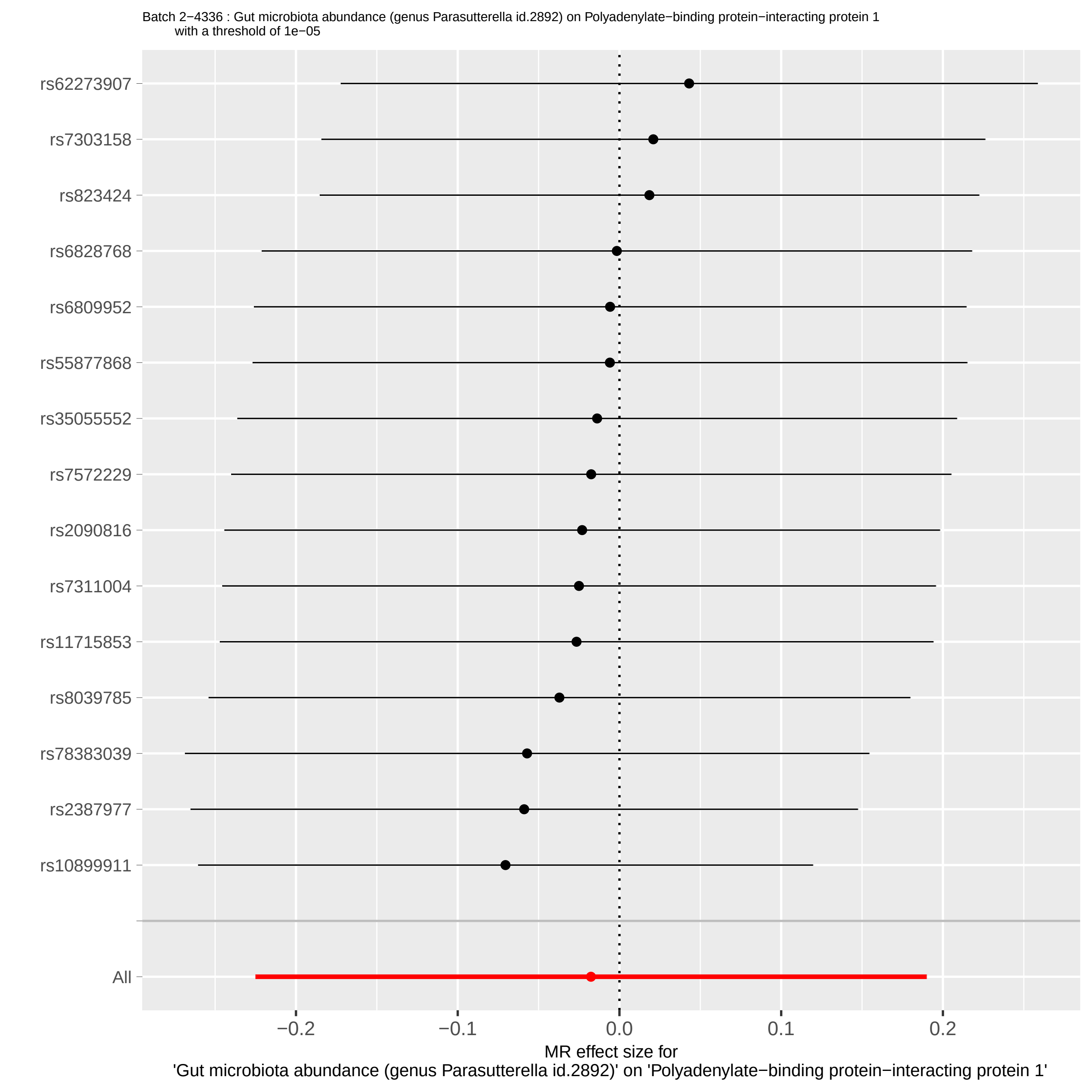
Batch 2−4336 : Gut microbiota abundance (genus Parasutterella id.2892) on Polyadenylate−binding protein−interacting protein 1
with a threshold of 1e−05
rs62273907
rs7303158
rs823424
rs6828768
rs6809952
rs55877868
rs35055552
rs7572229
rs2090816
rs7311004
rs11715853
rs8039785
rs78383039
rs2387977
rs10899911
All
−0.2
−0.1
0.0
0.1
0.2
MR effect size for
'Gut microbiota abundance (genus Parasutterella id.2892)' on 'Polyadenylate−binding protein−interacting protein 1'

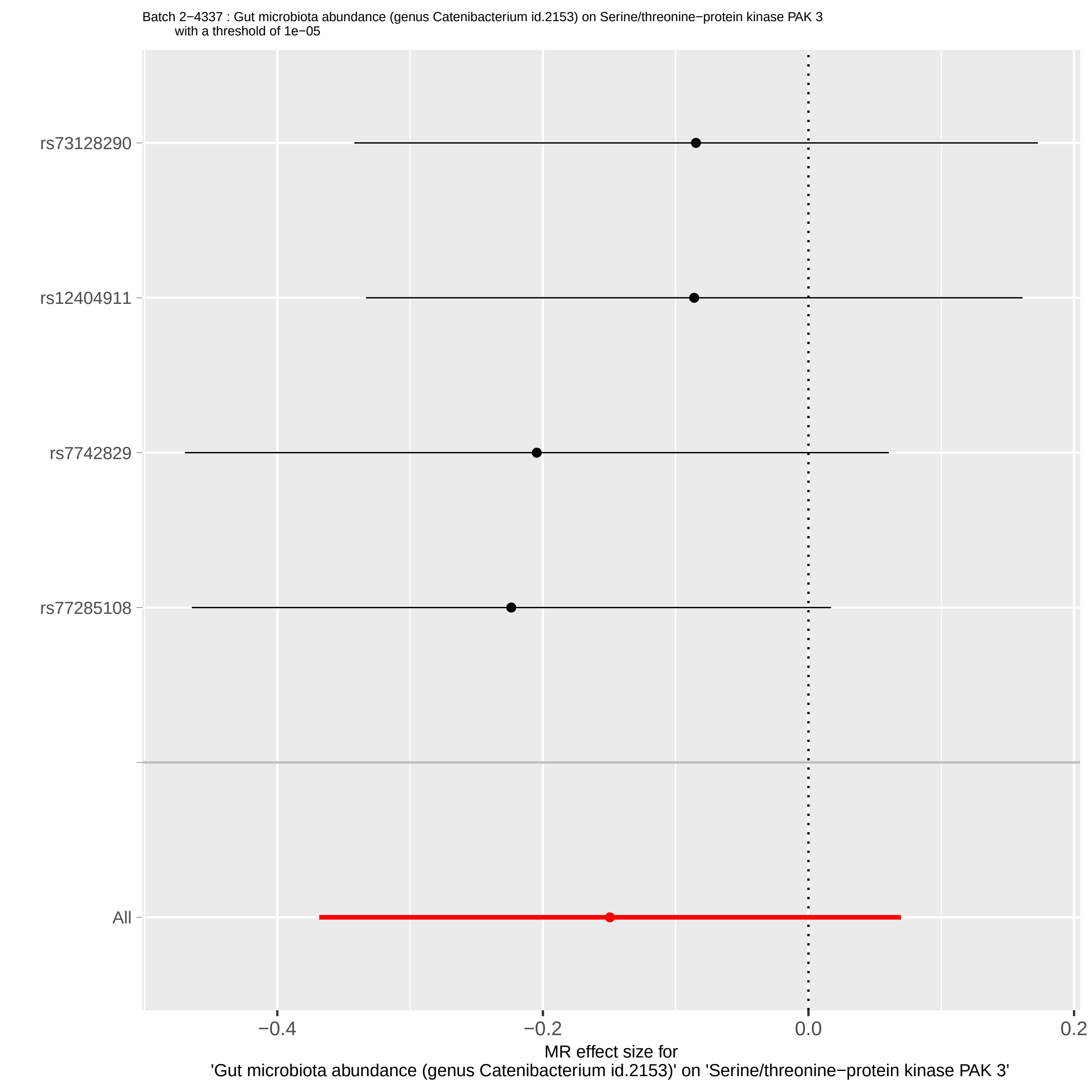

Batch 2−4337 : Gut microbiota abundance (genus Catenibacterium id.2153) on Serine/threonine−protein kinase PAK 3
with a threshold of 1e−05
rs73128290
rs12404911
rs7742829
rs77285108
All
−0.4
−0.2
0.0
0.2
MR effect size for
'Gut microbiota abundance (genus Catenibacterium id.2153)' on 'Serine/threonine−protein kinase PAK 3'

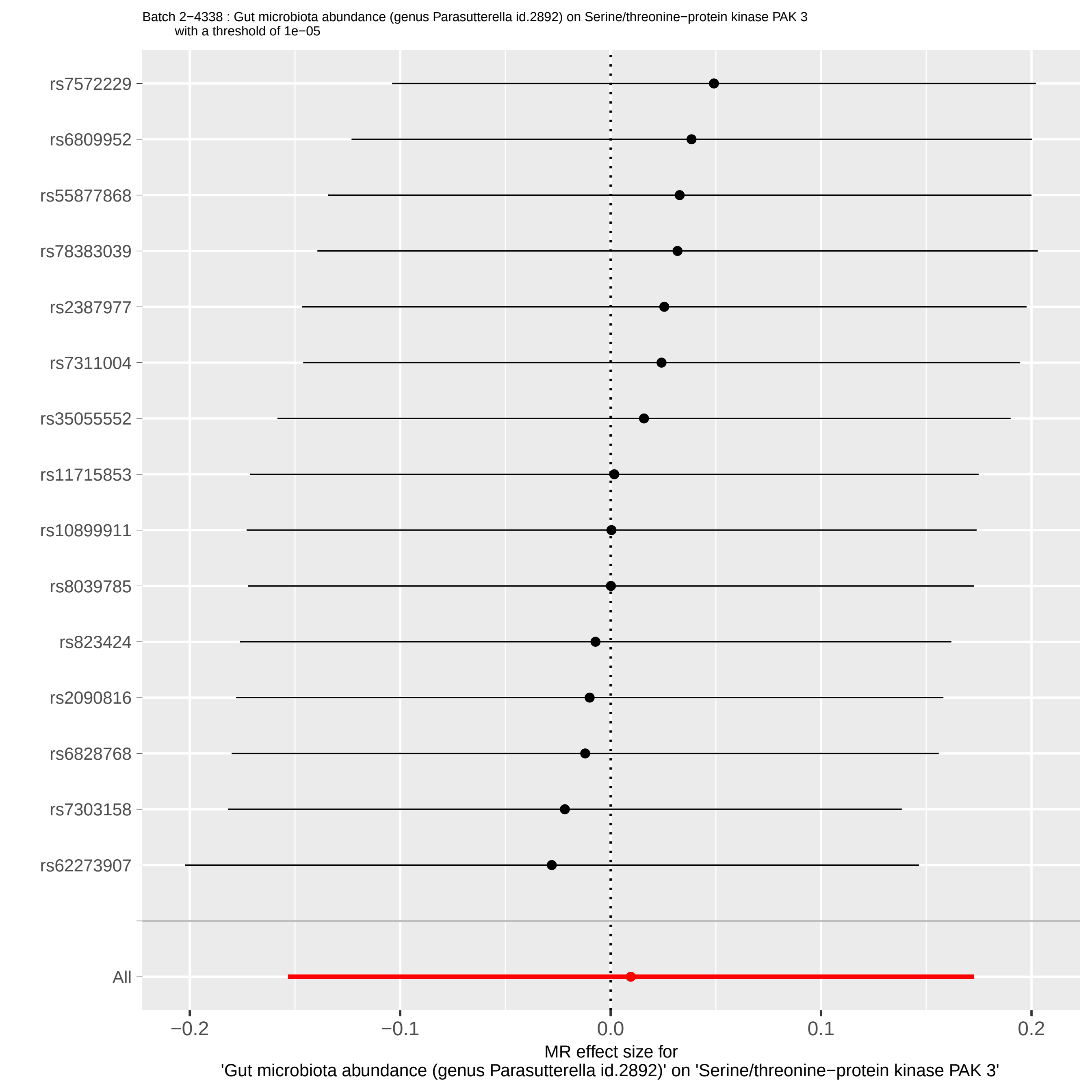

Batch 2−4338 : Gut microbiota abundance (genus Parasutterella id.2892) on Serine/threonine−protein kinase PAK 3
with a threshold of 1e−05
rs7572229
rs6809952
rs55877868
rs78383039
rs2387977
rs7311004
rs35055552
rs11715853
rs10899911
rs8039785
rs823424
rs2090816
rs6828768
rs7303158
rs62273907
All
−0.2
−0.1
0.0
0.1
0.2
MR effect size for
'Gut microbiota abundance (genus Parasutterella id.2892)' on 'Serine/threonine−protein kinase PAK 3'

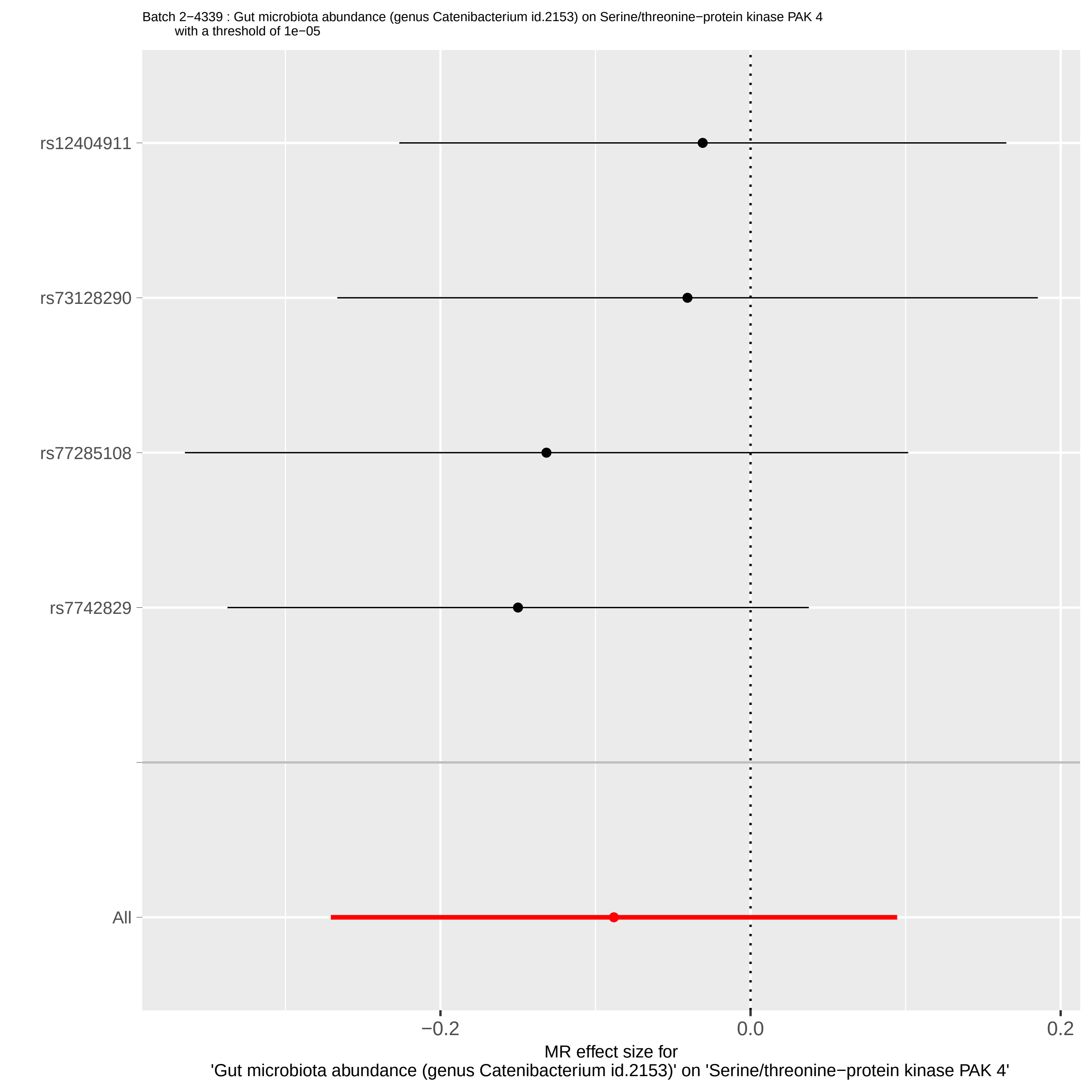
Batch 2−4339 : Gut microbiota abundance (genus Catenibacterium id.2153) on Serine/threonine−protein kinase PAK 4
with a threshold of 1e−05
rs12404911
rs73128290
rs77285108
rs7742829
All
−0.2
0.0
0.2
MR effect size for
'Gut microbiota abundance (genus Catenibacterium id.2153)' on 'Serine/threonine−protein kinase PAK 4'

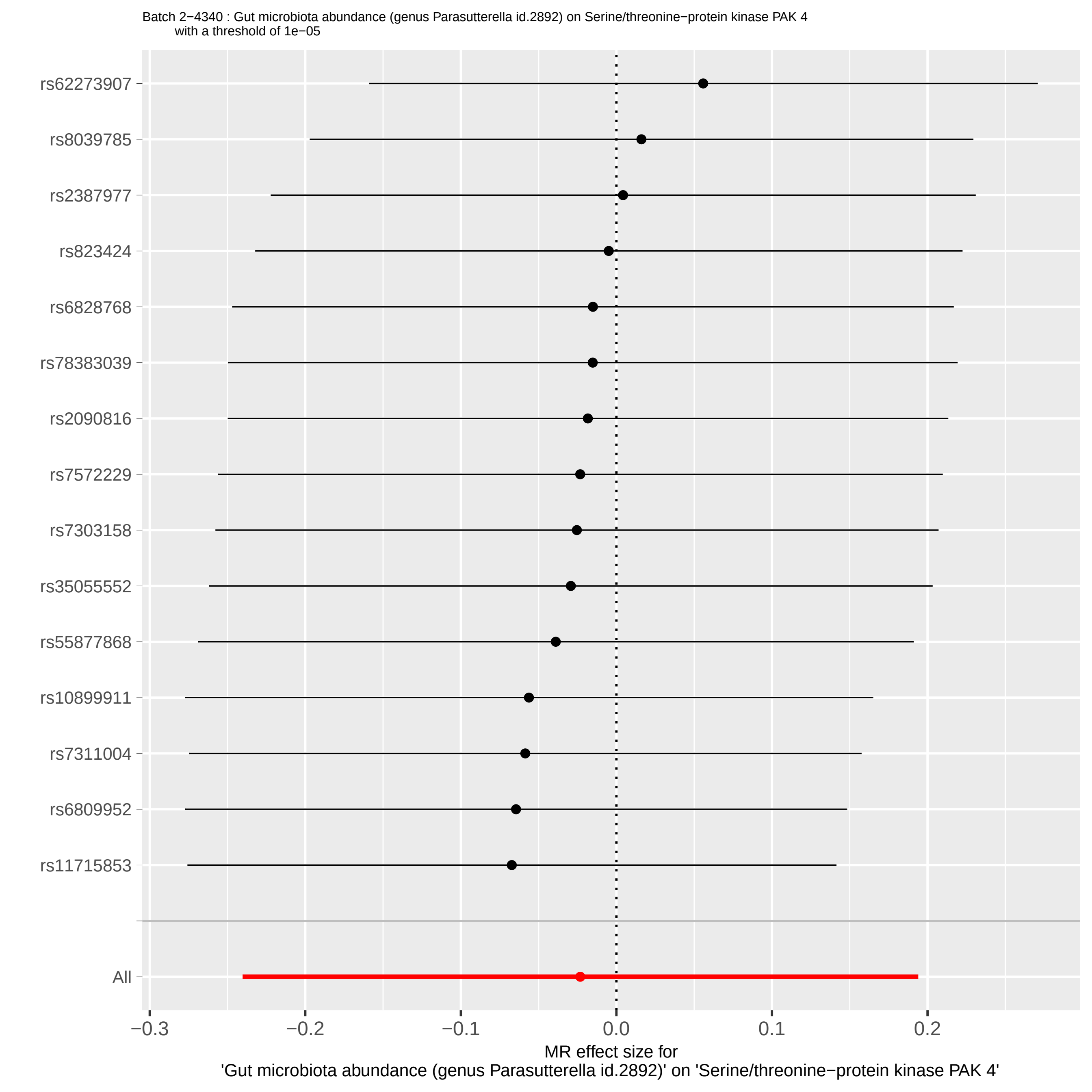
Batch 2−4340 : Gut microbiota abundance (genus Parasutterella id.2892) on Serine/threonine−protein kinase PAK 4
with a threshold of 1e−05
rs62273907
rs8039785
rs2387977
rs823424
rs6828768
rs78383039
rs2090816
rs7572229
rs7303158
rs35055552
rs55877868
rs10899911
rs7311004
rs6809952
rs11715853
All
−0.3
−0.2
−0.1
0.0
0.1
0.2
MR effect size for
'Gut microbiota abundance (genus Parasutterella id.2892)' on 'Serine/threonine−protein kinase PAK 4'

Batch 2−4341 : Gut microbiota abundance (genus Catenibacterium id.2153) on Serine/threonine−protein kinase PAK 7
with a threshold of 1e−05

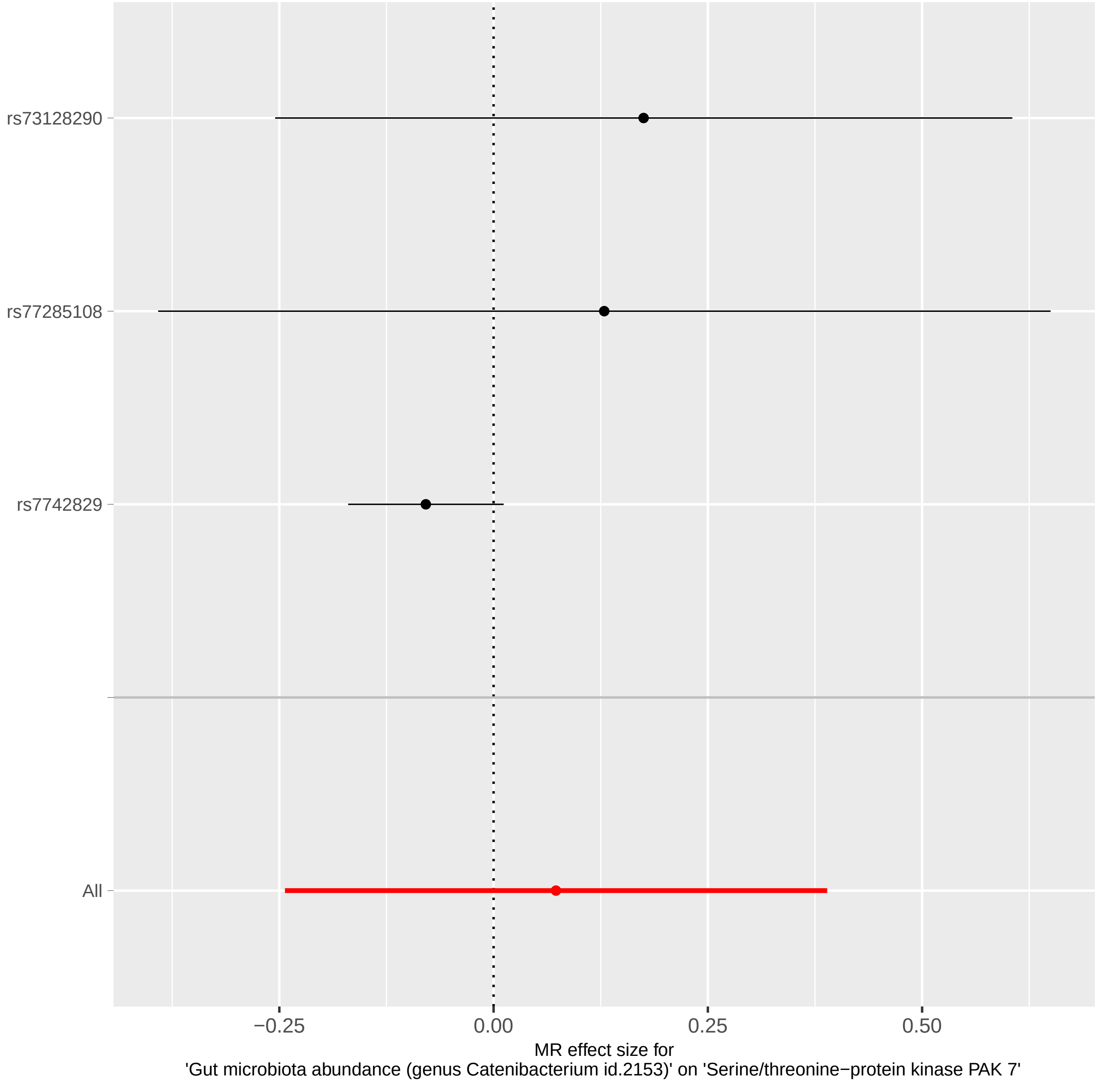

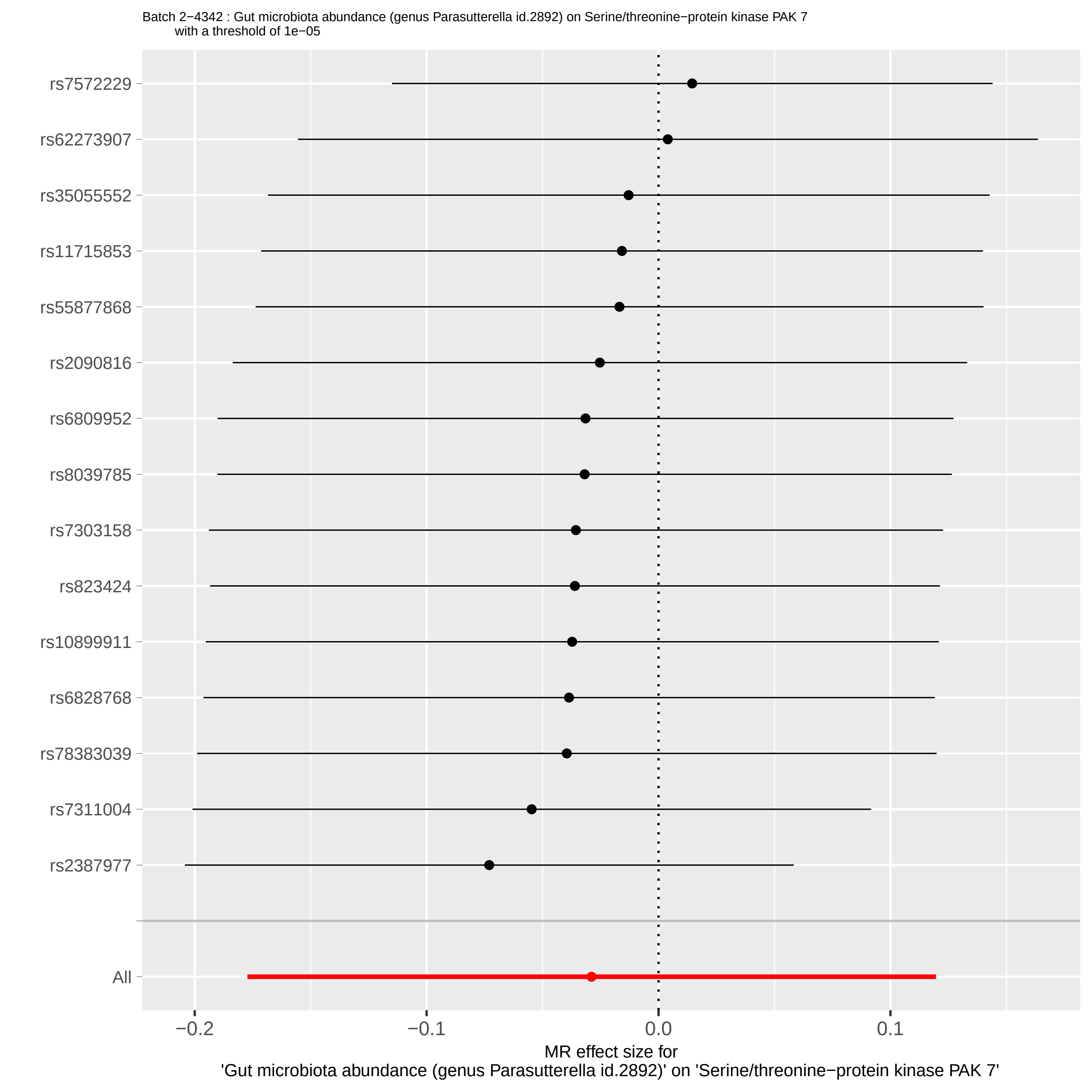

Batch 2−4342 : Gut microbiota abundance (genus Parasutterella id.2892) on Serine/threonine−protein kinase PAK 7
with a threshold of 1e−05
rs7572229
rs62273907
rs35055552
rs11715853
rs55877868
rs2090816
rs6809952
rs8039785
rs7303158
rs823424
rs10899911
rs6828768
rs78383039
rs7311004
rs2387977
All
−0.2
−0.1
0.0
0.1
MR effect size for
'Gut microbiota abundance (genus Parasutterella id.2892)' on 'Serine/threonine−protein kinase PAK 7'

Batch 2−4343 : Gut microbiota abundance (genus Catenibacterium id.2153) on Peptidyl−glycine alpha−amidating monooxygenase
with a threshold of 1e−05

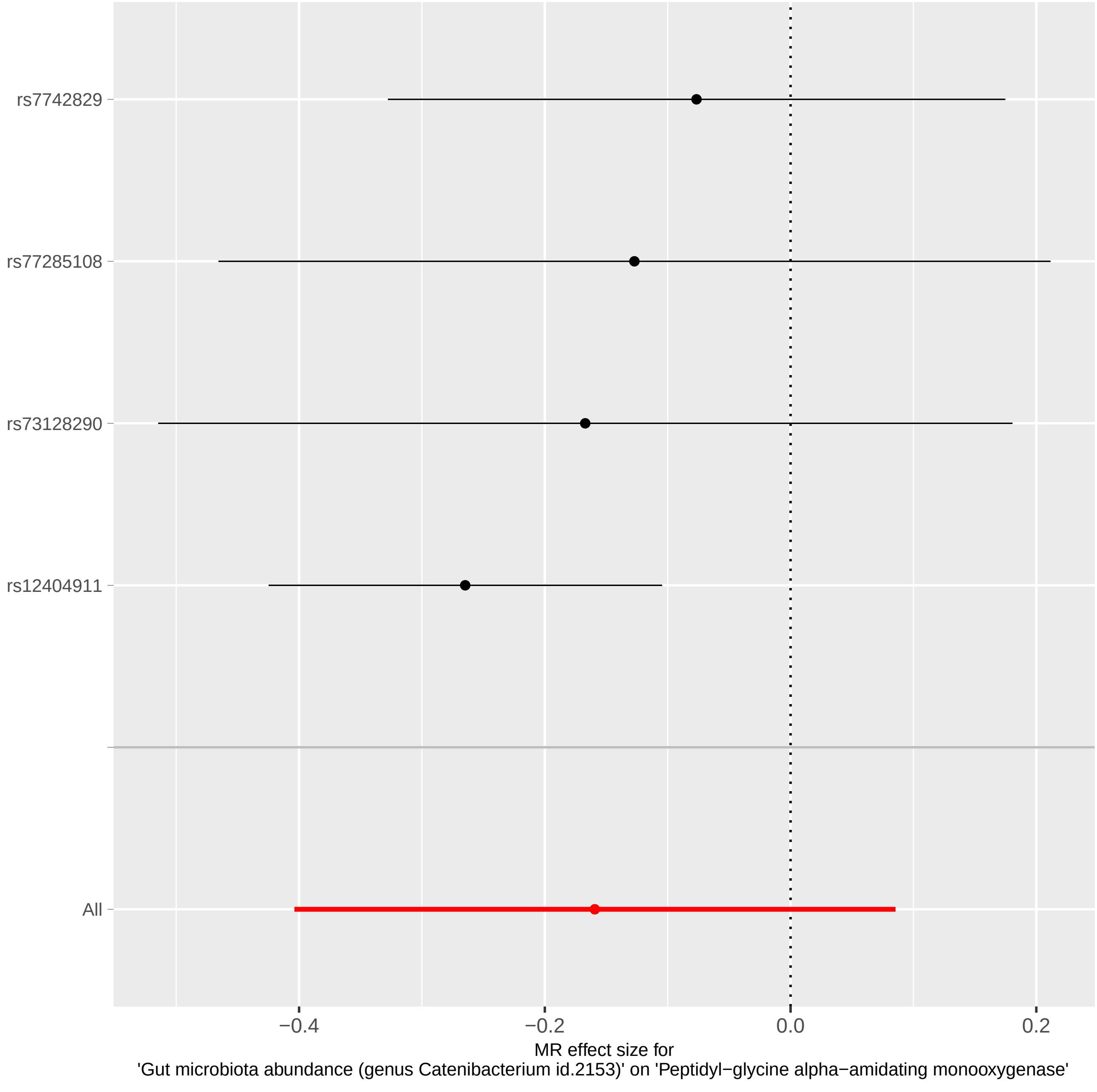

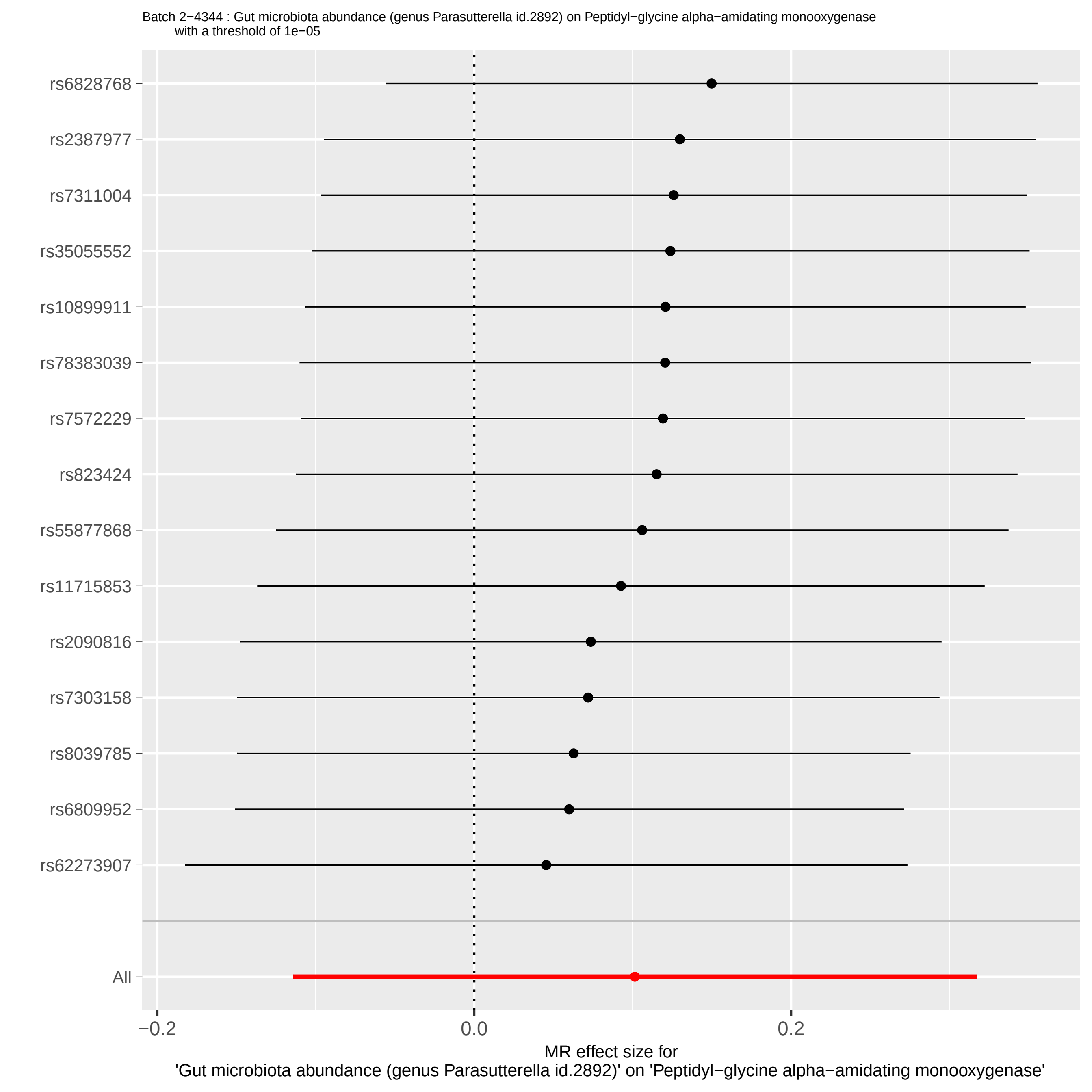

Batch 2−4344 : Gut microbiota abundance (genus Parasutterella id.2892) on Peptidyl−glycine alpha−amidating monooxygenase with a threshold of 1e−05
rs6828768
rs2387977
rs7311004
rs35055552
rs10899911
rs78383039
rs7572229
rs823424
rs55877868
rs11715853
rs2090816
rs7303158
rs8039785
rs6809952
rs62273907
All
−0.2
0.0
0.2
MR effect size for
'Gut microbiota abundance (genus Parasutterella id.2892)' on 'Peptidyl−glycine alpha−amidating monooxygenase'

Batch 2−4345 : Gut microbiota abundance (genus Catenibacterium id.2153) on Pantothenate kinase 3
with a threshold of 1e−05

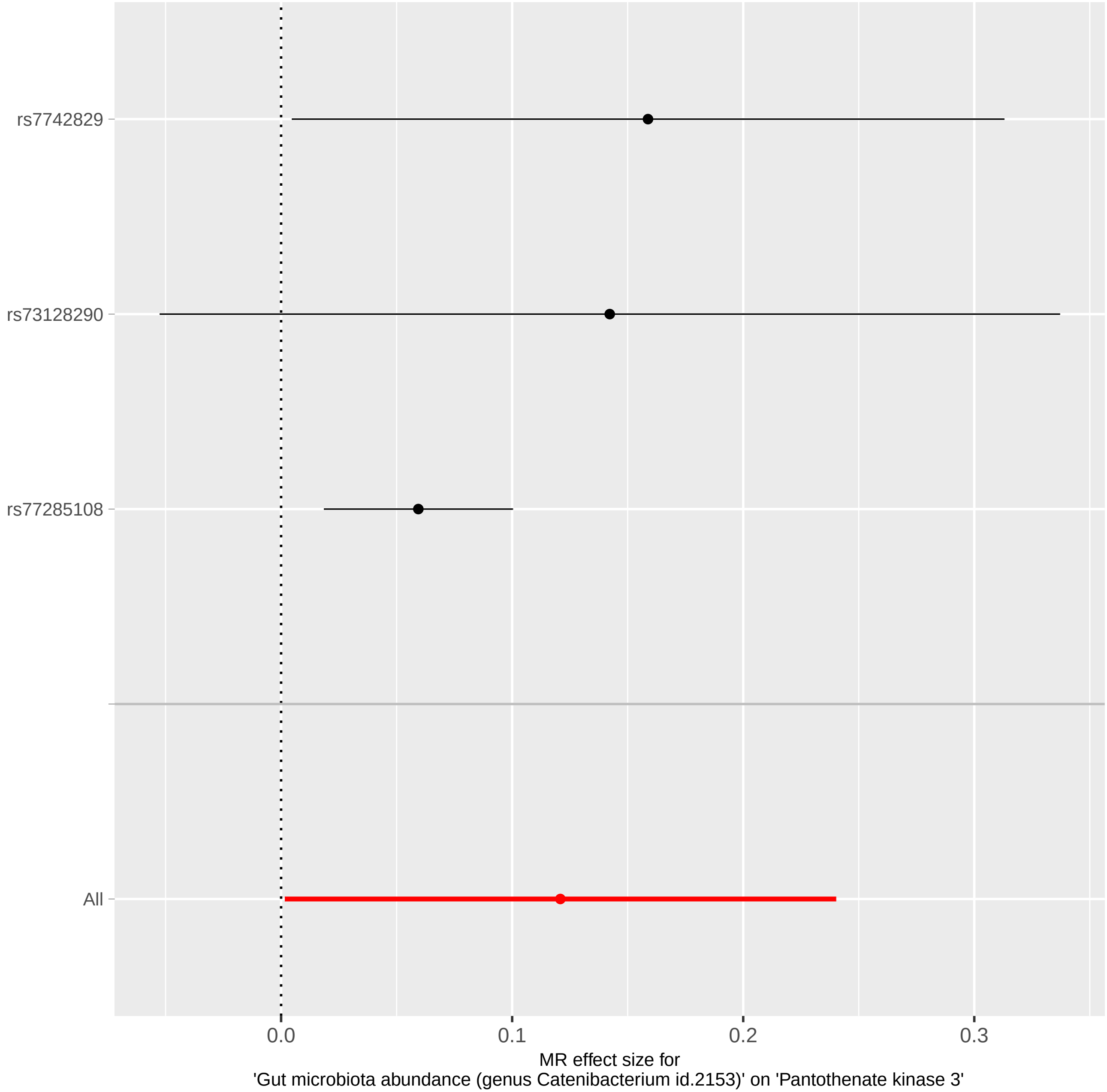

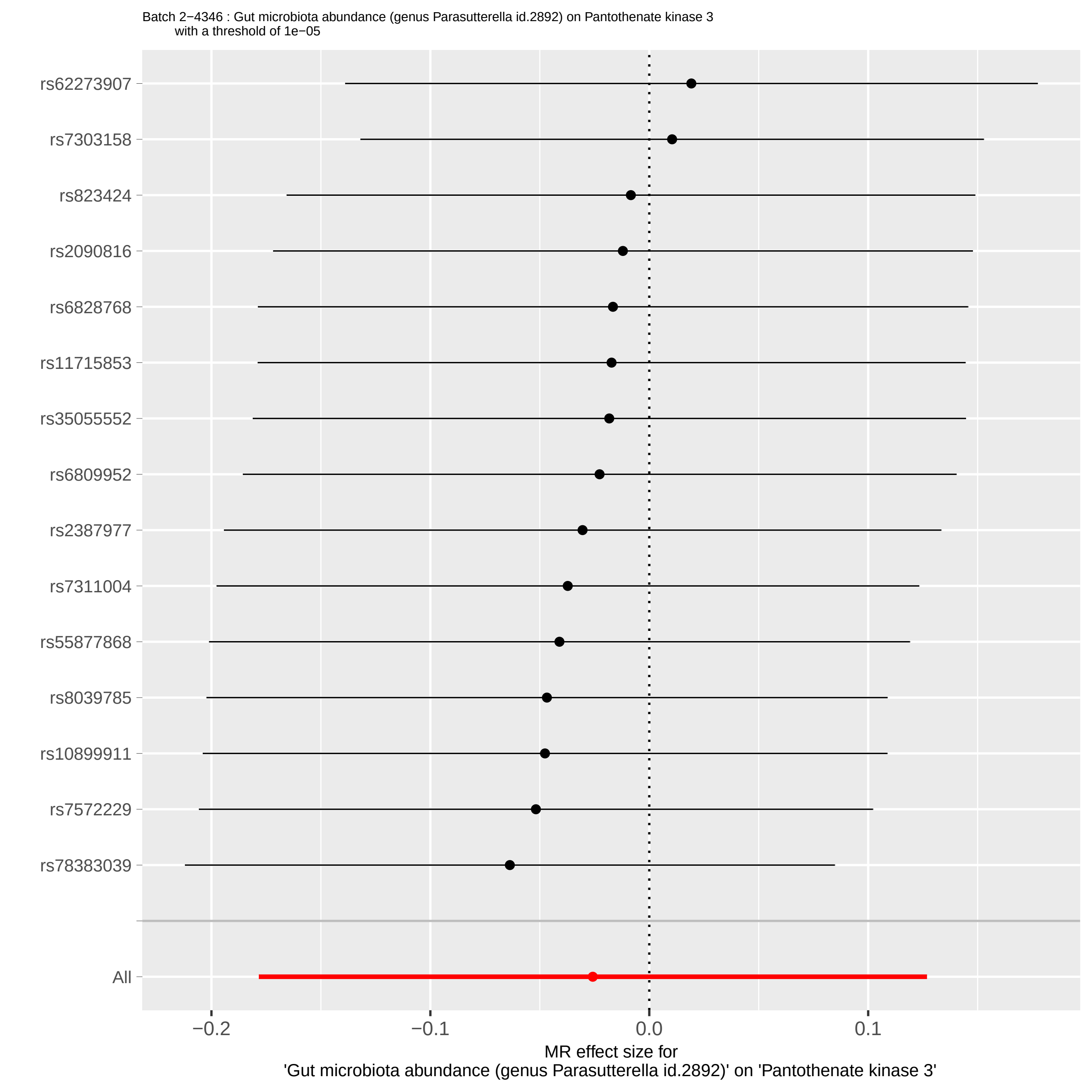
Batch 2−4346 : Gut microbiota abundance (genus Parasutterella id.2892) on Pantothenate kinase 3
with a threshold of 1e−05
rs62273907
rs7303158
rs823424
rs2090816
rs6828768
rs11715853
rs35055552
rs6809952
rs2387977
rs7311004
rs55877868
rs8039785
rs10899911
rs7572229
rs78383039
All
−0.2
−0.1
0.0
0.1
MR effect size for
'Gut microbiota abundance (genus Parasutterella id.2892)' on 'Pantothenate kinase 3'

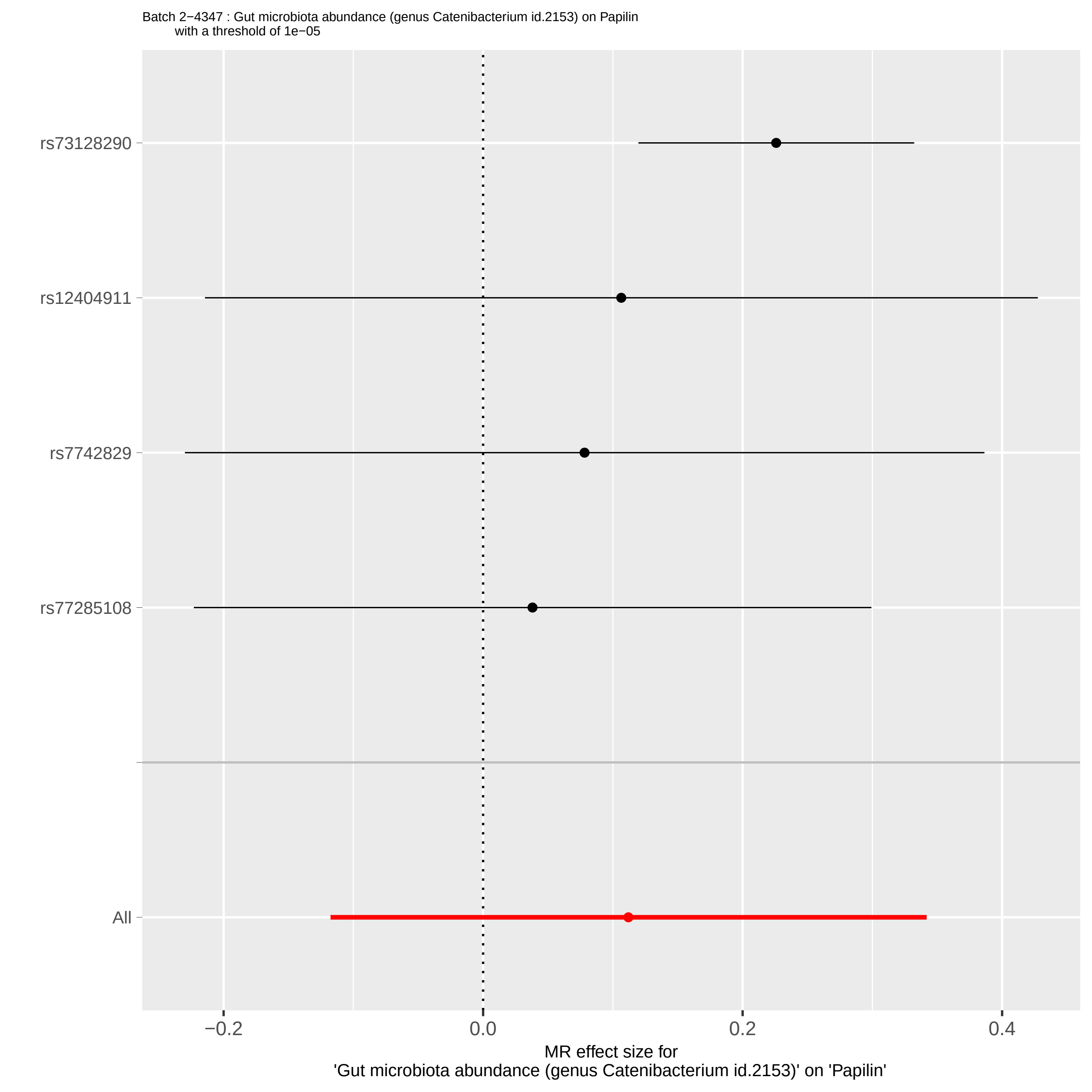
Batch 2−4347 : Gut microbiota abundance (genus Catenibacterium id.2153) on Papilin
with a threshold of 1e−05
rs73128290
rs12404911
rs7742829
rs77285108
All
−0.2
0.0
0.2
0.4
MR effect size for
'Gut microbiota abundance (genus Catenibacterium id.2153)' on 'Papilin'

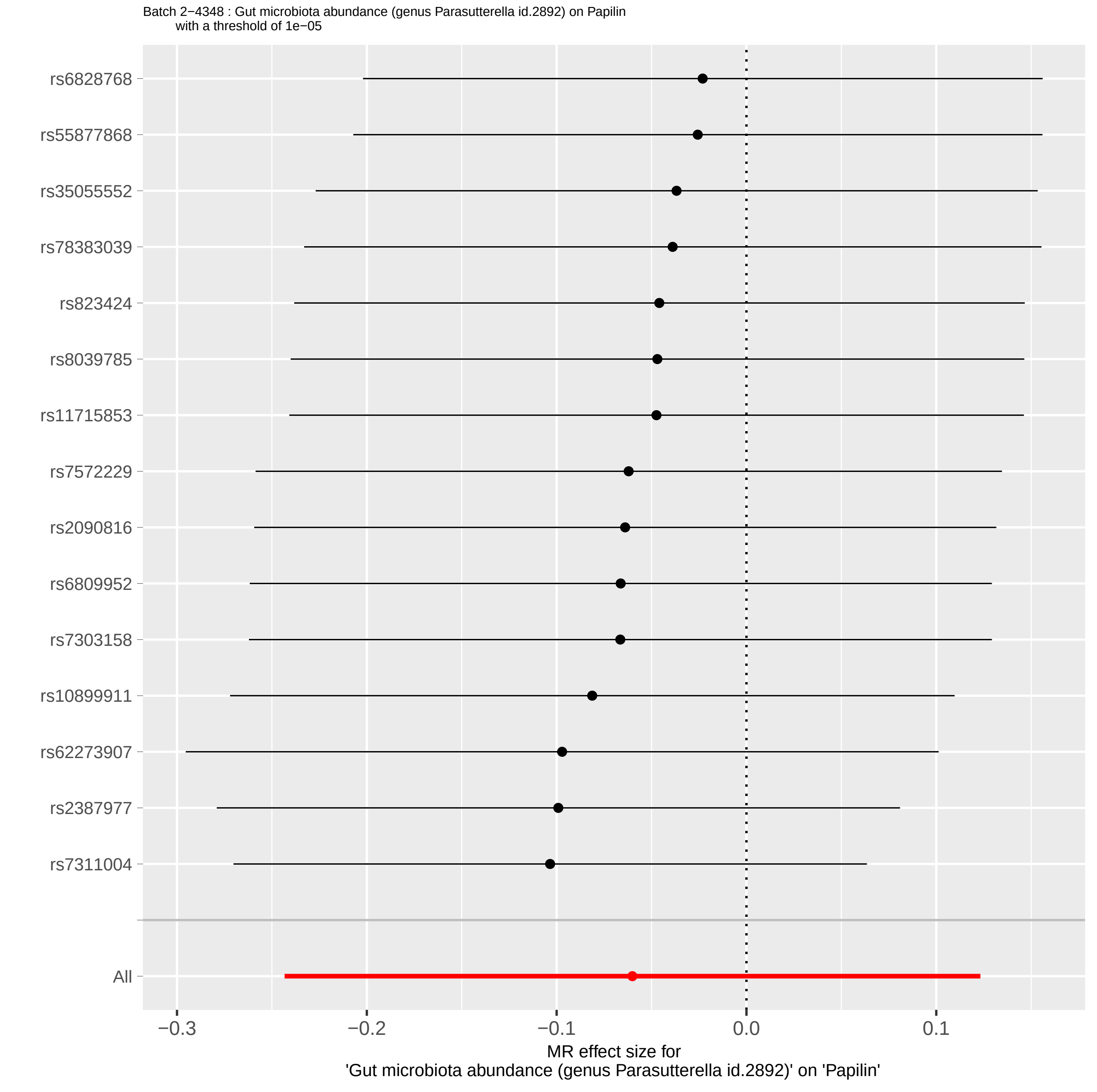

Batch 2−4348 : Gut microbiota abundance (genus Parasutterella id.2892) on Papilin
with a threshold of 1e−05
rs6828768
rs55877868
rs35055552
rs78383039
rs823424
rs8039785
rs11715853
rs7572229
rs2090816
rs6809952
rs7303158
rs10899911
rs62273907
rs2387977
rs7311004
All
−0.3
−0.2
−0.1
0.0
0.1
MR effect size for
'Gut microbiota abundance (genus Parasutterella id.2892)' on 'Papilin'

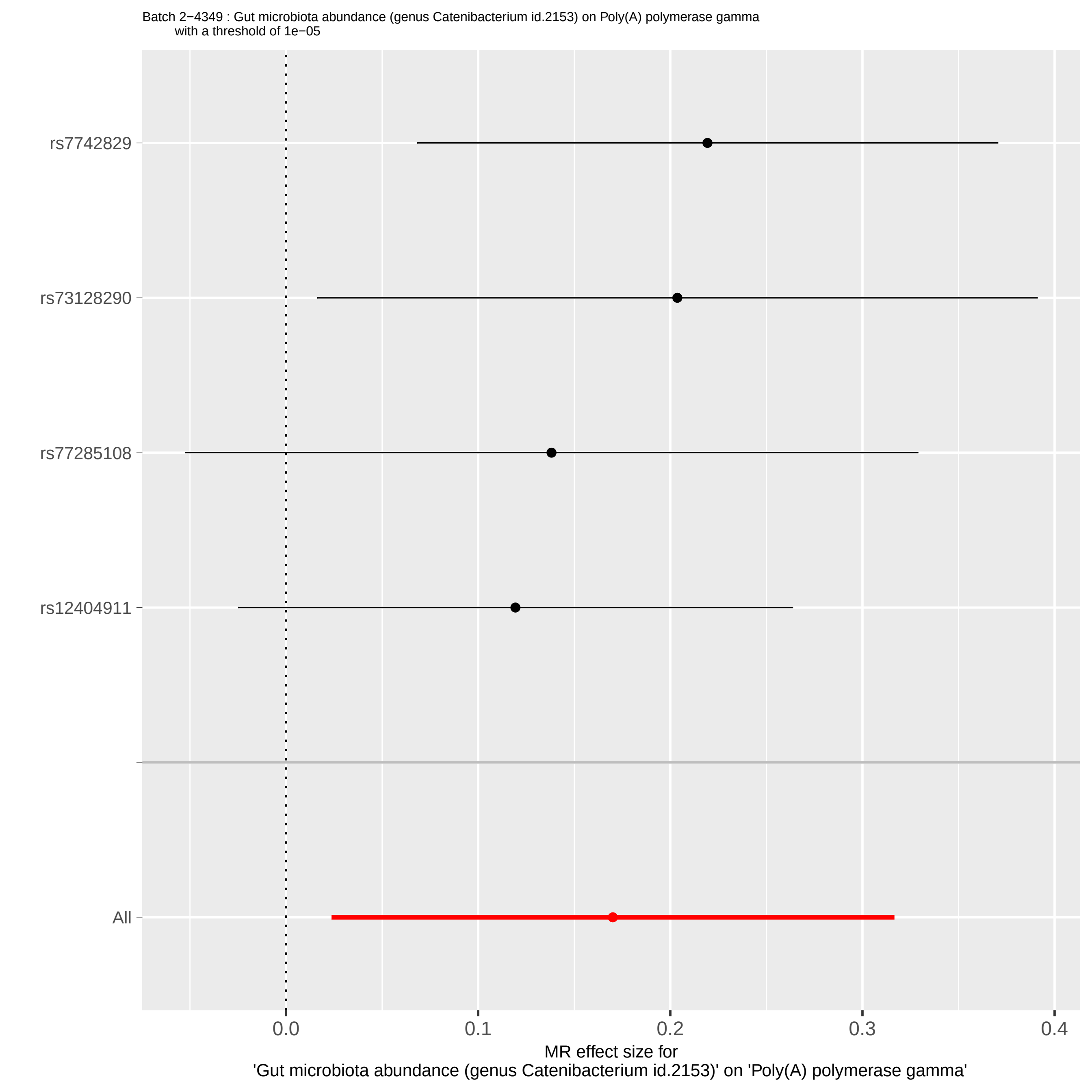
Batch 2−4349 : Gut microbiota abundance (genus Catenibacterium id.2153) on Poly(A) polymerase gamma
with a threshold of 1e−05
rs7742829
rs73128290
rs77285108
rs12404911
All
0.0
0.1
0.2
0.3
0.4
MR effect size for
'Gut microbiota abundance (genus Catenibacterium id.2153)' on 'Poly(A) polymerase gamma'

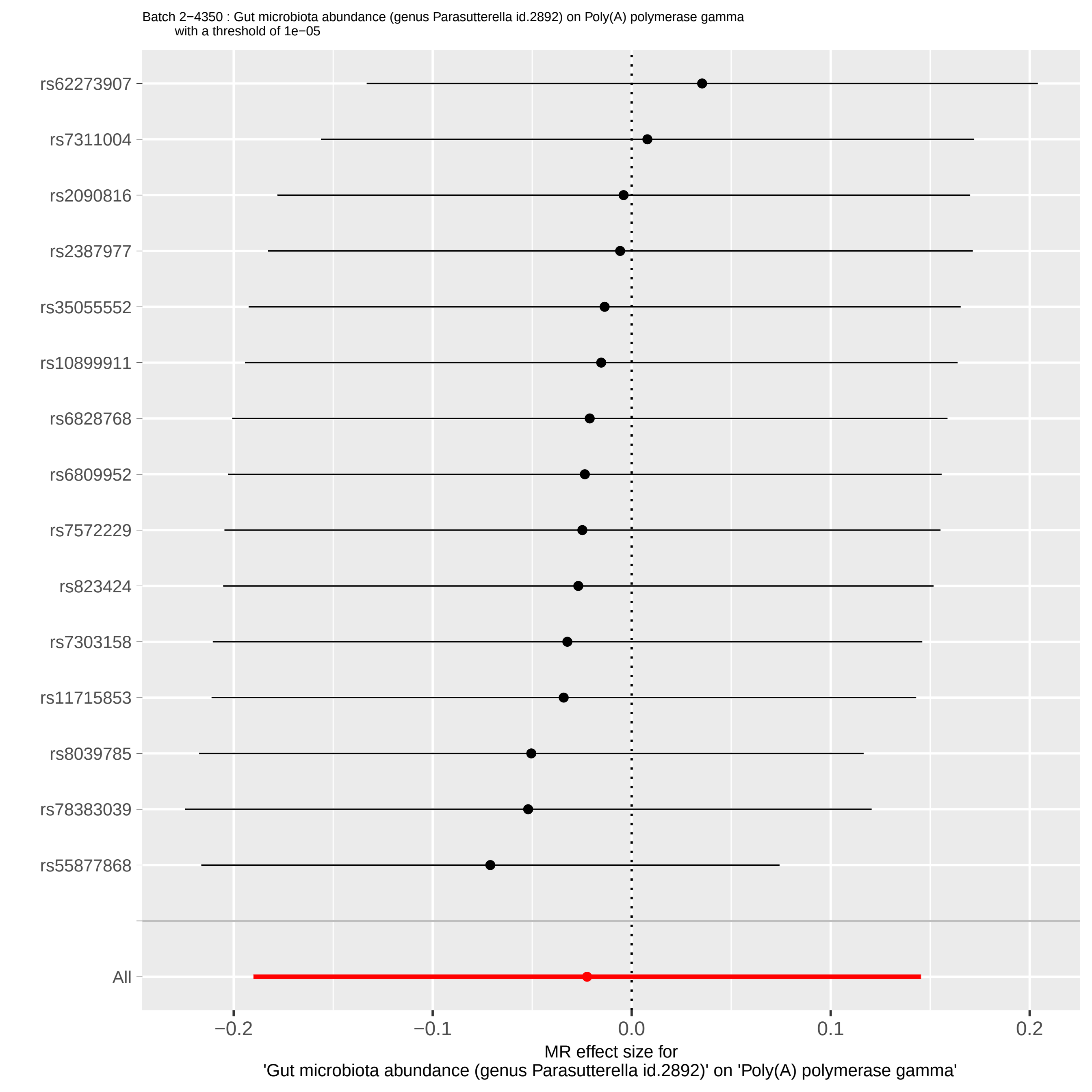
Batch 2−4350 : Gut microbiota abundance (genus Parasutterella id.2892) on Poly(A) polymerase gamma
with a threshold of 1e−05
rs62273907
rs7311004
rs2090816
rs2387977
rs35055552
rs10899911
rs6828768
rs6809952
rs7572229
rs823424
rs7303158
rs11715853
rs8039785
rs78383039
rs55877868
All
−0.2
−0.1
0.0
0.1
0.2
MR effect size for
'Gut microbiota abundance (genus Parasutterella id.2892)' on 'Poly(A) polymerase gamma'

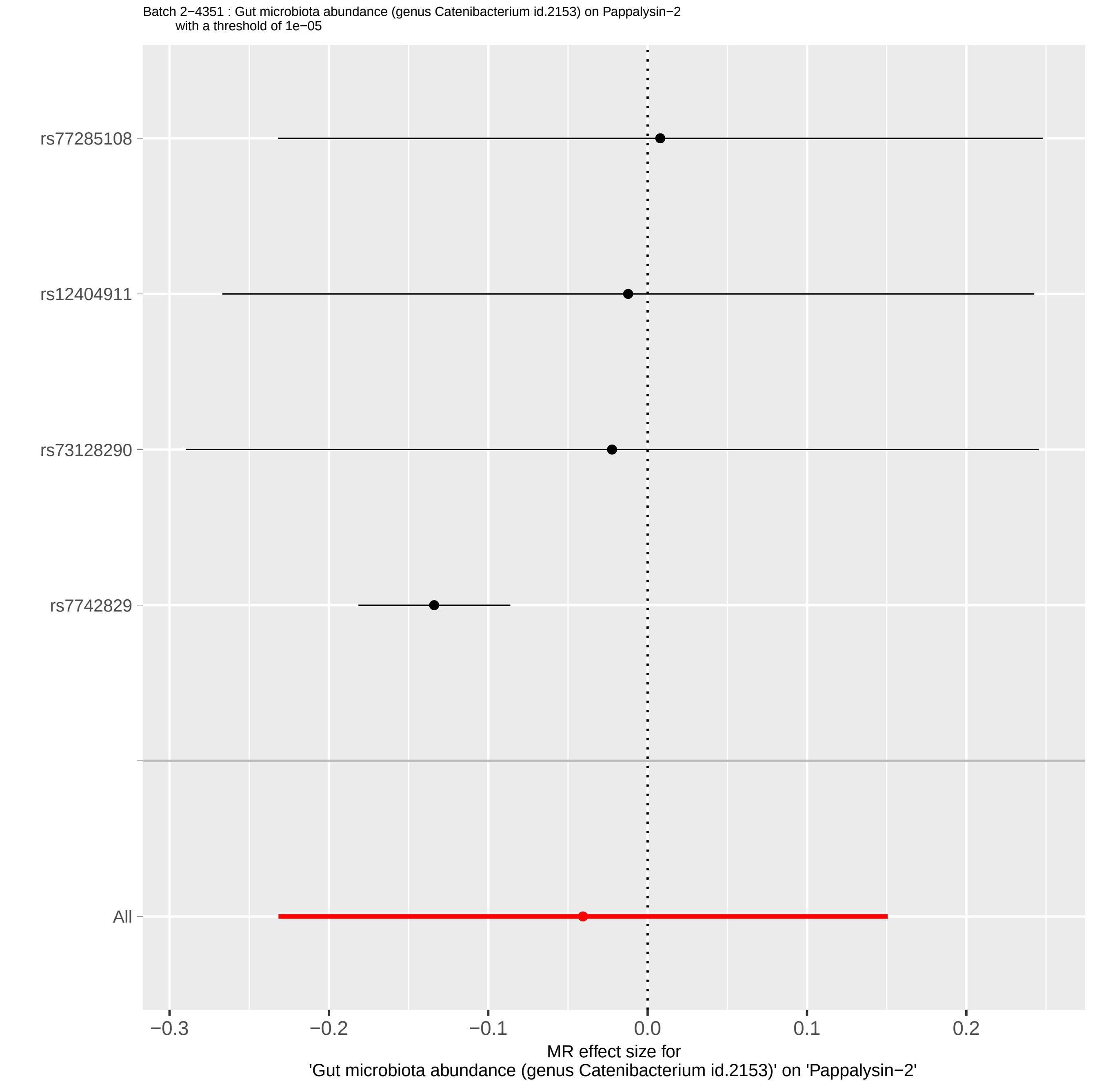

Batch 2−4351 : Gut microbiota abundance (genus Catenibacterium id.2153) on Pappalysin−2
with a threshold of 1e−05
rs77285108
rs12404911
rs73128290
rs7742829
All
−0.3
−0.2
−0.1
0.0
0.1
0.2
MR effect size for
'Gut microbiota abundance (genus Catenibacterium id.2153)' on 'Pappalysin−2'

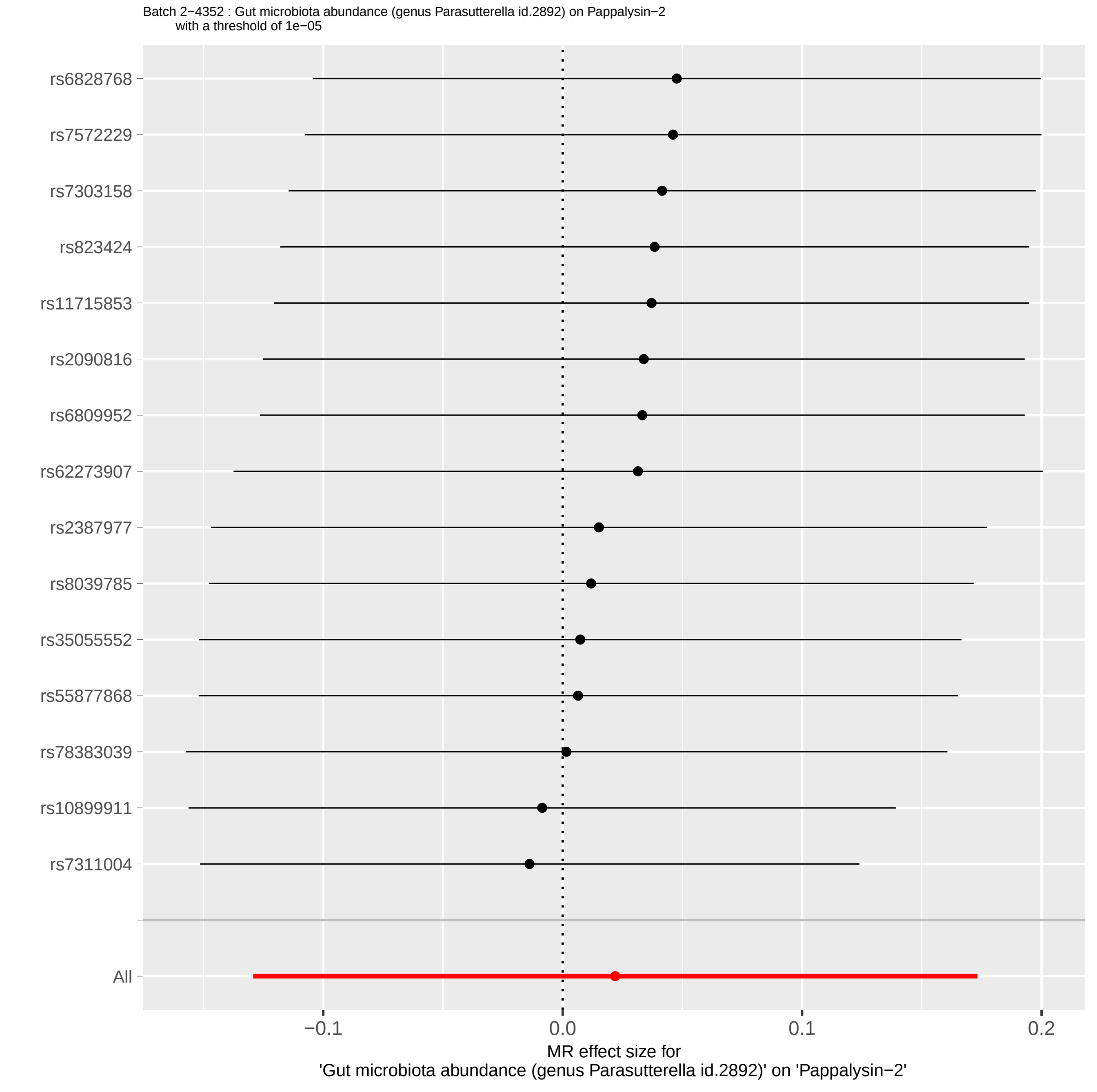

Batch 2−4352 : Gut microbiota abundance (genus Parasutterella id.2892) on Pappalysin−2
with a threshold of 1e−05
rs6828768
rs7572229
rs7303158
rs823424
rs11715853
rs2090816
rs6809952
rs62273907
rs2387977
rs8039785
rs35055552
rs55877868
rs78383039
rs10899911
rs7311004
All
−0.1
0.0
0.1
0.2
MR effect size for
'Gut microbiota abundance (genus Parasutterella id.2892)' on 'Pappalysin−2'

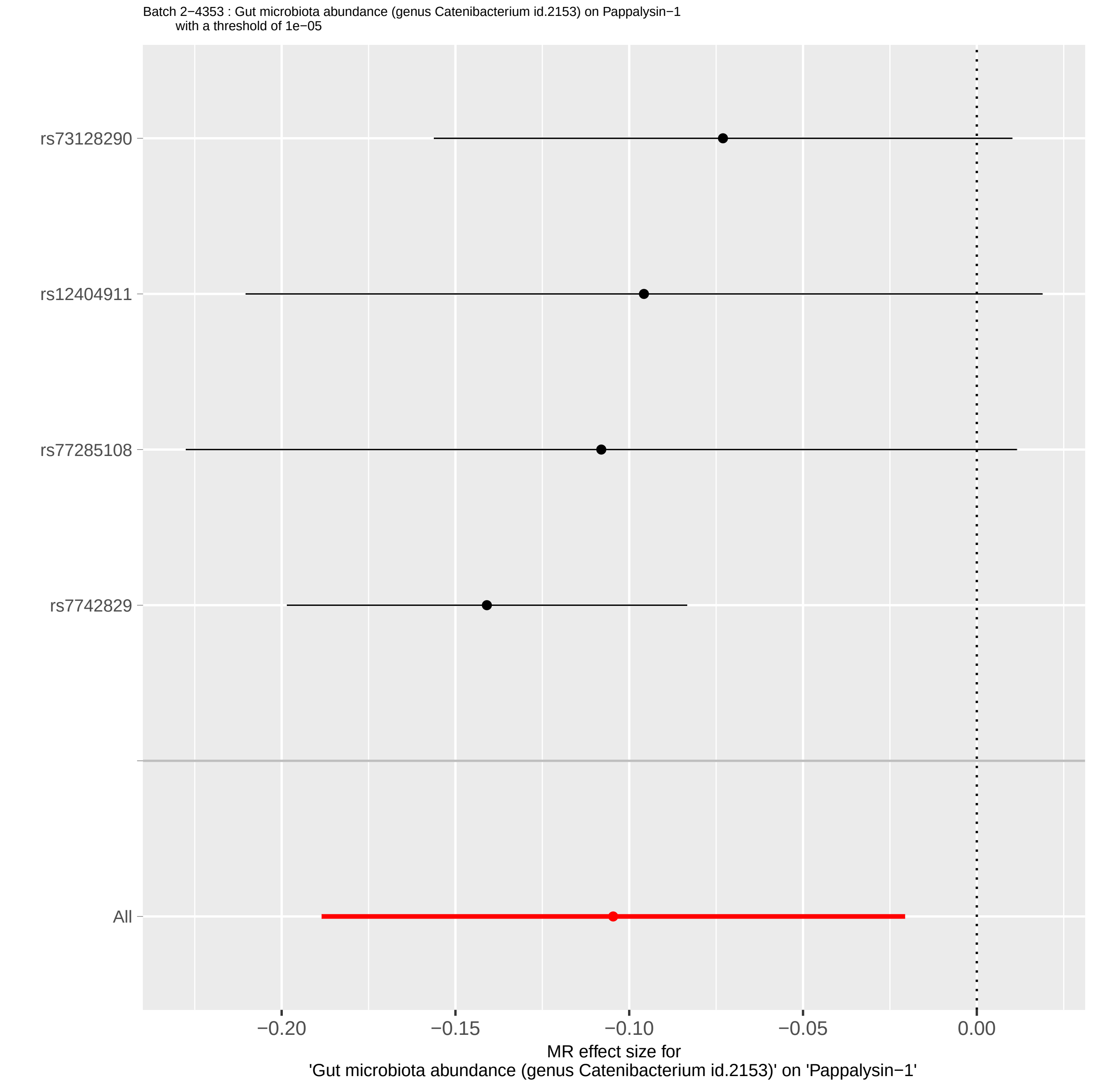

Batch 2−4353 : Gut microbiota abundance (genus Catenibacterium id.2153) on Pappalysin−1
with a threshold of 1e−05
rs73128290
rs12404911
rs77285108
rs7742829
All
−0.20
−0.15
−0.10
−0.05
0.00
MR effect size for
'Gut microbiota abundance (genus Catenibacterium id.2153)' on 'Pappalysin−1'

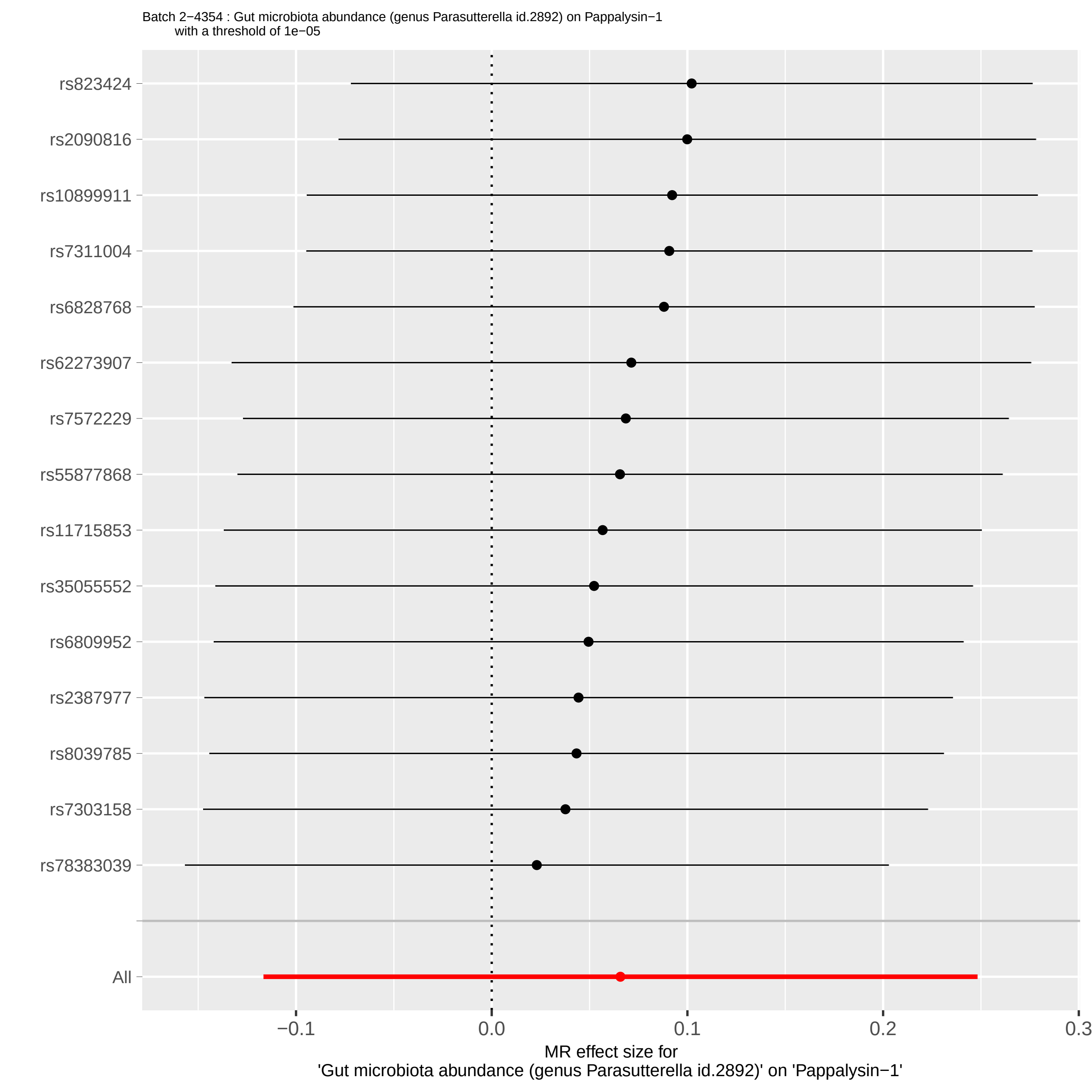

Batch 2−4354 : Gut microbiota abundance (genus Parasutterella id.2892) on Pappalysin−1
with a threshold of 1e−05
rs823424
rs2090816
rs10899911
rs7311004
rs6828768
rs62273907
rs7572229
rs55877868
rs11715853
rs35055552
rs6809952
rs2387977
rs8039785
rs7303158
rs78383039
All
−0.1
0.0
0.1
0.2
0.3
MR effect size for
'Gut microbiota abundance (genus Parasutterella id.2892)' on 'Pappalysin−1'

Batch 2−4355 : Gut microbiota abundance (genus Catenibacterium id.2153) on Bifunctional 3'−phosphoadenosine 5'−phosphosulfate synthase 1 with a threshold of 1e−05

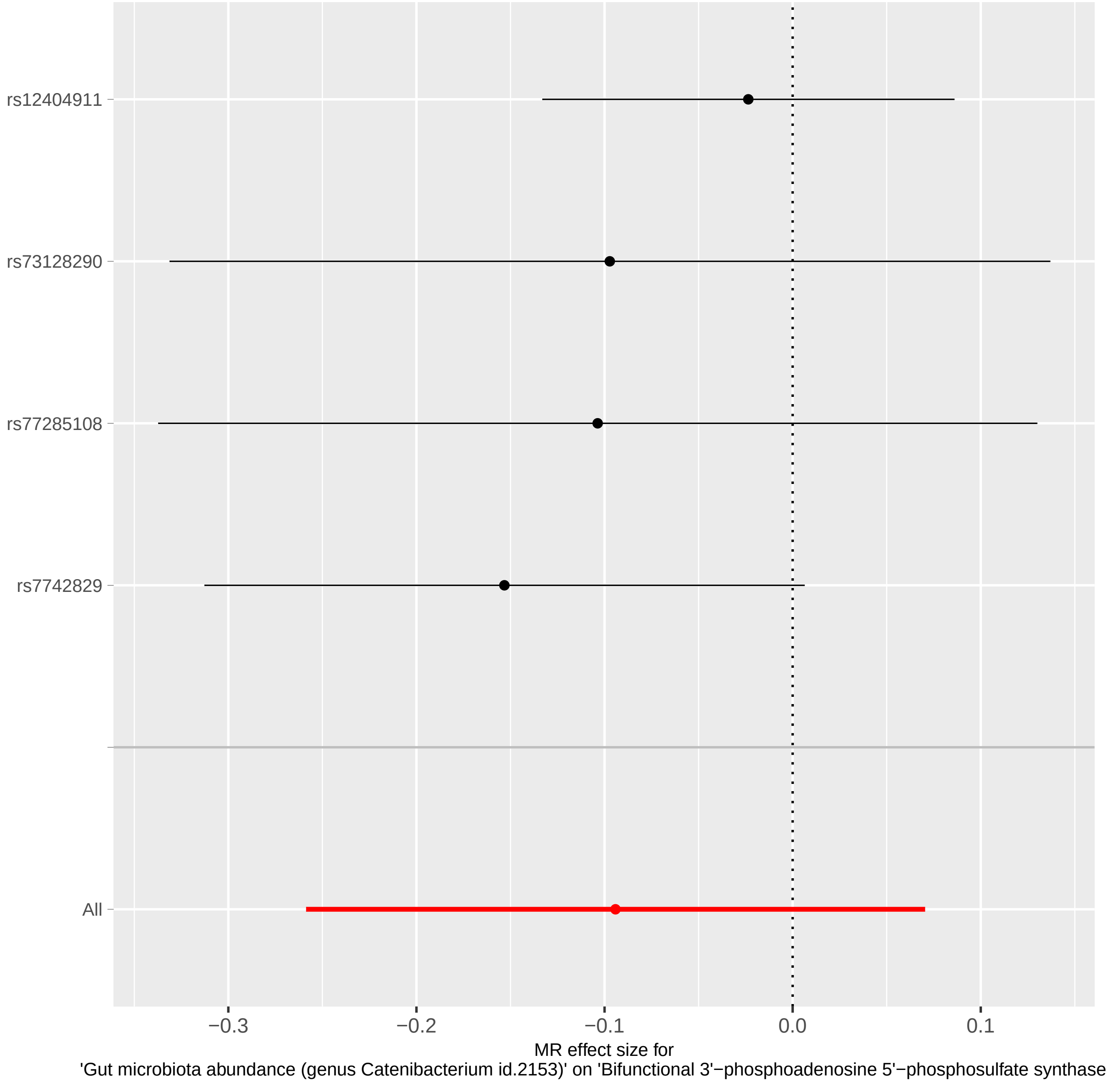

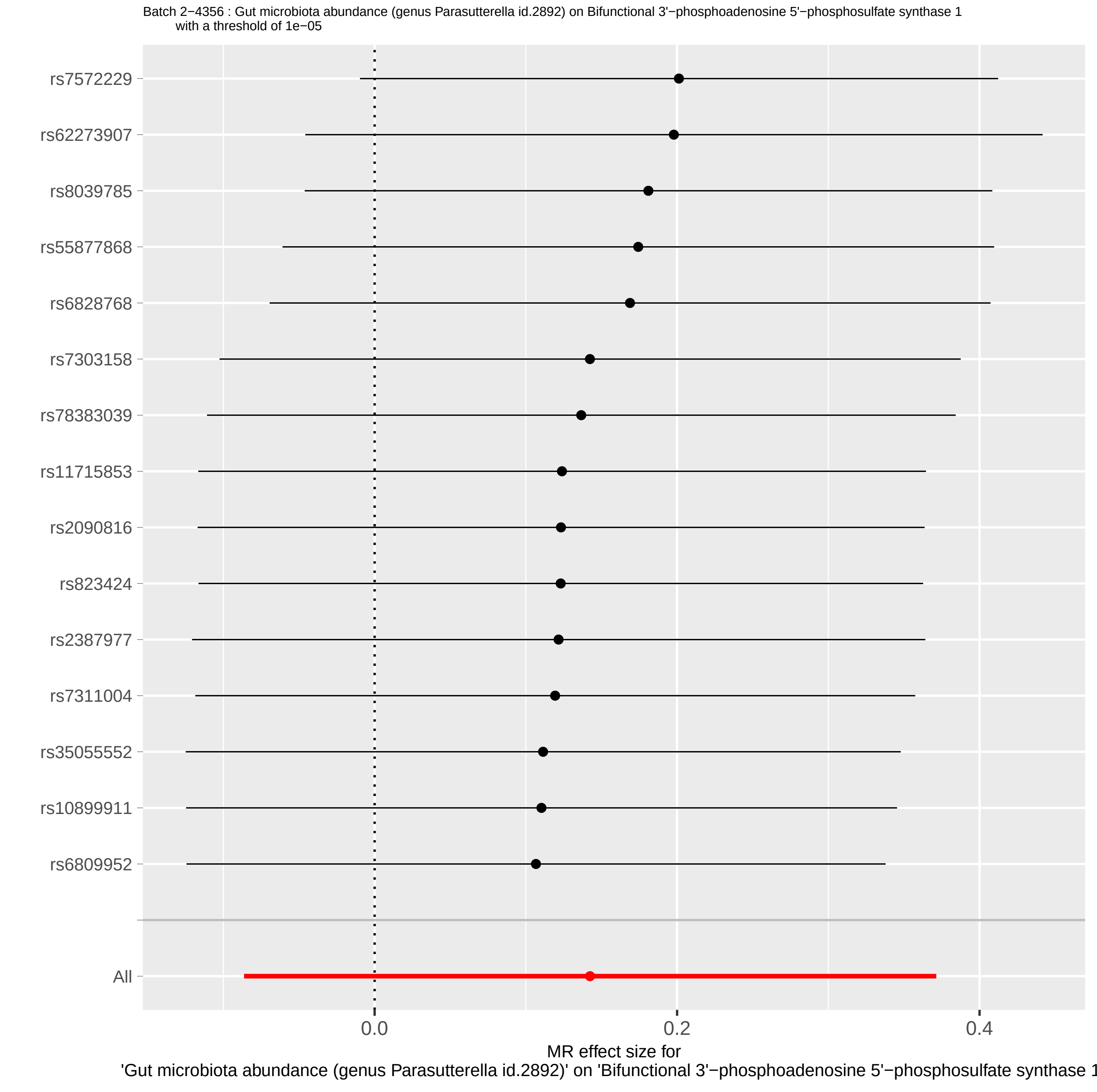

Batch 2−4356 : Gut microbiota abundance (genus Parasutterella id.2892) on Bifunctional 3'−phosphoadenosine 5'−phosphosulfate synthase 1
with a threshold of 1e−05
rs7572229
rs62273907
rs8039785
rs55877868
rs6828768
rs7303158
rs78383039
rs11715853
rs2090816
rs823424
rs2387977
rs7311004
rs35055552
rs10899911
rs6809952
All
0.0
0.2
0.4
MR effect size for
'Gut microbiota abundance (genus Parasutterella id.2892)' on 'Bifunctional 3'−phosphoadenosine 5'−phosphosulfate synthase 1

Batch 2−4357 : Gut microbiota abundance (genus Catenibacterium id.2153) on E3 ubiquitin−protein ligase parkin
with a threshold of 1e−05

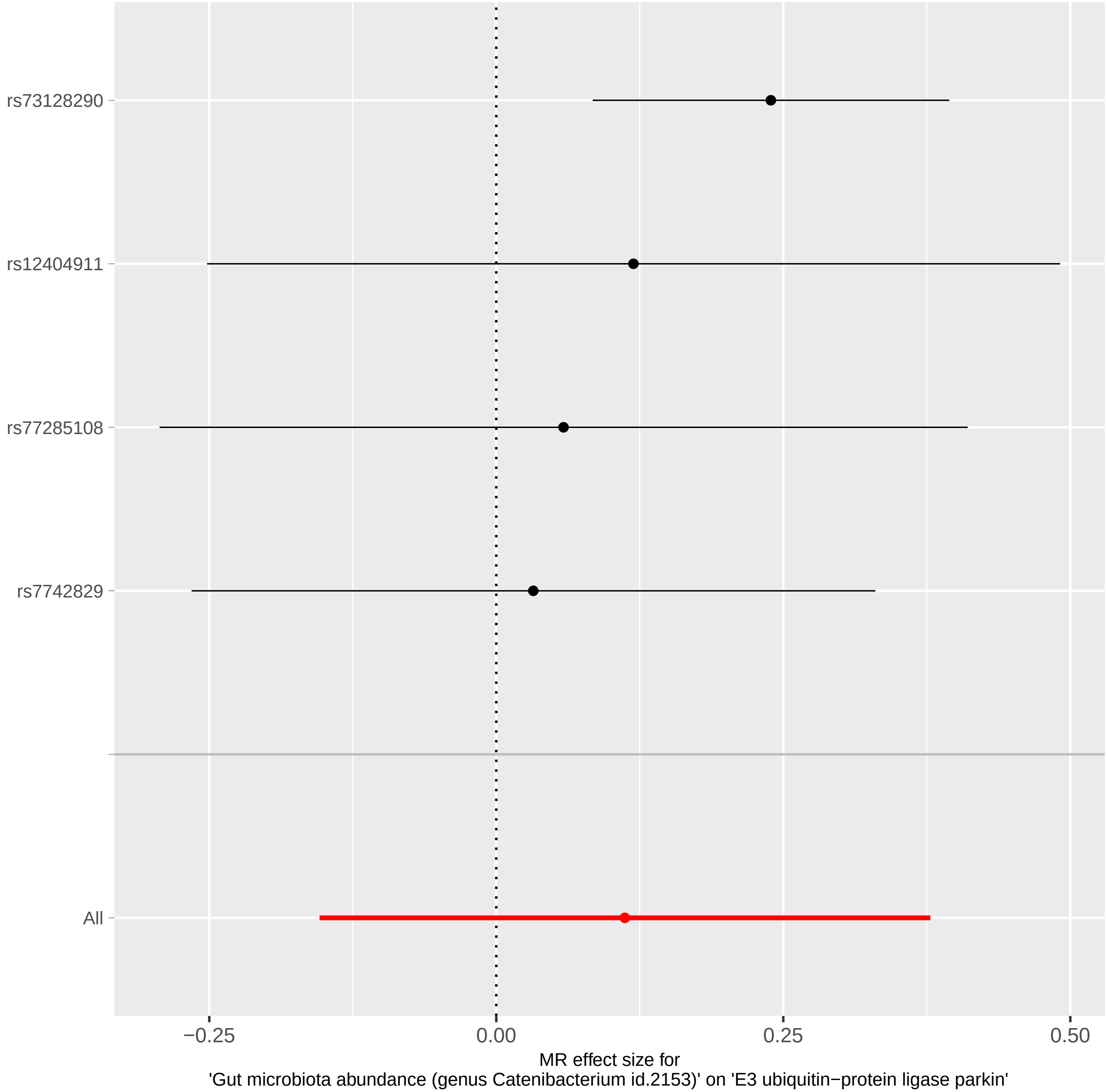

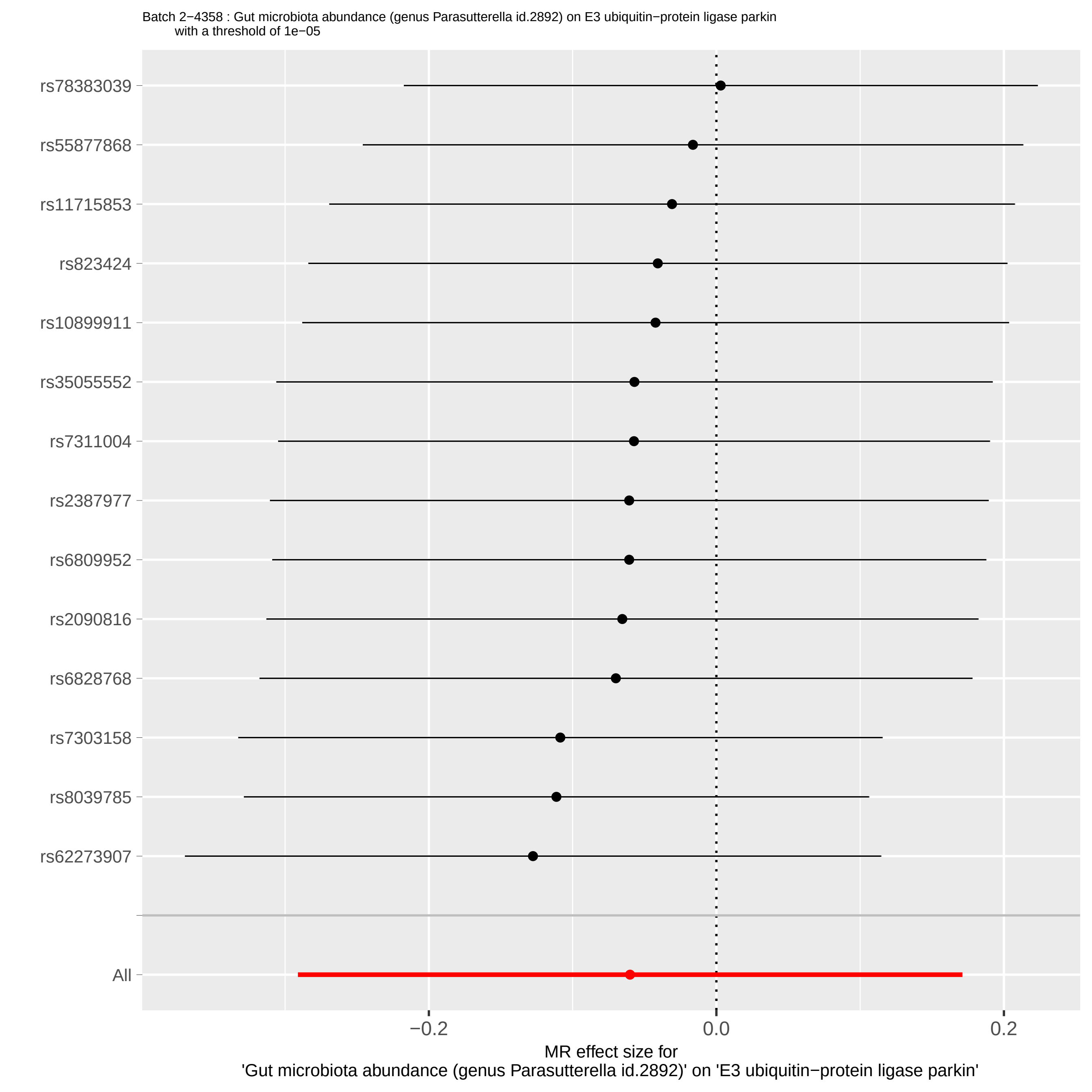
Batch 2−4358 : Gut microbiota abundance (genus Parasutterella id.2892) on E3 ubiquitin−protein ligase parkin
with a threshold of 1e−05
rs78383039
rs55877868
rs11715853
rs823424
rs10899911
rs35055552
rs7311004
rs2387977
rs6809952
rs2090816
rs6828768
rs7303158
rs8039785
rs62273907
All
−0.2
0.0
0.2
MR effect size for
'Gut microbiota abundance (genus Parasutterella id.2892)' on 'E3 ubiquitin−protein ligase parkin'

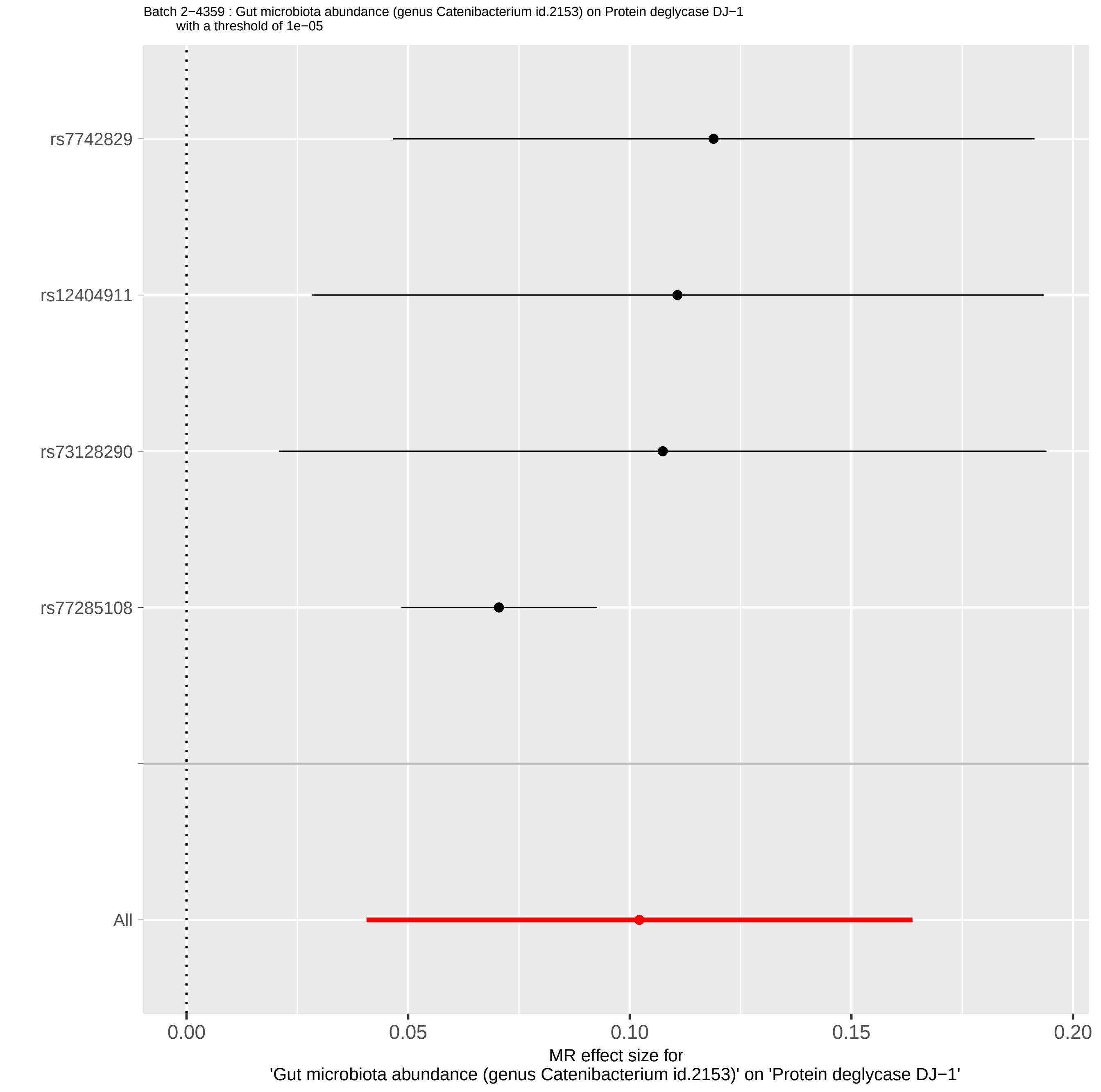
Batch 2−4359 : Gut microbiota abundance (genus Catenibacterium id.2153) on Protein deglycase DJ−1
with a threshold of 1e−05
rs7742829
rs12404911
rs73128290
rs77285108
All
0.00
0.05
0.10
0.15
0.20
MR effect size for
'Gut microbiota abundance (genus Catenibacterium id.2153)' on 'Protein deglycase DJ−1'

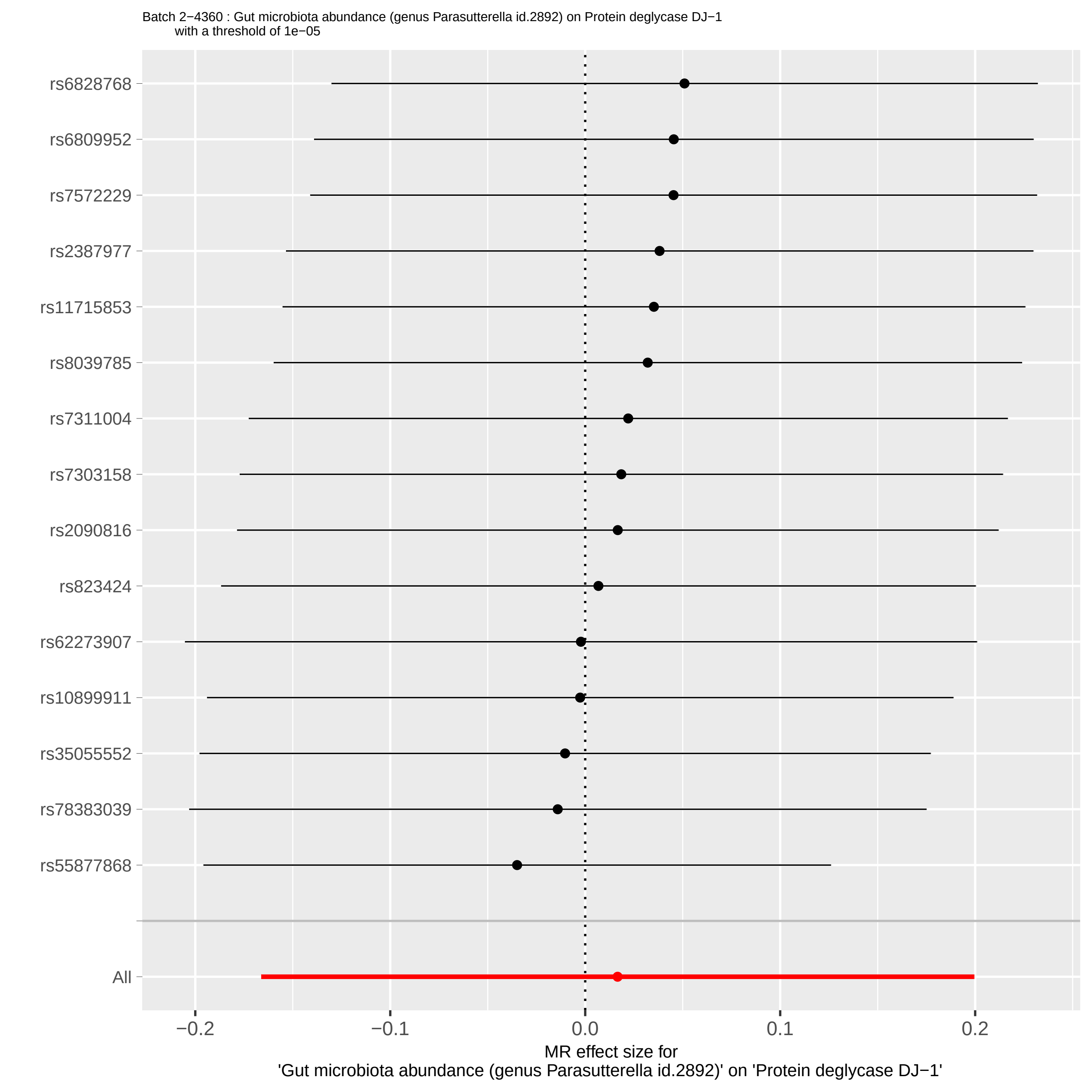
Batch 2−4360 : Gut microbiota abundance (genus Parasutterella id.2892) on Protein deglycase DJ−1
with a threshold of 1e−05
rs6828768
rs6809952
rs7572229
rs2387977
rs11715853
rs8039785
rs7311004
rs7303158
rs2090816
rs823424
rs62273907
rs10899911
rs35055552
rs78383039
rs55877868
All
−0.2
−0.1
0.0
0.1
0.2
MR effect size for
'Gut microbiota abundance (genus Parasutterella id.2892)' on 'Protein deglycase DJ−1'

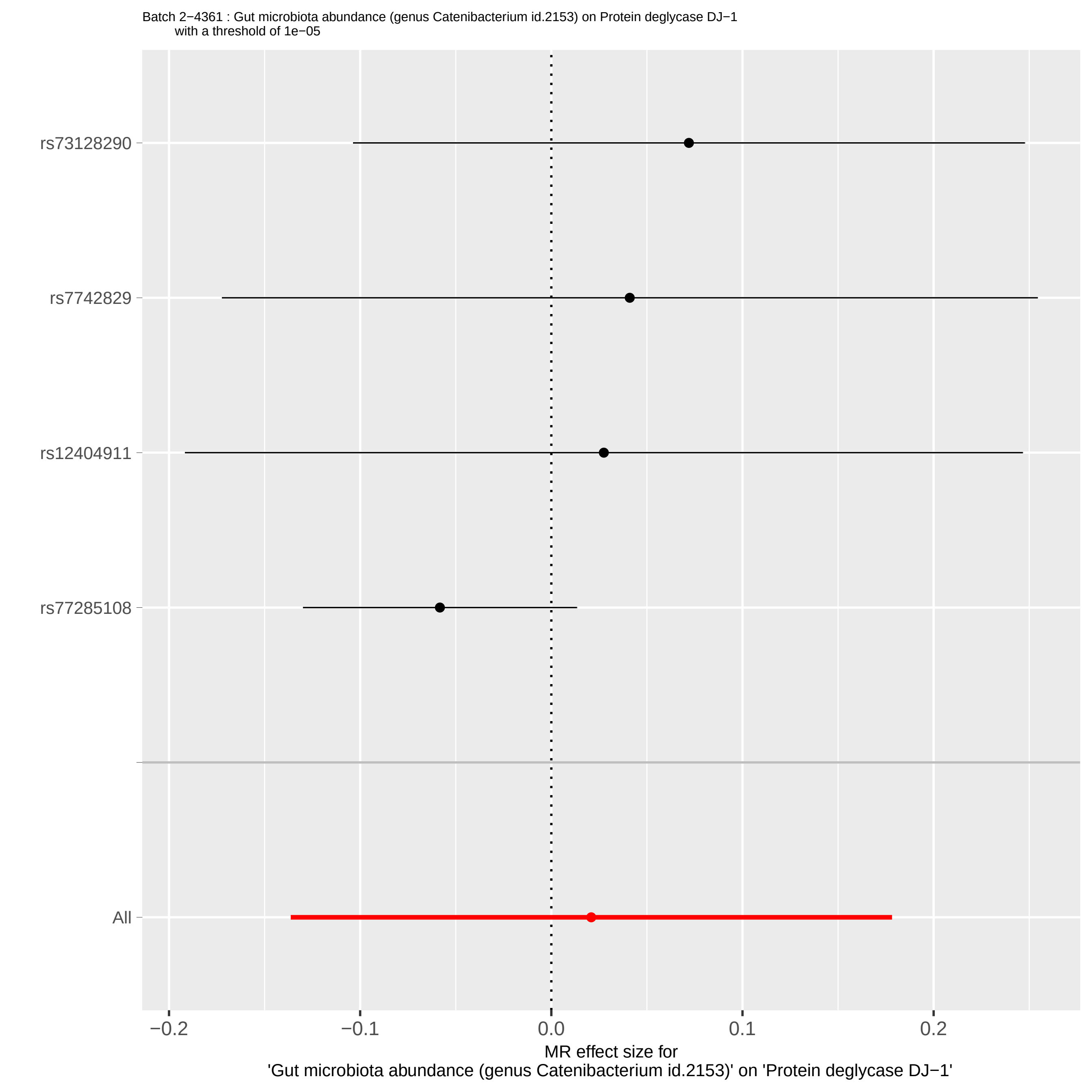
Batch 2−4361 : Gut microbiota abundance (genus Catenibacterium id.2153) on Protein deglycase DJ−1
with a threshold of 1e−05
rs73128290
rs7742829
rs12404911
rs77285108
All
−0.2
−0.1
0.0
0.1
0.2
MR effect size for
'Gut microbiota abundance (genus Catenibacterium id.2153)' on 'Protein deglycase DJ−1'

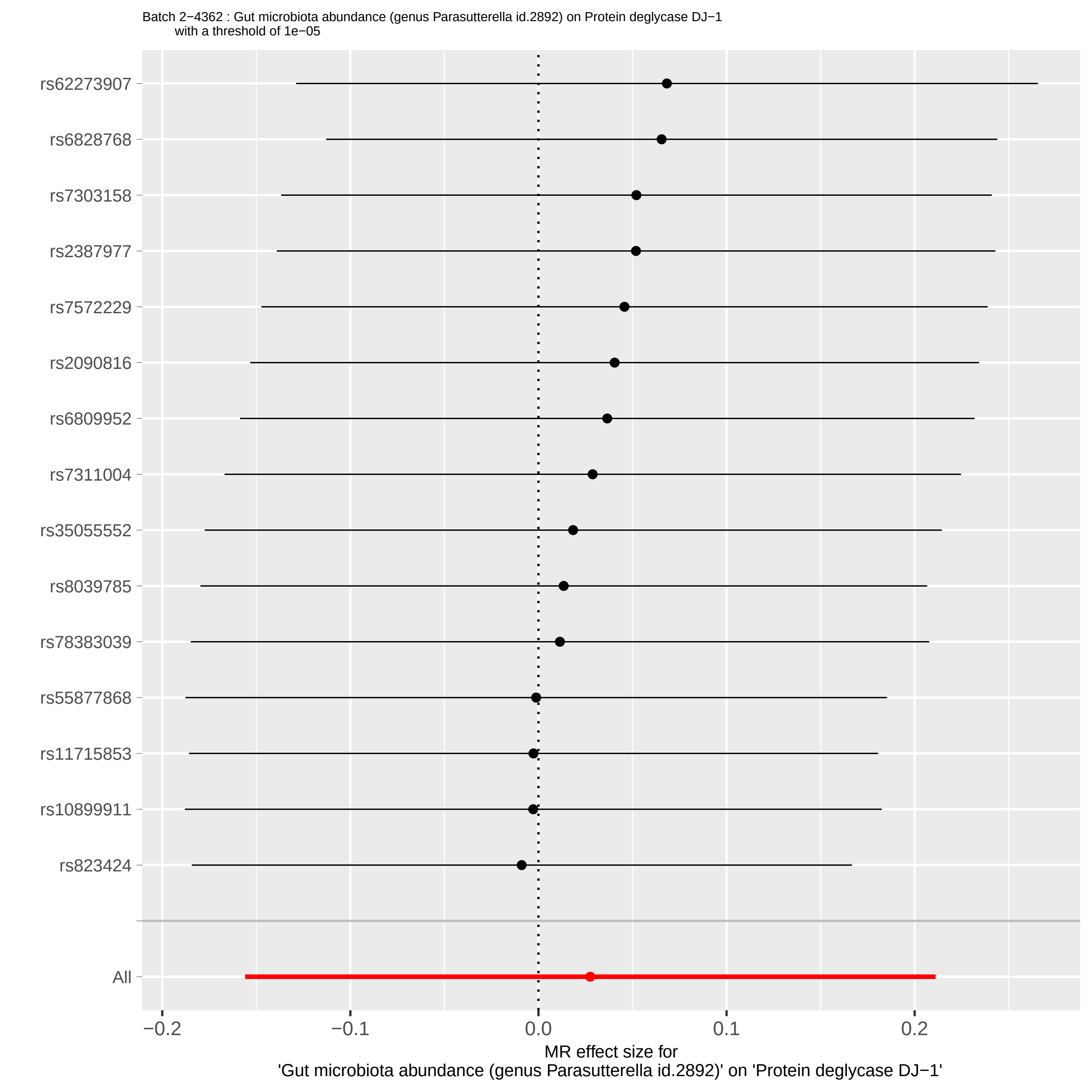

Batch 2−4362 : Gut microbiota abundance (genus Parasutterella id.2892) on Protein deglycase DJ−1
with a threshold of 1e−05
rs62273907
rs6828768
rs7303158
rs2387977
rs7572229
rs2090816
rs6809952
rs7311004
rs35055552
rs8039785
rs78383039
rs55877868
rs11715853
rs10899911
rs823424
All
−0.2
−0.1
0.0
0.1
0.2
MR effect size for
'Gut microbiota abundance (genus Parasutterella id.2892)' on 'Protein deglycase DJ−1'

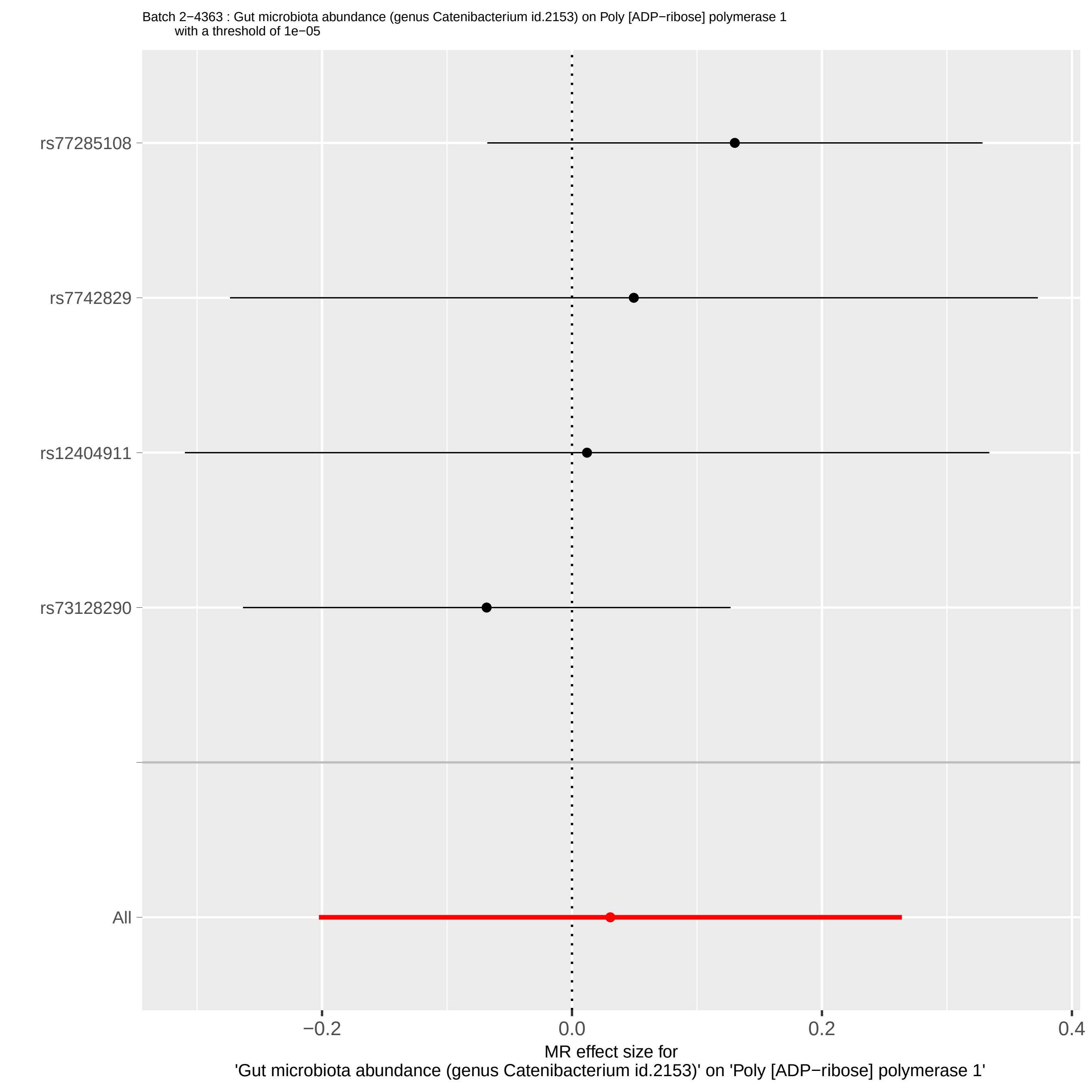
Batch 2−4363 : Gut microbiota abundance (genus Catenibacterium id.2153) on Poly [ADP−ribose] polymerase 1
with a threshold of 1e−05
rs77285108
rs7742829
rs12404911
rs73128290
All
−0.2
0.0
0.2
0.4
MR effect size for
'Gut microbiota abundance (genus Catenibacterium id.2153)' on 'Poly [ADP−ribose] polymerase 1'

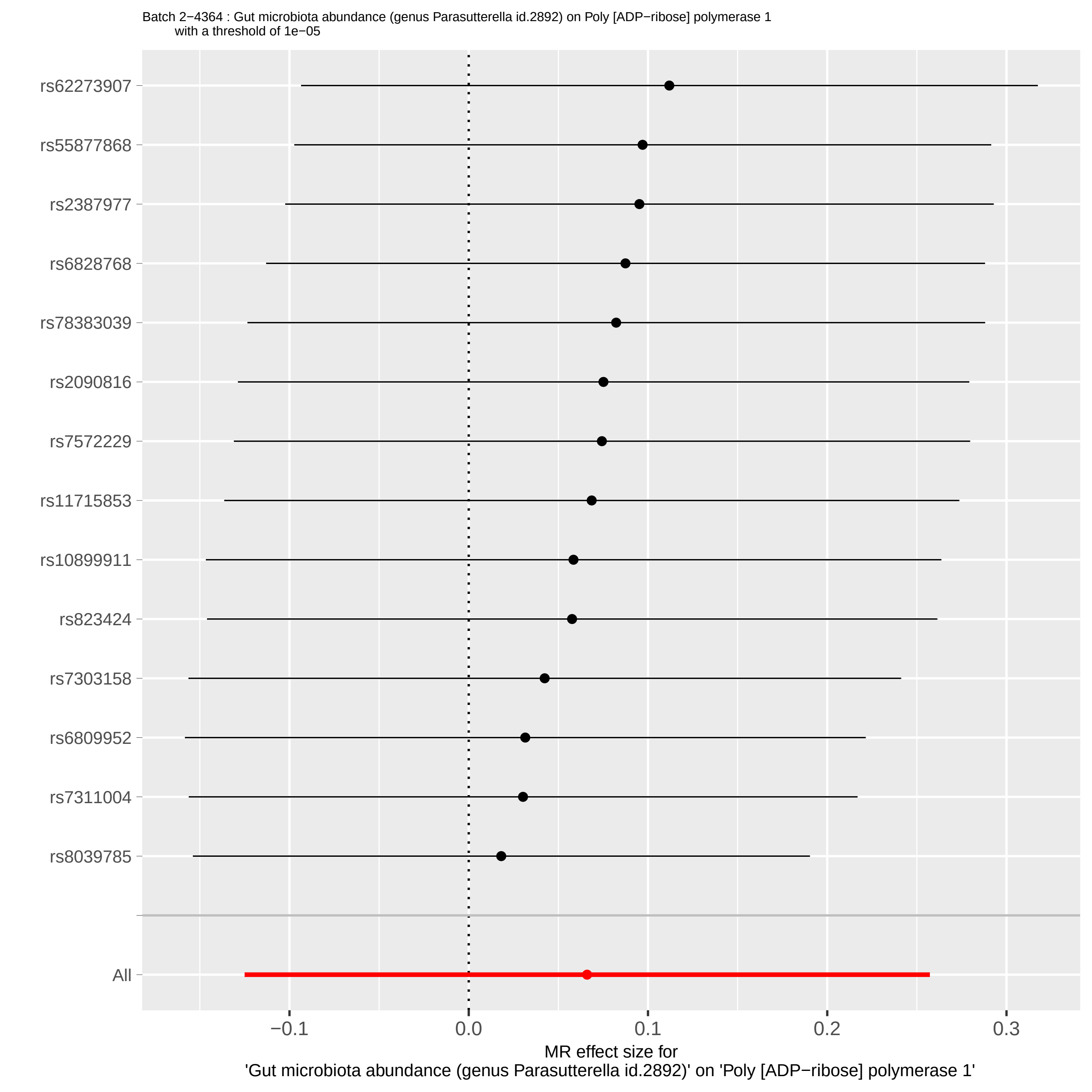
Batch 2−4364 : Gut microbiota abundance (genus Parasutterella id.2892) on Poly [ADP−ribose] polymerase 1
with a threshold of 1e−05
rs62273907
rs55877868
rs2387977
rs6828768
rs78383039
rs2090816
rs7572229
rs11715853
rs10899911
rs823424
rs7303158
rs6809952
rs7311004
rs8039785
All
−0.1
0.0
0.1
0.2
0.3
MR effect size for
'Gut microbiota abundance (genus Parasutterella id.2892)' on 'Poly [ADP−ribose] polymerase 1'

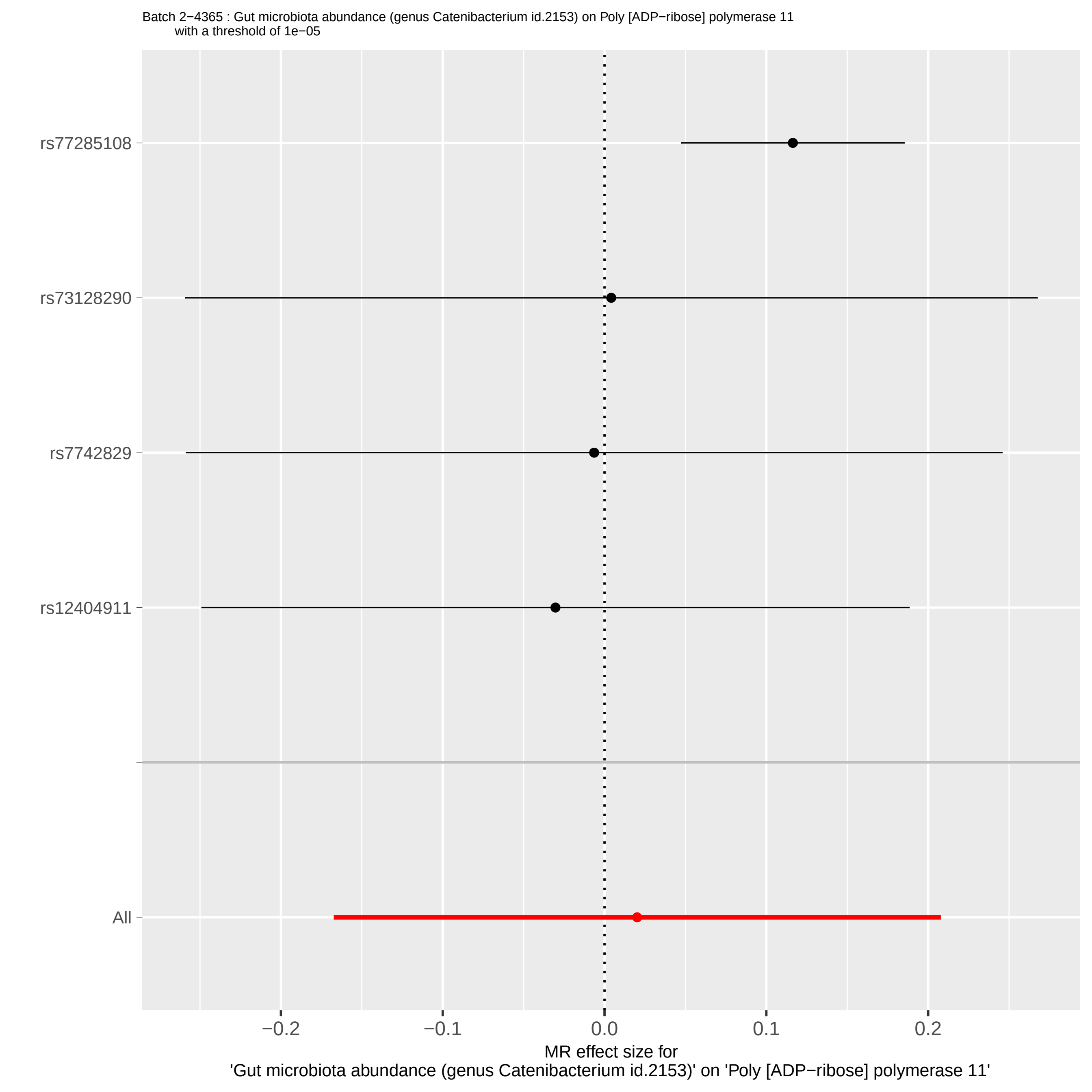
Batch 2−4365 : Gut microbiota abundance (genus Catenibacterium id.2153) on Poly [ADP−ribose] polymerase 11
with a threshold of 1e−05
rs77285108
rs73128290
rs7742829
rs12404911
All
−0.2
−0.1
0.0
0.1
0.2
MR effect size for
'Gut microbiota abundance (genus Catenibacterium id.2153)' on 'Poly [ADP−ribose] polymerase 11'

Batch 2−4366 : Gut microbiota abundance (genus Parasutterella id.2892) on Poly [ADP−ribose] polymerase 11
with a threshold of 1e−05

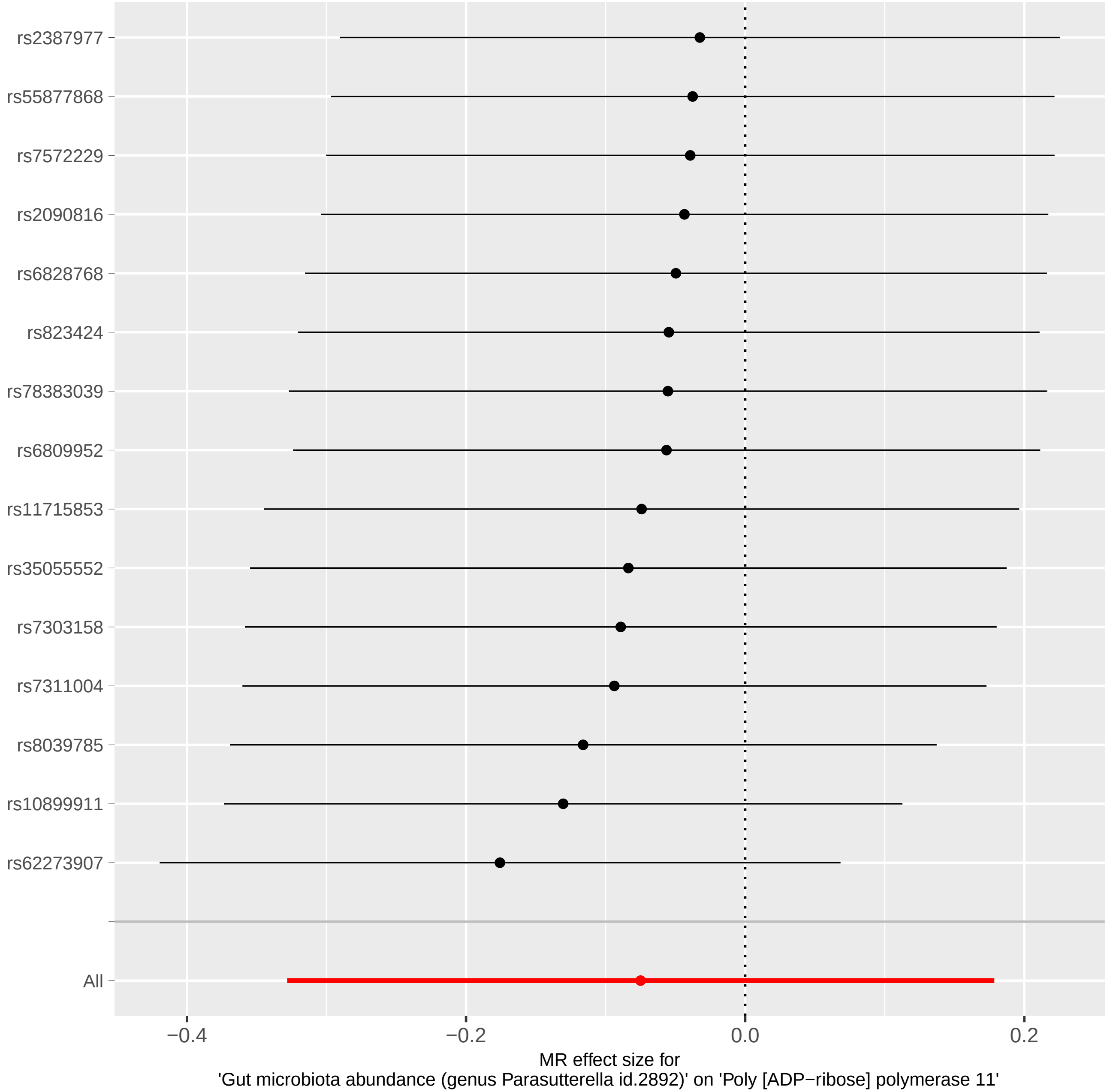

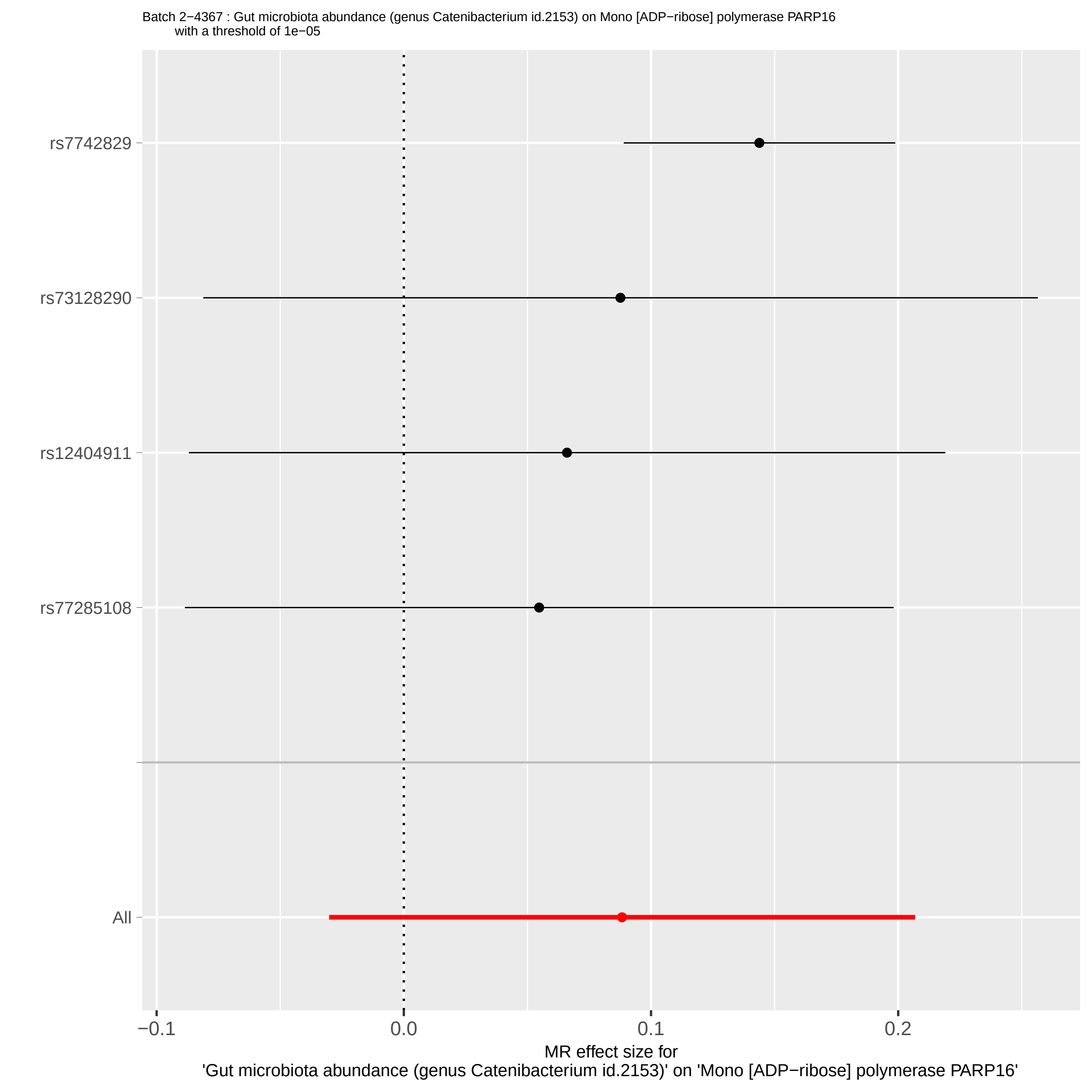
Batch 2−4367 : Gut microbiota abundance (genus Catenibacterium id.2153) on Mono [ADP−ribose] polymerase PARP16
with a threshold of 1e−05
rs7742829
rs73128290
rs12404911
rs77285108
All
−0.1
0.0
0.1
0.2
MR effect size for
'Gut microbiota abundance (genus Catenibacterium id.2153)' on 'Mono [ADP−ribose] polymerase PARP16'

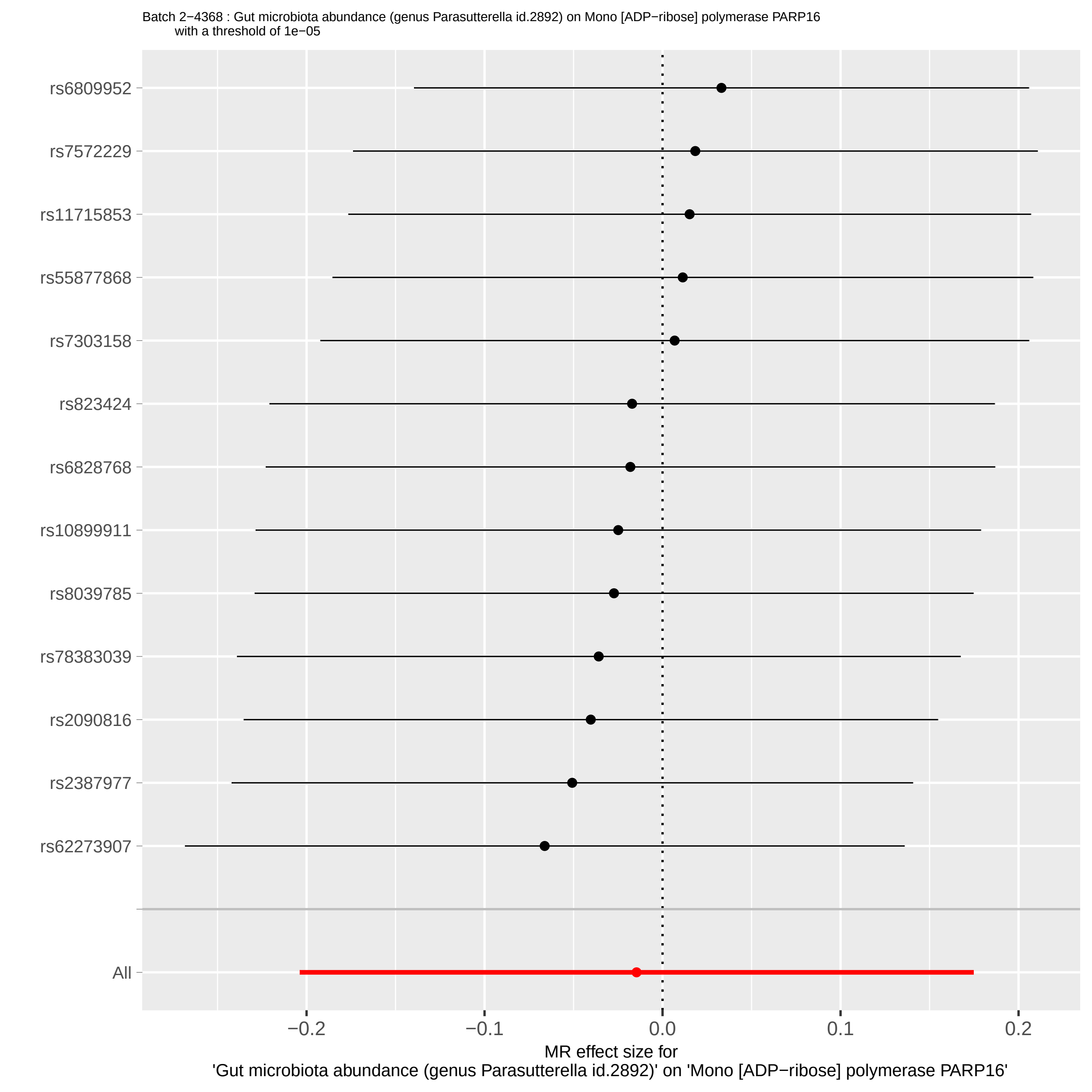

Batch 2−4368 : Gut microbiota abundance (genus Parasutterella id.2892) on Mono [ADP−ribose] polymerase PARP16
with a threshold of 1e−05
rs6809952
rs7572229
rs11715853
rs55877868
rs7303158
rs823424
rs6828768
rs10899911
rs8039785
rs78383039
rs2090816
rs2387977
rs62273907
All
−0.2
−0.1
0.0
0.1
0.2
MR effect size for
'Gut microbiota abundance (genus Parasutterella id.2892)' on 'Mono [ADP−ribose] polymerase PARP16'

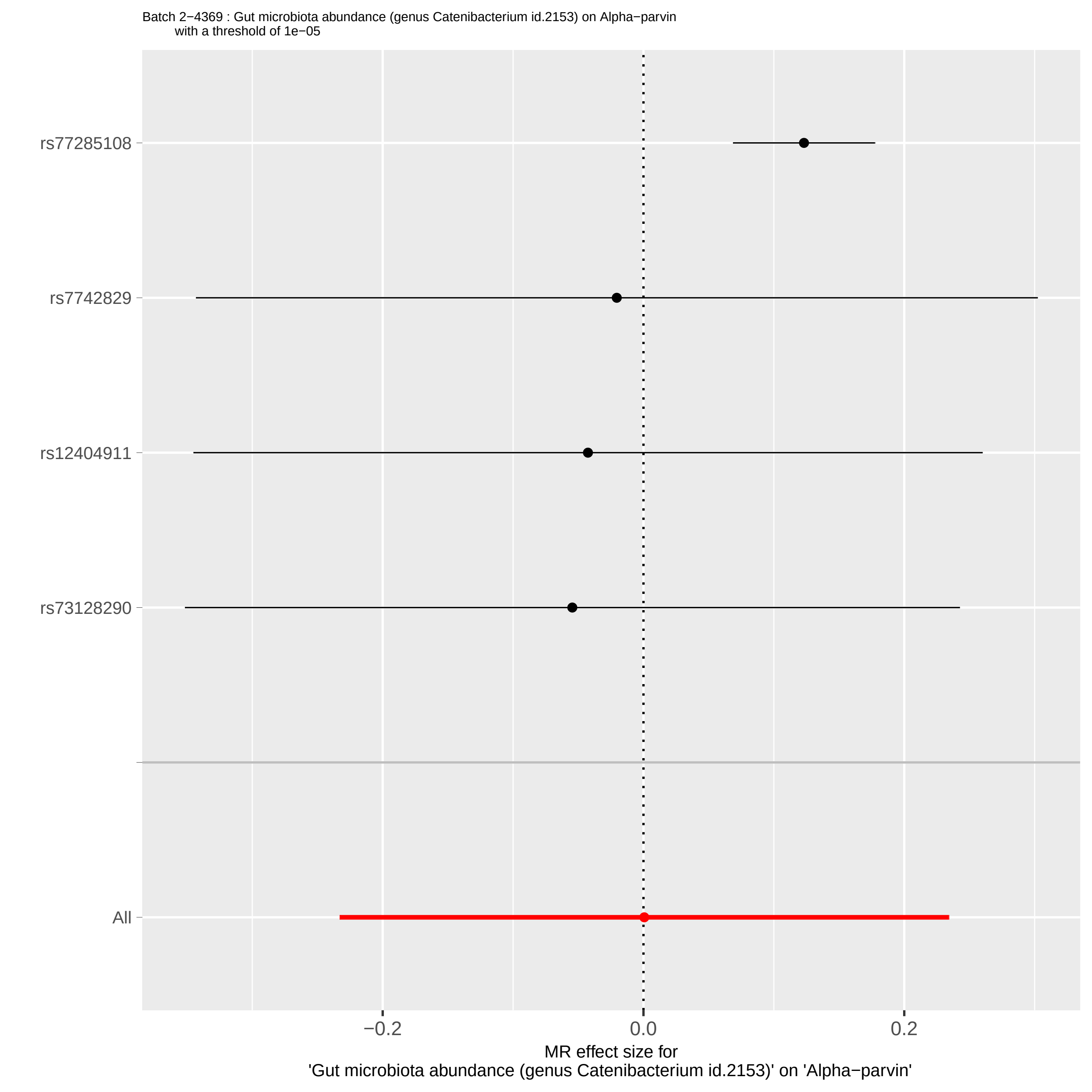

Batch 2−4369 : Gut microbiota abundance (genus Catenibacterium id.2153) on Alpha−parvin
with a threshold of 1e−05
rs77285108
rs7742829
rs12404911
rs73128290
All
−0.2
0.0
0.2
MR effect size for
'Gut microbiota abundance (genus Catenibacterium id.2153)' on 'Alpha−parvin'

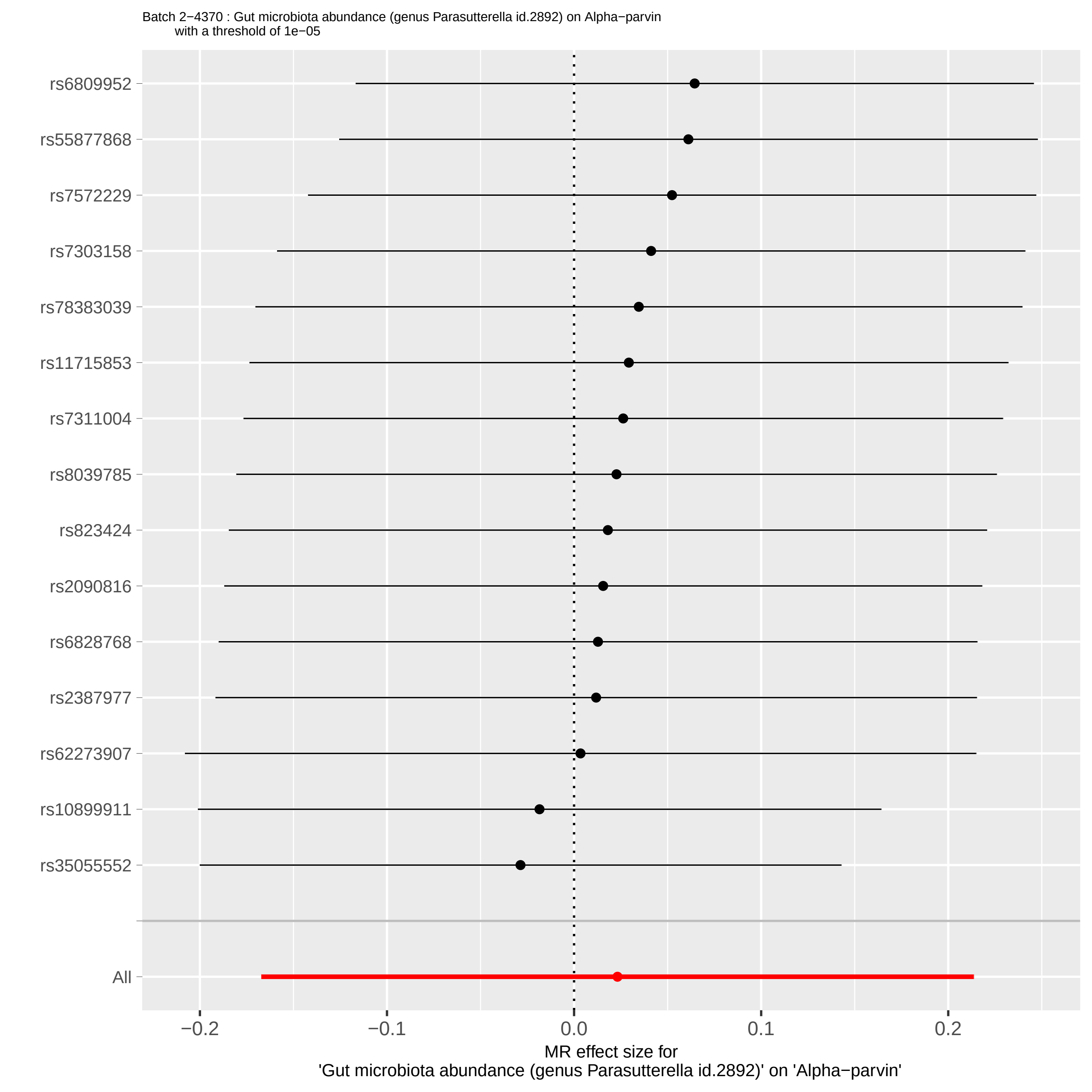

Batch 2−4370 : Gut microbiota abundance (genus Parasutterella id.2892) on Alpha−parvin
with a threshold of 1e−05
rs6809952
rs55877868
rs7572229
rs7303158
rs78383039
rs11715853
rs7311004
rs8039785
rs823424
rs2090816
rs6828768
rs2387977
rs62273907
rs10899911
rs35055552
All
−0.2
−0.1
0.0
0.1
0.2
MR effect size for
'Gut microbiota abundance (genus Parasutterella id.2892)' on 'Alpha−parvin'

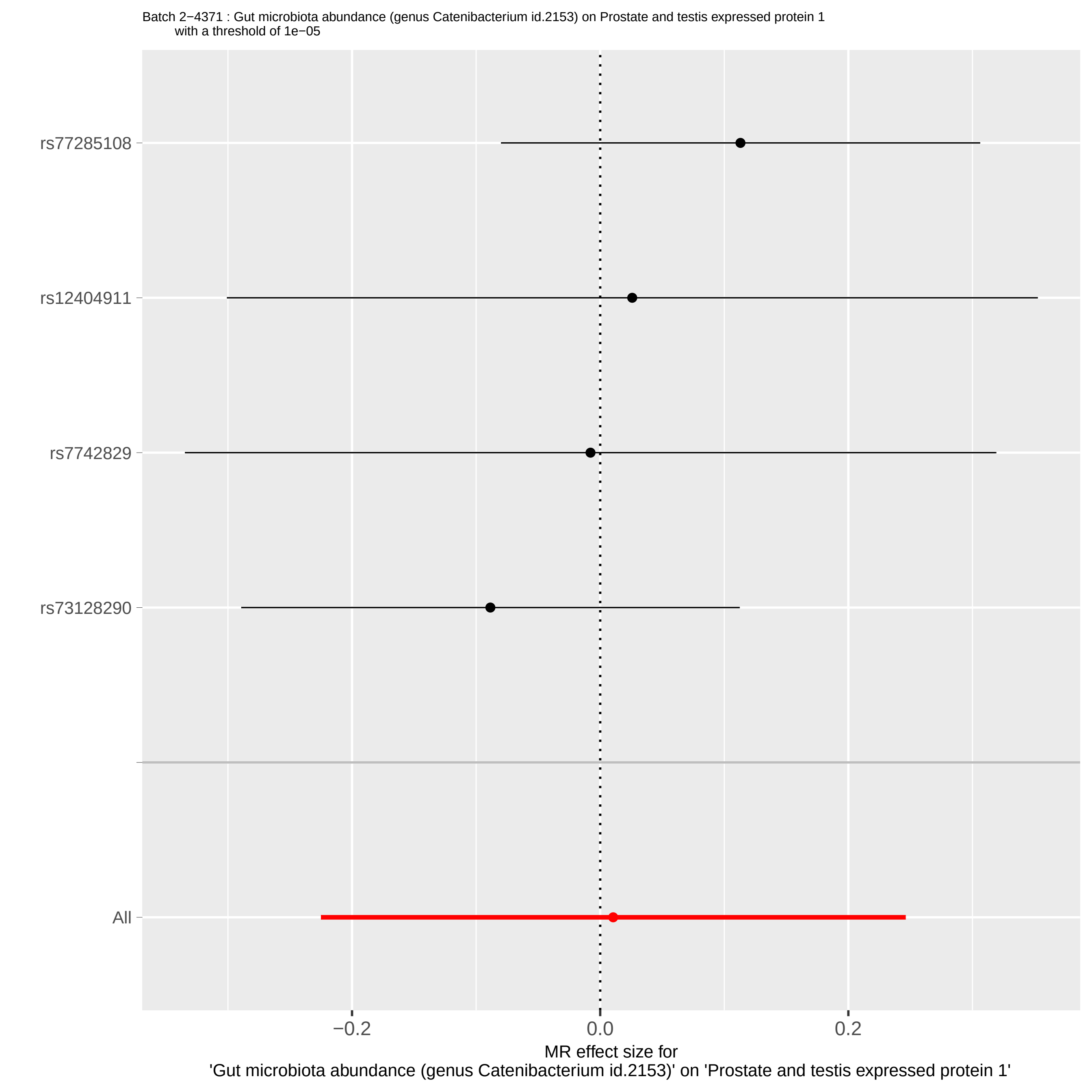
Batch 2−4371 : Gut microbiota abundance (genus Catenibacterium id.2153) on Prostate and testis expressed protein 1
with a threshold of 1e−05
rs77285108
rs12404911
rs7742829
rs73128290
All
−0.2
0.0
0.2
MR effect size for
'Gut microbiota abundance (genus Catenibacterium id.2153)' on 'Prostate and testis expressed protein 1'

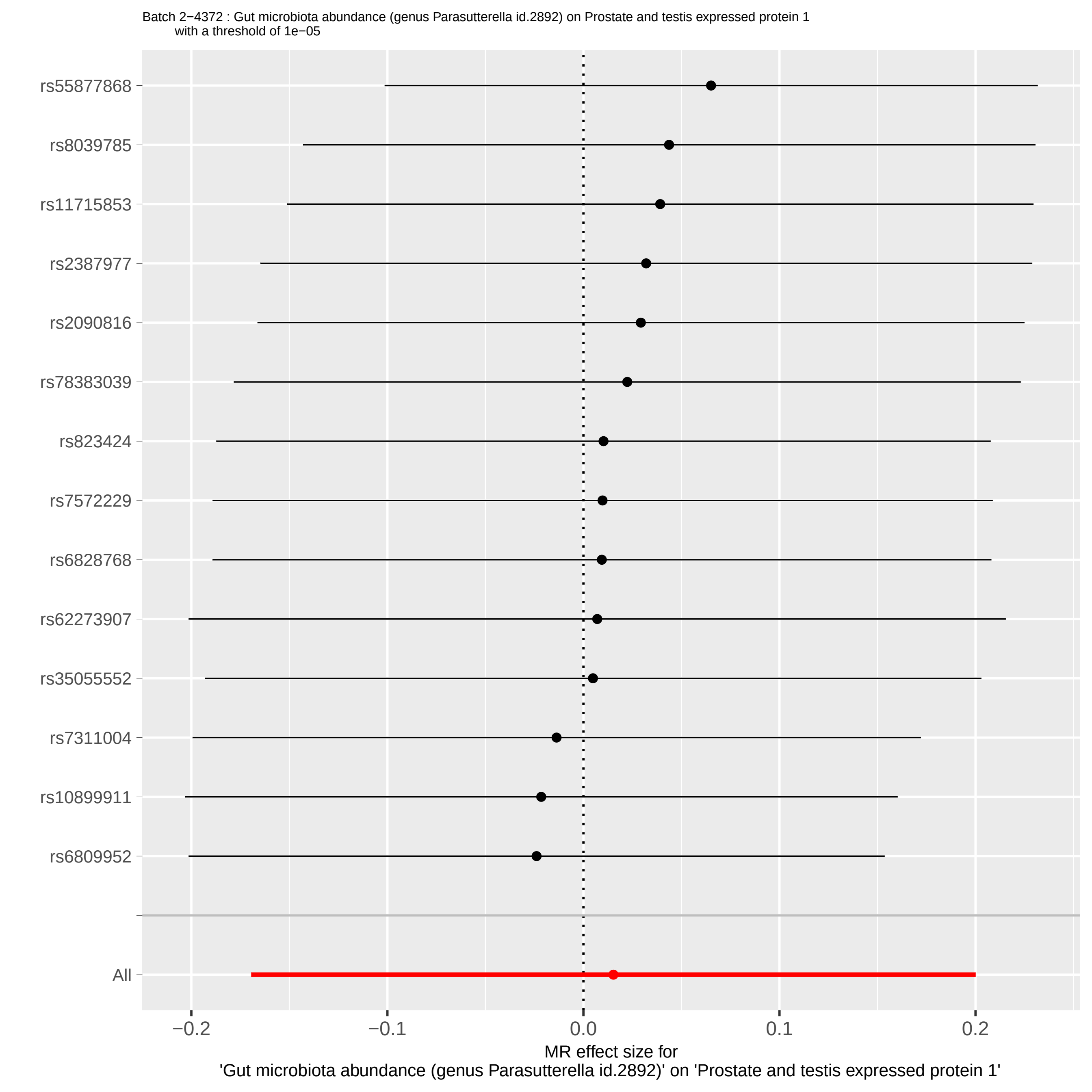
Batch 2−4372 : Gut microbiota abundance (genus Parasutterella id.2892) on Prostate and testis expressed protein 1
with a threshold of 1e−05
rs55877868
rs8039785
rs11715853
rs2387977
rs2090816
rs78383039
rs823424
rs7572229
rs6828768
rs62273907
rs35055552
rs7311004
rs10899911
rs6809952
All
−0.2
−0.1
0.0
0.1
0.2
MR effect size for
'Gut microbiota abundance (genus Parasutterella id.2892)' on 'Prostate and testis expressed protein 1'

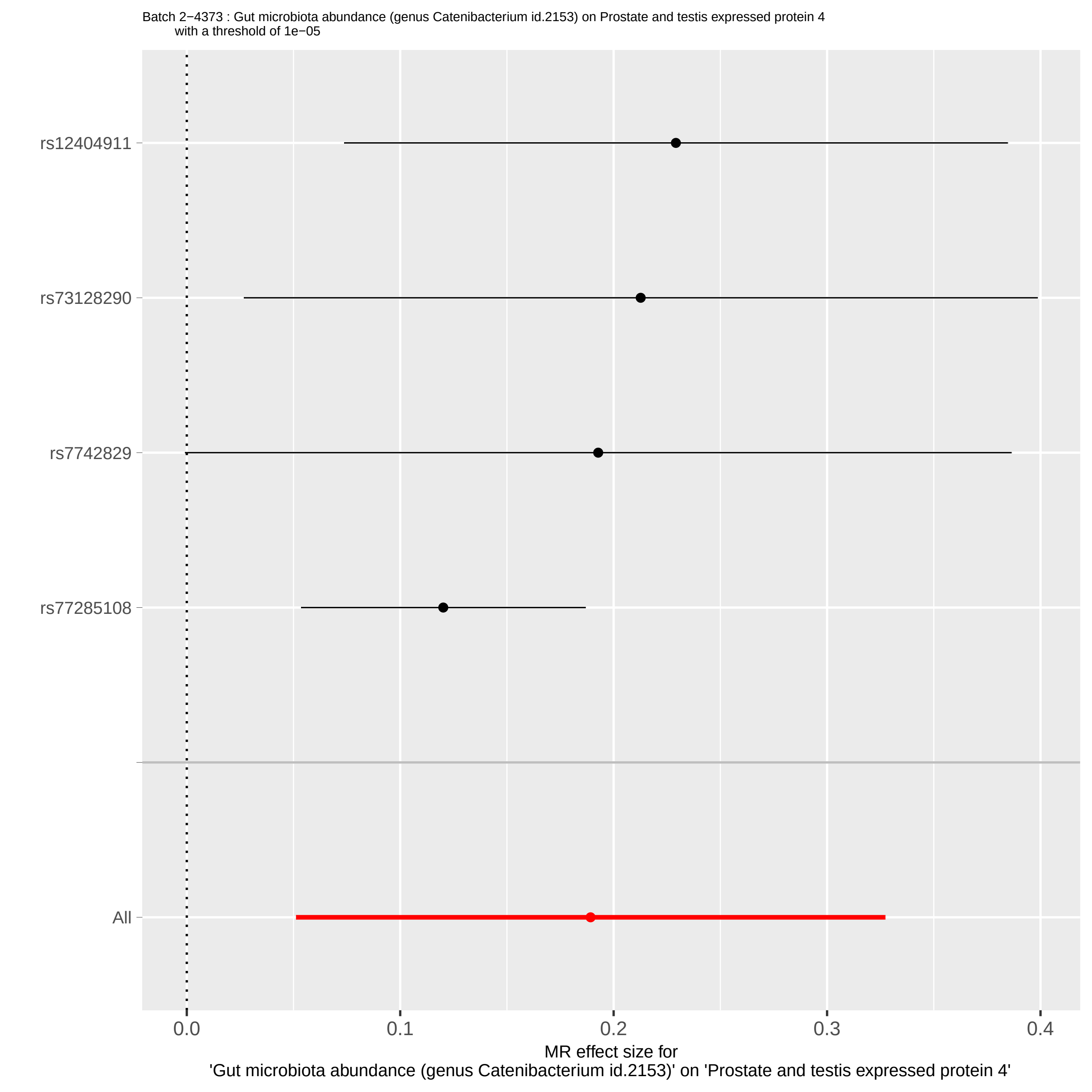
Batch 2−4373 : Gut microbiota abundance (genus Catenibacterium id.2153) on Prostate and testis expressed protein 4
with a threshold of 1e−05
rs12404911
rs73128290
rs7742829
rs77285108
All
0.0
0.1
0.2
0.3
0.4
MR effect size for
'Gut microbiota abundance (genus Catenibacterium id.2153)' on 'Prostate and testis expressed protein 4'

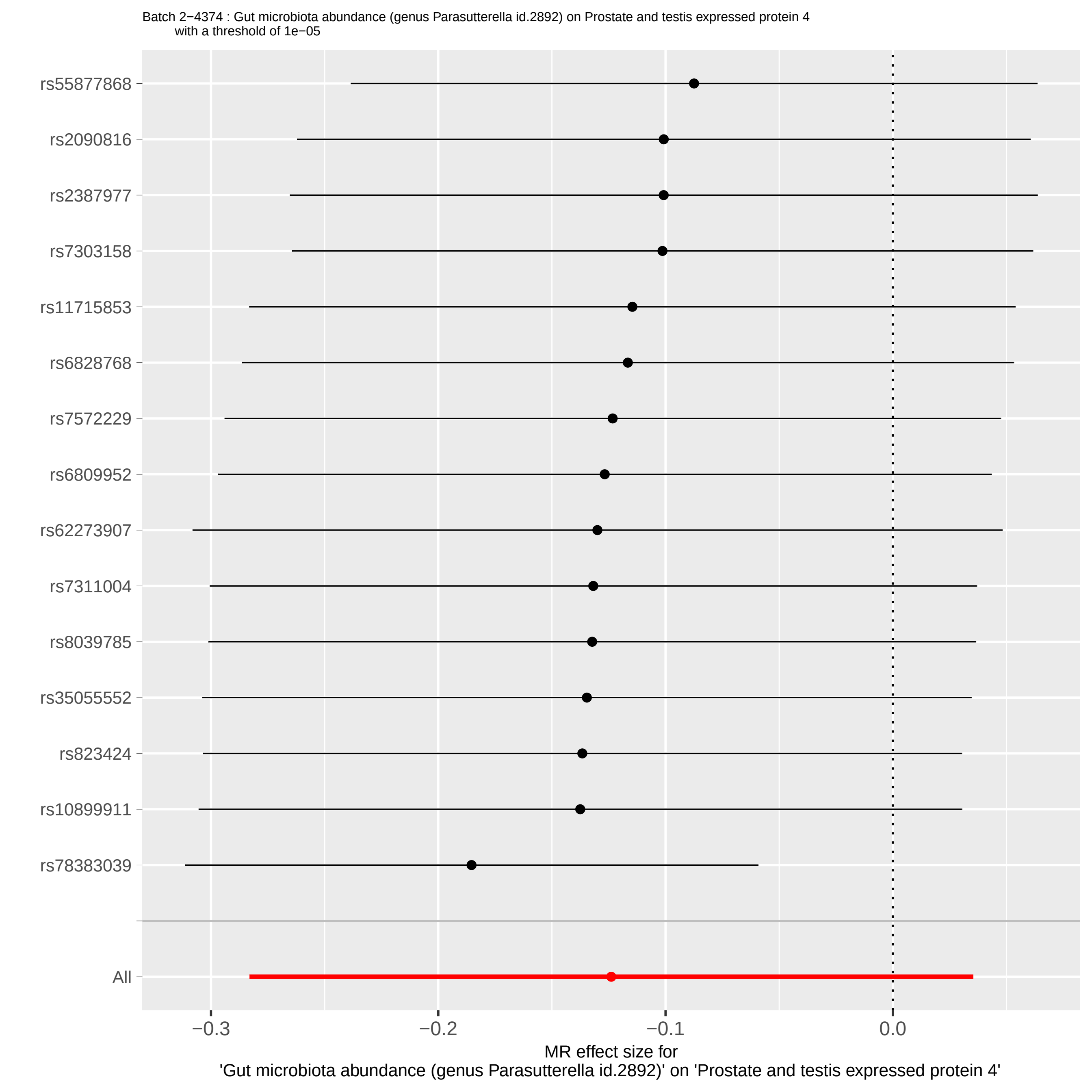

Batch 2−4374 : Gut microbiota abundance (genus Parasutterella id.2892) on Prostate and testis expressed protein 4
with a threshold of 1e−05
rs55877868
rs2090816
rs2387977
rs7303158
rs11715853
rs6828768
rs7572229
rs6809952
rs62273907
rs7311004
rs8039785
rs35055552
rs823424
rs10899911
rs78383039
All
−0.3
−0.2
−0.1
0.0
MR effect size for
'Gut microbiota abundance (genus Parasutterella id.2892)' on 'Prostate and testis expressed protein 4'

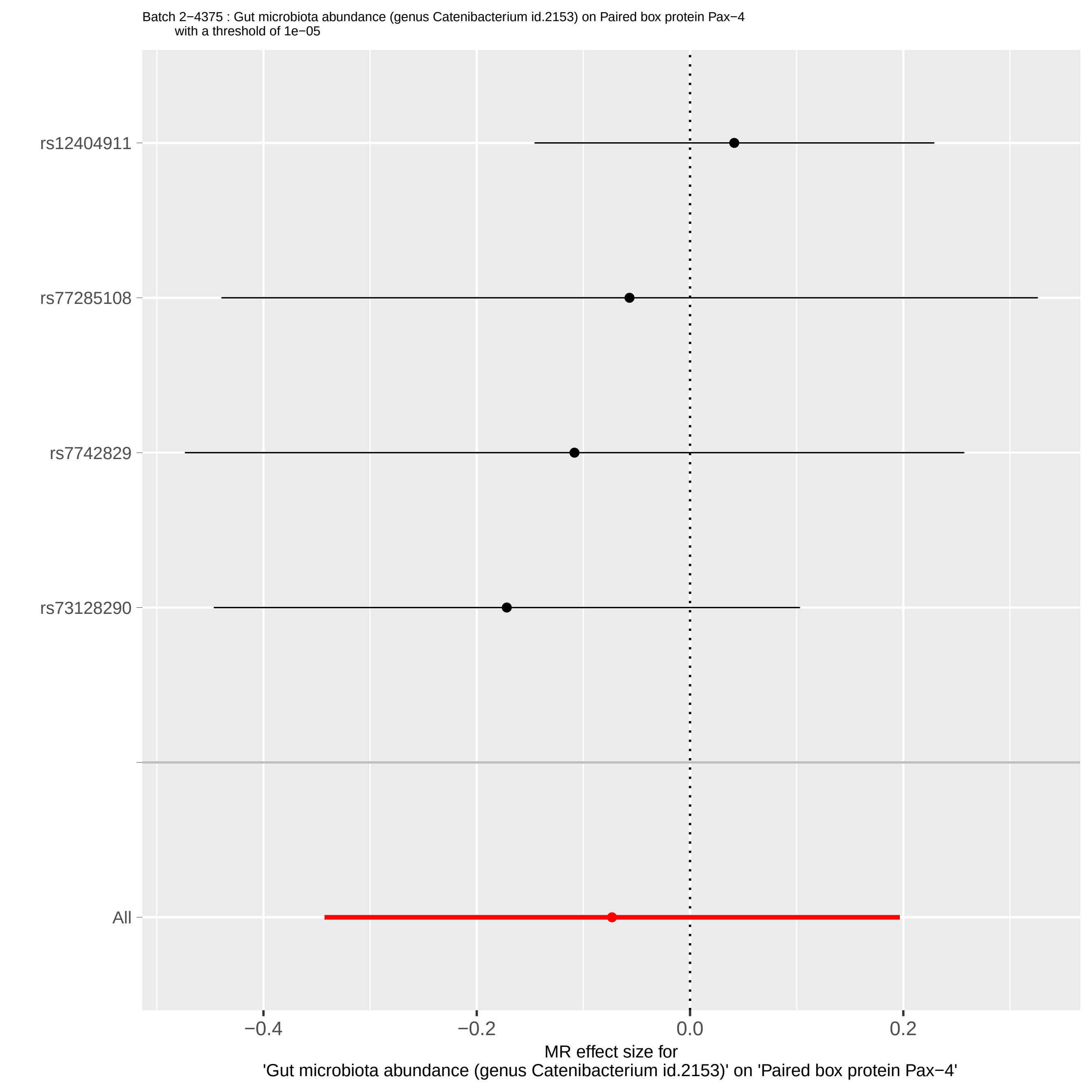
Batch 2−4375 : Gut microbiota abundance (genus Catenibacterium id.2153) on Paired box protein Pax−4
with a threshold of 1e−05
rs12404911
rs77285108
rs7742829
rs73128290
All
−0.4
−0.2
0.0
0.2
MR effect size for
'Gut microbiota abundance (genus Catenibacterium id.2153)' on 'Paired box protein Pax−4'

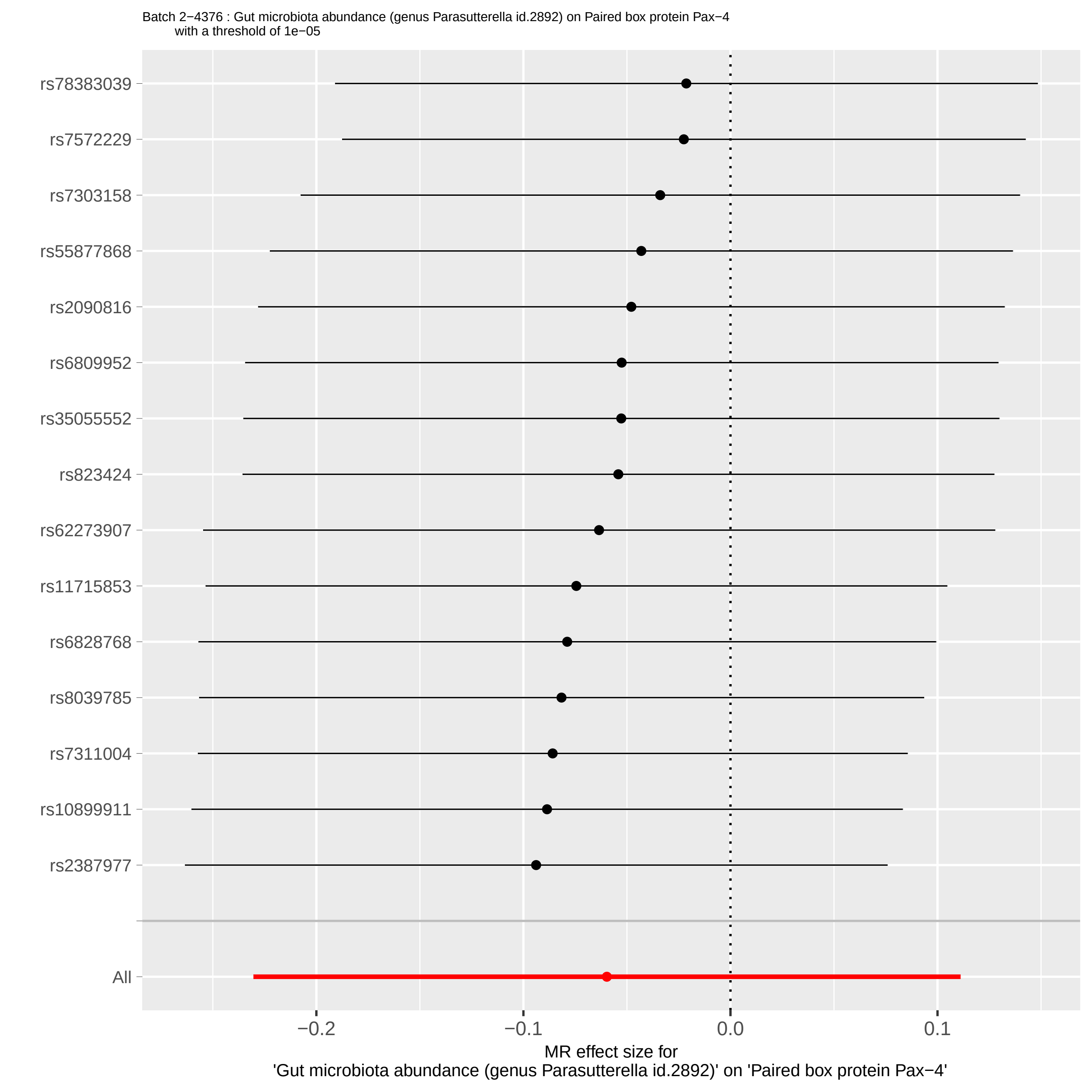
Batch 2−4376 : Gut microbiota abundance (genus Parasutterella id.2892) on Paired box protein Pax−4
with a threshold of 1e−05
rs78383039
rs7572229
rs7303158
rs55877868
rs2090816
rs6809952
rs35055552
rs823424
rs62273907
rs11715853
rs6828768
rs8039785
rs7311004
rs10899911
rs2387977
All
−0.2
−0.1
0.0
0.1
MR effect size for
'Gut microbiota abundance (genus Parasutterella id.2892)' on 'Paired box protein Pax−4'

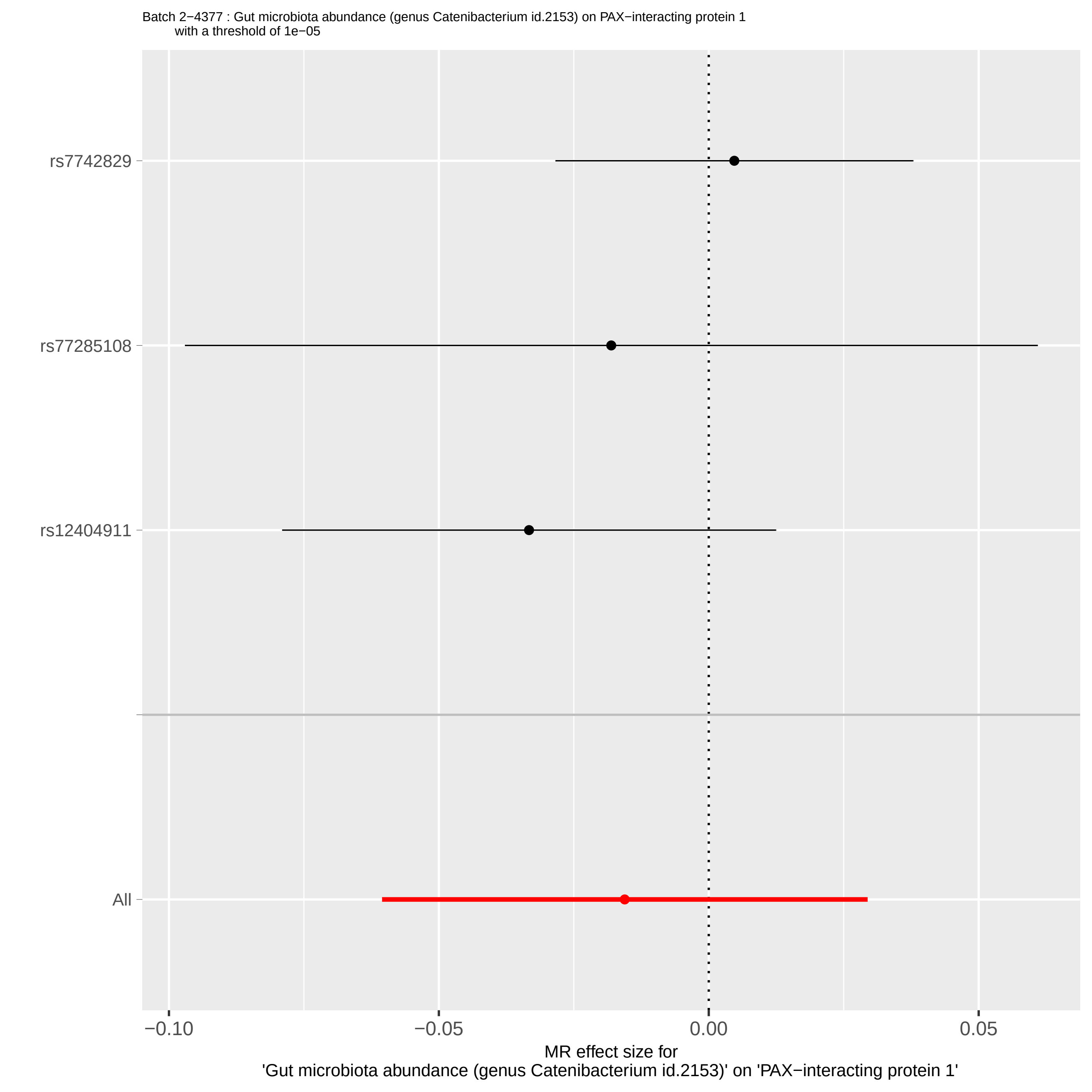
Batch 2−4377 : Gut microbiota abundance (genus Catenibacterium id.2153) on PAX−interacting protein 1
with a threshold of 1e−05
rs7742829
rs77285108
rs12404911
All
−0.10
−0.05
0.00
0.05
MR effect size for
'Gut microbiota abundance (genus Catenibacterium id.2153)' on 'PAX−interacting protein 1'

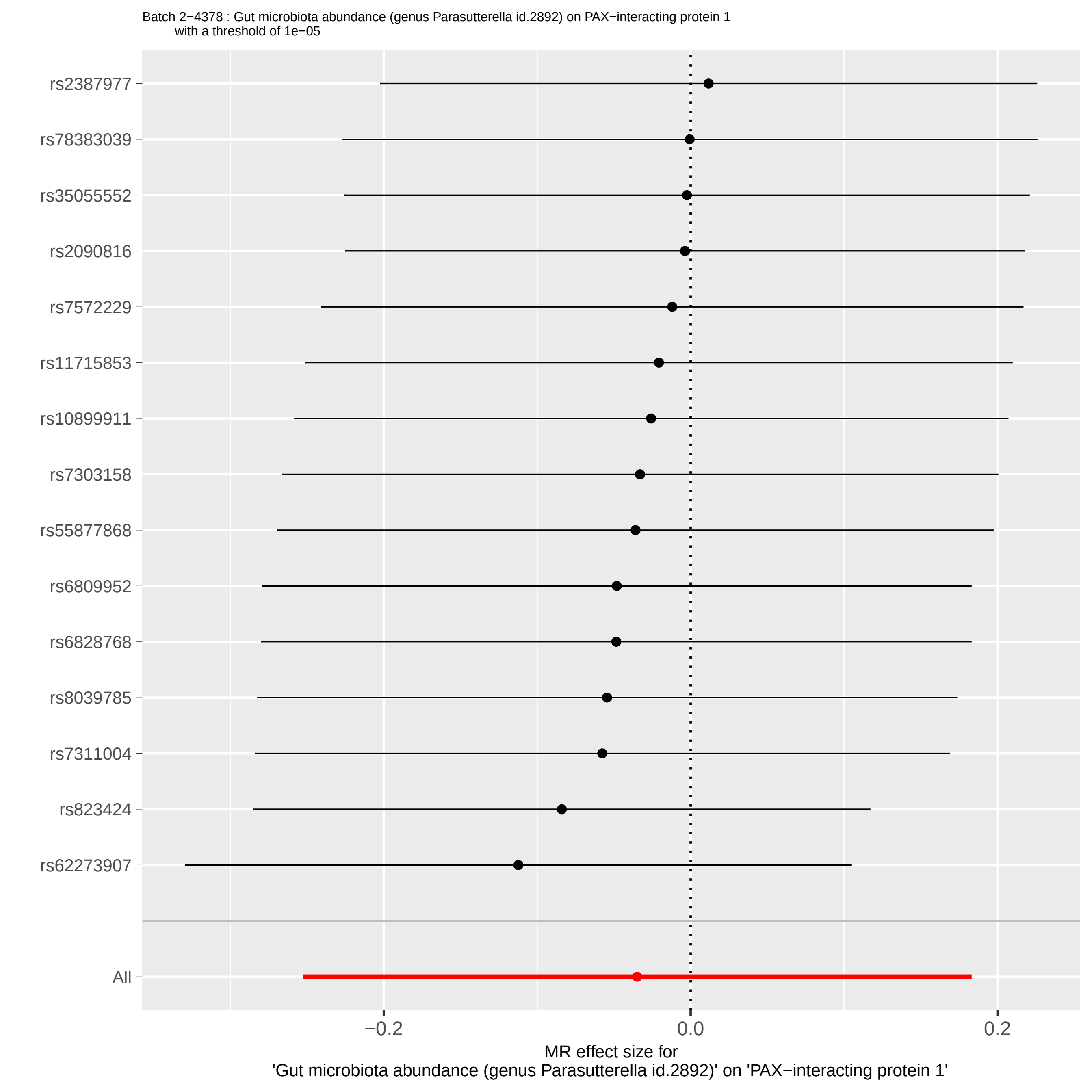
Batch 2−4378 : Gut microbiota abundance (genus Parasutterella id.2892) on PAX−interacting protein 1
with a threshold of 1e−05
rs2387977
rs78383039
rs35055552
rs2090816
rs7572229
rs11715853
rs10899911
rs7303158
rs55877868
rs6809952
rs6828768
rs8039785
rs7311004
rs823424
rs62273907
All
−0.2
0.0
0.2
MR effect size for
'Gut microbiota abundance (genus Parasutterella id.2892)' on 'PAX−interacting protein 1'

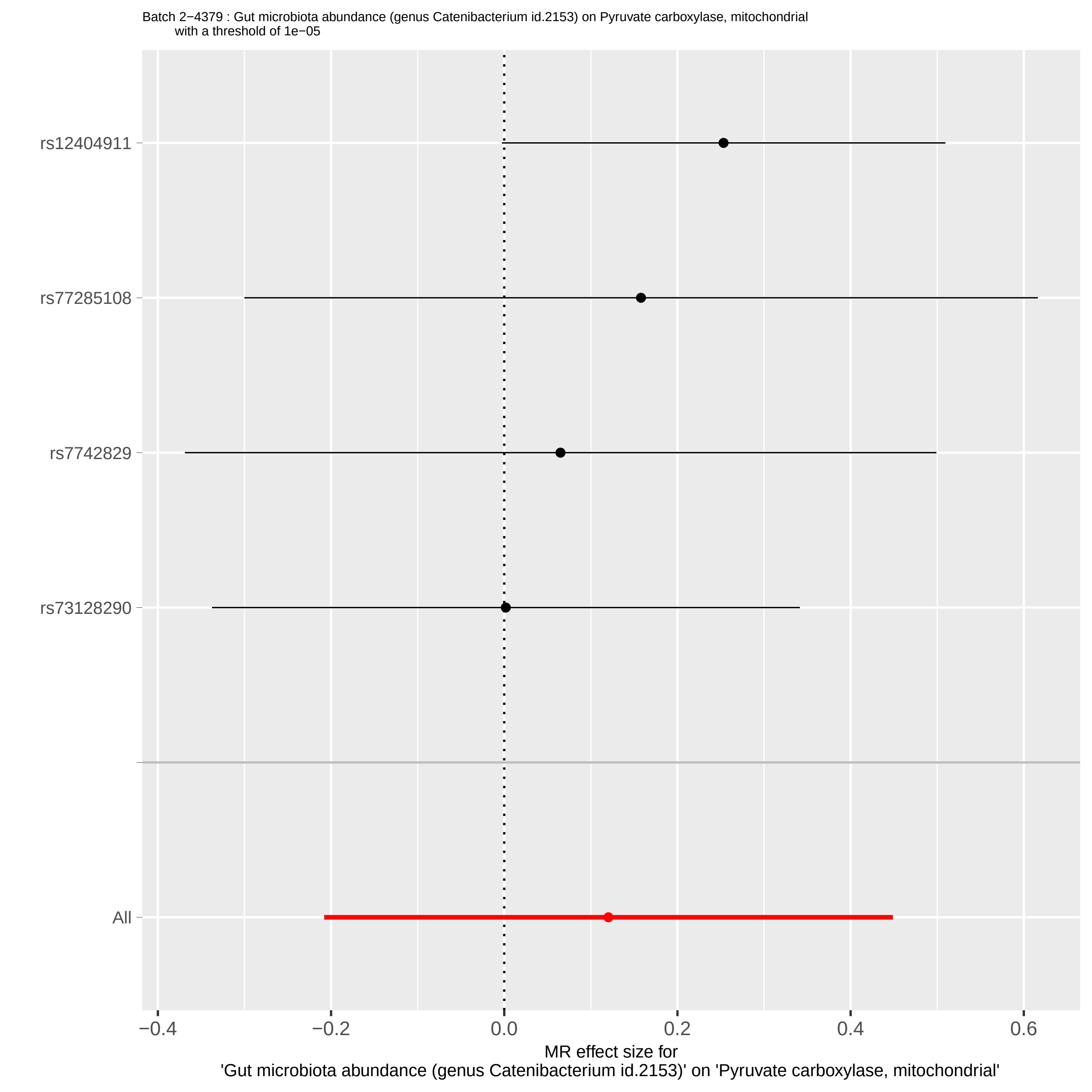
Batch 2−4379 : Gut microbiota abundance (genus Catenibacterium id.2153) on Pyruvate carboxylase, mitochondrial
with a threshold of 1e−05
rs12404911
rs77285108
rs7742829
rs73128290
All
−0.4
−0.2
0.0
0.2
0.4
0.6
MR effect size for
'Gut microbiota abundance (genus Catenibacterium id.2153)' on 'Pyruvate carboxylase, mitochondrial'

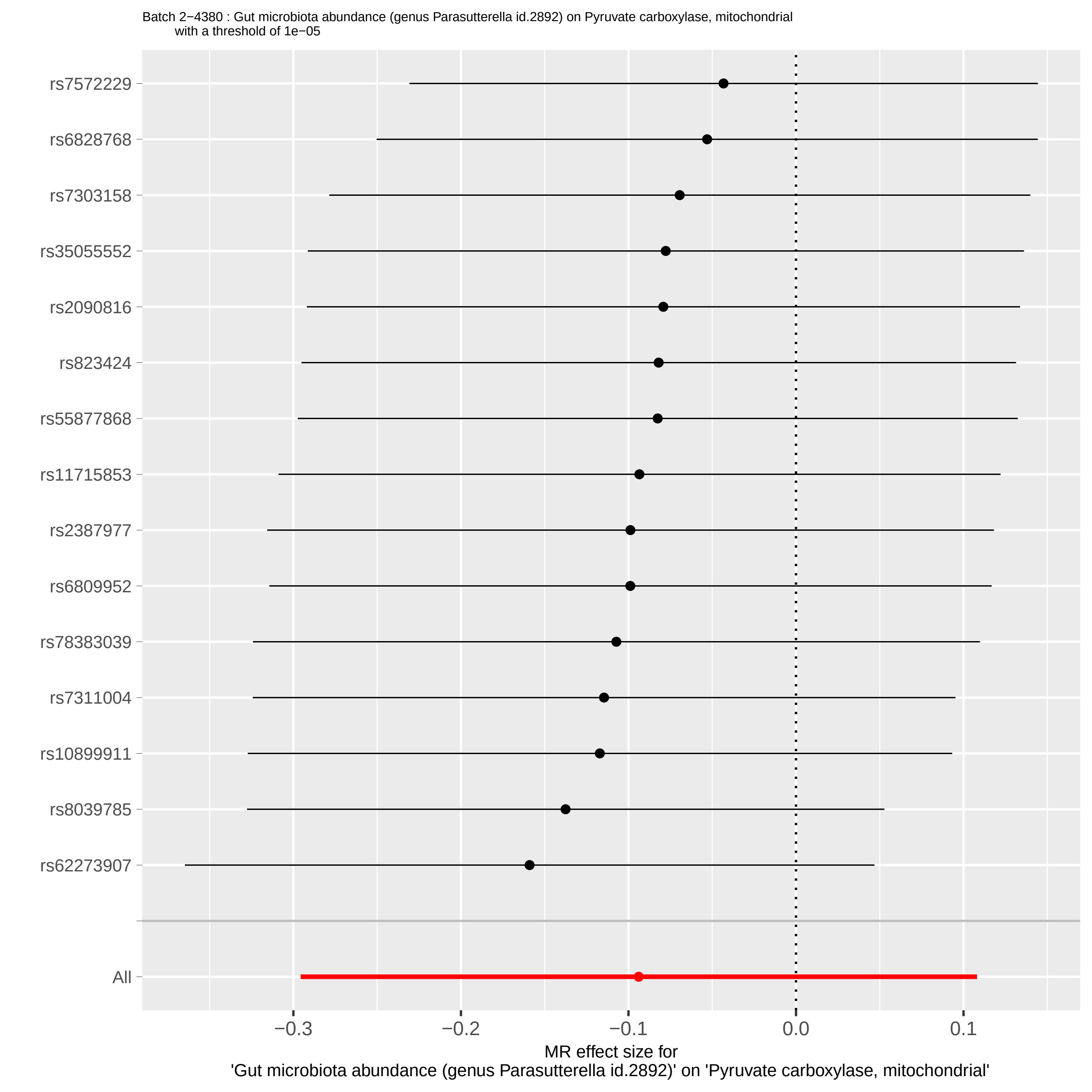

Batch 2−4380 : Gut microbiota abundance (genus Parasutterella id.2892) on Pyruvate carboxylase, mitochondrial
with a threshold of 1e−05
rs7572229
rs6828768
rs7303158
rs35055552
rs2090816
rs823424
rs55877868
rs11715853
rs2387977
rs6809952
rs78383039
rs7311004
rs10899911
rs8039785
rs62273907
All
−0.3
−0.2
−0.1
0.0
0.1
MR effect size for
'Gut microbiota abundance (genus Parasutterella id.2892)' on 'Pyruvate carboxylase, mitochondrial'

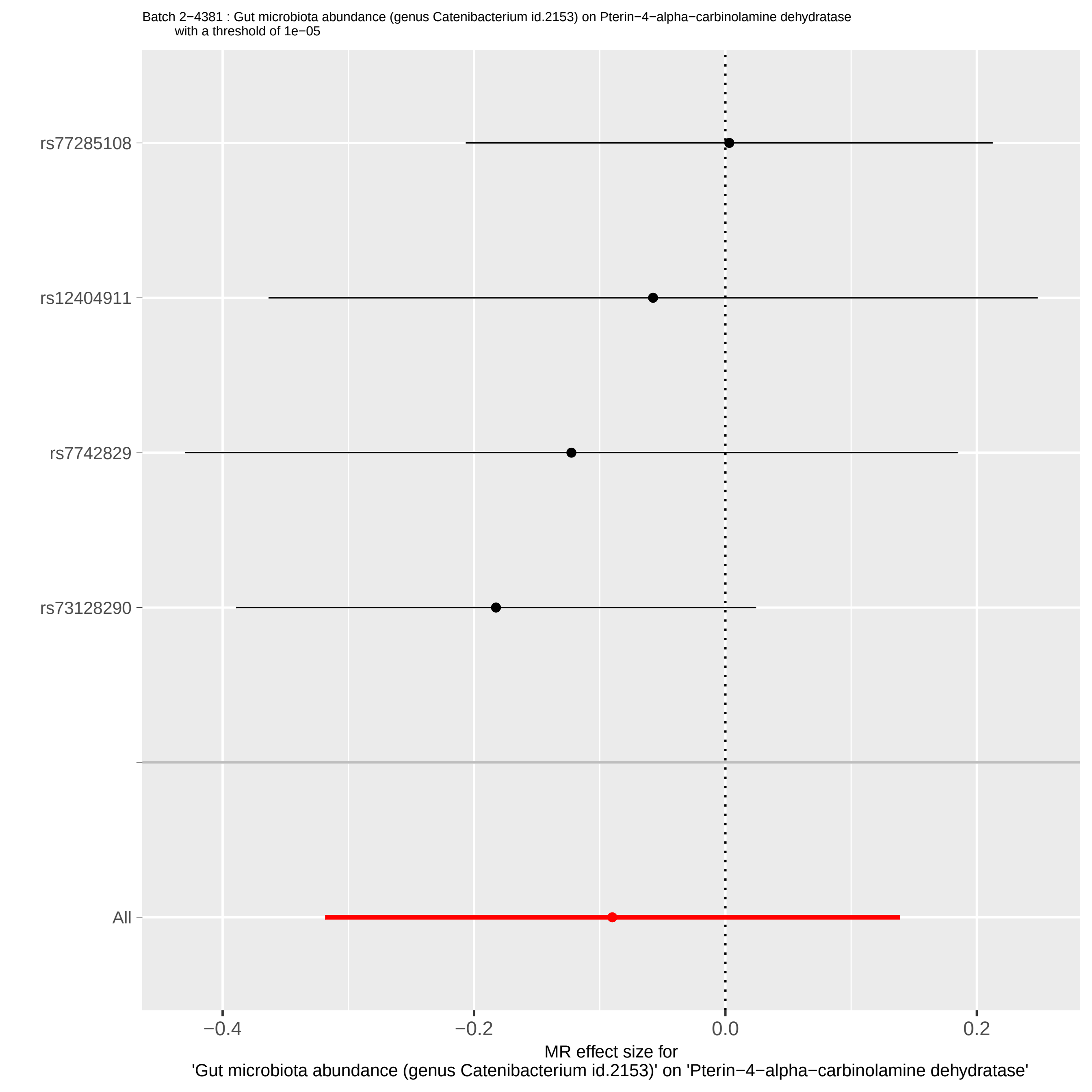

Batch 2−4381 : Gut microbiota abundance (genus Catenibacterium id.2153) on Pterin−4−alpha−carbinolamine dehydratase
with a threshold of 1e−05
rs77285108
rs12404911
rs7742829
rs73128290
All
−0.4
−0.2
0.0
0.2
MR effect size for
'Gut microbiota abundance (genus Catenibacterium id.2153)' on 'Pterin−4−alpha−carbinolamine dehydratase'

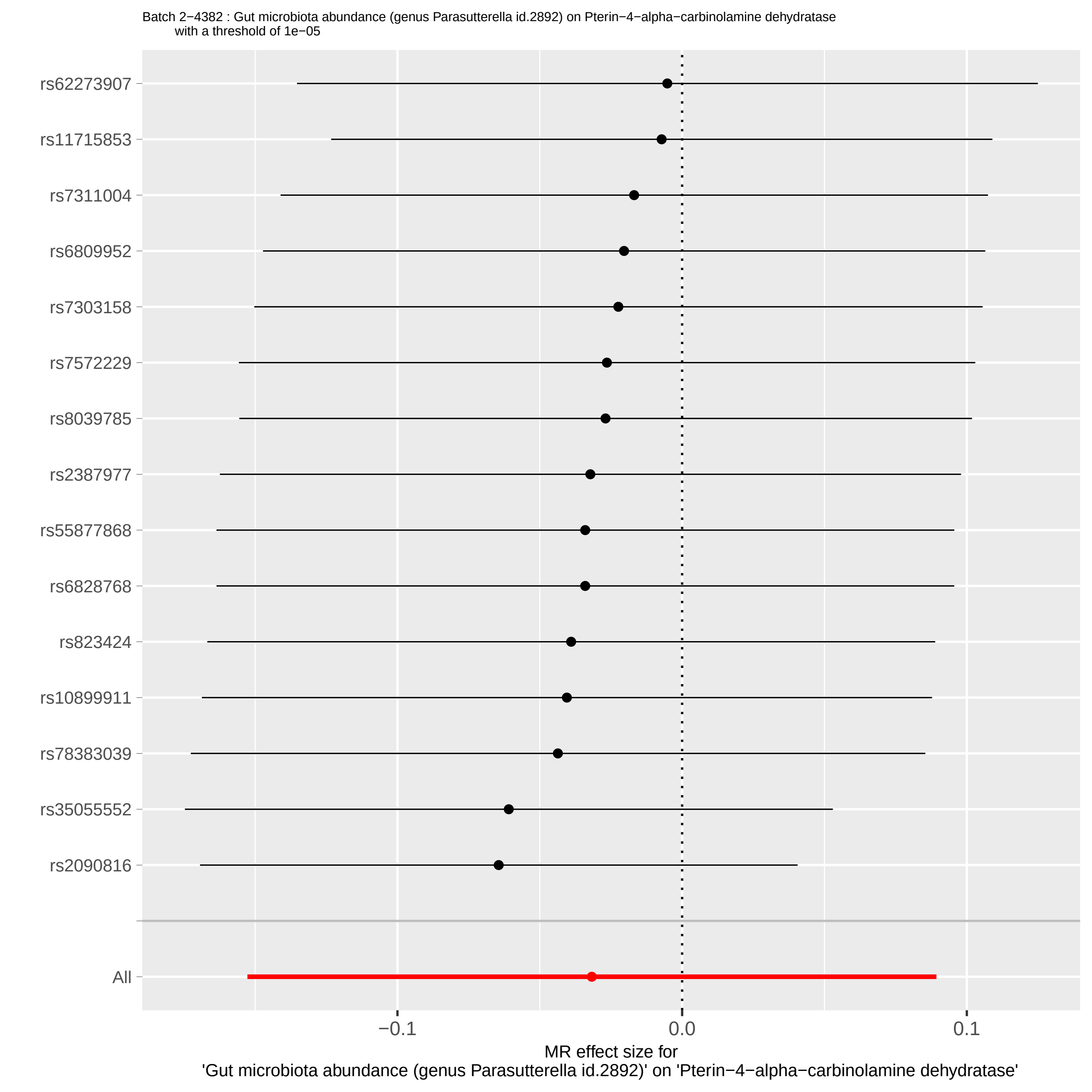

Batch 2−4382 : Gut microbiota abundance (genus Parasutterella id.2892) on Pterin−4−alpha−carbinolamine dehydratase
with a threshold of 1e−05
rs62273907
rs11715853
rs7311004
rs6809952
rs7303158
rs7572229
rs8039785
rs2387977
rs55877868
rs6828768
rs823424
rs10899911
rs78383039
rs35055552
rs2090816
All
−0.1
0.0
0.1
MR effect size for
'Gut microbiota abundance (genus Parasutterella id.2892)' on 'Pterin−4−alpha−carbinolamine dehydratase'

Batch 2−4383 : Gut microbiota abundance (genus Catenibacterium id.2153) on Pterin−4−alpha−carbinolamine dehydratase 2
with a threshold of 1e−05

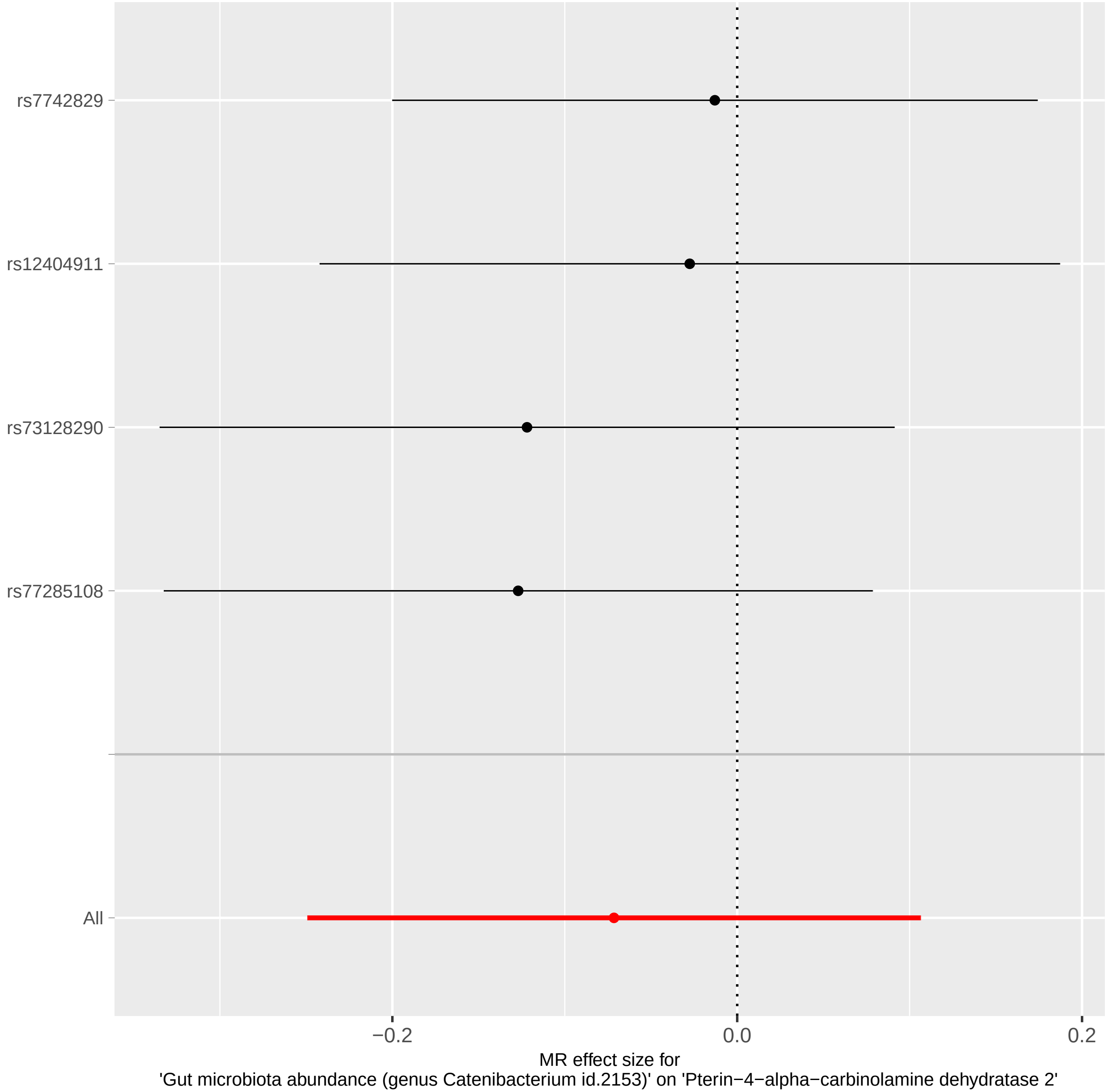

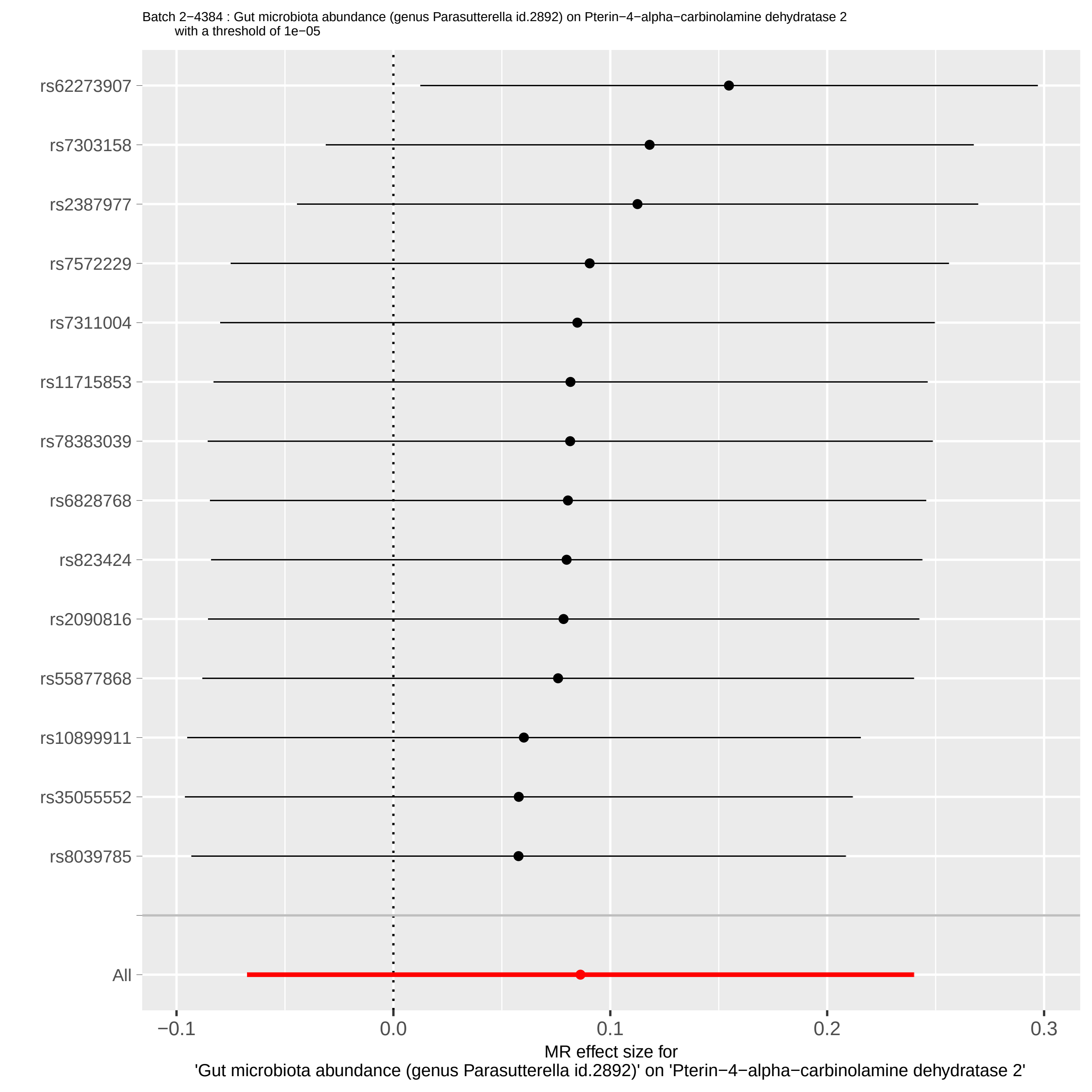

Batch 2−4384 : Gut microbiota abundance (genus Parasutterella id.2892) on Pterin−4−alpha−carbinolamine dehydratase 2
with a threshold of 1e−05
rs62273907
rs7303158
rs2387977
rs7572229
rs7311004
rs11715853
rs78383039
rs6828768
rs823424
rs2090816
rs55877868
rs10899911
rs35055552
rs8039785
All
−0.1
0.0
0.1
0.2
0.3
MR effect size for
'Gut microbiota abundance (genus Parasutterella id.2892)' on 'Pterin−4−alpha−carbinolamine dehydratase 2'

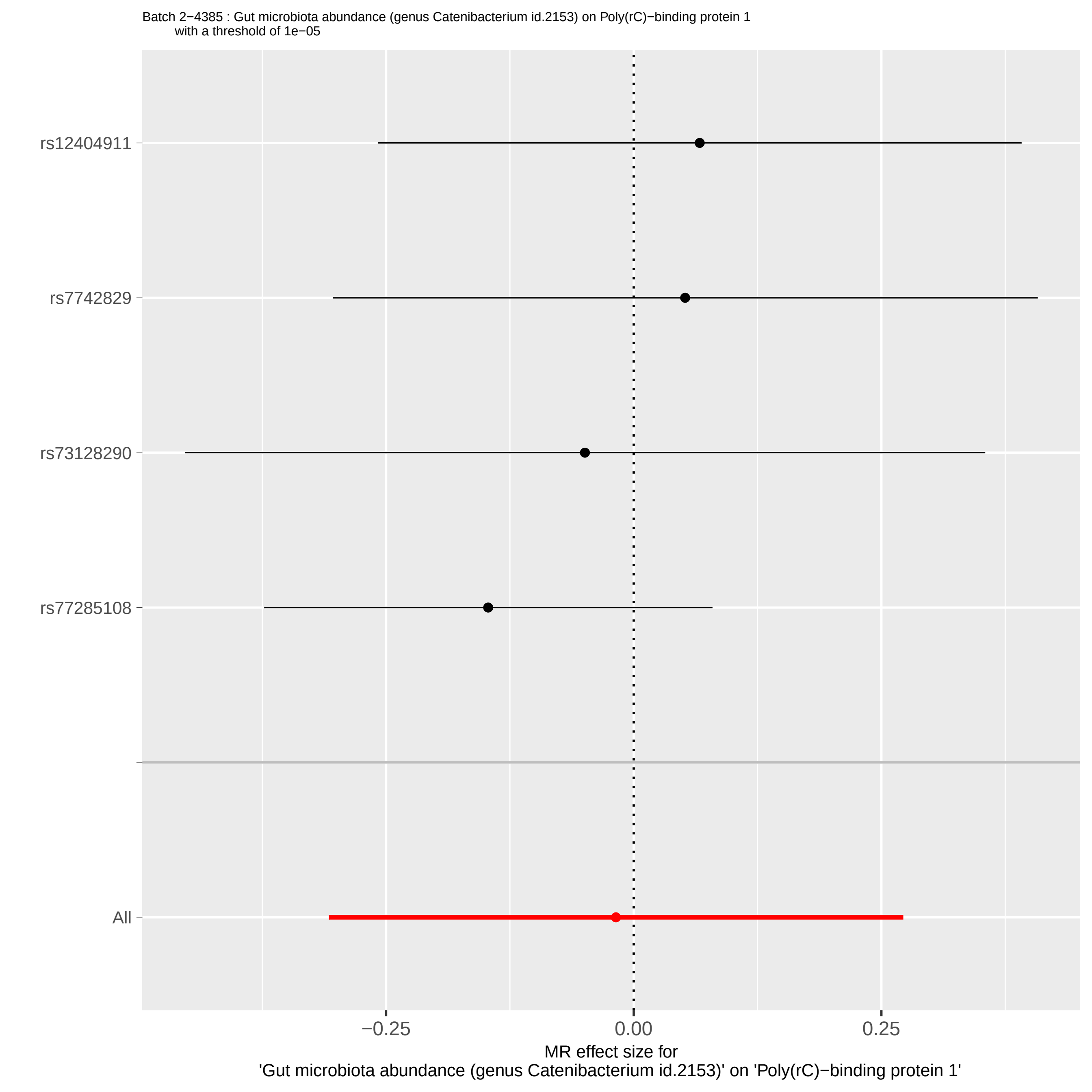

Batch 2−4385 : Gut microbiota abundance (genus Catenibacterium id.2153) on Poly(rC)−binding protein 1
with a threshold of 1e−05
rs12404911
rs7742829
rs73128290
rs77285108
All
−0.25
0.00
0.25
MR effect size for
'Gut microbiota abundance (genus Catenibacterium id.2153)' on 'Poly(rC)−binding protein 1'

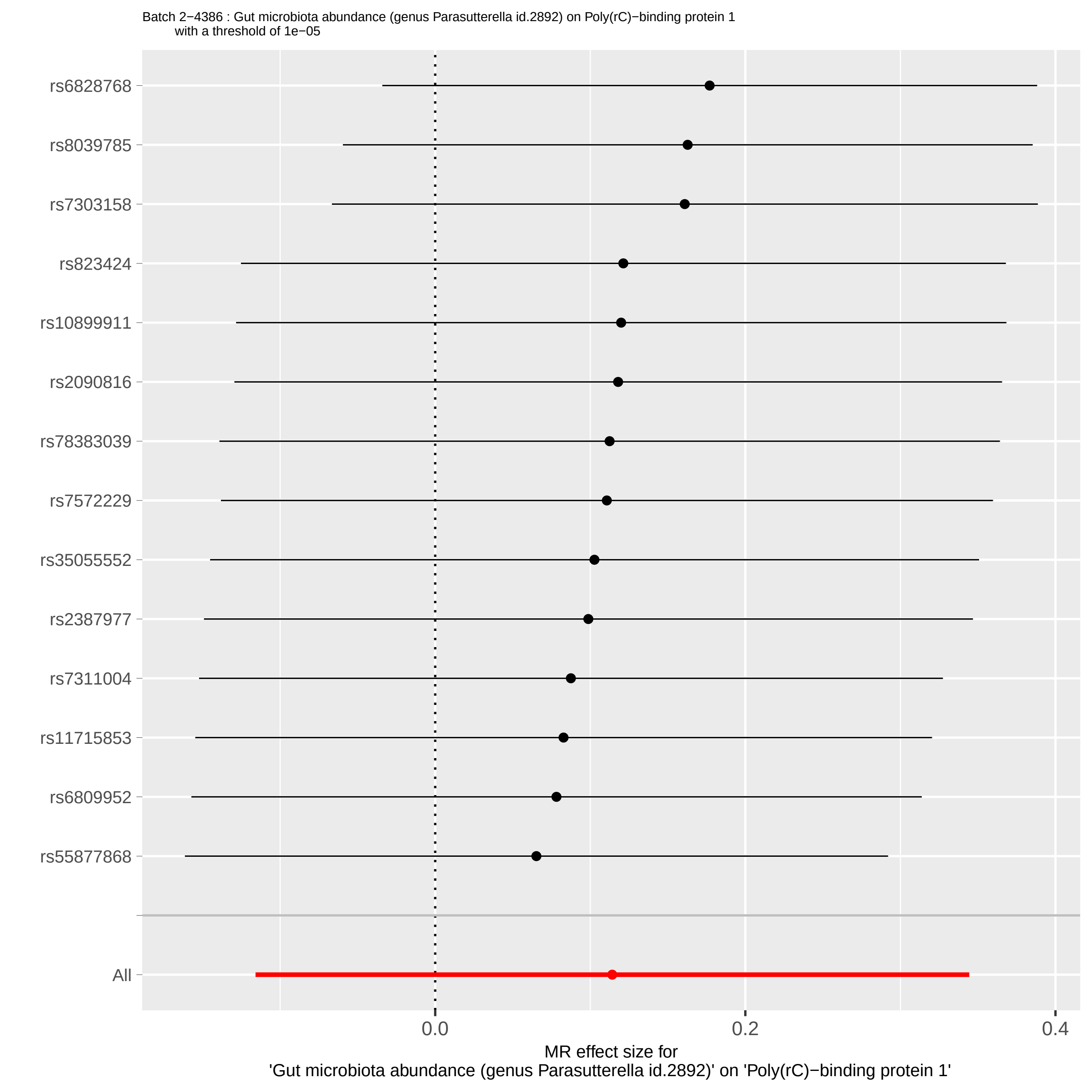

Batch 2−4386 : Gut microbiota abundance (genus Parasutterella id.2892) on Poly(rC)−binding protein 1
with a threshold of 1e−05
rs6828768
rs8039785
rs7303158
rs823424
rs10899911
rs2090816
rs78383039
rs7572229
rs35055552
rs2387977
rs7311004
rs11715853
rs6809952
rs55877868
All
0.0
0.2
0.4
MR effect size for
'Gut microbiota abundance (genus Parasutterella id.2892)' on 'Poly(rC)−binding protein 1'

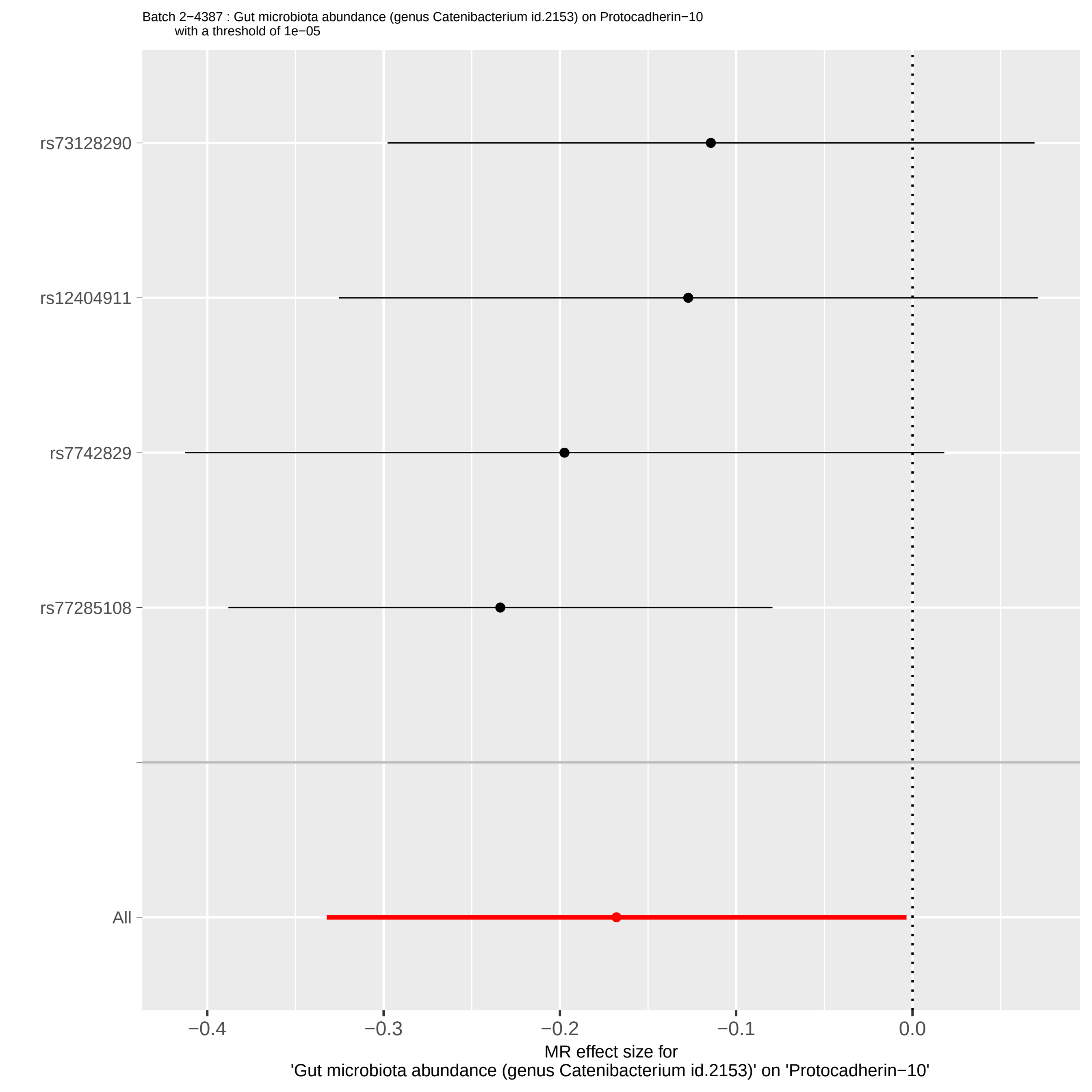
Batch 2−4387 : Gut microbiota abundance (genus Catenibacterium id.2153) on Protocadherin−10
with a threshold of 1e−05
rs73128290
rs12404911
rs7742829
rs77285108
All
−0.4
−0.3
−0.2
−0.1
0.0
MR effect size for
'Gut microbiota abundance (genus Catenibacterium id.2153)' on 'Protocadherin−10'

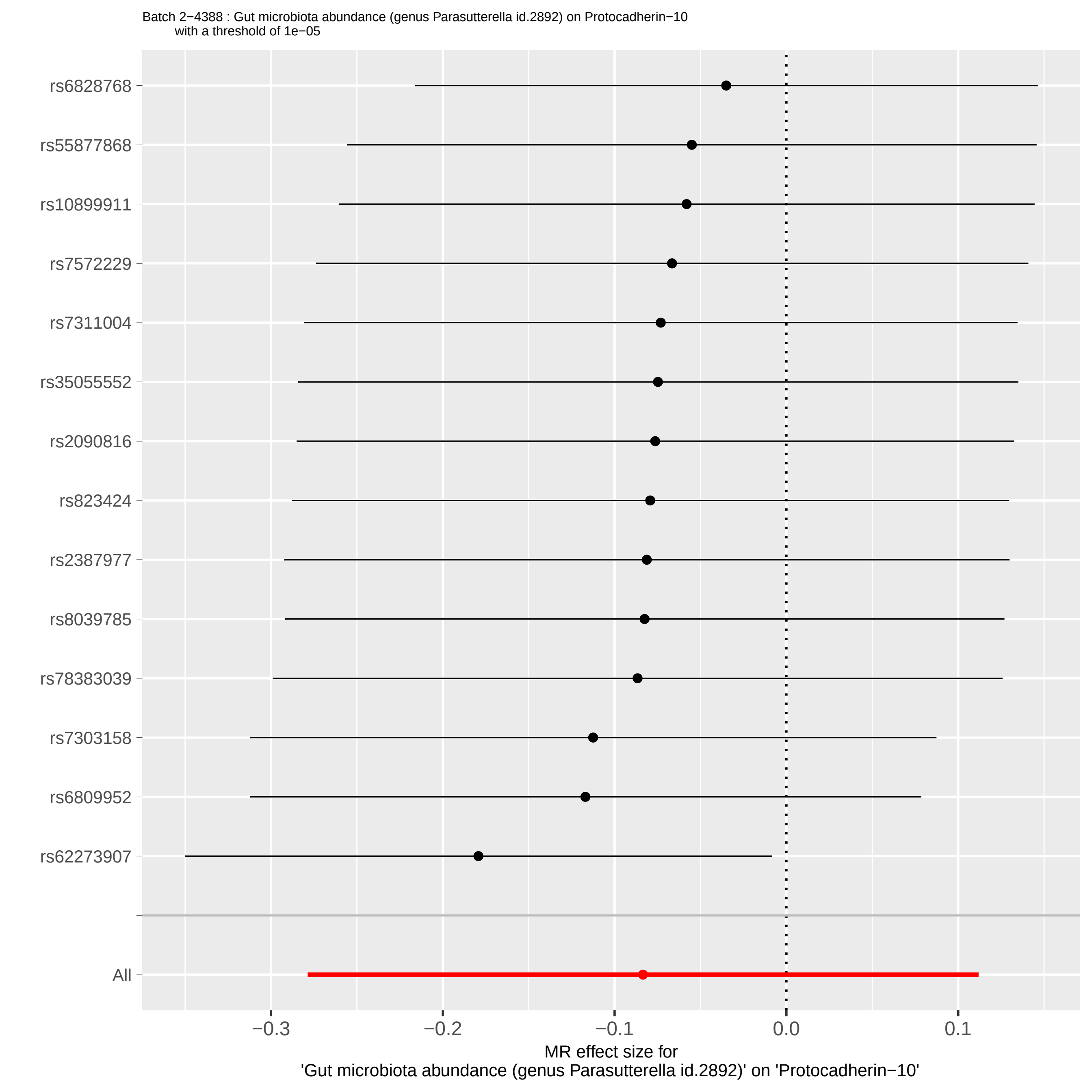

Batch 2−4388 : Gut microbiota abundance (genus Parasutterella id.2892) on Protocadherin−10
with a threshold of 1e−05
rs6828768
rs55877868
rs10899911
rs7572229
rs7311004
rs35055552
rs2090816
rs823424
rs2387977
rs8039785
rs78383039
rs7303158
rs6809952
rs62273907
All
−0.3
−0.2
−0.1
0.0
0.1
MR effect size for
'Gut microbiota abundance (genus Parasutterella id.2892)' on 'Protocadherin−10'

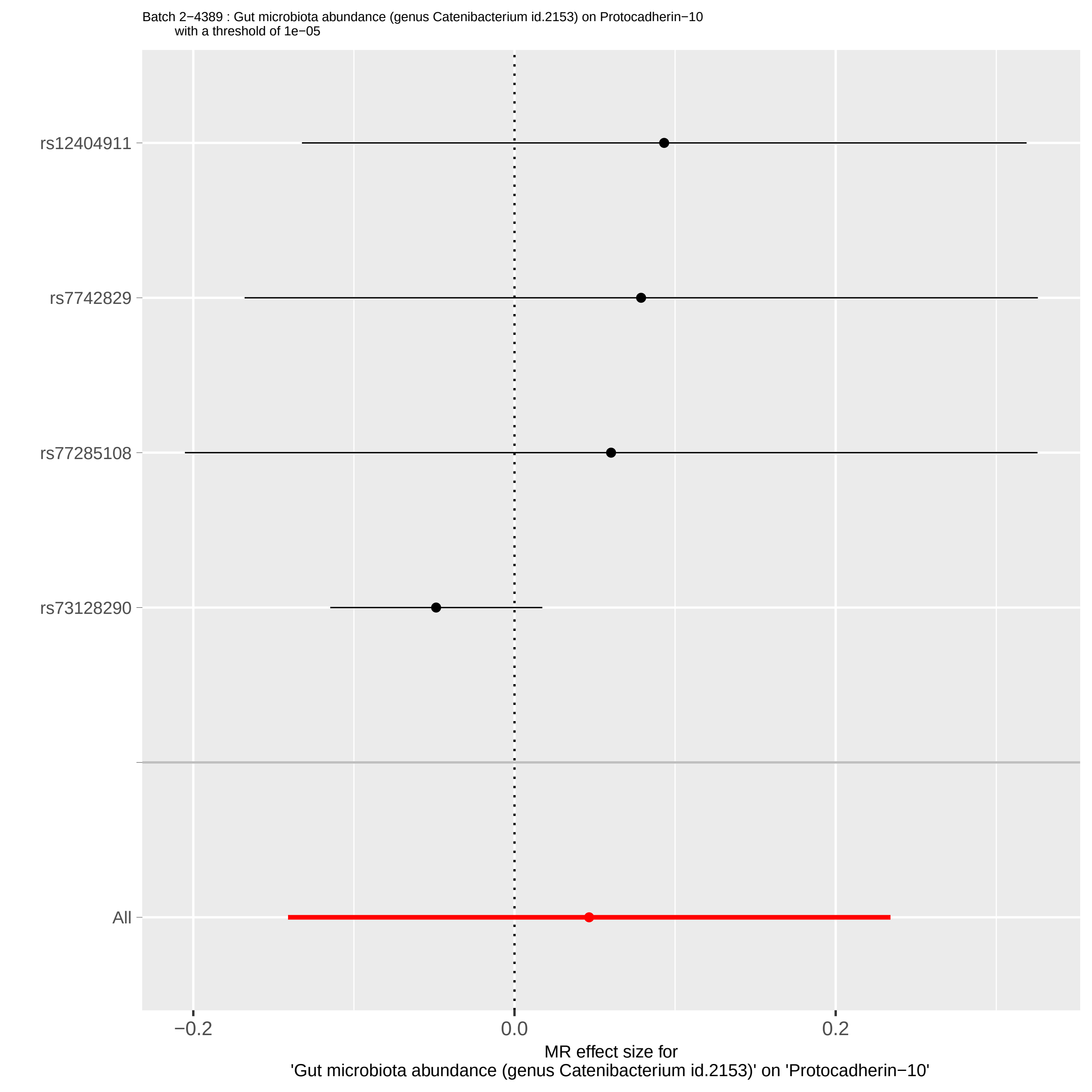
Batch 2−4389 : Gut microbiota abundance (genus Catenibacterium id.2153) on Protocadherin−10
with a threshold of 1e−05
rs12404911
rs7742829
rs77285108
rs73128290
All
−0.2
0.0
0.2
MR effect size for
'Gut microbiota abundance (genus Catenibacterium id.2153)' on 'Protocadherin−10'

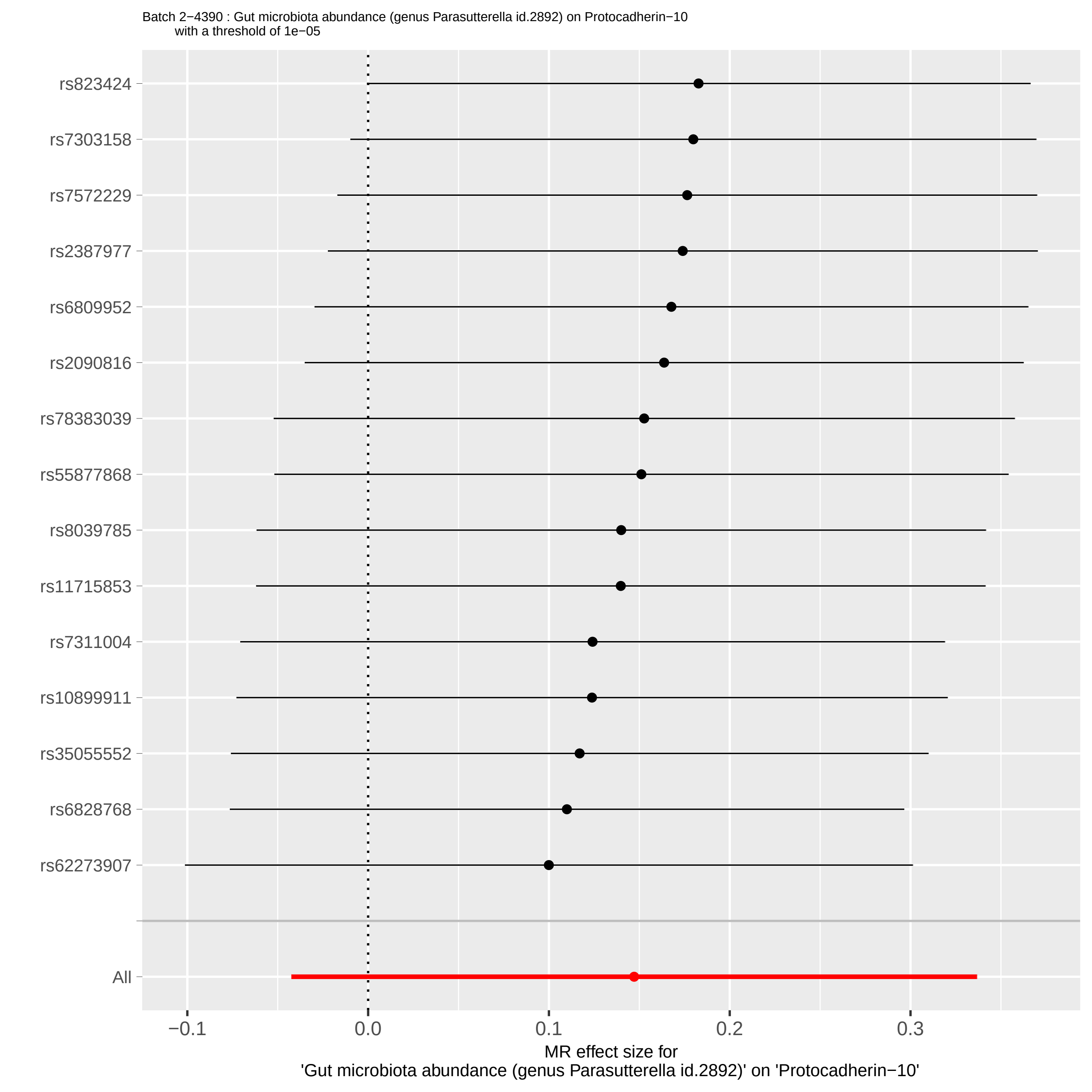

Batch 2−4390 : Gut microbiota abundance (genus Parasutterella id.2892) on Protocadherin−10
with a threshold of 1e−05
rs823424
rs7303158
rs7572229
rs2387977
rs6809952
rs2090816
rs78383039
rs55877868
rs8039785
rs11715853
rs7311004
rs10899911
rs35055552
rs6828768
rs62273907
All
−0.1
0.0
0.1
0.2
0.3
MR effect size for
'Gut microbiota abundance (genus Parasutterella id.2892)' on 'Protocadherin−10'

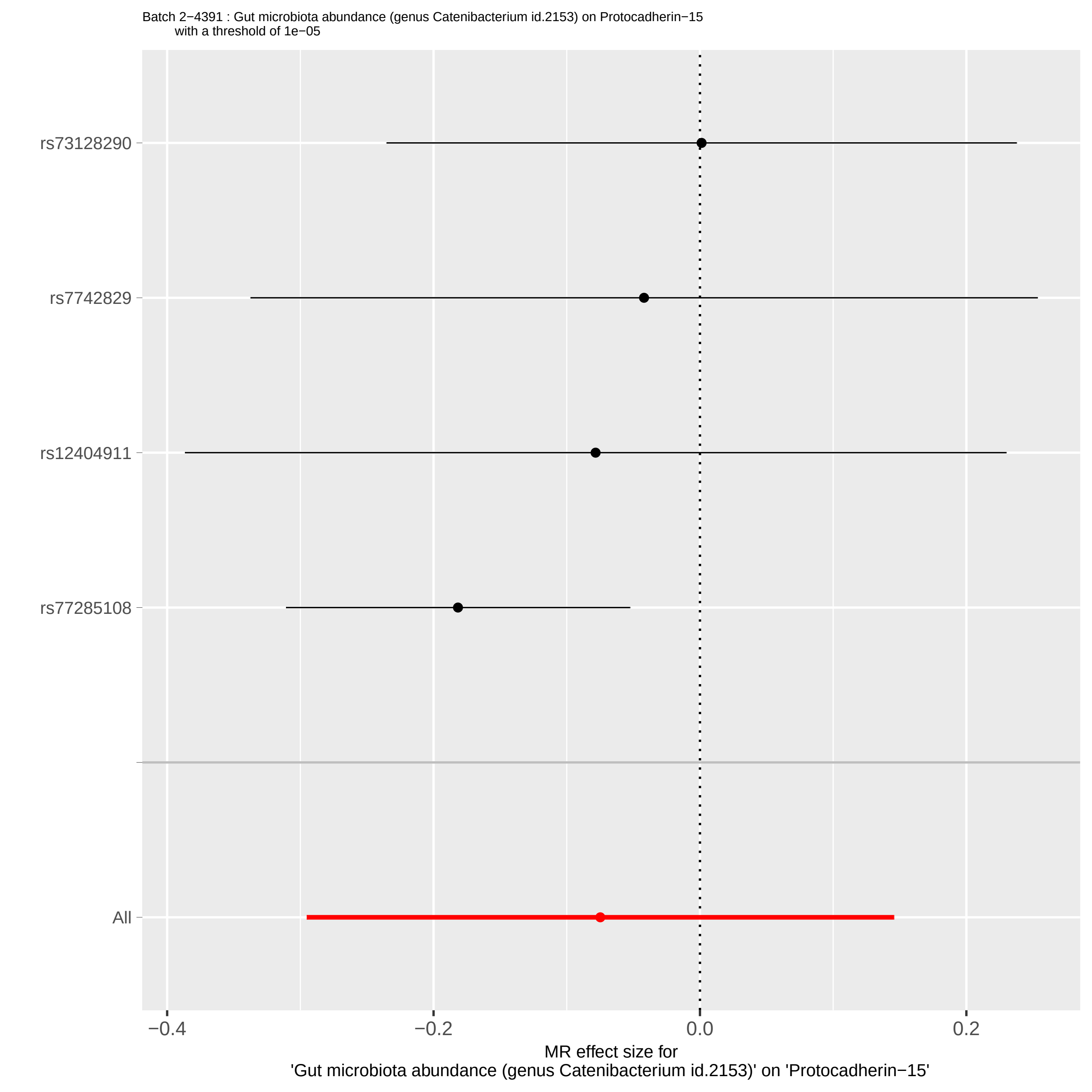
Batch 2−4391 : Gut microbiota abundance (genus Catenibacterium id.2153) on Protocadherin−15
with a threshold of 1e−05
rs73128290
rs7742829
rs12404911
rs77285108
All
−0.4
−0.2
0.0
0.2
MR effect size for
'Gut microbiota abundance (genus Catenibacterium id.2153)' on 'Protocadherin−15'

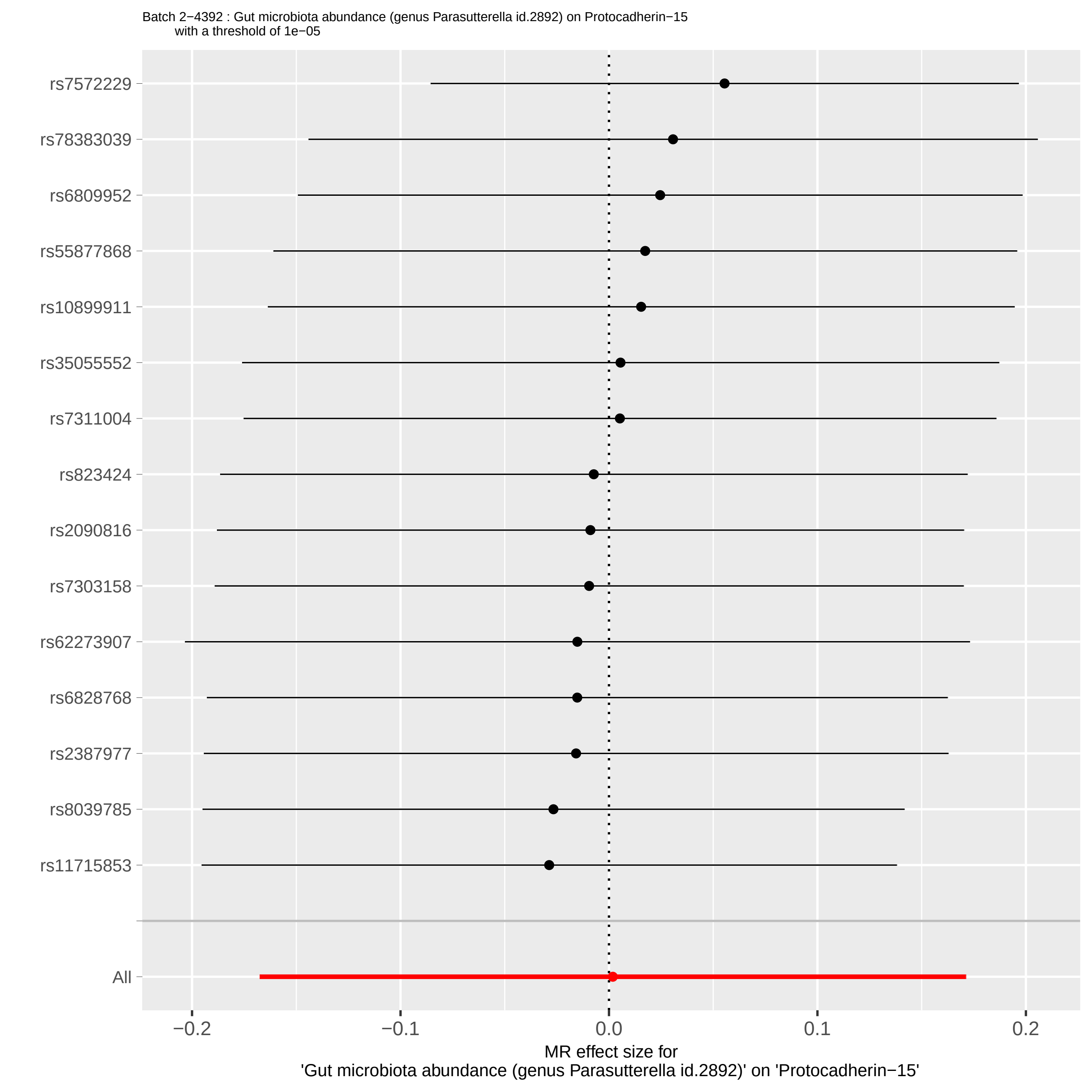

Batch 2−4392 : Gut microbiota abundance (genus Parasutterella id.2892) on Protocadherin−15
with a threshold of 1e−05
rs7572229
rs78383039
rs6809952
rs55877868
rs10899911
rs35055552
rs7311004
rs823424
rs2090816
rs7303158
rs62273907
rs6828768
rs2387977
rs8039785
rs11715853
All
−0.2
−0.1
0.0
0.1
0.2
MR effect size for
'Gut microbiota abundance (genus Parasutterella id.2892)' on 'Protocadherin−15'

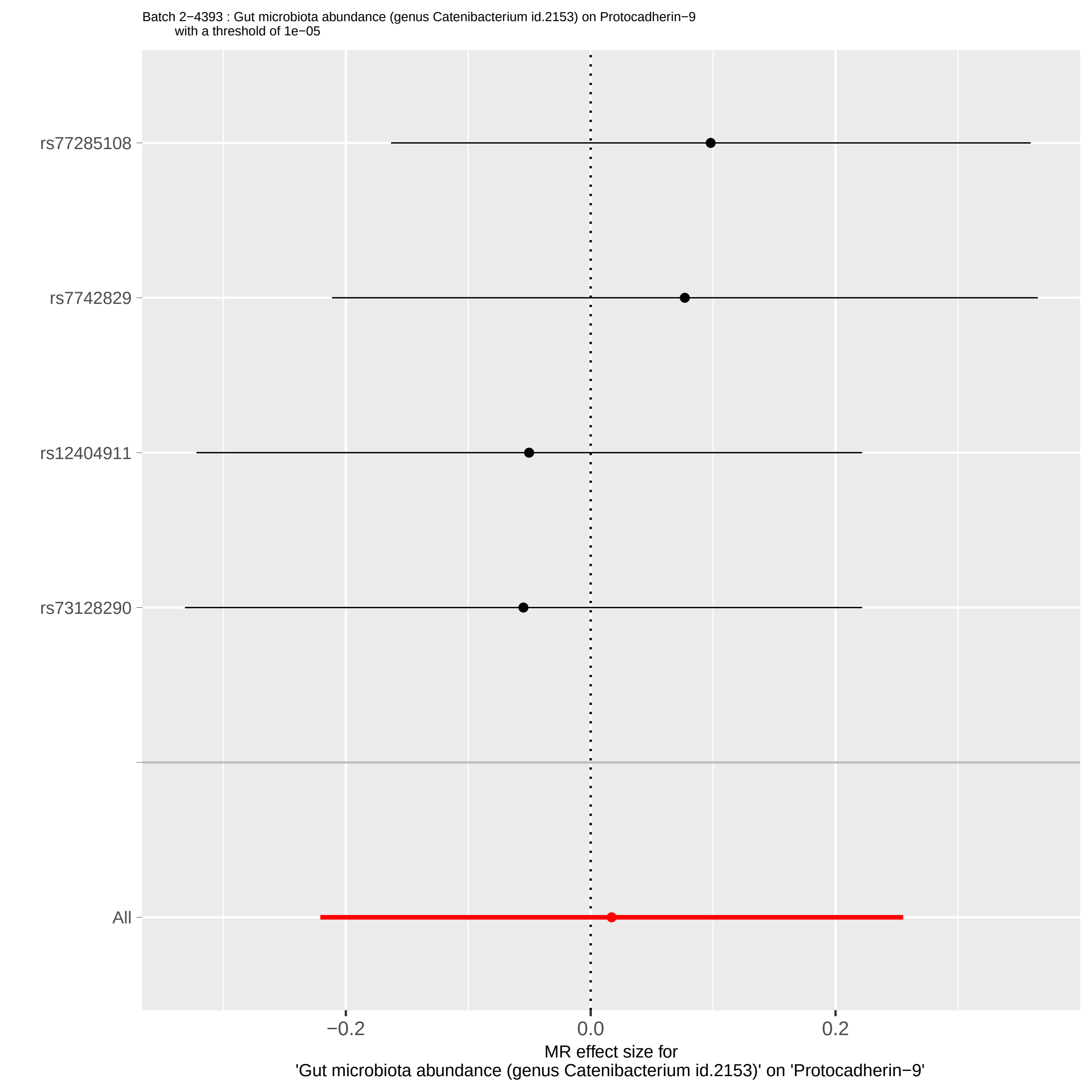
Batch 2−4393 : Gut microbiota abundance (genus Catenibacterium id.2153) on Protocadherin−9
with a threshold of 1e−05
rs77285108
rs7742829
rs12404911
rs73128290
All
−0.2
0.0
0.2
MR effect size for
'Gut microbiota abundance (genus Catenibacterium id.2153)' on 'Protocadherin−9'

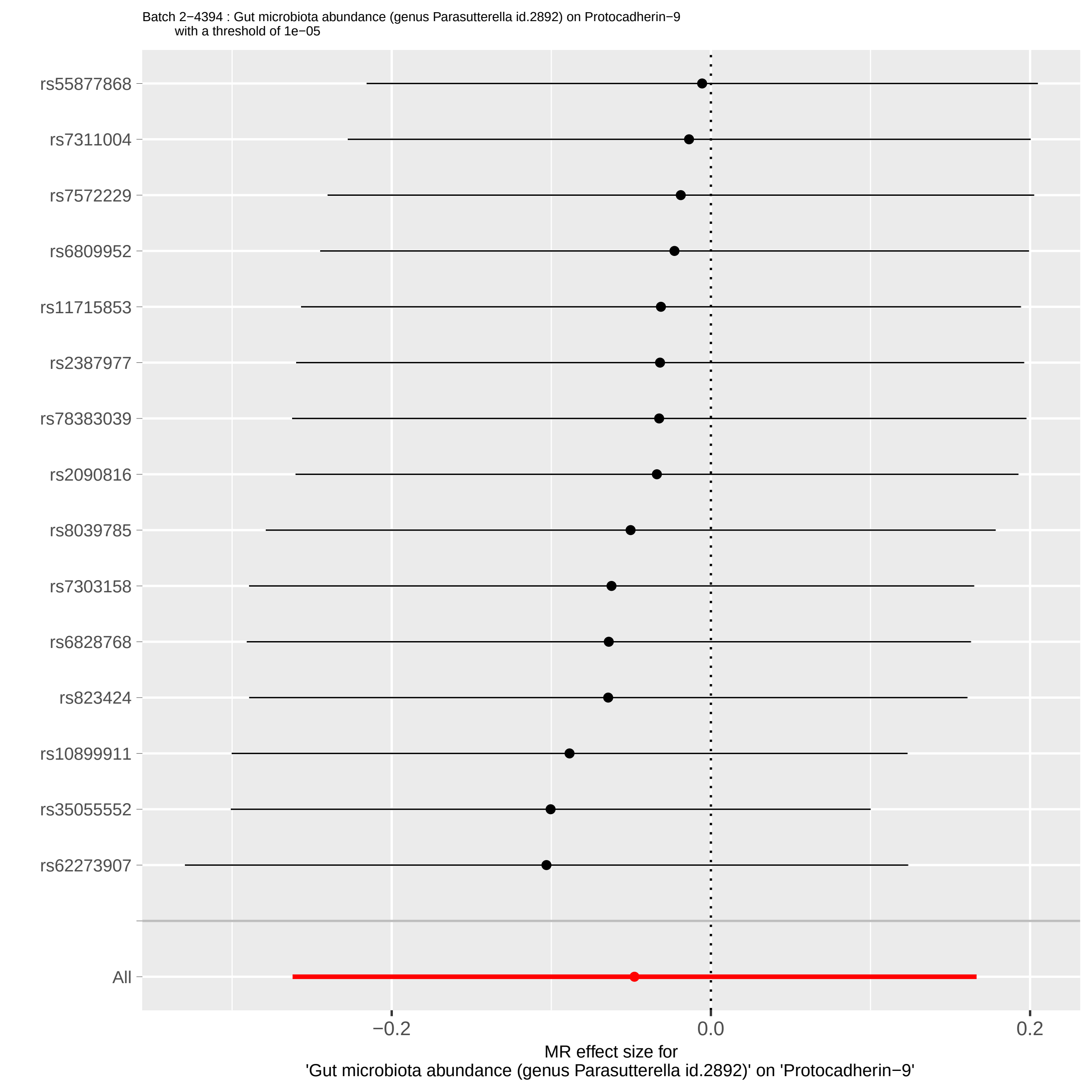

Batch 2−4394 : Gut microbiota abundance (genus Parasutterella id.2892) on Protocadherin−9
with a threshold of 1e−05
rs55877868
rs7311004
rs7572229
rs6809952
rs11715853
rs2387977
rs78383039
rs2090816
rs8039785
rs7303158
rs6828768
rs823424
rs10899911
rs35055552
rs62273907
All
−0.2
0.0
0.2
MR effect size for
'Gut microbiota abundance (genus Parasutterella id.2892)' on 'Protocadherin−9'

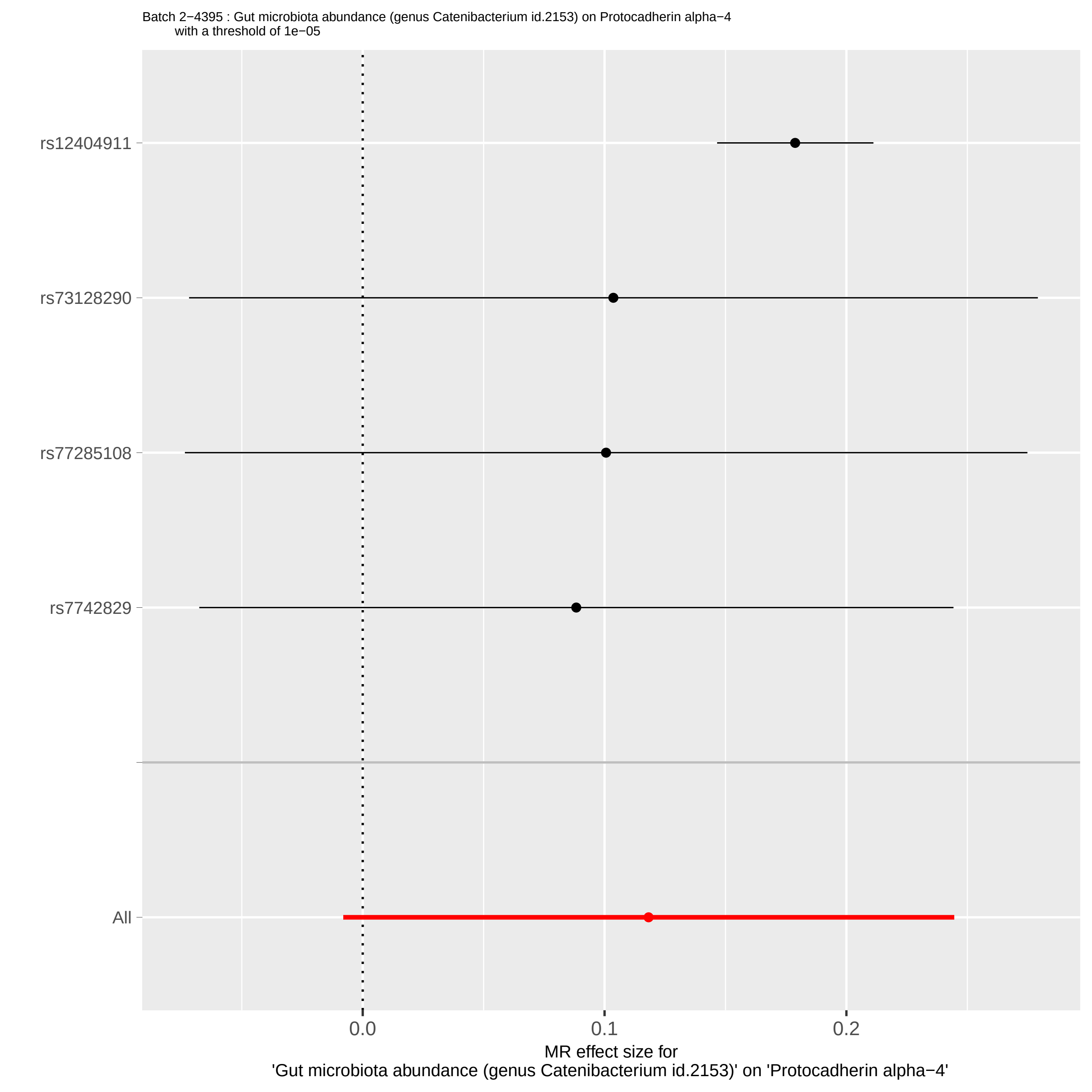
Batch 2−4395 : Gut microbiota abundance (genus Catenibacterium id.2153) on Protocadherin alpha−4
with a threshold of 1e−05
rs12404911
rs73128290
rs77285108
rs7742829
All
0.0
0.1
0.2
MR effect size for
'Gut microbiota abundance (genus Catenibacterium id.2153)' on 'Protocadherin alpha−4'

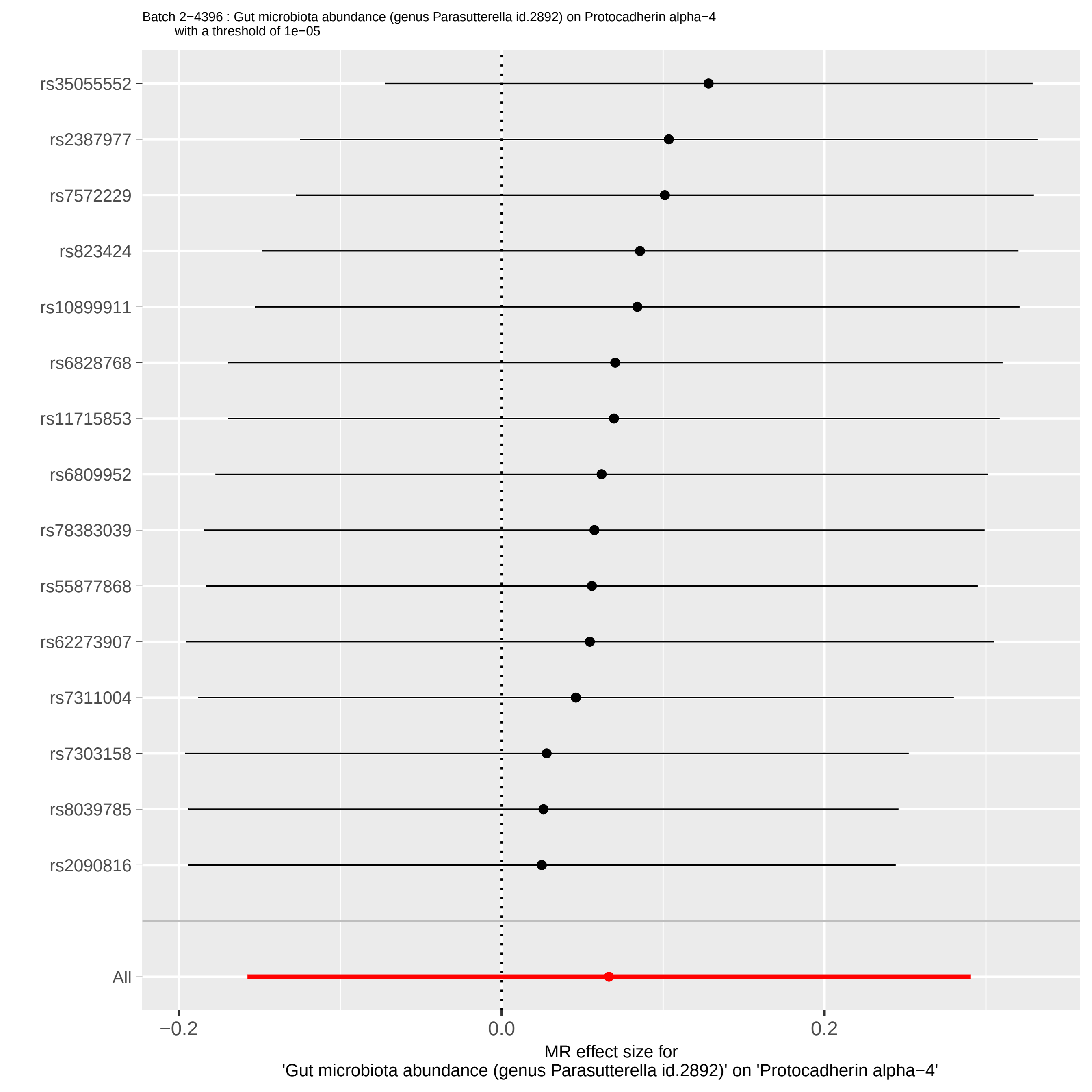

Batch 2−4396 : Gut microbiota abundance (genus Parasutterella id.2892) on Protocadherin alpha−4
with a threshold of 1e−05
rs35055552
rs2387977
rs7572229
rs823424
rs10899911
rs6828768
rs11715853
rs6809952
rs78383039
rs55877868
rs62273907
rs7311004
rs7303158
rs8039785
rs2090816
All
−0.2
0.0
0.2
MR effect size for
'Gut microbiota abundance (genus Parasutterella id.2892)' on 'Protocadherin alpha−4'

Batch 2−4397 : Gut microbiota abundance (genus Catenibacterium id.2153) on Protocadherin alpha−4
with a threshold of 1e−05

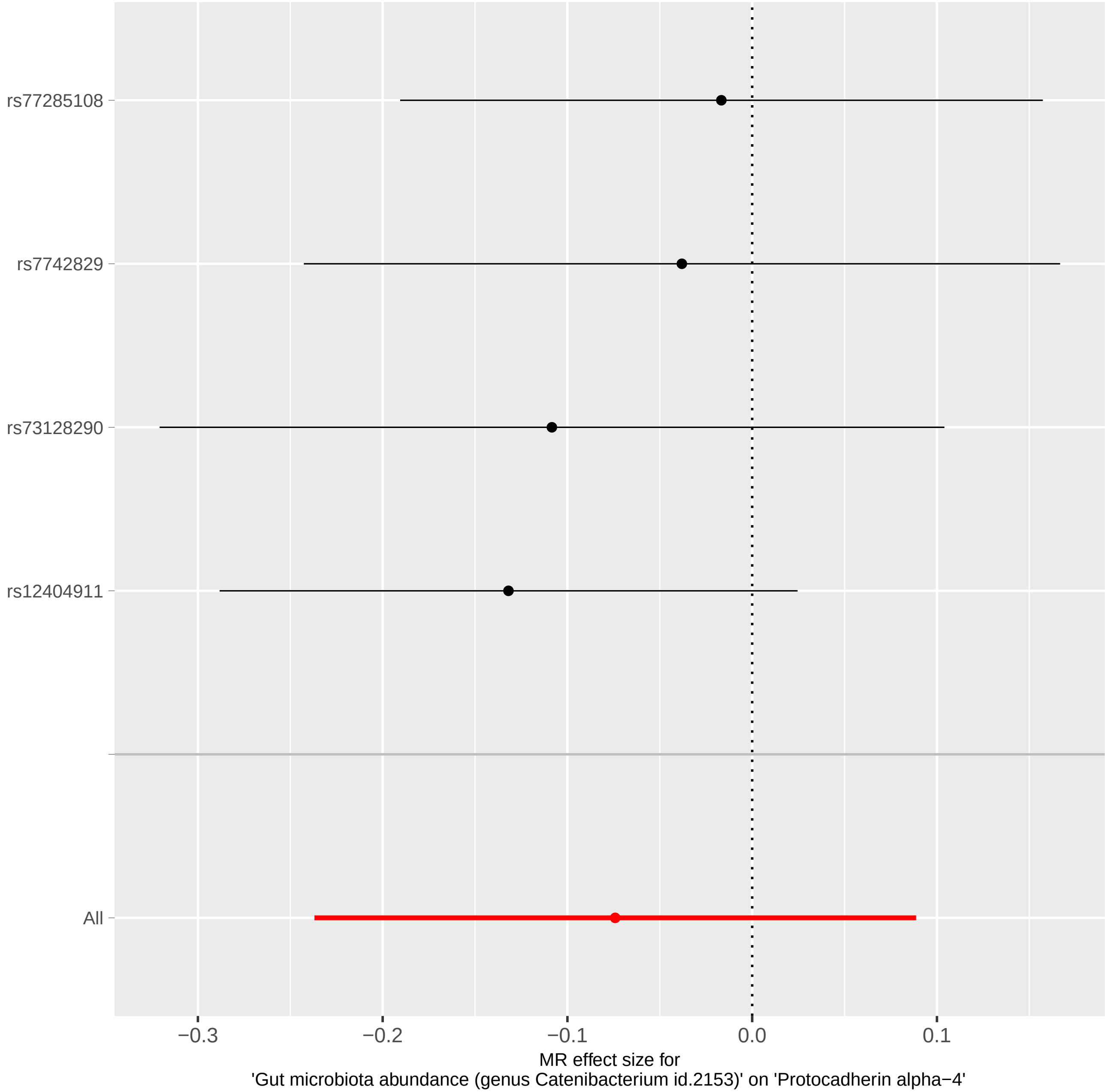

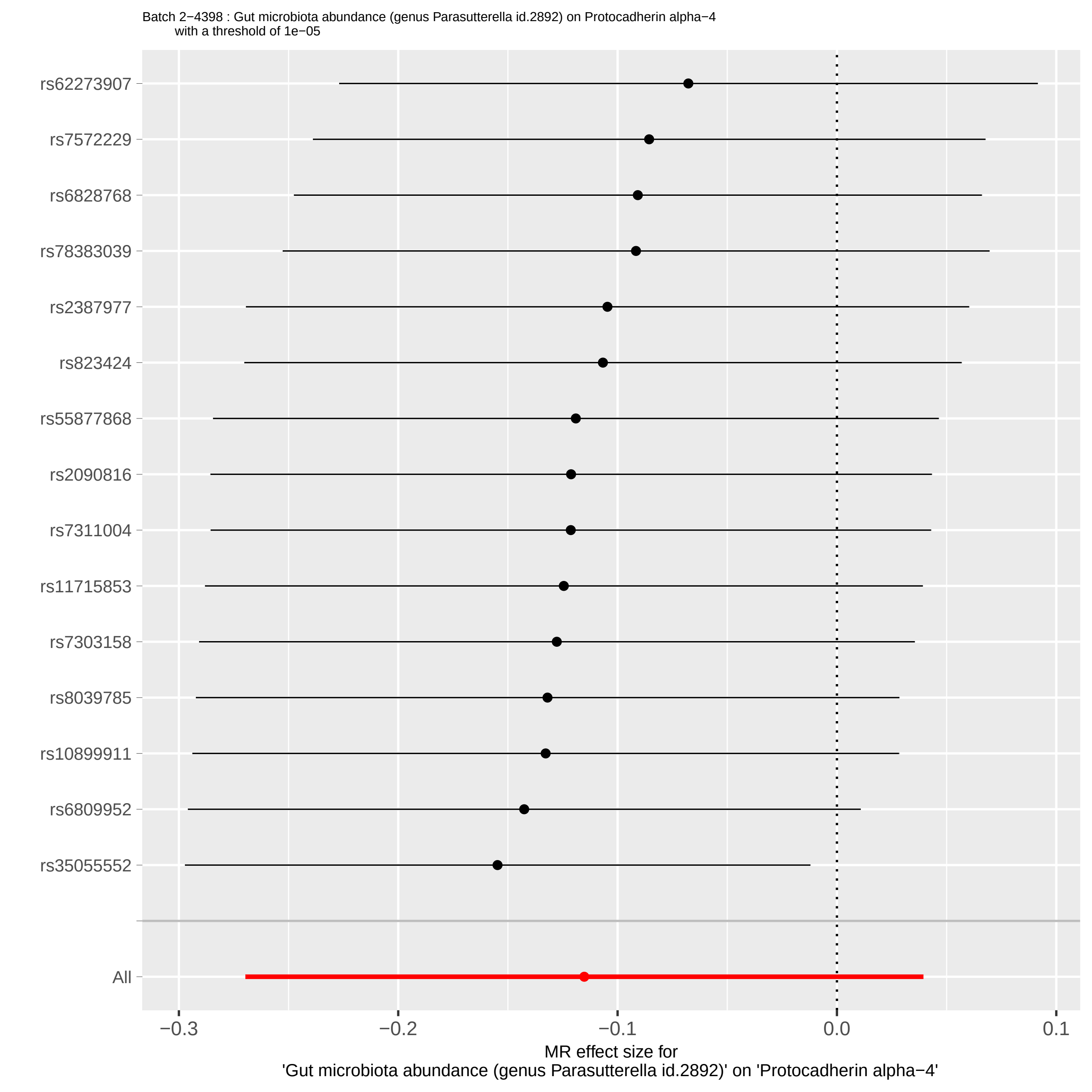
Batch 2−4398 : Gut microbiota abundance (genus Parasutterella id.2892) on Protocadherin alpha−4
with a threshold of 1e−05
rs62273907
rs7572229
rs6828768
rs78383039
rs2387977
rs823424
rs55877868
rs2090816
rs7311004
rs11715853
rs7303158
rs8039785
rs10899911
rs6809952
rs35055552
All
−0.3
−0.2
−0.1
0.0
0.1
MR effect size for
'Gut microbiota abundance (genus Parasutterella id.2892)' on 'Protocadherin alpha−4'

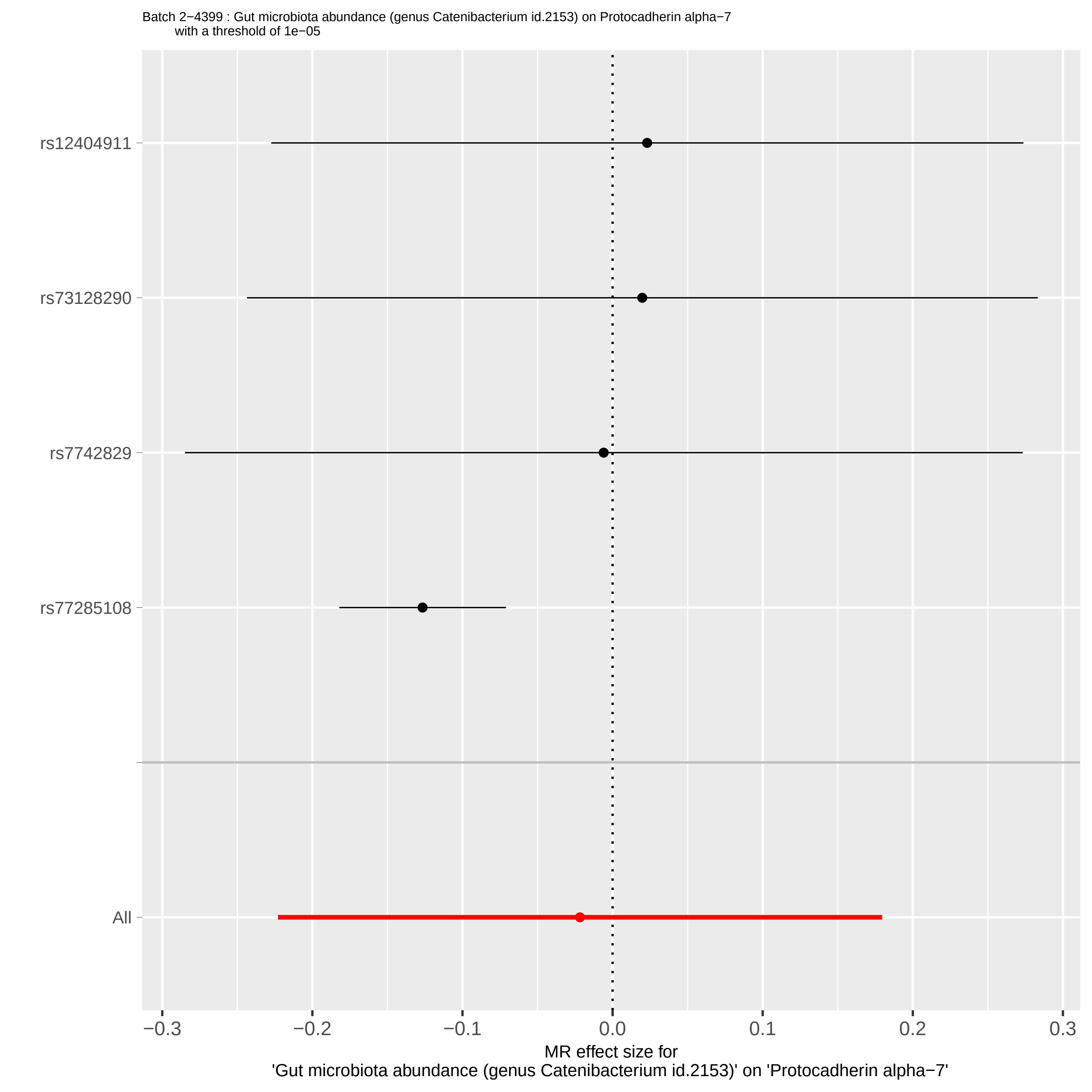
Batch 2−4399 : Gut microbiota abundance (genus Catenibacterium id.2153) on Protocadherin alpha−7
with a threshold of 1e−05
rs12404911
rs73128290
rs7742829
rs77285108
All
−0.3
−0.2
−0.1
0.0
0.1
0.2
0.3
MR effect size for
'Gut microbiota abundance (genus Catenibacterium id.2153)' on 'Protocadherin alpha−7'

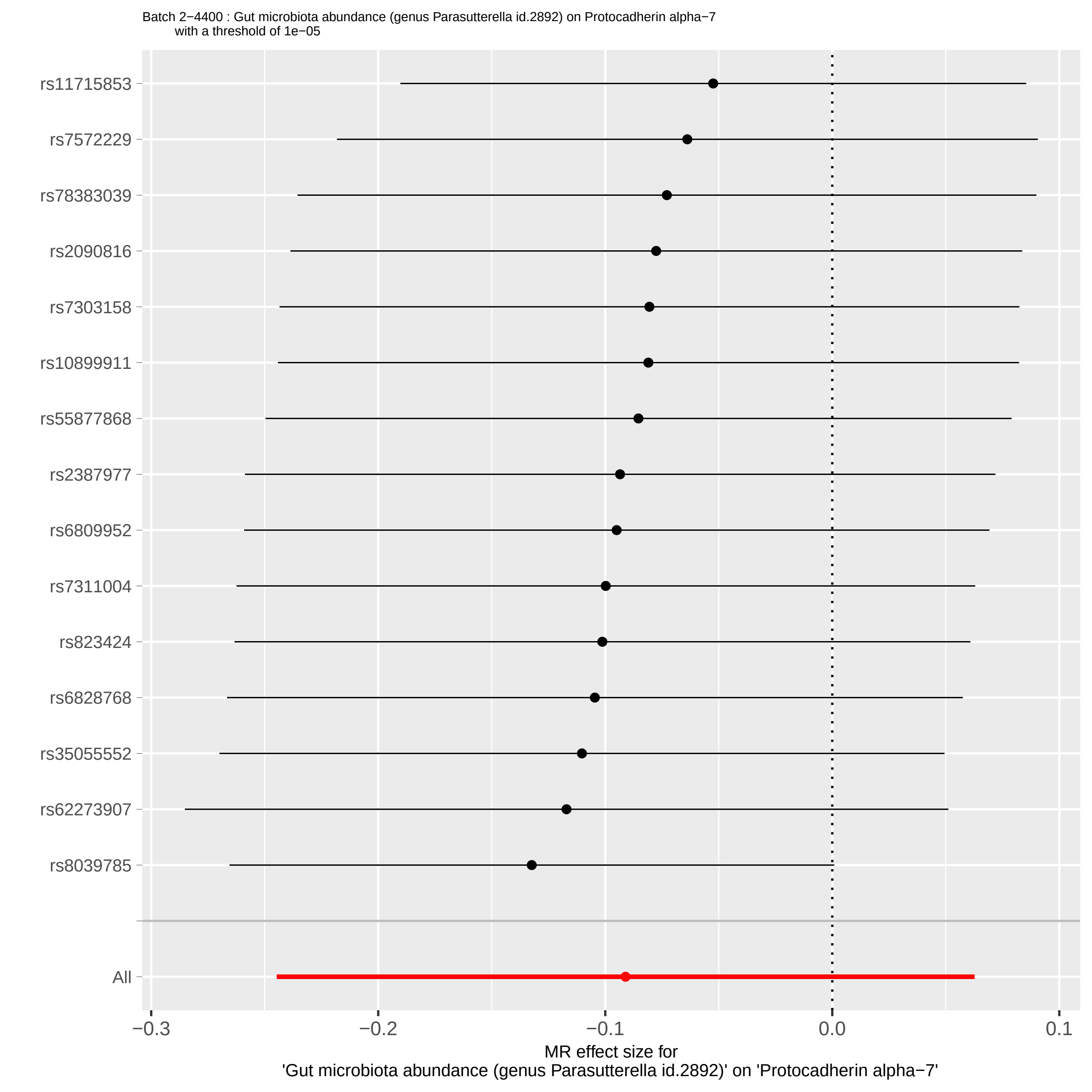
Batch 2−4400 : Gut microbiota abundance (genus Parasutterella id.2892) on Protocadherin alpha−7
with a threshold of 1e−05
rs11715853
rs7572229
rs78383039
rs2090816
rs7303158
rs10899911
rs55877868
rs2387977
rs6809952
rs7311004
rs823424
rs6828768
rs35055552
rs62273907
rs8039785
All
−0.3
−0.2
−0.1
0.0
0.1
MR effect size for
'Gut microbiota abundance (genus Parasutterella id.2892)' on 'Protocadherin alpha−7'

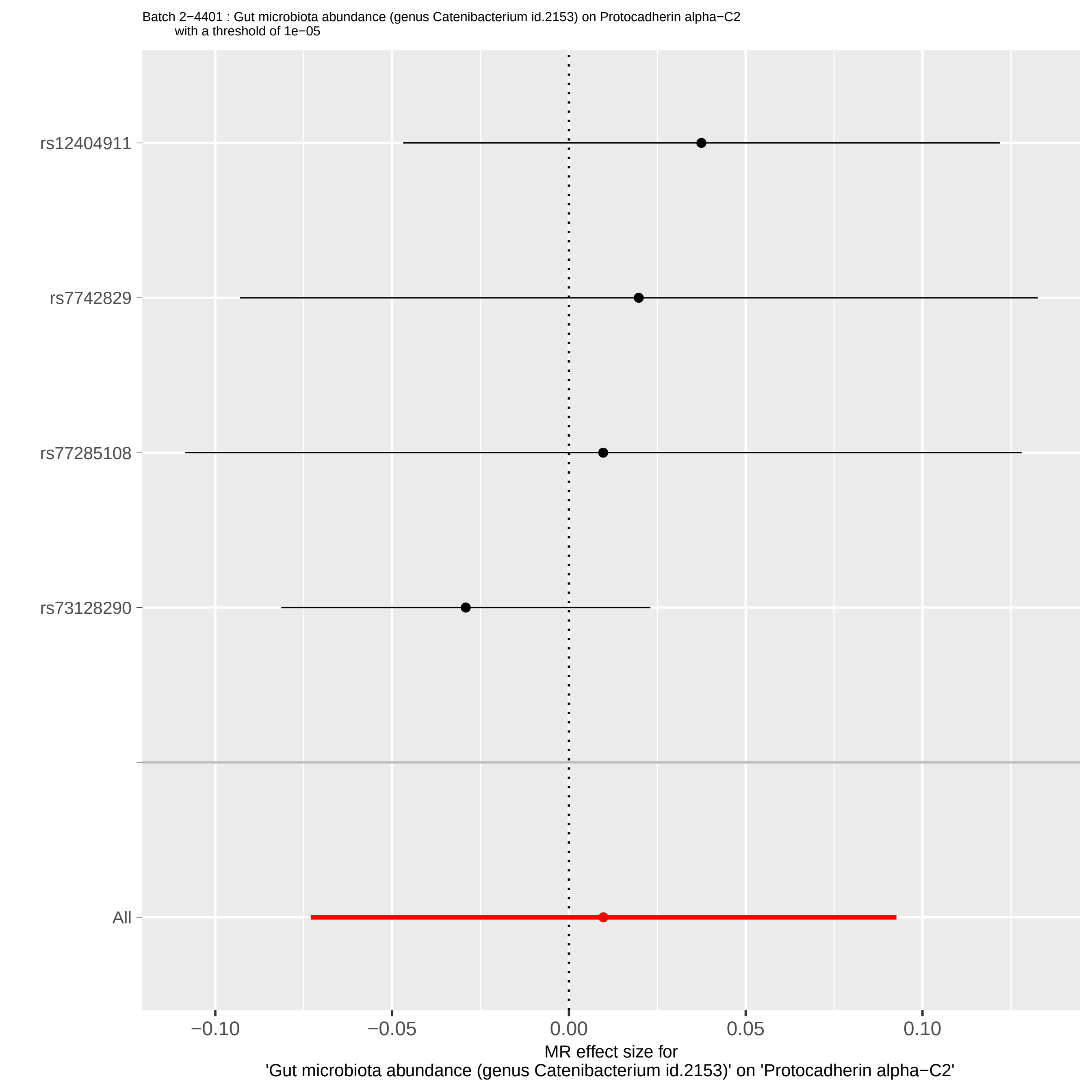
Batch 2−4401 : Gut microbiota abundance (genus Catenibacterium id.2153) on Protocadherin alpha−C2
with a threshold of 1e−05
rs12404911
rs7742829
rs77285108
rs73128290
All
−0.10
−0.05
0.00
0.05
0.10
MR effect size for
'Gut microbiota abundance (genus Catenibacterium id.2153)' on 'Protocadherin alpha−C2'

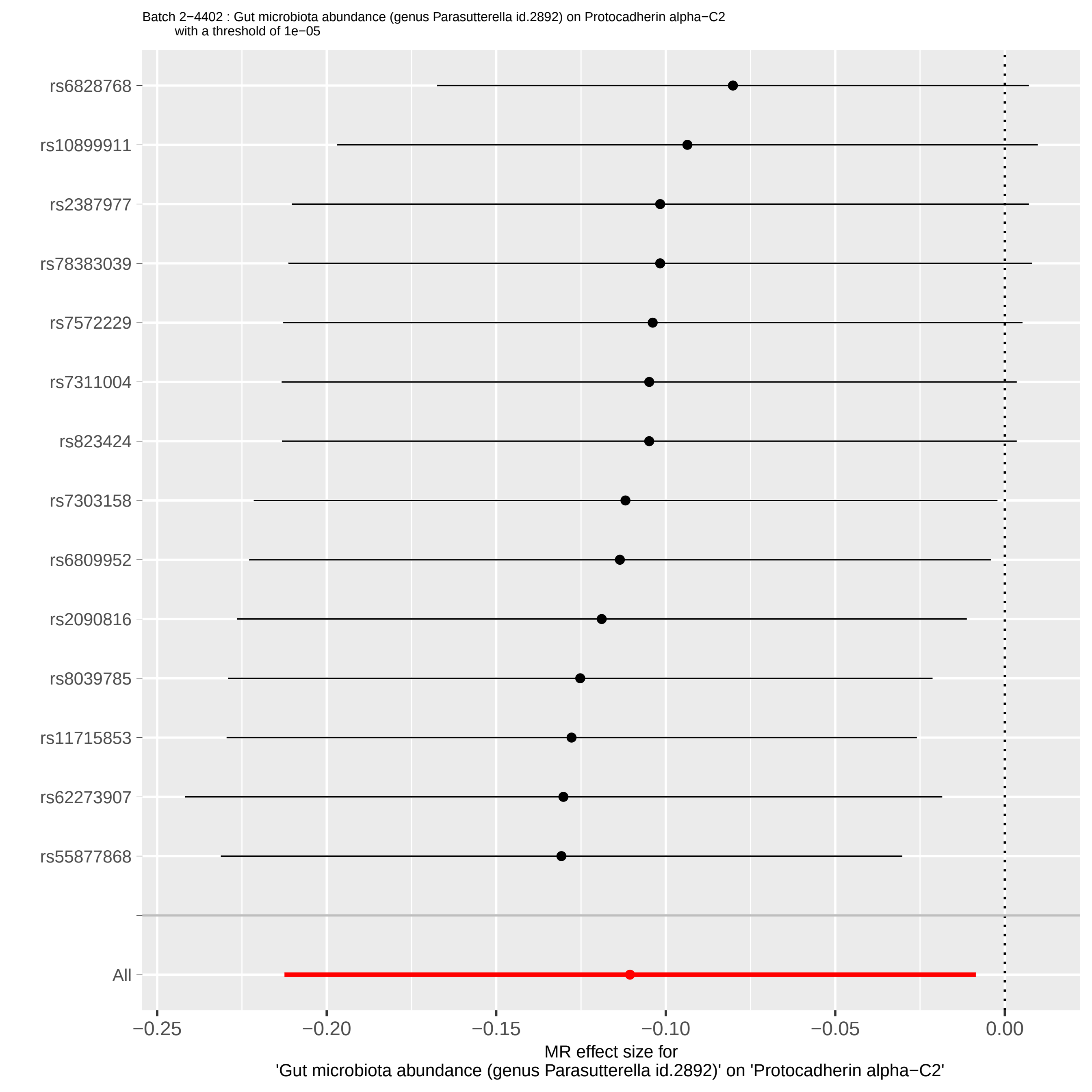
Batch 2−4402 : Gut microbiota abundance (genus Parasutterella id.2892) on Protocadherin alpha−C2
with a threshold of 1e−05
rs6828768
rs10899911
rs2387977
rs78383039
rs7572229
rs7311004
rs823424
rs7303158
rs6809952
rs2090816
rs8039785
rs11715853
rs62273907
rs55877868
All
−0.25
−0.20
−0.15
−0.10
−0.05
0.00
MR effect size for
'Gut microbiota abundance (genus Parasutterella id.2892)' on 'Protocadherin alpha−C2'

Batch 2−4403 : Gut microbiota abundance (genus Catenibacterium id.2153) on Protocadherin beta−10
with a threshold of 1e−05

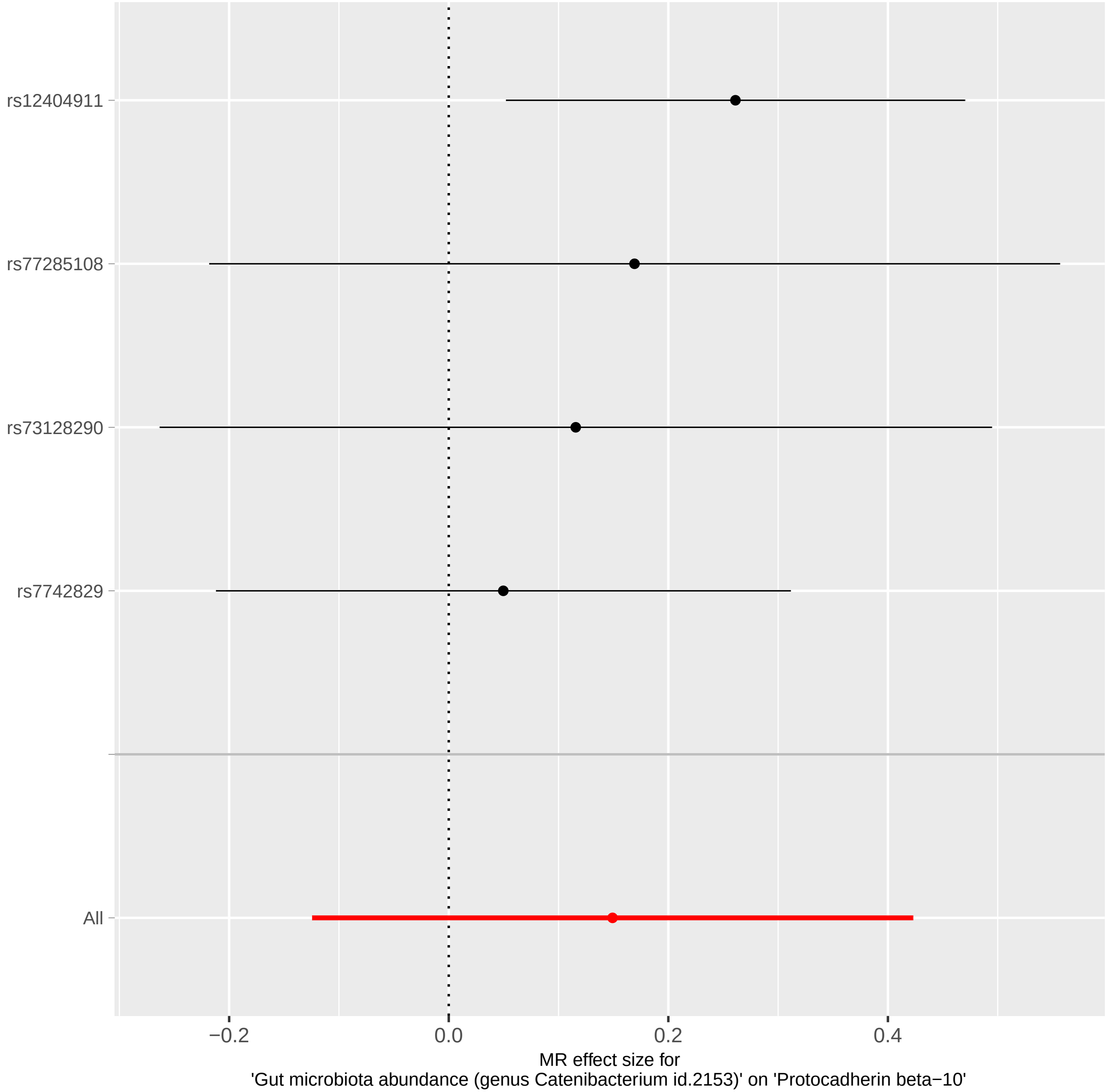

Batch 2−4404 : Gut microbiota abundance (genus Parasutterella id.2892) on Protocadherin beta−10
with a threshold of 1e−05

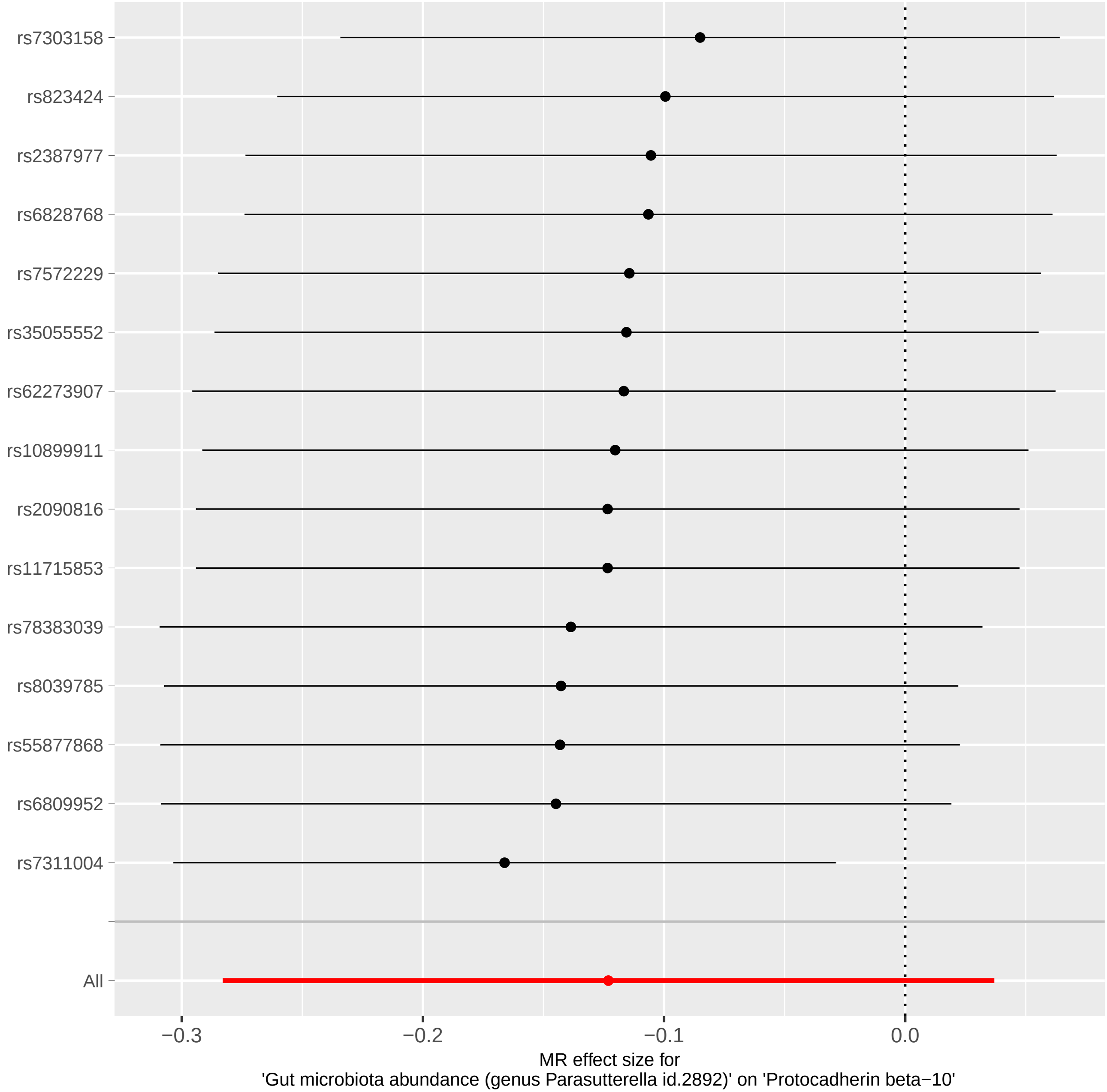

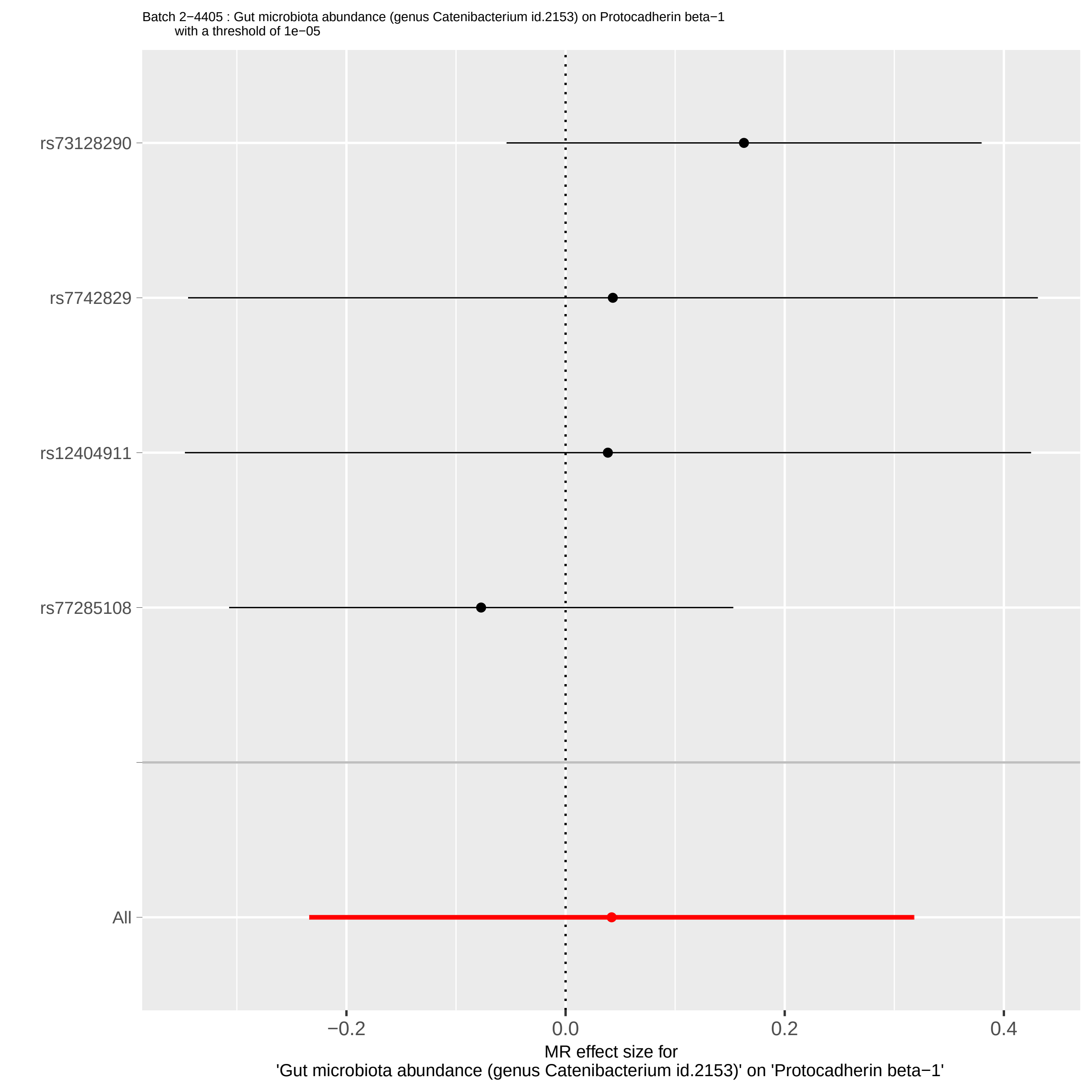
Batch 2−4405 : Gut microbiota abundance (genus Catenibacterium id.2153) on Protocadherin beta−1
with a threshold of 1e−05
rs73128290
rs7742829
rs12404911
rs77285108
All
−0.2
0.0
0.2
0.4
MR effect size for
'Gut microbiota abundance (genus Catenibacterium id.2153)' on 'Protocadherin beta−1'

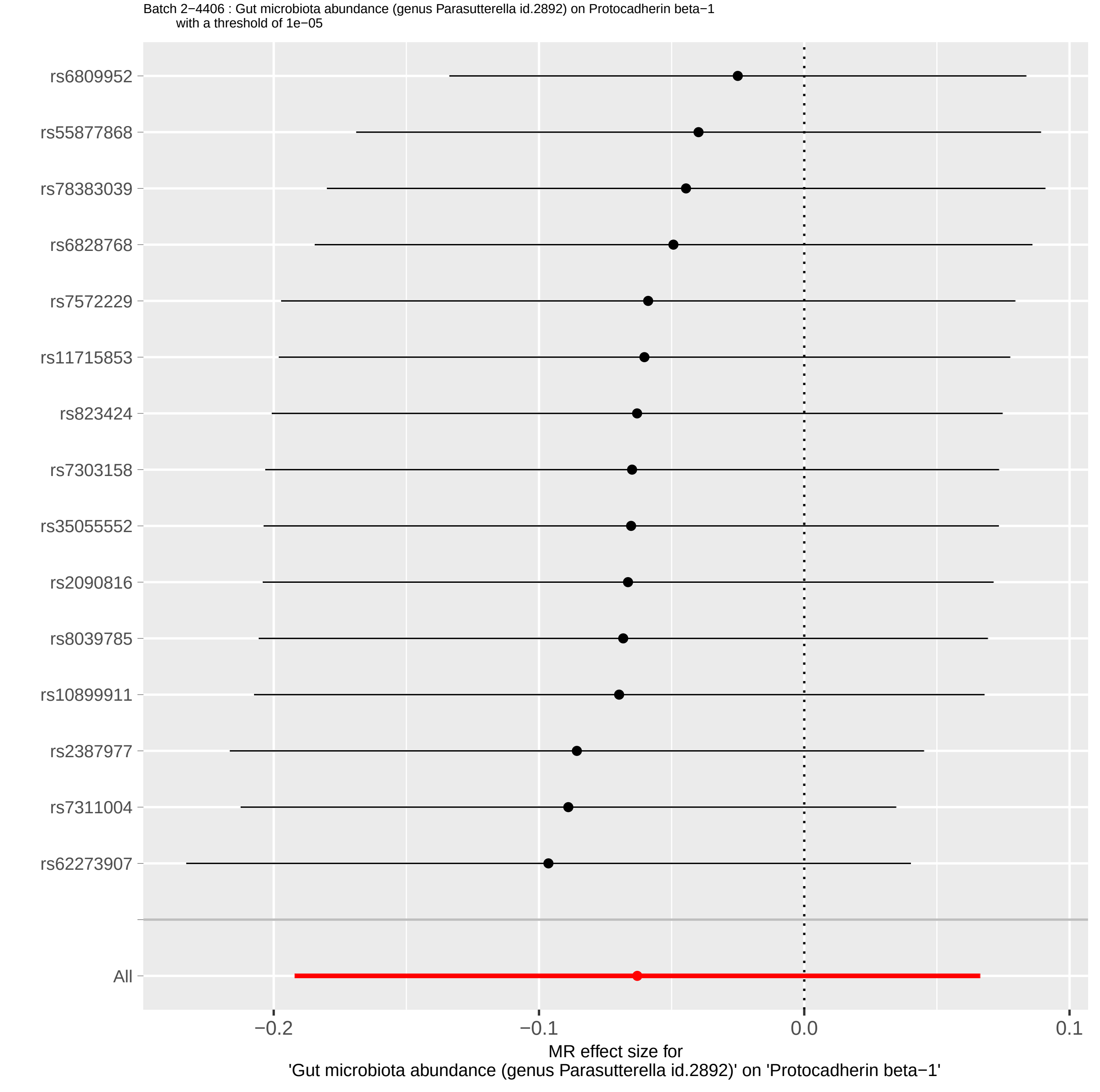
Batch 2−4406 : Gut microbiota abundance (genus Parasutterella id.2892) on Protocadherin beta−1
with a threshold of 1e−05
rs6809952
rs55877868
rs78383039
rs6828768
rs7572229
rs11715853
rs823424
rs7303158
rs35055552
rs2090816
rs8039785
rs10899911
rs2387977
rs7311004
rs62273907
All
−0.2
−0.1
0.0
0.1
MR effect size for
'Gut microbiota abundance (genus Parasutterella id.2892)' on 'Protocadherin beta−1'

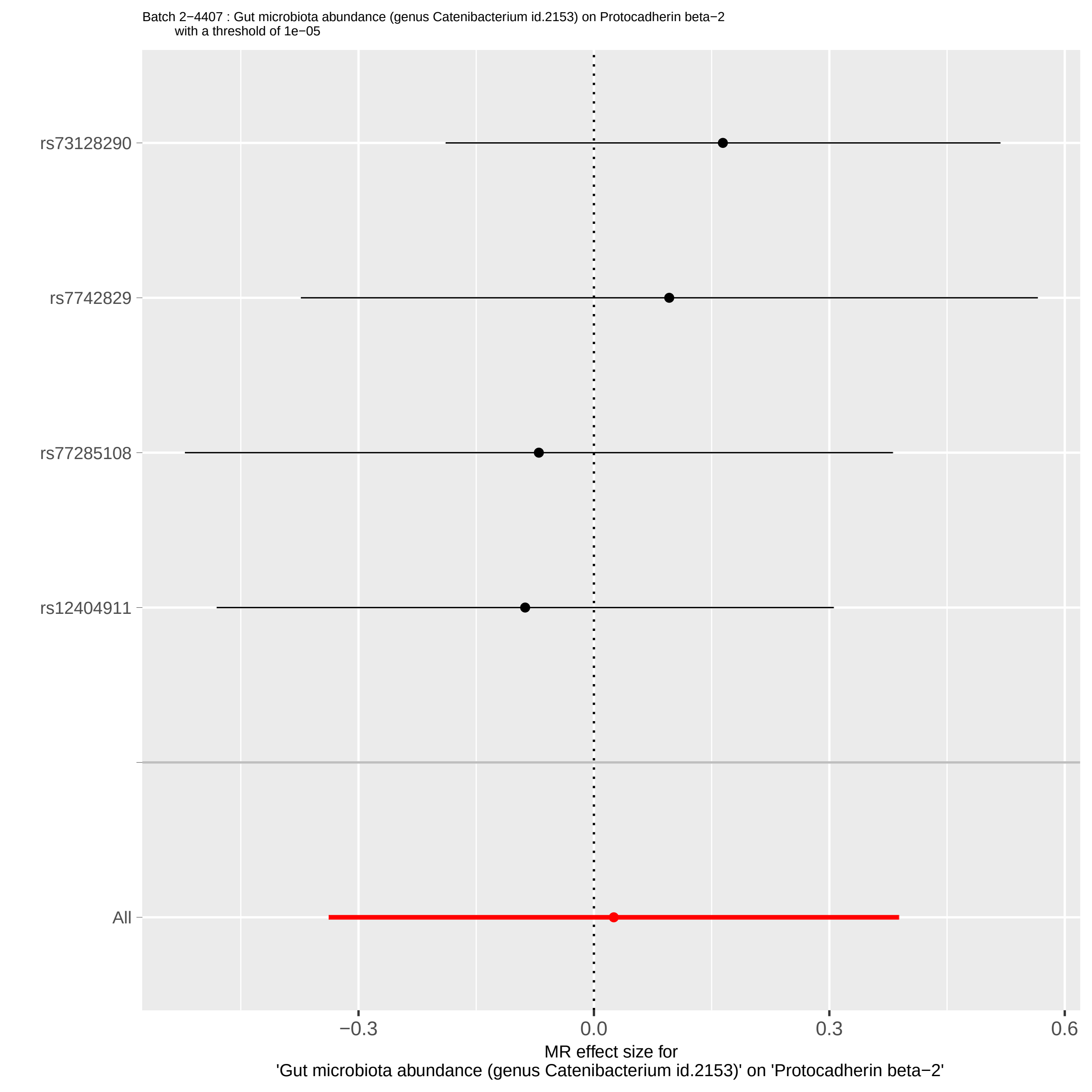
Batch 2−4407 : Gut microbiota abundance (genus Catenibacterium id.2153) on Protocadherin beta−2
with a threshold of 1e−05
rs73128290
rs7742829
rs77285108
rs12404911
All
−0.3
0.0
0.3
0.6
MR effect size for
'Gut microbiota abundance (genus Catenibacterium id.2153)' on 'Protocadherin beta−2'

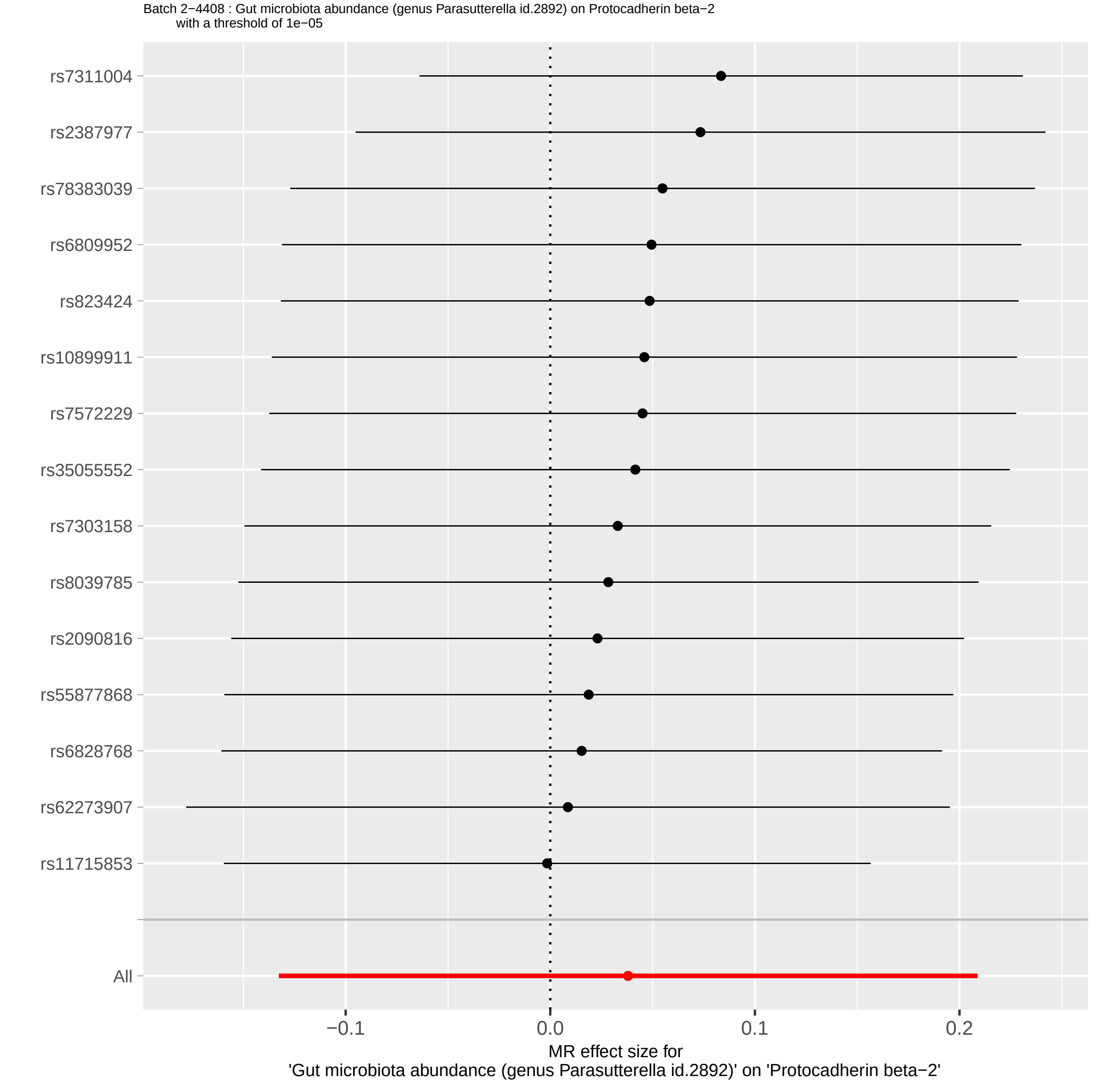

Batch 2−4408 : Gut microbiota abundance (genus Parasutterella id.2892) on Protocadherin beta−2
with a threshold of 1e−05
rs7311004
rs2387977
rs78383039
rs6809952
rs823424
rs10899911
rs7572229
rs35055552
rs7303158
rs8039785
rs2090816
rs55877868
rs6828768
rs62273907
rs11715853
All
−0.1
0.0
0.1
0.2
MR effect size for
'Gut microbiota abundance (genus Parasutterella id.2892)' on 'Protocadherin beta−2'

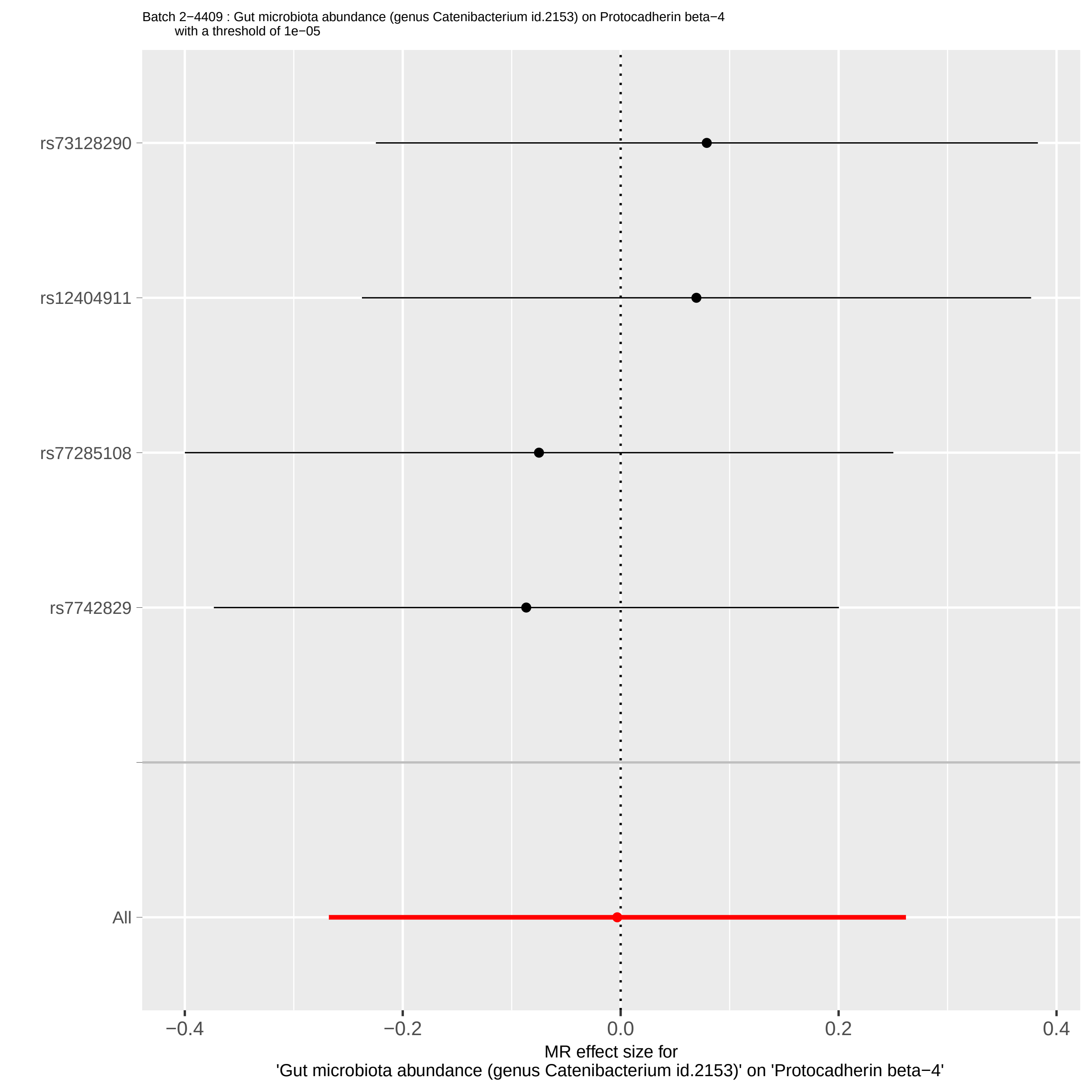
Batch 2−4409 : Gut microbiota abundance (genus Catenibacterium id.2153) on Protocadherin beta−4
with a threshold of 1e−05
rs73128290
rs12404911
rs77285108
rs7742829
All
−0.4
−0.2
0.0
0.2
0.4
MR effect size for
'Gut microbiota abundance (genus Catenibacterium id.2153)' on 'Protocadherin beta−4'

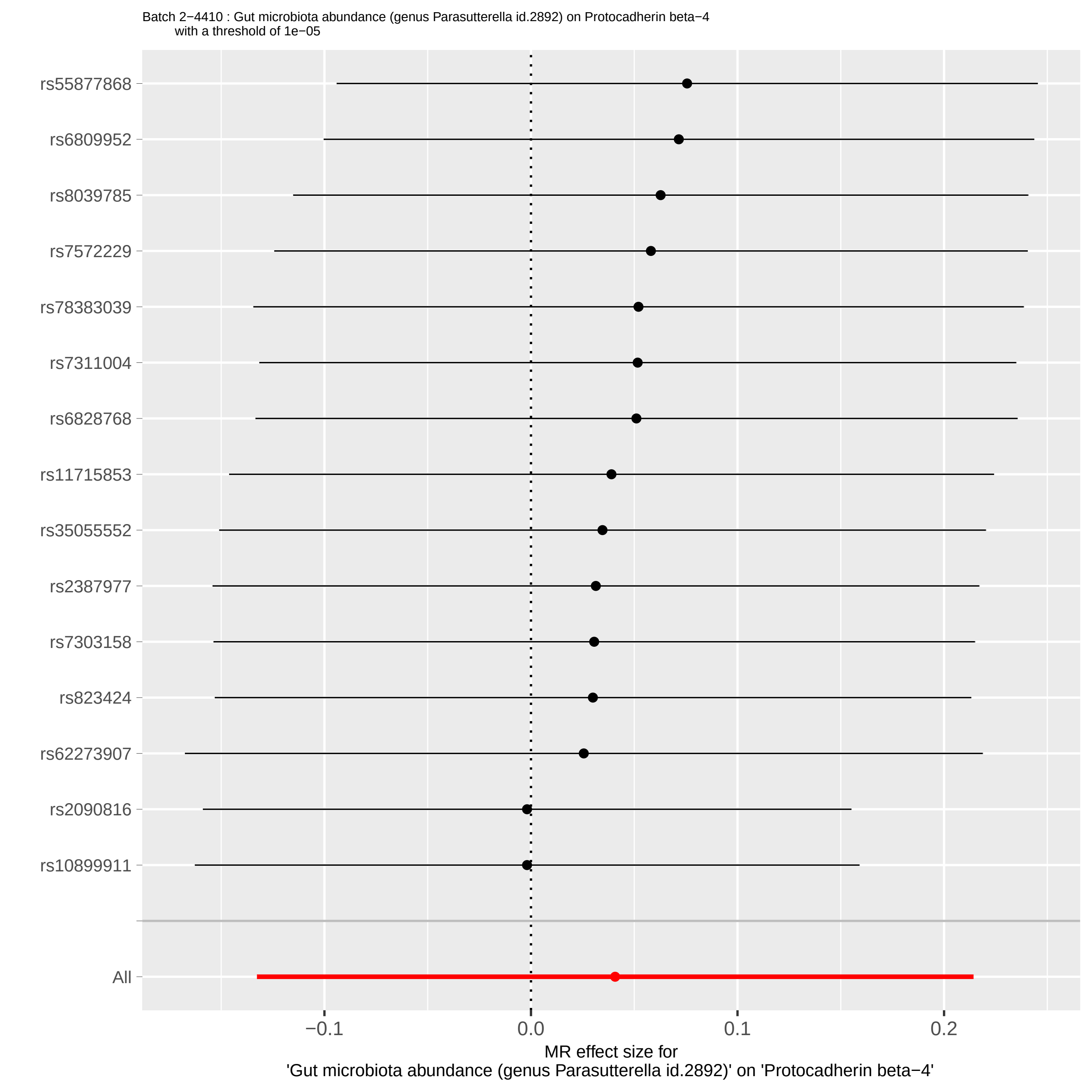
Batch 2−4410 : Gut microbiota abundance (genus Parasutterella id.2892) on Protocadherin beta−4
with a threshold of 1e−05
rs55877868
rs6809952
rs8039785
rs7572229
rs78383039
rs7311004
rs6828768
rs11715853
rs35055552
rs2387977
rs7303158
rs823424
rs62273907
rs2090816
rs10899911
All
−0.1
0.0
0.1
0.2
MR effect size for
'Gut microbiota abundance (genus Parasutterella id.2892)' on 'Protocadherin beta−4'

Batch 2−4411 : Gut microbiota abundance (genus Catenibacterium id.2153) on Protocadherin gamma−A8
with a threshold of 1e−05

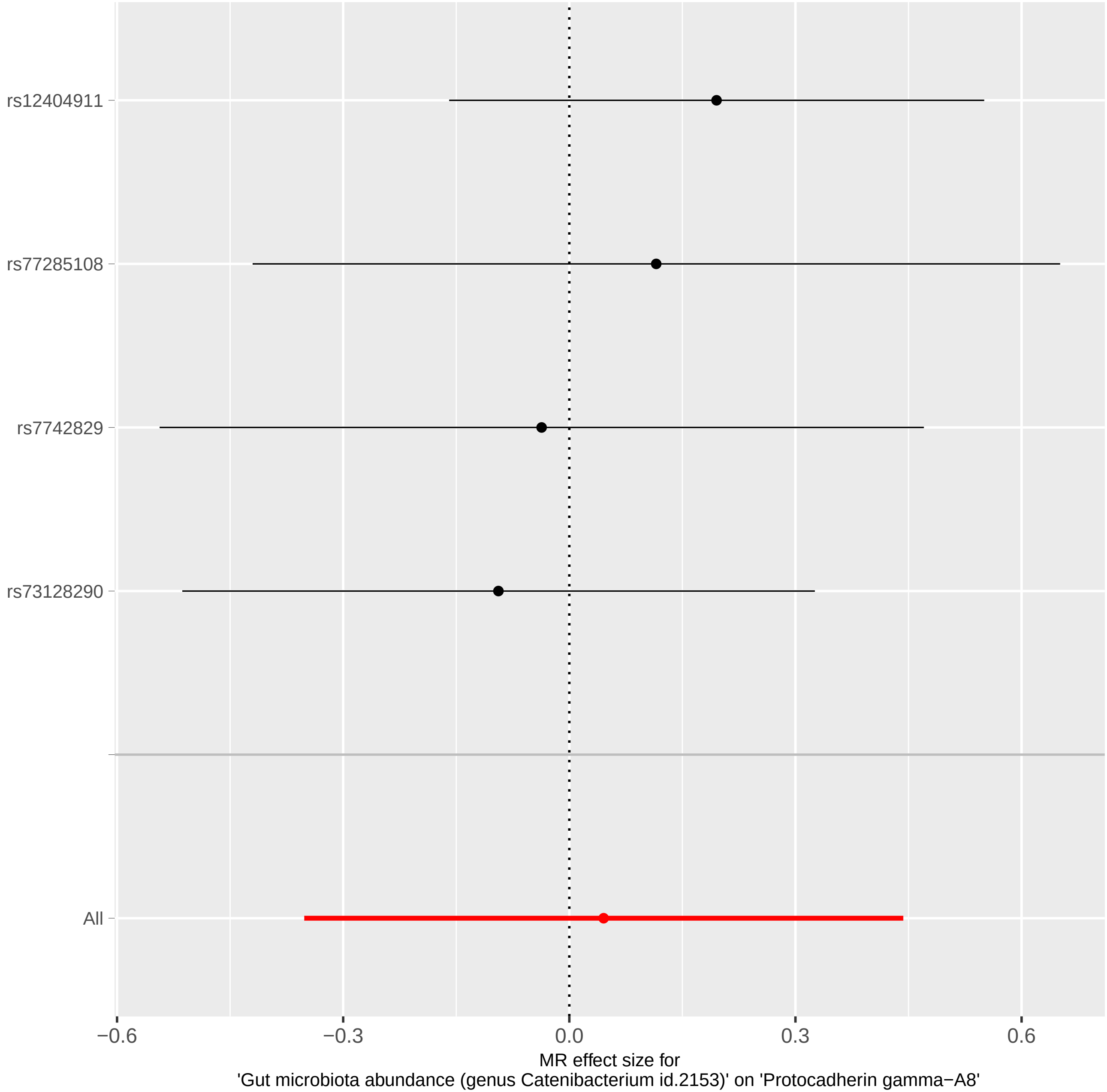

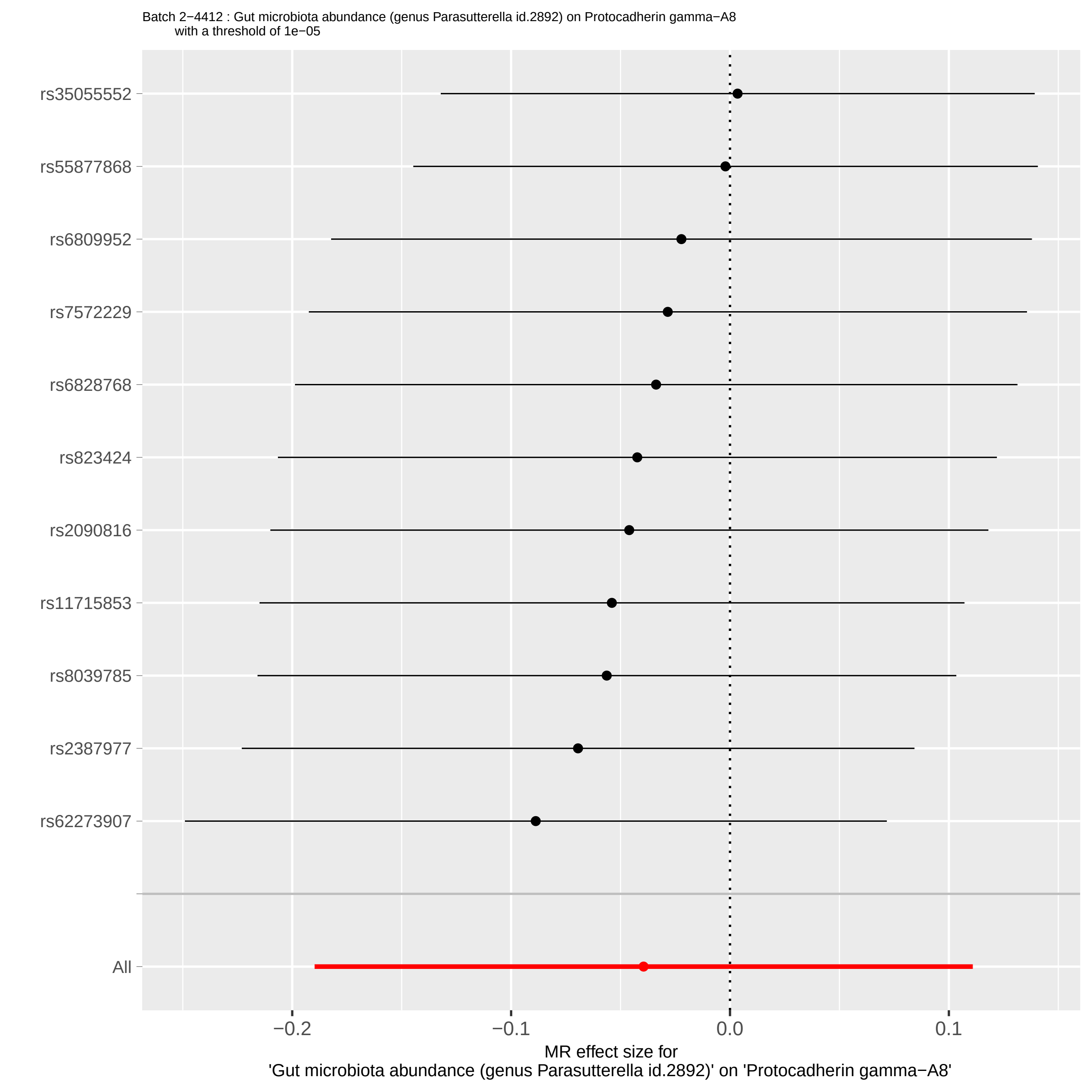
Batch 2−4412 : Gut microbiota abundance (genus Parasutterella id.2892) on Protocadherin gamma−A8
with a threshold of 1e−05
rs35055552
rs55877868
rs6809952
rs7572229
rs6828768
rs823424
rs2090816
rs11715853
rs8039785
rs2387977
rs62273907
All
−0.2
−0.1
0.0
0.1
MR effect size for
'Gut microbiota abundance (genus Parasutterella id.2892)' on 'Protocadherin gamma−A8'

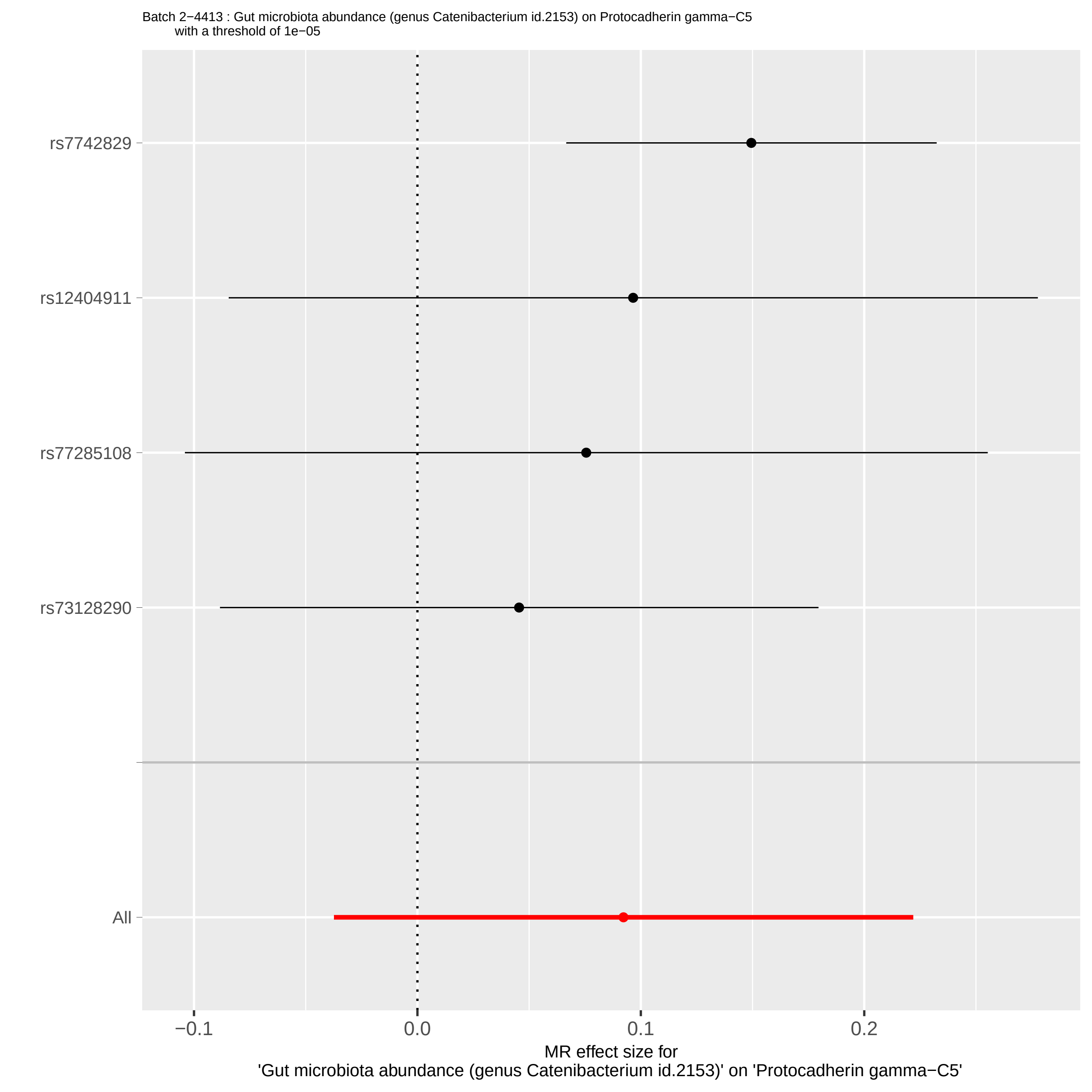

Batch 2−4413 : Gut microbiota abundance (genus Catenibacterium id.2153) on Protocadherin gamma−C5
with a threshold of 1e−05
rs7742829
rs12404911
rs77285108
rs73128290
All
−0.1
0.0
0.1
0.2
MR effect size for
'Gut microbiota abundance (genus Catenibacterium id.2153)' on 'Protocadherin gamma−C5'

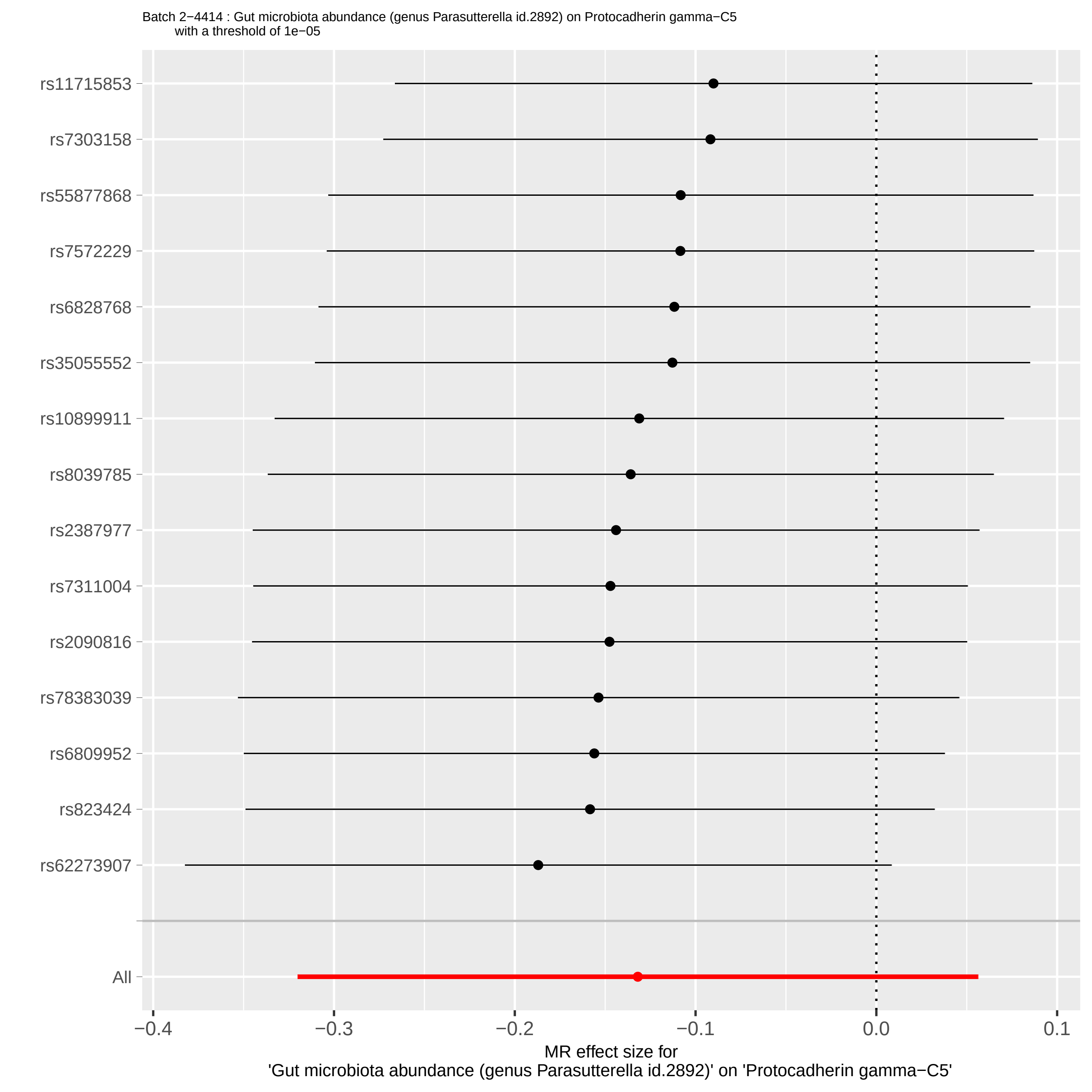

Batch 2−4414 : Gut microbiota abundance (genus Parasutterella id.2892) on Protocadherin gamma−C5
with a threshold of 1e−05
rs11715853
rs7303158
rs55877868
rs7572229
rs6828768
rs35055552
rs10899911
rs8039785
rs2387977
rs7311004
rs2090816
rs78383039
rs6809952
rs823424
rs62273907
All
−0.4
−0.3
−0.2
−0.1
0.0
0.1
MR effect size for
'Gut microbiota abundance (genus Parasutterella id.2892)' on 'Protocadherin gamma−C5'

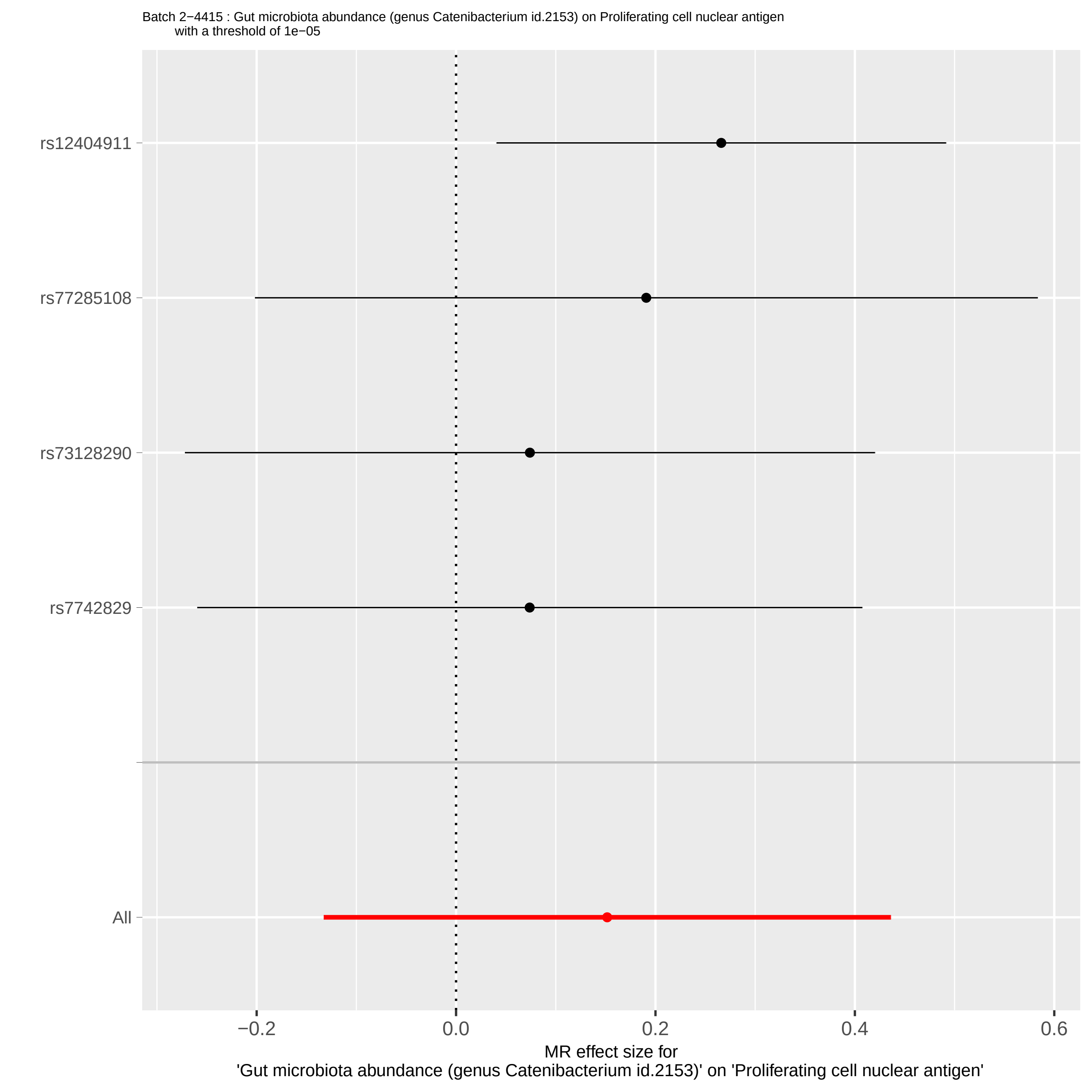
Batch 2−4415 : Gut microbiota abundance (genus Catenibacterium id.2153) on Proliferating cell nuclear antigen
with a threshold of 1e−05
rs12404911
rs77285108
rs73128290
rs7742829
All
−0.2
0.0
0.2
0.4
0.6
MR effect size for
'Gut microbiota abundance (genus Catenibacterium id.2153)' on 'Proliferating cell nuclear antigen'

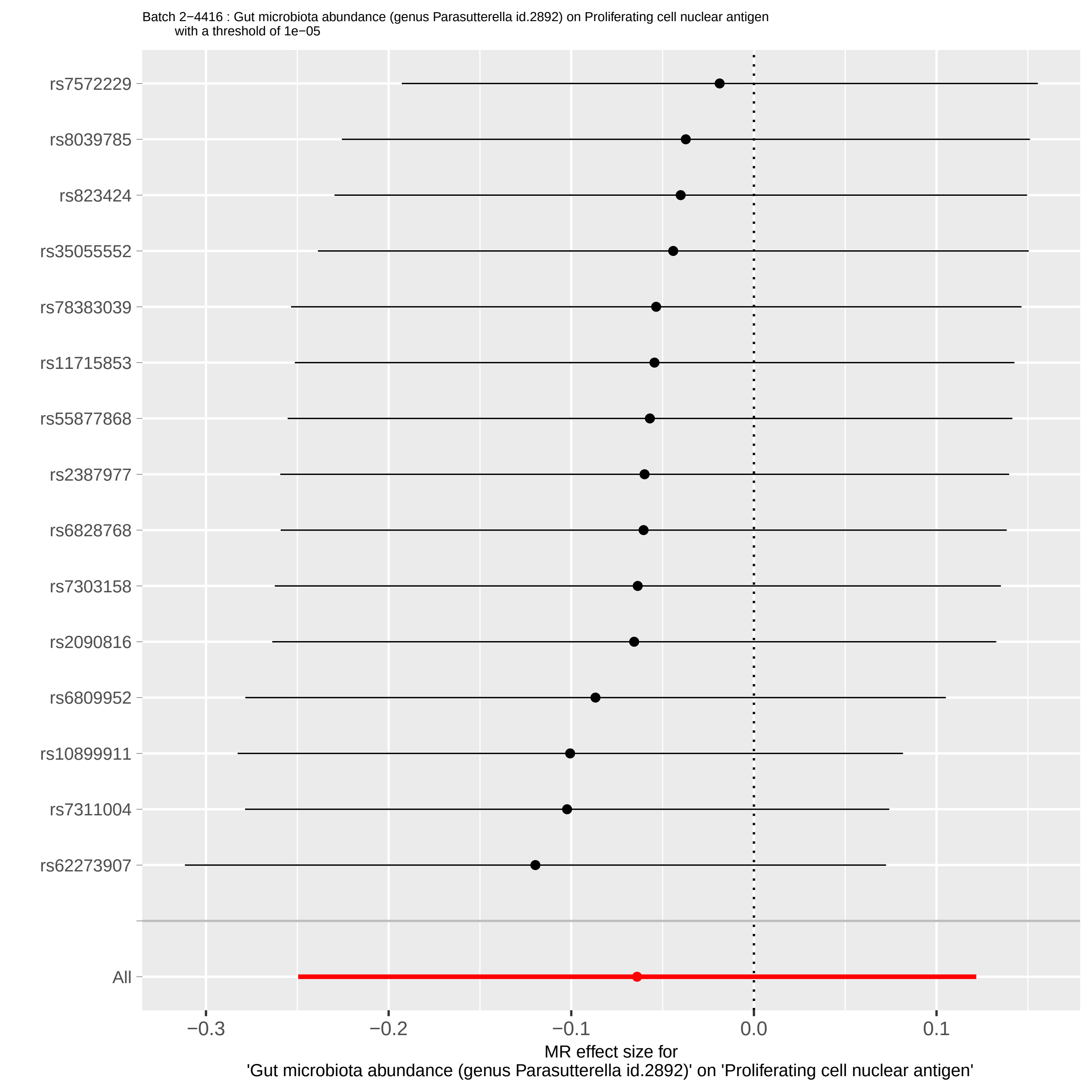

Batch 2−4416 : Gut microbiota abundance (genus Parasutterella id.2892) on Proliferating cell nuclear antigen
with a threshold of 1e−05
rs7572229
rs8039785
rs823424
rs35055552
rs78383039
rs11715853
rs55877868
rs2387977
rs6828768
rs7303158
rs2090816
rs6809952
rs10899911
rs7311004
rs62273907
All
−0.3
−0.2
−0.1
0.0
0.1
MR effect size for
'Gut microbiota abundance (genus Parasutterella id.2892)' on 'Proliferating cell nuclear antigen'

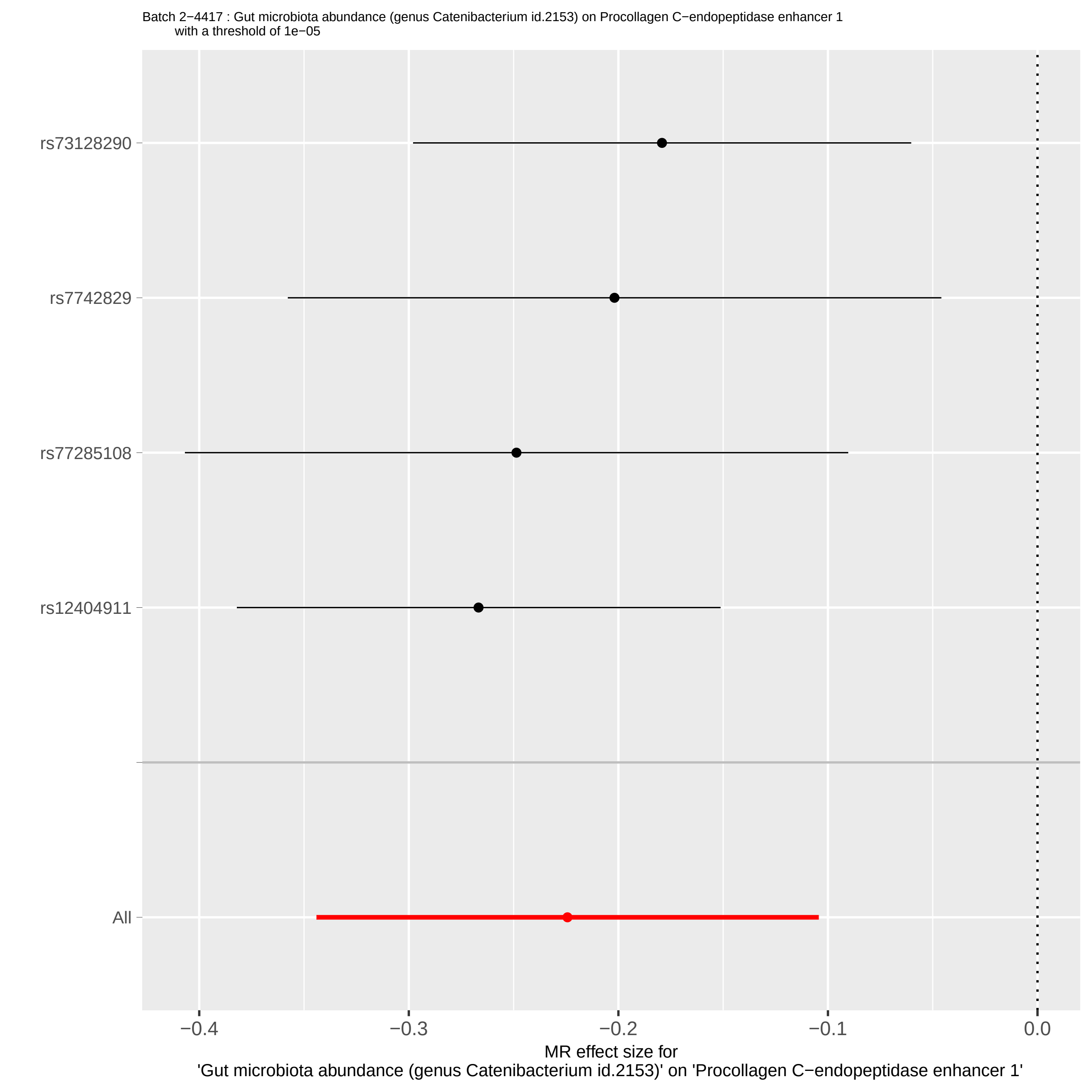
Batch 2−4417 : Gut microbiota abundance (genus Catenibacterium id.2153) on Procollagen C−endopeptidase enhancer 1
with a threshold of 1e−05
rs73128290
rs7742829
rs77285108
rs12404911
All
−0.4
−0.3
−0.2
−0.1
0.0
MR effect size for
'Gut microbiota abundance (genus Catenibacterium id.2153)' on 'Procollagen C−endopeptidase enhancer 1'

Batch 2−4418 : Gut microbiota abundance (genus Parasutterella id.2892) on Procollagen C−endopeptidase enhancer 1
with a threshold of 1e−05

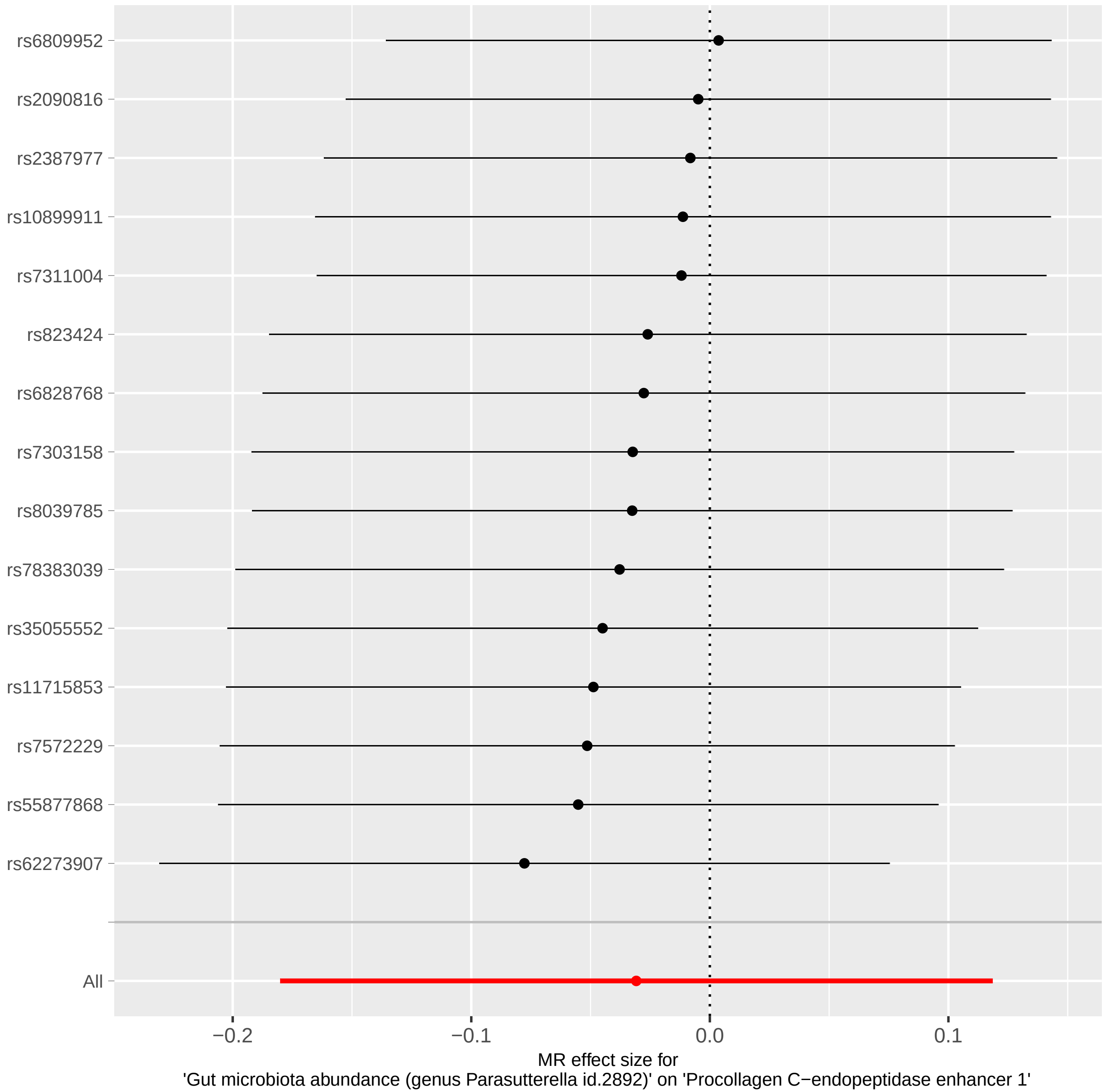

Batch 2−4419 : Gut microbiota abundance (genus Catenibacterium id.2153) on Procollagen C−endopeptidase enhancer 2
with a threshold of 1e−05

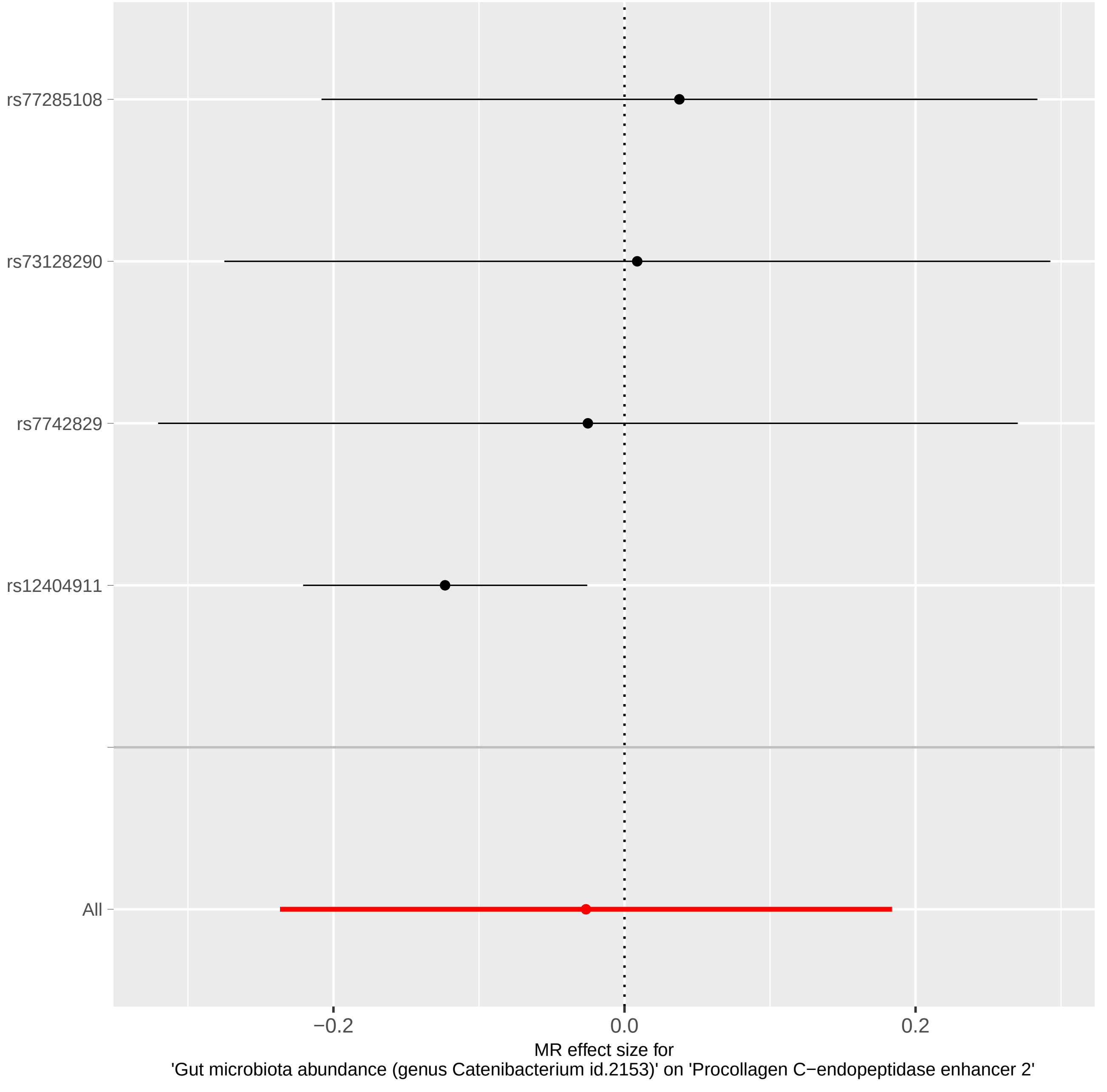

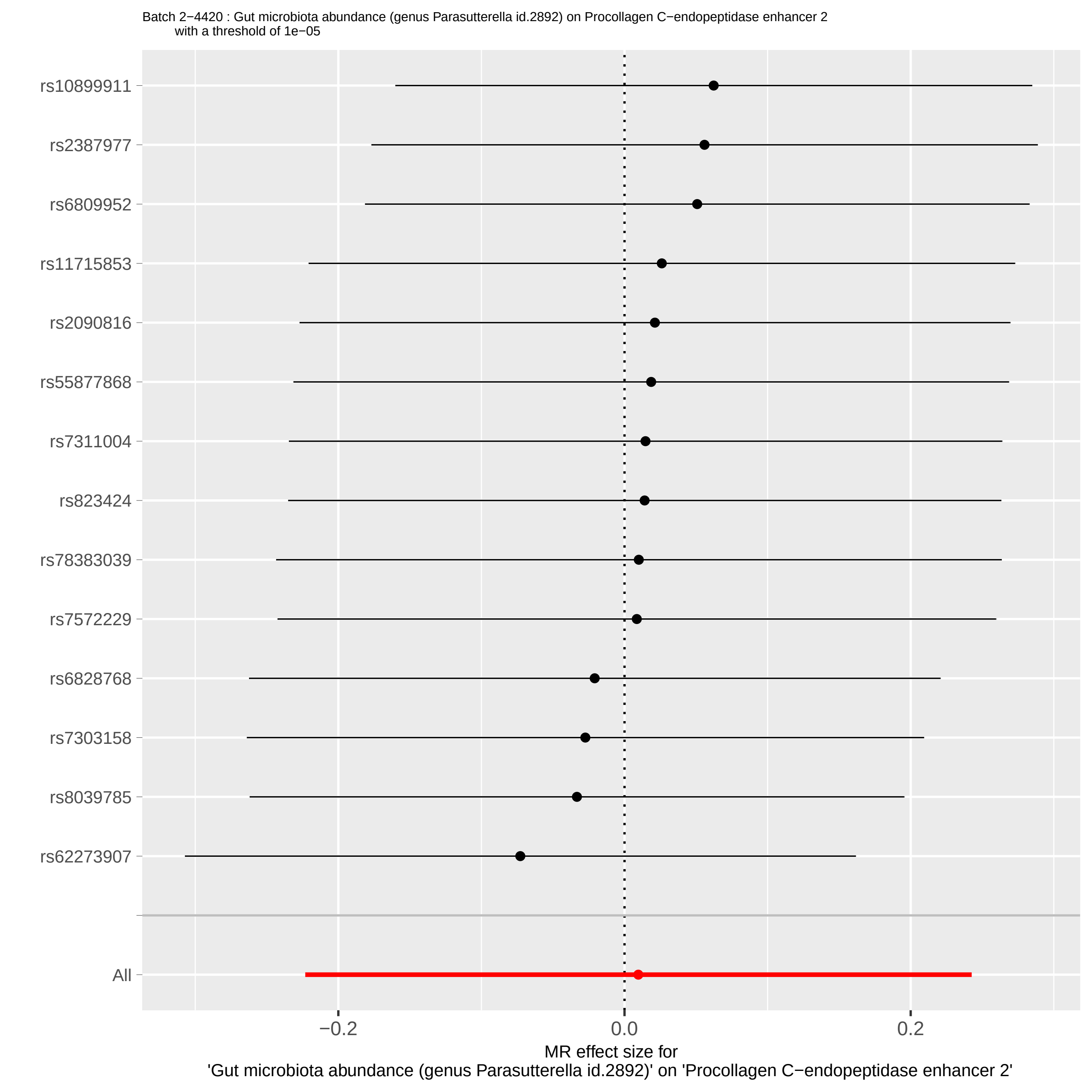
Batch 2−4420 : Gut microbiota abundance (genus Parasutterella id.2892) on Procollagen C−endopeptidase enhancer 2
with a threshold of 1e−05
rs10899911
rs2387977
rs6809952
rs11715853
rs2090816
rs55877868
rs7311004
rs823424
rs78383039
rs7572229
rs6828768
rs7303158
rs8039785
rs62273907
All
−0.2
0.0
0.2
MR effect size for
'Gut microbiota abundance (genus Parasutterella id.2892)' on 'Procollagen C−endopeptidase enhancer 2'

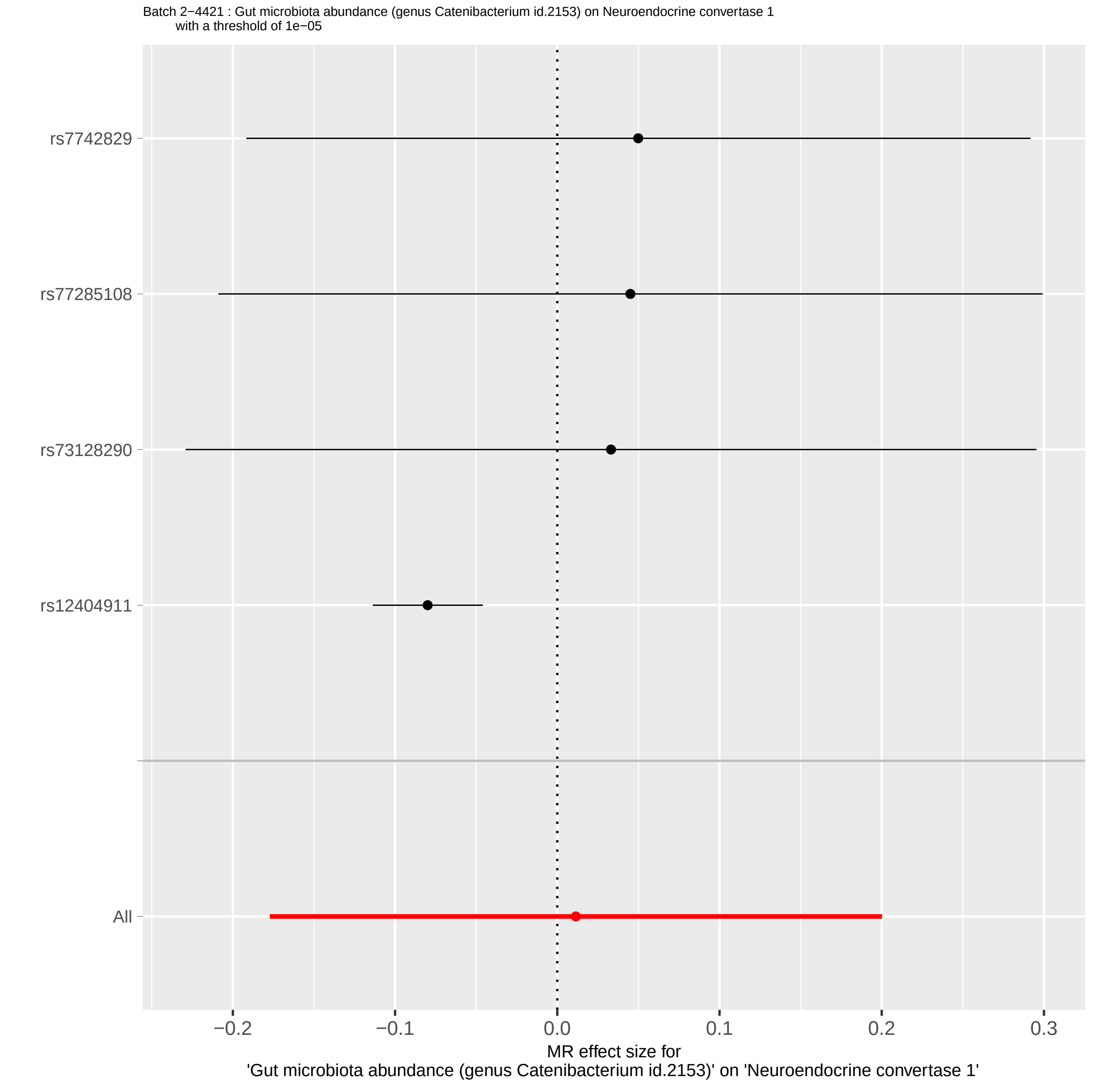
Batch 2−4421 : Gut microbiota abundance (genus Catenibacterium id.2153) on Neuroendocrine convertase 1
with a threshold of 1e−05
rs7742829
rs77285108
rs73128290
rs12404911
All
−0.2
−0.1
0.0
0.1
0.2
0.3
MR effect size for
'Gut microbiota abundance (genus Catenibacterium id.2153)' on 'Neuroendocrine convertase 1'

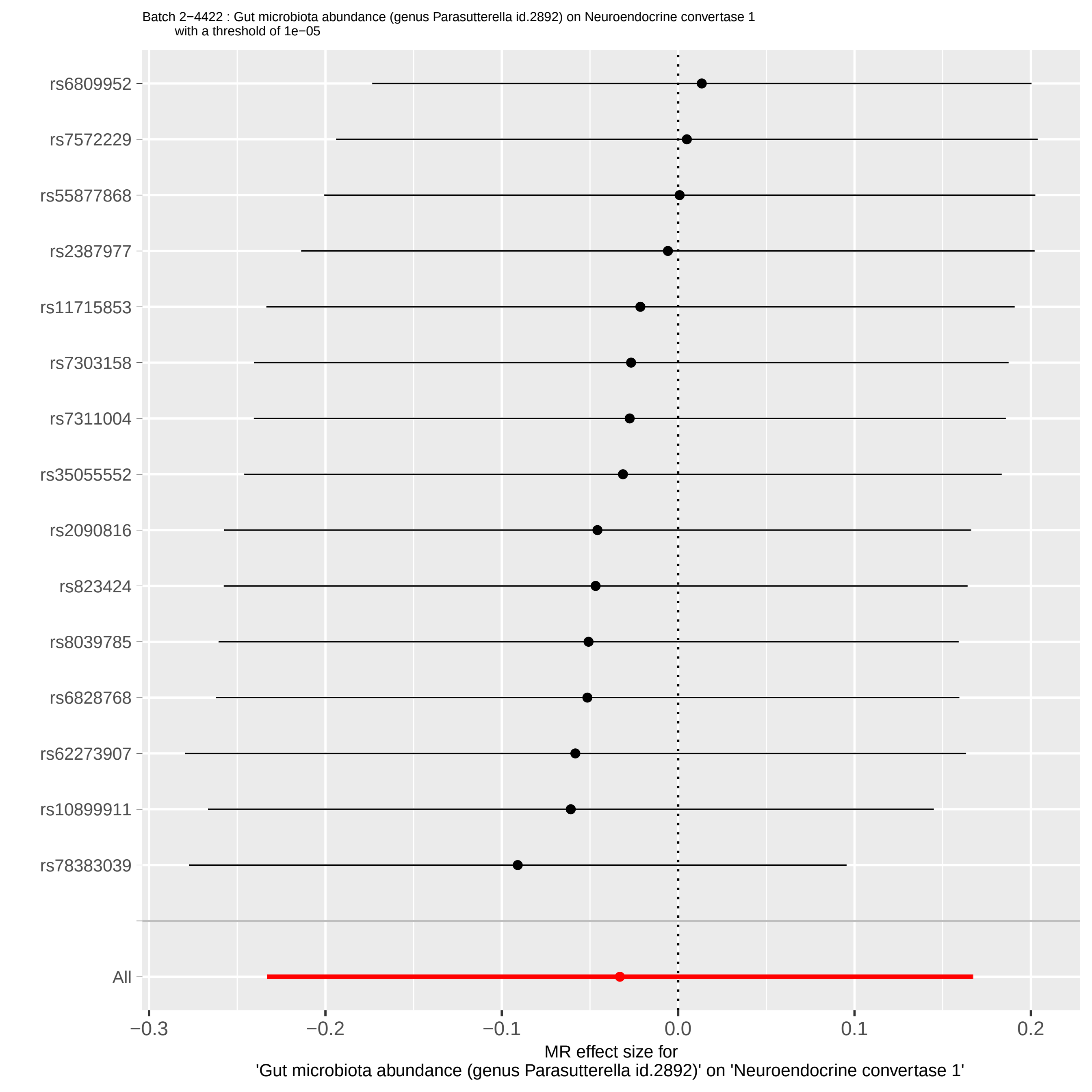
Batch 2−4422 : Gut microbiota abundance (genus Parasutterella id.2892) on Neuroendocrine convertase 1
with a threshold of 1e−05
rs6809952
rs7572229
rs55877868
rs2387977
rs11715853
rs7303158
rs7311004
rs35055552
rs2090816
rs823424
rs8039785
rs6828768
rs62273907
rs10899911
rs78383039
All
−0.3
−0.2
−0.1
0.0
0.1
0.2
MR effect size for
'Gut microbiota abundance (genus Parasutterella id.2892)' on 'Neuroendocrine convertase 1'

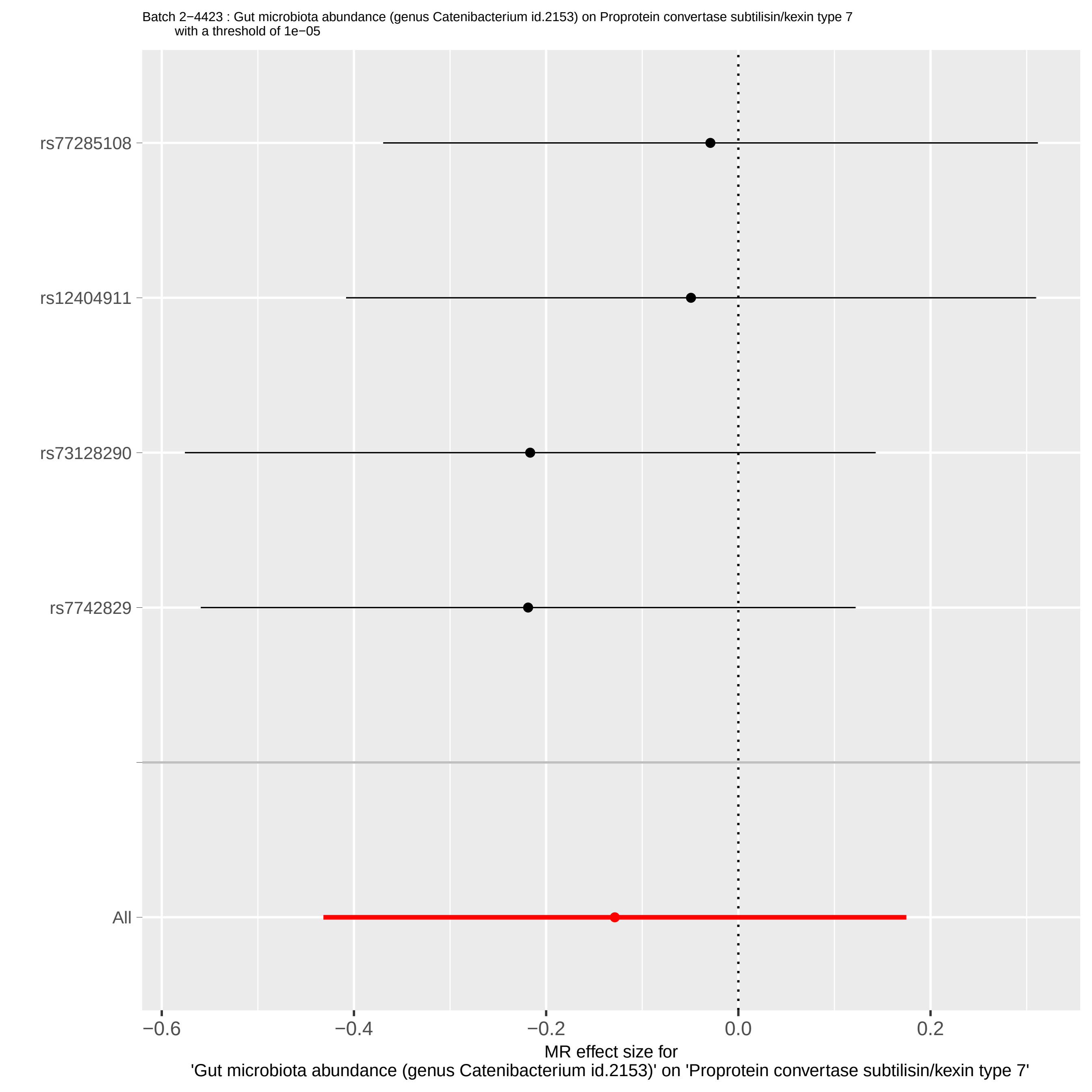
Batch 2−4423 : Gut microbiota abundance (genus Catenibacterium id.2153) on Proprotein convertase subtilisin/kexin type 7
with a threshold of 1e−05
rs77285108
rs12404911
rs73128290
rs7742829
All
−0.6
−0.4
−0.2
0.0
0.2
MR effect size for
'Gut microbiota abundance (genus Catenibacterium id.2153)' on 'Proprotein convertase subtilisin/kexin type 7'

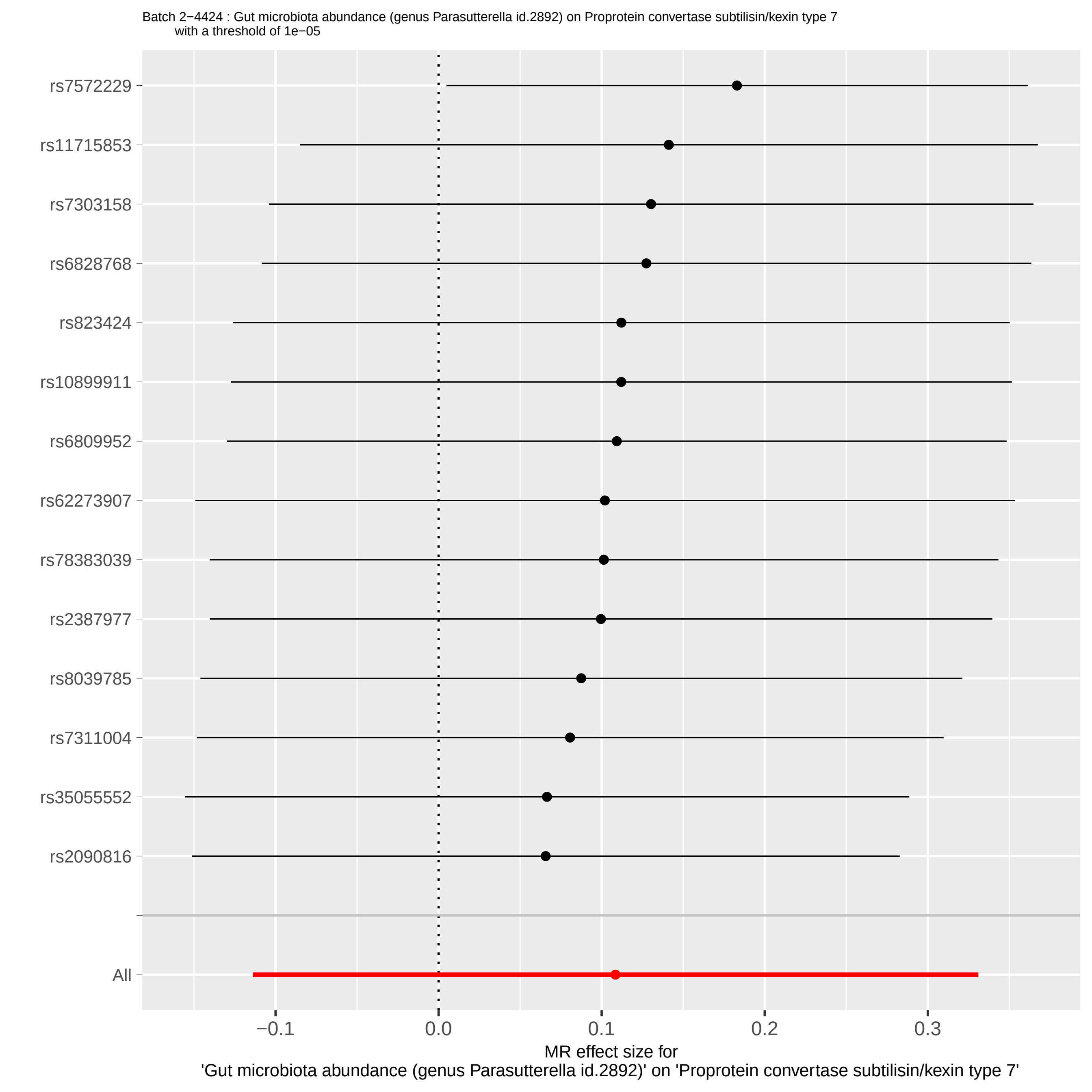
Batch 2−4424 : Gut microbiota abundance (genus Parasutterella id.2892) on Proprotein convertase subtilisin/kexin type 7
with a threshold of 1e−05
rs7572229
rs11715853
rs7303158
rs6828768
rs823424
rs10899911
rs6809952
rs62273907
rs78383039
rs2387977
rs8039785
rs7311004
rs35055552
rs2090816
All
−0.1
0.0
0.1
0.2
0.3
MR effect size for
'Gut microbiota abundance (genus Parasutterella id.2892)' on 'Proprotein convertase subtilisin/kexin type 7'

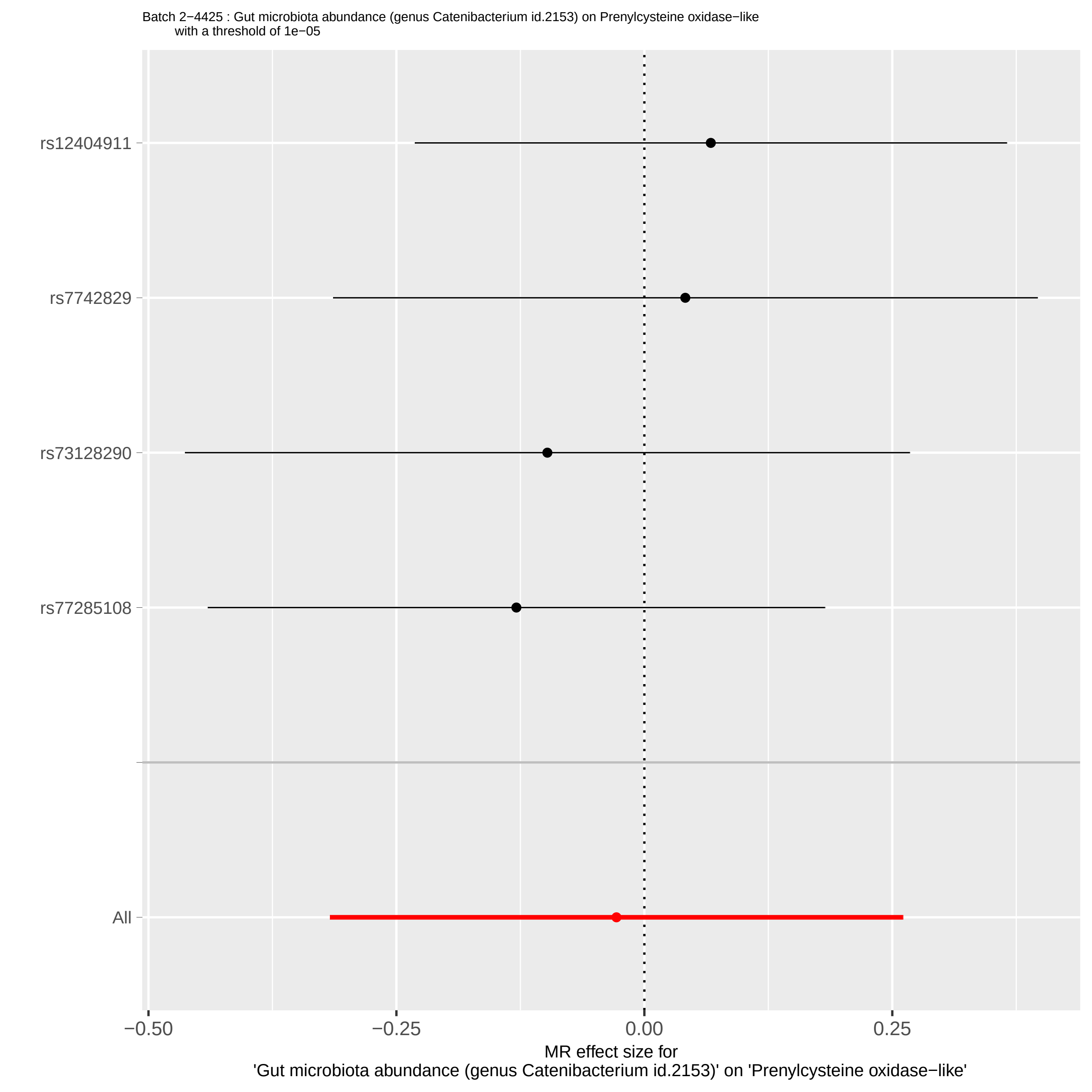

Batch 2−4425 : Gut microbiota abundance (genus Catenibacterium id.2153) on Prenylcysteine oxidase−like
with a threshold of 1e−05
rs12404911
rs7742829
rs73128290
rs77285108
All
−0.50
−0.25
0.00
0.25
MR effect size for
'Gut microbiota abundance (genus Catenibacterium id.2153)' on 'Prenylcysteine oxidase−like'

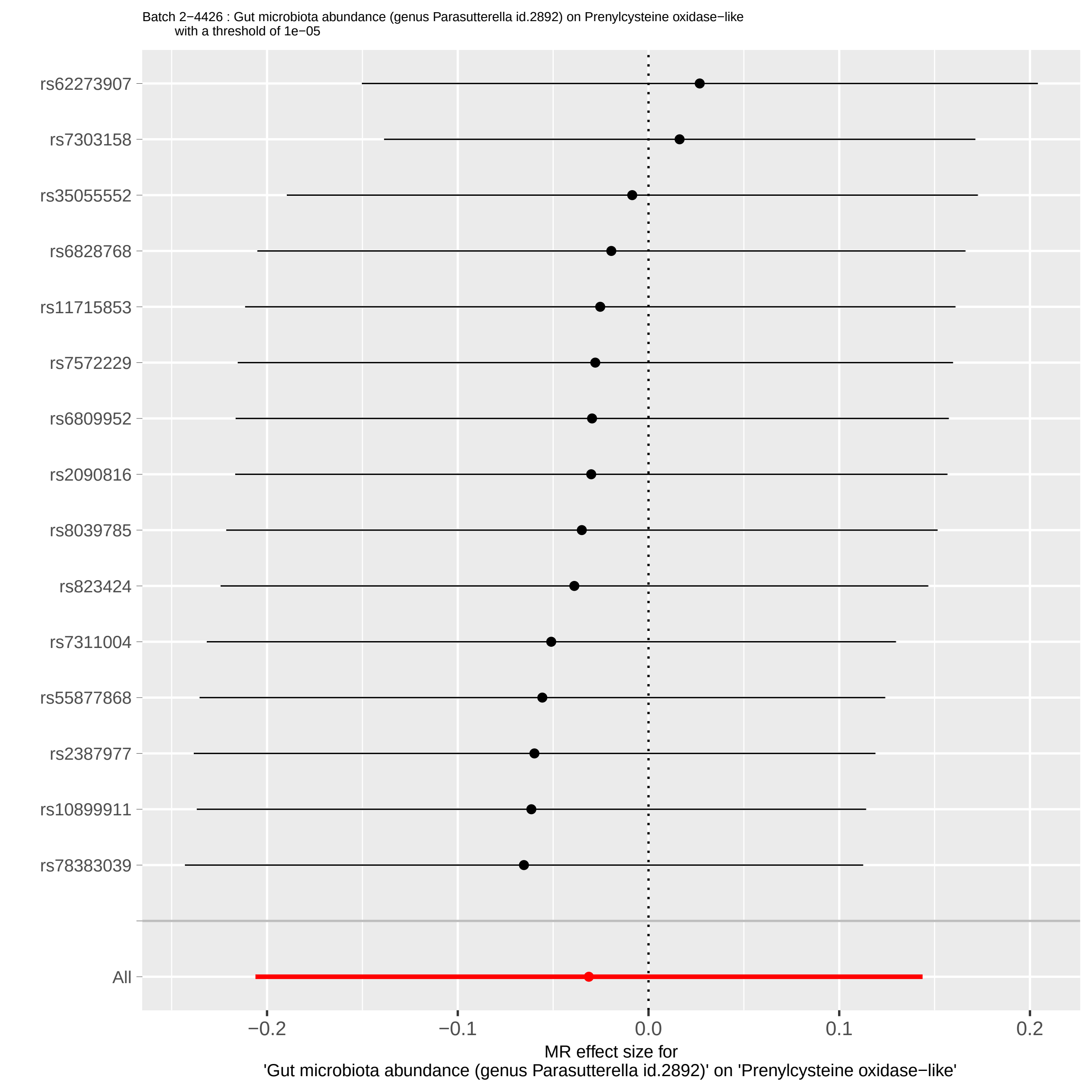
Batch 2−4426 : Gut microbiota abundance (genus Parasutterella id.2892) on Prenylcysteine oxidase−like
with a threshold of 1e−05
rs62273907
rs7303158
rs35055552
rs6828768
rs11715853
rs7572229
rs6809952
rs2090816
rs8039785
rs823424
rs7311004
rs55877868
rs2387977
rs10899911
rs78383039
All
−0.2
−0.1
0.0
0.1
0.2
MR effect size for
'Gut microbiota abundance (genus Parasutterella id.2892)' on 'Prenylcysteine oxidase−like'

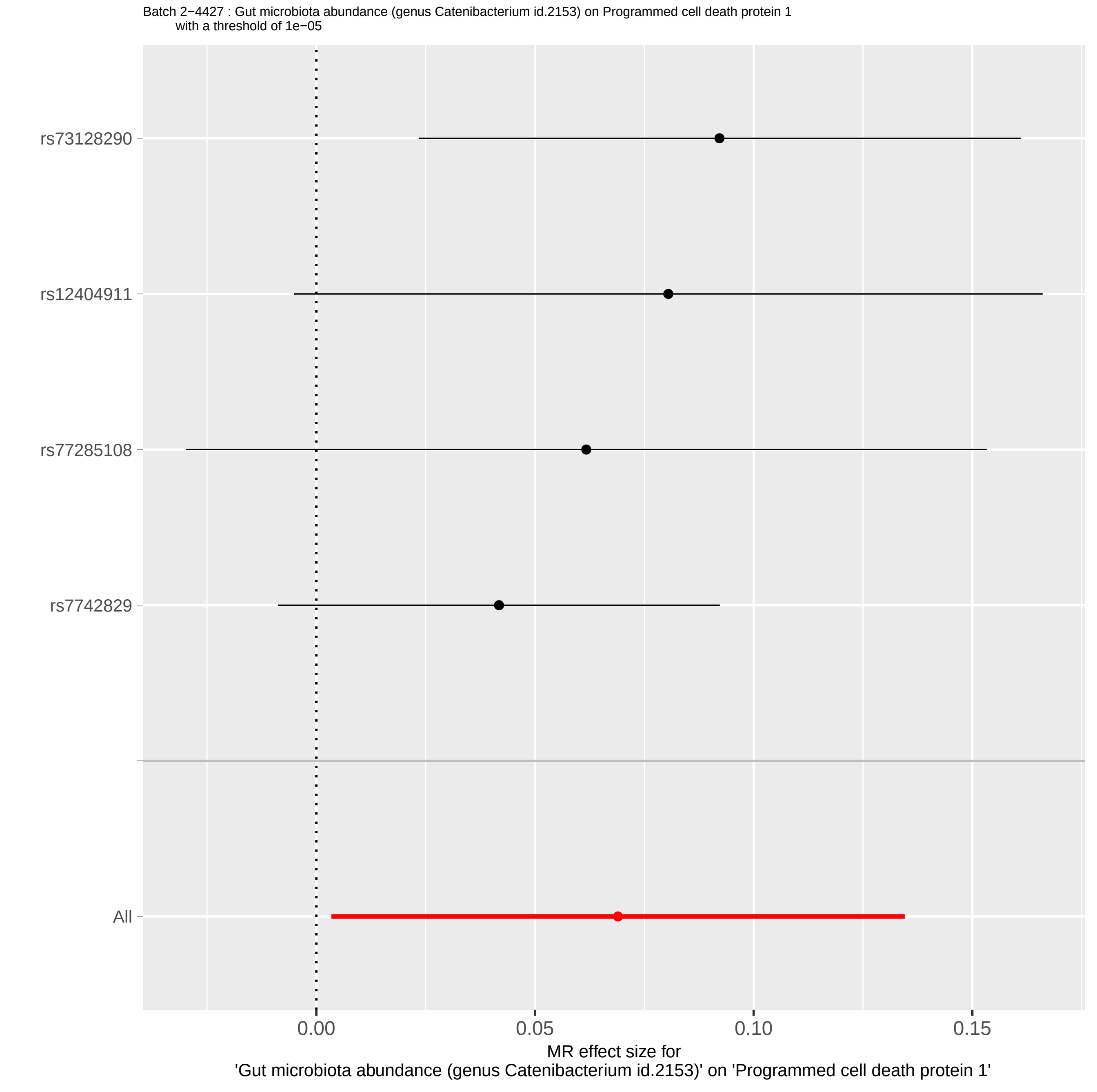
Batch 2−4427 : Gut microbiota abundance (genus Catenibacterium id.2153) on Programmed cell death protein 1
with a threshold of 1e−05
rs73128290
rs12404911
rs77285108
rs7742829
All
0.00
0.05
0.10
0.15
MR effect size for
'Gut microbiota abundance (genus Catenibacterium id.2153)' on 'Programmed cell death protein 1'

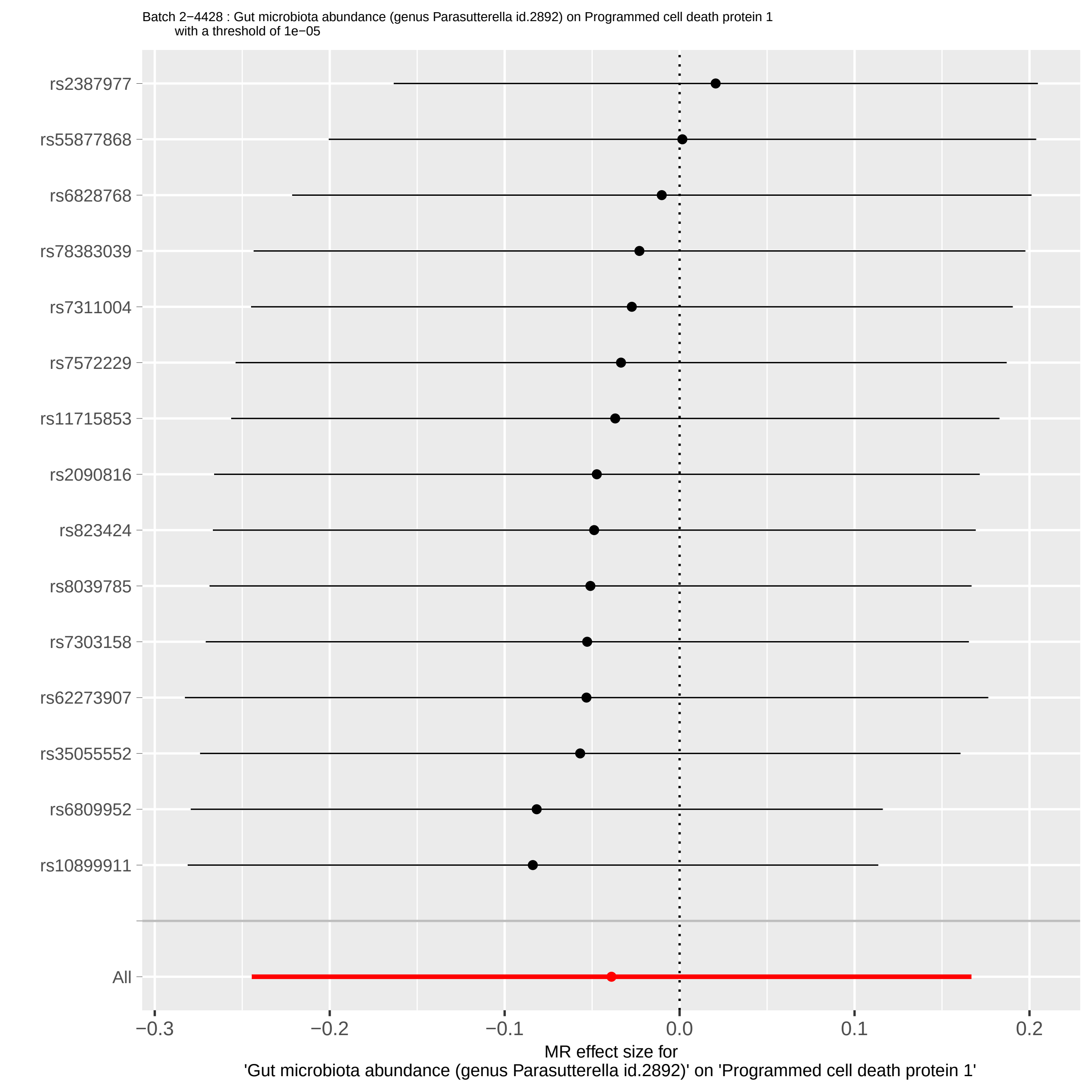

Batch 2−4428 : Gut microbiota abundance (genus Parasutterella id.2892) on Programmed cell death protein 1
with a threshold of 1e−05
rs2387977
rs55877868
rs6828768
rs78383039
rs7311004
rs7572229
rs11715853
rs2090816
rs823424
rs8039785
rs7303158
rs62273907
rs35055552
rs6809952
rs10899911
All
−0.3
−0.2
−0.1
0.0
0.1
0.2
MR effect size for
'Gut microbiota abundance (genus Parasutterella id.2892)' on 'Programmed cell death protein 1'

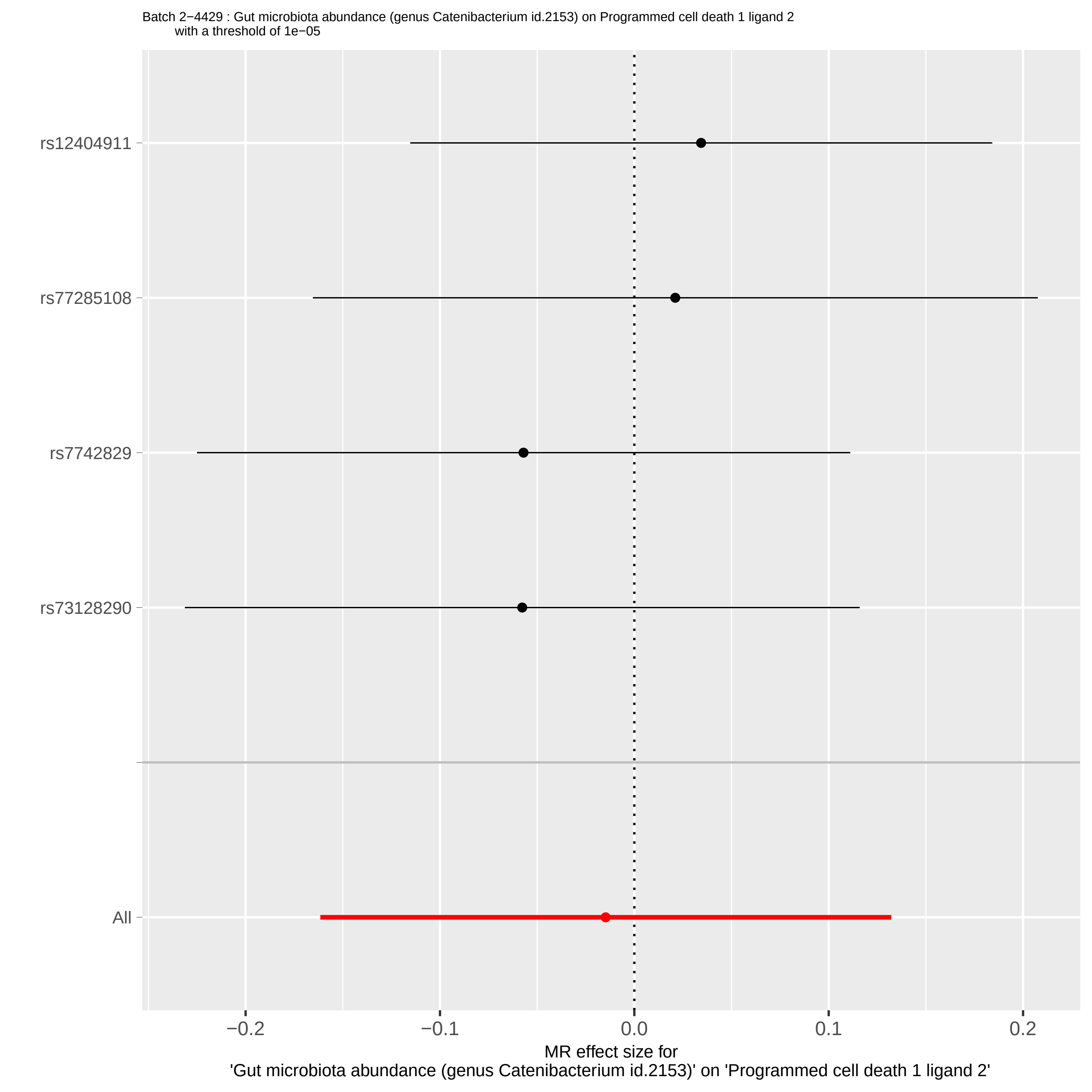
Batch 2−4429 : Gut microbiota abundance (genus Catenibacterium id.2153) on Programmed cell death 1 ligand 2
with a threshold of 1e−05
rs12404911
rs77285108
rs7742829
rs73128290
All
−0.2
−0.1
0.0
0.1
0.2
MR effect size for
'Gut microbiota abundance (genus Catenibacterium id.2153)' on 'Programmed cell death 1 ligand 2'

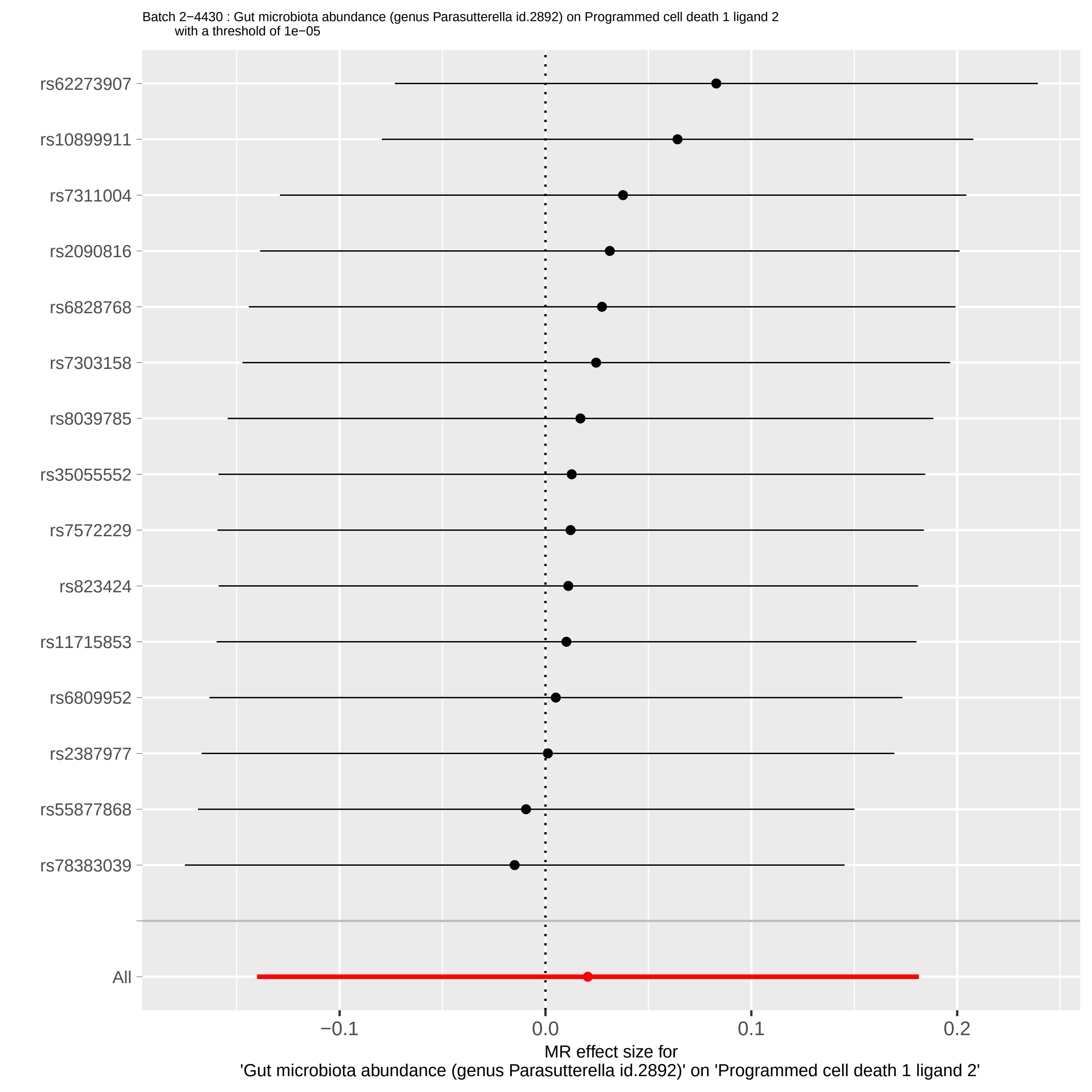
Batch 2−4430 : Gut microbiota abundance (genus Parasutterella id.2892) on Programmed cell death 1 ligand 2
with a threshold of 1e−05
rs62273907
rs10899911
rs7311004
rs2090816
rs6828768
rs7303158
rs8039785
rs35055552
rs7572229
rs823424
rs11715853
rs6809952
rs2387977
rs55877868
rs78383039
All
−0.1
0.0
0.1
0.2
MR effect size for
'Gut microbiota abundance (genus Parasutterella id.2892)' on 'Programmed cell death 1 ligand 2'

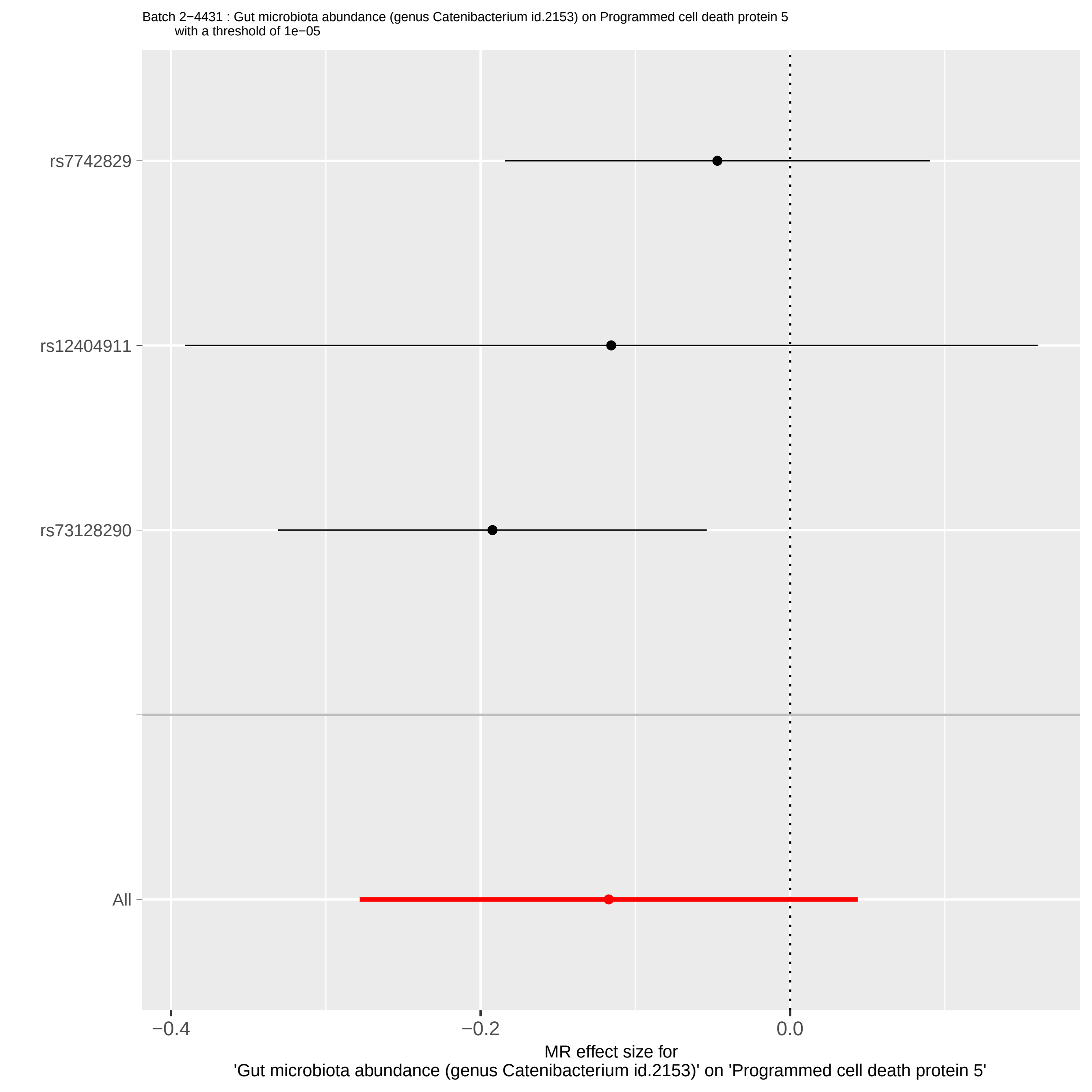

Batch 2−4431 : Gut microbiota abundance (genus Catenibacterium id.2153) on Programmed cell death protein 5
with a threshold of 1e−05
rs7742829
rs12404911
rs73128290
All
−0.4
−0.2
0.0
MR effect size for
'Gut microbiota abundance (genus Catenibacterium id.2153)' on 'Programmed cell death protein 5'

Batch 2−4432 : Gut microbiota abundance (genus Parasutterella id.2892) on Programmed cell death protein 5
with a threshold of 1e−05

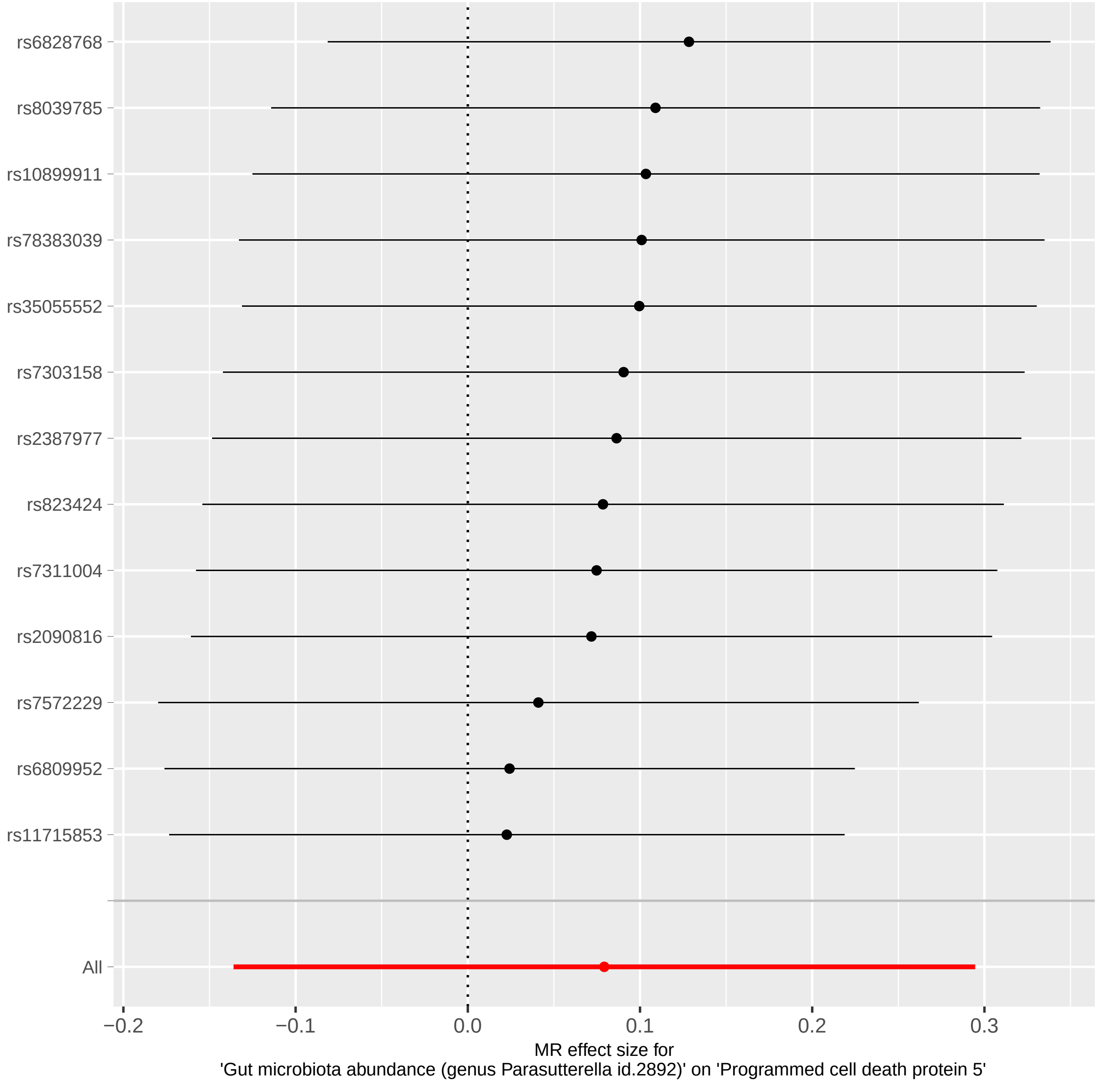

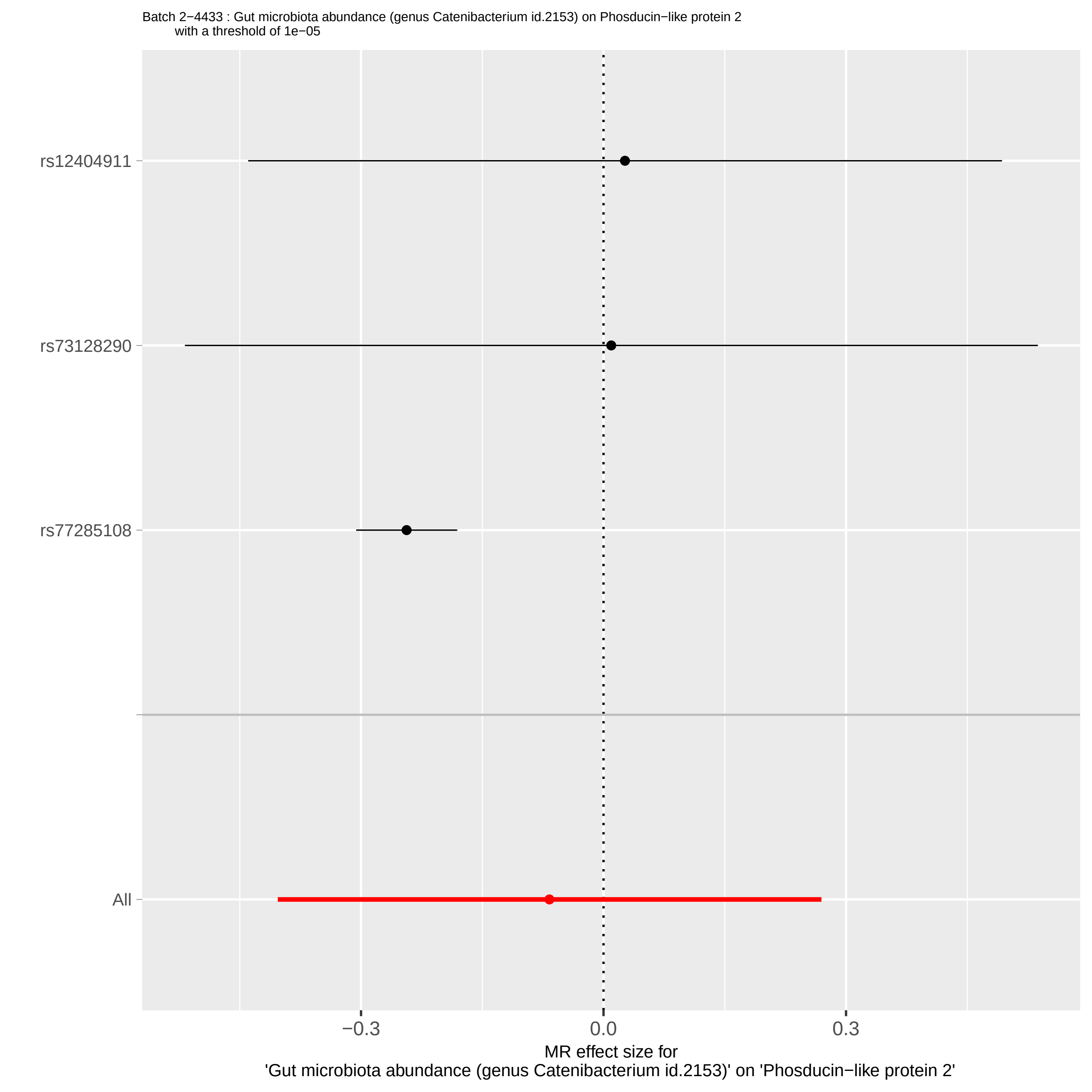
Batch 2−4433 : Gut microbiota abundance (genus Catenibacterium id.2153) on Phosducin−like protein 2
with a threshold of 1e−05
rs12404911
rs73128290
rs77285108
All
−0.3
0.0
0.3
MR effect size for
'Gut microbiota abundance (genus Catenibacterium id.2153)' on 'Phosducin−like protein 2'

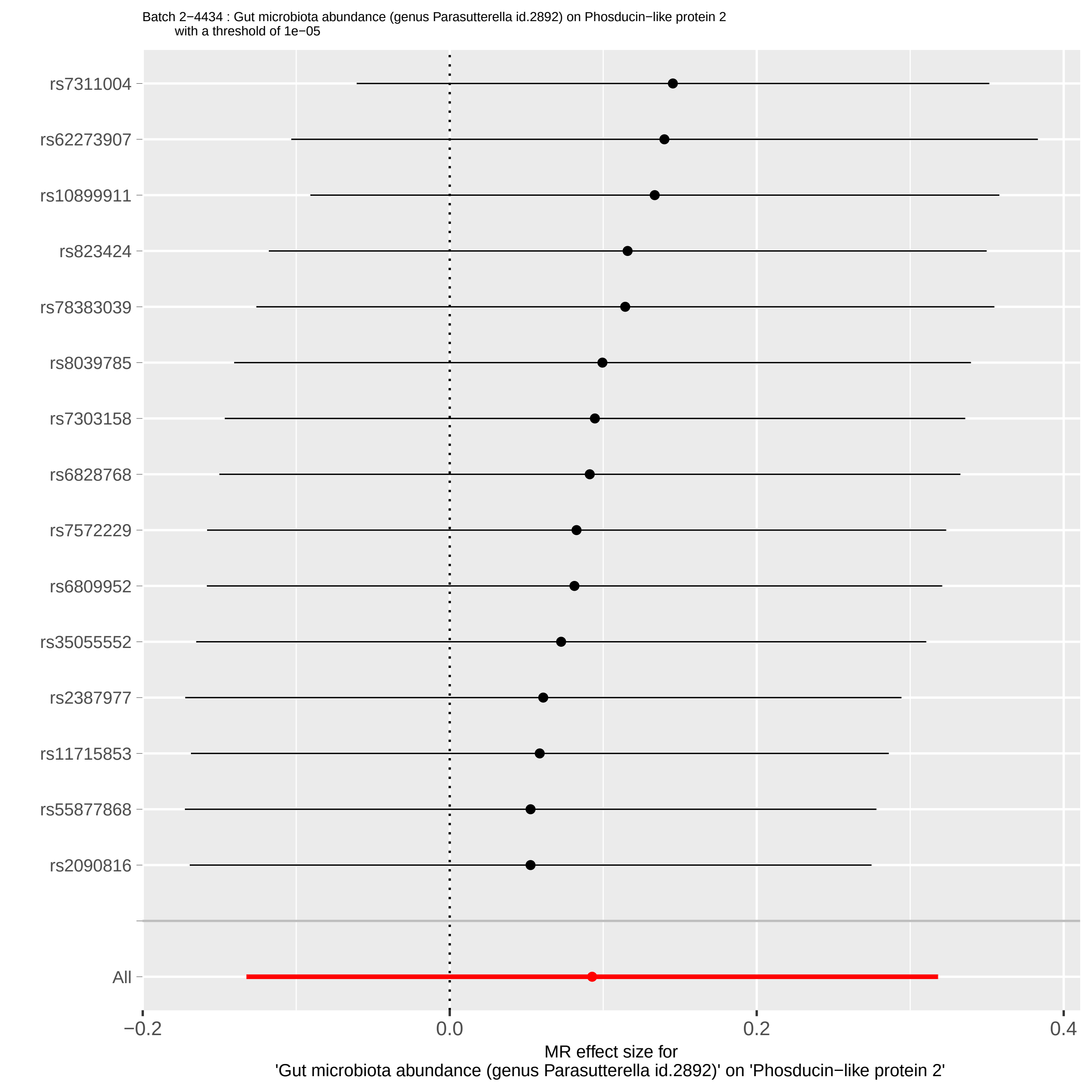

Batch 2−4434 : Gut microbiota abundance (genus Parasutterella id.2892) on Phosducin−like protein 2
with a threshold of 1e−05
rs7311004
rs62273907
rs10899911
rs823424
rs78383039
rs8039785
rs7303158
rs6828768
rs7572229
rs6809952
rs35055552
rs2387977
rs11715853
rs55877868
rs2090816
All
−0.2
0.0
0.2
0.4
MR effect size for
'Gut microbiota abundance (genus Parasutterella id.2892)' on 'Phosducin−like protein 2'

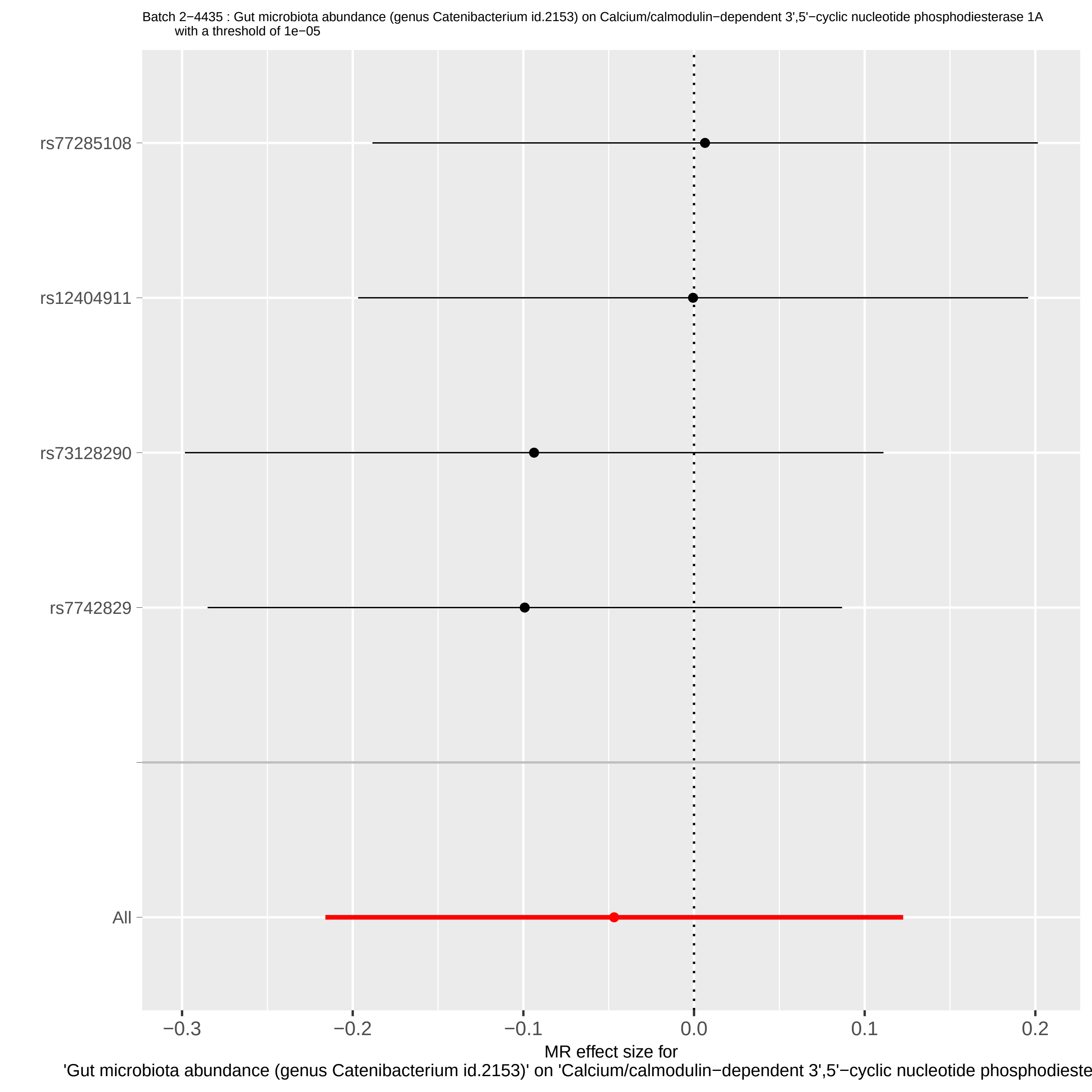

Batch 2−4435 : Gut microbiota abundance (genus Catenibacterium id.2153) on Calcium/calmodulin−dependent 3',5'−cyclic nucleotide phosphodiesterase 1A
with a threshold of 1e−05
rs77285108
rs12404911
rs73128290
rs7742829
All
−0.3
−0.2
−0.1
0.0
0.1
0.2
MR effect size for
'Gut microbiota abundance (genus Catenibacterium id.2153)' on 'Calcium/calmodulin−dependent 3',5'−cyclic nucleotide phosphodieste

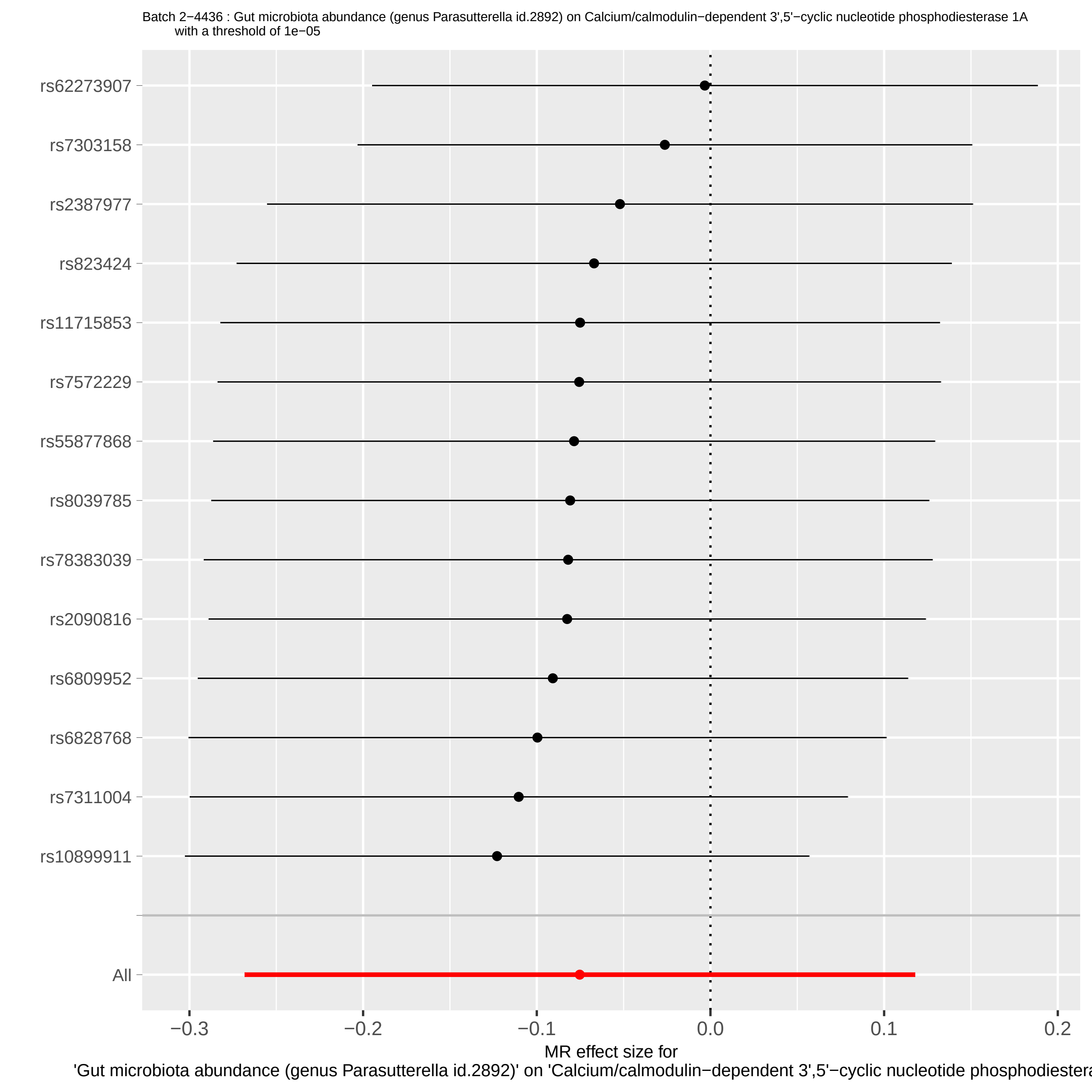
Batch 2−4436 : Gut microbiota abundance (genus Parasutterella id.2892) on Calcium/calmodulin−dependent 3',5'−cyclic nucleotide phosphodiesterase 1A with a threshold of 1e−05
rs62273907
rs7303158
rs2387977
rs823424
rs11715853
rs7572229
rs55877868
rs8039785
rs78383039
rs2090816
rs6809952
rs6828768
rs7311004
rs10899911
All
−0.3
−0.2
−0.1
0.0
0.1
0.2
MR effect size for
'Gut microbiota abundance (genus Parasutterella id.2892)' on 'Calcium/calmodulin−dependent 3',5'−cyclic nucleotide phosphodiestera

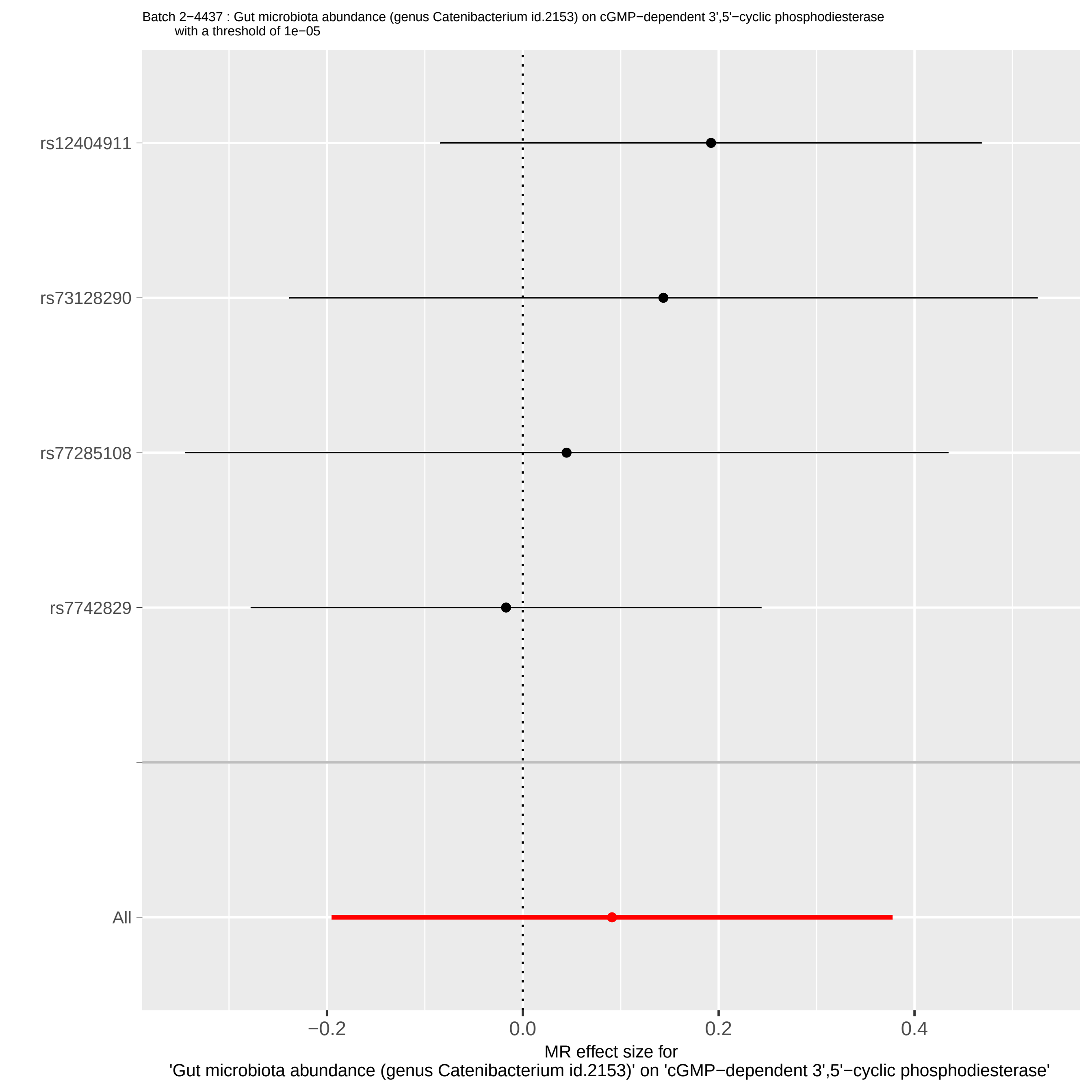

Batch 2−4437 : Gut microbiota abundance (genus Catenibacterium id.2153) on cGMP−dependent 3',5'−cyclic phosphodiesterase
with a threshold of 1e−05
rs12404911
rs73128290
rs77285108
rs7742829
All
−0.2
0.0
0.2
0.4
MR effect size for
'Gut microbiota abundance (genus Catenibacterium id.2153)' on 'cGMP−dependent 3',5'−cyclic phosphodiesterase'

Batch 2−4438 : Gut microbiota abundance (genus Parasutterella id.2892) on cGMP−dependent 3',5'−cyclic phosphodiesterase with a threshold of 1e−05

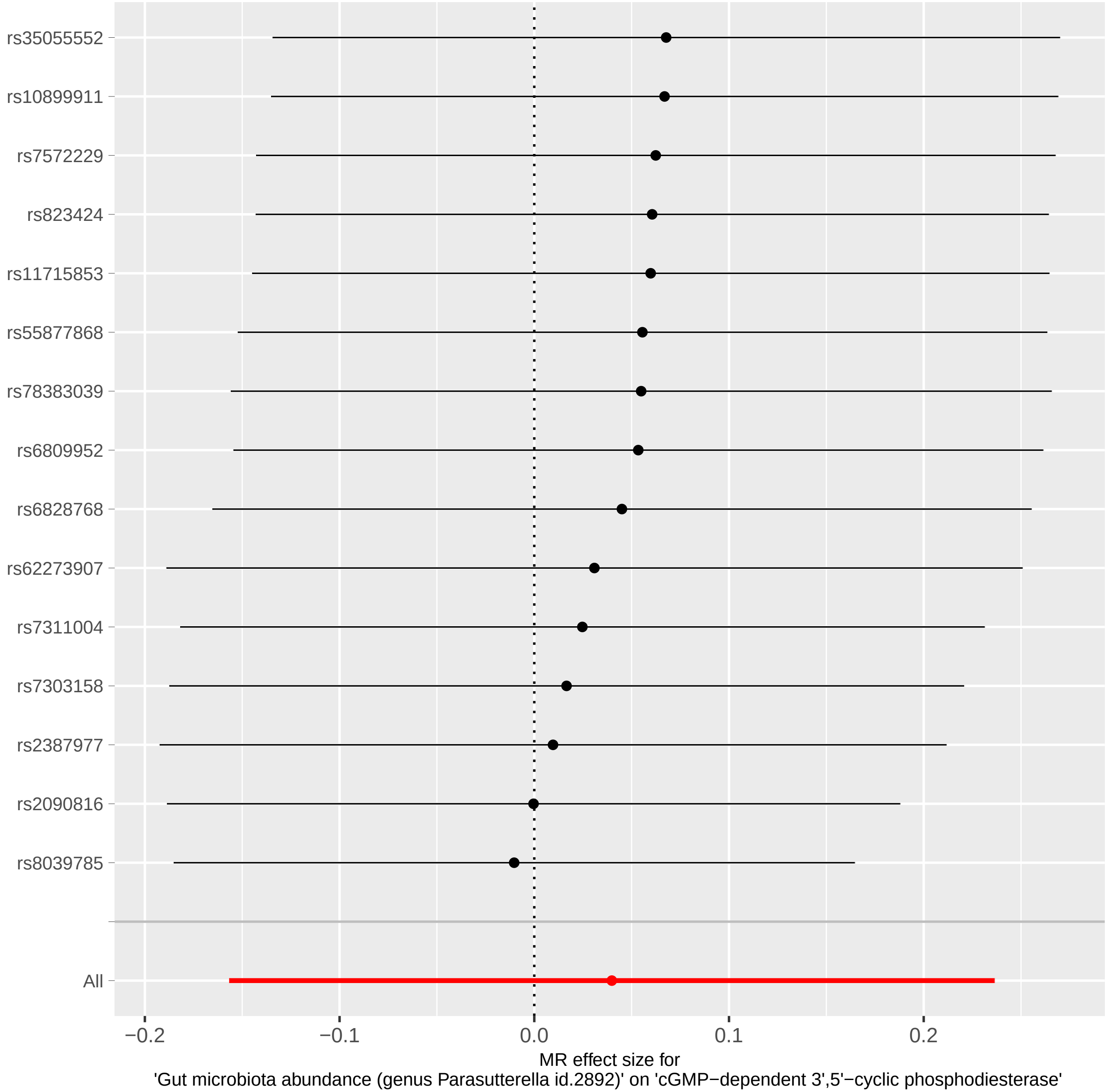

Batch 2−4439 : Gut microbiota abundance (genus Catenibacterium id.2153) on cGMP−inhibited 3',5'−cyclic phosphodiesterase A
with a threshold of 1e−05

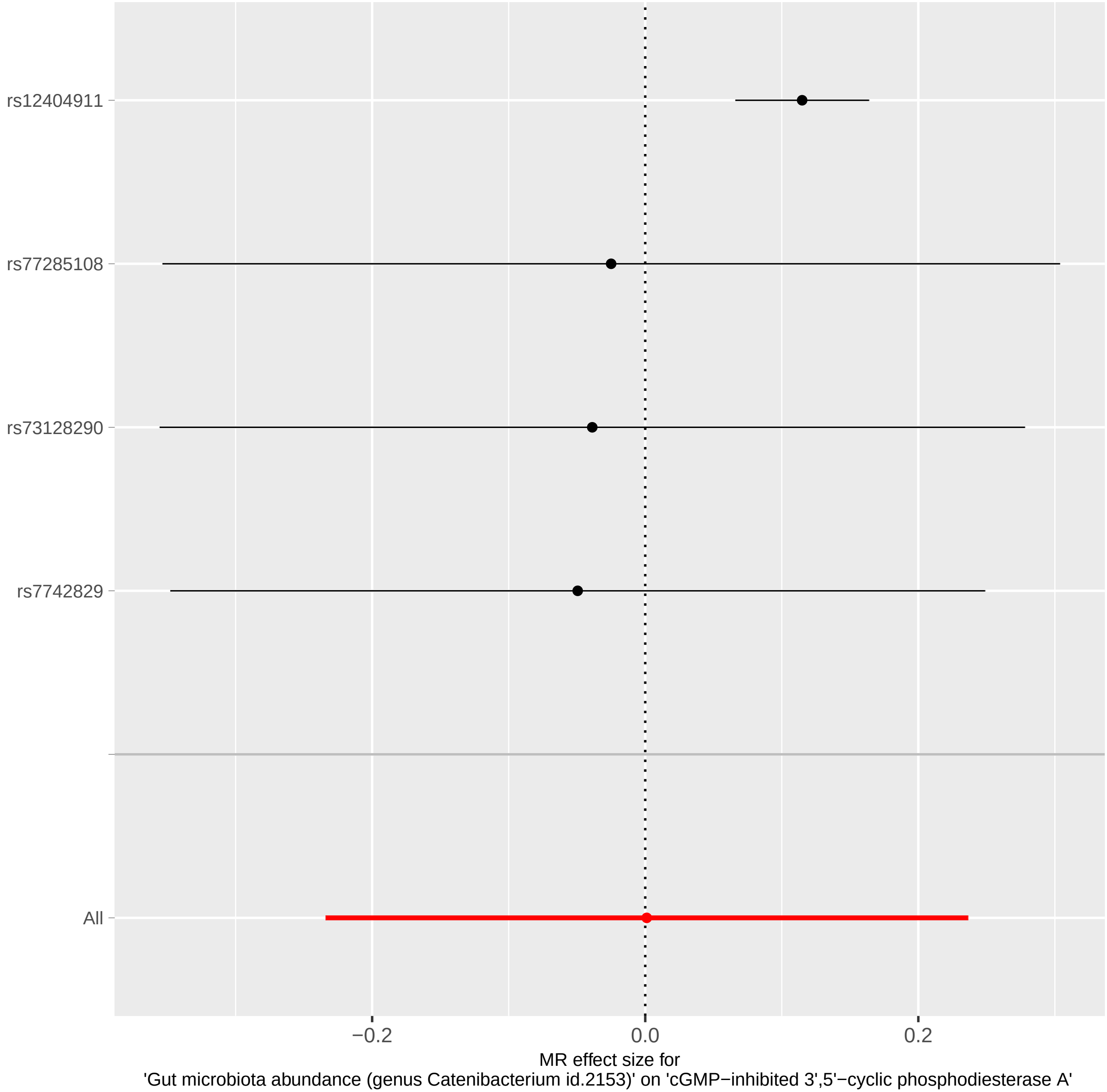

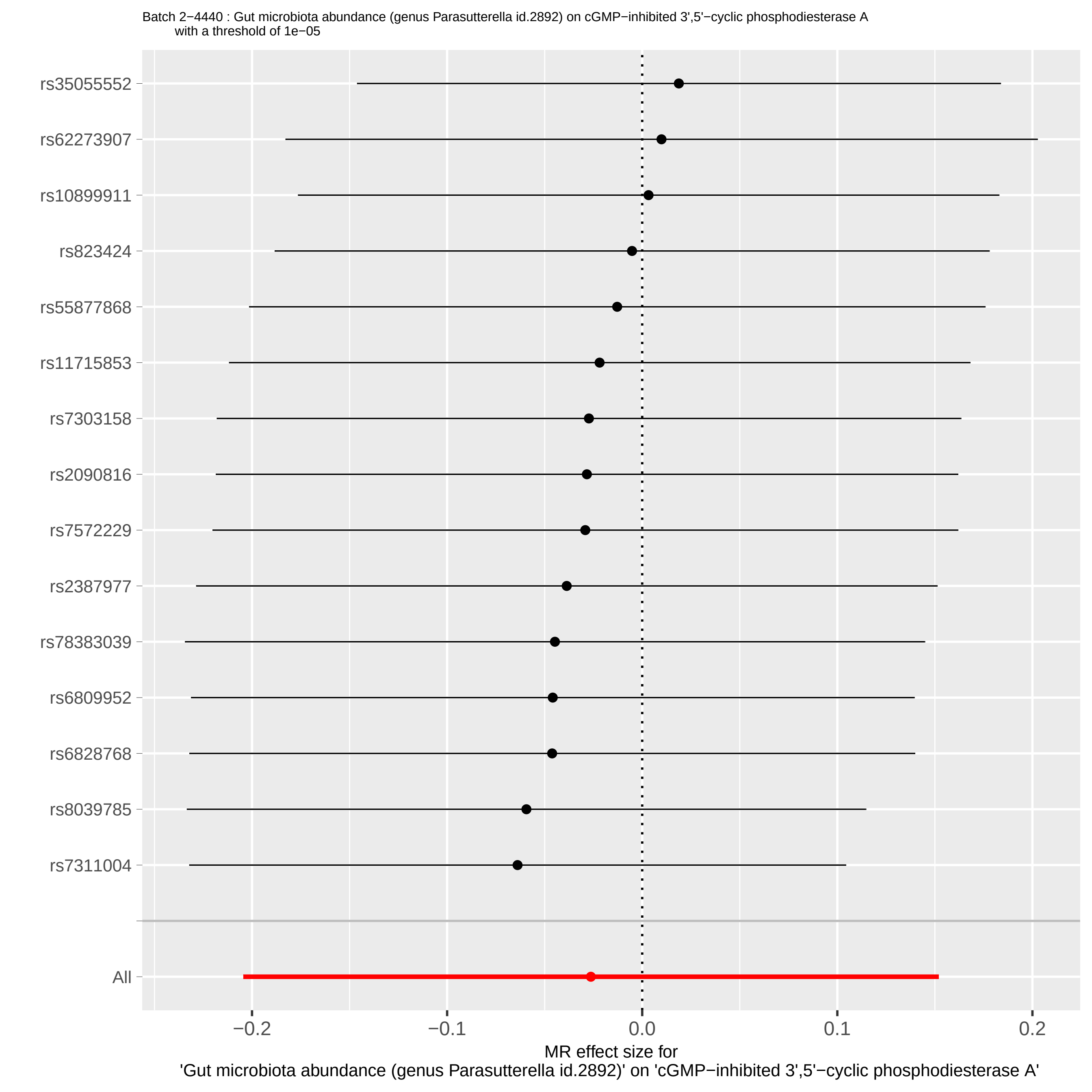

Batch 2−4440 : Gut microbiota abundance (genus Parasutterella id.2892) on cGMP−inhibited 3',5'−cyclic phosphodiesterase A
with a threshold of 1e−05
rs35055552
rs62273907
rs10899911
rs823424
rs55877868
rs11715853
rs7303158
rs2090816
rs7572229
rs2387977
rs78383039
rs6809952
rs6828768
rs8039785
rs7311004
All
−0.2
−0.1
0.0
0.1
0.2
MR effect size for
'Gut microbiota abundance (genus Parasutterella id.2892)' on 'cGMP−inhibited 3',5'−cyclic phosphodiesterase A'

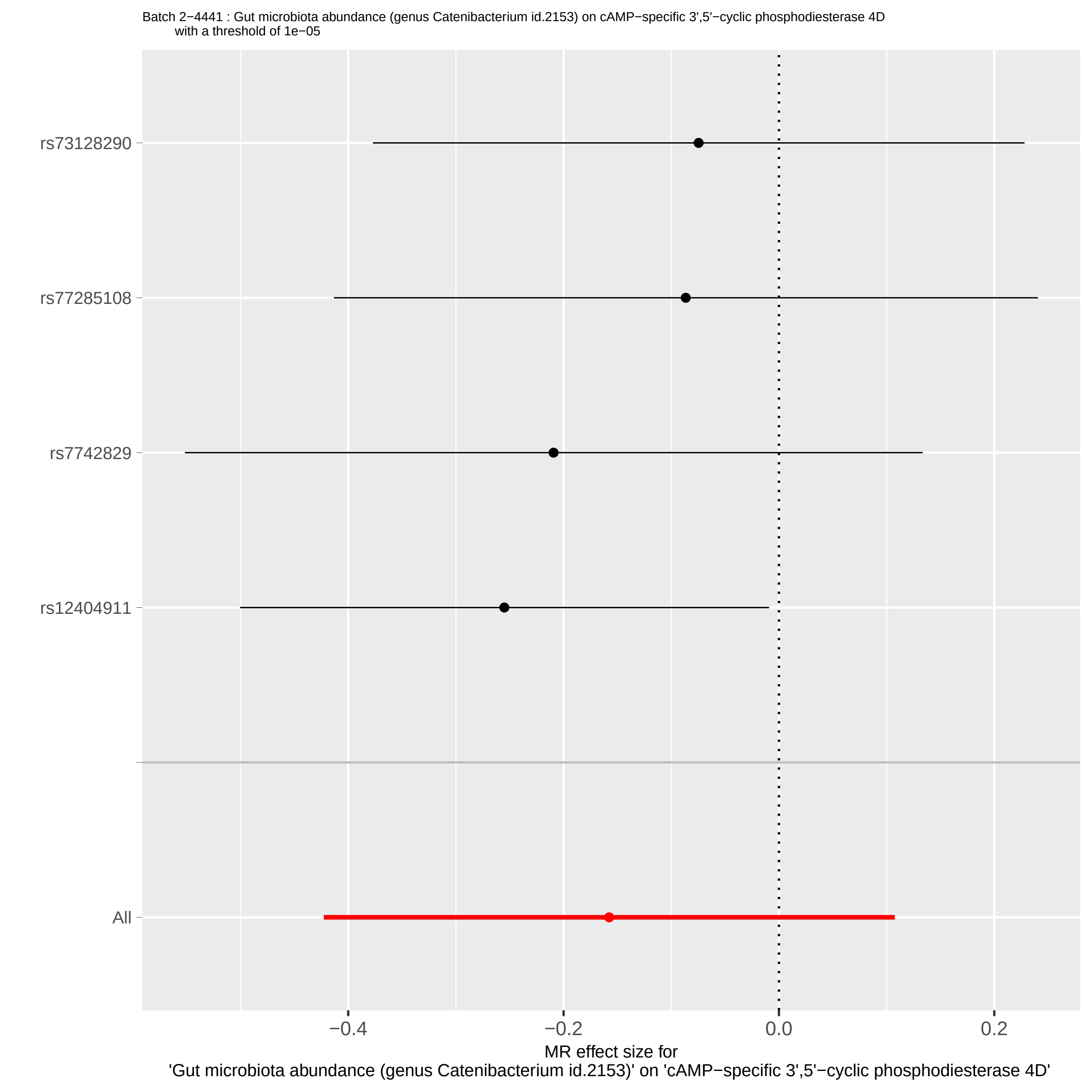
Batch 2−4441 : Gut microbiota abundance (genus Catenibacterium id.2153) on cAMP−specific 3',5'−cyclic phosphodiesterase 4D
with a threshold of 1e−05
rs73128290
rs77285108
rs7742829
rs12404911
All
−0.4
−0.2
0.0
0.2
MR effect size for
'Gut microbiota abundance (genus Catenibacterium id.2153)' on 'cAMP−specific 3',5'−cyclic phosphodiesterase 4D'

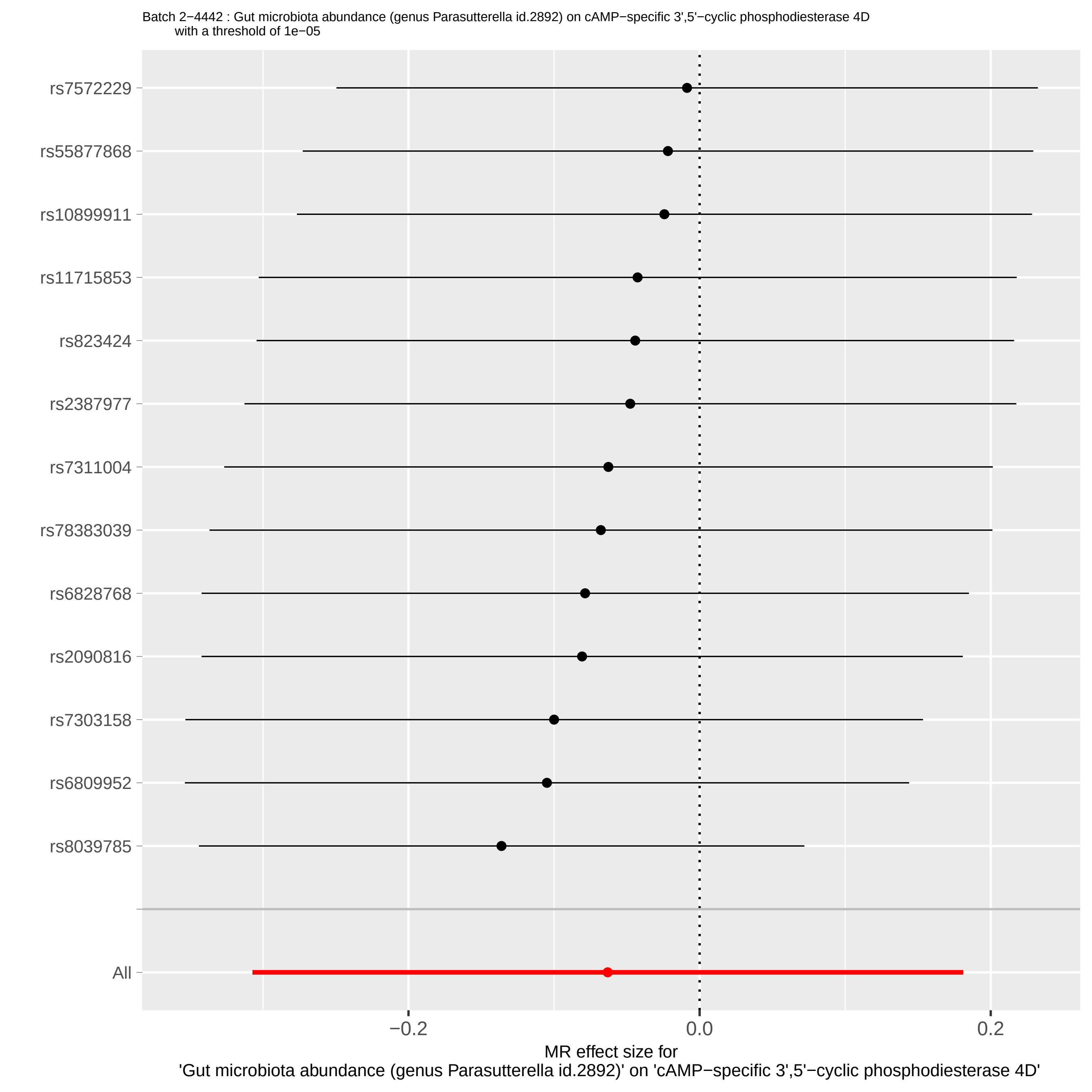
Batch 2−4442 : Gut microbiota abundance (genus Parasutterella id.2892) on cAMP−specific 3',5'−cyclic phosphodiesterase 4D
with a threshold of 1e−05
rs7572229
rs55877868
rs10899911
rs11715853
rs823424
rs2387977
rs7311004
rs78383039
rs6828768
rs2090816
rs7303158
rs6809952
rs8039785
All
−0.2
0.0
0.2
MR effect size for
'Gut microbiota abundance (genus Parasutterella id.2892)' on 'cAMP−specific 3',5'−cyclic phosphodiesterase 4D'

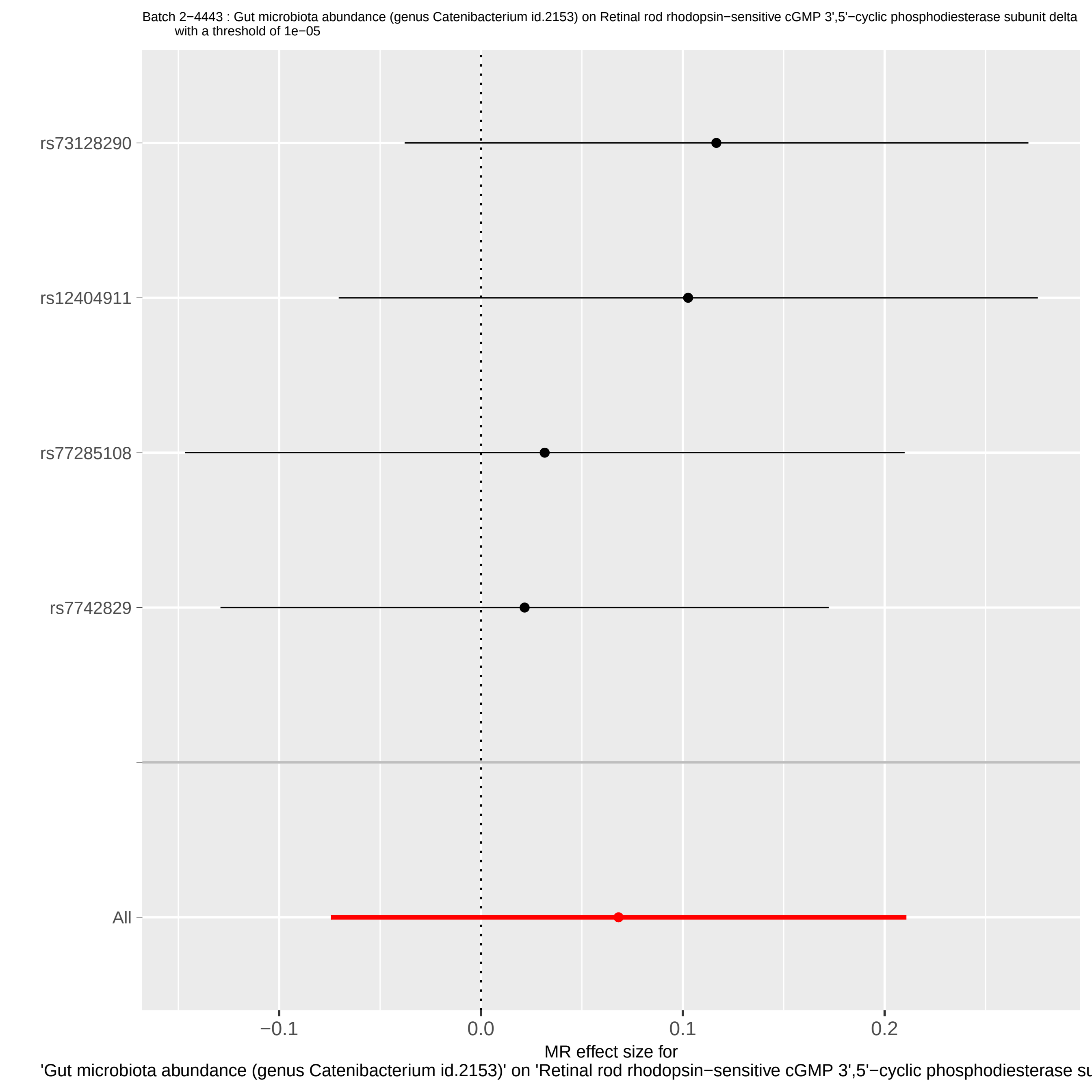
Batch 2−4443 : Gut microbiota abundance (genus Catenibacterium id.2153) on Retinal rod rhodopsin−sensitive cGMP 3',5'−cyclic phosphodiesterase subunit delta with a threshold of 1e−05
rs73128290
rs12404911
rs77285108
rs7742829
All
−0.1
0.0
0.1
0.2
MR effect size for
'Gut microbiota abundance (genus Catenibacterium id.2153)' on 'Retinal rod rhodopsin−sensitive cGMP 3',5'−cyclic phosphodiesterase su

Batch 2−4444 : Gut microbiota abundance (genus Parasutterella id.2892) on Retinal rod rhodopsin−sensitive cGMP 3',5'−cyclic phosphodiesterase subunit delta with a threshold of 1e−05

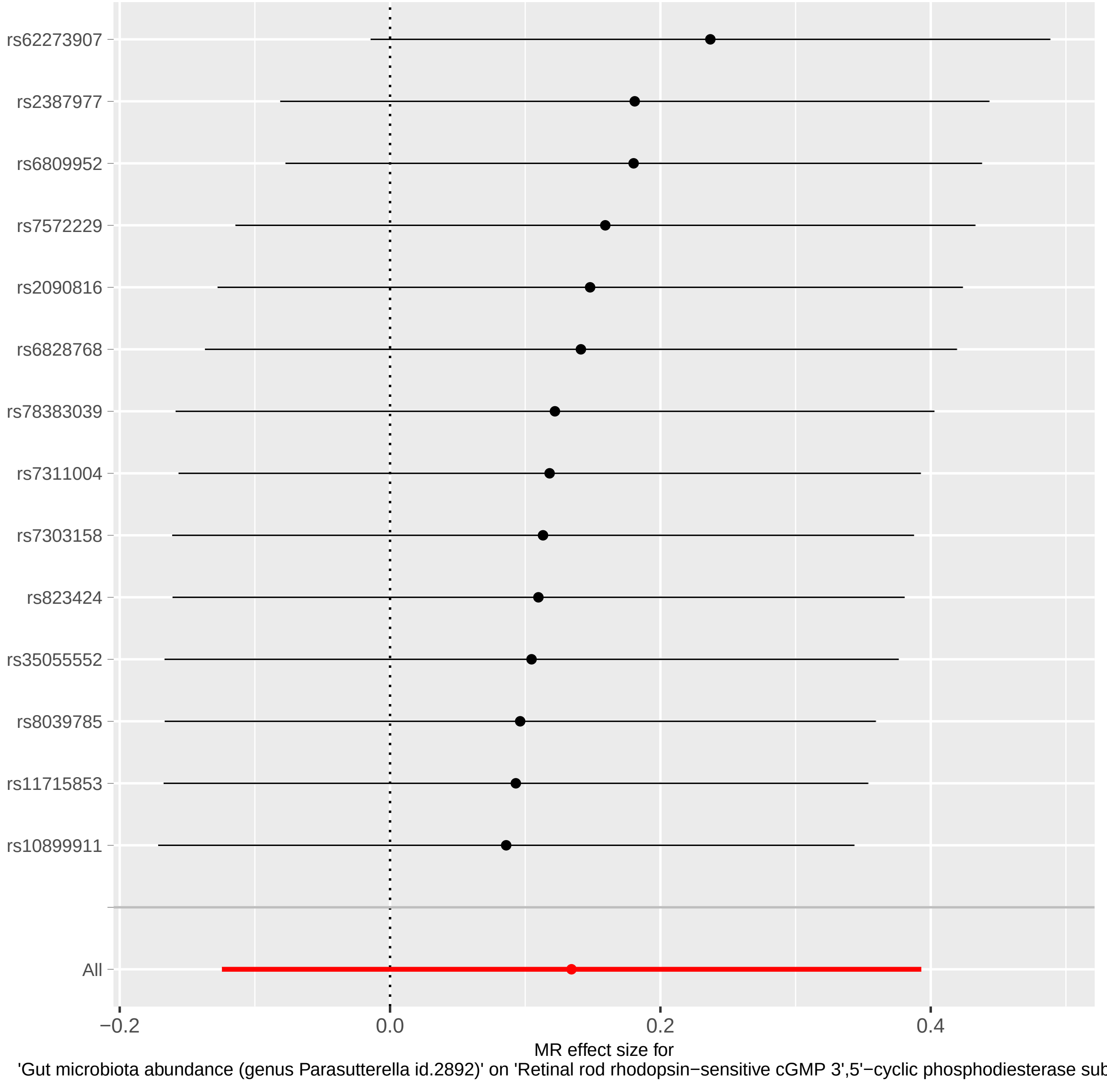

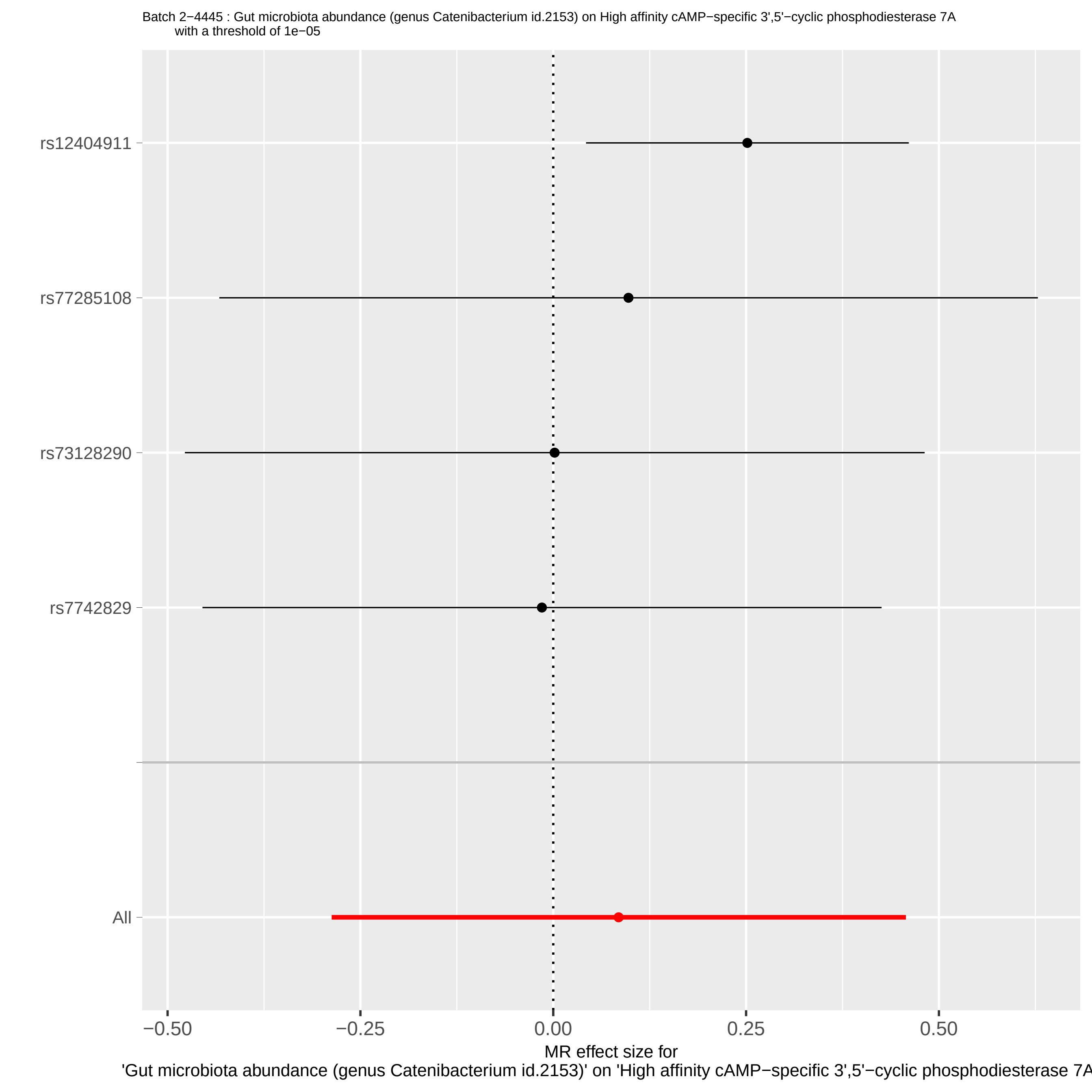
Batch 2−4445 : Gut microbiota abundance (genus Catenibacterium id.2153) on High affinity cAMP−specific 3',5'−cyclic phosphodiesterase 7A
with a threshold of 1e−05
rs12404911
rs77285108
rs73128290
rs7742829
All
−0.50
−0.25
0.00
0.25
0.50
MR effect size for
'Gut microbiota abundance (genus Catenibacterium id.2153)' on 'High affinity cAMP−specific 3',5'−cyclic phosphodiesterase 7A

Batch 2−4446 : Gut microbiota abundance (genus Parasutterella id.2892) on High affinity cAMP−specific 3',5'−cyclic phosphodiesterase 7A
with a threshold of 1e−05

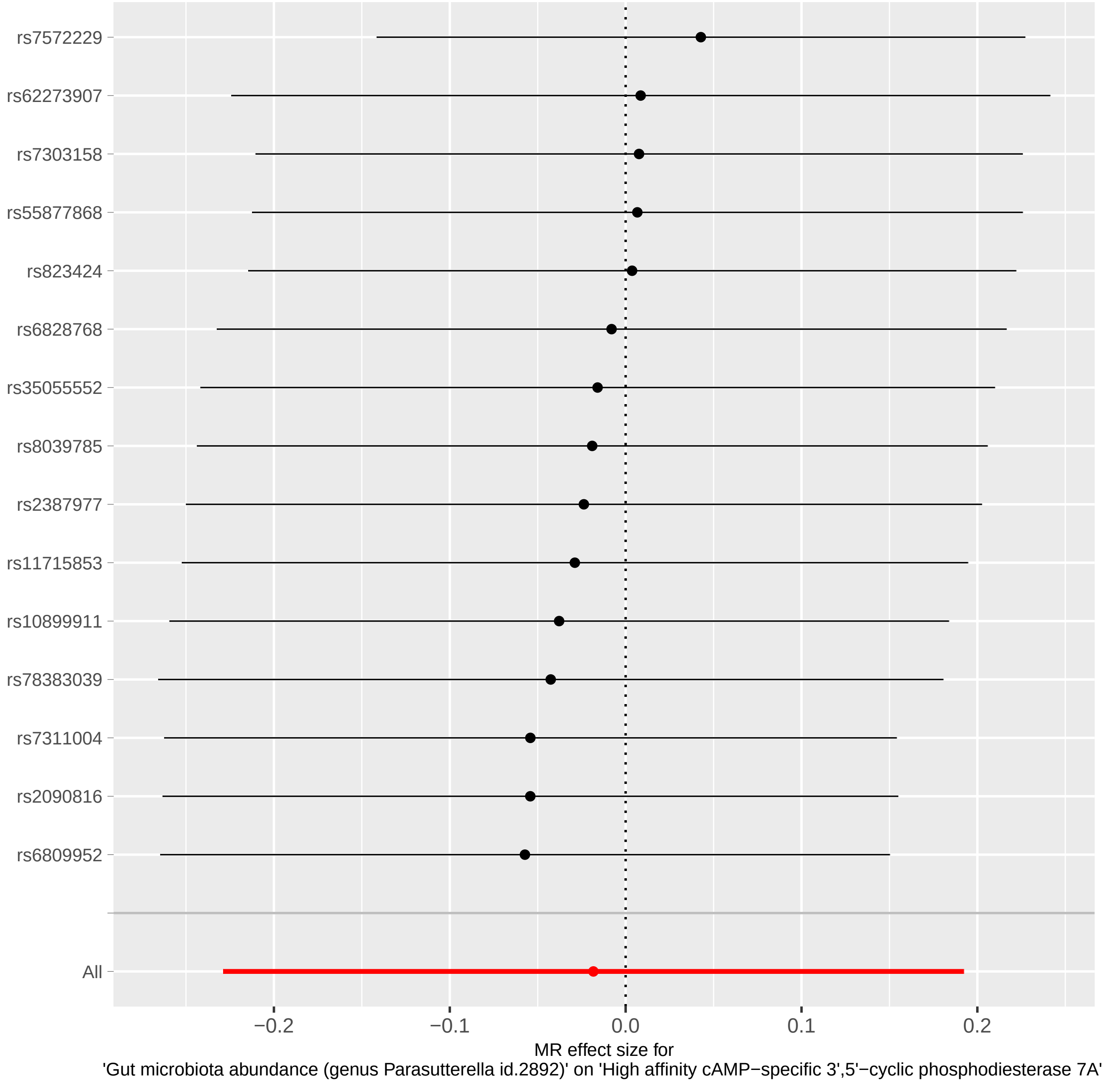

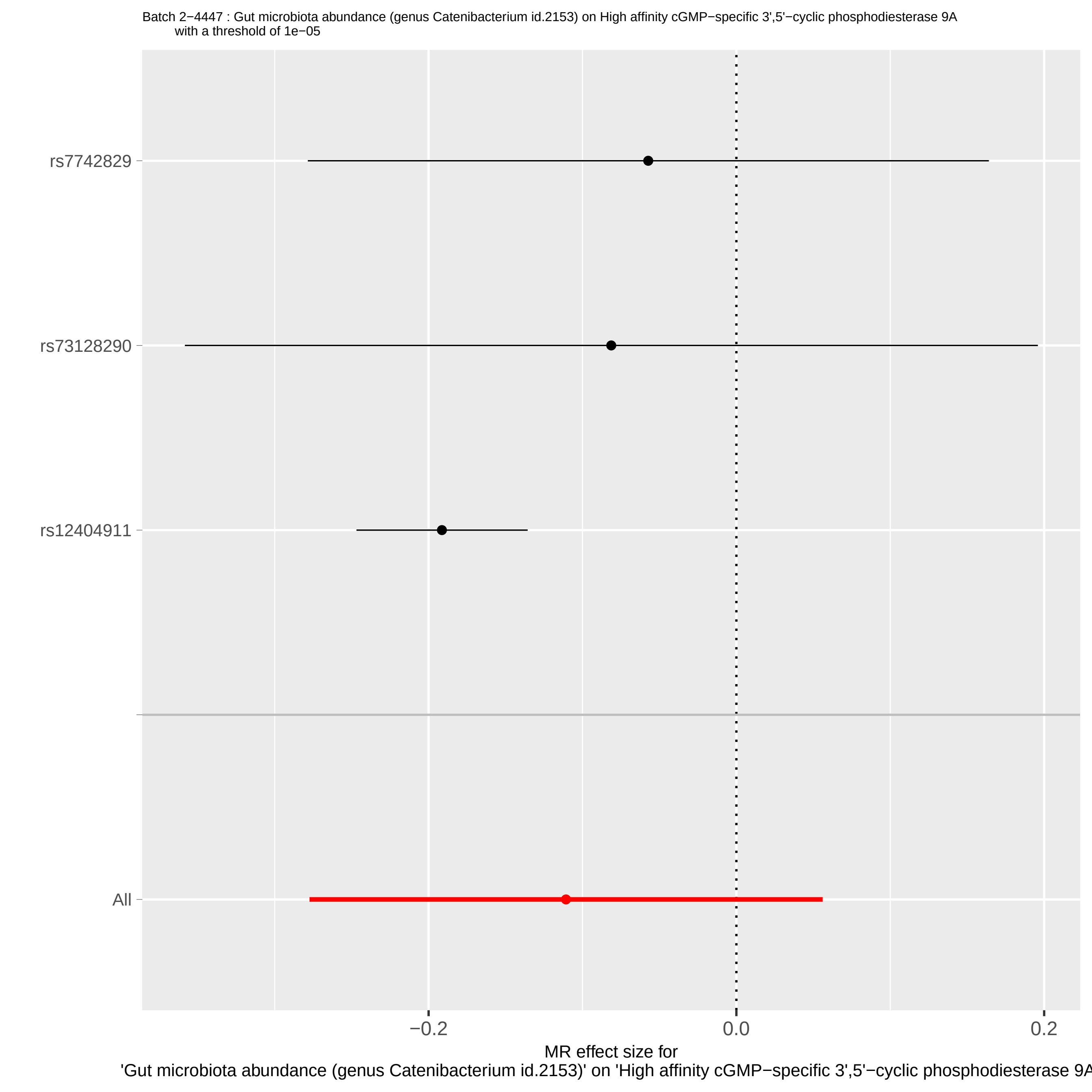
Batch 2−4447 : Gut microbiota abundance (genus Catenibacterium id.2153) on High affinity cGMP−specific 3',5'−cyclic phosphodiesterase 9A
with a threshold of 1e−05
rs7742829
rs73128290
rs12404911
All
−0.2
0.0
0.2
MR effect size for
'Gut microbiota abundance (genus Catenibacterium id.2153)' on 'High affinity cGMP−specific 3',5'−cyclic phosphodiesterase 9A

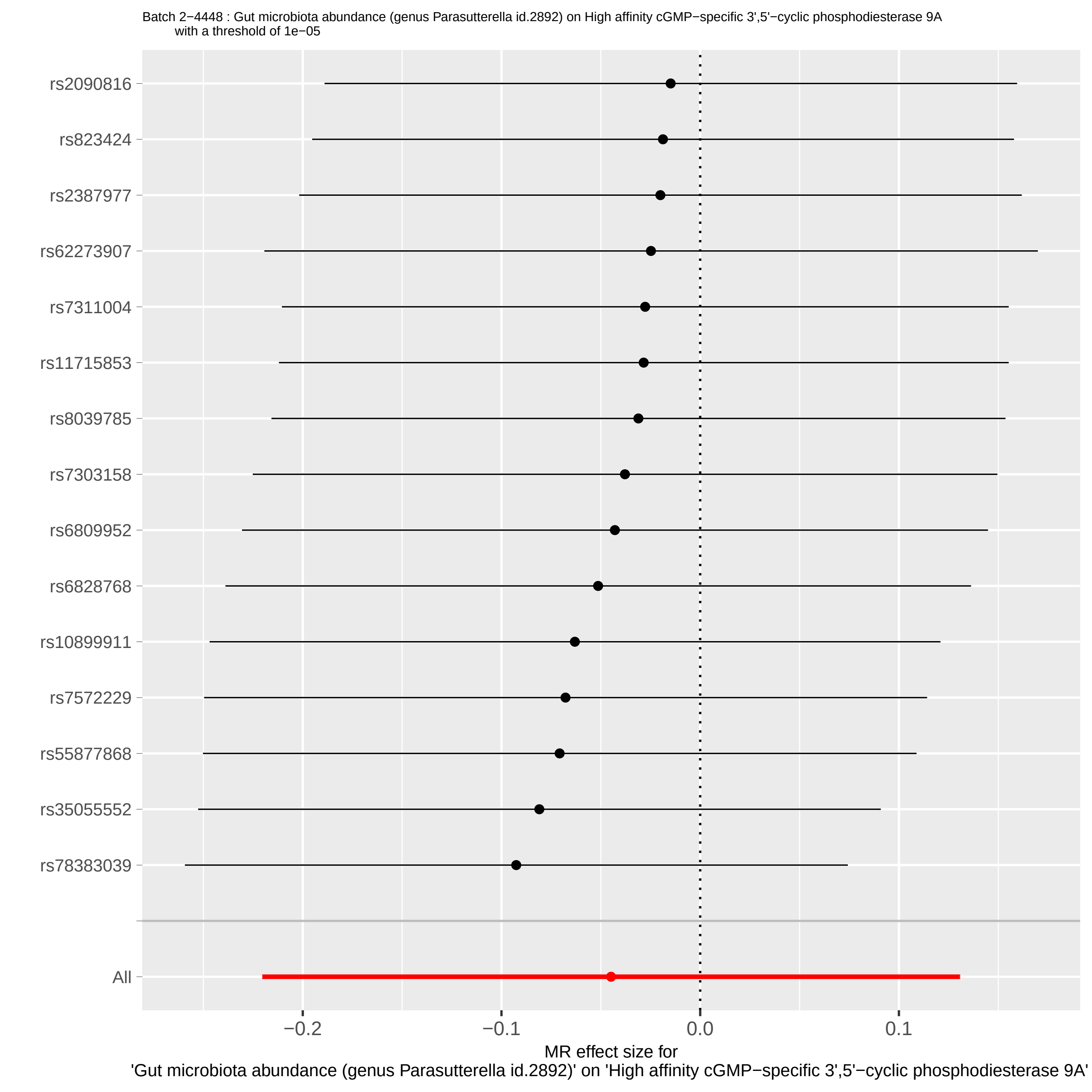
Batch 2−4448 : Gut microbiota abundance (genus Parasutterella id.2892) on High affinity cGMP−specific 3',5'−cyclic phosphodiesterase 9A
with a threshold of 1e−05
rs2090816
rs823424
rs2387977
rs62273907
rs7311004
rs11715853
rs8039785
rs7303158
rs6809952
rs6828768
rs10899911
rs7572229
rs55877868
rs35055552
rs78383039
All
−0.2
−0.1
0.0
0.1
MR effect size for
'Gut microbiota abundance (genus Parasutterella id.2892)' on 'High affinity cGMP−specific 3',5'−cyclic phosphodiesterase 9A'

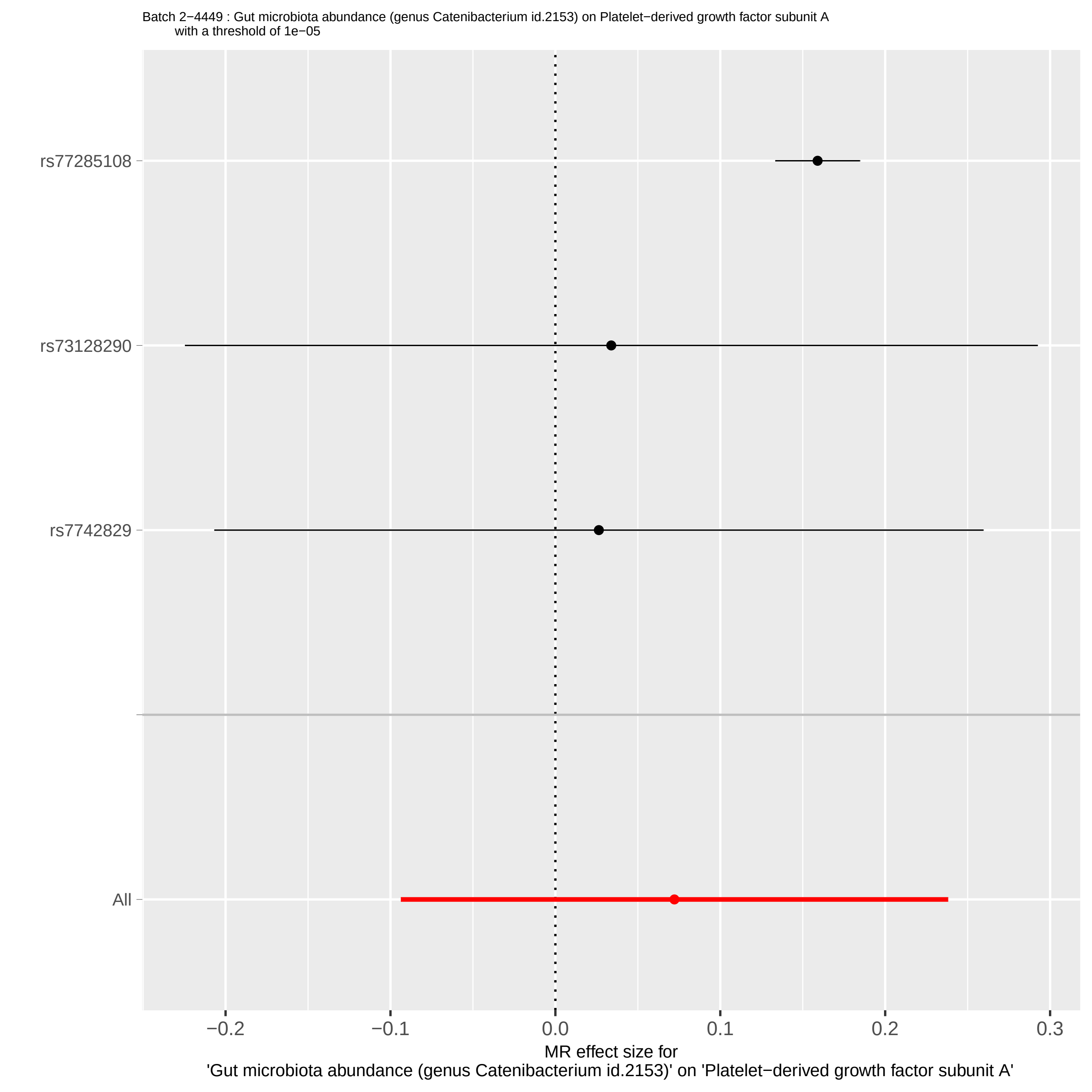
Batch 2−4449 : Gut microbiota abundance (genus Catenibacterium id.2153) on Platelet−derived growth factor subunit A
with a threshold of 1e−05
rs77285108
rs73128290
rs7742829
All
−0.2
−0.1
0.0
0.1
0.2
0.3
MR effect size for
'Gut microbiota abundance (genus Catenibacterium id.2153)' on 'Platelet−derived growth factor subunit A'

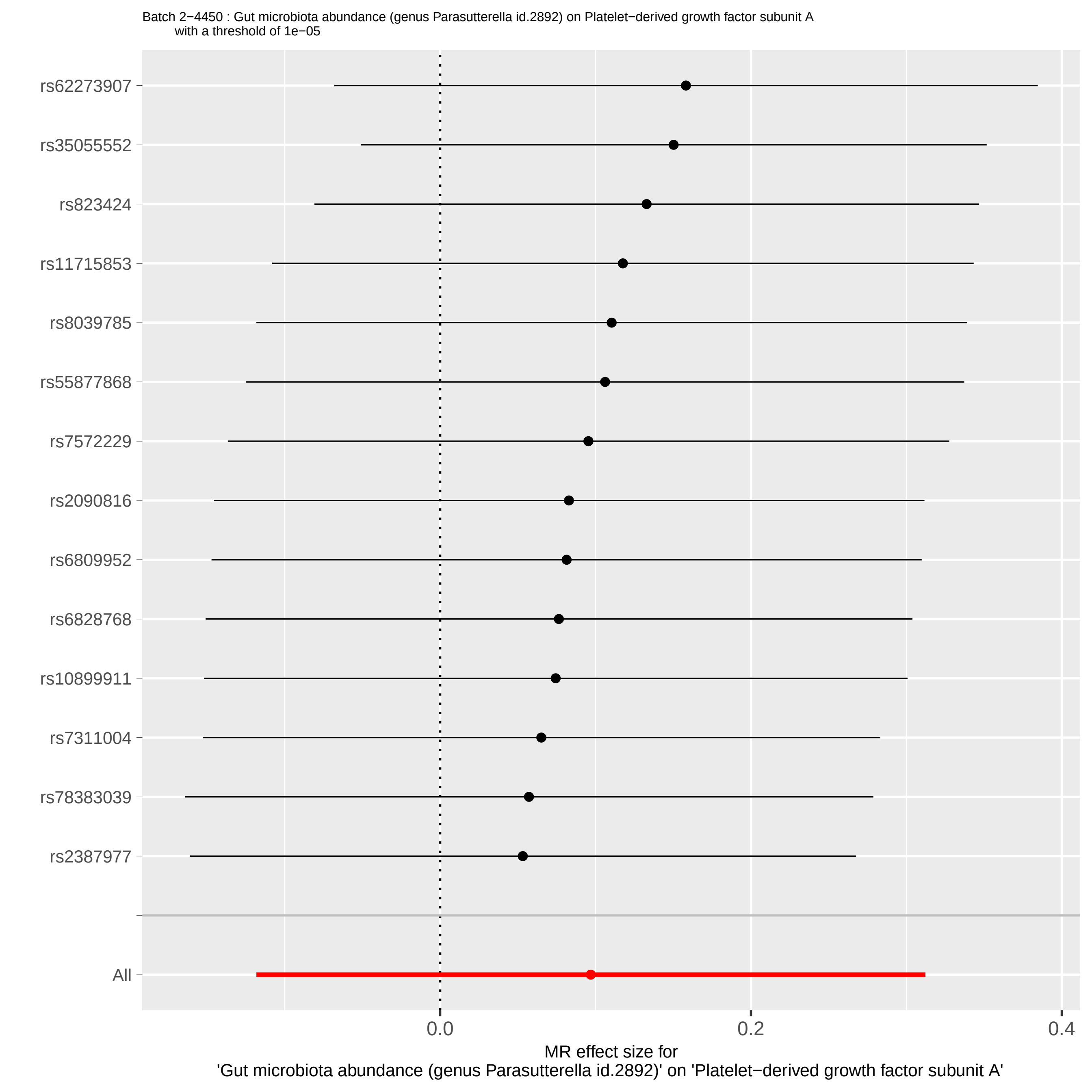
Batch 2−4450 : Gut microbiota abundance (genus Parasutterella id.2892) on Platelet−derived growth factor subunit A
with a threshold of 1e−05
rs62273907
rs35055552
rs823424
rs11715853
rs8039785
rs55877868
rs7572229
rs2090816
rs6809952
rs6828768
rs10899911
rs7311004
rs78383039
rs2387977
All
0.0
0.2
0.4
MR effect size for
'Gut microbiota abundance (genus Parasutterella id.2892)' on 'Platelet−derived growth factor subunit A'

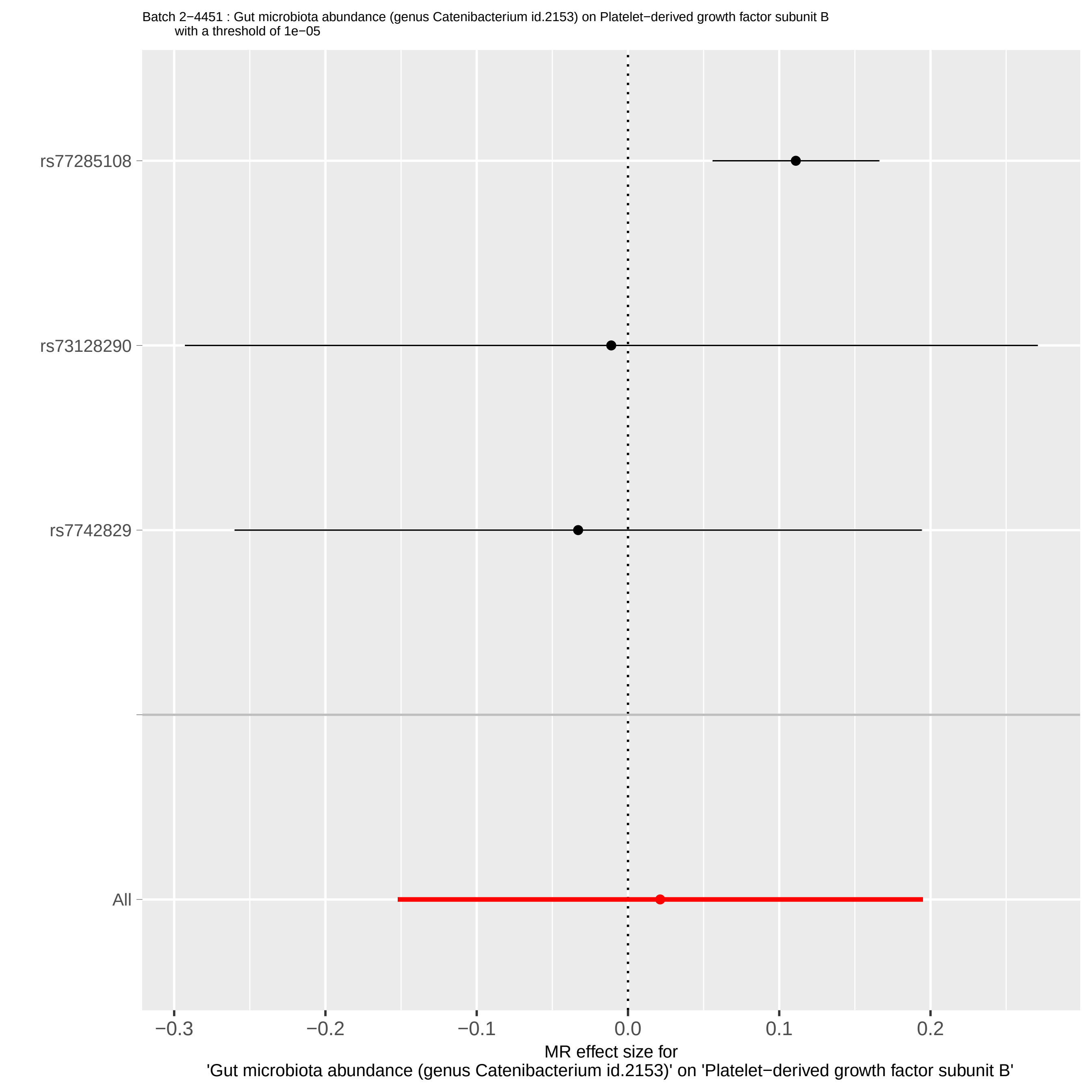
Batch 2−4451 : Gut microbiota abundance (genus Catenibacterium id.2153) on Platelet−derived growth factor subunit B
with a threshold of 1e−05
rs77285108
rs73128290
rs7742829
All
−0.3
−0.2
−0.1
0.0
0.1
0.2
MR effect size for
'Gut microbiota abundance (genus Catenibacterium id.2153)' on 'Platelet−derived growth factor subunit B'

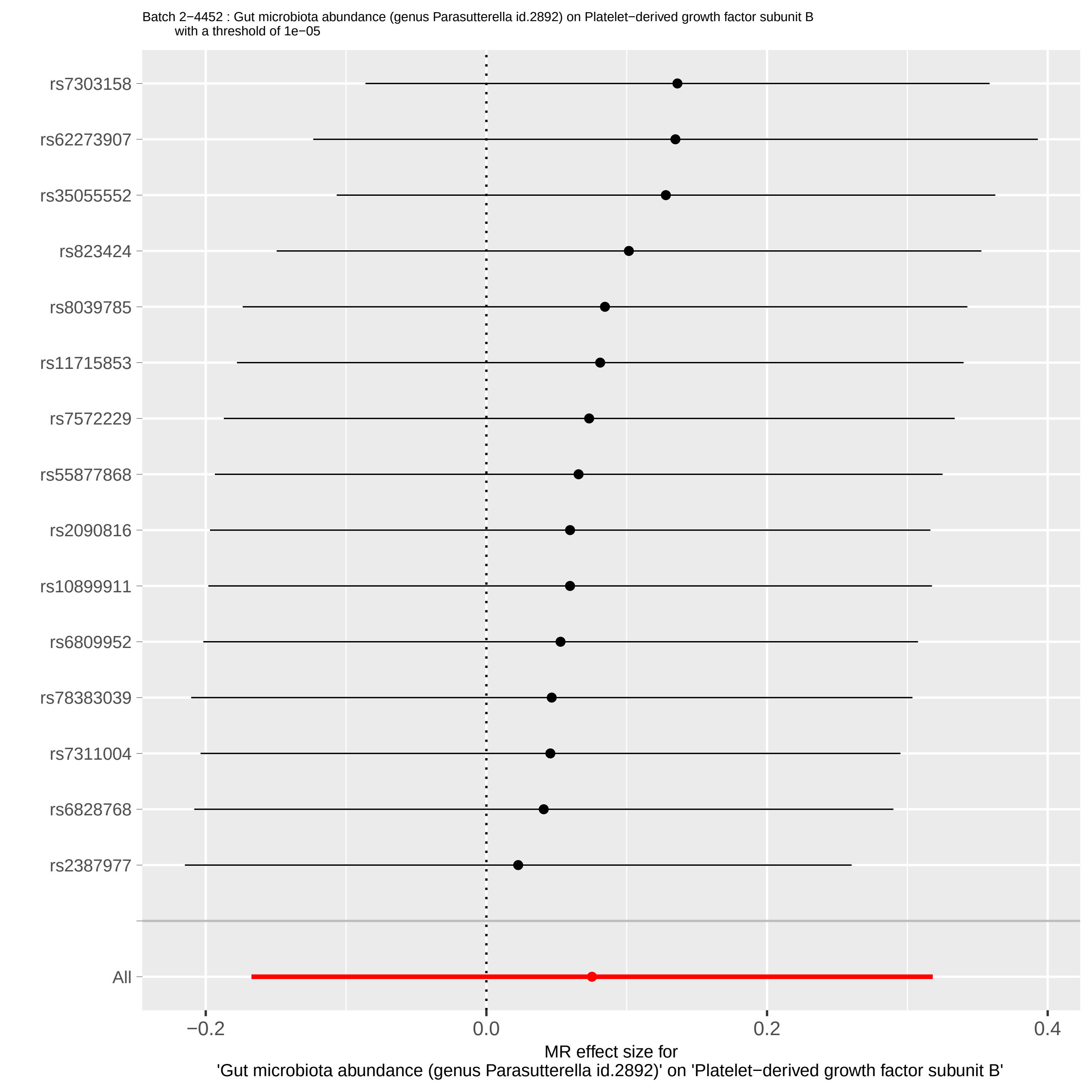

Batch 2−4452 : Gut microbiota abundance (genus Parasutterella id.2892) on Platelet−derived growth factor subunit B
with a threshold of 1e−05
rs7303158
rs62273907
rs35055552
rs823424
rs8039785
rs11715853
rs7572229
rs55877868
rs2090816
rs10899911
rs6809952
rs78383039
rs7311004
rs6828768
rs2387977
All
−0.2
0.0
0.2
0.4
MR effect size for
'Gut microbiota abundance (genus Parasutterella id.2892)' on 'Platelet−derived growth factor subunit B'

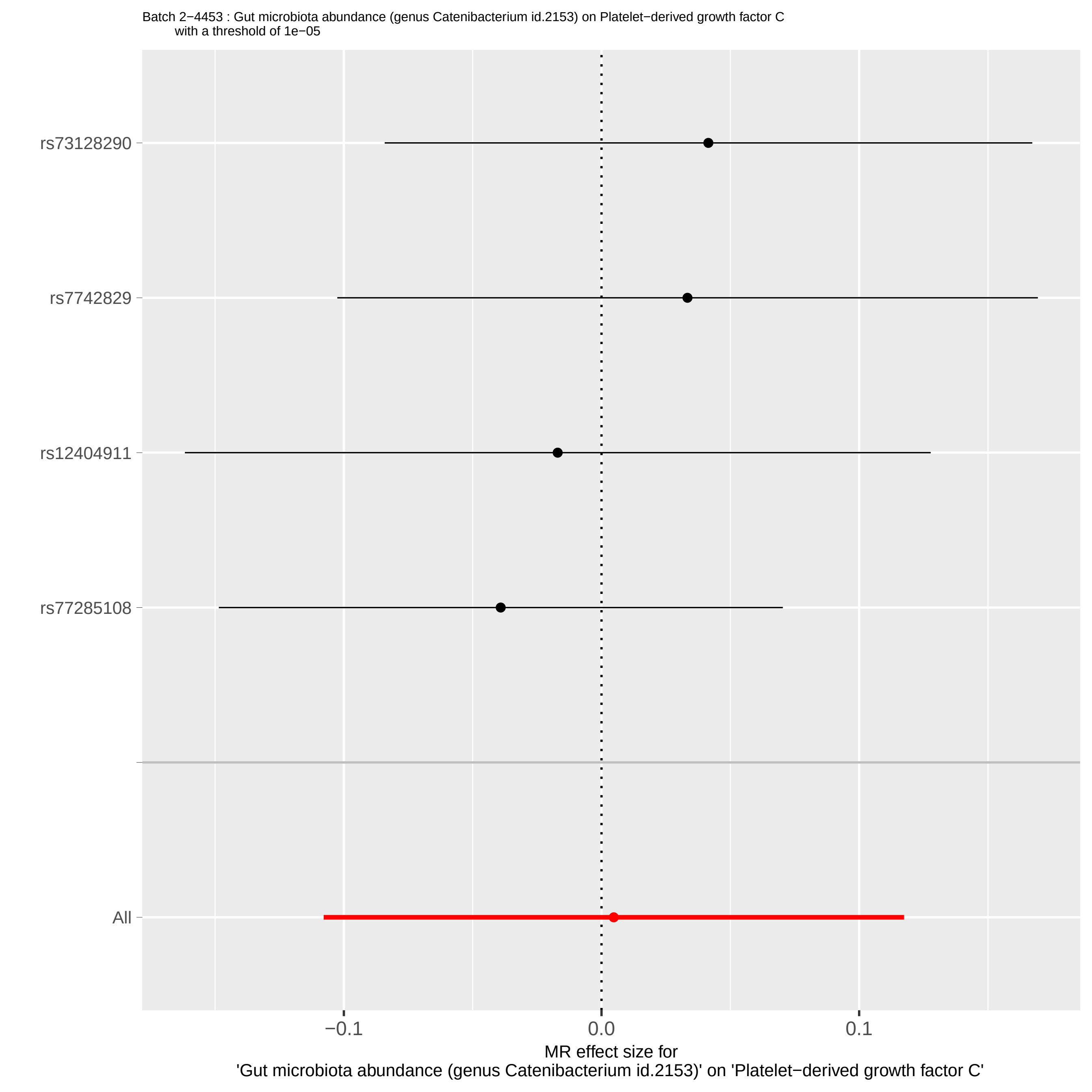
Batch 2−4453 : Gut microbiota abundance (genus Catenibacterium id.2153) on Platelet−derived growth factor C
with a threshold of 1e−05
rs73128290
rs7742829
rs12404911
rs77285108
All
−0.1
0.0
0.1
MR effect size for
'Gut microbiota abundance (genus Catenibacterium id.2153)' on 'Platelet−derived growth factor C'

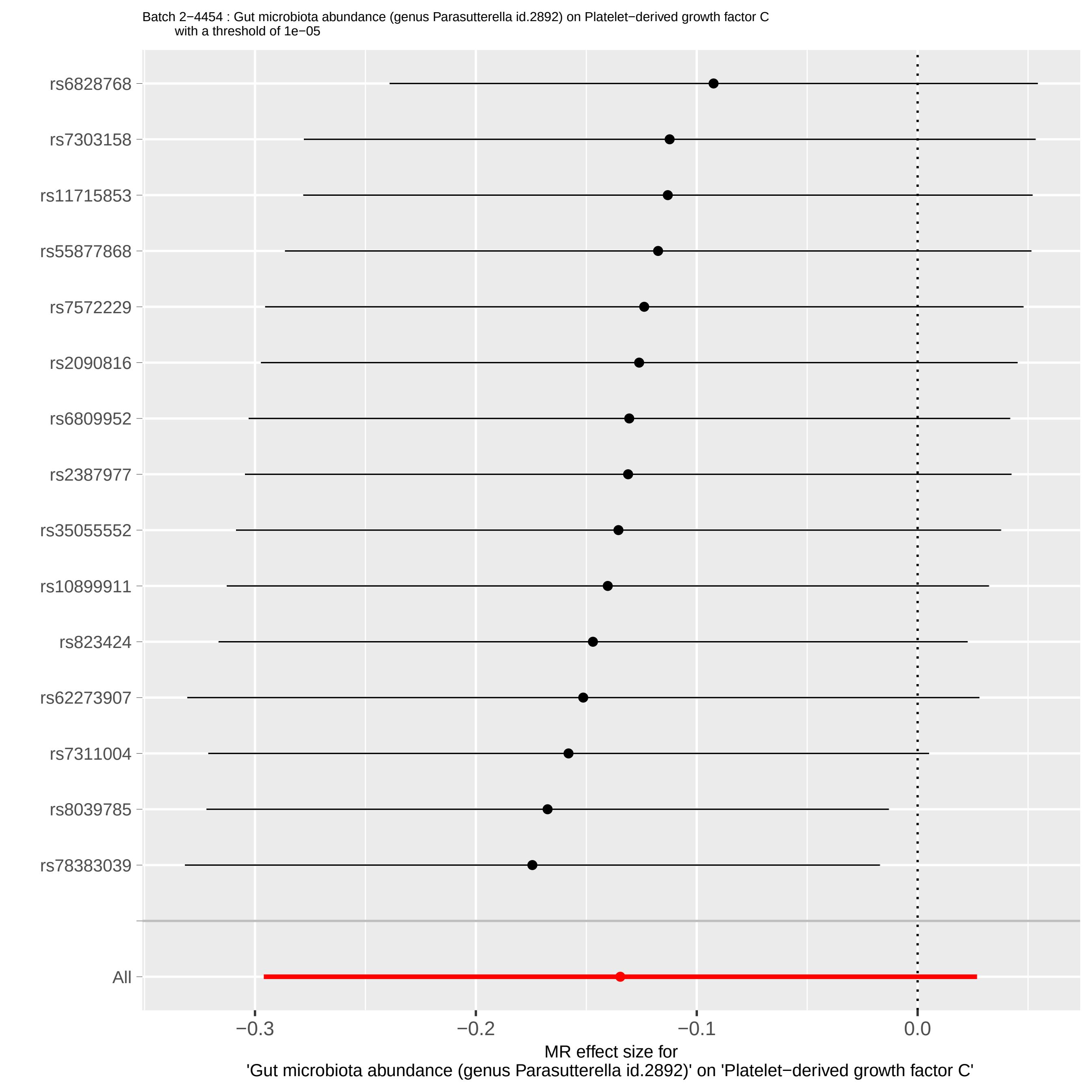
Batch 2−4454 : Gut microbiota abundance (genus Parasutterella id.2892) on Platelet−derived growth factor C
with a threshold of 1e−05
rs6828768
rs7303158
rs11715853
rs55877868
rs7572229
rs2090816
rs6809952
rs2387977
rs35055552
rs10899911
rs823424
rs62273907
rs7311004
rs8039785
rs78383039
All
−0.3
−0.2
−0.1
0.0
MR effect size for
'Gut microbiota abundance (genus Parasutterella id.2892)' on 'Platelet−derived growth factor C'

Batch 2−4455 : Gut microbiota abundance (genus Catenibacterium id.2153) on Platelet−derived growth factor D
with a threshold of 1e−05

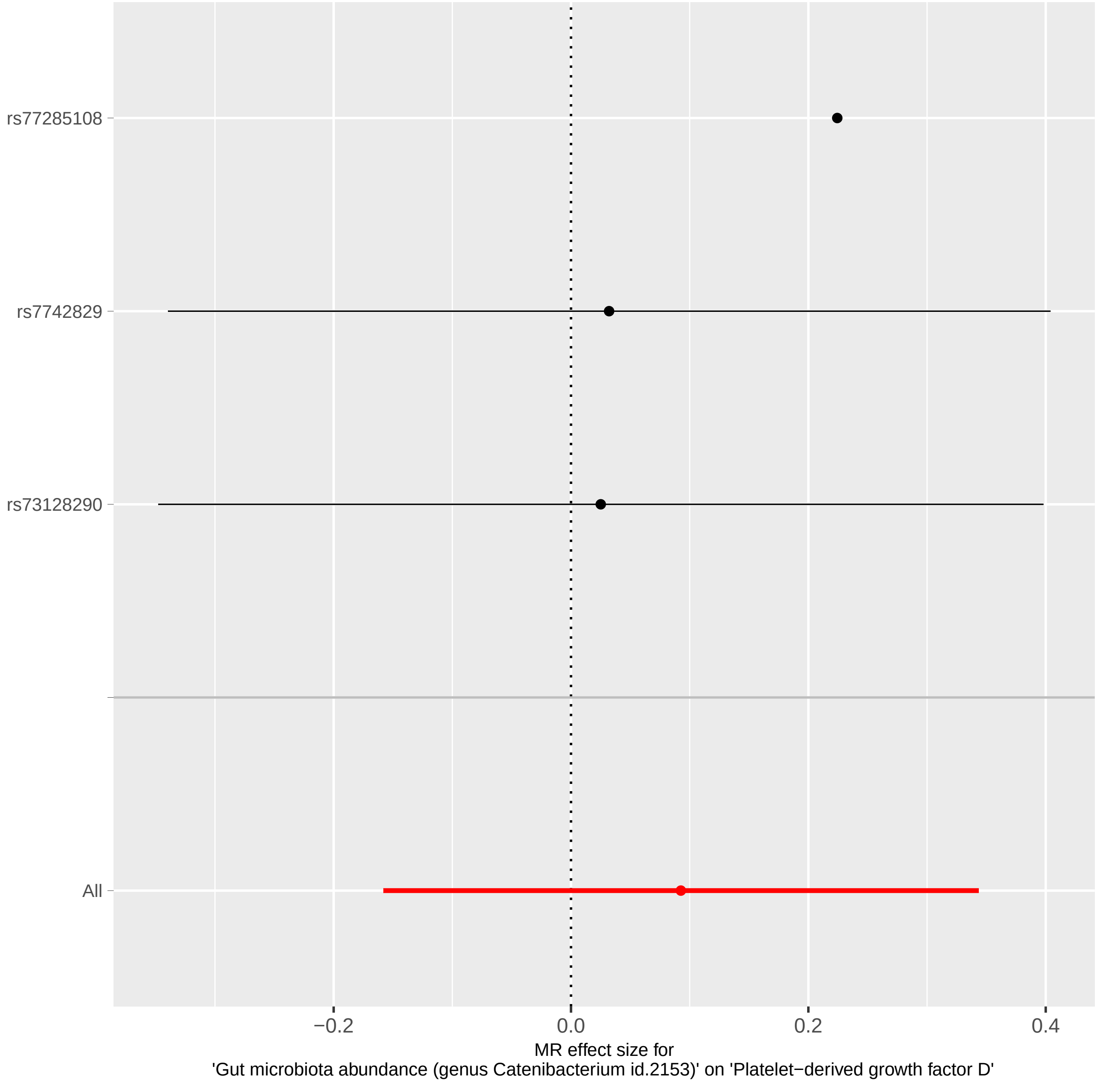

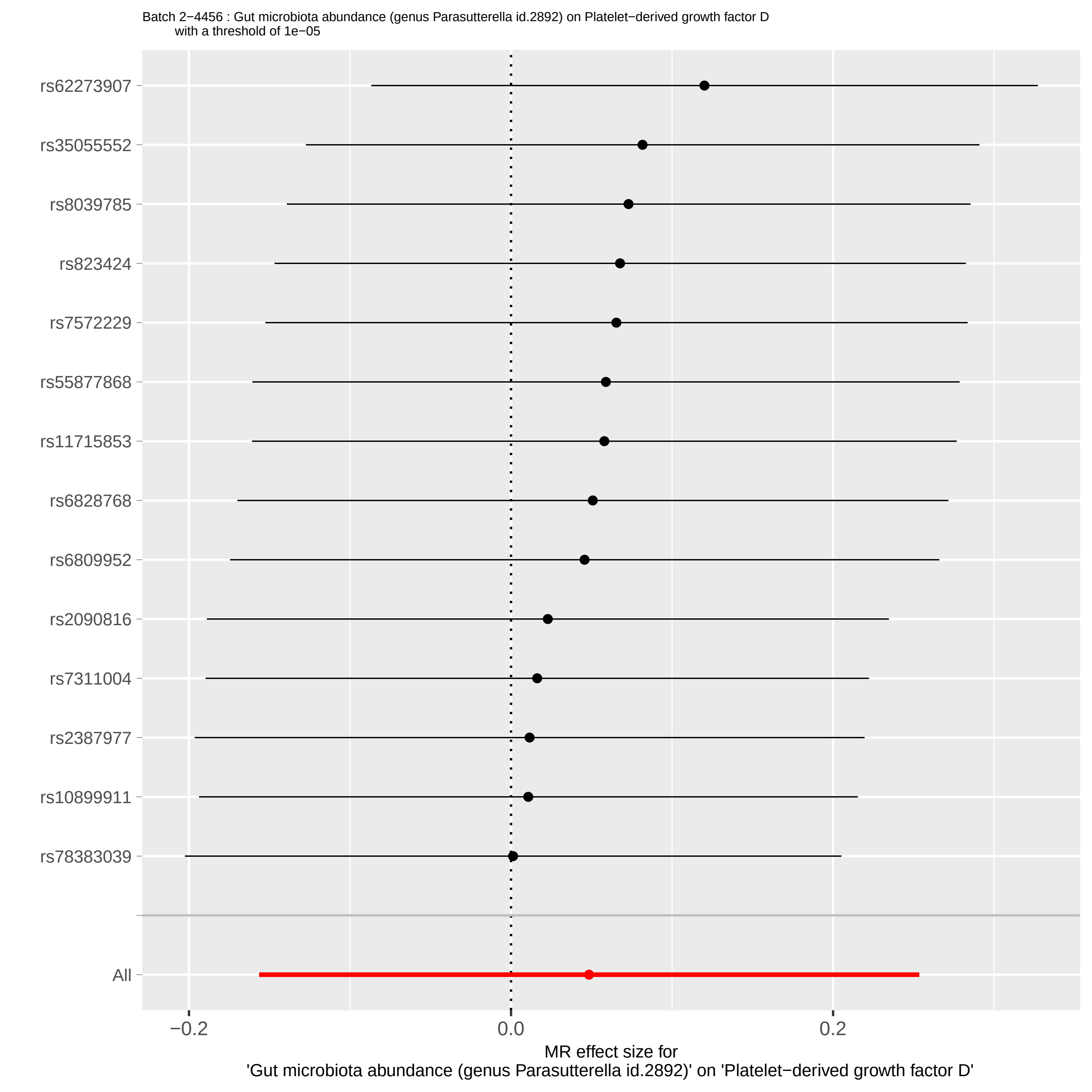
Batch 2−4456 : Gut microbiota abundance (genus Parasutterella id.2892) on Platelet−derived growth factor D
with a threshold of 1e−05
rs62273907
rs35055552
rs8039785
rs823424
rs7572229
rs55877868
rs11715853
rs6828768
rs6809952
rs2090816
rs7311004
rs2387977
rs10899911
rs78383039
All
−0.2
0.0
0.2
MR effect size for
'Gut microbiota abundance (genus Parasutterella id.2892)' on 'Platelet−derived growth factor D'

Batch 2−4457 : Gut microbiota abundance (genus Catenibacterium id.2153) on Platelet−derived growth factor receptor alpha
with a threshold of 1e−05

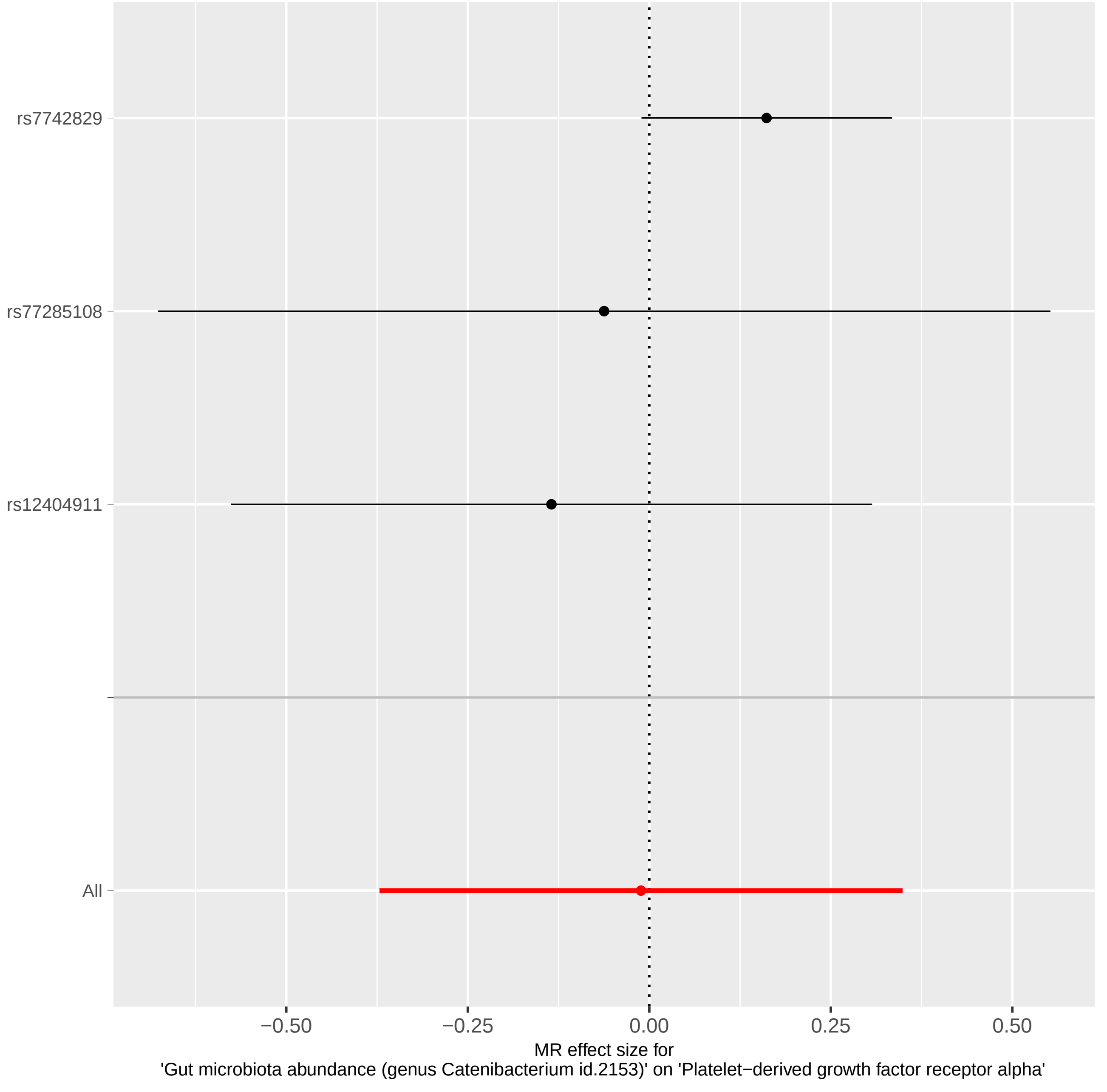

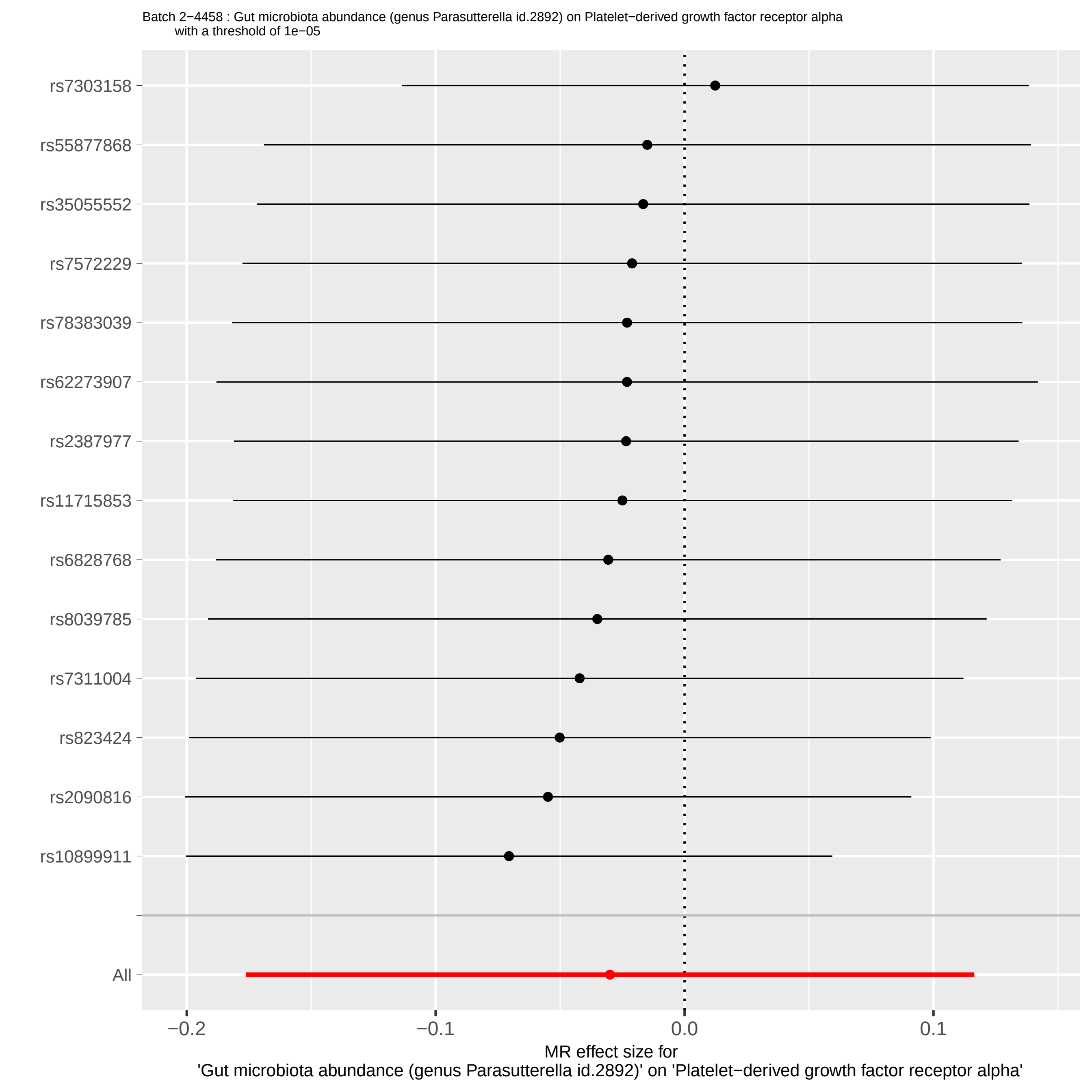

Batch 2−4458 : Gut microbiota abundance (genus Parasutterella id.2892) on Platelet−derived growth factor receptor alpha
with a threshold of 1e−05
rs7303158
rs55877868
rs35055552
rs7572229
rs78383039
rs62273907
rs2387977
rs11715853
rs6828768
rs8039785
rs7311004
rs823424
rs2090816
rs10899911
All
−0.2
−0.1
0.0
0.1
MR effect size for
'Gut microbiota abundance (genus Parasutterella id.2892)' on 'Platelet−derived growth factor receptor alpha'

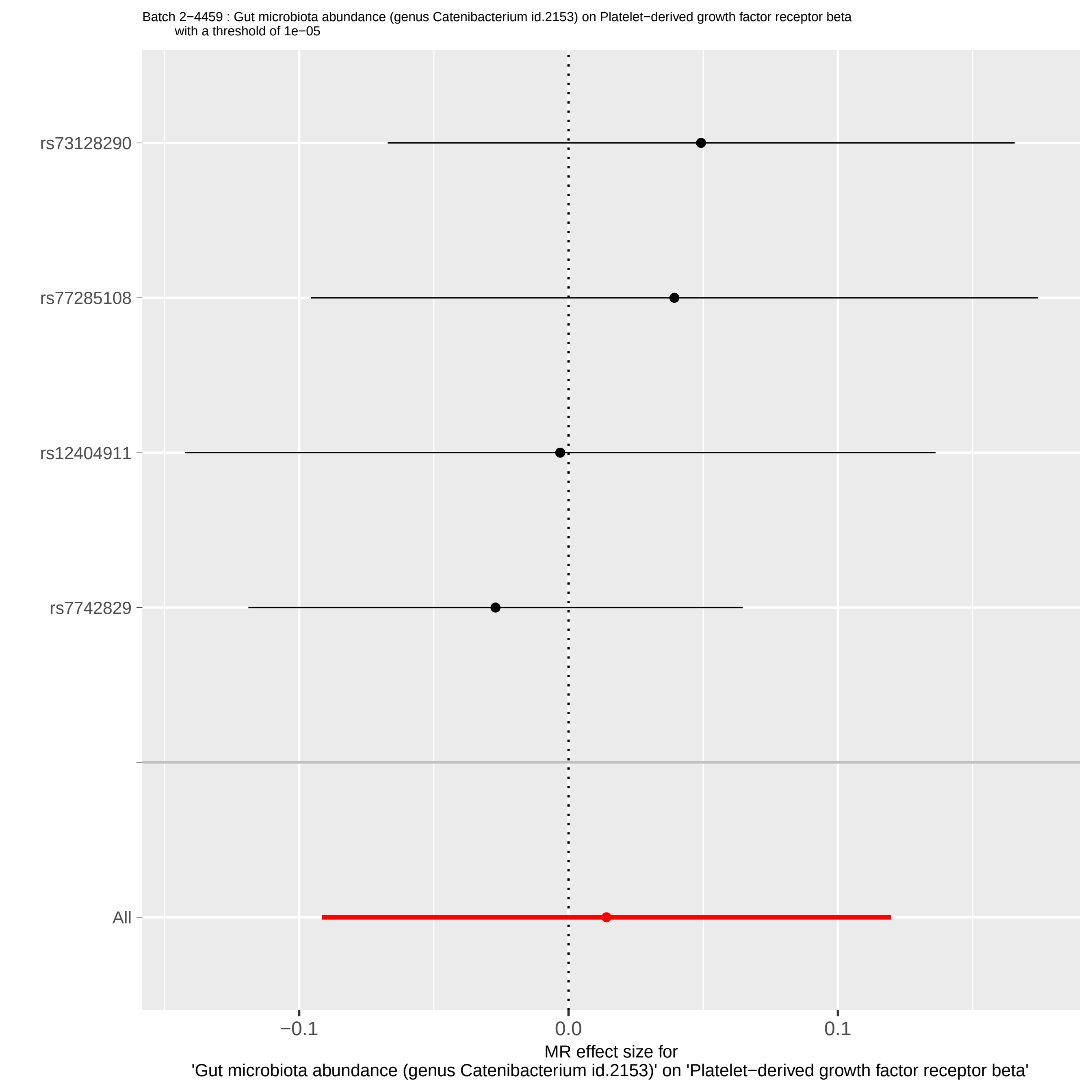
Batch 2−4459 : Gut microbiota abundance (genus Catenibacterium id.2153) on Platelet−derived growth factor receptor beta
with a threshold of 1e−05
rs73128290
rs77285108
rs12404911
rs7742829
All
−0.1
0.0
0.1
MR effect size for
'Gut microbiota abundance (genus Catenibacterium id.2153)' on 'Platelet−derived growth factor receptor beta'

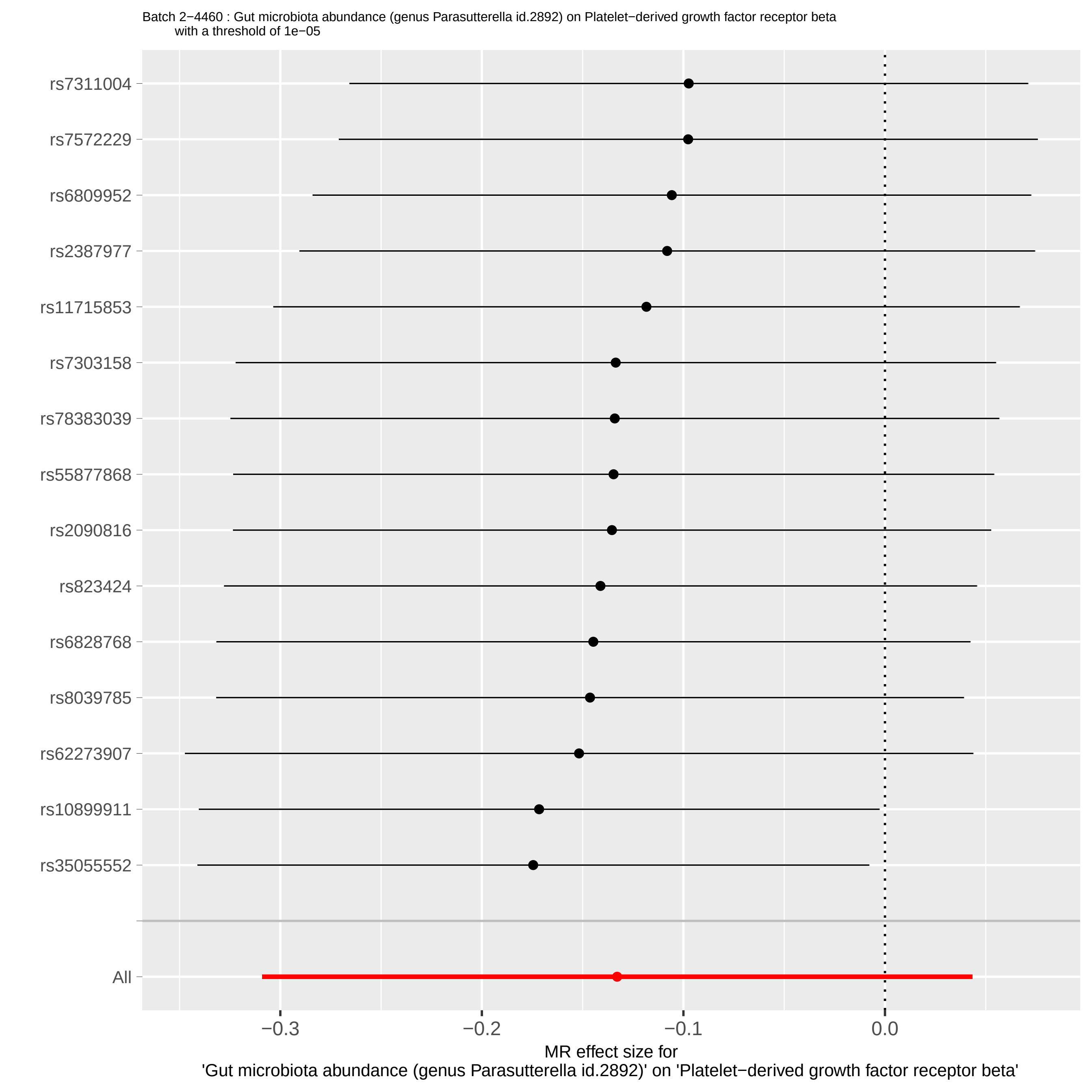
Batch 2−4460 : Gut microbiota abundance (genus Parasutterella id.2892) on Platelet−derived growth factor receptor beta
with a threshold of 1e−05
rs7311004
rs7572229
rs6809952
rs2387977
rs11715853
rs7303158
rs78383039
rs55877868
rs2090816
rs823424
rs6828768
rs8039785
rs62273907
rs10899911
rs35055552
All
−0.3
−0.2
−0.1
0.0
MR effect size for
'Gut microbiota abundance (genus Parasutterella id.2892)' on 'Platelet−derived growth factor receptor beta'

Batch 2−4461 : Gut microbiota abundance (genus Catenibacterium id.2153) on Platelet−derived growth factor receptor−like protein
with a threshold of 1e−05

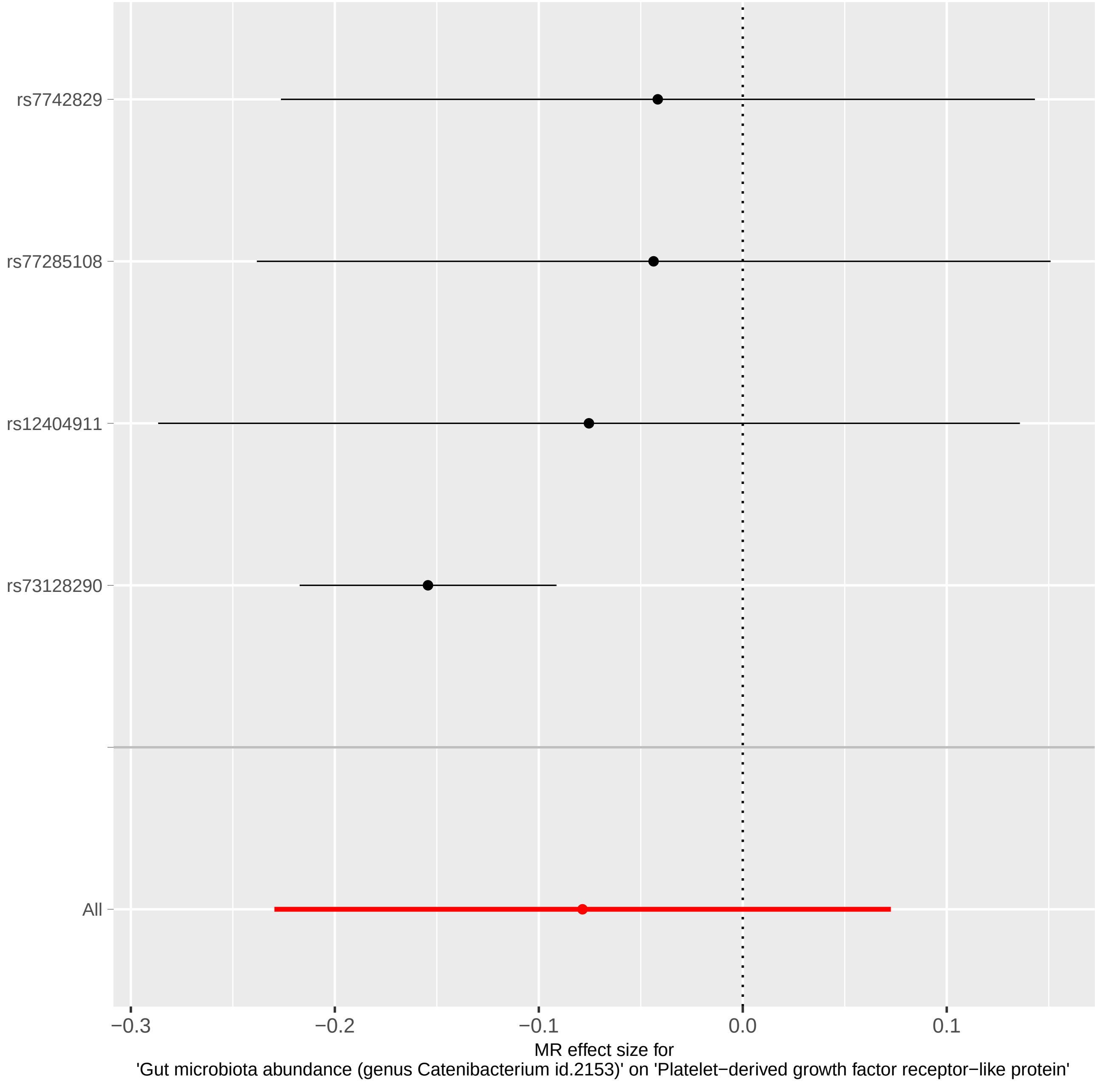

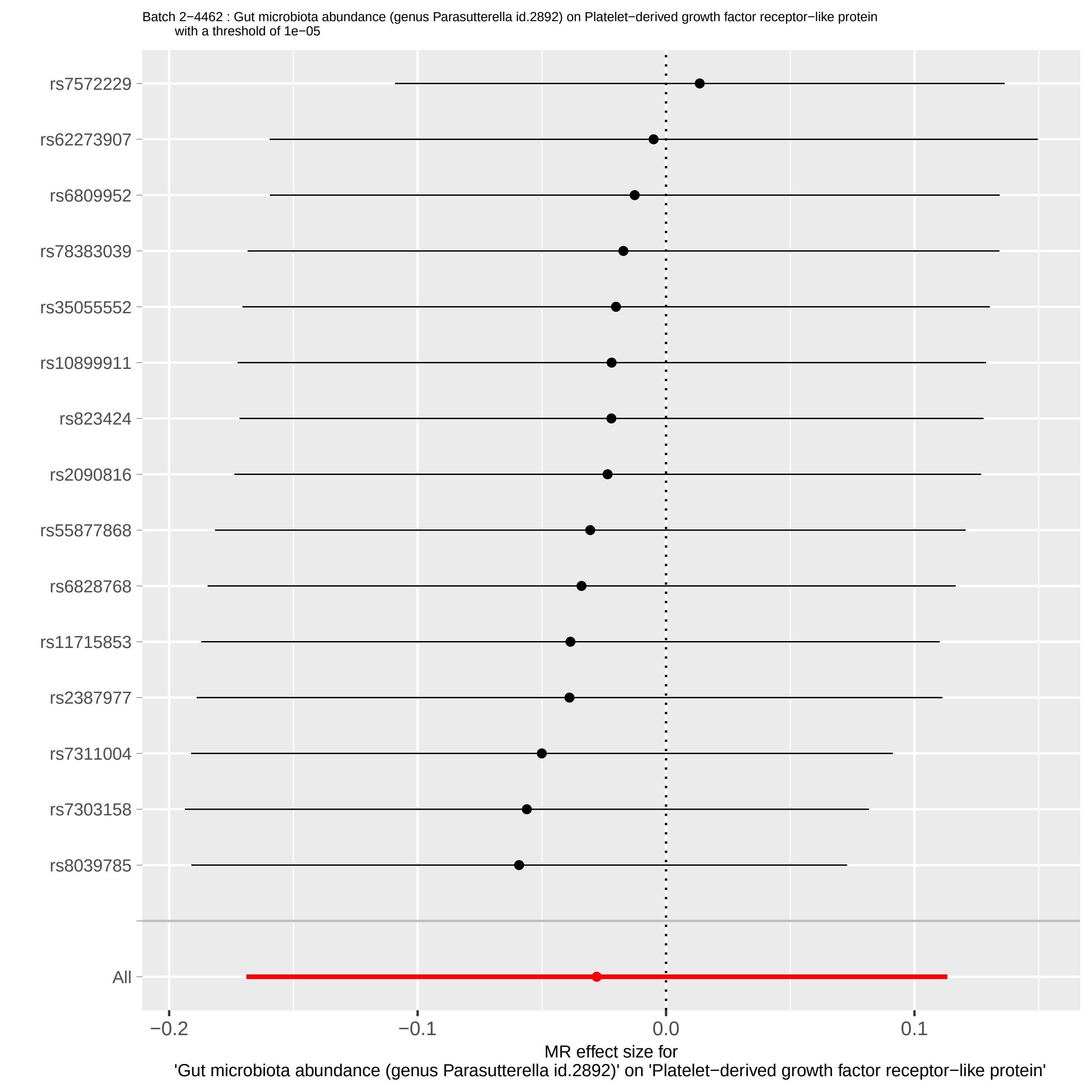
Batch 2−4462 : Gut microbiota abundance (genus Parasutterella id.2892) on Platelet−derived growth factor receptor−like protein
with a threshold of 1e−05
rs7572229
rs62273907
rs6809952
rs78383039
rs35055552
rs10899911
rs823424
rs2090816
rs55877868
rs6828768
rs11715853
rs2387977
rs7311004
rs7303158
rs8039785
All
−0.2
−0.1
0.0
0.1
MR effect size for
'Gut microbiota abundance (genus Parasutterella id.2892)' on 'Platelet−derived growth factor receptor−like protein'

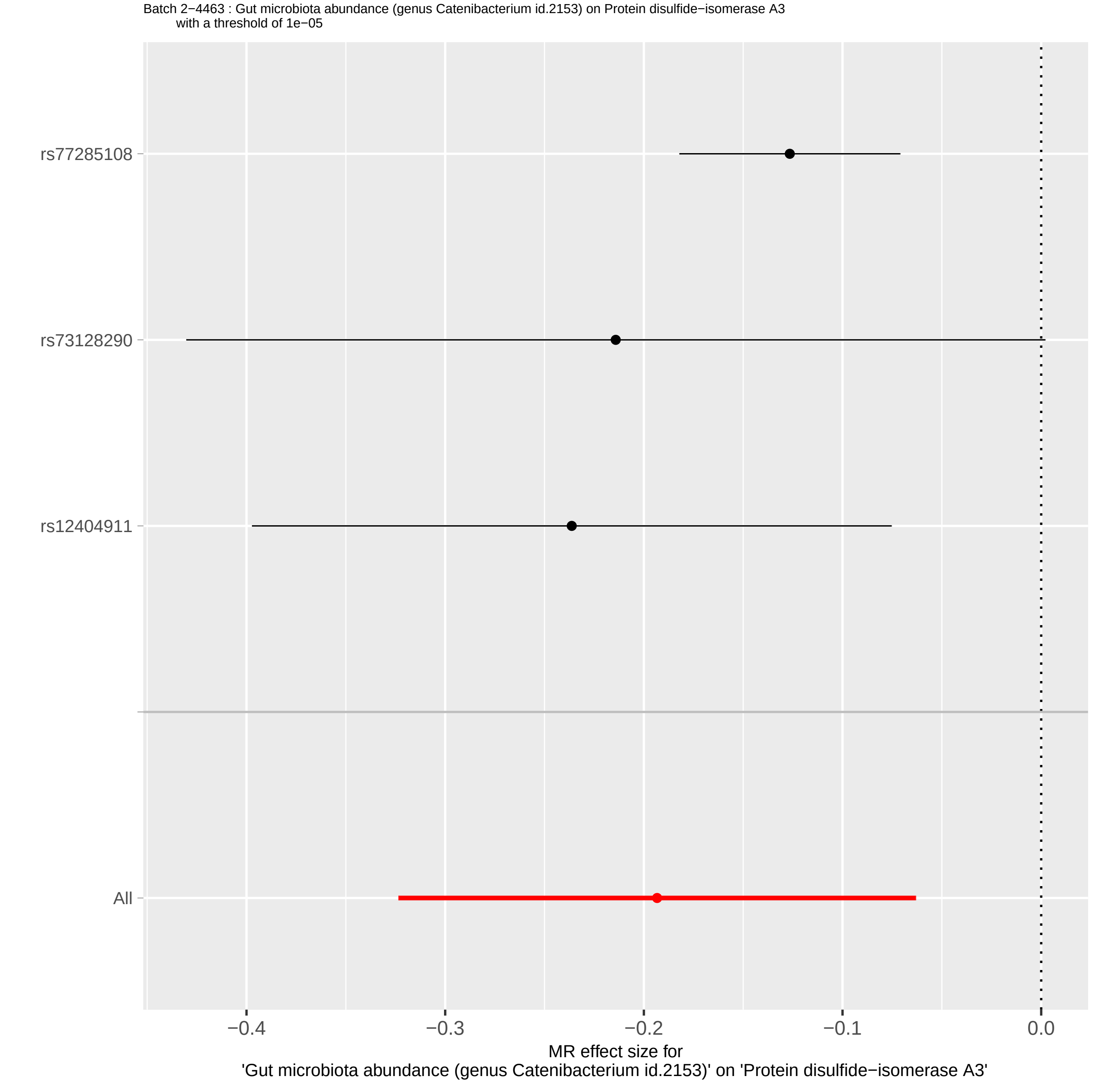
Batch 2−4463 : Gut microbiota abundance (genus Catenibacterium id.2153) on Protein disulfide−isomerase A3
with a threshold of 1e−05
rs77285108
rs73128290
rs12404911
All
−0.4
−0.3
−0.2
−0.1
0.0
MR effect size for
'Gut microbiota abundance (genus Catenibacterium id.2153)' on 'Protein disulfide−isomerase A3'

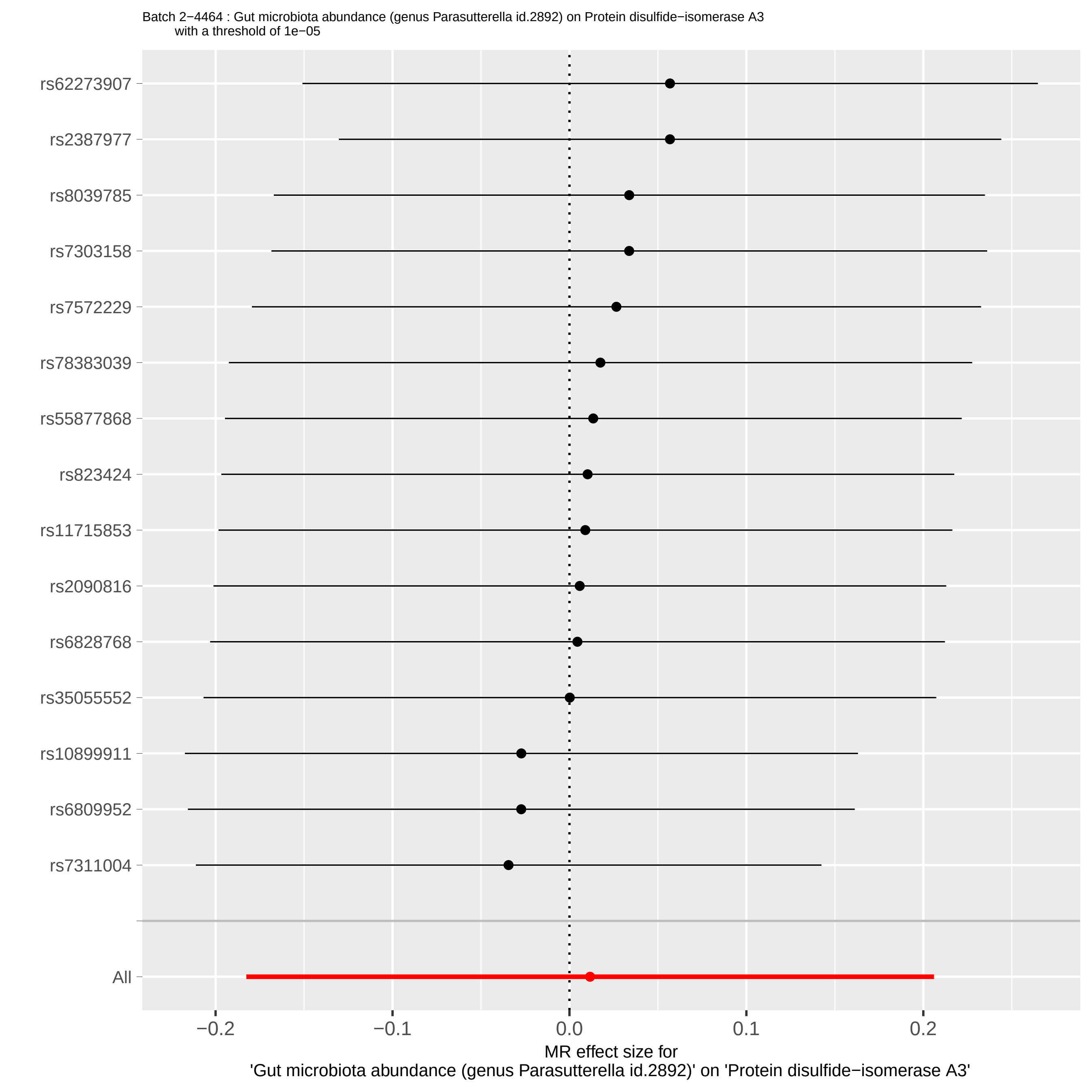
Batch 2−4464 : Gut microbiota abundance (genus Parasutterella id.2892) on Protein disulfide−isomerase A3
with a threshold of 1e−05
rs62273907
rs2387977
rs8039785
rs7303158
rs7572229
rs78383039
rs55877868
rs823424
rs11715853
rs2090816
rs6828768
rs35055552
rs10899911
rs6809952
rs7311004
All
−0.2
−0.1
0.0
0.1
0.2
MR effect size for
'Gut microbiota abundance (genus Parasutterella id.2892)' on 'Protein disulfide−isomerase A3'

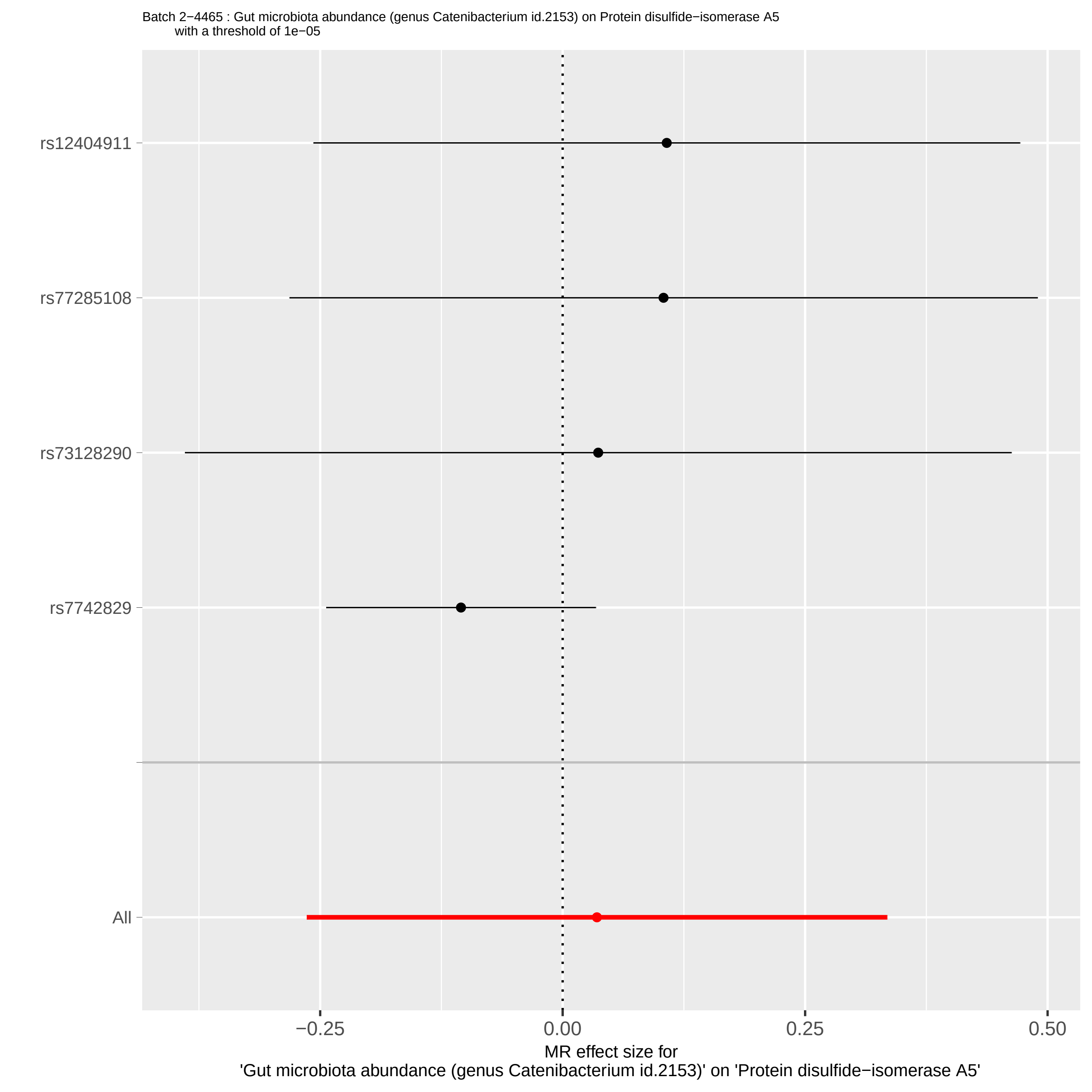
Batch 2−4465 : Gut microbiota abundance (genus Catenibacterium id.2153) on Protein disulfide−isomerase A5
with a threshold of 1e−05
rs12404911
rs77285108
rs73128290
rs7742829
All
−0.25
0.00
0.25
0.50
MR effect size for
'Gut microbiota abundance (genus Catenibacterium id.2153)' on 'Protein disulfide−isomerase A5'

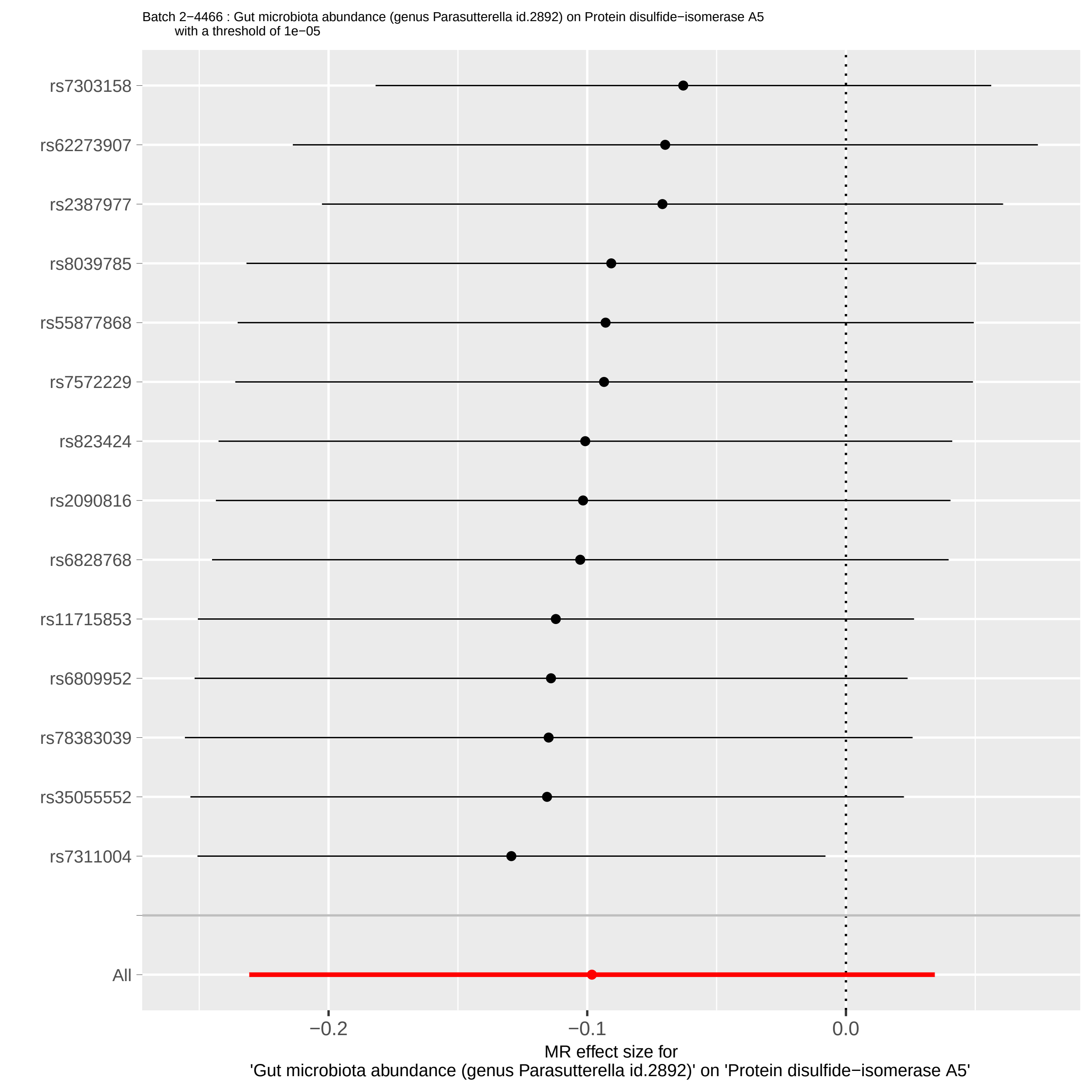
Batch 2−4466 : Gut microbiota abundance (genus Parasutterella id.2892) on Protein disulfide−isomerase A5
with a threshold of 1e−05
rs7303158
rs62273907
rs2387977
rs8039785
rs55877868
rs7572229
rs823424
rs2090816
rs6828768
rs11715853
rs6809952
rs78383039
rs35055552
rs7311004
All
−0.2
−0.1
0.0
MR effect size for
'Gut microbiota abundance (genus Parasutterella id.2892)' on 'Protein disulfide−isomerase A5'

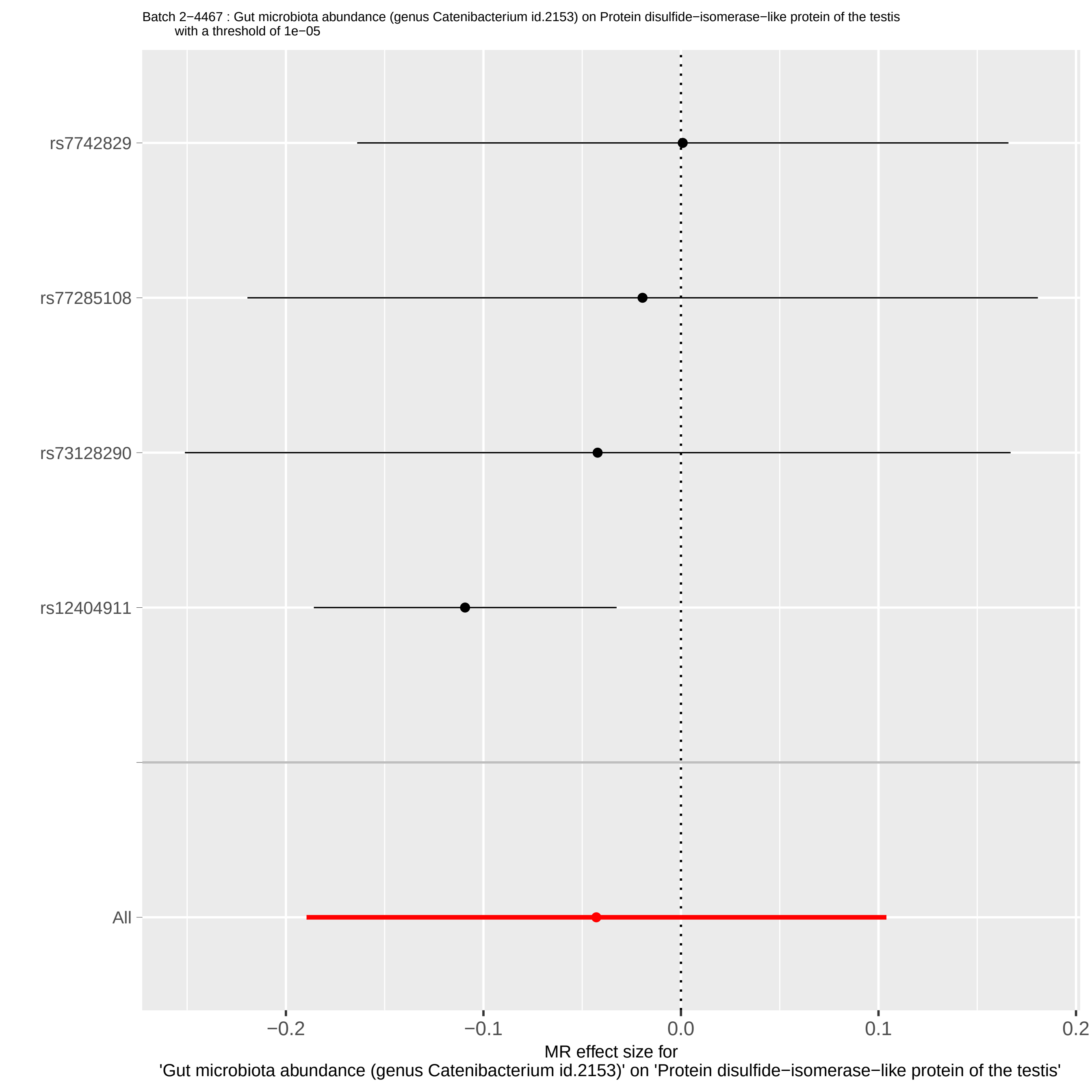

Batch 2−4467 : Gut microbiota abundance (genus Catenibacterium id.2153) on Protein disulfide−isomerase−like protein of the testis
with a threshold of 1e−05
rs7742829
rs77285108
rs73128290
rs12404911
All
−0.2
−0.1
0.0
0.1
0.2
MR effect size for
'Gut microbiota abundance (genus Catenibacterium id.2153)' on 'Protein disulfide−isomerase−like protein of the testis'

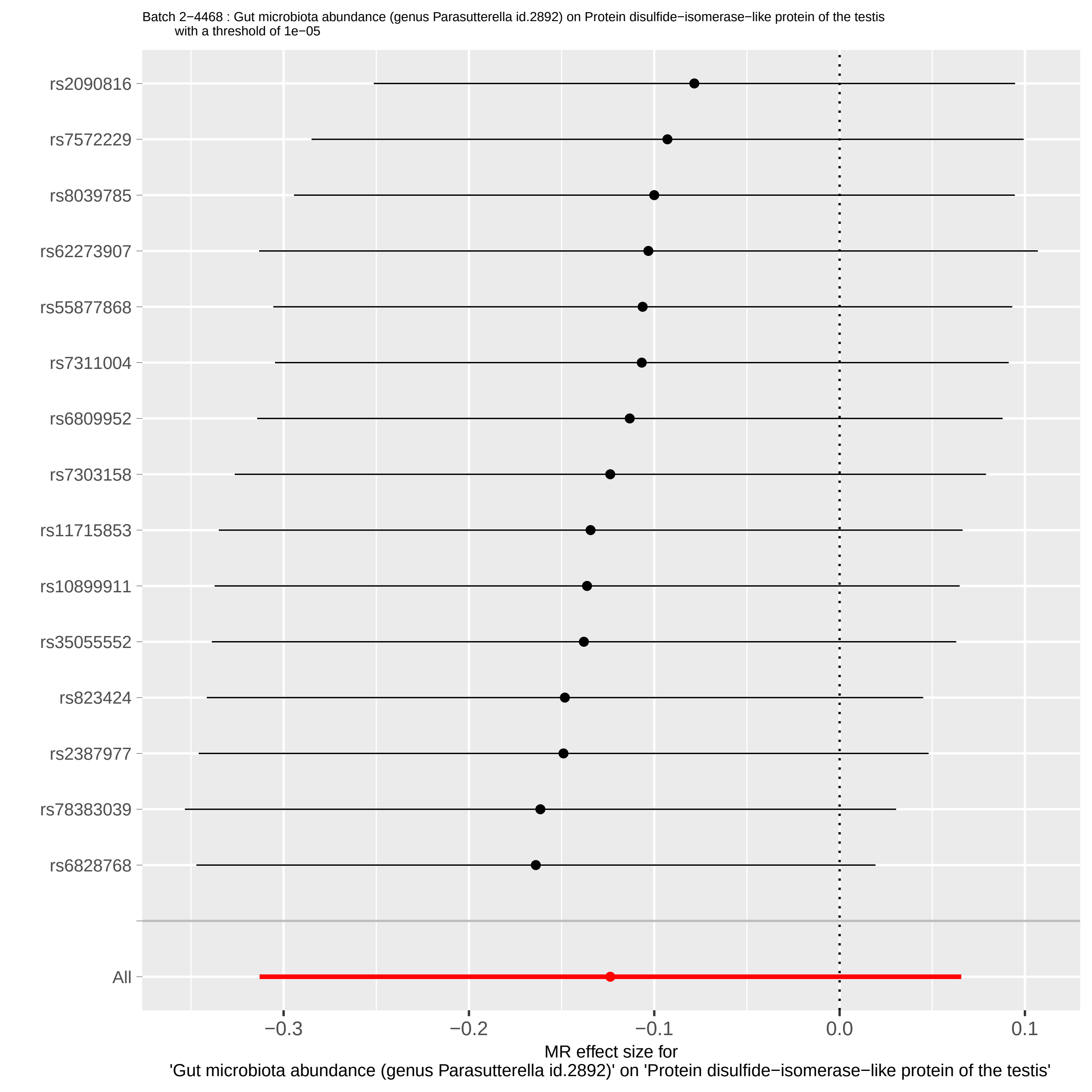
Batch 2−4468 : Gut microbiota abundance (genus Parasutterella id.2892) on Protein disulfide−isomerase−like protein of the testis
with a threshold of 1e−05
rs2090816
rs7572229
rs8039785
rs62273907
rs55877868
rs7311004
rs6809952
rs7303158
rs11715853
rs10899911
rs35055552
rs823424
rs2387977
rs78383039
rs6828768
All
−0.3
−0.2
−0.1
0.0
0.1
MR effect size for
'Gut microbiota abundance (genus Parasutterella id.2892)' on 'Protein disulfide−isomerase−like protein of the testis'

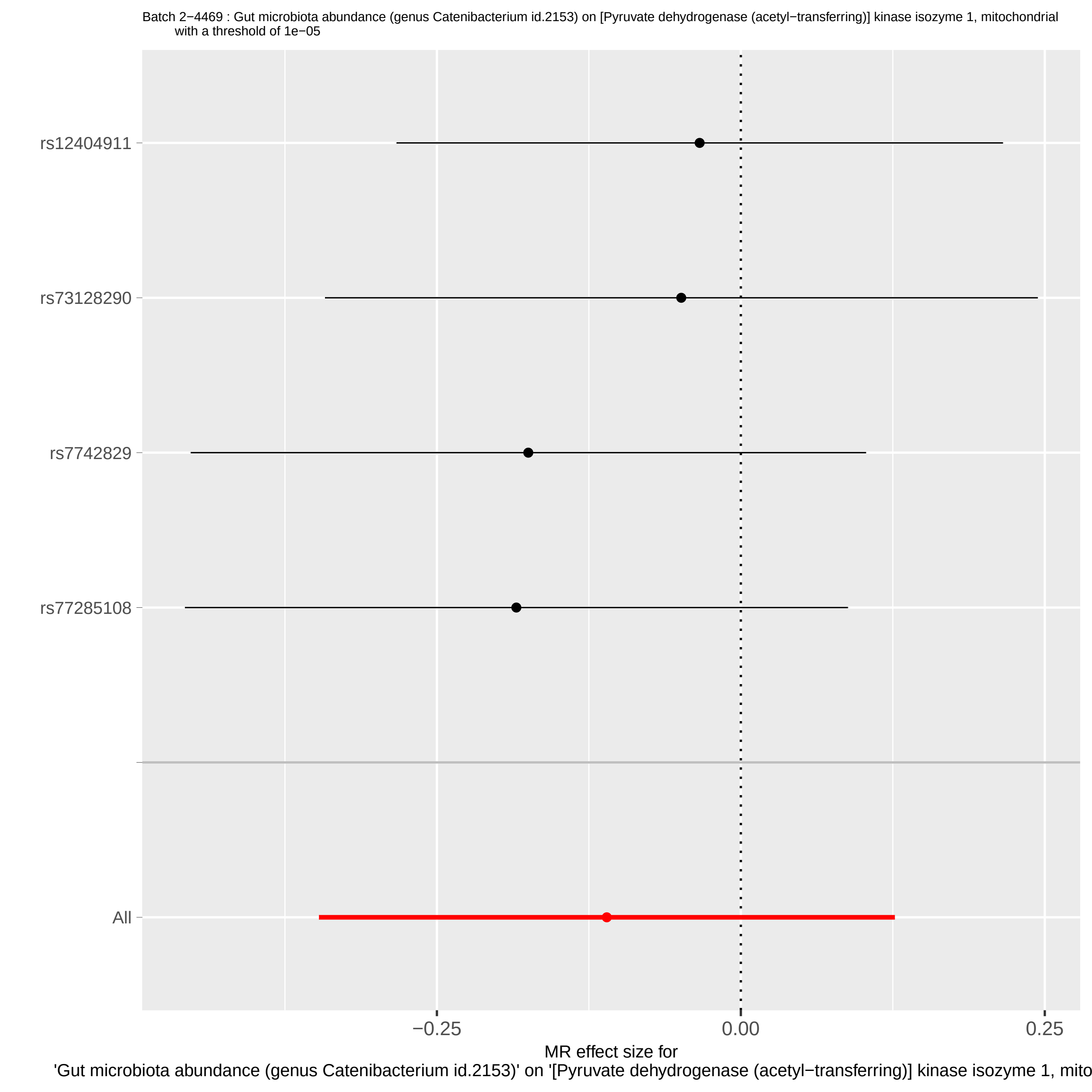

Batch 2−4469 : Gut microbiota abundance (genus Catenibacterium id.2153) on [Pyruvate dehydrogenase (acetyl−transferring)] kinase isozyme 1, mitochondrial
with a threshold of 1e−05
rs12404911
rs73128290
rs7742829
rs77285108
All
−0.25
0.00
0.25
MR effect size for
'Gut microbiota abundance (genus Catenibacterium id.2153)' on '[Pyruvate dehydrogenase (acetyl−transferring)] kinase isozyme 1, mito

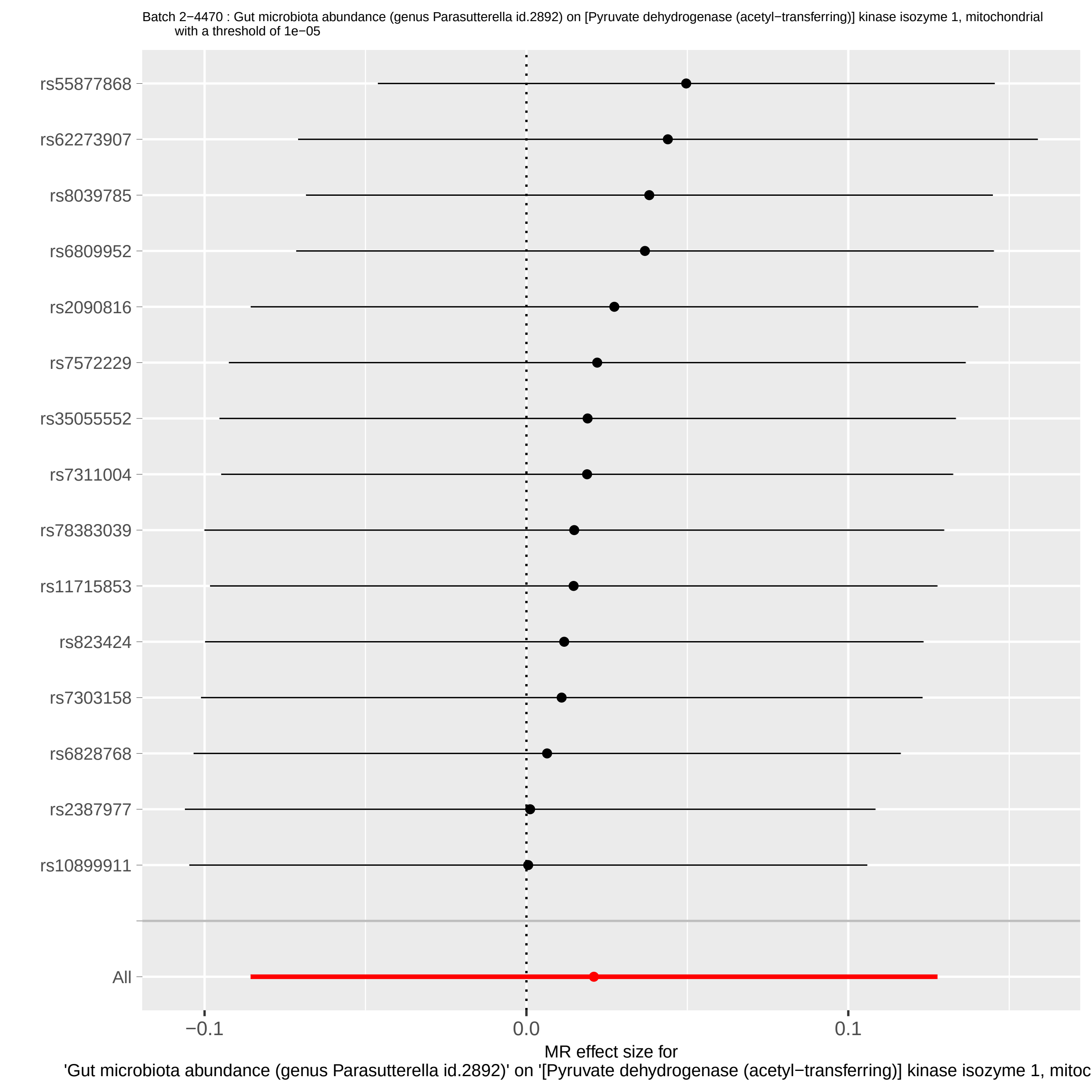

Batch 2−4470 : Gut microbiota abundance (genus Parasutterella id.2892) on [Pyruvate dehydrogenase (acetyl−transferring)] kinase isozyme 1, mitochondrial
with a threshold of 1e−05
rs55877868
rs62273907
rs8039785
rs6809952
rs2090816
rs7572229
rs35055552
rs7311004
rs78383039
rs11715853
rs823424
rs7303158
rs6828768
rs2387977
rs10899911
All
−0.1
0.0
0.1
MR effect size for
'Gut microbiota abundance (genus Parasutterella id.2892)' on '[Pyruvate dehydrogenase (acetyl−transferring)] kinase isozyme 1, mitoc

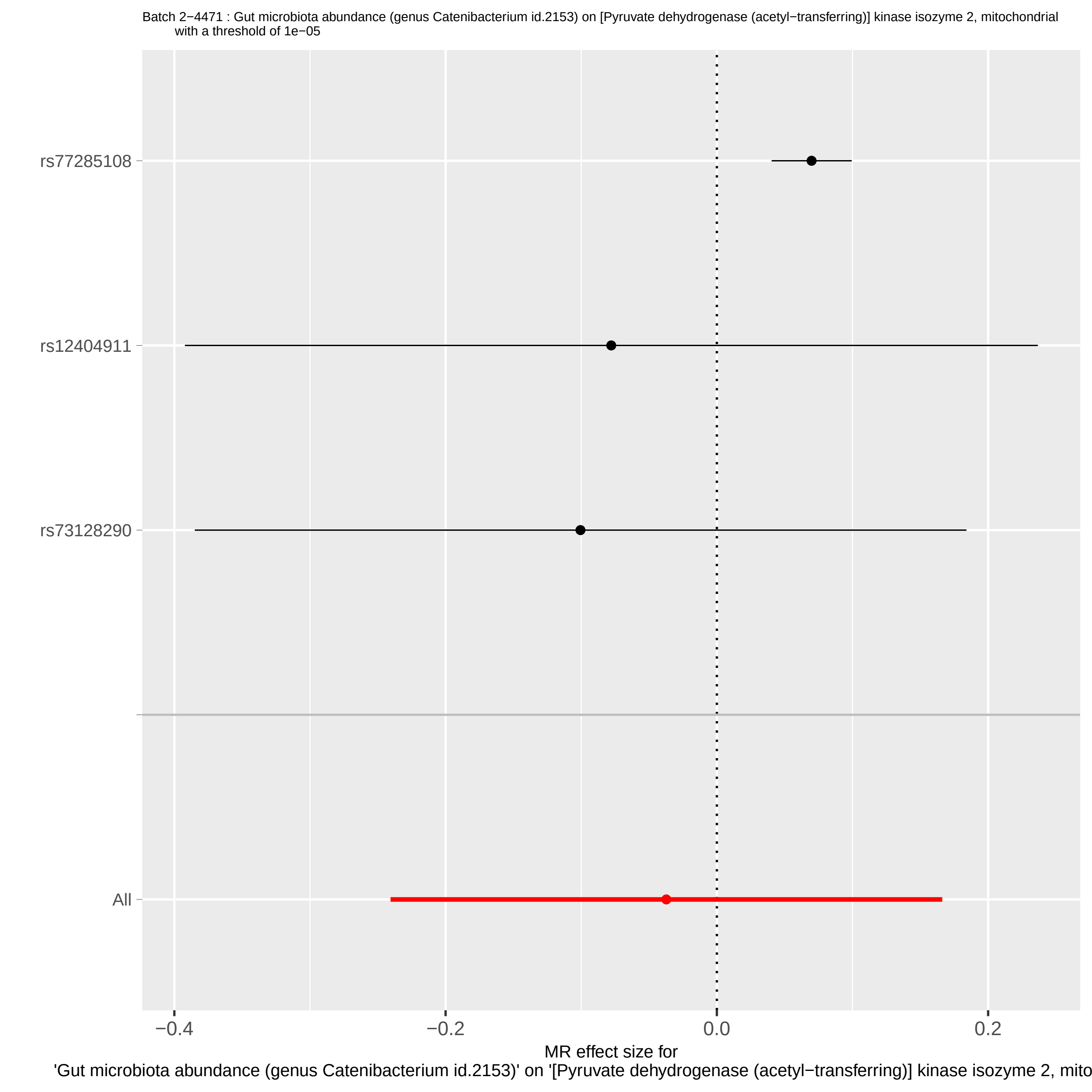
Batch 2−4471 : Gut microbiota abundance (genus Catenibacterium id.2153) on [Pyruvate dehydrogenase (acetyl−transferring)] kinase isozyme 2, mitochondrial
with a threshold of 1e−05
rs77285108
rs12404911
rs73128290
All
−0.4
−0.2
0.0
0.2
MR effect size for
'Gut microbiota abundance (genus Catenibacterium id.2153)' on '[Pyruvate dehydrogenase (acetyl−transferring)] kinase isozyme 2, mito

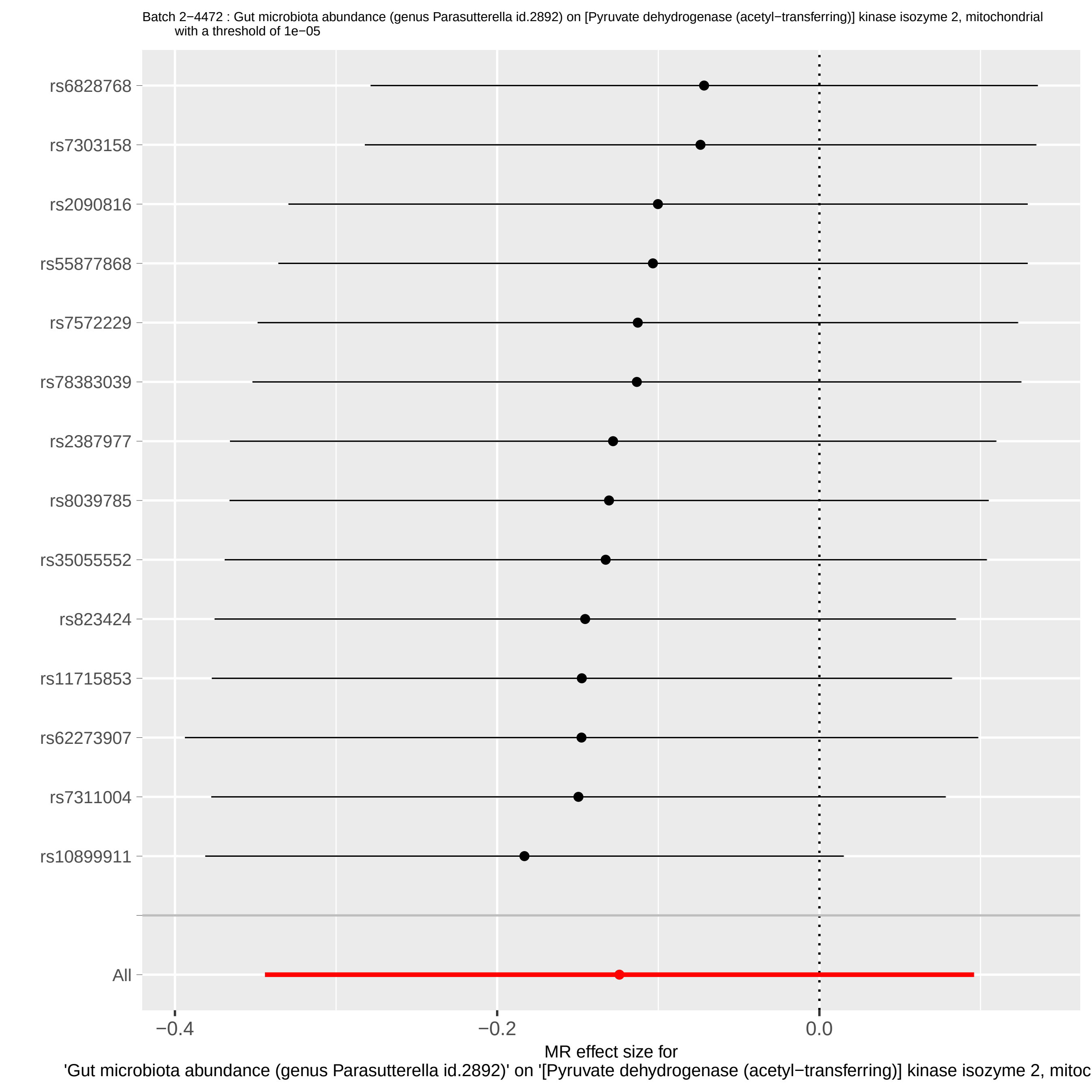
Batch 2−4472 : Gut microbiota abundance (genus Parasutterella id.2892) on [Pyruvate dehydrogenase (acetyl−transferring)] kinase isozyme 2, mitochondrial
with a threshold of 1e−05
rs6828768
rs7303158
rs2090816
rs55877868
rs7572229
rs78383039
rs2387977
rs8039785
rs35055552
rs823424
rs11715853
rs62273907
rs7311004
rs10899911
All
−0.4
−0.2
0.0
MR effect size for
'Gut microbiota abundance (genus Parasutterella id.2892)' on '[Pyruvate dehydrogenase (acetyl−transferring)] kinase isozyme 2, mitoc

Batch 2−4473 : Gut microbiota abundance (genus Catenibacterium id.2153) on PDZ and LIM domain protein 4
with a threshold of 1e−05

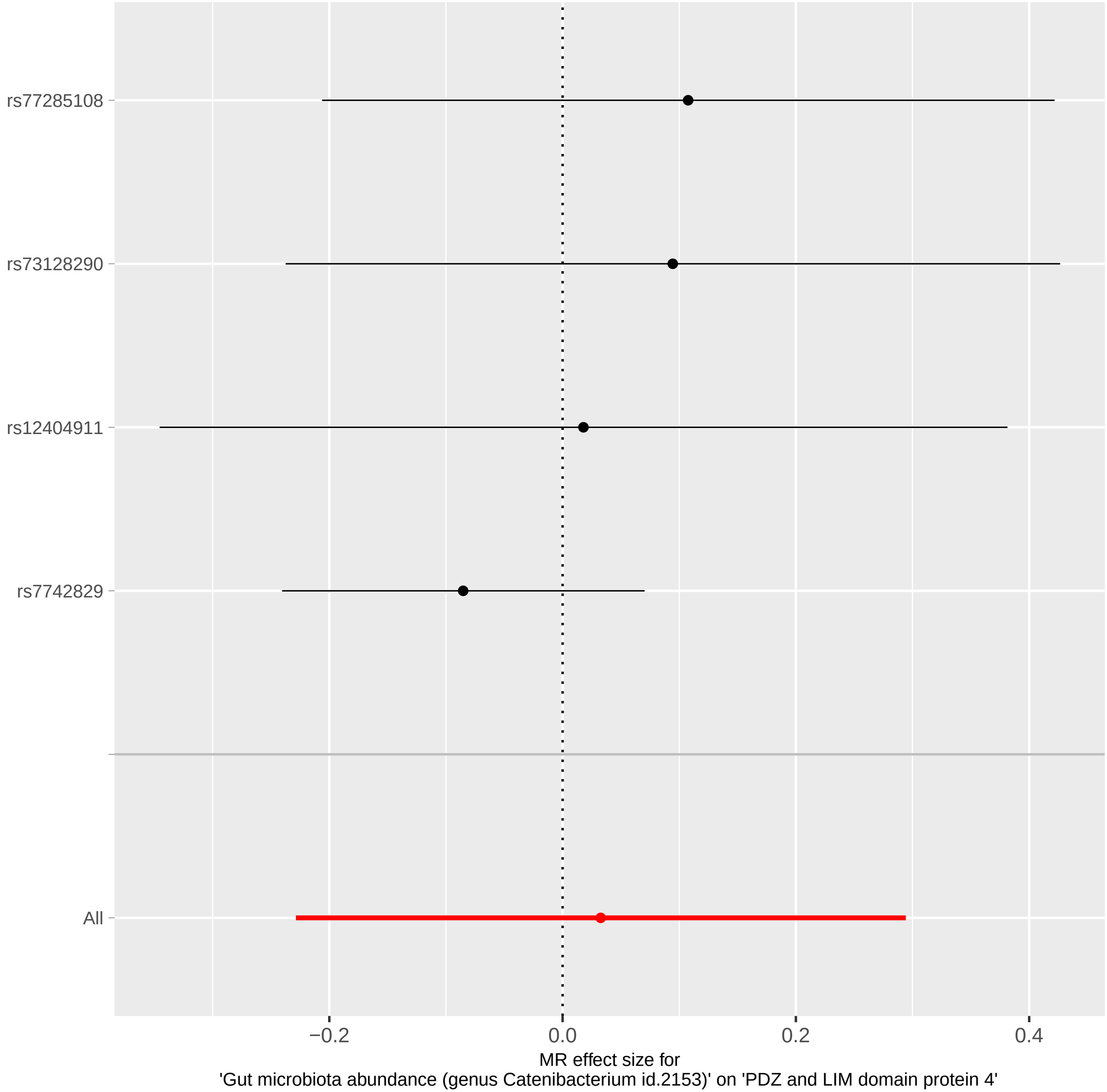

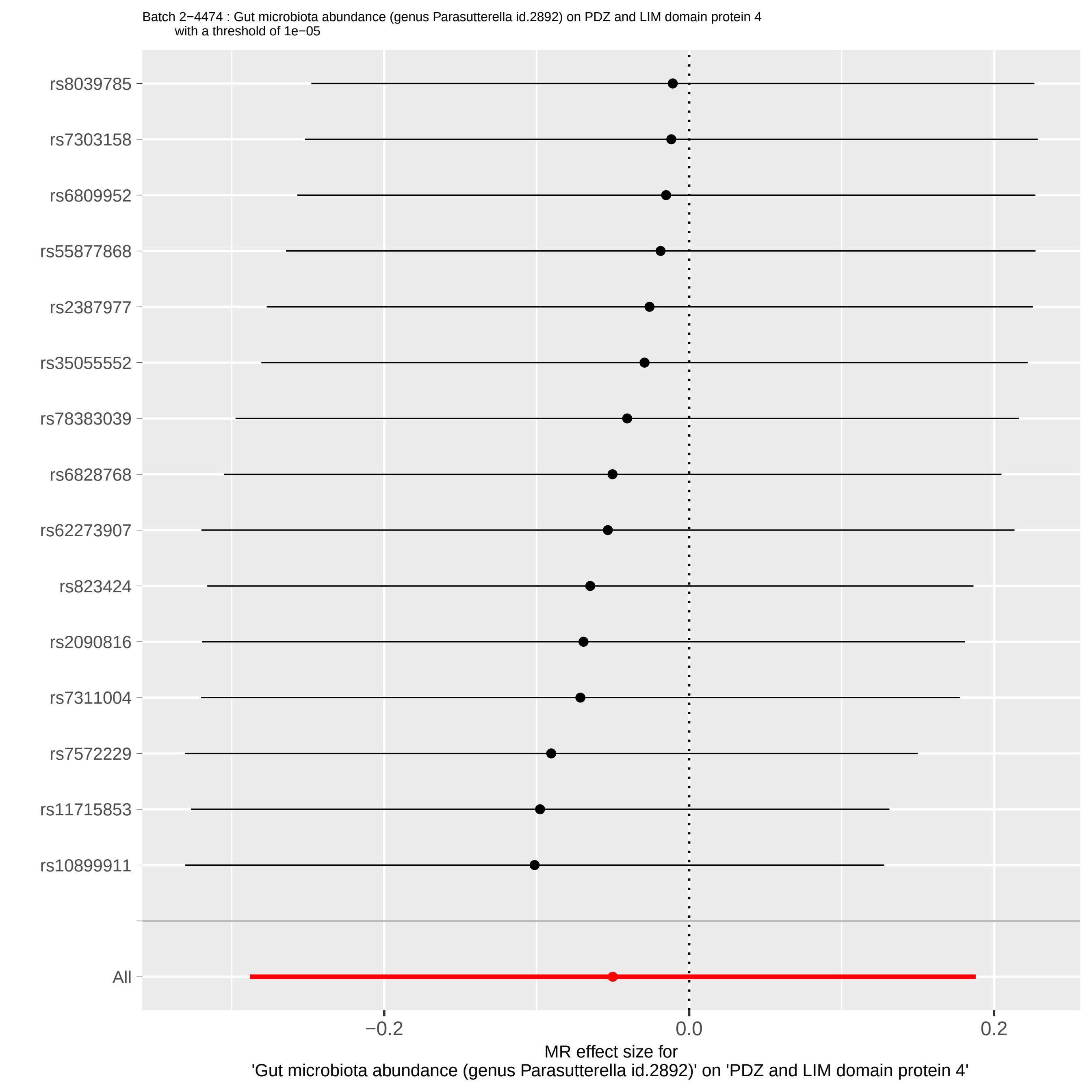

Batch 2−4474 : Gut microbiota abundance (genus Parasutterella id.2892) on PDZ and LIM domain protein 4
with a threshold of 1e−05
rs8039785
rs7303158
rs6809952
rs55877868
rs2387977
rs35055552
rs78383039
rs6828768
rs62273907
rs823424
rs2090816
rs7311004
rs7572229
rs11715853
rs10899911
All
−0.2
0.0
0.2
MR effect size for
'Gut microbiota abundance (genus Parasutterella id.2892)' on 'PDZ and LIM domain protein 4'

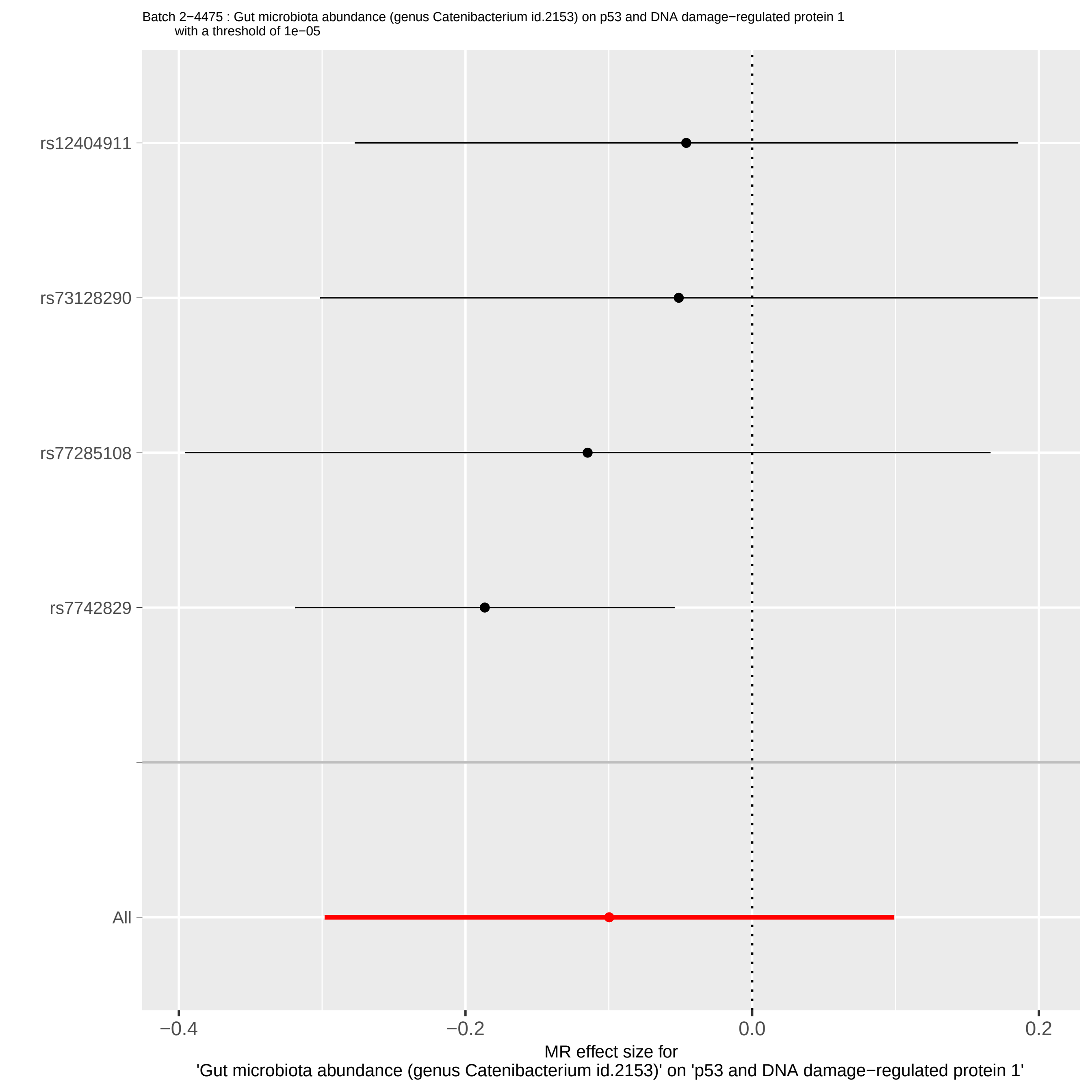
Batch 2−4475 : Gut microbiota abundance (genus Catenibacterium id.2153) on p53 and DNA damage−regulated protein 1
with a threshold of 1e−05
rs12404911
rs73128290
rs77285108
rs7742829
All
−0.4
−0.2
0.0
0.2
MR effect size for
'Gut microbiota abundance (genus Catenibacterium id.2153)' on 'p53 and DNA damage−regulated protein 1'

Batch 2−4476 : Gut microbiota abundance (genus Parasutterella id.2892) on p53 and DNA damage−regulated protein 1
with a threshold of 1e−05

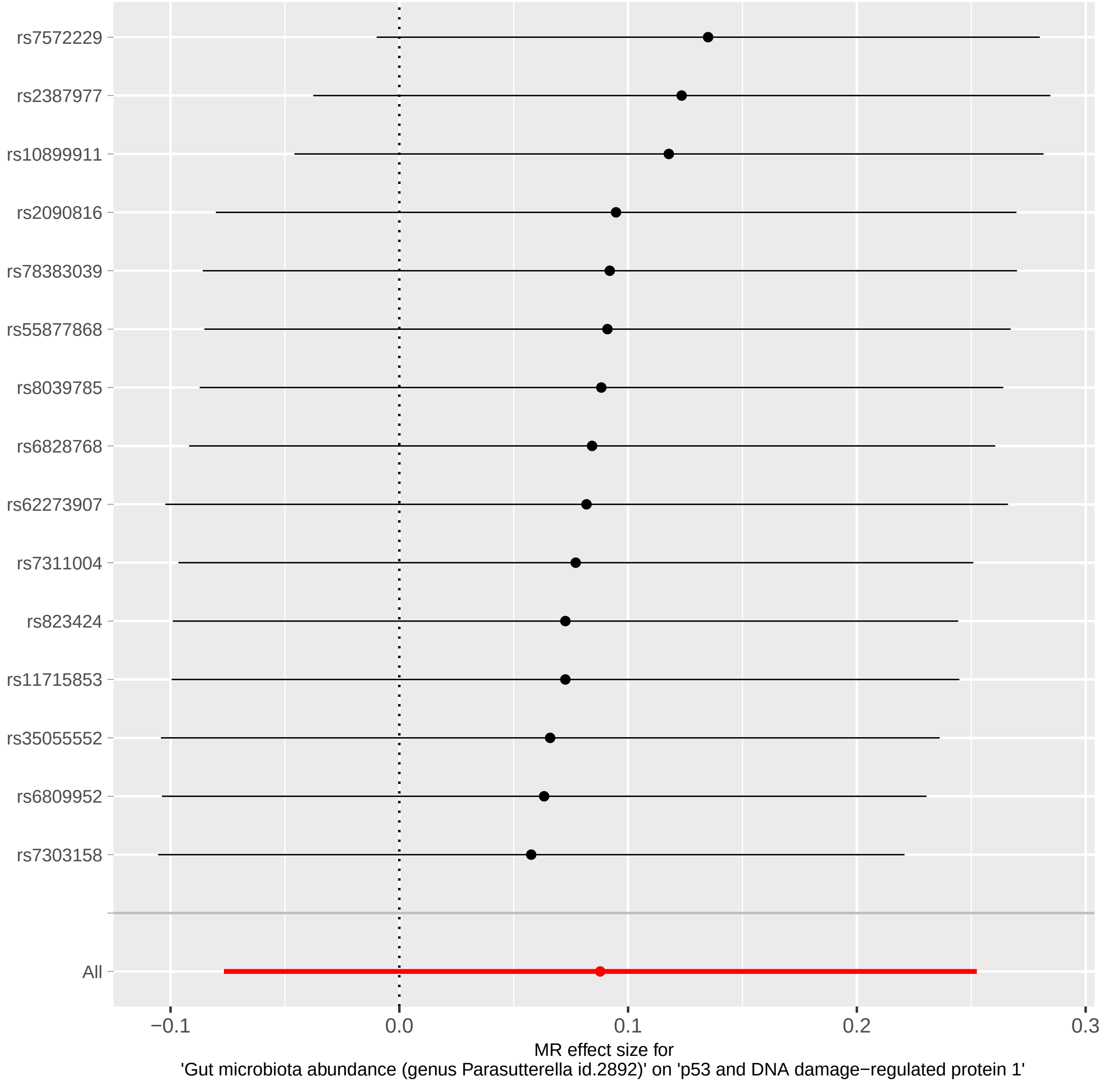

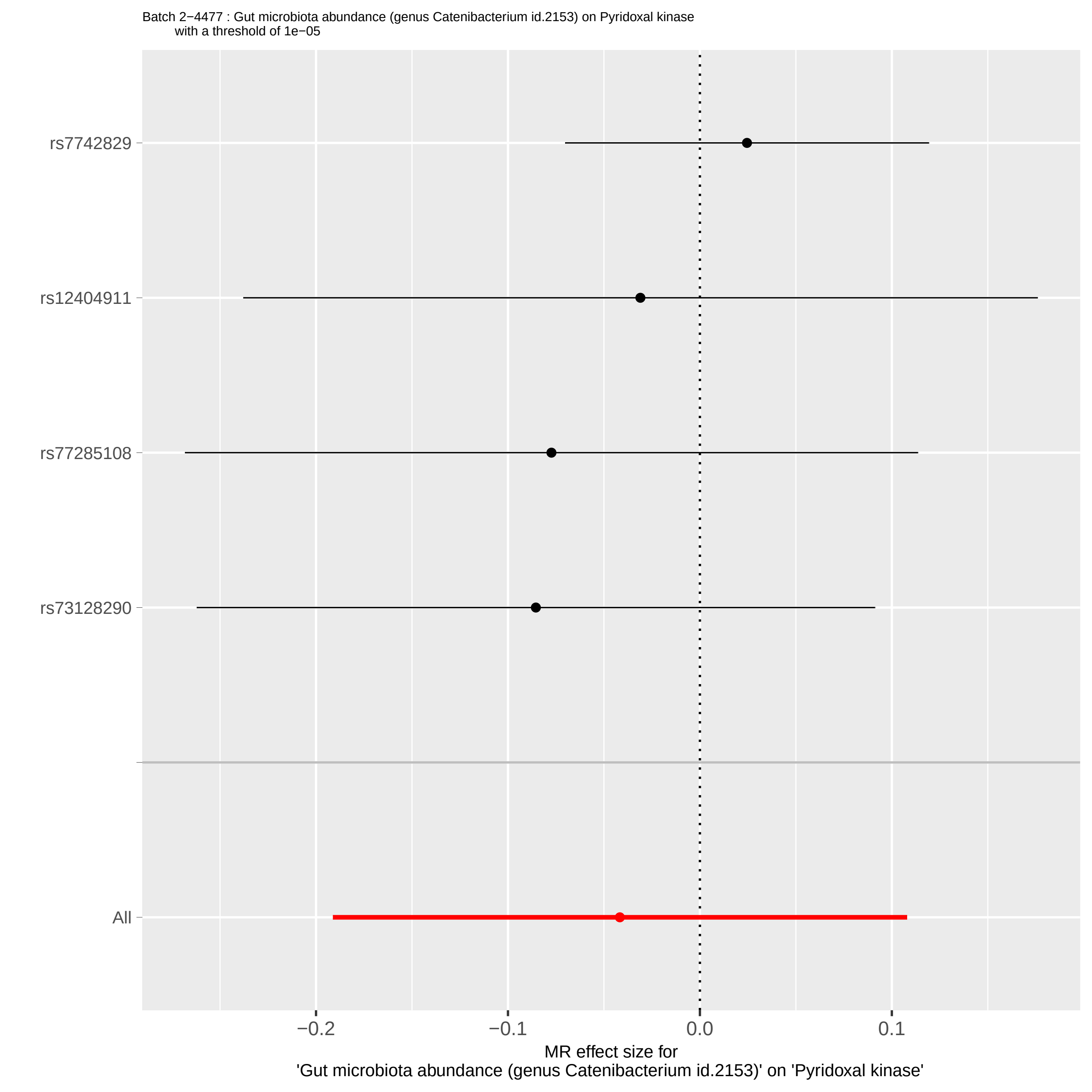

Batch 2−4477 : Gut microbiota abundance (genus Catenibacterium id.2153) on Pyridoxal kinase
with a threshold of 1e−05
rs7742829
rs12404911
rs77285108
rs73128290
All
−0.2
−0.1
0.0
0.1
MR effect size for
'Gut microbiota abundance (genus Catenibacterium id.2153)' on 'Pyridoxal kinase'

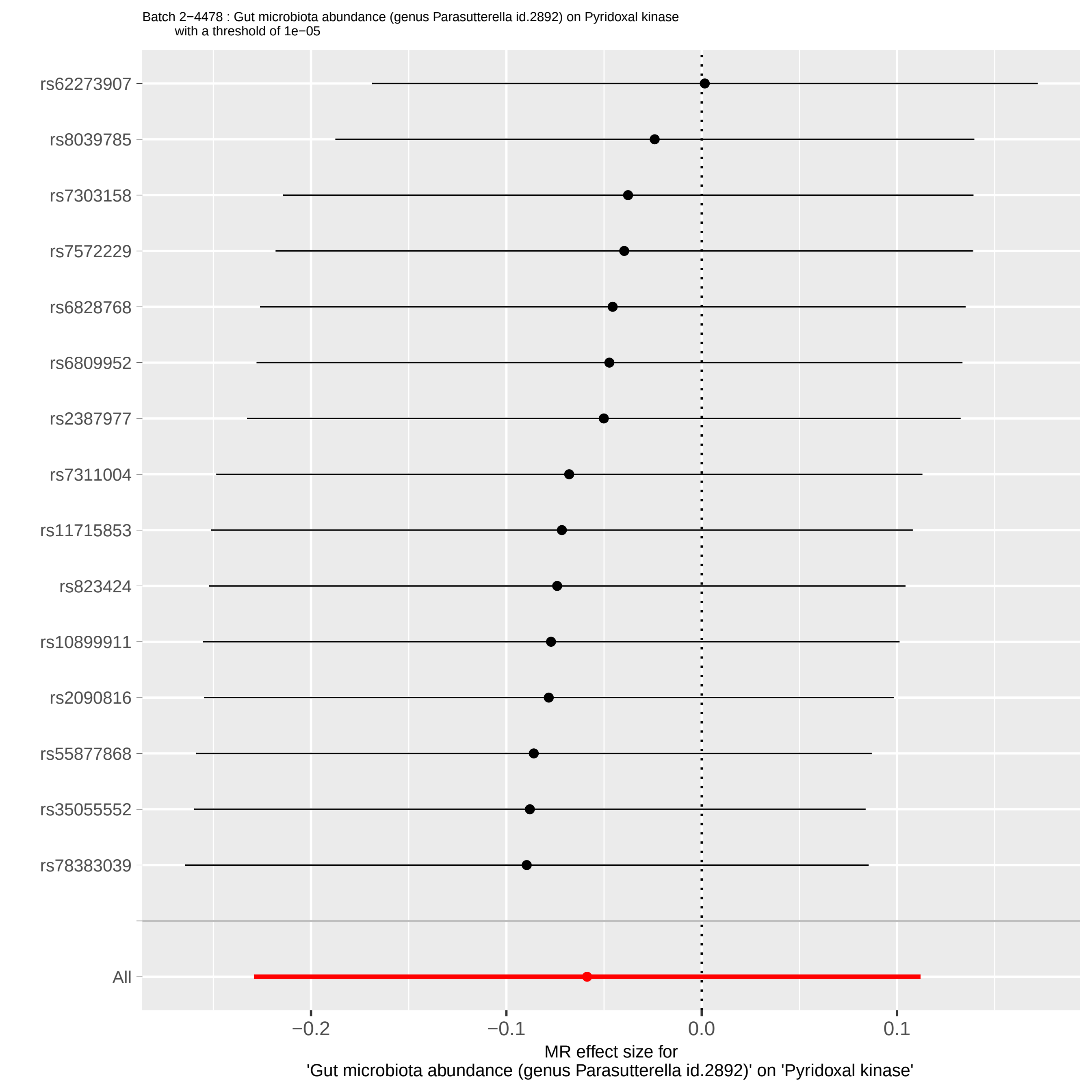

Batch 2−4478 : Gut microbiota abundance (genus Parasutterella id.2892) on Pyridoxal kinase
with a threshold of 1e−05
rs62273907
rs8039785
rs7303158
rs7572229
rs6828768
rs6809952
rs2387977
rs7311004
rs11715853
rs823424
rs10899911
rs2090816
rs55877868
rs35055552
rs78383039
All
−0.2
−0.1
0.0
0.1
MR effect size for
'Gut microbiota abundance (genus Parasutterella id.2892)' on 'Pyridoxal kinase'

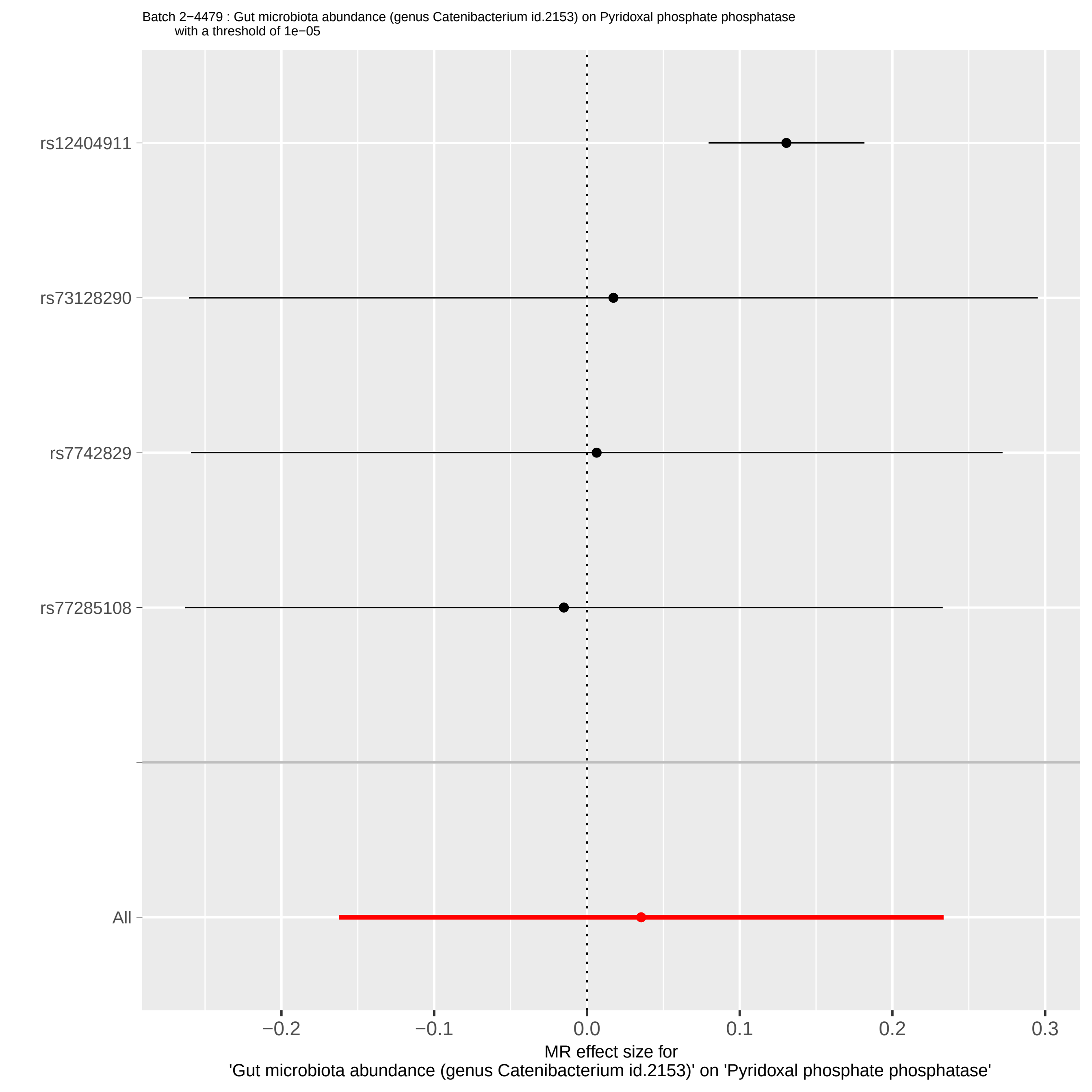

Batch 2−4479 : Gut microbiota abundance (genus Catenibacterium id.2153) on Pyridoxal phosphate phosphatase
with a threshold of 1e−05
rs12404911
rs73128290
rs7742829
rs77285108
All
−0.2
−0.1
0.0
0.1
0.2
0.3
MR effect size for
'Gut microbiota abundance (genus Catenibacterium id.2153)' on 'Pyridoxal phosphate phosphatase'

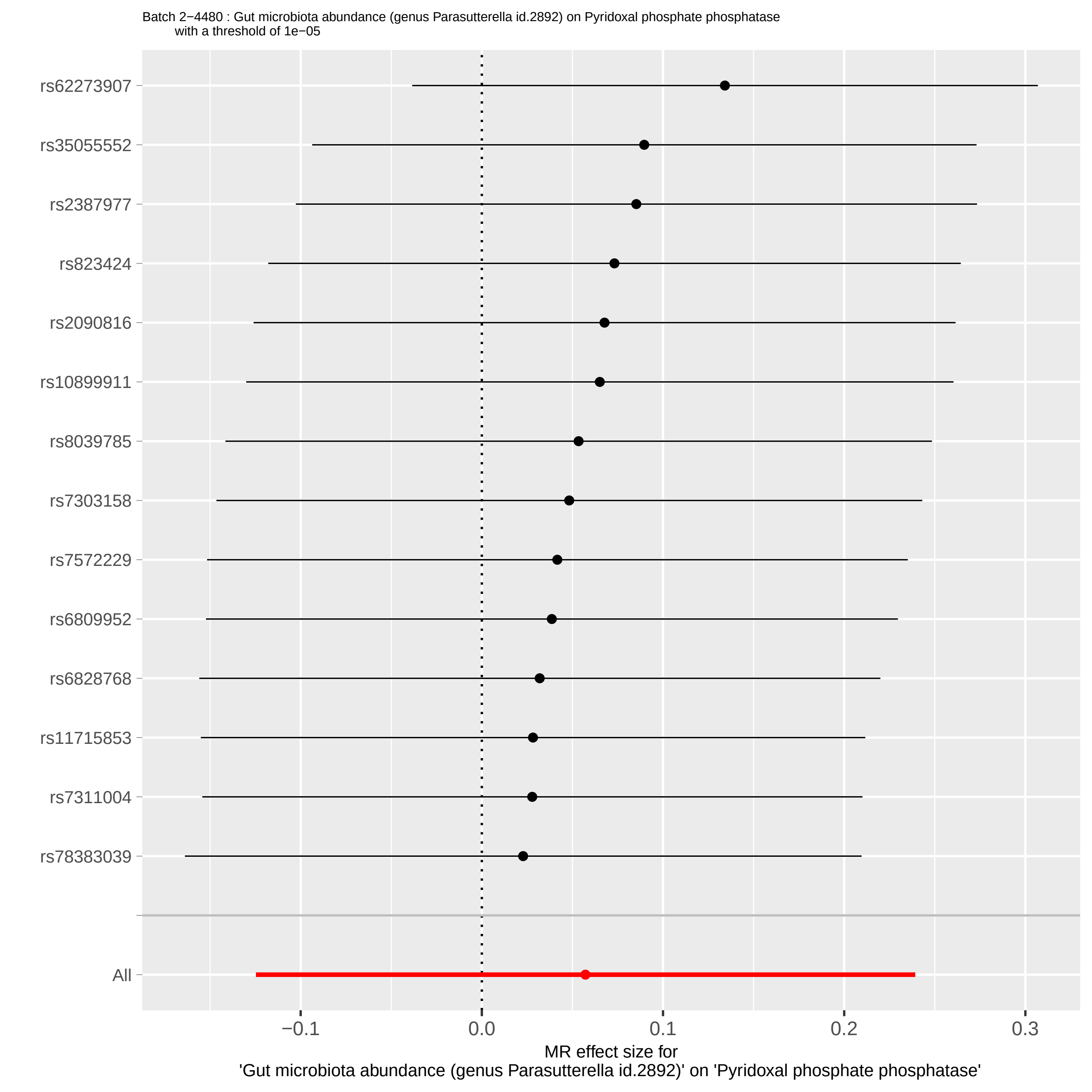

Batch 2−4480 : Gut microbiota abundance (genus Parasutterella id.2892) on Pyridoxal phosphate phosphatase
with a threshold of 1e−05
rs62273907
rs35055552
rs2387977
rs823424
rs2090816
rs10899911
rs8039785
rs7303158
rs7572229
rs6809952
rs6828768
rs11715853
rs7311004
rs78383039
All
−0.1
0.0
0.1
0.2
0.3
MR effect size for
'Gut microbiota abundance (genus Parasutterella id.2892)' on 'Pyridoxal phosphate phosphatase'

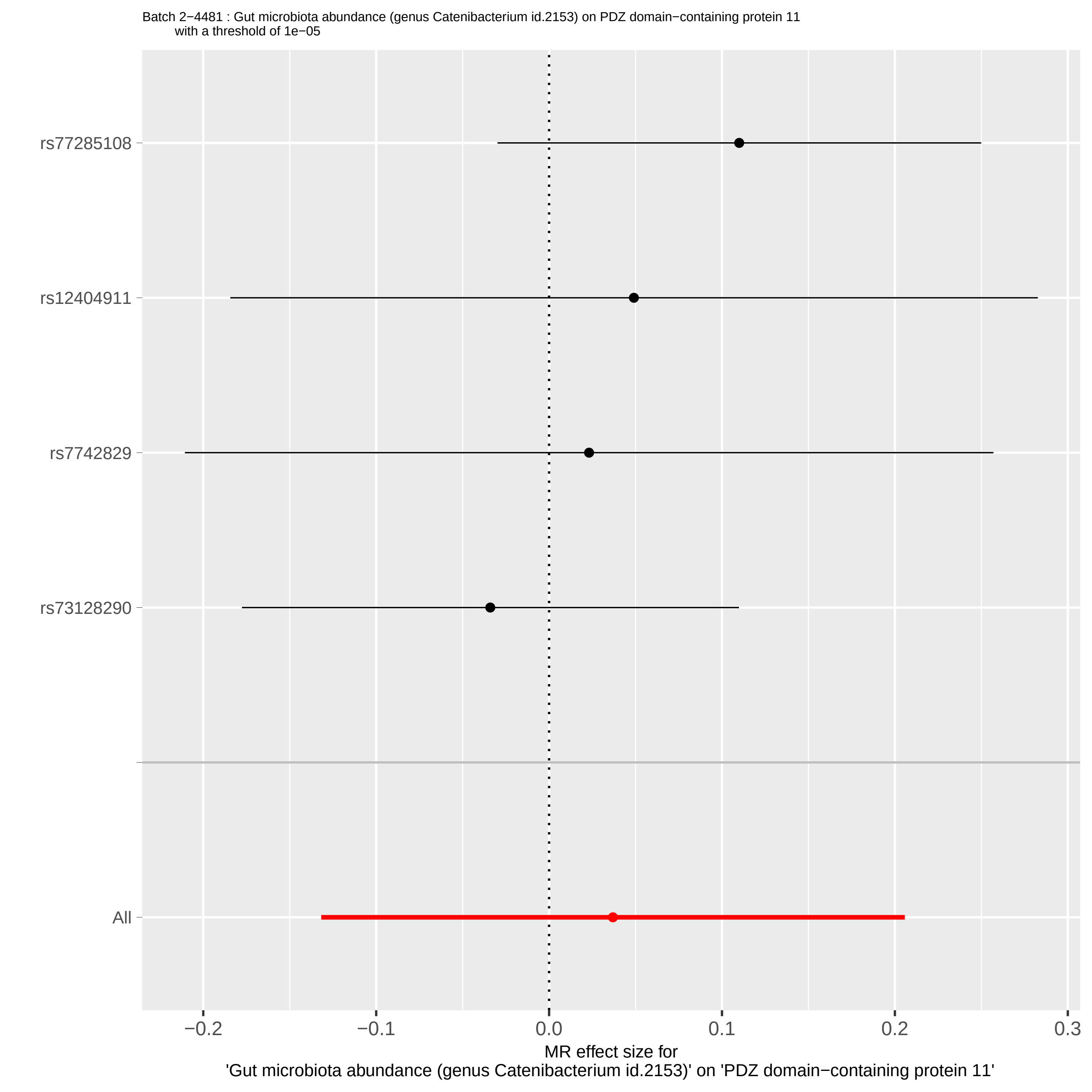
Batch 2−4481 : Gut microbiota abundance (genus Catenibacterium id.2153) on PDZ domain−containing protein 11
with a threshold of 1e−05
rs77285108
rs12404911
rs7742829
rs73128290
All
−0.2
−0.1
0.0
0.1
0.2
0.3
MR effect size for
'Gut microbiota abundance (genus Catenibacterium id.2153)' on 'PDZ domain−containing protein 11'

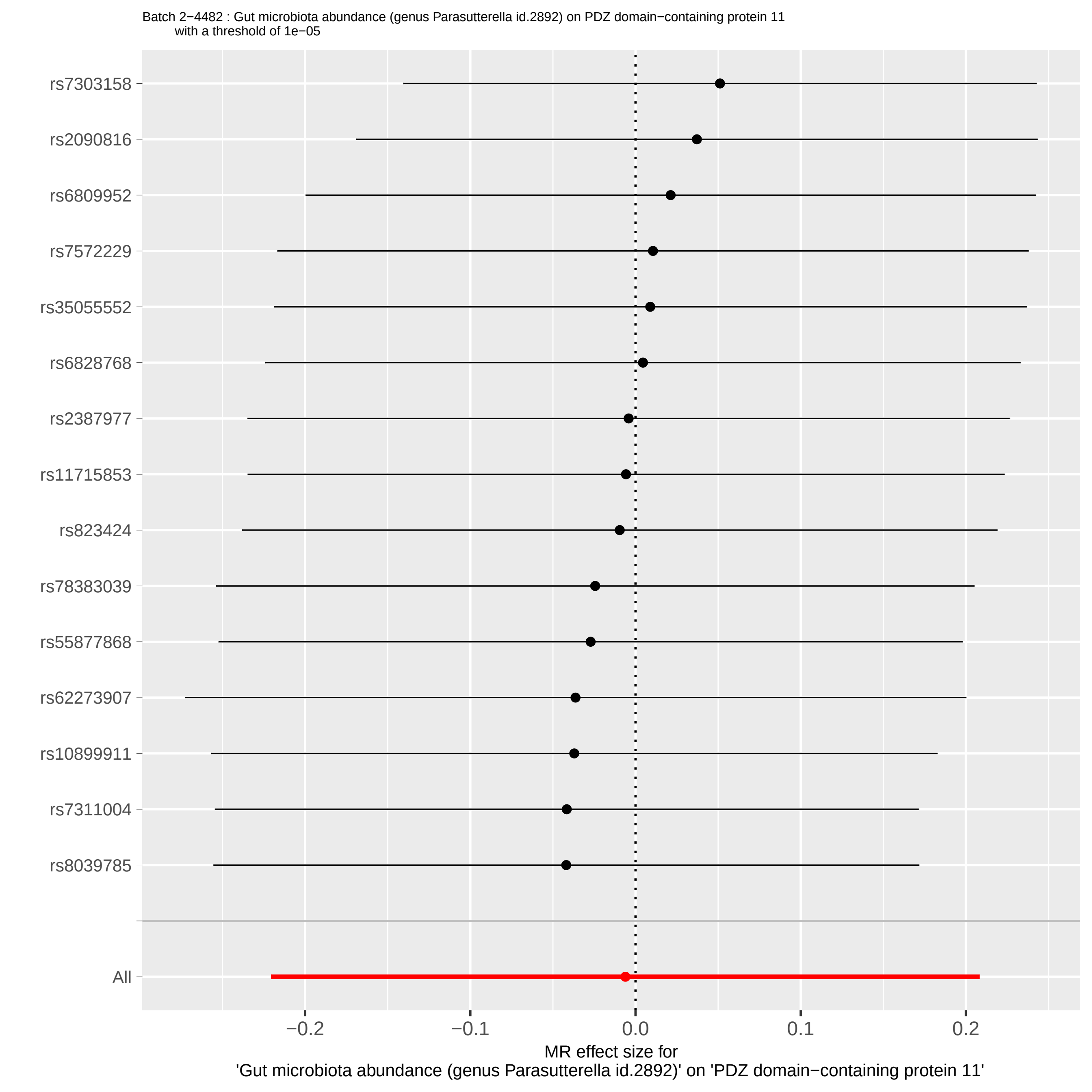

Batch 2−4482 : Gut microbiota abundance (genus Parasutterella id.2892) on PDZ domain−containing protein 11
with a threshold of 1e−05
rs7303158
rs2090816
rs6809952
rs7572229
rs35055552
rs6828768
rs2387977
rs11715853
rs823424
rs78383039
rs55877868
rs62273907
rs10899911
rs7311004
rs8039785
All
−0.2
−0.1
0.0
0.1
0.2
MR effect size for
'Gut microbiota abundance (genus Parasutterella id.2892)' on 'PDZ domain−containing protein 11'

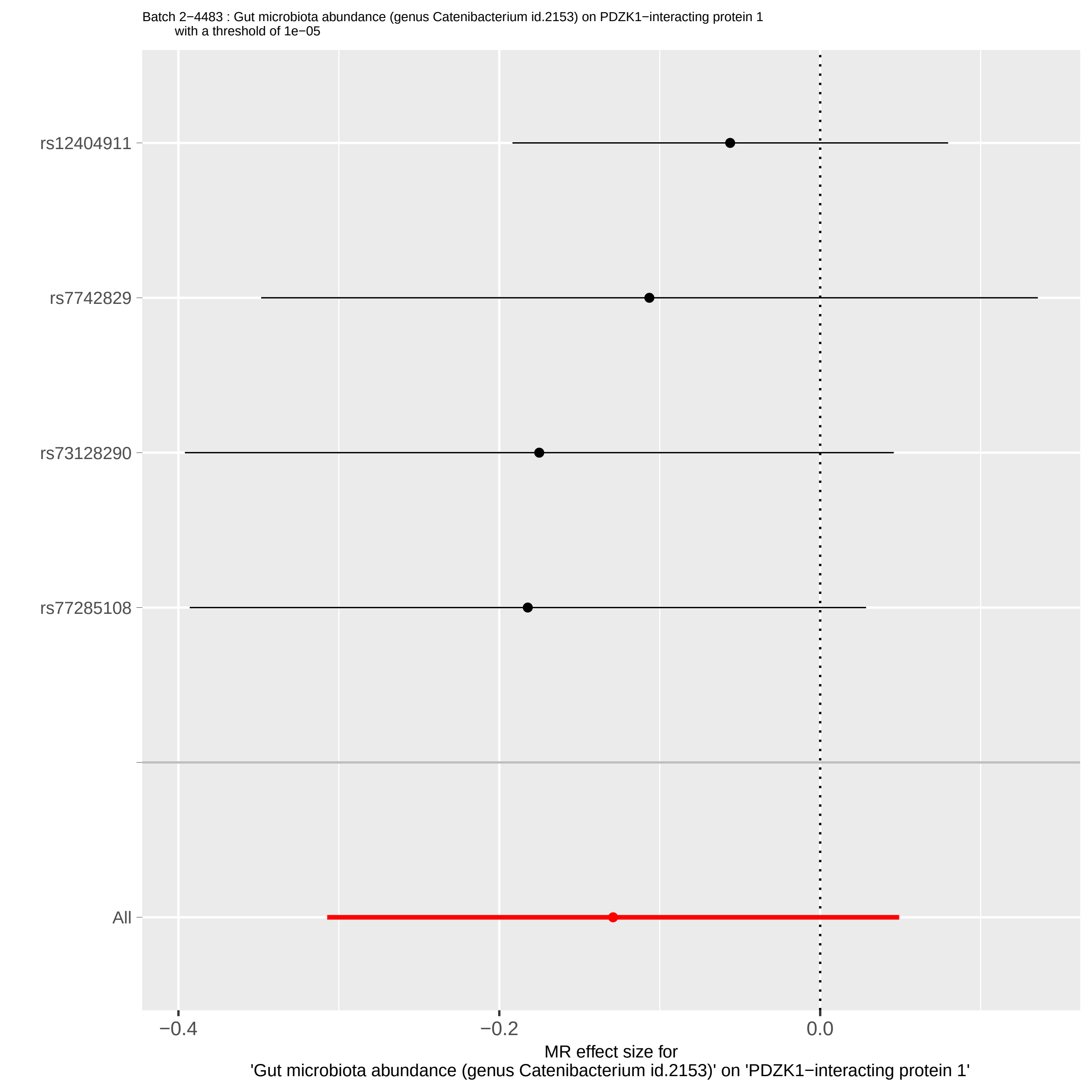

Batch 2−4483 : Gut microbiota abundance (genus Catenibacterium id.2153) on PDZK1−interacting protein 1
with a threshold of 1e−05
rs12404911
rs7742829
rs73128290
rs77285108
All
−0.4
−0.2
0.0
MR effect size for
'Gut microbiota abundance (genus Catenibacterium id.2153)' on 'PDZK1−interacting protein 1'

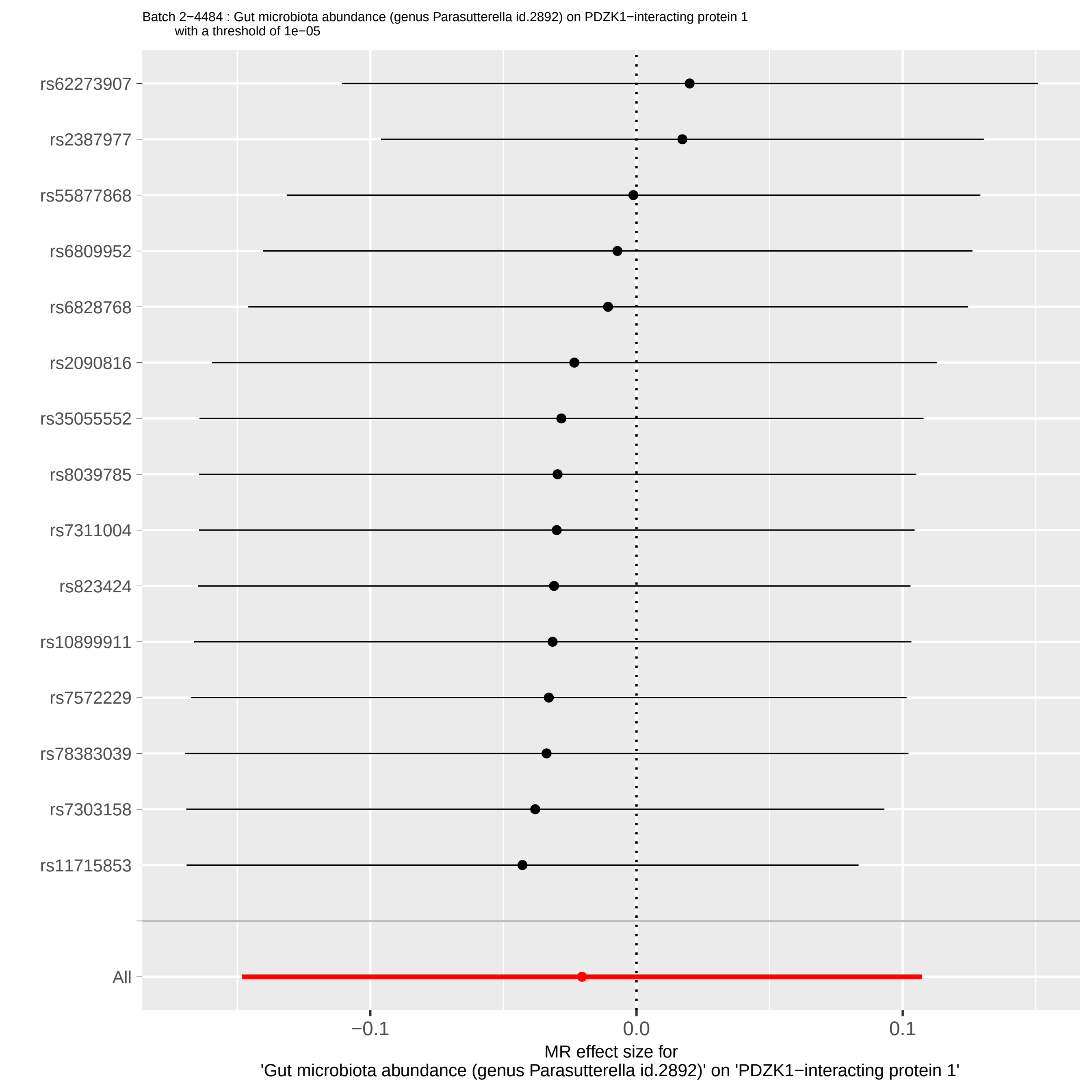
Batch 2−4484 : Gut microbiota abundance (genus Parasutterella id.2892) on PDZK1−interacting protein 1
with a threshold of 1e−05
rs62273907
rs2387977
rs55877868
rs6809952
rs6828768
rs2090816
rs35055552
rs8039785
rs7311004
rs823424
rs10899911
rs7572229
rs78383039
rs7303158
rs11715853
All
−0.1
0.0
0.1
MR effect size for
'Gut microbiota abundance (genus Parasutterella id.2892)' on 'PDZK1−interacting protein 1'

Batch 2−4485 : Gut microbiota abundance (genus Catenibacterium id.2153) on Platelet endothelial aggregation receptor 1
with a threshold of 1e−05

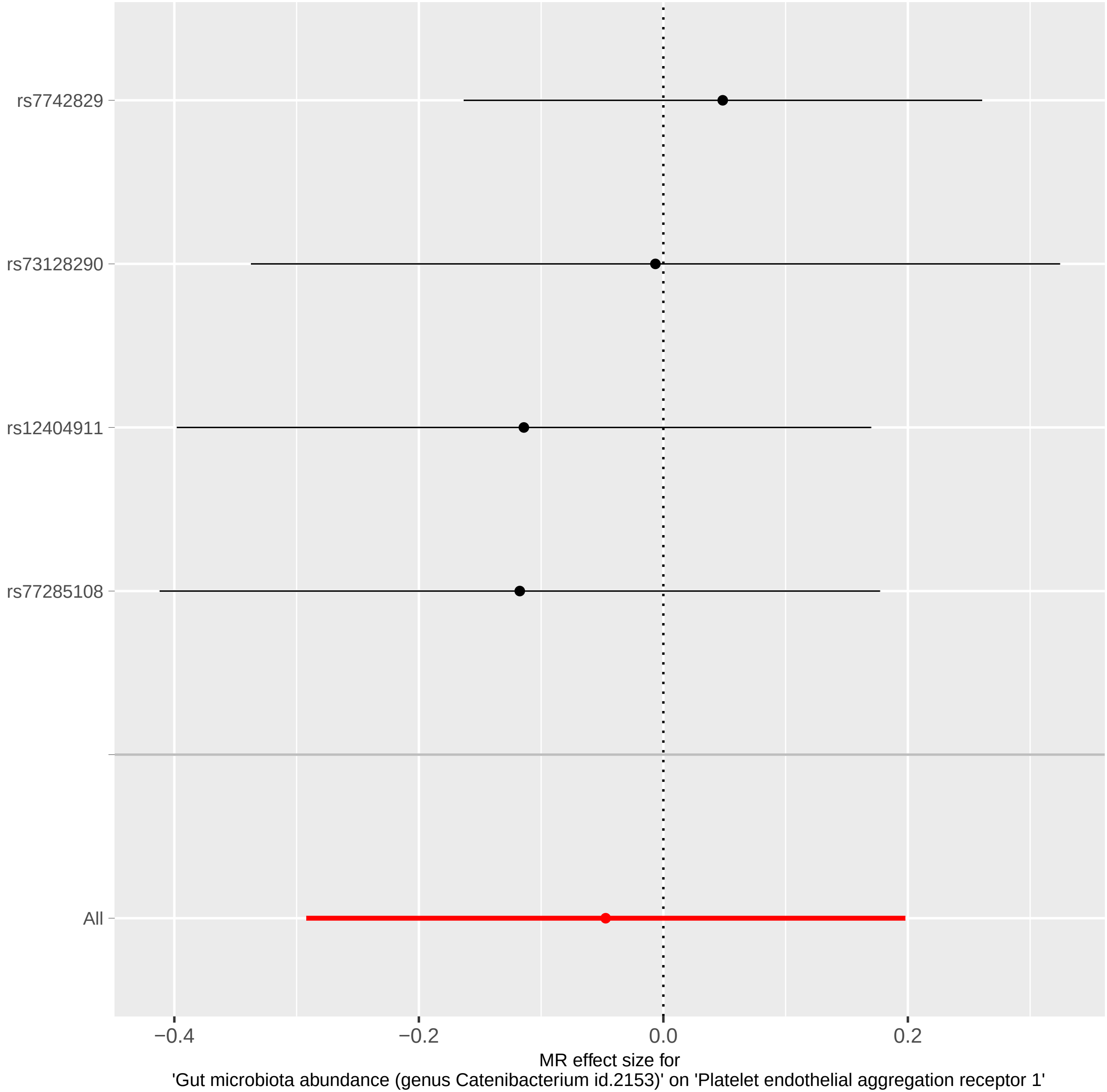

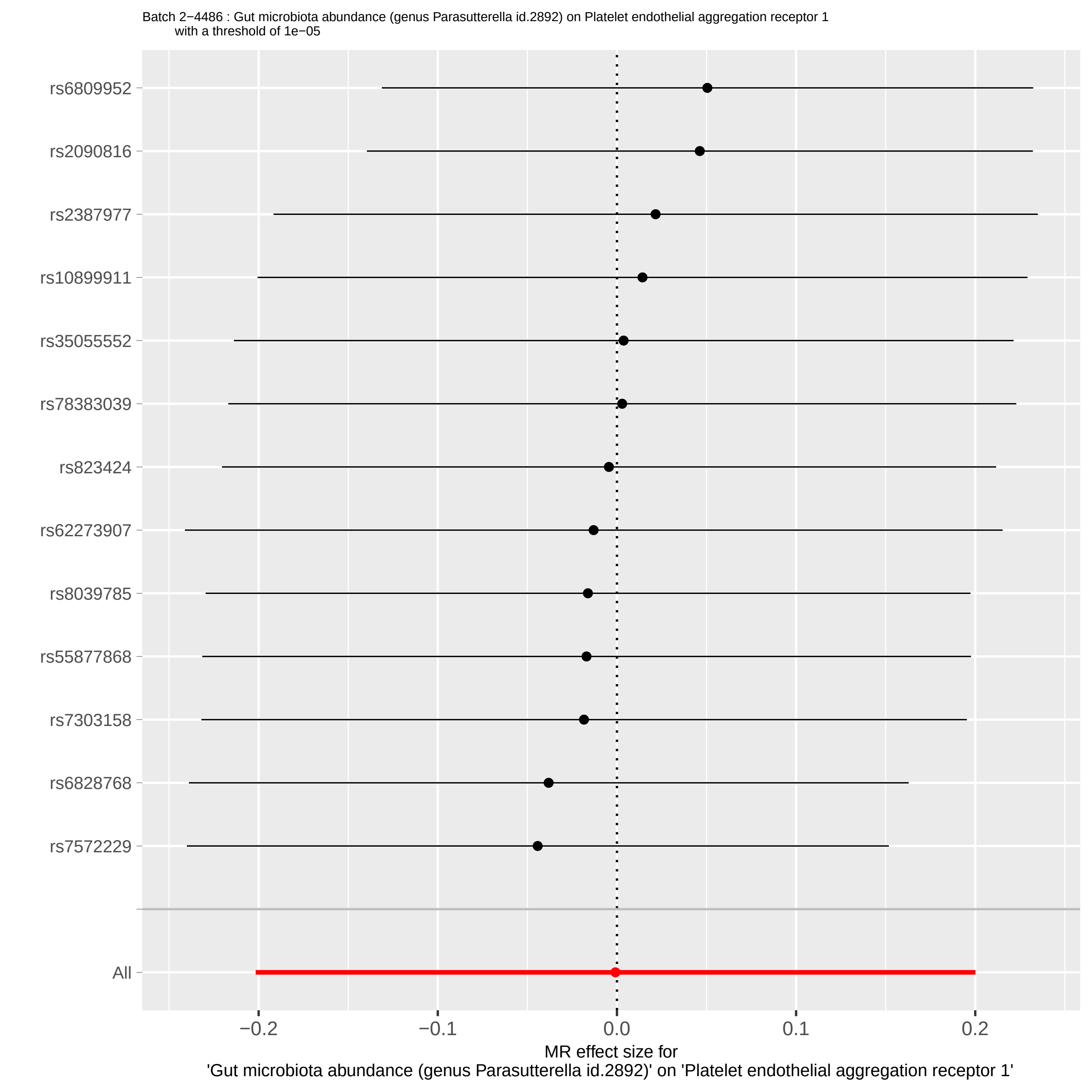
Batch 2−4486 : Gut microbiota abundance (genus Parasutterella id.2892) on Platelet endothelial aggregation receptor 1
with a threshold of 1e−05
rs6809952
rs2090816
rs2387977
rs10899911
rs35055552
rs78383039
rs823424
rs62273907
rs8039785
rs55877868
rs7303158
rs6828768
rs7572229
All
−0.2
−0.1
0.0
0.1
0.2
MR effect size for
'Gut microbiota abundance (genus Parasutterella id.2892)' on 'Platelet endothelial aggregation receptor 1'

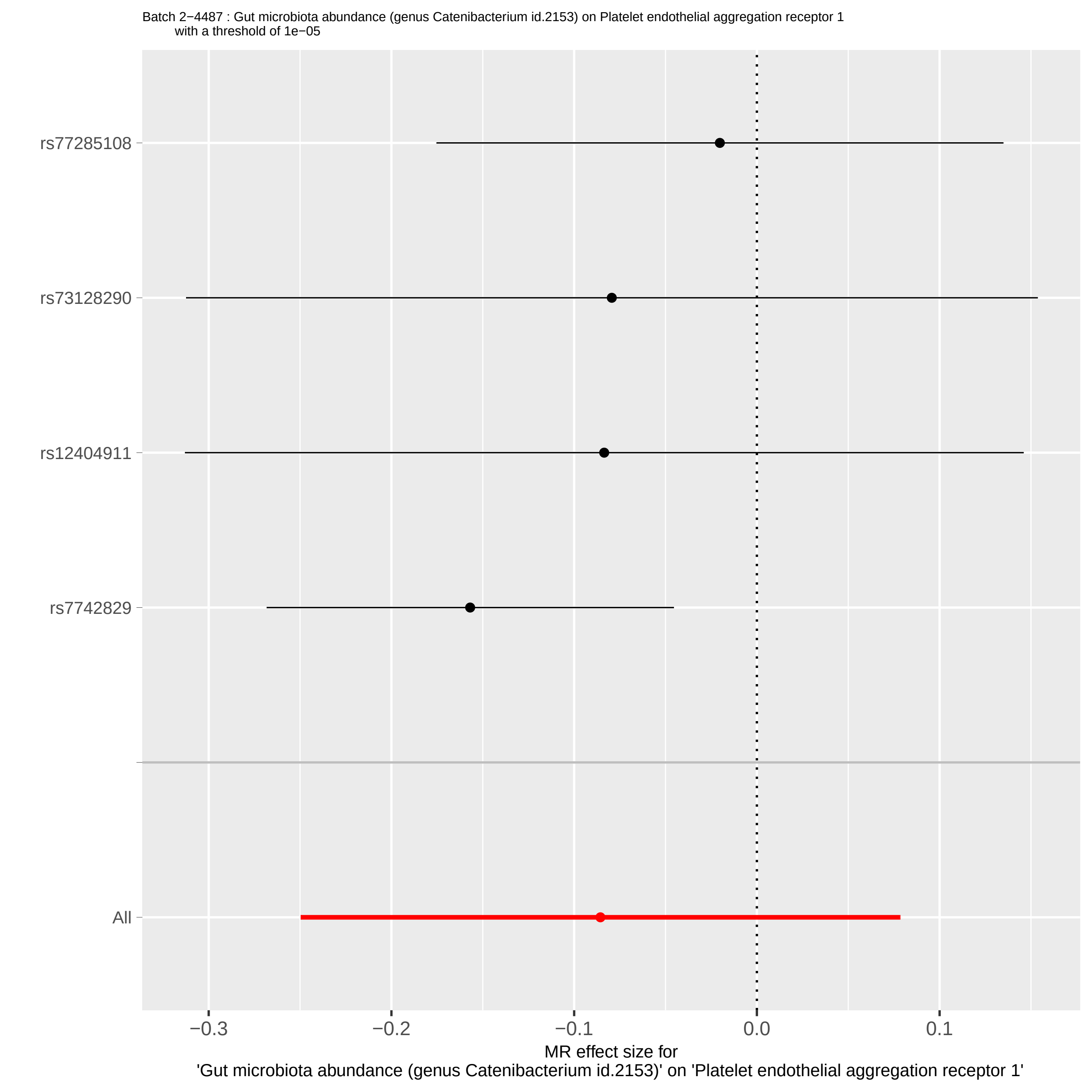

Batch 2−4487 : Gut microbiota abundance (genus Catenibacterium id.2153) on Platelet endothelial aggregation receptor 1
with a threshold of 1e−05
rs77285108
rs73128290
rs12404911
rs7742829
All
−0.3
−0.2
−0.1
0.0
0.1
MR effect size for
'Gut microbiota abundance (genus Catenibacterium id.2153)' on 'Platelet endothelial aggregation receptor 1'

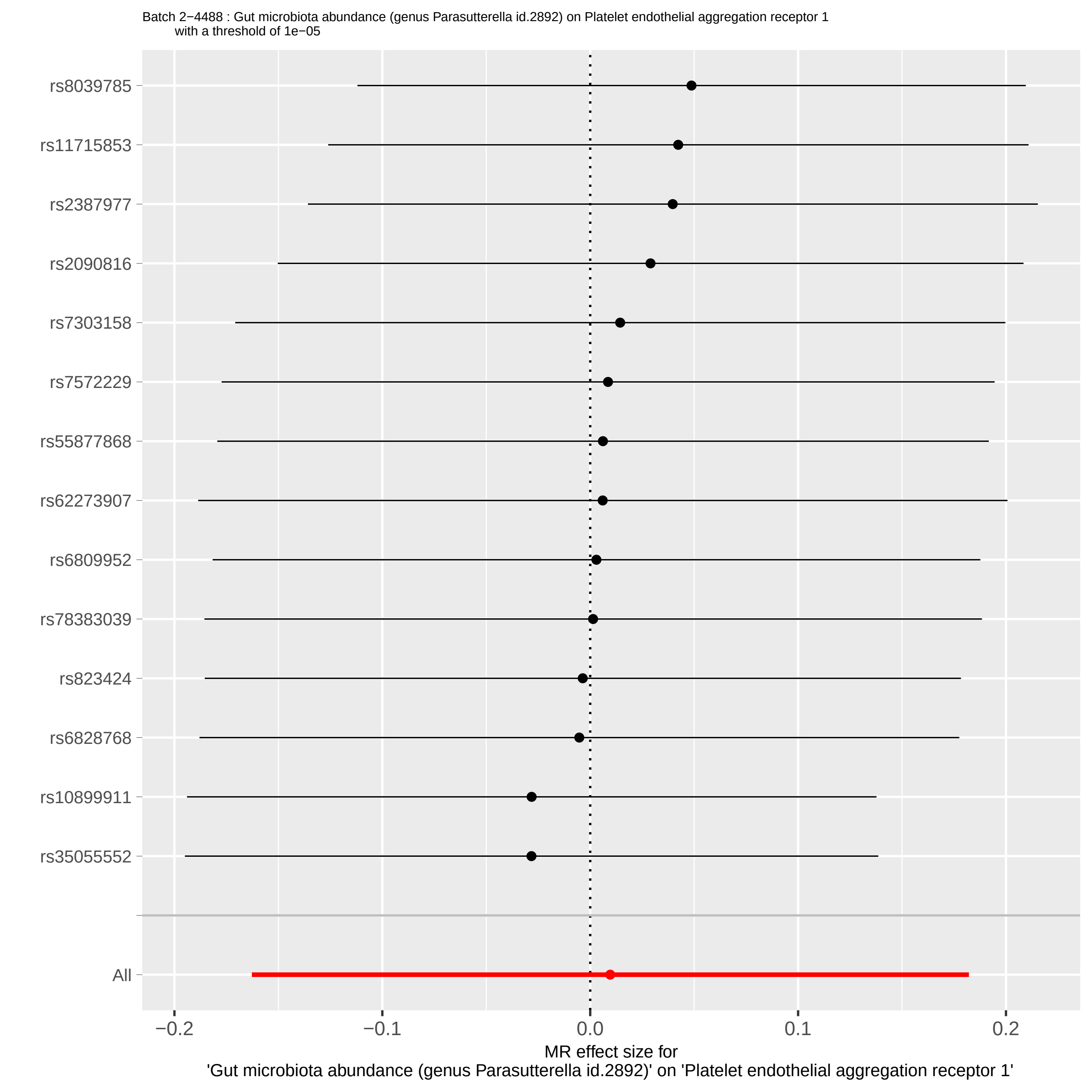
Batch 2−4488 : Gut microbiota abundance (genus Parasutterella id.2892) on Platelet endothelial aggregation receptor 1
with a threshold of 1e−05
rs8039785
rs11715853
rs2387977
rs2090816
rs7303158
rs7572229
rs55877868
rs62273907
rs6809952
rs78383039
rs823424
rs6828768
rs10899911
rs35055552
All
−0.2
−0.1
0.0
0.1
0.2
MR effect size for
'Gut microbiota abundance (genus Parasutterella id.2892)' on 'Platelet endothelial aggregation receptor 1'

Batch 2−4489 : Gut microbiota abundance (genus Catenibacterium id.2153) on Platelet endothelial cell adhesion molecule
with a threshold of 1e−05

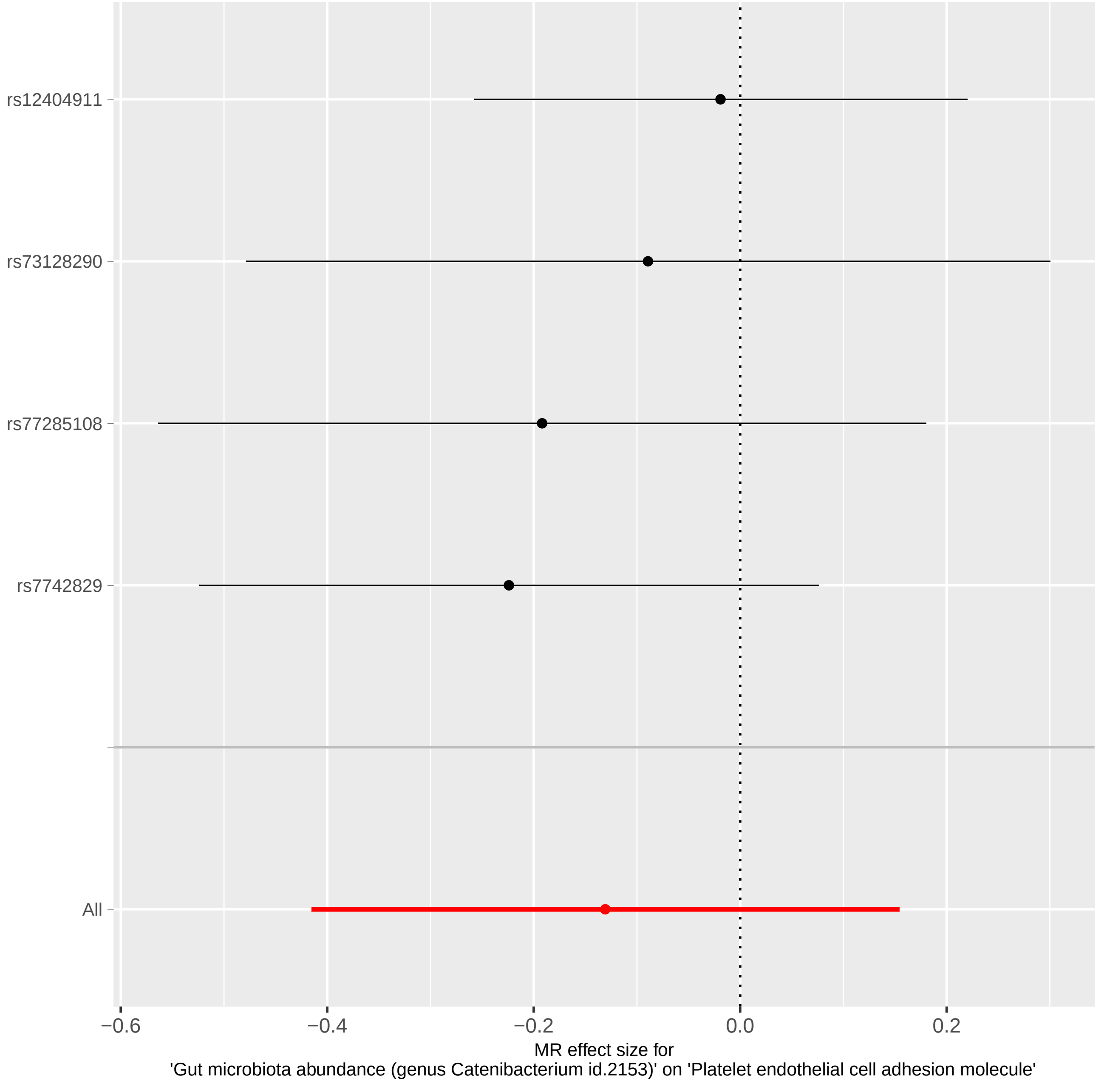

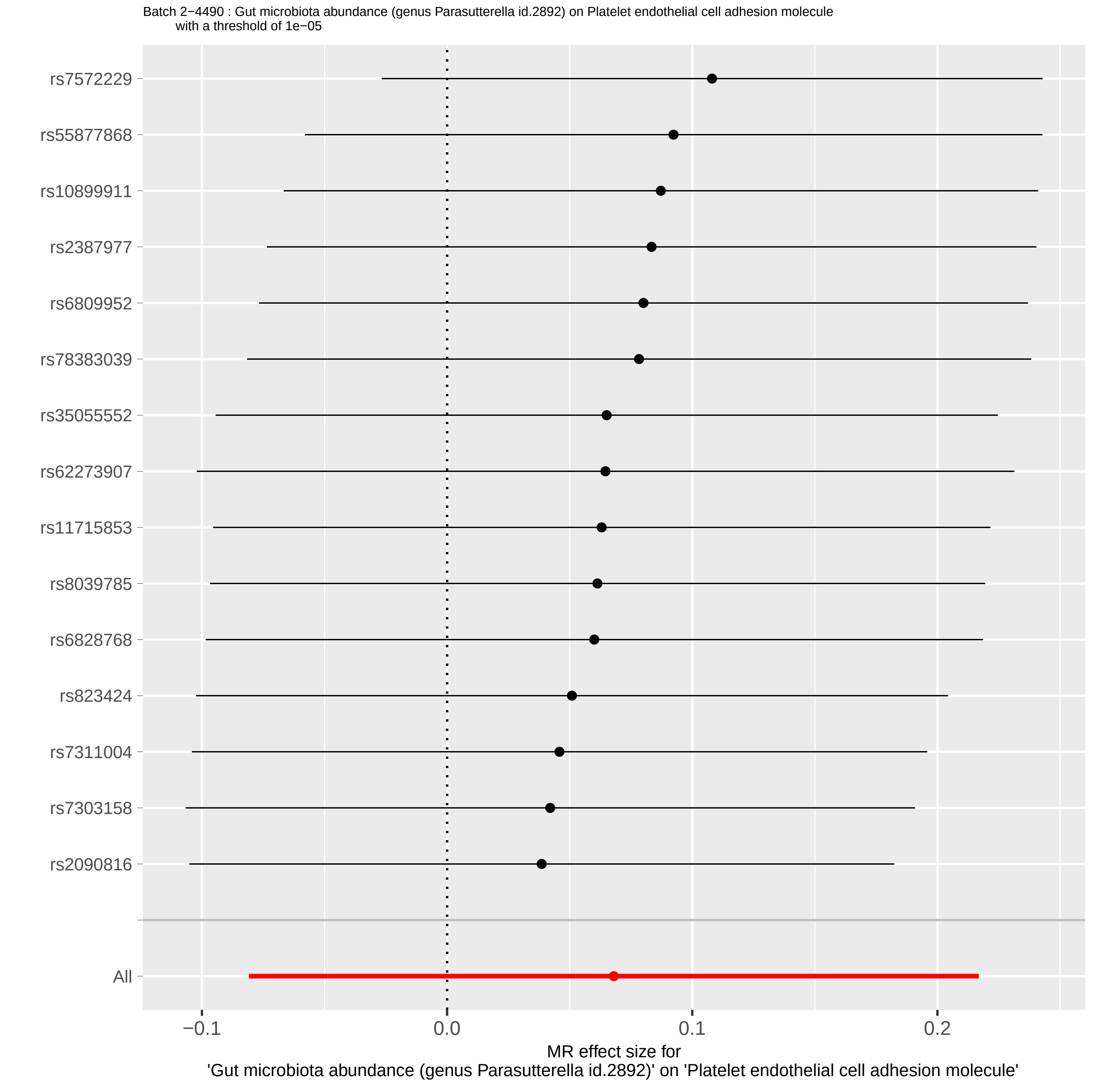

Batch 2−4490 : Gut microbiota abundance (genus Parasutterella id.2892) on Platelet endothelial cell adhesion molecule
with a threshold of 1e−05
rs7572229
rs55877868
rs10899911
rs2387977
rs6809952
rs78383039
rs35055552
rs62273907
rs11715853
rs8039785
rs6828768
rs823424
rs7311004
rs7303158
rs2090816
All
−0.1
0.0
0.1
0.2
MR effect size for
'Gut microbiota abundance (genus Parasutterella id.2892)' on 'Platelet endothelial cell adhesion molecule'

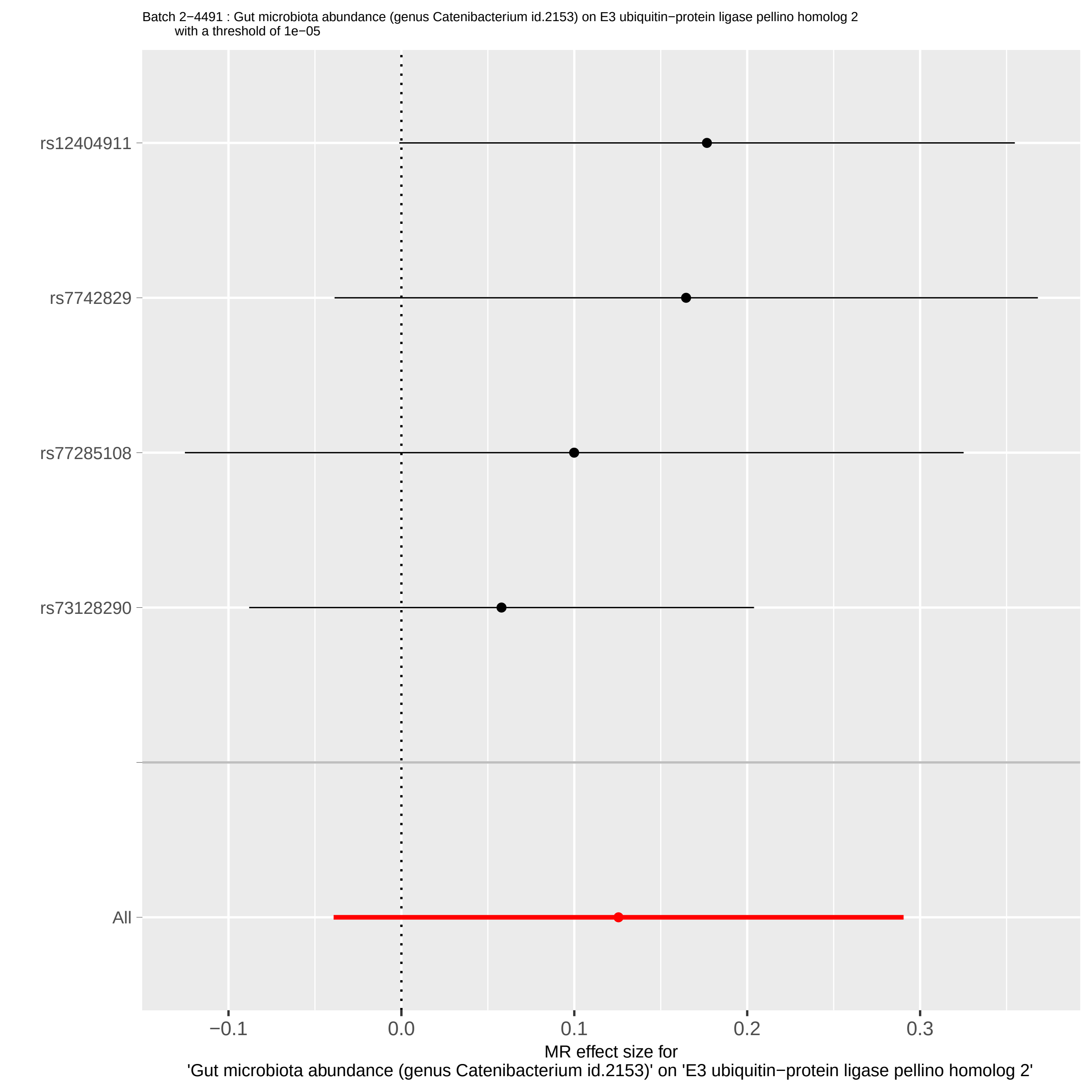
Batch 2−4491 : Gut microbiota abundance (genus Catenibacterium id.2153) on E3 ubiquitin−protein ligase pellino homolog 2
with a threshold of 1e−05
rs12404911
rs7742829
rs77285108
rs73128290
All
−0.1
0.0
0.1
0.2
0.3
MR effect size for
'Gut microbiota abundance (genus Catenibacterium id.2153)' on 'E3 ubiquitin−protein ligase pellino homolog 2'

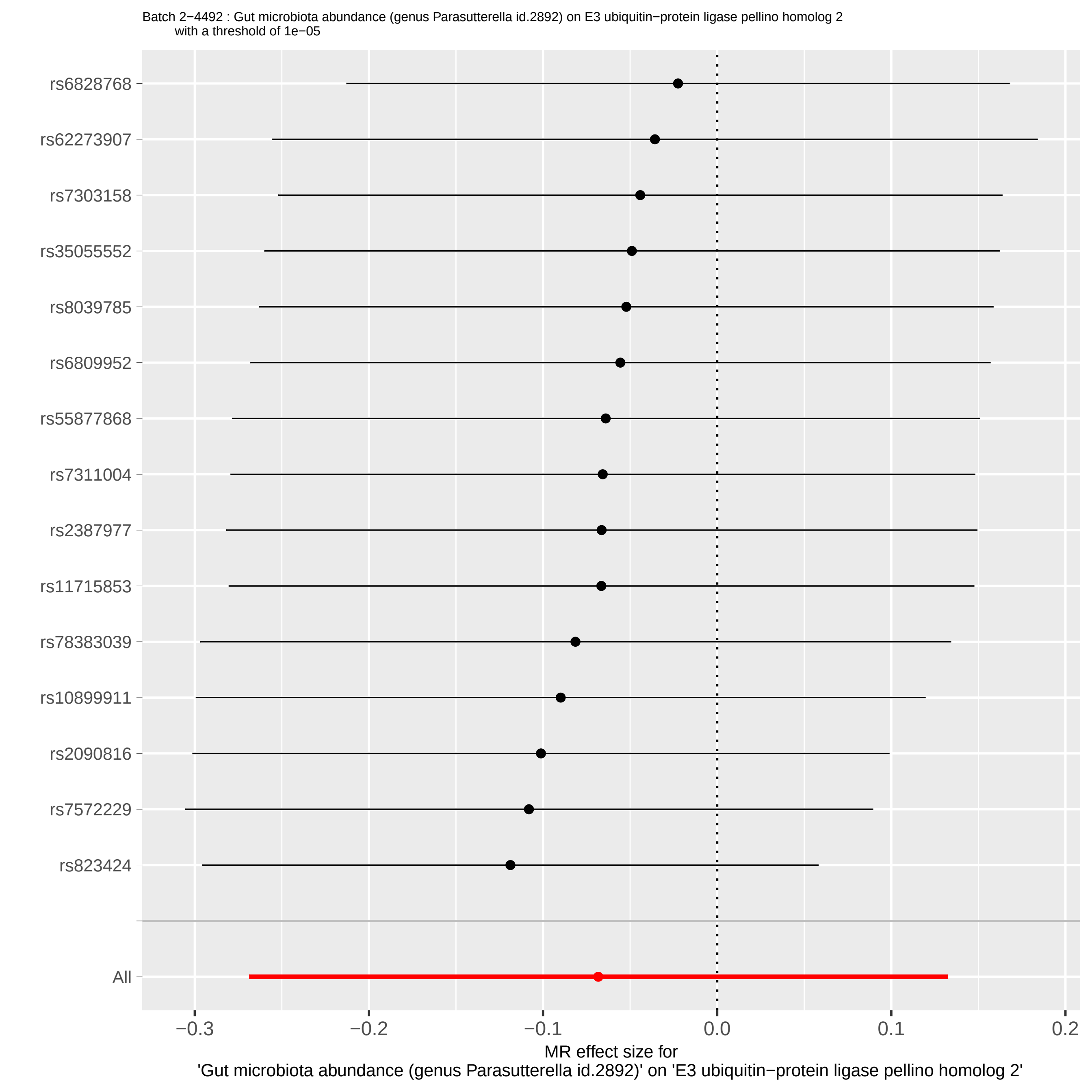
Batch 2−4492 : Gut microbiota abundance (genus Parasutterella id.2892) on E3 ubiquitin−protein ligase pellino homolog 2
with a threshold of 1e−05
rs6828768
rs62273907
rs7303158
rs35055552
rs8039785
rs6809952
rs55877868
rs7311004
rs2387977
rs11715853
rs78383039
rs10899911
rs2090816
rs7572229
rs823424
All
−0.3
−0.2
−0.1
0.0
0.1
0.2
MR effect size for
'Gut microbiota abundance (genus Parasutterella id.2892)' on 'E3 ubiquitin−protein ligase pellino homolog 2'

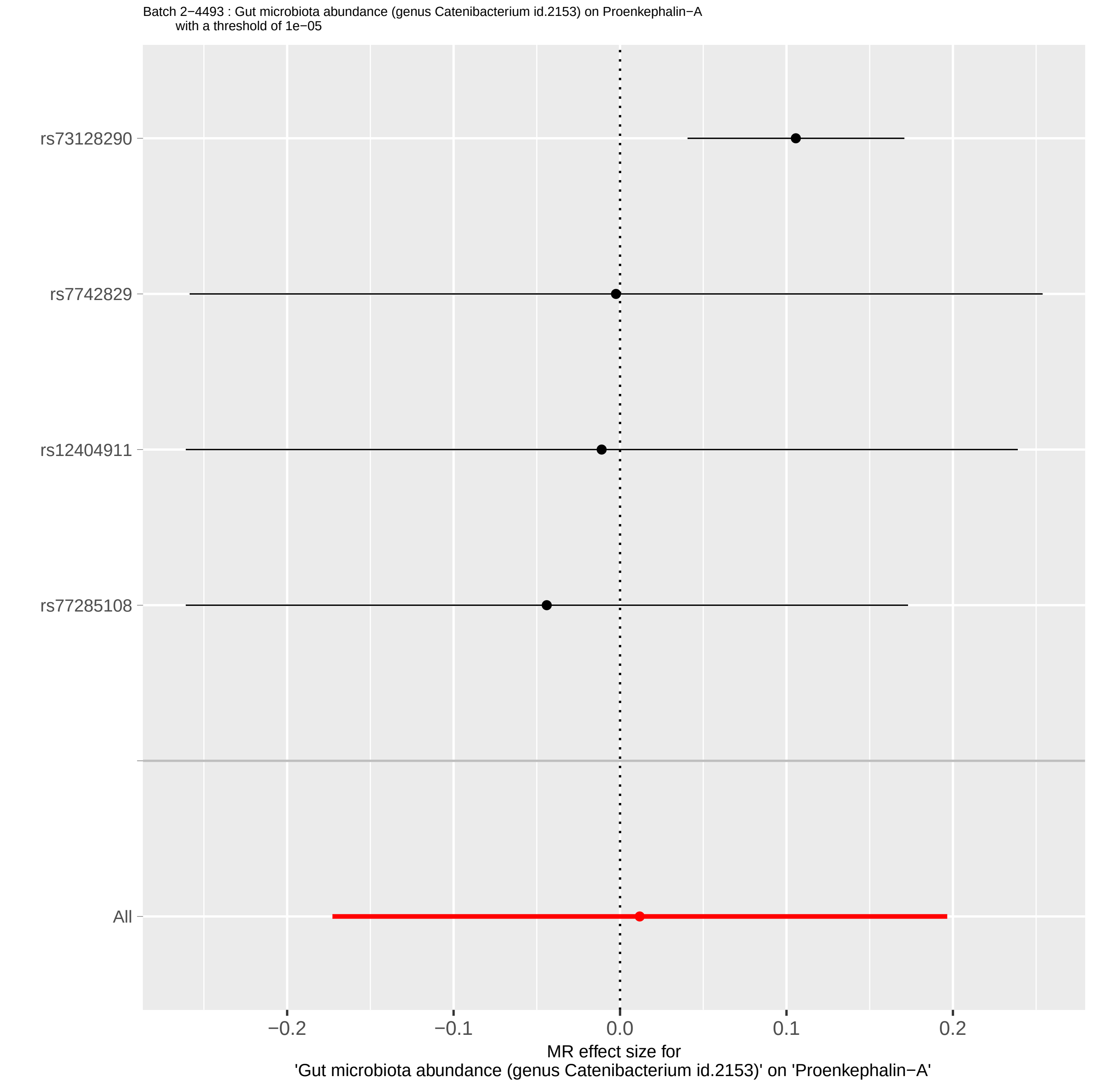
Batch 2−4493 : Gut microbiota abundance (genus Catenibacterium id.2153) on Proenkephalin−A
with a threshold of 1e−05
rs73128290
rs7742829
rs12404911
rs77285108
All
−0.2
−0.1
0.0
0.1
0.2
MR effect size for
'Gut microbiota abundance (genus Catenibacterium id.2153)' on 'Proenkephalin−A'

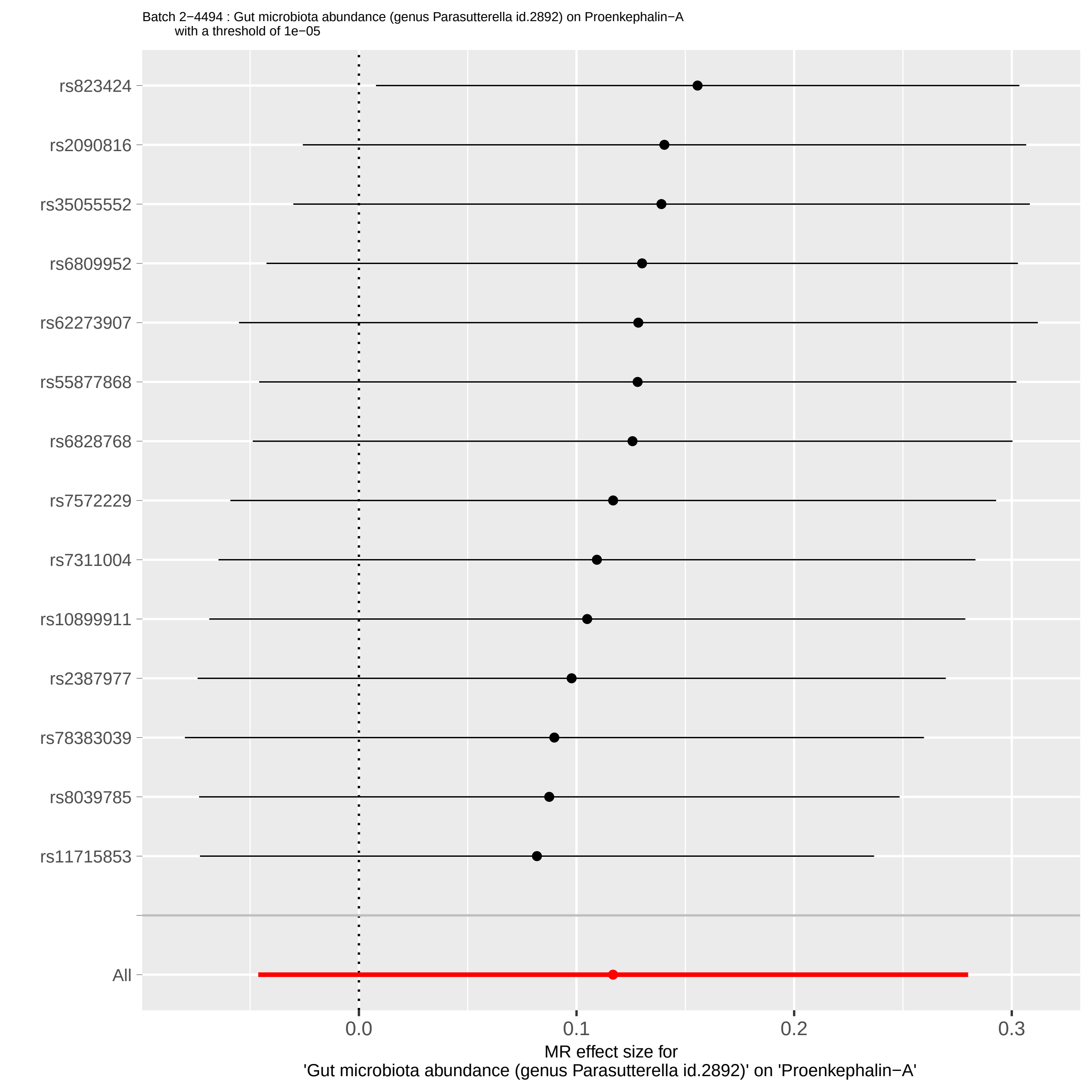

Batch 2−4494 : Gut microbiota abundance (genus Parasutterella id.2892) on Proenkephalin−A
with a threshold of 1e−05
rs823424
rs2090816
rs35055552
rs6809952
rs62273907
rs55877868
rs6828768
rs7572229
rs7311004
rs10899911
rs2387977
rs78383039
rs8039785
rs11715853
All
0.0
0.1
0.2
0.3
MR effect size for
'Gut microbiota abundance (genus Parasutterella id.2892)' on 'Proenkephalin−A'

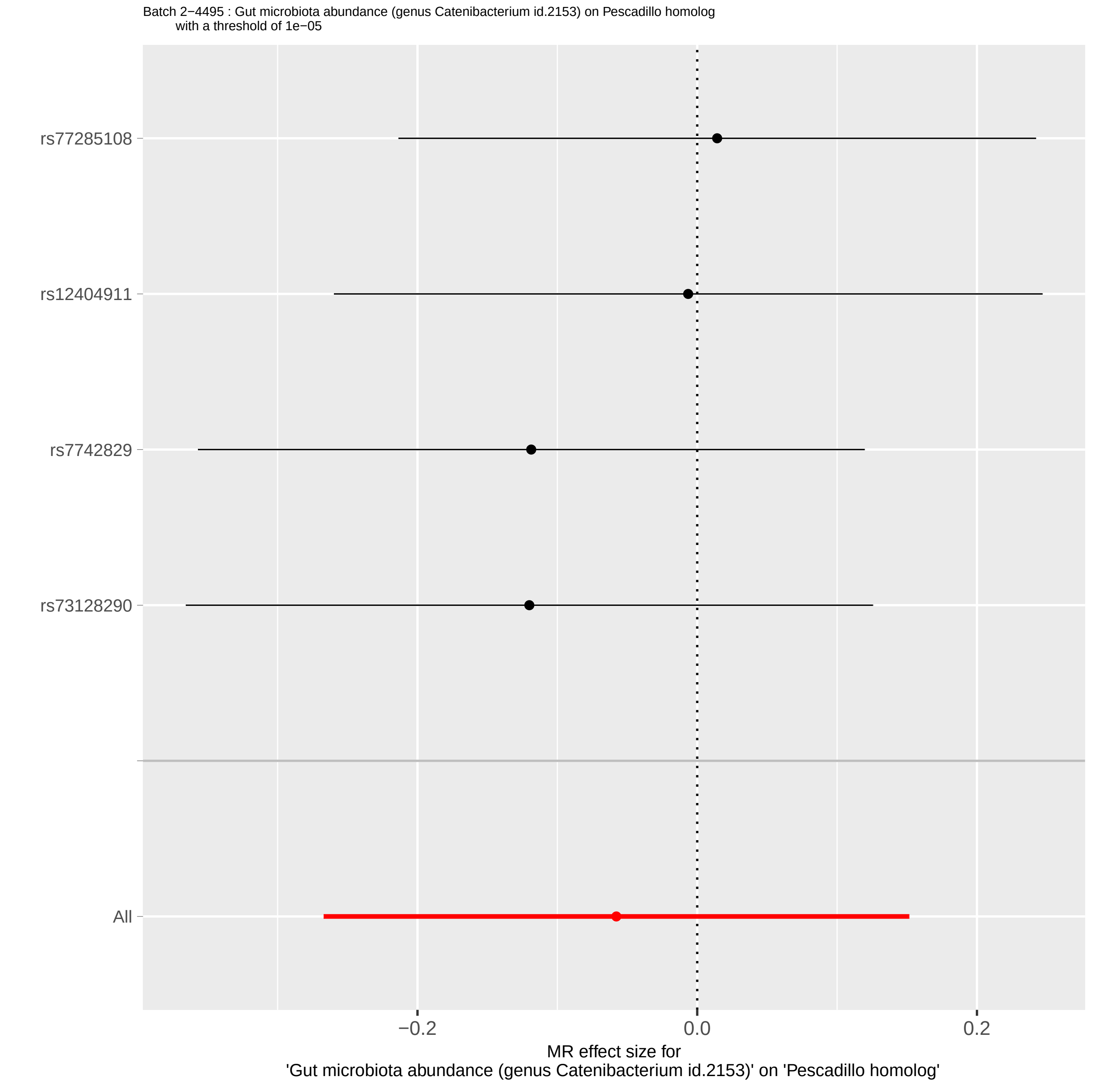
Batch 2−4495 : Gut microbiota abundance (genus Catenibacterium id.2153) on Pescadillo homolog
with a threshold of 1e−05
rs77285108
rs12404911
rs7742829
rs73128290
All
−0.2
0.0
0.2
MR effect size for
'Gut microbiota abundance (genus Catenibacterium id.2153)' on 'Pescadillo homolog'

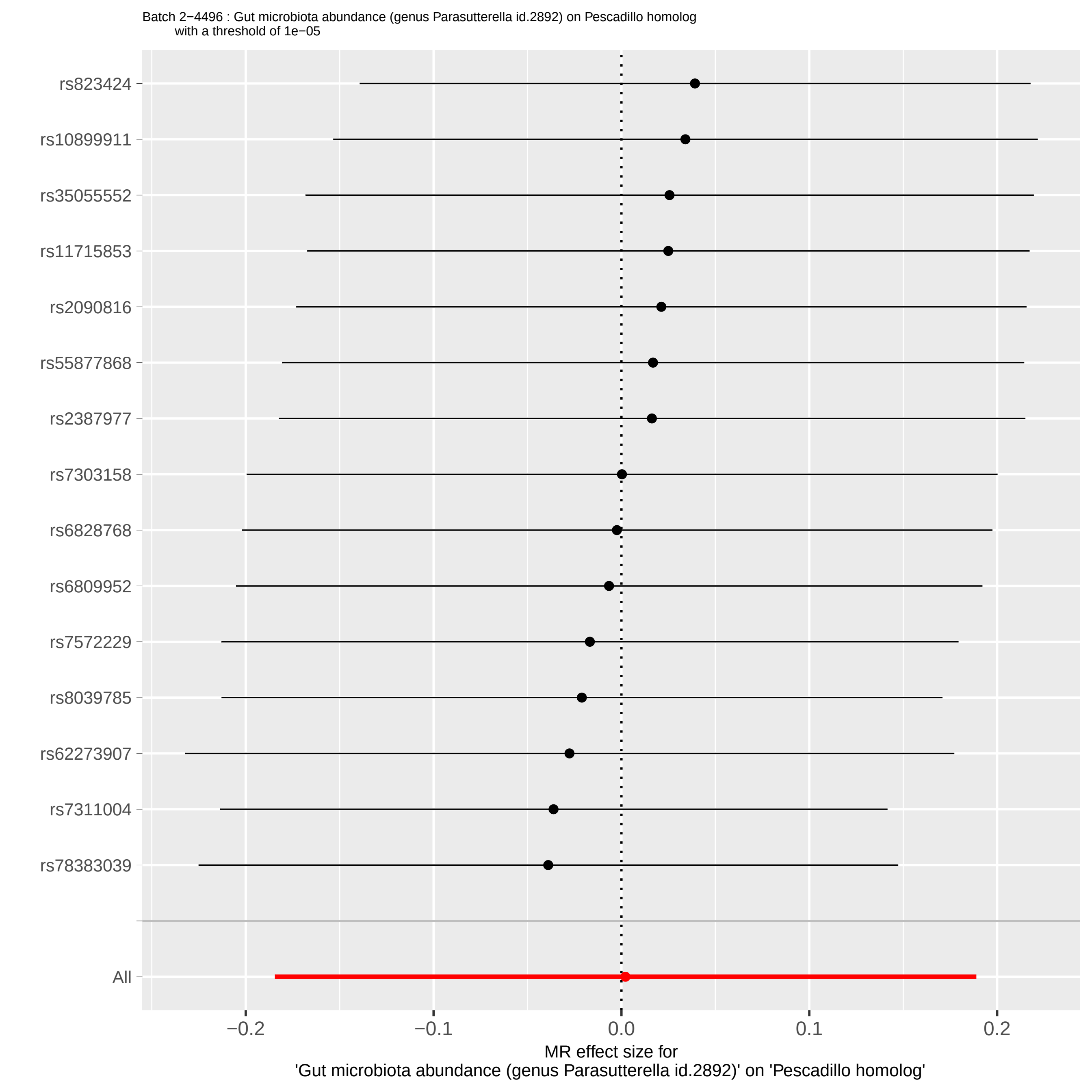

Batch 2−4496 : Gut microbiota abundance (genus Parasutterella id.2892) on Pescadillo homolog
with a threshold of 1e−05
rs823424
rs10899911
rs35055552
rs11715853
rs2090816
rs55877868
rs2387977
rs7303158
rs6828768
rs6809952
rs7572229
rs8039785
rs62273907
rs7311004
rs78383039
All
−0.2
−0.1
0.0
0.1
0.2
MR effect size for
'Gut microbiota abundance (genus Parasutterella id.2892)' on 'Pescadillo homolog'

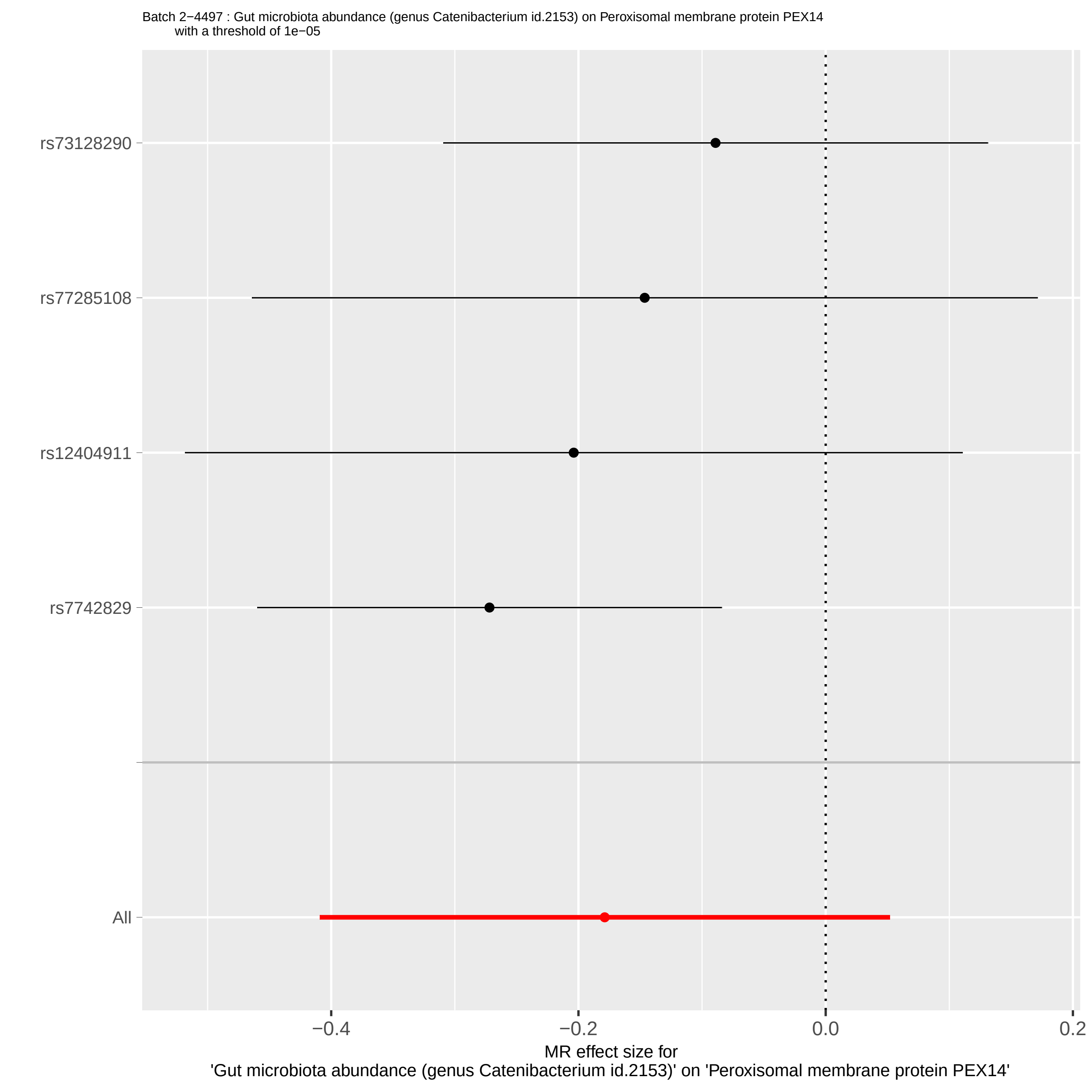
Batch 2−4497 : Gut microbiota abundance (genus Catenibacterium id.2153) on Peroxisomal membrane protein PEX14
with a threshold of 1e−05
rs73128290
rs77285108
rs12404911
rs7742829
All
−0.4
−0.2
0.0
0.2
MR effect size for
'Gut microbiota abundance (genus Catenibacterium id.2153)' on 'Peroxisomal membrane protein PEX14'

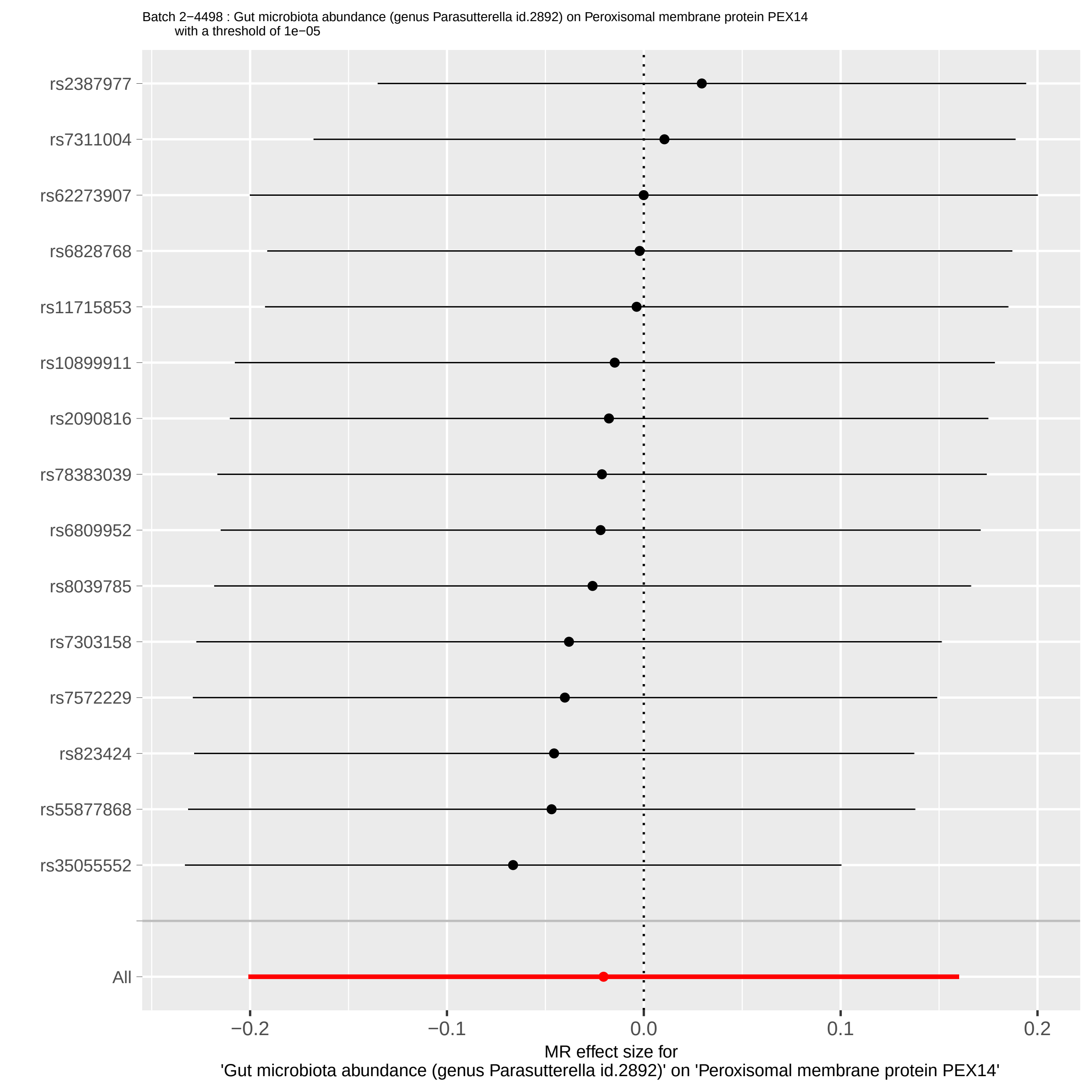

Batch 2−4498 : Gut microbiota abundance (genus Parasutterella id.2892) on Peroxisomal membrane protein PEX14
with a threshold of 1e−05
rs2387977
rs7311004
rs62273907
rs6828768
rs11715853
rs10899911
rs2090816
rs78383039
rs6809952
rs8039785
rs7303158
rs7572229
rs823424
rs55877868
rs35055552
All
−0.2
−0.1
0.0
0.1
0.2
MR effect size for
'Gut microbiota abundance (genus Parasutterella id.2892)' on 'Peroxisomal membrane protein PEX14'

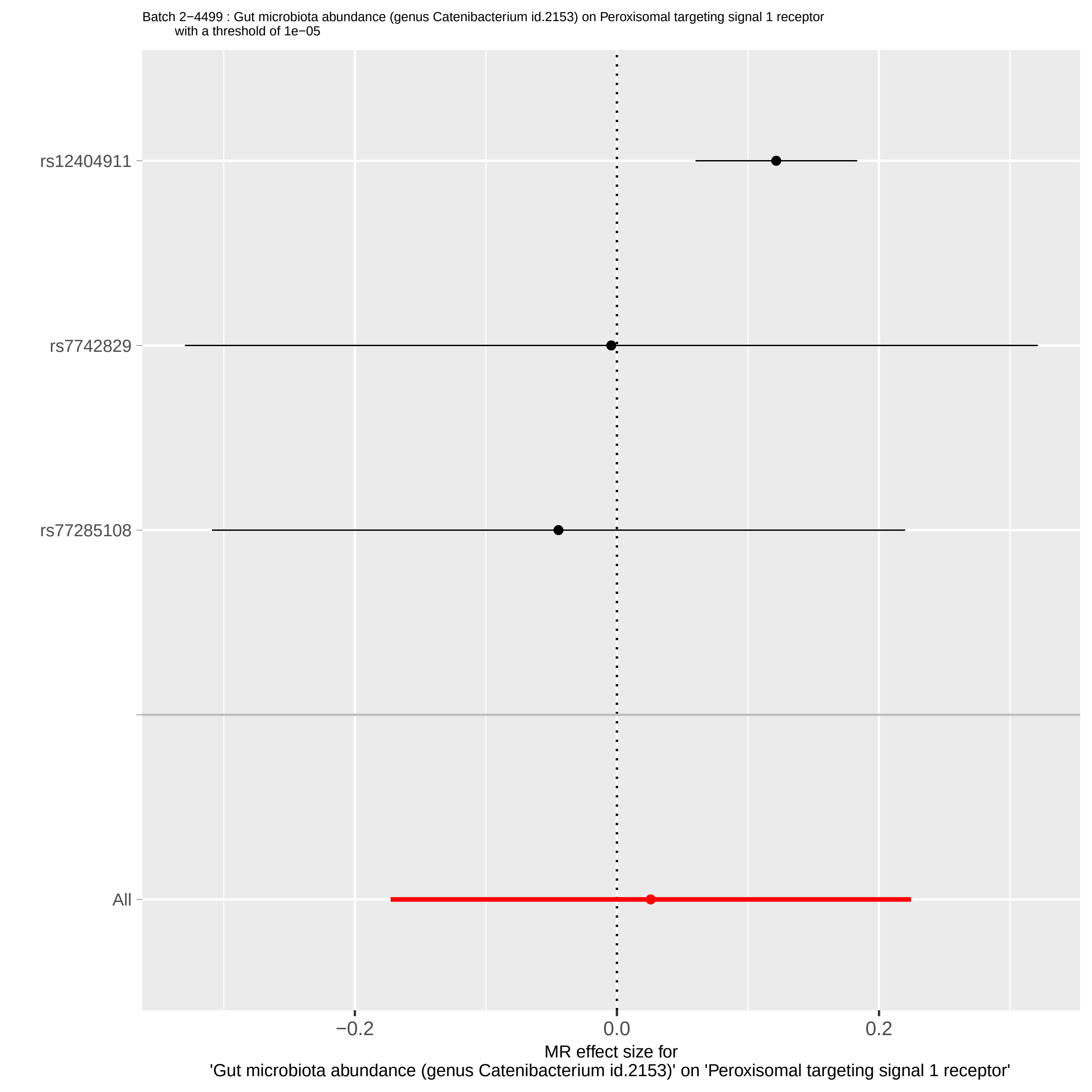
Batch 2−4499 : Gut microbiota abundance (genus Catenibacterium id.2153) on Peroxisomal targeting signal 1 receptor
with a threshold of 1e−05
rs12404911
rs7742829
rs77285108
All
−0.2
0.0
0.2
MR effect size for
'Gut microbiota abundance (genus Catenibacterium id.2153)' on 'Peroxisomal targeting signal 1 receptor'

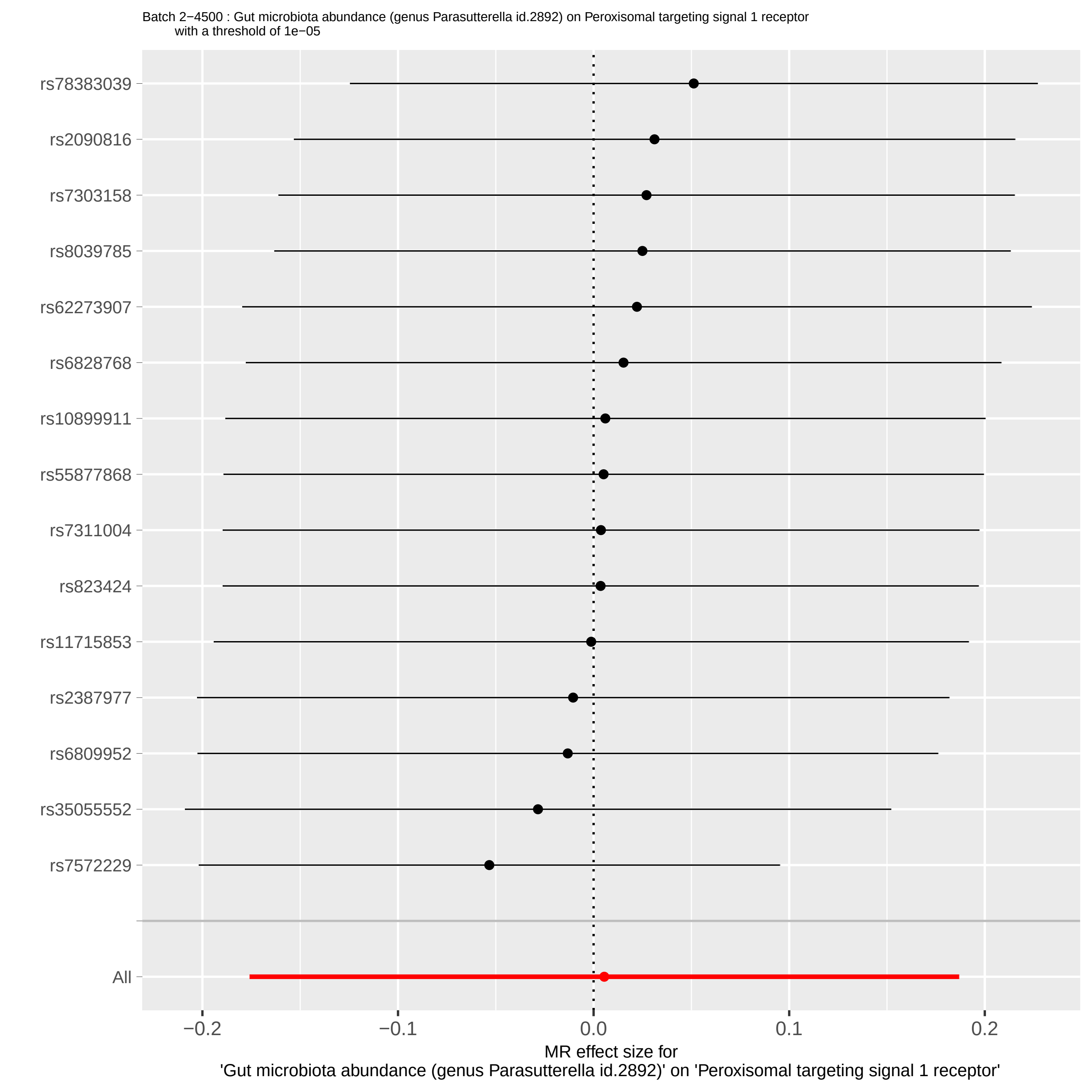
Batch 2−4500 : Gut microbiota abundance (genus Parasutterella id.2892) on Peroxisomal targeting signal 1 receptor
with a threshold of 1e−05
rs78383039
rs2090816
rs7303158
rs8039785
rs62273907
rs6828768
rs10899911
rs55877868
rs7311004
rs823424
rs11715853
rs2387977
rs6809952
rs35055552
rs7572229
All
−0.2
−0.1
0.0
0.1
0.2
MR effect size for
'Gut microbiota abundance (genus Parasutterella id.2892)' on 'Peroxisomal targeting signal 1 receptor'

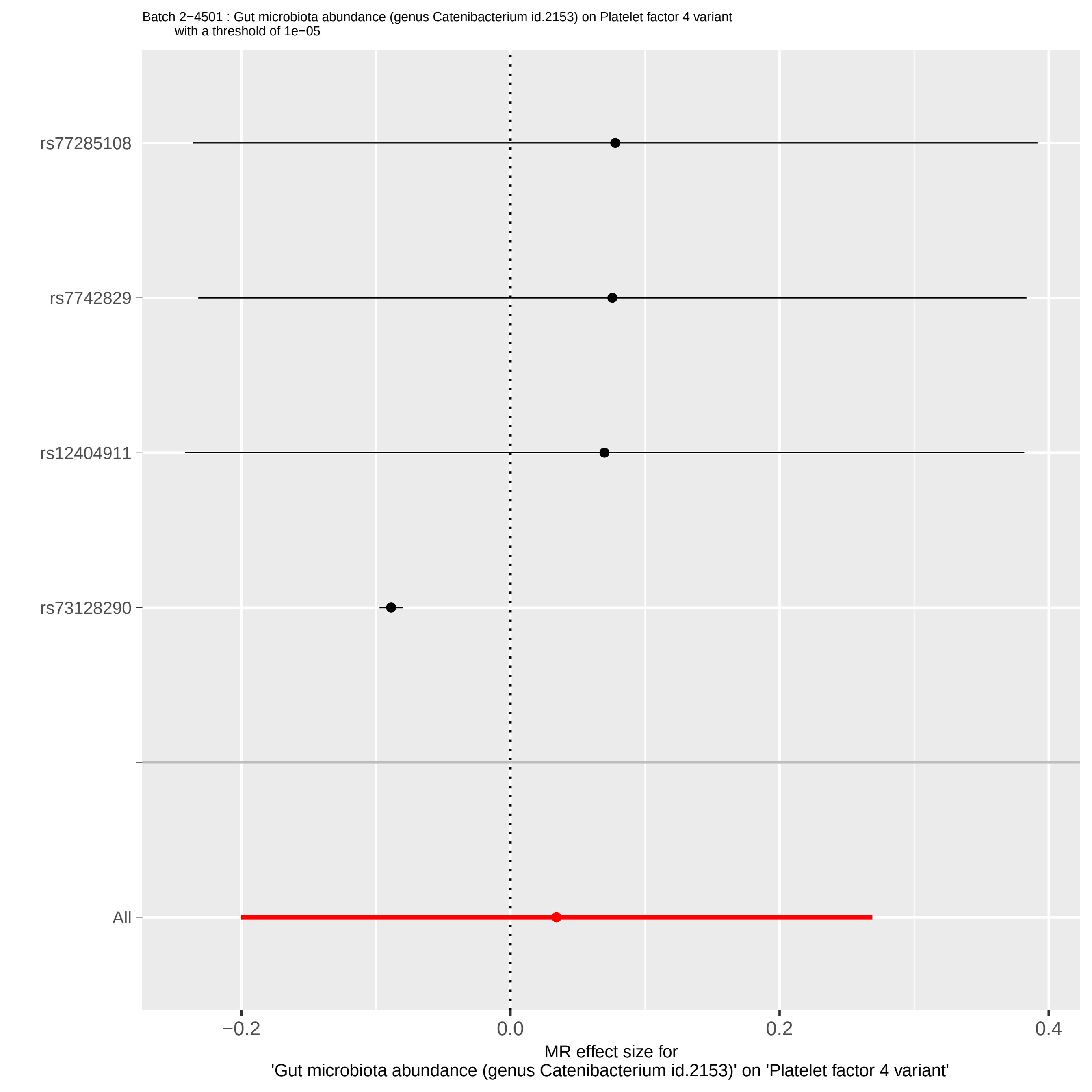
Batch 2−4501 : Gut microbiota abundance (genus Catenibacterium id.2153) on Platelet factor 4 variant
with a threshold of 1e−05
rs77285108
rs7742829
rs12404911
rs73128290
All
−0.2
0.0
0.2
0.4
MR effect size for
'Gut microbiota abundance (genus Catenibacterium id.2153)' on 'Platelet factor 4 variant'

Batch 2−4502 : Gut microbiota abundance (genus Parasutterella id.2892) on Platelet factor 4 variant
with a threshold of 1e−05

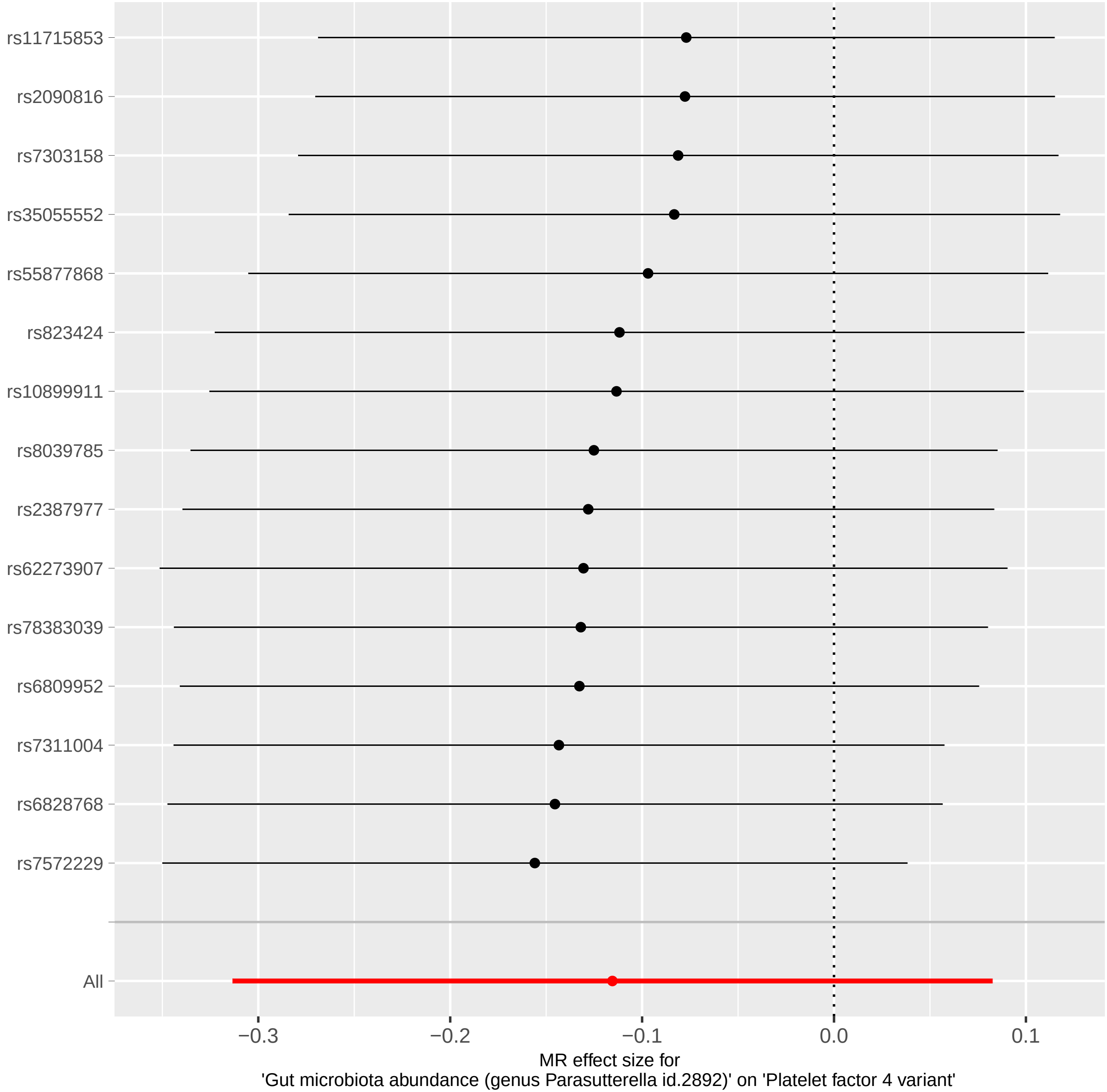

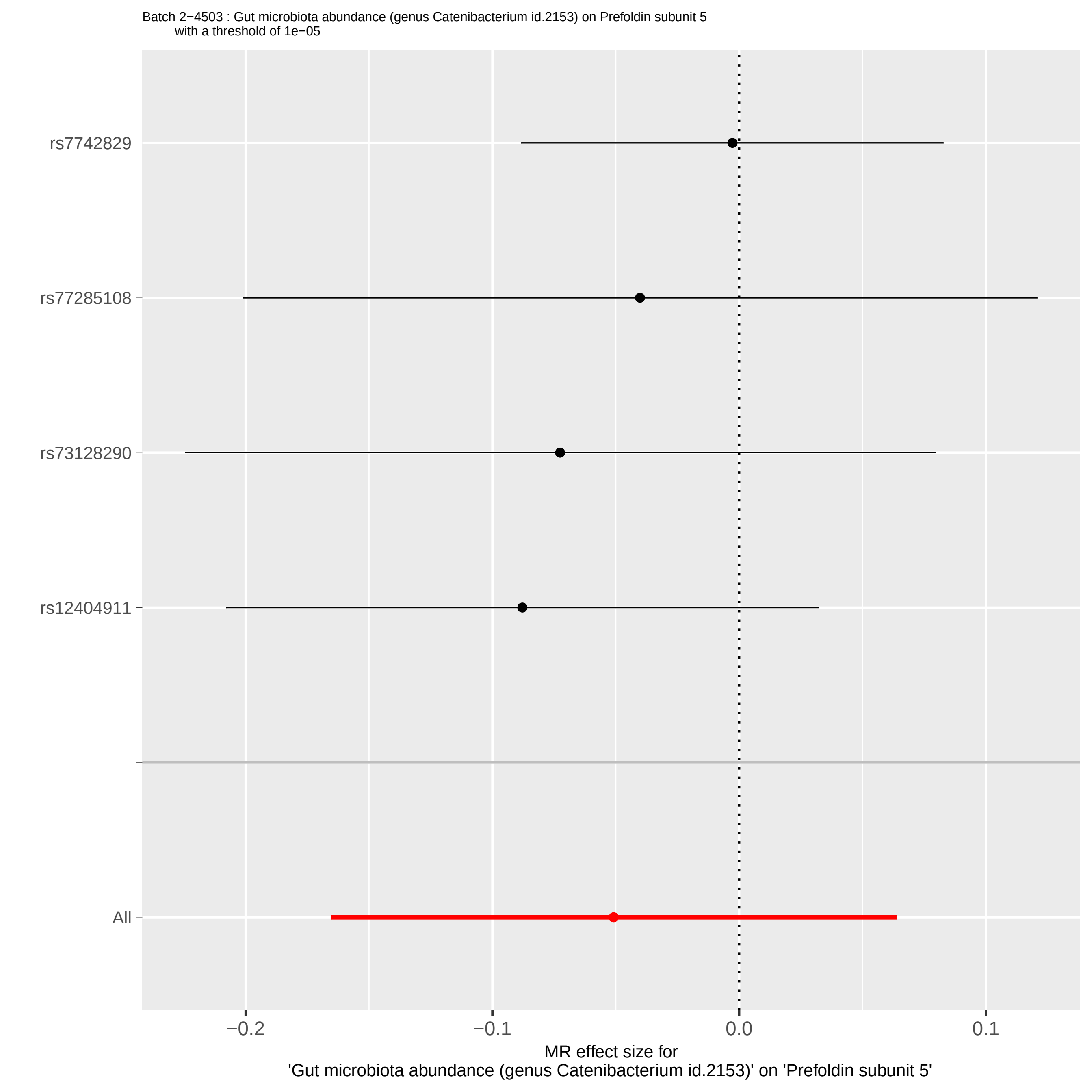

Batch 2−4503 : Gut microbiota abundance (genus Catenibacterium id.2153) on Prefoldin subunit 5
with a threshold of 1e−05
rs7742829
rs77285108
rs73128290
rs12404911
All
−0.2
−0.1
0.0
0.1
MR effect size for
'Gut microbiota abundance (genus Catenibacterium id.2153)' on 'Prefoldin subunit 5'

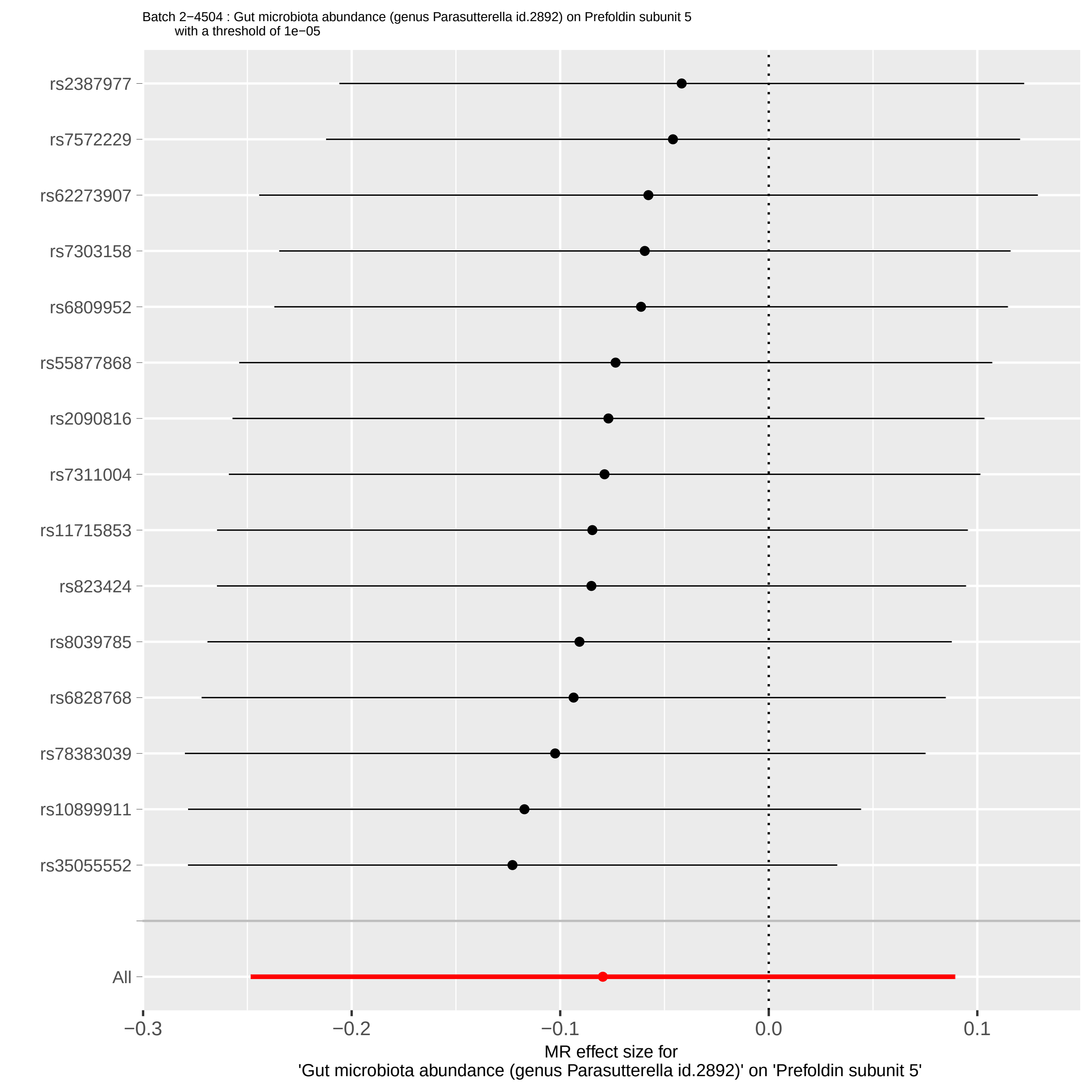
Batch 2−4504 : Gut microbiota abundance (genus Parasutterella id.2892) on Prefoldin subunit 5
with a threshold of 1e−05
rs2387977
rs7572229
rs62273907
rs7303158
rs6809952
rs55877868
rs2090816
rs7311004
rs11715853
rs823424
rs8039785
rs6828768
rs78383039
rs10899911
rs35055552
All
−0.3
−0.2
−0.1
0.0
0.1
MR effect size for
'Gut microbiota abundance (genus Parasutterella id.2892)' on 'Prefoldin subunit 5'

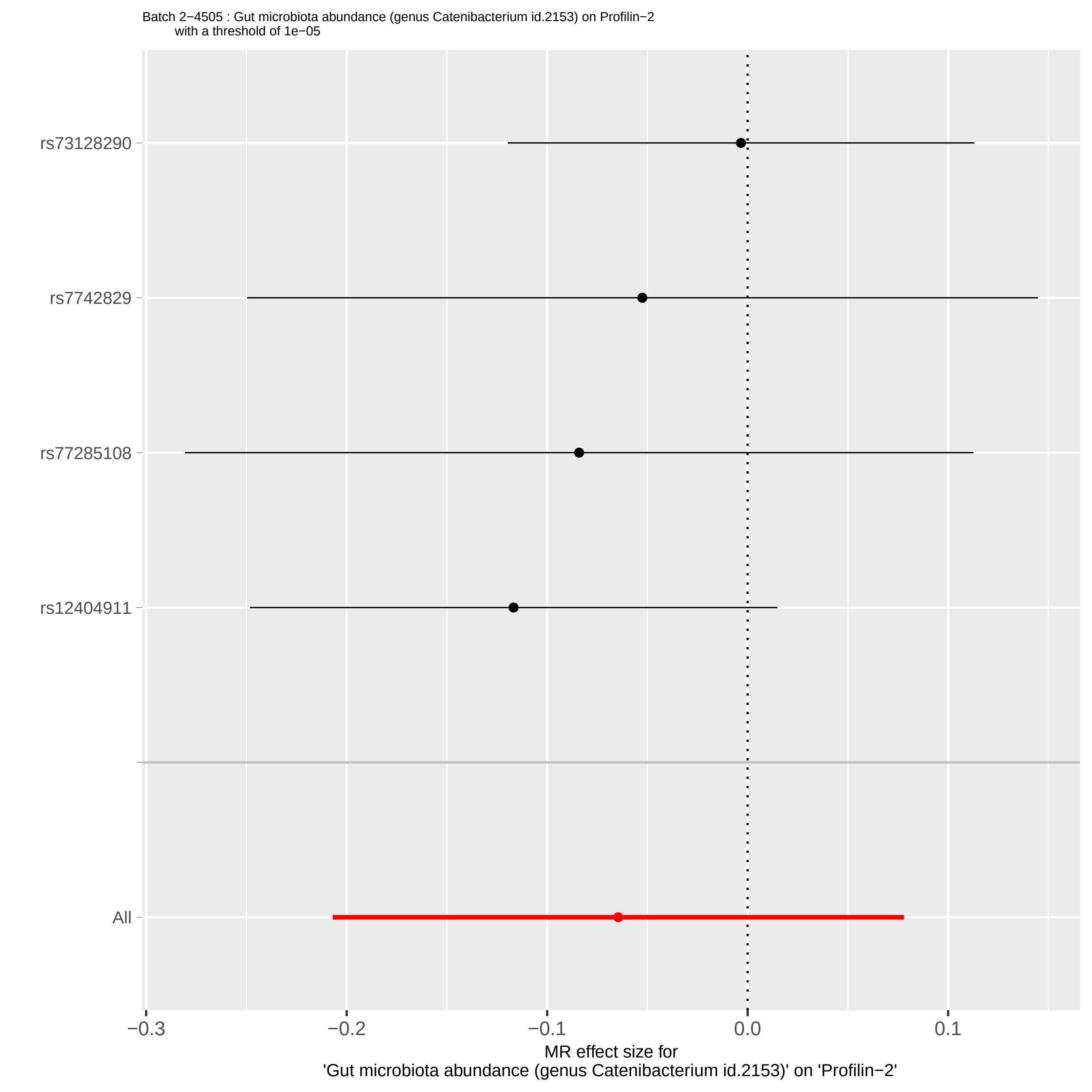
Batch 2−4505 : Gut microbiota abundance (genus Catenibacterium id.2153) on Profilin−2
with a threshold of 1e−05
rs73128290
rs7742829
rs77285108
rs12404911
All
−0.3
−0.2
−0.1
0.0
0.1
MR effect size for
'Gut microbiota abundance (genus Catenibacterium id.2153)' on 'Profilin−2'

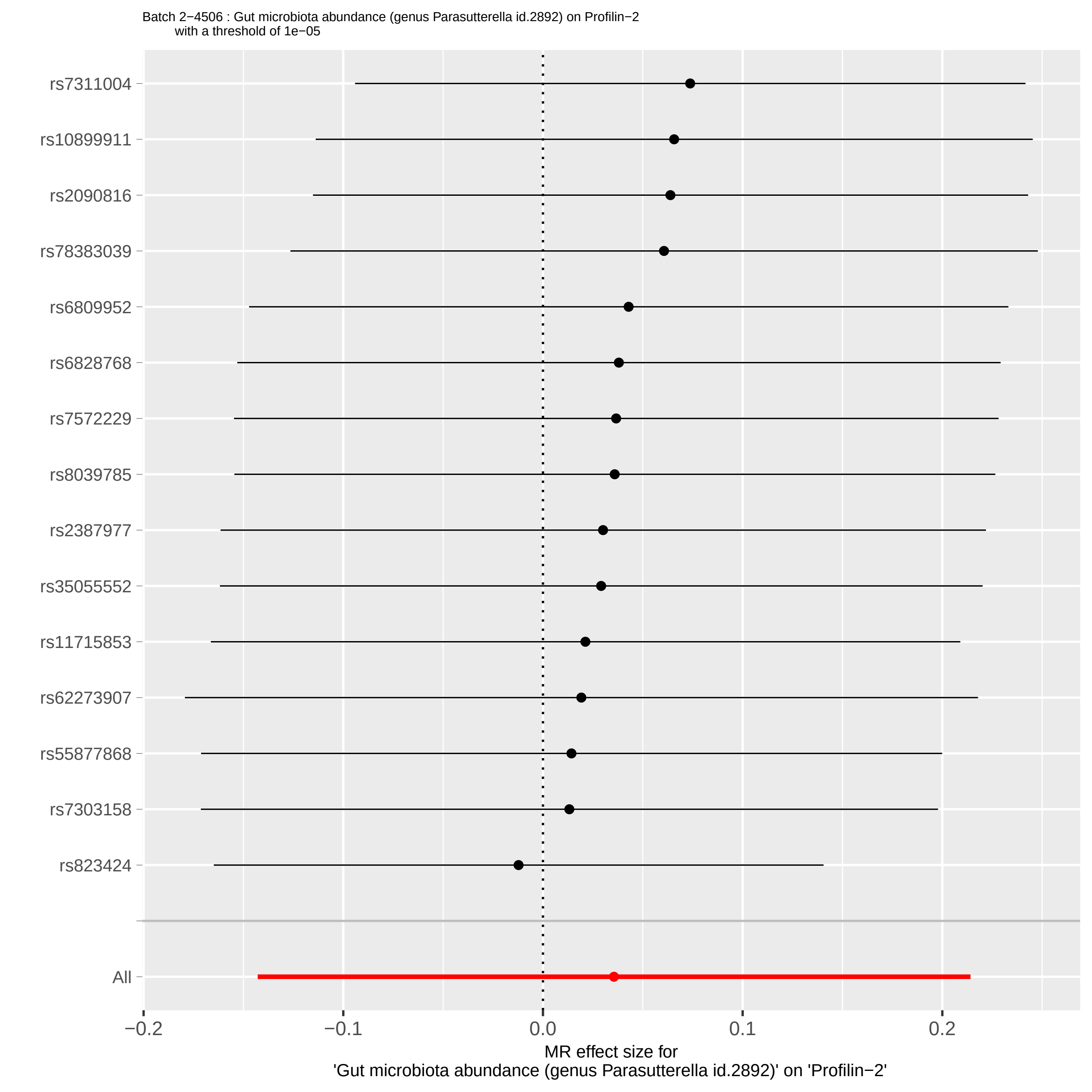
Batch 2−4506 : Gut microbiota abundance (genus Parasutterella id.2892) on Profilin−2
with a threshold of 1e−05
rs7311004
rs10899911
rs2090816
rs78383039
rs6809952
rs6828768
rs7572229
rs8039785
rs2387977
rs35055552
rs11715853
rs62273907
rs55877868
rs7303158
rs823424
All
−0.2
−0.1
0.0
0.1
0.2
MR effect size for
'Gut microbiota abundance (genus Parasutterella id.2892)' on 'Profilin−2'

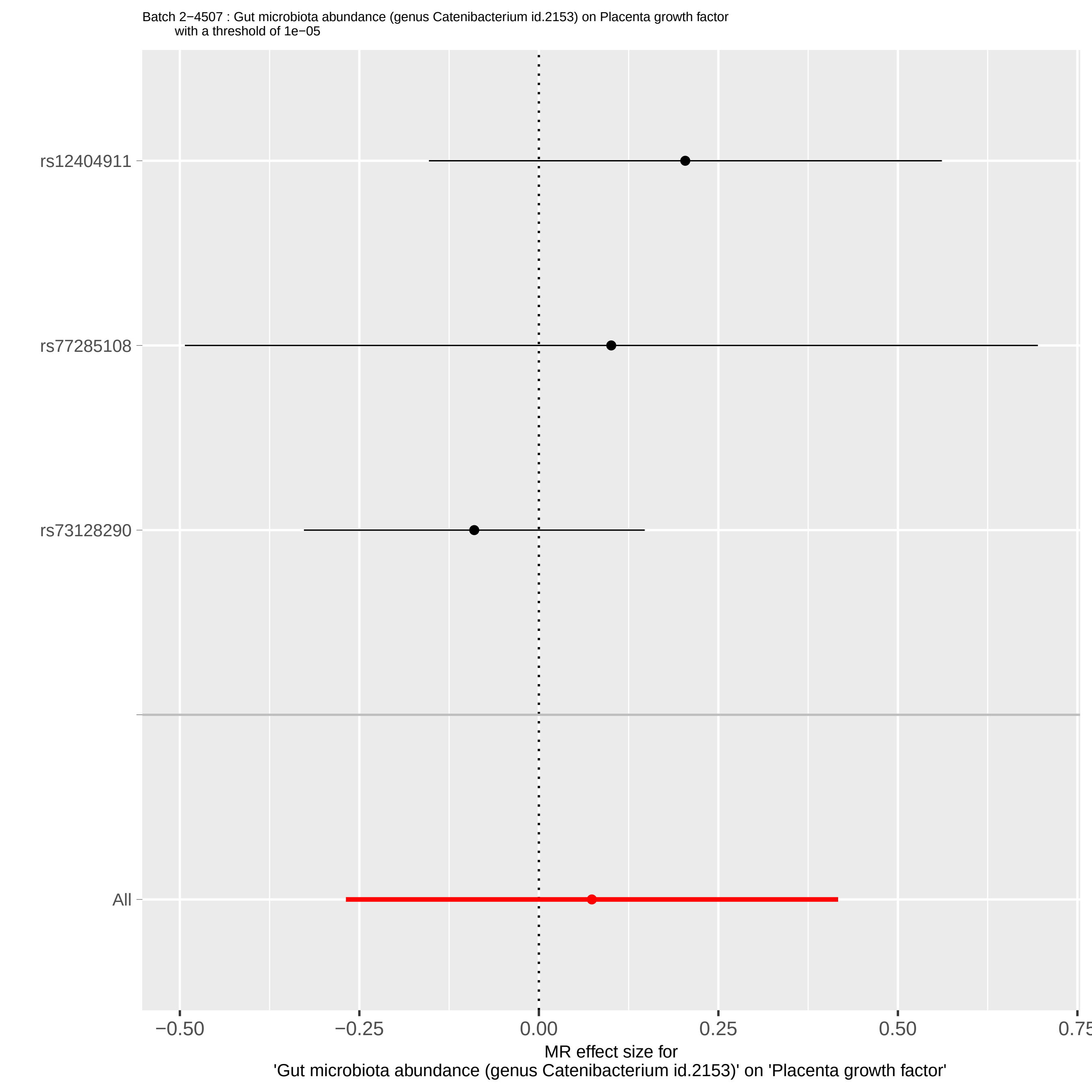
Batch 2−4507 : Gut microbiota abundance (genus Catenibacterium id.2153) on Placenta growth factor
with a threshold of 1e−05
rs12404911
rs77285108
rs73128290
All
−0.50
−0.25
0.00
0.25
0.50
0.75
MR effect size for
'Gut microbiota abundance (genus Catenibacterium id.2153)' on 'Placenta growth factor'

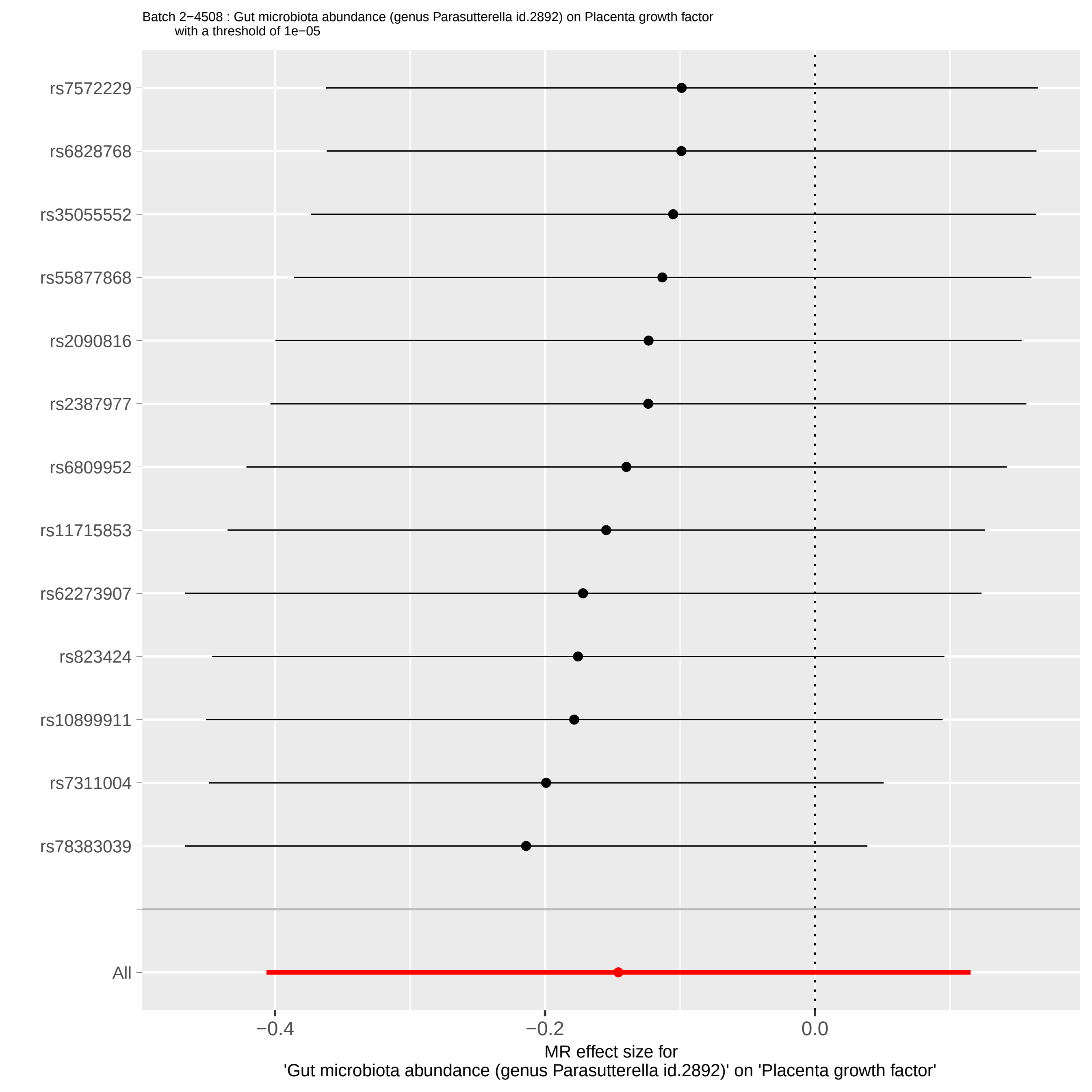
Batch 2−4508 : Gut microbiota abundance (genus Parasutterella id.2892) on Placenta growth factor
with a threshold of 1e−05
rs7572229
rs6828768
rs35055552
rs55877868
rs2090816
rs2387977
rs6809952
rs11715853
rs62273907
rs823424
rs10899911
rs7311004
rs78383039
All
−0.4
−0.2
0.0
MR effect size for
'Gut microbiota abundance (genus Parasutterella id.2892)' on 'Placenta growth factor'

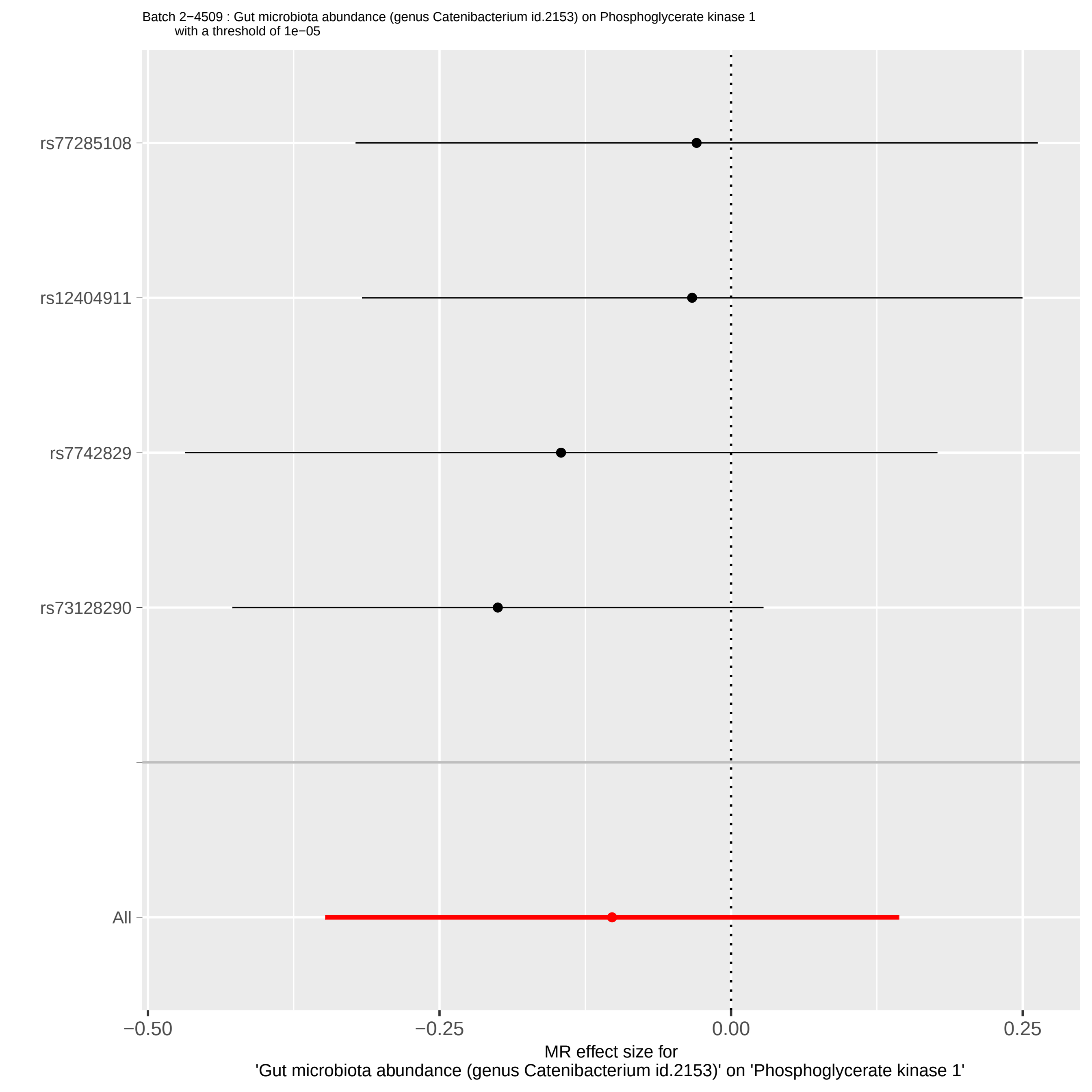
Batch 2−4509 : Gut microbiota abundance (genus Catenibacterium id.2153) on Phosphoglycerate kinase 1
with a threshold of 1e−05
rs77285108
rs12404911
rs7742829
rs73128290
All
−0.50
−0.25
0.00
0.25
MR effect size for
'Gut microbiota abundance (genus Catenibacterium id.2153)' on 'Phosphoglycerate kinase 1'

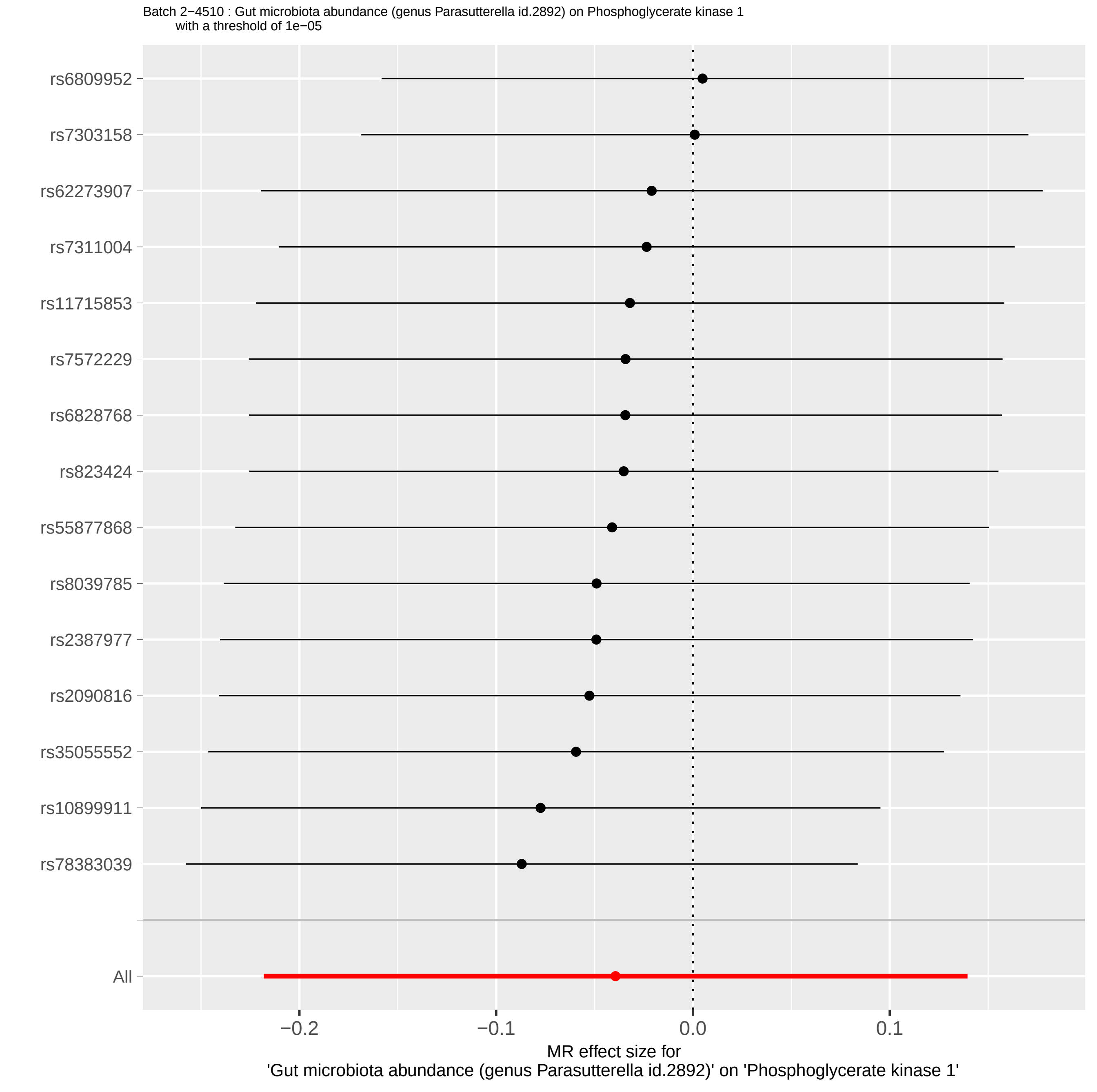

Batch 2−4510 : Gut microbiota abundance (genus Parasutterella id.2892) on Phosphoglycerate kinase 1
with a threshold of 1e−05
rs6809952
rs7303158
rs62273907
rs7311004
rs11715853
rs7572229
rs6828768
rs823424
rs55877868
rs8039785
rs2387977
rs2090816
rs35055552
rs10899911
rs78383039
All
−0.2
−0.1
0.0
0.1
MR effect size for
'Gut microbiota abundance (genus Parasutterella id.2892)' on 'Phosphoglycerate kinase 1'

Batch 2−4511 : Gut microbiota abundance (genus Catenibacterium id.2153) on Phosphoglycerate kinase 2
with a threshold of 1e−05

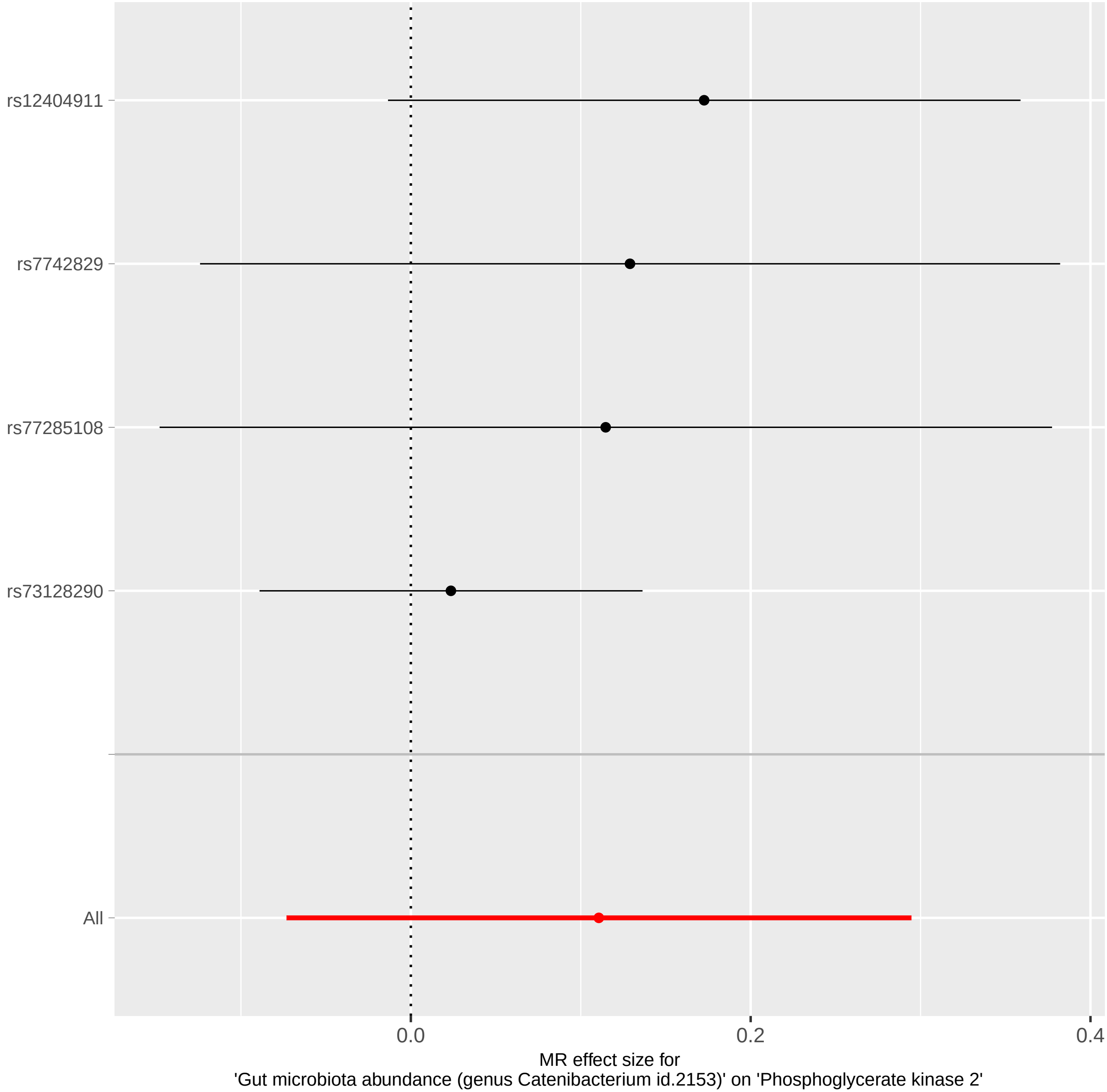

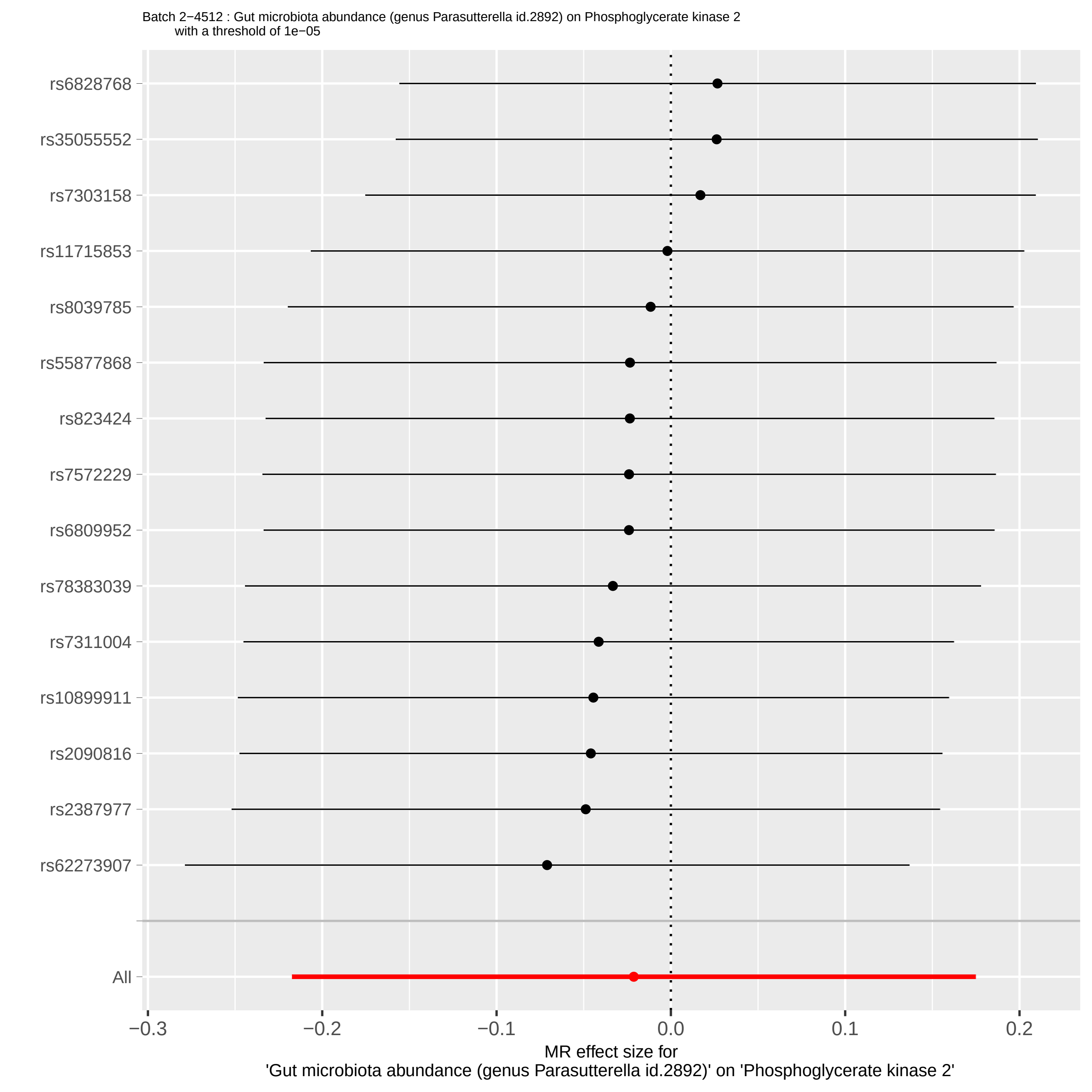

Batch 2−4512 : Gut microbiota abundance (genus Parasutterella id.2892) on Phosphoglycerate kinase 2
with a threshold of 1e−05
rs6828768
rs35055552
rs7303158
rs11715853
rs8039785
rs55877868
rs823424
rs7572229
rs6809952
rs78383039
rs7311004
rs10899911
rs2090816
rs2387977
rs62273907
All
−0.3
−0.2
−0.1
0.0
0.1
0.2
MR effect size for
'Gut microbiota abundance (genus Parasutterella id.2892)' on 'Phosphoglycerate kinase 2'

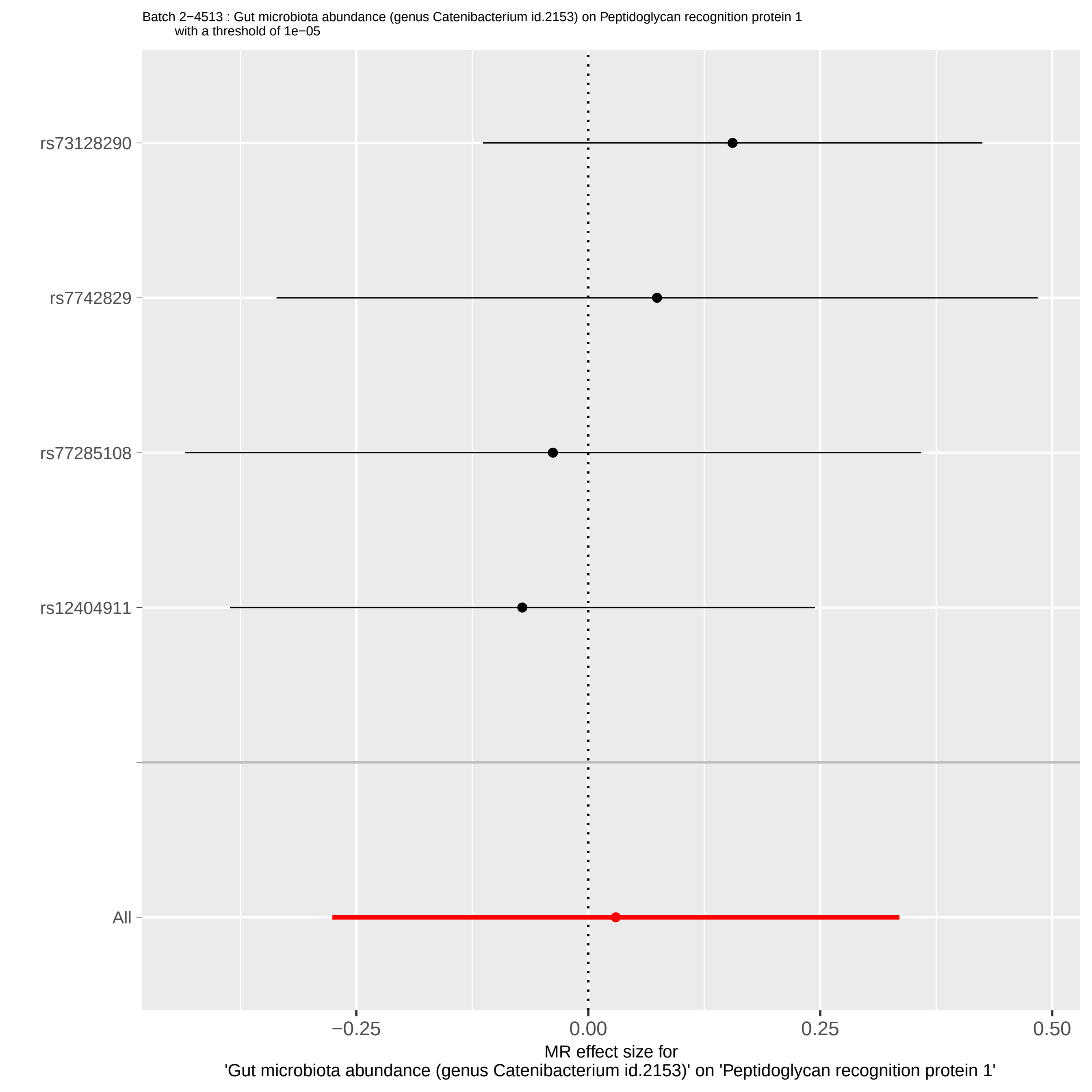
Batch 2−4513 : Gut microbiota abundance (genus Catenibacterium id.2153) on Peptidoglycan recognition protein 1
with a threshold of 1e−05
rs73128290
rs7742829
rs77285108
rs12404911
All
−0.25
0.00
0.25
0.50
MR effect size for
'Gut microbiota abundance (genus Catenibacterium id.2153)' on 'Peptidoglycan recognition protein 1'

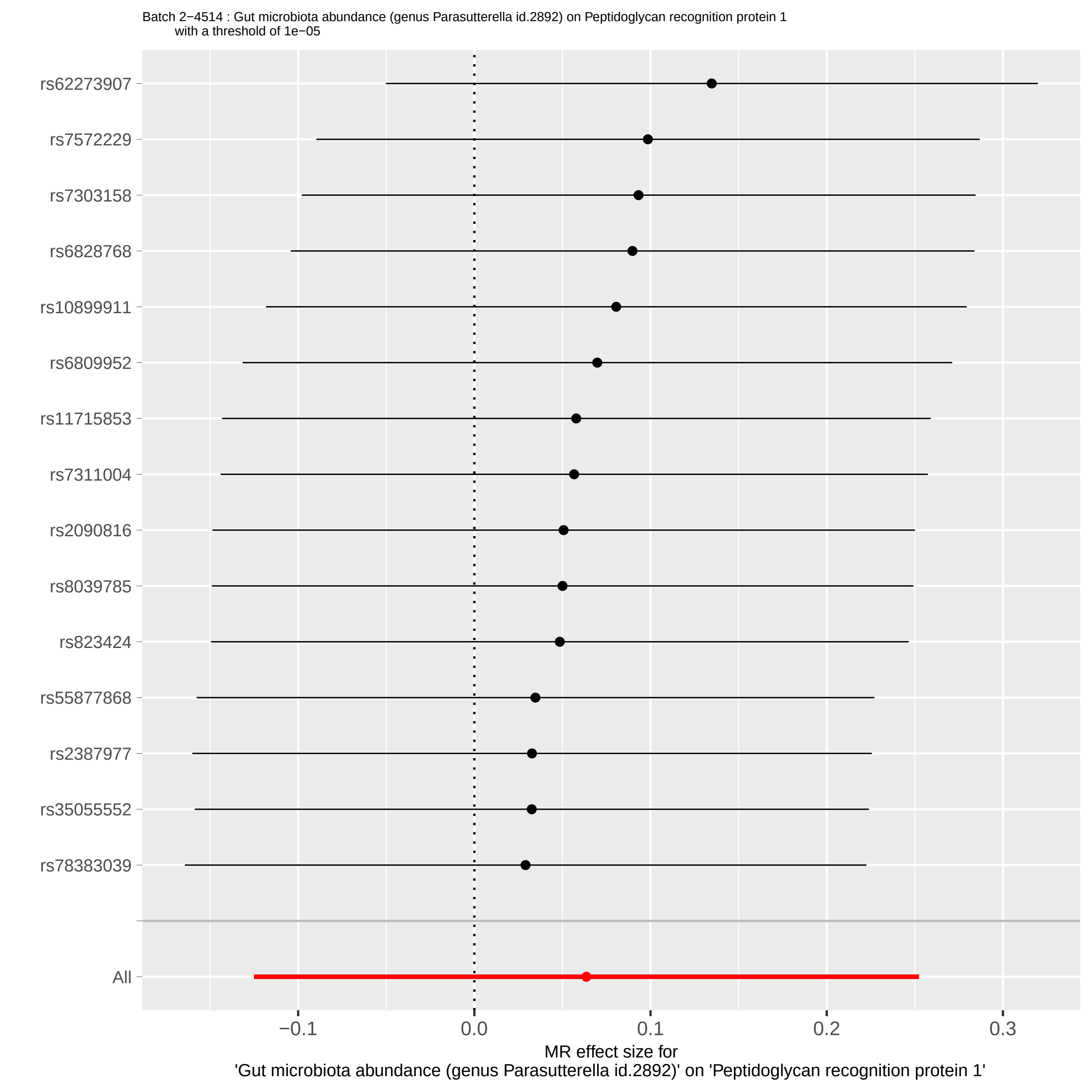

Batch 2−4514 : Gut microbiota abundance (genus Parasutterella id.2892) on Peptidoglycan recognition protein 1
with a threshold of 1e−05
rs62273907
rs7572229
rs7303158
rs6828768
rs10899911
rs6809952
rs11715853
rs7311004
rs2090816
rs8039785
rs823424
rs55877868
rs2387977
rs35055552
rs78383039
All
−0.1
0.0
0.1
0.2
0.3
MR effect size for
'Gut microbiota abundance (genus Parasutterella id.2892)' on 'Peptidoglycan recognition protein 1'

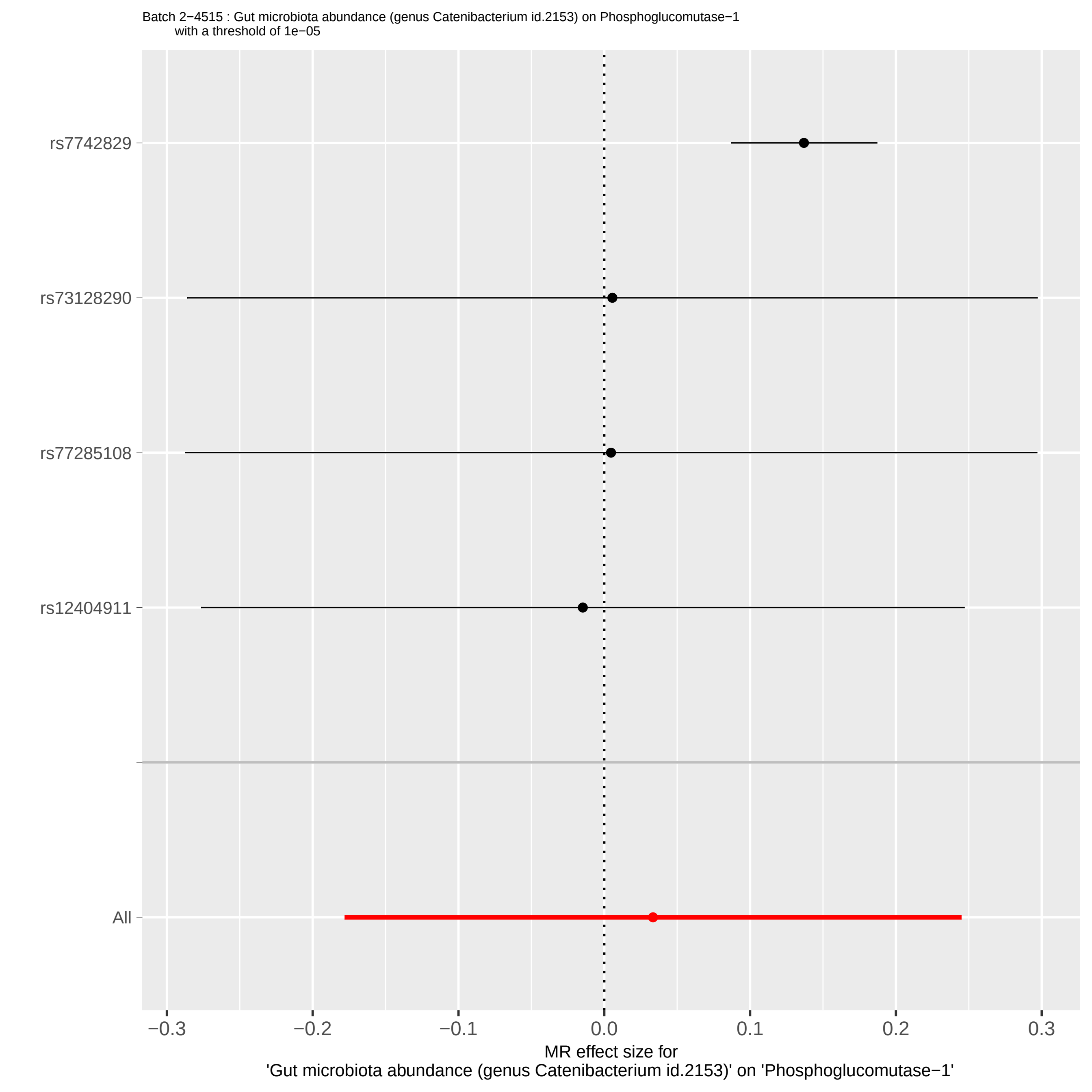
Batch 2−4515 : Gut microbiota abundance (genus Catenibacterium id.2153) on Phosphoglucomutase−1
with a threshold of 1e−05
rs7742829
rs73128290
rs77285108
rs12404911
All
−0.3
−0.2
−0.1
0.0
0.1
0.2
0.3
MR effect size for
'Gut microbiota abundance (genus Catenibacterium id.2153)' on 'Phosphoglucomutase−1'

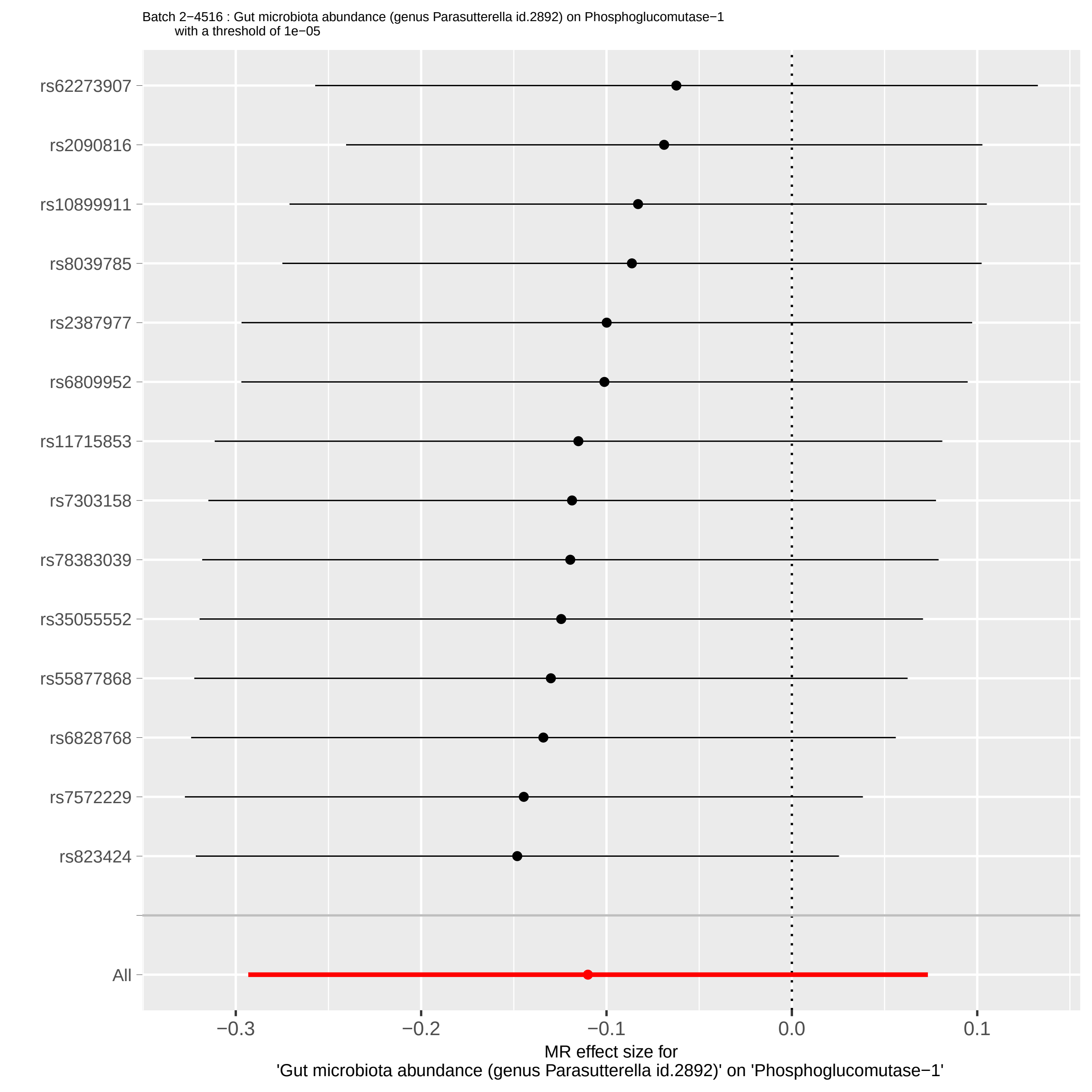

Batch 2−4516 : Gut microbiota abundance (genus Parasutterella id.2892) on Phosphoglucomutase−1
with a threshold of 1e−05
rs62273907
rs2090816
rs10899911
rs8039785
rs2387977
rs6809952
rs11715853
rs7303158
rs78383039
rs35055552
rs55877868
rs6828768
rs7572229
rs823424
All
−0.3
−0.2
−0.1
0.0
0.1
MR effect size for
'Gut microbiota abundance (genus Parasutterella id.2892)' on 'Phosphoglucomutase−1'

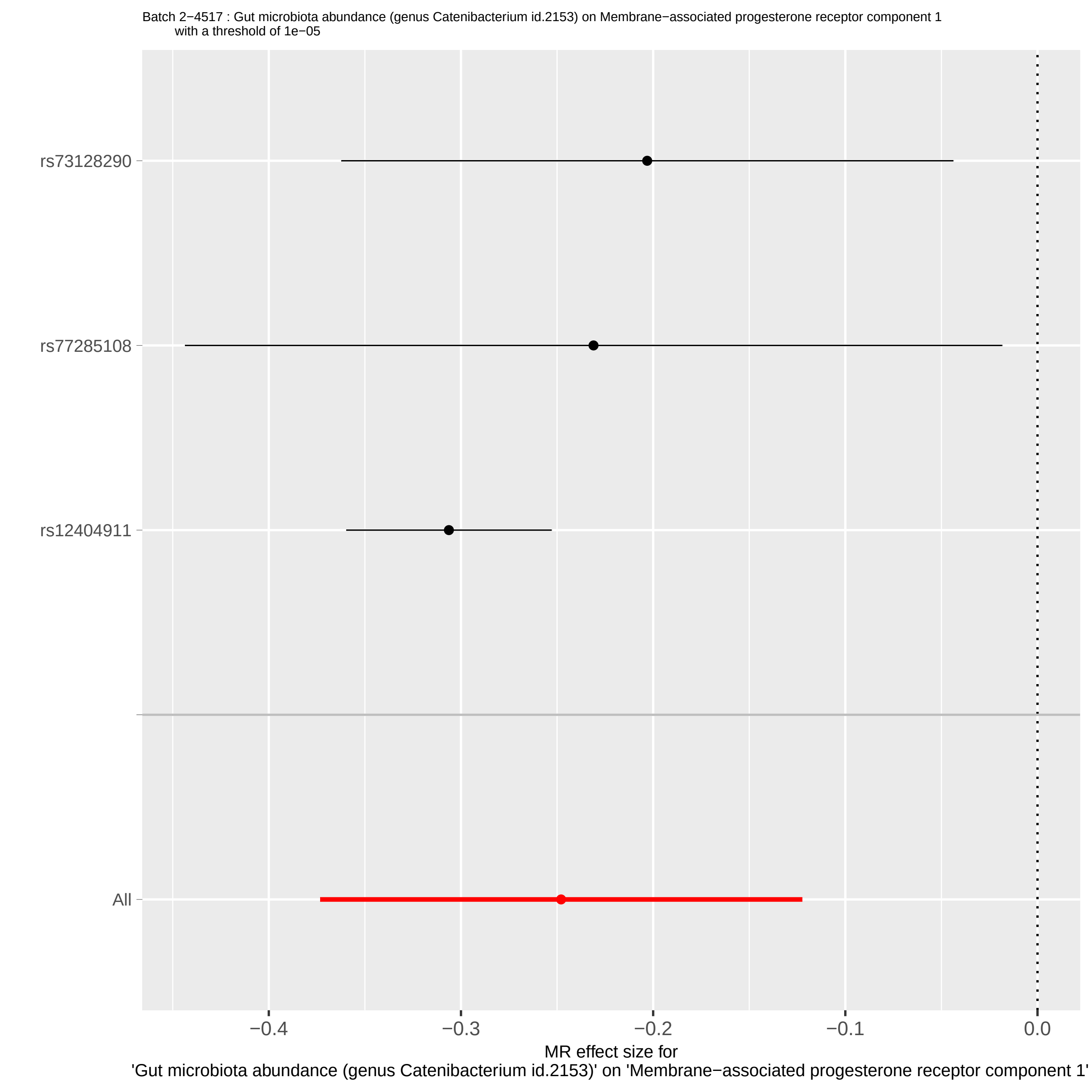

Batch 2−4517 : Gut microbiota abundance (genus Catenibacterium id.2153) on Membrane−associated progesterone receptor component 1
with a threshold of 1e−05
rs73128290
rs77285108
rs12404911
All
−0.4
−0.3
−0.2
−0.1
0.0
MR effect size for
'Gut microbiota abundance (genus Catenibacterium id.2153)' on 'Membrane−associated progesterone receptor component 1'

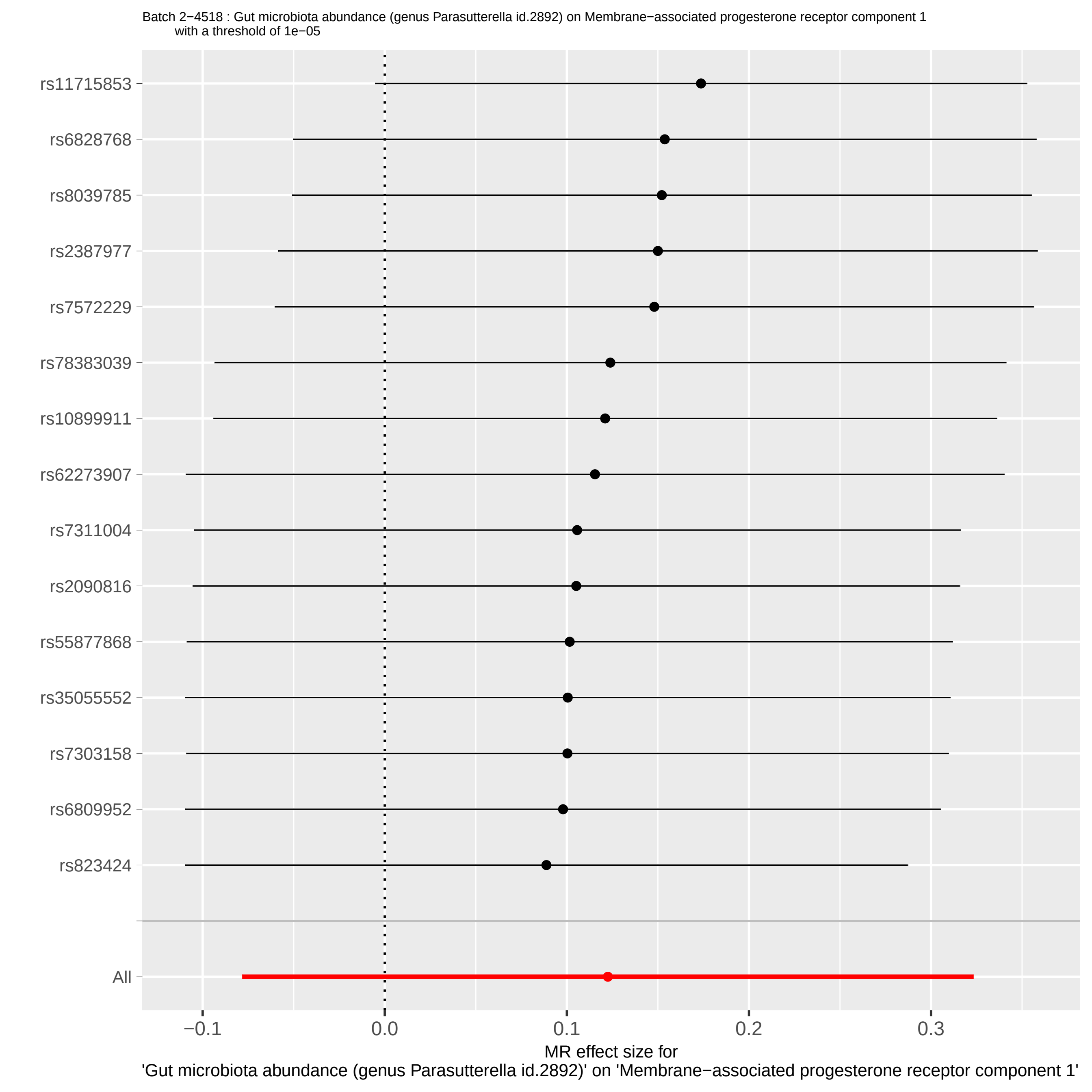
Batch 2−4518 : Gut microbiota abundance (genus Parasutterella id.2892) on Membrane−associated progesterone receptor component 1
with a threshold of 1e−05
rs11715853
rs6828768
rs8039785
rs2387977
rs7572229
rs78383039
rs10899911
rs62273907
rs7311004
rs2090816
rs55877868
rs35055552
rs7303158
rs6809952
rs823424
All
−0.1
0.0
0.1
0.2
0.3
MR effect size for
'Gut microbiota abundance (genus Parasutterella id.2892)' on 'Membrane−associated progesterone receptor component 1'

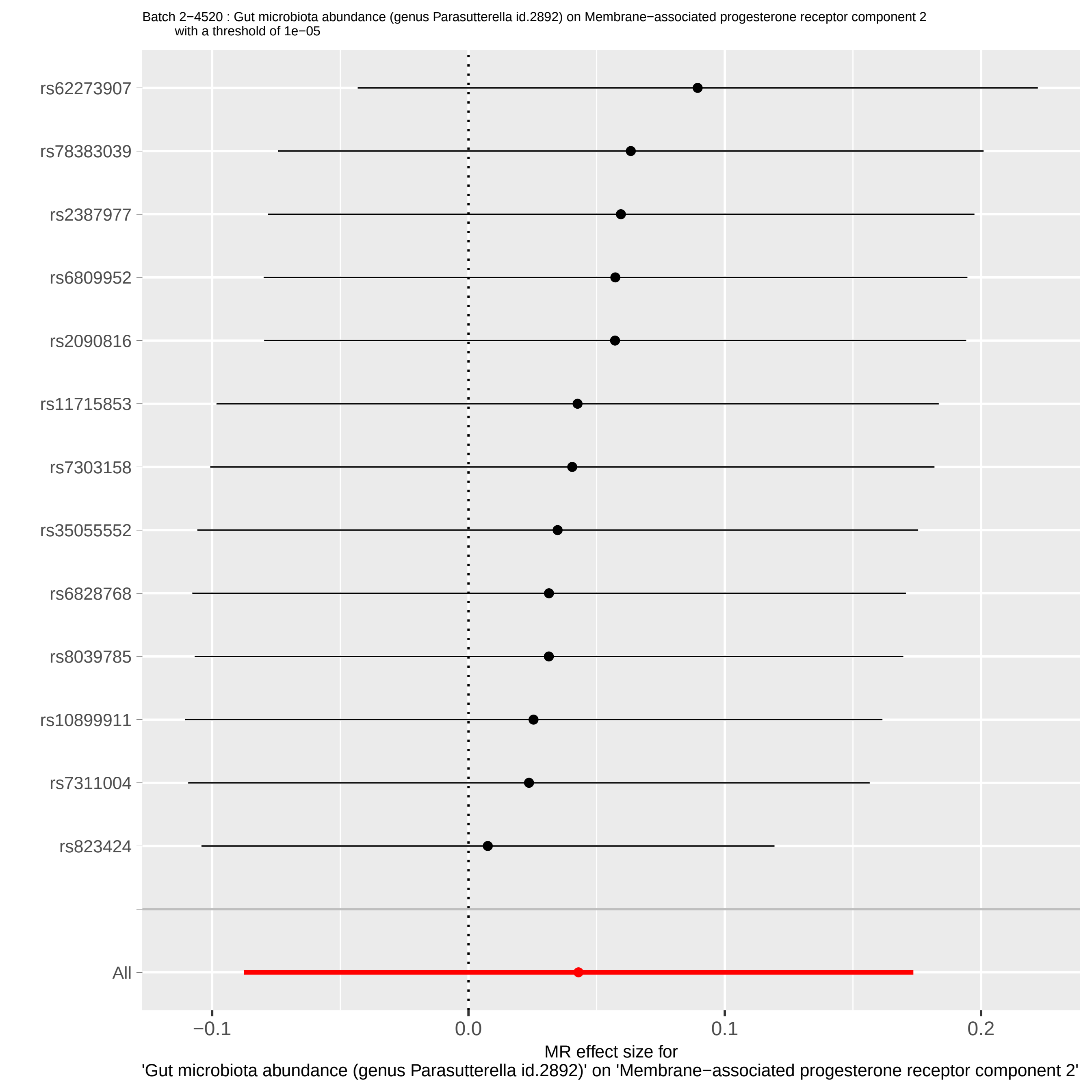

Batch 2−4520 : Gut microbiota abundance (genus Parasutterella id.2892) on Membrane−associated progesterone receptor component 2
with a threshold of 1e−05
rs62273907
rs78383039
rs2387977
rs6809952
rs2090816
rs11715853
rs7303158
rs35055552
rs6828768
rs8039785
rs10899911
rs7311004
rs823424
All
−0.1
0.0
0.1
0.2
MR effect size for
'Gut microbiota abundance (genus Parasutterella id.2892)' on 'Membrane−associated progesterone receptor component 2'

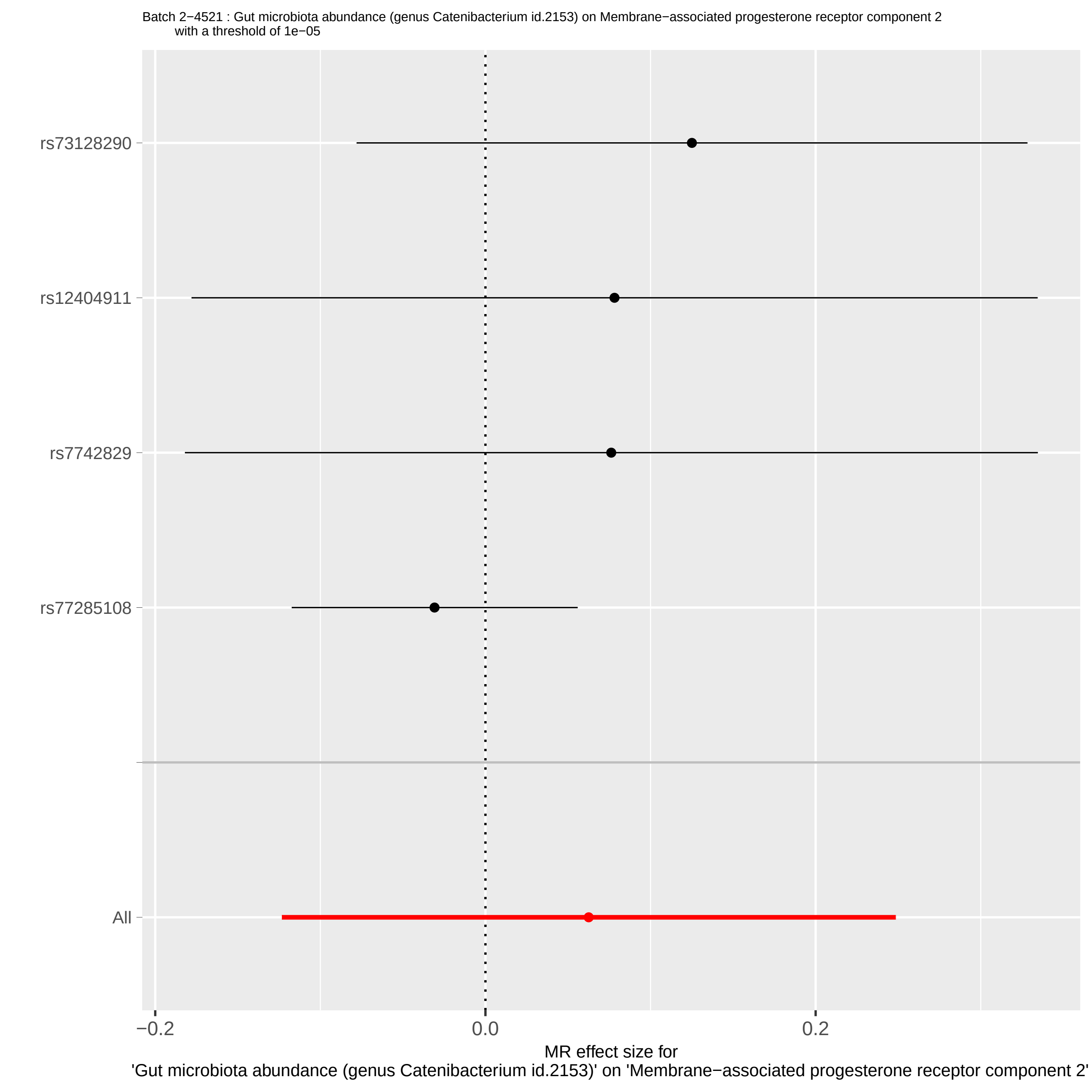

Batch 2−4521 : Gut microbiota abundance (genus Catenibacterium id.2153) on Membrane−associated progesterone receptor component 2
with a threshold of 1e−05
rs73128290
rs12404911
rs7742829
rs77285108
All
−0.2
0.0
0.2
MR effect size for
'Gut microbiota abundance (genus Catenibacterium id.2153)' on 'Membrane−associated progesterone receptor component 2'

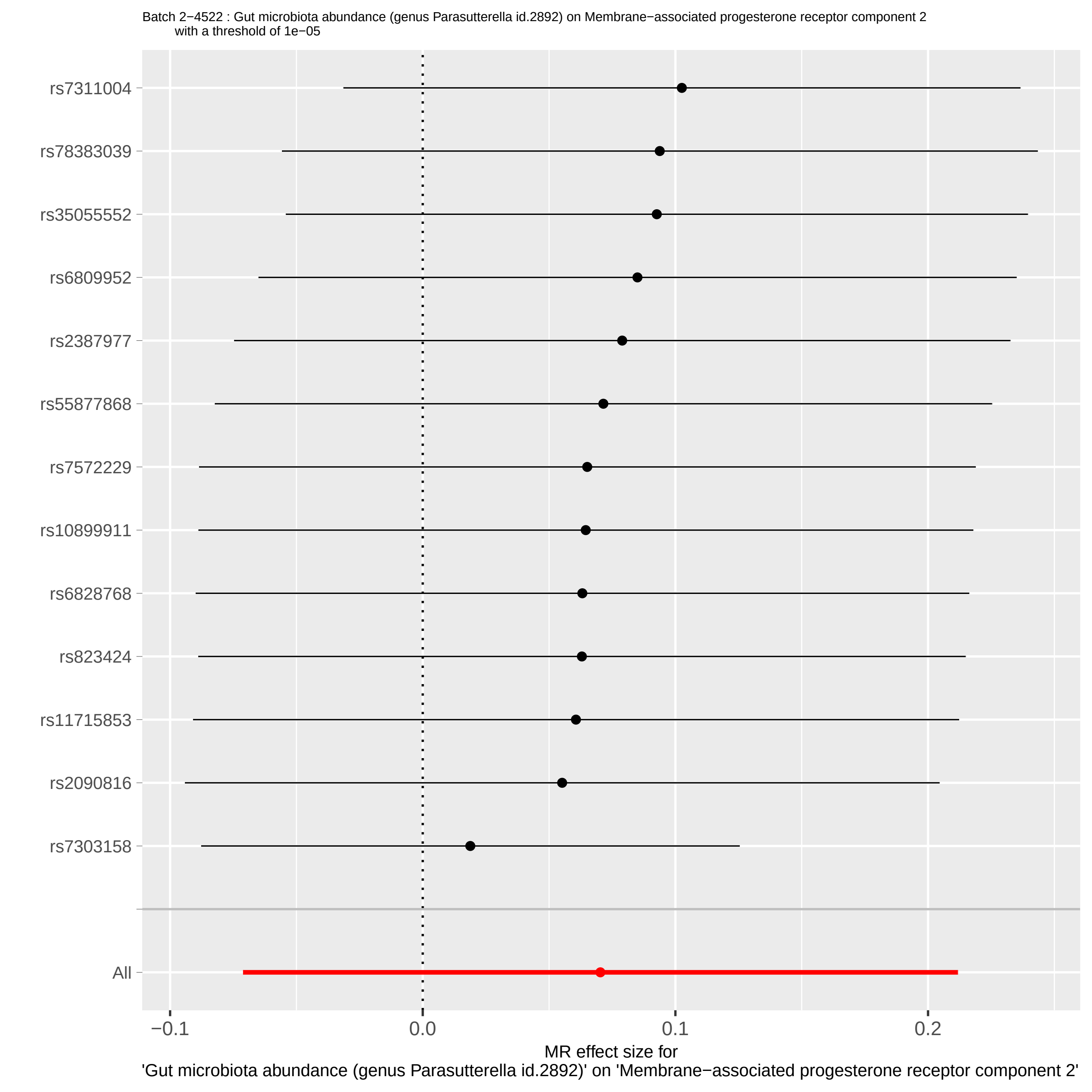
Batch 2−4522 : Gut microbiota abundance (genus Parasutterella id.2892) on Membrane−associated progesterone receptor component 2
with a threshold of 1e−05
rs7311004
rs78383039
rs35055552
rs6809952
rs2387977
rs55877868
rs7572229
rs10899911
rs6828768
rs823424
rs11715853
rs2090816
rs7303158
All
−0.1
0.0
0.1
0.2
MR effect size for
'Gut microbiota abundance (genus Parasutterella id.2892)' on 'Membrane−associated progesterone receptor component 2'

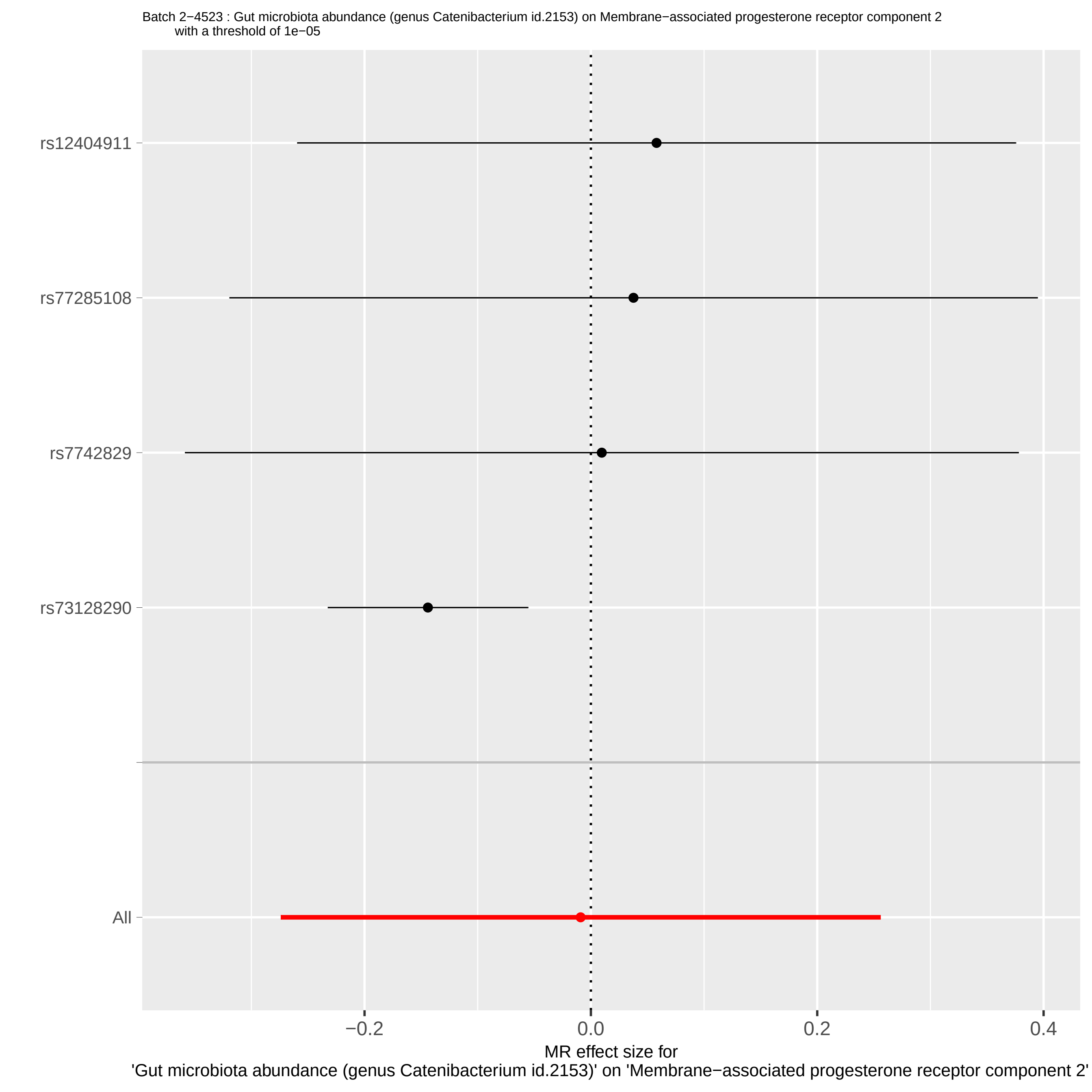

Batch 2−4523 : Gut microbiota abundance (genus Catenibacterium id.2153) on Membrane−associated progesterone receptor component 2
with a threshold of 1e−05
rs12404911
rs77285108
rs7742829
rs73128290
All
−0.2
0.0
0.2
0.4
MR effect size for
'Gut microbiota abundance (genus Catenibacterium id.2153)' on 'Membrane−associated progesterone receptor component 2'

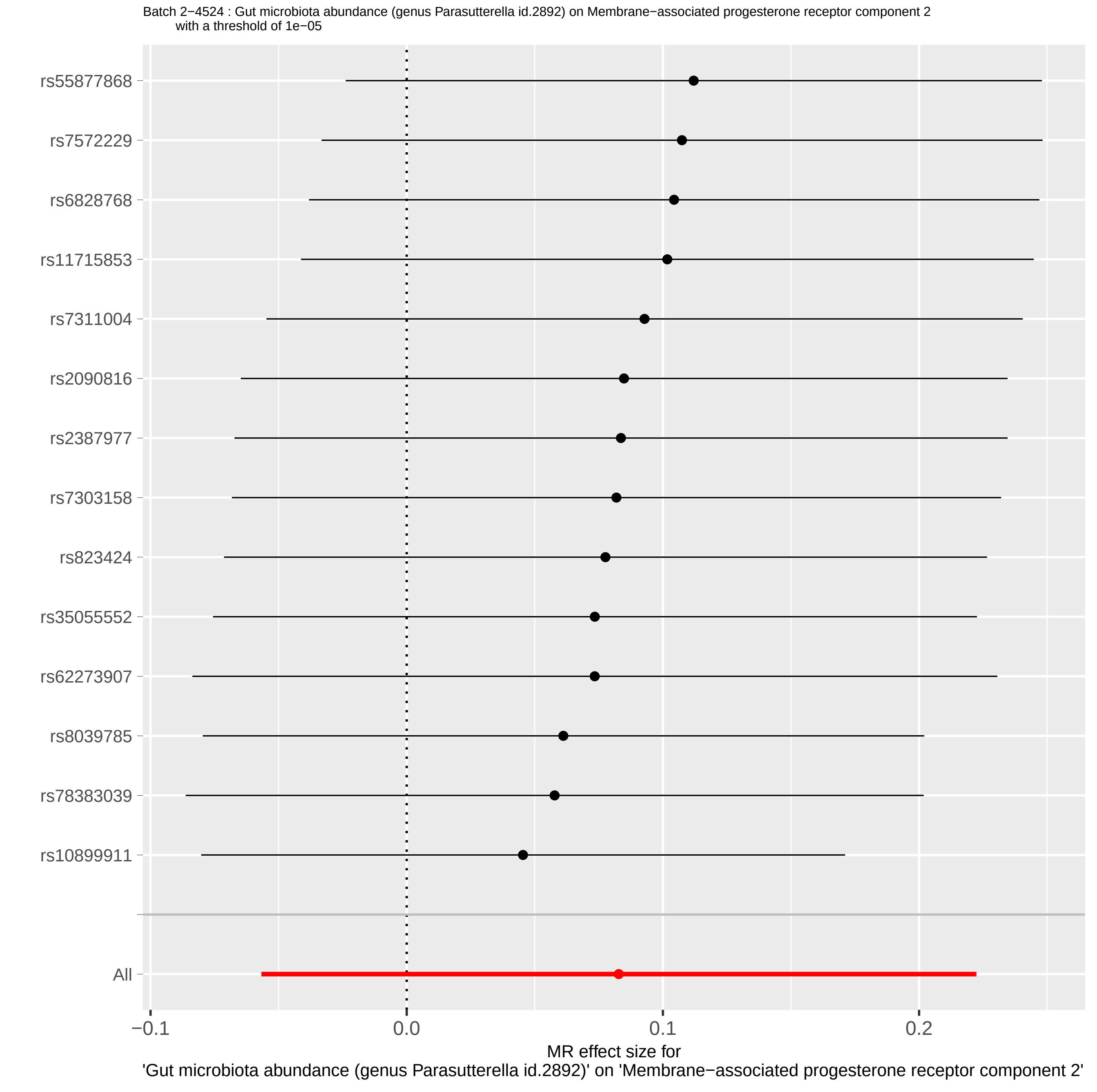

Batch 2−4524 : Gut microbiota abundance (genus Parasutterella id.2892) on Membrane−associated progesterone receptor component 2
with a threshold of 1e−05
rs55877868
rs7572229
rs6828768
rs11715853
rs7311004
rs2090816
rs2387977
rs7303158
rs823424
rs35055552
rs62273907
rs8039785
rs78383039
rs10899911
All
−0.1
0.0
0.1
0.2
MR effect size for
'Gut microbiota abundance (genus Parasutterella id.2892)' on 'Membrane−associated progesterone receptor component 2'

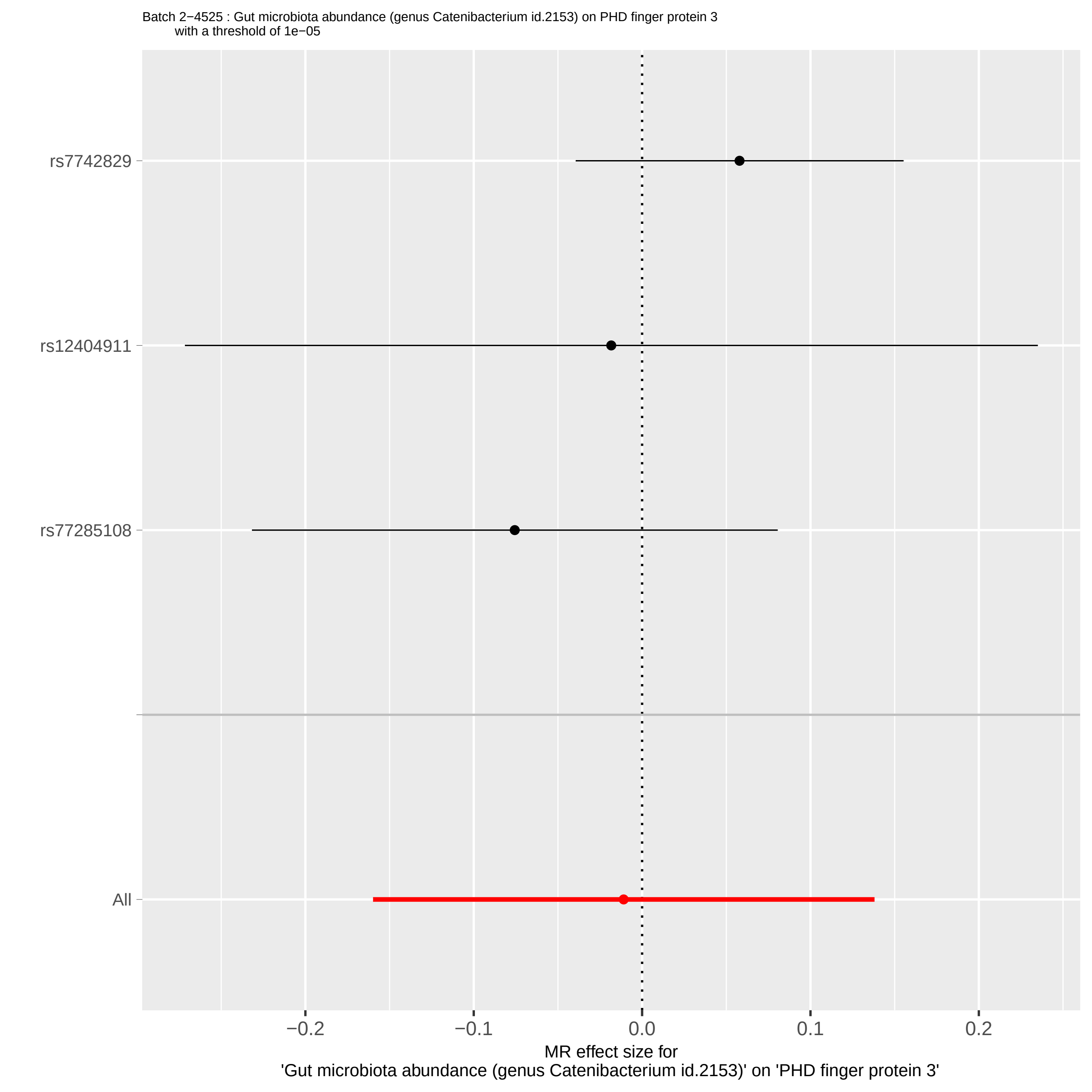
Batch 2−4525 : Gut microbiota abundance (genus Catenibacterium id.2153) on PHD finger protein 3
with a threshold of 1e−05
rs7742829
rs12404911
rs77285108
All
−0.2
−0.1
0.0
0.1
0.2
MR effect size for
'Gut microbiota abundance (genus Catenibacterium id.2153)' on 'PHD finger protein 3'

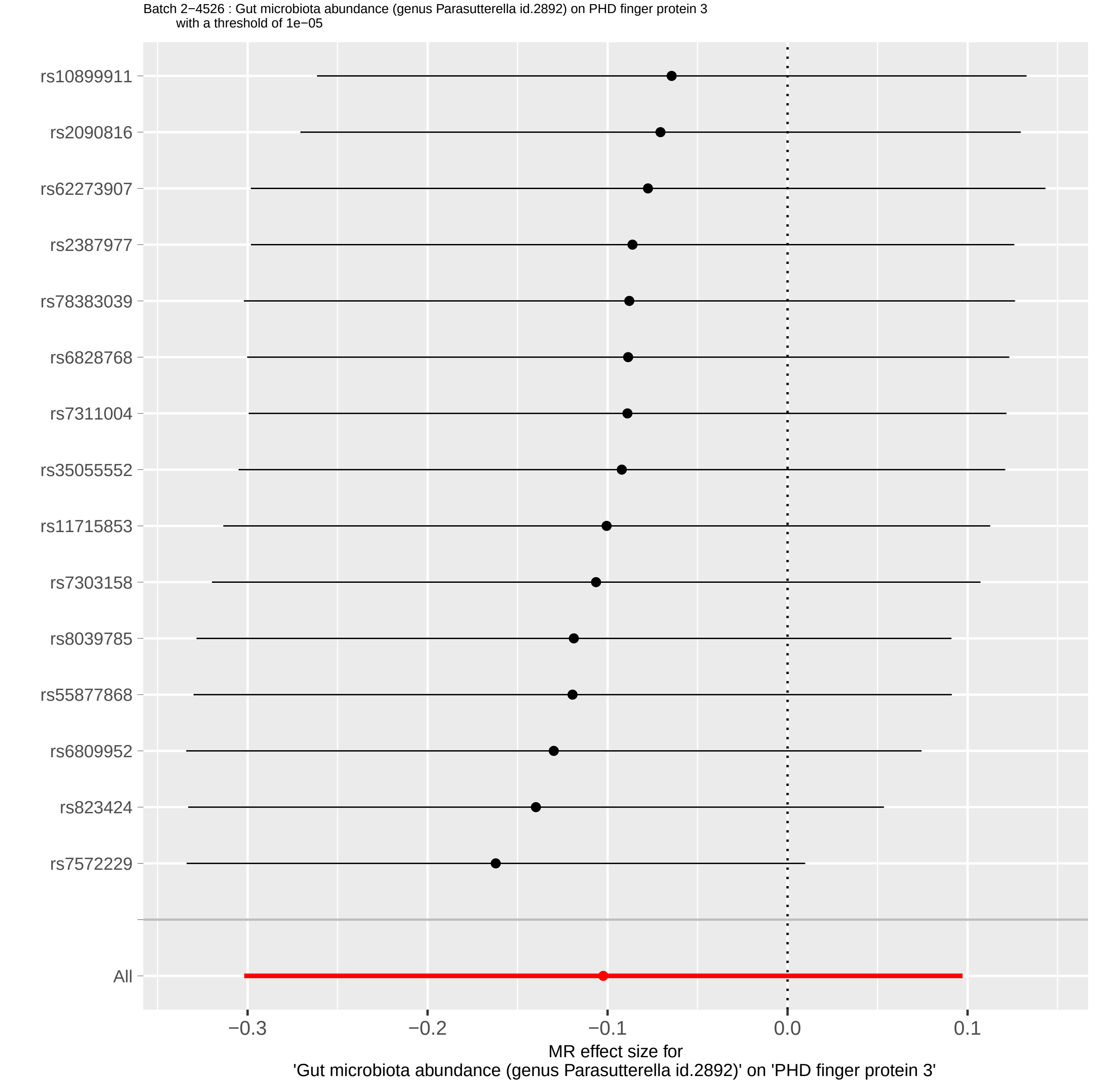
Batch 2−4526 : Gut microbiota abundance (genus Parasutterella id.2892) on PHD finger protein 3
with a threshold of 1e−05
rs10899911
rs2090816
rs62273907
rs2387977
rs78383039
rs6828768
rs7311004
rs35055552
rs11715853
rs7303158
rs8039785
rs55877868
rs6809952
rs823424
rs7572229
All
−0.3
−0.2
−0.1
0.0
0.1
MR effect size for
'Gut microbiota abundance (genus Parasutterella id.2892)' on 'PHD finger protein 3'

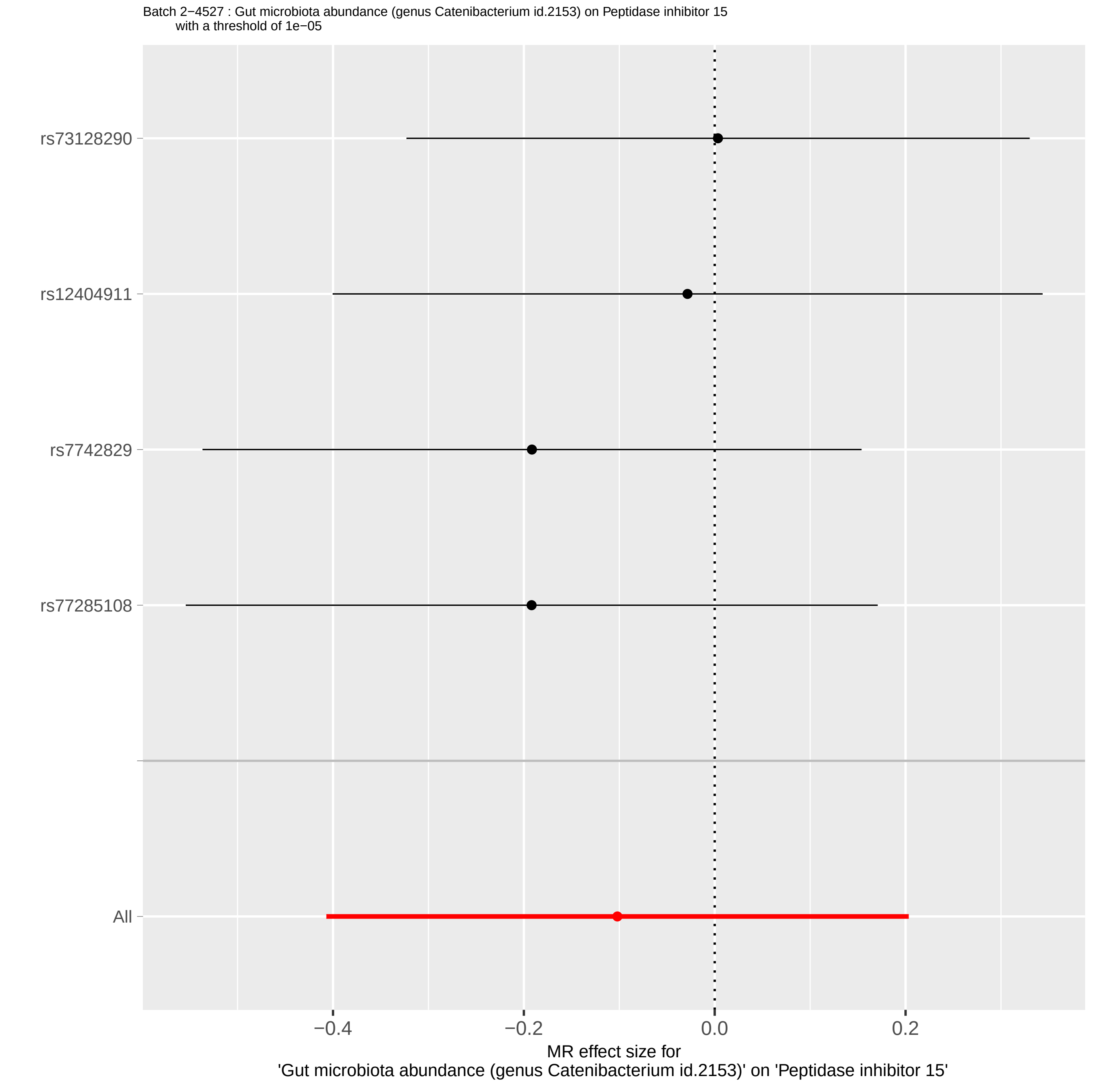

Batch 2−4527 : Gut microbiota abundance (genus Catenibacterium id.2153) on Peptidase inhibitor 15
with a threshold of 1e−05
rs73128290
rs12404911
rs7742829
rs77285108
All
−0.4
−0.2
0.0
0.2
MR effect size for
'Gut microbiota abundance (genus Catenibacterium id.2153)' on 'Peptidase inhibitor 15'

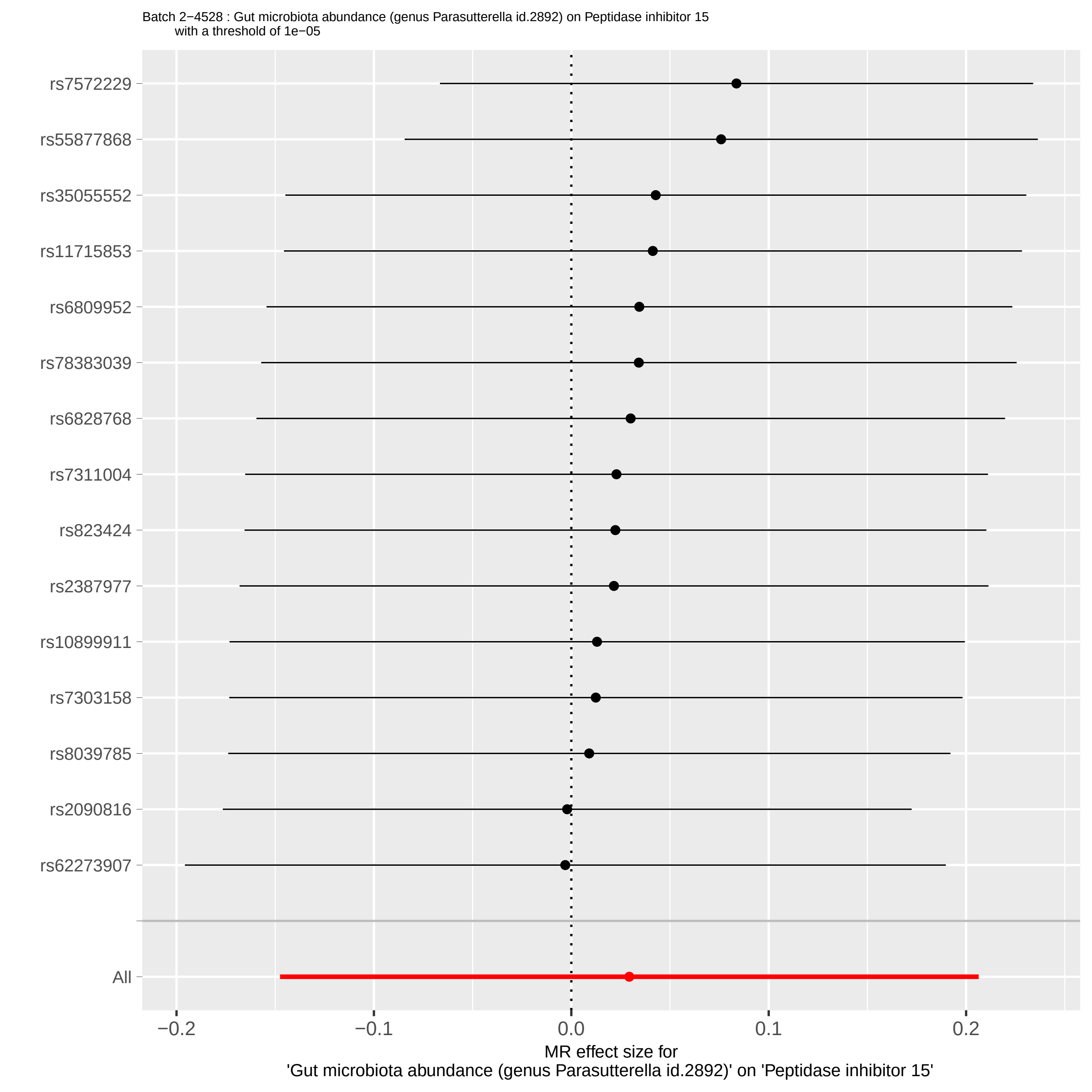
Batch 2−4528 : Gut microbiota abundance (genus Parasutterella id.2892) on Peptidase inhibitor 15
with a threshold of 1e−05
rs7572229
rs55877868
rs35055552
rs11715853
rs6809952
rs78383039
rs6828768
rs7311004
rs823424
rs2387977
rs10899911
rs7303158
rs8039785
rs2090816
rs62273907
All
−0.2
−0.1
0.0
0.1
0.2
MR effect size for
'Gut microbiota abundance (genus Parasutterella id.2892)' on 'Peptidase inhibitor 15'

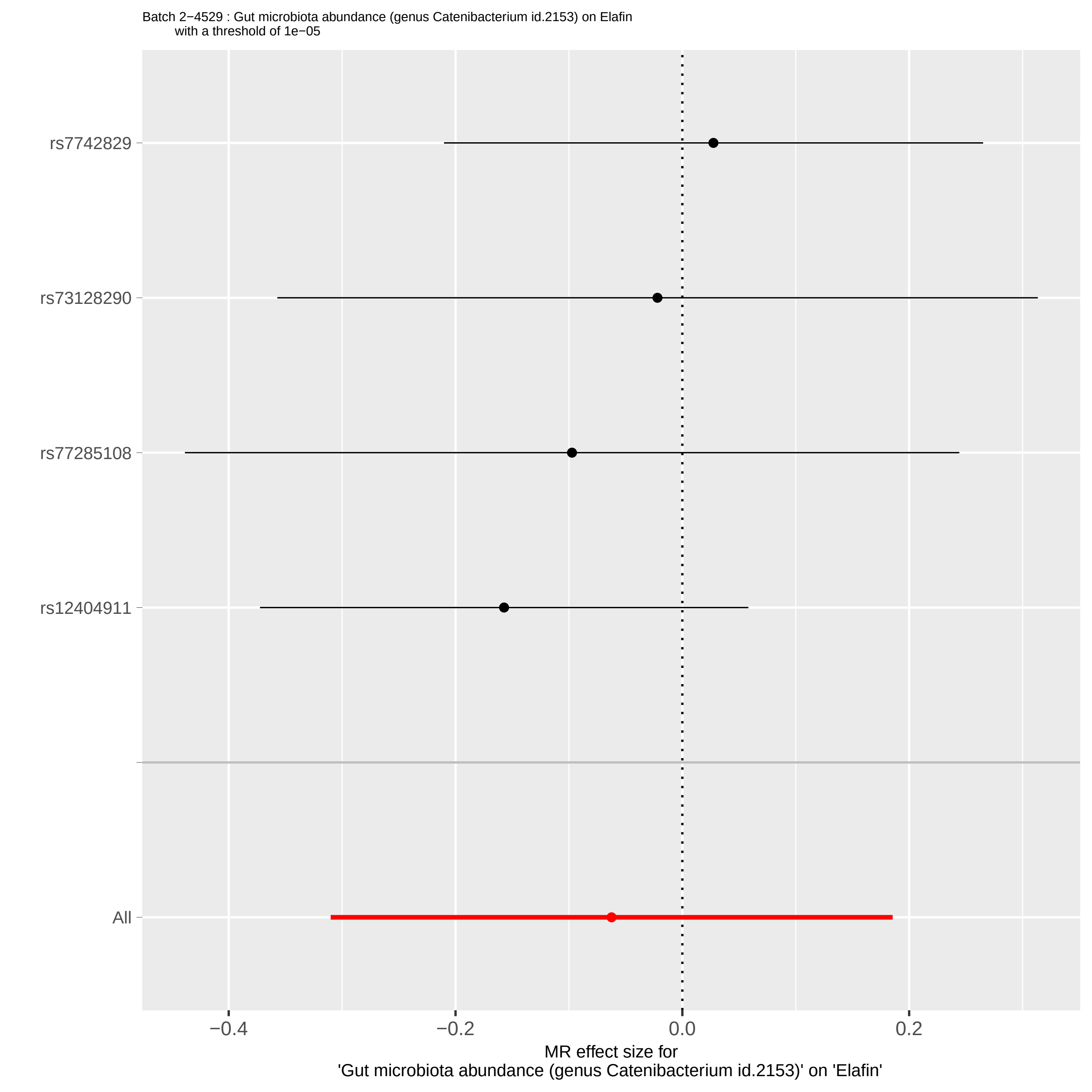
Batch 2−4529 : Gut microbiota abundance (genus Catenibacterium id.2153) on Elafin
with a threshold of 1e−05
rs7742829
rs73128290
rs77285108
rs12404911
All
−0.4
−0.2
0.0
0.2
MR effect size for
'Gut microbiota abundance (genus Catenibacterium id.2153)' on 'Elafin'

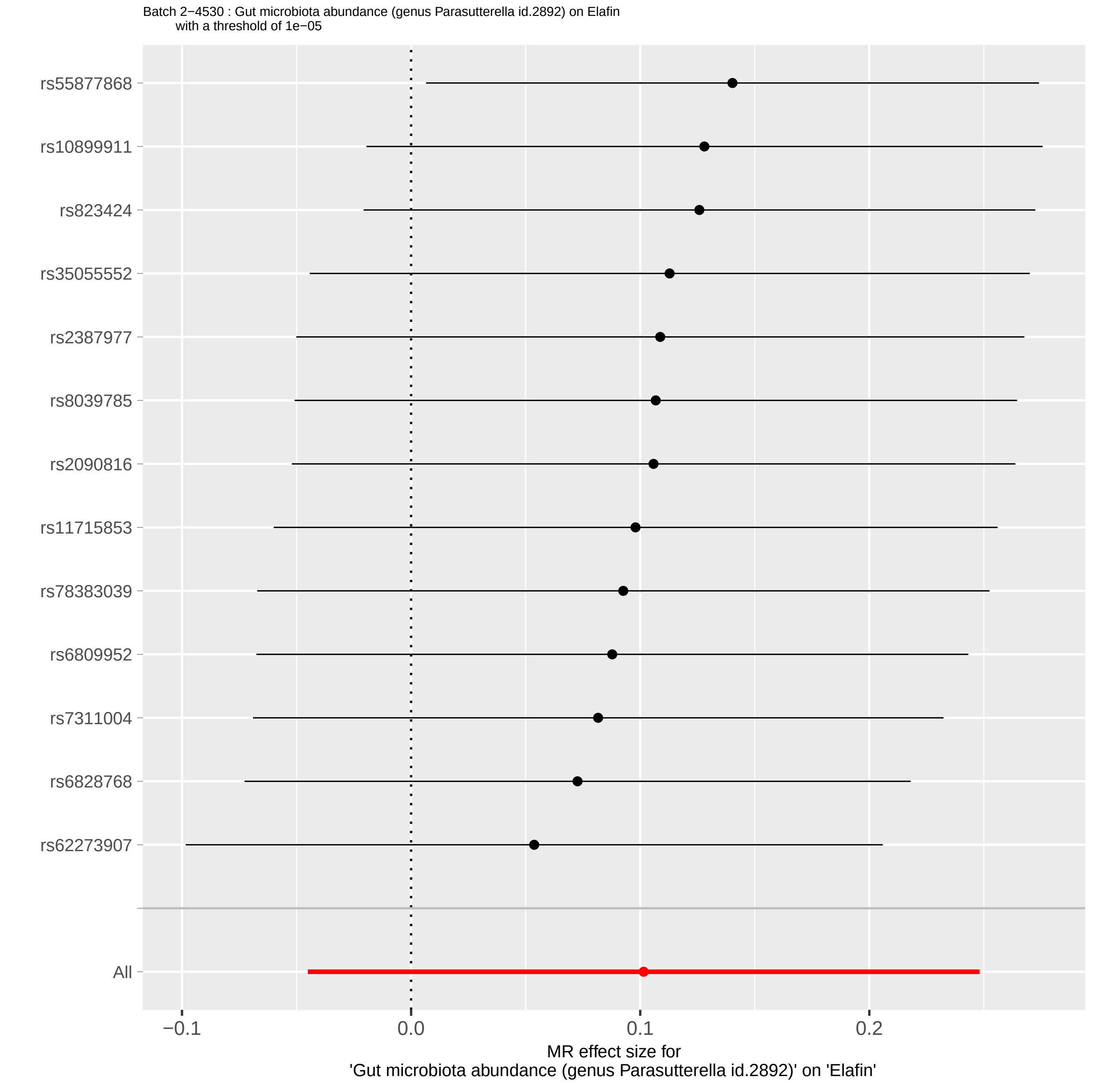
Batch 2−4530 : Gut microbiota abundance (genus Parasutterella id.2892) on Elafin
with a threshold of 1e−05
rs55877868
rs10899911
rs823424
rs35055552
rs2387977
rs8039785
rs2090816
rs11715853
rs78383039
rs6809952
rs7311004
rs6828768
rs62273907
All
−0.1
0.0
0.1
0.2
MR effect size for
'Gut microbiota abundance (genus Parasutterella id.2892)' on 'Elafin'

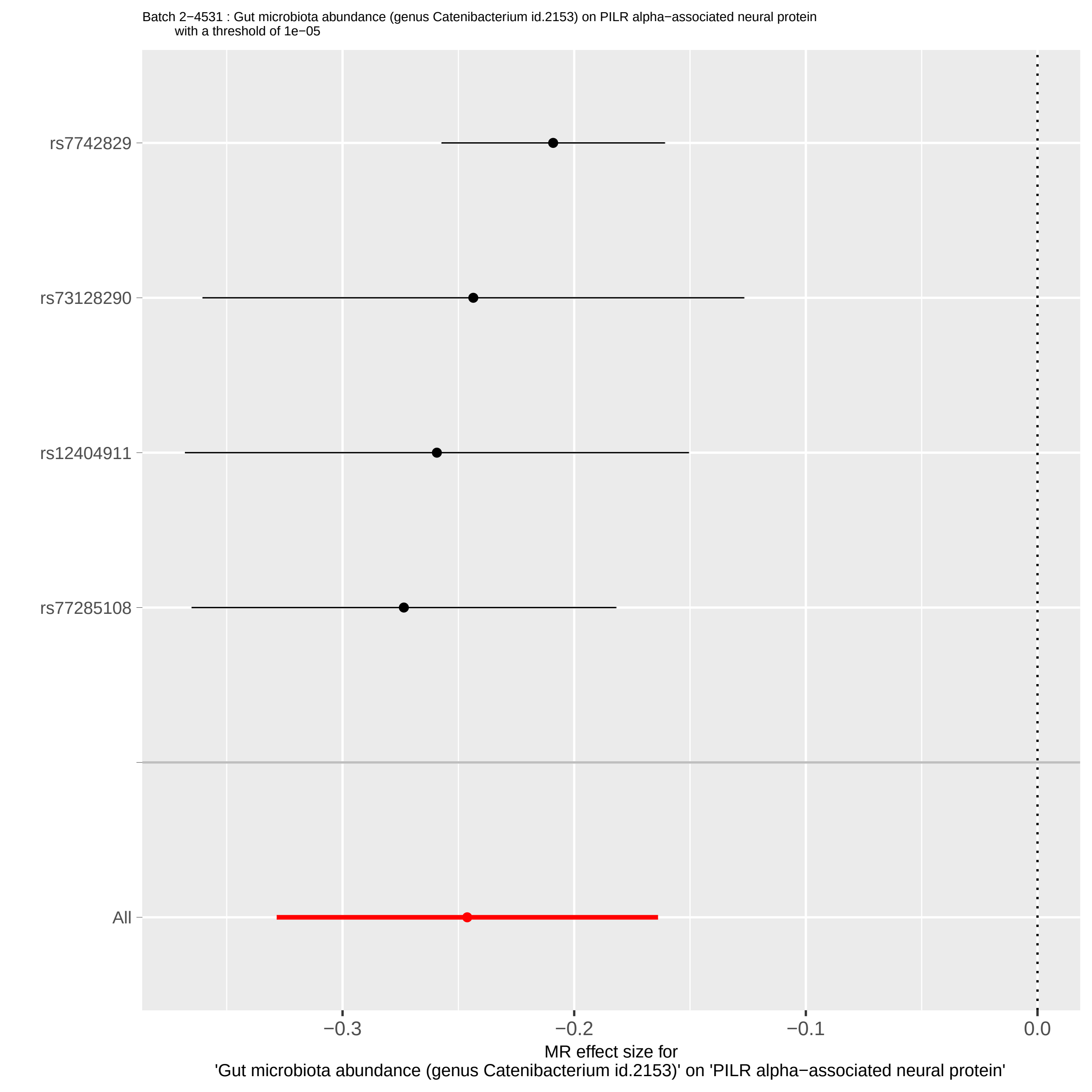

Batch 2−4531 : Gut microbiota abundance (genus Catenibacterium id.2153) on PILR alpha−associated neural protein
with a threshold of 1e−05
rs7742829
rs73128290
rs12404911
rs77285108
All
−0.3
−0.2
−0.1
0.0
MR effect size for
'Gut microbiota abundance (genus Catenibacterium id.2153)' on 'PILR alpha−associated neural protein'

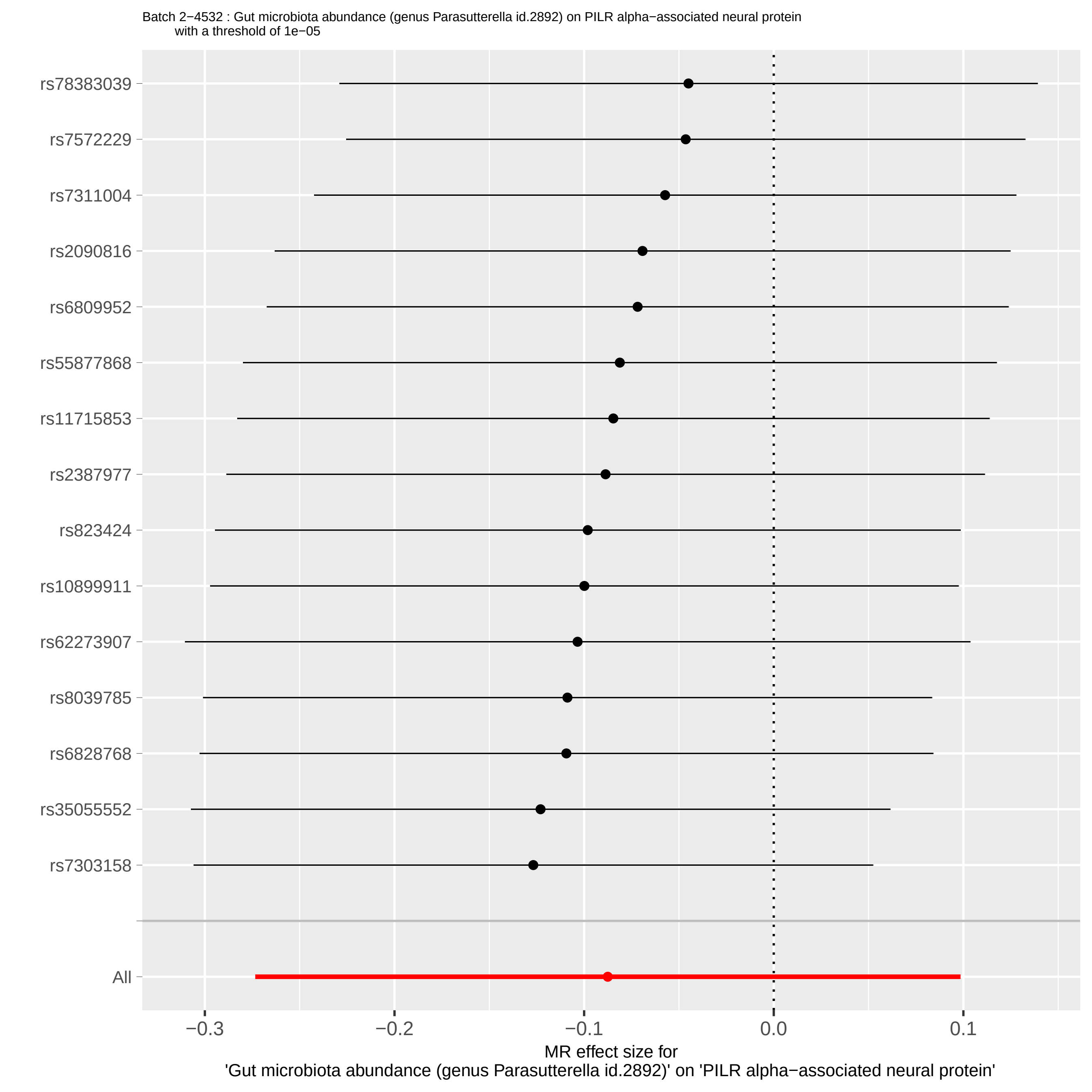

Batch 2−4532 : Gut microbiota abundance (genus Parasutterella id.2892) on PILR alpha−associated neural protein
with a threshold of 1e−05
rs78383039
rs7572229
rs7311004
rs2090816
rs6809952
rs55877868
rs11715853
rs2387977
rs823424
rs10899911
rs62273907
rs8039785
rs6828768
rs35055552
rs7303158
All
−0.3
−0.2
−0.1
0.0
0.1
MR effect size for
'Gut microbiota abundance (genus Parasutterella id.2892)' on 'PILR alpha−associated neural protein'

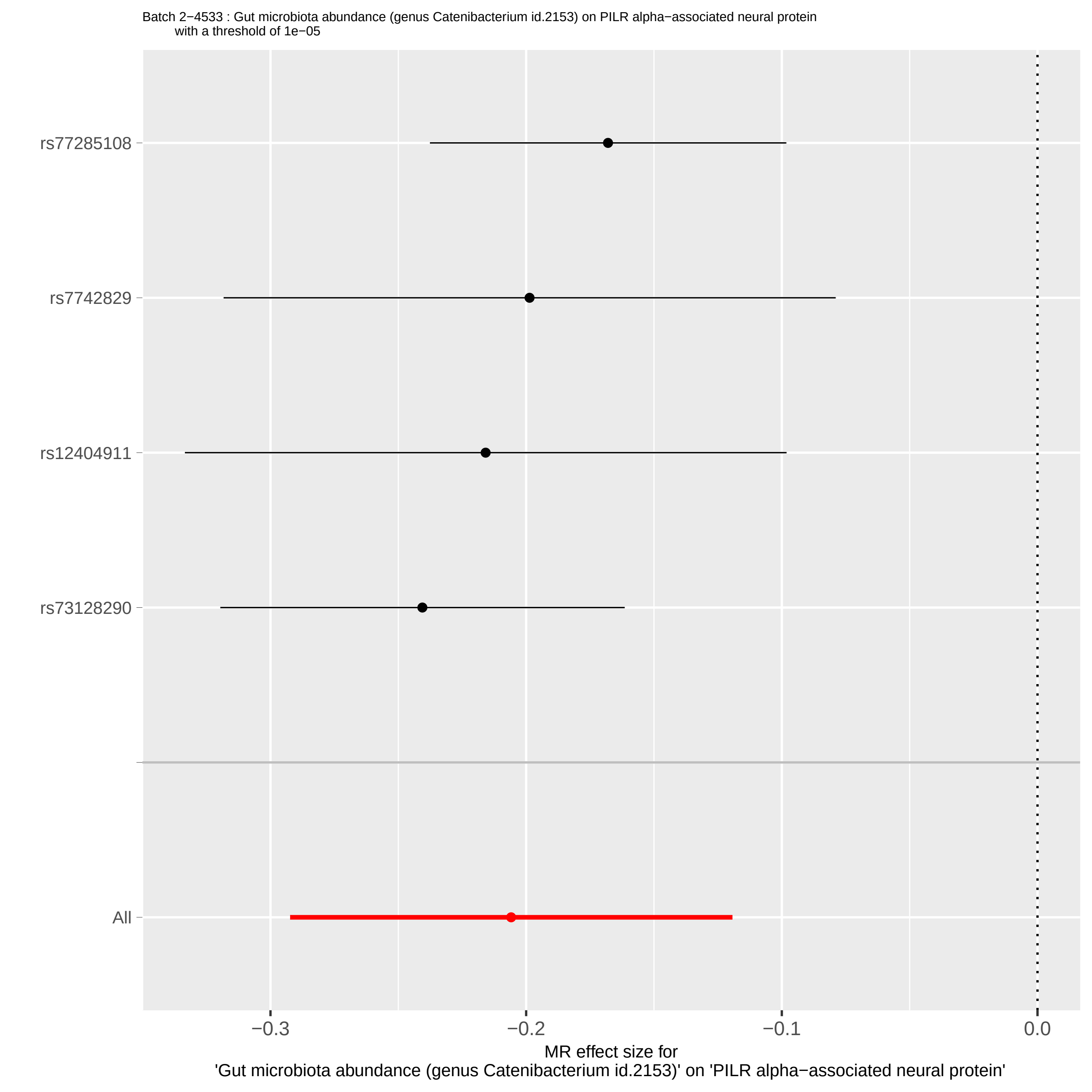
Batch 2−4533 : Gut microbiota abundance (genus Catenibacterium id.2153) on PILR alpha−associated neural protein
with a threshold of 1e−05
rs77285108
rs7742829
rs12404911
rs73128290
All
−0.3
−0.2
−0.1
0.0
MR effect size for
'Gut microbiota abundance (genus Catenibacterium id.2153)' on 'PILR alpha−associated neural protein'

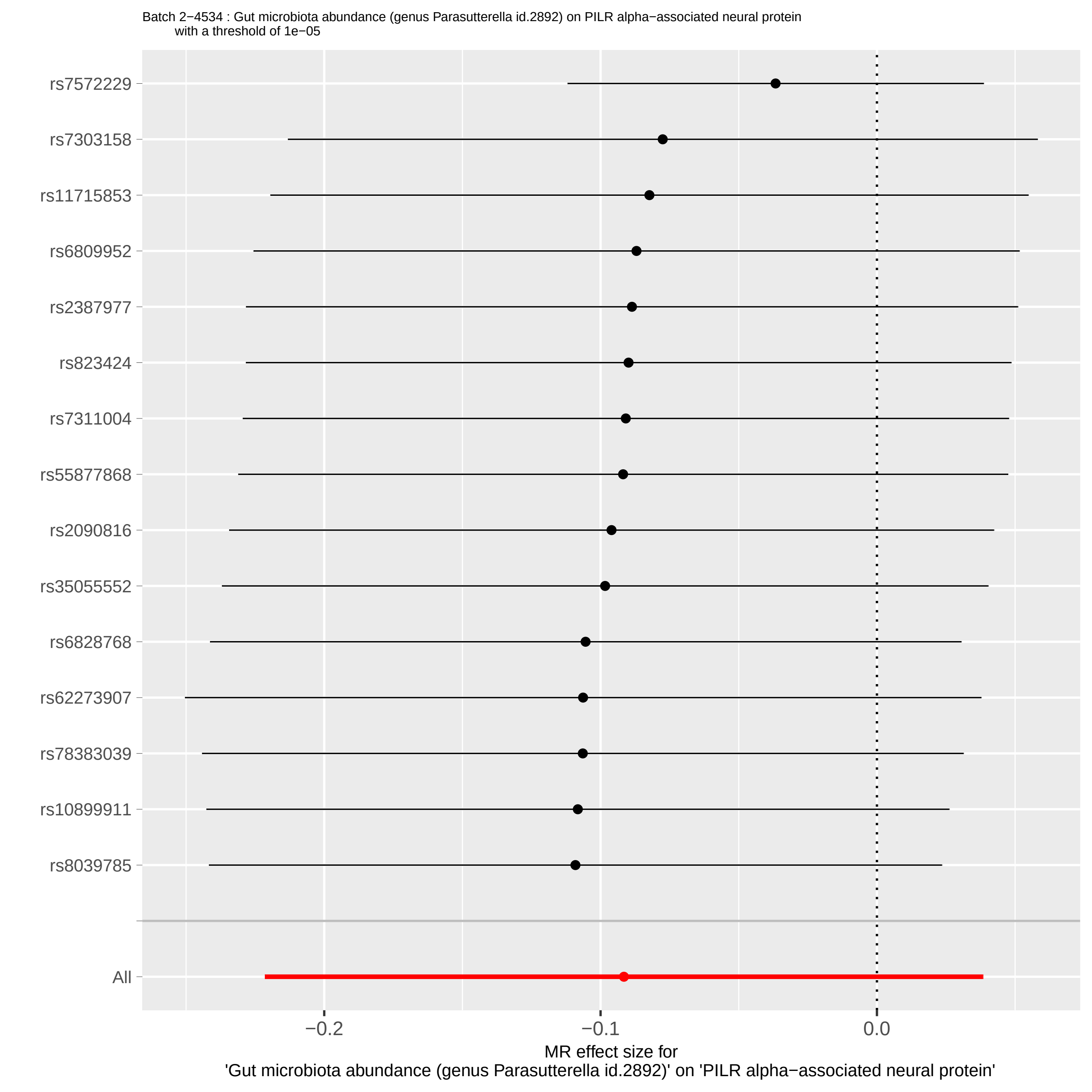

Batch 2−4534 : Gut microbiota abundance (genus Parasutterella id.2892) on PILR alpha−associated neural protein
with a threshold of 1e−05
rs7572229
rs7303158
rs11715853
rs6809952
rs2387977
rs823424
rs7311004
rs55877868
rs2090816
rs35055552
rs6828768
rs62273907
rs78383039
rs10899911
rs8039785
All
−0.2
−0.1
0.0
MR effect size for
'Gut microbiota abundance (genus Parasutterella id.2892)' on 'PILR alpha−associated neural protein'

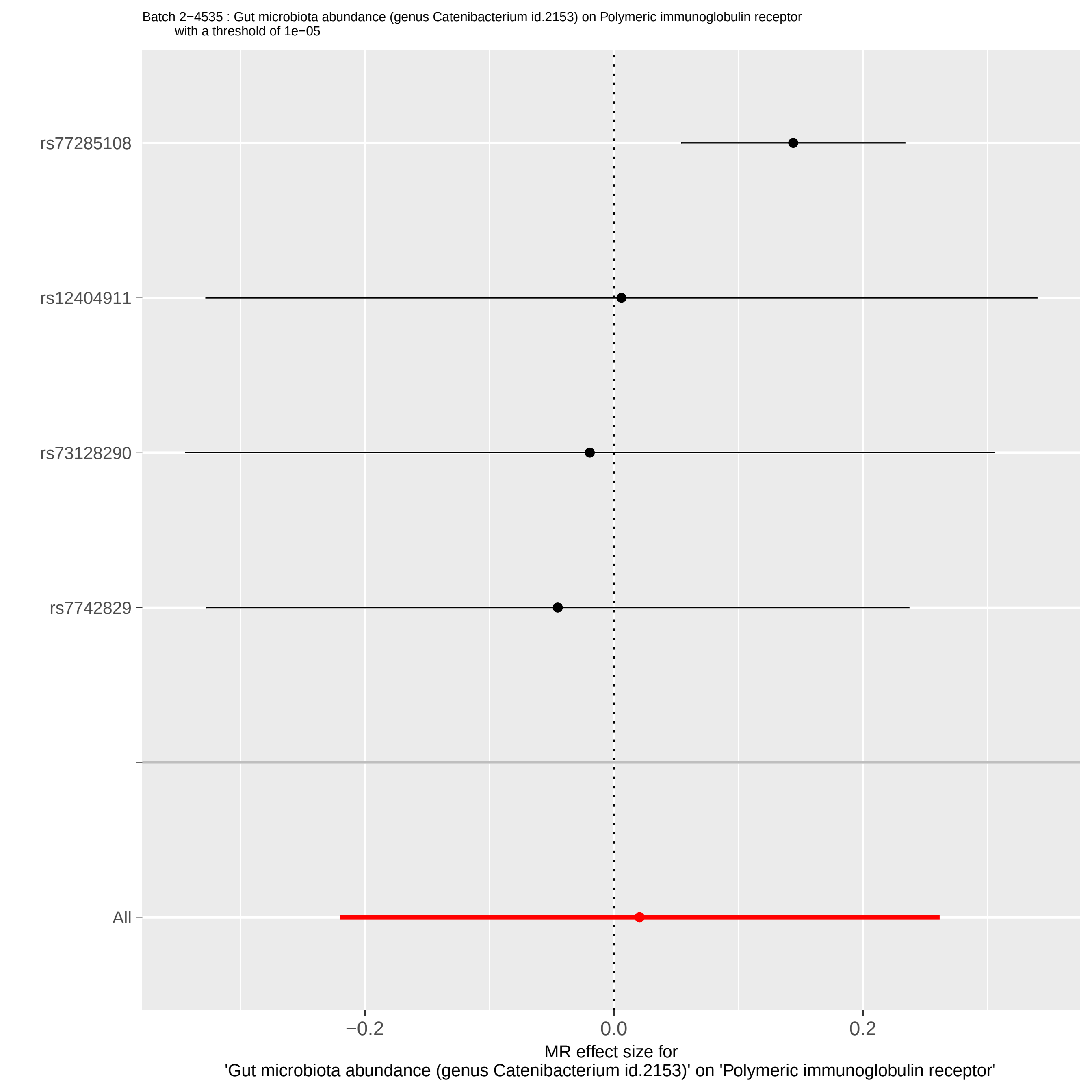
Batch 2−4535 : Gut microbiota abundance (genus Catenibacterium id.2153) on Polymeric immunoglobulin receptor
with a threshold of 1e−05
rs77285108
rs12404911
rs73128290
rs7742829
All
−0.2
0.0
0.2
MR effect size for
'Gut microbiota abundance (genus Catenibacterium id.2153)' on 'Polymeric immunoglobulin receptor'

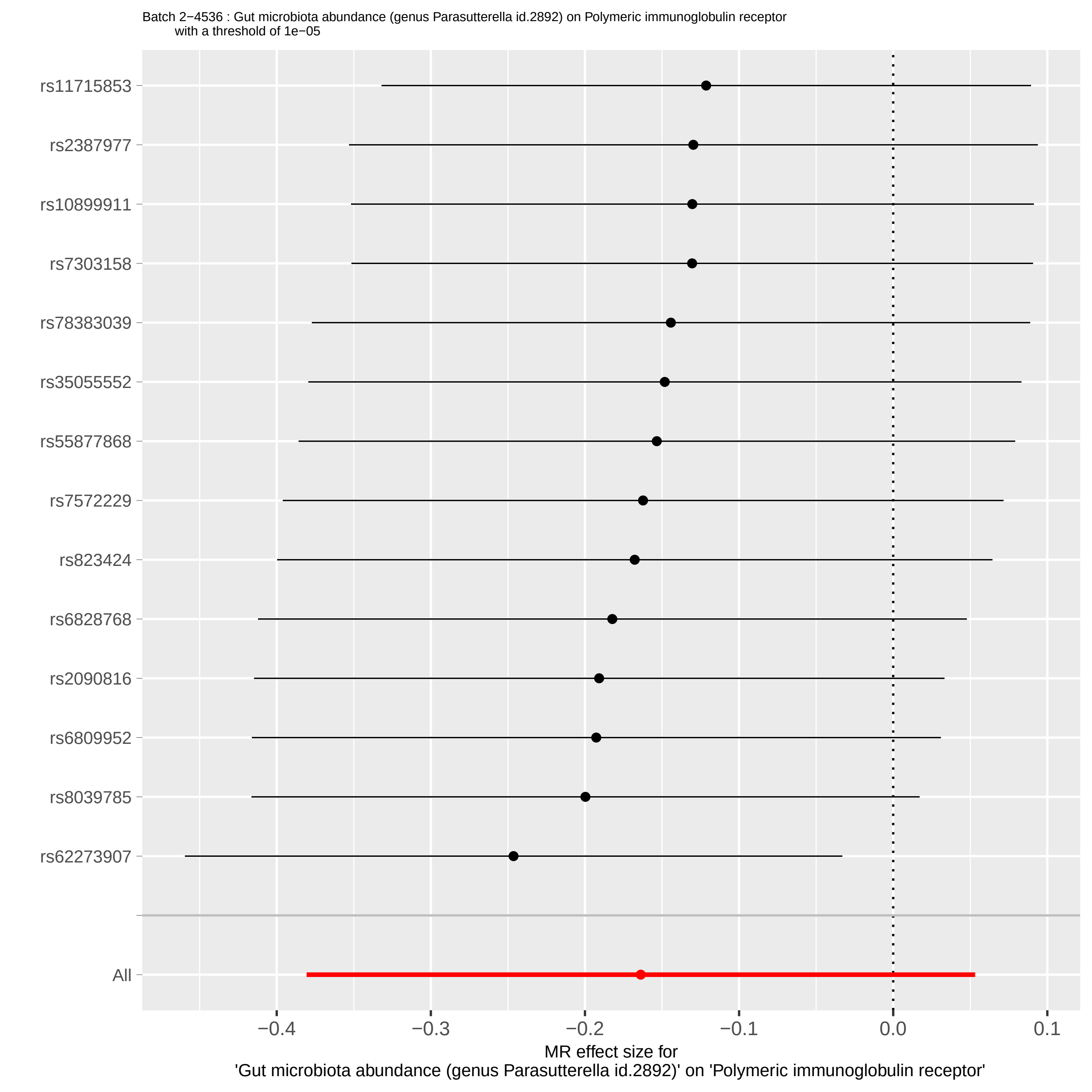
Batch 2−4536 : Gut microbiota abundance (genus Parasutterella id.2892) on Polymeric immunoglobulin receptor
with a threshold of 1e−05
rs11715853
rs2387977
rs10899911
rs7303158
rs78383039
rs35055552
rs55877868
rs7572229
rs823424
rs6828768
rs2090816
rs6809952
rs8039785
rs62273907
All
−0.4
−0.3
−0.2
−0.1
0.0
0.1
MR effect size for
'Gut microbiota abundance (genus Parasutterella id.2892)' on 'Polymeric immunoglobulin receptor

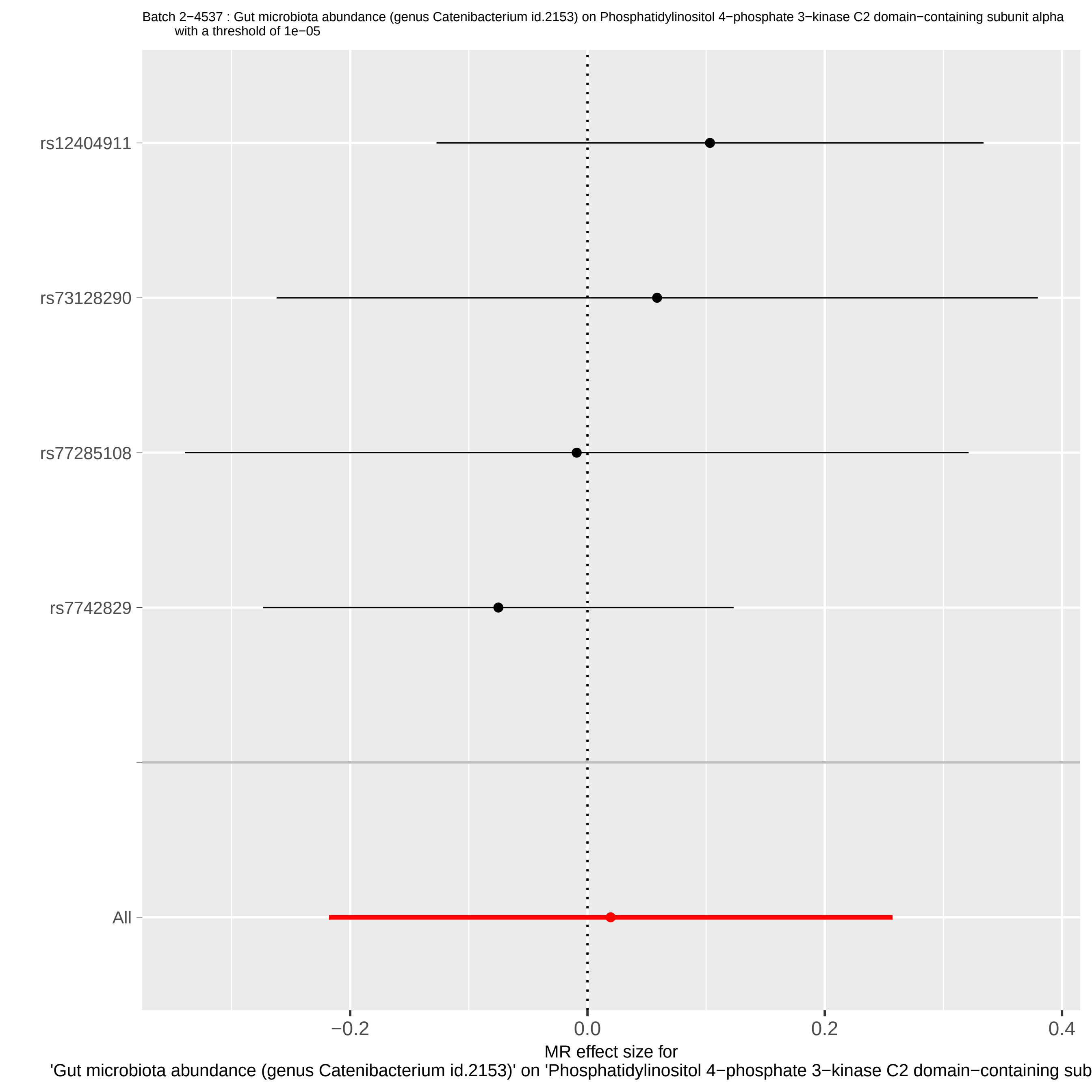

Batch 2−4537 : Gut microbiota abundance (genus Catenibacterium id.2153) on Phosphatidylinositol 4−phosphate 3−kinase C2 domain−containing subunit alpha with a threshold of 1e−05
rs12404911
rs73128290
rs77285108
rs7742829
All
−0.2
0.0
0.2
0.4
MR effect size for
'Gut microbiota abundance (genus Catenibacterium id.2153)' on 'Phosphatidylinositol 4−phosphate 3−kinase C2 domain−containing sub

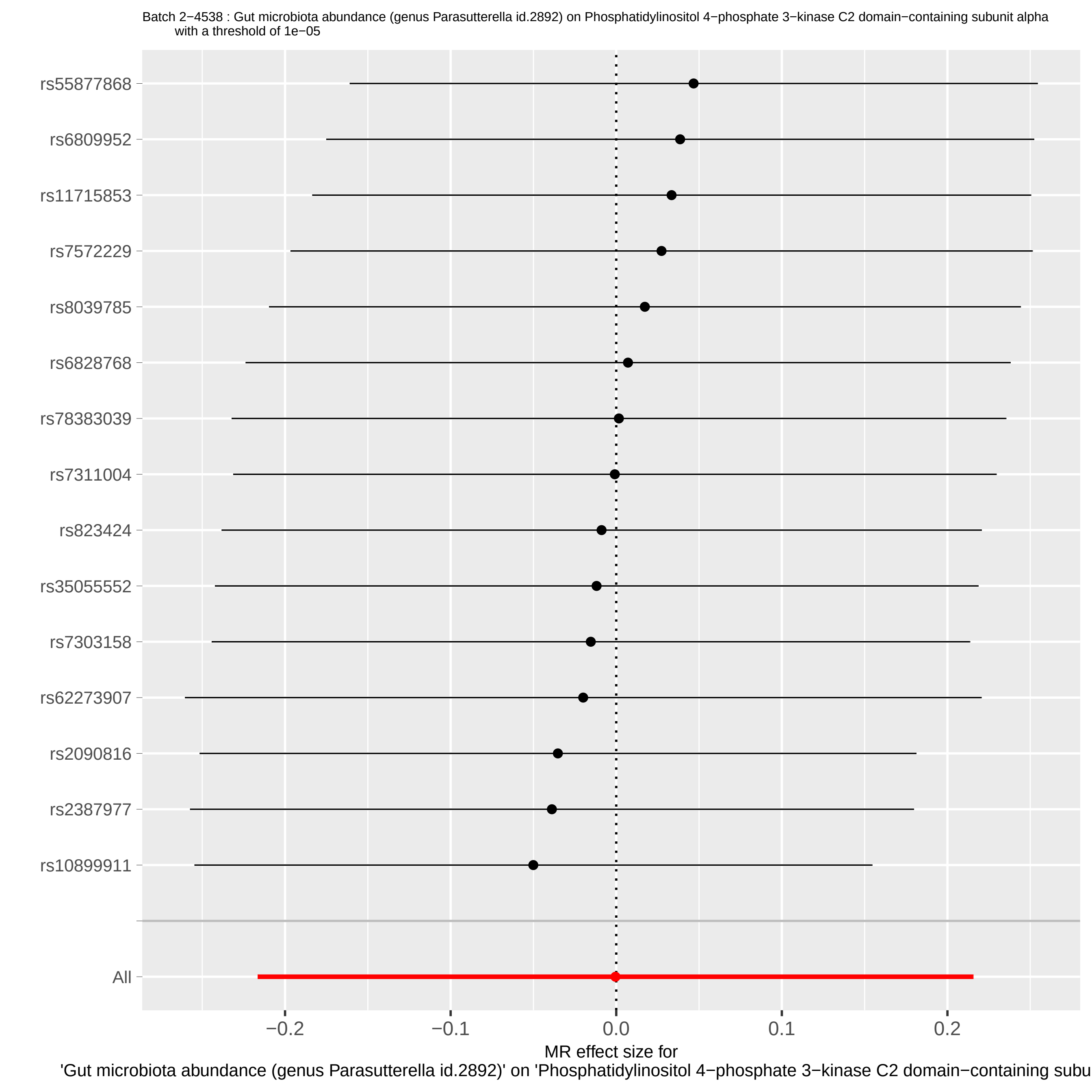

Batch 2−4538 : Gut microbiota abundance (genus Parasutterella id.2892) on Phosphatidylinositol 4−phosphate 3−kinase C2 domain−containing subunit alpha with a threshold of 1e−05
rs55877868
rs6809952
rs11715853
rs7572229
rs8039785
rs6828768
rs78383039
rs7311004
rs823424
rs35055552
rs7303158
rs62273907
rs2090816
rs2387977
rs10899911
All
−0.2
−0.1
0.0
0.1
0.2
MR effect size for
'Gut microbiota abundance (genus Parasutterella id.2892)' on 'Phosphatidylinositol 4−phosphate 3−kinase C2 domain−containing subu

Batch 2−4539 : Gut microbiota abundance (genus Catenibacterium id.2153) on PIK3CA/PIK3R1
with a threshold of 1e−05

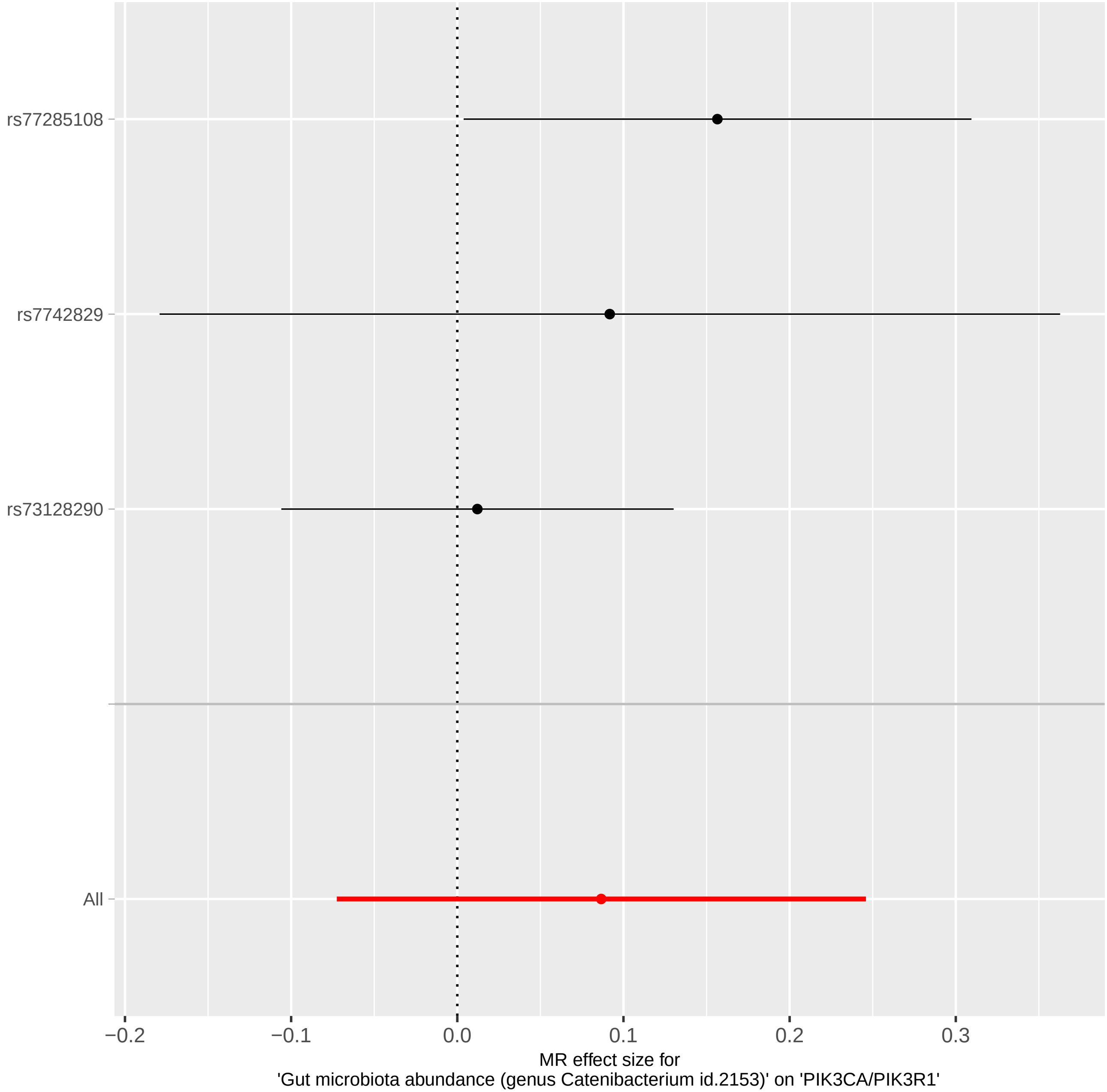

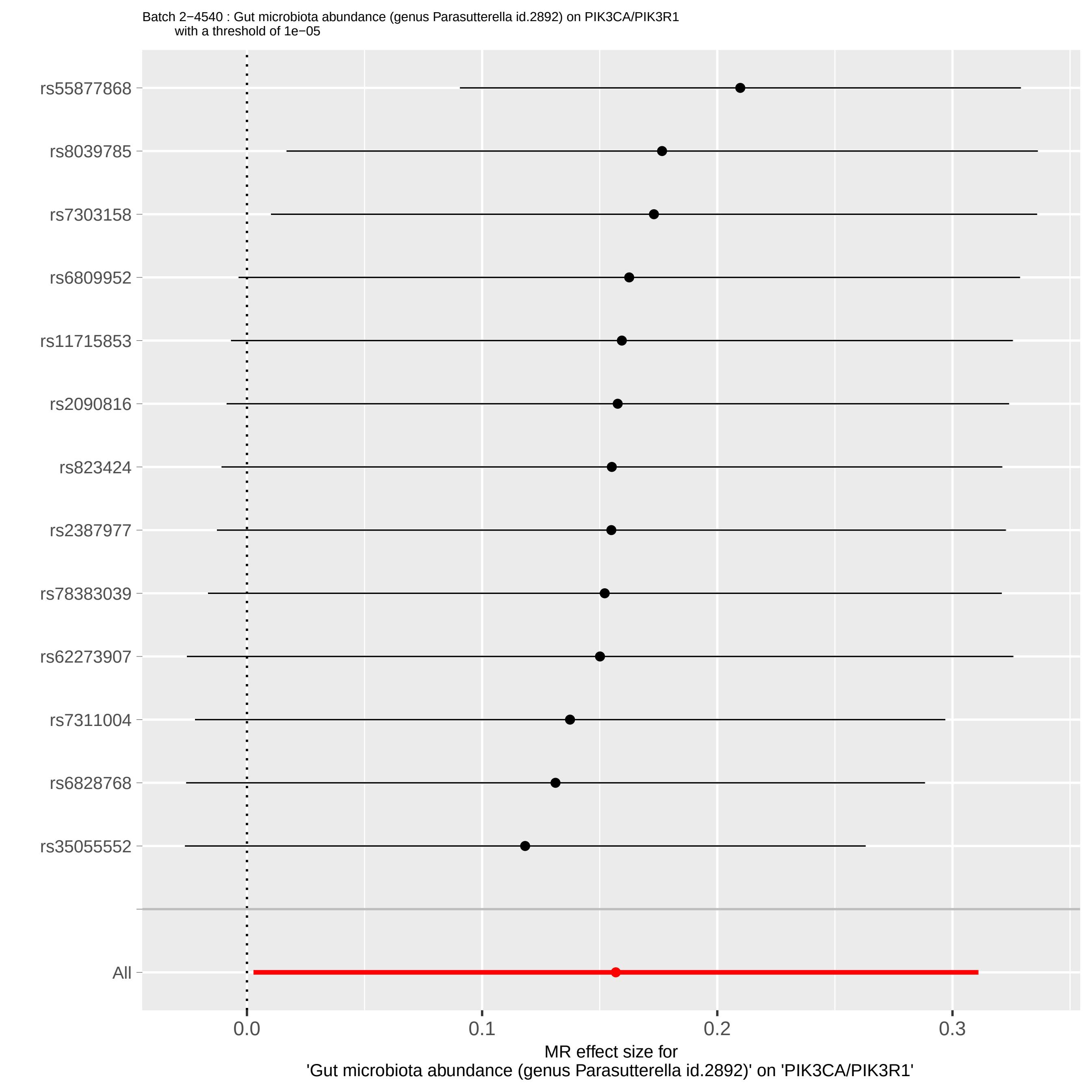
Batch 2−4540 : Gut microbiota abundance (genus Parasutterella id.2892) on PIK3CA/PIK3R1
with a threshold of 1e−05
rs55877868
rs8039785
rs7303158
rs6809952
rs11715853
rs2090816
rs823424
rs2387977
rs78383039
rs62273907
rs7311004
rs6828768
rs35055552
All
0.0
0.1
0.2
0.3
MR effect size for
'Gut microbiota abundance (genus Parasutterella id.2892)' on 'PIK3CA/PIK3R1'

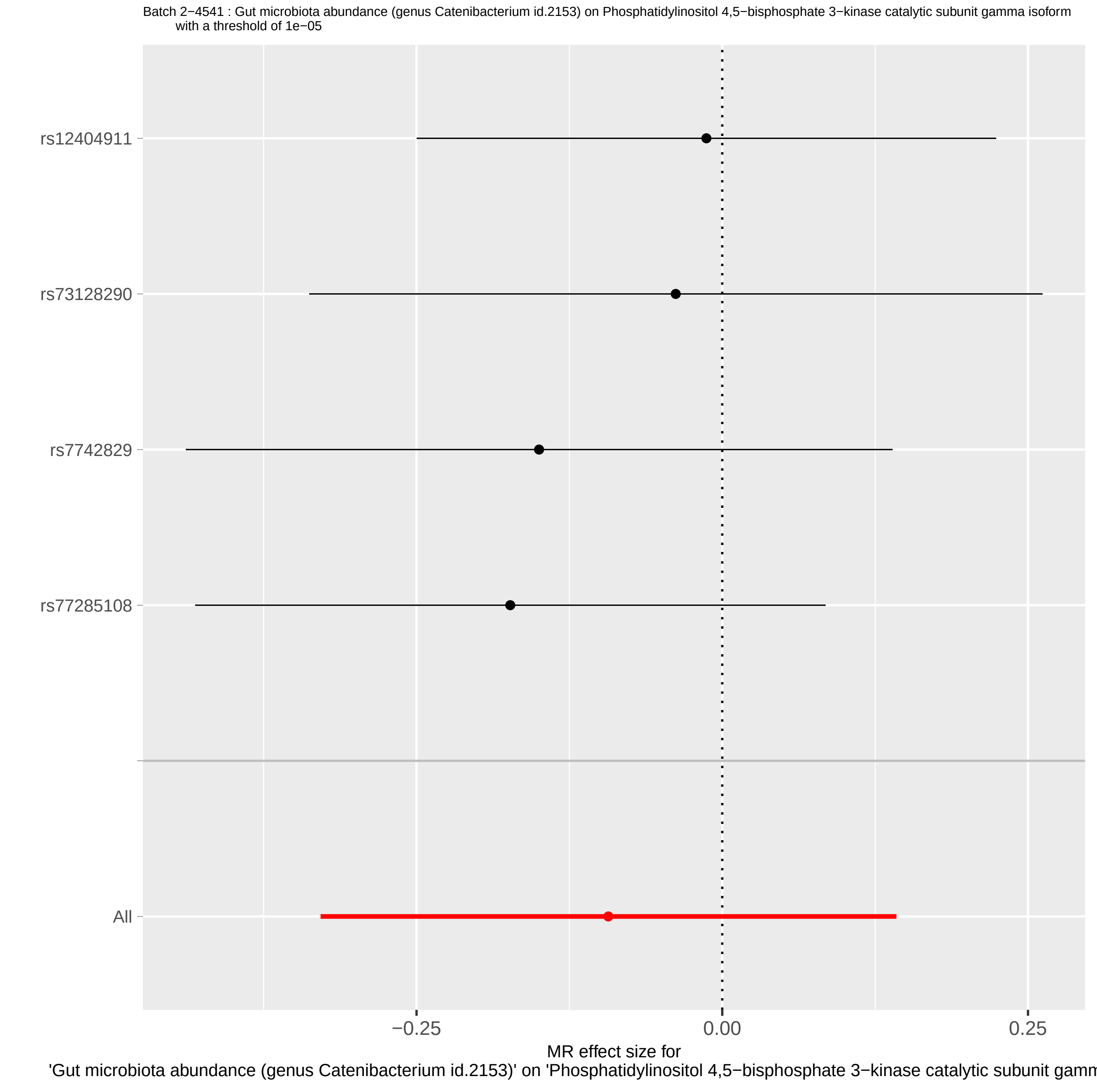
Batch 2−4541 : Gut microbiota abundance (genus Catenibacterium id.2153) on Phosphatidylinositol 4,5−bisphosphate 3−kinase catalytic subunit gamma isoform with a threshold of 1e−05
rs12404911
rs73128290
rs7742829
rs77285108
All
−0.25
0.00
0.25
MR effect size for
'Gut microbiota abundance (genus Catenibacterium id.2153)' on 'Phosphatidylinositol 4,5−bisphosphate 3−kinase catalytic subunit gamm

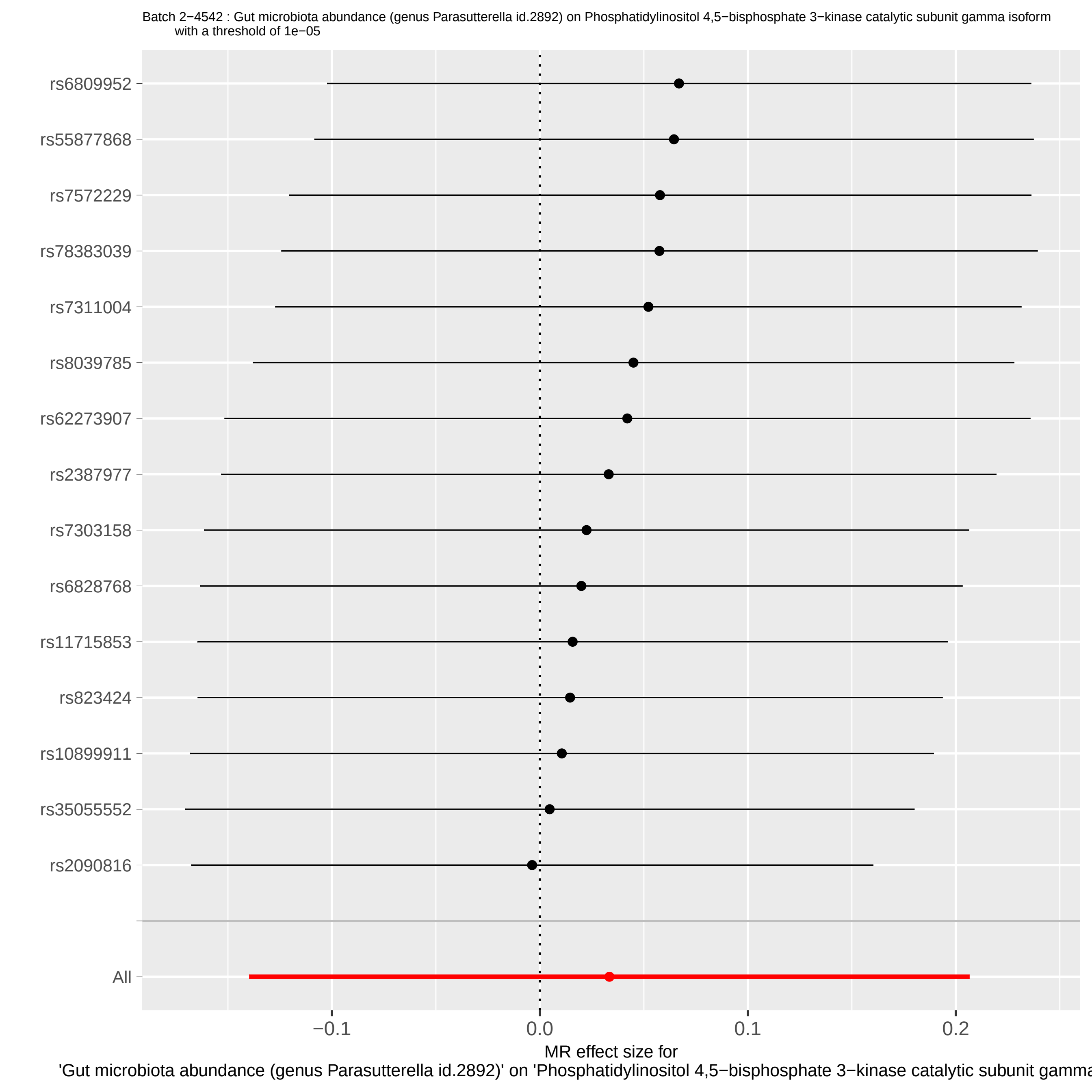
Batch 2−4542 : Gut microbiota abundance (genus Parasutterella id.2892) on Phosphatidylinositol 4,5−bisphosphate 3−kinase catalytic subunit gamma isoform with a threshold of 1e−05
rs6809952
rs55877868
rs7572229
rs78383039
rs7311004
rs8039785
rs62273907
rs2387977
rs7303158
rs6828768
rs11715853
rs823424
rs10899911
rs35055552
rs2090816
All
−0.1
0.0
0.1
0.2
MR effect size for
'Gut microbiota abundance (genus Parasutterella id.2892)' on 'Phosphatidylinositol 4,5−bisphosphate 3−kinase catalytic subunit gamma

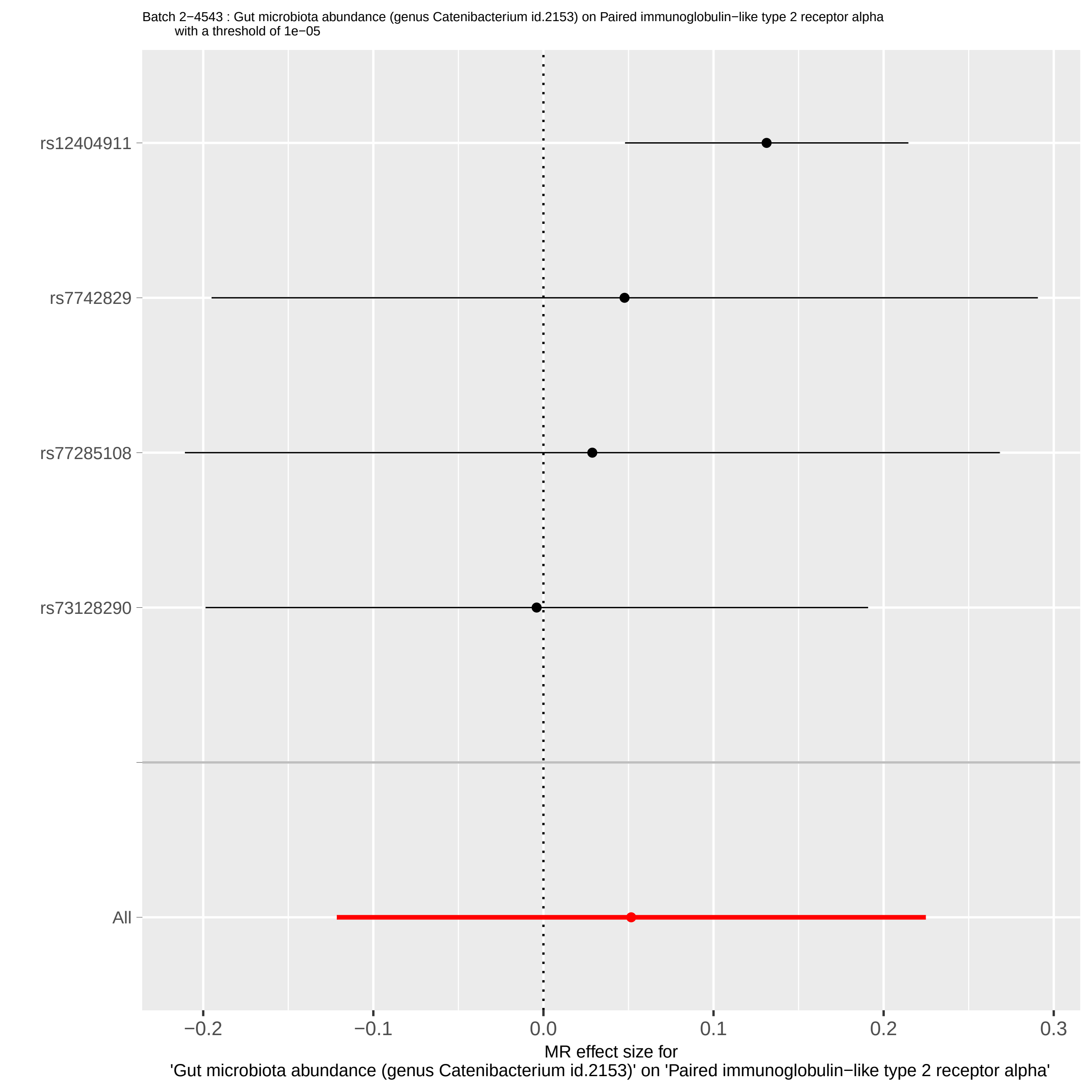

Batch 2−4543 : Gut microbiota abundance (genus Catenibacterium id.2153) on Paired immunoglobulin−like type 2 receptor alpha
with a threshold of 1e−05
rs12404911
rs7742829
rs77285108
rs73128290
All
−0.2
−0.1
0.0
0.1
0.2
0.3
MR effect size for
'Gut microbiota abundance (genus Catenibacterium id.2153)' on 'Paired immunoglobulin−like type 2 receptor alpha'

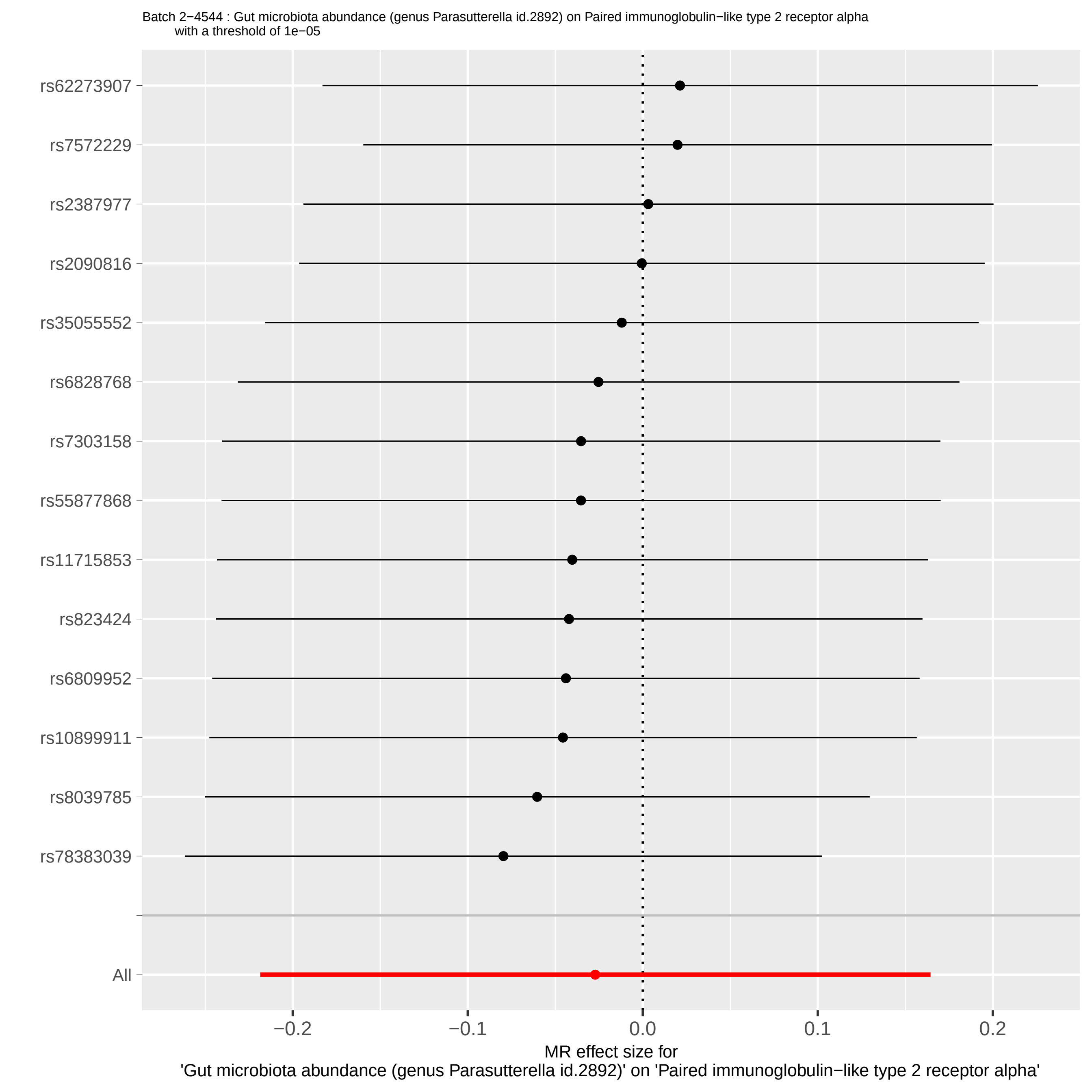
Batch 2−4544 : Gut microbiota abundance (genus Parasutterella id.2892) on Paired immunoglobulin−like type 2 receptor alpha with a threshold of 1e−05
rs62273907
rs7572229
rs2387977
rs2090816
rs35055552
rs6828768
rs7303158
rs55877868
rs11715853
rs823424
rs6809952
rs10899911
rs8039785
rs78383039
All
−0.2
−0.1
0.0
0.1
0.2
MR effect size for
'Gut microbiota abundance (genus Parasutterella id.2892)' on 'Paired immunoglobulin−like type 2 receptor alpha'

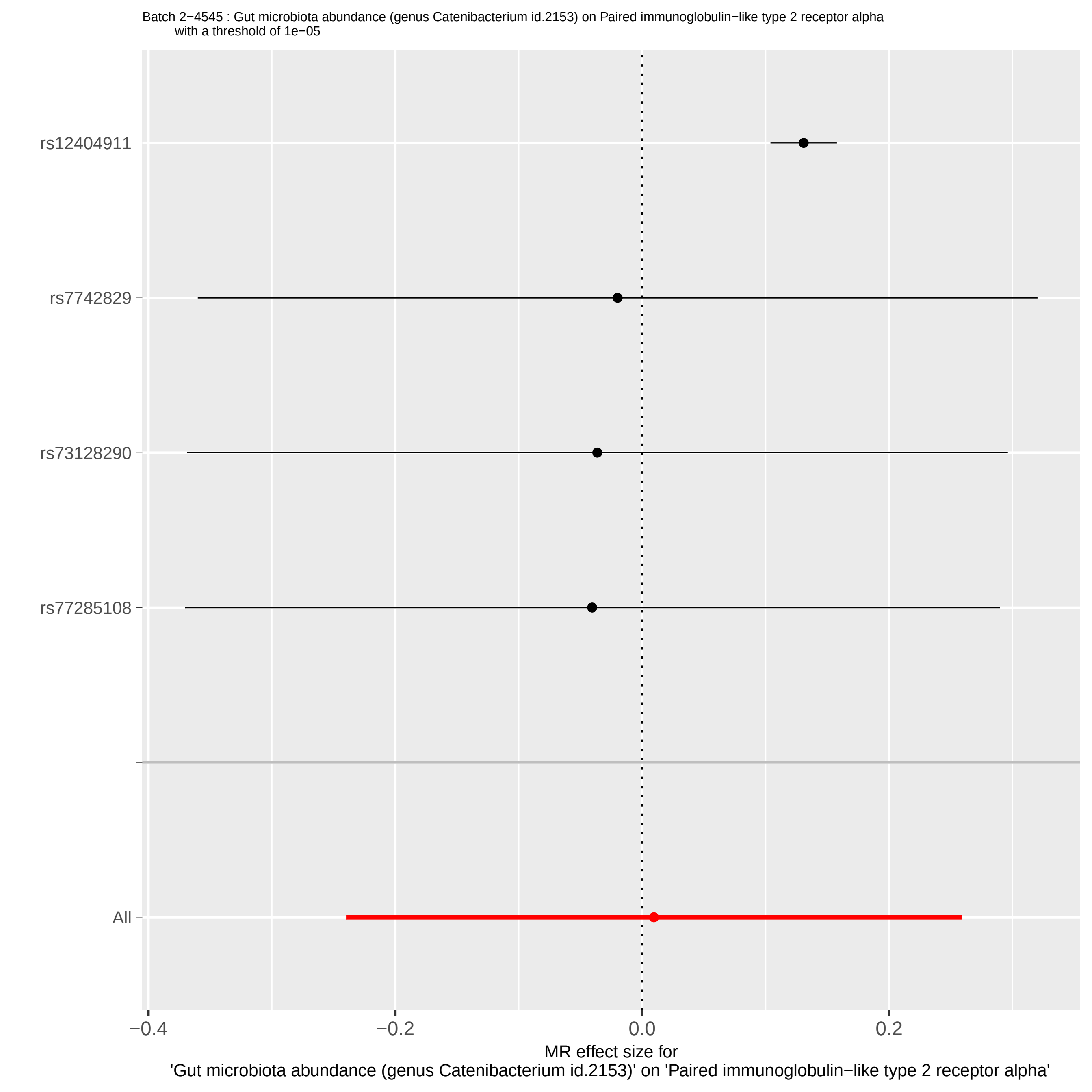

Batch 2−4545 : Gut microbiota abundance (genus Catenibacterium id.2153) on Paired immunoglobulin−like type 2 receptor alpha
with a threshold of 1e−05
rs12404911
rs7742829
rs73128290
rs77285108
All
−0.4
−0.2
0.0
0.2
MR effect size for
'Gut microbiota abundance (genus Catenibacterium id.2153)' on 'Paired immunoglobulin−like type 2 receptor alpha'

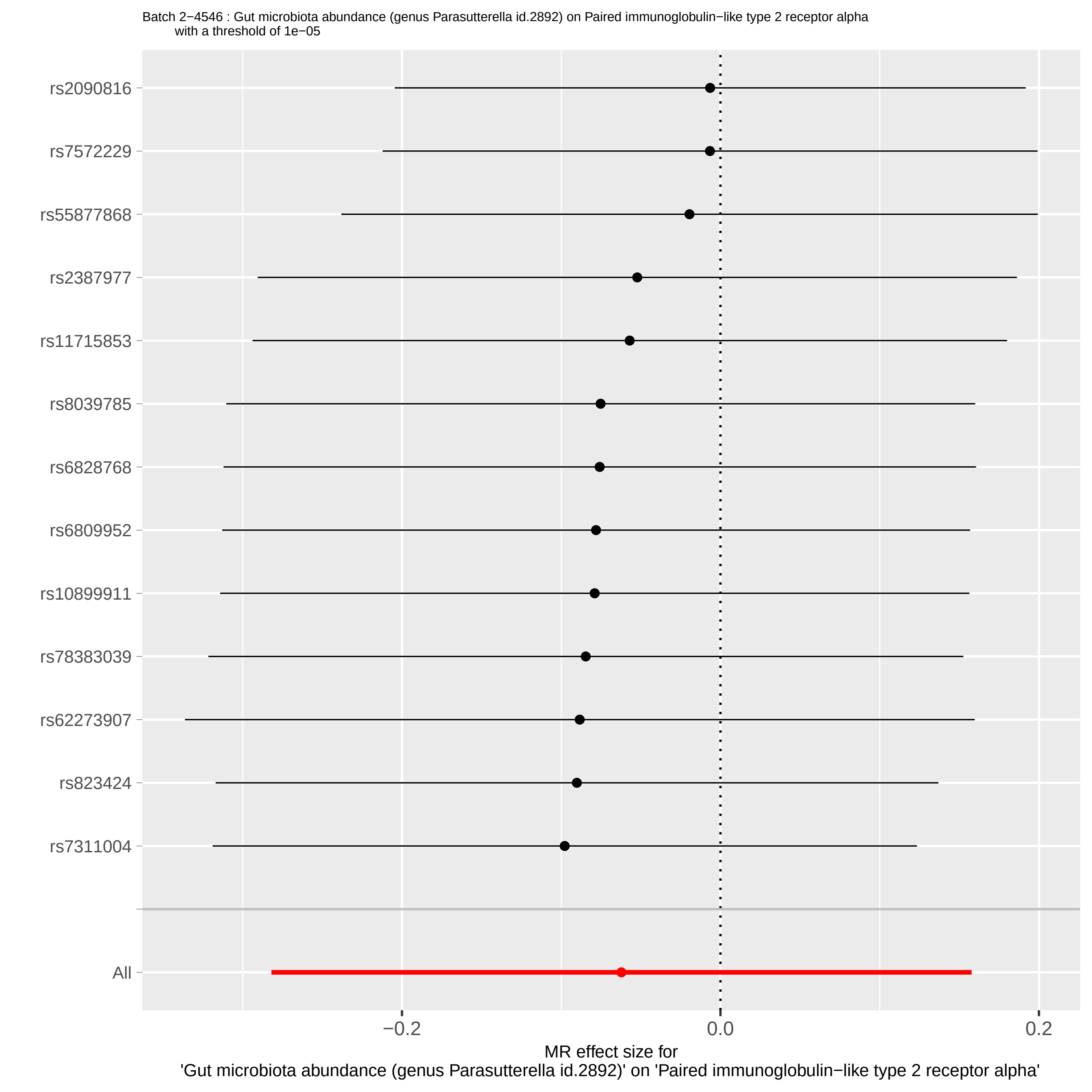

Batch 2−4546 : Gut microbiota abundance (genus Parasutterella id.2892) on Paired immunoglobulin−like type 2 receptor alpha
with a threshold of 1e−05
rs2090816
rs7572229
rs55877868
rs2387977
rs11715853
rs8039785
rs6828768
rs6809952
rs10899911
rs78383039
rs62273907
rs823424
rs7311004
All
−0.2
0.0
0.2
MR effect size for
'Gut microbiota abundance (genus Parasutterella id.2892)' on 'Paired immunoglobulin−like type 2 receptor alpha'

Batch 2−4547 : Gut microbiota abundance (genus Catenibacterium id.2153) on Serine/threonine−protein kinase pim−1
with a threshold of 1e−05

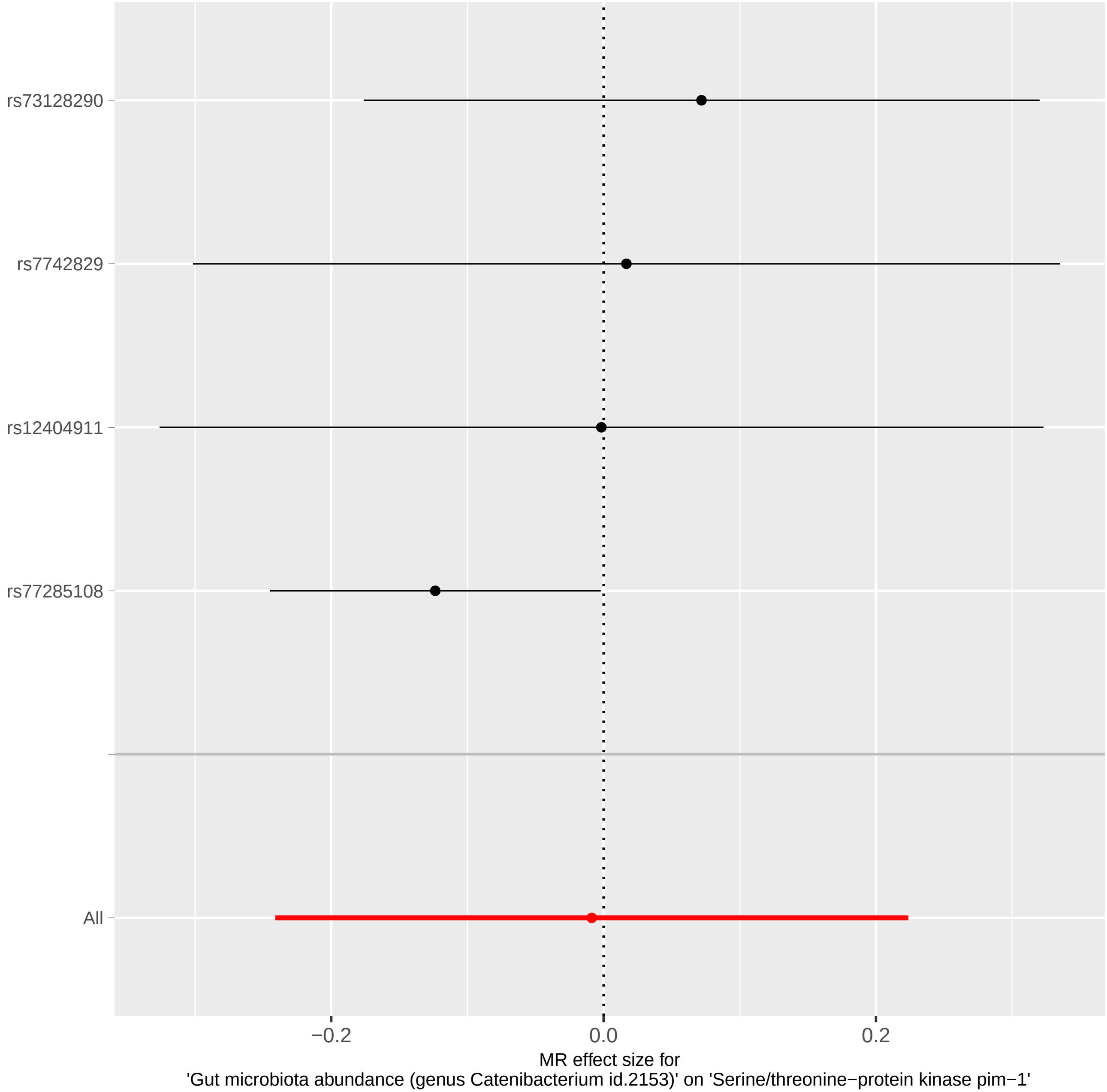

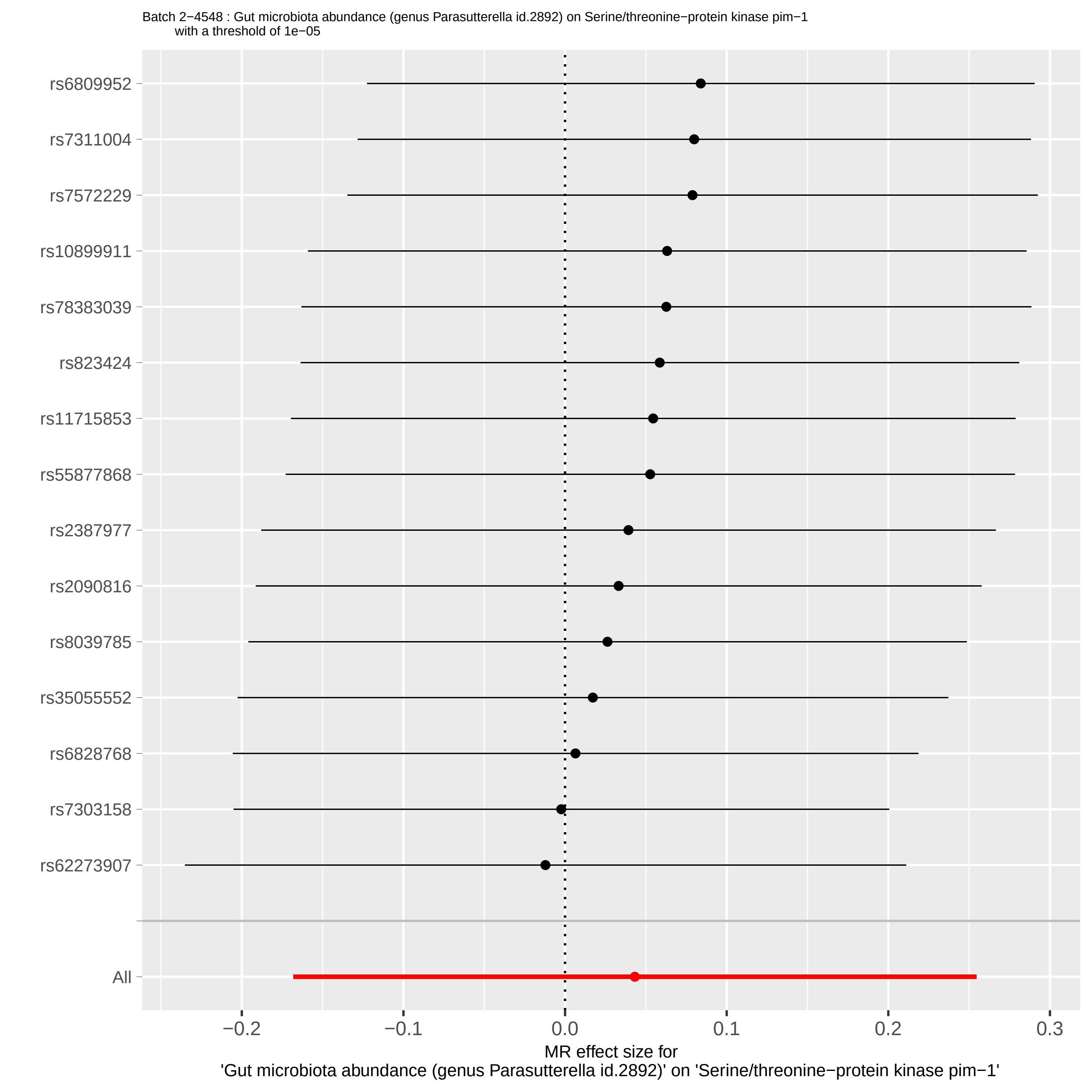

Batch 2−4548 : Gut microbiota abundance (genus Parasutterella id.2892) on Serine/threonine−protein kinase pim−1
with a threshold of 1e−05
rs6809952
rs7311004
rs7572229
rs10899911
rs78383039
rs823424
rs11715853
rs55877868
rs2387977
rs2090816
rs8039785
rs35055552
rs6828768
rs7303158
rs62273907
All
−0.2
−0.1
0.0
0.1
0.2
0.3
MR effect size for
'Gut microbiota abundance (genus Parasutterella id.2892)' on 'Serine/threonine−protein kinase pim−1'

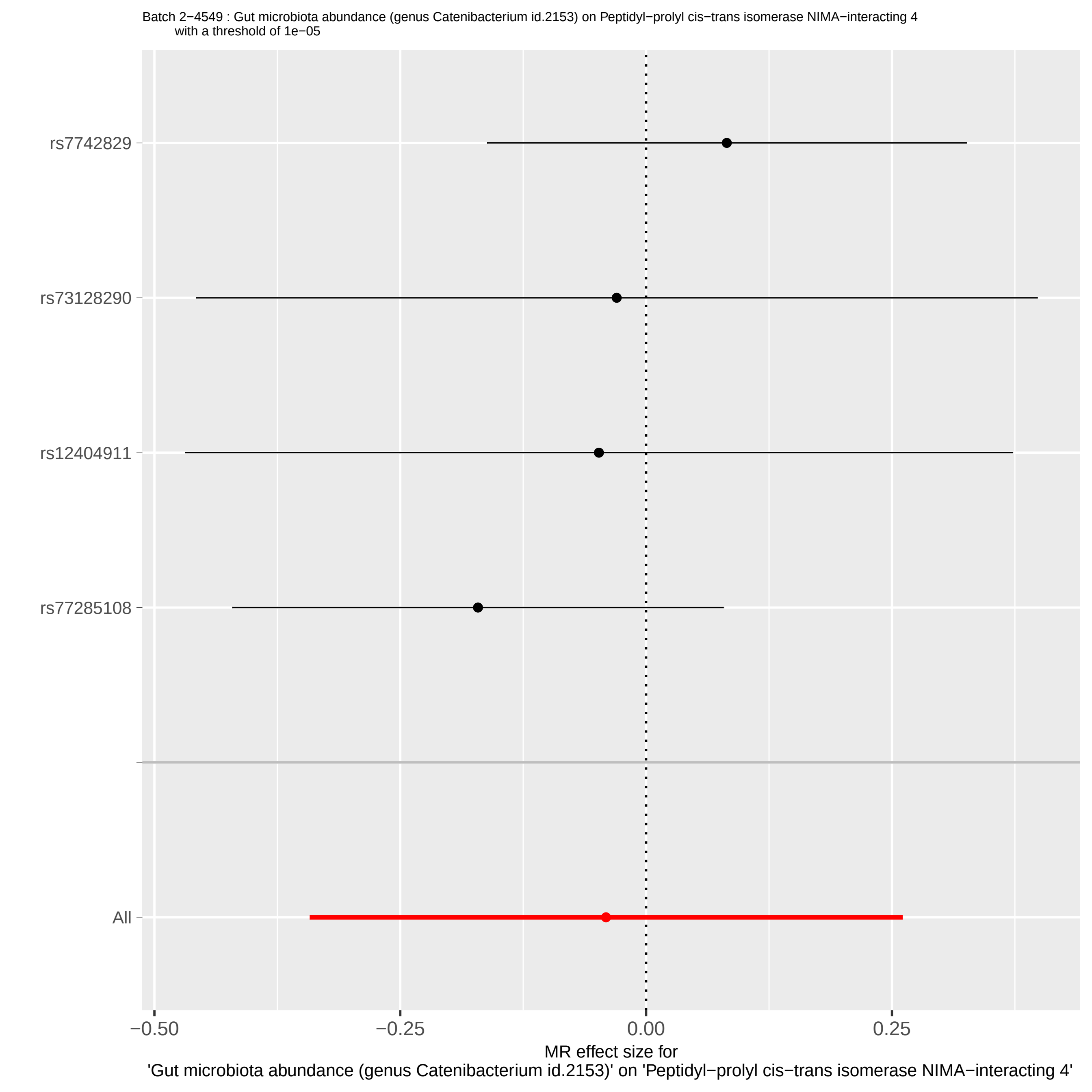
Batch 2−4549 : Gut microbiota abundance (genus Catenibacterium id.2153) on Peptidyl−prolyl cis−trans isomerase NIMA−interacting 4
with a threshold of 1e−05
rs7742829
rs73128290
rs12404911
rs77285108
All
−0.50
−0.25
0.00
0.25
MR effect size for
'Gut microbiota abundance (genus Catenibacterium id.2153)' on 'Peptidyl−prolyl cis−trans isomerase NIMA−interacting 4'

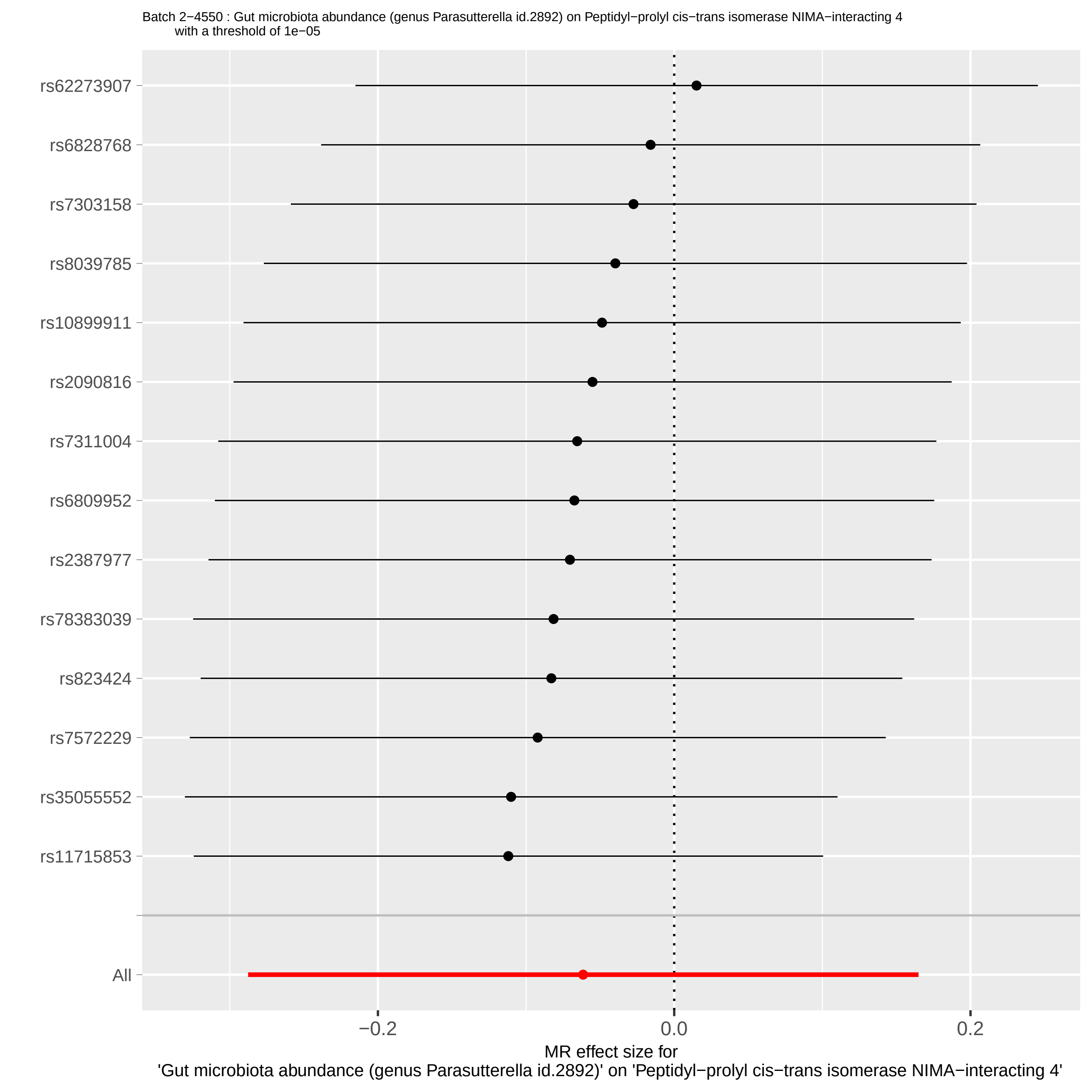

Batch 2−4550 : Gut microbiota abundance (genus Parasutterella id.2892) on Peptidyl−prolyl cis−trans isomerase NIMA−interacting 4
with a threshold of 1e−05
rs62273907
rs6828768
rs7303158
rs8039785
rs10899911
rs2090816
rs7311004
rs6809952
rs2387977
rs78383039
rs823424
rs7572229
rs35055552
rs11715853
All
−0.2
0.0
0.2
MR effect size for
'Gut microbiota abundance (genus Parasutterella id.2892)' on 'Peptidyl−prolyl cis−trans isomerase NIMA−interacting 4'

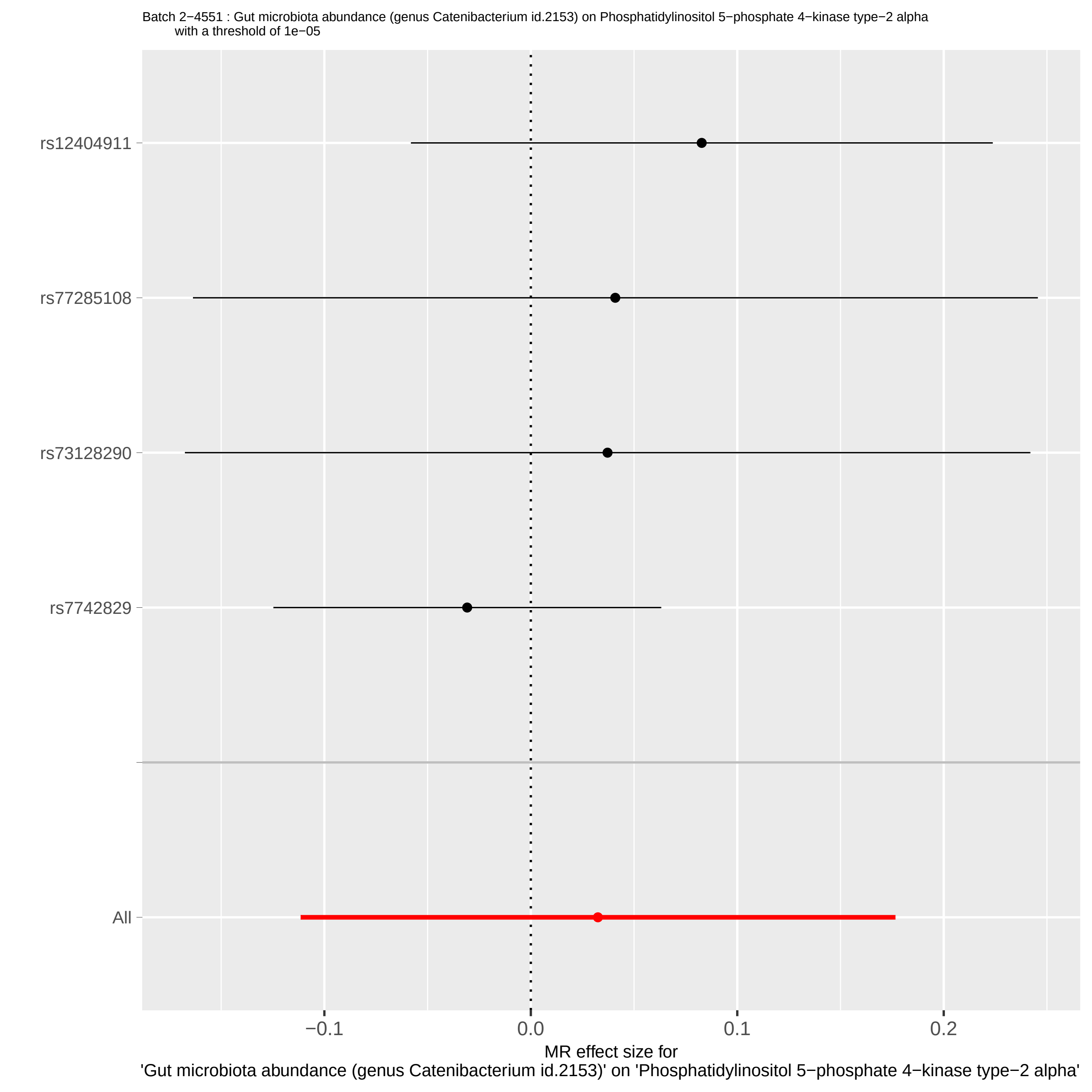
Batch 2−4551 : Gut microbiota abundance (genus Catenibacterium id.2153) on Phosphatidylinositol 5−phosphate 4−kinase type−2 alpha
with a threshold of 1e−05
rs12404911
rs77285108
rs73128290
rs7742829
All
−0.1
0.0
0.1
0.2
MR effect size for
'Gut microbiota abundance (genus Catenibacterium id.2153)' on 'Phosphatidylinositol 5−phosphate 4−kinase type−2 alpha'

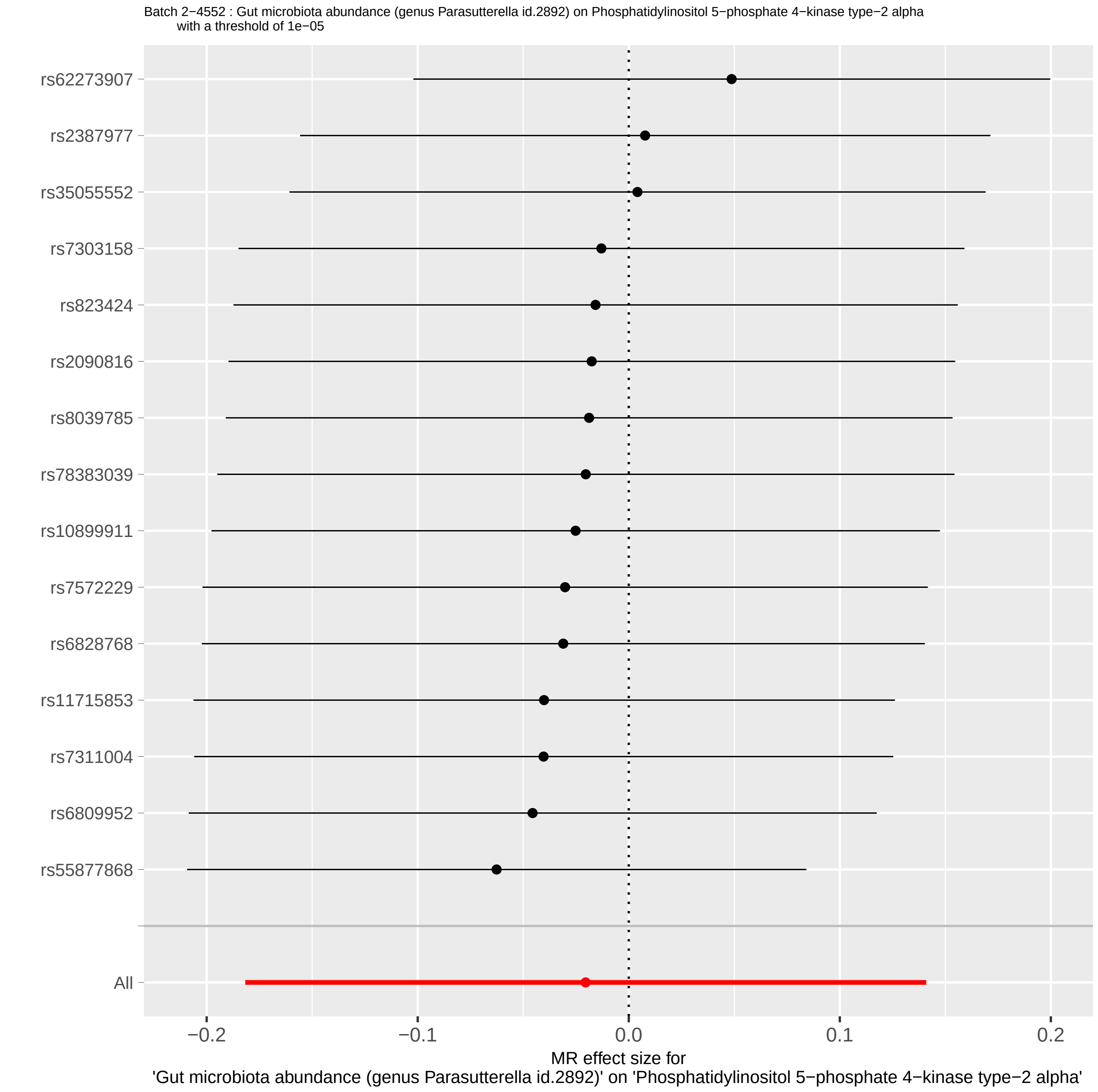
Batch 2−4552 : Gut microbiota abundance (genus Parasutterella id.2892) on Phosphatidylinositol 5−phosphate 4−kinase type−2 alpha with a threshold of 1e−05
rs62273907
rs2387977
rs35055552
rs7303158
rs823424
rs2090816
rs8039785
rs78383039
rs10899911
rs7572229
rs6828768
rs11715853
rs7311004
rs6809952
rs55877868
All
−0.2
−0.1
0.0
0.1
0.2
MR effect size for
'Gut microbiota abundance (genus Parasutterella id.2892)' on 'Phosphatidylinositol 5−phosphate 4−kinase type−2 alpha'

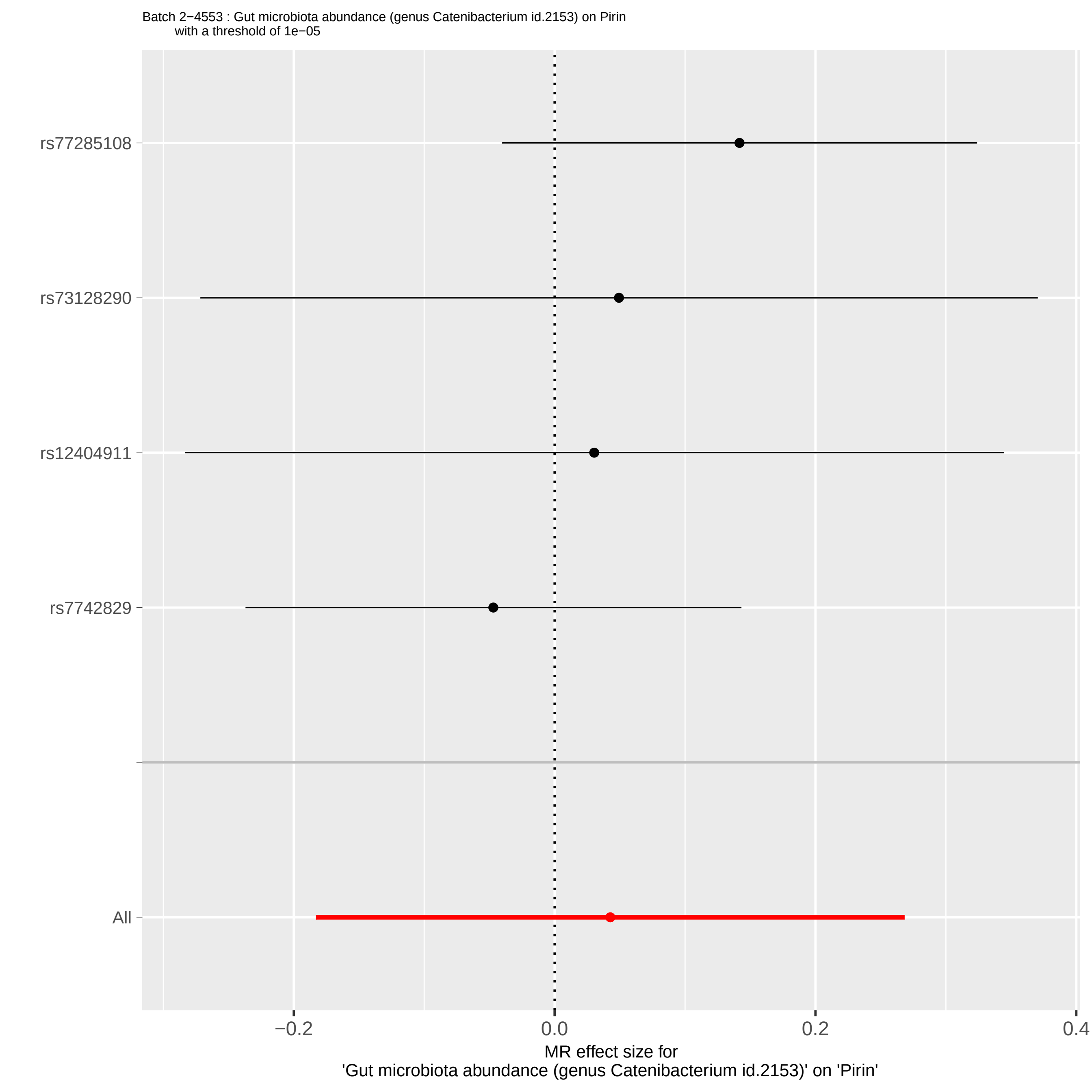
Batch 2−4553 : Gut microbiota abundance (genus Catenibacterium id.2153) on Pirin
with a threshold of 1e−05
rs77285108
rs73128290
rs12404911
rs7742829
All
−0.2
0.0
0.2
0.4
MR effect size for
'Gut microbiota abundance (genus Catenibacterium id.2153)' on 'Pirin'

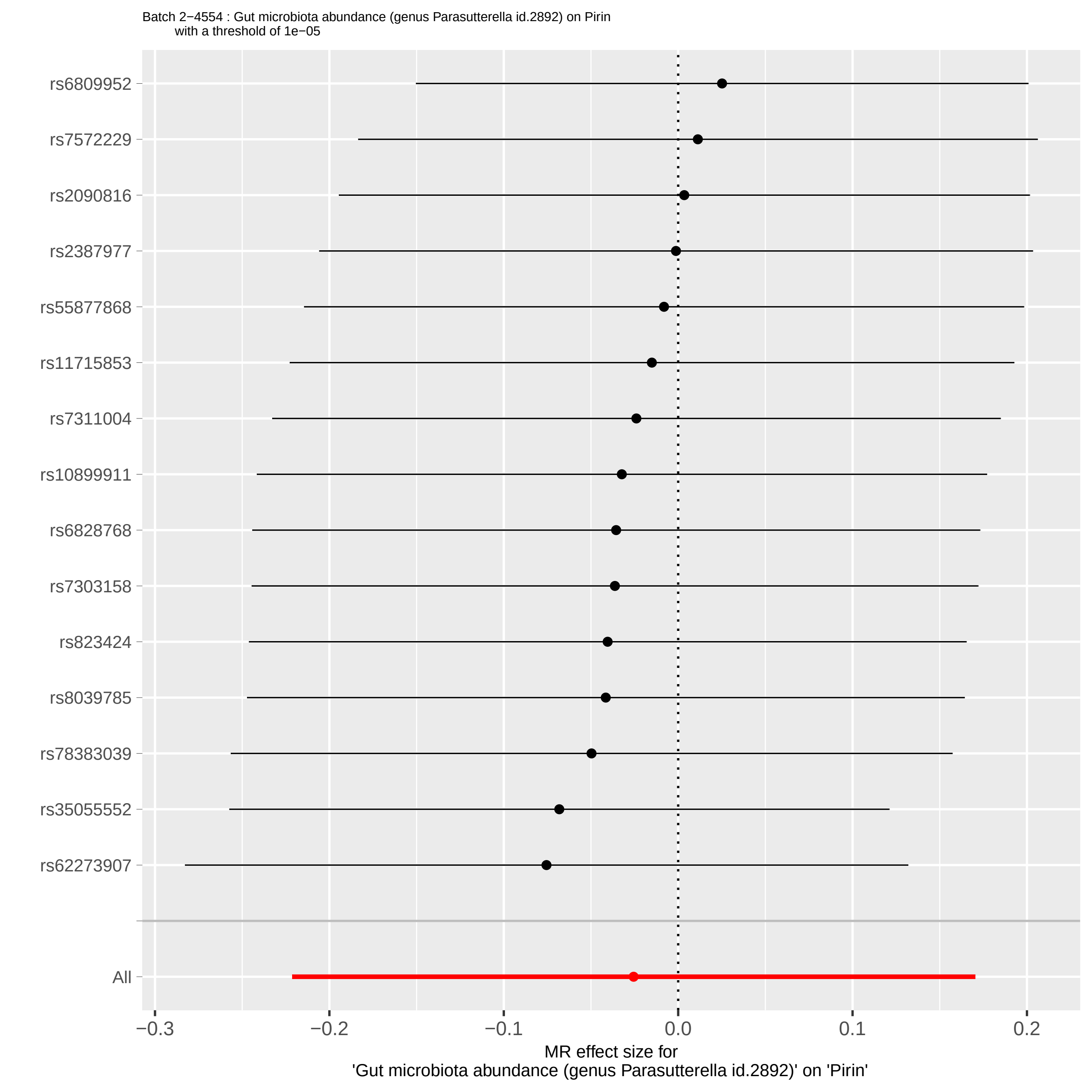
Batch 2−4554 : Gut microbiota abundance (genus Parasutterella id.2892) on Pirin
with a threshold of 1e−05
rs6809952
rs7572229
rs2090816
rs2387977
rs55877868
rs11715853
rs7311004
rs10899911
rs6828768
rs7303158
rs823424
rs8039785
rs78383039
rs35055552
rs62273907
All
−0.3
−0.2
−0.1
0.0
0.1
0.2
MR effect size for
'Gut microbiota abundance (genus Parasutterella id.2892)' on 'Pirin'

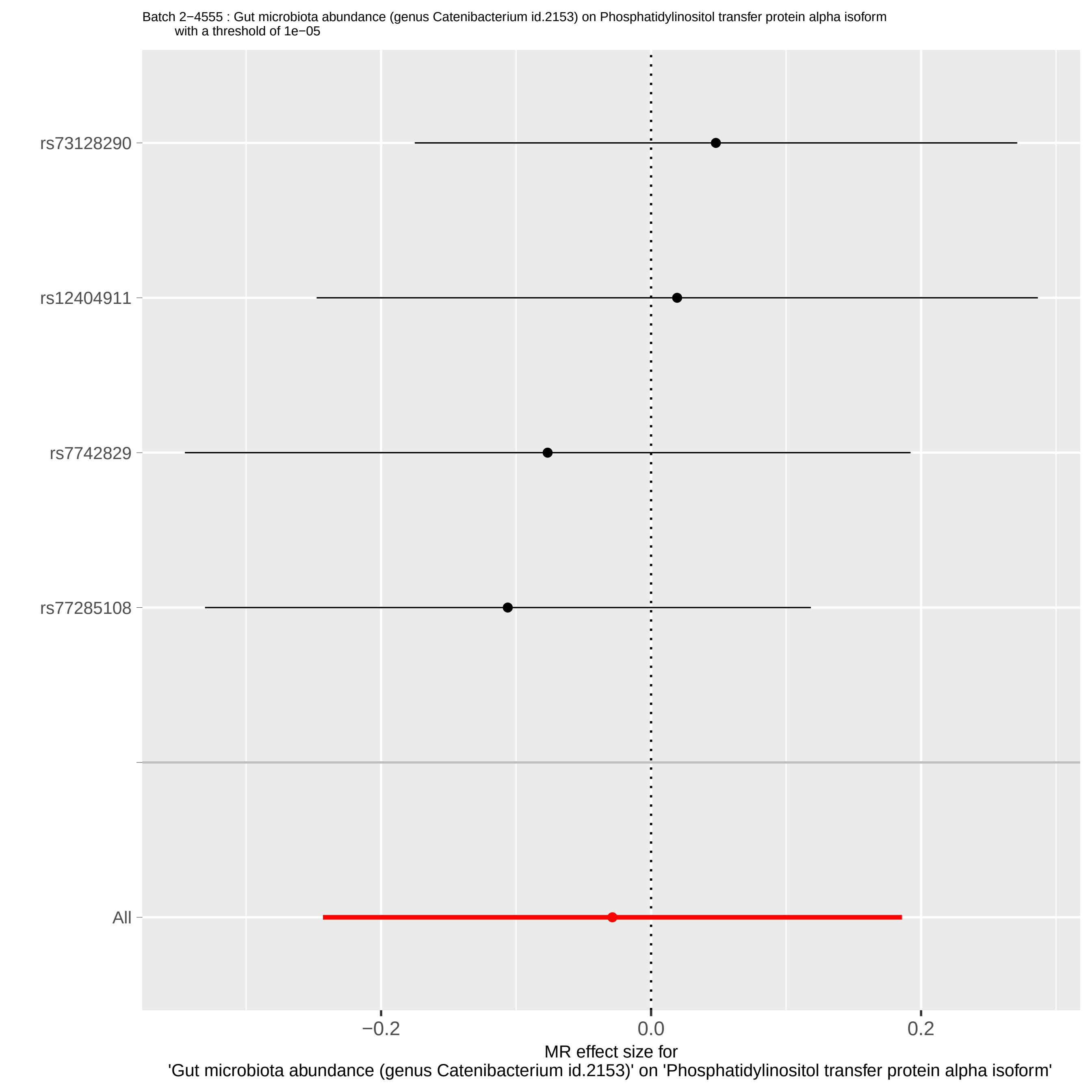
Batch 2−4555 : Gut microbiota abundance (genus Catenibacterium id.2153) on Phosphatidylinositol transfer protein alpha isoform
with a threshold of 1e−05
rs73128290
rs12404911
rs7742829
rs77285108
All
−0.2
0.0
0.2
MR effect size for
'Gut microbiota abundance (genus Catenibacterium id.2153)' on 'Phosphatidylinositol transfer protein alpha isoform'

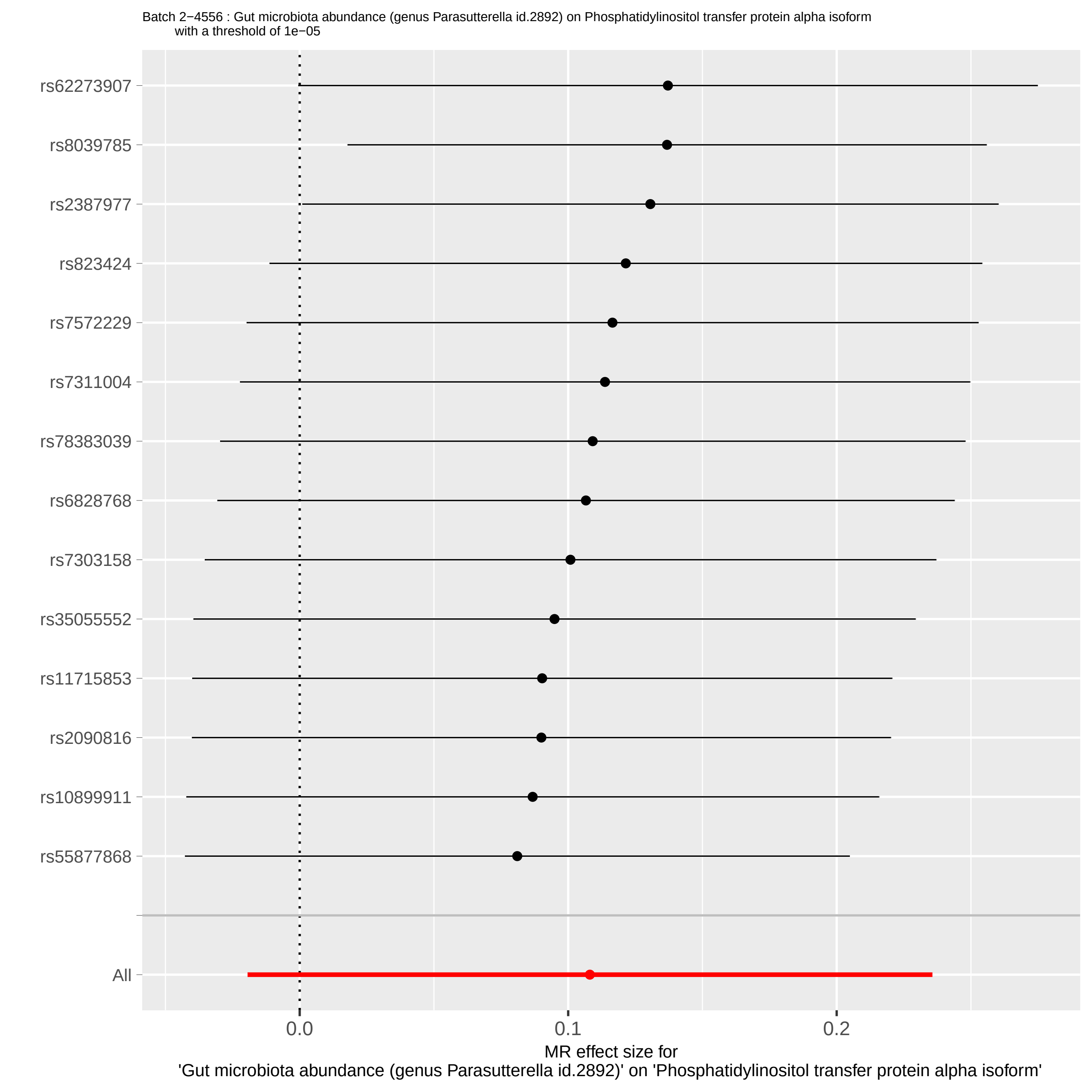

Batch 2−4556 : Gut microbiota abundance (genus Parasutterella id.2892) on Phosphatidylinositol transfer protein alpha isoform
with a threshold of 1e−05
rs62273907
rs8039785
rs2387977
rs823424
rs7572229
rs7311004
rs78383039
rs6828768
rs7303158
rs35055552
rs11715853
rs2090816
rs10899911
rs55877868
All
0.0
0.1
0.2
MR effect size for
'Gut microbiota abundance (genus Parasutterella id.2892)' on 'Phosphatidylinositol transfer protein alpha isoform'

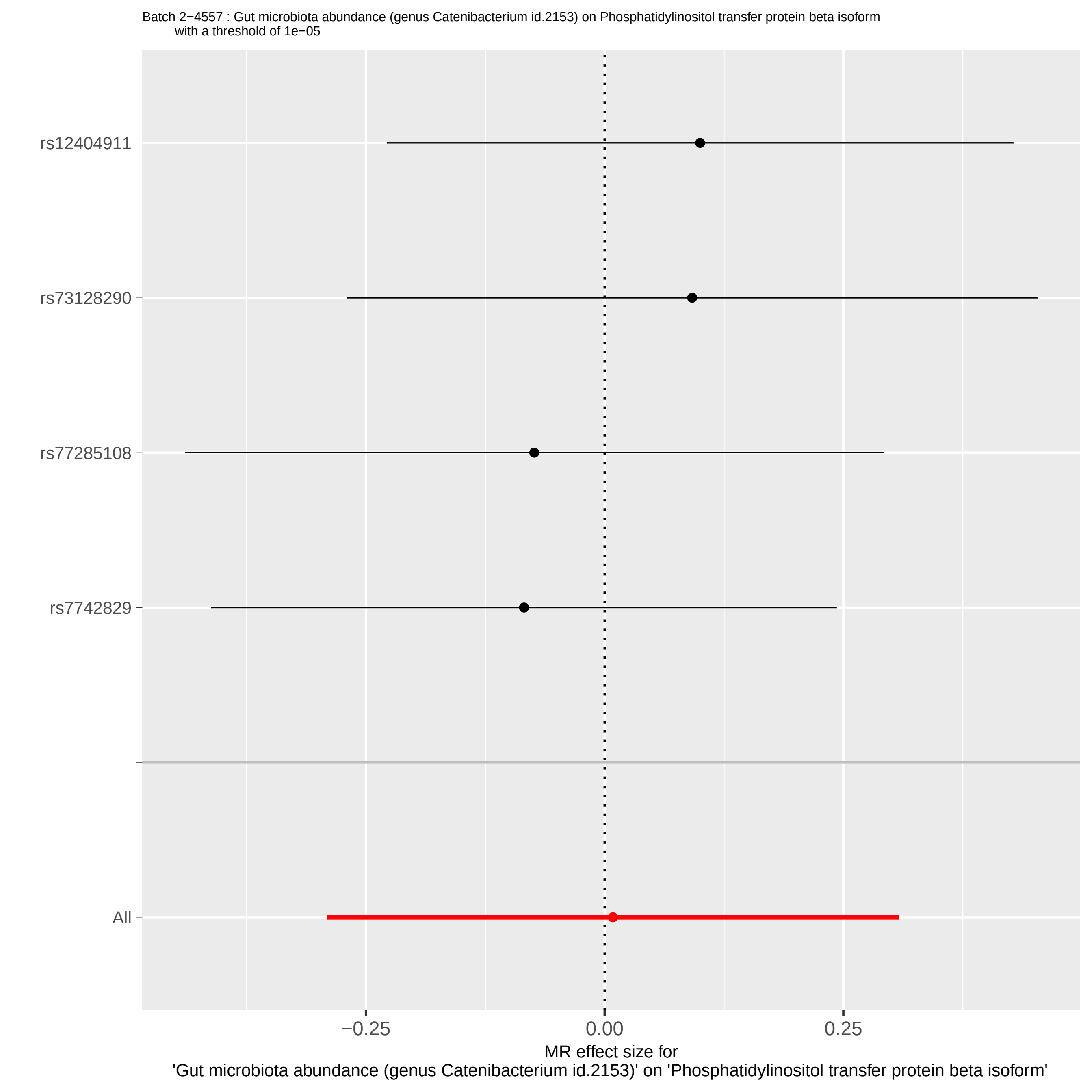
Batch 2−4557 : Gut microbiota abundance (genus Catenibacterium id.2153) on Phosphatidylinositol transfer protein beta isoform
with a threshold of 1e−05
rs12404911
rs73128290
rs77285108
rs7742829
All
−0.25
0.00
0.25
MR effect size for
'Gut microbiota abundance (genus Catenibacterium id.2153)' on 'Phosphatidylinositol transfer protein beta isoform'

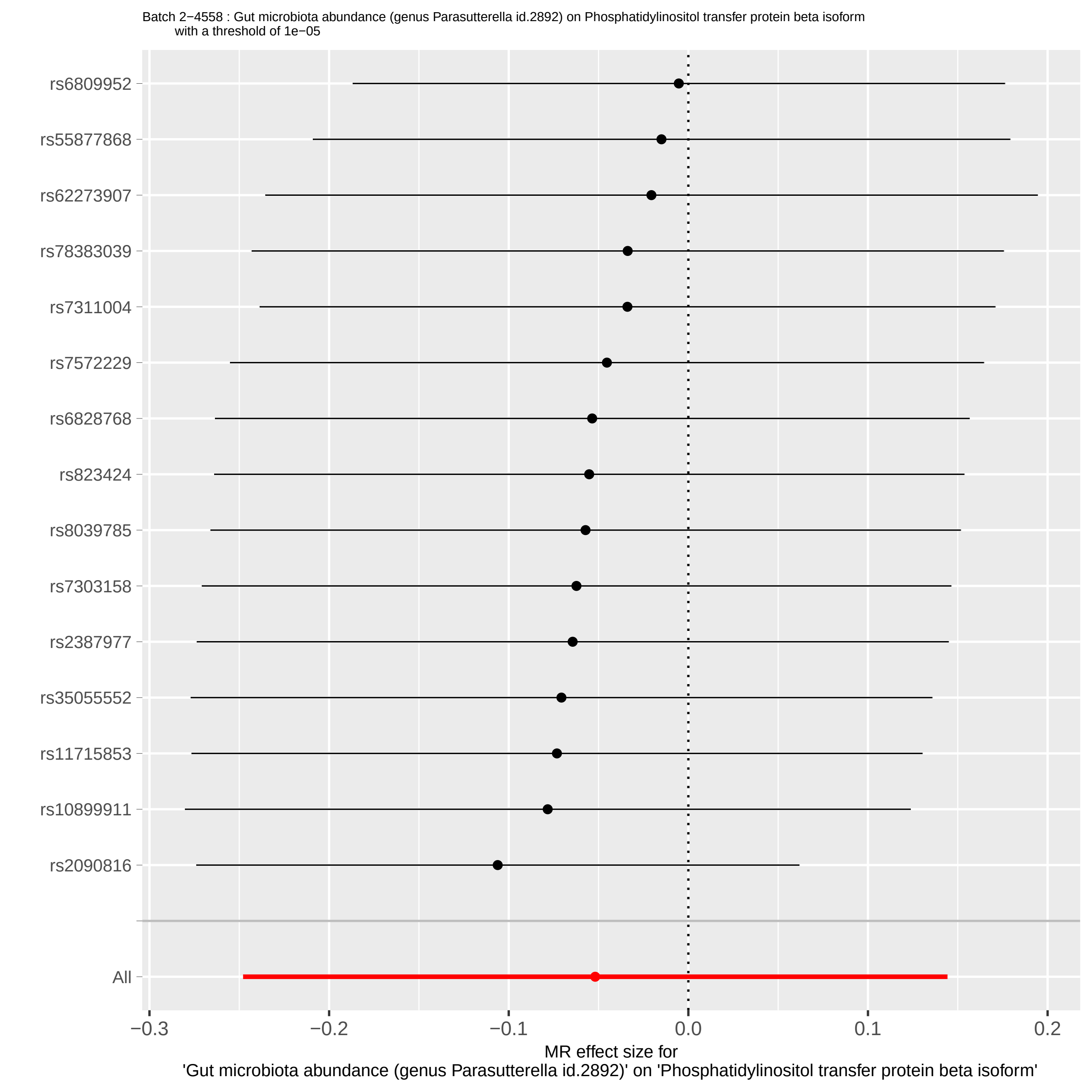
Batch 2−4558 : Gut microbiota abundance (genus Parasutterella id.2892) on Phosphatidylinositol transfer protein beta isoform
with a threshold of 1e−05
rs6809952
rs55877868
rs62273907
rs78383039
rs7311004
rs7572229
rs6828768
rs823424
rs8039785
rs7303158
rs2387977
rs35055552
rs11715853
rs10899911
rs2090816
All
−0.3
−0.2
−0.1
0.0
0.1
0.2
MR effect size for
'Gut microbiota abundance (genus Parasutterella id.2892)' on 'Phosphatidylinositol transfer protein beta isoform'

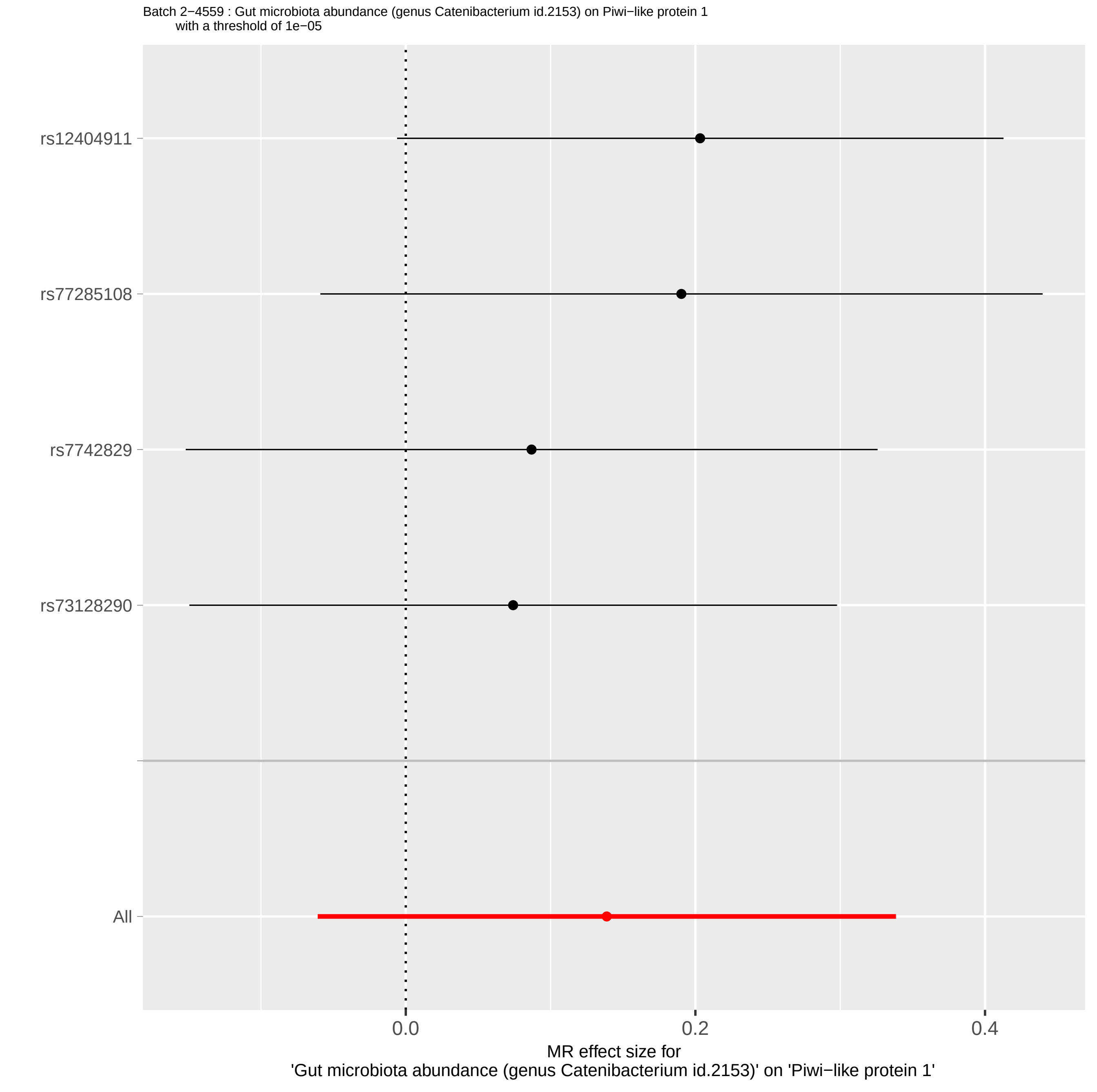
Batch 2−4559 : Gut microbiota abundance (genus Catenibacterium id.2153) on Piwi−like protein 1
with a threshold of 1e−05
rs12404911
rs77285108
rs7742829
rs73128290
All
0.0
0.2
0.4
MR effect size for
'Gut microbiota abundance (genus Catenibacterium id.2153)' on 'Piwi−like protein 1'

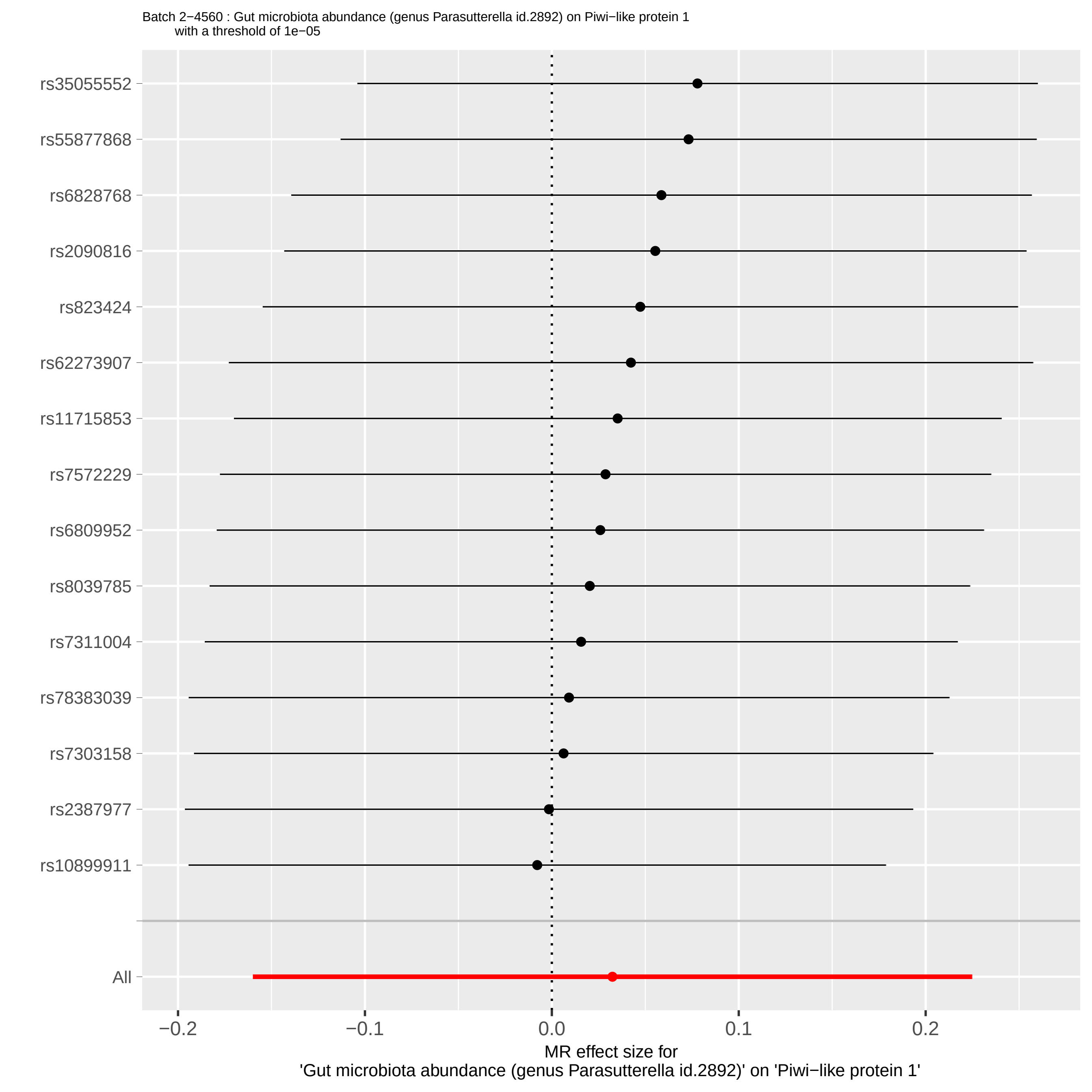
Batch 2−4560 : Gut microbiota abundance (genus Parasutterella id.2892) on Piwi−like protein 1
with a threshold of 1e−05
rs35055552
rs55877868
rs6828768
rs2090816
rs823424
rs62273907
rs11715853
rs7572229
rs6809952
rs8039785
rs7311004
rs78383039
rs7303158
rs2387977
rs10899911
All
−0.2
−0.1
0.0
0.1
0.2
MR effect size for
'Gut microbiota abundance (genus Parasutterella id.2892)' on 'Piwi−like protein 1'

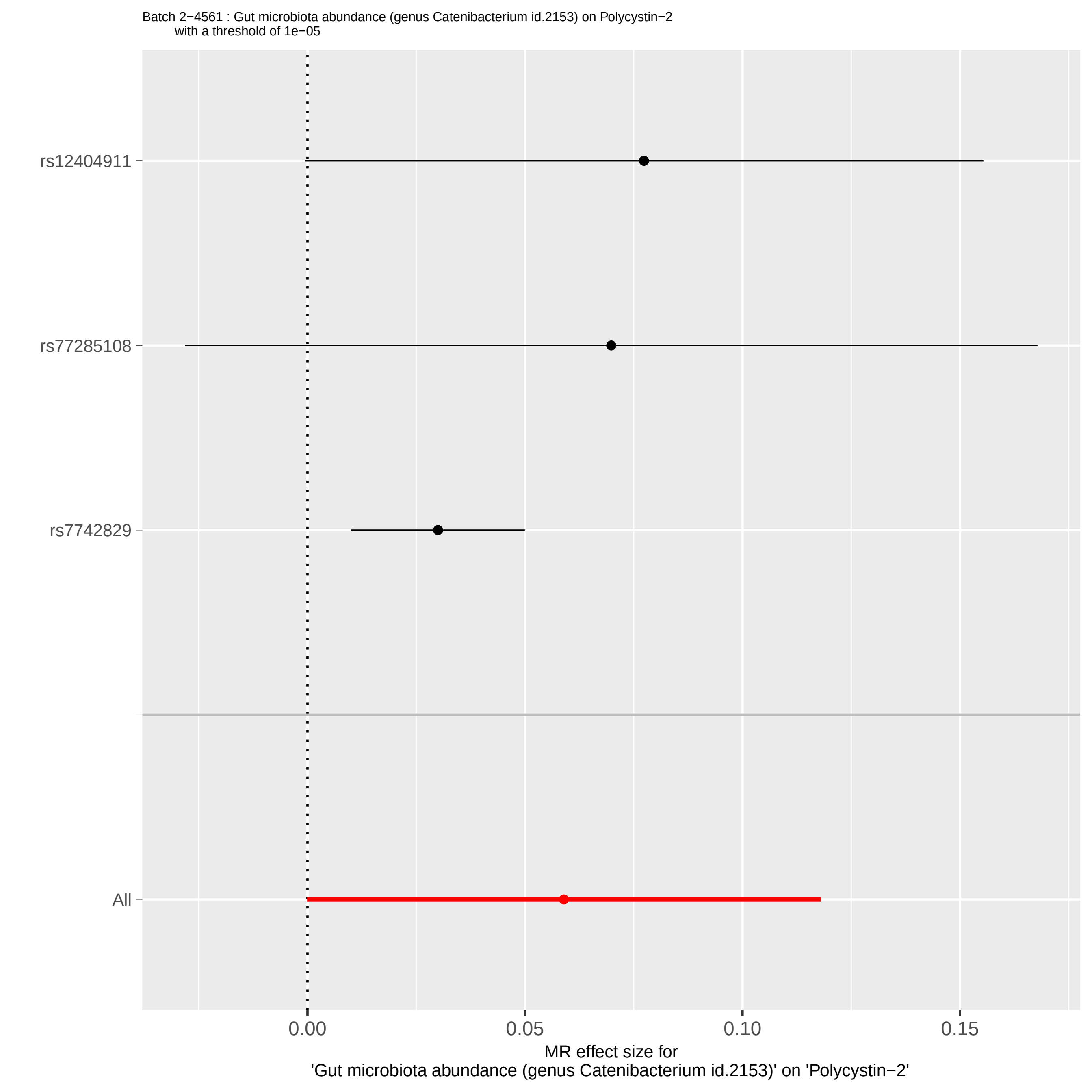
Batch 2−4561 : Gut microbiota abundance (genus Catenibacterium id.2153) on Polycystin−2
with a threshold of 1e−05
rs12404911
rs77285108
rs7742829
All
0.00
0.05
0.10
0.15
MR effect size for
'Gut microbiota abundance (genus Catenibacterium id.2153)' on 'Polycystin−2'

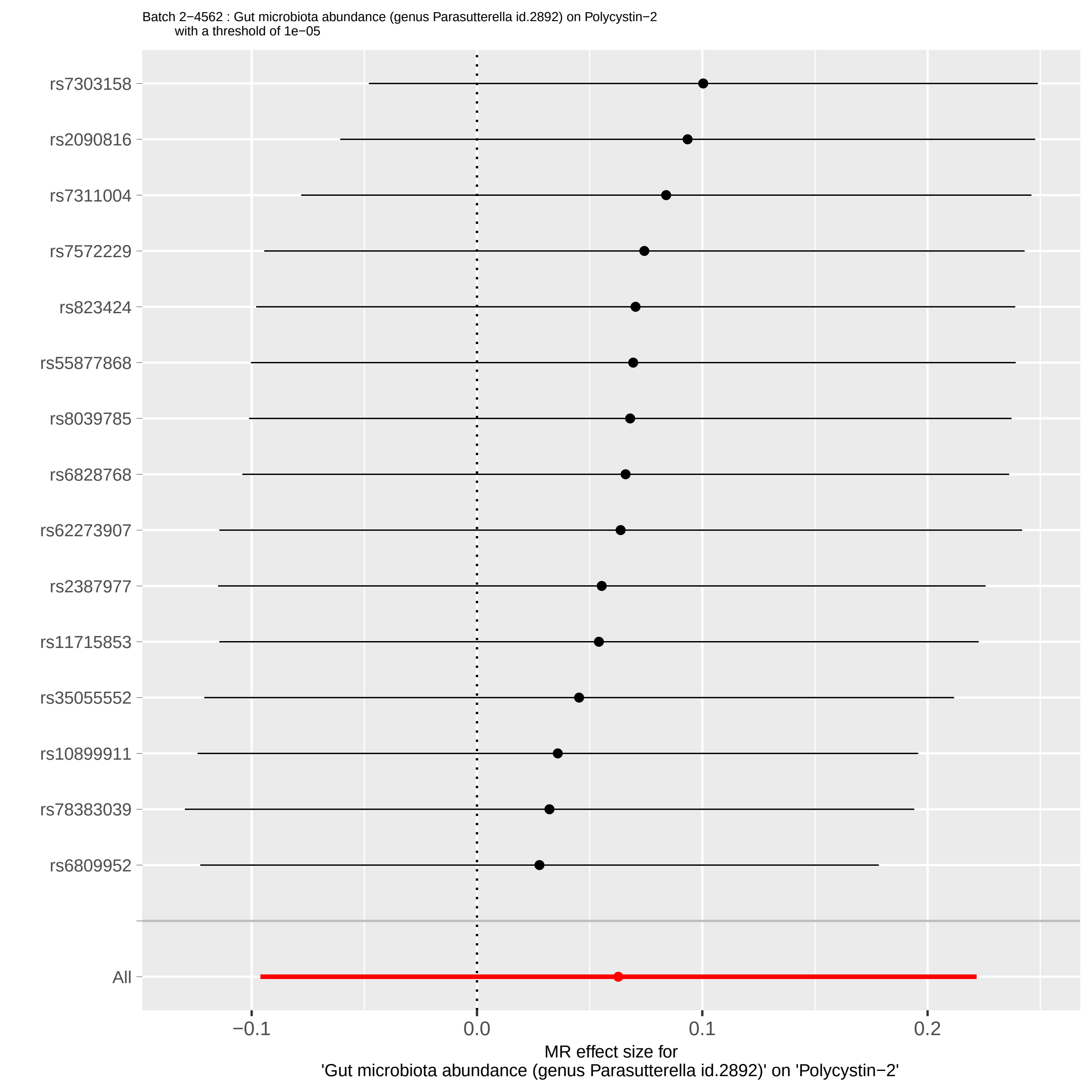
Batch 2−4562 : Gut microbiota abundance (genus Parasutterella id.2892) on Polycystin−2
with a threshold of 1e−05
rs7303158
rs2090816
rs7311004
rs7572229
rs823424
rs55877868
rs8039785
rs6828768
rs62273907
rs2387977
rs11715853
rs35055552
rs10899911
rs78383039
rs6809952
All
−0.1
0.0
0.1
0.2
MR effect size for
'Gut microbiota abundance (genus Parasutterella id.2892)' on 'Polycystin−2'

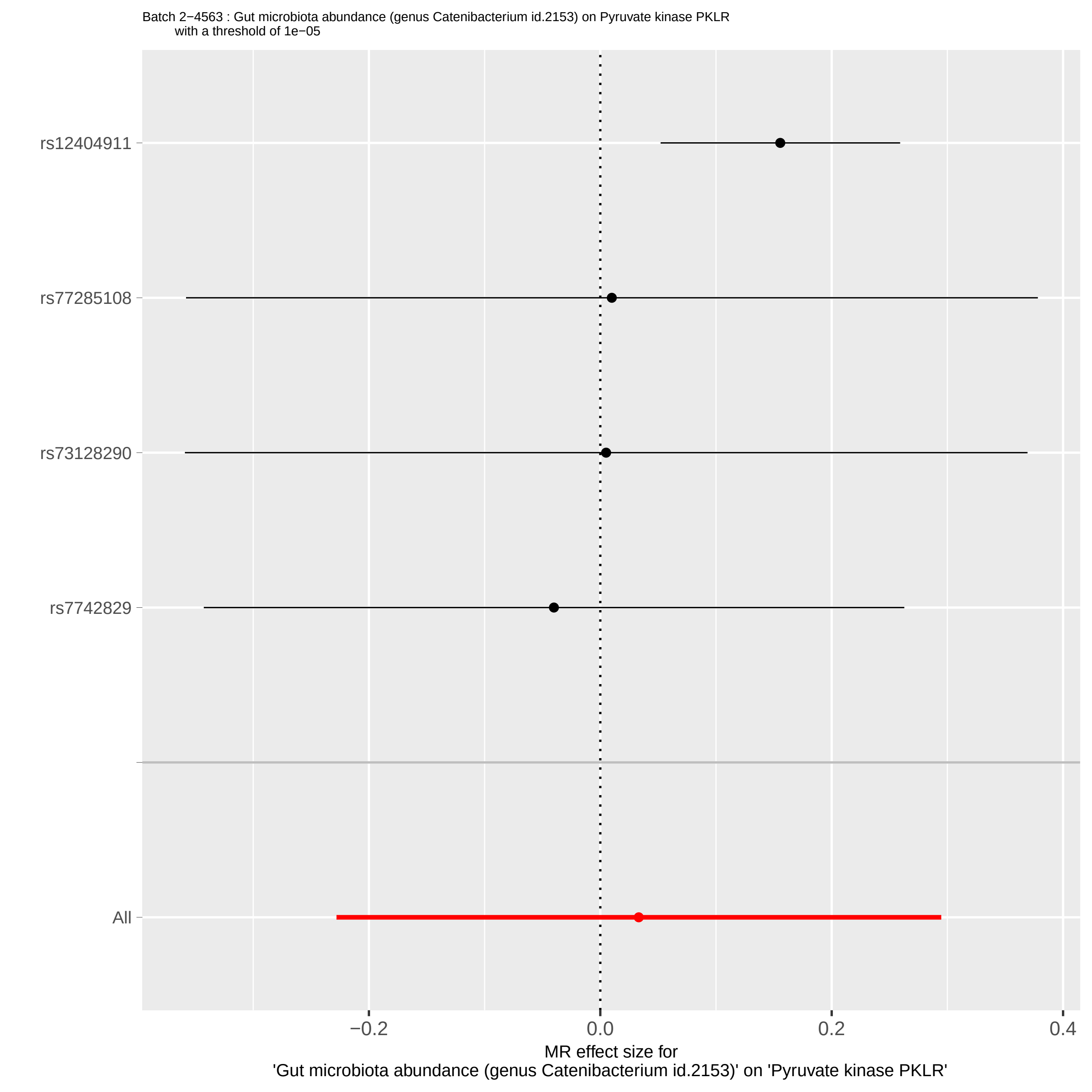
Batch 2−4563 : Gut microbiota abundance (genus Catenibacterium id.2153) on Pyruvate kinase PKLR
with a threshold of 1e−05
rs12404911
rs77285108
rs73128290
rs7742829
All
−0.2
0.0
0.2
0.4
MR effect size for
'Gut microbiota abundance (genus Catenibacterium id.2153)' on 'Pyruvate kinase PKLR'

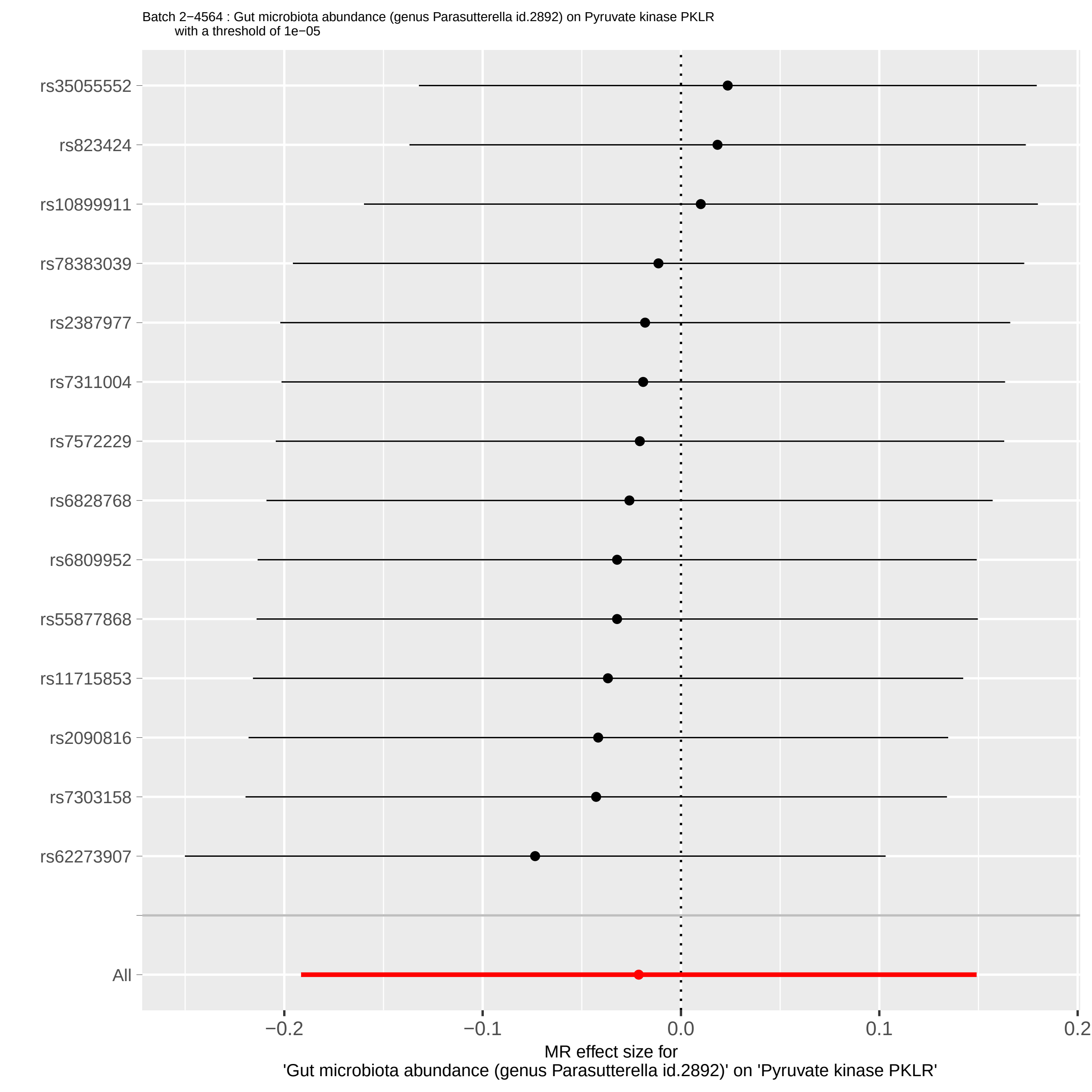
Batch 2−4564 : Gut microbiota abundance (genus Parasutterella id.2892) on Pyruvate kinase PKLR
with a threshold of 1e−05
rs35055552
rs823424
rs10899911
rs78383039
rs2387977
rs7311004
rs7572229
rs6828768
rs6809952
rs55877868
rs11715853
rs2090816
rs7303158
rs62273907
All
−0.2
−0.1
0.0
0.1
0.2
MR effect size for
'Gut microbiota abundance (genus Parasutterella id.2892)' on 'Pyruvate kinase PKLR'

Batch 2−4565 : Gut microbiota abundance (genus Catenibacterium id.2153) on Group 10 secretory phospholipase A2
with a threshold of 1e−05

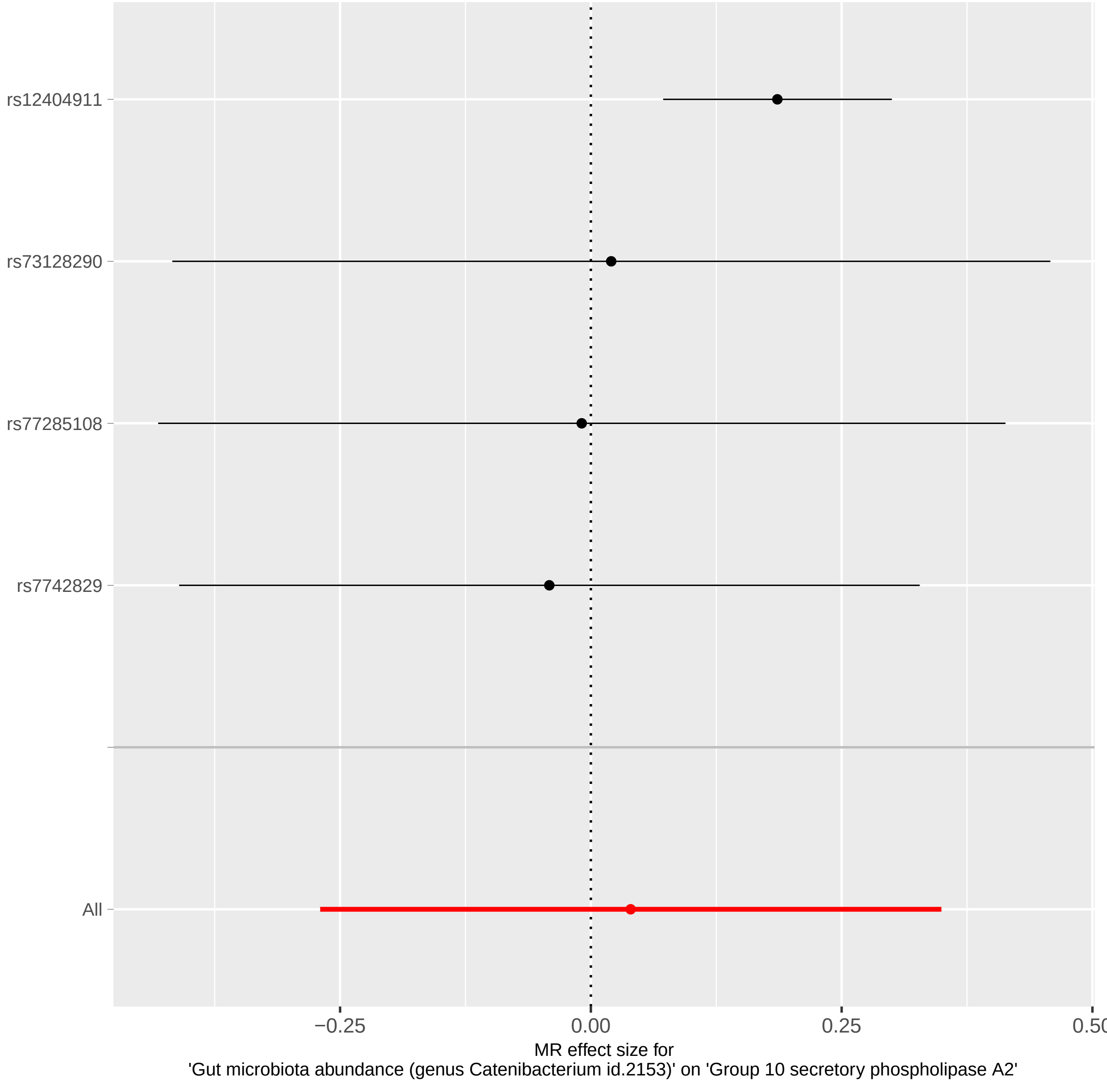

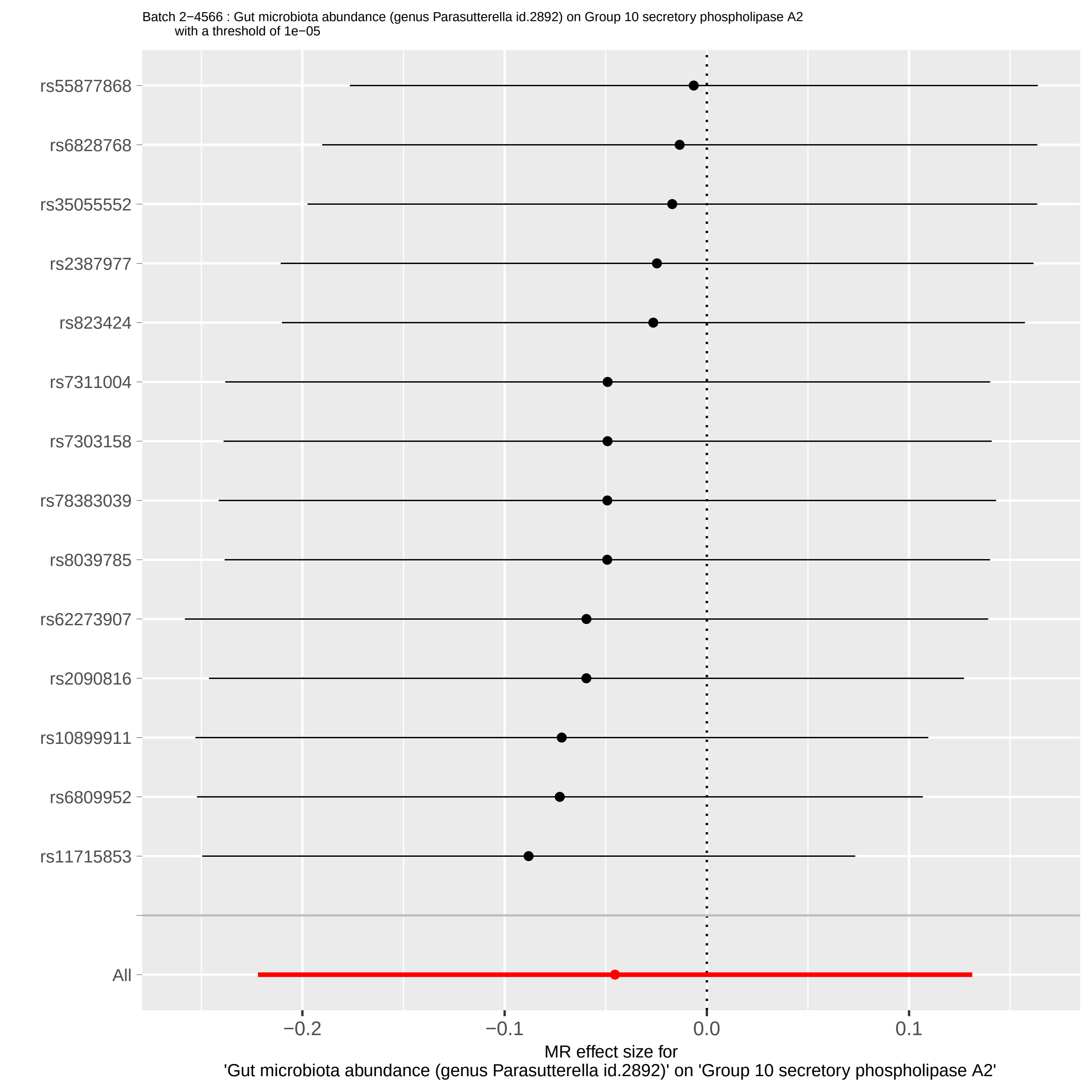

Batch 2−4566 : Gut microbiota abundance (genus Parasutterella id.2892) on Group 10 secretory phospholipase A2
with a threshold of 1e−05
rs55877868
rs6828768
rs35055552
rs2387977
rs823424
rs7311004
rs7303158
rs78383039
rs8039785
rs62273907
rs2090816
rs10899911
rs6809952
rs11715853
All
−0.2
−0.1
0.0
0.1
MR effect size for
'Gut microbiota abundance (genus Parasutterella id.2892)' on 'Group 10 secretory phospholipase A2'

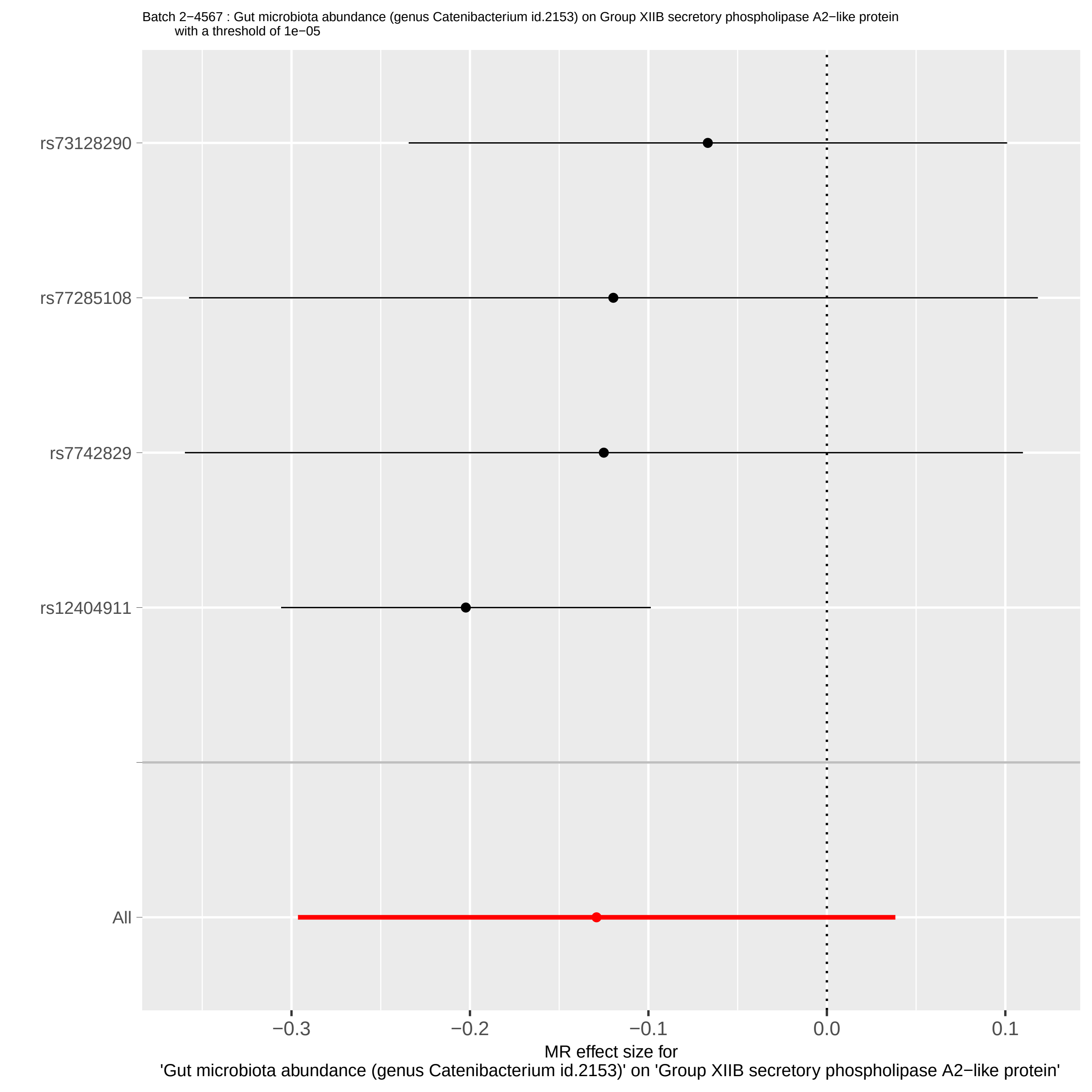
Batch 2−4567 : Gut microbiota abundance (genus Catenibacterium id.2153) on Group XIIB secretory phospholipase A2−like protein
with a threshold of 1e−05
rs73128290
rs77285108
rs7742829
rs12404911
All
−0.3
−0.2
−0.1
0.0
0.1
MR effect size for
'Gut microbiota abundance (genus Catenibacterium id.2153)' on 'Group XIIB secretory phospholipase A2−like protein'

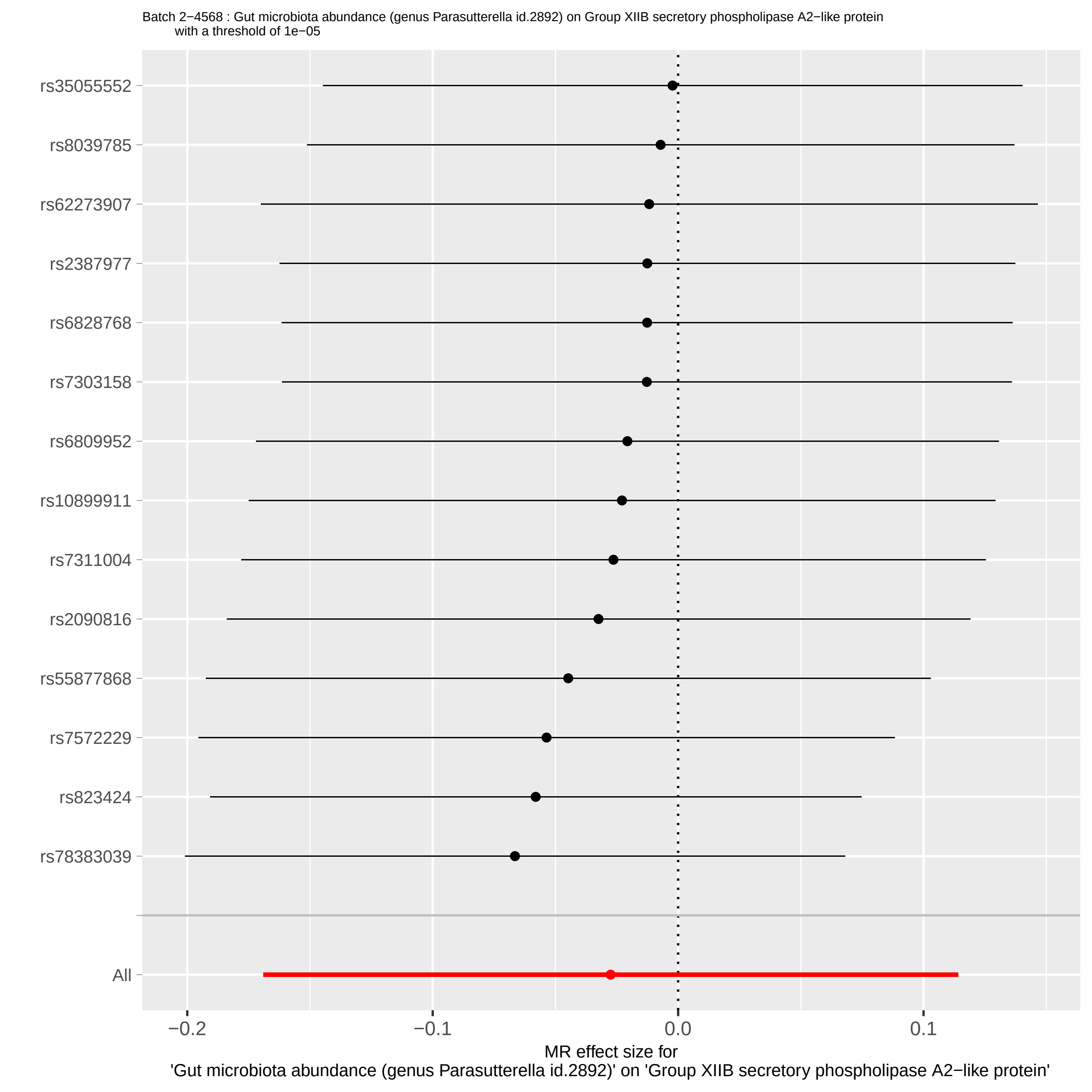
Batch 2−4568 : Gut microbiota abundance (genus Parasutterella id.2892) on Group XIIB secretory phospholipase A2−like protein
with a threshold of 1e−05
rs35055552
rs8039785
rs62273907
rs2387977
rs6828768
rs7303158
rs6809952
rs10899911
rs7311004
rs2090816
rs55877868
rs7572229
rs823424
rs78383039
All
−0.2
−0.1
0.0
0.1
MR effect size for
'Gut microbiota abundance (genus Parasutterella id.2892)' on 'Group XIIB secretory phospholipase A2−like protein'

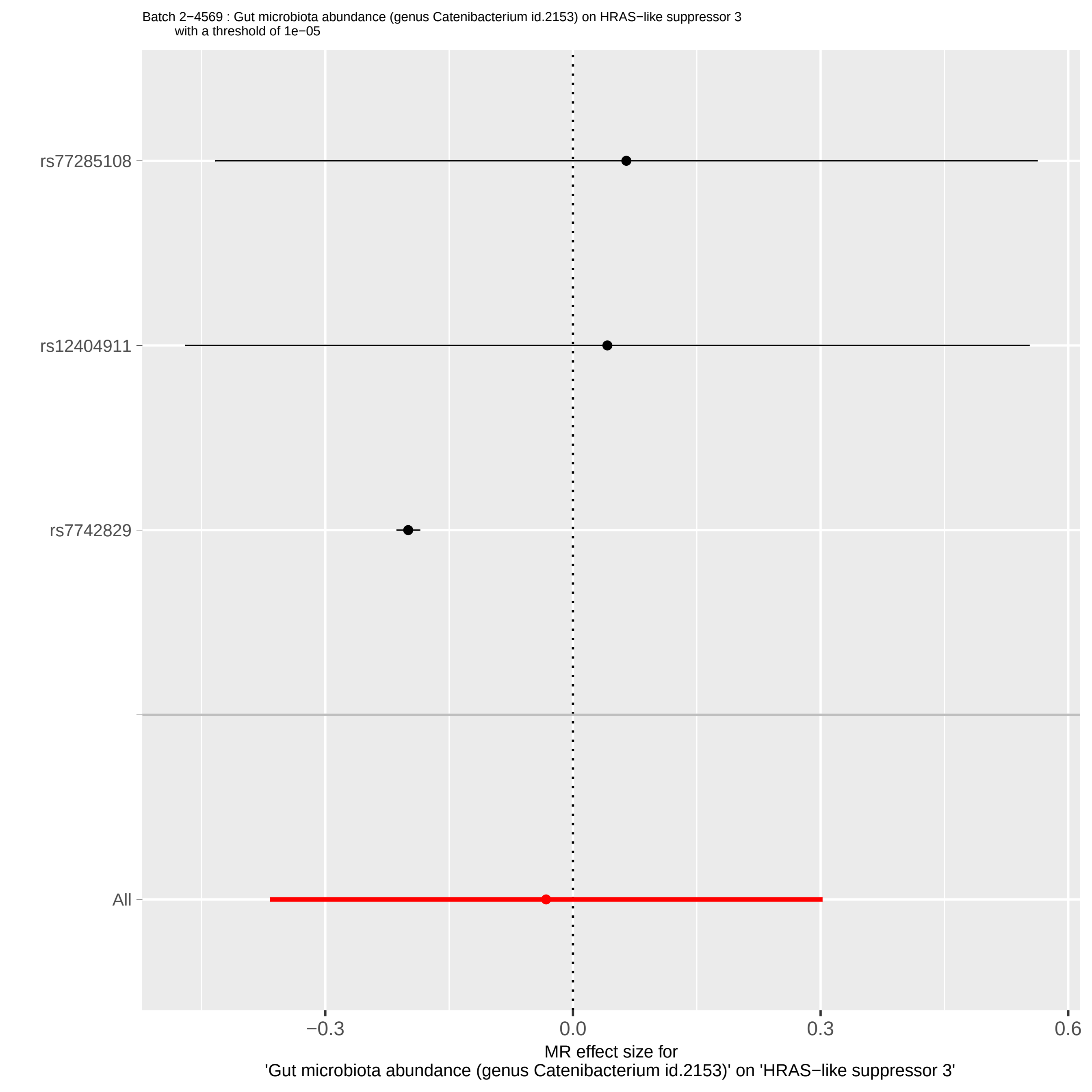

Batch 2−4569 : Gut microbiota abundance (genus Catenibacterium id.2153) on HRAS−like suppressor 3
with a threshold of 1e−05
rs77285108
rs12404911
rs7742829
All
−0.3
0.0
0.3
0.6
MR effect size for
'Gut microbiota abundance (genus Catenibacterium id.2153)' on 'HRAS−like suppressor 3'

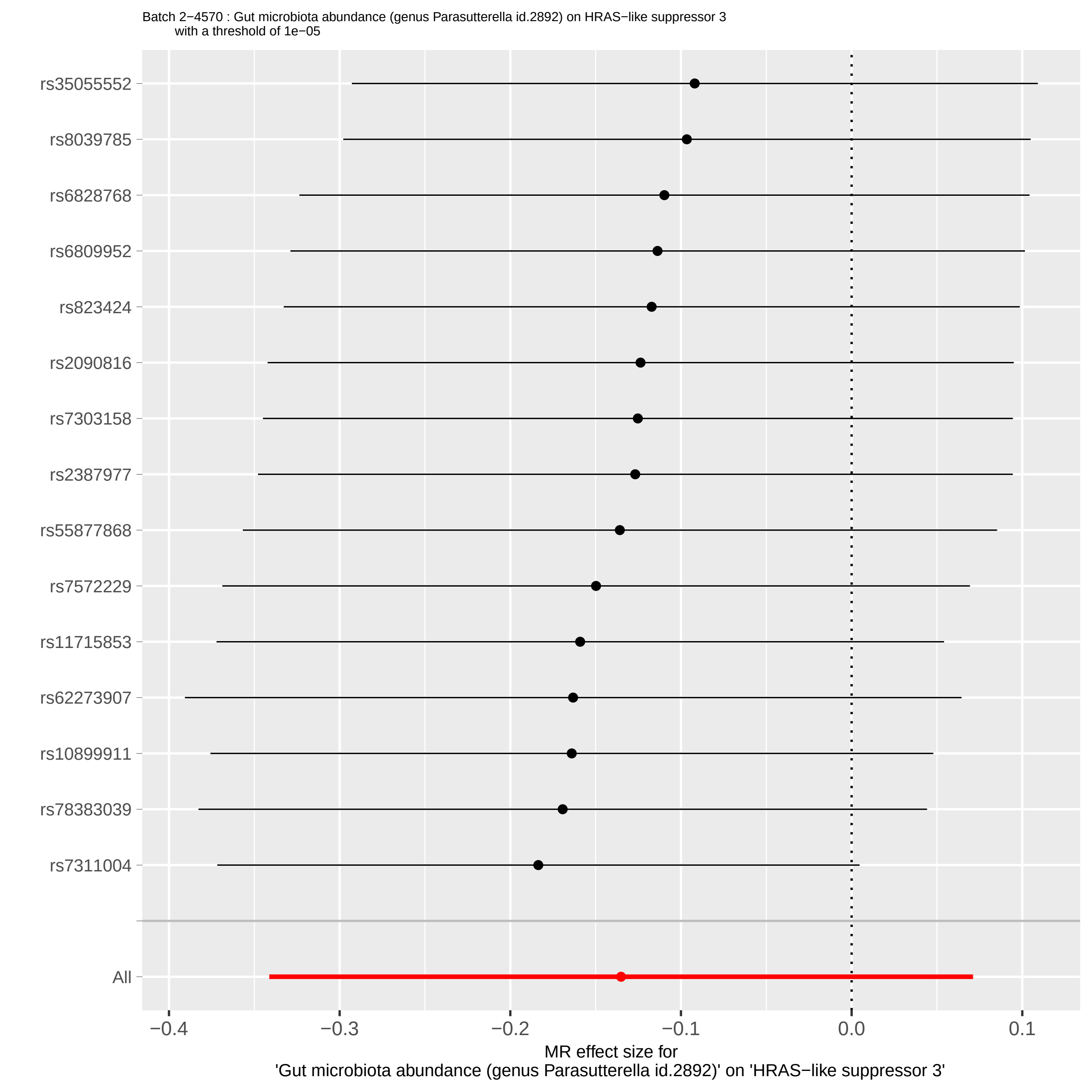
Batch 2−4570 : Gut microbiota abundance (genus Parasutterella id.2892) on HRAS−like suppressor 3
with a threshold of 1e−05
rs35055552
rs8039785
rs6828768
rs6809952
rs823424
rs2090816
rs7303158
rs2387977
rs55877868
rs7572229
rs11715853
rs62273907
rs10899911
rs78383039
rs7311004
All
−0.4
−0.3
−0.2
−0.1
0.0
0.1
MR effect size for
'Gut microbiota abundance (genus Parasutterella id.2892)' on 'HRAS−like suppressor 3'

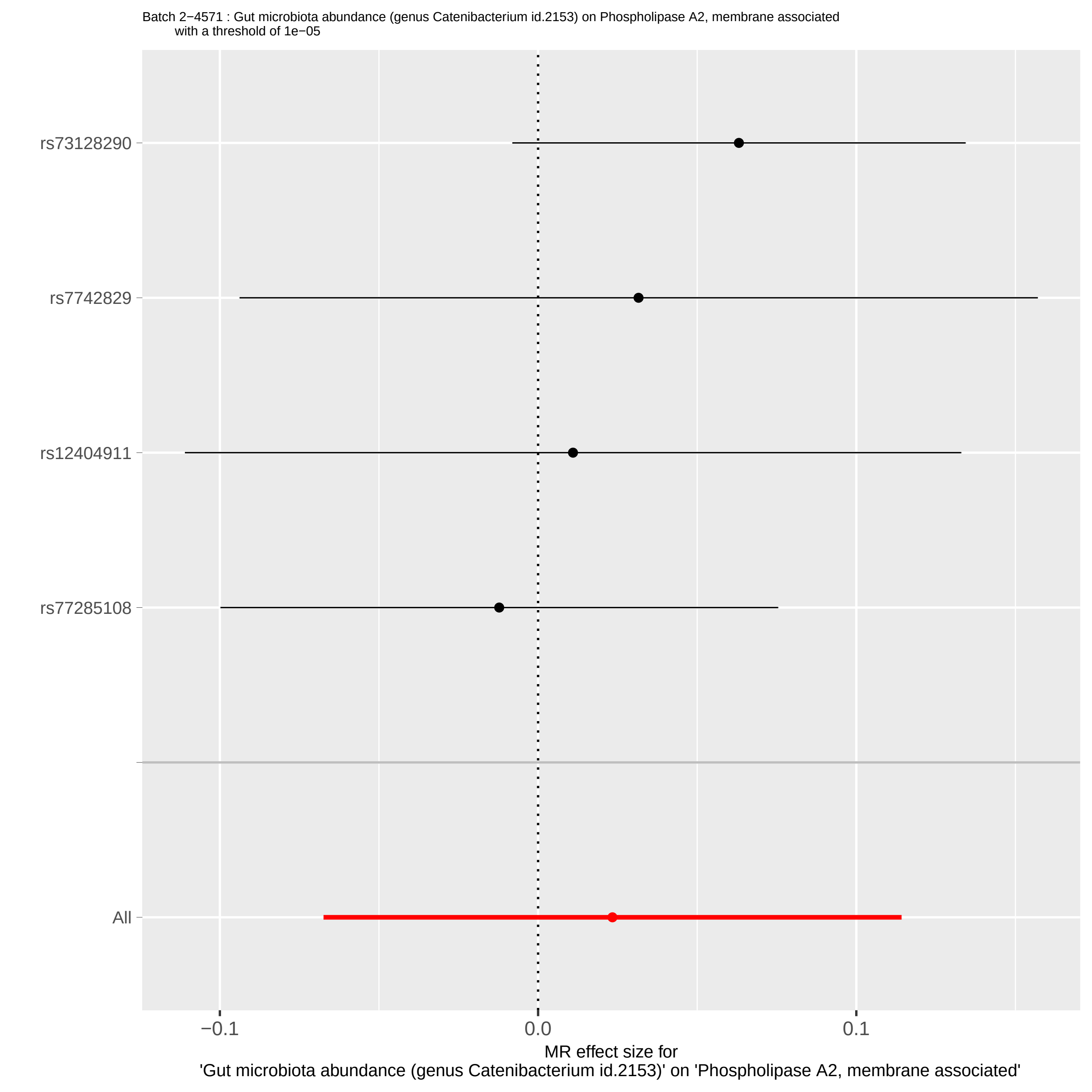

Batch 2−4571 : Gut microbiota abundance (genus Catenibacterium id.2153) on Phospholipase A2, membrane associated
with a threshold of 1e−05
rs73128290
rs7742829
rs12404911
rs77285108
All
−0.1
0.0
0.1
MR effect size for
'Gut microbiota abundance (genus Catenibacterium id.2153)' on 'Phospholipase A2, membrane associated'

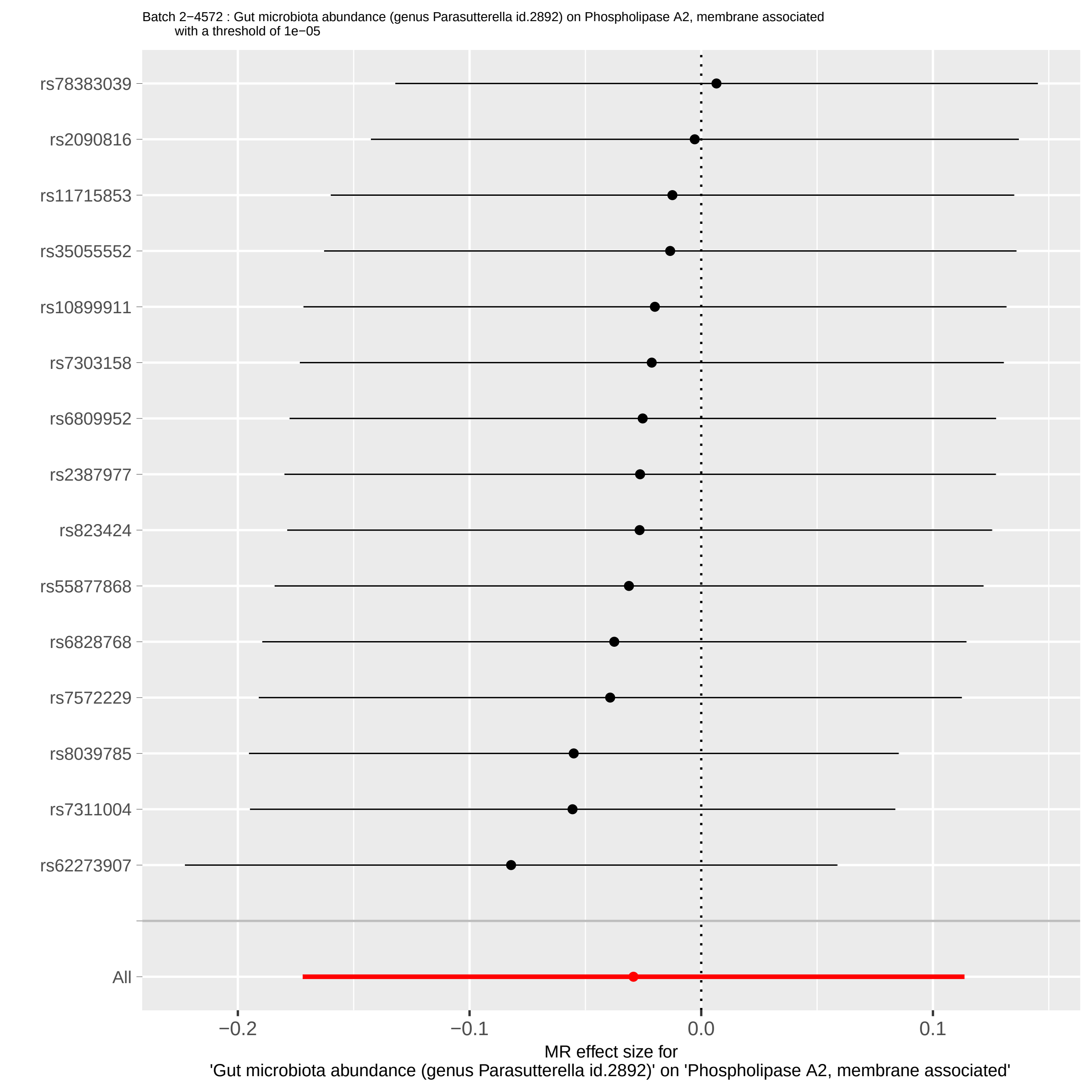

Batch 2−4572 : Gut microbiota abundance (genus Parasutterella id.2892) on Phospholipase A2, membrane associated
with a threshold of 1e−05
rs78383039
rs2090816
rs11715853
rs35055552
rs10899911
rs7303158
rs6809952
rs2387977
rs823424
rs55877868
rs6828768
rs7572229
rs8039785
rs7311004
rs62273907
All
−0.2
−0.1
0.0
0.1
MR effect size for
'Gut microbiota abundance (genus Parasutterella id.2892)' on 'Phospholipase A2, membrane associated'

Batch 2−4573 : Gut microbiota abundance (genus Catenibacterium id.2153) on Putative inactive group IIC secretory phospholipase A2
with a threshold of 1e−05

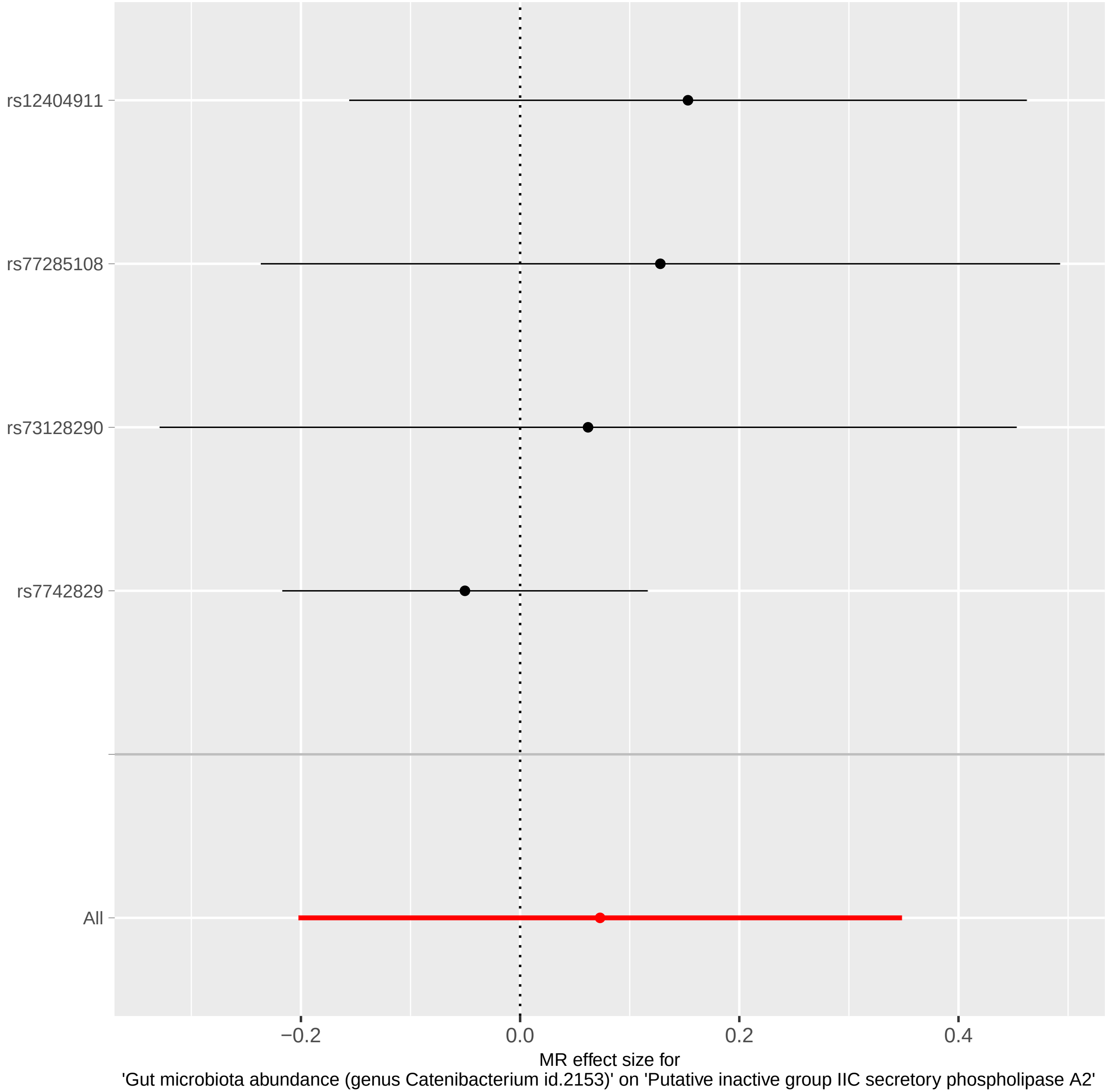

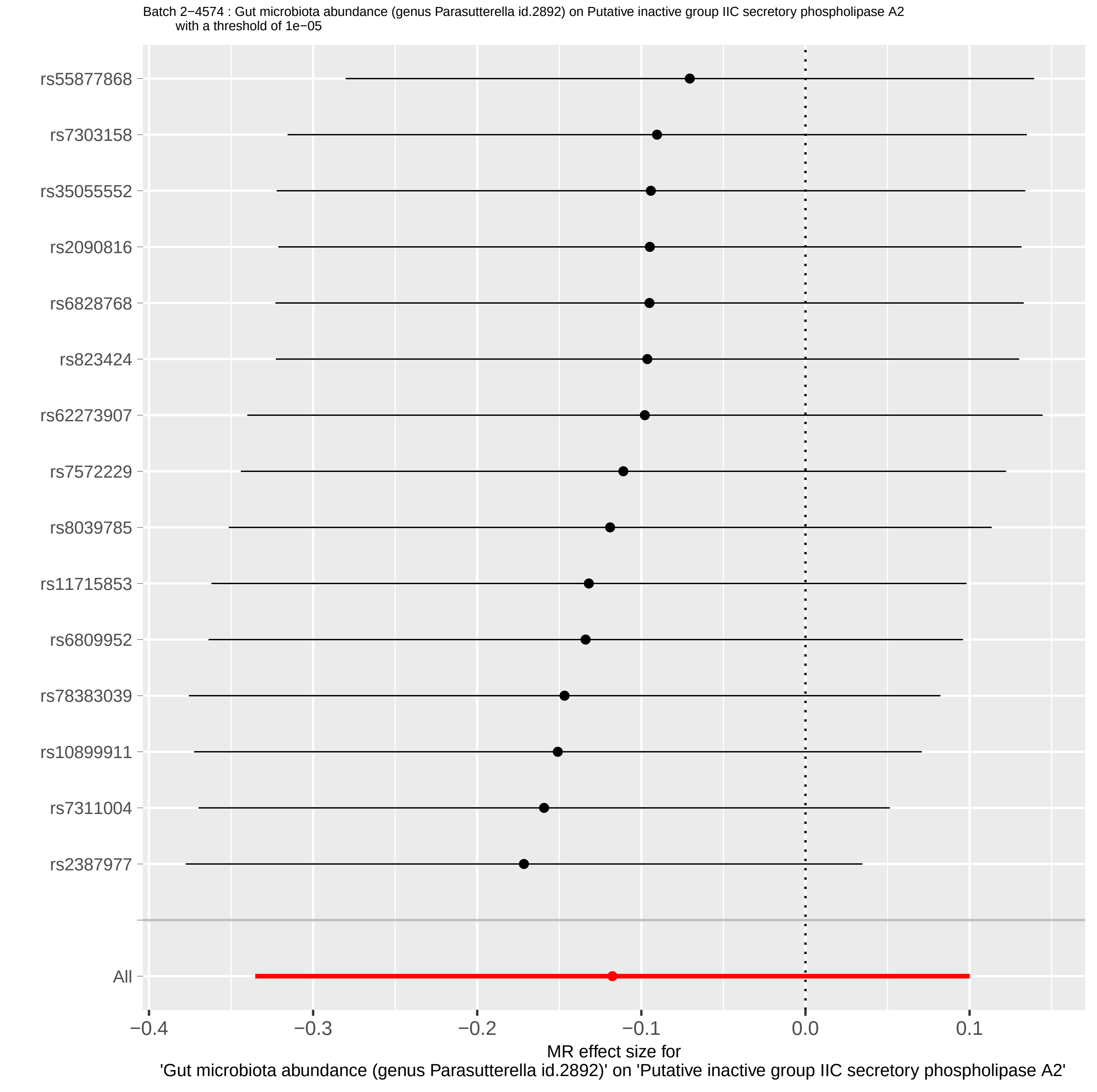
Batch 2−4574 : Gut microbiota abundance (genus Parasutterella id.2892) on Putative inactive group IIC secretory phospholipase A2
with a threshold of 1e−05
rs55877868
rs7303158
rs35055552
rs2090816
rs6828768
rs823424
rs62273907
rs7572229
rs8039785
rs11715853
rs6809952
rs78383039
rs10899911
rs7311004
rs2387977
All
−0.4
−0.3
−0.2
−0.1
0.0
0.1
MR effect size for
'Gut microbiota abundance (genus Parasutterella id.2892)' on 'Putative inactive group IIC secretory phospholipase A2'

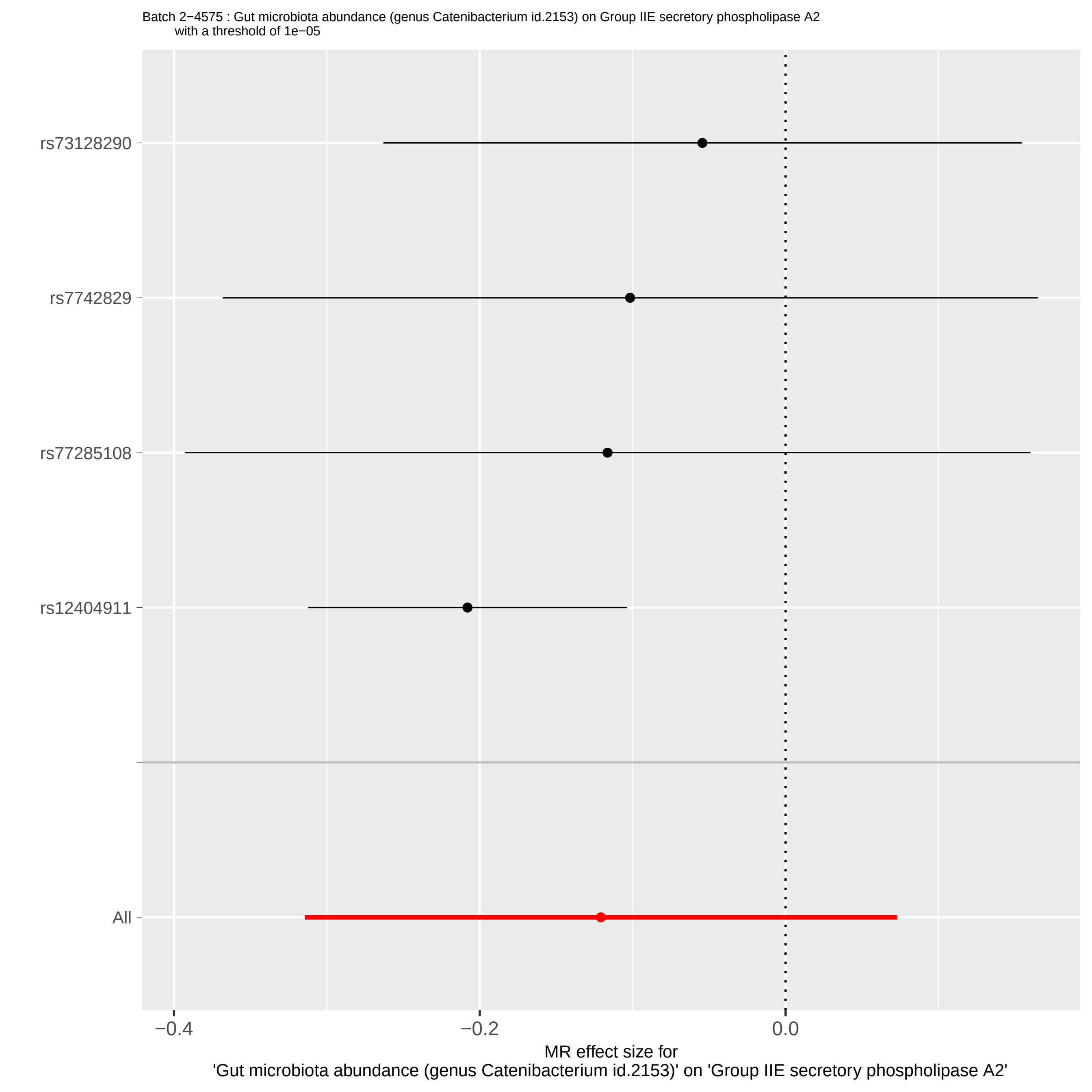
Batch 2−4575 : Gut microbiota abundance (genus Catenibacterium id.2153) on Group IIE secretory phospholipase A2
with a threshold of 1e−05
rs73128290
rs7742829
rs77285108
rs12404911
All
−0.4
−0.2
0.0
MR effect size for
'Gut microbiota abundance (genus Catenibacterium id.2153)' on 'Group IIE secretory phospholipase A2'

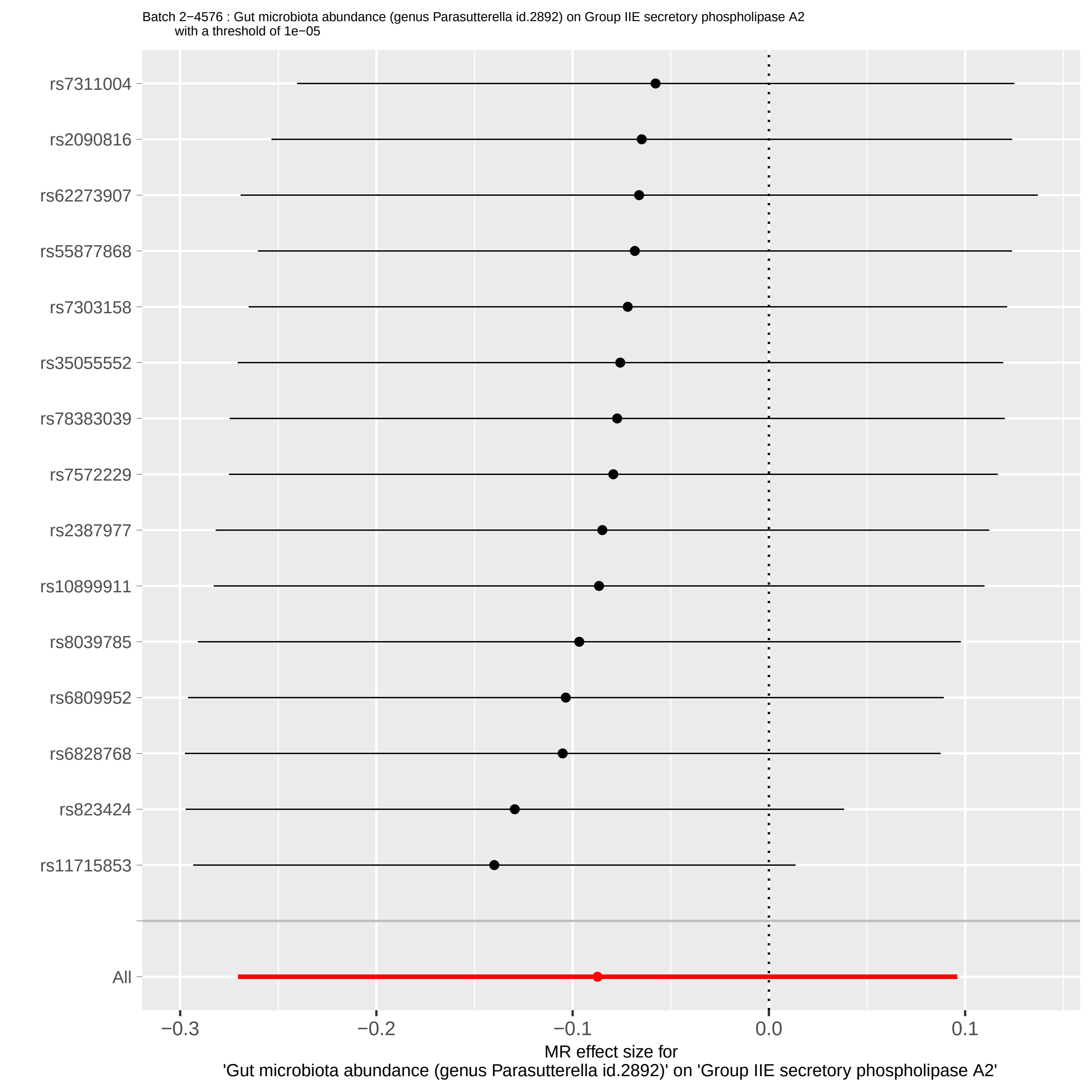
Batch 2−4576 : Gut microbiota abundance (genus Parasutterella id.2892) on Group IIE secretory phospholipase A2
with a threshold of 1e−05
rs7311004
rs2090816
rs62273907
rs55877868
rs7303158
rs35055552
rs78383039
rs7572229
rs2387977
rs10899911
rs8039785
rs6809952
rs6828768
rs823424
rs11715853
All
−0.3
−0.2
−0.1
0.0
0.1
MR effect size for
'Gut microbiota abundance (genus Parasutterella id.2892)' on 'Group IIE secretory phospholipase A2'

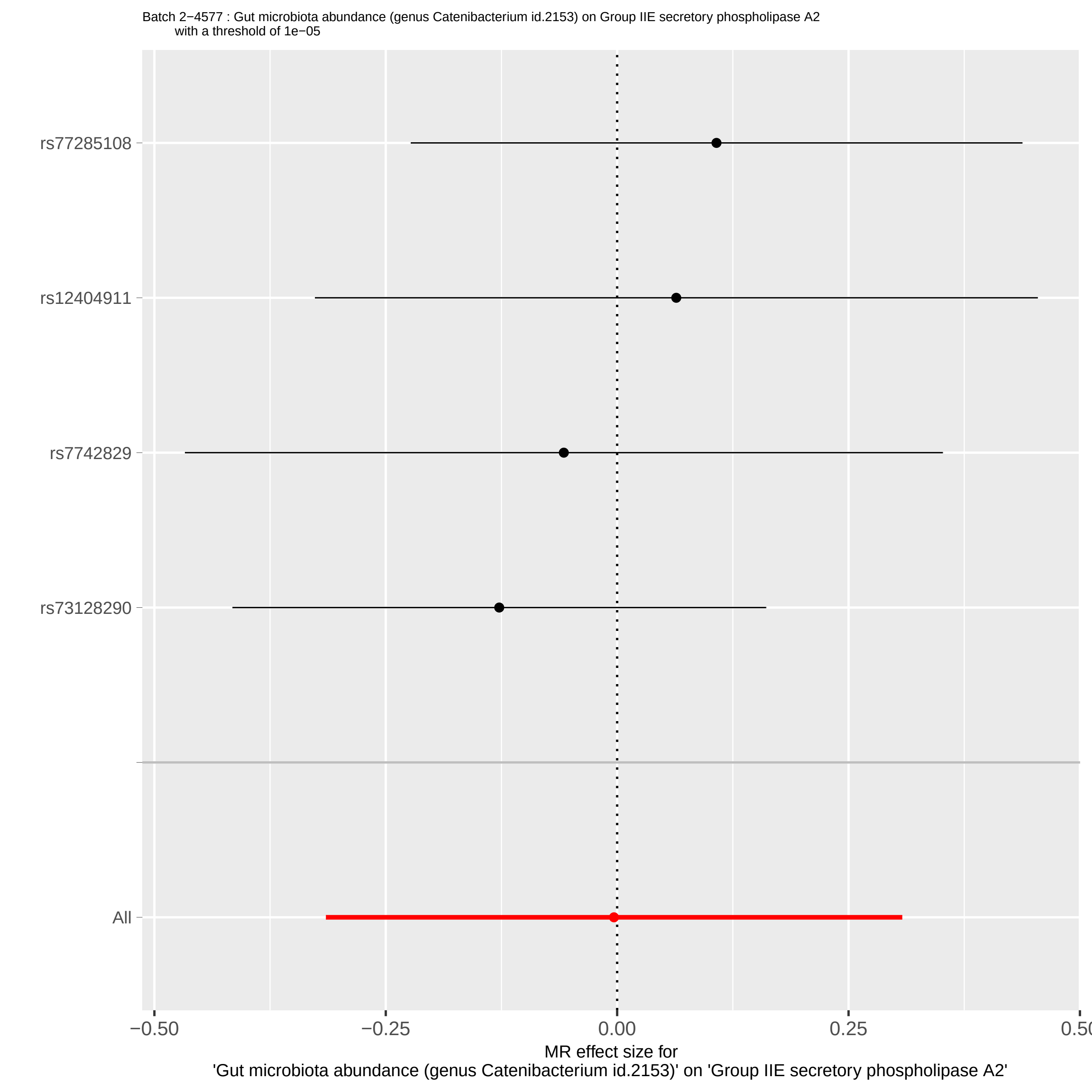
Batch 2−4577 : Gut microbiota abundance (genus Catenibacterium id.2153) on Group IIE secretory phospholipase A2
with a threshold of 1e−05
rs77285108
rs12404911
rs7742829
rs73128290
All
−0.50
−0.25
0.00
0.25
0.50
MR effect size for
'Gut microbiota abundance (genus Catenibacterium id.2153)' on 'Group IIE secretory phospholipase A2'

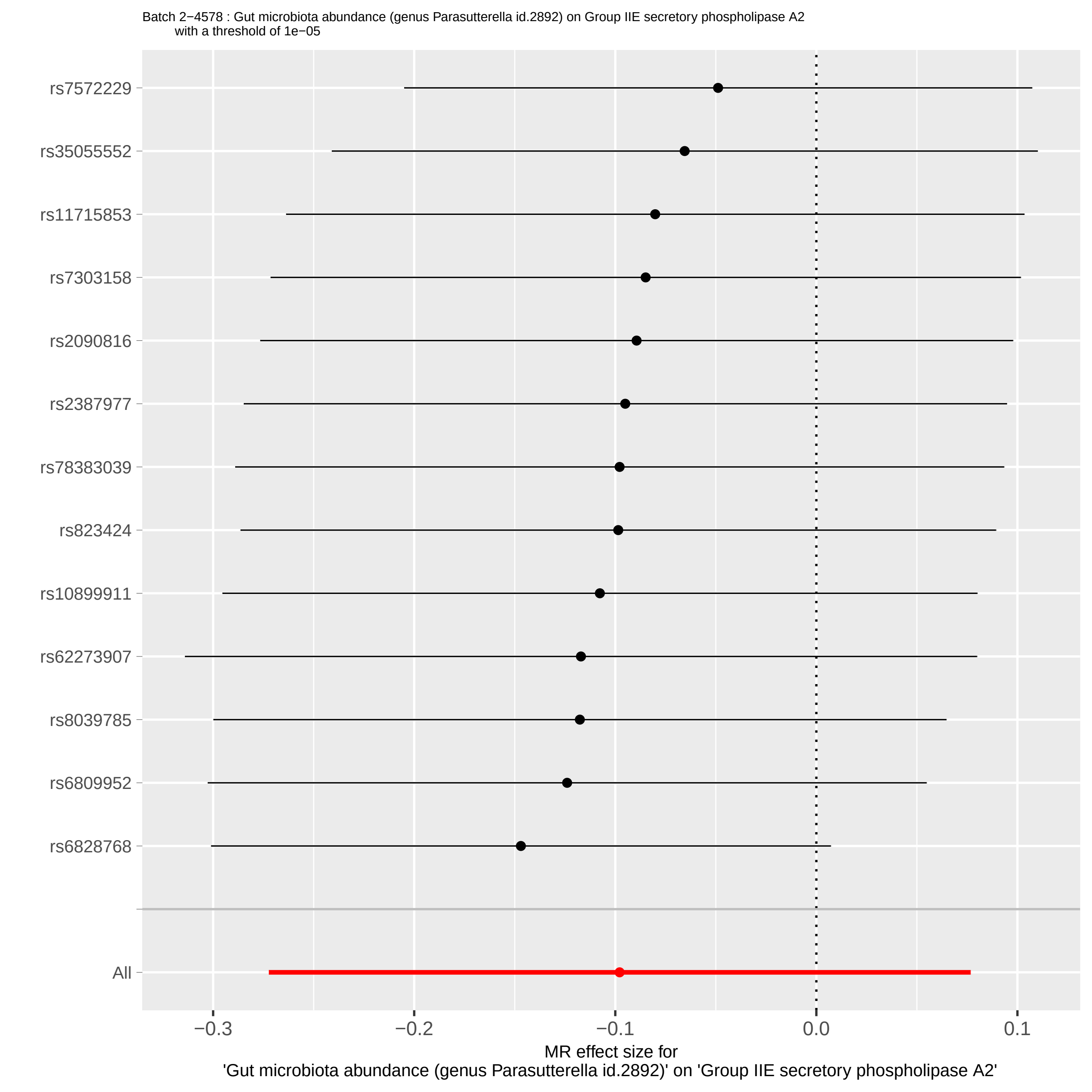

Batch 2−4578 : Gut microbiota abundance (genus Parasutterella id.2892) on Group IIE secretory phospholipase A2
with a threshold of 1e−05
rs7572229
rs35055552
rs11715853
rs7303158
rs2090816
rs2387977
rs78383039
rs823424
rs10899911
rs62273907
rs8039785
rs6809952
rs6828768
All
−0.3
−0.2
−0.1
0.0
0.1
MR effect size for
'Gut microbiota abundance (genus Parasutterella id.2892)' on 'Group IIE secretory phospholipase A2'

Batch 2−4579 : Gut microbiota abundance (genus Catenibacterium id.2153) on Calcium−dependent phospholipase A2
with a threshold of 1e−05

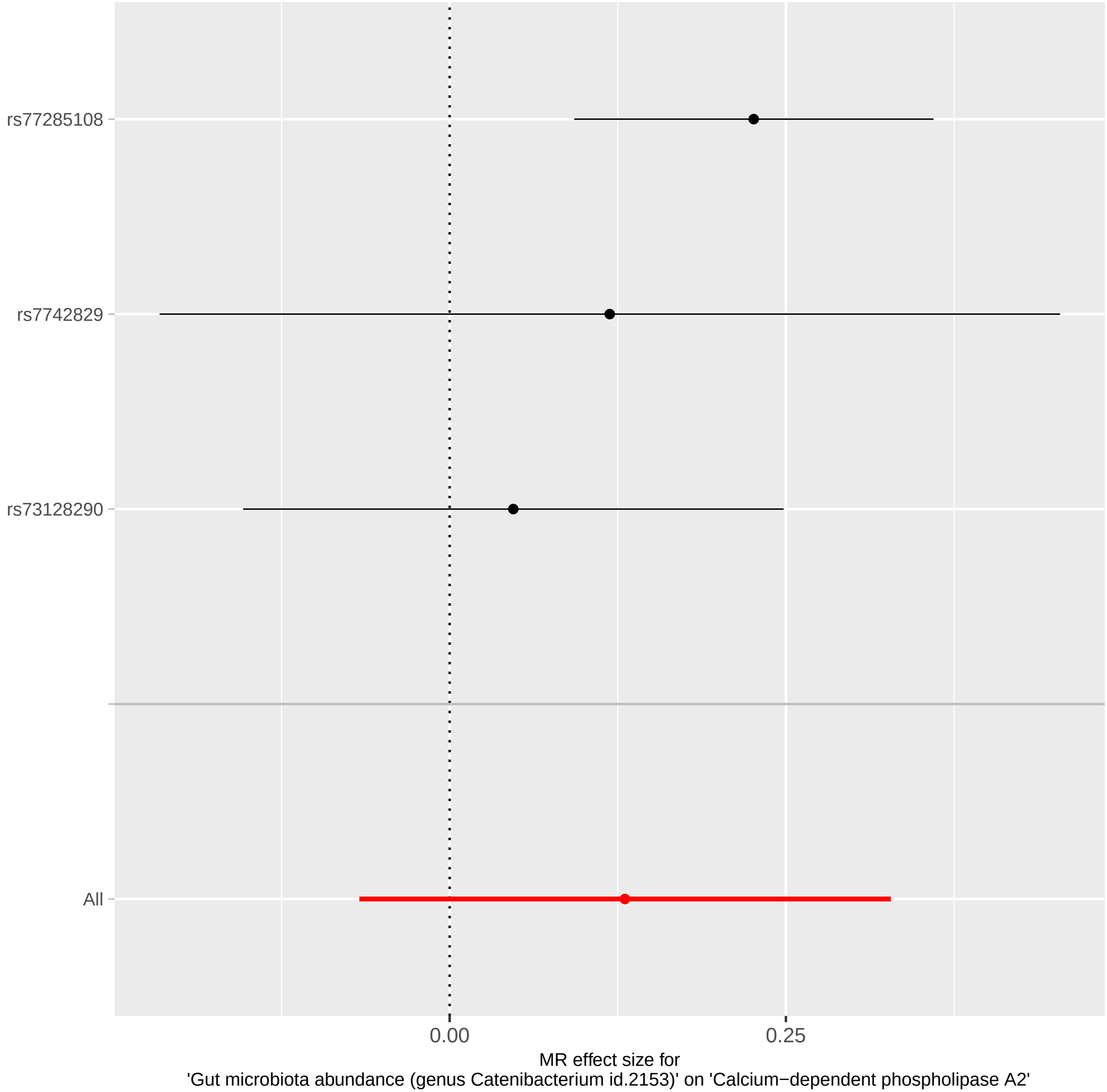

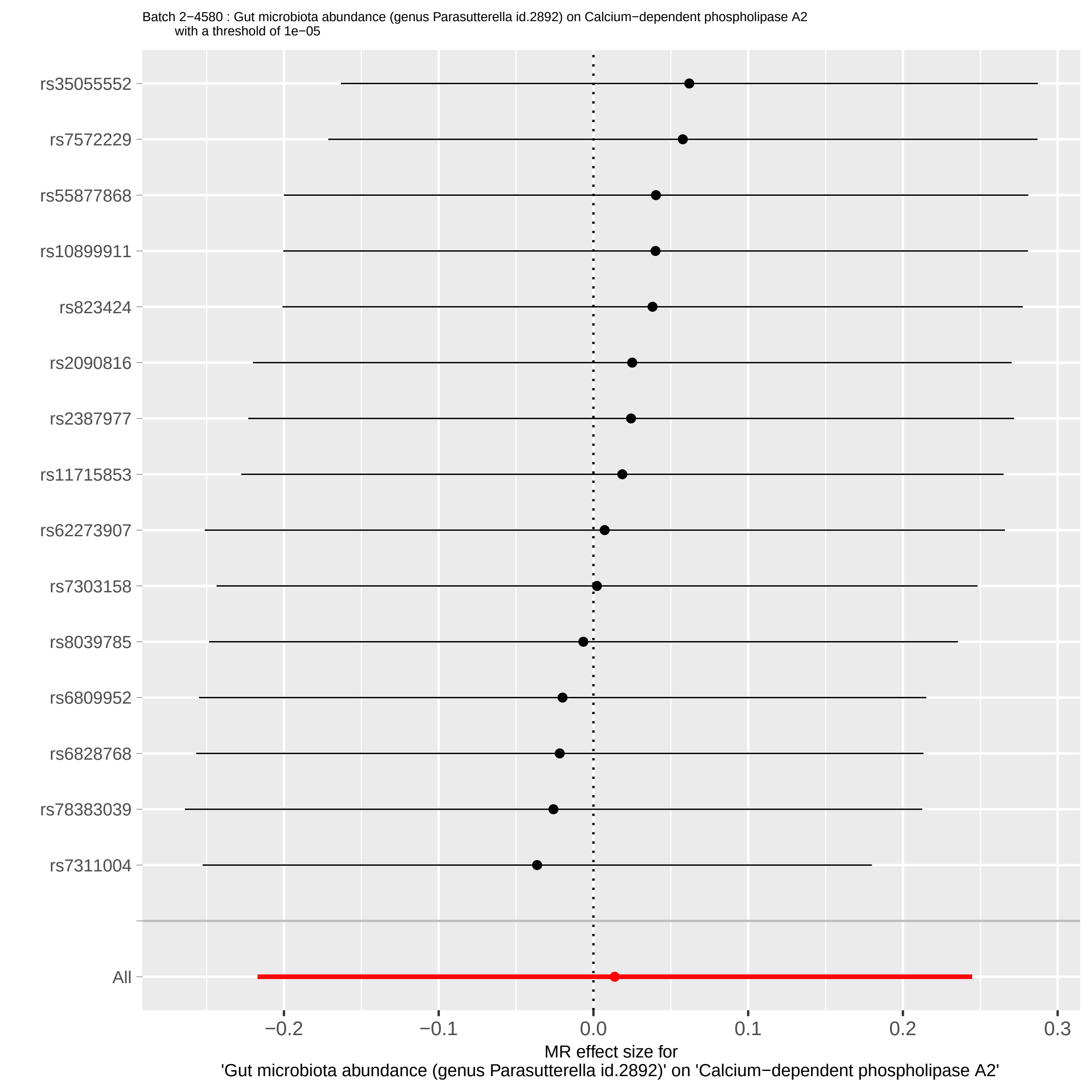

Batch 2−4580 : Gut microbiota abundance (genus Parasutterella id.2892) on Calcium−dependent phospholipase A2
with a threshold of 1e−05
rs35055552
rs7572229
rs55877868
rs10899911
rs823424
rs2090816
rs2387977
rs11715853
rs62273907
rs7303158
rs8039785
rs6809952
rs6828768
rs78383039
rs7311004
All
−0.2
−0.1
0.0
0.1
0.2
0.3
MR effect size for
'Gut microbiota abundance (genus Parasutterella id.2892)' on 'Calcium−dependent phospholipase A2'

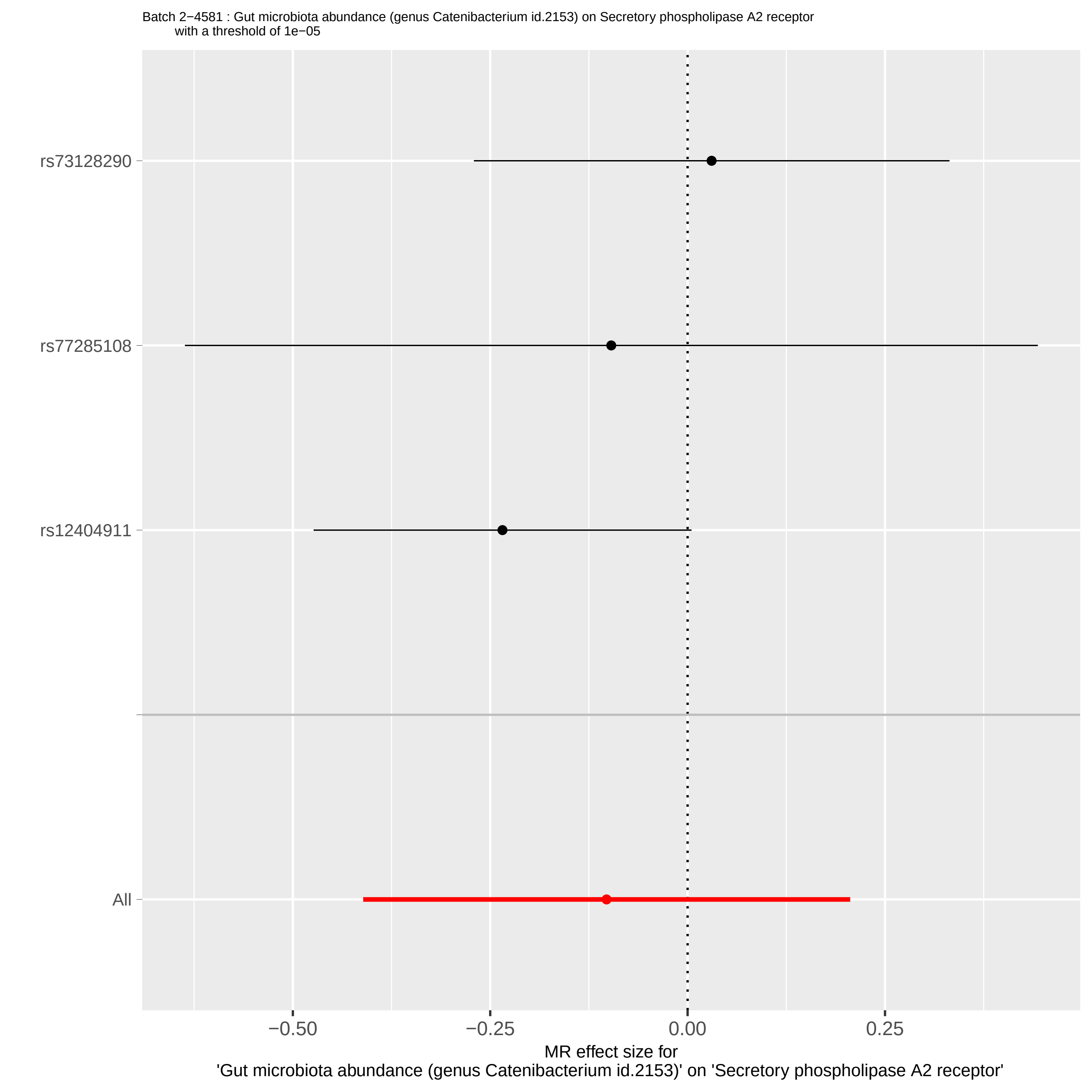
Batch 2−4581 : Gut microbiota abundance (genus Catenibacterium id.2153) on Secretory phospholipase A2 receptor
with a threshold of 1e−05
rs73128290
rs77285108
rs12404911
All
−0.50
−0.25
0.00
0.25
MR effect size for
'Gut microbiota abundance (genus Catenibacterium id.2153)' on 'Secretory phospholipase A2 receptor'

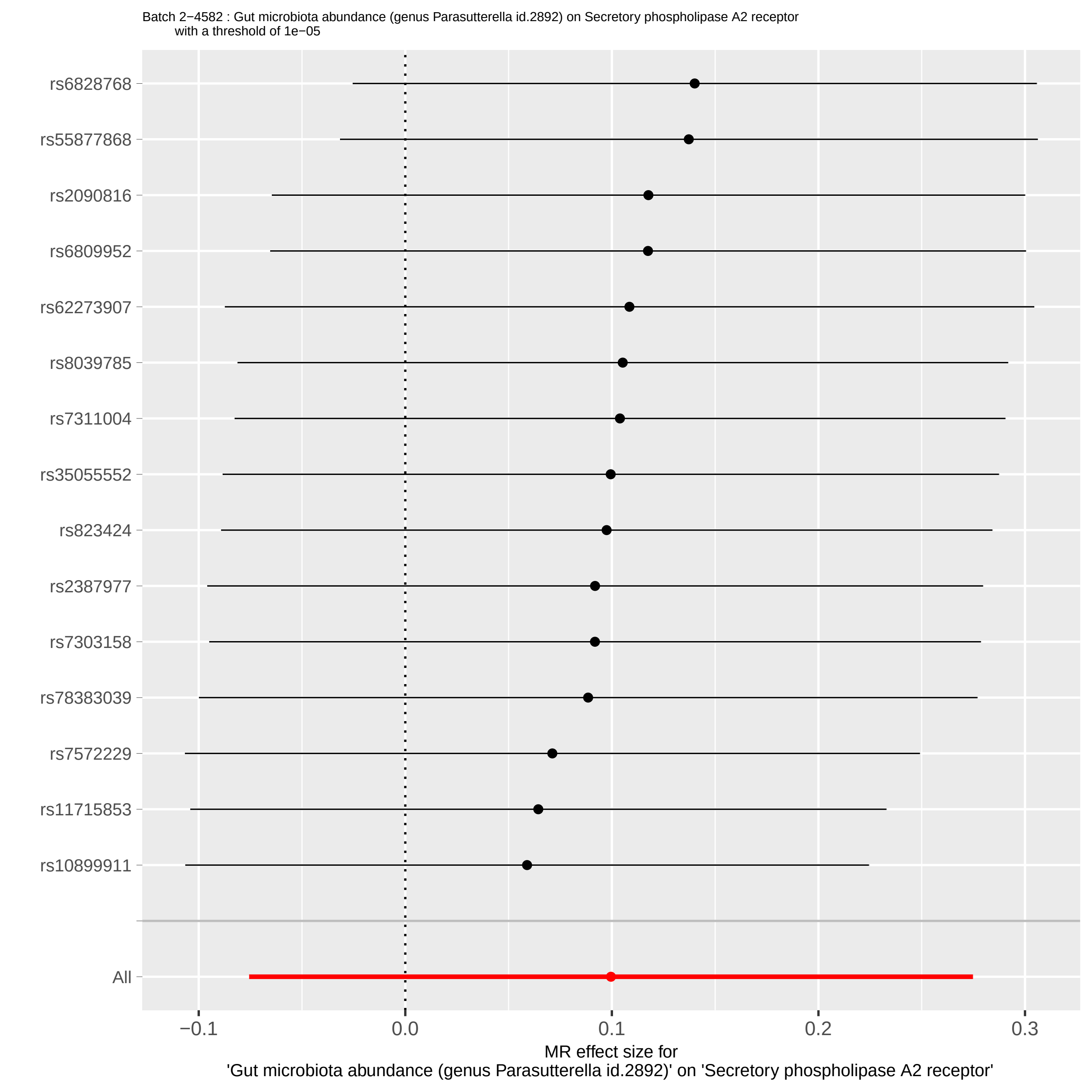
Batch 2−4582 : Gut microbiota abundance (genus Parasutterella id.2892) on Secretory phospholipase A2 receptor
with a threshold of 1e−05
rs6828768
rs55877868
rs2090816
rs6809952
rs62273907
rs8039785
rs7311004
rs35055552
rs823424
rs2387977
rs7303158
rs78383039
rs7572229
rs11715853
rs10899911
All
−0.1
0.0
0.1
0.2
0.3
MR effect size for
'Gut microbiota abundance (genus Parasutterella id.2892)' on 'Secretory phospholipase A2 receptor'

Batch 2−4583 : Gut microbiota abundance (genus Catenibacterium id.2153) on Urokinase−type plasminogen activator
with a threshold of 1e−05

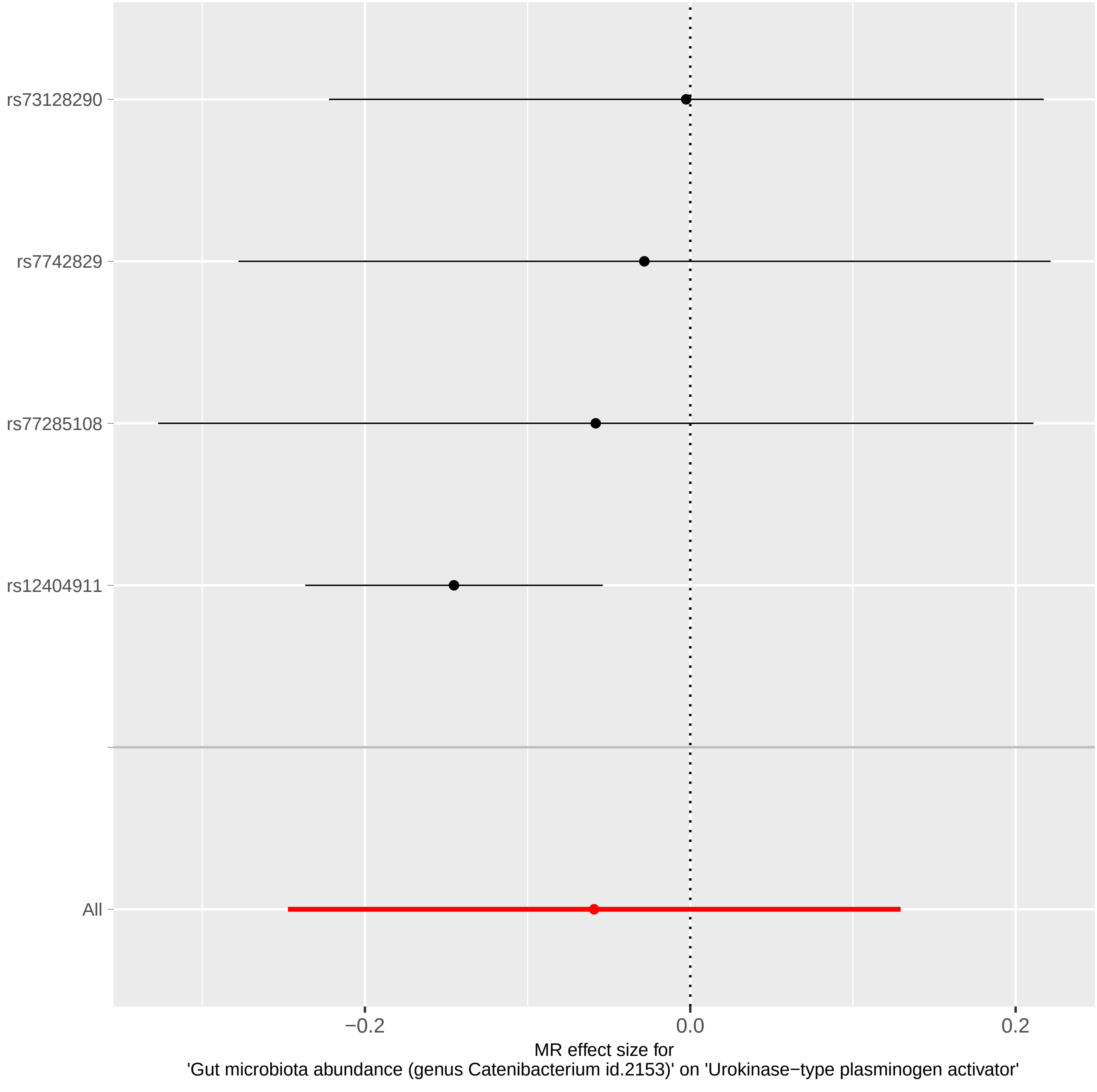

Batch 2−4584 : Gut microbiota abundance (genus Parasutterella id.2892) on Urokinase−type plasminogen activator
with a threshold of 1e−05

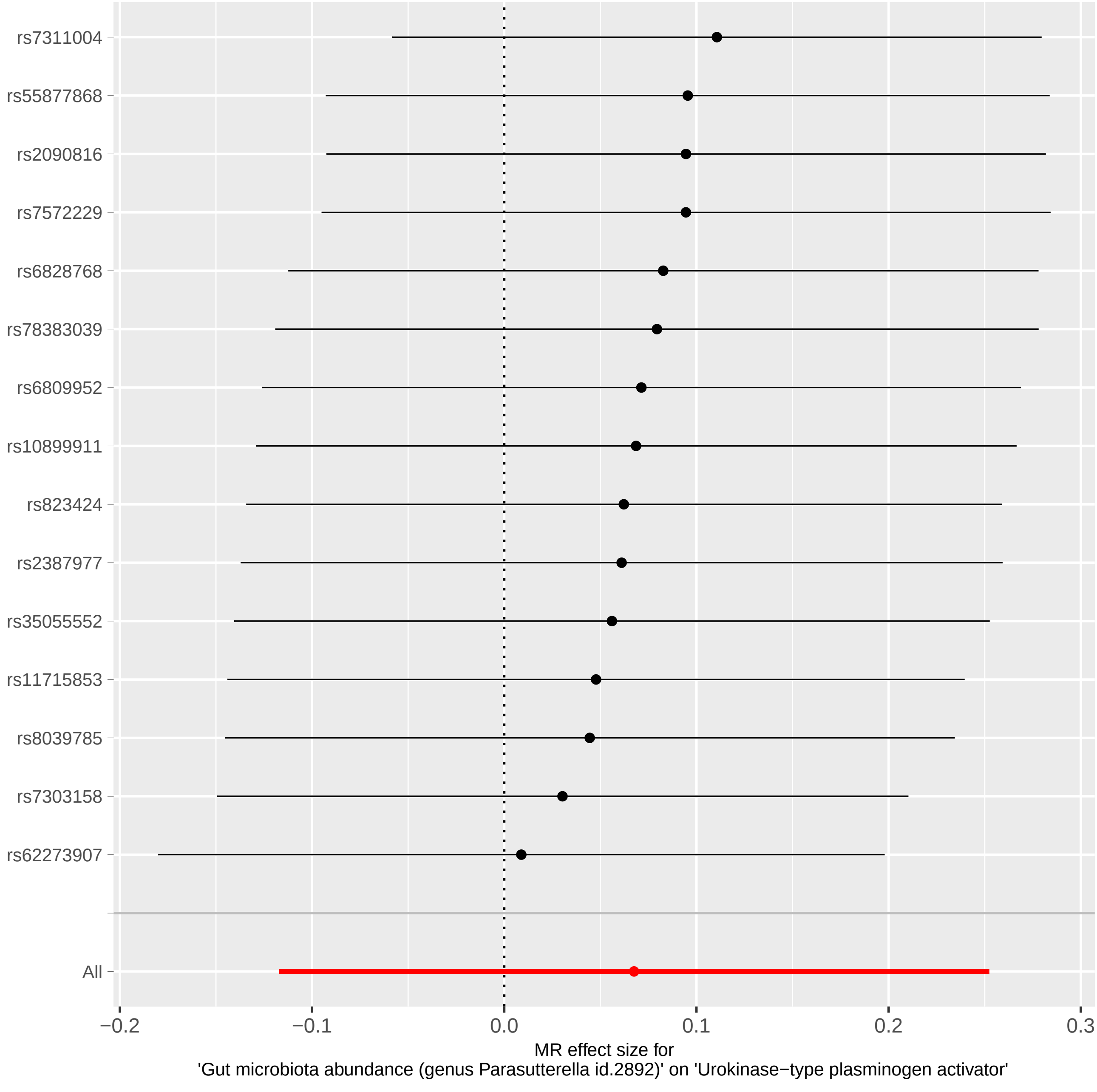

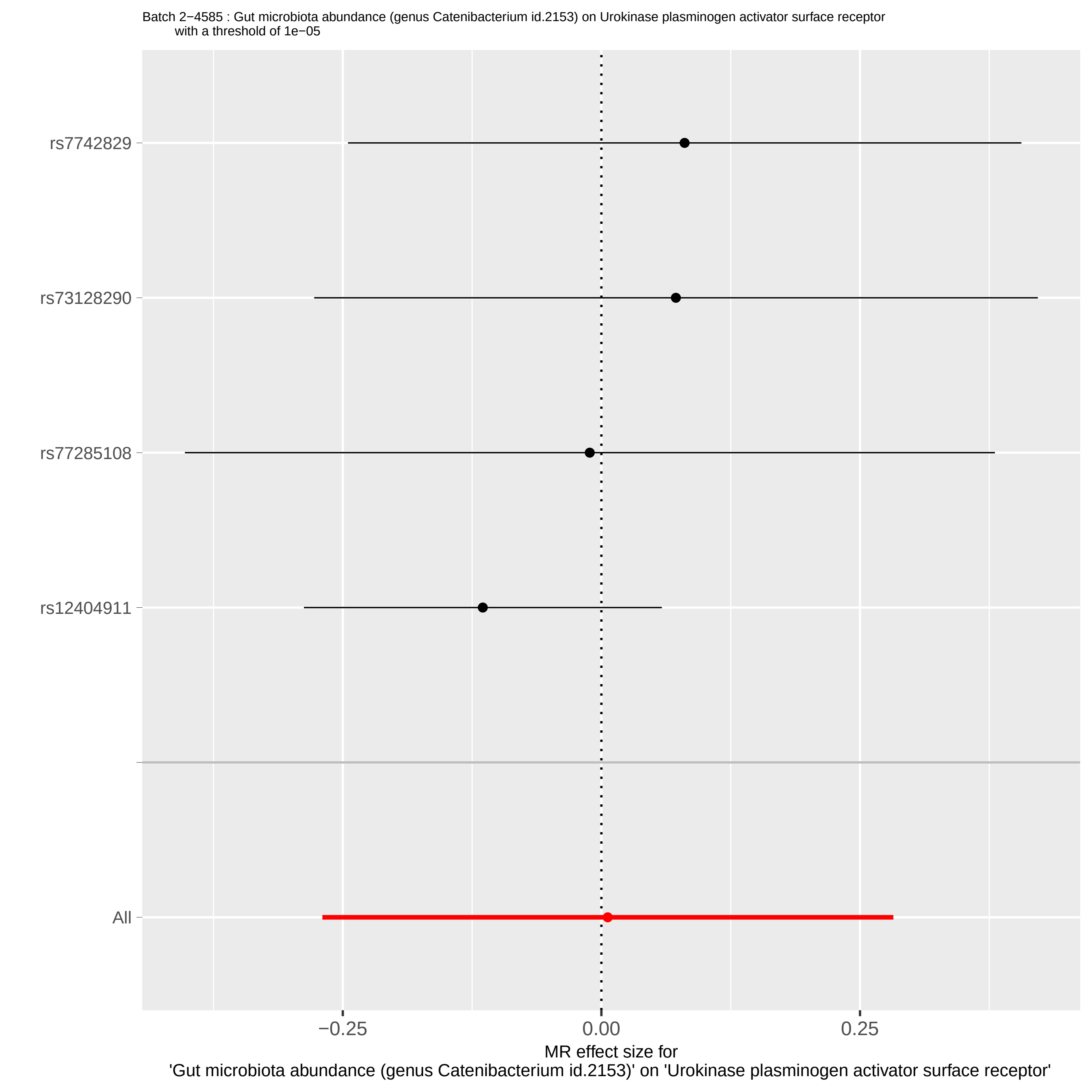

Batch 2−4585 : Gut microbiota abundance (genus Catenibacterium id.2153) on Urokinase plasminogen activator surface receptor
with a threshold of 1e−05
rs7742829
rs73128290
rs77285108
rs12404911
All
−0.25
0.00
0.25
MR effect size for
'Gut microbiota abundance (genus Catenibacterium id.2153)' on 'Urokinase plasminogen activator surface receptor'

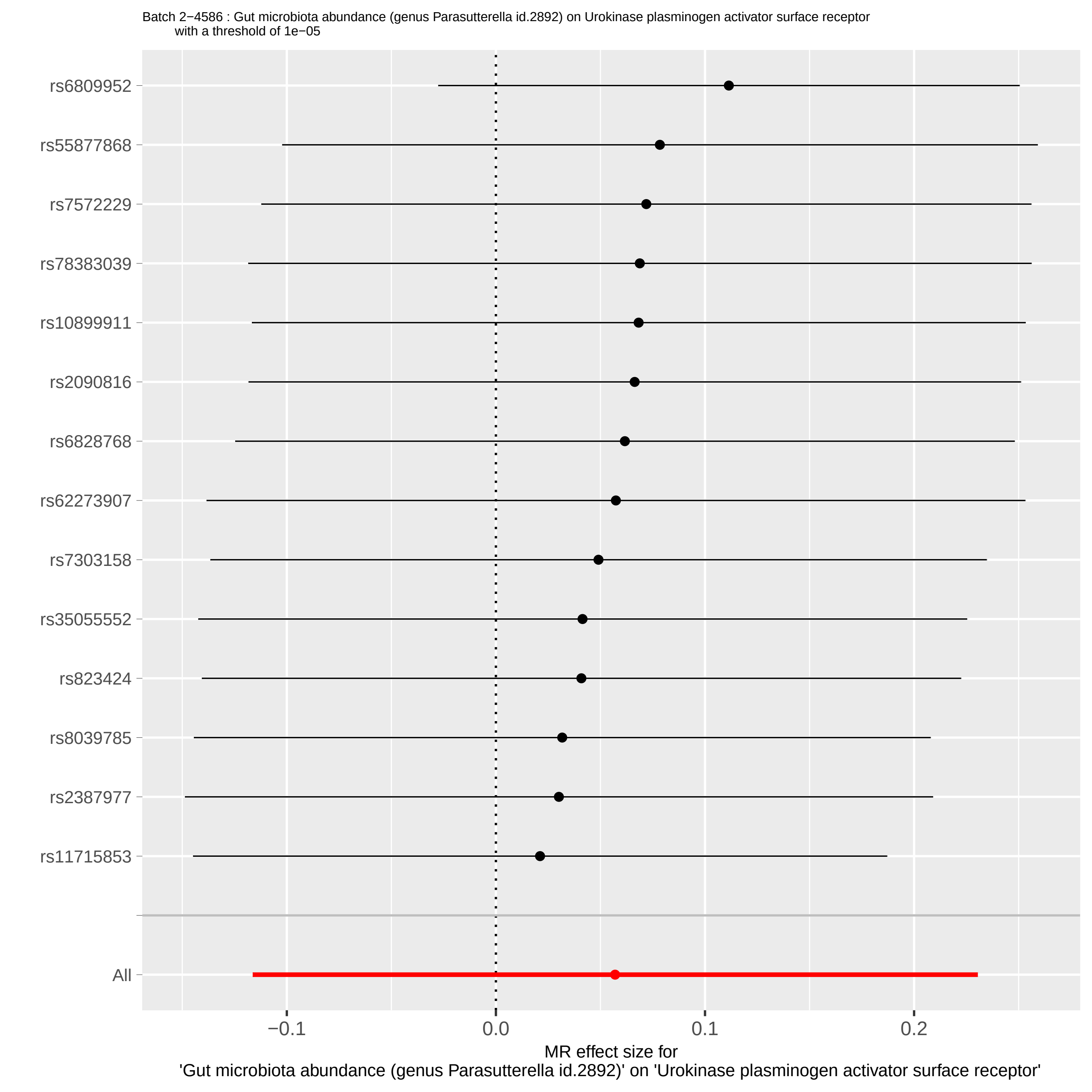

Batch 2−4586 : Gut microbiota abundance (genus Parasutterella id.2892) on Urokinase plasminogen activator surface receptor with a threshold of 1e−05
rs6809952
rs55877868
rs7572229
rs78383039
rs10899911
rs2090816
rs6828768
rs62273907
rs7303158
rs35055552
rs823424
rs8039785
rs2387977
rs11715853
All
−0.1
0.0
0.1
0.2
MR effect size for
'Gut microbiota abundance (genus Parasutterella id.2892)' on 'Urokinase plasminogen activator surface receptor'

Batch 2−4587 : Gut microbiota abundance (genus Catenibacterium id.2153) on Phospholipase B−like 1
with a threshold of 1e−05

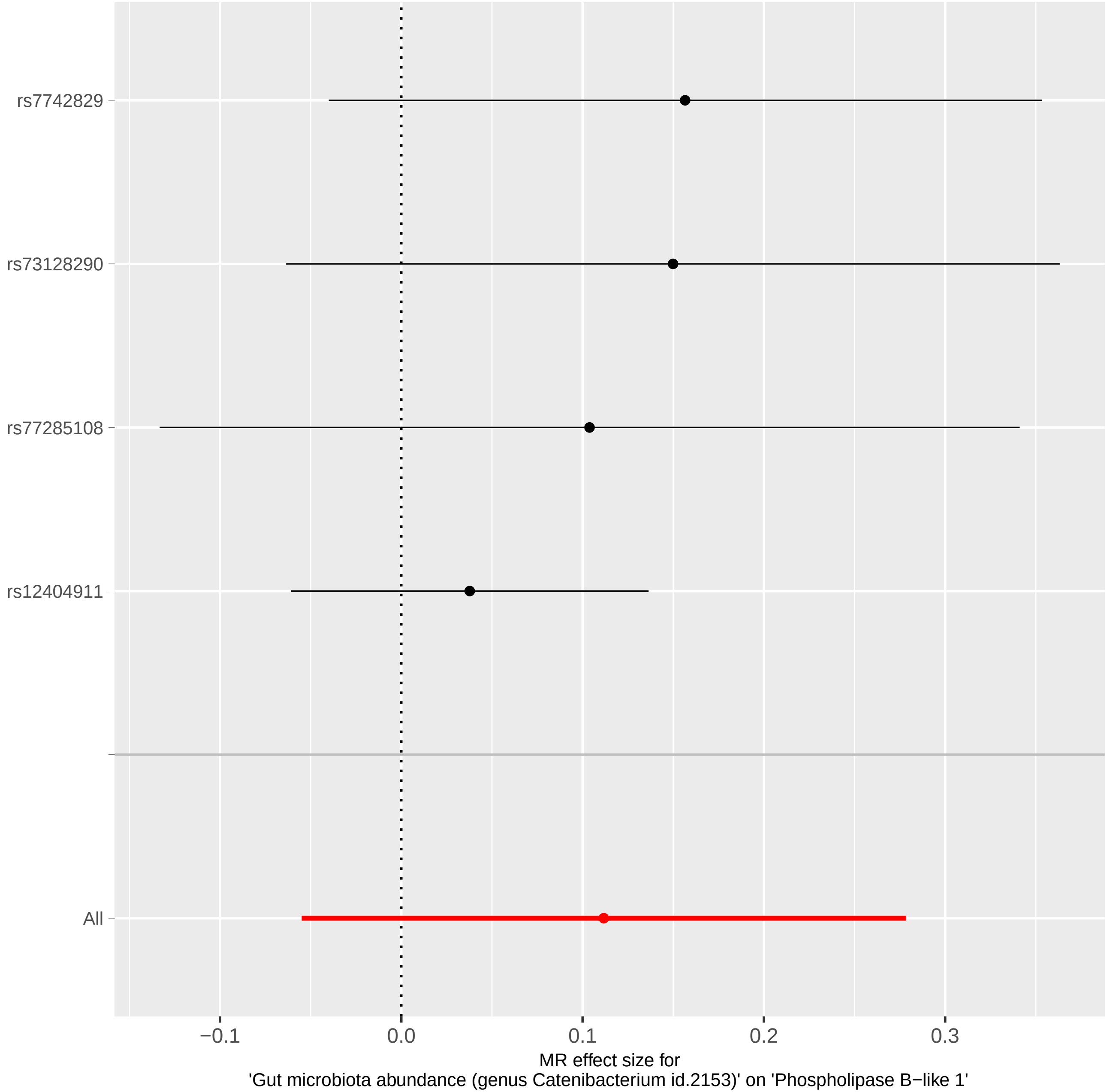

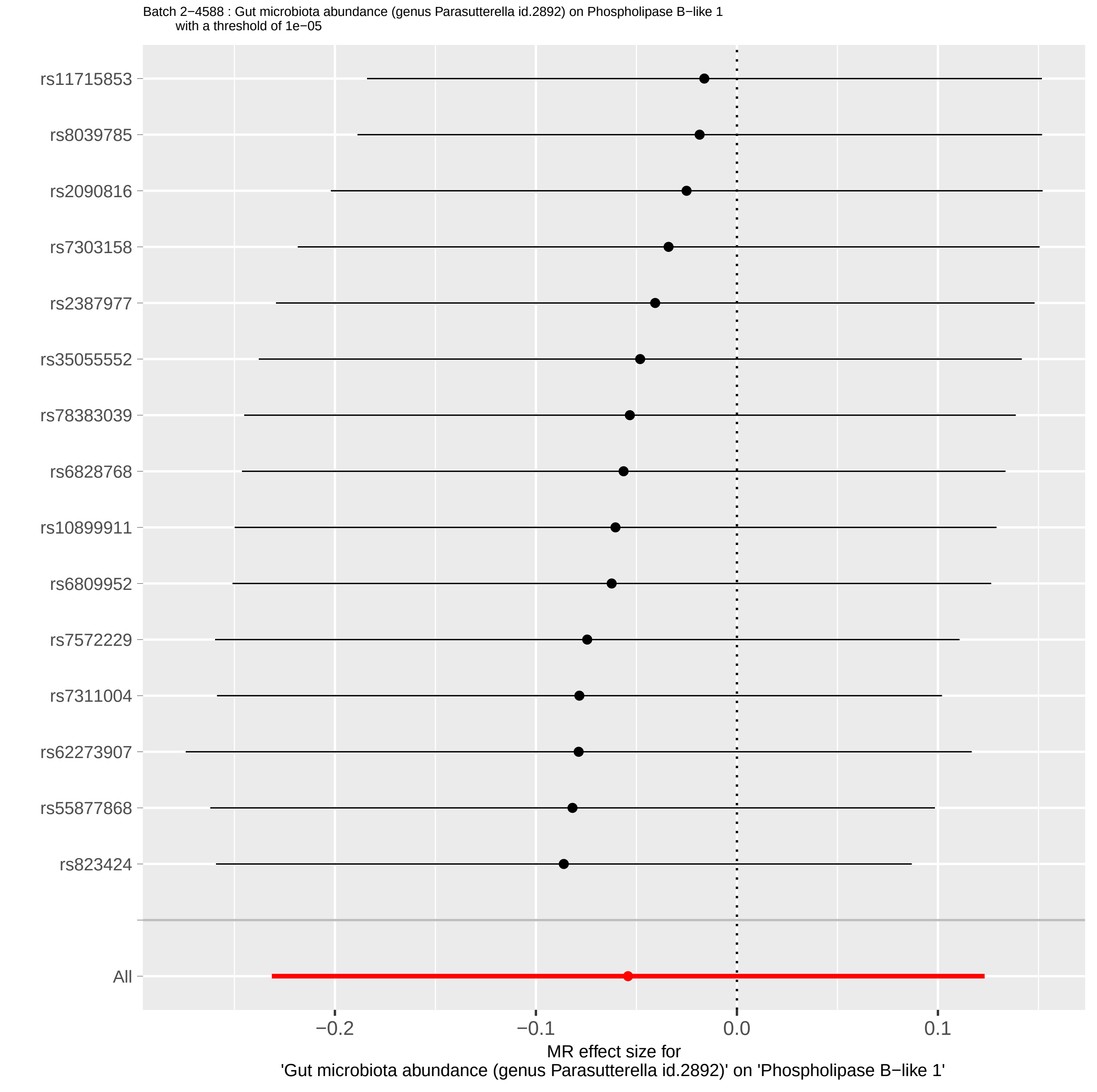
Batch 2−4588 : Gut microbiota abundance (genus Parasutterella id.2892) on Phospholipase B−like 1
with a threshold of 1e−05
rs11715853
rs8039785
rs2090816
rs7303158
rs2387977
rs35055552
rs78383039
rs6828768
rs10899911
rs6809952
rs7572229
rs7311004
rs62273907
rs55877868
rs823424
All
−0.2
−0.1
0.0
0.1
MR effect size for
'Gut microbiota abundance (genus Parasutterella id.2892)' on 'Phospholipase B−like 1'

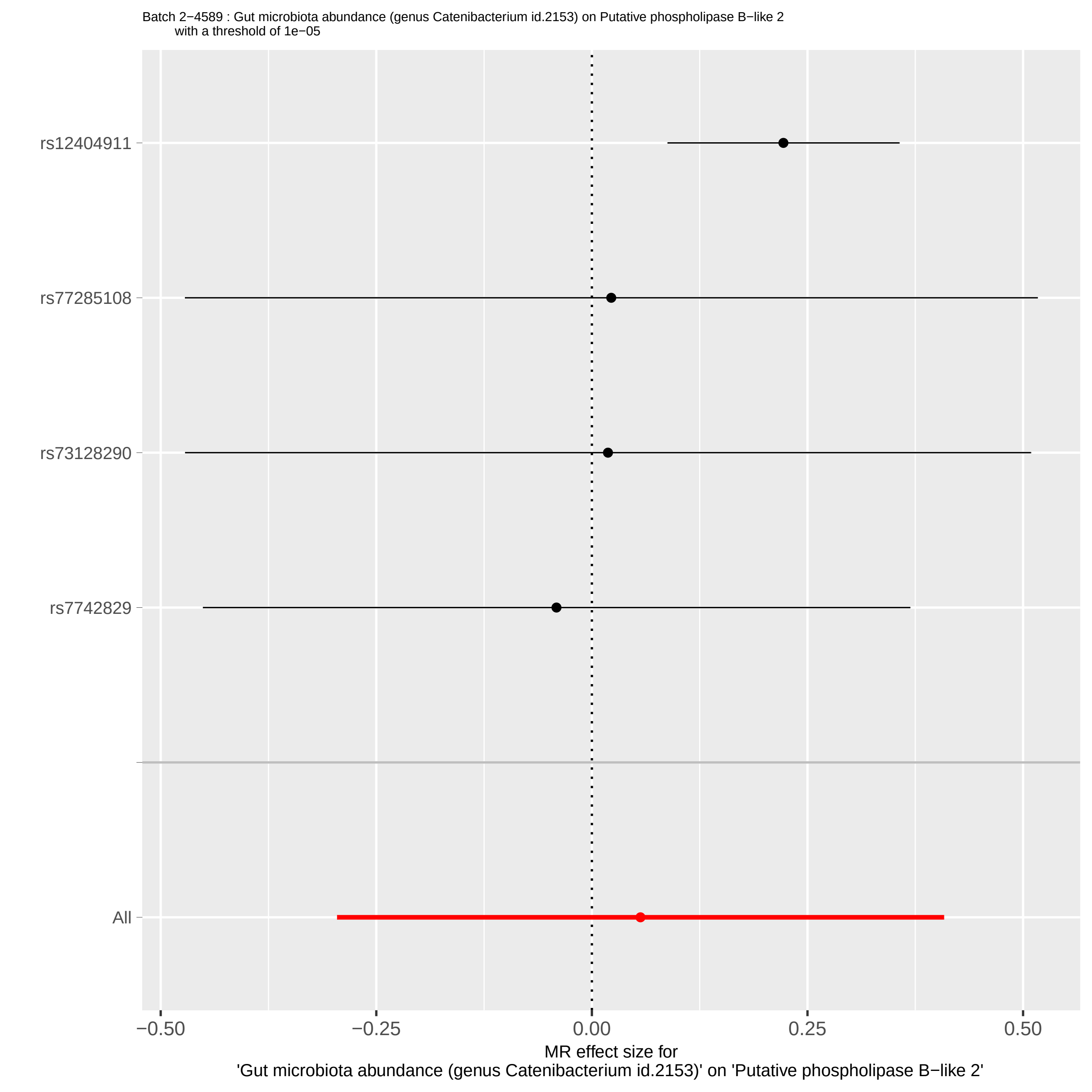
Batch 2−4589 : Gut microbiota abundance (genus Catenibacterium id.2153) on Putative phospholipase B−like 2
with a threshold of 1e−05
rs12404911
rs77285108
rs73128290
rs7742829
All
−0.50
−0.25
0.00
0.25
0.50
MR effect size for
'Gut microbiota abundance (genus Catenibacterium id.2153)' on 'Putative phospholipase B−like 2'

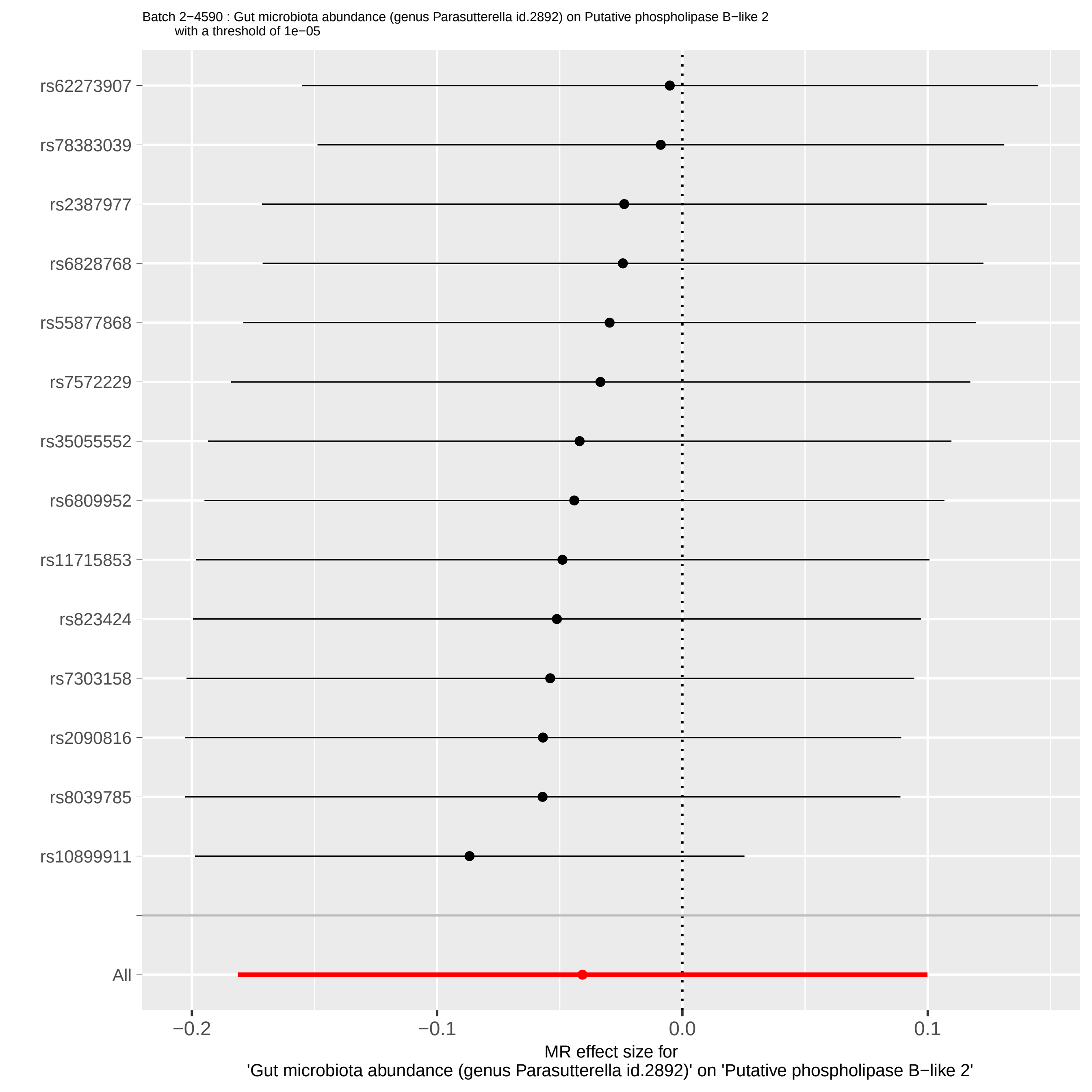

Batch 2−4590 : Gut microbiota abundance (genus Parasutterella id.2892) on Putative phospholipase B−like 2
with a threshold of 1e−05
rs62273907
rs78383039
rs2387977
rs6828768
rs55877868
rs7572229
rs35055552
rs6809952
rs11715853
rs823424
rs7303158
rs2090816
rs8039785
rs10899911
All
−0.2
−0.1
0.0
0.1
MR effect size for
'Gut microbiota abundance (genus Parasutterella id.2892)' on 'Putative phospholipase B−like 2'

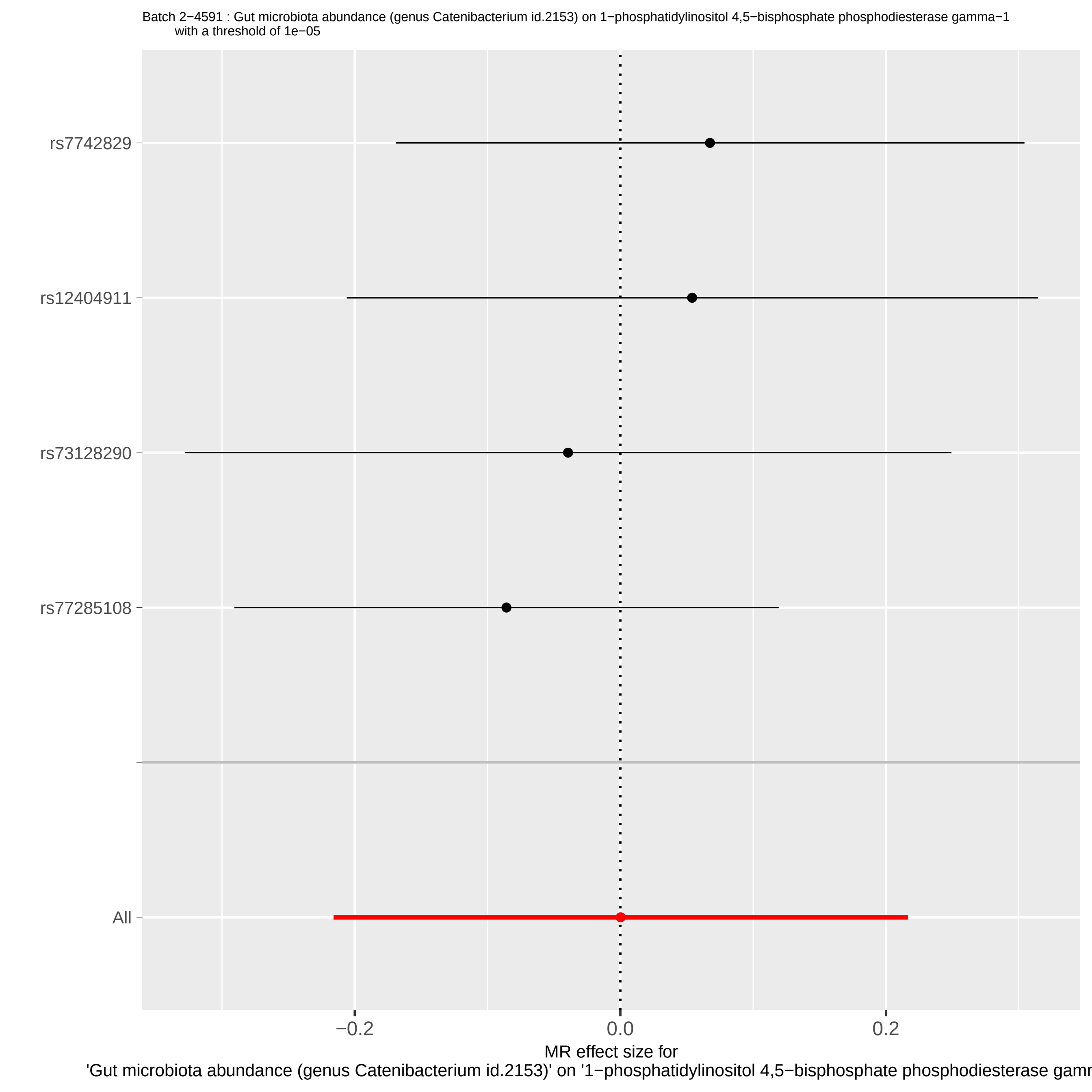

Batch 2−4591 : Gut microbiota abundance (genus Catenibacterium id.2153) on 1−phosphatidylinositol 4,5−bisphosphate phosphodiesterase gamma−1
with a threshold of 1e−05
rs7742829
rs12404911
rs73128290
rs77285108
All
−0.2
0.0
0.2
MR effect size for
'Gut microbiota abundance (genus Catenibacterium id.2153)' on '1−phosphatidylinositol 4,5−bisphosphate phosphodiesterase gamm

Batch 2−4592 : Gut microbiota abundance (genus Parasutterella id.2892) on 1−phosphatidylinositol 4,5−bisphosphate phosphodiesterase gamma−1 with a threshold of 1e−05

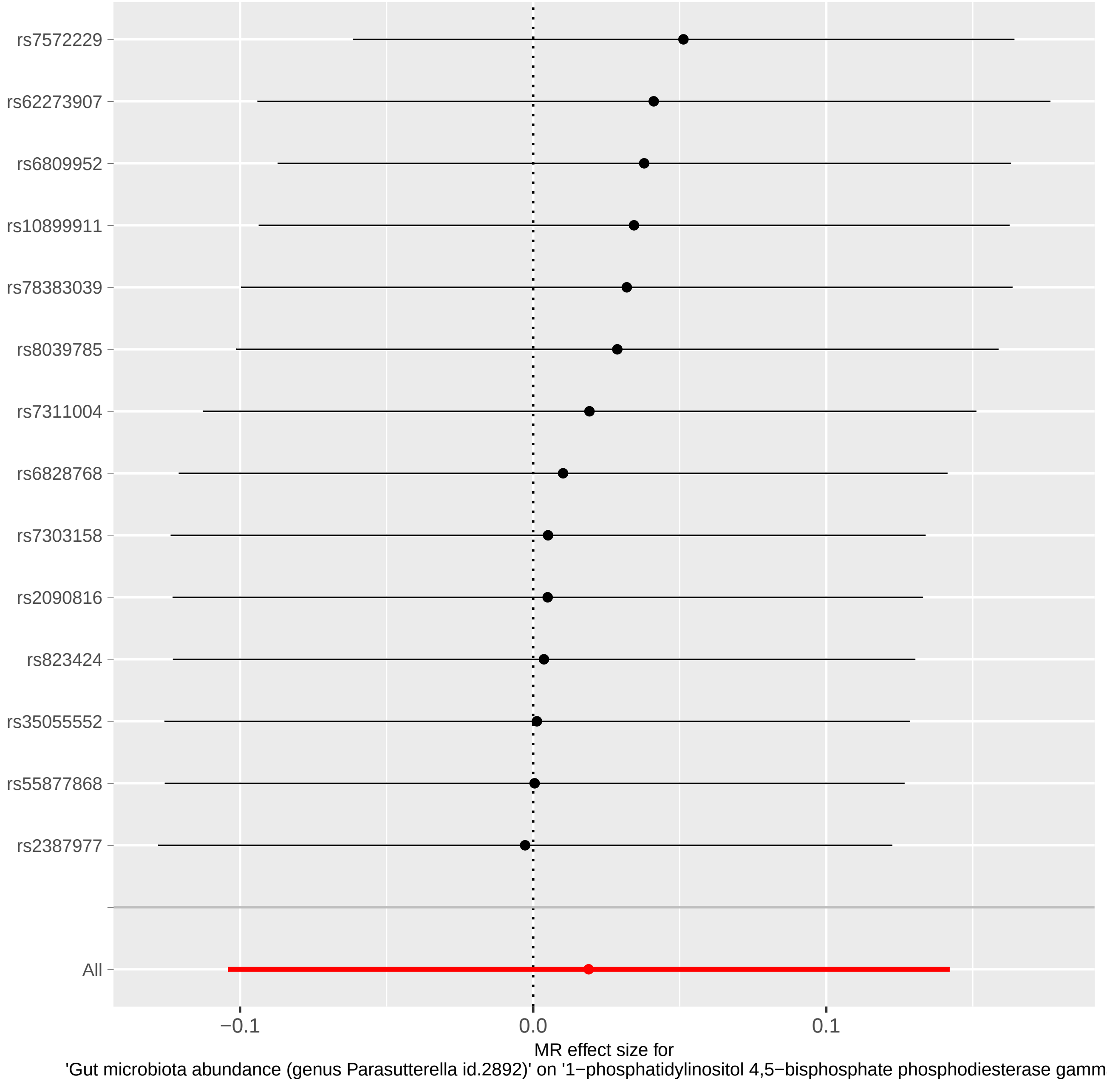

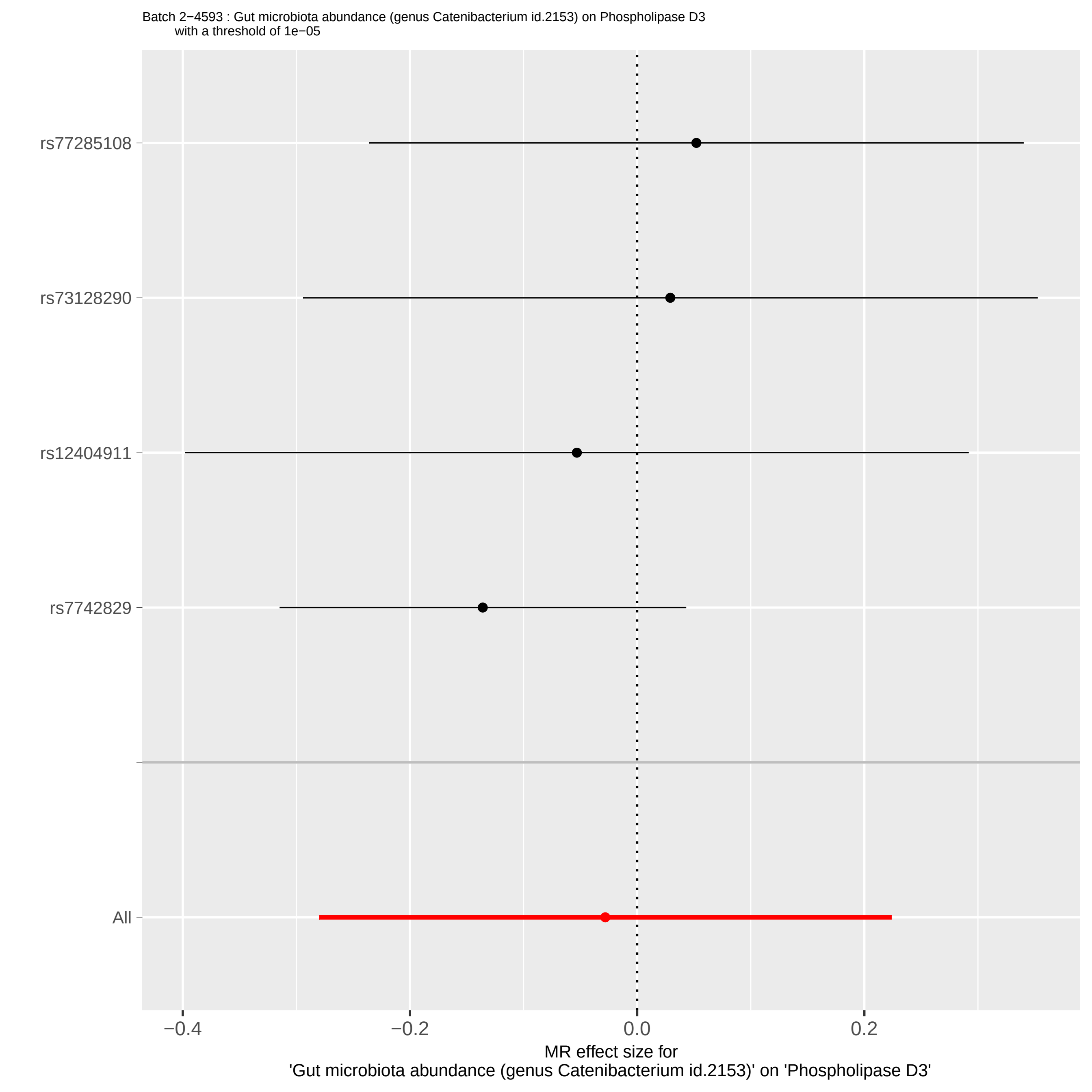
Batch 2−4593 : Gut microbiota abundance (genus Catenibacterium id.2153) on Phospholipase D3
with a threshold of 1e−05
rs77285108
rs73128290
rs12404911
rs7742829
All
−0.4
−0.2
0.0
0.2
MR effect size for
'Gut microbiota abundance (genus Catenibacterium id.2153)' on 'Phospholipase D3'

Batch 2−4594 : Gut microbiota abundance (genus Parasutterella id.2892) on Phospholipase D3
with a threshold of 1e−05

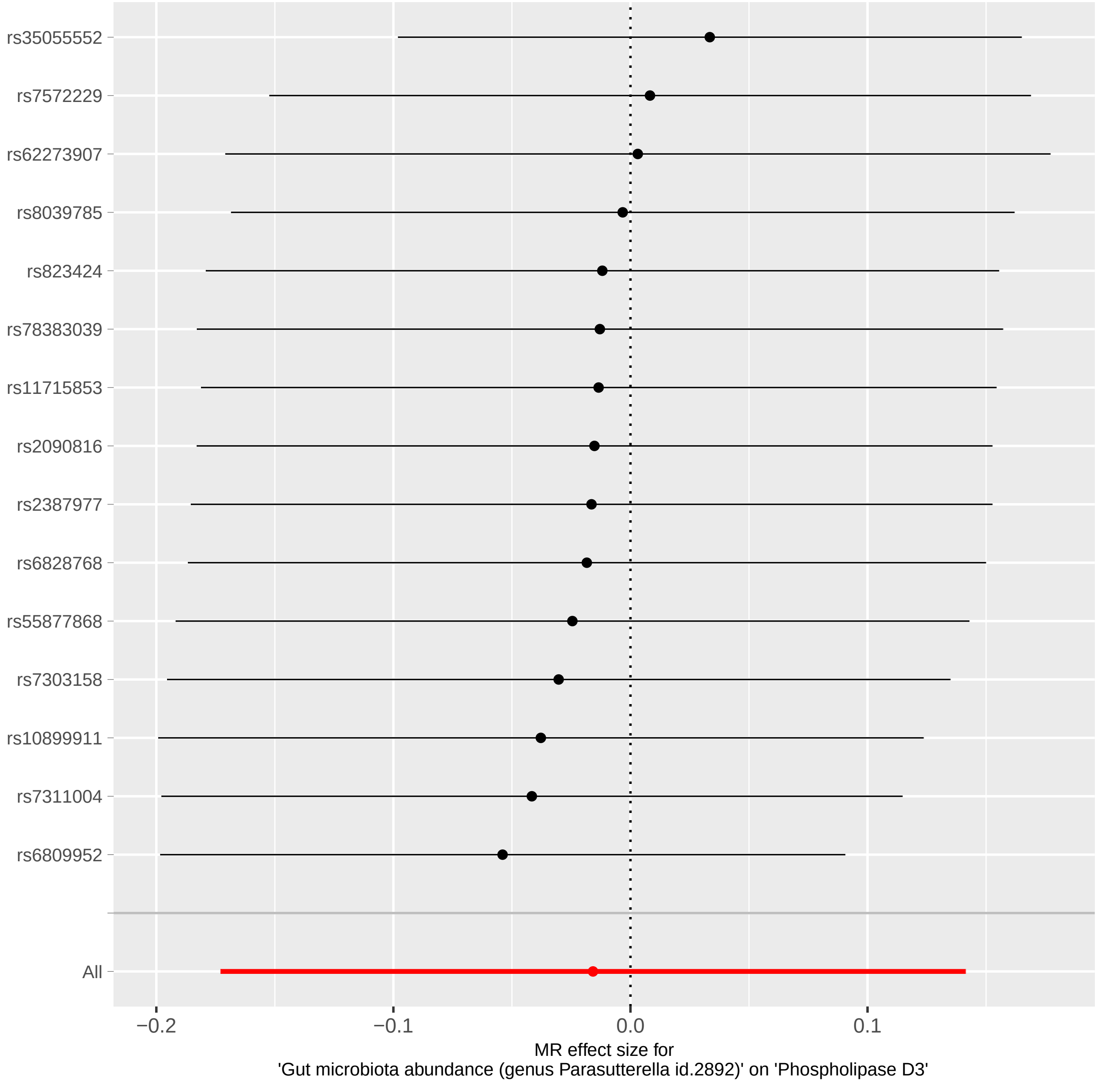

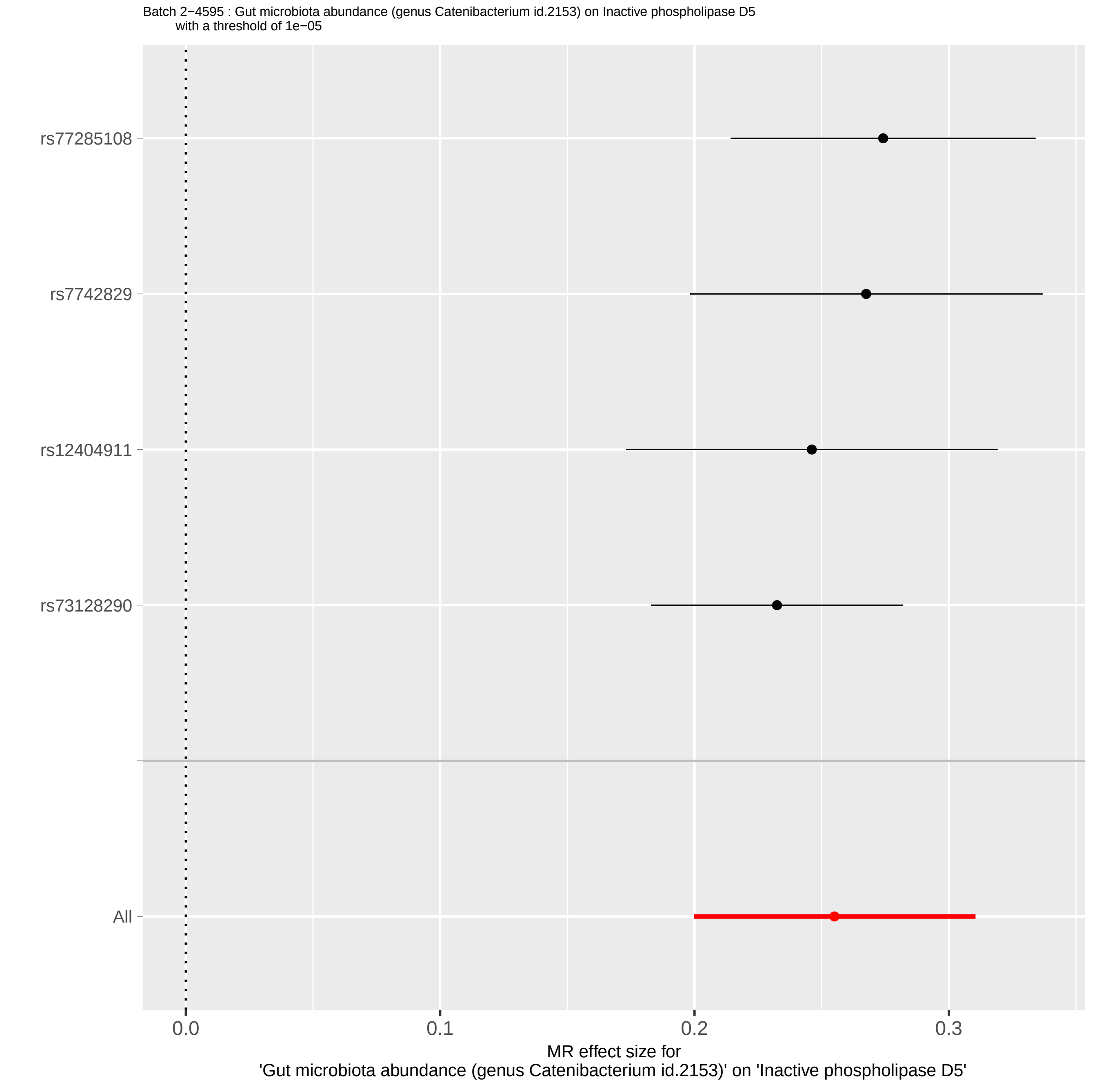
Batch 2−4595 : Gut microbiota abundance (genus Catenibacterium id.2153) on Inactive phospholipase D5
with a threshold of 1e−05
rs77285108
rs7742829
rs12404911
rs73128290
All
0.0
0.1
0.2
0.3
MR effect size for
'Gut microbiota abundance (genus Catenibacterium id.2153)' on 'Inactive phospholipase D5'

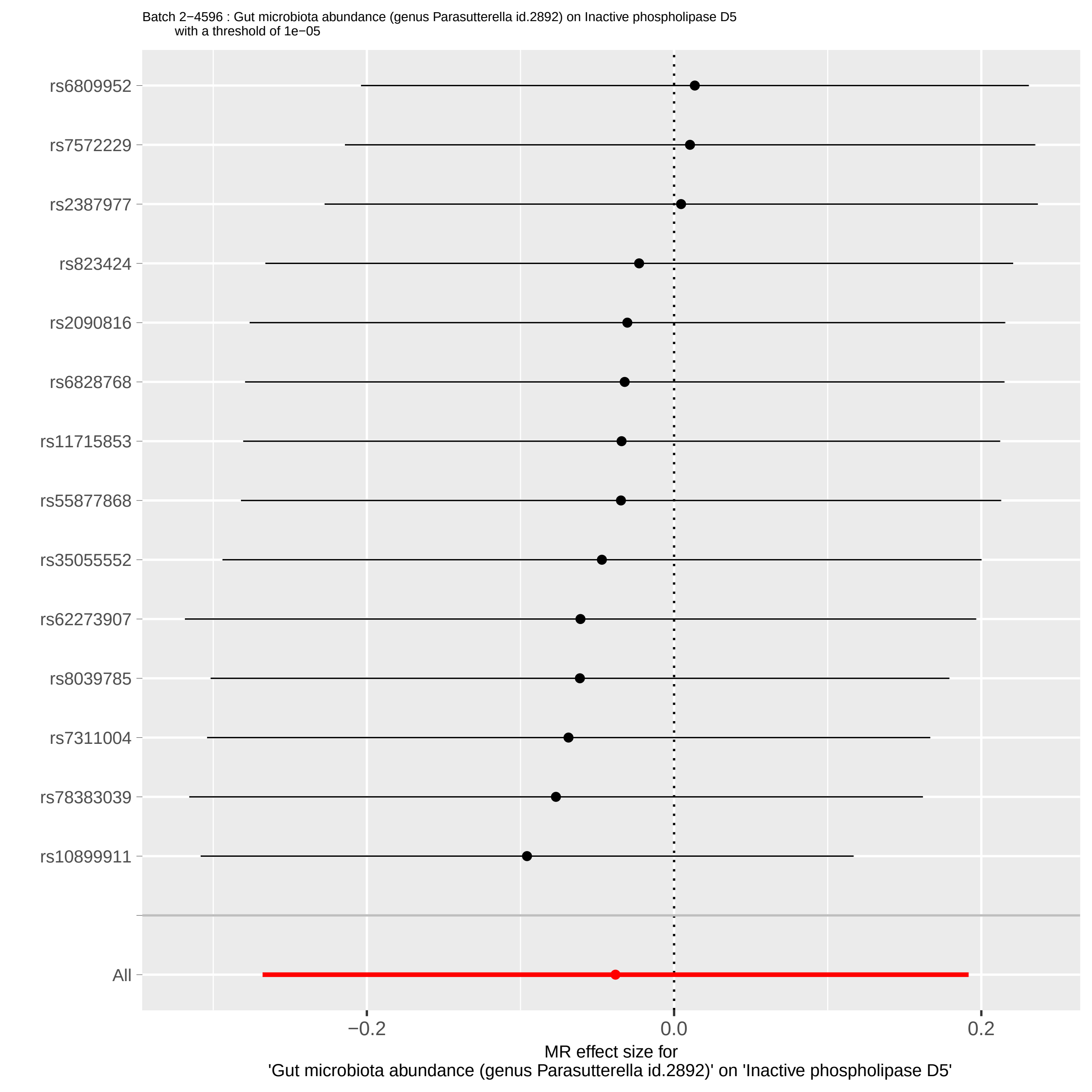

Batch 2−4596 : Gut microbiota abundance (genus Parasutterella id.2892) on Inactive phospholipase D5
with a threshold of 1e−05
rs6809952
rs7572229
rs2387977
rs823424
rs2090816
rs6828768
rs11715853
rs55877868
rs35055552
rs62273907
rs8039785
rs7311004
rs78383039
rs10899911
All
−0.2
0.0
0.2
MR effect size for
'Gut microbiota abundance (genus Parasutterella id.2892)' on 'Inactive phospholipase D5'

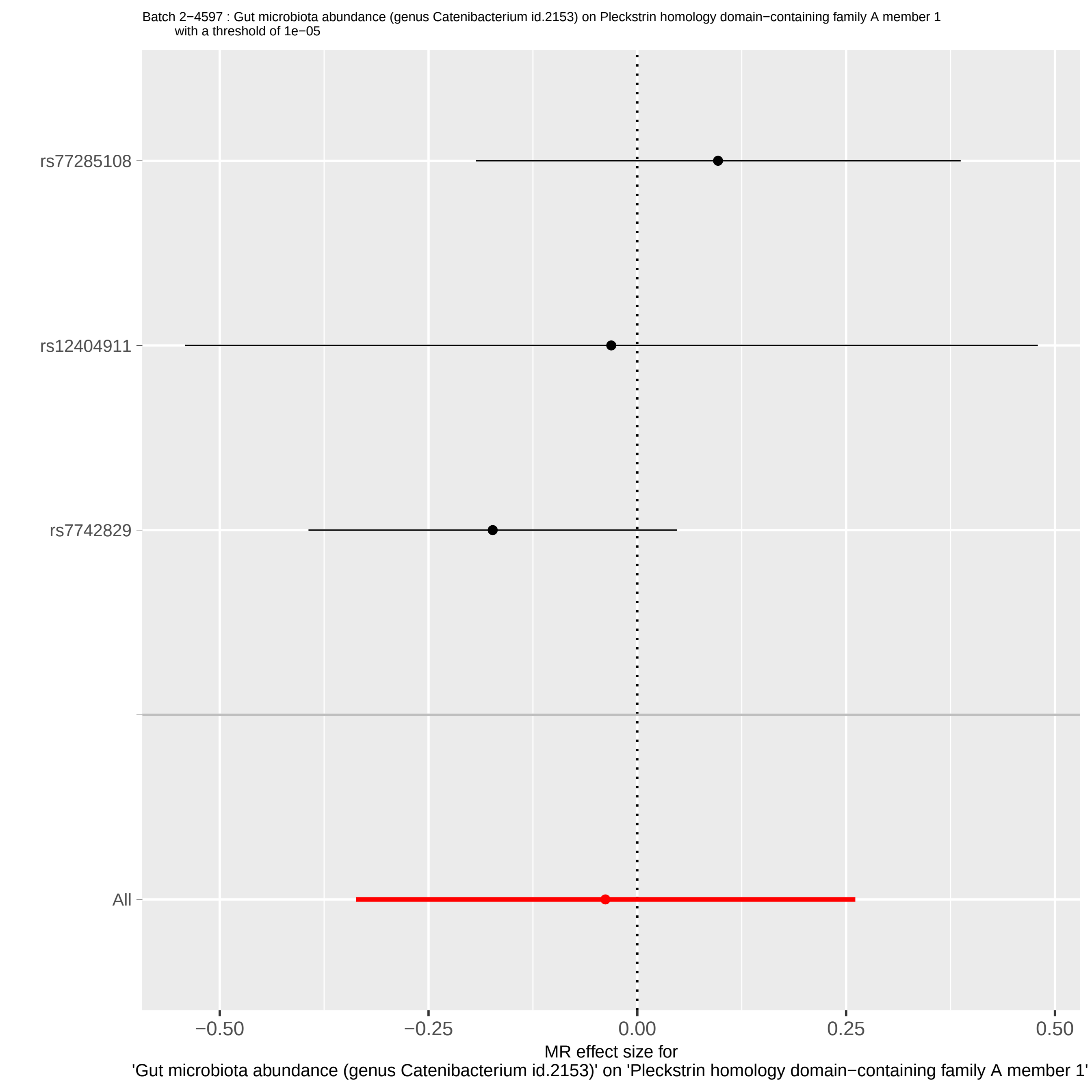

Batch 2−4597 : Gut microbiota abundance (genus Catenibacterium id.2153) on Pleckstrin homology domain−containing family A member 1
with a threshold of 1e−05
rs77285108
rs12404911
rs7742829
All
−0.50
−0.25
0.00
0.25
0.50
MR effect size for
'Gut microbiota abundance (genus Catenibacterium id.2153)' on 'Pleckstrin homology domain−containing family A member 1'

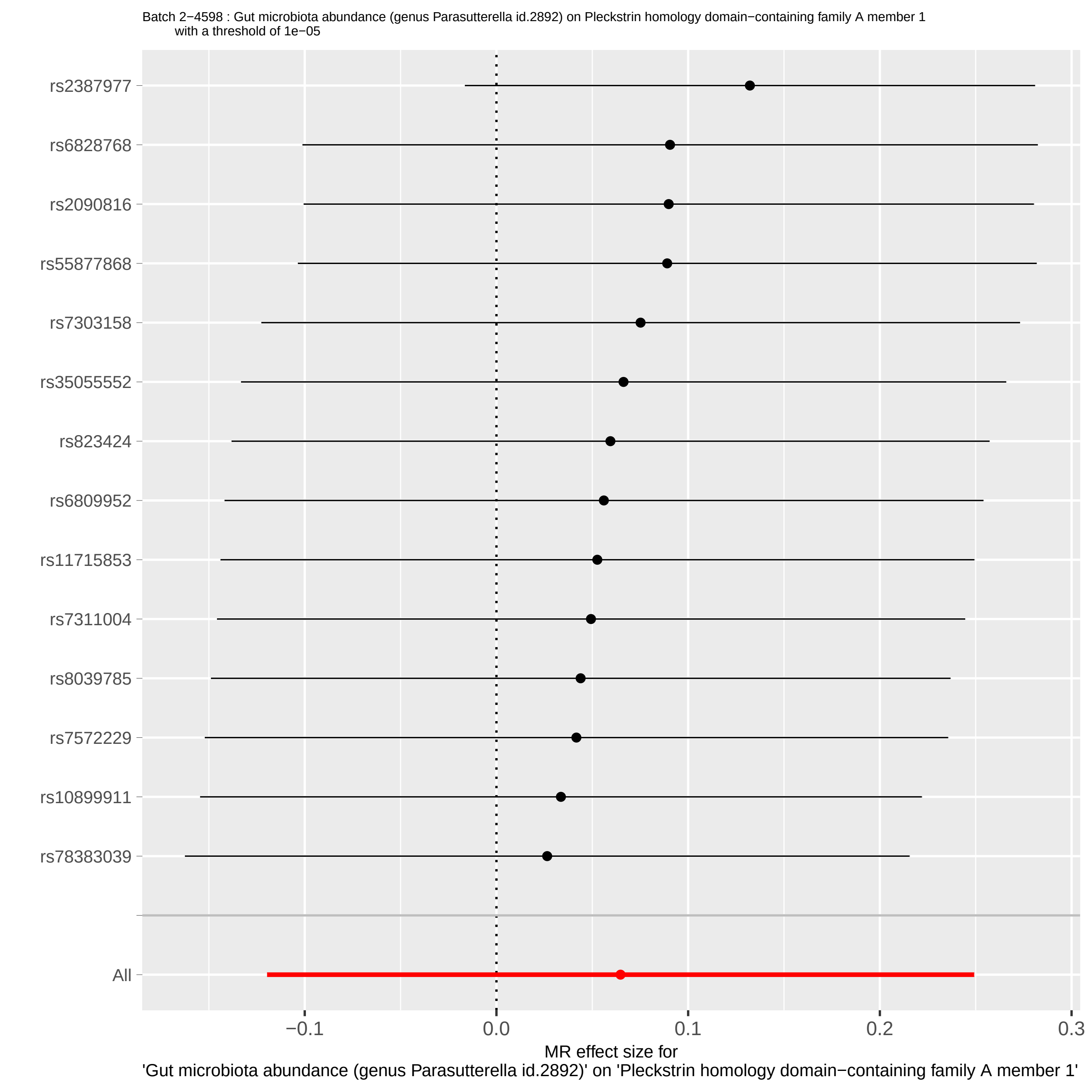

Batch 2−4598 : Gut microbiota abundance (genus Parasutterella id.2892) on Pleckstrin homology domain−containing family A member 1
with a threshold of 1e−05
rs2387977
rs6828768
rs2090816
rs55877868
rs7303158
rs35055552
rs823424
rs6809952
rs11715853
rs7311004
rs8039785
rs7572229
rs10899911
rs78383039
All
−0.1
0.0
0.1
0.2
0.3
MR effect size for
'Gut microbiota abundance (genus Parasutterella id.2892)' on 'Pleckstrin homology domain−containing family A member 1'

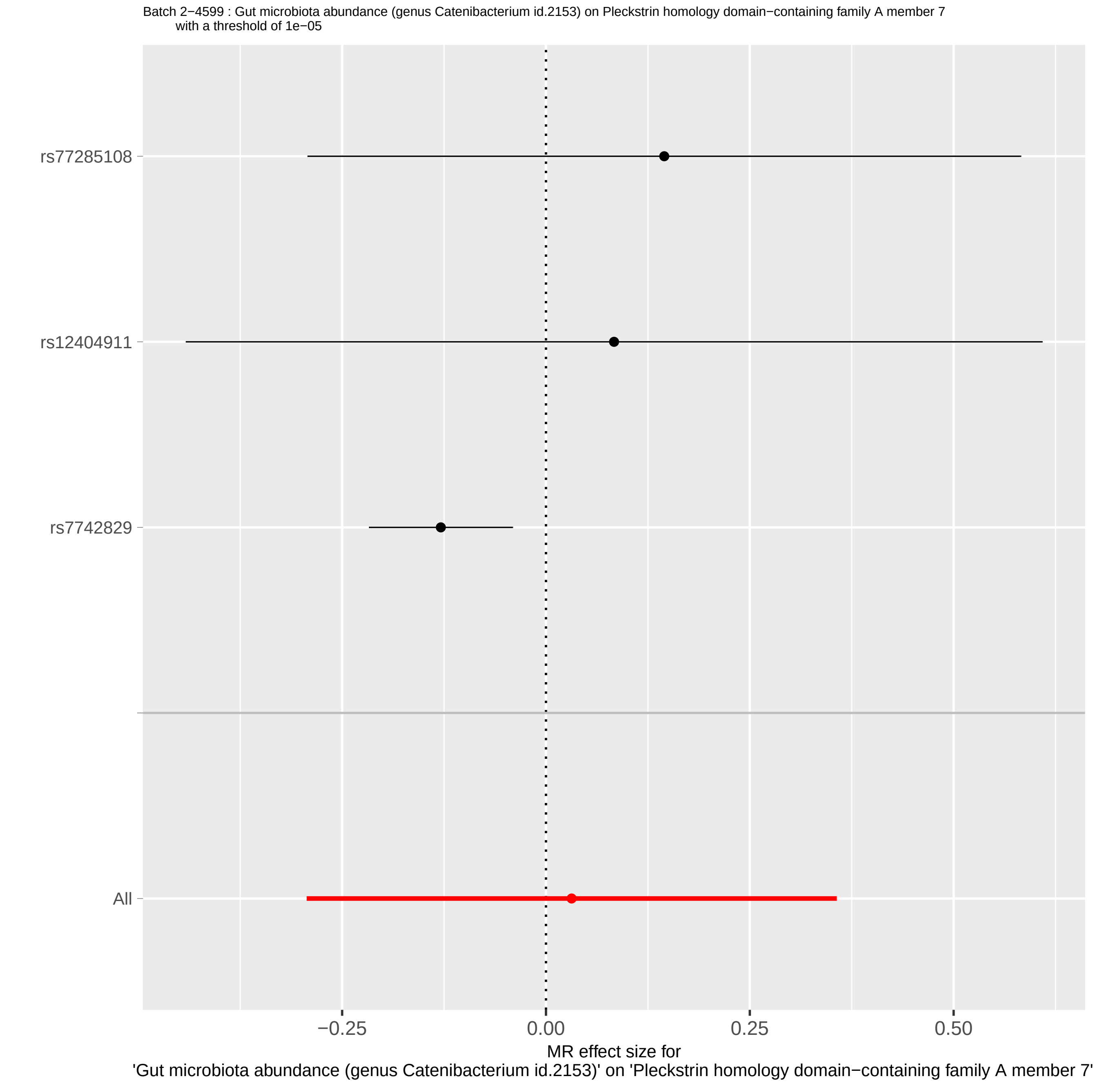

Batch 2−4599 : Gut microbiota abundance (genus Catenibacterium id.2153) on Pleckstrin homology domain−containing family A member 7
with a threshold of 1e−05
rs77285108
rs12404911
rs7742829
All
−0.25
0.00
0.25
0.50
MR effect size for
'Gut microbiota abundance (genus Catenibacterium id.2153)' on 'Pleckstrin homology domain−containing family A member 7'

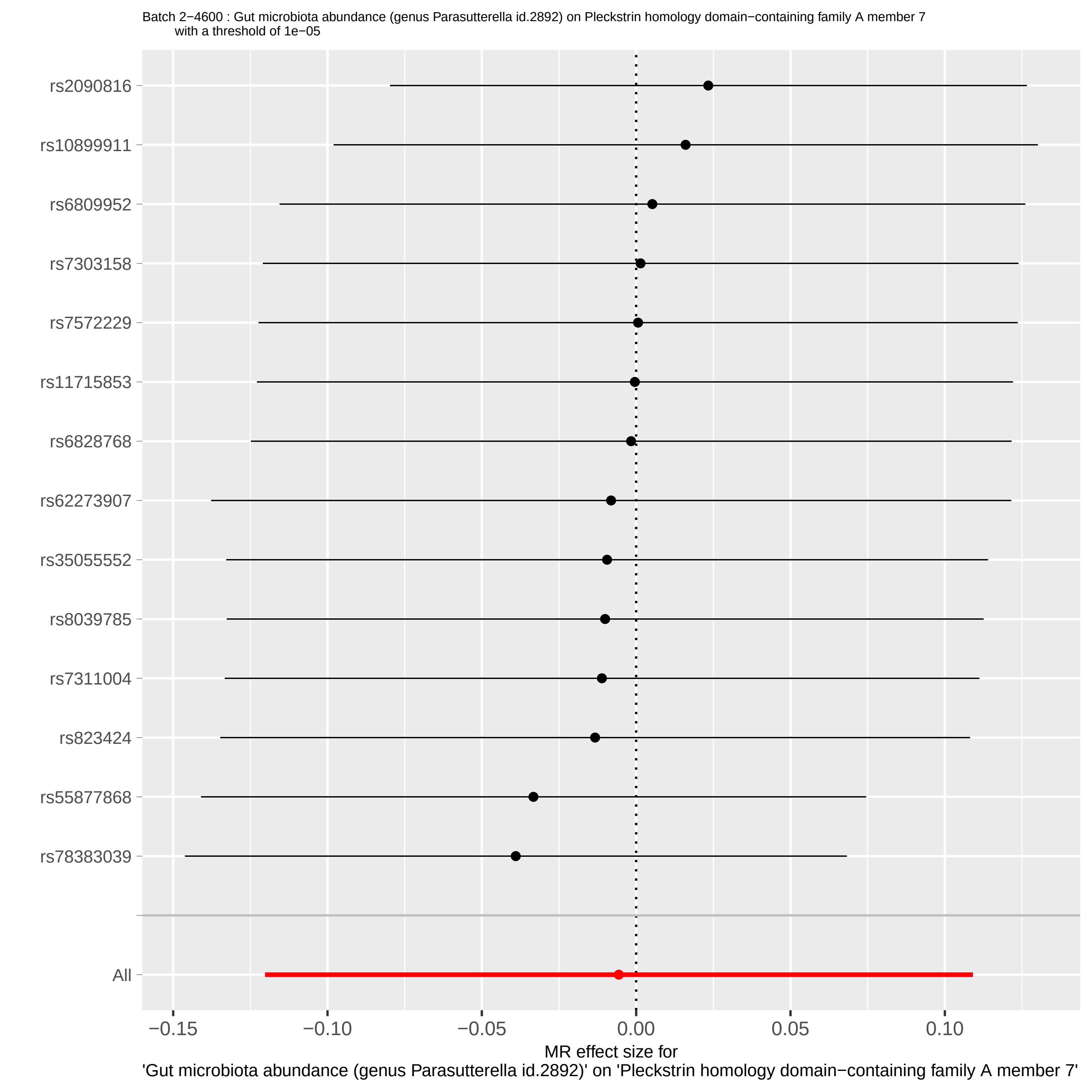

Batch 2−4600 : Gut microbiota abundance (genus Parasutterella id.2892) on Pleckstrin homology domain−containing family A member 7
with a threshold of 1e−05
rs2090816
rs10899911
rs6809952
rs7303158
rs7572229
rs11715853
rs6828768
rs62273907
rs35055552
rs8039785
rs7311004
rs823424
rs55877868
rs78383039
All
−0.15
−0.10
−0.05
0.00
0.05
0.10
MR effect size for
'Gut microbiota abundance (genus Parasutterella id.2892)' on 'Pleckstrin homology domain−containing family A member 7'

Batch 2−4601 : Gut microbiota abundance (genus Catenibacterium id.2153) on Angiostatin
with a threshold of 1e−05

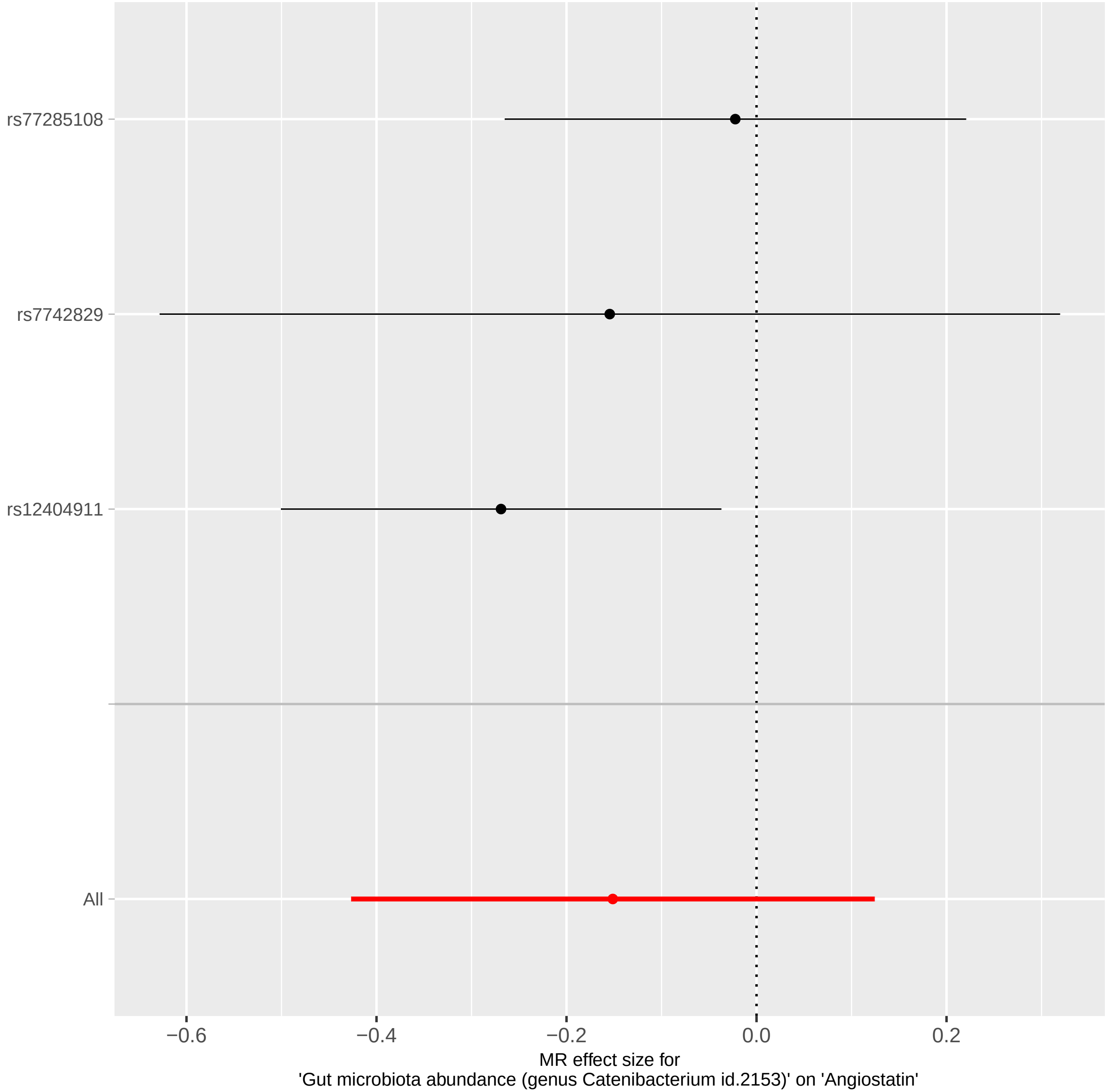

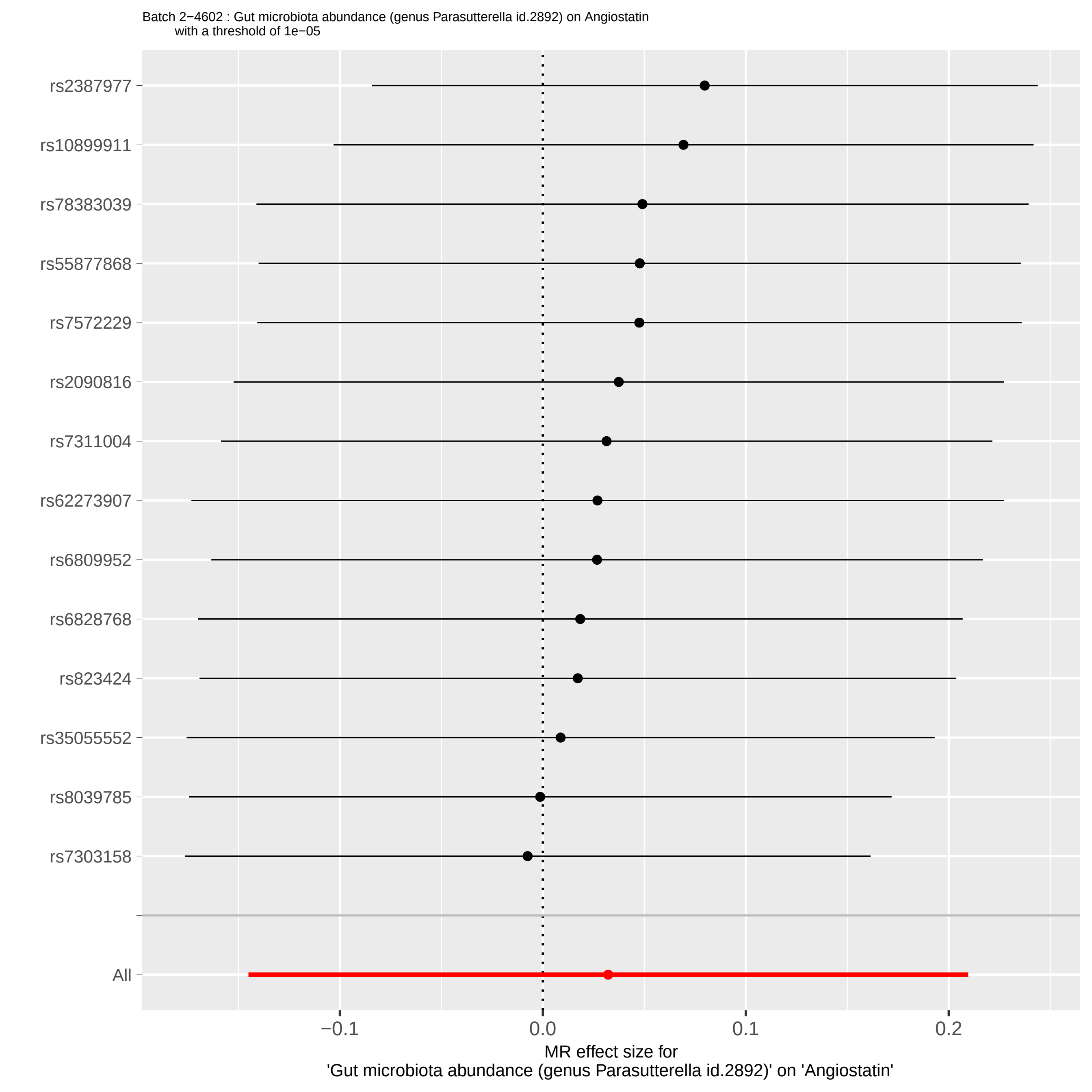

Batch 2−4602 : Gut microbiota abundance (genus Parasutterella id.2892) on Angiostatin
with a threshold of 1e−05
rs2387977
rs10899911
rs78383039
rs55877868
rs7572229
rs2090816
rs7311004
rs62273907
rs6809952
rs6828768
rs823424
rs35055552
rs8039785
rs7303158
All
−0.1
0.0
0.1
0.2
MR effect size for
'Gut microbiota abundance (genus Parasutterella id.2892)' on 'Angiostatin'

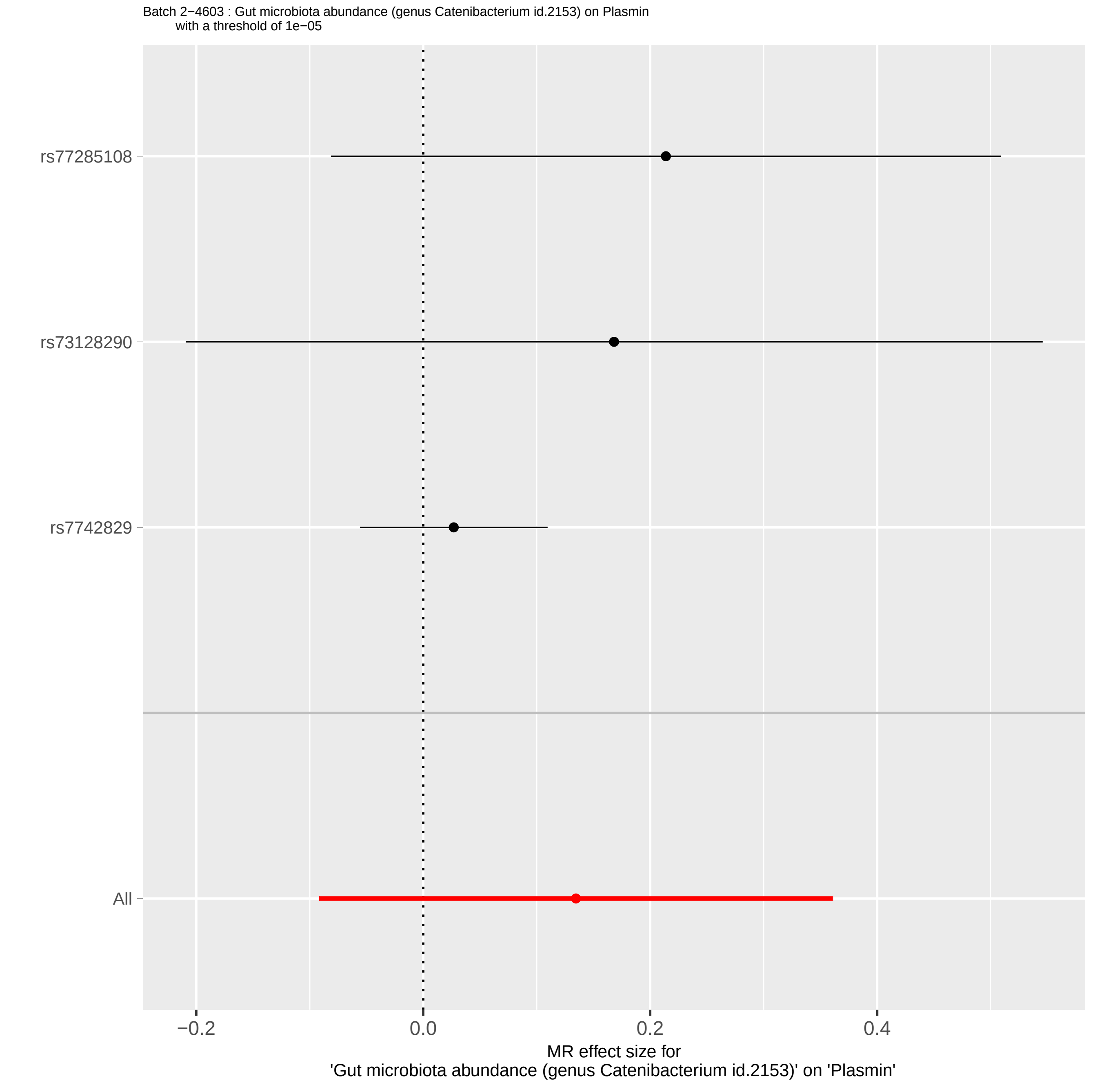
Batch 2−4603 : Gut microbiota abundance (genus Catenibacterium id.2153) on Plasmin
with a threshold of 1e−05
rs77285108
rs73128290
rs7742829
All
−0.2
0.0
0.2
0.4
MR effect size for
'Gut microbiota abundance (genus Catenibacterium id.2153)' on 'Plasmin'

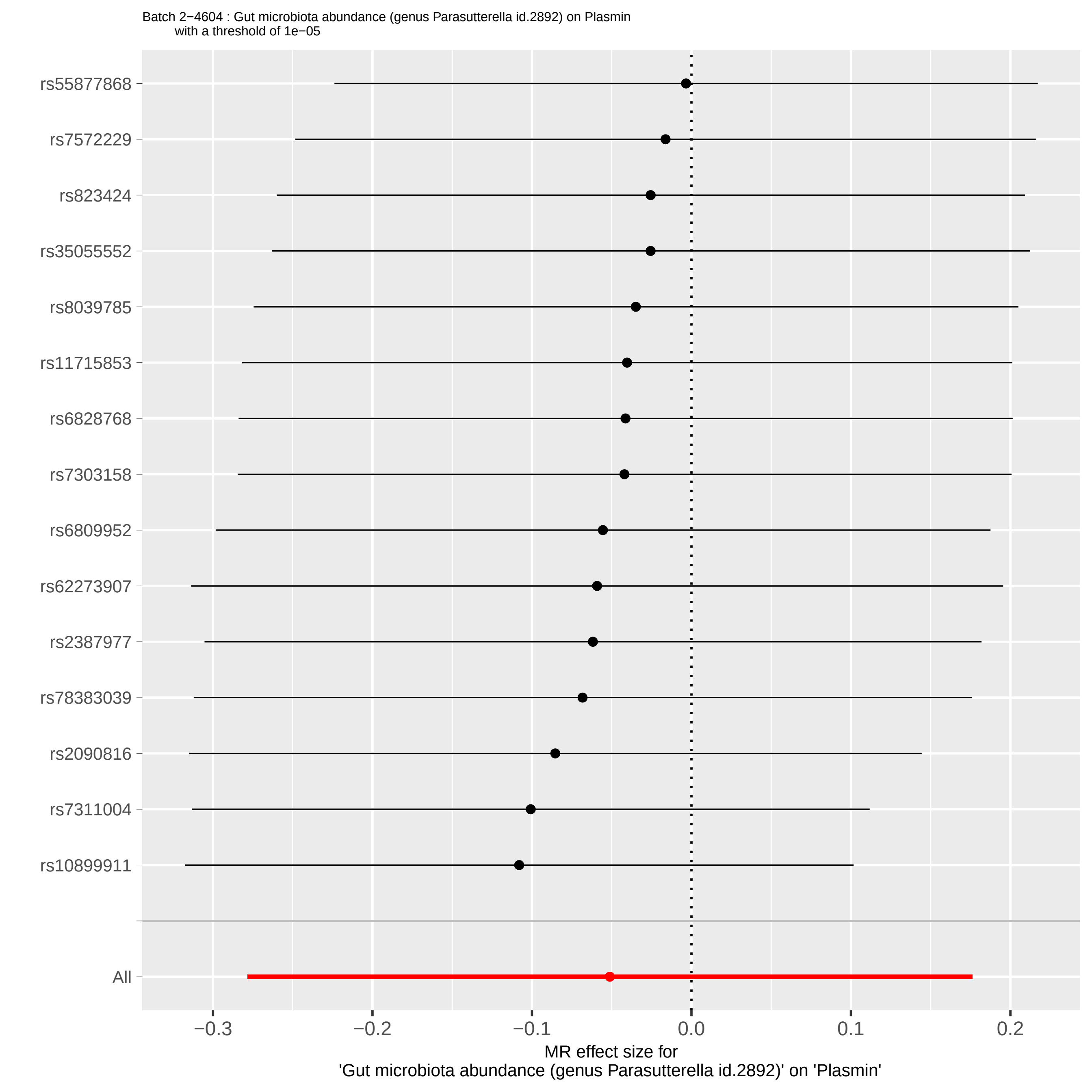

Batch 2−4604 : Gut microbiota abundance (genus Parasutterella id.2892) on Plasmin
with a threshold of 1e−05
rs55877868
rs7572229
rs823424
rs35055552
rs8039785
rs11715853
rs6828768
rs7303158
rs6809952
rs62273907
rs2387977
rs78383039
rs2090816
rs7311004
rs10899911
All
−0.3
−0.2
−0.1
0.0
0.1
0.2
MR effect size for
'Gut microbiota abundance (genus Parasutterella id.2892)' on 'Plasmin'

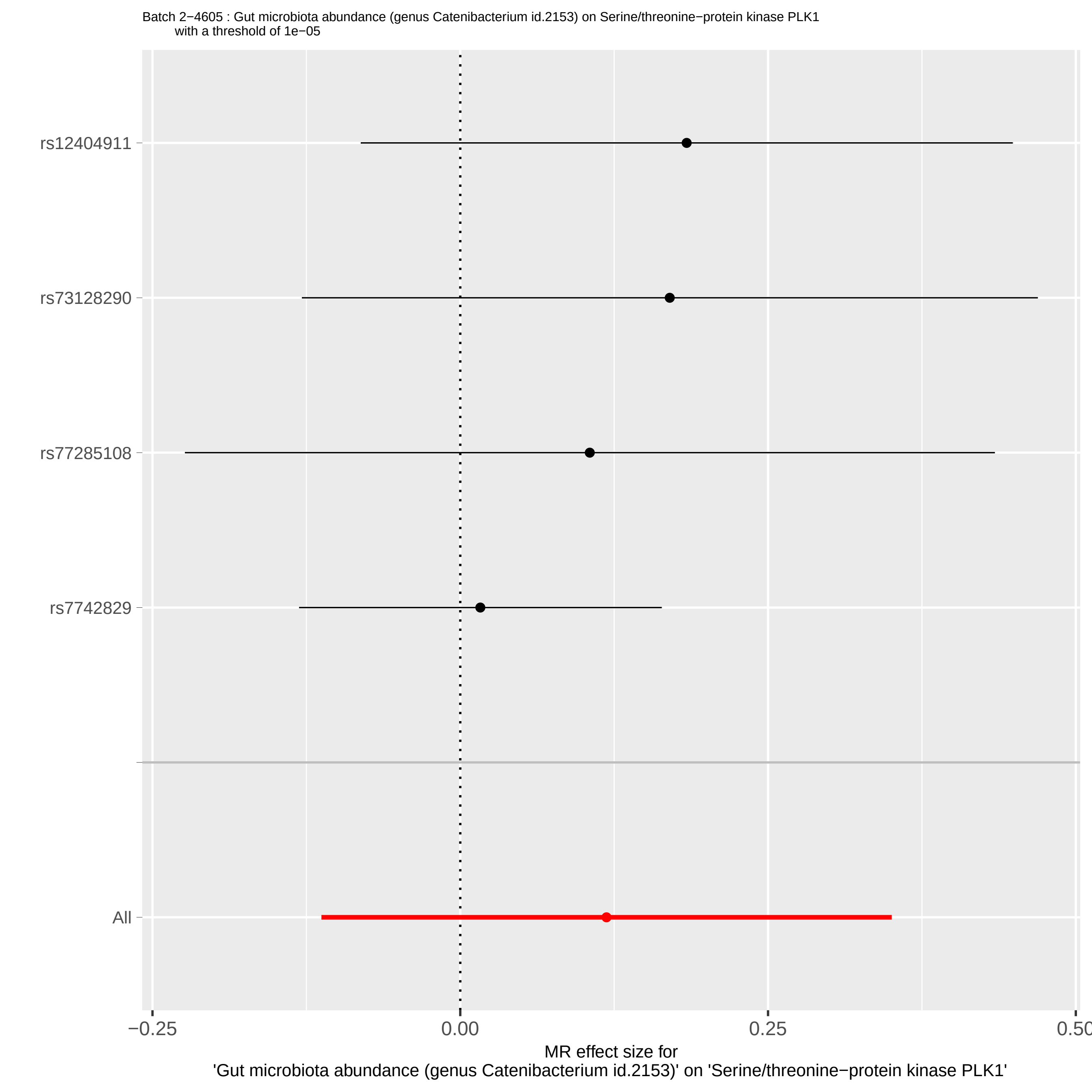
Batch 2−4605 : Gut microbiota abundance (genus Catenibacterium id.2153) on Serine/threonine−protein kinase PLK1
with a threshold of 1e−05
rs12404911
rs73128290
rs77285108
rs7742829
All
−0.25
0.00
0.25
0.50
MR effect size for
'Gut microbiota abundance (genus Catenibacterium id.2153)' on 'Serine/threonine−protein kinase PLK1'

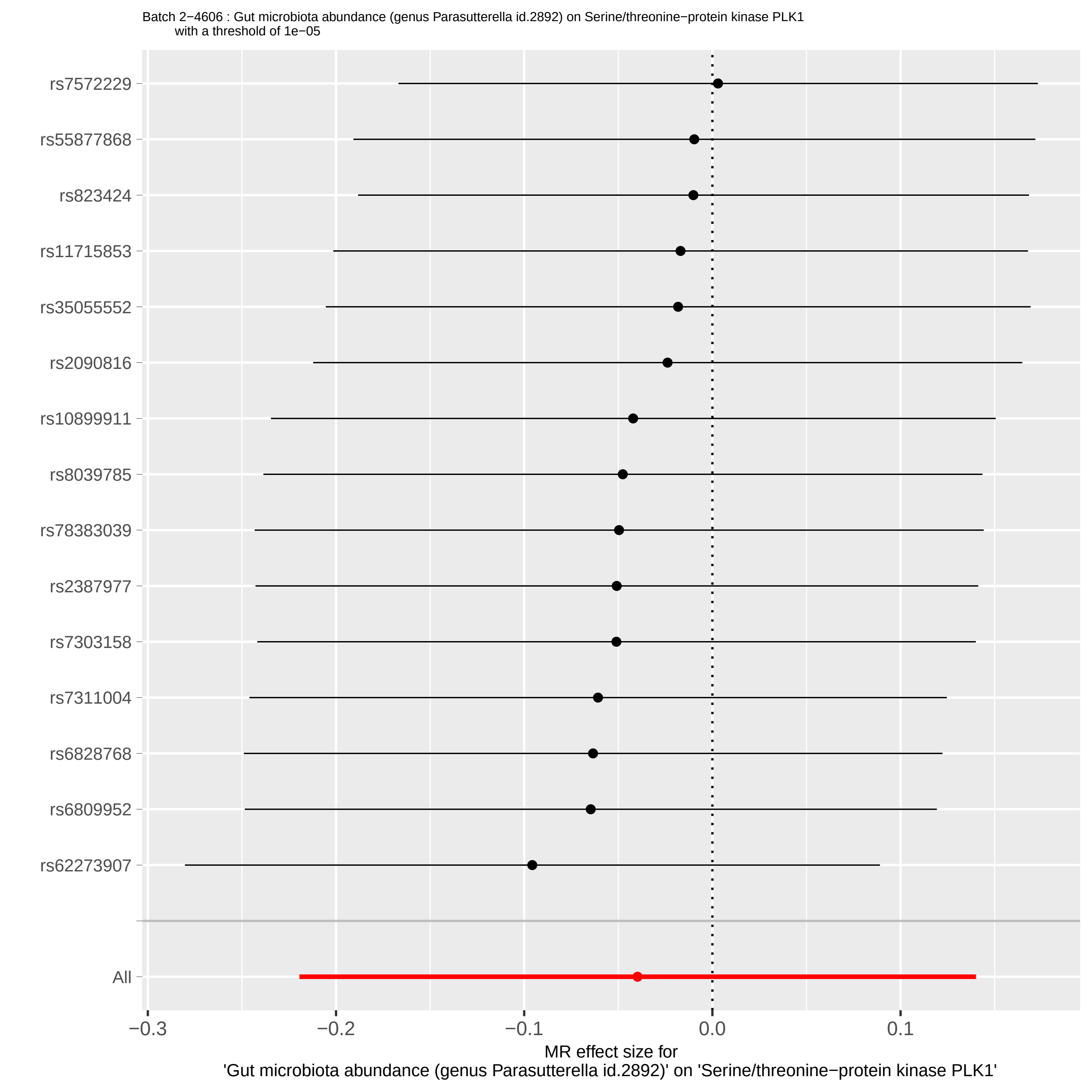

Batch 2−4606 : Gut microbiota abundance (genus Parasutterella id.2892) on Serine/threonine−protein kinase PLK1
with a threshold of 1e−05
rs7572229
rs55877868
rs823424
rs11715853
rs35055552
rs2090816
rs10899911
rs8039785
rs78383039
rs2387977
rs7303158
rs7311004
rs6828768
rs6809952
rs62273907
All
−0.3
−0.2
−0.1
0.0
0.1
MR effect size for
'Gut microbiota abundance (genus Parasutterella id.2892)' on 'Serine/threonine−protein kinase PLK1'

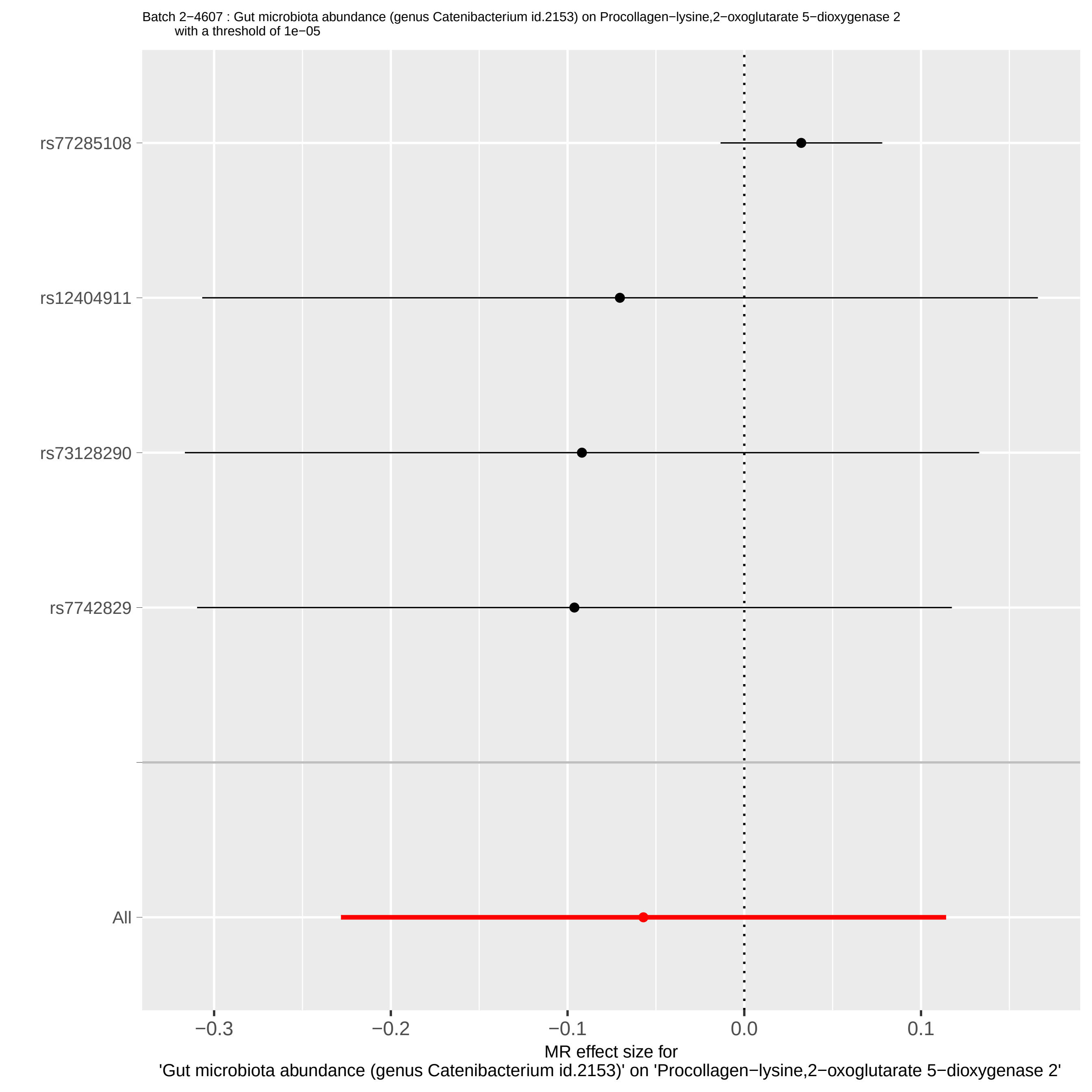

Batch 2−4607 : Gut microbiota abundance (genus Catenibacterium id.2153) on Procollagen−lysine,2−oxoglutarate 5−dioxygenase 2
with a threshold of 1e−05
rs77285108
rs12404911
rs73128290
rs7742829
All
−0.3
−0.2
−0.1
0.0
0.1
MR effect size for
'Gut microbiota abundance (genus Catenibacterium id.2153)' on 'Procollagen−lysine,2−oxoglutarate 5−dioxygenase 2'

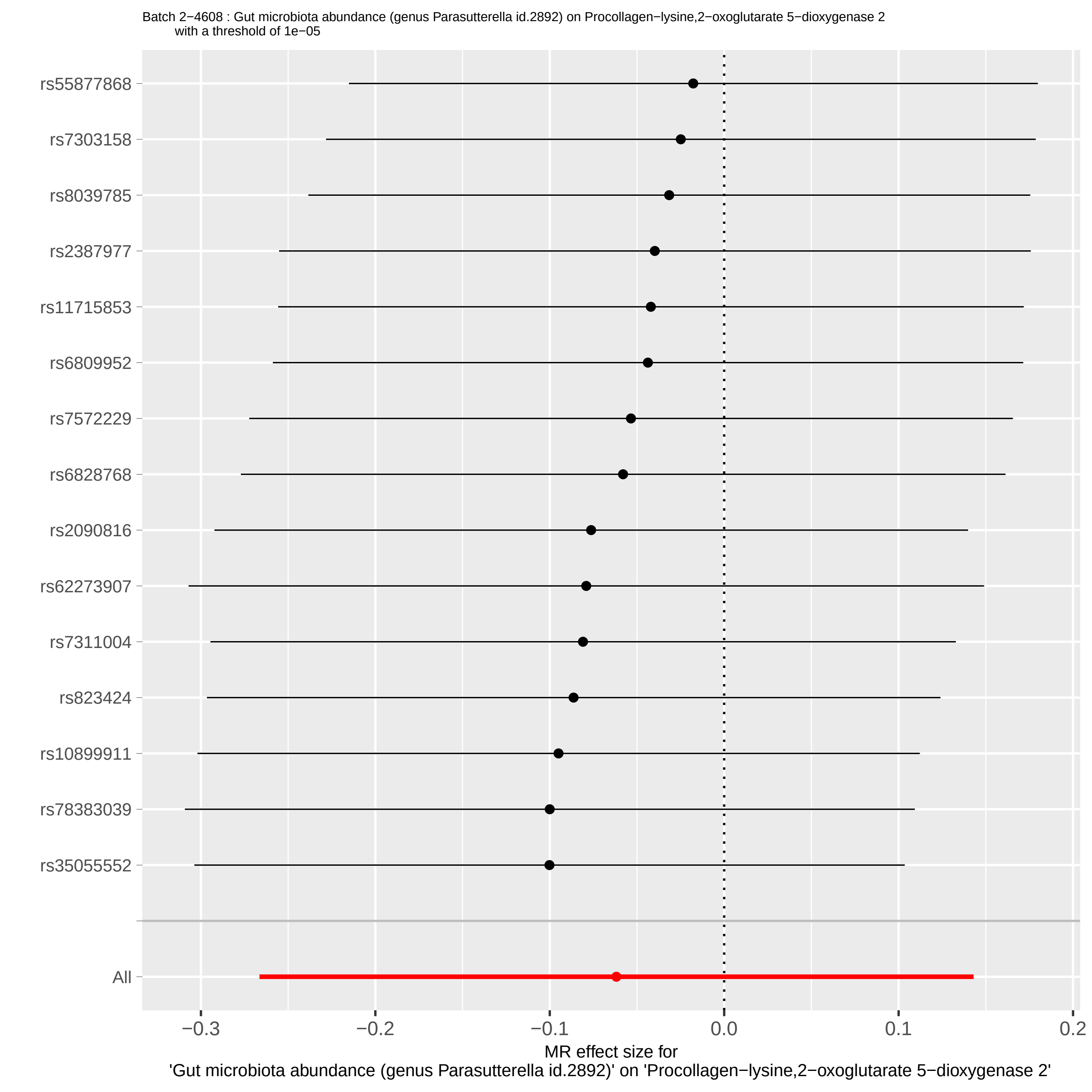

Batch 2−4608 : Gut microbiota abundance (genus Parasutterella id.2892) on Procollagen−lysine,2−oxoglutarate 5−dioxygenase 2
with a threshold of 1e−05
rs55877868
rs7303158
rs8039785
rs2387977
rs11715853
rs6809952
rs7572229
rs6828768
rs2090816
rs62273907
rs7311004
rs823424
rs10899911
rs78383039
rs35055552
All
−0.3
−0.2
−0.1
0.0
0.1
0.2
MR effect size for
'Gut microbiota abundance (genus Parasutterella id.2892)' on 'Procollagen−lysine,2−oxoglutarate 5−dioxygenase 2'

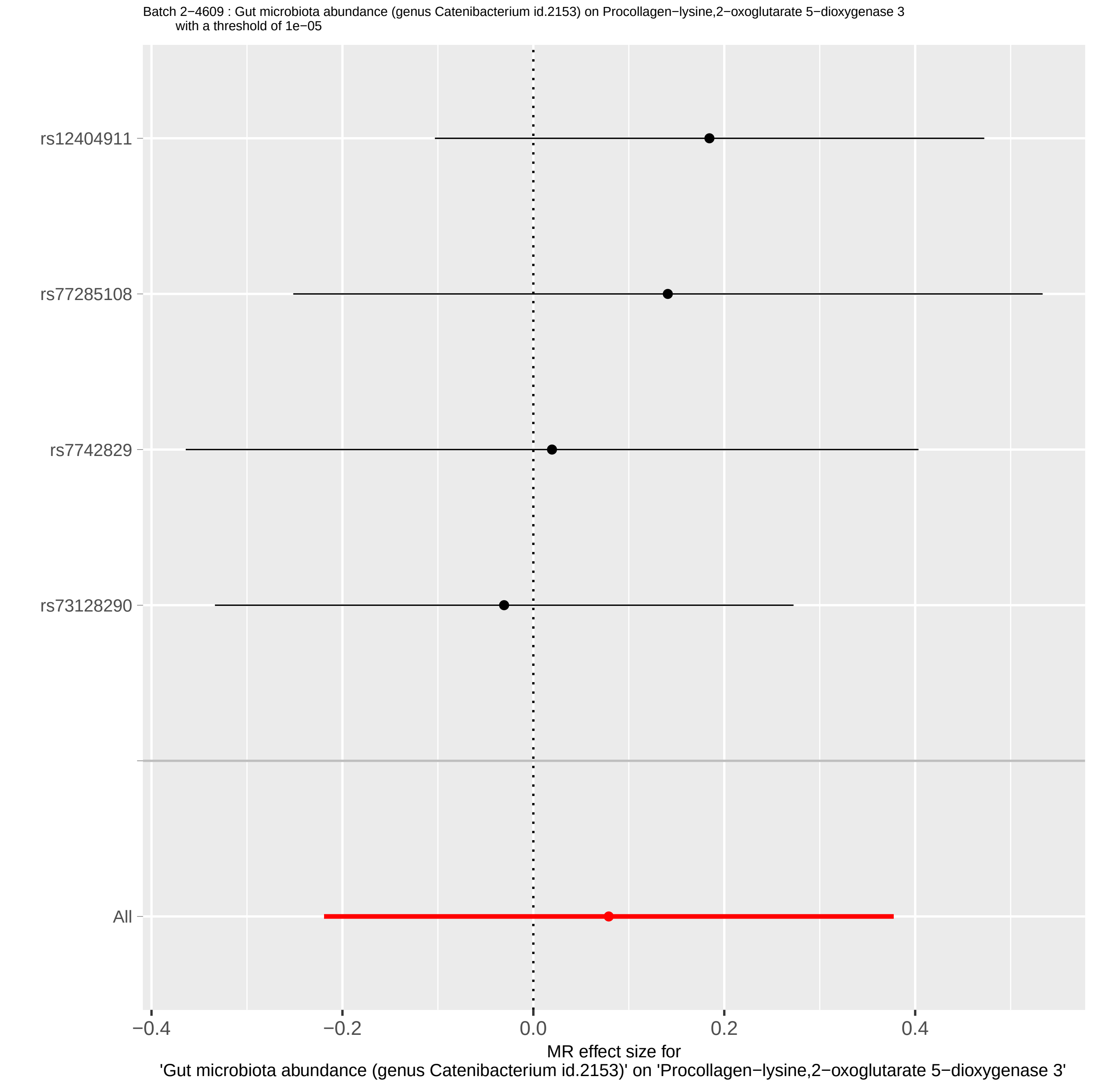
Batch 2−4609 : Gut microbiota abundance (genus Catenibacterium id.2153) on Procollagen−lysine,2−oxoglutarate 5−dioxygenase 3
with a threshold of 1e−05
rs12404911
rs77285108
rs7742829
rs73128290
All
−0.4
−0.2
0.0
0.2
0.4
MR effect size for
'Gut microbiota abundance (genus Catenibacterium id.2153)' on 'Procollagen−lysine,2−oxoglutarate 5−dioxygenase 3'

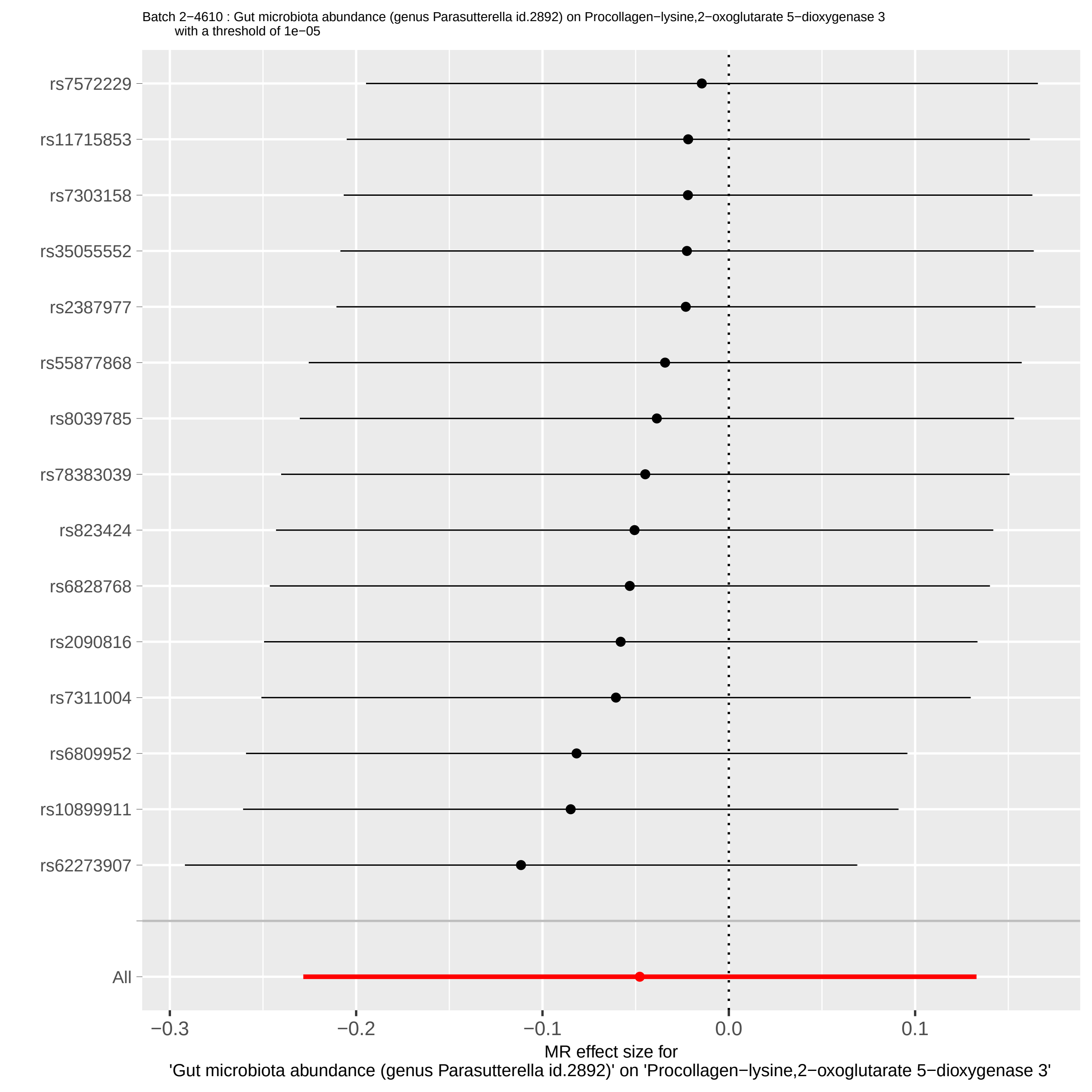

Batch 2−4610 : Gut microbiota abundance (genus Parasutterella id.2892) on Procollagen−lysine,2−oxoglutarate 5−dioxygenase 3
with a threshold of 1e−05
rs7572229
rs11715853
rs7303158
rs35055552
rs2387977
rs55877868
rs8039785
rs78383039
rs823424
rs6828768
rs2090816
rs7311004
rs6809952
rs10899911
rs62273907
All
−0.3
−0.2
−0.1
0.0
0.1
MR effect size for
'Gut microbiota abundance (genus Parasutterella id.2892)' on 'Procollagen−lysine,2−oxoglutarate 5−dioxygenase 3'

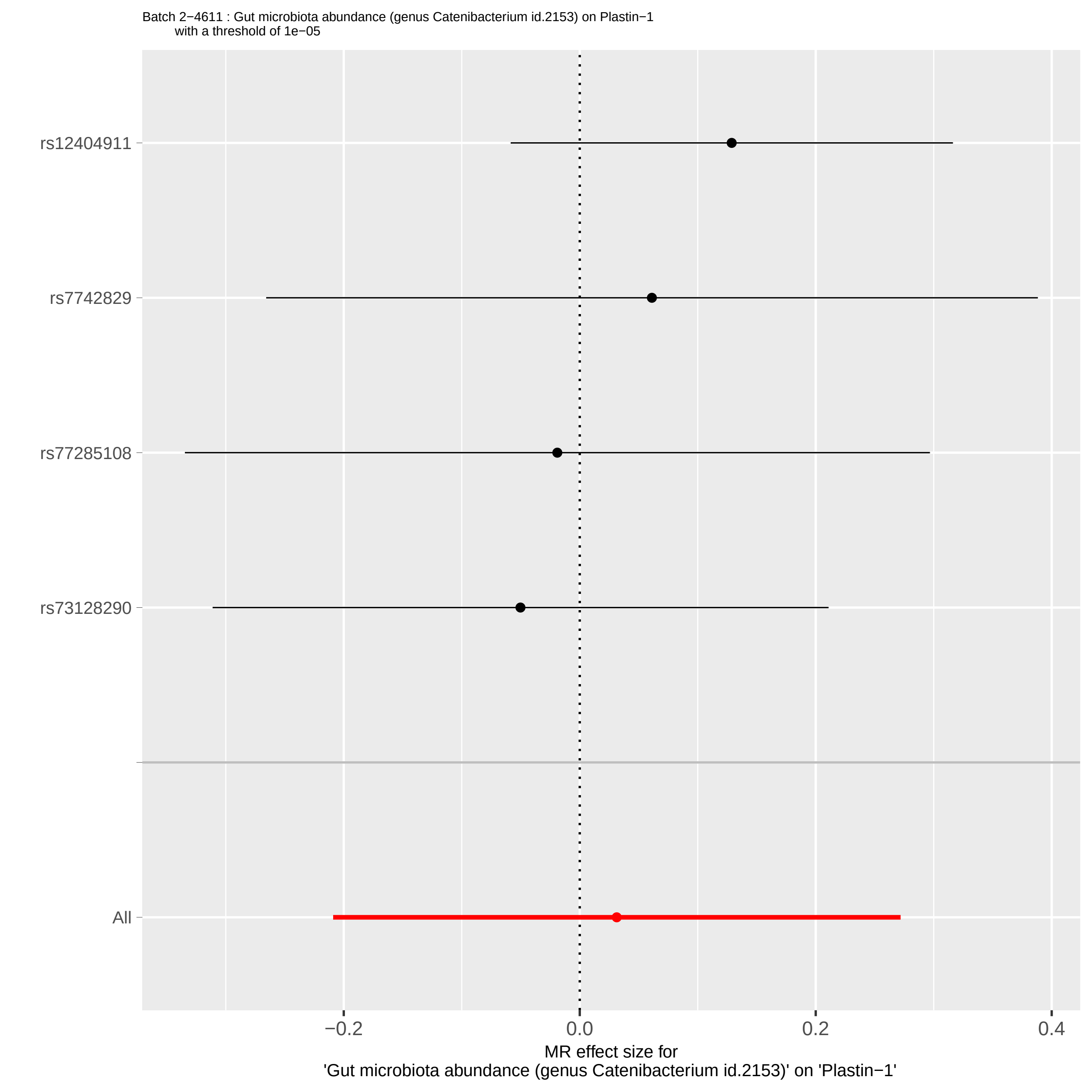

Batch 2−4611 : Gut microbiota abundance (genus Catenibacterium id.2153) on Plastin−1
with a threshold of 1e−05
rs12404911
rs7742829
rs77285108
rs73128290
All
−0.2
0.0
0.2
0.4
MR effect size for
'Gut microbiota abundance (genus Catenibacterium id.2153)' on 'Plastin−1'

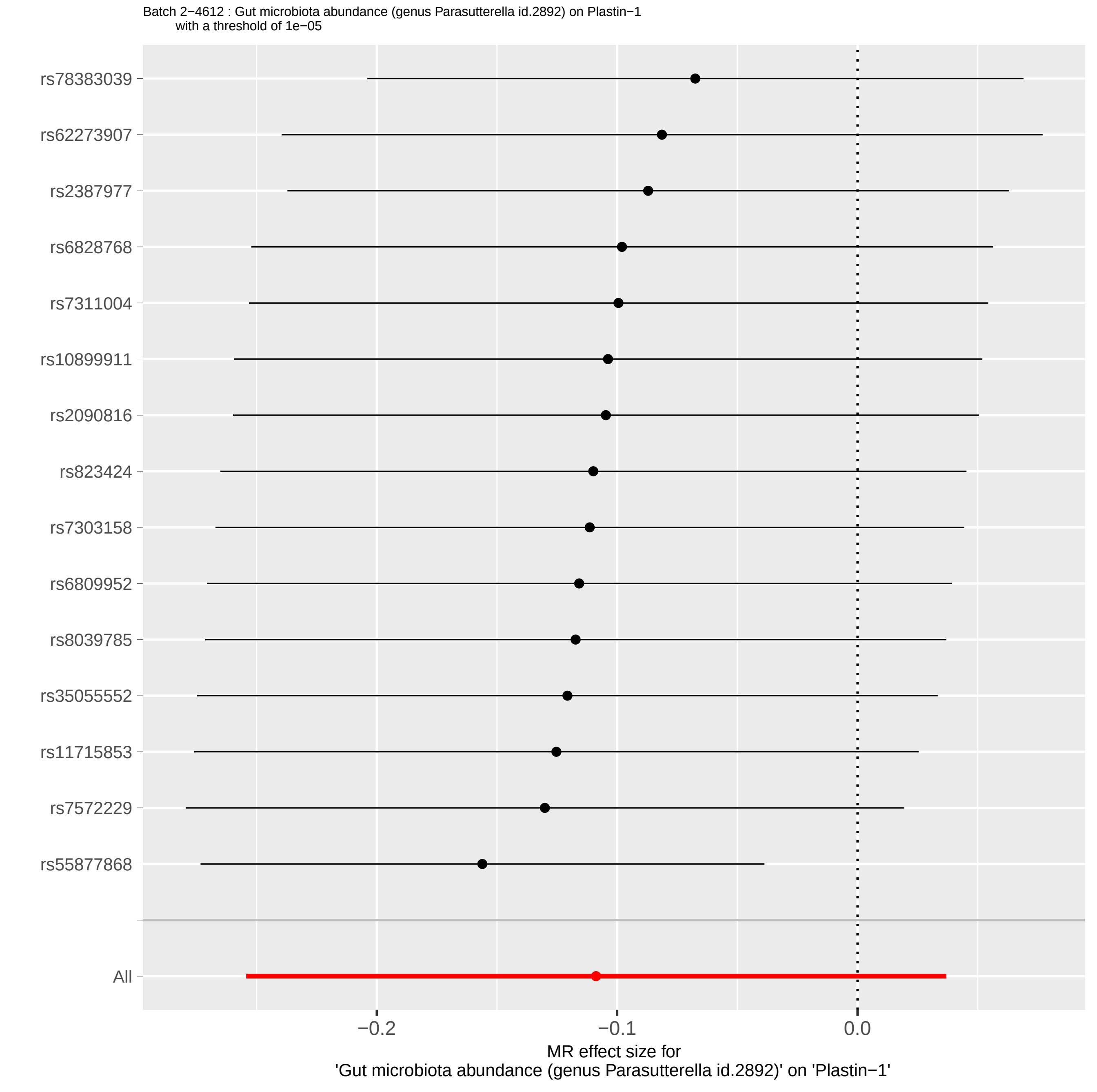
Batch 2−4612 : Gut microbiota abundance (genus Parasutterella id.2892) on Plastin−1
with a threshold of 1e−05
rs78383039
rs62273907
rs2387977
rs6828768
rs7311004
rs10899911
rs2090816
rs823424
rs7303158
rs6809952
rs8039785
rs35055552
rs11715853
rs7572229
rs55877868
All
−0.2
−0.1
0.0
MR effect size for
'Gut microbiota abundance (genus Parasutterella id.2892)' on 'Plastin−1'

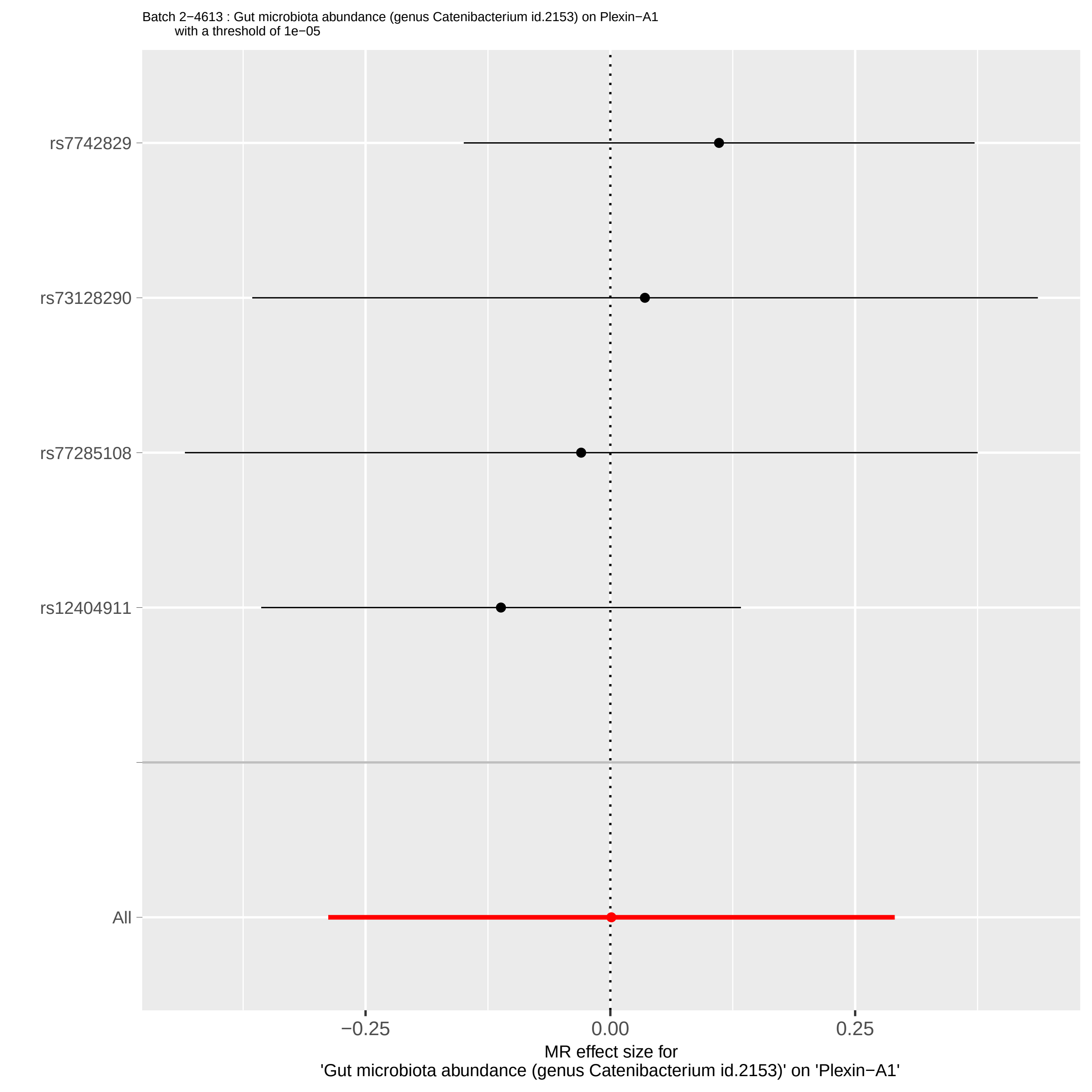

Batch 2−4613 : Gut microbiota abundance (genus Catenibacterium id.2153) on Plexin−A1
with a threshold of 1e−05
rs7742829
rs73128290
rs77285108
rs12404911
All
−0.25
0.00
0.25
MR effect size for
'Gut microbiota abundance (genus Catenibacterium id.2153)' on 'Plexin−A1'

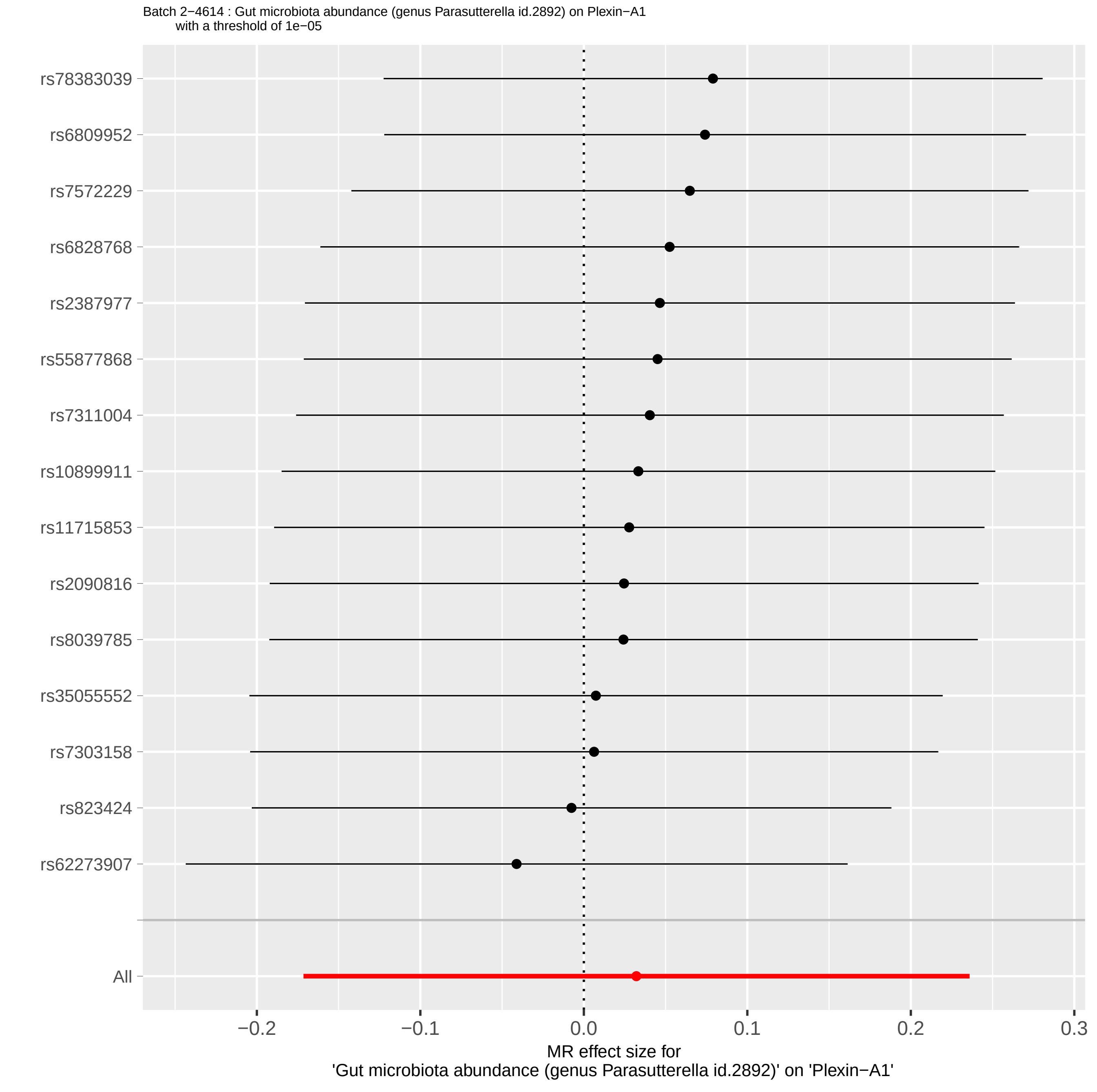
Batch 2−4614 : Gut microbiota abundance (genus Parasutterella id.2892) on Plexin−A1
with a threshold of 1e−05
rs78383039
rs6809952
rs7572229
rs6828768
rs2387977
rs55877868
rs7311004
rs10899911
rs11715853
rs2090816
rs8039785
rs35055552
rs7303158
rs823424
rs62273907
All
−0.2
−0.1
0.0
0.1
0.2
0.3
MR effect size for
'Gut microbiota abundance (genus Parasutterella id.2892)' on 'Plexin−A1'

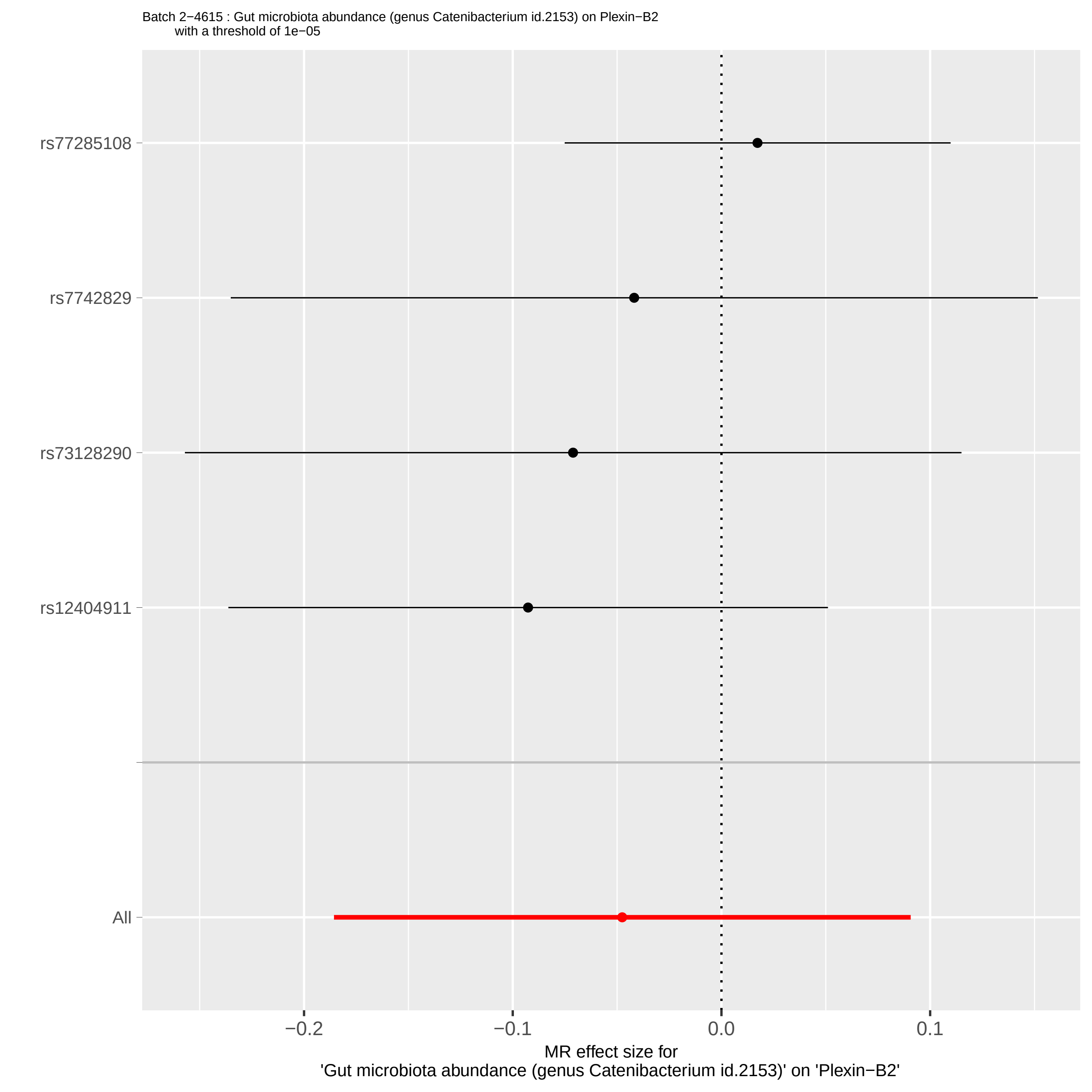
Batch 2−4615 : Gut microbiota abundance (genus Catenibacterium id.2153) on Plexin−B2
with a threshold of 1e−05
rs77285108
rs7742829
rs73128290
rs12404911
All
−0.2
−0.1
0.0
0.1
MR effect size for
'Gut microbiota abundance (genus Catenibacterium id.2153)' on 'Plexin−B2'

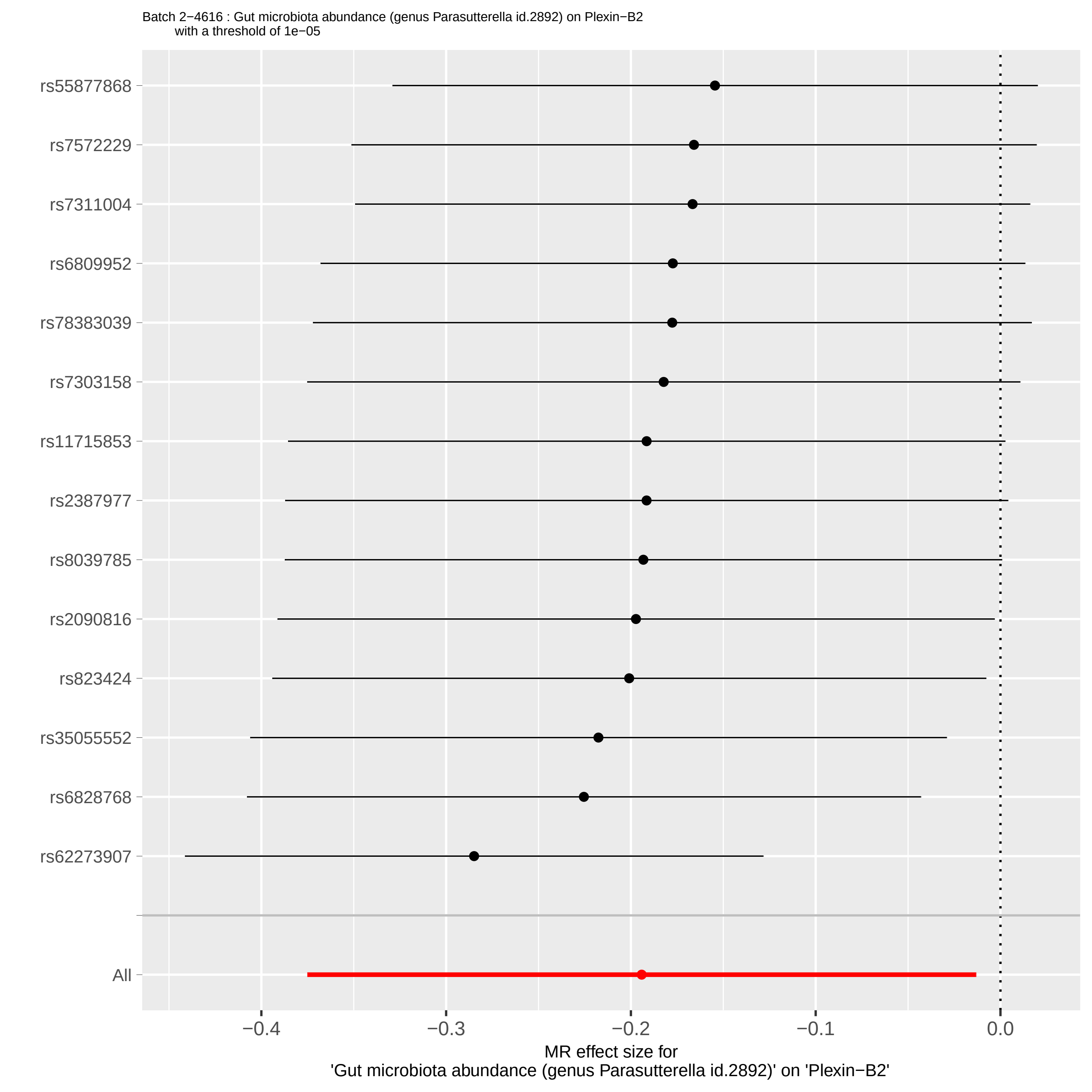

Batch 2−4616 : Gut microbiota abundance (genus Parasutterella id.2892) on Plexin−B2
with a threshold of 1e−05
rs55877868
rs7572229
rs7311004
rs6809952
rs78383039
rs7303158
rs11715853
rs2387977
rs8039785
rs2090816
rs823424
rs35055552
rs6828768
rs62273907
All
−0.4
−0.3
−0.2
−0.1
0.0
MR effect size for
'Gut microbiota abundance (genus Parasutterella id.2892)' on 'Plexin−B2'

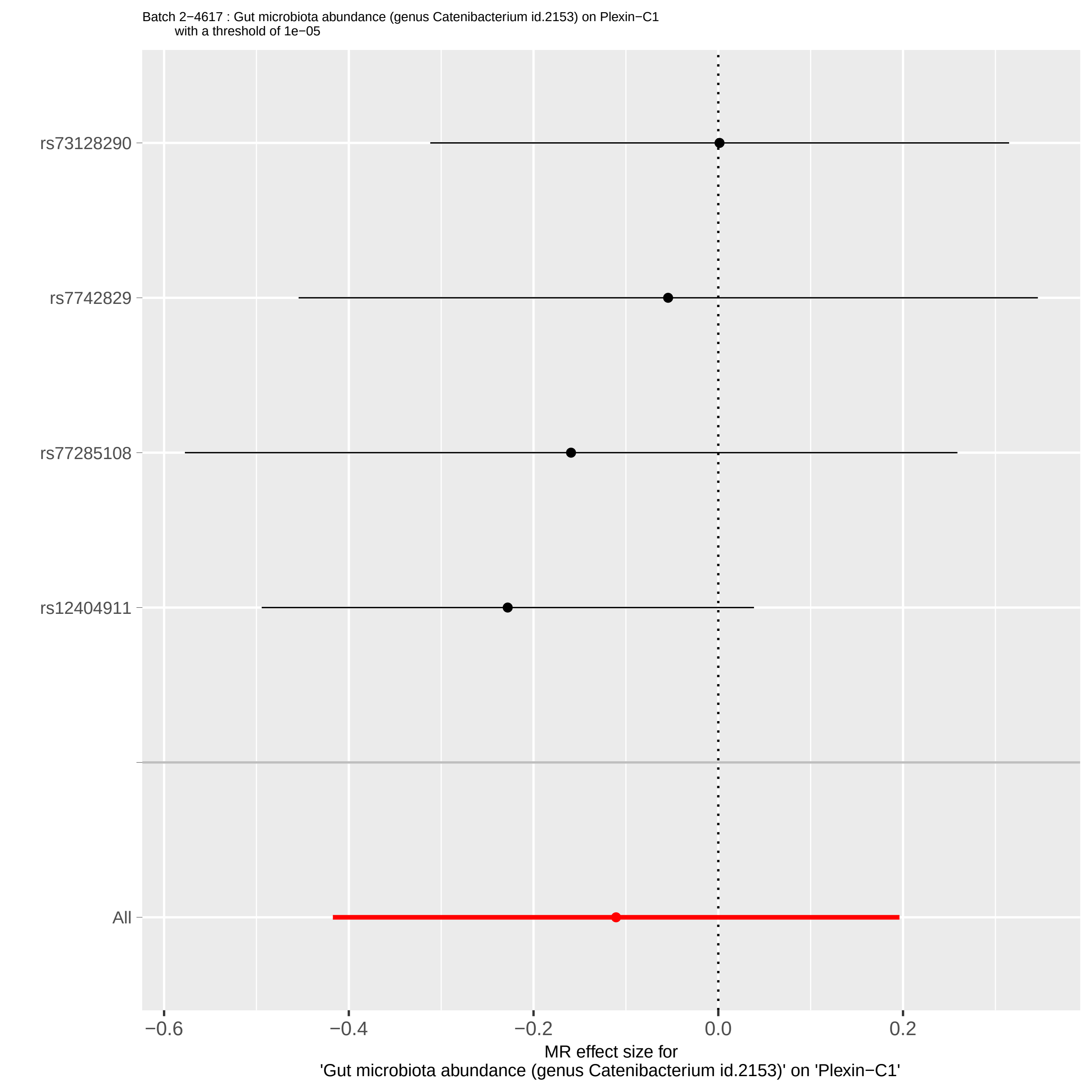
Batch 2−4617 : Gut microbiota abundance (genus Catenibacterium id.2153) on Plexin−C1
with a threshold of 1e−05
rs73128290
rs7742829
rs77285108
rs12404911
All
−0.6
−0.4
−0.2
0.0
0.2
MR effect size for
'Gut microbiota abundance (genus Catenibacterium id.2153)' on 'Plexin−C1'

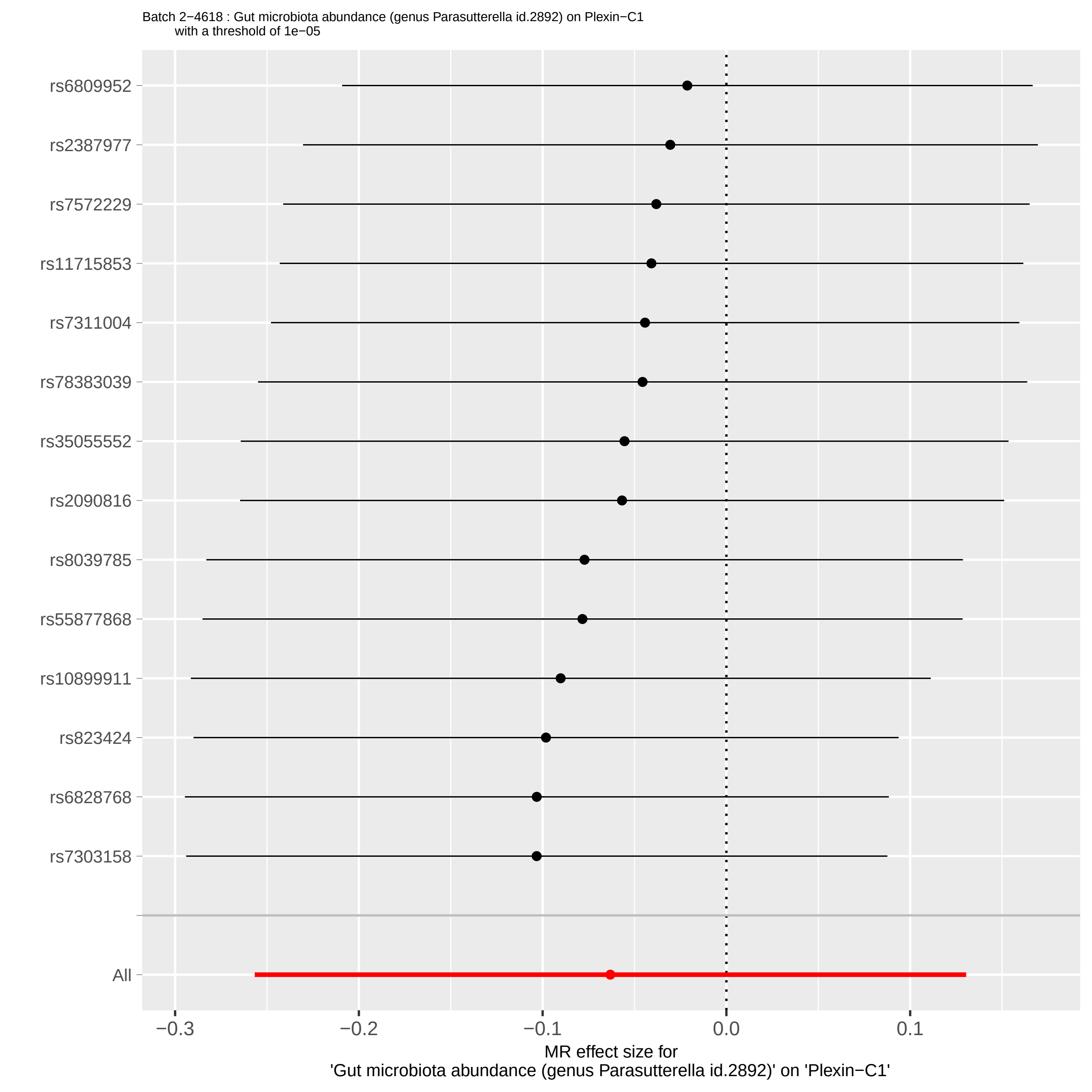
Batch 2−4618 : Gut microbiota abundance (genus Parasutterella id.2892) on Plexin−C1
with a threshold of 1e−05
rs6809952
rs2387977
rs7572229
rs11715853
rs7311004
rs78383039
rs35055552
rs2090816
rs8039785
rs55877868
rs10899911
rs823424
rs6828768
rs7303158
All
−0.3
−0.2
−0.1
0.0
0.1
MR effect size for
'Gut microbiota abundance (genus Parasutterella id.2892)' on 'Plexin−C1'

Batch 2−4619 : Gut microbiota abundance (genus Catenibacterium id.2153) on Melanocyte protein PMEL
with a threshold of 1e−05

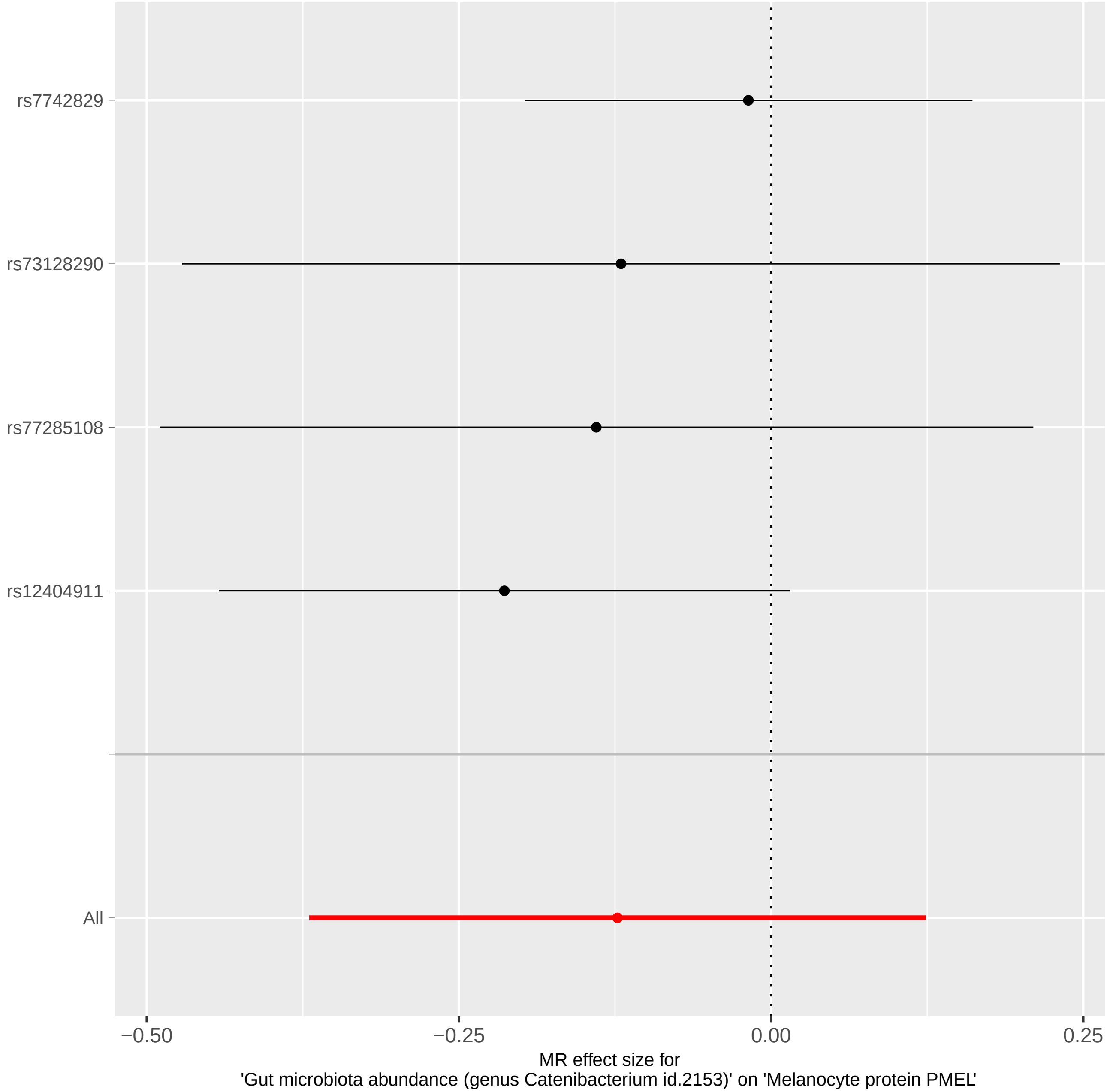

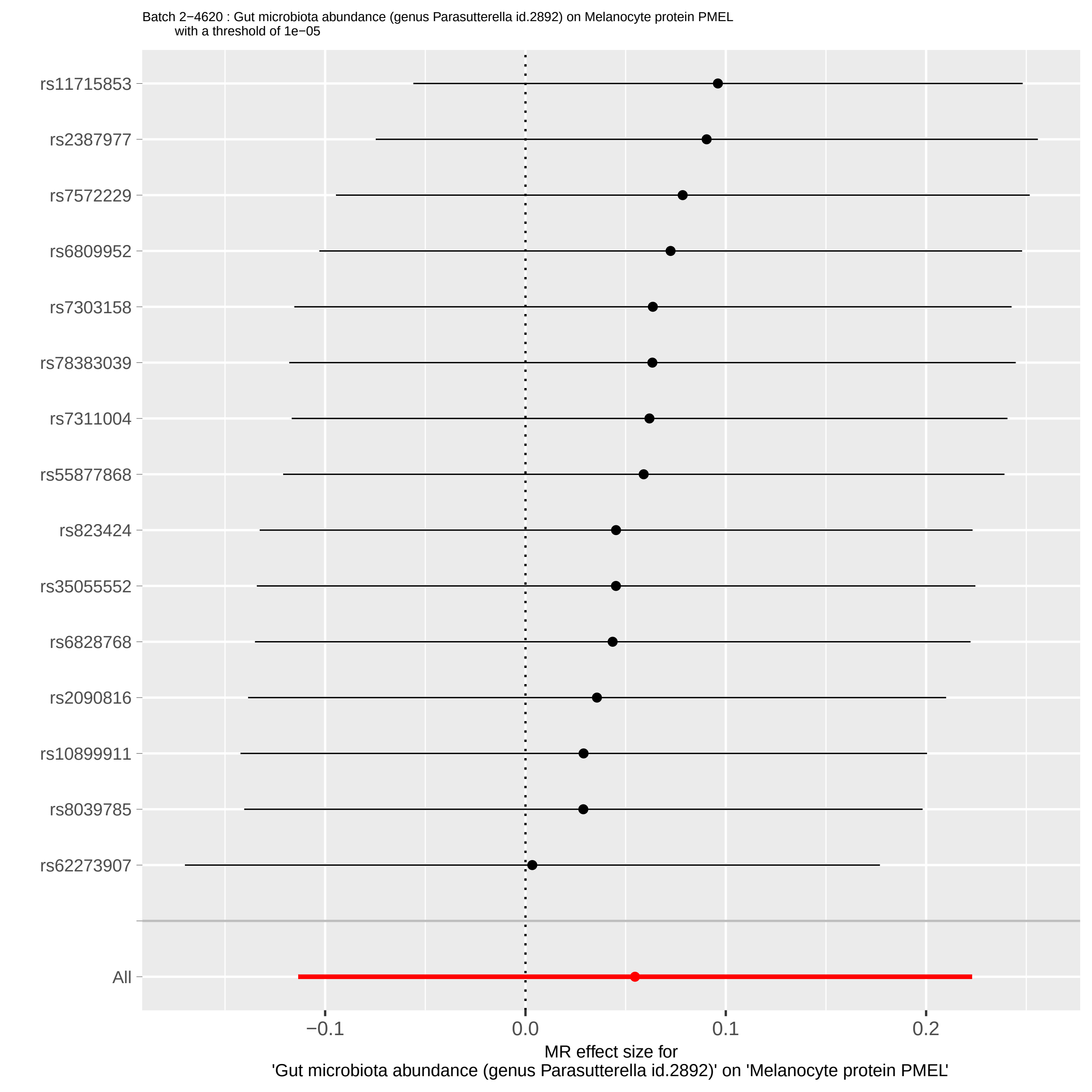

Batch 2−4620 : Gut microbiota abundance (genus Parasutterella id.2892) on Melanocyte protein PMEL
with a threshold of 1e−05
rs11715853
rs2387977
rs7572229
rs6809952
rs7303158
rs78383039
rs7311004
rs55877868
rs823424
rs35055552
rs6828768
rs2090816
rs10899911
rs8039785
rs62273907
All
−0.1
0.0
0.1
0.2
MR effect size for
'Gut microbiota abundance (genus Parasutterella id.2892)' on 'Melanocyte protein PMEL'

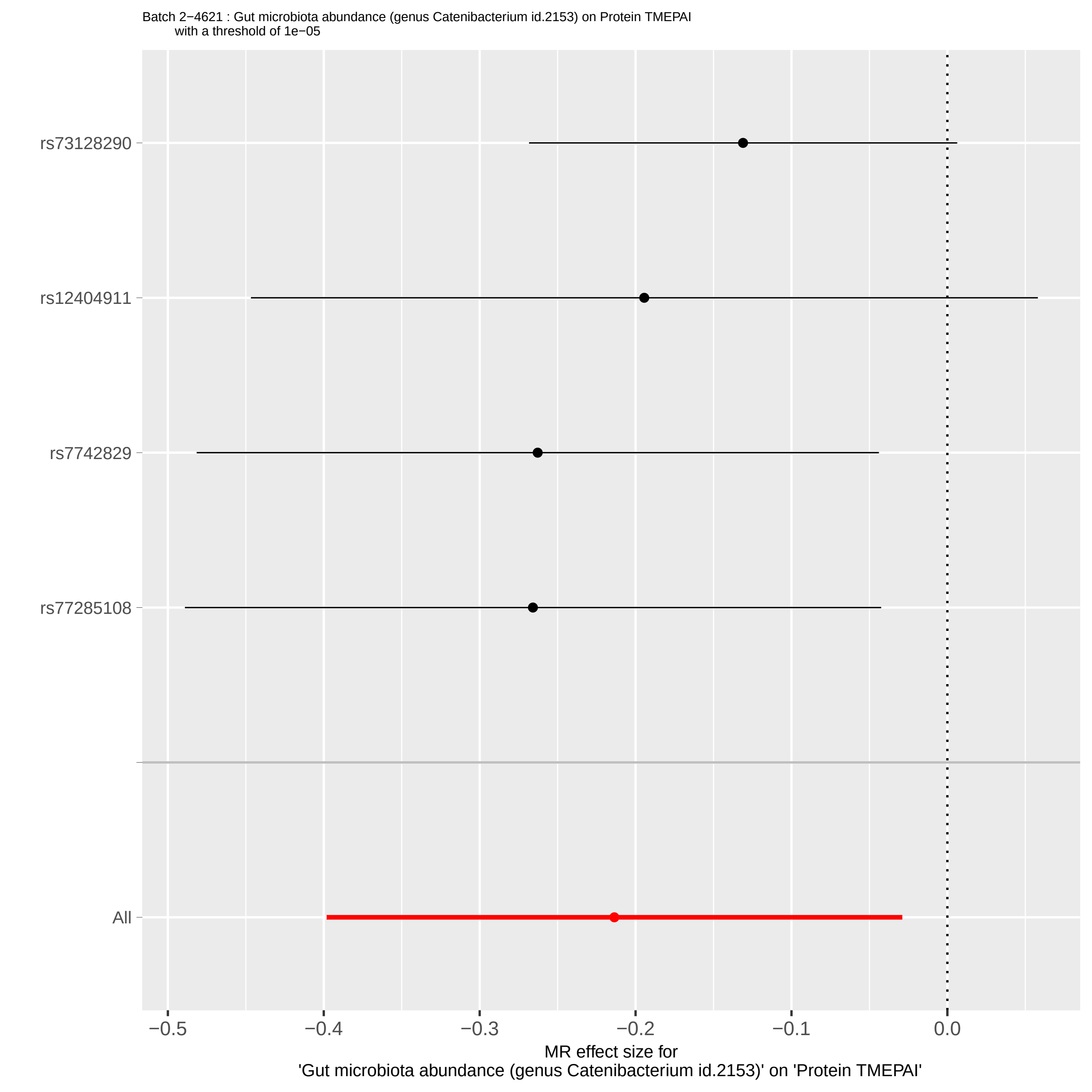
Batch 2−4621 : Gut microbiota abundance (genus Catenibacterium id.2153) on Protein TMEPAI
with a threshold of 1e−05
rs73128290
rs12404911
rs7742829
rs77285108
All
−0.5
−0.4
−0.3
−0.2
−0.1
0.0
MR effect size for
'Gut microbiota abundance (genus Catenibacterium id.2153)' on 'Protein TMEPAI'

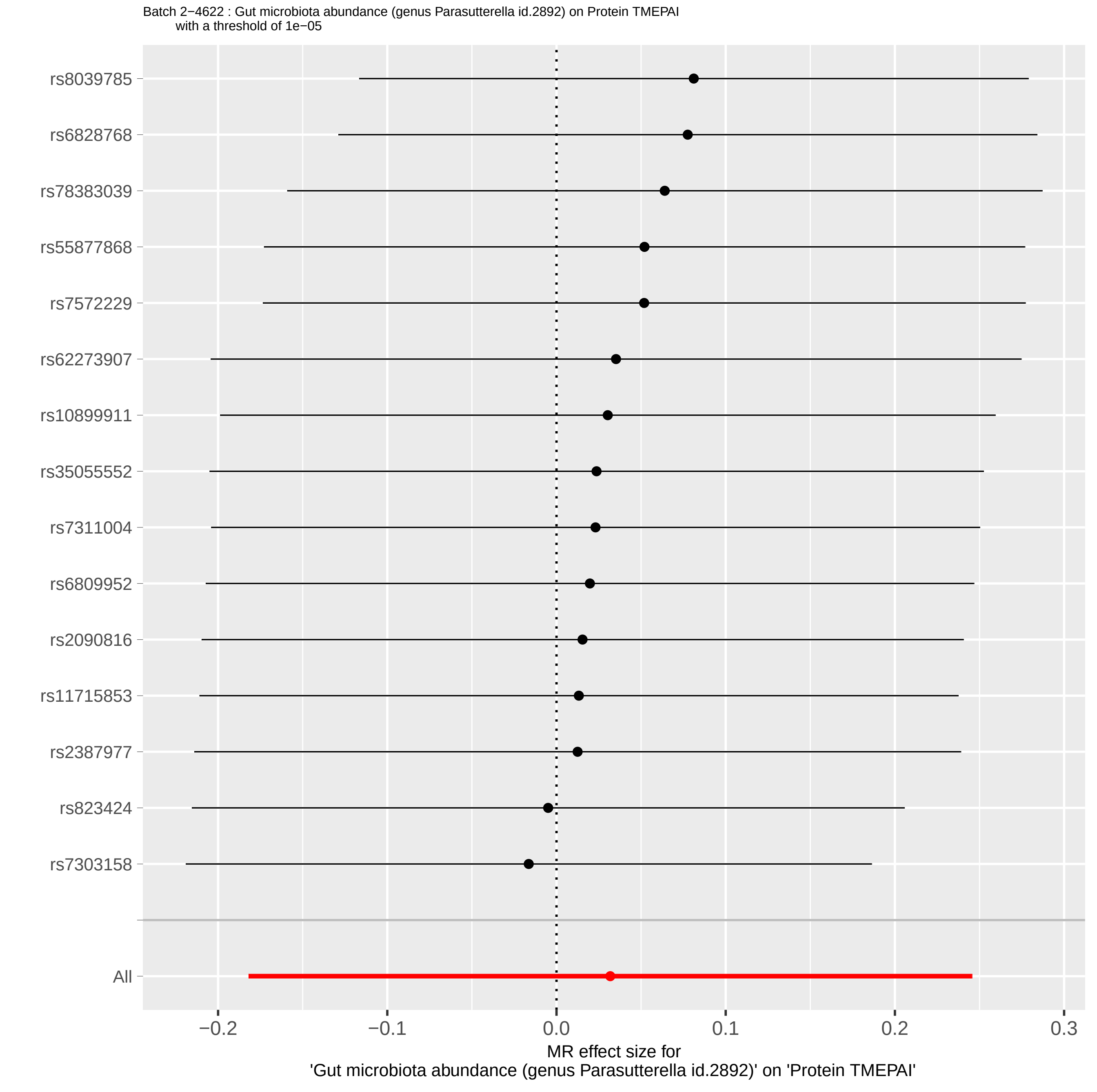

Batch 2−4622 : Gut microbiota abundance (genus Parasutterella id.2892) on Protein TMEPAI
with a threshold of 1e−05
rs8039785
rs6828768
rs78383039
rs55877868
rs7572229
rs62273907
rs10899911
rs35055552
rs7311004
rs6809952
rs2090816
rs11715853
rs2387977
rs823424
rs7303158
All
−0.2
−0.1
0.0
0.1
0.2
0.3
MR effect size for
'Gut microbiota abundance (genus Parasutterella id.2892)' on 'Protein TMEPAI'

Batch 2−4623 : Gut microbiota abundance (genus Catenibacterium id.2153) on Mismatch repair endonuclease PMS2
with a threshold of 1e−05

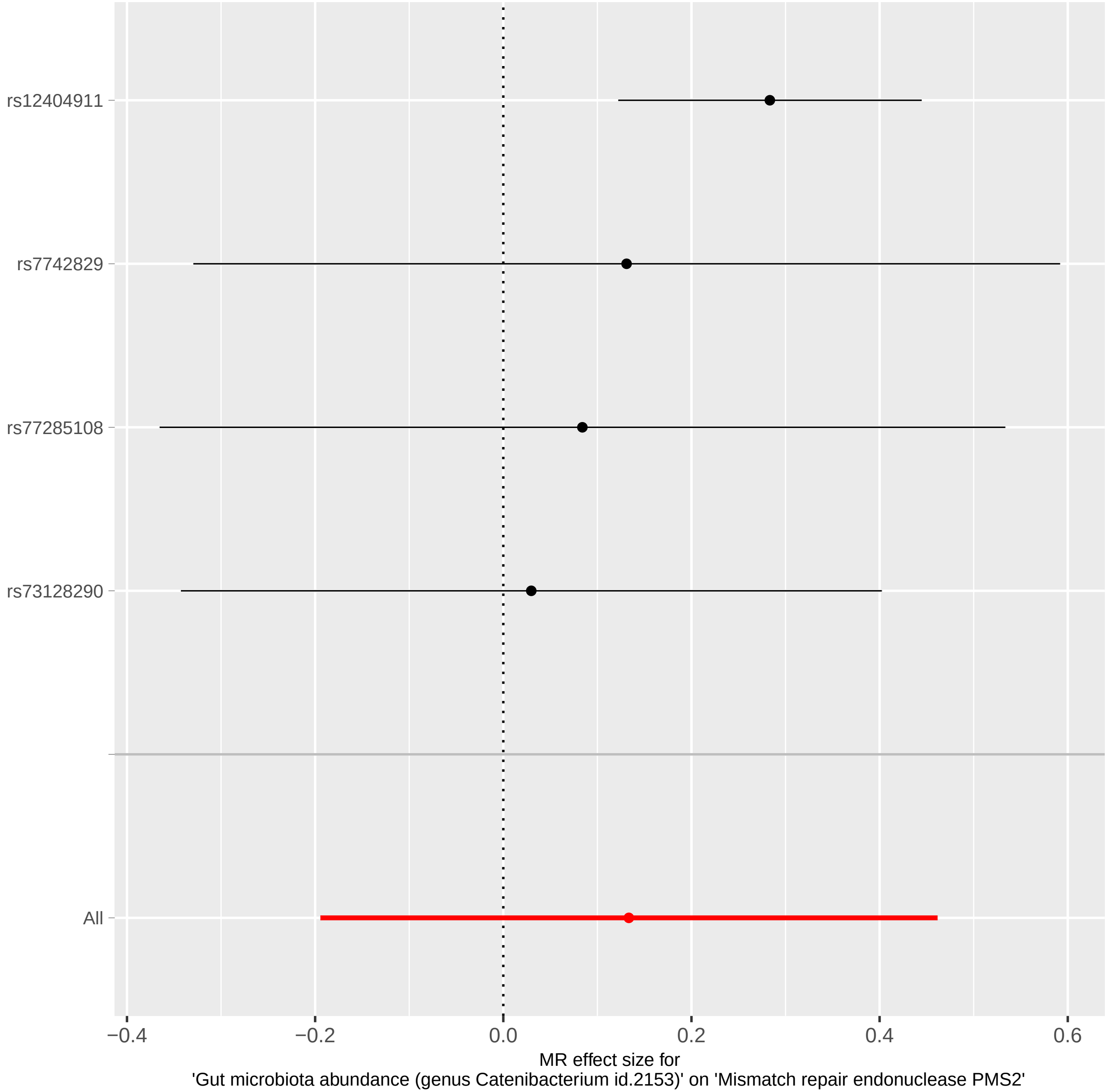

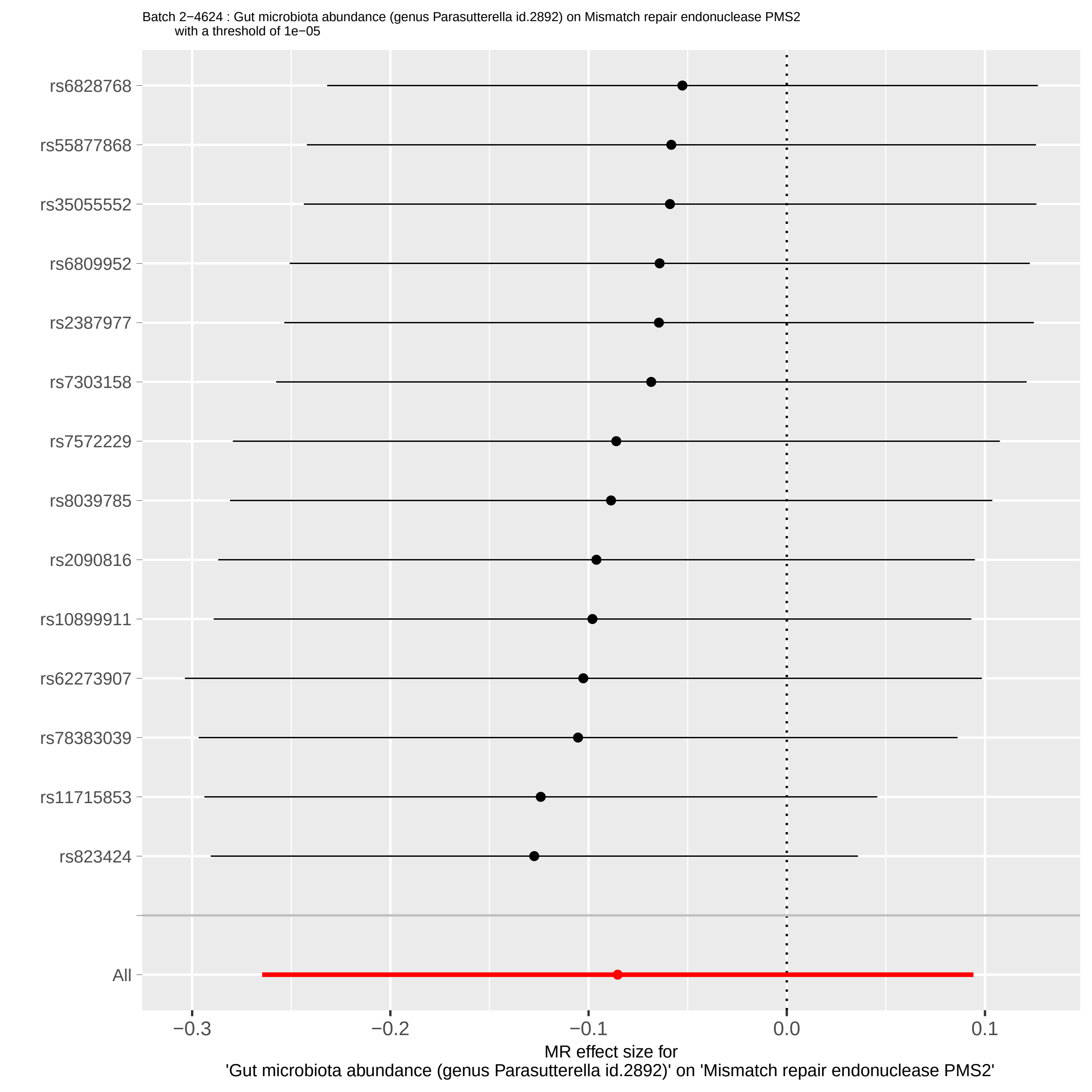

Batch 2−4624 : Gut microbiota abundance (genus Parasutterella id.2892) on Mismatch repair endonuclease PMS2
with a threshold of 1e−05
rs6828768
rs55877868
rs35055552
rs6809952
rs2387977
rs7303158
rs7572229
rs8039785
rs2090816
rs10899911
rs62273907
rs78383039
rs11715853
rs823424
All
−0.3
−0.2
−0.1
0.0
0.1
MR effect size for
'Gut microbiota abundance (genus Parasutterella id.2892)' on 'Mismatch repair endonuclease PMS2'

Batch 2−4625 : Gut microbiota abundance (genus Catenibacterium id.2153) on Phosphomevalonate kinase
with a threshold of 1e−05

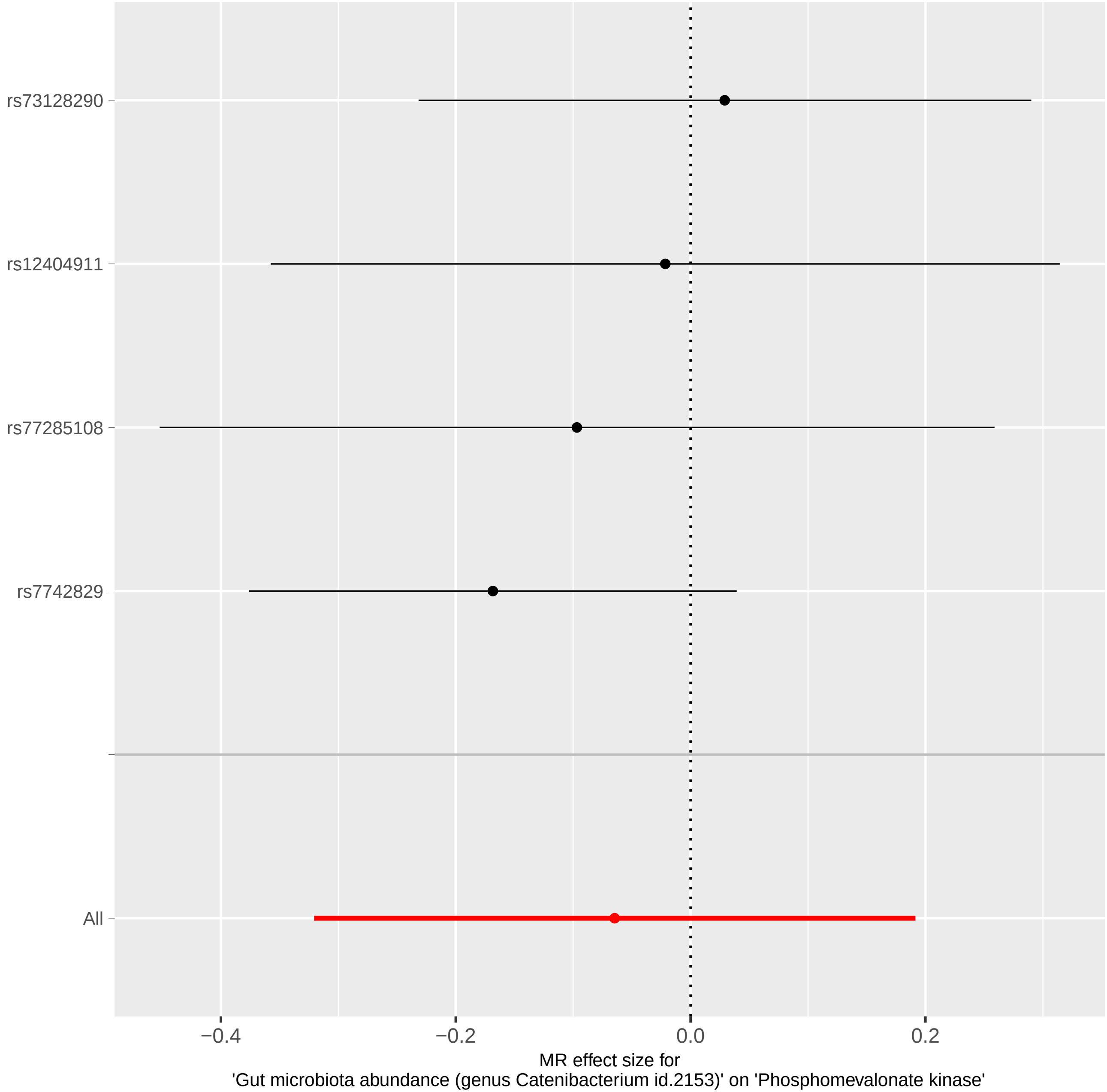

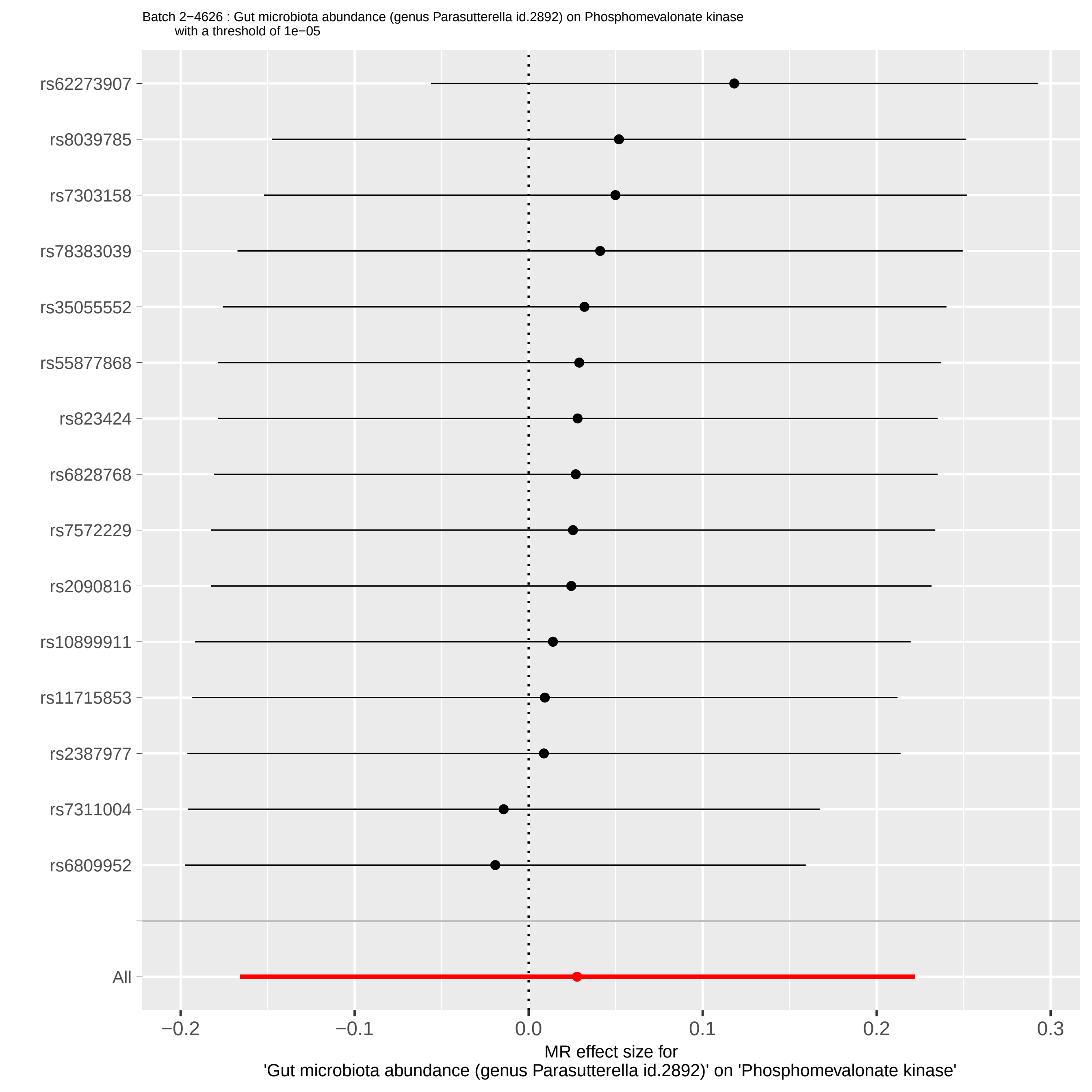
Batch 2−4626 : Gut microbiota abundance (genus Parasutterella id.2892) on Phosphomevalonate kinase
with a threshold of 1e−05
rs62273907
rs8039785
rs7303158
rs78383039
rs35055552
rs55877868
rs823424
rs6828768
rs7572229
rs2090816
rs10899911
rs11715853
rs2387977
rs7311004
rs6809952
All
−0.2
−0.1
0.0
0.1
0.2
0.3
MR effect size for
'Gut microbiota abundance (genus Parasutterella id.2892)' on 'Phosphomevalonate kinase'

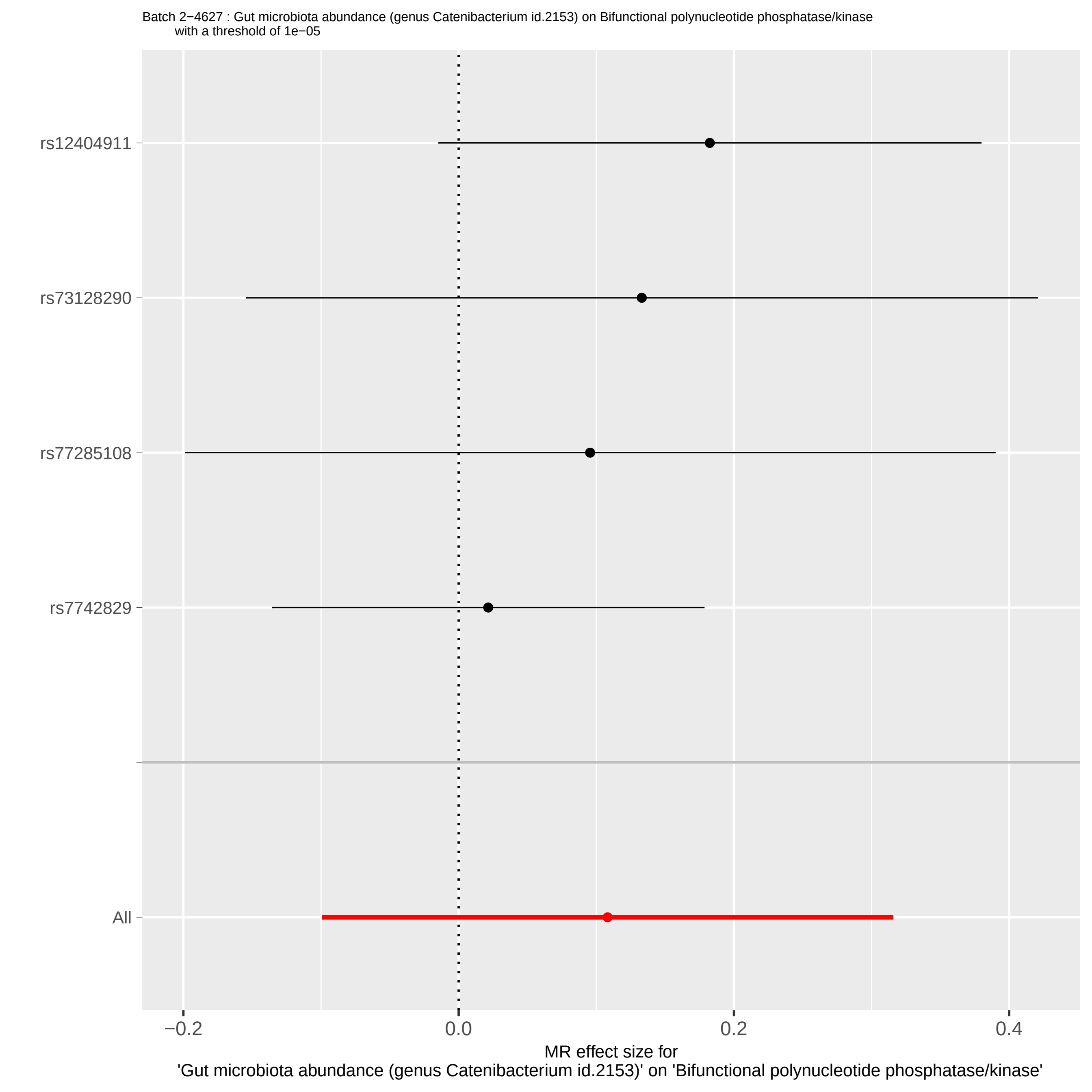
Batch 2−4627 : Gut microbiota abundance (genus Catenibacterium id.2153) on Bifunctional polynucleotide phosphatase/kinase
with a threshold of 1e−05
rs12404911
rs73128290
rs77285108
rs7742829
All
−0.2
0.0
0.2
0.4
MR effect size for
'Gut microbiota abundance (genus Catenibacterium id.2153)' on 'Bifunctional polynucleotide phosphatase/kinase'

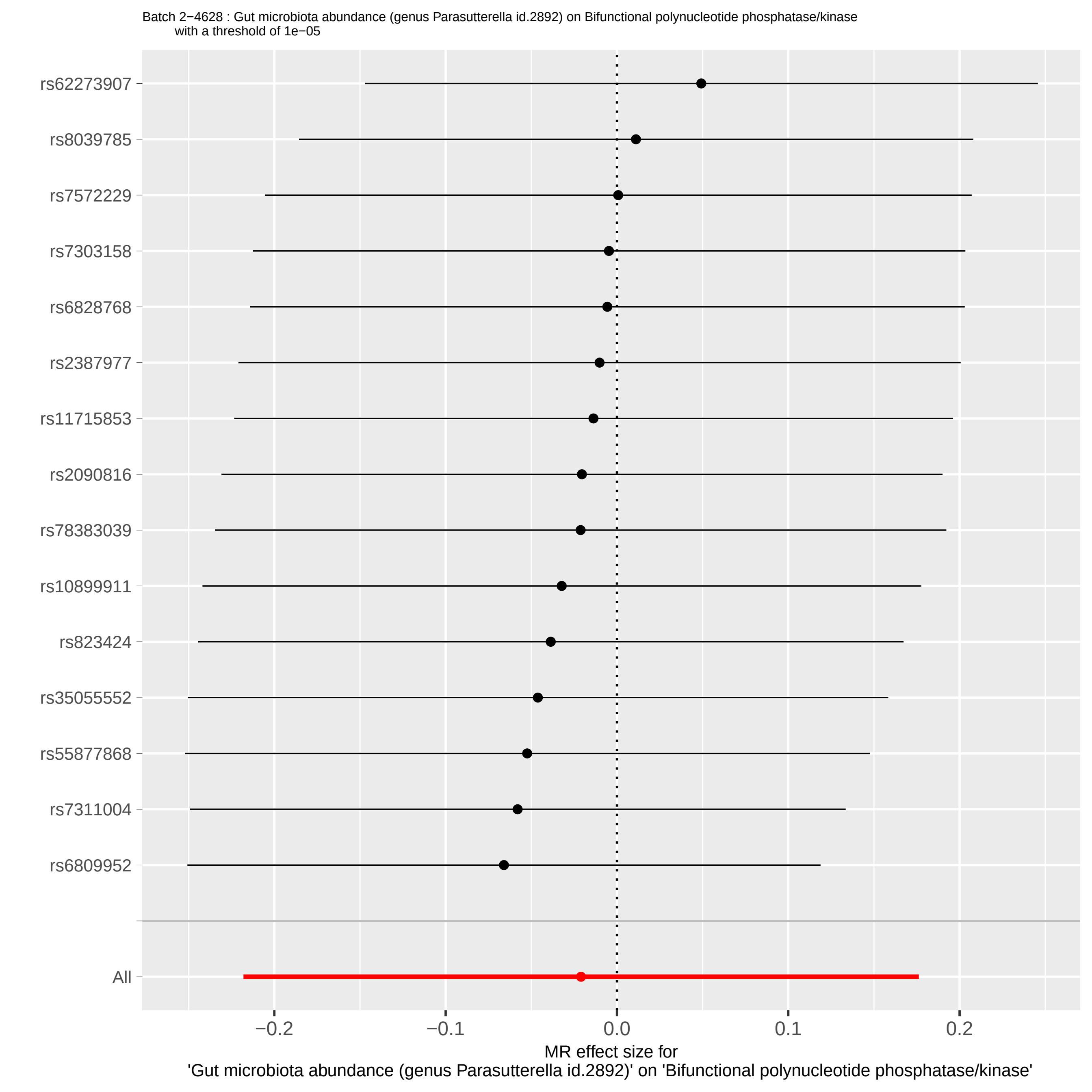
Batch 2−4628 : Gut microbiota abundance (genus Parasutterella id.2892) on Bifunctional polynucleotide phosphatase/kinase
with a threshold of 1e−05
rs62273907
rs8039785
rs7572229
rs7303158
rs6828768
rs2387977
rs11715853
rs2090816
rs78383039
rs10899911
rs823424
rs35055552
rs55877868
rs7311004
rs6809952
All
−0.2
−0.1
0.0
0.1
0.2
MR effect size for
'Gut microbiota abundance (genus Parasutterella id.2892)' on 'Bifunctional polynucleotide phosphatase/kinase'

Batch 2−4629 : Gut microbiota abundance (genus Catenibacterium id.2153) on Inactive pancreatic lipase−related protein 1
with a threshold of 1e−05

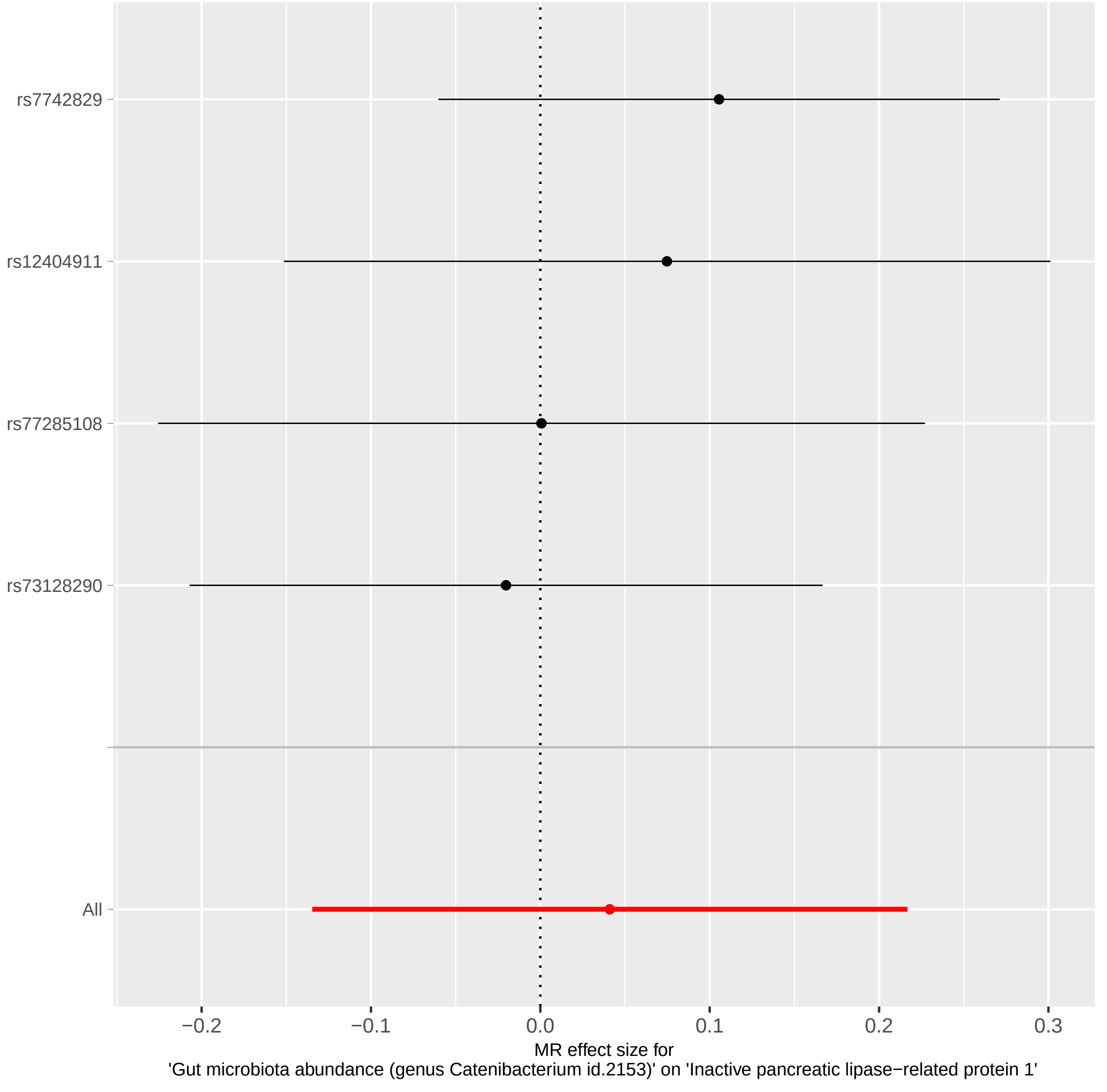

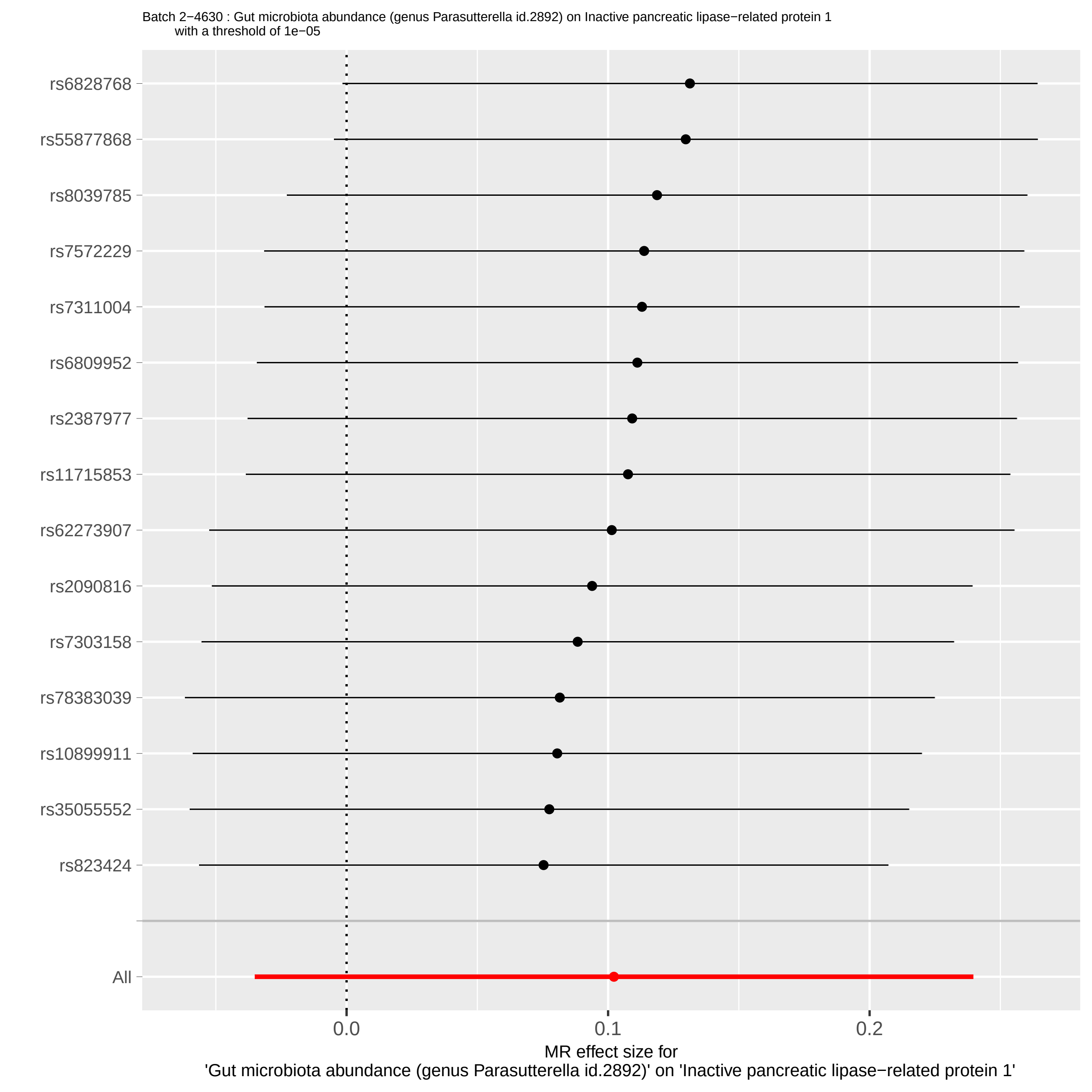

Batch 2−4630 : Gut microbiota abundance (genus Parasutterella id.2892) on Inactive pancreatic lipase−related protein 1
with a threshold of 1e−05
rs6828768
rs55877868
rs8039785
rs7572229
rs7311004
rs6809952
rs2387977
rs11715853
rs62273907
rs2090816
rs7303158
rs78383039
rs10899911
rs35055552
rs823424
All
0.0
0.1
0.2
MR effect size for
'Gut microbiota abundance (genus Parasutterella id.2892)' on 'Inactive pancreatic lipase−related protein 1'

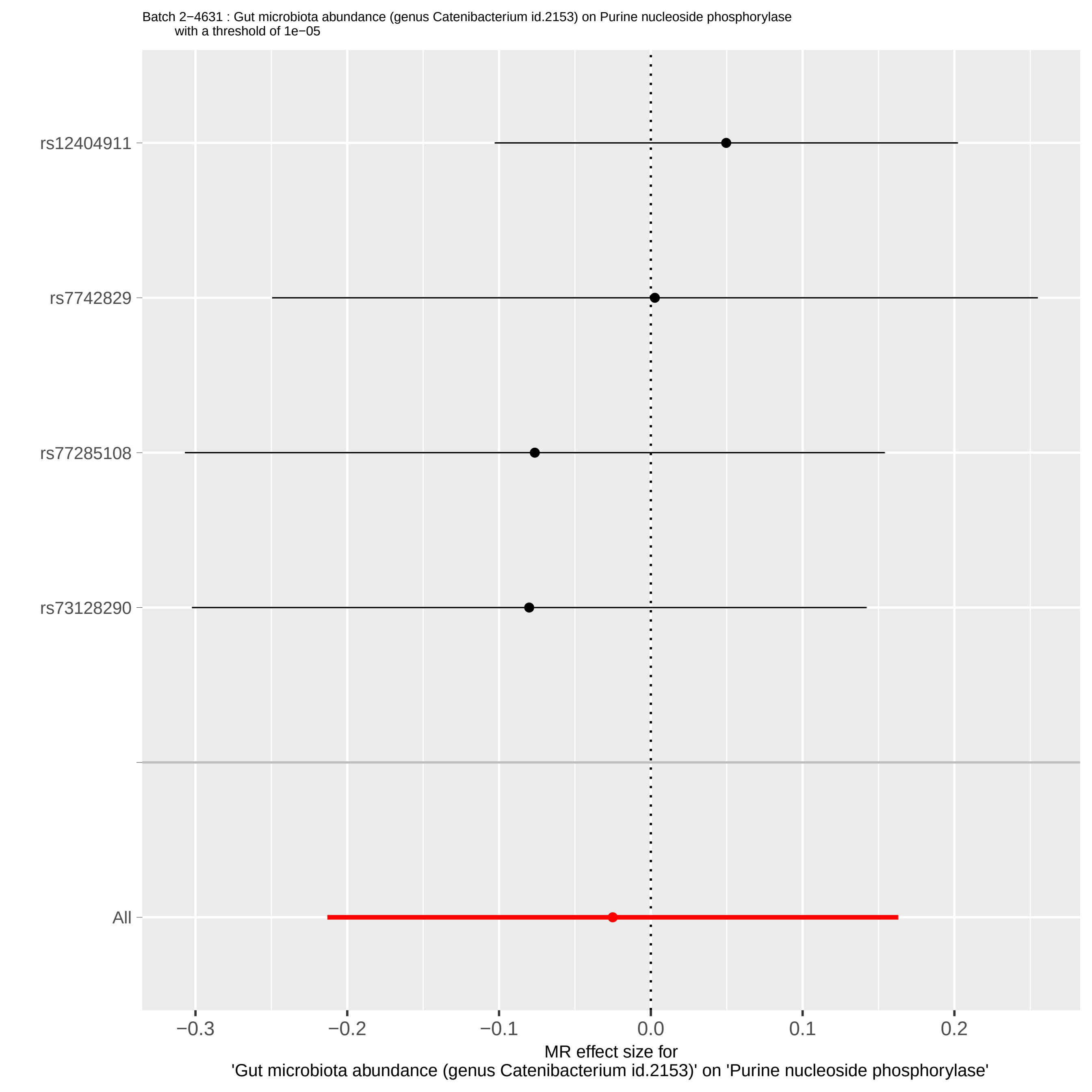
Batch 2−4631 : Gut microbiota abundance (genus Catenibacterium id.2153) on Purine nucleoside phosphorylase
with a threshold of 1e−05
rs12404911
rs7742829
rs77285108
rs73128290
All
−0.3
−0.2
−0.1
0.0
0.1
0.2
MR effect size for
'Gut microbiota abundance (genus Catenibacterium id.2153)' on 'Purine nucleoside phosphorylase'

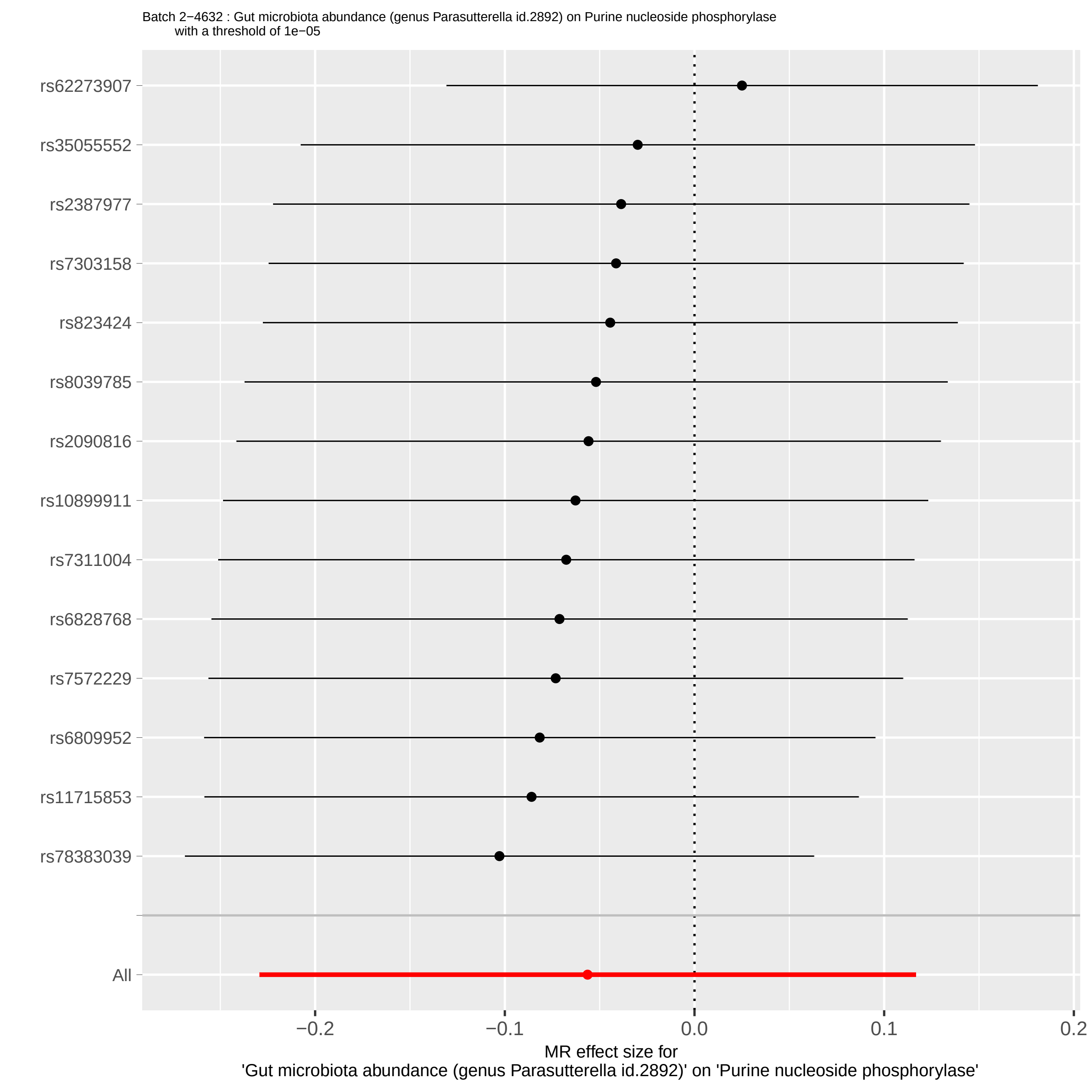
Batch 2−4632 : Gut microbiota abundance (genus Parasutterella id.2892) on Purine nucleoside phosphorylase
with a threshold of 1e−05
rs62273907
rs35055552
rs2387977
rs7303158
rs823424
rs8039785
rs2090816
rs10899911
rs7311004
rs6828768
rs7572229
rs6809952
rs11715853
rs78383039
All
−0.2
−0.1
0.0
0.1
0.2
MR effect size for
'Gut microbiota abundance (genus Parasutterella id.2892)' on 'Purine nucleoside phosphorylase'

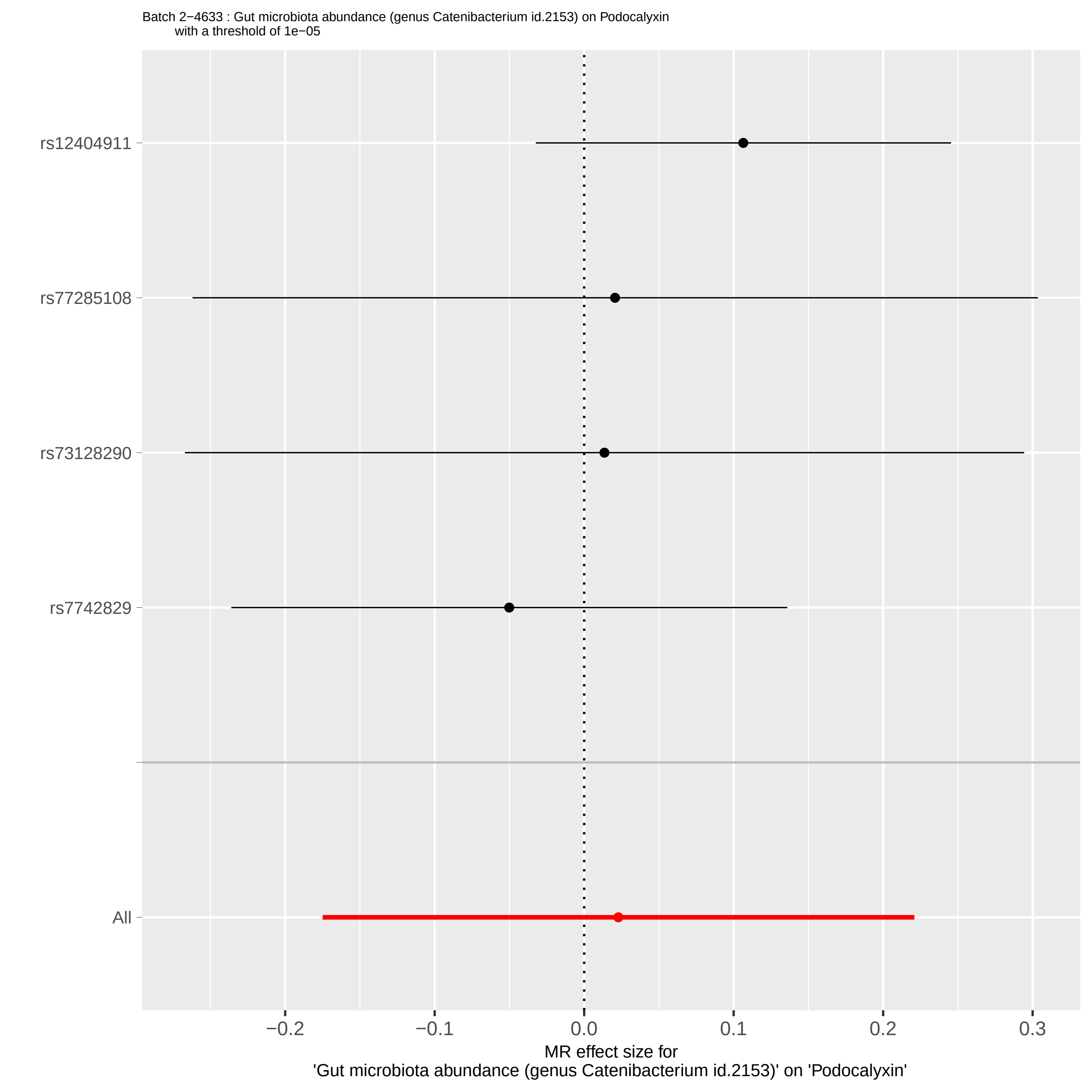
Batch 2−4633 : Gut microbiota abundance (genus Catenibacterium id.2153) on Podocalyxin
with a threshold of 1e−05
rs12404911
rs77285108
rs73128290
rs7742829
All
−0.2
−0.1
0.0
0.1
0.2
0.3
MR effect size for
'Gut microbiota abundance (genus Catenibacterium id.2153)' on 'Podocalyxin'

Batch 2−4634 : Gut microbiota abundance (genus Parasutterella id.2892) on Podocalyxin
with a threshold of 1e−05

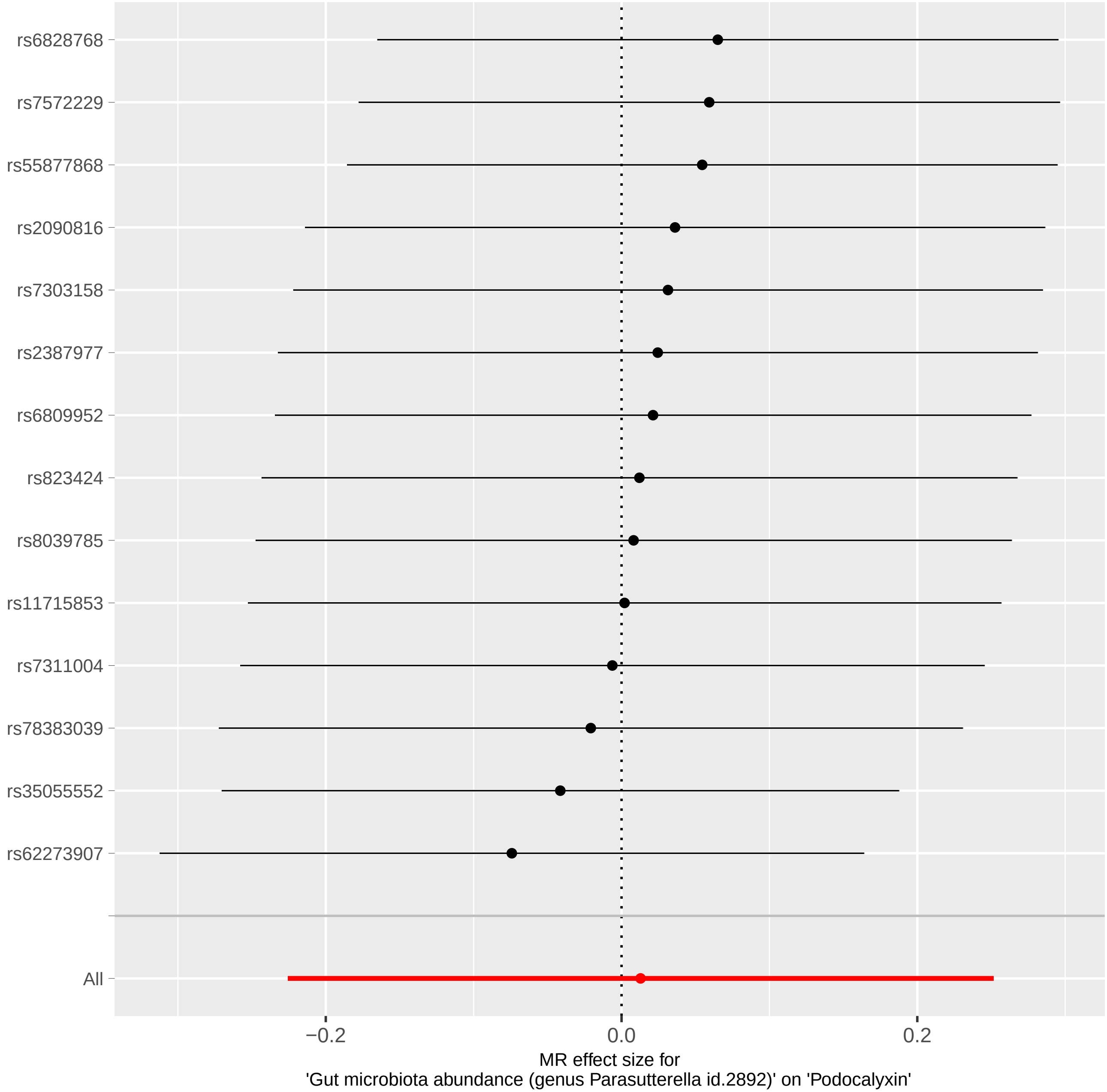

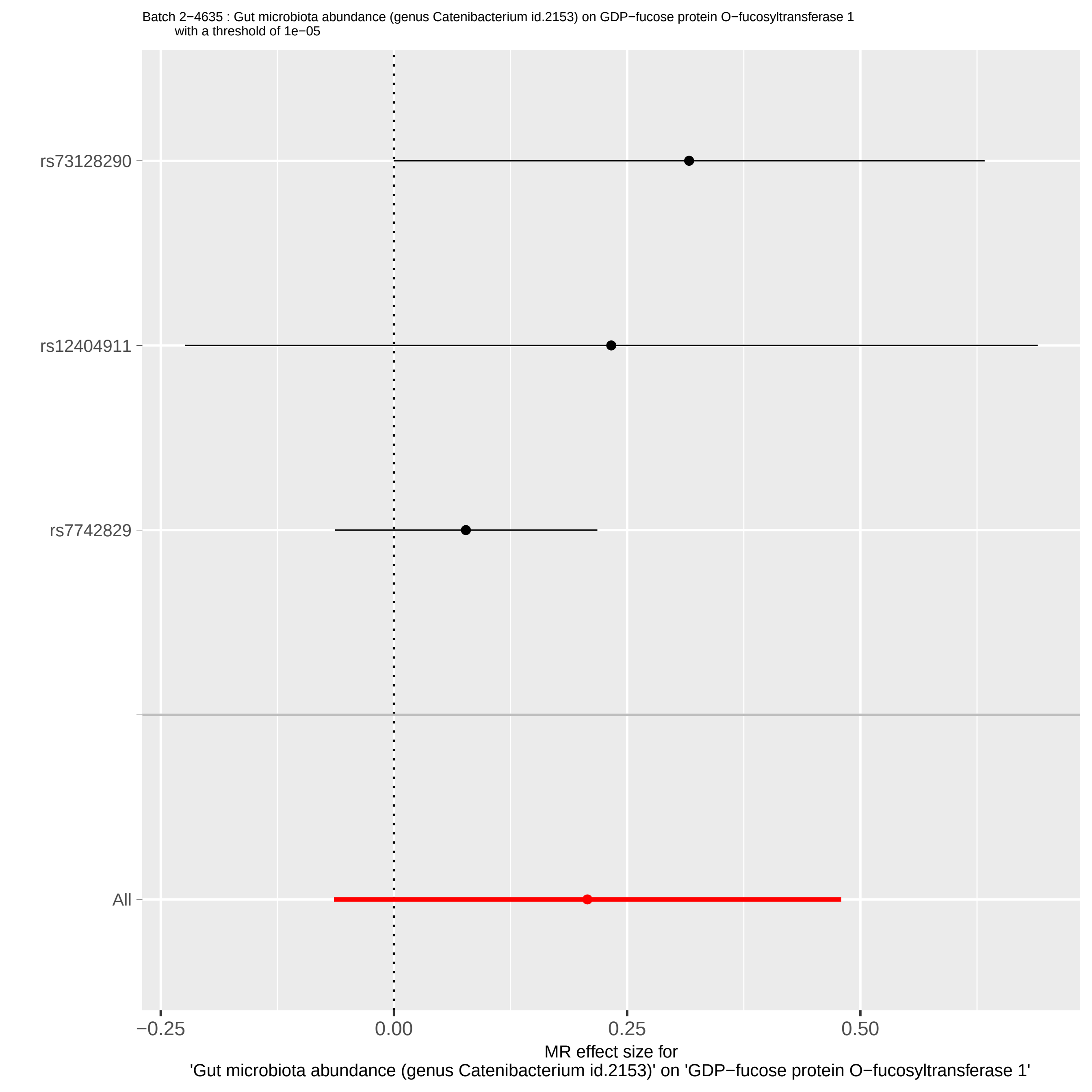

Batch 2−4635 : Gut microbiota abundance (genus Catenibacterium id.2153) on GDP−fucose protein O−fucosyltransferase 1
with a threshold of 1e−05
rs73128290
rs12404911
rs7742829
All
−0.25
0.00
0.25
0.50
MR effect size for
'Gut microbiota abundance (genus Catenibacterium id.2153)' on 'GDP−fucose protein O−fucosyltransferase 1'

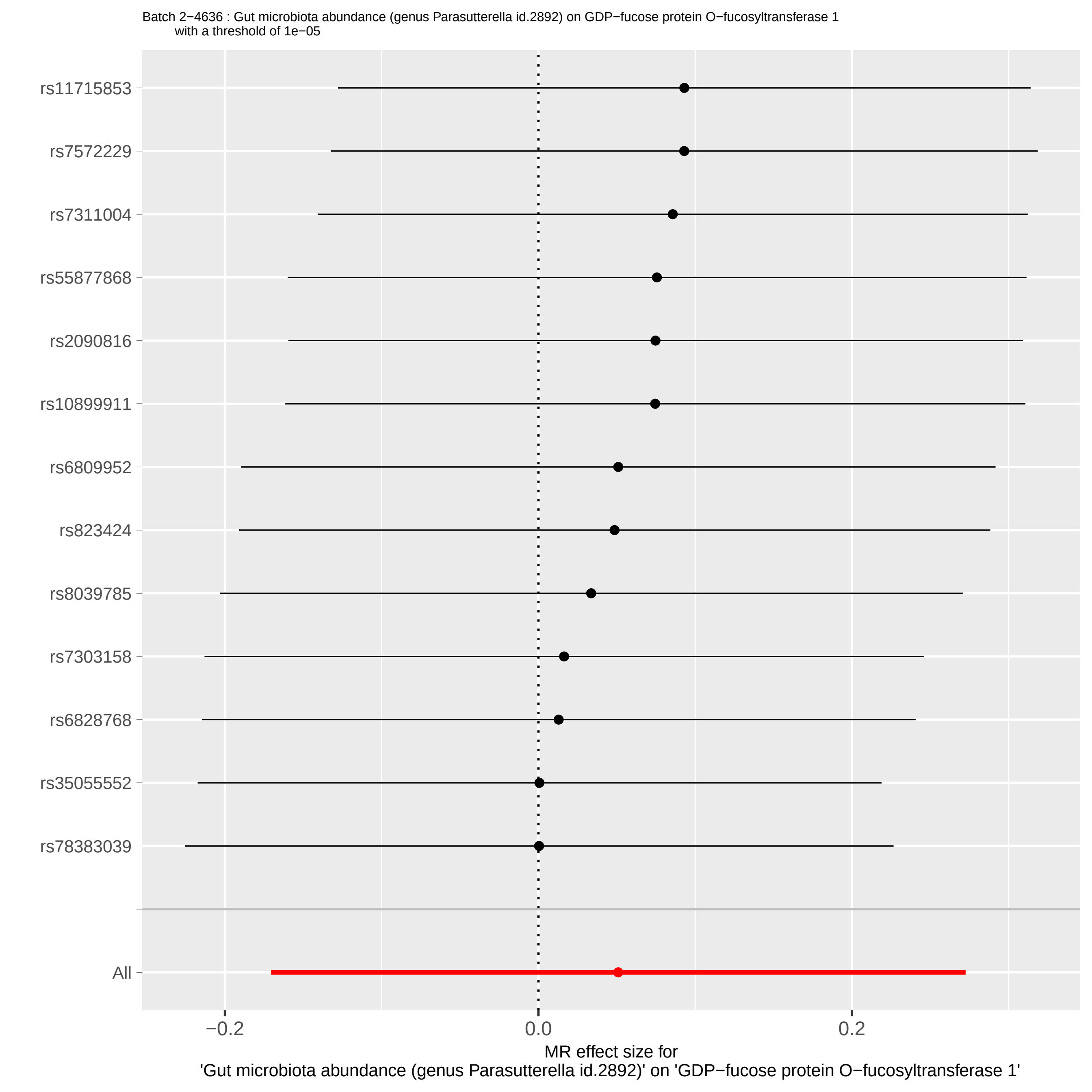

Batch 2−4636 : Gut microbiota abundance (genus Parasutterella id.2892) on GDP−fucose protein O−fucosyltransferase 1
with a threshold of 1e−05
rs11715853
rs7572229
rs7311004
rs55877868
rs2090816
rs10899911
rs6809952
rs823424
rs8039785
rs7303158
rs6828768
rs35055552
rs78383039
All
−0.2
0.0
0.2
MR effect size for
'Gut microbiota abundance (genus Parasutterella id.2892)' on 'GDP−fucose protein O−fucosyltransferase 1'

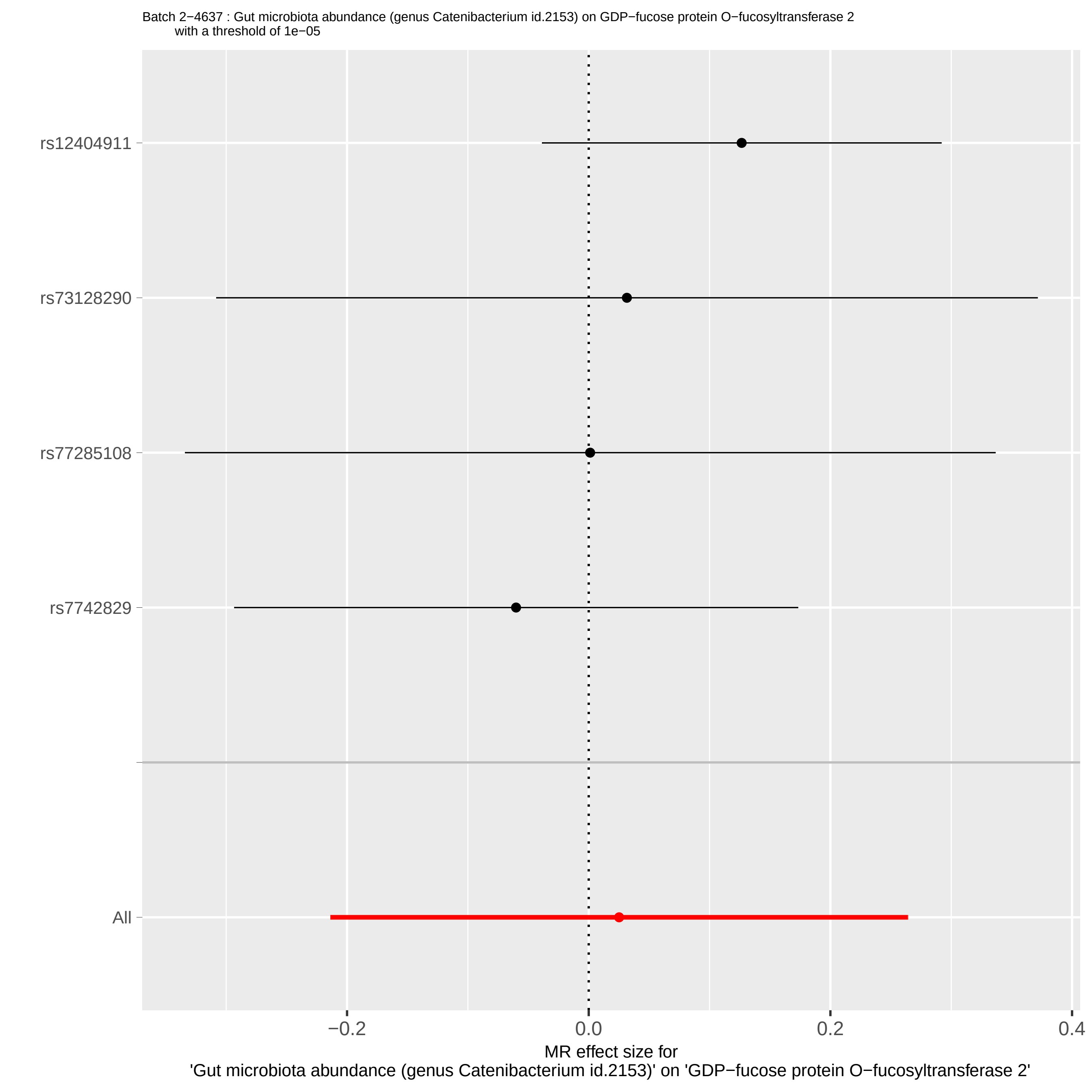

Batch 2−4637 : Gut microbiota abundance (genus Catenibacterium id.2153) on GDP−fucose protein O−fucosyltransferase 2
with a threshold of 1e−05
rs12404911
rs73128290
rs77285108
rs7742829
All
−0.2
0.0
0.2
0.4
MR effect size for
'Gut microbiota abundance (genus Catenibacterium id.2153)' on 'GDP−fucose protein O−fucosyltransferase 2'

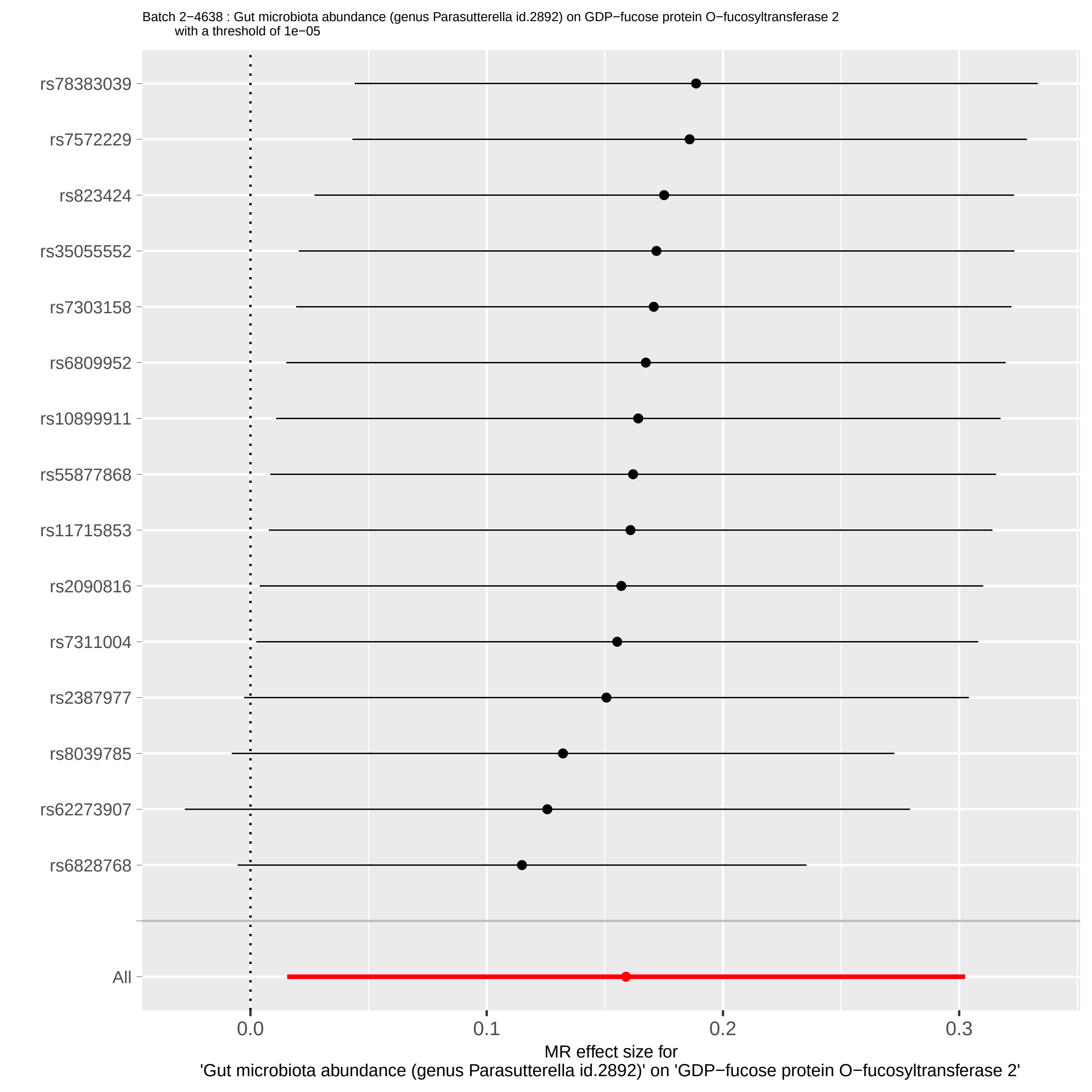
Batch 2−4638 : Gut microbiota abundance (genus Parasutterella id.2892) on GDP−fucose protein O−fucosyltransferase 2
with a threshold of 1e−05
rs78383039
rs7572229
rs823424
rs35055552
rs7303158
rs6809952
rs10899911
rs55877868
rs11715853
rs2090816
rs7311004
rs2387977
rs8039785
rs62273907
rs6828768
All
0.0
0.1
0.2
0.3
MR effect size for
'Gut microbiota abundance (genus Parasutterella id.2892)' on 'GDP−fucose protein O−fucosyltransferase 2'

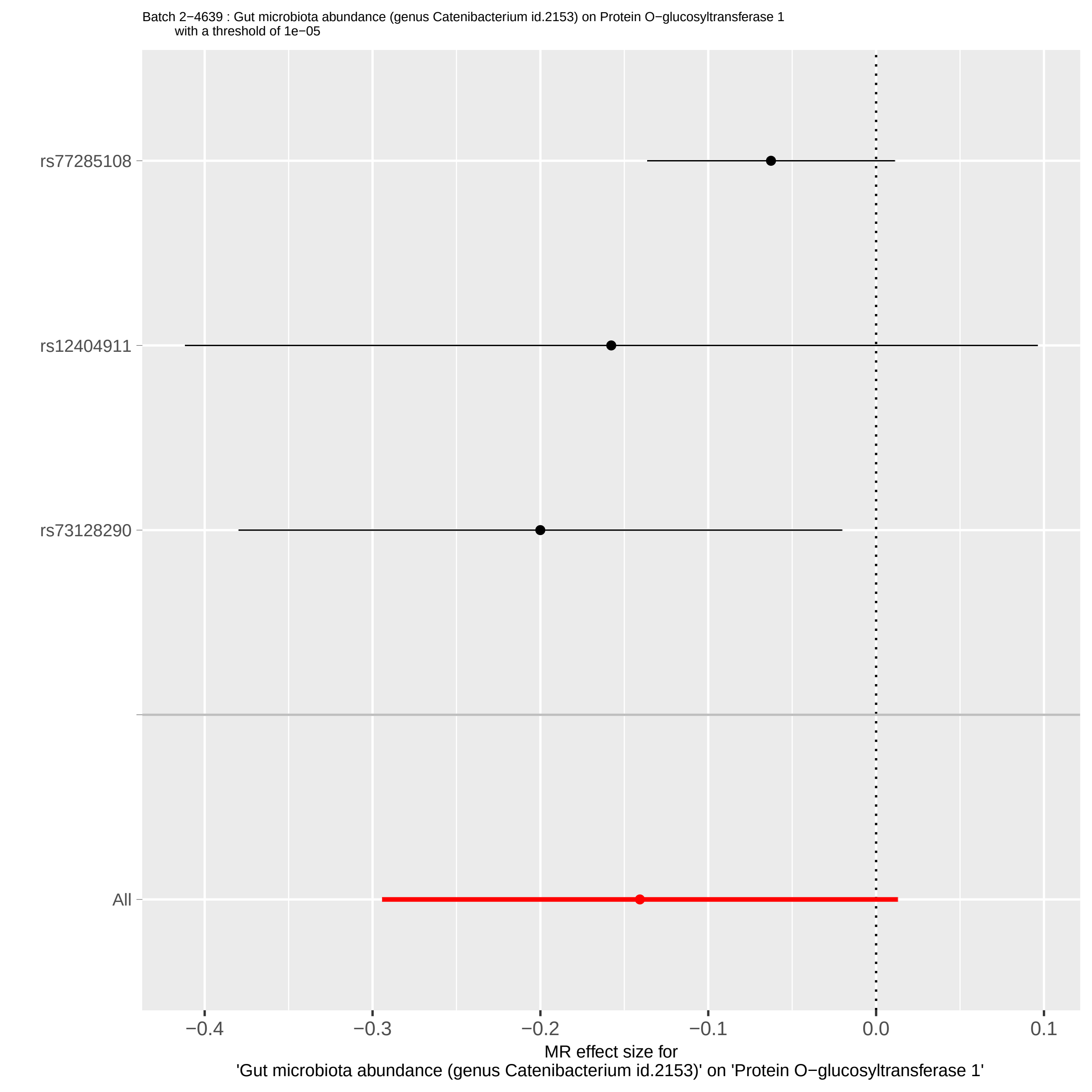
Batch 2−4639 : Gut microbiota abundance (genus Catenibacterium id.2153) on Protein O−glucosyltransferase 1
with a threshold of 1e−05
rs77285108
rs12404911
rs73128290
All
−0.4
−0.3
−0.2
−0.1
0.0
0.1
MR effect size for
'Gut microbiota abundance (genus Catenibacterium id.2153)' on 'Protein O−glucosyltransferase 1'

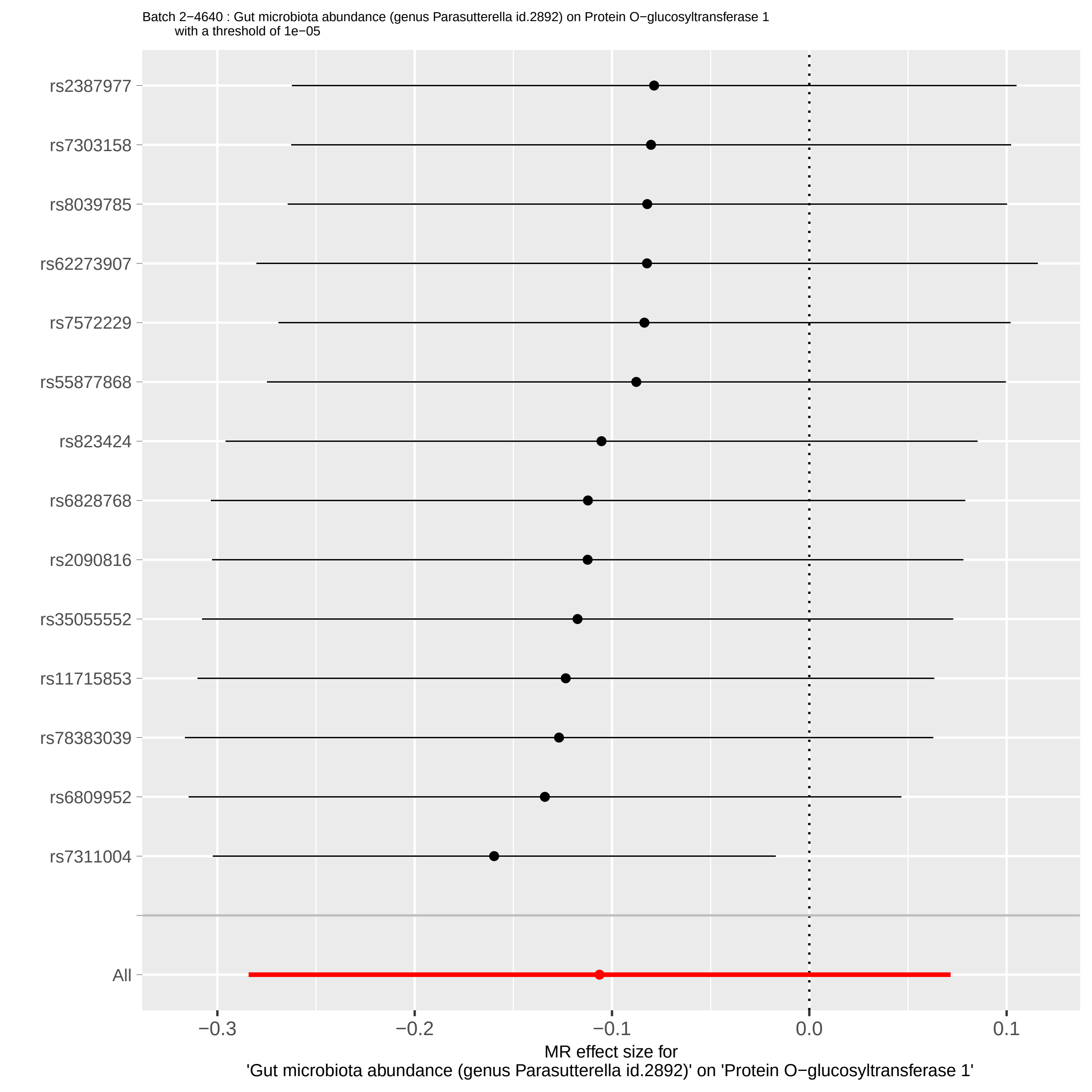

Batch 2−4640 : Gut microbiota abundance (genus Parasutterella id.2892) on Protein O−glucosyltransferase 1
with a threshold of 1e−05
rs2387977
rs7303158
rs8039785
rs62273907
rs7572229
rs55877868
rs823424
rs6828768
rs2090816
rs35055552
rs11715853
rs78383039
rs6809952
rs7311004
All
−0.3
−0.2
−0.1
0.0
0.1
MR effect size for
'Gut microbiota abundance (genus Parasutterella id.2892)' on 'Protein O−glucosyltransferase 1'

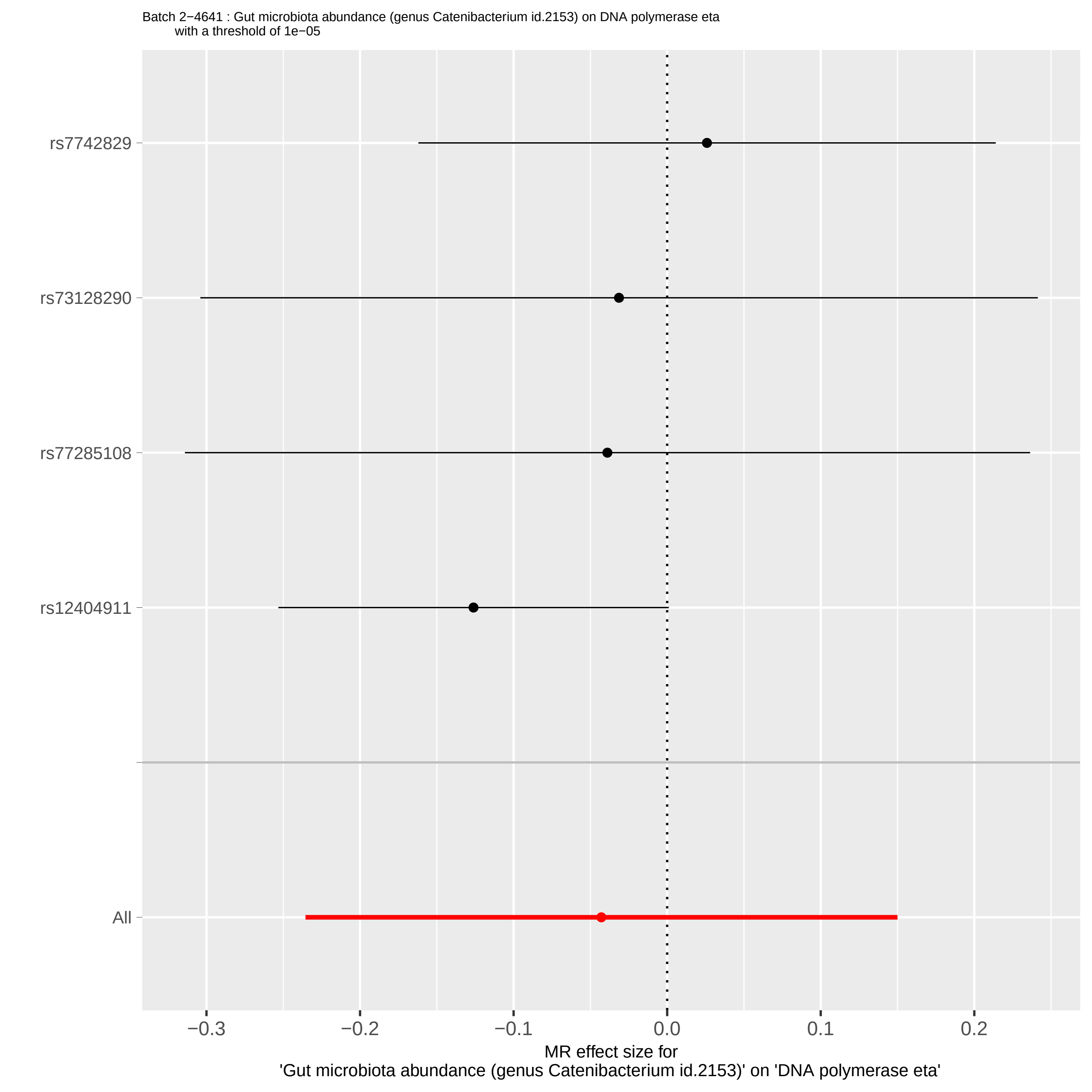
Batch 2−4641 : Gut microbiota abundance (genus Catenibacterium id.2153) on DNA polymerase eta
with a threshold of 1e−05
rs7742829
rs73128290
rs77285108
rs12404911
All
−0.3
−0.2
−0.1
0.0
0.1
0.2
MR effect size for
'Gut microbiota abundance (genus Catenibacterium id.2153)' on 'DNA polymerase eta'

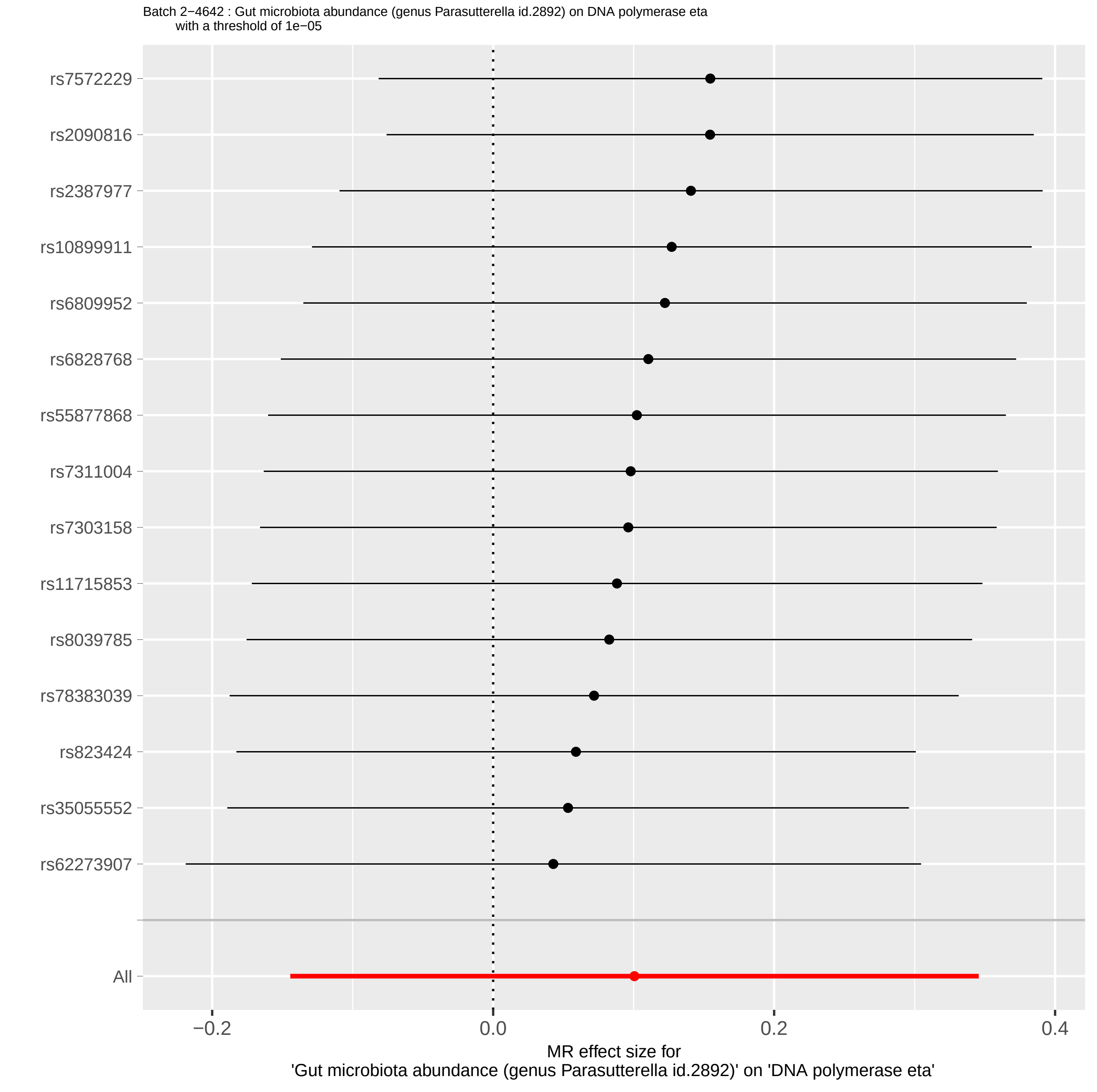

Batch 2−4642 : Gut microbiota abundance (genus Parasutterella id.2892) on DNA polymerase eta
with a threshold of 1e−05
rs7572229
rs2090816
rs2387977
rs10899911
rs6809952
rs6828768
rs55877868
rs7311004
rs7303158
rs11715853
rs8039785
rs78383039
rs823424
rs35055552
rs62273907
All
−0.2
0.0
0.2
0.4
MR effect size for
'Gut microbiota abundance (genus Parasutterella id.2892)' on 'DNA polymerase eta'

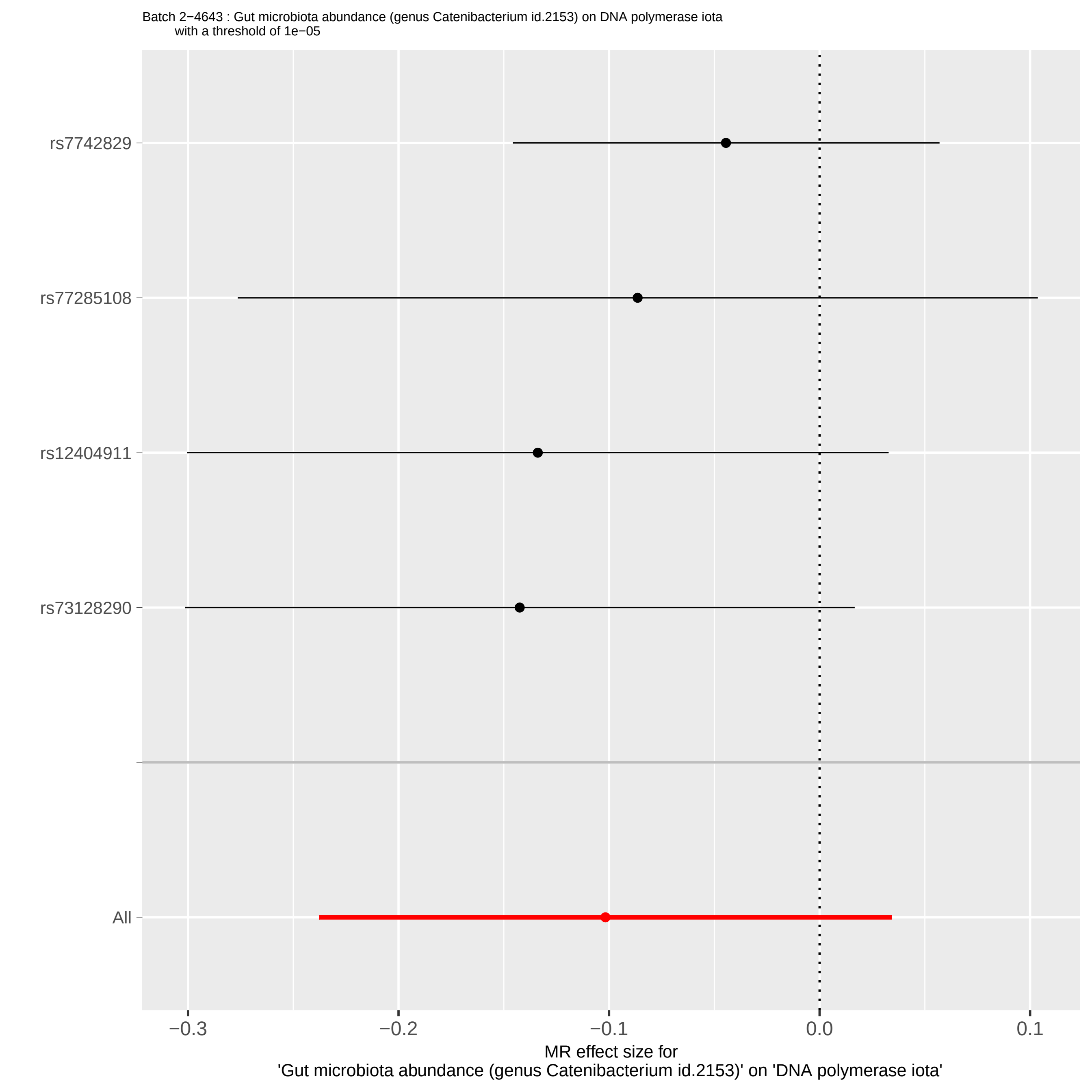

Batch 2−4643 : Gut microbiota abundance (genus Catenibacterium id.2153) on DNA polymerase iota
with a threshold of 1e−05
rs7742829
rs77285108
rs12404911
rs73128290
All
−0.3
−0.2
−0.1
0.0
0.1
MR effect size for
'Gut microbiota abundance (genus Catenibacterium id.2153)' on 'DNA polymerase iota'

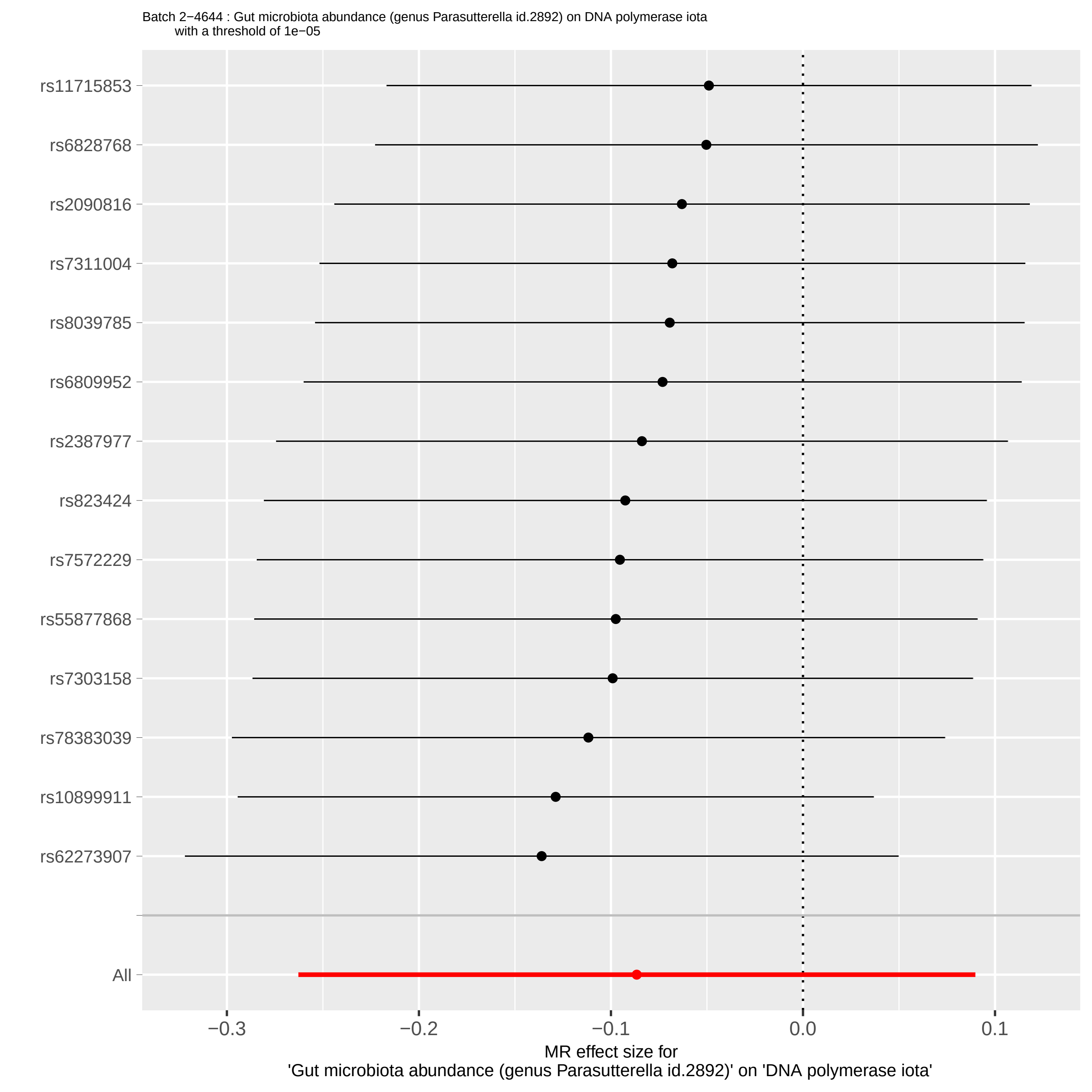
Batch 2−4644 : Gut microbiota abundance (genus Parasutterella id.2892) on DNA polymerase iota
with a threshold of 1e−05
rs11715853
rs6828768
rs2090816
rs7311004
rs8039785
rs6809952
rs2387977
rs823424
rs7572229
rs55877868
rs7303158
rs78383039
rs10899911
rs62273907
All
−0.3
−0.2
−0.1
0.0
0.1
MR effect size for
'Gut microbiota abundance (genus Parasutterella id.2892)' on 'DNA polymerase iota'

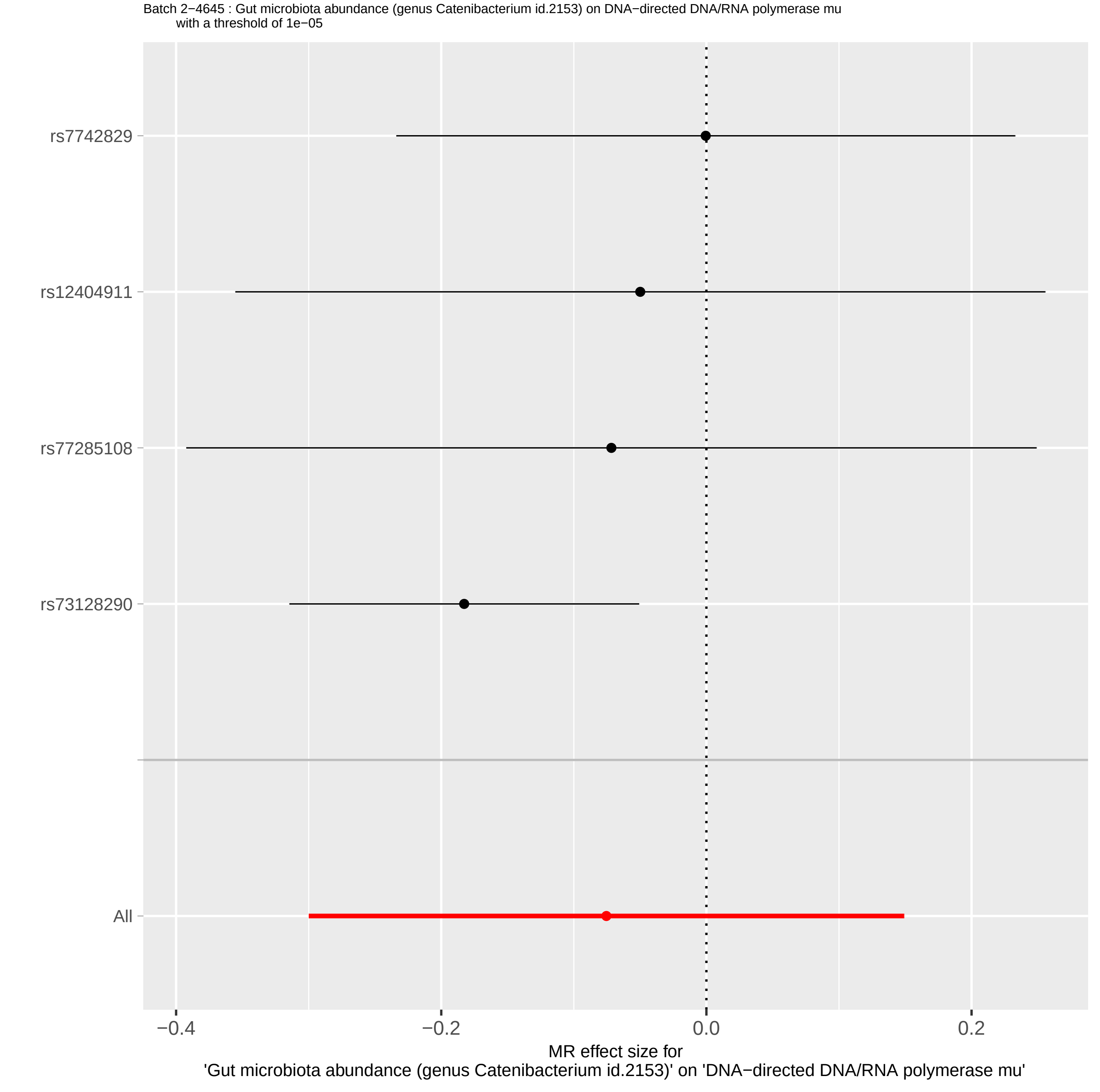

Batch 2−4645 : Gut microbiota abundance (genus Catenibacterium id.2153) on DNA−directed DNA/RNA polymerase mu
with a threshold of 1e−05
rs7742829
rs12404911
rs77285108
rs73128290
All
−0.4
−0.2
0.0
0.2
MR effect size for
'Gut microbiota abundance (genus Catenibacterium id.2153)' on 'DNA−directed DNA/RNA polymerase mu'

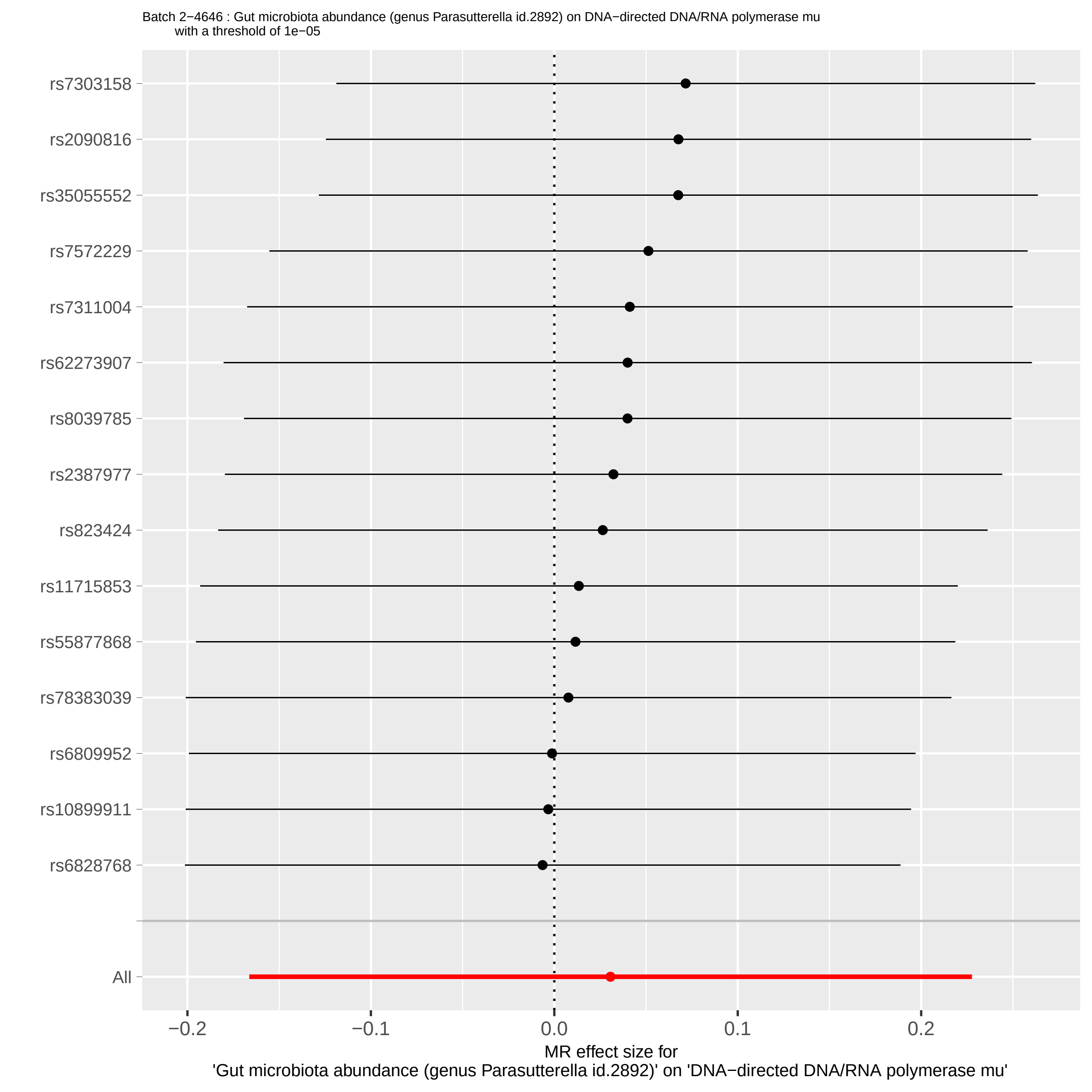

Batch 2−4646 : Gut microbiota abundance (genus Parasutterella id.2892) on DNA−directed DNA/RNA polymerase mu
with a threshold of 1e−05
rs7303158
rs2090816
rs35055552
rs7572229
rs7311004
rs62273907
rs8039785
rs2387977
rs823424
rs11715853
rs55877868
rs78383039
rs6809952
rs10899911
rs6828768
All
−0.2
−0.1
0.0
0.1
0.2
MR effect size for
'Gut microbiota abundance (genus Parasutterella id.2892)' on 'DNA−directed DNA/RNA polymerase mu'

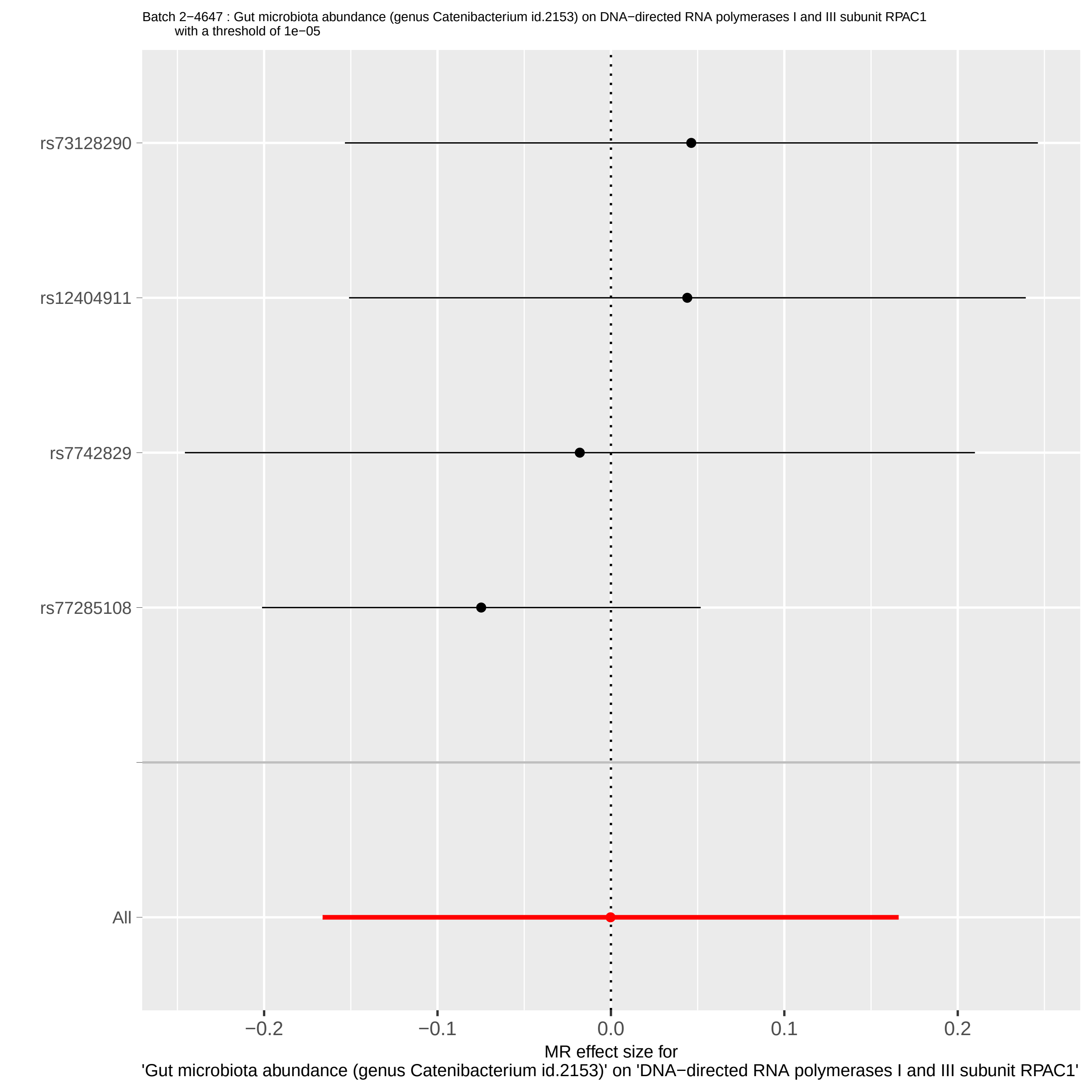

Batch 2−4647 : Gut microbiota abundance (genus Catenibacterium id.2153) on DNA−directed RNA polymerases I and III subunit RPAC1
with a threshold of 1e−05
rs73128290
rs12404911
rs7742829
rs77285108
All
−0.2
−0.1
0.0
0.1
0.2
MR effect size for
'Gut microbiota abundance (genus Catenibacterium id.2153)' on 'DNA−directed RNA polymerases I and III subunit RPAC1'

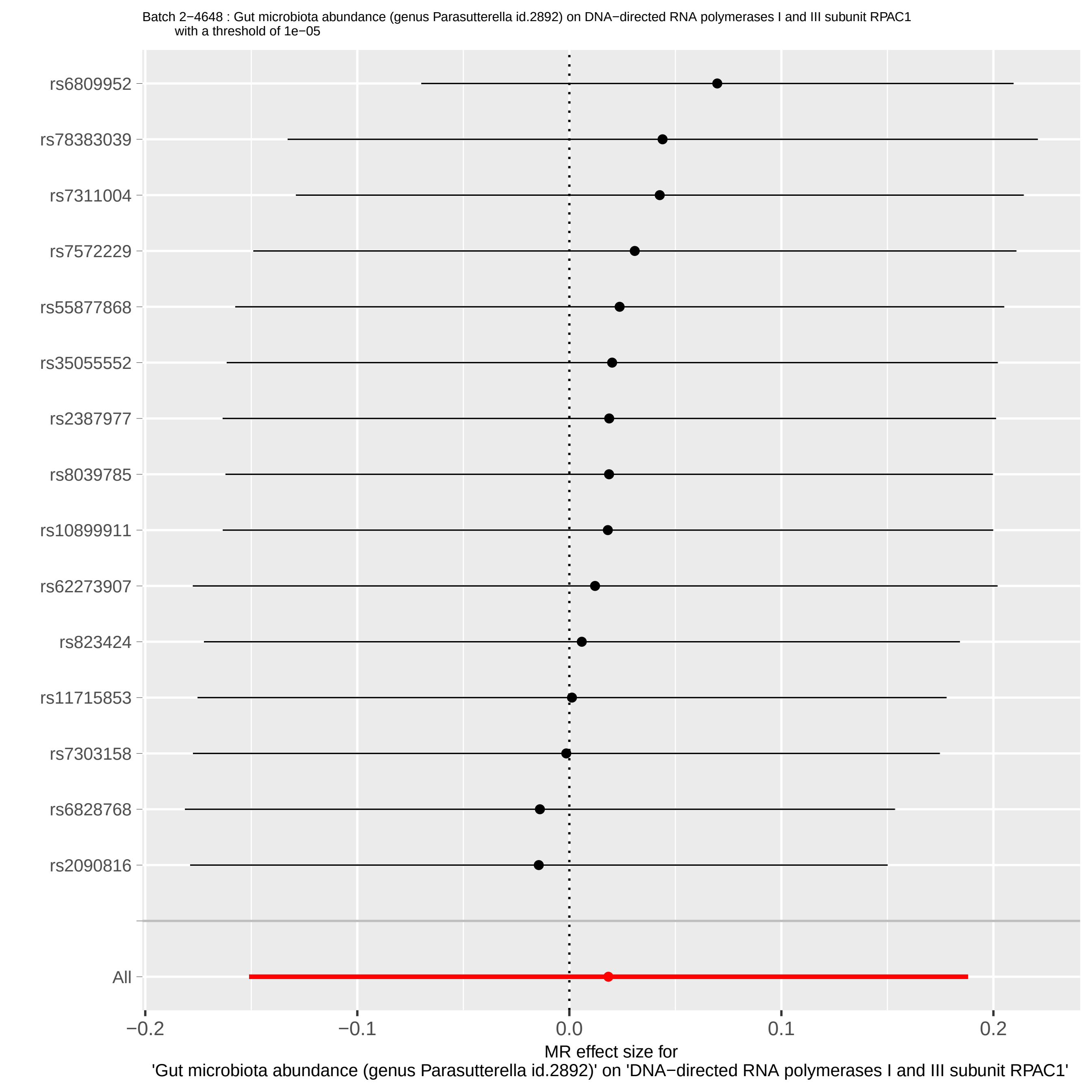
Batch 2−4648 : Gut microbiota abundance (genus Parasutterella id.2892) on DNA−directed RNA polymerases I and III subunit RPAC1
with a threshold of 1e−05
rs6809952
rs78383039
rs7311004
rs7572229
rs55877868
rs35055552
rs2387977
rs8039785
rs10899911
rs62273907
rs823424
rs11715853
rs7303158
rs6828768
rs2090816
All
−0.2
−0.1
0.0
0.1
0.2
MR effect size for
'Gut microbiota abundance (genus Parasutterella id.2892)' on 'DNA−directed RNA polymerases I and III subunit RPAC1'

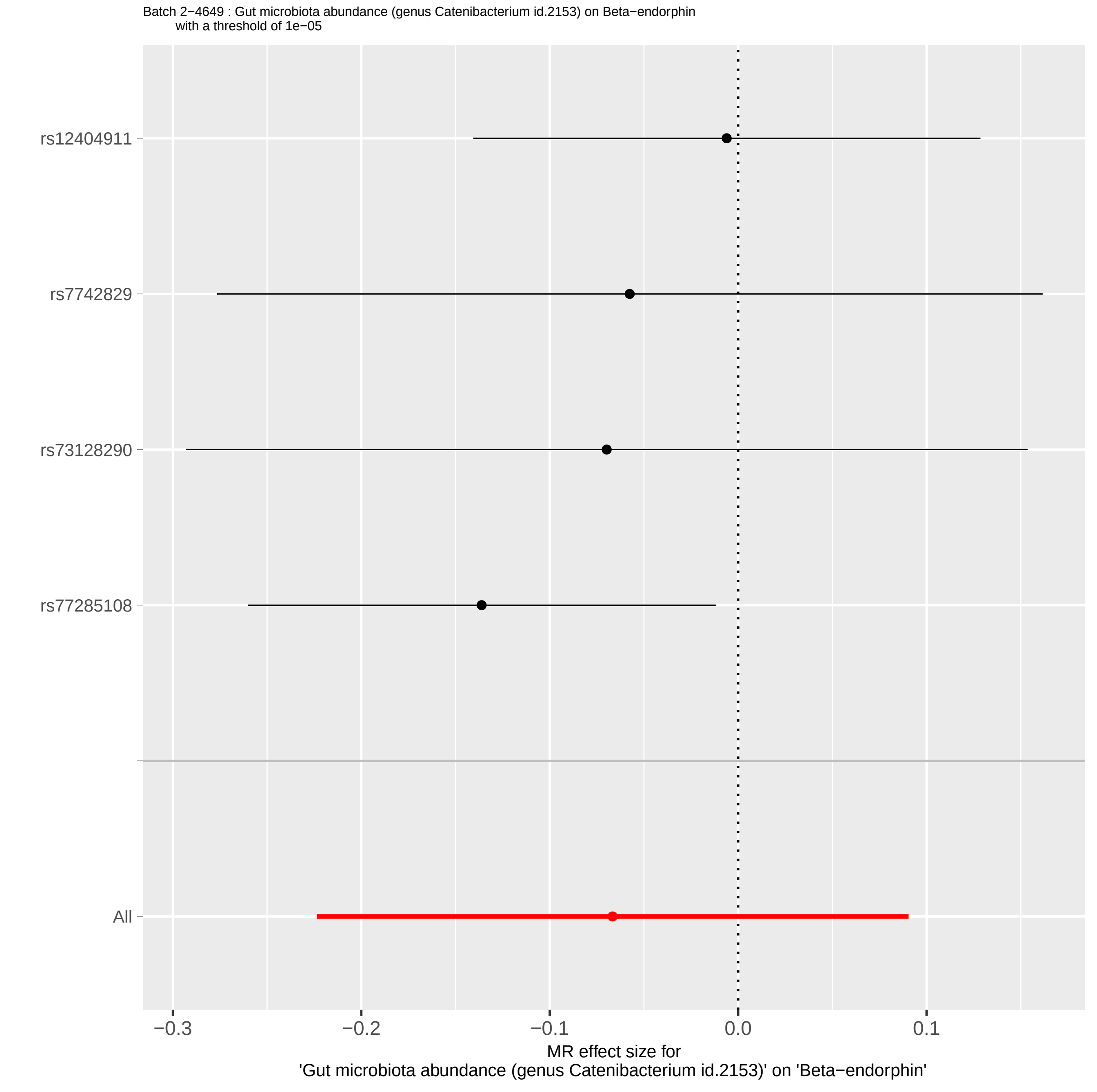
Batch 2−4649 : Gut microbiota abundance (genus Catenibacterium id.2153) on Beta−endorphin
with a threshold of 1e−05
rs12404911
rs7742829
rs73128290
rs77285108
All
−0.3
−0.2
−0.1
0.0
0.1
MR effect size for
'Gut microbiota abundance (genus Catenibacterium id.2153)' on 'Beta−endorphin'

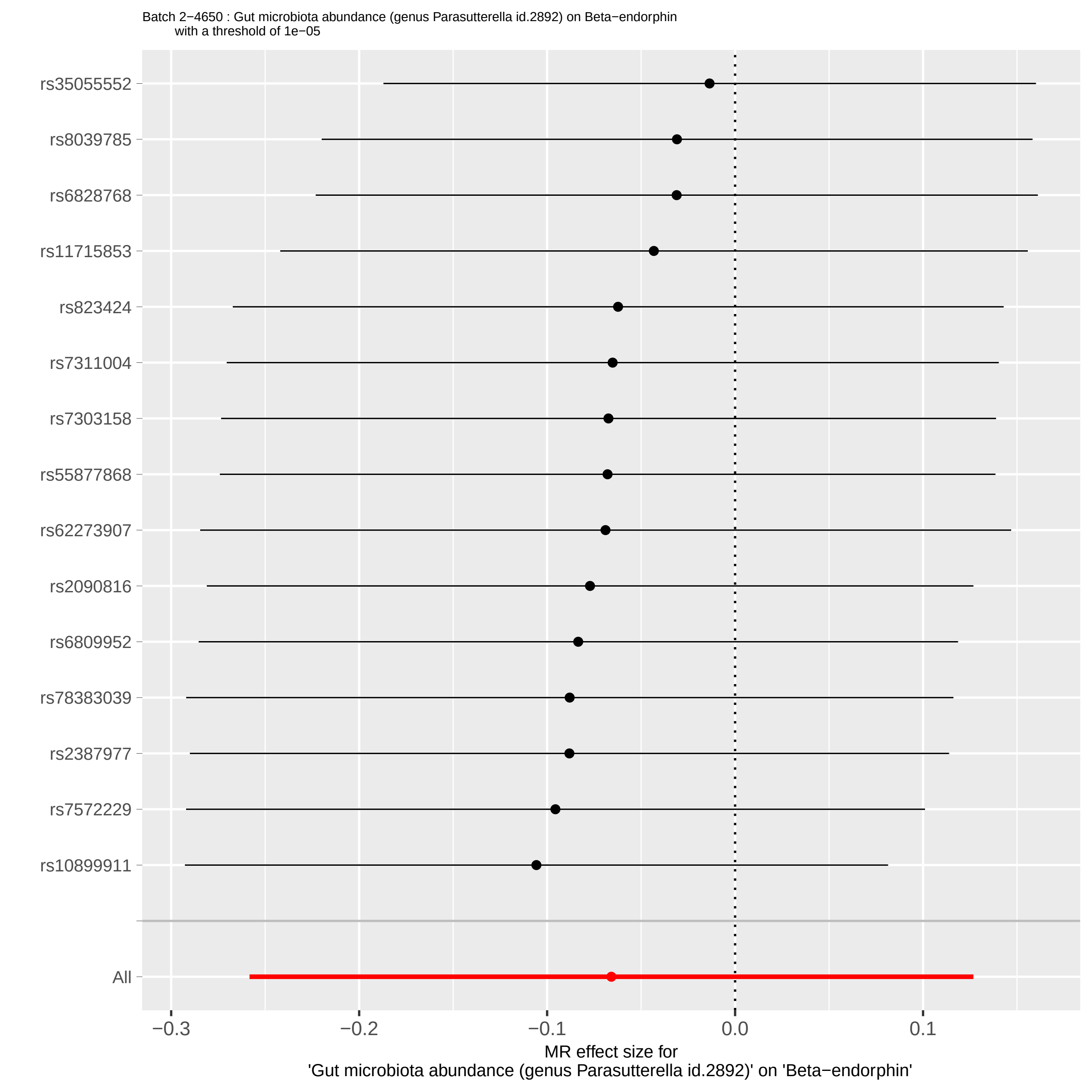
Batch 2−4650 : Gut microbiota abundance (genus Parasutterella id.2892) on Beta−endorphin
with a threshold of 1e−05
rs35055552
rs8039785
rs6828768
rs11715853
rs823424
rs7311004
rs7303158
rs55877868
rs62273907
rs2090816
rs6809952
rs78383039
rs2387977
rs7572229
rs10899911
All
−0.3
−0.2
−0.1
0.0
0.1
MR effect size for
'Gut microbiota abundance (genus Parasutterella id.2892)' on 'Beta−endorphin'

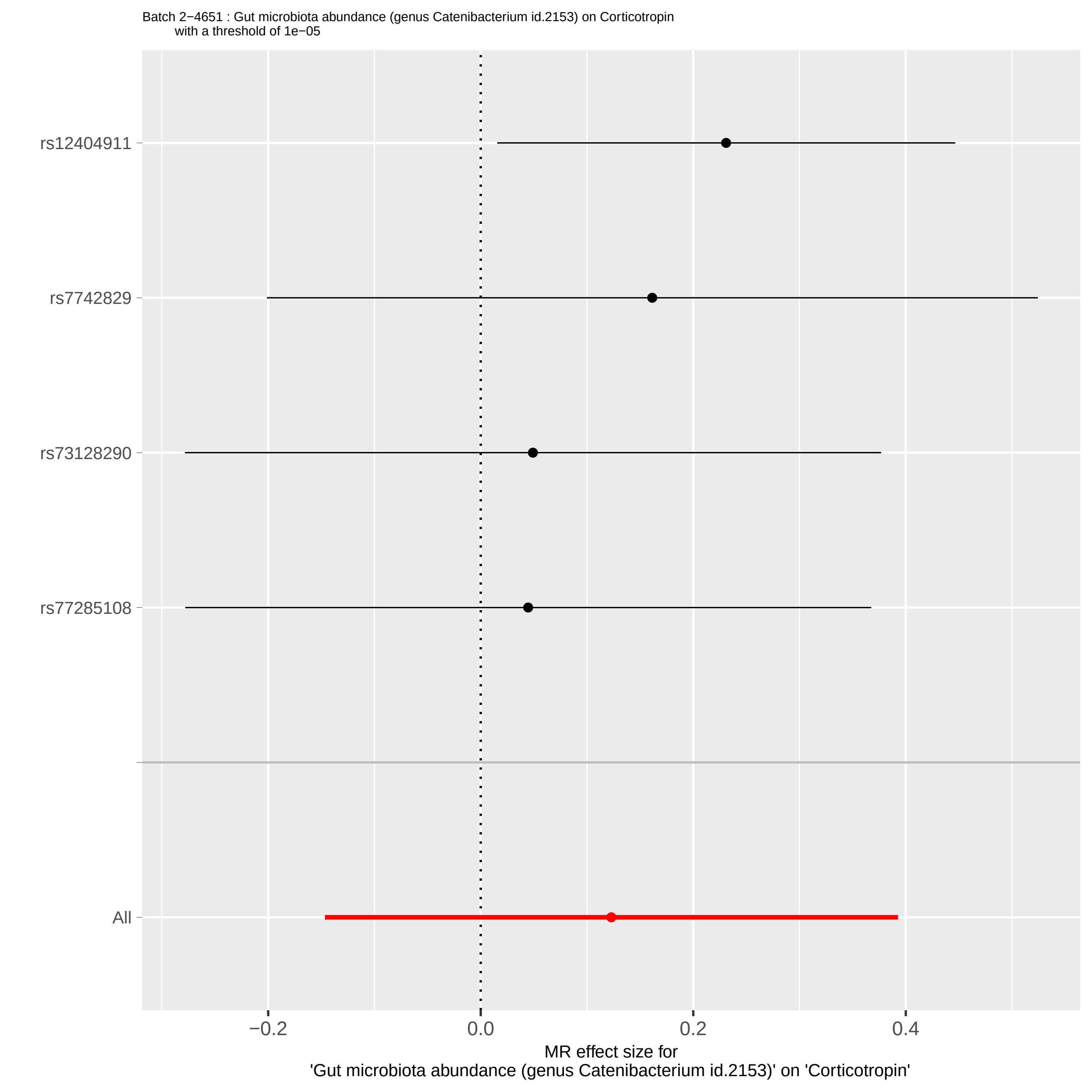
Batch 2−4651 : Gut microbiota abundance (genus Catenibacterium id.2153) on Corticotropin
with a threshold of 1e−05
rs12404911
rs7742829
rs73128290
rs77285108
All
−0.2
0.0
0.2
0.4
MR effect size for
'Gut microbiota abundance (genus Catenibacterium id.2153)' on 'Corticotropin'

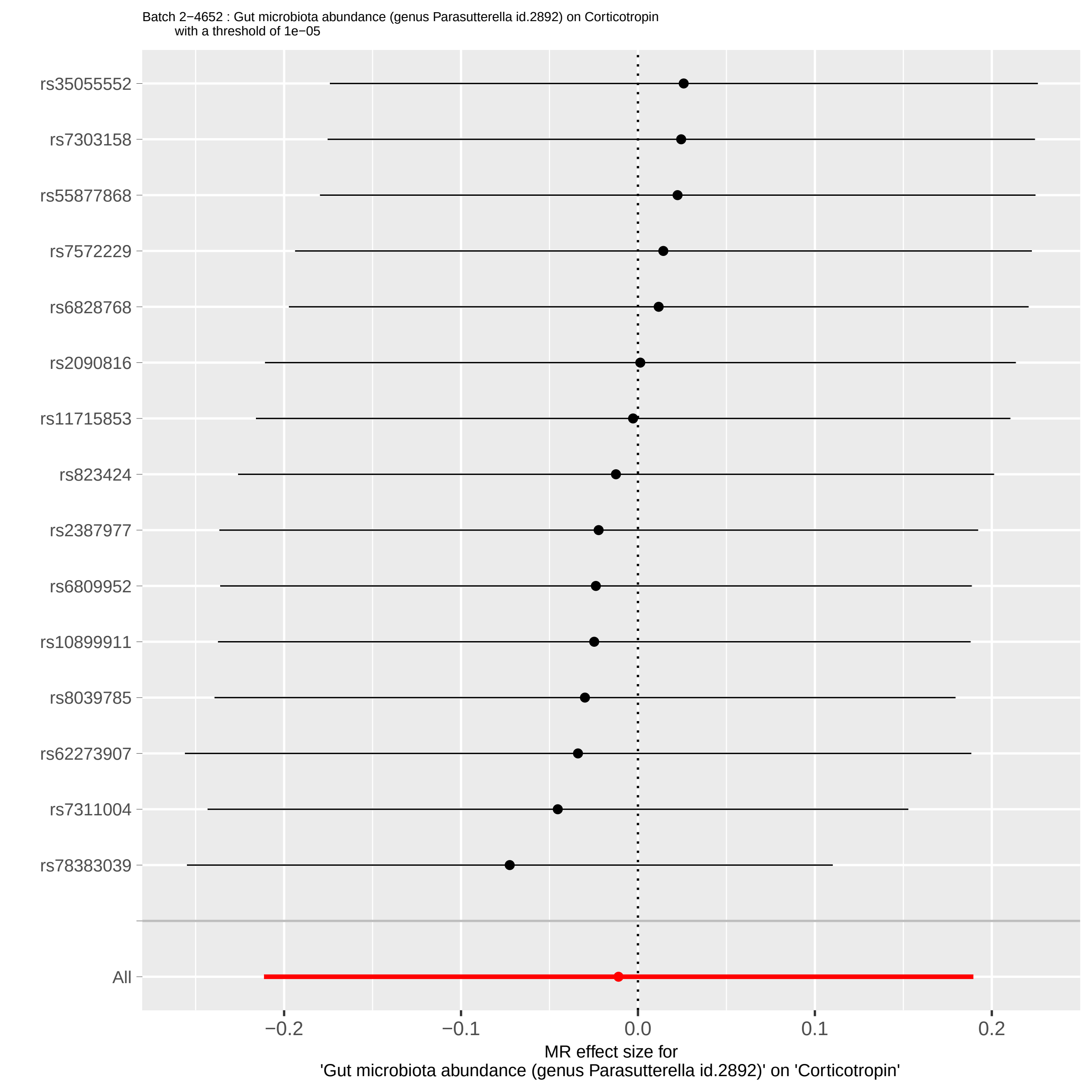

Batch 2−4652 : Gut microbiota abundance (genus Parasutterella id.2892) on Corticotropin
with a threshold of 1e−05
rs35055552
rs7303158
rs55877868
rs7572229
rs6828768
rs2090816
rs11715853
rs823424
rs2387977
rs6809952
rs10899911
rs8039785
rs62273907
rs7311004
rs78383039
All
−0.2
−0.1
0.0
0.1
0.2
MR effect size for
'Gut microbiota abundance (genus Parasutterella id.2892)' on 'Corticotropin'

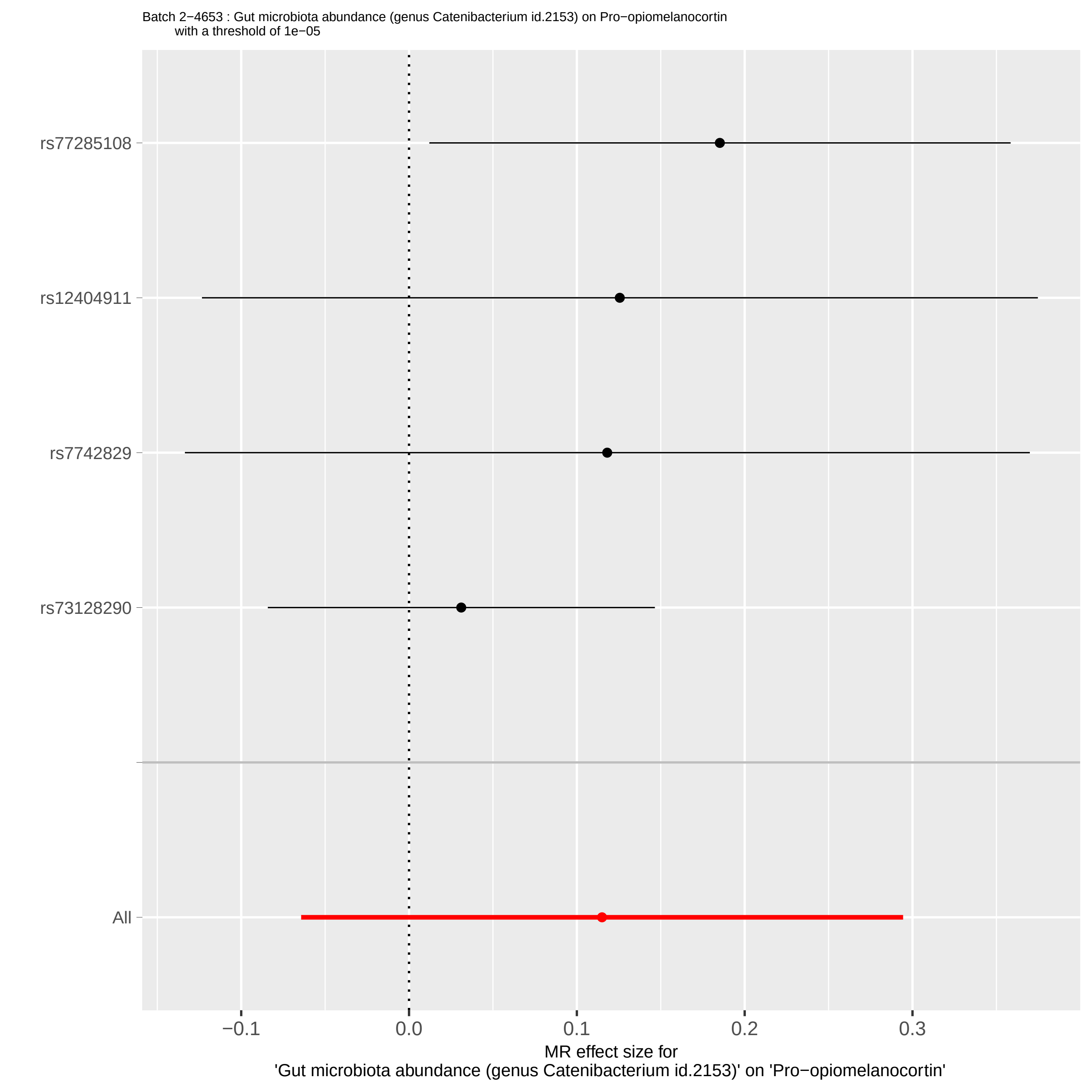
Batch 2−4653 : Gut microbiota abundance (genus Catenibacterium id.2153) on Pro−opiomelanocortin
with a threshold of 1e−05
rs77285108
rs12404911
rs7742829
rs73128290
All
−0.1
0.0
0.1
0.2
0.3
MR effect size for
'Gut microbiota abundance (genus Catenibacterium id.2153)' on 'Pro−opiomelanocortin'

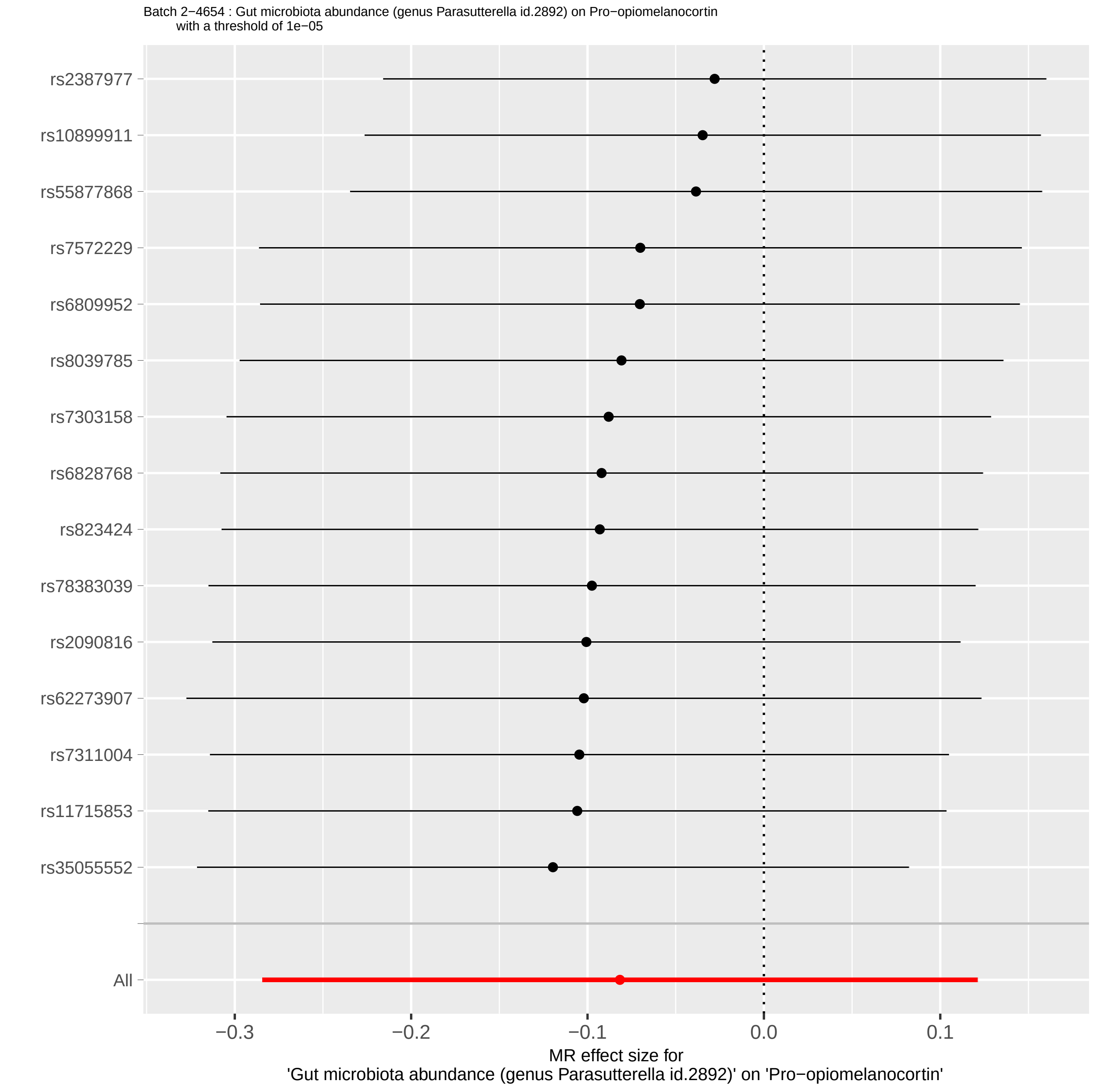
Batch 2−4654 : Gut microbiota abundance (genus Parasutterella id.2892) on Pro−opiomelanocortin
with a threshold of 1e−05
rs2387977
rs10899911
rs55877868
rs7572229
rs6809952
rs8039785
rs7303158
rs6828768
rs823424
rs78383039
rs2090816
rs62273907
rs7311004
rs11715853
rs35055552
All
−0.3
−0.2
−0.1
0.0
0.1
MR effect size for
'Gut microbiota abundance (genus Parasutterella id.2892)' on 'Pro−opiomelanocortin'

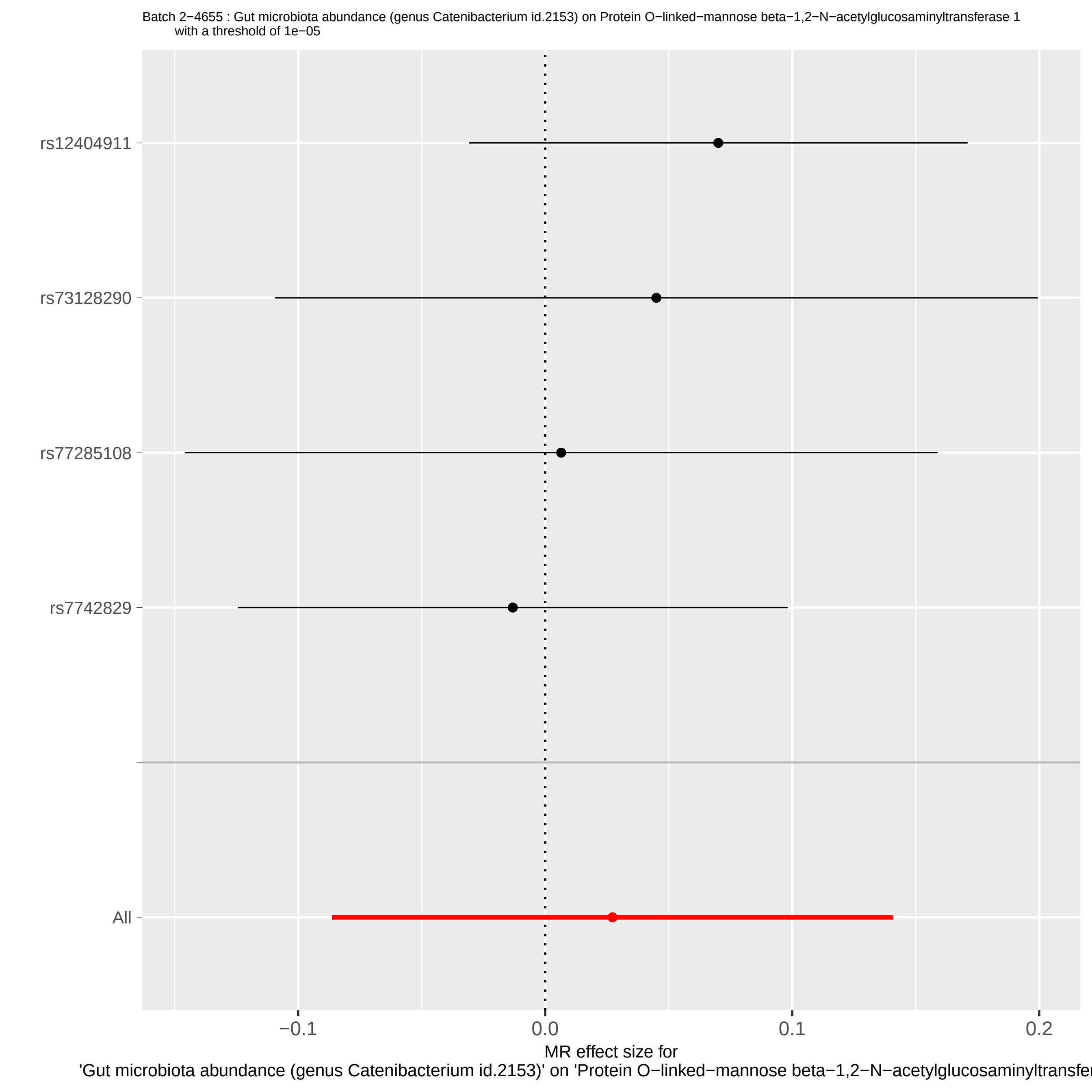

Batch 2−4655 : Gut microbiota abundance (genus Catenibacterium id.2153) on Protein O−linked−mannose beta−1,2−N−acetylglucosaminyltransferase 1
with a threshold of 1e−05
rs12404911
rs73128290
rs77285108
rs7742829
All
−0.1
0.0
0.1
0.2
MR effect size for
'Gut microbiota abundance (genus Catenibacterium id.2153)' on 'Protein O−linked−mannose beta−1,2−N−acetylglucosaminyltransfer

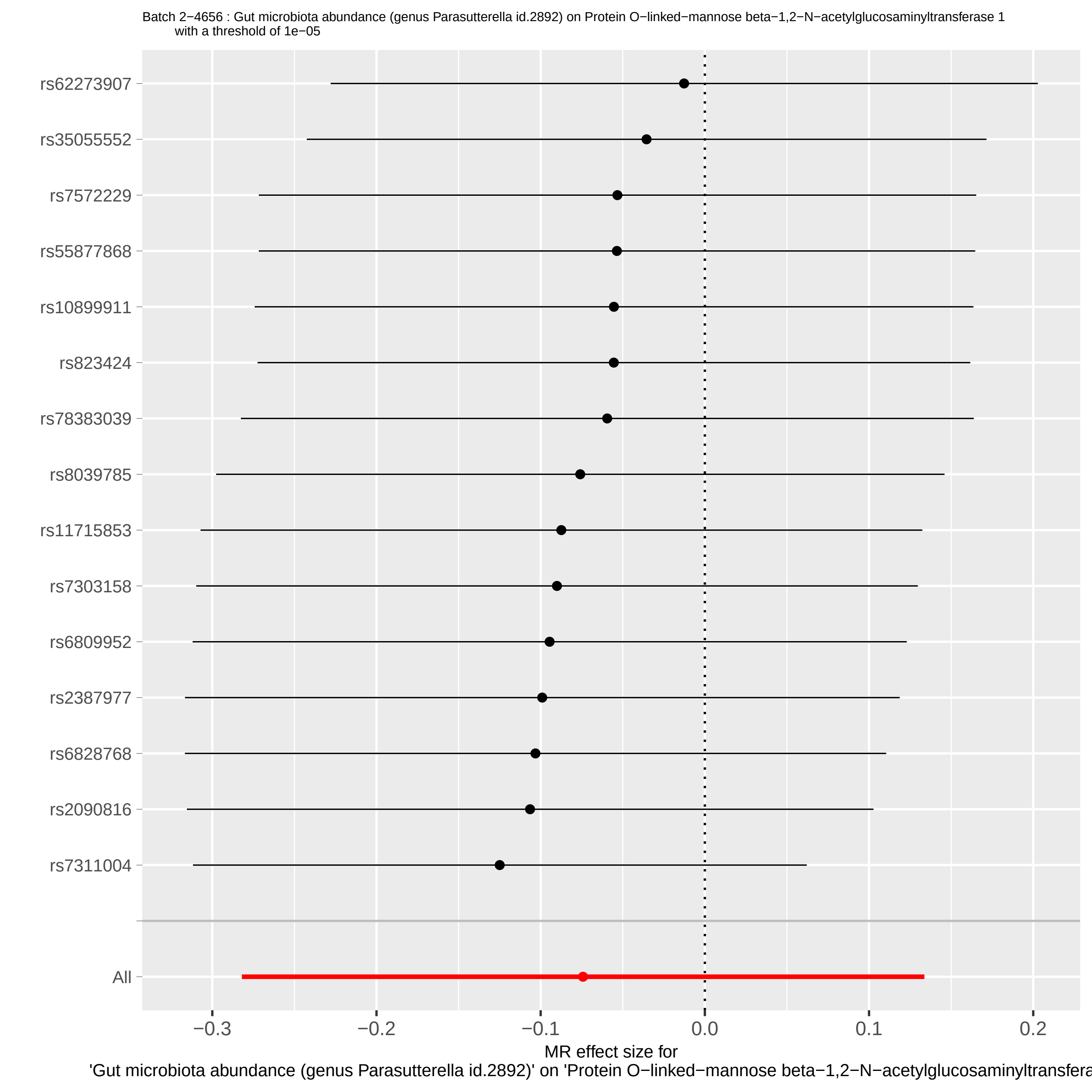

Batch 2−4656 : Gut microbiota abundance (genus Parasutterella id.2892) on Protein O−linked−mannose beta−1,2−N−acetylglucosaminyltransferase 1
with a threshold of 1e−05
rs62273907
rs35055552
rs7572229
rs55877868
rs10899911
rs823424
rs78383039
rs8039785
rs11715853
rs7303158
rs6809952
rs2387977
rs6828768
rs2090816
rs7311004
All
−0.3
−0.2
−0.1
0.0
0.1
0.2
MR effect size for
'Gut microbiota abundance (genus Parasutterella id.2892)' on 'Protein O−linked−mannose beta−1,2−N−acetylglucosaminyltransfera

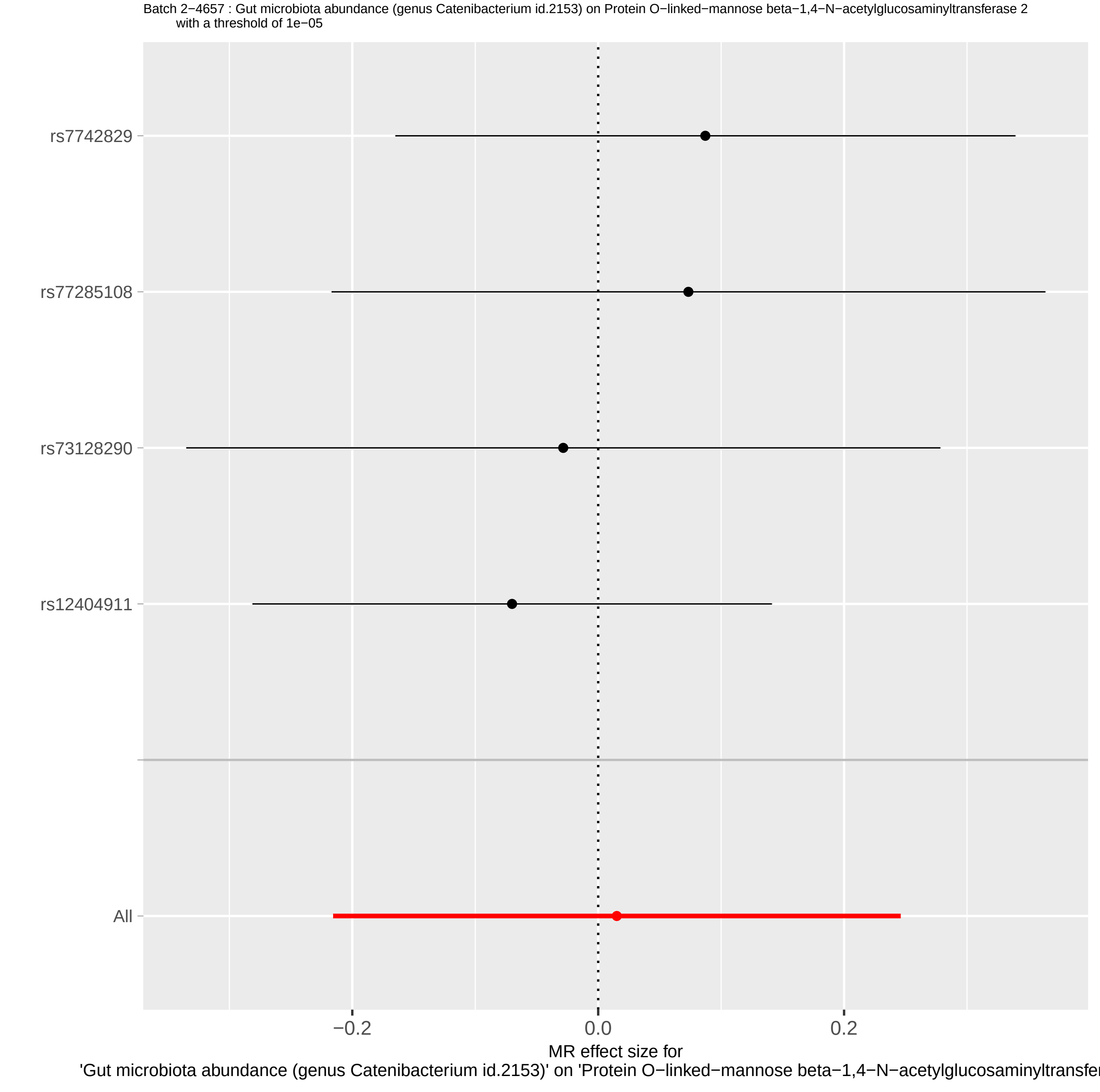

Batch 2−4657 : Gut microbiota abundance (genus Catenibacterium id.2153) on Protein O−linked−mannose beta−1,4−N−acetylglucosaminyltransferase 2
with a threshold of 1e−05
rs7742829
rs77285108
rs73128290
rs12404911
All
−0.2
0.0
0.2
MR effect size for
'Gut microbiota abundance (genus Catenibacterium id.2153)' on 'Protein O−linked−mannose beta−1,4−N−acetylglucosaminyltransfer

Batch 2−4658 : Gut microbiota abundance (genus Parasutterella id.2892) on Protein O−linked−mannose beta−1,4−N−acetylglucosaminyltransferase 2
with a threshold of 1e−05

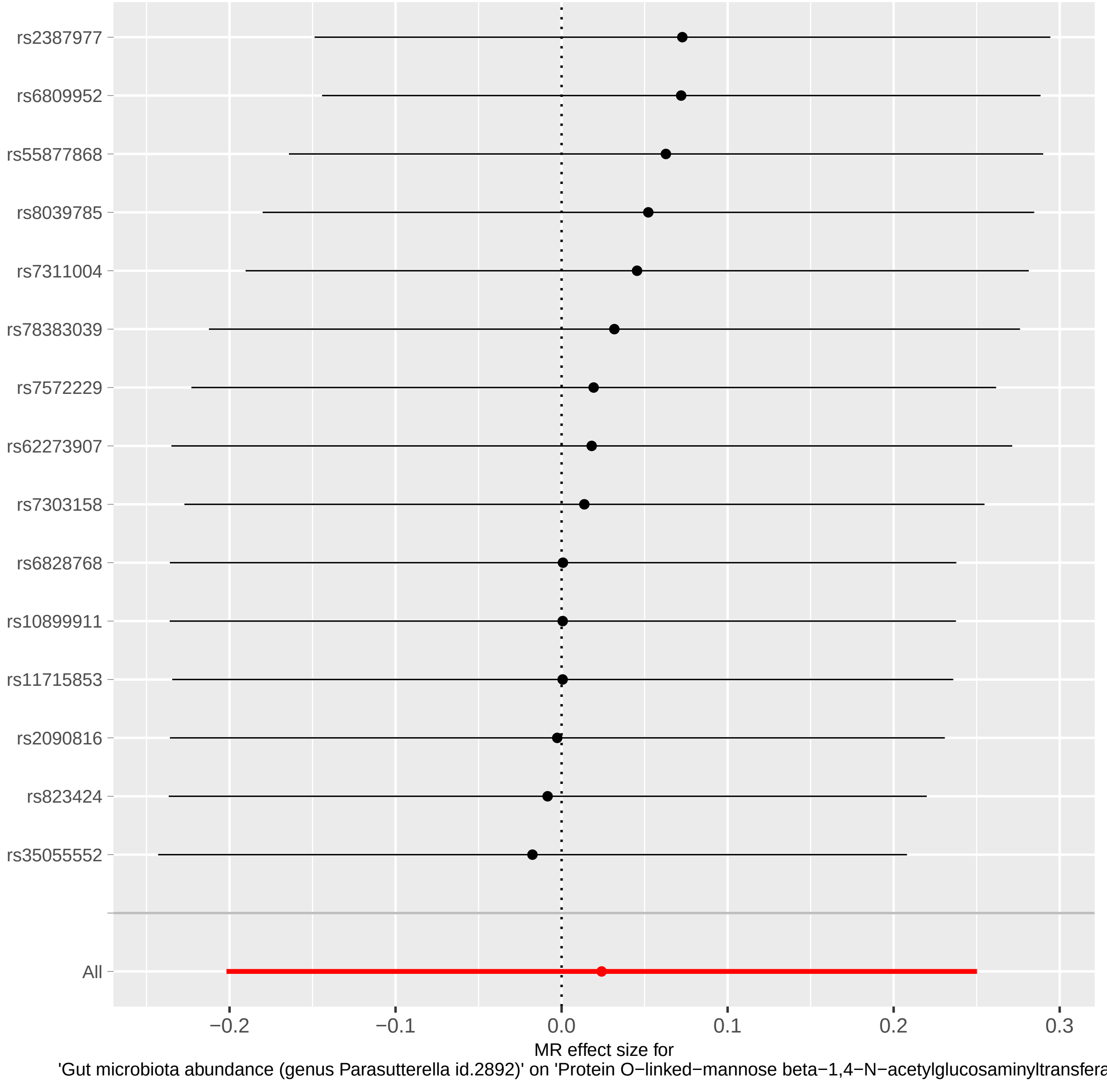

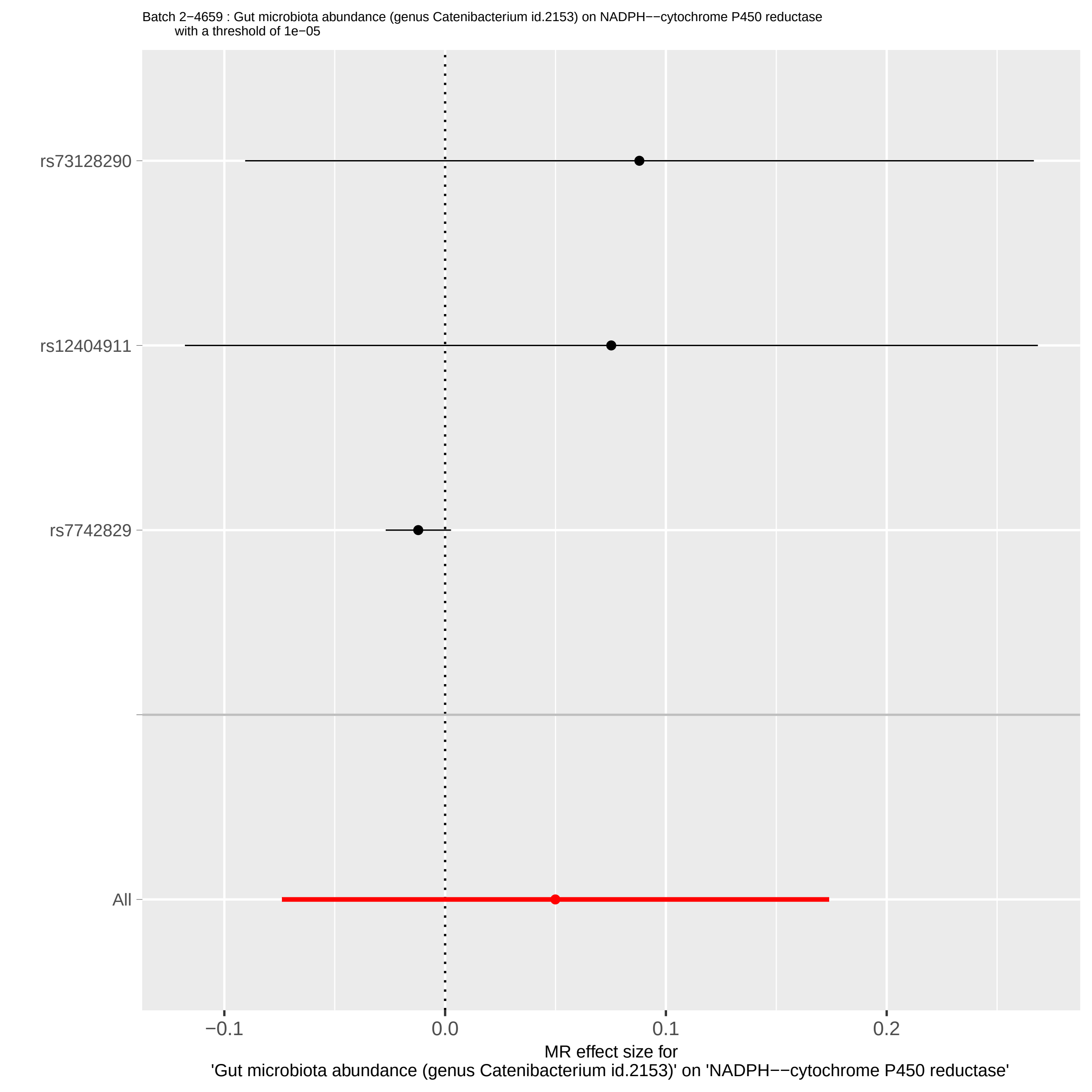
Batch 2−4659 : Gut microbiota abundance (genus Catenibacterium id.2153) on NADPH−−cytochrome P450 reductase
with a threshold of 1e−05
rs73128290
rs12404911
rs7742829
All
−0.1
0.0
0.1
0.2
MR effect size for
'Gut microbiota abundance (genus Catenibacterium id.2153)' on 'NADPH−−cytochrome P450 reductase'

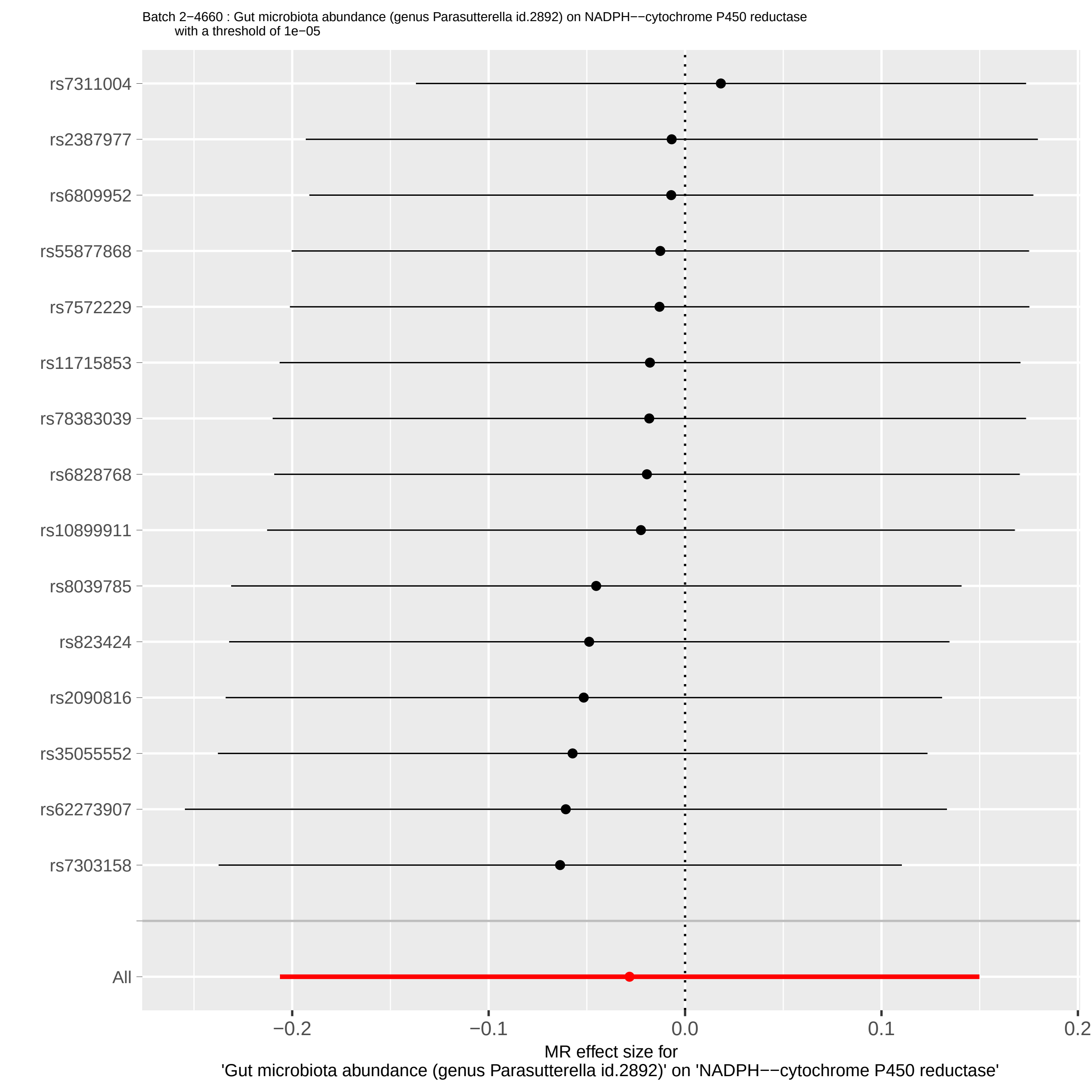
Batch 2−4660 : Gut microbiota abundance (genus Parasutterella id.2892) on NADPH−−cytochrome P450 reductase
with a threshold of 1e−05
rs7311004
rs2387977
rs6809952
rs55877868
rs7572229
rs11715853
rs78383039
rs6828768
rs10899911
rs8039785
rs823424
rs2090816
rs35055552
rs62273907
rs7303158
All
−0.2
−0.1
0.0
0.1
0.2
MR effect size for
'Gut microbiota abundance (genus Parasutterella id.2892)' on 'NADPH−−cytochrome P450 reductase'

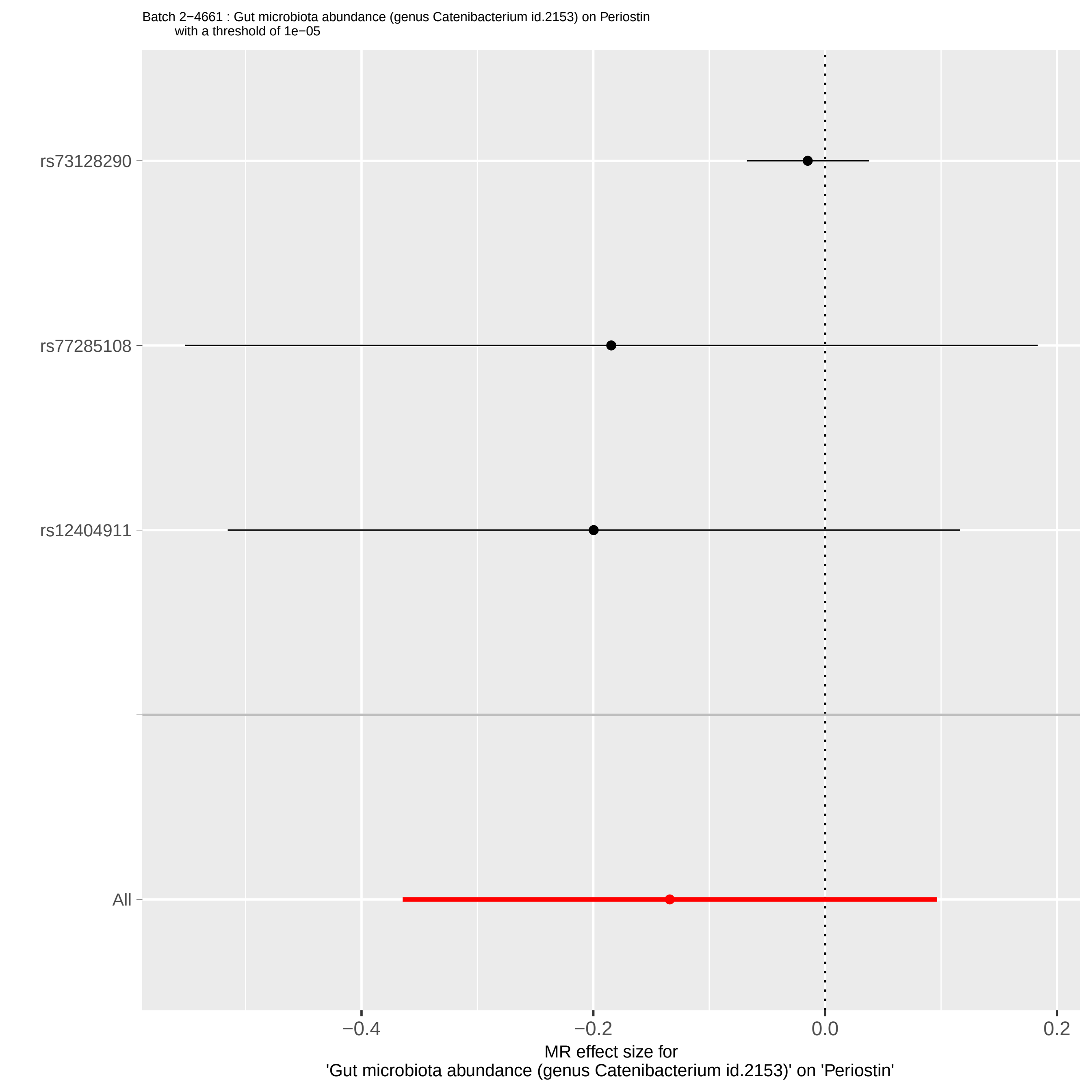

Batch 2−4661 : Gut microbiota abundance (genus Catenibacterium id.2153) on Periostin
with a threshold of 1e−05
rs73128290
rs77285108
rs12404911
All
−0.4
−0.2
0.0
0.2
MR effect size for
'Gut microbiota abundance (genus Catenibacterium id.2153)' on 'Periostin'

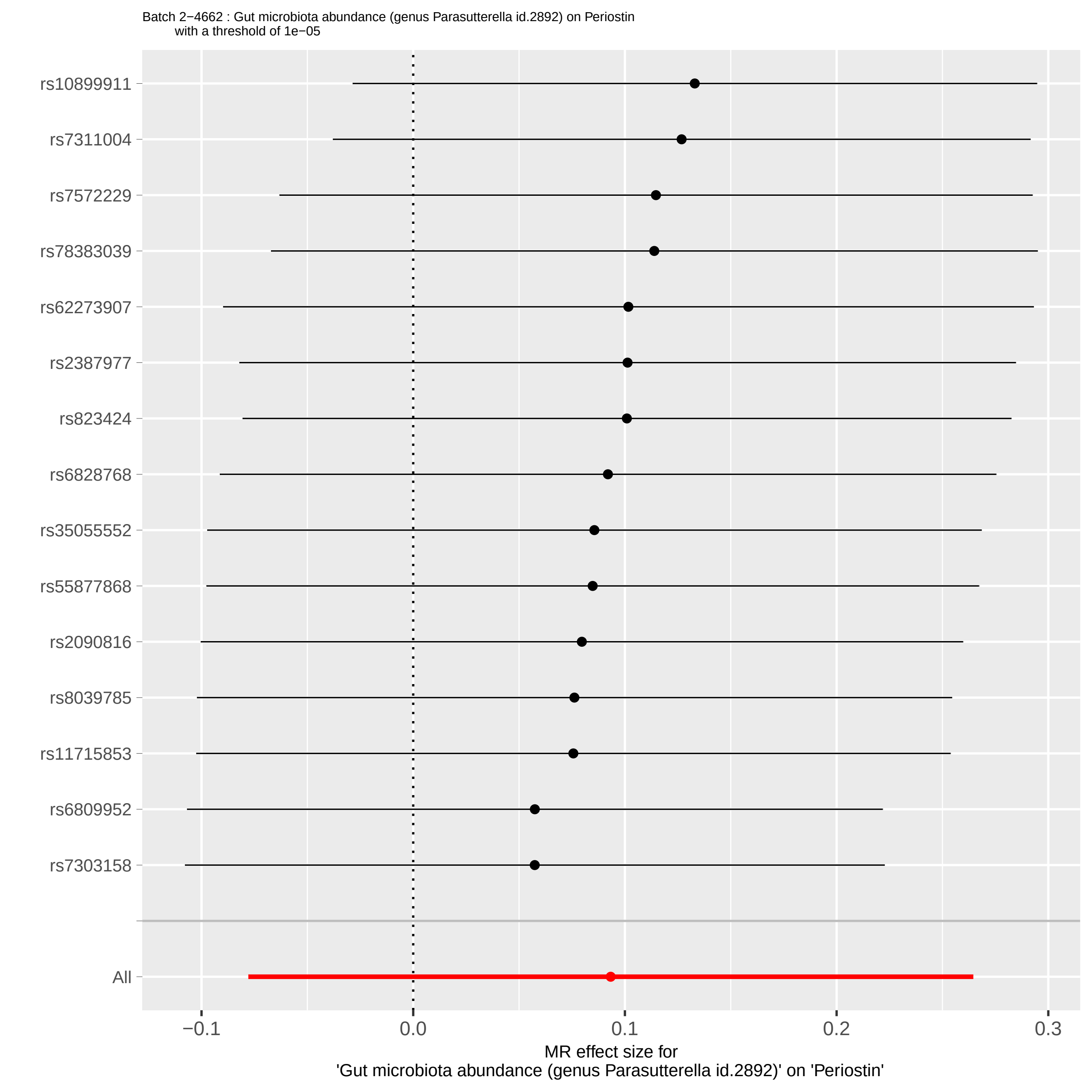
Batch 2−4662 : Gut microbiota abundance (genus Parasutterella id.2892) on Periostin
with a threshold of 1e−05
rs10899911
rs7311004
rs7572229
rs78383039
rs62273907
rs2387977
rs823424
rs6828768
rs35055552
rs55877868
rs2090816
rs8039785
rs11715853
rs6809952
rs7303158
All
−0.1
0.0
0.1
0.2
0.3
MR effect size for
'Gut microbiota abundance (genus Parasutterella id.2892)' on 'Periostin'

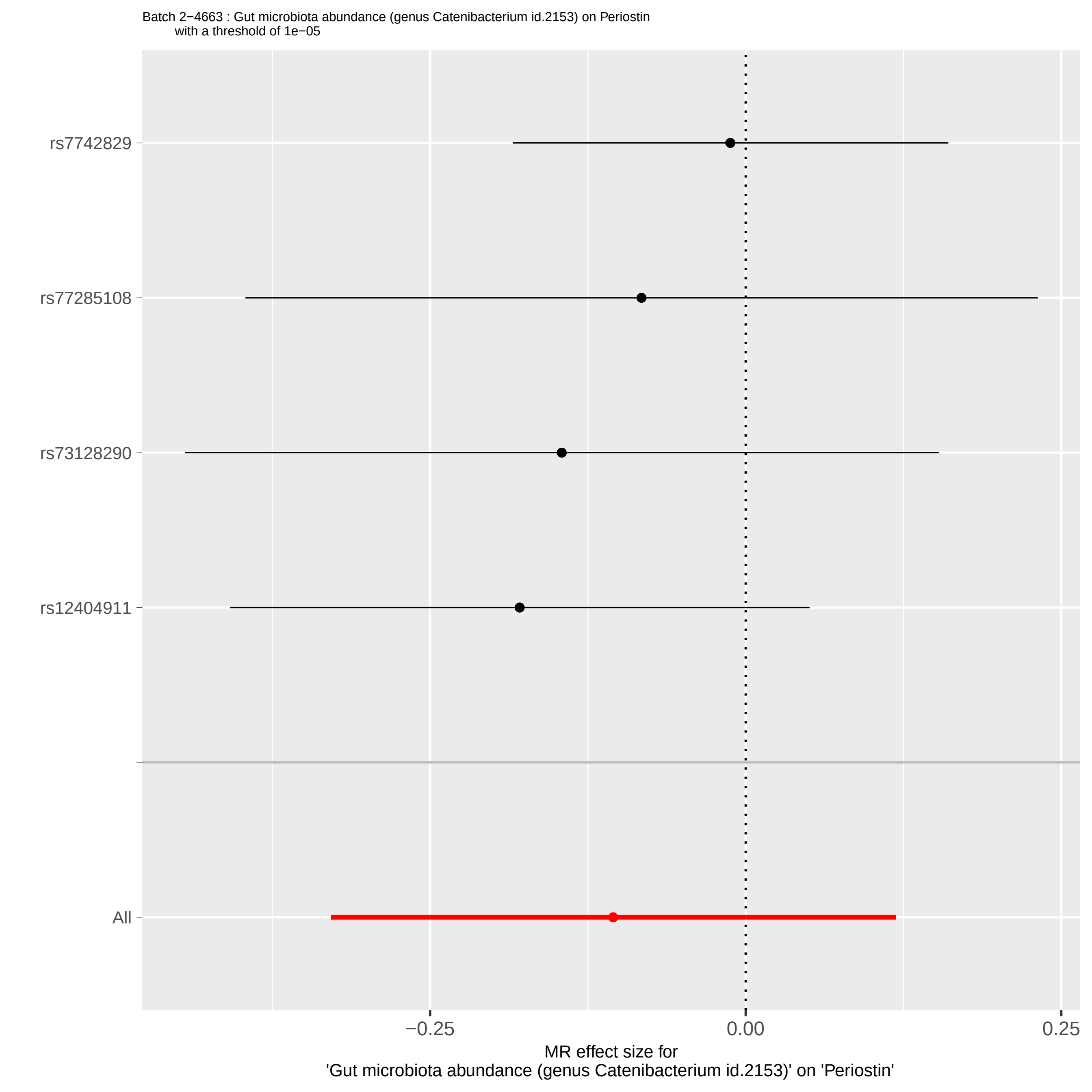
Batch 2−4663 : Gut microbiota abundance (genus Catenibacterium id.2153) on Periostin
with a threshold of 1e−05
rs7742829
rs77285108
rs73128290
rs12404911
All
−0.25
0.00
0.25
MR effect size for
'Gut microbiota abundance (genus Catenibacterium id.2153)' on 'Periostin'

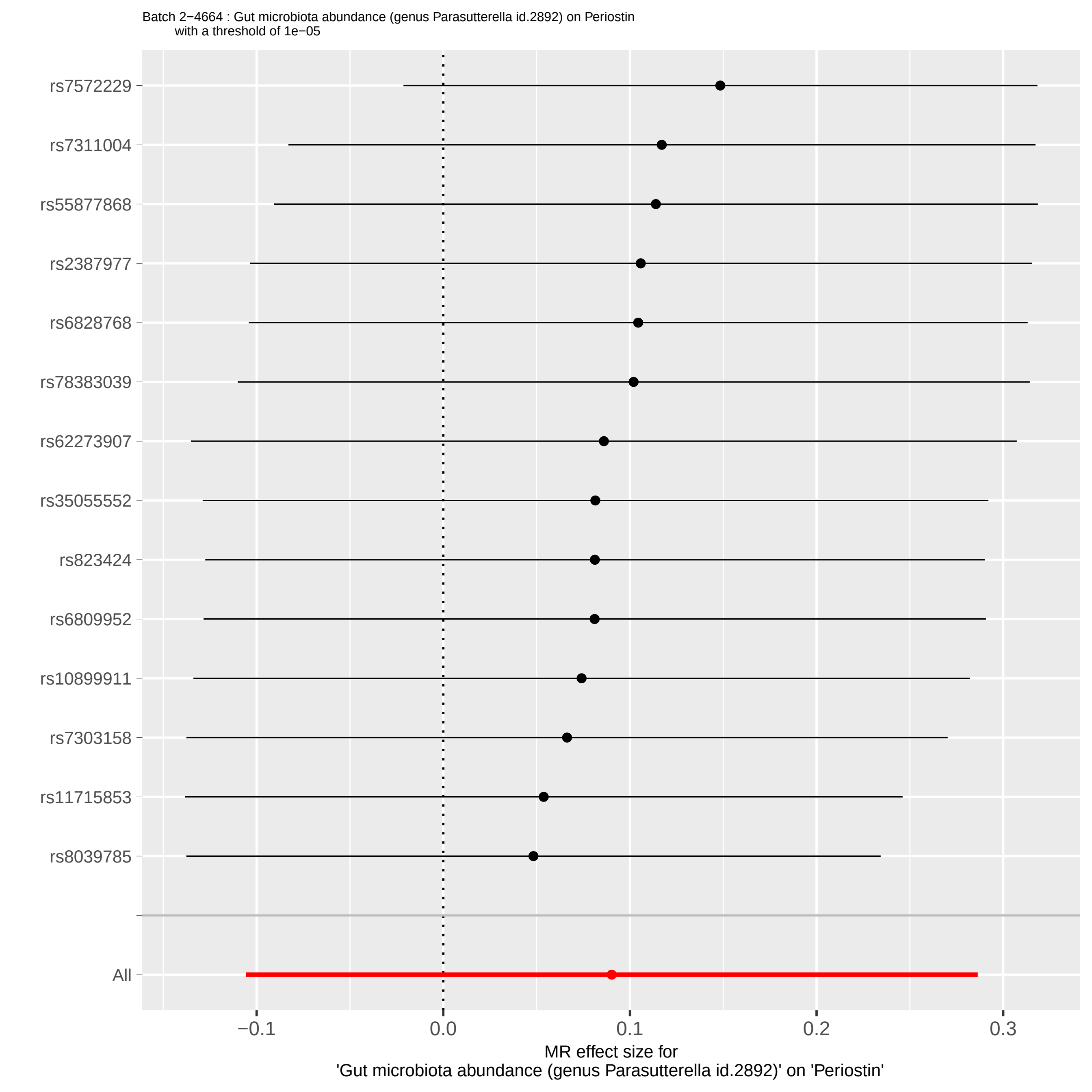
Batch 2−4664 : Gut microbiota abundance (genus Parasutterella id.2892) on Periostin
with a threshold of 1e−05
rs7572229
rs7311004
rs55877868
rs2387977
rs6828768
rs78383039
rs62273907
rs35055552
rs823424
rs6809952
rs10899911
rs7303158
rs11715853
rs8039785
All
−0.1
0.0
0.1
0.2
0.3
MR effect size for
'Gut microbiota abundance (genus Parasutterella id.2892)' on 'Periostin'

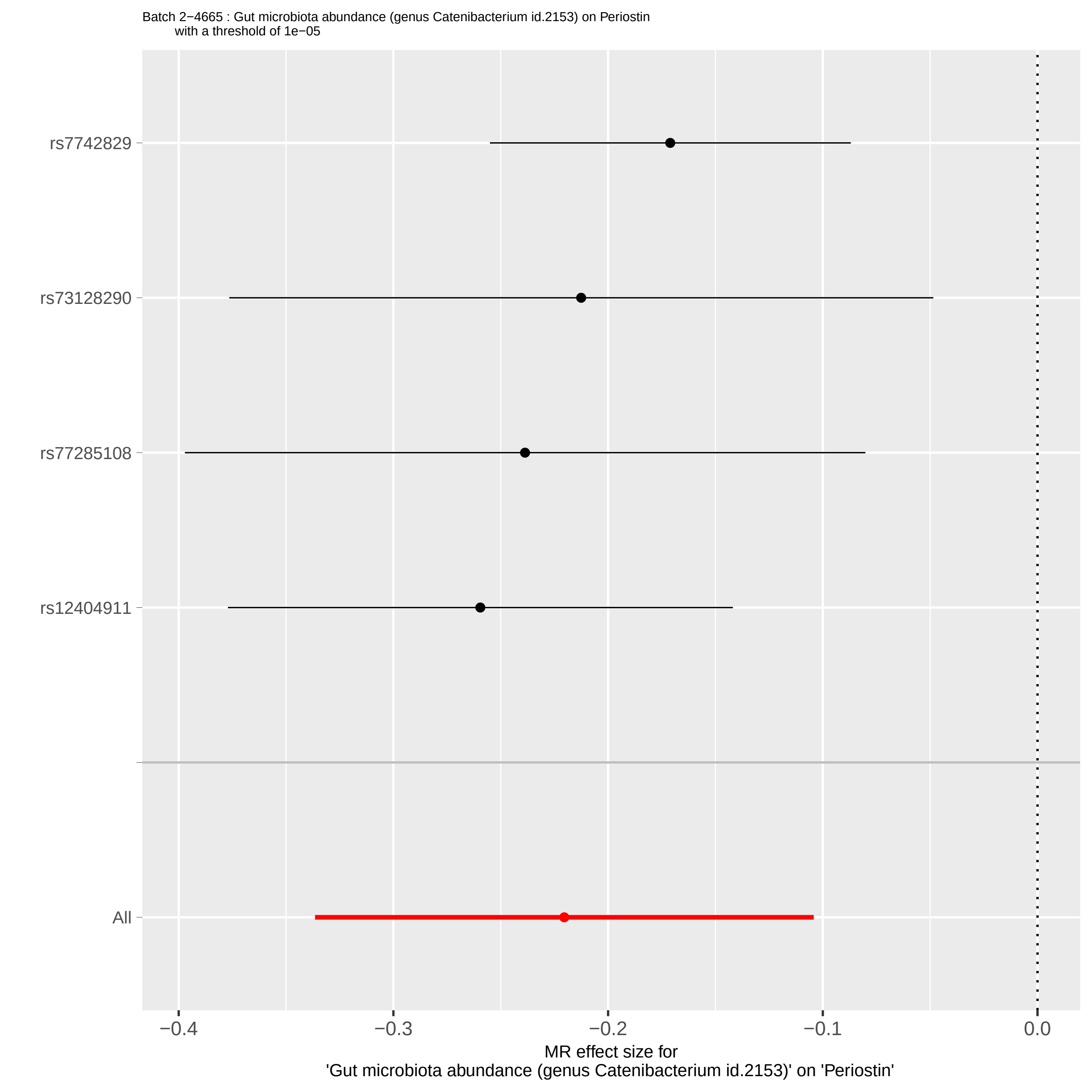
Batch 2−4665 : Gut microbiota abundance (genus Catenibacterium id.2153) on Periostin
with a threshold of 1e−05
rs7742829
rs73128290
rs77285108
rs12404911
All
−0.4
−0.3
−0.2
−0.1
0.0
MR effect size for
'Gut microbiota abundance (genus Catenibacterium id.2153)' on 'Periostin'

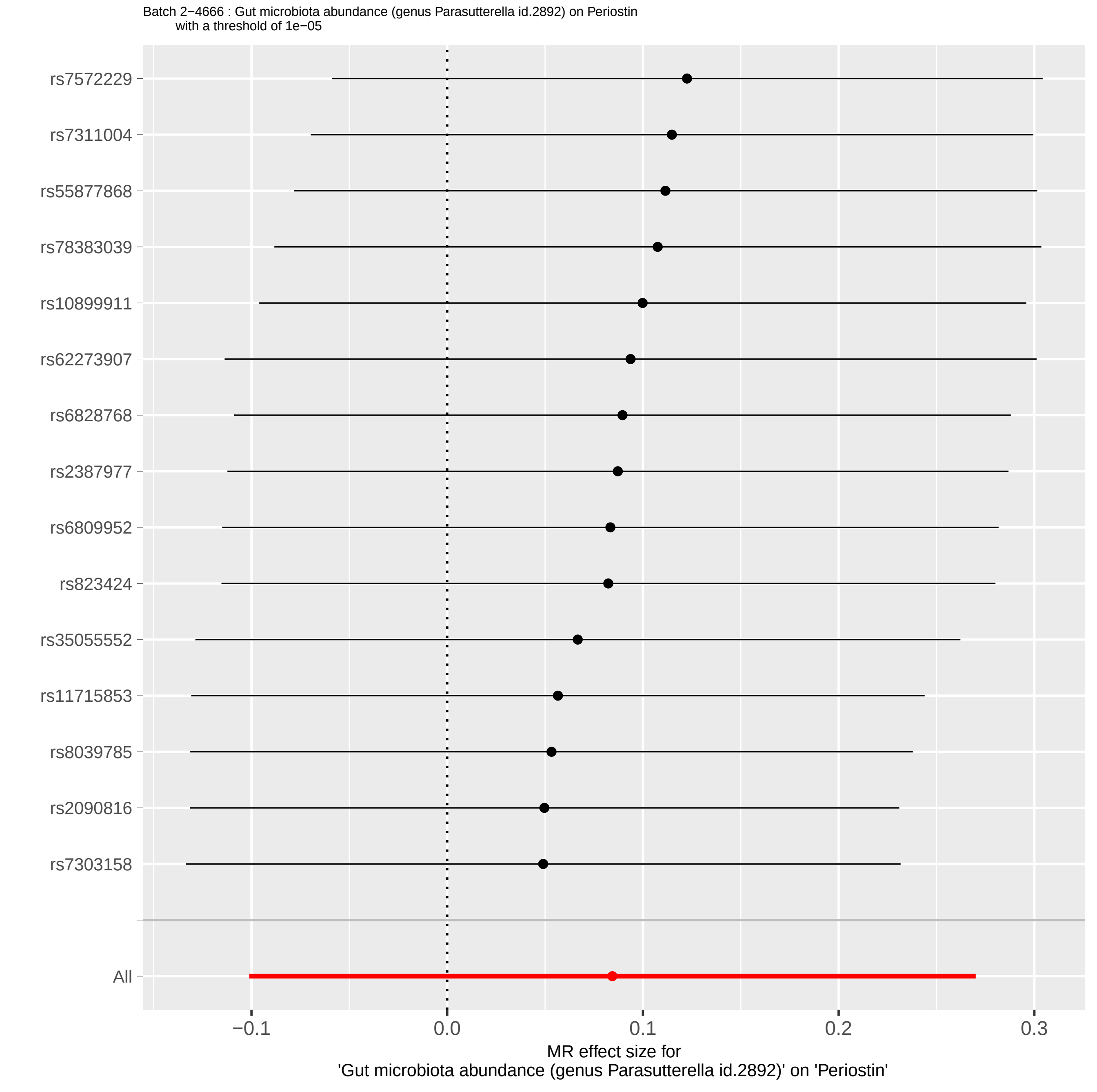

Batch 2−4666 : Gut microbiota abundance (genus Parasutterella id.2892) on Periostin
with a threshold of 1e−05
rs7572229
rs7311004
rs55877868
rs78383039
rs10899911
rs62273907
rs6828768
rs2387977
rs6809952
rs823424
rs35055552
rs11715853
rs8039785
rs2090816
rs7303158
All
−0.1
0.0
0.1
0.2
0.3
MR effect size for
'Gut microbiota abundance (genus Parasutterella id.2892)' on 'Periostin'

Batch 2−4667 : Gut microbiota abundance (genus Catenibacterium id.2153) on POU domain, class 2, transcription factor 1
with a threshold of 1e−05

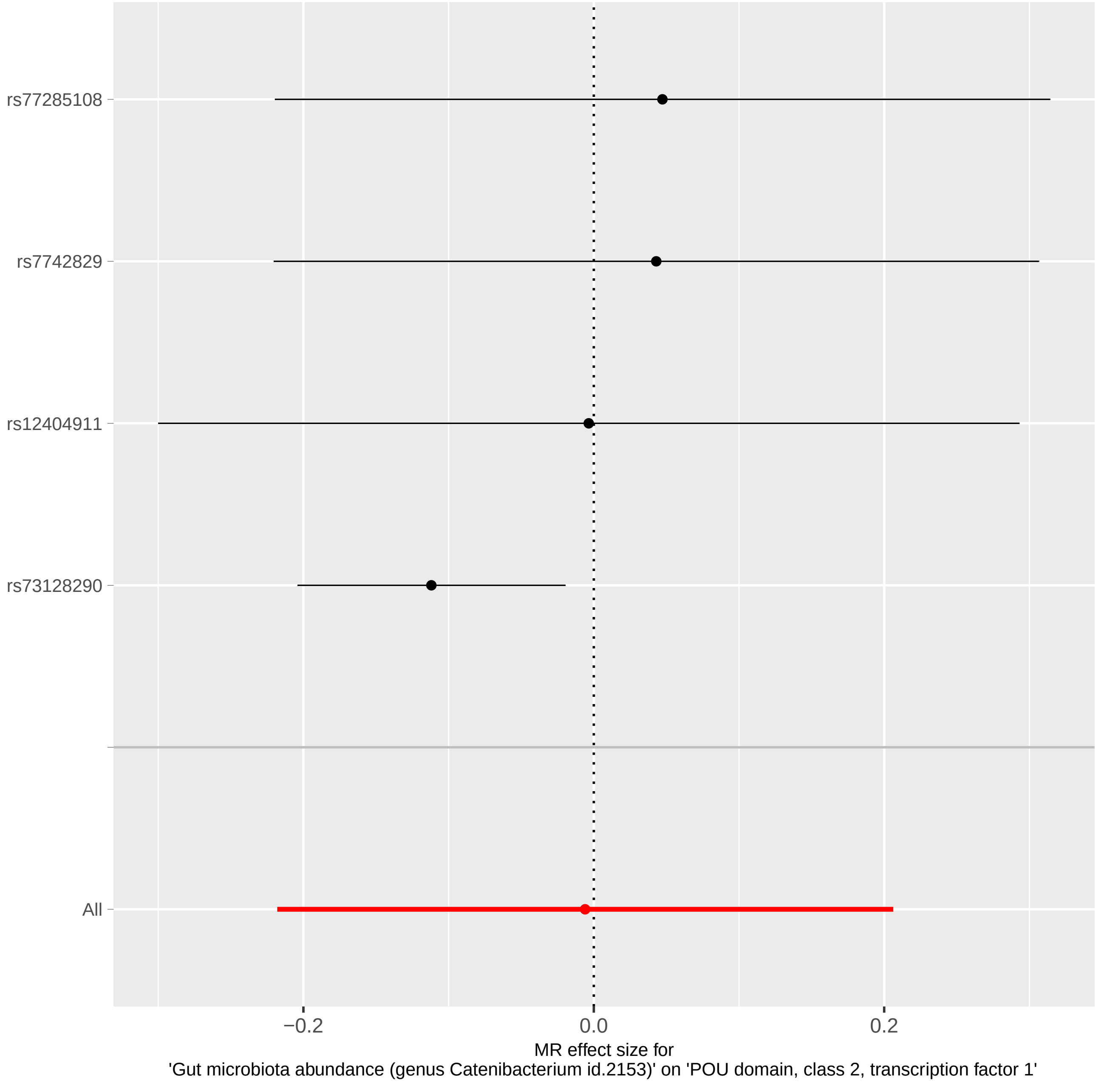

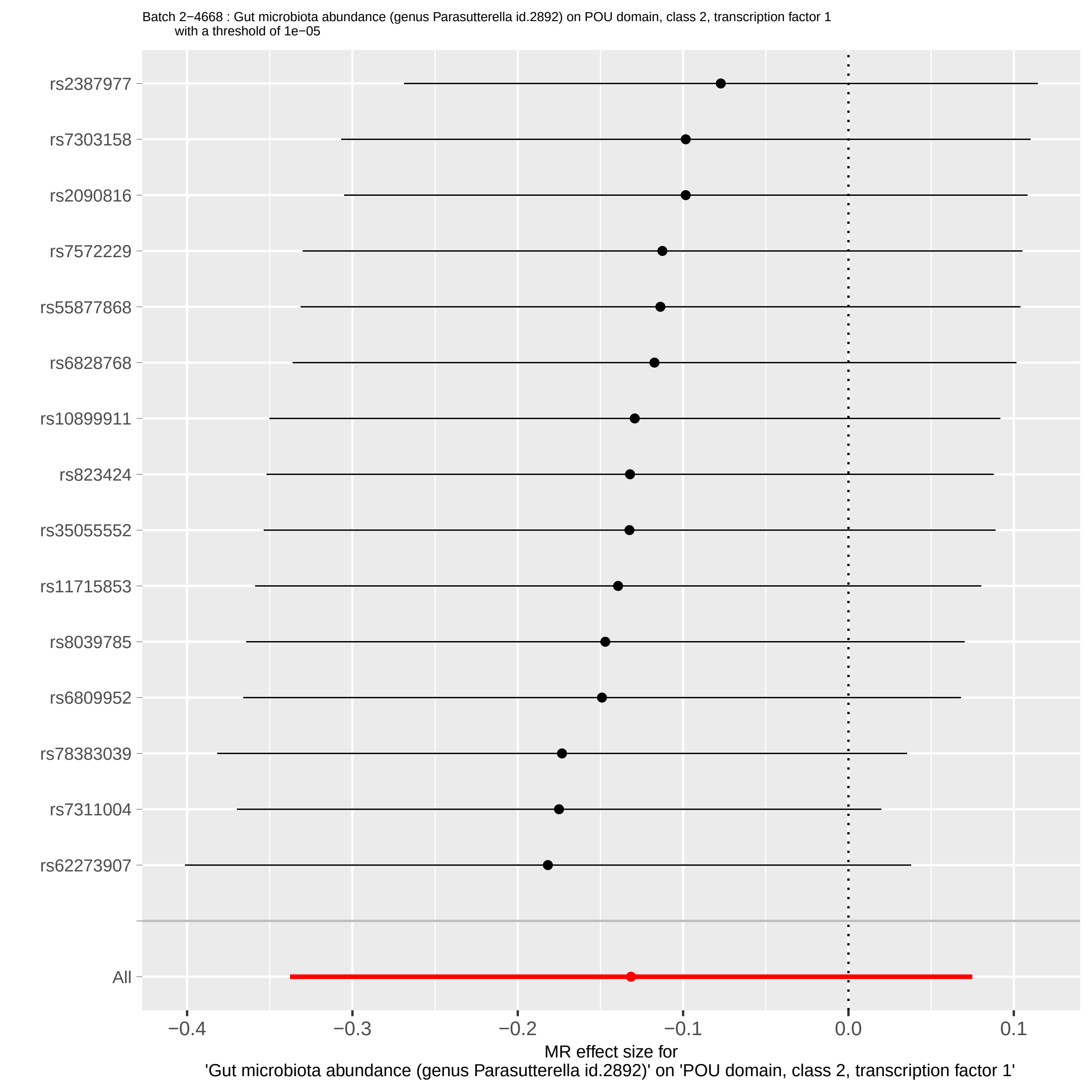
Batch 2−4668 : Gut microbiota abundance (genus Parasutterella id.2892) on POU domain, class 2, transcription factor 1
with a threshold of 1e−05
rs2387977
rs7303158
rs2090816
rs7572229
rs55877868
rs6828768
rs10899911
rs823424
rs35055552
rs11715853
rs8039785
rs6809952
rs78383039
rs7311004
rs62273907
All
−0.4
−0.3
−0.2
−0.1
0.0
0.1
MR effect size for
'Gut microbiota abundance (genus Parasutterella id.2892)' on 'POU domain, class 2, transcription factor 1'

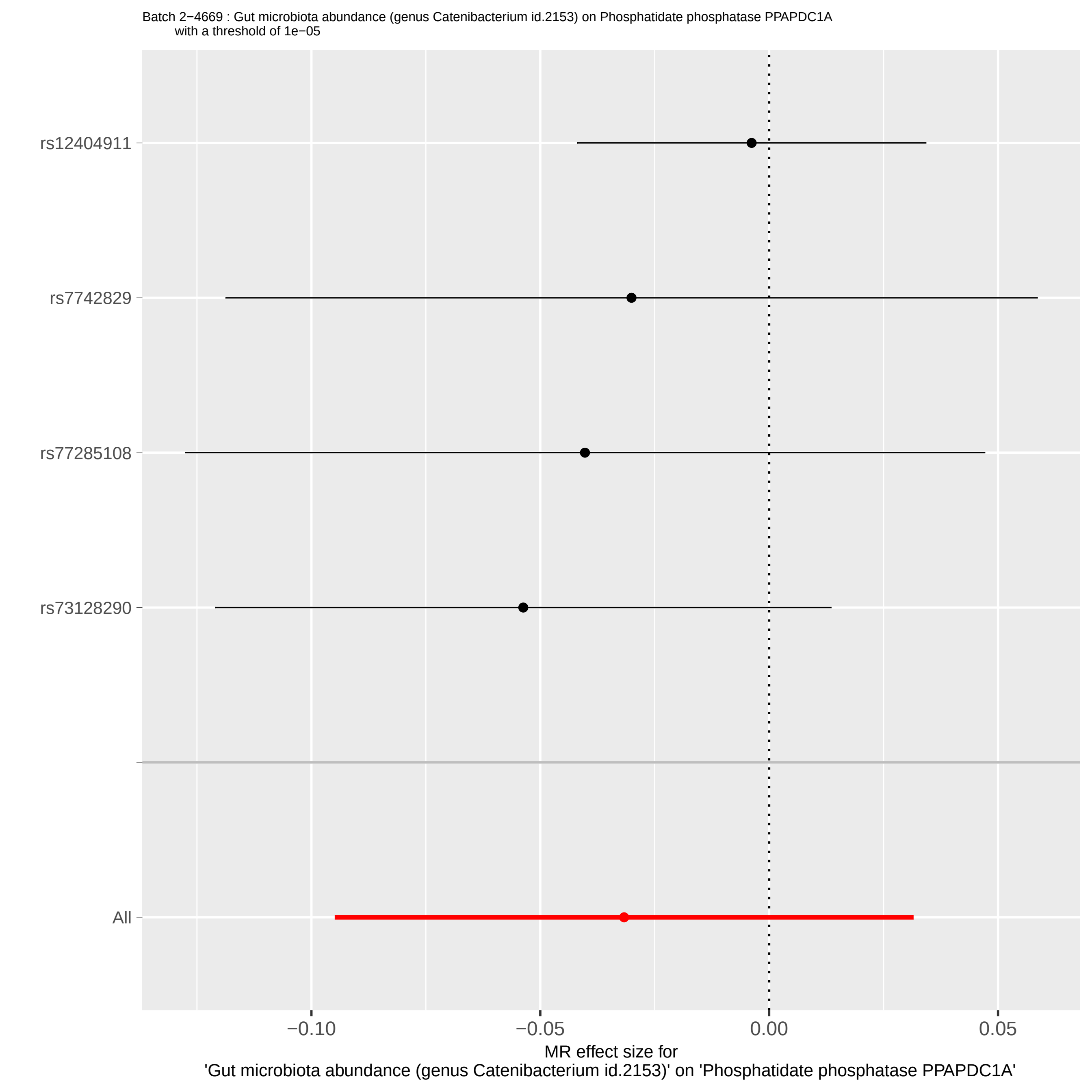

Batch 2−4669 : Gut microbiota abundance (genus Catenibacterium id.2153) on Phosphatidate phosphatase PPAPDC1A
with a threshold of 1e−05
rs12404911
rs7742829
rs77285108
rs73128290
All
−0.10
−0.05
0.00
0.05
MR effect size for
'Gut microbiota abundance (genus Catenibacterium id.2153)' on 'Phosphatidate phosphatase PPAPDC1A'

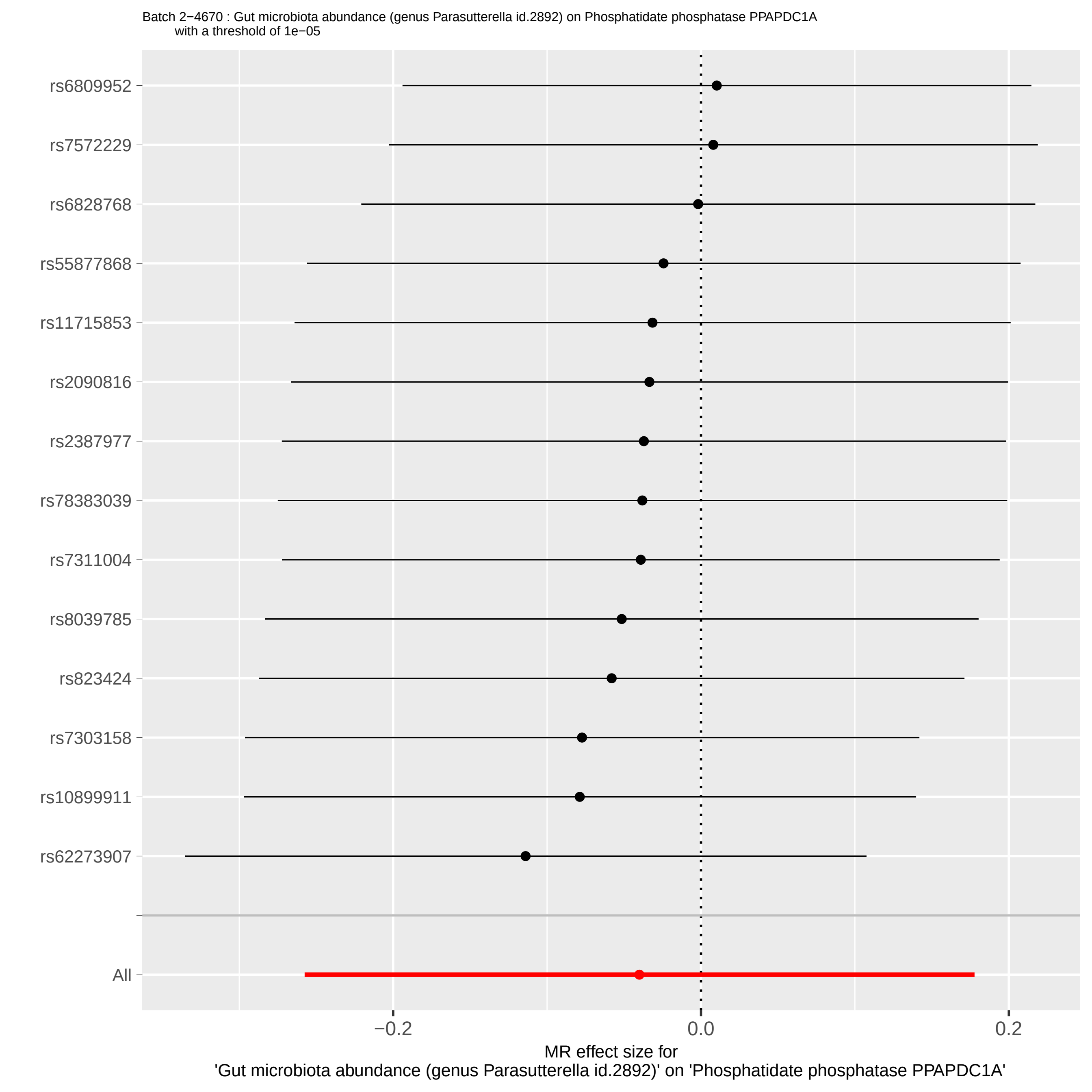

Batch 2−4670 : Gut microbiota abundance (genus Parasutterella id.2892) on Phosphatidate phosphatase PPAPDC1A
with a threshold of 1e−05
rs6809952
rs7572229
rs6828768
rs55877868
rs11715853
rs2090816
rs2387977
rs78383039
rs7311004
rs8039785
rs823424
rs7303158
rs10899911
rs62273907
All
−0.2
0.0
0.2
MR effect size for
'Gut microbiota abundance (genus Parasutterella id.2892)' on 'Phosphatidate phosphatase PPAPDC1A'

Batch 2−4671 : Gut microbiota abundance (genus Catenibacterium id.2153) on Peroxisome proliferator−activated receptor alpha
with a threshold of 1e−05

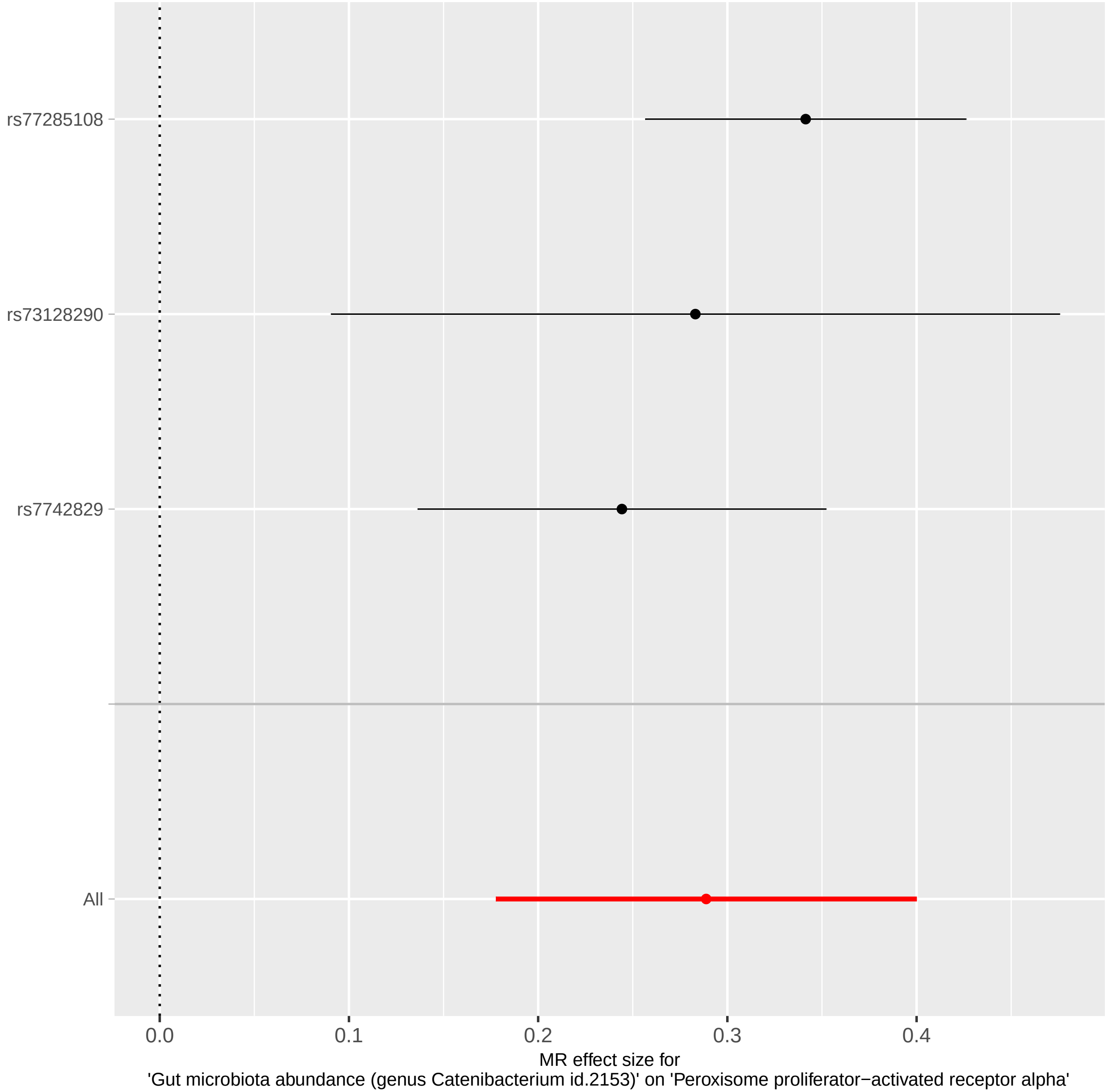

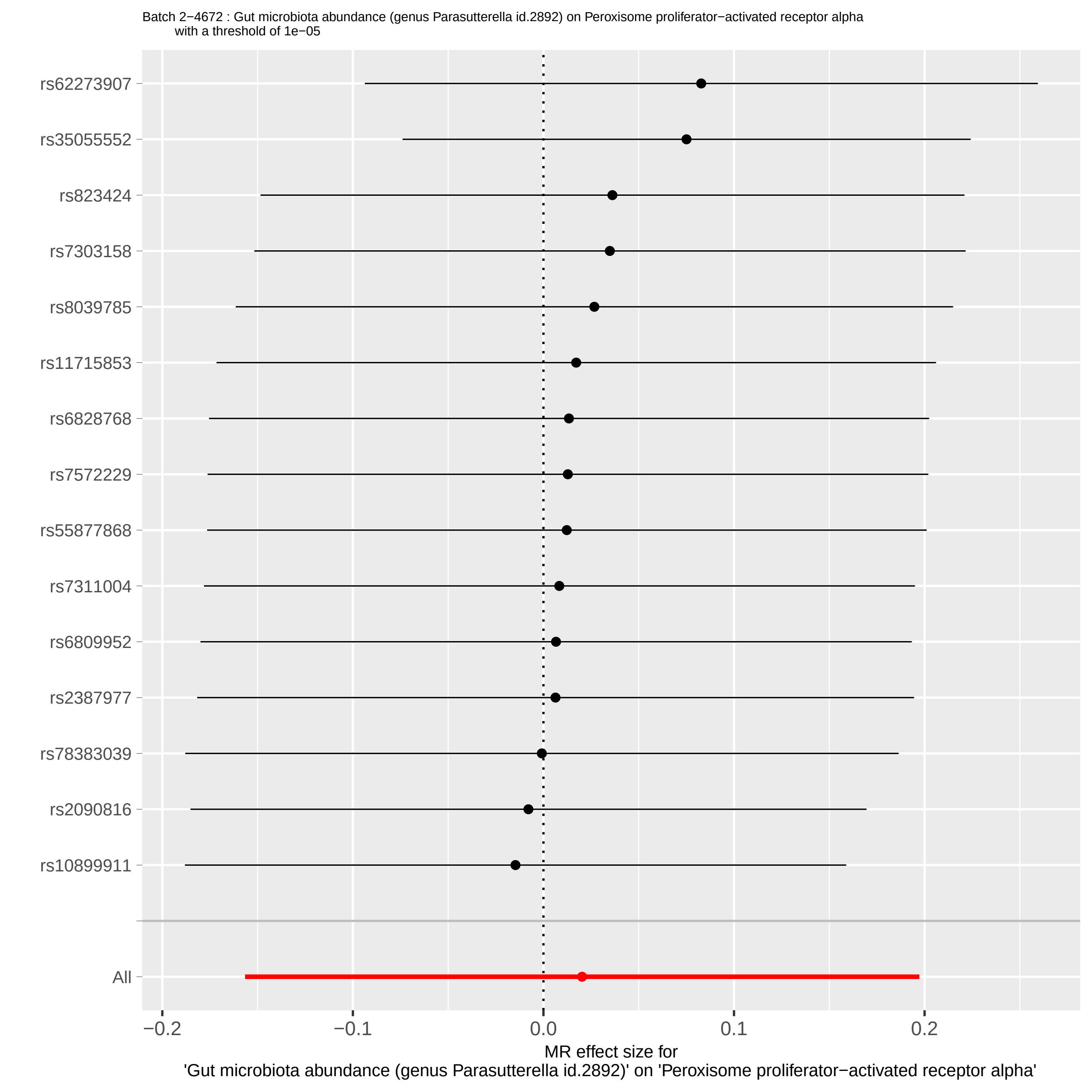
Batch 2−4672 : Gut microbiota abundance (genus Parasutterella id.2892) on Peroxisome proliferator−activated receptor alpha
with a threshold of 1e−05
rs62273907
rs35055552
rs823424
rs7303158
rs8039785
rs11715853
rs6828768
rs7572229
rs55877868
rs7311004
rs6809952
rs2387977
rs78383039
rs2090816
rs10899911
All
−0.2
−0.1
0.0
0.1
0.2
MR effect size for
'Gut microbiota abundance (genus Parasutterella id.2892)' on 'Peroxisome proliferator−activated receptor alpha'

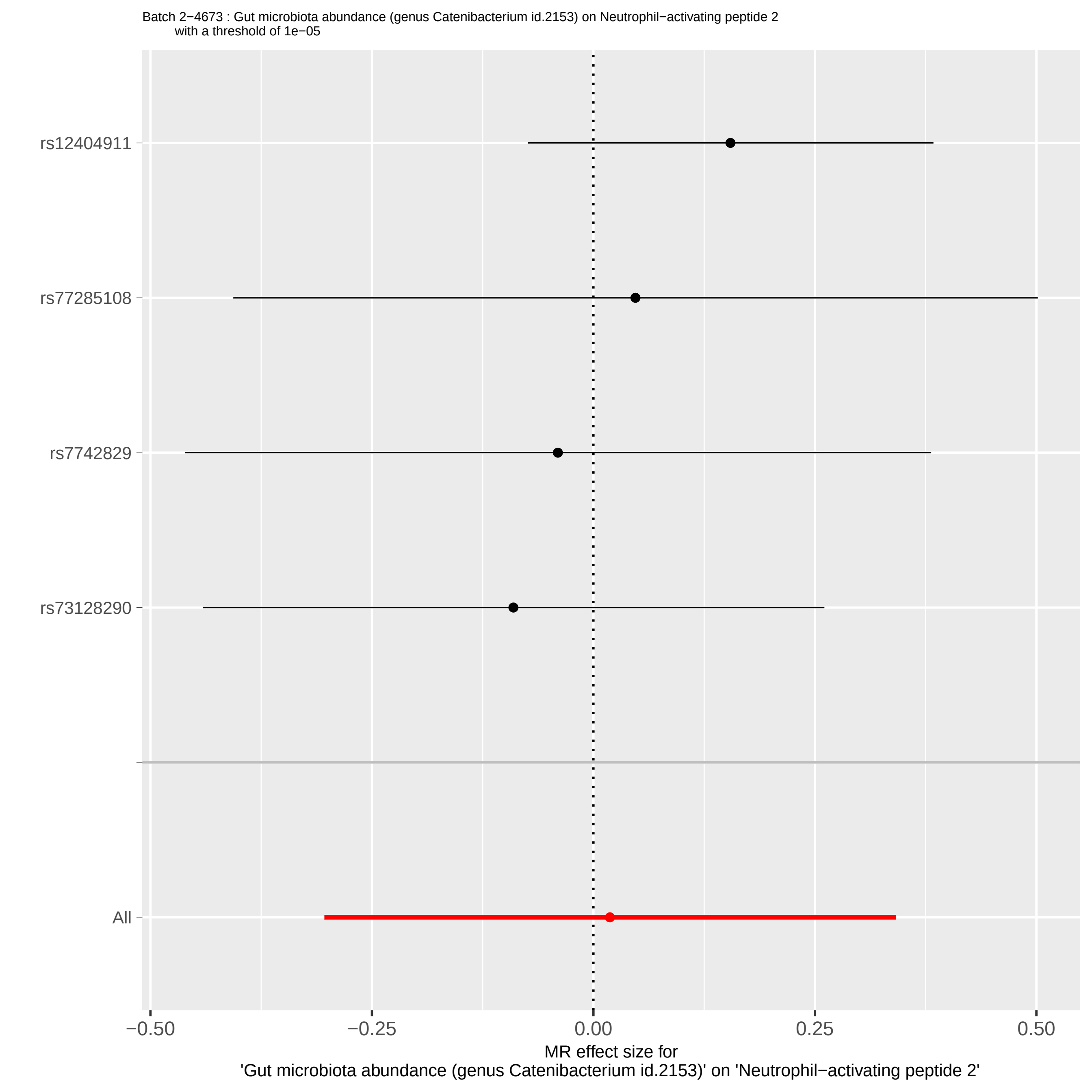

Batch 2−4673 : Gut microbiota abundance (genus Catenibacterium id.2153) on Neutrophil−activating peptide 2
with a threshold of 1e−05
rs12404911
rs77285108
rs7742829
rs73128290
All
−0.50
−0.25
0.00
0.25
0.50
MR effect size for
'Gut microbiota abundance (genus Catenibacterium id.2153)' on 'Neutrophil−activating peptide 2'

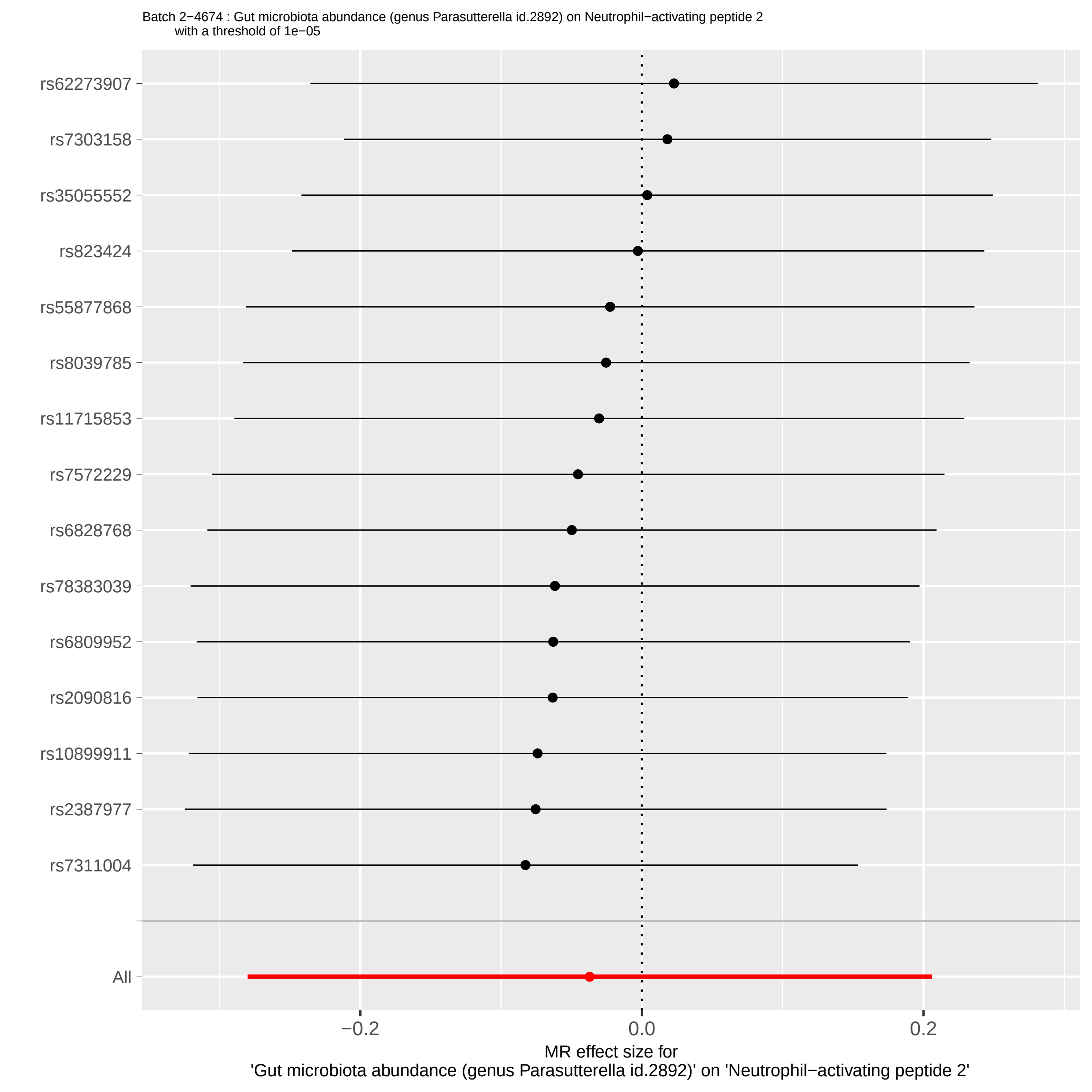

Batch 2−4674 : Gut microbiota abundance (genus Parasutterella id.2892) on Neutrophil−activating peptide 2
with a threshold of 1e−05
rs62273907
rs7303158
rs35055552
rs823424
rs55877868
rs8039785
rs11715853
rs7572229
rs6828768
rs78383039
rs6809952
rs2090816
rs10899911
rs2387977
rs7311004
All
−0.2
0.0
0.2
MR effect size for
'Gut microbiota abundance (genus Parasutterella id.2892)' on 'Neutrophil−activating peptide 2'

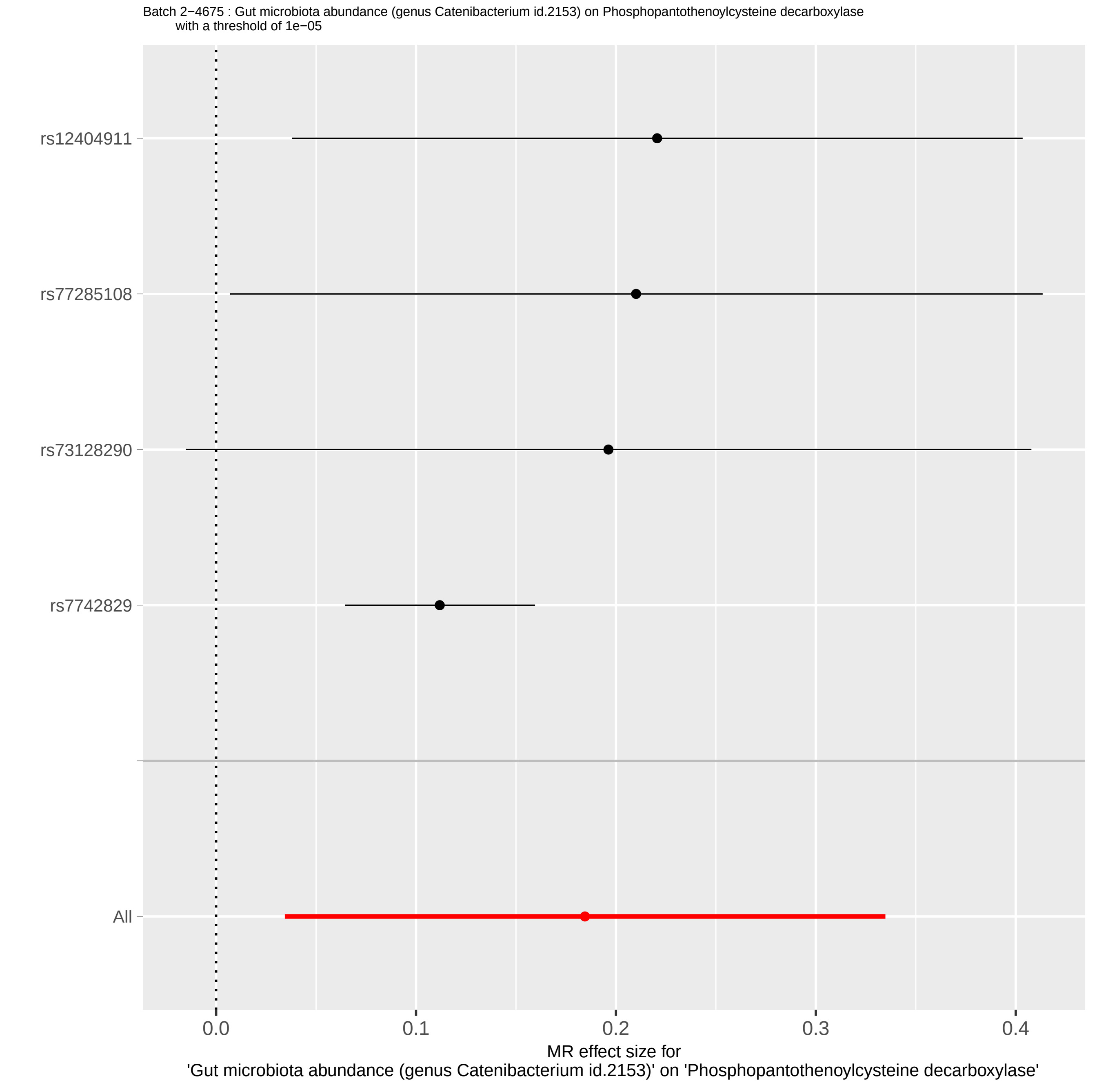
Batch 2−4675 : Gut microbiota abundance (genus Catenibacterium id.2153) on Phosphopantothenoylcysteine decarboxylase
with a threshold of 1e−05
rs12404911
rs77285108
rs73128290
rs7742829
All
0.0
0.1
0.2
0.3
0.4
MR effect size for
'Gut microbiota abundance (genus Catenibacterium id.2153)' on 'Phosphopantothenoylcysteine decarboxylase'

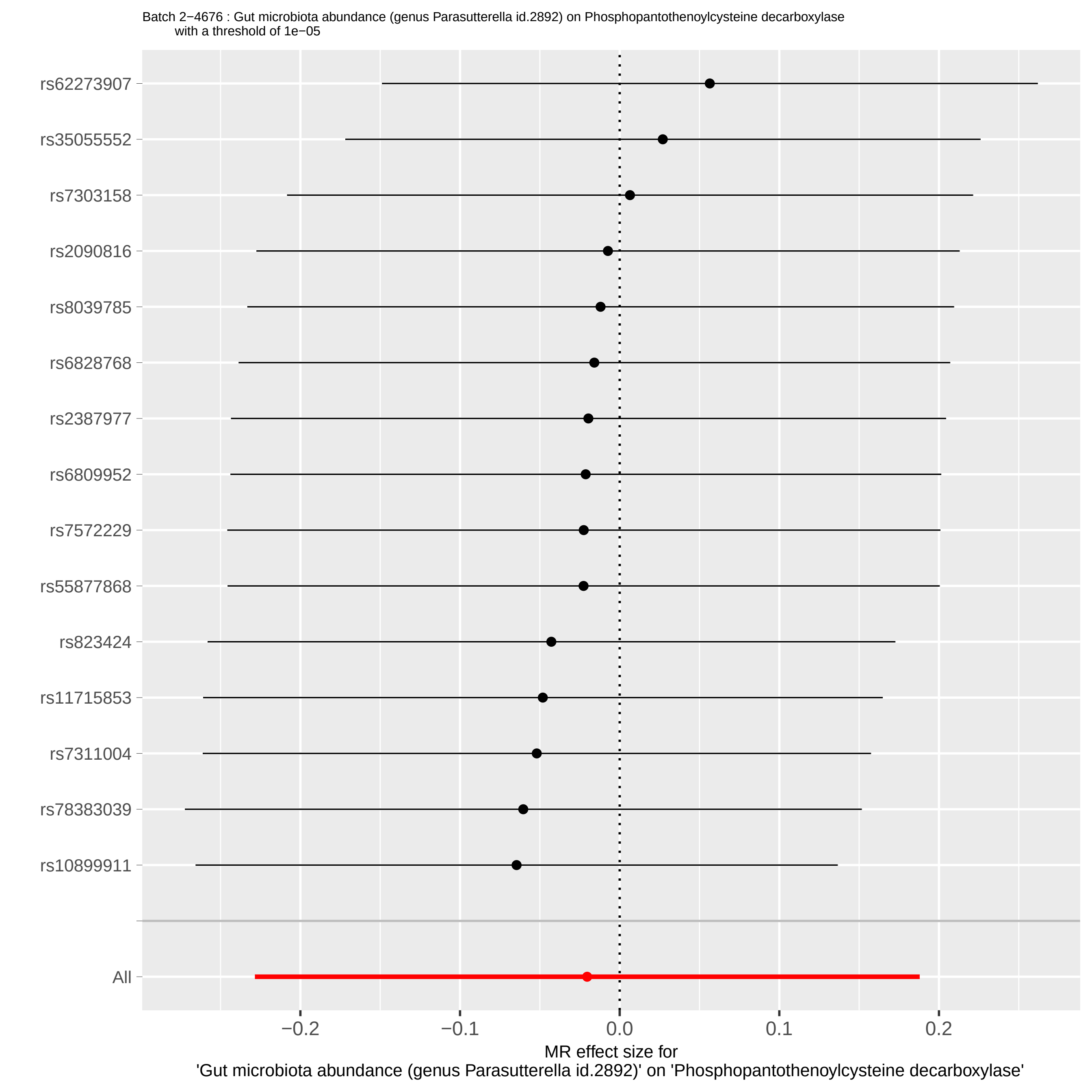

Batch 2−4676 : Gut microbiota abundance (genus Parasutterella id.2892) on Phosphopantothenoylcysteine decarboxylase
with a threshold of 1e−05
rs62273907
rs35055552
rs7303158
rs2090816
rs8039785
rs6828768
rs2387977
rs6809952
rs7572229
rs55877868
rs823424
rs11715853
rs7311004
rs78383039
rs10899911
All
−0.2
−0.1
0.0
0.1
0.2
MR effect size for
'Gut microbiota abundance (genus Parasutterella id.2892)' on 'Phosphopantothenoylcysteine decarboxylase'

Batch 2−4677 : Gut microbiota abundance (genus Catenibacterium id.2153) on Peptidyl−prolyl cis−trans isomerase A
with a threshold of 1e−05

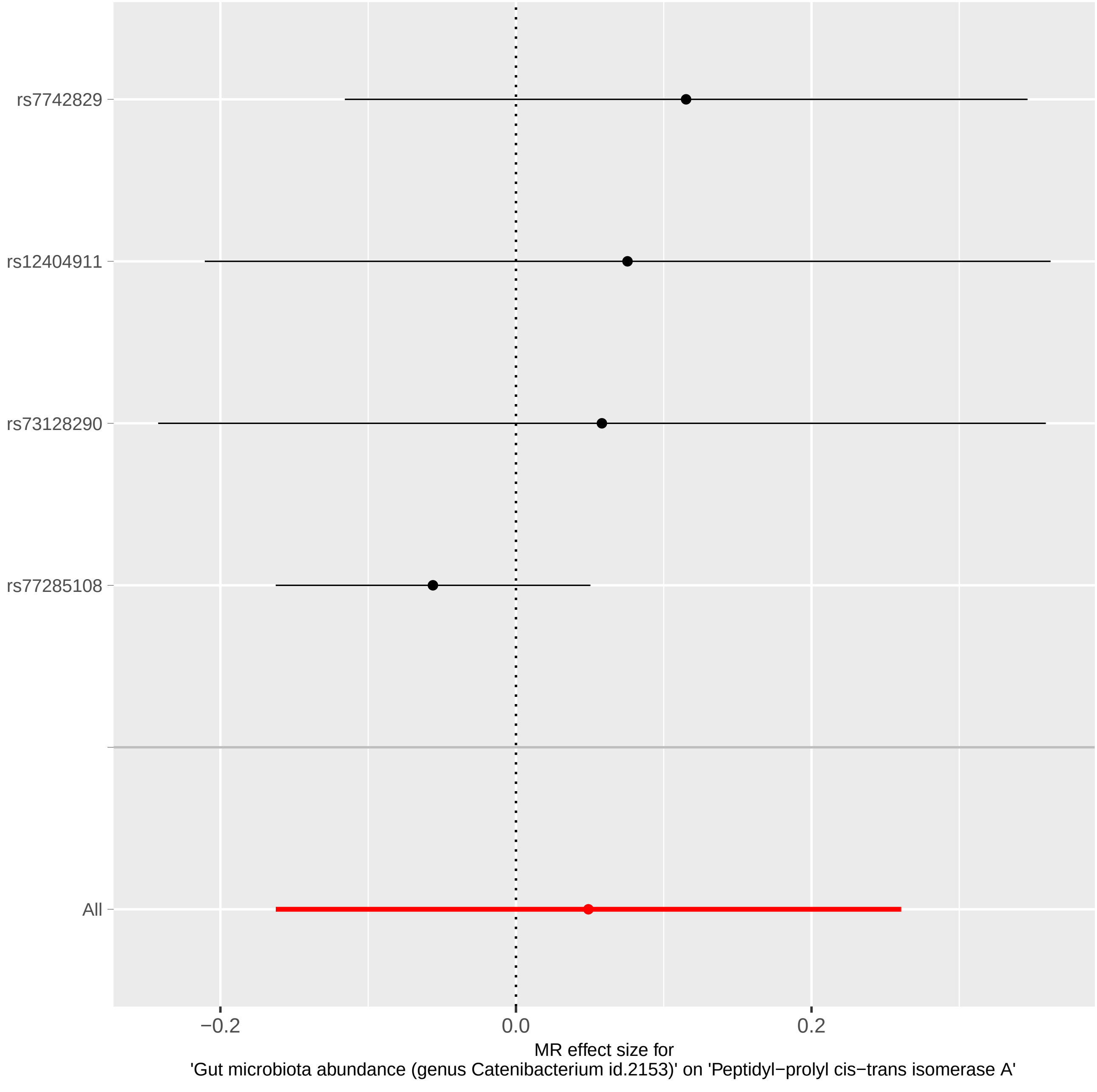

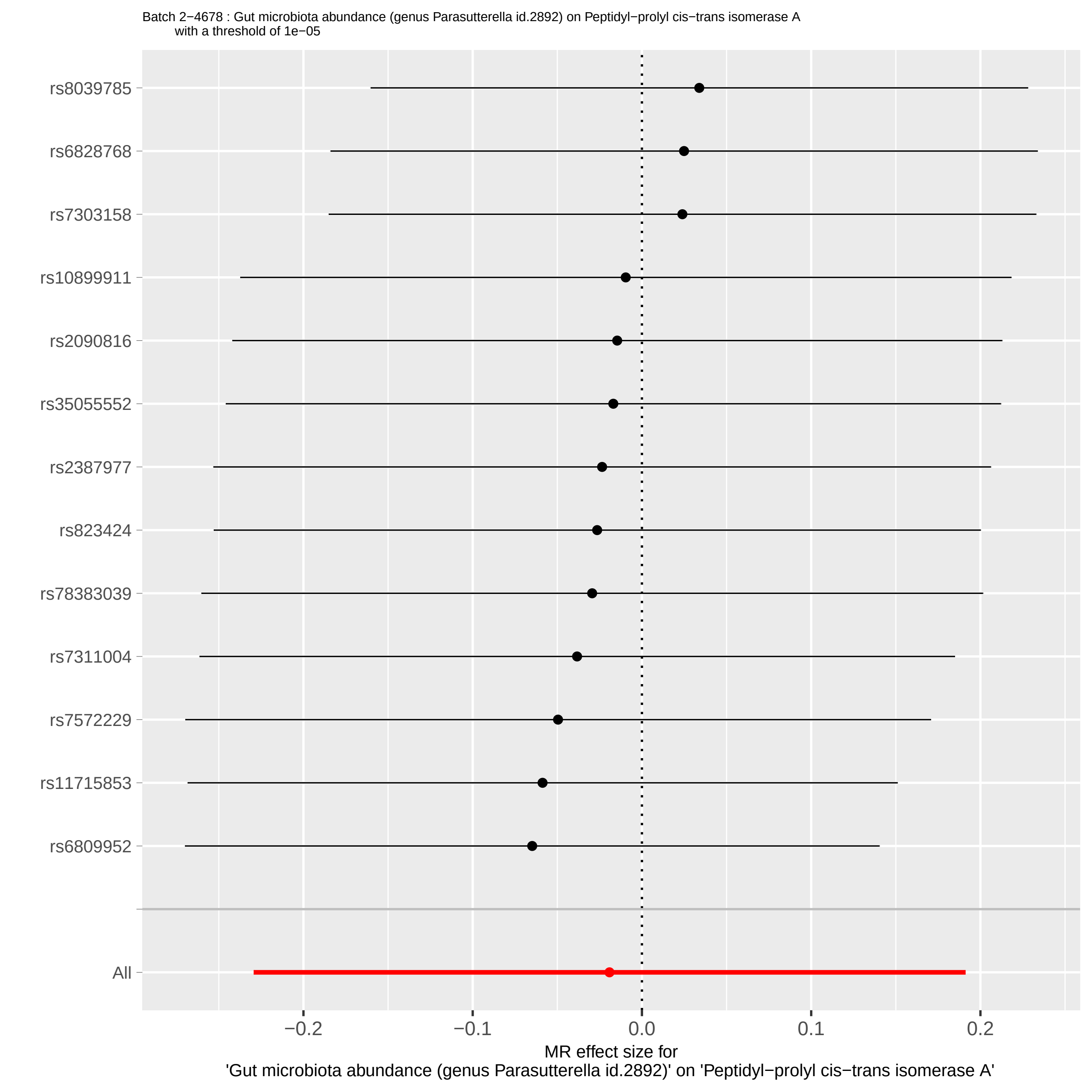
Batch 2−4678 : Gut microbiota abundance (genus Parasutterella id.2892) on Peptidyl−prolyl cis−trans isomerase A
with a threshold of 1e−05
rs8039785
rs6828768
rs7303158
rs10899911
rs2090816
rs35055552
rs2387977
rs823424
rs78383039
rs7311004
rs7572229
rs11715853
rs6809952
All
−0.2
−0.1
0.0
0.1
0.2
MR effect size for
'Gut microbiota abundance (genus Parasutterella id.2892)' on 'Peptidyl−prolyl cis−trans isomerase A'

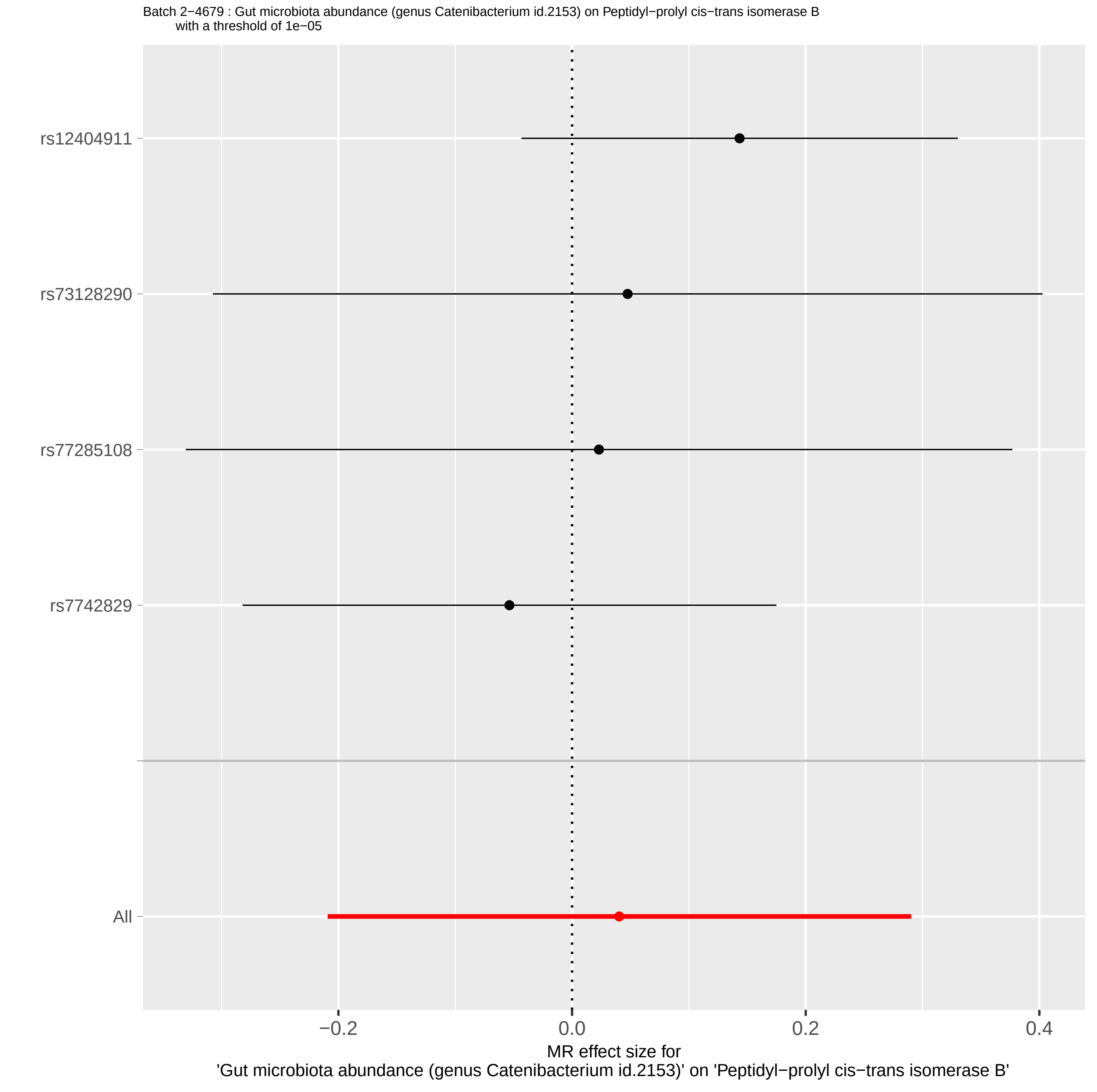
Batch 2−4679 : Gut microbiota abundance (genus Catenibacterium id.2153) on Peptidyl−prolyl cis−trans isomerase B
with a threshold of 1e−05
rs12404911
rs73128290
rs77285108
rs7742829
All
−0.2
0.0
0.2
0.4
MR effect size for
'Gut microbiota abundance (genus Catenibacterium id.2153)' on 'Peptidyl−prolyl cis−trans isomerase B'

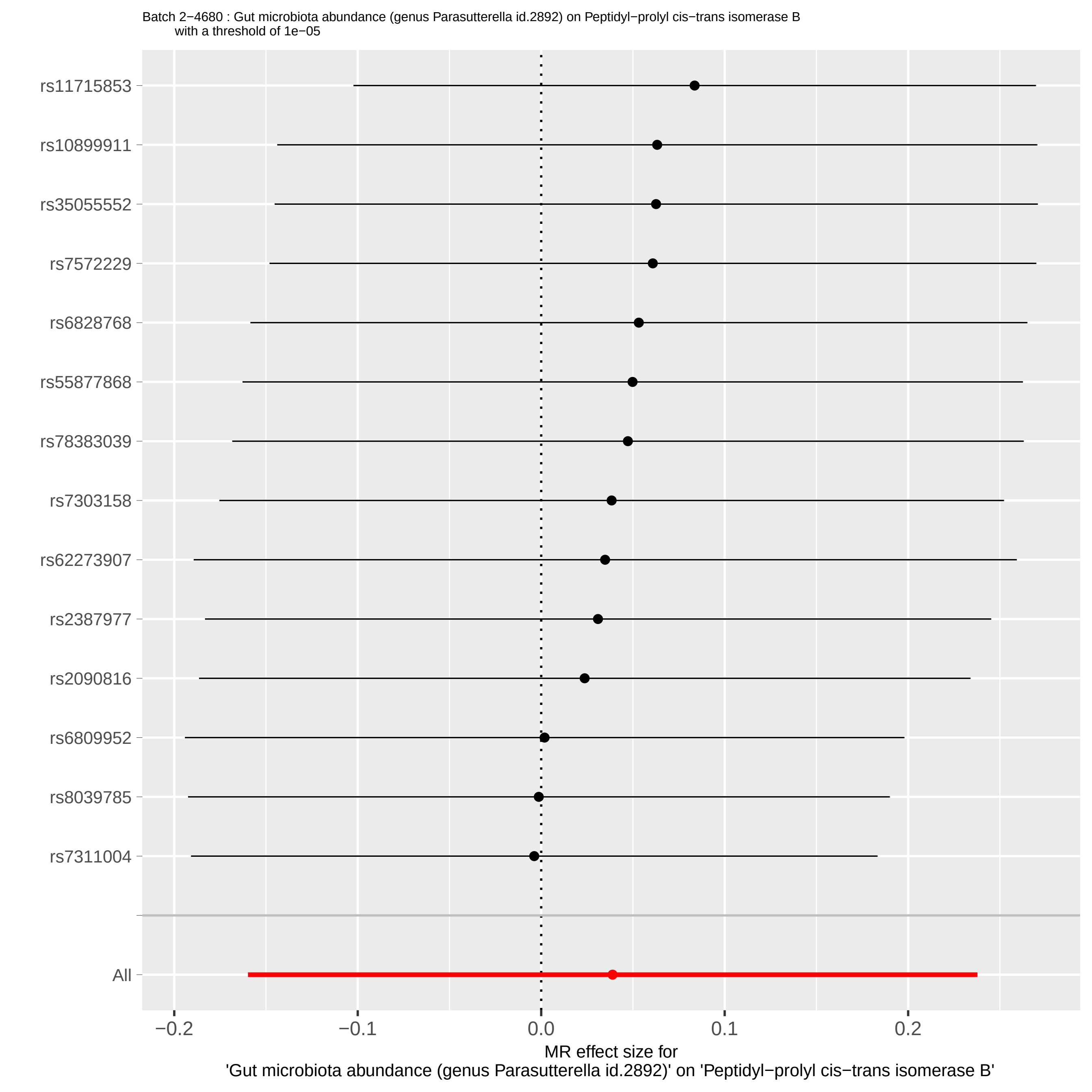
Batch 2−4680 : Gut microbiota abundance (genus Parasutterella id.2892) on Peptidyl−prolyl cis−trans isomerase B
with a threshold of 1e−05
rs11715853
rs10899911
rs35055552
rs7572229
rs6828768
rs55877868
rs78383039
rs7303158
rs62273907
rs2387977
rs2090816
rs6809952
rs8039785
rs7311004
All
−0.2
−0.1
0.0
0.1
0.2
MR effect size for
'Gut microbiota abundance (genus Parasutterella id.2892)' on 'Peptidyl−prolyl cis−trans isomerase B'

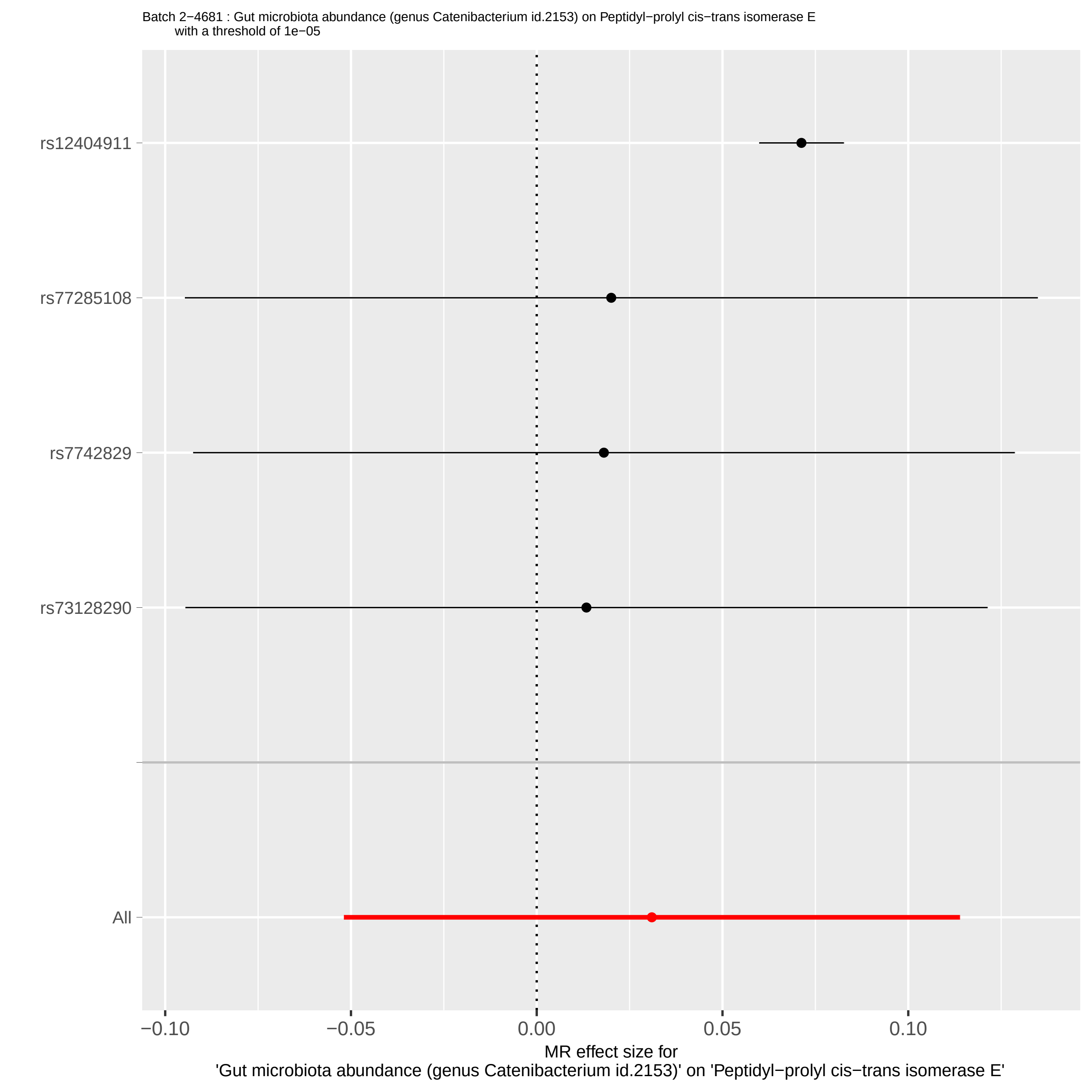

Batch 2−4681 : Gut microbiota abundance (genus Catenibacterium id.2153) on Peptidyl−prolyl cis−trans isomerase E
with a threshold of 1e−05
rs12404911
rs77285108
rs7742829
rs73128290
All
−0.10
−0.05
0.00
0.05
0.10
MR effect size for
'Gut microbiota abundance (genus Catenibacterium id.2153)' on 'Peptidyl−prolyl cis−trans isomerase E'

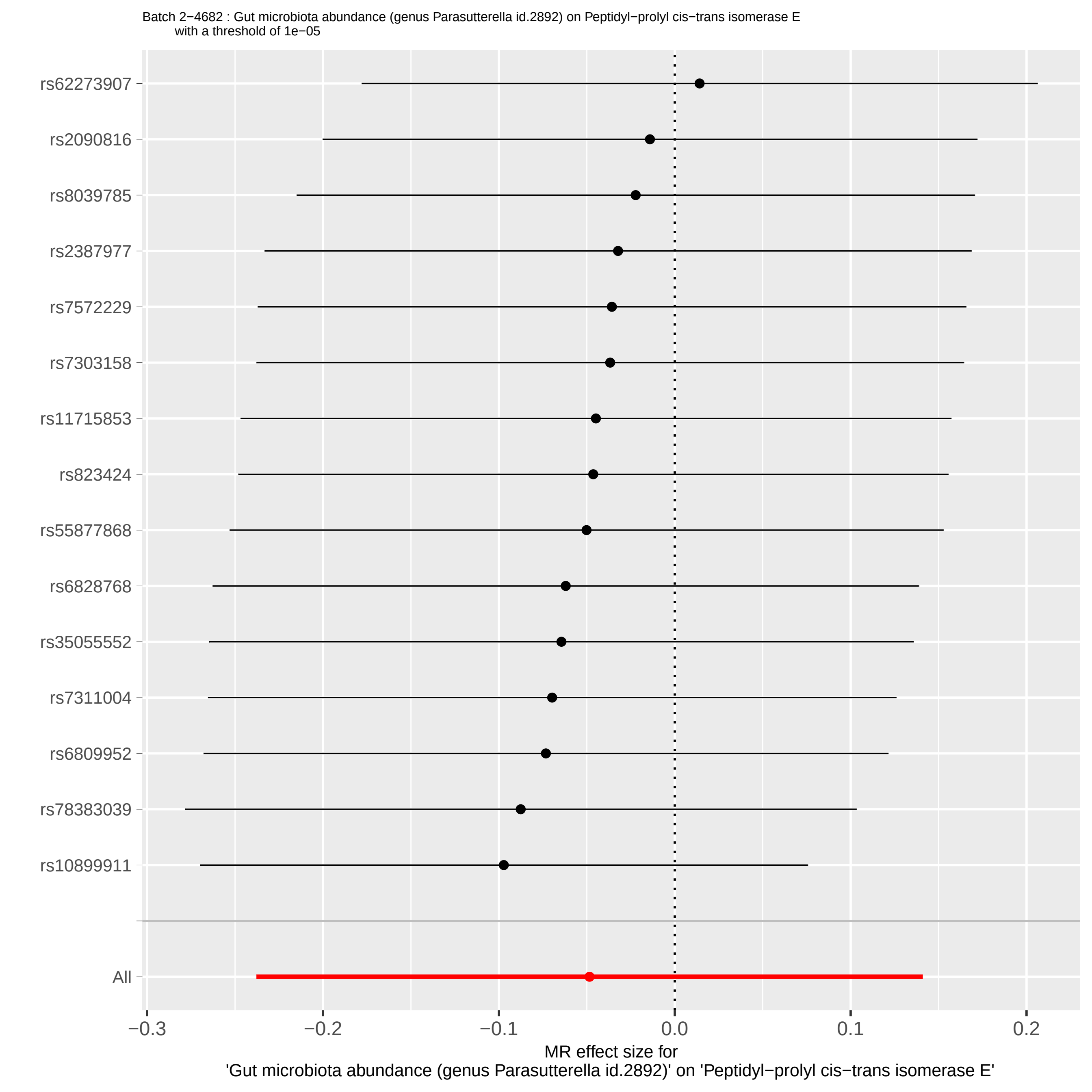
Batch 2−4682 : Gut microbiota abundance (genus Parasutterella id.2892) on Peptidyl−prolyl cis−trans isomerase E
with a threshold of 1e−05
rs62273907
rs2090816
rs8039785
rs2387977
rs7572229
rs7303158
rs11715853
rs823424
rs55877868
rs6828768
rs35055552
rs7311004
rs6809952
rs78383039
rs10899911
All
−0.3
−0.2
−0.1
0.0
0.1
0.2
MR effect size for
'Gut microbiota abundance (genus Parasutterella id.2892)' on 'Peptidyl−prolyl cis−trans isomerase E'

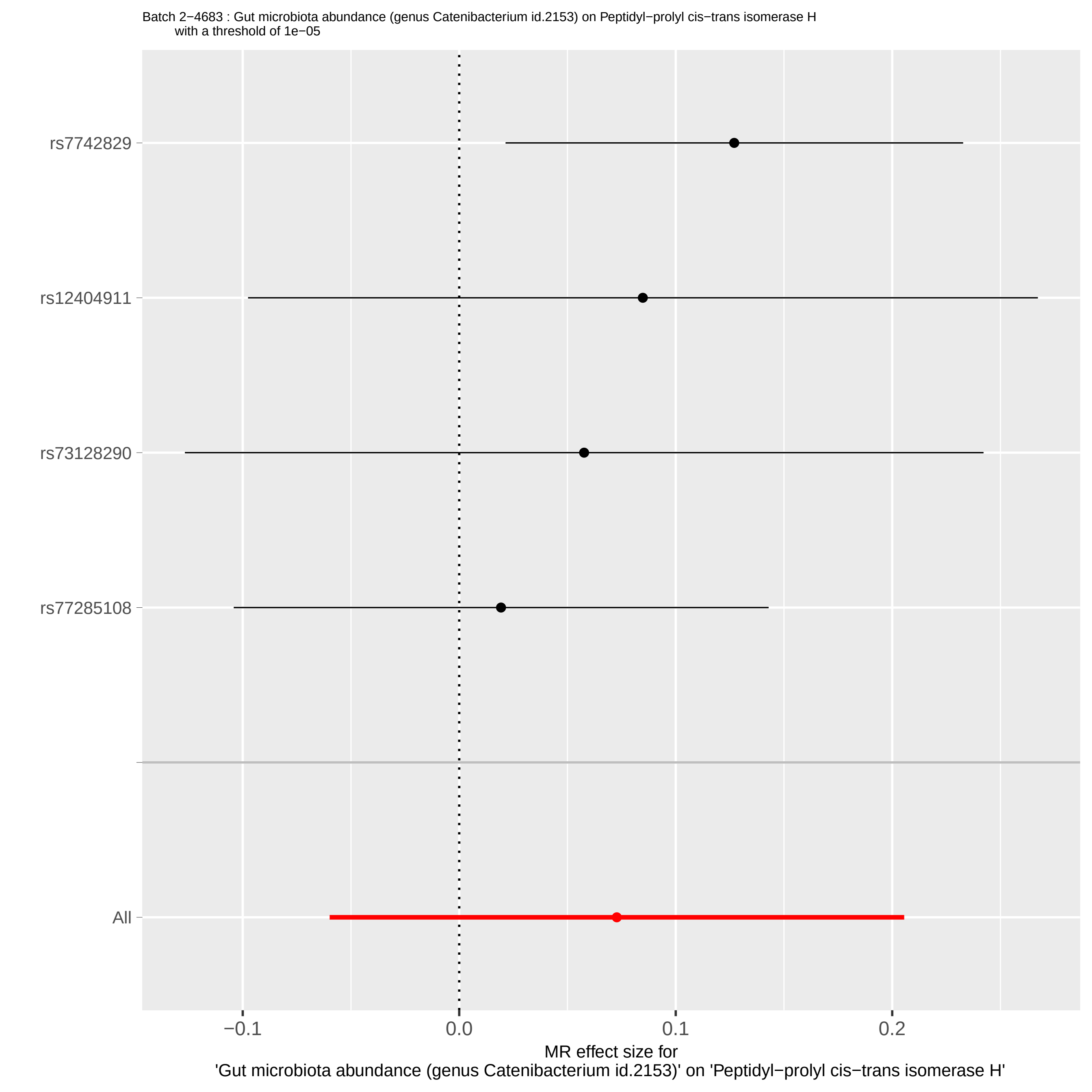

Batch 2−4683 : Gut microbiota abundance (genus Catenibacterium id.2153) on Peptidyl−prolyl cis−trans isomerase H
with a threshold of 1e−05
rs7742829
rs12404911
rs73128290
rs77285108
All
−0.1
0.0
0.1
0.2
MR effect size for
'Gut microbiota abundance (genus Catenibacterium id.2153)' on 'Peptidyl−prolyl cis−trans isomerase H'

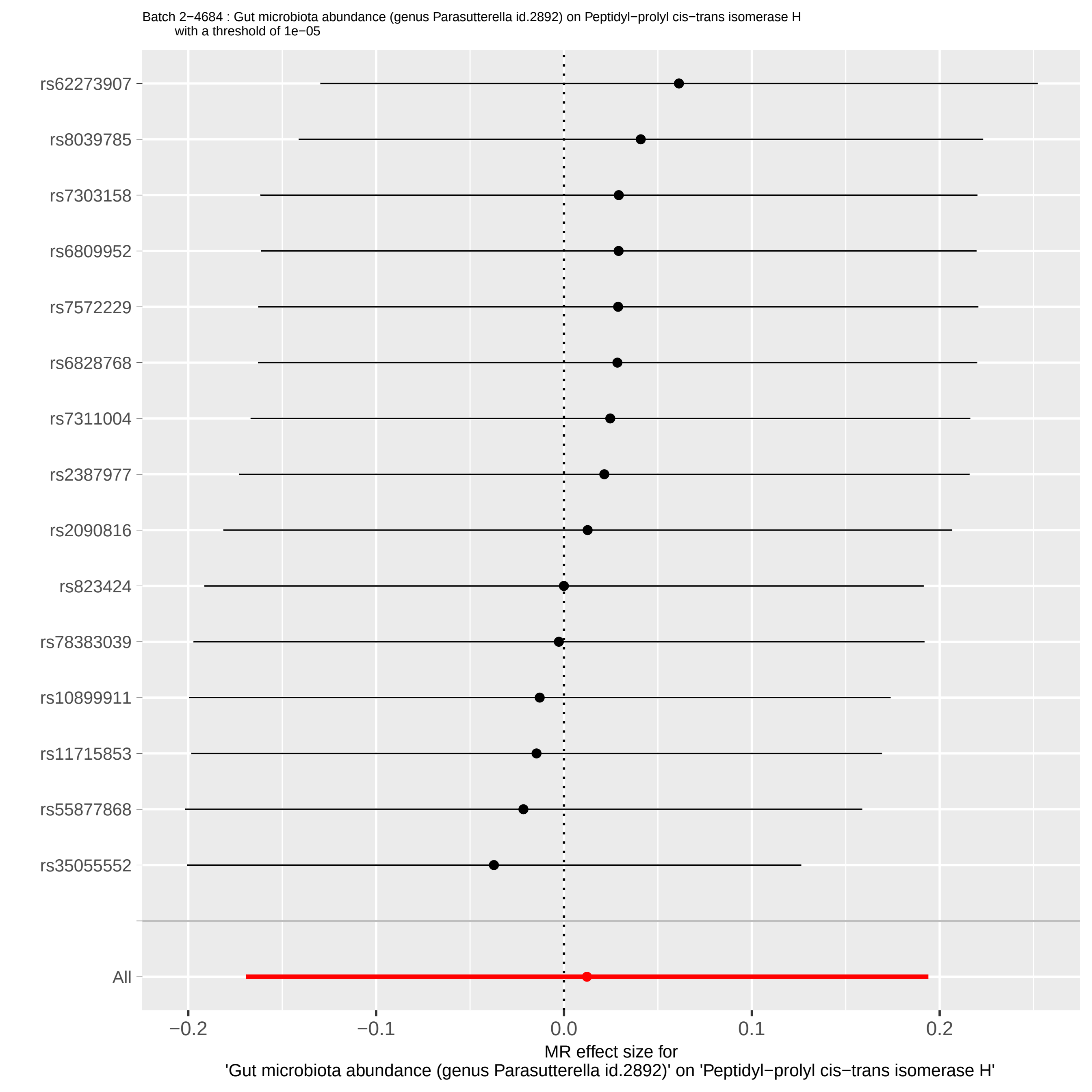

Batch 2−4684 : Gut microbiota abundance (genus Parasutterella id.2892) on Peptidyl−prolyl cis−trans isomerase H
with a threshold of 1e−05
rs62273907
rs8039785
rs7303158
rs6809952
rs7572229
rs6828768
rs7311004
rs2387977
rs2090816
rs823424
rs78383039
rs10899911
rs11715853
rs55877868
rs35055552
All
−0.2
−0.1
0.0
0.1
0.2
MR effect size for
'Gut microbiota abundance (genus Parasutterella id.2892)' on 'Peptidyl−prolyl cis−trans isomerase H'

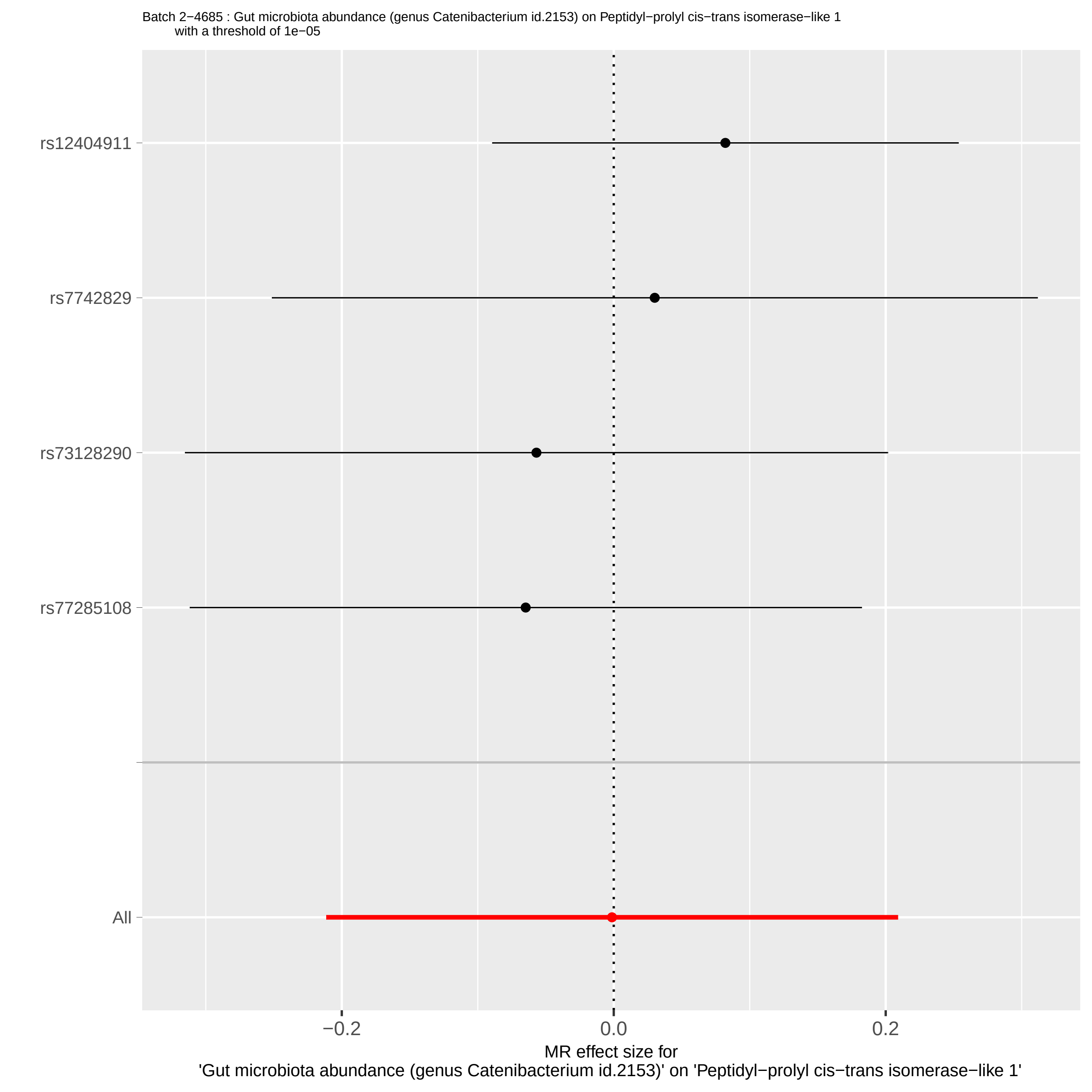
Batch 2−4685 : Gut microbiota abundance (genus Catenibacterium id.2153) on Peptidyl−prolyl cis−trans isomerase−like 1
with a threshold of 1e−05
rs12404911
rs7742829
rs73128290
rs77285108
All
−0.2
0.0
0.2
MR effect size for
'Gut microbiota abundance (genus Catenibacterium id.2153)' on 'Peptidyl−prolyl cis−trans isomerase−like 1'

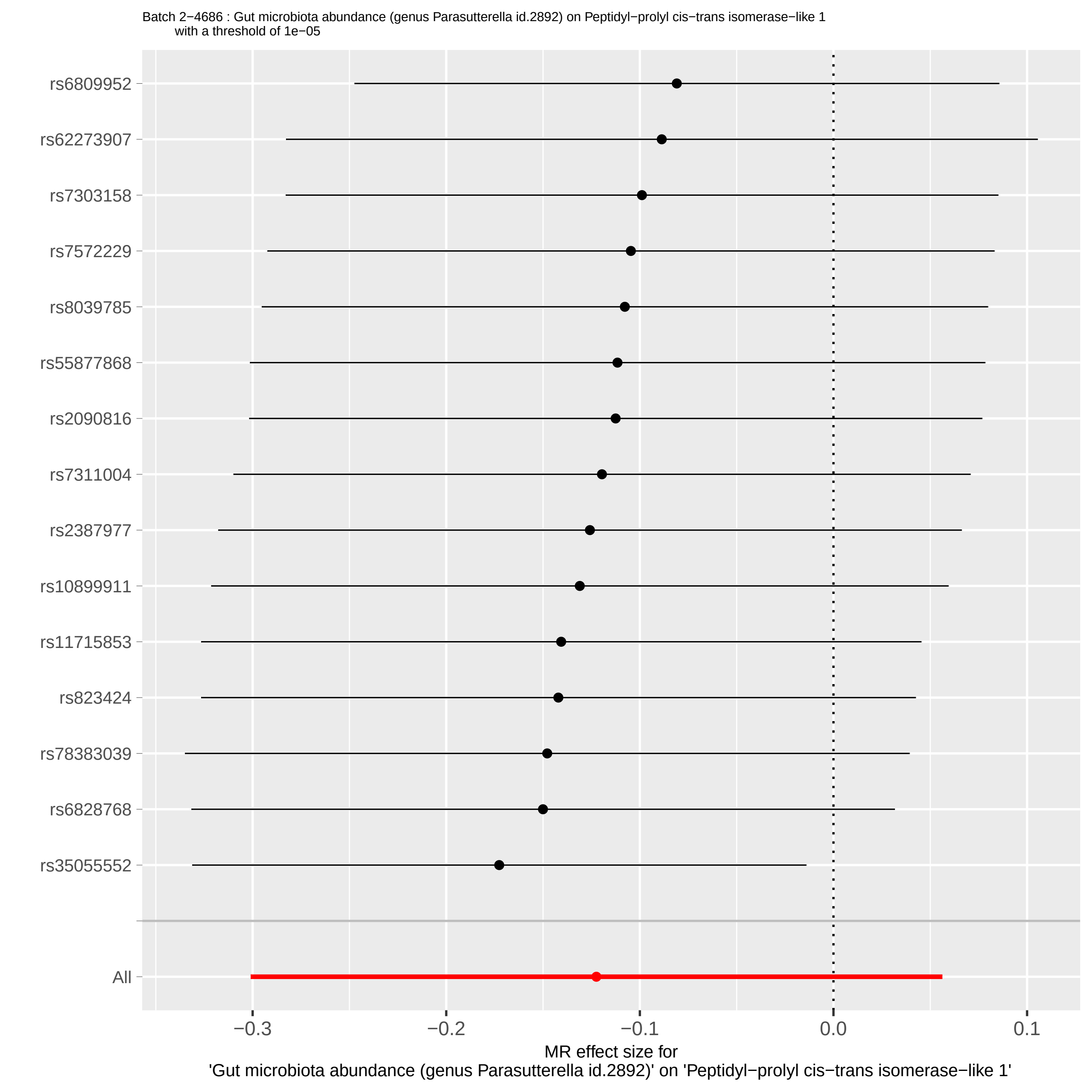
Batch 2−4686 : Gut microbiota abundance (genus Parasutterella id.2892) on Peptidyl−prolyl cis−trans isomerase−like 1
with a threshold of 1e−05
rs6809952
rs62273907
rs7303158
rs7572229
rs8039785
rs55877868
rs2090816
rs7311004
rs2387977
rs10899911
rs11715853
rs823424
rs78383039
rs6828768
rs35055552
All
−0.3
−0.2
−0.1
0.0
0.1
MR effect size for
'Gut microbiota abundance (genus Parasutterella id.2892)' on 'Peptidyl−prolyl cis−trans isomerase−like 1'

Batch 2−4687 : Gut microbiota abundance (genus Catenibacterium id.2153) on Peptidyl−prolyl cis−trans isomerase−like 2
with a threshold of 1e−05

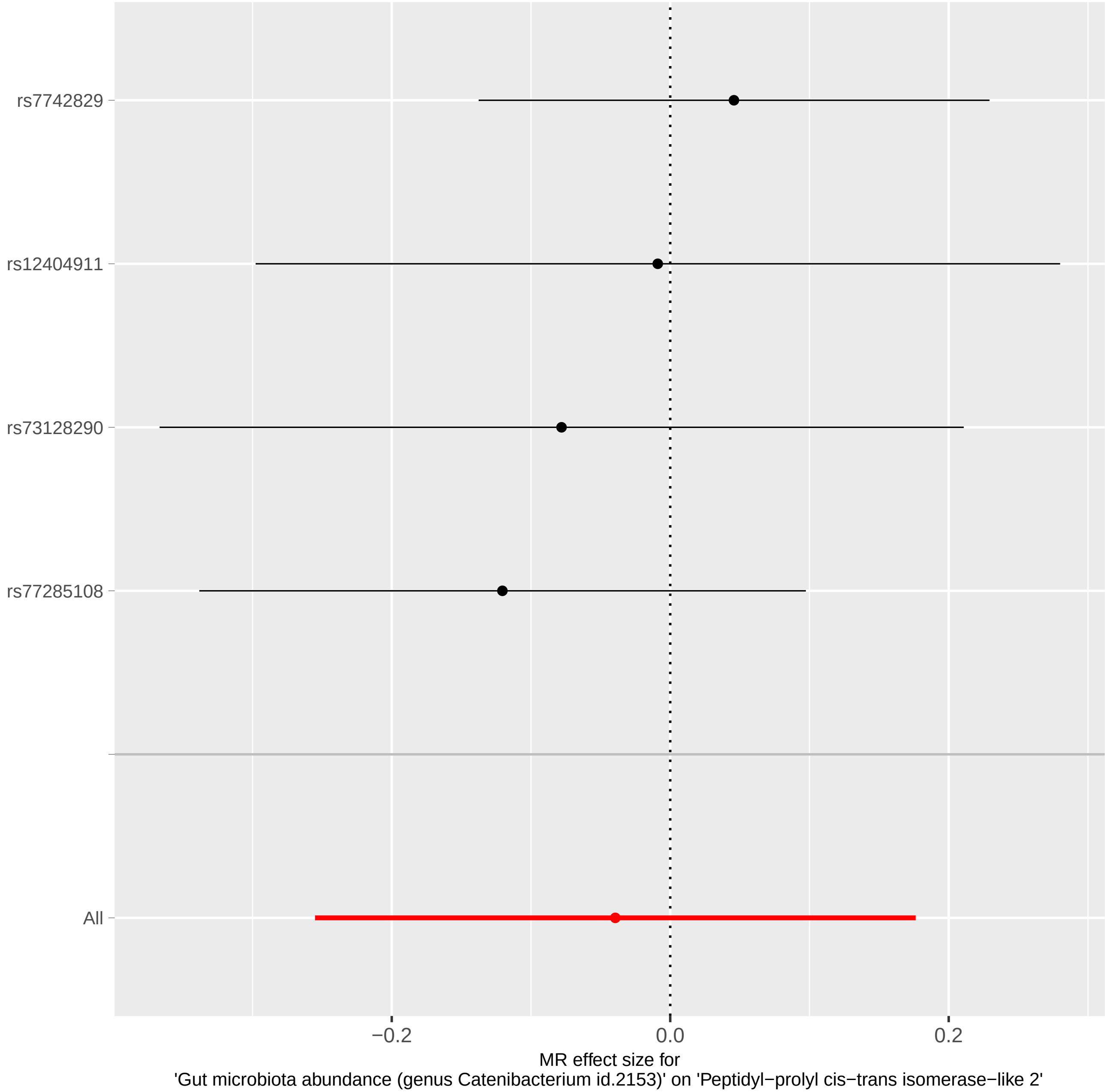

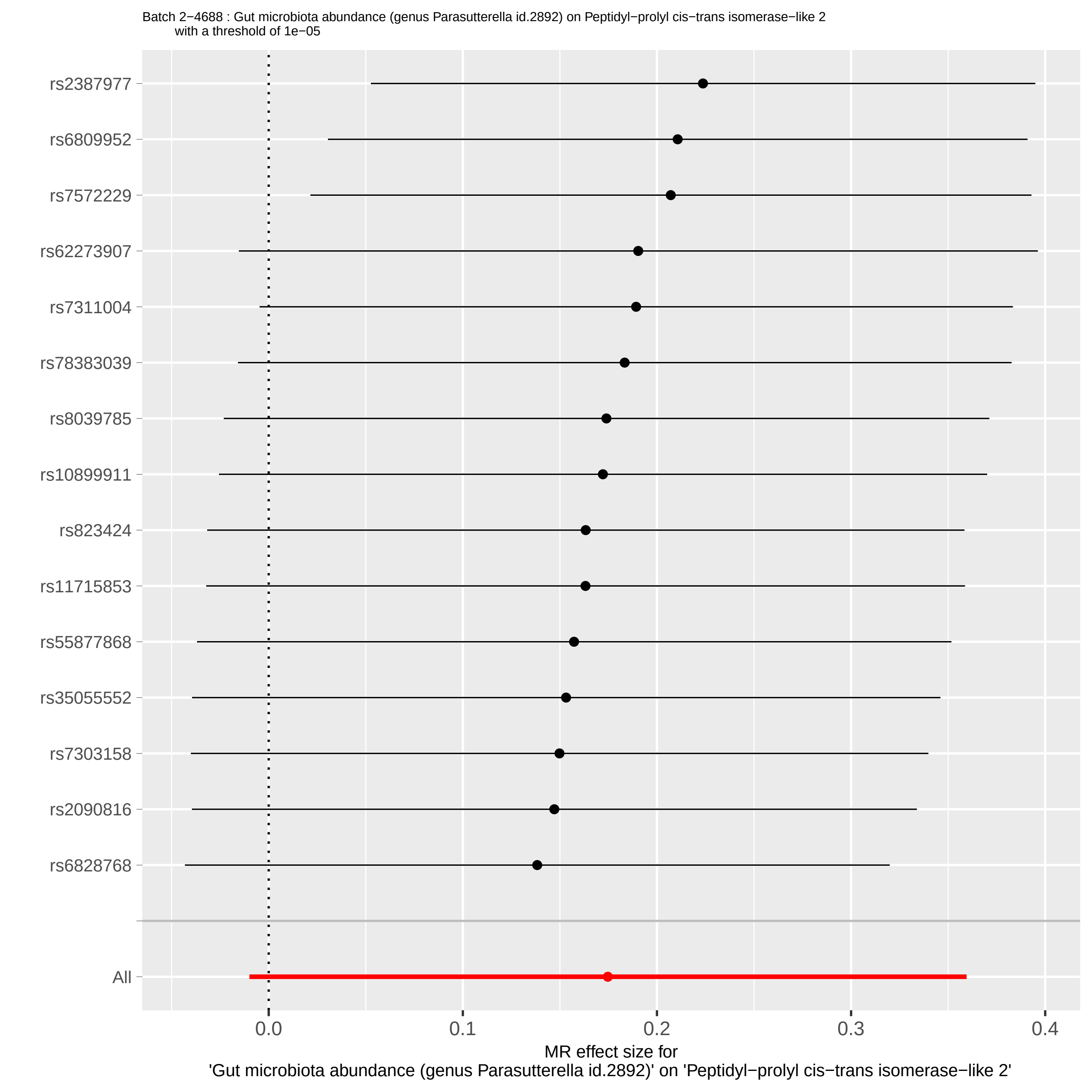

Batch 2−4688 : Gut microbiota abundance (genus Parasutterella id.2892) on Peptidyl−prolyl cis−trans isomerase−like 2
with a threshold of 1e−05
rs2387977
rs6809952
rs7572229
rs62273907
rs7311004
rs78383039
rs8039785
rs10899911
rs823424
rs11715853
rs55877868
rs35055552
rs7303158
rs2090816
rs6828768
All
0.0
0.1
0.2
0.3
0.4
MR effect size for
'Gut microbiota abundance (genus Parasutterella id.2892)' on 'Peptidyl−prolyl cis−trans isomerase−like 2'

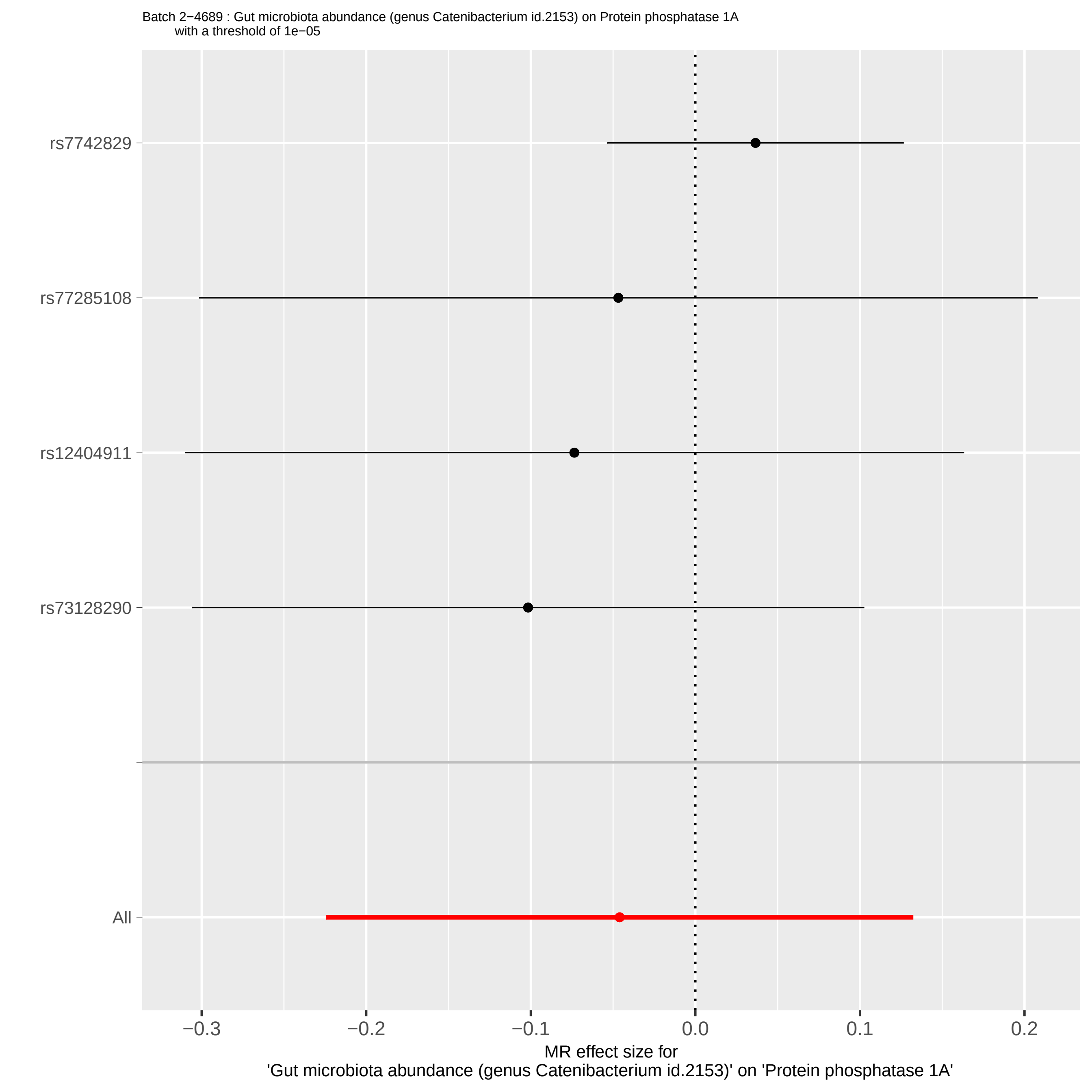
Batch 2−4689 : Gut microbiota abundance (genus Catenibacterium id.2153) on Protein phosphatase 1A
with a threshold of 1e−05
rs7742829
rs77285108
rs12404911
rs73128290
All
−0.3
−0.2
−0.1
0.0
0.1
0.2
MR effect size for
'Gut microbiota abundance (genus Catenibacterium id.2153)' on 'Protein phosphatase 1A'

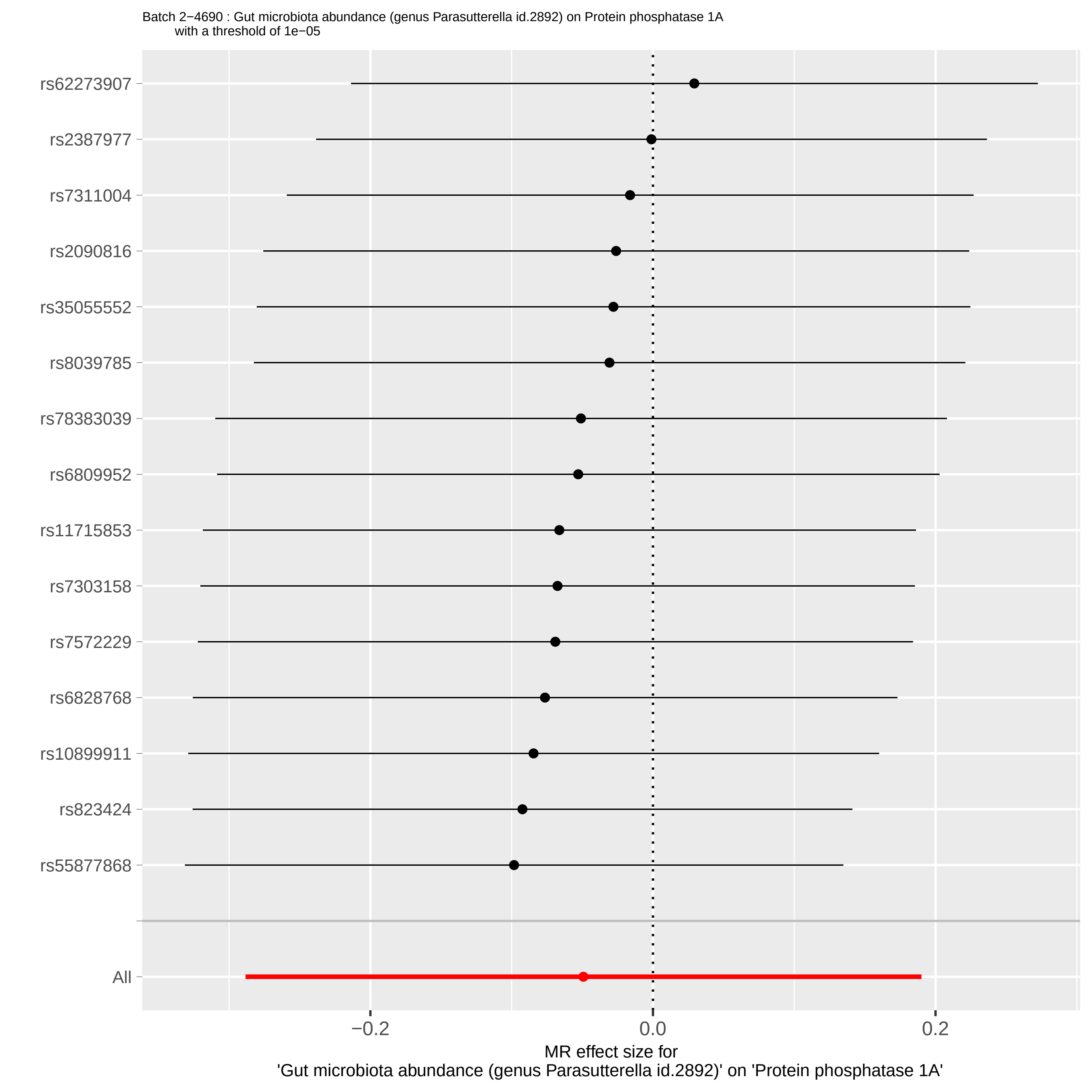

Batch 2−4690 : Gut microbiota abundance (genus Parasutterella id.2892) on Protein phosphatase 1A
with a threshold of 1e−05
rs62273907
rs2387977
rs7311004
rs2090816
rs35055552
rs8039785
rs78383039
rs6809952
rs11715853
rs7303158
rs7572229
rs6828768
rs10899911
rs823424
rs55877868
All
−0.2
0.0
0.2
MR effect size for
'Gut microbiota abundance (genus Parasutterella id.2892)' on 'Protein phosphatase 1A'

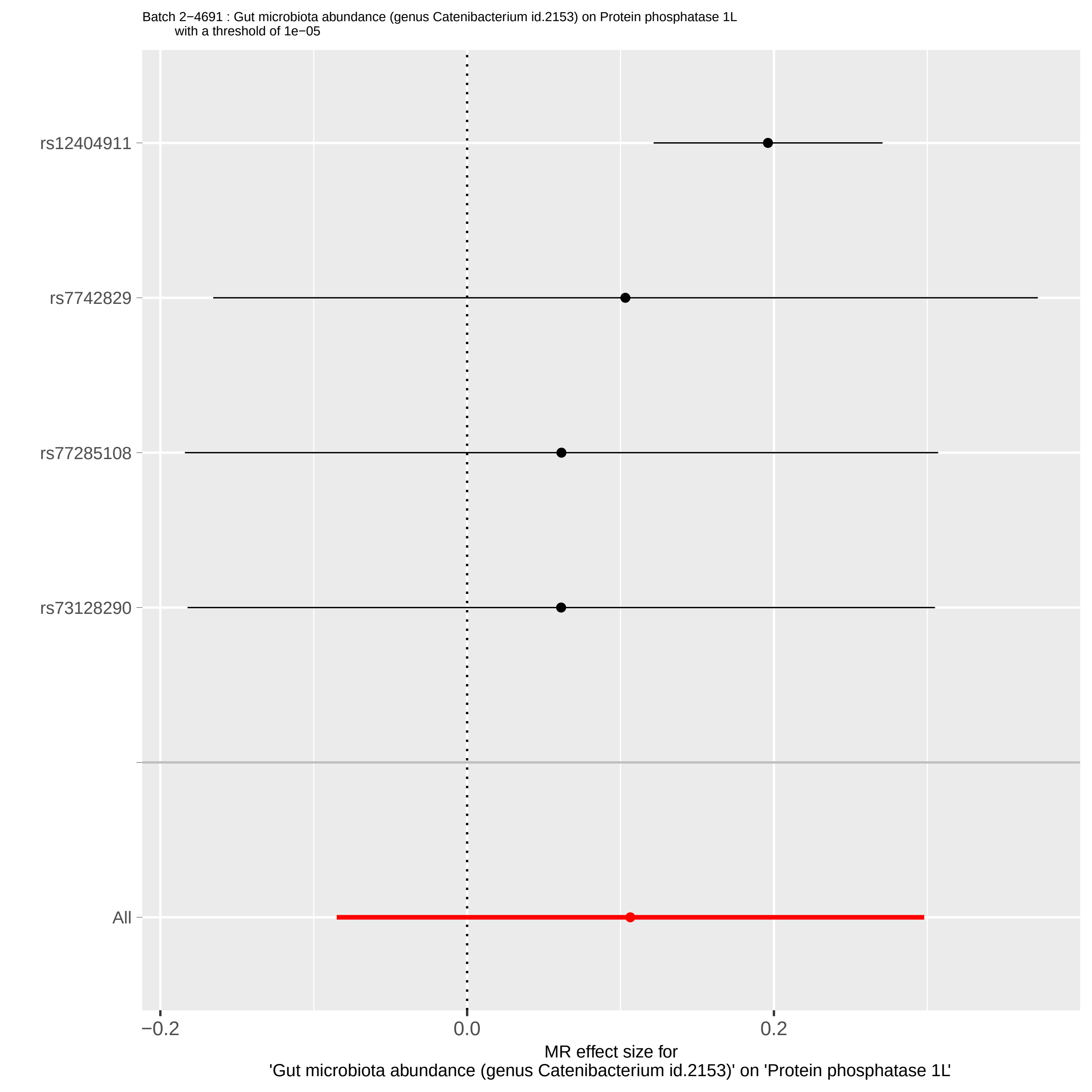
Batch 2−4691 : Gut microbiota abundance (genus Catenibacterium id.2153) on Protein phosphatase 1L
with a threshold of 1e−05
rs12404911
rs7742829
rs77285108
rs73128290
All
−0.2
0.0
0.2
MR effect size for
'Gut microbiota abundance (genus Catenibacterium id.2153)' on 'Protein phosphatase 1L'

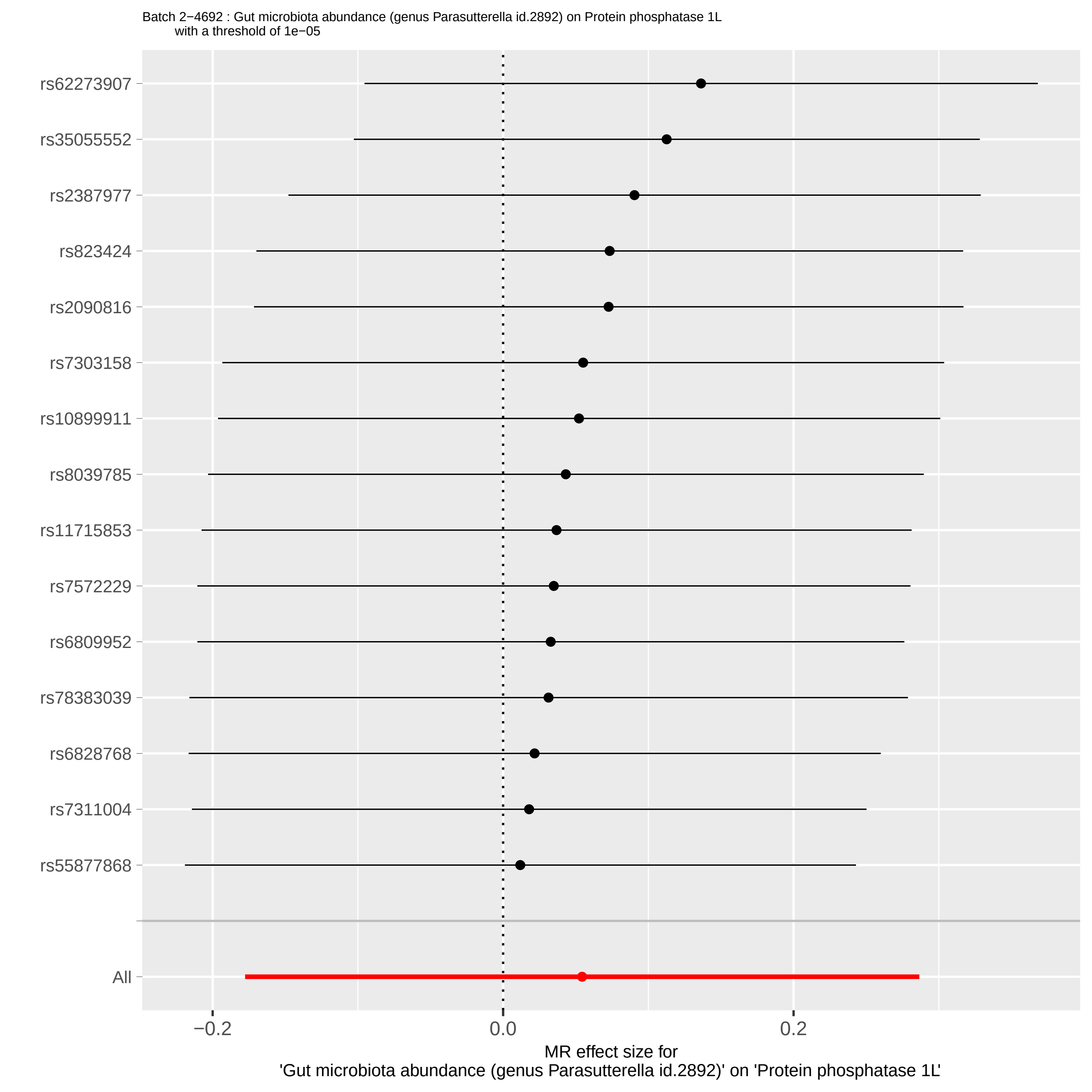

Batch 2−4692 : Gut microbiota abundance (genus Parasutterella id.2892) on Protein phosphatase 1L
with a threshold of 1e−05
rs62273907
rs35055552
rs2387977
rs823424
rs2090816
rs7303158
rs10899911
rs8039785
rs11715853
rs7572229
rs6809952
rs78383039
rs6828768
rs7311004
rs55877868
All
−0.2
0.0
0.2
MR effect size for
'Gut microbiota abundance (genus Parasutterella id.2892)' on 'Protein phosphatase 1L'

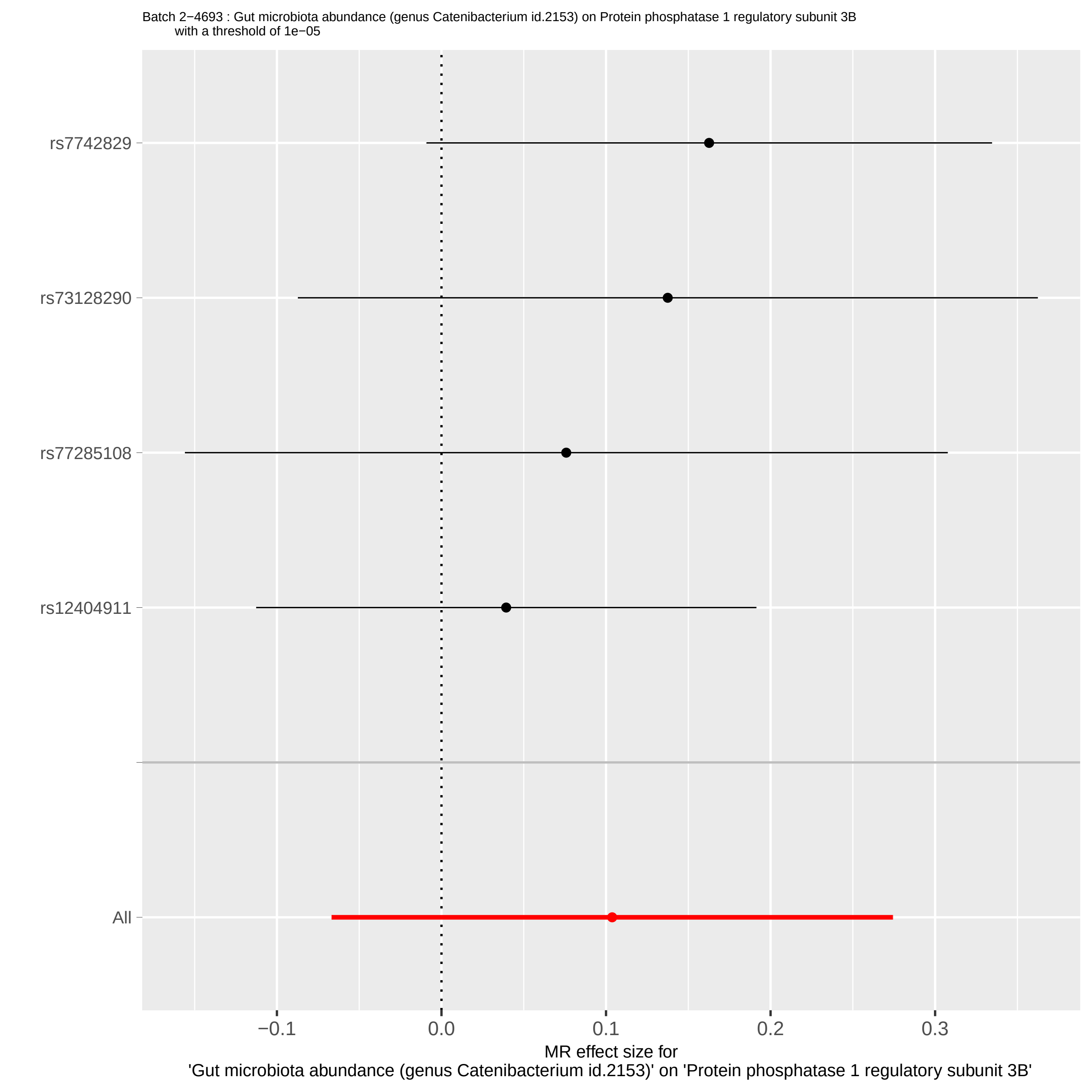

Batch 2−4693 : Gut microbiota abundance (genus Catenibacterium id.2153) on Protein phosphatase 1 regulatory subunit 3B
with a threshold of 1e−05
rs7742829
rs73128290
rs77285108
rs12404911
All
−0.1
0.0
0.1
0.2
0.3
MR effect size for
'Gut microbiota abundance (genus Catenibacterium id.2153)' on 'Protein phosphatase 1 regulatory subunit 3B'

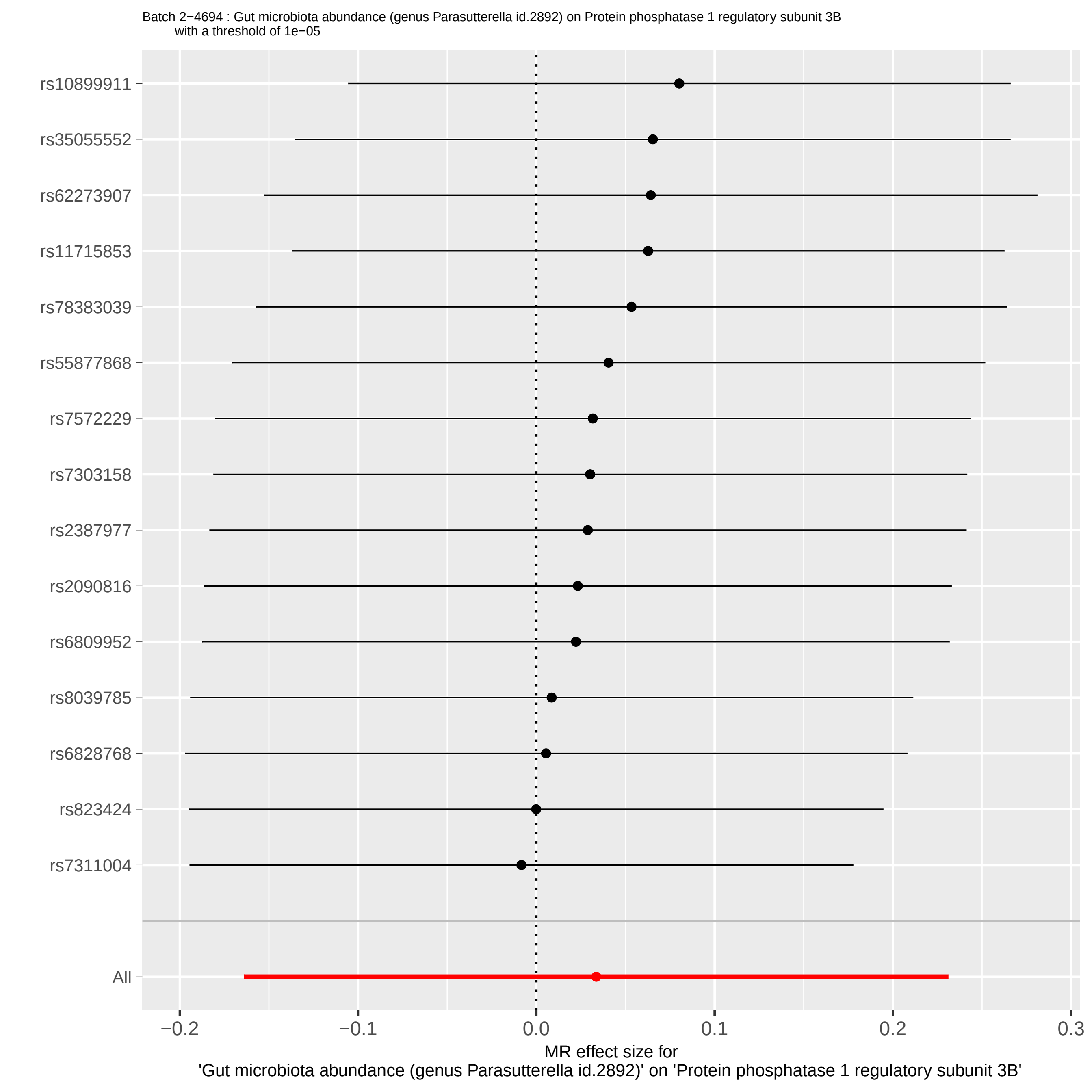

Batch 2−4694 : Gut microbiota abundance (genus Parasutterella id.2892) on Protein phosphatase 1 regulatory subunit 3B
with a threshold of 1e−05
rs10899911
rs35055552
rs62273907
rs11715853
rs78383039
rs55877868
rs7572229
rs7303158
rs2387977
rs2090816
rs6809952
rs8039785
rs6828768
rs823424
rs7311004
All
−0.2
−0.1
0.0
0.1
0.2
0.3
MR effect size for
'Gut microbiota abundance (genus Parasutterella id.2892)' on 'Protein phosphatase 1 regulatory subunit 3B'

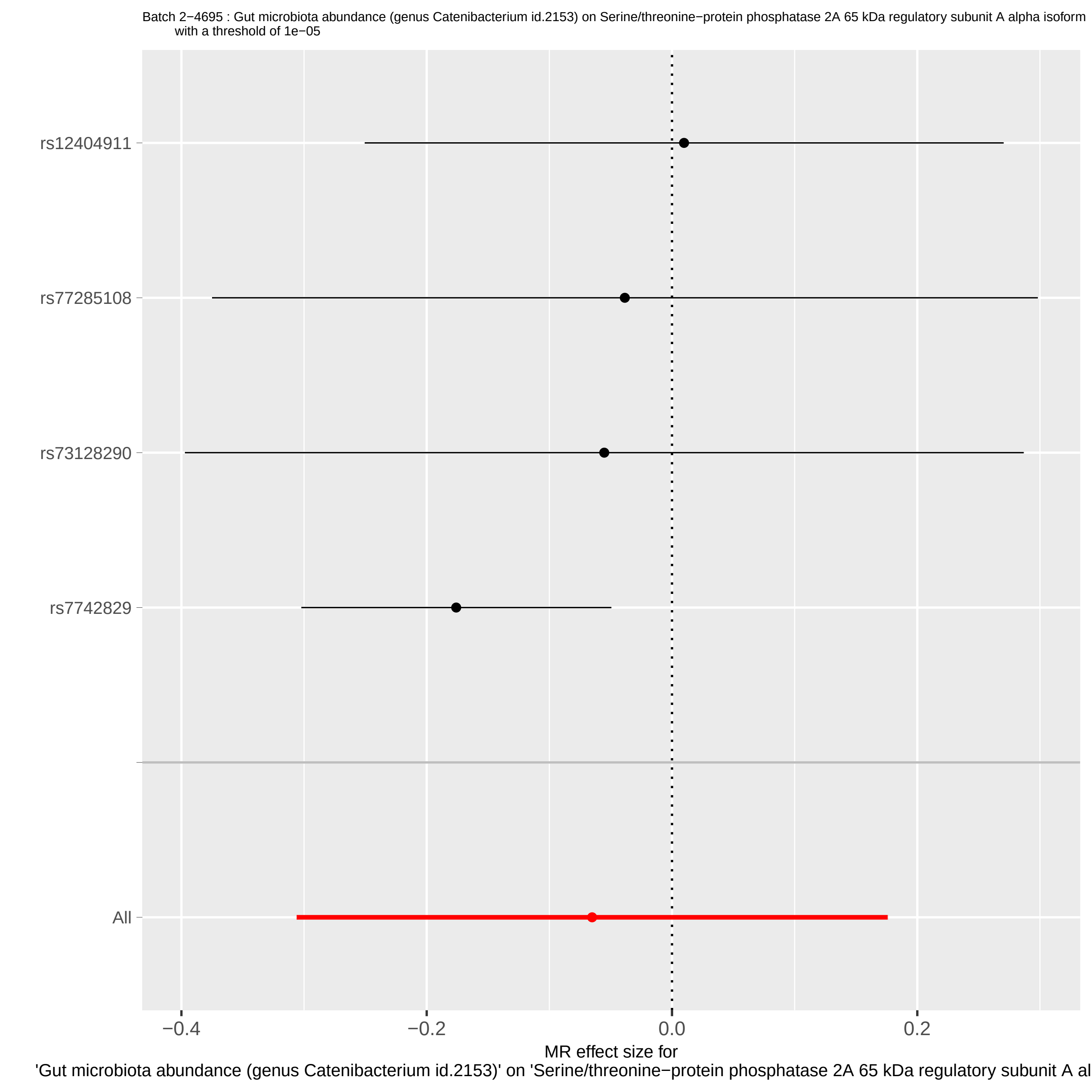

Batch 2−4695 : Gut microbiota abundance (genus Catenibacterium id.2153) on Serine/threonine−protein phosphatase 2A 65 kDa regulatory subunit A alpha isoform
with a threshold of 1e−05
rs12404911
rs77285108
rs73128290
rs7742829
All
−0.4
−0.2
0.0
0.2
MR effect size for
'Gut microbiota abundance (genus Catenibacterium id.2153)' on 'Serine/threonine−protein phosphatase 2A 65 kDa regulatory subunit A al

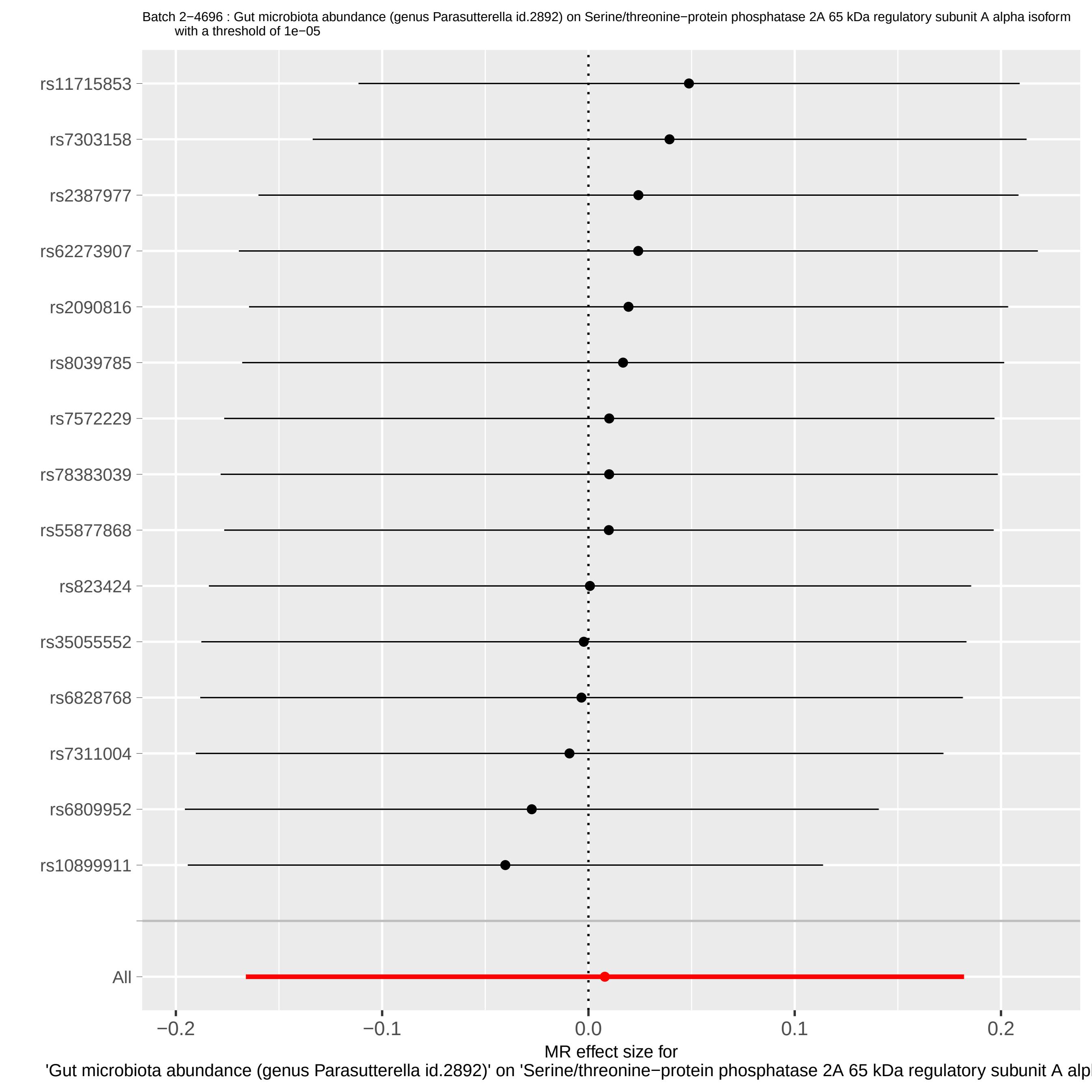
Batch 2−4696 : Gut microbiota abundance (genus Parasutterella id.2892) on Serine/threonine−protein phosphatase 2A 65 kDa regulatory subunit A alpha isoform with a threshold of 1e−05
rs11715853
rs7303158
rs2387977
rs62273907
rs2090816
rs8039785
rs7572229
rs78383039
rs55877868
rs823424
rs35055552
rs6828768
rs7311004
rs6809952
rs10899911
All
−0.2
−0.1
0.0
0.1
0.2
MR effect size for
'Gut microbiota abundance (genus Parasutterella id.2892)' on 'Serine/threonine−protein phosphatase 2A 65 kDa regulatory subunit A alp

Batch 2−4697 : Gut microbiota abundance (genus Catenibacterium id.2153) on Serine/threonine−protein phosphatase 2A regulatory subunit B" subunit alpha
with a threshold of 1e−05

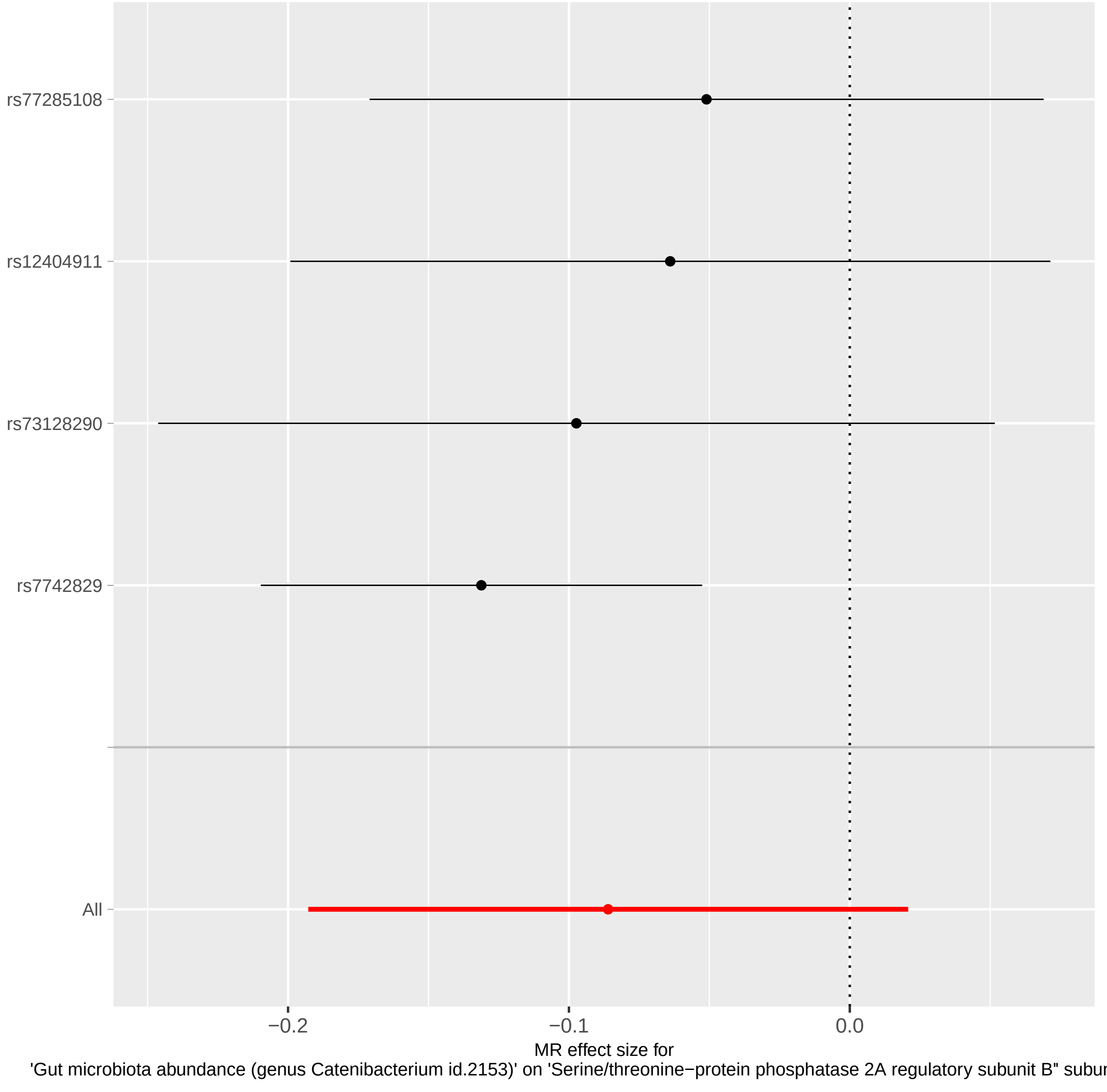

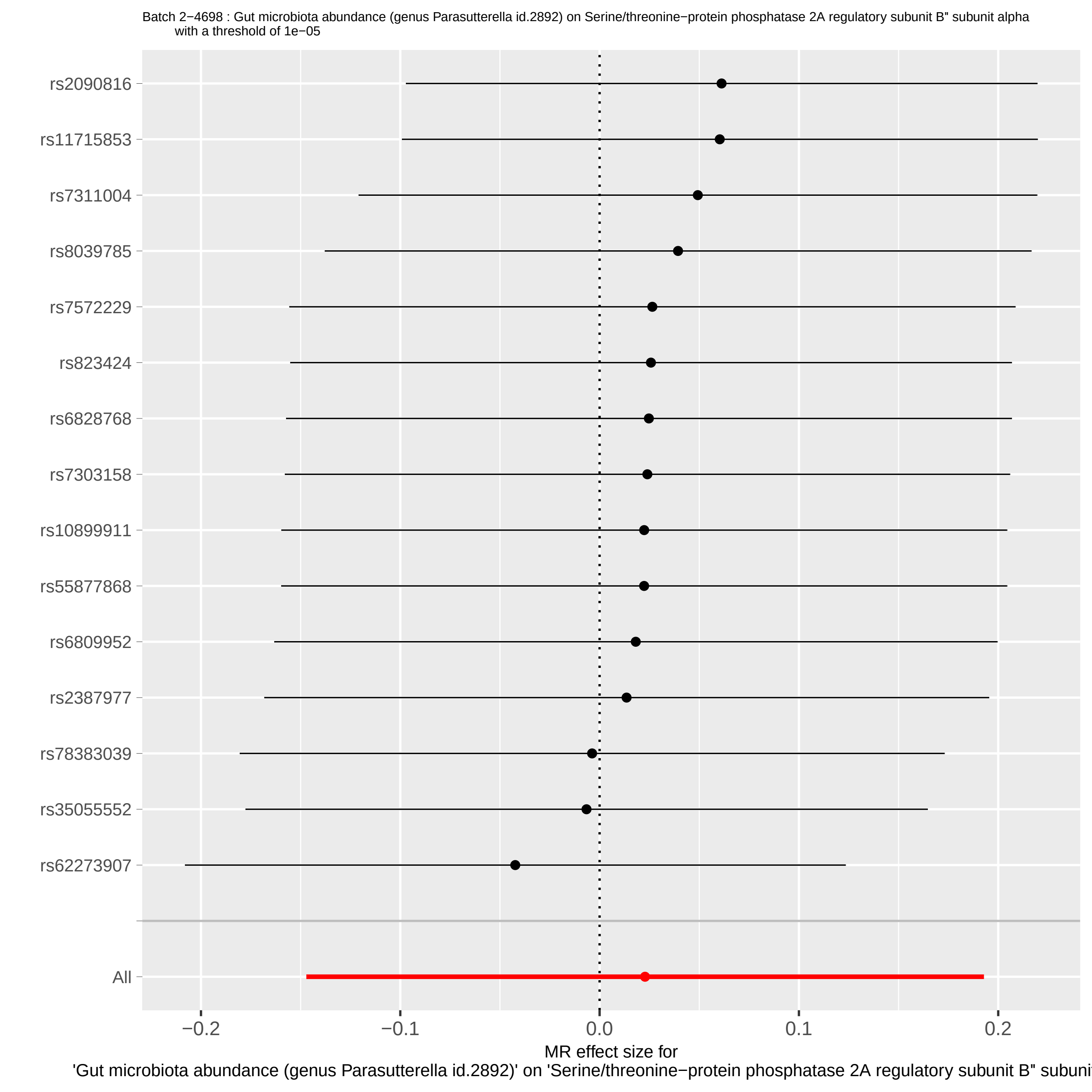
Batch 2−4698 : Gut microbiota abundance (genus Parasutterella id.2892) on Serine/threonine−protein phosphatase 2A regulatory subunit B" subunit alpha with a threshold of 1e−05
rs2090816
rs11715853
rs7311004
rs8039785
rs7572229
rs823424
rs6828768
rs7303158
rs10899911
rs55877868
rs6809952
rs2387977
rs78383039
rs35055552
rs62273907
All
−0.2
−0.1
0.0
0.1
0.2
MR effect size for
'Gut microbiota abundance (genus Parasutterella id.2892)' on 'Serine/threonine−protein phosphatase 2A regulatory subunit B" subuni

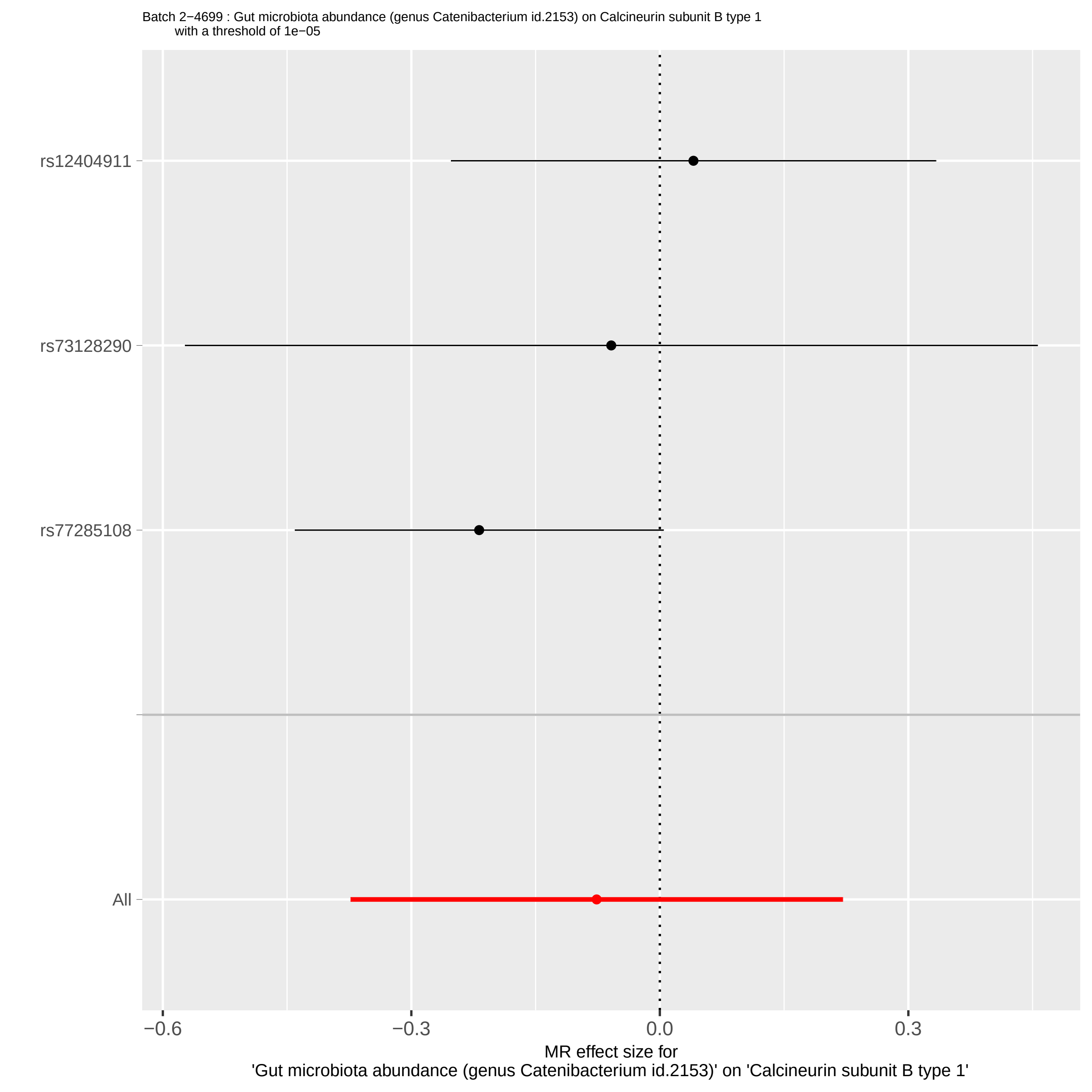
Batch 2−4699 : Gut microbiota abundance (genus Catenibacterium id.2153) on Calcineurin subunit B type 1
with a threshold of 1e−05
rs12404911
rs73128290
rs77285108
All
−0.6
−0.3
0.0
0.3
MR effect size for
'Gut microbiota abundance (genus Catenibacterium id.2153)' on 'Calcineurin subunit B type 1'

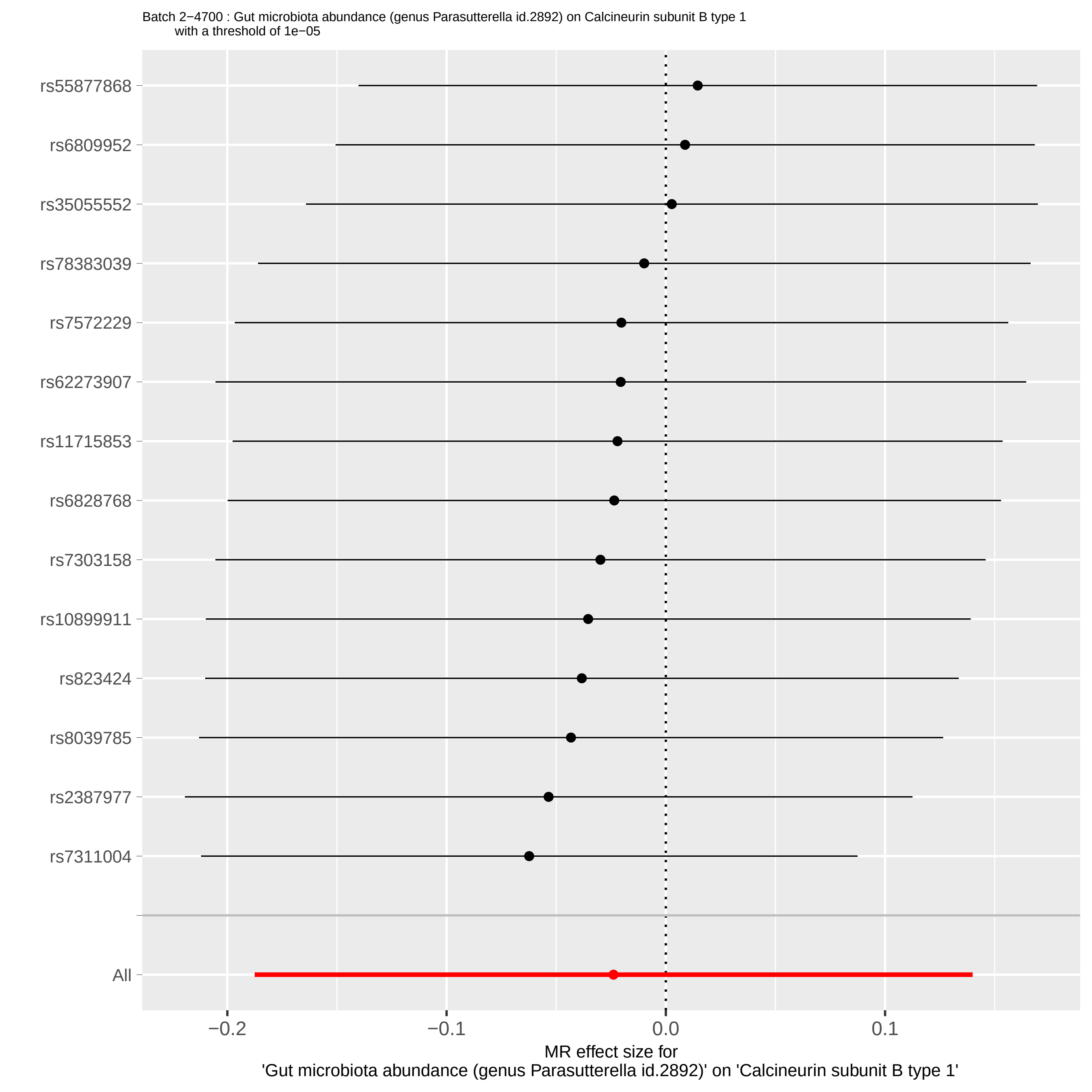
Batch 2−4700 : Gut microbiota abundance (genus Parasutterella id.2892) on Calcineurin subunit B type 1
with a threshold of 1e−05
rs55877868
rs6809952
rs35055552
rs78383039
rs7572229
rs62273907
rs11715853
rs6828768
rs7303158
rs10899911
rs823424
rs8039785
rs2387977
rs7311004
All
−0.2
−0.1
0.0
0.1
MR effect size for
'Gut microbiota abundance (genus Parasutterella id.2892)' on 'Calcineurin subunit B type 1'

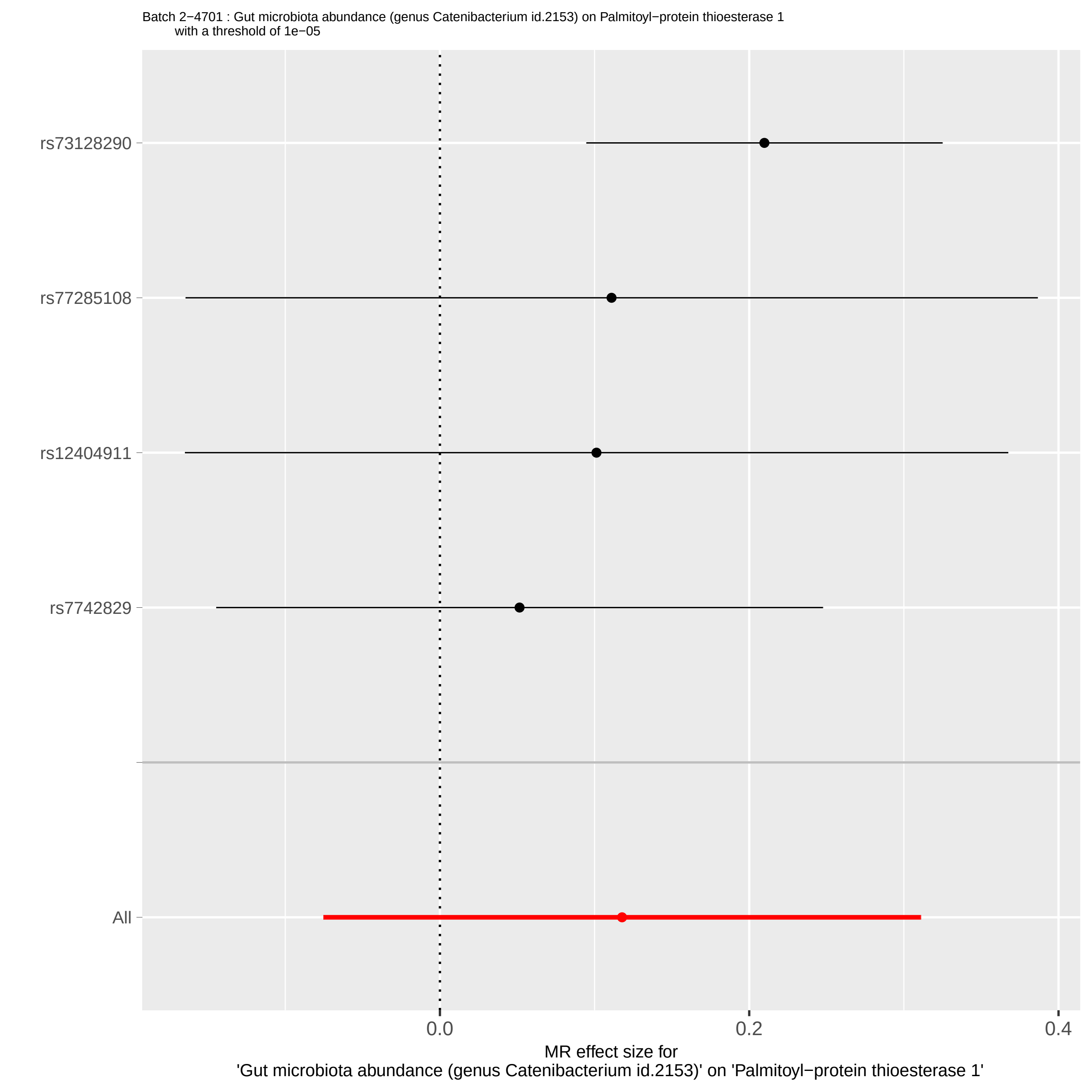
Batch 2−4701 : Gut microbiota abundance (genus Catenibacterium id.2153) on Palmitoyl−protein thioesterase 1
with a threshold of 1e−05
rs73128290
rs77285108
rs12404911
rs7742829
All
0.0
0.2
0.4
MR effect size for
'Gut microbiota abundance (genus Catenibacterium id.2153)' on 'Palmitoyl−protein thioesterase 1'

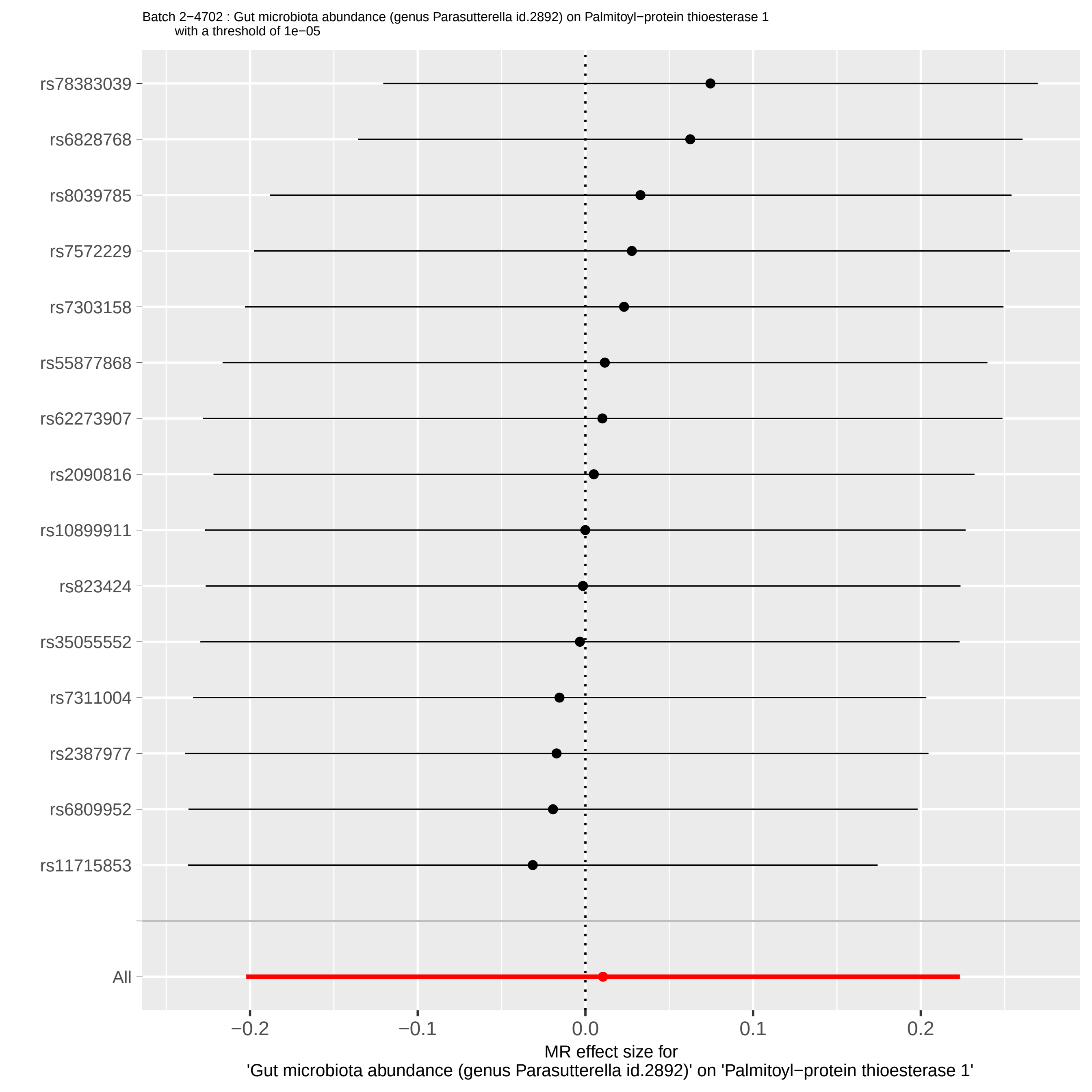
Batch 2−4702 : Gut microbiota abundance (genus Parasutterella id.2892) on Palmitoyl−protein thioesterase 1
with a threshold of 1e−05
rs78383039
rs6828768
rs8039785
rs7572229
rs7303158
rs55877868
rs62273907
rs2090816
rs10899911
rs823424
rs35055552
rs7311004
rs2387977
rs6809952
rs11715853
All
−0.2
−0.1
0.0
0.1
0.2
MR effect size for
'Gut microbiota abundance (genus Parasutterella id.2892)' on 'Palmitoyl−protein thioesterase 1'

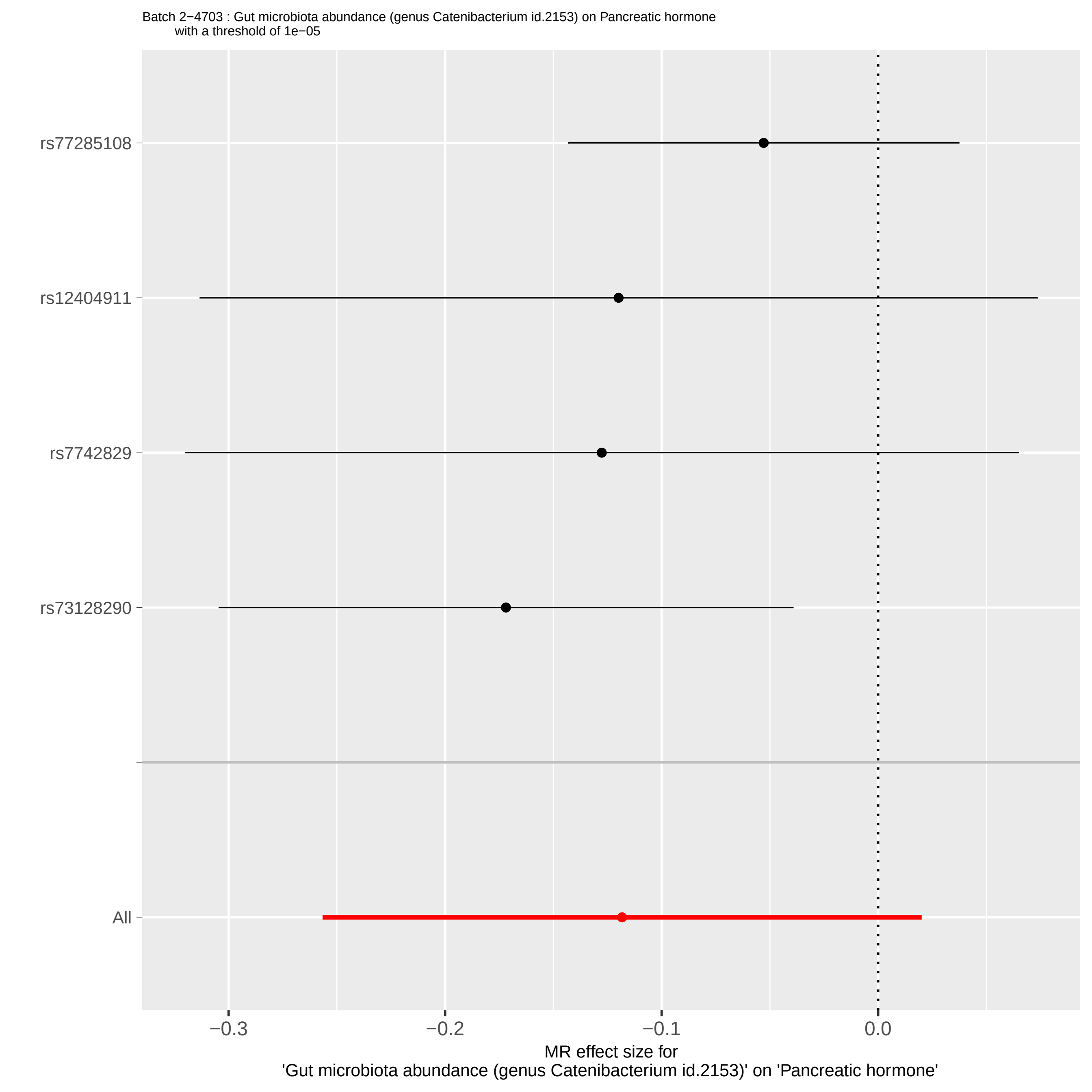
Batch 2−4703 : Gut microbiota abundance (genus Catenibacterium id.2153) on Pancreatic hormone
with a threshold of 1e−05
rs77285108
rs12404911
rs7742829
rs73128290
All
−0.3
−0.2
−0.1
0.0
MR effect size for
'Gut microbiota abundance (genus Catenibacterium id.2153)' on 'Pancreatic hormone'

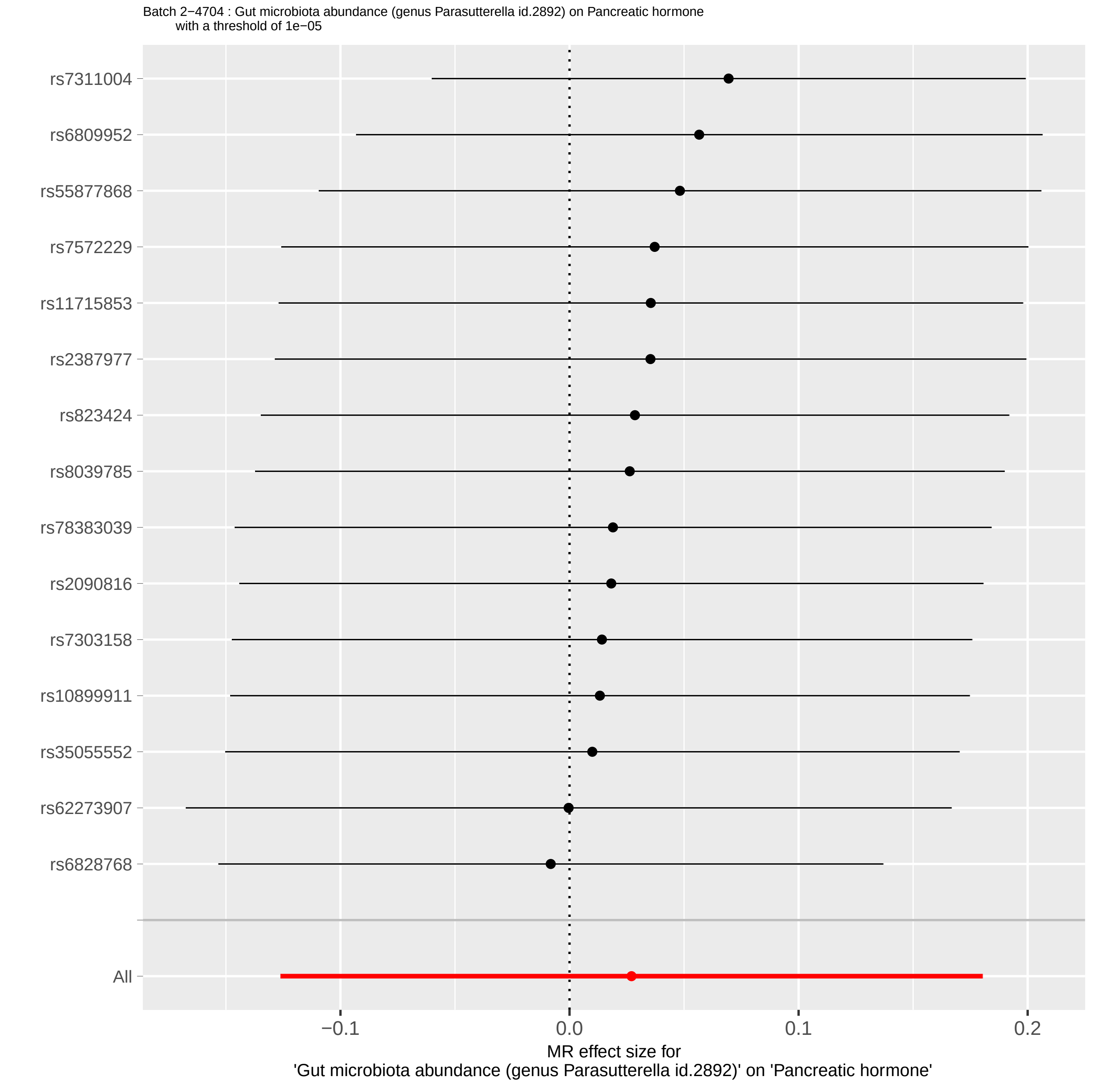

Batch 2−4704 : Gut microbiota abundance (genus Parasutterella id.2892) on Pancreatic hormone
with a threshold of 1e−05
rs7311004
rs6809952
rs55877868
rs7572229
rs11715853
rs2387977
rs823424
rs8039785
rs78383039
rs2090816
rs7303158
rs10899911
rs35055552
rs62273907
rs6828768
All
−0.1
0.0
0.1
0.2
MR effect size for
'Gut microbiota abundance (genus Parasutterella id.2892)' on 'Pancreatic hormone'

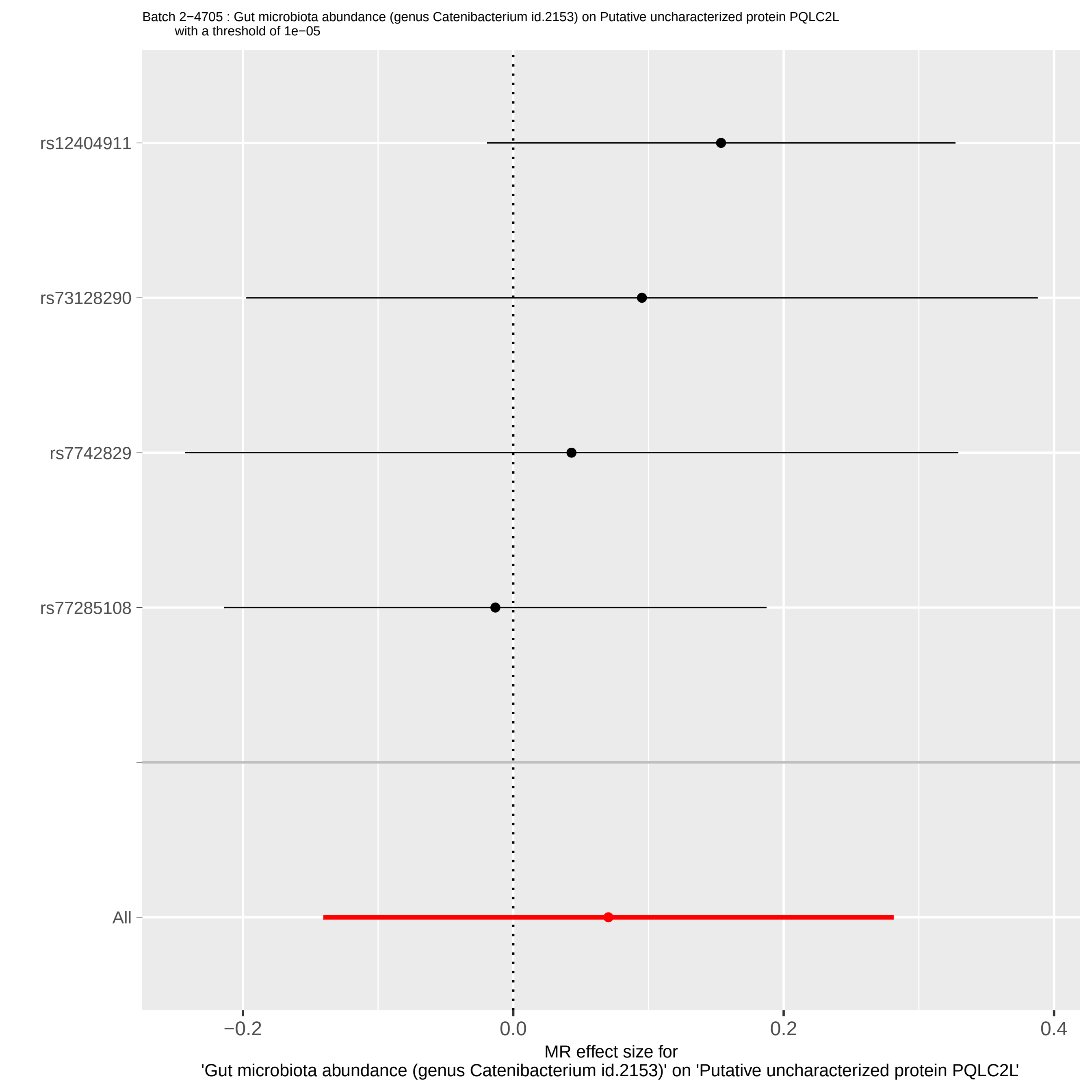

Batch 2−4705 : Gut microbiota abundance (genus Catenibacterium id.2153) on Putative uncharacterized protein PQLC2L
with a threshold of 1e−05
rs12404911
rs73128290
rs7742829
rs77285108
All
−0.2
0.0
0.2
0.4
MR effect size for
'Gut microbiota abundance (genus Catenibacterium id.2153)' on 'Putative uncharacterized protein PQLC2L'

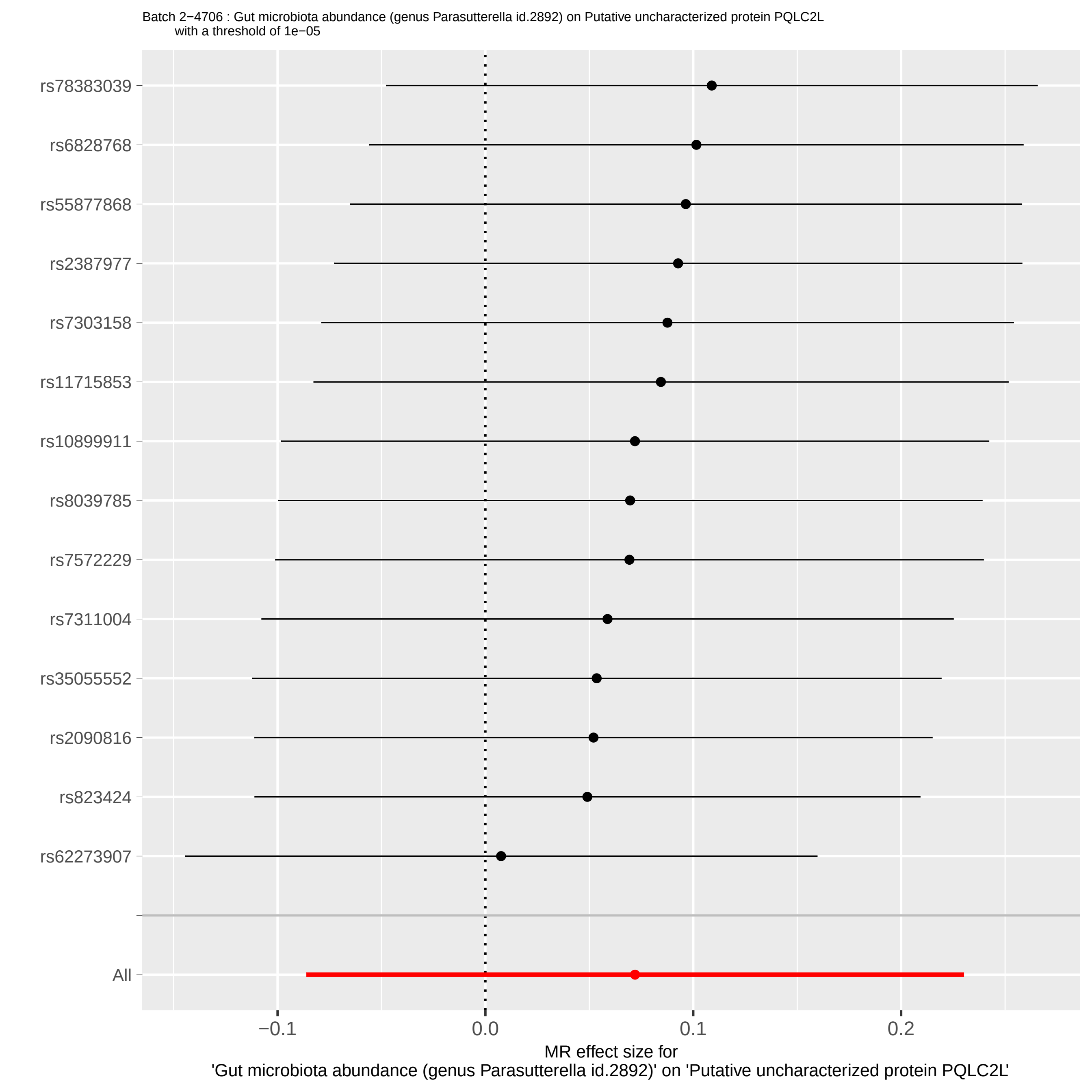

Batch 2−4706 : Gut microbiota abundance (genus Parasutterella id.2892) on Putative uncharacterized protein PQLC2L
with a threshold of 1e−05
rs78383039
rs6828768
rs55877868
rs2387977
rs7303158
rs11715853
rs10899911
rs8039785
rs7572229
rs7311004
rs35055552
rs2090816
rs823424
rs62273907
All
−0.1
0.0
0.1
0.2
MR effect size for
'Gut microbiota abundance (genus Parasutterella id.2892)' on 'Putative uncharacterized protein PQLC2L'

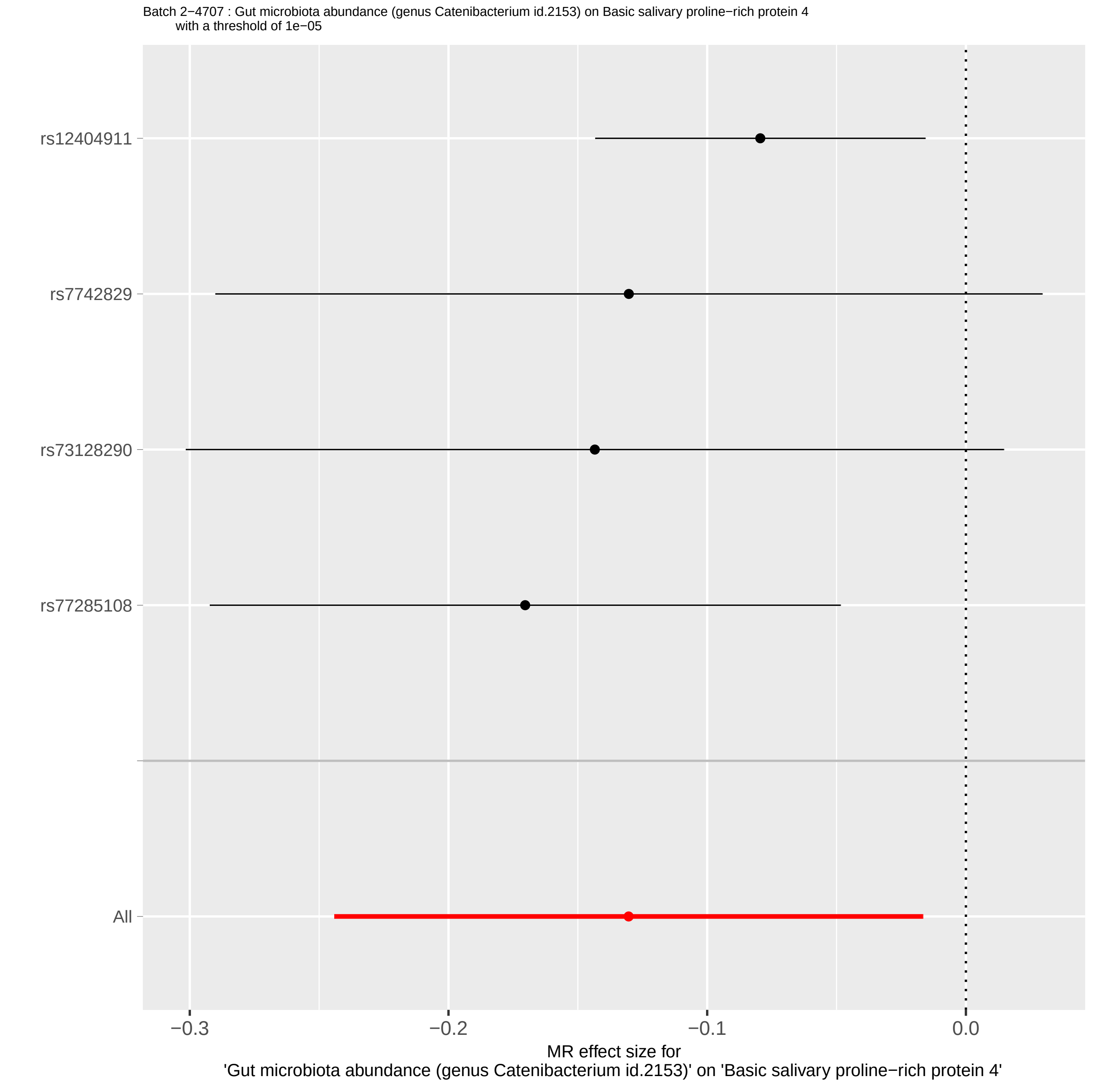
Batch 2−4707 : Gut microbiota abundance (genus Catenibacterium id.2153) on Basic salivary proline−rich protein 4
with a threshold of 1e−05
rs12404911
rs7742829
rs73128290
rs77285108
All
−0.3
−0.2
−0.1
0.0
MR effect size for
'Gut microbiota abundance (genus Catenibacterium id.2153)' on 'Basic salivary proline−rich protein 4'

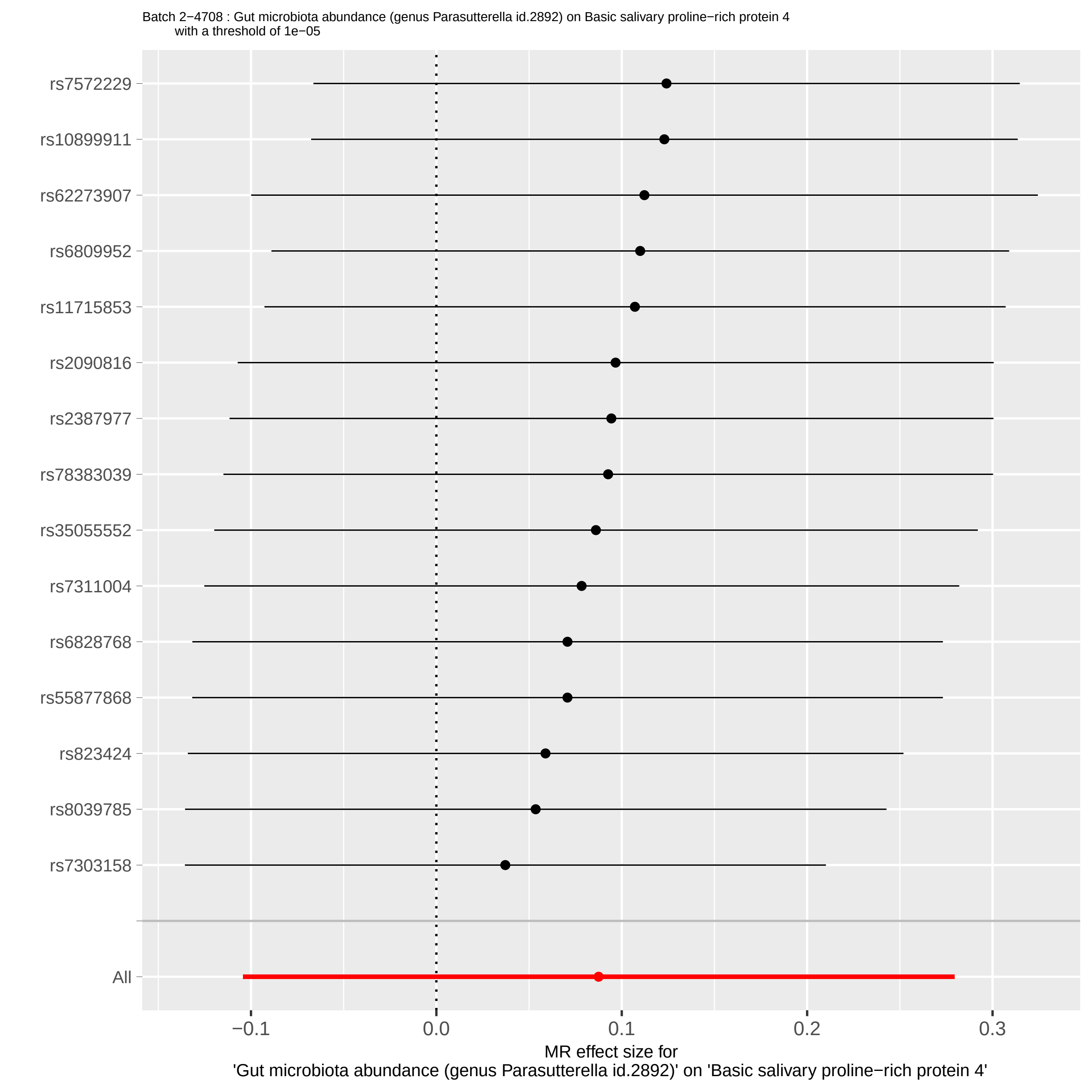

Batch 2−4708 : Gut microbiota abundance (genus Parasutterella id.2892) on Basic salivary proline−rich protein 4
with a threshold of 1e−05
rs7572229
rs10899911
rs62273907
rs6809952
rs11715853
rs2090816
rs2387977
rs78383039
rs35055552
rs7311004
rs6828768
rs55877868
rs823424
rs8039785
rs7303158
All
−0.1
0.0
0.1
0.2
0.3
MR effect size for
'Gut microbiota abundance (genus Parasutterella id.2892)' on 'Basic salivary proline−rich protein 4'

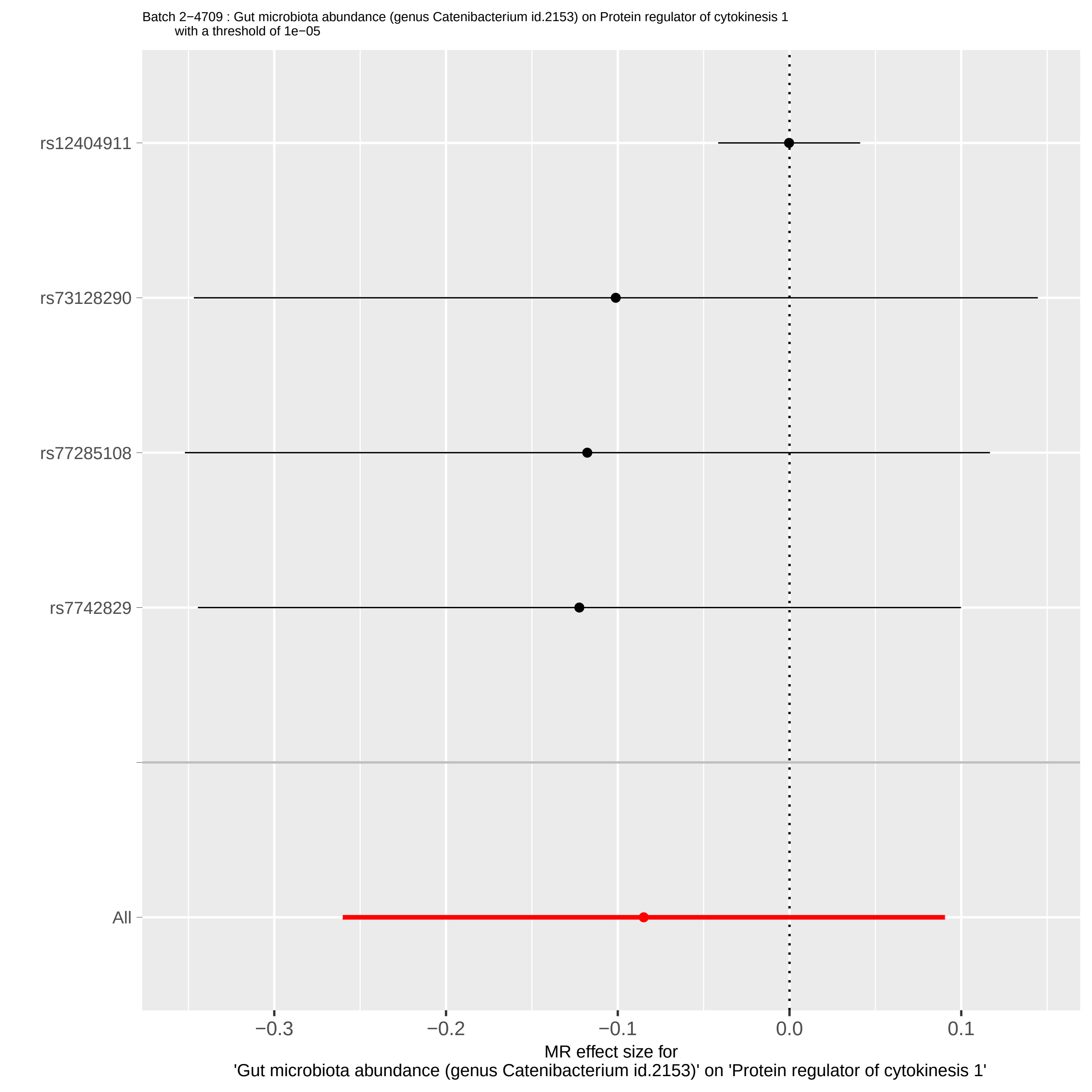

Batch 2−4709 : Gut microbiota abundance (genus Catenibacterium id.2153) on Protein regulator of cytokinesis 1
with a threshold of 1e−05
rs12404911
rs73128290
rs77285108
rs7742829
All
−0.3
−0.2
−0.1
0.0
0.1
MR effect size for
'Gut microbiota abundance (genus Catenibacterium id.2153)' on 'Protein regulator of cytokinesis 1'

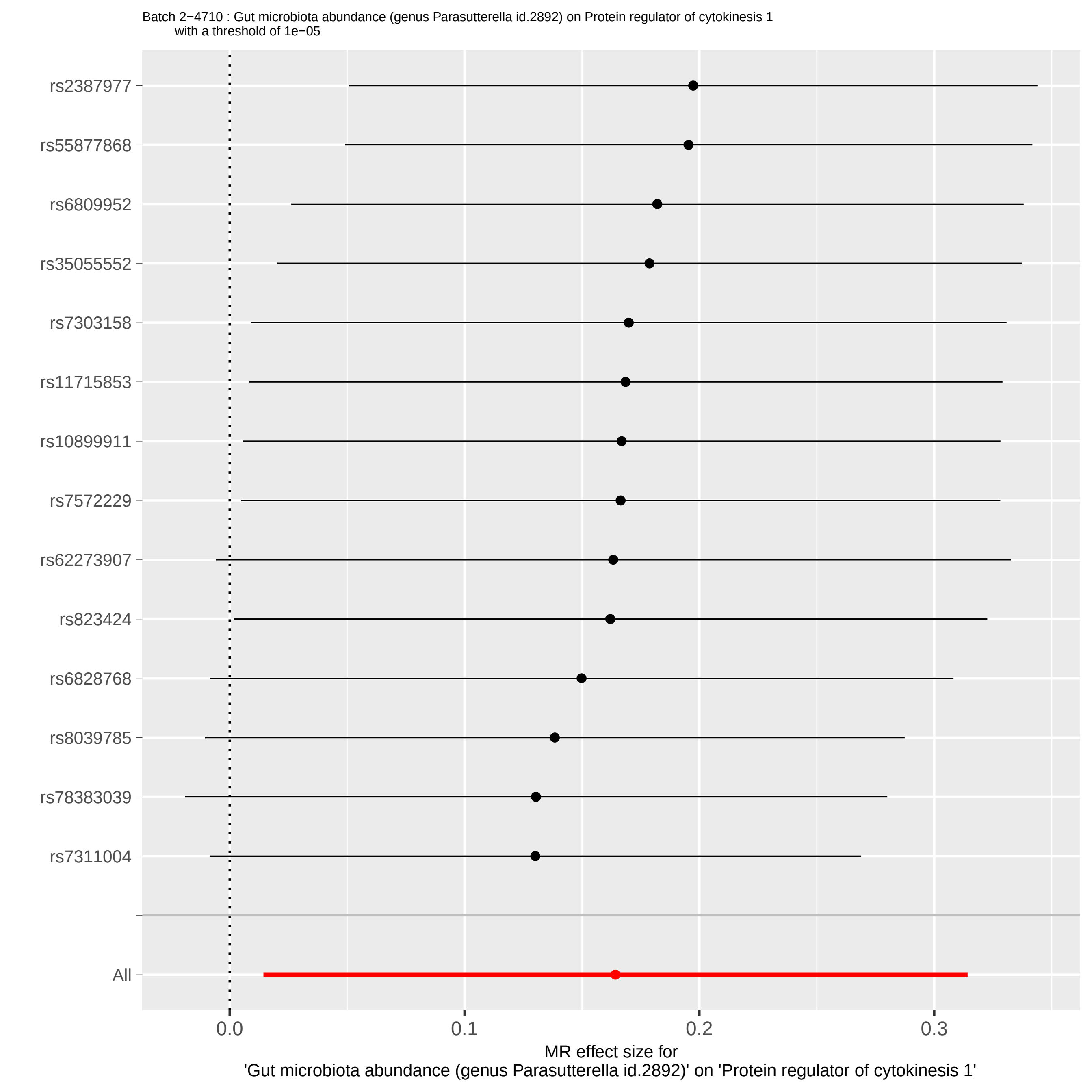

Batch 2−4710 : Gut microbiota abundance (genus Parasutterella id.2892) on Protein regulator of cytokinesis 1
with a threshold of 1e−05
rs2387977
rs55877868
rs6809952
rs35055552
rs7303158
rs11715853
rs10899911
rs7572229
rs62273907
rs823424
rs6828768
rs8039785
rs78383039
rs7311004
All
0.0
0.1
0.2
0.3
MR effect size for
'Gut microbiota abundance (genus Parasutterella id.2892)' on 'Protein regulator of cytokinesis 1'

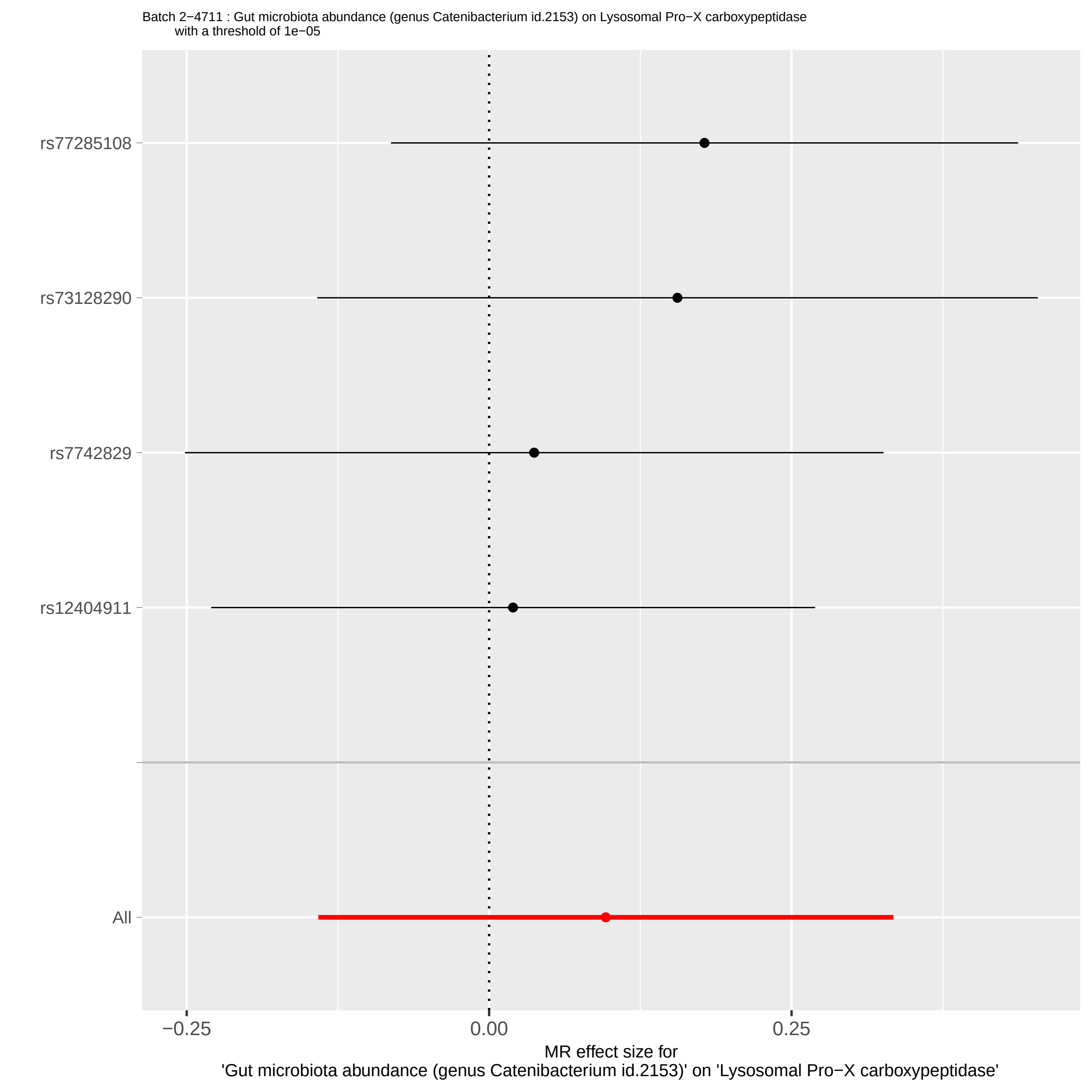

Batch 2−4711 : Gut microbiota abundance (genus Catenibacterium id.2153) on Lysosomal Pro−X carboxypeptidase
with a threshold of 1e−05
rs77285108
rs73128290
rs7742829
rs12404911
All
−0.25
0.00
0.25
MR effect size for
'Gut microbiota abundance (genus Catenibacterium id.2153)' on 'Lysosomal Pro−X carboxypeptidase'

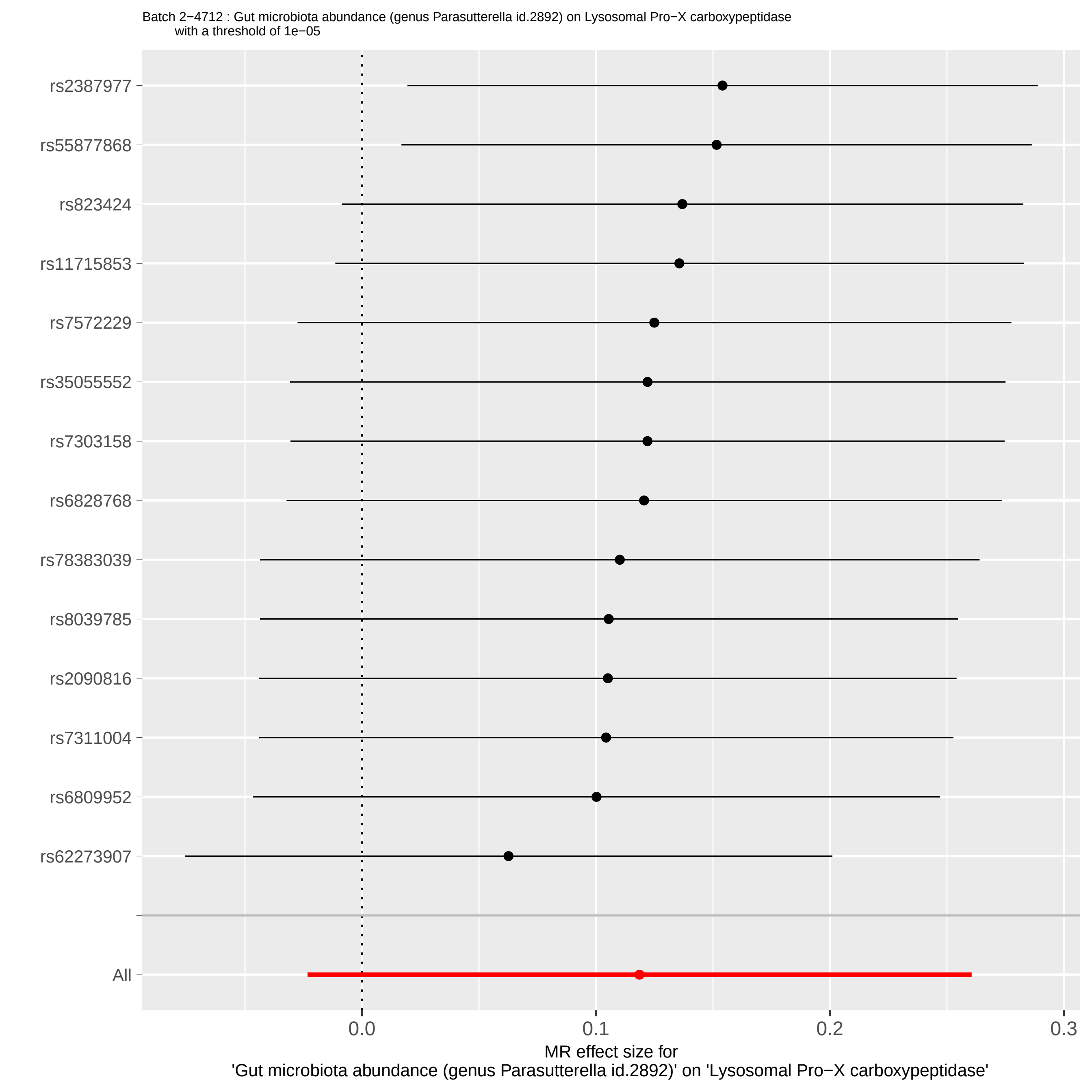
Batch 2−4712 : Gut microbiota abundance (genus Parasutterella id.2892) on Lysosomal Pro−X carboxypeptidase
with a threshold of 1e−05
rs2387977
rs55877868
rs823424
rs11715853
rs7572229
rs35055552
rs7303158
rs6828768
rs78383039
rs8039785
rs2090816
rs7311004
rs6809952
rs62273907
All
0.0
0.1
0.2
0.3
MR effect size for
'Gut microbiota abundance (genus Parasutterella id.2892)' on 'Lysosomal Pro−X carboxypeptidase'

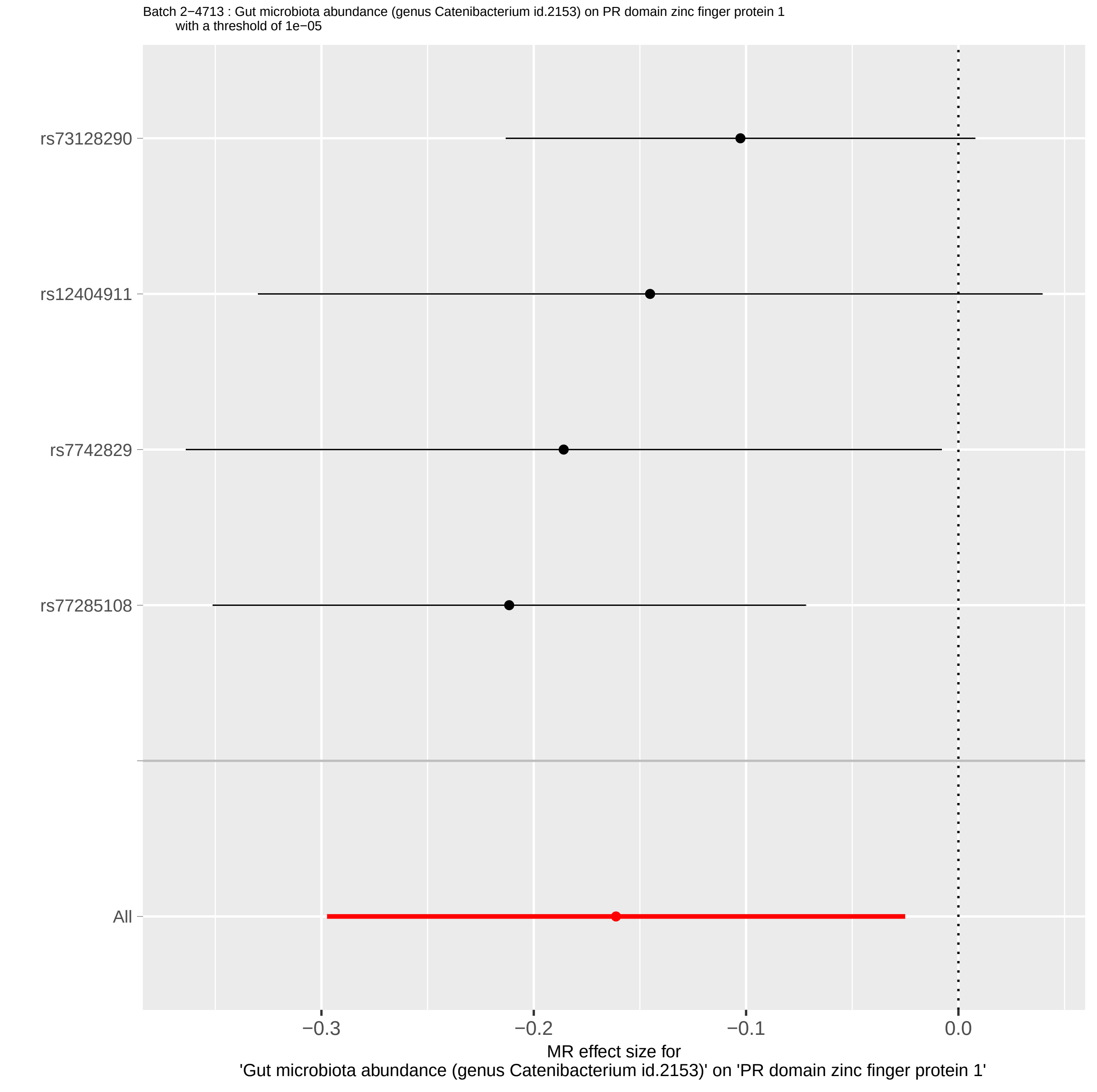

Batch 2−4713 : Gut microbiota abundance (genus Catenibacterium id.2153) on PR domain zinc finger protein 1
with a threshold of 1e−05
rs73128290
rs12404911
rs7742829
rs77285108
All
−0.3
−0.2
−0.1
0.0
MR effect size for
'Gut microbiota abundance (genus Catenibacterium id.2153)' on 'PR domain zinc finger protein 1'

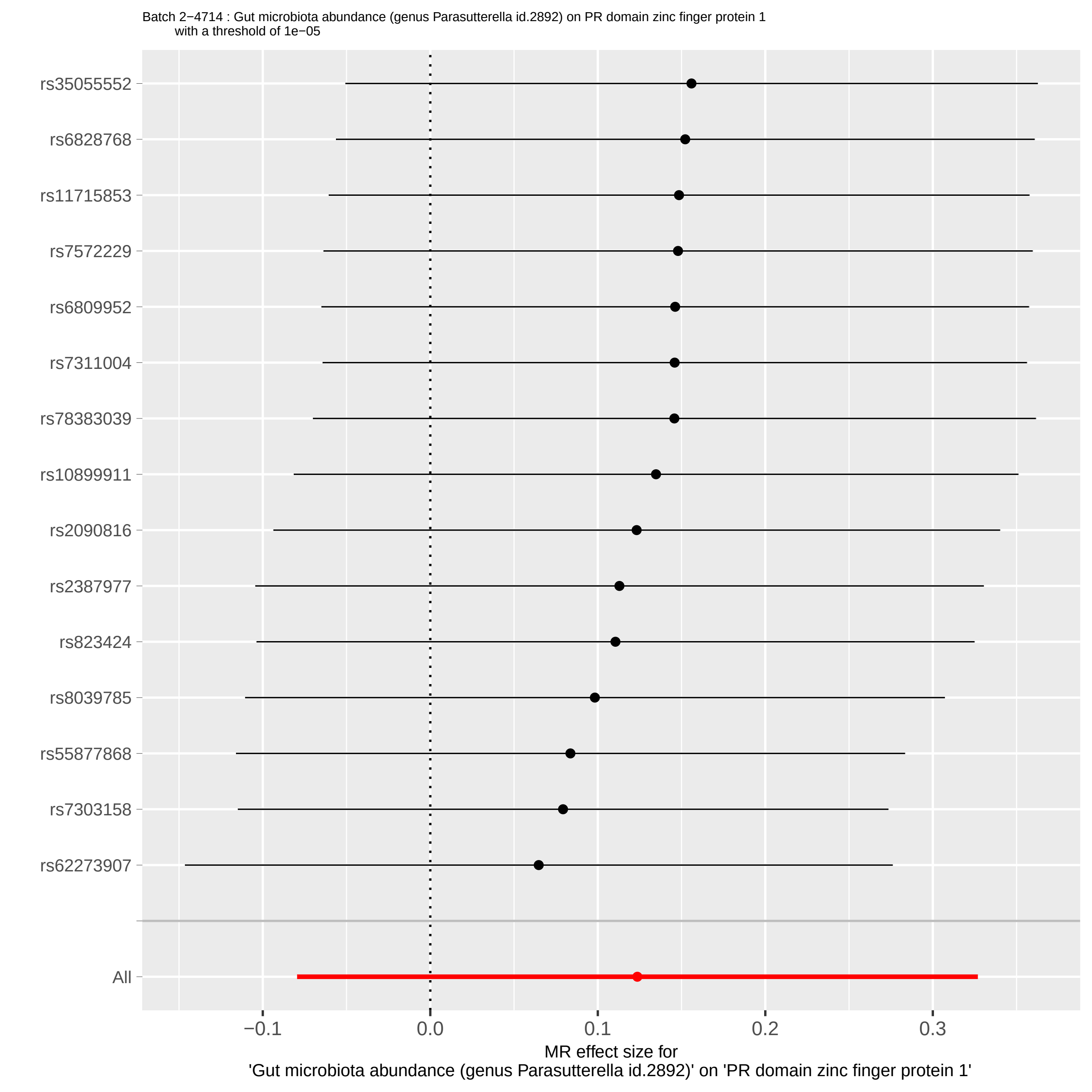

Batch 2−4714 : Gut microbiota abundance (genus Parasutterella id.2892) on PR domain zinc finger protein 1
with a threshold of 1e−05
rs35055552
rs6828768
rs11715853
rs7572229
rs6809952
rs7311004
rs78383039
rs10899911
rs2090816
rs2387977
rs823424
rs8039785
rs55877868
rs7303158
rs62273907
All
−0.1
0.0
0.1
0.2
0.3
MR effect size for
'Gut microbiota abundance (genus Parasutterella id.2892)' on 'PR domain zinc finger protein 1'

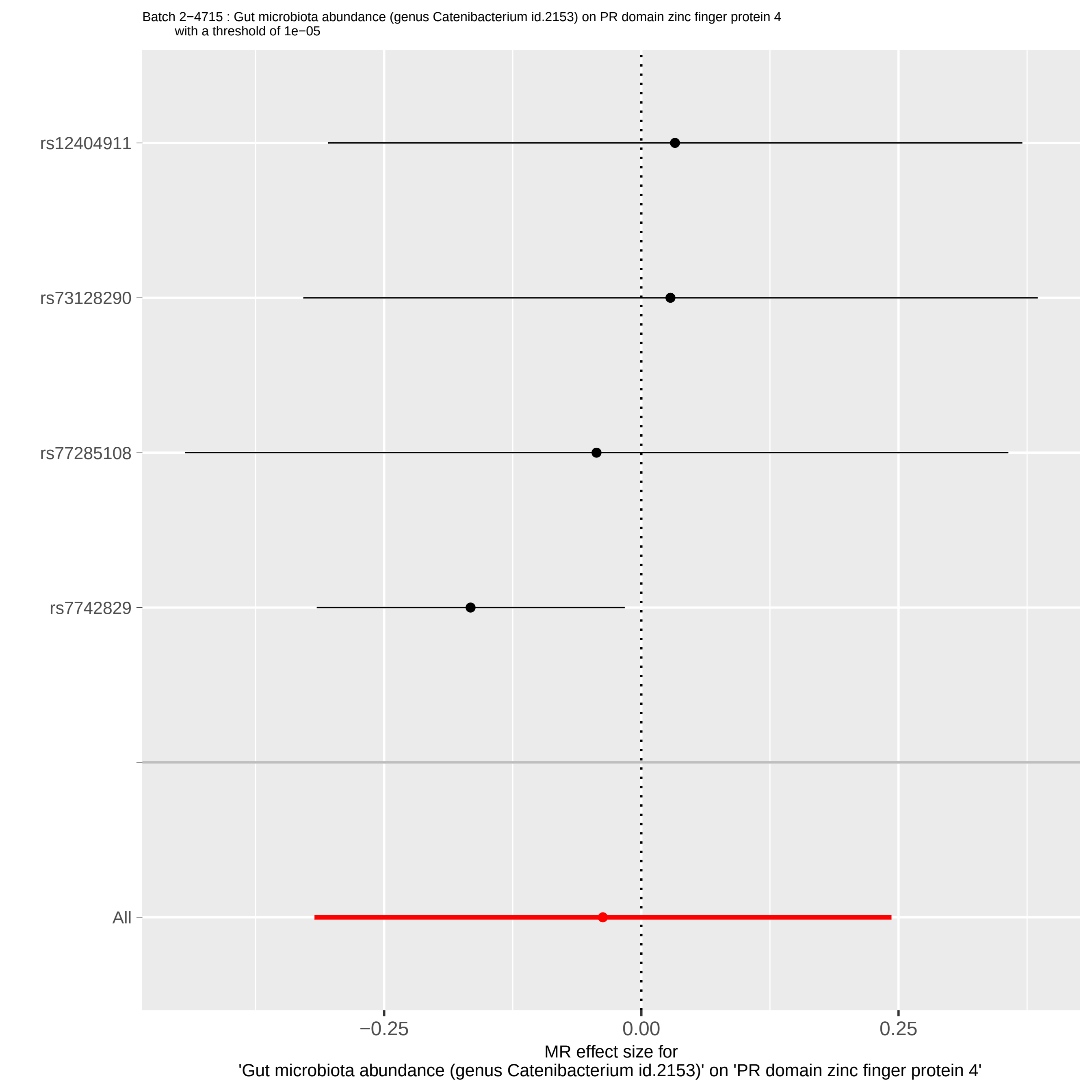
Batch 2−4715 : Gut microbiota abundance (genus Catenibacterium id.2153) on PR domain zinc finger protein 4
with a threshold of 1e−05
rs12404911
rs73128290
rs77285108
rs7742829
All
−0.25
0.00
0.25
MR effect size for
'Gut microbiota abundance (genus Catenibacterium id.2153)' on 'PR domain zinc finger protein 4'

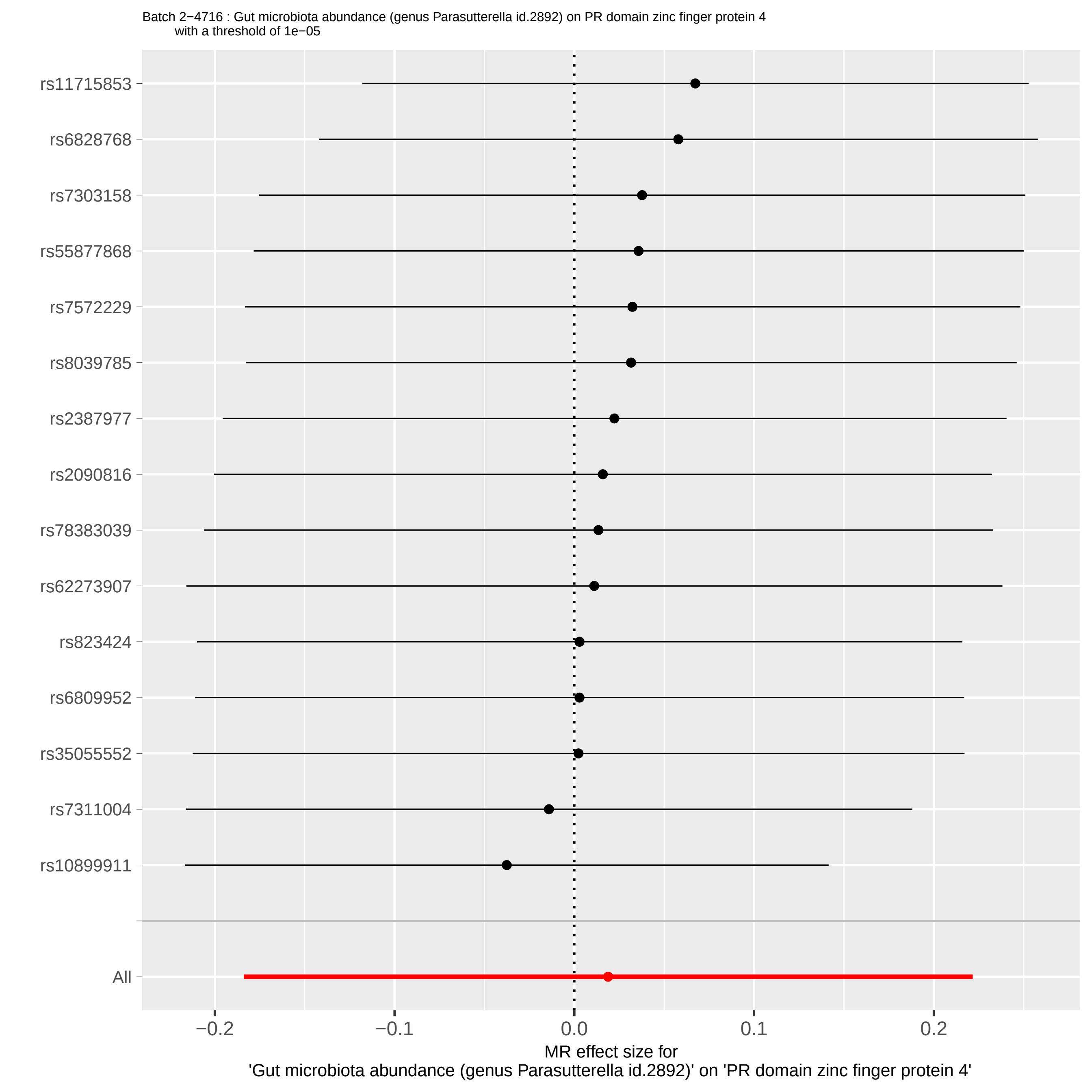

Batch 2−4716 : Gut microbiota abundance (genus Parasutterella id.2892) on PR domain zinc finger protein 4
with a threshold of 1e−05
rs11715853
rs6828768
rs7303158
rs55877868
rs7572229
rs8039785
rs2387977
rs2090816
rs78383039
rs62273907
rs823424
rs6809952
rs35055552
rs7311004
rs10899911
All
−0.2
−0.1
0.0
0.1
0.2
MR effect size for
'Gut microbiota abundance (genus Parasutterella id.2892)' on 'PR domain zinc finger protein 4'

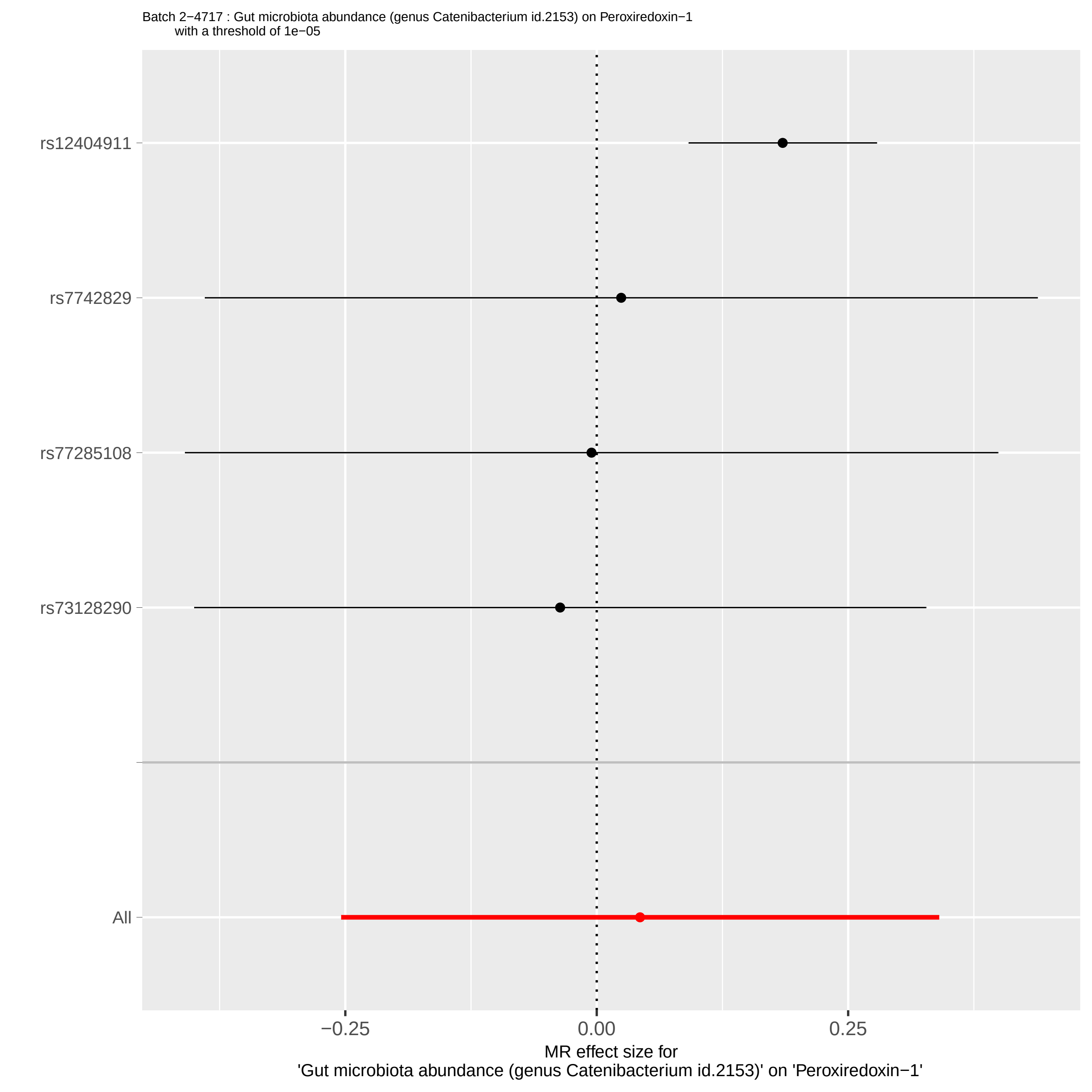
Batch 2−4717 : Gut microbiota abundance (genus Catenibacterium id.2153) on Peroxiredoxin−1
with a threshold of 1e−05
rs12404911
rs7742829
rs77285108
rs73128290
All
−0.25
0.00
0.25
MR effect size for
'Gut microbiota abundance (genus Catenibacterium id.2153)' on 'Peroxiredoxin−1'

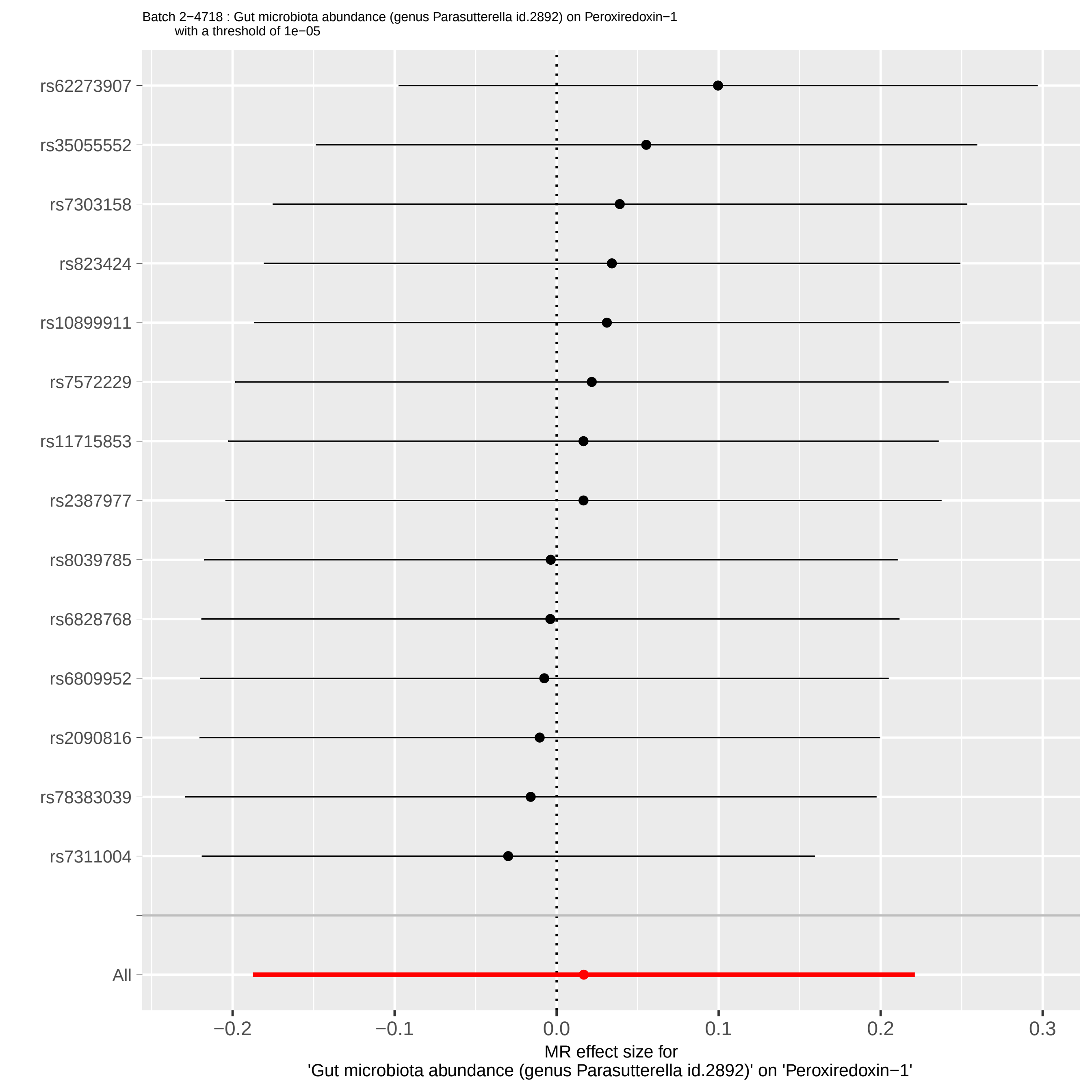
Batch 2−4718 : Gut microbiota abundance (genus Parasutterella id.2892) on Peroxiredoxin−1
with a threshold of 1e−05
rs62273907
rs35055552
rs7303158
rs823424
rs10899911
rs7572229
rs11715853
rs2387977
rs8039785
rs6828768
rs6809952
rs2090816
rs78383039
rs7311004
All
−0.2
−0.1
0.0
0.1
0.2
0.3
MR effect size for
'Gut microbiota abundance (genus Parasutterella id.2892)' on 'Peroxiredoxin−1'

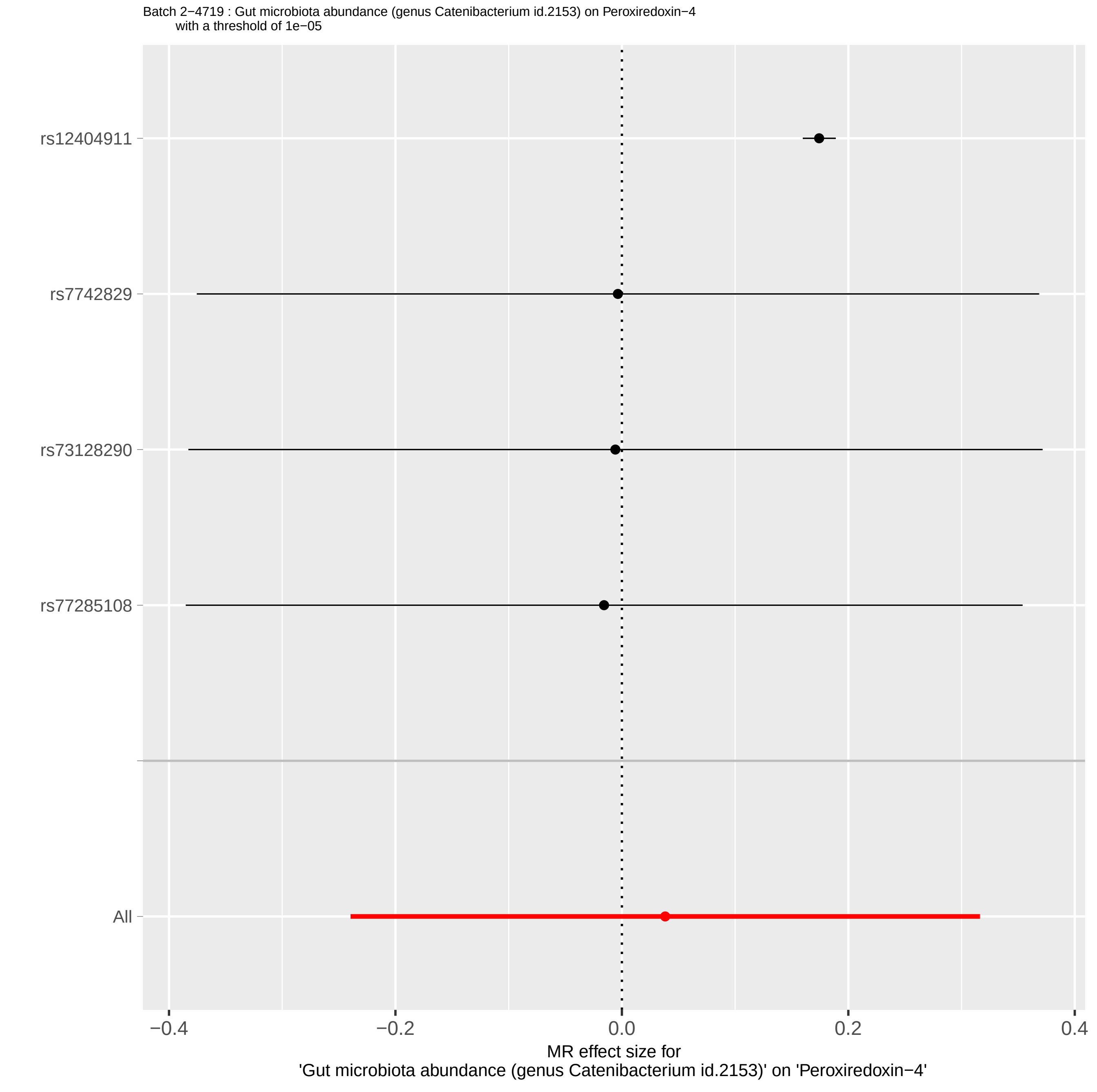

Batch 2−4719 : Gut microbiota abundance (genus Catenibacterium id.2153) on Peroxiredoxin−4
with a threshold of 1e−05
rs12404911
rs7742829
rs73128290
rs77285108
All
−0.4
−0.2
0.0
0.2
0.4
MR effect size for
'Gut microbiota abundance (genus Catenibacterium id.2153)' on 'Peroxiredoxin−4'

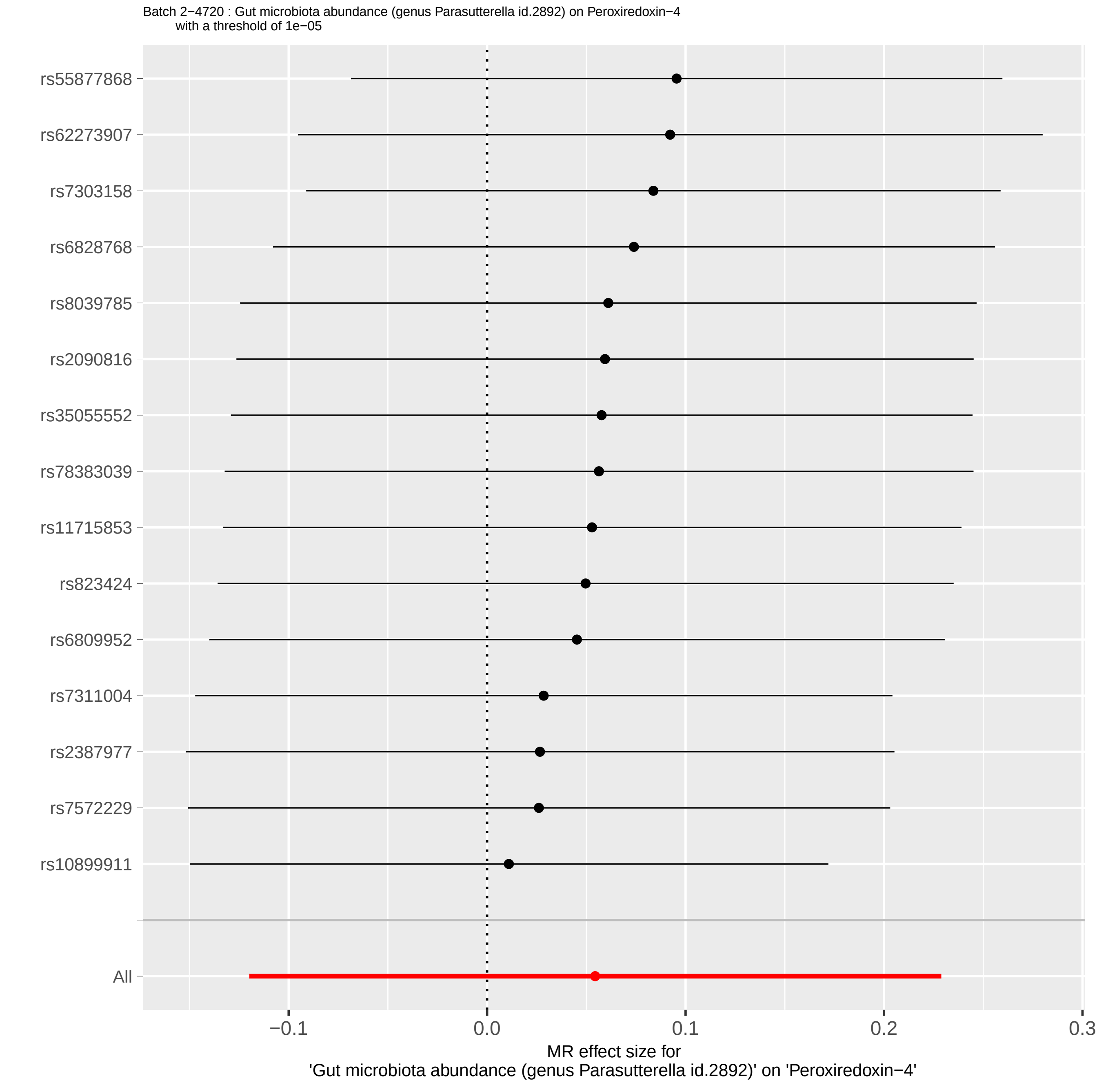
Batch 2−4720 : Gut microbiota abundance (genus Parasutterella id.2892) on Peroxiredoxin−4
with a threshold of 1e−05
rs55877868
rs62273907
rs7303158
rs6828768
rs8039785
rs2090816
rs35055552
rs78383039
rs11715853
rs823424
rs6809952
rs7311004
rs2387977
rs7572229
rs10899911
All
−0.1
0.0
0.1
0.2
0.3
MR effect size for
'Gut microbiota abundance (genus Parasutterella id.2892)' on 'Peroxiredoxin−4'

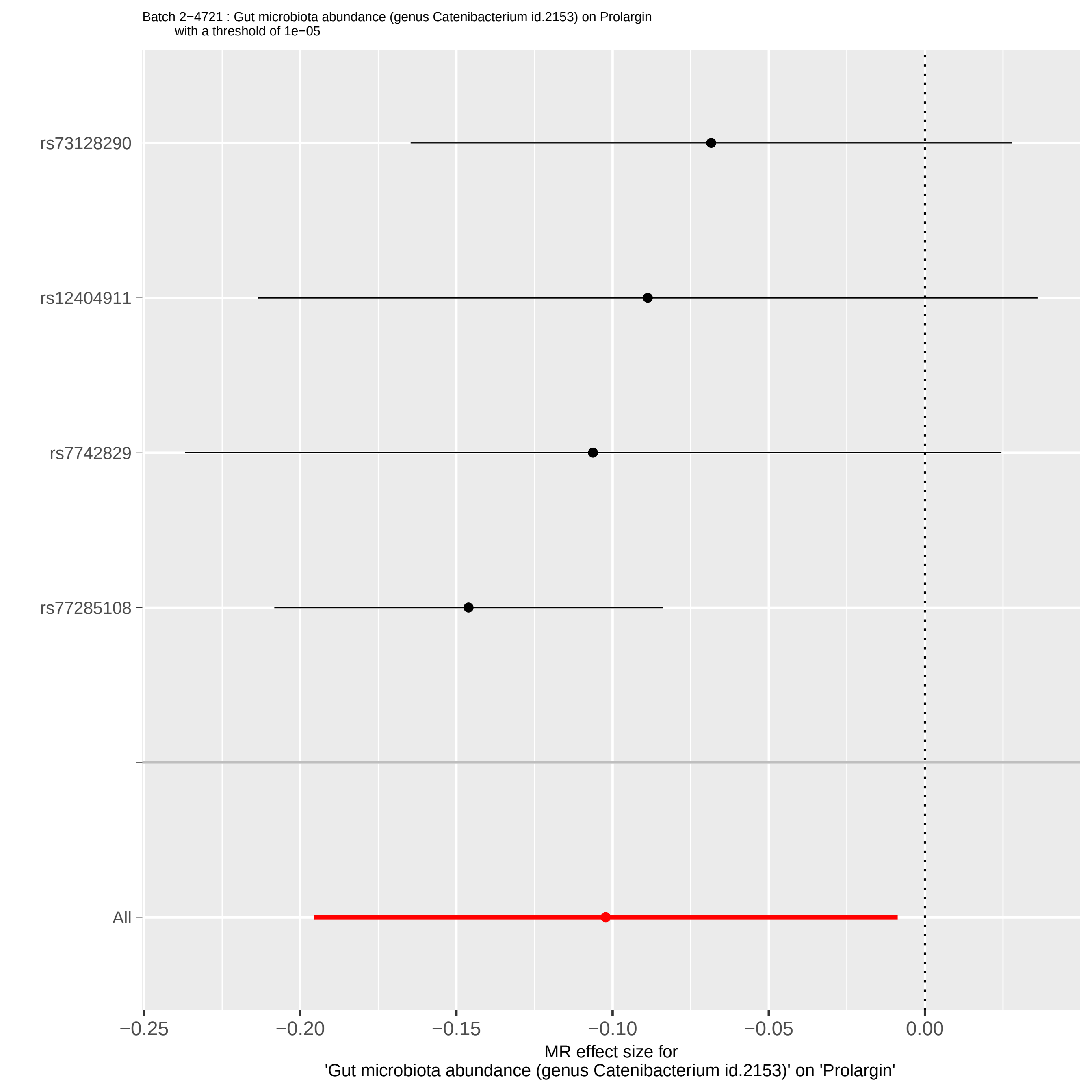

Batch 2−4721 : Gut microbiota abundance (genus Catenibacterium id.2153) on Prolargin
with a threshold of 1e−05
rs73128290
rs12404911
rs7742829
rs77285108
All
−0.25
−0.20
−0.15
−0.10
−0.05
0.00
MR effect size for
'Gut microbiota abundance (genus Catenibacterium id.2153)' on 'Prolargin'

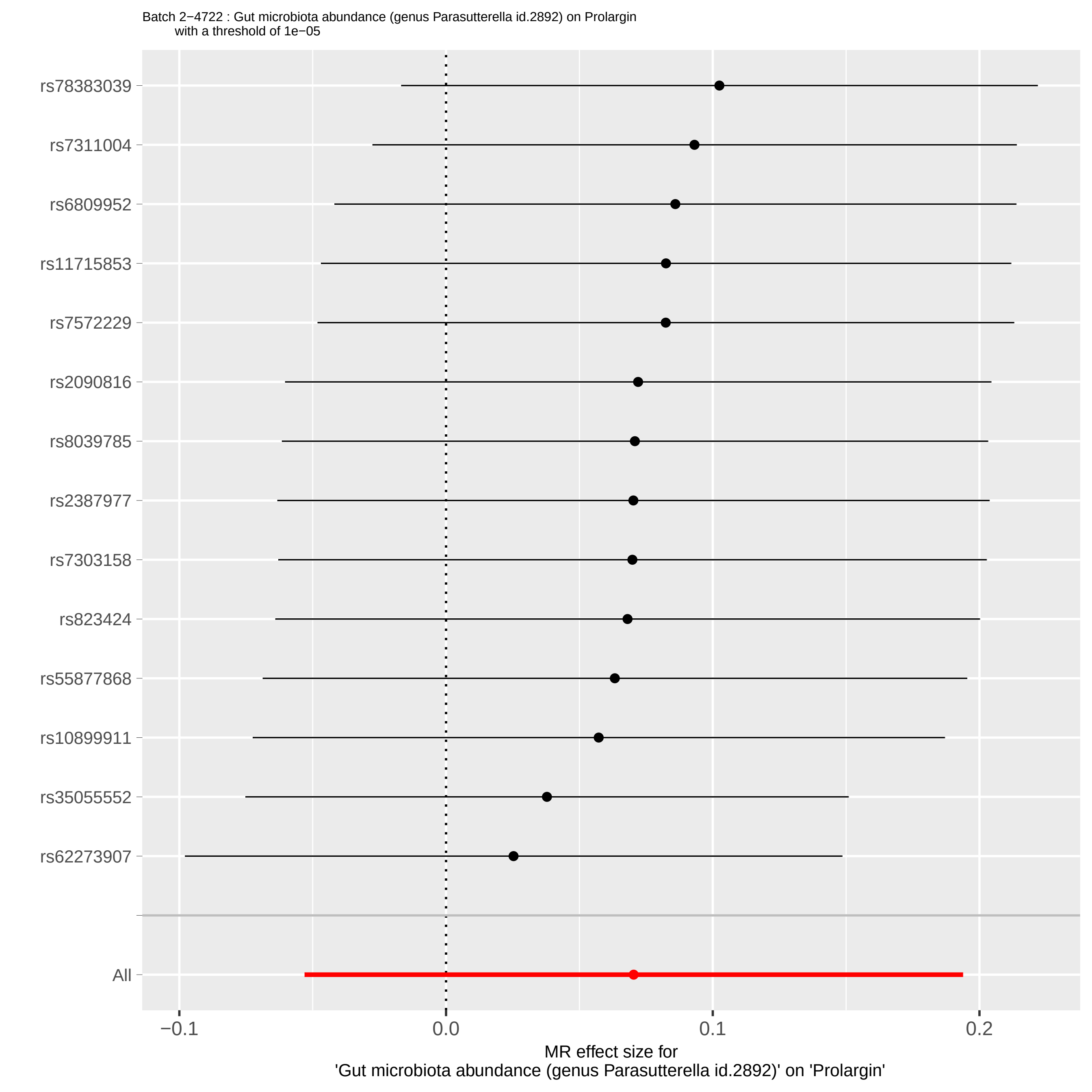
Batch 2−4722 : Gut microbiota abundance (genus Parasutterella id.2892) on Prolargin
with a threshold of 1e−05
rs78383039
rs7311004
rs6809952
rs11715853
rs7572229
rs2090816
rs8039785
rs2387977
rs7303158
rs823424
rs55877868
rs10899911
rs35055552
rs62273907
All
−0.1
0.0
0.1
0.2
MR effect size for
'Gut microbiota abundance (genus Parasutterella id.2892)' on 'Prolargin'

Batch 2−4723 : Gut microbiota abundance (genus Catenibacterium id.2153) on Proteoglycan 3
with a threshold of 1e−05

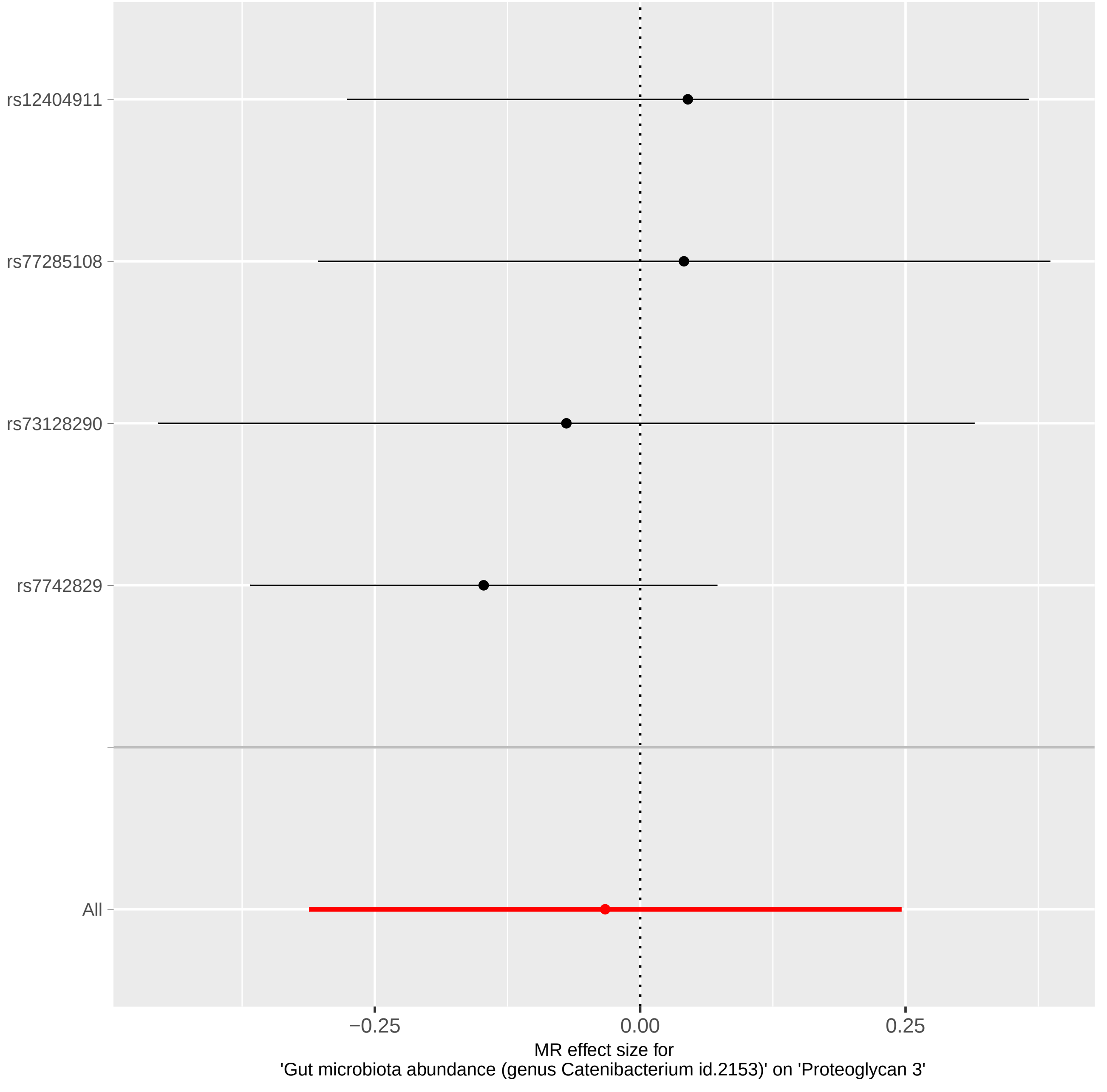

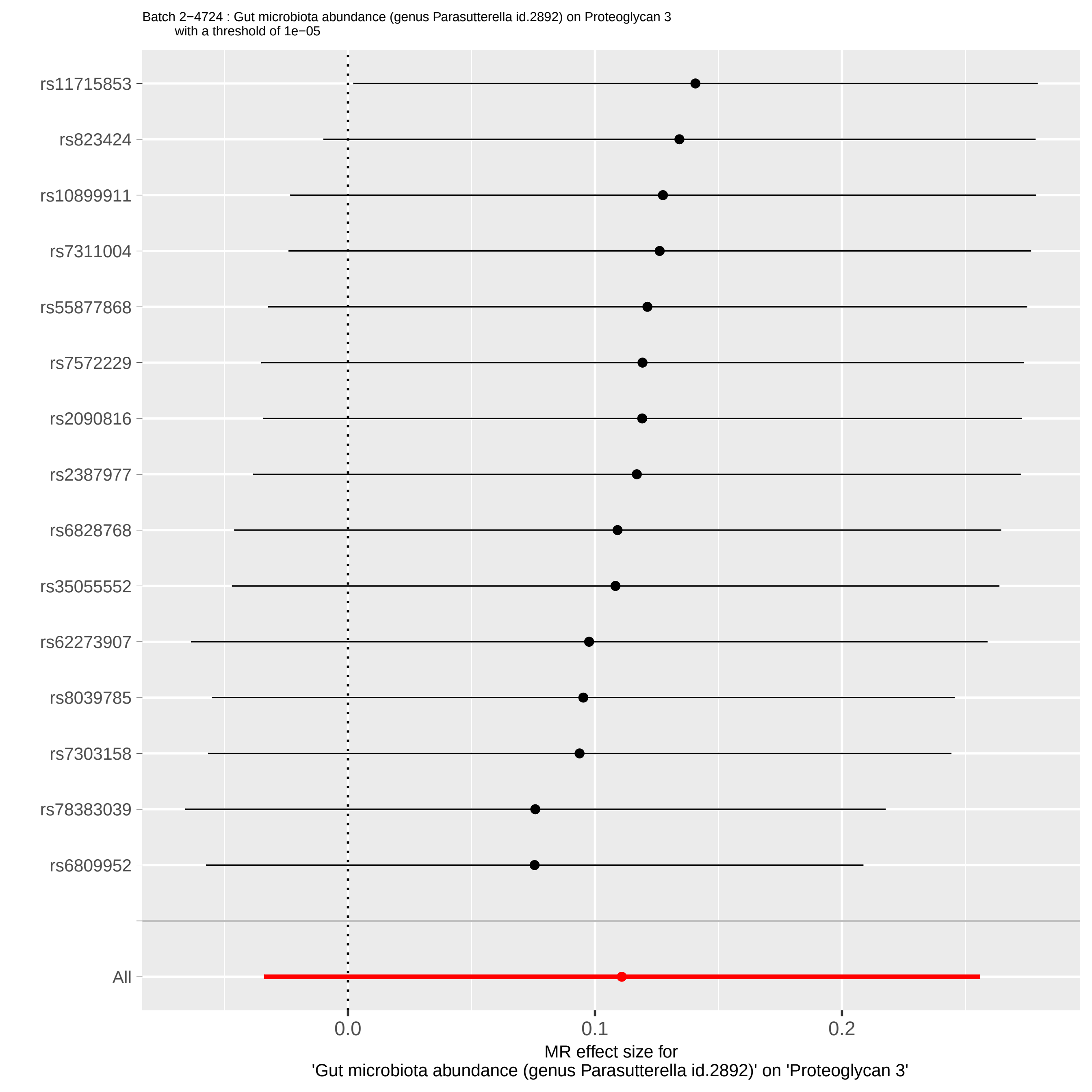

Batch 2−4724 : Gut microbiota abundance (genus Parasutterella id.2892) on Proteoglycan 3
with a threshold of 1e−05
rs11715853
rs823424
rs10899911
rs7311004
rs55877868
rs7572229
rs2090816
rs2387977
rs6828768
rs35055552
rs62273907
rs8039785
rs7303158
rs78383039
rs6809952
All
0.0
0.1
0.2
MR effect size for
'Gut microbiota abundance (genus Parasutterella id.2892)' on 'Proteoglycan 3'

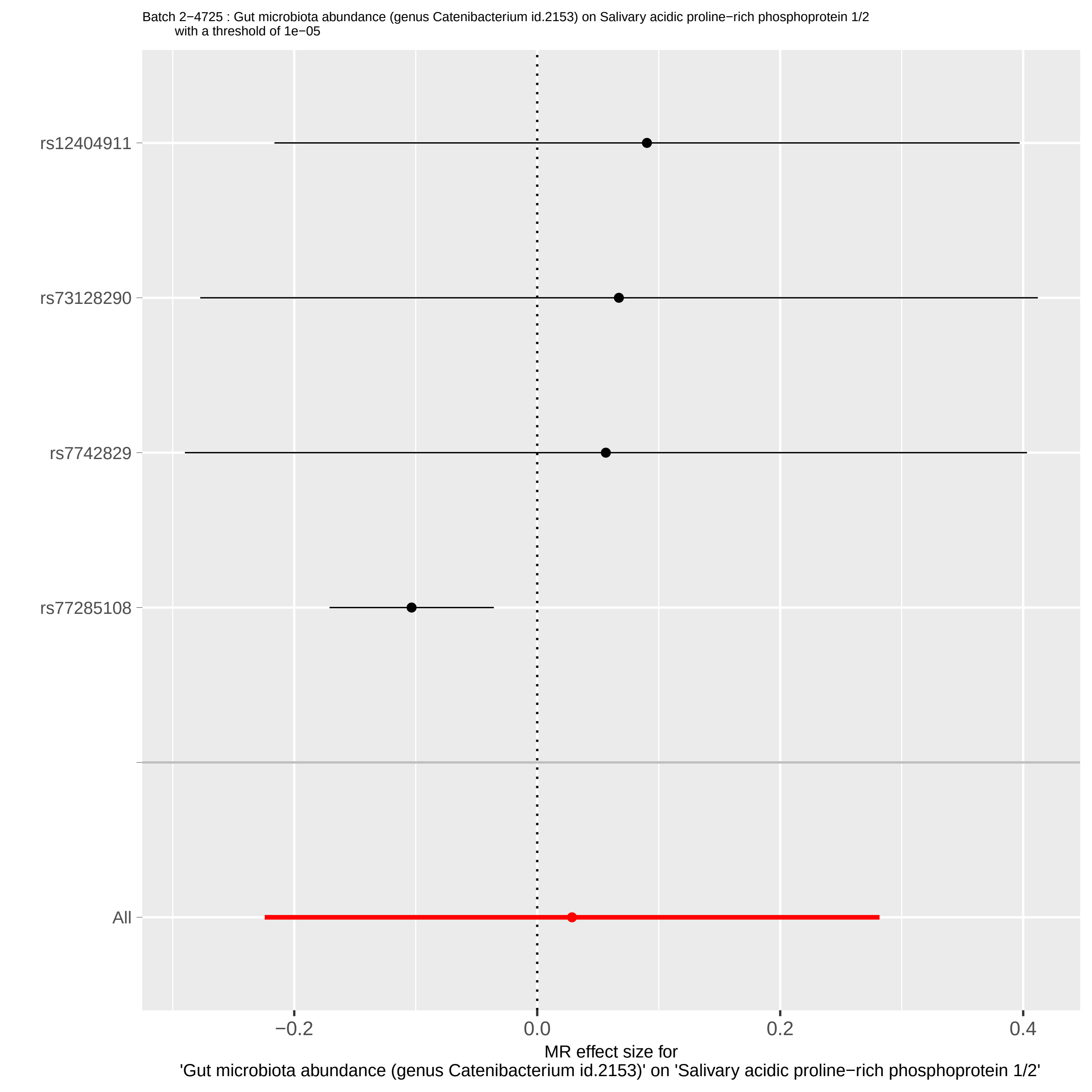
Batch 2−4725 : Gut microbiota abundance (genus Catenibacterium id.2153) on Salivary acidic proline−rich phosphoprotein 1/2
with a threshold of 1e−05
rs12404911
rs73128290
rs7742829
rs77285108
All
−0.2
0.0
0.2
0.4
MR effect size for
'Gut microbiota abundance (genus Catenibacterium id.2153)' on 'Salivary acidic proline−rich phosphoprotein 1/2'

Batch 2−4726 : Gut microbiota abundance (genus Parasutterella id.2892) on Salivary acidic proline−rich phosphoprotein 1/2
with a threshold of 1e−05

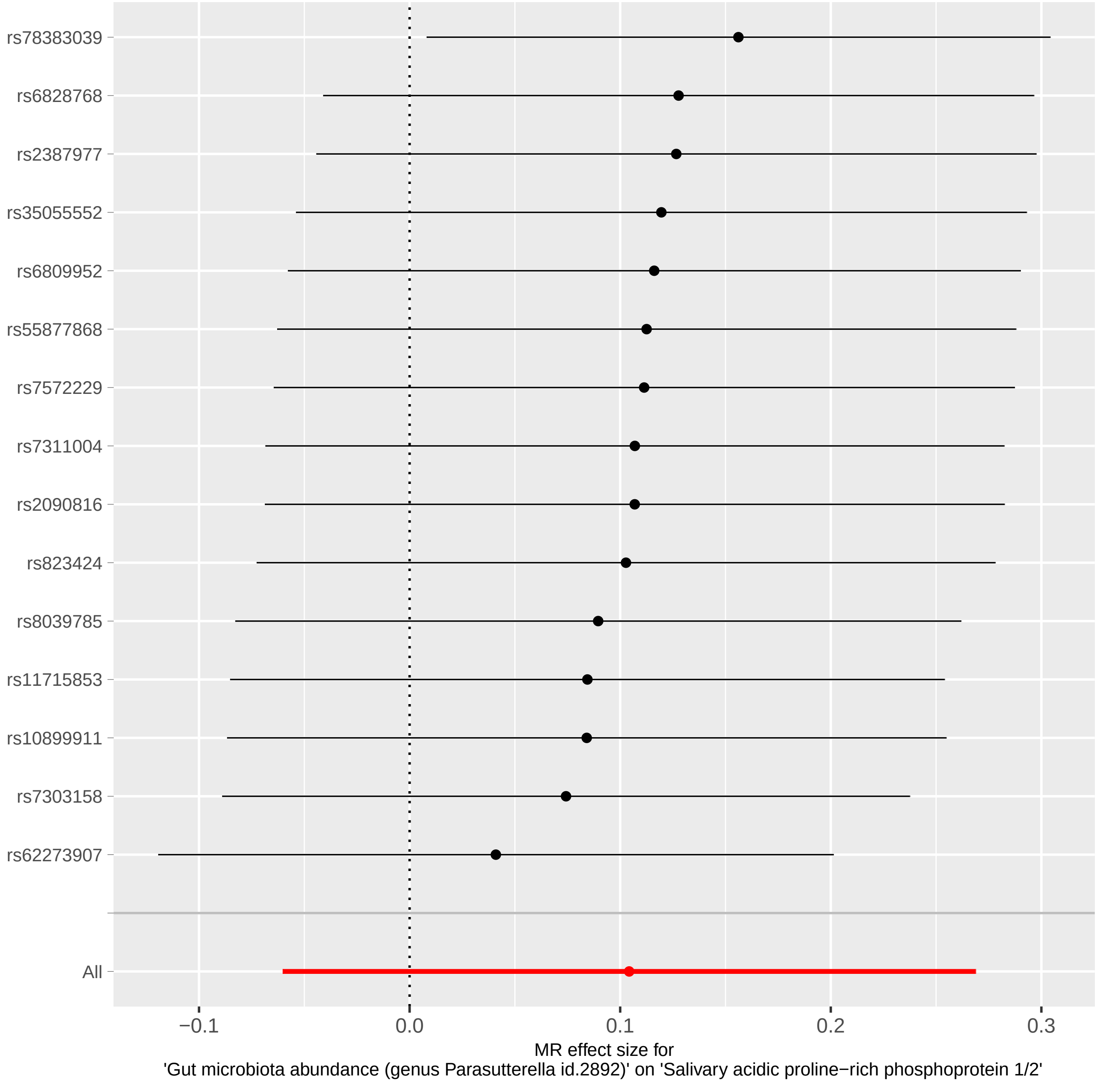

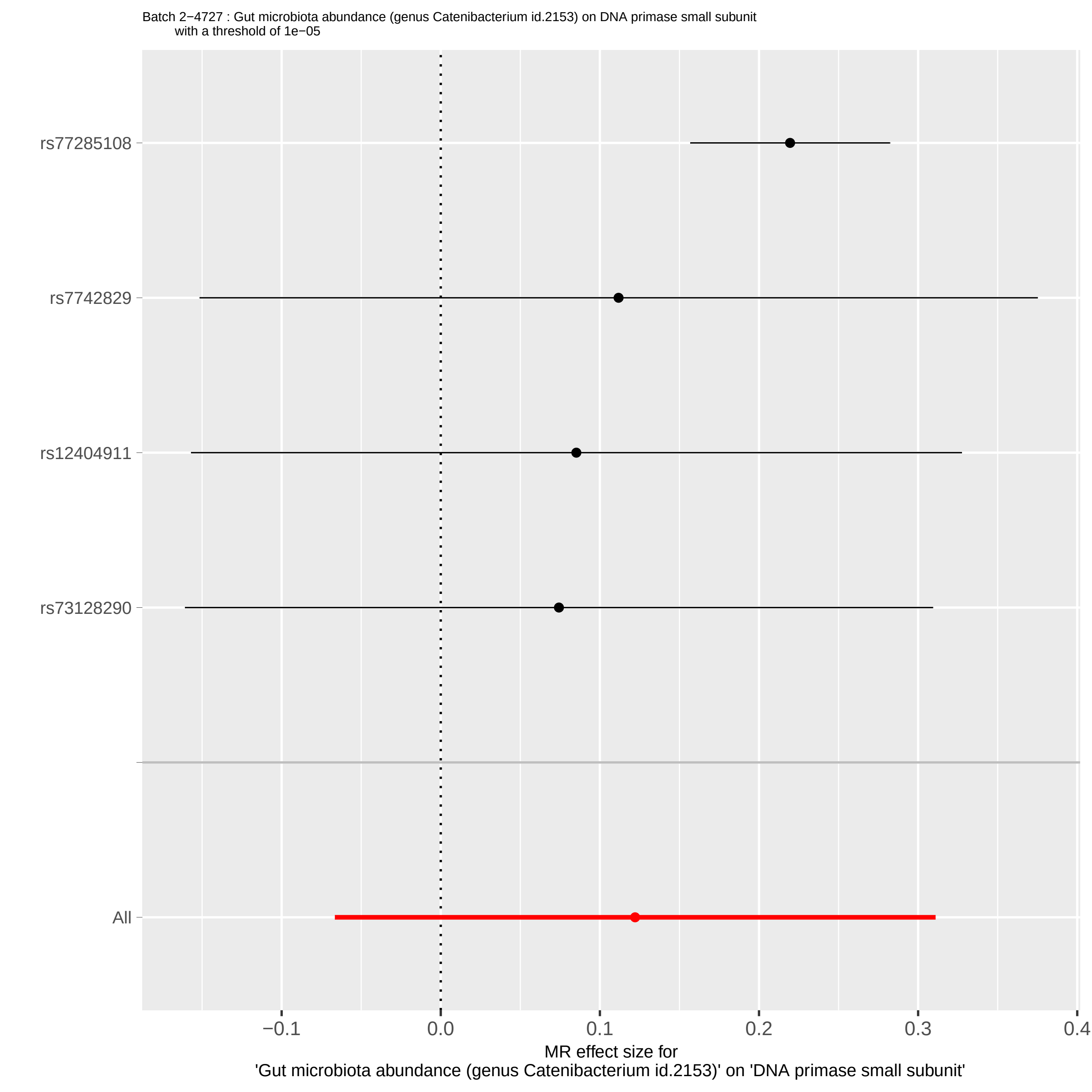

Batch 2−4727 : Gut microbiota abundance (genus Catenibacterium id.2153) on DNA primase small subunit
with a threshold of 1e−05
rs77285108
rs7742829
rs12404911
rs73128290
All
−0.1
0.0
0.1
0.2
0.3
0.4
MR effect size for
'Gut microbiota abundance (genus Catenibacterium id.2153)' on 'DNA primase small subunit'

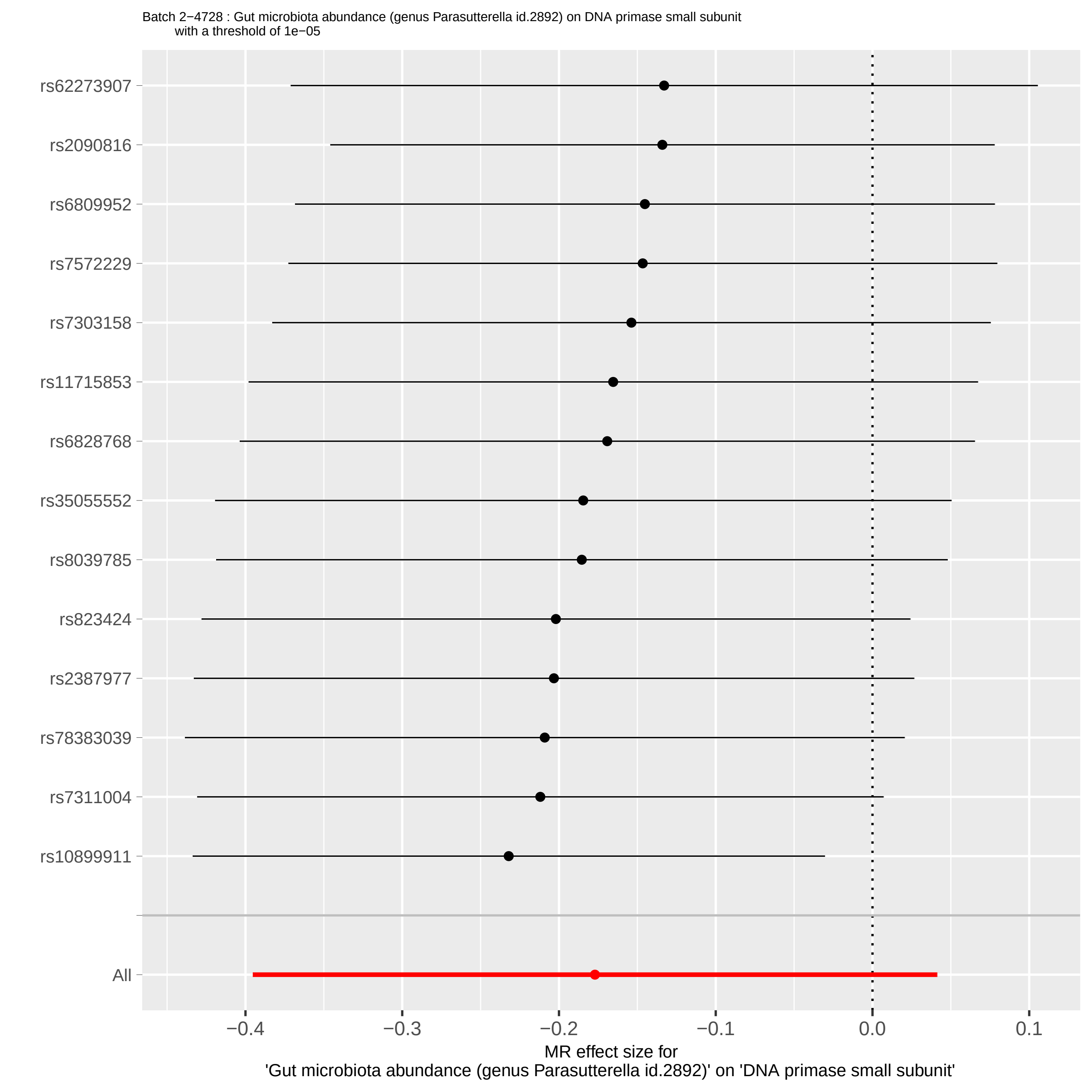
Batch 2−4728 : Gut microbiota abundance (genus Parasutterella id.2892) on DNA primase small subunit
with a threshold of 1e−05
rs62273907
rs2090816
rs6809952
rs7572229
rs7303158
rs11715853
rs6828768
rs35055552
rs8039785
rs823424
rs2387977
rs78383039
rs7311004
rs10899911
All
−0.4
−0.3
−0.2
−0.1
0.0
0.1
MR effect size for
'Gut microbiota abundance (genus Parasutterella id.2892)' on 'DNA primase small subunit'

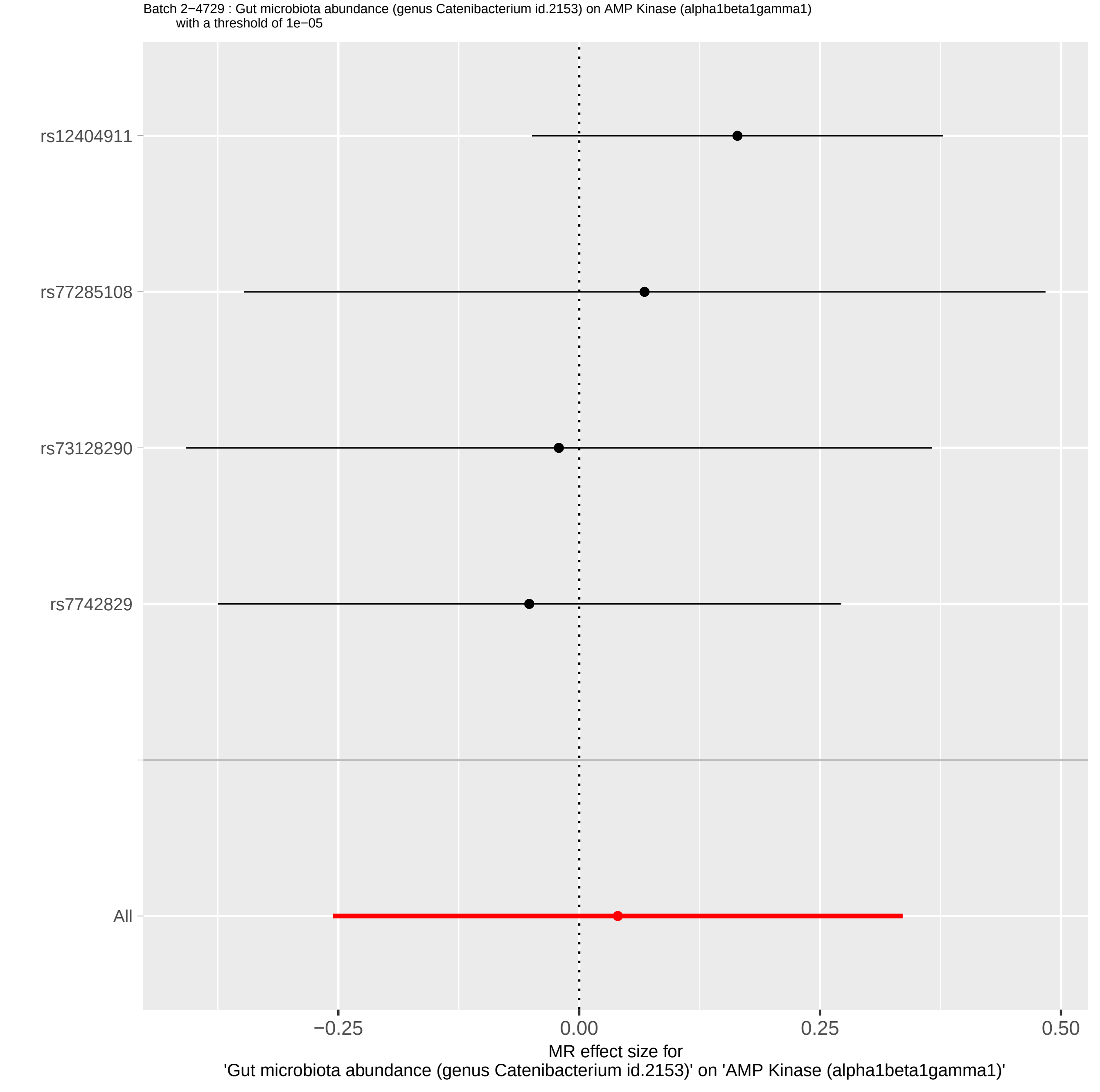

Batch 2−4729 : Gut microbiota abundance (genus Catenibacterium id.2153) on AMP Kinase (alpha1beta1gamma1)
with a threshold of 1e−05
rs12404911
rs77285108
rs73128290
rs7742829
All
−0.25
0.00
0.25
0.50
MR effect size for
'Gut microbiota abundance (genus Catenibacterium id.2153)' on 'AMP Kinase (alpha1beta1gamma1)'

Batch 2−4730 : Gut microbiota abundance (genus Parasutterella id.2892) on AMP Kinase (alpha1beta1gamma1) with a threshold of 1e−05

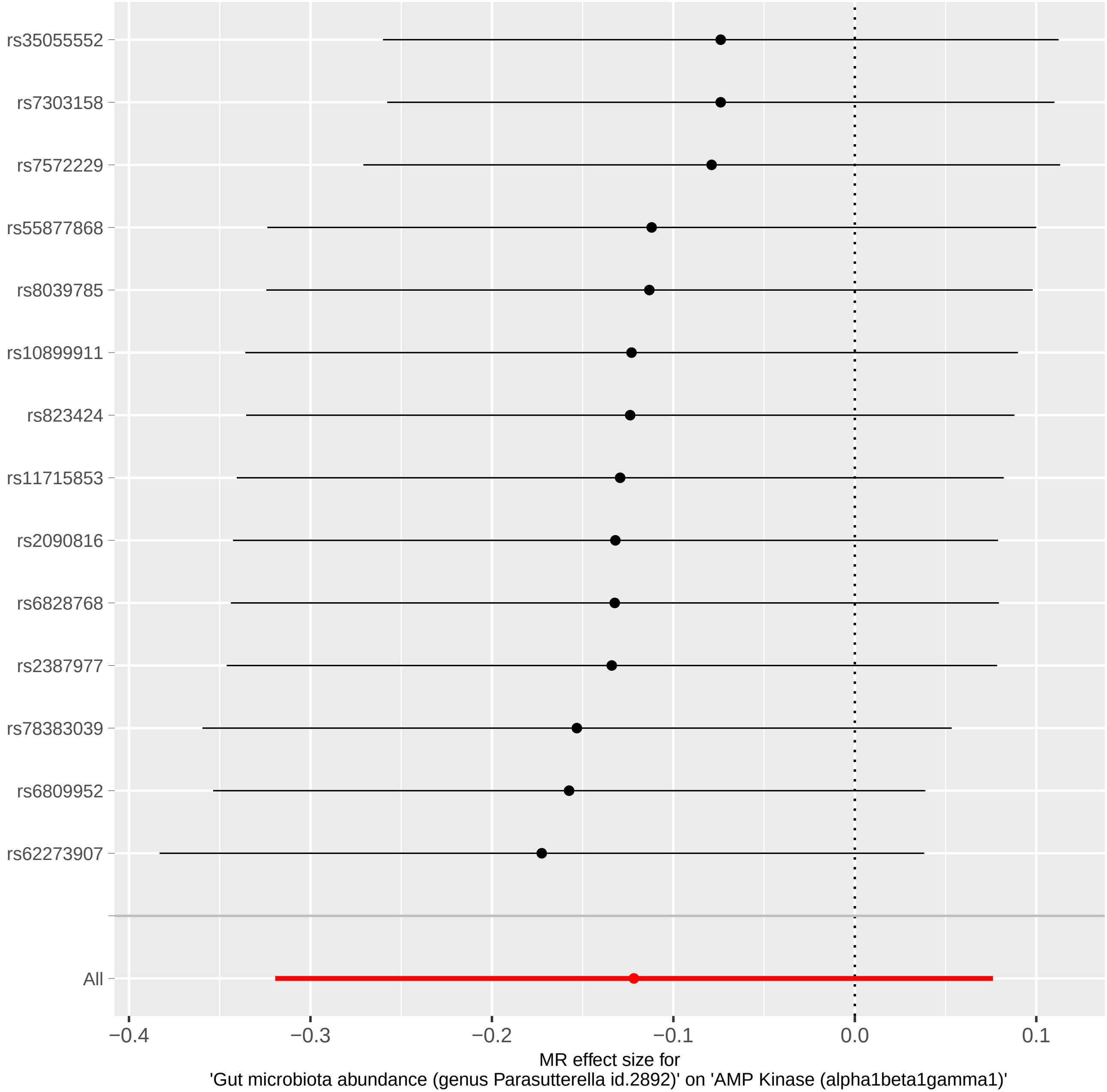

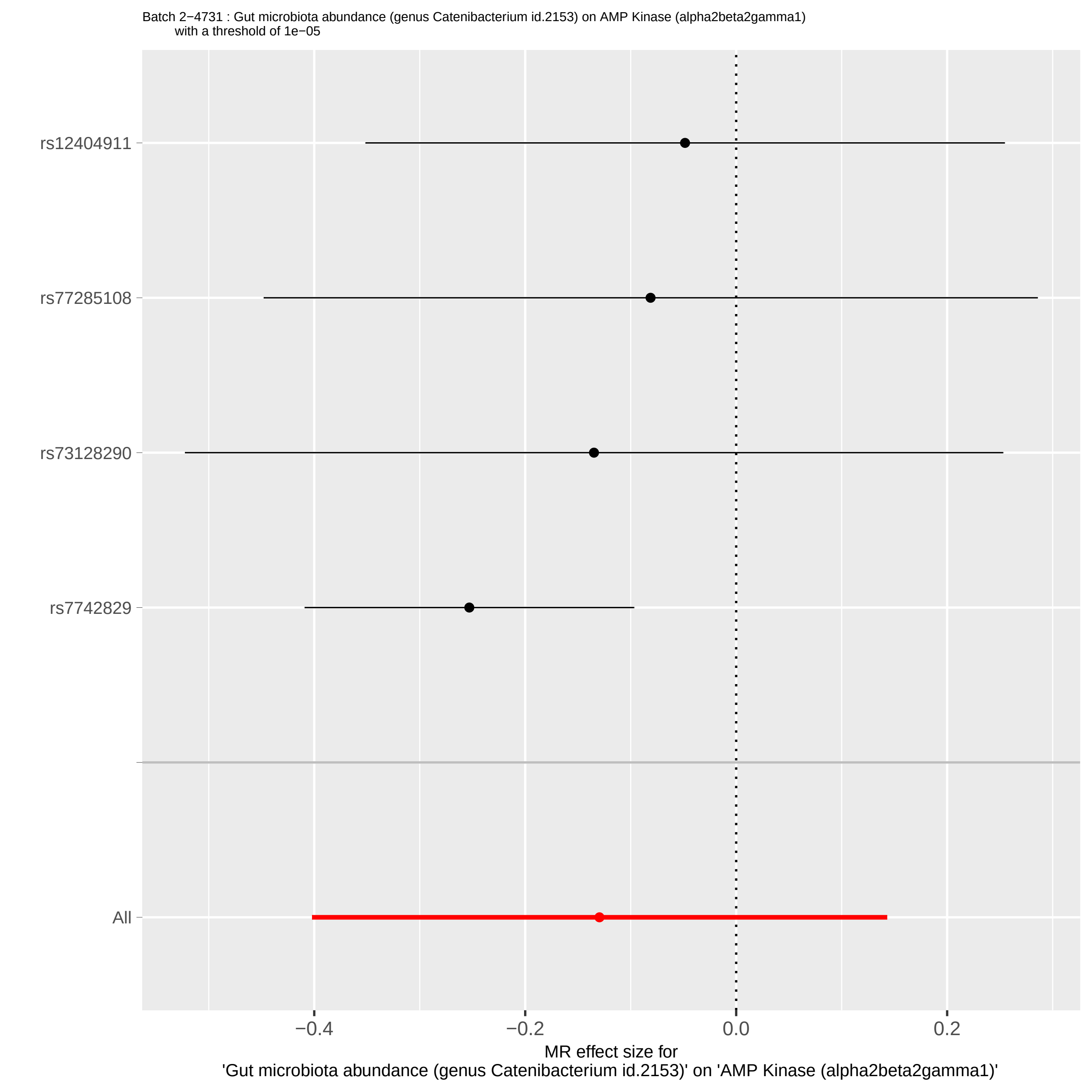

Batch 2−4731 : Gut microbiota abundance (genus Catenibacterium id.2153) on AMP Kinase (alpha2beta2gamma1)
with a threshold of 1e−05
rs12404911
rs77285108
rs73128290
rs7742829
All
−0.4
−0.2
0.0
0.2
MR effect size for
'Gut microbiota abundance (genus Catenibacterium id.2153)' on 'AMP Kinase (alpha2beta2gamma1)'

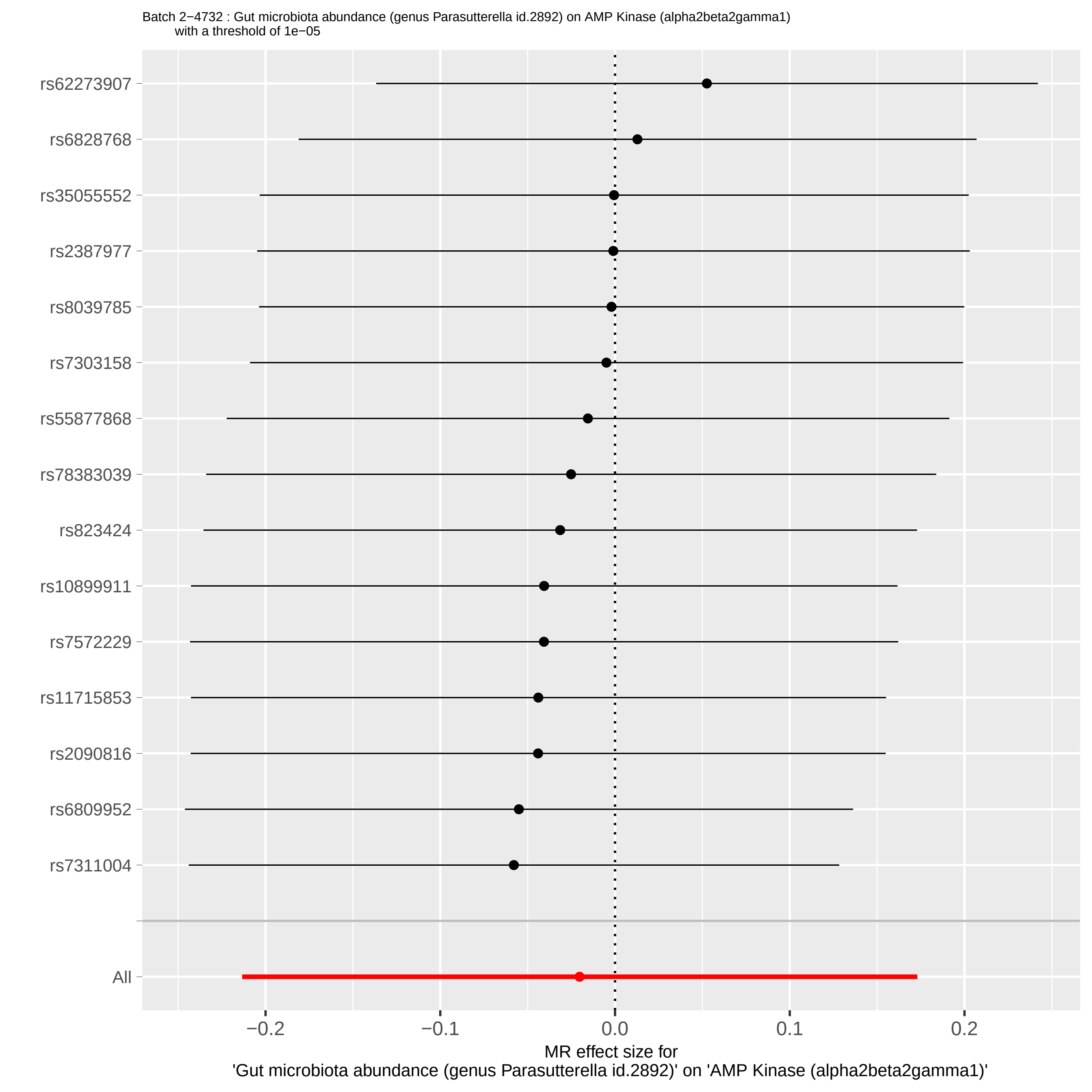
Batch 2−4732 : Gut microbiota abundance (genus Parasutterella id.2892) on AMP Kinase (alpha2beta2gamma1)
with a threshold of 1e−05
rs62273907
rs6828768
rs35055552
rs2387977
rs8039785
rs7303158
rs55877868
rs78383039
rs823424
rs10899911
rs7572229
rs11715853
rs2090816
rs6809952
rs7311004
All
−0.2
−0.1
0.0
0.1
0.2
MR effect size for
'Gut microbiota abundance (genus Parasutterella id.2892)' on 'AMP Kinase (alpha2beta2gamma1)'

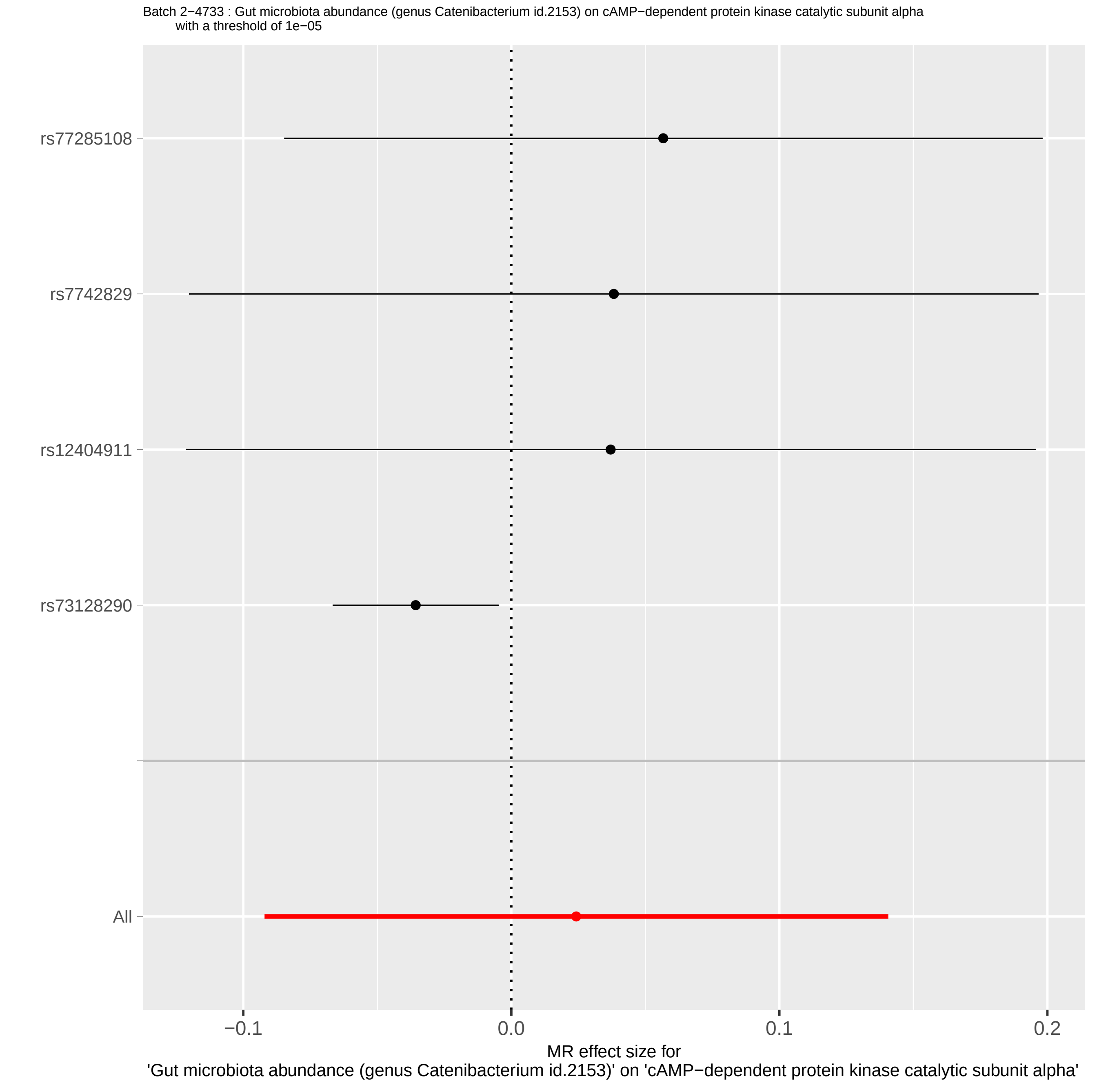

Batch 2−4733 : Gut microbiota abundance (genus Catenibacterium id.2153) on cAMP−dependent protein kinase catalytic subunit alpha
with a threshold of 1e−05
rs77285108
rs7742829
rs12404911
rs73128290
All
−0.1
0.0
0.1
0.2
MR effect size for
'Gut microbiota abundance (genus Catenibacterium id.2153)' on 'cAMP−dependent protein kinase catalytic subunit alpha'

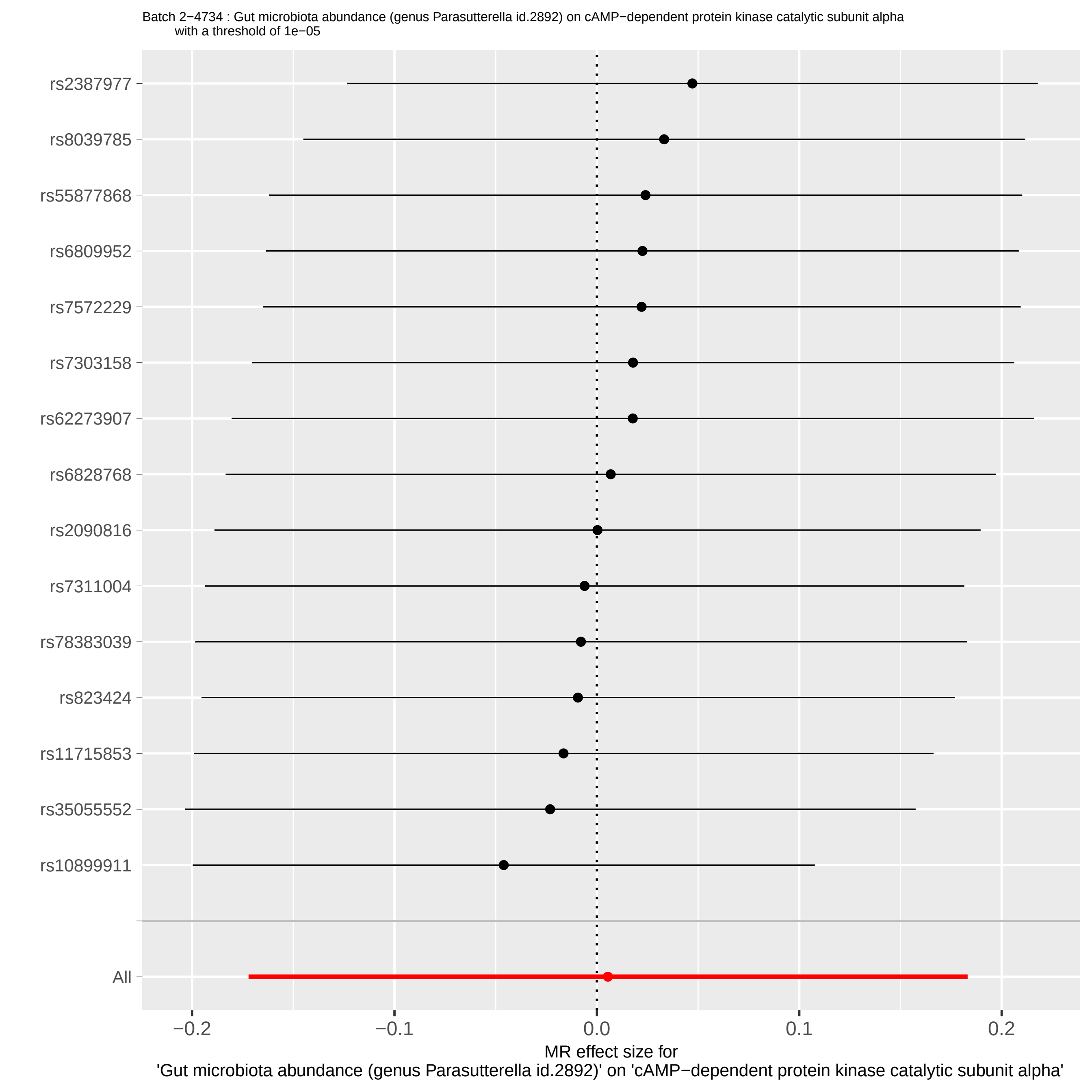
Batch 2−4734 : Gut microbiota abundance (genus Parasutterella id.2892) on cAMP−dependent protein kinase catalytic subunit alpha
with a threshold of 1e−05
rs2387977
rs8039785
rs55877868
rs6809952
rs7572229
rs7303158
rs62273907
rs6828768
rs2090816
rs7311004
rs78383039
rs823424
rs11715853
rs35055552
rs10899911
All
−0.2
−0.1
0.0
0.1
0.2
MR effect size for
'Gut microbiota abundance (genus Parasutterella id.2892)' on 'cAMP−dependent protein kinase catalytic subunit alpha'

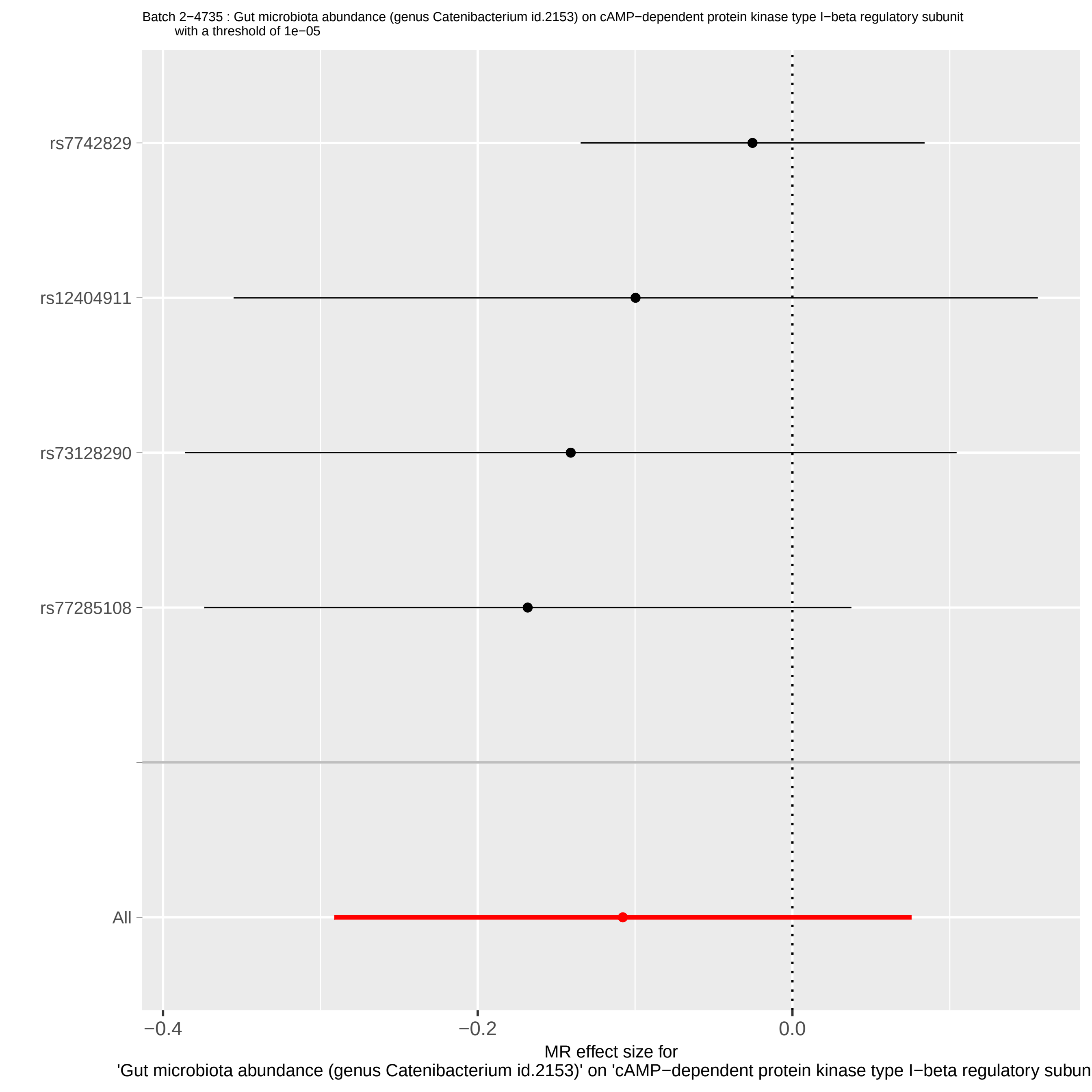
Batch 2−4735 : Gut microbiota abundance (genus Catenibacterium id.2153) on cAMP−dependent protein kinase type I−beta regulatory subunit
with a threshold of 1e−05
rs7742829
rs12404911
rs73128290
rs77285108
All
−0.4
−0.2
0.0
MR effect size for
'Gut microbiota abundance (genus Catenibacterium id.2153)' on 'cAMP−dependent protein kinase type I−beta regulatory subun

Batch 2−4736 : Gut microbiota abundance (genus Parasutterella id.2892) on cAMP−dependent protein kinase type I−beta regulatory subunit
with a threshold of 1e−05

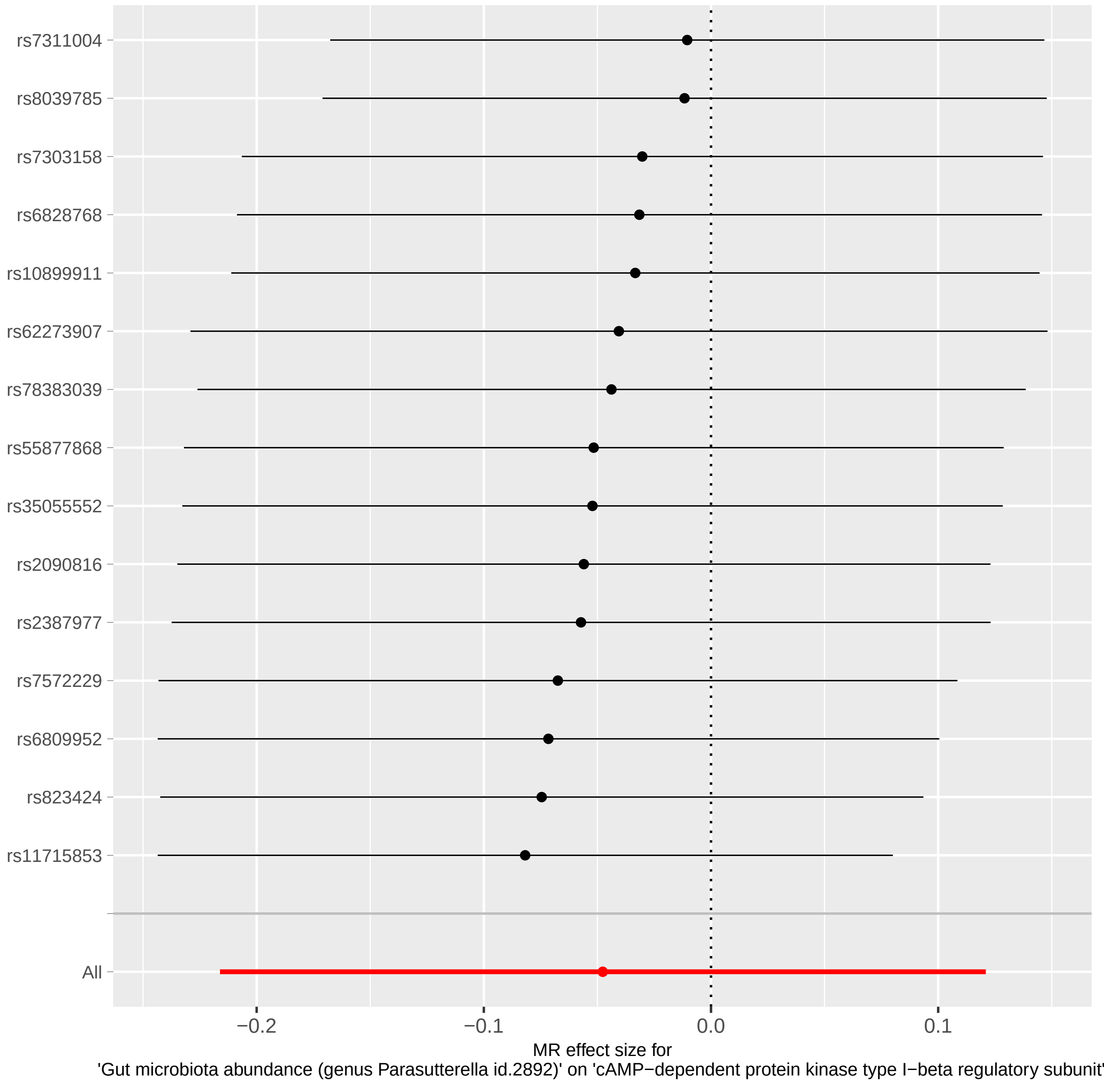

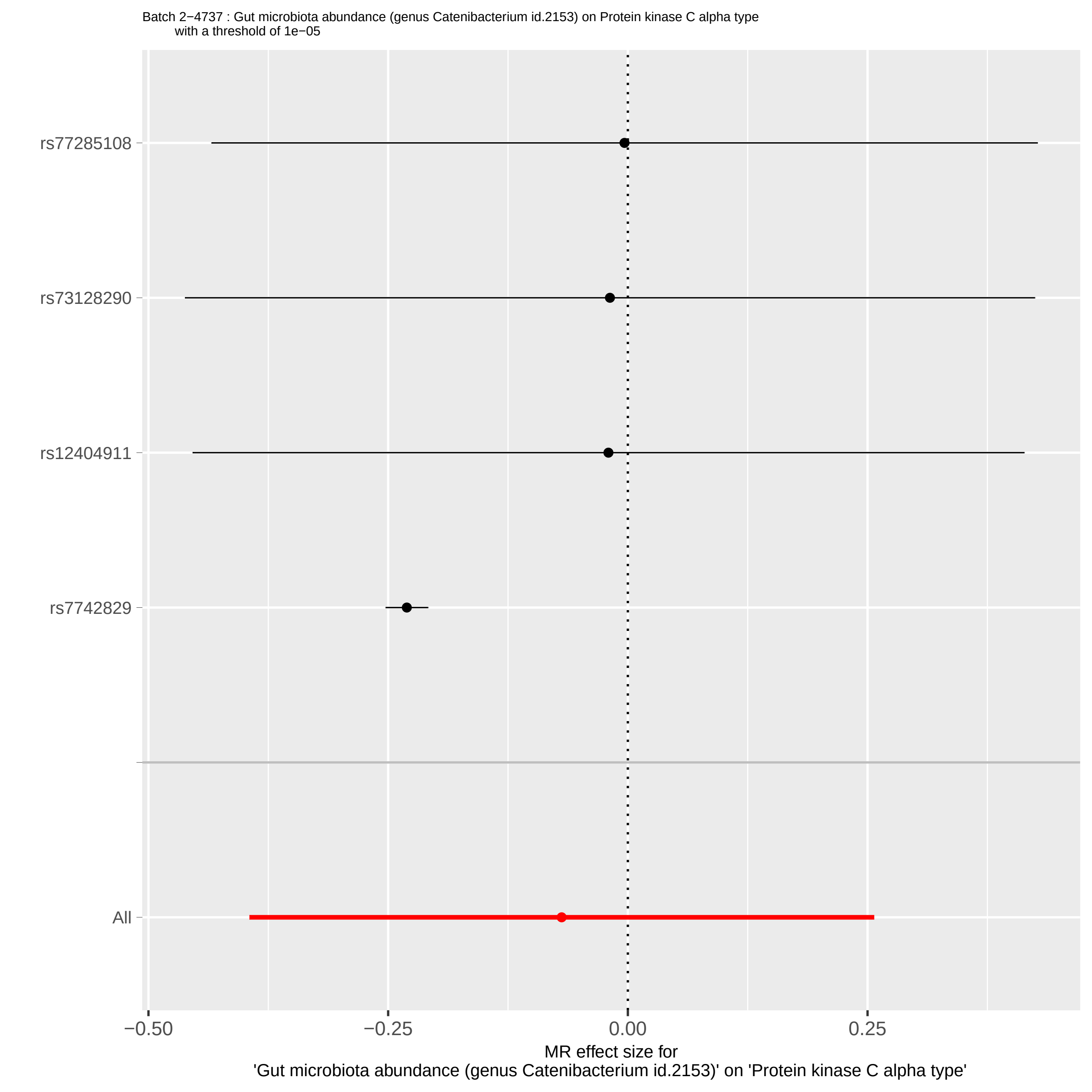
Batch 2−4737 : Gut microbiota abundance (genus Catenibacterium id.2153) on Protein kinase C alpha type
with a threshold of 1e−05
rs77285108
rs73128290
rs12404911
rs7742829
All
−0.50
−0.25
0.00
0.25
MR effect size for
'Gut microbiota abundance (genus Catenibacterium id.2153)' on 'Protein kinase C alpha type'

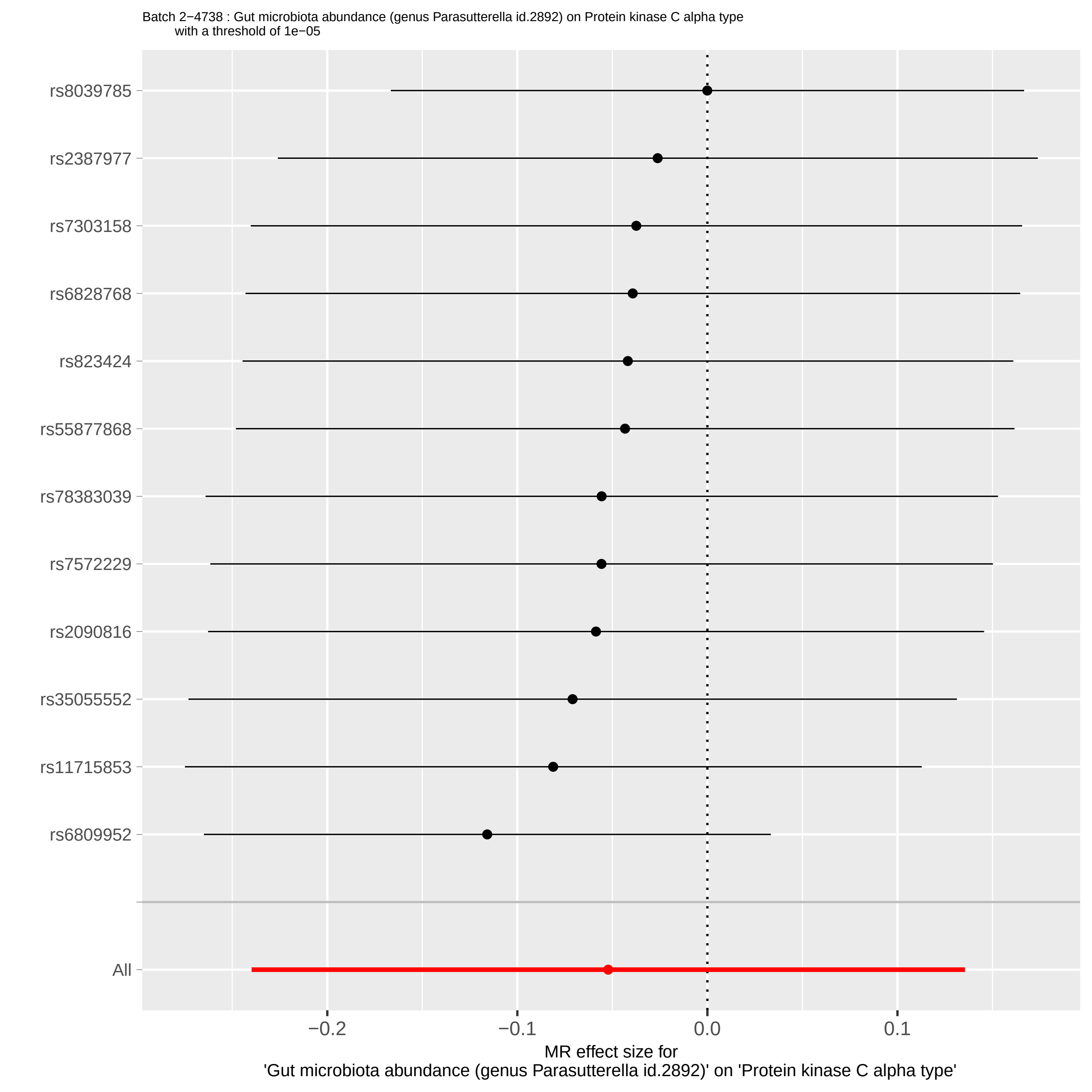

Batch 2−4738 : Gut microbiota abundance (genus Parasutterella id.2892) on Protein kinase C alpha type
with a threshold of 1e−05
rs8039785
rs2387977
rs7303158
rs6828768
rs823424
rs55877868
rs78383039
rs7572229
rs2090816
rs35055552
rs11715853
rs6809952
All
−0.2
−0.1
0.0
0.1
MR effect size for
'Gut microbiota abundance (genus Parasutterella id.2892)' on 'Protein kinase C alpha type'

Batch 2−4739 : Gut microbiota abundance (genus Catenibacterium id.2153) on Protein kinase C beta type (splice variant beta−II) with a threshold of 1e−05

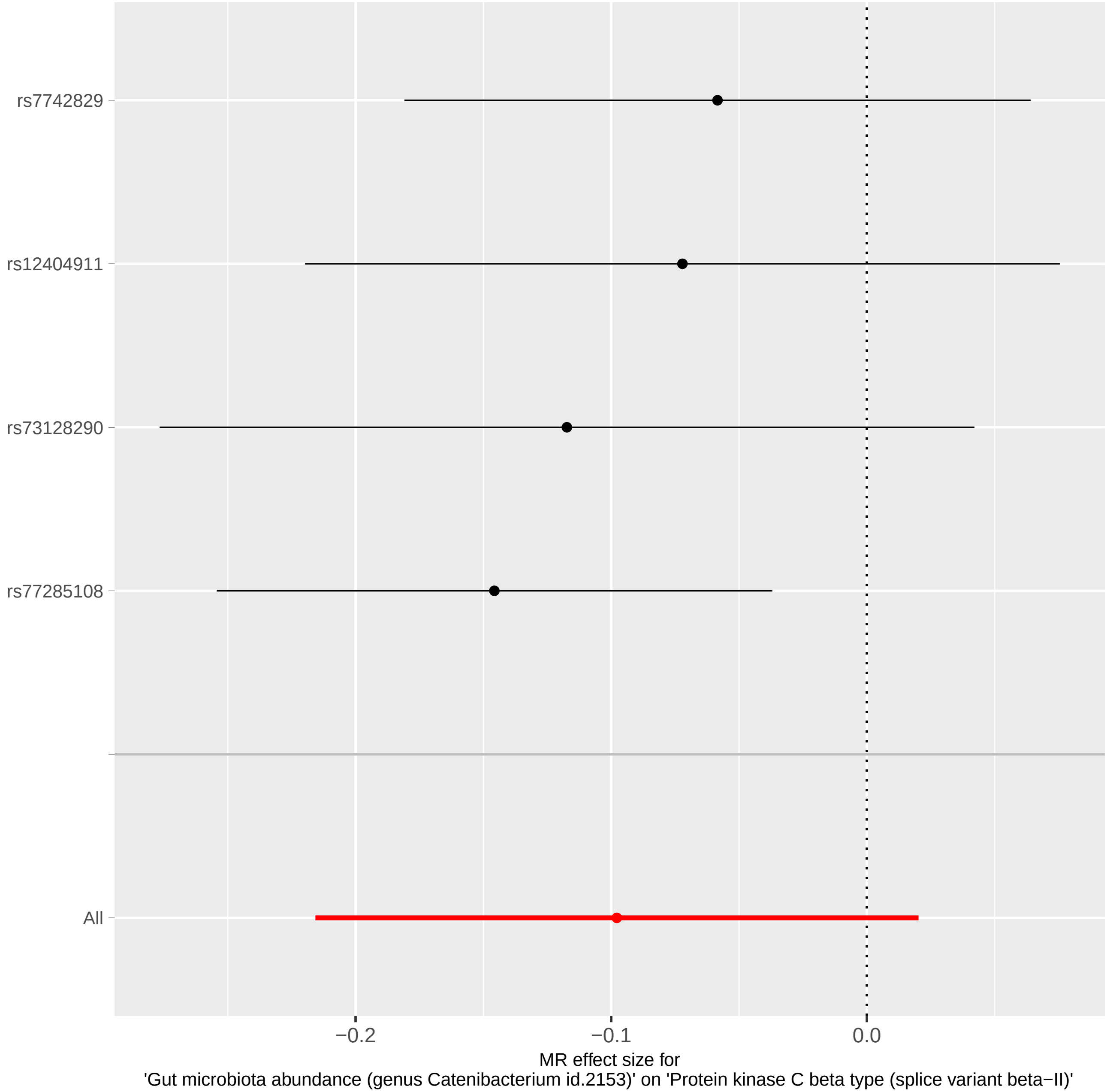

Batch 2−4740 : Gut microbiota abundance (genus Parasutterella id.2892) on Protein kinase C beta type (splice variant beta−II) with a threshold of 1e−05

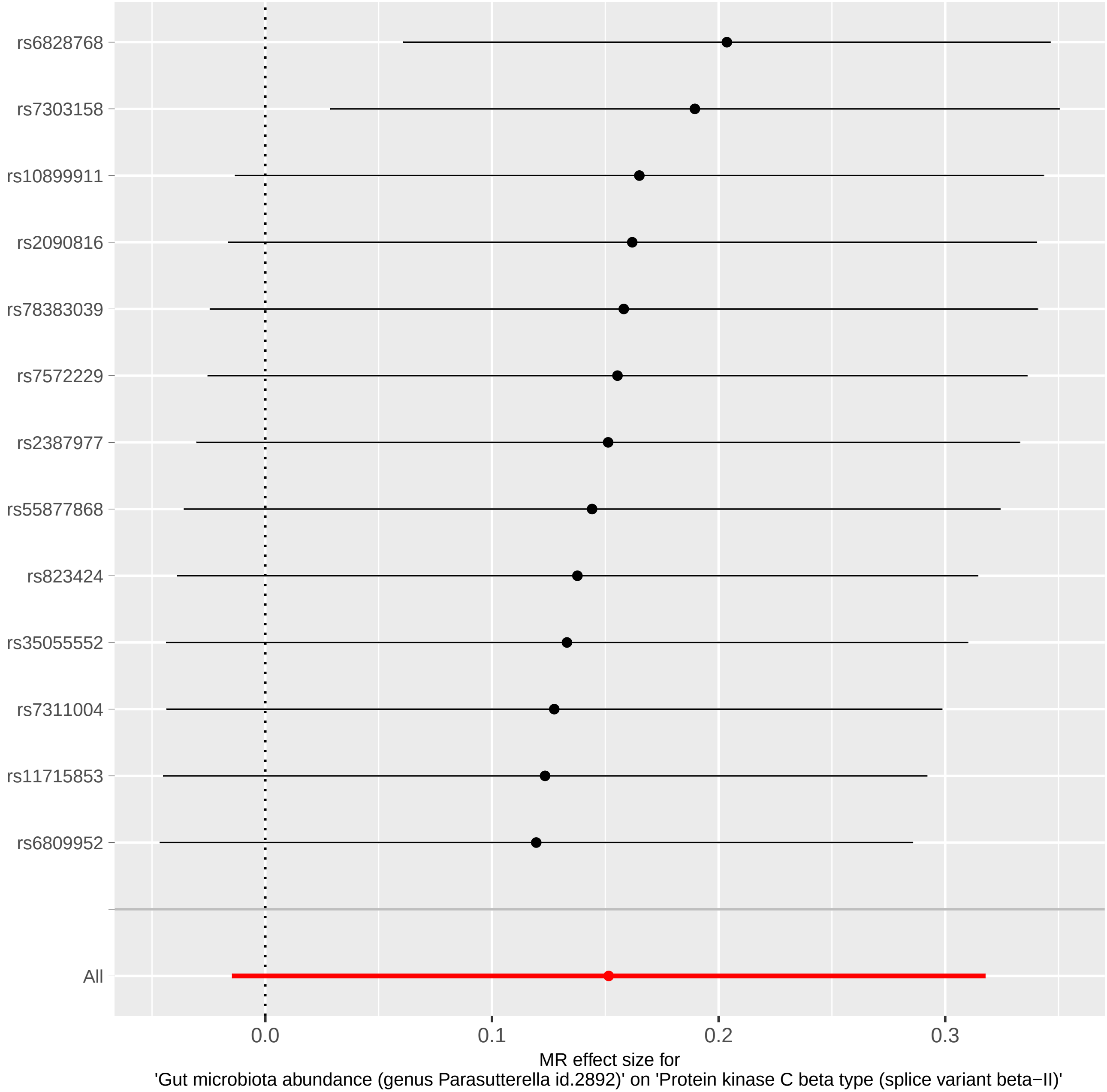

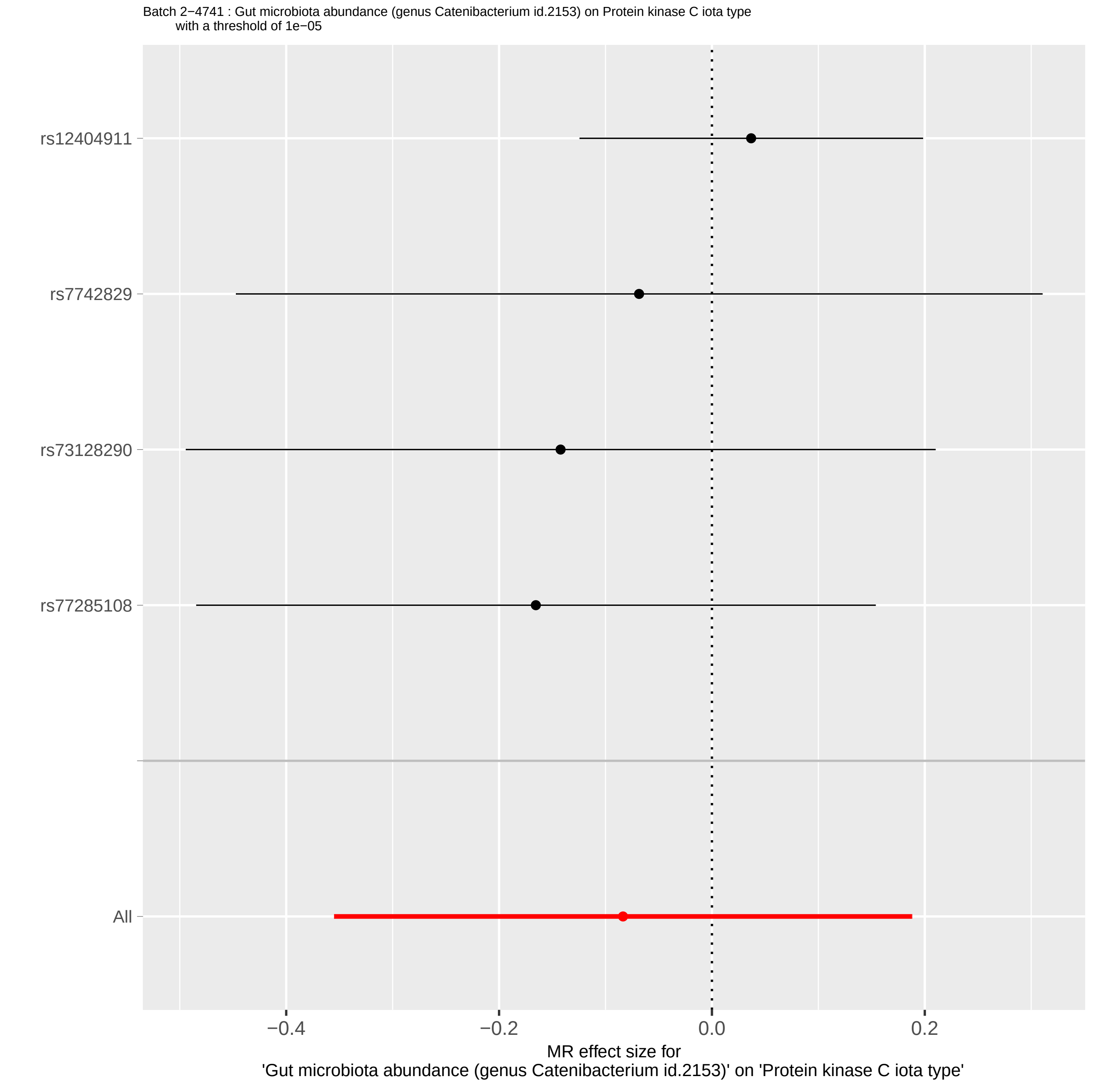

Batch 2−4741 : Gut microbiota abundance (genus Catenibacterium id.2153) on Protein kinase C iota type
with a threshold of 1e−05
rs12404911
rs7742829
rs73128290
rs77285108
All
−0.4
−0.2
0.0
0.2
MR effect size for
'Gut microbiota abundance (genus Catenibacterium id.2153)' on 'Protein kinase C iota type'

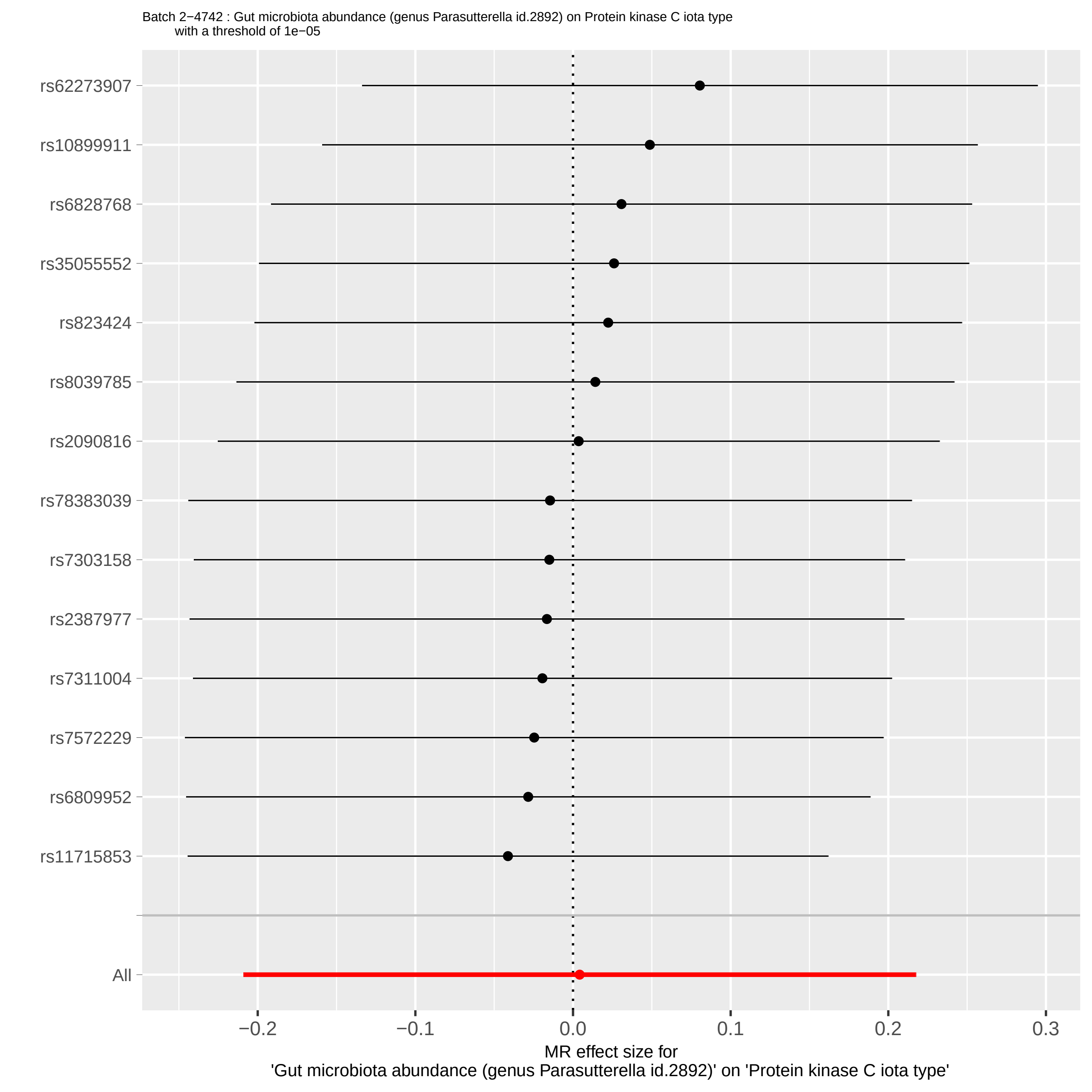
Batch 2−4742 : Gut microbiota abundance (genus Parasutterella id.2892) on Protein kinase C iota type
with a threshold of 1e−05
rs62273907
rs10899911
rs6828768
rs35055552
rs823424
rs8039785
rs2090816
rs78383039
rs7303158
rs2387977
rs7311004
rs7572229
rs6809952
rs11715853
All
−0.2
−0.1
0.0
0.1
0.2
0.3
MR effect size for
'Gut microbiota abundance (genus Parasutterella id.2892)' on 'Protein kinase C iota type'

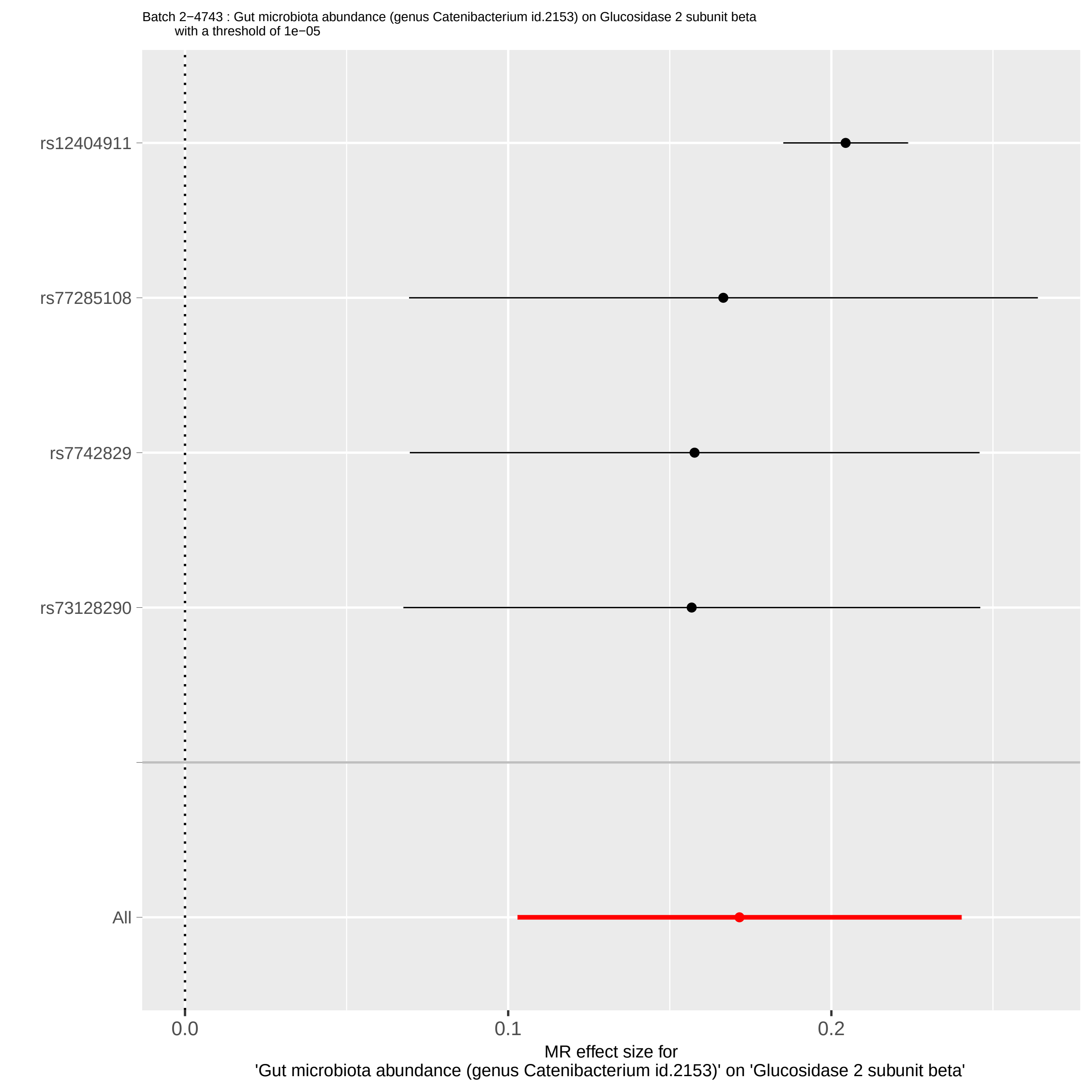

Batch 2−4743 : Gut microbiota abundance (genus Catenibacterium id.2153) on Glucosidase 2 subunit beta
with a threshold of 1e−05
rs12404911
rs77285108
rs7742829
rs73128290
All
0.0
0.1
0.2
MR effect size for
'Gut microbiota abundance (genus Catenibacterium id.2153)' on 'Glucosidase 2 subunit beta'

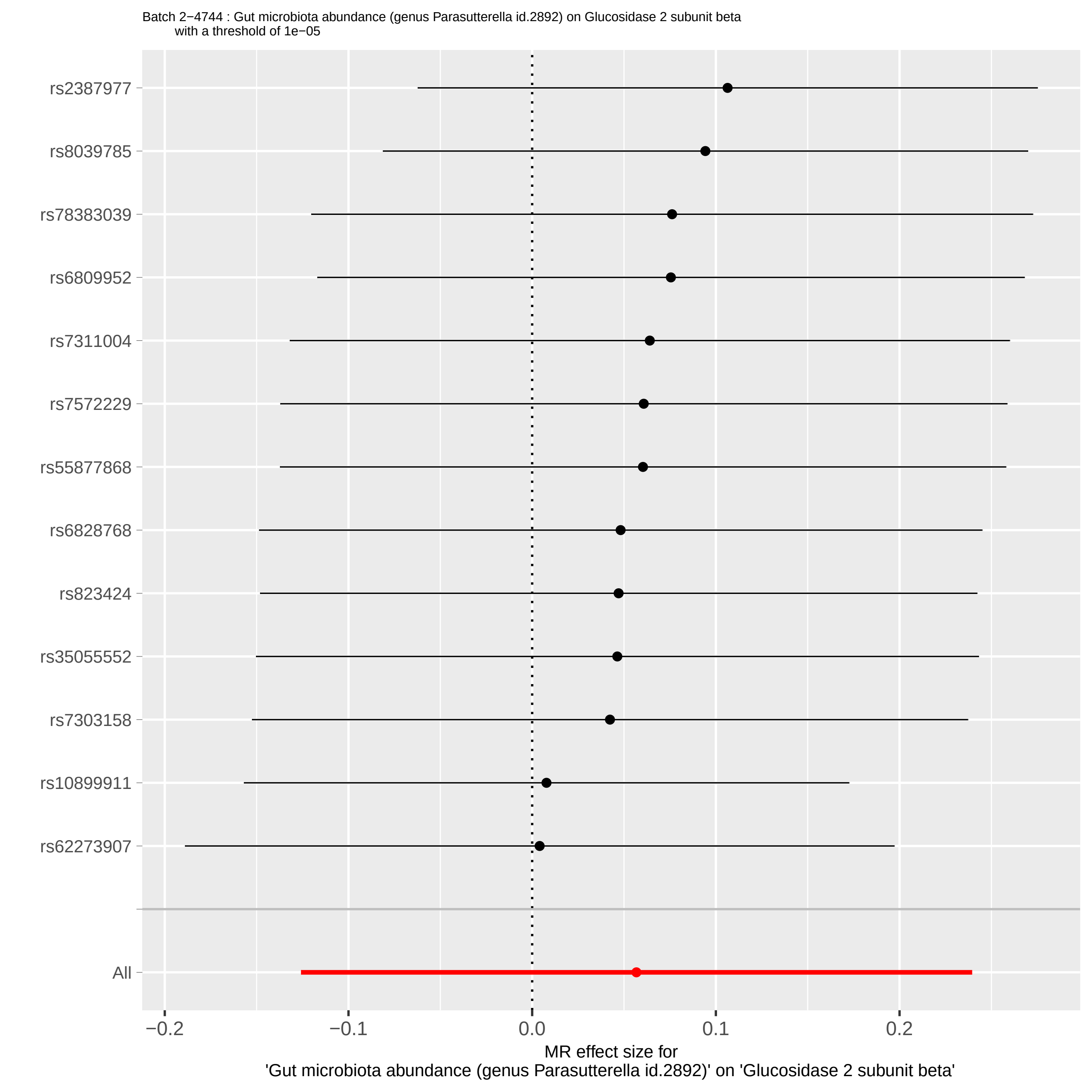
Batch 2−4744 : Gut microbiota abundance (genus Parasutterella id.2892) on Glucosidase 2 subunit beta
with a threshold of 1e−05
rs2387977
rs8039785
rs78383039
rs6809952
rs7311004
rs7572229
rs55877868
rs6828768
rs823424
rs35055552
rs7303158
rs10899911
rs62273907
All
−0.2
−0.1
0.0
0.1
0.2
MR effect size for
'Gut microbiota abundance (genus Parasutterella id.2892)' on 'Glucosidase 2 subunit beta'

Batch 2−4745 : Gut microbiota abundance (genus Catenibacterium id.2153) on Protein kinase C zeta type
with a threshold of 1e−05

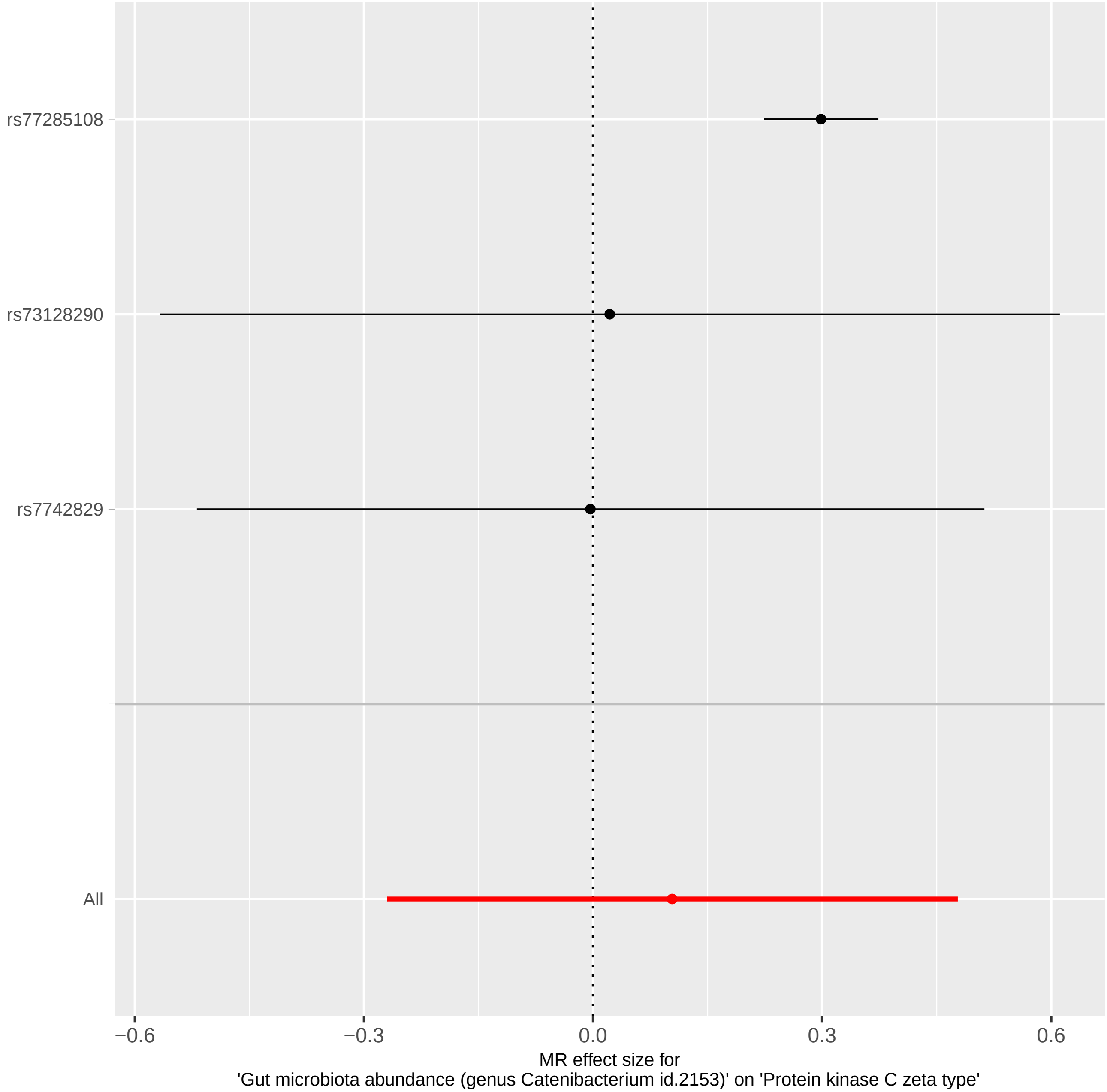

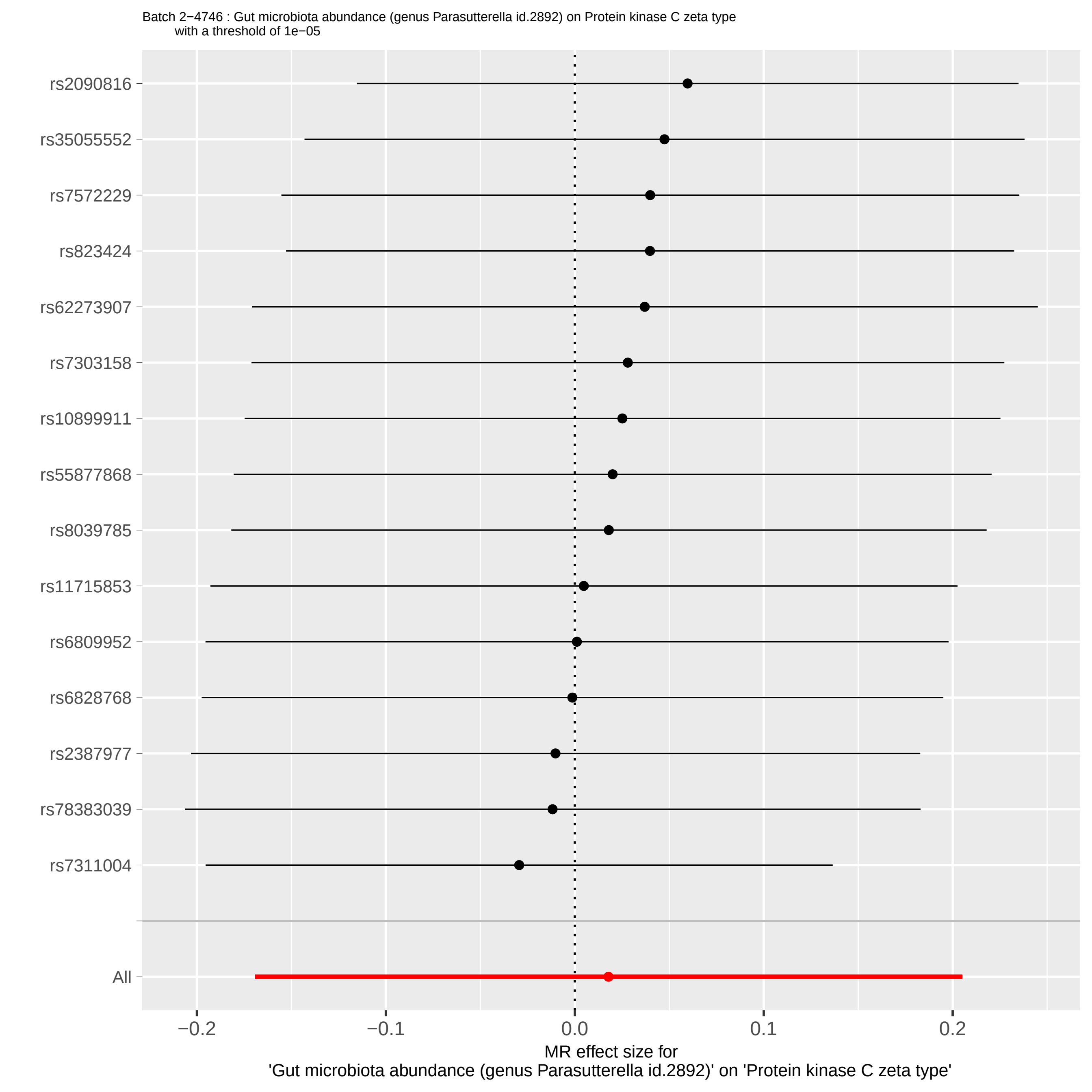

Batch 2−4746 : Gut microbiota abundance (genus Parasutterella id.2892) on Protein kinase C zeta type
with a threshold of 1e−05
rs2090816
rs35055552
rs7572229
rs823424
rs62273907
rs7303158
rs10899911
rs55877868
rs8039785
rs11715853
rs6809952
rs6828768
rs2387977
rs78383039
rs7311004
All
−0.2
−0.1
0.0
0.1
0.2
MR effect size for
'Gut microbiota abundance (genus Parasutterella id.2892)' on 'Protein kinase C zeta type'

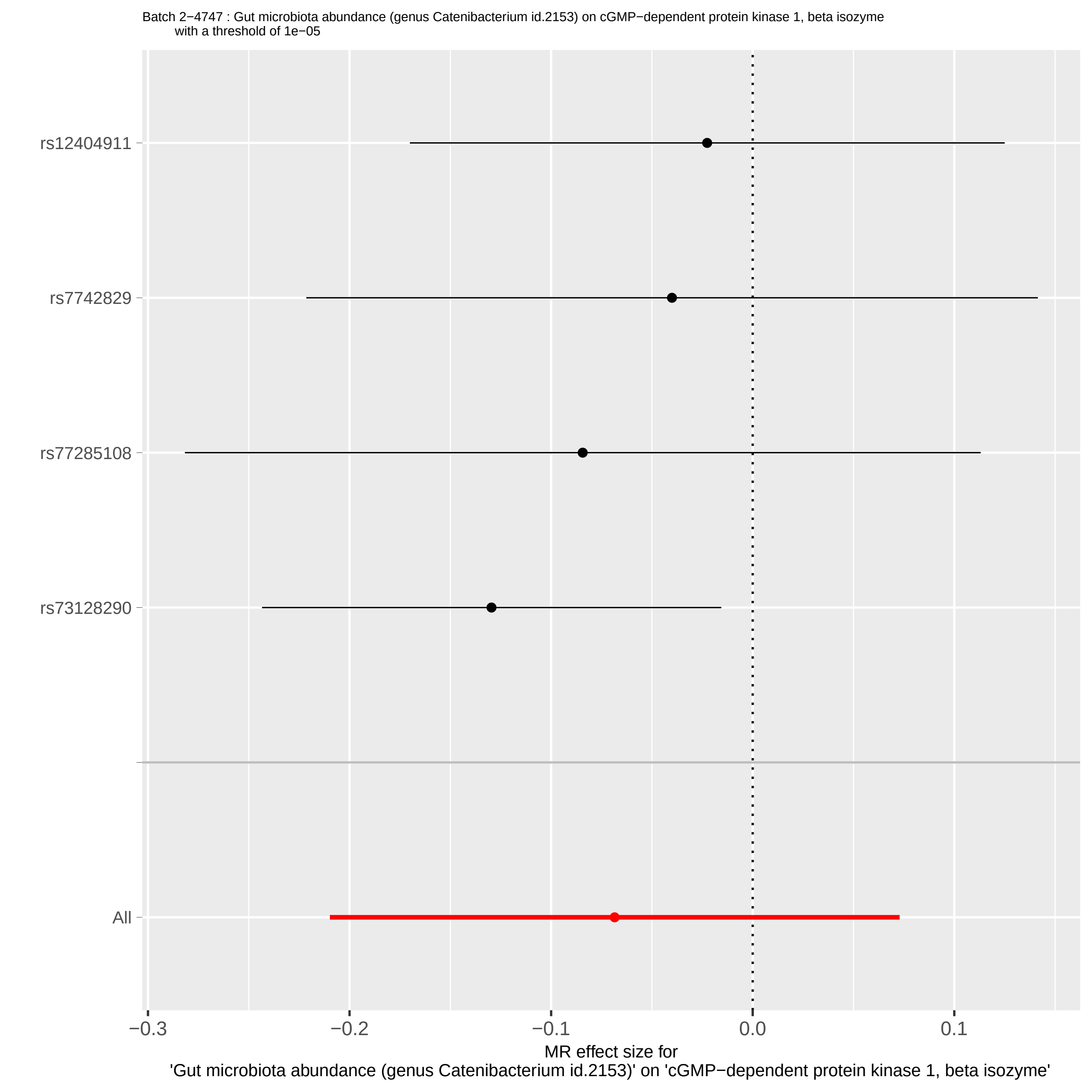

Batch 2−4747 : Gut microbiota abundance (genus Catenibacterium id.2153) on cGMP−dependent protein kinase 1, beta isozyme
with a threshold of 1e−05
rs12404911
rs7742829
rs77285108
rs73128290
All
−0.3
−0.2
−0.1
0.0
0.1
MR effect size for
'Gut microbiota abundance (genus Catenibacterium id.2153)' on 'cGMP−dependent protein kinase 1, beta isozyme'

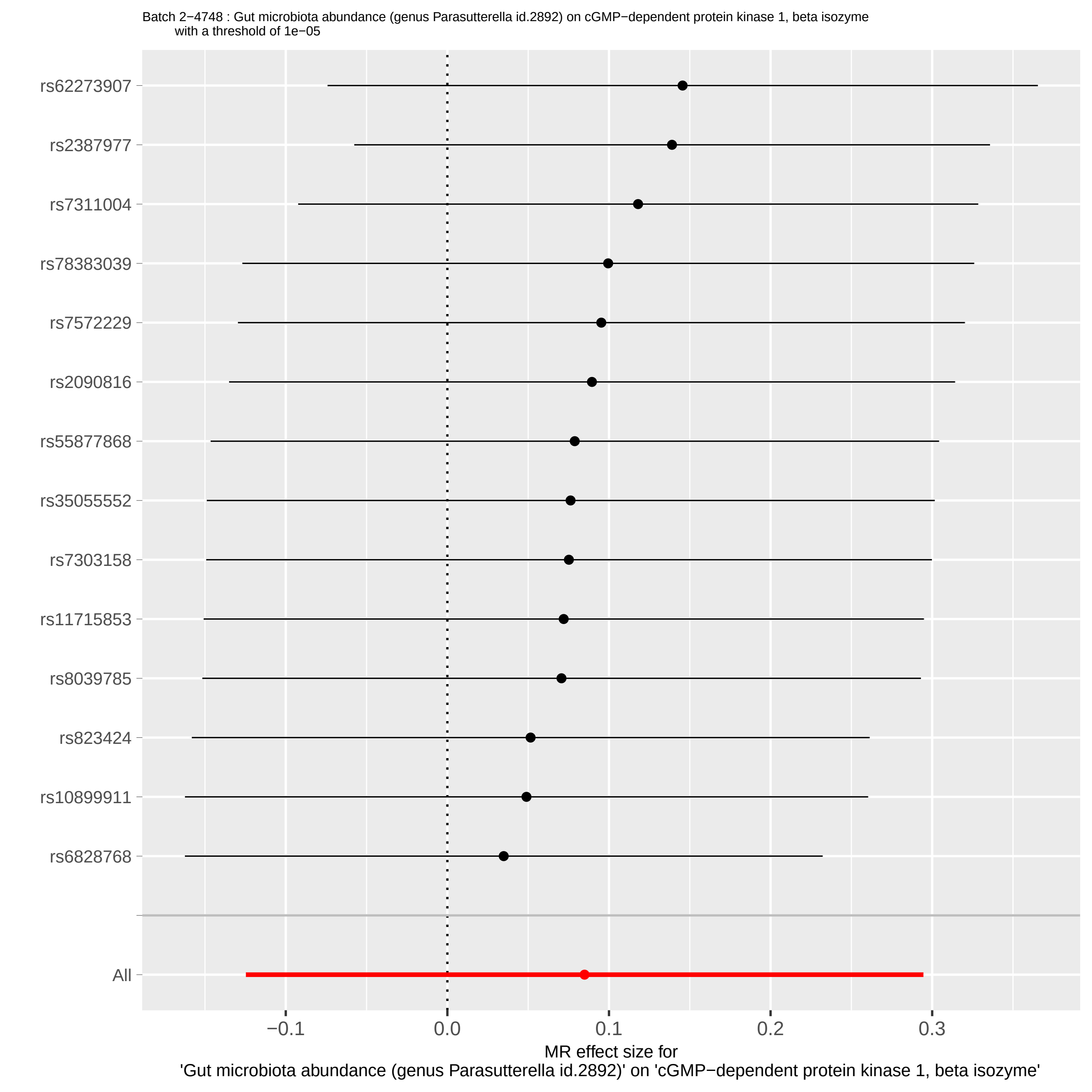
Batch 2−4748 : Gut microbiota abundance (genus Parasutterella id.2892) on cGMP−dependent protein kinase 1, beta isozyme
with a threshold of 1e−05
rs62273907
rs2387977
rs7311004
rs78383039
rs7572229
rs2090816
rs55877868
rs35055552
rs7303158
rs11715853
rs8039785
rs823424
rs10899911
rs6828768
All
−0.1
0.0
0.1
0.2
0.3
MR effect size for
'Gut microbiota abundance (genus Parasutterella id.2892)' on 'cGMP−dependent protein kinase 1, beta isozyme'

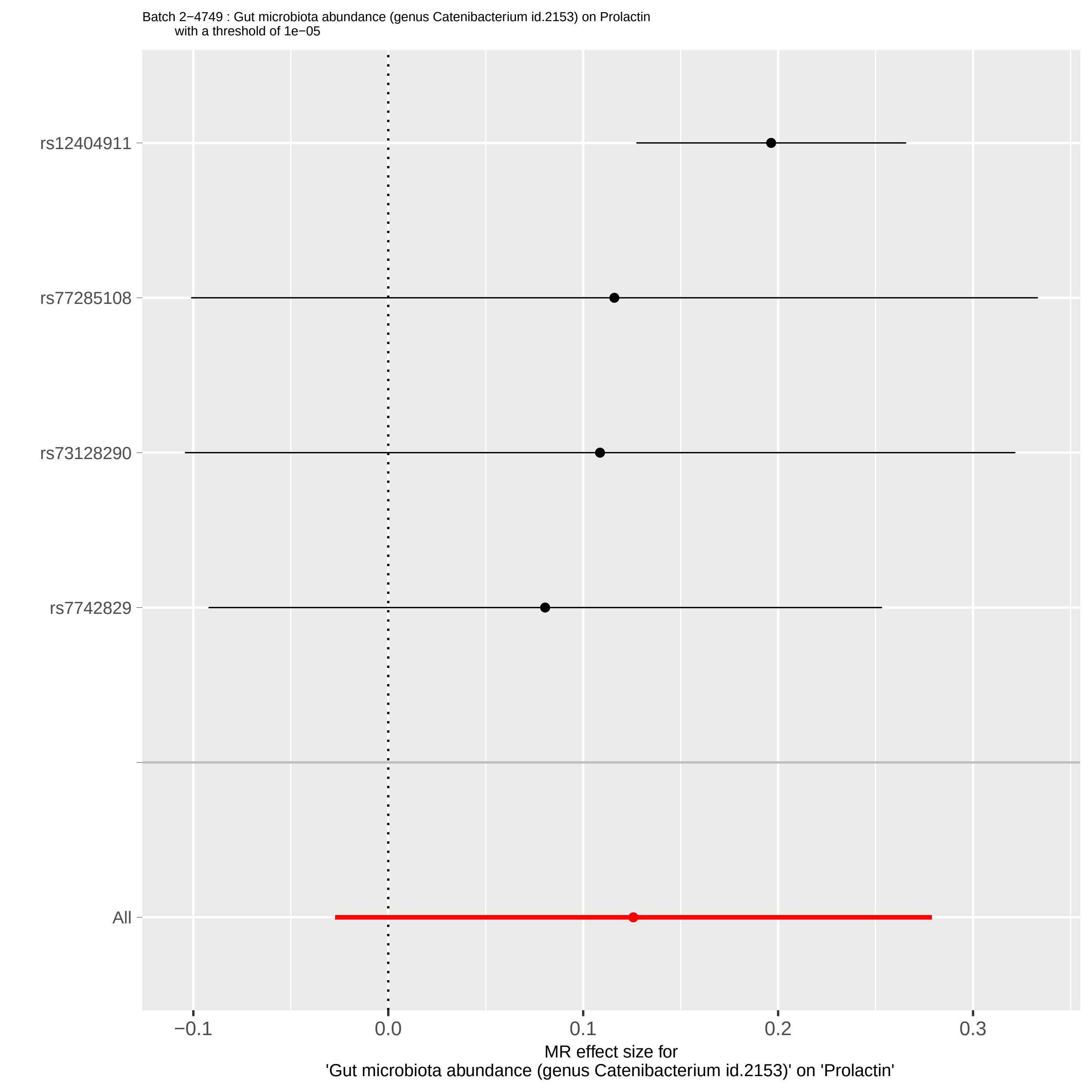
Batch 2−4749 : Gut microbiota abundance (genus Catenibacterium id.2153) on Prolactin
with a threshold of 1e−05
rs12404911
rs77285108
rs73128290
rs7742829
All
−0.1
0.0
0.1
0.2
0.3
MR effect size for
'Gut microbiota abundance (genus Catenibacterium id.2153)' on 'Prolactin'

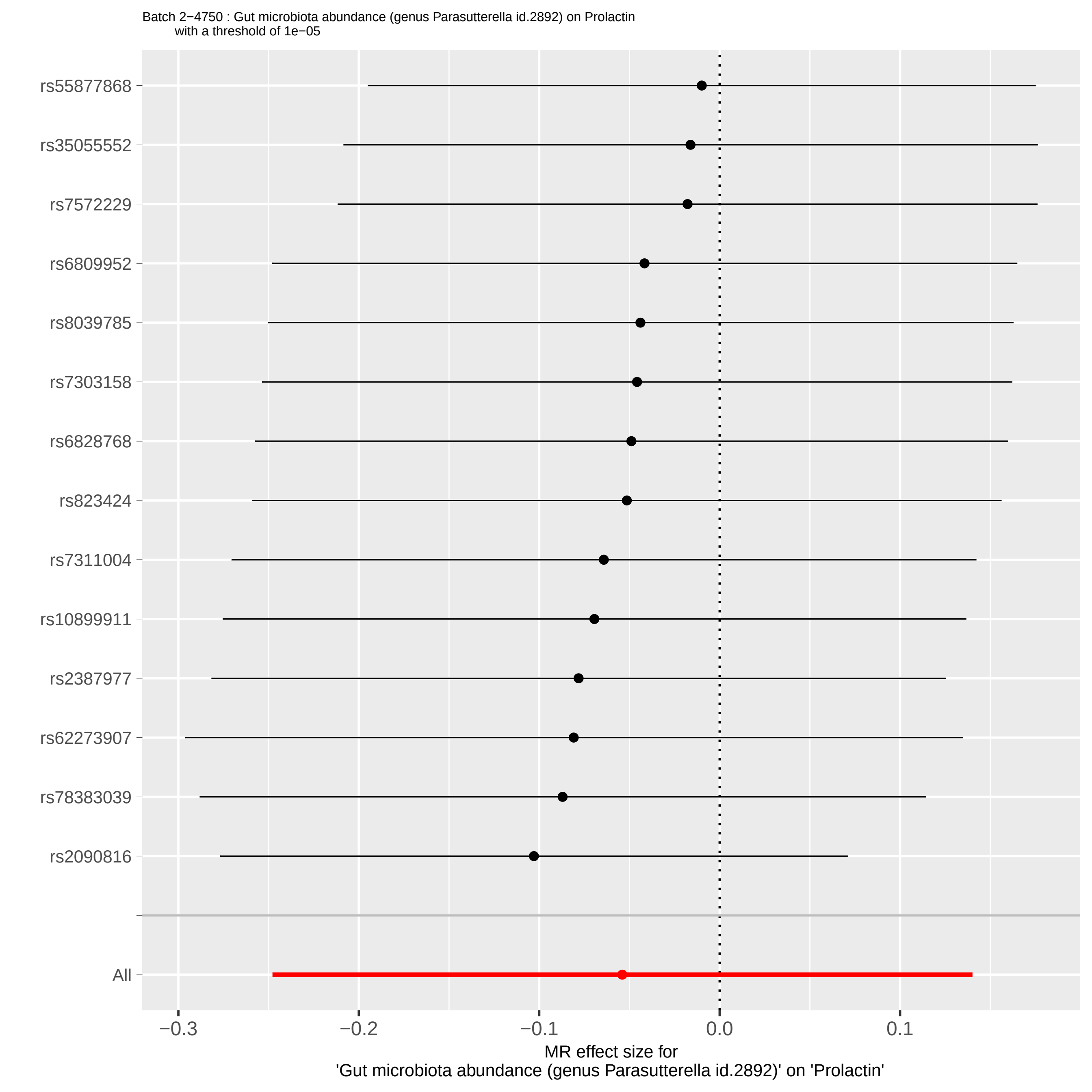
Batch 2−4750 : Gut microbiota abundance (genus Parasutterella id.2892) on Prolactin
with a threshold of 1e−05
rs55877868
rs35055552
rs7572229
rs6809952
rs8039785
rs7303158
rs6828768
rs823424
rs7311004
rs10899911
rs2387977
rs62273907
rs78383039
rs2090816
All
−0.3
−0.2
−0.1
0.0
0.1
MR effect size for
'Gut microbiota abundance (genus Parasutterella id.2892)' on 'Prolactin'

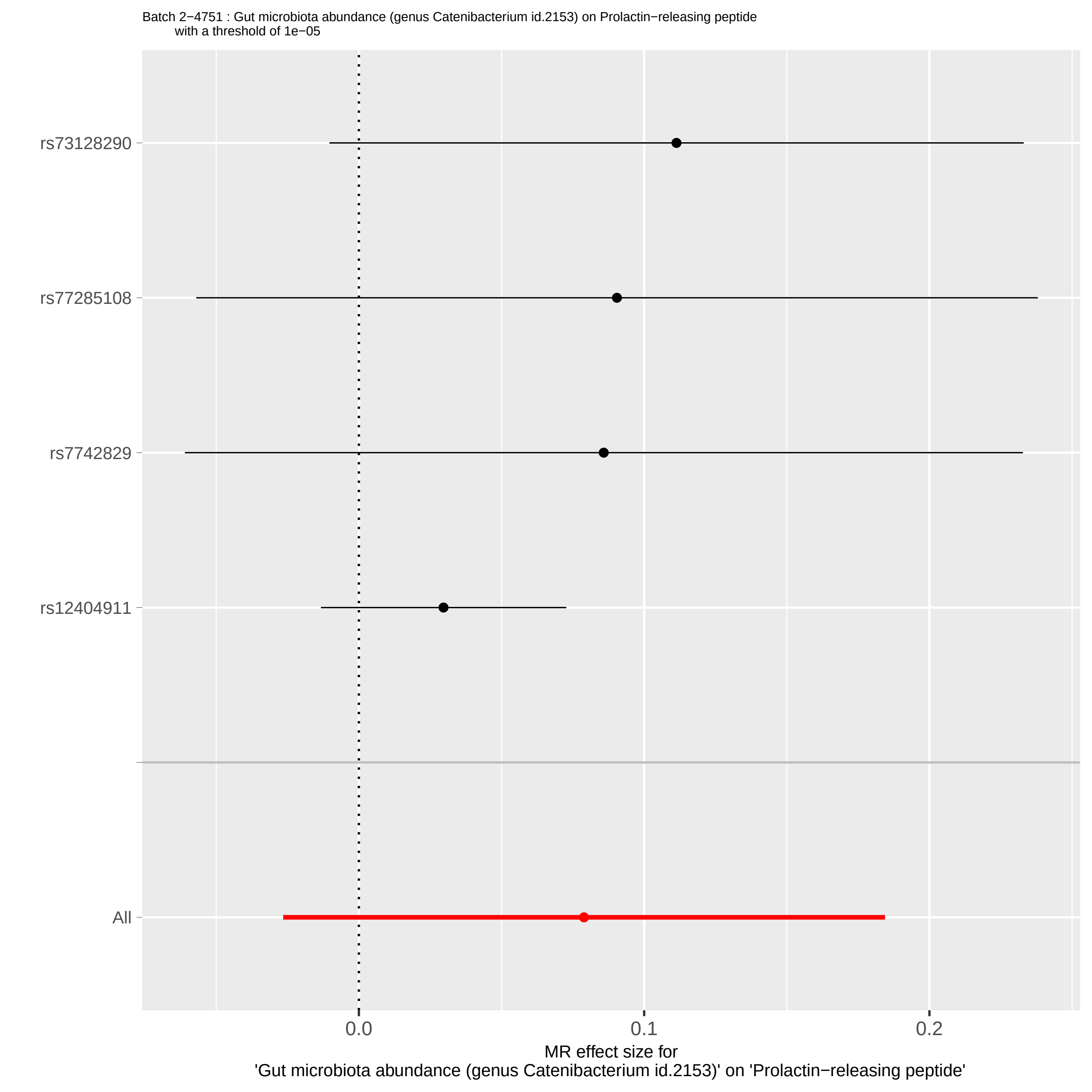
Batch 2−4751 : Gut microbiota abundance (genus Catenibacterium id.2153) on Prolactin−releasing peptide
with a threshold of 1e−05
rs73128290
rs77285108
rs7742829
rs12404911
All
0.0
0.1
0.2
MR effect size for
'Gut microbiota abundance (genus Catenibacterium id.2153)' on 'Prolactin−releasing peptide'

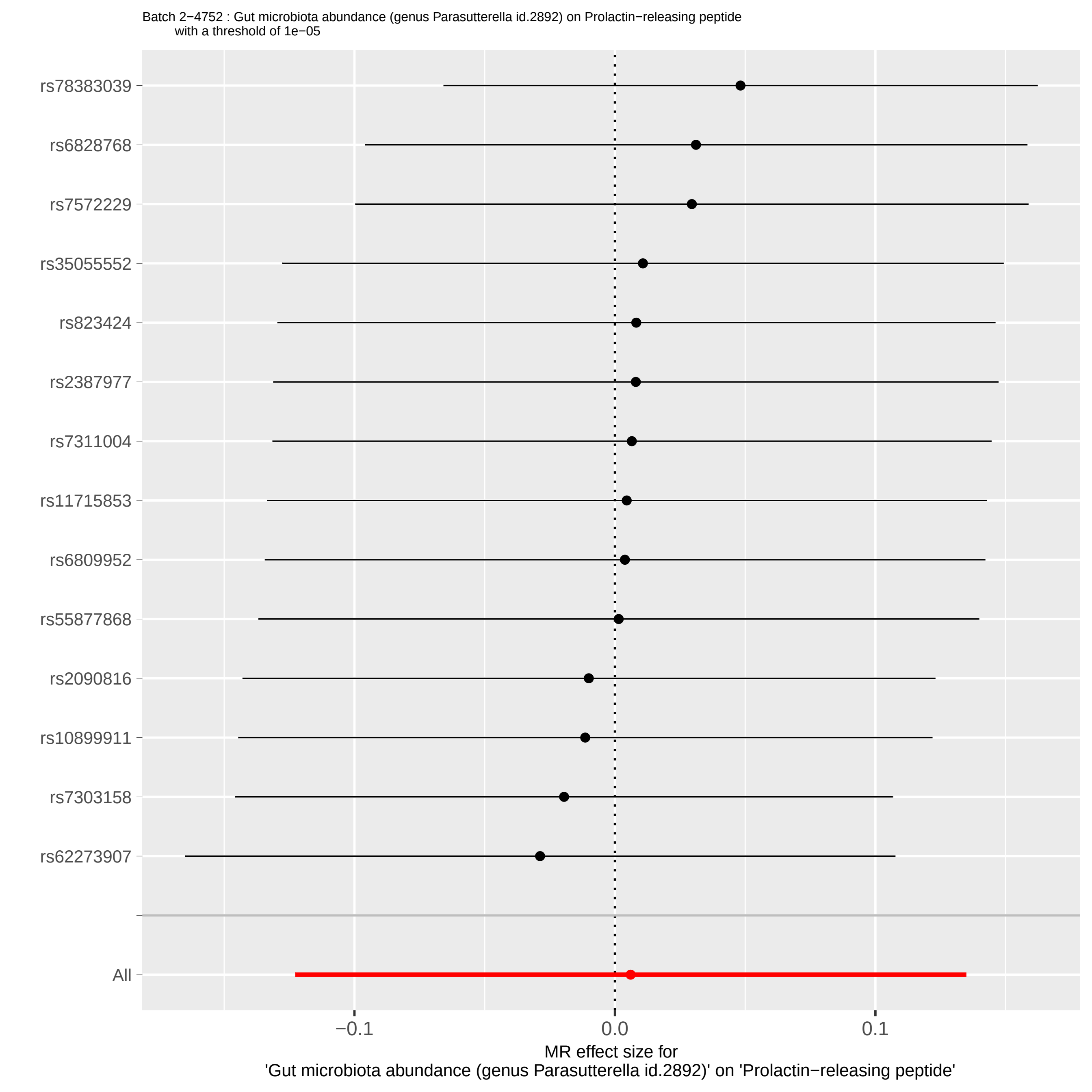

Batch 2−4752 : Gut microbiota abundance (genus Parasutterella id.2892) on Prolactin−releasing peptide
with a threshold of 1e−05
rs78383039
rs6828768
rs7572229
rs35055552
rs823424
rs2387977
rs7311004
rs11715853
rs6809952
rs55877868
rs2090816
rs10899911
rs7303158
rs62273907
All
−0.1
0.0
0.1
MR effect size for
'Gut microbiota abundance (genus Parasutterella id.2892)' on 'Prolactin−releasing peptide'

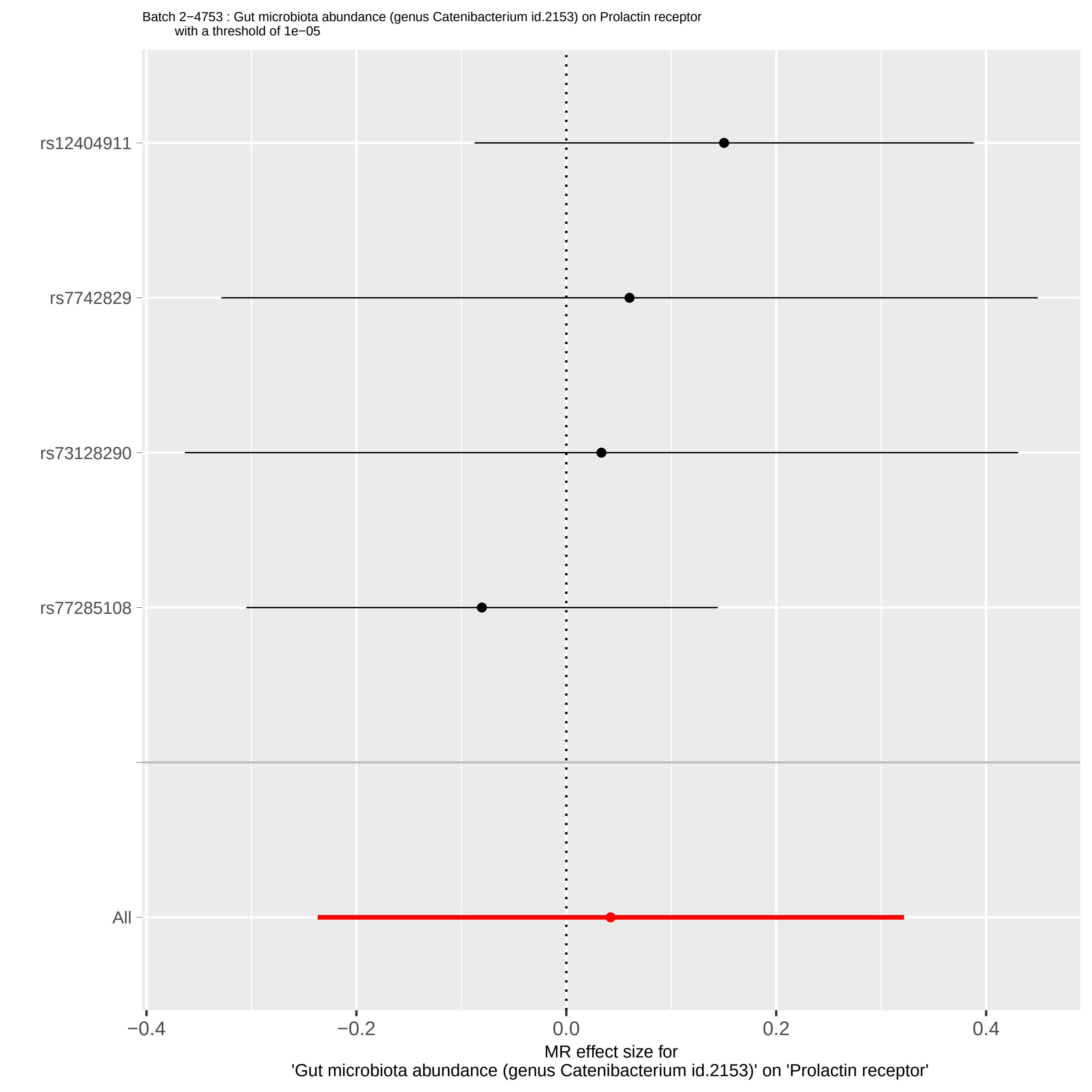
Batch 2−4753 : Gut microbiota abundance (genus Catenibacterium id.2153) on Prolactin receptor
with a threshold of 1e−05
rs12404911
rs7742829
rs73128290
rs77285108
All
−0.4
−0.2
0.0
0.2
0.4
MR effect size for
'Gut microbiota abundance (genus Catenibacterium id.2153)' on 'Prolactin receptor'

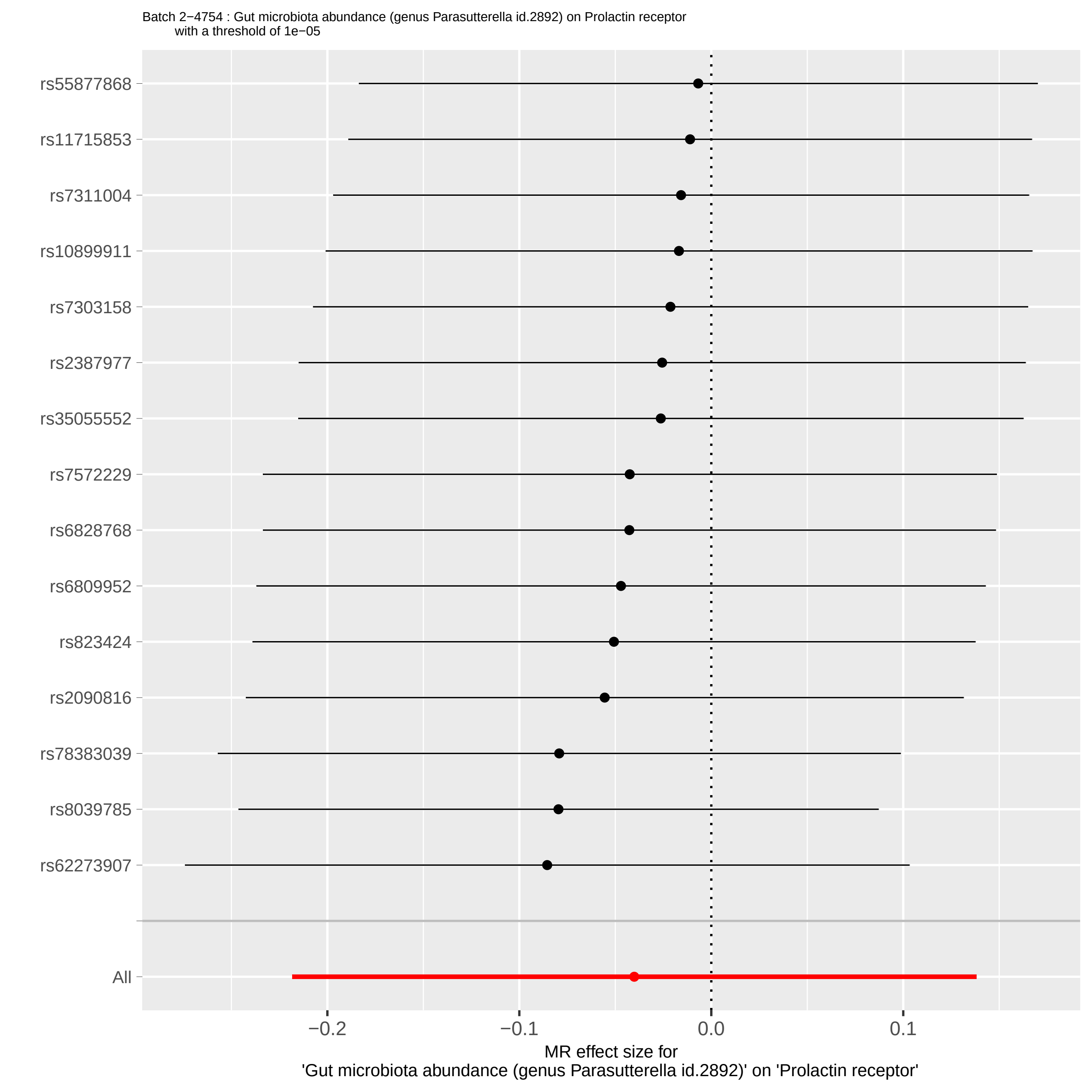
Batch 2−4754 : Gut microbiota abundance (genus Parasutterella id.2892) on Prolactin receptor
with a threshold of 1e−05
rs55877868
rs11715853
rs7311004
rs10899911
rs7303158
rs2387977
rs35055552
rs7572229
rs6828768
rs6809952
rs823424
rs2090816
rs78383039
rs8039785
rs62273907
All
−0.2
−0.1
0.0
0.1
MR effect size for
'Gut microbiota abundance (genus Parasutterella id.2892)' on 'Prolactin receptor'

Batch 2−4755 : Gut microbiota abundance (genus Catenibacterium id.2153) on Protein arginine N−methyltransferase 3
with a threshold of 1e−05

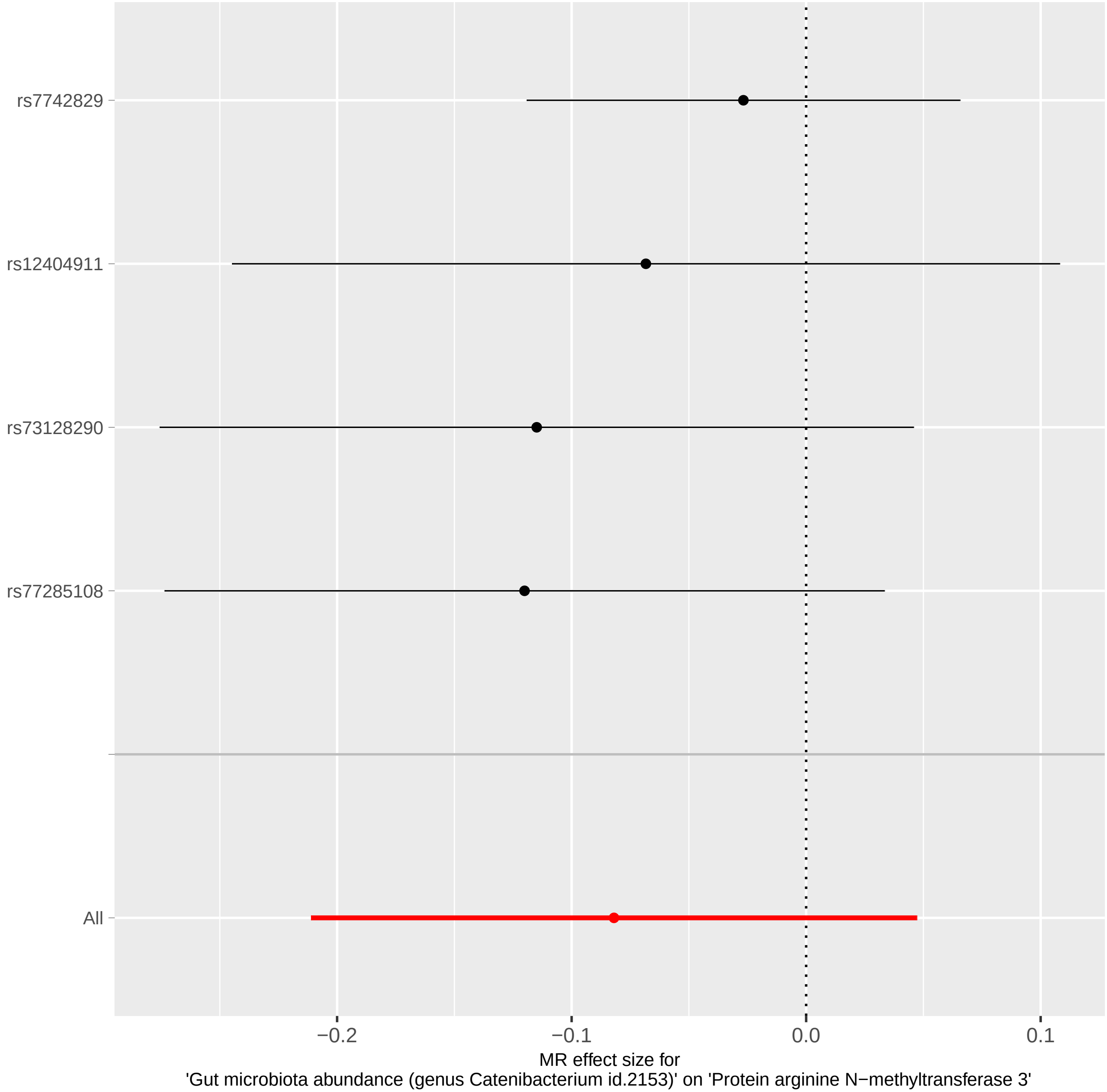

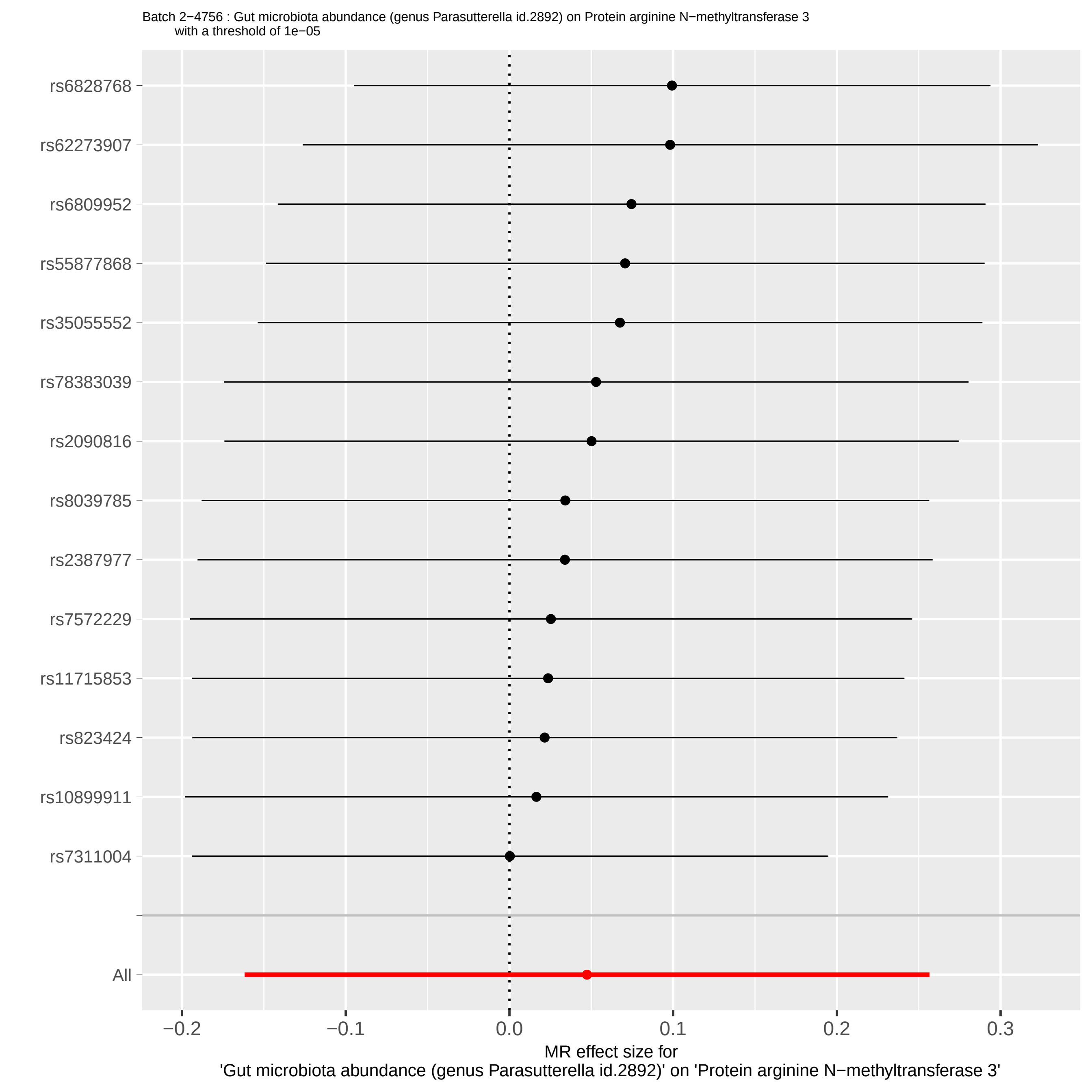

Batch 2−4756 : Gut microbiota abundance (genus Parasutterella id.2892) on Protein arginine N−methyltransferase 3
with a threshold of 1e−05
rs6828768
rs62273907
rs6809952
rs55877868
rs35055552
rs78383039
rs2090816
rs8039785
rs2387977
rs7572229
rs11715853
rs823424
rs10899911
rs7311004
All
−0.2
−0.1
0.0
0.1
0.2
0.3
MR effect size for
'Gut microbiota abundance (genus Parasutterella id.2892)' on 'Protein arginine N−methyltransferase 3'

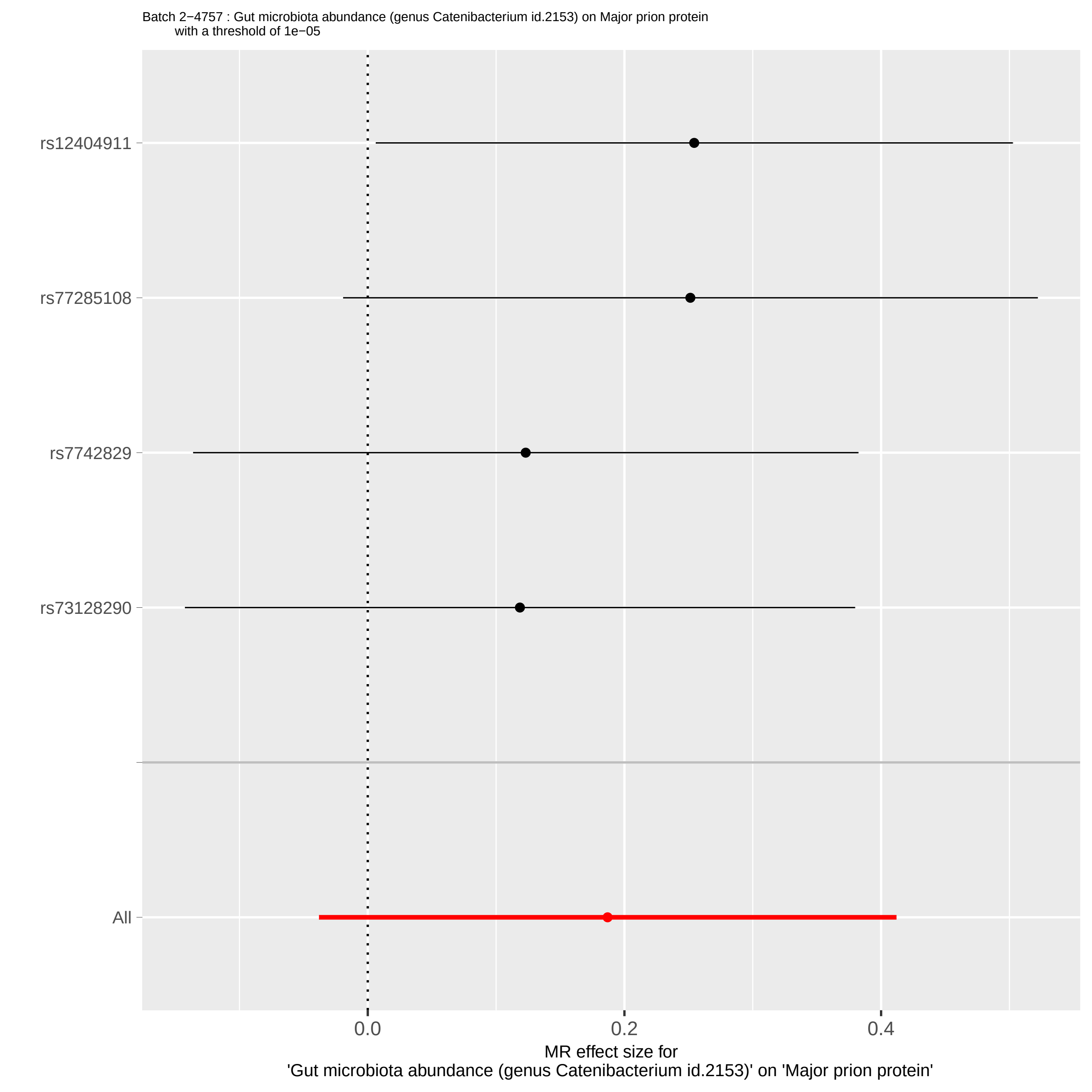
Batch 2−4757 : Gut microbiota abundance (genus Catenibacterium id.2153) on Major prion protein
with a threshold of 1e−05
rs12404911
rs77285108
rs7742829
rs73128290
All
0.0
0.2
0.4
MR effect size for
'Gut microbiota abundance (genus Catenibacterium id.2153)' on 'Major prion protein'

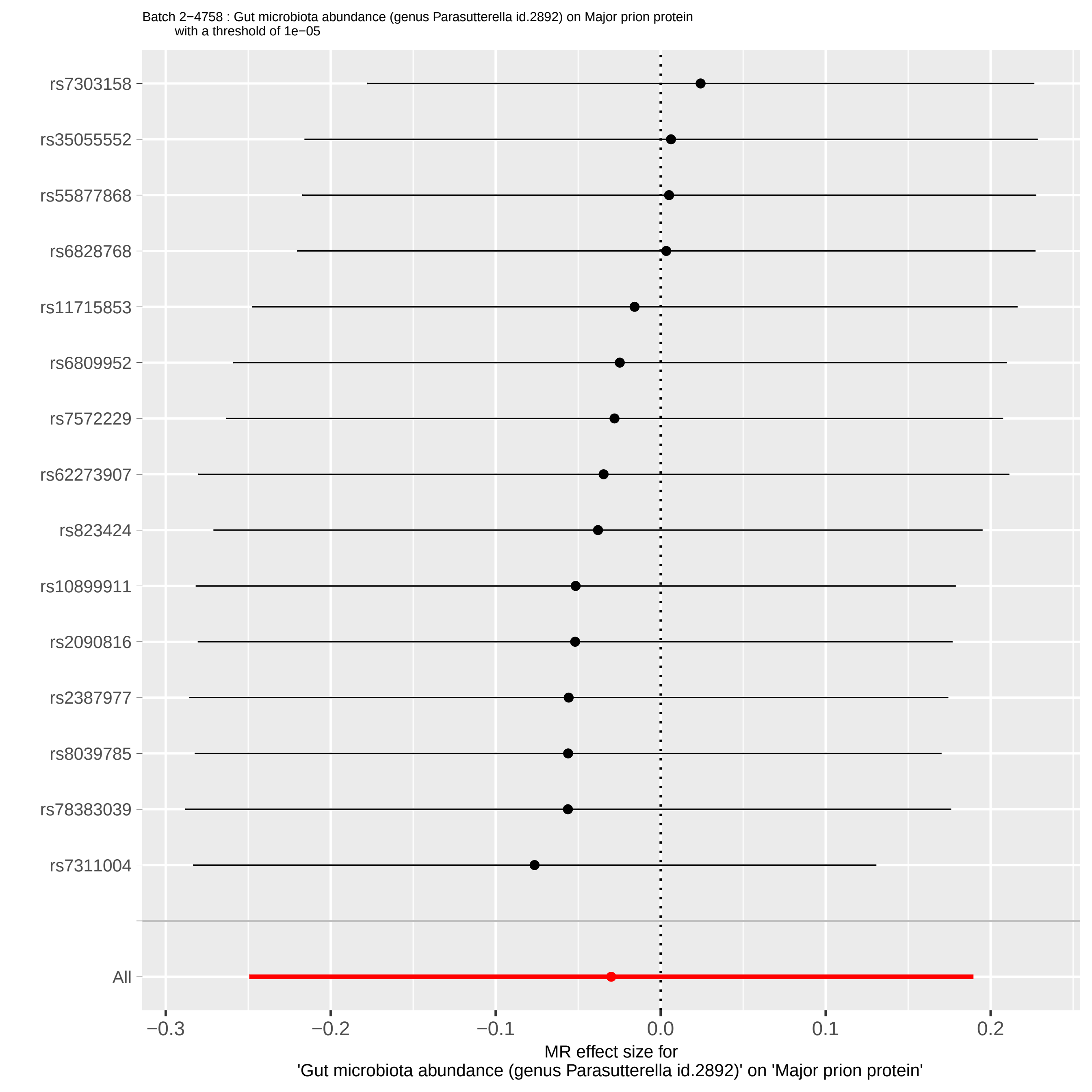
Batch 2−4758 : Gut microbiota abundance (genus Parasutterella id.2892) on Major prion protein
with a threshold of 1e−05
rs7303158
rs35055552
rs55877868
rs6828768
rs11715853
rs6809952
rs7572229
rs62273907
rs823424
rs10899911
rs2090816
rs2387977
rs8039785
rs78383039
rs7311004
All
−0.3
−0.2
−0.1
0.0
0.1
0.2
MR effect size for
'Gut microbiota abundance (genus Parasutterella id.2892)' on 'Major prion protein'

Batch 2−4759 : Gut microbiota abundance (genus Catenibacterium id.2153) on Activated Protein C
with a threshold of 1e−05

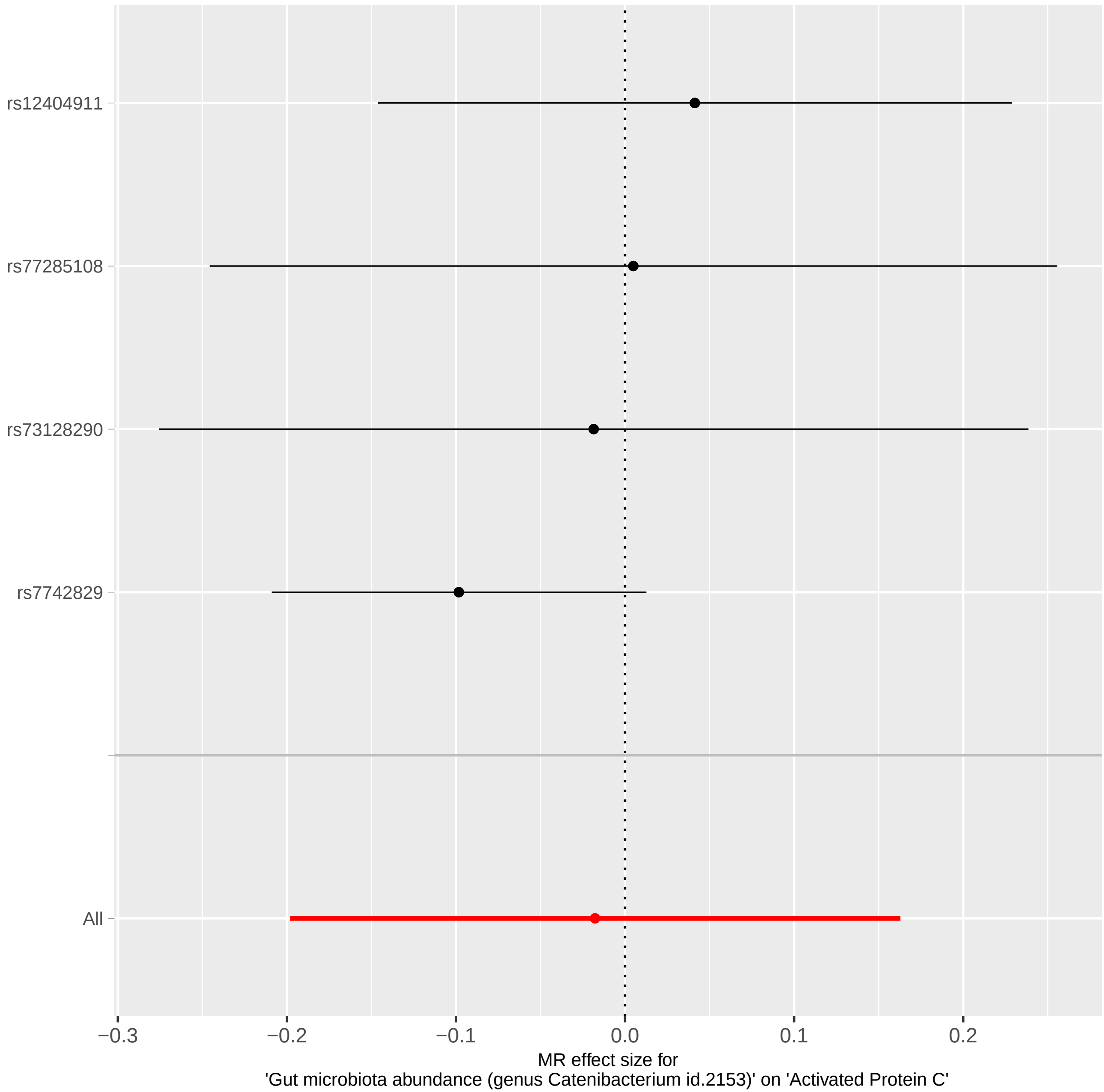

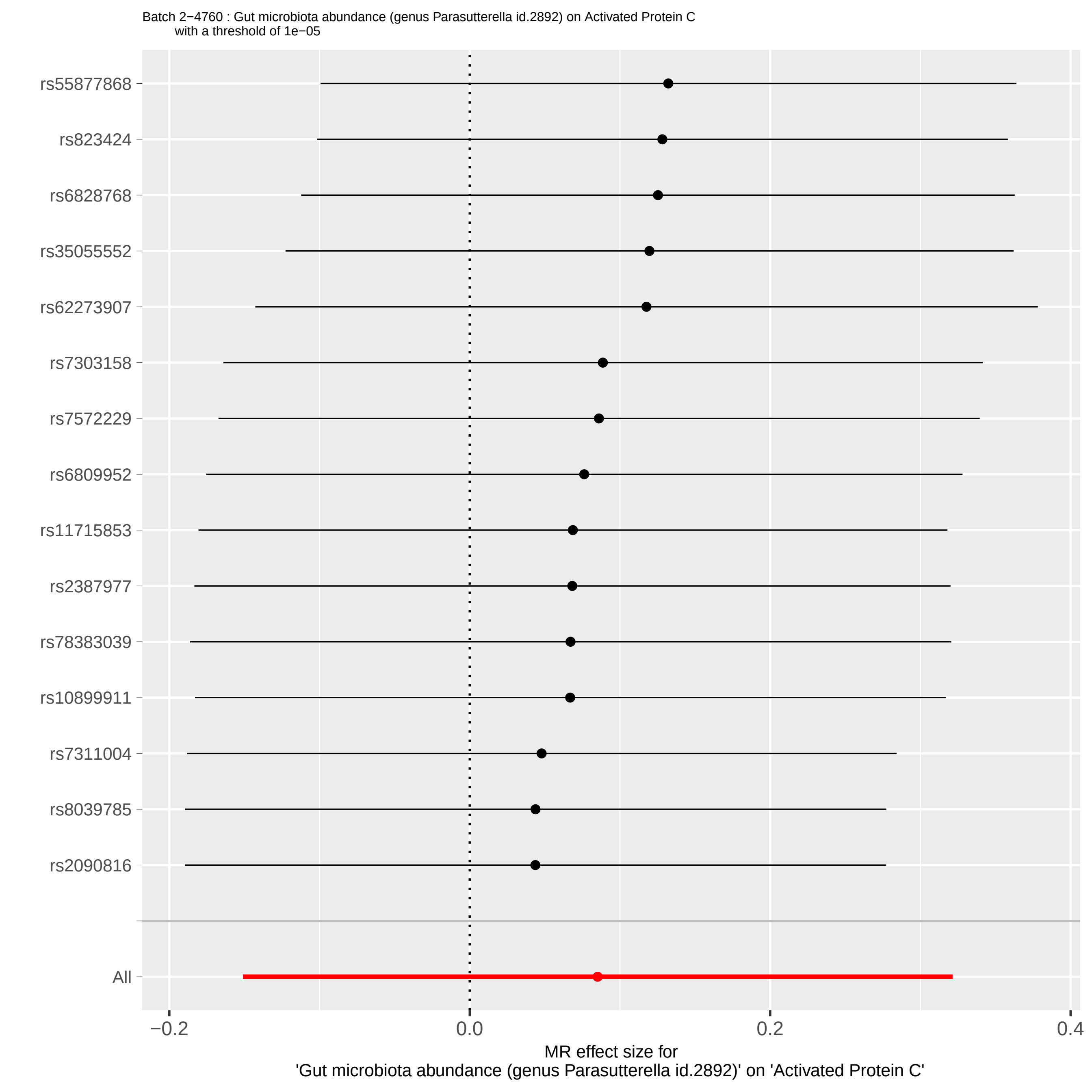

Batch 2−4760 : Gut microbiota abundance (genus Parasutterella id.2892) on Activated Protein C
with a threshold of 1e−05
rs55877868
rs823424
rs6828768
rs35055552
rs62273907
rs7303158
rs7572229
rs6809952
rs11715853
rs2387977
rs78383039
rs10899911
rs7311004
rs8039785
rs2090816
All
−0.2
0.0
0.2
0.4
MR effect size for
'Gut microbiota abundance (genus Parasutterella id.2892)' on 'Activated Protein C'

Batch 2−4761 : Gut microbiota abundance (genus Catenibacterium id.2153) on Activated Protein C
with a threshold of 1e−05

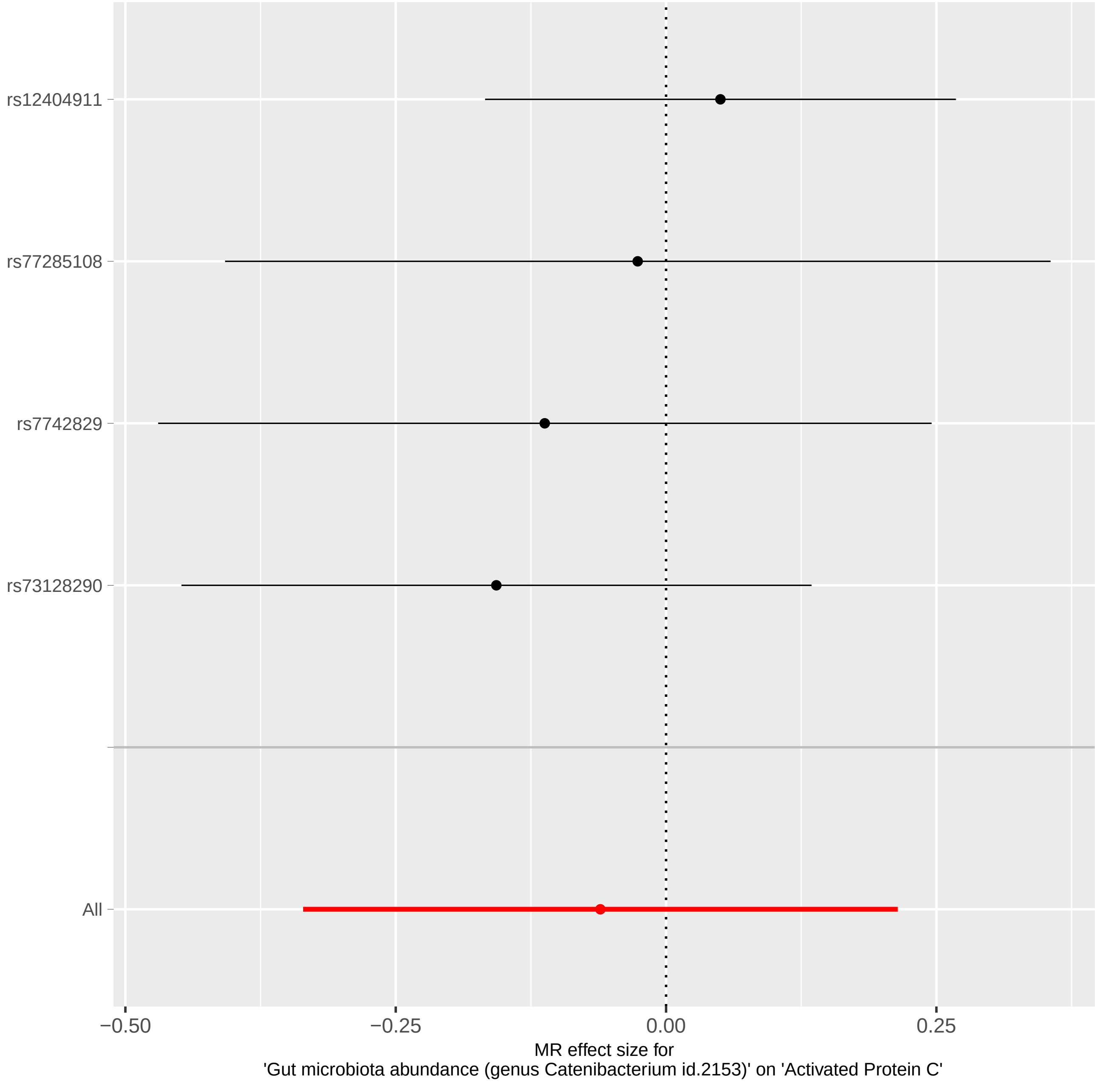

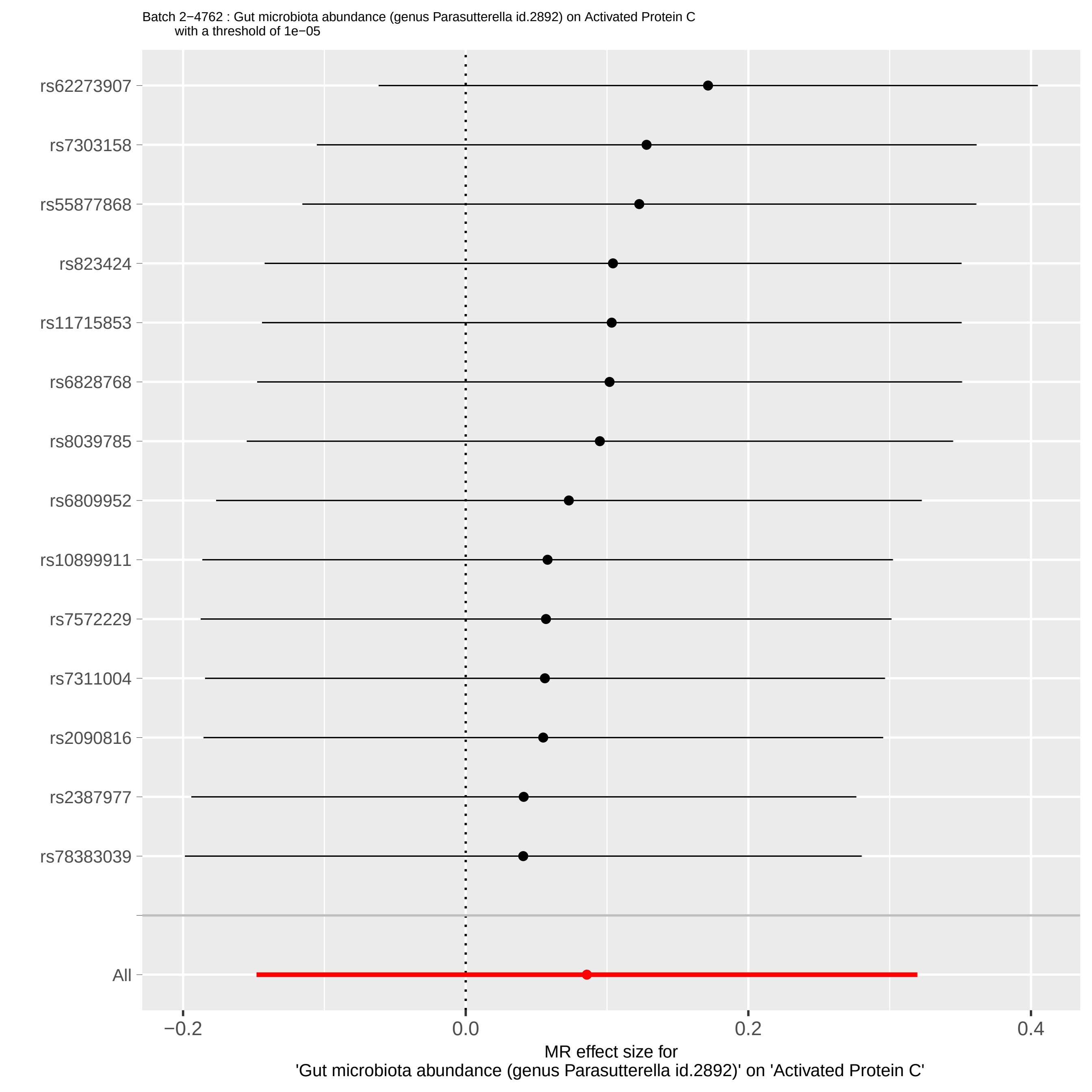

Batch 2−4762 : Gut microbiota abundance (genus Parasutterella id.2892) on Activated Protein C
with a threshold of 1e−05
rs62273907
rs7303158
rs55877868
rs823424
rs11715853
rs6828768
rs8039785
rs6809952
rs10899911
rs7572229
rs7311004
rs2090816
rs2387977
rs78383039
All
−0.2
0.0
0.2
0.4
MR effect size for
'Gut microbiota abundance (genus Parasutterella id.2892)' on 'Activated Protein C'

Batch 2−4763 : Gut microbiota abundance (genus Catenibacterium id.2153) on Prokineticin−1
with a threshold of 1e−05

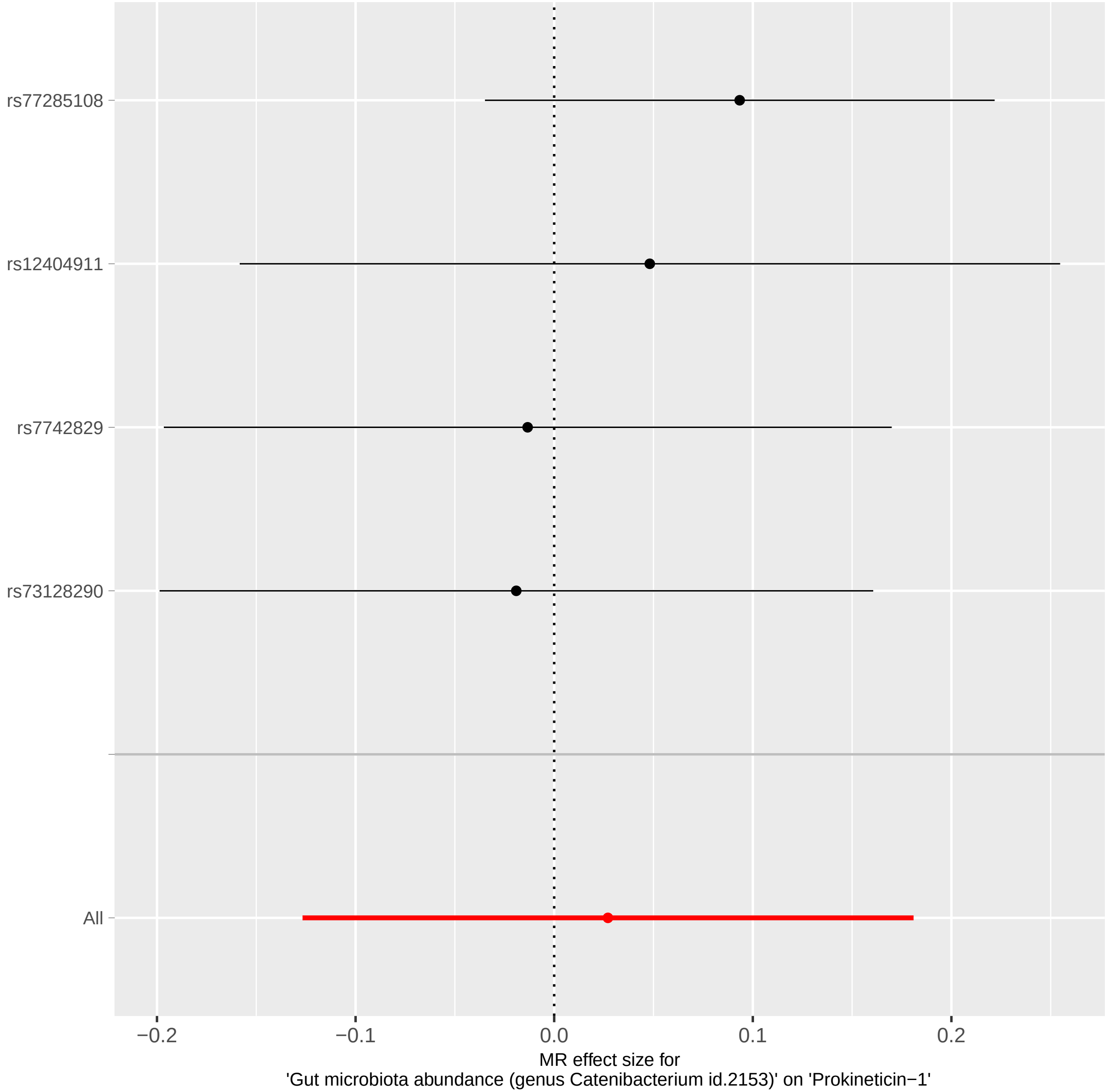

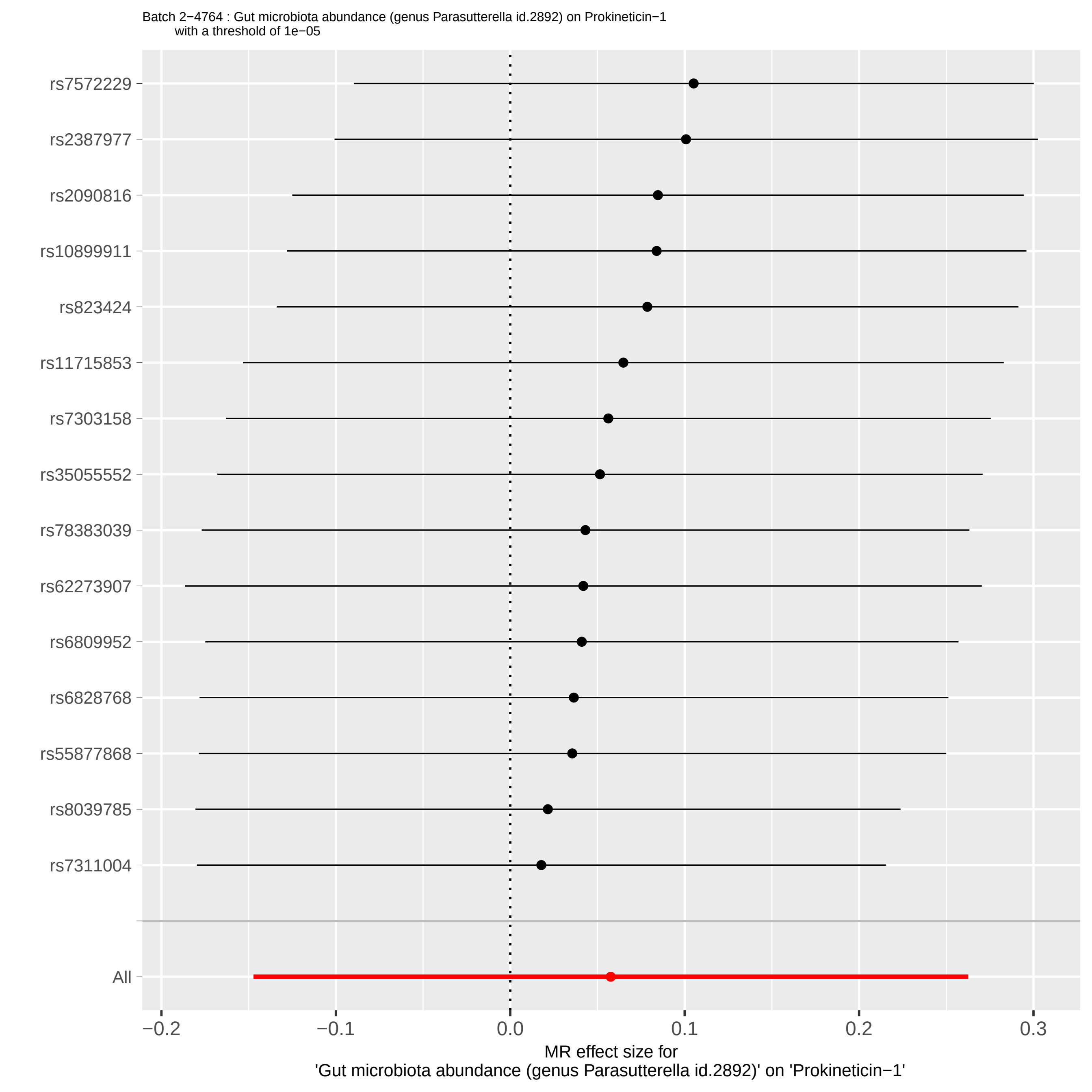
Batch 2−4764 : Gut microbiota abundance (genus Parasutterella id.2892) on Prokineticin−1
with a threshold of 1e−05
rs7572229
rs2387977
rs2090816
rs10899911
rs823424
rs11715853
rs7303158
rs35055552
rs78383039
rs62273907
rs6809952
rs6828768
rs55877868
rs8039785
rs7311004
All
−0.2
−0.1
0.0
0.1
0.2
0.3
MR effect size for
'Gut microbiota abundance (genus Parasutterella id.2892)' on 'Prokineticin−1'

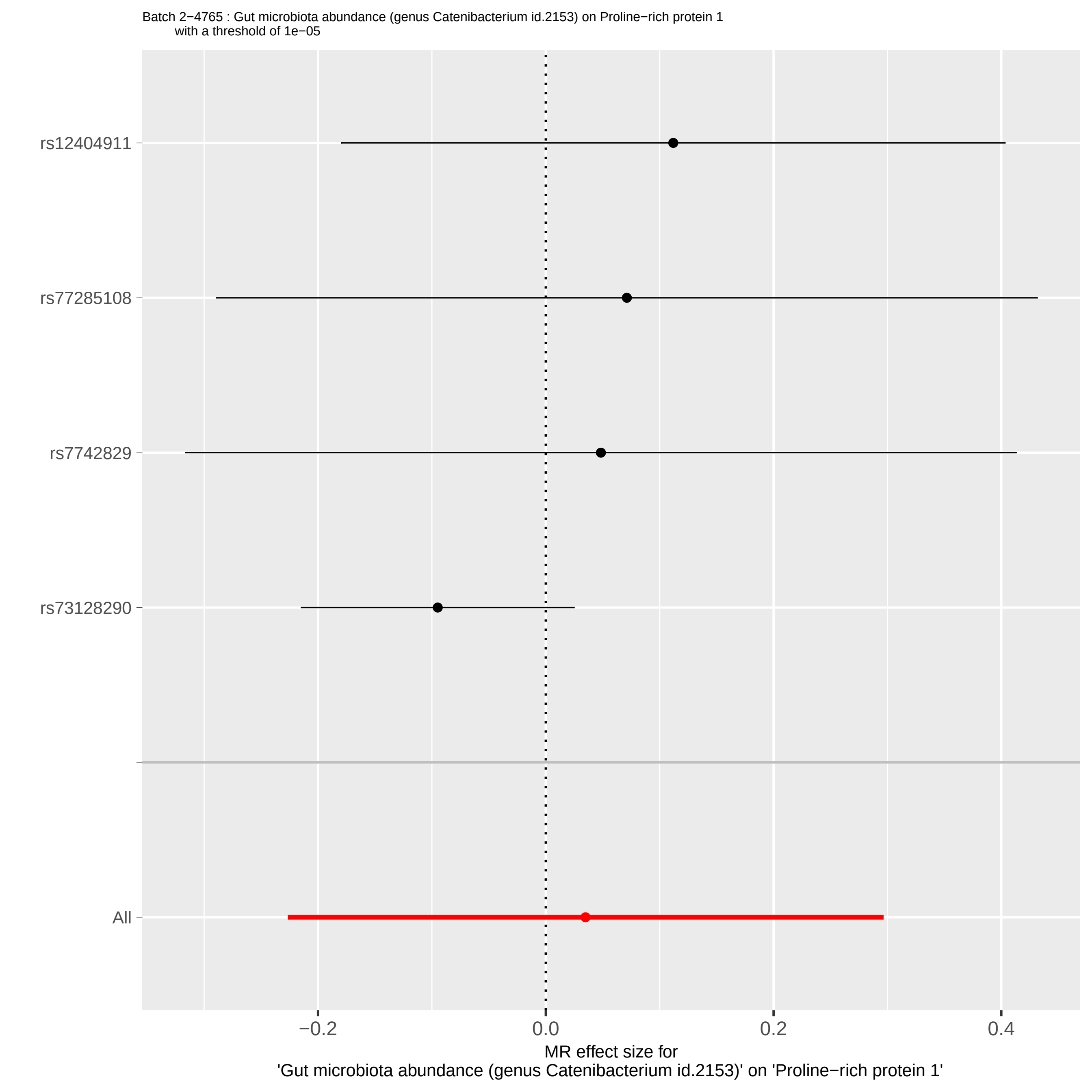

Batch 2−4765 : Gut microbiota abundance (genus Catenibacterium id.2153) on Proline−rich protein 1
with a threshold of 1e−05
rs12404911
rs77285108
rs7742829
rs73128290
All
−0.2
0.0
0.2
0.4
MR effect size for
'Gut microbiota abundance (genus Catenibacterium id.2153)' on 'Proline−rich protein 1'

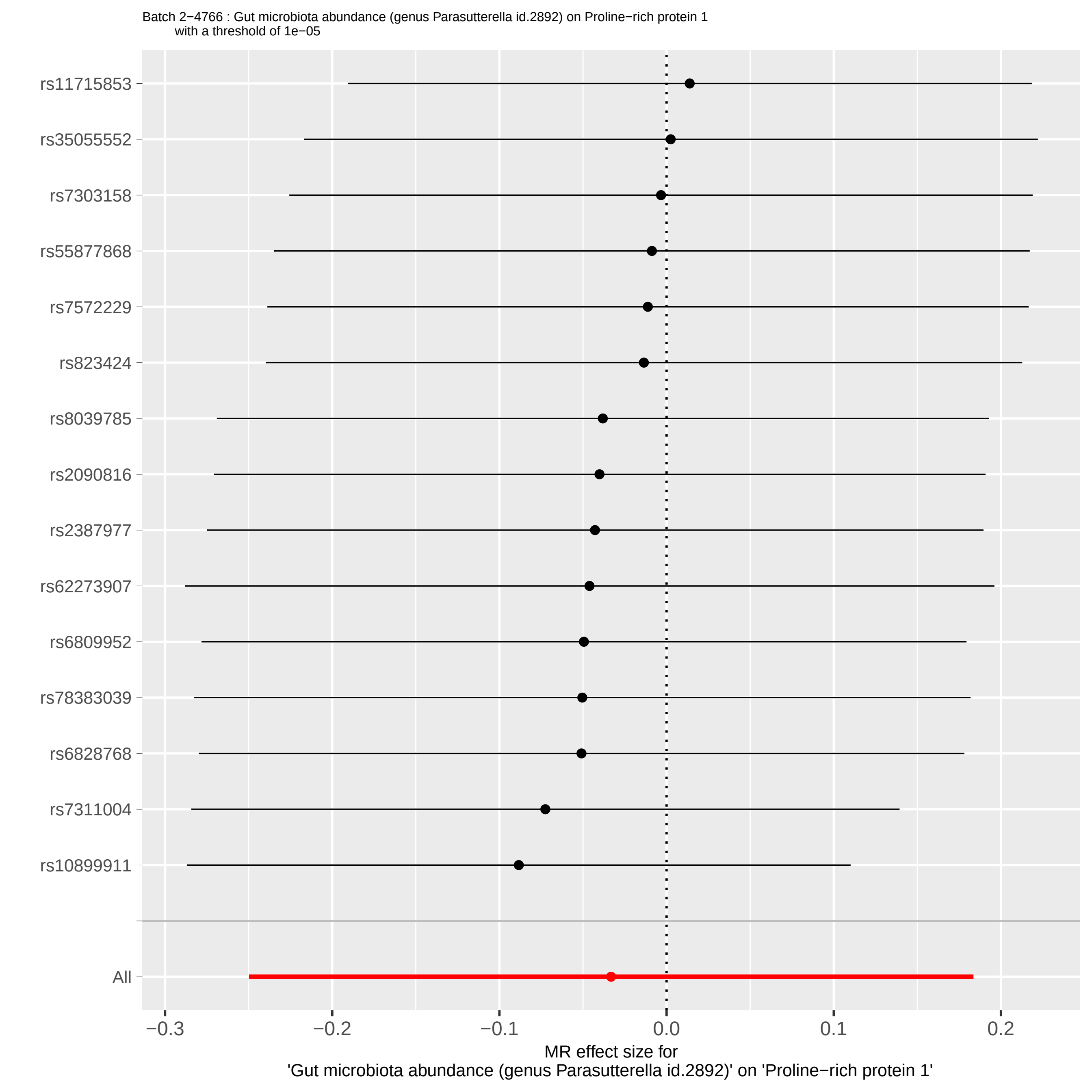
Batch 2−4766 : Gut microbiota abundance (genus Parasutterella id.2892) on Proline−rich protein 1
with a threshold of 1e−05
rs11715853
rs35055552
rs7303158
rs55877868
rs7572229
rs823424
rs8039785
rs2090816
rs2387977
rs62273907
rs6809952
rs78383039
rs6828768
rs7311004
rs10899911
All
−0.3
−0.2
−0.1
0.0
0.1
0.2
MR effect size for
'Gut microbiota abundance (genus Parasutterella id.2892)' on 'Proline−rich protein 1'

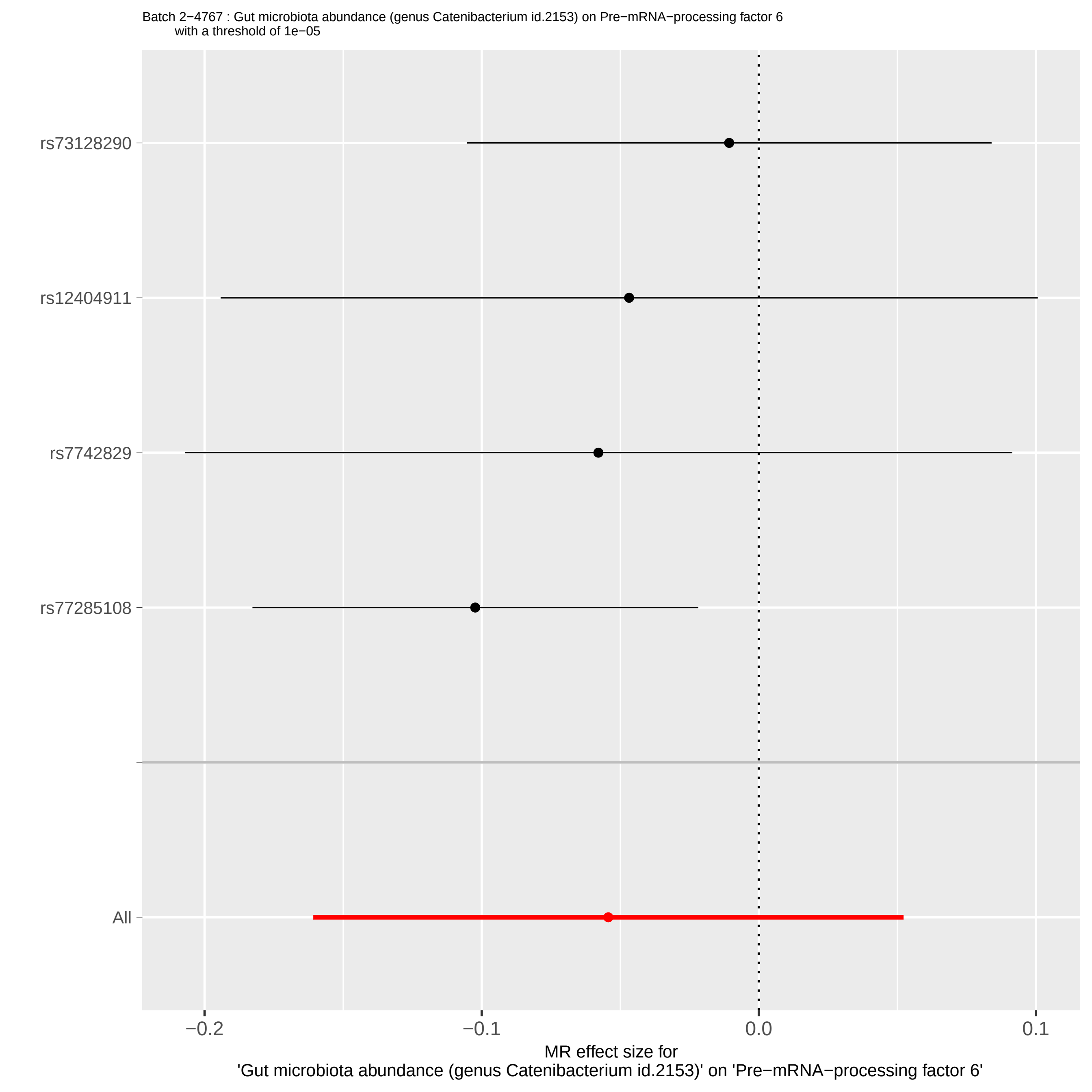

Batch 2−4767 : Gut microbiota abundance (genus Catenibacterium id.2153) on Pre−mRNA−processing factor 6
with a threshold of 1e−05
rs73128290
rs12404911
rs7742829
rs77285108
All
−0.2
−0.1
0.0
0.1
MR effect size for
'Gut microbiota abundance (genus Catenibacterium id.2153)' on 'Pre−mRNA−processing factor 6'

Batch 2−4768 : Gut microbiota abundance (genus Parasutterella id.2892) on Pre−mRNA−processing factor 6
with a threshold of 1e−05

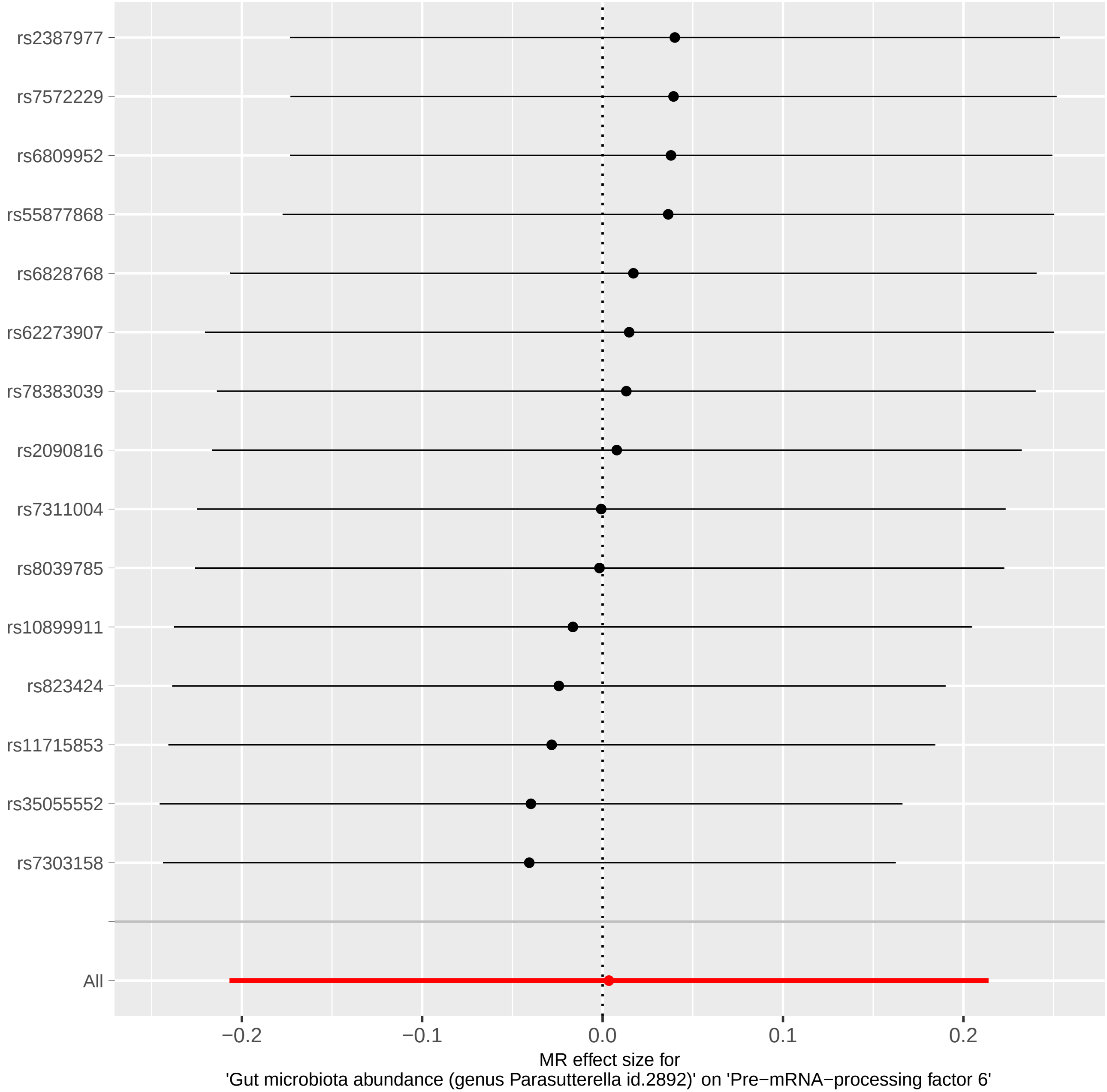

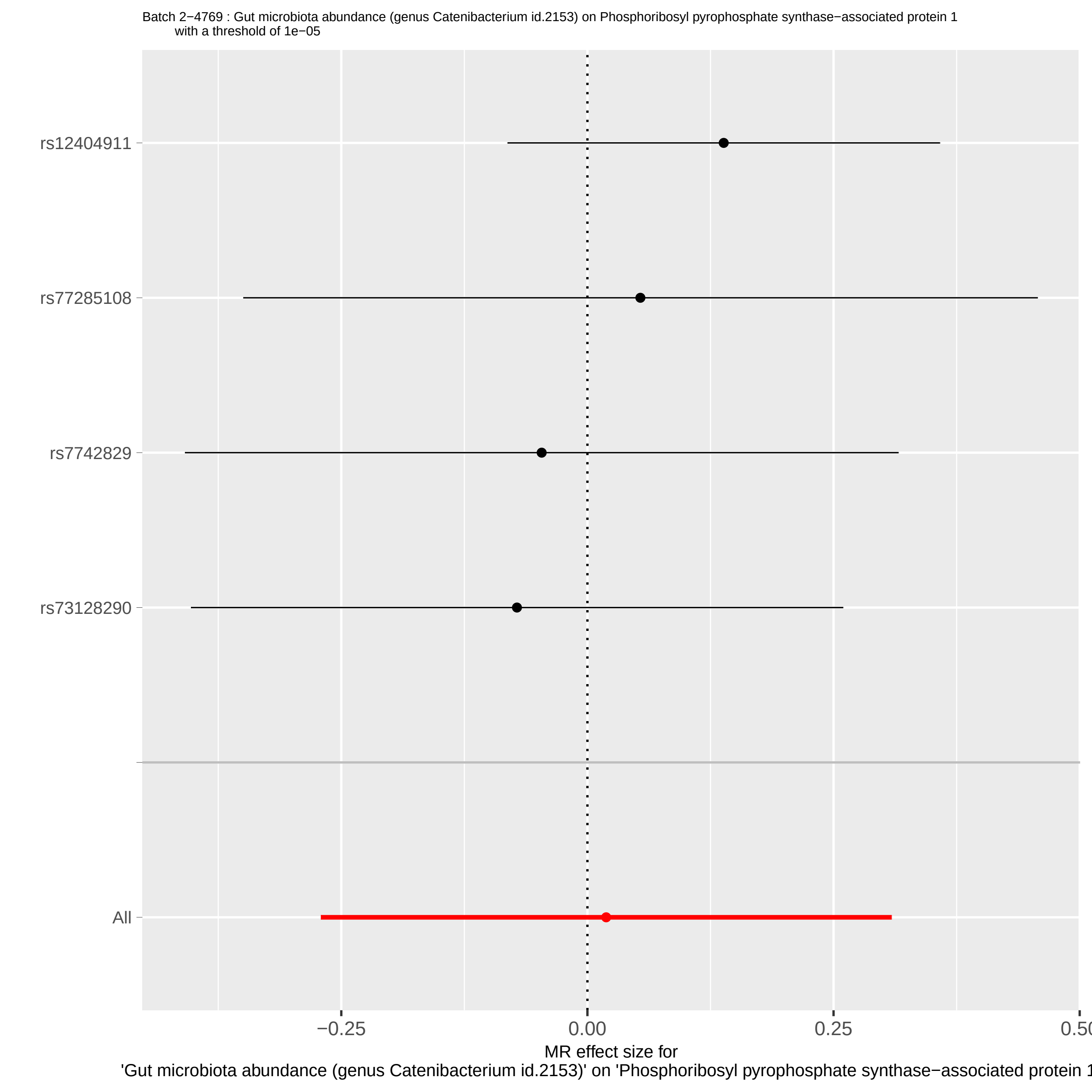

Batch 2−4769 : Gut microbiota abundance (genus Catenibacterium id.2153) on Phosphoribosyl pyrophosphate synthase−associated protein 1
with a threshold of 1e−05
rs12404911
rs77285108
rs7742829
rs73128290
All
−0.25
0.00
0.25
0.50
MR effect size for
'Gut microbiota abundance (genus Catenibacterium id.2153)' on 'Phosphoribosyl pyrophosphate synthase−associated protein 1

Batch 2−4770 : Gut microbiota abundance (genus Parasutterella id.2892) on Phosphoribosyl pyrophosphate synthase−associated protein 1
with a threshold of 1e−05

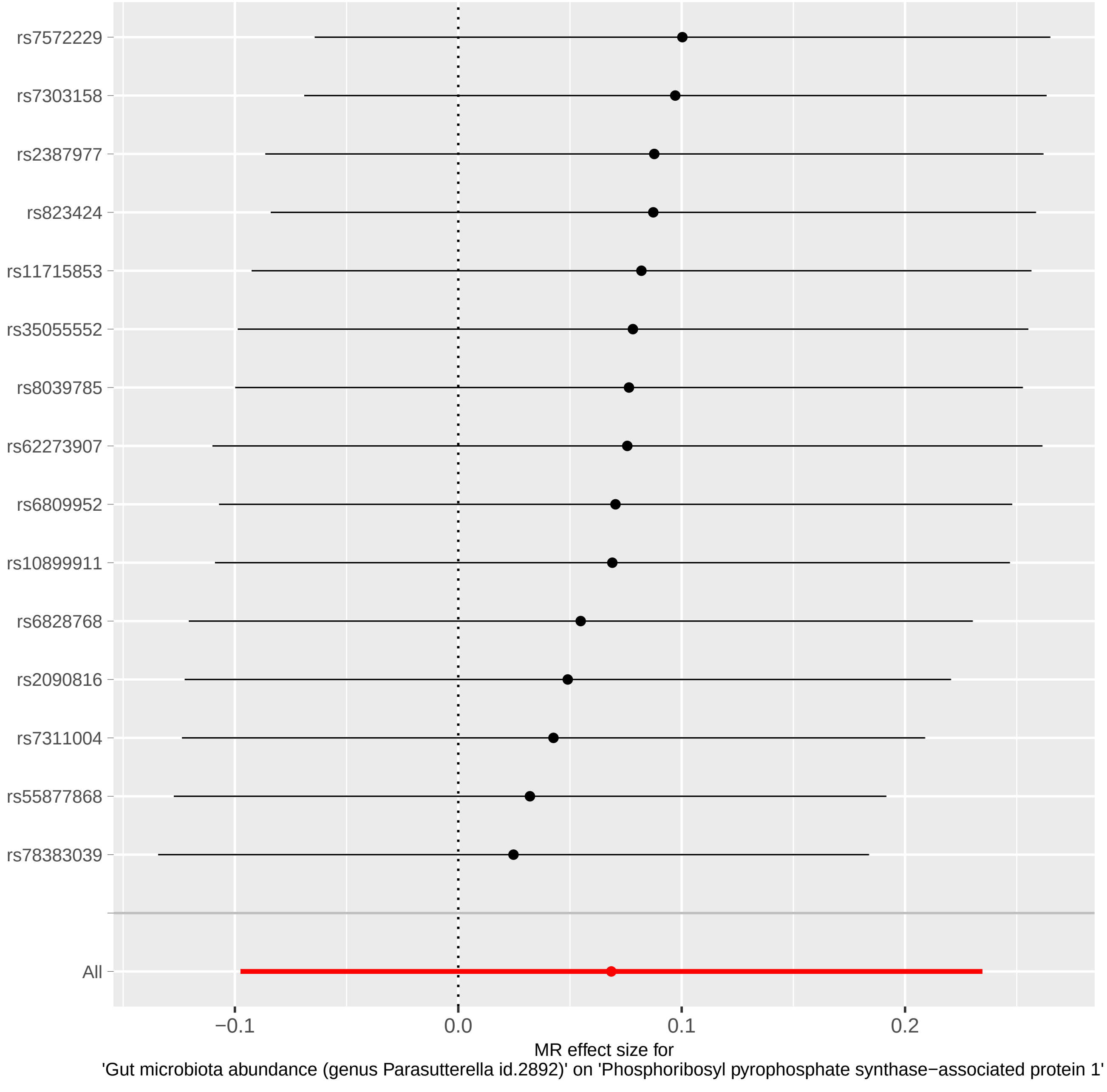

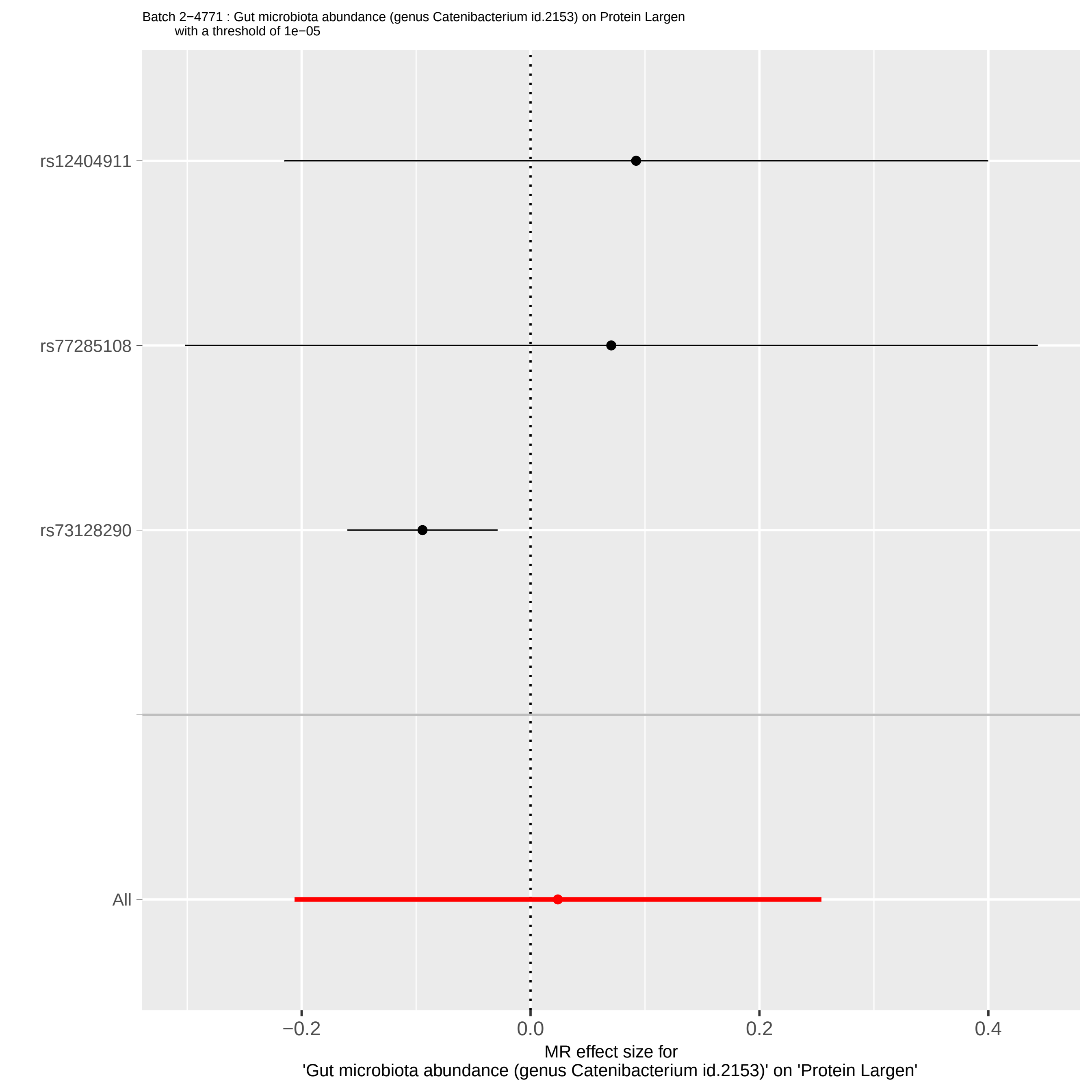
Batch 2−4771 : Gut microbiota abundance (genus Catenibacterium id.2153) on Protein Largen
with a threshold of 1e−05
rs12404911
rs77285108
rs73128290
All
−0.2
0.0
0.2
0.4
MR effect size for
'Gut microbiota abundance (genus Catenibacterium id.2153)' on 'Protein Largen'

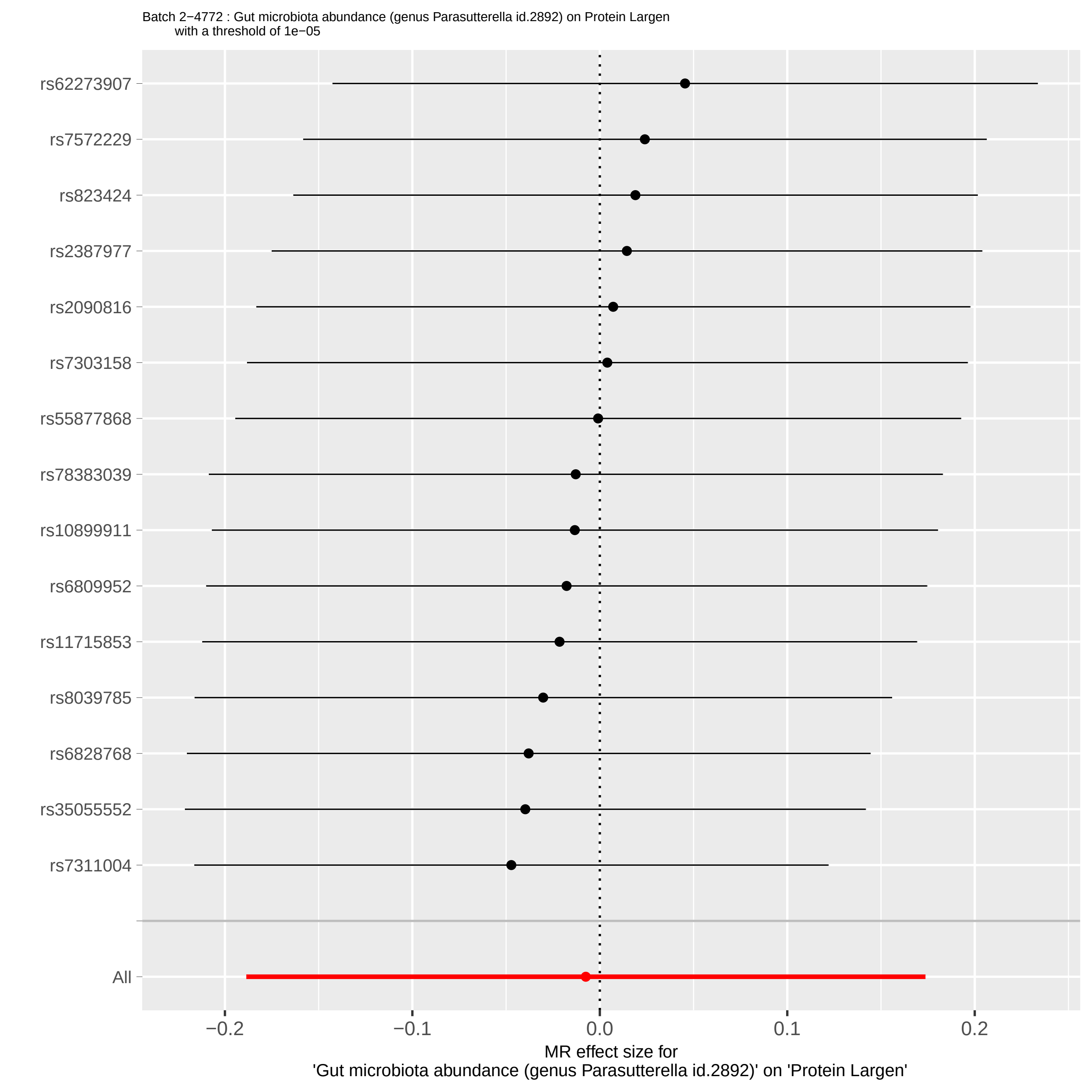
Batch 2−4772 : Gut microbiota abundance (genus Parasutterella id.2892) on Protein Largen
with a threshold of 1e−05
rs62273907
rs7572229
rs823424
rs2387977
rs2090816
rs7303158
rs55877868
rs78383039
rs10899911
rs6809952
rs11715853
rs8039785
rs6828768
rs35055552
rs7311004
All
−0.2
−0.1
0.0
0.1
0.2
MR effect size for
'Gut microbiota abundance (genus Parasutterella id.2892)' on 'Protein Largen'

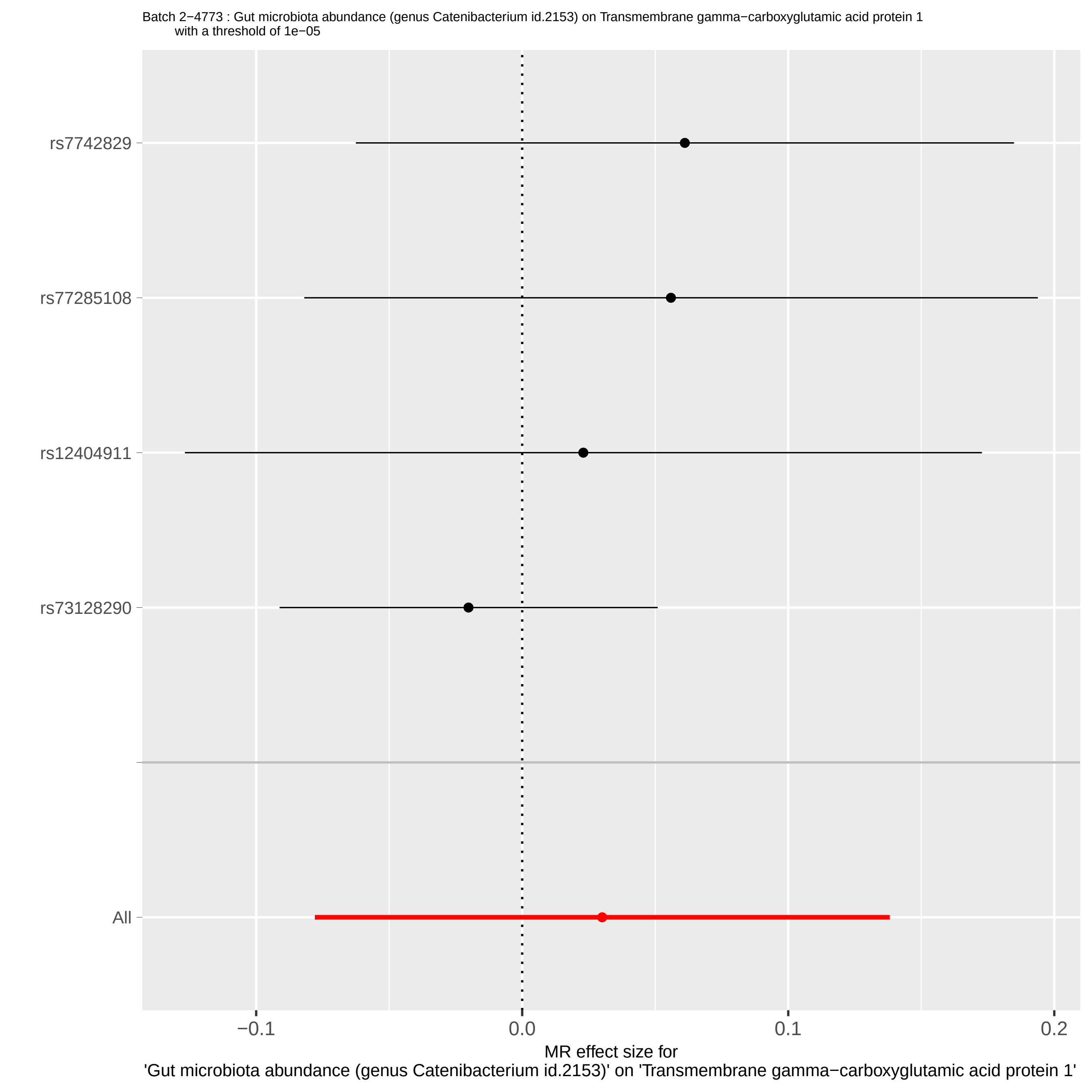

Batch 2−4773 : Gut microbiota abundance (genus Catenibacterium id.2153) on Transmembrane gamma−carboxyglutamic acid protein 1
with a threshold of 1e−05
rs7742829
rs77285108
rs12404911
rs73128290
All
−0.1
0.0
0.1
0.2
MR effect size for
'Gut microbiota abundance (genus Catenibacterium id.2153)' on 'Transmembrane gamma−carboxyglutamic acid protein 1'

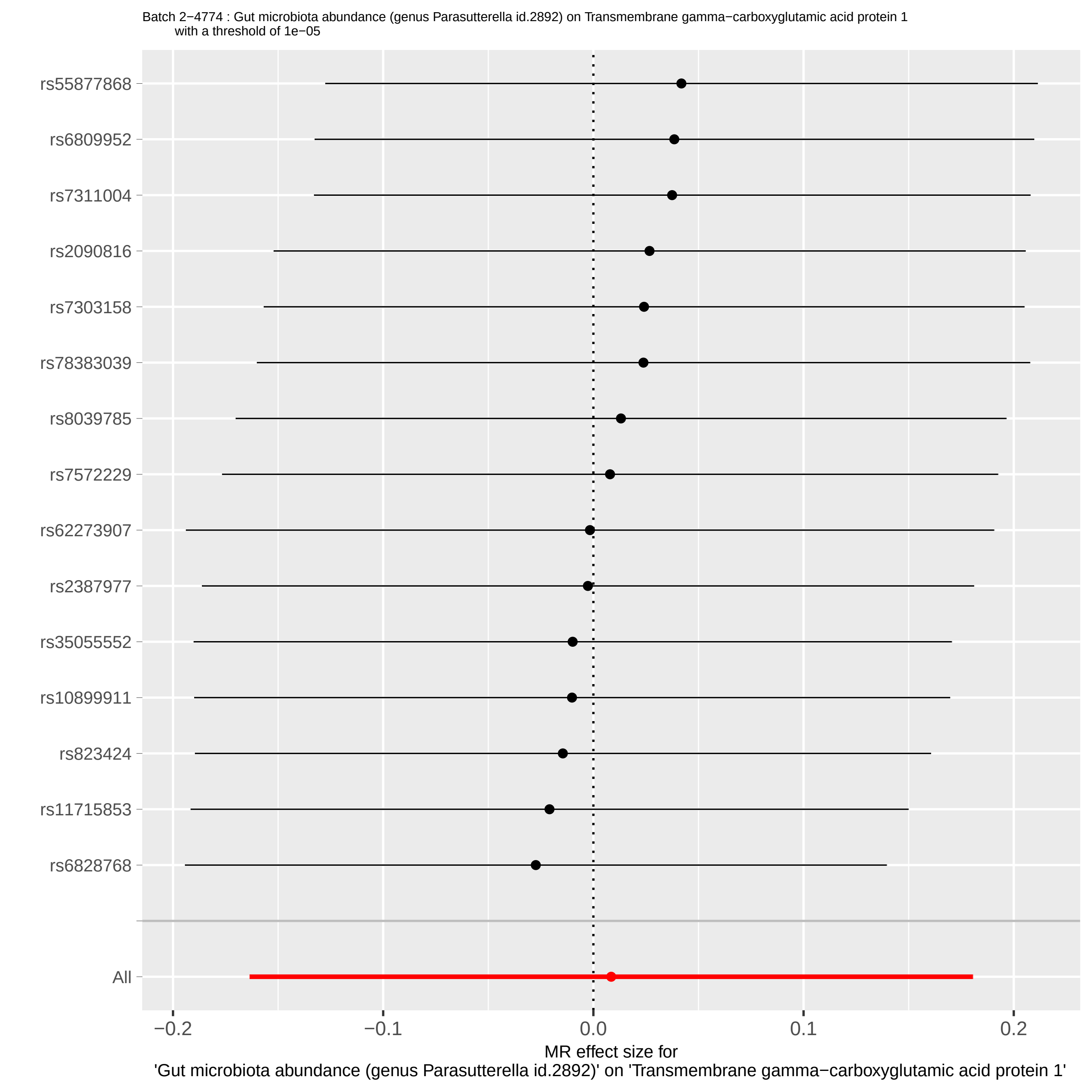
Batch 2−4774 : Gut microbiota abundance (genus Parasutterella id.2892) on Transmembrane gamma−carboxyglutamic acid protein 1
with a threshold of 1e−05
rs55877868
rs6809952
rs7311004
rs2090816
rs7303158
rs78383039
rs8039785
rs7572229
rs62273907
rs2387977
rs35055552
rs10899911
rs823424
rs11715853
rs6828768
All
−0.2
−0.1
0.0
0.1
0.2
MR effect size for
'Gut microbiota abundance (genus Parasutterella id.2892)' on 'Transmembrane gamma−carboxyglutamic acid protein 1'

Batch 2−4775 : Gut microbiota abundance (genus Catenibacterium id.2153) on Transmembrane gamma−carboxyglutamic acid protein 1 with a threshold of 1e−05

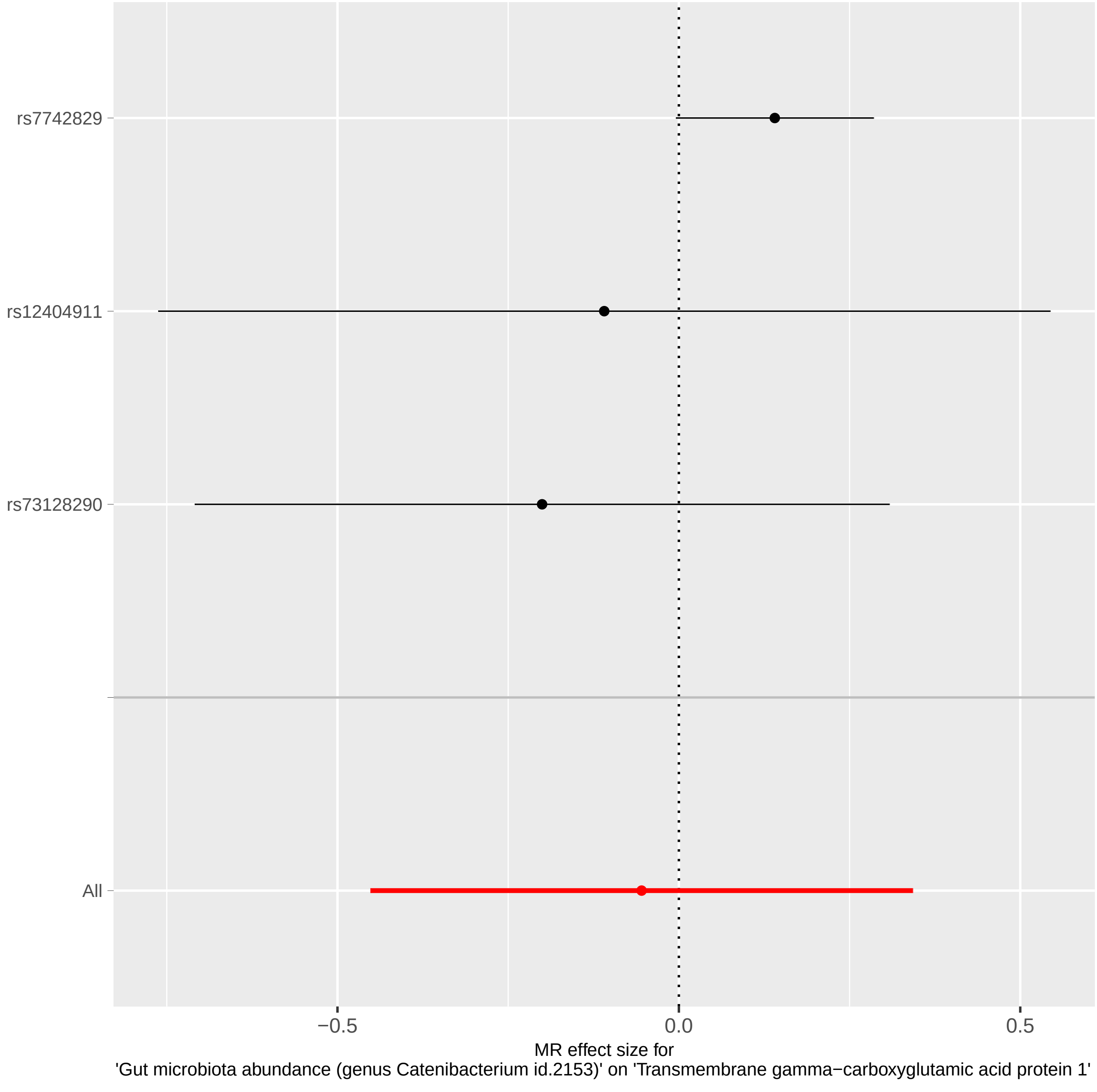

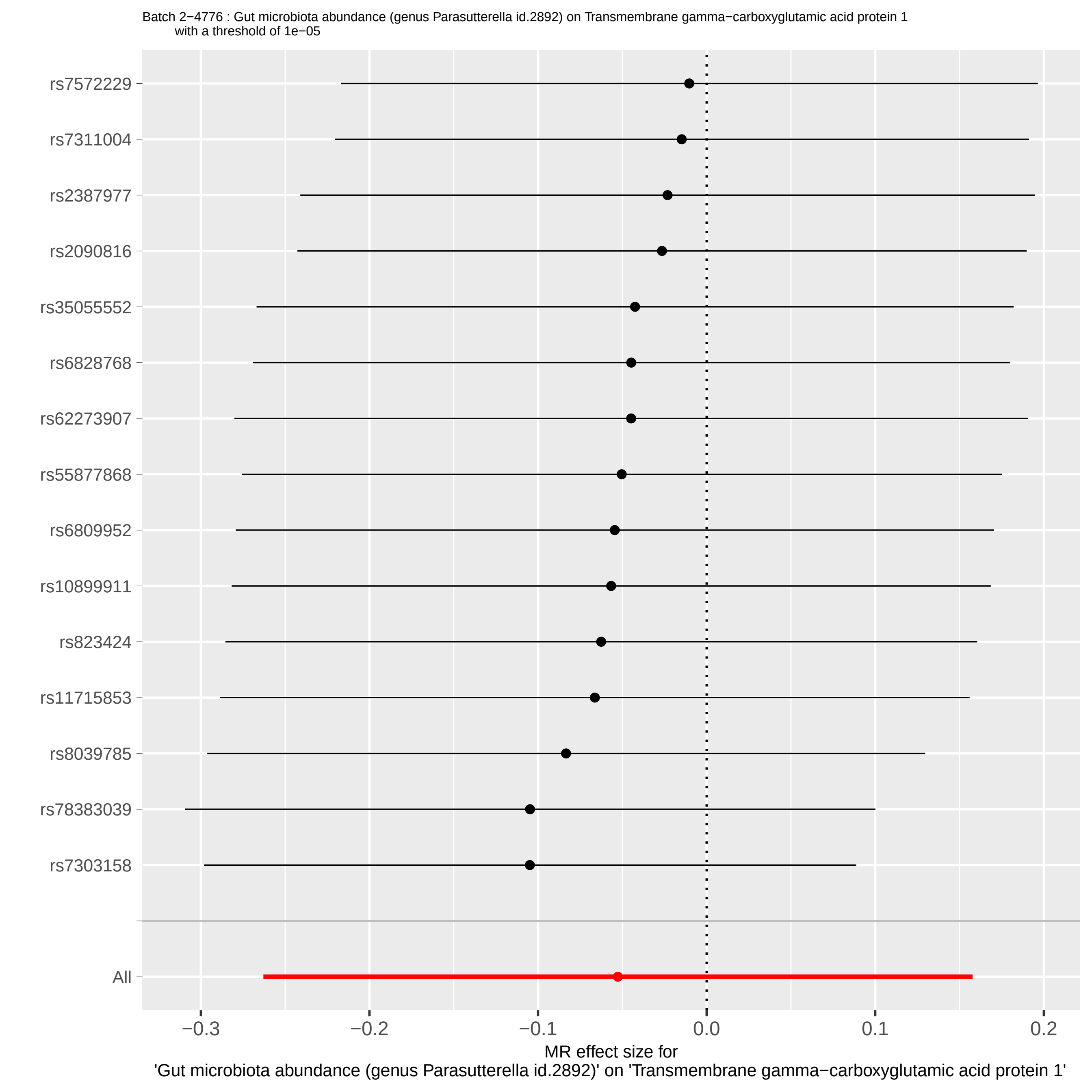

Batch 2−4776 : Gut microbiota abundance (genus Parasutterella id.2892) on Transmembrane gamma−carboxyglutamic acid protein 1 with a threshold of 1e−05
rs7572229
rs7311004
rs2387977
rs2090816
rs35055552
rs6828768
rs62273907
rs55877868
rs6809952
rs10899911
rs823424
rs11715853
rs8039785
rs78383039
rs7303158
All
−0.3
−0.2
−0.1
0.0
0.1
0.2
MR effect size for
'Gut microbiota abundance (genus Parasutterella id.2892)' on 'Transmembrane gamma−carboxyglutamic acid protein 1'

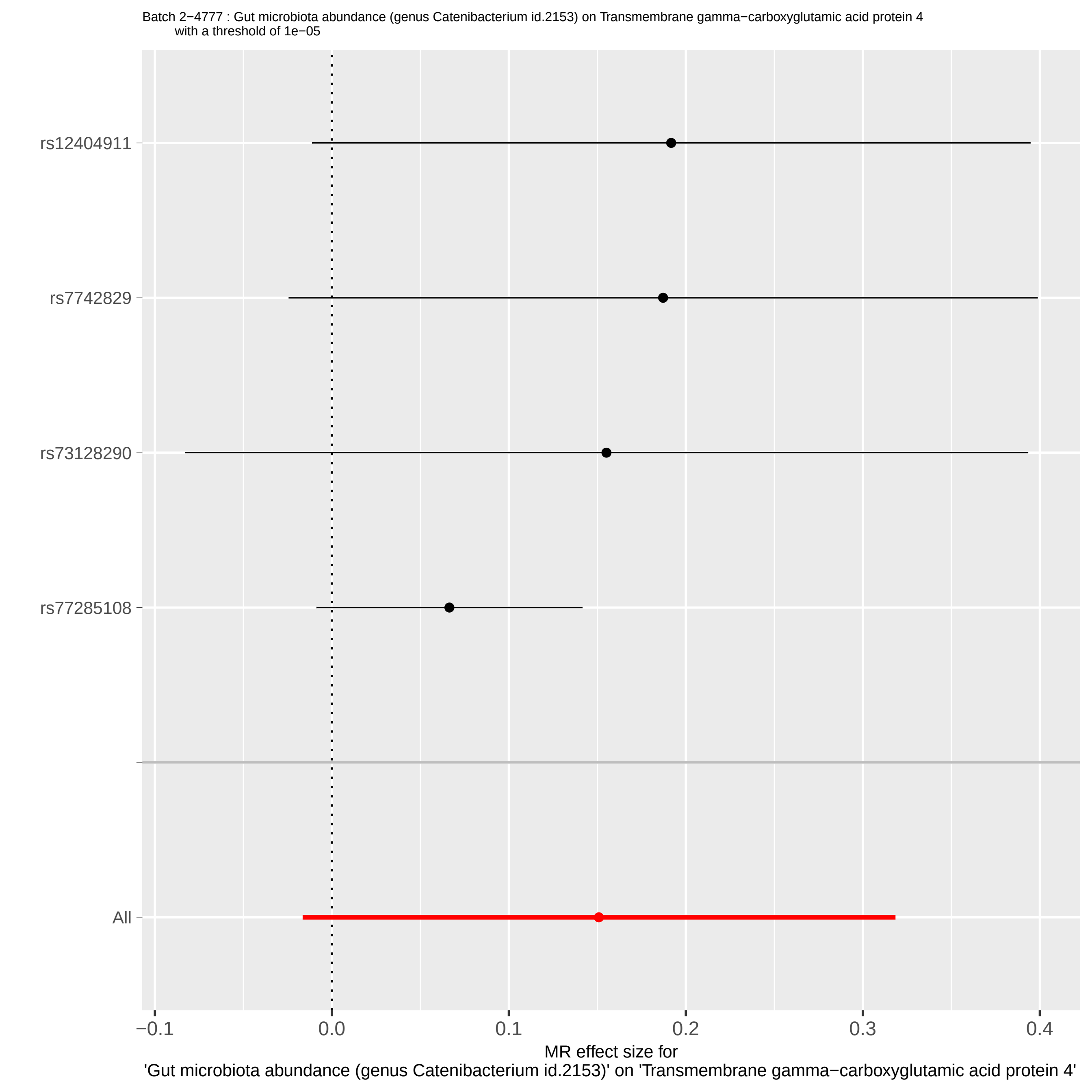
Batch 2−4777 : Gut microbiota abundance (genus Catenibacterium id.2153) on Transmembrane gamma−carboxyglutamic acid protein 4
with a threshold of 1e−05
rs12404911
rs7742829
rs73128290
rs77285108
All
−0.1
0.0
0.1
0.2
0.3
0.4
MR effect size for
'Gut microbiota abundance (genus Catenibacterium id.2153)' on 'Transmembrane gamma−carboxyglutamic acid protein 4'

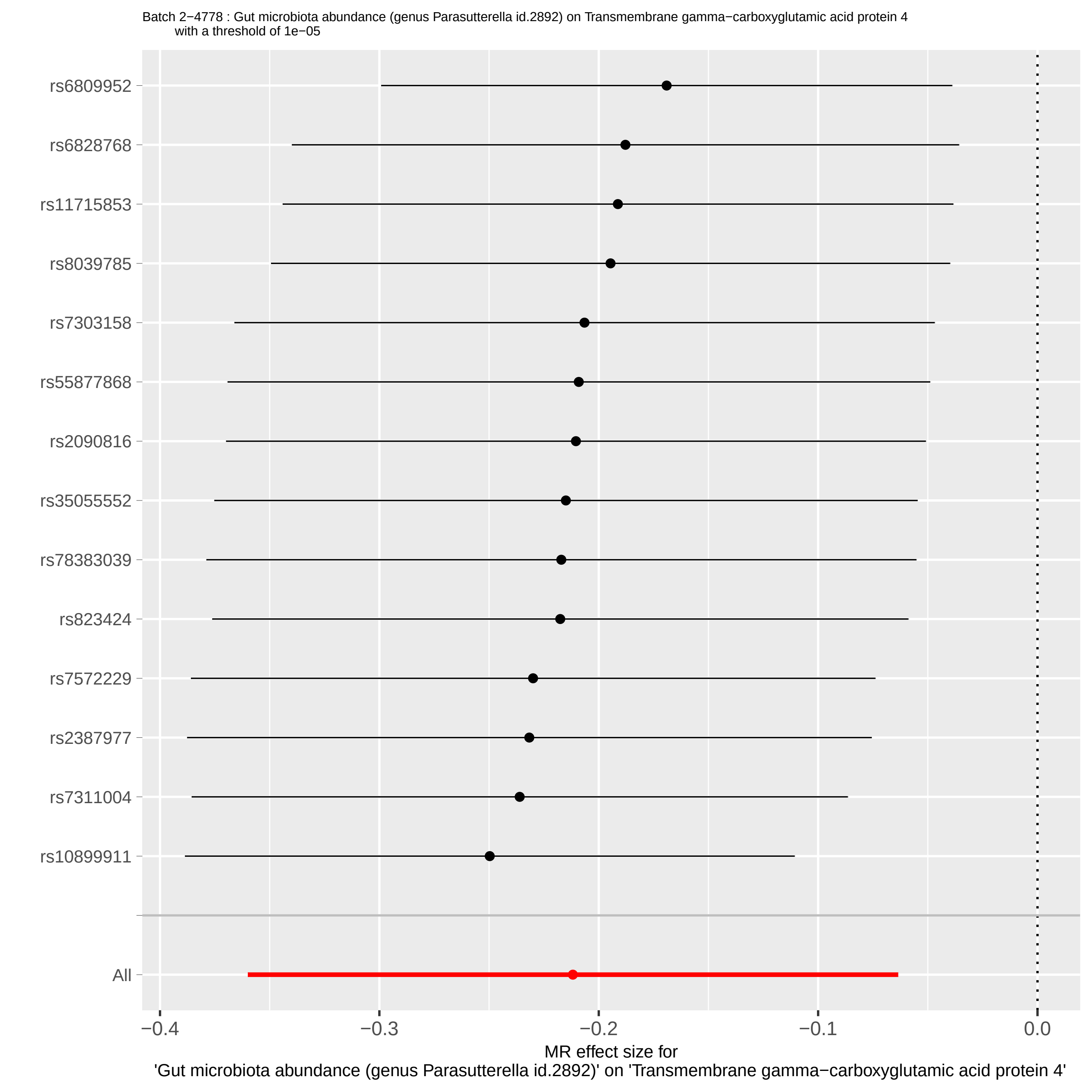
Batch 2−4778 : Gut microbiota abundance (genus Parasutterella id.2892) on Transmembrane gamma−carboxyglutamic acid protein 4
with a threshold of 1e−05
rs6809952
rs6828768
rs11715853
rs8039785
rs7303158
rs55877868
rs2090816
rs35055552
rs78383039
rs823424
rs7572229
rs2387977
rs7311004
rs10899911
All
−0.4
−0.3
−0.2
−0.1
0.0
MR effect size for
'Gut microbiota abundance (genus Parasutterella id.2892)' on 'Transmembrane gamma−carboxyglutamic acid protein 4'

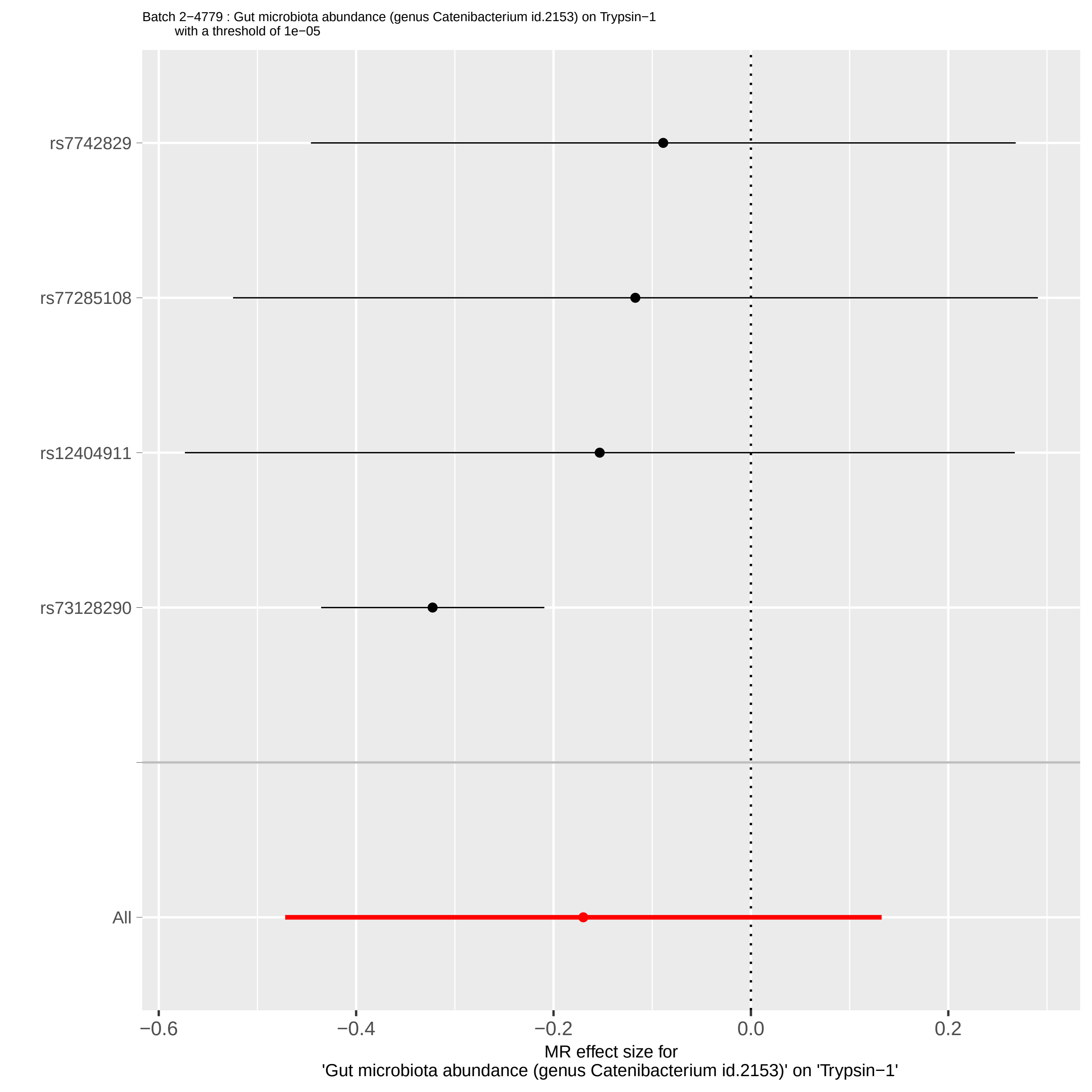
Batch 2−4779 : Gut microbiota abundance (genus Catenibacterium id.2153) on Trypsin−1
with a threshold of 1e−05
rs7742829
rs77285108
rs12404911
rs73128290
All
−0.6
−0.4
−0.2
0.0
0.2
MR effect size for
'Gut microbiota abundance (genus Catenibacterium id.2153)' on 'Trypsin−1'

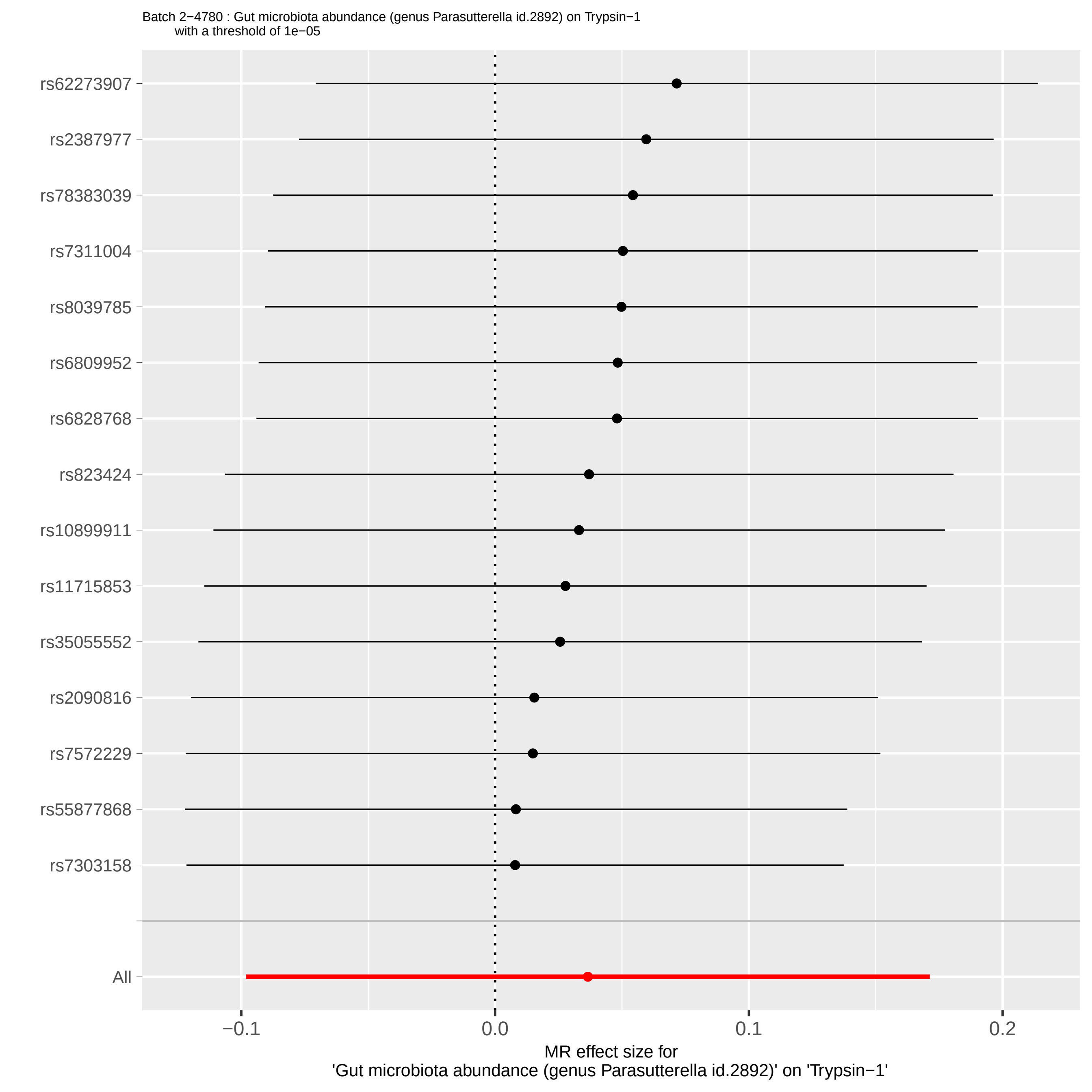

Batch 2−4780 : Gut microbiota abundance (genus Parasutterella id.2892) on Trypsin−1
with a threshold of 1e−05
rs62273907
rs2387977
rs78383039
rs7311004
rs8039785
rs6809952
rs6828768
rs823424
rs10899911
rs11715853
rs35055552
rs2090816
rs7572229
rs55877868
rs7303158
All
−0.1
0.0
0.1
0.2
MR effect size for
'Gut microbiota abundance (genus Parasutterella id.2892)' on 'Trypsin−1'

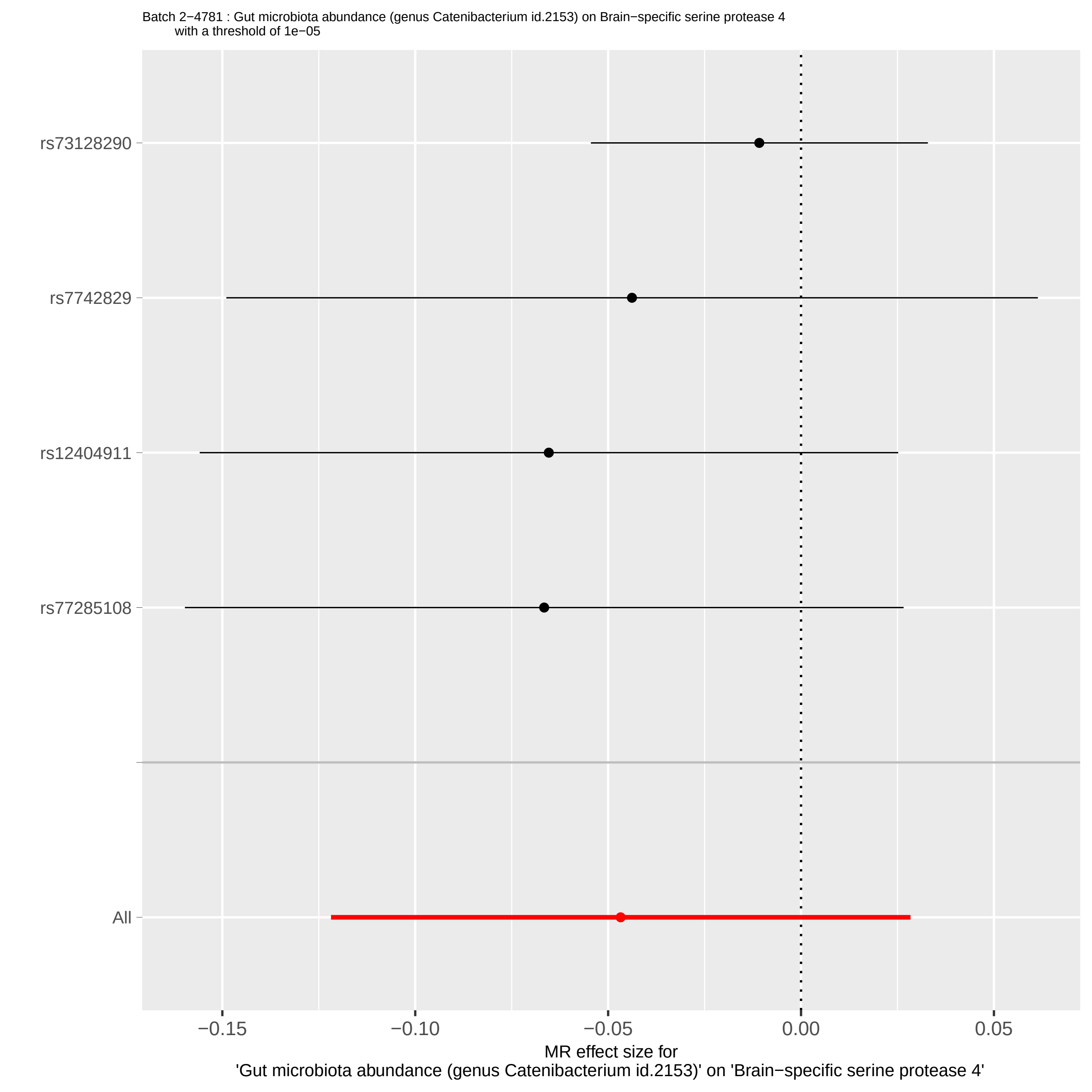
Batch 2−4781 : Gut microbiota abundance (genus Catenibacterium id.2153) on Brain−specific serine protease 4
with a threshold of 1e−05
rs73128290
rs7742829
rs12404911
rs77285108
All
−0.15
−0.10
−0.05
0.00
0.05
MR effect size for
'Gut microbiota abundance (genus Catenibacterium id.2153)' on 'Brain−specific serine protease 4'

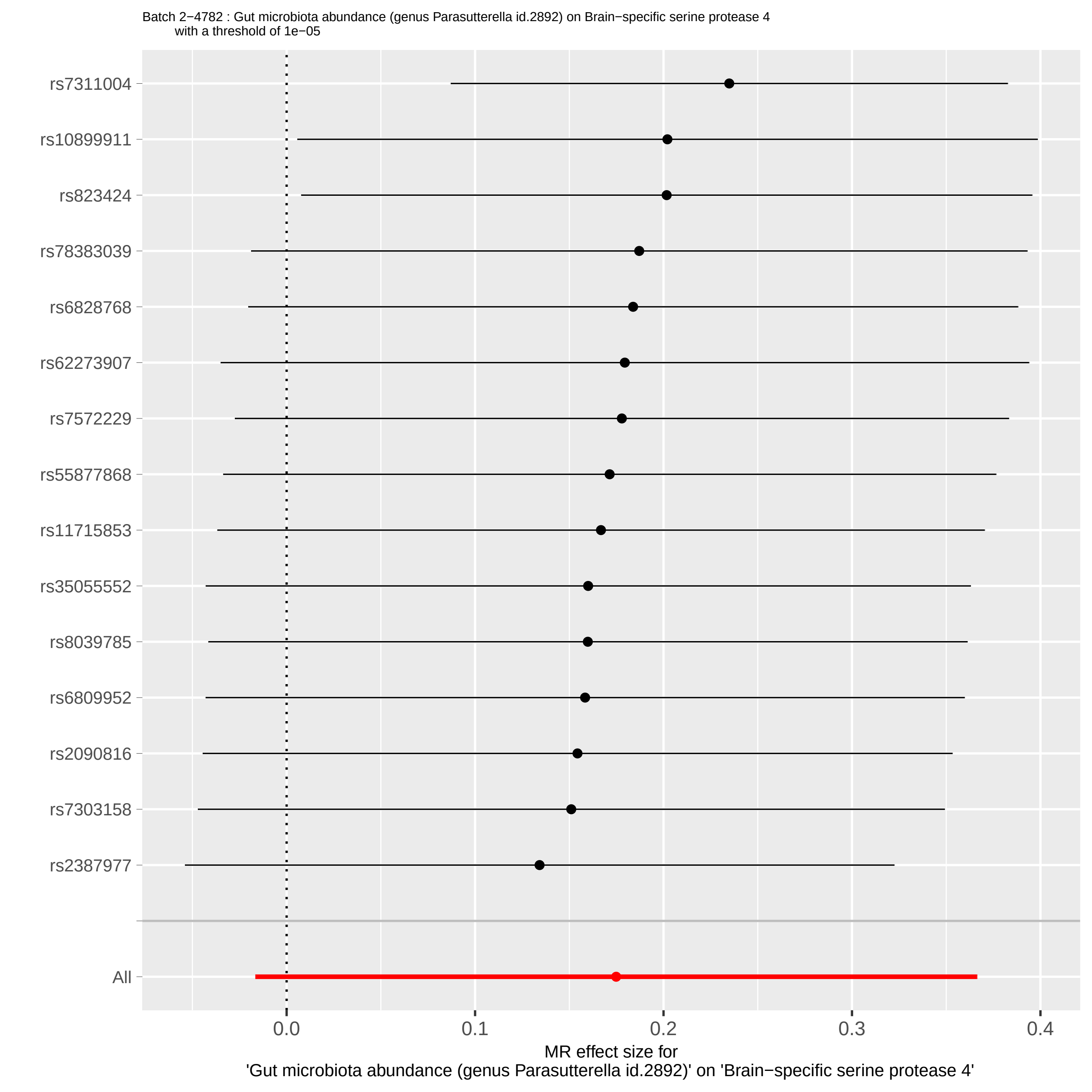

Batch 2−4782 : Gut microbiota abundance (genus Parasutterella id.2892) on Brain−specific serine protease 4
with a threshold of 1e−05
rs7311004
rs10899911
rs823424
rs78383039
rs6828768
rs62273907
rs7572229
rs55877868
rs11715853
rs35055552
rs8039785
rs6809952
rs2090816
rs7303158
rs2387977
All
0.0
0.1
0.2
0.3
0.4
MR effect size for
'Gut microbiota abundance (genus Parasutterella id.2892)' on 'Brain−specific serine protease 4'

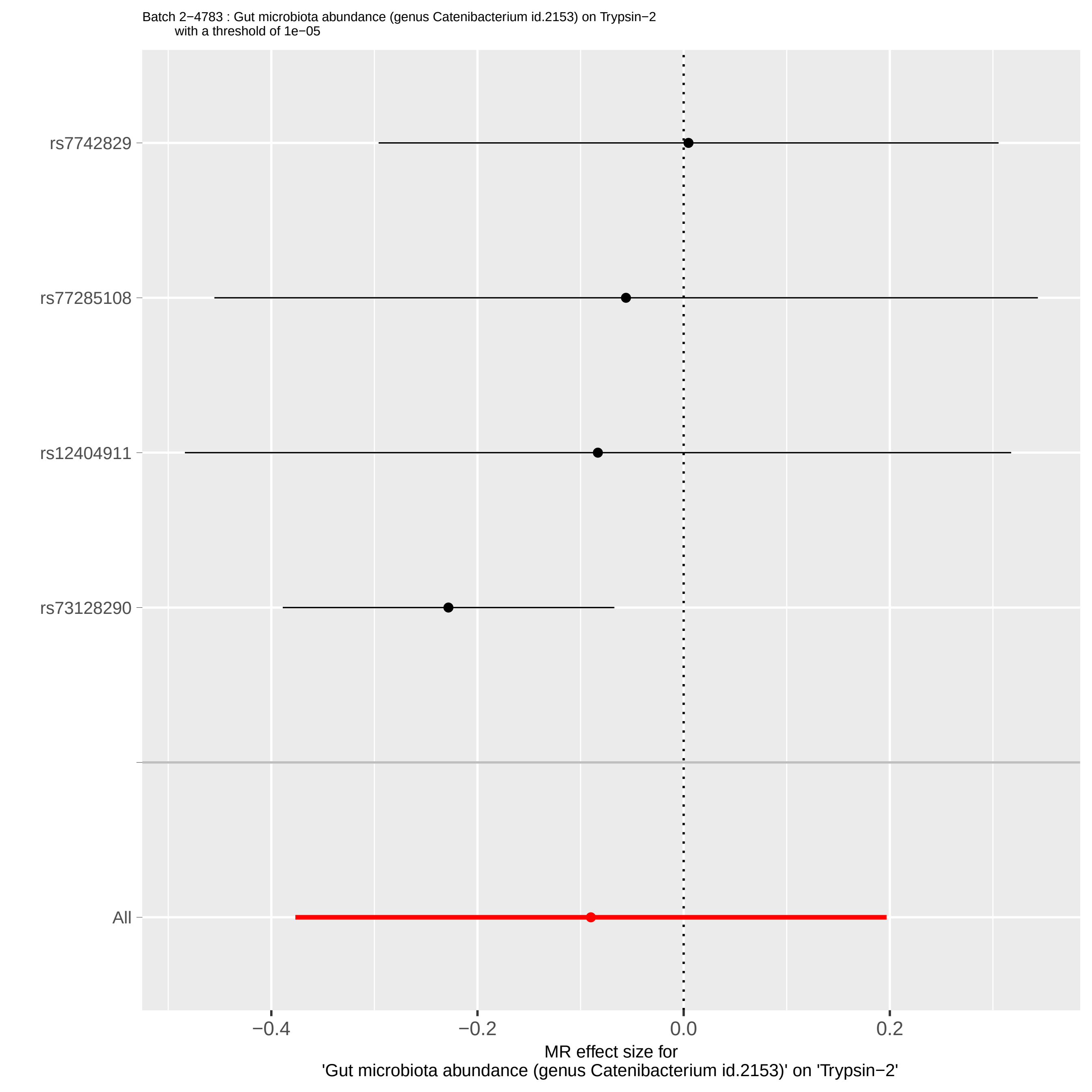
Batch 2−4783 : Gut microbiota abundance (genus Catenibacterium id.2153) on Trypsin−2
with a threshold of 1e−05
rs7742829
rs77285108
rs12404911
rs73128290
All
−0.4
−0.2
0.0
0.2
MR effect size for
'Gut microbiota abundance (genus Catenibacterium id.2153)' on 'Trypsin−2'

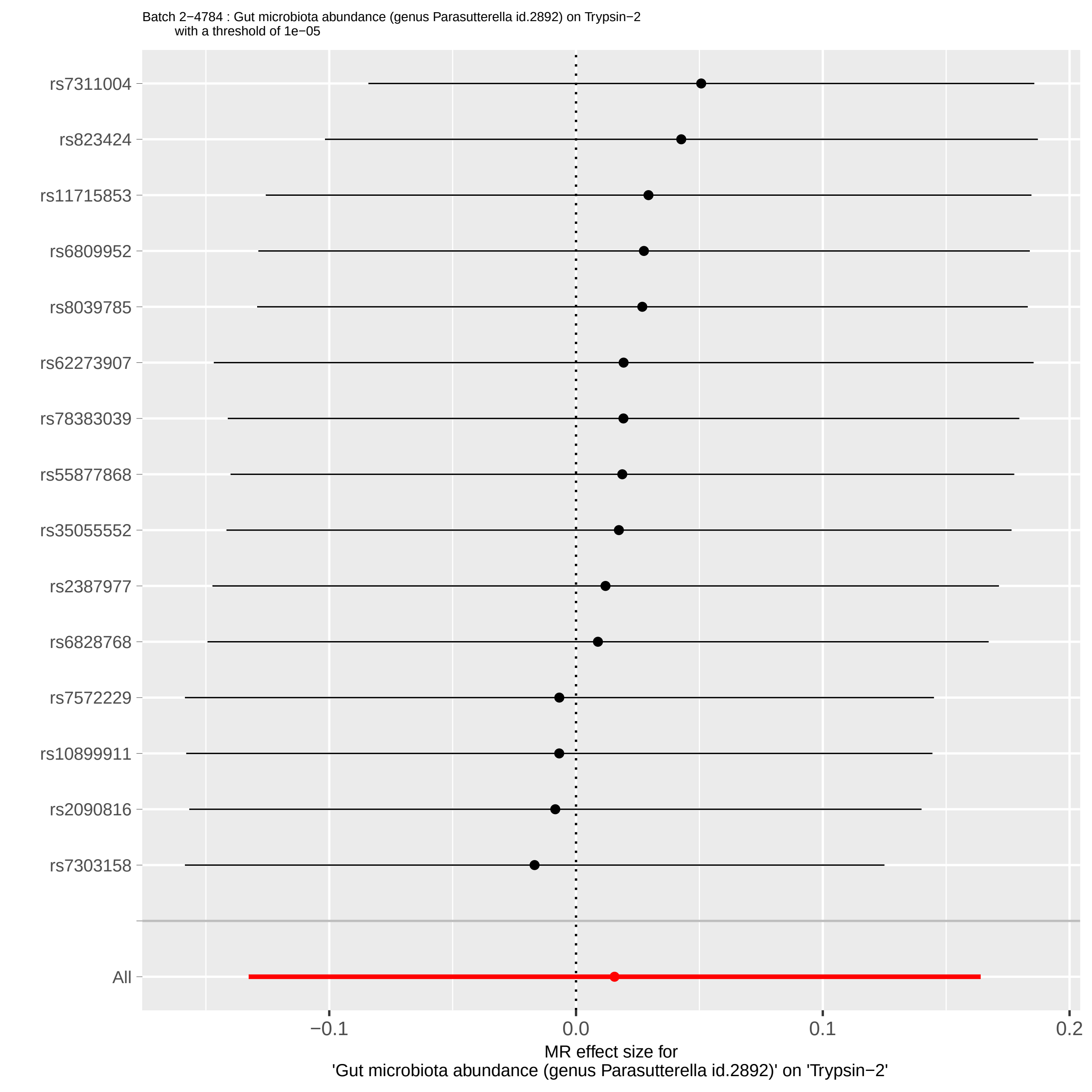

Batch 2−4784 : Gut microbiota abundance (genus Parasutterella id.2892) on Trypsin−2
with a threshold of 1e−05
rs7311004
rs823424
rs11715853
rs6809952
rs8039785
rs62273907
rs78383039
rs55877868
rs35055552
rs2387977
rs6828768
rs7572229
rs10899911
rs2090816
rs7303158
All
−0.1
0.0
0.1
0.2
MR effect size for
'Gut microbiota abundance (genus Parasutterella id.2892)' on 'Trypsin−2'

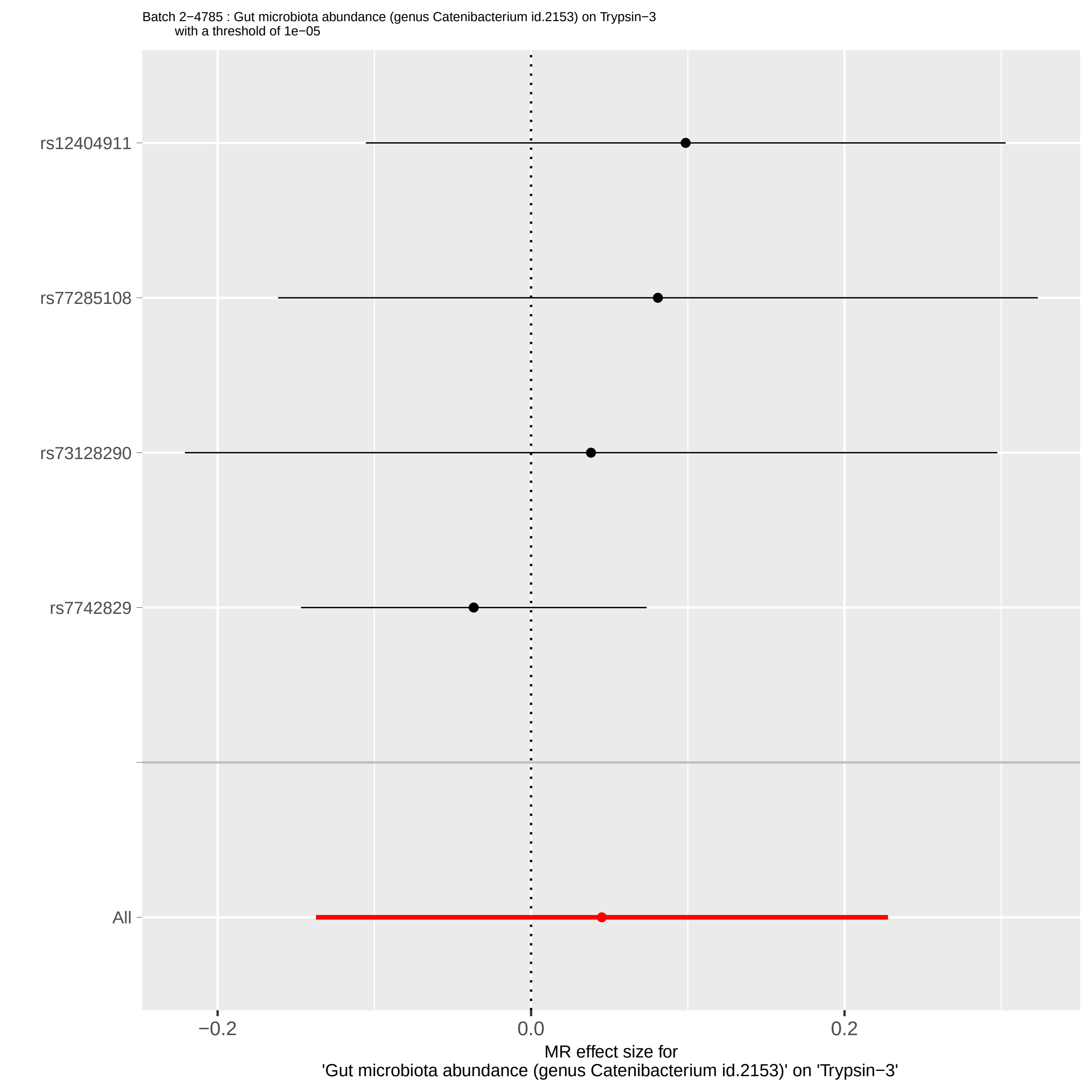
Batch 2−4785 : Gut microbiota abundance (genus Catenibacterium id.2153) on Trypsin−3
with a threshold of 1e−05
rs12404911
rs77285108
rs73128290
rs7742829
All
−0.2
0.0
0.2
MR effect size for
'Gut microbiota abundance (genus Catenibacterium id.2153)' on 'Trypsin−3'

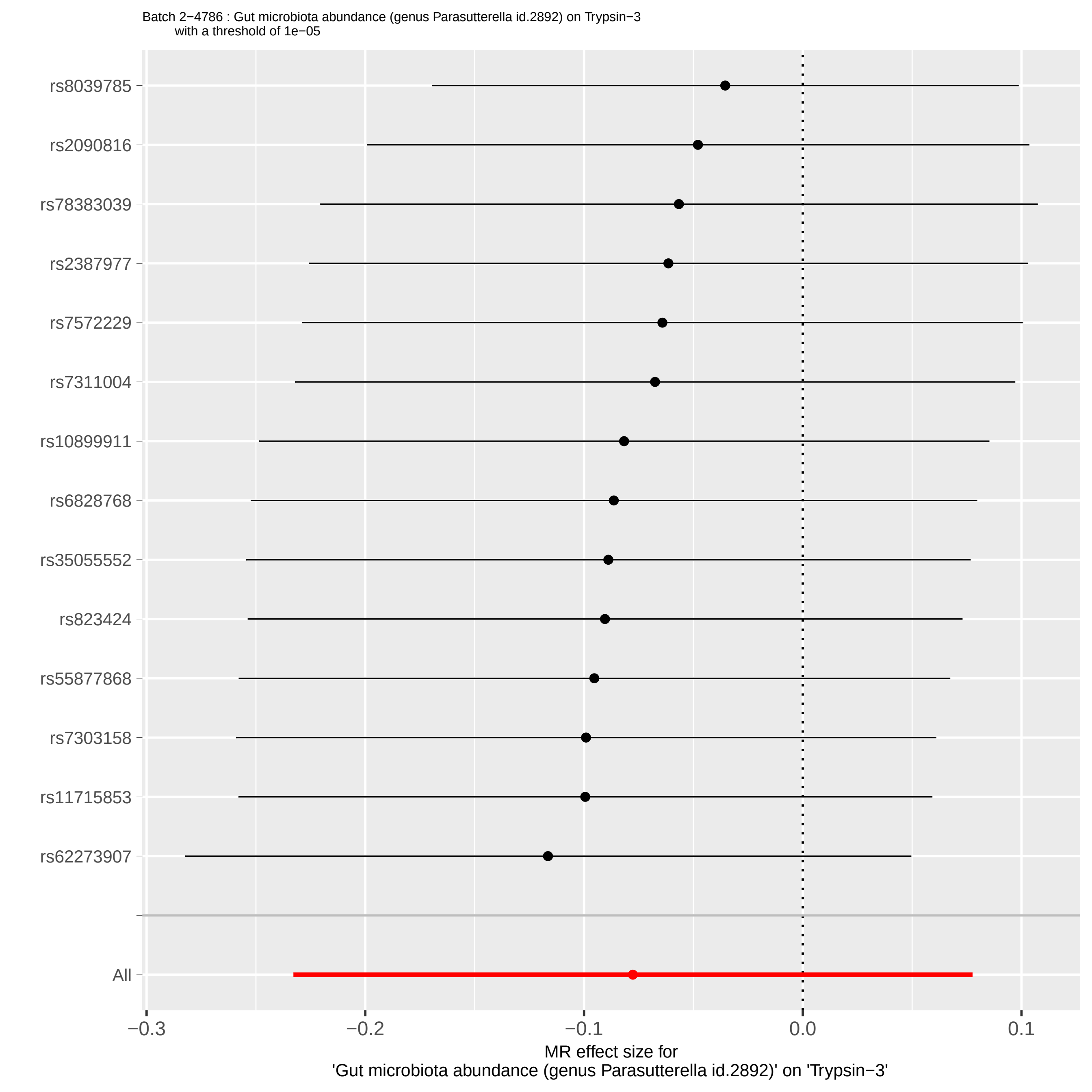
Batch 2−4786 : Gut microbiota abundance (genus Parasutterella id.2892) on Trypsin−3
with a threshold of 1e−05
rs8039785
rs2090816
rs78383039
rs2387977
rs7572229
rs7311004
rs10899911
rs6828768
rs35055552
rs823424
rs55877868
rs7303158
rs11715853
rs62273907
All
−0.3
−0.2
−0.1
0.0
0.1
MR effect size for
'Gut microbiota abundance (genus Parasutterella id.2892)' on 'Trypsin−3'

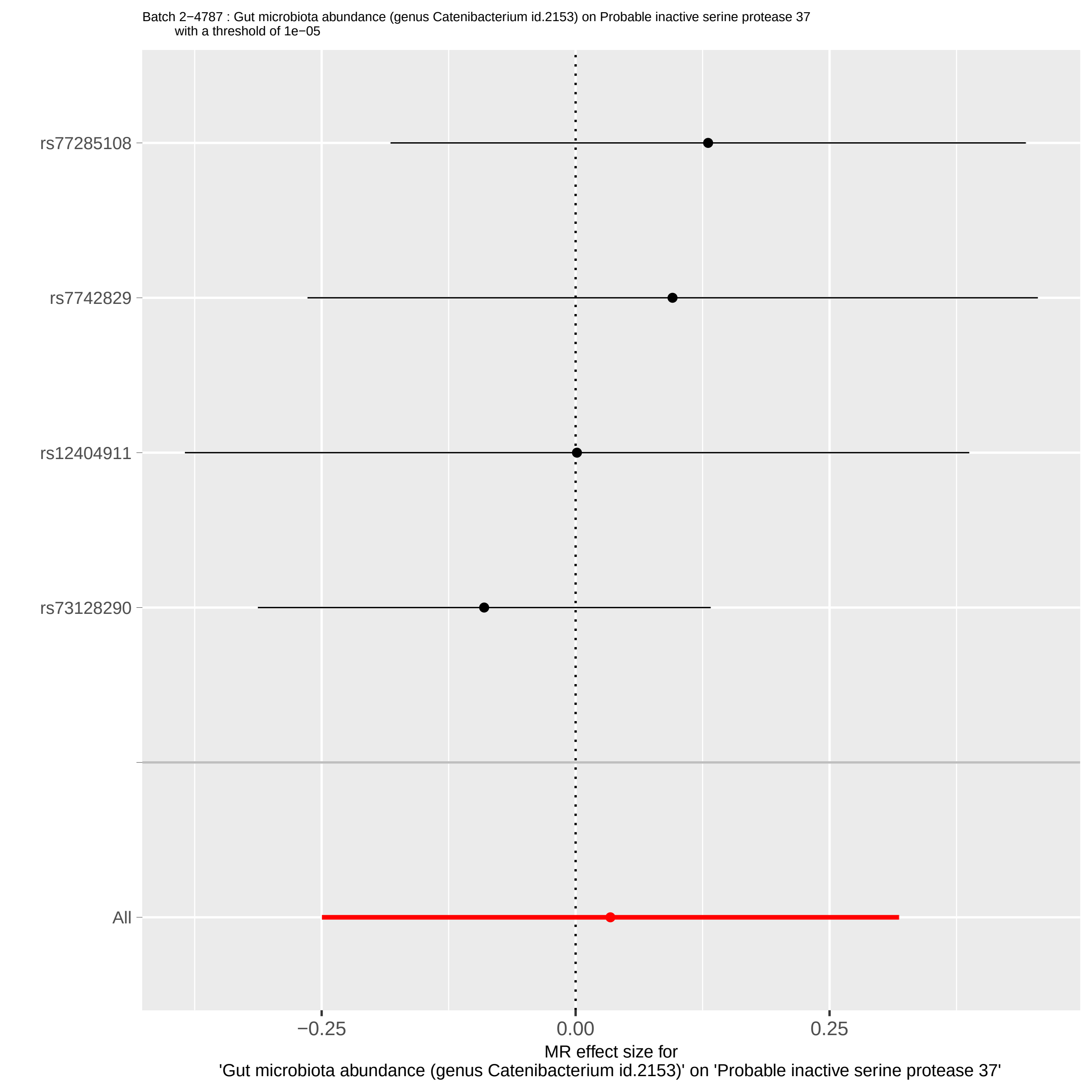
Batch 2−4787 : Gut microbiota abundance (genus Catenibacterium id.2153) on Probable inactive serine protease 37
with a threshold of 1e−05
rs77285108
rs7742829
rs12404911
rs73128290
All
−0.25
0.00
0.25
MR effect size for
'Gut microbiota abundance (genus Catenibacterium id.2153)' on 'Probable inactive serine protease 37'

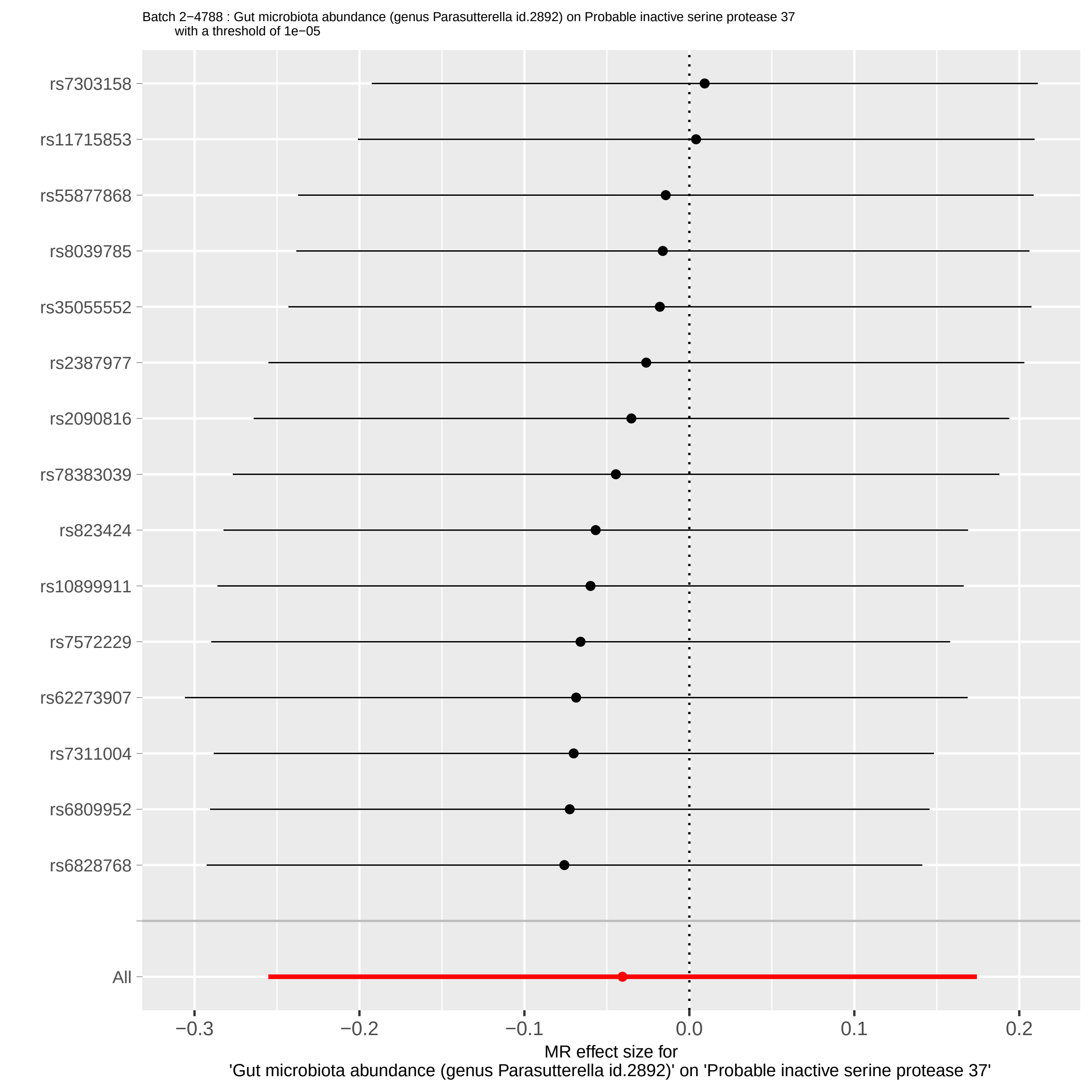

Batch 2−4788 : Gut microbiota abundance (genus Parasutterella id.2892) on Probable inactive serine protease 37
with a threshold of 1e−05
rs7303158
rs11715853
rs55877868
rs8039785
rs35055552
rs2387977
rs2090816
rs78383039
rs823424
rs10899911
rs7572229
rs62273907
rs7311004
rs6809952
rs6828768
All
−0.3
−0.2
−0.1
0.0
0.1
0.2
MR effect size for
'Gut microbiota abundance (genus Parasutterella id.2892)' on 'Probable inactive serine protease 37'

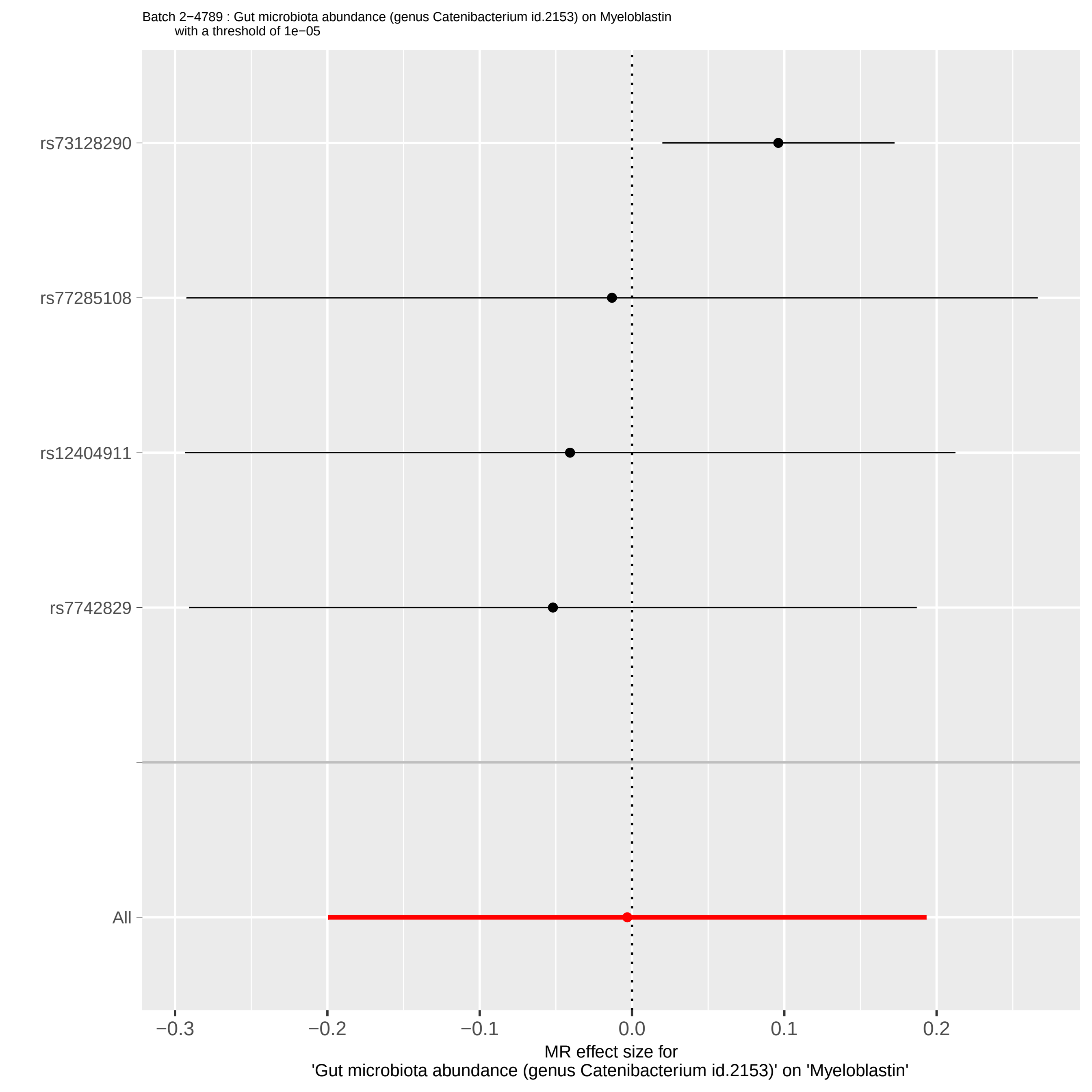

Batch 2−4789 : Gut microbiota abundance (genus Catenibacterium id.2153) on Myeloblastin
with a threshold of 1e−05
rs73128290
rs77285108
rs12404911
rs7742829
All
−0.3
−0.2
−0.1
0.0
0.1
0.2
MR effect size for
'Gut microbiota abundance (genus Catenibacterium id.2153)' on 'Myeloblastin'

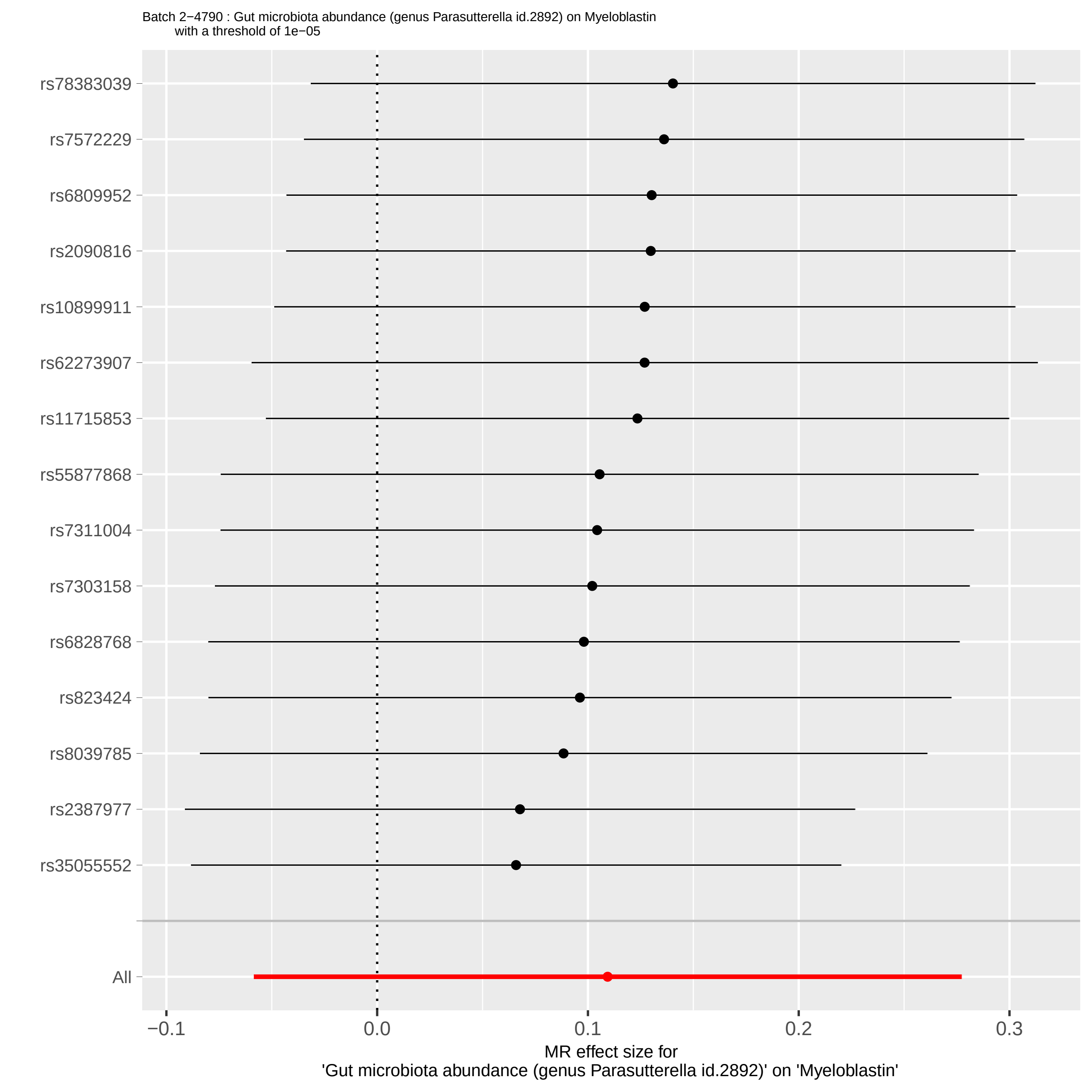

Batch 2−4790 : Gut microbiota abundance (genus Parasutterella id.2892) on Myeloblastin
with a threshold of 1e−05
rs78383039
rs7572229
rs6809952
rs2090816
rs10899911
rs62273907
rs11715853
rs55877868
rs7311004
rs7303158
rs6828768
rs823424
rs8039785
rs2387977
rs35055552
All
−0.1
0.0
0.1
0.2
0.3
MR effect size for
'Gut microbiota abundance (genus Parasutterella id.2892)' on 'Myeloblastin'

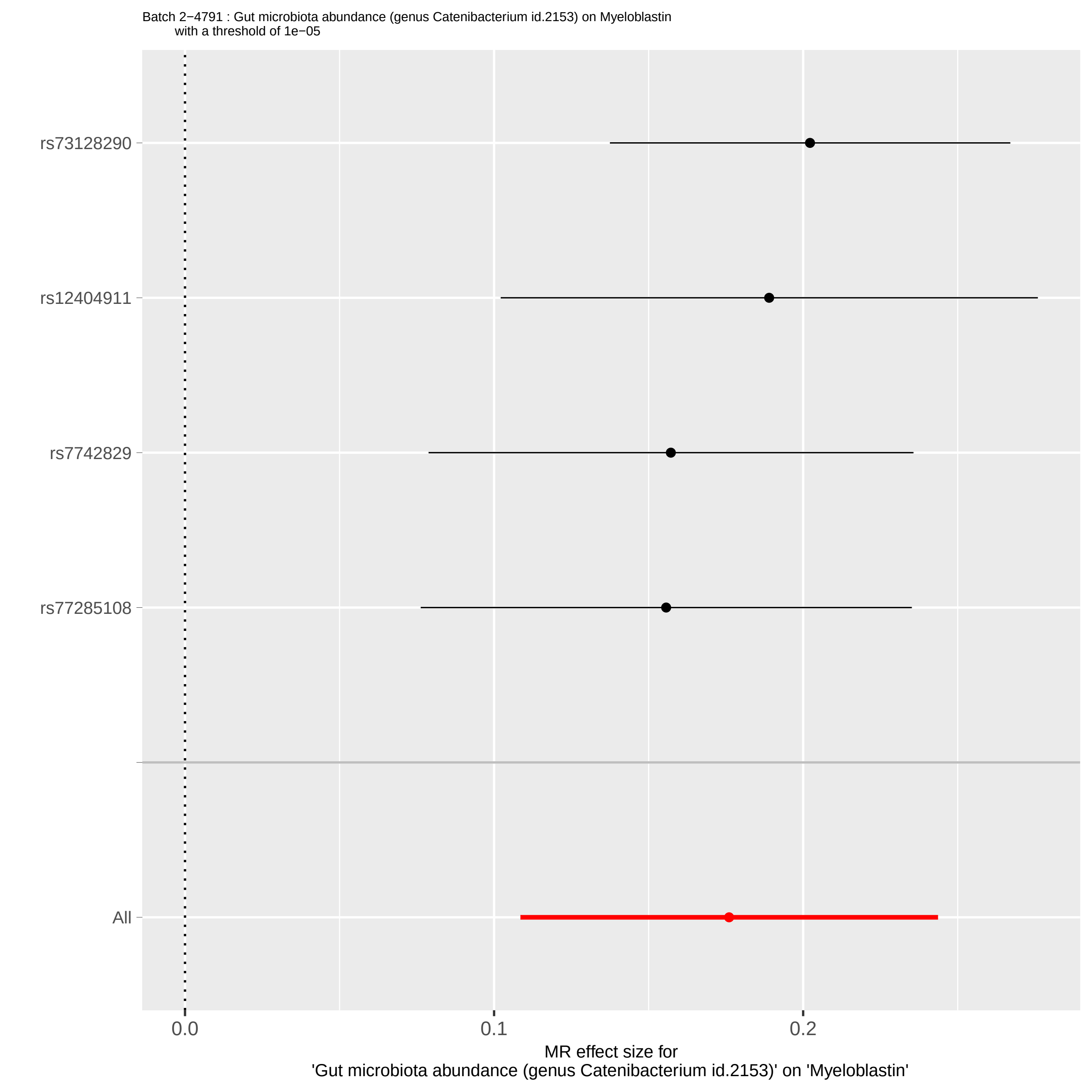
Batch 2−4791 : Gut microbiota abundance (genus Catenibacterium id.2153) on Myeloblastin
with a threshold of 1e−05
rs73128290
rs12404911
rs7742829
rs77285108
All
0.0
0.1
0.2
MR effect size for
'Gut microbiota abundance (genus Catenibacterium id.2153)' on 'Myeloblastin'

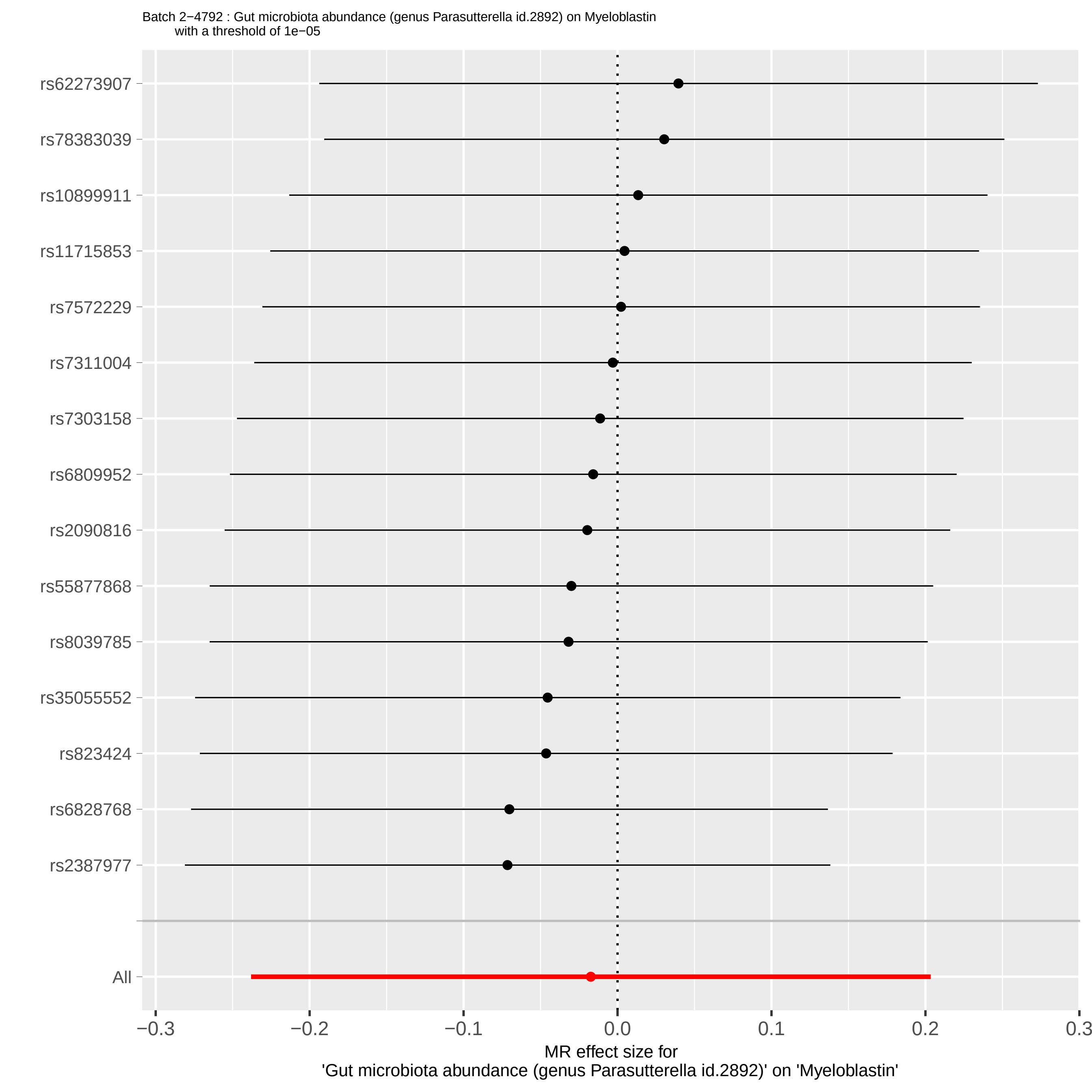

Batch 2−4792 : Gut microbiota abundance (genus Parasutterella id.2892) on Myeloblastin
with a threshold of 1e−05
rs62273907
rs78383039
rs10899911
rs11715853
rs7572229
rs7311004
rs7303158
rs6809952
rs2090816
rs55877868
rs8039785
rs35055552
rs823424
rs6828768
rs2387977
All
−0.3
−0.2
−0.1
0.0
0.1
0.2
0.3
MR effect size for
'Gut microbiota abundance (genus Parasutterella id.2892)' on 'Myeloblastin'

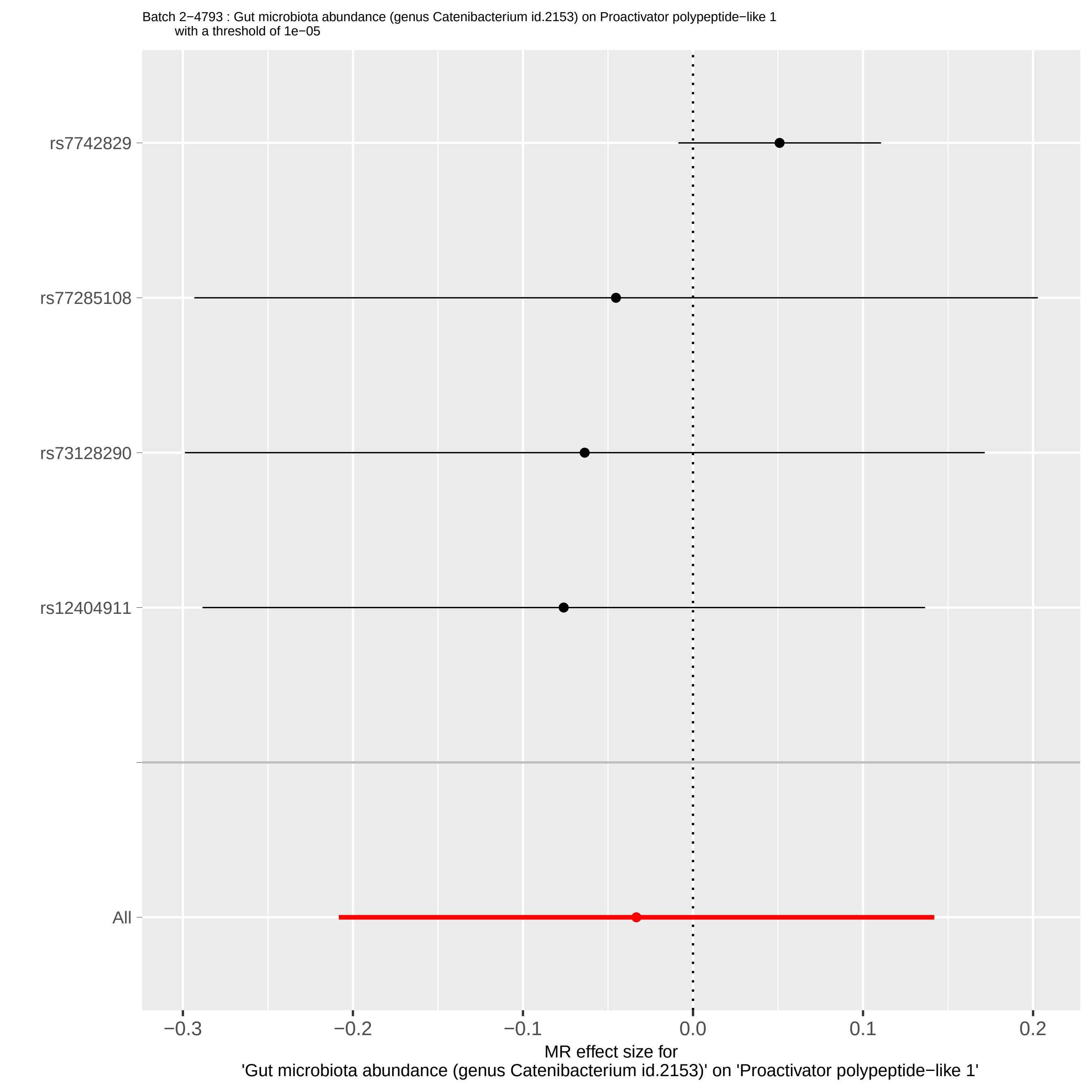
Batch 2−4793 : Gut microbiota abundance (genus Catenibacterium id.2153) on Proactivator polypeptide−like 1
with a threshold of 1e−05
rs7742829
rs77285108
rs73128290
rs12404911
All
−0.3
−0.2
−0.1
0.0
0.1
0.2
MR effect size for
'Gut microbiota abundance (genus Catenibacterium id.2153)' on 'Proactivator polypeptide−like 1'

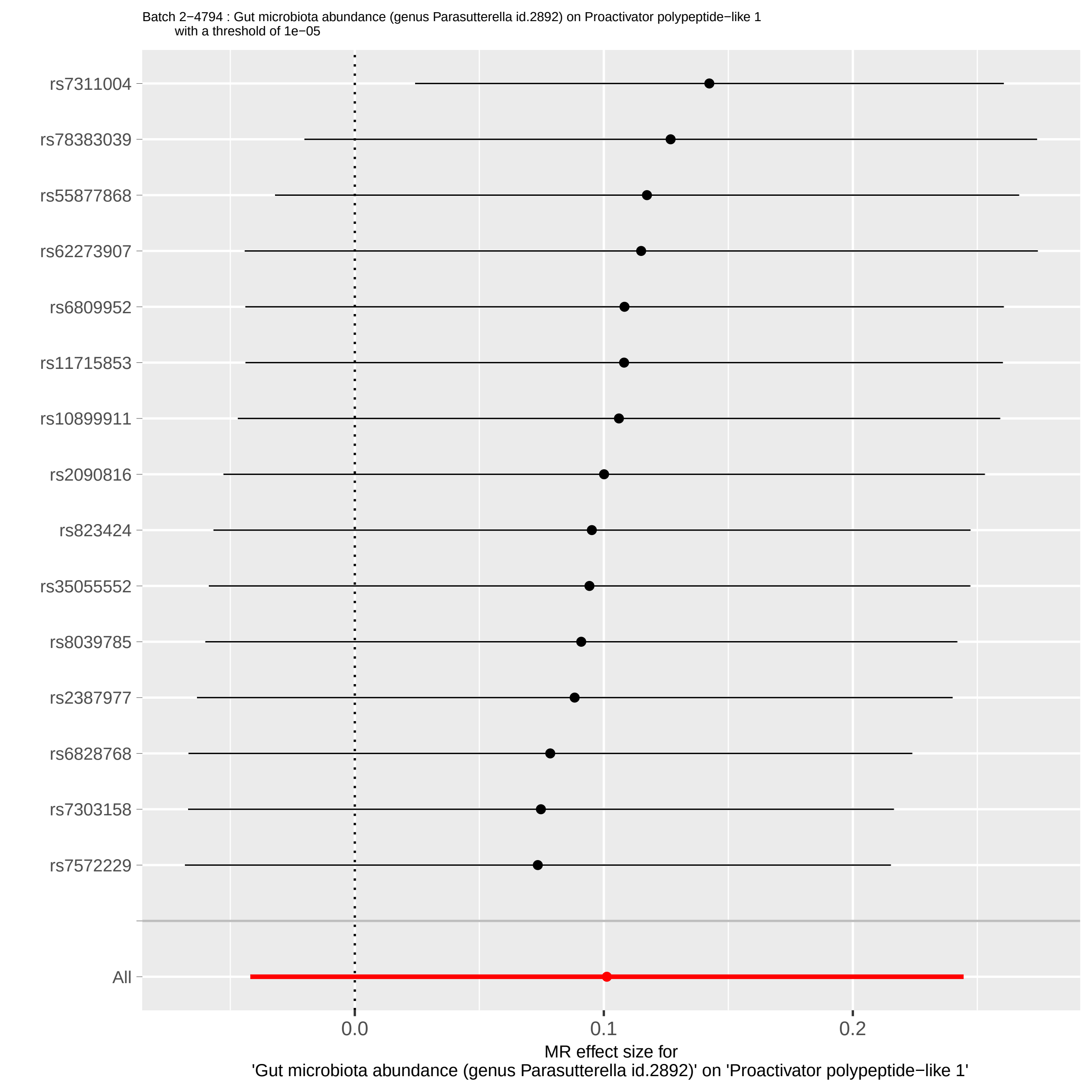

Batch 2−4794 : Gut microbiota abundance (genus Parasutterella id.2892) on Proactivator polypeptide−like 1
with a threshold of 1e−05
rs7311004
rs78383039
rs55877868
rs62273907
rs6809952
rs11715853
rs10899911
rs2090816
rs823424
rs35055552
rs8039785
rs2387977
rs6828768
rs7303158
rs7572229
All
0.0
0.1
0.2
MR effect size for
'Gut microbiota abundance (genus Parasutterella id.2892)' on 'Proactivator polypeptide−like 1'

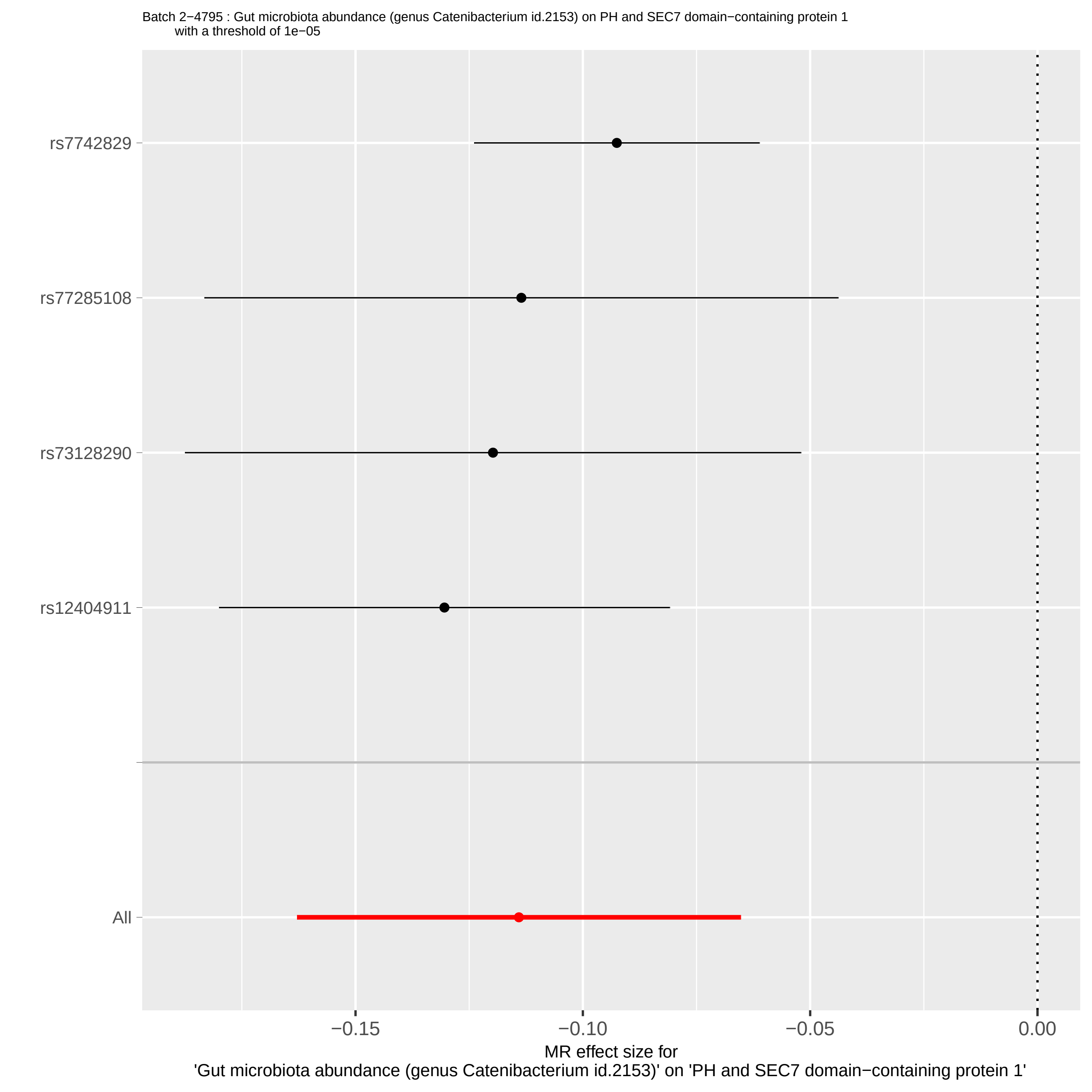

Batch 2−4795 : Gut microbiota abundance (genus Catenibacterium id.2153) on PH and SEC7 domain−containing protein 1
with a threshold of 1e−05
rs7742829
rs77285108
rs73128290
rs12404911
All
−0.15
−0.10
−0.05
0.00
MR effect size for
'Gut microbiota abundance (genus Catenibacterium id.2153)' on 'PH and SEC7 domain−containing protein 1'

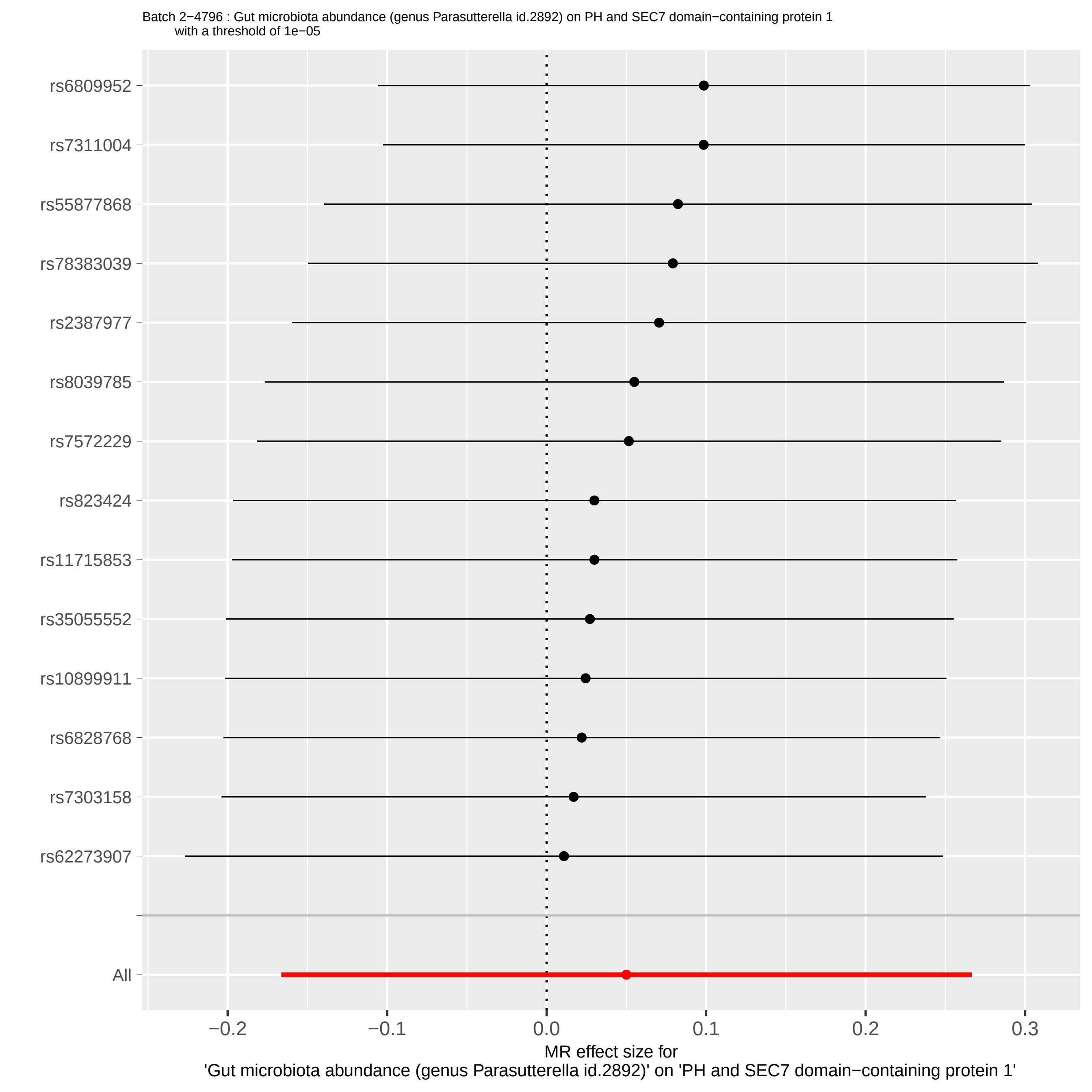
Batch 2−4796 : Gut microbiota abundance (genus Parasutterella id.2892) on PH and SEC7 domain−containing protein 1
with a threshold of 1e−05
rs6809952
rs7311004
rs55877868
rs78383039
rs2387977
rs8039785
rs7572229
rs823424
rs11715853
rs35055552
rs10899911
rs6828768
rs7303158
rs62273907
All
−0.2
−0.1
0.0
0.1
0.2
0.3
MR effect size for
'Gut microbiota abundance (genus Parasutterella id.2892)' on 'PH and SEC7 domain−containing protein 1'

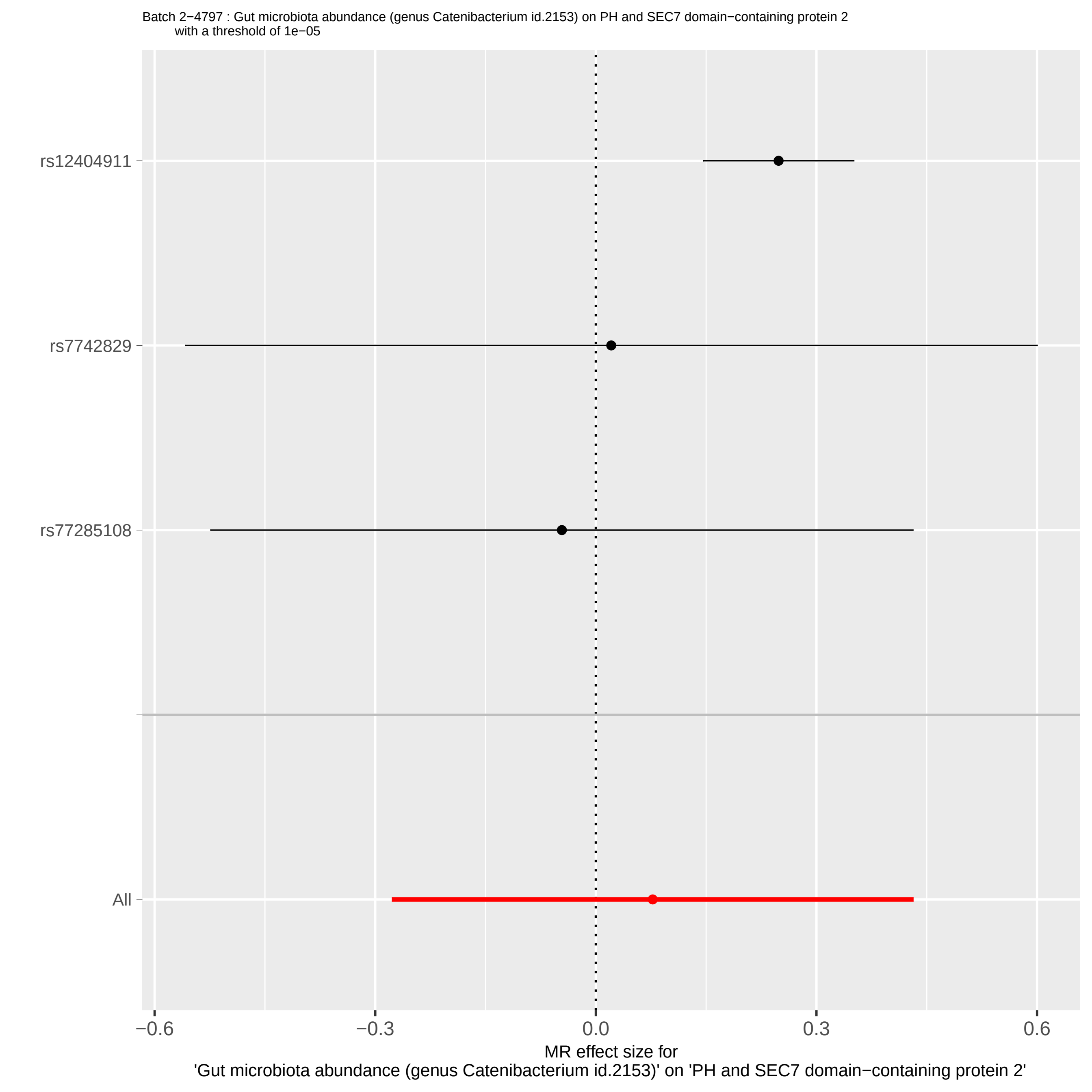
Batch 2−4797 : Gut microbiota abundance (genus Catenibacterium id.2153) on PH and SEC7 domain−containing protein 2
with a threshold of 1e−05
rs12404911
rs7742829
rs77285108
All
−0.6
−0.3
0.0
0.3
0.6
MR effect size for
'Gut microbiota abundance (genus Catenibacterium id.2153)' on 'PH and SEC7 domain−containing protein 2'

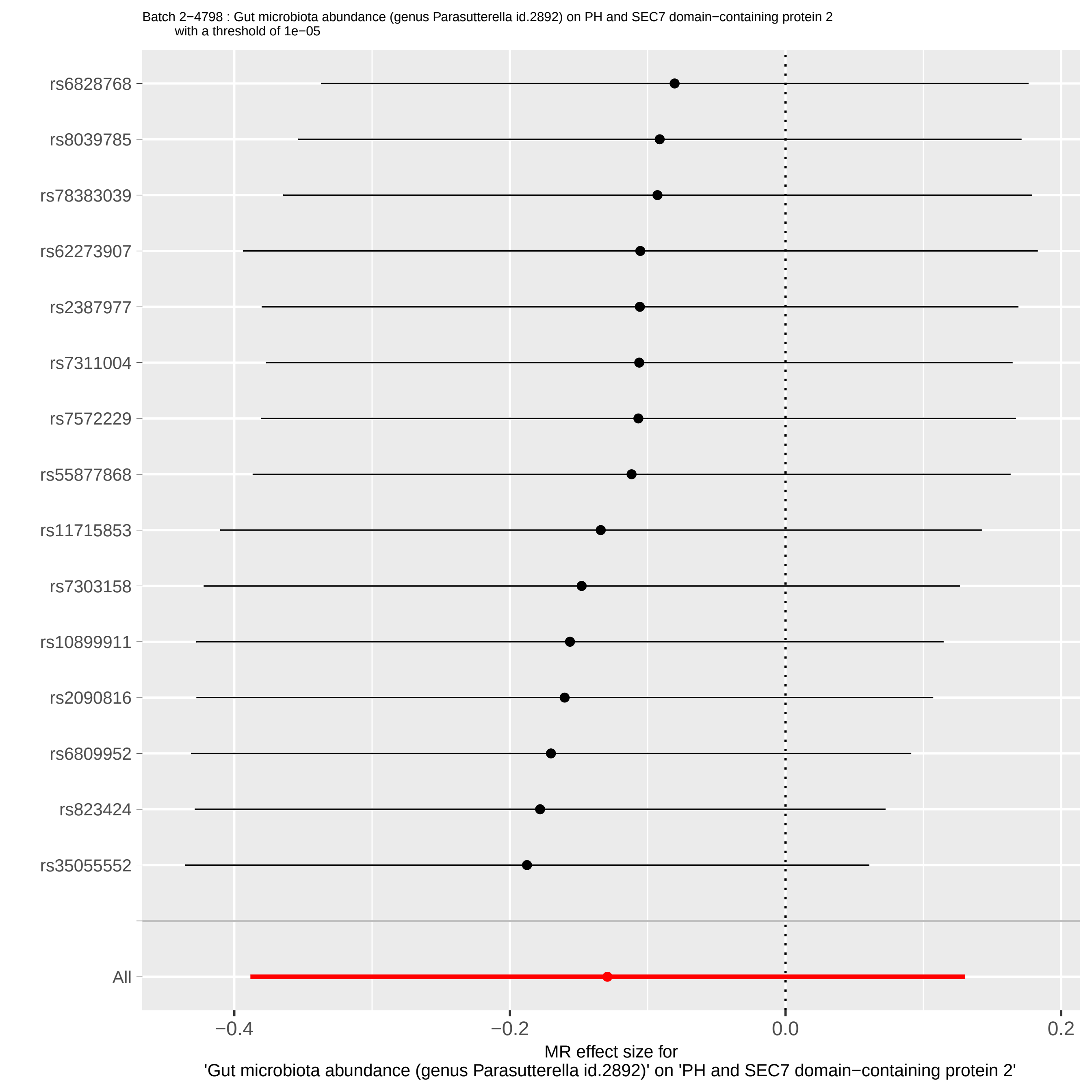

Batch 2−4798 : Gut microbiota abundance (genus Parasutterella id.2892) on PH and SEC7 domain−containing protein 2
with a threshold of 1e−05
rs6828768
rs8039785
rs78383039
rs62273907
rs2387977
rs7311004
rs7572229
rs55877868
rs11715853
rs7303158
rs10899911
rs2090816
rs6809952
rs823424
rs35055552
All
−0.4
−0.2
0.0
0.2
MR effect size for
'Gut microbiota abundance (genus Parasutterella id.2892)' on 'PH and SEC7 domain−containing protein 2'

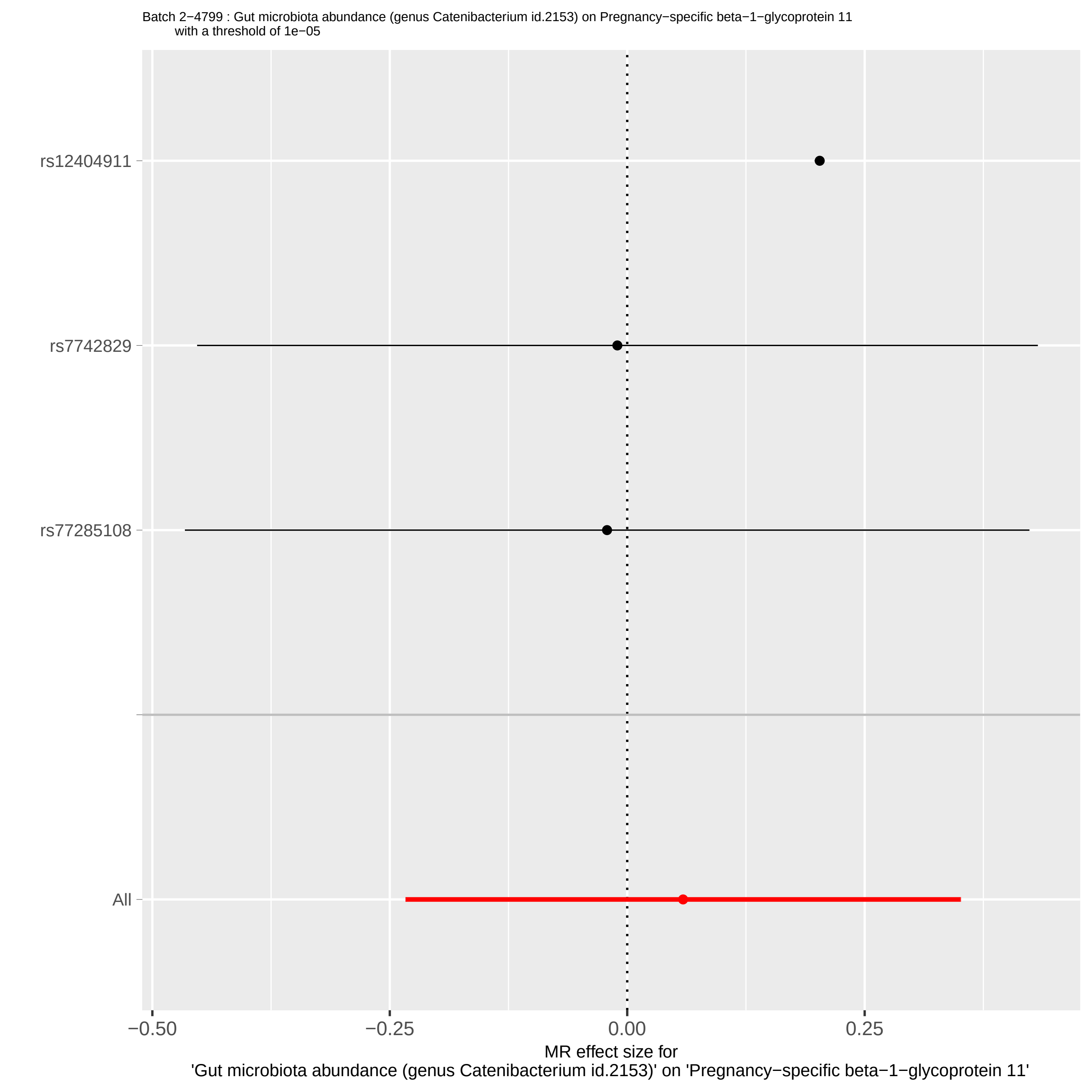
Batch 2−4799 : Gut microbiota abundance (genus Catenibacterium id.2153) on Pregnancy−specific beta−1−glycoprotein 11
with a threshold of 1e−05
rs12404911
rs7742829
rs77285108
All
−0.50
−0.25
0.00
0.25
MR effect size for
'Gut microbiota abundance (genus Catenibacterium id.2153)' on 'Pregnancy−specific beta−1−glycoprotein 11'

Batch 2−4800 : Gut microbiota abundance (genus Parasutterella id.2892) on Pregnancy−specific beta−1−glycoprotein 11
with a threshold of 1e−05

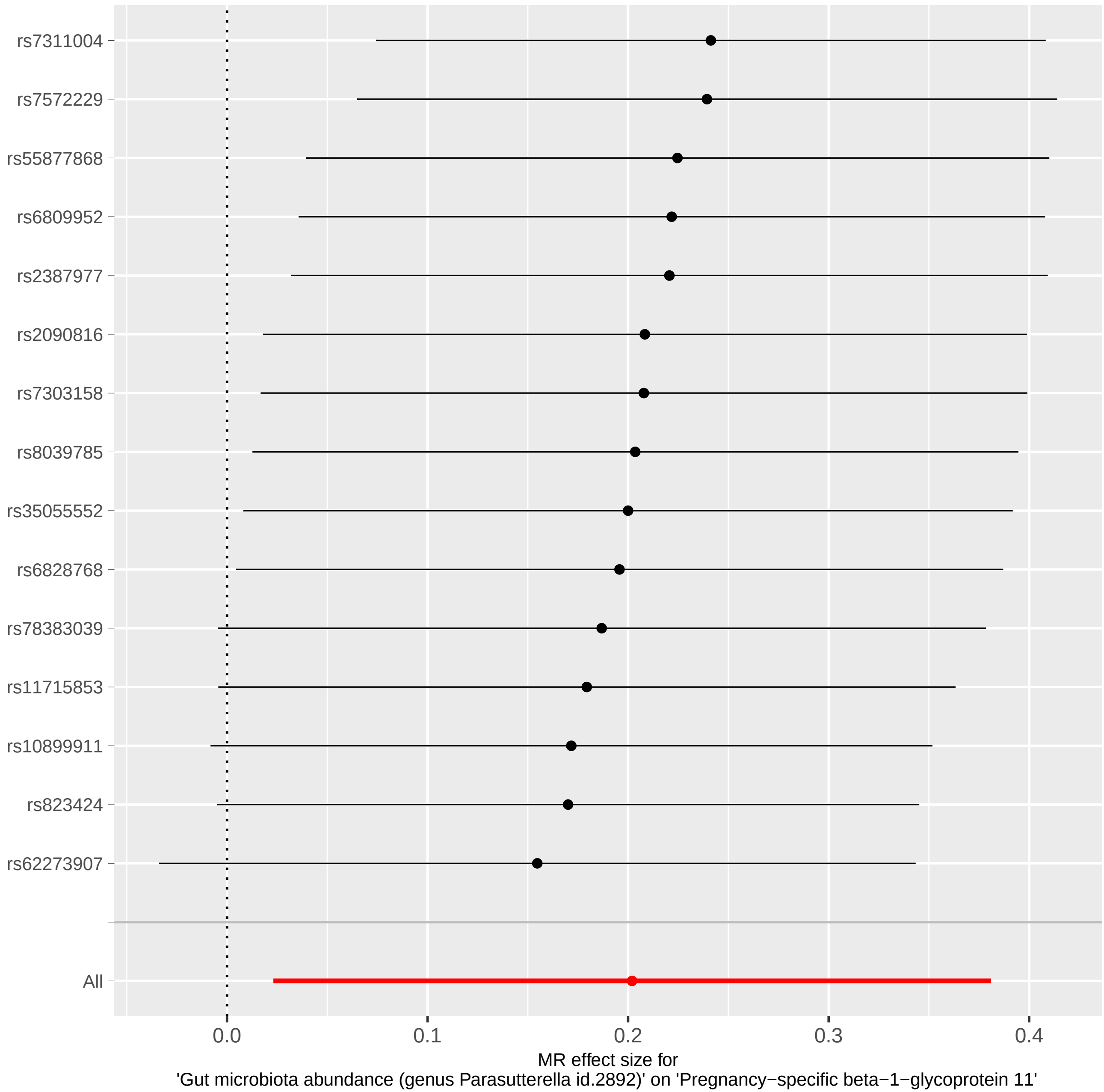

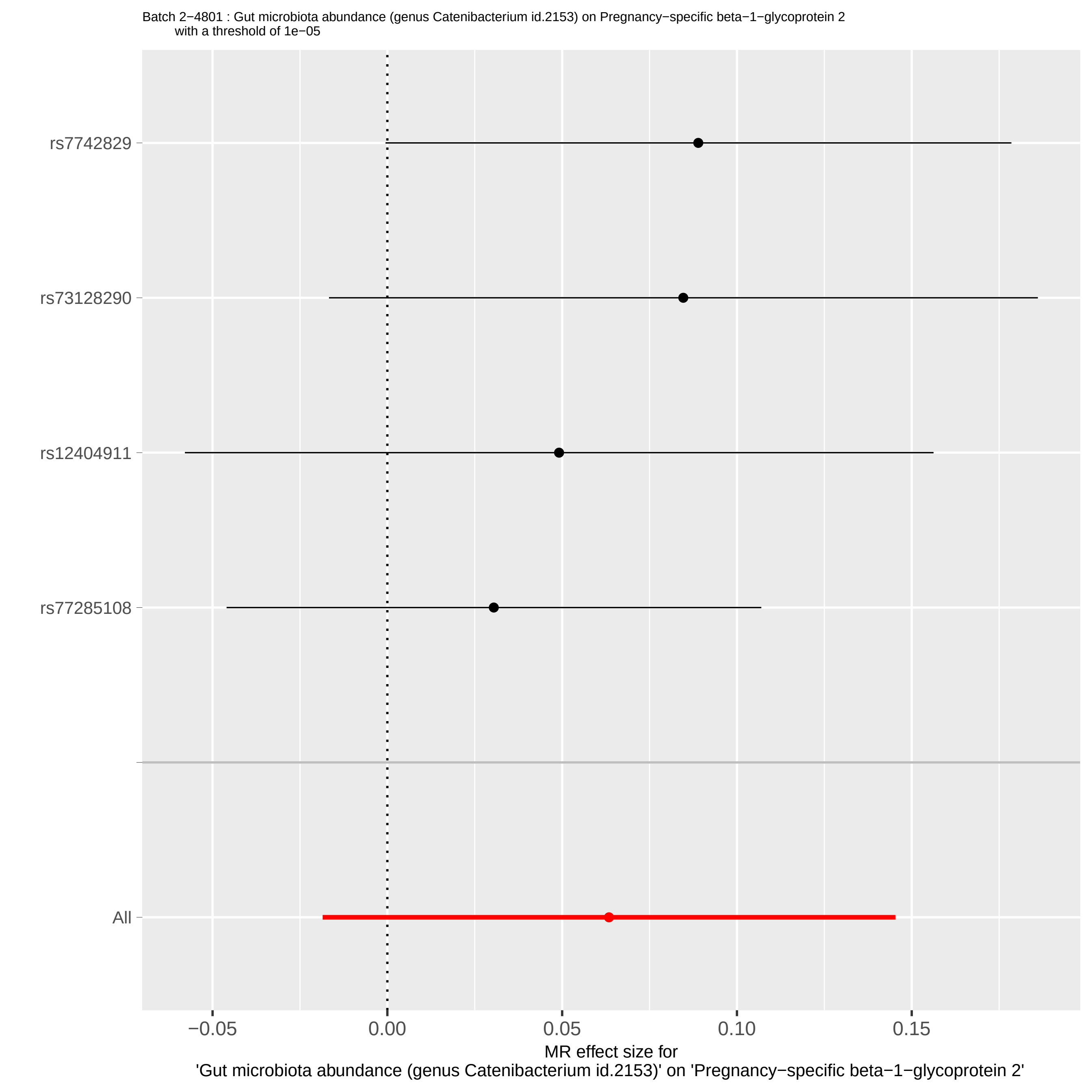

Batch 2−4801 : Gut microbiota abundance (genus Catenibacterium id.2153) on Pregnancy−specific beta−1−glycoprotein 2
with a threshold of 1e−05
rs7742829
rs73128290
rs12404911
rs77285108
All
−0.05
0.00
0.05
0.10
0.15
MR effect size for
'Gut microbiota abundance (genus Catenibacterium id.2153)' on 'Pregnancy−specific beta−1−glycoprotein 2'

Batch 2−4802 : Gut microbiota abundance (genus Parasutterella id.2892) on Pregnancy−specific beta−1−glycoprotein 2
with a threshold of 1e−05

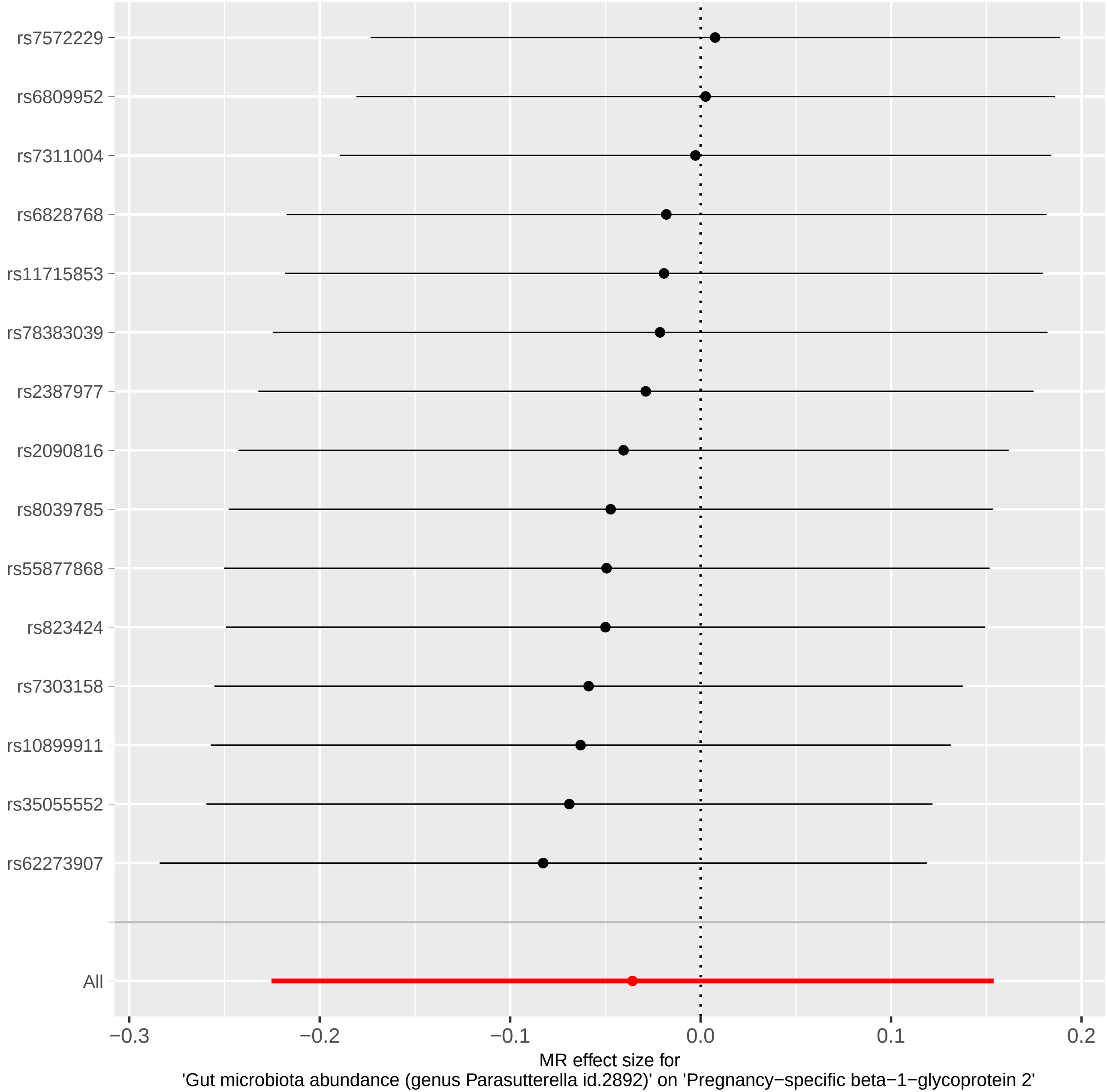

Batch 2−4803 : Gut microbiota abundance (genus Catenibacterium id.2153) on Pregnancy−specific beta−1−glycoprotein 3
with a threshold of 1e−05

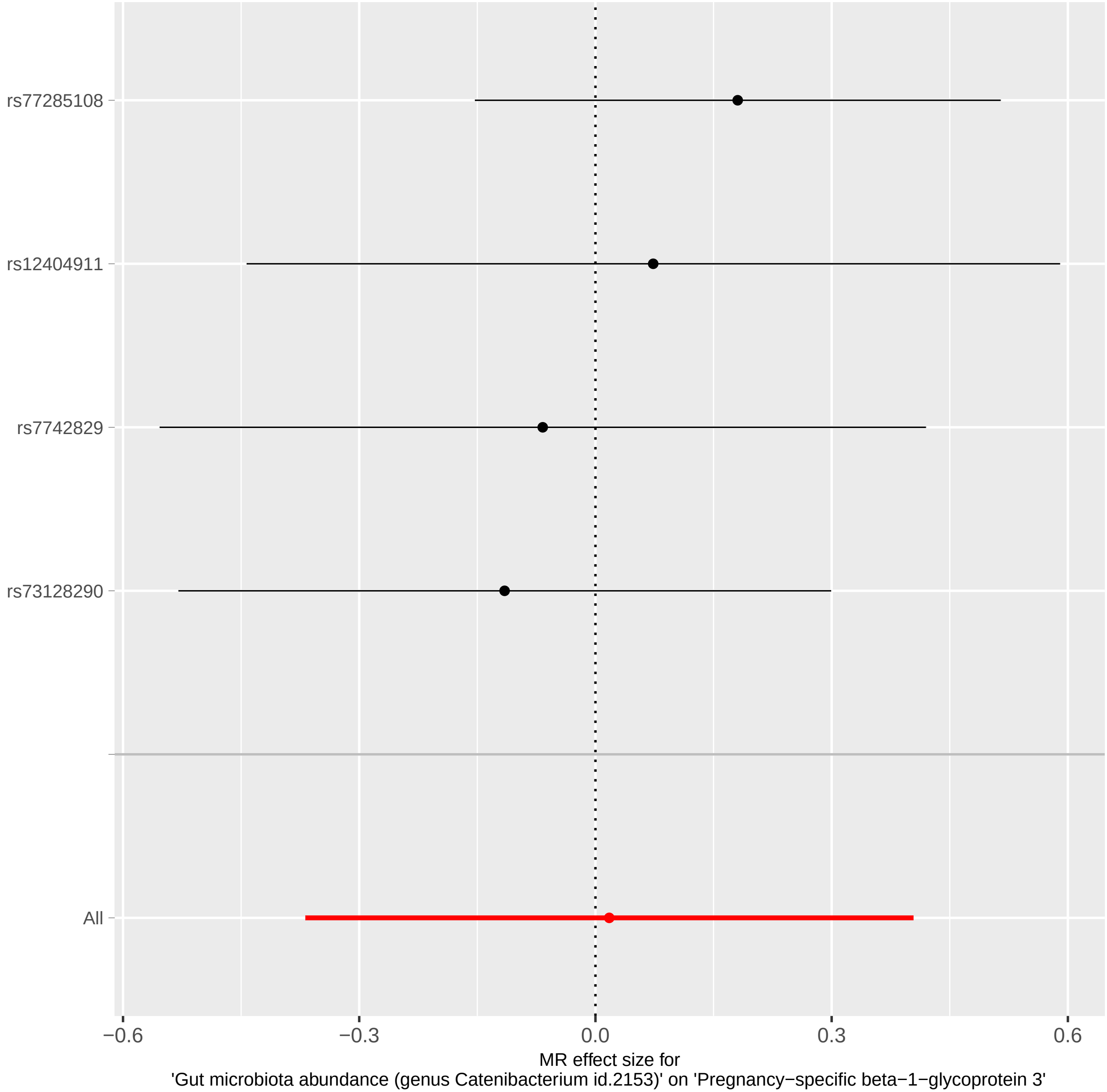

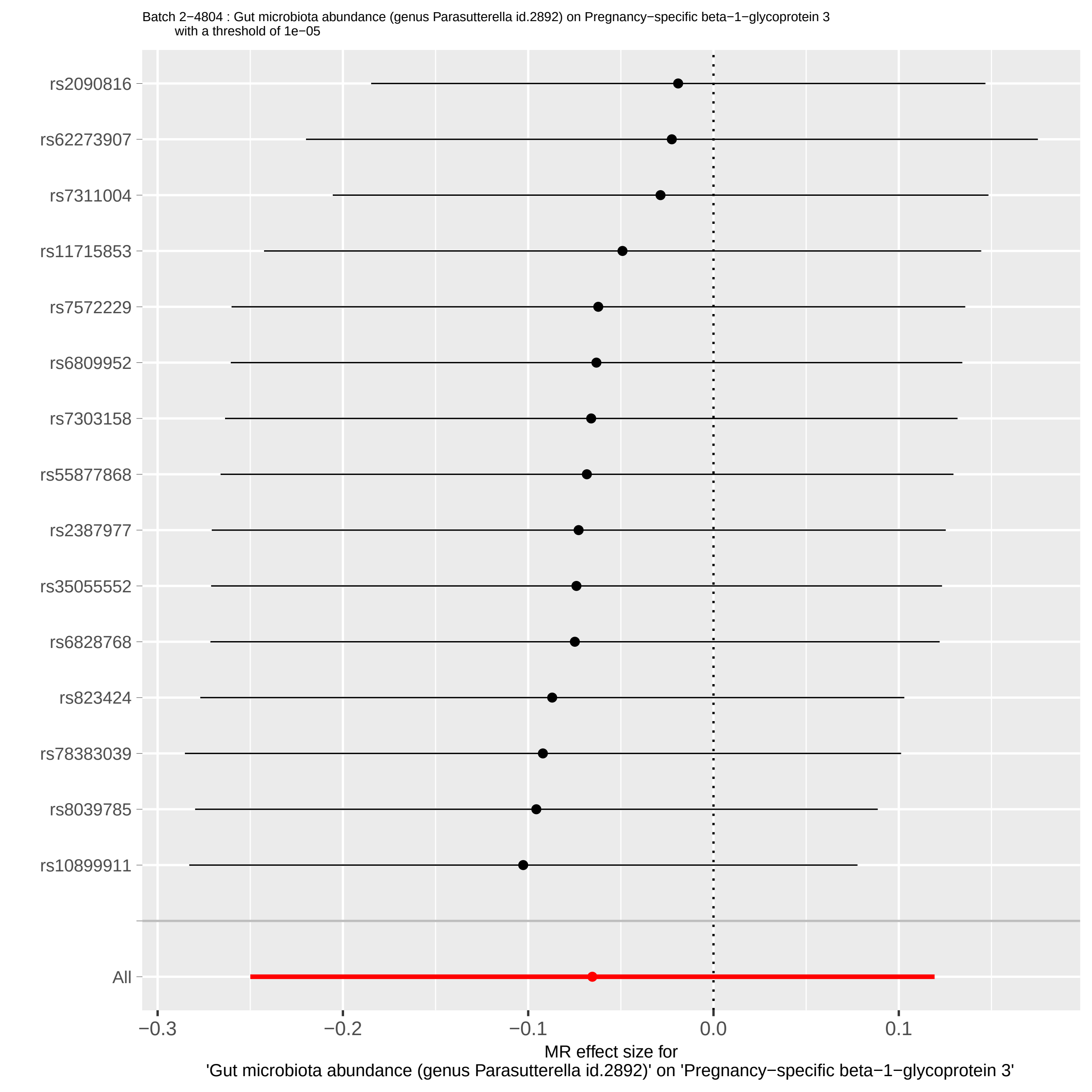
Batch 2−4804 : Gut microbiota abundance (genus Parasutterella id.2892) on Pregnancy−specific beta−1−glycoprotein 3
with a threshold of 1e−05
rs2090816
rs62273907
rs7311004
rs11715853
rs7572229
rs6809952
rs7303158
rs55877868
rs2387977
rs35055552
rs6828768
rs823424
rs78383039
rs8039785
rs10899911
All
−0.3
−0.2
−0.1
0.0
0.1
MR effect size for
'Gut microbiota abundance (genus Parasutterella id.2892)' on 'Pregnancy−specific beta−1−glycoprotein 3'

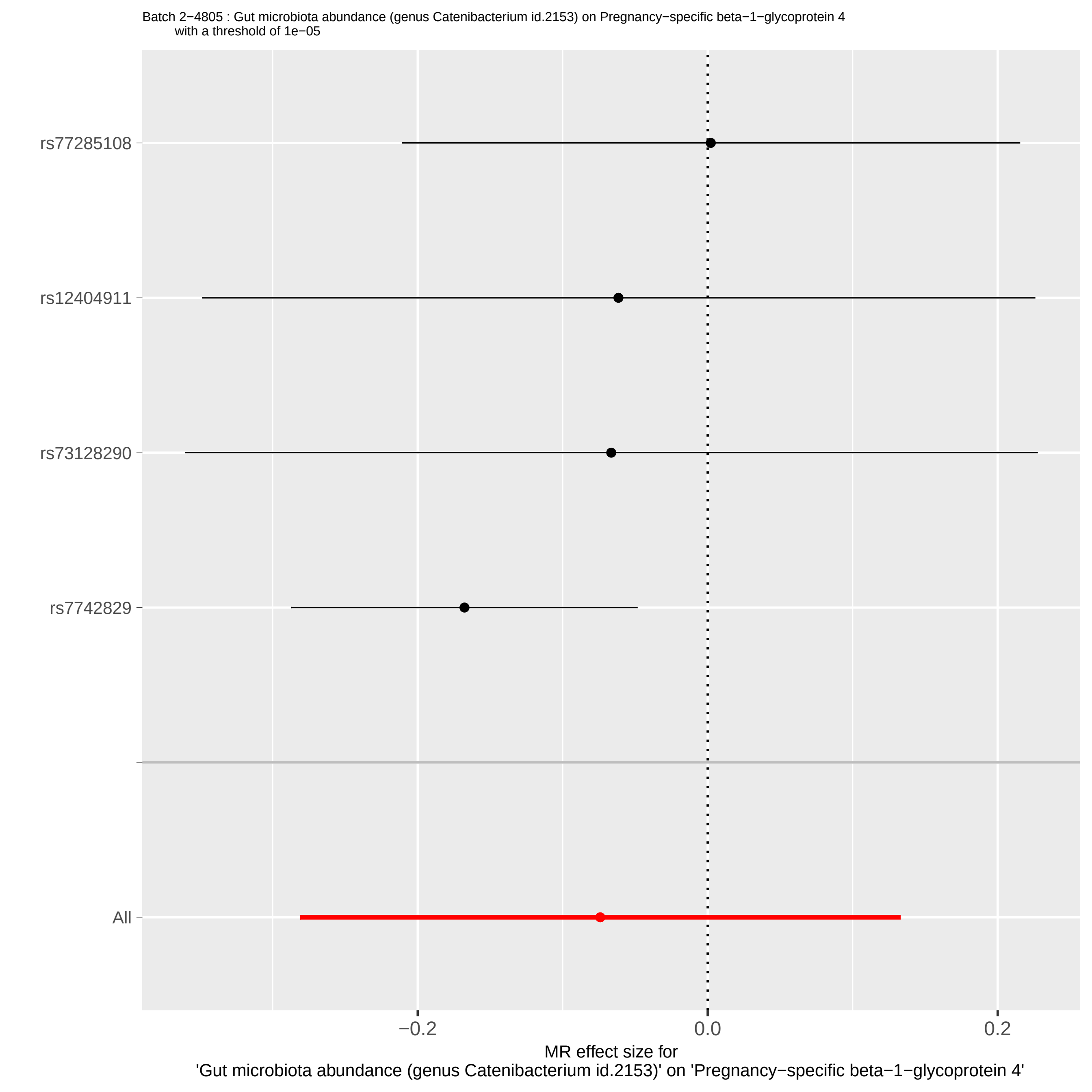

Batch 2−4805 : Gut microbiota abundance (genus Catenibacterium id.2153) on Pregnancy−specific beta−1−glycoprotein 4
with a threshold of 1e−05
rs77285108
rs12404911
rs73128290
rs7742829
All
−0.2
0.0
0.2
MR effect size for
'Gut microbiota abundance (genus Catenibacterium id.2153)' on 'Pregnancy−specific beta−1−glycoprotein 4'

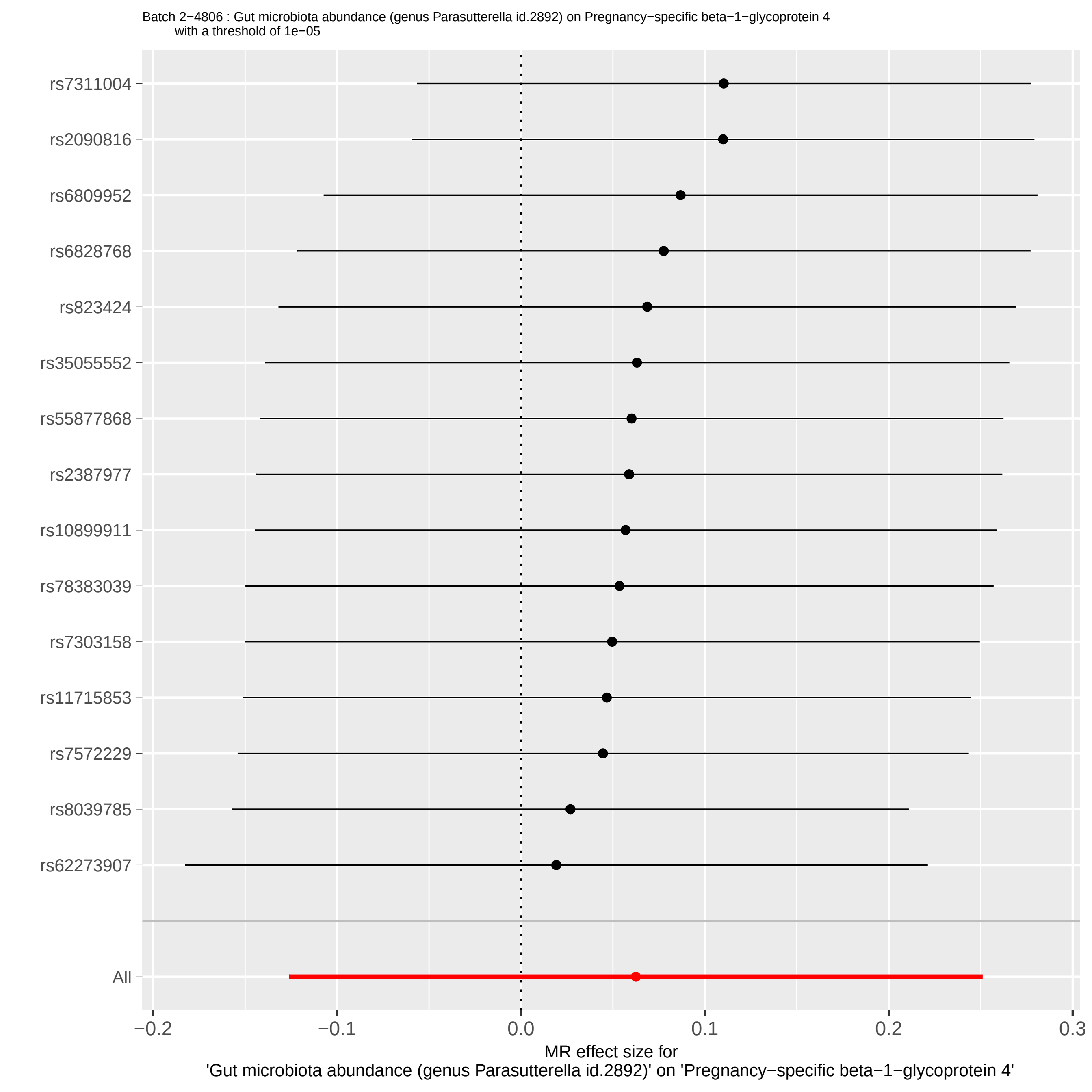
Batch 2−4806 : Gut microbiota abundance (genus Parasutterella id.2892) on Pregnancy−specific beta−1−glycoprotein 4
with a threshold of 1e−05
rs7311004
rs2090816
rs6809952
rs6828768
rs823424
rs35055552
rs55877868
rs2387977
rs10899911
rs78383039
rs7303158
rs11715853
rs7572229
rs8039785
rs62273907
All
−0.2
−0.1
0.0
0.1
0.2
0.3
MR effect size for
'Gut microbiota abundance (genus Parasutterella id.2892)' on 'Pregnancy−specific beta−1−glycoprotein 4'

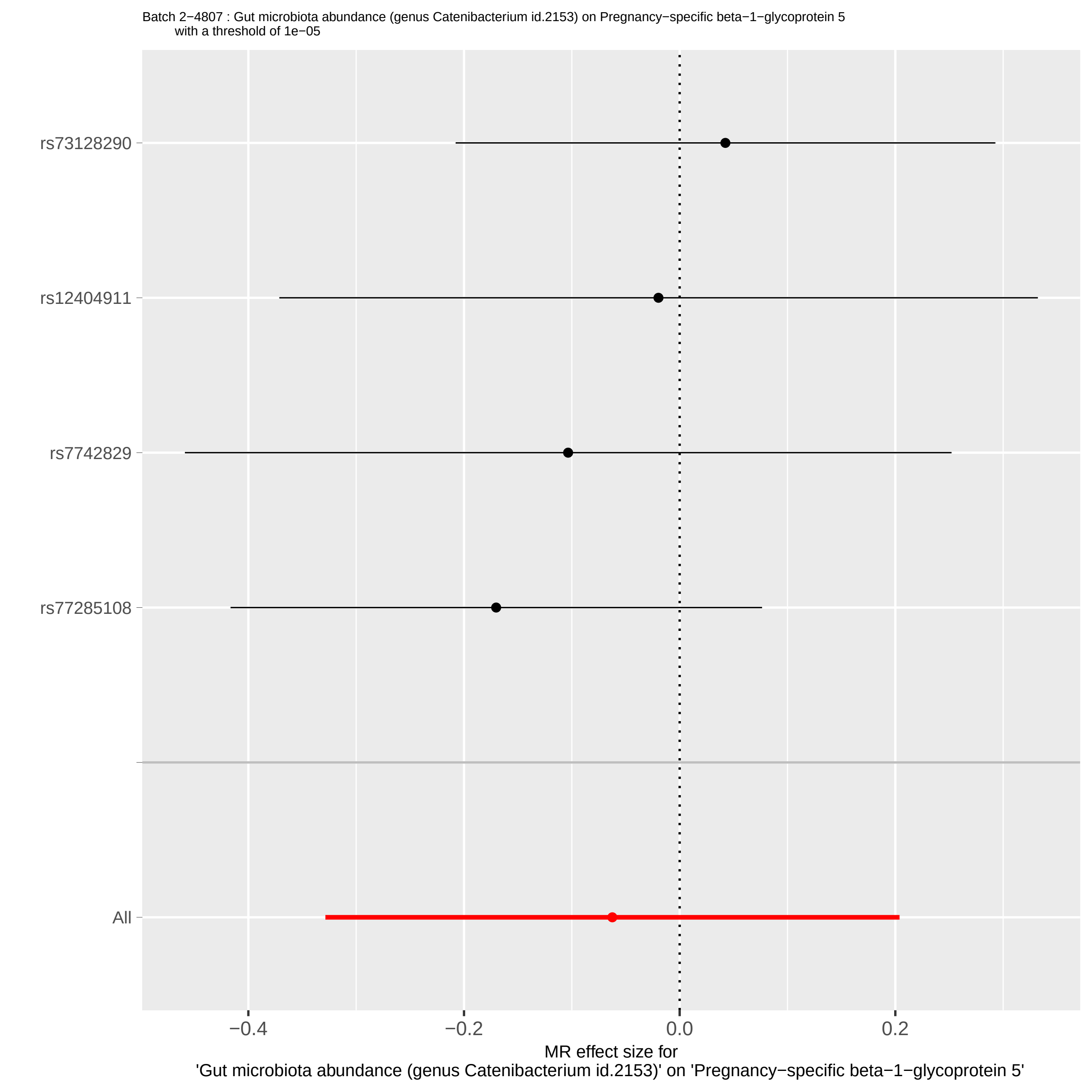

Batch 2−4807 : Gut microbiota abundance (genus Catenibacterium id.2153) on Pregnancy−specific beta−1−glycoprotein 5
with a threshold of 1e−05
rs73128290
rs12404911
rs7742829
rs77285108
All
−0.4
−0.2
0.0
0.2
MR effect size for
'Gut microbiota abundance (genus Catenibacterium id.2153)' on 'Pregnancy−specific beta−1−glycoprotein 5'

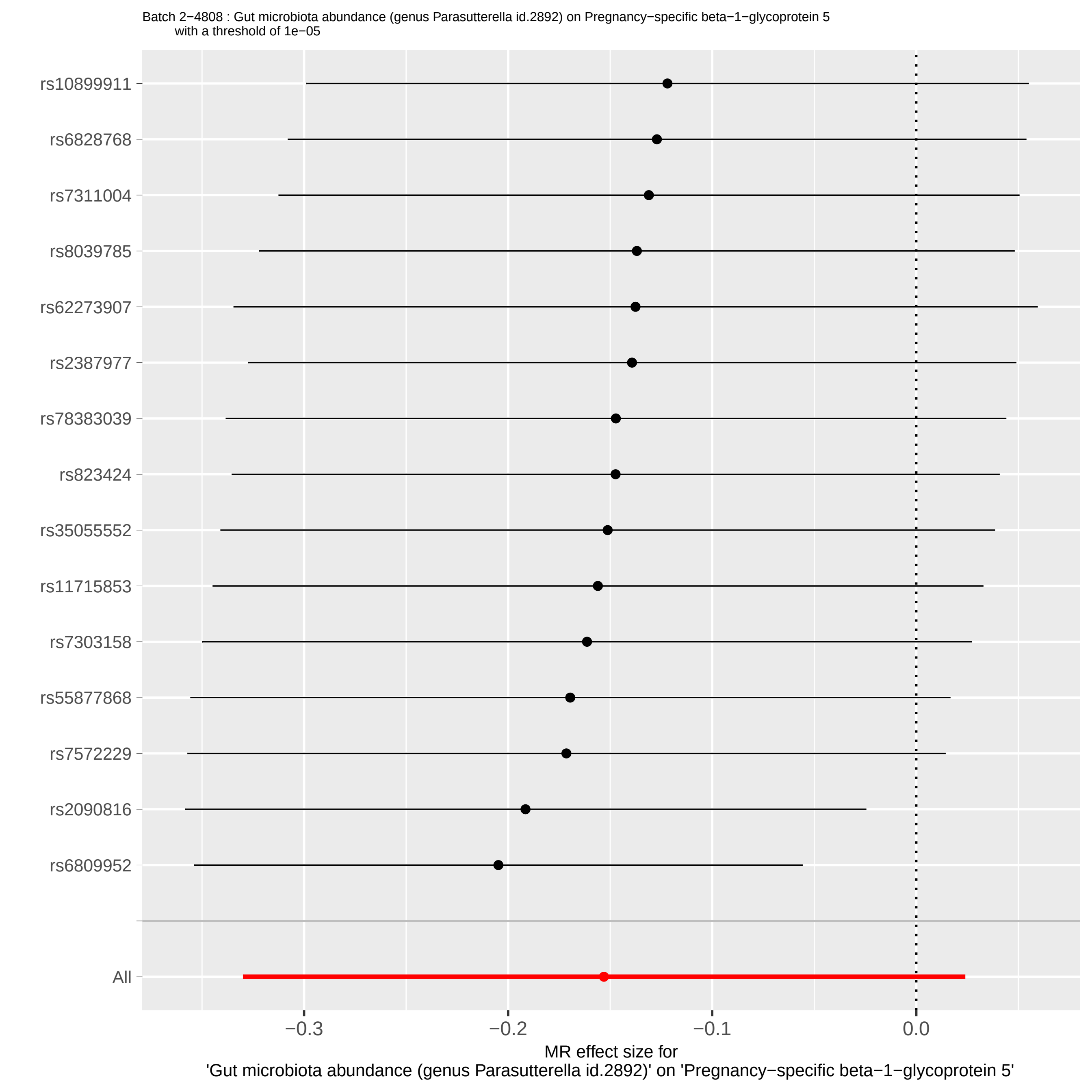
Batch 2−4808 : Gut microbiota abundance (genus Parasutterella id.2892) on Pregnancy−specific beta−1−glycoprotein 5
with a threshold of 1e−05
rs10899911
rs6828768
rs7311004
rs8039785
rs62273907
rs2387977
rs78383039
rs823424
rs35055552
rs11715853
rs7303158
rs55877868
rs7572229
rs2090816
rs6809952
All
−0.3
−0.2
−0.1
0.0
MR effect size for
'Gut microbiota abundance (genus Parasutterella id.2892)' on 'Pregnancy−specific beta−1−glycoprotein 5'

Batch 2−4809 : Gut microbiota abundance (genus Catenibacterium id.2153) on Pregnancy−specific beta−1−glycoprotein 6
with a threshold of 1e−05

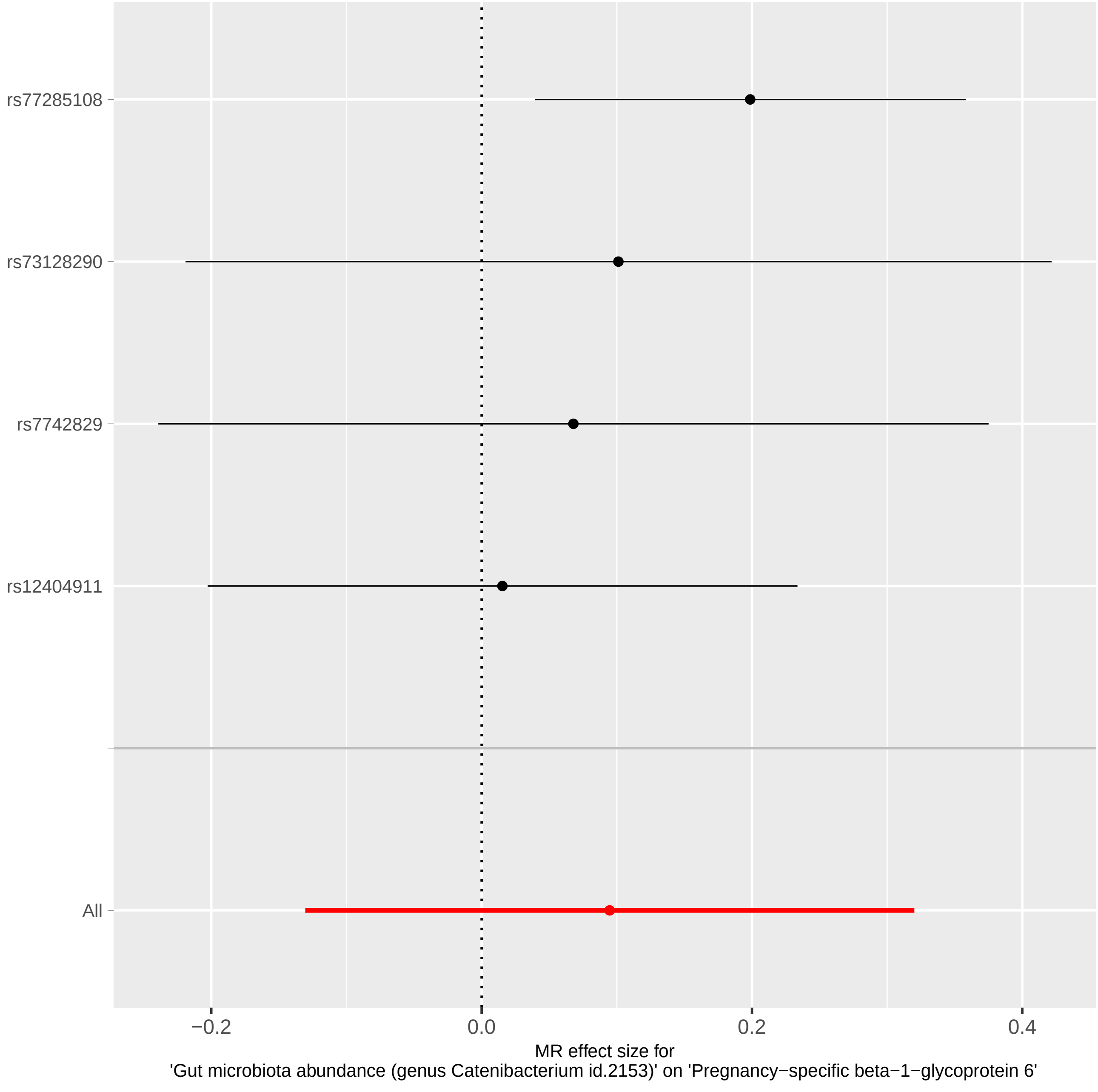

Batch 2−4810 : Gut microbiota abundance (genus Parasutterella id.2892) on Pregnancy−specific beta−1−glycoprotein 6
with a threshold of 1e−05

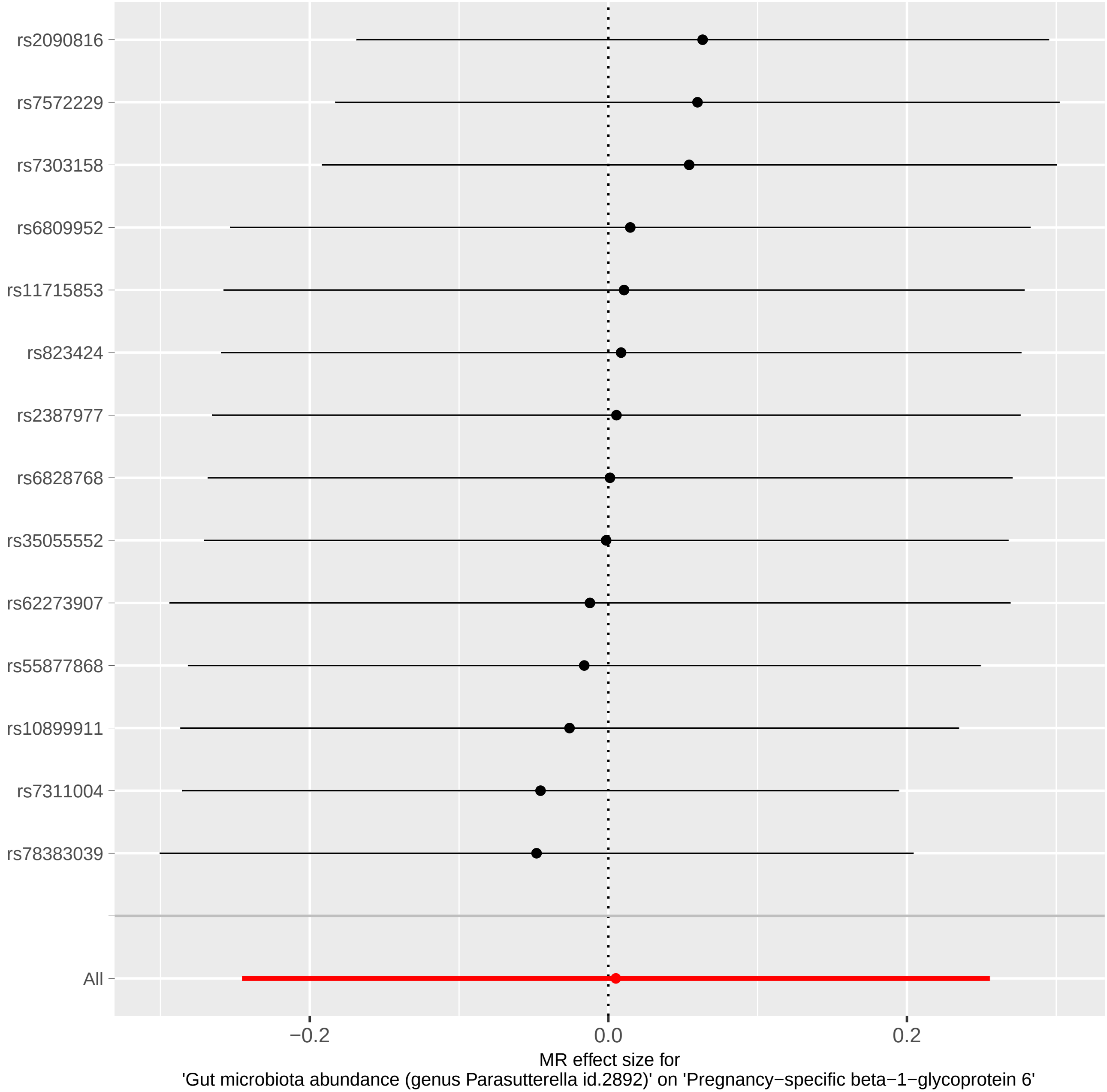

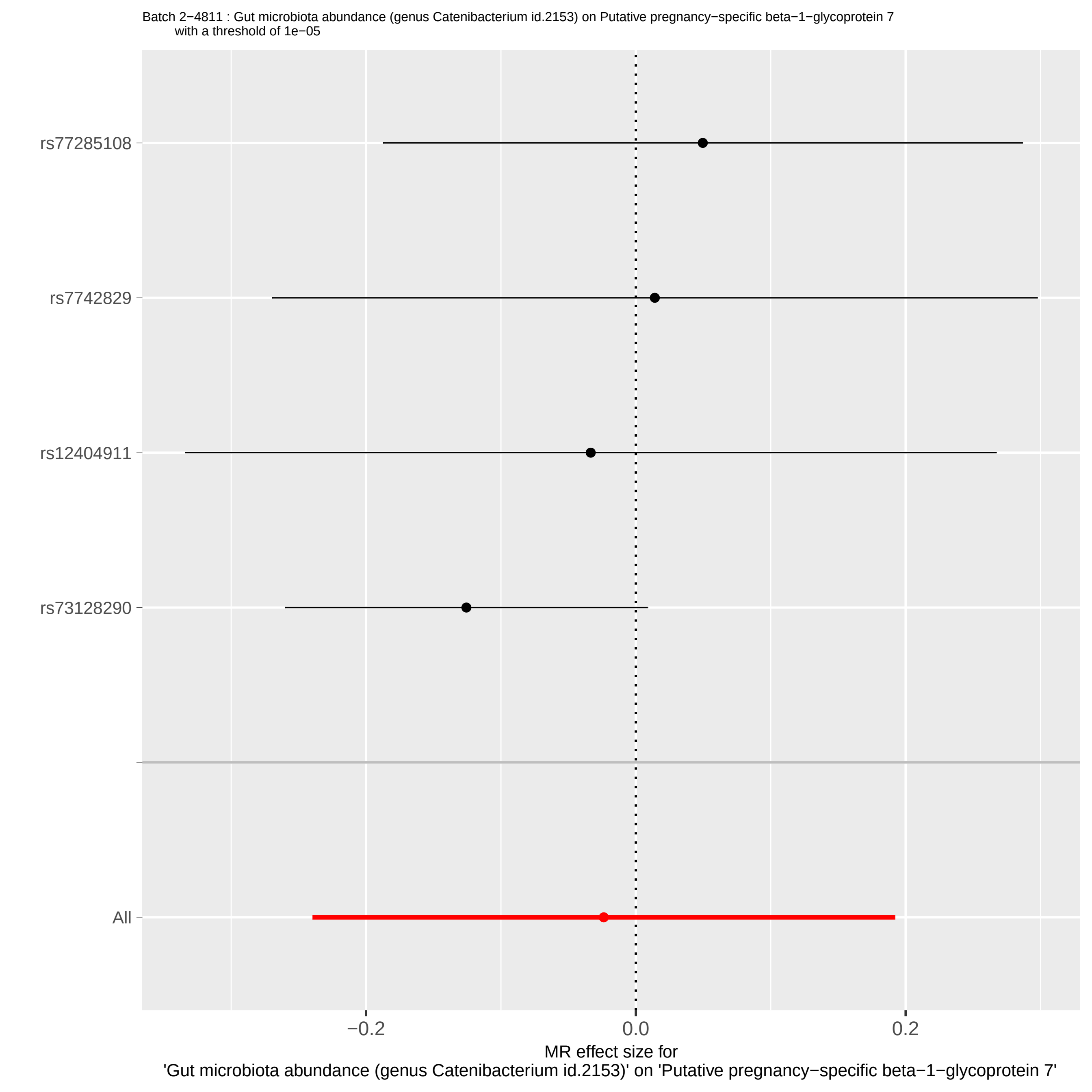
Batch 2−4811 : Gut microbiota abundance (genus Catenibacterium id.2153) on Putative pregnancy−specific beta−1−glycoprotein 7
with a threshold of 1e−05
rs77285108
rs7742829
rs12404911
rs73128290
All
−0.2
0.0
0.2
MR effect size for
'Gut microbiota abundance (genus Catenibacterium id.2153)' on 'Putative pregnancy−specific beta−1−glycoprotein 7'

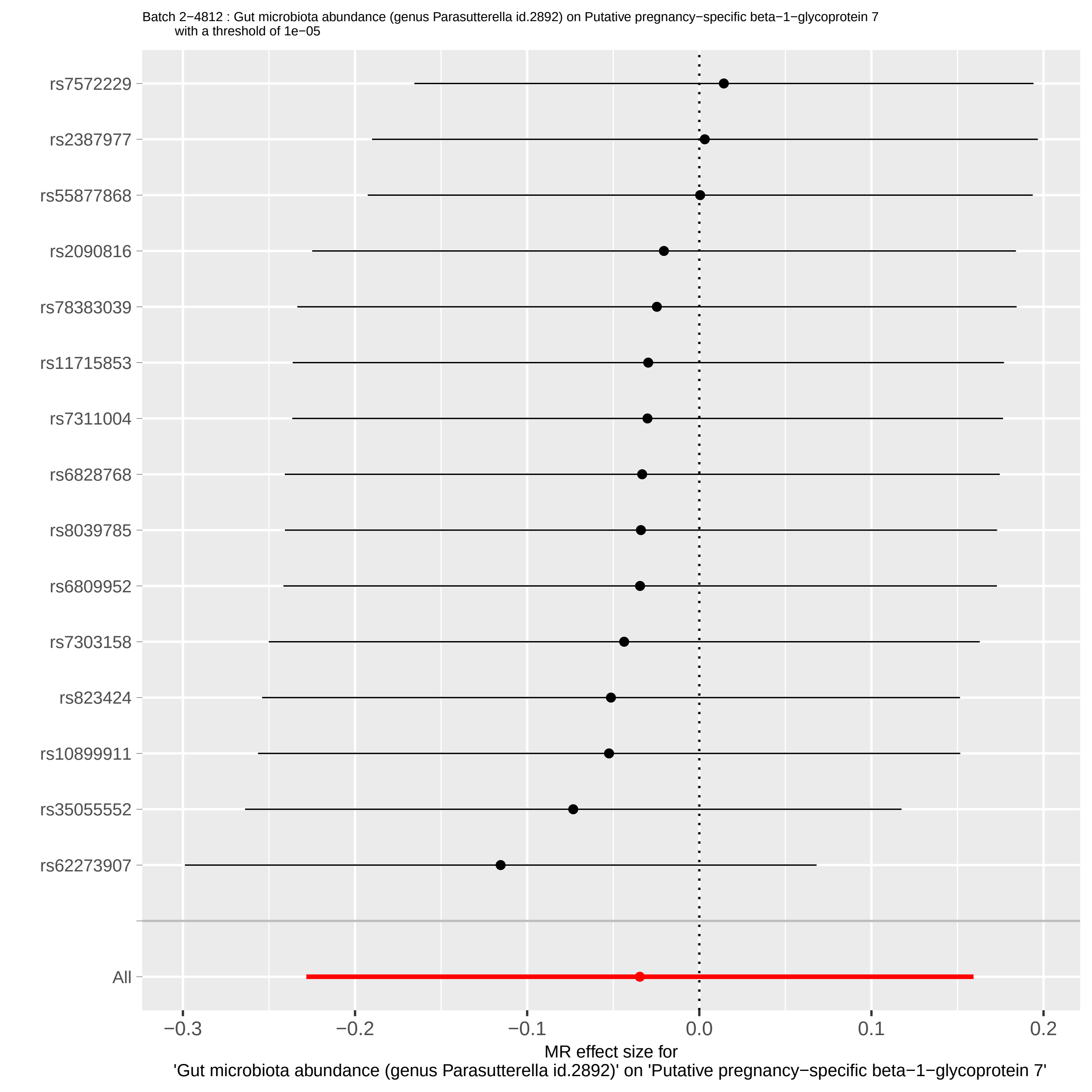
Batch 2−4812 : Gut microbiota abundance (genus Parasutterella id.2892) on Putative pregnancy−specific beta−1−glycoprotein 7
with a threshold of 1e−05
rs7572229
rs2387977
rs55877868
rs2090816
rs78383039
rs11715853
rs7311004
rs6828768
rs8039785
rs6809952
rs7303158
rs823424
rs10899911
rs35055552
rs62273907
All
−0.3
−0.2
−0.1
0.0
0.1
0.2
MR effect size for
'Gut microbiota abundance (genus Parasutterella id.2892)' on 'Putative pregnancy−specific beta−1−glycoprotein 7'

Batch 2−4813 : Gut microbiota abundance (genus Catenibacterium id.2153) on Pregnancy−specific beta−1−glycoprotein 8
with a threshold of 1e−05

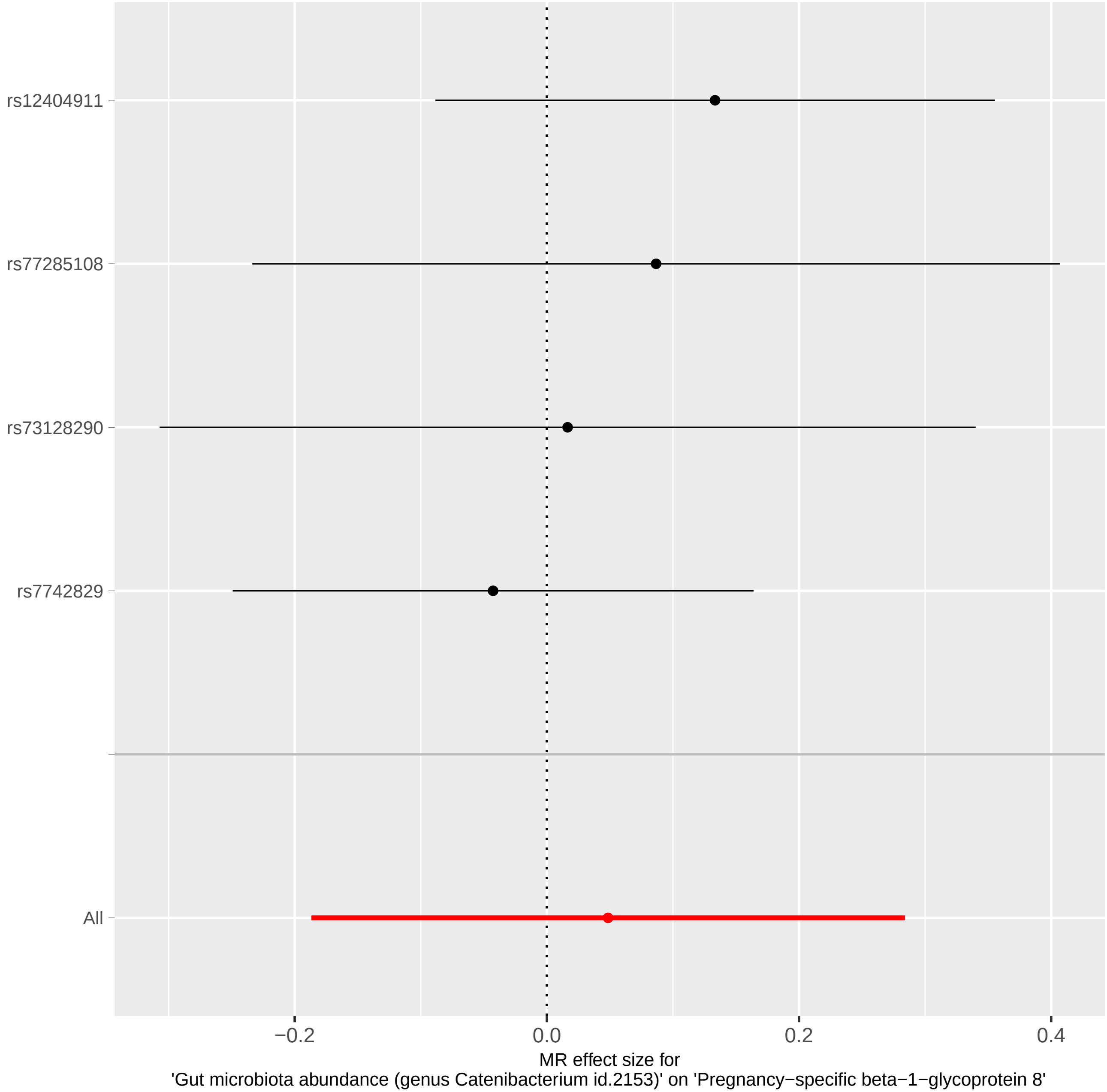

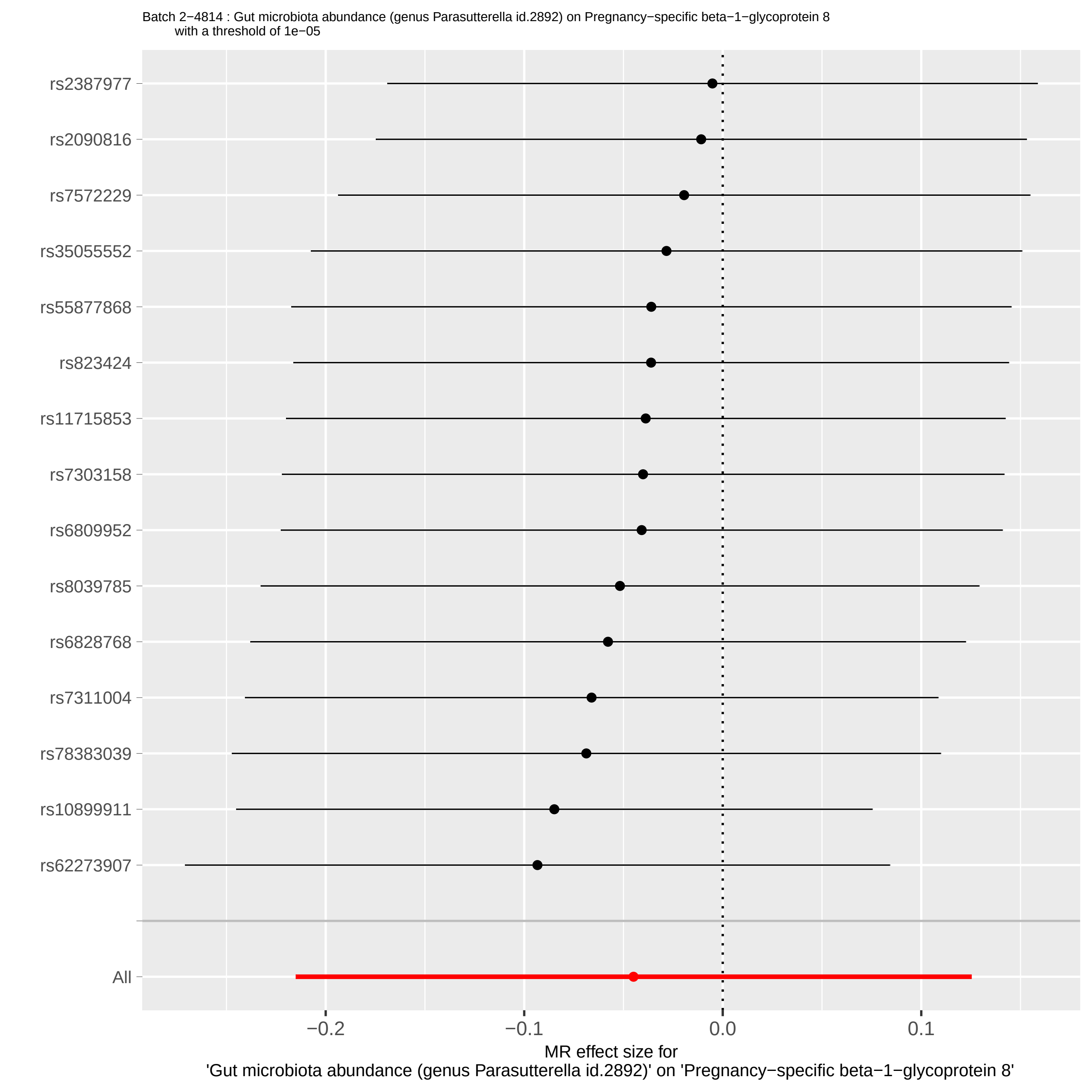
Batch 2−4814 : Gut microbiota abundance (genus Parasutterella id.2892) on Pregnancy−specific beta−1−glycoprotein 8
with a threshold of 1e−05
rs2387977
rs2090816
rs7572229
rs35055552
rs55877868
rs823424
rs11715853
rs7303158
rs6809952
rs8039785
rs6828768
rs7311004
rs78383039
rs10899911
rs62273907
All
−0.2
−0.1
0.0
0.1
MR effect size for
'Gut microbiota abundance (genus Parasutterella id.2892)' on 'Pregnancy−specific beta−1−glycoprotein 8'

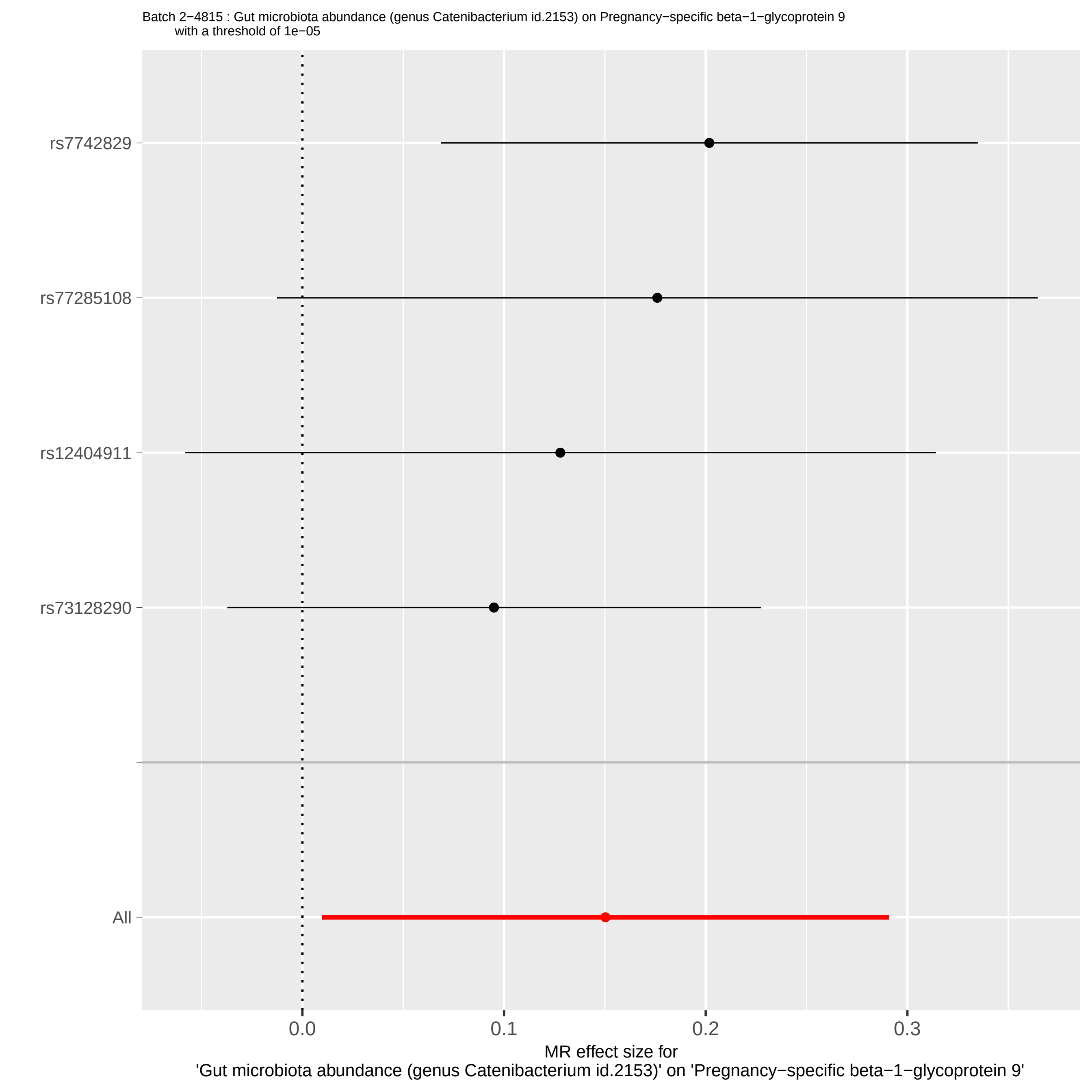
Batch 2−4815 : Gut microbiota abundance (genus Catenibacterium id.2153) on Pregnancy−specific beta−1−glycoprotein 9
with a threshold of 1e−05
rs7742829
rs77285108
rs12404911
rs73128290
All
0.0
0.1
0.2
0.3
MR effect size for
'Gut microbiota abundance (genus Catenibacterium id.2153)' on 'Pregnancy−specific beta−1−glycoprotein 9'

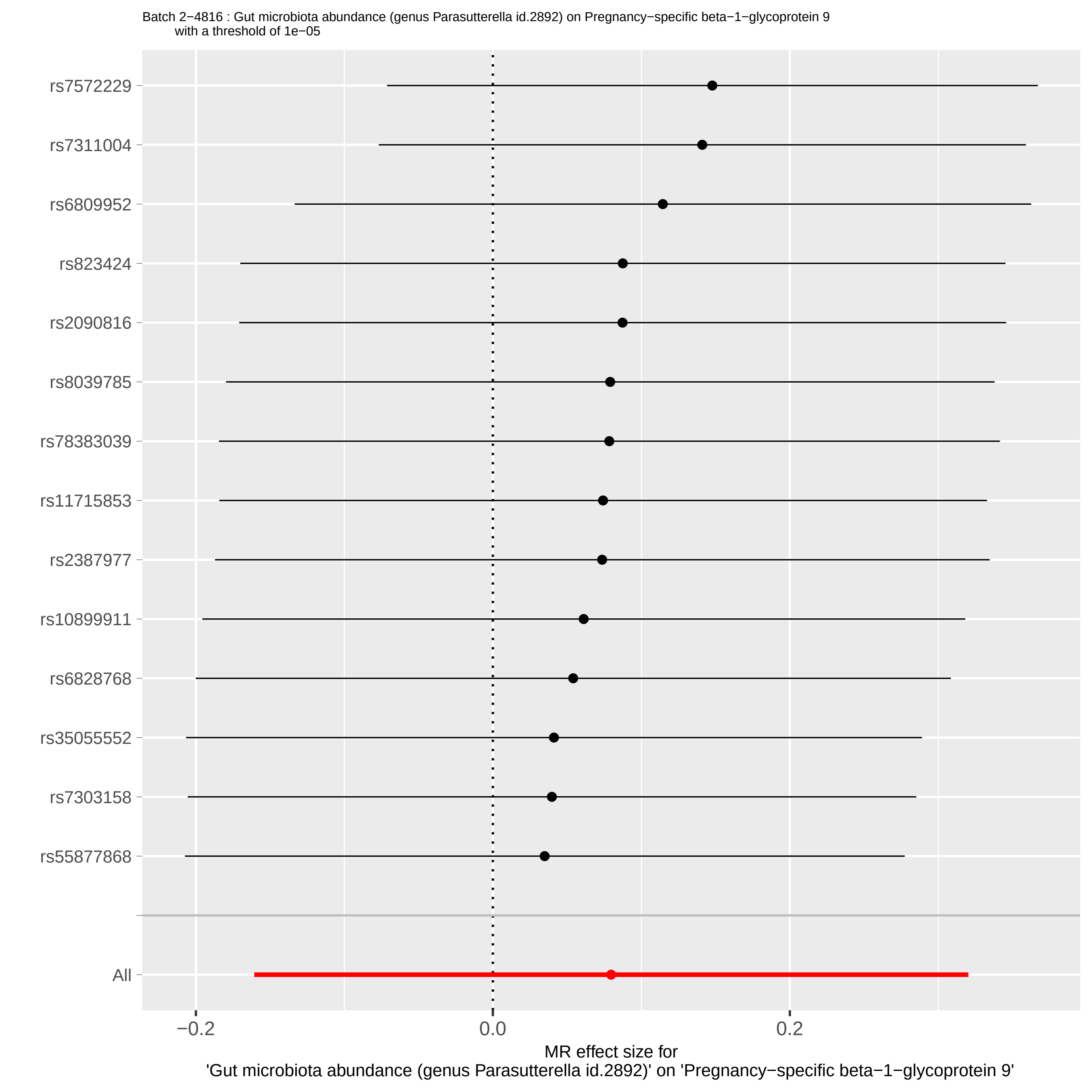
Batch 2−4816 : Gut microbiota abundance (genus Parasutterella id.2892) on Pregnancy−specific beta−1−glycoprotein 9
with a threshold of 1e−05
rs7572229
rs7311004
rs6809952
rs823424
rs2090816
rs8039785
rs78383039
rs11715853
rs2387977
rs10899911
rs6828768
rs35055552
rs7303158
rs55877868
All
−0.2
0.0
0.2
MR effect size for
'Gut microbiota abundance (genus Parasutterella id.2892)' on 'Pregnancy−specific beta−1−glycoprotein 9'

Batch 2−4817 : Gut microbiota abundance (genus Catenibacterium id.2153) on Proteasome subunit alpha type−1
with a threshold of 1e−05

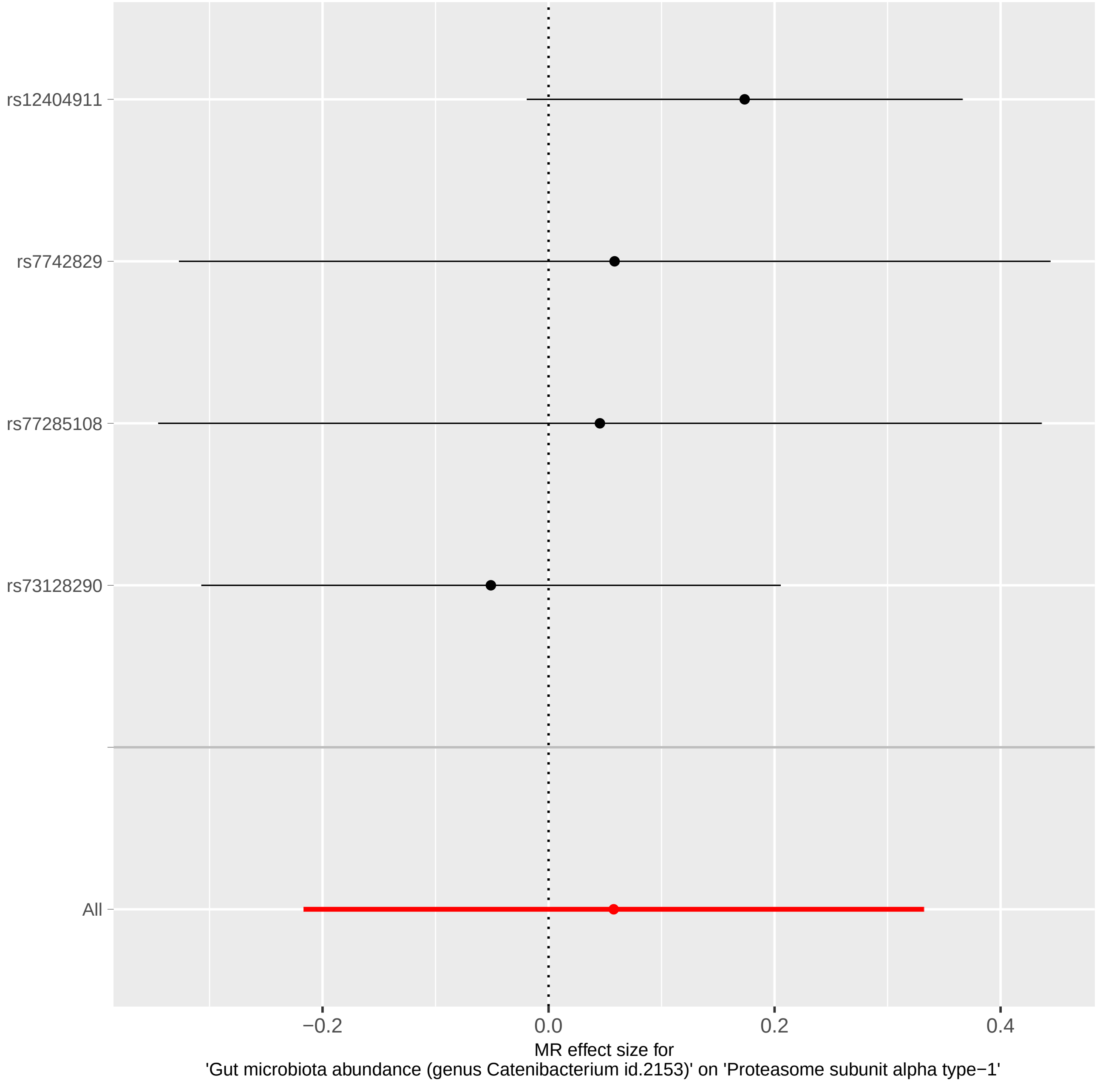

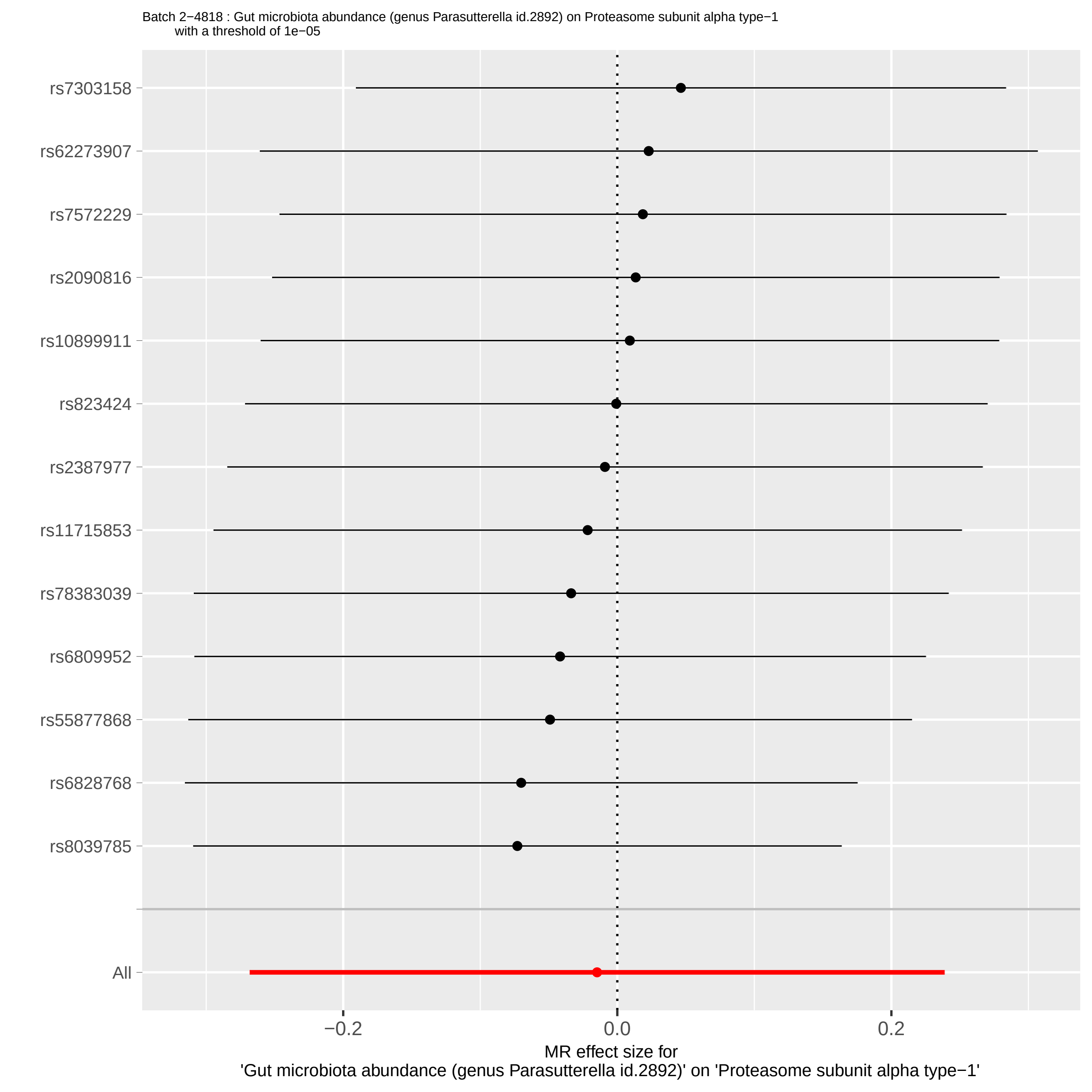
Batch 2−4818 : Gut microbiota abundance (genus Parasutterella id.2892) on Proteasome subunit alpha type−1
with a threshold of 1e−05
rs7303158
rs62273907
rs7572229
rs2090816
rs10899911
rs823424
rs2387977
rs11715853
rs78383039
rs6809952
rs55877868
rs6828768
rs8039785
All
−0.2
0.0
0.2
MR effect size for
'Gut microbiota abundance (genus Parasutterella id.2892)' on 'Proteasome subunit alpha type−1'

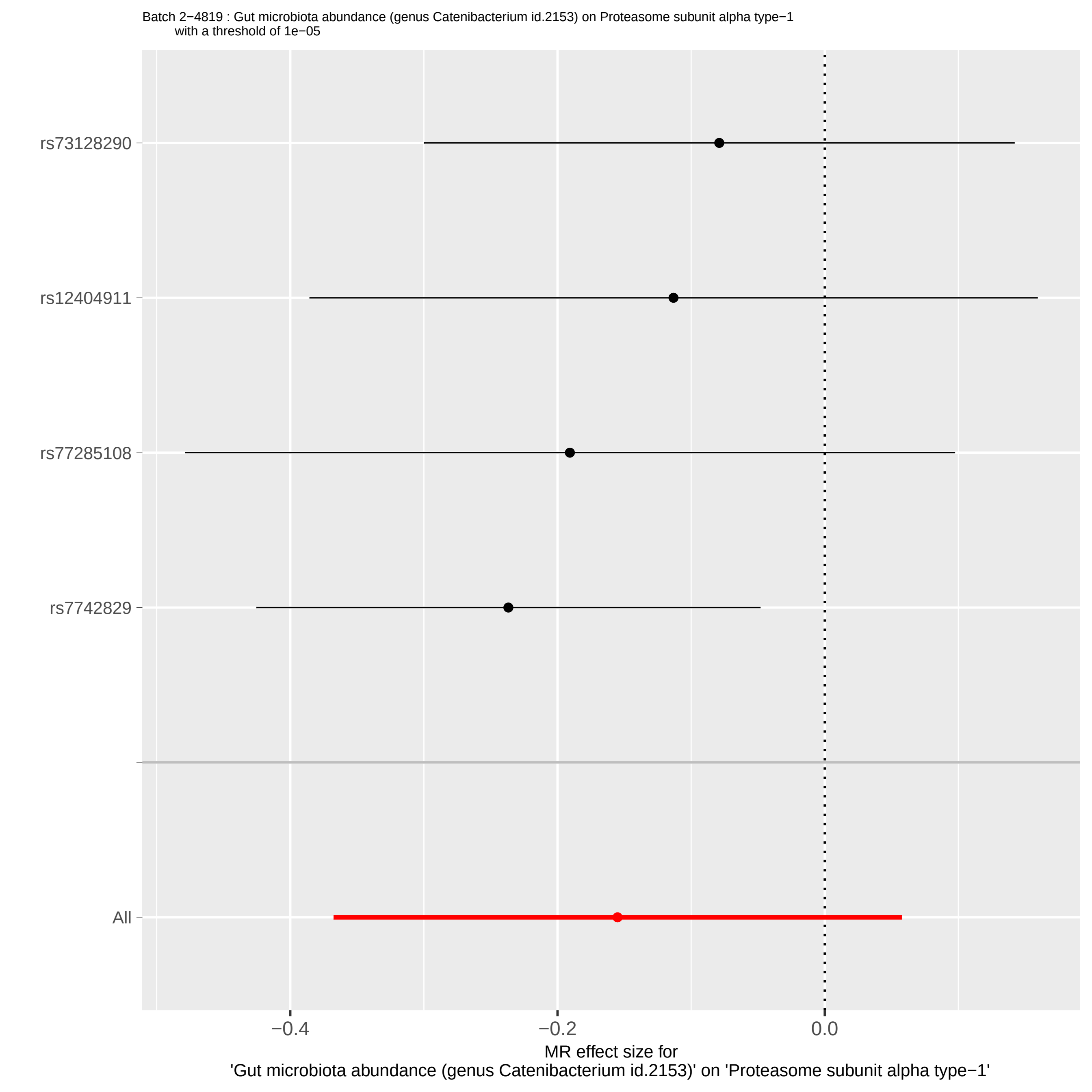
Batch 2−4819 : Gut microbiota abundance (genus Catenibacterium id.2153) on Proteasome subunit alpha type−1
with a threshold of 1e−05
rs73128290
rs12404911
rs77285108
rs7742829
All
−0.4
−0.2
0.0
MR effect size for
'Gut microbiota abundance (genus Catenibacterium id.2153)' on 'Proteasome subunit alpha type−1'

Batch 2−4820 : Gut microbiota abundance (genus Parasutterella id.2892) on Proteasome subunit alpha type−1
with a threshold of 1e−05

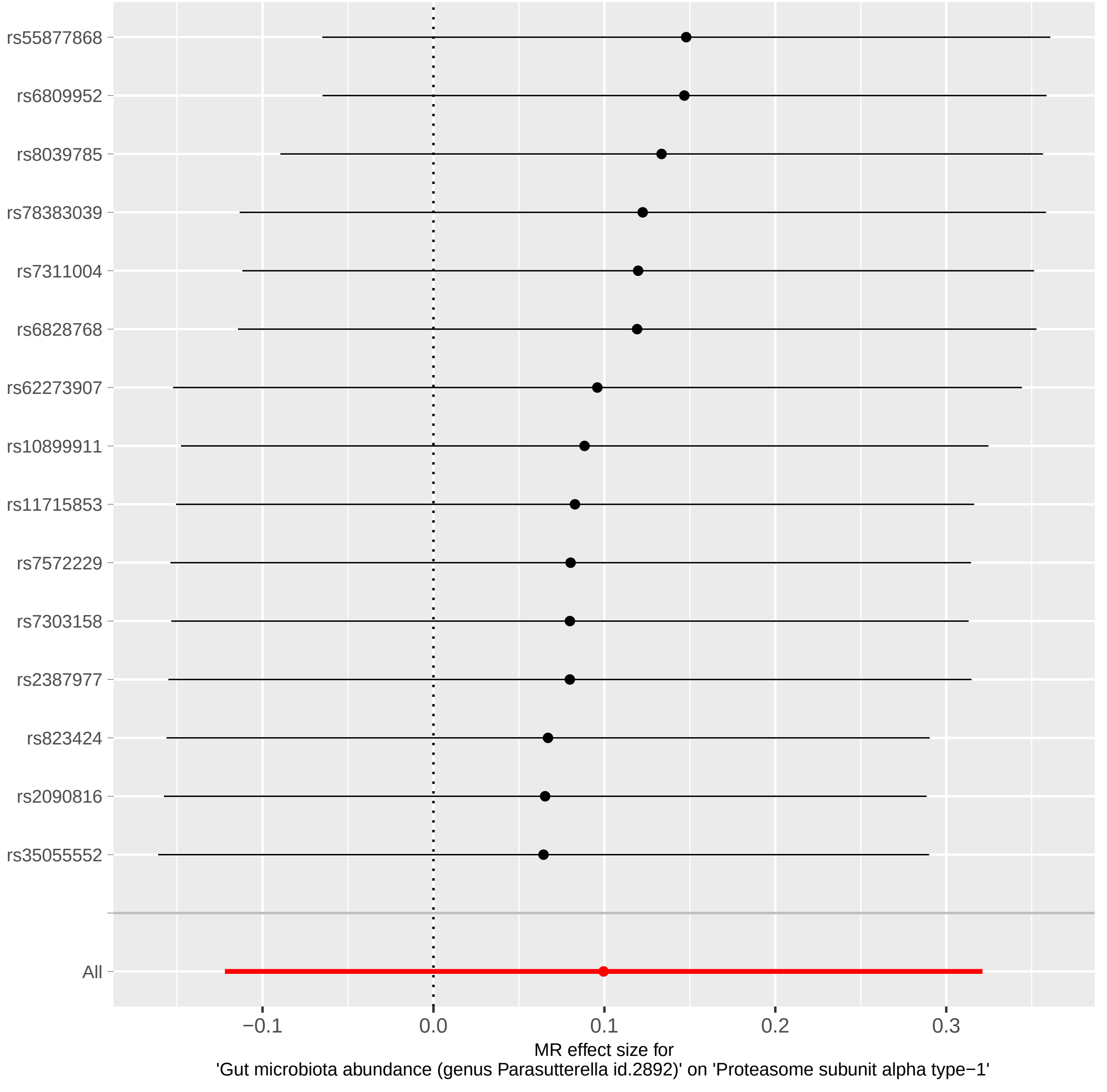

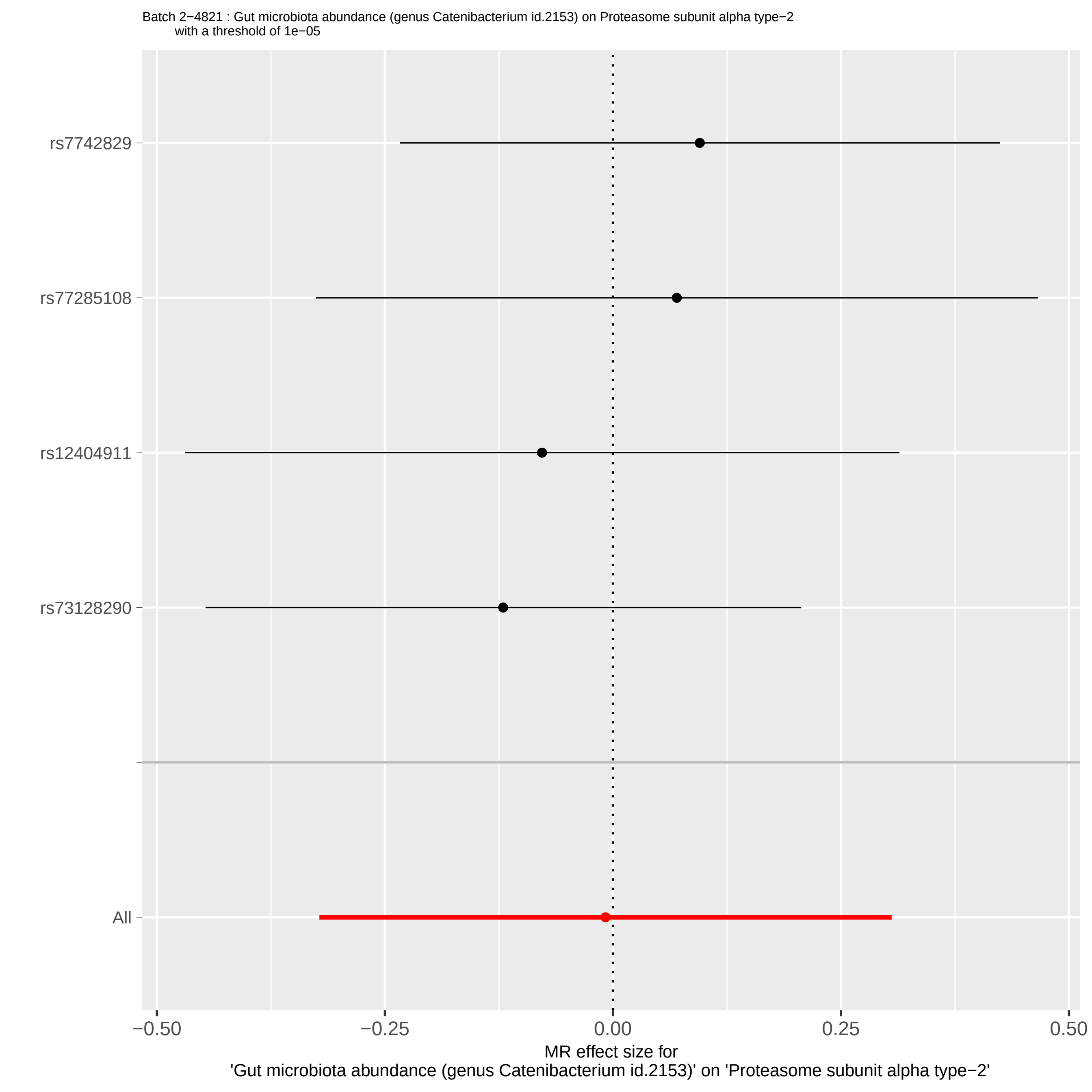

Batch 2−4821 : Gut microbiota abundance (genus Catenibacterium id.2153) on Proteasome subunit alpha type−2
with a threshold of 1e−05
rs7742829
rs77285108
rs12404911
rs73128290
All
−0.50
−0.25
0.00
0.25
0.50
MR effect size for
'Gut microbiota abundance (genus Catenibacterium id.2153)' on 'Proteasome subunit alpha type−2'

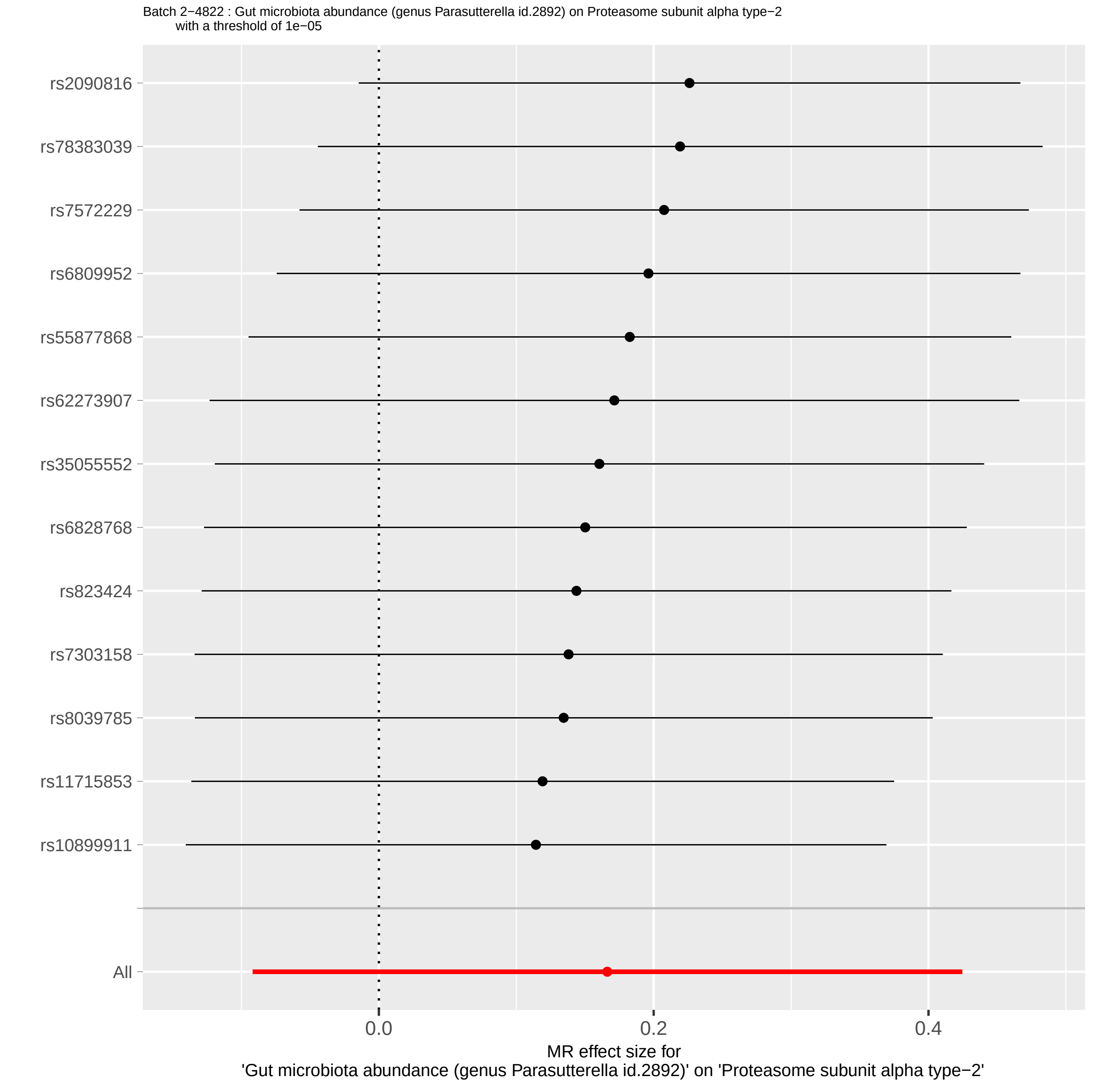
Batch 2−4822 : Gut microbiota abundance (genus Parasutterella id.2892) on Proteasome subunit alpha type−2
with a threshold of 1e−05
rs2090816
rs78383039
rs7572229
rs6809952
rs55877868
rs62273907
rs35055552
rs6828768
rs823424
rs7303158
rs8039785
rs11715853
rs10899911
All
0.0
0.2
0.4
MR effect size for
'Gut microbiota abundance (genus Parasutterella id.2892)' on 'Proteasome subunit alpha type−2'

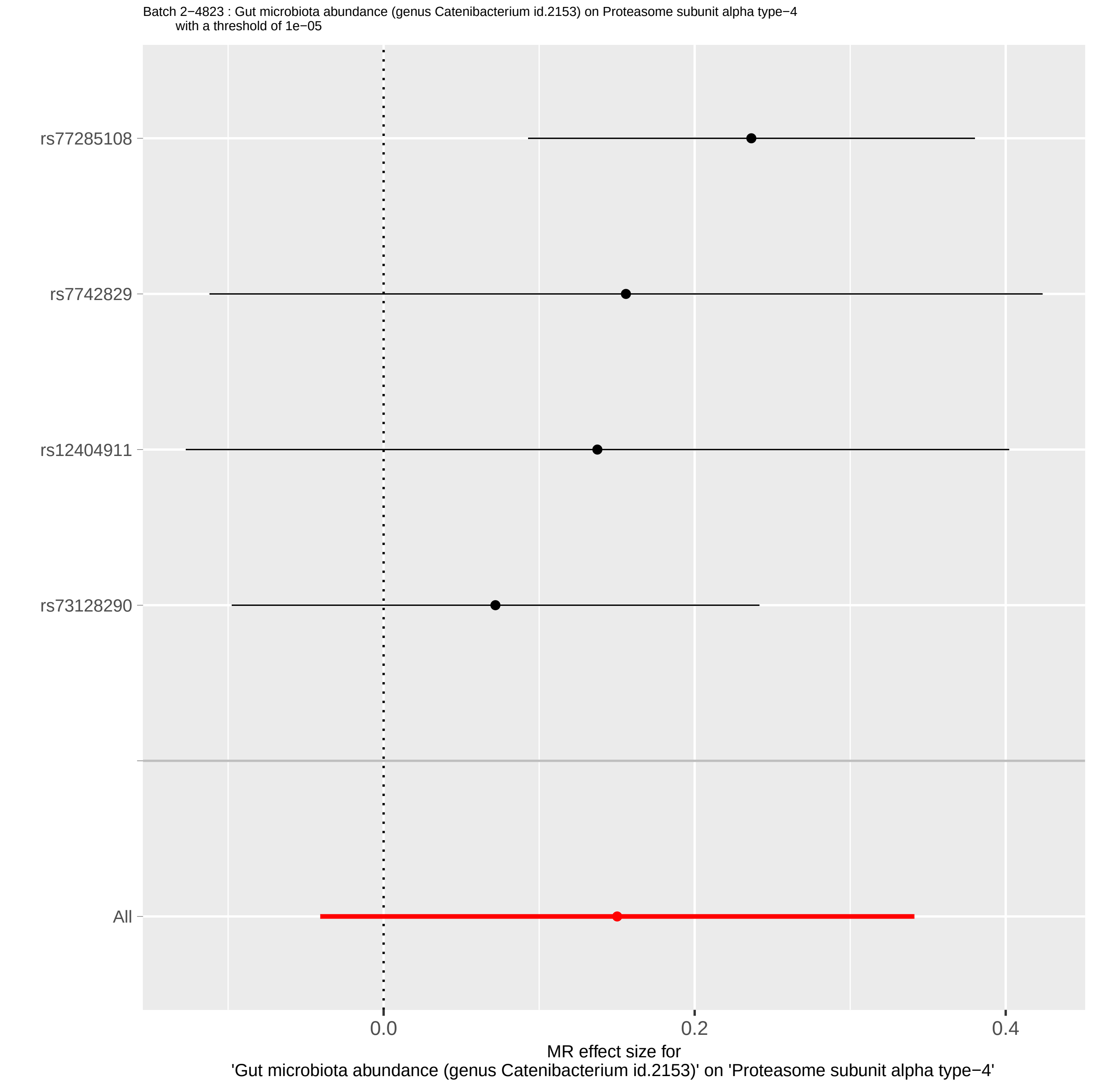
Batch 2−4823 : Gut microbiota abundance (genus Catenibacterium id.2153) on Proteasome subunit alpha type−4
with a threshold of 1e−05
rs77285108
rs7742829
rs12404911
rs73128290
All
0.0
0.2
0.4
MR effect size for
'Gut microbiota abundance (genus Catenibacterium id.2153)' on 'Proteasome subunit alpha type−4'

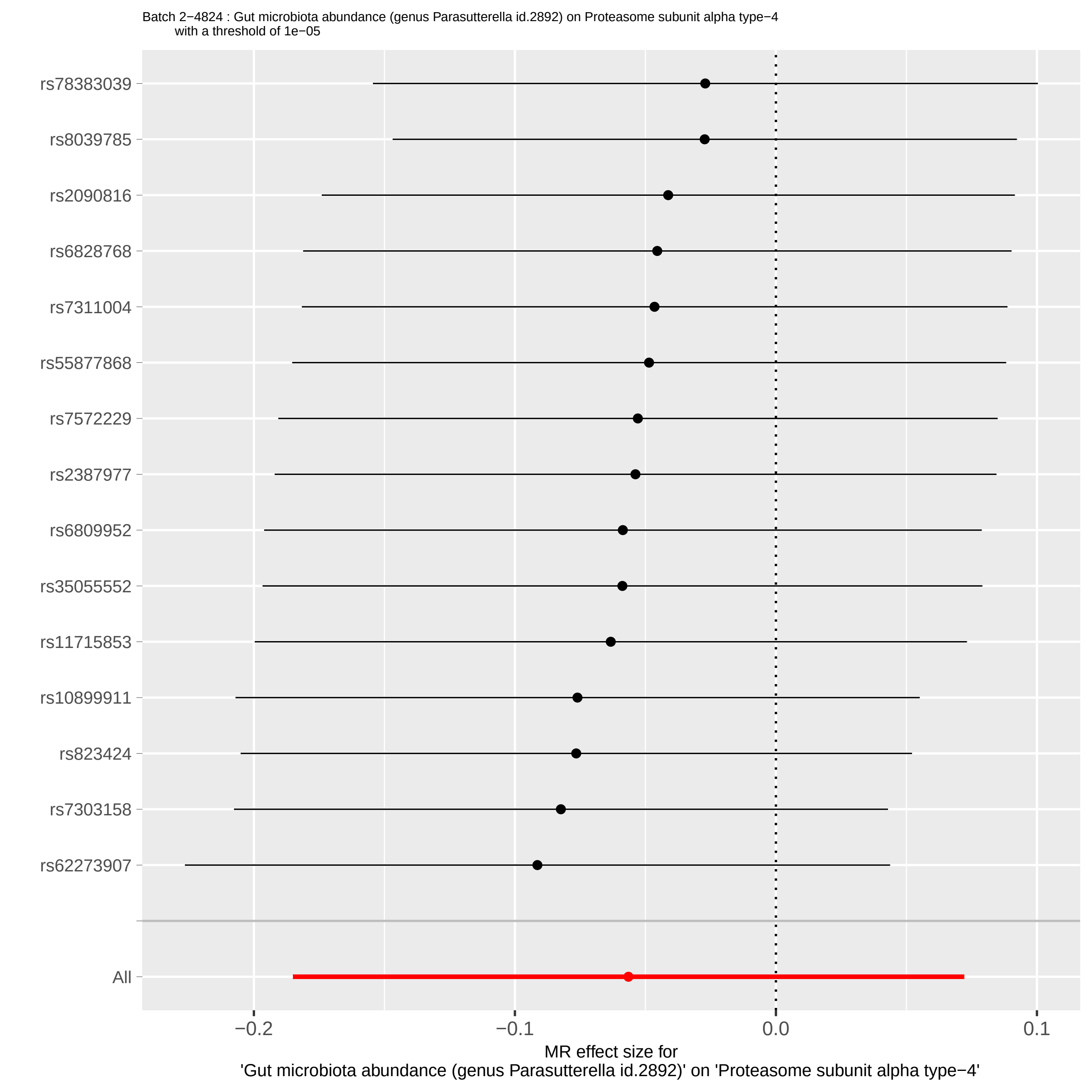
Batch 2−4824 : Gut microbiota abundance (genus Parasutterella id.2892) on Proteasome subunit alpha type−4
with a threshold of 1e−05
rs78383039
rs8039785
rs2090816
rs6828768
rs7311004
rs55877868
rs7572229
rs2387977
rs6809952
rs35055552
rs11715853
rs10899911
rs823424
rs7303158
rs62273907
All
−0.2
−0.1
0.0
0.1
MR effect size for
'Gut microbiota abundance (genus Parasutterella id.2892)' on 'Proteasome subunit alpha type−4'

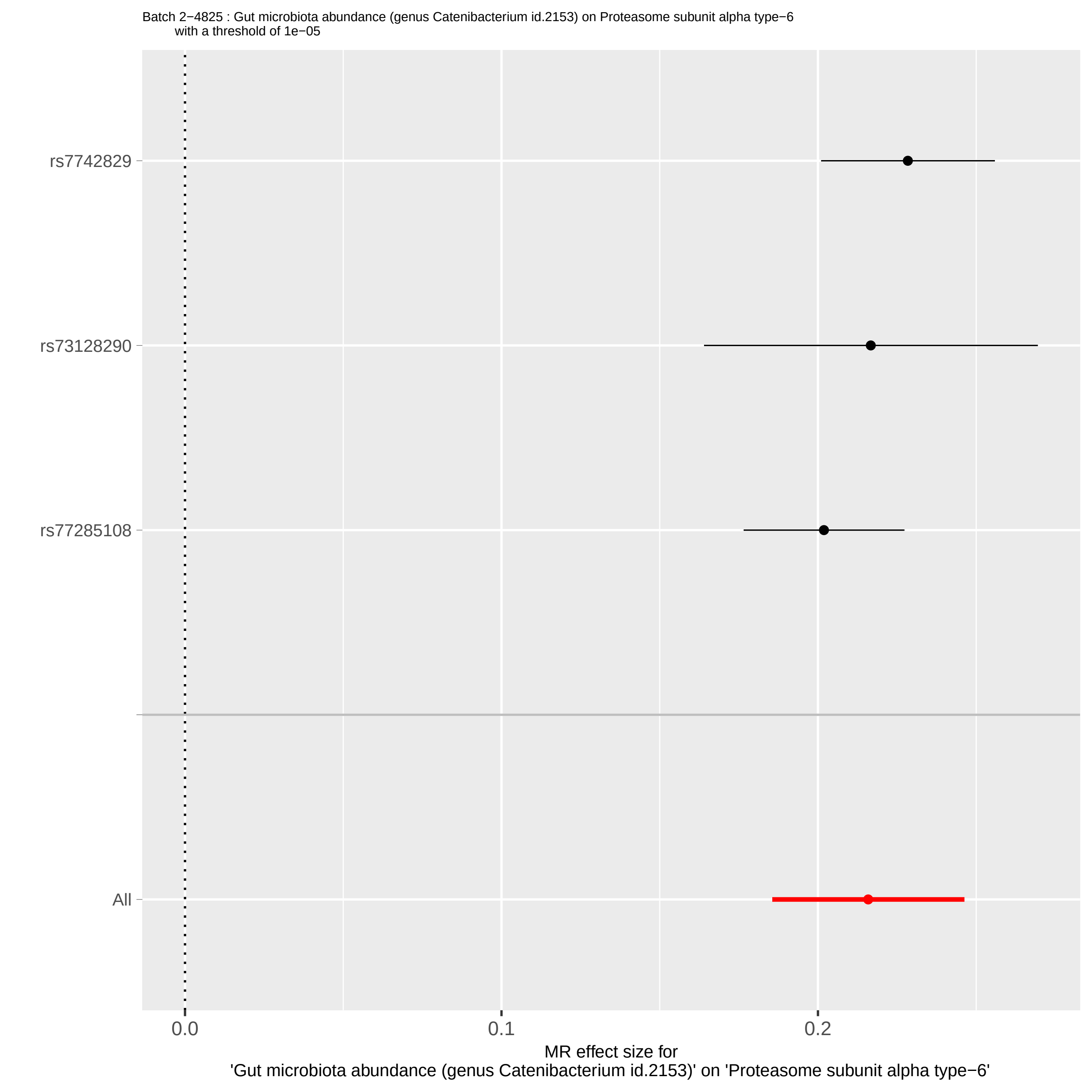

Batch 2−4825 : Gut microbiota abundance (genus Catenibacterium id.2153) on Proteasome subunit alpha type−6
with a threshold of 1e−05
rs7742829
rs73128290
rs77285108
All
0.0
0.1
0.2
MR effect size for
'Gut microbiota abundance (genus Catenibacterium id.2153)' on 'Proteasome subunit alpha type−6'

Batch 2−4826 : Gut microbiota abundance (genus Parasutterella id.2892) on Proteasome subunit alpha type−6
with a threshold of 1e−05

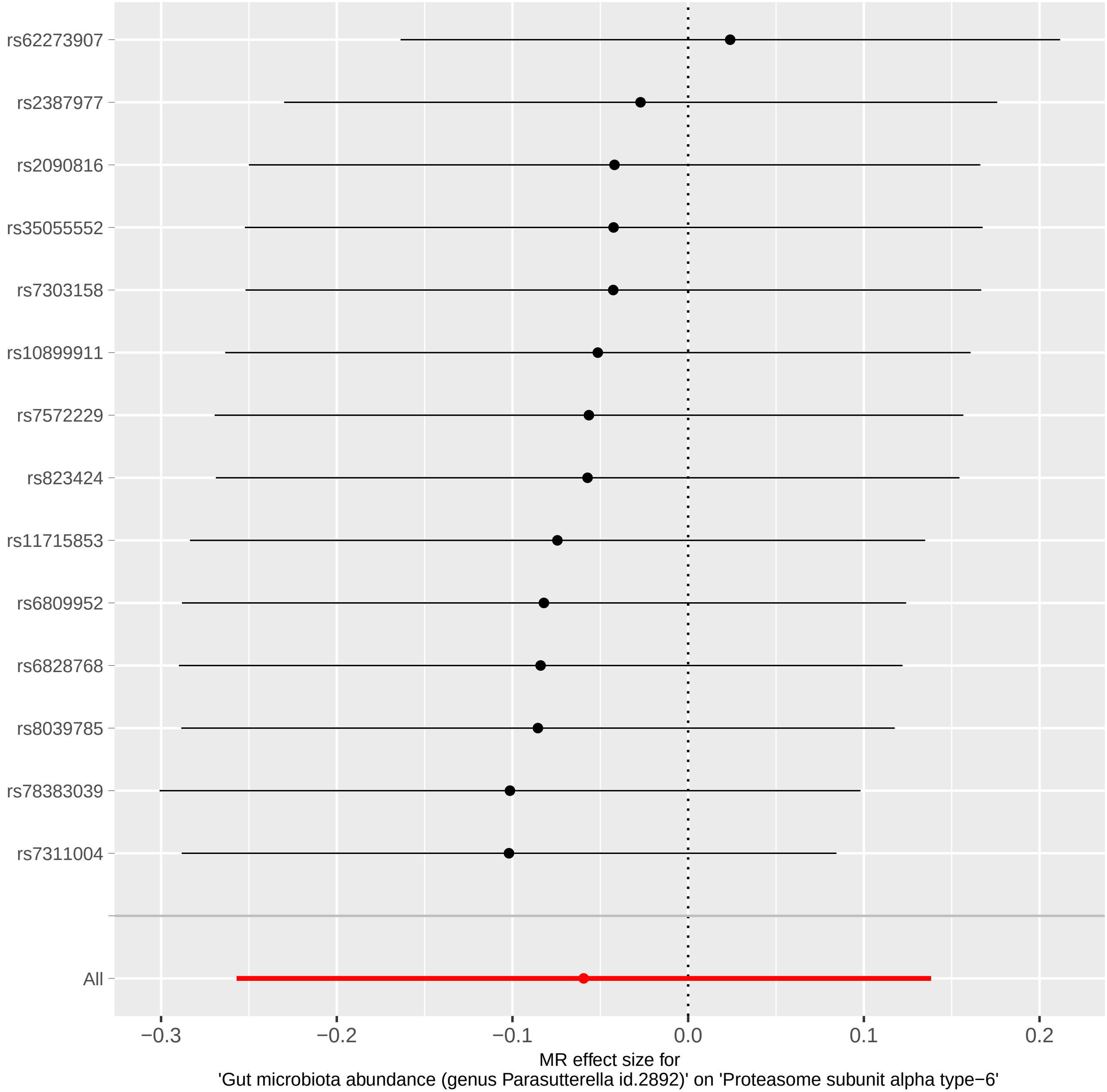

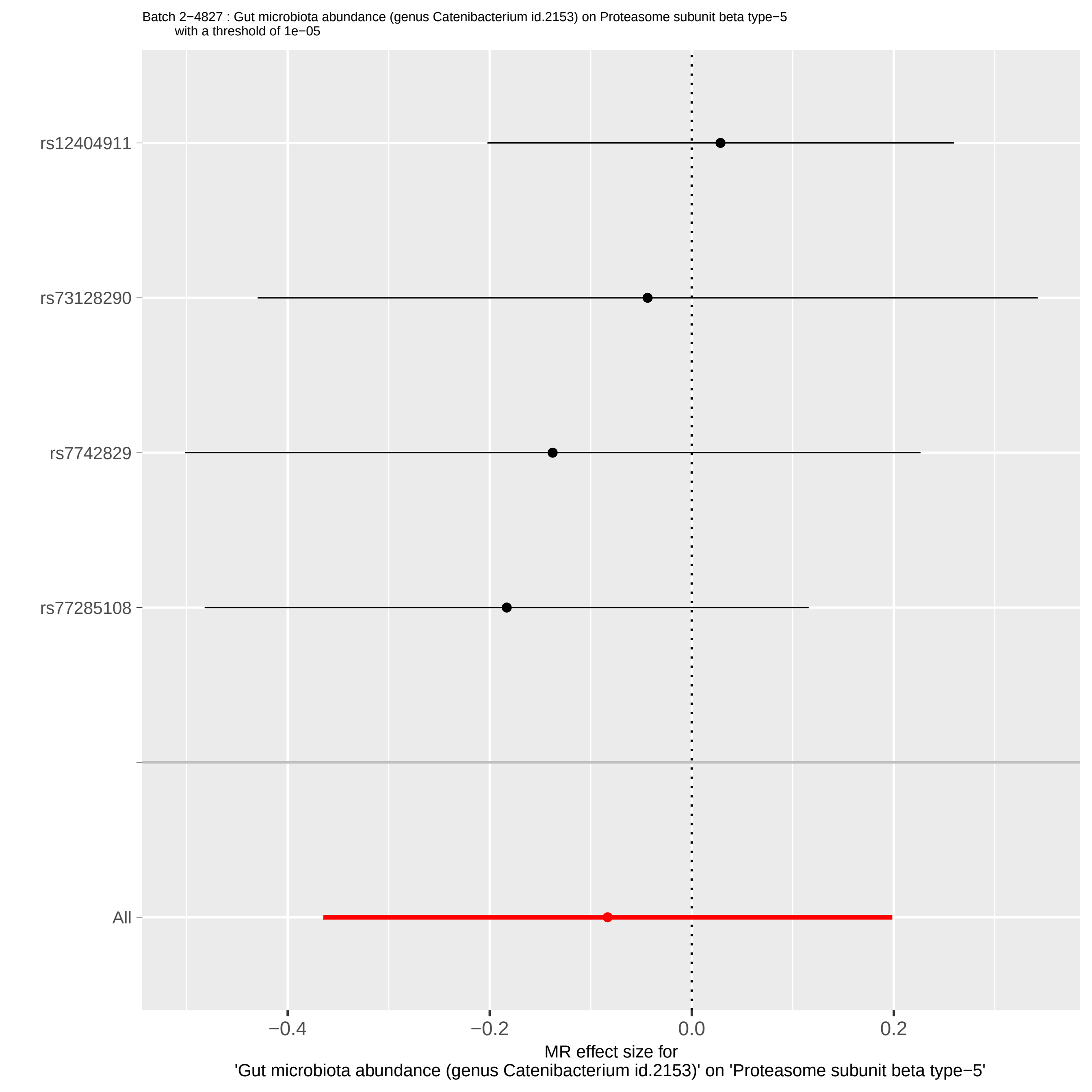

Batch 2−4827 : Gut microbiota abundance (genus Catenibacterium id.2153) on Proteasome subunit beta type−5
with a threshold of 1e−05
rs12404911
rs73128290
rs7742829
rs77285108
All
−0.4
−0.2
0.0
0.2
MR effect size for
'Gut microbiota abundance (genus Catenibacterium id.2153)' on 'Proteasome subunit beta type−5'

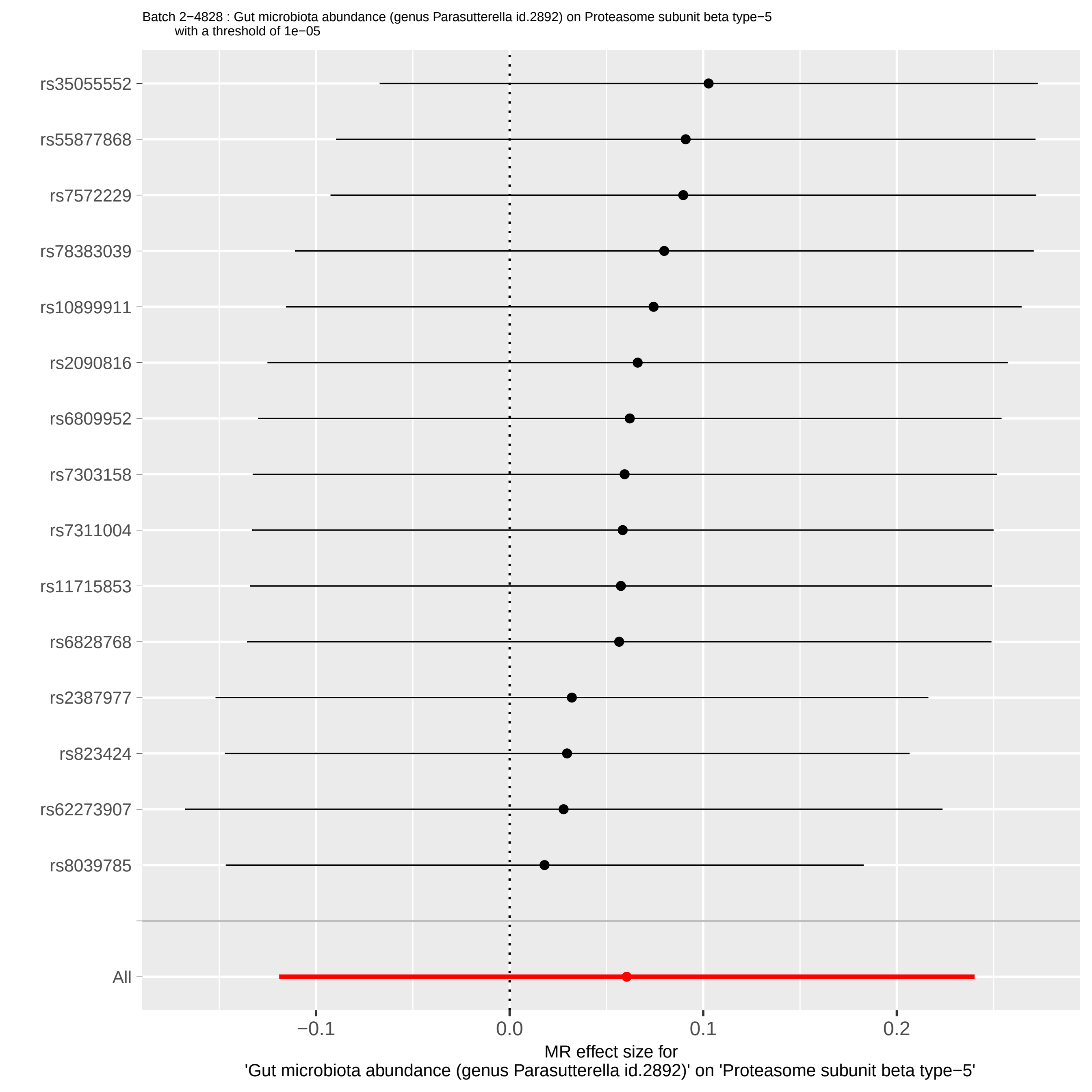

Batch 2−4828 : Gut microbiota abundance (genus Parasutterella id.2892) on Proteasome subunit beta type−5
with a threshold of 1e−05
rs35055552
rs55877868
rs7572229
rs78383039
rs10899911
rs2090816
rs6809952
rs7303158
rs7311004
rs11715853
rs6828768
rs2387977
rs823424
rs62273907
rs8039785
All
−0.1
0.0
0.1
0.2
MR effect size for
'Gut microbiota abundance (genus Parasutterella id.2892)' on 'Proteasome subunit beta type−5'

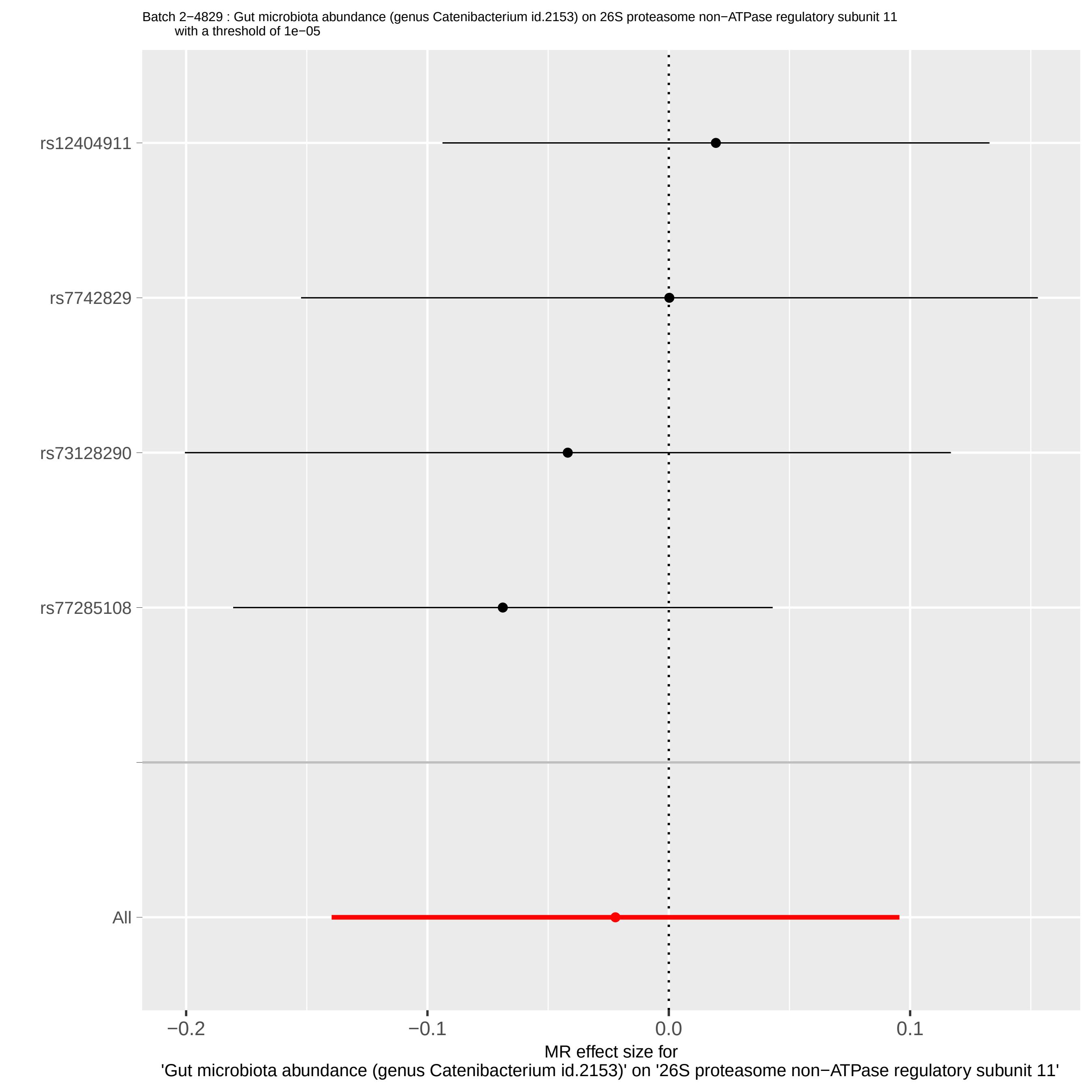

Batch 2−4829 : Gut microbiota abundance (genus Catenibacterium id.2153) on 26S proteasome non−ATPase regulatory subunit 11
with a threshold of 1e−05
rs12404911
rs7742829
rs73128290
rs77285108
All
−0.2
−0.1
0.0
0.1
MR effect size for
'Gut microbiota abundance (genus Catenibacterium id.2153)' on '26S proteasome non−ATPase regulatory subunit 11'

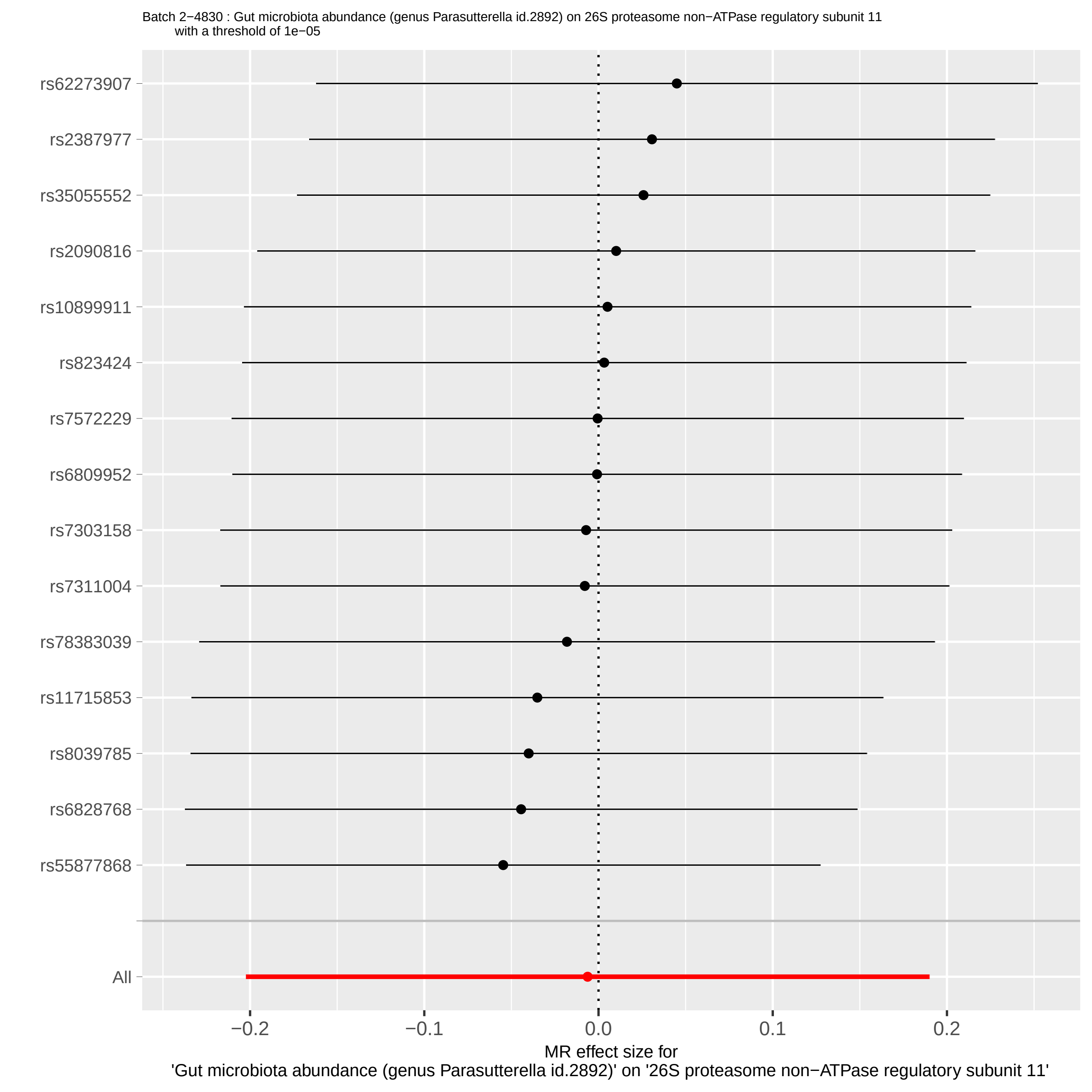

Batch 2−4830 : Gut microbiota abundance (genus Parasutterella id.2892) on 26S proteasome non−ATPase regulatory subunit 11
with a threshold of 1e−05
rs62273907
rs2387977
rs35055552
rs2090816
rs10899911
rs823424
rs7572229
rs6809952
rs7303158
rs7311004
rs78383039
rs11715853
rs8039785
rs6828768
rs55877868
All
−0.2
−0.1
0.0
0.1
0.2
MR effect size for
'Gut microbiota abundance (genus Parasutterella id.2892)' on '26S proteasome non−ATPase regulatory subunit 11'

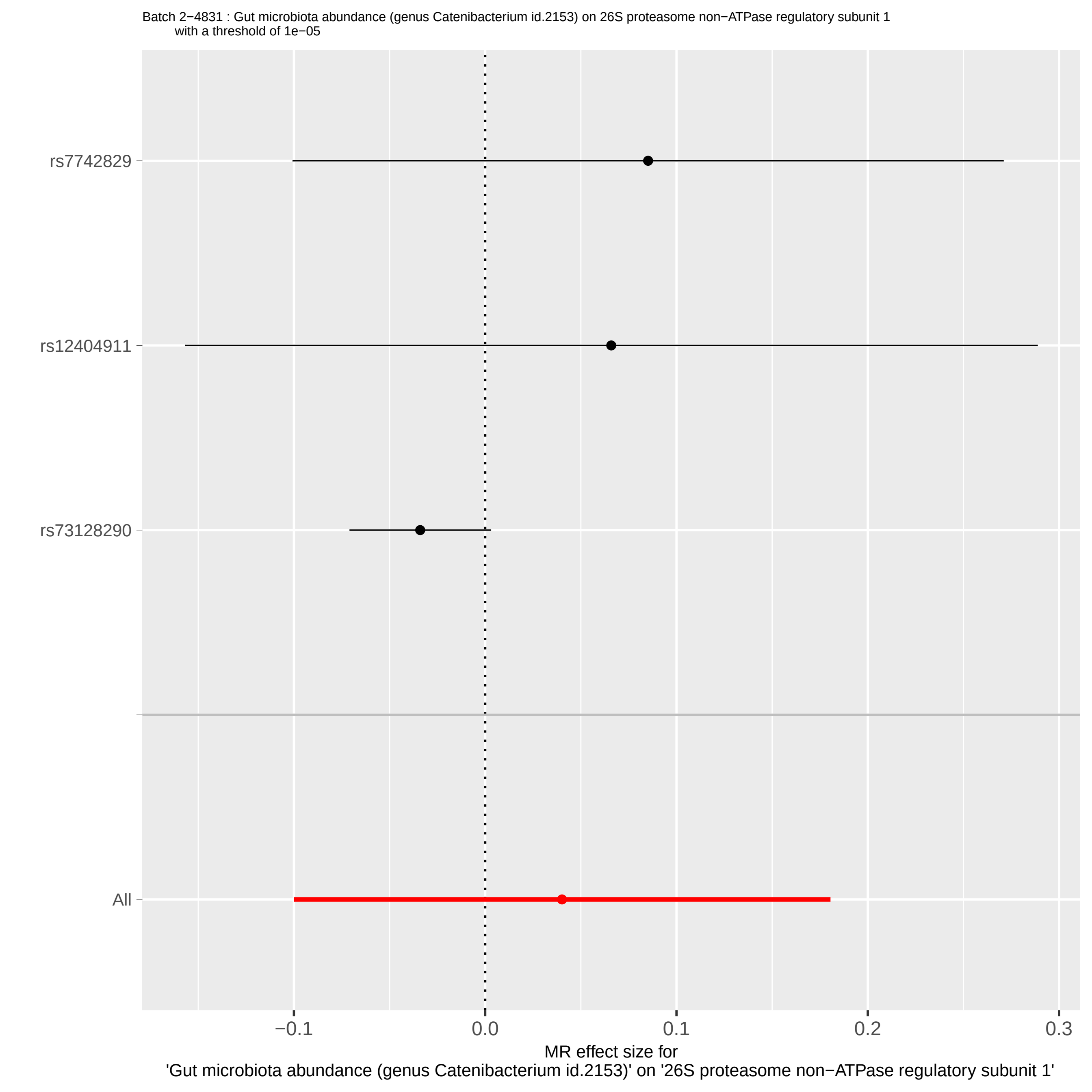

Batch 2−4831 : Gut microbiota abundance (genus Catenibacterium id.2153) on 26S proteasome non−ATPase regulatory subunit 1
with a threshold of 1e−05
rs7742829
rs12404911
rs73128290
All
−0.1
0.0
0.1
0.2
0.3
MR effect size for
'Gut microbiota abundance (genus Catenibacterium id.2153)' on '26S proteasome non−ATPase regulatory subunit 1'

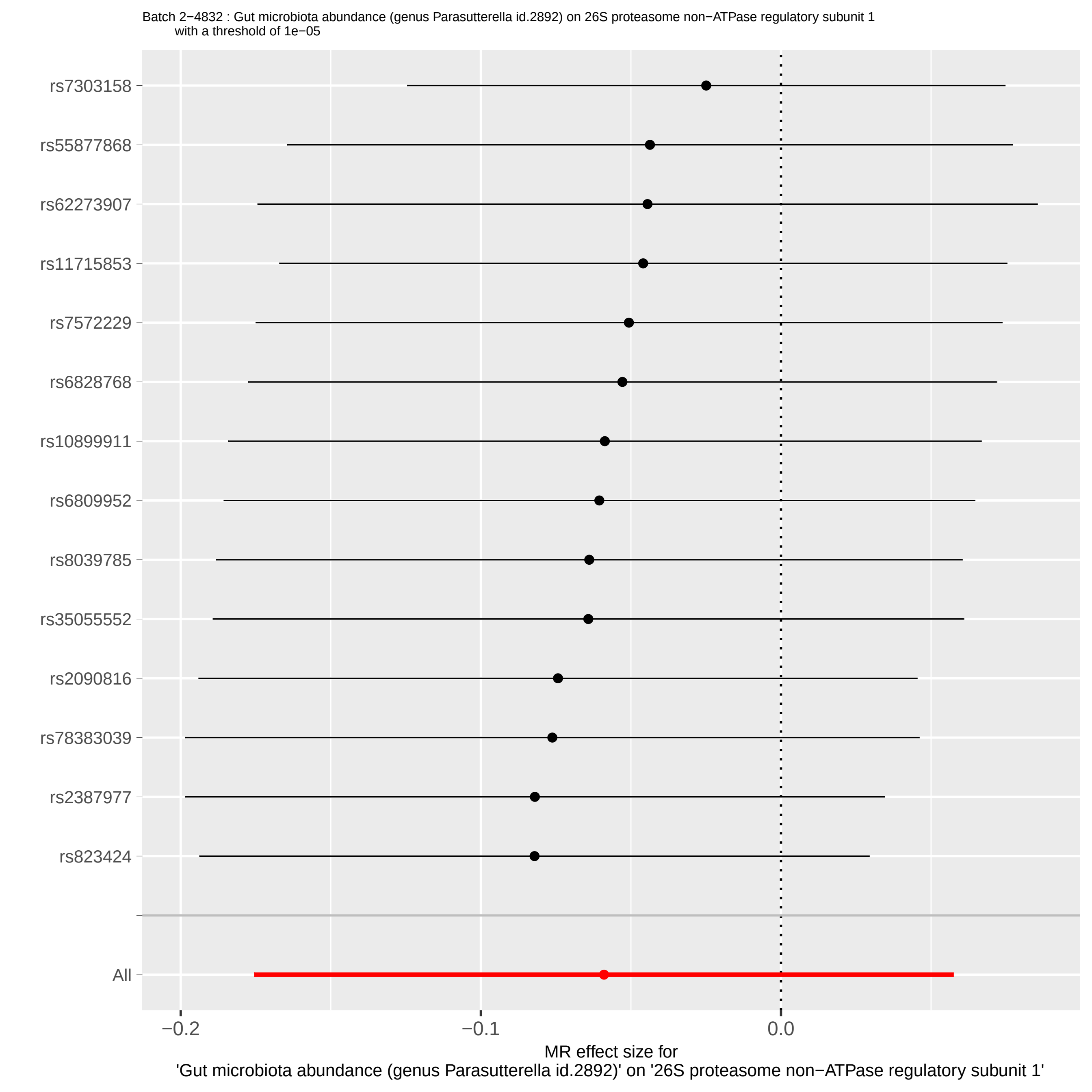

Batch 2−4832 : Gut microbiota abundance (genus Parasutterella id.2892) on 26S proteasome non−ATPase regulatory subunit 1
with a threshold of 1e−05
rs7303158
rs55877868
rs62273907
rs11715853
rs7572229
rs6828768
rs10899911
rs6809952
rs8039785
rs35055552
rs2090816
rs78383039
rs2387977
rs823424
All
−0.2
−0.1
0.0
MR effect size for
'Gut microbiota abundance (genus Parasutterella id.2892)' on '26S proteasome non−ATPase regulatory subunit 1'

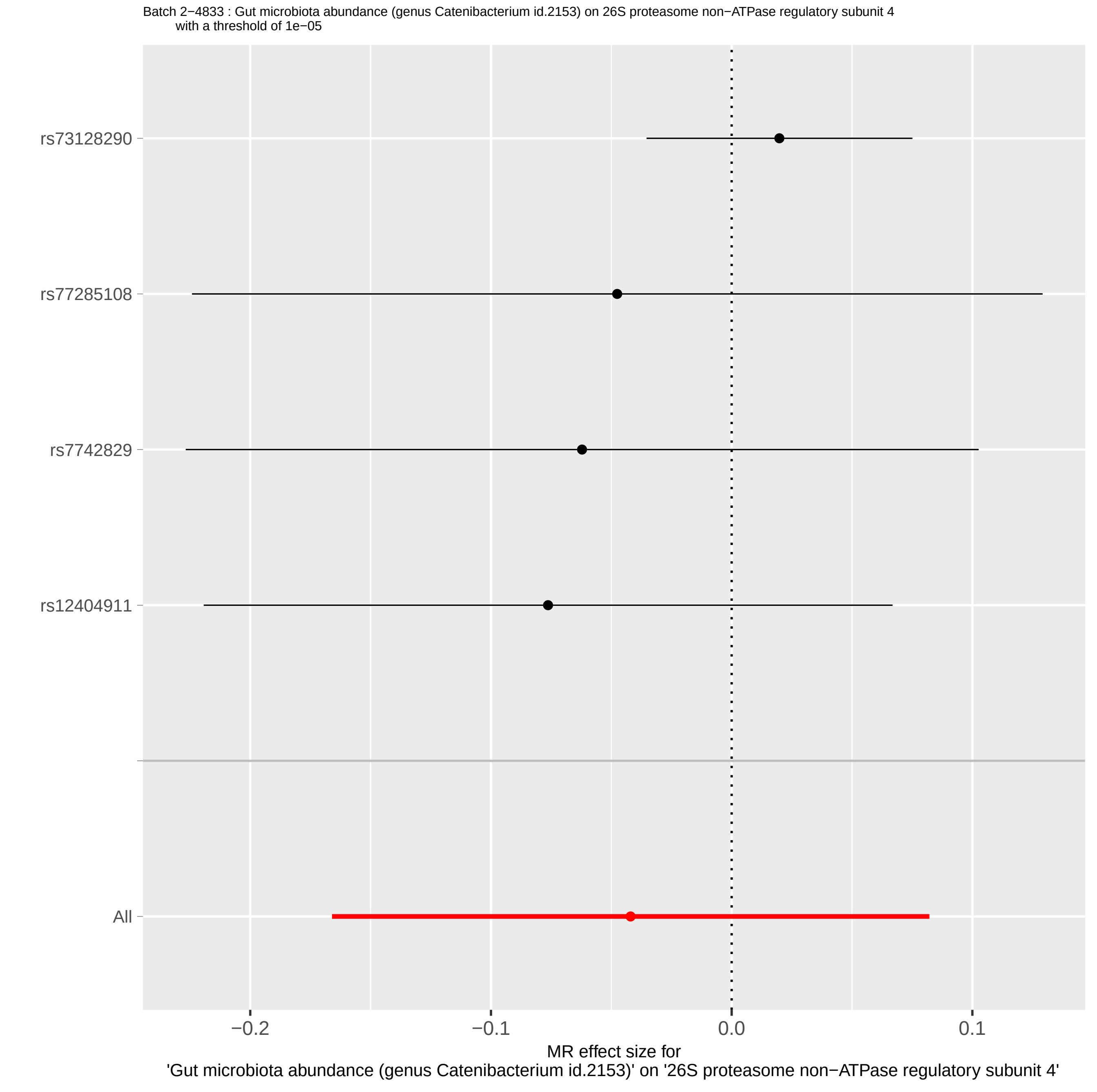
Batch 2−4833 : Gut microbiota abundance (genus Catenibacterium id.2153) on 26S proteasome non−ATPase regulatory subunit 4
with a threshold of 1e−05
rs73128290
rs77285108
rs7742829
rs12404911
All
−0.2
−0.1
0.0
0.1
MR effect size for
'Gut microbiota abundance (genus Catenibacterium id.2153)' on '26S proteasome non−ATPase regulatory subunit 4'

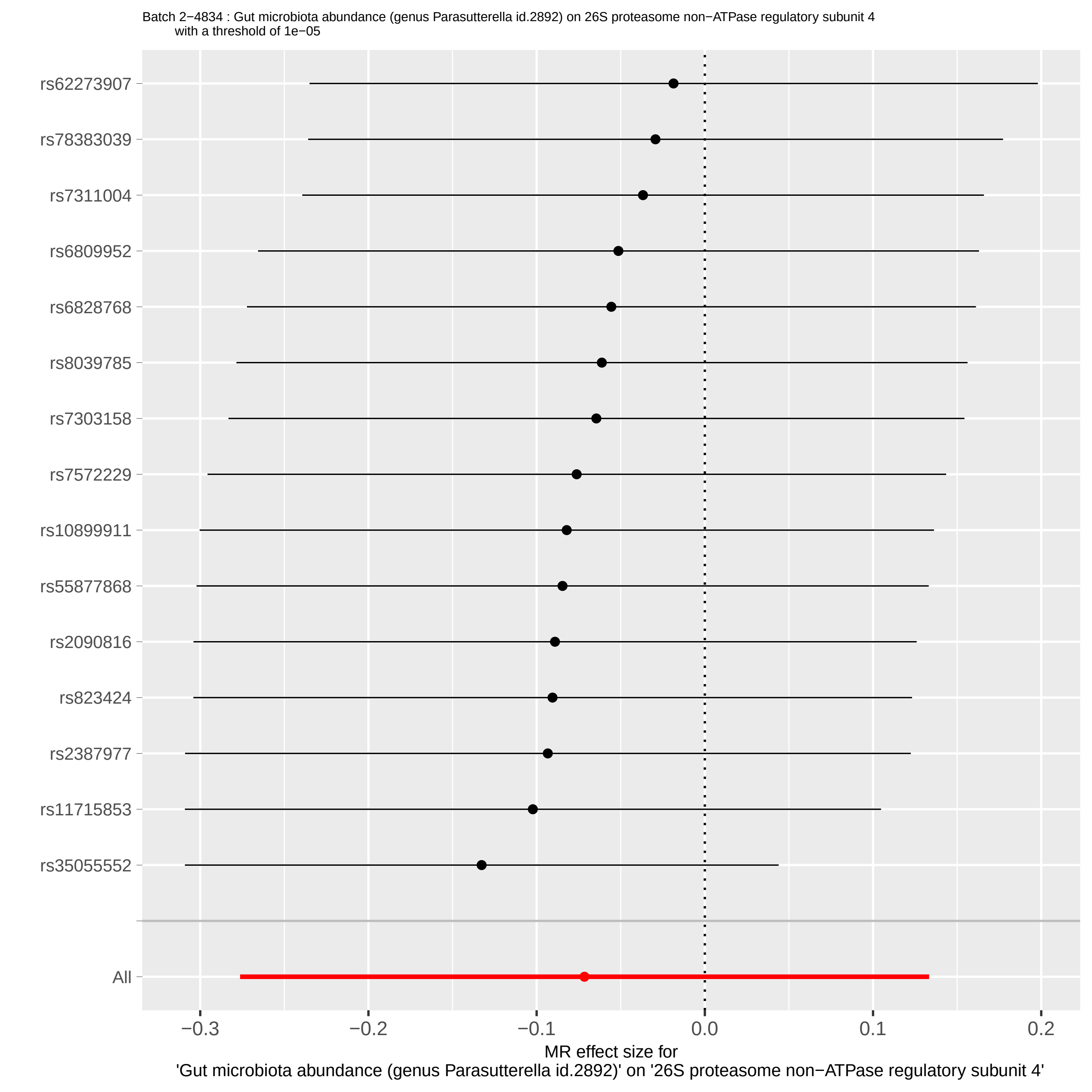

Batch 2−4834 : Gut microbiota abundance (genus Parasutterella id.2892) on 26S proteasome non−ATPase regulatory subunit 4
with a threshold of 1e−05
rs62273907
rs78383039
rs7311004
rs6809952
rs6828768
rs8039785
rs7303158
rs7572229
rs10899911
rs55877868
rs2090816
rs823424
rs2387977
rs11715853
rs35055552
All
−0.3
−0.2
−0.1
0.0
0.1
0.2
MR effect size for
'Gut microbiota abundance (genus Parasutterella id.2892)' on '26S proteasome non−ATPase regulatory subunit 4'

Batch 2−4835 : Gut microbiota abundance (genus Catenibacterium id.2153) on 26S proteasome non−ATPase regulatory subunit 5
with a threshold of 1e−05

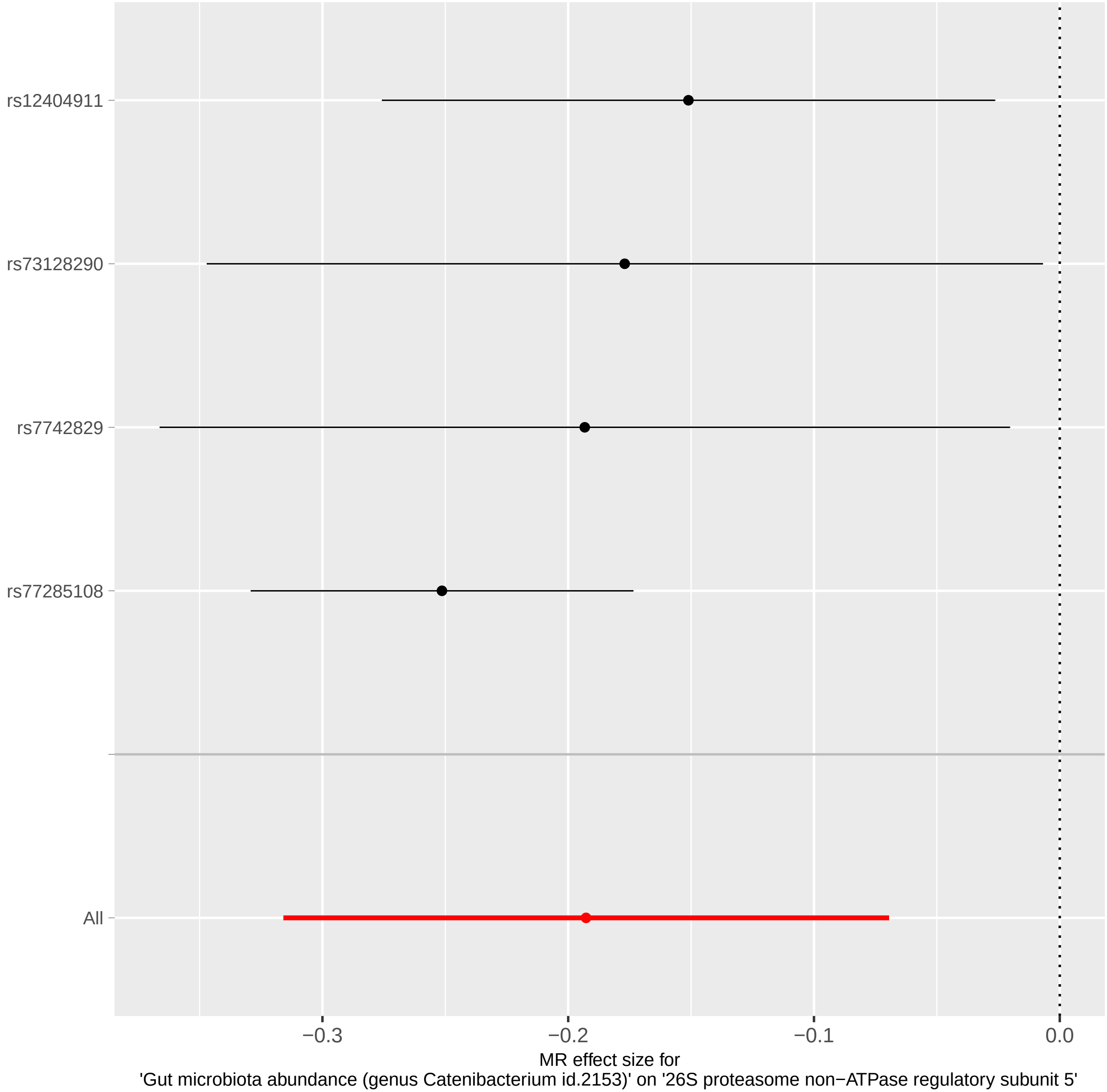

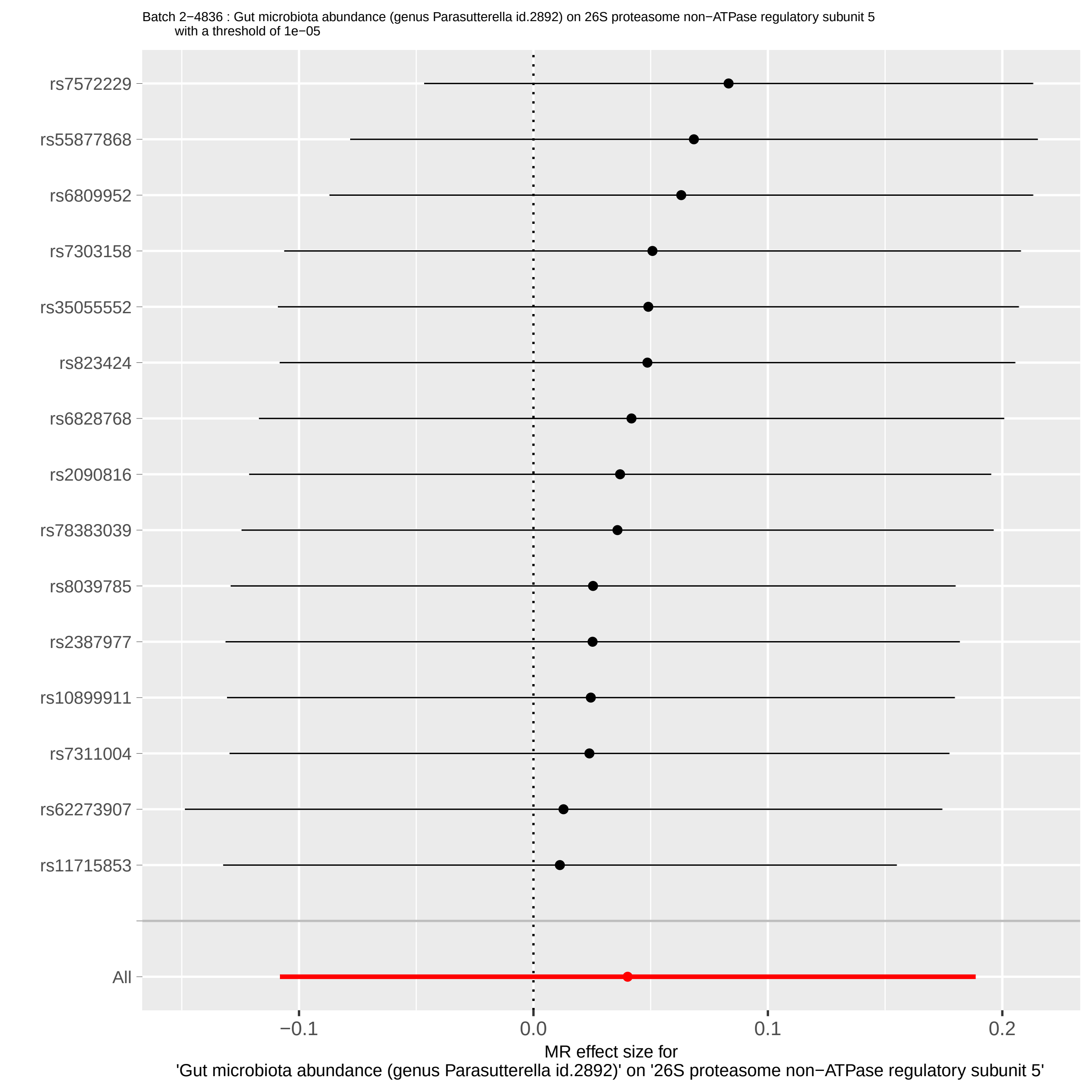
Batch 2−4836 : Gut microbiota abundance (genus Parasutterella id.2892) on 26S proteasome non−ATPase regulatory subunit 5
with a threshold of 1e−05
rs7572229
rs55877868
rs6809952
rs7303158
rs35055552
rs823424
rs6828768
rs2090816
rs78383039
rs8039785
rs2387977
rs10899911
rs7311004
rs62273907
rs11715853
All
−0.1
0.0
0.1
0.2
MR effect size for
'Gut microbiota abundance (genus Parasutterella id.2892)' on '26S proteasome non−ATPase regulatory subunit 5'

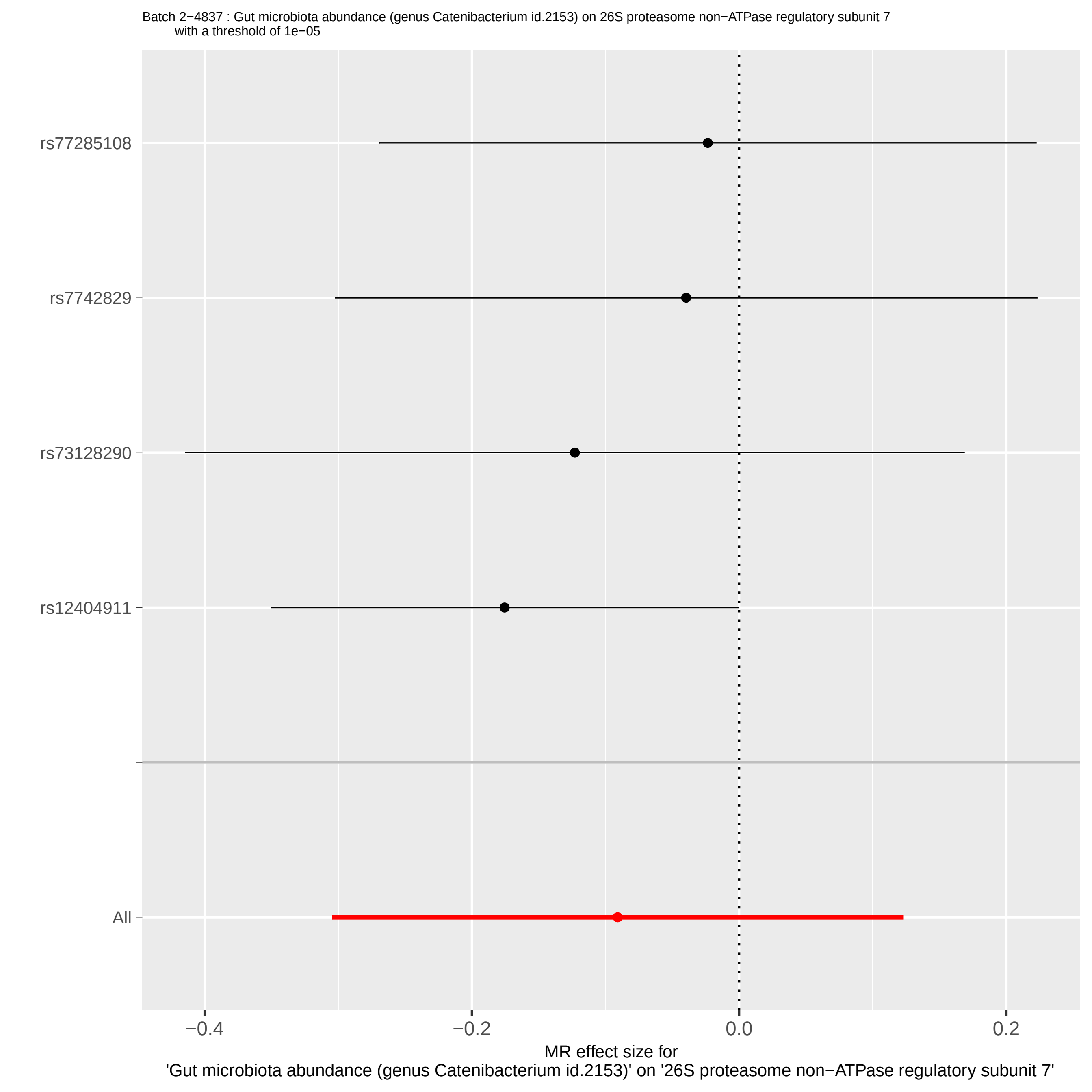
Batch 2−4837 : Gut microbiota abundance (genus Catenibacterium id.2153) on 26S proteasome non−ATPase regulatory subunit 7
with a threshold of 1e−05
rs77285108
rs7742829
rs73128290
rs12404911
All
−0.4
−0.2
0.0
0.2
MR effect size for
'Gut microbiota abundance (genus Catenibacterium id.2153)' on '26S proteasome non−ATPase regulatory subunit 7'

Batch 2−4838 : Gut microbiota abundance (genus Parasutterella id.2892) on 26S proteasome non−ATPase regulatory subunit 7
with a threshold of 1e−05

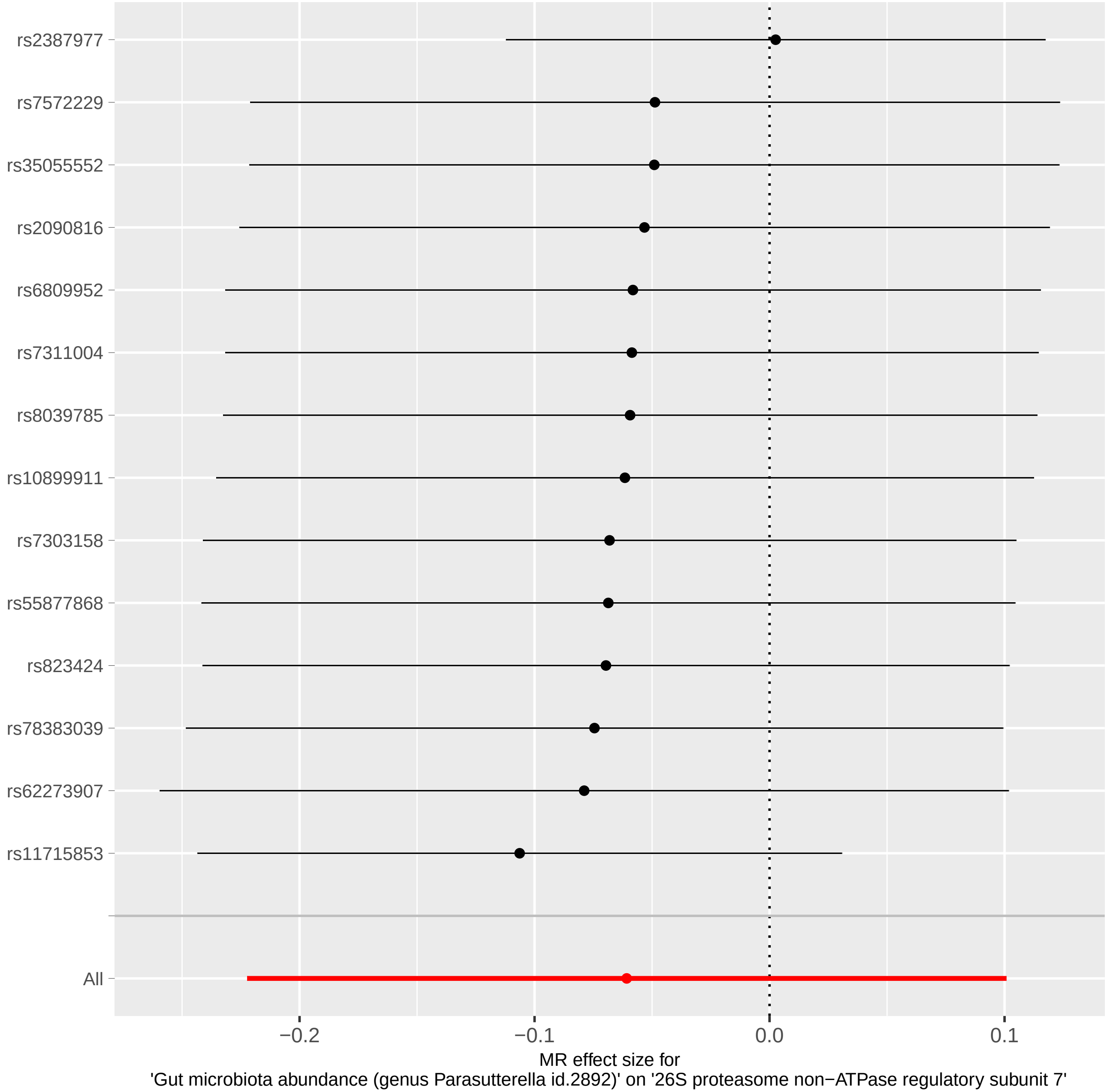

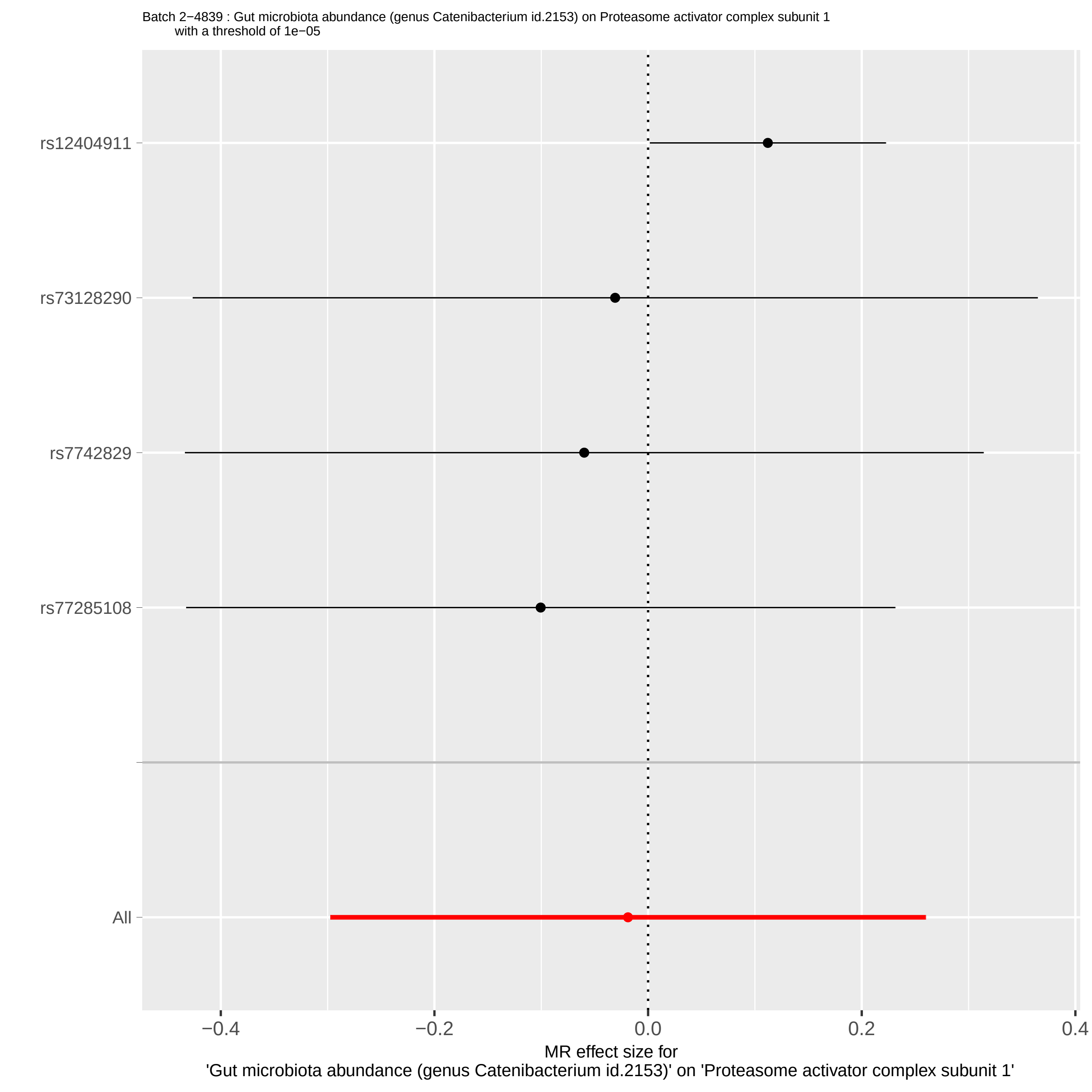
Batch 2−4839 : Gut microbiota abundance (genus Catenibacterium id.2153) on Proteasome activator complex subunit 1
with a threshold of 1e−05
rs12404911
rs73128290
rs7742829
rs77285108
All
−0.4
−0.2
0.0
0.2
0.4
MR effect size for
'Gut microbiota abundance (genus Catenibacterium id.2153)' on 'Proteasome activator complex subunit 1'

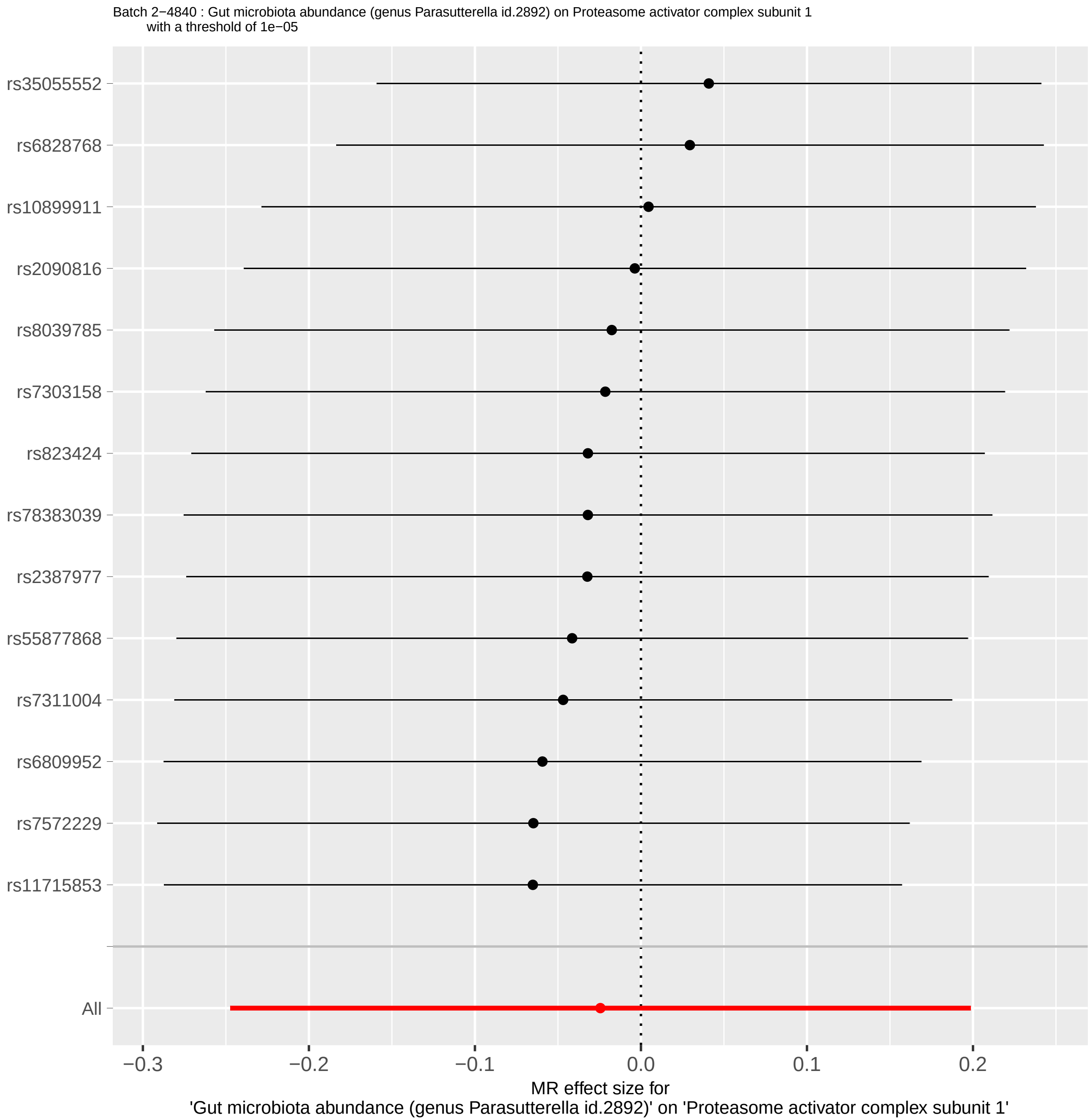

Batch 2−4840 : Gut microbiota abundance (genus Parasutterella id.2892) on Proteasome activator complex subunit 1
with a threshold of 1e−05
rs35055552
rs6828768
rs10899911
rs2090816
rs8039785
rs7303158
rs823424
rs78383039
rs2387977
rs55877868
rs7311004
rs6809952
rs7572229
rs11715853
All
−0.3
−0.2
−0.1
0.0
0.1
0.2
MR effect size for
'Gut microbiota abundance (genus Parasutterella id.2892)' on 'Proteasome activator complex subunit 1'

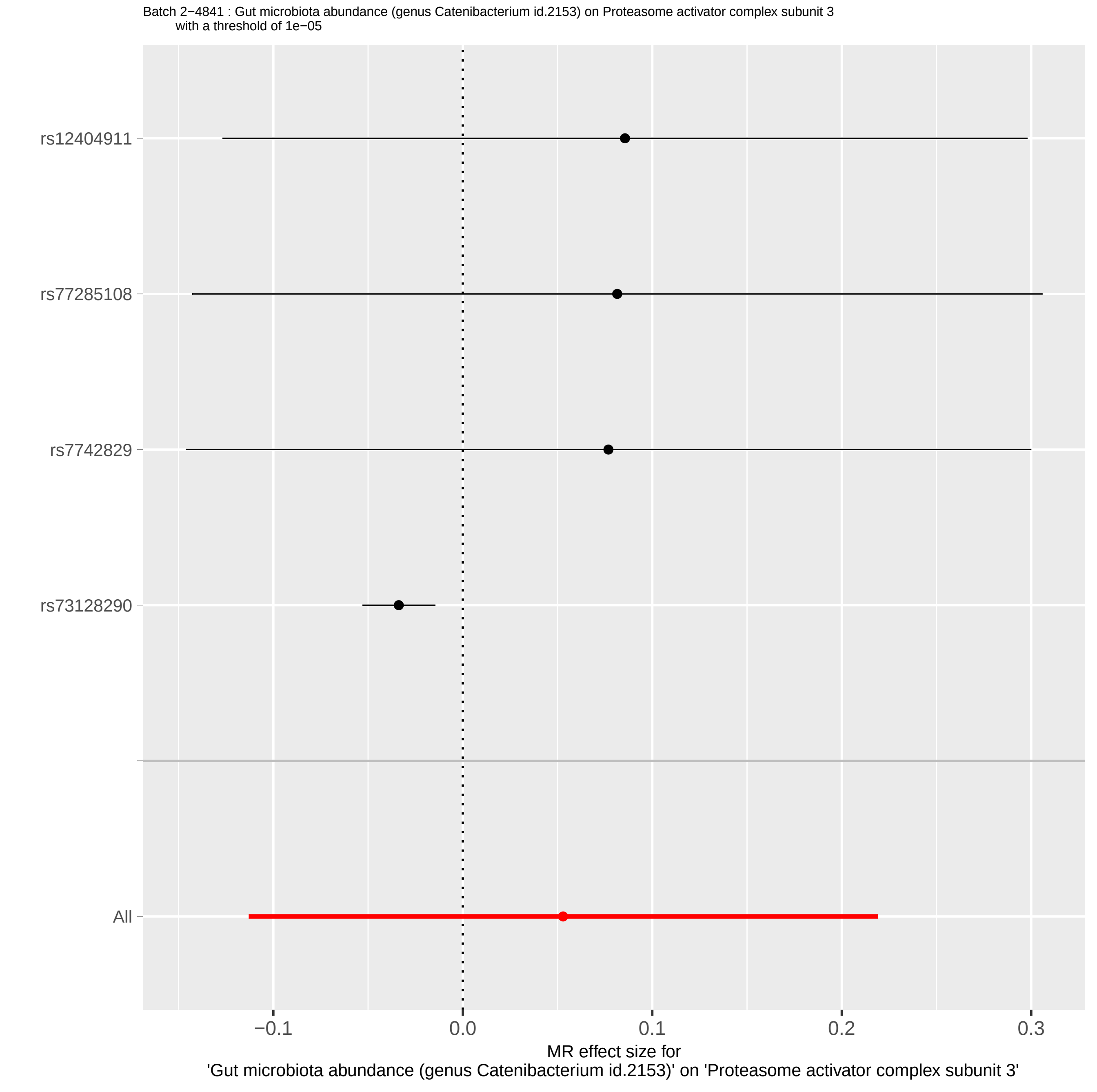
Batch 2−4841 : Gut microbiota abundance (genus Catenibacterium id.2153) on Proteasome activator complex subunit 3
with a threshold of 1e−05
rs12404911
rs77285108
rs7742829
rs73128290
All
−0.1
0.0
0.1
0.2
0.3
MR effect size for
'Gut microbiota abundance (genus Catenibacterium id.2153)' on 'Proteasome activator complex subunit 3'

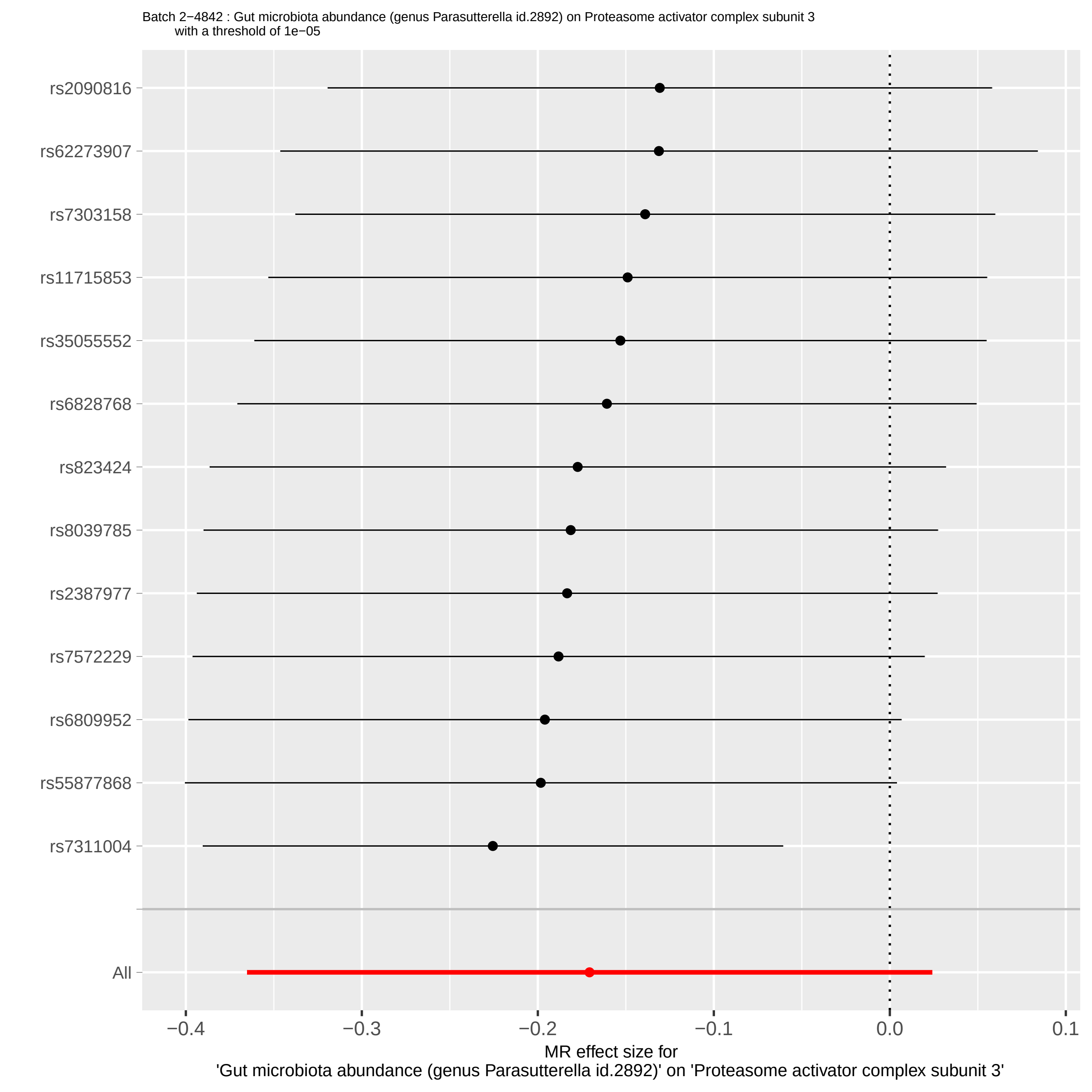

Batch 2−4842 : Gut microbiota abundance (genus Parasutterella id.2892) on Proteasome activator complex subunit 3
with a threshold of 1e−05
rs2090816
rs62273907
rs7303158
rs11715853
rs35055552
rs6828768
rs823424
rs8039785
rs2387977
rs7572229
rs6809952
rs55877868
rs7311004
All
−0.4
−0.3
−0.2
−0.1
0.0
0.1
MR effect size for
'Gut microbiota abundance (genus Parasutterella id.2892)' on 'Proteasome activator complex subunit 3'

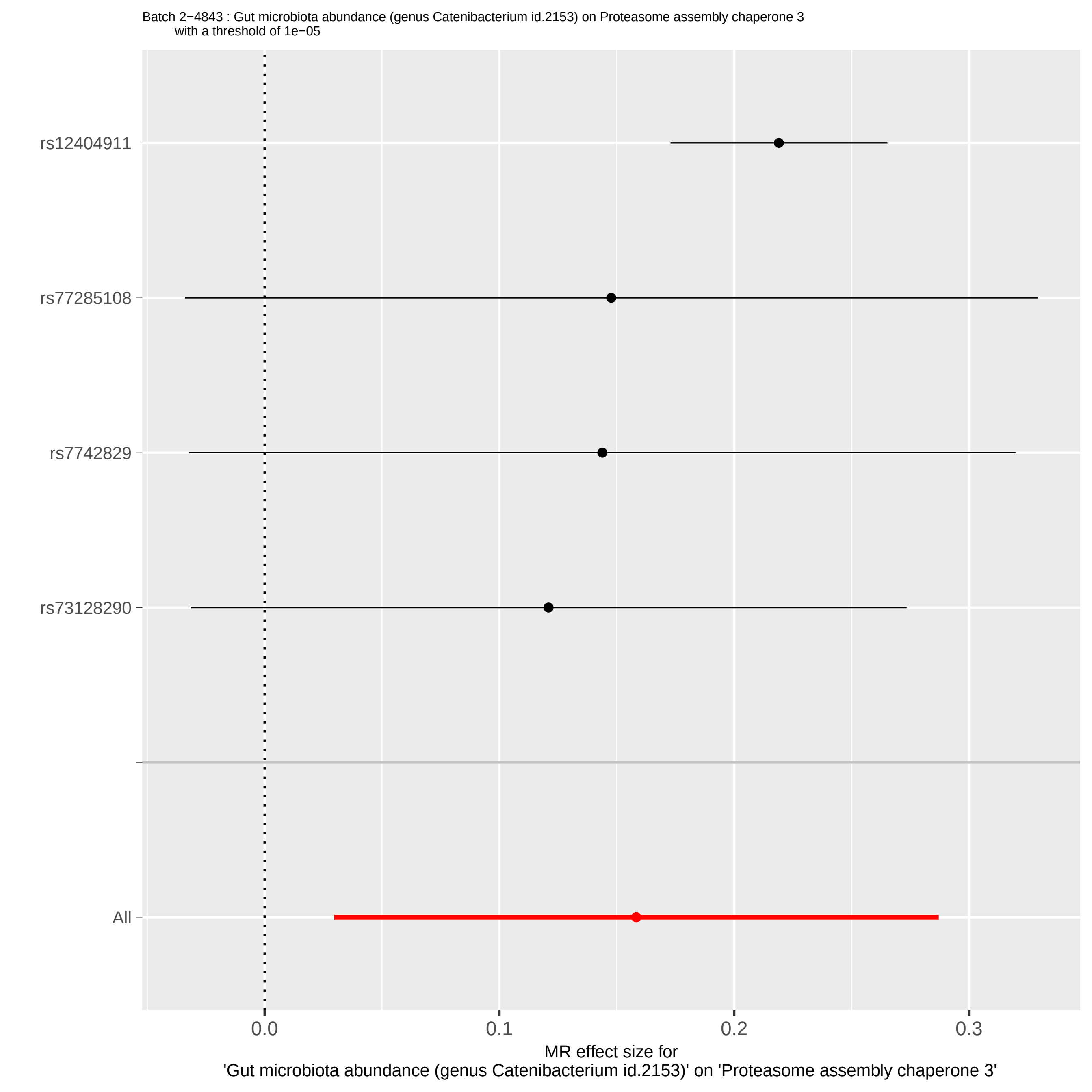
Batch 2−4843 : Gut microbiota abundance (genus Catenibacterium id.2153) on Proteasome assembly chaperone 3
with a threshold of 1e−05
rs12404911
rs77285108
rs7742829
rs73128290
All
0.0
0.1
0.2
0.3
MR effect size for
'Gut microbiota abundance (genus Catenibacterium id.2153)' on 'Proteasome assembly chaperone 3'

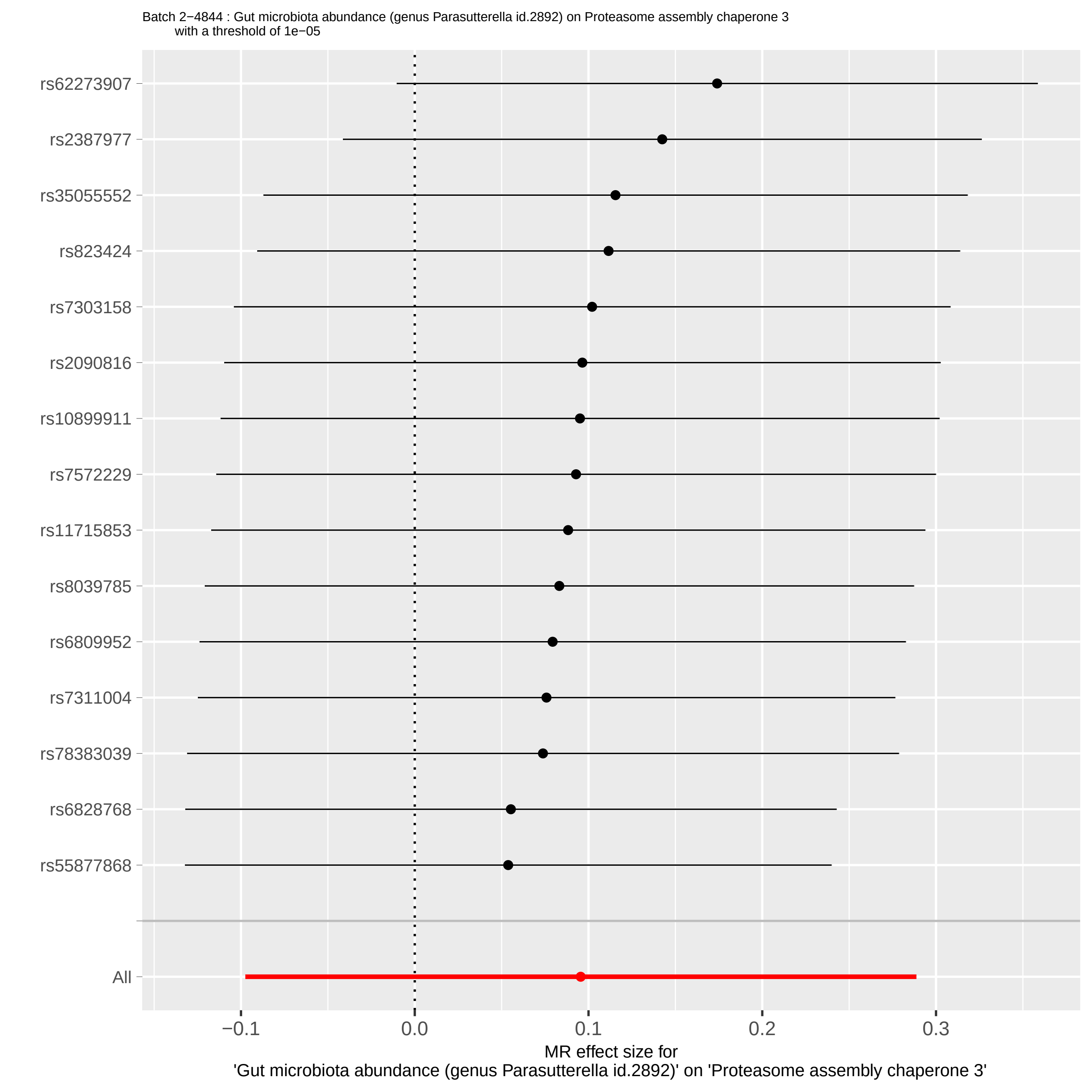

Batch 2−4844 : Gut microbiota abundance (genus Parasutterella id.2892) on Proteasome assembly chaperone 3
with a threshold of 1e−05
rs62273907
rs2387977
rs35055552
rs823424
rs7303158
rs2090816
rs10899911
rs7572229
rs11715853
rs8039785
rs6809952
rs7311004
rs78383039
rs6828768
rs55877868
All
−0.1
0.0
0.1
0.2
0.3
MR effect size for
'Gut microbiota abundance (genus Parasutterella id.2892)' on 'Proteasome assembly chaperone 3'

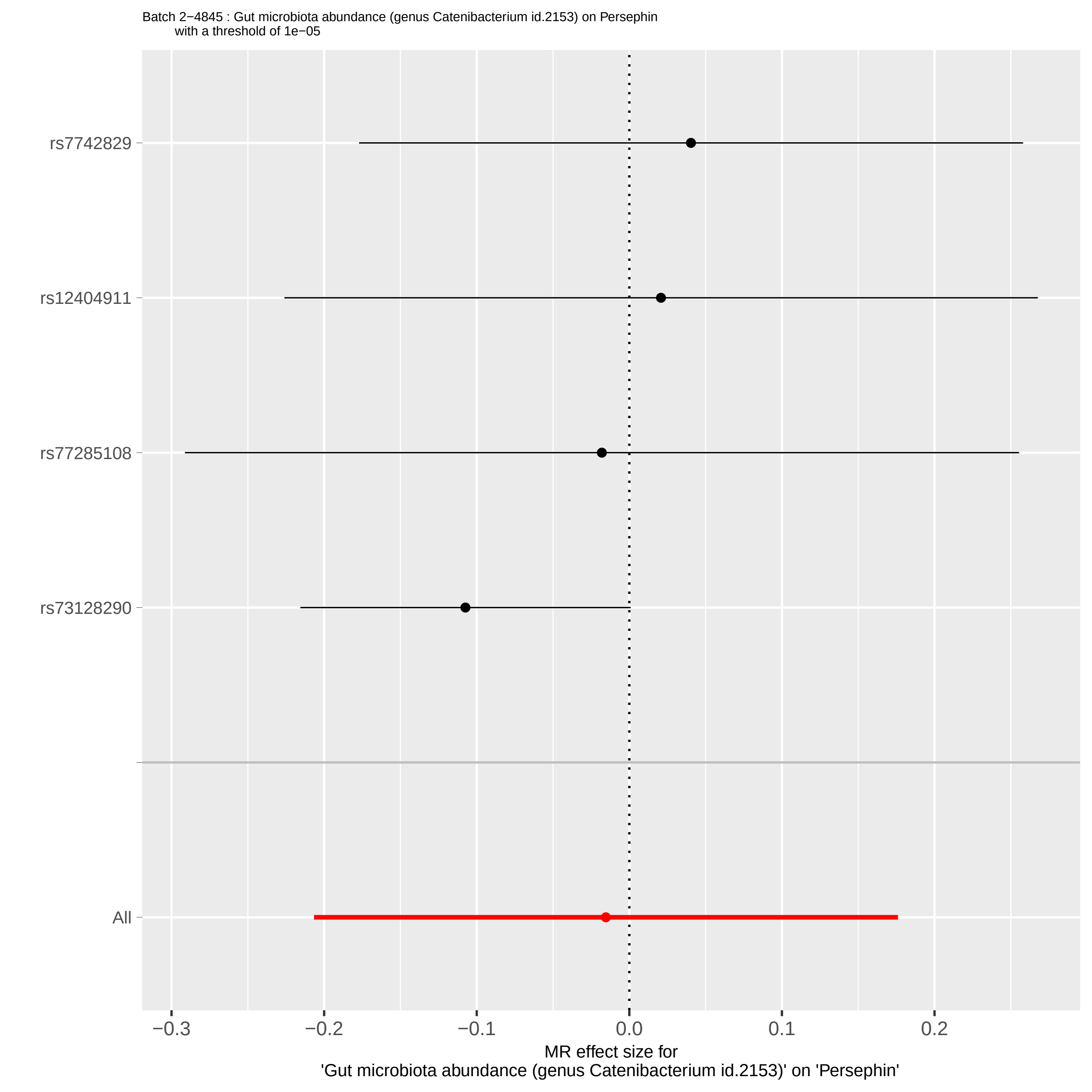
Batch 2−4845 : Gut microbiota abundance (genus Catenibacterium id.2153) on Persephin
with a threshold of 1e−05
rs7742829
rs12404911
rs77285108
rs73128290
All
−0.3
−0.2
−0.1
0.0
0.1
0.2
MR effect size for
'Gut microbiota abundance (genus Catenibacterium id.2153)' on 'Persephin'

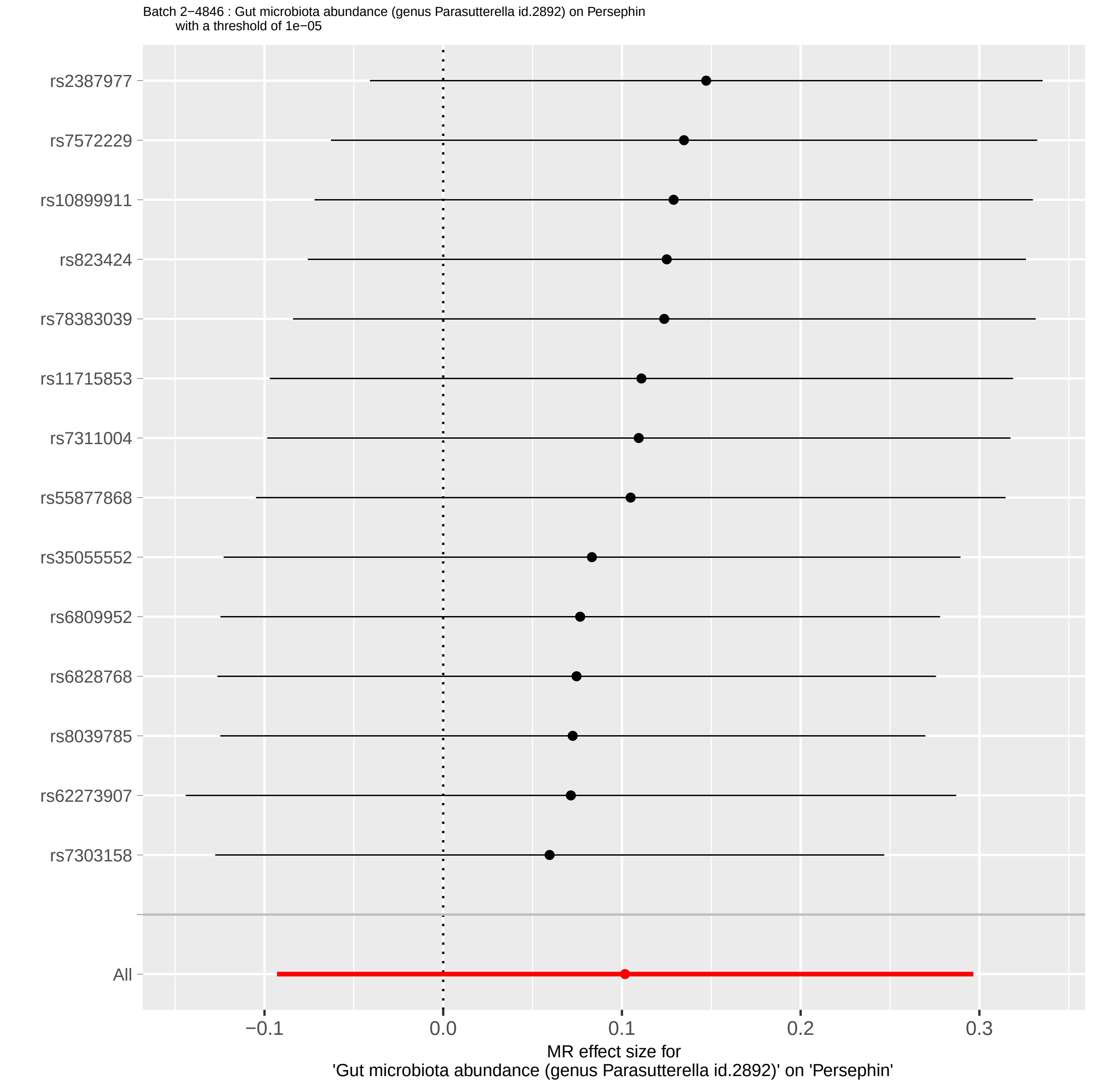

Batch 2−4846 : Gut microbiota abundance (genus Parasutterella id.2892) on Persephin
with a threshold of 1e−05
rs2387977
rs7572229
rs10899911
rs823424
rs78383039
rs11715853
rs7311004
rs55877868
rs35055552
rs6809952
rs6828768
rs8039785
rs62273907
rs7303158
All
−0.1
0.0
0.1
0.2
0.3
MR effect size for
'Gut microbiota abundance (genus Parasutterella id.2892)' on 'Persephin'

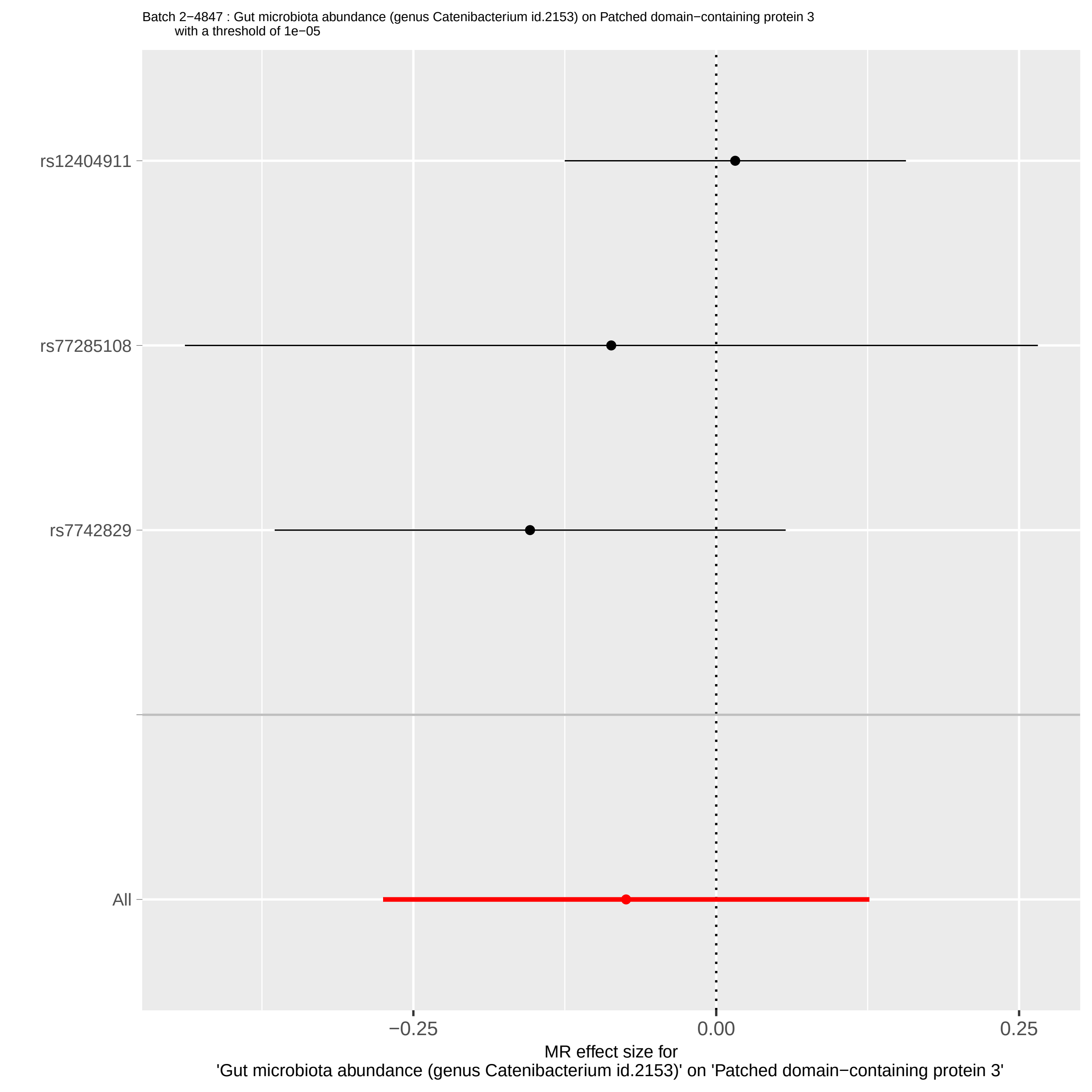

Batch 2−4847 : Gut microbiota abundance (genus Catenibacterium id.2153) on Patched domain−containing protein 3
with a threshold of 1e−05
rs12404911
rs77285108
rs7742829
All
−0.25
0.00
0.25
MR effect size for
'Gut microbiota abundance (genus Catenibacterium id.2153)' on 'Patched domain−containing protein 3'

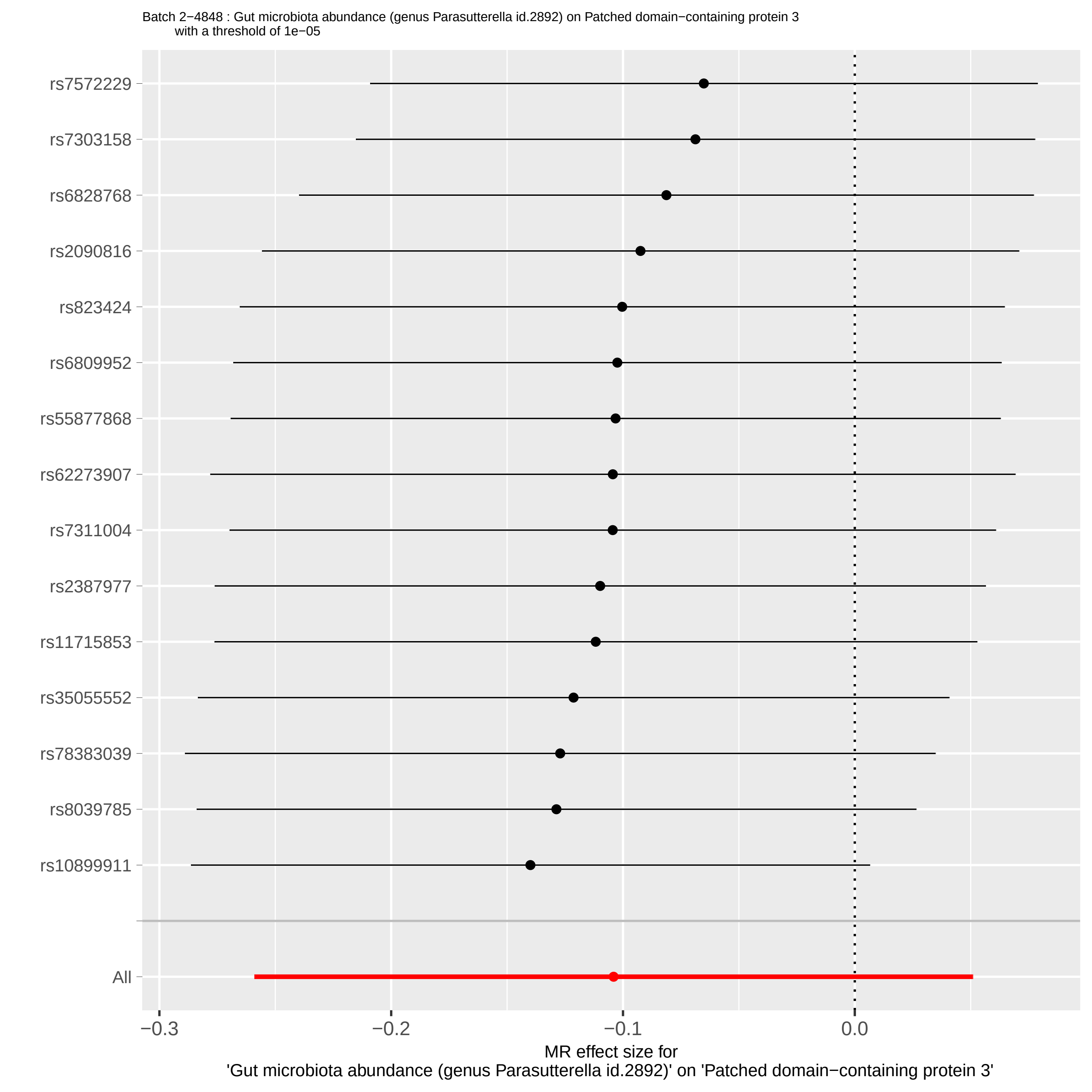
Batch 2−4848 : Gut microbiota abundance (genus Parasutterella id.2892) on Patched domain−containing protein 3
with a threshold of 1e−05
rs7572229
rs7303158
rs6828768
rs2090816
rs823424
rs6809952
rs55877868
rs62273907
rs7311004
rs2387977
rs11715853
rs35055552
rs78383039
rs8039785
rs10899911
All
−0.3
−0.2
−0.1
0.0
MR effect size for
'Gut microbiota abundance (genus Parasutterella id.2892)' on 'Patched domain−containing protein 3'

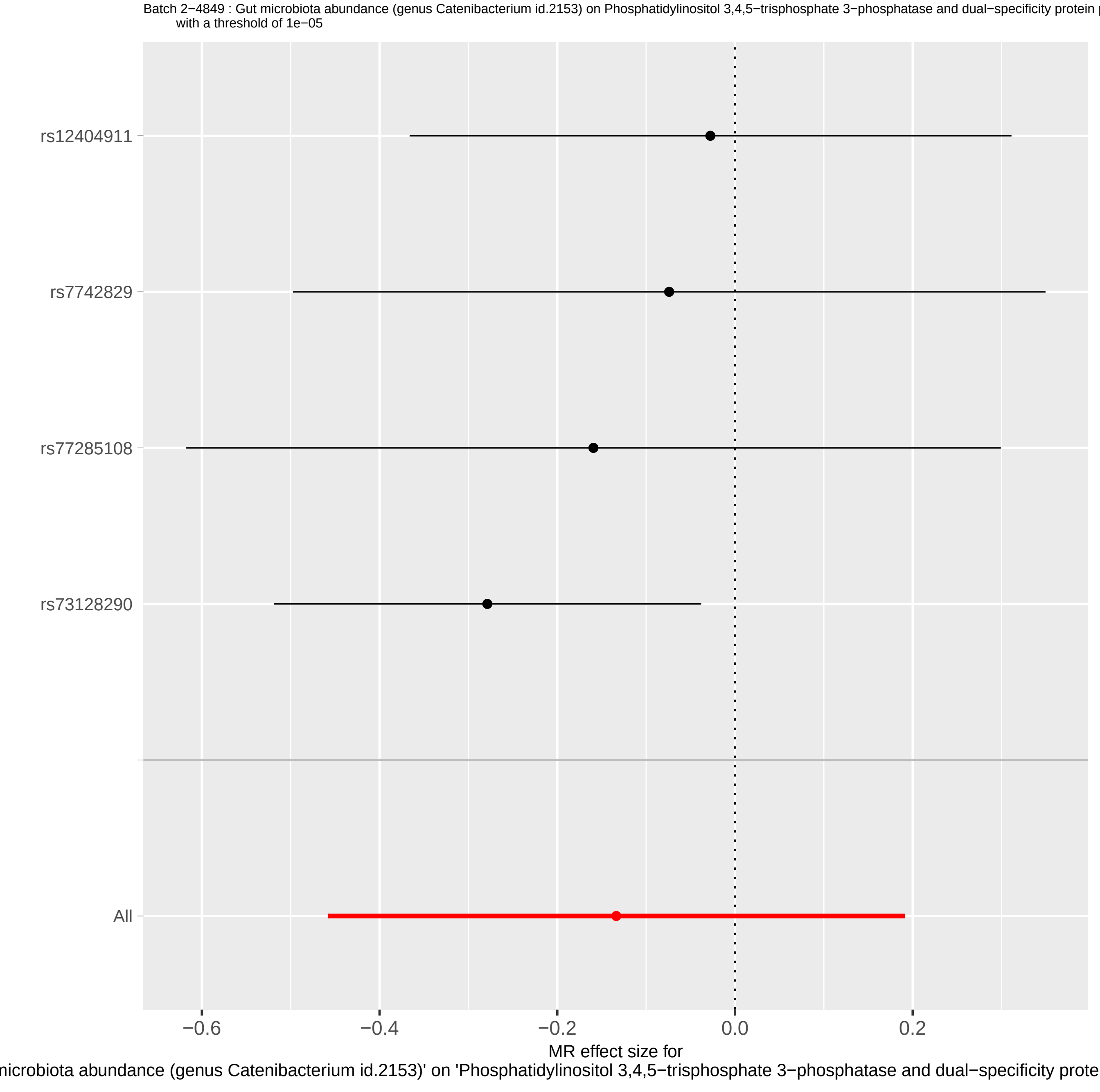

Batch 2−4849 : Gut microbiota abundance (genus Catenibacterium id.2153) on Phosphatidylinositol 3,4,5−trisphosphate 3−phosphatase and dual−specificity protein p
with a threshold of 1e−05
rs12404911
rs7742829
rs77285108
rs73128290
All
−0.6
−0.4
−0.2
0.0
0.2
MR effect size for
microbiota abundance (genus Catenibacterium id.2153)' on 'Phosphatidylinositol 3,4,5−trisphosphate 3−phosphatase and dual−specificity prote

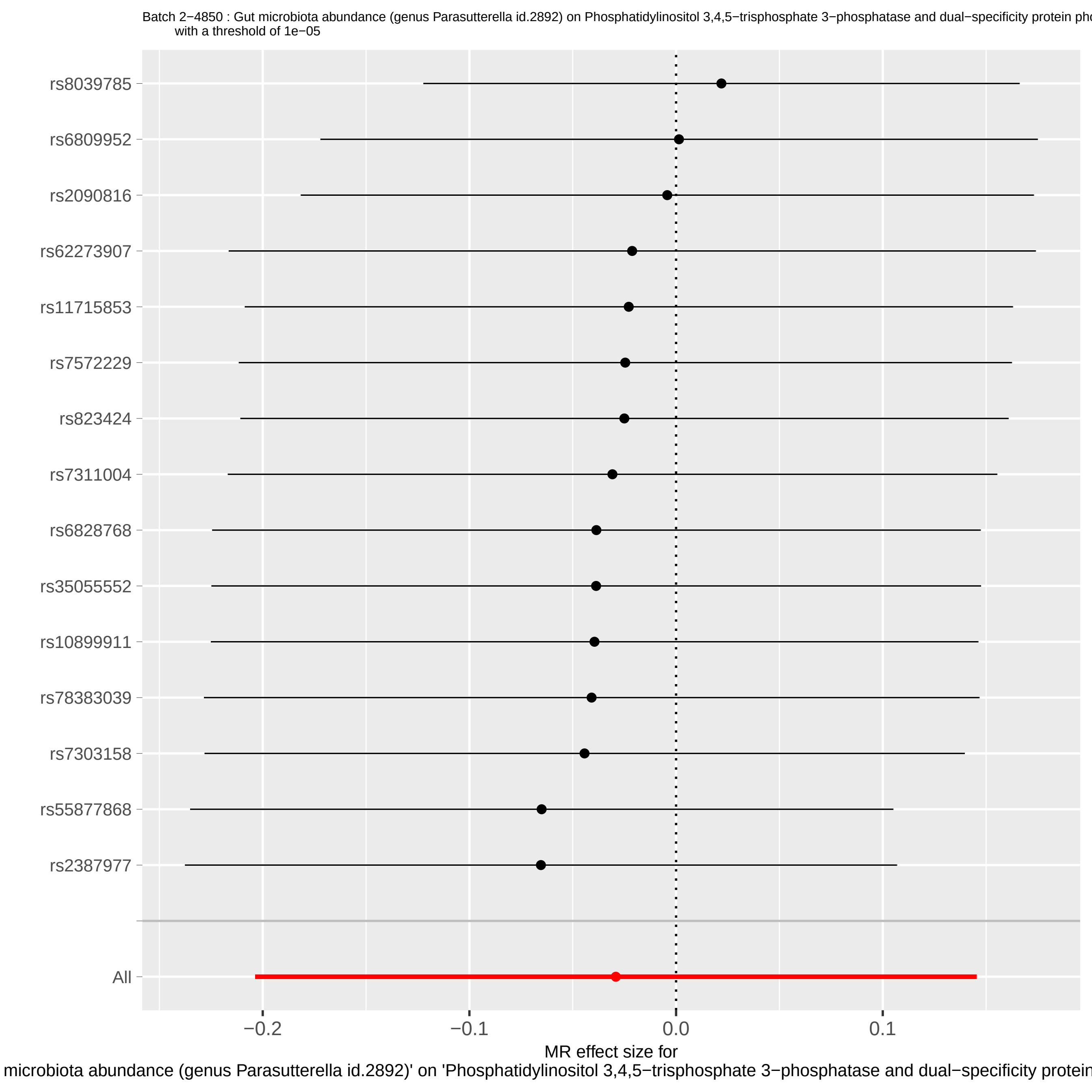
Batch 2−4850 : Gut microbiota abundance (genus Parasutterella id.2892) on Phosphatidylinositol 3,4,5−trisphosphate 3−phosphatase and dual−specificity protein pho
with a threshold of 1e−05
rs8039785
rs6809952
rs2090816
rs62273907
rs11715853
rs7572229
rs823424
rs7311004
rs6828768
rs35055552
rs10899911
rs78383039
rs7303158
rs55877868
rs2387977
All
−0.2
−0.1
0.0
0.1
MR effect size for
microbiota abundance (genus Parasutterella id.2892)' on 'Phosphatidylinositol 3,4,5−trisphosphate 3−phosphatase and dual−specificity protein

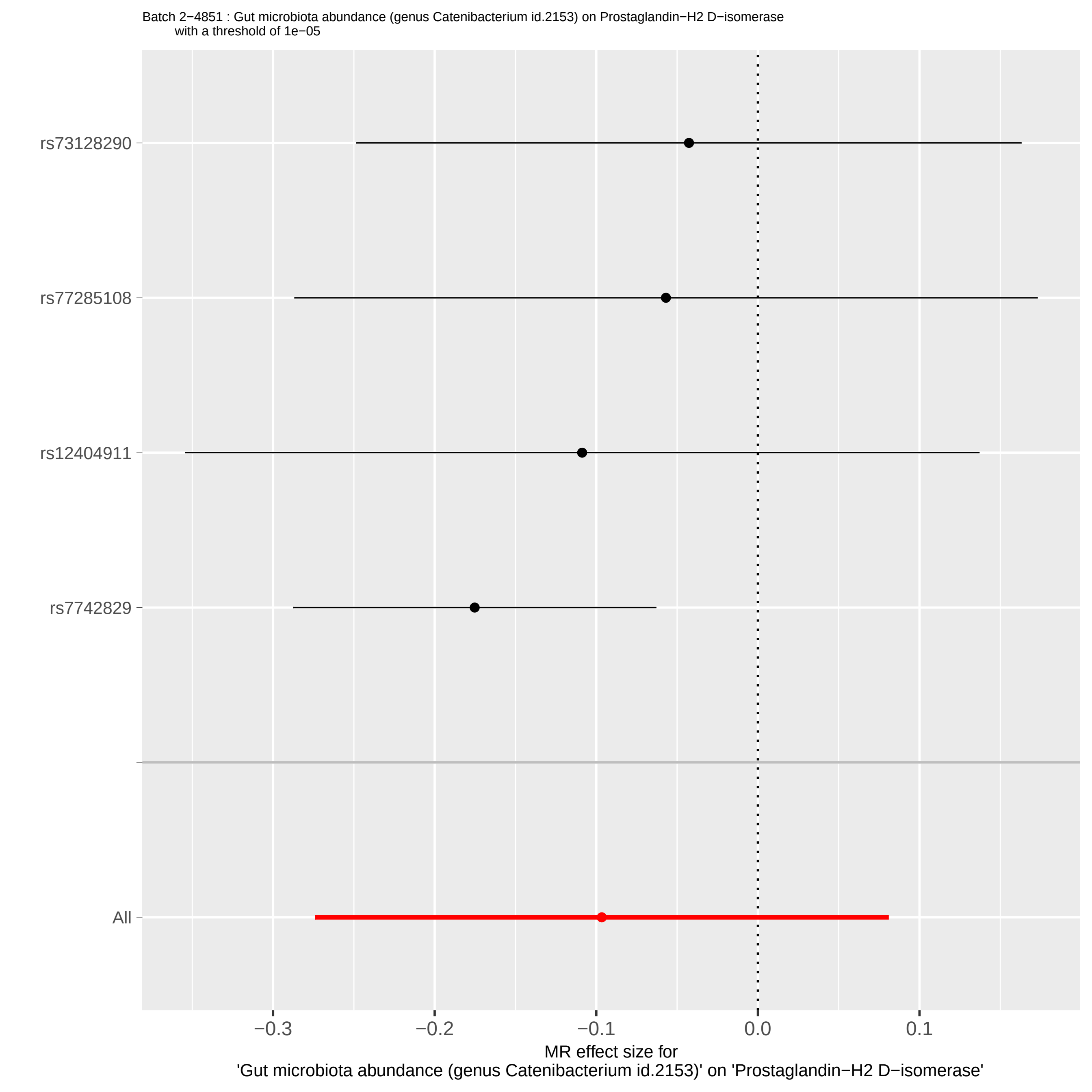
Batch 2−4851 : Gut microbiota abundance (genus Catenibacterium id.2153) on Prostaglandin−H2 D−isomerase
with a threshold of 1e−05
rs73128290
rs77285108
rs12404911
rs7742829
All
−0.3
−0.2
−0.1
0.0
0.1
MR effect size for
'Gut microbiota abundance (genus Catenibacterium id.2153)' on 'Prostaglandin−H2 D−isomerase'

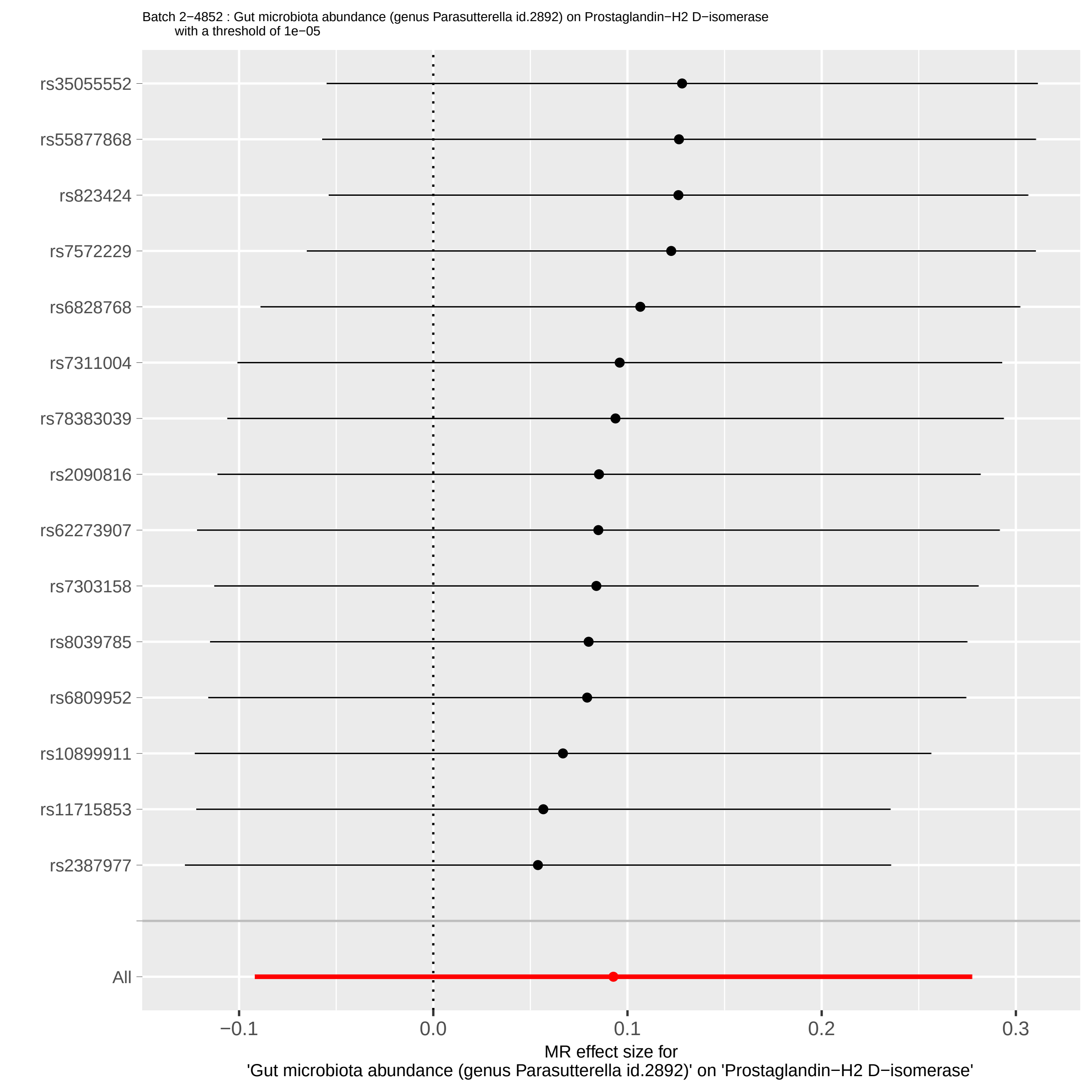

Batch 2−4852 : Gut microbiota abundance (genus Parasutterella id.2892) on Prostaglandin−H2 D−isomerase
with a threshold of 1e−05
rs35055552
rs55877868
rs823424
rs7572229
rs6828768
rs7311004
rs78383039
rs2090816
rs62273907
rs7303158
rs8039785
rs6809952
rs10899911
rs11715853
rs2387977
All
−0.1
0.0
0.1
0.2
0.3
MR effect size for
'Gut microbiota abundance (genus Parasutterella id.2892)' on 'Prostaglandin−H2 D−isomerase'

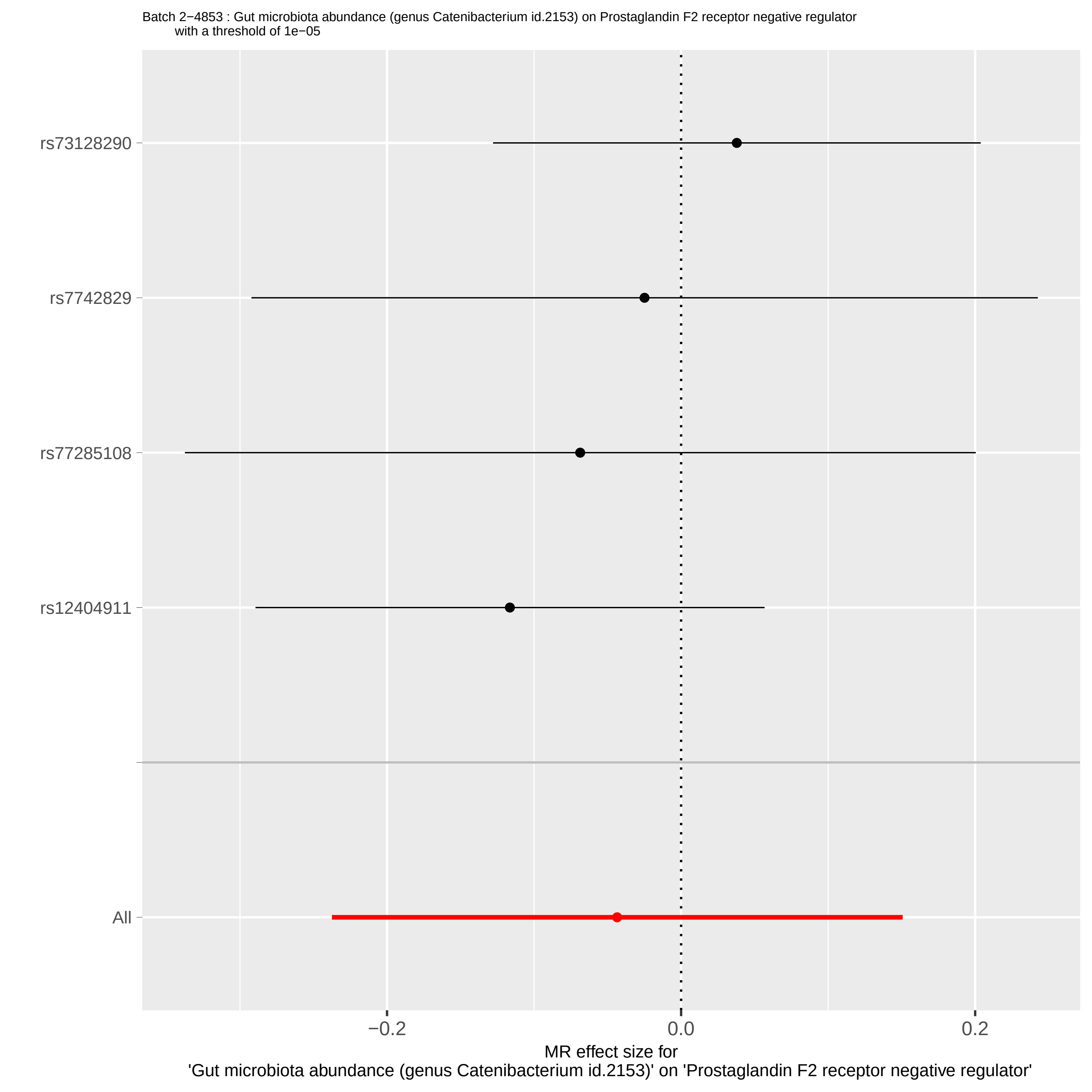
Batch 2−4853 : Gut microbiota abundance (genus Catenibacterium id.2153) on Prostaglandin F2 receptor negative regulator
with a threshold of 1e−05
rs73128290
rs7742829
rs77285108
rs12404911
All
−0.2
0.0
0.2
MR effect size for
'Gut microbiota abundance (genus Catenibacterium id.2153)' on 'Prostaglandin F2 receptor negative regulator'

Batch 2−4854 : Gut microbiota abundance (genus Parasutterella id.2892) on Prostaglandin F2 receptor negative regulator
with a threshold of 1e−05

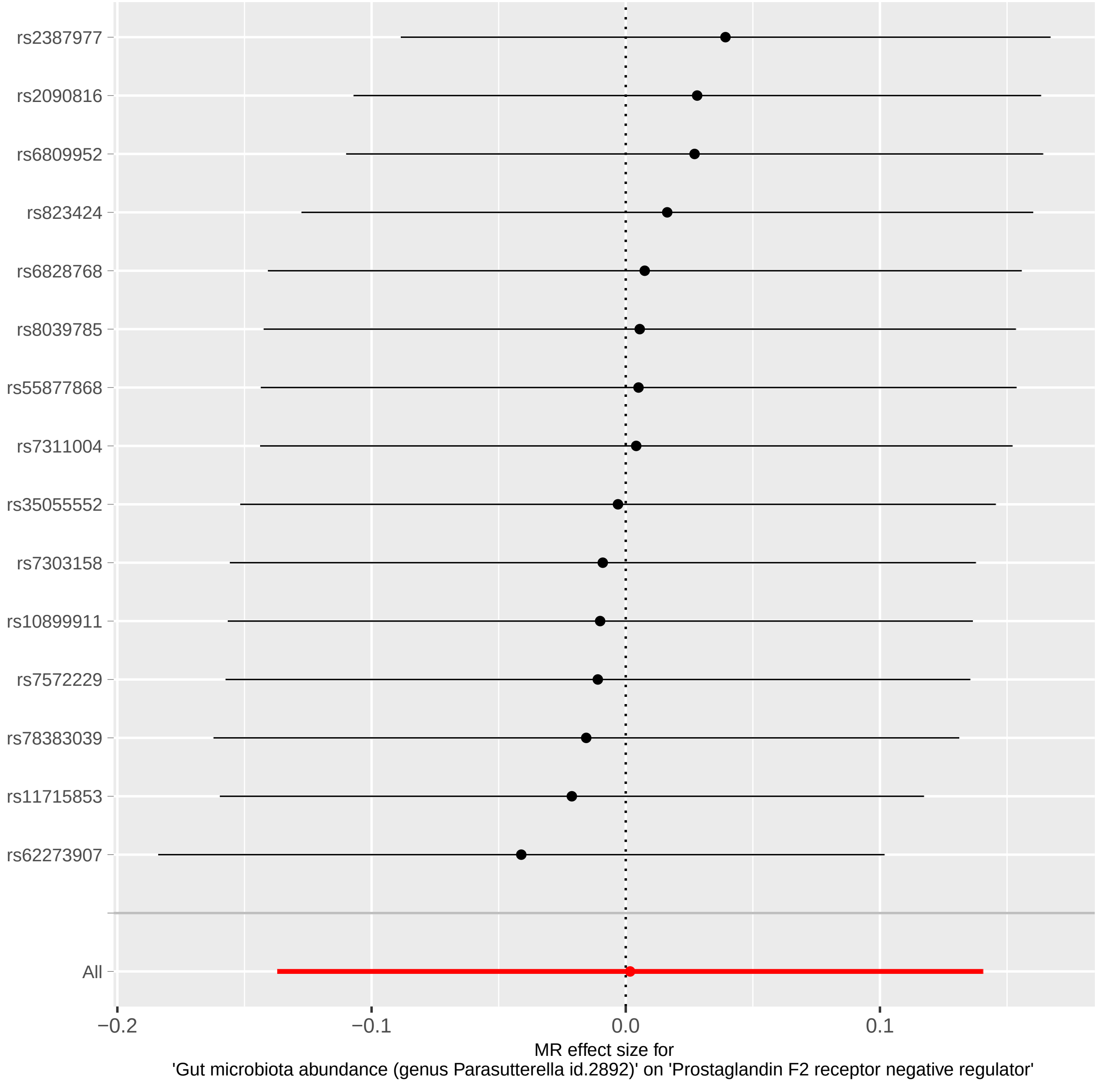

Batch 2−4855 : Gut microbiota abundance (genus Catenibacterium id.2153) on Prostaglandin reductase 1
with a threshold of 1e−05

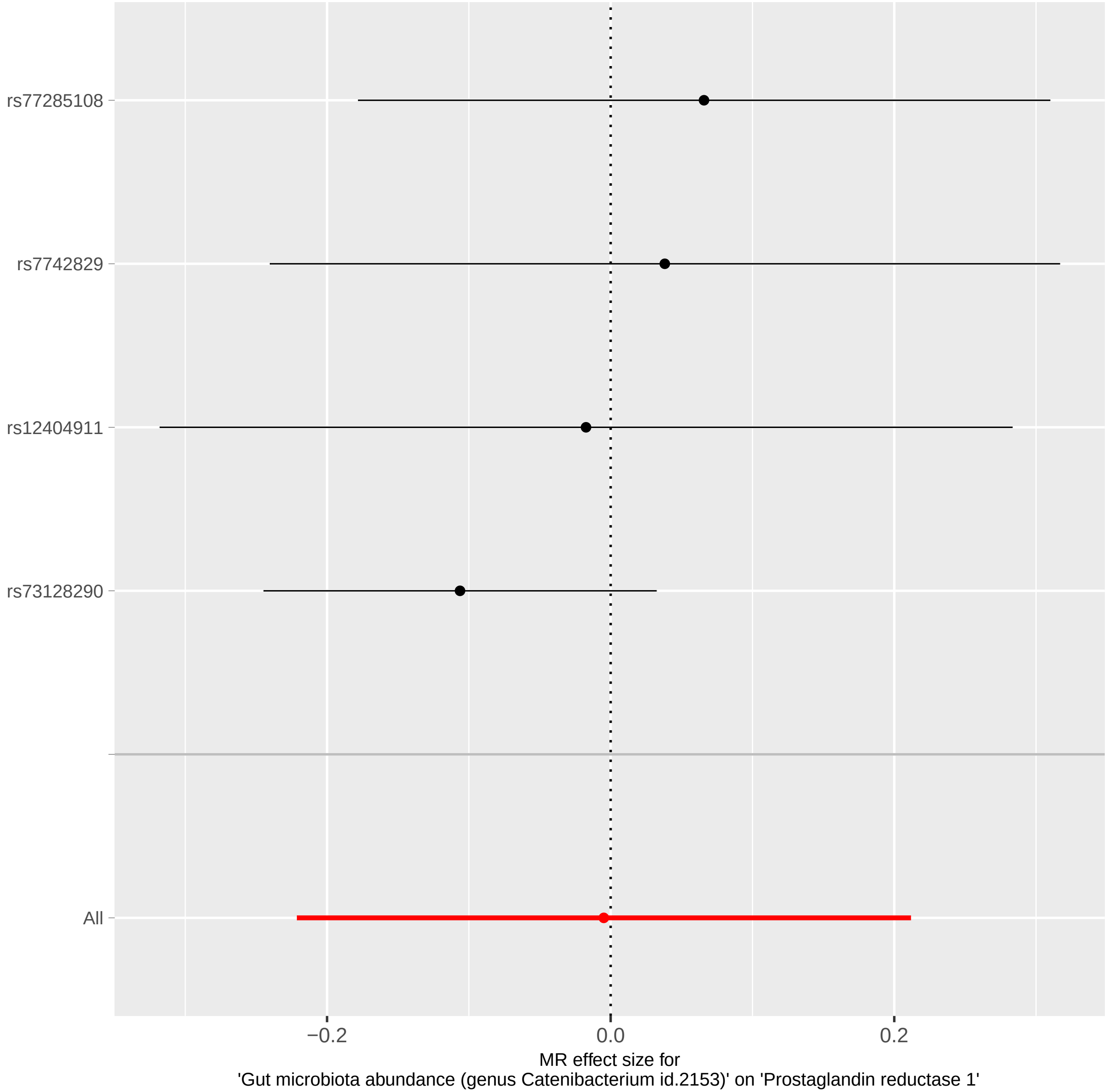

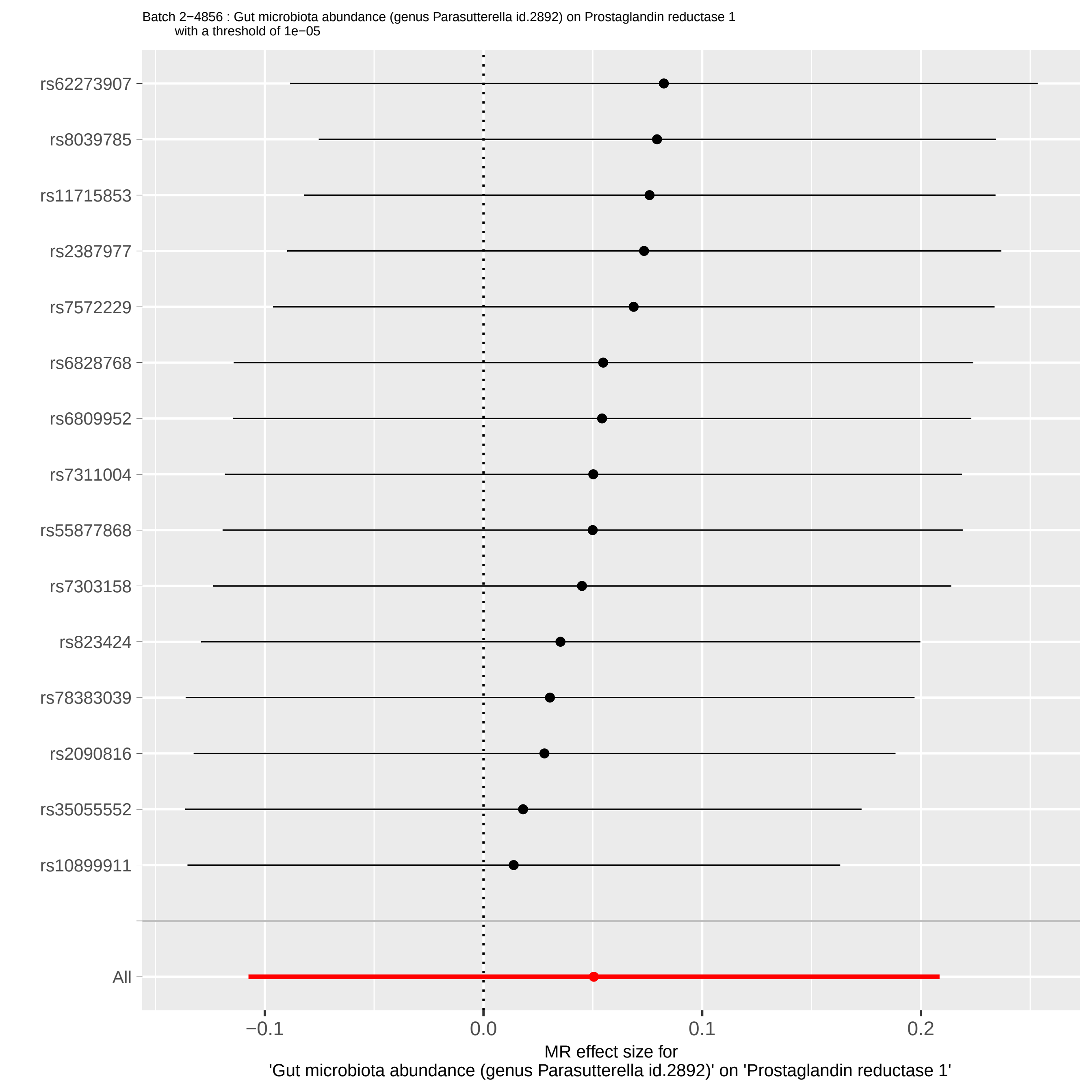
Batch 2−4856 : Gut microbiota abundance (genus Parasutterella id.2892) on Prostaglandin reductase 1
with a threshold of 1e−05
rs62273907
rs8039785
rs11715853
rs2387977
rs7572229
rs6828768
rs6809952
rs7311004
rs55877868
rs7303158
rs823424
rs78383039
rs2090816
rs35055552
rs10899911
All
−0.1
0.0
0.1
0.2
MR effect size for
'Gut microbiota abundance (genus Parasutterella id.2892)' on 'Prostaglandin reductase 1'

Batch 2−4857 : Gut microbiota abundance (genus Catenibacterium id.2153) on Parathyroid hormone/parathyroid hormone−related peptide receptor
with a threshold of 1e−05

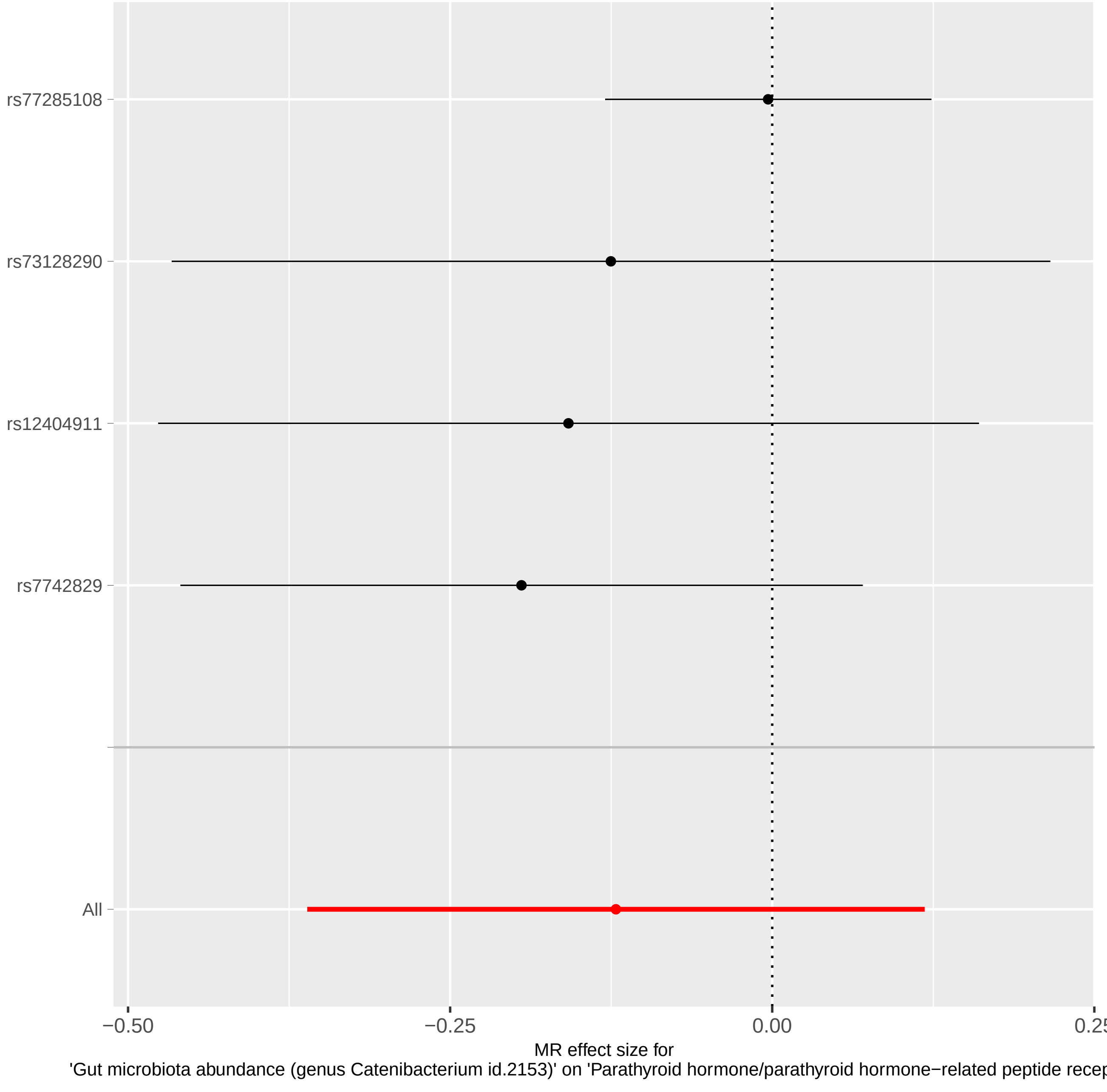

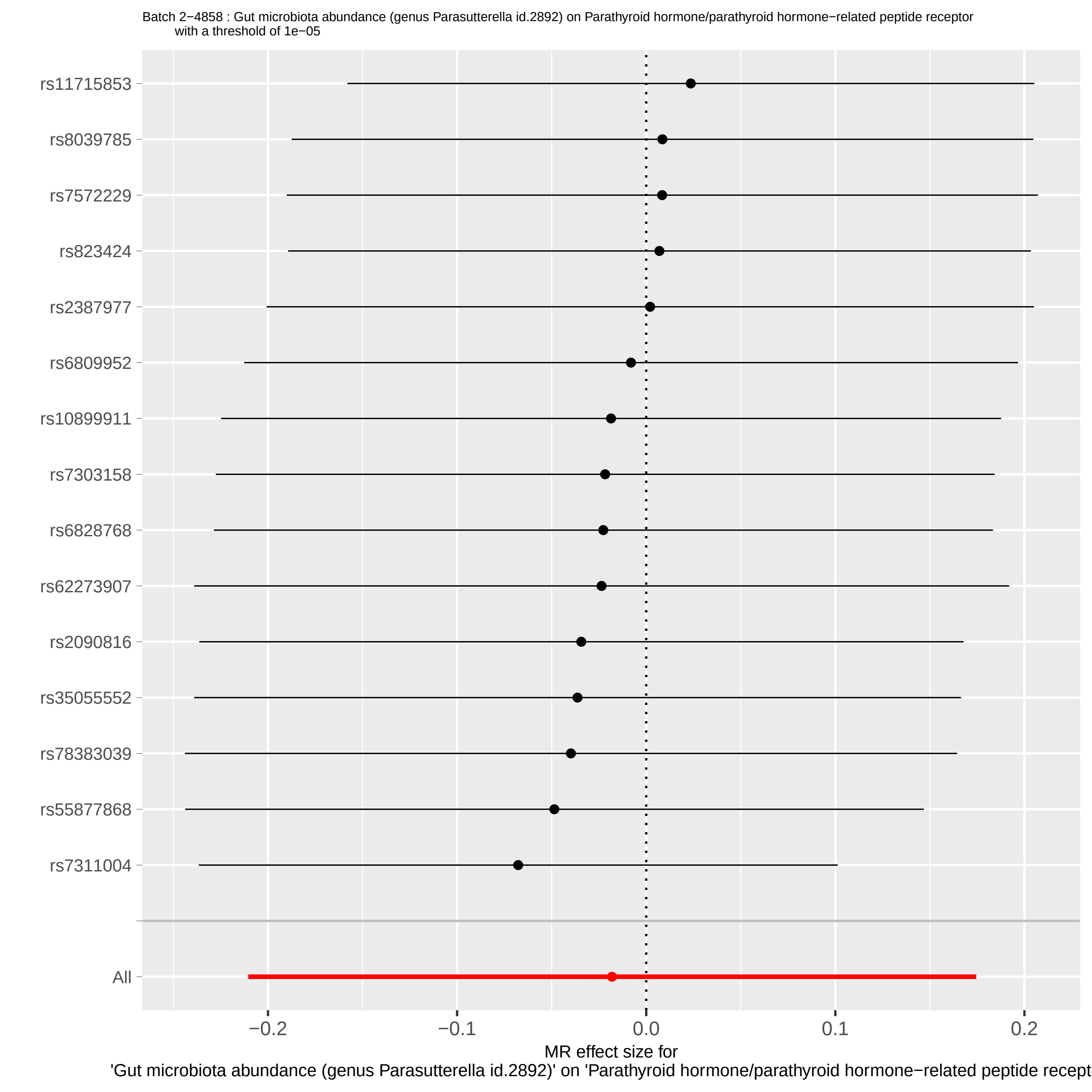
Batch 2−4858 : Gut microbiota abundance (genus Parasutterella id.2892) on Parathyroid hormone/parathyroid hormone−related peptide receptor with a threshold of 1e−05
rs11715853
rs8039785
rs7572229
rs823424
rs2387977
rs6809952
rs10899911
rs7303158
rs6828768
rs62273907
rs2090816
rs35055552
rs78383039
rs55877868
rs7311004
All
−0.2
−0.1
0.0
0.1
0.2
MR effect size for
'Gut microbiota abundance (genus Parasutterella id.2892)' on 'Parathyroid hormone/parathyroid hormone−related peptide recept

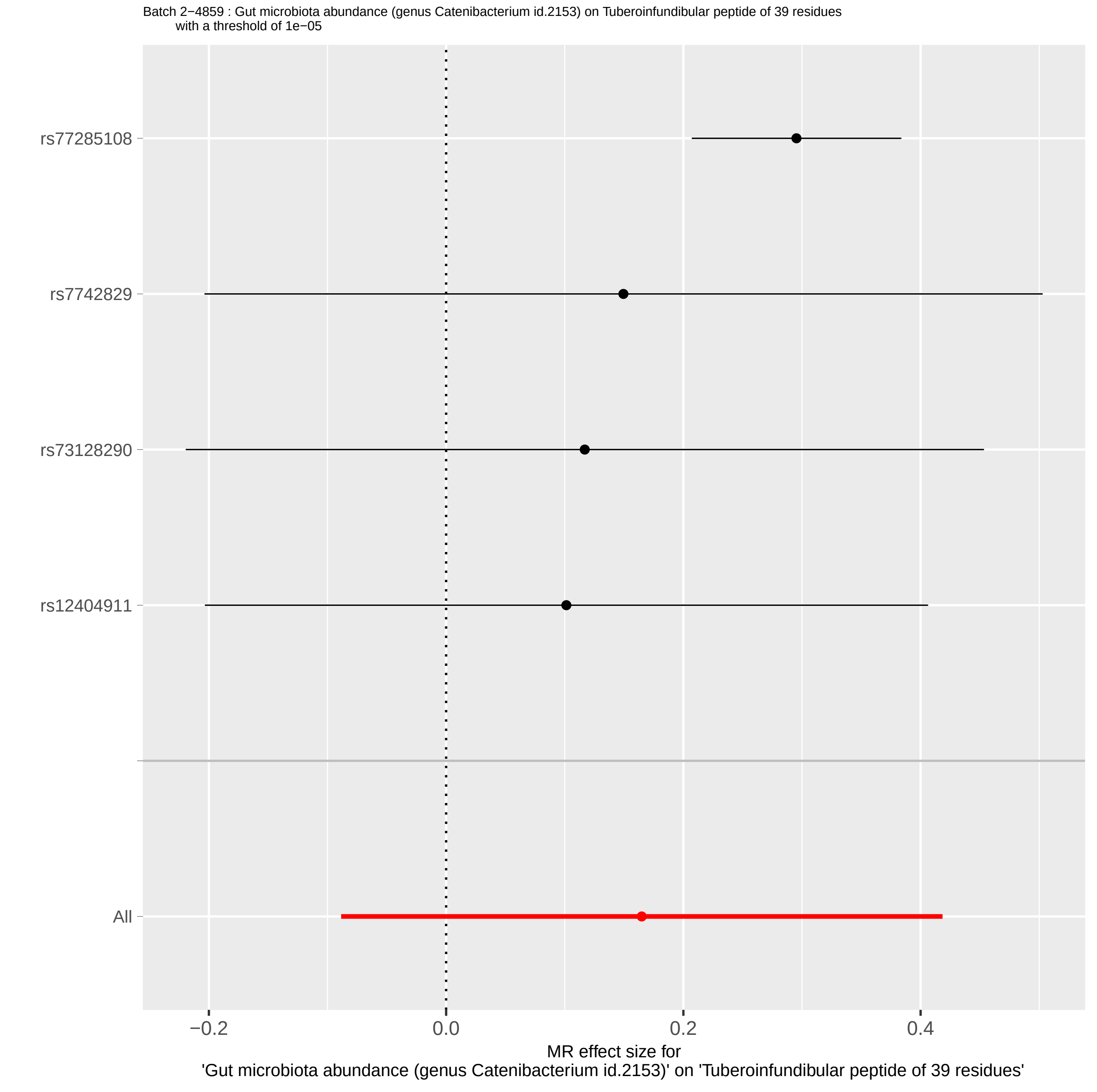

Batch 2−4859 : Gut microbiota abundance (genus Catenibacterium id.2153) on Tuberoinfundibular peptide of 39 residues
with a threshold of 1e−05
rs77285108
rs7742829
rs73128290
rs12404911
All
−0.2
0.0
0.2
0.4
MR effect size for
'Gut microbiota abundance (genus Catenibacterium id.2153)' on 'Tuberoinfundibular peptide of 39 residues'

Batch 2−4860 : Gut microbiota abundance (genus Parasutterella id.2892) on Tuberoinfundibular peptide of 39 residues
with a threshold of 1e−05

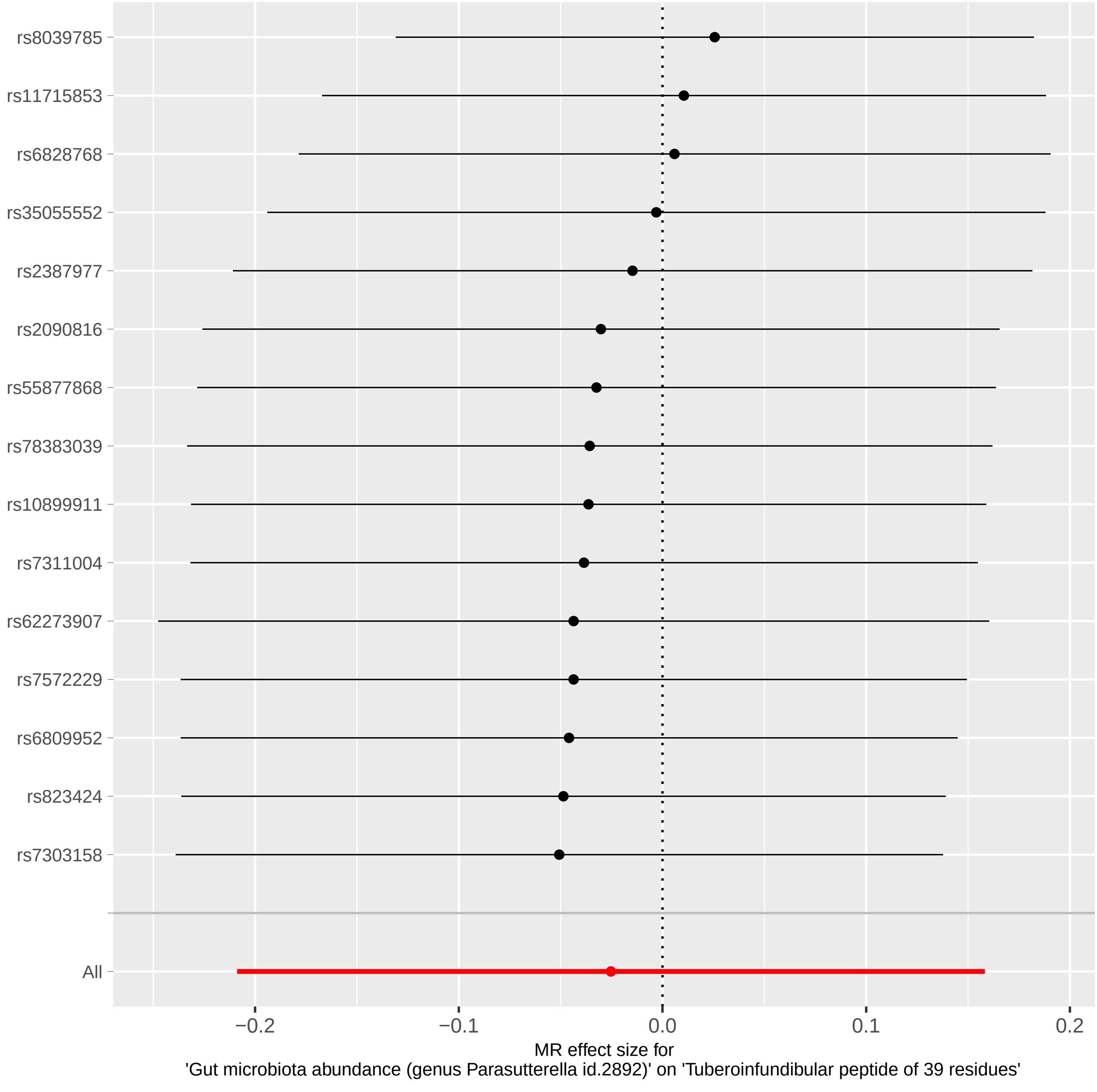

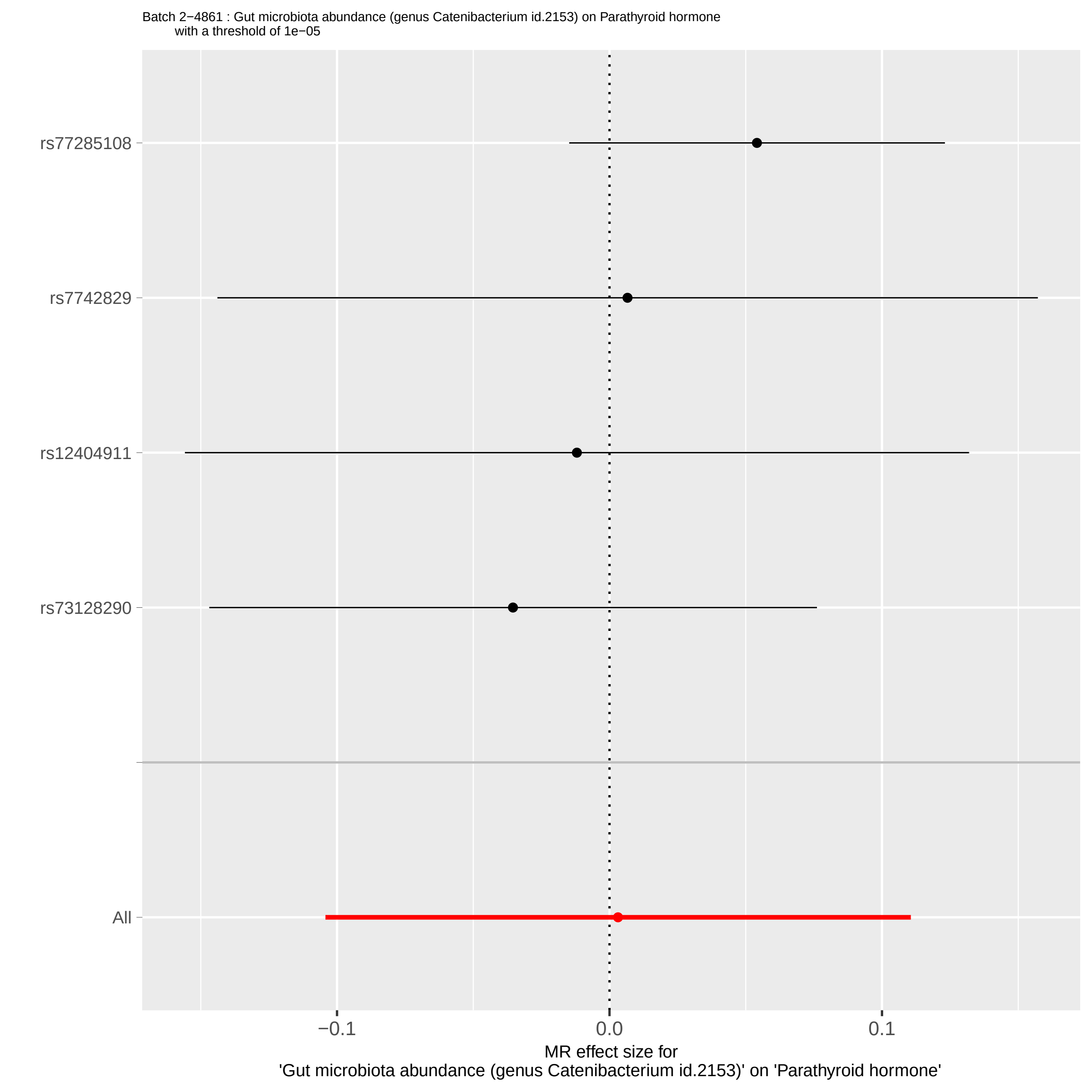
Batch 2−4861 : Gut microbiota abundance (genus Catenibacterium id.2153) on Parathyroid hormone
with a threshold of 1e−05
rs77285108
rs7742829
rs12404911
rs73128290
All
−0.1
0.0
0.1
MR effect size for
'Gut microbiota abundance (genus Catenibacterium id.2153)' on 'Parathyroid hormone'

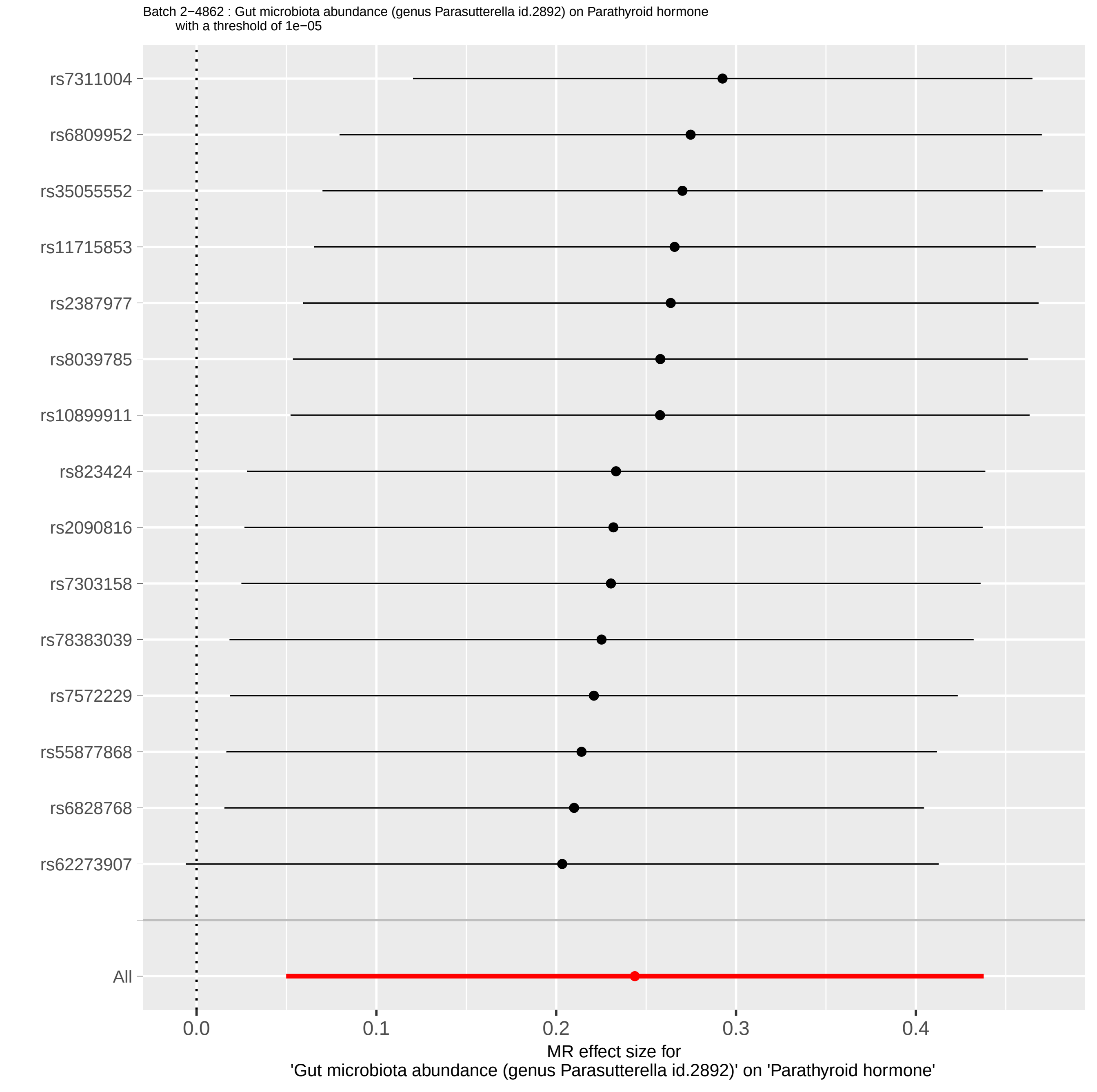

Batch 2−4862 : Gut microbiota abundance (genus Parasutterella id.2892) on Parathyroid hormone
with a threshold of 1e−05
rs7311004
rs6809952
rs35055552
rs11715853
rs2387977
rs8039785
rs10899911
rs823424
rs2090816
rs7303158
rs78383039
rs7572229
rs55877868
rs6828768
rs62273907
All
0.0
0.1
0.2
0.3
0.4
MR effect size for
'Gut microbiota abundance (genus Parasutterella id.2892)' on 'Parathyroid hormone'

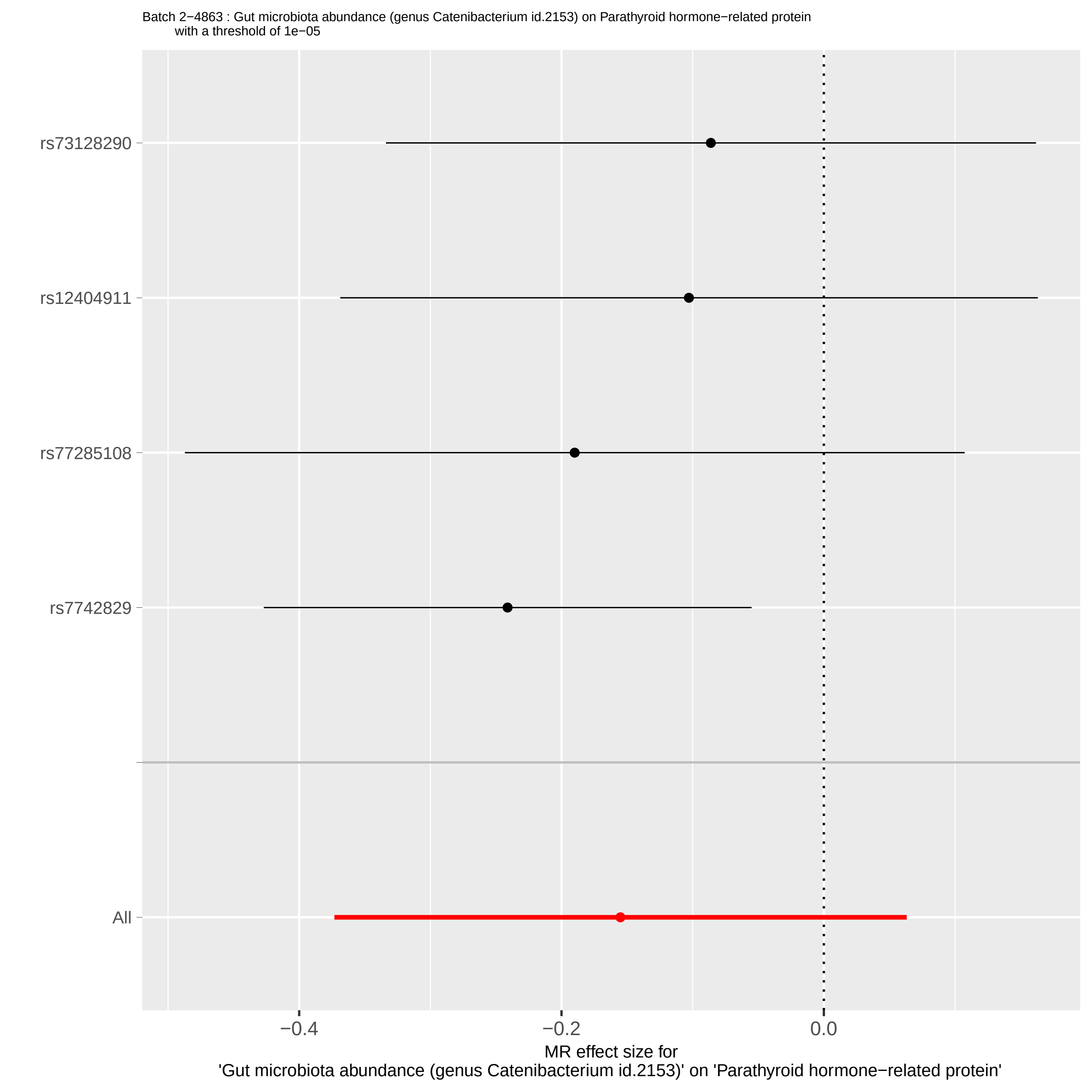
Batch 2−4863 : Gut microbiota abundance (genus Catenibacterium id.2153) on Parathyroid hormone−related protein
with a threshold of 1e−05
rs73128290
rs12404911
rs77285108
rs7742829
All
−0.4
−0.2
0.0
MR effect size for
'Gut microbiota abundance (genus Catenibacterium id.2153)' on 'Parathyroid hormone−related protein'

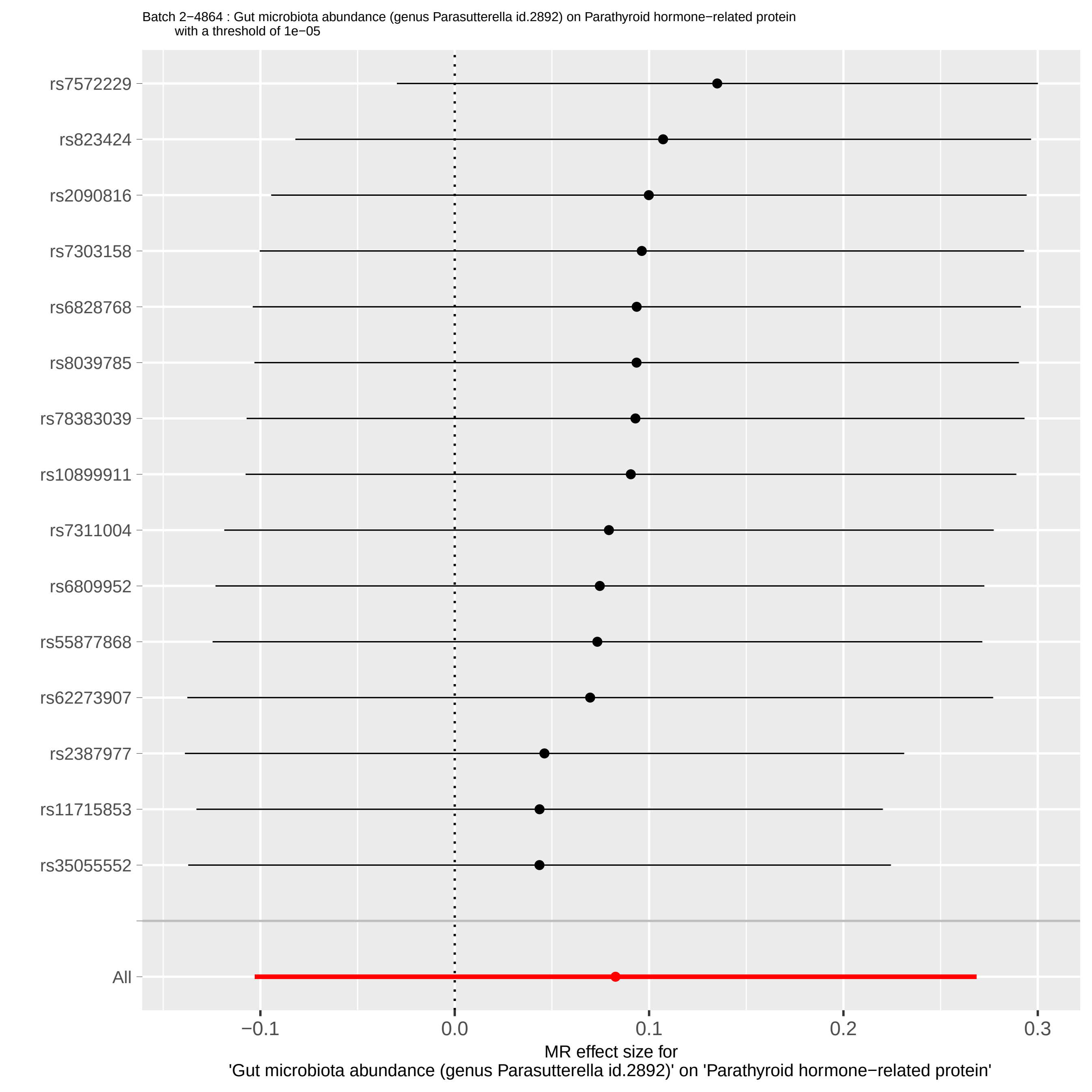

Batch 2−4864 : Gut microbiota abundance (genus Parasutterella id.2892) on Parathyroid hormone−related protein
with a threshold of 1e−05
rs7572229
rs823424
rs2090816
rs7303158
rs6828768
rs8039785
rs78383039
rs10899911
rs7311004
rs6809952
rs55877868
rs62273907
rs2387977
rs11715853
rs35055552
All
−0.1
0.0
0.1
0.2
0.3
MR effect size for
'Gut microbiota abundance (genus Parasutterella id.2892)' on 'Parathyroid hormone−related protein'

Batch 2−4865 : Gut microbiota abundance (genus Catenibacterium id.2153) on Protein−tyrosine kinase 2−beta
with a threshold of 1e−05

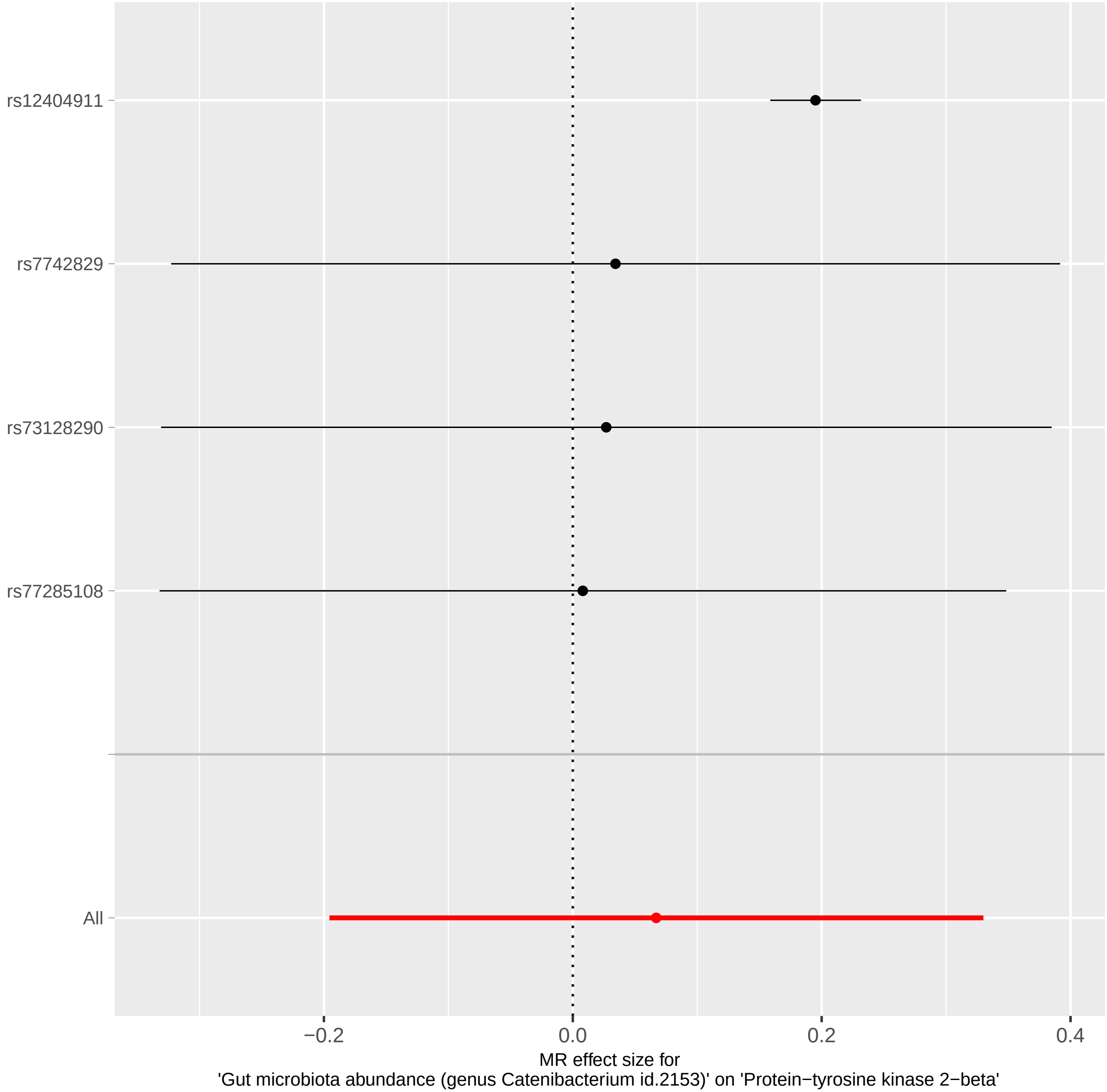

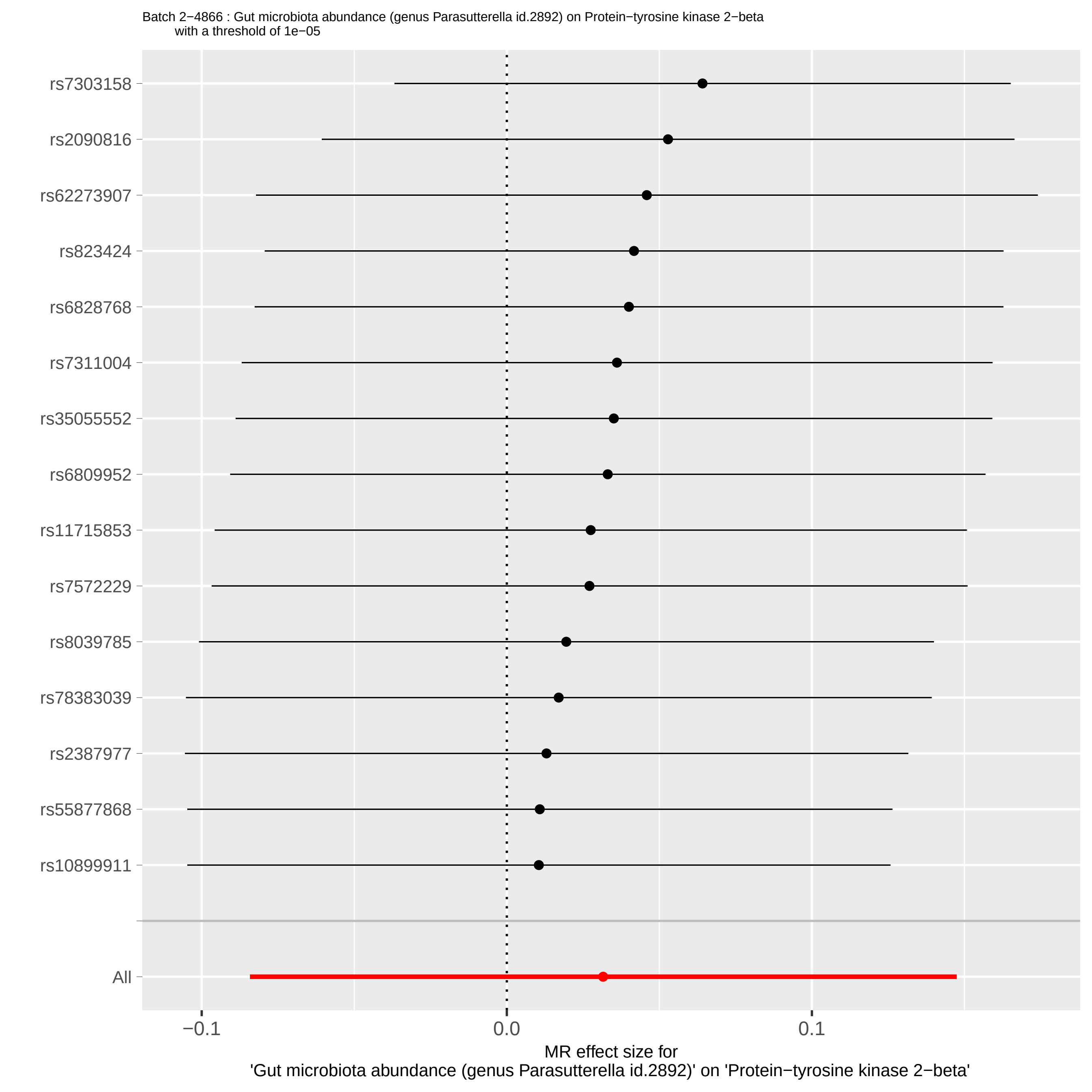
Batch 2−4866 : Gut microbiota abundance (genus Parasutterella id.2892) on Protein−tyrosine kinase 2−beta
with a threshold of 1e−05
rs7303158
rs2090816
rs62273907
rs823424
rs6828768
rs7311004
rs35055552
rs6809952
rs11715853
rs7572229
rs8039785
rs78383039
rs2387977
rs55877868
rs10899911
All
−0.1
0.0
0.1
MR effect size for
'Gut microbiota abundance (genus Parasutterella id.2892)' on 'Protein−tyrosine kinase 2−beta'

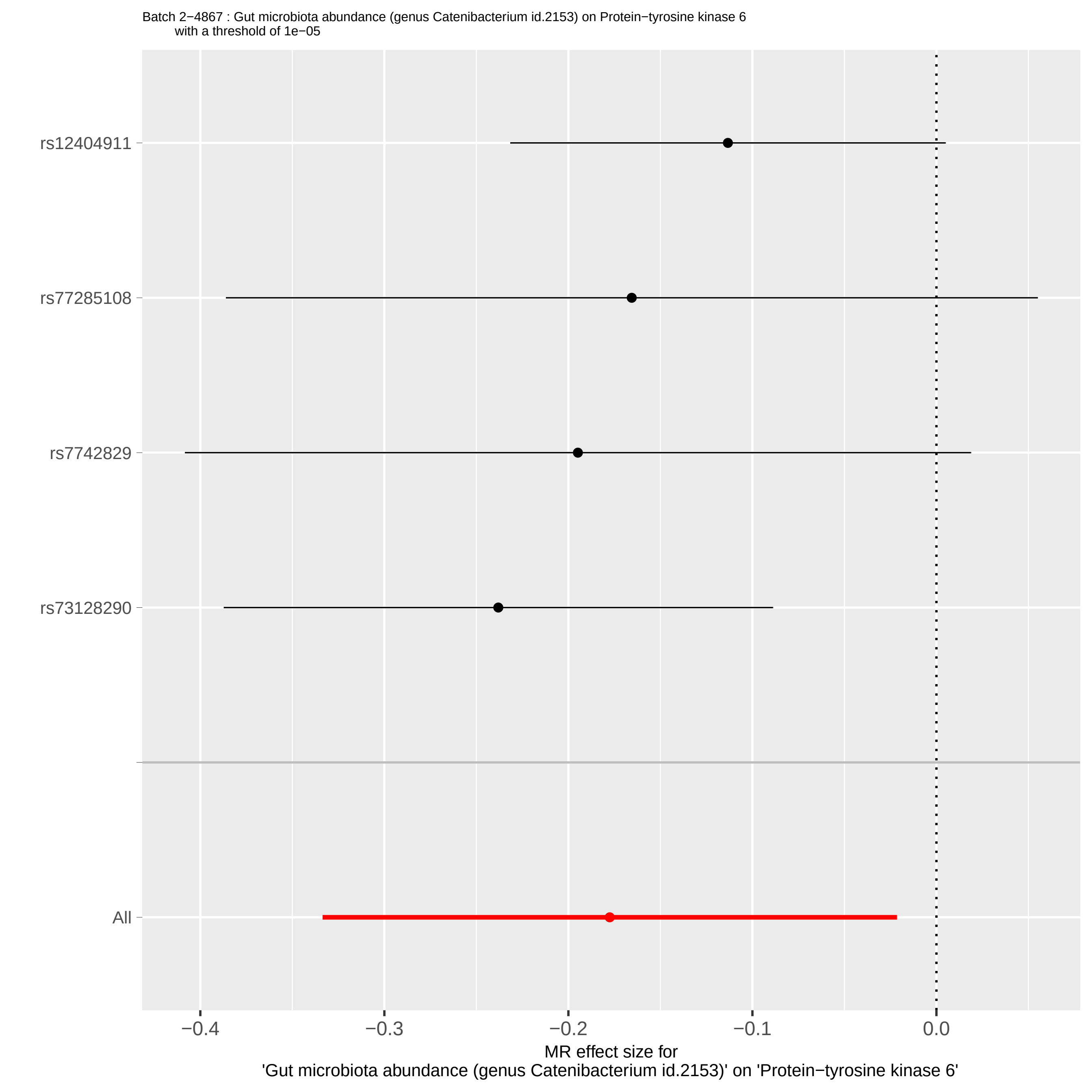

Batch 2−4867 : Gut microbiota abundance (genus Catenibacterium id.2153) on Protein−tyrosine kinase 6
with a threshold of 1e−05
rs12404911
rs77285108
rs7742829
rs73128290
All
−0.4
−0.3
−0.2
−0.1
0.0
MR effect size for
'Gut microbiota abundance (genus Catenibacterium id.2153)' on 'Protein−tyrosine kinase 6'

Batch 2−4868 : Gut microbiota abundance (genus Parasutterella id.2892) on Protein−tyrosine kinase 6
with a threshold of 1e−05

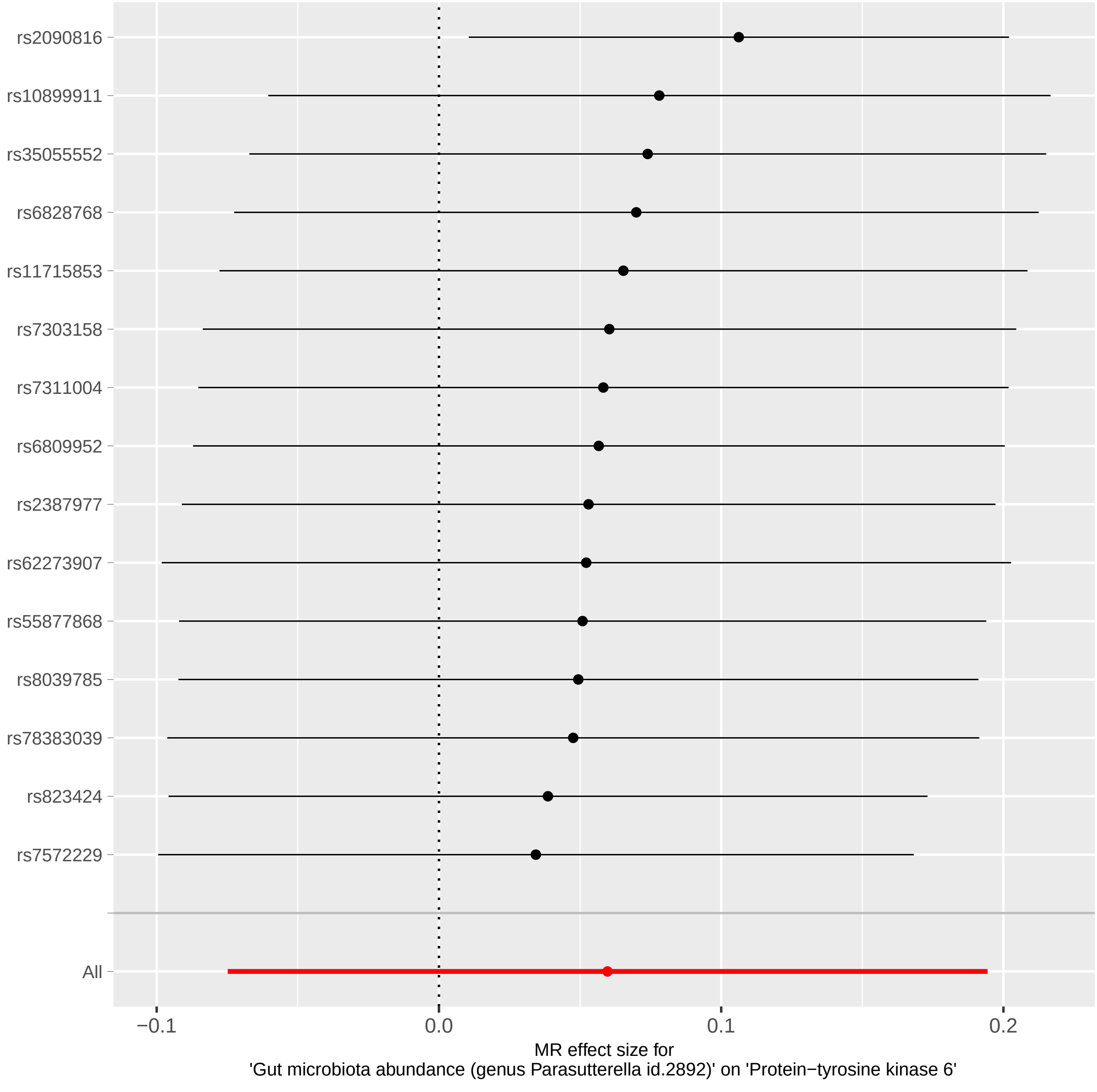

Batch 2−4869 : Gut microbiota abundance (genus Catenibacterium id.2153) on Pleiotrophin
with a threshold of 1e−05

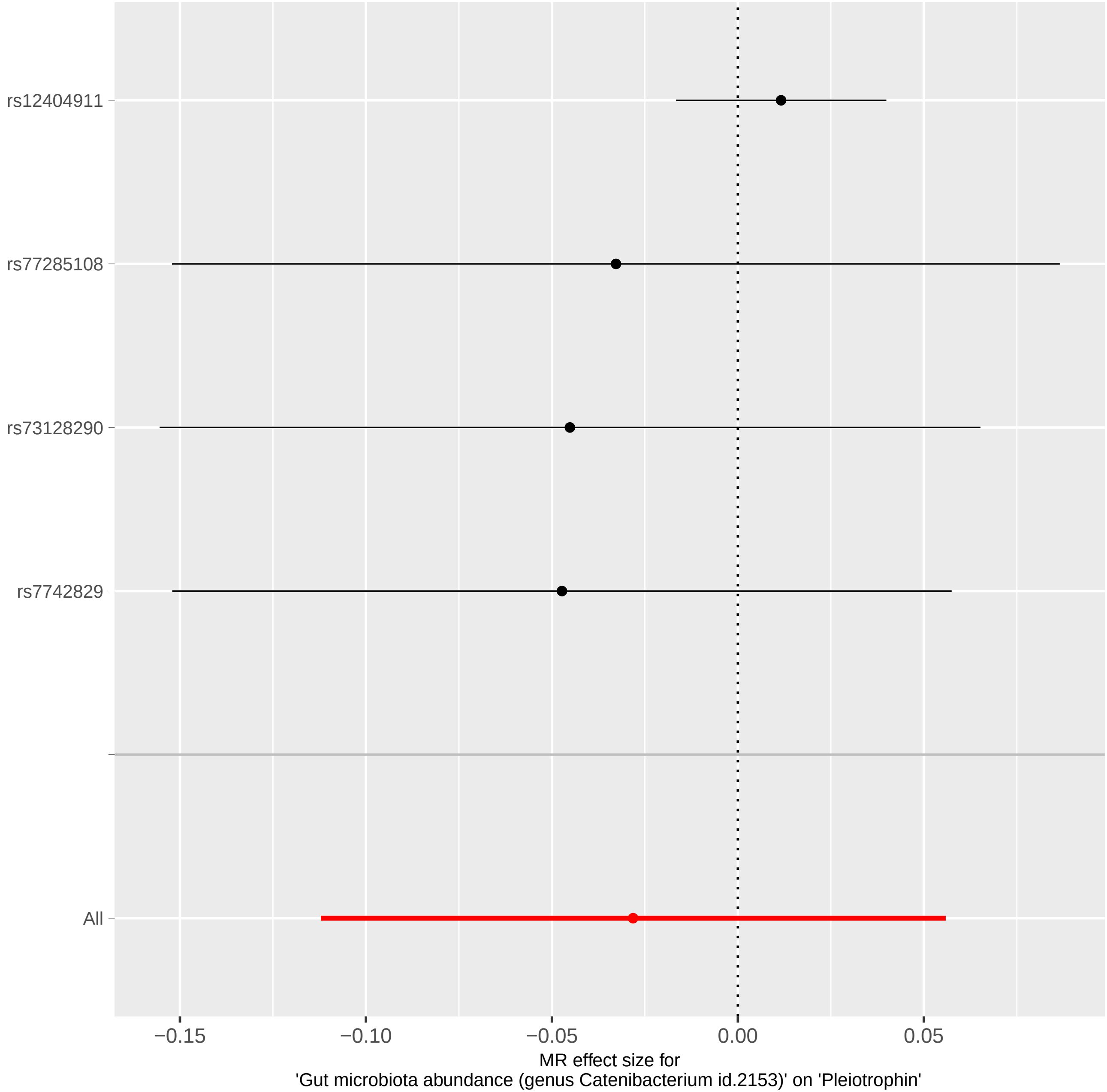

Batch 2−4870 : Gut microbiota abundance (genus Parasutterella id.2892) on Pleiotrophin
with a threshold of 1e−05

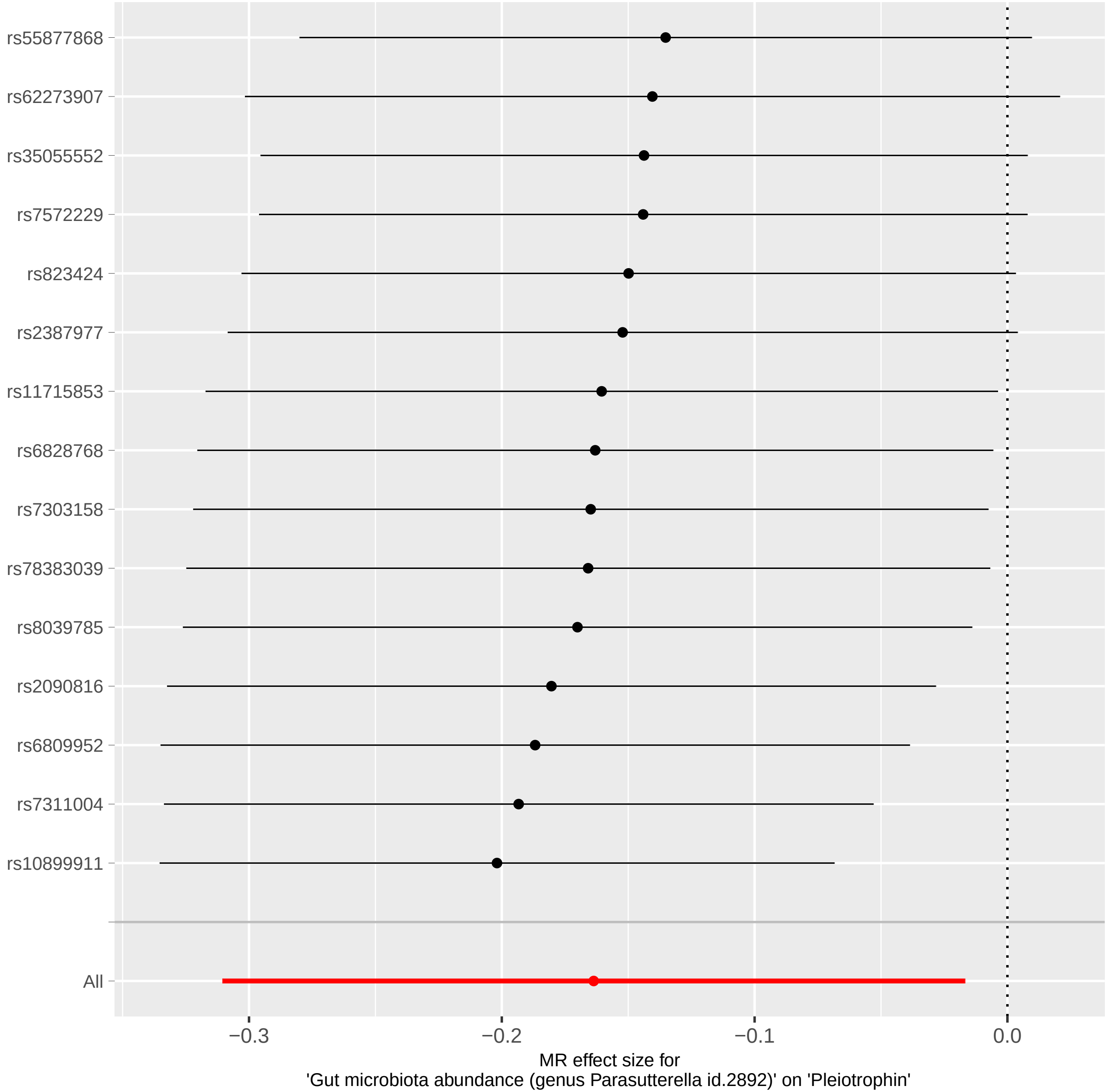

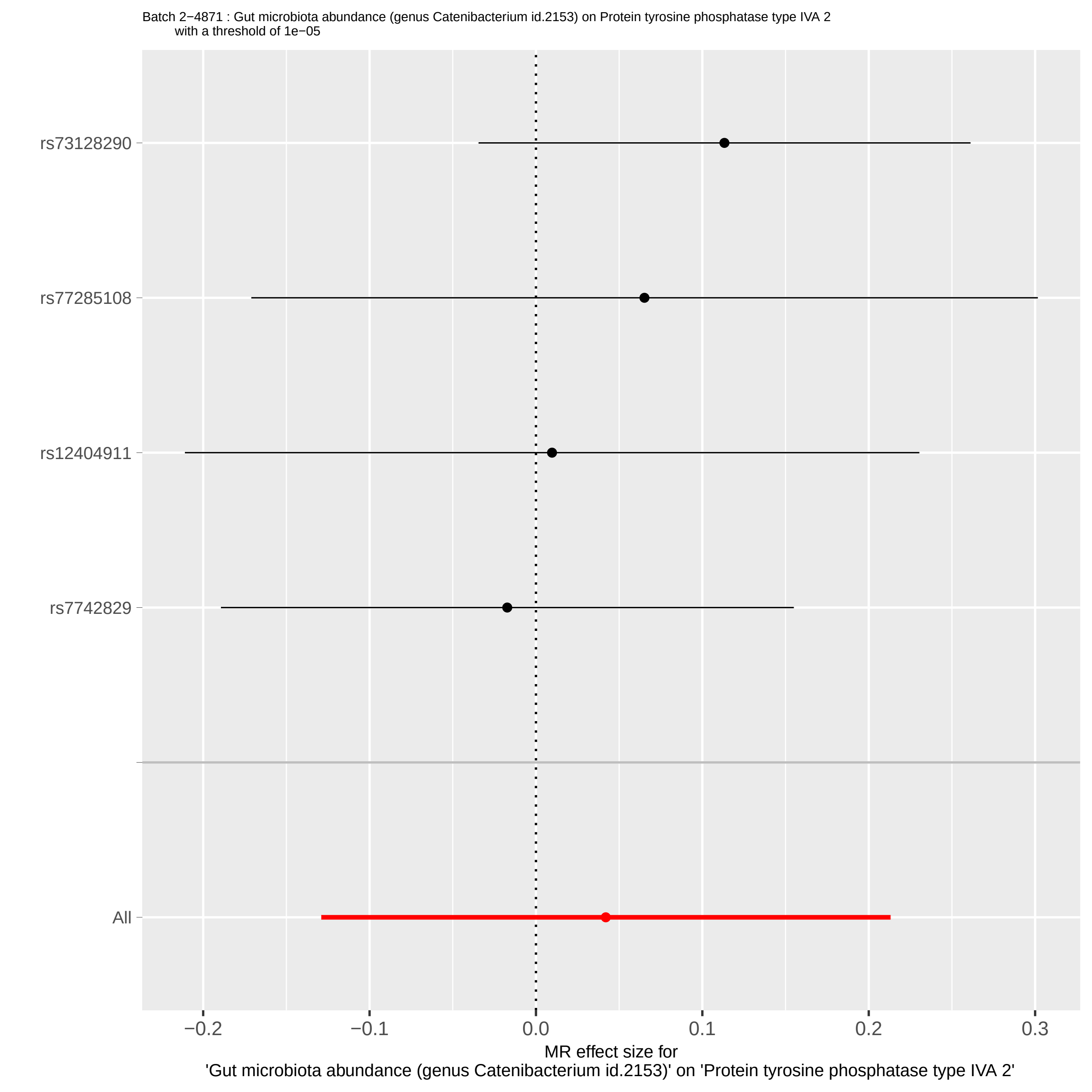

Batch 2−4871 : Gut microbiota abundance (genus Catenibacterium id.2153) on Protein tyrosine phosphatase type IVA 2
with a threshold of 1e−05
rs73128290
rs77285108
rs12404911
rs7742829
All
−0.2
−0.1
0.0
0.1
0.2
0.3
MR effect size for
'Gut microbiota abundance (genus Catenibacterium id.2153)' on 'Protein tyrosine phosphatase type IVA 2'

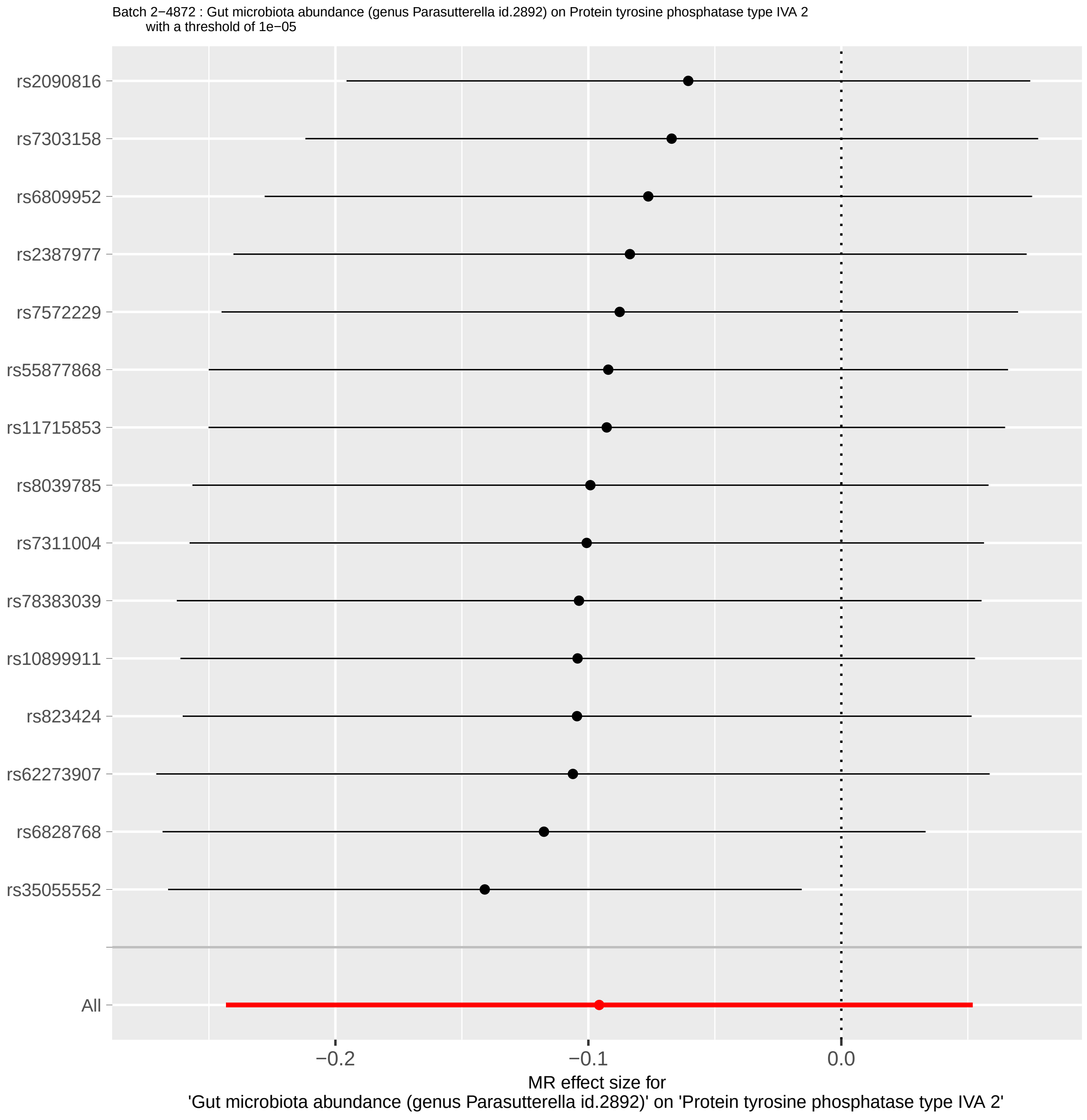

Batch 2−4872 : Gut microbiota abundance (genus Parasutterella id.2892) on Protein tyrosine phosphatase type IVA 2
with a threshold of 1e−05
rs2090816
rs7303158
rs6809952
rs2387977
rs7572229
rs55877868
rs11715853
rs8039785
rs7311004
rs78383039
rs10899911
rs823424
rs62273907
rs6828768
rs35055552
All
−0.2
−0.1
0.0
MR effect size for
'Gut microbiota abundance (genus Parasutterella id.2892)' on 'Protein tyrosine phosphatase type IVA 2'

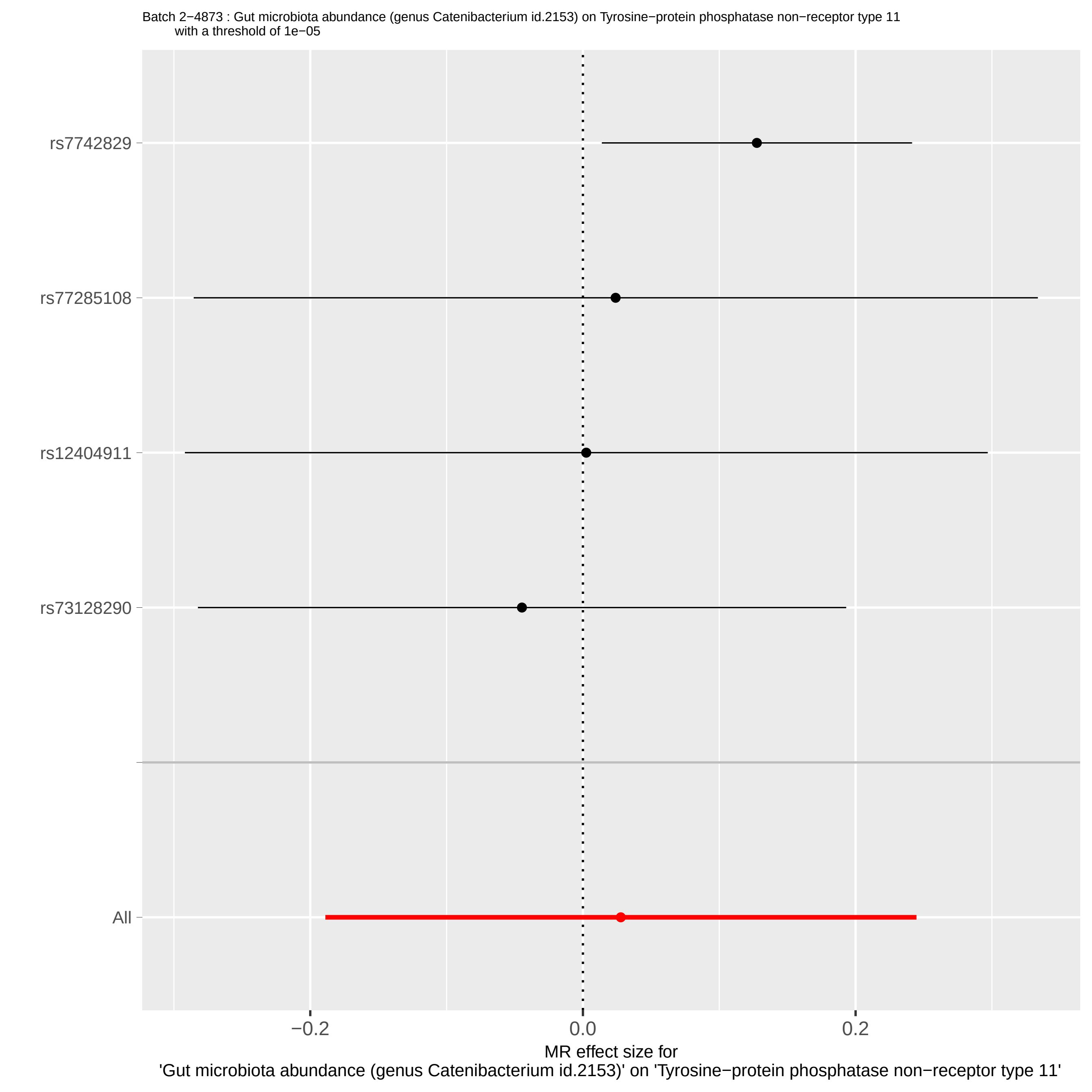
Batch 2−4873 : Gut microbiota abundance (genus Catenibacterium id.2153) on Tyrosine−protein phosphatase non−receptor type 11
with a threshold of 1e−05
rs7742829
rs77285108
rs12404911
rs73128290
All
−0.2
0.0
0.2
MR effect size for
'Gut microbiota abundance (genus Catenibacterium id.2153)' on 'Tyrosine−protein phosphatase non−receptor type 11'

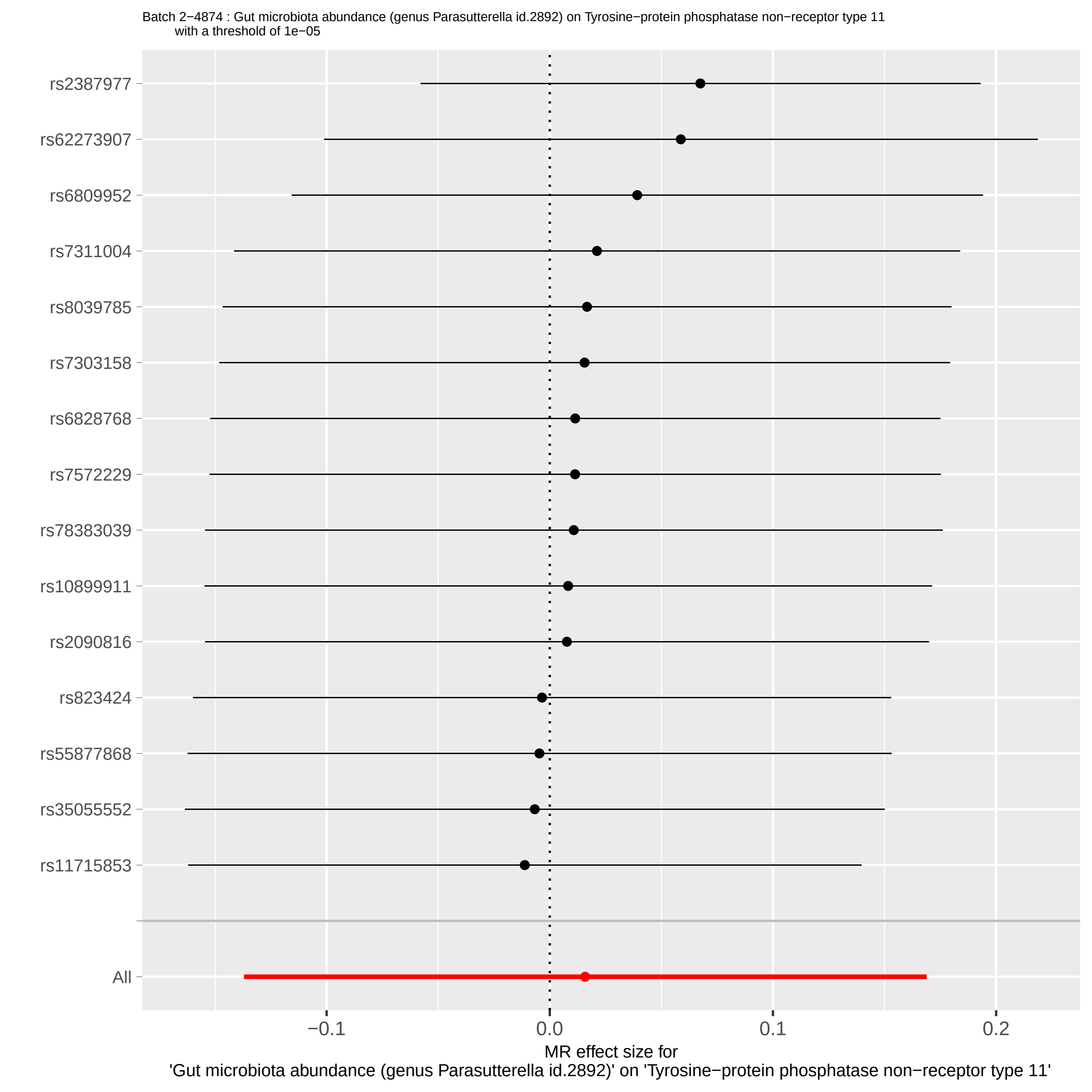

Batch 2−4874 : Gut microbiota abundance (genus Parasutterella id.2892) on Tyrosine−protein phosphatase non−receptor type 11
with a threshold of 1e−05
rs2387977
rs62273907
rs6809952
rs7311004
rs8039785
rs7303158
rs6828768
rs7572229
rs78383039
rs10899911
rs2090816
rs823424
rs55877868
rs35055552
rs11715853
All
−0.1
0.0
0.1
0.2
MR effect size for
'Gut microbiota abundance (genus Parasutterella id.2892)' on 'Tyrosine−protein phosphatase non−receptor type 11'

Batch 2−4875 : Gut microbiota abundance (genus Catenibacterium id.2153) on Tyrosine−protein phosphatase non−receptor type 1
with a threshold of 1e−05

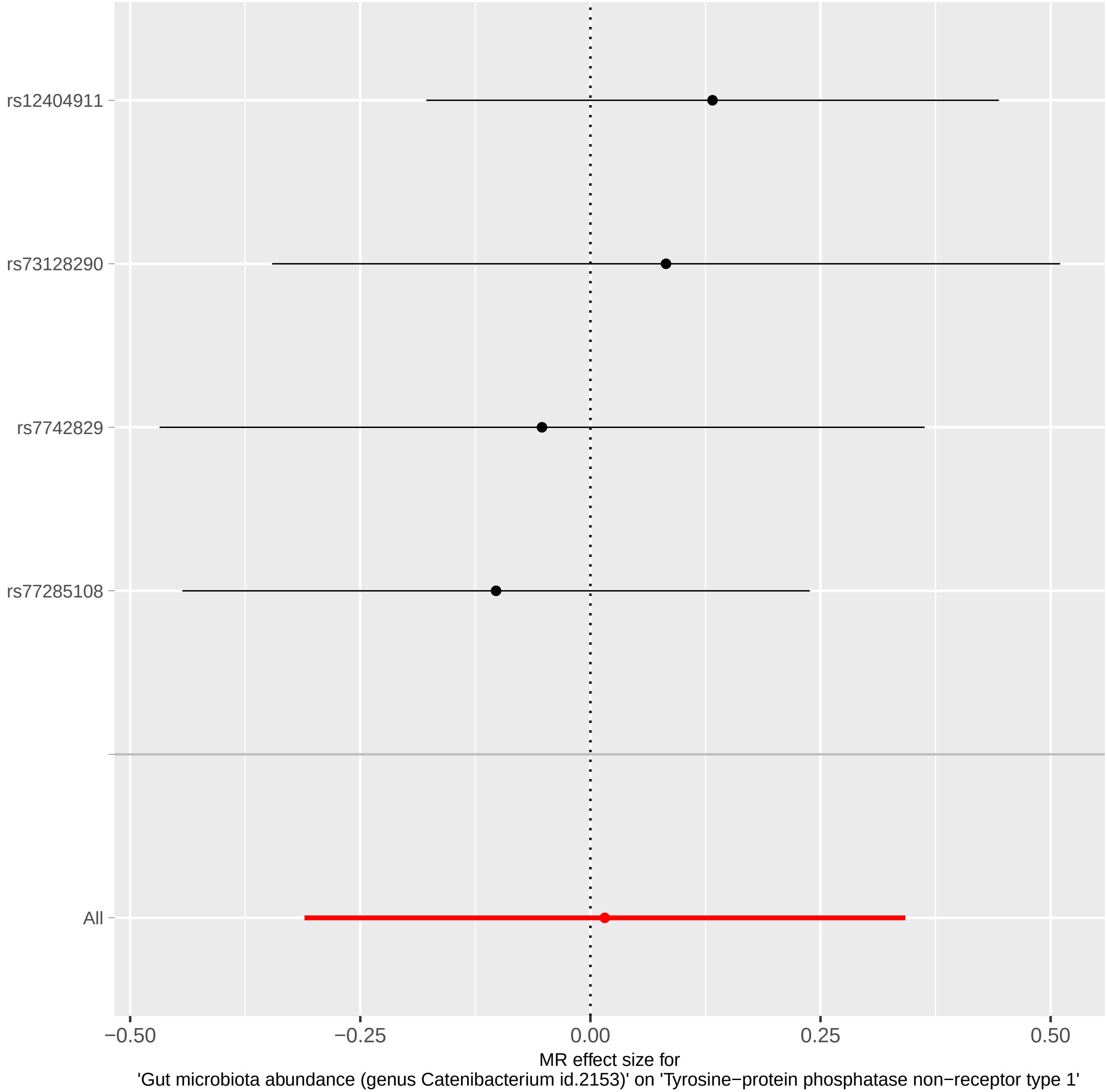

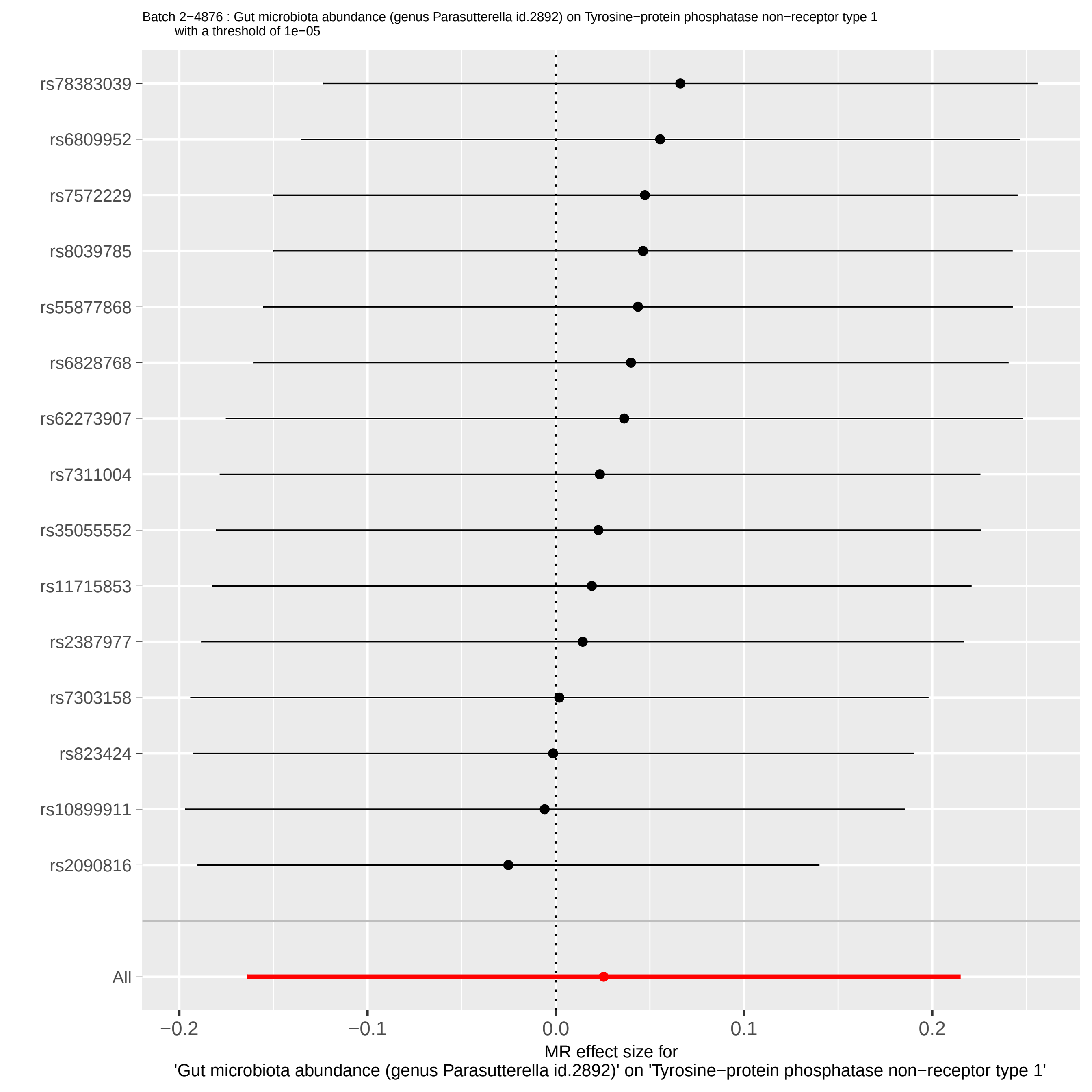

Batch 2−4876 : Gut microbiota abundance (genus Parasutterella id.2892) on Tyrosine−protein phosphatase non−receptor type 1
with a threshold of 1e−05
rs78383039
rs6809952
rs7572229
rs8039785
rs55877868
rs6828768
rs62273907
rs7311004
rs35055552
rs11715853
rs2387977
rs7303158
rs823424
rs10899911
rs2090816
All
−0.2
−0.1
0.0
0.1
0.2
MR effect size for
'Gut microbiota abundance (genus Parasutterella id.2892)' on 'Tyrosine−protein phosphatase non−receptor type 1'

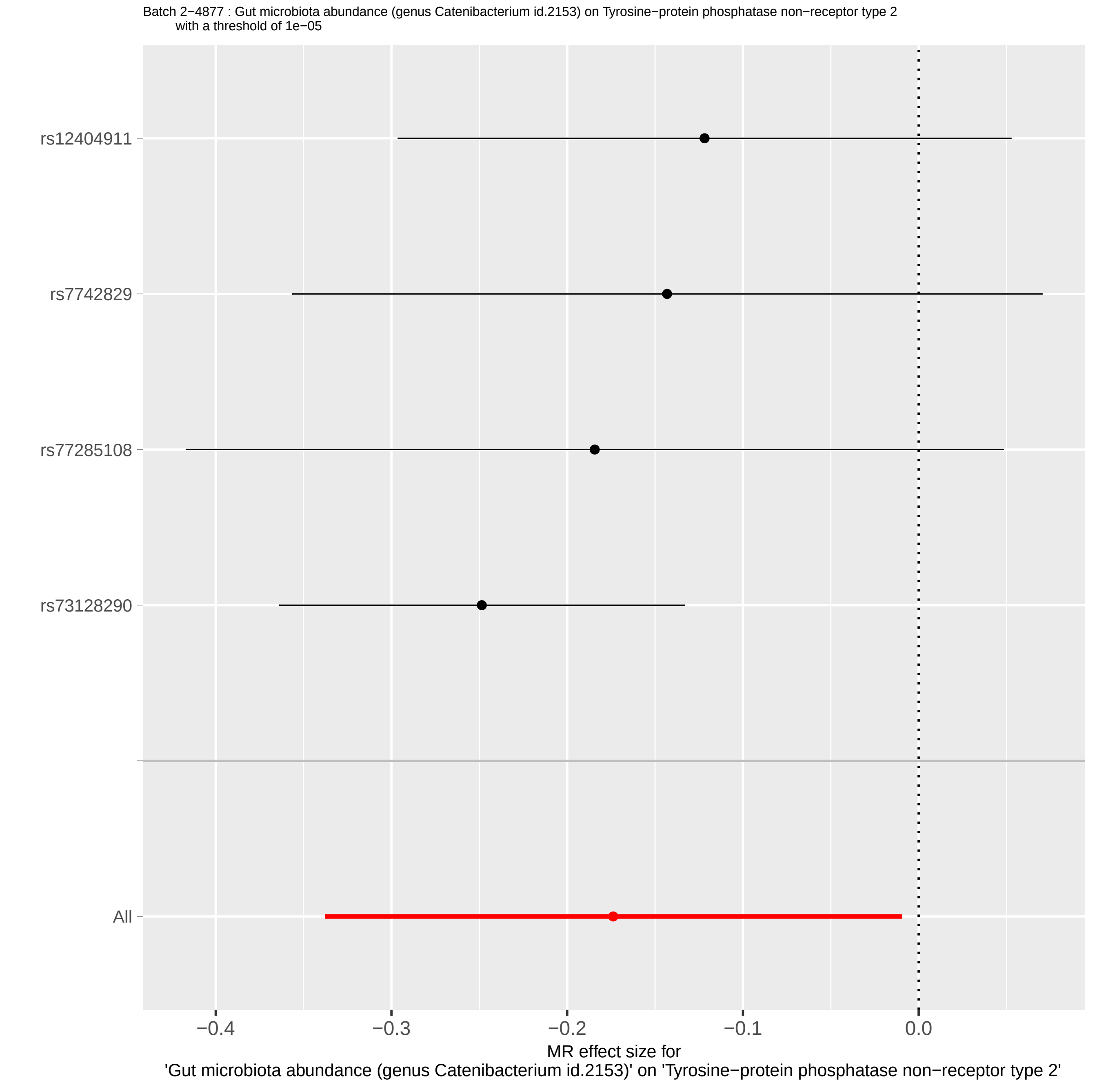

Batch 2−4877 : Gut microbiota abundance (genus Catenibacterium id.2153) on Tyrosine−protein phosphatase non−receptor type 2
with a threshold of 1e−05
rs12404911
rs7742829
rs77285108
rs73128290
All
−0.4
−0.3
−0.2
−0.1
0.0
MR effect size for
'Gut microbiota abundance (genus Catenibacterium id.2153)' on 'Tyrosine−protein phosphatase non−receptor type 2'

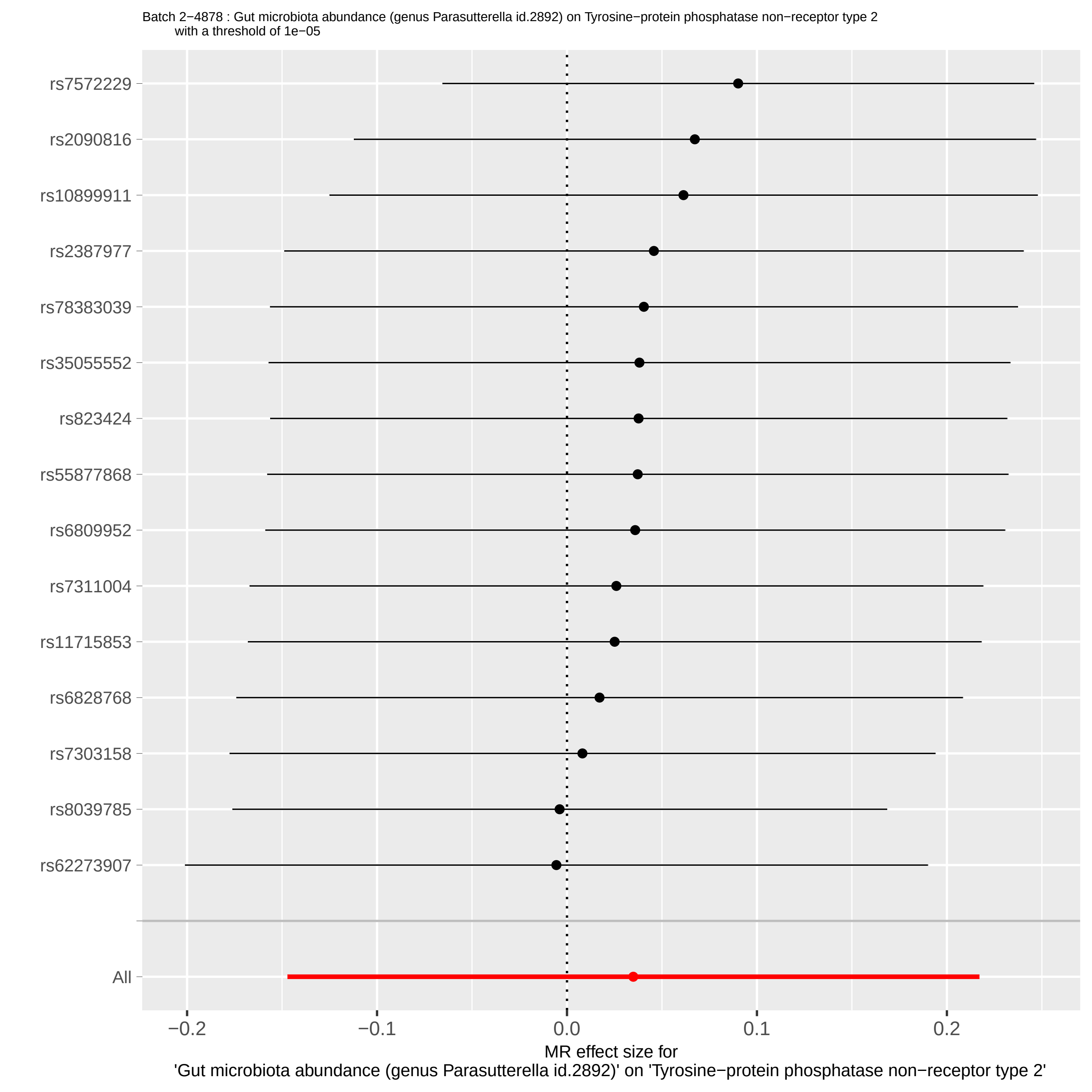

Batch 2−4878 : Gut microbiota abundance (genus Parasutterella id.2892) on Tyrosine−protein phosphatase non−receptor type 2
with a threshold of 1e−05
rs7572229
rs2090816
rs10899911
rs2387977
rs78383039
rs35055552
rs823424
rs55877868
rs6809952
rs7311004
rs11715853
rs6828768
rs7303158
rs8039785
rs62273907
All
−0.2
−0.1
0.0
0.1
0.2
MR effect size for
'Gut microbiota abundance (genus Parasutterella id.2892)' on 'Tyrosine−protein phosphatase non−receptor type 2'

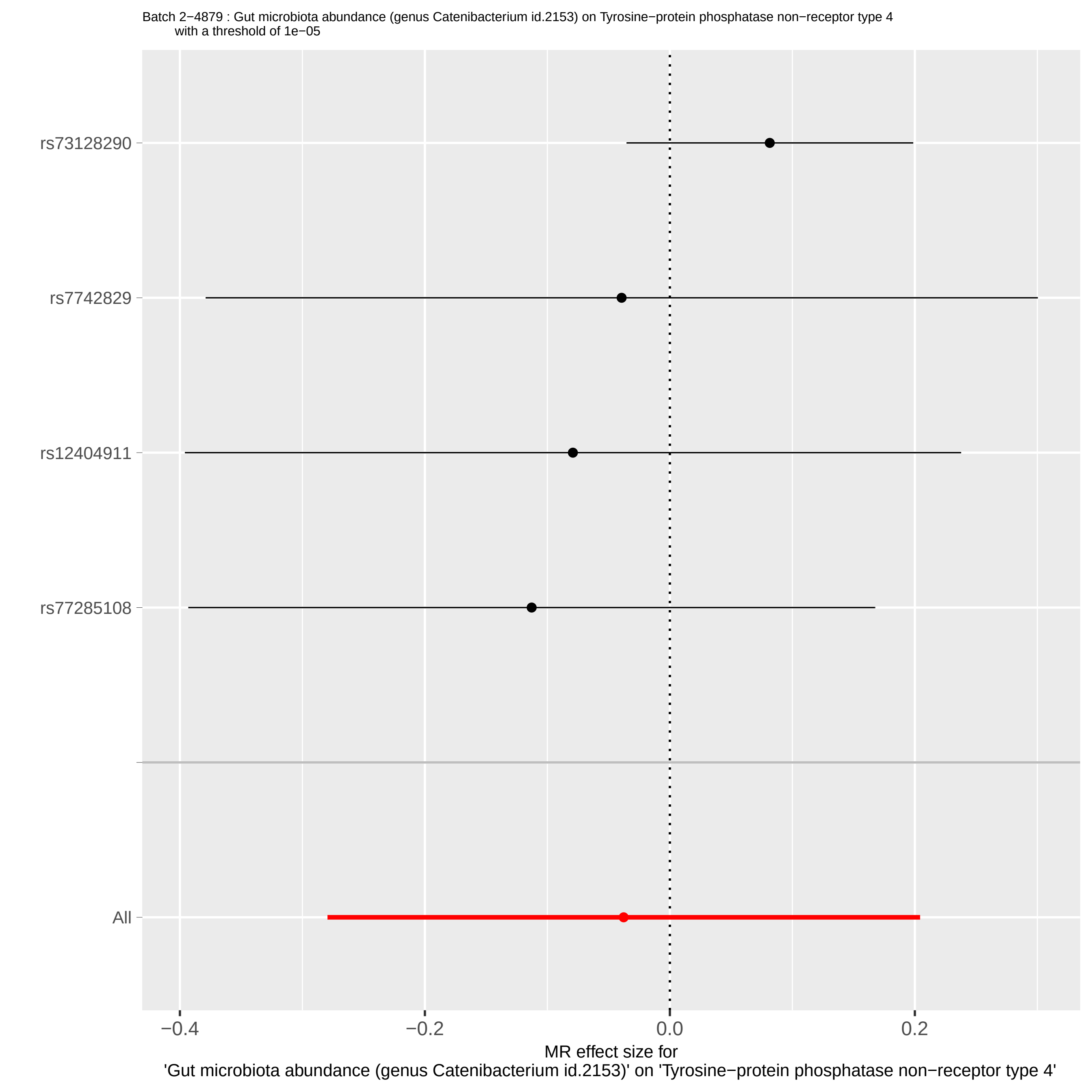
Batch 2−4879 : Gut microbiota abundance (genus Catenibacterium id.2153) on Tyrosine−protein phosphatase non−receptor type 4
with a threshold of 1e−05
rs73128290
rs7742829
rs12404911
rs77285108
All
−0.4
−0.2
0.0
0.2
MR effect size for
'Gut microbiota abundance (genus Catenibacterium id.2153)' on 'Tyrosine−protein phosphatase non−receptor type 4'

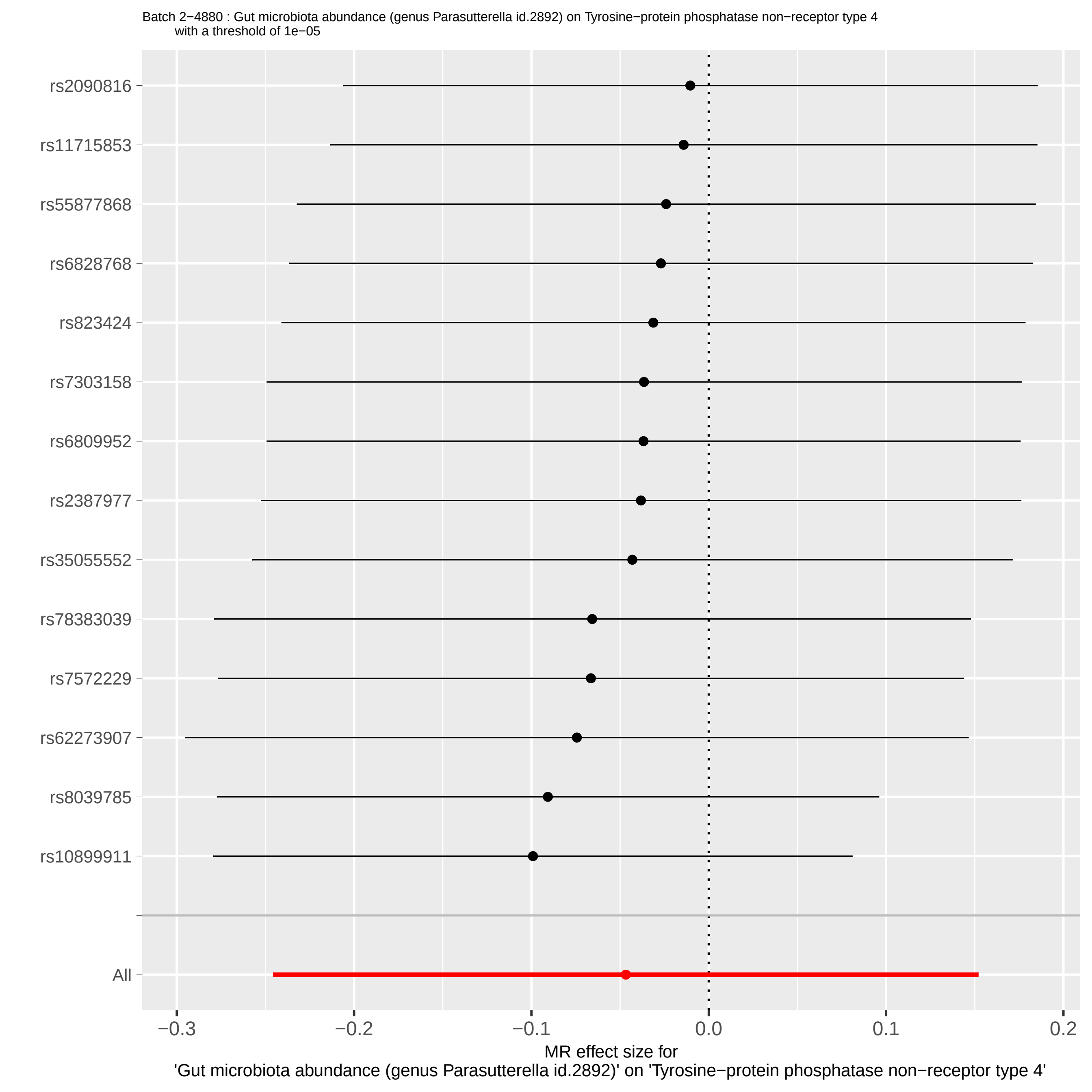
Batch 2−4880 : Gut microbiota abundance (genus Parasutterella id.2892) on Tyrosine−protein phosphatase non−receptor type 4
with a threshold of 1e−05
rs2090816
rs11715853
rs55877868
rs6828768
rs823424
rs7303158
rs6809952
rs2387977
rs35055552
rs78383039
rs7572229
rs62273907
rs8039785
rs10899911
All
−0.3
−0.2
−0.1
0.0
0.1
0.2
MR effect size for
'Gut microbiota abundance (genus Parasutterella id.2892)' on 'Tyrosine−protein phosphatase non−receptor type 4'

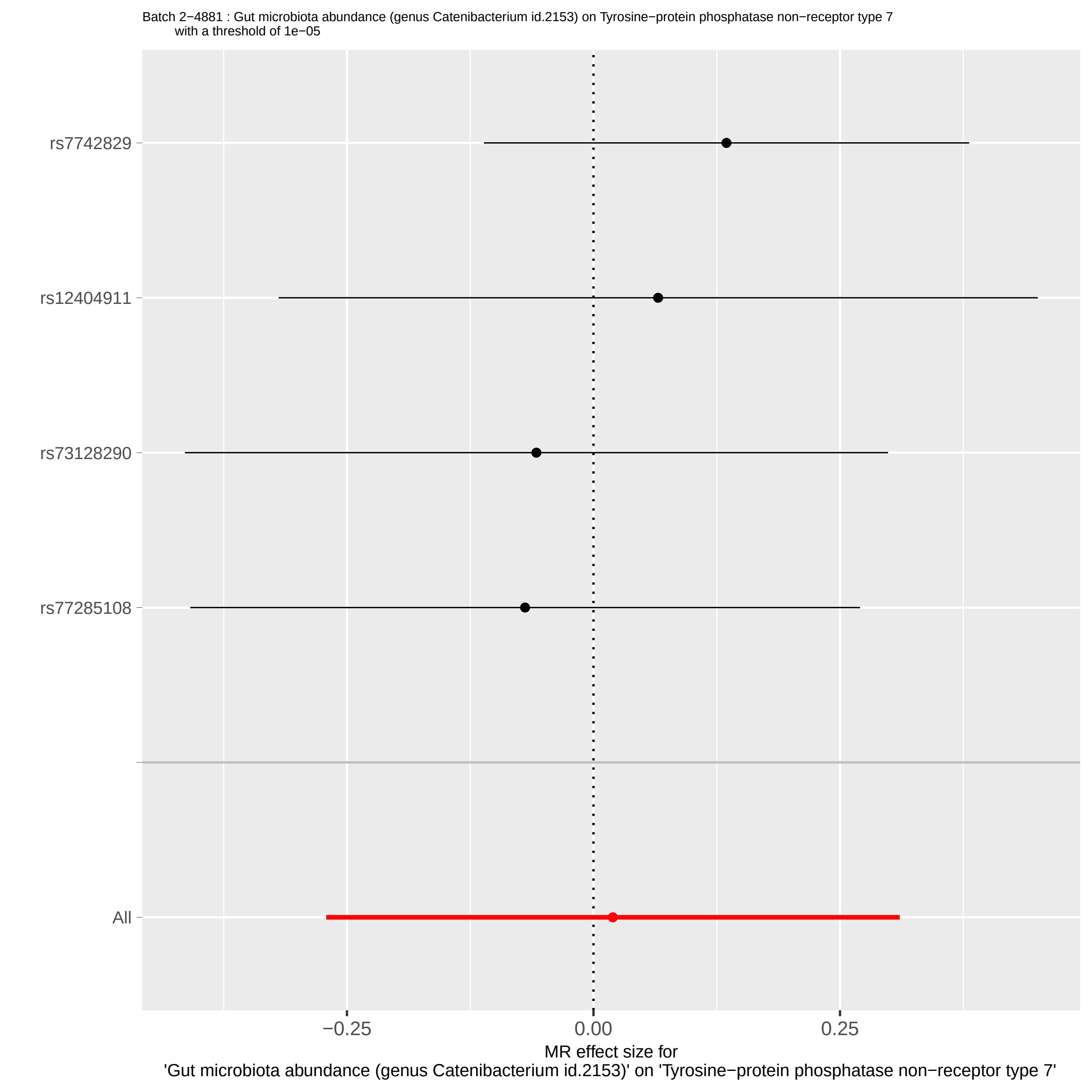
Batch 2−4881 : Gut microbiota abundance (genus Catenibacterium id.2153) on Tyrosine−protein phosphatase non−receptor type 7
with a threshold of 1e−05
rs7742829
rs12404911
rs73128290
rs77285108
All
−0.25
0.00
0.25
MR effect size for
'Gut microbiota abundance (genus Catenibacterium id.2153)' on 'Tyrosine−protein phosphatase non−receptor type 7'

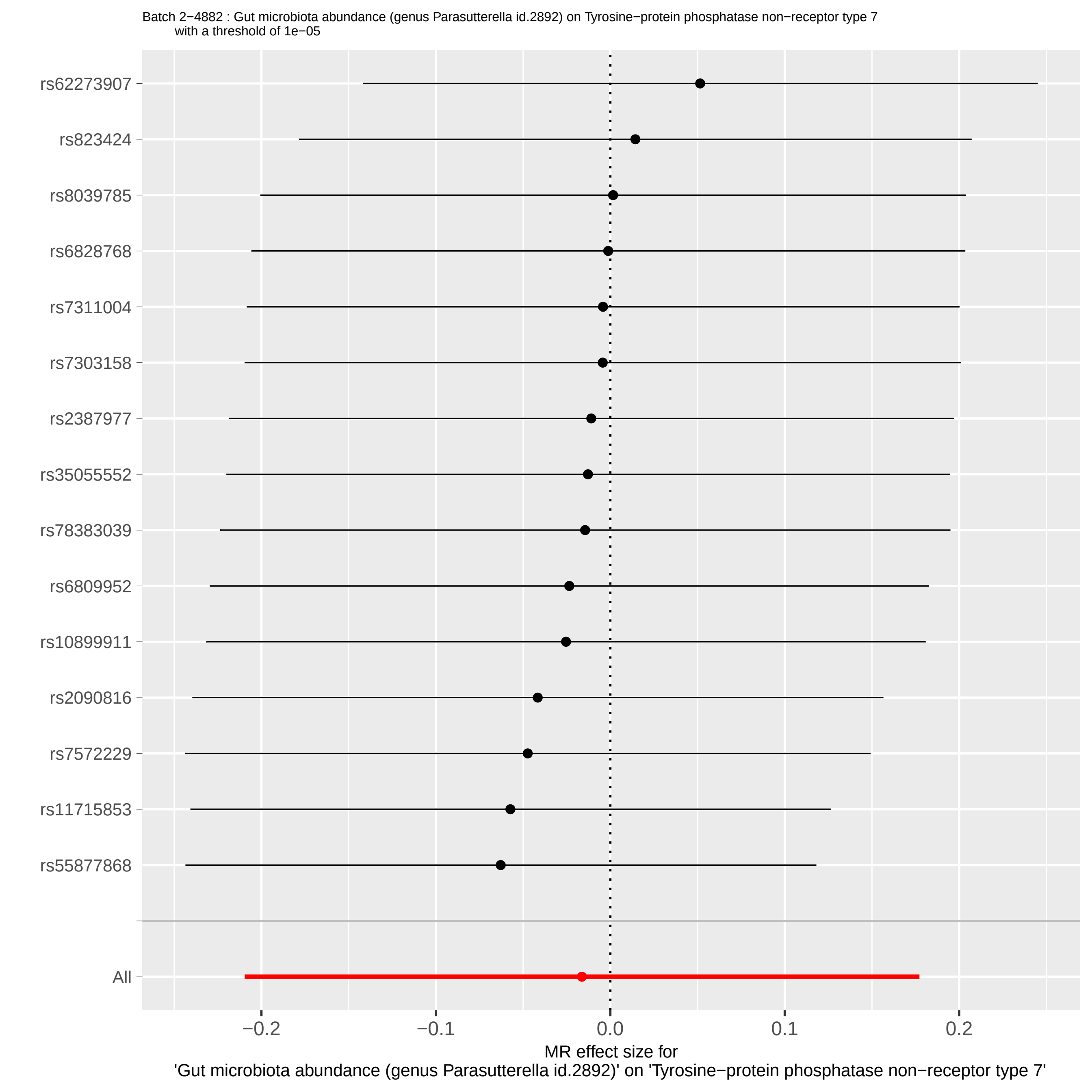

Batch 2−4882 : Gut microbiota abundance (genus Parasutterella id.2892) on Tyrosine−protein phosphatase non−receptor type 7
with a threshold of 1e−05
rs62273907
rs823424
rs8039785
rs6828768
rs7311004
rs7303158
rs2387977
rs35055552
rs78383039
rs6809952
rs10899911
rs2090816
rs7572229
rs11715853
rs55877868
All
−0.2
−0.1
0.0
0.1
0.2
MR effect size for
'Gut microbiota abundance (genus Parasutterella id.2892)' on 'Tyrosine−protein phosphatase non−receptor type 7'

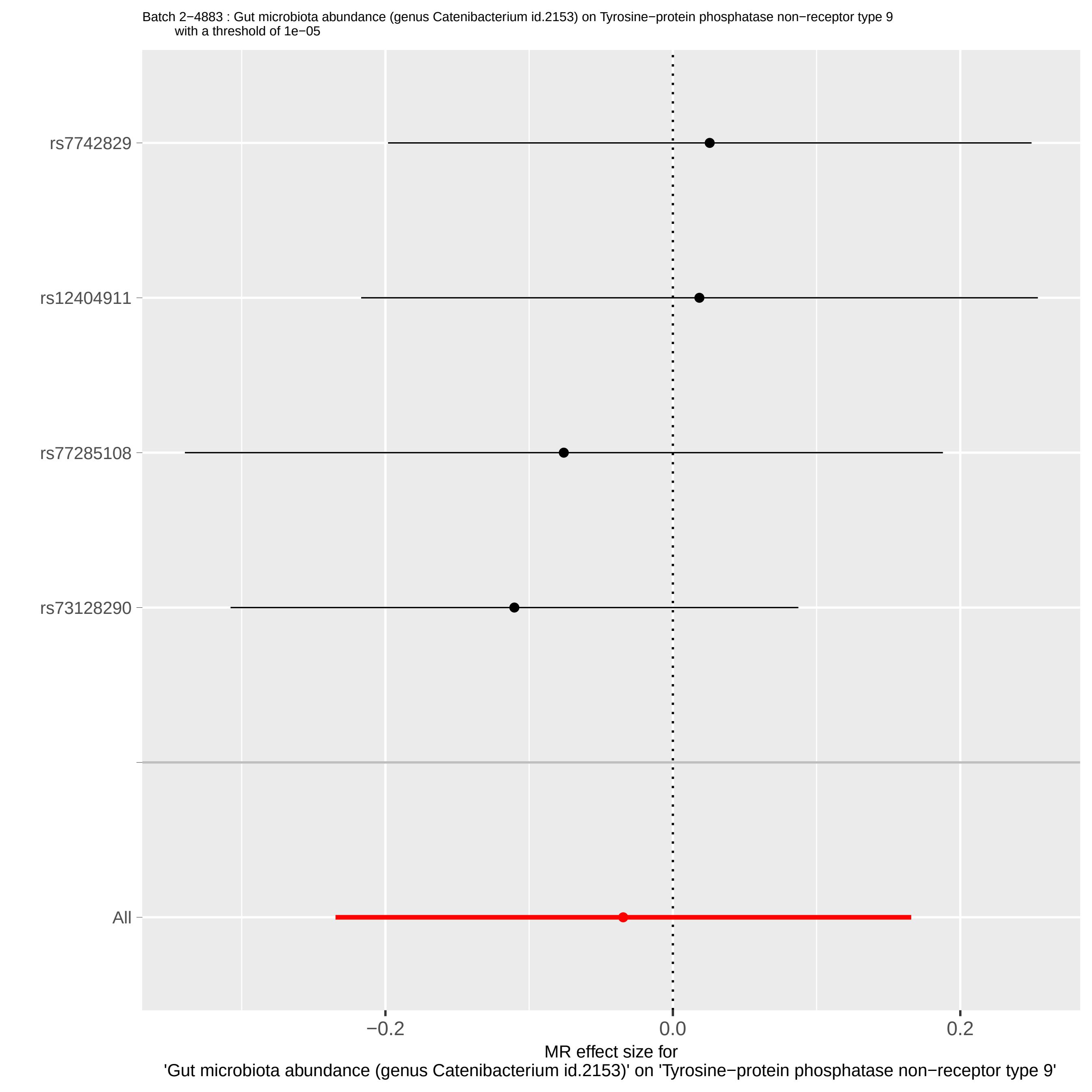
Batch 2−4883 : Gut microbiota abundance (genus Catenibacterium id.2153) on Tyrosine−protein phosphatase non−receptor type 9
with a threshold of 1e−05
rs7742829
rs12404911
rs77285108
rs73128290
All
−0.2
0.0
0.2
MR effect size for
'Gut microbiota abundance (genus Catenibacterium id.2153)' on 'Tyrosine−protein phosphatase non−receptor type 9'

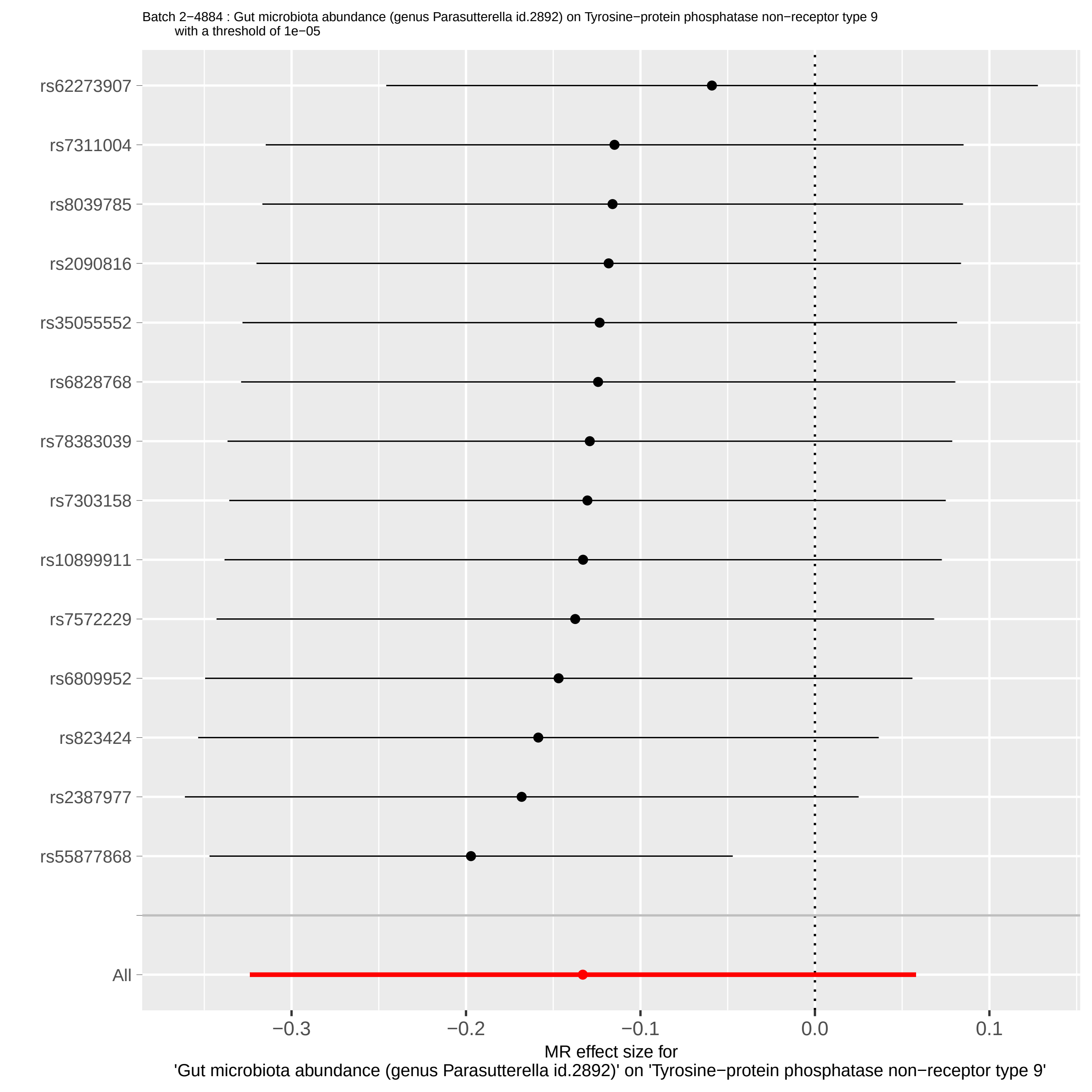

Batch 2−4884 : Gut microbiota abundance (genus Parasutterella id.2892) on Tyrosine−protein phosphatase non−receptor type 9
with a threshold of 1e−05
rs62273907
rs7311004
rs8039785
rs2090816
rs35055552
rs6828768
rs78383039
rs7303158
rs10899911
rs7572229
rs6809952
rs823424
rs2387977
rs55877868
All
−0.3
−0.2
−0.1
0.0
0.1
MR effect size for
'Gut microbiota abundance (genus Parasutterella id.2892)' on 'Tyrosine−protein phosphatase non−receptor type 9'

Batch 2−4885 : Gut microbiota abundance (genus Catenibacterium id.2153) on Receptor−type tyrosine−protein phosphatase delta
with a threshold of 1e−05

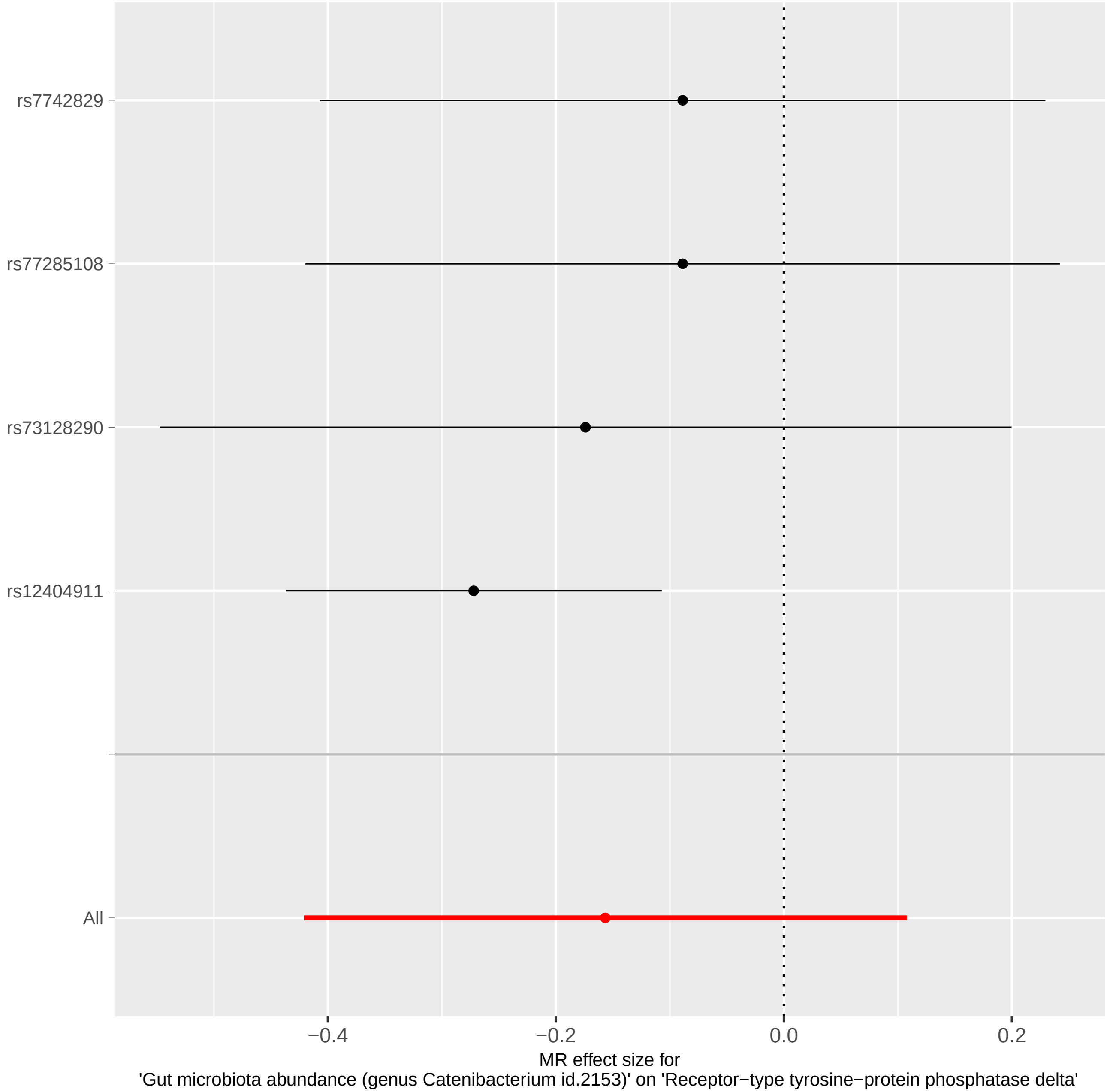

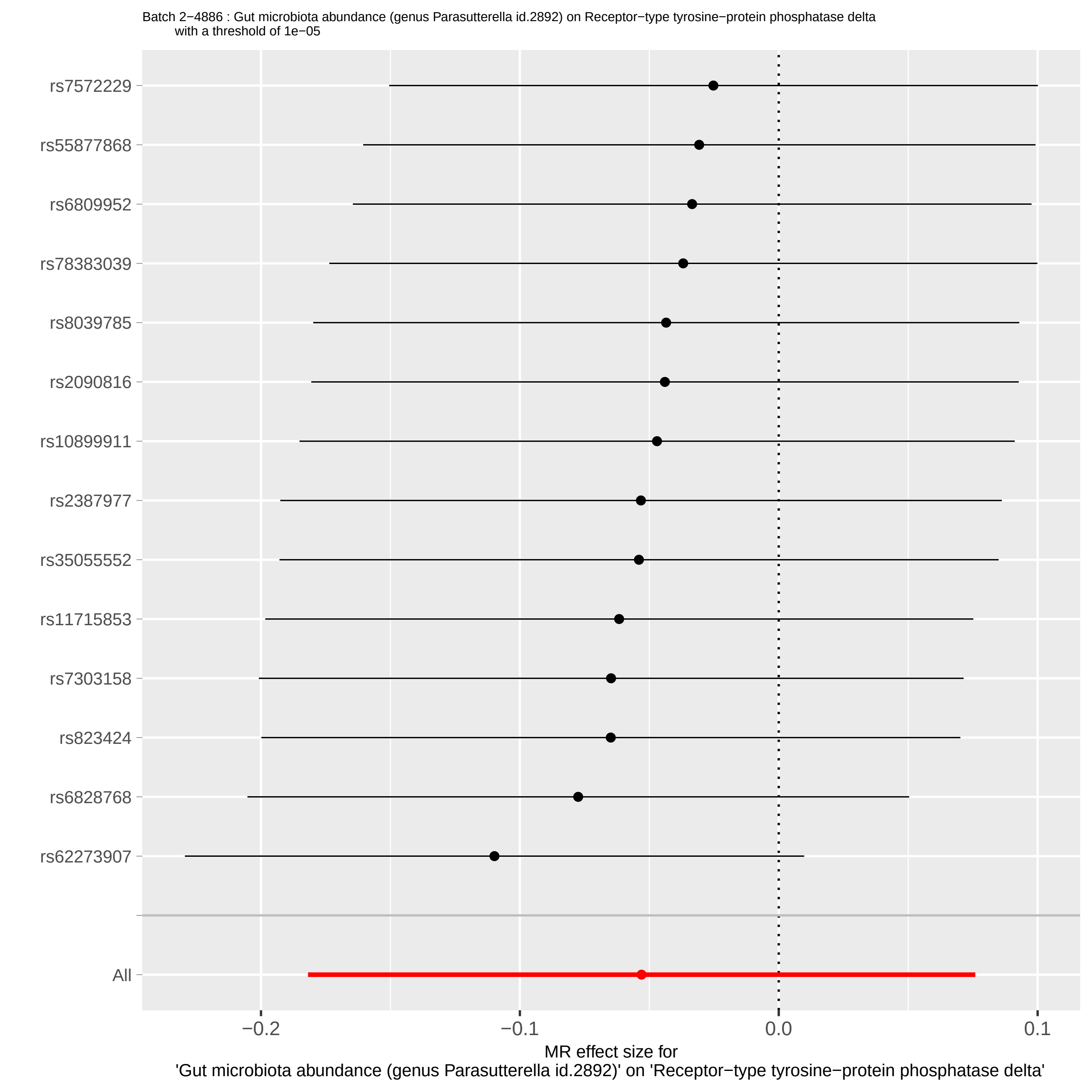
Batch 2−4886 : Gut microbiota abundance (genus Parasutterella id.2892) on Receptor−type tyrosine−protein phosphatase delta
with a threshold of 1e−05
rs7572229
rs55877868
rs6809952
rs78383039
rs8039785
rs2090816
rs10899911
rs2387977
rs35055552
rs11715853
rs7303158
rs823424
rs6828768
rs62273907
All
−0.2
−0.1
0.0
0.1
MR effect size for
'Gut microbiota abundance (genus Parasutterella id.2892)' on 'Receptor−type tyrosine−protein phosphatase delta'

Batch 2−4887 : Gut microbiota abundance (genus Catenibacterium id.2153) on Receptor−type tyrosine−protein phosphatase H
with a threshold of 1e−05

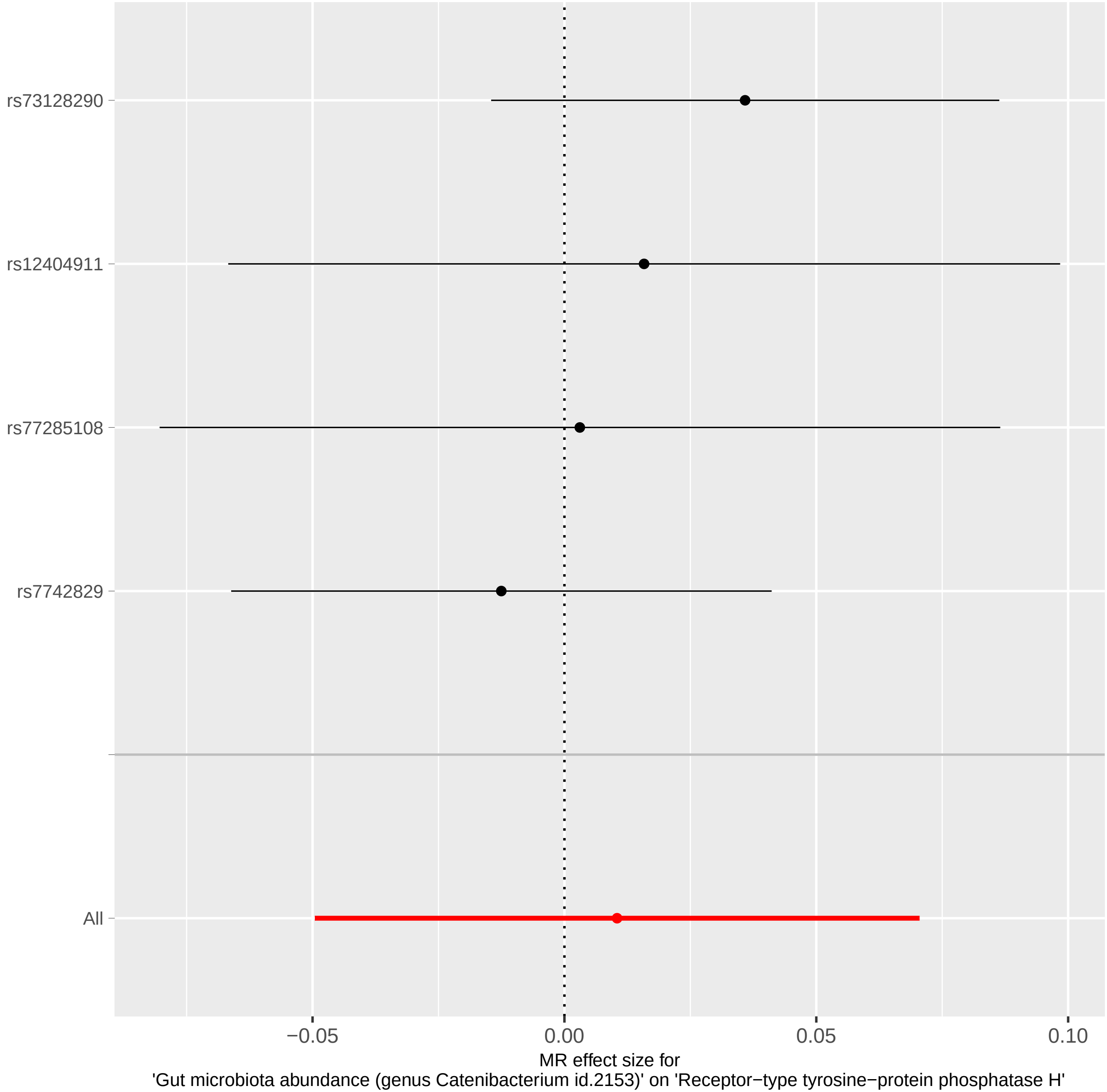

Batch 2−4888 : Gut microbiota abundance (genus Parasutterella id.2892) on Receptor−type tyrosine−protein phosphatase H
with a threshold of 1e−05

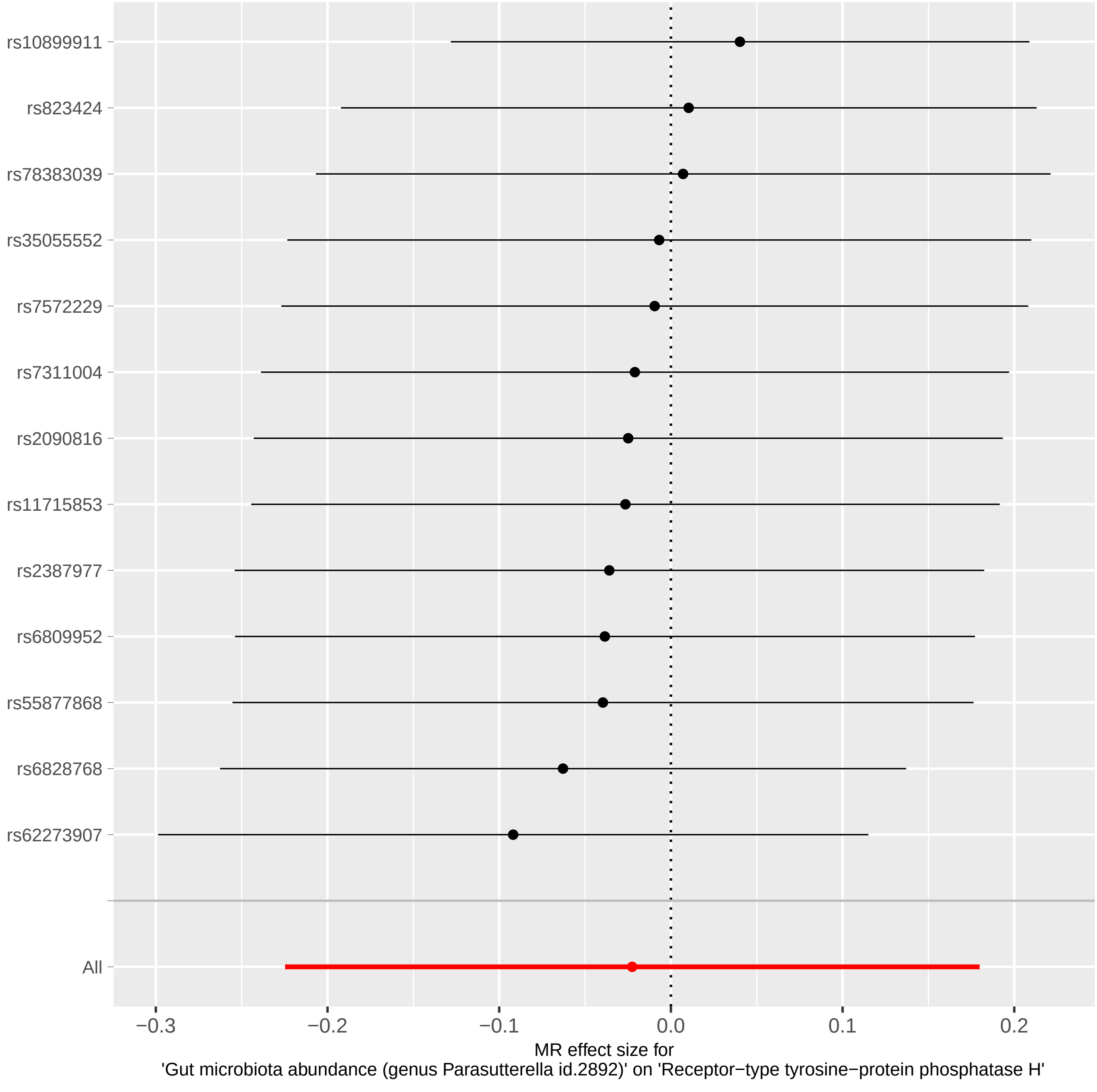

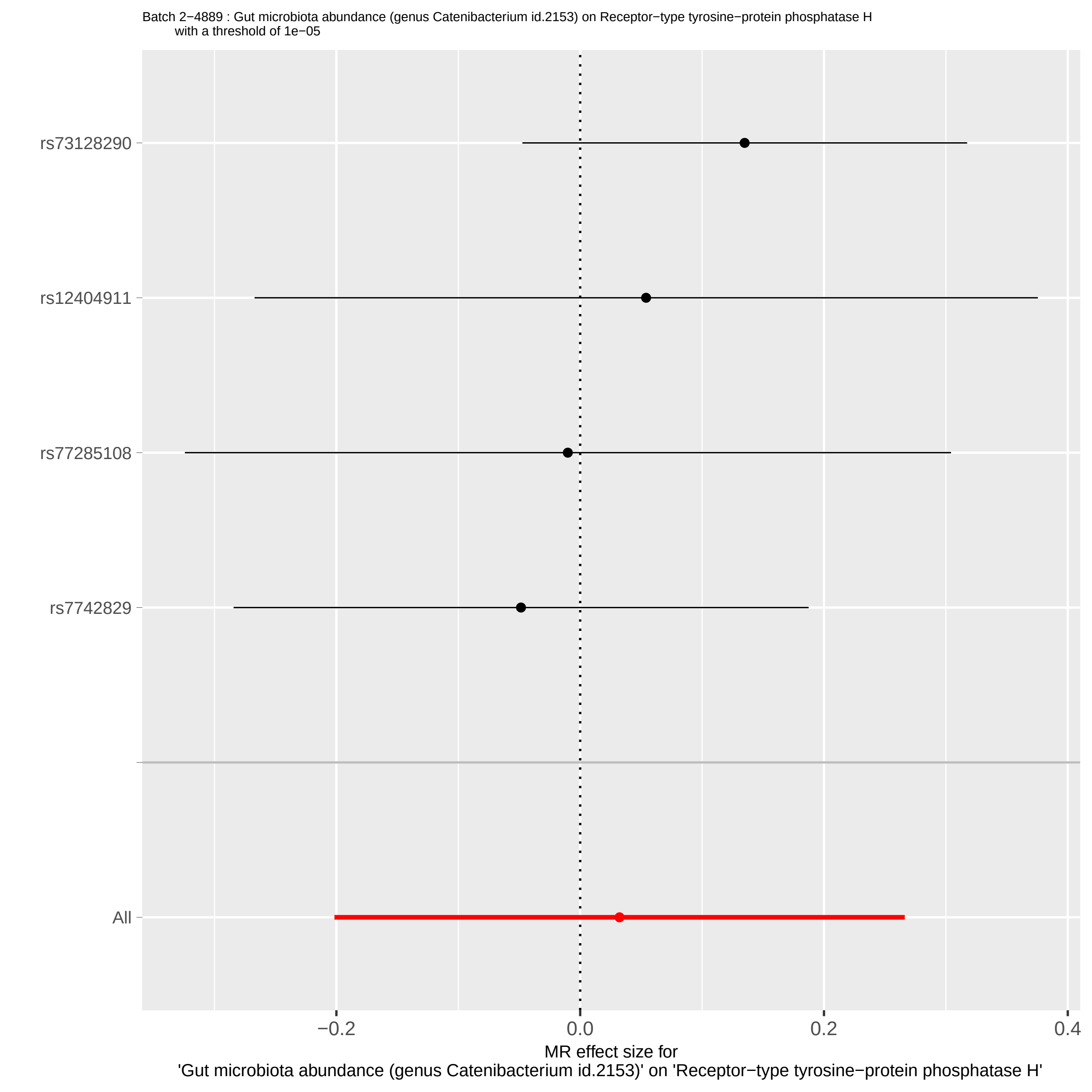
Batch 2−4889 : Gut microbiota abundance (genus Catenibacterium id.2153) on Receptor−type tyrosine−protein phosphatase H
with a threshold of 1e−05
rs73128290
rs12404911
rs77285108
rs7742829
All
−0.2
0.0
0.2
0.4
MR effect size for
'Gut microbiota abundance (genus Catenibacterium id.2153)' on 'Receptor−type tyrosine−protein phosphatase H'

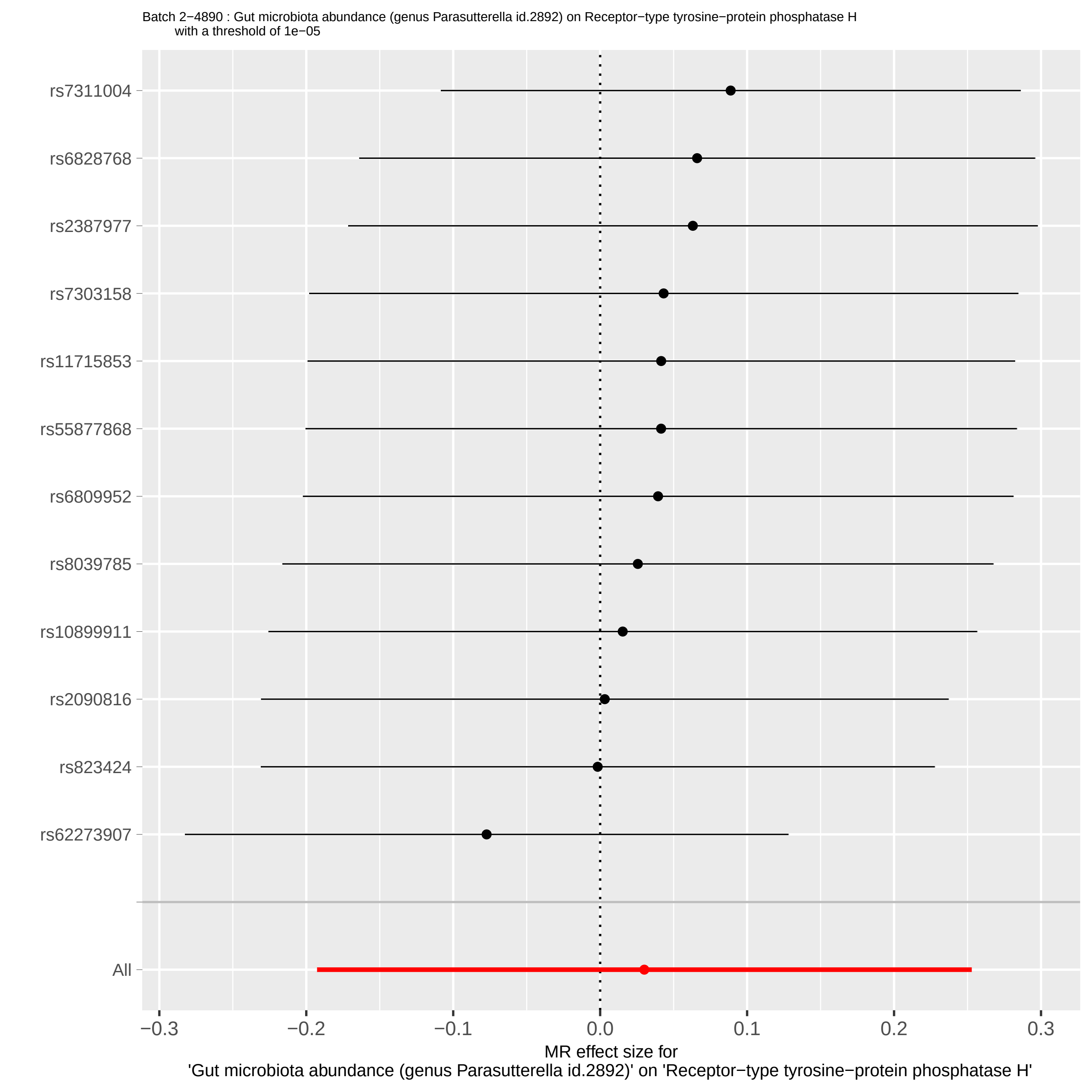
Batch 2−4890 : Gut microbiota abundance (genus Parasutterella id.2892) on Receptor−type tyrosine−protein phosphatase H
with a threshold of 1e−05
rs7311004
rs6828768
rs2387977
rs7303158
rs11715853
rs55877868
rs6809952
rs8039785
rs10899911
rs2090816
rs823424
rs62273907
All
−0.3
−0.2
−0.1
0.0
0.1
0.2
0.3
MR effect size for
'Gut microbiota abundance (genus Parasutterella id.2892)' on 'Receptor−type tyrosine−protein phosphatase H'

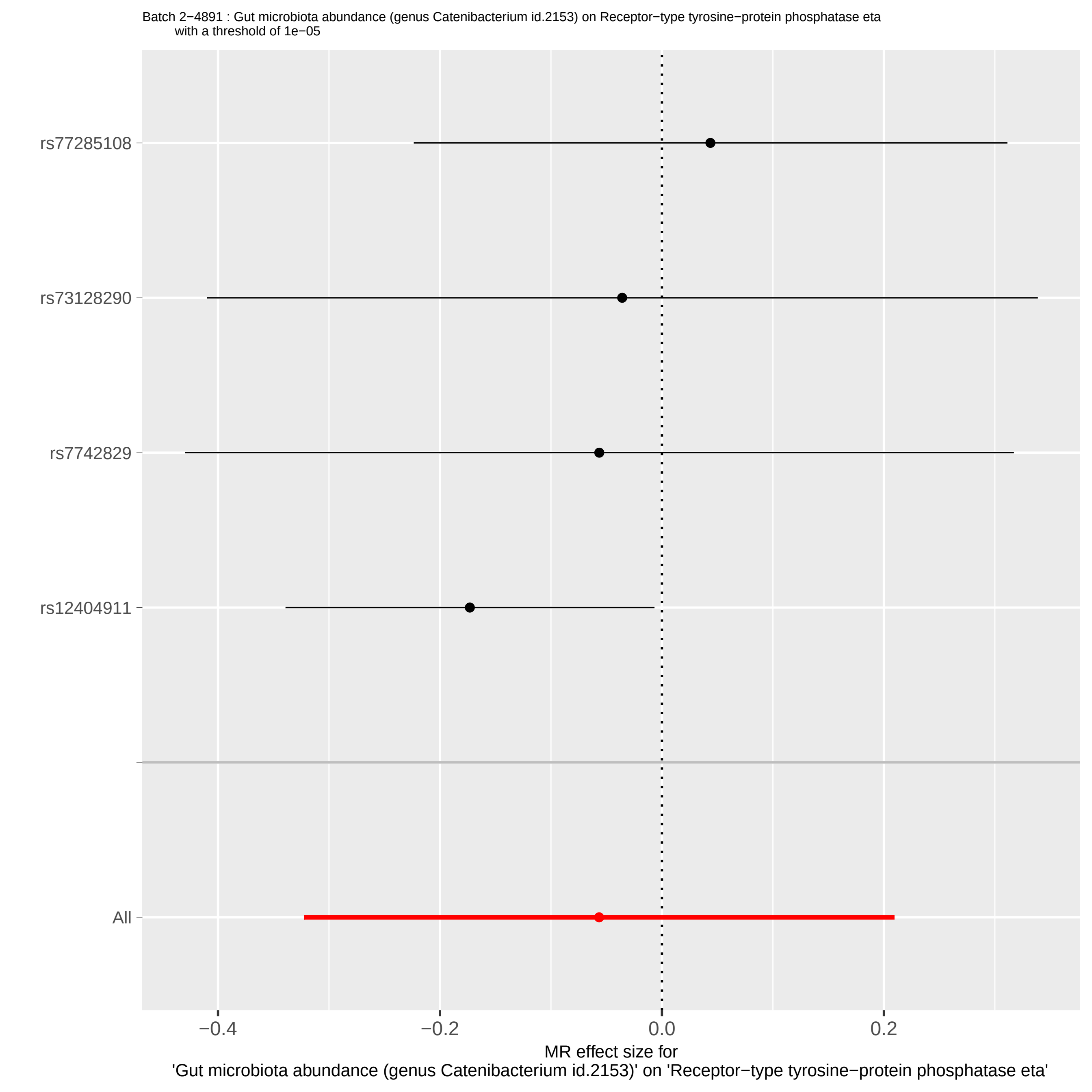

Batch 2−4891 : Gut microbiota abundance (genus Catenibacterium id.2153) on Receptor−type tyrosine−protein phosphatase eta
with a threshold of 1e−05
rs77285108
rs73128290
rs7742829
rs12404911
All
−0.4
−0.2
0.0
0.2
MR effect size for
'Gut microbiota abundance (genus Catenibacterium id.2153)' on 'Receptor−type tyrosine−protein phosphatase eta'

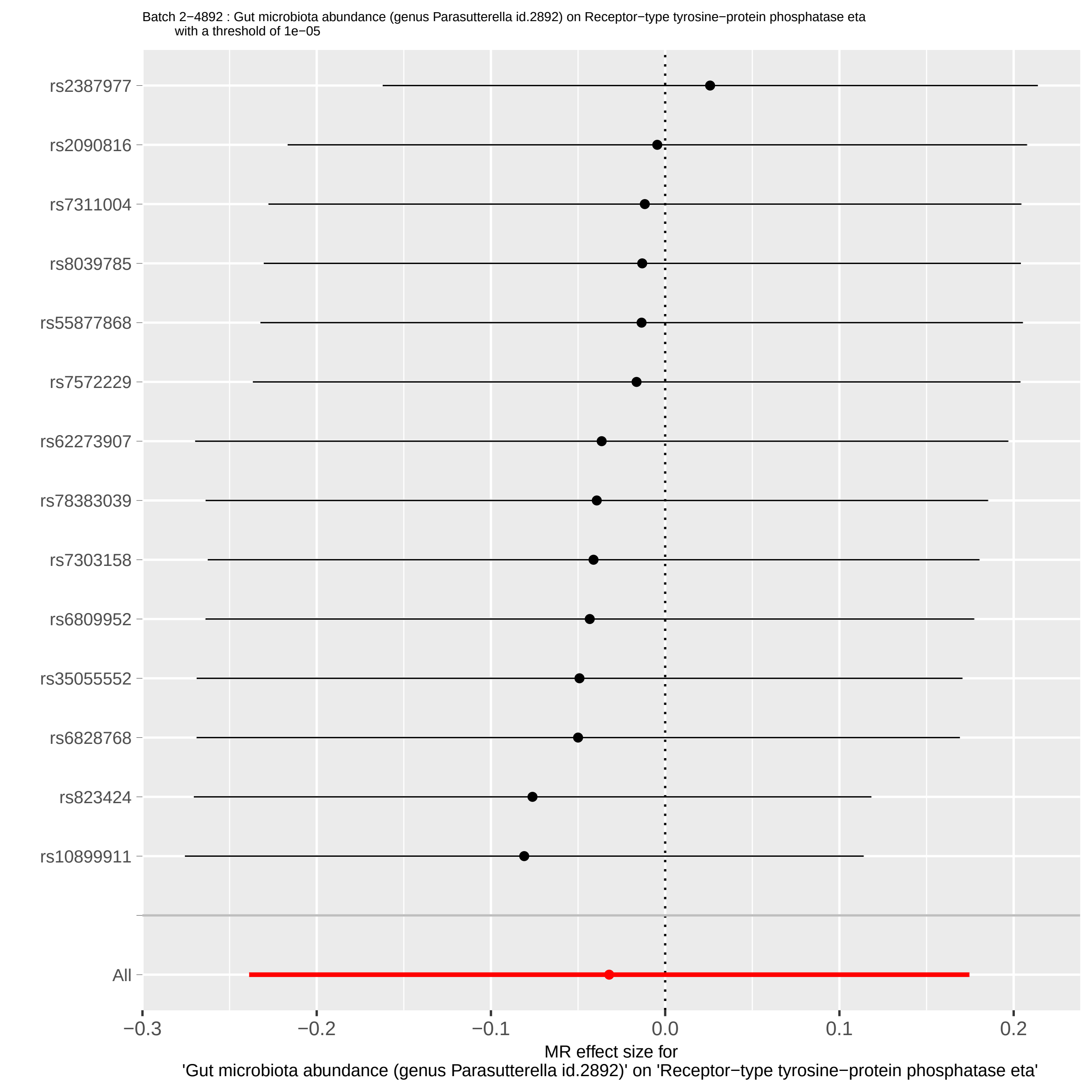
Batch 2−4892 : Gut microbiota abundance (genus Parasutterella id.2892) on Receptor−type tyrosine−protein phosphatase eta
with a threshold of 1e−05
rs2387977
rs2090816
rs7311004
rs8039785
rs55877868
rs7572229
rs62273907
rs78383039
rs7303158
rs6809952
rs35055552
rs6828768
rs823424
rs10899911
All
−0.3
−0.2
−0.1
0.0
0.1
0.2
MR effect size for
'Gut microbiota abundance (genus Parasutterella id.2892)' on 'Receptor−type tyrosine−protein phosphatase eta'

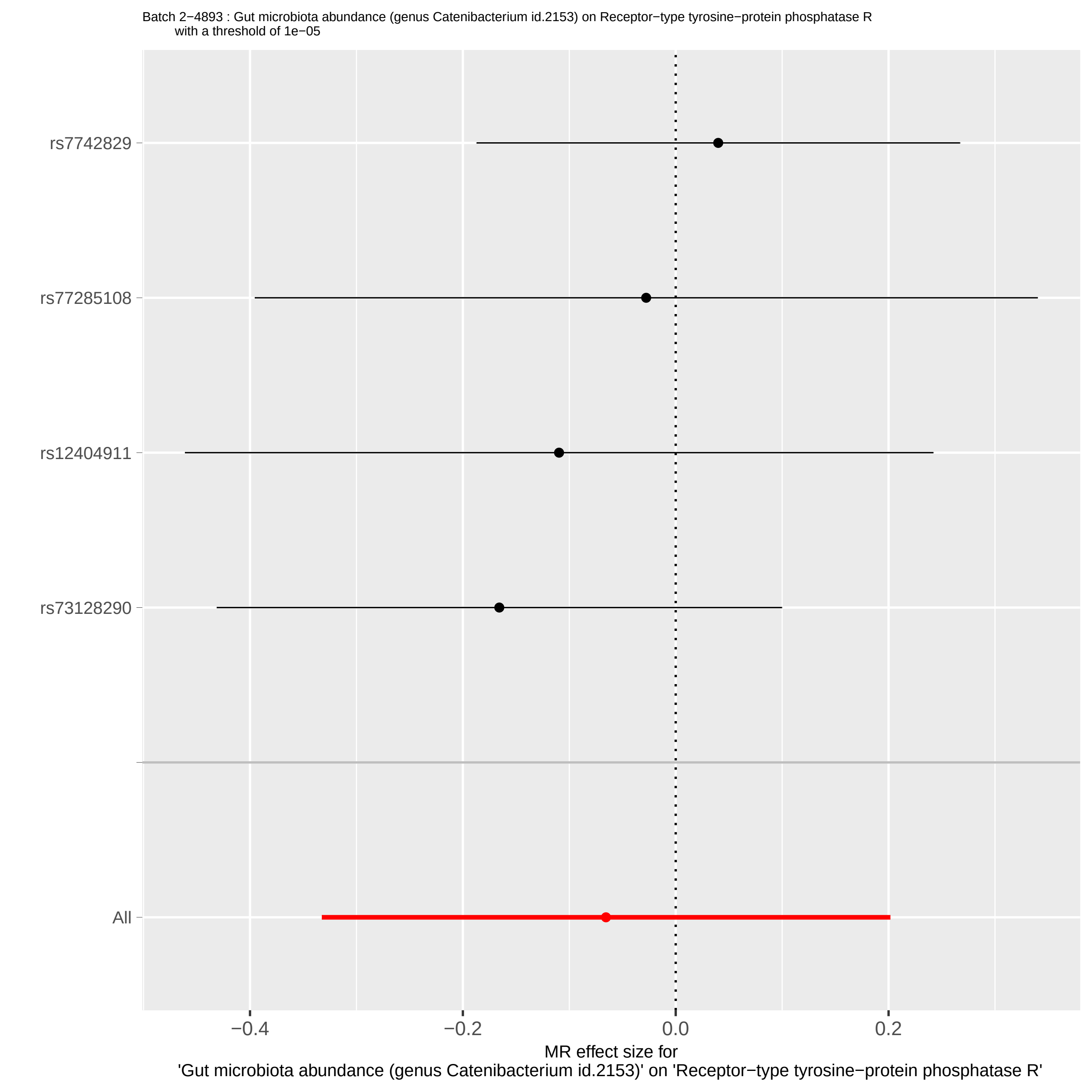
Batch 2−4893 : Gut microbiota abundance (genus Catenibacterium id.2153) on Receptor−type tyrosine−protein phosphatase R
with a threshold of 1e−05
rs7742829
rs77285108
rs12404911
rs73128290
All
−0.4
−0.2
0.0
0.2
MR effect size for
'Gut microbiota abundance (genus Catenibacterium id.2153)' on 'Receptor−type tyrosine−protein phosphatase R'

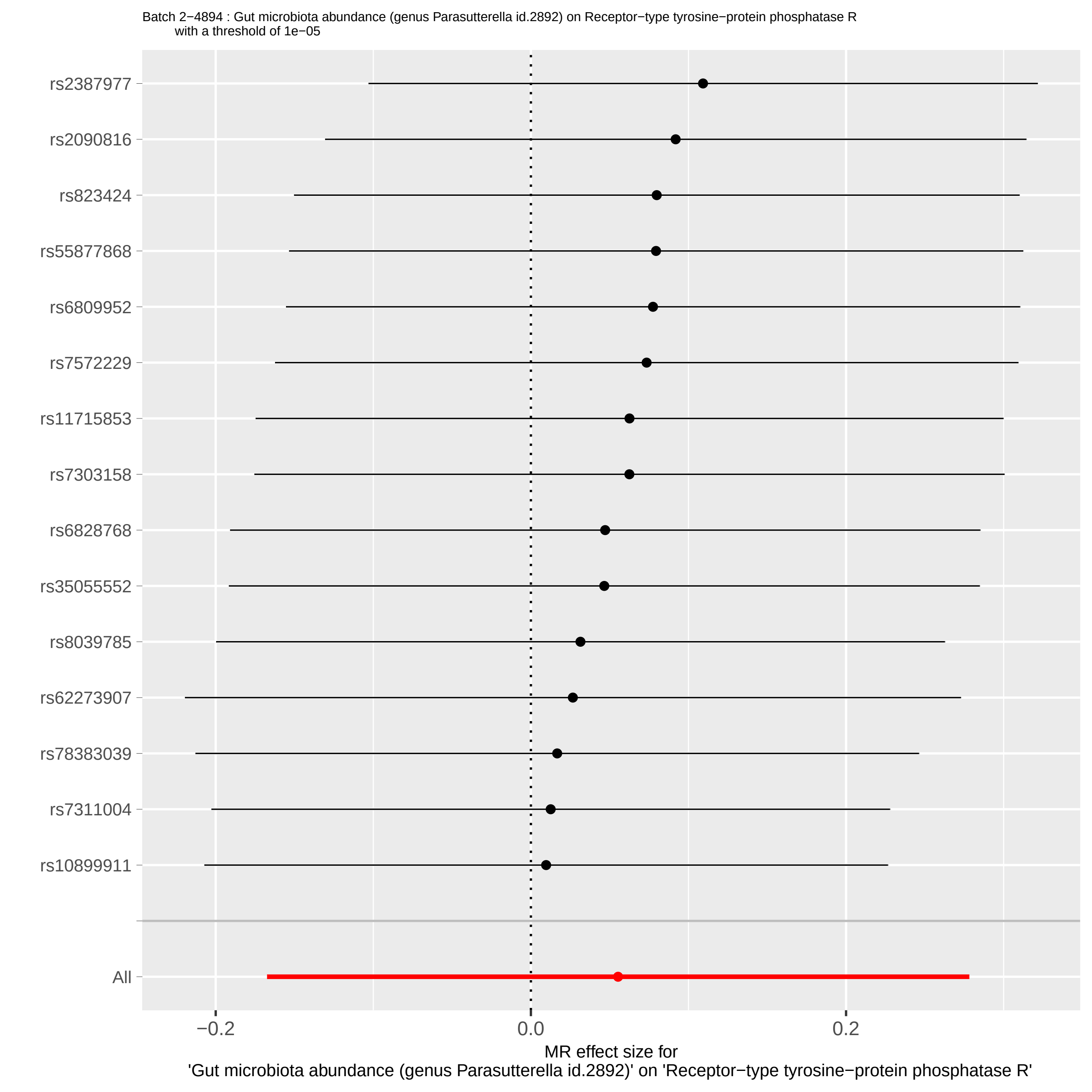
Batch 2−4894 : Gut microbiota abundance (genus Parasutterella id.2892) on Receptor−type tyrosine−protein phosphatase R
with a threshold of 1e−05
rs2387977
rs2090816
rs823424
rs55877868
rs6809952
rs7572229
rs11715853
rs7303158
rs6828768
rs35055552
rs8039785
rs62273907
rs78383039
rs7311004
rs10899911
All
−0.2
0.0
0.2
MR effect size for
'Gut microbiota abundance (genus Parasutterella id.2892)' on 'Receptor−type tyrosine−protein phosphatase R'

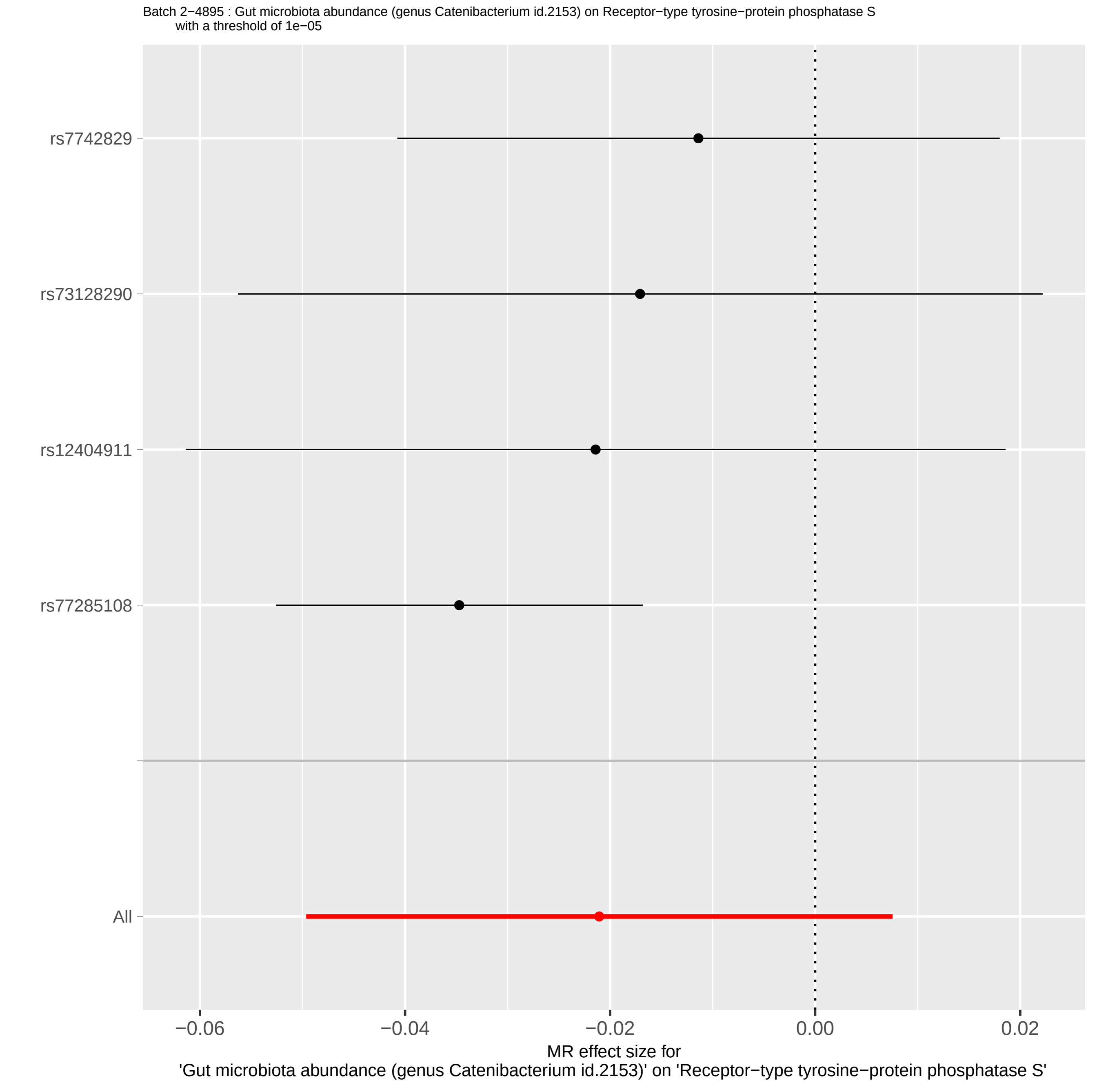
Batch 2−4895 : Gut microbiota abundance (genus Catenibacterium id.2153) on Receptor−type tyrosine−protein phosphatase S
with a threshold of 1e−05
rs7742829
rs73128290
rs12404911
rs77285108
All
−0.06
−0.04
−0.02
0.00
0.02
MR effect size for
'Gut microbiota abundance (genus Catenibacterium id.2153)' on 'Receptor−type tyrosine−protein phosphatase S'

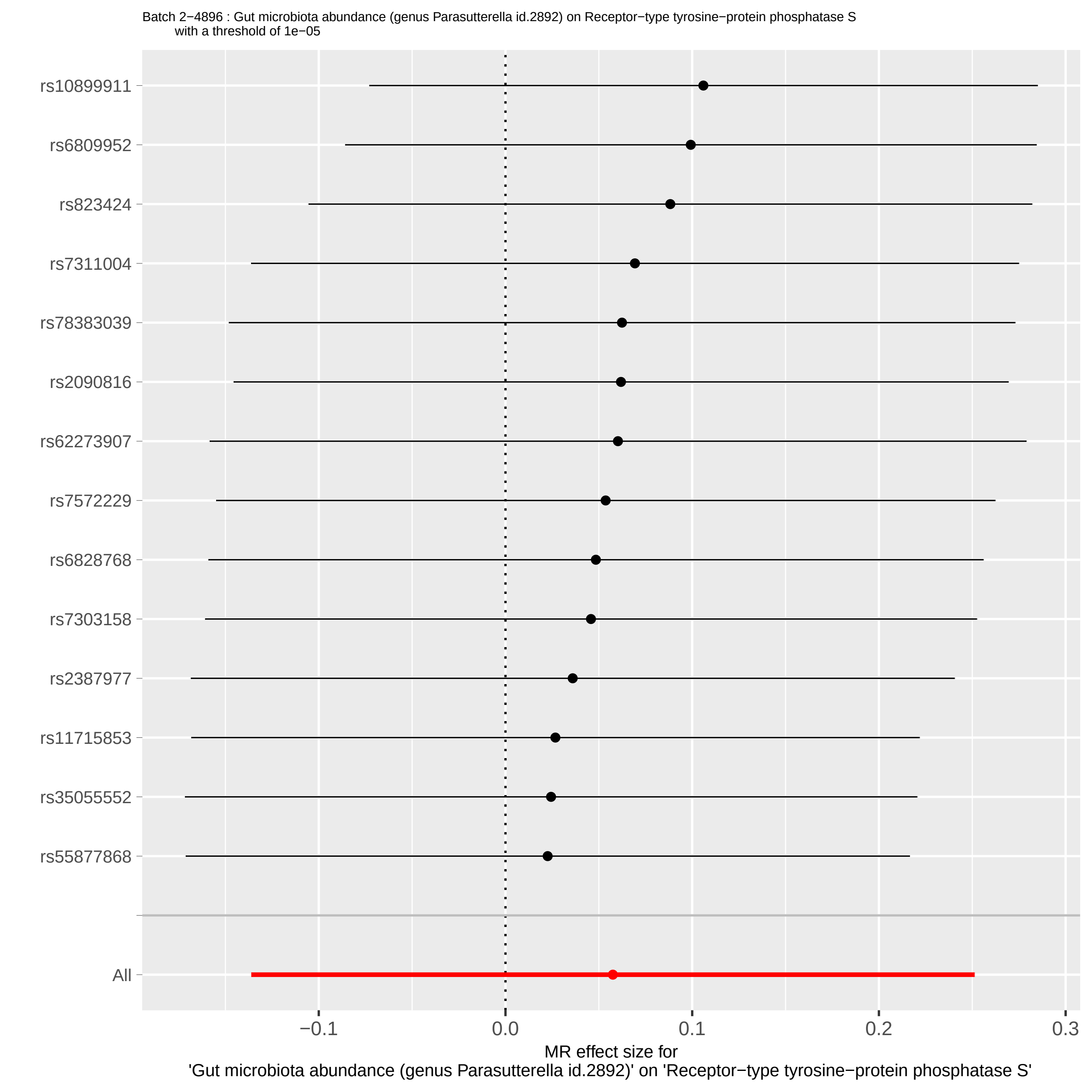

Batch 2−4896 : Gut microbiota abundance (genus Parasutterella id.2892) on Receptor−type tyrosine−protein phosphatase S
with a threshold of 1e−05
rs10899911
rs6809952
rs823424
rs7311004
rs78383039
rs2090816
rs62273907
rs7572229
rs6828768
rs7303158
rs2387977
rs11715853
rs35055552
rs55877868
All
−0.1
0.0
0.1
0.2
0.3
MR effect size for
'Gut microbiota abundance (genus Parasutterella id.2892)' on 'Receptor−type tyrosine−protein phosphatase S'

Batch 2−4897 : Gut microbiota abundance (genus Catenibacterium id.2153) on Receptor−type tyrosine−protein phosphatase U
with a threshold of 1e−05

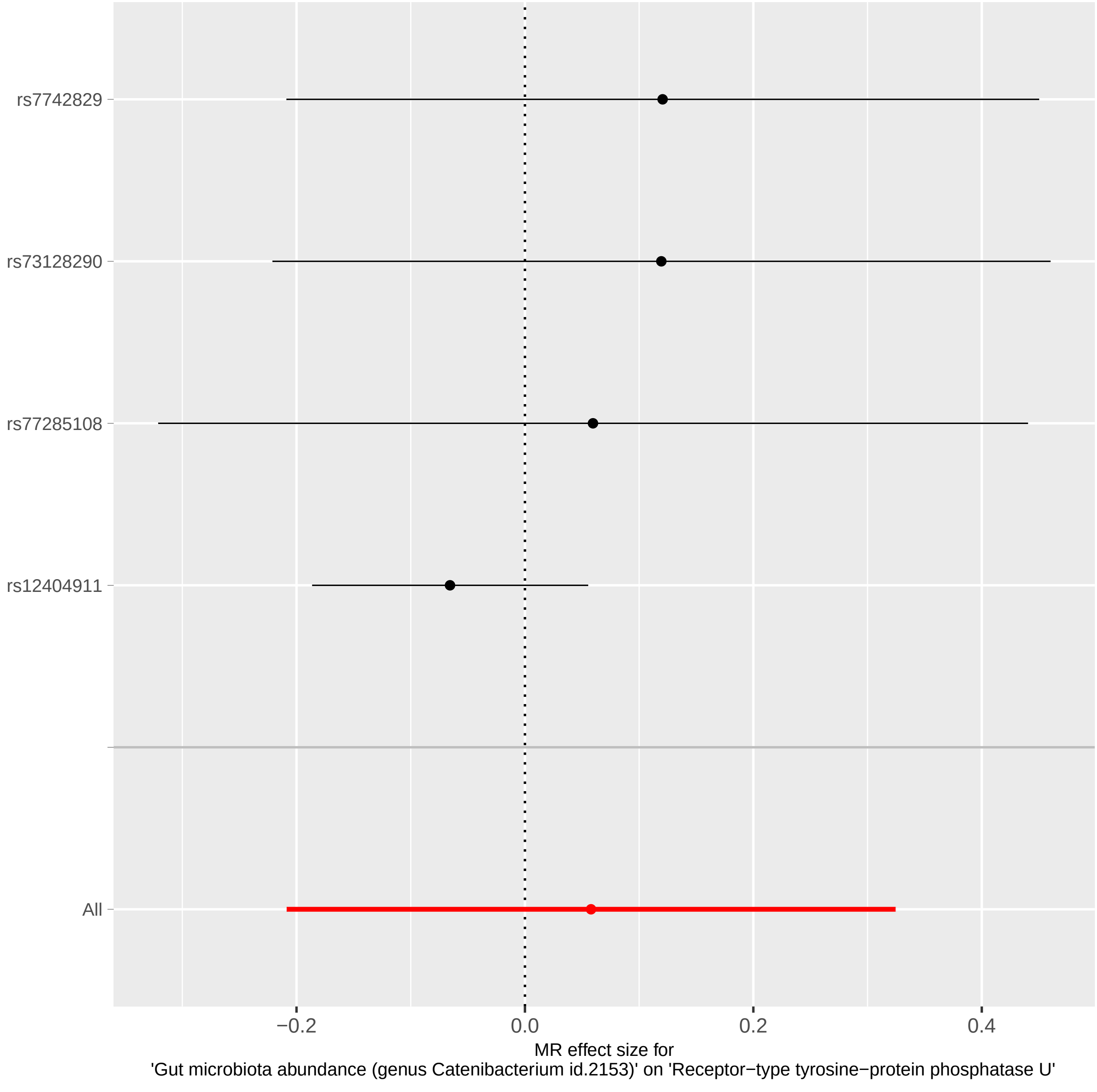

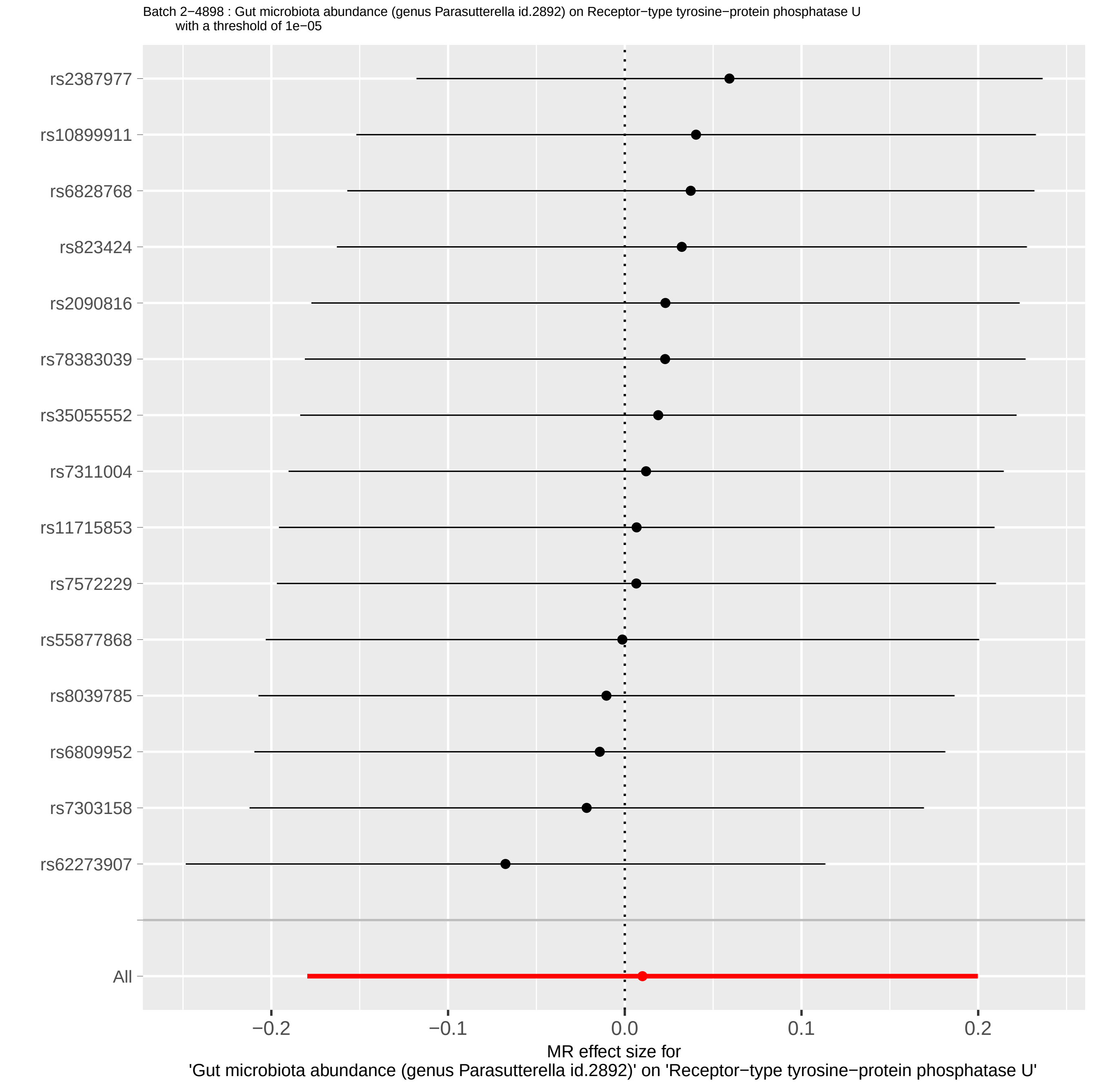

Batch 2−4898 : Gut microbiota abundance (genus Parasutterella id.2892) on Receptor−type tyrosine−protein phosphatase U
with a threshold of 1e−05
rs2387977
rs10899911
rs6828768
rs823424
rs2090816
rs78383039
rs35055552
rs7311004
rs11715853
rs7572229
rs55877868
rs8039785
rs6809952
rs7303158
rs62273907
All
−0.2
−0.1
0.0
0.1
0.2
MR effect size for
'Gut microbiota abundance (genus Parasutterella id.2892)' on 'Receptor−type tyrosine−protein phosphatase U'

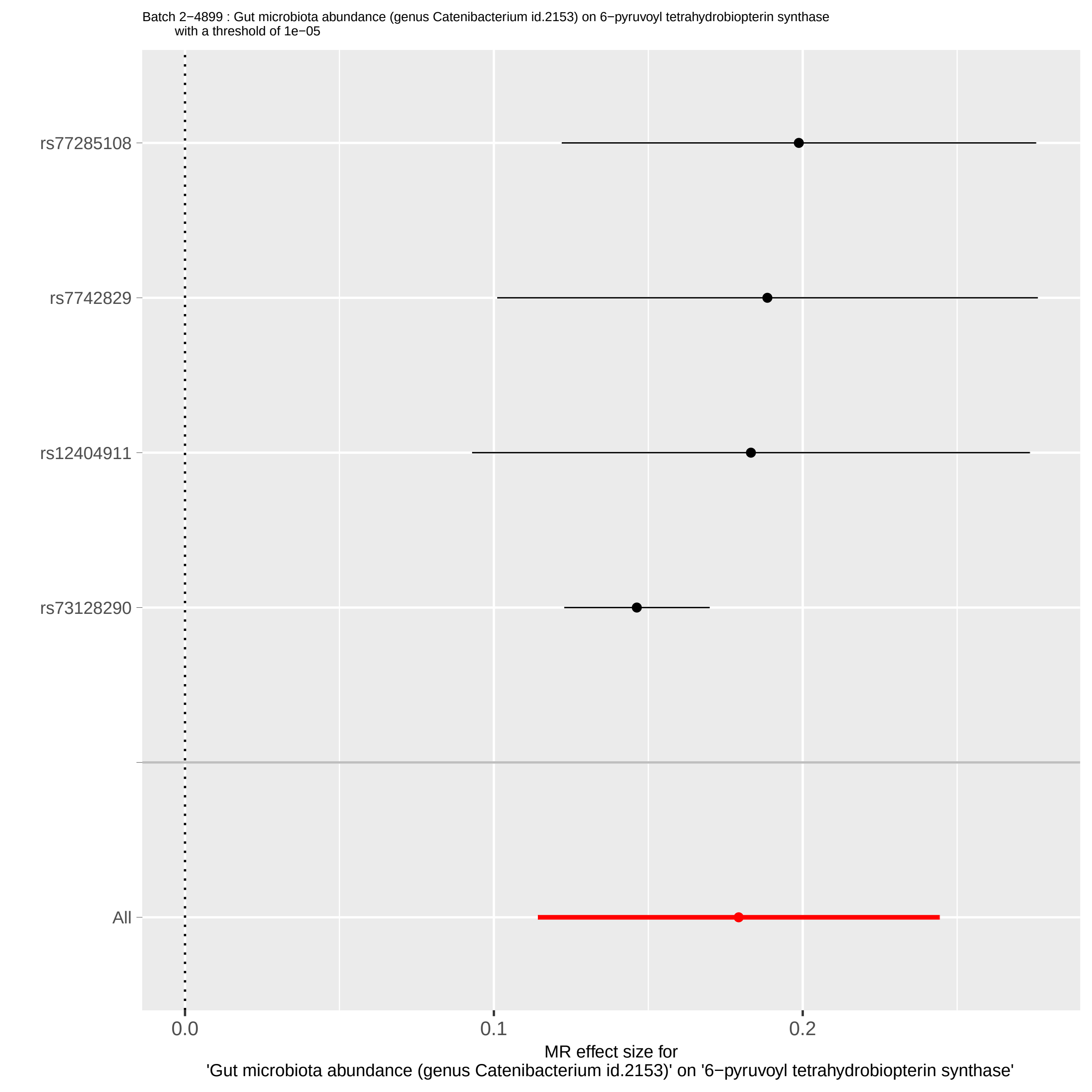
Batch 2−4899 : Gut microbiota abundance (genus Catenibacterium id.2153) on 6−pyruvoyl tetrahydrobiopterin synthase
with a threshold of 1e−05
rs77285108
rs7742829
rs12404911
rs73128290
All
0.0
0.1
0.2
MR effect size for
'Gut microbiota abundance (genus Catenibacterium id.2153)' on '6−pyruvoyl tetrahydrobiopterin synthase'

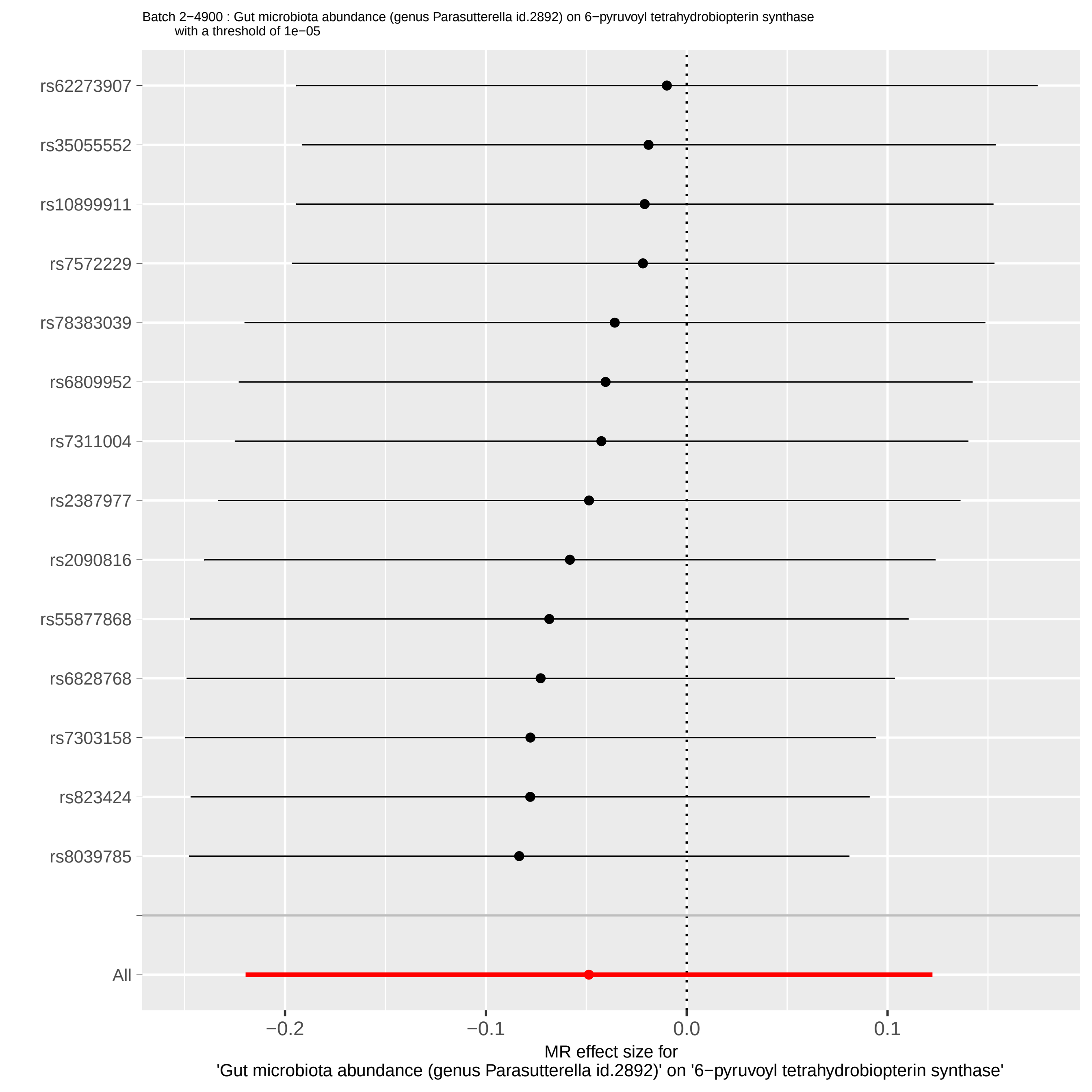

Batch 2−4900 : Gut microbiota abundance (genus Parasutterella id.2892) on 6−pyruvoyl tetrahydrobiopterin synthase
with a threshold of 1e−05
rs62273907
rs35055552
rs10899911
rs7572229
rs78383039
rs6809952
rs7311004
rs2387977
rs2090816
rs55877868
rs6828768
rs7303158
rs823424
rs8039785
All
−0.2
−0.1
0.0
0.1
MR effect size for
'Gut microbiota abundance (genus Parasutterella id.2892)' on '6−pyruvoyl tetrahydrobiopterin synthase'

Batch 2−4901 : Gut microbiota abundance (genus Catenibacterium id.2153) on Pentraxin−related protein PTX3
with a threshold of 1e−05

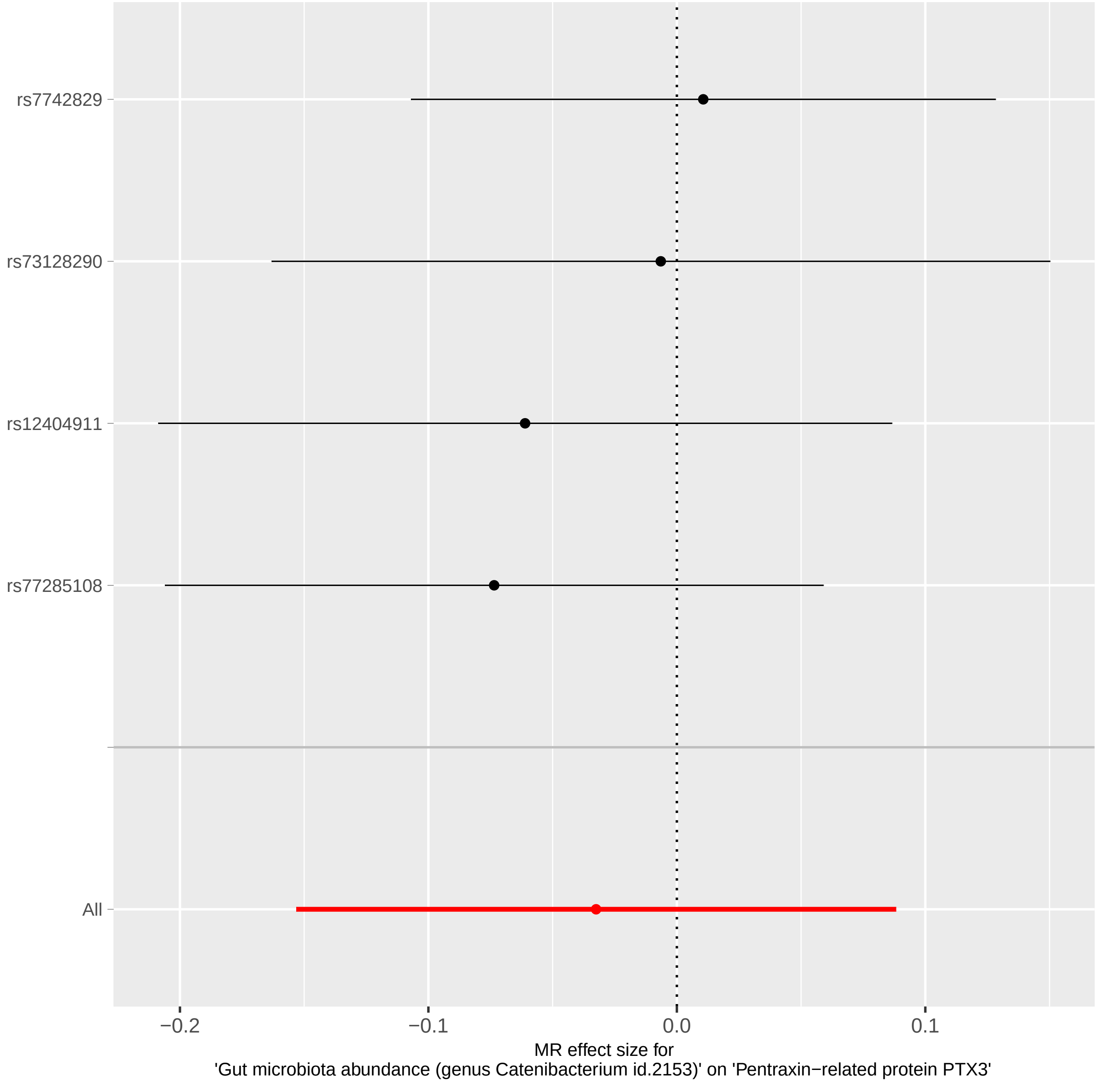

Batch 2−4902 : Gut microbiota abundance (genus Parasutterella id.2892) on Pentraxin−related protein PTX3
with a threshold of 1e−05

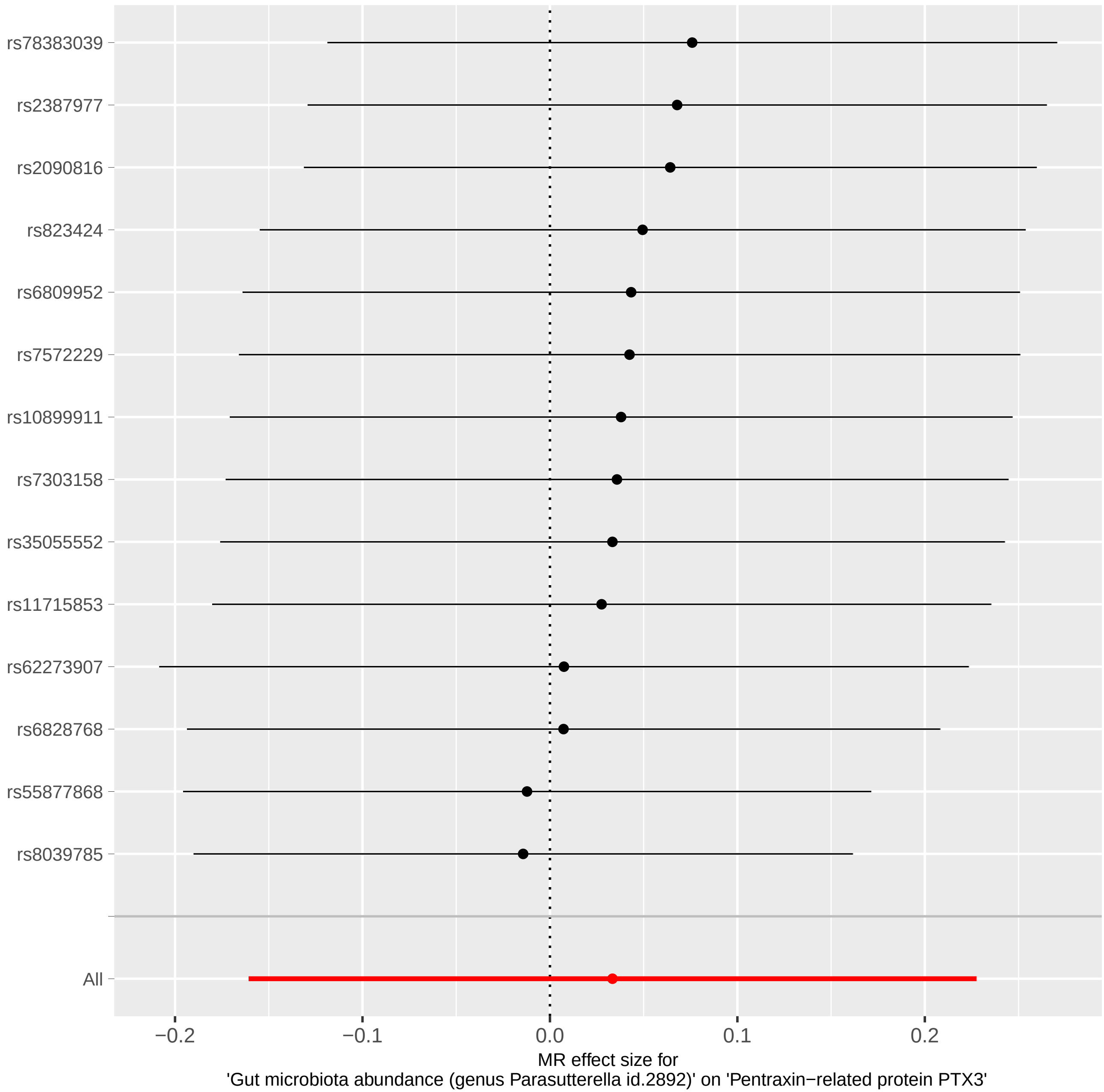

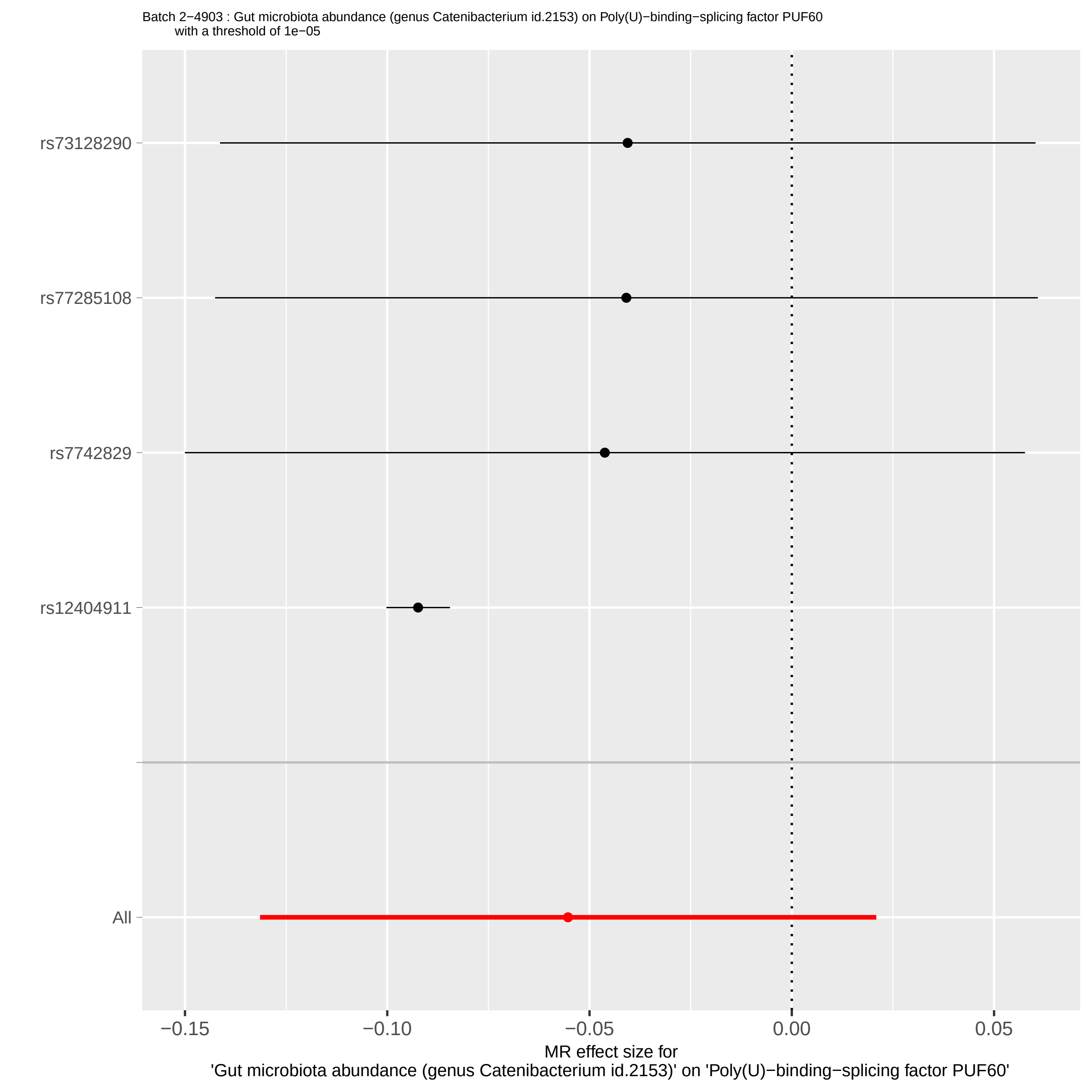
Batch 2−4903 : Gut microbiota abundance (genus Catenibacterium id.2153) on Poly(U)−binding−splicing factor PUF60
with a threshold of 1e−05
rs73128290
rs77285108
rs7742829
rs12404911
All
−0.15
−0.10
−0.05
0.00
0.05
MR effect size for
'Gut microbiota abundance (genus Catenibacterium id.2153)' on 'Poly(U)−binding−splicing factor PUF60'

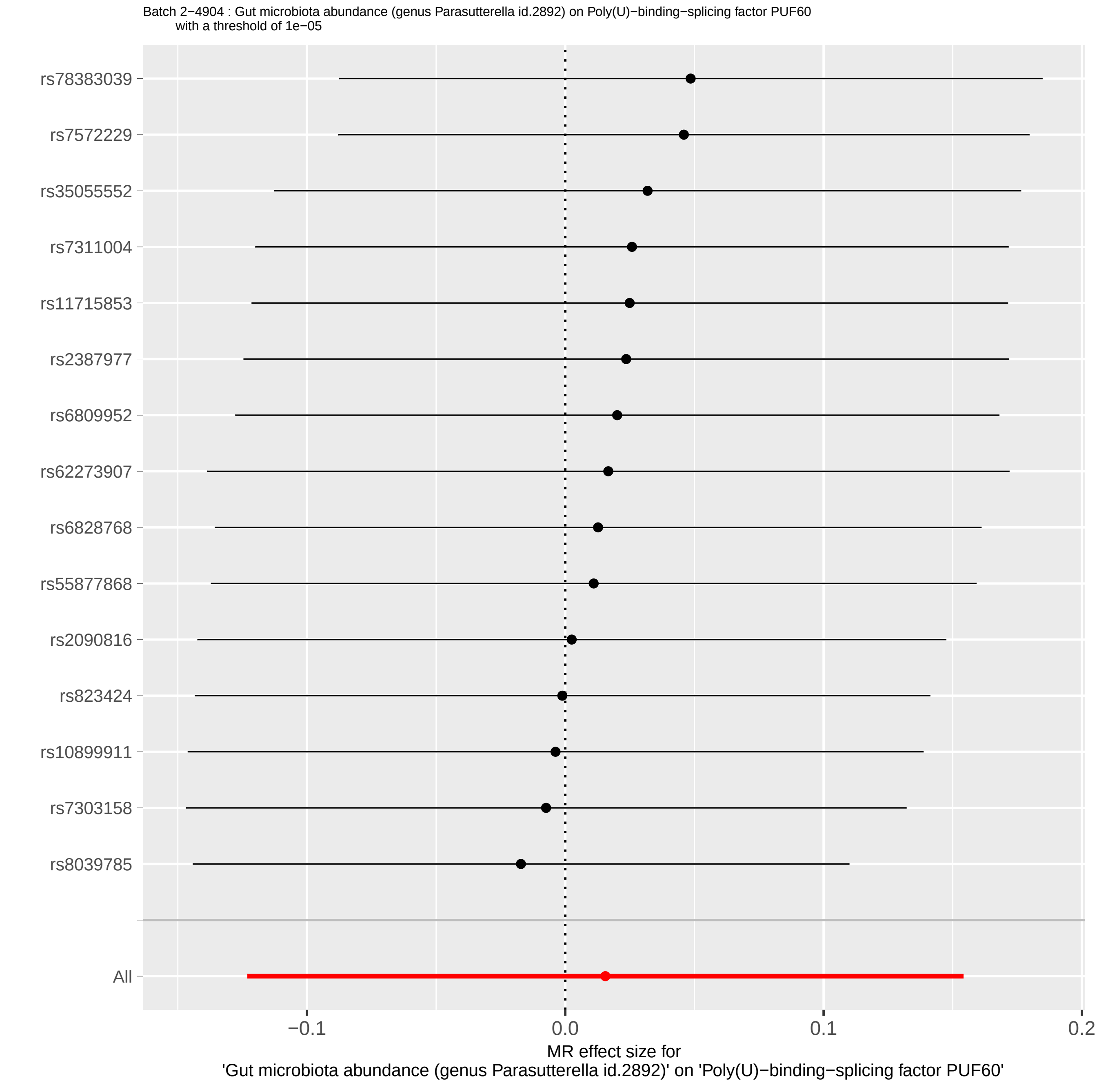
Batch 2−4904 : Gut microbiota abundance (genus Parasutterella id.2892) on Poly(U)−binding−splicing factor PUF60
with a threshold of 1e−05
rs78383039
rs7572229
rs35055552
rs7311004
rs11715853
rs2387977
rs6809952
rs62273907
rs6828768
rs55877868
rs2090816
rs823424
rs10899911
rs7303158
rs8039785
All
−0.1
0.0
0.1
0.2
MR effect size for
'Gut microbiota abundance (genus Parasutterella id.2892)' on 'Poly(U)−binding−splicing factor PUF60'

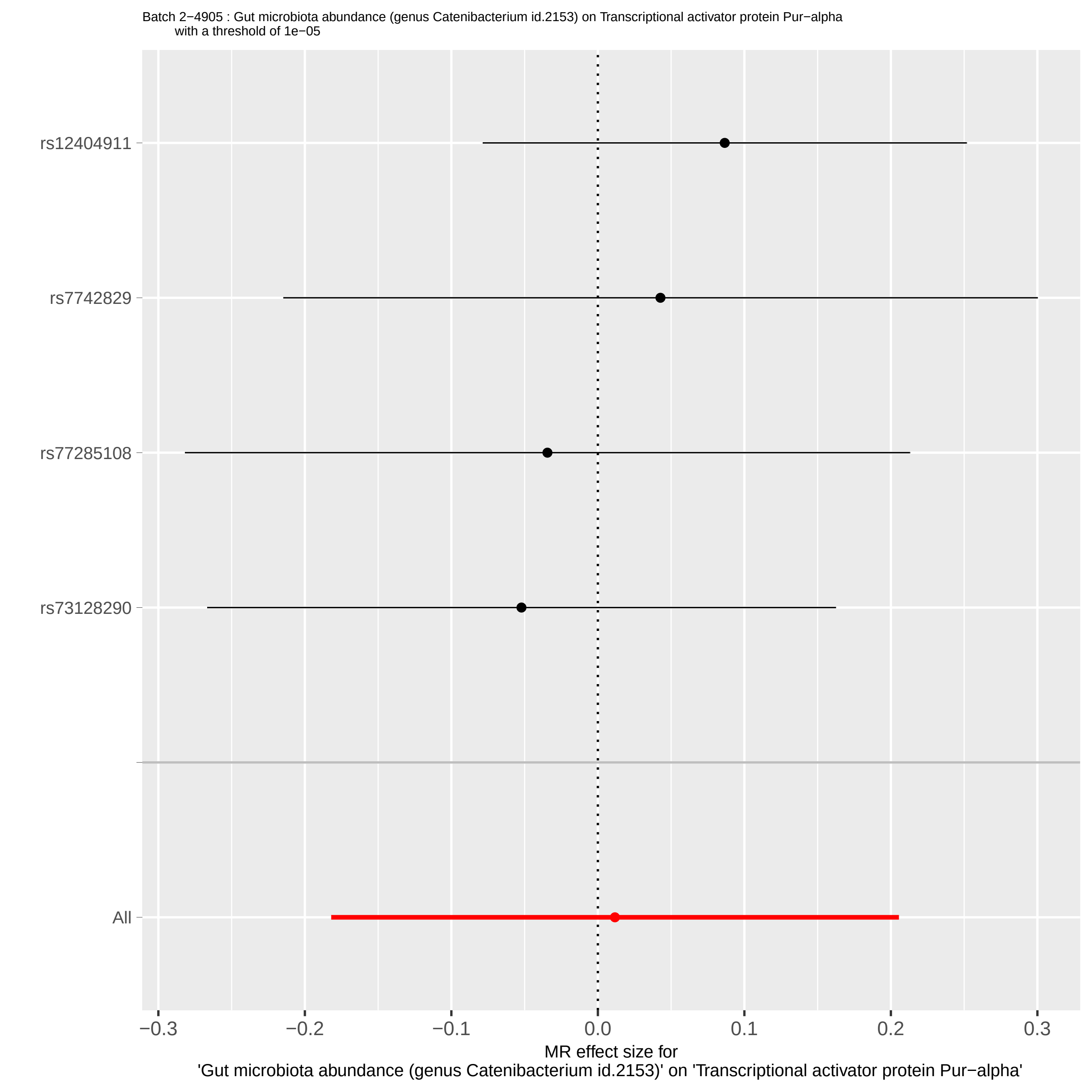
Batch 2−4905 : Gut microbiota abundance (genus Catenibacterium id.2153) on Transcriptional activator protein Pur−alpha
with a threshold of 1e−05
rs12404911
rs7742829
rs77285108
rs73128290
All
−0.3
−0.2
−0.1
0.0
0.1
0.2
0.3
MR effect size for
'Gut microbiota abundance (genus Catenibacterium id.2153)' on 'Transcriptional activator protein Pur−alpha'

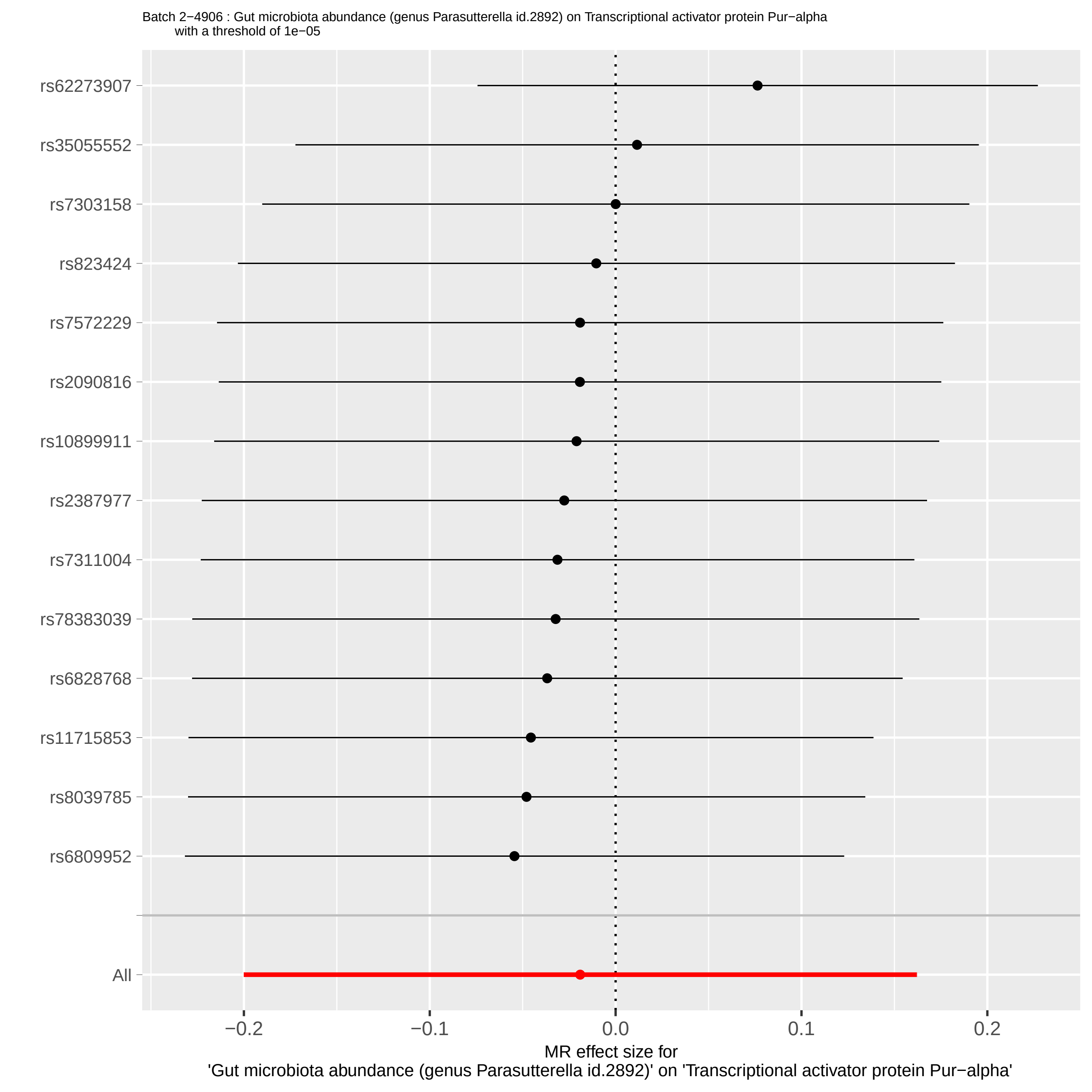

Batch 2−4906 : Gut microbiota abundance (genus Parasutterella id.2892) on Transcriptional activator protein Pur−alpha
with a threshold of 1e−05
rs62273907
rs35055552
rs7303158
rs823424
rs7572229
rs2090816
rs10899911
rs2387977
rs7311004
rs78383039
rs6828768
rs11715853
rs8039785
rs6809952
All
−0.2
−0.1
0.0
0.1
0.2
MR effect size for
'Gut microbiota abundance (genus Parasutterella id.2892)' on 'Transcriptional activator protein Pur−alpha'

Batch 2−4907 : Gut microbiota abundance (genus Catenibacterium id.2153) on tRNA pseudouridine synthase A, mitochondrial
with a threshold of 1e−05

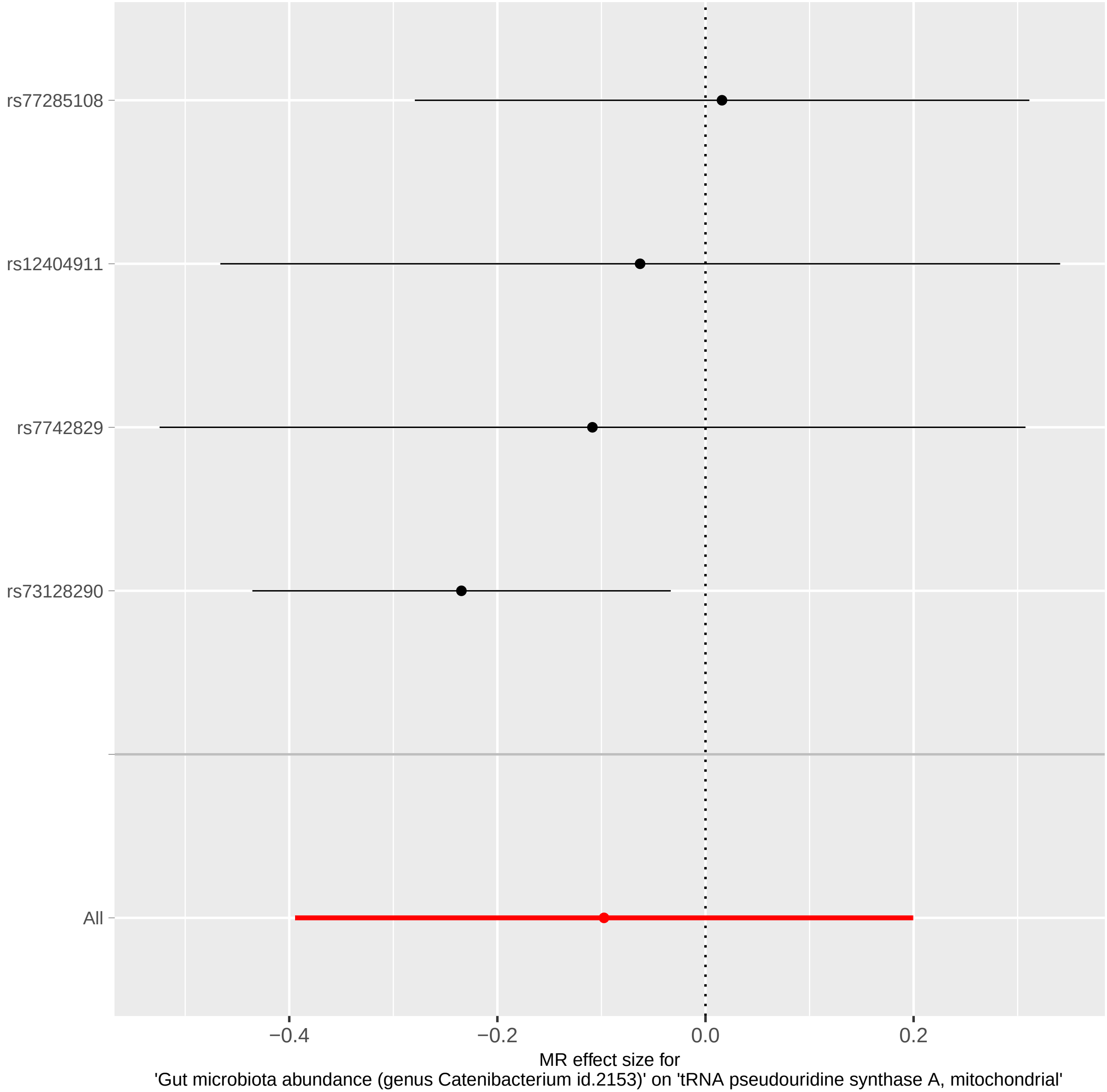

Batch 2−4908 : Gut microbiota abundance (genus Parasutterella id.2892) on tRNA pseudouridine synthase A, mitochondrial
with a threshold of 1e−05

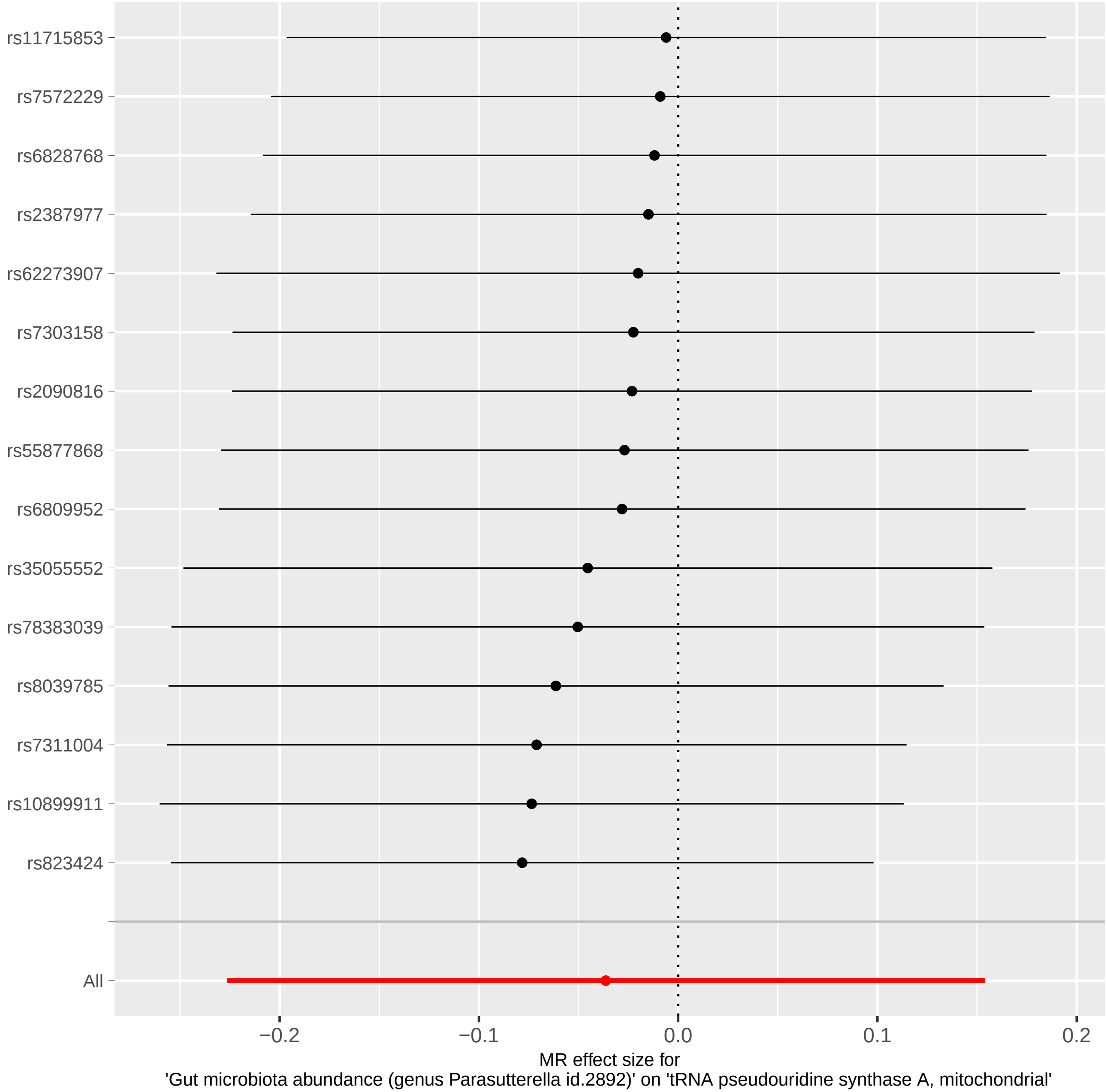

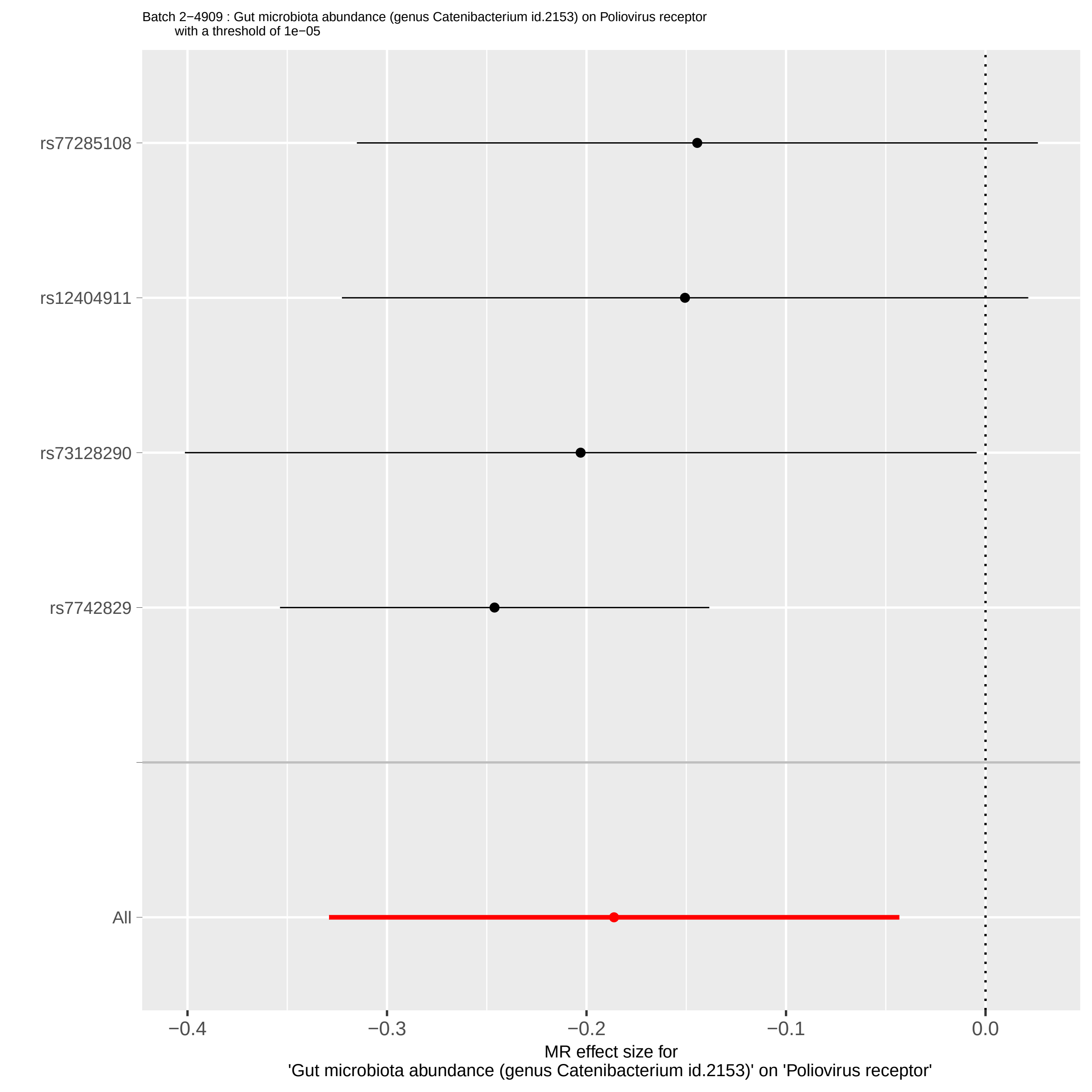
Batch 2−4909 : Gut microbiota abundance (genus Catenibacterium id.2153) on Poliovirus receptor
with a threshold of 1e−05
rs77285108
rs12404911
rs73128290
rs7742829
All
−0.4
−0.3
−0.2
−0.1
0.0
MR effect size for
'Gut microbiota abundance (genus Catenibacterium id.2153)' on 'Poliovirus receptor'

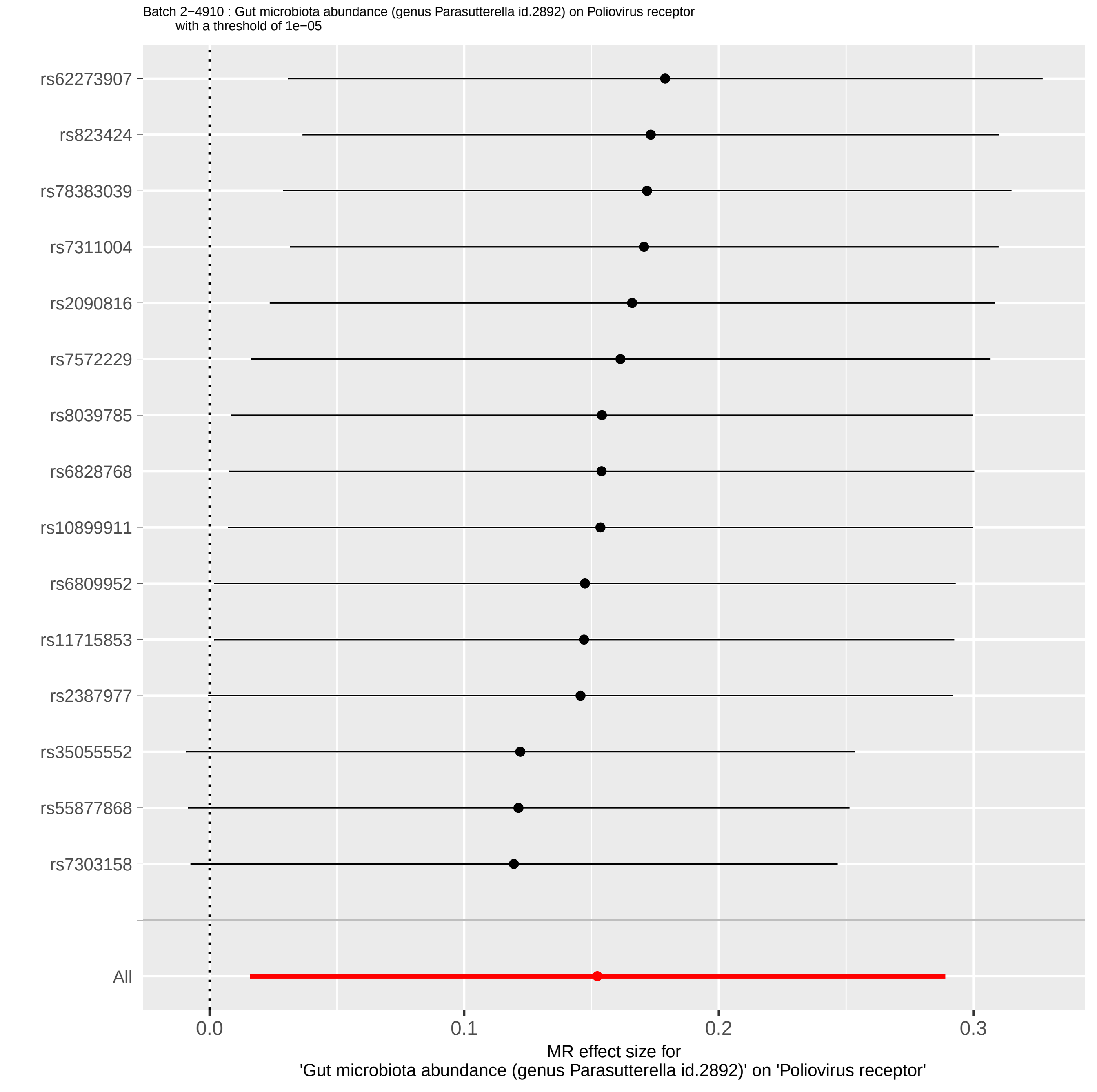

Batch 2−4910 : Gut microbiota abundance (genus Parasutterella id.2892) on Poliovirus receptor
with a threshold of 1e−05
rs62273907
rs823424
rs78383039
rs7311004
rs2090816
rs7572229
rs8039785
rs6828768
rs10899911
rs6809952
rs11715853
rs2387977
rs35055552
rs55877868
rs7303158
All
0.0
0.1
0.2
0.3
MR effect size for
'Gut microbiota abundance (genus Parasutterella id.2892)' on 'Poliovirus receptor'

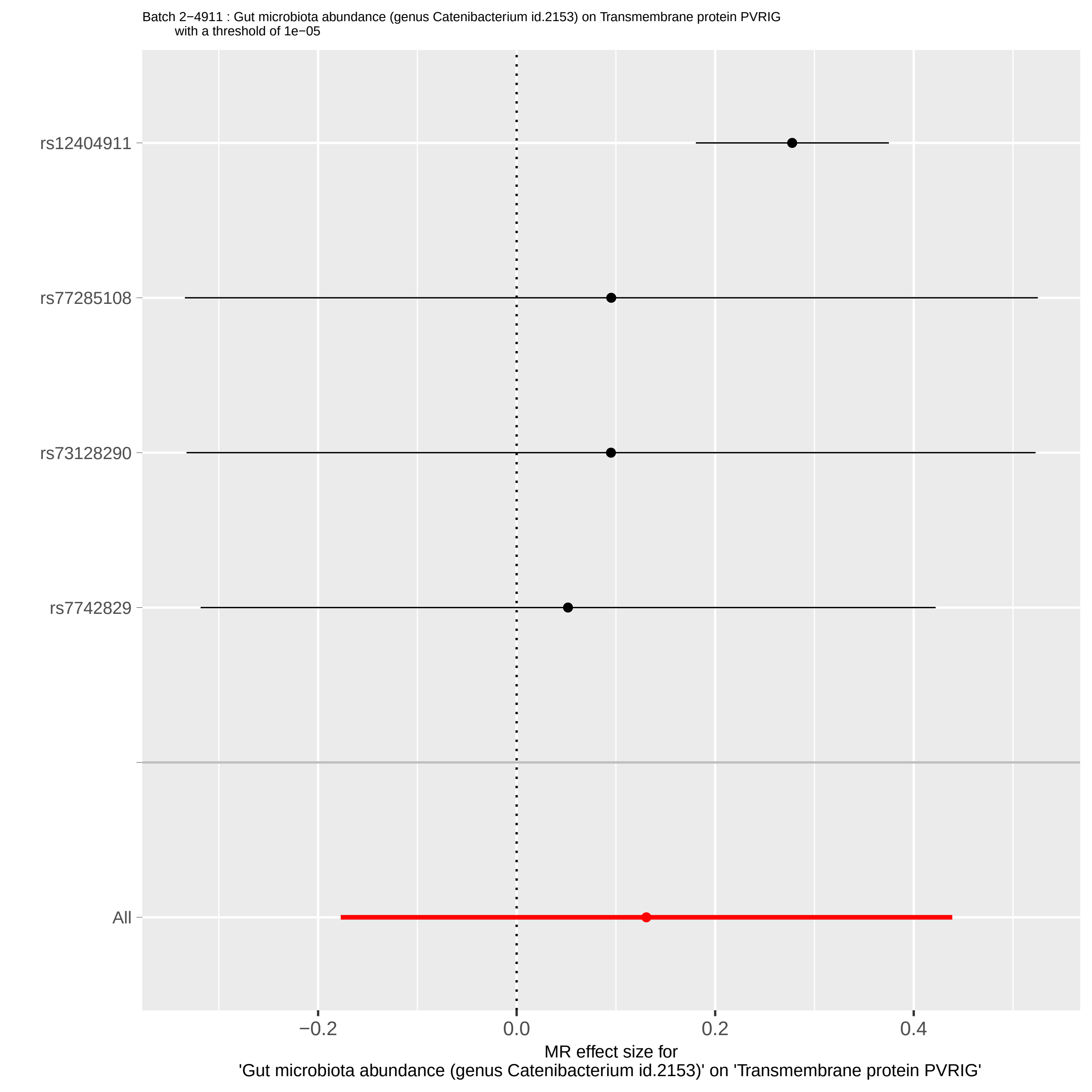

Batch 2−4911 : Gut microbiota abundance (genus Catenibacterium id.2153) on Transmembrane protein PVRIG
with a threshold of 1e−05
rs12404911
rs77285108
rs73128290
rs7742829
All
−0.2
0.0
0.2
0.4
MR effect size for
'Gut microbiota abundance (genus Catenibacterium id.2153)' on 'Transmembrane protein PVRIG'

Batch 2−4912 : Gut microbiota abundance (genus Parasutterella id.2892) on Transmembrane protein PVRIG
with a threshold of 1e−05

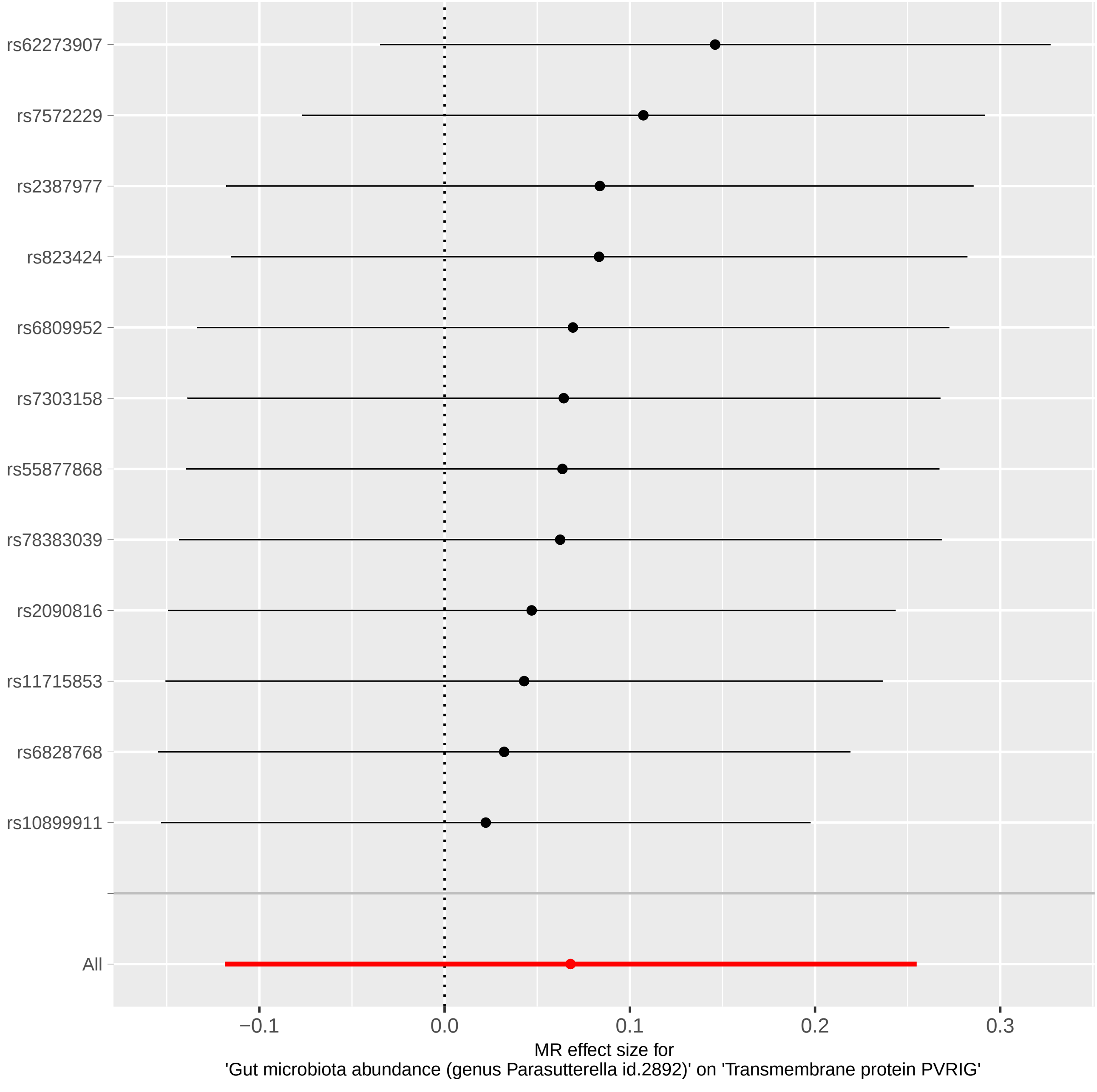

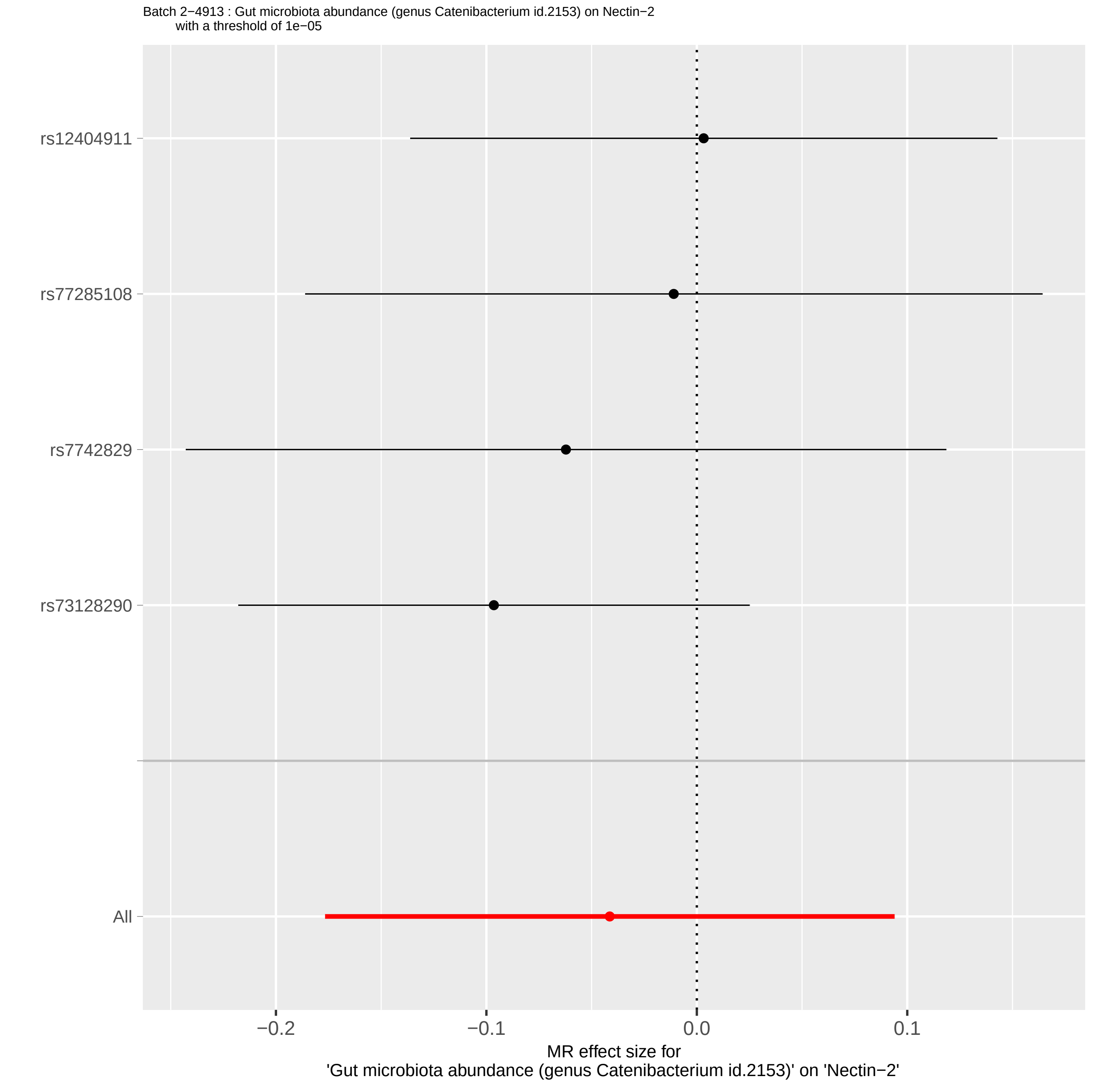
Batch 2−4913 : Gut microbiota abundance (genus Catenibacterium id.2153) on Nectin−2
with a threshold of 1e−05
rs12404911
rs77285108
rs7742829
rs73128290
All
−0.2
−0.1
0.0
0.1
MR effect size for
'Gut microbiota abundance (genus Catenibacterium id.2153)' on 'Nectin−2'

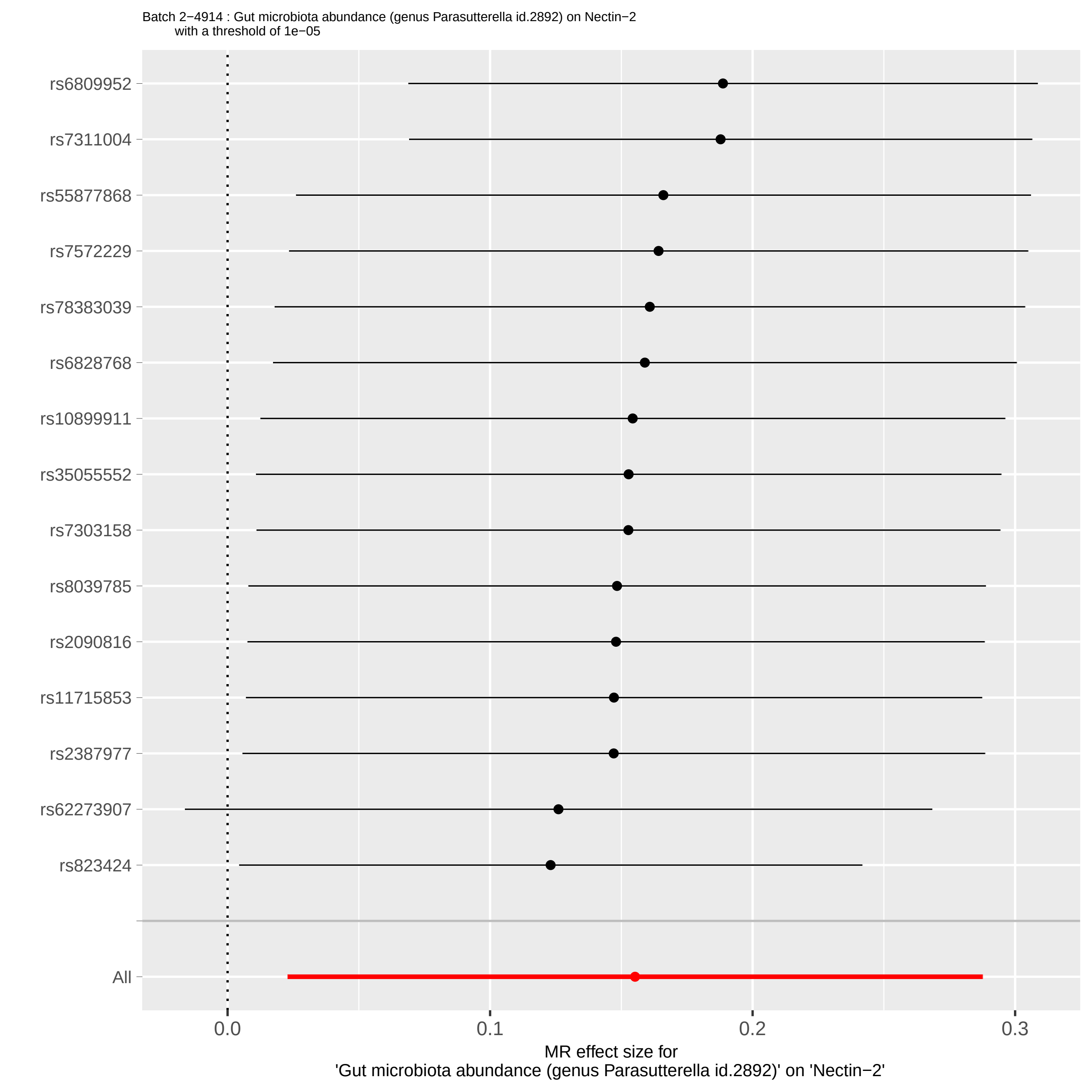

Batch 2−4914 : Gut microbiota abundance (genus Parasutterella id.2892) on Nectin−2
with a threshold of 1e−05
rs6809952
rs7311004
rs55877868
rs7572229
rs78383039
rs6828768
rs10899911
rs35055552
rs7303158
rs8039785
rs2090816
rs11715853
rs2387977
rs62273907
rs823424
All
0.0
0.1
0.2
0.3
MR effect size for
'Gut microbiota abundance (genus Parasutterella id.2892)' on 'Nectin−2'

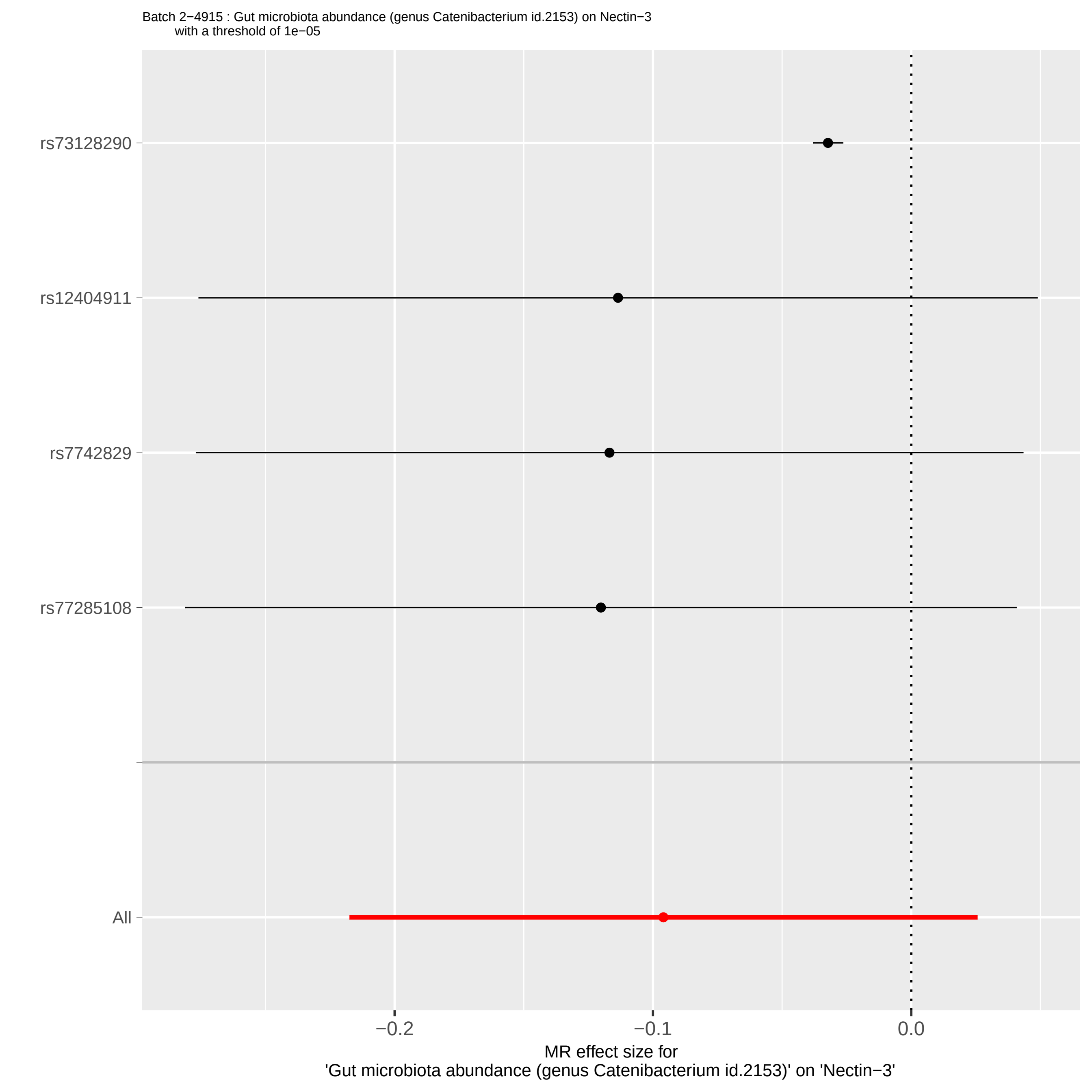
Batch 2−4915 : Gut microbiota abundance (genus Catenibacterium id.2153) on Nectin−3
with a threshold of 1e−05
rs73128290
rs12404911
rs7742829
rs77285108
All
−0.2
−0.1
0.0
MR effect size for
'Gut microbiota abundance (genus Catenibacterium id.2153)' on 'Nectin−3'

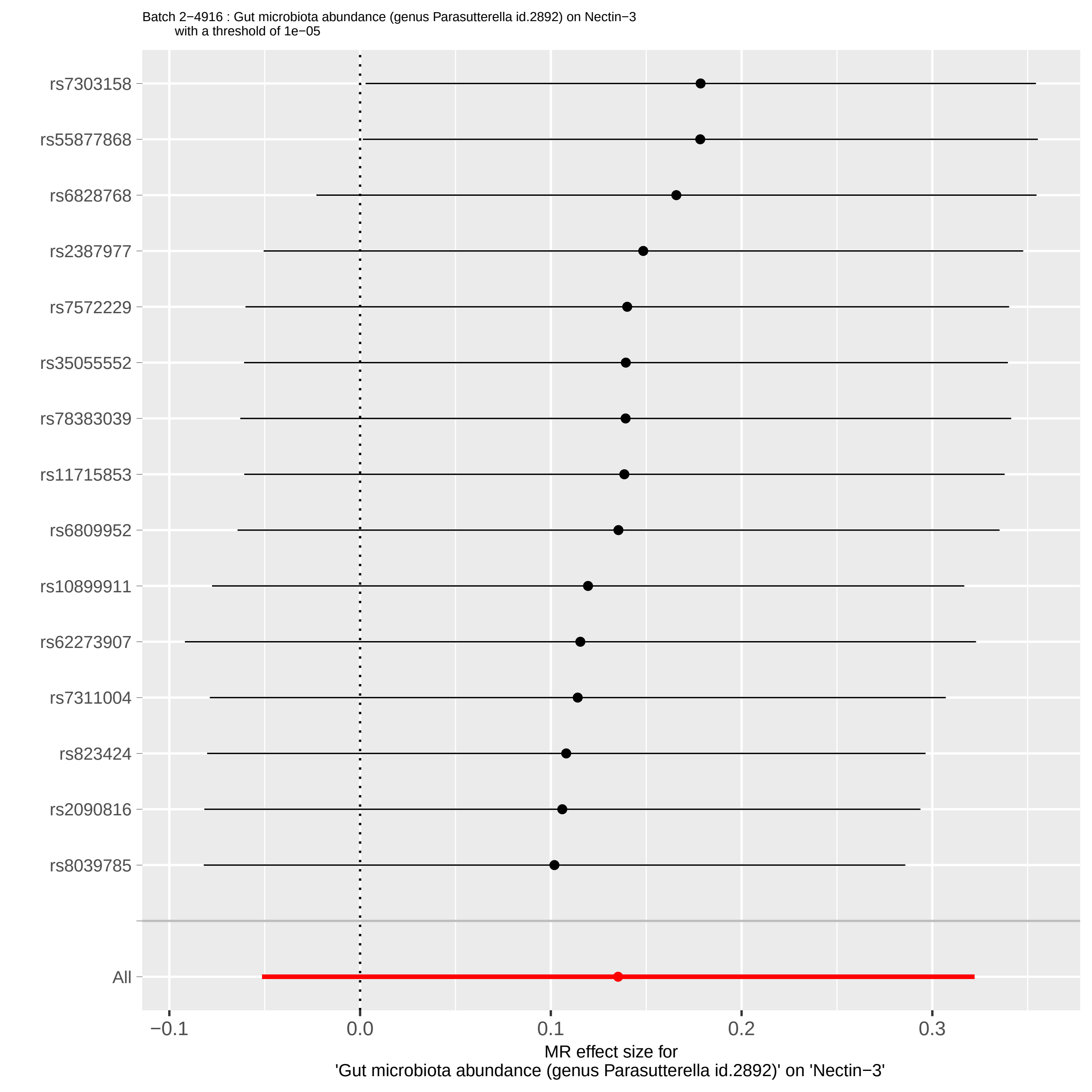

Batch 2−4916 : Gut microbiota abundance (genus Parasutterella id.2892) on Nectin−3
with a threshold of 1e−05
rs7303158
rs55877868
rs6828768
rs2387977
rs7572229
rs35055552
rs78383039
rs11715853
rs6809952
rs10899911
rs62273907
rs7311004
rs823424
rs2090816
rs8039785
All
−0.1
0.0
0.1
0.2
0.3
MR effect size for
'Gut microbiota abundance (genus Parasutterella id.2892)' on 'Nectin−3'

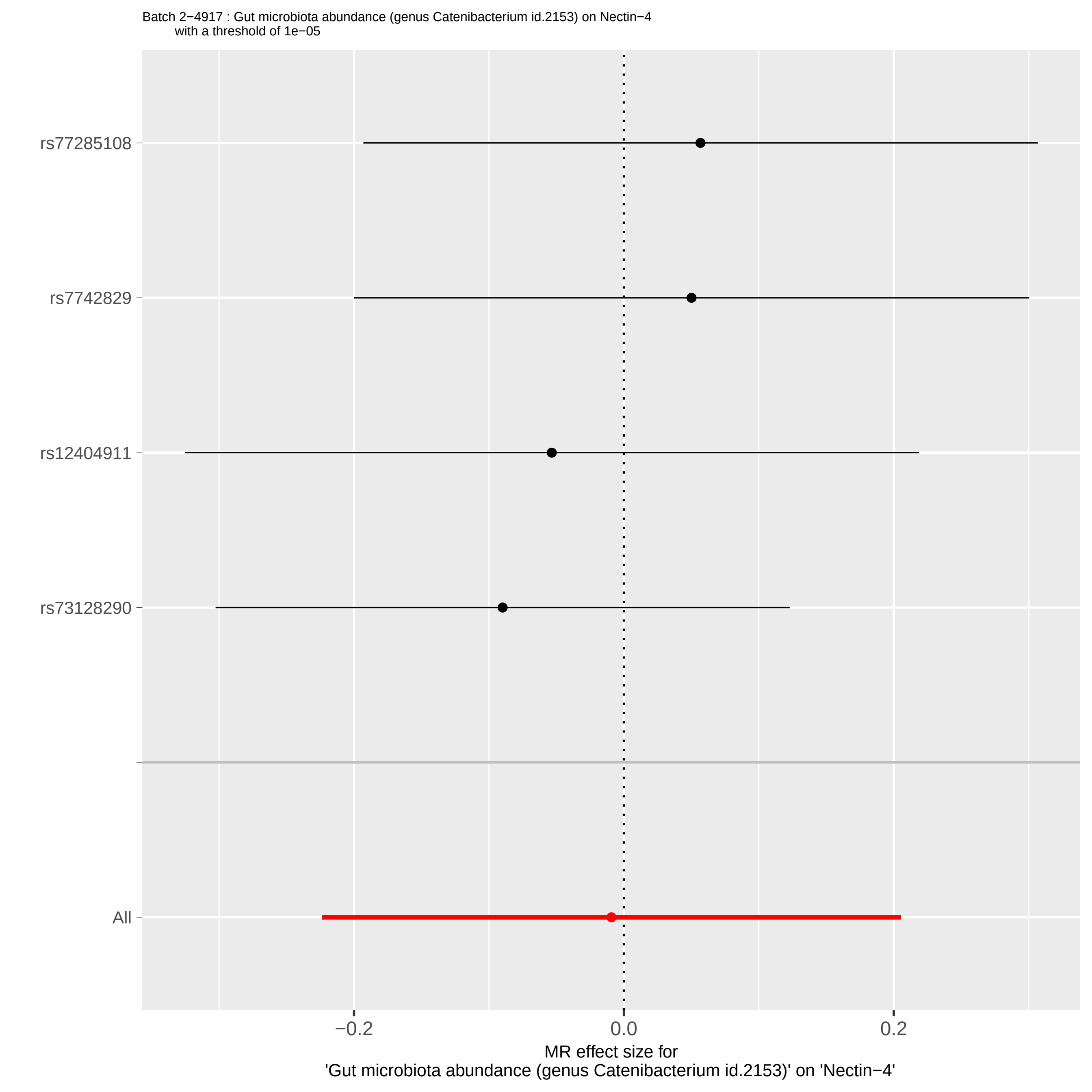

Batch 2−4917 : Gut microbiota abundance (genus Catenibacterium id.2153) on Nectin−4
with a threshold of 1e−05
rs77285108
rs7742829
rs12404911
rs73128290
All
−0.2
0.0
0.2
MR effect size for
'Gut microbiota abundance (genus Catenibacterium id.2153)' on 'Nectin−4'

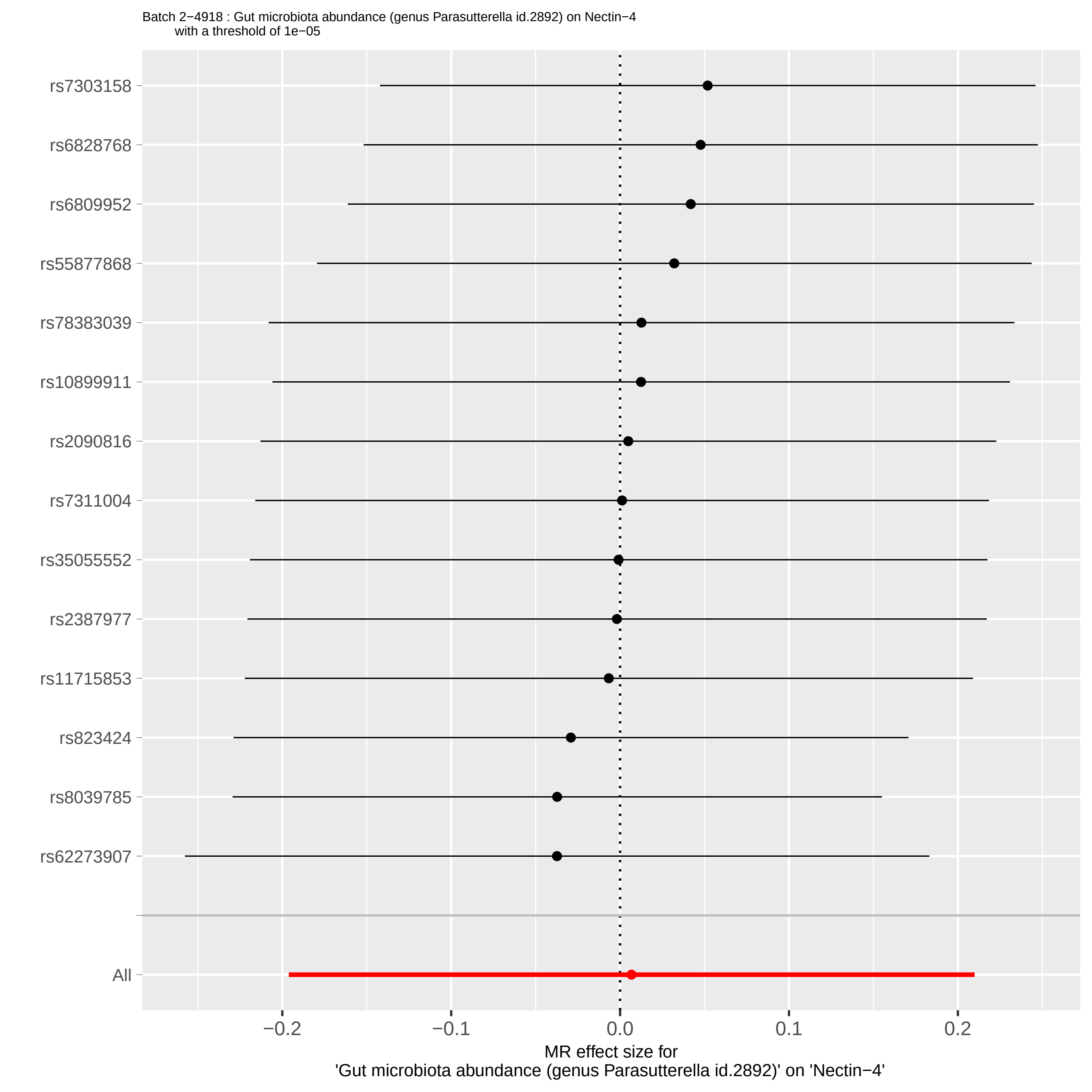

Batch 2−4918 : Gut microbiota abundance (genus Parasutterella id.2892) on Nectin−4
with a threshold of 1e−05
rs7303158
rs6828768
rs6809952
rs55877868
rs78383039
rs10899911
rs2090816
rs7311004
rs35055552
rs2387977
rs11715853
rs823424
rs8039785
rs62273907
All
−0.2
−0.1
0.0
0.1
0.2
MR effect size for
'Gut microbiota abundance (genus Parasutterella id.2892)' on 'Nectin−4'

Batch 2−4919 : Gut microbiota abundance (genus Catenibacterium id.2153) on Peroxidasin−like protein
with a threshold of 1e−05

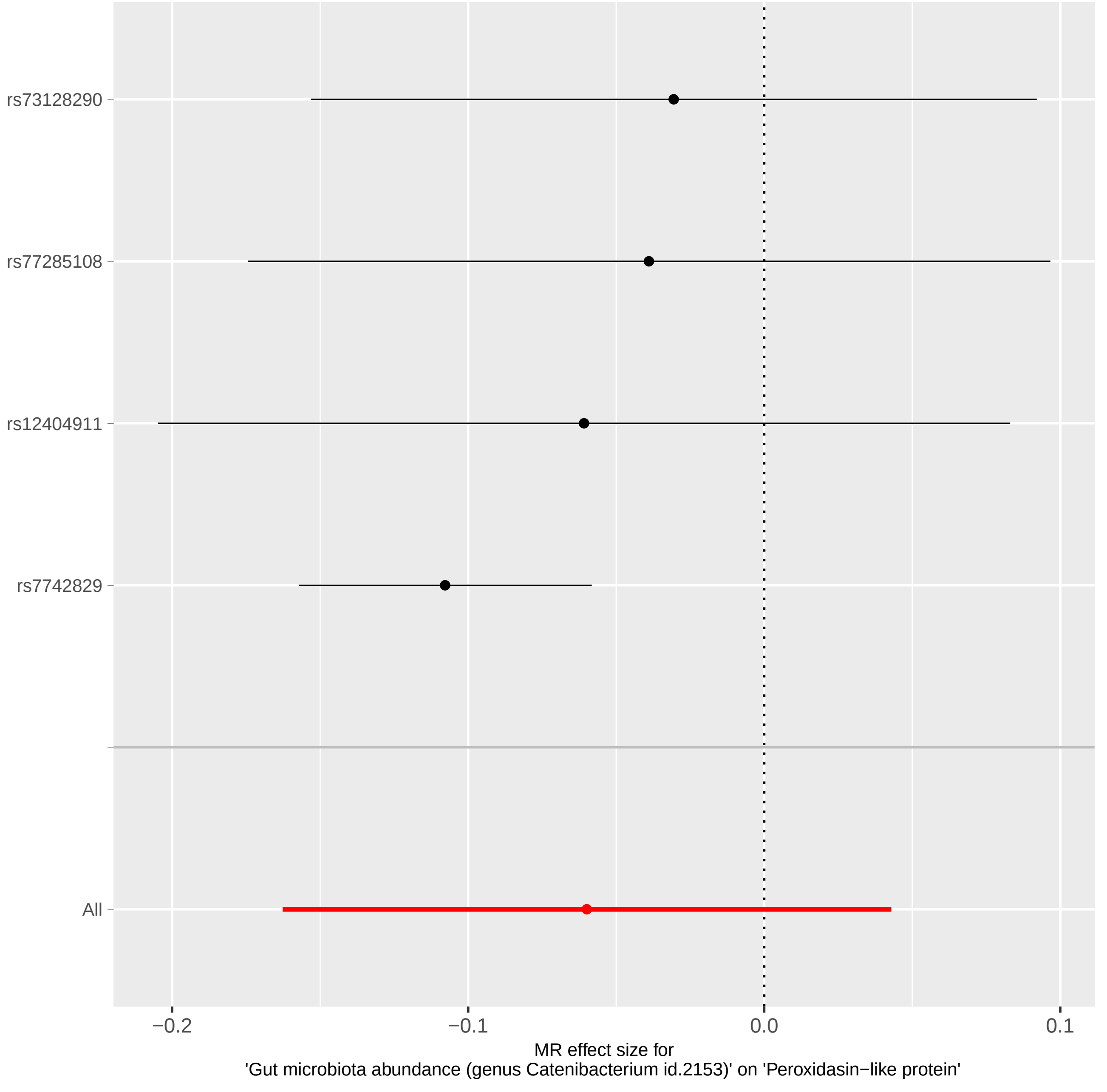

Batch 2−4920 : Gut microbiota abundance (genus Parasutterella id.2892) on Peroxidasin−like protein
with a threshold of 1e−05

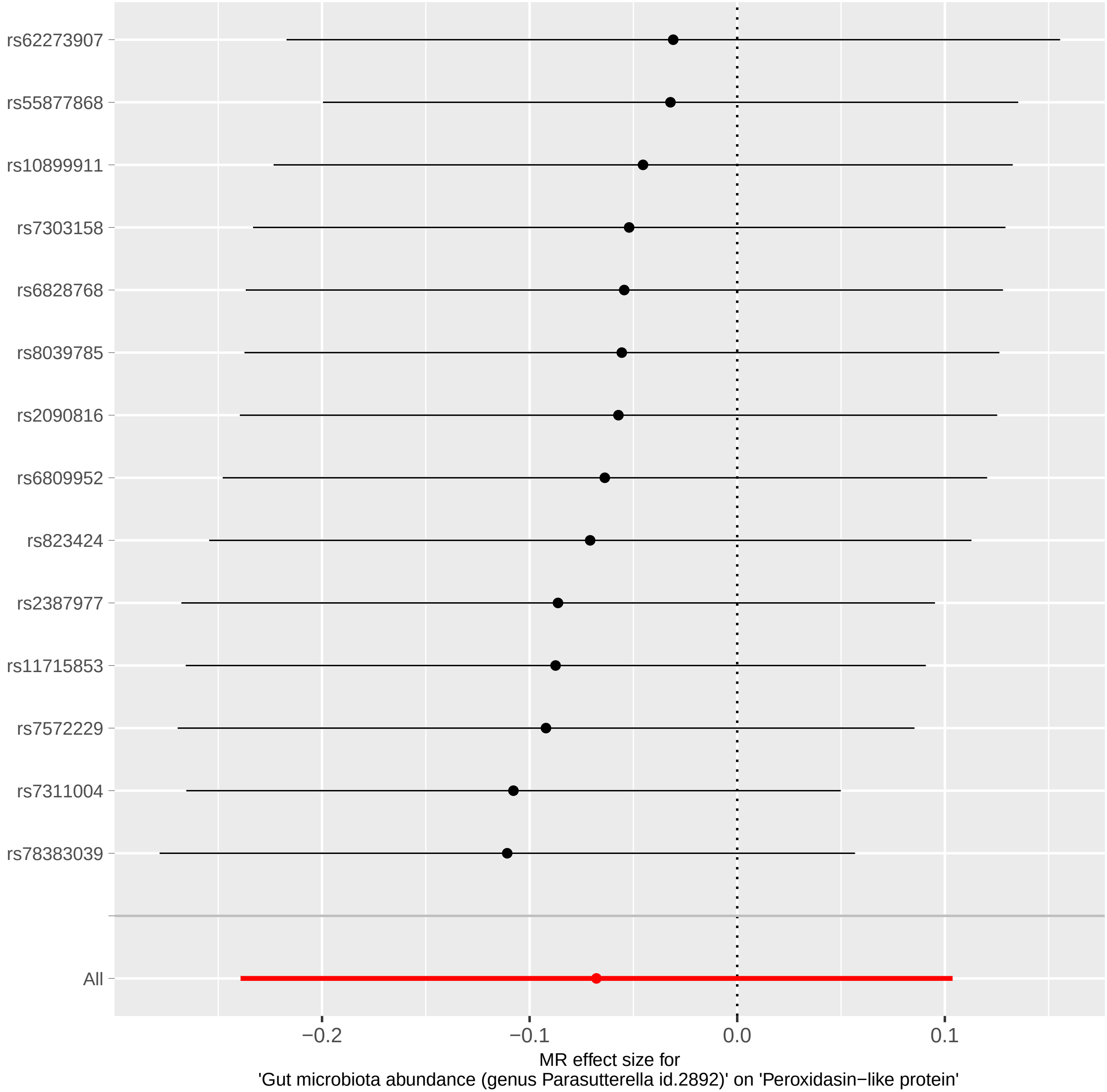

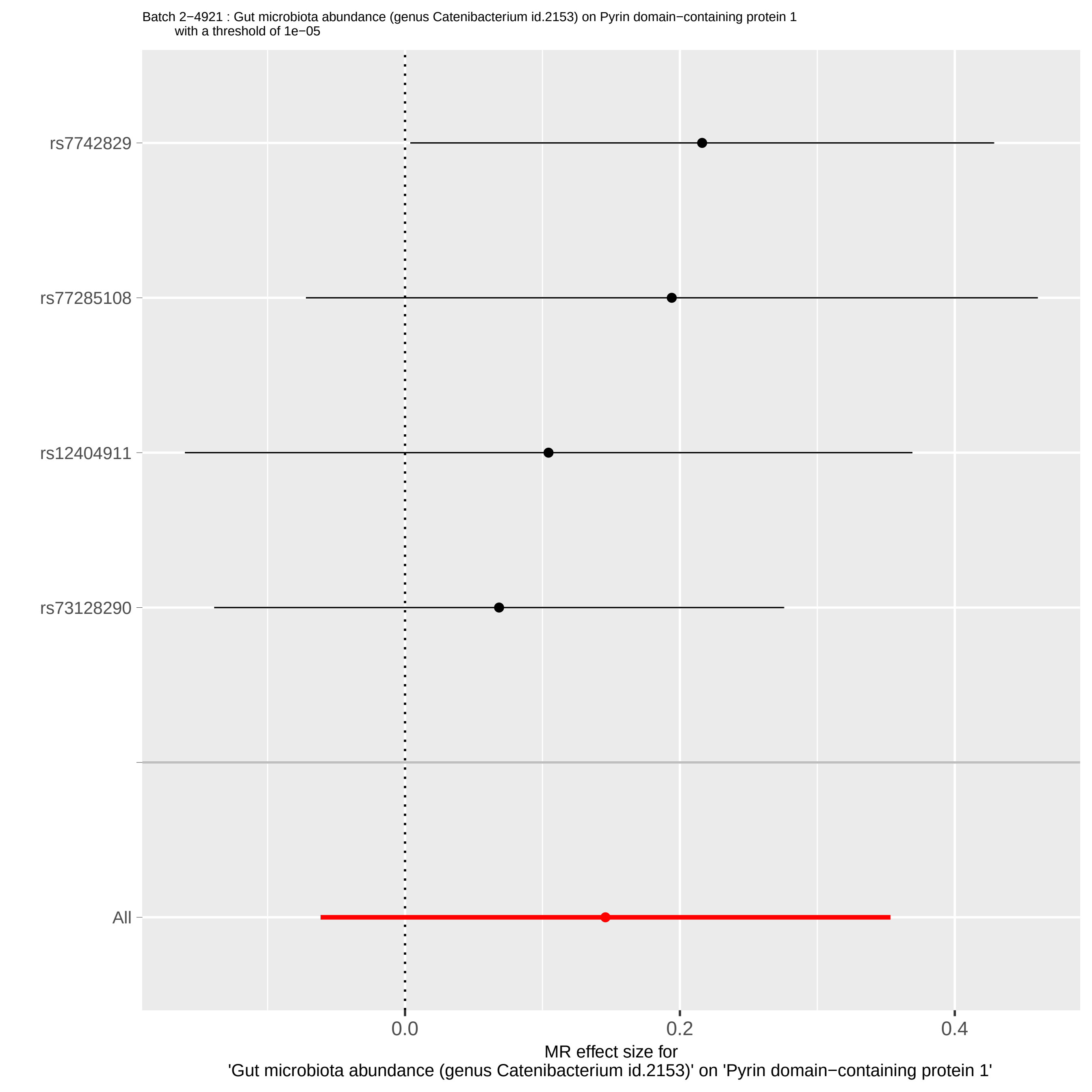
Batch 2−4921 : Gut microbiota abundance (genus Catenibacterium id.2153) on Pyrin domain−containing protein 1
with a threshold of 1e−05
rs7742829
rs77285108
rs12404911
rs73128290
All
0.0
0.2
0.4
MR effect size for
'Gut microbiota abundance (genus Catenibacterium id.2153)' on 'Pyrin domain−containing protein 1'

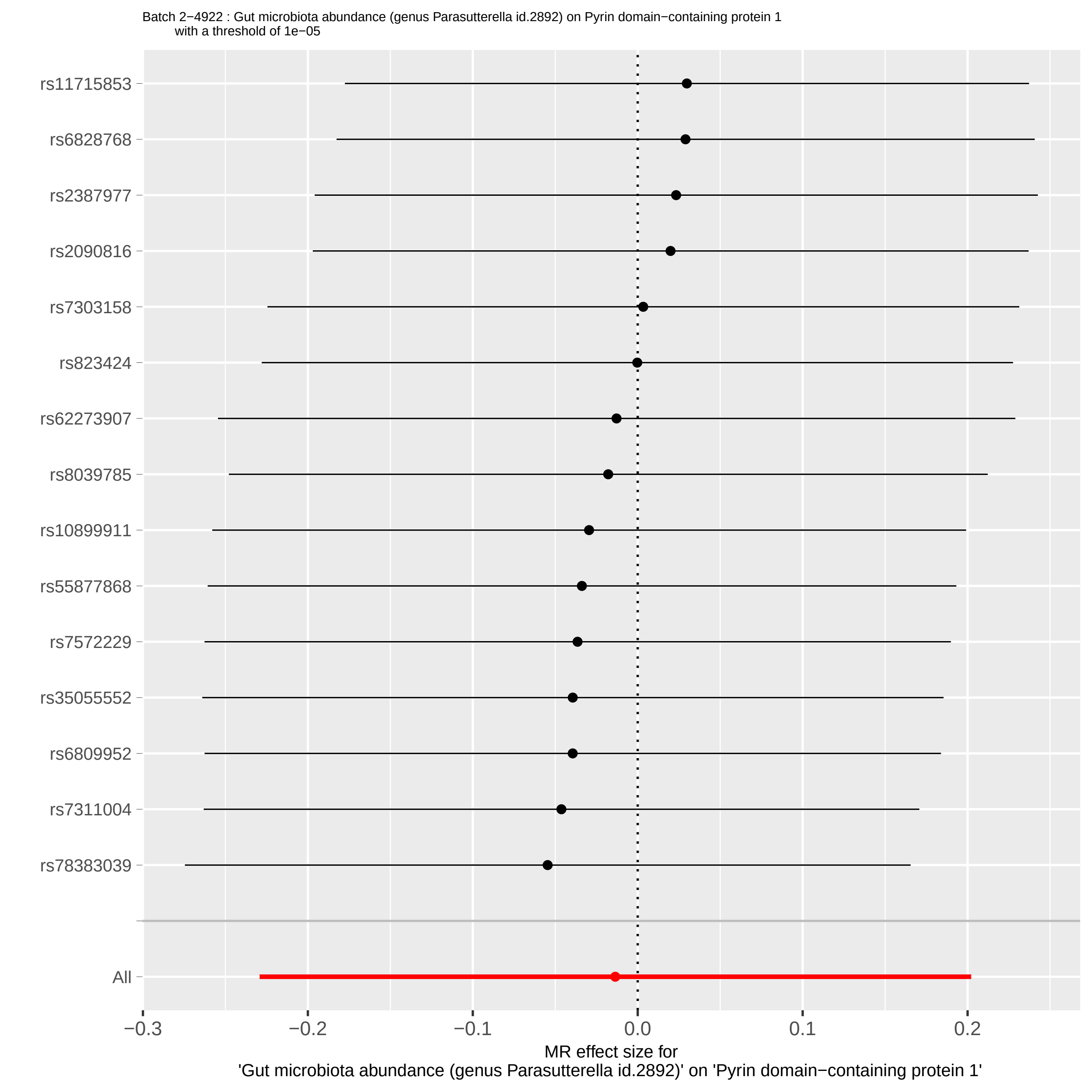

Batch 2−4922 : Gut microbiota abundance (genus Parasutterella id.2892) on Pyrin domain−containing protein 1
with a threshold of 1e−05
rs11715853
rs6828768
rs2387977
rs2090816
rs7303158
rs823424
rs62273907
rs8039785
rs10899911
rs55877868
rs7572229
rs35055552
rs6809952
rs7311004
rs78383039
All
−0.3
−0.2
−0.1
0.0
0.1
0.2
MR effect size for
'Gut microbiota abundance (genus Parasutterella id.2892)' on 'Pyrin domain−containing protein 1'

Batch 2−4923 : Gut microbiota abundance (genus Catenibacterium id.2153) on Glycogen phosphorylase, liver form
with a threshold of 1e−05

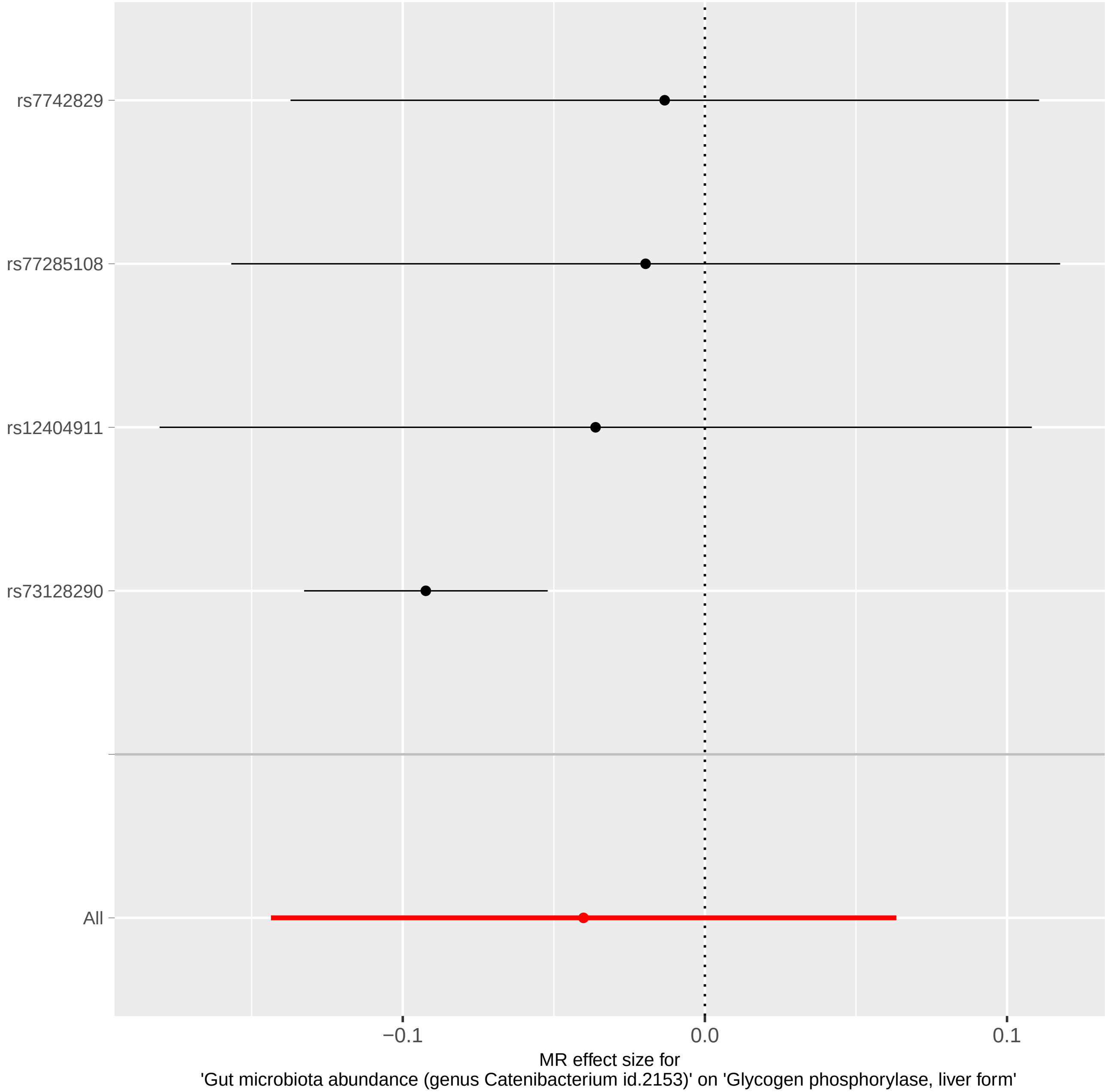

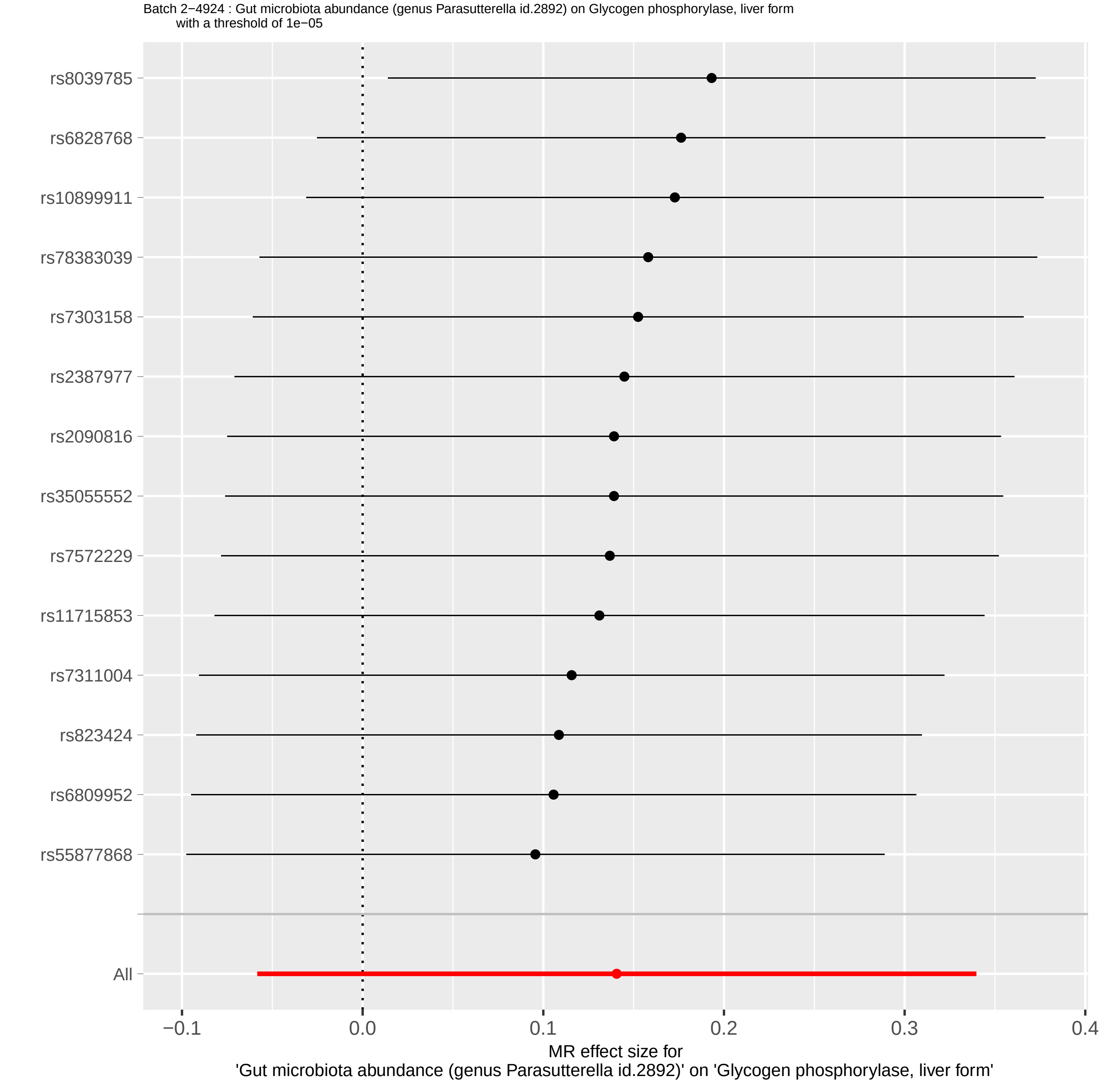
Batch 2−4924 : Gut microbiota abundance (genus Parasutterella id.2892) on Glycogen phosphorylase, liver form
with a threshold of 1e−05
rs8039785
rs6828768
rs10899911
rs78383039
rs7303158
rs2387977
rs2090816
rs35055552
rs7572229
rs11715853
rs7311004
rs823424
rs6809952
rs55877868
All
−0.1
0.0
0.1
0.2
0.3
0.4
MR effect size for
'Gut microbiota abundance (genus Parasutterella id.2892)' on 'Glycogen phosphorylase, liver form'

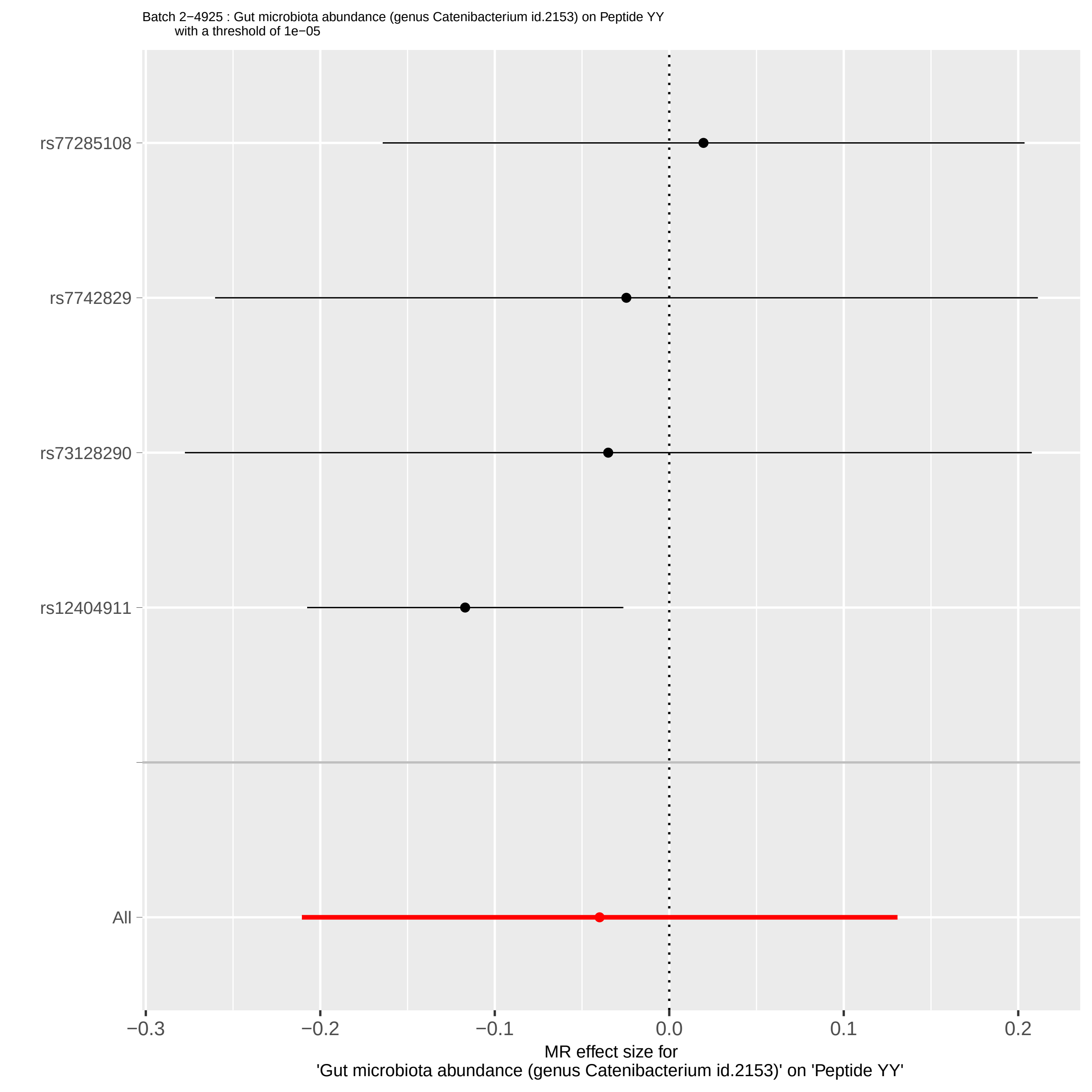

Batch 2−4925 : Gut microbiota abundance (genus Catenibacterium id.2153) on Peptide YY
with a threshold of 1e−05
rs77285108
rs7742829
rs73128290
rs12404911
All
−0.3
−0.2
−0.1
0.0
0.1
0.2
MR effect size for
'Gut microbiota abundance (genus Catenibacterium id.2153)' on 'Peptide YY'

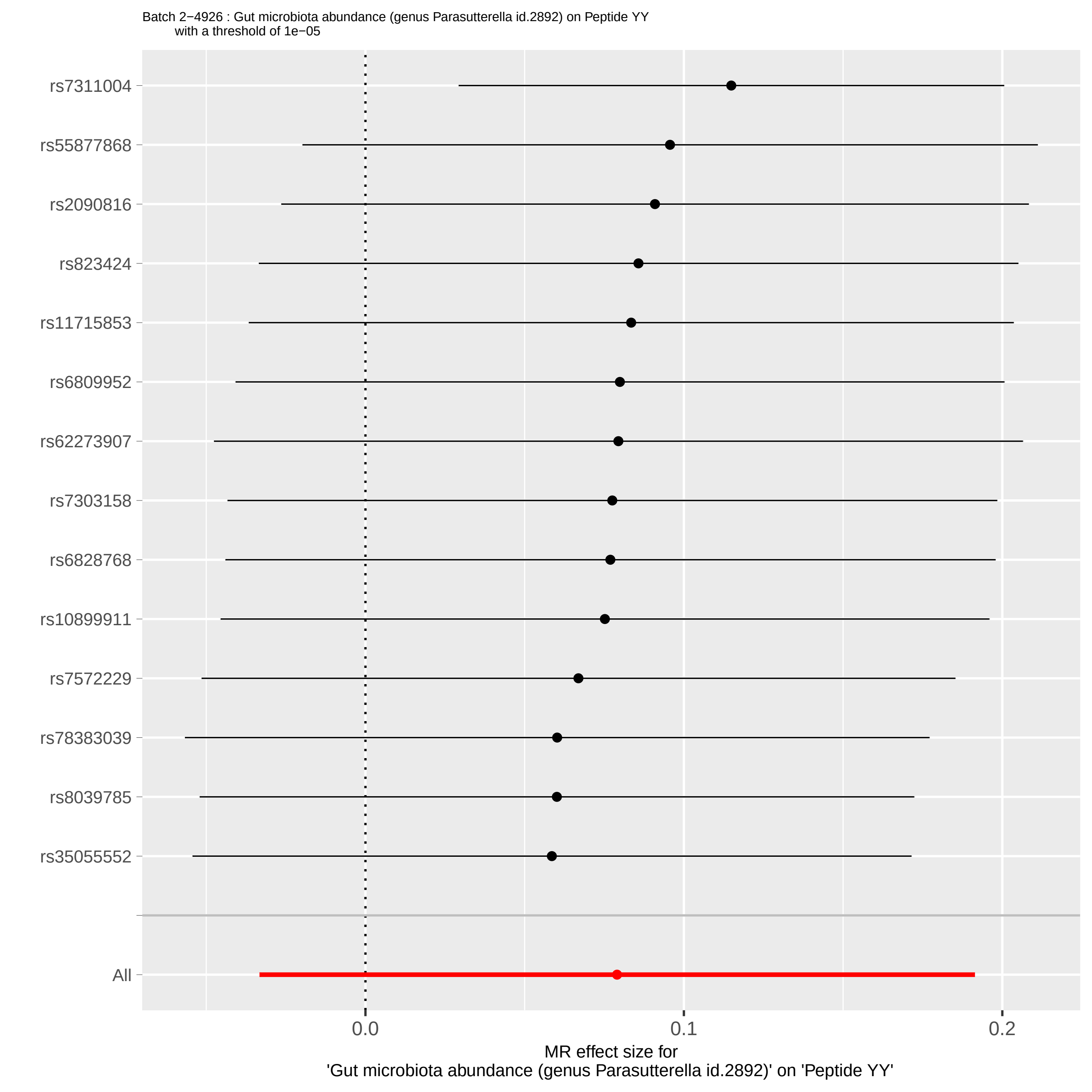

Batch 2−4926 : Gut microbiota abundance (genus Parasutterella id.2892) on Peptide YY
with a threshold of 1e−05
rs7311004
rs55877868
rs2090816
rs823424
rs11715853
rs6809952
rs62273907
rs7303158
rs6828768
rs10899911
rs7572229
rs78383039
rs8039785
rs35055552
All
0.0
0.1
0.2
MR effect size for
'Gut microbiota abundance (genus Parasutterella id.2892)' on 'Peptide YY'

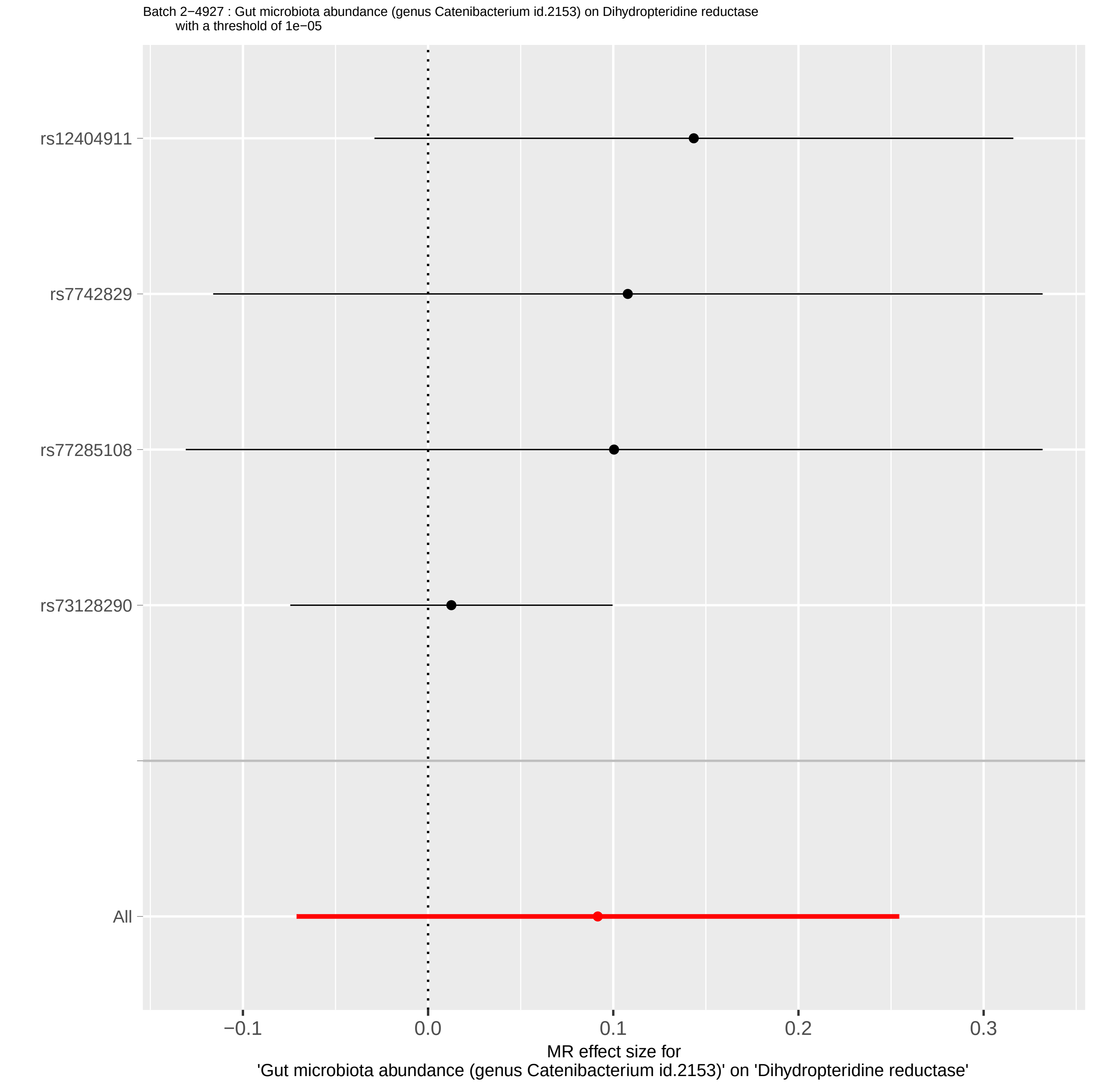
Batch 2−4927 : Gut microbiota abundance (genus Catenibacterium id.2153) on Dihydropteridine reductase
with a threshold of 1e−05
rs12404911
rs7742829
rs77285108
rs73128290
All
−0.1
0.0
0.1
0.2
0.3
MR effect size for
'Gut microbiota abundance (genus Catenibacterium id.2153)' on 'Dihydropteridine reductase'

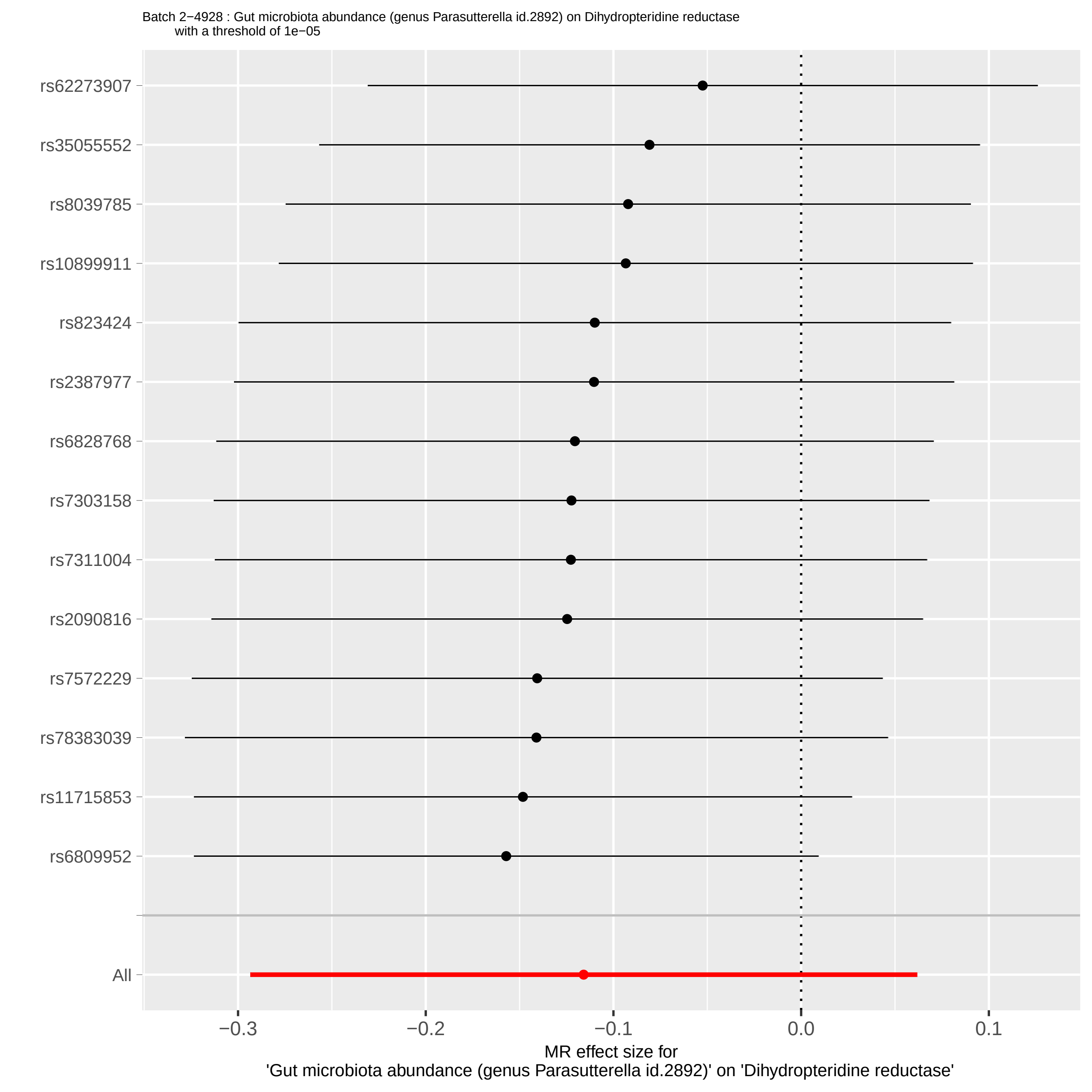
Batch 2−4928 : Gut microbiota abundance (genus Parasutterella id.2892) on Dihydropteridine reductase
with a threshold of 1e−05
rs62273907
rs35055552
rs8039785
rs10899911
rs823424
rs2387977
rs6828768
rs7303158
rs7311004
rs2090816
rs7572229
rs78383039
rs11715853
rs6809952
All
−0.3
−0.2
−0.1
0.0
0.1
MR effect size for
'Gut microbiota abundance (genus Parasutterella id.2892)' on 'Dihydropteridine reductase'

Batch 2−4929 : Gut microbiota abundance (genus Catenibacterium id.2153) on Glutaminyl−peptide cyclotransferase
with a threshold of 1e−05

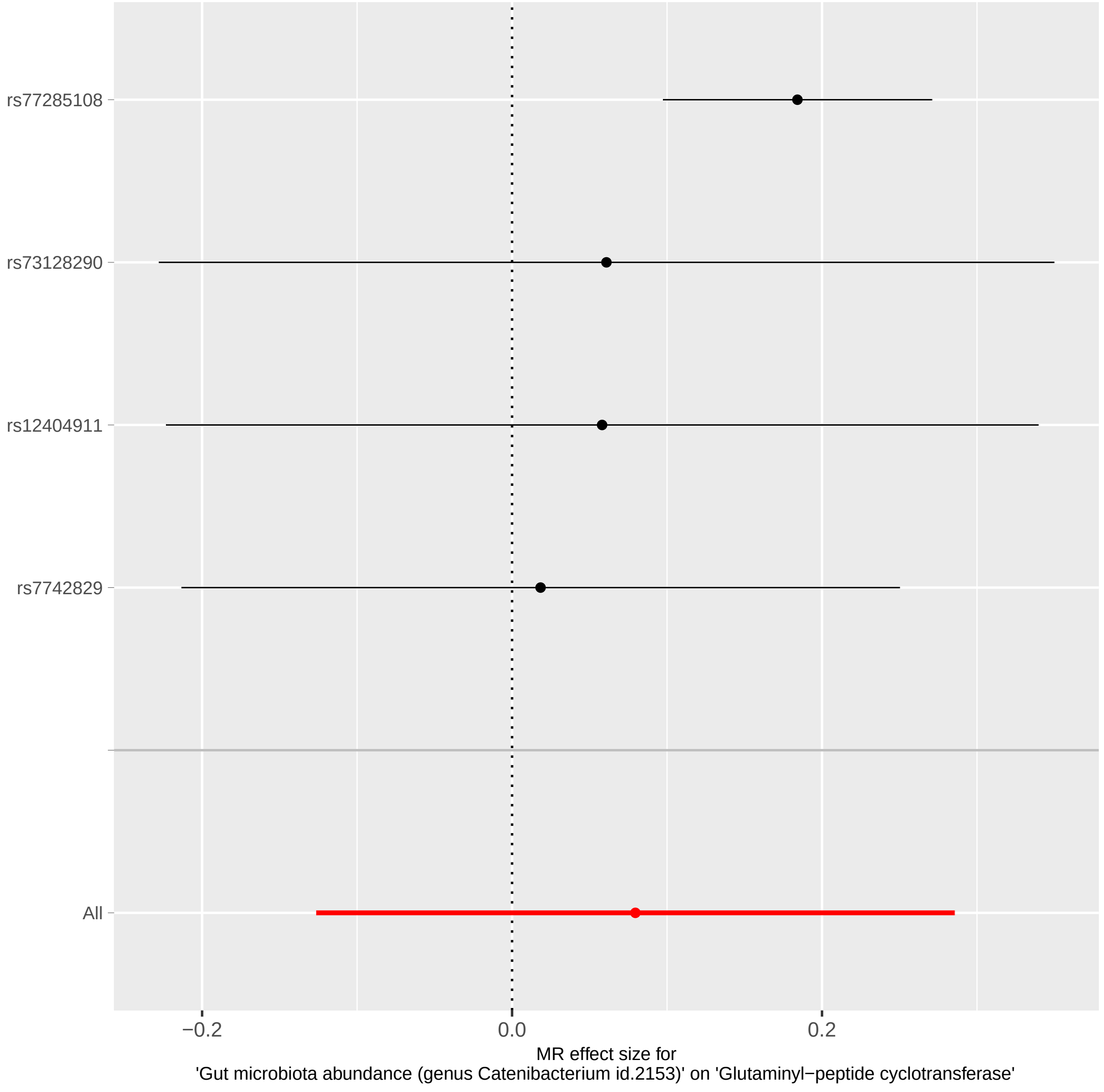

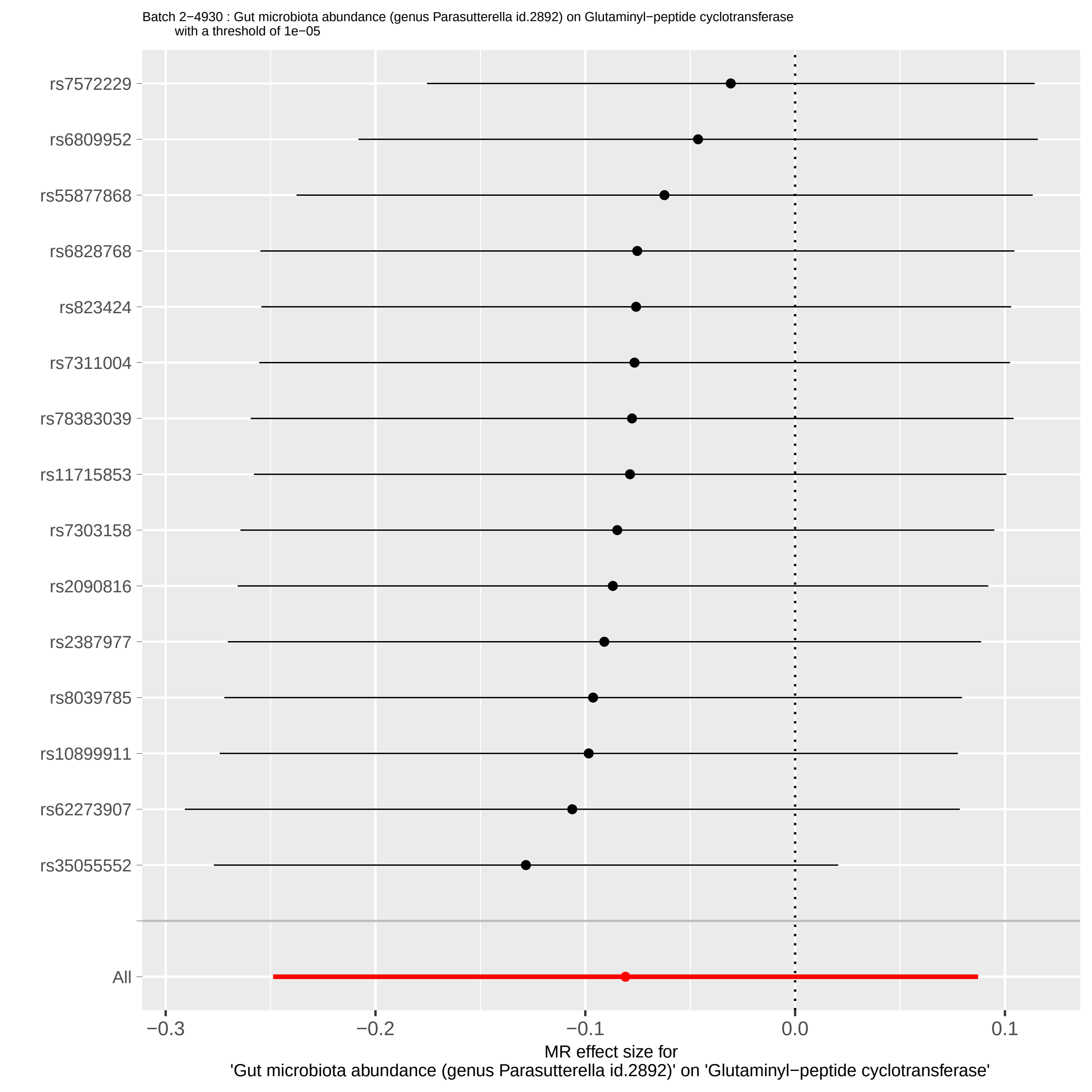
Batch 2−4930 : Gut microbiota abundance (genus Parasutterella id.2892) on Glutaminyl−peptide cyclotransferase
with a threshold of 1e−05
rs7572229
rs6809952
rs55877868
rs6828768
rs823424
rs7311004
rs78383039
rs11715853
rs7303158
rs2090816
rs2387977
rs8039785
rs10899911
rs62273907
rs35055552
All
−0.3
−0.2
−0.1
0.0
0.1
MR effect size for
'Gut microbiota abundance (genus Parasutterella id.2892)' on 'Glutaminyl−peptide cyclotransferase'

Batch 2−4931 : Gut microbiota abundance (genus Catenibacterium id.2153) on Glutaminyl−peptide cyclotransferase−like protein
with a threshold of 1e−05

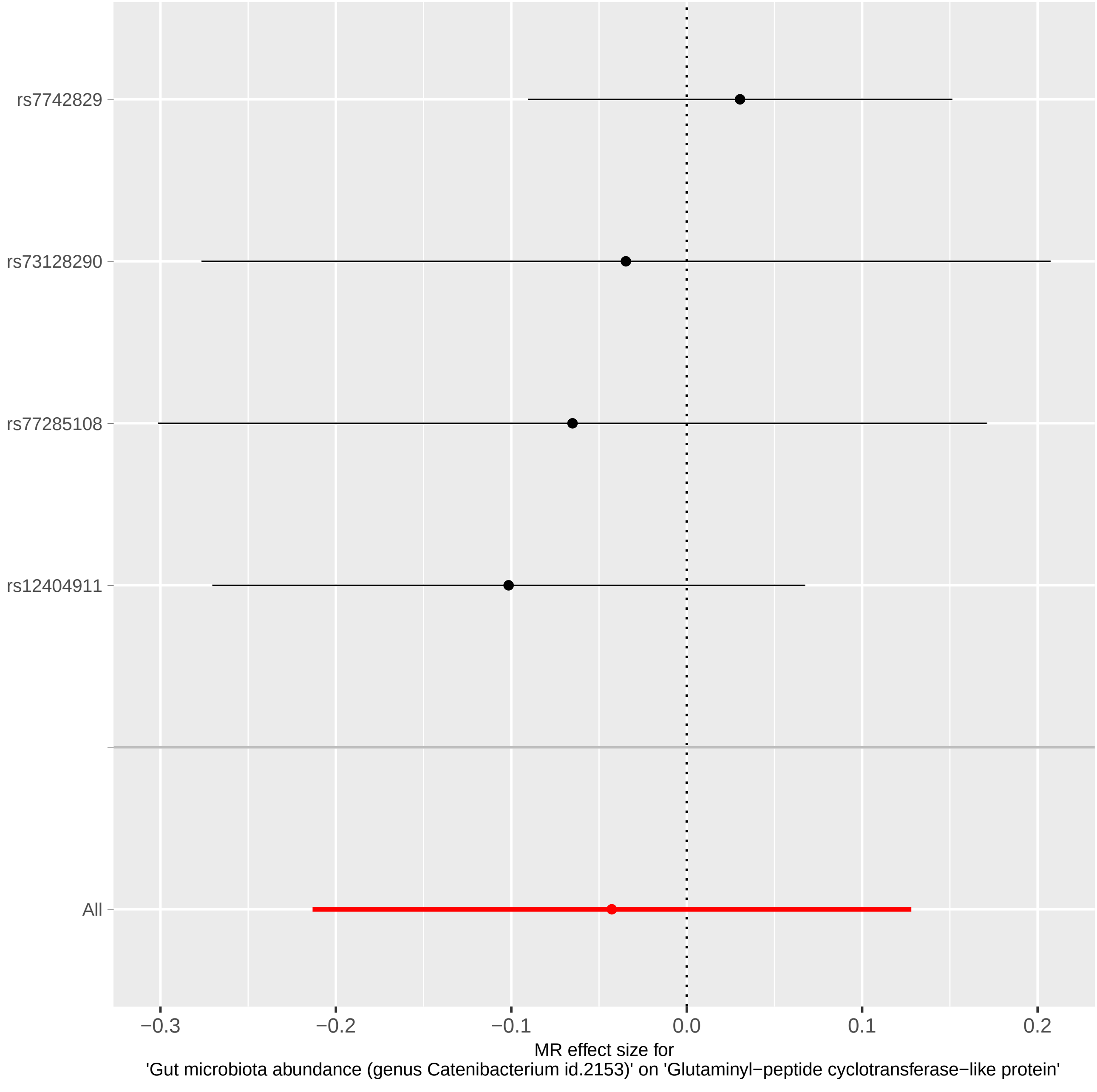

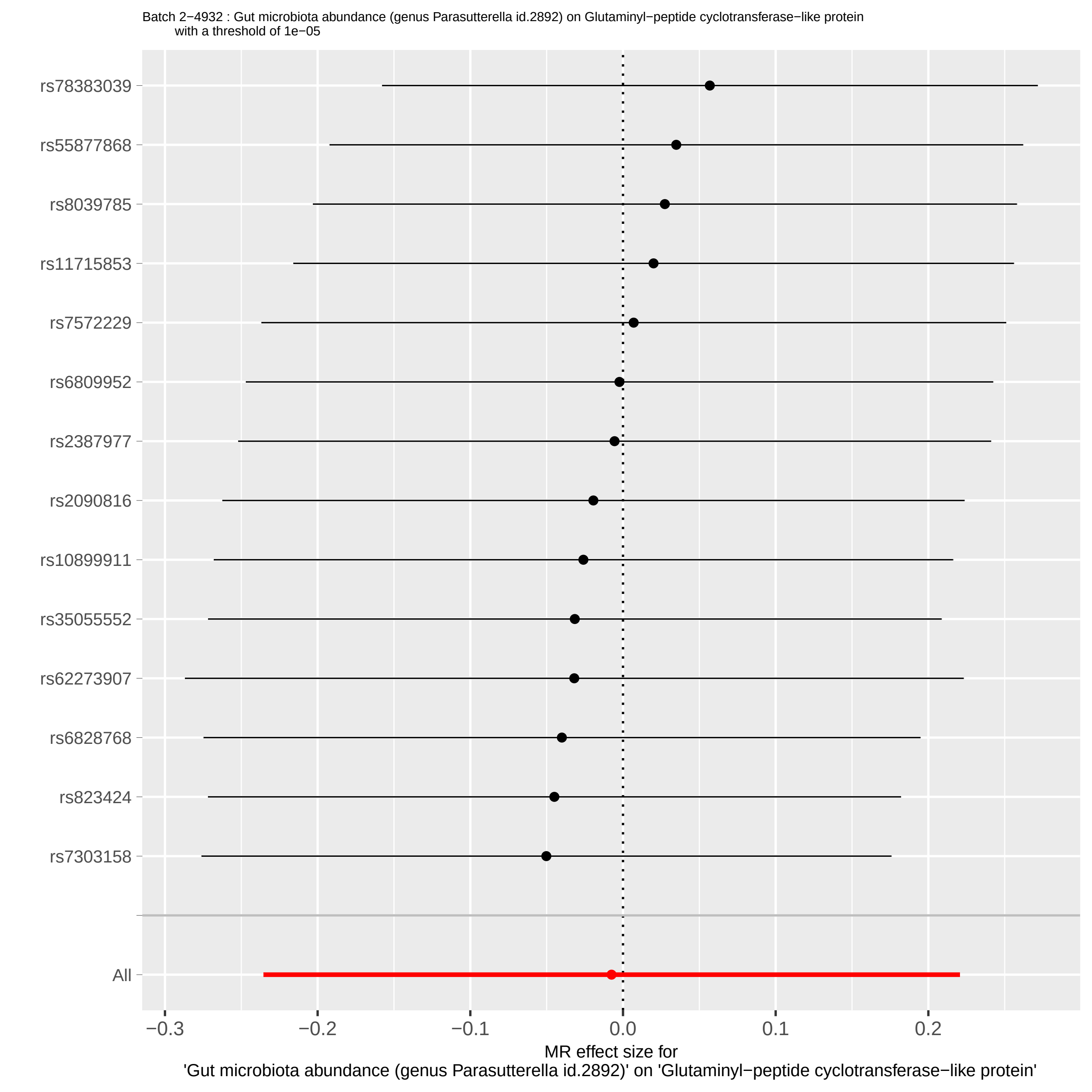

Batch 2−4932 : Gut microbiota abundance (genus Parasutterella id.2892) on Glutaminyl−peptide cyclotransferase−like protein
with a threshold of 1e−05
rs78383039
rs55877868
rs8039785
rs11715853
rs7572229
rs6809952
rs2387977
rs2090816
rs10899911
rs35055552
rs62273907
rs6828768
rs823424
rs7303158
All
−0.3
−0.2
−0.1
0.0
0.1
0.2
MR effect size for
'Gut microbiota abundance (genus Parasutterella id.2892)' on 'Glutaminyl−peptide cyclotransferase−like protein'

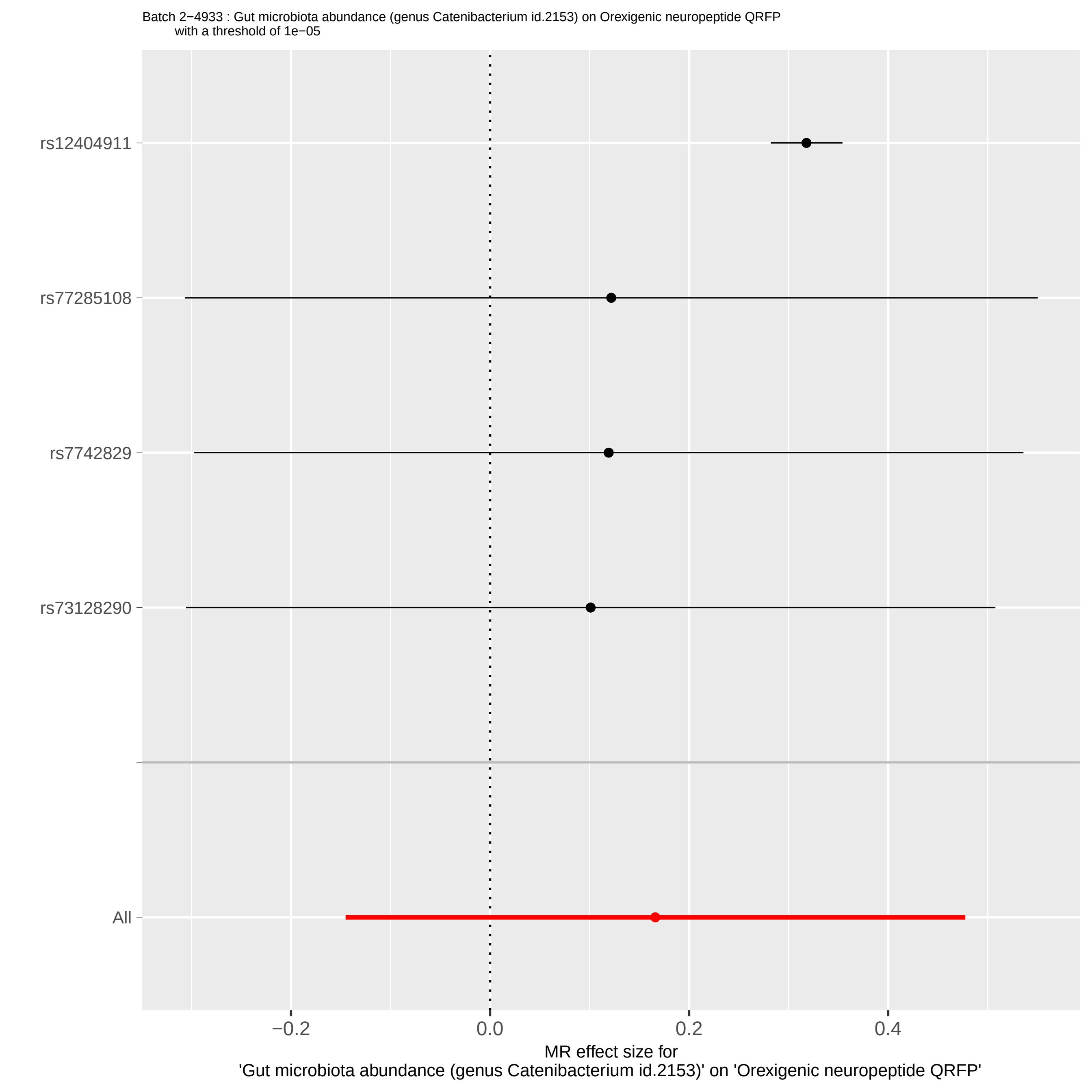
Batch 2−4933 : Gut microbiota abundance (genus Catenibacterium id.2153) on Orexigenic neuropeptide QRFP
with a threshold of 1e−05
rs12404911
rs77285108
rs7742829
rs73128290
All
−0.2
0.0
0.2
0.4
MR effect size for
'Gut microbiota abundance (genus Catenibacterium id.2153)' on 'Orexigenic neuropeptide QRFP'

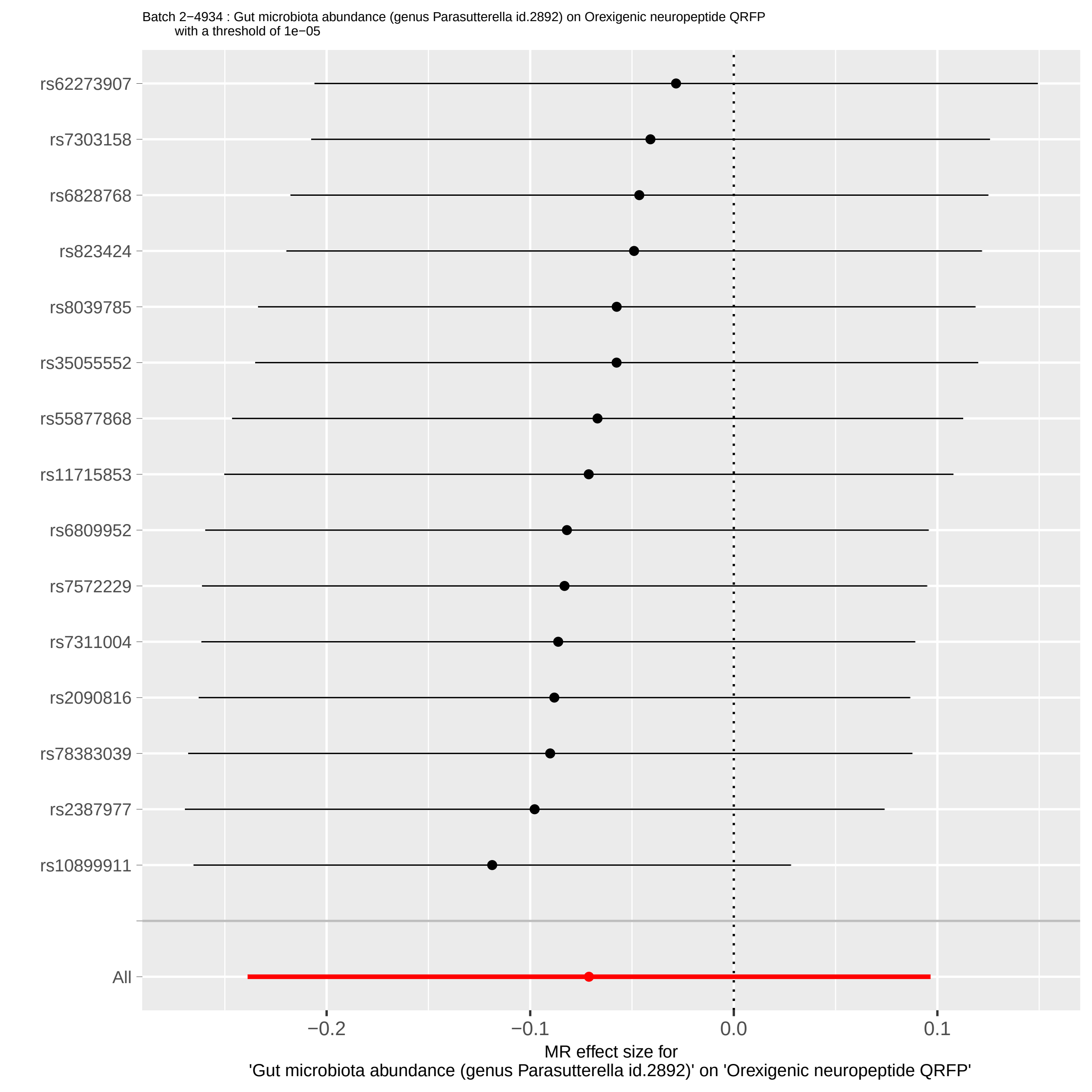

Batch 2−4934 : Gut microbiota abundance (genus Parasutterella id.2892) on Orexigenic neuropeptide QRFP
with a threshold of 1e−05
rs62273907
rs7303158
rs6828768
rs823424
rs8039785
rs35055552
rs55877868
rs11715853
rs6809952
rs7572229
rs7311004
rs2090816
rs78383039
rs2387977
rs10899911
All
−0.2
−0.1
0.0
0.1
MR effect size for
'Gut microbiota abundance (genus Parasutterella id.2892)' on 'Orexigenic neuropeptide QRFP'

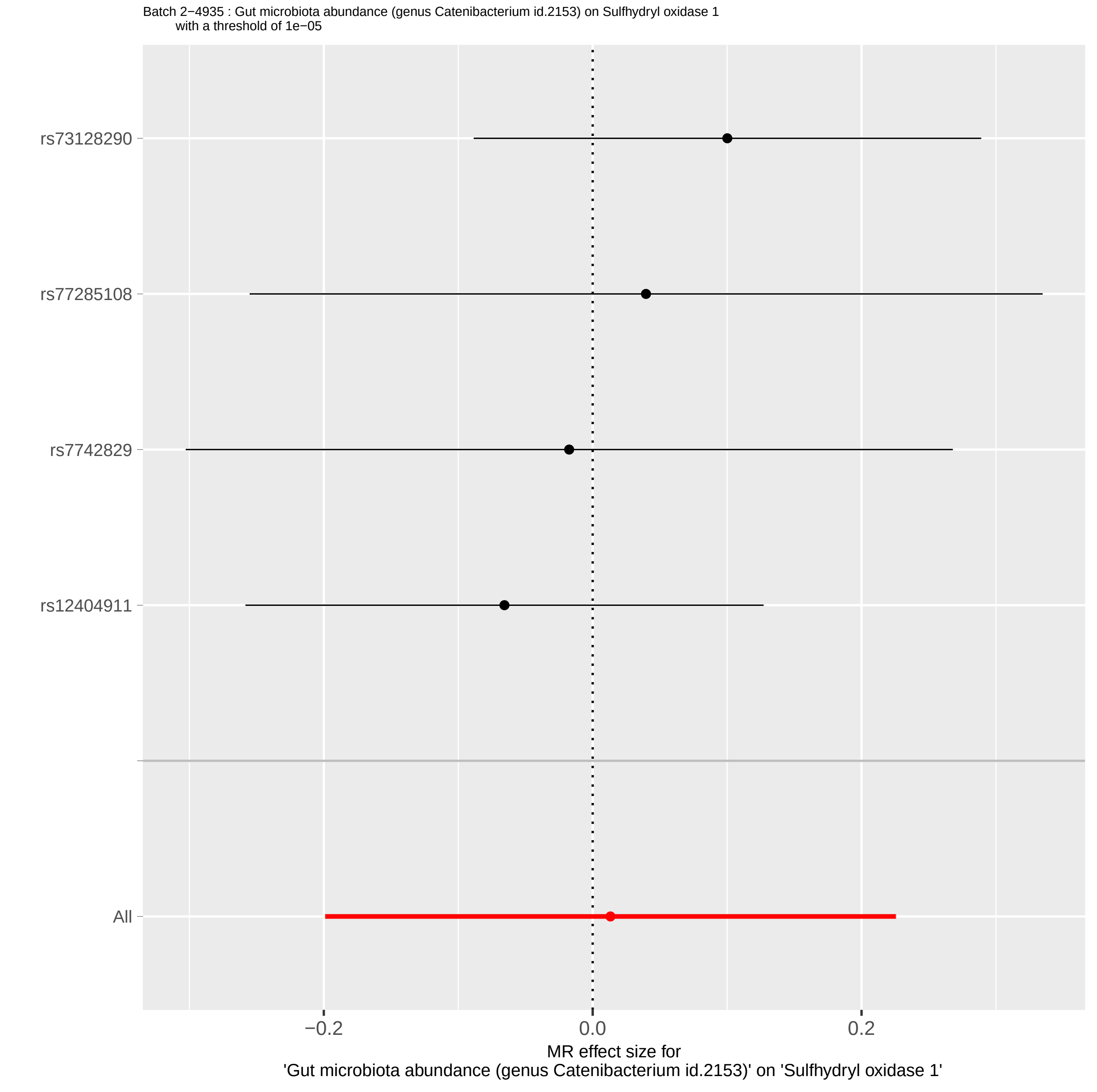
Batch 2−4935 : Gut microbiota abundance (genus Catenibacterium id.2153) on Sulfhydryl oxidase 1
with a threshold of 1e−05
rs73128290
rs77285108
rs7742829
rs12404911
All
−0.2
0.0
0.2
MR effect size for
'Gut microbiota abundance (genus Catenibacterium id.2153)' on 'Sulfhydryl oxidase 1'

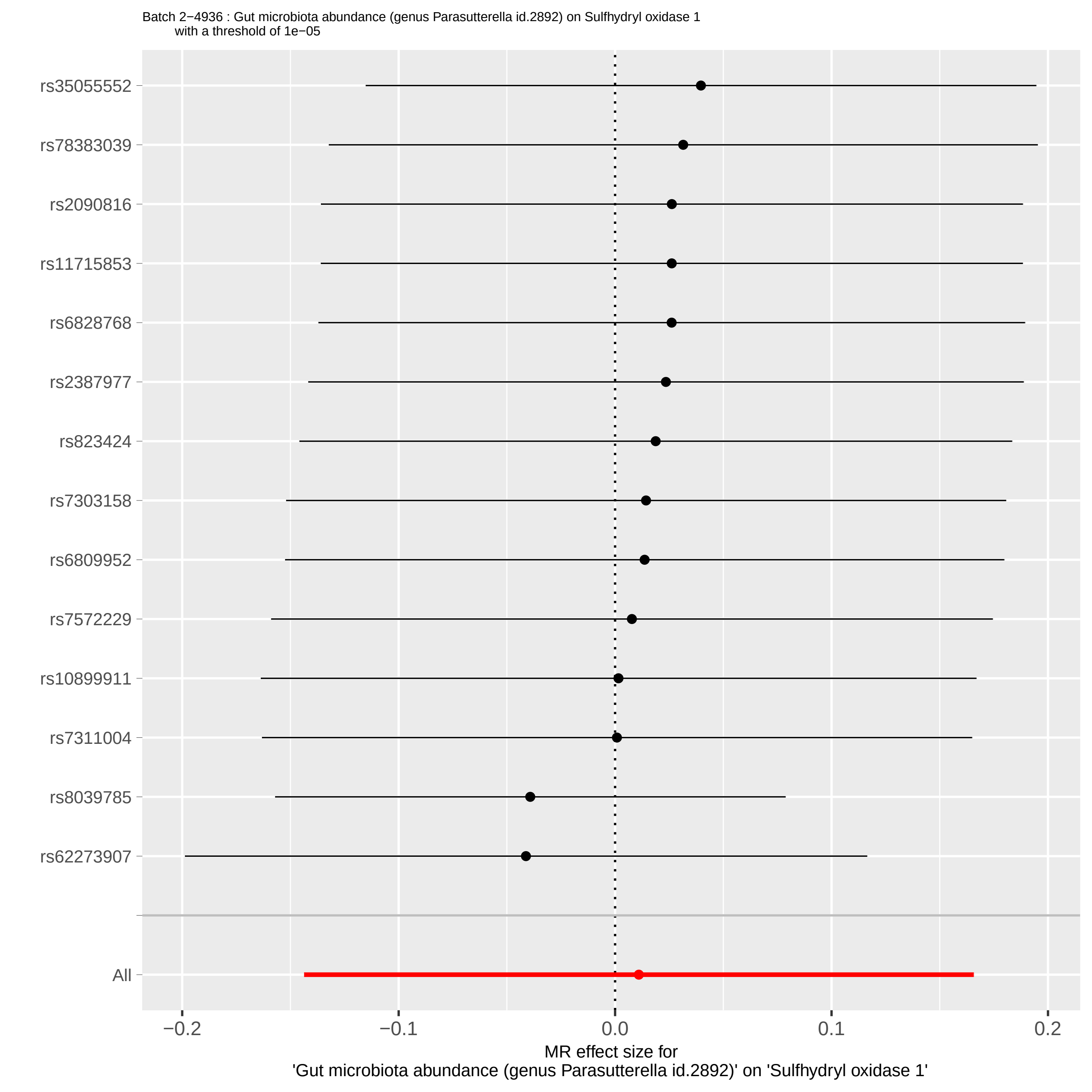
Batch 2−4936 : Gut microbiota abundance (genus Parasutterella id.2892) on Sulfhydryl oxidase 1
with a threshold of 1e−05
rs35055552
rs78383039
rs2090816
rs11715853
rs6828768
rs2387977
rs823424
rs7303158
rs6809952
rs7572229
rs10899911
rs7311004
rs8039785
rs62273907
All
−0.2
−0.1
0.0
0.1
0.2
MR effect size for
'Gut microbiota abundance (genus Parasutterella id.2892)' on 'Sulfhydryl oxidase 1'

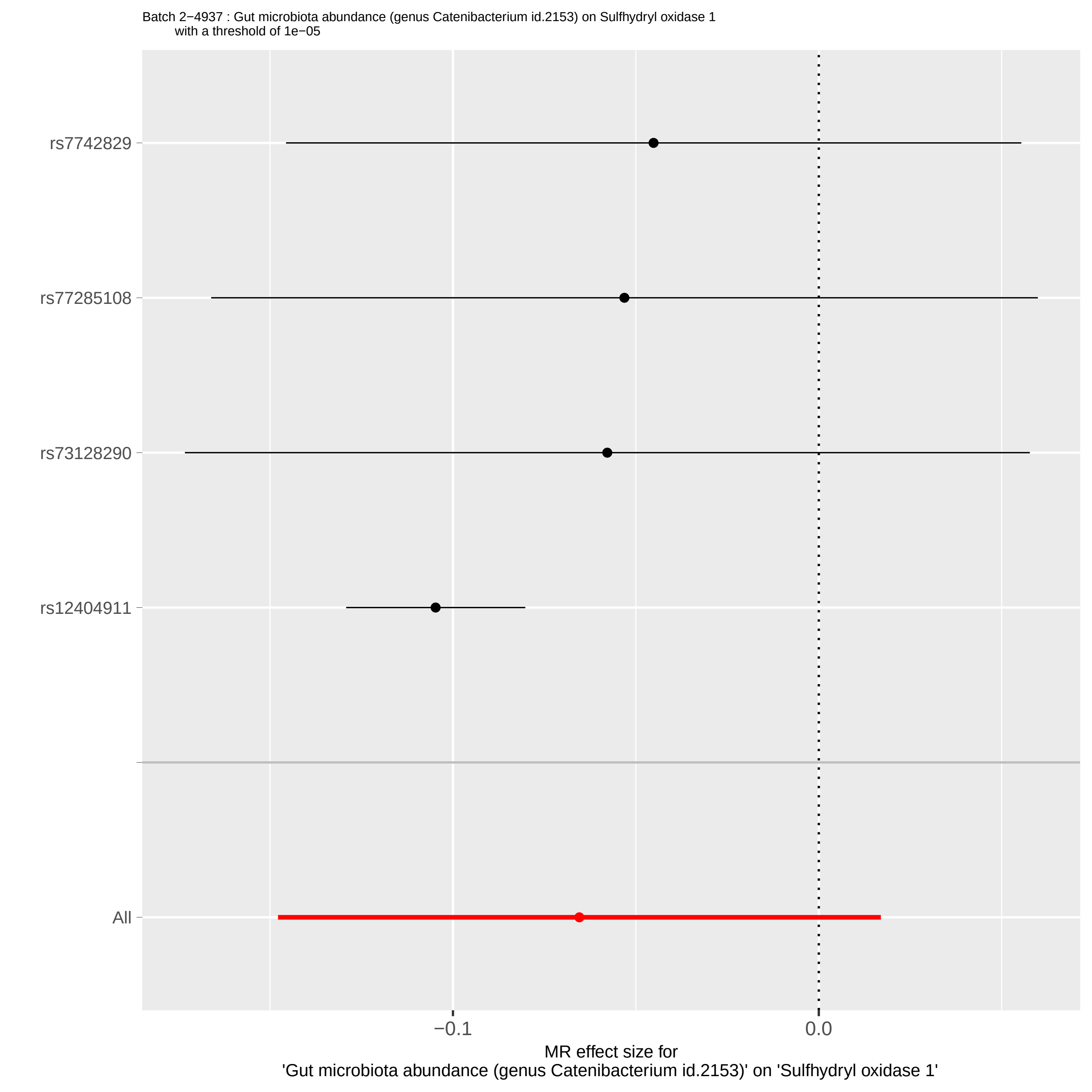
Batch 2−4937 : Gut microbiota abundance (genus Catenibacterium id.2153) on Sulfhydryl oxidase 1
with a threshold of 1e−05
rs7742829
rs77285108
rs73128290
rs12404911
All
−0.1
0.0
MR effect size for
'Gut microbiota abundance (genus Catenibacterium id.2153)' on 'Sulfhydryl oxidase 1'

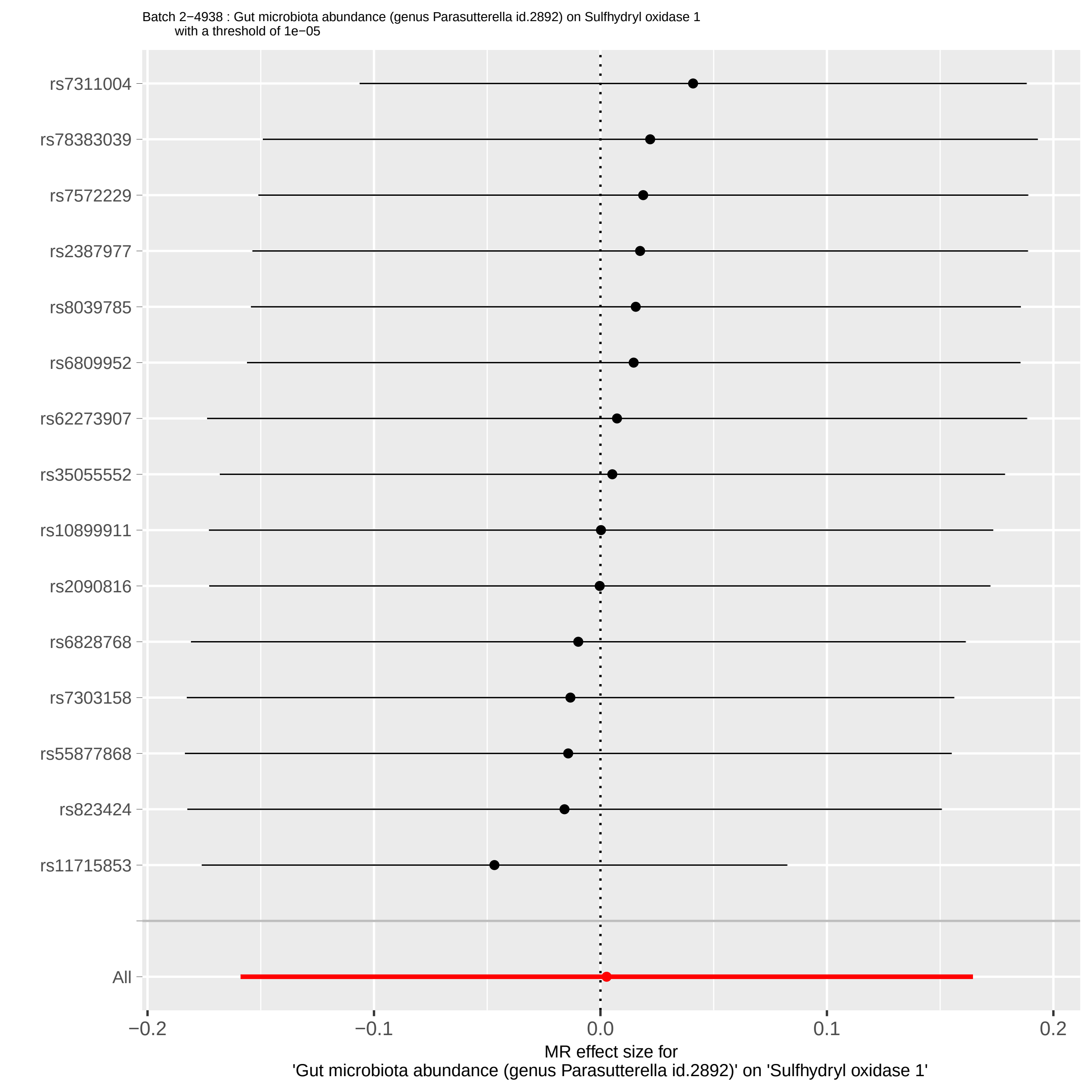

Batch 2−4938 : Gut microbiota abundance (genus Parasutterella id.2892) on Sulfhydryl oxidase 1
with a threshold of 1e−05
rs7311004
rs78383039
rs7572229
rs2387977
rs8039785
rs6809952
rs62273907
rs35055552
rs10899911
rs2090816
rs6828768
rs7303158
rs55877868
rs823424
rs11715853
All
−0.2
−0.1
0.0
0.1
0.2
MR effect size for
'Gut microbiota abundance (genus Parasutterella id.2892)' on 'Sulfhydryl oxidase 1'

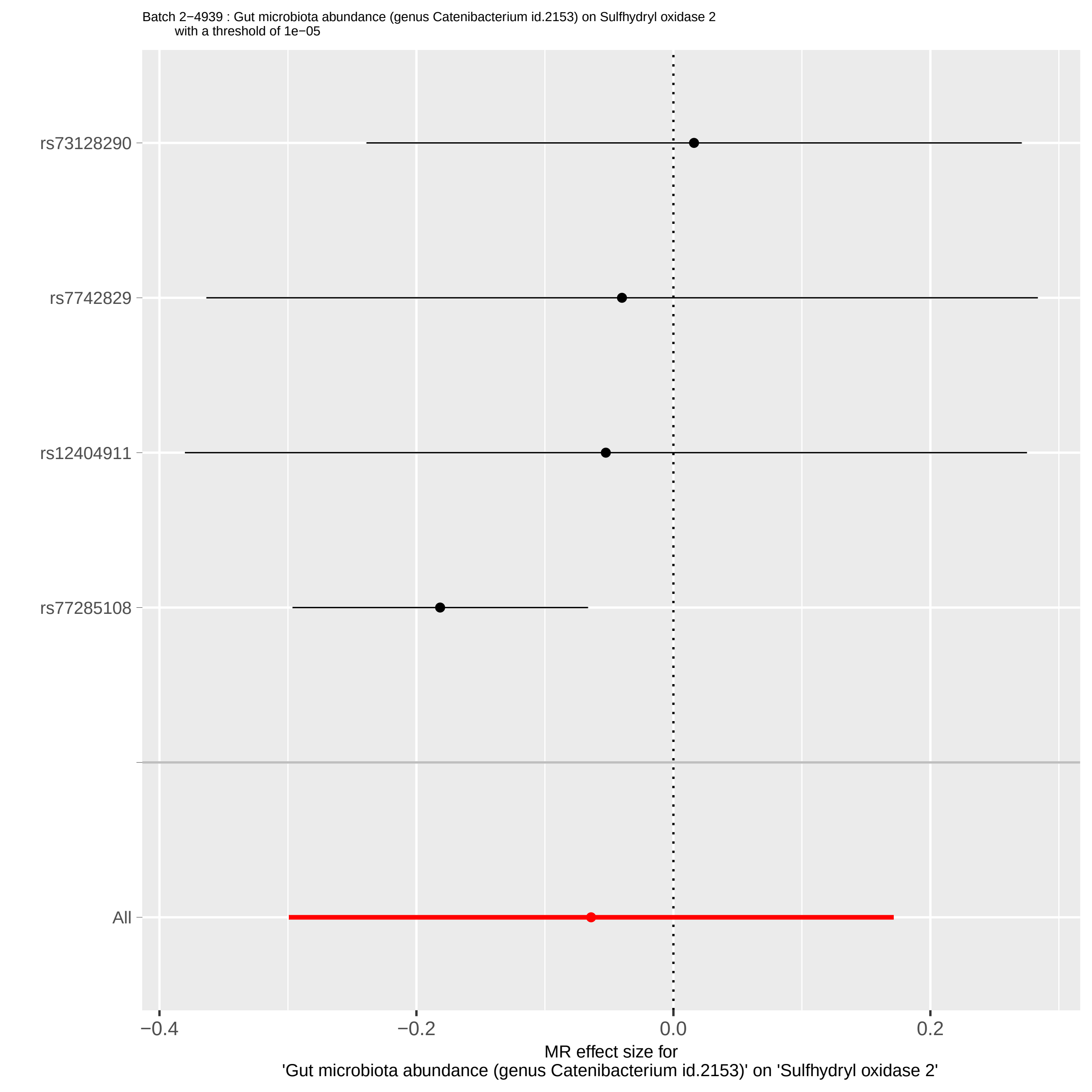
Batch 2−4939 : Gut microbiota abundance (genus Catenibacterium id.2153) on Sulfhydryl oxidase 2
with a threshold of 1e−05
rs73128290
rs7742829
rs12404911
rs77285108
All
−0.4
−0.2
0.0
0.2
MR effect size for
'Gut microbiota abundance (genus Catenibacterium id.2153)' on 'Sulfhydryl oxidase 2'

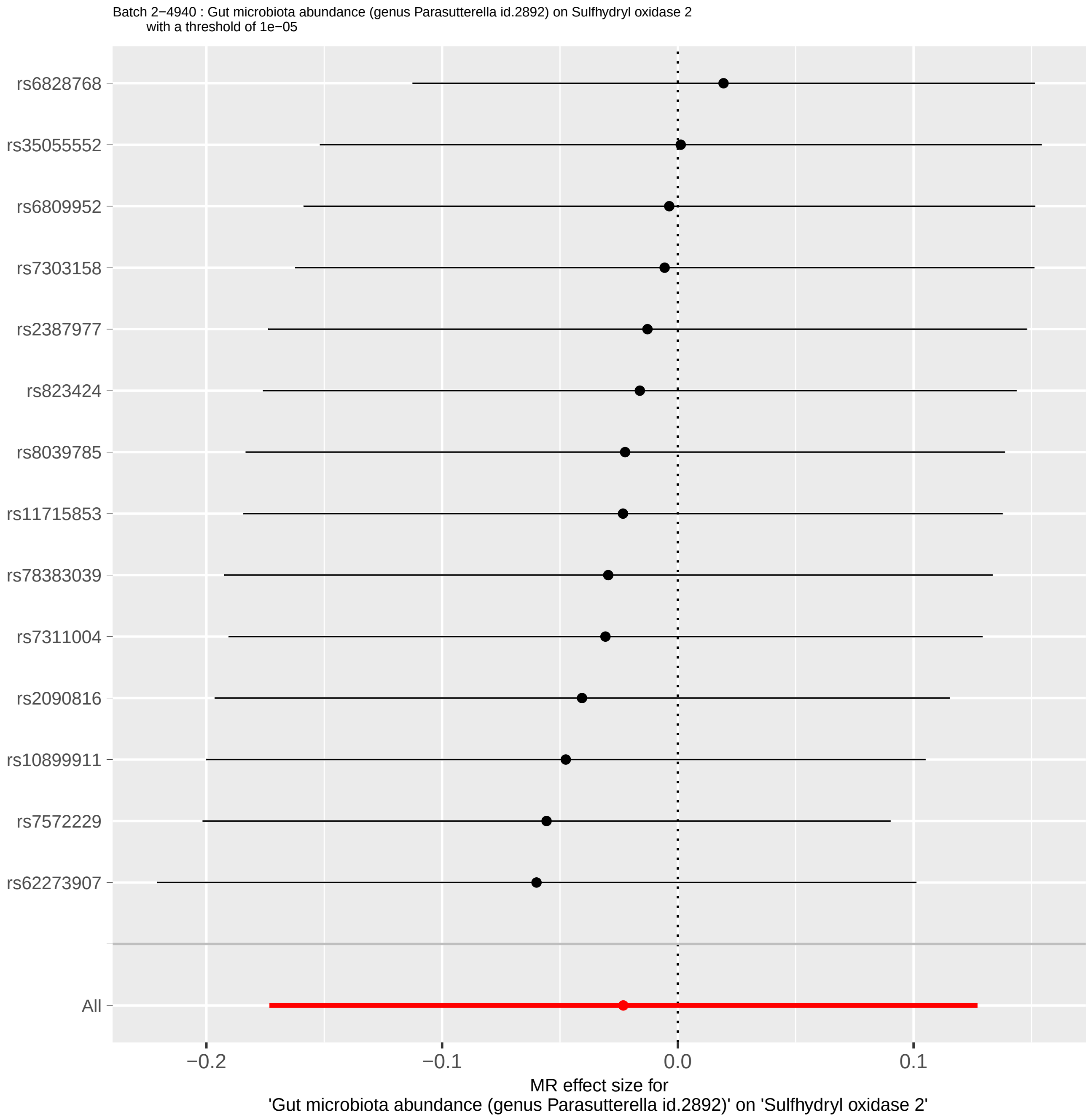
Batch 2−4940 : Gut microbiota abundance (genus Parasutterella id.2892) on Sulfhydryl oxidase 2
with a threshold of 1e−05
rs6828768
rs35055552
rs6809952
rs7303158
rs2387977
rs823424
rs8039785
rs11715853
rs78383039
rs7311004
rs2090816
rs10899911
rs7572229
rs62273907
All
−0.2
−0.1
0.0
0.1
MR effect size for
'Gut microbiota abundance (genus Parasutterella id.2892)' on 'Sulfhydryl oxidase 2'

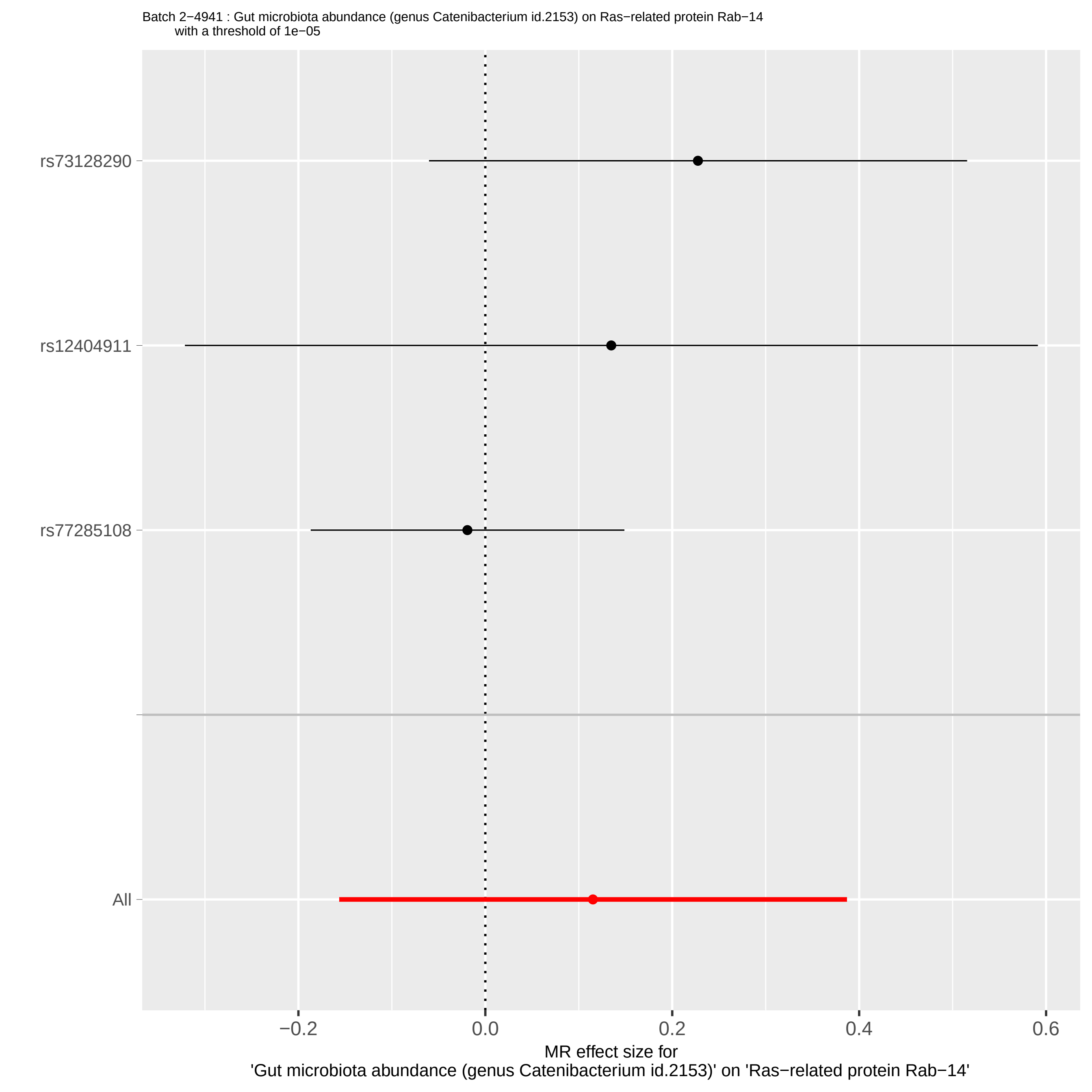
Batch 2−4941 : Gut microbiota abundance (genus Catenibacterium id.2153) on Ras−related protein Rab−14
with a threshold of 1e−05
rs73128290
rs12404911
rs77285108
All
−0.2
0.0
0.2
0.4
0.6
MR effect size for
'Gut microbiota abundance (genus Catenibacterium id.2153)' on 'Ras−related protein Rab−14'

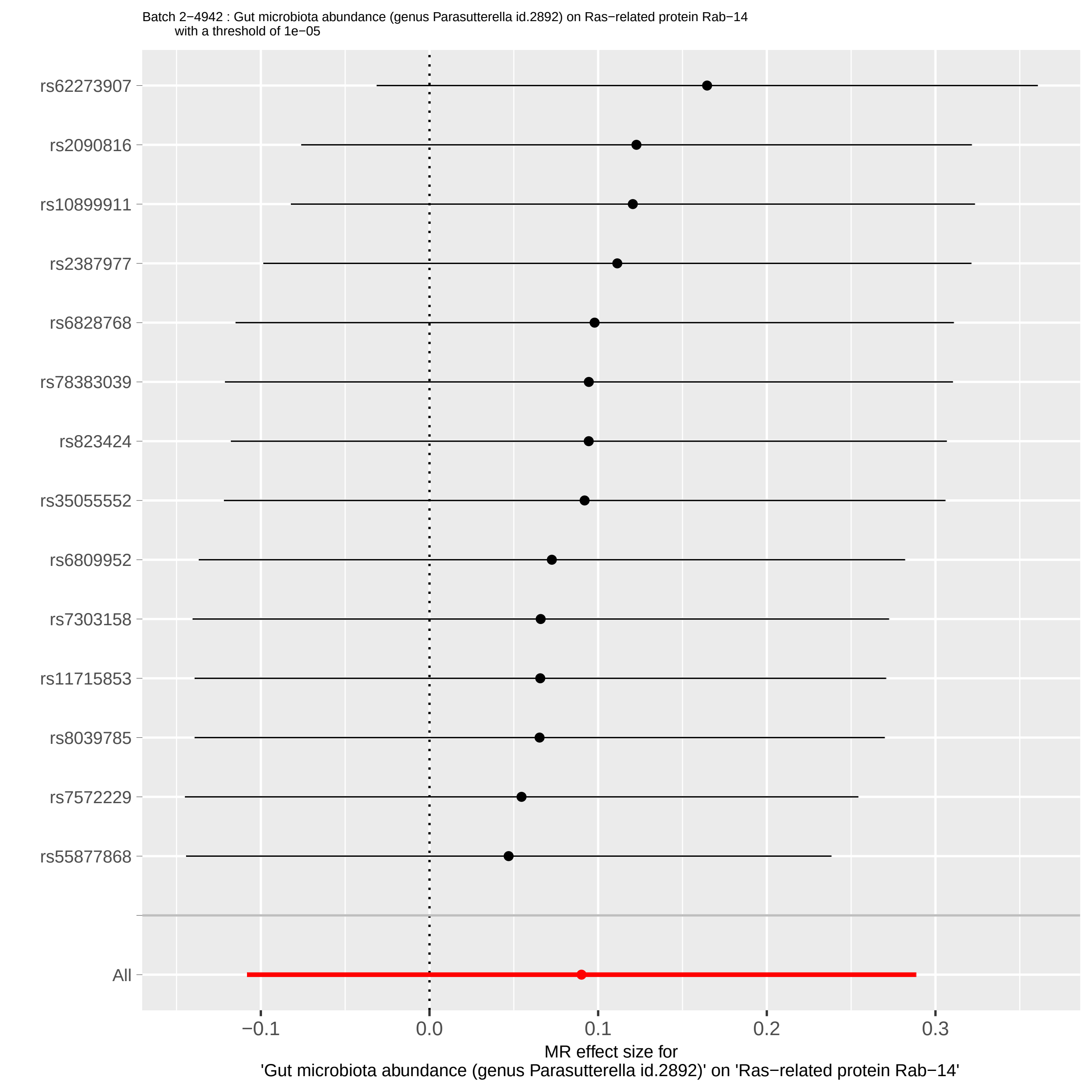

Batch 2−4942 : Gut microbiota abundance (genus Parasutterella id.2892) on Ras−related protein Rab−14
with a threshold of 1e−05
rs62273907
rs2090816
rs10899911
rs2387977
rs6828768
rs78383039
rs823424
rs35055552
rs6809952
rs7303158
rs11715853
rs8039785
rs7572229
rs55877868
All
−0.1
0.0
0.1
0.2
0.3
MR effect size for
'Gut microbiota abundance (genus Parasutterella id.2892)' on 'Ras−related protein Rab−14'

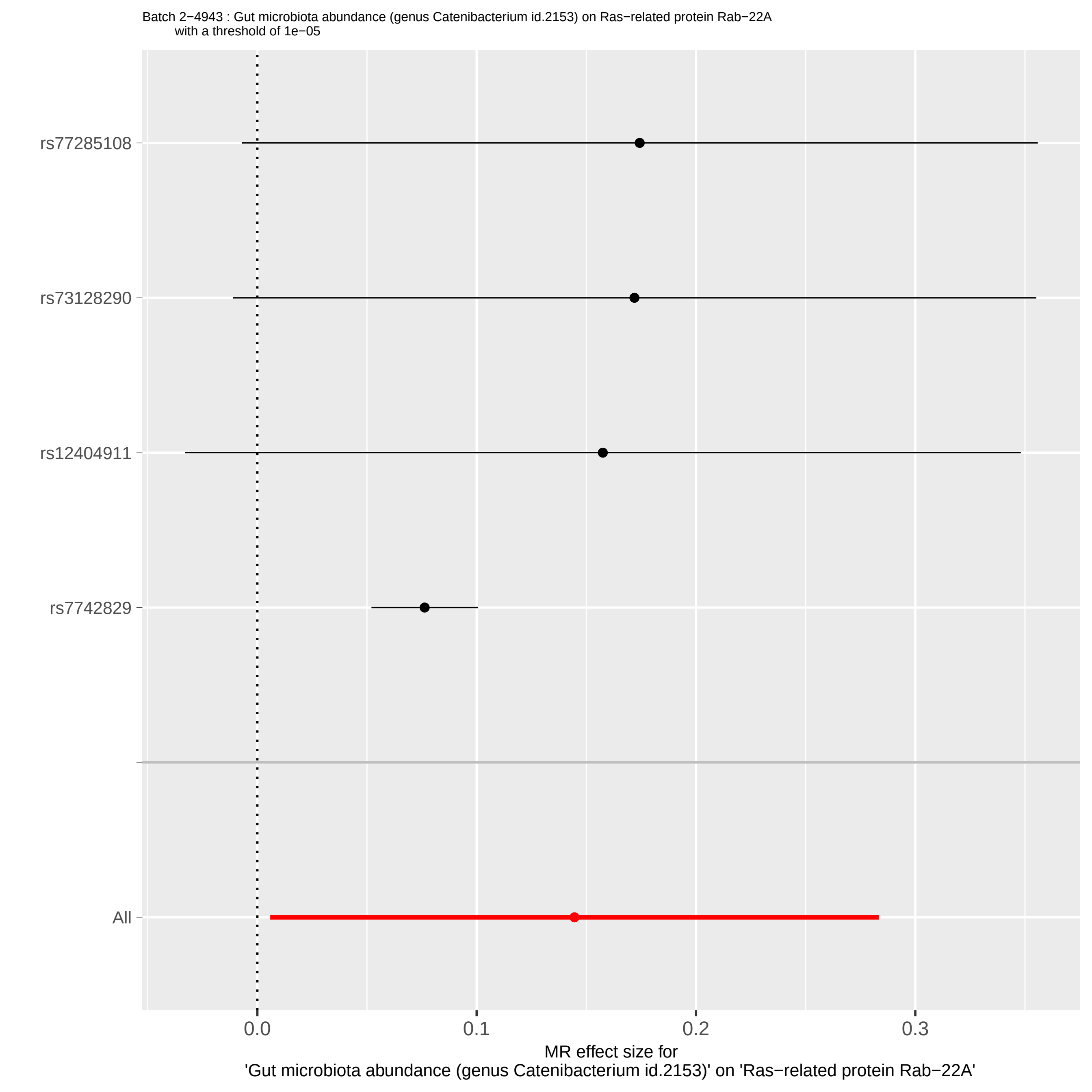
Batch 2−4943 : Gut microbiota abundance (genus Catenibacterium id.2153) on Ras−related protein Rab−22A
with a threshold of 1e−05
rs77285108
rs73128290
rs12404911
rs7742829
All
0.0
0.1
0.2
0.3
MR effect size for
'Gut microbiota abundance (genus Catenibacterium id.2153)' on 'Ras−related protein Rab−22A'

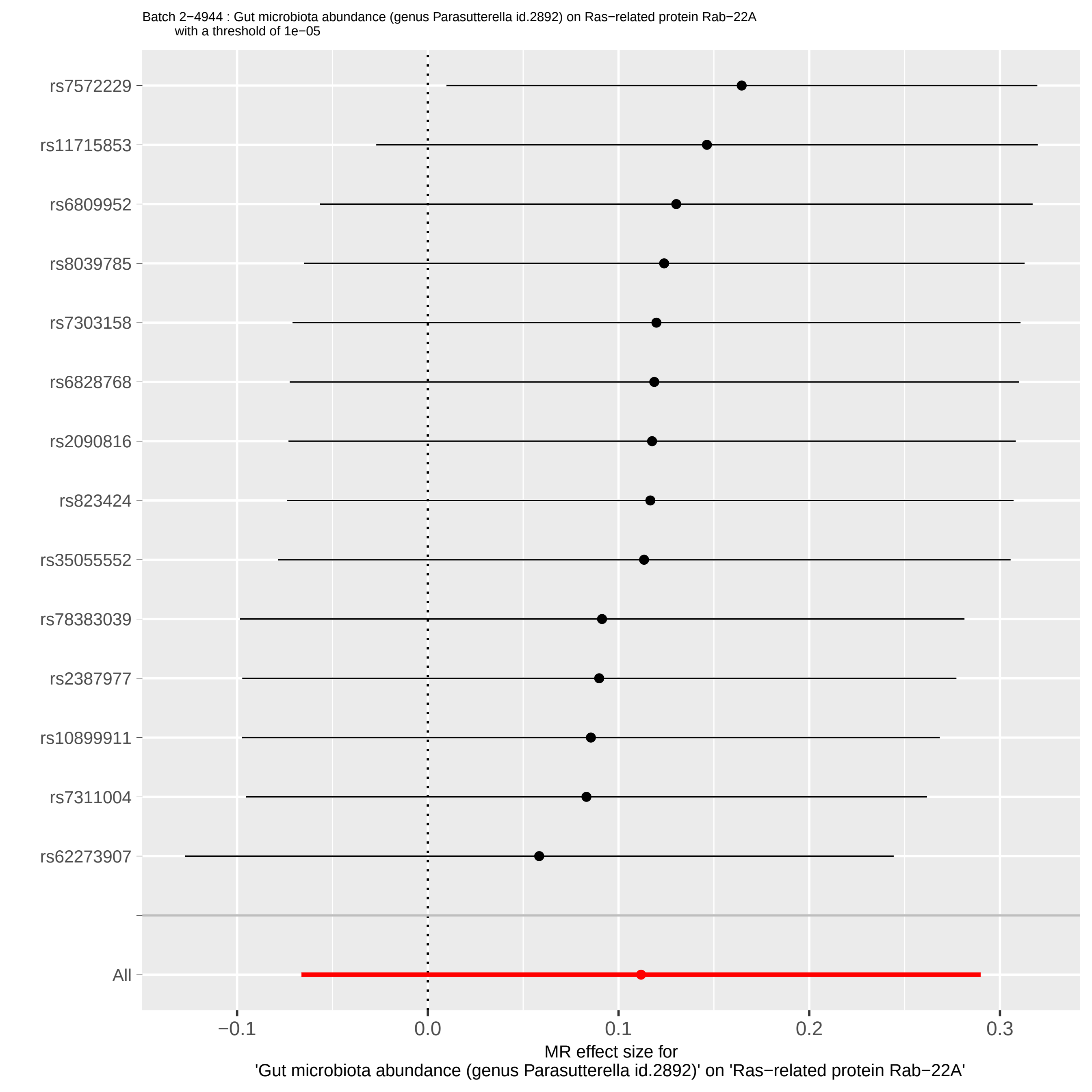
Batch 2−4944 : Gut microbiota abundance (genus Parasutterella id.2892) on Ras−related protein Rab−22A
with a threshold of 1e−05
rs7572229
rs11715853
rs6809952
rs8039785
rs7303158
rs6828768
rs2090816
rs823424
rs35055552
rs78383039
rs2387977
rs10899911
rs7311004
rs62273907
All
−0.1
0.0
0.1
0.2
0.3
MR effect size for
'Gut microbiota abundance (genus Parasutterella id.2892)' on 'Ras−related protein Rab−22A'

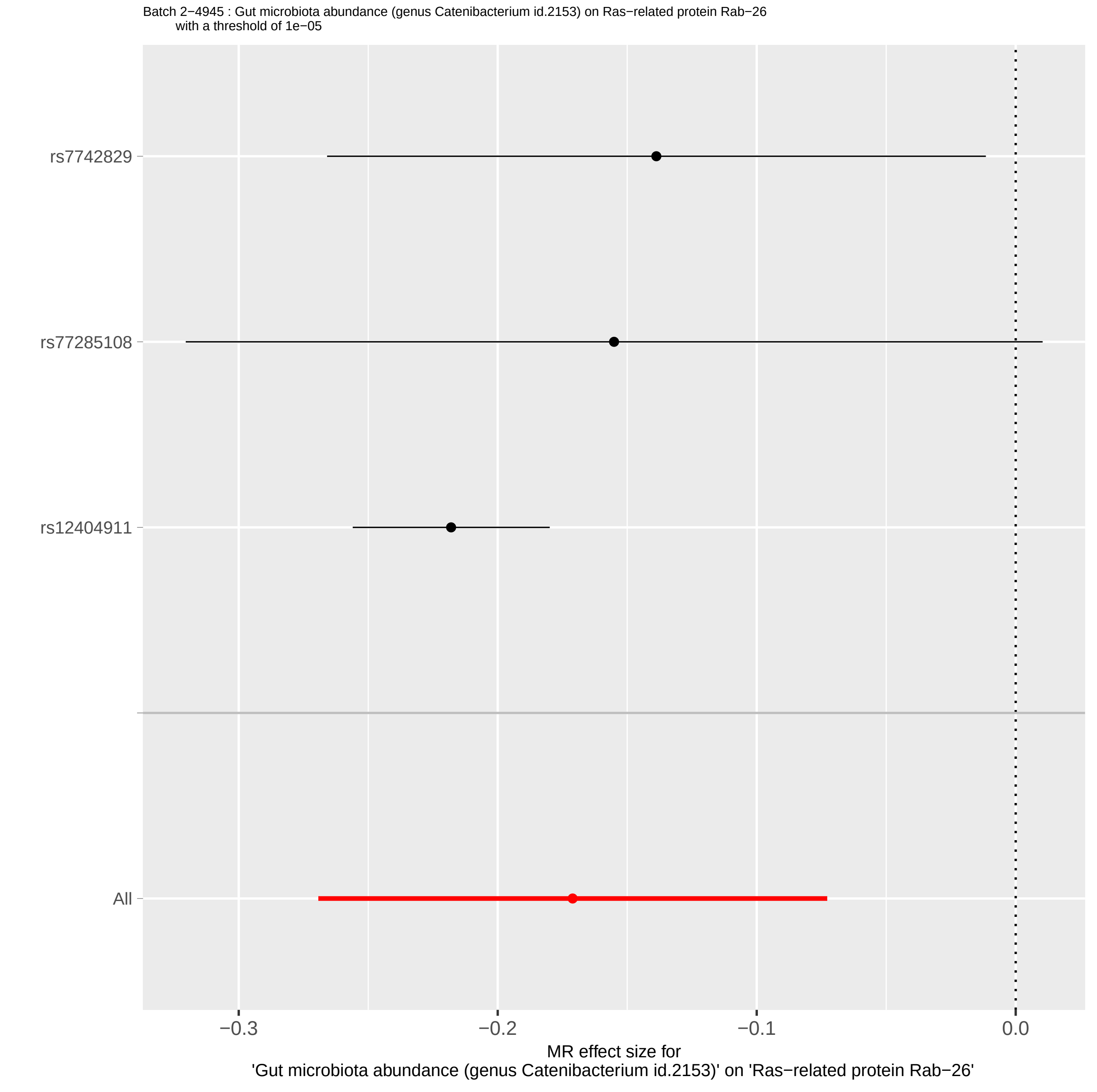
Batch 2−4945 : Gut microbiota abundance (genus Catenibacterium id.2153) on Ras−related protein Rab−26
with a threshold of 1e−05
rs7742829
rs77285108
rs12404911
All
−0.3
−0.2
−0.1
0.0
MR effect size for
'Gut microbiota abundance (genus Catenibacterium id.2153)' on 'Ras−related protein Rab−26'

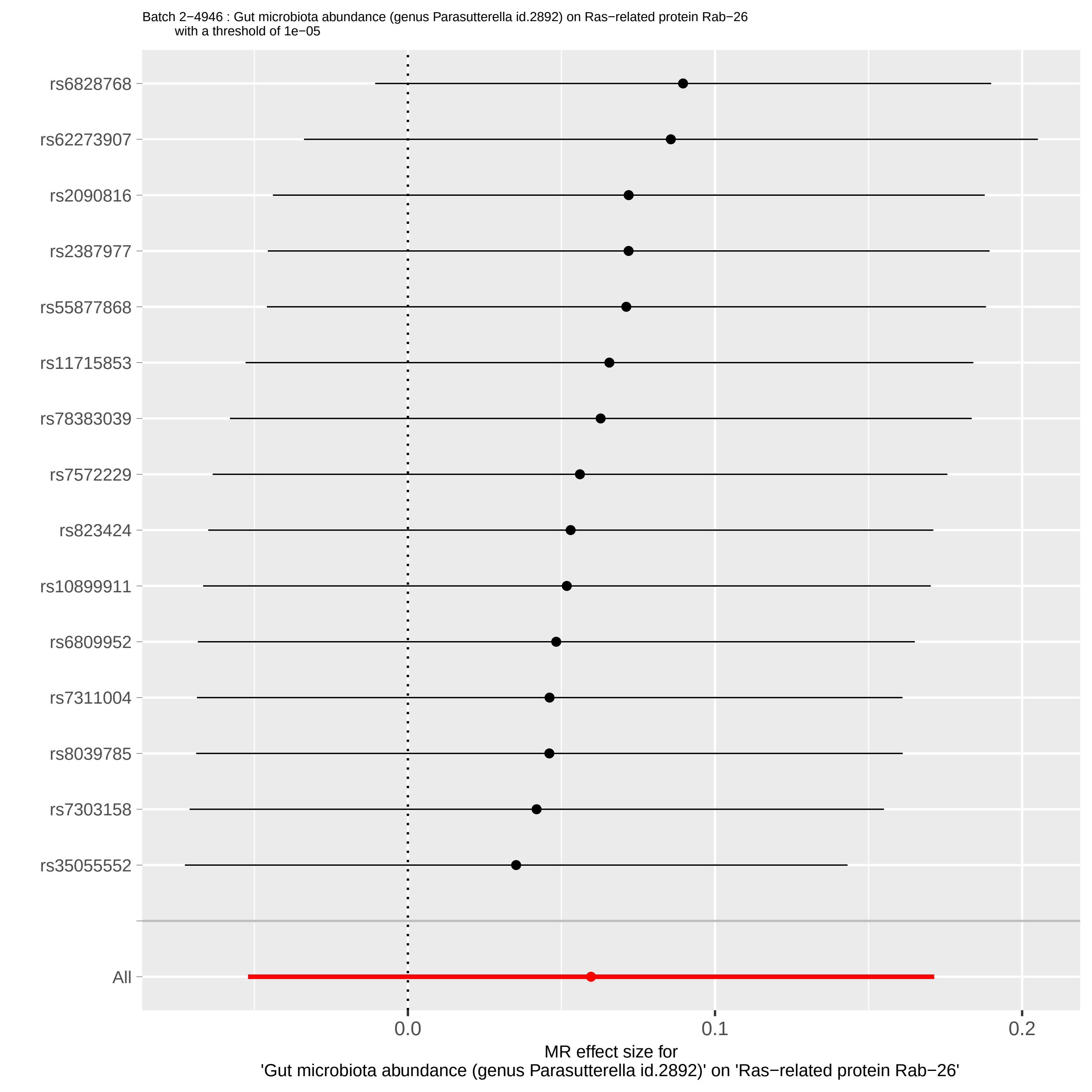
Batch 2−4946 : Gut microbiota abundance (genus Parasutterella id.2892) on Ras−related protein Rab−26
with a threshold of 1e−05
rs6828768
rs62273907
rs2090816
rs2387977
rs55877868
rs11715853
rs78383039
rs7572229
rs823424
rs10899911
rs6809952
rs7311004
rs8039785
rs7303158
rs35055552
All
0.0
0.1
0.2
MR effect size for
'Gut microbiota abundance (genus Parasutterella id.2892)' on 'Ras−related protein Rab−26'

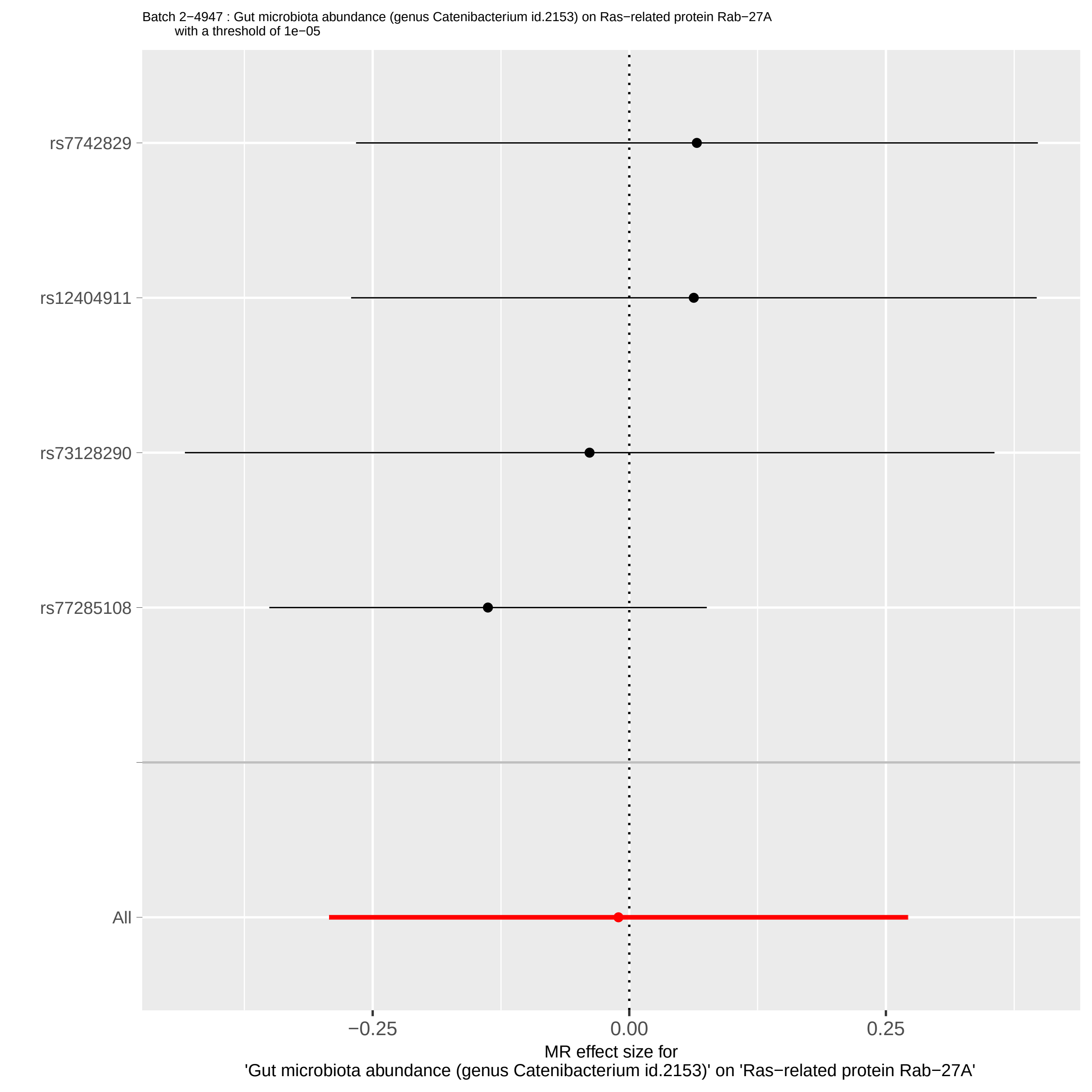
Batch 2−4947 : Gut microbiota abundance (genus Catenibacterium id.2153) on Ras−related protein Rab−27A
with a threshold of 1e−05
rs7742829
rs12404911
rs73128290
rs77285108
All
−0.25
0.00
0.25
MR effect size for
'Gut microbiota abundance (genus Catenibacterium id.2153)' on 'Ras−related protein Rab−27A'

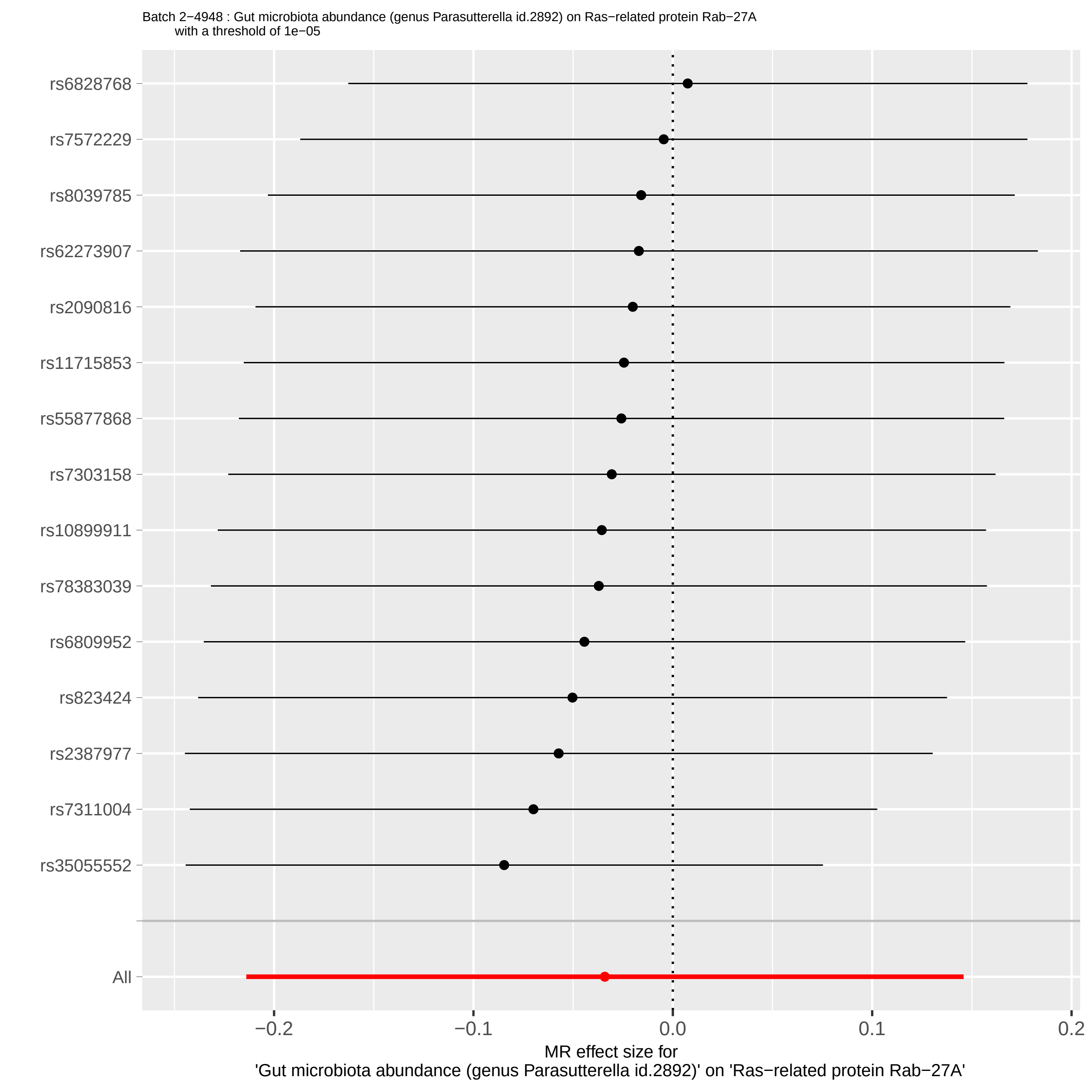
Batch 2−4948 : Gut microbiota abundance (genus Parasutterella id.2892) on Ras−related protein Rab−27A
with a threshold of 1e−05
rs6828768
rs7572229
rs8039785
rs62273907
rs2090816
rs11715853
rs55877868
rs7303158
rs10899911
rs78383039
rs6809952
rs823424
rs2387977
rs7311004
rs35055552
All
−0.2
−0.1
0.0
0.1
0.2
MR effect size for
'Gut microbiota abundance (genus Parasutterella id.2892)' on 'Ras−related protein Rab−27A'

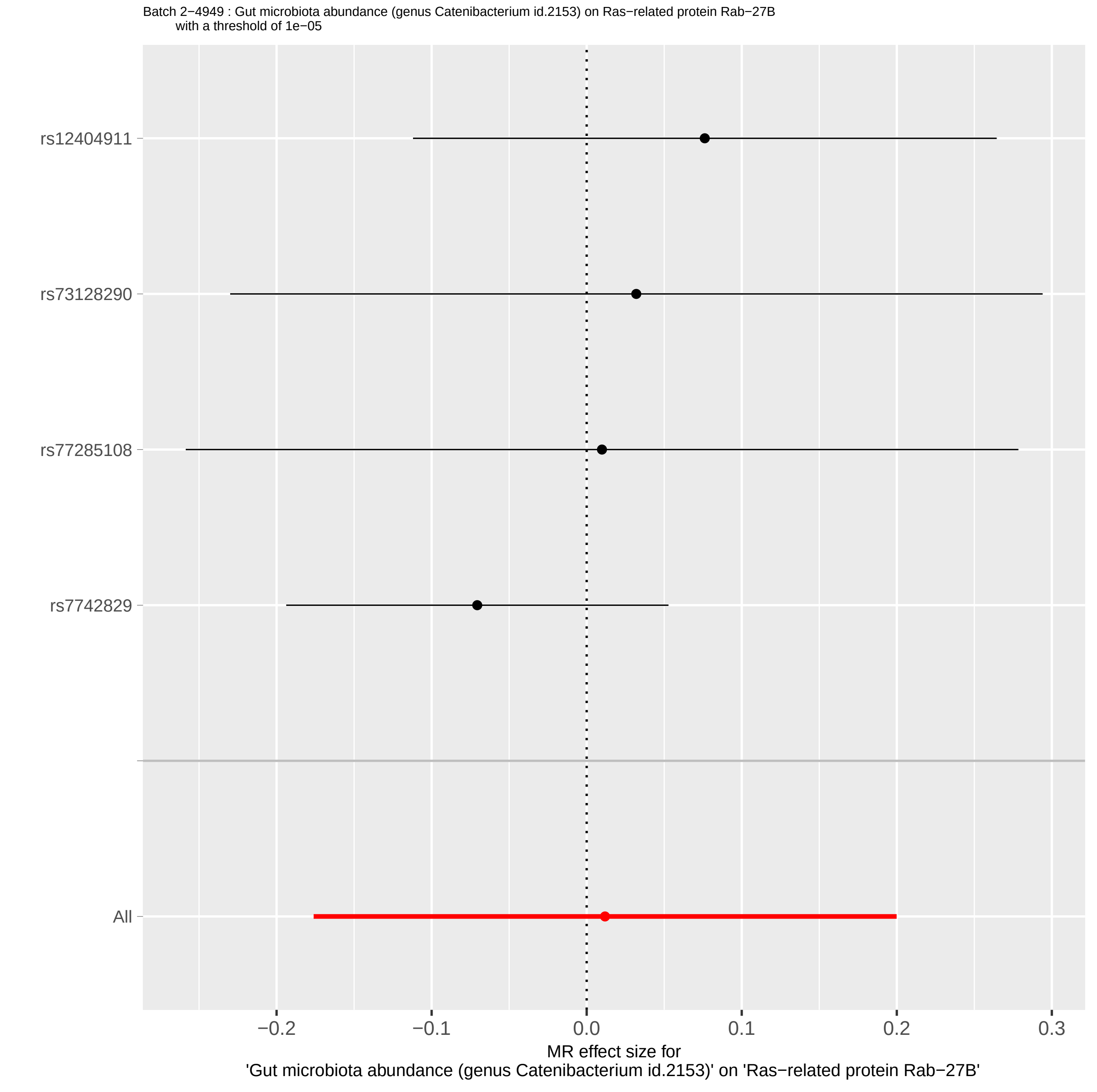
Batch 2−4949 : Gut microbiota abundance (genus Catenibacterium id.2153) on Ras−related protein Rab−27B
with a threshold of 1e−05
rs12404911
rs73128290
rs77285108
rs7742829
All
−0.2
−0.1
0.0
0.1
0.2
0.3
MR effect size for
'Gut microbiota abundance (genus Catenibacterium id.2153)' on 'Ras−related protein Rab−27B'

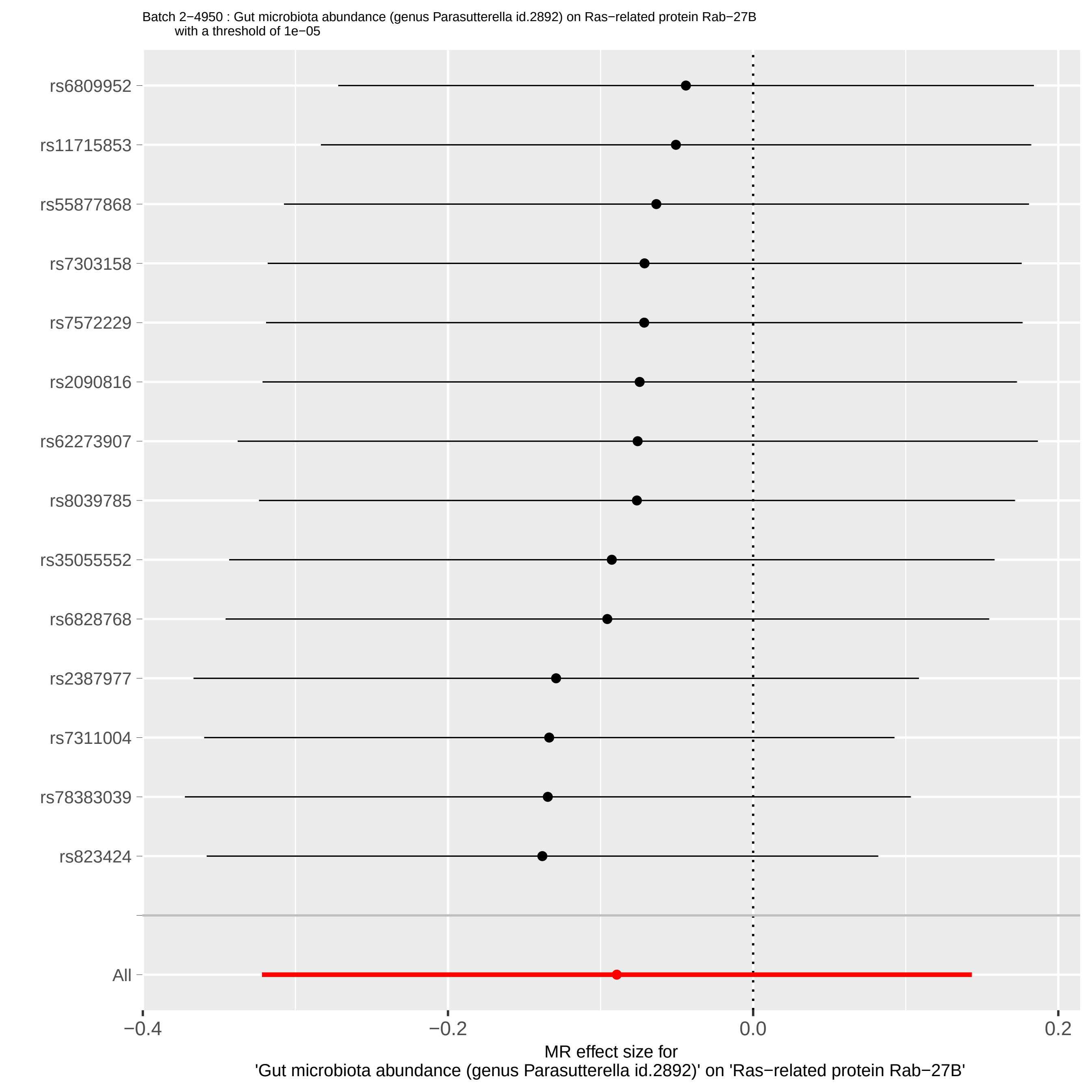

Batch 2−4950 : Gut microbiota abundance (genus Parasutterella id.2892) on Ras−related protein Rab−27B
with a threshold of 1e−05
rs6809952
rs11715853
rs55877868
rs7303158
rs7572229
rs2090816
rs62273907
rs8039785
rs35055552
rs6828768
rs2387977
rs7311004
rs78383039
rs823424
All
−0.4
−0.2
0.0
0.2
MR effect size for
'Gut microbiota abundance (genus Parasutterella id.2892)' on 'Ras−related protein Rab−27B'

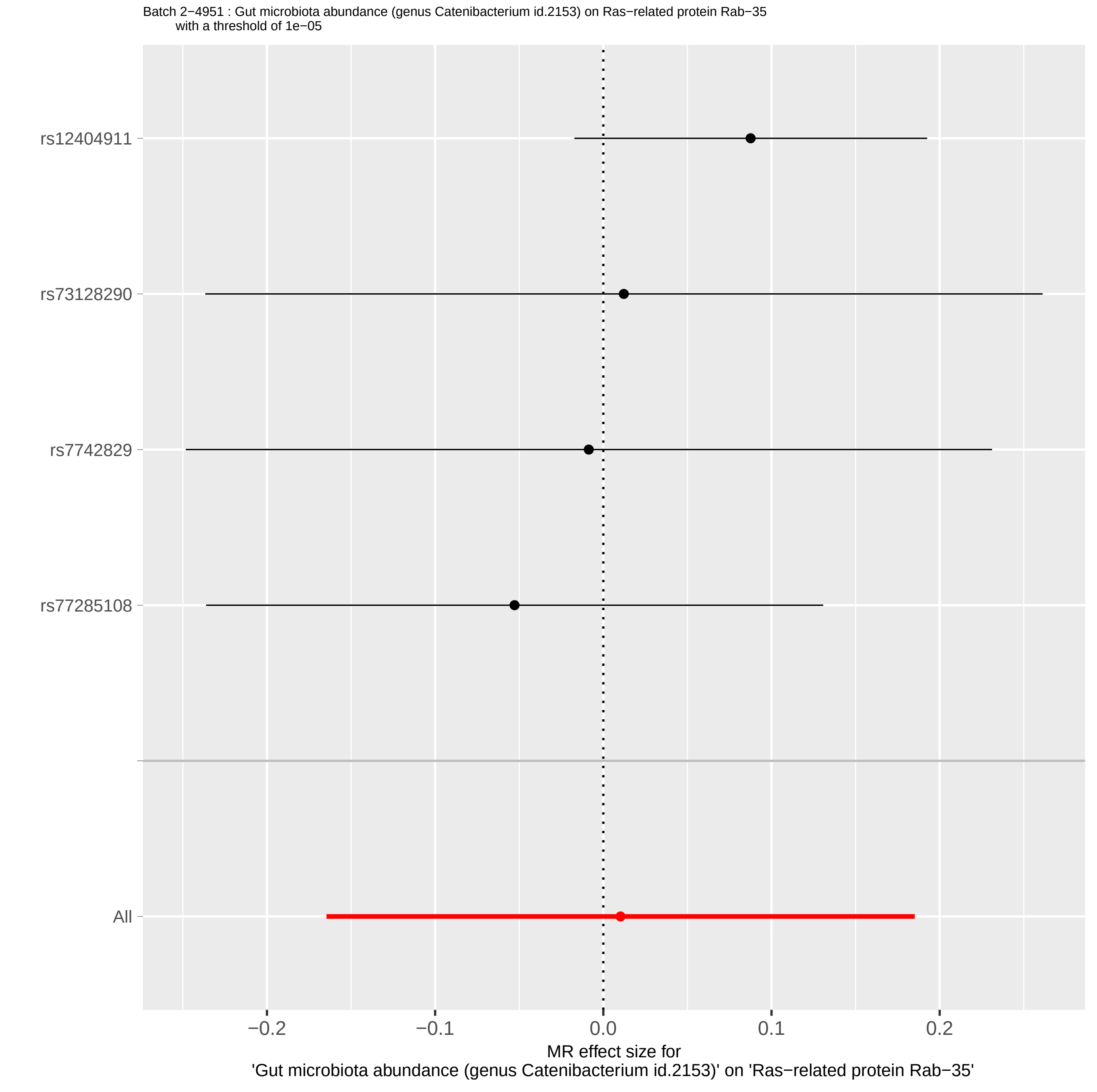
Batch 2−4951 : Gut microbiota abundance (genus Catenibacterium id.2153) on Ras−related protein Rab−35
with a threshold of 1e−05
rs12404911
rs73128290
rs7742829
rs77285108
All
−0.2
−0.1
0.0
0.1
0.2
MR effect size for
'Gut microbiota abundance (genus Catenibacterium id.2153)' on 'Ras−related protein Rab−35'

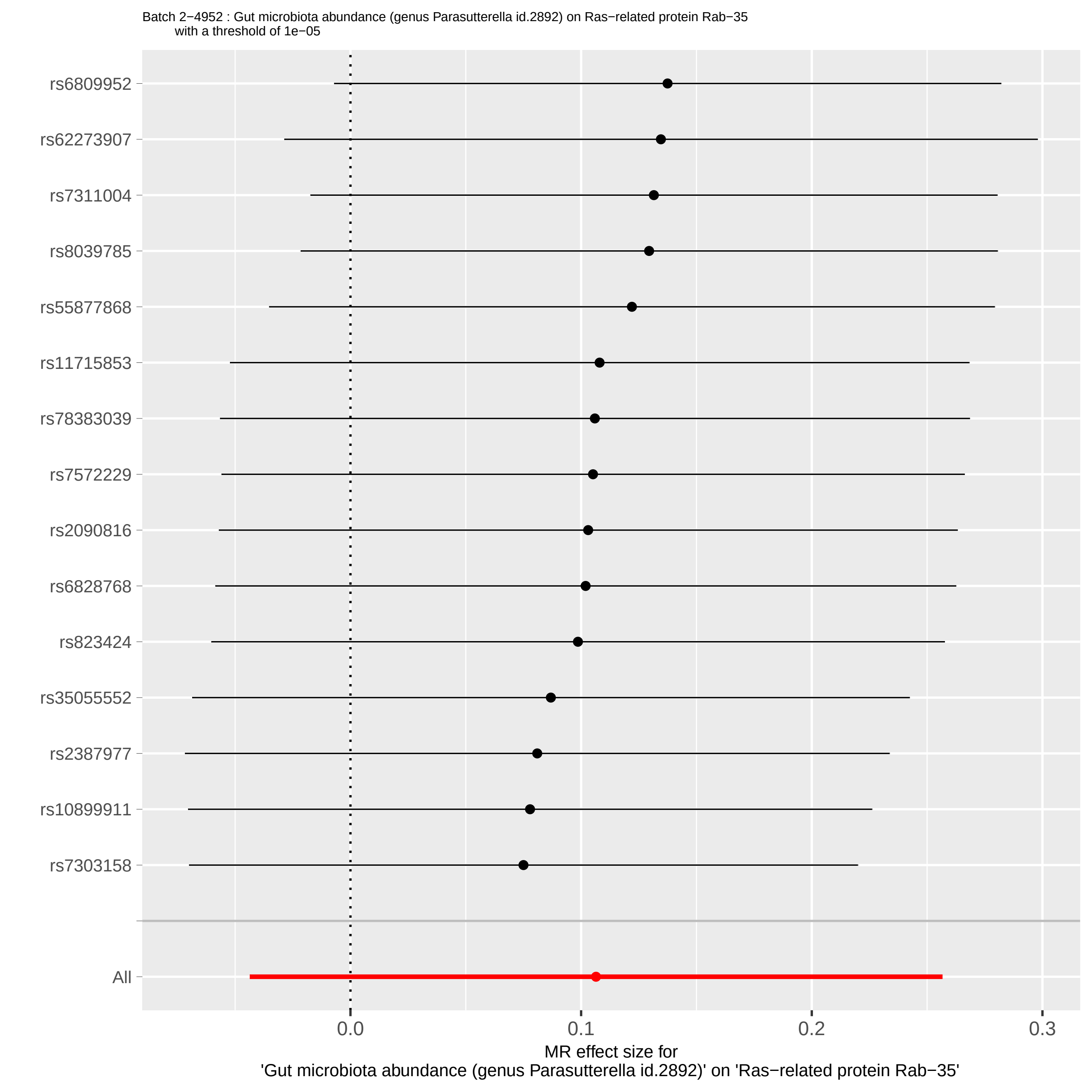

Batch 2−4952 : Gut microbiota abundance (genus Parasutterella id.2892) on Ras−related protein Rab−35
with a threshold of 1e−05
rs6809952
rs62273907
rs7311004
rs8039785
rs55877868
rs11715853
rs78383039
rs7572229
rs2090816
rs6828768
rs823424
rs35055552
rs2387977
rs10899911
rs7303158
All
0.0
0.1
0.2
0.3
MR effect size for
'Gut microbiota abundance (genus Parasutterella id.2892)' on 'Ras−related protein Rab−35'

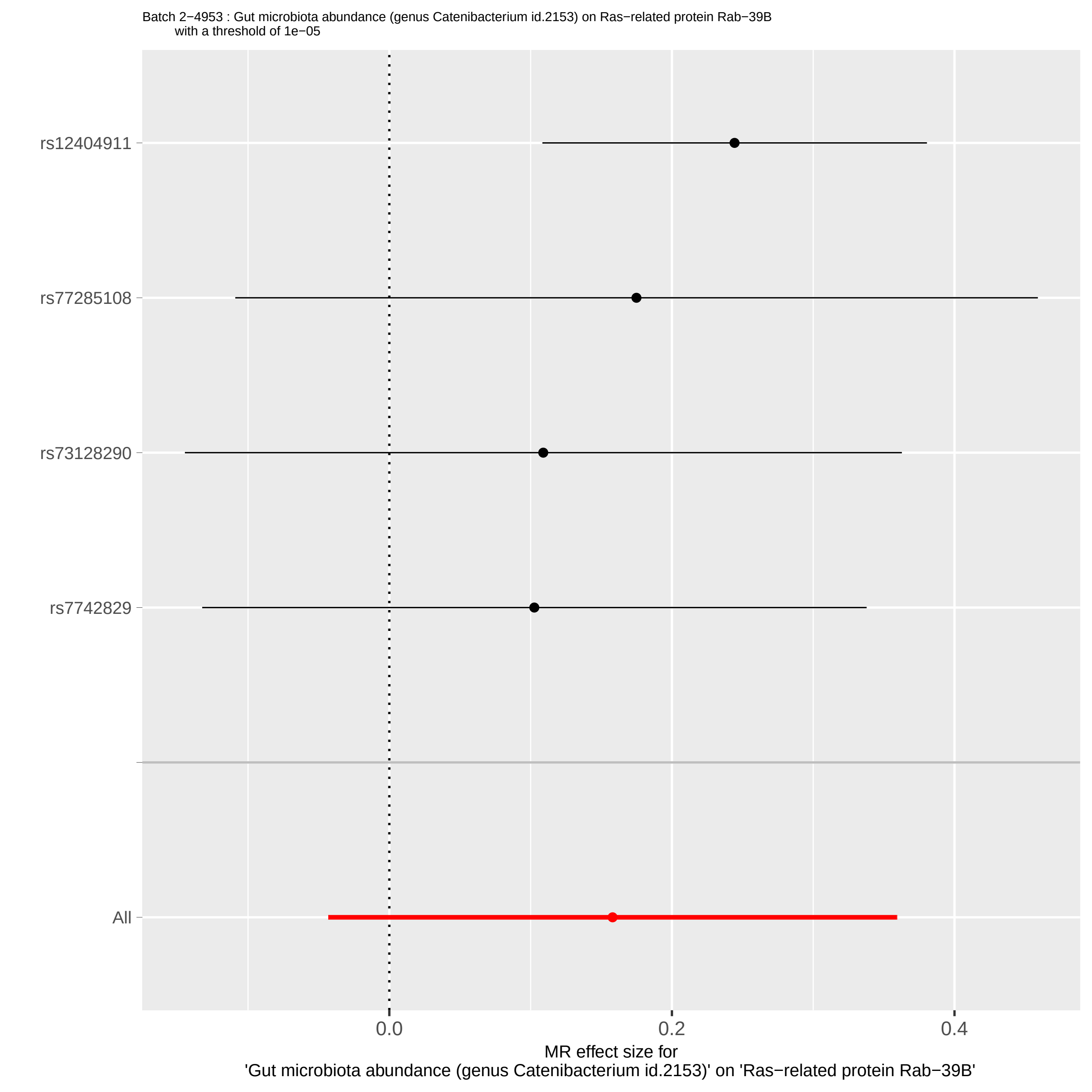
Batch 2−4953 : Gut microbiota abundance (genus Catenibacterium id.2153) on Ras−related protein Rab−39B
with a threshold of 1e−05
rs12404911
rs77285108
rs73128290
rs7742829
All
0.0
0.2
0.4
MR effect size for
'Gut microbiota abundance (genus Catenibacterium id.2153)' on 'Ras−related protein Rab−39B'

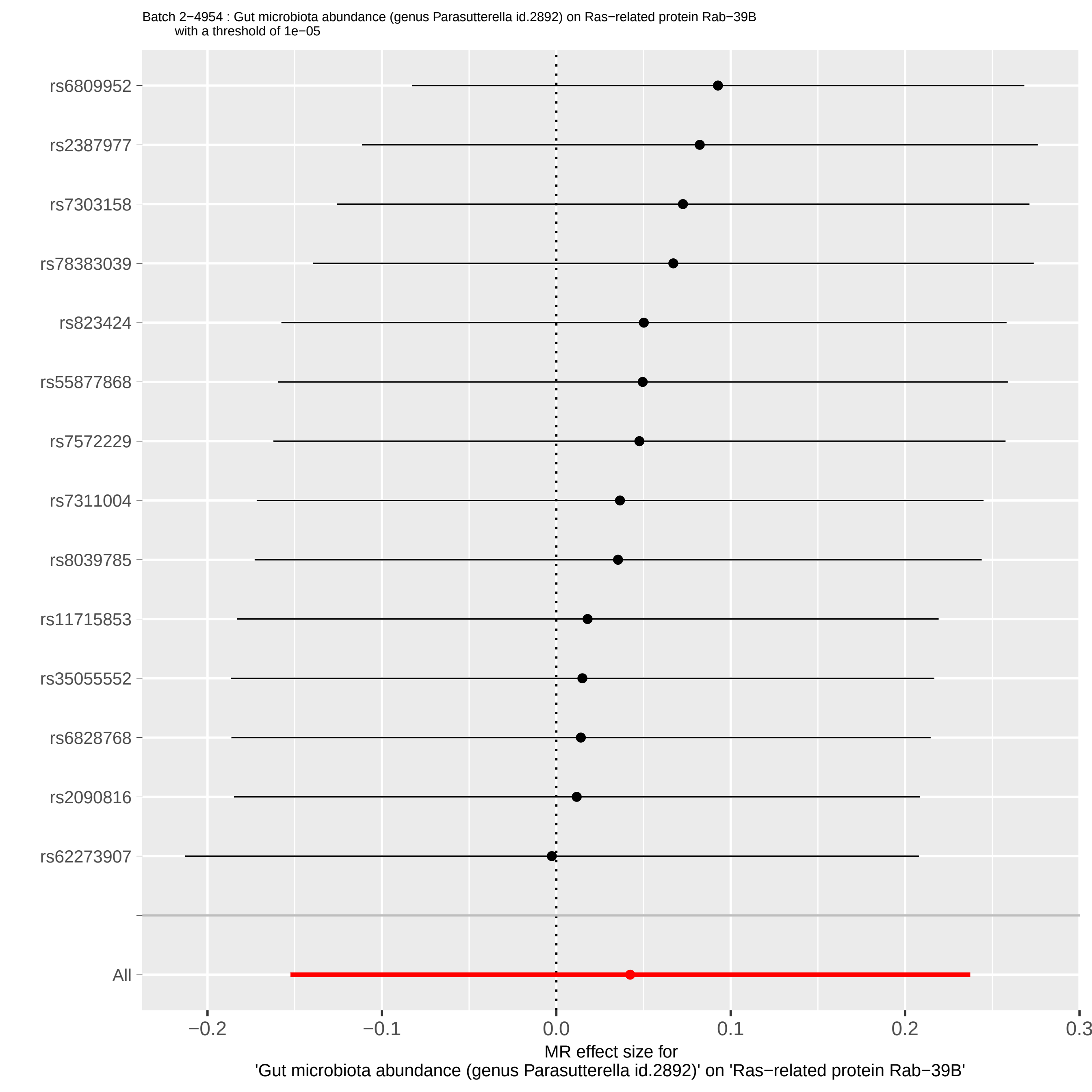

Batch 2−4954 : Gut microbiota abundance (genus Parasutterella id.2892) on Ras−related protein Rab−39B
with a threshold of 1e−05
rs6809952
rs2387977
rs7303158
rs78383039
rs823424
rs55877868
rs7572229
rs7311004
rs8039785
rs11715853
rs35055552
rs6828768
rs2090816
rs62273907
All
−0.2
−0.1
0.0
0.1
0.2
0.3
MR effect size for
'Gut microbiota abundance (genus Parasutterella id.2892)' on 'Ras−related protein Rab−39B'

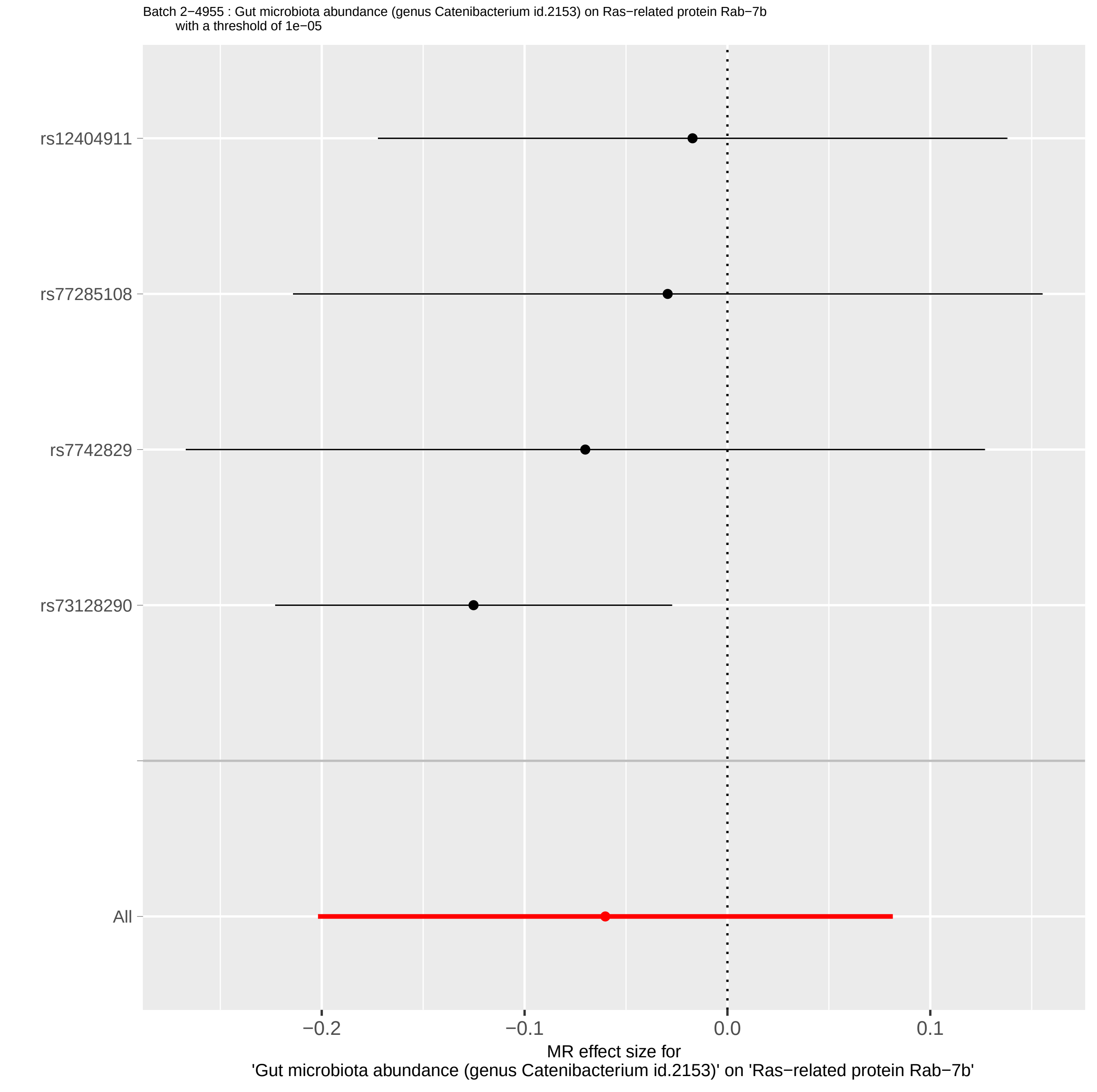
Batch 2−4955 : Gut microbiota abundance (genus Catenibacterium id.2153) on Ras−related protein Rab−7b
with a threshold of 1e−05
rs12404911
rs77285108
rs7742829
rs73128290
All
−0.2
−0.1
0.0
0.1
MR effect size for
'Gut microbiota abundance (genus Catenibacterium id.2153)' on 'Ras−related protein Rab−7b'

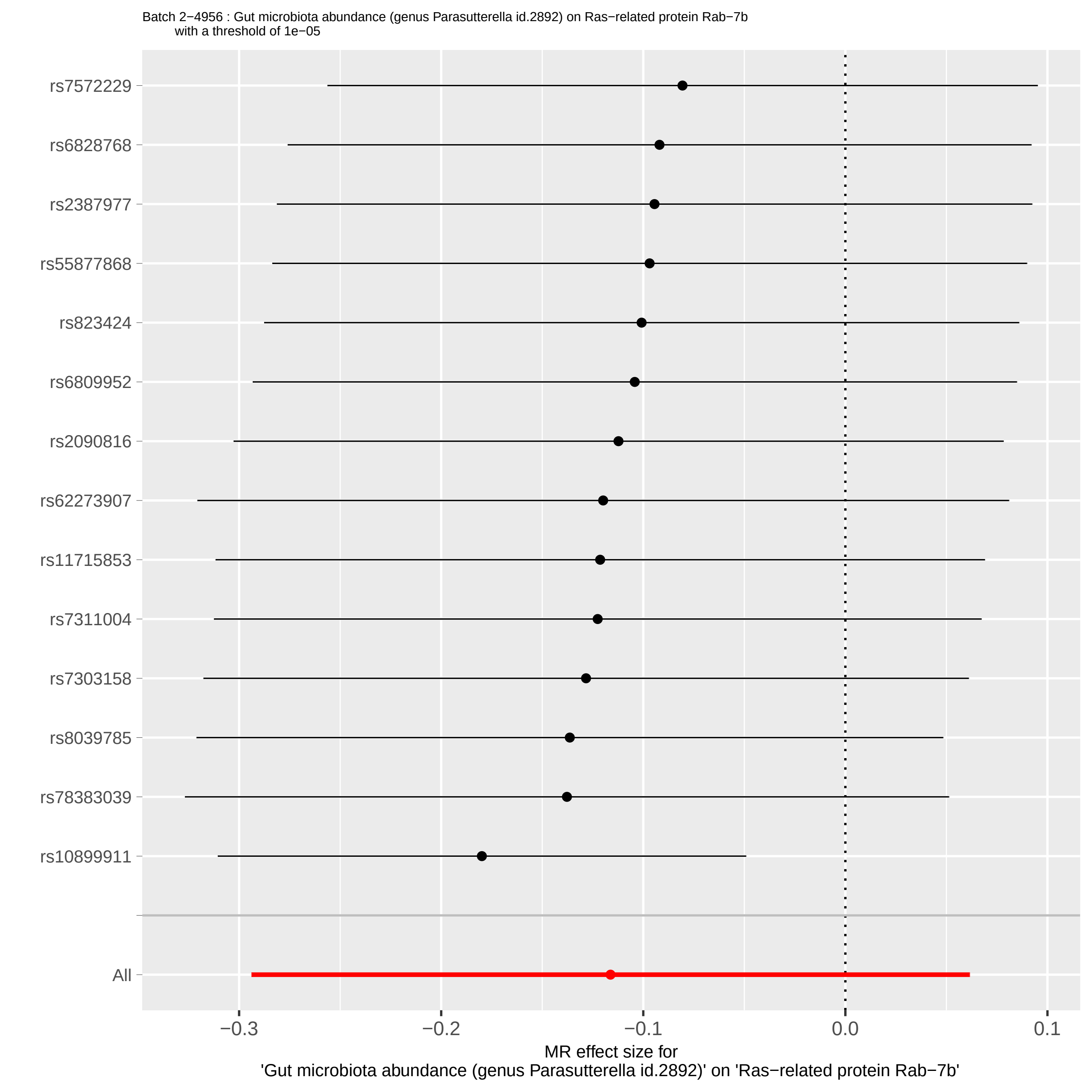

Batch 2−4956 : Gut microbiota abundance (genus Parasutterella id.2892) on Ras−related protein Rab−7b
with a threshold of 1e−05
rs7572229
rs6828768
rs2387977
rs55877868
rs823424
rs6809952
rs2090816
rs62273907
rs11715853
rs7311004
rs7303158
rs8039785
rs78383039
rs10899911
All
−0.3
−0.2
−0.1
0.0
0.1
MR effect size for
'Gut microbiota abundance (genus Parasutterella id.2892)' on 'Ras−related protein Rab−7b'

Batch 2−4957 : Gut microbiota abundance (genus Catenibacterium id.2153) on Rab9 effector protein with kelch motifs
with a threshold of 1e−05

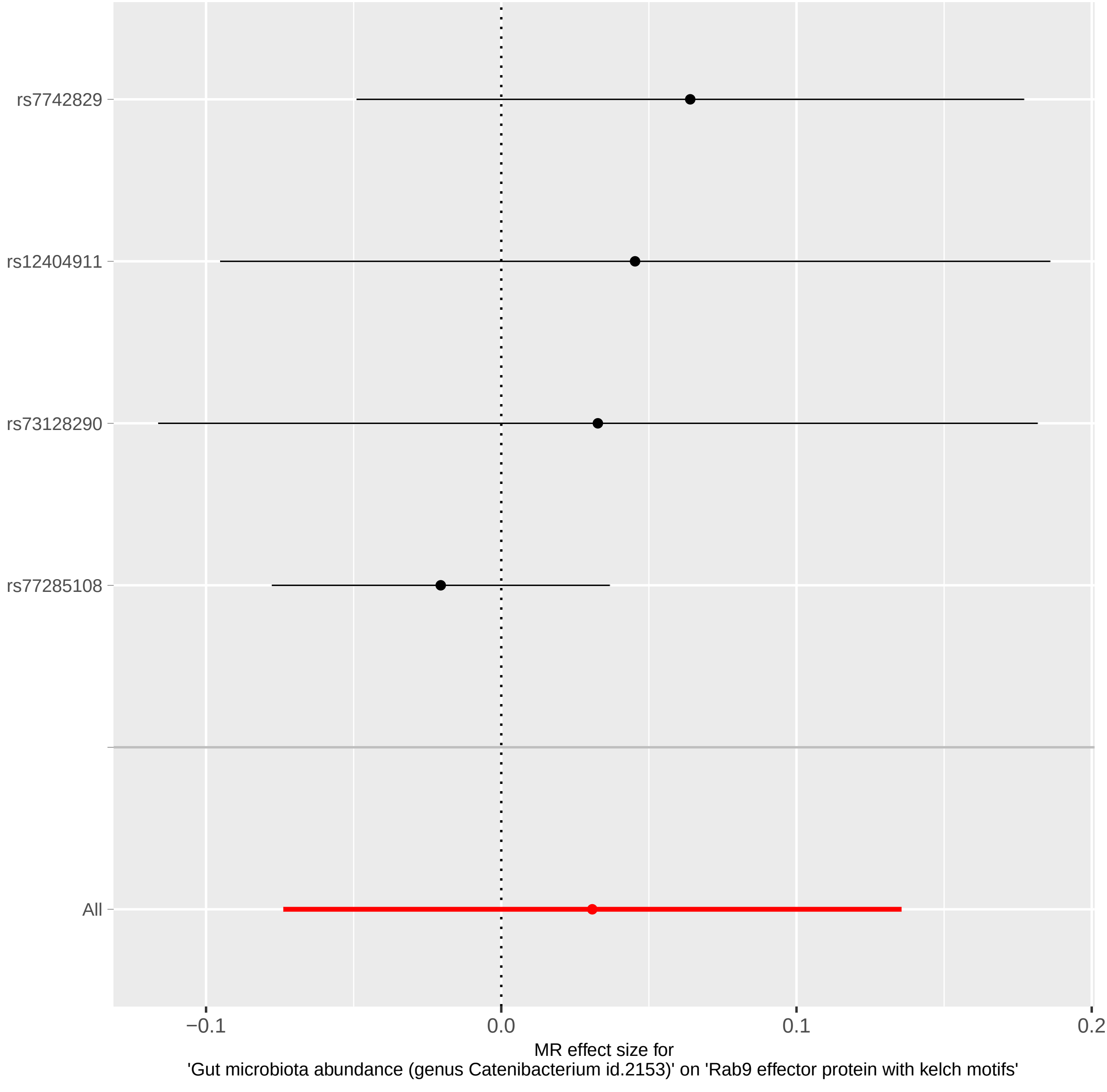

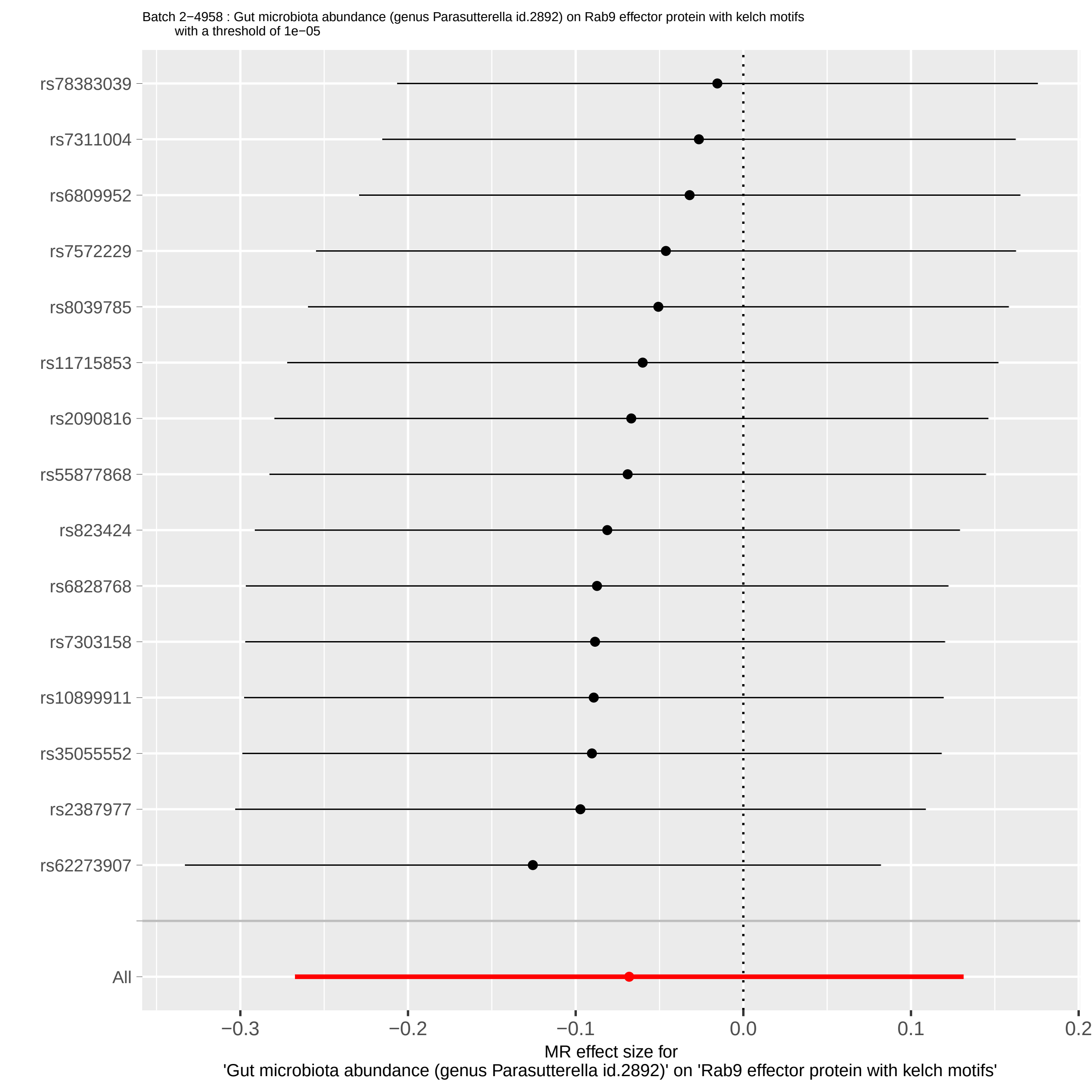

Batch 2−4958 : Gut microbiota abundance (genus Parasutterella id.2892) on Rab9 effector protein with kelch motifs
with a threshold of 1e−05
rs78383039
rs7311004
rs6809952
rs7572229
rs8039785
rs11715853
rs2090816
rs55877868
rs823424
rs6828768
rs7303158
rs10899911
rs35055552
rs2387977
rs62273907
All
−0.3
−0.2
−0.1
0.0
0.1
0.2
MR effect size for
'Gut microbiota abundance (genus Parasutterella id.2892)' on 'Rab9 effector protein with kelch motifs'

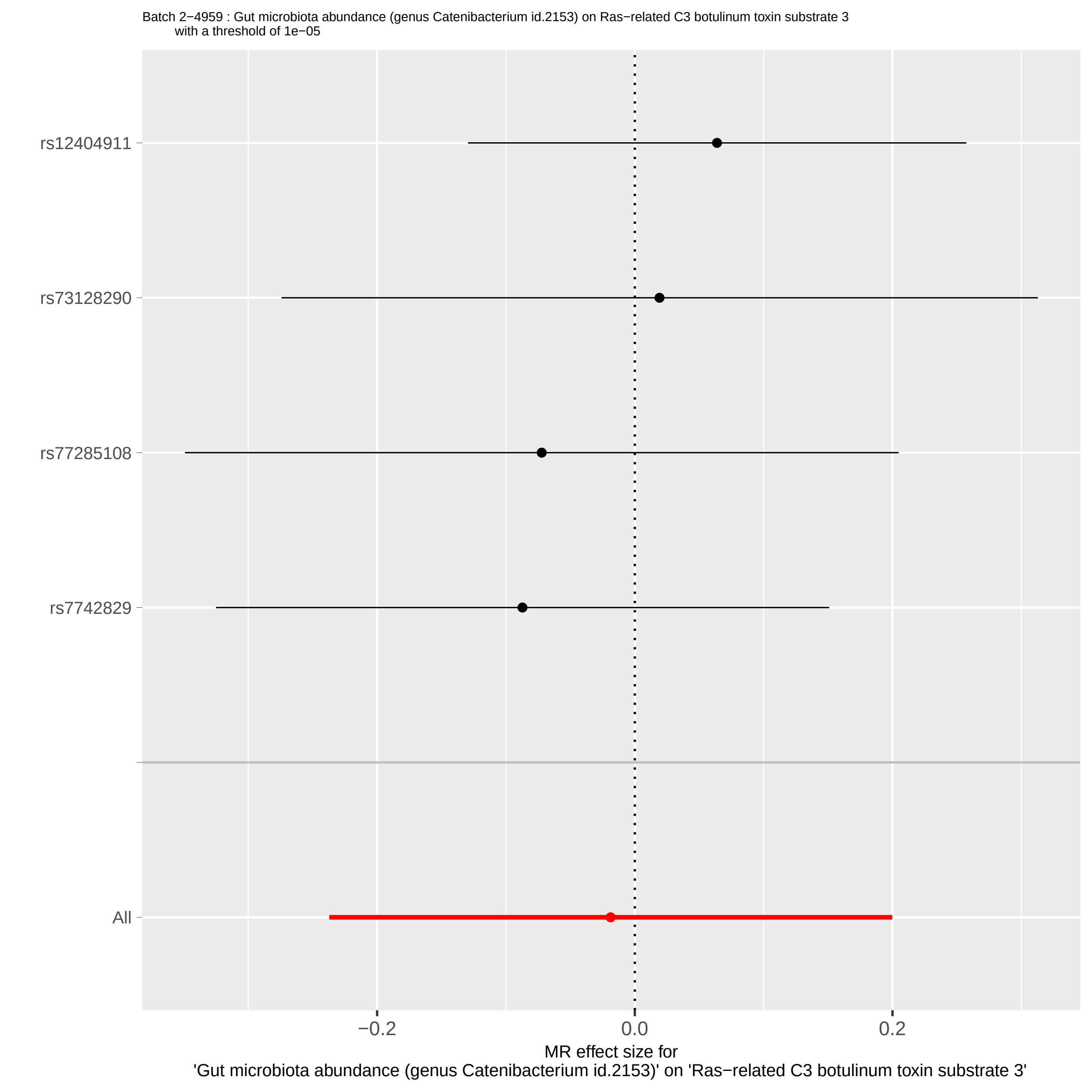

Batch 2−4959 : Gut microbiota abundance (genus Catenibacterium id.2153) on Ras−related C3 botulinum toxin substrate 3
with a threshold of 1e−05
rs12404911
rs73128290
rs77285108
rs7742829
All
−0.2
0.0
0.2
MR effect size for
'Gut microbiota abundance (genus Catenibacterium id.2153)' on 'Ras−related C3 botulinum toxin substrate 3'

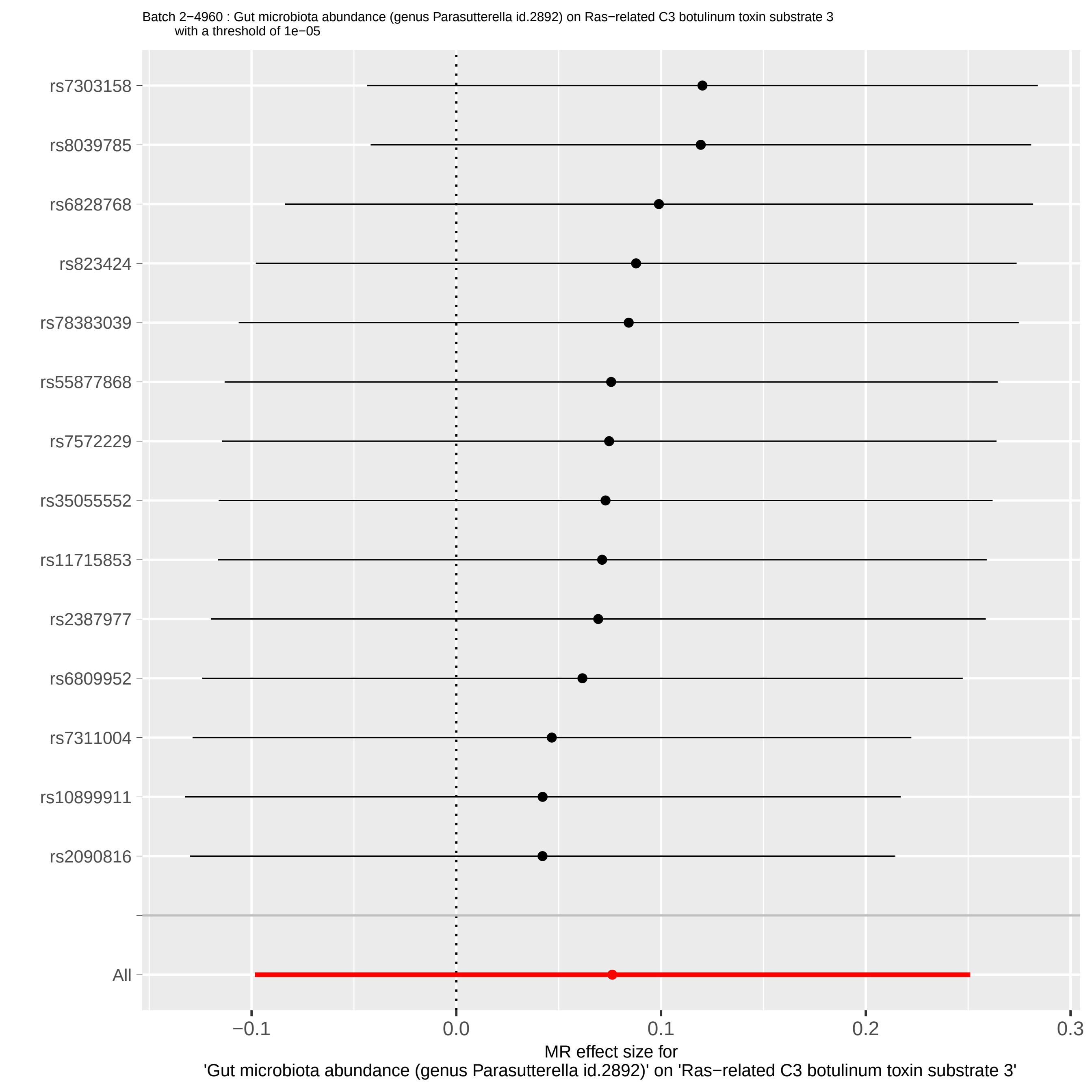

Batch 2−4960 : Gut microbiota abundance (genus Parasutterella id.2892) on Ras−related C3 botulinum toxin substrate 3
with a threshold of 1e−05
rs7303158
rs8039785
rs6828768
rs823424
rs78383039
rs55877868
rs7572229
rs35055552
rs11715853
rs2387977
rs6809952
rs7311004
rs10899911
rs2090816
All
−0.1
0.0
0.1
0.2
0.3
MR effect size for
'Gut microbiota abundance (genus Parasutterella id.2892)' on 'Ras−related C3 botulinum toxin substrate 3'

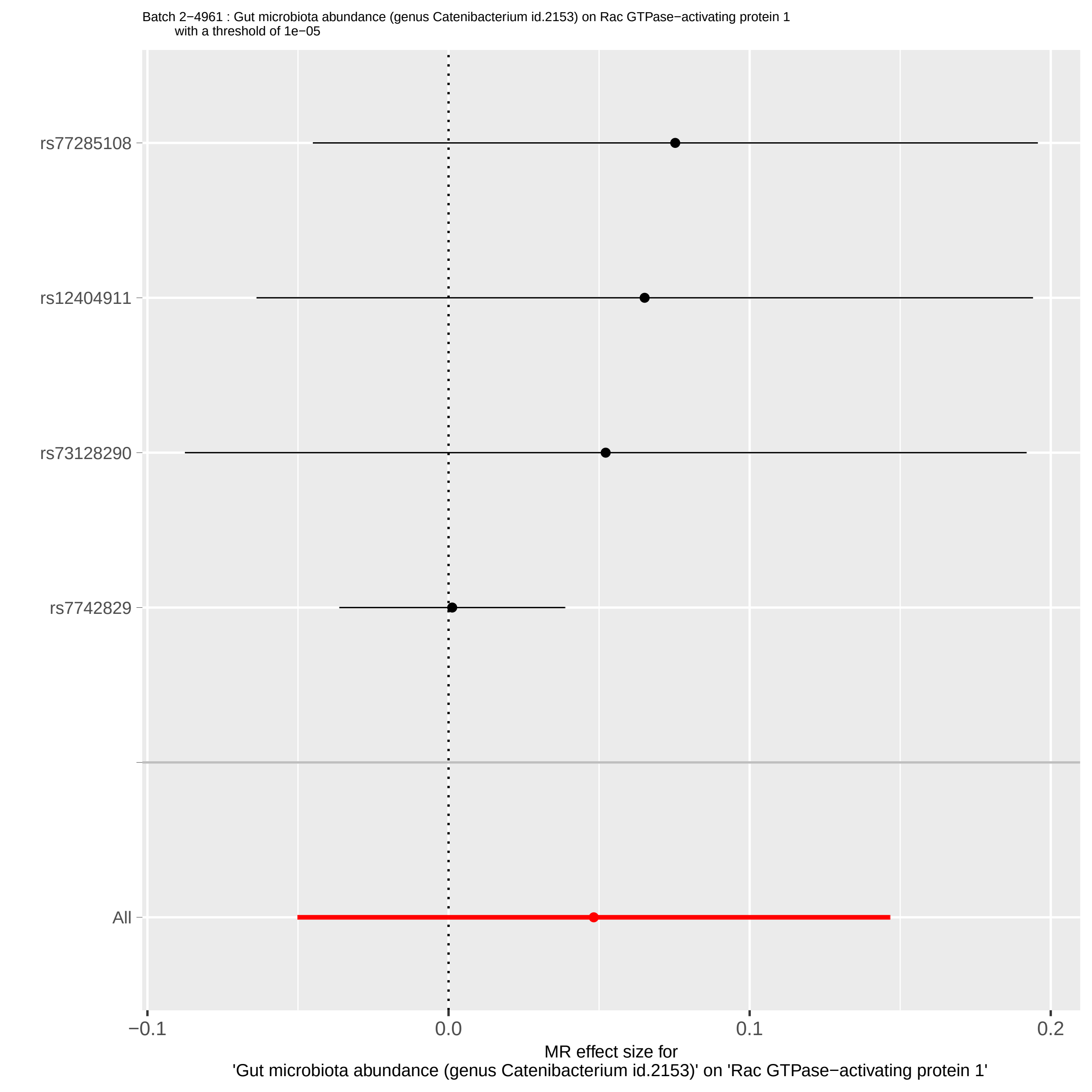

Batch 2−4961 : Gut microbiota abundance (genus Catenibacterium id.2153) on Rac GTPase−activating protein 1
with a threshold of 1e−05
rs77285108
rs12404911
rs73128290
rs7742829
All
−0.1
0.0
0.1
0.2
MR effect size for
'Gut microbiota abundance (genus Catenibacterium id.2153)' on 'Rac GTPase−activating protein 1'

Batch 2−4962 : Gut microbiota abundance (genus Parasutterella id.2892) on Rac GTPase−activating protein 1 with a threshold of 1e−05

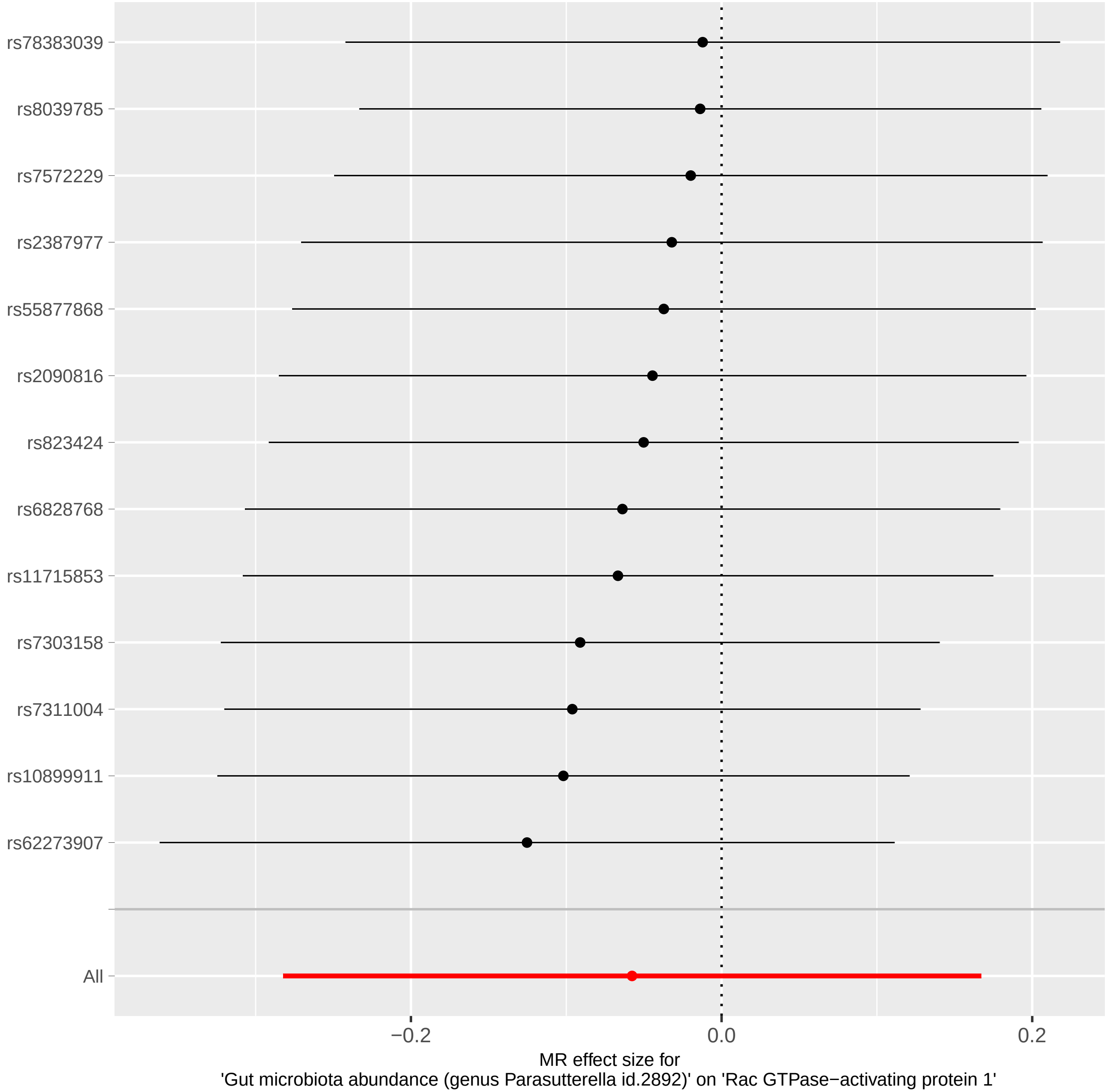

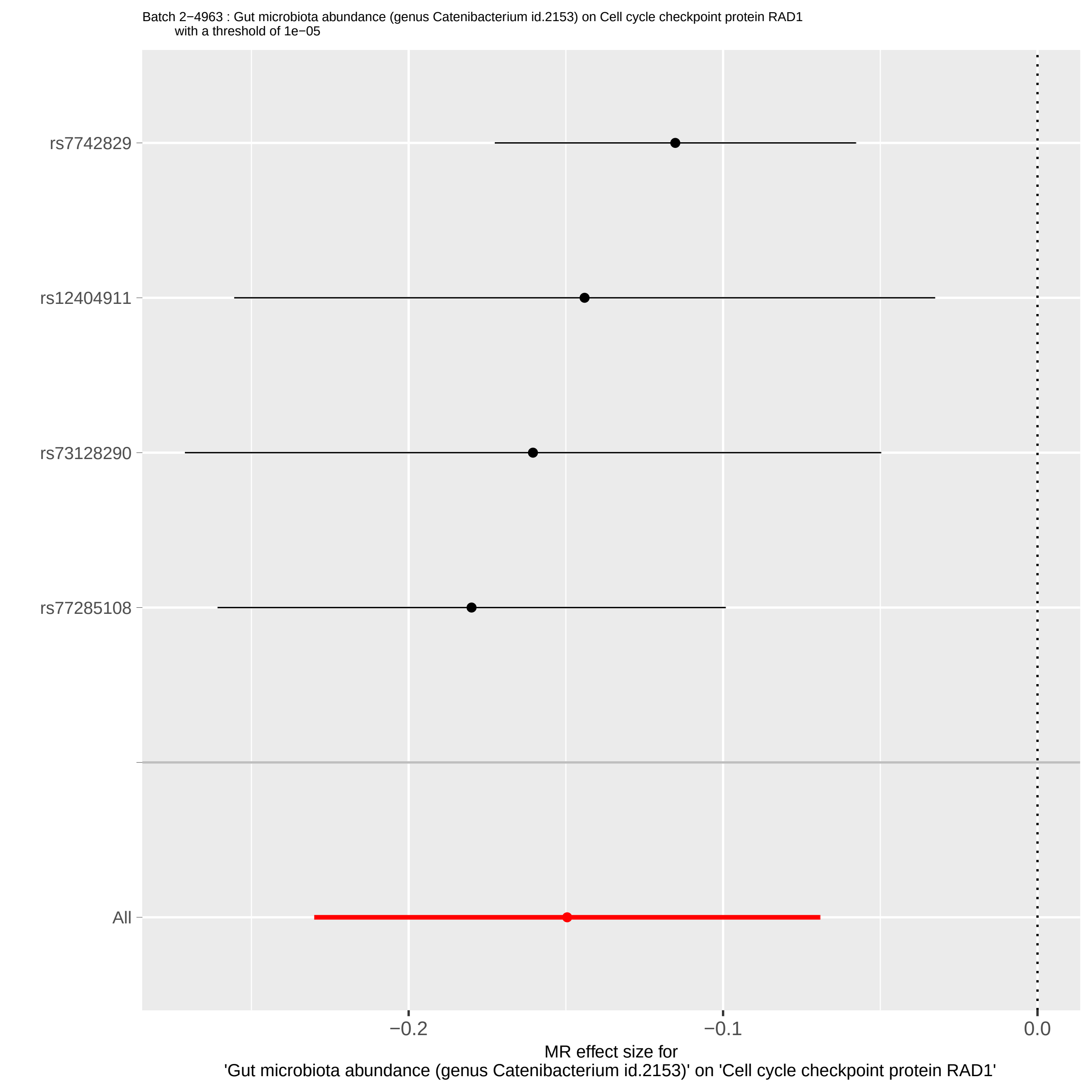
Batch 2−4963 : Gut microbiota abundance (genus Catenibacterium id.2153) on Cell cycle checkpoint protein RAD1
with a threshold of 1e−05
rs7742829
rs12404911
rs73128290
rs77285108
All
−0.2
−0.1
0.0
MR effect size for
'Gut microbiota abundance (genus Catenibacterium id.2153)' on 'Cell cycle checkpoint protein RAD1'

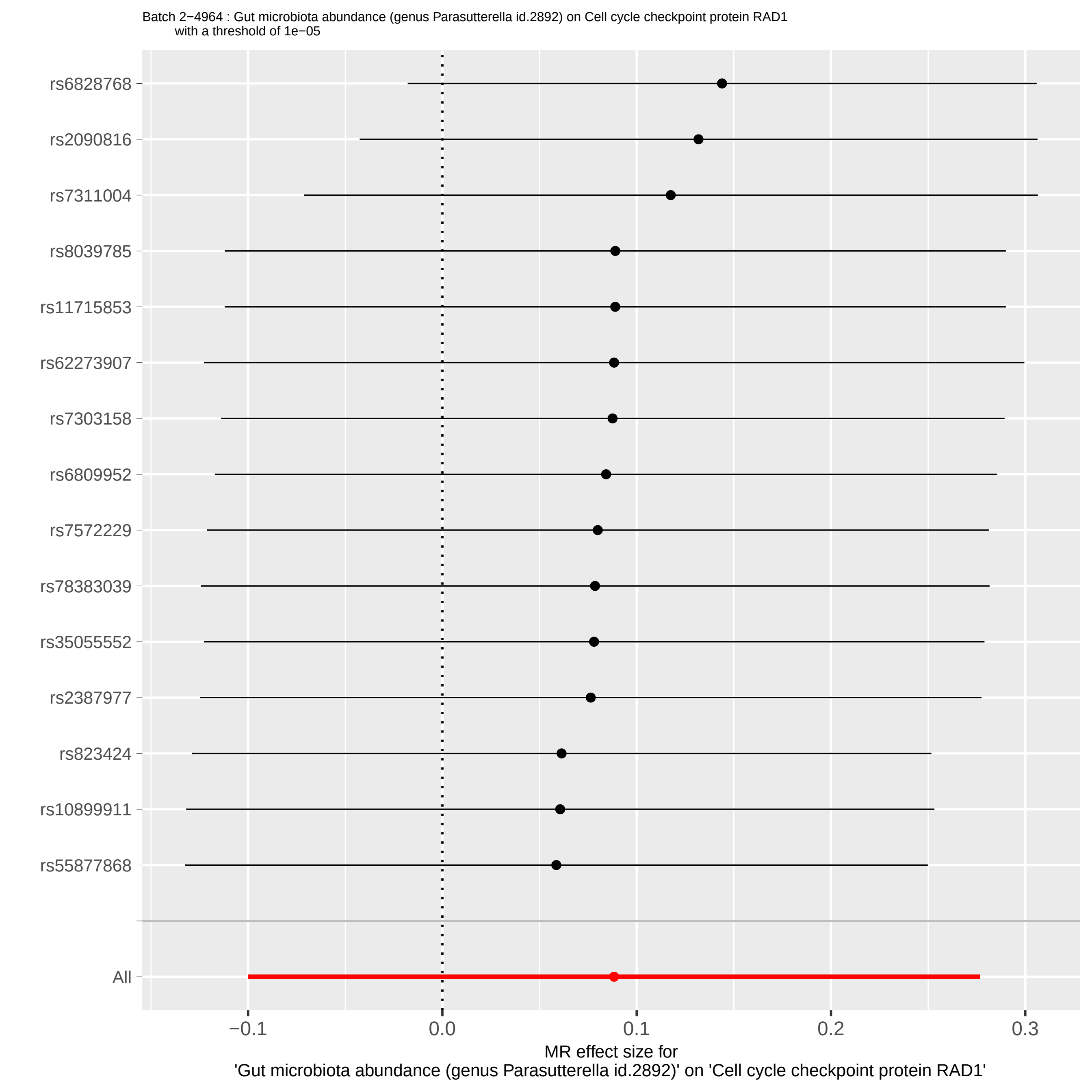
Batch 2−4964 : Gut microbiota abundance (genus Parasutterella id.2892) on Cell cycle checkpoint protein RAD1
with a threshold of 1e−05
rs6828768
rs2090816
rs7311004
rs8039785
rs11715853
rs62273907
rs7303158
rs6809952
rs7572229
rs78383039
rs35055552
rs2387977
rs823424
rs10899911
rs55877868
All
−0.1
0.0
0.1
0.2
0.3
MR effect size for
'Gut microbiota abundance (genus Parasutterella id.2892)' on 'Cell cycle checkpoint protein RAD1'

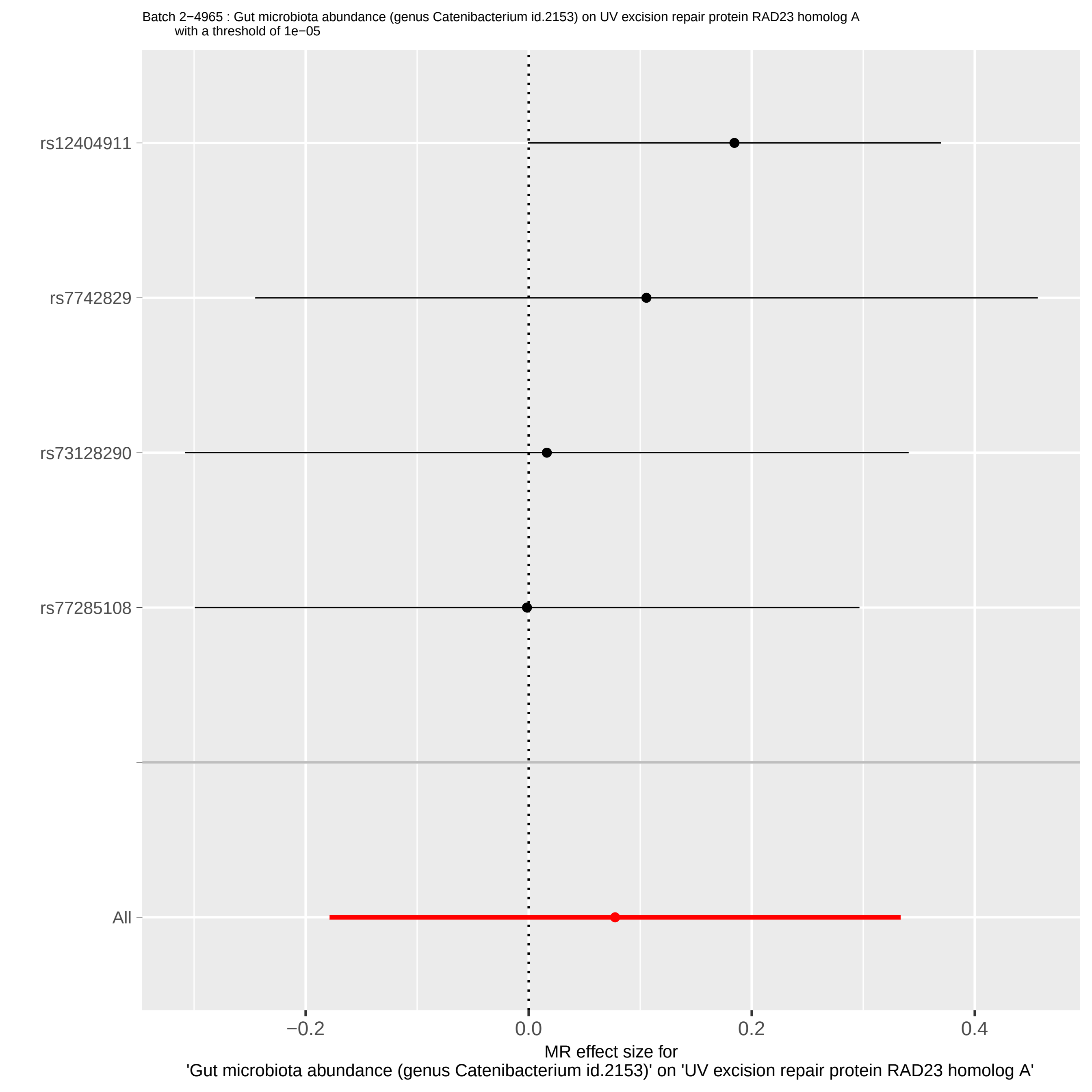
Batch 2−4965 : Gut microbiota abundance (genus Catenibacterium id.2153) on UV excision repair protein RAD23 homolog A
with a threshold of 1e−05
rs12404911
rs7742829
rs73128290
rs77285108
All
−0.2
0.0
0.2
0.4
MR effect size for
'Gut microbiota abundance (genus Catenibacterium id.2153)' on 'UV excision repair protein RAD23 homolog A'

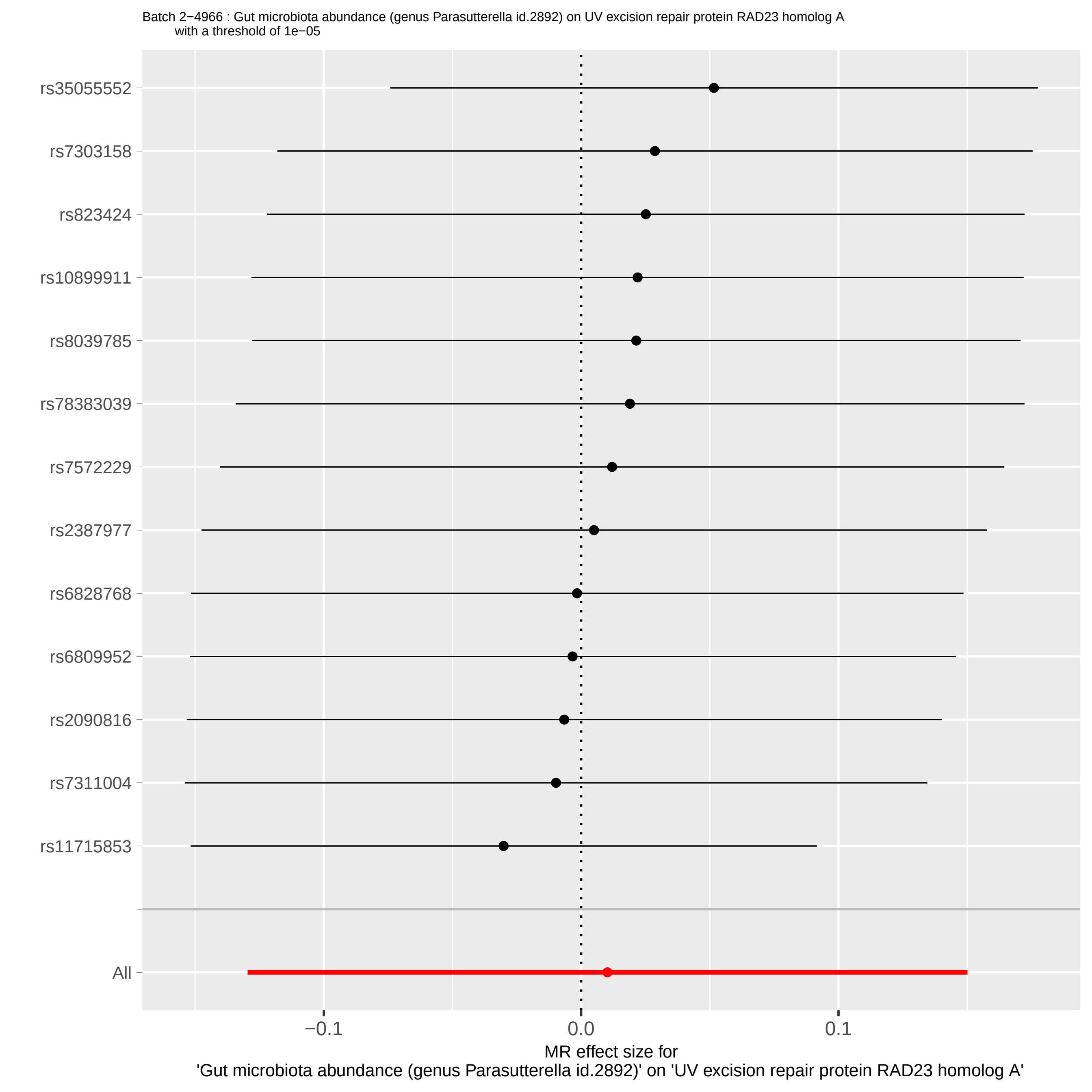
Batch 2−4966 : Gut microbiota abundance (genus Parasutterella id.2892) on UV excision repair protein RAD23 homolog A
with a threshold of 1e−05
rs35055552
rs7303158
rs823424
rs10899911
rs8039785
rs78383039
rs7572229
rs2387977
rs6828768
rs6809952
rs2090816
rs7311004
rs11715853
All
−0.1
0.0
0.1
MR effect size for
'Gut microbiota abundance (genus Parasutterella id.2892)' on 'UV excision repair protein RAD23 homolog A'

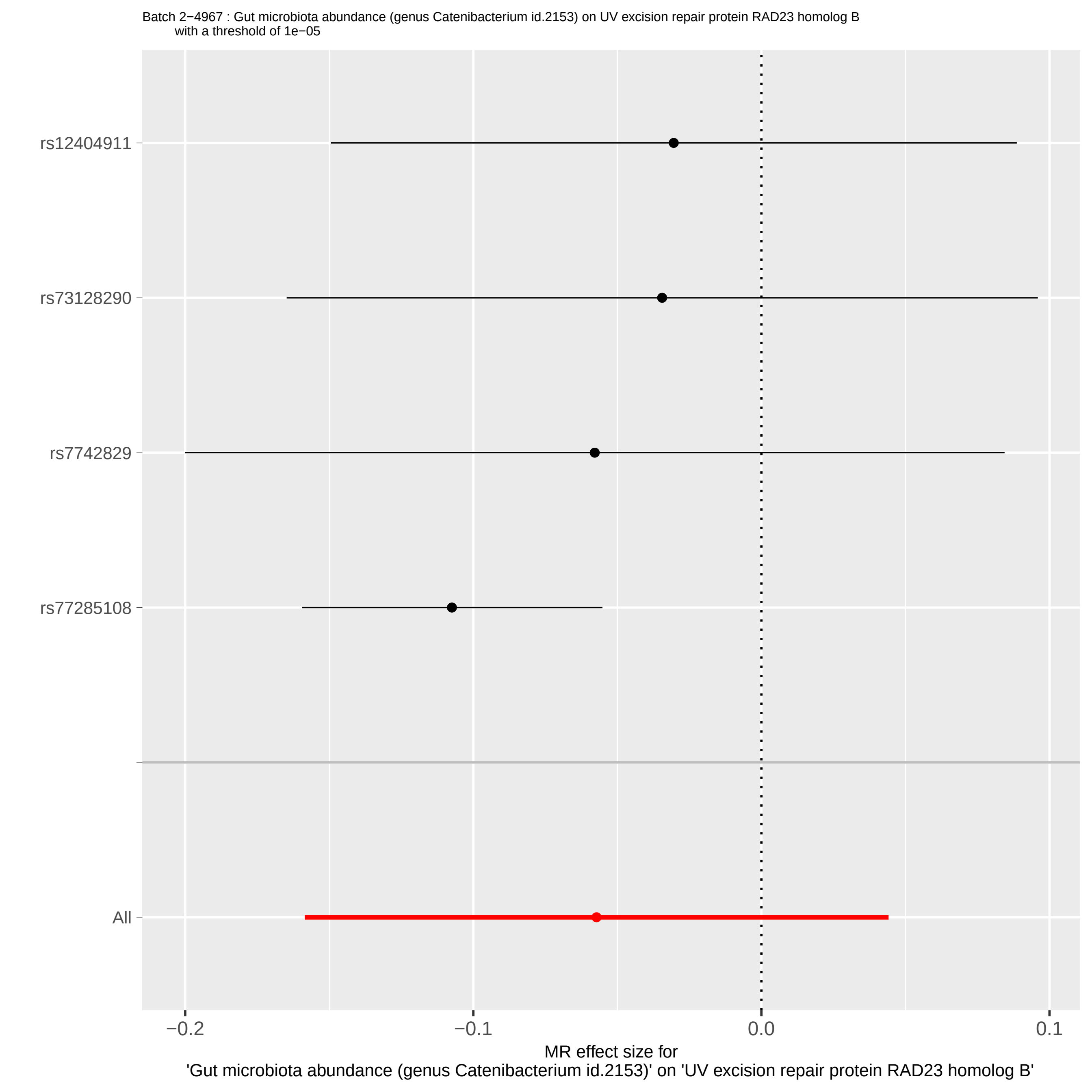
Batch 2−4967 : Gut microbiota abundance (genus Catenibacterium id.2153) on UV excision repair protein RAD23 homolog B
with a threshold of 1e−05
rs12404911
rs73128290
rs7742829
rs77285108
All
−0.2
−0.1
0.0
0.1
MR effect size for
'Gut microbiota abundance (genus Catenibacterium id.2153)' on 'UV excision repair protein RAD23 homolog B'

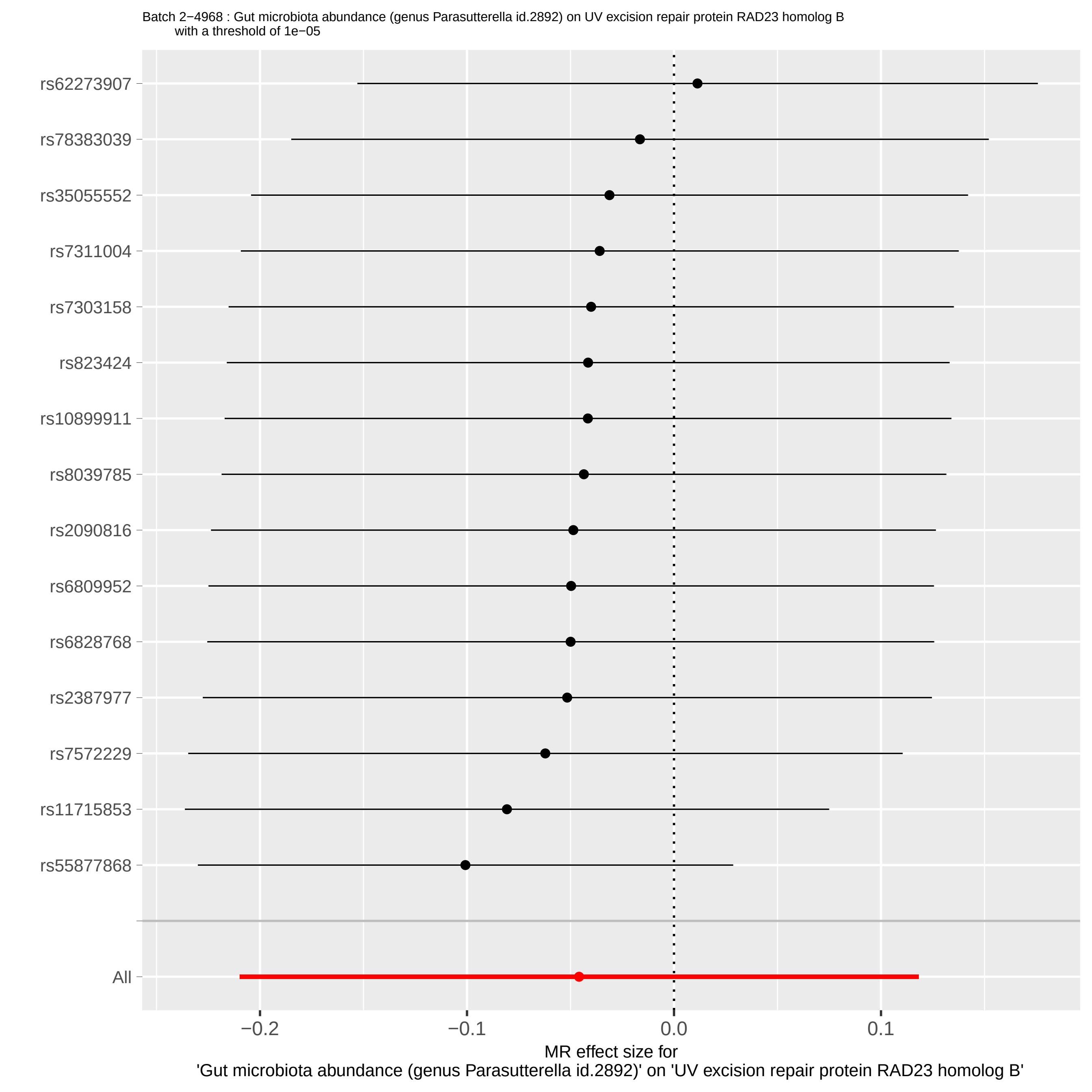
Batch 2−4968 : Gut microbiota abundance (genus Parasutterella id.2892) on UV excision repair protein RAD23 homolog B
with a threshold of 1e−05
rs62273907
rs78383039
rs35055552
rs7311004
rs7303158
rs823424
rs10899911
rs8039785
rs2090816
rs6809952
rs6828768
rs2387977
rs7572229
rs11715853
rs55877868
All
−0.2
−0.1
0.0
0.1
MR effect size for
'Gut microbiota abundance (genus Parasutterella id.2892)' on 'UV excision repair protein RAD23 homolog B'

Batch 2−4969 : Gut microbiota abundance (genus Catenibacterium id.2153) on DNA repair protein RAD51 homolog 1
with a threshold of 1e−05

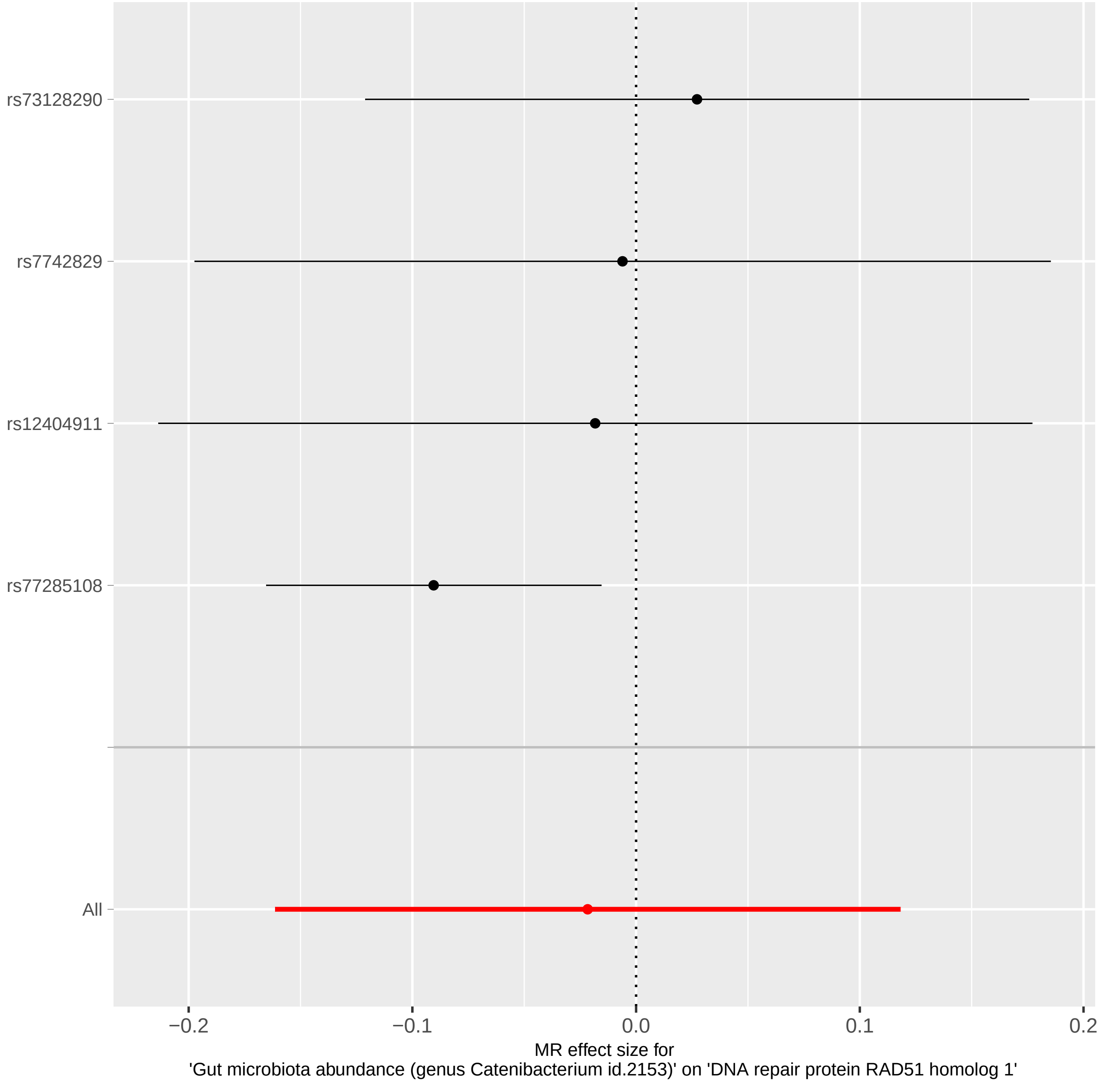

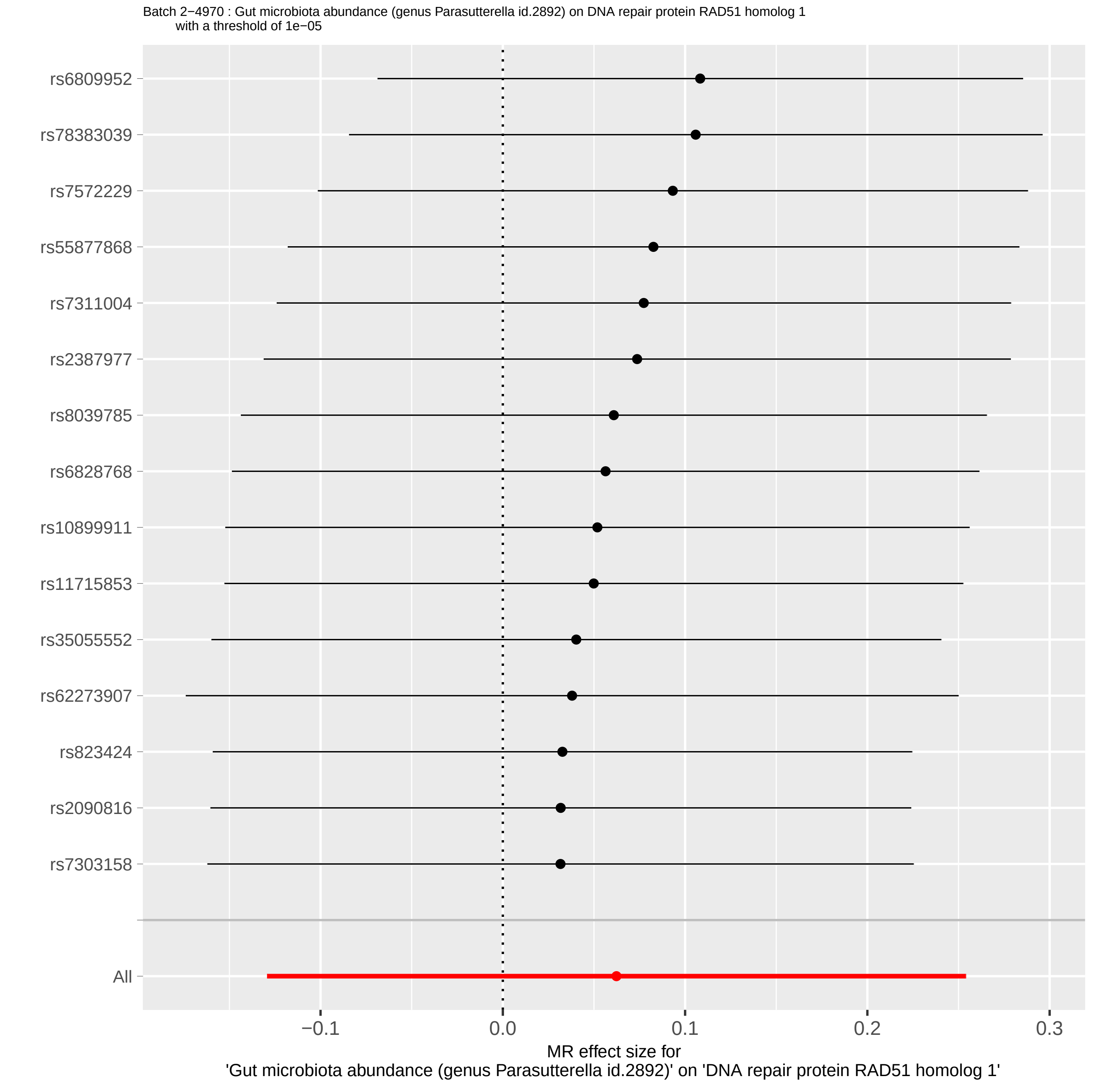
Batch 2−4970 : Gut microbiota abundance (genus Parasutterella id.2892) on DNA repair protein RAD51 homolog 1
with a threshold of 1e−05
rs6809952
rs78383039
rs7572229
rs55877868
rs7311004
rs2387977
rs8039785
rs6828768
rs10899911
rs11715853
rs35055552
rs62273907
rs823424
rs2090816
rs7303158
All
−0.1
0.0
0.1
0.2
0.3
MR effect size for
'Gut microbiota abundance (genus Parasutterella id.2892)' on 'DNA repair protein RAD51 homolog 1'

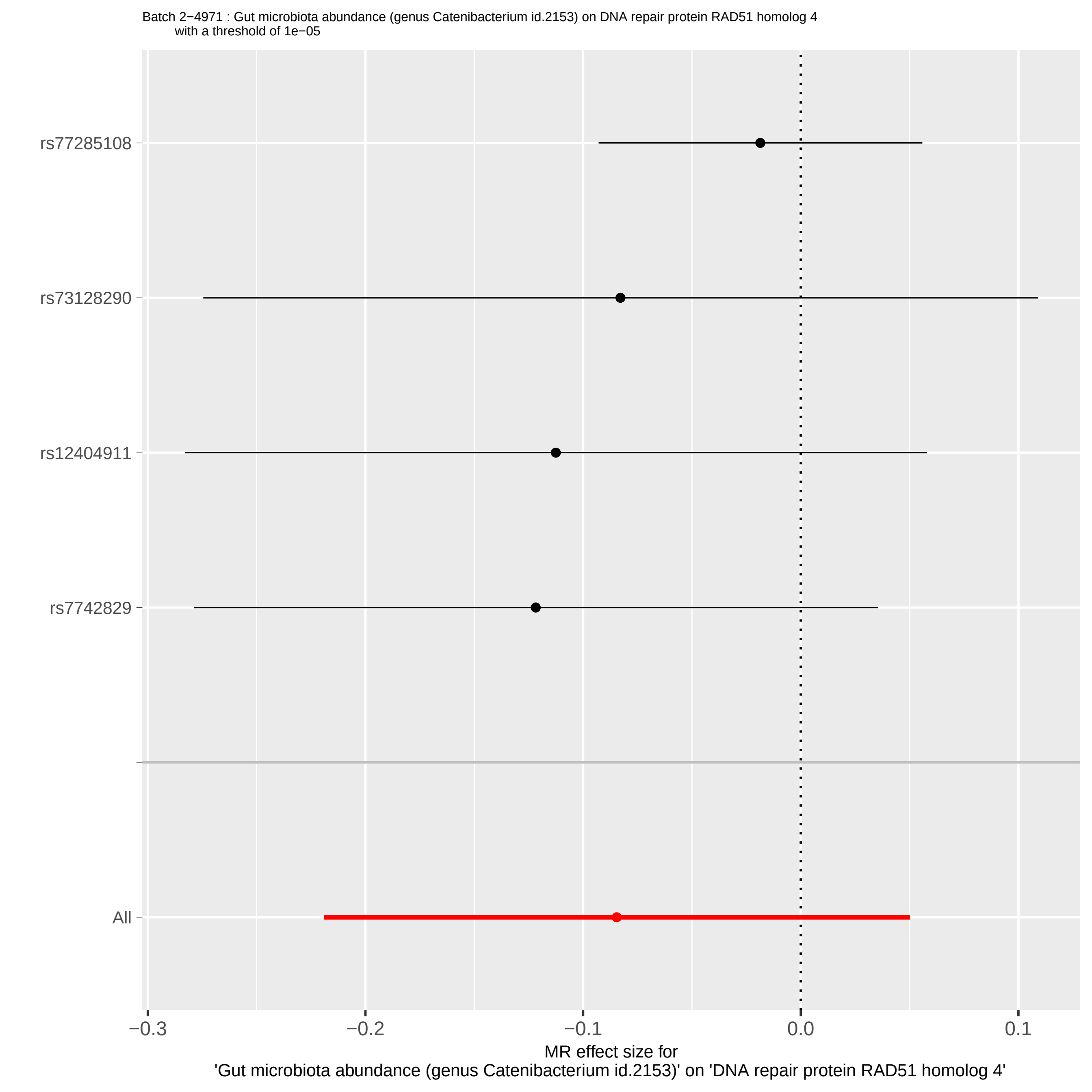

Batch 2−4971 : Gut microbiota abundance (genus Catenibacterium id.2153) on DNA repair protein RAD51 homolog 4
with a threshold of 1e−05
rs77285108
rs73128290
rs12404911
rs7742829
All
−0.3
−0.2
−0.1
0.0
0.1
MR effect size for
'Gut microbiota abundance (genus Catenibacterium id.2153)' on 'DNA repair protein RAD51 homolog 4'

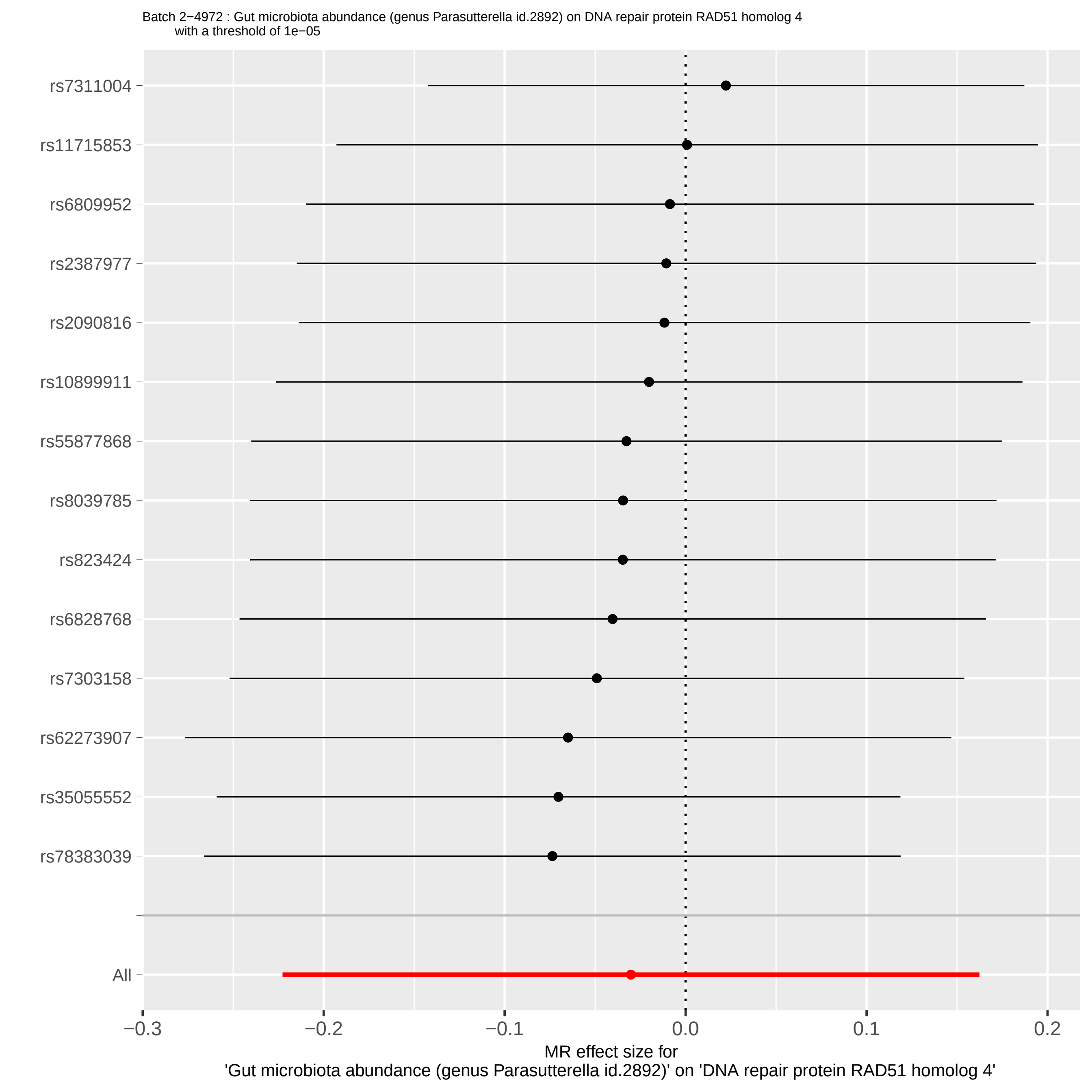

Batch 2−4972 : Gut microbiota abundance (genus Parasutterella id.2892) on DNA repair protein RAD51 homolog 4
with a threshold of 1e−05
rs7311004
rs11715853
rs6809952
rs2387977
rs2090816
rs10899911
rs55877868
rs8039785
rs823424
rs6828768
rs7303158
rs62273907
rs35055552
rs78383039
All
−0.3
−0.2
−0.1
0.0
0.1
0.2
MR effect size for
'Gut microbiota abundance (genus Parasutterella id.2892)' on 'DNA repair protein RAD51 homolog 4'

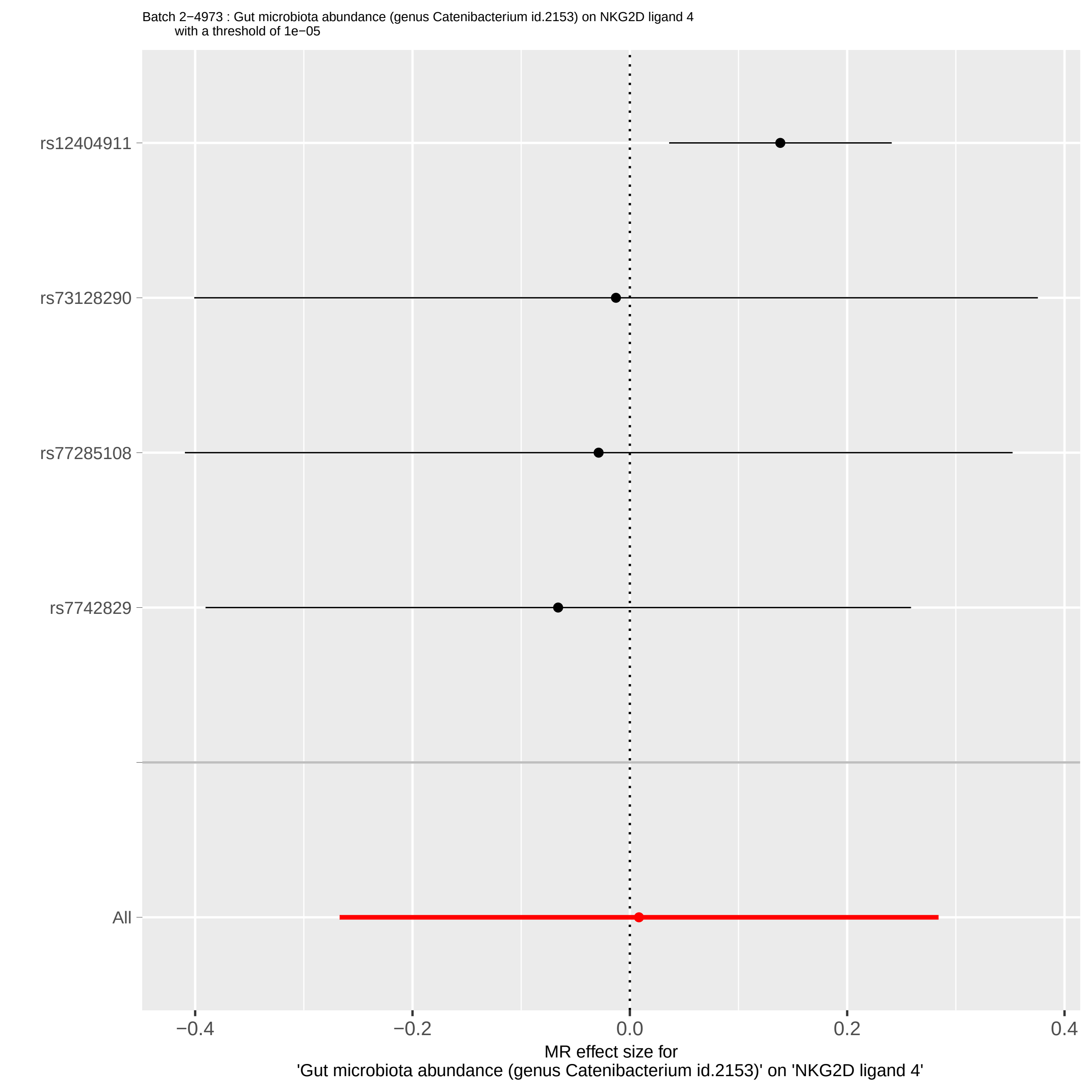
Batch 2−4973 : Gut microbiota abundance (genus Catenibacterium id.2153) on NKG2D ligand 4
with a threshold of 1e−05
rs12404911
rs73128290
rs77285108
rs7742829
All
−0.4
−0.2
0.0
0.2
0.4
MR effect size for
'Gut microbiota abundance (genus Catenibacterium id.2153)' on 'NKG2D ligand 4'

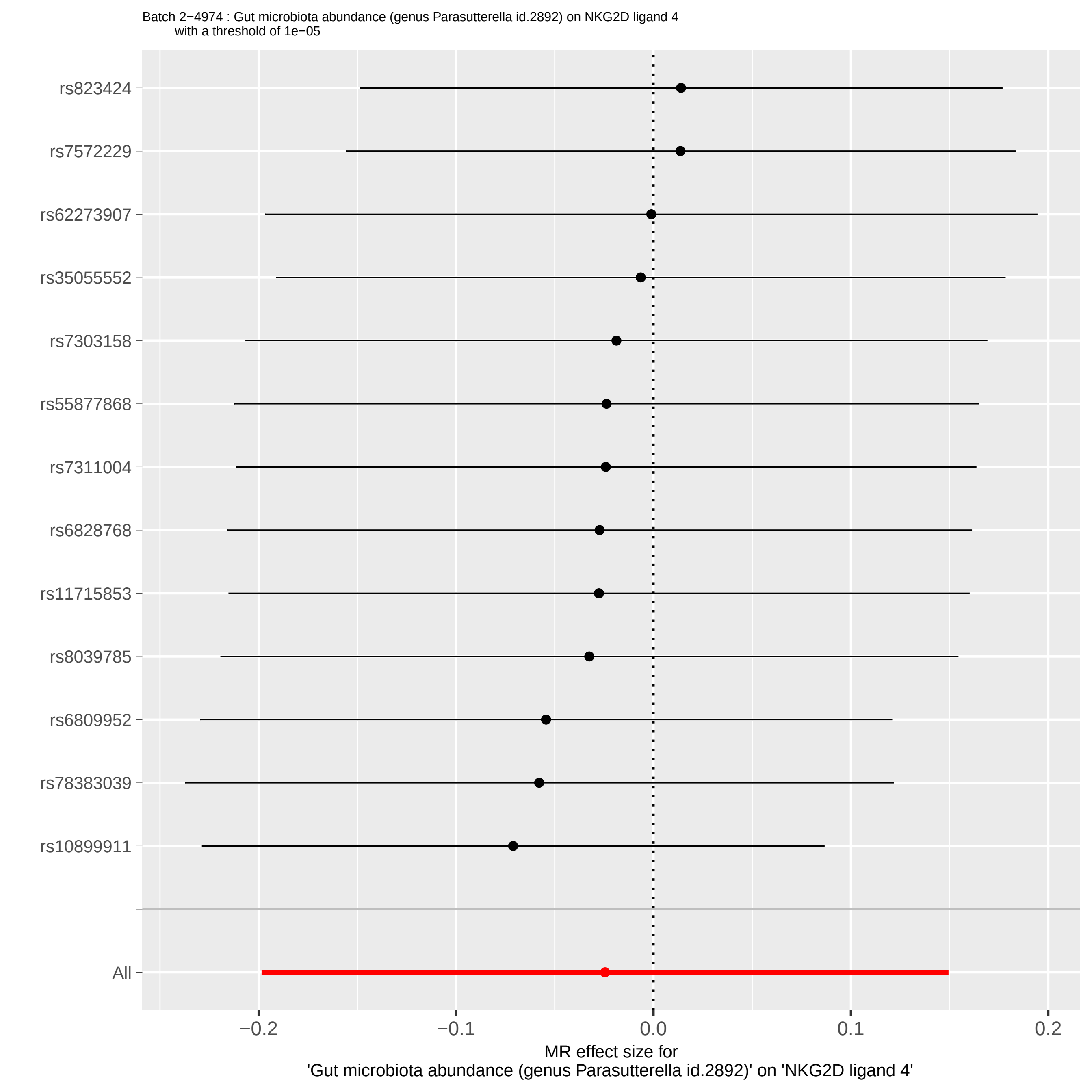

Batch 2−4974 : Gut microbiota abundance (genus Parasutterella id.2892) on NKG2D ligand 4
with a threshold of 1e−05
rs823424
rs7572229
rs62273907
rs35055552
rs7303158
rs55877868
rs7311004
rs6828768
rs11715853
rs8039785
rs6809952
rs78383039
rs10899911
All
−0.2
−0.1
0.0
0.1
0.2
MR effect size for
'Gut microbiota abundance (genus Parasutterella id.2892)' on 'NKG2D ligand 4'

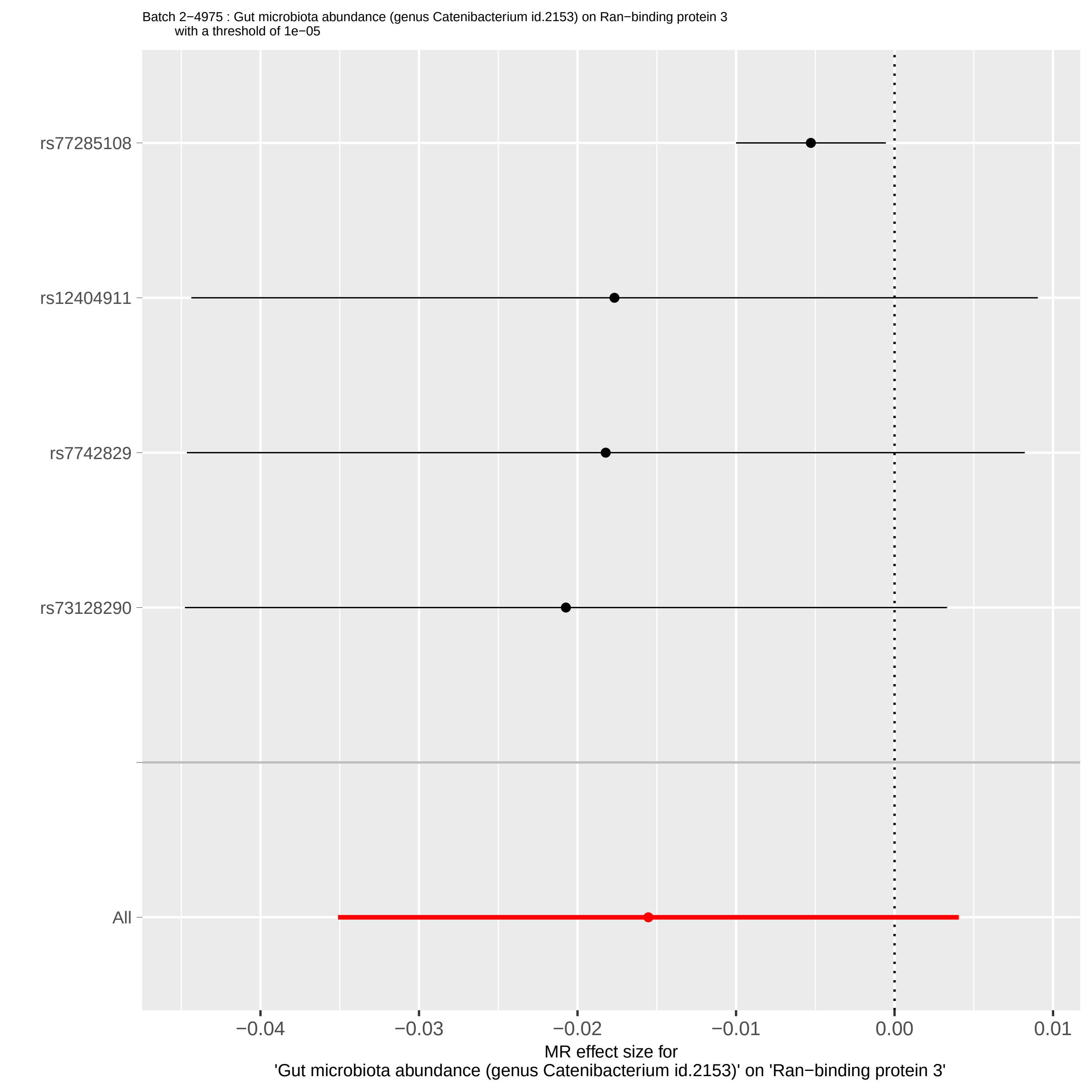

Batch 2−4975 : Gut microbiota abundance (genus Catenibacterium id.2153) on Ran−binding protein 3
with a threshold of 1e−05
rs77285108
rs12404911
rs7742829
rs73128290
All
−0.04
−0.03
−0.02
−0.01
0.00
0.01
MR effect size for
'Gut microbiota abundance (genus Catenibacterium id.2153)' on 'Ran−binding protein 3'

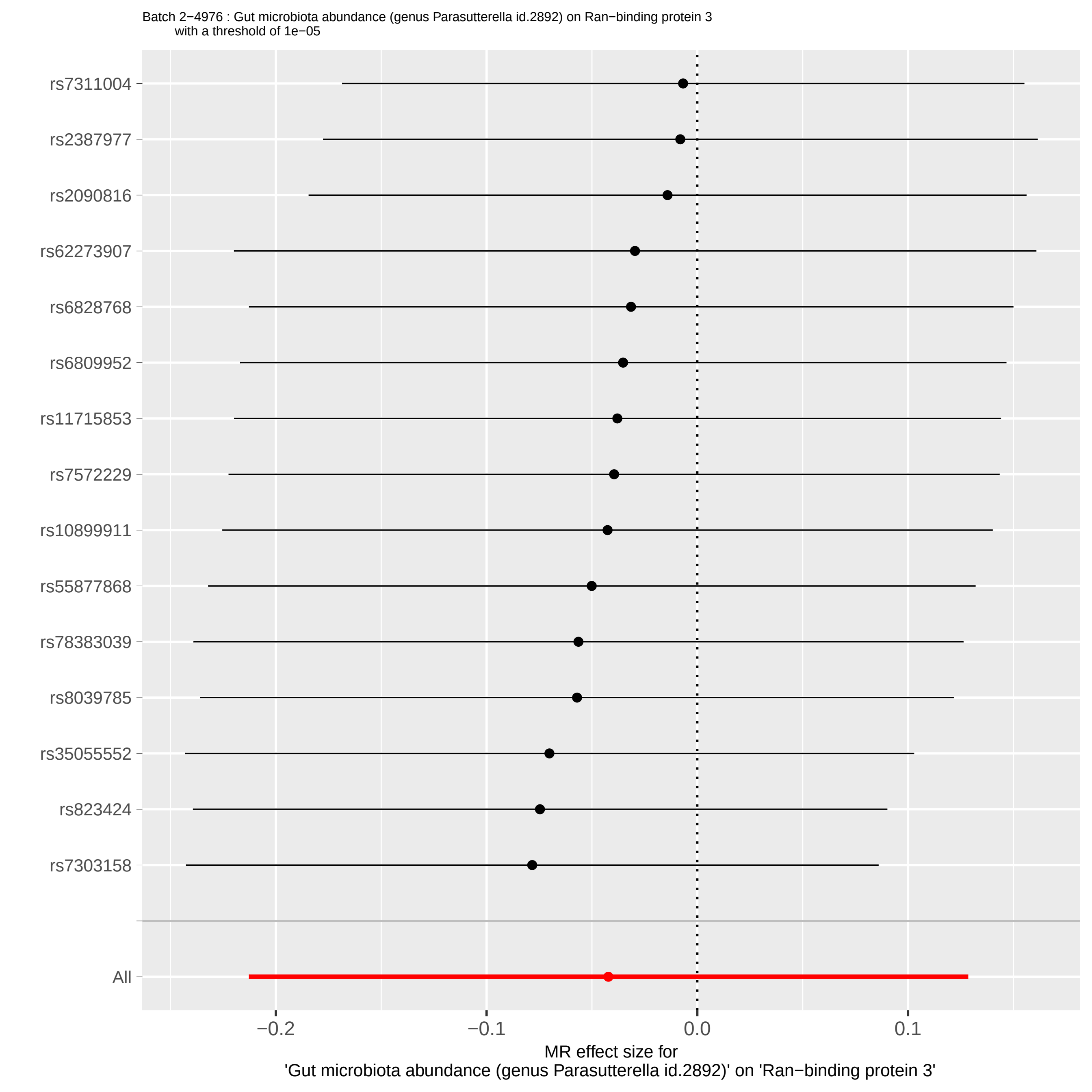
Batch 2−4976 : Gut microbiota abundance (genus Parasutterella id.2892) on Ran−binding protein 3
with a threshold of 1e−05
rs7311004
rs2387977
rs2090816
rs62273907
rs6828768
rs6809952
rs11715853
rs7572229
rs10899911
rs55877868
rs78383039
rs8039785
rs35055552
rs823424
rs7303158
All
−0.2
−0.1
0.0
0.1
MR effect size for
'Gut microbiota abundance (genus Parasutterella id.2892)' on 'Ran−binding protein 3'

Batch 2−4977 : Gut microbiota abundance (genus Catenibacterium id.2153) on Rap1 GTPase−activating protein 1
with a threshold of 1e−05

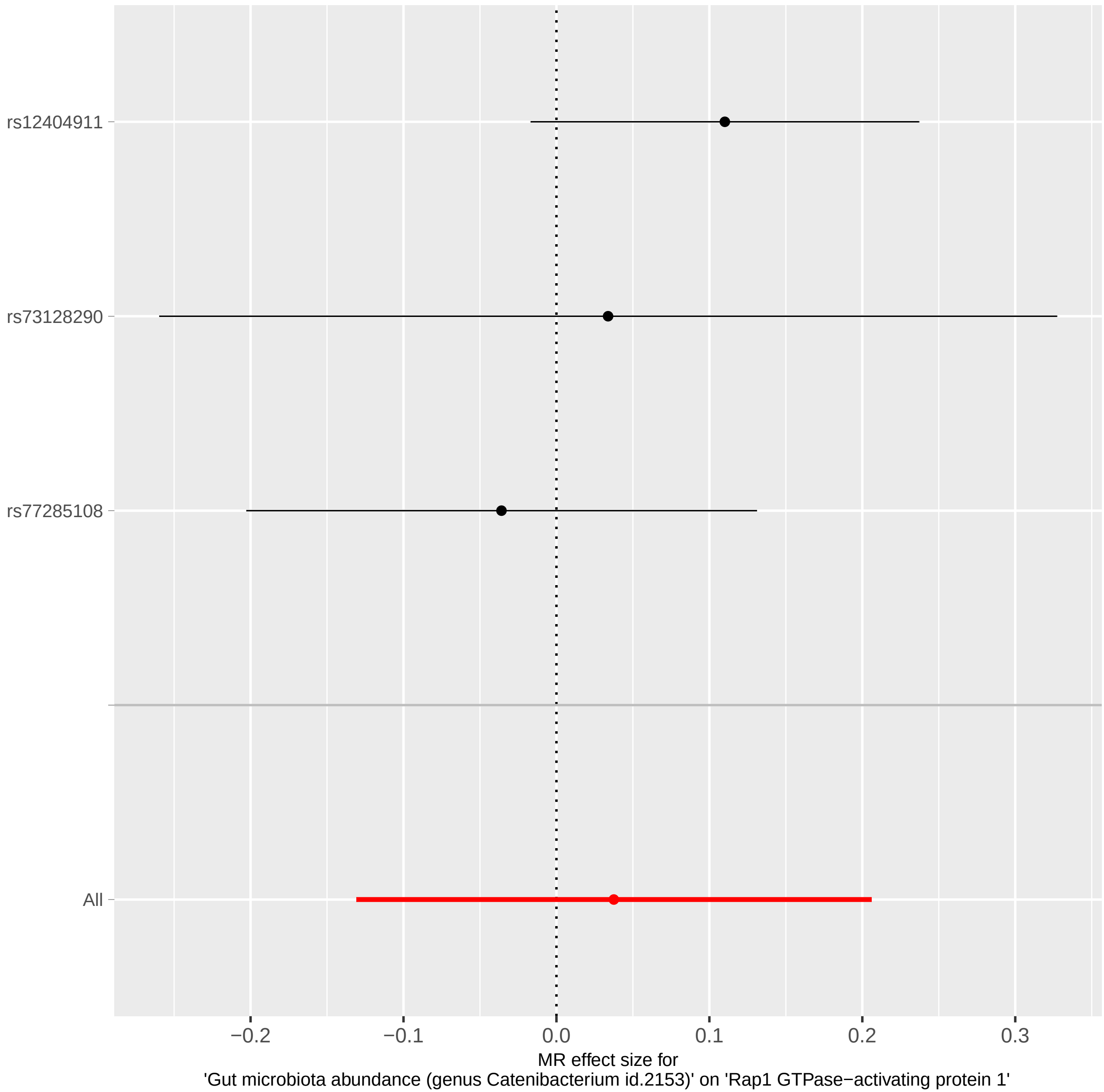

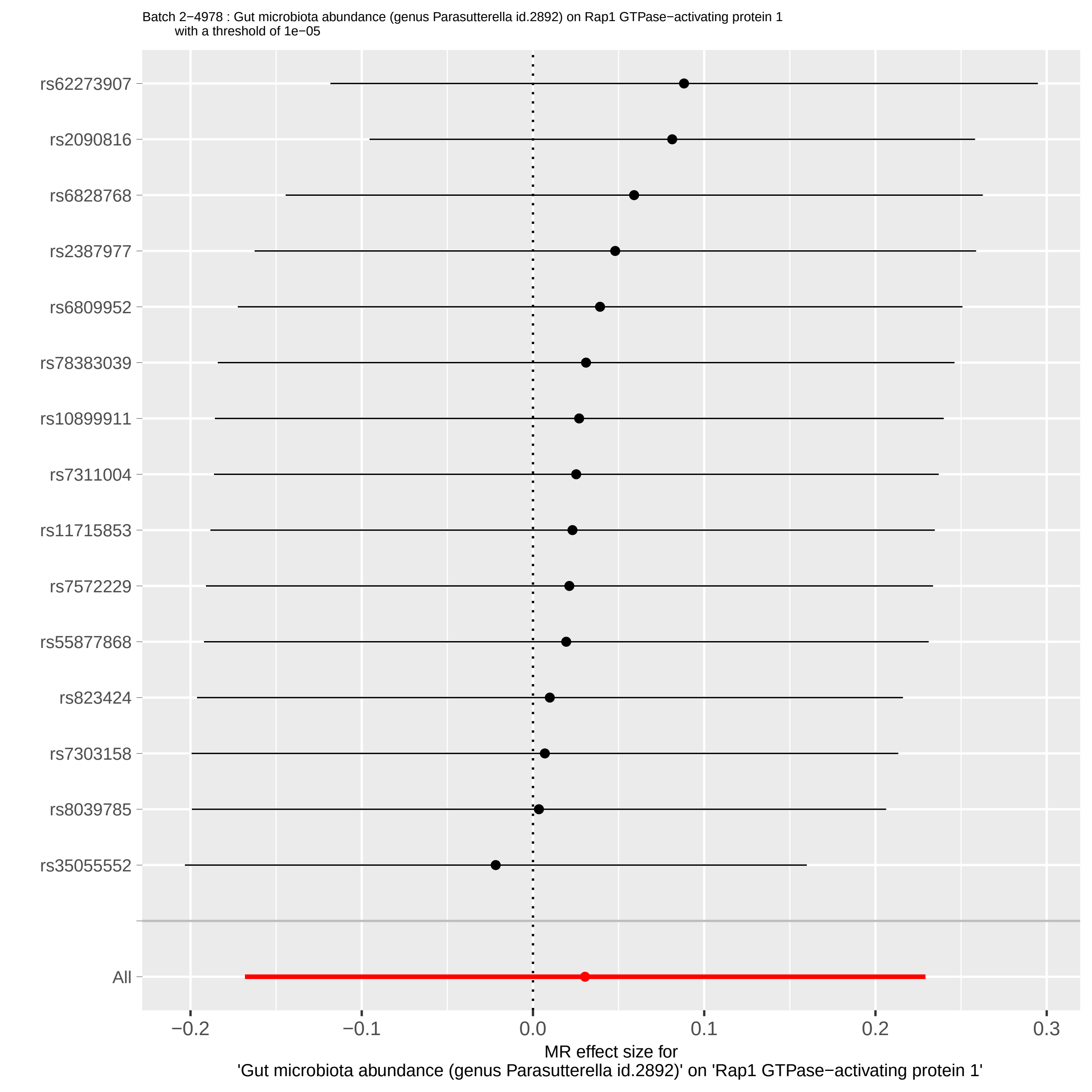
Batch 2−4978 : Gut microbiota abundance (genus Parasutterella id.2892) on Rap1 GTPase−activating protein 1
with a threshold of 1e−05
rs62273907
rs2090816
rs6828768
rs2387977
rs6809952
rs78383039
rs10899911
rs7311004
rs11715853
rs7572229
rs55877868
rs823424
rs7303158
rs8039785
rs35055552
All
−0.2
−0.1
0.0
0.1
0.2
0.3
MR effect size for
'Gut microbiota abundance (genus Parasutterella id.2892)' on 'Rap1 GTPase−activating protein 1'

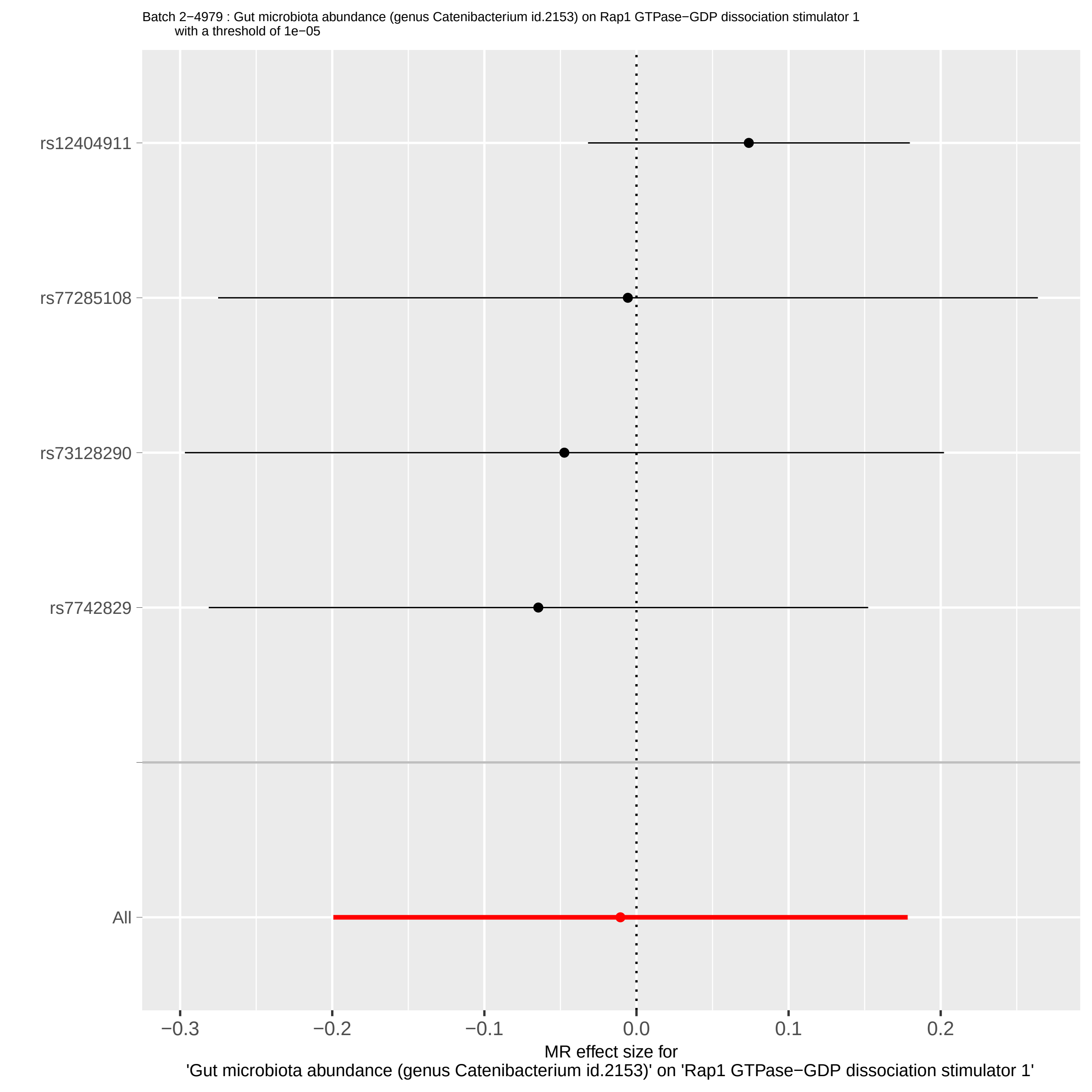

Batch 2−4979 : Gut microbiota abundance (genus Catenibacterium id.2153) on Rap1 GTPase−GDP dissociation stimulator 1
with a threshold of 1e−05
rs12404911
rs77285108
rs73128290
rs7742829
All
−0.3
−0.2
−0.1
0.0
0.1
0.2
MR effect size for
'Gut microbiota abundance (genus Catenibacterium id.2153)' on 'Rap1 GTPase−GDP dissociation stimulator 1'

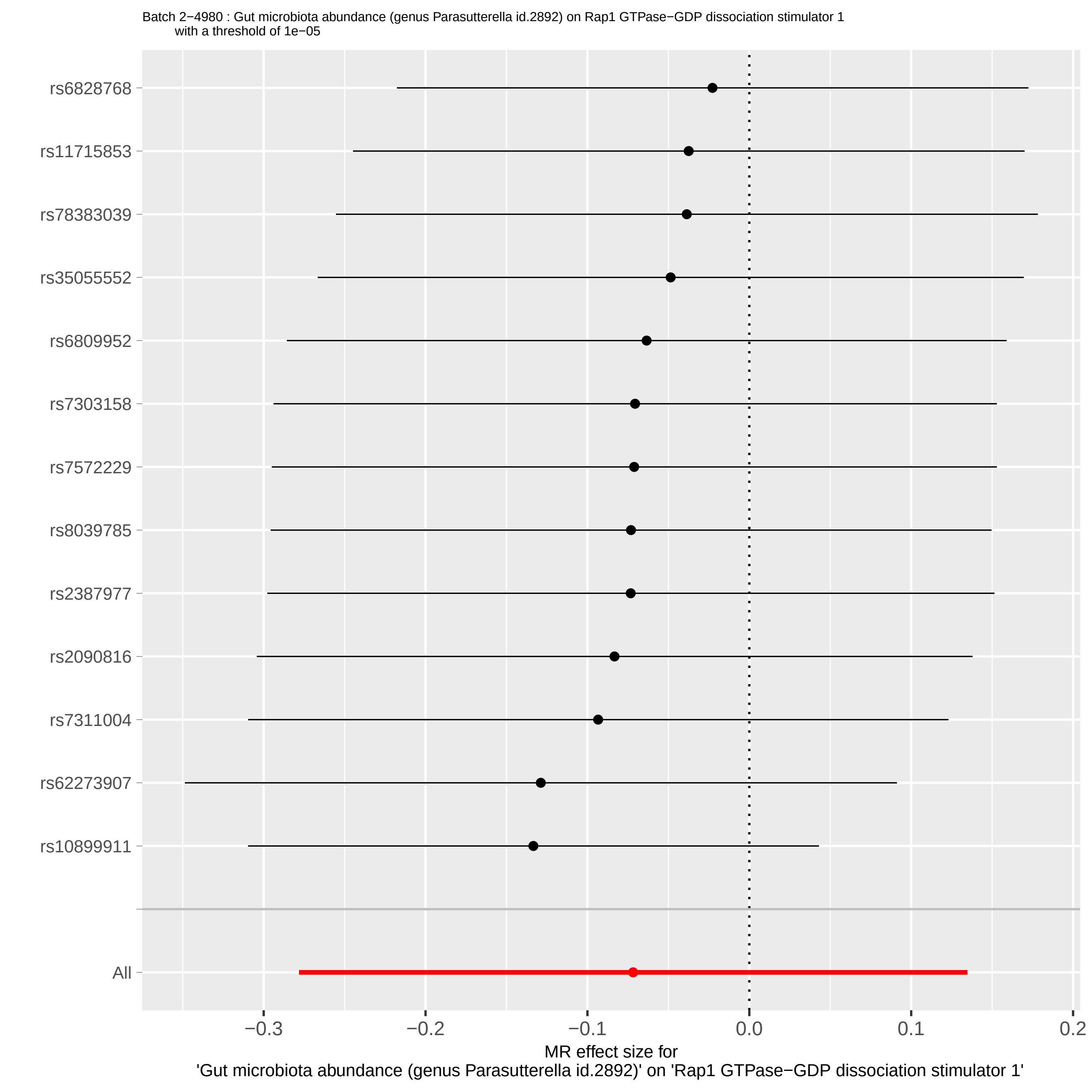
Batch 2−4980 : Gut microbiota abundance (genus Parasutterella id.2892) on Rap1 GTPase−GDP dissociation stimulator 1
with a threshold of 1e−05
rs6828768
rs11715853
rs78383039
rs35055552
rs6809952
rs7303158
rs7572229
rs8039785
rs2387977
rs2090816
rs7311004
rs62273907
rs10899911
All
−0.3
−0.2
−0.1
0.0
0.1
0.2
MR effect size for
'Gut microbiota abundance (genus Parasutterella id.2892)' on 'Rap1 GTPase−GDP dissociation stimulator 1'

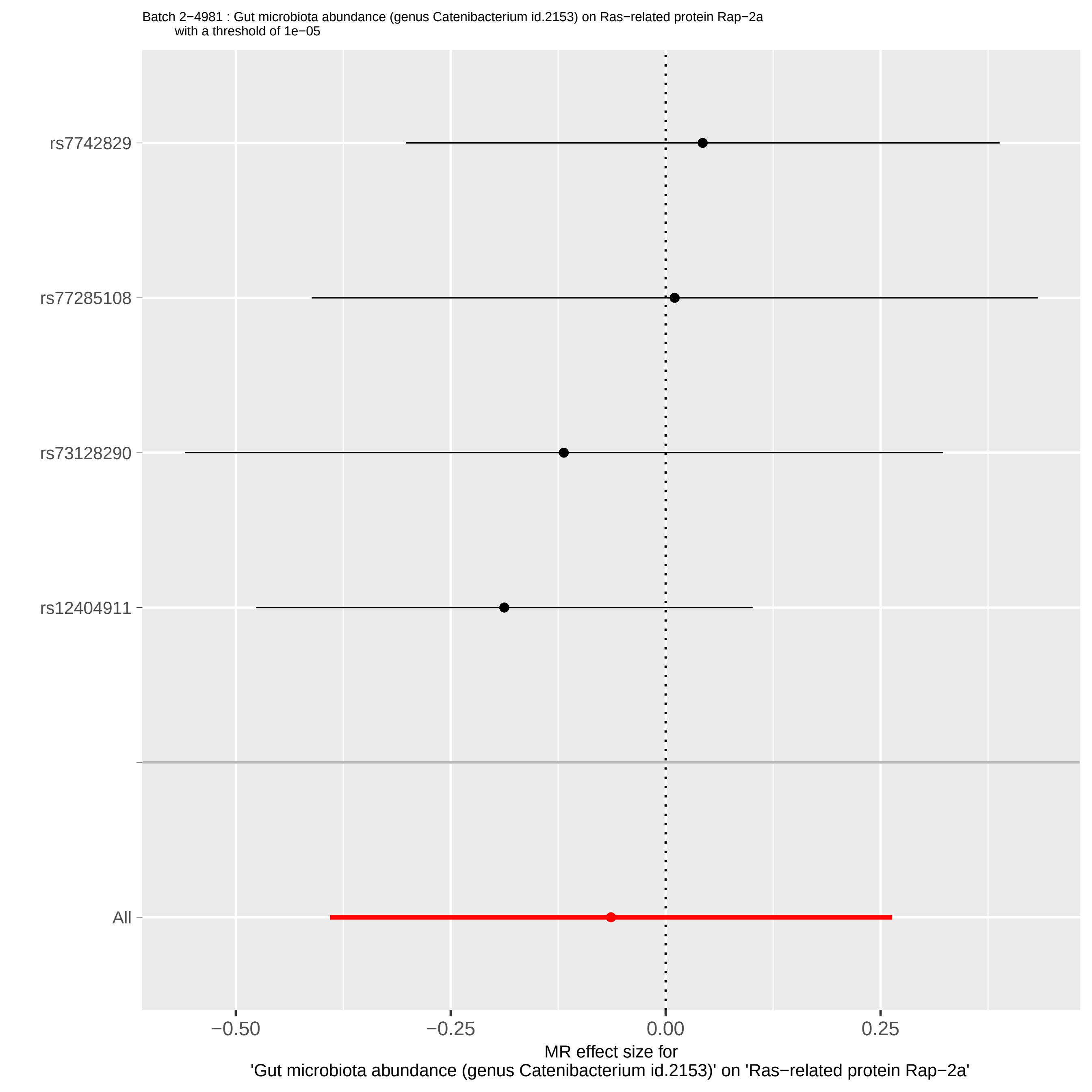
Batch 2−4981 : Gut microbiota abundance (genus Catenibacterium id.2153) on Ras−related protein Rap−2a
with a threshold of 1e−05
rs7742829
rs77285108
rs73128290
rs12404911
All
−0.50
−0.25
0.00
0.25
MR effect size for
'Gut microbiota abundance (genus Catenibacterium id.2153)' on 'Ras−related protein Rap−2a'

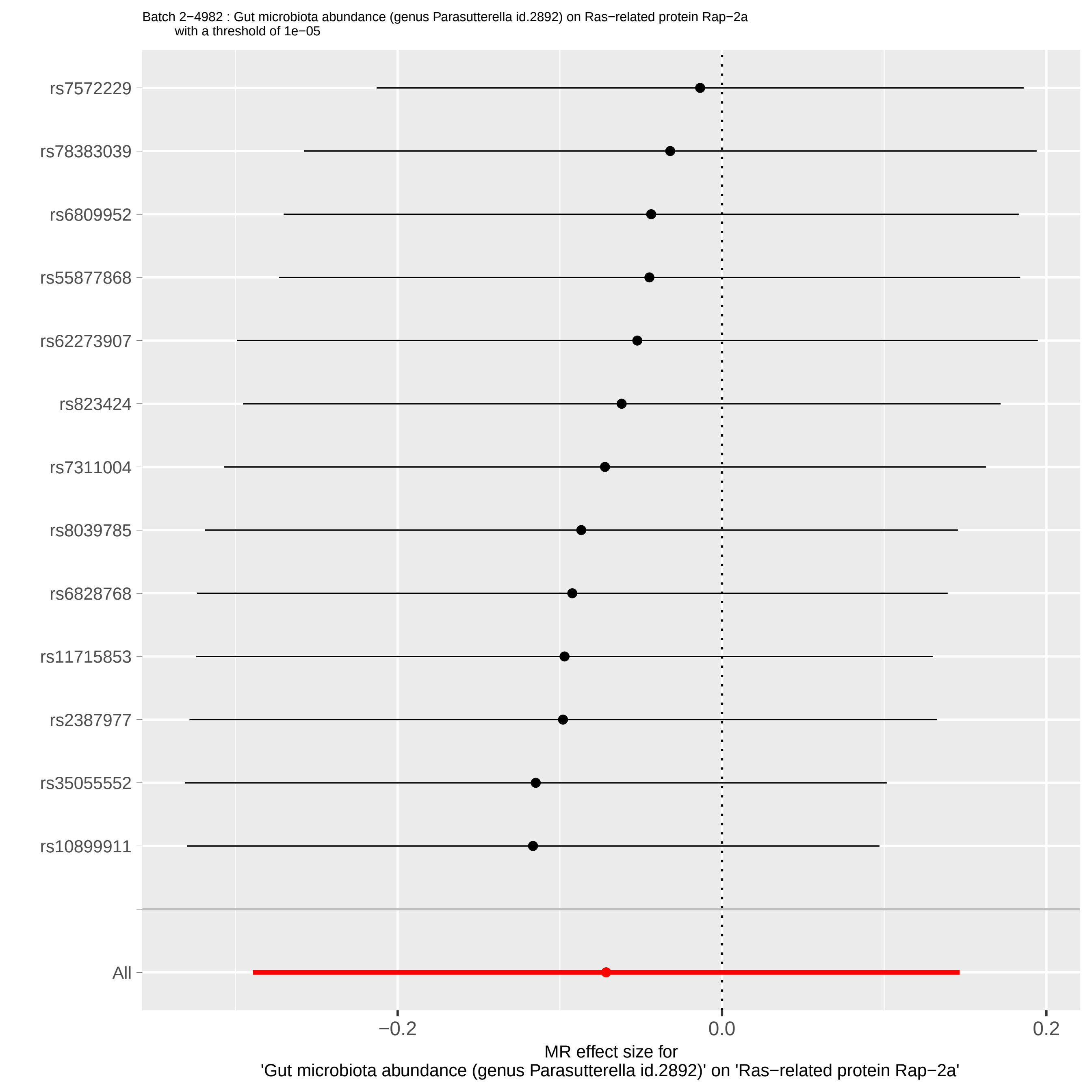
Batch 2−4982 : Gut microbiota abundance (genus Parasutterella id.2892) on Ras−related protein Rap−2a
with a threshold of 1e−05
rs7572229
rs78383039
rs6809952
rs55877868
rs62273907
rs823424
rs7311004
rs8039785
rs6828768
rs11715853
rs2387977
rs35055552
rs10899911
All
−0.2
0.0
0.2
MR effect size for
'Gut microbiota abundance (genus Parasutterella id.2892)' on 'Ras−related protein Rap−2a'

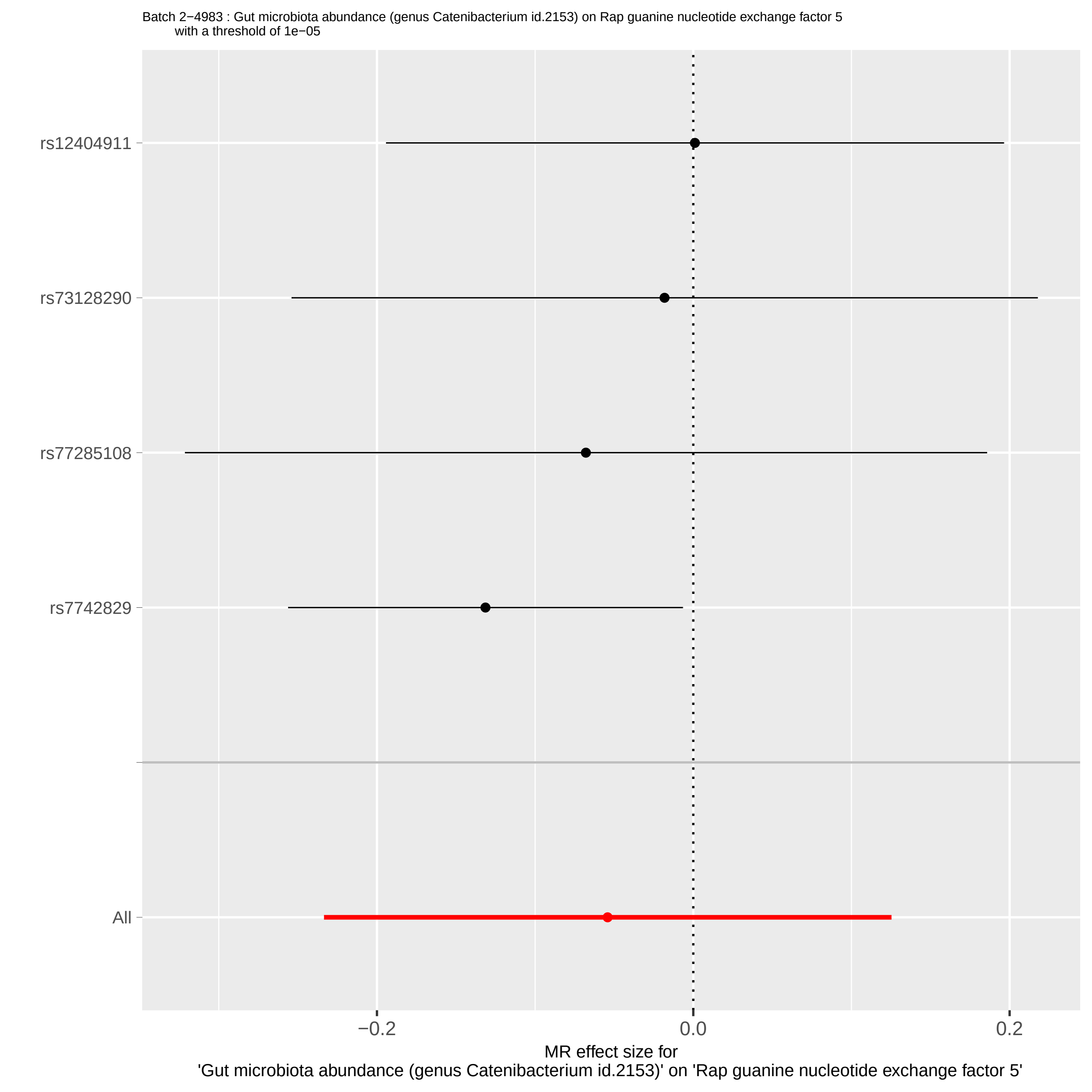

Batch 2−4983 : Gut microbiota abundance (genus Catenibacterium id.2153) on Rap guanine nucleotide exchange factor 5
with a threshold of 1e−05
rs12404911
rs73128290
rs77285108
rs7742829
All
−0.2
0.0
0.2
MR effect size for
'Gut microbiota abundance (genus Catenibacterium id.2153)' on 'Rap guanine nucleotide exchange factor 5'

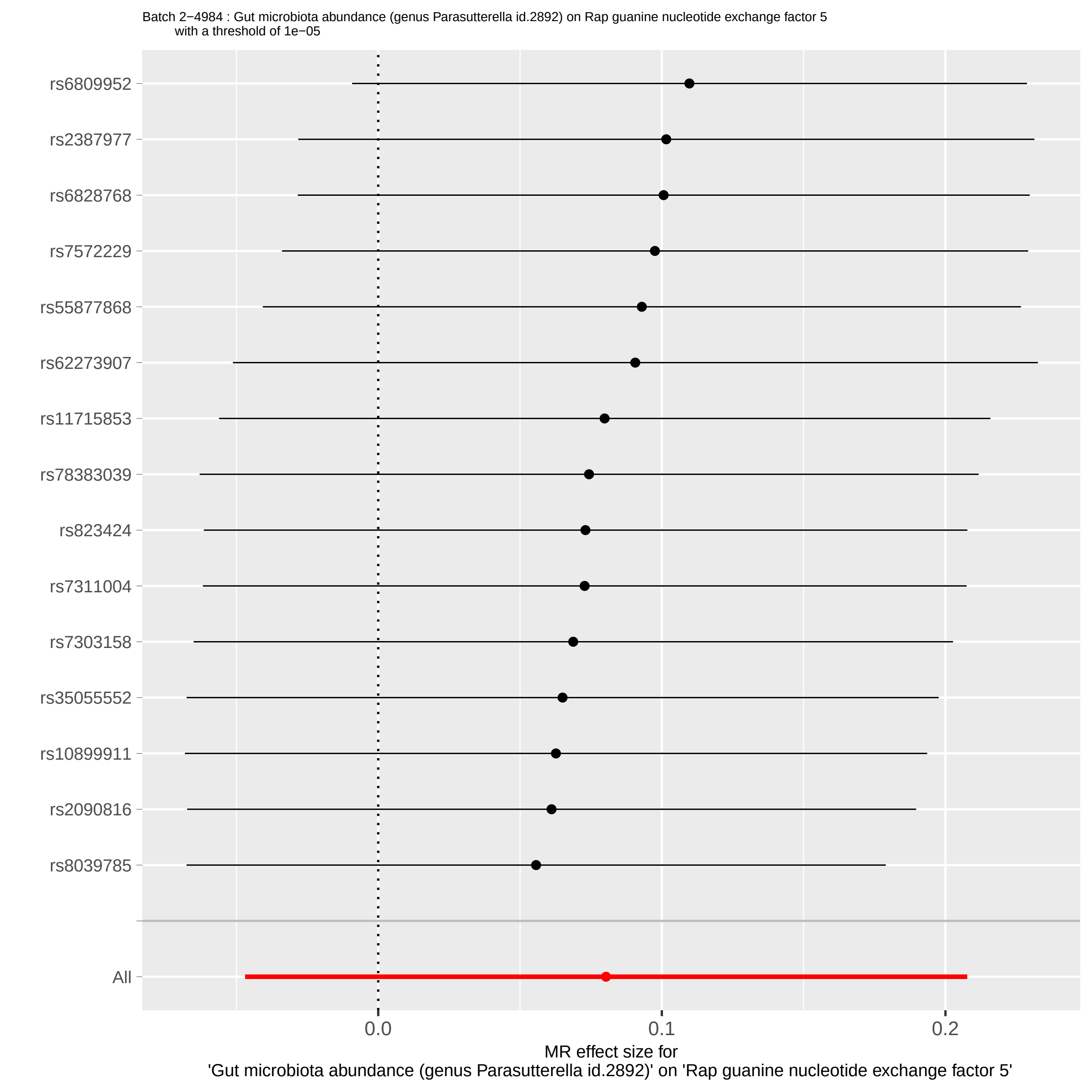
Batch 2−4984 : Gut microbiota abundance (genus Parasutterella id.2892) on Rap guanine nucleotide exchange factor 5
with a threshold of 1e−05
rs6809952
rs2387977
rs6828768
rs7572229
rs55877868
rs62273907
rs11715853
rs78383039
rs823424
rs7311004
rs7303158
rs35055552
rs10899911
rs2090816
rs8039785
All
0.0
0.1
0.2
MR effect size for
'Gut microbiota abundance (genus Parasutterella id.2892)' on 'Rap guanine nucleotide exchange factor 5'

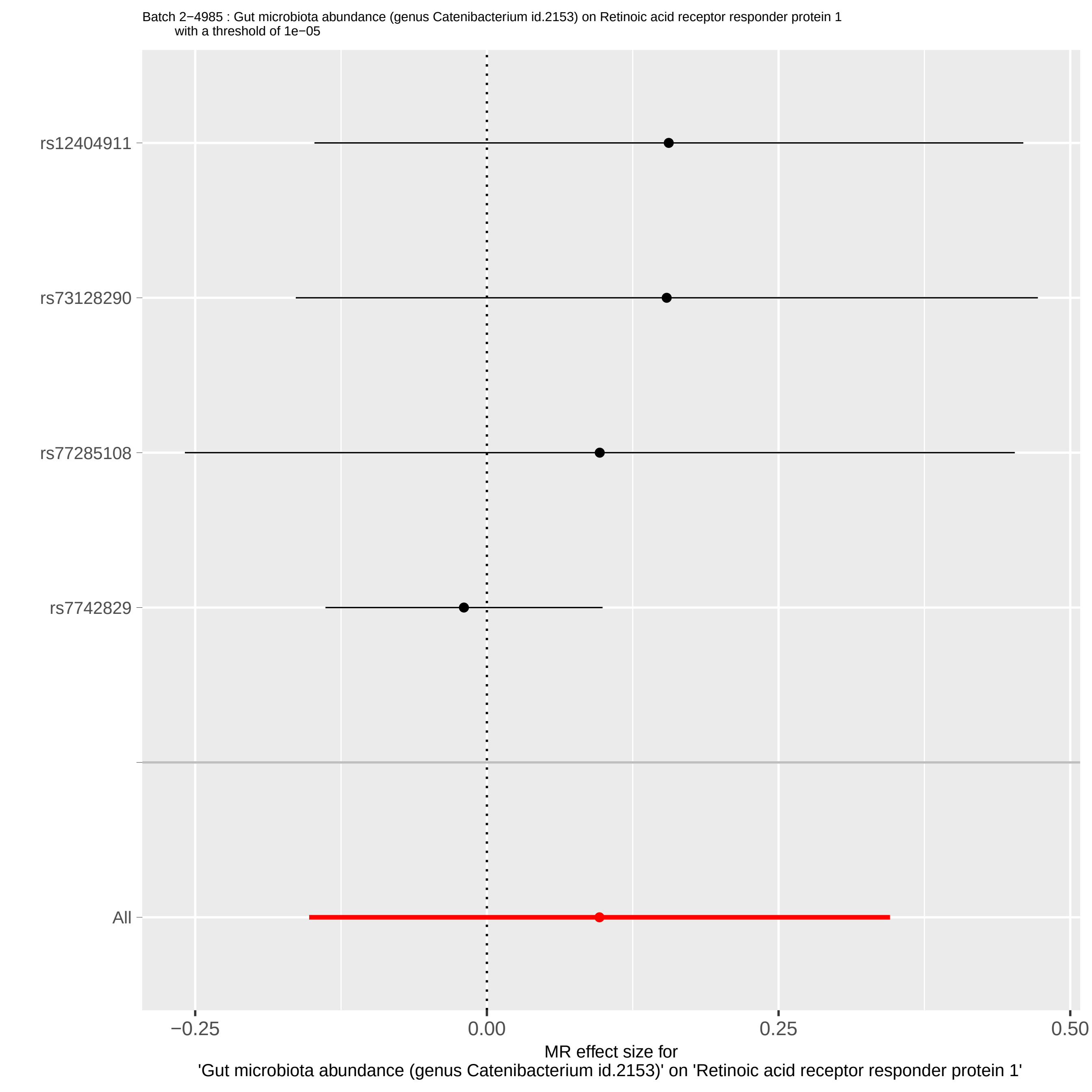
Batch 2−4985 : Gut microbiota abundance (genus Catenibacterium id.2153) on Retinoic acid receptor responder protein 1
with a threshold of 1e−05
rs12404911
rs73128290
rs77285108
rs7742829
All
−0.25
0.00
0.25
0.50
MR effect size for
'Gut microbiota abundance (genus Catenibacterium id.2153)' on 'Retinoic acid receptor responder protein 1'

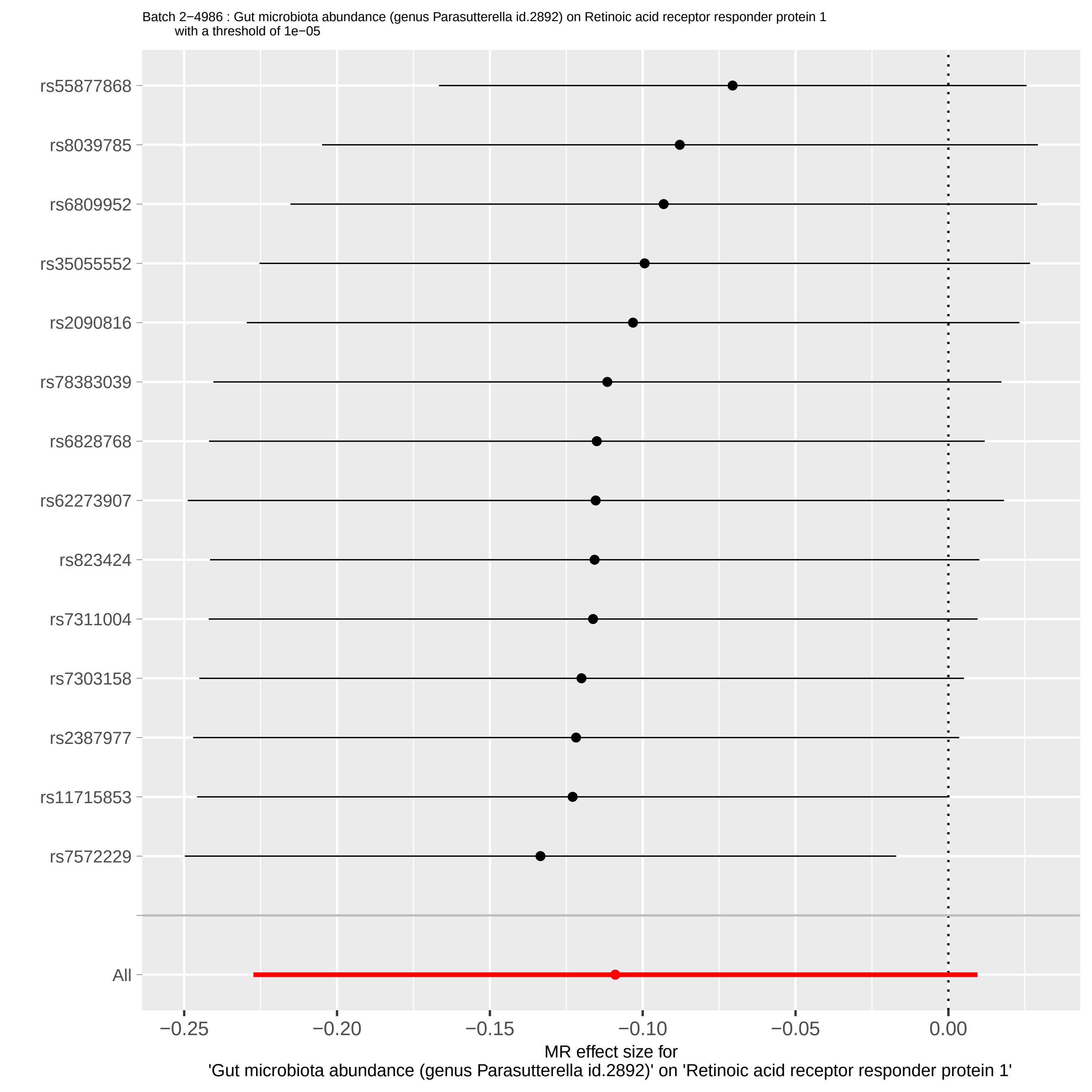
Batch 2−4986 : Gut microbiota abundance (genus Parasutterella id.2892) on Retinoic acid receptor responder protein 1
with a threshold of 1e−05
rs55877868
rs8039785
rs6809952
rs35055552
rs2090816
rs78383039
rs6828768
rs62273907
rs823424
rs7311004
rs7303158
rs2387977
rs11715853
rs7572229
All
−0.25
−0.20
−0.15
−0.10
−0.05
0.00
MR effect size for
'Gut microbiota abundance (genus Parasutterella id.2892)' on 'Retinoic acid receptor responder protein 1'

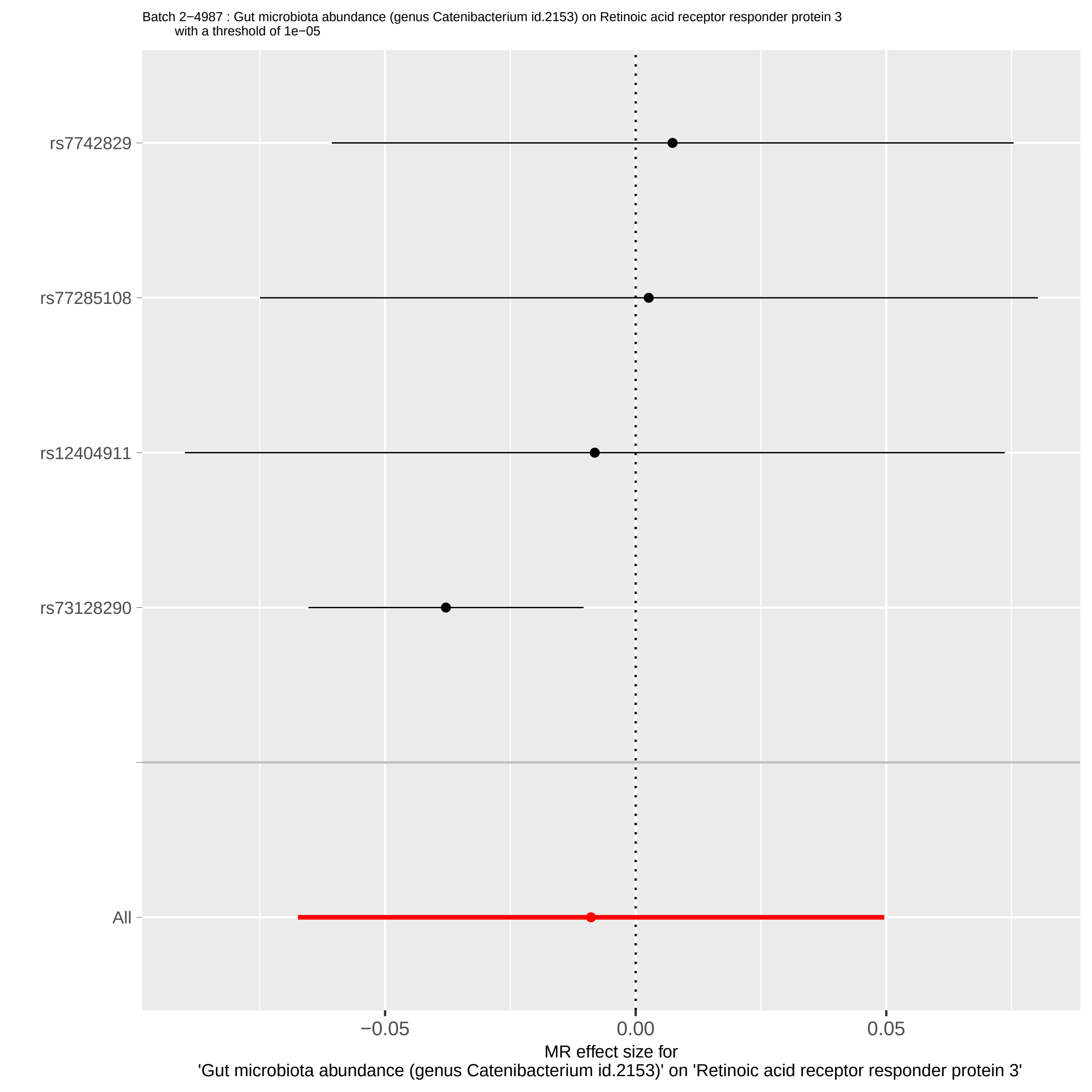

Batch 2−4987 : Gut microbiota abundance (genus Catenibacterium id.2153) on Retinoic acid receptor responder protein 3
with a threshold of 1e−05
rs7742829
rs77285108
rs12404911
rs73128290
All
−0.05
0.00
0.05
MR effect size for
'Gut microbiota abundance (genus Catenibacterium id.2153)' on 'Retinoic acid receptor responder protein 3'

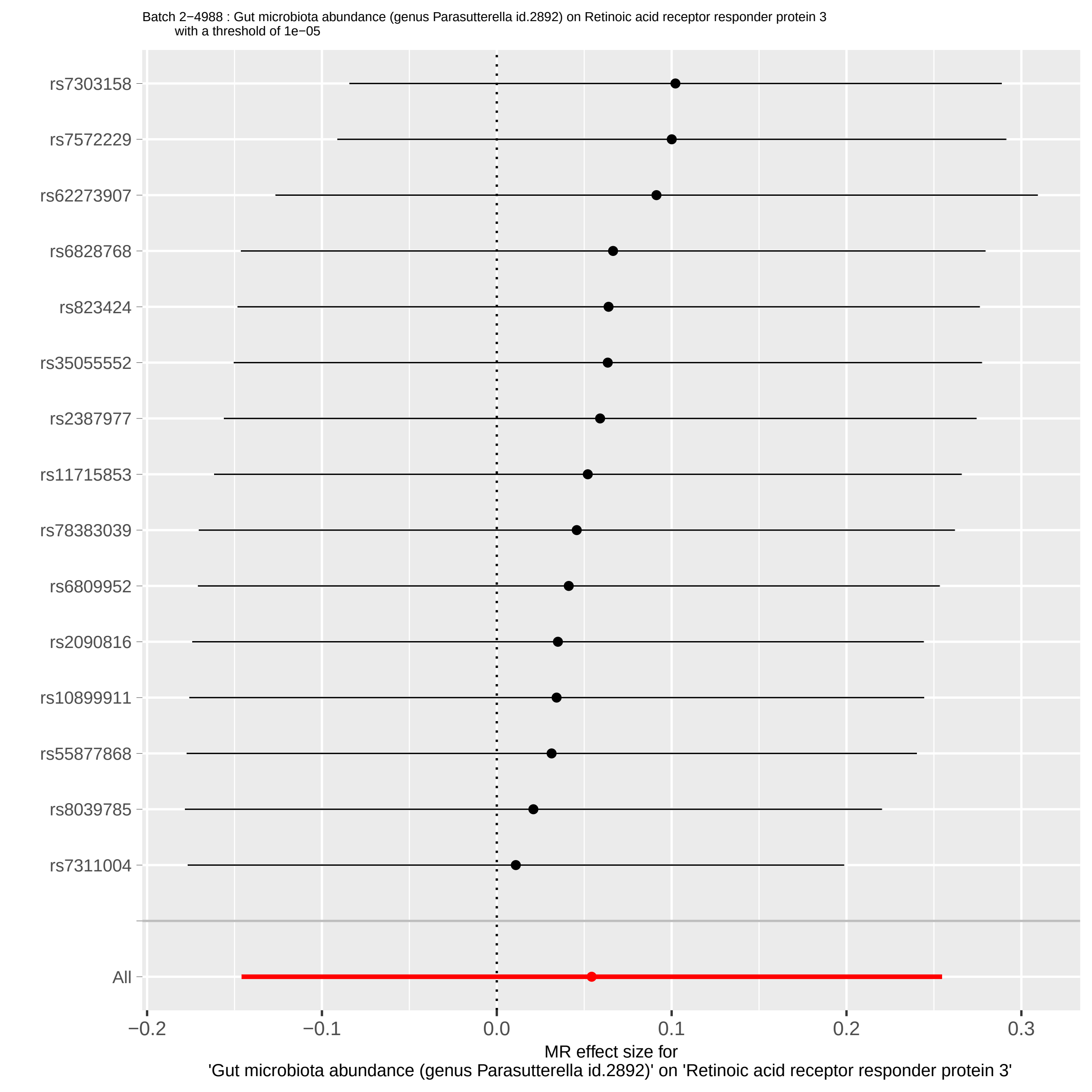

Batch 2−4988 : Gut microbiota abundance (genus Parasutterella id.2892) on Retinoic acid receptor responder protein 3
with a threshold of 1e−05
rs7303158
rs7572229
rs62273907
rs6828768
rs823424
rs35055552
rs2387977
rs11715853
rs78383039
rs6809952
rs2090816
rs10899911
rs55877868
rs8039785
rs7311004
All
−0.2
−0.1
0.0
0.1
0.2
0.3
MR effect size for
'Gut microbiota abundance (genus Parasutterella id.2892)' on 'Retinoic acid receptor responder protein 3'

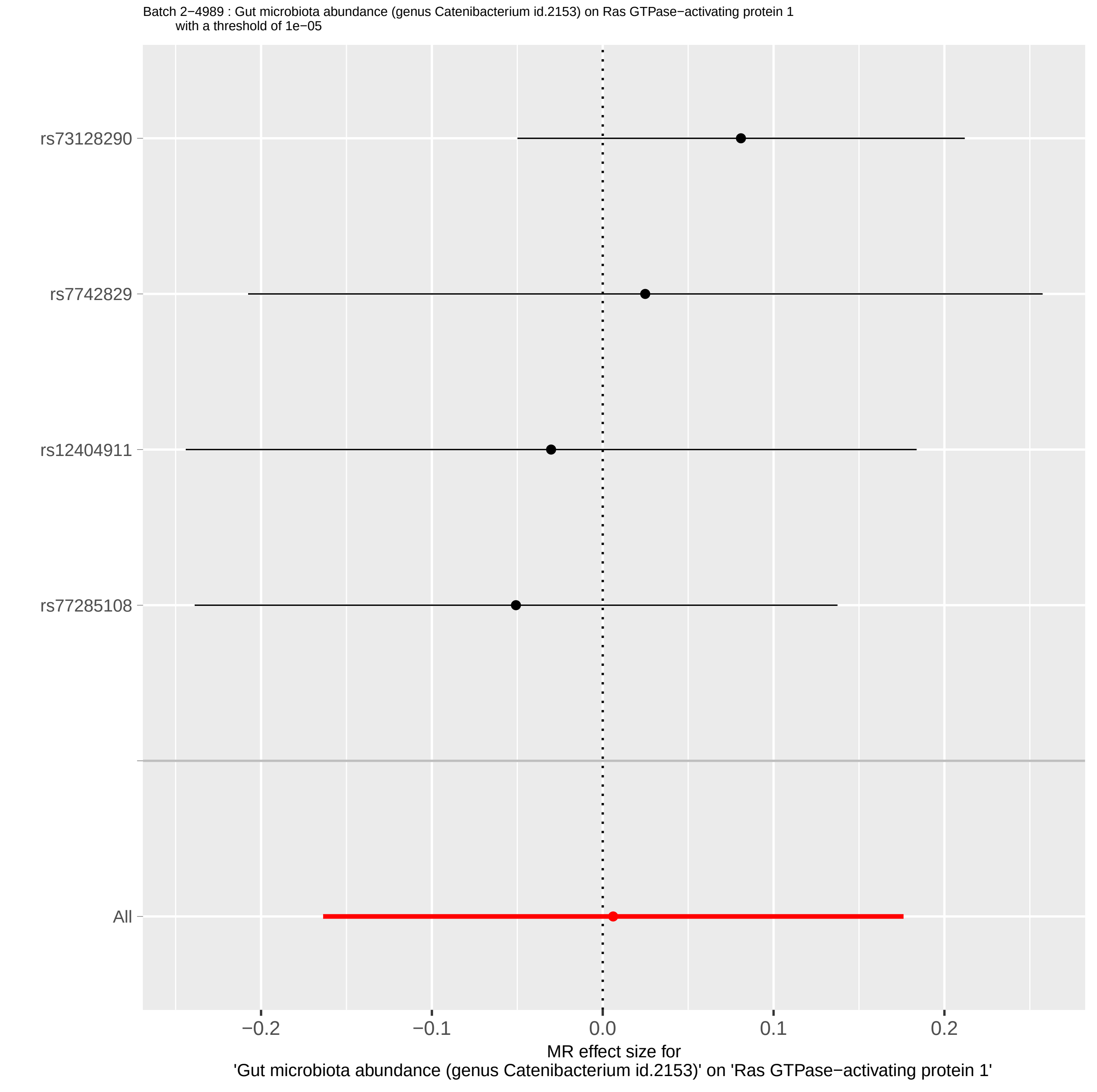
Batch 2−4989 : Gut microbiota abundance (genus Catenibacterium id.2153) on Ras GTPase−activating protein 1
with a threshold of 1e−05
rs73128290
rs7742829
rs12404911
rs77285108
All
−0.2
−0.1
0.0
0.1
0.2
MR effect size for
'Gut microbiota abundance (genus Catenibacterium id.2153)' on 'Ras GTPase−activating protein 1'

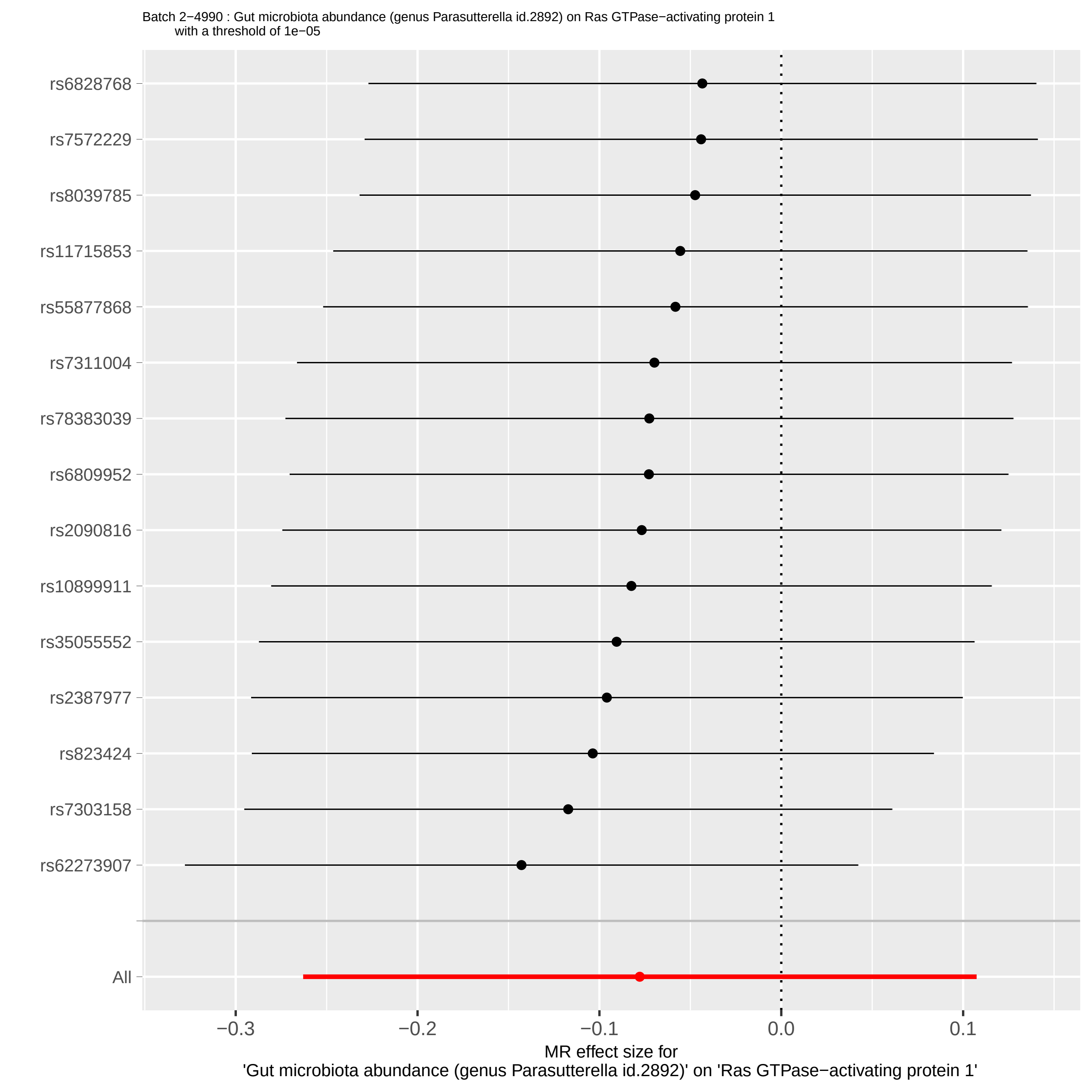

Batch 2−4990 : Gut microbiota abundance (genus Parasutterella id.2892) on Ras GTPase−activating protein 1
with a threshold of 1e−05
rs6828768
rs7572229
rs8039785
rs11715853
rs55877868
rs7311004
rs78383039
rs6809952
rs2090816
rs10899911
rs35055552
rs2387977
rs823424
rs7303158
rs62273907
All
−0.3
−0.2
−0.1
0.0
0.1
MR effect size for
'Gut microbiota abundance (genus Parasutterella id.2892)' on 'Ras GTPase−activating protein 1'

Batch 2−4991 : Gut microbiota abundance (genus Catenibacterium id.2153) on Retinoblastoma−associated protein
with a threshold of 1e−05

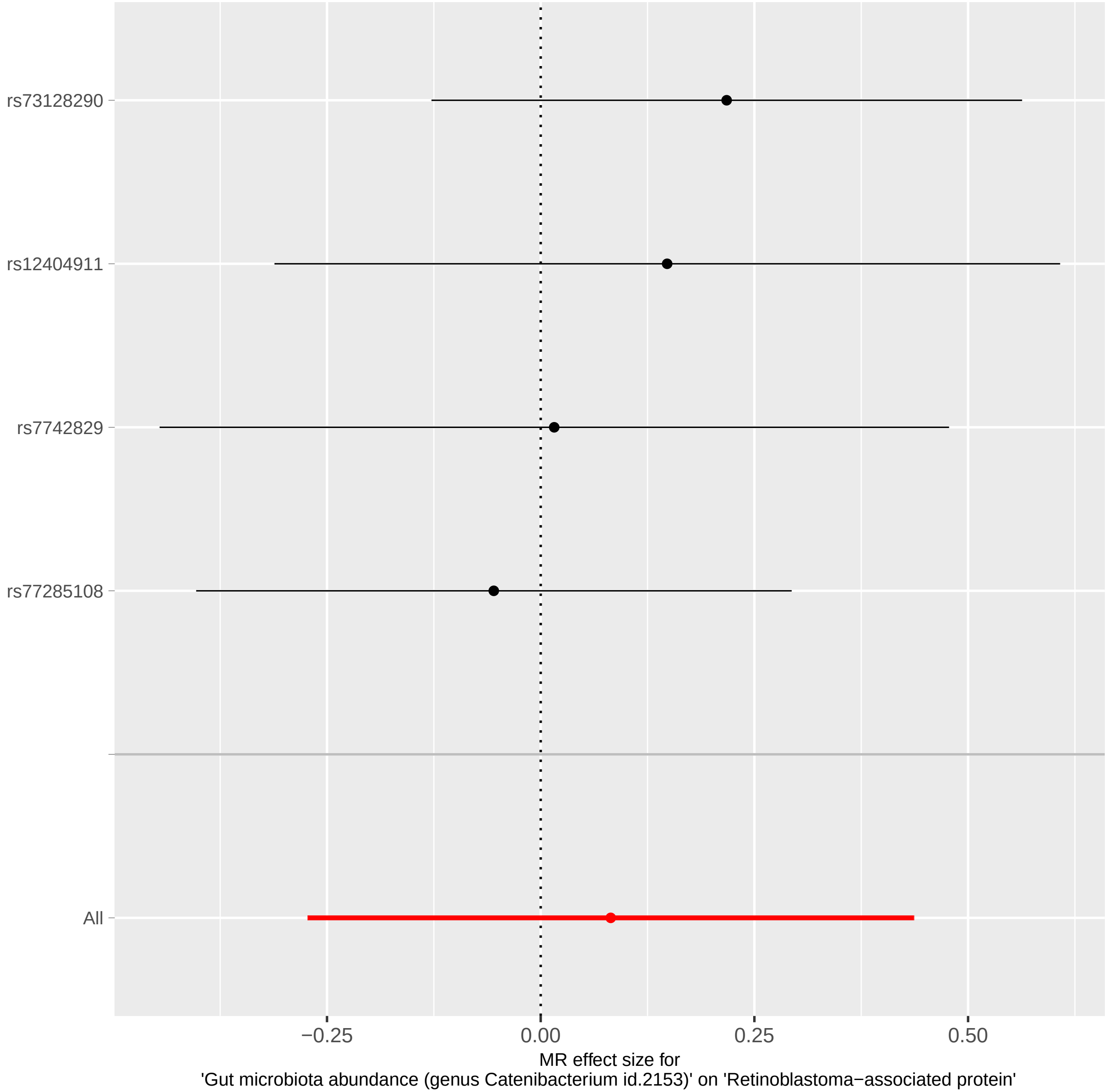

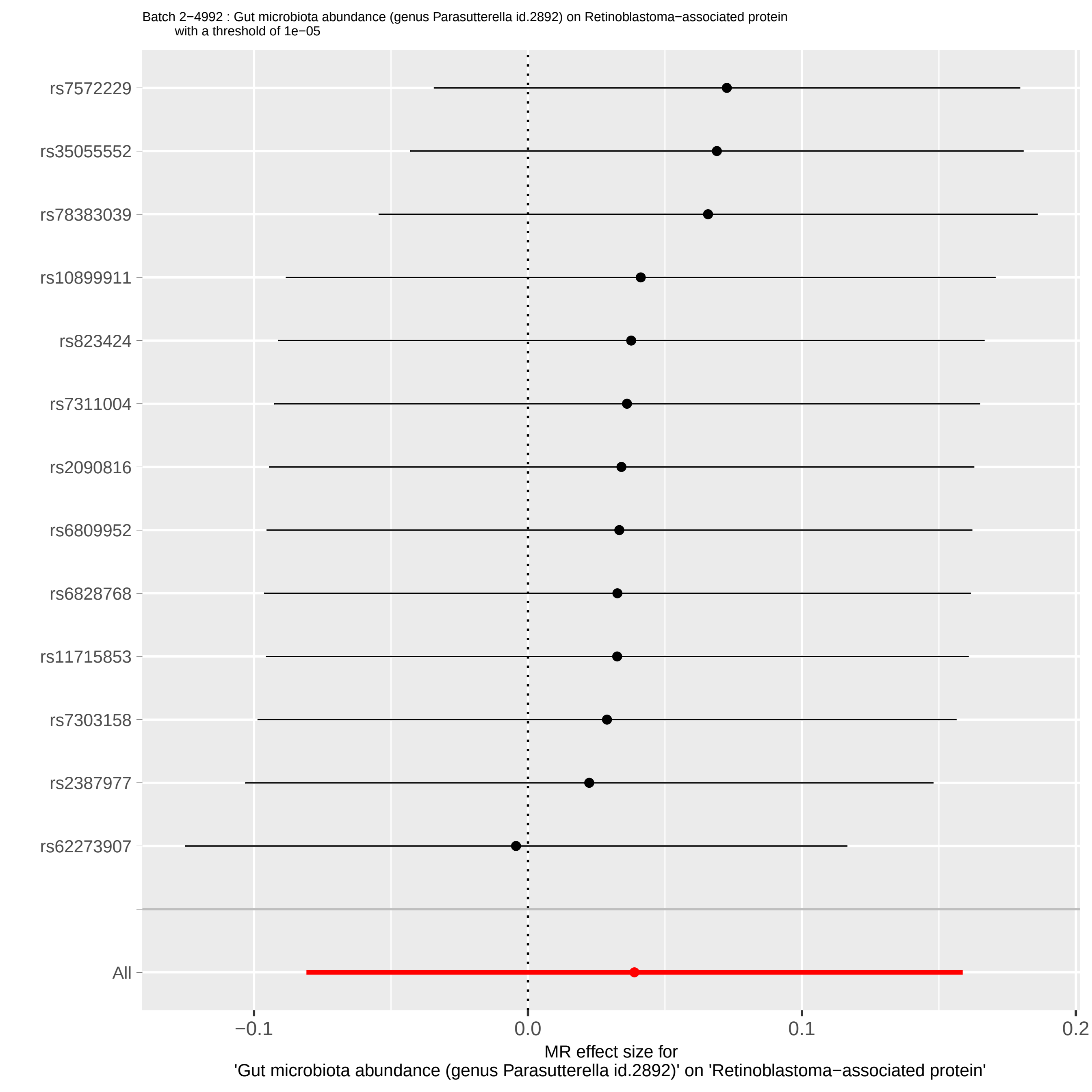

Batch 2−4992 : Gut microbiota abundance (genus Parasutterella id.2892) on Retinoblastoma−associated protein
with a threshold of 1e−05
rs7572229
rs35055552
rs78383039
rs10899911
rs823424
rs7311004
rs2090816
rs6809952
rs6828768
rs11715853
rs7303158
rs2387977
rs62273907
All
−0.1
0.0
0.1
0.2
MR effect size for
'Gut microbiota abundance (genus Parasutterella id.2892)' on 'Retinoblastoma−associated protein'

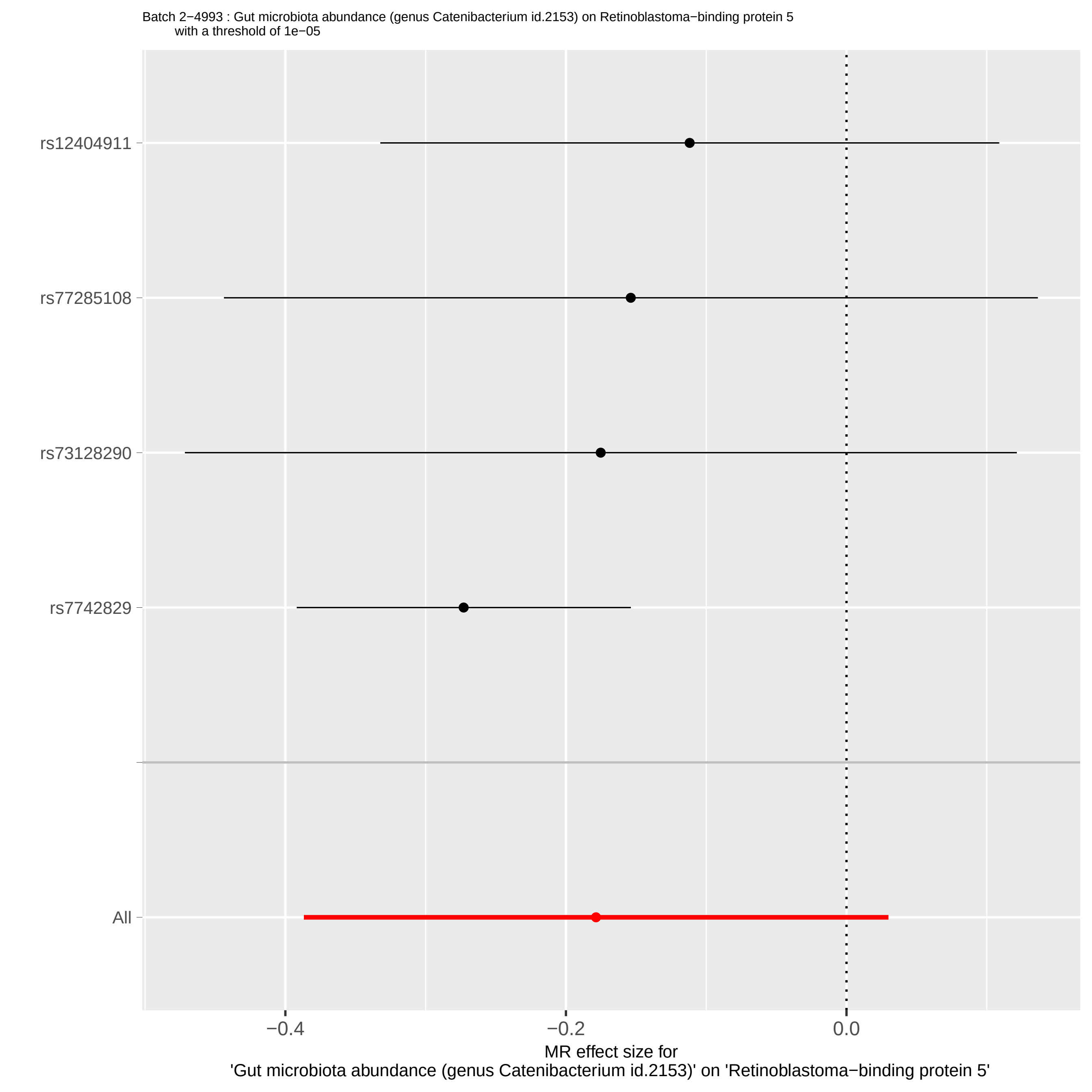

Batch 2−4993 : Gut microbiota abundance (genus Catenibacterium id.2153) on Retinoblastoma−binding protein 5
with a threshold of 1e−05
rs12404911
rs77285108
rs73128290
rs7742829
All
−0.4
−0.2
0.0
MR effect size for
'Gut microbiota abundance (genus Catenibacterium id.2153)' on 'Retinoblastoma−binding protein 5'

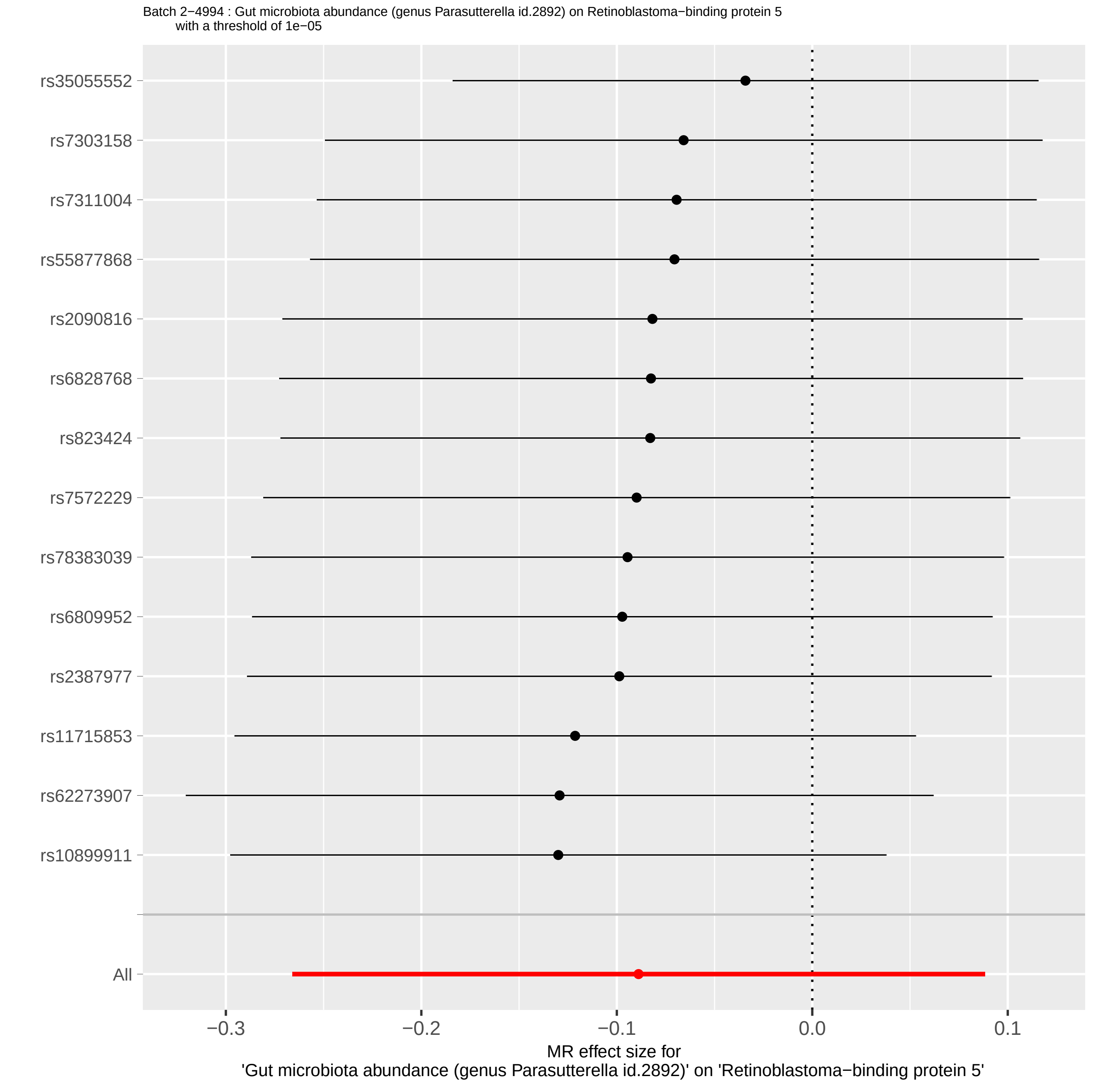
Batch 2−4994 : Gut microbiota abundance (genus Parasutterella id.2892) on Retinoblastoma−binding protein 5
with a threshold of 1e−05
rs35055552
rs7303158
rs7311004
rs55877868
rs2090816
rs6828768
rs823424
rs7572229
rs78383039
rs6809952
rs2387977
rs11715853
rs62273907
rs10899911
All
−0.3
−0.2
−0.1
0.0
0.1
MR effect size for
'Gut microbiota abundance (genus Parasutterella id.2892)' on 'Retinoblastoma−binding protein 5'

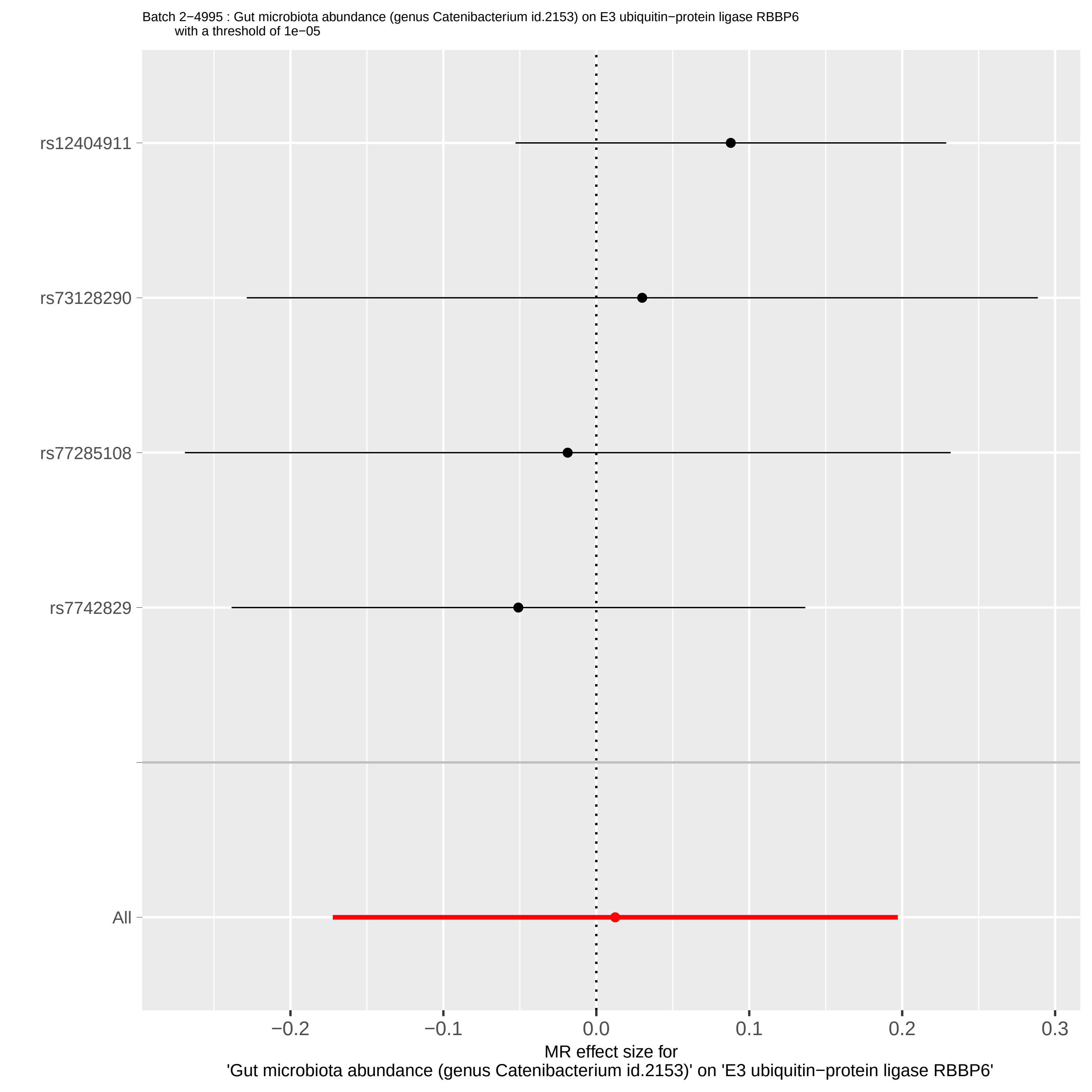

Batch 2−4995 : Gut microbiota abundance (genus Catenibacterium id.2153) on E3 ubiquitin−protein ligase RBBP6
with a threshold of 1e−05
rs12404911
rs73128290
rs77285108
rs7742829
All
−0.2
−0.1
0.0
0.1
0.2
0.3
MR effect size for
'Gut microbiota abundance (genus Catenibacterium id.2153)' on 'E3 ubiquitin−protein ligase RBBP6'

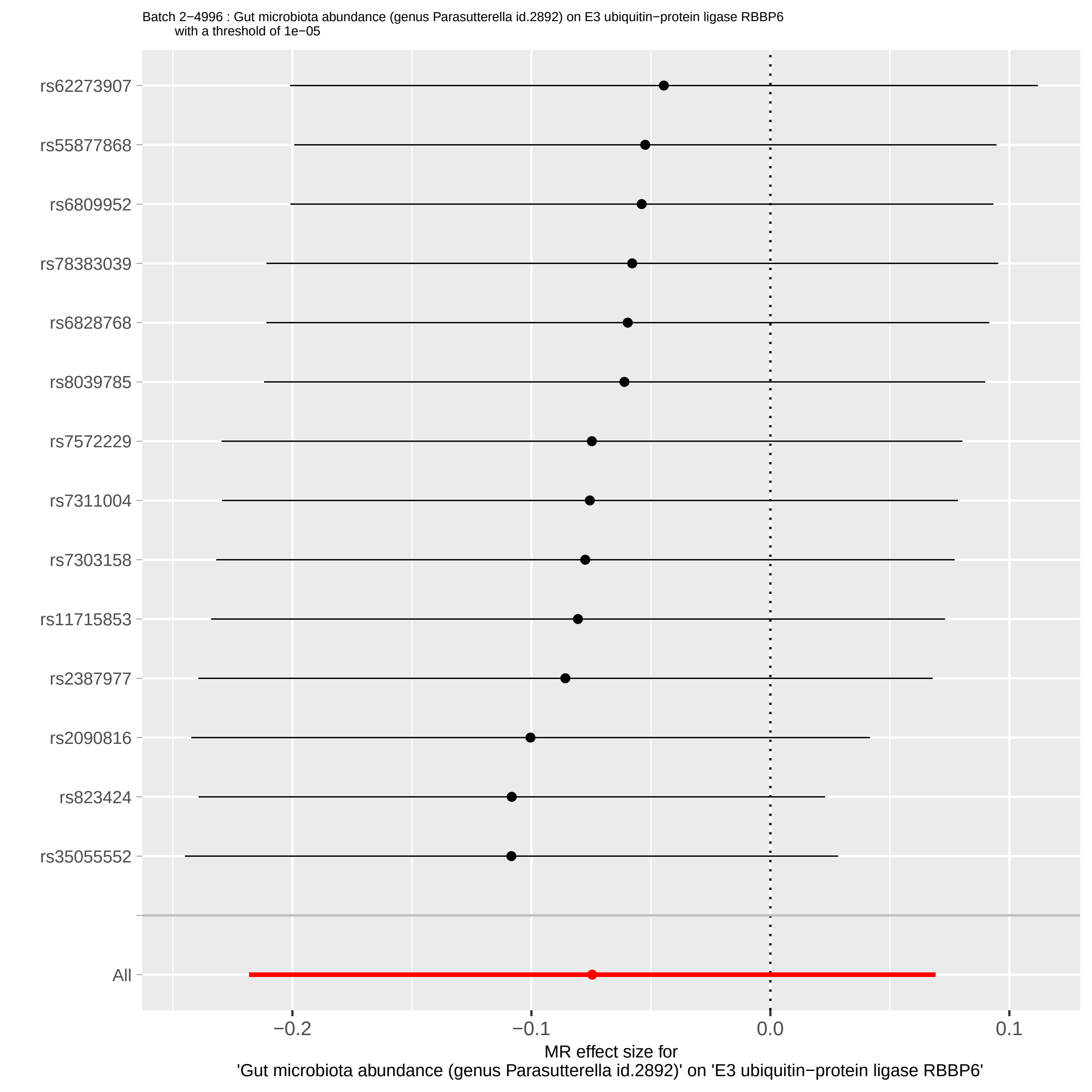
Batch 2−4996 : Gut microbiota abundance (genus Parasutterella id.2892) on E3 ubiquitin−protein ligase RBBP6
with a threshold of 1e−05
rs62273907
rs55877868
rs6809952
rs78383039
rs6828768
rs8039785
rs7572229
rs7311004
rs7303158
rs11715853
rs2387977
rs2090816
rs823424
rs35055552
All
−0.2
−0.1
0.0
0.1
MR effect size for
'Gut microbiota abundance (genus Parasutterella id.2892)' on 'E3 ubiquitin−protein ligase RBBP6'

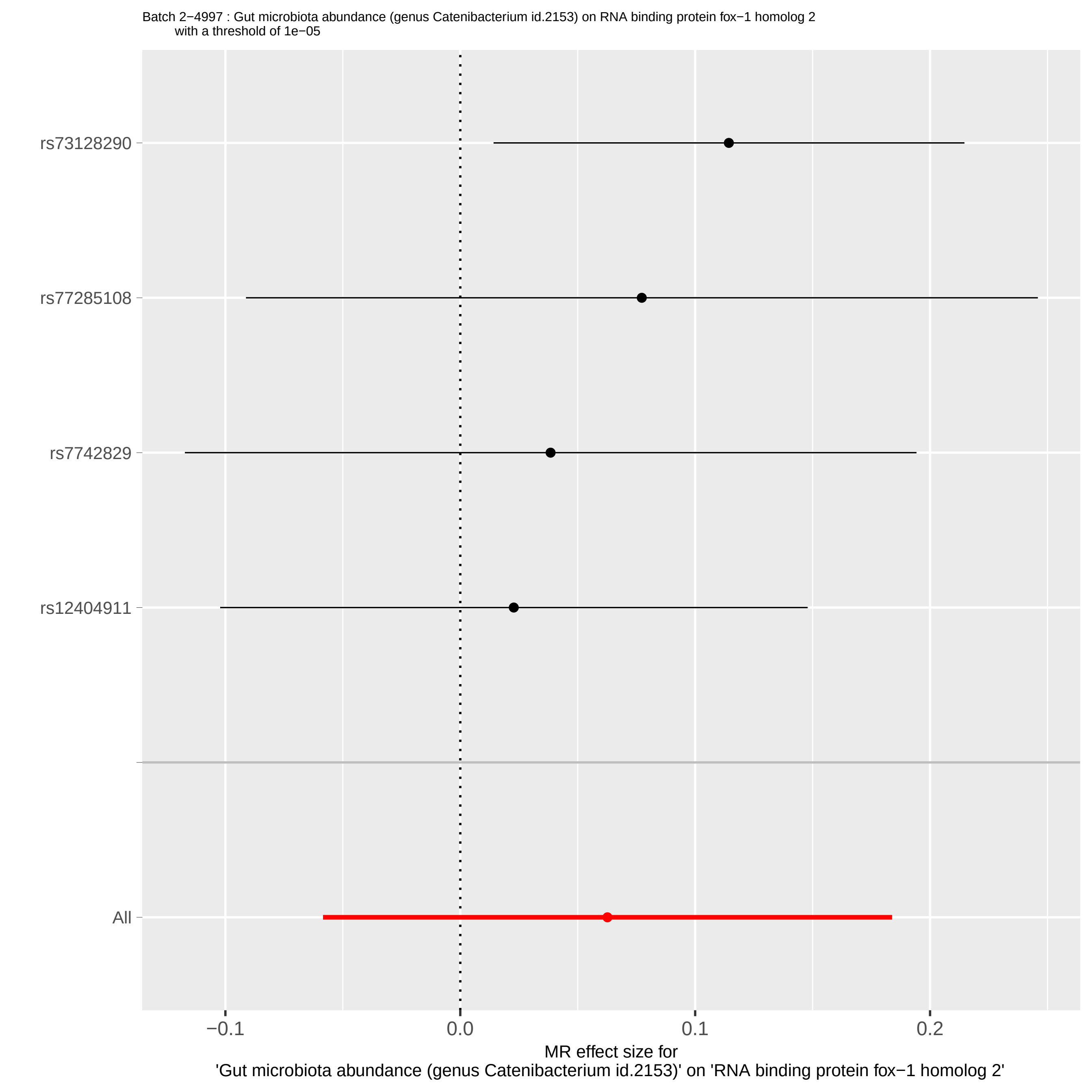
Batch 2−4997 : Gut microbiota abundance (genus Catenibacterium id.2153) on RNA binding protein fox−1 homolog 2
with a threshold of 1e−05
rs73128290
rs77285108
rs7742829
rs12404911
All
−0.1
0.0
0.1
0.2
MR effect size for
'Gut microbiota abundance (genus Catenibacterium id.2153)' on 'RNA binding protein fox−1 homolog 2'

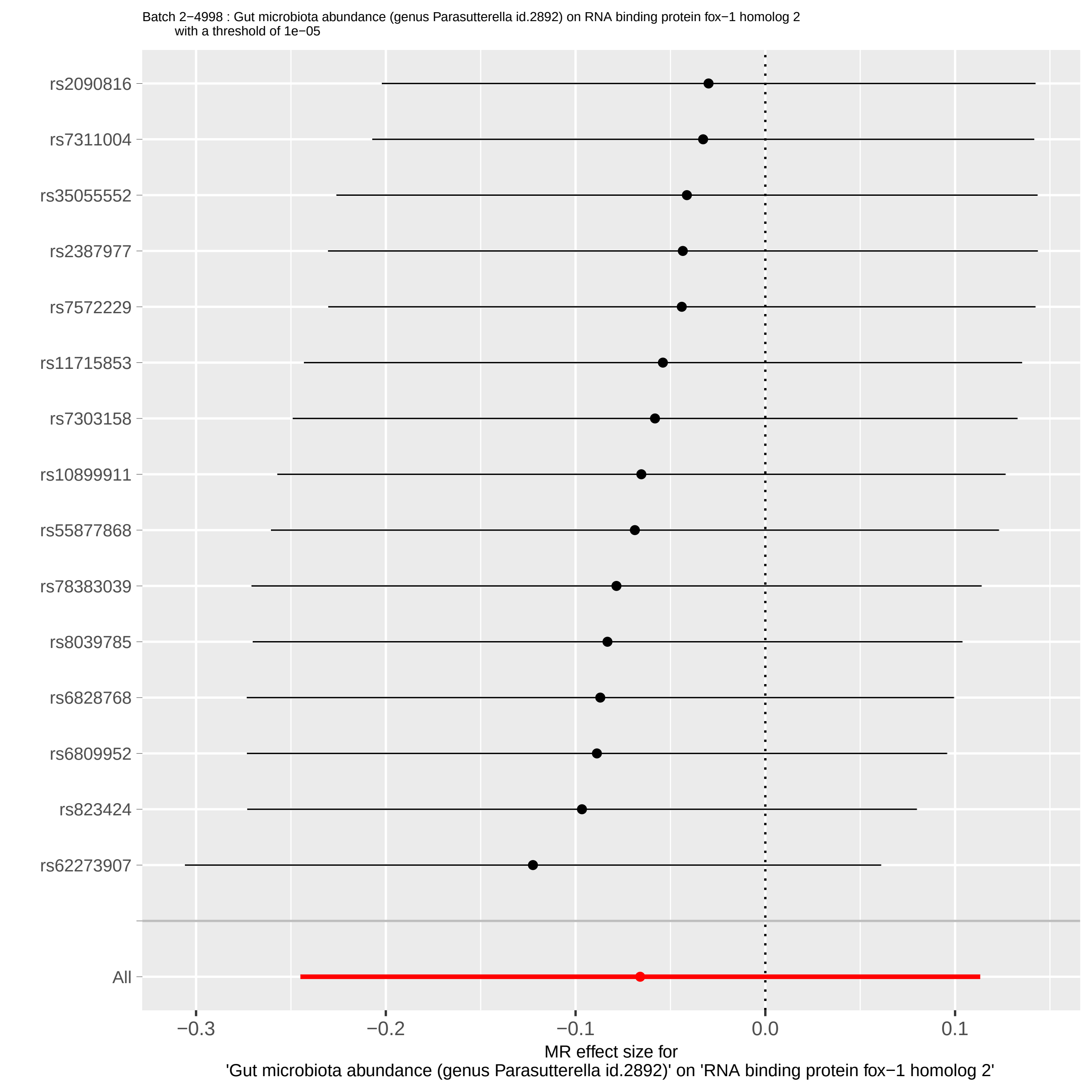

Batch 2−4998 : Gut microbiota abundance (genus Parasutterella id.2892) on RNA binding protein fox−1 homolog 2
with a threshold of 1e−05
rs2090816
rs7311004
rs35055552
rs2387977
rs7572229
rs11715853
rs7303158
rs10899911
rs55877868
rs78383039
rs8039785
rs6828768
rs6809952
rs823424
rs62273907
All
−0.3
−0.2
−0.1
0.0
0.1
MR effect size for
'Gut microbiota abundance (genus Parasutterella id.2892)' on 'RNA binding protein fox−1 homolog 2'

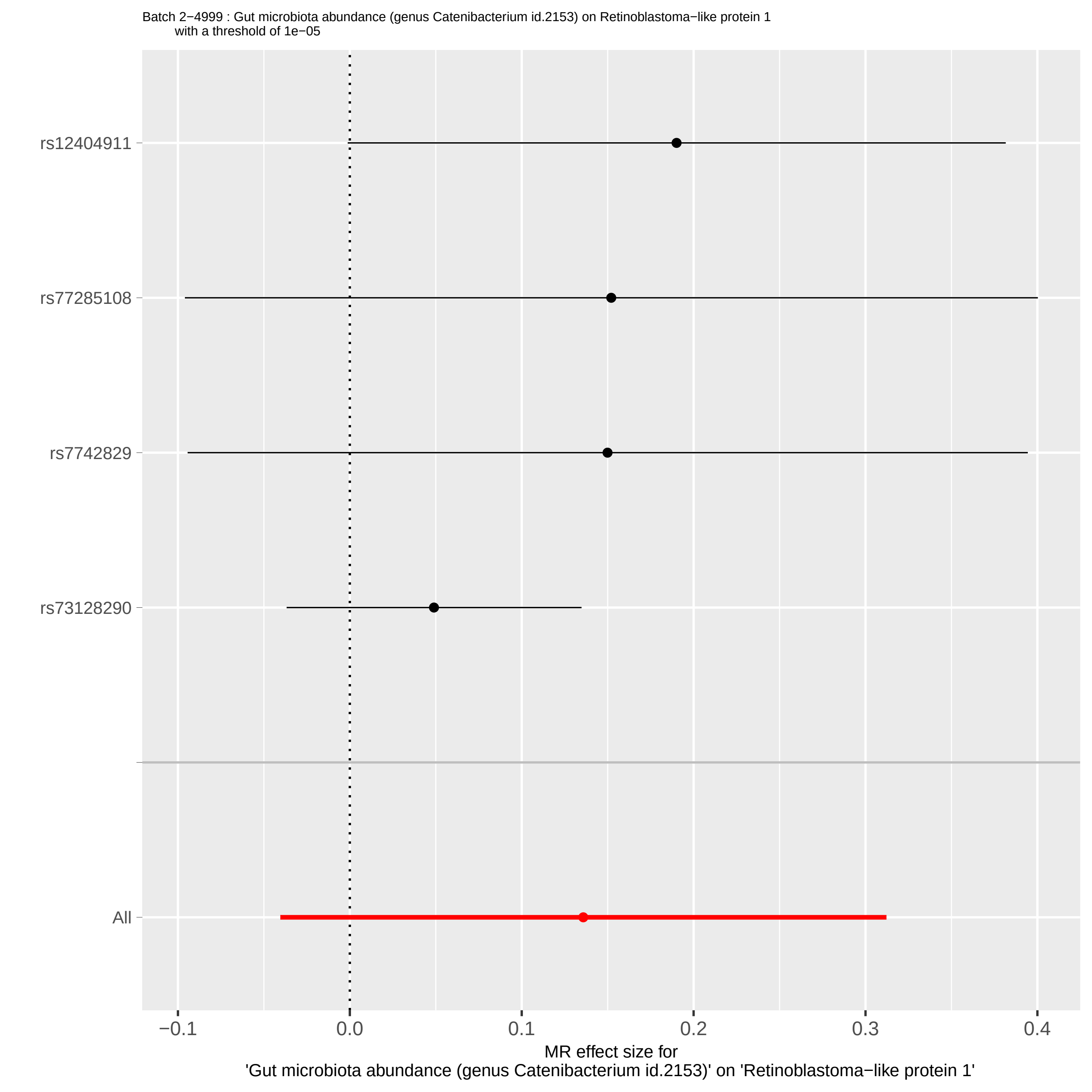
Batch 2−4999 : Gut microbiota abundance (genus Catenibacterium id.2153) on Retinoblastoma−like protein 1
with a threshold of 1e−05
rs12404911
rs77285108
rs7742829
rs73128290
All
−0.1
0.0
0.1
0.2
0.3
0.4
MR effect size for
'Gut microbiota abundance (genus Catenibacterium id.2153)' on 'Retinoblastoma−like protein 1'

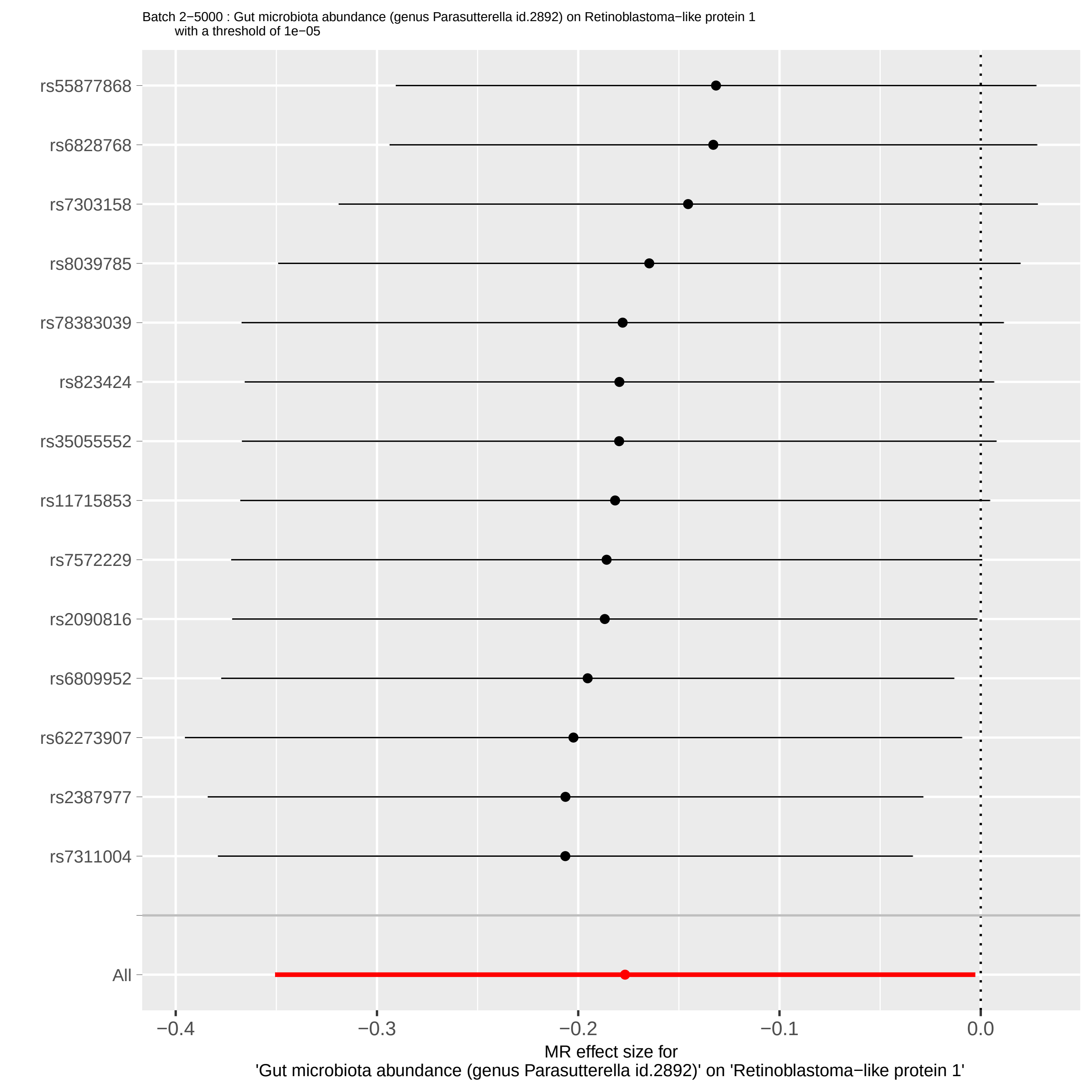
Batch 2−5000 : Gut microbiota abundance (genus Parasutterella id.2892) on Retinoblastoma−like protein 1
with a threshold of 1e−05
rs55877868
rs6828768
rs7303158
rs8039785
rs78383039
rs823424
rs35055552
rs11715853
rs7572229
rs2090816
rs6809952
rs62273907
rs2387977
rs7311004
All
−0.4
−0.3
−0.2
−0.1
0.0
MR effect size for
'Gut microbiota abundance (genus Parasutterella id.2892)' on 'Retinoblastoma−like protein 1'

Batch 2−5001 : Gut microbiota abundance (genus Catenibacterium id.2153) on Probable RNA−binding protein 19
with a threshold of 1e−05

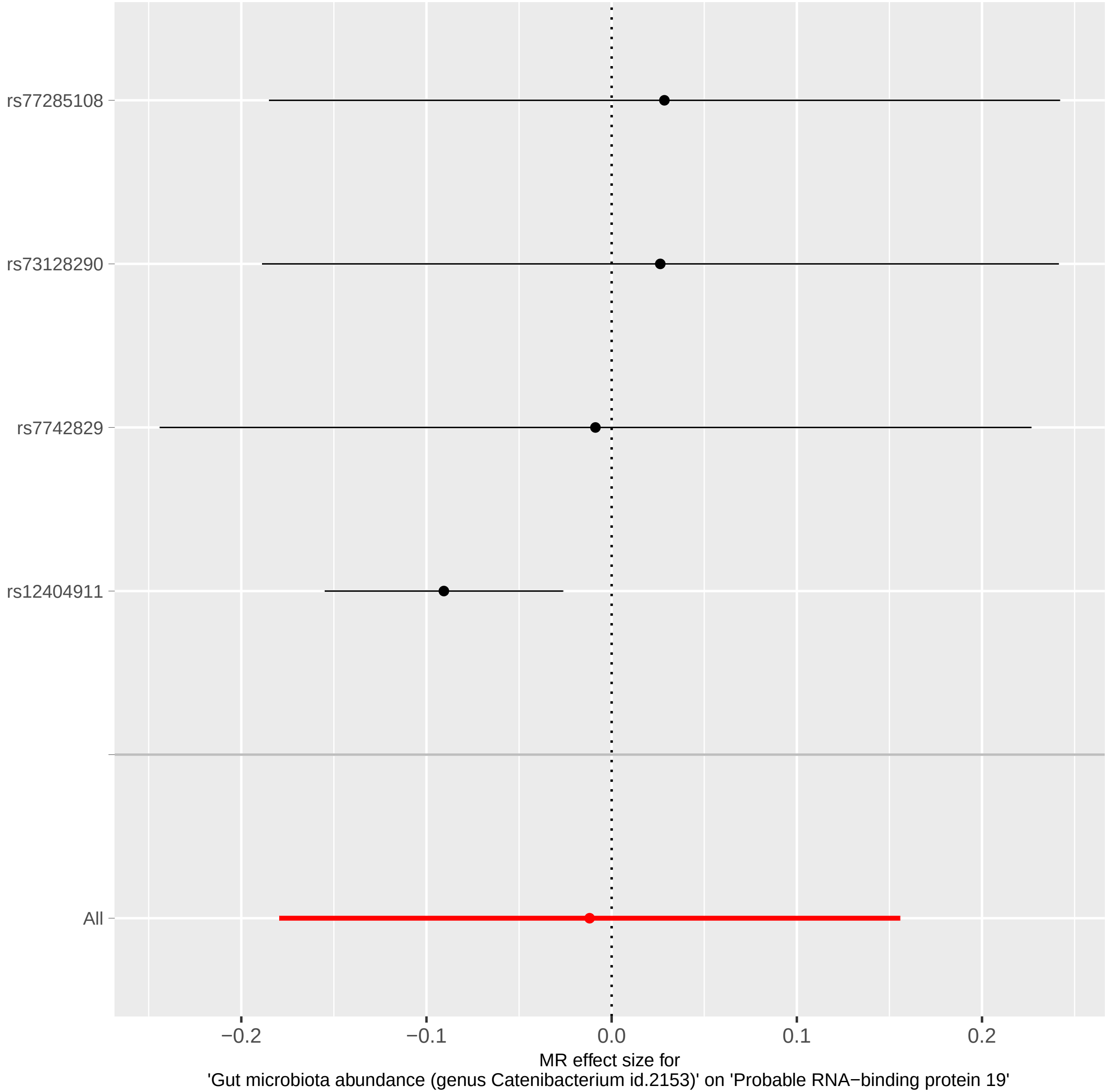

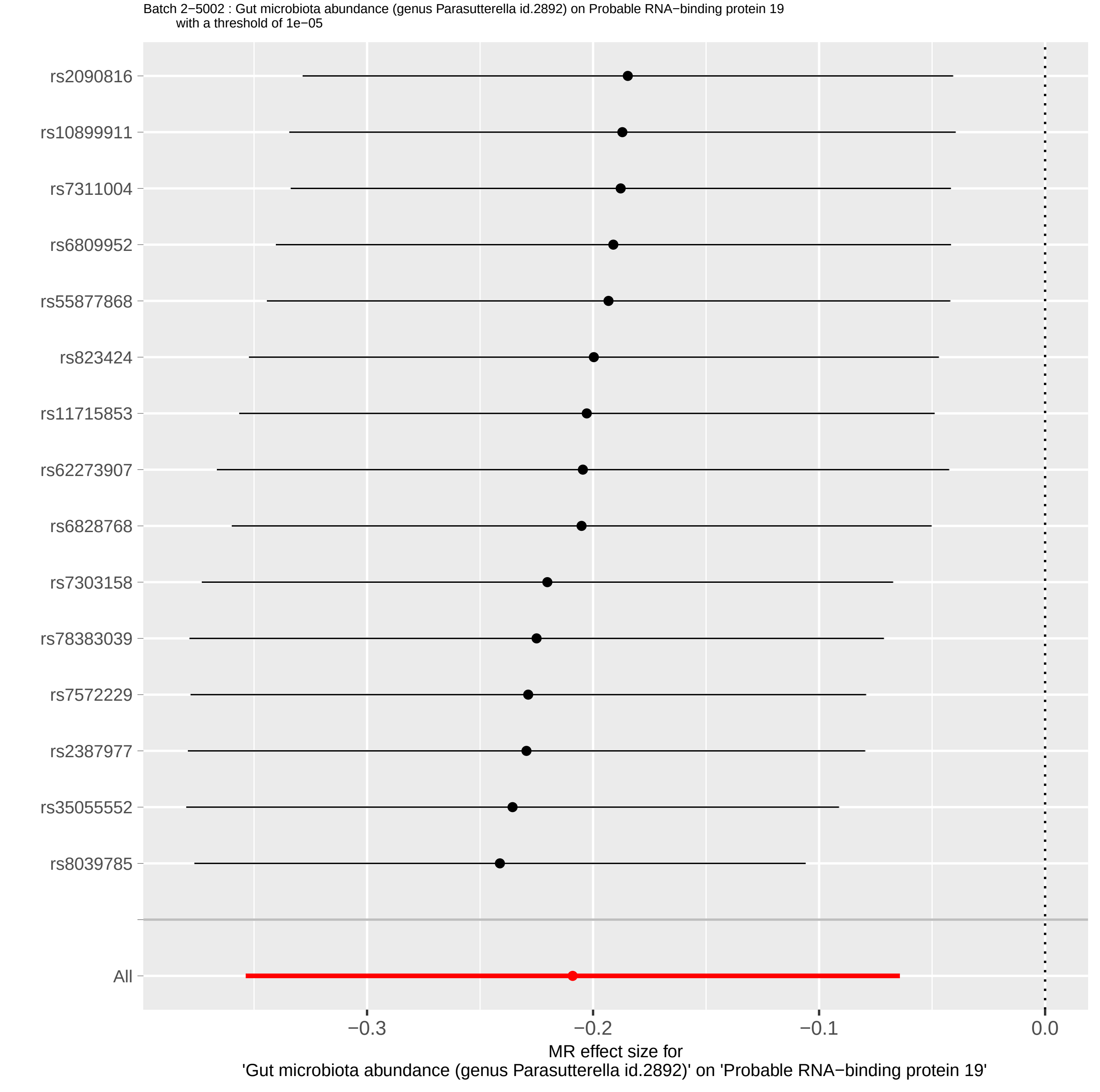

Batch 2−5002 : Gut microbiota abundance (genus Parasutterella id.2892) on Probable RNA−binding protein 19
with a threshold of 1e−05
rs2090816
rs10899911
rs7311004
rs6809952
rs55877868
rs823424
rs11715853
rs62273907
rs6828768
rs7303158
rs78383039
rs7572229
rs2387977
rs35055552
rs8039785
All
−0.3
−0.2
−0.1
0.0
MR effect size for
'Gut microbiota abundance (genus Parasutterella id.2892)' on 'Probable RNA−binding protein 19'

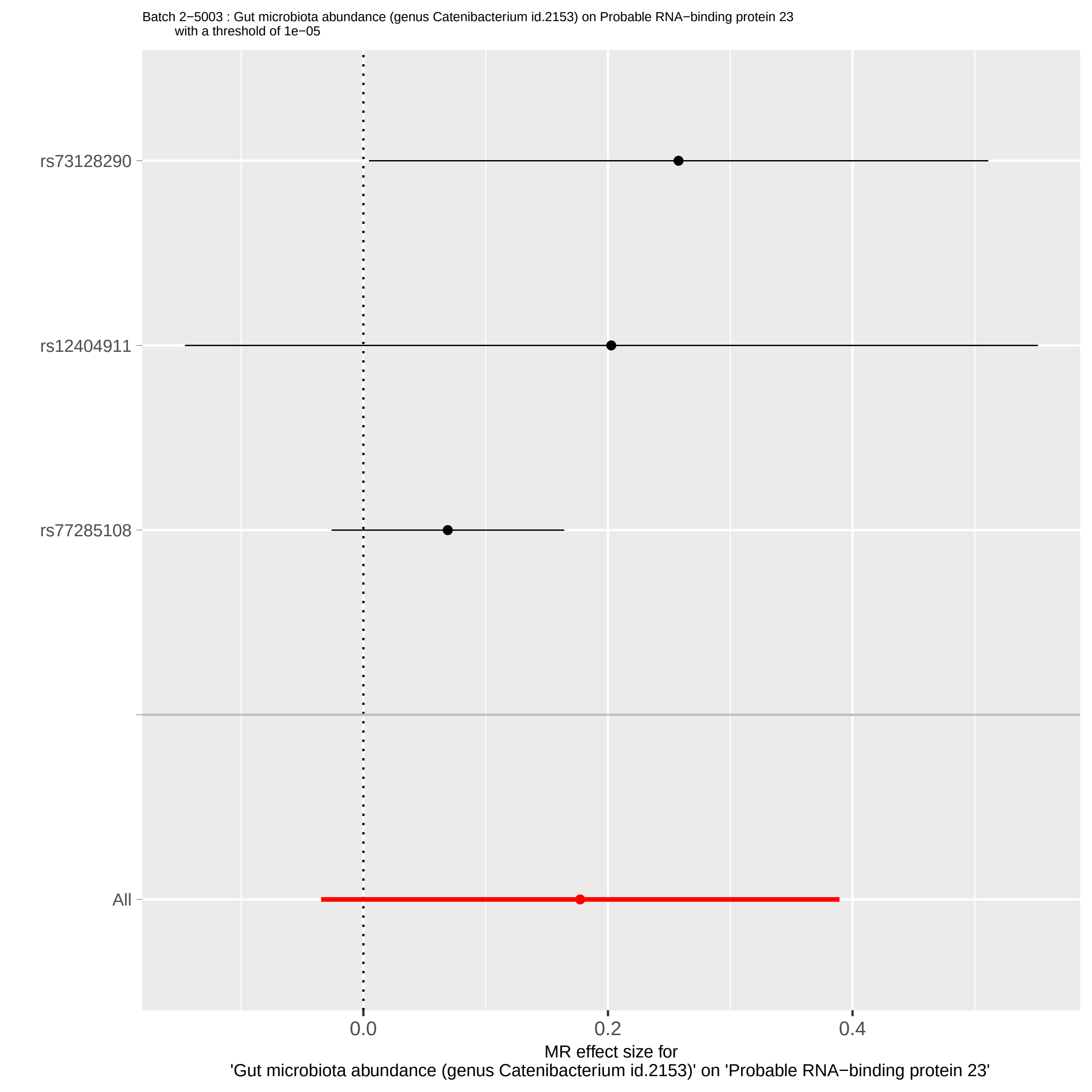
Batch 2−5003 : Gut microbiota abundance (genus Catenibacterium id.2153) on Probable RNA−binding protein 23
with a threshold of 1e−05
rs73128290
rs12404911
rs77285108
All
0.0
0.2
0.4
MR effect size for
'Gut microbiota abundance (genus Catenibacterium id.2153)' on 'Probable RNA−binding protein 23'

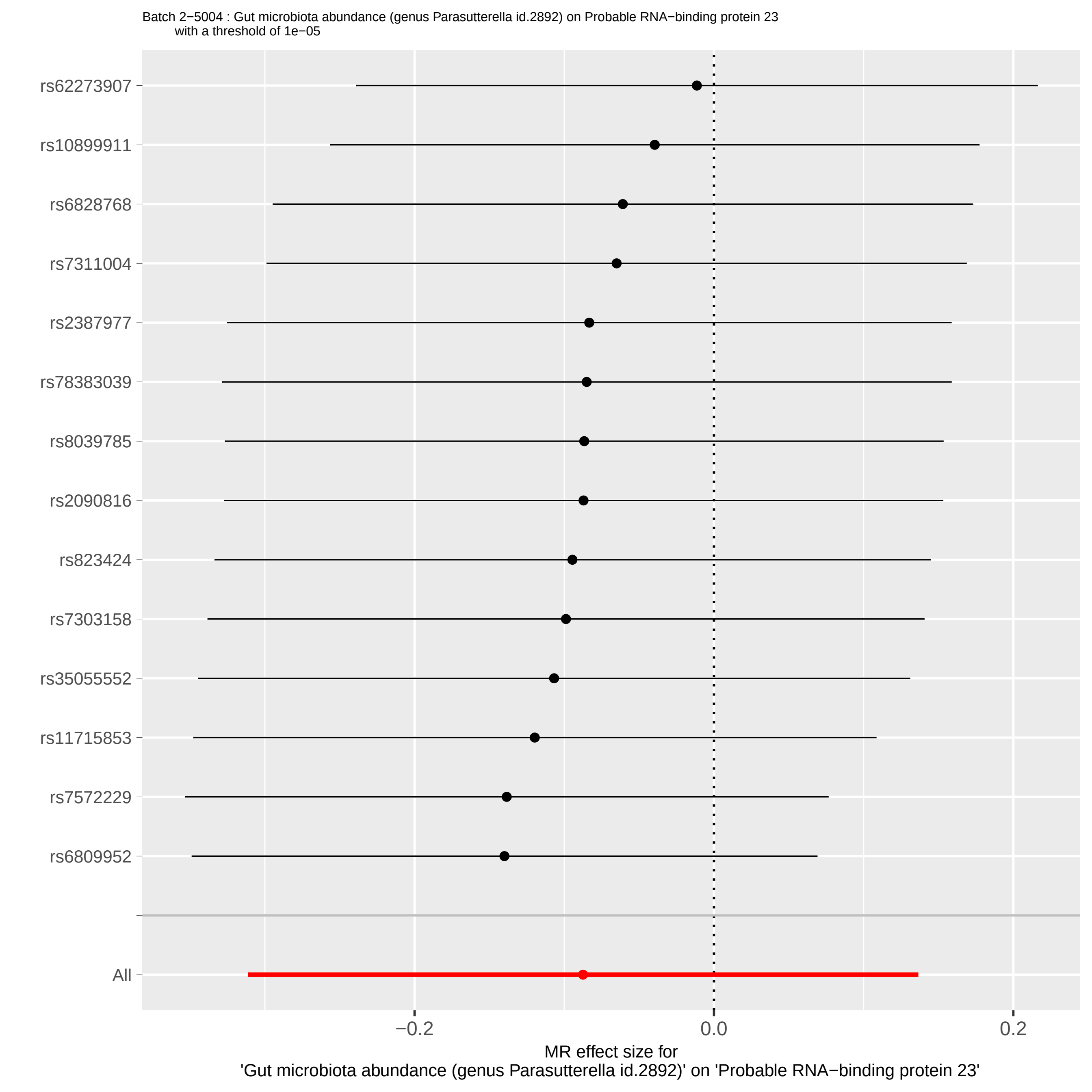
Batch 2−5004 : Gut microbiota abundance (genus Parasutterella id.2892) on Probable RNA−binding protein 23
with a threshold of 1e−05
rs62273907
rs10899911
rs6828768
rs7311004
rs2387977
rs78383039
rs8039785
rs2090816
rs823424
rs7303158
rs35055552
rs11715853
rs7572229
rs6809952
All
−0.2
0.0
0.2
MR effect size for
'Gut microbiota abundance (genus Parasutterella id.2892)' on 'Probable RNA−binding protein 23'

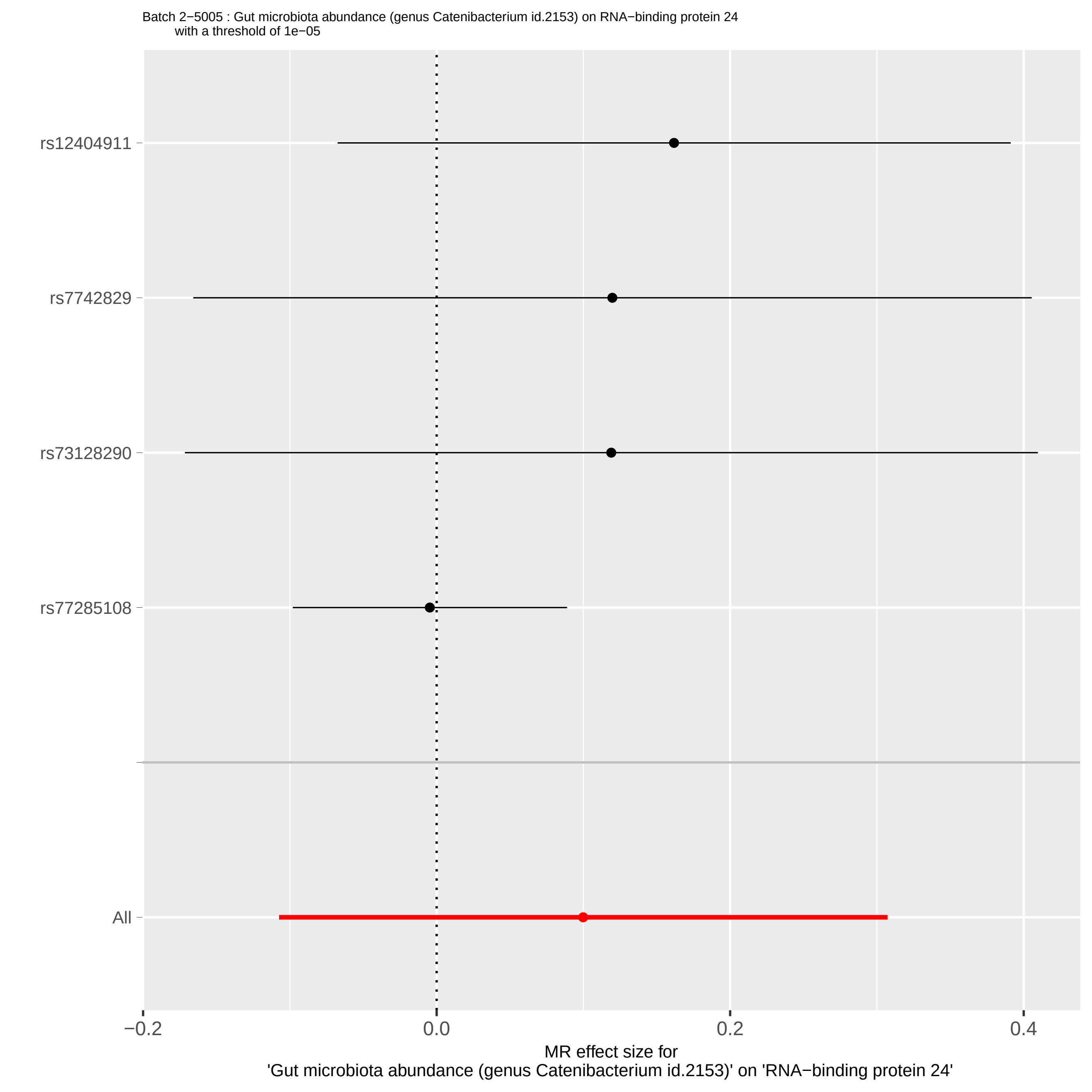
Batch 2−5005 : Gut microbiota abundance (genus Catenibacterium id.2153) on RNA−binding protein 24
with a threshold of 1e−05
rs12404911
rs7742829
rs73128290
rs77285108
All
−0.2
0.0
0.2
0.4
MR effect size for
'Gut microbiota abundance (genus Catenibacterium id.2153)' on 'RNA−binding protein 24'

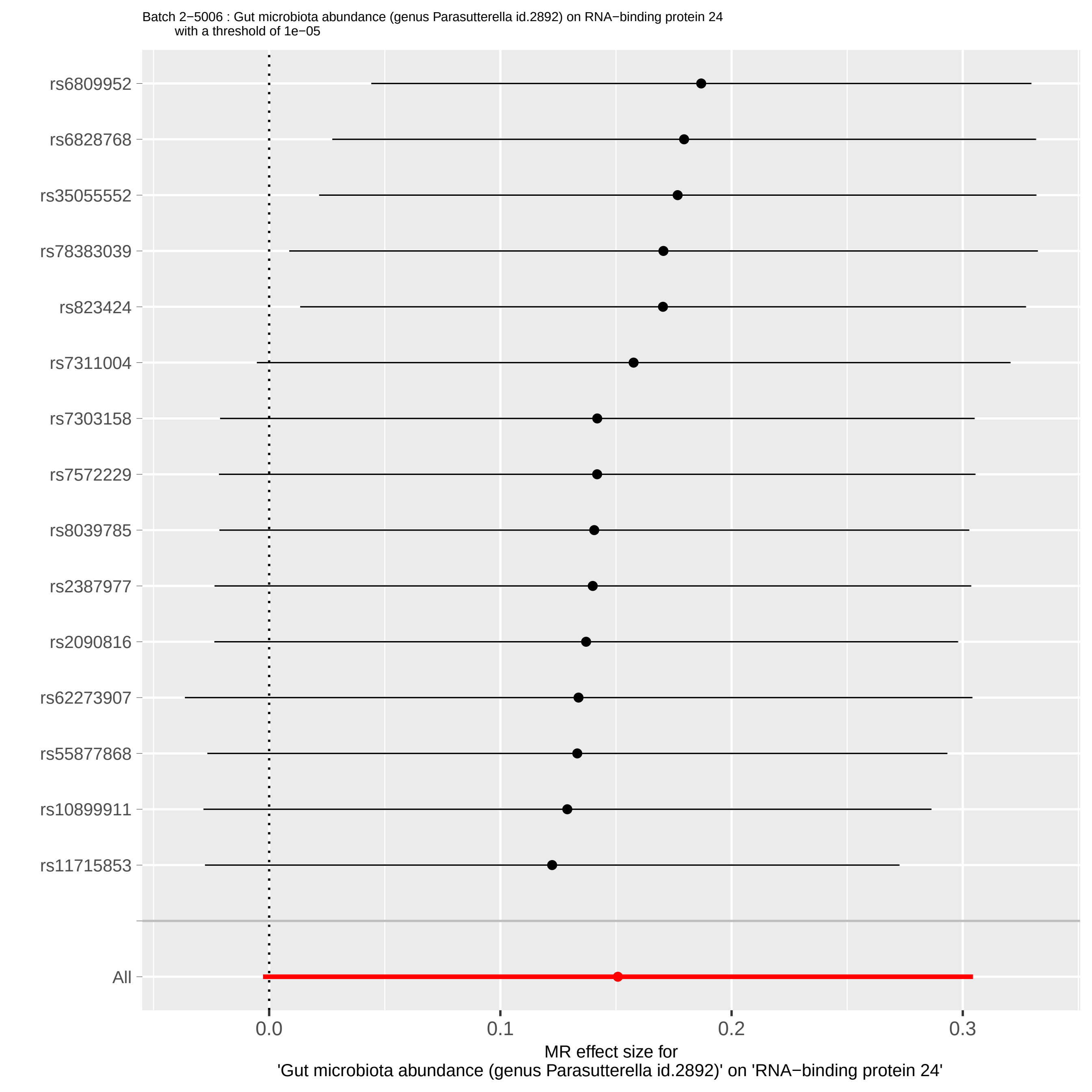

Batch 2−5006 : Gut microbiota abundance (genus Parasutterella id.2892) on RNA−binding protein 24
with a threshold of 1e−05
rs6809952
rs6828768
rs35055552
rs78383039
rs823424
rs7311004
rs7303158
rs7572229
rs8039785
rs2387977
rs2090816
rs62273907
rs55877868
rs10899911
rs11715853
All
0.0
0.1
0.2
0.3
MR effect size for
'Gut microbiota abundance (genus Parasutterella id.2892)' on 'RNA−binding protein 24'

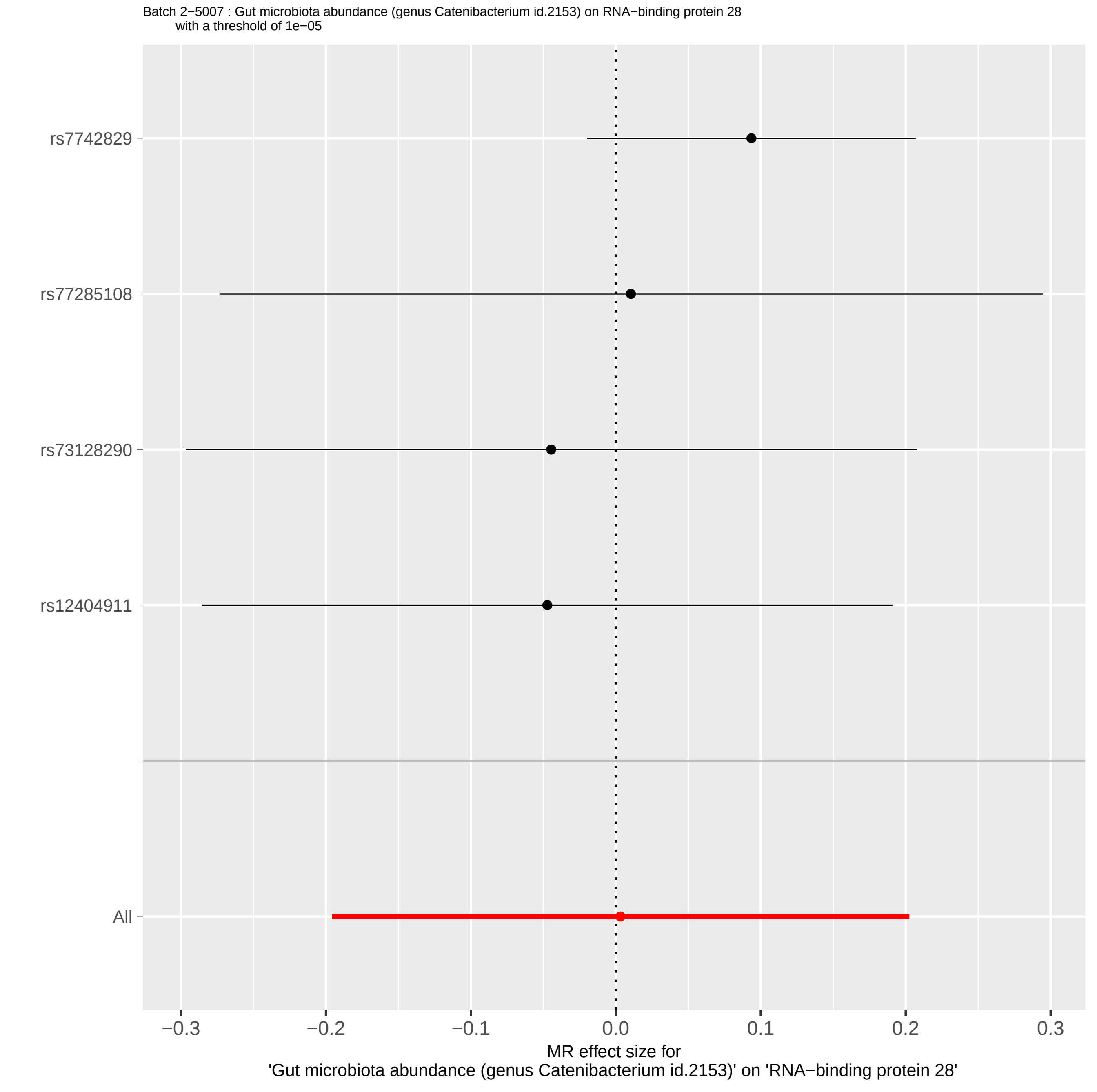
Batch 2−5007 : Gut microbiota abundance (genus Catenibacterium id.2153) on RNA−binding protein 28
with a threshold of 1e−05
rs7742829
rs77285108
rs73128290
rs12404911
All
−0.3
−0.2
−0.1
0.0
0.1
0.2
0.3
MR effect size for
'Gut microbiota abundance (genus Catenibacterium id.2153)' on 'RNA−binding protein 28'

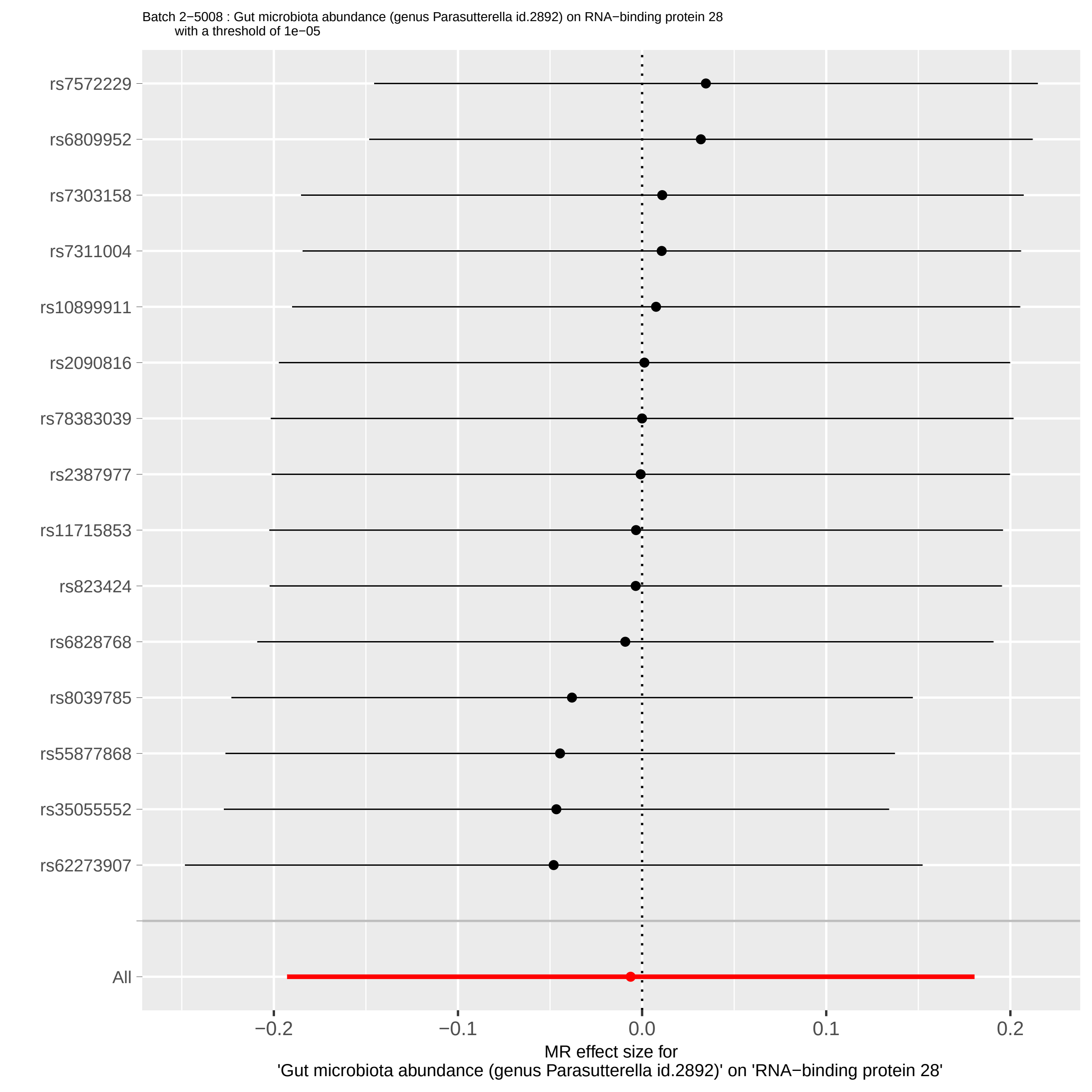
Batch 2−5008 : Gut microbiota abundance (genus Parasutterella id.2892) on RNA−binding protein 28
with a threshold of 1e−05
rs7572229
rs6809952
rs7303158
rs7311004
rs10899911
rs2090816
rs78383039
rs2387977
rs11715853
rs823424
rs6828768
rs8039785
rs55877868
rs35055552
rs62273907
All
−0.2
−0.1
0.0
0.1
0.2
MR effect size for
'Gut microbiota abundance (genus Parasutterella id.2892)' on 'RNA−binding protein 28'

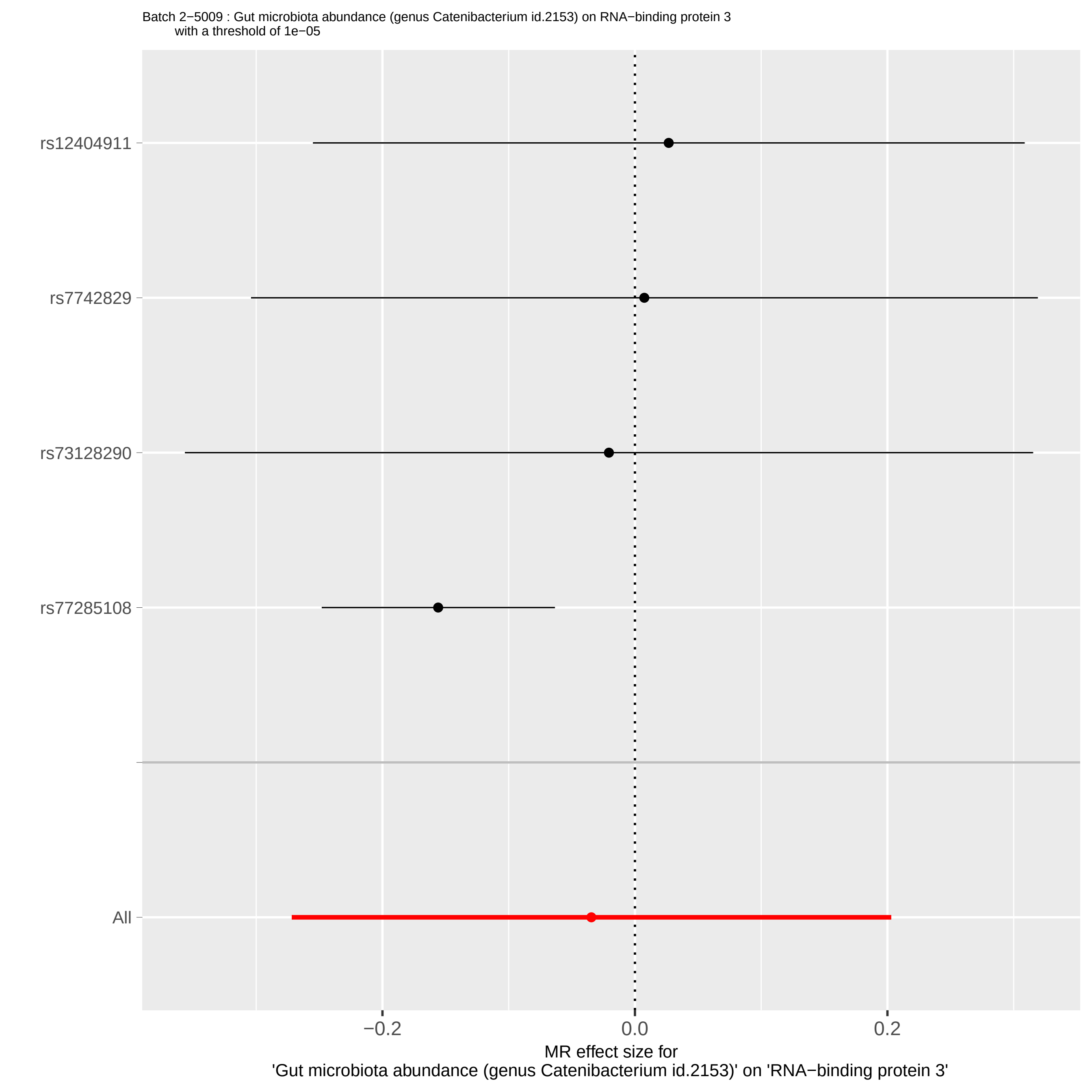

Batch 2−5009 : Gut microbiota abundance (genus Catenibacterium id.2153) on RNA−binding protein 3
with a threshold of 1e−05
rs12404911
rs7742829
rs73128290
rs77285108
All
−0.2
0.0
0.2
MR effect size for
'Gut microbiota abundance (genus Catenibacterium id.2153)' on 'RNA−binding protein 3'

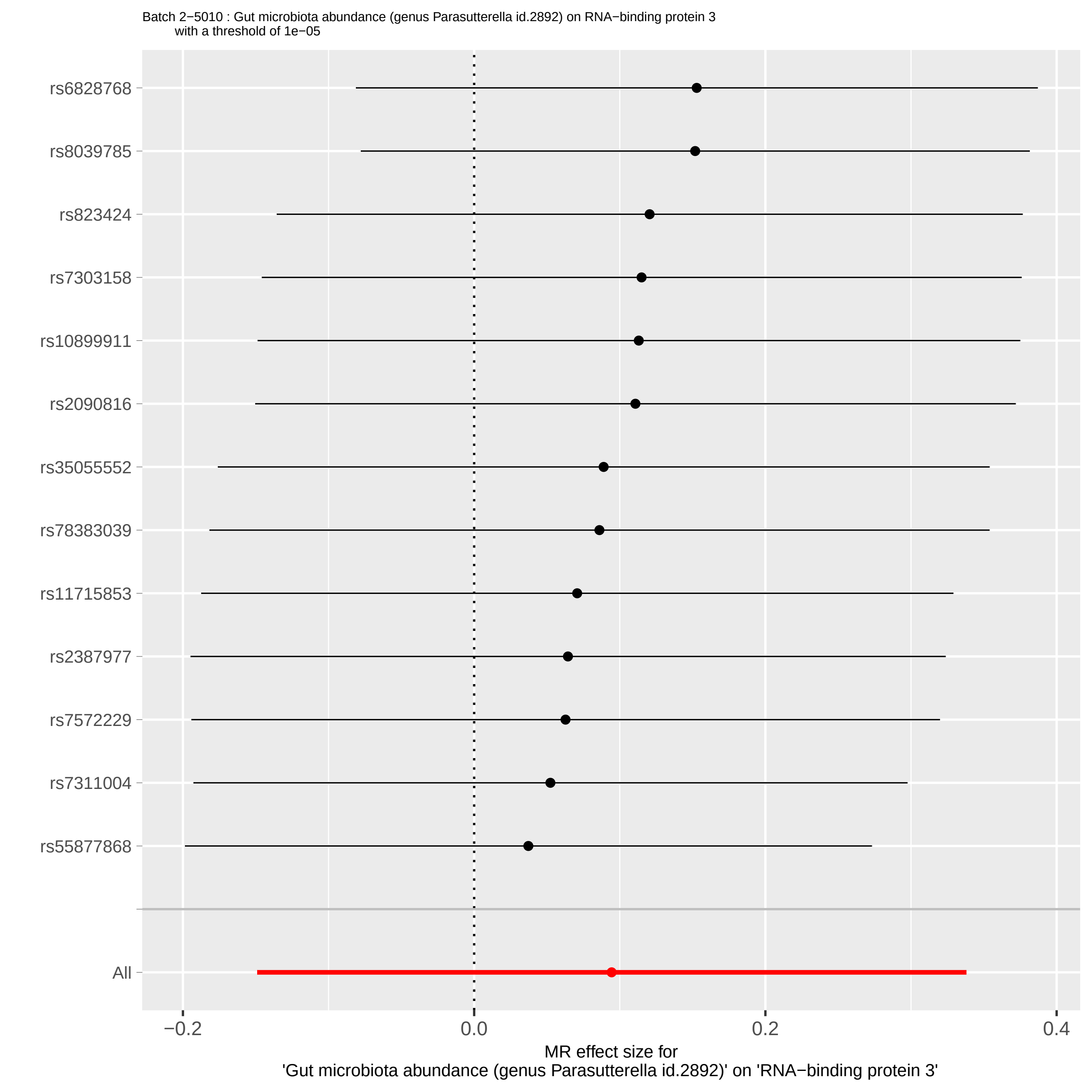
Batch 2−5010 : Gut microbiota abundance (genus Parasutterella id.2892) on RNA−binding protein 3
with a threshold of 1e−05
rs6828768
rs8039785
rs823424
rs7303158
rs10899911
rs2090816
rs35055552
rs78383039
rs11715853
rs2387977
rs7572229
rs7311004
rs55877868
All
−0.2
0.0
0.2
0.4
MR effect size for
'Gut microbiota abundance (genus Parasutterella id.2892)' on 'RNA−binding protein 3'

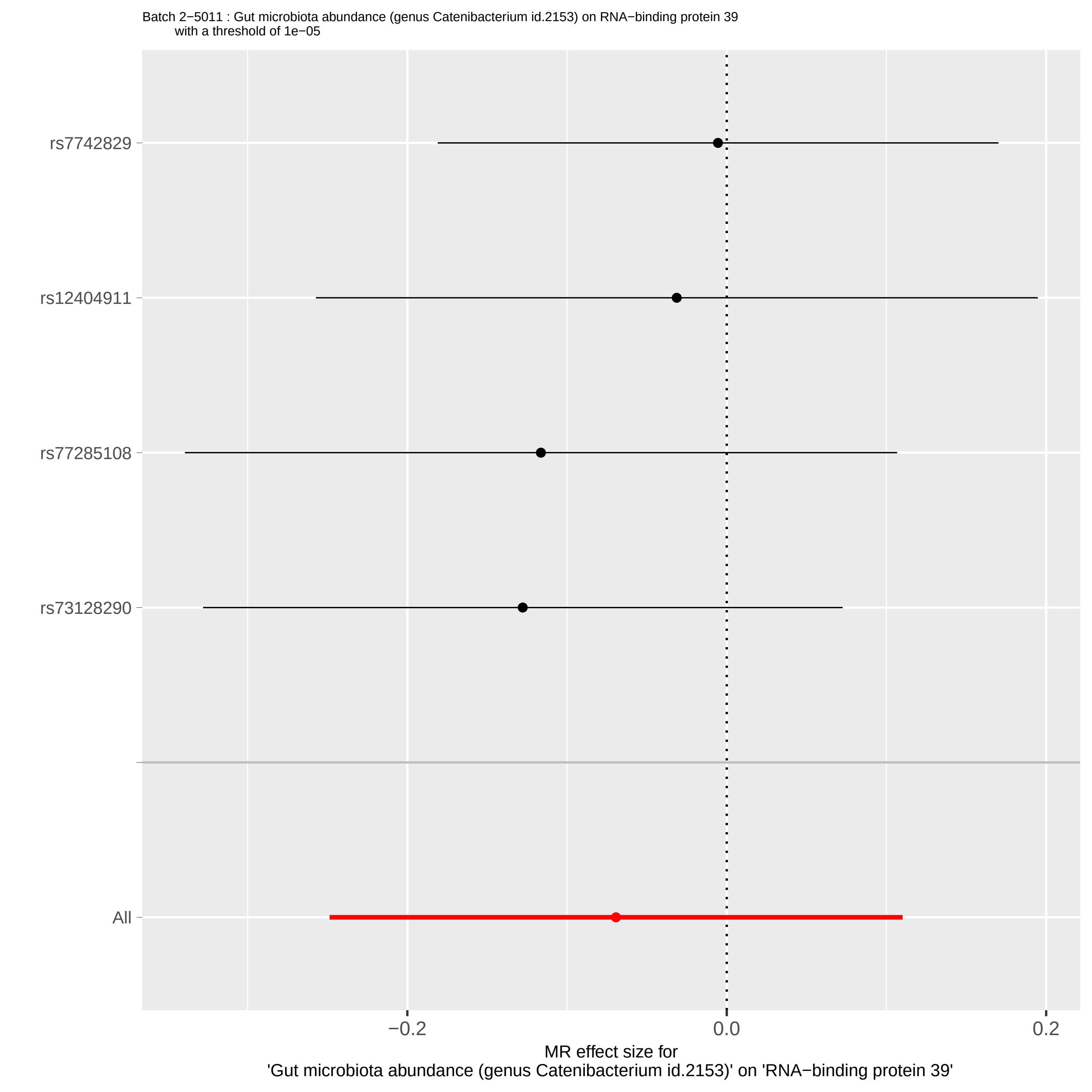
Batch 2−5011 : Gut microbiota abundance (genus Catenibacterium id.2153) on RNA−binding protein 39
with a threshold of 1e−05
rs7742829
rs12404911
rs77285108
rs73128290
All
−0.2
0.0
0.2
MR effect size for
'Gut microbiota abundance (genus Catenibacterium id.2153)' on 'RNA−binding protein 39'

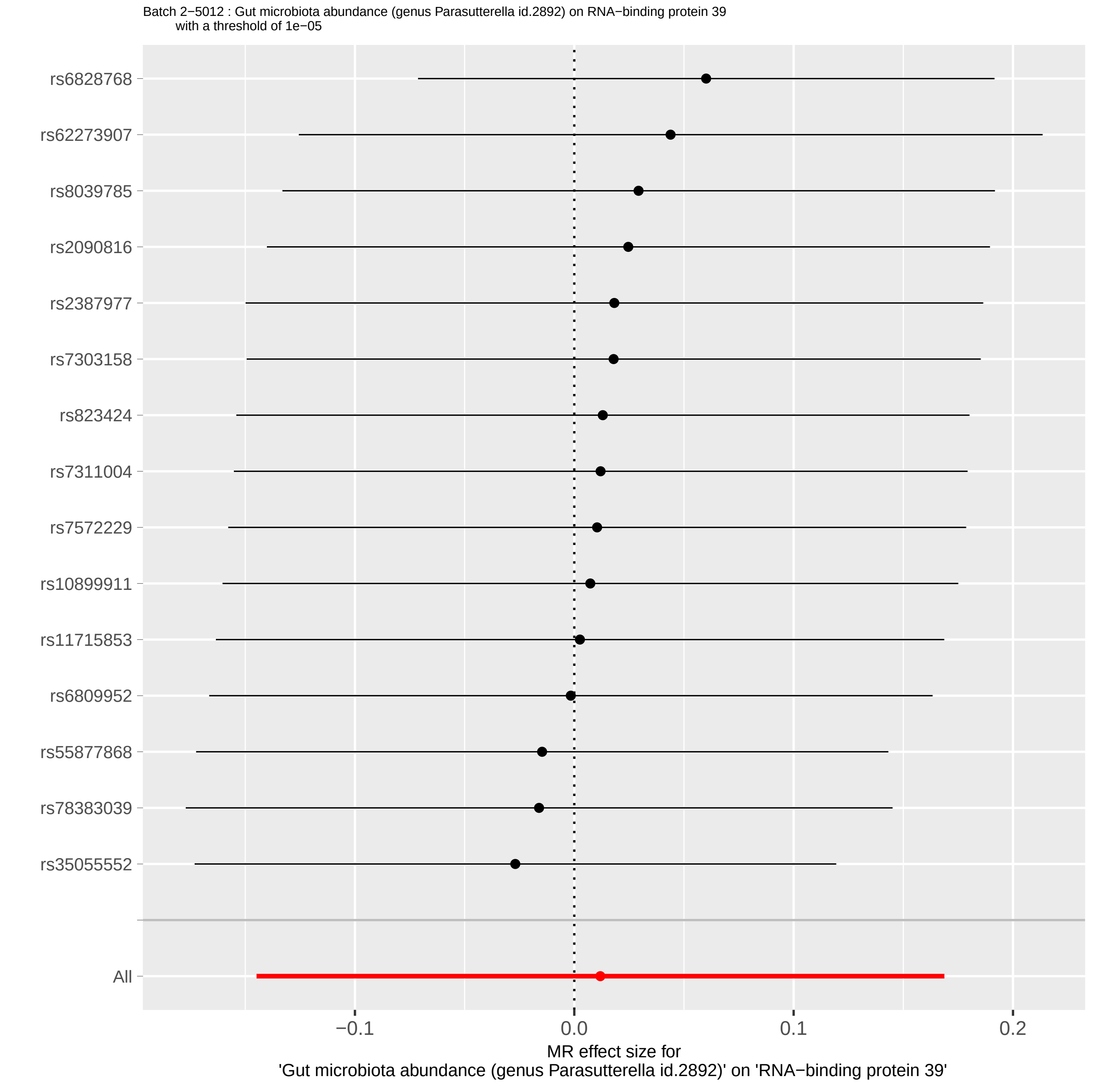

Batch 2−5012 : Gut microbiota abundance (genus Parasutterella id.2892) on RNA−binding protein 39
with a threshold of 1e−05
rs6828768
rs62273907
rs8039785
rs2090816
rs2387977
rs7303158
rs823424
rs7311004
rs7572229
rs10899911
rs11715853
rs6809952
rs55877868
rs78383039
rs35055552
All
−0.1
0.0
0.1
0.2
MR effect size for
'Gut microbiota abundance (genus Parasutterella id.2892)' on 'RNA−binding protein 39'

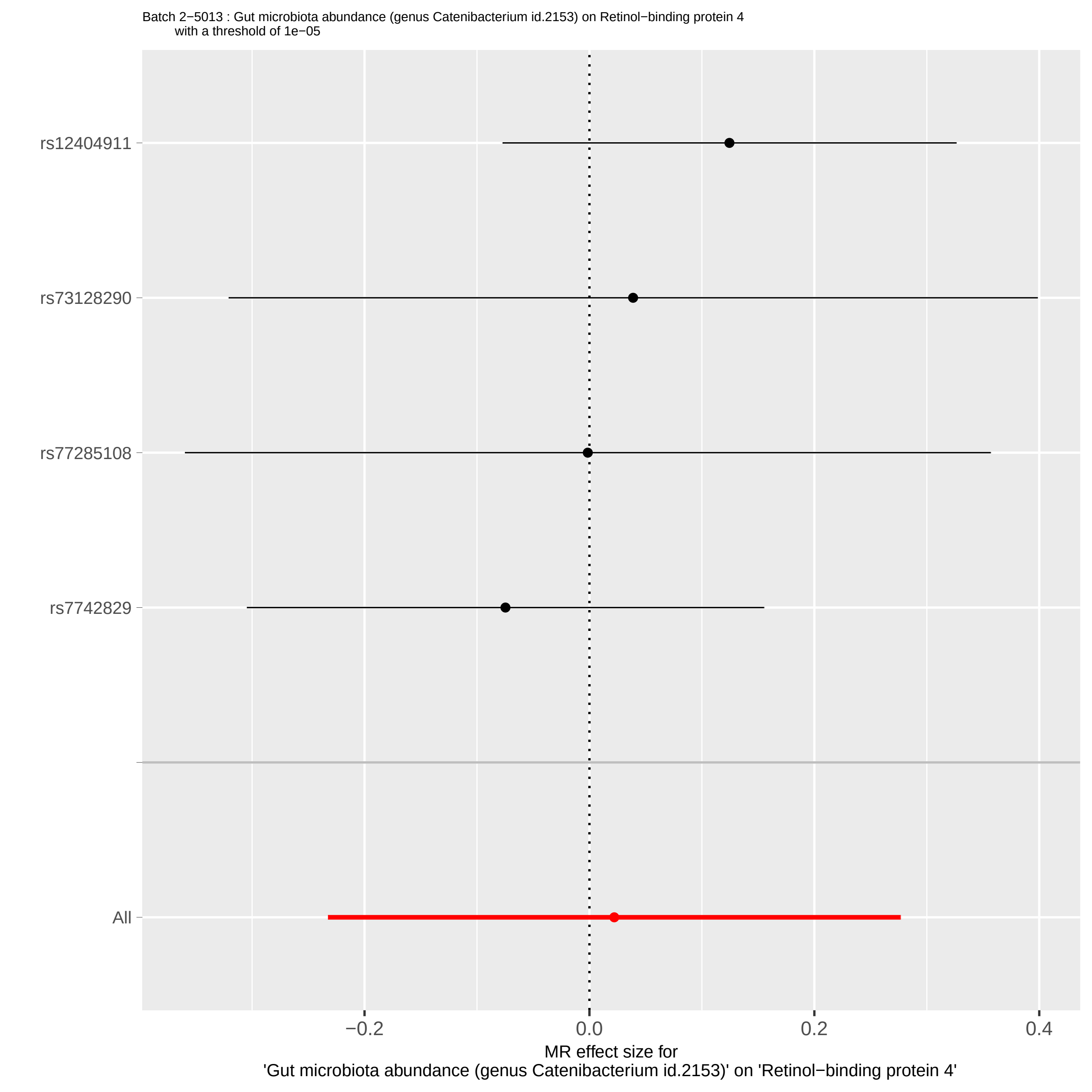
Batch 2−5013 : Gut microbiota abundance (genus Catenibacterium id.2153) on Retinol−binding protein 4
with a threshold of 1e−05
rs12404911
rs73128290
rs77285108
rs7742829
All
−0.2
0.0
0.2
0.4
MR effect size for
'Gut microbiota abundance (genus Catenibacterium id.2153)' on 'Retinol−binding protein 4'

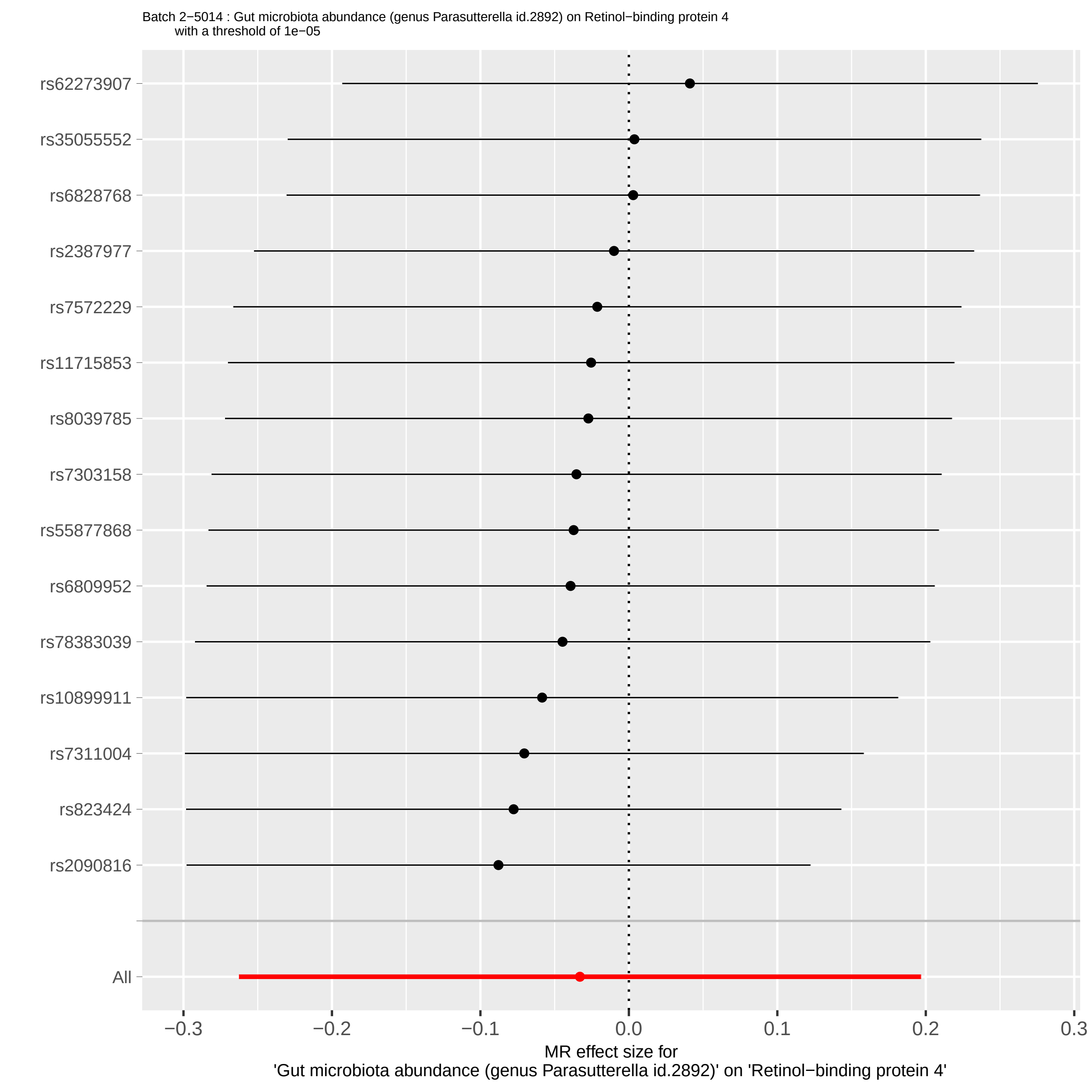
Batch 2−5014 : Gut microbiota abundance (genus Parasutterella id.2892) on Retinol−binding protein 4
with a threshold of 1e−05
rs62273907
rs35055552
rs6828768
rs2387977
rs7572229
rs11715853
rs8039785
rs7303158
rs55877868
rs6809952
rs78383039
rs10899911
rs7311004
rs823424
rs2090816
All
−0.3
−0.2
−0.1
0.0
0.1
0.2
0.3
MR effect size for
'Gut microbiota abundance (genus Parasutterella id.2892)' on 'Retinol−binding protein 4'

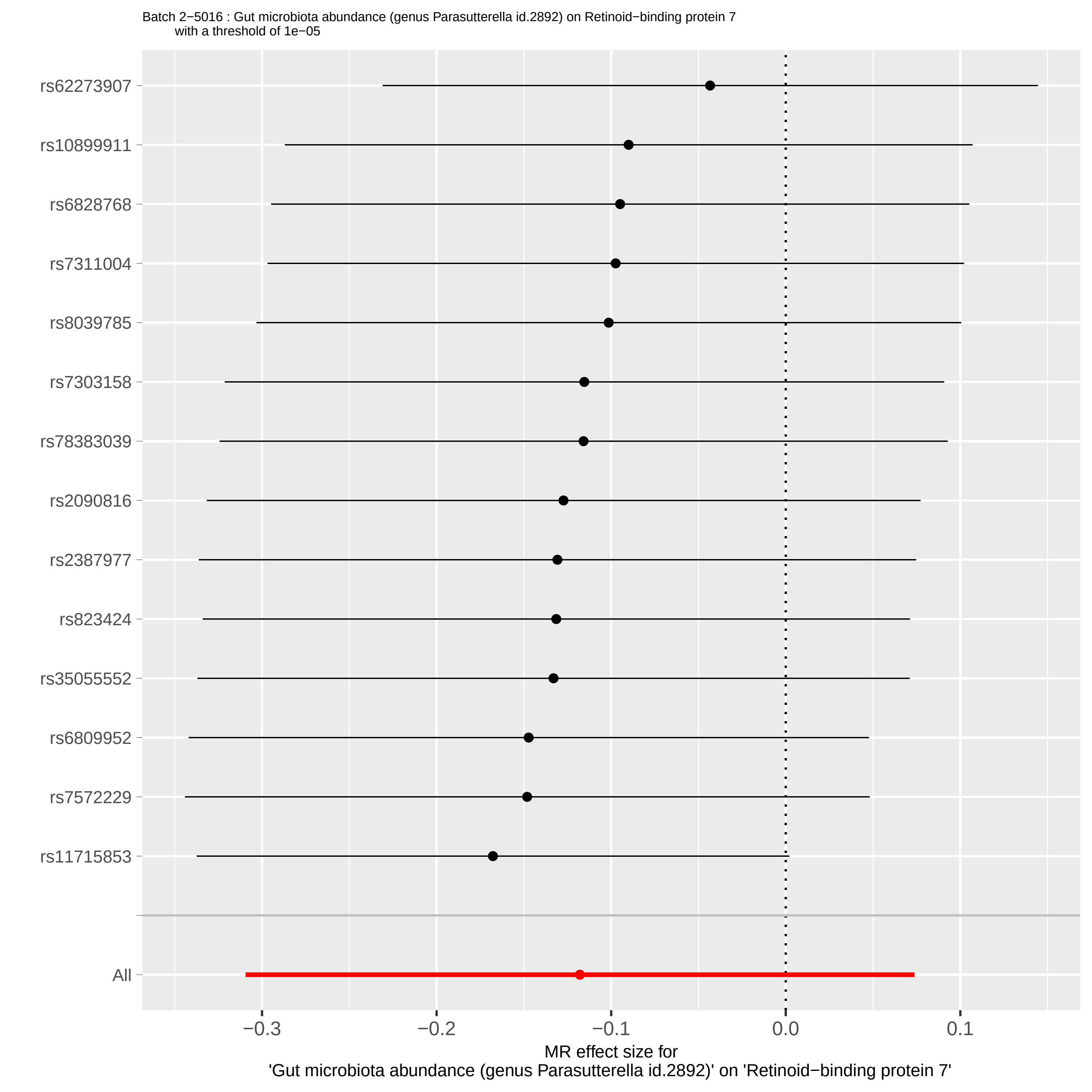
Batch 2−5016 : Gut microbiota abundance (genus Parasutterella id.2892) on Retinoid−binding protein 7
with a threshold of 1e−05
rs62273907
rs10899911
rs6828768
rs7311004
rs8039785
rs7303158
rs78383039
rs2090816
rs2387977
rs823424
rs35055552
rs6809952
rs7572229
rs11715853
All
−0.3
−0.2
−0.1
0.0
0.1
MR effect size for
'Gut microbiota abundance (genus Parasutterella id.2892)' on 'Retinoid−binding protein 7'

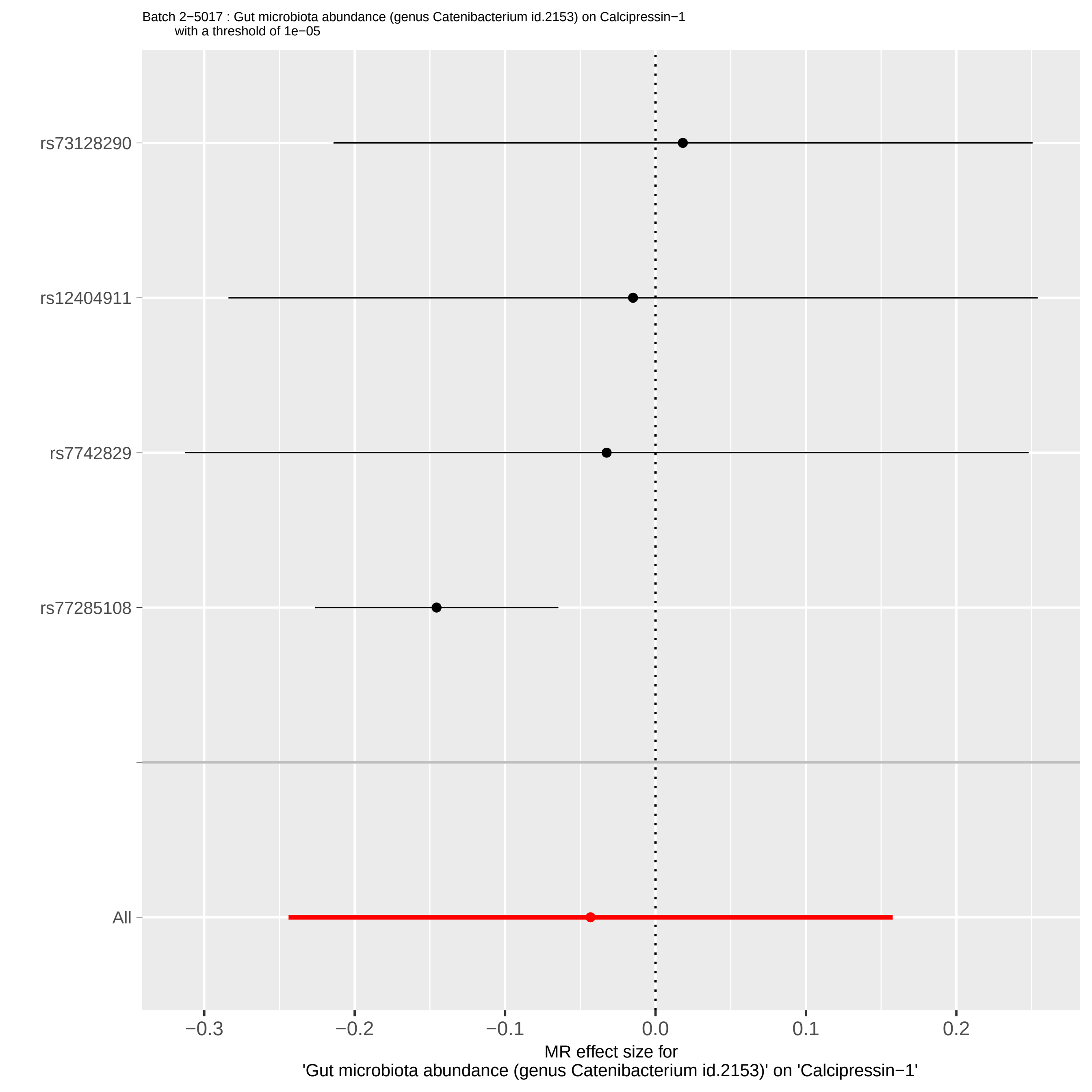

Batch 2−5017 : Gut microbiota abundance (genus Catenibacterium id.2153) on Calcipressin−1
with a threshold of 1e−05
rs73128290
rs12404911
rs7742829
rs77285108
All
−0.3
−0.2
−0.1
0.0
0.1
0.2
MR effect size for
'Gut microbiota abundance (genus Catenibacterium id.2153)' on 'Calcipressin−1'

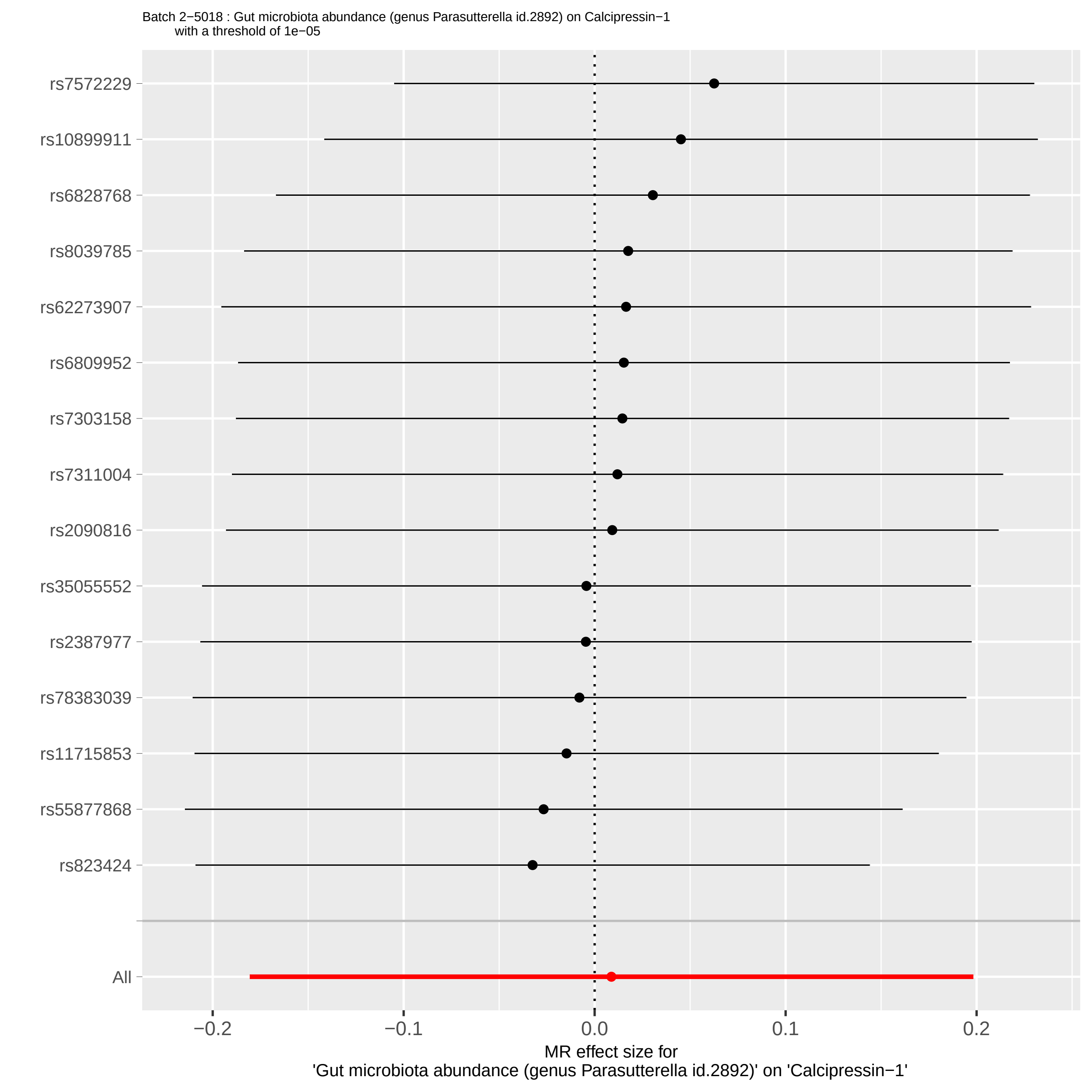
Batch 2−5018 : Gut microbiota abundance (genus Parasutterella id.2892) on Calcipressin−1
with a threshold of 1e−05
rs7572229
rs10899911
rs6828768
rs8039785
rs62273907
rs6809952
rs7303158
rs7311004
rs2090816
rs35055552
rs2387977
rs78383039
rs11715853
rs55877868
rs823424
All
−0.2
−0.1
0.0
0.1
0.2
MR effect size for
'Gut microbiota abundance (genus Parasutterella id.2892)' on 'Calcipressin−1'

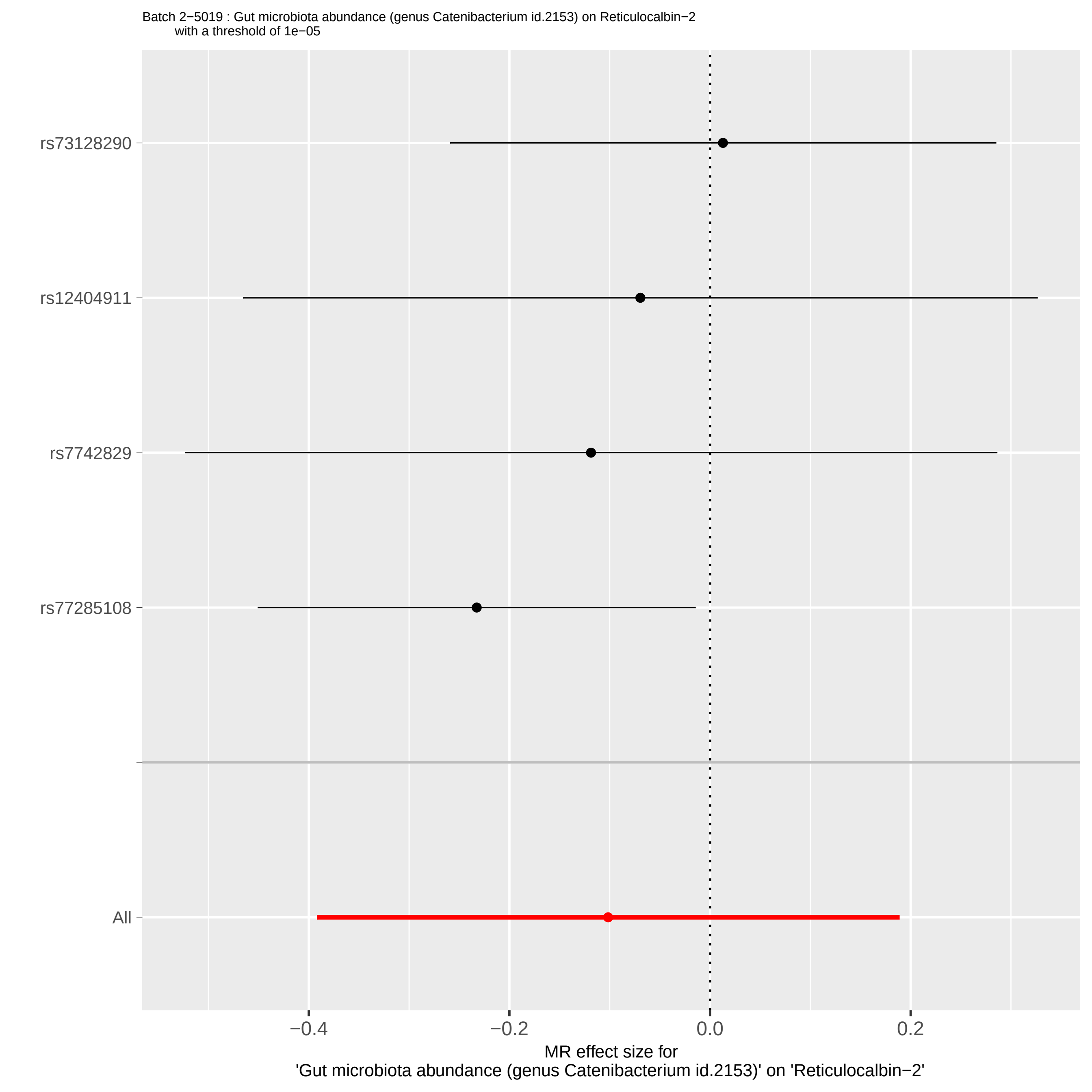
Batch 2−5019 : Gut microbiota abundance (genus Catenibacterium id.2153) on Reticulocalbin−2
with a threshold of 1e−05
rs73128290
rs12404911
rs7742829
rs77285108
All
−0.4
−0.2
0.0
0.2
MR effect size for
'Gut microbiota abundance (genus Catenibacterium id.2153)' on 'Reticulocalbin−2'

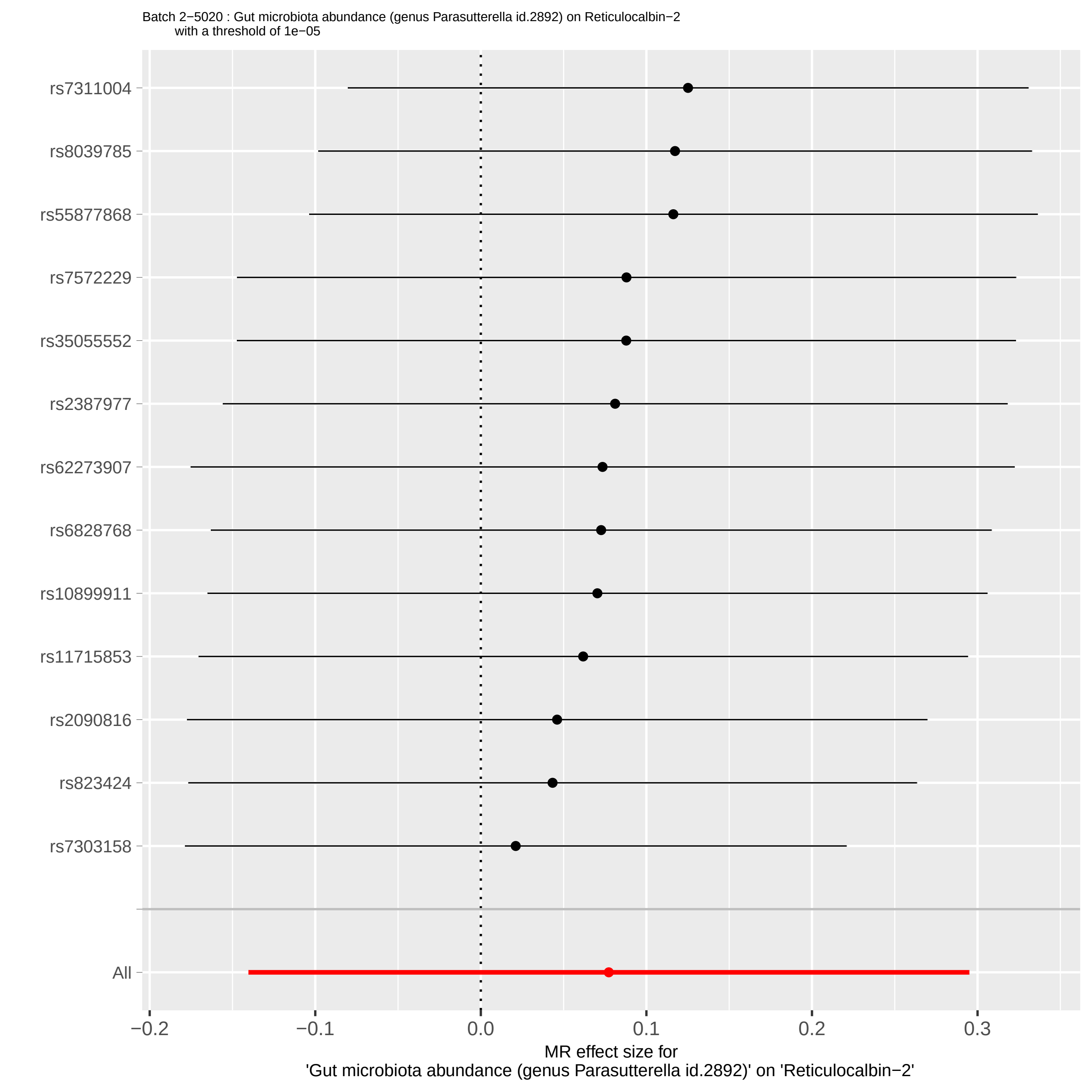

Batch 2−5020 : Gut microbiota abundance (genus Parasutterella id.2892) on Reticulocalbin−2
with a threshold of 1e−05
rs7311004
rs8039785
rs55877868
rs7572229
rs35055552
rs2387977
rs62273907
rs6828768
rs10899911
rs11715853
rs2090816
rs823424
rs7303158
All
−0.2
−0.1
0.0
0.1
0.2
0.3
MR effect size for
'Gut microbiota abundance (genus Parasutterella id.2892)' on 'Reticulocalbin−2'

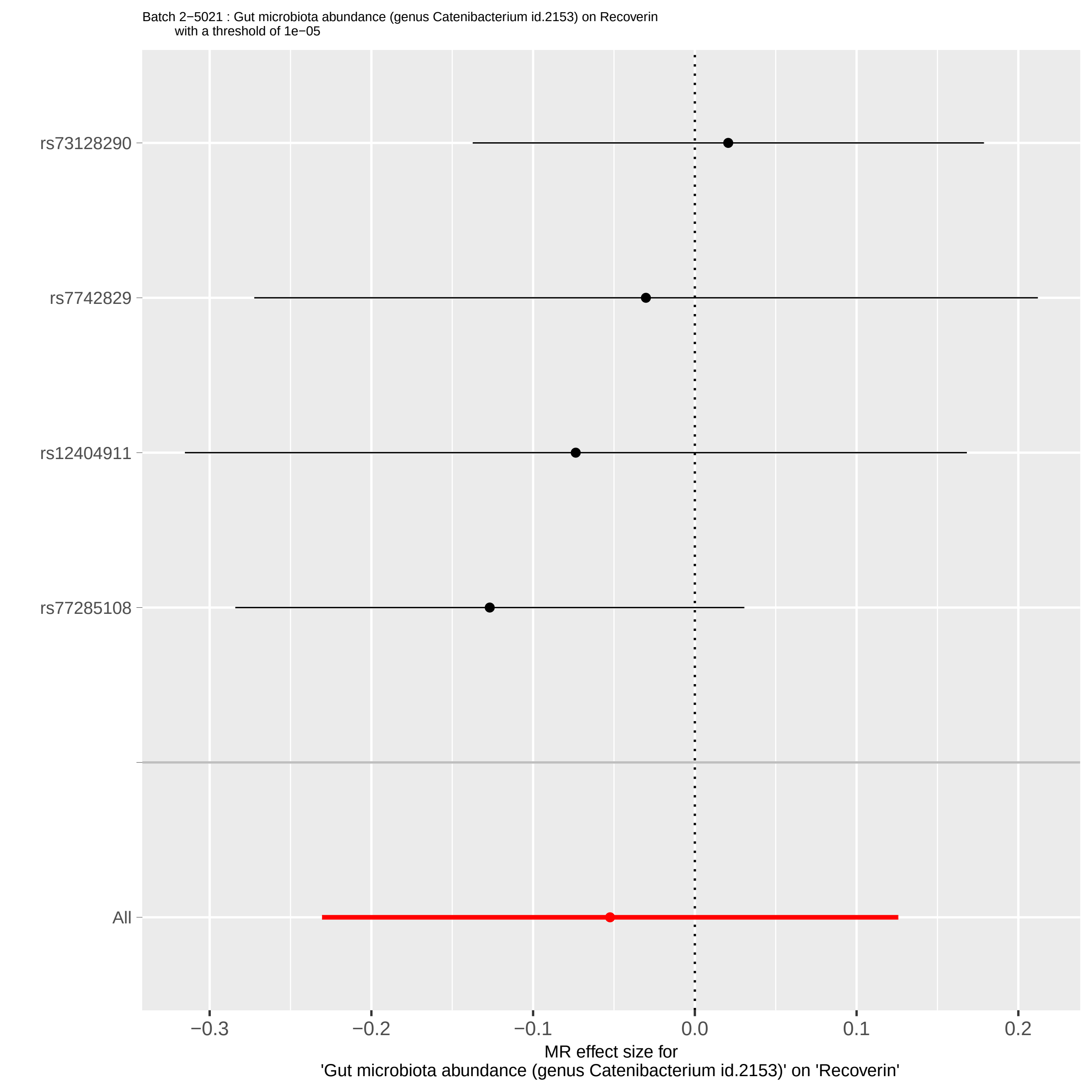
Batch 2−5021 : Gut microbiota abundance (genus Catenibacterium id.2153) on Recoverin
with a threshold of 1e−05
rs73128290
rs7742829
rs12404911
rs77285108
All
−0.3
−0.2
−0.1
0.0
0.1
0.2
MR effect size for
'Gut microbiota abundance (genus Catenibacterium id.2153)' on 'Recoverin'

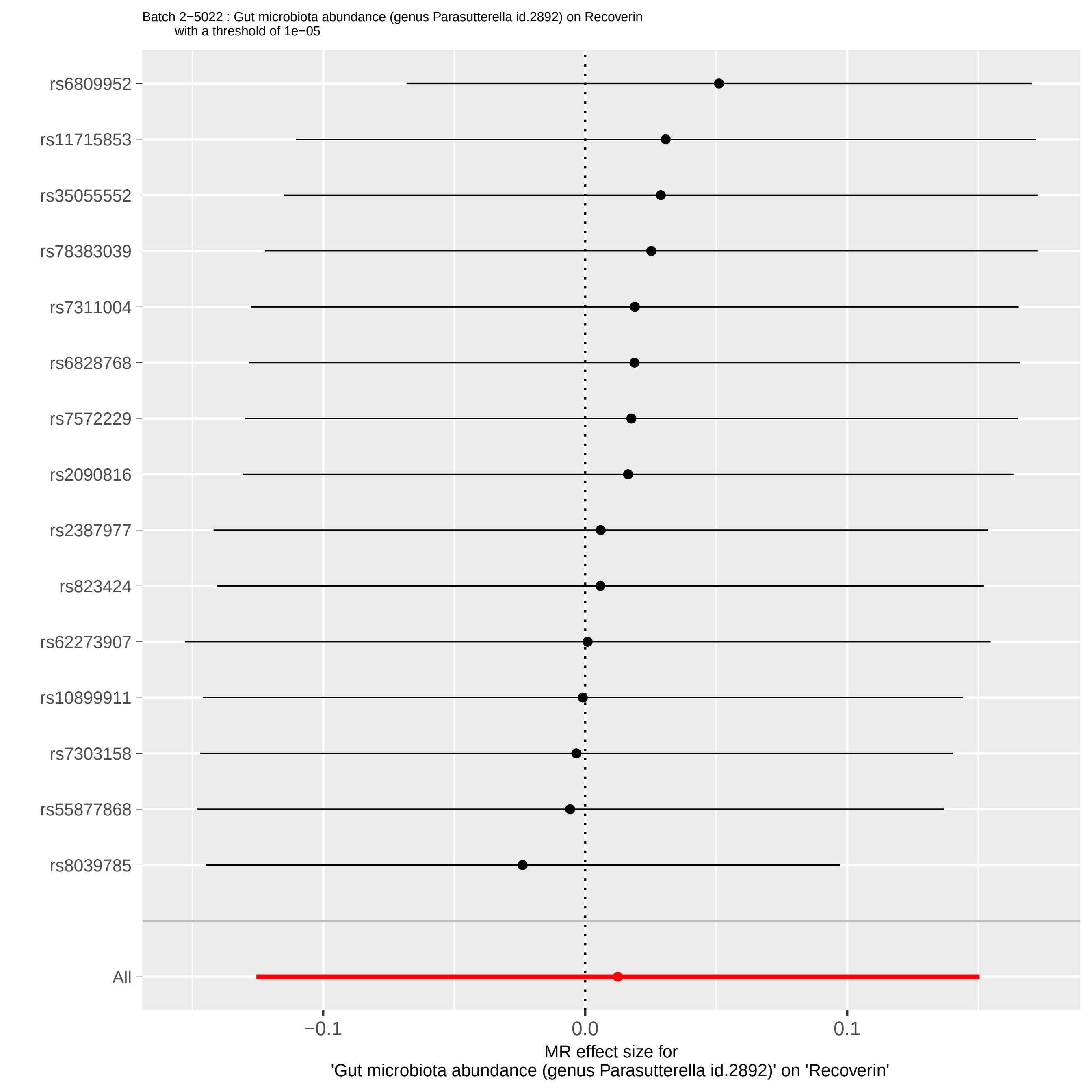
Batch 2−5022 : Gut microbiota abundance (genus Parasutterella id.2892) on Recoverin
with a threshold of 1e−05
rs6809952
rs11715853
rs35055552
rs78383039
rs7311004
rs6828768
rs7572229
rs2090816
rs2387977
rs823424
rs62273907
rs10899911
rs7303158
rs55877868
rs8039785
All
−0.1
0.0
0.1
MR effect size for
'Gut microbiota abundance (genus Parasutterella id.2892)' on 'Recoverin'

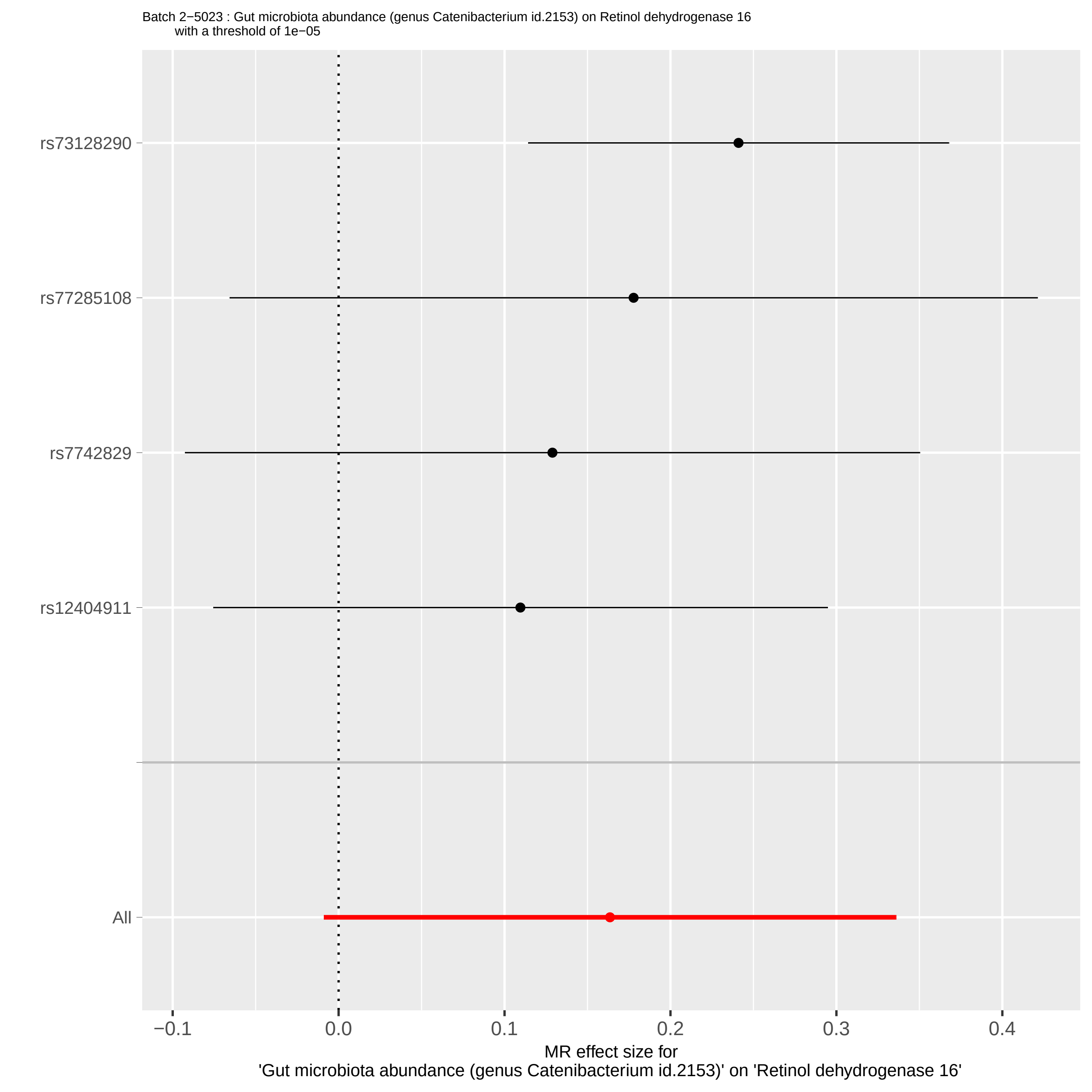

Batch 2−5023 : Gut microbiota abundance (genus Catenibacterium id.2153) on Retinol dehydrogenase 16
with a threshold of 1e−05
rs73128290
rs77285108
rs7742829
rs12404911
All
−0.1
0.0
0.1
0.2
0.3
0.4
MR effect size for
'Gut microbiota abundance (genus Catenibacterium id.2153)' on 'Retinol dehydrogenase 16'

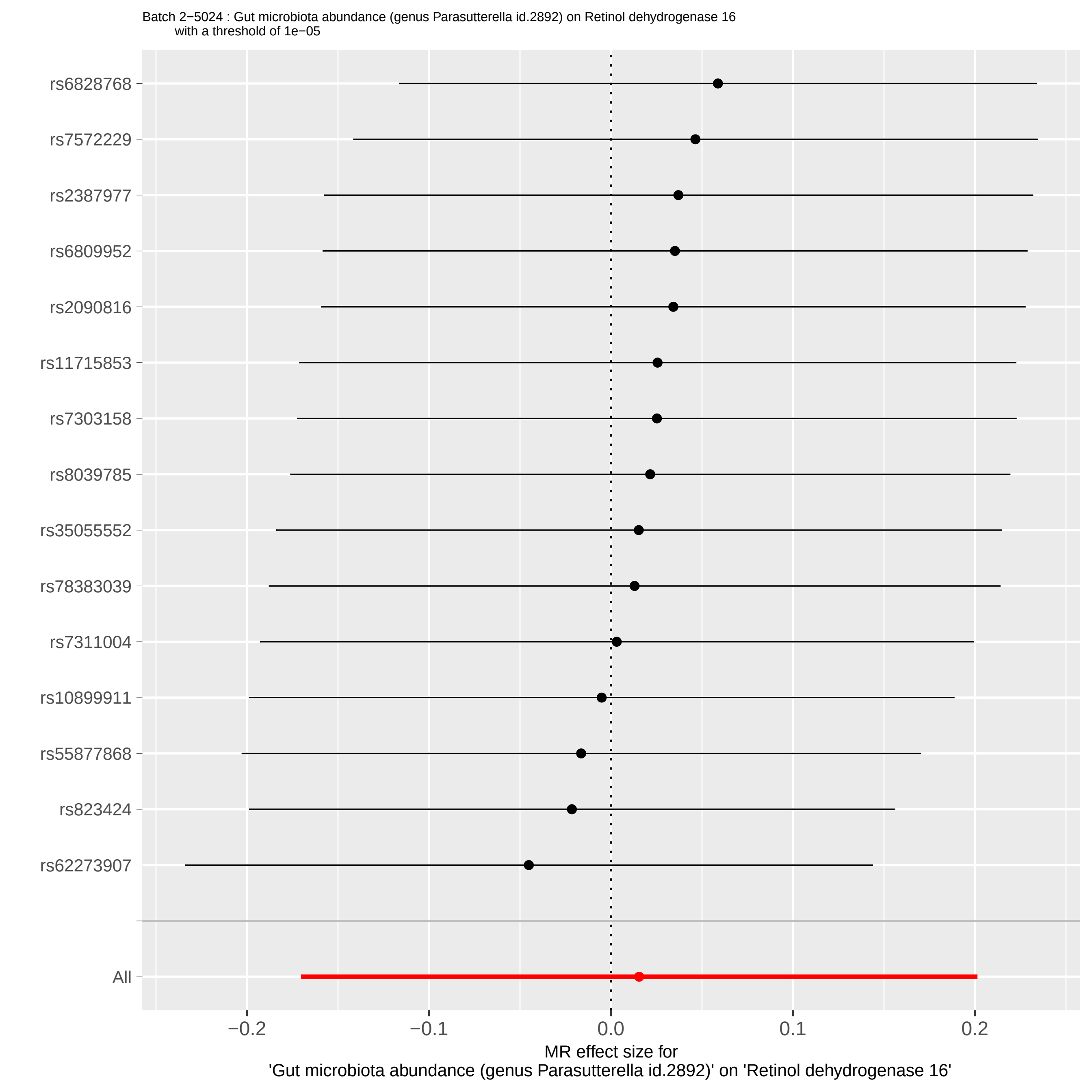
Batch 2−5024 : Gut microbiota abundance (genus Parasutterella id.2892) on Retinol dehydrogenase 16
with a threshold of 1e−05
rs6828768
rs7572229
rs2387977
rs6809952
rs2090816
rs11715853
rs7303158
rs8039785
rs35055552
rs78383039
rs7311004
rs10899911
rs55877868
rs823424
rs62273907
All
−0.2
−0.1
0.0
0.1
0.2
MR effect size for
'Gut microbiota abundance (genus Parasutterella id.2892)' on 'Retinol dehydrogenase 16'

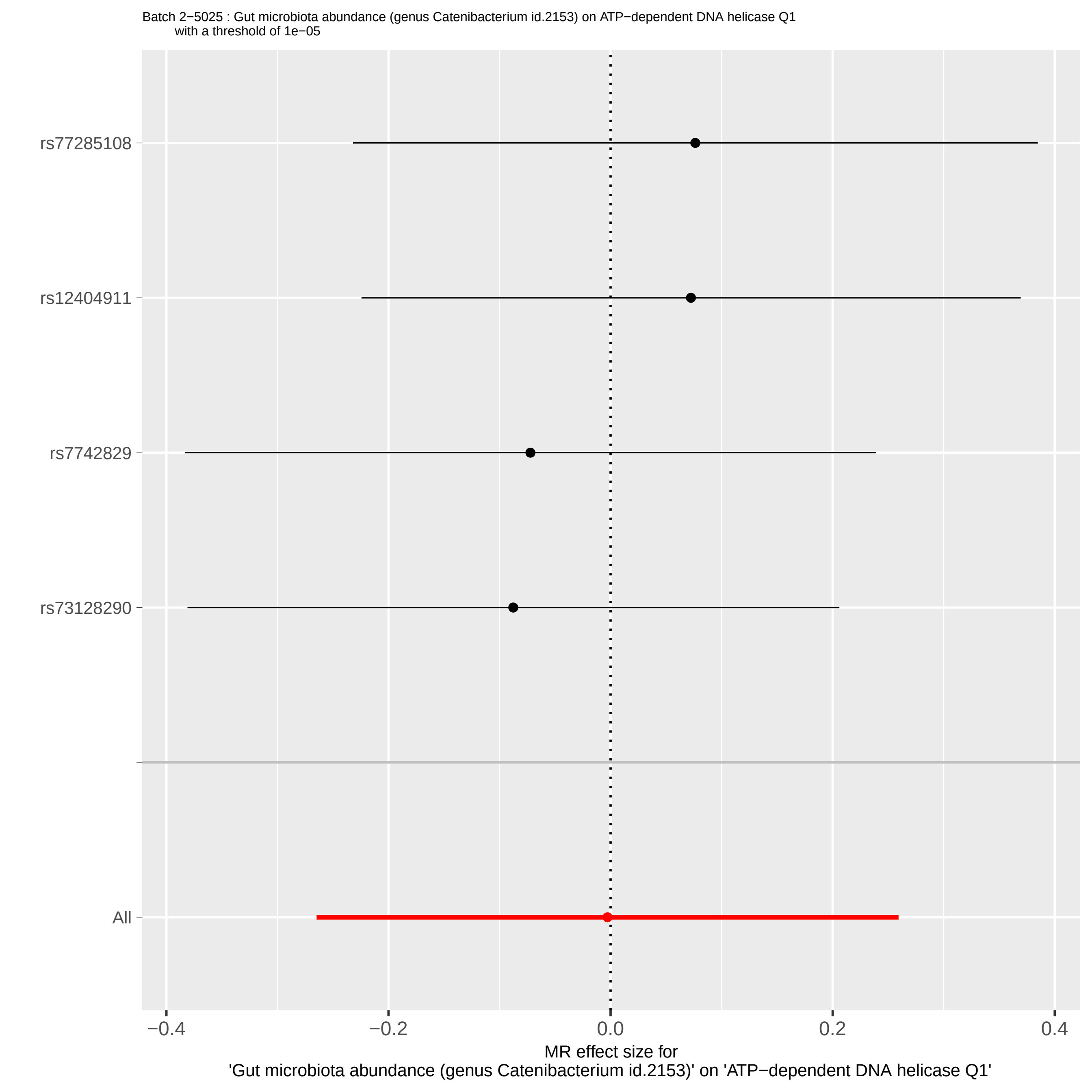

Batch 2−5025 : Gut microbiota abundance (genus Catenibacterium id.2153) on ATP−dependent DNA helicase Q1
with a threshold of 1e−05
rs77285108
rs12404911
rs7742829
rs73128290
All
−0.4
−0.2
0.0
0.2
0.4
MR effect size for
'Gut microbiota abundance (genus Catenibacterium id.2153)' on 'ATP−dependent DNA helicase Q1'

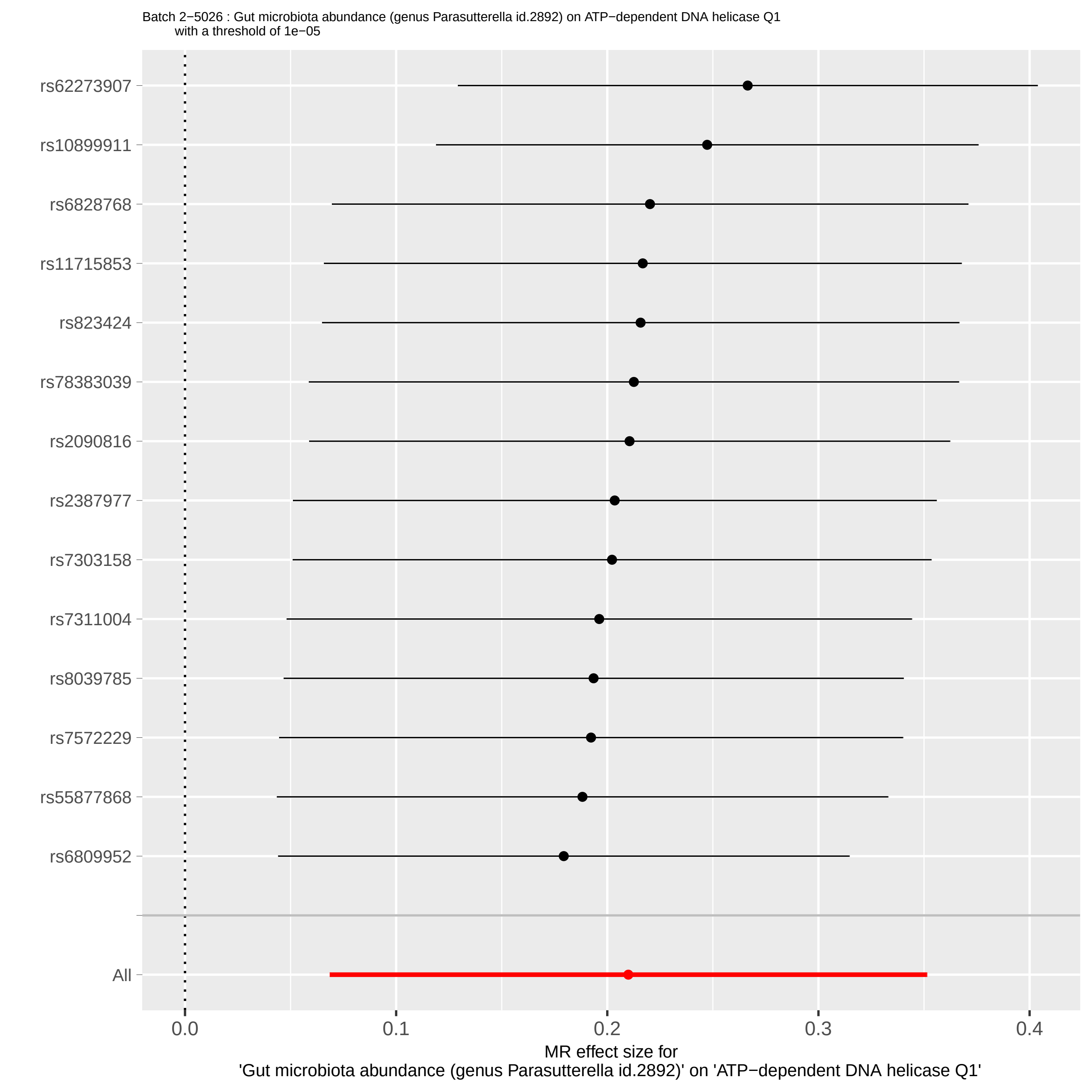
Batch 2−5026 : Gut microbiota abundance (genus Parasutterella id.2892) on ATP−dependent DNA helicase Q1
with a threshold of 1e−05
rs62273907
rs10899911
rs6828768
rs11715853
rs823424
rs78383039
rs2090816
rs2387977
rs7303158
rs7311004
rs8039785
rs7572229
rs55877868
rs6809952
All
0.0
0.1
0.2
0.3
0.4
MR effect size for
'Gut microbiota abundance (genus Parasutterella id.2892)' on 'ATP−dependent DNA helicase Q1'

Batch 2−5027 : Gut microbiota abundance (genus Catenibacterium id.2153) on Regenerating islet−derived protein 3−alpha
with a threshold of 1e−05

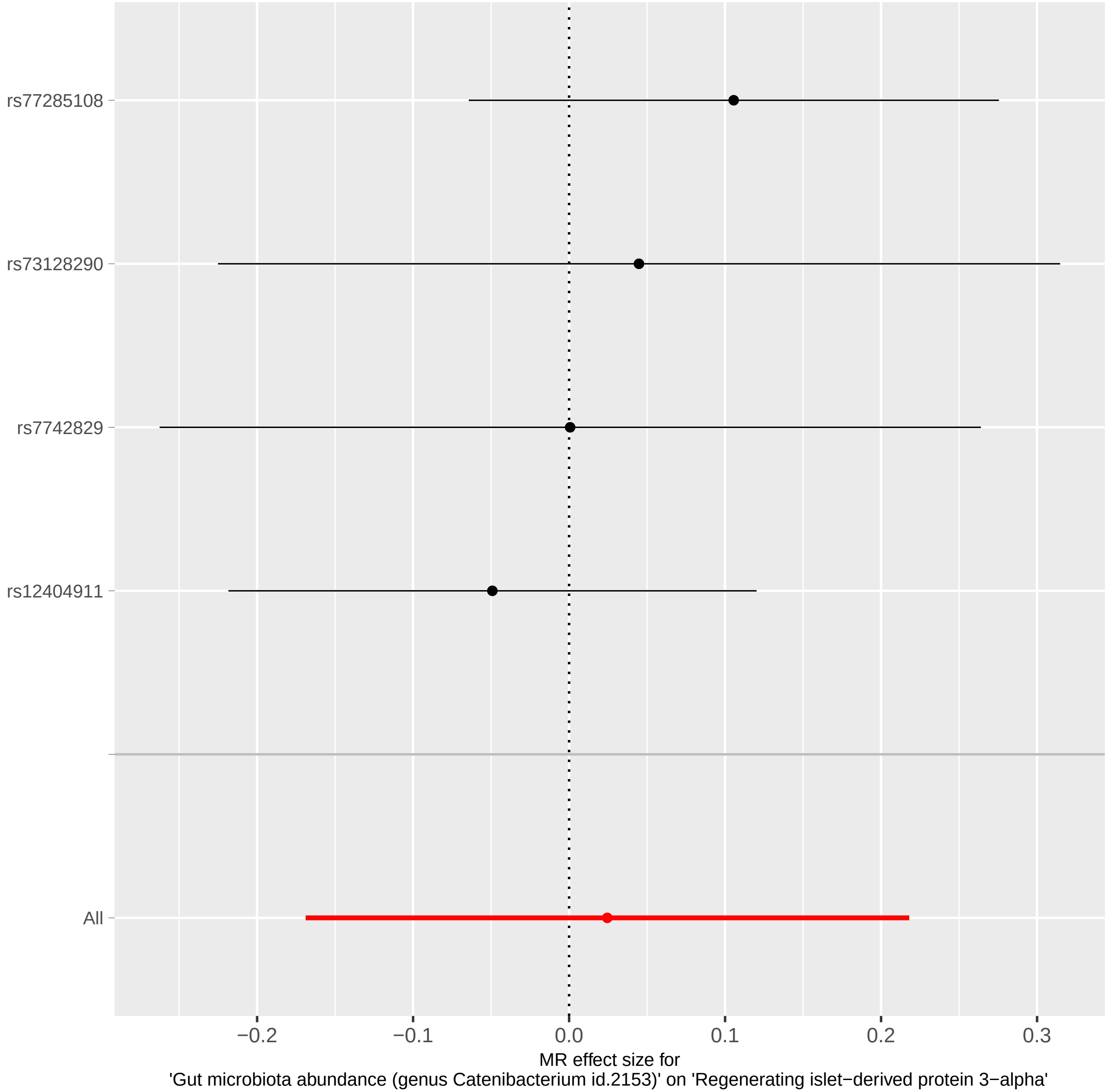

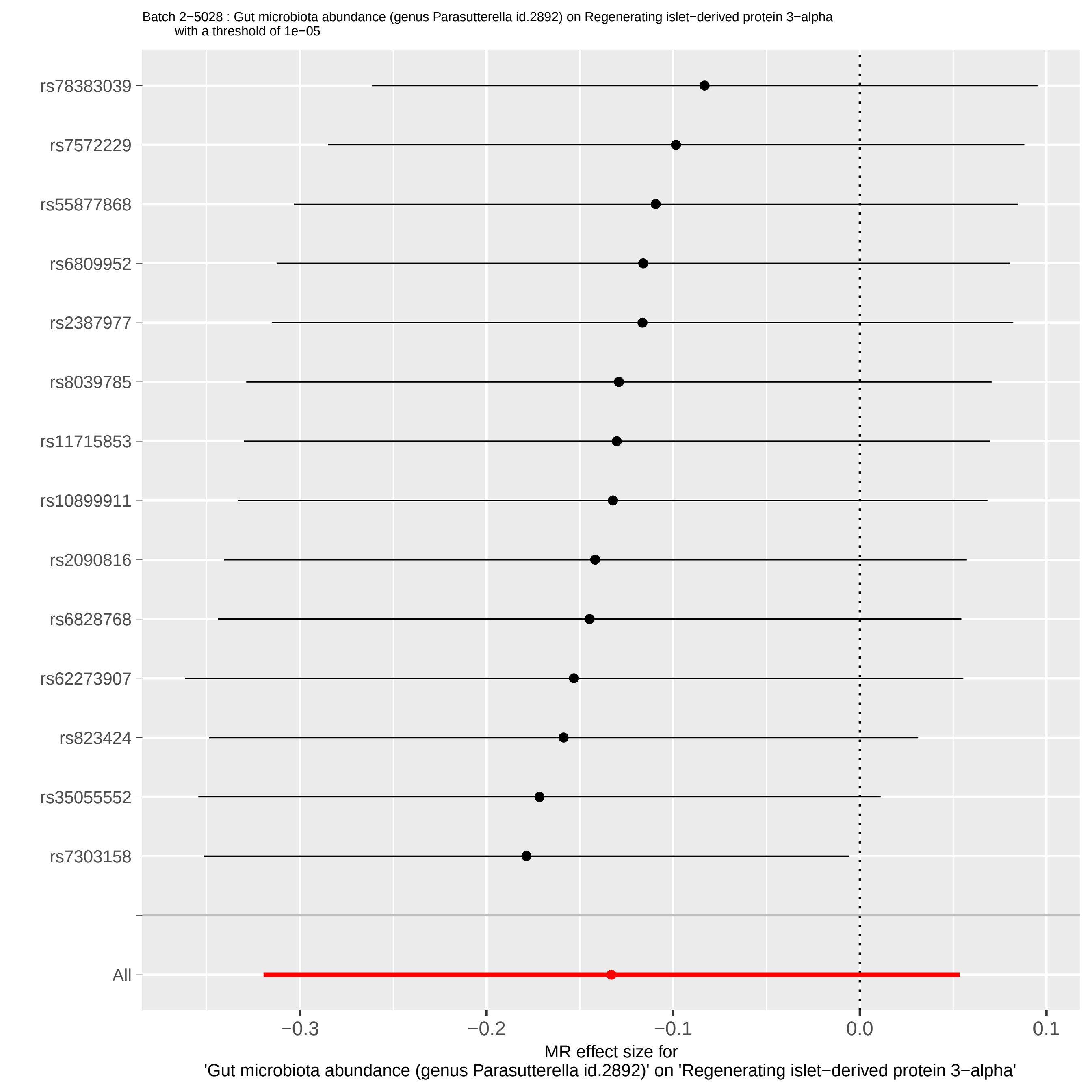
Batch 2−5028 : Gut microbiota abundance (genus Parasutterella id.2892) on Regenerating islet−derived protein 3−alpha
with a threshold of 1e−05
rs78383039
rs7572229
rs55877868
rs6809952
rs2387977
rs8039785
rs11715853
rs10899911
rs2090816
rs6828768
rs62273907
rs823424
rs35055552
rs7303158
All
−0.3
−0.2
−0.1
0.0
0.1
MR effect size for
'Gut microbiota abundance (genus Parasutterella id.2892)' on 'Regenerating islet−derived protein 3−alpha'

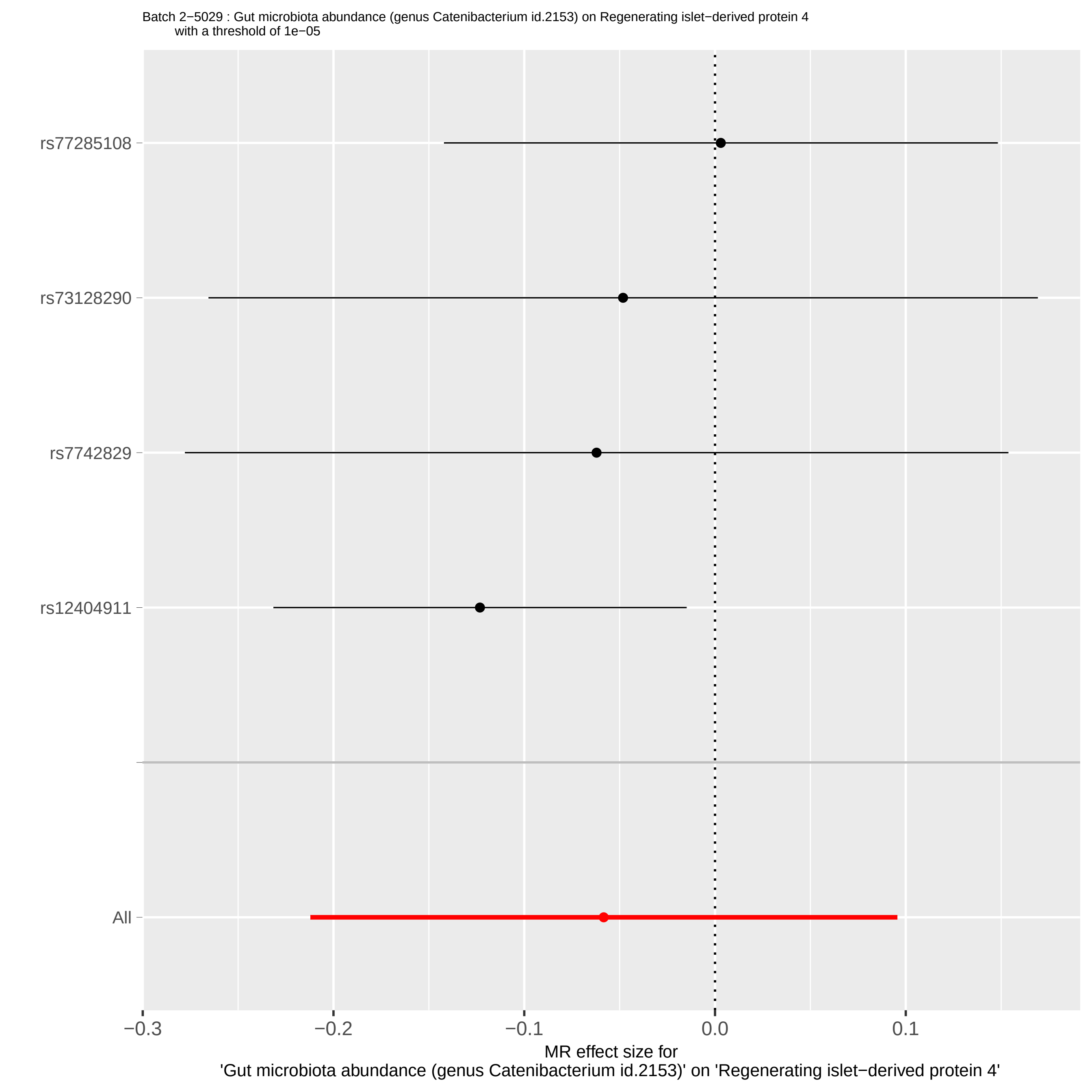

Batch 2−5029 : Gut microbiota abundance (genus Catenibacterium id.2153) on Regenerating islet−derived protein 4
with a threshold of 1e−05
rs77285108
rs73128290
rs7742829
rs12404911
All
−0.3
−0.2
−0.1
0.0
0.1
MR effect size for
'Gut microbiota abundance (genus Catenibacterium id.2153)' on 'Regenerating islet−derived protein 4'

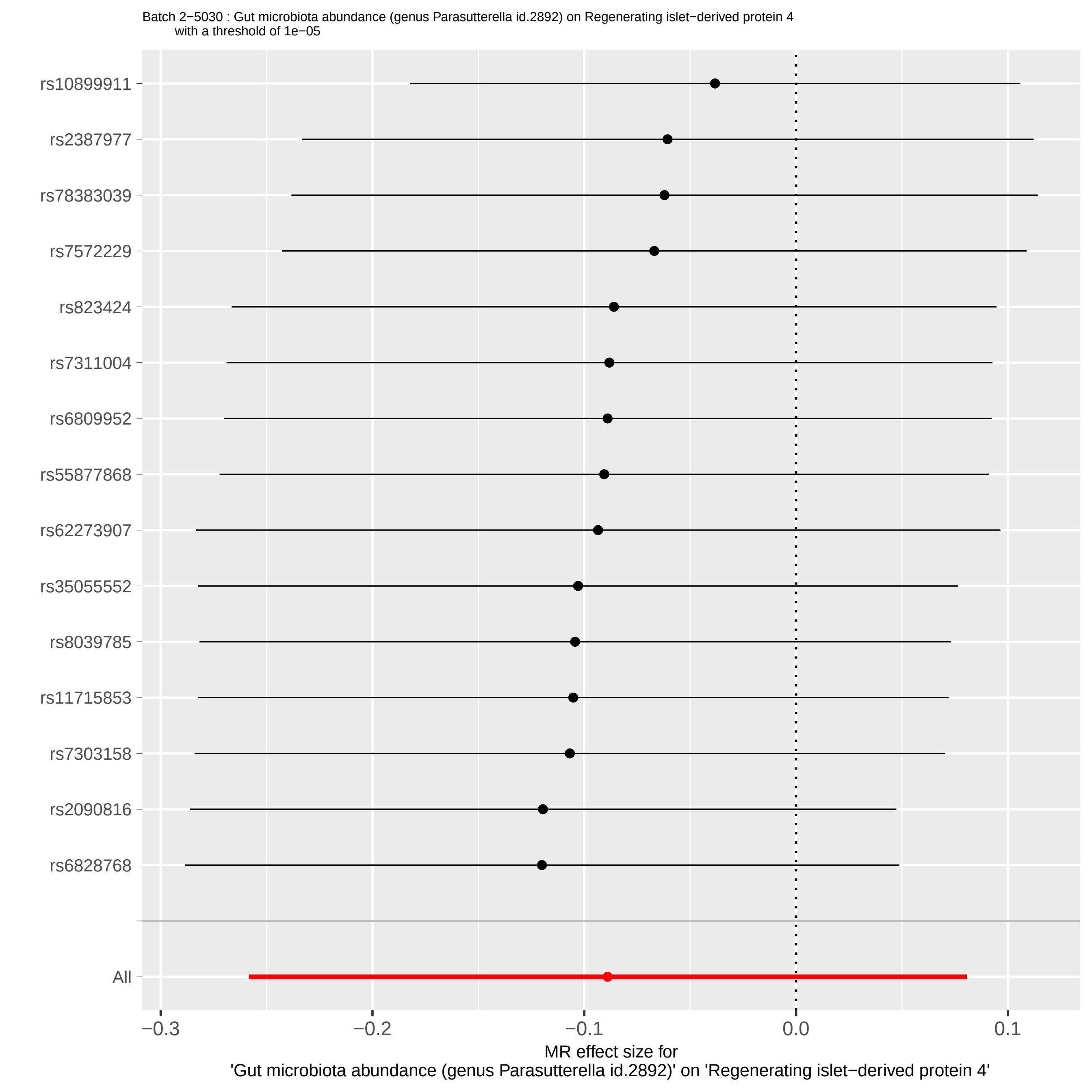

Batch 2−5030 : Gut microbiota abundance (genus Parasutterella id.2892) on Regenerating islet−derived protein 4
with a threshold of 1e−05
rs10899911
rs2387977
rs78383039
rs7572229
rs823424
rs7311004
rs6809952
rs55877868
rs62273907
rs35055552
rs8039785
rs11715853
rs7303158
rs2090816
rs6828768
All
−0.3
−0.2
−0.1
0.0
0.1
MR effect size for
'Gut microbiota abundance (genus Parasutterella id.2892)' on 'Regenerating islet−derived protein 4'

Batch 2−5031 : Gut microbiota abundance (genus Catenibacterium id.2153) on Transcription factor RelB
with a threshold of 1e−05

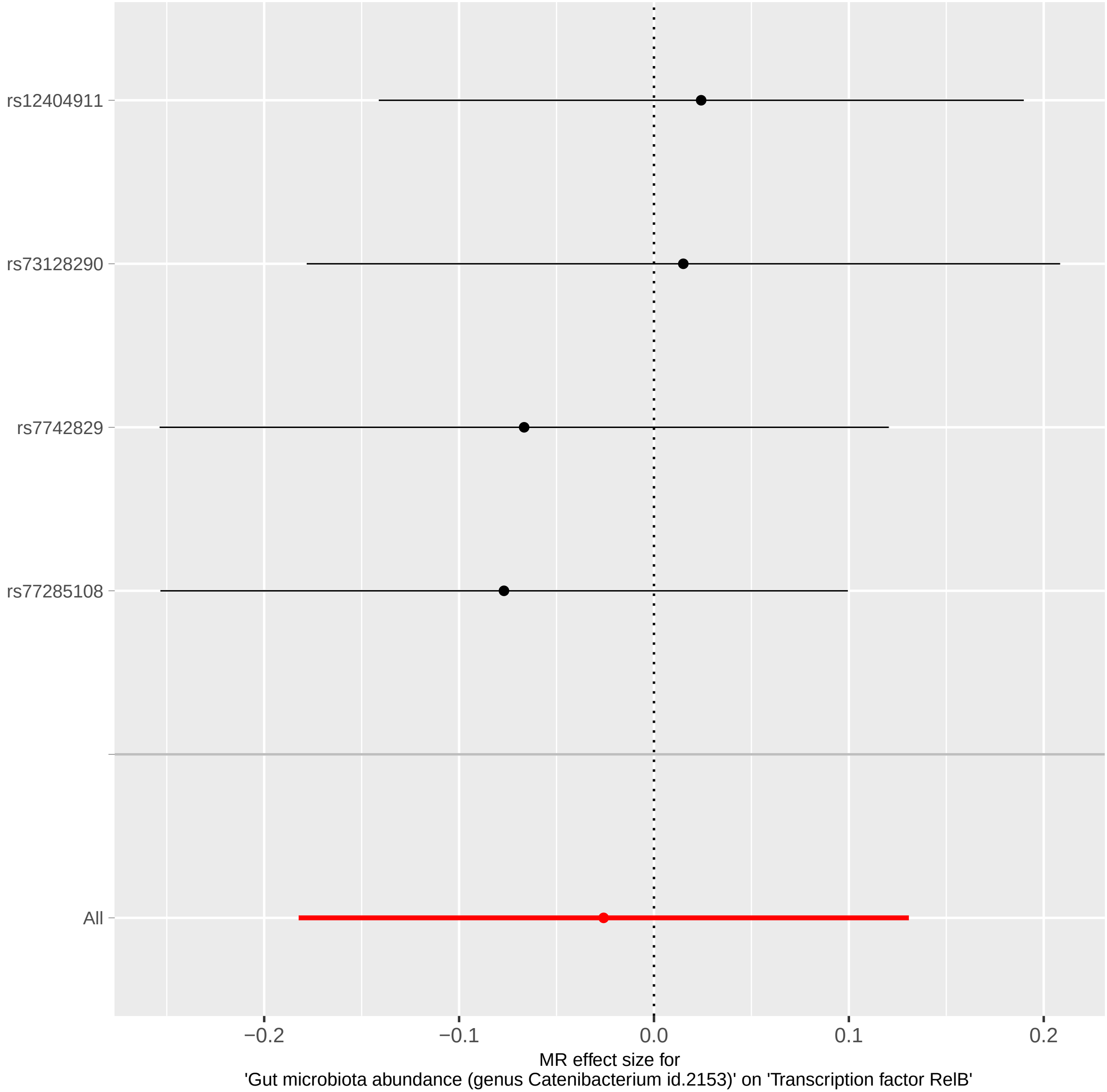

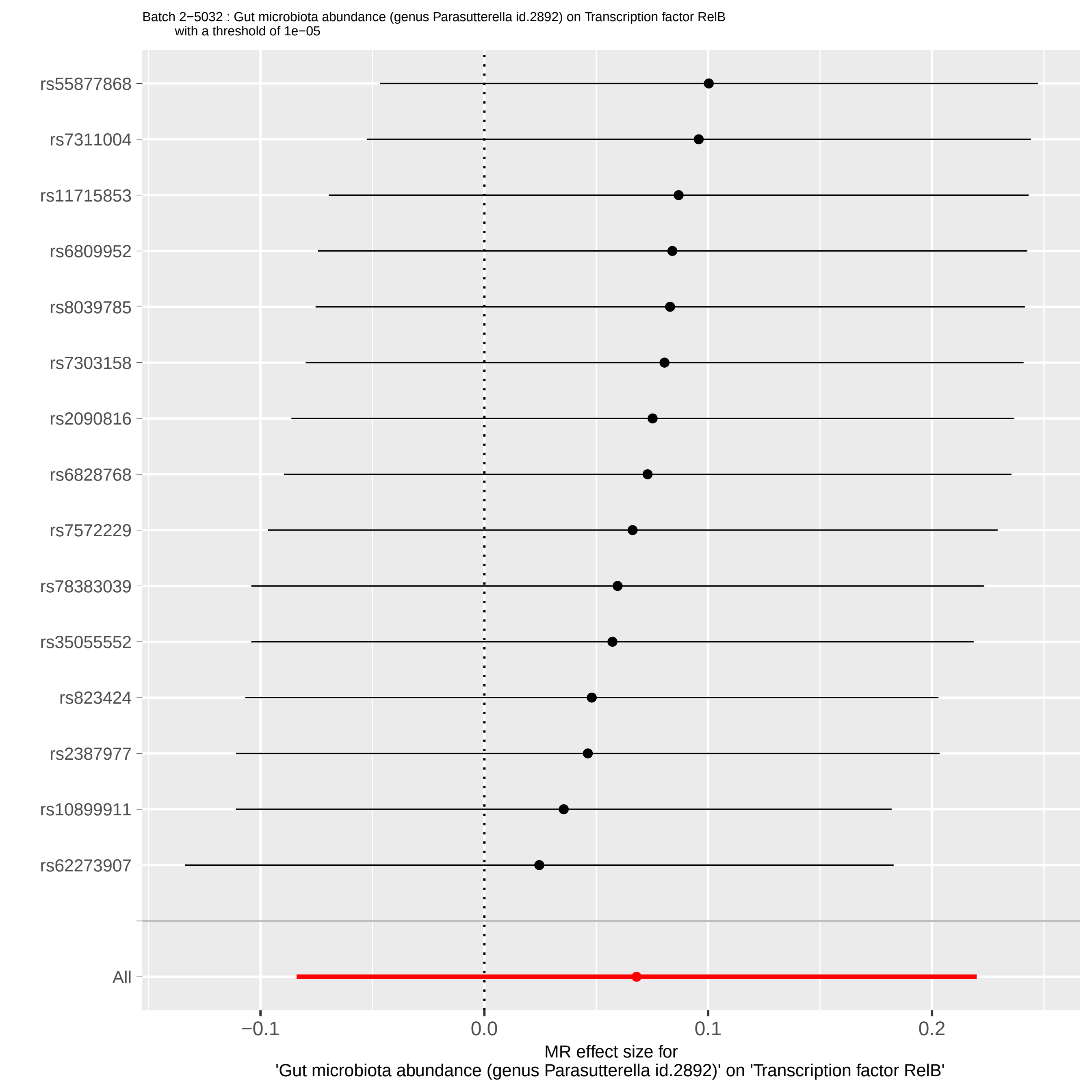
Batch 2−5032 : Gut microbiota abundance (genus Parasutterella id.2892) on Transcription factor RelB
with a threshold of 1e−05
rs55877868
rs7311004
rs11715853
rs6809952
rs8039785
rs7303158
rs2090816
rs6828768
rs7572229
rs78383039
rs35055552
rs823424
rs2387977
rs10899911
rs62273907
All
−0.1
0.0
0.1
0.2
MR effect size for
'Gut microbiota abundance (genus Parasutterella id.2892)' on 'Transcription factor RelB'

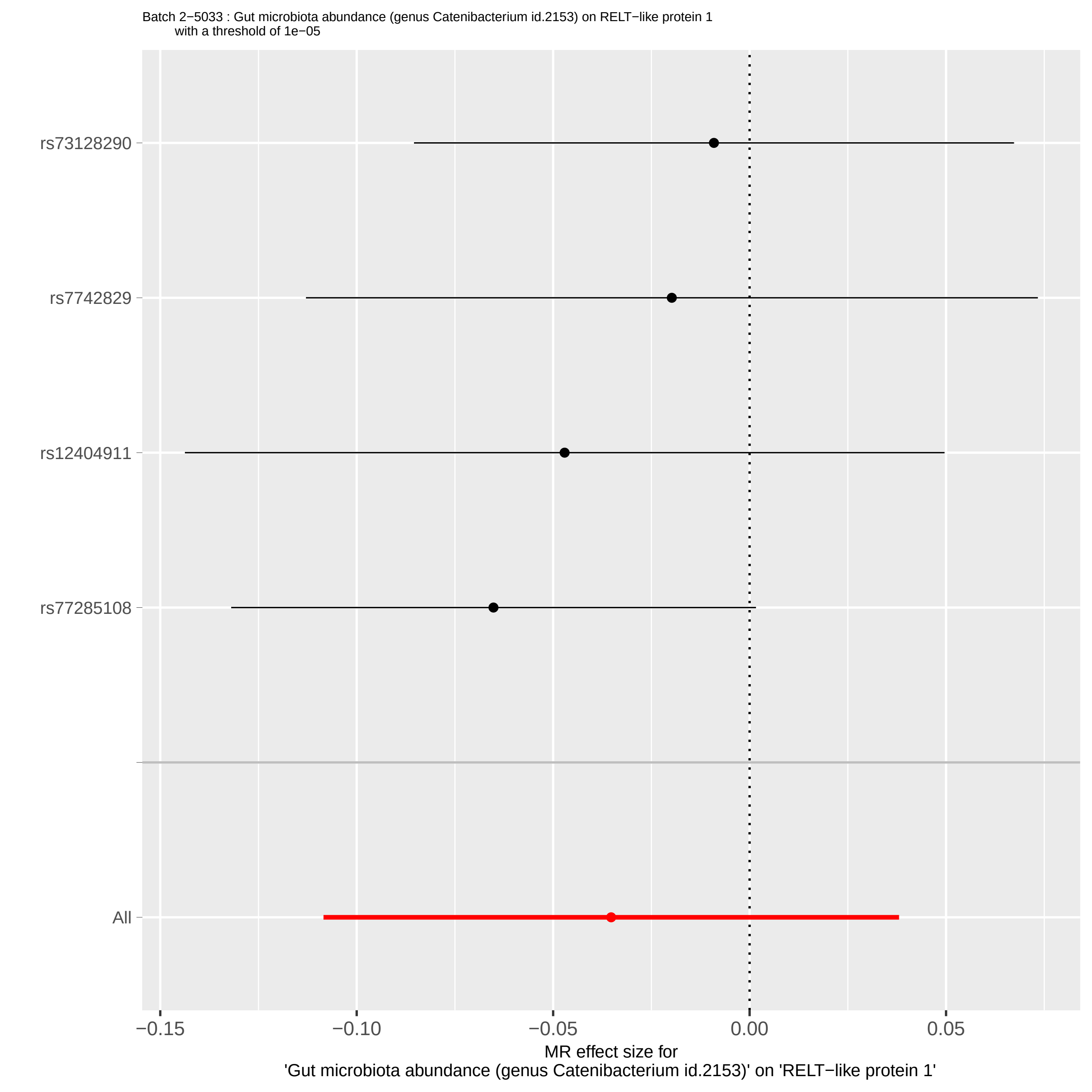
Batch 2−5033 : Gut microbiota abundance (genus Catenibacterium id.2153) on RELT−like protein 1
with a threshold of 1e−05
rs73128290
rs7742829
rs12404911
rs77285108
All
−0.15
−0.10
−0.05
0.00
0.05
MR effect size for
'Gut microbiota abundance (genus Catenibacterium id.2153)' on 'RELT−like protein 1'

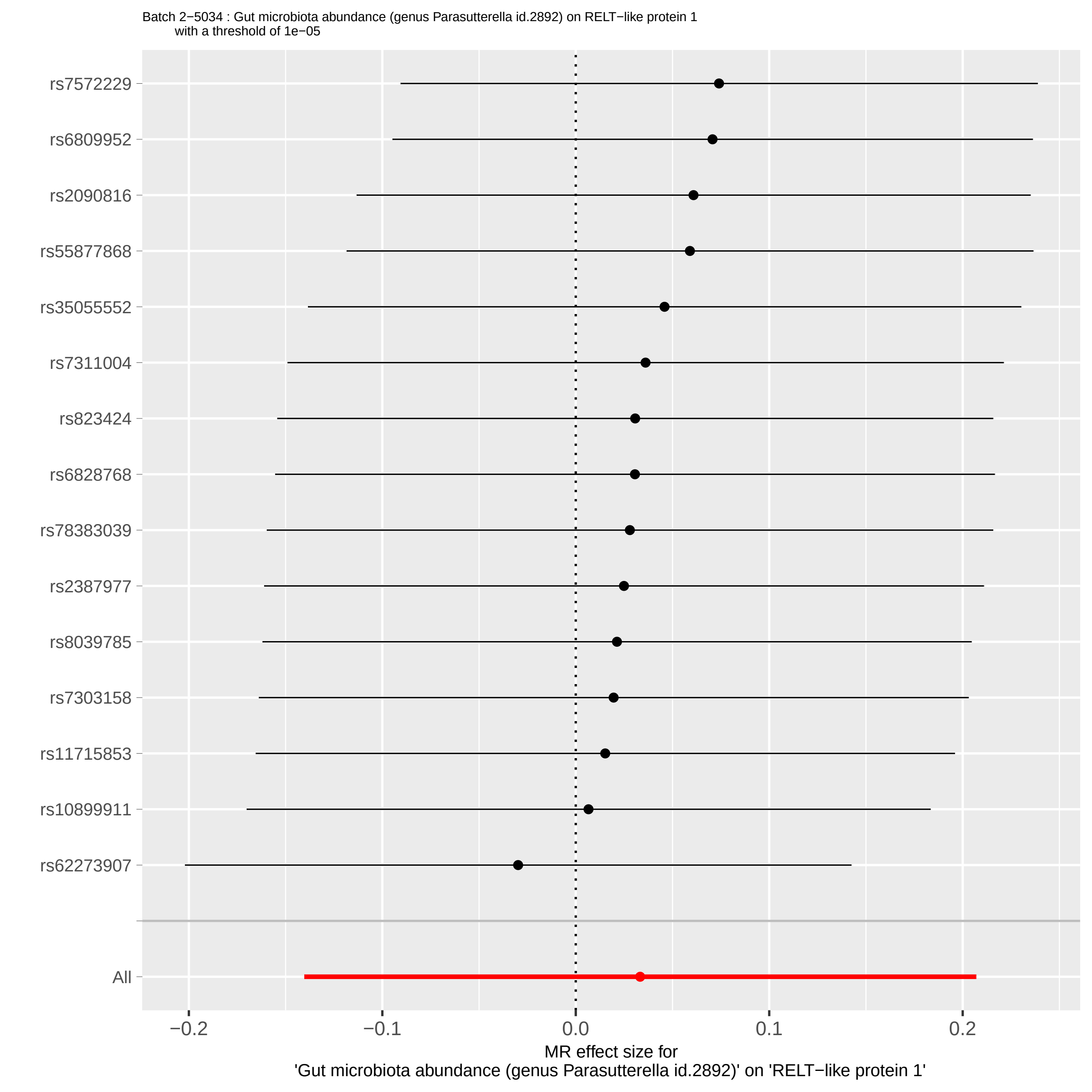

Batch 2−5034 : Gut microbiota abundance (genus Parasutterella id.2892) on RELT−like protein 1
with a threshold of 1e−05
rs7572229
rs6809952
rs2090816
rs55877868
rs35055552
rs7311004
rs823424
rs6828768
rs78383039
rs2387977
rs8039785
rs7303158
rs11715853
rs10899911
rs62273907
All
−0.2
−0.1
0.0
0.1
0.2
MR effect size for
'Gut microbiota abundance (genus Parasutterella id.2892)' on 'RELT−like protein 1'

Batch 2−5035 : Gut microbiota abundance (genus Catenibacterium id.2153) on RELT−like protein 2
with a threshold of 1e−05

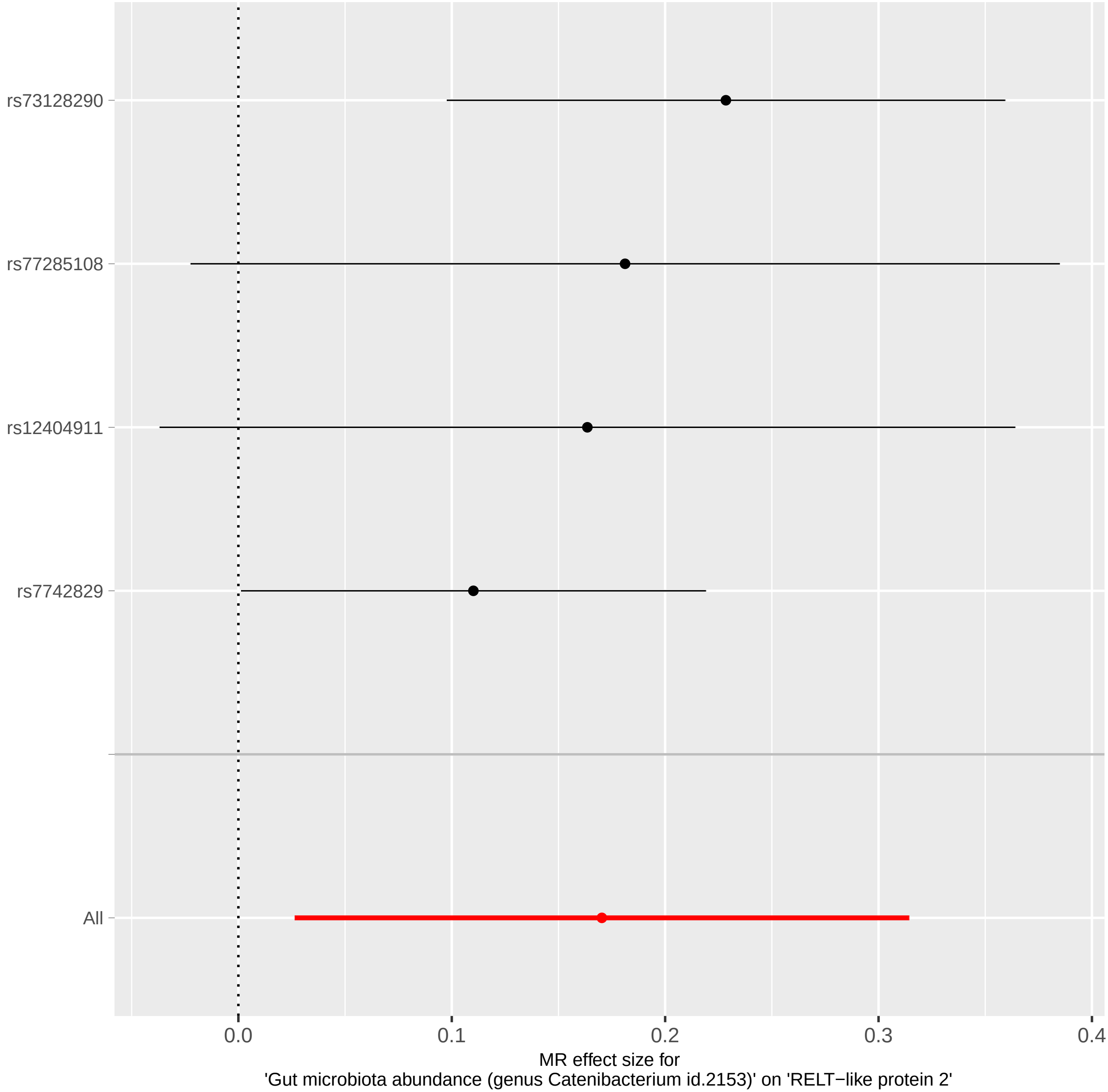

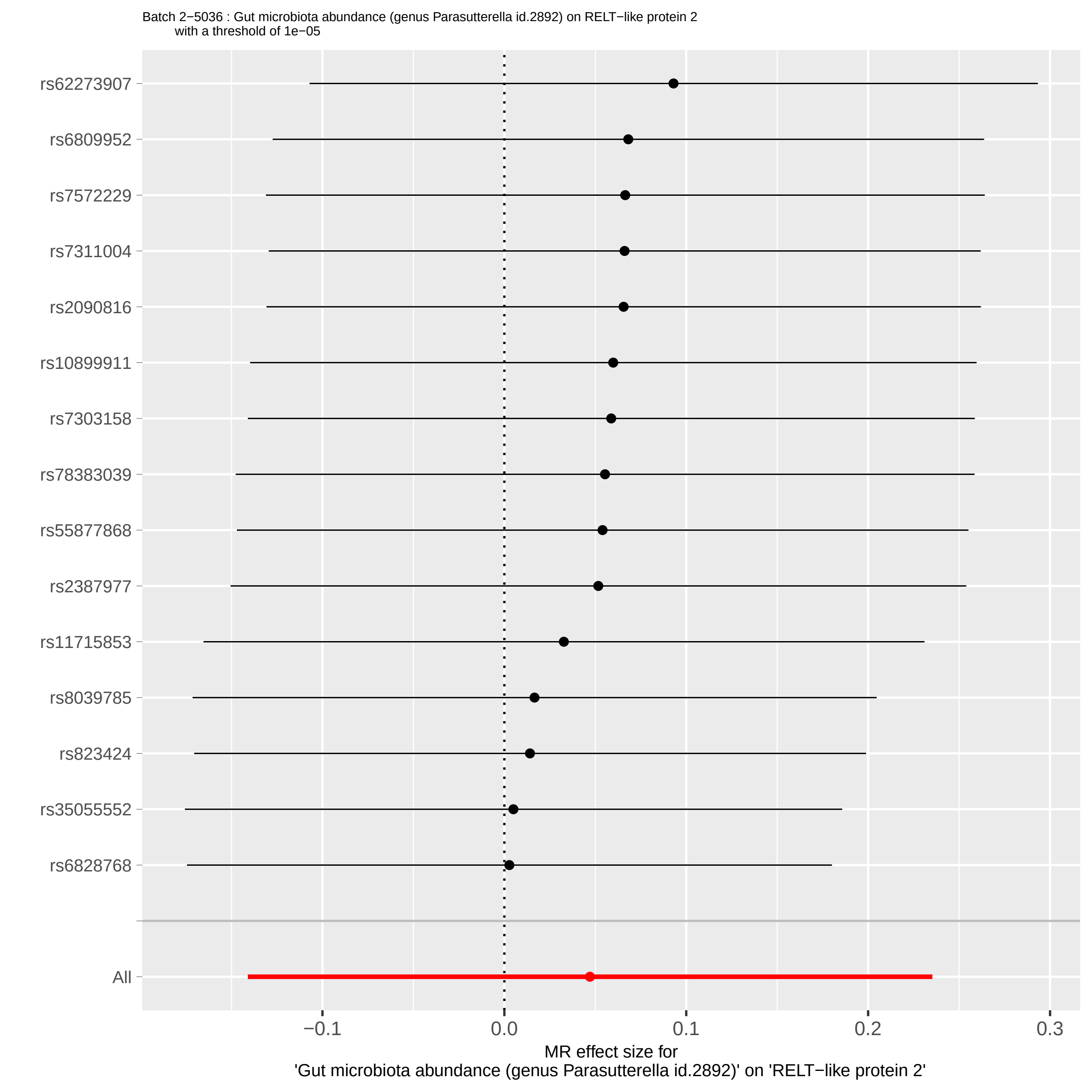

Batch 2−5036 : Gut microbiota abundance (genus Parasutterella id.2892) on RELT−like protein 2
with a threshold of 1e−05
rs62273907
rs6809952
rs7572229
rs7311004
rs2090816
rs10899911
rs7303158
rs78383039
rs55877868
rs2387977
rs11715853
rs8039785
rs823424
rs35055552
rs6828768
All
−0.1
0.0
0.1
0.2
0.3
MR effect size for
'Gut microbiota abundance (genus Parasutterella id.2892)' on 'RELT−like protein 2'

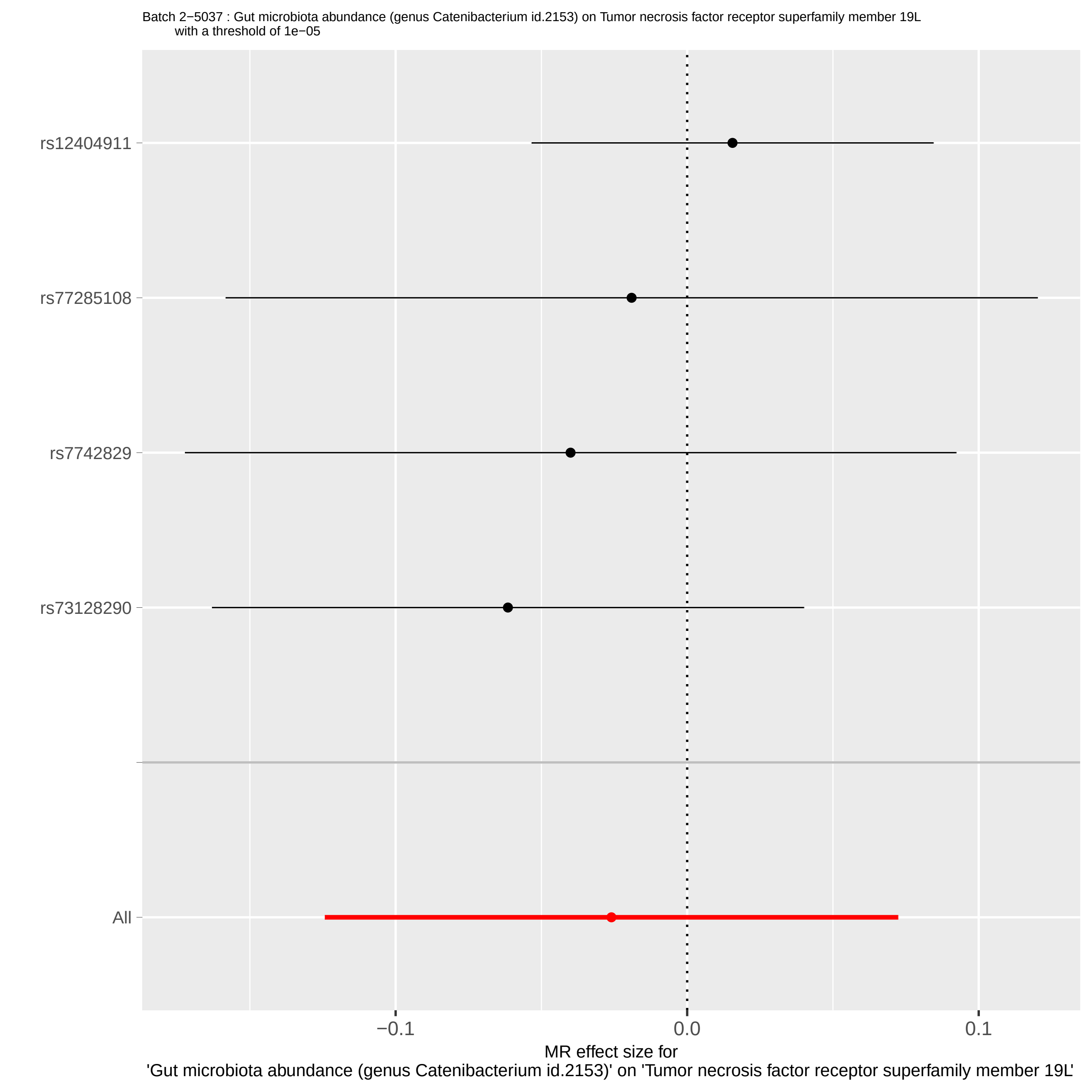

Batch 2−5037 : Gut microbiota abundance (genus Catenibacterium id.2153) on Tumor necrosis factor receptor superfamily member 19L
with a threshold of 1e−05
rs12404911
rs77285108
rs7742829
rs73128290
All
−0.1
0.0
0.1
MR effect size for
'Gut microbiota abundance (genus Catenibacterium id.2153)' on 'Tumor necrosis factor receptor superfamily member 19L'

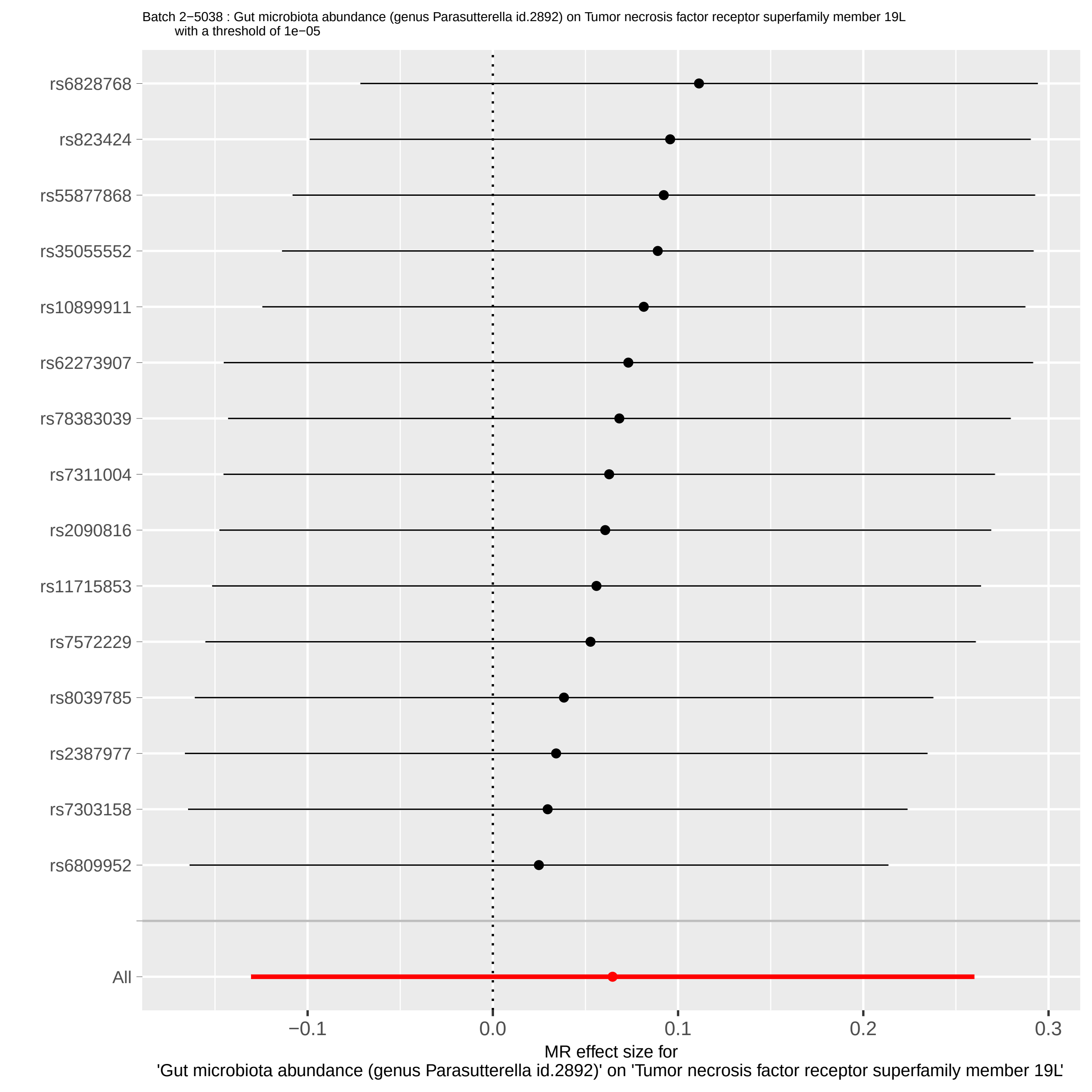

Batch 2−5038 : Gut microbiota abundance (genus Parasutterella id.2892) on Tumor necrosis factor receptor superfamily member 19L
with a threshold of 1e−05
rs6828768
rs823424
rs55877868
rs35055552
rs10899911
rs62273907
rs78383039
rs7311004
rs2090816
rs11715853
rs7572229
rs8039785
rs2387977
rs7303158
rs6809952
All
−0.1
0.0
0.1
0.2
0.3
MR effect size for
'Gut microbiota abundance (genus Parasutterella id.2892)' on 'Tumor necrosis factor receptor superfamily member 19L'

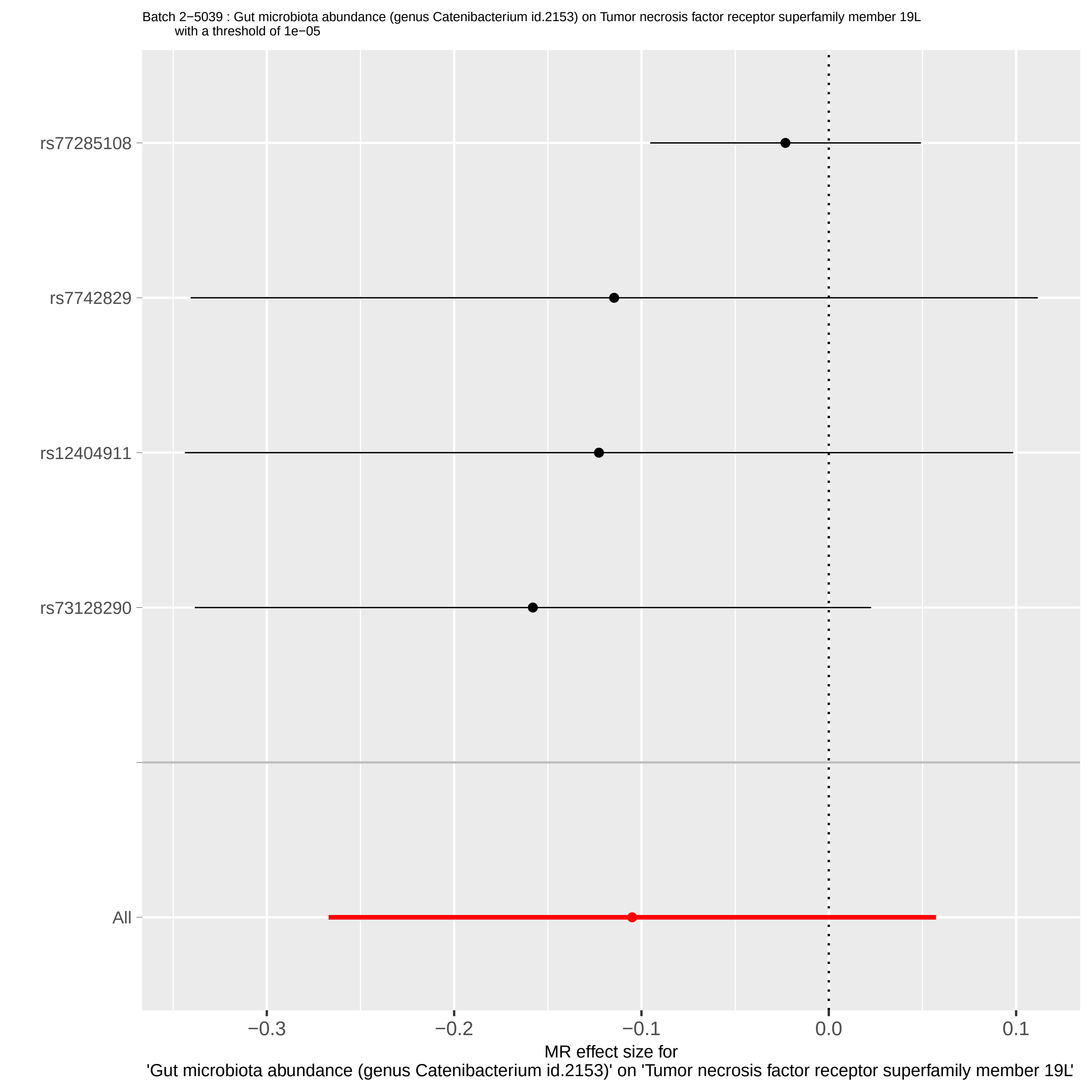

Batch 2−5039 : Gut microbiota abundance (genus Catenibacterium id.2153) on Tumor necrosis factor receptor superfamily member 19L
with a threshold of 1e−05
rs77285108
rs7742829
rs12404911
rs73128290
All
−0.3
−0.2
−0.1
0.0
0.1
MR effect size for
'Gut microbiota abundance (genus Catenibacterium id.2153)' on 'Tumor necrosis factor receptor superfamily member 19L'

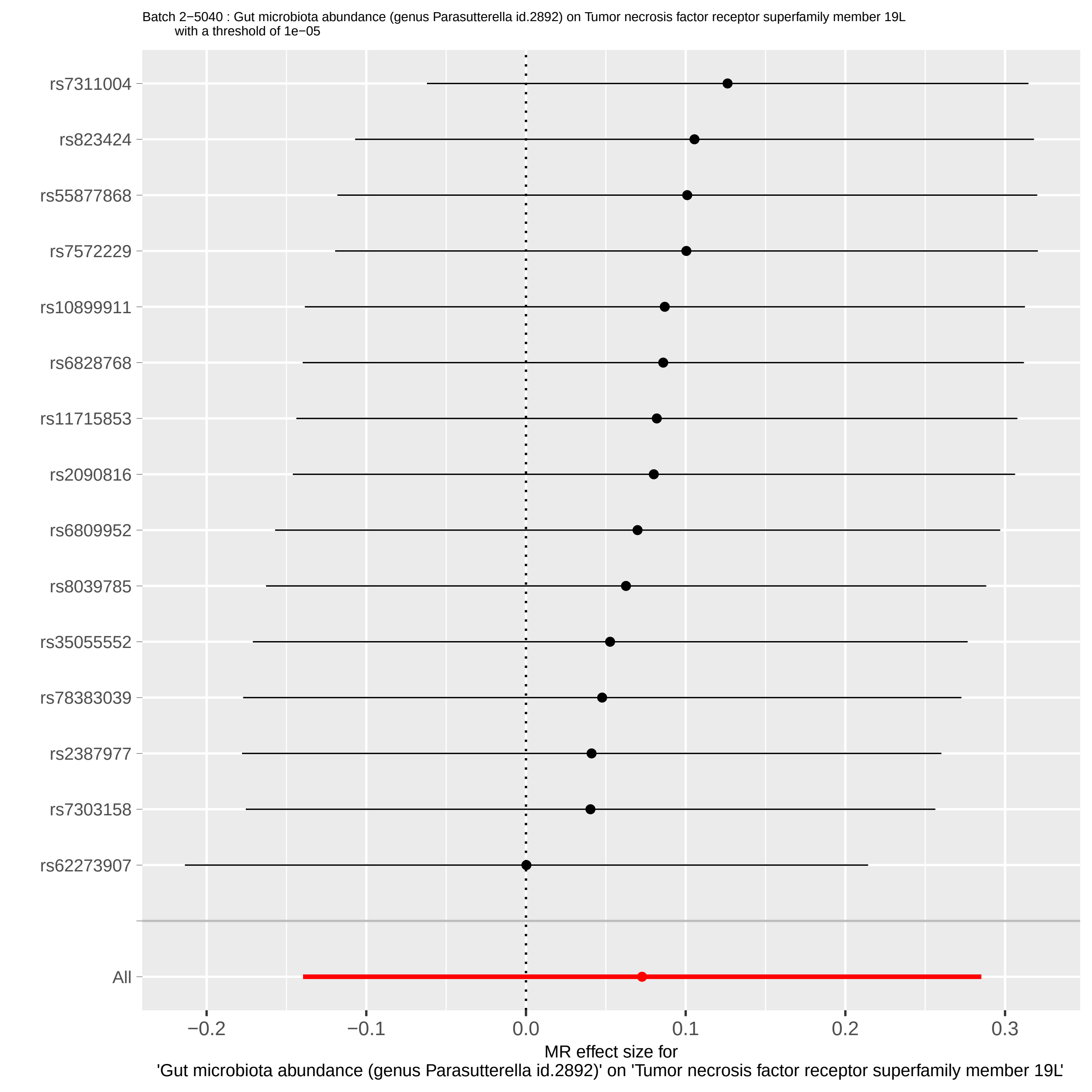
Batch 2−5040 : Gut microbiota abundance (genus Parasutterella id.2892) on Tumor necrosis factor receptor superfamily member 19L
with a threshold of 1e−05
rs7311004
rs823424
rs55877868
rs7572229
rs10899911
rs6828768
rs11715853
rs2090816
rs6809952
rs8039785
rs35055552
rs78383039
rs2387977
rs7303158
rs62273907
All
−0.2
−0.1
0.0
0.1
0.2
0.3
MR effect size for
'Gut microbiota abundance (genus Parasutterella id.2892)' on 'Tumor necrosis factor receptor superfamily member 19L'

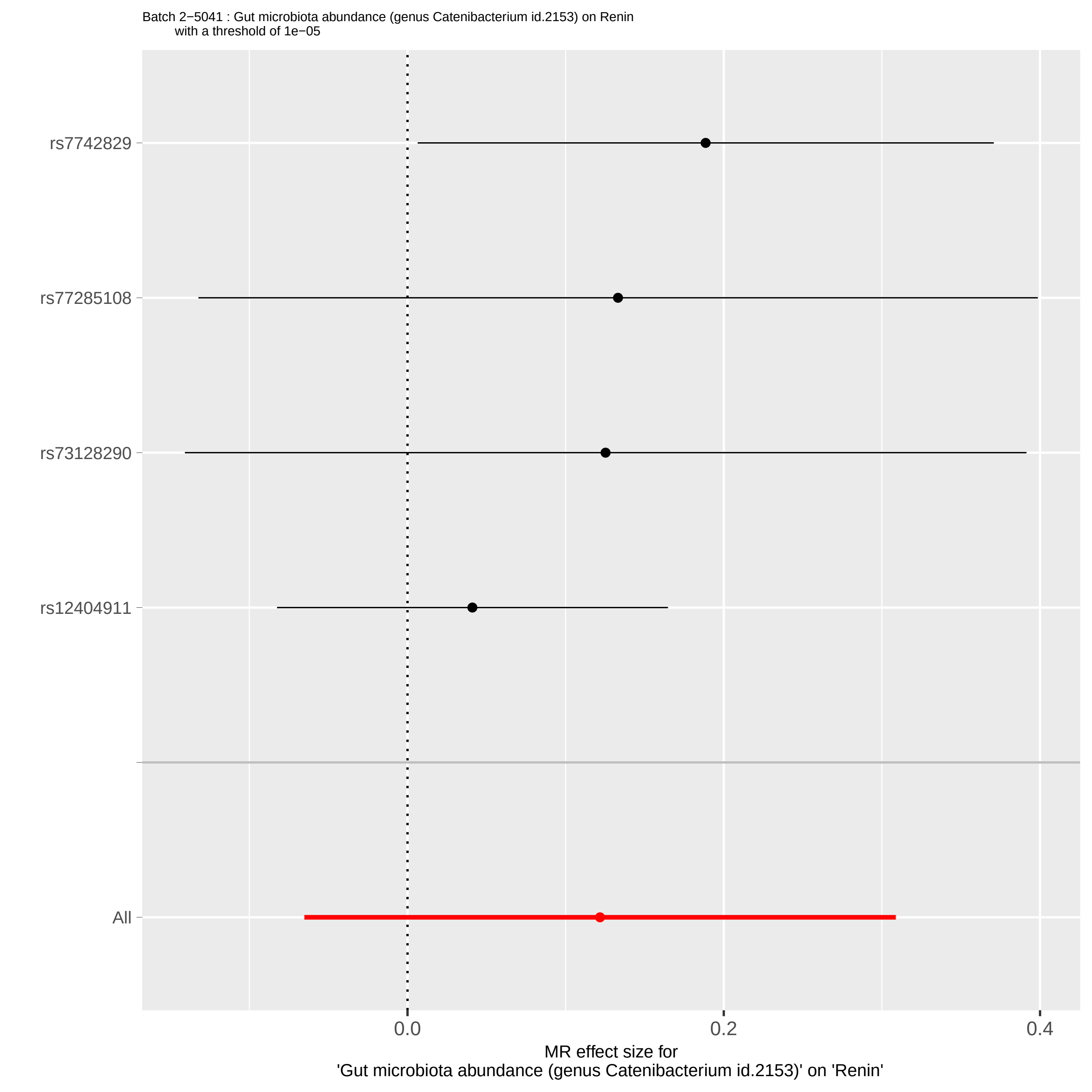
Batch 2−5041 : Gut microbiota abundance (genus Catenibacterium id.2153) on Renin
with a threshold of 1e−05
rs7742829
rs77285108
rs73128290
rs12404911
All
0.0
0.2
0.4
MR effect size for
'Gut microbiota abundance (genus Catenibacterium id.2153)' on 'Renin'

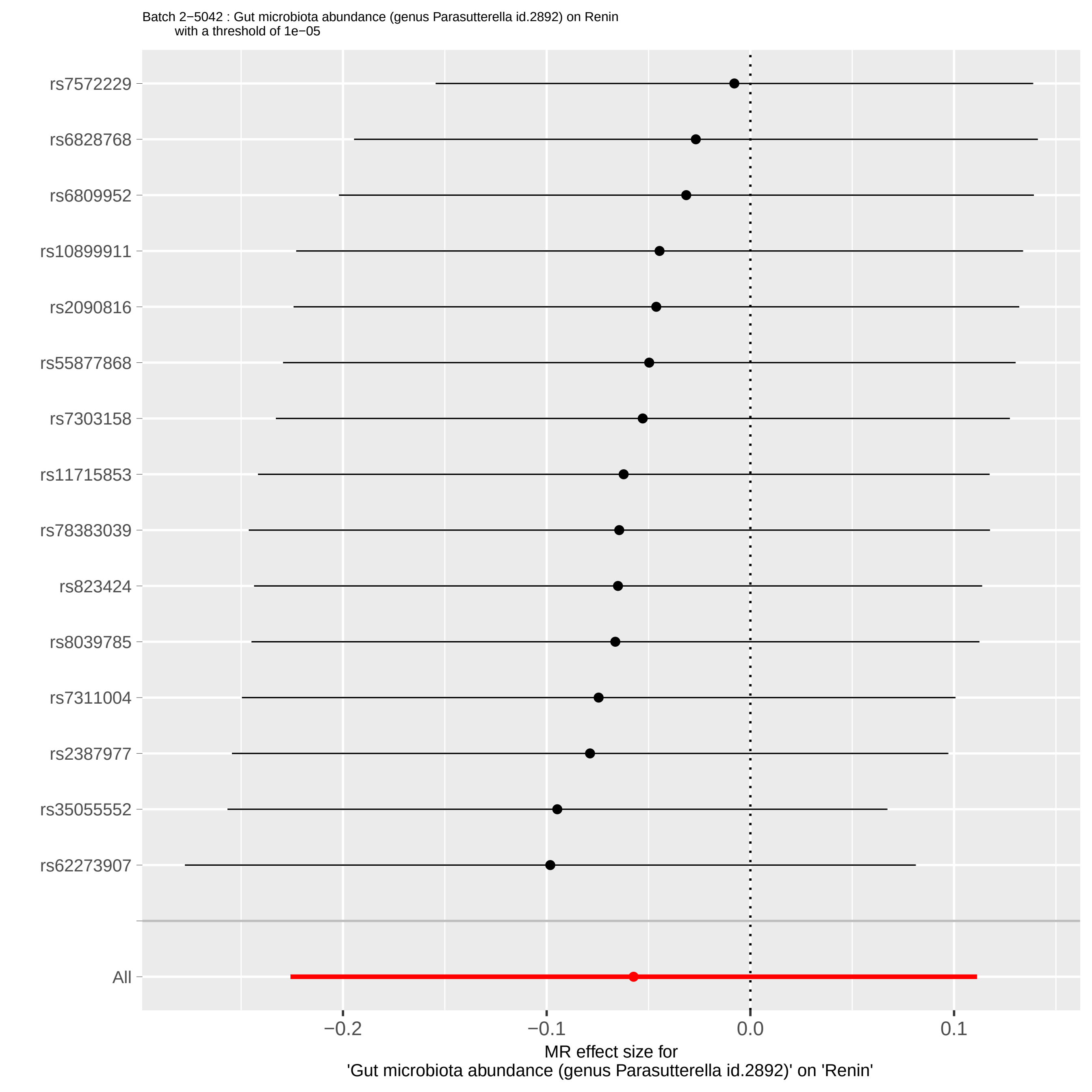

Batch 2−5042 : Gut microbiota abundance (genus Parasutterella id.2892) on Renin
with a threshold of 1e−05
rs7572229
rs6828768
rs6809952
rs10899911
rs2090816
rs55877868
rs7303158
rs11715853
rs78383039
rs823424
rs8039785
rs7311004
rs2387977
rs35055552
rs62273907
All
−0.2
−0.1
0.0
0.1
MR effect size for
'Gut microbiota abundance (genus Parasutterella id.2892)' on 'Renin'

Batch 2−5043 : Gut microbiota abundance (genus Catenibacterium id.2153) on Replication initiator 1
with a threshold of 1e−05

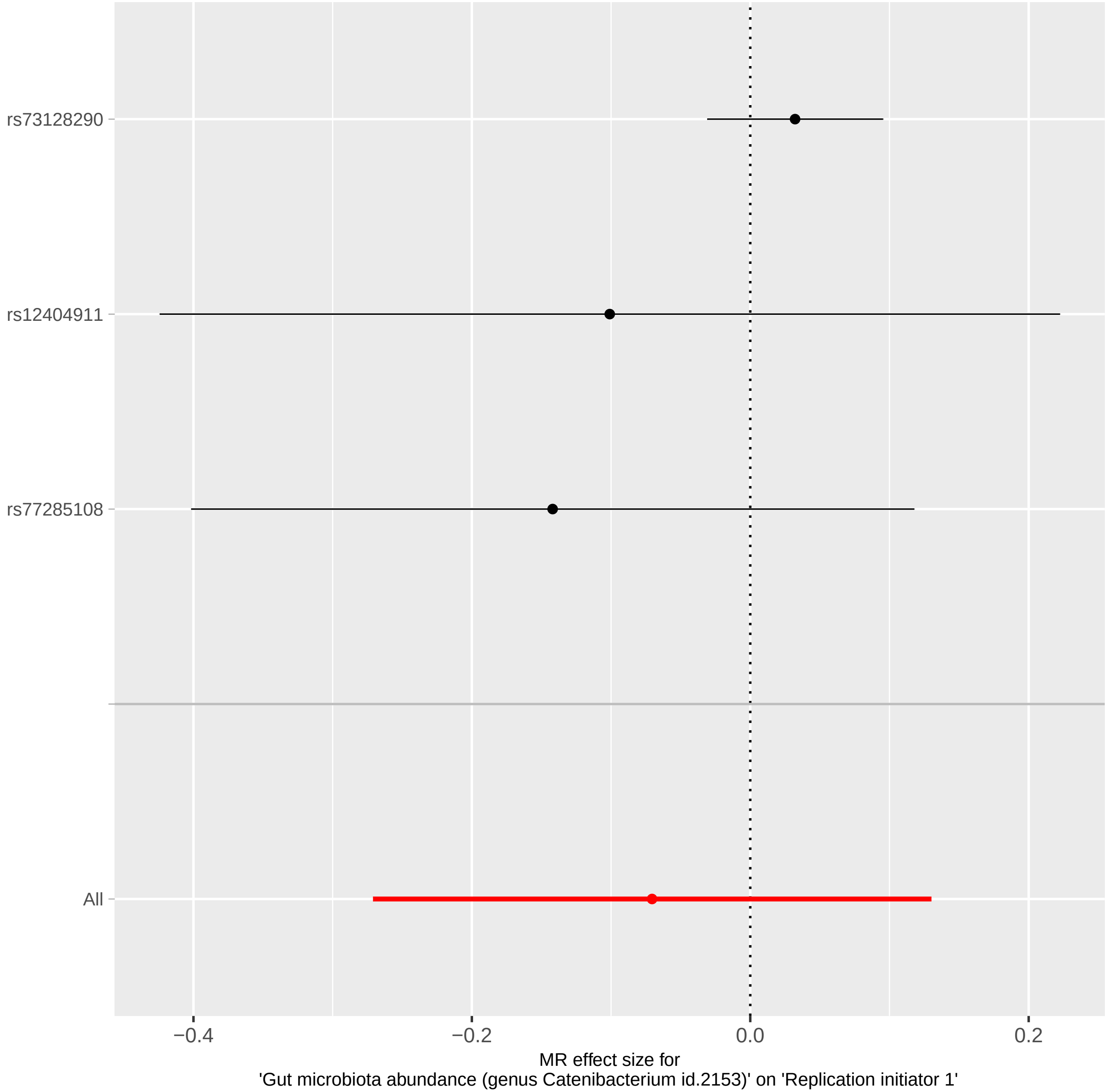

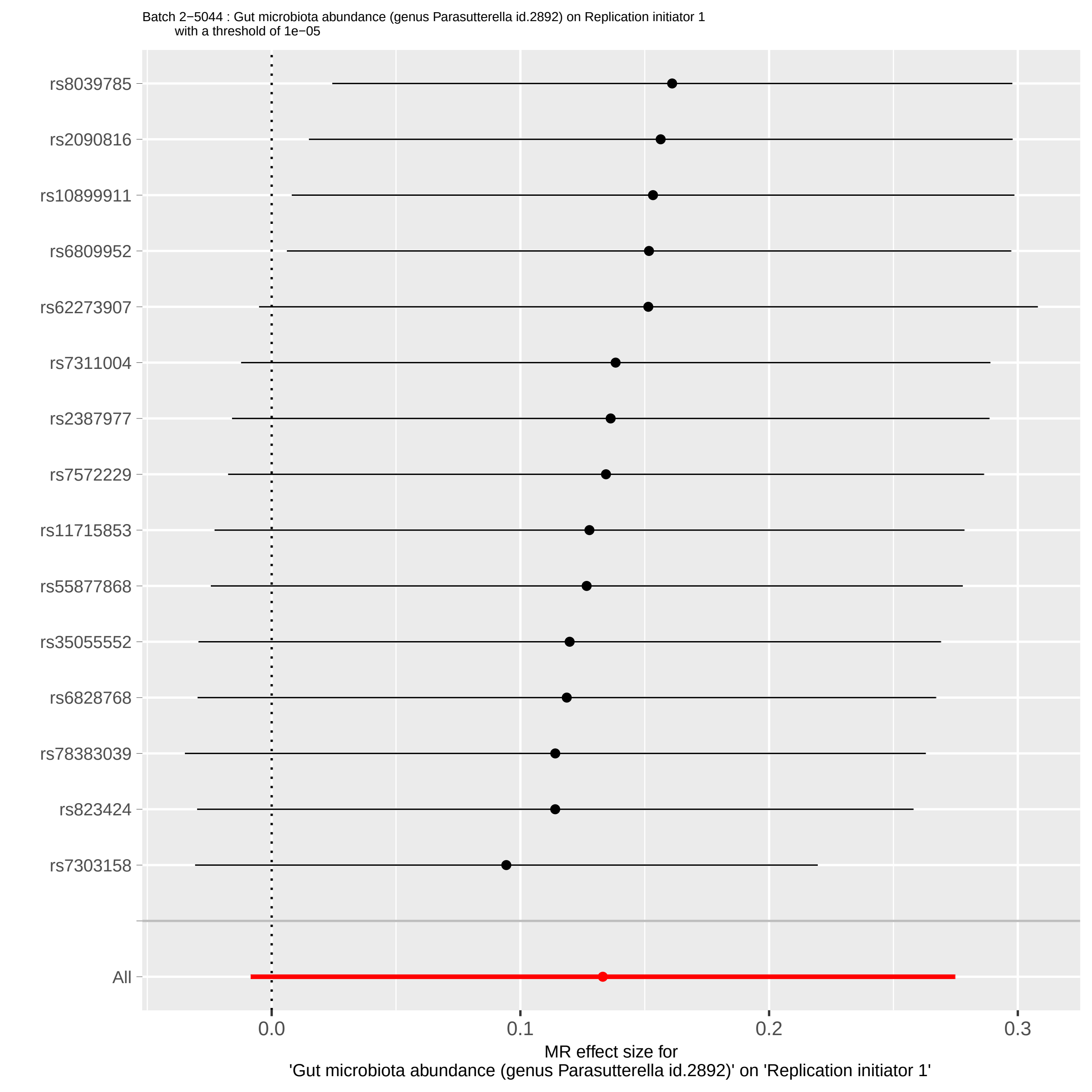
Batch 2−5044 : Gut microbiota abundance (genus Parasutterella id.2892) on Replication initiator 1
with a threshold of 1e−05
rs8039785
rs2090816
rs10899911
rs6809952
rs62273907
rs7311004
rs2387977
rs7572229
rs11715853
rs55877868
rs35055552
rs6828768
rs78383039
rs823424
rs7303158
All
0.0
0.1
0.2
0.3
MR effect size for
'Gut microbiota abundance (genus Parasutterella id.2892)' on 'Replication initiator 1'

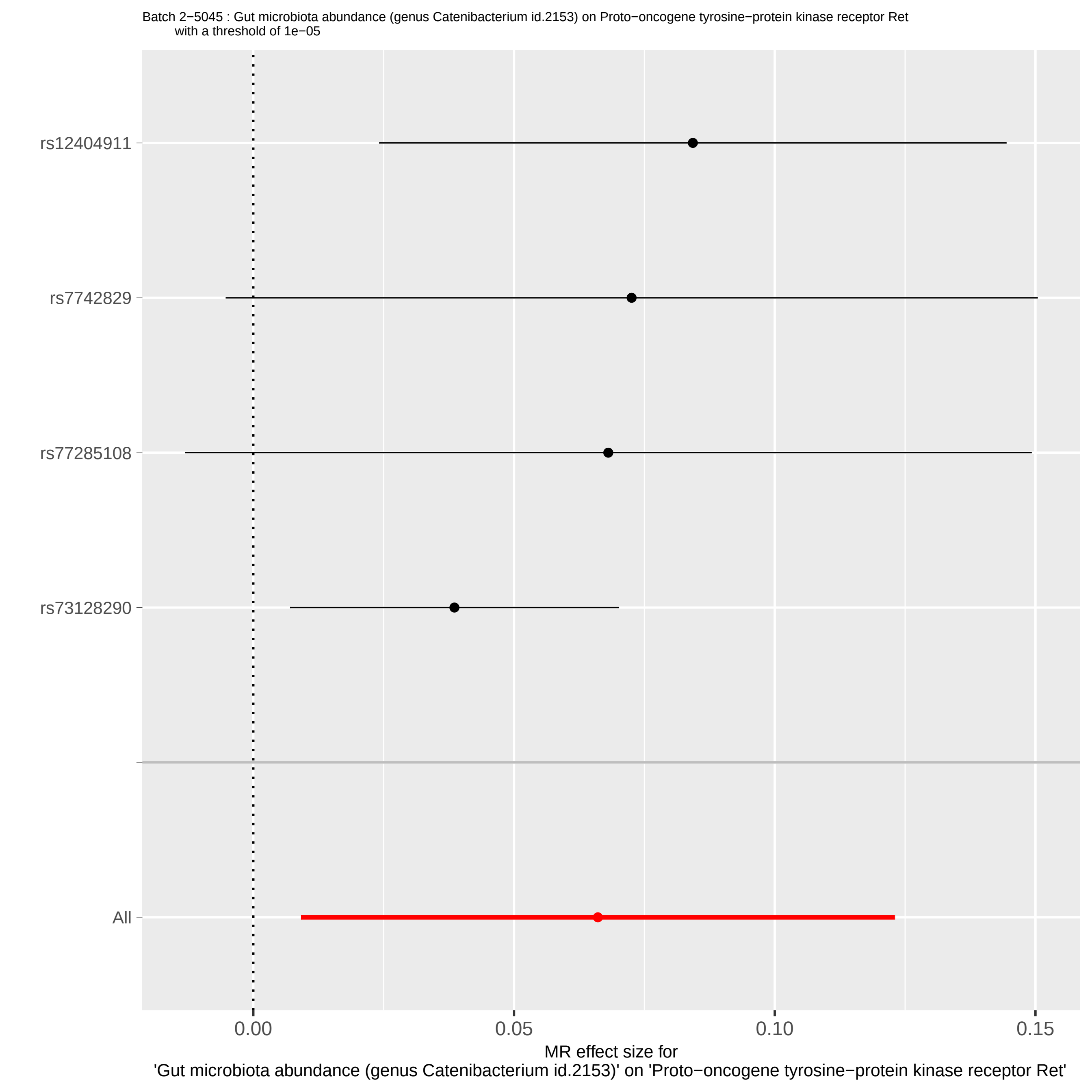
Batch 2−5045 : Gut microbiota abundance (genus Catenibacterium id.2153) on Proto−oncogene tyrosine−protein kinase receptor Ret
with a threshold of 1e−05
rs12404911
rs7742829
rs77285108
rs73128290
All
0.00
0.05
0.10
0.15
MR effect size for
'Gut microbiota abundance (genus Catenibacterium id.2153)' on 'Proto−oncogene tyrosine−protein kinase receptor Ret'

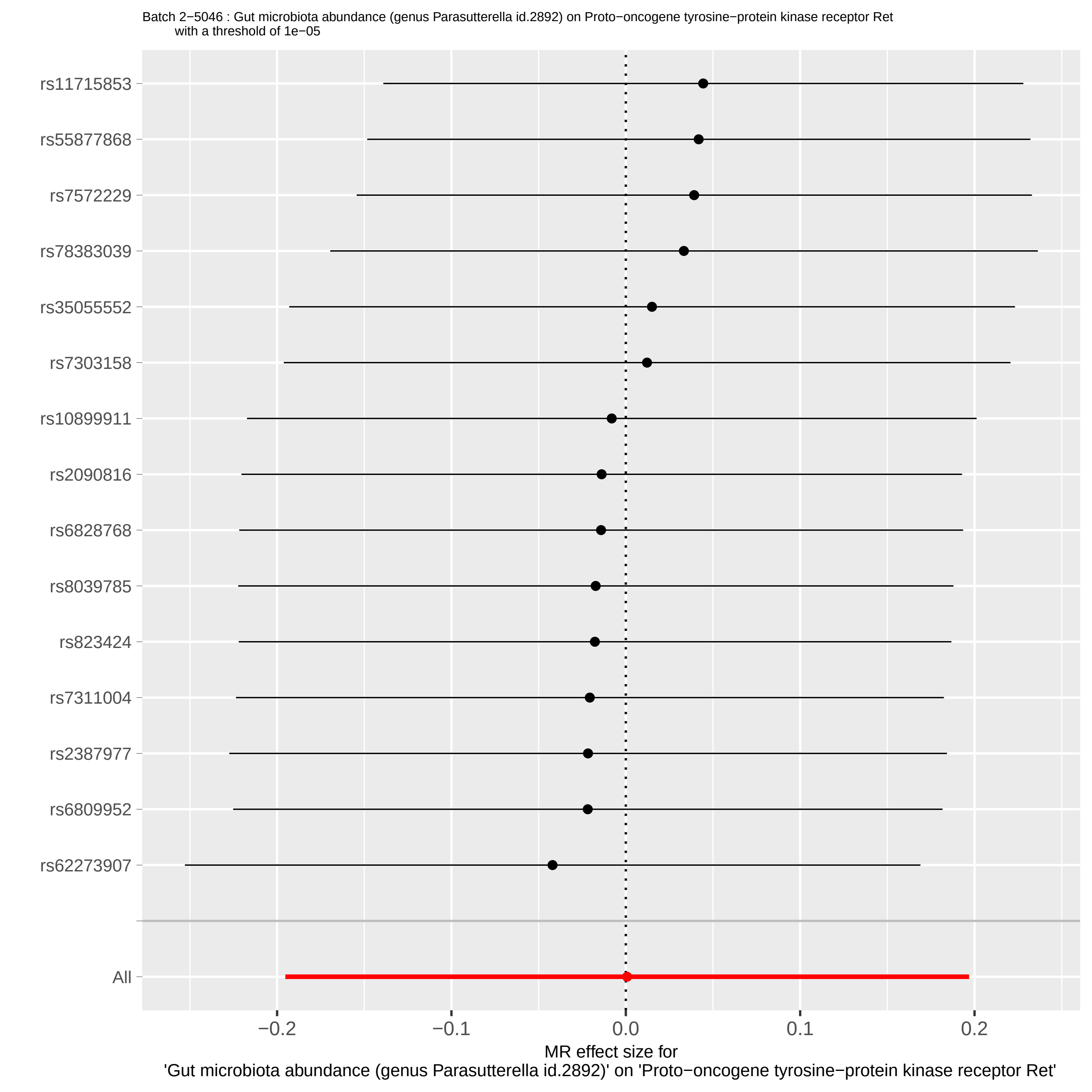

Batch 2−5046 : Gut microbiota abundance (genus Parasutterella id.2892) on Proto−oncogene tyrosine−protein kinase receptor Ret
with a threshold of 1e−05
rs11715853
rs55877868
rs7572229
rs78383039
rs35055552
rs7303158
rs10899911
rs2090816
rs6828768
rs8039785
rs823424
rs7311004
rs2387977
rs6809952
rs62273907
All
−0.2
−0.1
0.0
0.1
0.2
MR effect size for
'Gut microbiota abundance (genus Parasutterella id.2892)' on 'Proto−oncogene tyrosine−protein kinase receptor Ret'

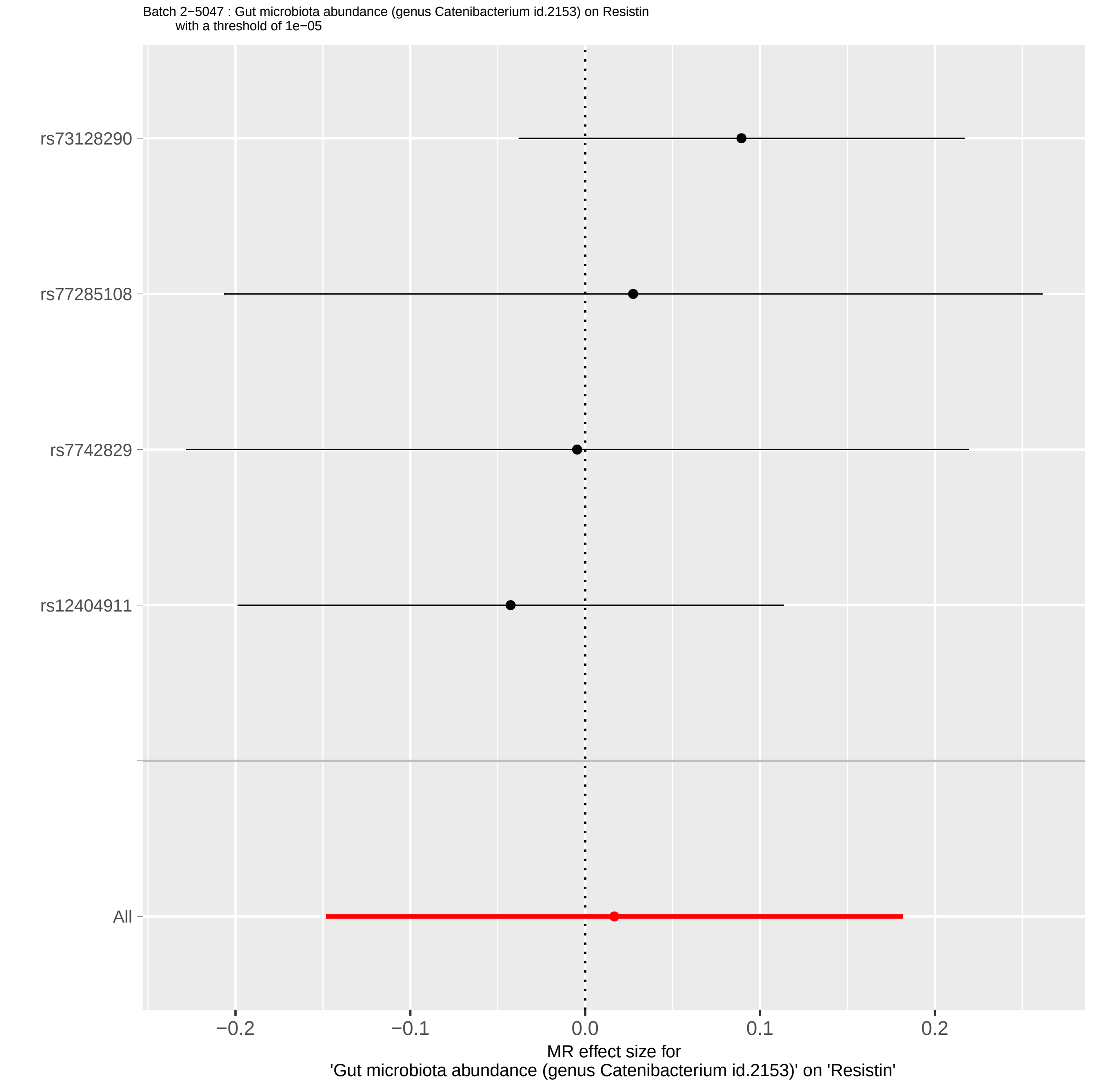

Batch 2−5047 : Gut microbiota abundance (genus Catenibacterium id.2153) on Resistin
with a threshold of 1e−05
rs73128290
rs77285108
rs7742829
rs12404911
All
−0.2
−0.1
0.0
0.1
0.2
MR effect size for
'Gut microbiota abundance (genus Catenibacterium id.2153)' on 'Resistin'

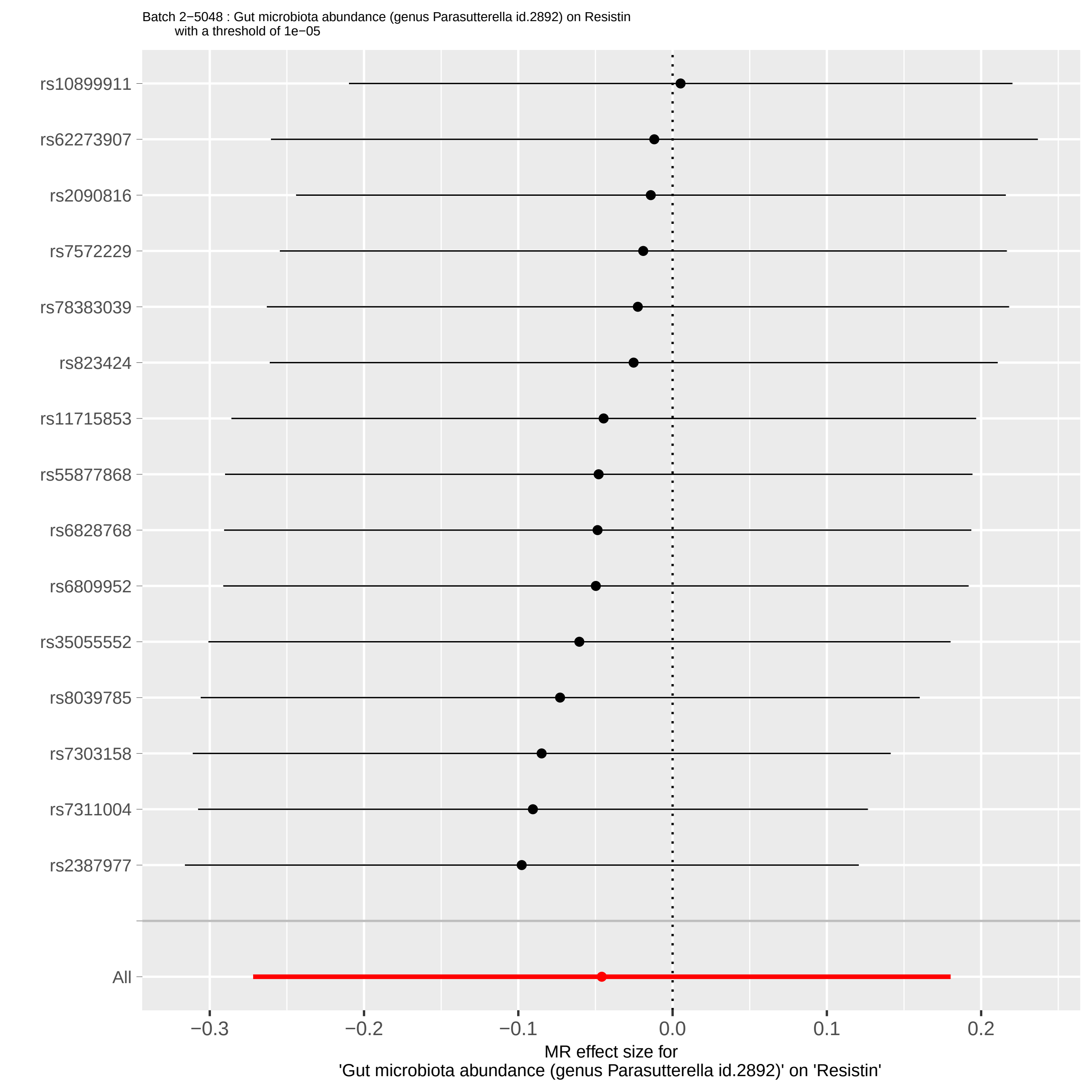

Batch 2−5048 : Gut microbiota abundance (genus Parasutterella id.2892) on Resistin
with a threshold of 1e−05
rs10899911
rs62273907
rs2090816
rs7572229
rs78383039
rs823424
rs11715853
rs55877868
rs6828768
rs6809952
rs35055552
rs8039785
rs7303158
rs7311004
rs2387977
All
−0.3
−0.2
−0.1
0.0
0.1
0.2
MR effect size for
'Gut microbiota abundance (genus Parasutterella id.2892)' on 'Resistin'

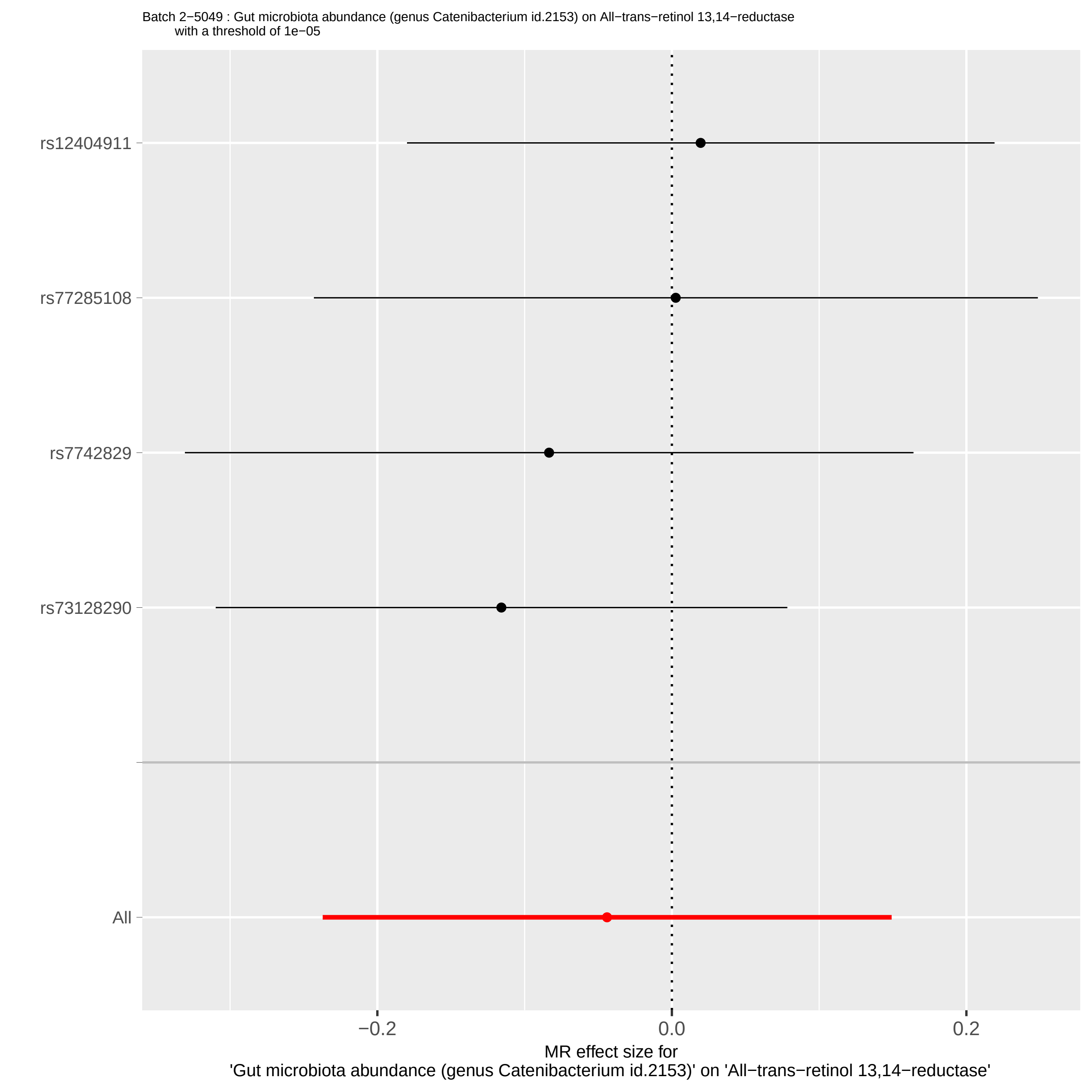
Batch 2−5049 : Gut microbiota abundance (genus Catenibacterium id.2153) on All−trans−retinol 13,14−reductase
with a threshold of 1e−05
rs12404911
rs77285108
rs7742829
rs73128290
All
−0.2
0.0
0.2
MR effect size for
'Gut microbiota abundance (genus Catenibacterium id.2153)' on 'All−trans−retinol 13,14−reductase'

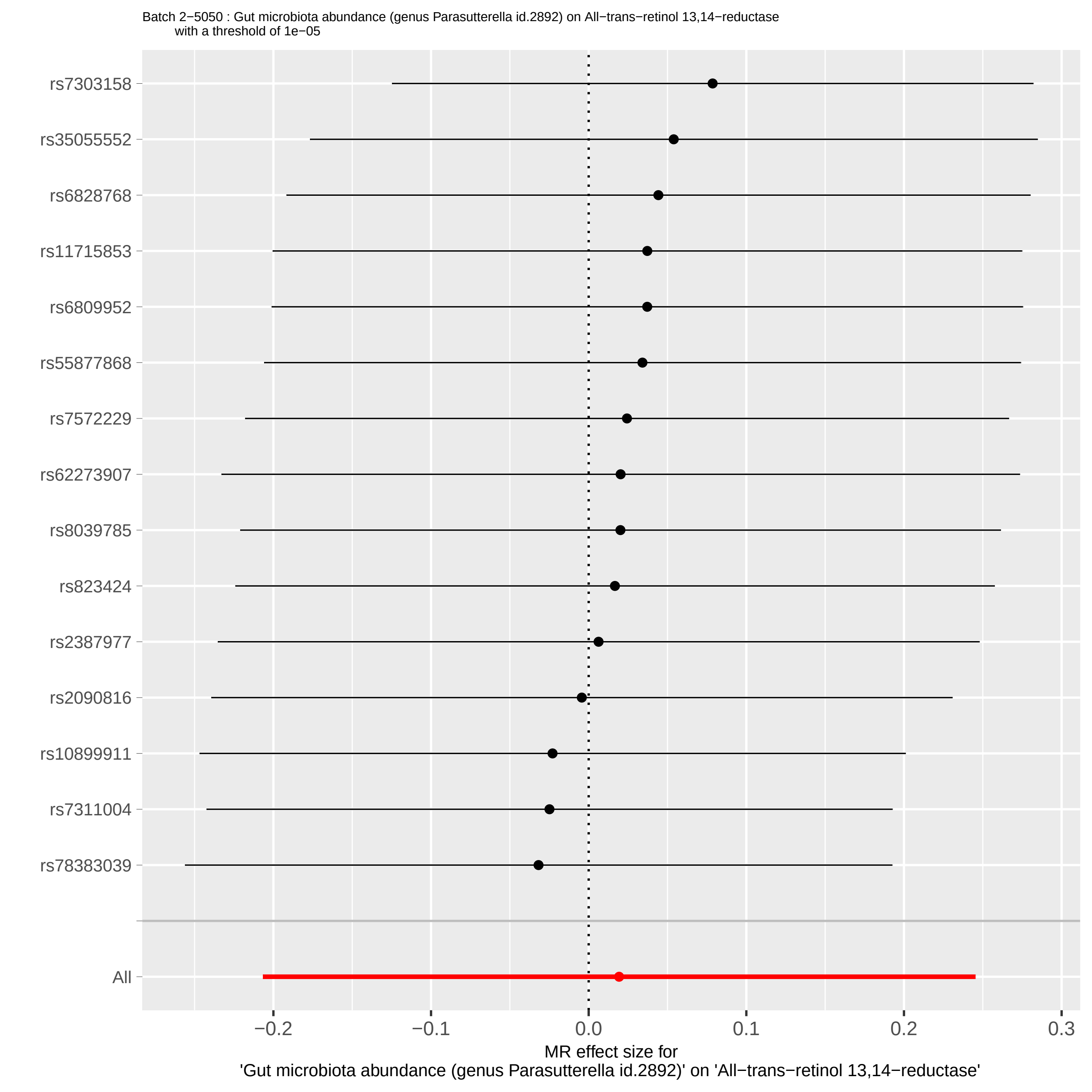
Batch 2−5050 : Gut microbiota abundance (genus Parasutterella id.2892) on All−trans−retinol 13,14−reductase
with a threshold of 1e−05
rs7303158
rs35055552
rs6828768
rs11715853
rs6809952
rs55877868
rs7572229
rs62273907
rs8039785
rs823424
rs2387977
rs2090816
rs10899911
rs7311004
rs78383039
All
−0.2
−0.1
0.0
0.1
0.2
0.3
MR effect size for
'Gut microbiota abundance (genus Parasutterella id.2892)' on 'All−trans−retinol 13,14−reductase'

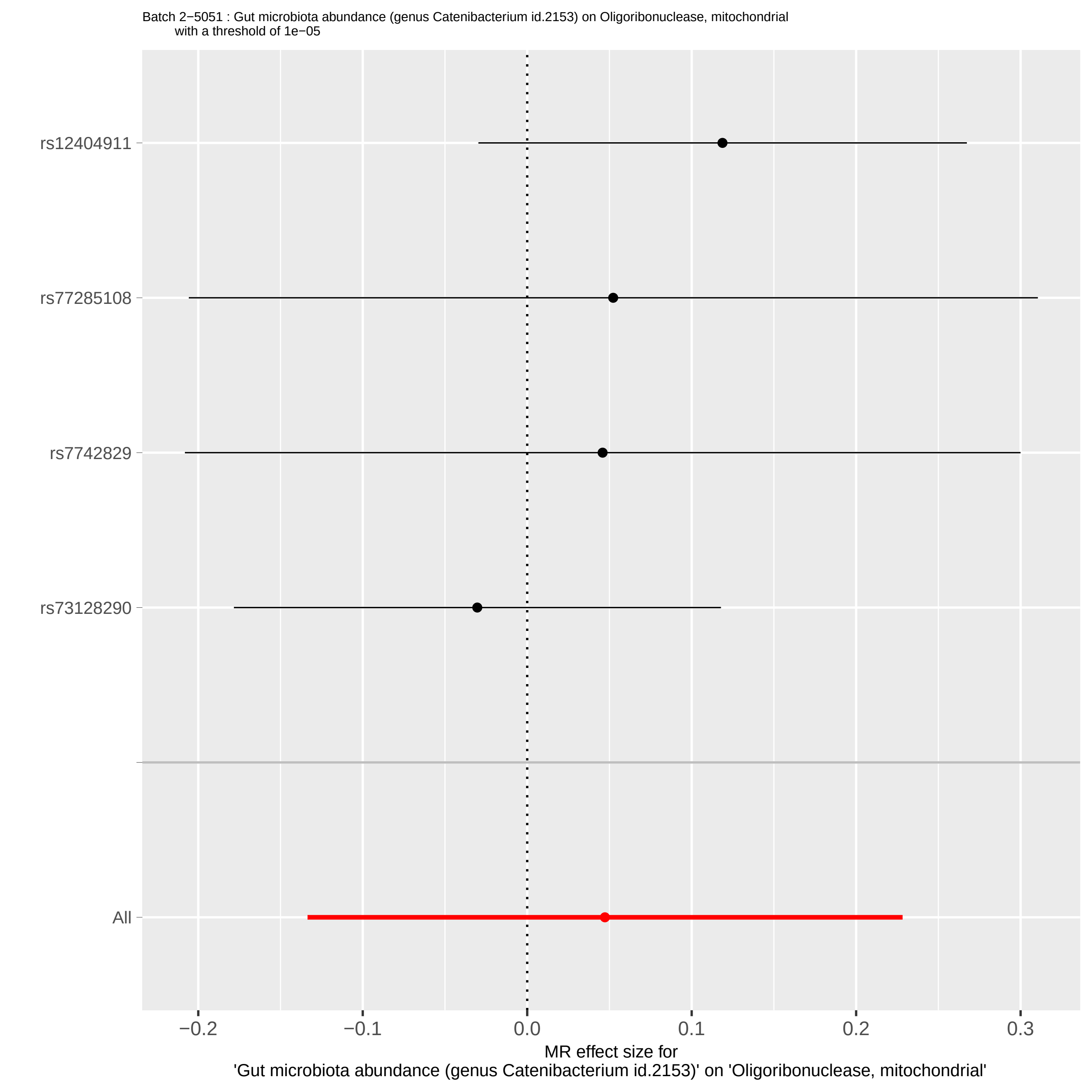
Batch 2−5051 : Gut microbiota abundance (genus Catenibacterium id.2153) on Oligoribonuclease, mitochondrial
with a threshold of 1e−05
rs12404911
rs77285108
rs7742829
rs73128290
All
−0.2
−0.1
0.0
0.1
0.2
0.3
MR effect size for
'Gut microbiota abundance (genus Catenibacterium id.2153)' on 'Oligoribonuclease, mitochondrial'

Batch 2−5052 : Gut microbiota abundance (genus Parasutterella id.2892) on Oligoribonuclease, mitochondrial
with a threshold of 1e−05

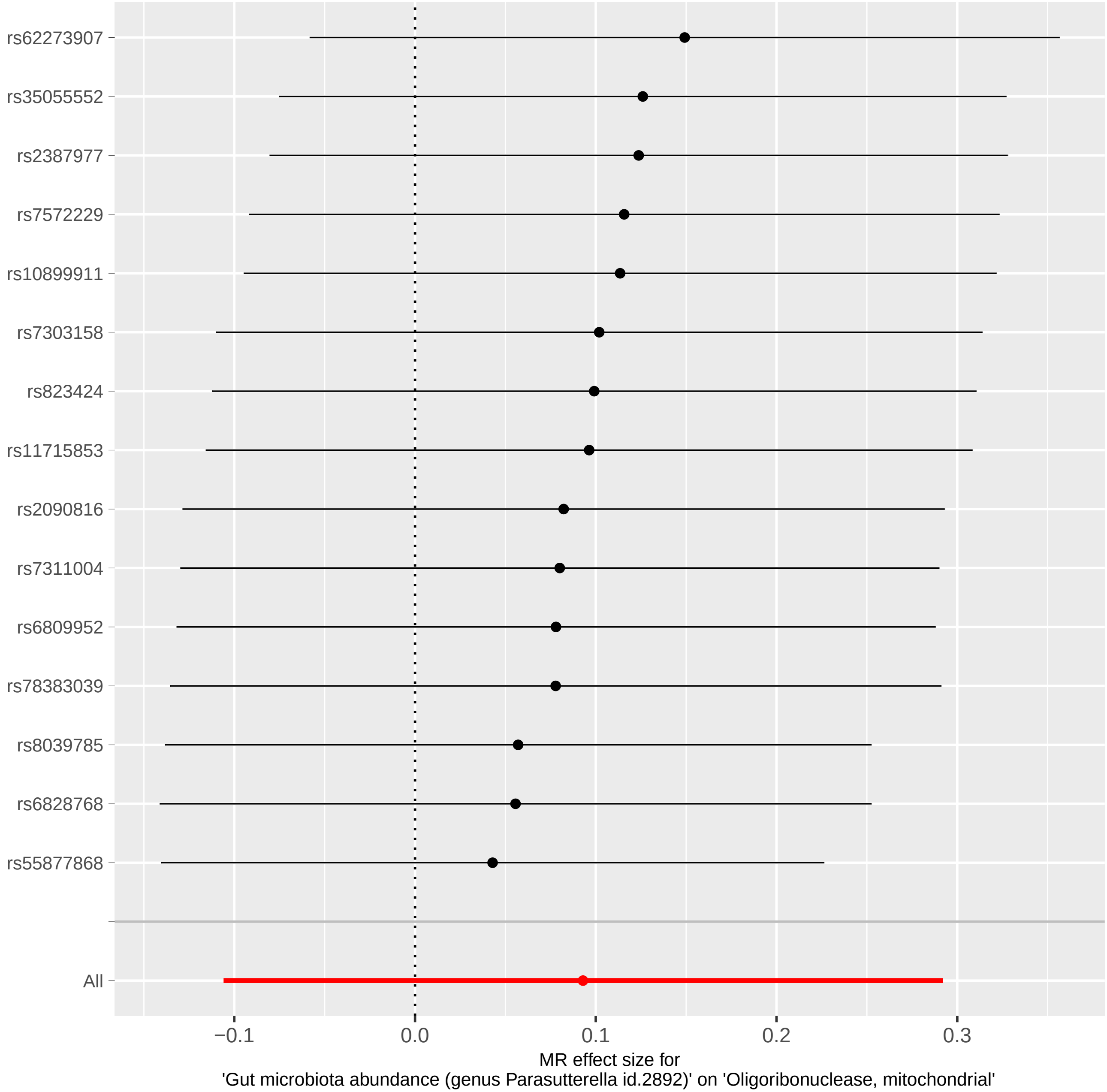

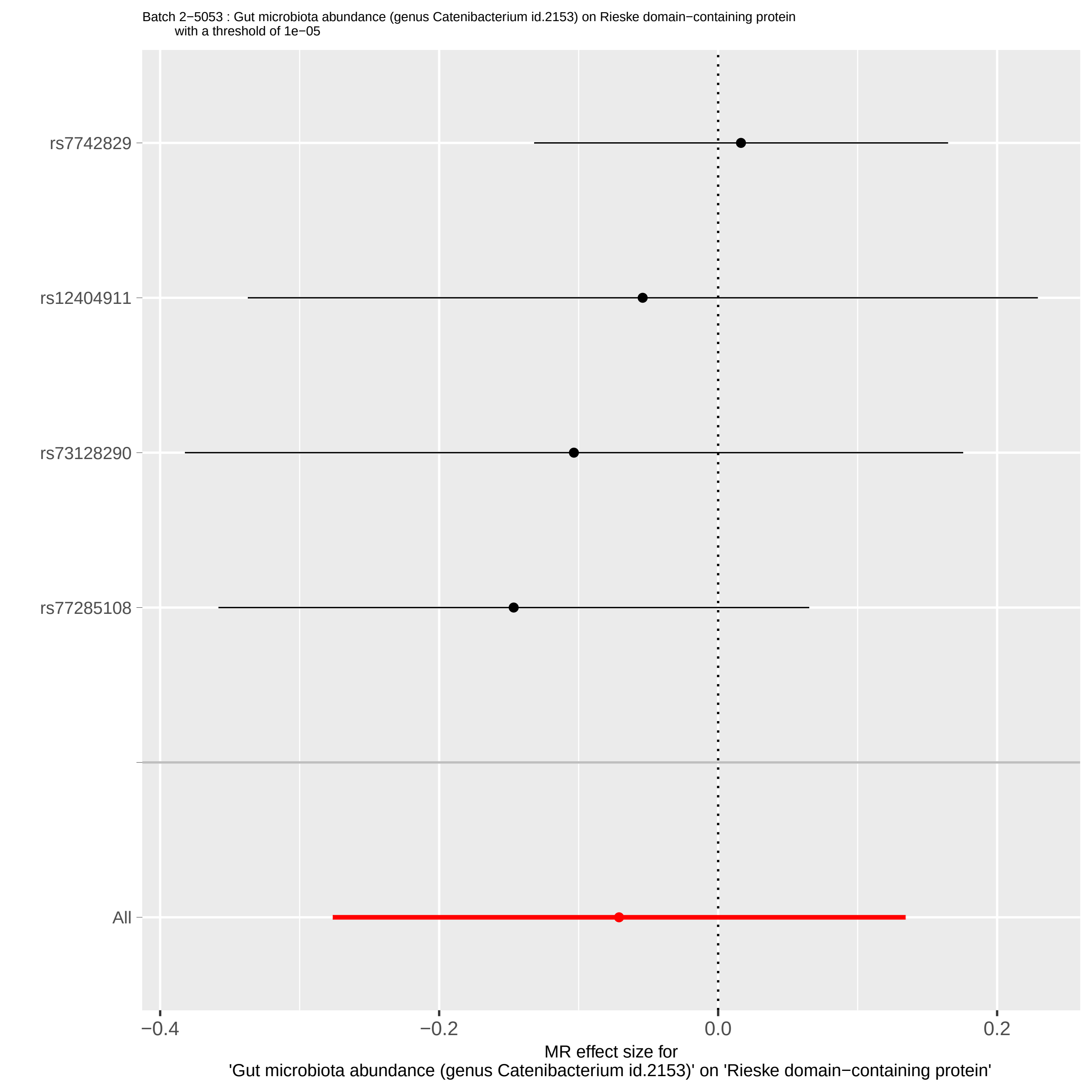
Batch 2−5053 : Gut microbiota abundance (genus Catenibacterium id.2153) on Rieske domain−containing protein
with a threshold of 1e−05
rs7742829
rs12404911
rs73128290
rs77285108
All
−0.4
−0.2
0.0
0.2
MR effect size for
'Gut microbiota abundance (genus Catenibacterium id.2153)' on 'Rieske domain−containing protein'

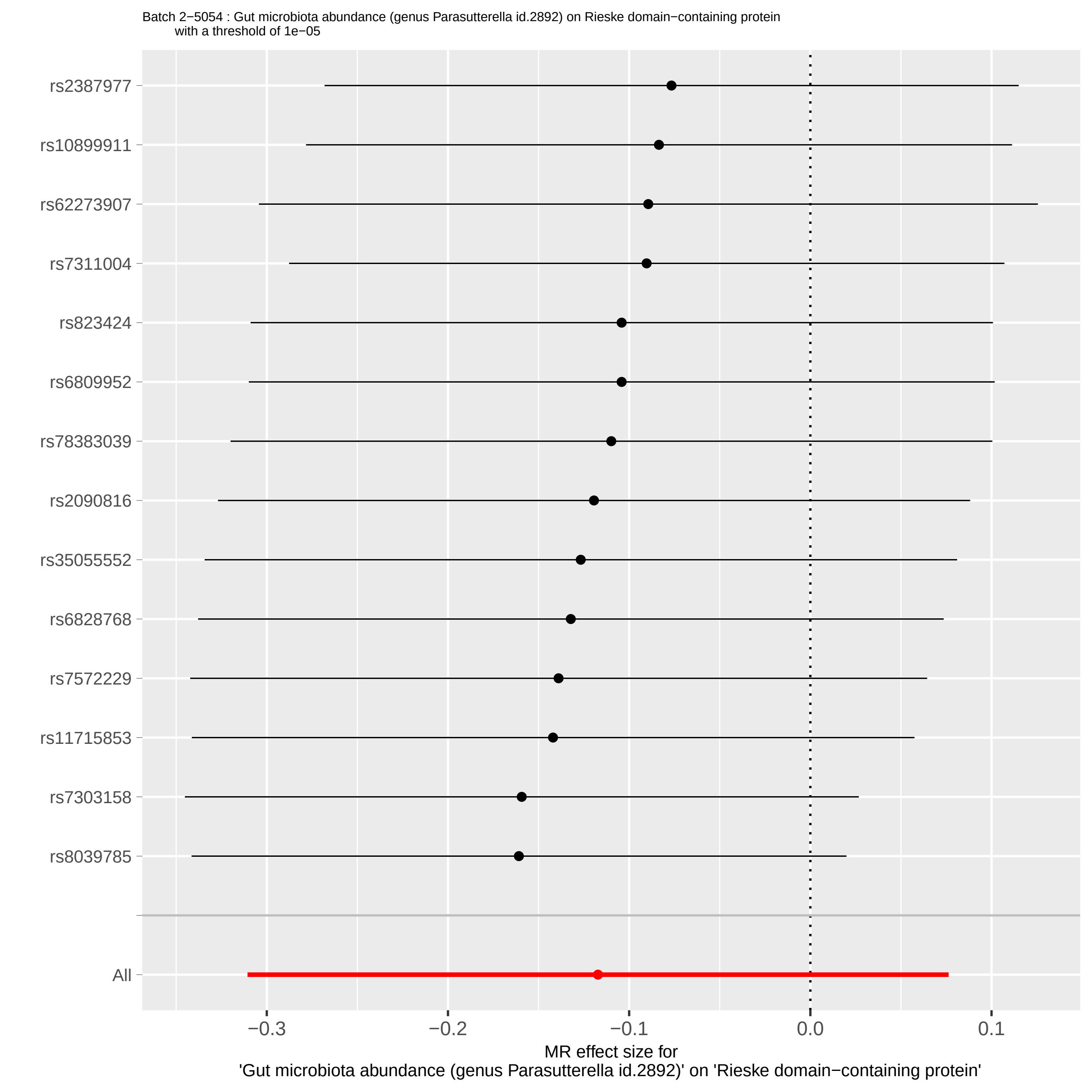
Batch 2−5054 : Gut microbiota abundance (genus Parasutterella id.2892) on Rieske domain−containing protein
with a threshold of 1e−05
rs2387977
rs10899911
rs62273907
rs7311004
rs823424
rs6809952
rs78383039
rs2090816
rs35055552
rs6828768
rs7572229
rs11715853
rs7303158
rs8039785
All
−0.3
−0.2
−0.1
0.0
0.1
MR effect size for
'Gut microbiota abundance (genus Parasutterella id.2892)' on 'Rieske domain−containing protein

Batch 2−5055 : Gut microbiota abundance (genus Catenibacterium id.2153) on E3 ubiquitin−protein ligase rififylin
with a threshold of 1e−05

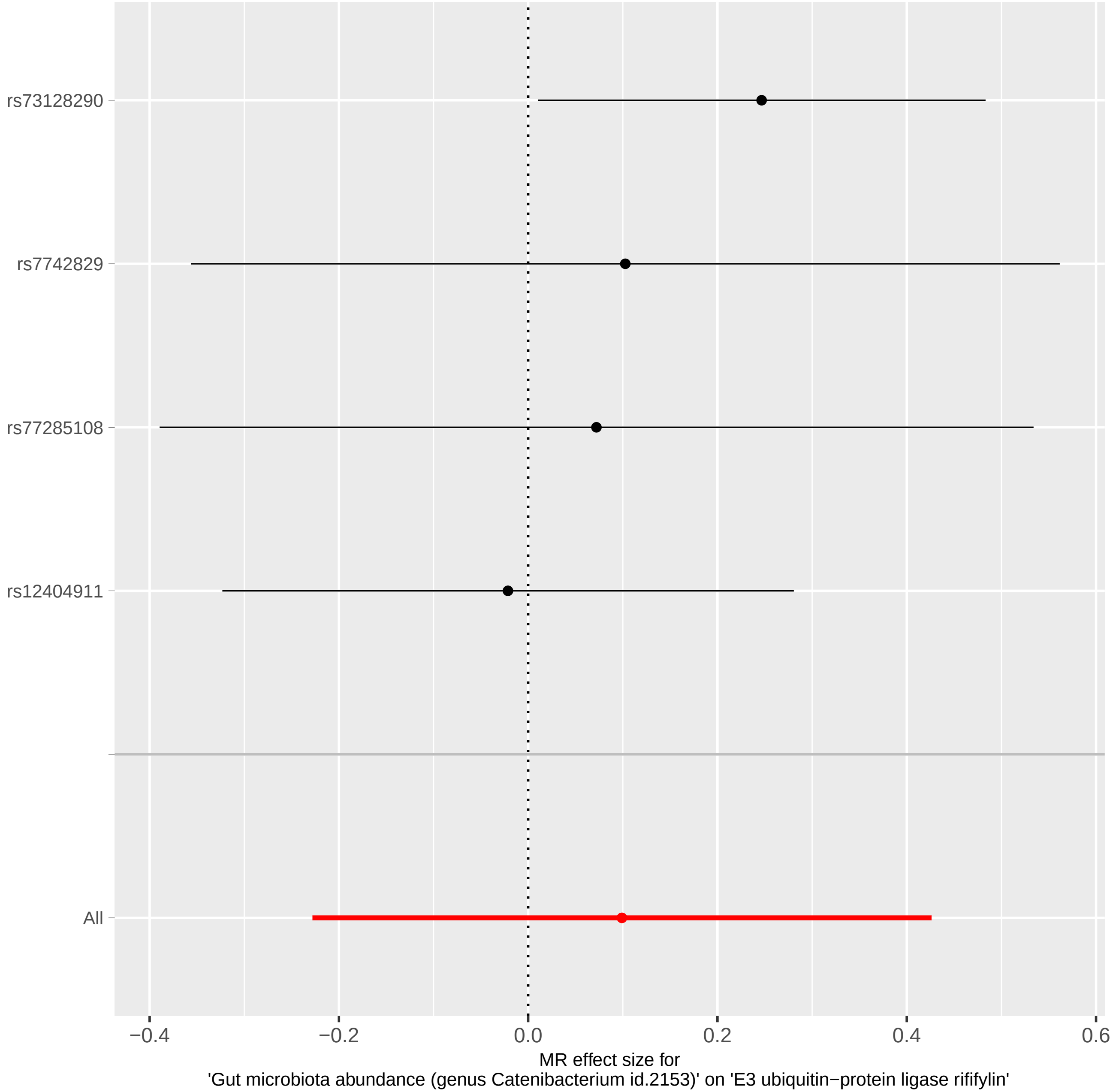

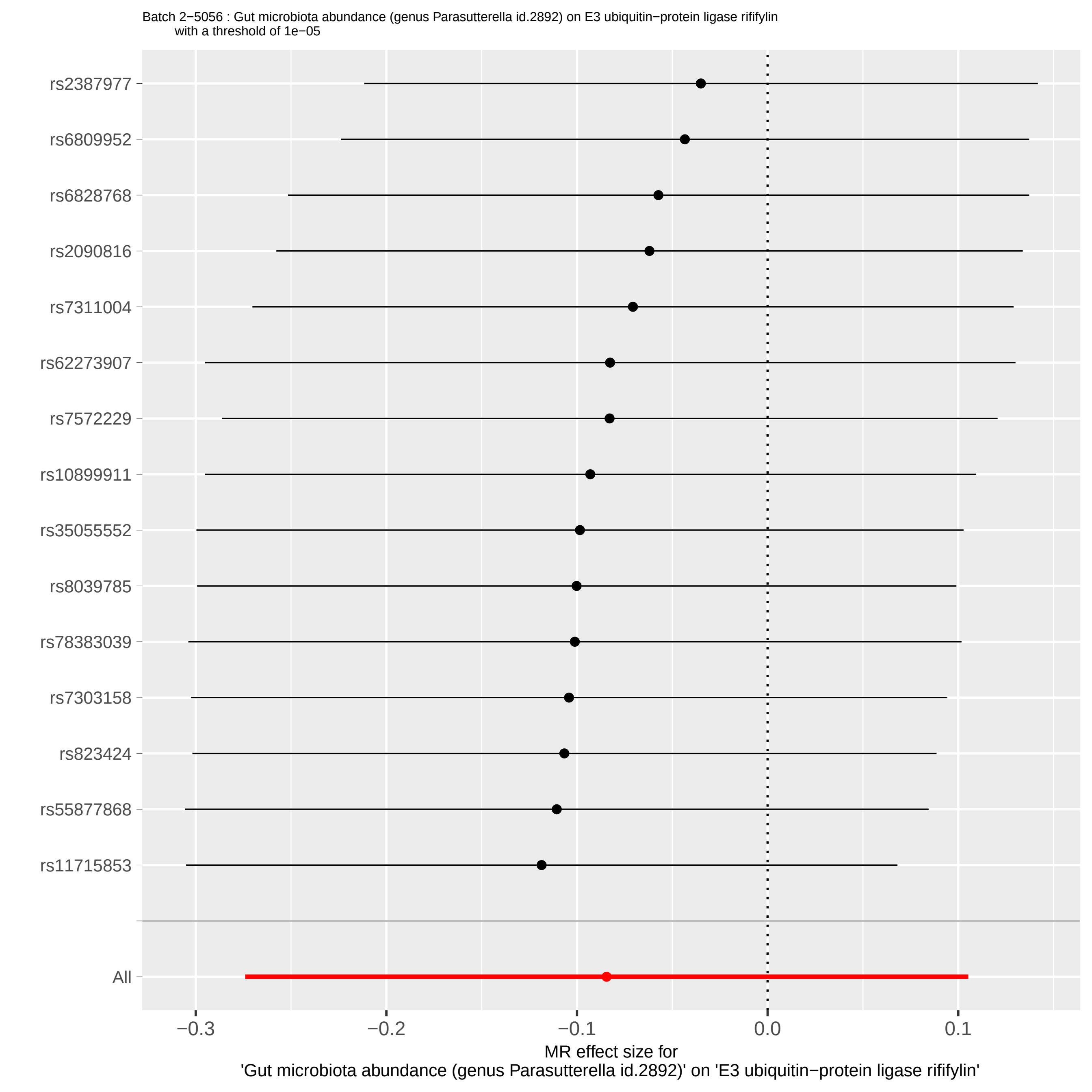

Batch 2−5056 : Gut microbiota abundance (genus Parasutterella id.2892) on E3 ubiquitin−protein ligase rififylin
with a threshold of 1e−05
rs2387977
rs6809952
rs6828768
rs2090816
rs7311004
rs62273907
rs7572229
rs10899911
rs35055552
rs8039785
rs78383039
rs7303158
rs823424
rs55877868
rs11715853
All
−0.3
−0.2
−0.1
0.0
0.1
MR effect size for
'Gut microbiota abundance (genus Parasutterella id.2892)' on 'E3 ubiquitin−protein ligase rififylin'

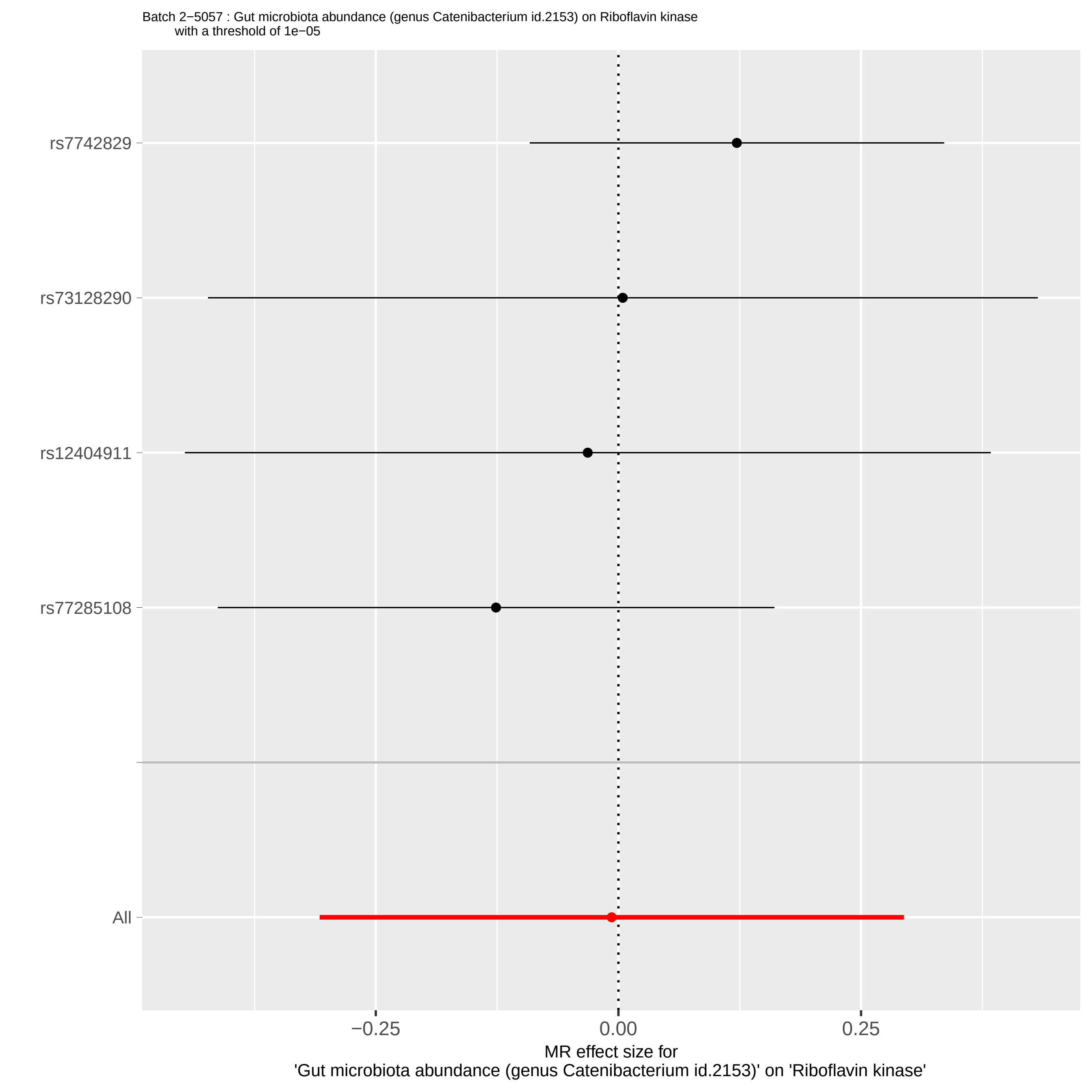
Batch 2−5057 : Gut microbiota abundance (genus Catenibacterium id.2153) on Riboflavin kinase
with a threshold of 1e−05
rs7742829
rs73128290
rs12404911
rs77285108
All
−0.25
0.00
0.25
MR effect size for
'Gut microbiota abundance (genus Catenibacterium id.2153)' on 'Riboflavin kinase'

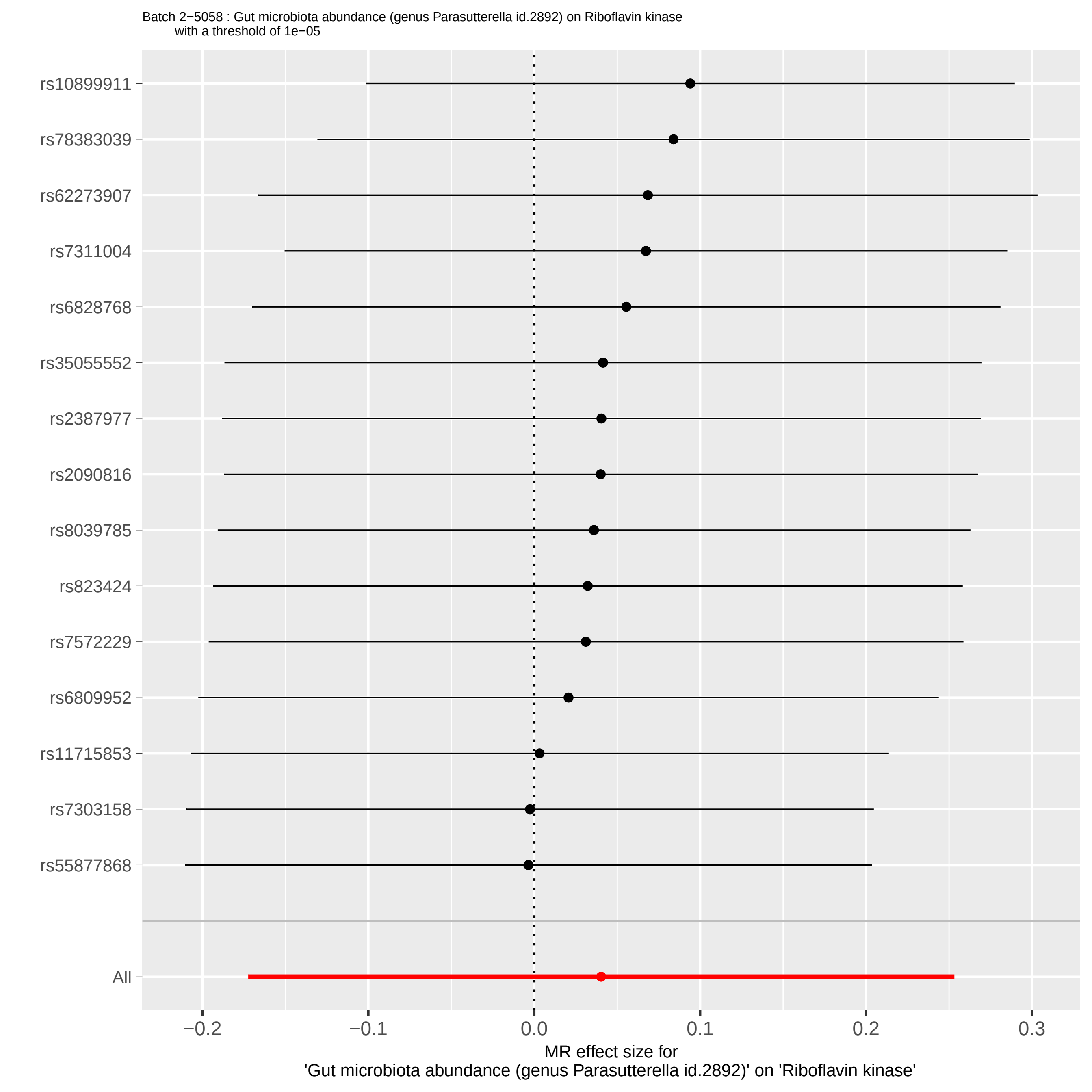
Batch 2−5058 : Gut microbiota abundance (genus Parasutterella id.2892) on Riboflavin kinase
with a threshold of 1e−05
rs10899911
rs78383039
rs62273907
rs7311004
rs6828768
rs35055552
rs2387977
rs2090816
rs8039785
rs823424
rs7572229
rs6809952
rs11715853
rs7303158
rs55877868
All
−0.2
−0.1
0.0
0.1
0.2
0.3
MR effect size for
'Gut microbiota abundance (genus Parasutterella id.2892)' on 'Riboflavin kinase'

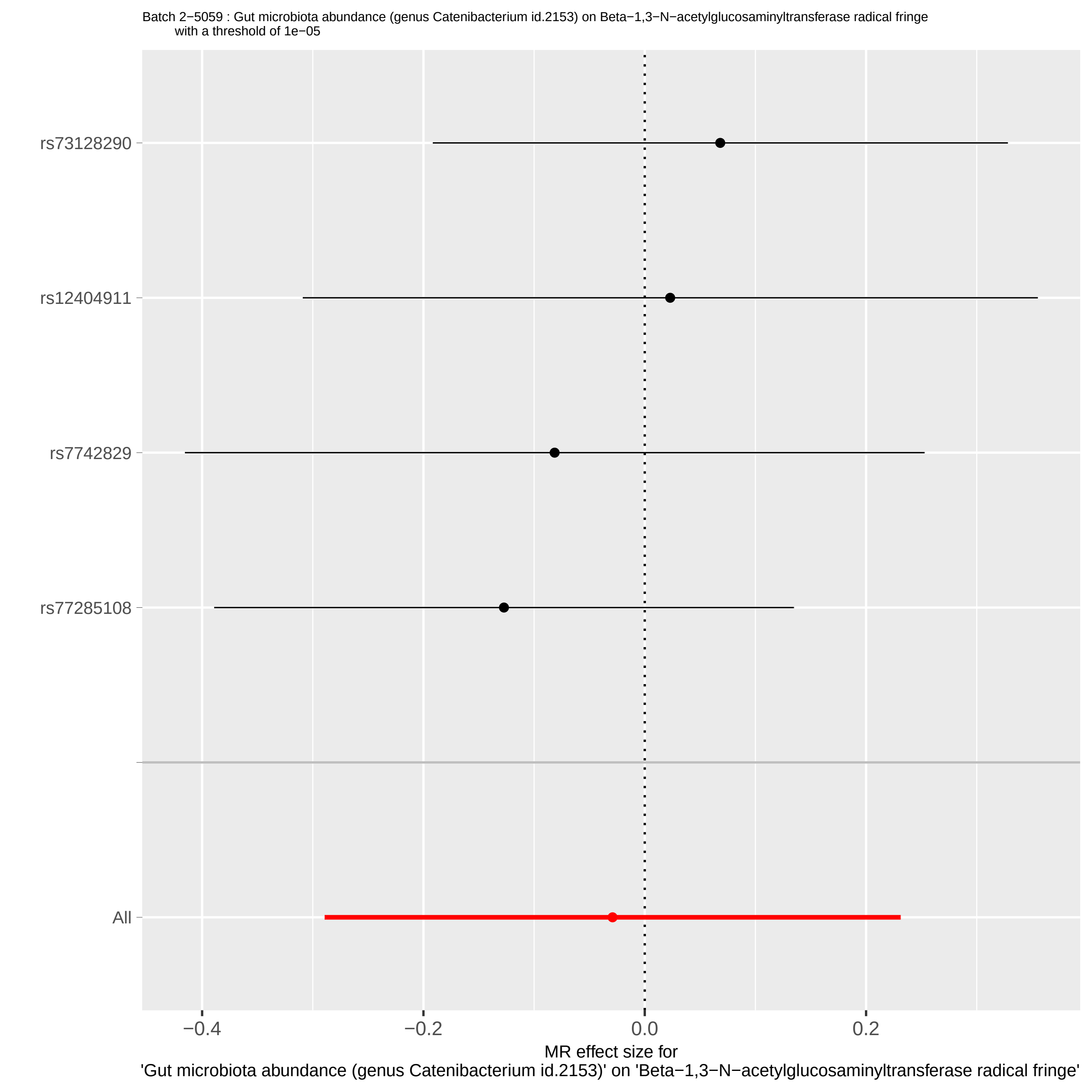

Batch 2−5059 : Gut microbiota abundance (genus Catenibacterium id.2153) on Beta−1,3−N−acetylglucosaminyltransferase radical fringe
with a threshold of 1e−05
rs73128290
rs12404911
rs7742829
rs77285108
All
−0.4
−0.2
0.0
0.2
MR effect size for
'Gut microbiota abundance (genus Catenibacterium id.2153)' on 'Beta−1,3−N−acetylglucosaminyltransferase radical fringe'

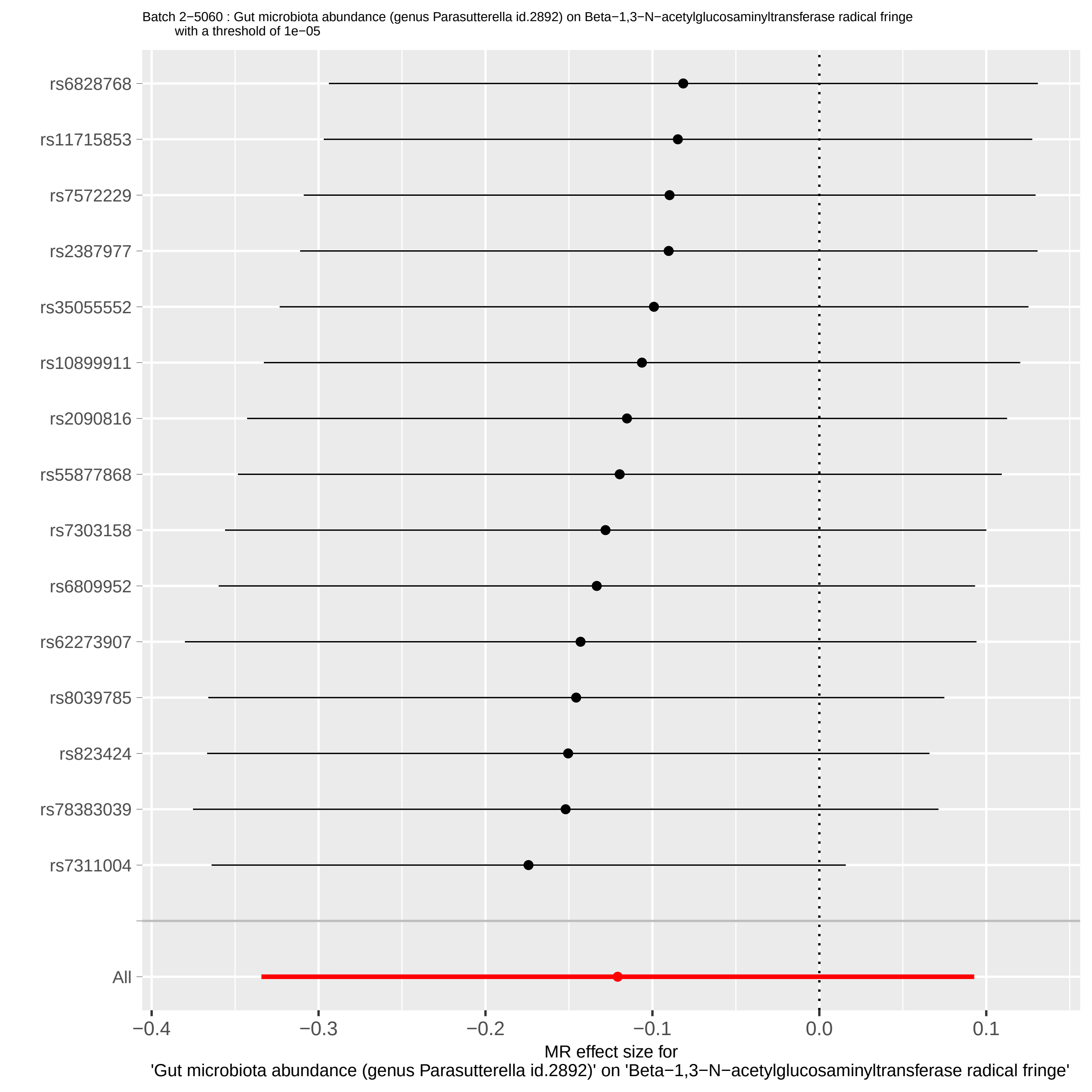

Batch 2−5060 : Gut microbiota abundance (genus Parasutterella id.2892) on Beta−1,3−N−acetylglucosaminyltransferase radical fringe
with a threshold of 1e−05
rs6828768
rs11715853
rs7572229
rs2387977
rs35055552
rs10899911
rs2090816
rs55877868
rs7303158
rs6809952
rs62273907
rs8039785
rs823424
rs78383039
rs7311004
All
−0.4
−0.3
−0.2
−0.1
0.0
0.1
MR effect size for
'Gut microbiota abundance (genus Parasutterella id.2892)' on 'Beta−1,3−N−acetylglucosaminyltransferase radical fringe'

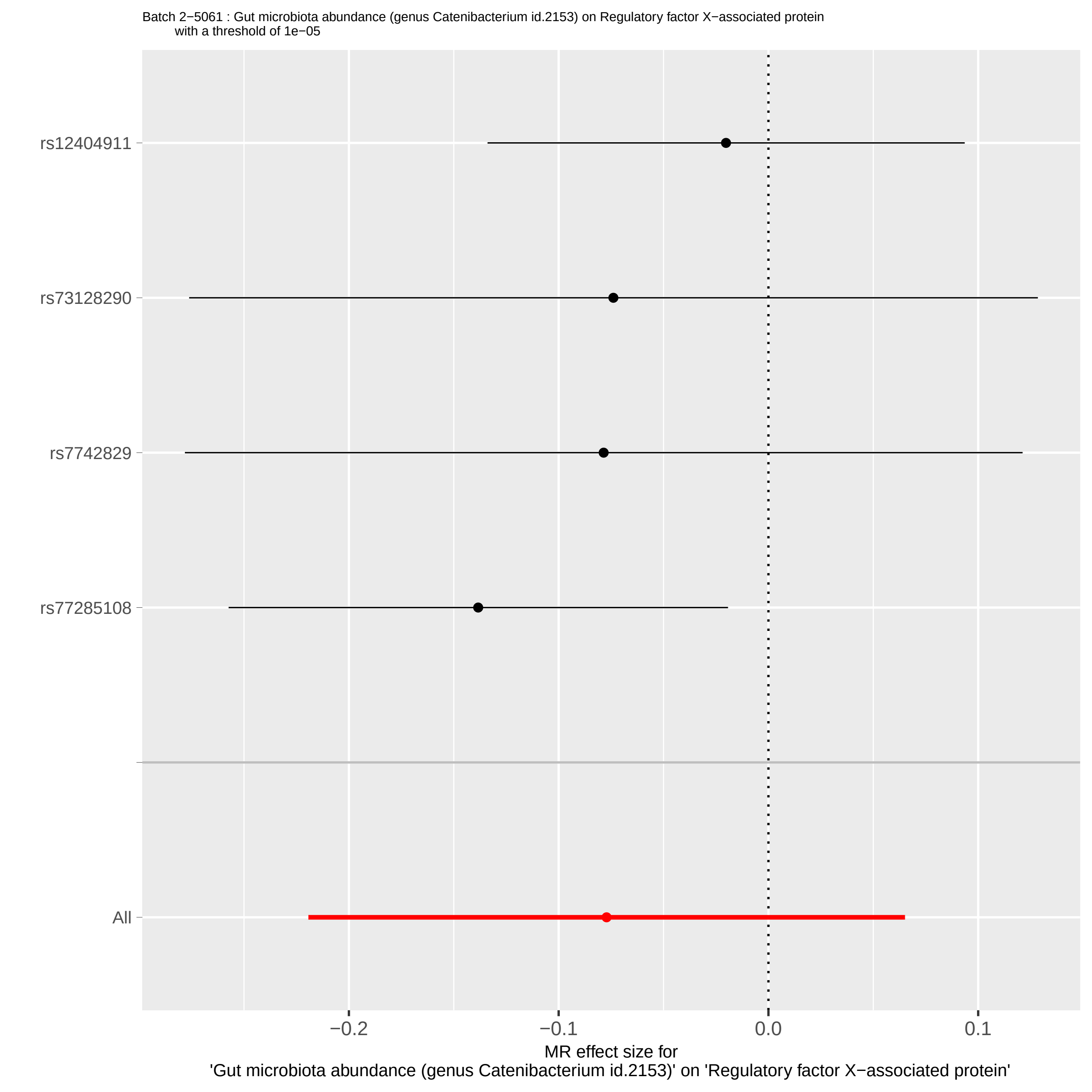
Batch 2−5061 : Gut microbiota abundance (genus Catenibacterium id.2153) on Regulatory factor X−associated protein
with a threshold of 1e−05
rs12404911
rs73128290
rs7742829
rs77285108
All
−0.2
−0.1
0.0
0.1
MR effect size for
'Gut microbiota abundance (genus Catenibacterium id.2153)' on 'Regulatory factor X−associated protein'

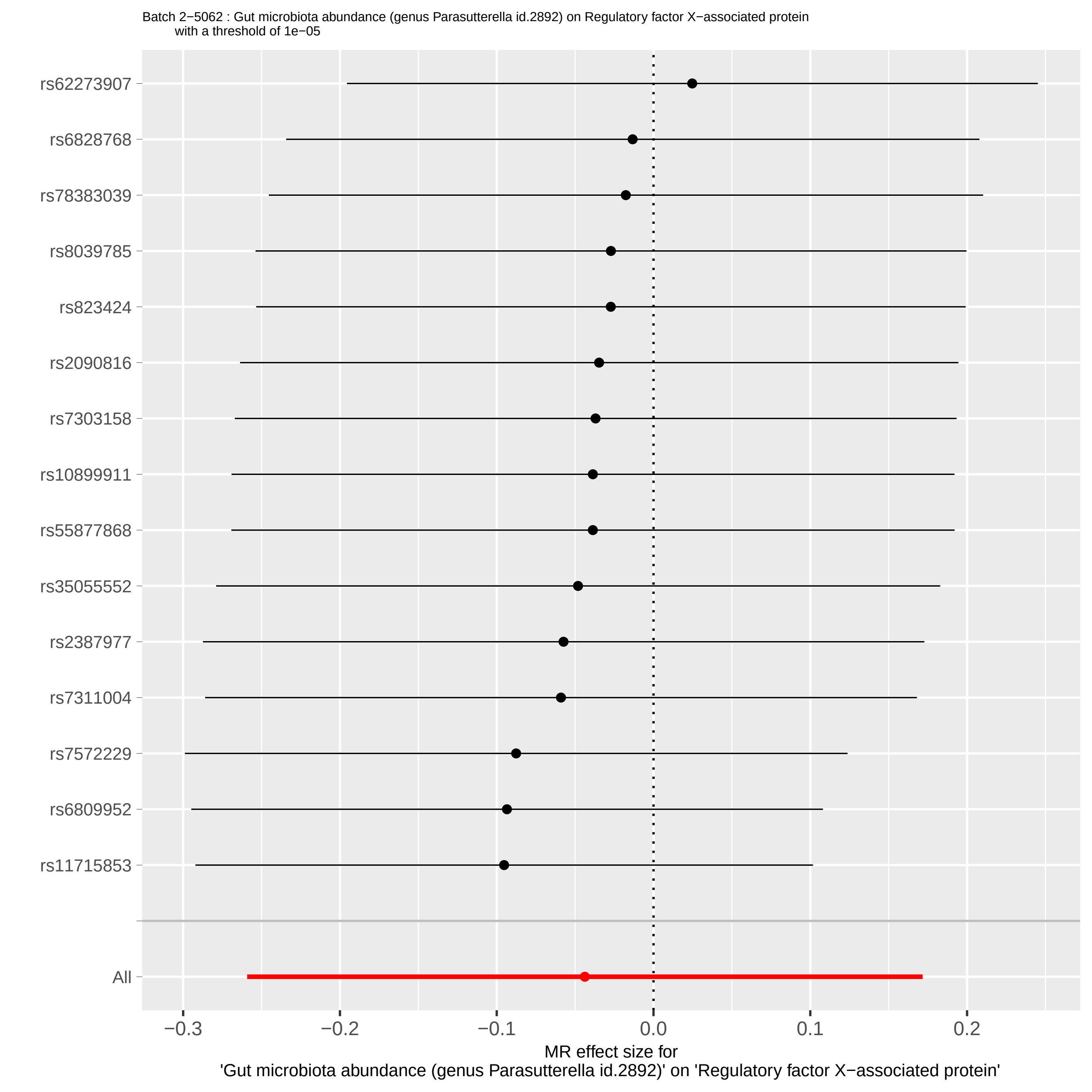

Batch 2−5062 : Gut microbiota abundance (genus Parasutterella id.2892) on Regulatory factor X−associated protein
with a threshold of 1e−05
rs62273907
rs6828768
rs78383039
rs8039785
rs823424
rs2090816
rs7303158
rs10899911
rs55877868
rs35055552
rs2387977
rs7311004
rs7572229
rs6809952
rs11715853
All
−0.3
−0.2
−0.1
0.0
0.1
0.2
MR effect size for
'Gut microbiota abundance (genus Parasutterella id.2892)' on 'Regulatory factor X−associated protein'

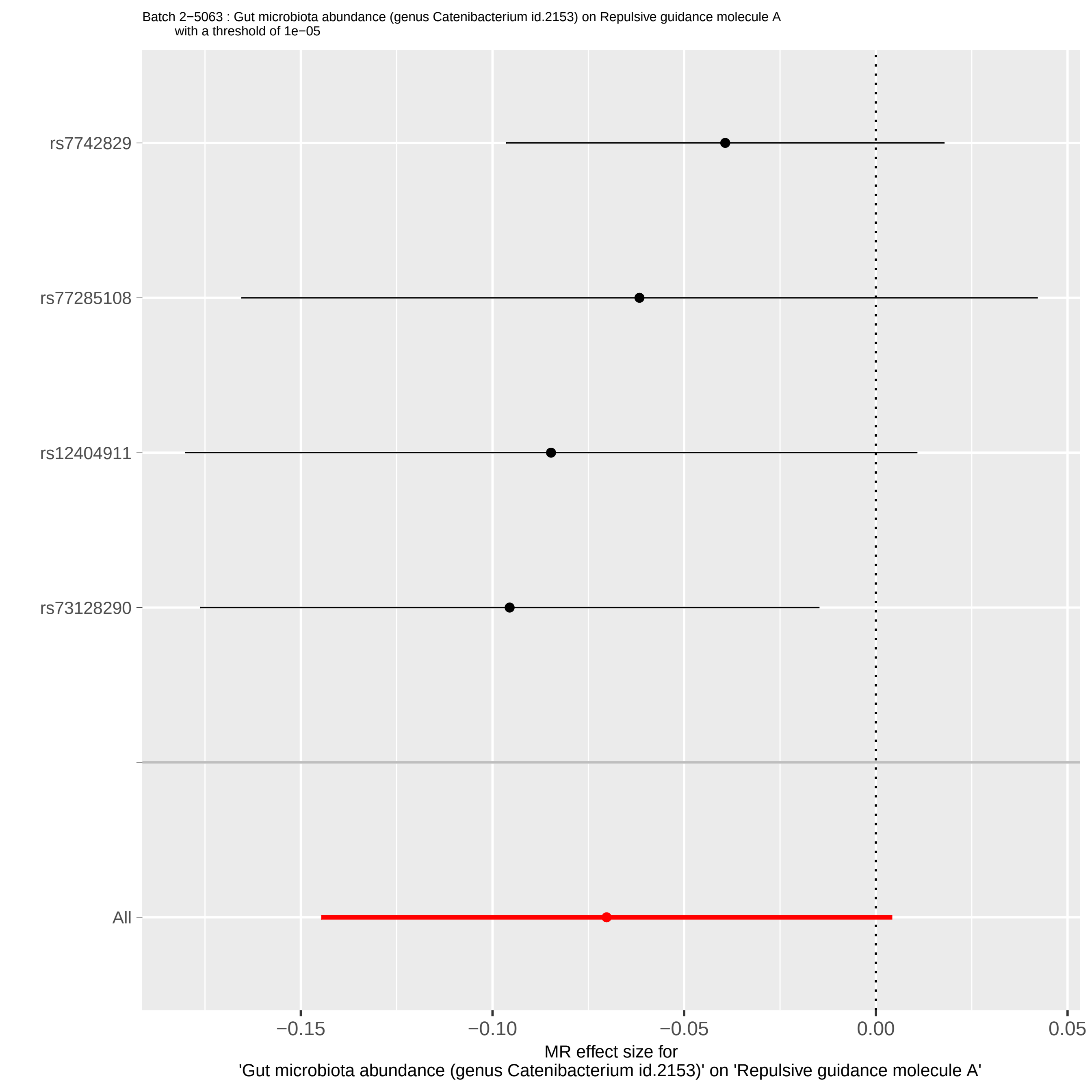
Batch 2−5063 : Gut microbiota abundance (genus Catenibacterium id.2153) on Repulsive guidance molecule A
with a threshold of 1e−05
rs7742829
rs77285108
rs12404911
rs73128290
All
−0.15
−0.10
−0.05
0.00
0.05
MR effect size for
'Gut microbiota abundance (genus Catenibacterium id.2153)' on 'Repulsive guidance molecule A'

Batch 2−5064 : Gut microbiota abundance (genus Parasutterella id.2892) on Repulsive guidance molecule A
with a threshold of 1e−05

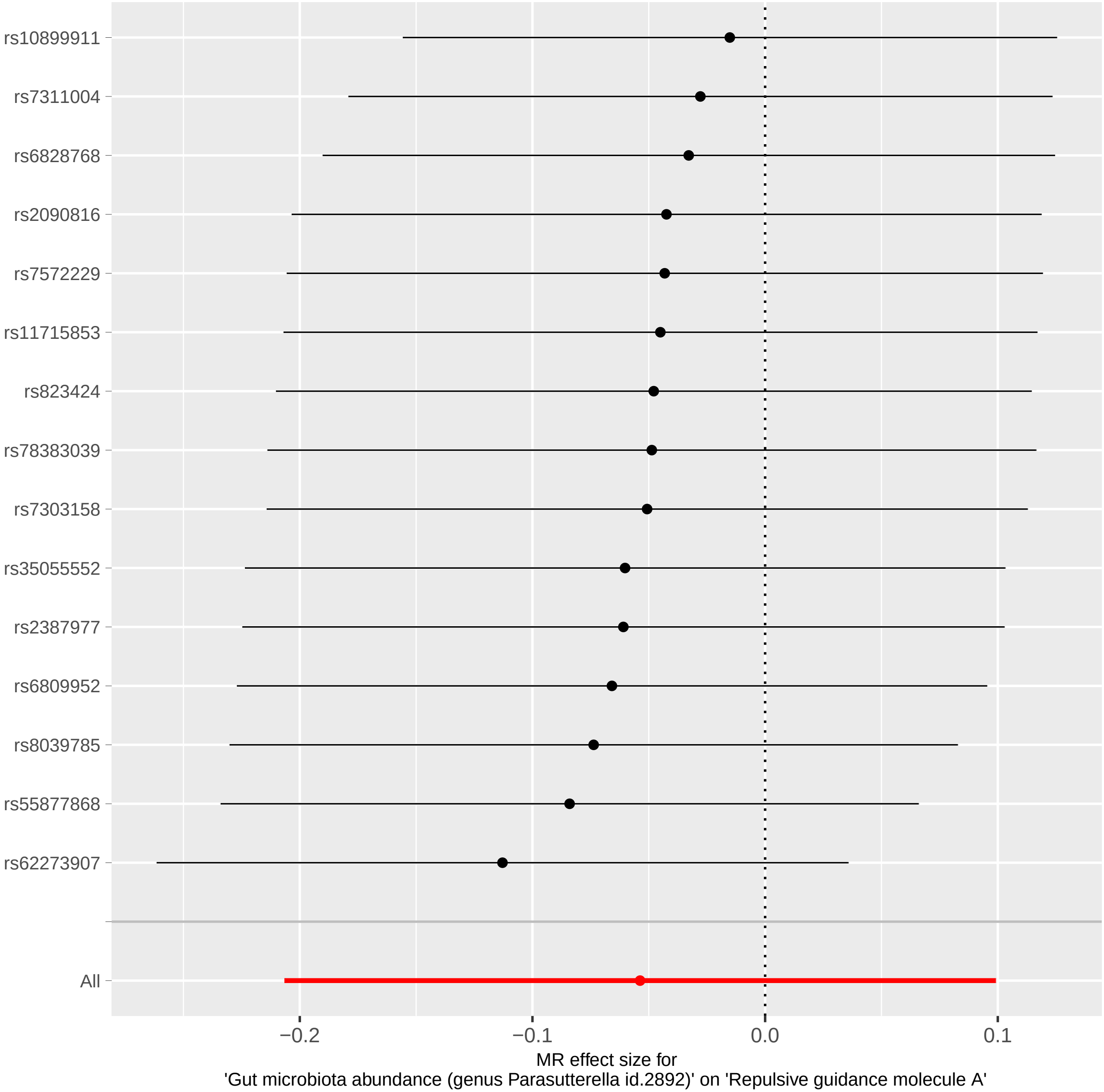

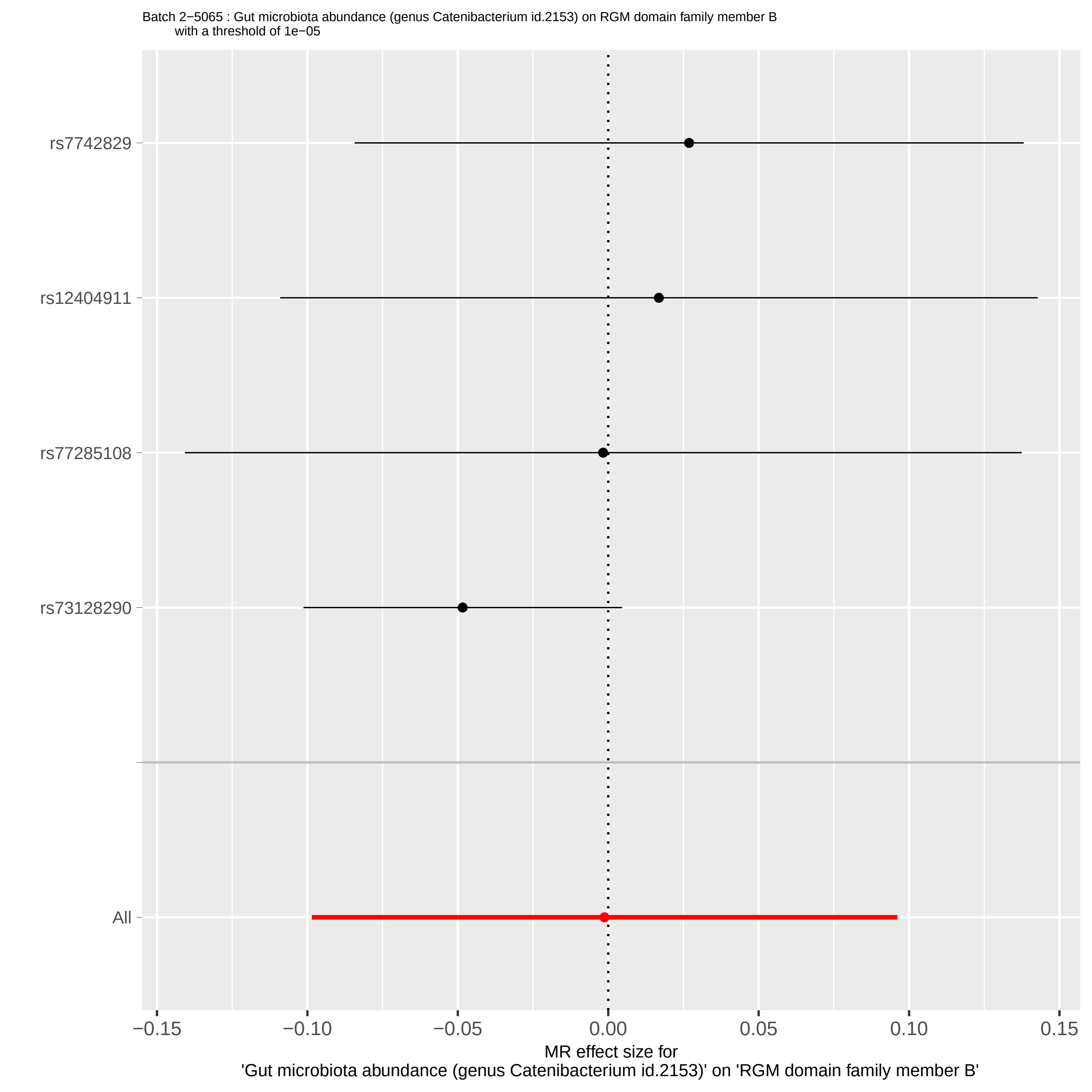
Batch 2−5065 : Gut microbiota abundance (genus Catenibacterium id.2153) on RGM domain family member B
with a threshold of 1e−05
rs7742829
rs12404911
rs77285108
rs73128290
All
−0.15
−0.10
−0.05
0.00
0.05
0.10
0.15
MR effect size for
'Gut microbiota abundance (genus Catenibacterium id.2153)' on 'RGM domain family member B'

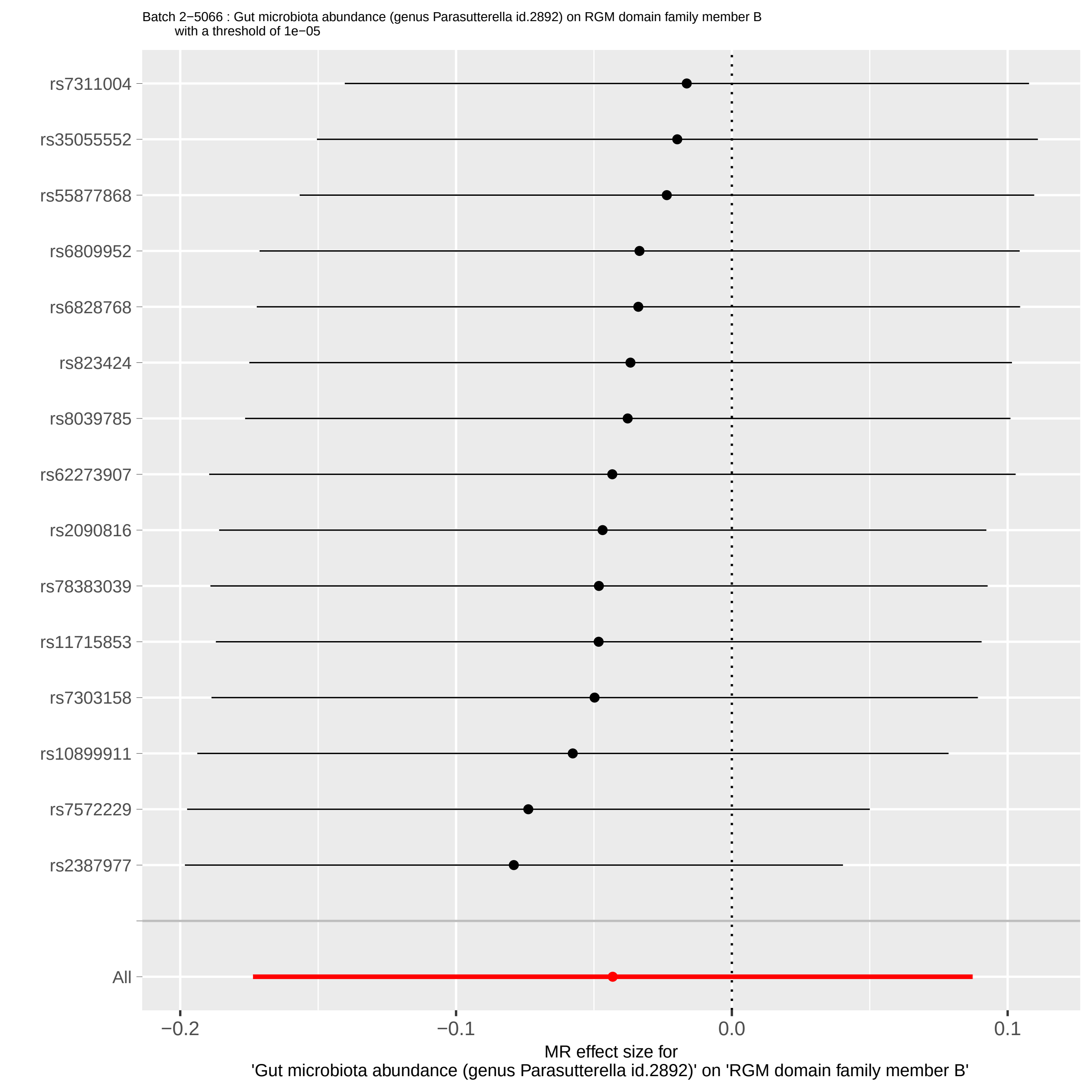

Batch 2−5066 : Gut microbiota abundance (genus Parasutterella id.2892) on RGM domain family member B
with a threshold of 1e−05
rs7311004
rs35055552
rs55877868
rs6809952
rs6828768
rs823424
rs8039785
rs62273907
rs2090816
rs78383039
rs11715853
rs7303158
rs10899911
rs7572229
rs2387977
All
−0.2
−0.1
0.0
0.1
MR effect size for
'Gut microbiota abundance (genus Parasutterella id.2892)' on 'RGM domain family member B'

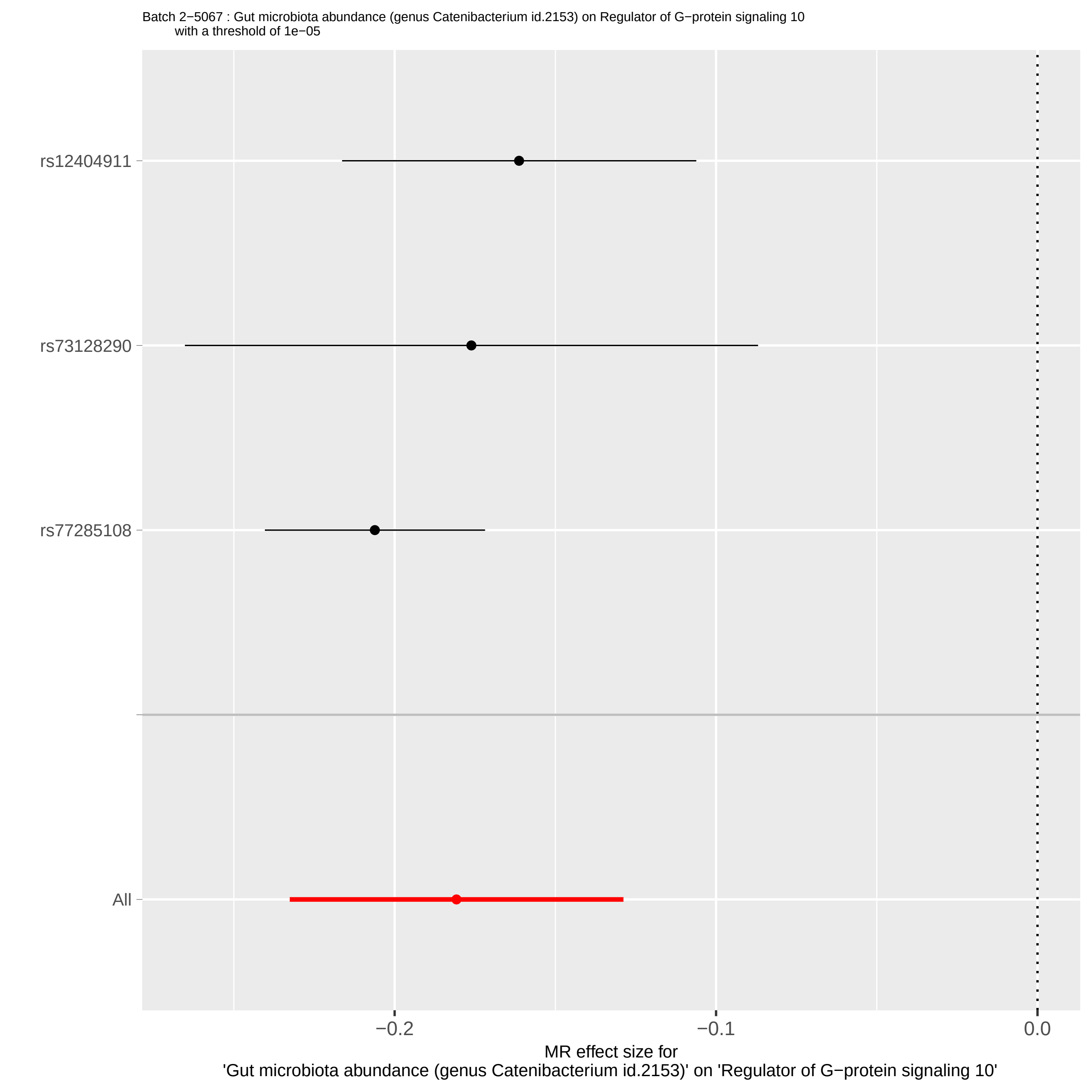

Batch 2−5067 : Gut microbiota abundance (genus Catenibacterium id.2153) on Regulator of G−protein signaling 10
with a threshold of 1e−05
rs12404911
rs73128290
rs77285108
All
−0.2
−0.1
0.0
MR effect size for
'Gut microbiota abundance (genus Catenibacterium id.2153)' on 'Regulator of G−protein signaling 10'

Batch 2−5068 : Gut microbiota abundance (genus Parasutterella id.2892) on Regulator of G−protein signaling 10
with a threshold of 1e−05

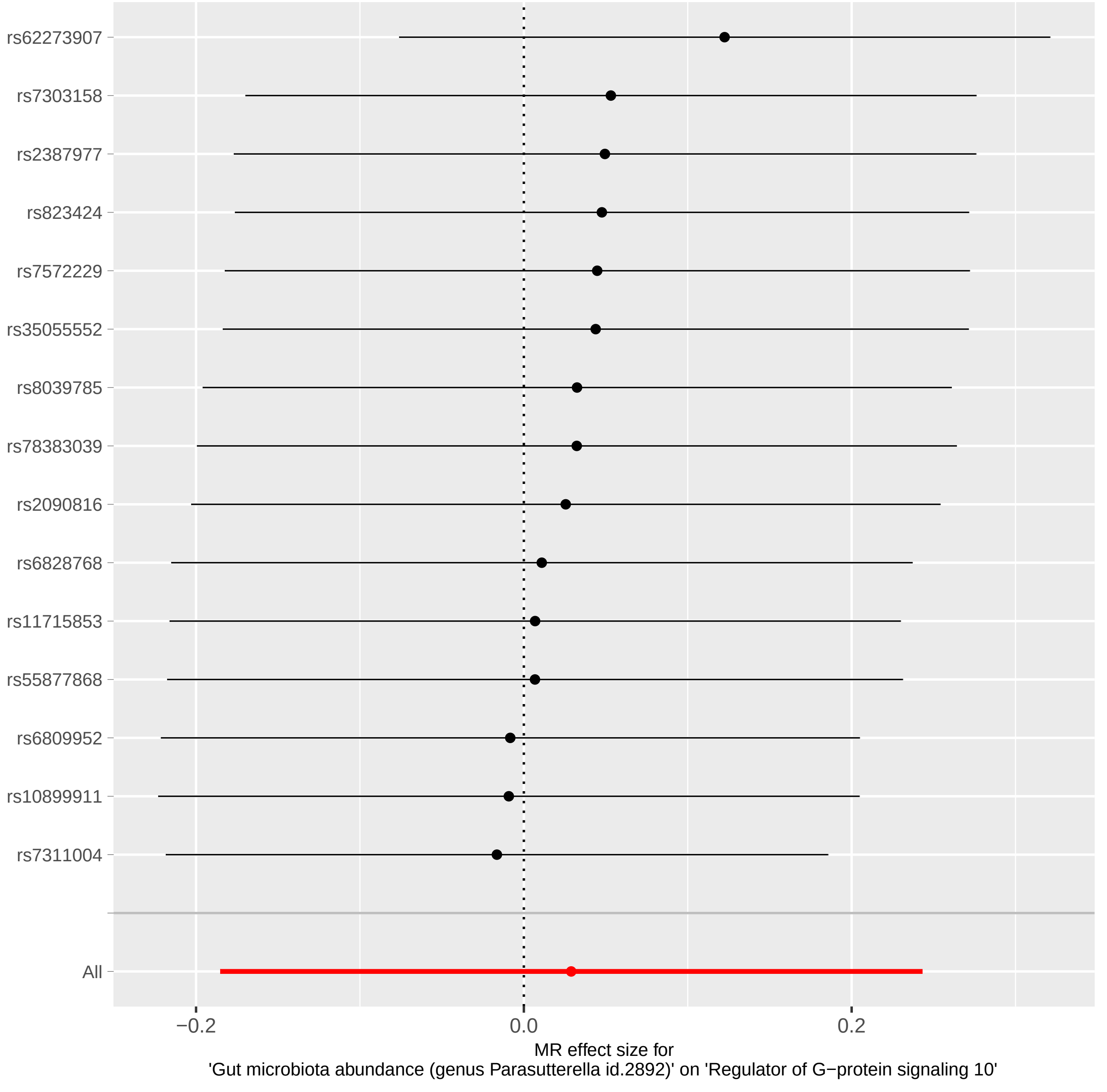

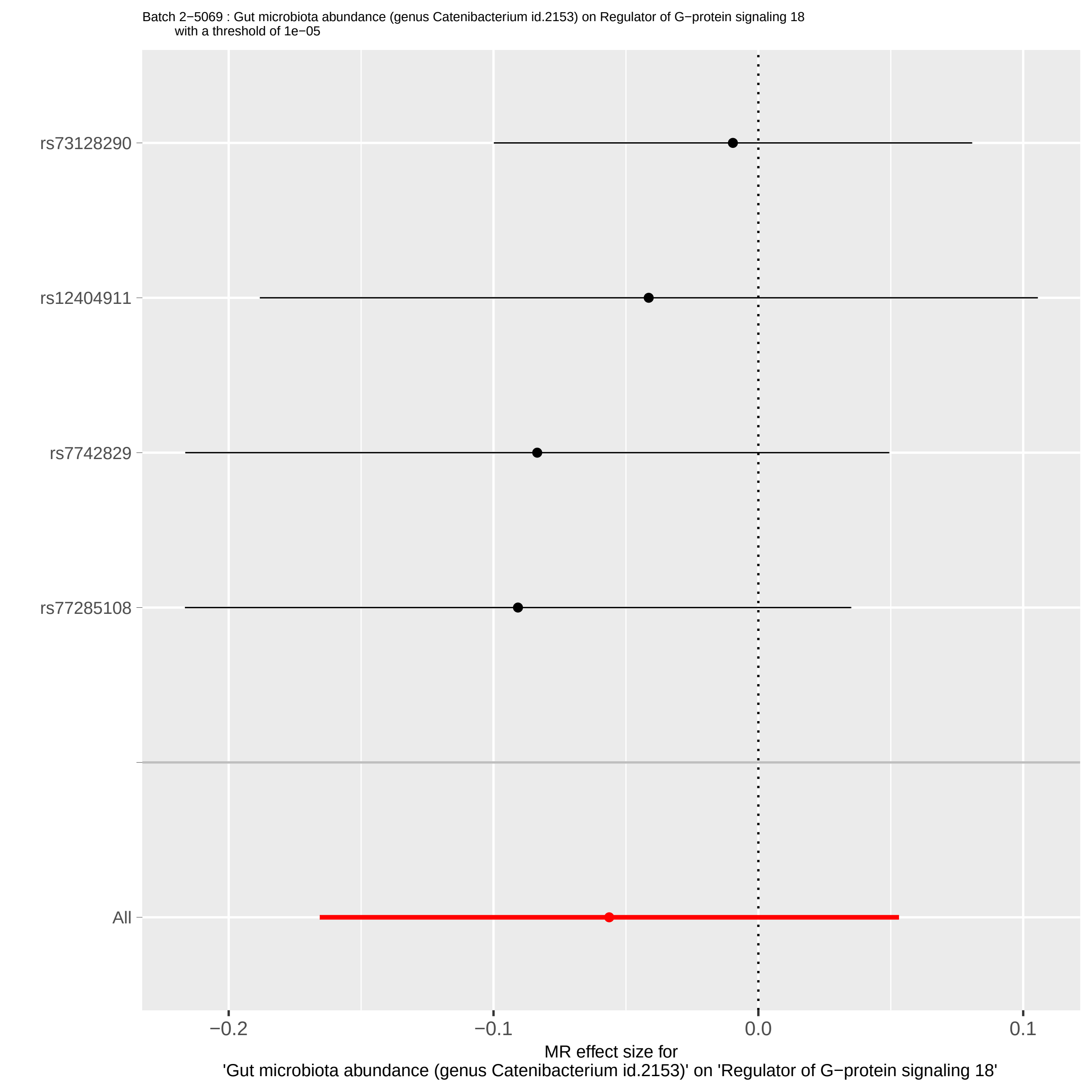
Batch 2−5069 : Gut microbiota abundance (genus Catenibacterium id.2153) on Regulator of G−protein signaling 18
with a threshold of 1e−05
rs73128290
rs12404911
rs7742829
rs77285108
All
−0.2
−0.1
0.0
0.1
MR effect size for
'Gut microbiota abundance (genus Catenibacterium id.2153)' on 'Regulator of G−protein signaling 18'

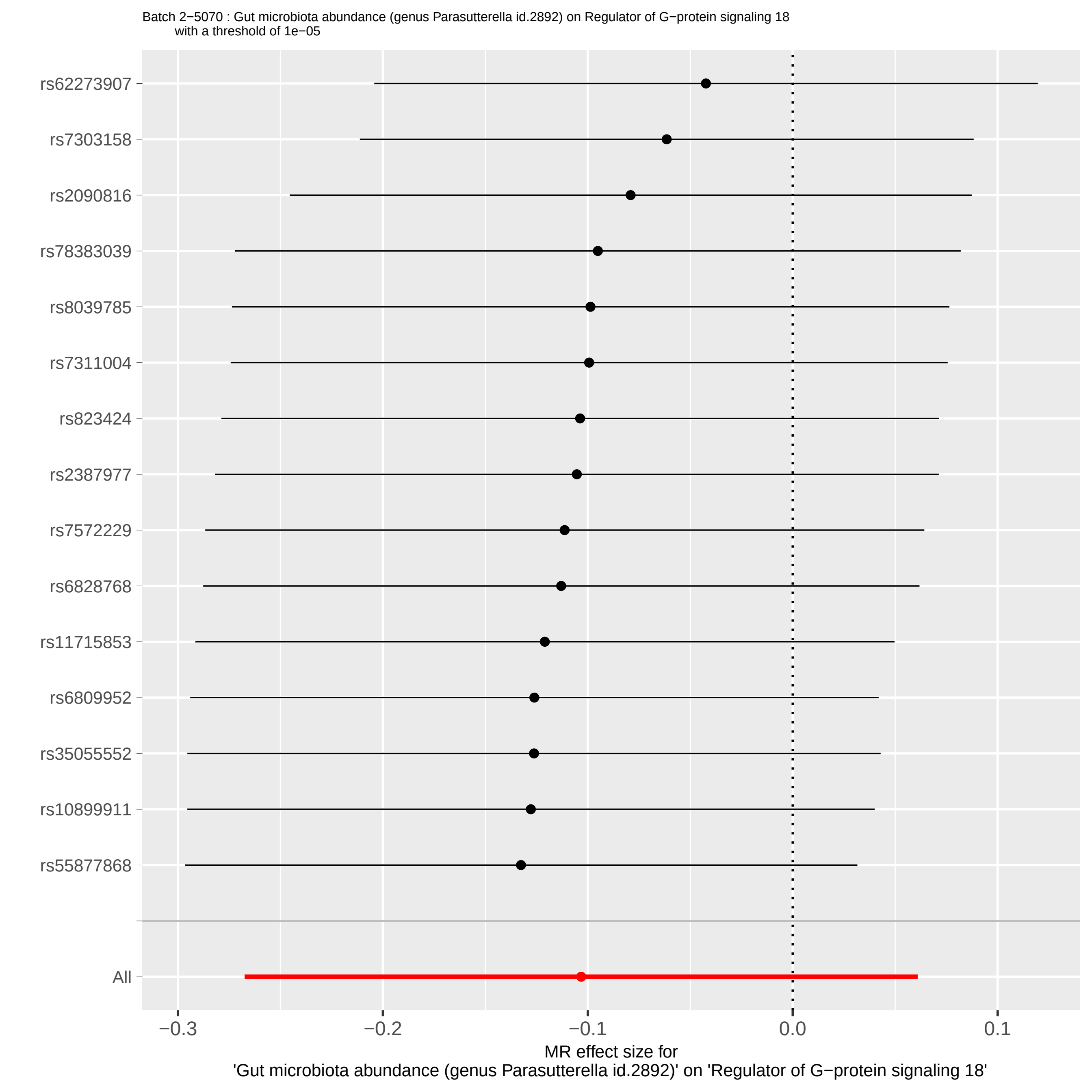
Batch 2−5070 : Gut microbiota abundance (genus Parasutterella id.2892) on Regulator of G−protein signaling 18
with a threshold of 1e−05
rs62273907
rs7303158
rs2090816
rs78383039
rs8039785
rs7311004
rs823424
rs2387977
rs7572229
rs6828768
rs11715853
rs6809952
rs35055552
rs10899911
rs55877868
All
−0.3
−0.2
−0.1
0.0
0.1
MR effect size for
'Gut microbiota abundance (genus Parasutterella id.2892)' on 'Regulator of G−protein signaling 18'

Batch 2−5071 : Gut microbiota abundance (genus Catenibacterium id.2153) on Regulator of G−protein signaling 19
with a threshold of 1e−05

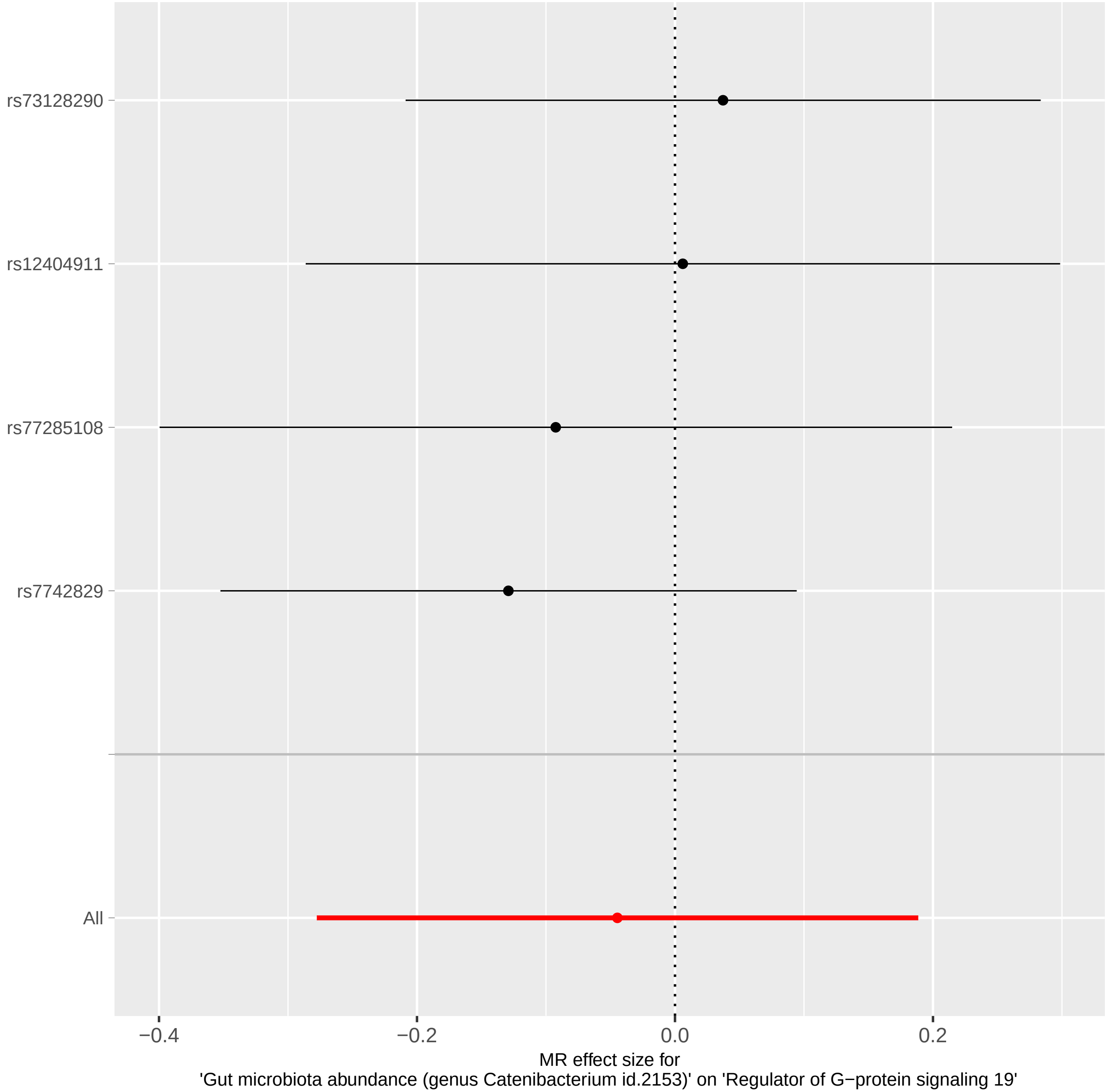

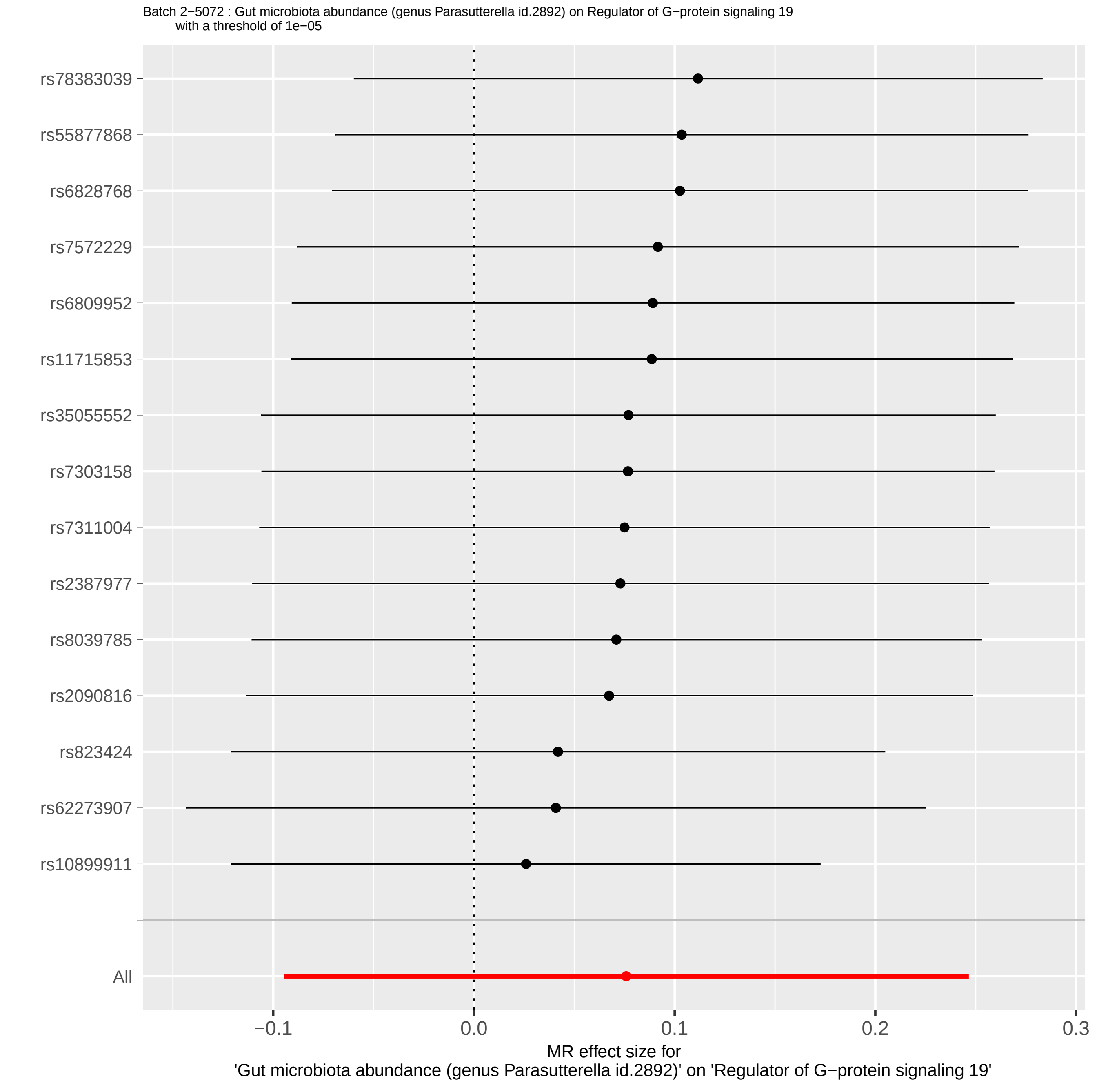
Batch 2−5072 : Gut microbiota abundance (genus Parasutterella id.2892) on Regulator of G−protein signaling 19
with a threshold of 1e−05
rs78383039
rs55877868
rs6828768
rs7572229
rs6809952
rs11715853
rs35055552
rs7303158
rs7311004
rs2387977
rs8039785
rs2090816
rs823424
rs62273907
rs10899911
All
−0.1
0.0
0.1
0.2
0.3
MR effect size for
'Gut microbiota abundance (genus Parasutterella id.2892)' on 'Regulator of G−protein signaling 19'

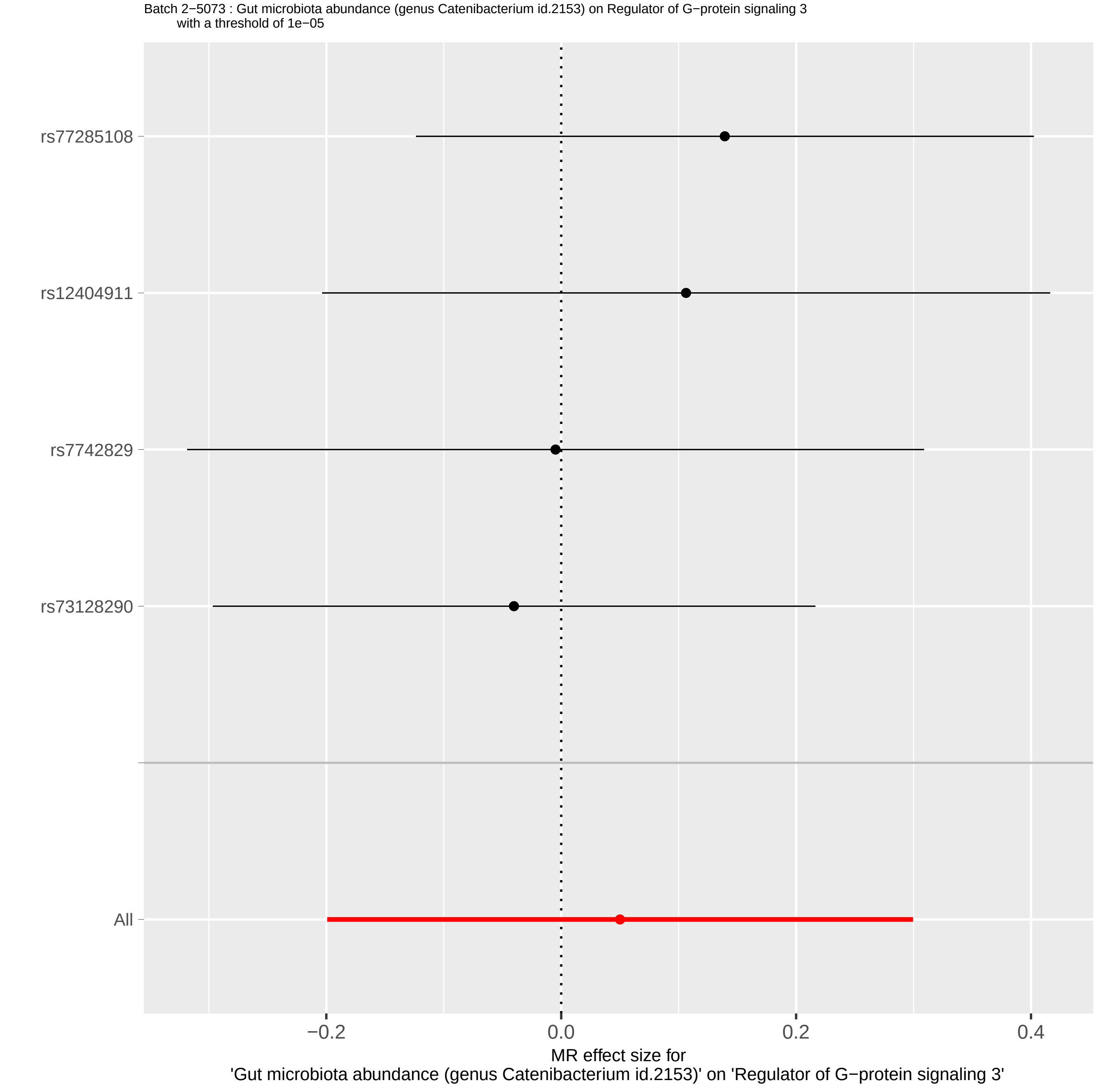
Batch 2−5073 : Gut microbiota abundance (genus Catenibacterium id.2153) on Regulator of G−protein signaling 3
with a threshold of 1e−05
rs77285108
rs12404911
rs7742829
rs73128290
All
−0.2
0.0
0.2
0.4
MR effect size for
'Gut microbiota abundance (genus Catenibacterium id.2153)' on 'Regulator of G−protein signaling 3'

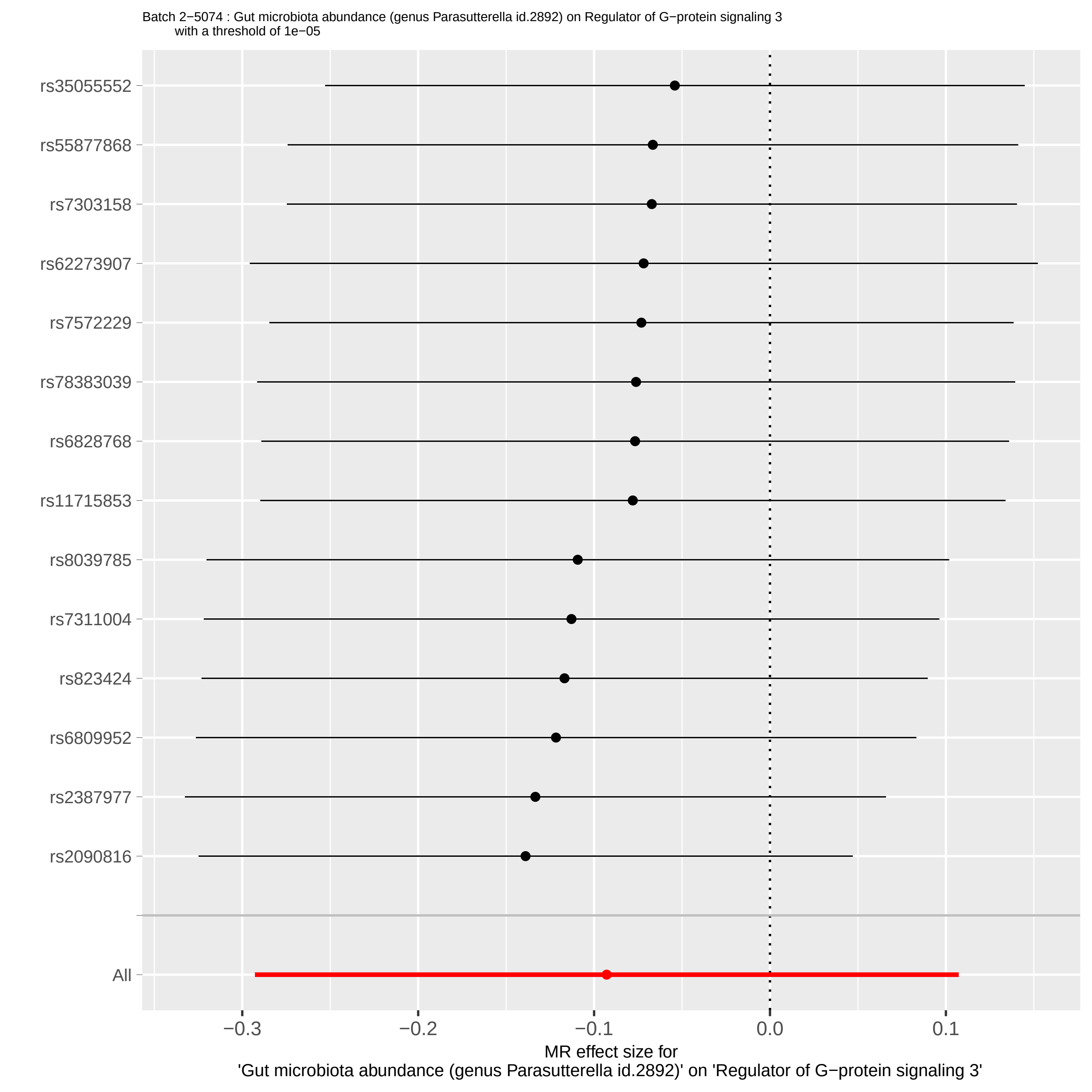
Batch 2−5074 : Gut microbiota abundance (genus Parasutterella id.2892) on Regulator of G−protein signaling 3
with a threshold of 1e−05
rs35055552
rs55877868
rs7303158
rs62273907
rs7572229
rs78383039
rs6828768
rs11715853
rs8039785
rs7311004
rs823424
rs6809952
rs2387977
rs2090816
All
−0.3
−0.2
−0.1
0.0
0.1
MR effect size for
'Gut microbiota abundance (genus Parasutterella id.2892)' on 'Regulator of G−protein signaling 3'

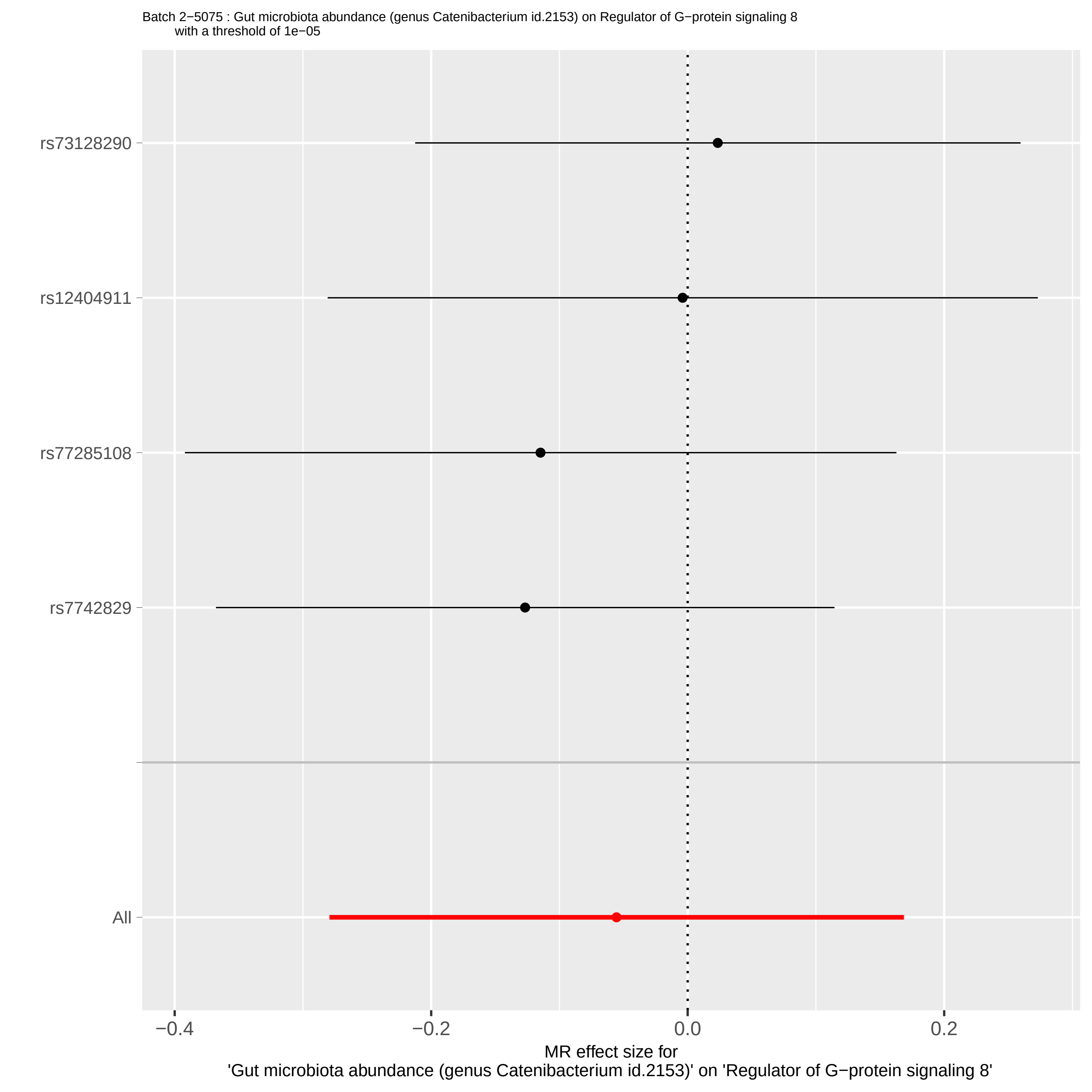
Batch 2−5075 : Gut microbiota abundance (genus Catenibacterium id.2153) on Regulator of G−protein signaling 8
with a threshold of 1e−05
rs73128290
rs12404911
rs77285108
rs7742829
All
−0.4
−0.2
0.0
0.2
MR effect size for
'Gut microbiota abundance (genus Catenibacterium id.2153)' on 'Regulator of G−protein signaling 8'

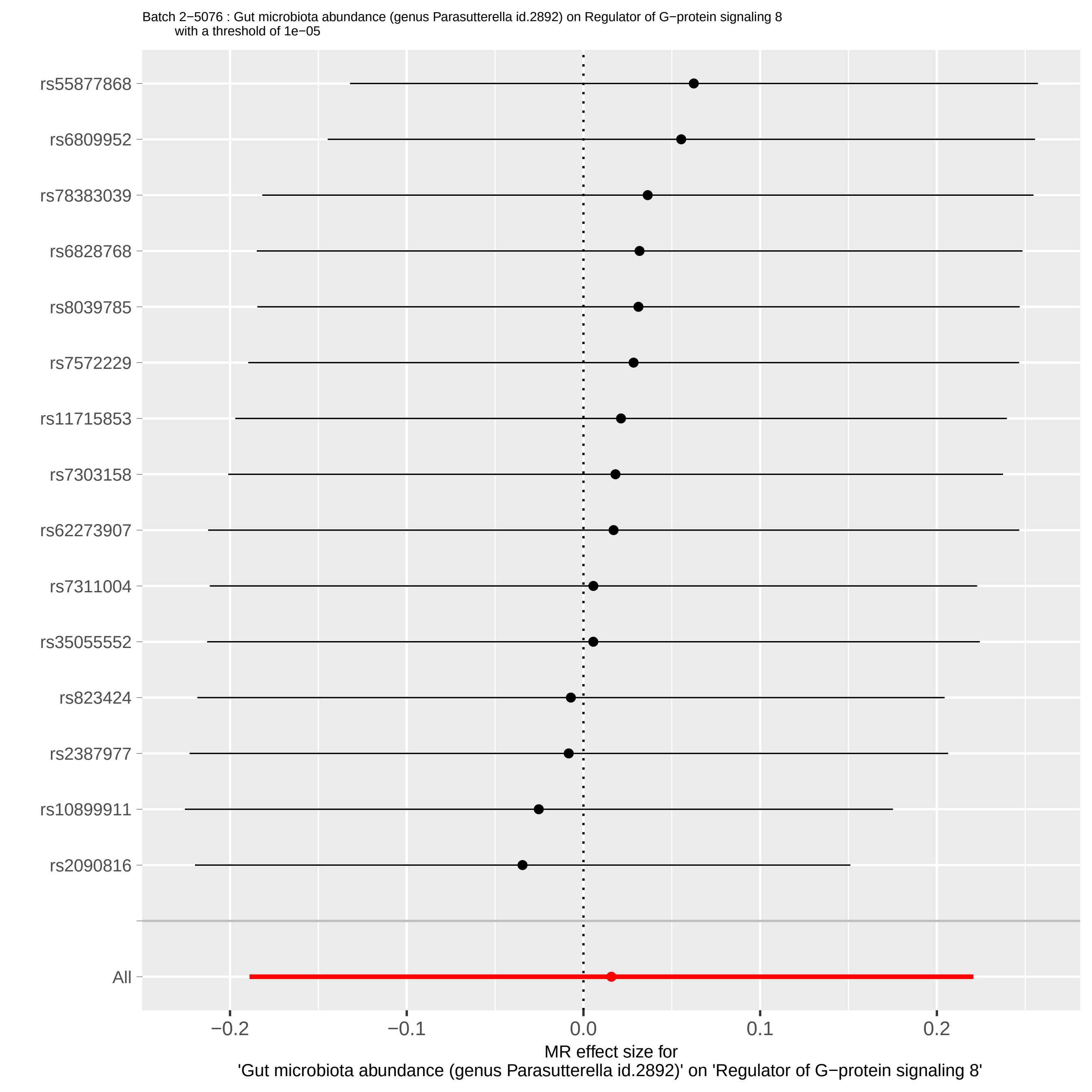

Batch 2−5076 : Gut microbiota abundance (genus Parasutterella id.2892) on Regulator of G−protein signaling 8
with a threshold of 1e−05
rs55877868
rs6809952
rs78383039
rs6828768
rs8039785
rs7572229
rs11715853
rs7303158
rs62273907
rs7311004
rs35055552
rs823424
rs2387977
rs10899911
rs2090816
All
−0.2
−0.1
0.0
0.1
0.2
MR effect size for
'Gut microbiota abundance (genus Parasutterella id.2892)' on 'Regulator of G−protein signaling 8'

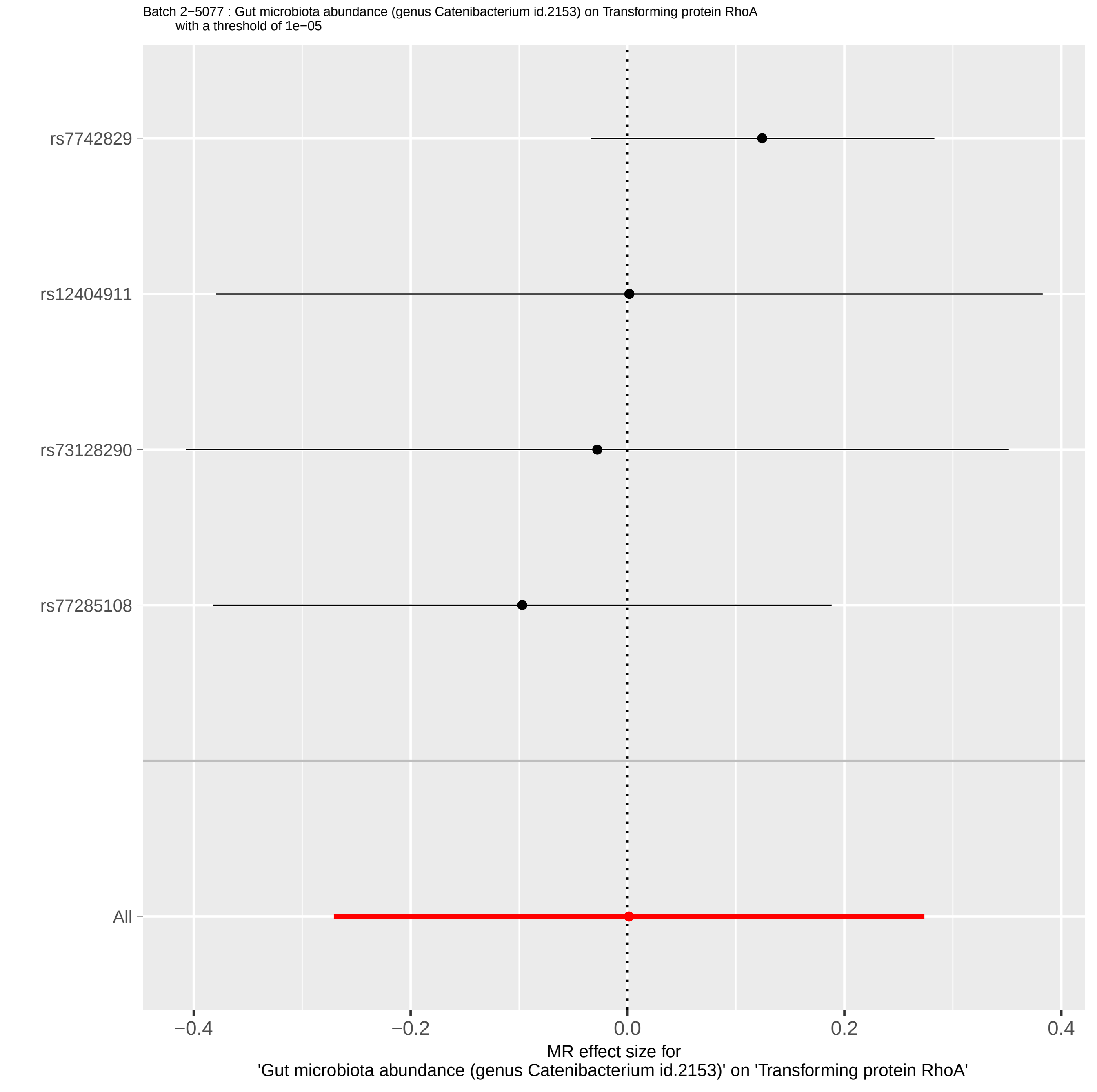
Batch 2−5077 : Gut microbiota abundance (genus Catenibacterium id.2153) on Transforming protein RhoA
with a threshold of 1e−05
rs7742829
rs12404911
rs73128290
rs77285108
All
−0.4
−0.2
0.0
0.2
0.4
MR effect size for
'Gut microbiota abundance (genus Catenibacterium id.2153)' on 'Transforming protein RhoA'

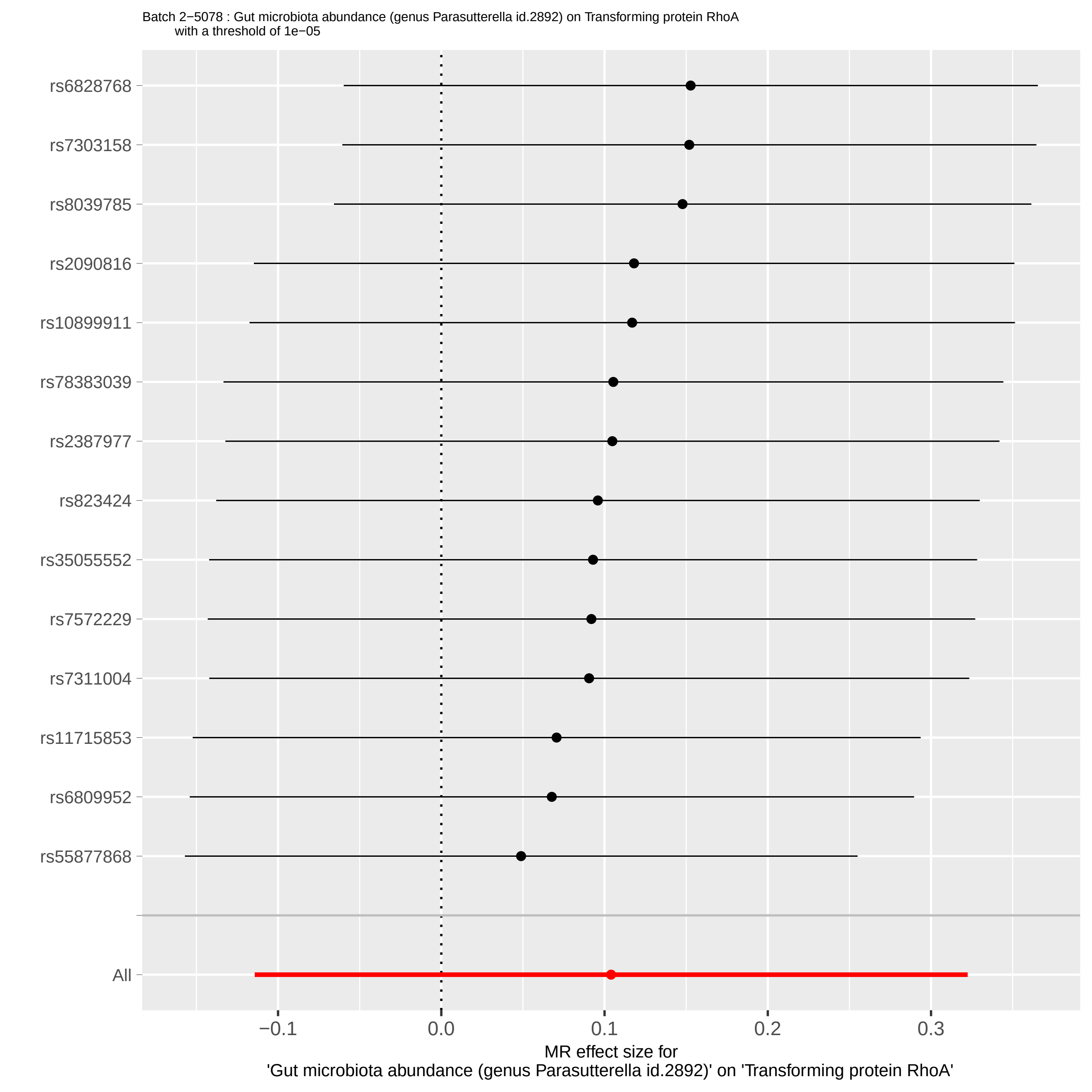
Batch 2−5078 : Gut microbiota abundance (genus Parasutterella id.2892) on Transforming protein RhoA
with a threshold of 1e−05
rs6828768
rs7303158
rs8039785
rs2090816
rs10899911
rs78383039
rs2387977
rs823424
rs35055552
rs7572229
rs7311004
rs11715853
rs6809952
rs55877868
All
−0.1
0.0
0.1
0.2
0.3
MR effect size for
'Gut microbiota abundance (genus Parasutterella id.2892)' on 'Transforming protein RhoA'

Batch 2−5079 : Gut microbiota abundance (genus Catenibacterium id.2153) on Rho−related GTP−binding protein RhoD
with a threshold of 1e−05

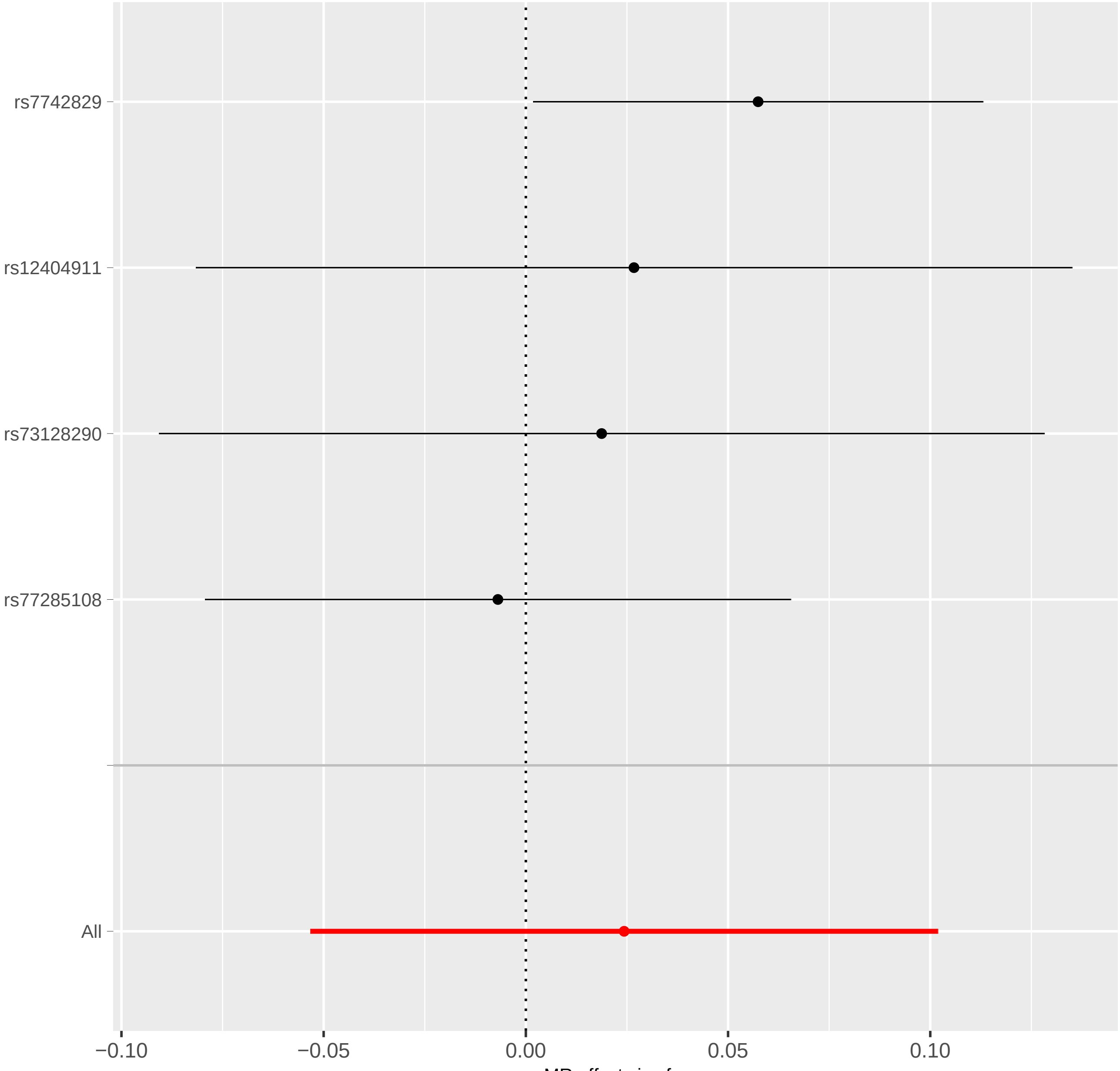

MR effect size for
'Gut microbiota abundance (genus Catenibacterium id.2153)' on 'Rho−related GTP−binding protein RhoD'

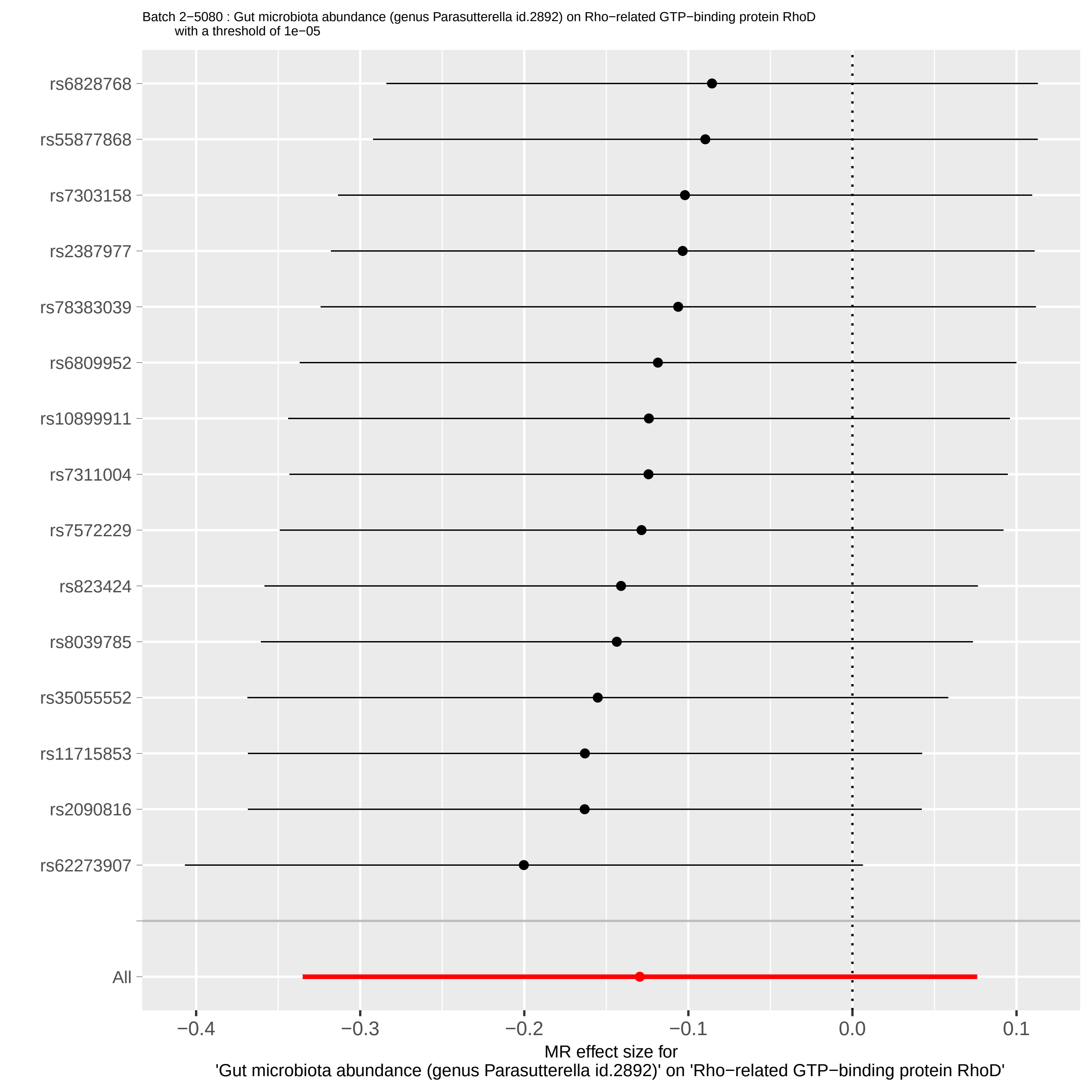
Batch 2−5080 : Gut microbiota abundance (genus Parasutterella id.2892) on Rho−related GTP−binding protein RhoD
with a threshold of 1e−05
rs6828768
rs55877868
rs7303158
rs2387977
rs78383039
rs6809952
rs10899911
rs7311004
rs7572229
rs823424
rs8039785
rs35055552
rs11715853
rs2090816
rs62273907
All
−0.4
−0.3
−0.2
−0.1
0.0
0.1
MR effect size for
'Gut microbiota abundance (genus Parasutterella id.2892)' on 'Rho−related GTP−binding protein RhoD'

Batch 2−5081 : Gut microbiota abundance (genus Catenibacterium id.2153) on Rho−related GTP−binding protein RhoG
with a threshold of 1e−05

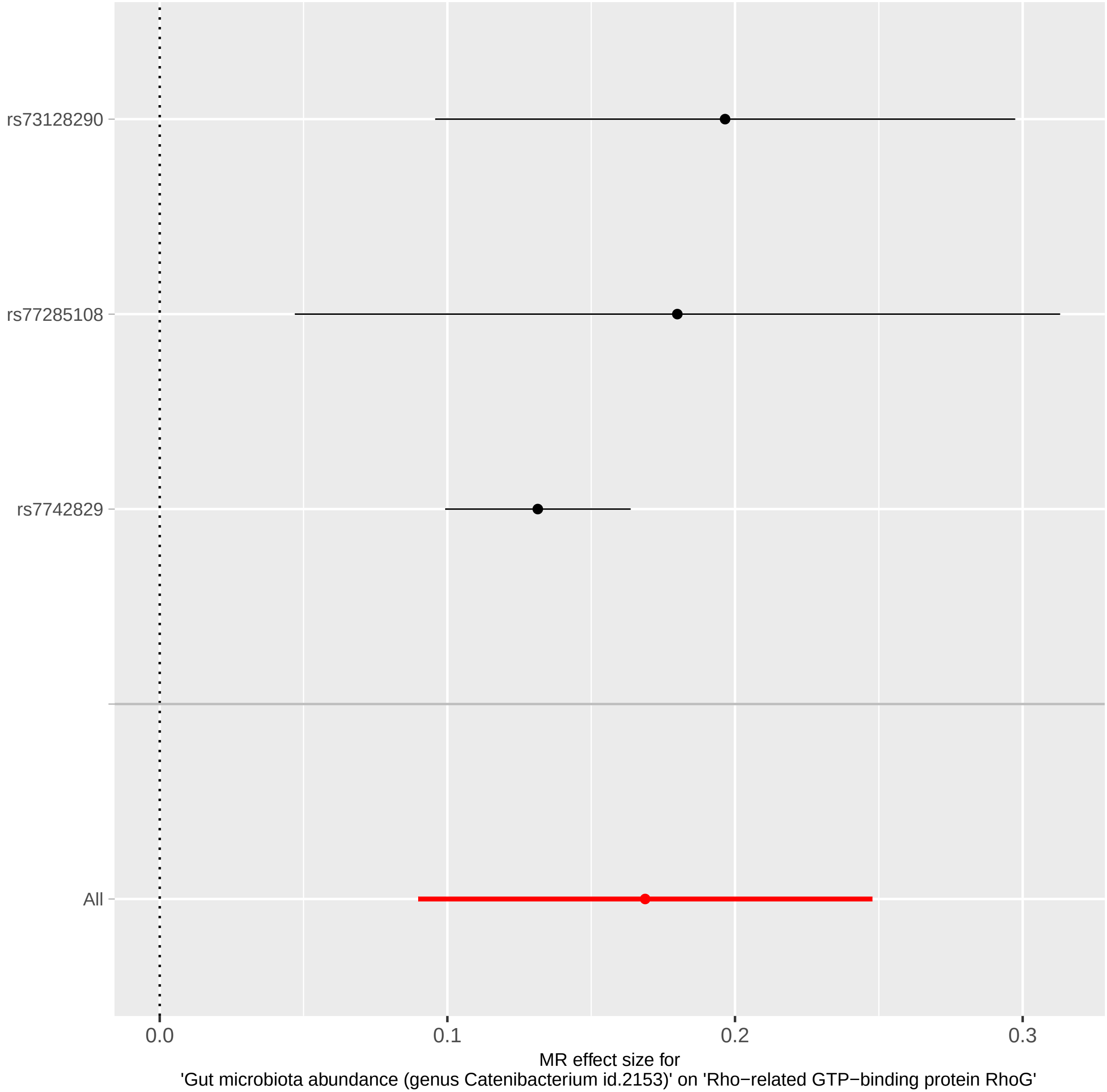

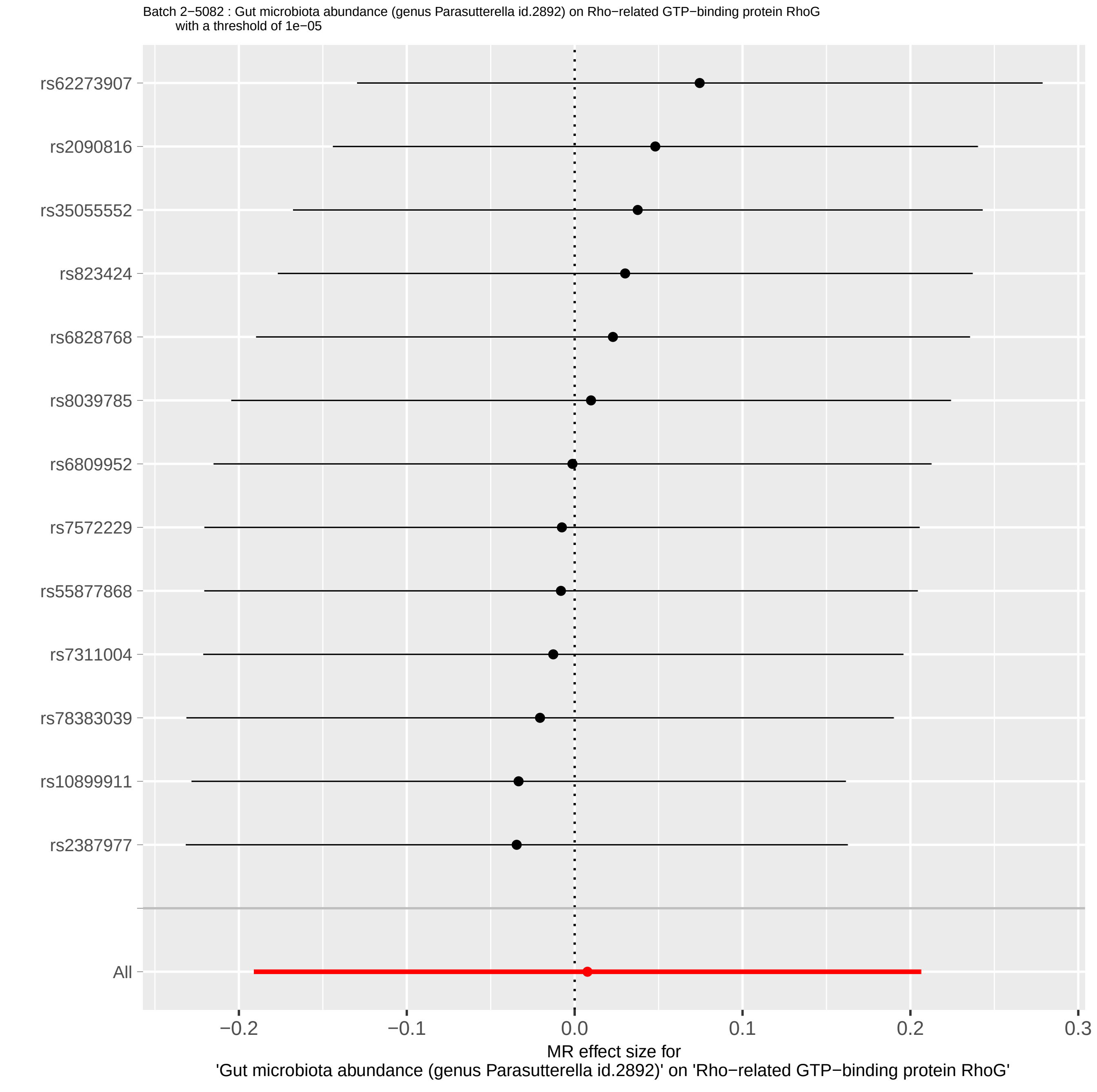
Batch 2−5082 : Gut microbiota abundance (genus Parasutterella id.2892) on Rho−related GTP−binding protein RhoG
with a threshold of 1e−05
rs62273907
rs2090816
rs35055552
rs823424
rs6828768
rs8039785
rs6809952
rs7572229
rs55877868
rs7311004
rs78383039
rs10899911
rs2387977
All
−0.2
−0.1
0.0
0.1
0.2
0.3
MR effect size for
'Gut microbiota abundance (genus Parasutterella id.2892)' on 'Rho−related GTP−binding protein RhoG'

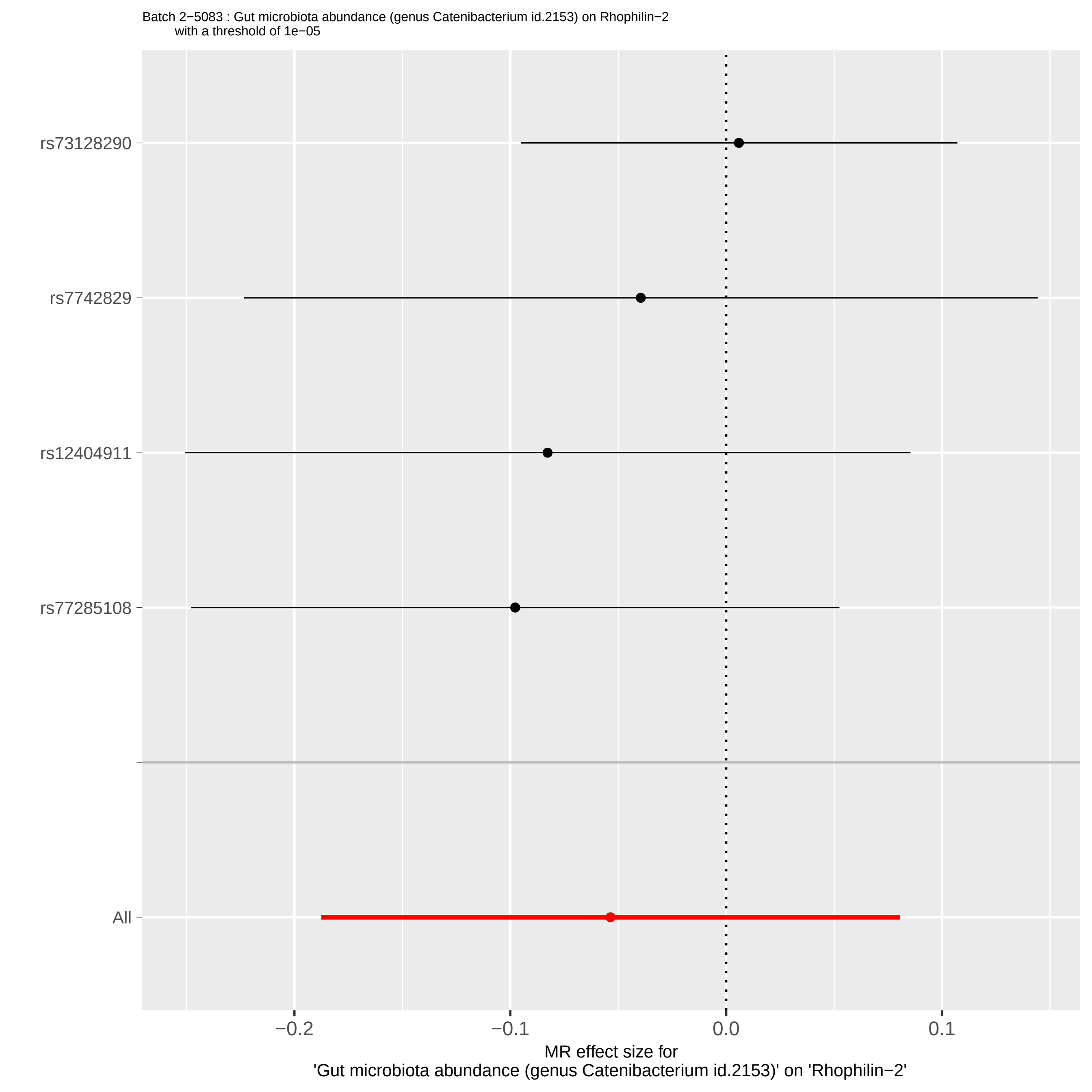

Batch 2−5083 : Gut microbiota abundance (genus Catenibacterium id.2153) on Rhophilin−2
with a threshold of 1e−05
rs73128290
rs7742829
rs12404911
rs77285108
All
−0.2
−0.1
0.0
0.1
MR effect size for
'Gut microbiota abundance (genus Catenibacterium id.2153)' on 'Rhophilin−2'

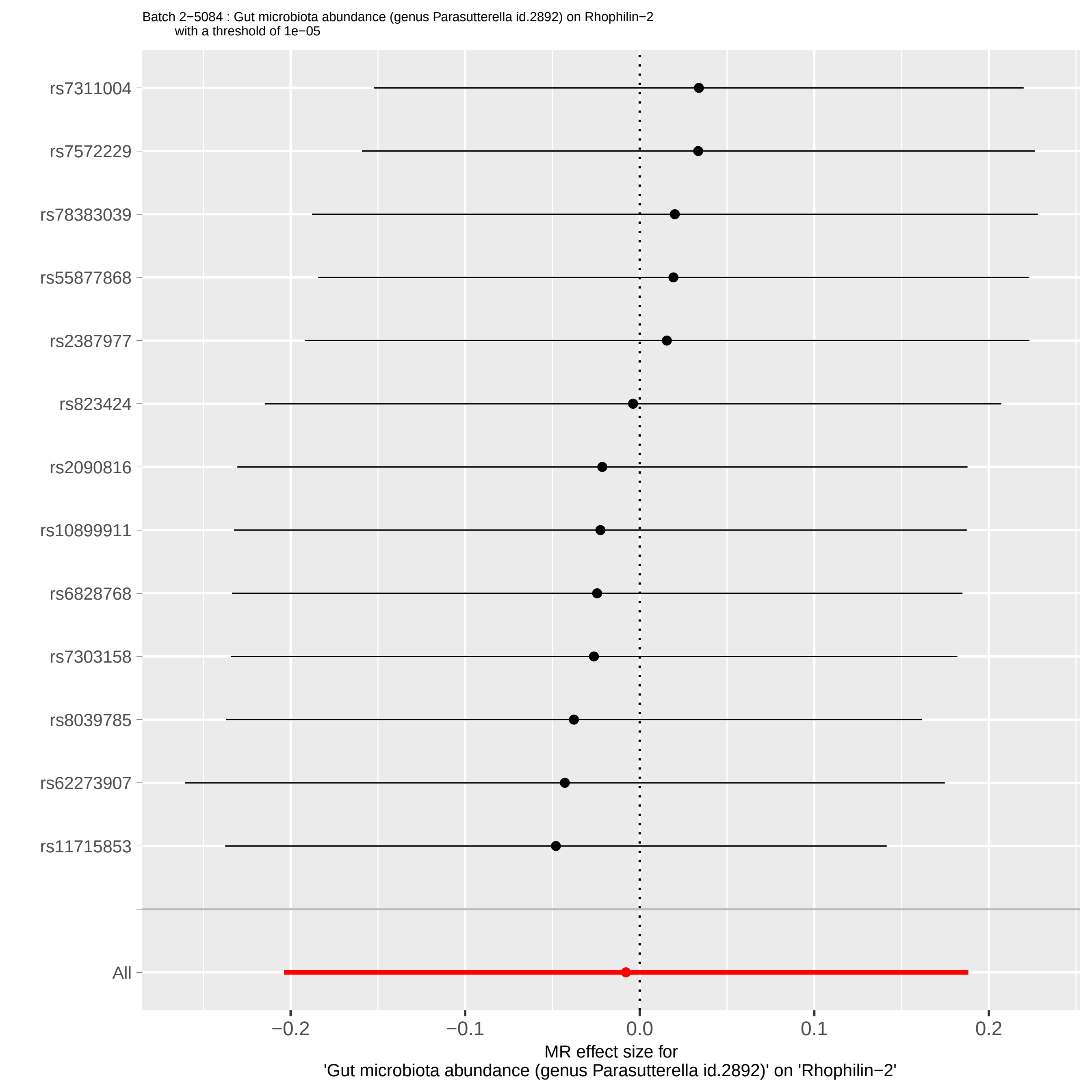

Batch 2−5084 : Gut microbiota abundance (genus Parasutterella id.2892) on Rhophilin−2
with a threshold of 1e−05
rs7311004
rs7572229
rs78383039
rs55877868
rs2387977
rs823424
rs2090816
rs10899911
rs6828768
rs7303158
rs8039785
rs62273907
rs11715853
All
−0.2
−0.1
0.0
0.1
0.2
MR effect size for
'Gut microbiota abundance (genus Parasutterella id.2892)' on 'Rhophilin−2'

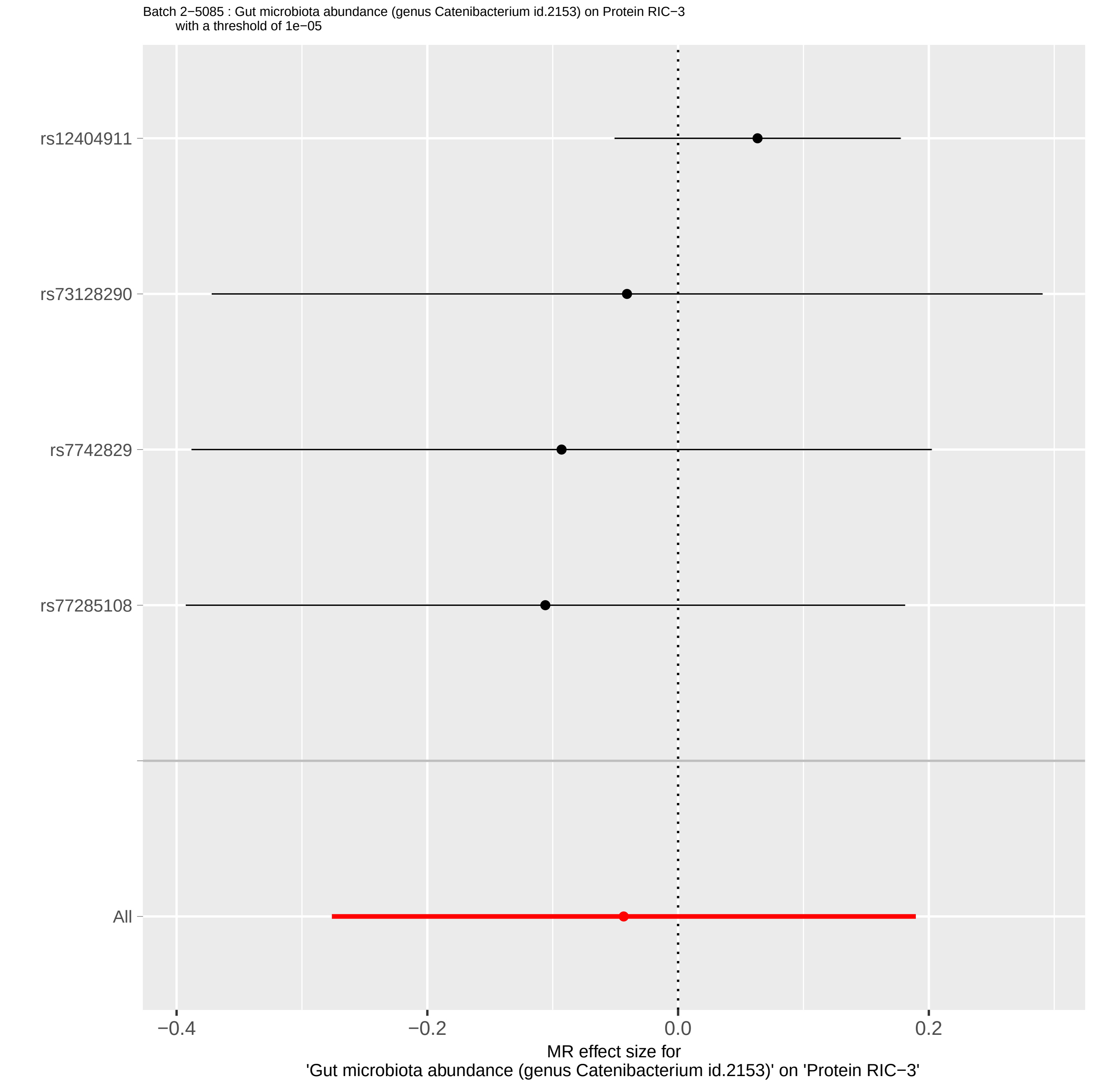

Batch 2−5085 : Gut microbiota abundance (genus Catenibacterium id.2153) on Protein RIC−3
with a threshold of 1e−05
rs12404911
rs73128290
rs7742829
rs77285108
All
−0.4
−0.2
0.0
0.2
MR effect size for
'Gut microbiota abundance (genus Catenibacterium id.2153)' on 'Protein RIC−3'

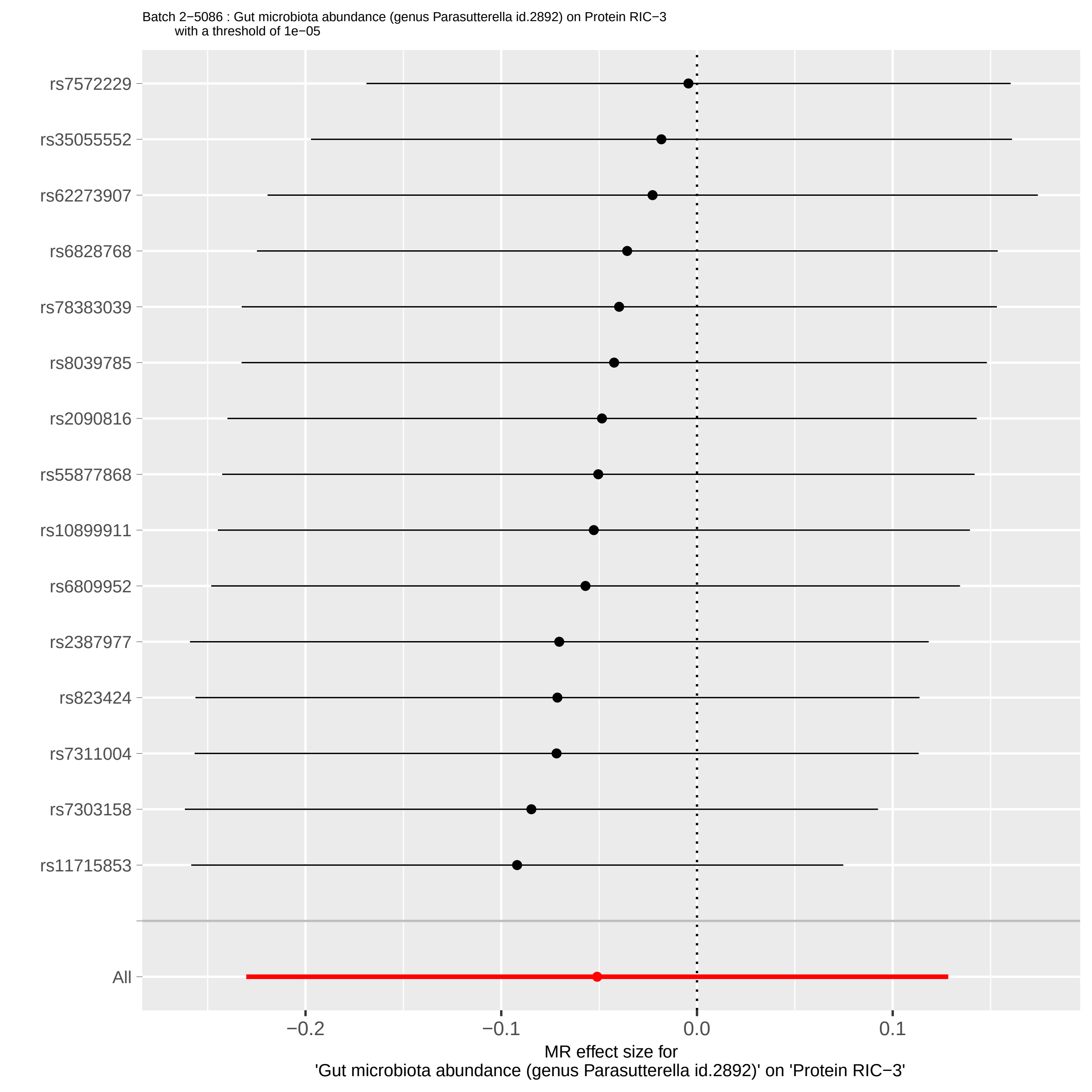

Batch 2−5086 : Gut microbiota abundance (genus Parasutterella id.2892) on Protein RIC−3
with a threshold of 1e−05
rs7572229
rs35055552
rs62273907
rs6828768
rs78383039
rs8039785
rs2090816
rs55877868
rs10899911
rs6809952
rs2387977
rs823424
rs7311004
rs7303158
rs11715853
All
−0.2
−0.1
0.0
0.1
MR effect size for
'Gut microbiota abundance (genus Parasutterella id.2892)' on 'Protein RIC−3'

Batch 2−5087 : Gut microbiota abundance (genus Catenibacterium id.2153) on Protein RIC−3
with a threshold of 1e−05

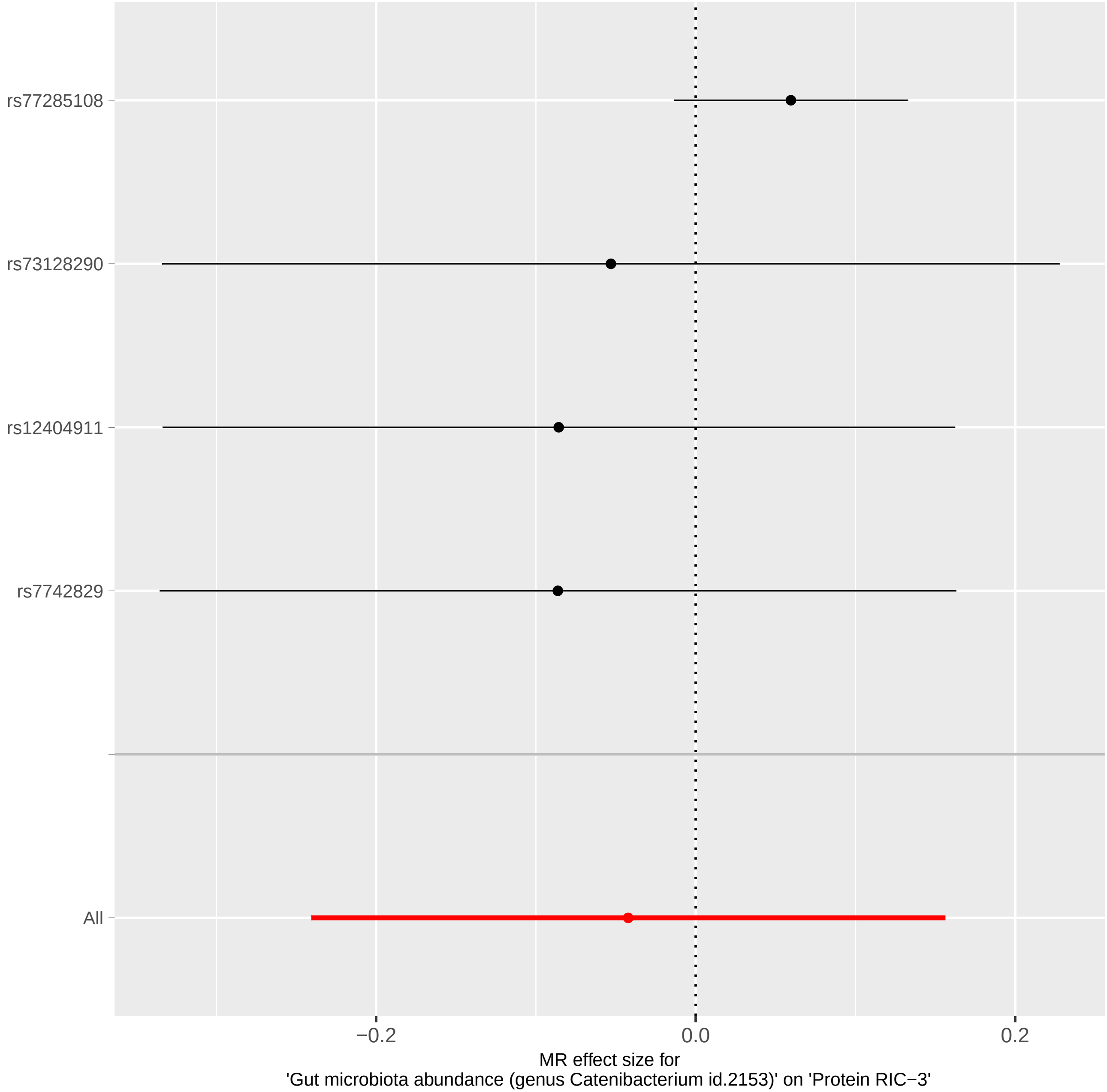

Batch 2−5088 : Gut microbiota abundance (genus Parasutterella id.2892) on Protein RIC−3
with a threshold of 1e−05

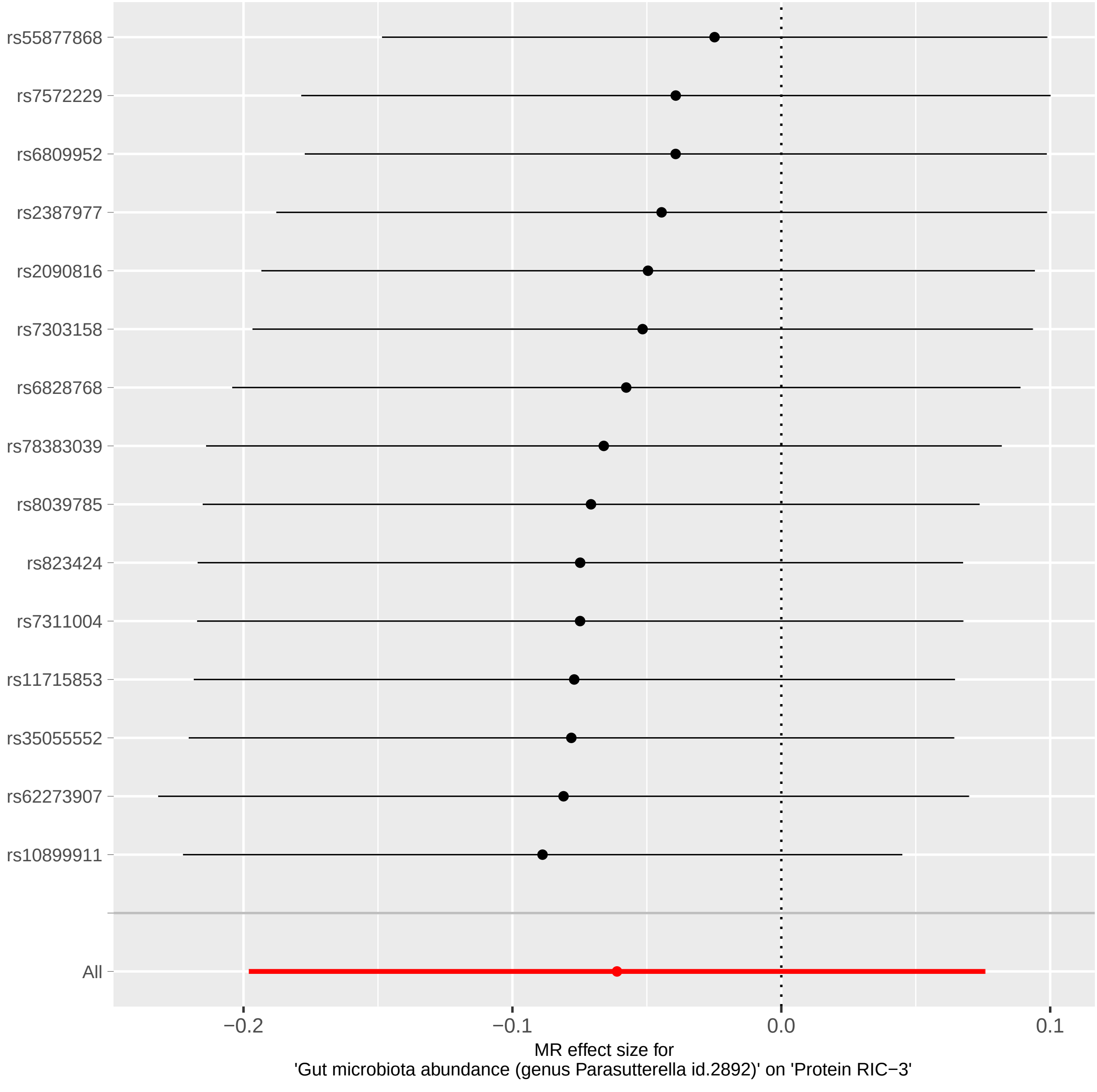

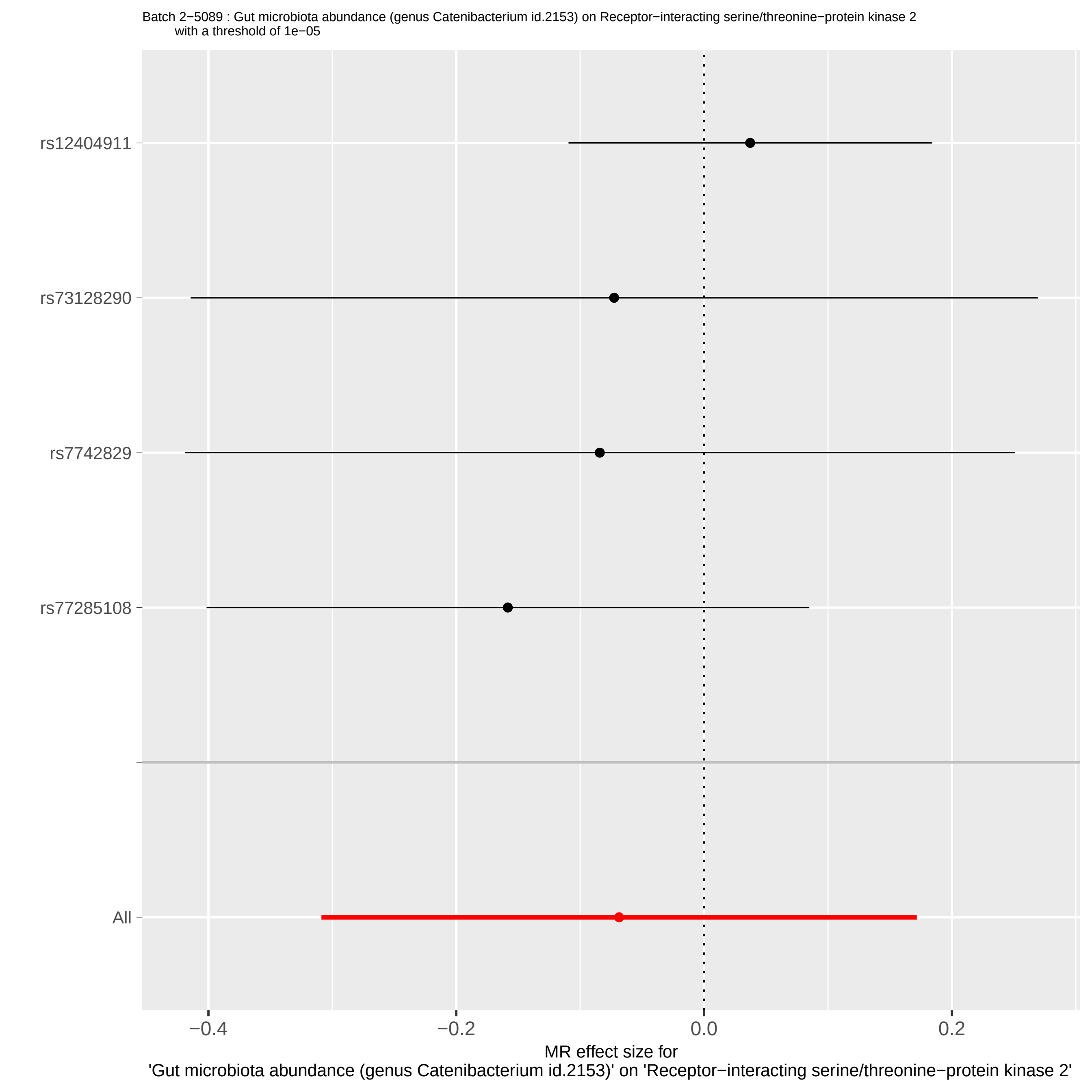

Batch 2−5089 : Gut microbiota abundance (genus Catenibacterium id.2153) on Receptor−interacting serine/threonine−protein kinase 2
with a threshold of 1e−05
rs12404911
rs73128290
rs7742829
rs77285108
All
−0.4
−0.2
0.0
0.2
MR effect size for
'Gut microbiota abundance (genus Catenibacterium id.2153)' on 'Receptor−interacting serine/threonine−protein kinase 2'

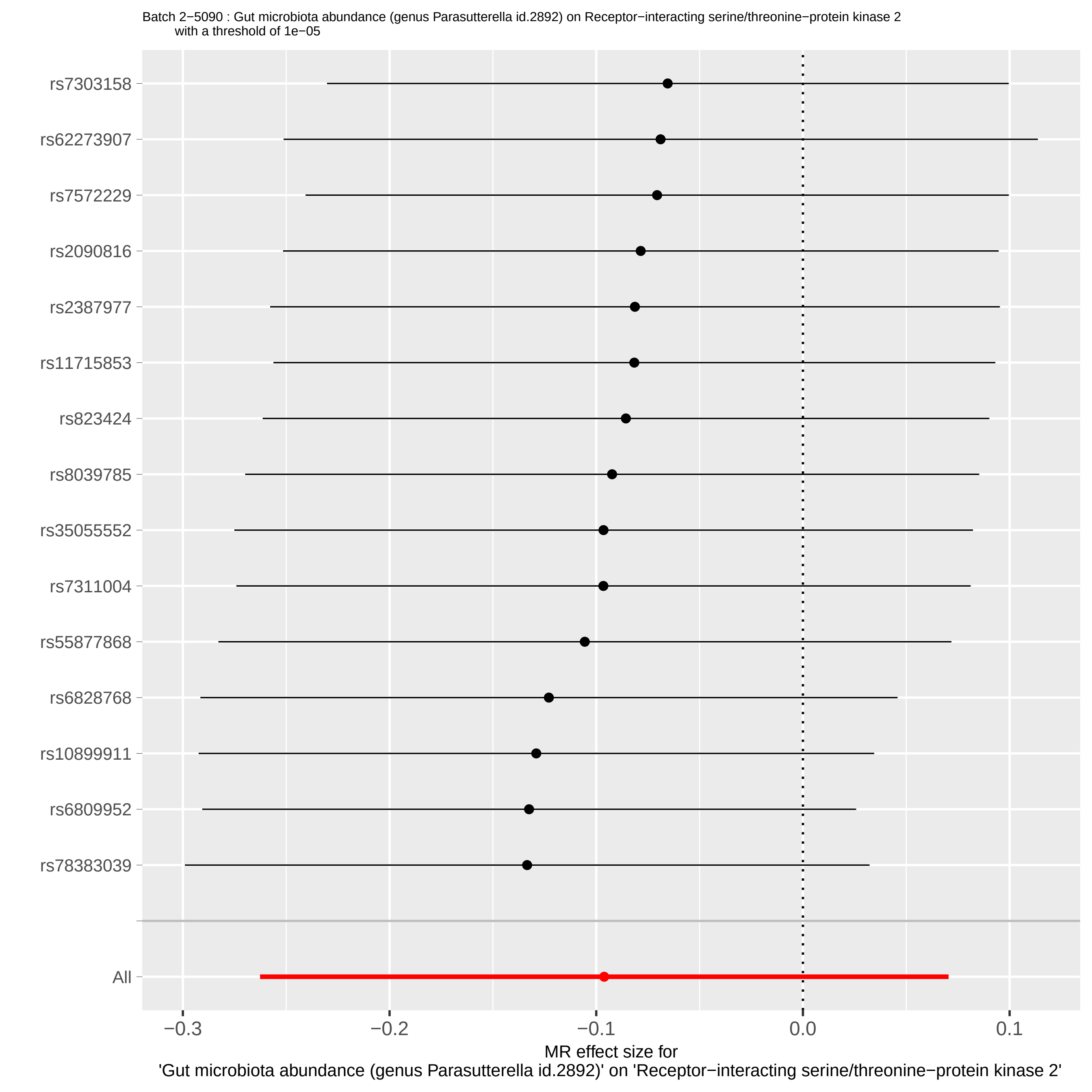
Batch 2−5090 : Gut microbiota abundance (genus Parasutterella id.2892) on Receptor−interacting serine/threonine−protein kinase 2
with a threshold of 1e−05
rs7303158
rs62273907
rs7572229
rs2090816
rs2387977
rs11715853
rs823424
rs8039785
rs35055552
rs7311004
rs55877868
rs6828768
rs10899911
rs6809952
rs78383039
All
−0.3
−0.2
−0.1
0.0
0.1
MR effect size for
'Gut microbiota abundance (genus Parasutterella id.2892)' on 'Receptor−interacting serine/threonine−protein kinase 2'

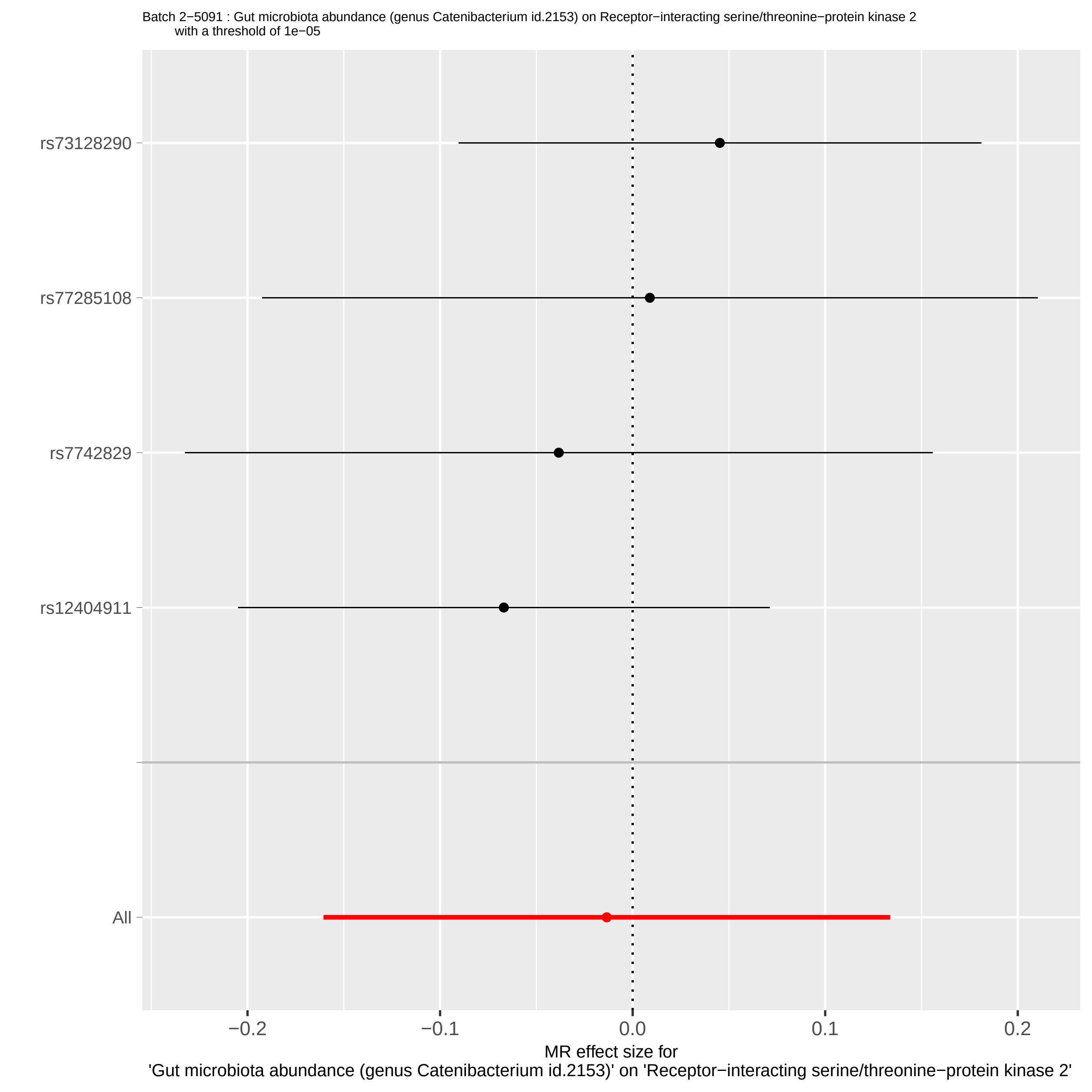

Batch 2−5091 : Gut microbiota abundance (genus Catenibacterium id.2153) on Receptor−interacting serine/threonine−protein kinase 2
with a threshold of 1e−05
rs73128290
rs77285108
rs7742829
rs12404911
All
−0.2
−0.1
0.0
0.1
0.2
MR effect size for
'Gut microbiota abundance (genus Catenibacterium id.2153)' on 'Receptor−interacting serine/threonine−protein kinase 2'

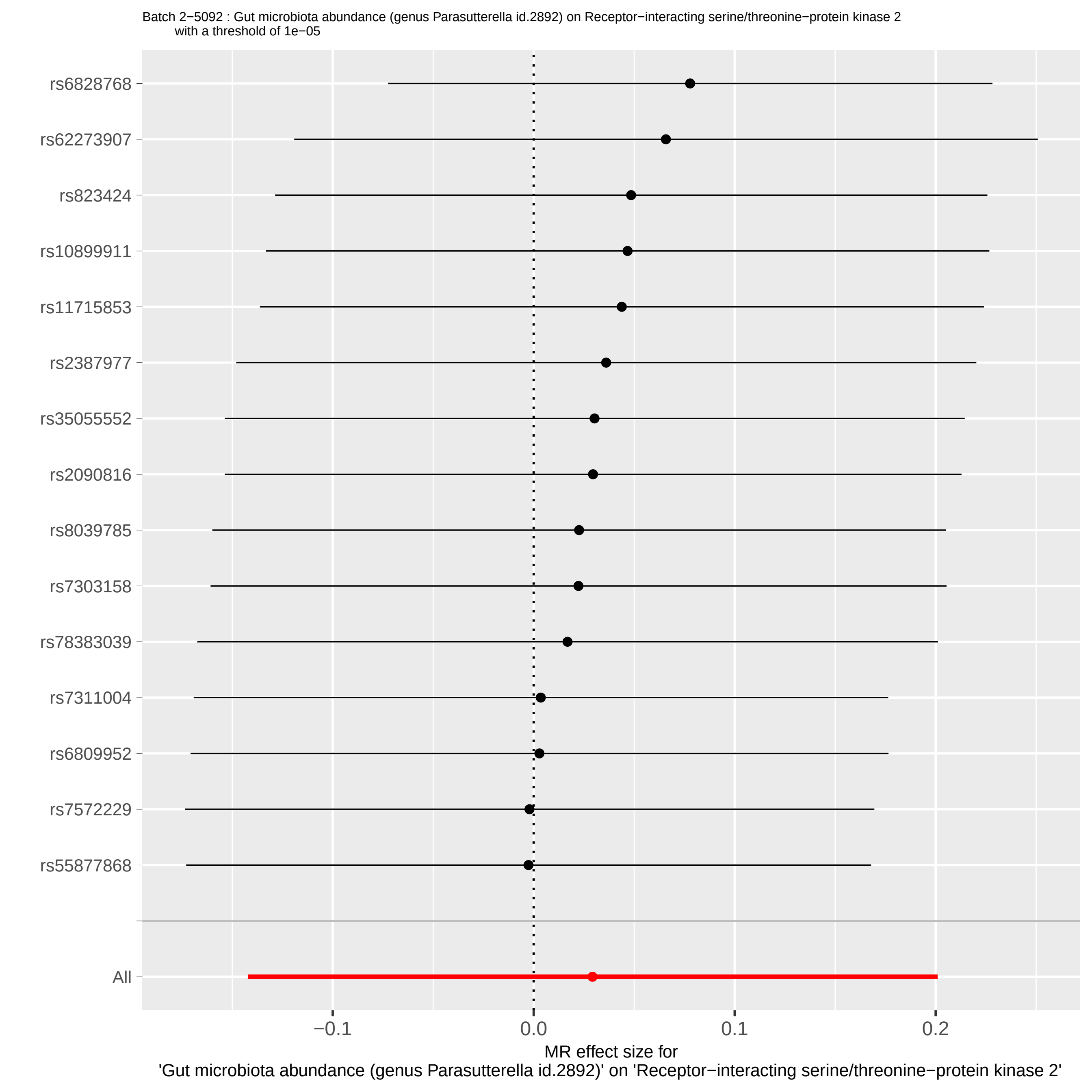
Batch 2−5092 : Gut microbiota abundance (genus Parasutterella id.2892) on Receptor−interacting serine/threonine−protein kinase 2
with a threshold of 1e−05
rs6828768
rs62273907
rs823424
rs10899911
rs11715853
rs2387977
rs35055552
rs2090816
rs8039785
rs7303158
rs78383039
rs7311004
rs6809952
rs7572229
rs55877868
All
−0.1
0.0
0.1
0.2
MR effect size for
'Gut microbiota abundance (genus Parasutterella id.2892)' on 'Receptor−interacting serine/threonine−protein kinase 2'

Batch 2−5093 : Gut microbiota abundance (genus Catenibacterium id.2153) on Protein ripply1
with a threshold of 1e−05

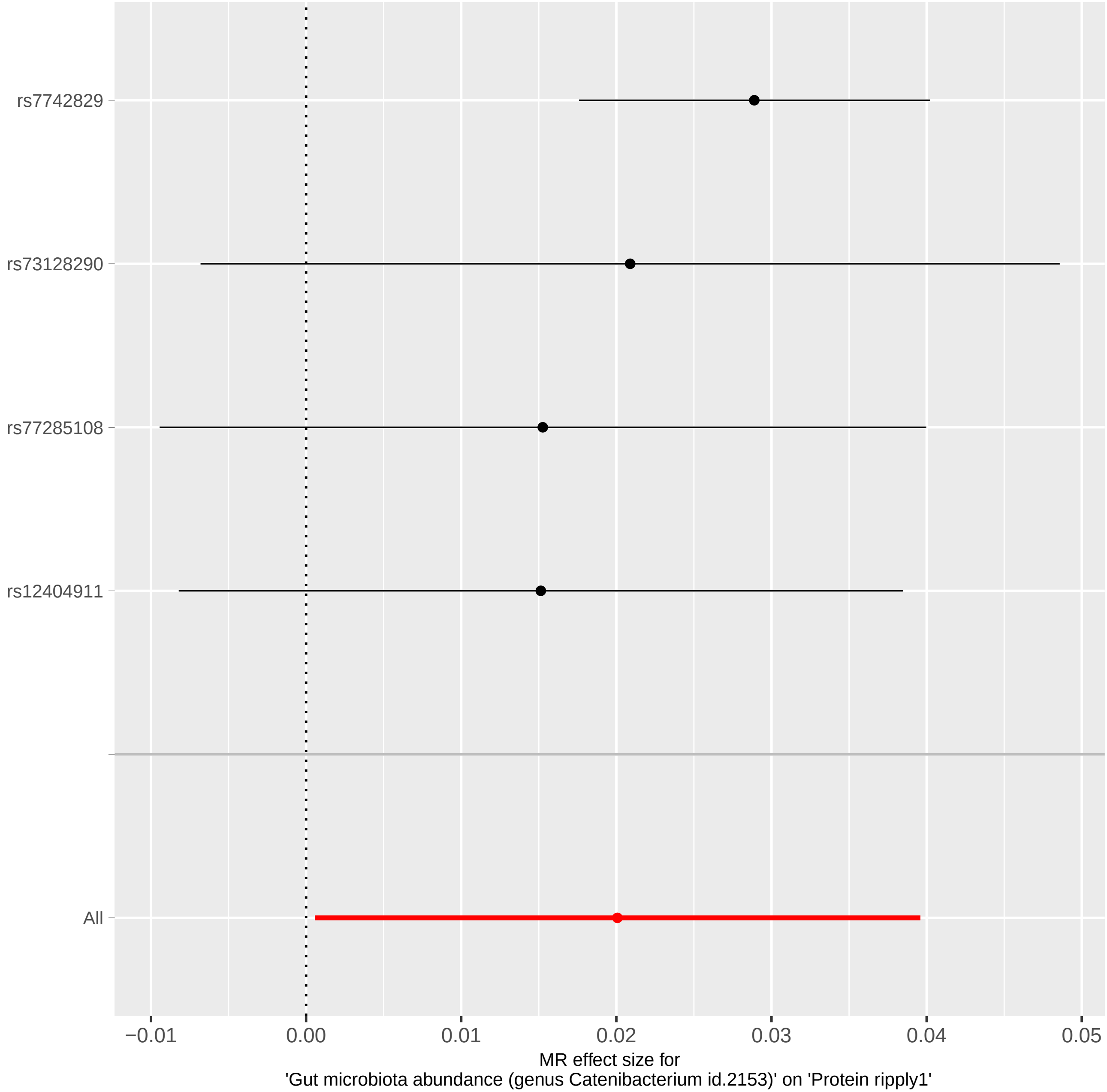

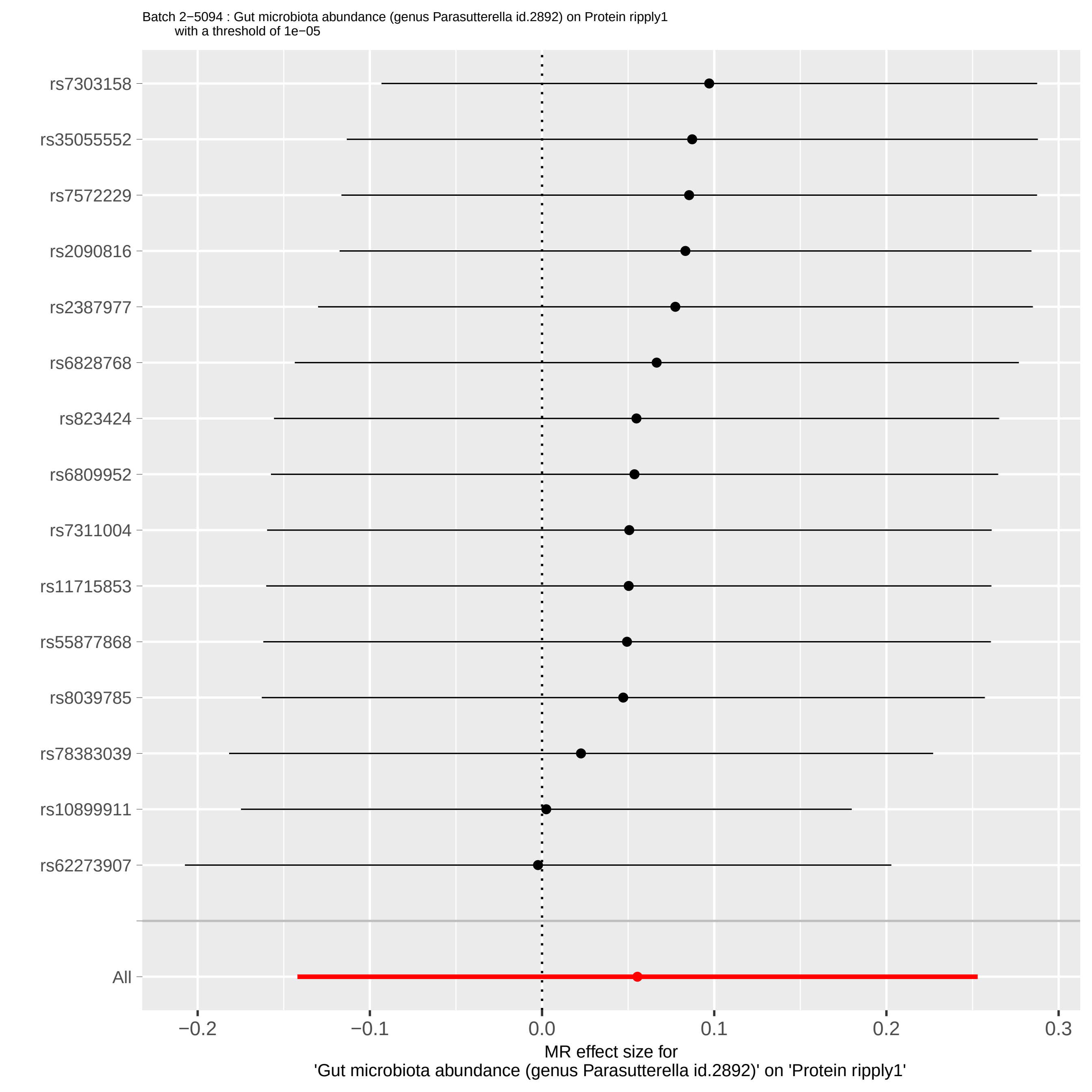
Batch 2−5094 : Gut microbiota abundance (genus Parasutterella id.2892) on Protein ripply1
with a threshold of 1e−05
rs7303158
rs35055552
rs7572229
rs2090816
rs2387977
rs6828768
rs823424
rs6809952
rs7311004
rs11715853
rs55877868
rs8039785
rs78383039
rs10899911
rs62273907
All
−0.2
−0.1
0.0
0.1
0.2
0.3
MR effect size for
'Gut microbiota abundance (genus Parasutterella id.2892)' on 'Protein ripply1'

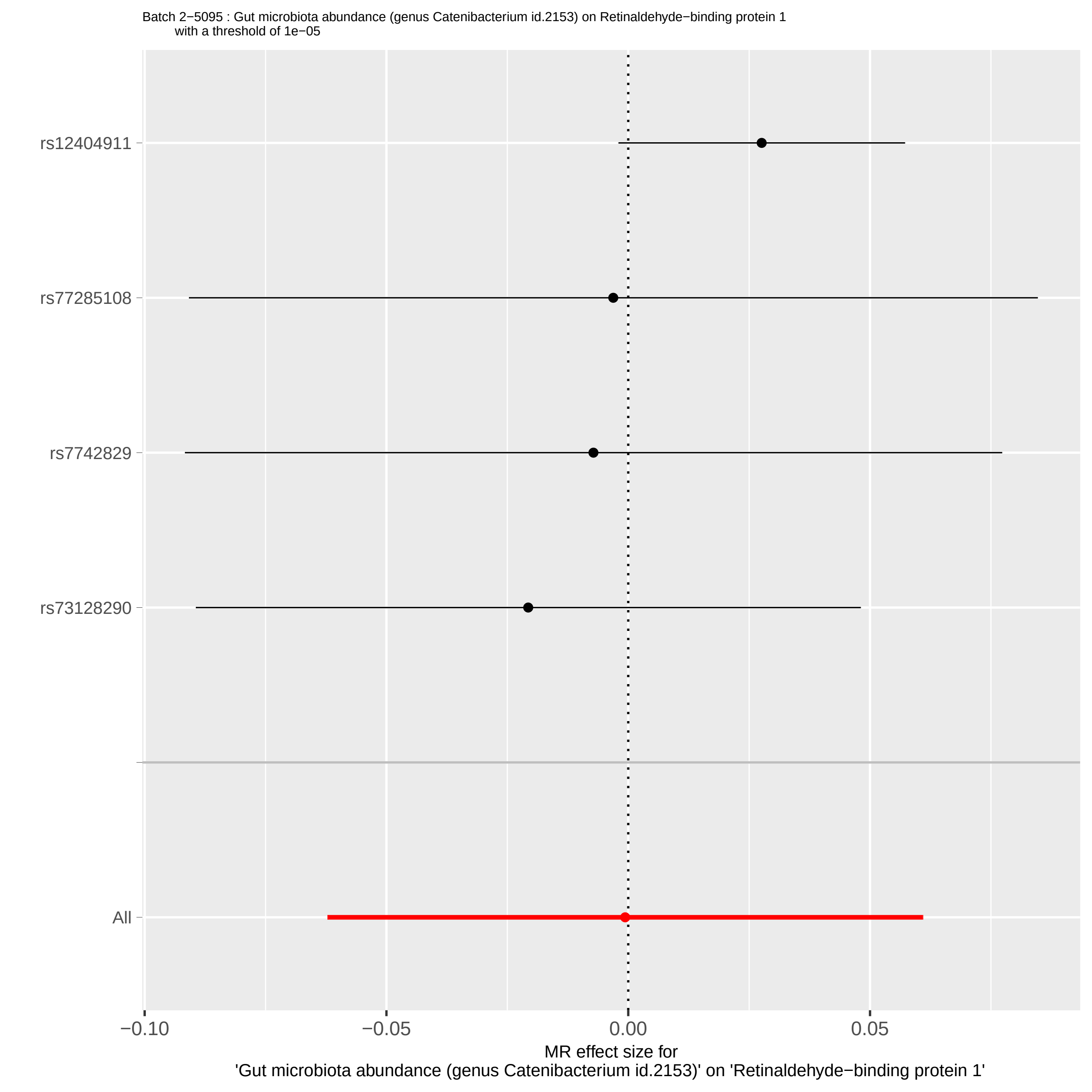

Batch 2−5095 : Gut microbiota abundance (genus Catenibacterium id.2153) on Retinaldehyde−binding protein 1
with a threshold of 1e−05
rs12404911
rs77285108
rs7742829
rs73128290
All
−0.10
−0.05
0.00
0.05
MR effect size for
'Gut microbiota abundance (genus Catenibacterium id.2153)' on 'Retinaldehyde−binding protein 1'

Batch 2−5096 : Gut microbiota abundance (genus Parasutterella id.2892) on Retinaldehyde−binding protein 1
with a threshold of 1e−05

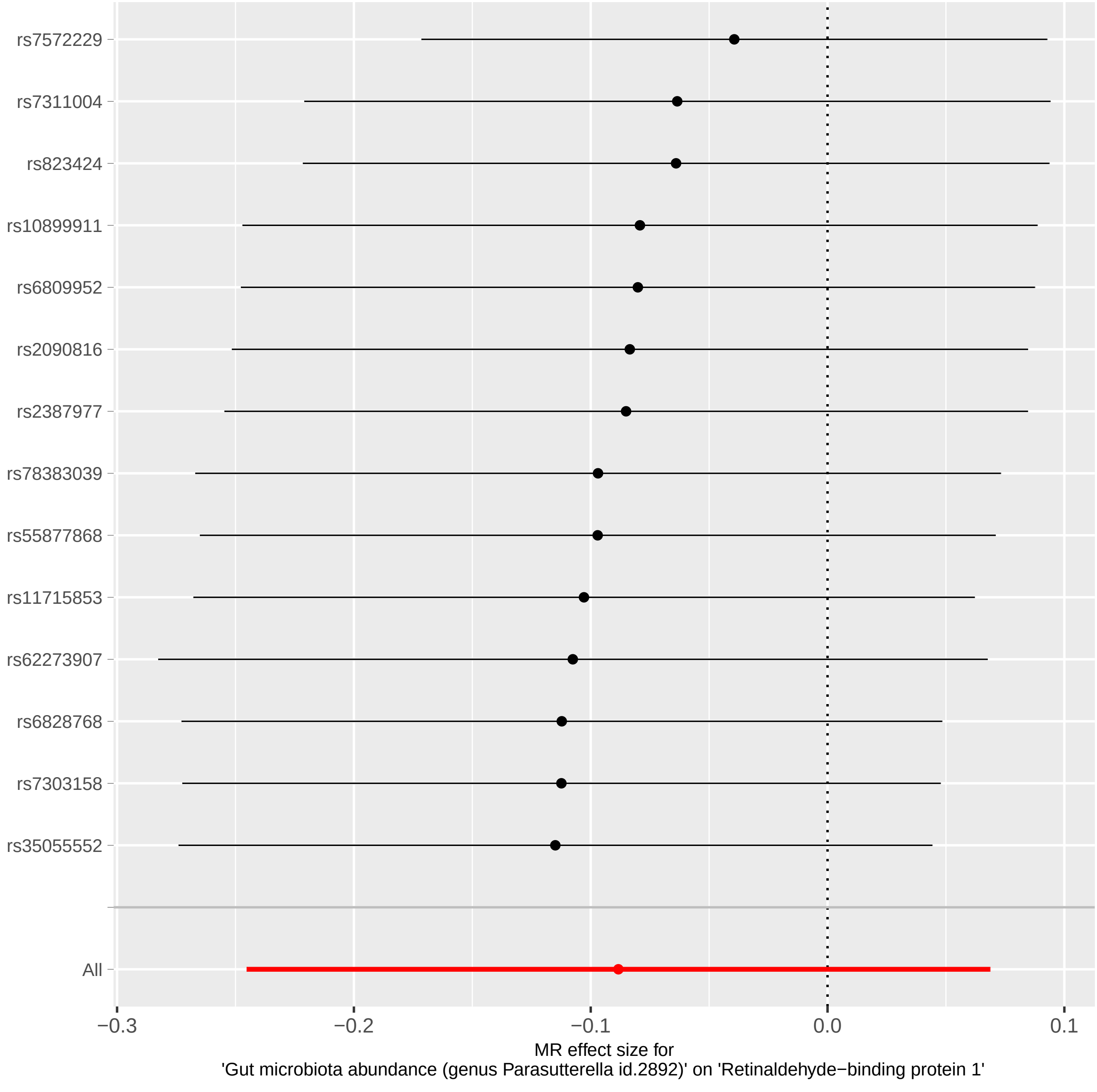

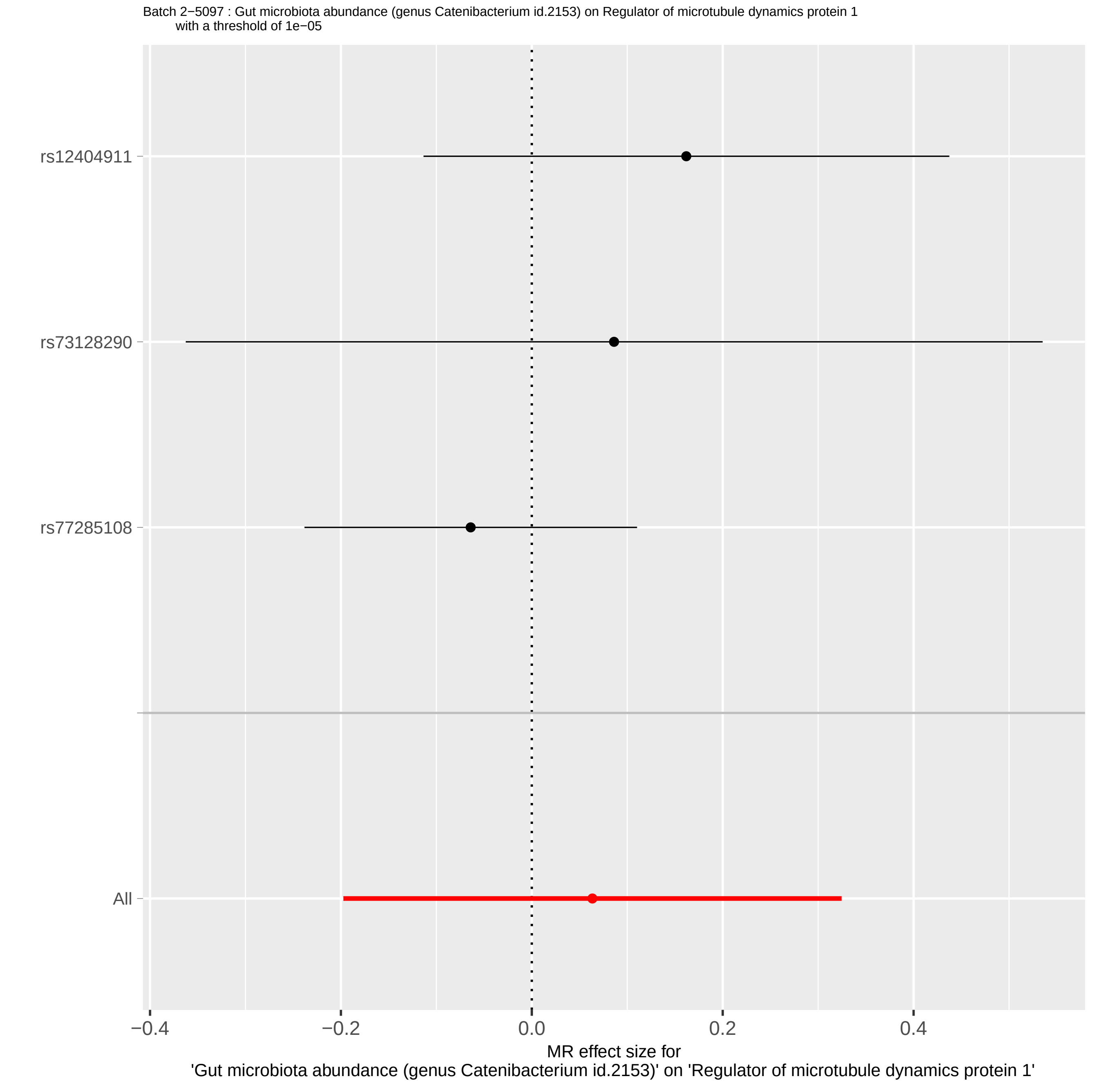
Batch 2−5097 : Gut microbiota abundance (genus Catenibacterium id.2153) on Regulator of microtubule dynamics protein 1
with a threshold of 1e−05
rs12404911
rs73128290
rs77285108
All
−0.4
−0.2
0.0
0.2
0.4
MR effect size for
'Gut microbiota abundance (genus Catenibacterium id.2153)' on 'Regulator of microtubule dynamics protein 1'

Batch 2−5098 : Gut microbiota abundance (genus Parasutterella id.2892) on Regulator of microtubule dynamics protein 1
with a threshold of 1e−05

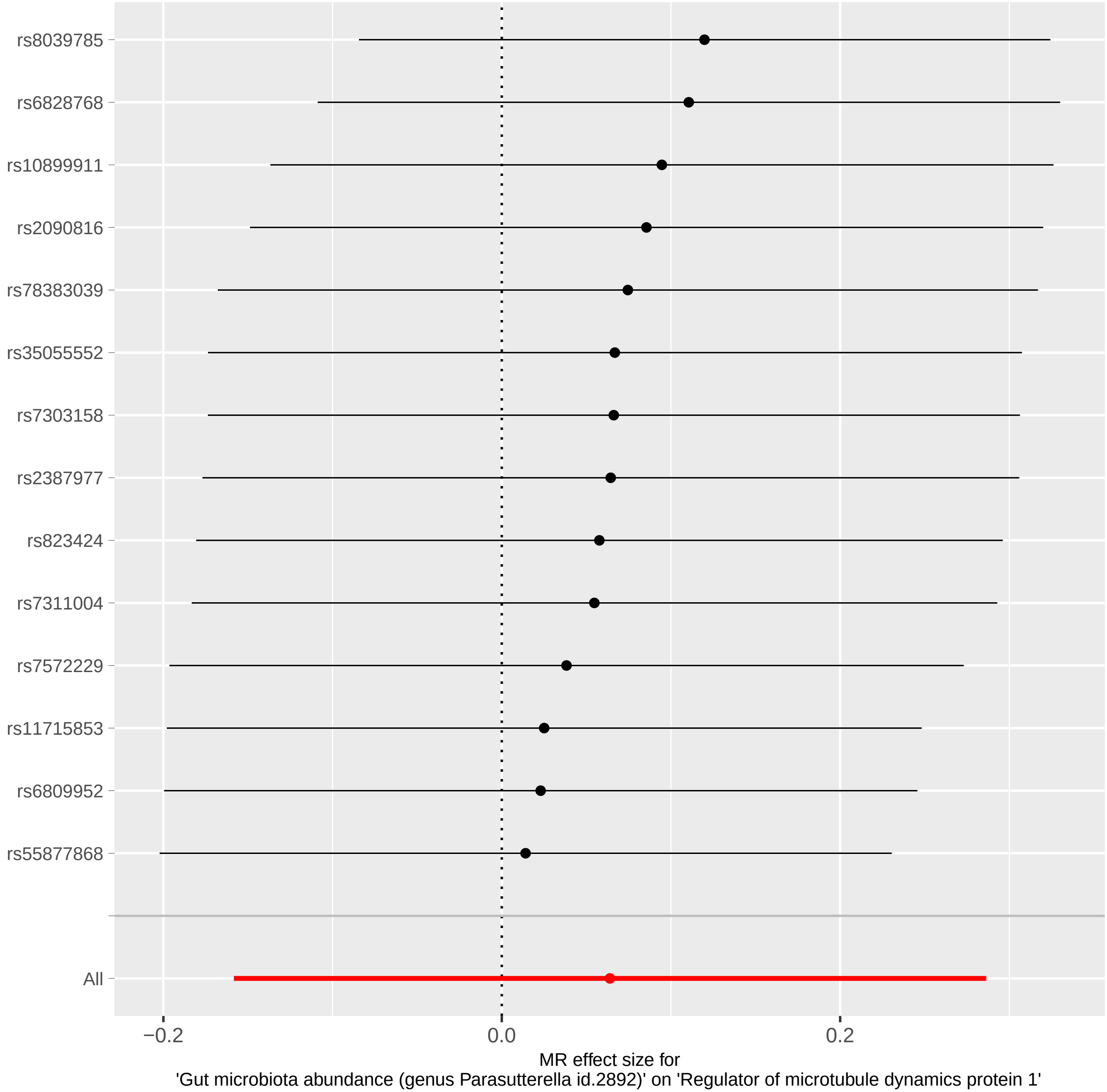

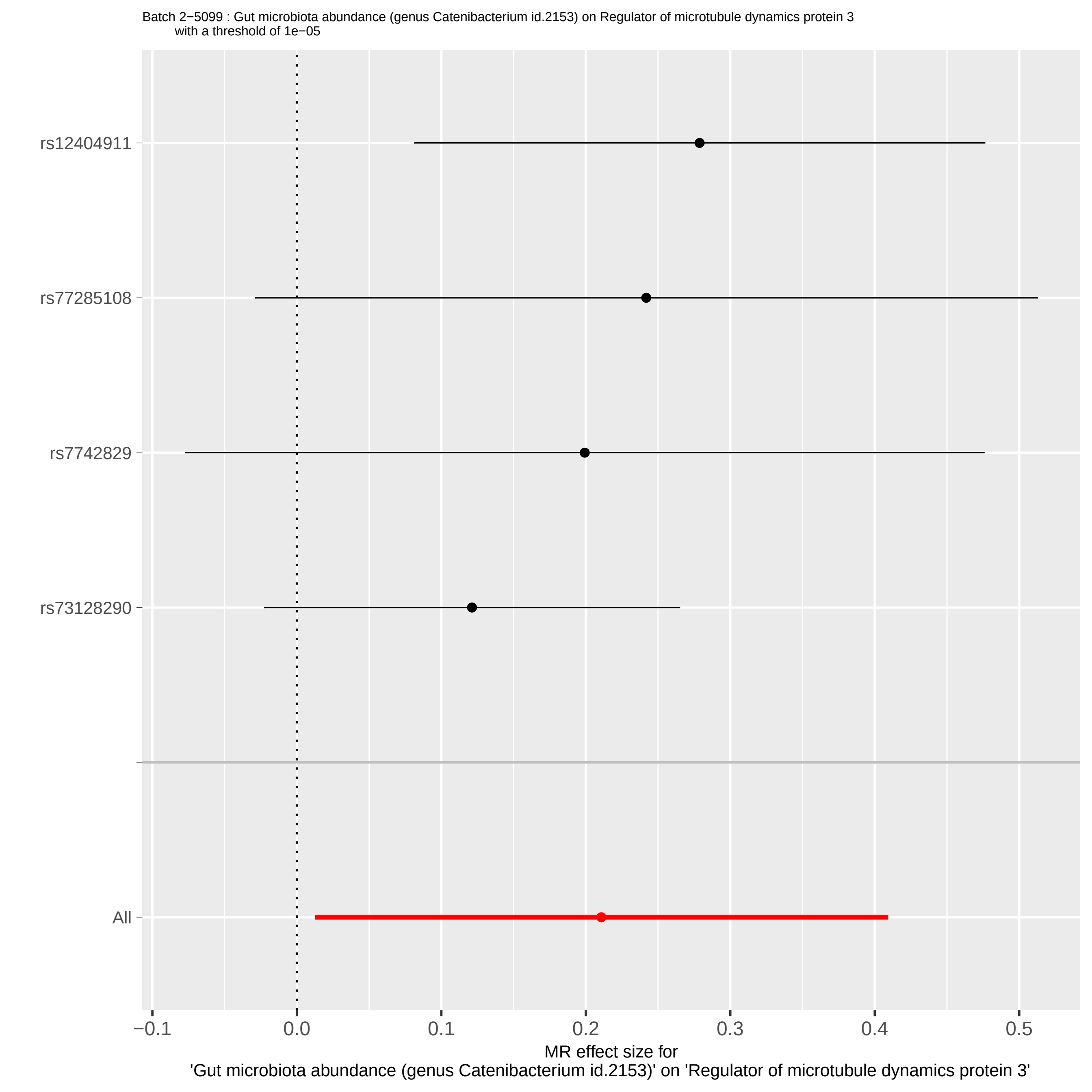

Batch 2−5099 : Gut microbiota abundance (genus Catenibacterium id.2153) on Regulator of microtubule dynamics protein 3
with a threshold of 1e−05
rs12404911
rs77285108
rs7742829
rs73128290
All
−0.1
0.0
0.1
0.2
0.3
0.4
0.5
MR effect size for
'Gut microbiota abundance (genus Catenibacterium id.2153)' on 'Regulator of microtubule dynamics protein 3'

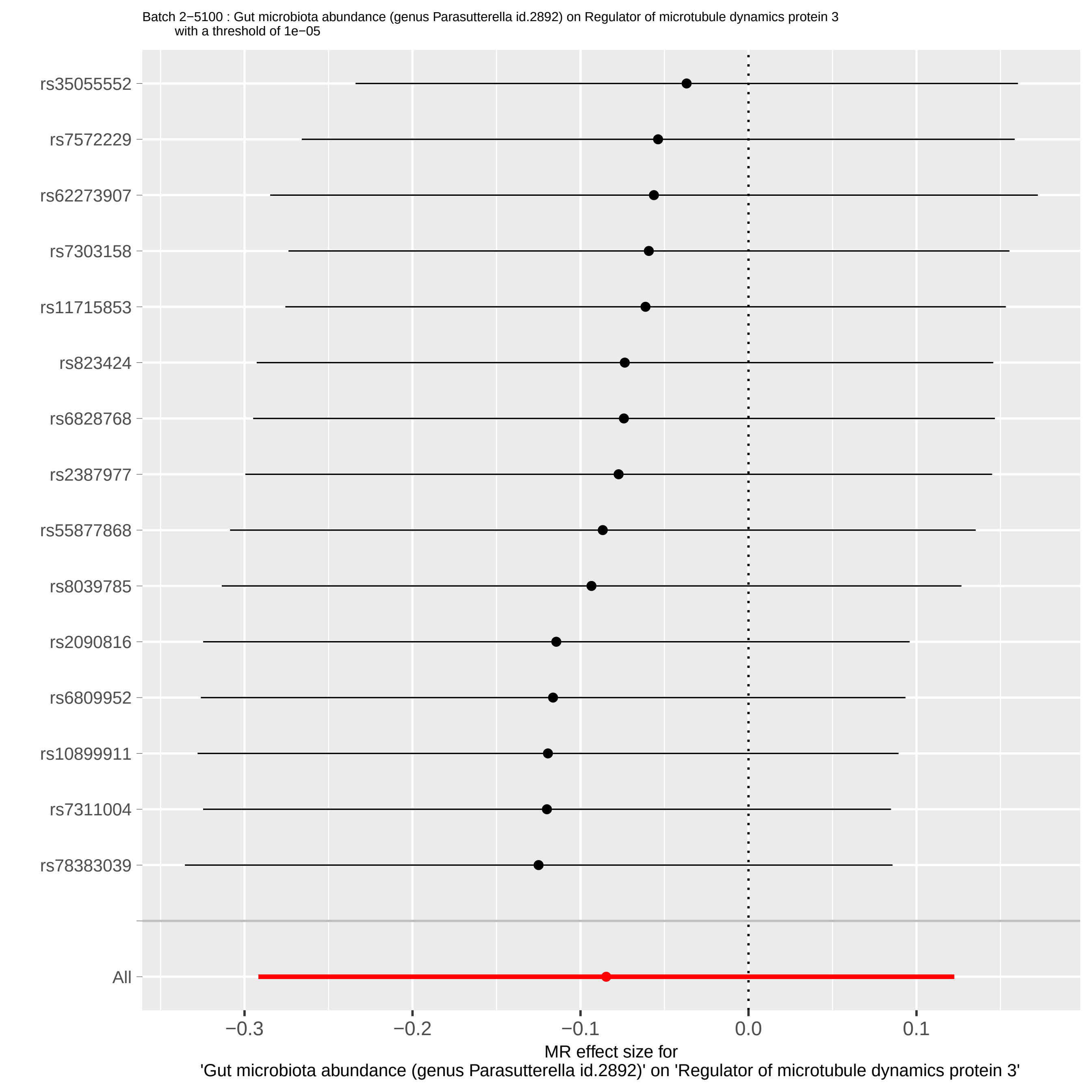

Batch 2−5100 : Gut microbiota abundance (genus Parasutterella id.2892) on Regulator of microtubule dynamics protein 3
with a threshold of 1e−05
rs35055552
rs7572229
rs62273907
rs7303158
rs11715853
rs823424
rs6828768
rs2387977
rs55877868
rs8039785
rs2090816
rs6809952
rs10899911
rs7311004
rs78383039
All
−0.3
−0.2
−0.1
0.0
0.1
MR effect size for
'Gut microbiota abundance (genus Parasutterella id.2892)' on 'Regulator of microtubule dynamics protein 3'

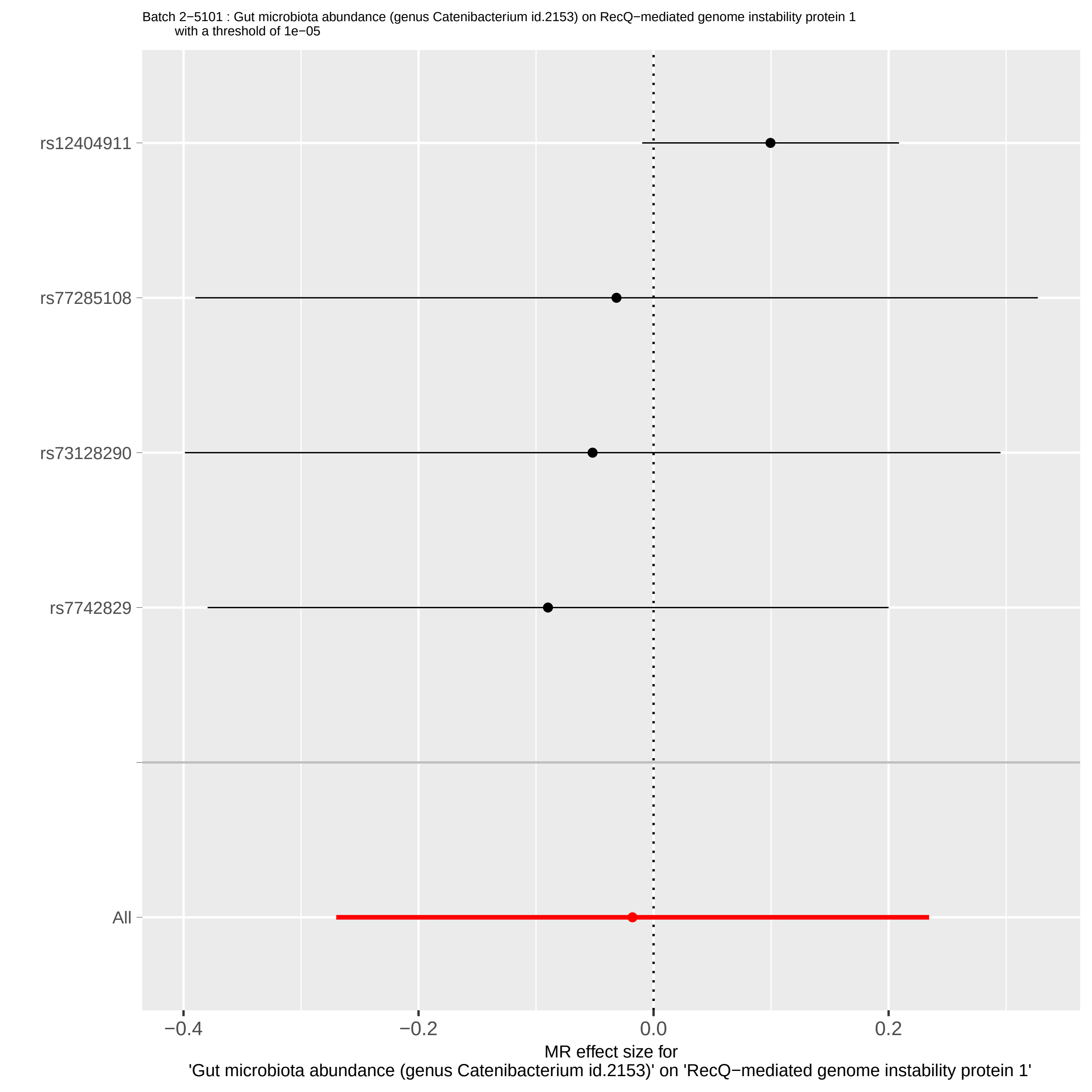
Batch 2−5101 : Gut microbiota abundance (genus Catenibacterium id.2153) on RecQ−mediated genome instability protein 1
with a threshold of 1e−05
rs12404911
rs77285108
rs73128290
rs7742829
All
−0.4
−0.2
0.0
0.2
MR effect size for
'Gut microbiota abundance (genus Catenibacterium id.2153)' on 'RecQ−mediated genome instability protein 1'

Batch 2−5102 : Gut microbiota abundance (genus Parasutterella id.2892) on RecQ−mediated genome instability protein 1
with a threshold of 1e−05

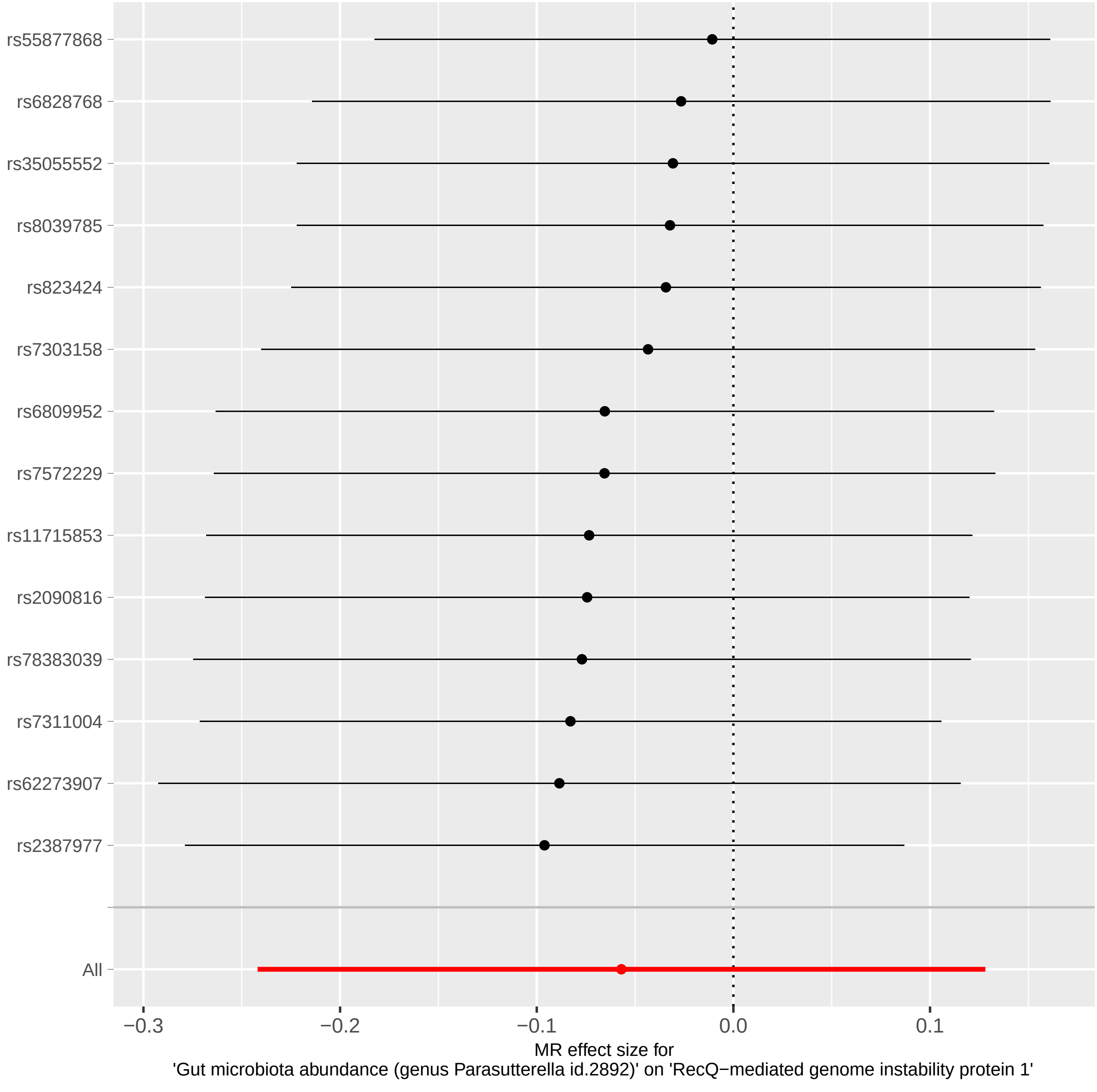

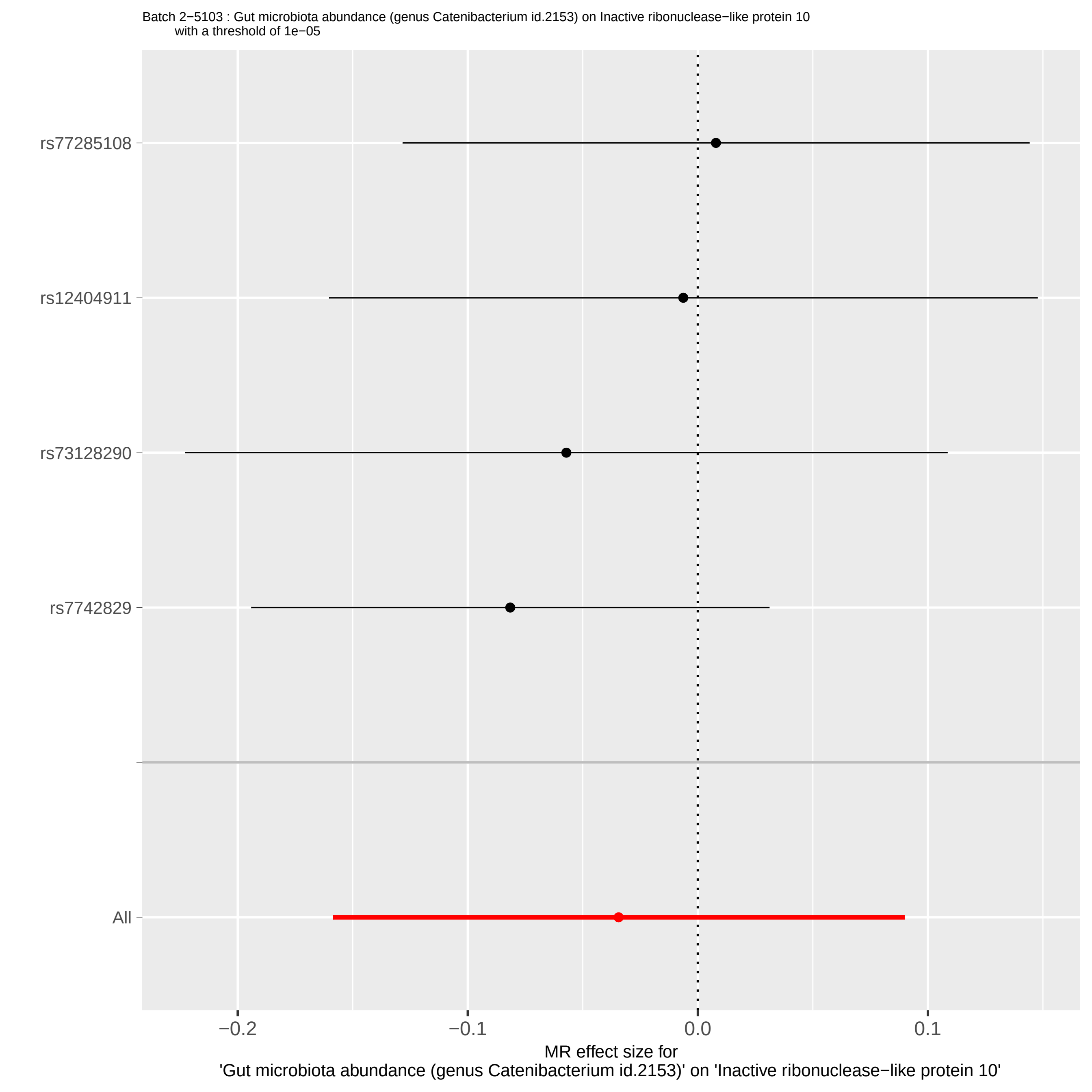
Batch 2−5103 : Gut microbiota abundance (genus Catenibacterium id.2153) on Inactive ribonuclease−like protein 10
with a threshold of 1e−05
rs77285108
rs12404911
rs73128290
rs7742829
All
−0.2
−0.1
0.0
0.1
MR effect size for
'Gut microbiota abundance (genus Catenibacterium id.2153)' on 'Inactive ribonuclease−like protein 10'

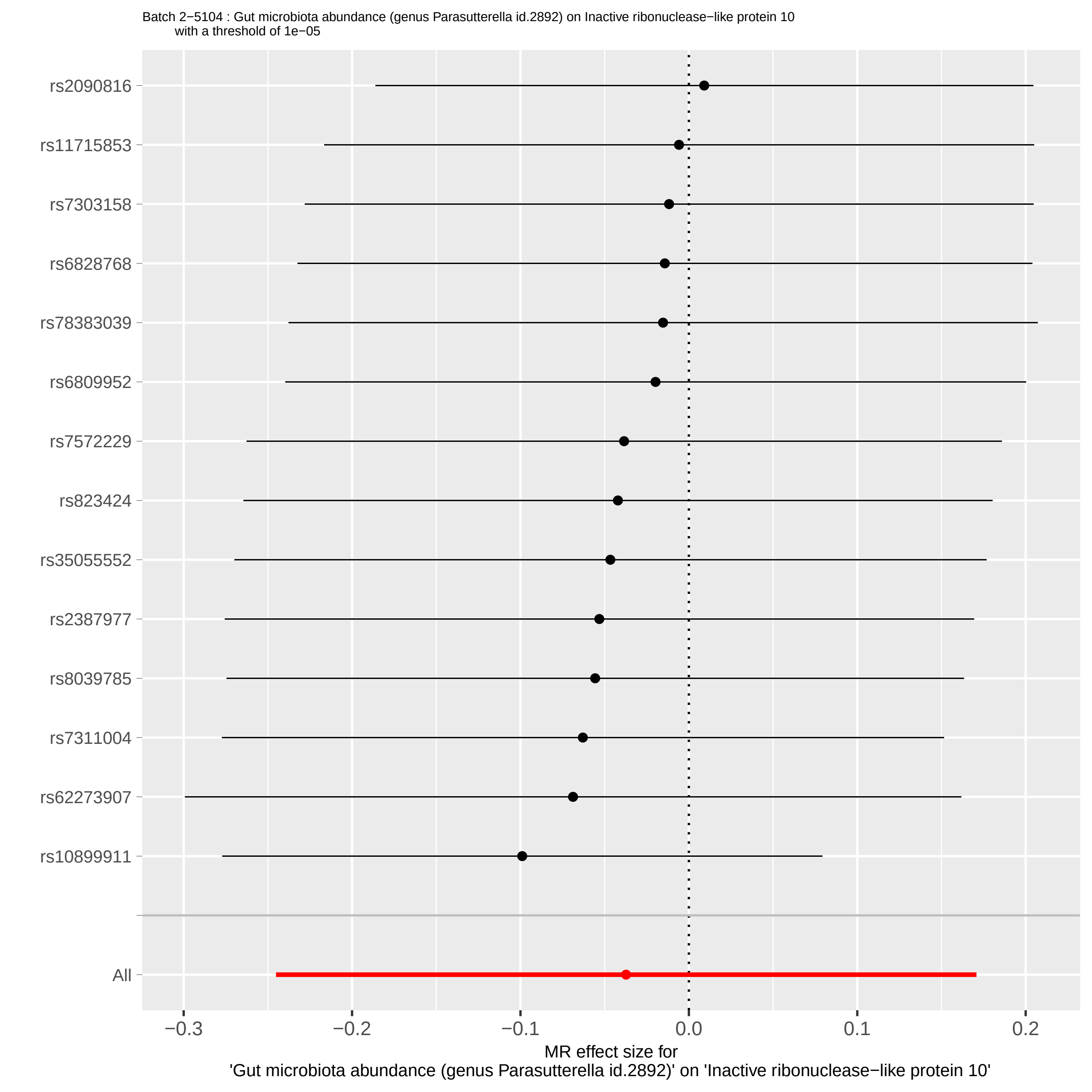

Batch 2−5104 : Gut microbiota abundance (genus Parasutterella id.2892) on Inactive ribonuclease−like protein 10
with a threshold of 1e−05
rs2090816
rs11715853
rs7303158
rs6828768
rs78383039
rs6809952
rs7572229
rs823424
rs35055552
rs2387977
rs8039785
rs7311004
rs62273907
rs10899911
All
−0.3
−0.2
−0.1
0.0
0.1
0.2
MR effect size for
'Gut microbiota abundance (genus Parasutterella id.2892)' on 'Inactive ribonuclease−like protein 10'

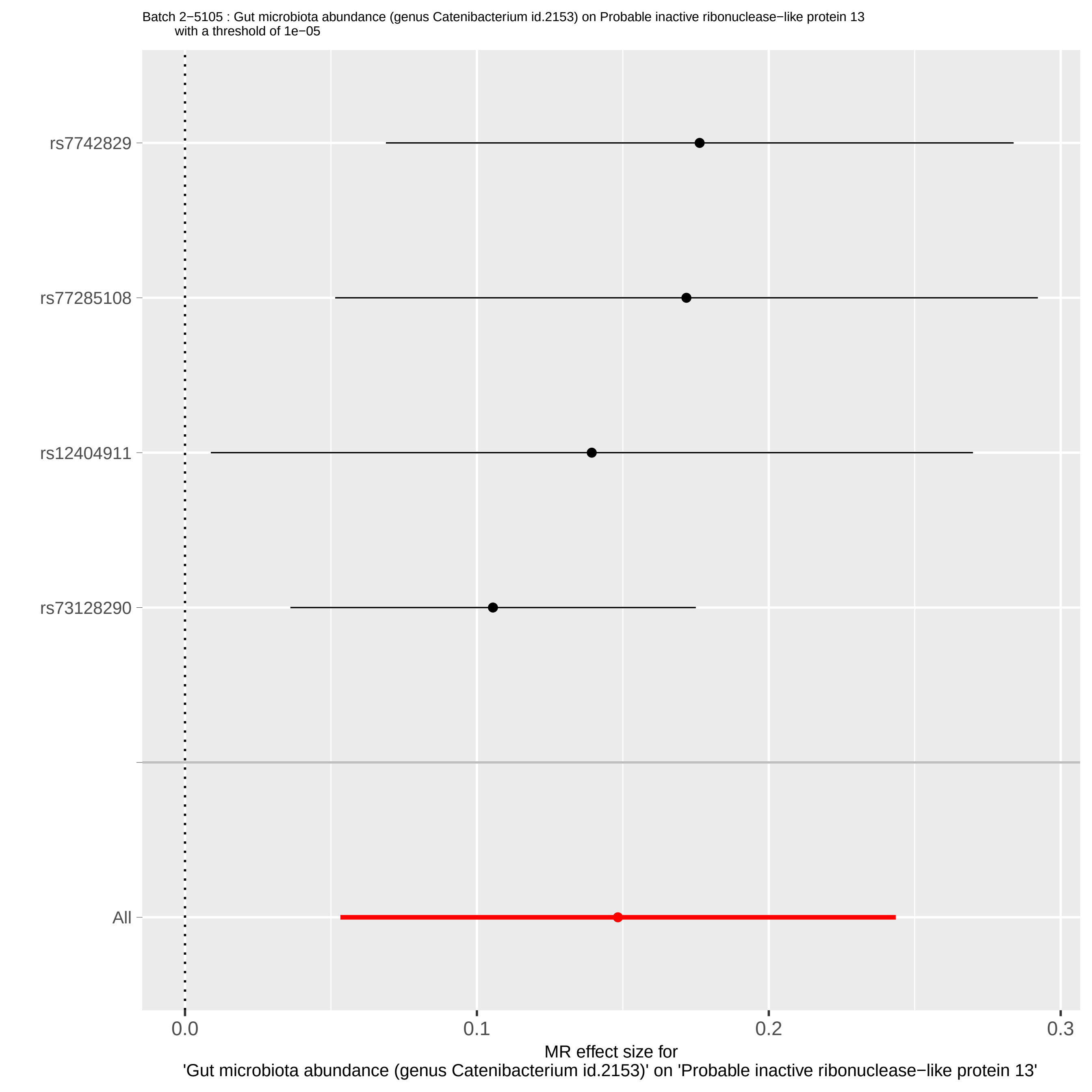
Batch 2−5105 : Gut microbiota abundance (genus Catenibacterium id.2153) on Probable inactive ribonuclease−like protein 13
with a threshold of 1e−05
rs7742829
rs77285108
rs12404911
rs73128290
All
0.0
0.1
0.2
0.3
MR effect size for
'Gut microbiota abundance (genus Catenibacterium id.2153)' on 'Probable inactive ribonuclease−like protein 13'

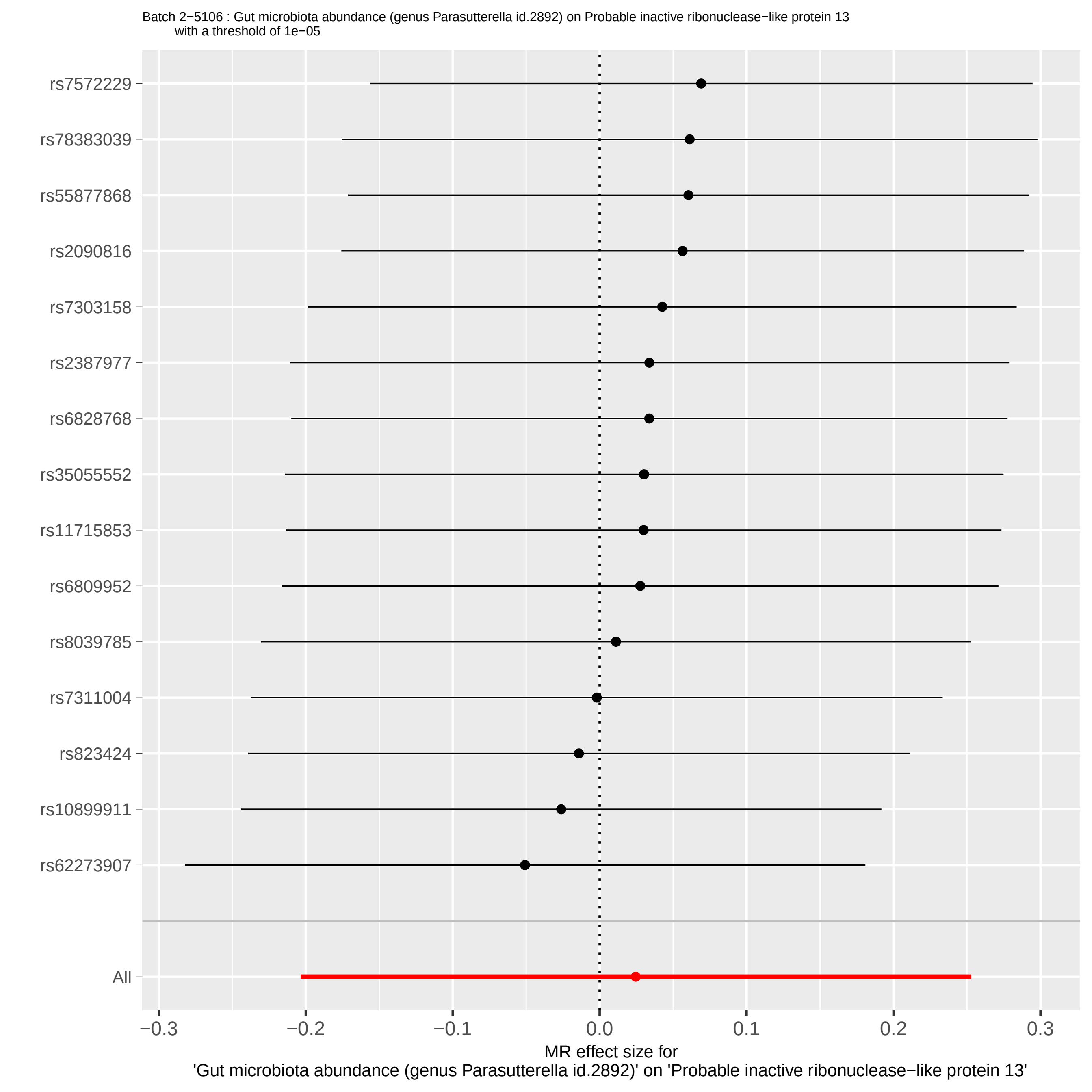

Batch 2−5106 : Gut microbiota abundance (genus Parasutterella id.2892) on Probable inactive ribonuclease−like protein 13
with a threshold of 1e−05
rs7572229
rs78383039
rs55877868
rs2090816
rs7303158
rs2387977
rs6828768
rs35055552
rs11715853
rs6809952
rs8039785
rs7311004
rs823424
rs10899911
rs62273907
All
−0.3
−0.2
−0.1
0.0
0.1
0.2
0.3
MR effect size for
'Gut microbiota abundance (genus Parasutterella id.2892)' on 'Probable inactive ribonuclease−like protein 13'

Batch 2−5107 : Gut microbiota abundance (genus Catenibacterium id.2153) on Ribonuclease pancreatic
with a threshold of 1e−05

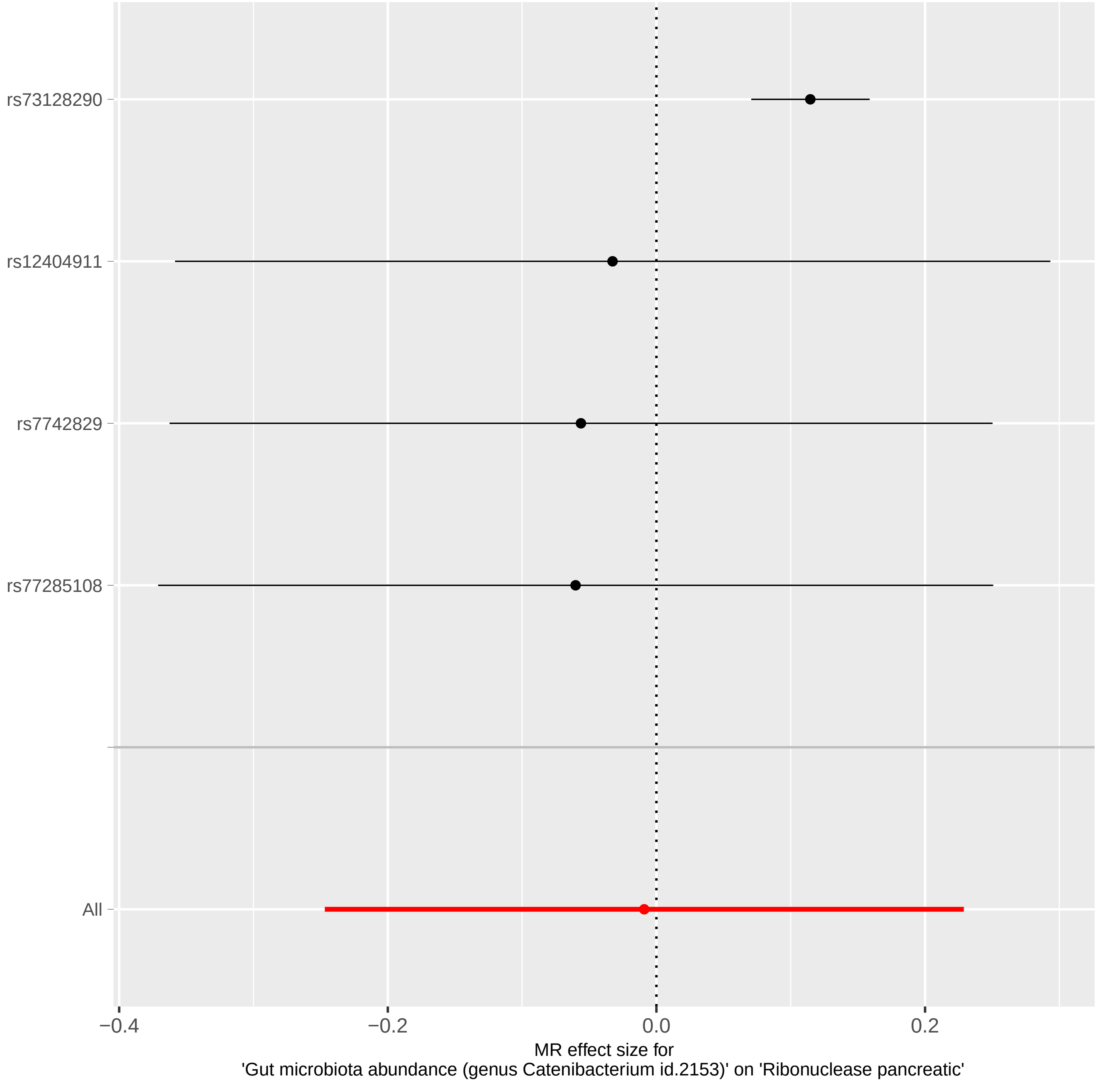

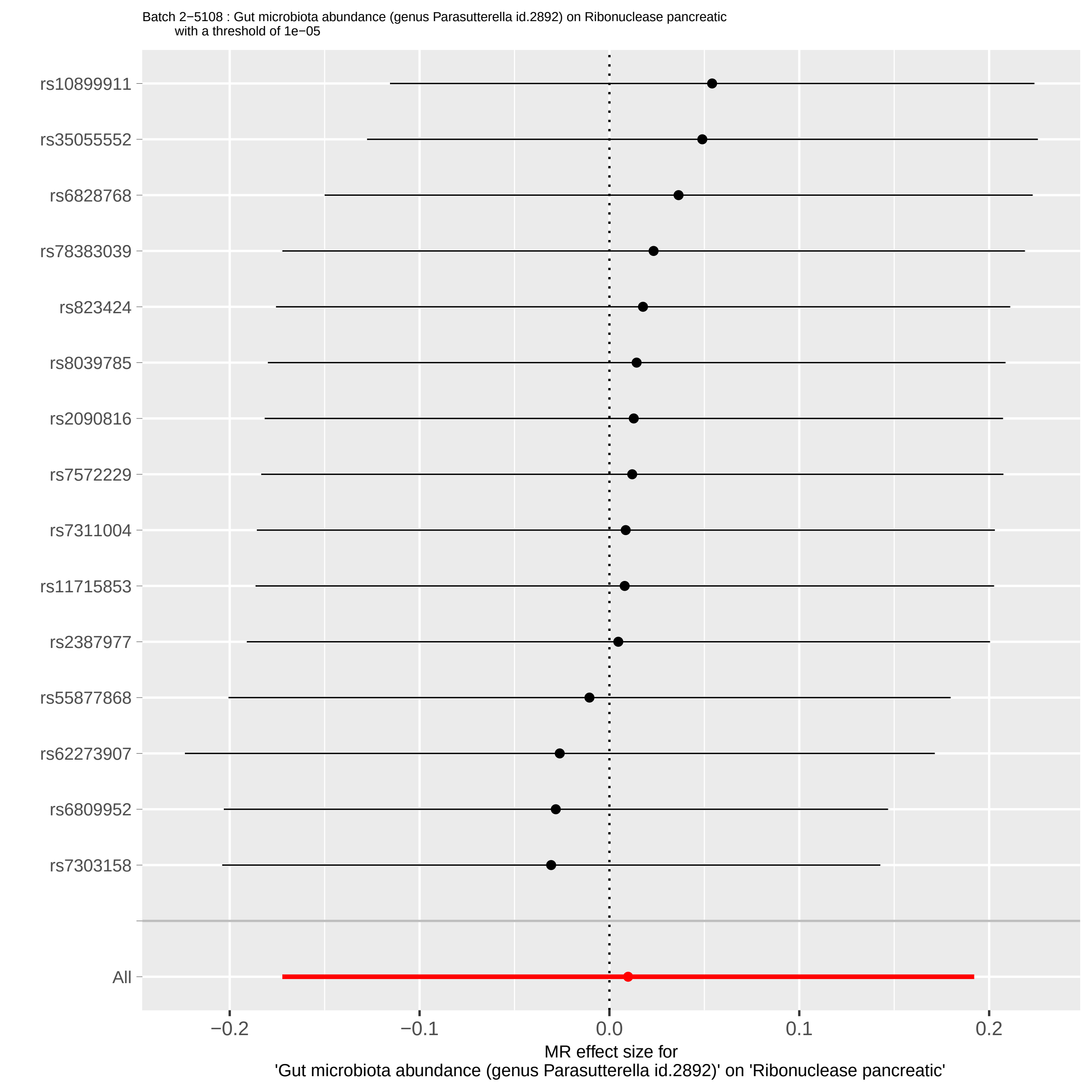
Batch 2−5108 : Gut microbiota abundance (genus Parasutterella id.2892) on Ribonuclease pancreatic
with a threshold of 1e−05
rs10899911
rs35055552
rs6828768
rs78383039
rs823424
rs8039785
rs2090816
rs7572229
rs7311004
rs11715853
rs2387977
rs55877868
rs62273907
rs6809952
rs7303158
All
−0.2
−0.1
0.0
0.1
0.2
MR effect size for
'Gut microbiota abundance (genus Parasutterella id.2892)' on 'Ribonuclease pancreatic'

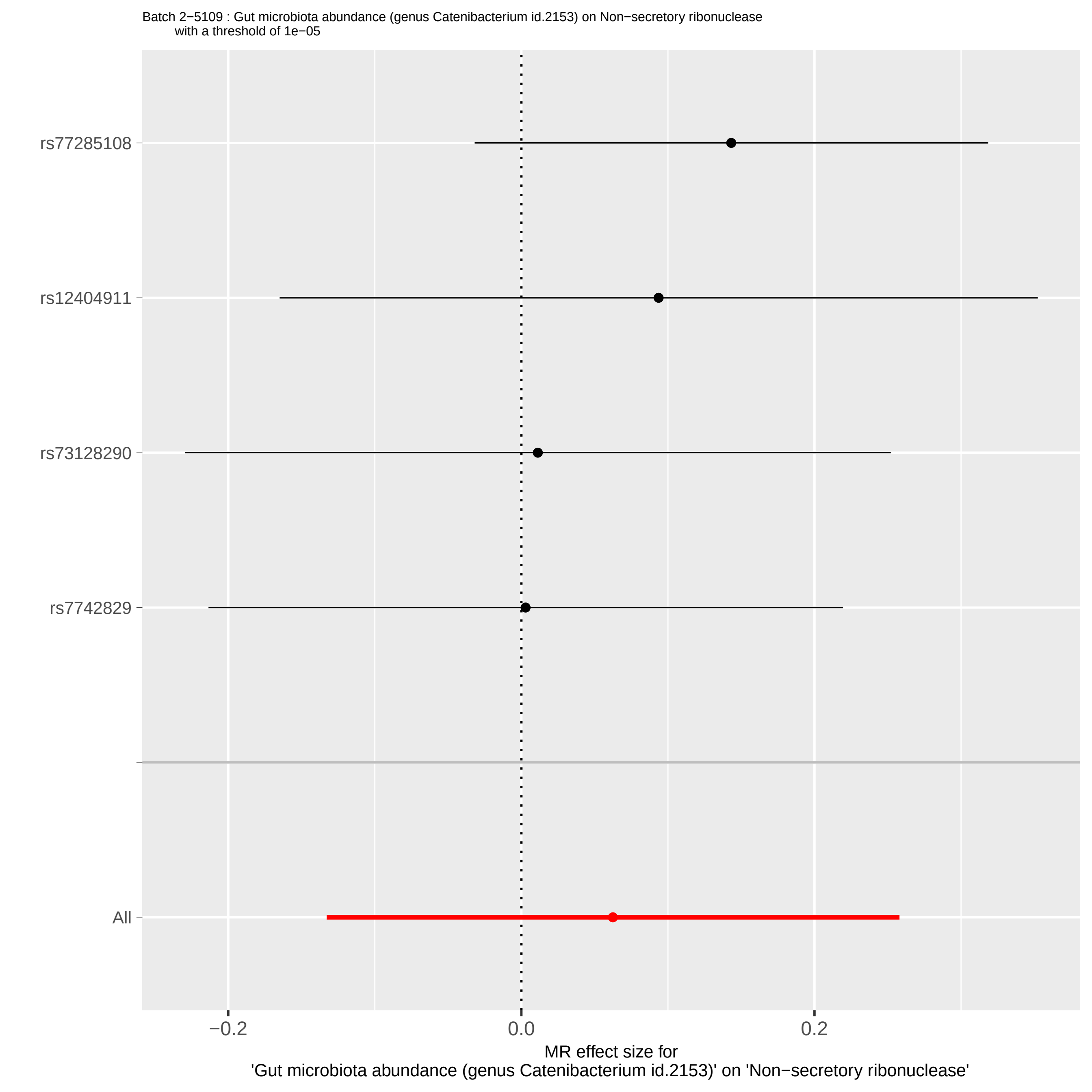
Batch 2−5109 : Gut microbiota abundance (genus Catenibacterium id.2153) on Non−secretory ribonuclease
with a threshold of 1e−05
rs77285108
rs12404911
rs73128290
rs7742829
All
−0.2
0.0
0.2
MR effect size for
'Gut microbiota abundance (genus Catenibacterium id.2153)' on 'Non−secretory ribonuclease'

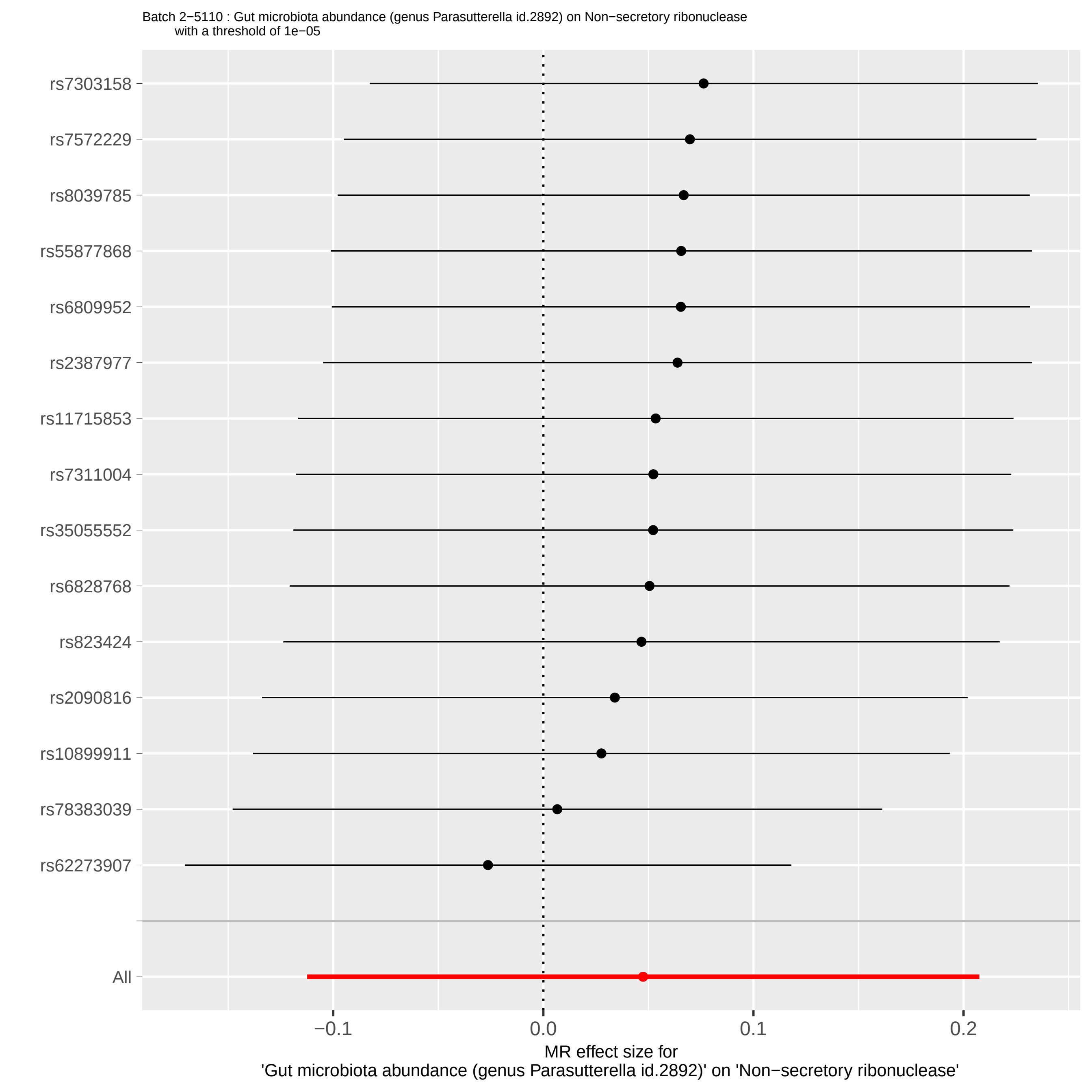

Batch 2−5110 : Gut microbiota abundance (genus Parasutterella id.2892) on Non−secretory ribonuclease
with a threshold of 1e−05
rs7303158
rs7572229
rs8039785
rs55877868
rs6809952
rs2387977
rs11715853
rs7311004
rs35055552
rs6828768
rs823424
rs2090816
rs10899911
rs78383039
rs62273907
All
−0.1
0.0
0.1
0.2
MR effect size for
'Gut microbiota abundance (genus Parasutterella id.2892)' on 'Non−secretory ribonuclease'

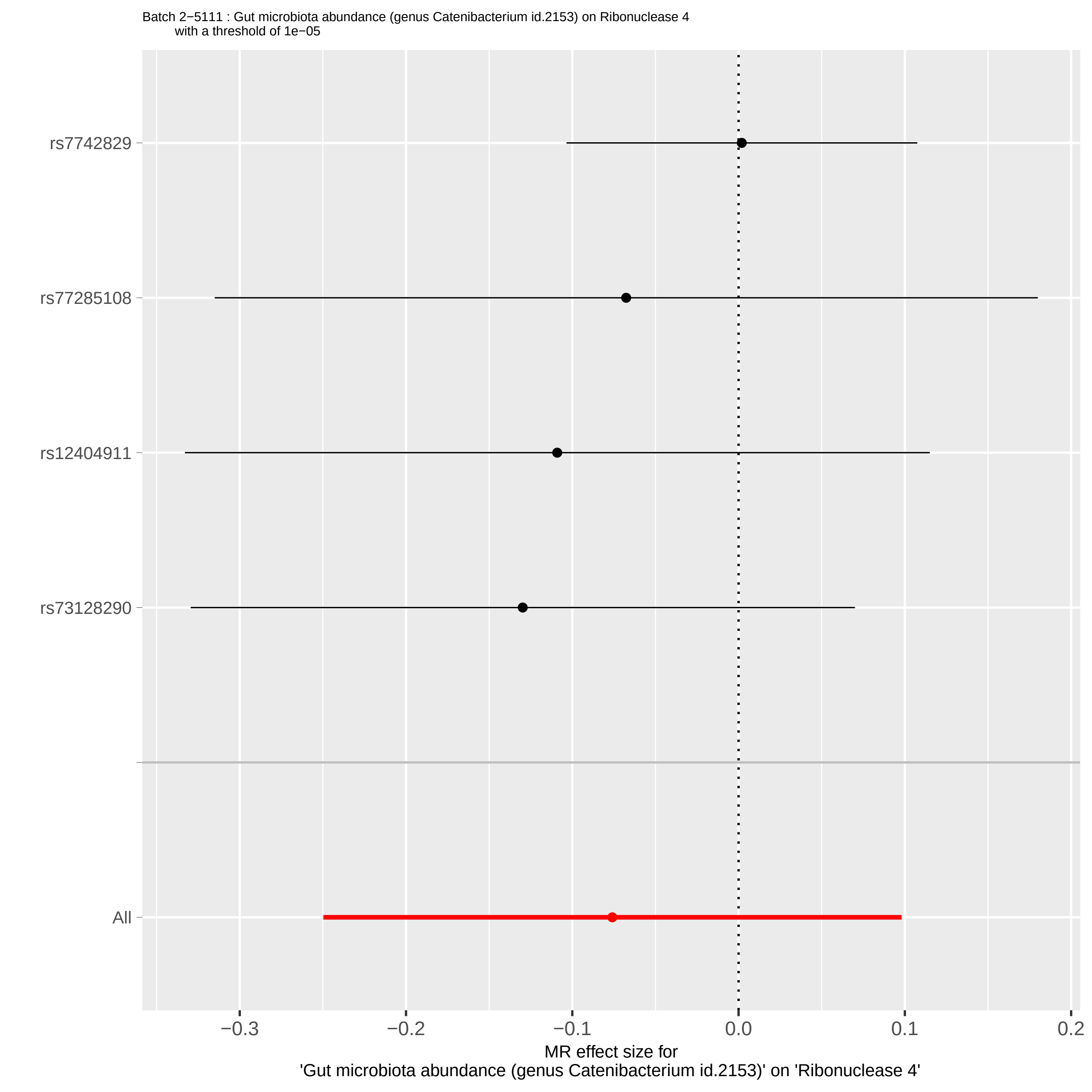

Batch 2−5111 : Gut microbiota abundance (genus Catenibacterium id.2153) on Ribonuclease 4
with a threshold of 1e−05
rs7742829
rs77285108
rs12404911
rs73128290
All
−0.3
−0.2
−0.1
0.0
0.1
0.2
MR effect size for
'Gut microbiota abundance (genus Catenibacterium id.2153)' on 'Ribonuclease 4'

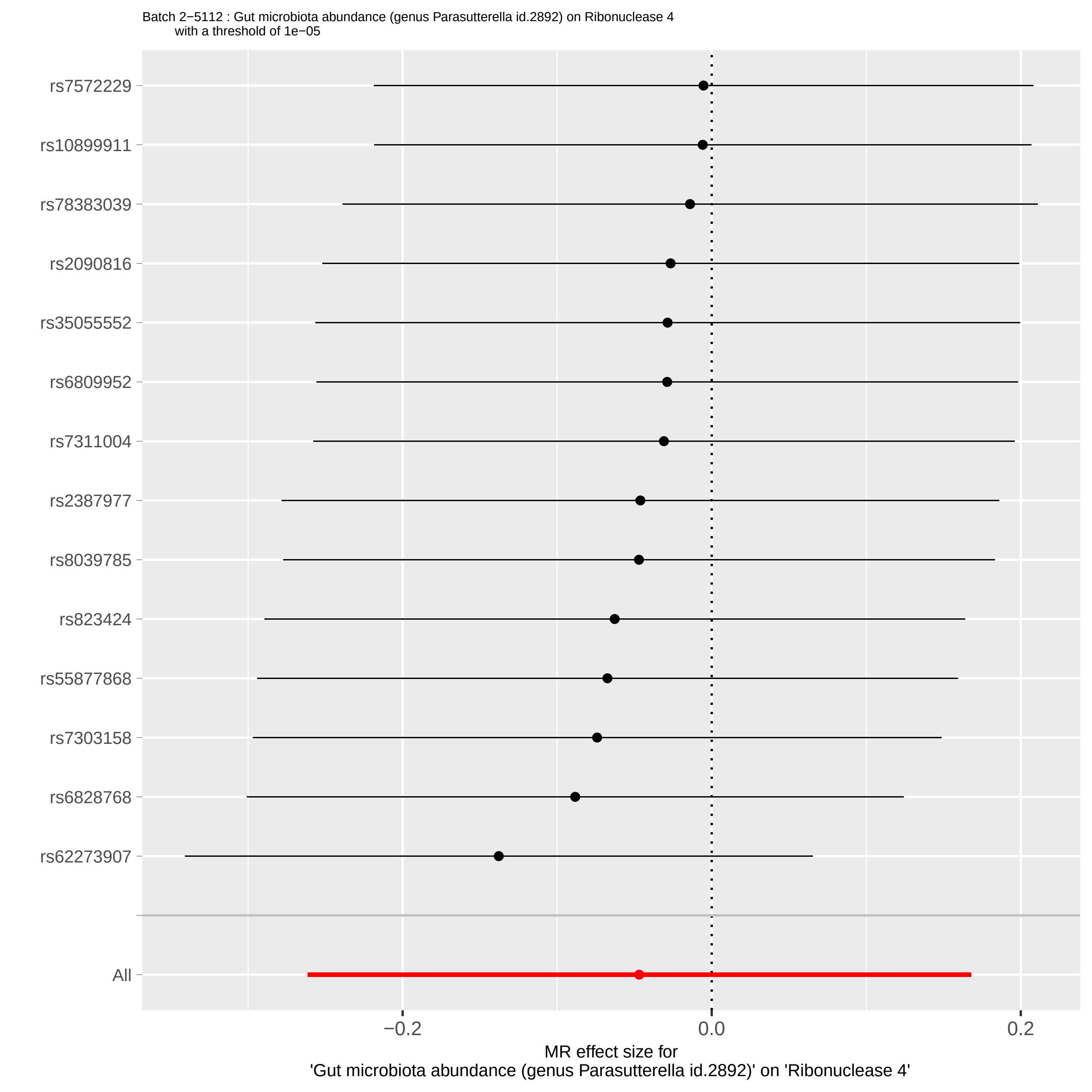

Batch 2−5112 : Gut microbiota abundance (genus Parasutterella id.2892) on Ribonuclease 4
with a threshold of 1e−05
rs7572229
rs10899911
rs78383039
rs2090816
rs35055552
rs6809952
rs7311004
rs2387977
rs8039785
rs823424
rs55877868
rs7303158
rs6828768
rs62273907
All
−0.2
0.0
0.2
MR effect size for
'Gut microbiota abundance (genus Parasutterella id.2892)' on 'Ribonuclease 4'

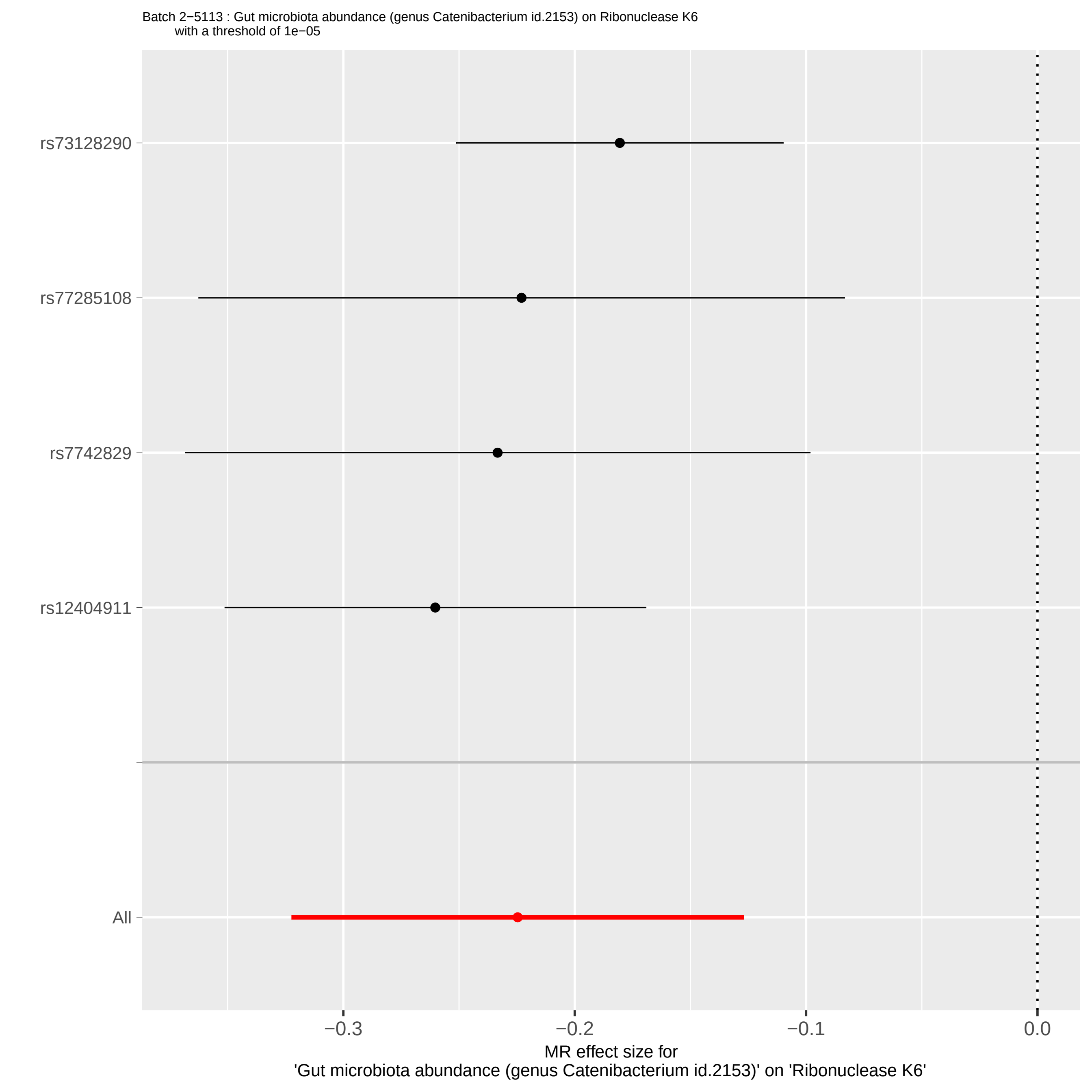
Batch 2−5113 : Gut microbiota abundance (genus Catenibacterium id.2153) on Ribonuclease K6
with a threshold of 1e−05
rs73128290
rs77285108
rs7742829
rs12404911
All
−0.3
−0.2
−0.1
0.0
MR effect size for
'Gut microbiota abundance (genus Catenibacterium id.2153)' on 'Ribonuclease K6'

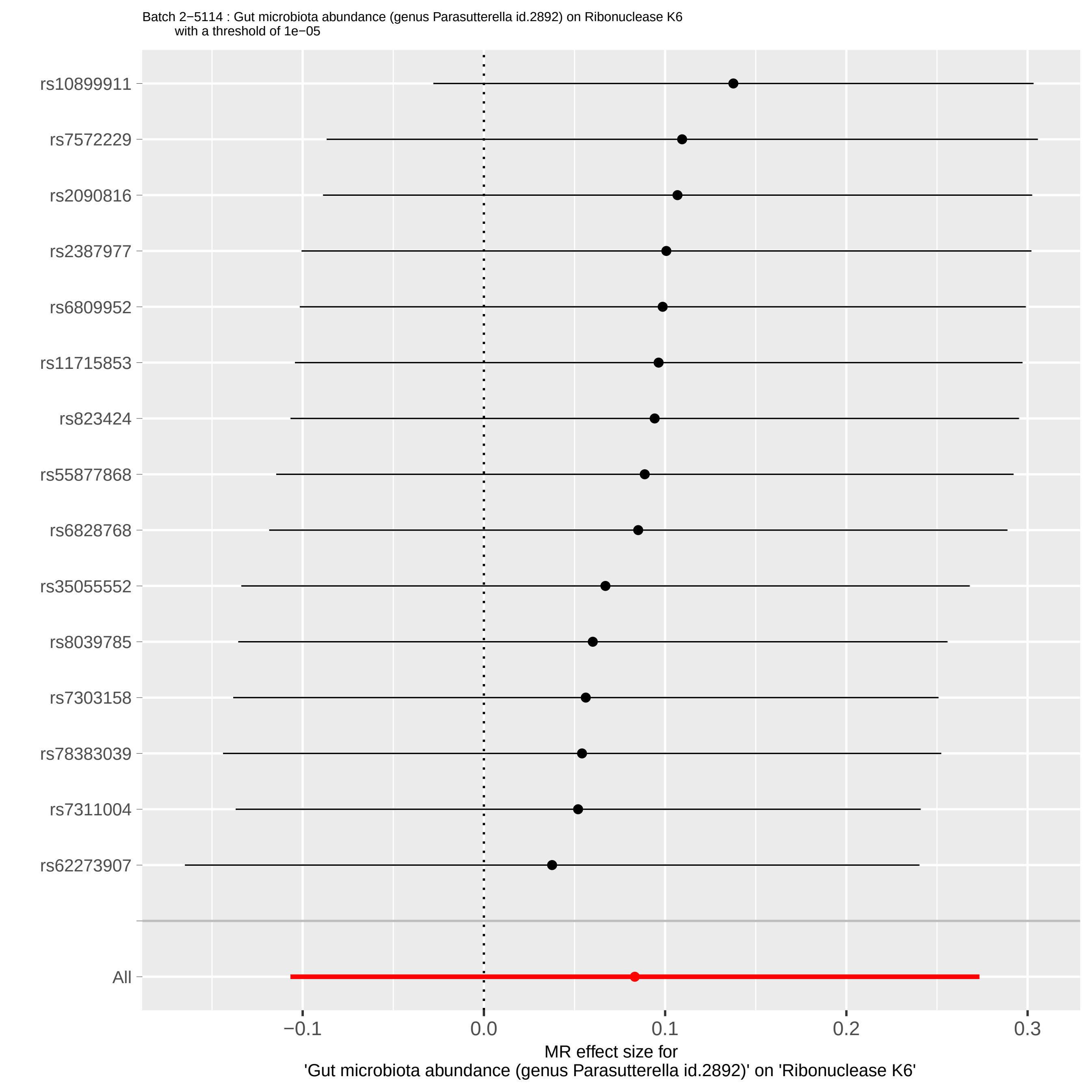
Batch 2−5114 : Gut microbiota abundance (genus Parasutterella id.2892) on Ribonuclease K6
with a threshold of 1e−05
rs10899911
rs7572229
rs2090816
rs2387977
rs6809952
rs11715853
rs823424
rs55877868
rs6828768
rs35055552
rs8039785
rs7303158
rs78383039
rs7311004
rs62273907
All
−0.1
0.0
0.1
0.2
0.3
MR effect size for
'Gut microbiota abundance (genus Parasutterella id.2892)' on 'Ribonuclease K6'

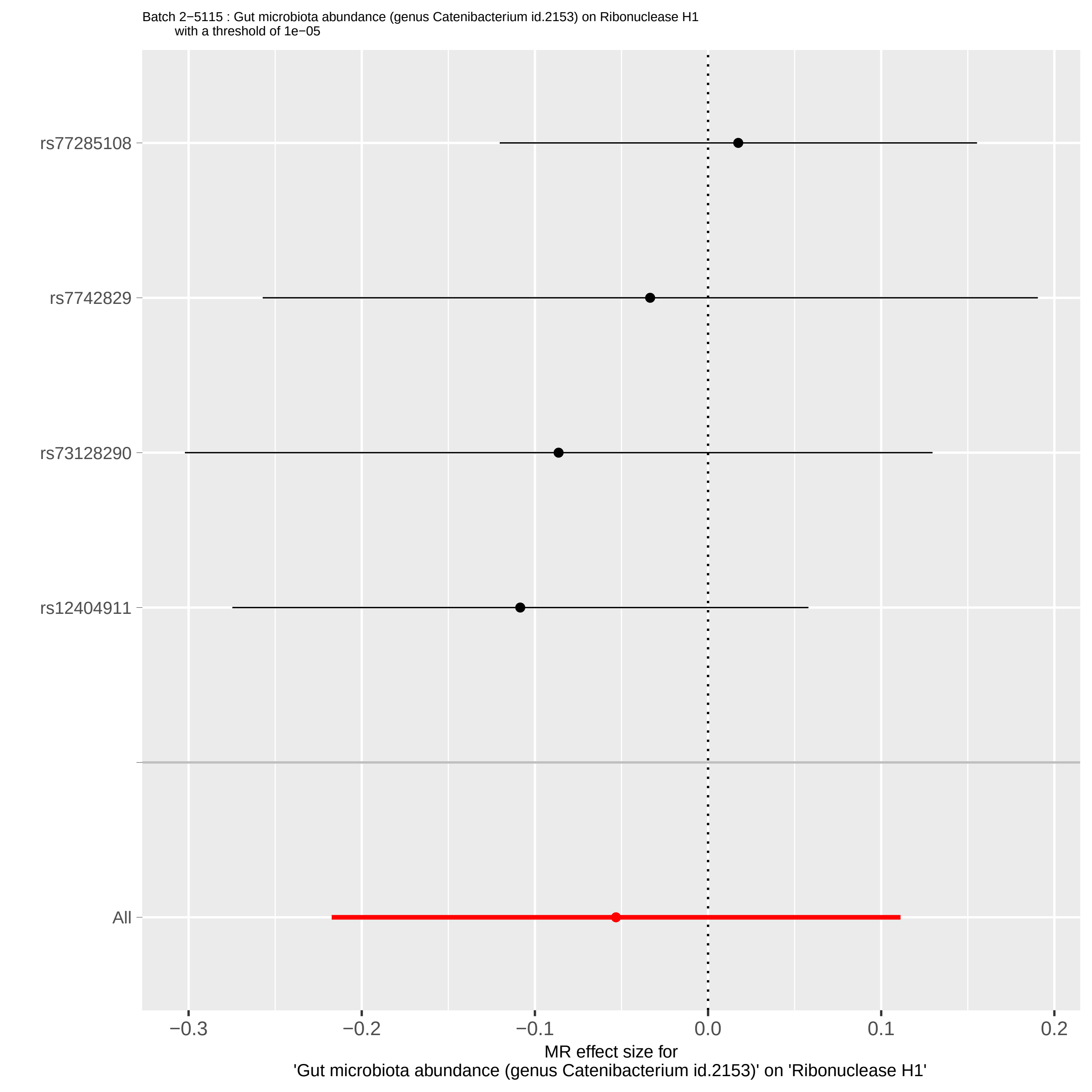

Batch 2−5115 : Gut microbiota abundance (genus Catenibacterium id.2153) on Ribonuclease H1
with a threshold of 1e−05
rs77285108
rs7742829
rs73128290
rs12404911
All
−0.3
−0.2
−0.1
0.0
0.1
0.2
MR effect size for
'Gut microbiota abundance (genus Catenibacterium id.2153)' on 'Ribonuclease H1'

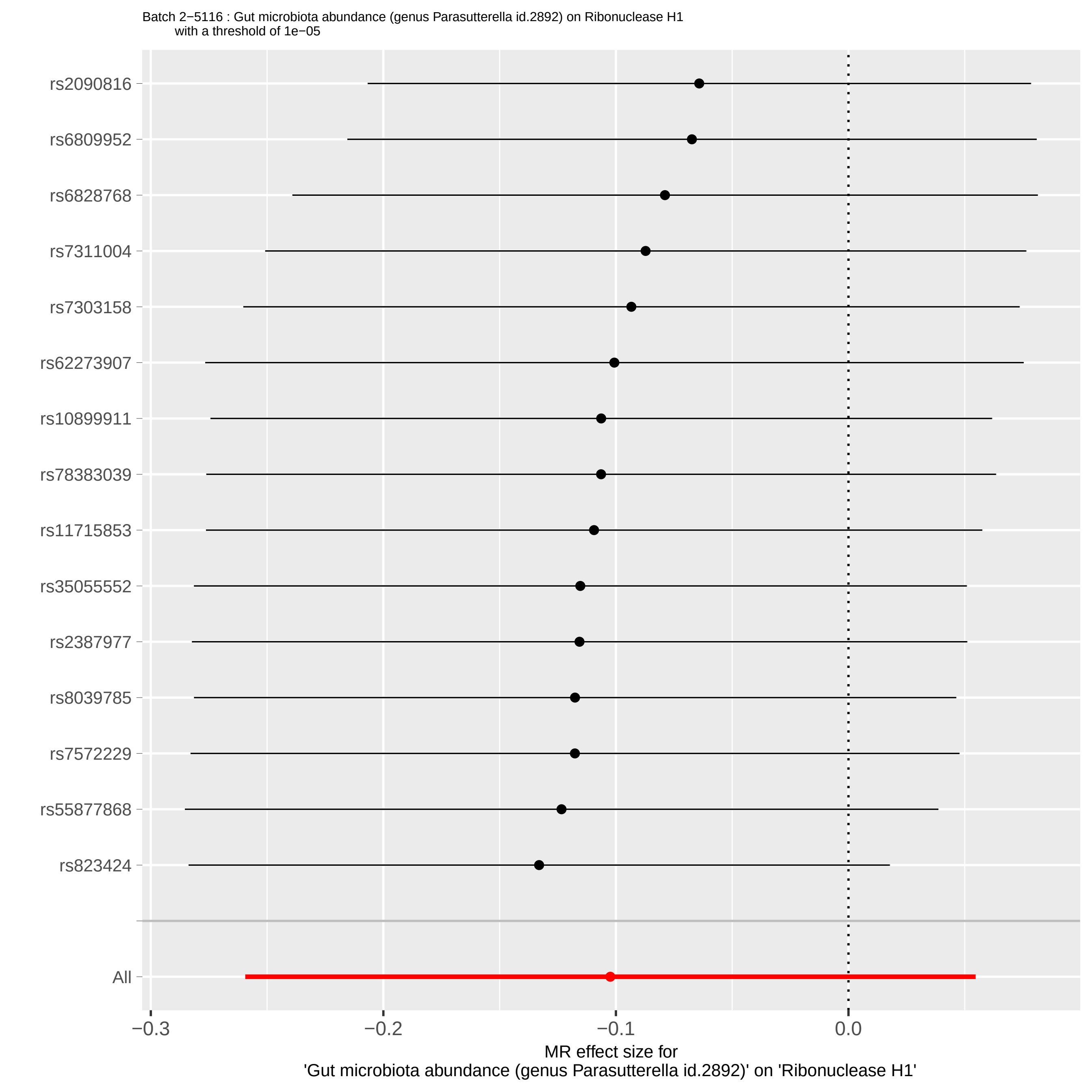
Batch 2−5116 : Gut microbiota abundance (genus Parasutterella id.2892) on Ribonuclease H1
with a threshold of 1e−05
rs2090816
rs6809952
rs6828768
rs7311004
rs7303158
rs62273907
rs10899911
rs78383039
rs11715853
rs35055552
rs2387977
rs8039785
rs7572229
rs55877868
rs823424
All
−0.3
−0.2
−0.1
0.0
MR effect size for
'Gut microbiota abundance (genus Parasutterella id.2892)' on 'Ribonuclease H1'

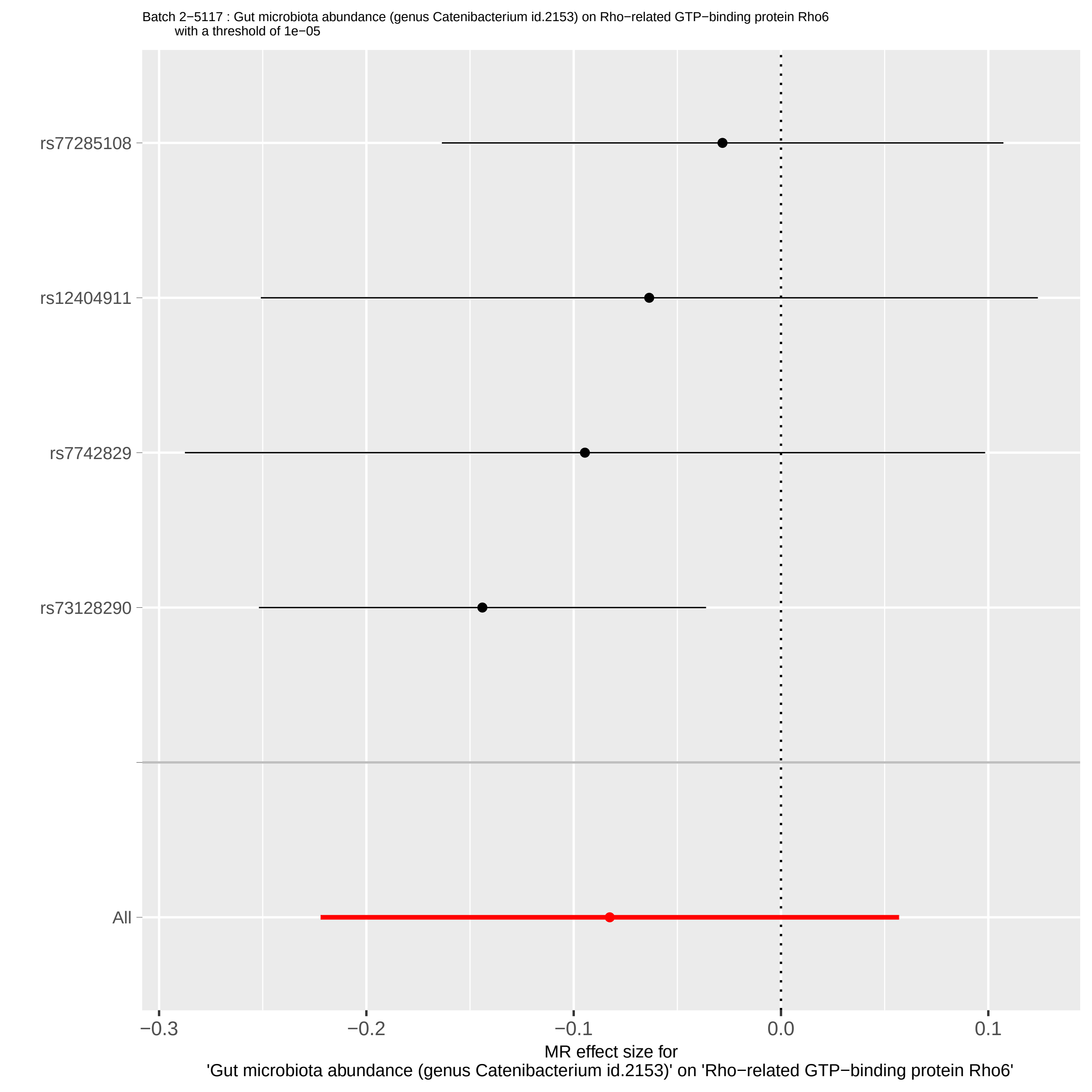

Batch 2−5117 : Gut microbiota abundance (genus Catenibacterium id.2153) on Rho−related GTP−binding protein Rho6
with a threshold of 1e−05
rs77285108
rs12404911
rs7742829
rs73128290
All
−0.3
−0.2
−0.1
0.0
0.1
MR effect size for
'Gut microbiota abundance (genus Catenibacterium id.2153)' on 'Rho−related GTP−binding protein Rho6'

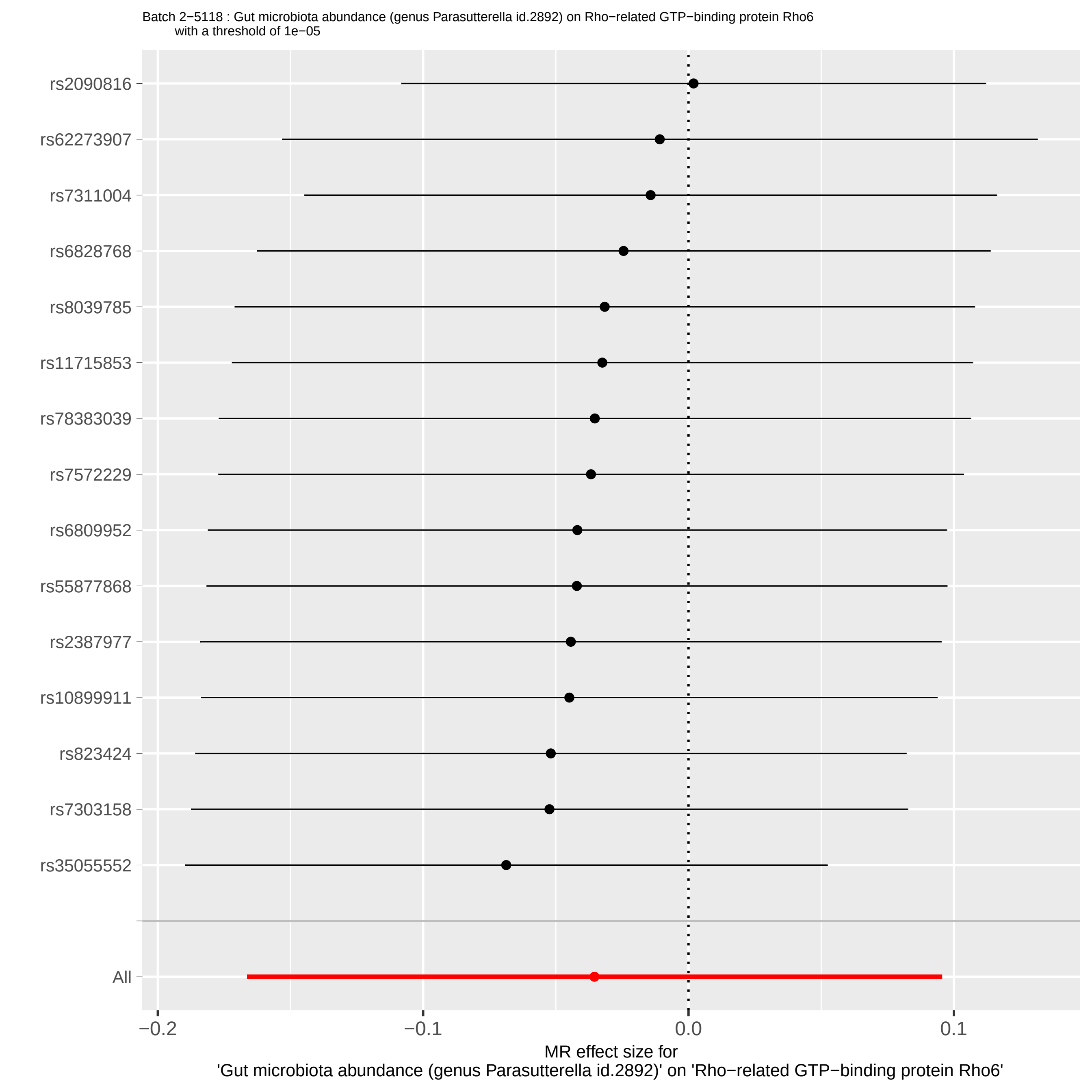
Batch 2−5118 : Gut microbiota abundance (genus Parasutterella id.2892) on Rho−related GTP−binding protein Rho6
with a threshold of 1e−05
rs2090816
rs62273907
rs7311004
rs6828768
rs8039785
rs11715853
rs78383039
rs7572229
rs6809952
rs55877868
rs2387977
rs10899911
rs823424
rs7303158
rs35055552
All
−0.2
−0.1
0.0
0.1
MR effect size for
'Gut microbiota abundance (genus Parasutterella id.2892)' on 'Rho−related GTP−binding protein Rho6'

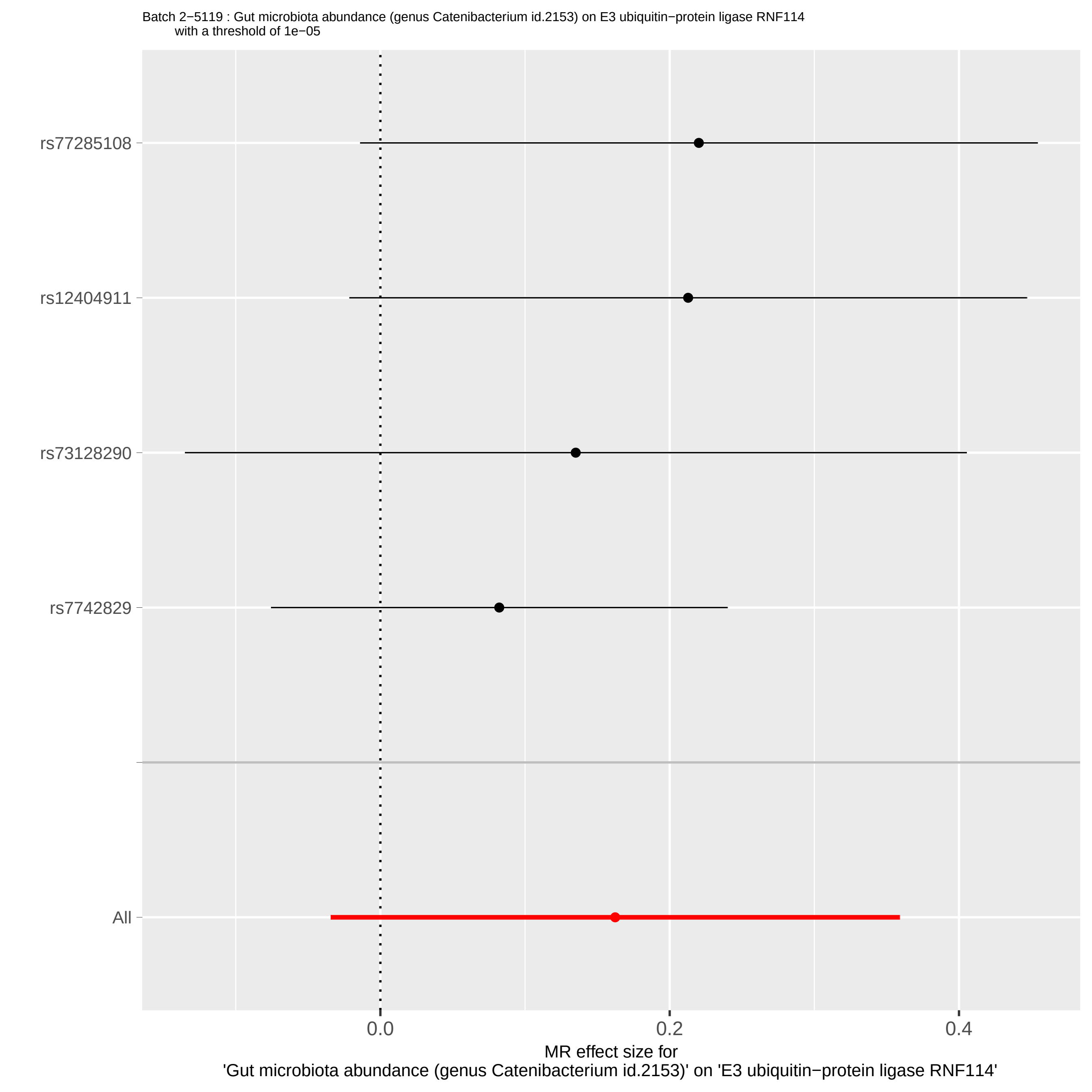
Batch 2−5119 : Gut microbiota abundance (genus Catenibacterium id.2153) on E3 ubiquitin−protein ligase RNF114
with a threshold of 1e−05
rs77285108
rs12404911
rs73128290
rs7742829
All
0.0
0.2
0.4
MR effect size for
'Gut microbiota abundance (genus Catenibacterium id.2153)' on 'E3 ubiquitin−protein ligase RNF114'

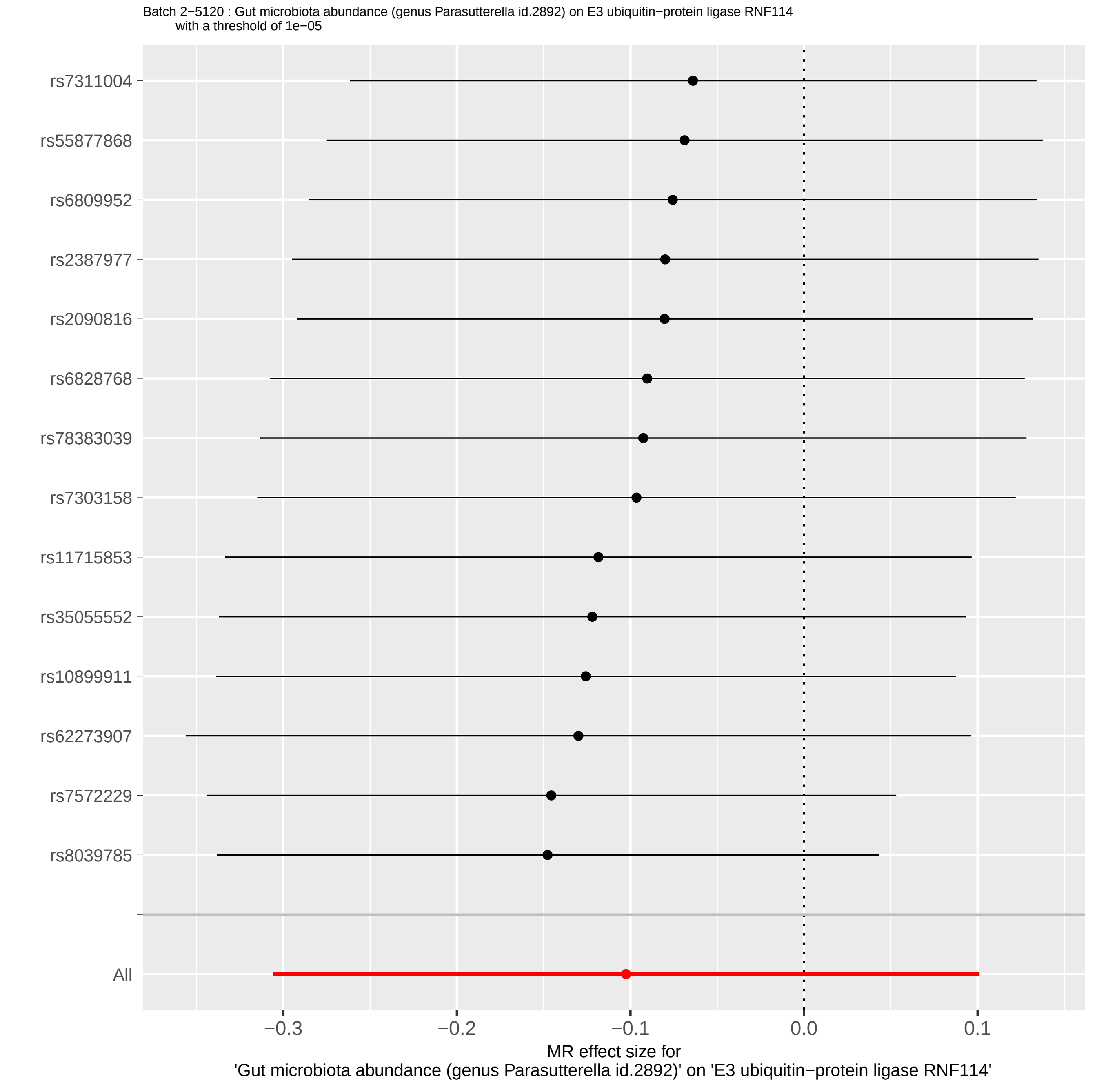
Batch 2−5120 : Gut microbiota abundance (genus Parasutterella id.2892) on E3 ubiquitin−protein ligase RNF114
with a threshold of 1e−05
rs7311004
rs55877868
rs6809952
rs2387977
rs2090816
rs6828768
rs78383039
rs7303158
rs11715853
rs35055552
rs10899911
rs62273907
rs7572229
rs8039785
All
−0.3
−0.2
−0.1
0.0
0.1
MR effect size for
'Gut microbiota abundance (genus Parasutterella id.2892)' on 'E3 ubiquitin−protein ligase RNF114'

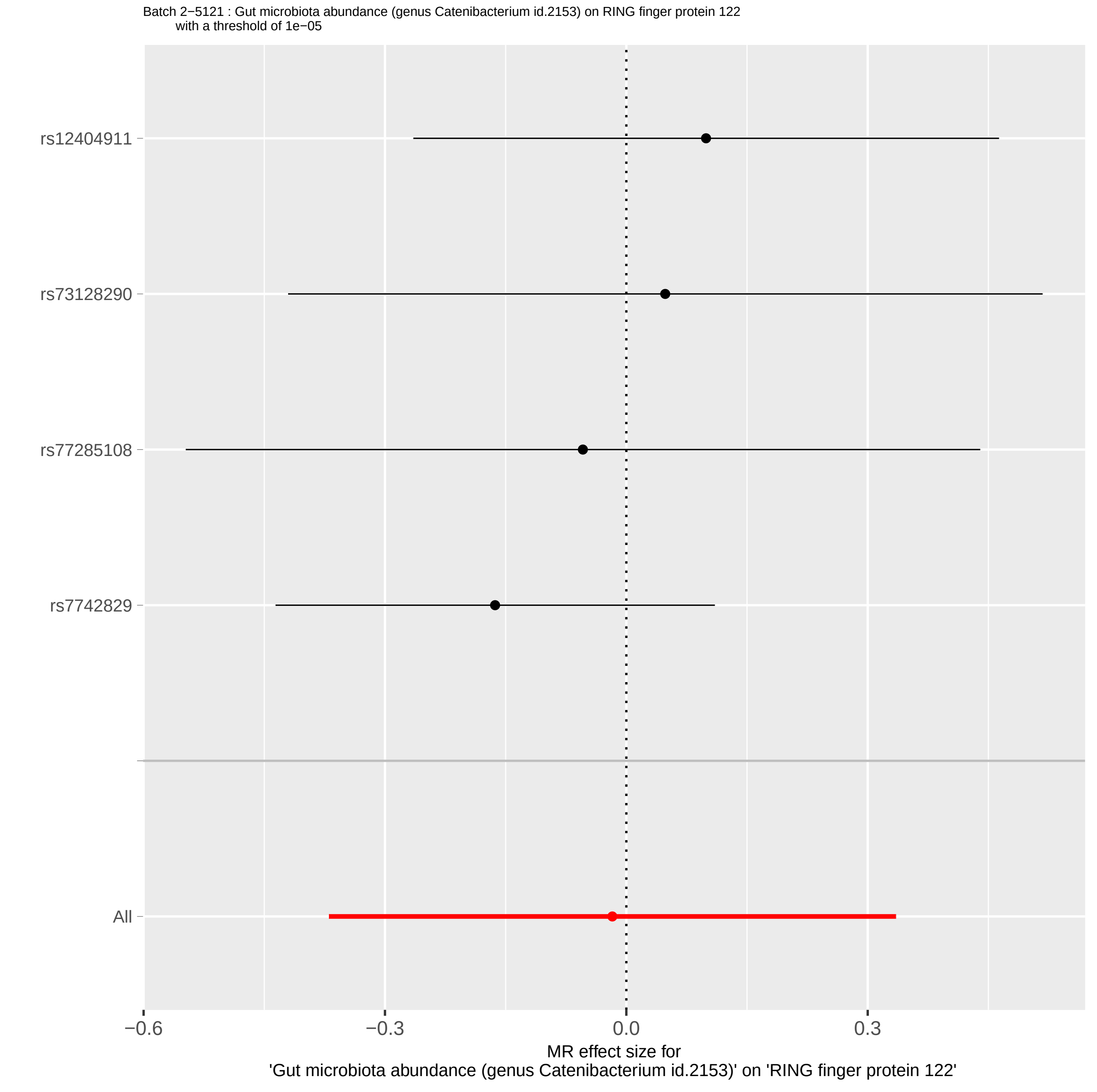

Batch 2−5121 : Gut microbiota abundance (genus Catenibacterium id.2153) on RING finger protein 122
with a threshold of 1e−05
rs12404911
rs73128290
rs77285108
rs7742829
All
−0.6
−0.3
0.0
0.3
MR effect size for
'Gut microbiota abundance (genus Catenibacterium id.2153)' on 'RING finger protein 122'

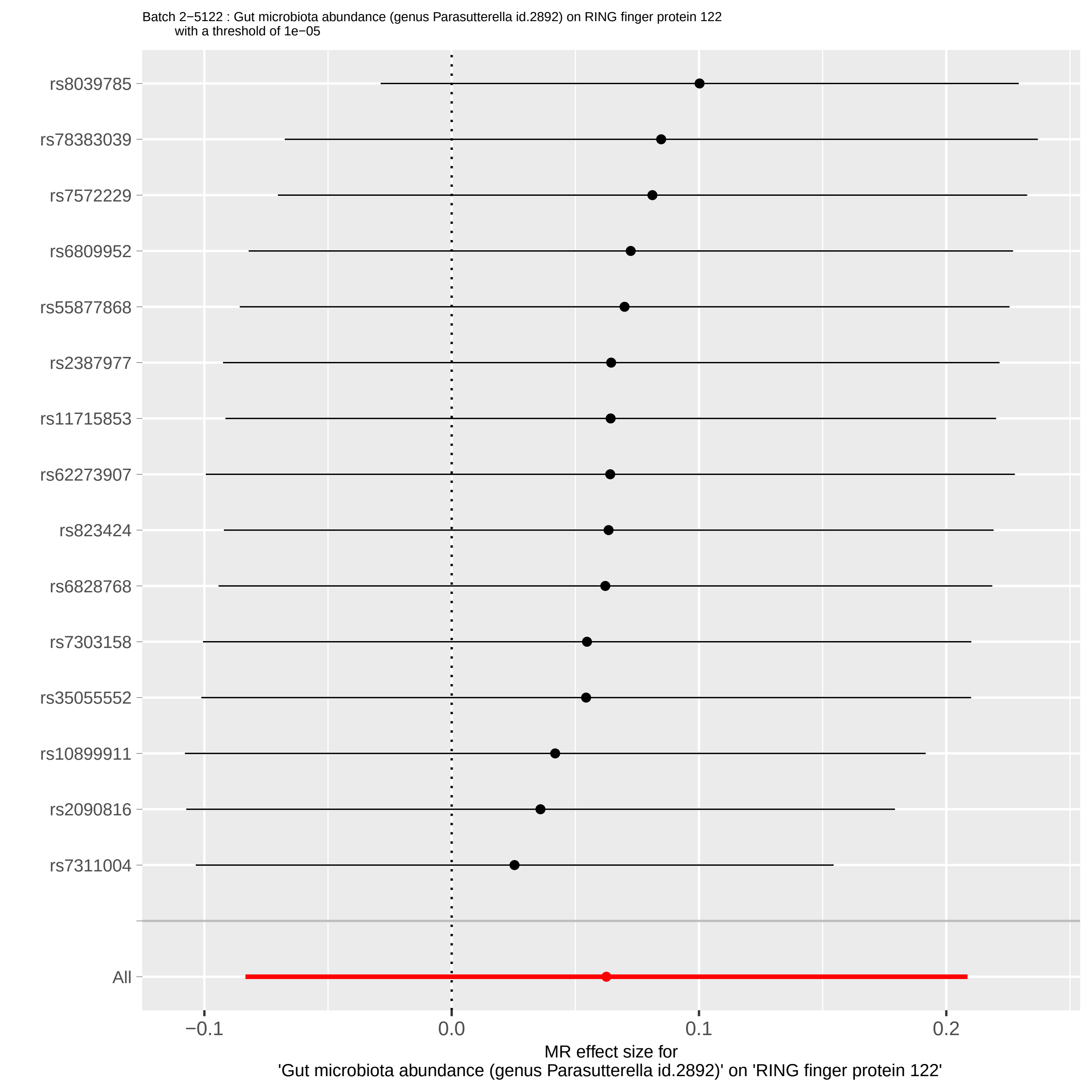
Batch 2−5122 : Gut microbiota abundance (genus Parasutterella id.2892) on RING finger protein 122
with a threshold of 1e−05
rs8039785
rs78383039
rs7572229
rs6809952
rs55877868
rs2387977
rs11715853
rs62273907
rs823424
rs6828768
rs7303158
rs35055552
rs10899911
rs2090816
rs7311004
All
−0.1
0.0
0.1
0.2
MR effect size for
'Gut microbiota abundance (genus Parasutterella id.2892)' on 'RING finger protein 122'

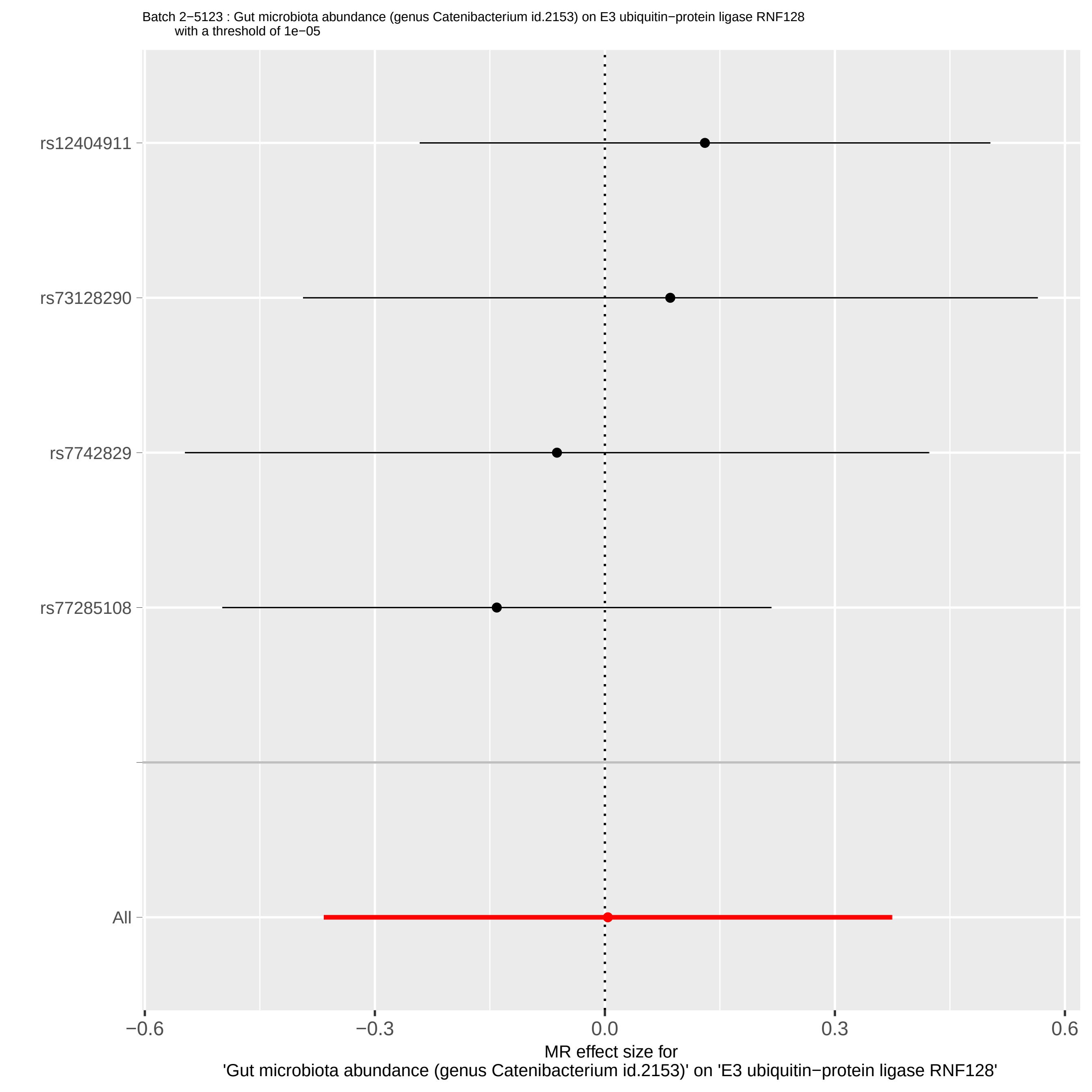
Batch 2−5123 : Gut microbiota abundance (genus Catenibacterium id.2153) on E3 ubiquitin−protein ligase RNF128
with a threshold of 1e−05
rs12404911
rs73128290
rs7742829
rs77285108
All
−0.6
−0.3
0.0
0.3
0.6
MR effect size for
'Gut microbiota abundance (genus Catenibacterium id.2153)' on 'E3 ubiquitin−protein ligase RNF128'

Batch 2−5124 : Gut microbiota abundance (genus Parasutterella id.2892) on E3 ubiquitin−protein ligase RNF128
with a threshold of 1e−05

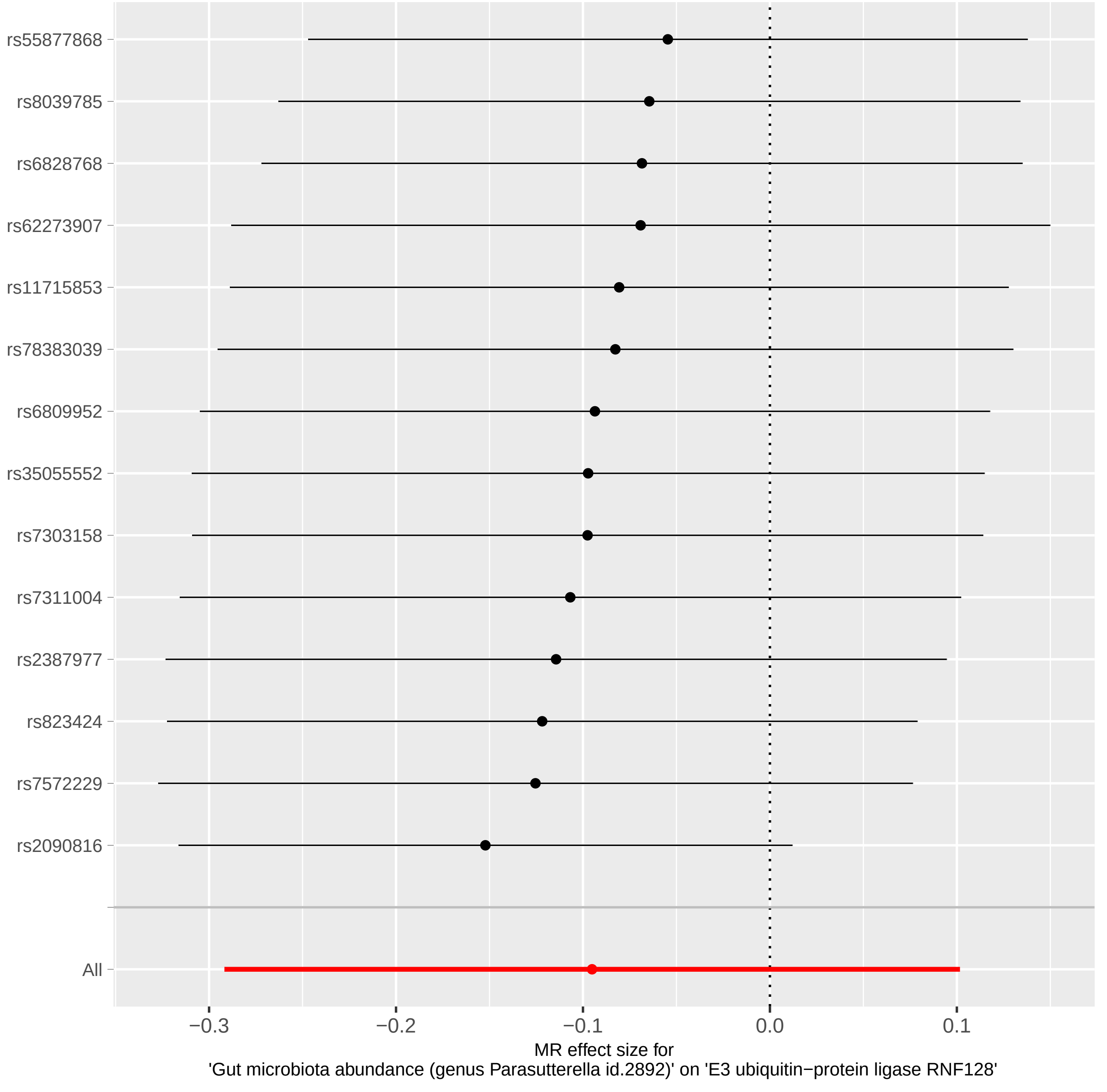

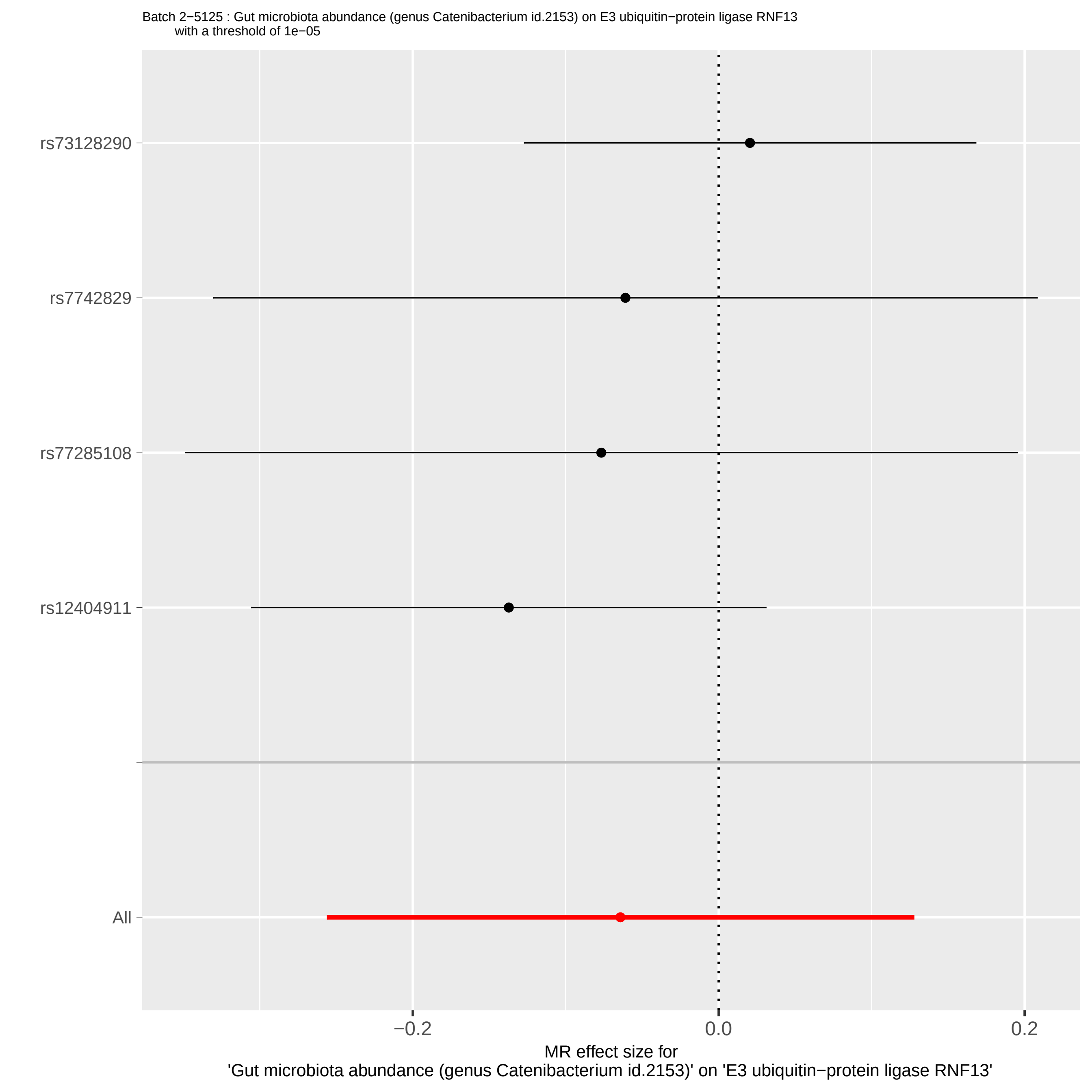
Batch 2−5125 : Gut microbiota abundance (genus Catenibacterium id.2153) on E3 ubiquitin−protein ligase RNF13
with a threshold of 1e−05
rs73128290
rs7742829
rs77285108
rs12404911
All
−0.2
0.0
0.2
MR effect size for
'Gut microbiota abundance (genus Catenibacterium id.2153)' on 'E3 ubiquitin−protein ligase RNF13'

Batch 2−5126 : Gut microbiota abundance (genus Parasutterella id.2892) on E3 ubiquitin−protein ligase RNF13
with a threshold of 1e−05

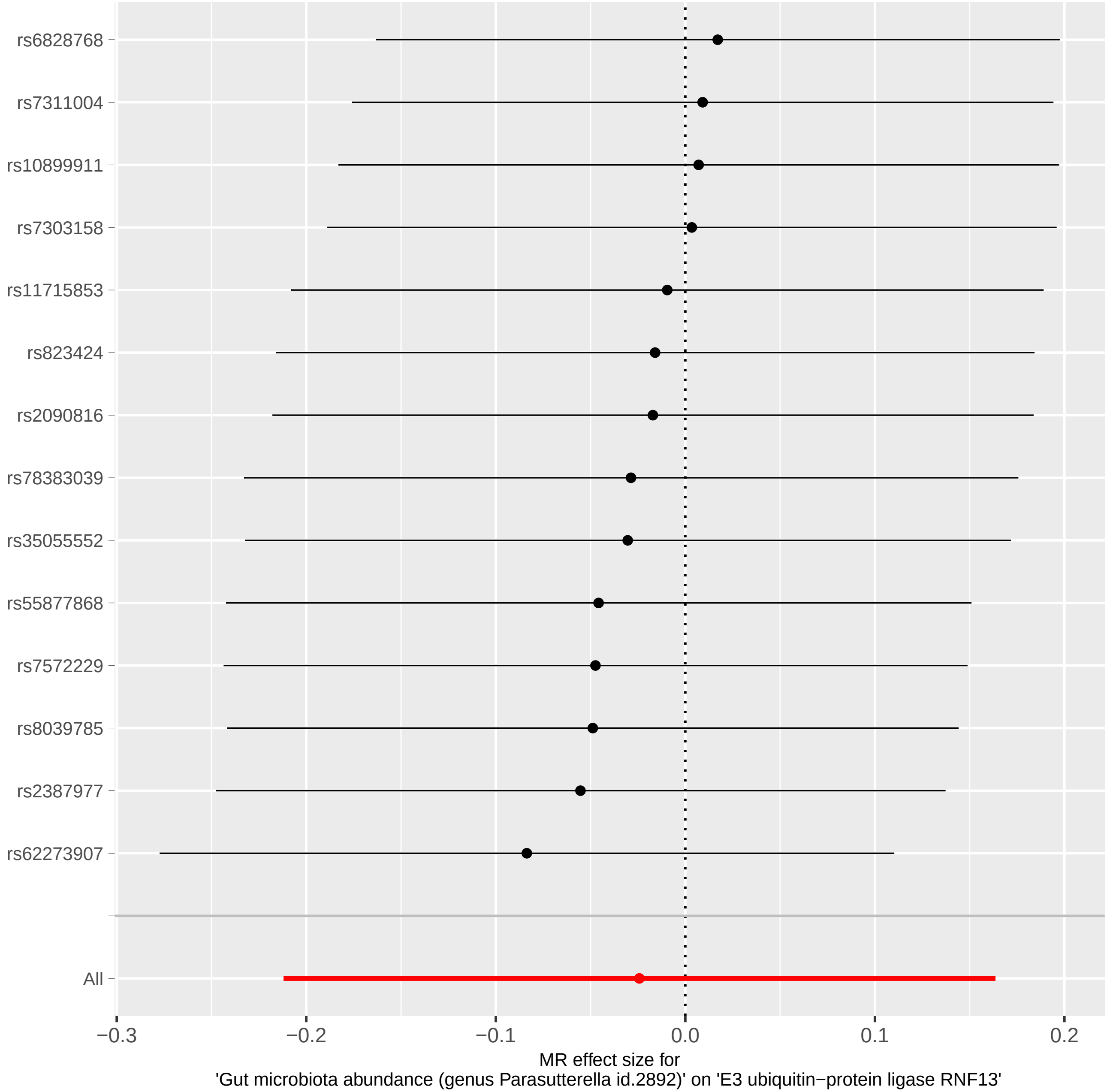

Batch 2−5127 : Gut microbiota abundance (genus Catenibacterium id.2153) on RING finger protein 148
with a threshold of 1e−05

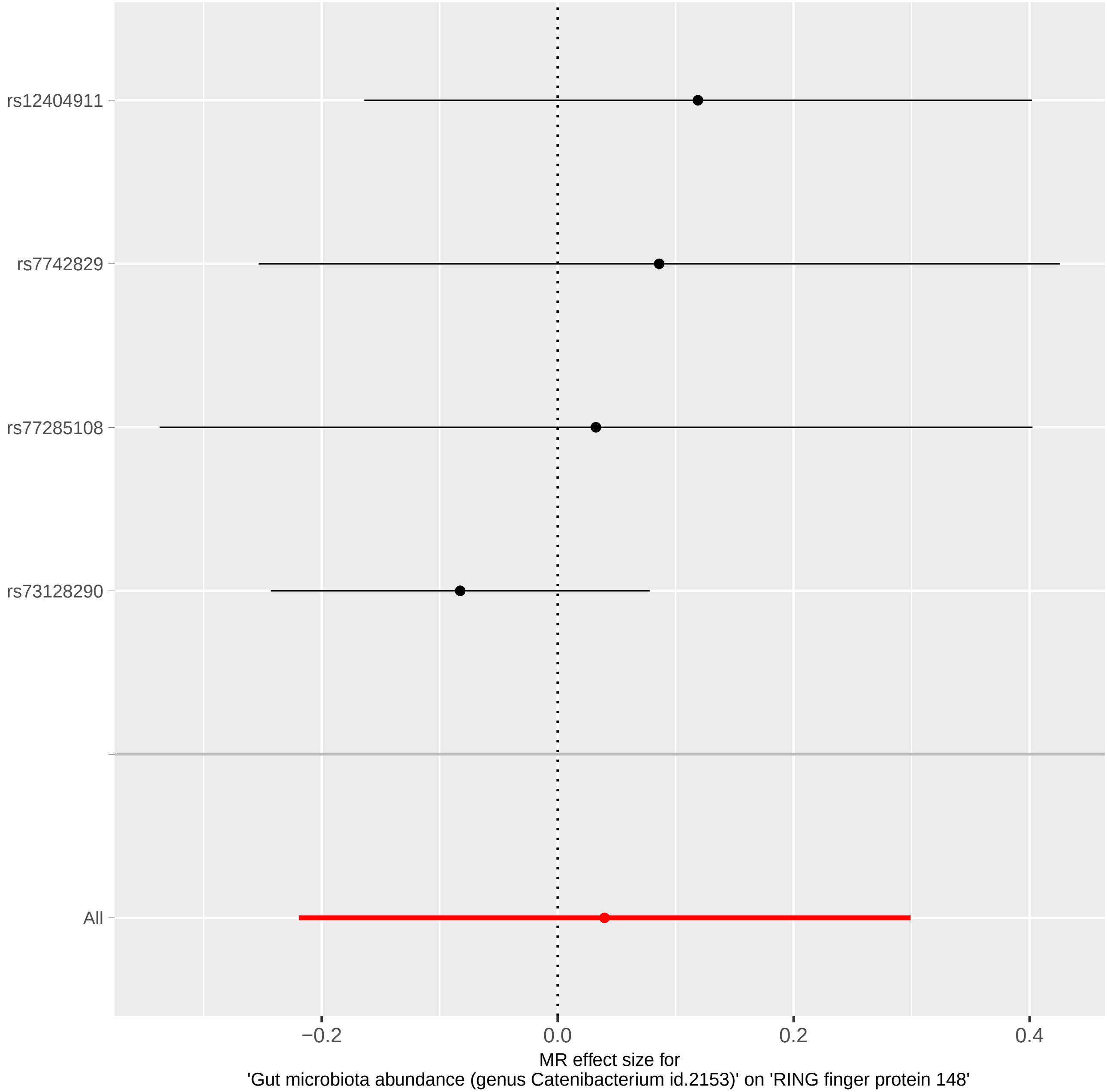

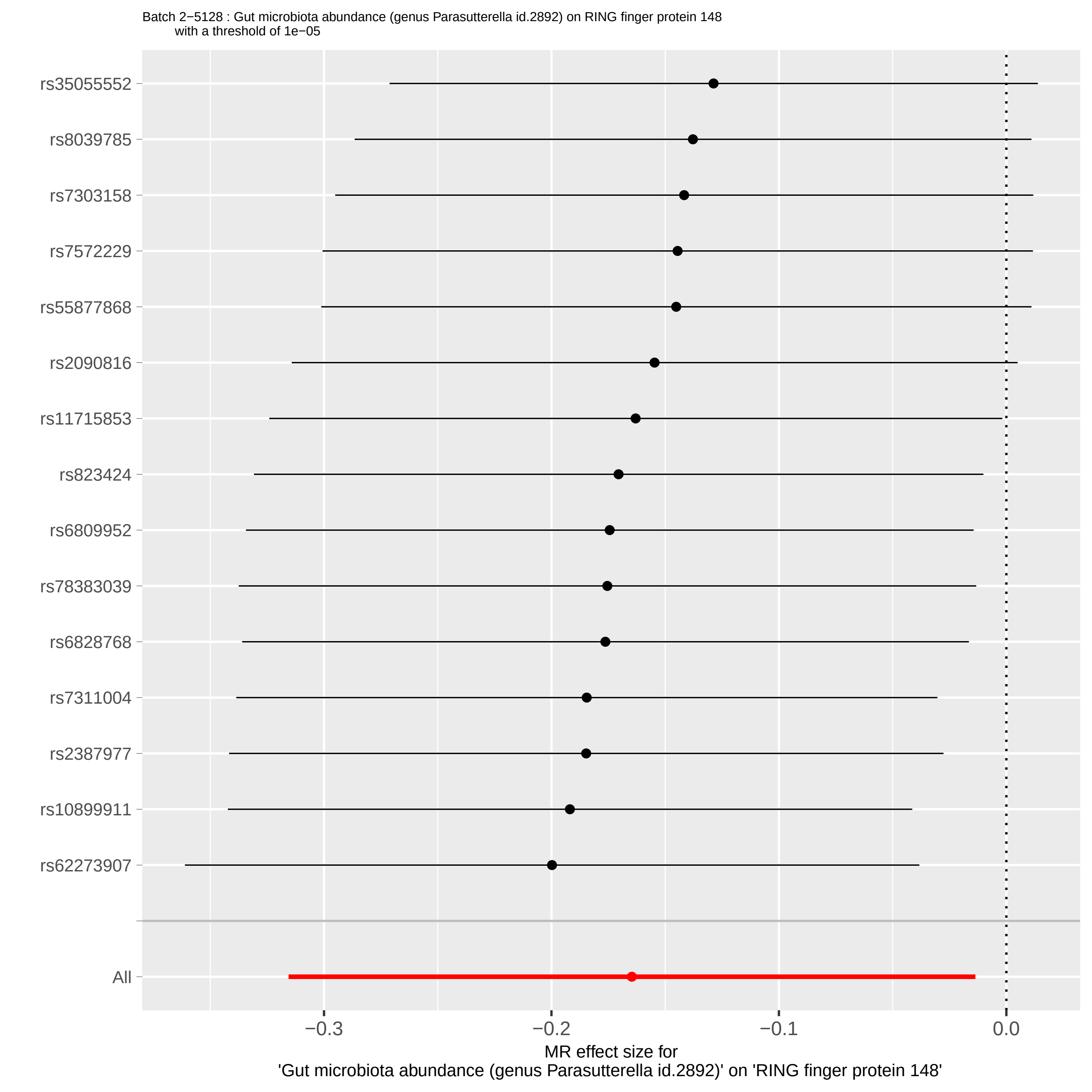
Batch 2−5128 : Gut microbiota abundance (genus Parasutterella id.2892) on RING finger protein 148
with a threshold of 1e−05
rs35055552
rs8039785
rs7303158
rs7572229
rs55877868
rs2090816
rs11715853
rs823424
rs6809952
rs78383039
rs6828768
rs7311004
rs2387977
rs10899911
rs62273907
All
−0.3
−0.2
−0.1
0.0
MR effect size for
'Gut microbiota abundance (genus Parasutterella id.2892)' on 'RING finger protein 148'

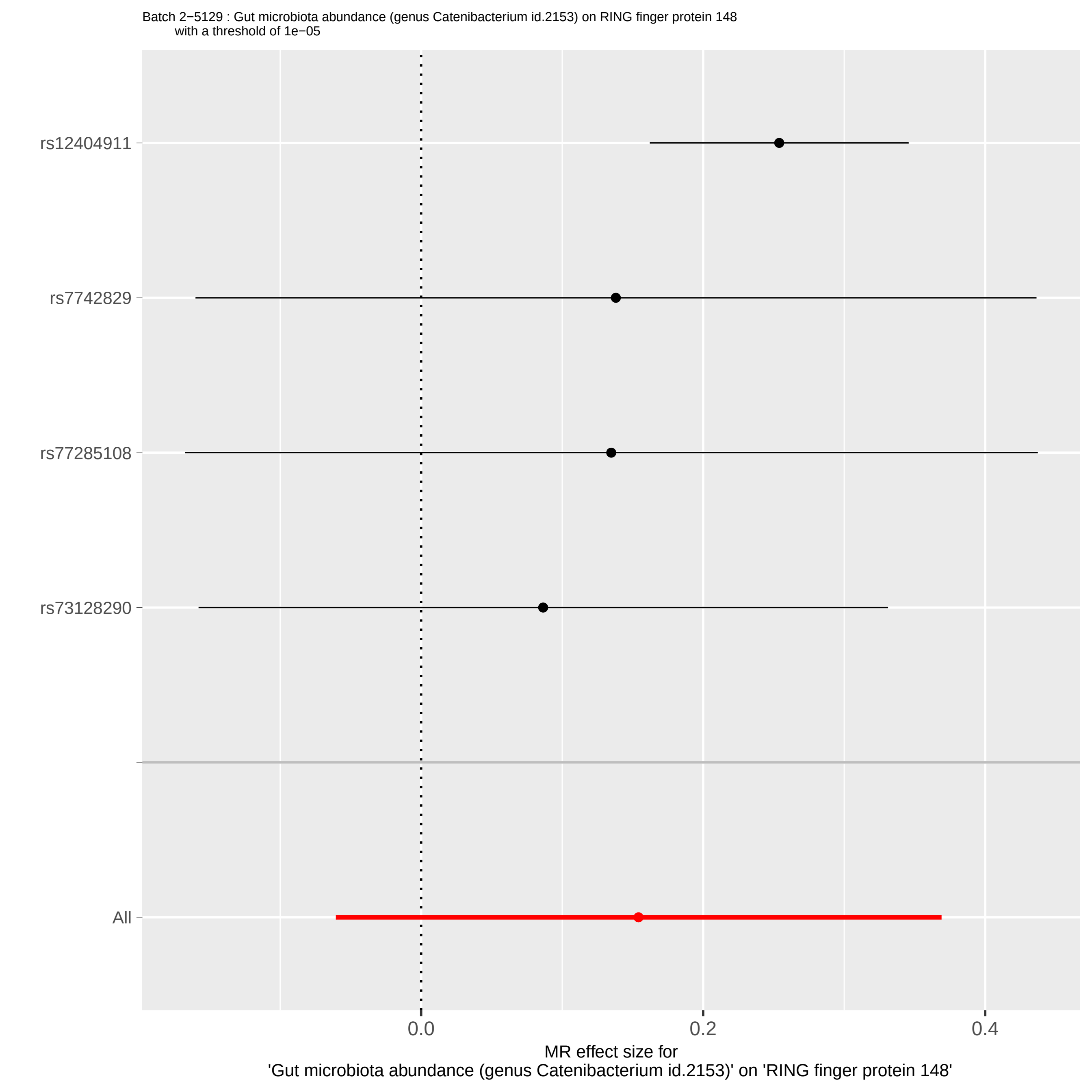
Batch 2−5129 : Gut microbiota abundance (genus Catenibacterium id.2153) on RING finger protein 148
with a threshold of 1e−05
rs12404911
rs7742829
rs77285108
rs73128290
All
0.0
0.2
0.4
MR effect size for
'Gut microbiota abundance (genus Catenibacterium id.2153)' on 'RING finger protein 148'

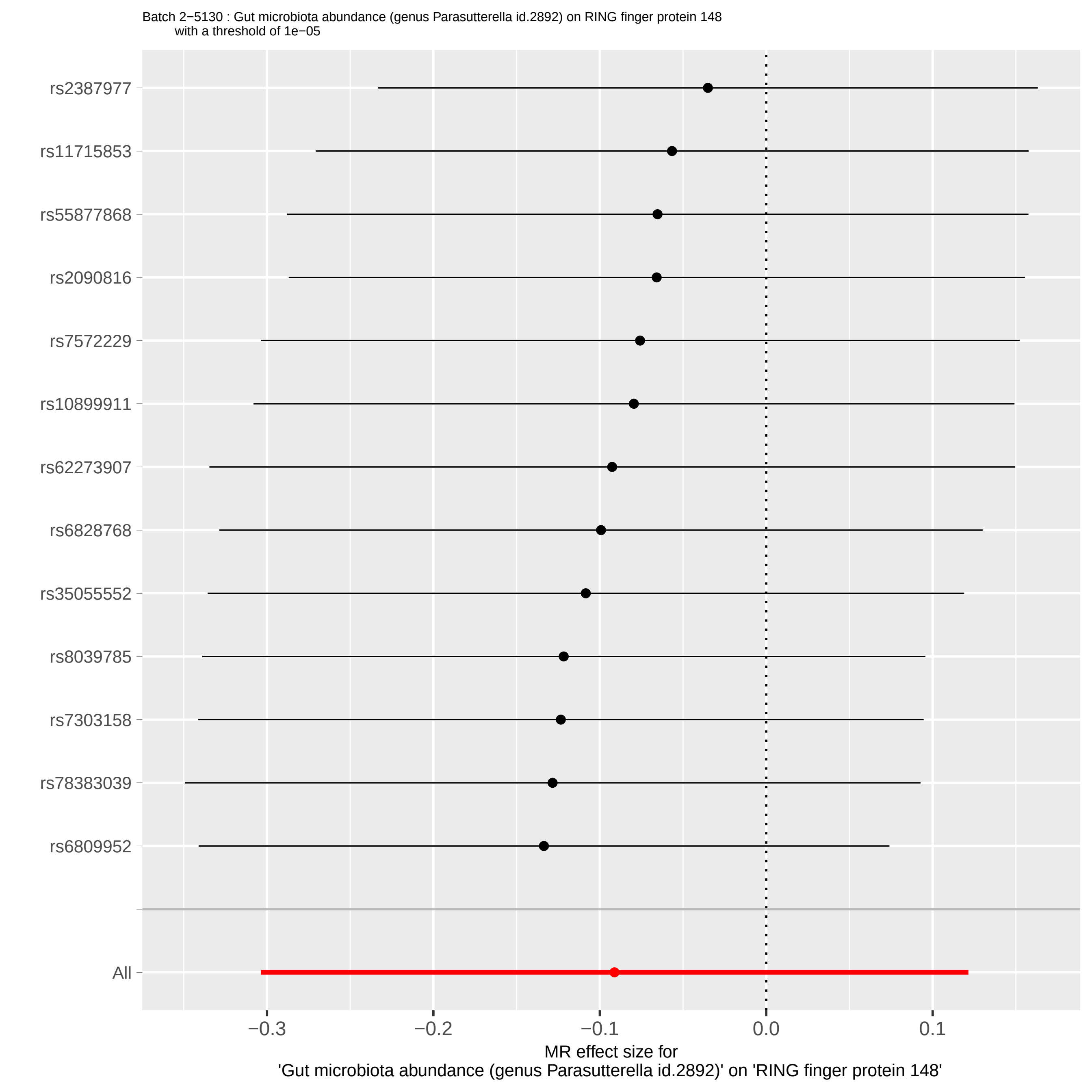
Batch 2−5130 : Gut microbiota abundance (genus Parasutterella id.2892) on RING finger protein 148
with a threshold of 1e−05
rs2387977
rs11715853
rs55877868
rs2090816
rs7572229
rs10899911
rs62273907
rs6828768
rs35055552
rs8039785
rs7303158
rs78383039
rs6809952
All
−0.3
−0.2
−0.1
0.0
0.1
MR effect size for
'Gut microbiota abundance (genus Parasutterella id.2892)' on 'RING finger protein 148'

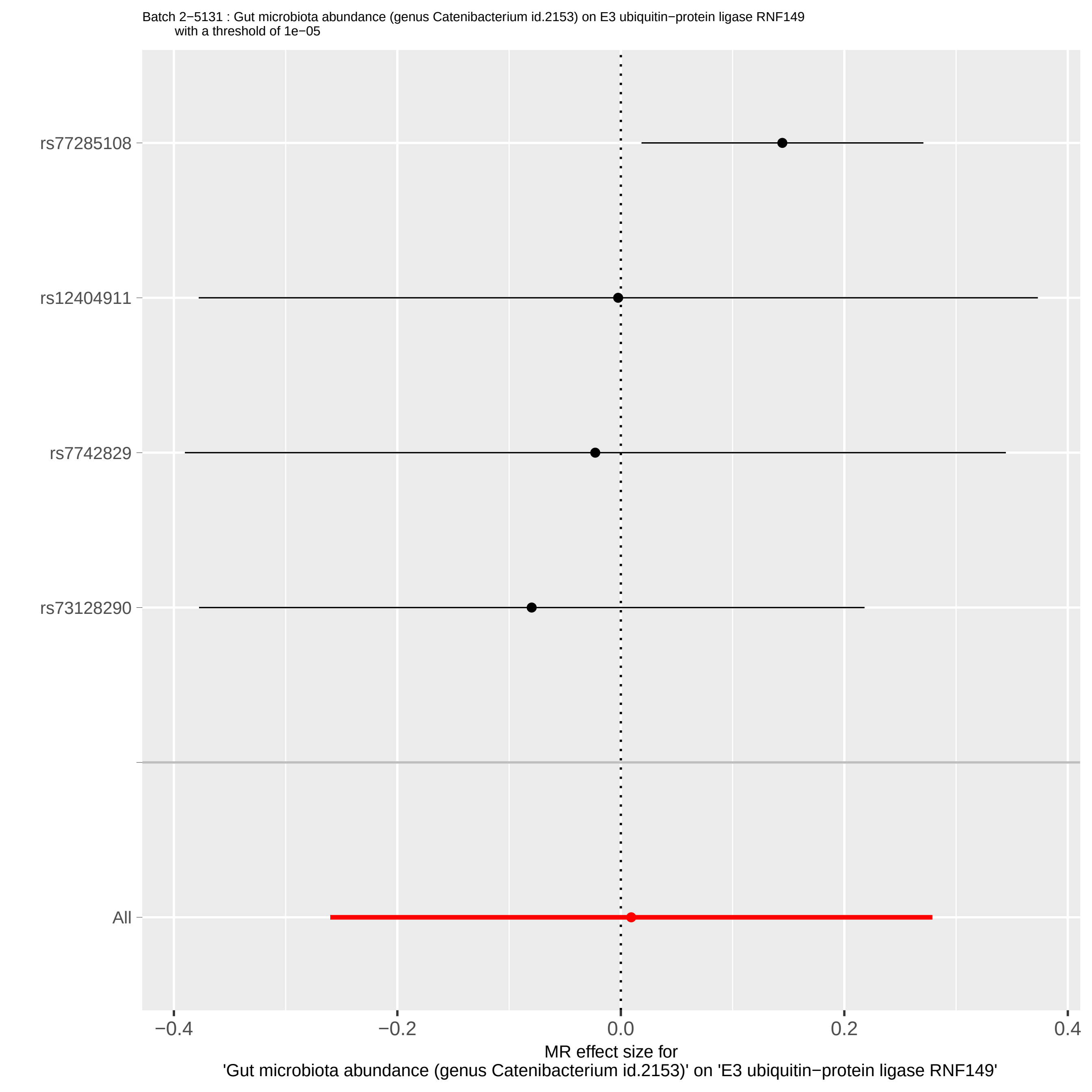
Batch 2−5131 : Gut microbiota abundance (genus Catenibacterium id.2153) on E3 ubiquitin−protein ligase RNF149
with a threshold of 1e−05
rs77285108
rs12404911
rs7742829
rs73128290
All
−0.4
−0.2
0.0
0.2
0.4
MR effect size for
'Gut microbiota abundance (genus Catenibacterium id.2153)' on 'E3 ubiquitin−protein ligase RNF149'

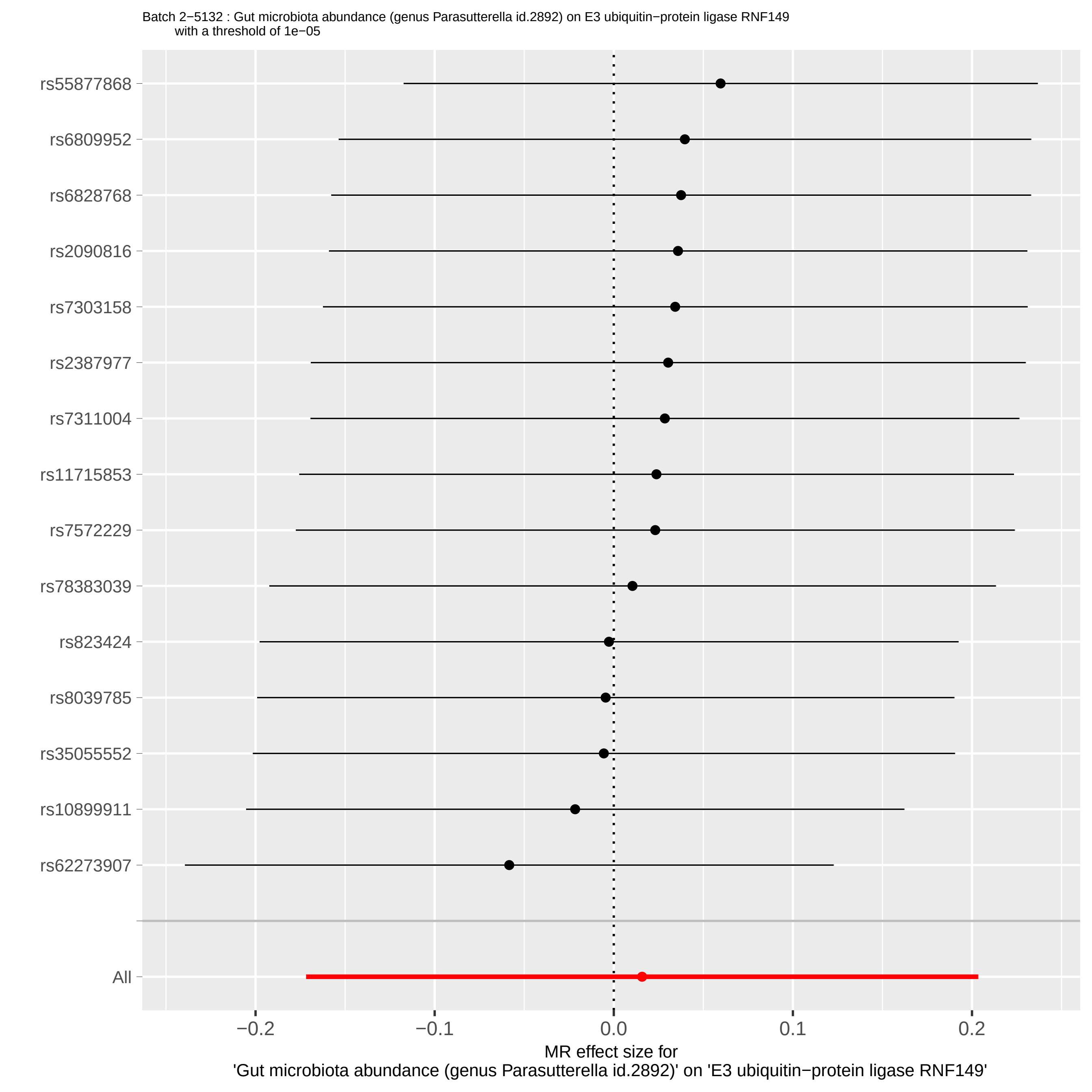
Batch 2−5132 : Gut microbiota abundance (genus Parasutterella id.2892) on E3 ubiquitin−protein ligase RNF149
with a threshold of 1e−05
rs55877868
rs6809952
rs6828768
rs2090816
rs7303158
rs2387977
rs7311004
rs11715853
rs7572229
rs78383039
rs823424
rs8039785
rs35055552
rs10899911
rs62273907
All
−0.2
−0.1
0.0
0.1
0.2
MR effect size for
'Gut microbiota abundance (genus Parasutterella id.2892)' on 'E3 ubiquitin−protein ligase RNF149'

Batch 2−5133 : Gut microbiota abundance (genus Catenibacterium id.2153) on RING finger protein 150
with a threshold of 1e−05

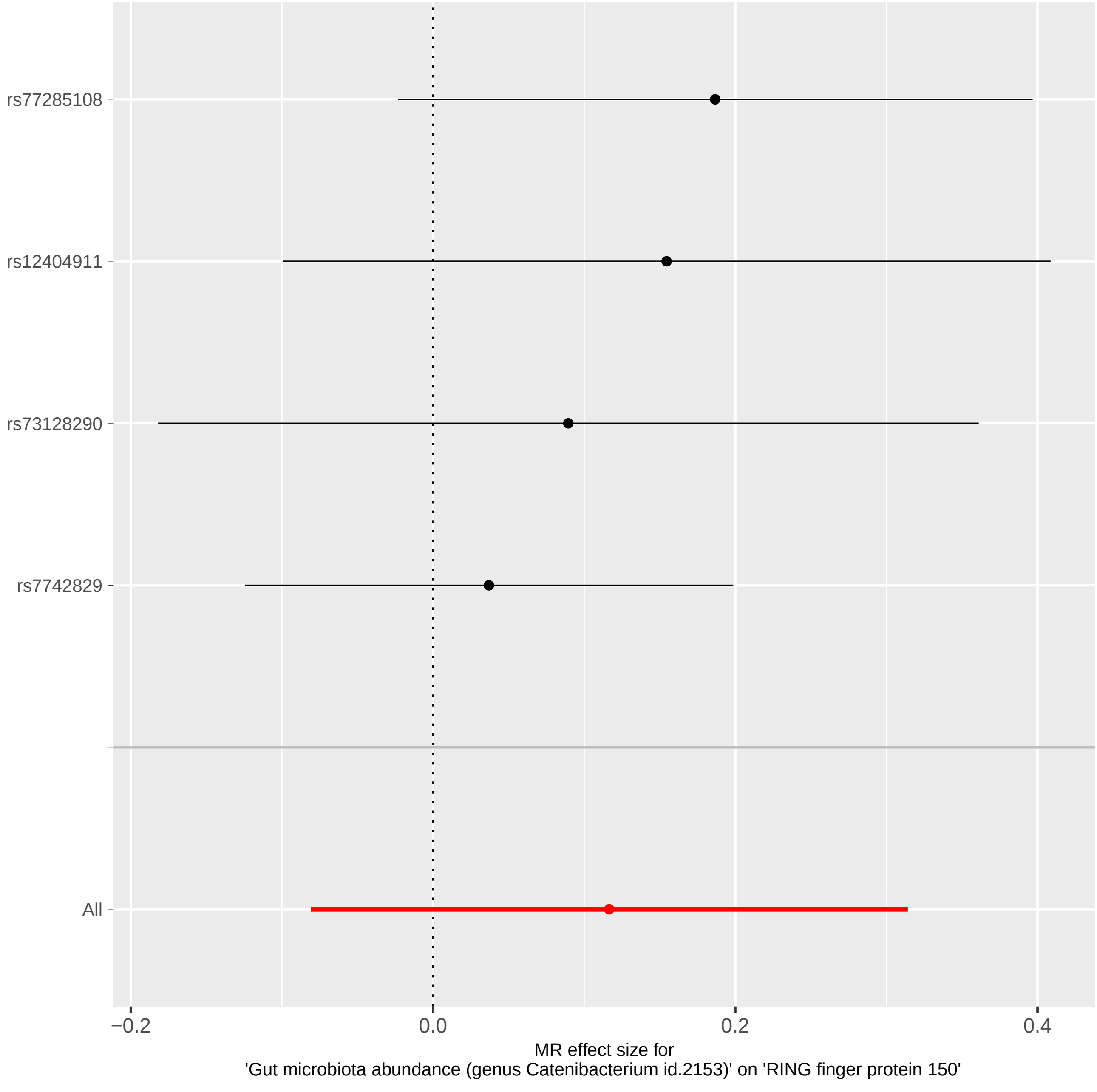

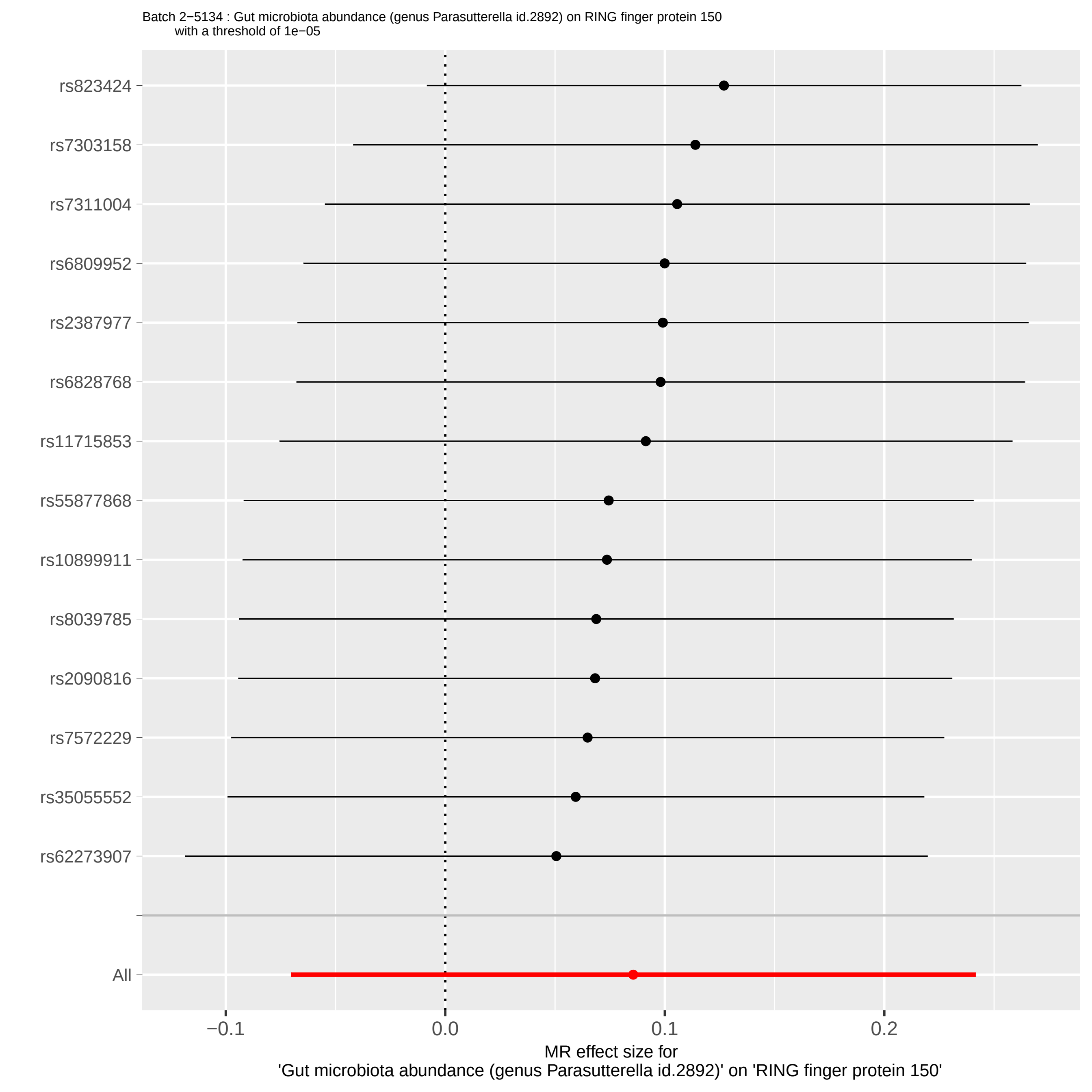

Batch 2−5134 : Gut microbiota abundance (genus Parasutterella id.2892) on RING finger protein 150
with a threshold of 1e−05
rs823424
rs7303158
rs7311004
rs6809952
rs2387977
rs6828768
rs11715853
rs55877868
rs10899911
rs8039785
rs2090816
rs7572229
rs35055552
rs62273907
All
−0.1
0.0
0.1
0.2
MR effect size for
'Gut microbiota abundance (genus Parasutterella id.2892)' on 'RING finger protein 150'

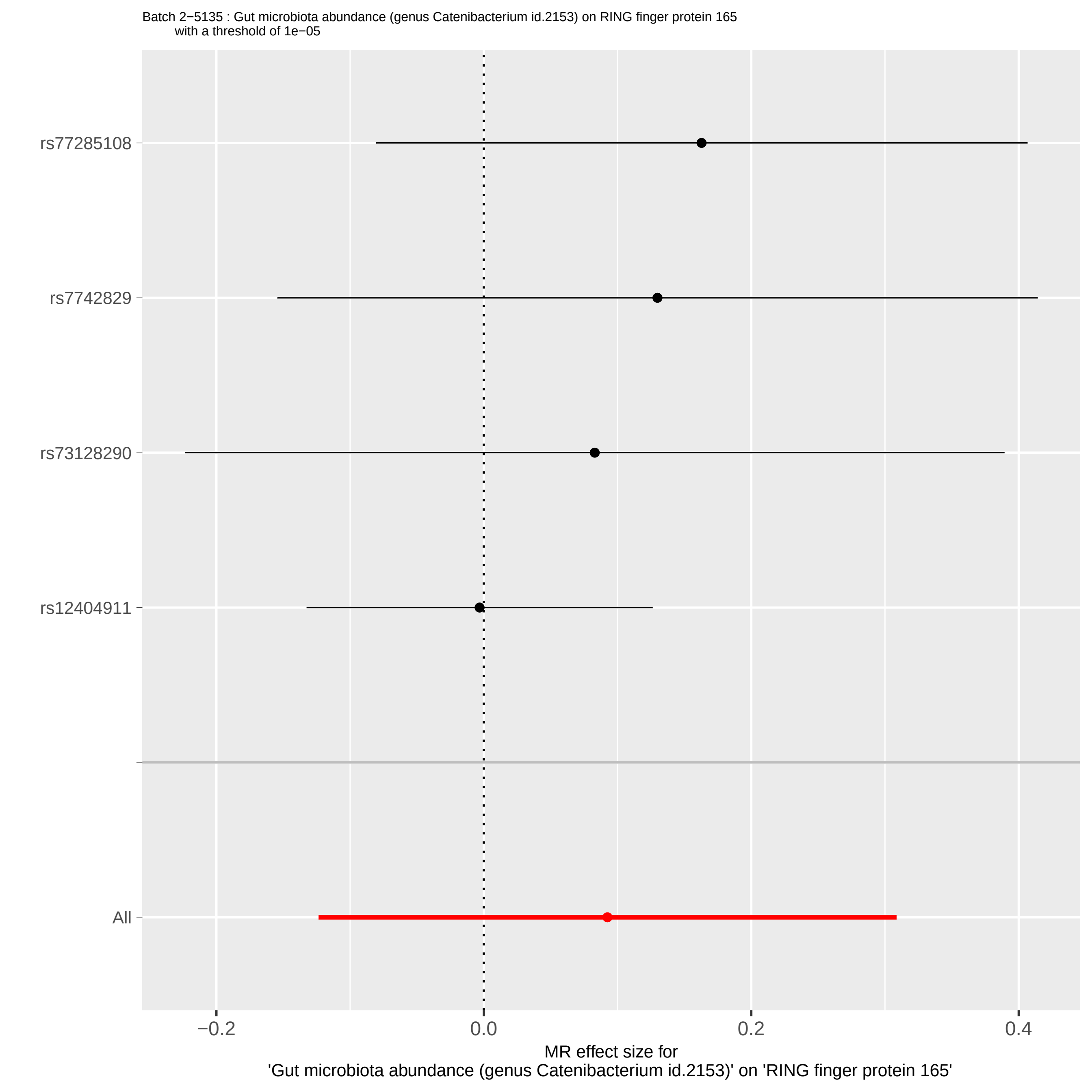
Batch 2−5135 : Gut microbiota abundance (genus Catenibacterium id.2153) on RING finger protein 165
with a threshold of 1e−05
rs77285108
rs7742829
rs73128290
rs12404911
All
−0.2
0.0
0.2
0.4
MR effect size for
'Gut microbiota abundance (genus Catenibacterium id.2153)' on 'RING finger protein 165'

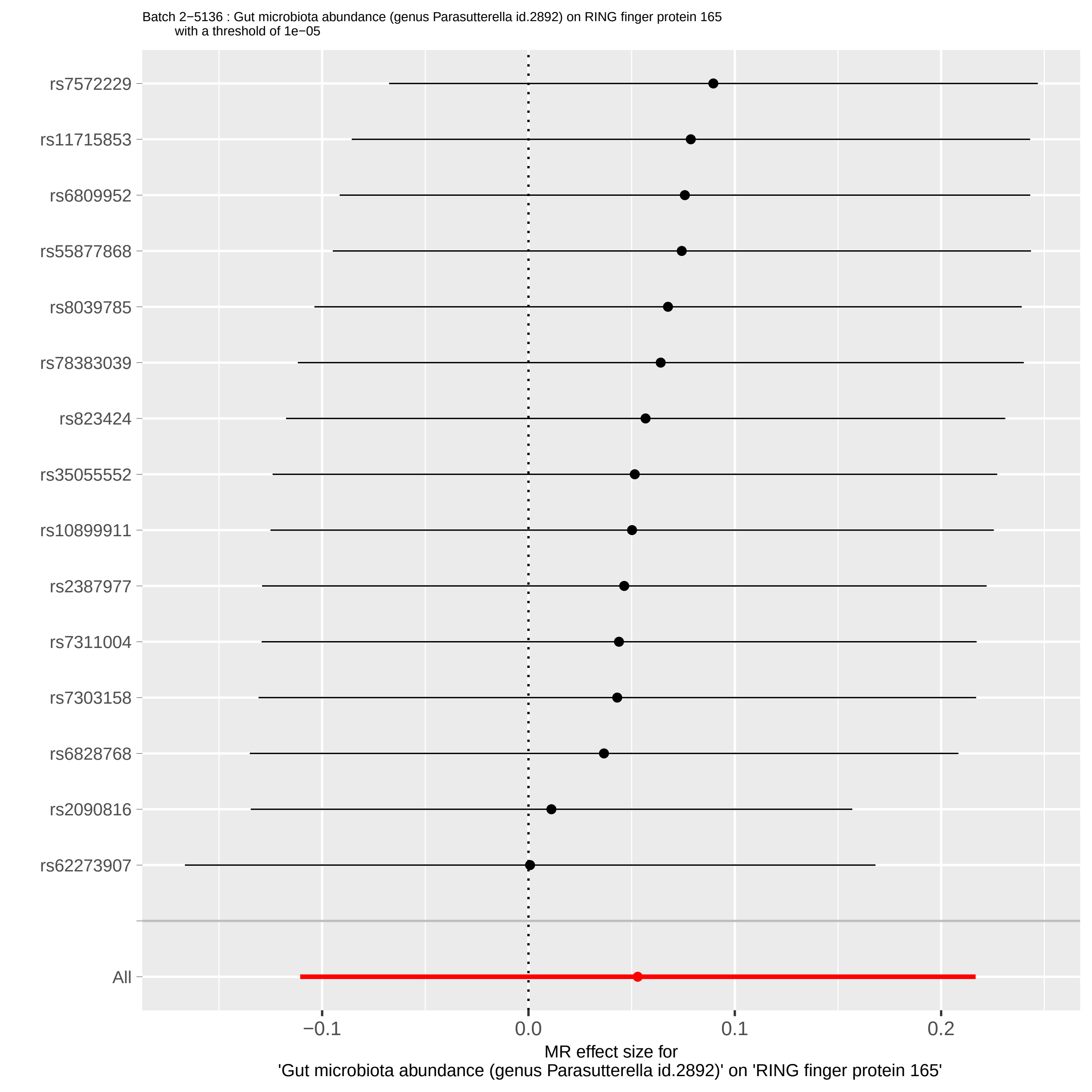
Batch 2−5136 : Gut microbiota abundance (genus Parasutterella id.2892) on RING finger protein 165
with a threshold of 1e−05
rs7572229
rs11715853
rs6809952
rs55877868
rs8039785
rs78383039
rs823424
rs35055552
rs10899911
rs2387977
rs7311004
rs7303158
rs6828768
rs2090816
rs62273907
All
−0.1
0.0
0.1
0.2
MR effect size for
'Gut microbiota abundance (genus Parasutterella id.2892)' on 'RING finger protein 165'

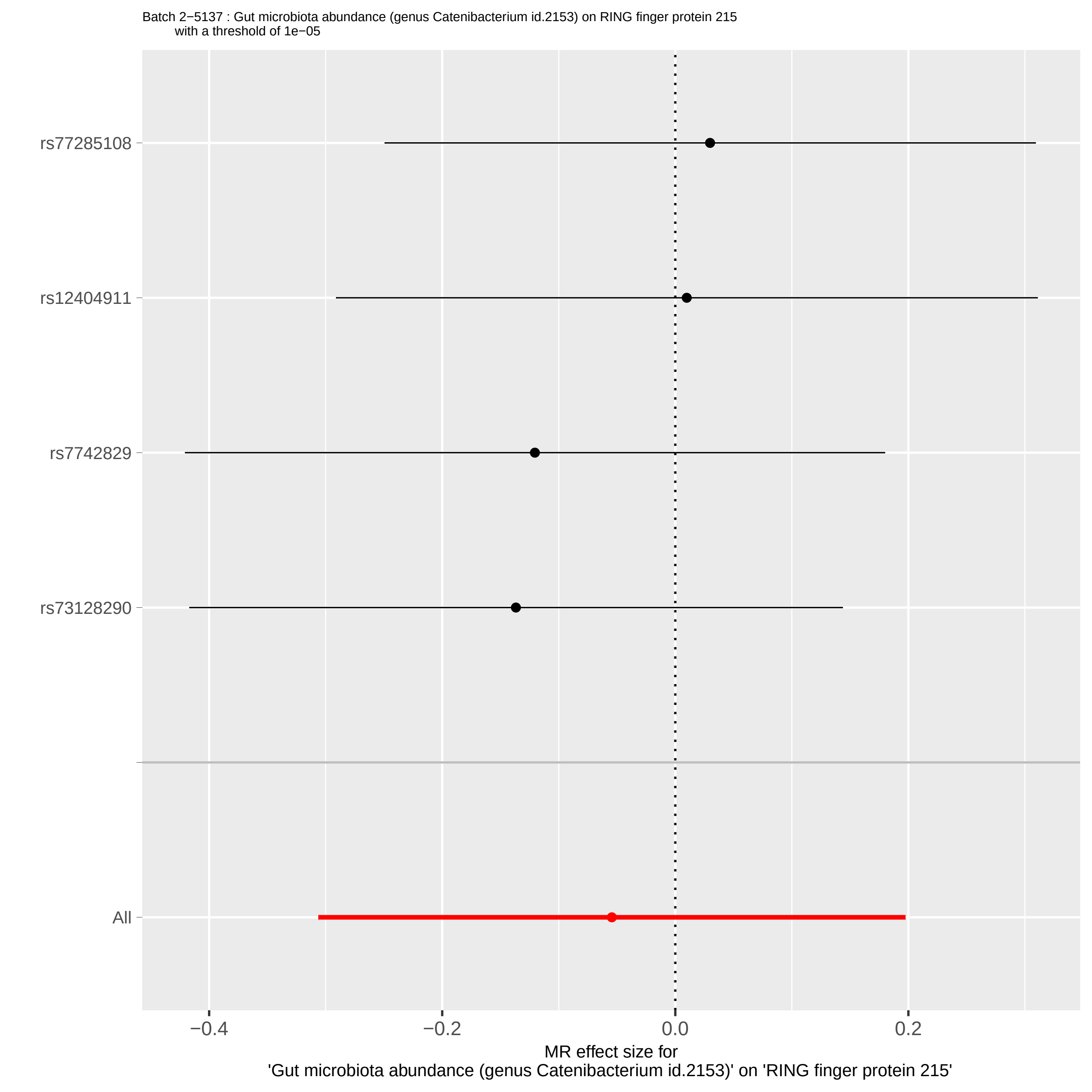
Batch 2−5137 : Gut microbiota abundance (genus Catenibacterium id.2153) on RING finger protein 215
with a threshold of 1e−05
rs77285108
rs12404911
rs7742829
rs73128290
All
−0.4
−0.2
0.0
0.2
MR effect size for
'Gut microbiota abundance (genus Catenibacterium id.2153)' on 'RING finger protein 215'

Batch 2−5138 : Gut microbiota abundance (genus Parasutterella id.2892) on RING finger protein 215
with a threshold of 1e−05

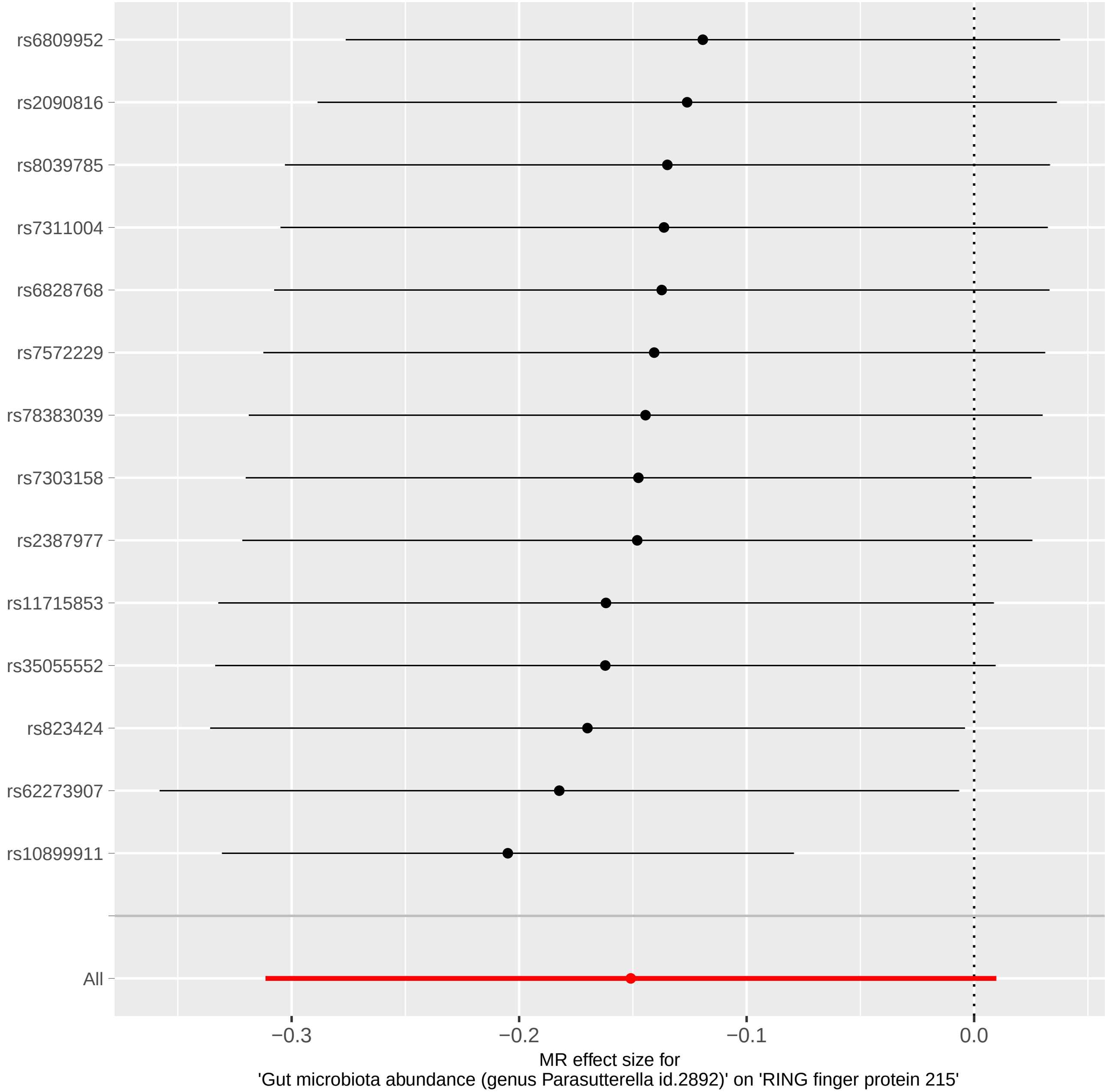

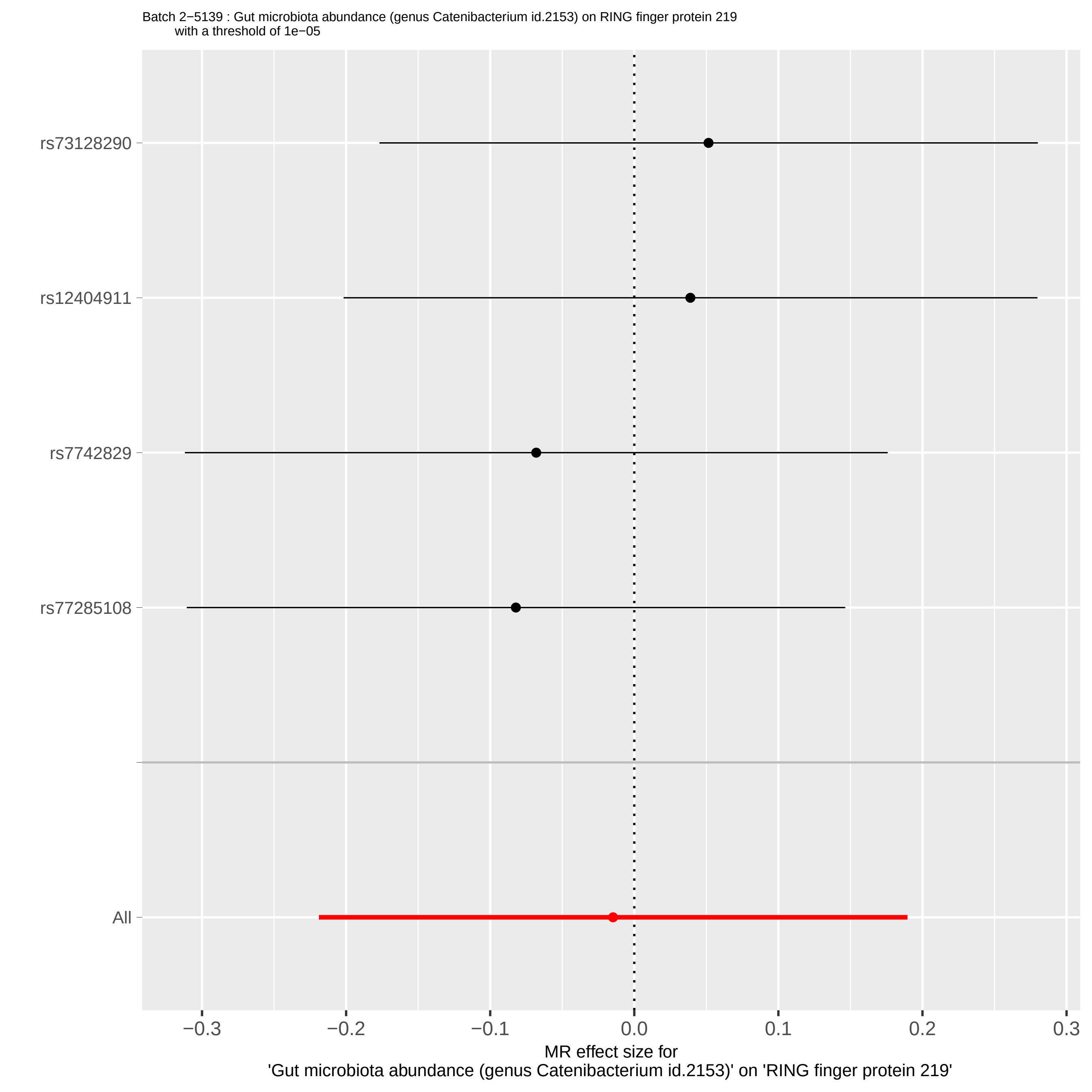

Batch 2−5139 : Gut microbiota abundance (genus Catenibacterium id.2153) on RING finger protein 219
with a threshold of 1e−05
rs73128290
rs12404911
rs7742829
rs77285108
All
−0.3
−0.2
−0.1
0.0
0.1
0.2
0.3
MR effect size for
'Gut microbiota abundance (genus Catenibacterium id.2153)' on 'RING finger protein 219'

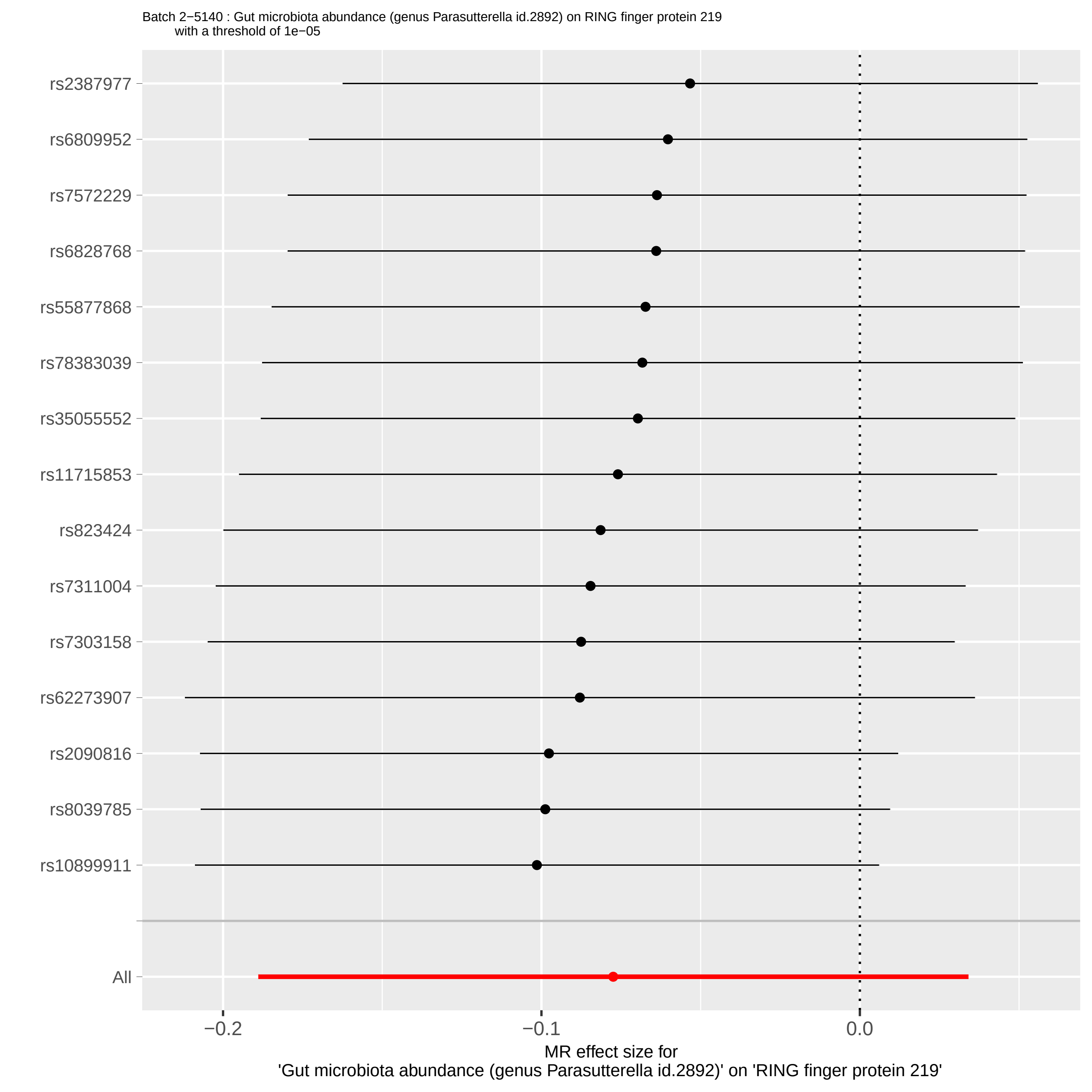
Batch 2−5140 : Gut microbiota abundance (genus Parasutterella id.2892) on RING finger protein 219
with a threshold of 1e−05
rs2387977
rs6809952
rs7572229
rs6828768
rs55877868
rs78383039
rs35055552
rs11715853
rs823424
rs7311004
rs7303158
rs62273907
rs2090816
rs8039785
rs10899911
All
−0.2
−0.1
0.0
MR effect size for
'Gut microbiota abundance (genus Parasutterella id.2892)' on 'RING finger protein 219'

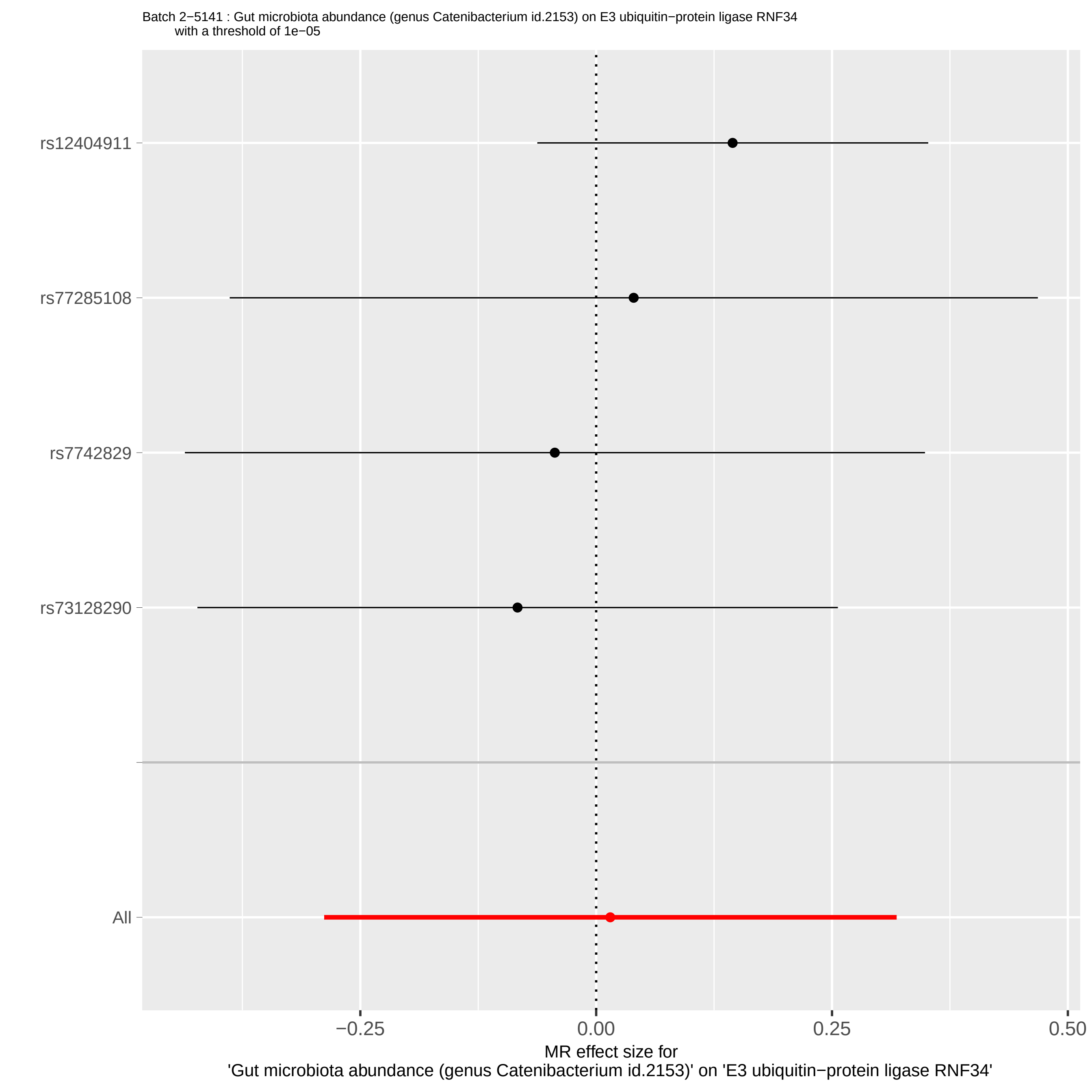
Batch 2−5141 : Gut microbiota abundance (genus Catenibacterium id.2153) on E3 ubiquitin−protein ligase RNF34
with a threshold of 1e−05
rs12404911
rs77285108
rs7742829
rs73128290
All
−0.25
0.00
0.25
0.50
MR effect size for
'Gut microbiota abundance (genus Catenibacterium id.2153)' on 'E3 ubiquitin−protein ligase RNF34'

Batch 2−5142 : Gut microbiota abundance (genus Parasutterella id.2892) on E3 ubiquitin−protein ligase RNF34
with a threshold of 1e−05

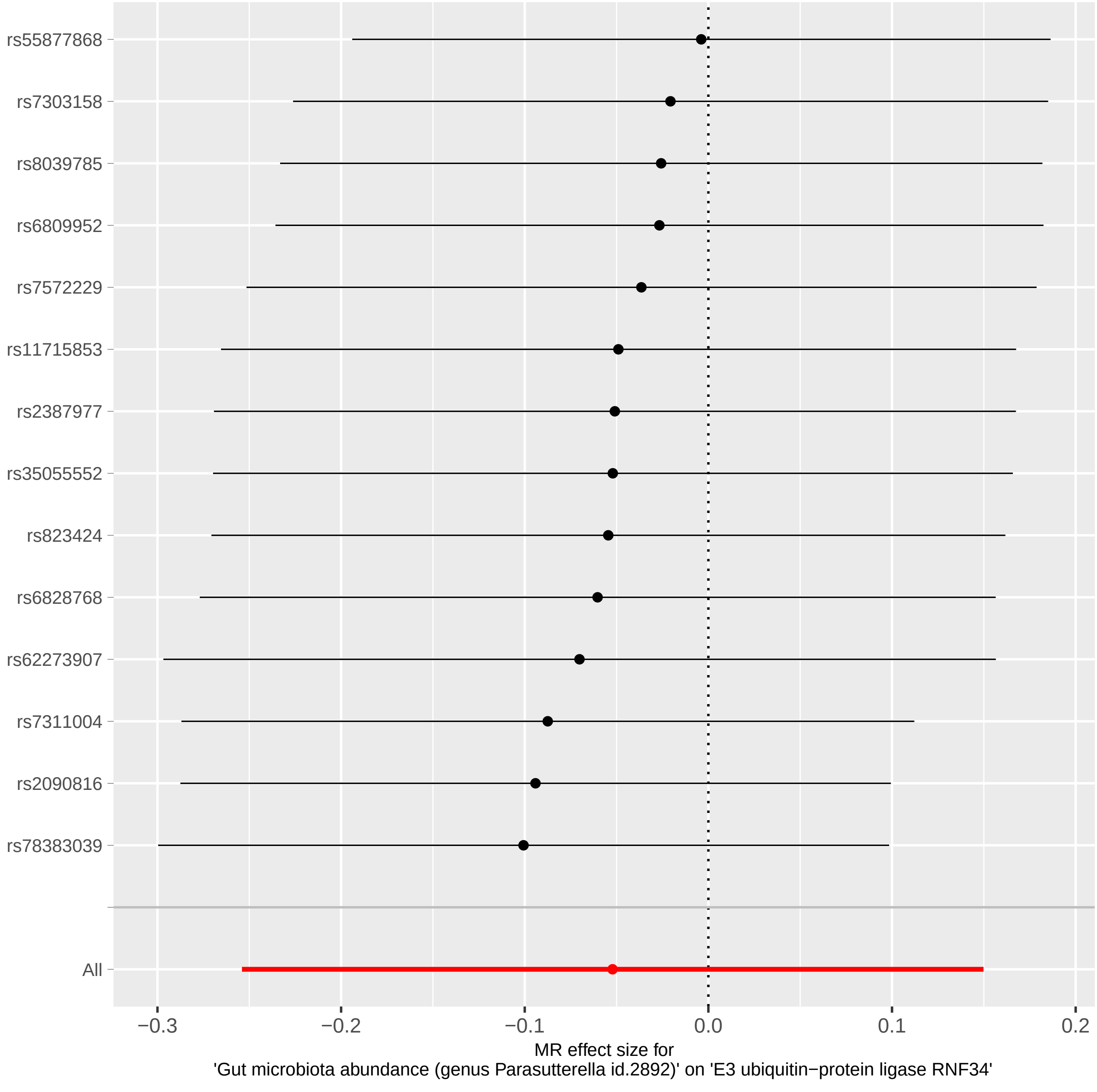

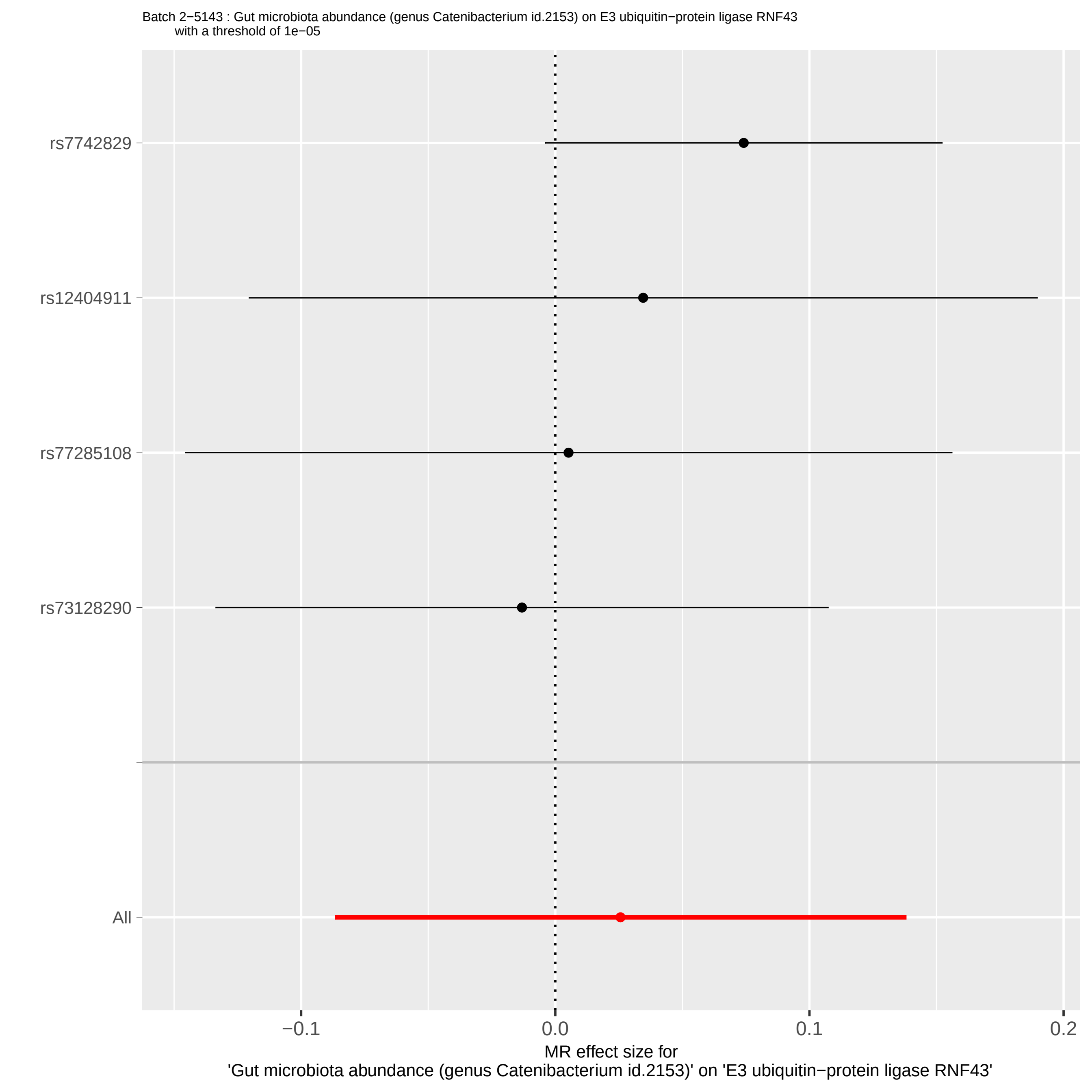
Batch 2−5143 : Gut microbiota abundance (genus Catenibacterium id.2153) on E3 ubiquitin−protein ligase RNF43
with a threshold of 1e−05
rs7742829
rs12404911
rs77285108
rs73128290
All
−0.1
0.0
0.1
0.2
MR effect size for
'Gut microbiota abundance (genus Catenibacterium id.2153)' on 'E3 ubiquitin−protein ligase RNF43'

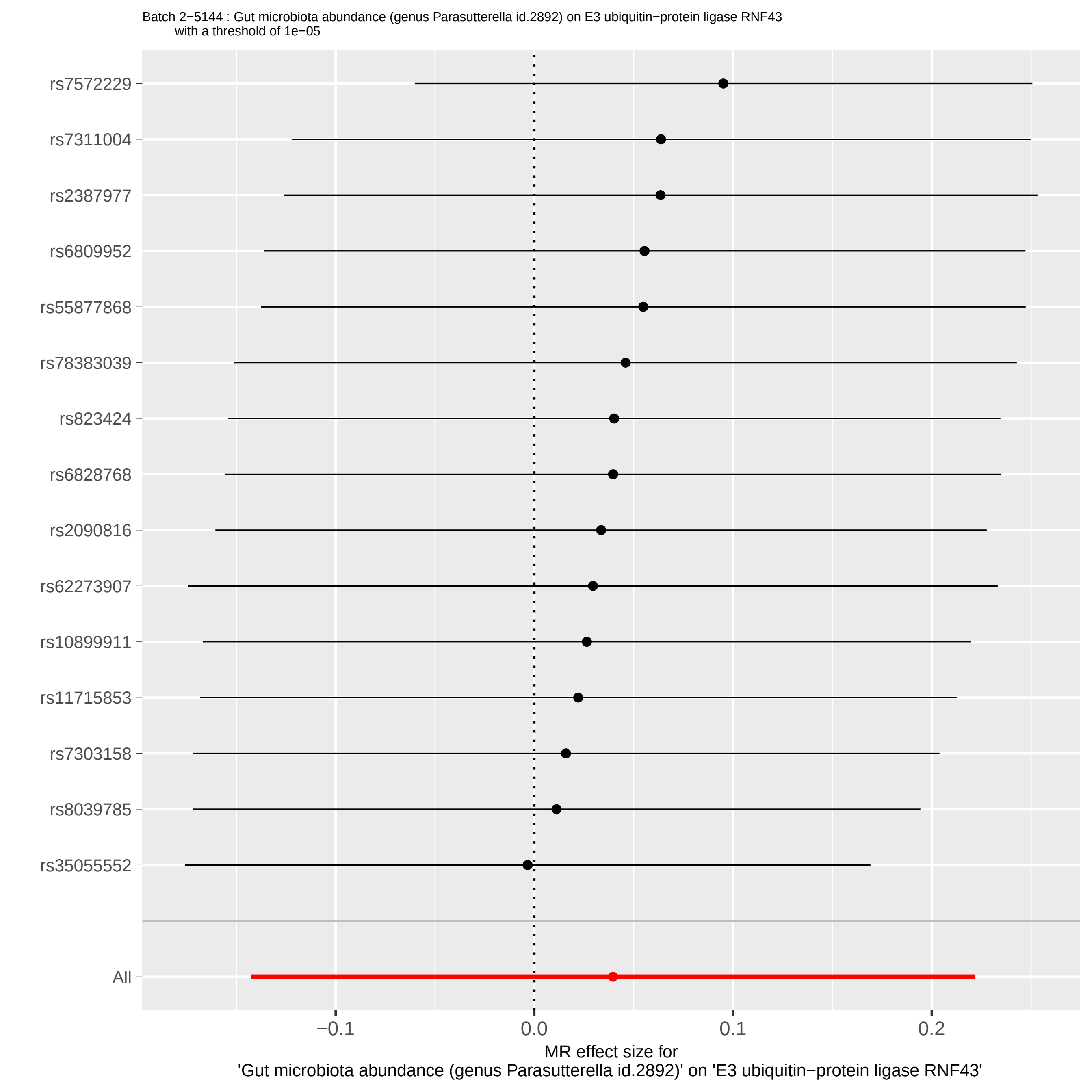
Batch 2−5144 : Gut microbiota abundance (genus Parasutterella id.2892) on E3 ubiquitin−protein ligase RNF43
with a threshold of 1e−05
rs7572229
rs7311004
rs2387977
rs6809952
rs55877868
rs78383039
rs823424
rs6828768
rs2090816
rs62273907
rs10899911
rs11715853
rs7303158
rs8039785
rs35055552
All
−0.1
0.0
0.1
0.2
MR effect size for
'Gut microbiota abundance (genus Parasutterella id.2892)' on 'E3 ubiquitin−protein ligase RNF43'

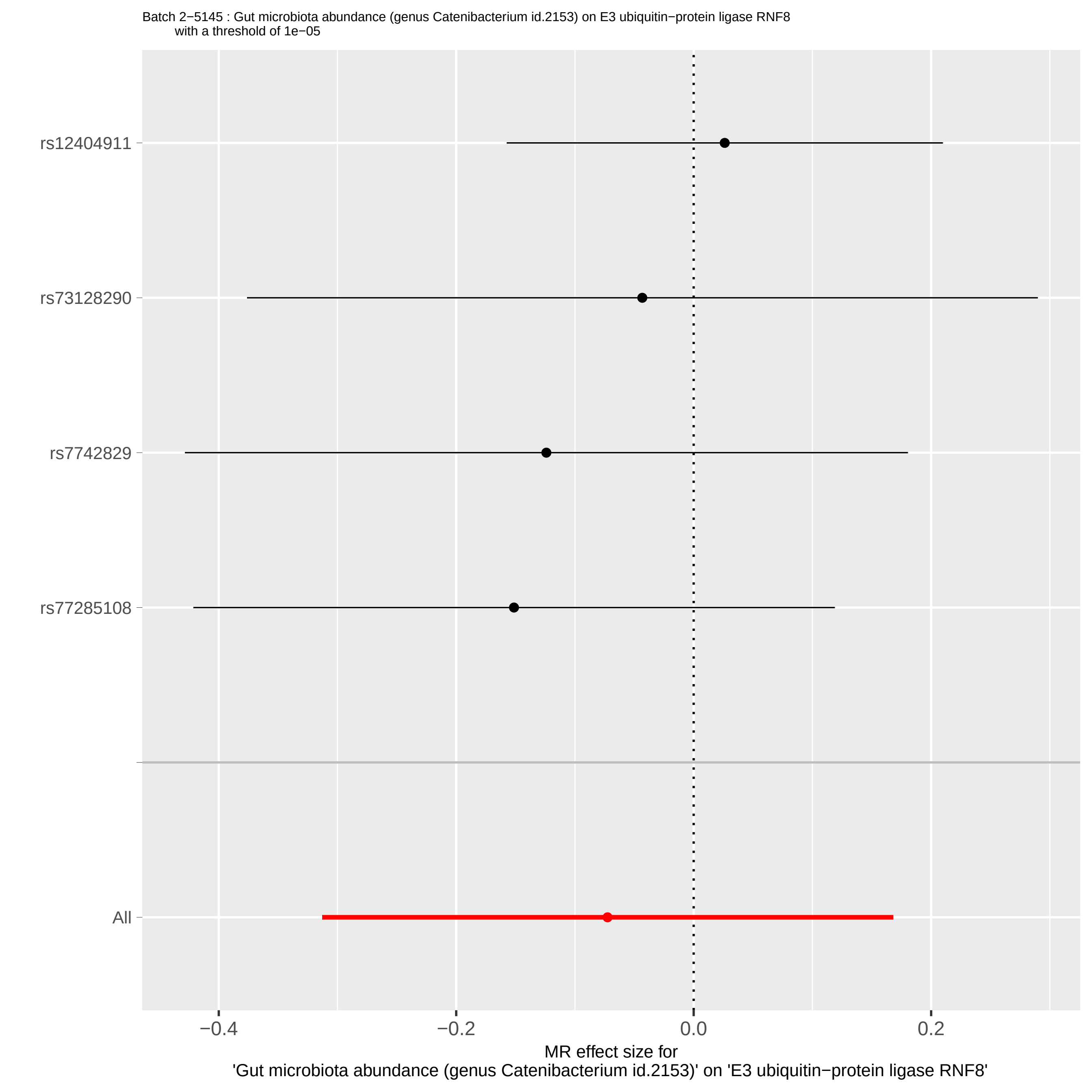
Batch 2−5145 : Gut microbiota abundance (genus Catenibacterium id.2153) on E3 ubiquitin−protein ligase RNF8
with a threshold of 1e−05
rs12404911
rs73128290
rs7742829
rs77285108
All
−0.4
−0.2
0.0
0.2
MR effect size for
'Gut microbiota abundance (genus Catenibacterium id.2153)' on 'E3 ubiquitin−protein ligase RNF8'

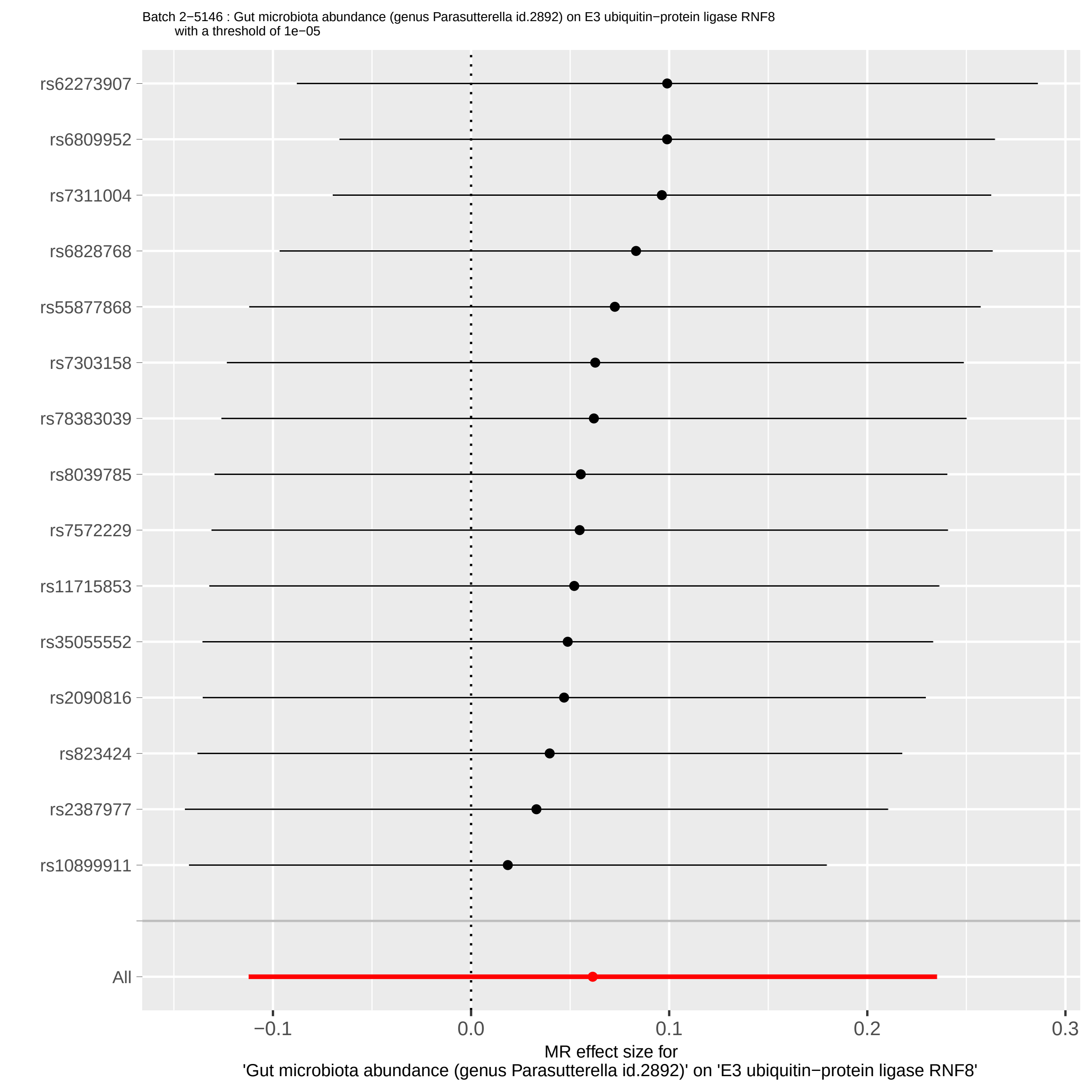
Batch 2−5146 : Gut microbiota abundance (genus Parasutterella id.2892) on E3 ubiquitin−protein ligase RNF8
with a threshold of 1e−05
rs62273907
rs6809952
rs7311004
rs6828768
rs55877868
rs7303158
rs78383039
rs8039785
rs7572229
rs11715853
rs35055552
rs2090816
rs823424
rs2387977
rs10899911
All
−0.1
0.0
0.1
0.2
0.3
MR effect size for
'Gut microbiota abundance (genus Parasutterella id.2892)' on 'E3 ubiquitin−protein ligase RNF8'

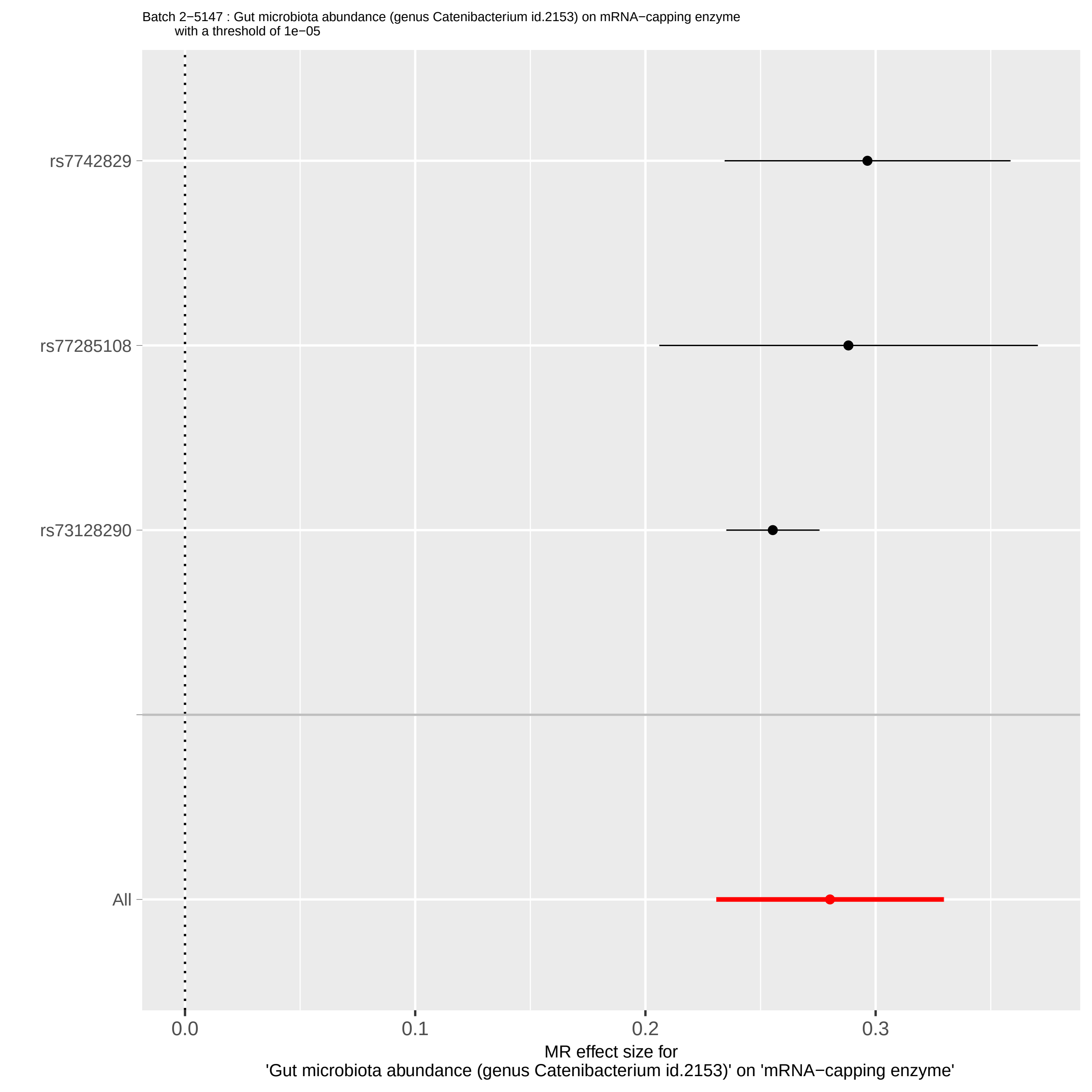

Batch 2−5147 : Gut microbiota abundance (genus Catenibacterium id.2153) on mRNA−capping enzyme
with a threshold of 1e−05
rs7742829
rs77285108
rs73128290
All
0.0
0.1
0.2
0.3
MR effect size for
'Gut microbiota abundance (genus Catenibacterium id.2153)' on 'mRNA−capping enzyme'

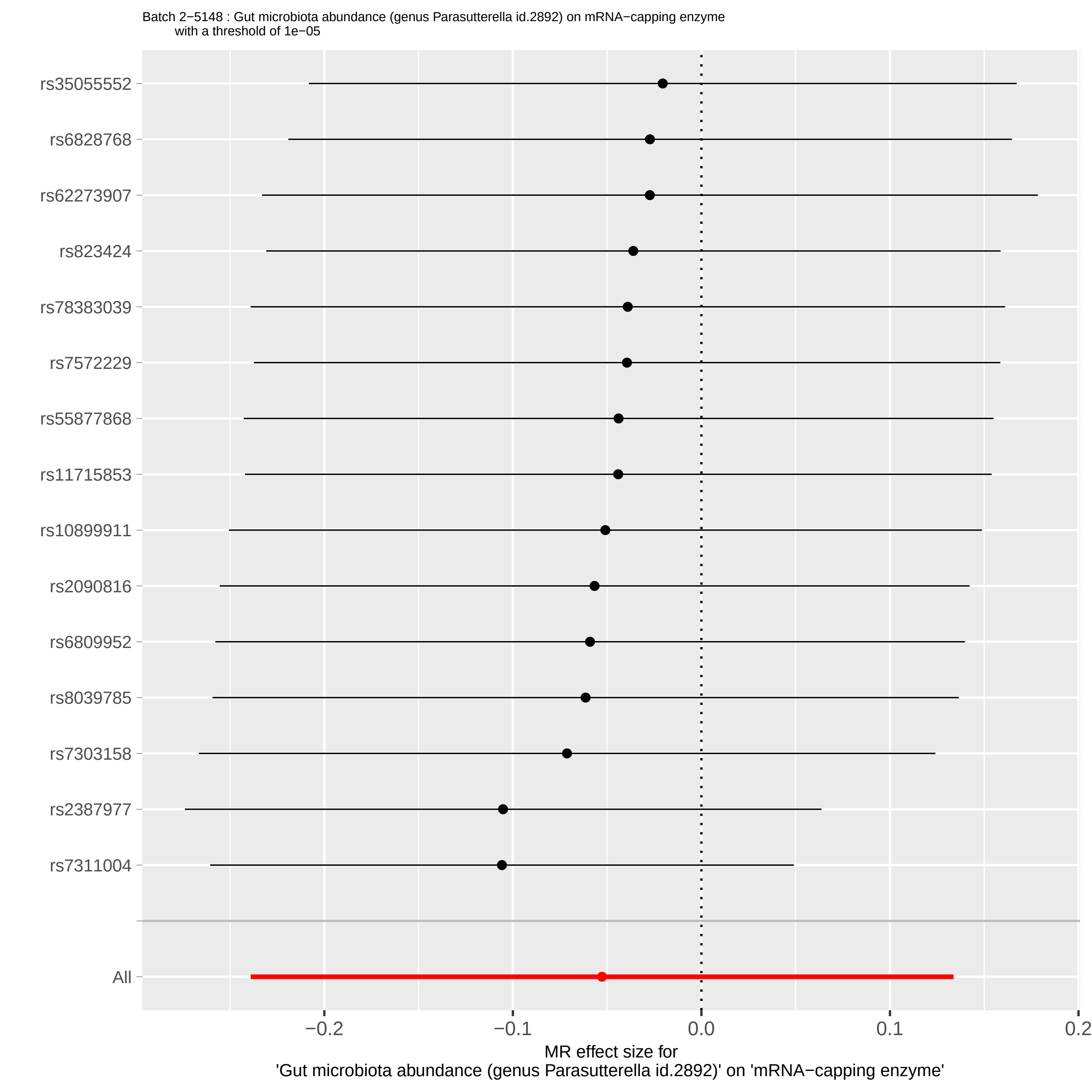

Batch 2−5148 : Gut microbiota abundance (genus Parasutterella id.2892) on mRNA−capping enzyme
with a threshold of 1e−05
rs35055552
rs6828768
rs62273907
rs823424
rs78383039
rs7572229
rs55877868
rs11715853
rs10899911
rs2090816
rs6809952
rs8039785
rs7303158
rs2387977
rs7311004
All
−0.2
−0.1
0.0
0.1
0.2
MR effect size for
'Gut microbiota abundance (genus Parasutterella id.2892)' on 'mRNA−capping enzyme'

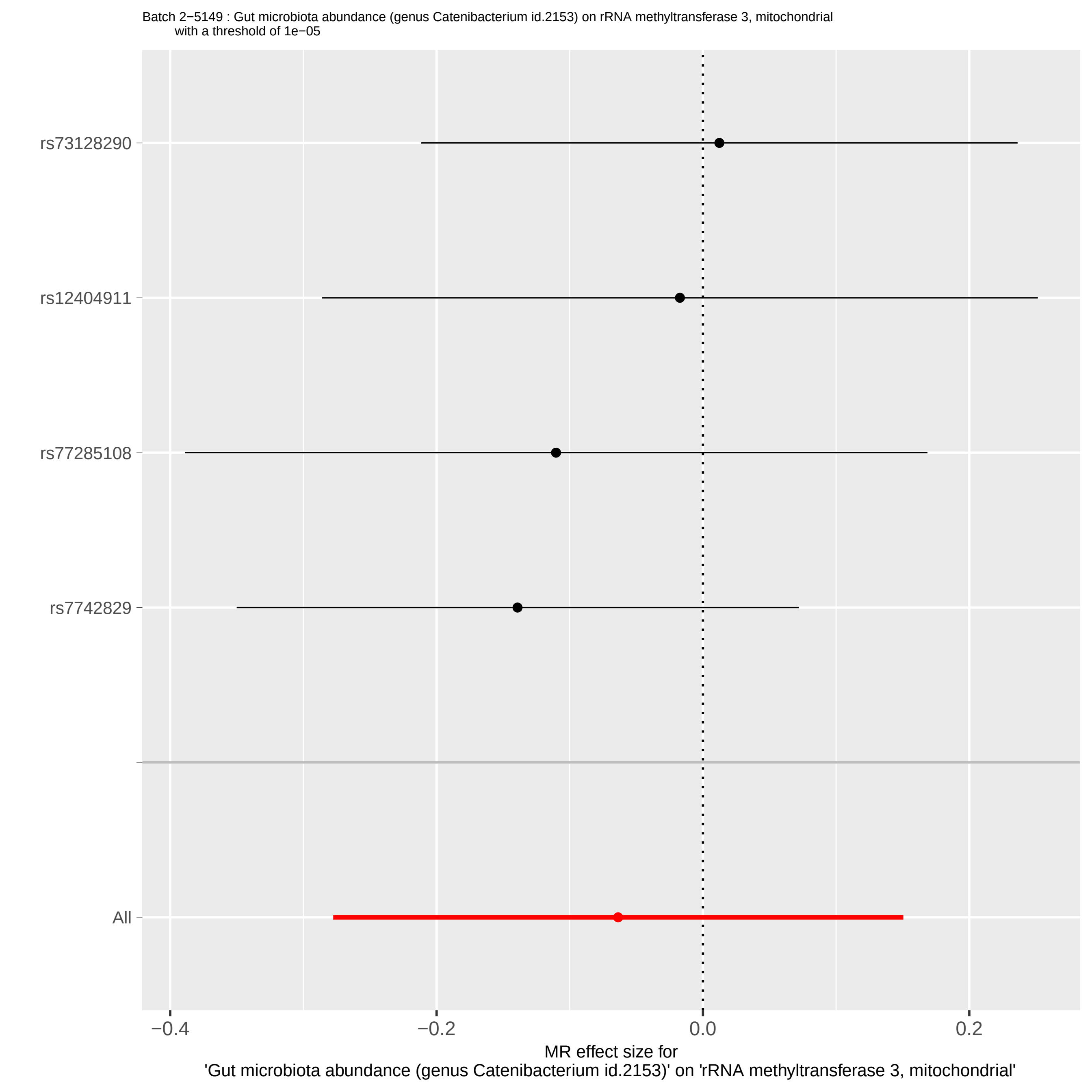
Batch 2−5149 : Gut microbiota abundance (genus Catenibacterium id.2153) on rRNA methyltransferase 3, mitochondrial
with a threshold of 1e−05
rs73128290
rs12404911
rs77285108
rs7742829
All
−0.4
−0.2
0.0
0.2
MR effect size for
'Gut microbiota abundance (genus Catenibacterium id.2153)' on 'rRNA methyltransferase 3, mitochondrial'

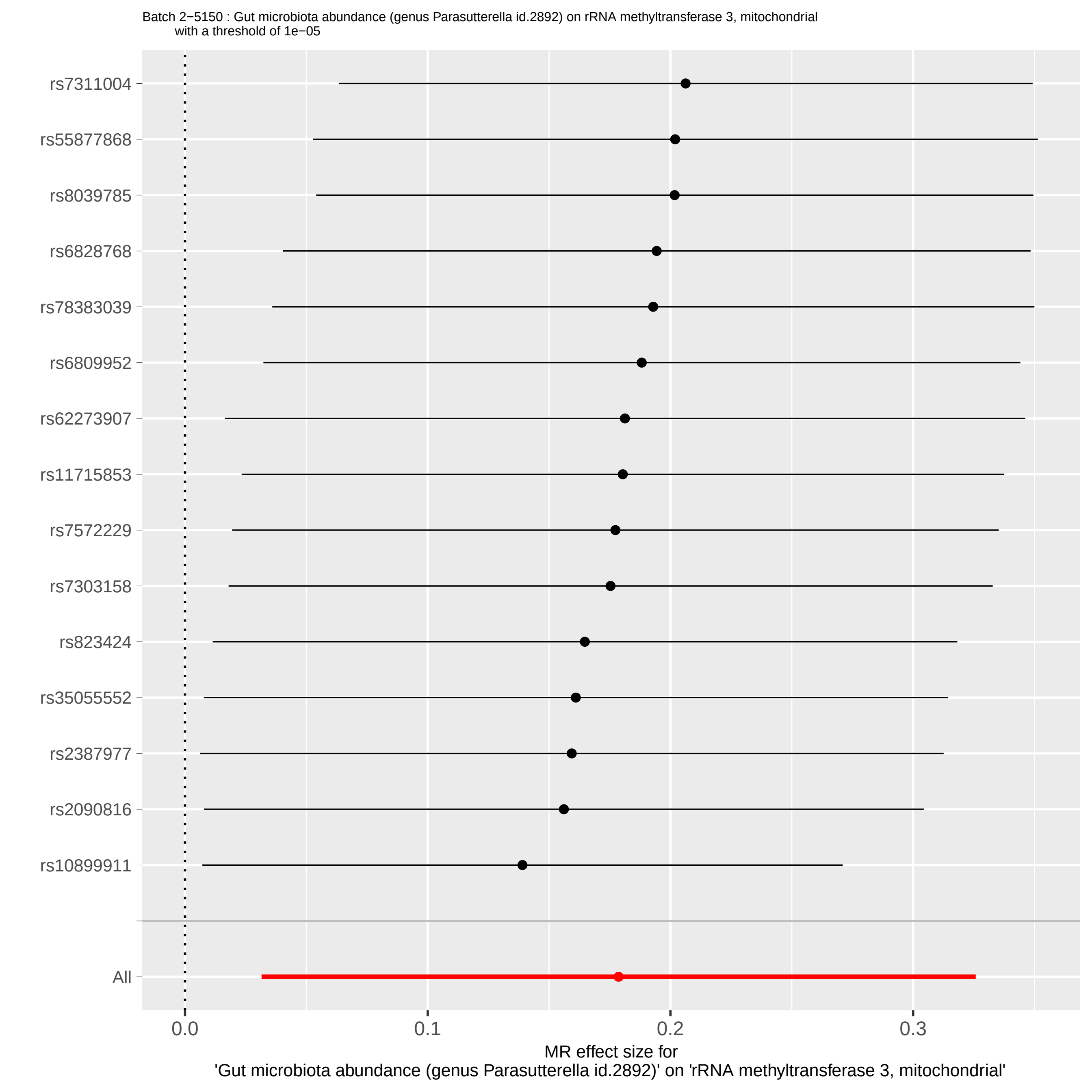

Batch 2−5150 : Gut microbiota abundance (genus Parasutterella id.2892) on rRNA methyltransferase 3, mitochondrial
with a threshold of 1e−05
rs7311004
rs55877868
rs8039785
rs6828768
rs78383039
rs6809952
rs62273907
rs11715853
rs7572229
rs7303158
rs823424
rs35055552
rs2387977
rs2090816
rs10899911
All
0.0
0.1
0.2
0.3
MR effect size for
'Gut microbiota abundance (genus Parasutterella id.2892)' on 'rRNA methyltransferase 3, mitochondrial'

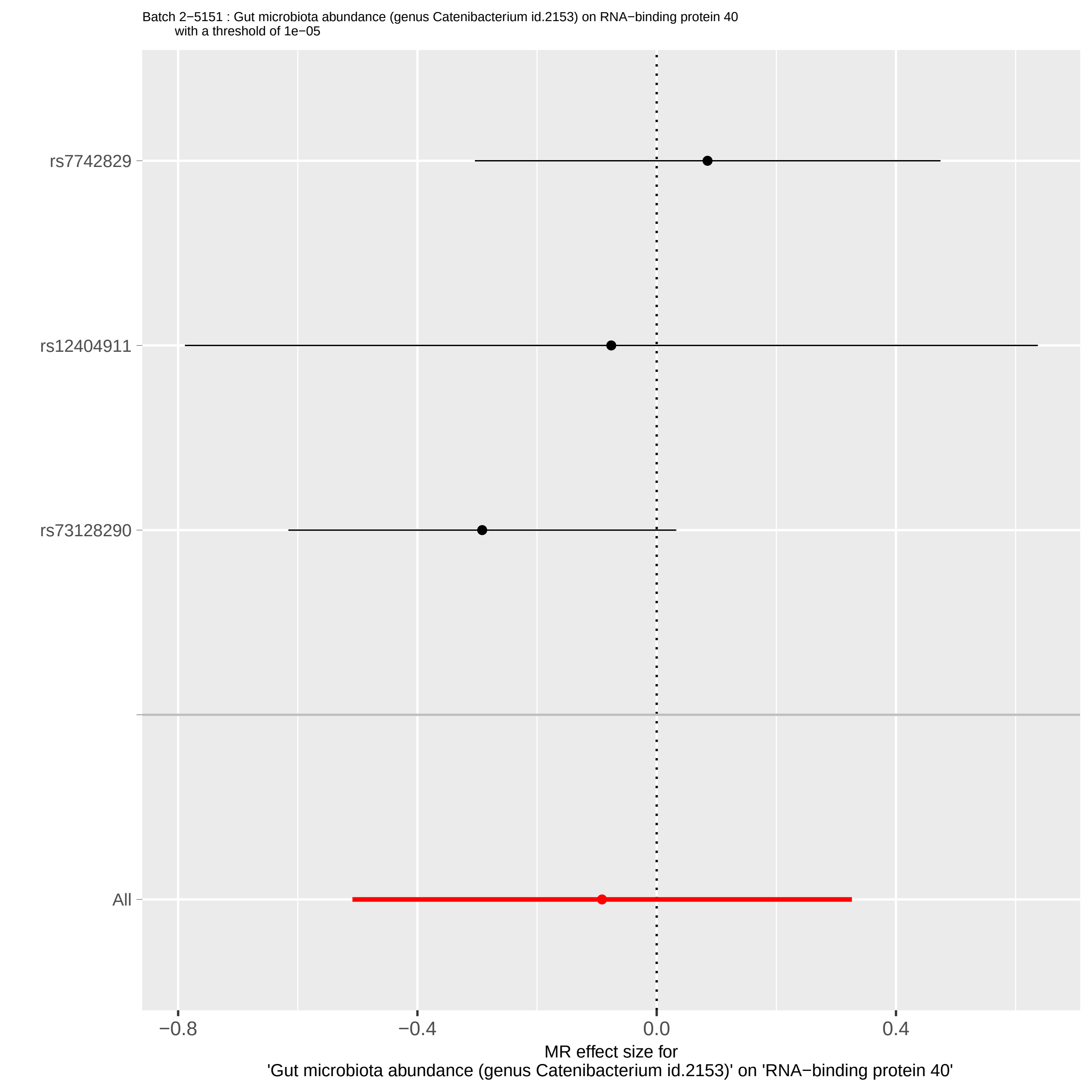

Batch 2−5151 : Gut microbiota abundance (genus Catenibacterium id.2153) on RNA−binding protein 40
with a threshold of 1e−05
rs7742829
rs12404911
rs73128290
All
−0.8
−0.4
0.0
0.4
MR effect size for
'Gut microbiota abundance (genus Catenibacterium id.2153)' on 'RNA−binding protein 40'

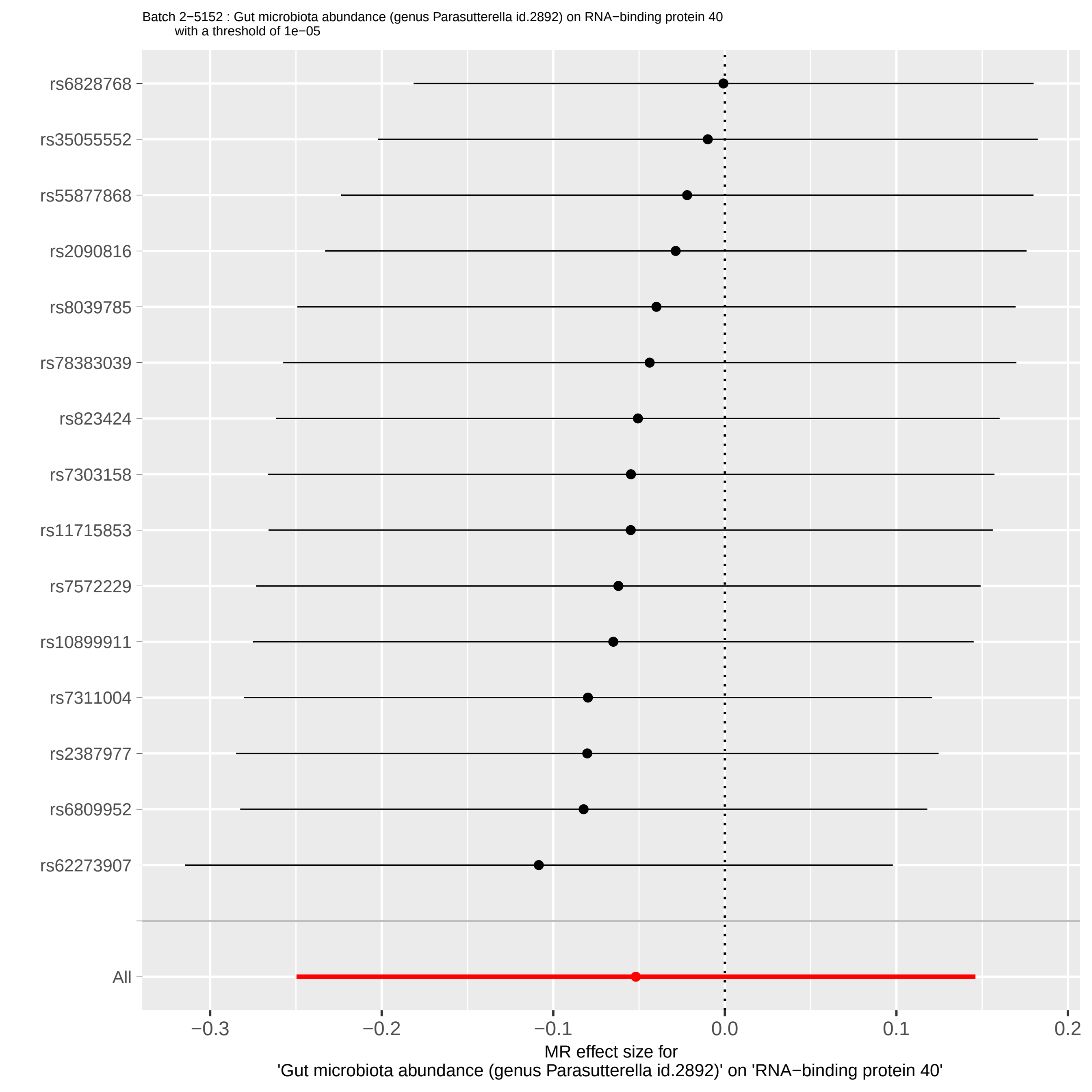
Batch 2−5152 : Gut microbiota abundance (genus Parasutterella id.2892) on RNA−binding protein 40
with a threshold of 1e−05
rs6828768
rs35055552
rs55877868
rs2090816
rs8039785
rs78383039
rs823424
rs7303158
rs11715853
rs7572229
rs10899911
rs7311004
rs2387977
rs6809952
rs62273907
All
−0.3
−0.2
−0.1
0.0
0.1
0.2
MR effect size for
'Gut microbiota abundance (genus Parasutterella id.2892)' on 'RNA−binding protein 40'

Batch 2−5153 : Gut microbiota abundance (genus Catenibacterium id.2153) on Roundabout homolog 1
with a threshold of 1e−05

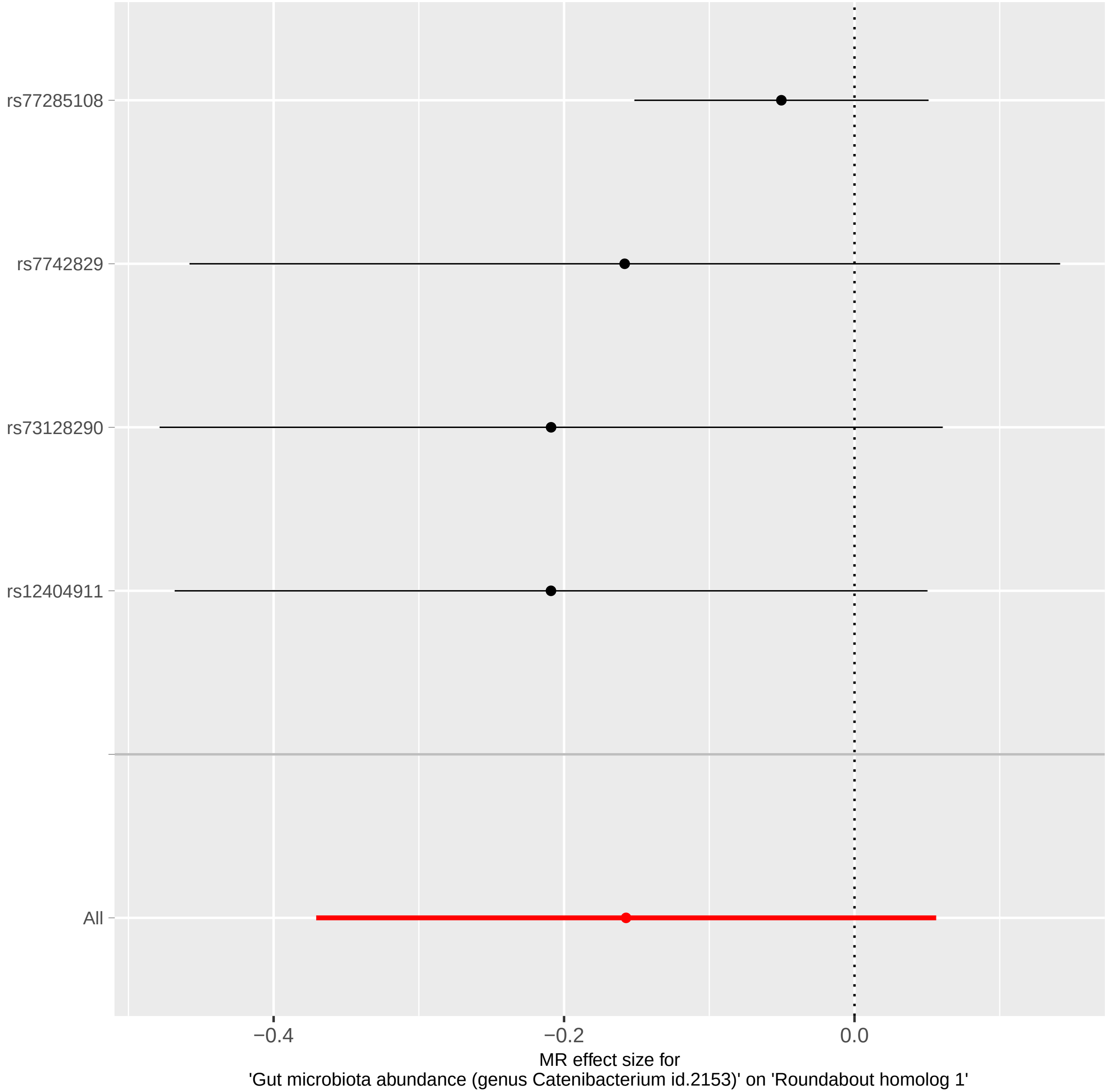

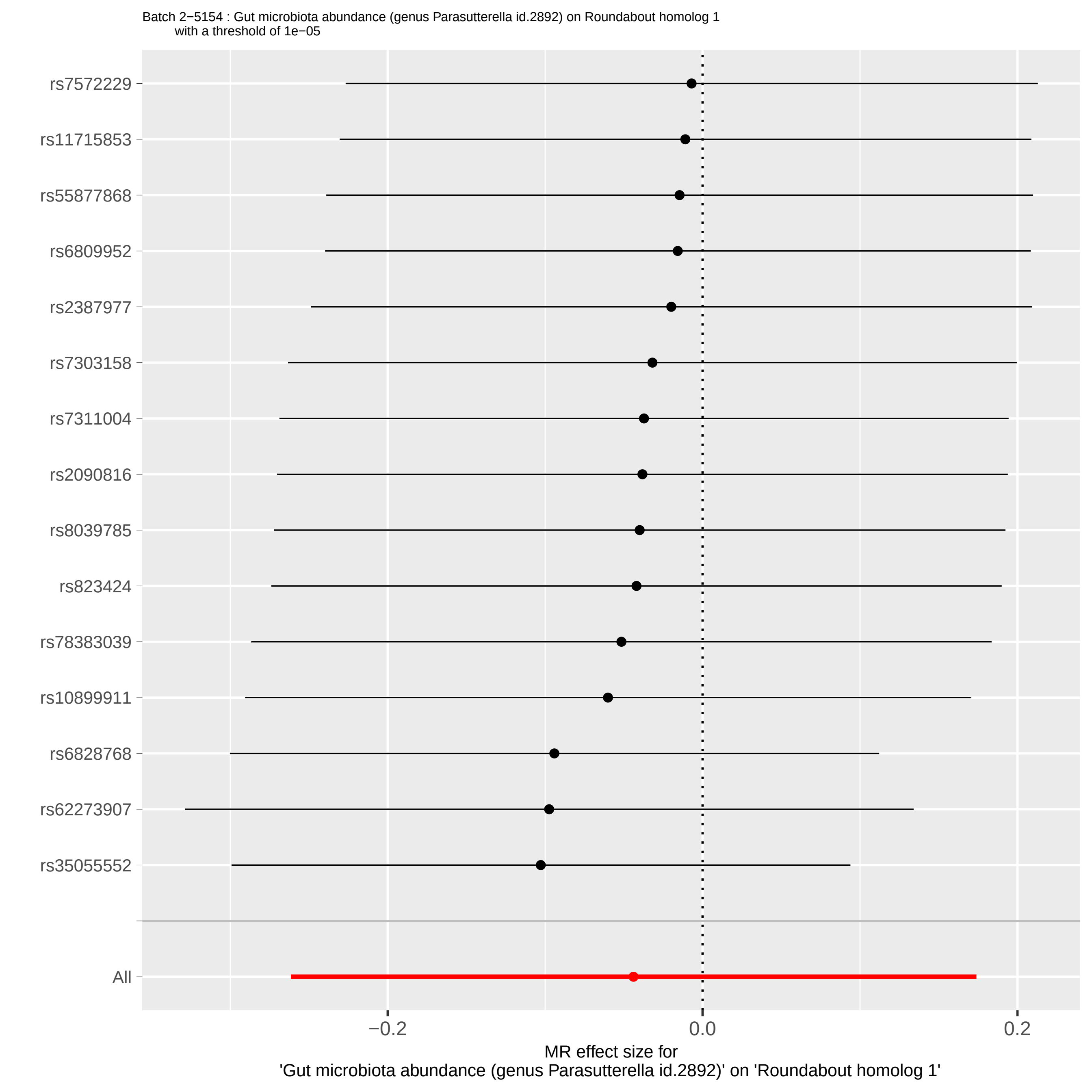

Batch 2−5154 : Gut microbiota abundance (genus Parasutterella id.2892) on Roundabout homolog 1
with a threshold of 1e−05
rs7572229
rs11715853
rs55877868
rs6809952
rs2387977
rs7303158
rs7311004
rs2090816
rs8039785
rs823424
rs78383039
rs10899911
rs6828768
rs62273907
rs35055552
All
−0.2
0.0
0.2
MR effect size for
'Gut microbiota abundance (genus Parasutterella id.2892)' on 'Roundabout homolog 1'

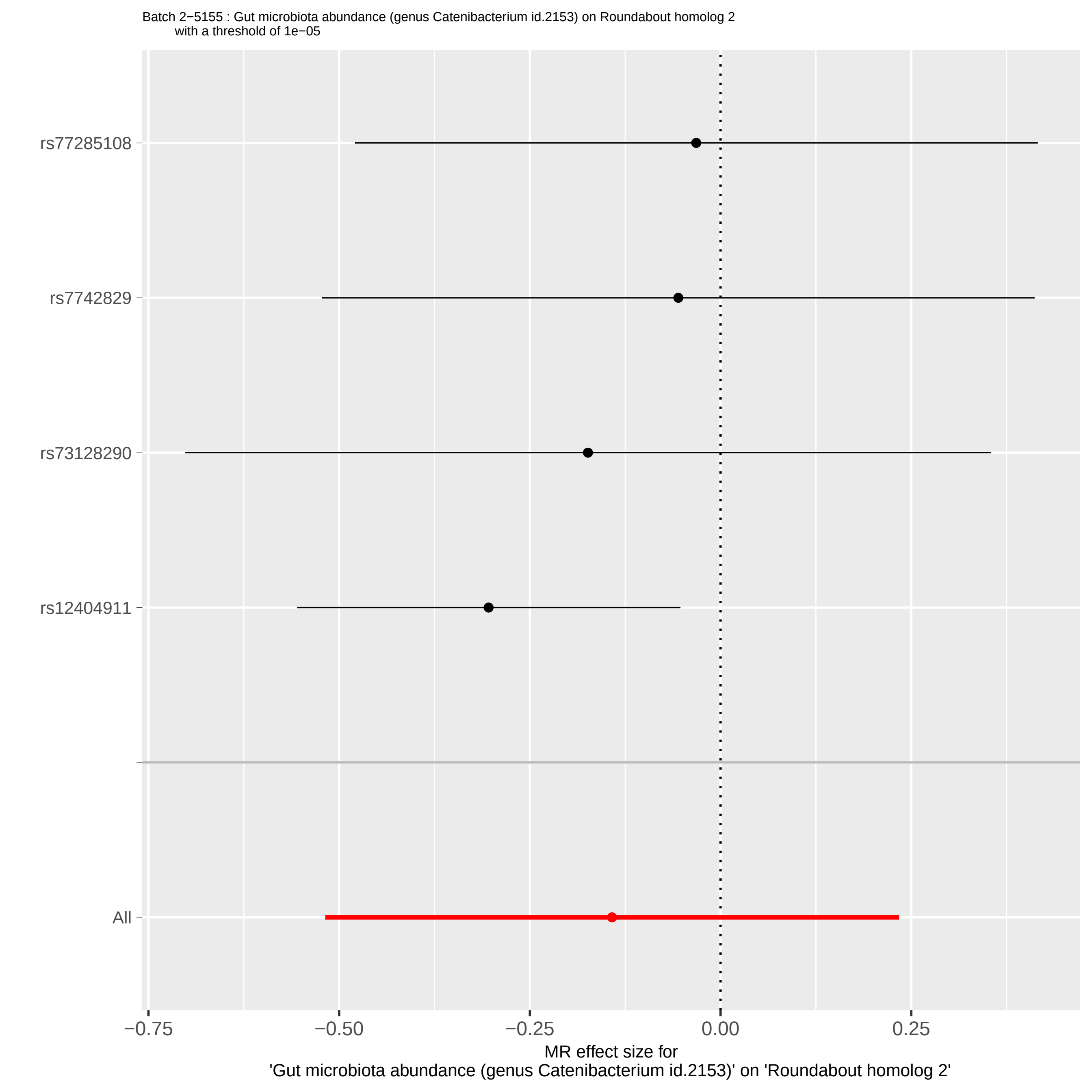
Batch 2−5155 : Gut microbiota abundance (genus Catenibacterium id.2153) on Roundabout homolog 2
with a threshold of 1e−05
rs77285108
rs7742829
rs73128290
rs12404911
All
−0.75
−0.50
−0.25
0.00
0.25
MR effect size for
'Gut microbiota abundance (genus Catenibacterium id.2153)' on 'Roundabout homolog 2'

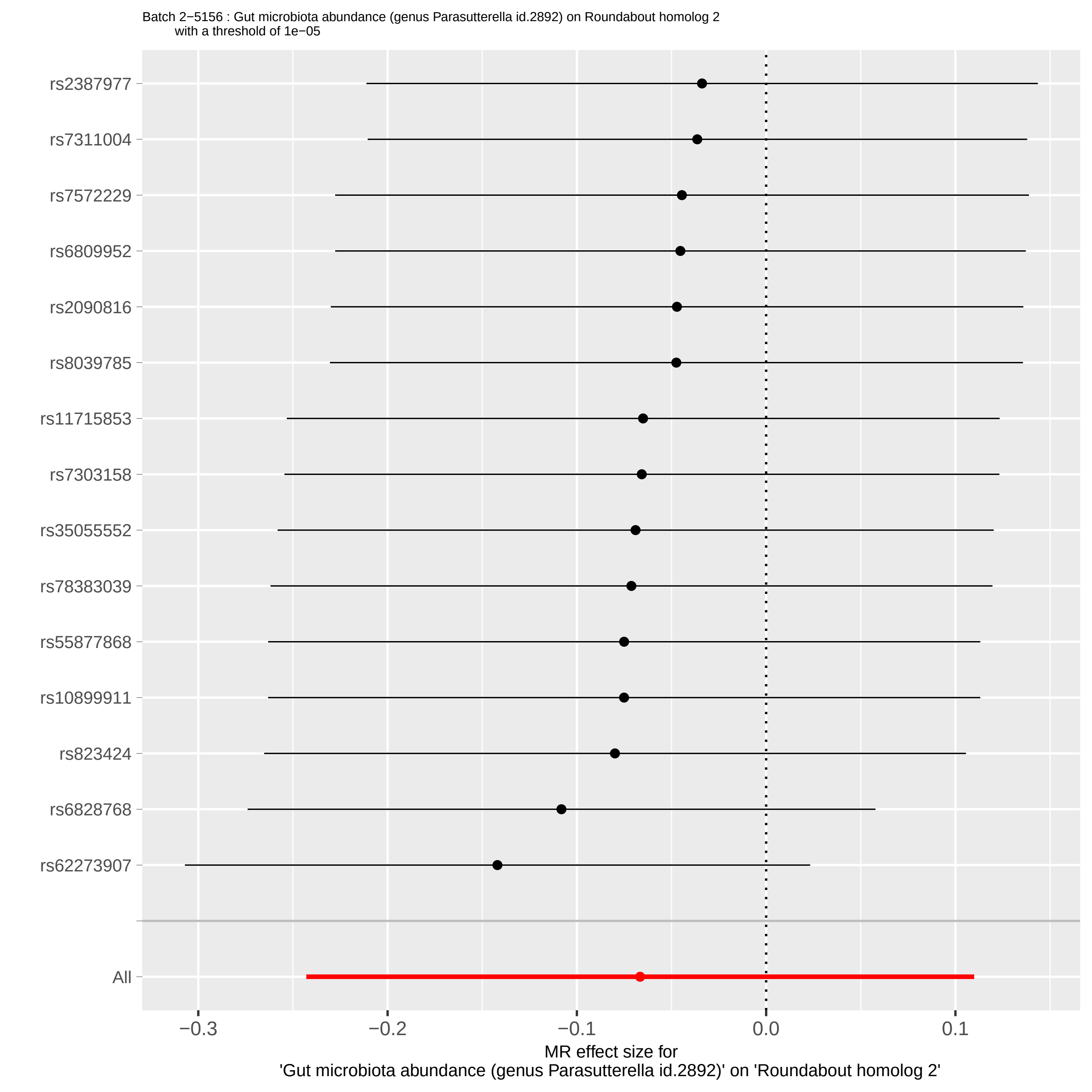

Batch 2−5156 : Gut microbiota abundance (genus Parasutterella id.2892) on Roundabout homolog 2
with a threshold of 1e−05
rs2387977
rs7311004
rs7572229
rs6809952
rs2090816
rs8039785
rs11715853
rs7303158
rs35055552
rs78383039
rs55877868
rs10899911
rs823424
rs6828768
rs62273907
All
−0.3
−0.2
−0.1
0.0
0.1
MR effect size for
'Gut microbiota abundance (genus Parasutterella id.2892)' on 'Roundabout homolog 2'

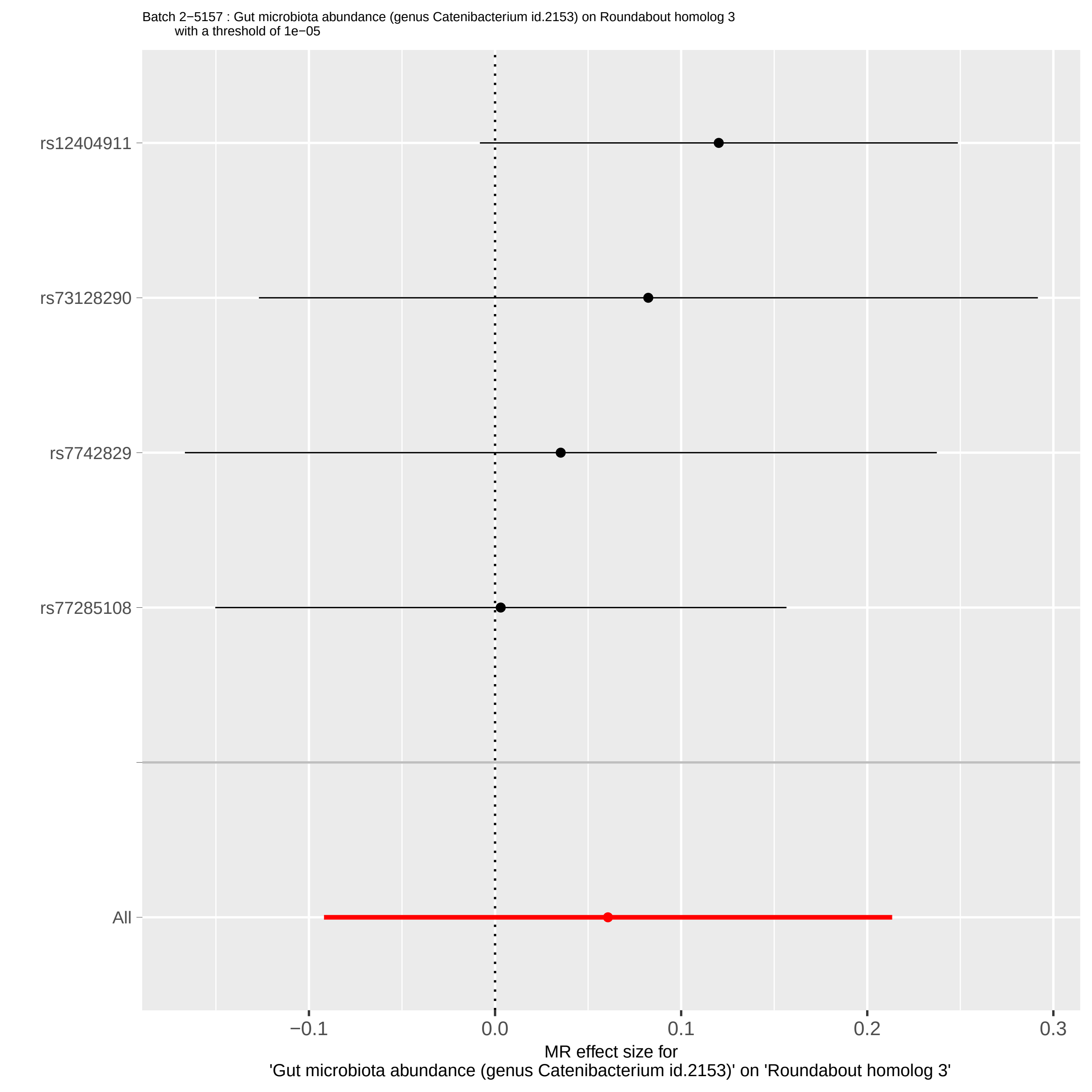
Batch 2−5157 : Gut microbiota abundance (genus Catenibacterium id.2153) on Roundabout homolog 3
with a threshold of 1e−05
rs12404911
rs73128290
rs7742829
rs77285108
All
−0.1
0.0
0.1
0.2
0.3
MR effect size for
'Gut microbiota abundance (genus Catenibacterium id.2153)' on 'Roundabout homolog 3'

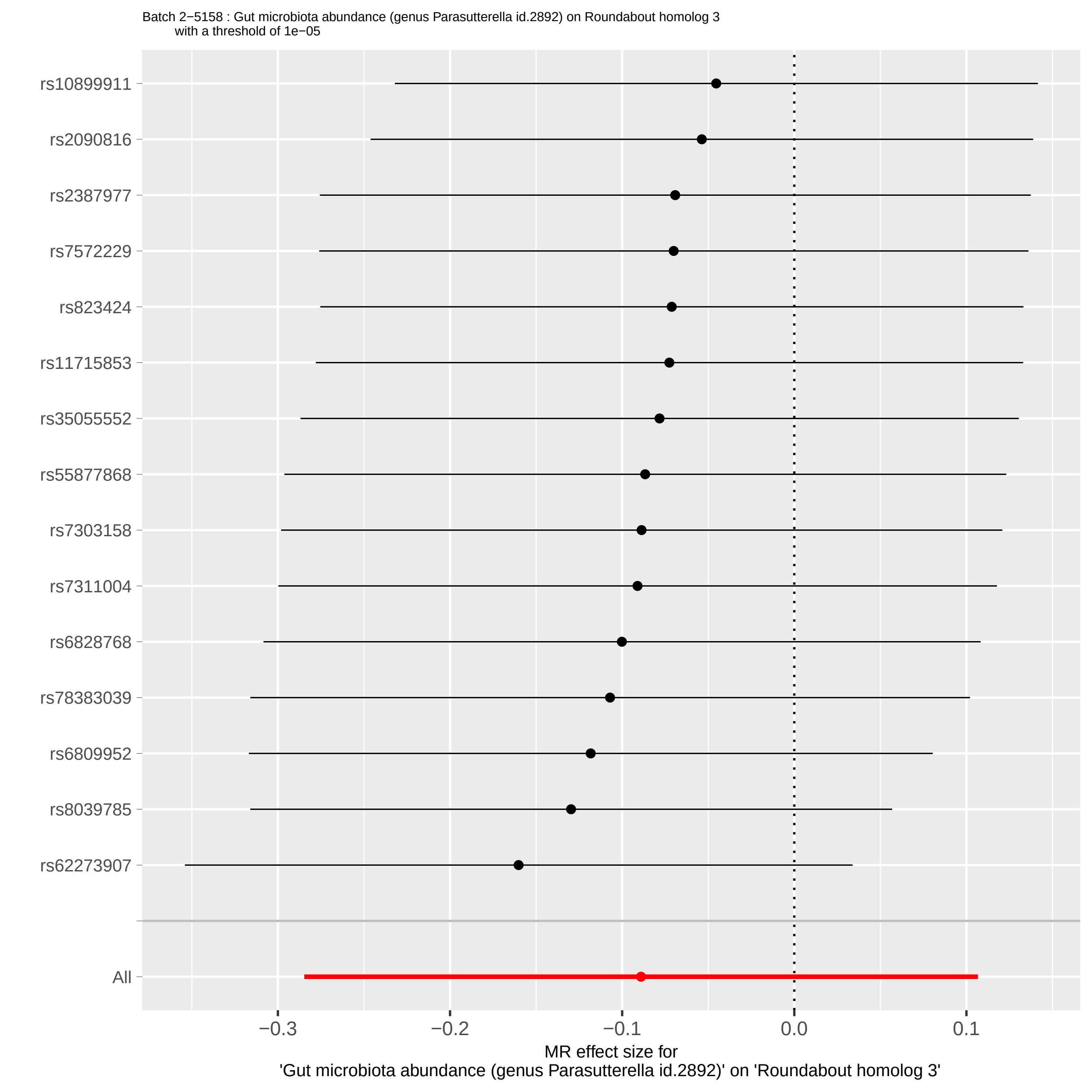
Batch 2−5158 : Gut microbiota abundance (genus Parasutterella id.2892) on Roundabout homolog 3
with a threshold of 1e−05
rs10899911
rs2090816
rs2387977
rs7572229
rs823424
rs11715853
rs35055552
rs55877868
rs7303158
rs7311004
rs6828768
rs78383039
rs6809952
rs8039785
rs62273907
All
−0.3
−0.2
−0.1
0.0
0.1
MR effect size for
'Gut microbiota abundance (genus Parasutterella id.2892)' on 'Roundabout homolog 3'

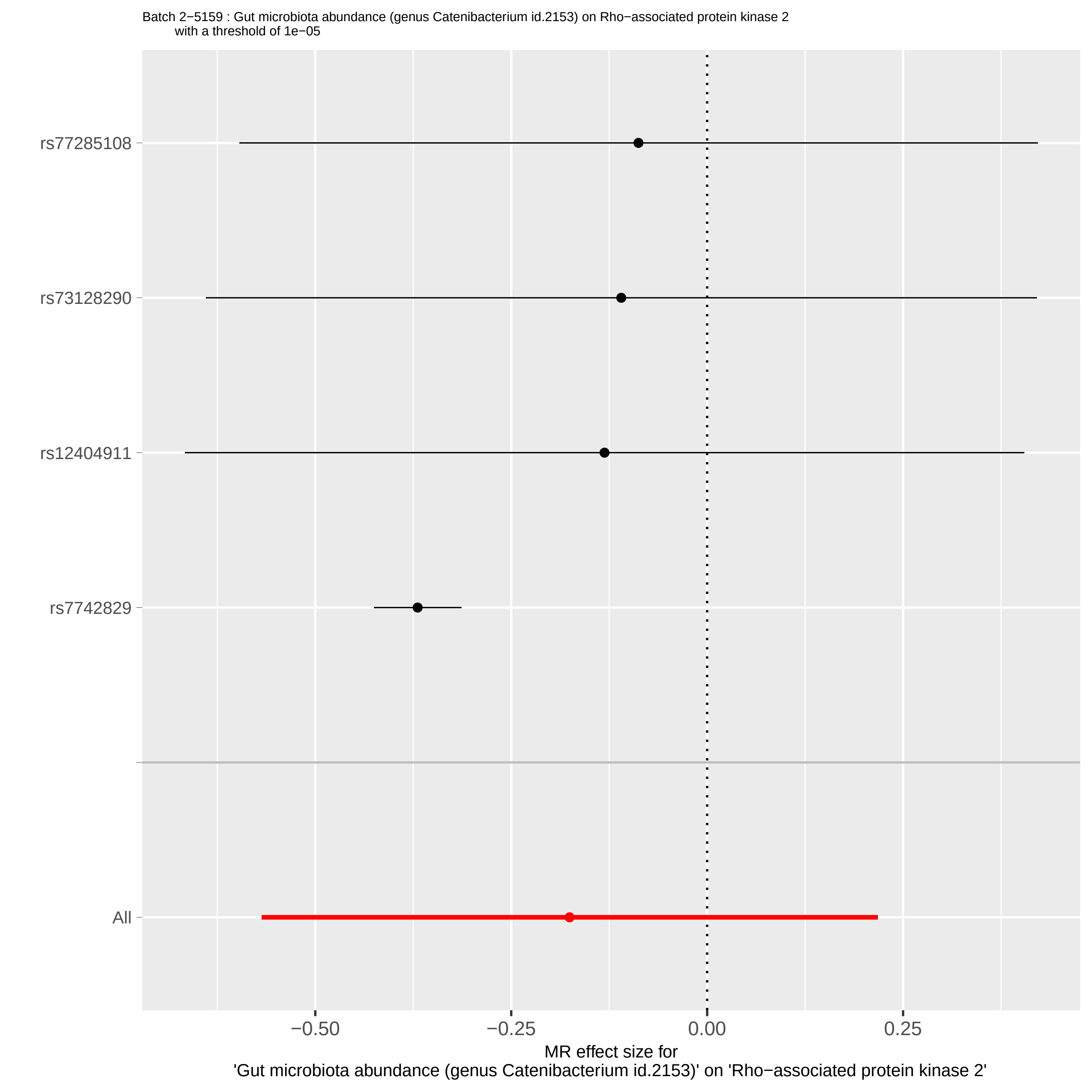
Batch 2−5159 : Gut microbiota abundance (genus Catenibacterium id.2153) on Rho−associated protein kinase 2
with a threshold of 1e−05
rs77285108
rs73128290
rs12404911
rs7742829
All
−0.50
−0.25
0.00
0.25
MR effect size for
'Gut microbiota abundance (genus Catenibacterium id.2153)' on 'Rho−associated protein kinase 2'

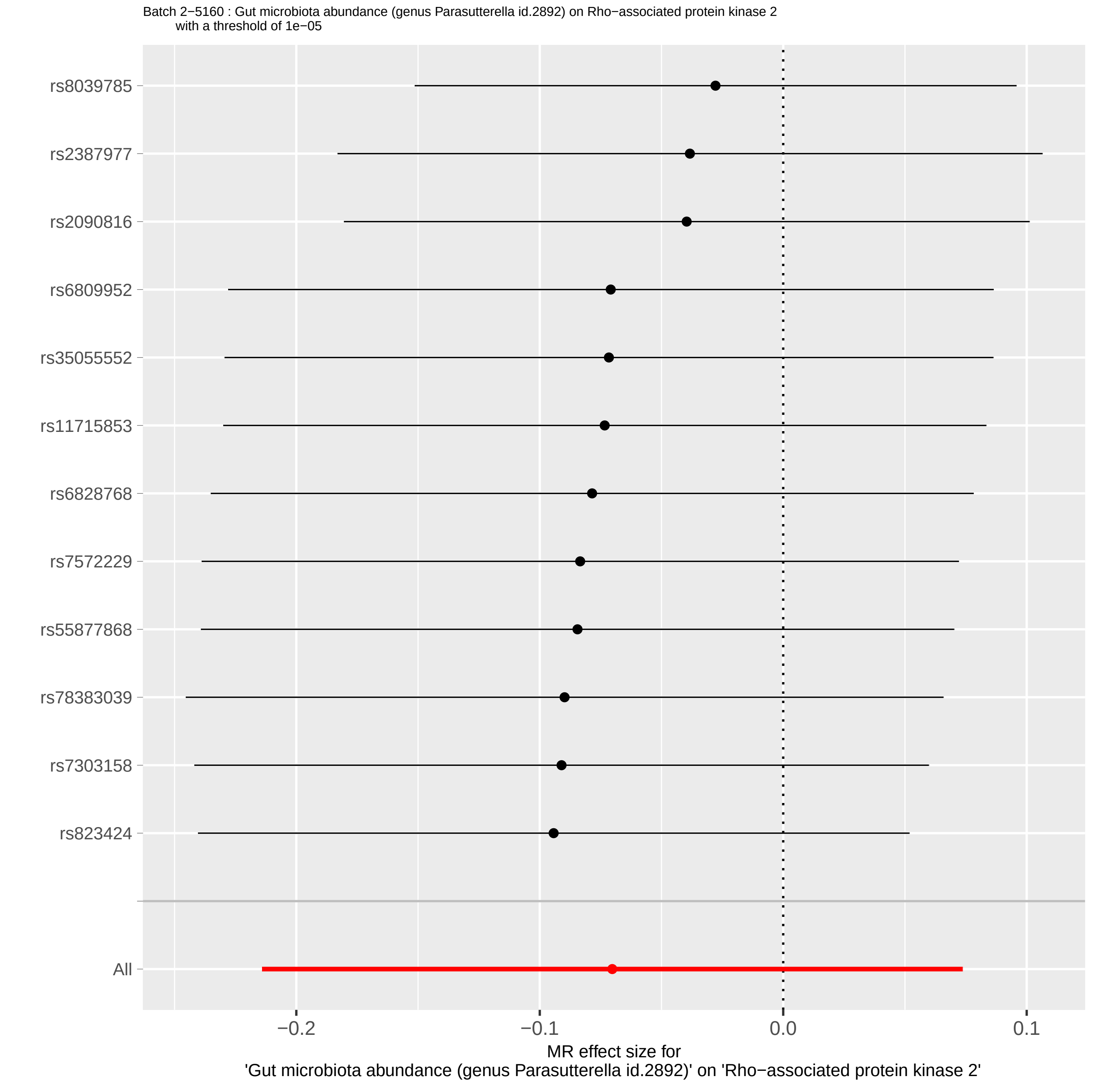

Batch 2−5160 : Gut microbiota abundance (genus Parasutterella id.2892) on Rho−associated protein kinase 2
with a threshold of 1e−05
rs8039785
rs2387977
rs2090816
rs6809952
rs35055552
rs11715853
rs6828768
rs7572229
rs55877868
rs78383039
rs7303158
rs823424
All
−0.2
−0.1
0.0
0.1
MR effect size for
'Gut microbiota abundance (genus Parasutterella id.2892)' on 'Rho−associated protein kinase 2'

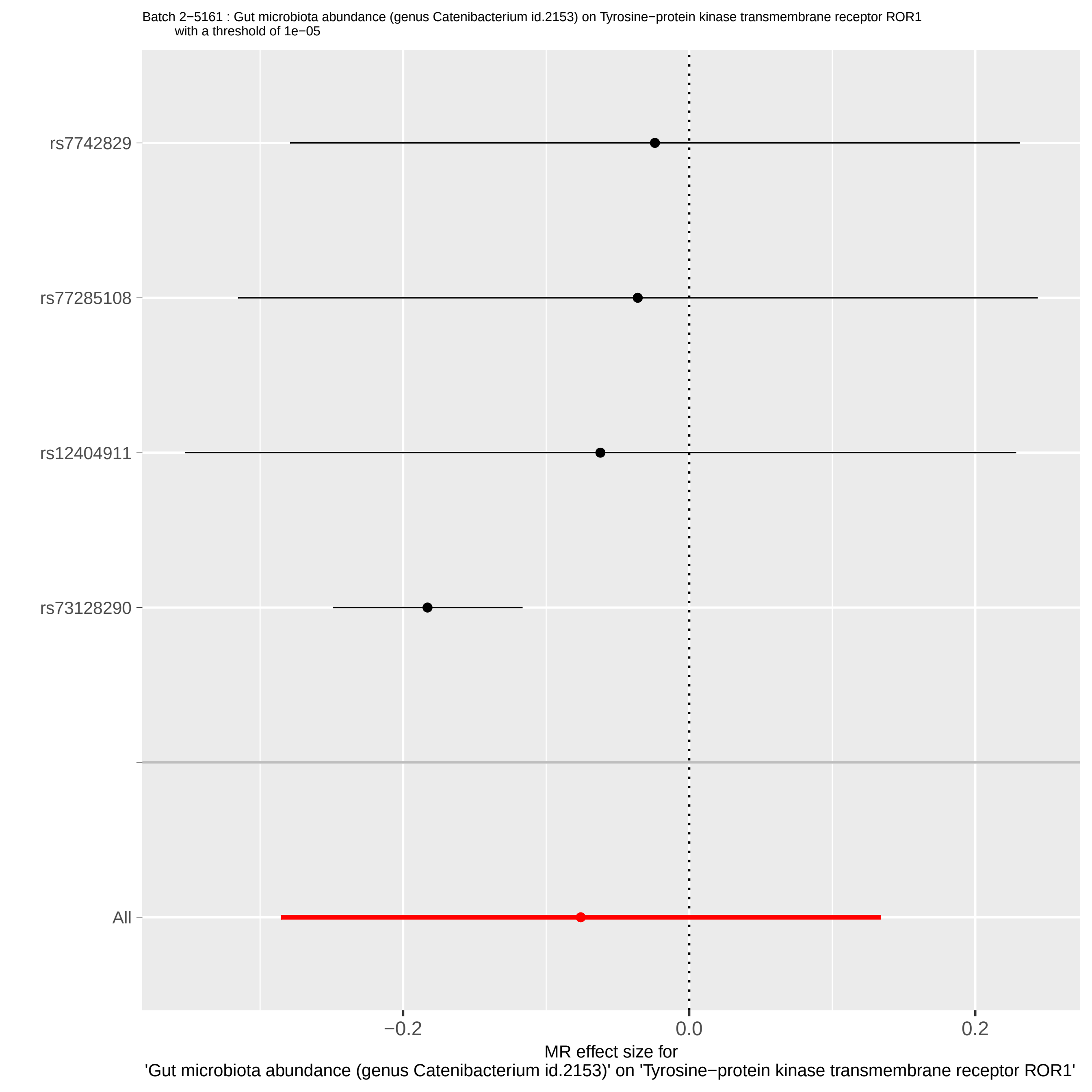
Batch 2−5161 : Gut microbiota abundance (genus Catenibacterium id.2153) on Tyrosine−protein kinase transmembrane receptor ROR1
with a threshold of 1e−05
rs7742829
rs77285108
rs12404911
rs73128290
All
−0.2
0.0
0.2
MR effect size for
'Gut microbiota abundance (genus Catenibacterium id.2153)' on 'Tyrosine−protein kinase transmembrane receptor ROR1'

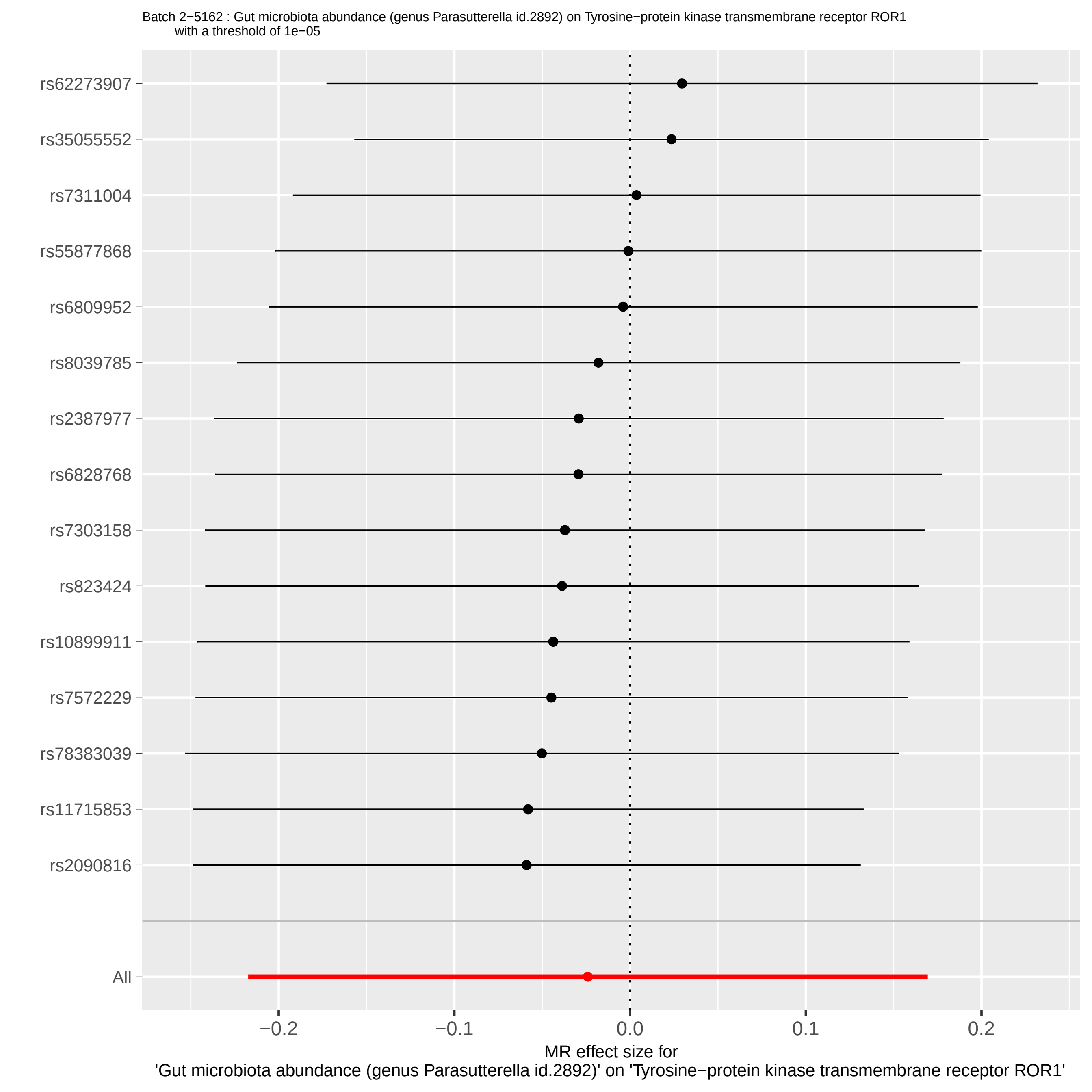

Batch 2−5162 : Gut microbiota abundance (genus Parasutterella id.2892) on Tyrosine−protein kinase transmembrane receptor ROR1
with a threshold of 1e−05
rs62273907
rs35055552
rs7311004
rs55877868
rs6809952
rs8039785
rs2387977
rs6828768
rs7303158
rs823424
rs10899911
rs7572229
rs78383039
rs11715853
rs2090816
All
−0.2
−0.1
0.0
0.1
0.2
MR effect size for
'Gut microbiota abundance (genus Parasutterella id.2892)' on 'Tyrosine−protein kinase transmembrane receptor ROR1'

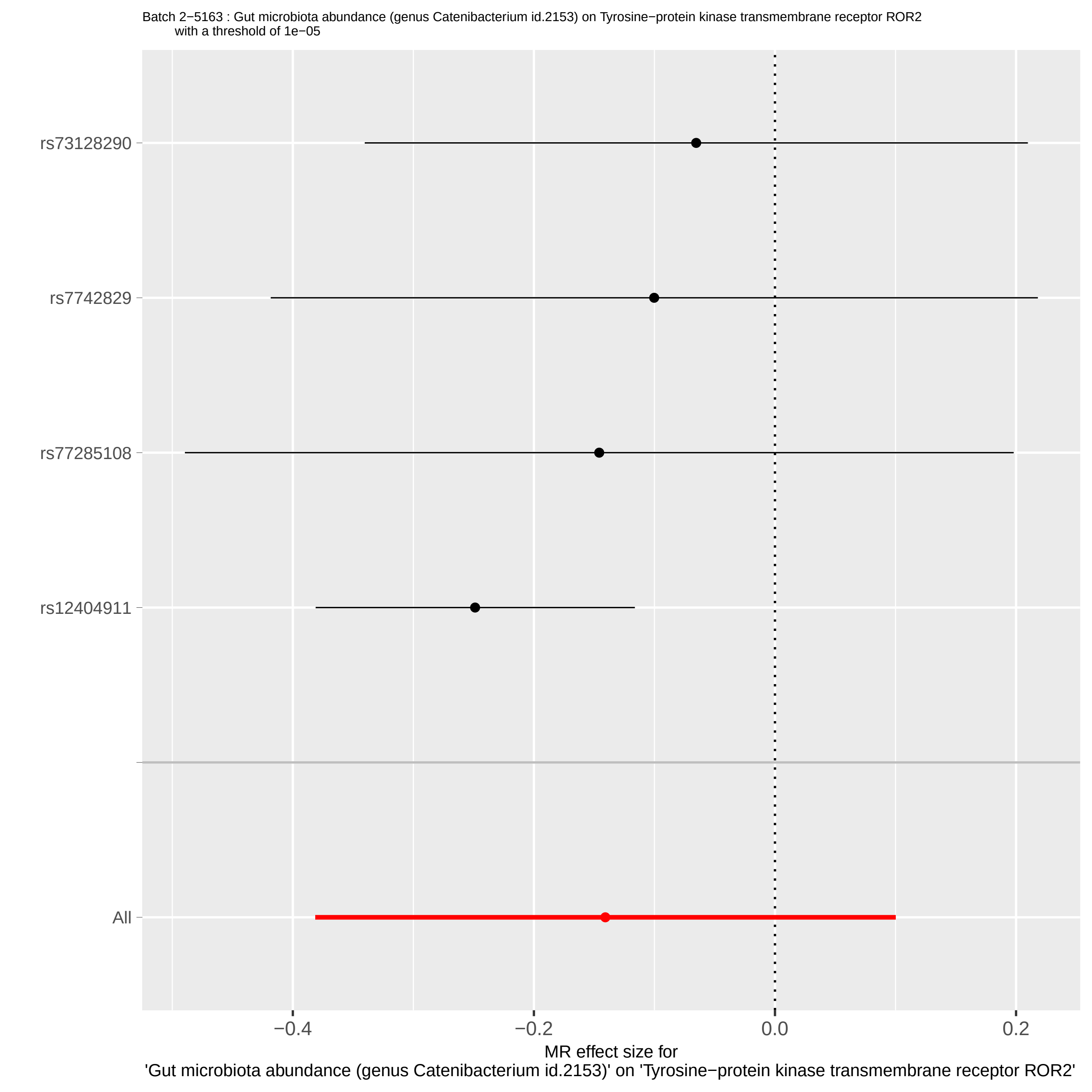

Batch 2−5163 : Gut microbiota abundance (genus Catenibacterium id.2153) on Tyrosine−protein kinase transmembrane receptor ROR2
with a threshold of 1e−05
rs73128290
rs7742829
rs77285108
rs12404911
All
−0.4
−0.2
0.0
0.2
MR effect size for
'Gut microbiota abundance (genus Catenibacterium id.2153)' on 'Tyrosine−protein kinase transmembrane receptor ROR2'

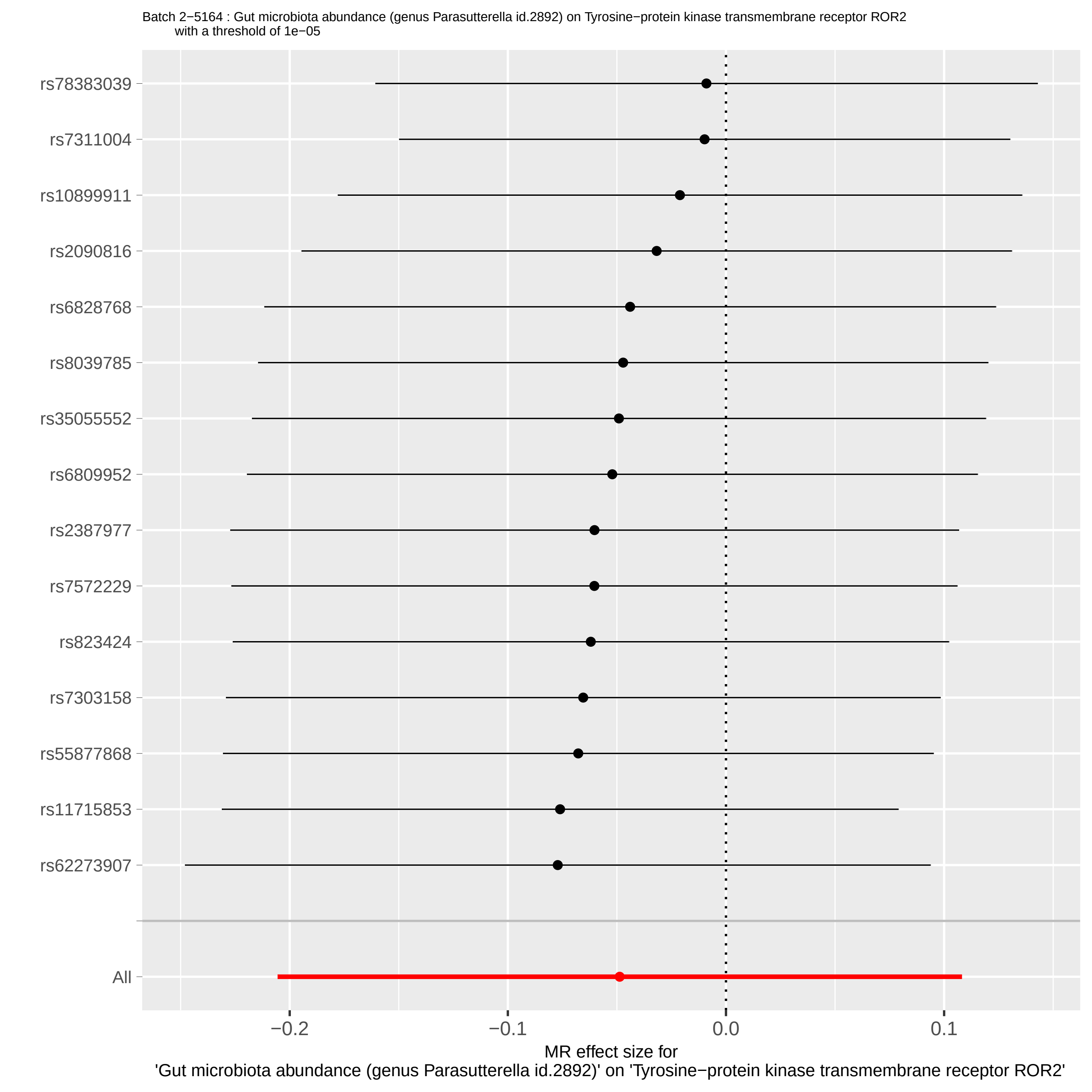

Batch 2−5164 : Gut microbiota abundance (genus Parasutterella id.2892) on Tyrosine−protein kinase transmembrane receptor ROR2
with a threshold of 1e−05
rs78383039
rs7311004
rs10899911
rs2090816
rs6828768
rs8039785
rs35055552
rs6809952
rs2387977
rs7572229
rs823424
rs7303158
rs55877868
rs11715853
rs62273907
All
−0.2
−0.1
0.0
0.1
MR effect size for
'Gut microbiota abundance (genus Parasutterella id.2892)' on 'Tyrosine−protein kinase transmembrane receptor ROR2'

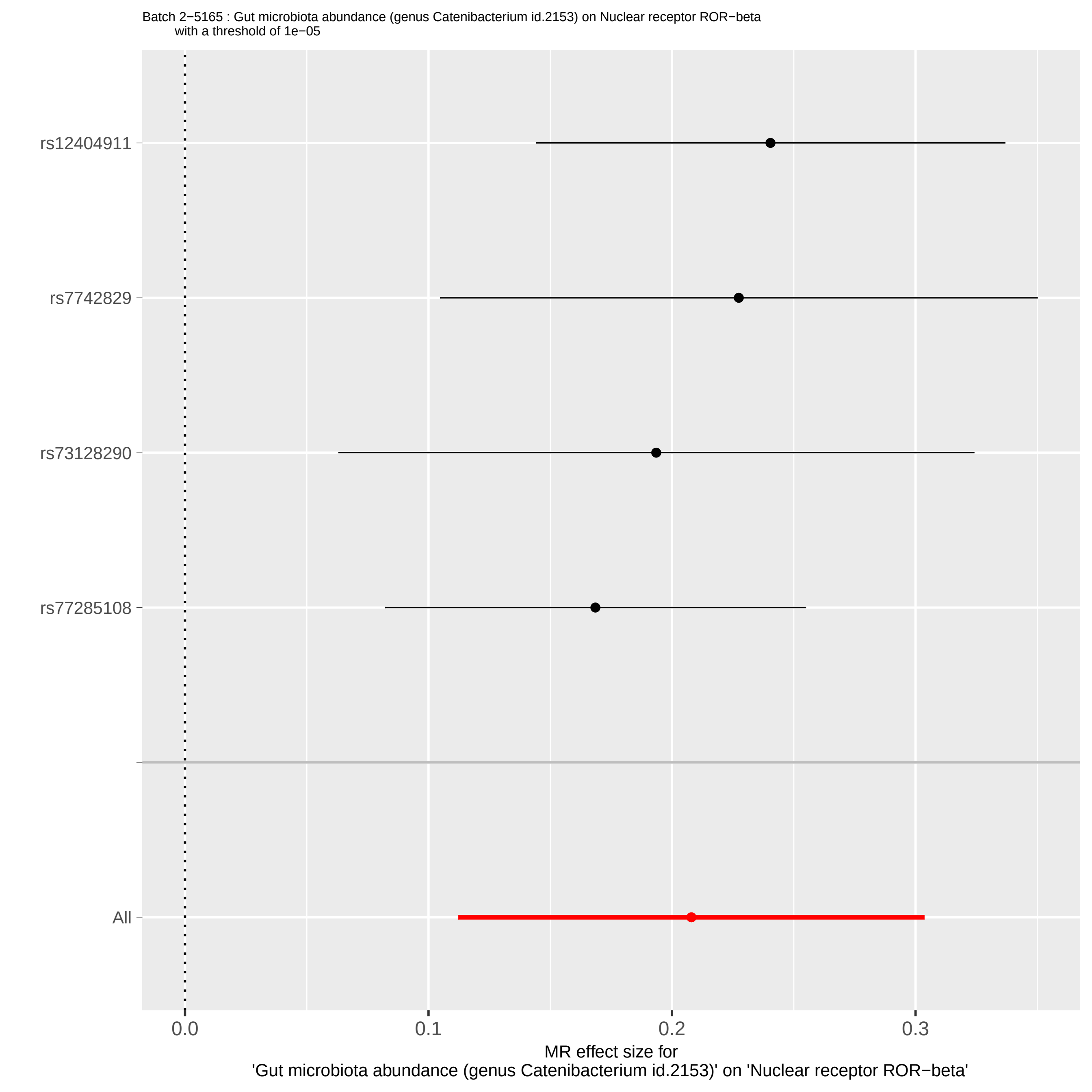
Batch 2−5165 : Gut microbiota abundance (genus Catenibacterium id.2153) on Nuclear receptor ROR−beta
with a threshold of 1e−05
rs12404911
rs7742829
rs73128290
rs77285108
All
0.0
0.1
0.2
0.3
MR effect size for
'Gut microbiota abundance (genus Catenibacterium id.2153)' on 'Nuclear receptor ROR−beta'

Batch 2−5166 : Gut microbiota abundance (genus Parasutterella id.2892) on Nuclear receptor ROR−beta
with a threshold of 1e−05

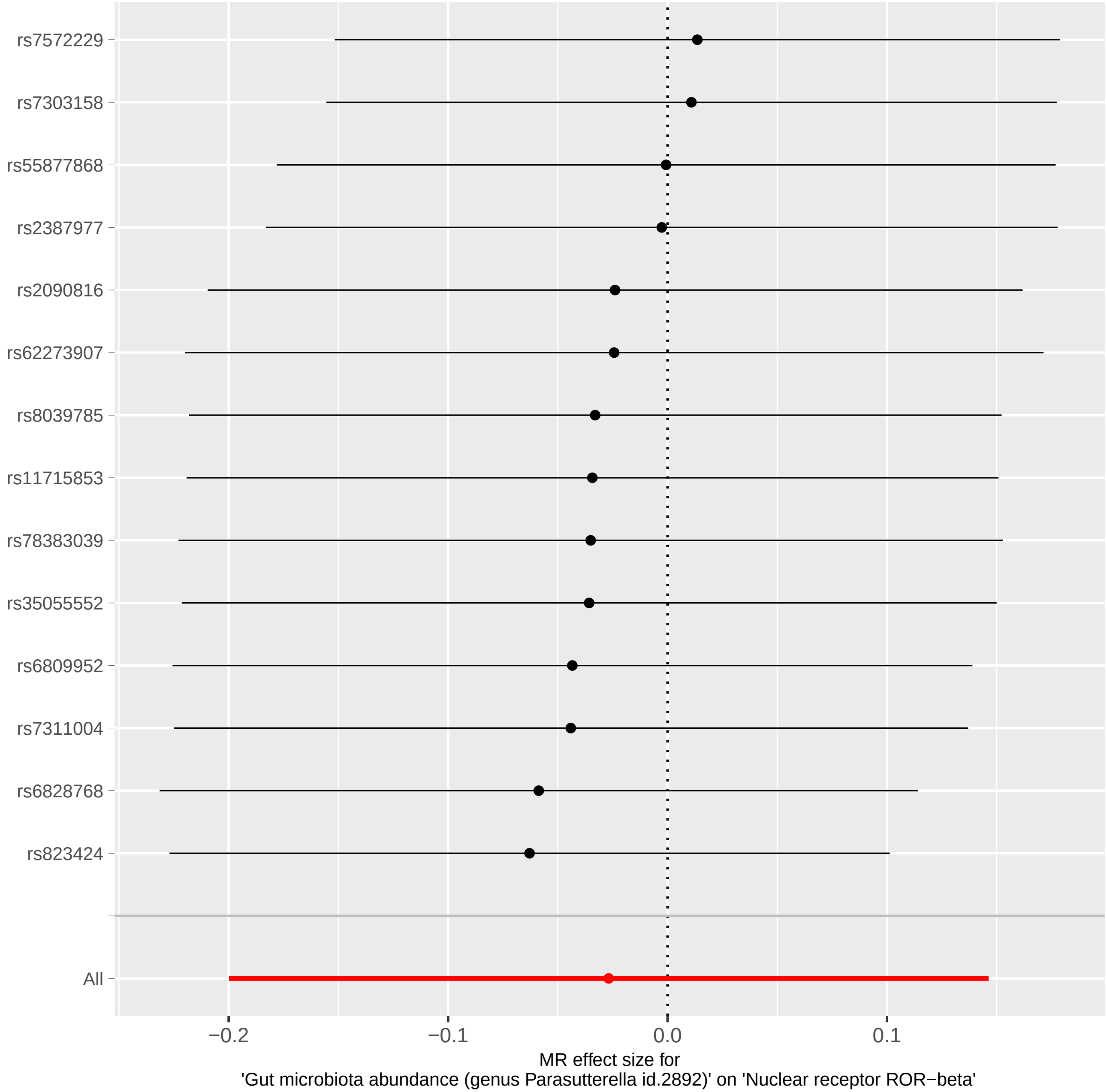

Batch 2−5167 : Gut microbiota abundance (genus Catenibacterium id.2153) on Ribulose−phosphate 3−epimerase with a threshold of 1e−05

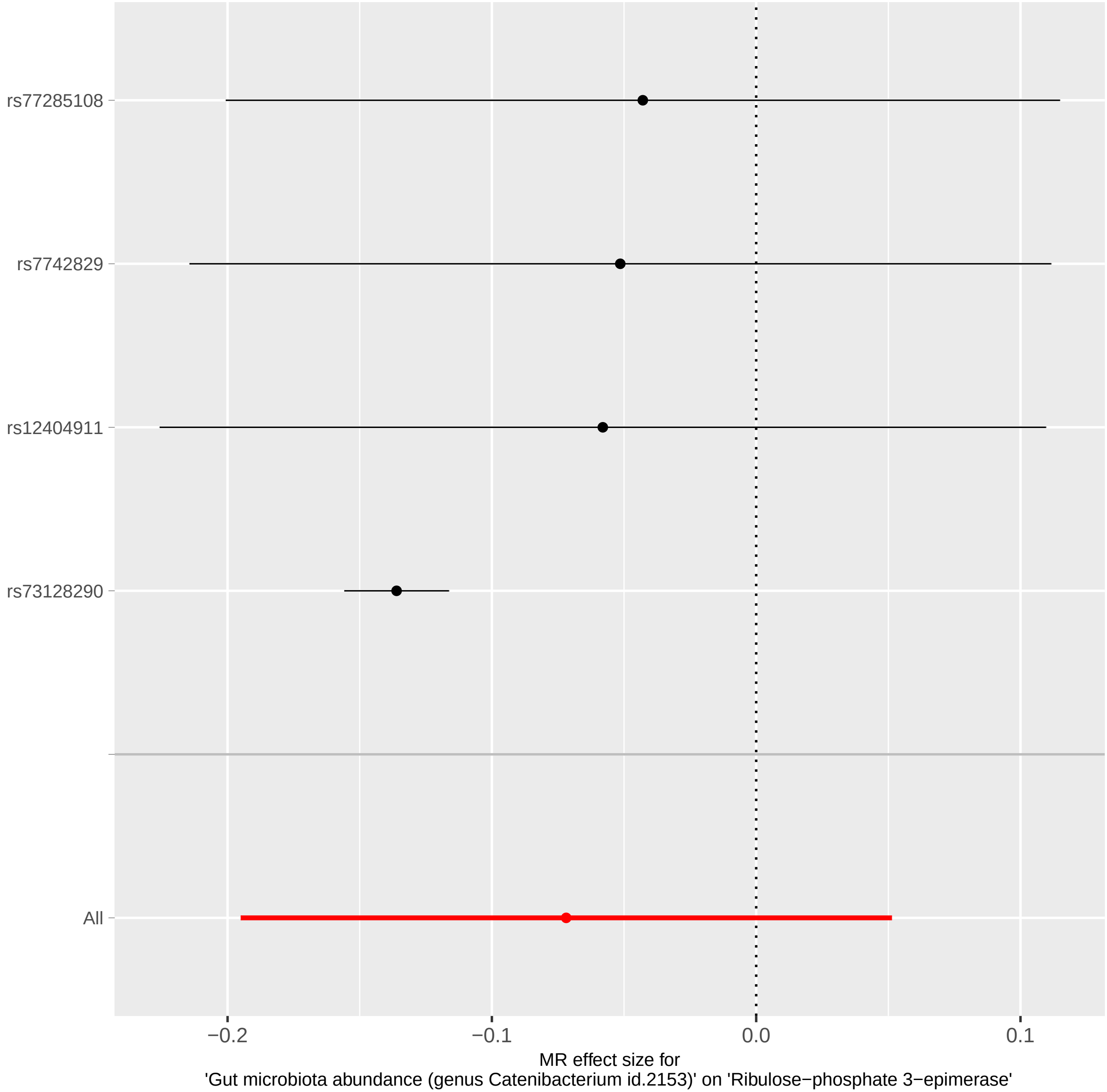

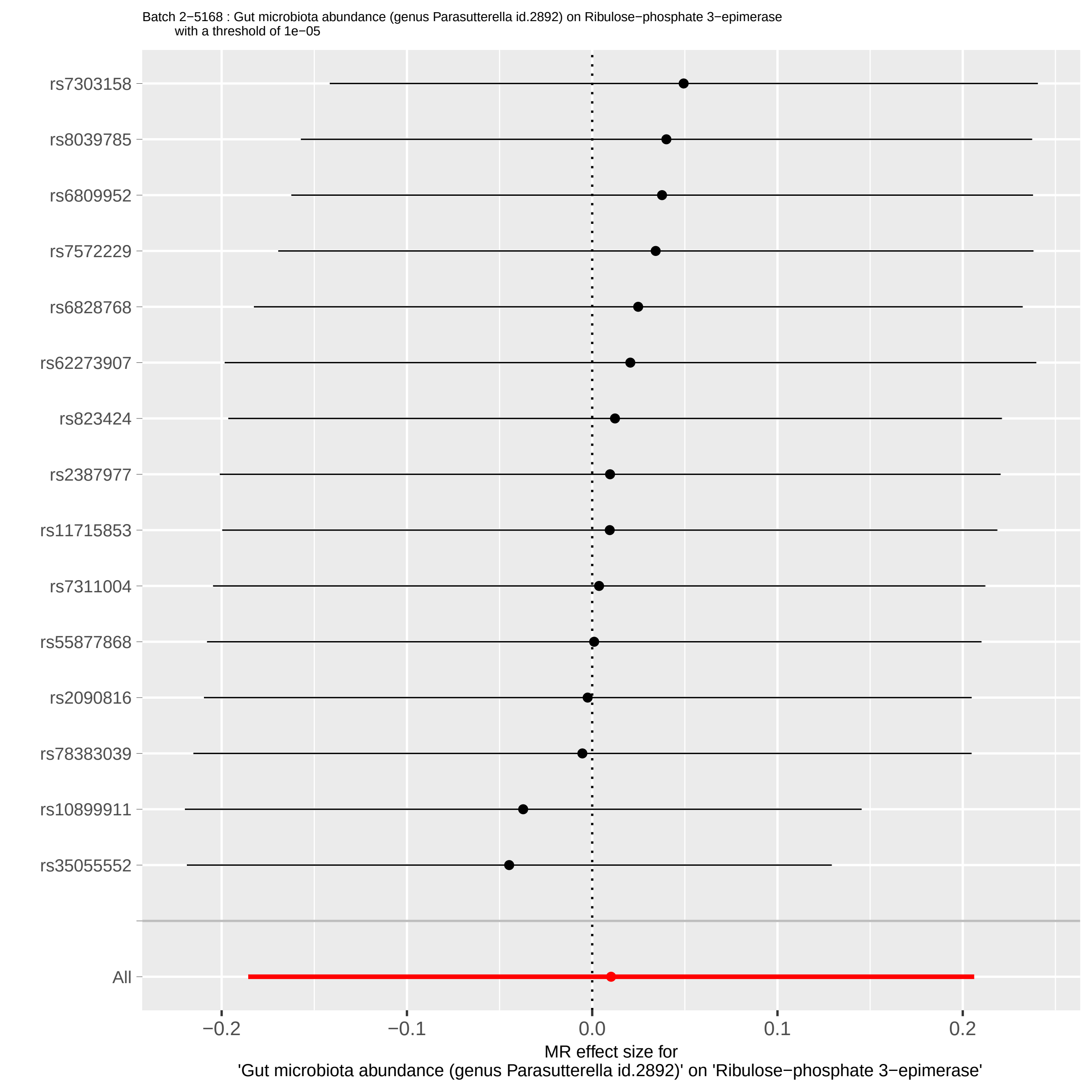

Batch 2−5168 : Gut microbiota abundance (genus Parasutterella id.2892) on Ribulose−phosphate 3−epimerase
with a threshold of 1e−05
rs7303158
rs8039785
rs6809952
rs7572229
rs6828768
rs62273907
rs823424
rs2387977
rs11715853
rs7311004
rs55877868
rs2090816
rs78383039
rs10899911
rs35055552
All
−0.2
−0.1
0.0
0.1
0.2
MR effect size for
'Gut microbiota abundance (genus Parasutterella id.2892)' on 'Ribulose−phosphate 3−epimerase'

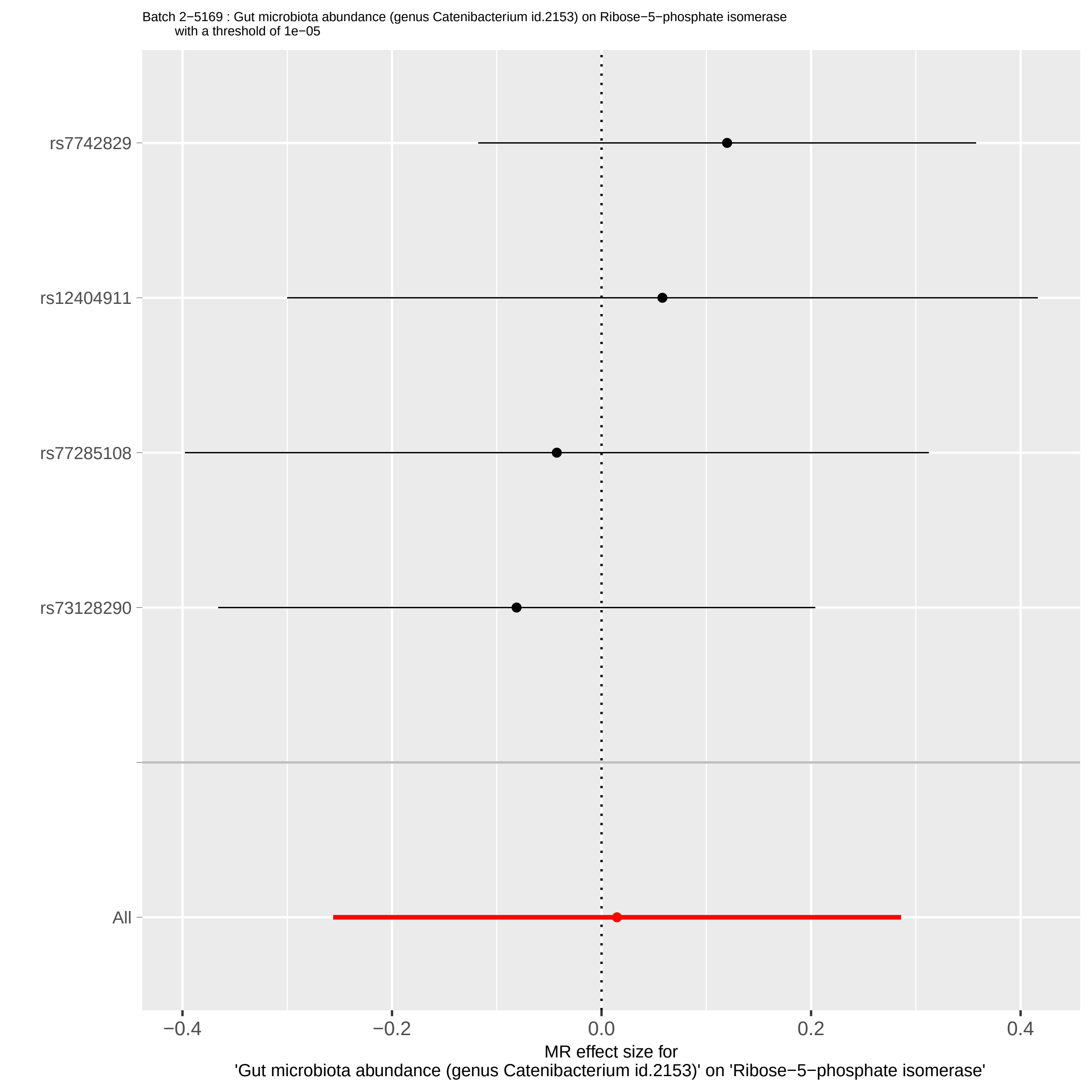
Batch 2−5169 : Gut microbiota abundance (genus Catenibacterium id.2153) on Ribose−5−phosphate isomerase
with a threshold of 1e−05
rs7742829
rs12404911
rs77285108
rs73128290
All
−0.4
−0.2
0.0
0.2
0.4
MR effect size for
'Gut microbiota abundance (genus Catenibacterium id.2153)' on 'Ribose−5−phosphate isomerase'

Batch 2−5170 : Gut microbiota abundance (genus Parasutterella id.2892) on Ribose−5−phosphate isomerase
with a threshold of 1e−05

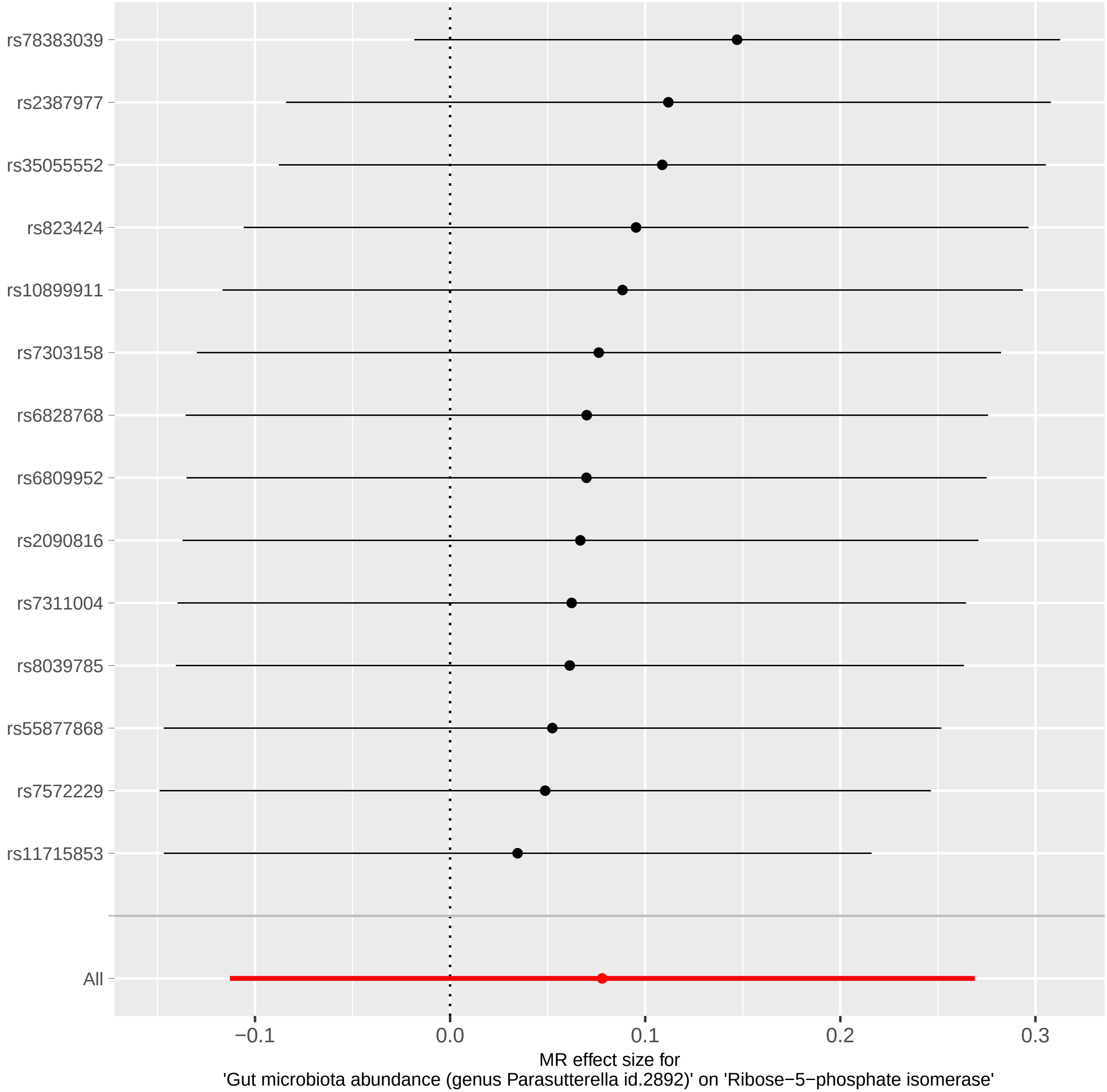

Batch 2−5171 : Gut microbiota abundance (genus Catenibacterium id.2153) on 60S ribosomal protein L30
with a threshold of 1e−05

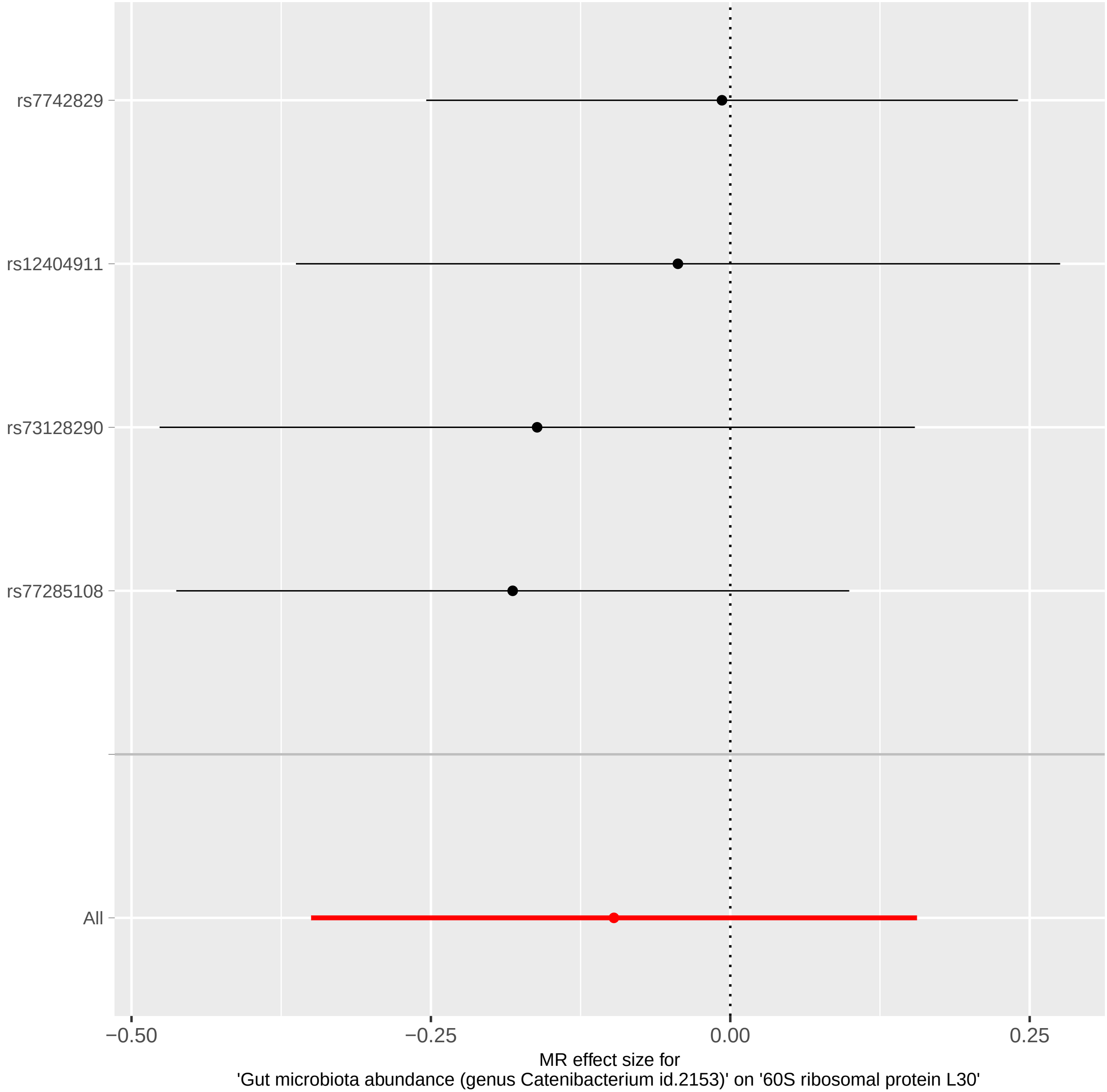

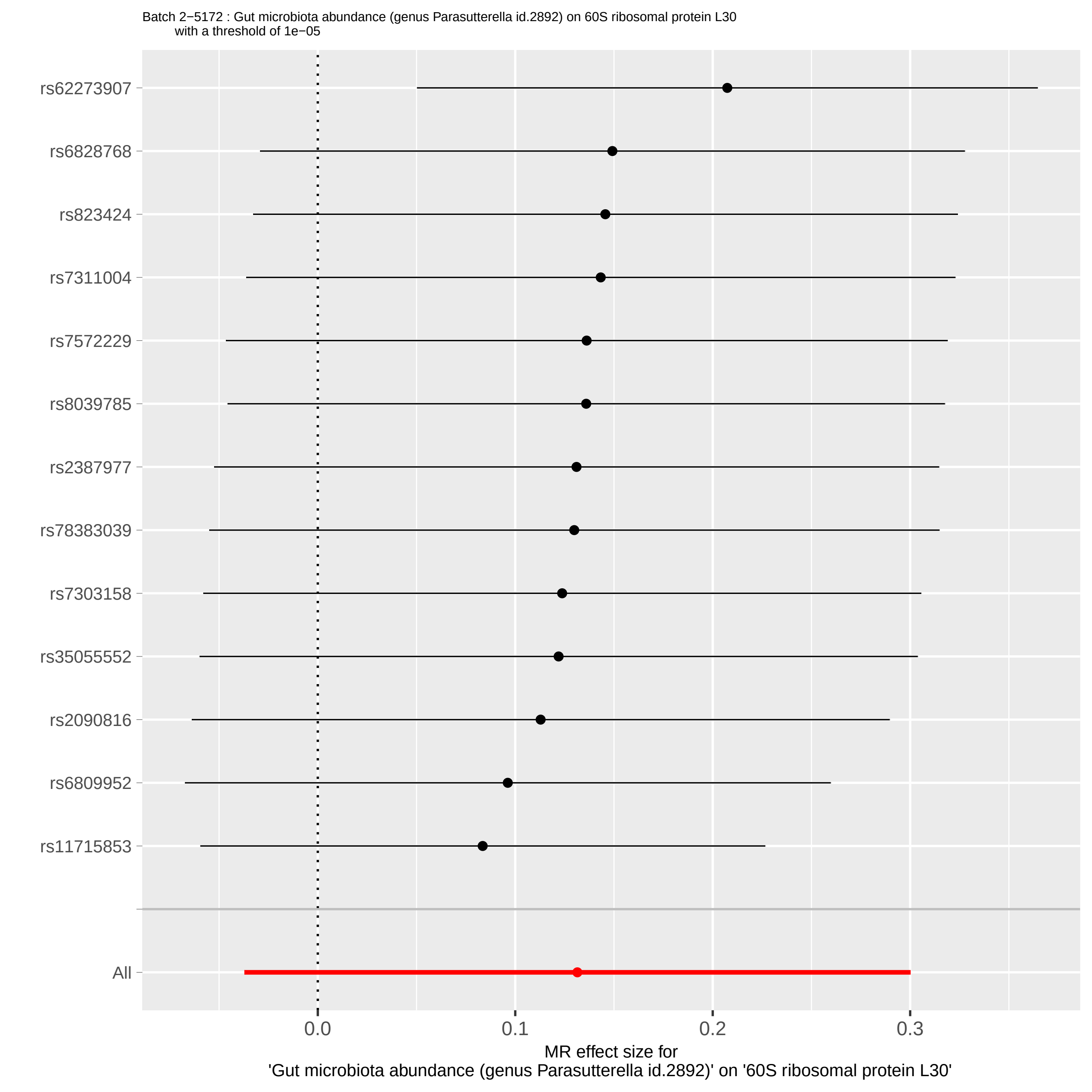

Batch 2−5172 : Gut microbiota abundance (genus Parasutterella id.2892) on 60S ribosomal protein L30
with a threshold of 1e−05
rs62273907
rs6828768
rs823424
rs7311004
rs7572229
rs8039785
rs2387977
rs78383039
rs7303158
rs35055552
rs2090816
rs6809952
rs11715853
All
0.0
0.1
0.2
0.3
MR effect size for
'Gut microbiota abundance (genus Parasutterella id.2892)' on '60S ribosomal protein L30'

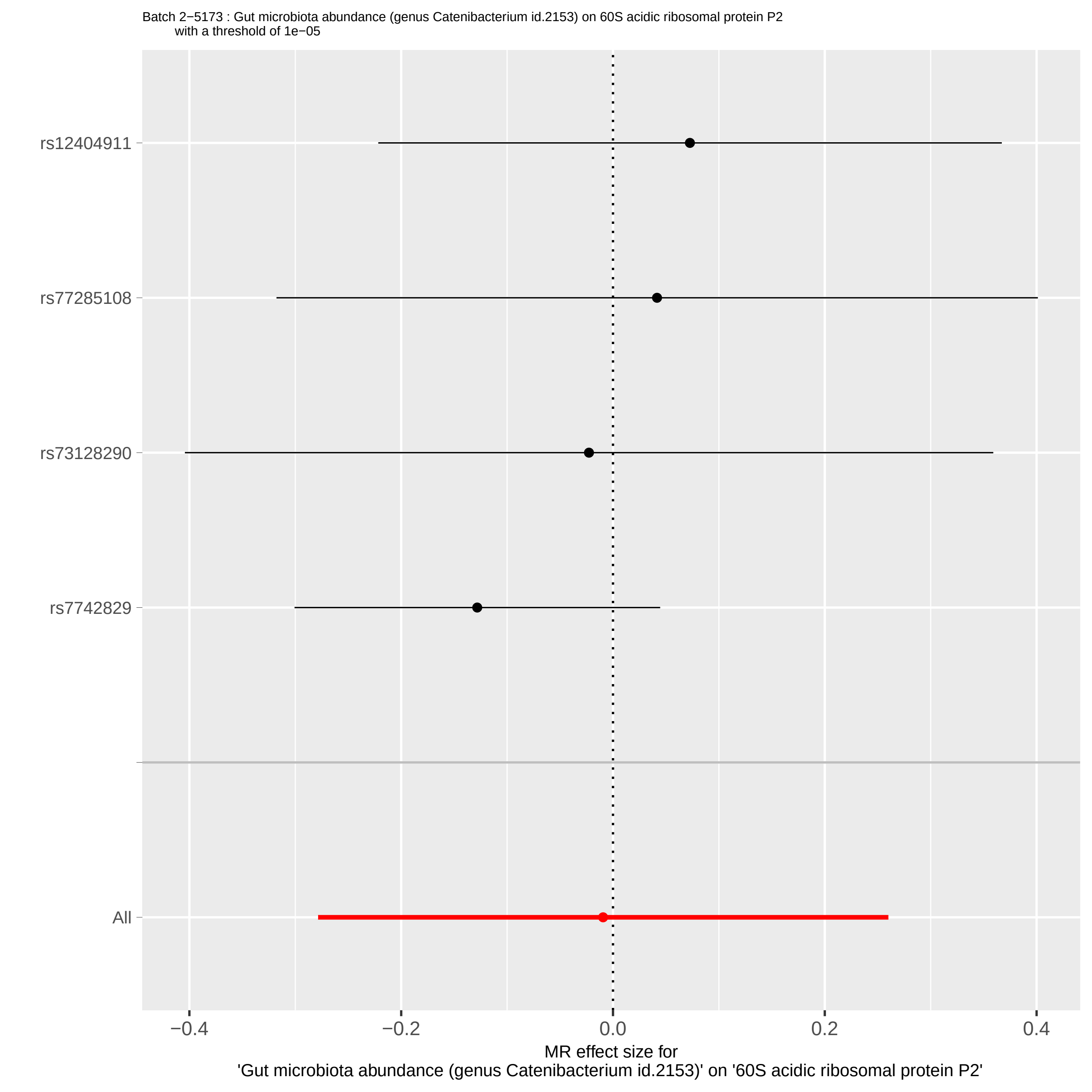
Batch 2−5173 : Gut microbiota abundance (genus Catenibacterium id.2153) on 60S acidic ribosomal protein P2
with a threshold of 1e−05
rs12404911
rs77285108
rs73128290
rs7742829
All
−0.4
−0.2
0.0
0.2
0.4
MR effect size for
'Gut microbiota abundance (genus Catenibacterium id.2153)' on '60S acidic ribosomal protein P2'

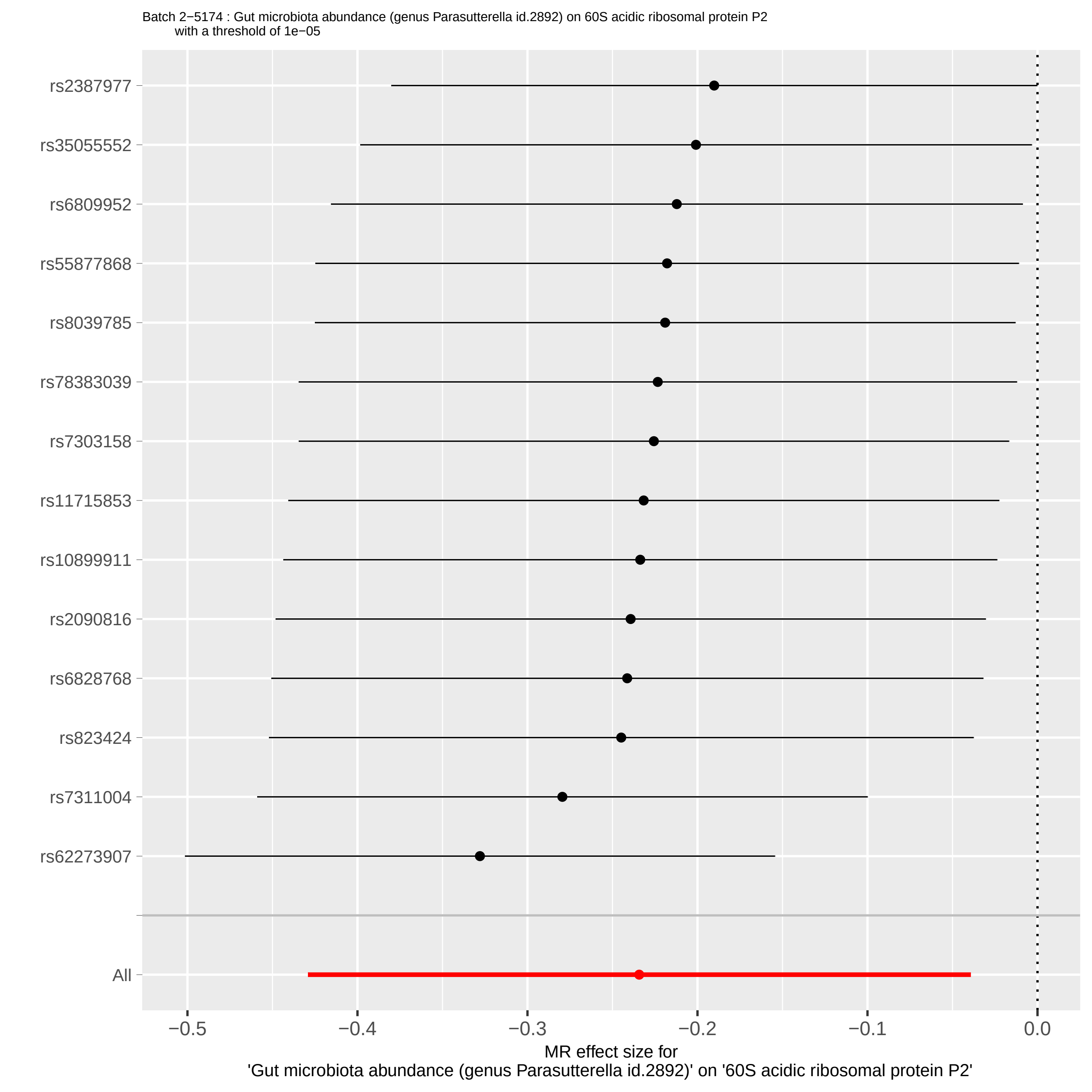

Batch 2−5174 : Gut microbiota abundance (genus Parasutterella id.2892) on 60S acidic ribosomal protein P2
with a threshold of 1e−05
rs2387977
rs35055552
rs6809952
rs55877868
rs8039785
rs78383039
rs7303158
rs11715853
rs10899911
rs2090816
rs6828768
rs823424
rs7311004
rs62273907
All
−0.5
−0.4
−0.3
−0.2
−0.1
0.0
MR effect size for
'Gut microbiota abundance (genus Parasutterella id.2892)' on '60S acidic ribosomal protein P2'

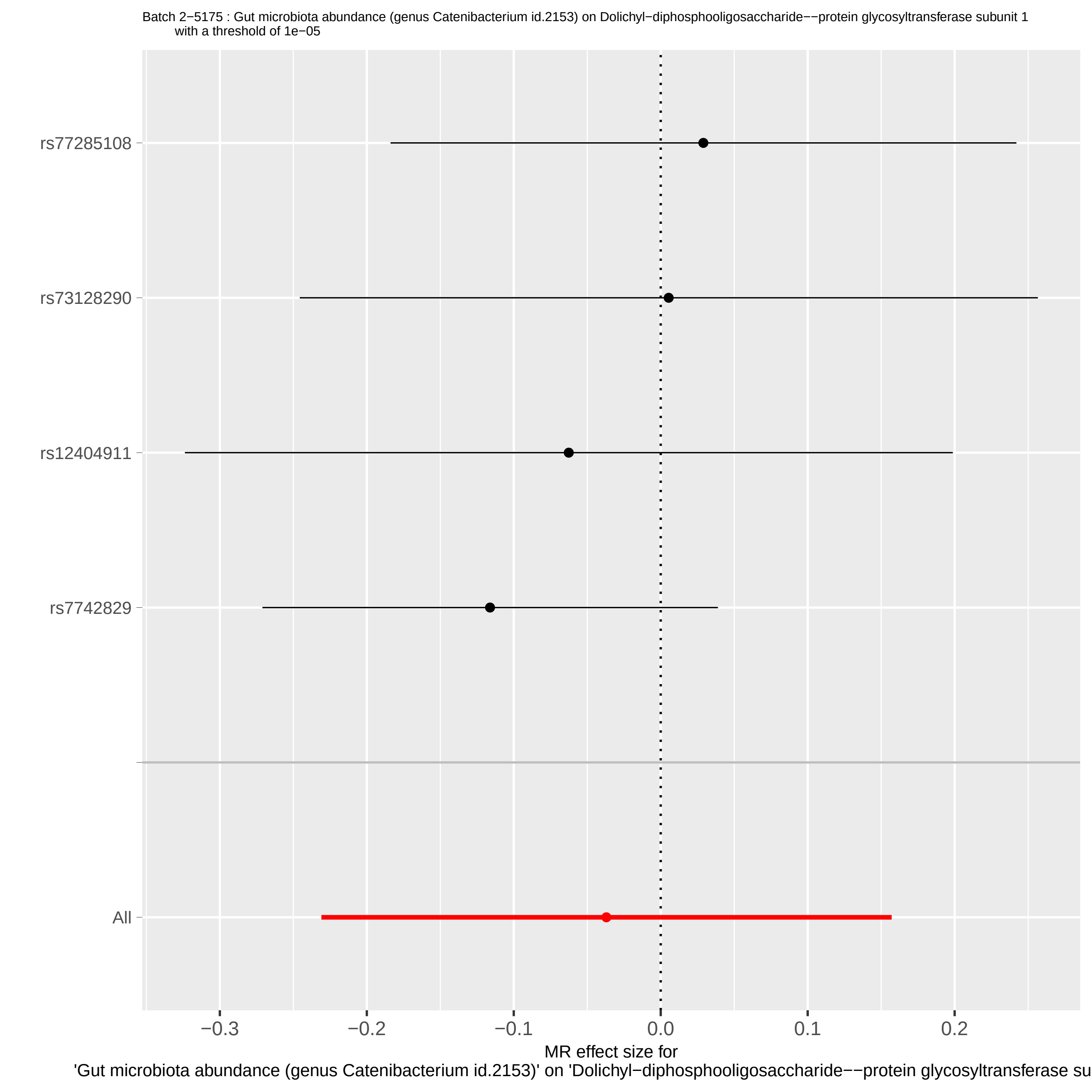
Batch 2−5175 : Gut microbiota abundance (genus Catenibacterium id.2153) on Dolichyl−diphosphooligosaccharide−−protein glycosyltransferase subunit 1
with a threshold of 1e−05
rs77285108
rs73128290
rs12404911
rs7742829
All
−0.3
−0.2
−0.1
0.0
0.1
0.2
MR effect size for
'Gut microbiota abundance (genus Catenibacterium id.2153)' on 'Dolichyl−diphosphooligosaccharide−−protein glycosyltransferase su

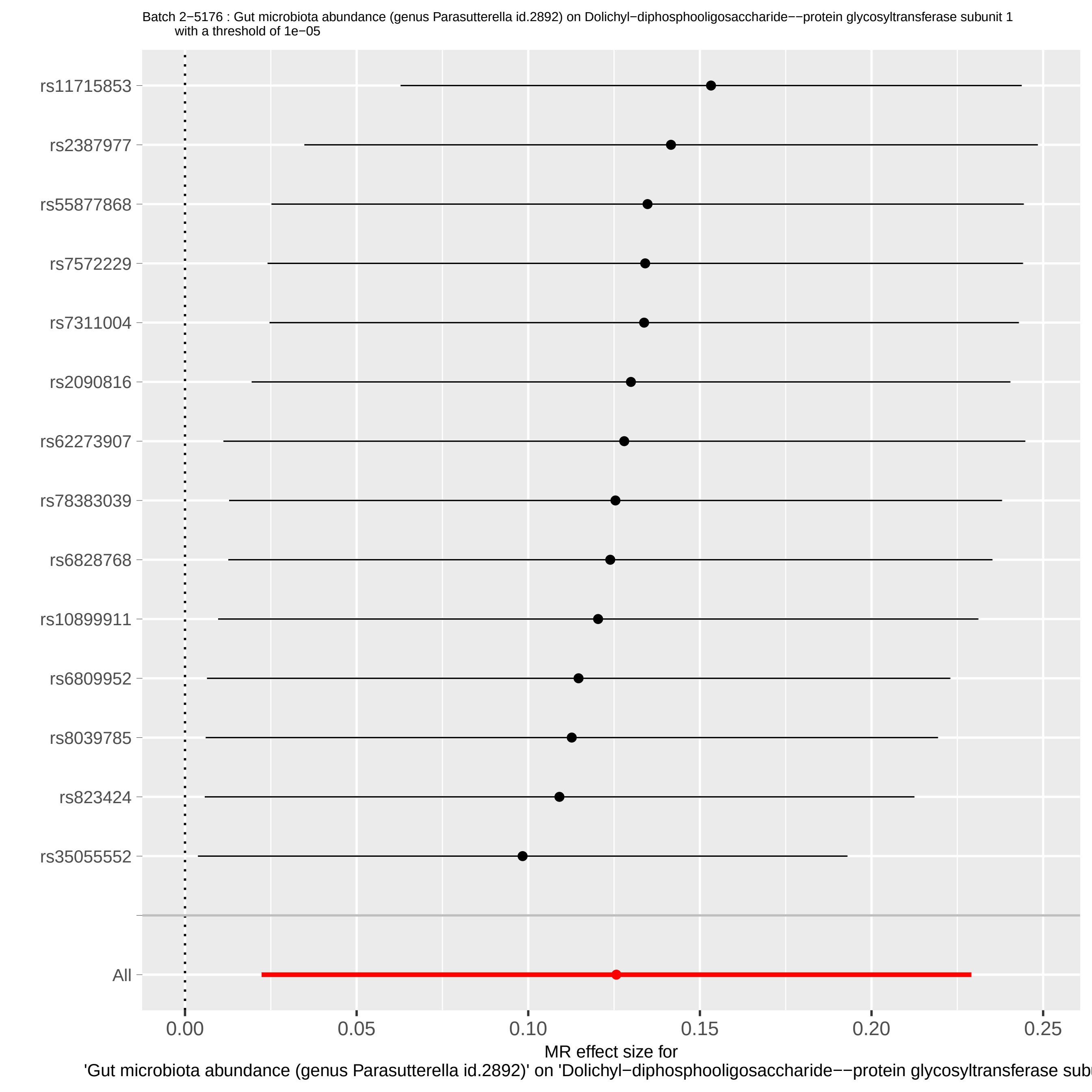

Batch 2−5176 : Gut microbiota abundance (genus Parasutterella id.2892) on Dolichyl−diphosphooligosaccharide−−protein glycosyltransferase subunit 1 with a threshold of 1e−05
rs11715853
rs2387977
rs55877868
rs7572229
rs7311004
rs2090816
rs62273907
rs78383039
rs6828768
rs10899911
rs6809952
rs8039785
rs823424
rs35055552
All
0.00
0.05
0.10
0.15
0.20
0.25
MR effect size for
'Gut microbiota abundance (genus Parasutterella id.2892)' on 'Dolichyl−diphosphooligosaccharide−−protein glycosyltransferase sub

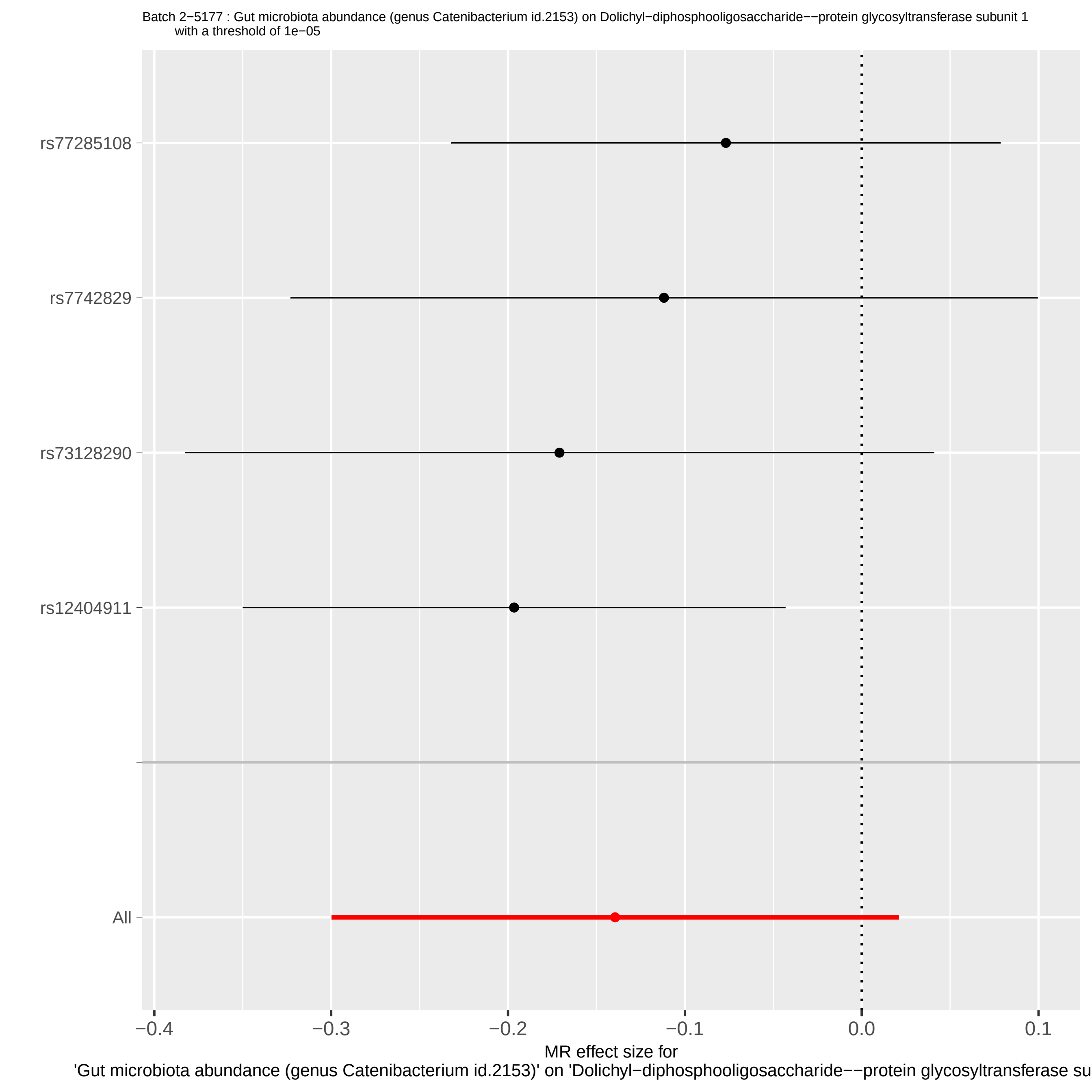

Batch 2−5177 : Gut microbiota abundance (genus Catenibacterium id.2153) on Dolichyl−diphosphooligosaccharide−−protein glycosyltransferase subunit 1
with a threshold of 1e−05
rs77285108
rs7742829
rs73128290
rs12404911
All
−0.4
−0.3
−0.2
−0.1
0.0
0.1
MR effect size for
'Gut microbiota abundance (genus Catenibacterium id.2153)' on 'Dolichyl−diphosphooligosaccharide−−protein glycosyltransferase su

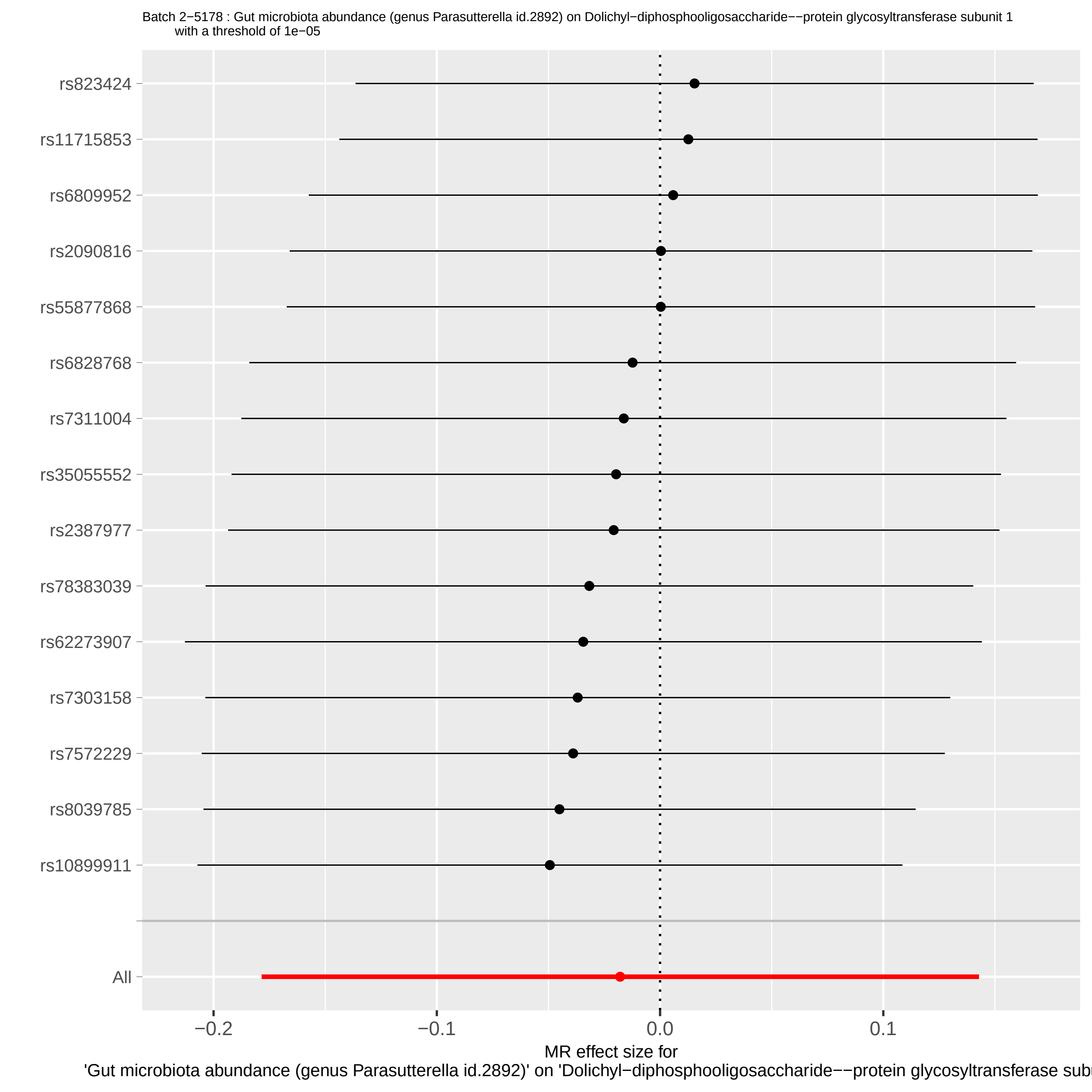

Batch 2−5178 : Gut microbiota abundance (genus Parasutterella id.2892) on Dolichyl−diphosphooligosaccharide−−protein glycosyltransferase subunit 1
with a threshold of 1e−05
rs823424
rs11715853
rs6809952
rs2090816
rs55877868
rs6828768
rs7311004
rs35055552
rs2387977
rs78383039
rs62273907
rs7303158
rs7572229
rs8039785
rs10899911
All
−0.2
−0.1
0.0
0.1
MR effect size for
'Gut microbiota abundance (genus Parasutterella id.2892)' on 'Dolichyl−diphosphooligosaccharide−−protein glycosyltransferase sub

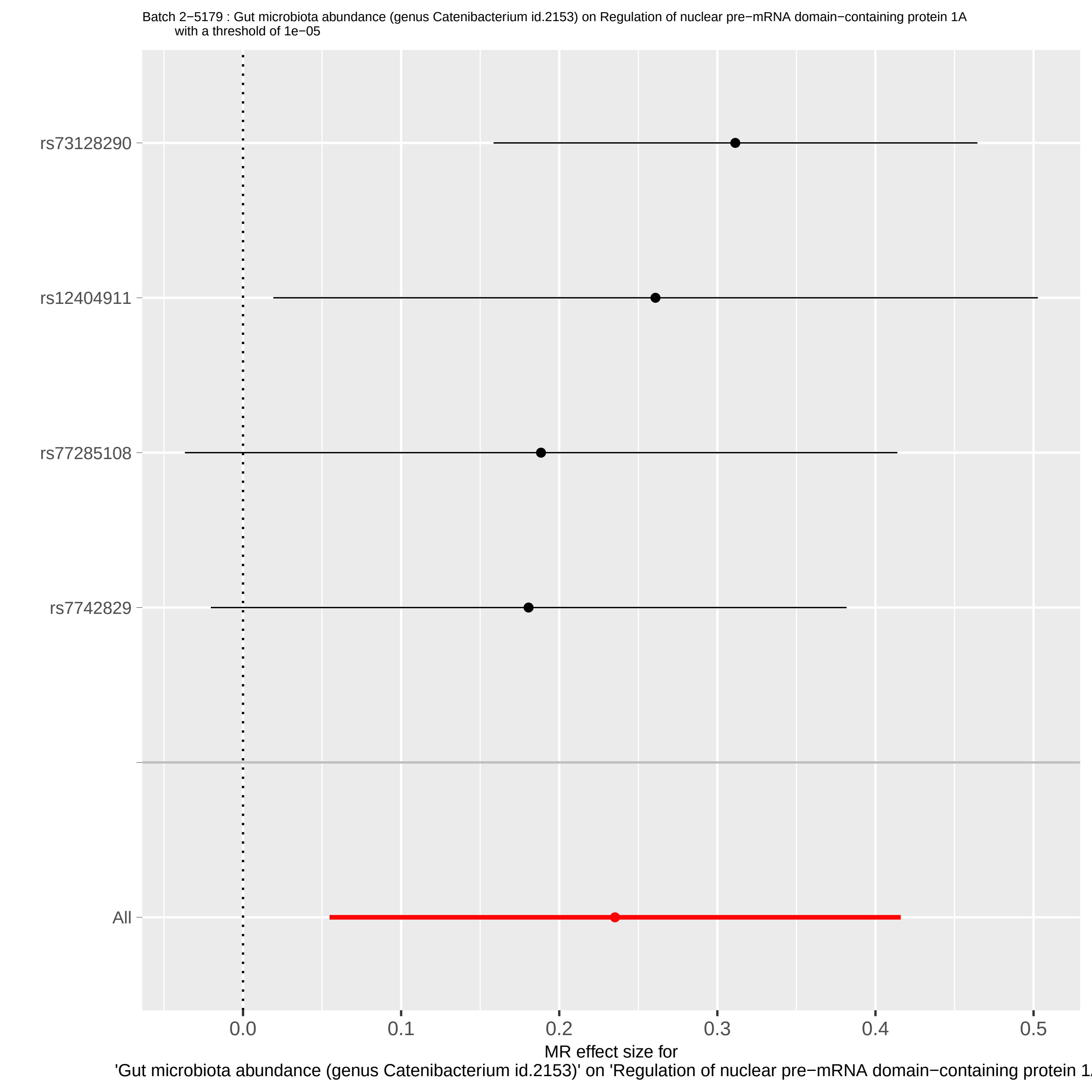
Batch 2−5179 : Gut microbiota abundance (genus Catenibacterium id.2153) on Regulation of nuclear pre−mRNA domain−containing protein 1A
with a threshold of 1e−05
rs73128290
rs12404911
rs77285108
rs7742829
All
0.0
0.1
0.2
0.3
0.4
0.5
MR effect size for
'Gut microbiota abundance (genus Catenibacterium id.2153)' on 'Regulation of nuclear pre−mRNA domain−containing protein 1

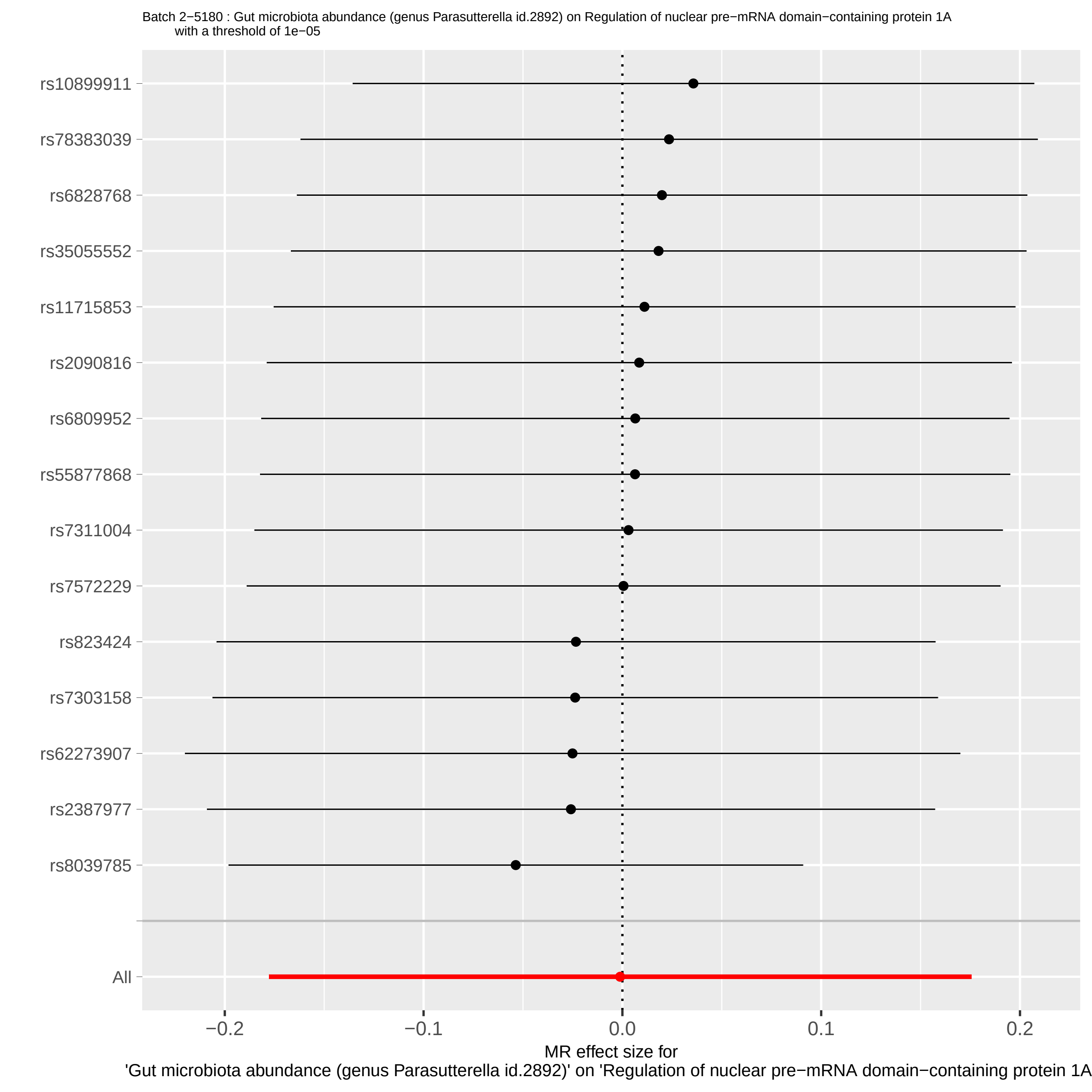
Batch 2−5180 : Gut microbiota abundance (genus Parasutterella id.2892) on Regulation of nuclear pre−mRNA domain−containing protein 1A
with a threshold of 1e−05
rs10899911
rs78383039
rs6828768
rs35055552
rs11715853
rs2090816
rs6809952
rs55877868
rs7311004
rs7572229
rs823424
rs7303158
rs62273907
rs2387977
rs8039785
All
−0.2
−0.1
0.0
0.1
0.2
MR effect size for
'Gut microbiota abundance (genus Parasutterella id.2892)' on 'Regulation of nuclear pre−mRNA domain−containing protein 1A

Batch 2−5181 : Gut microbiota abundance (genus Catenibacterium id.2153) on Ubiquitin+1, truncated mutation for UbB
with a threshold of 1e−05

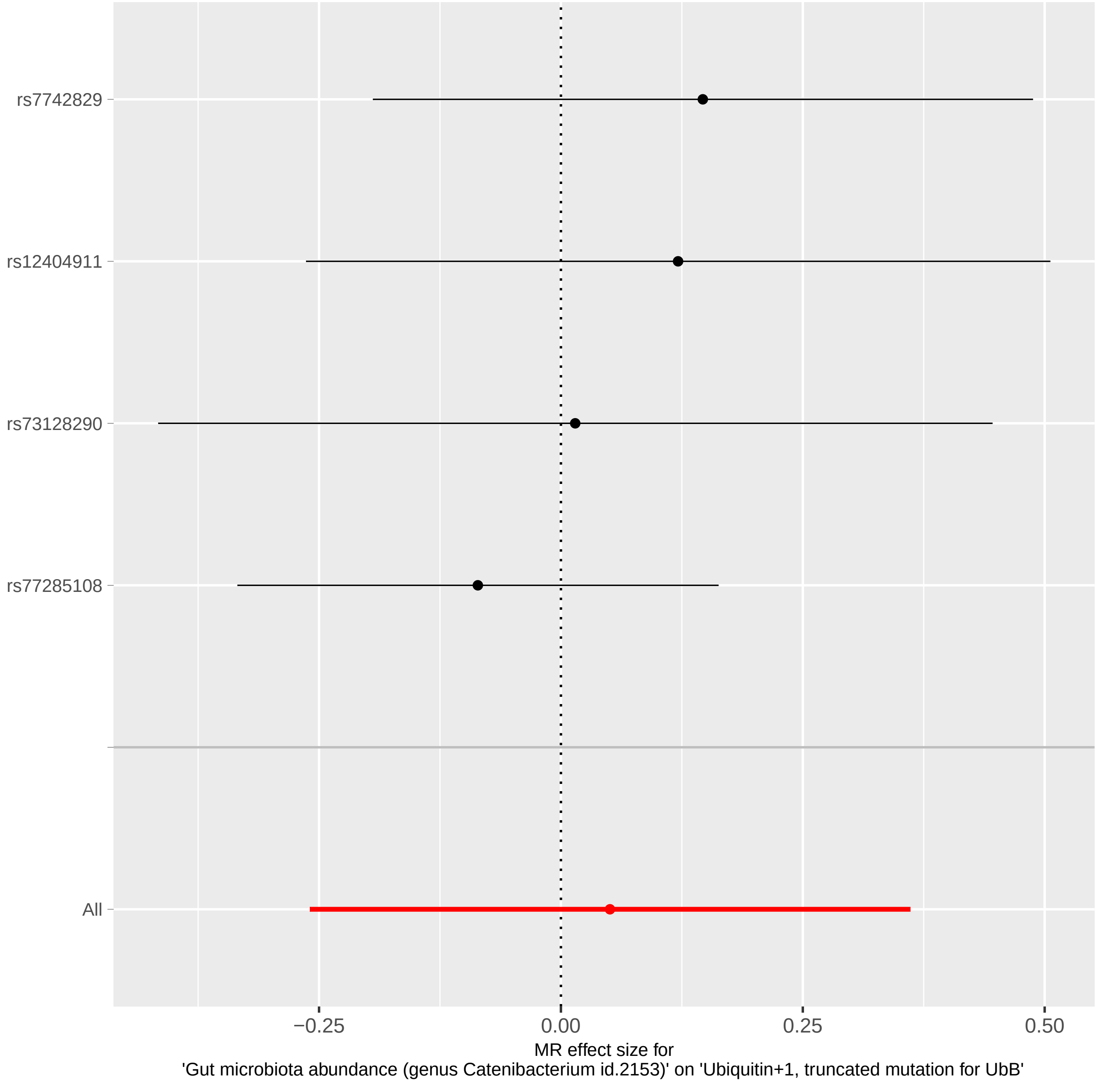

Batch 2−5182 : Gut microbiota abundance (genus Parasutterella id.2892) on Ubiquitin+1, truncated mutation for UbB
with a threshold of 1e−05

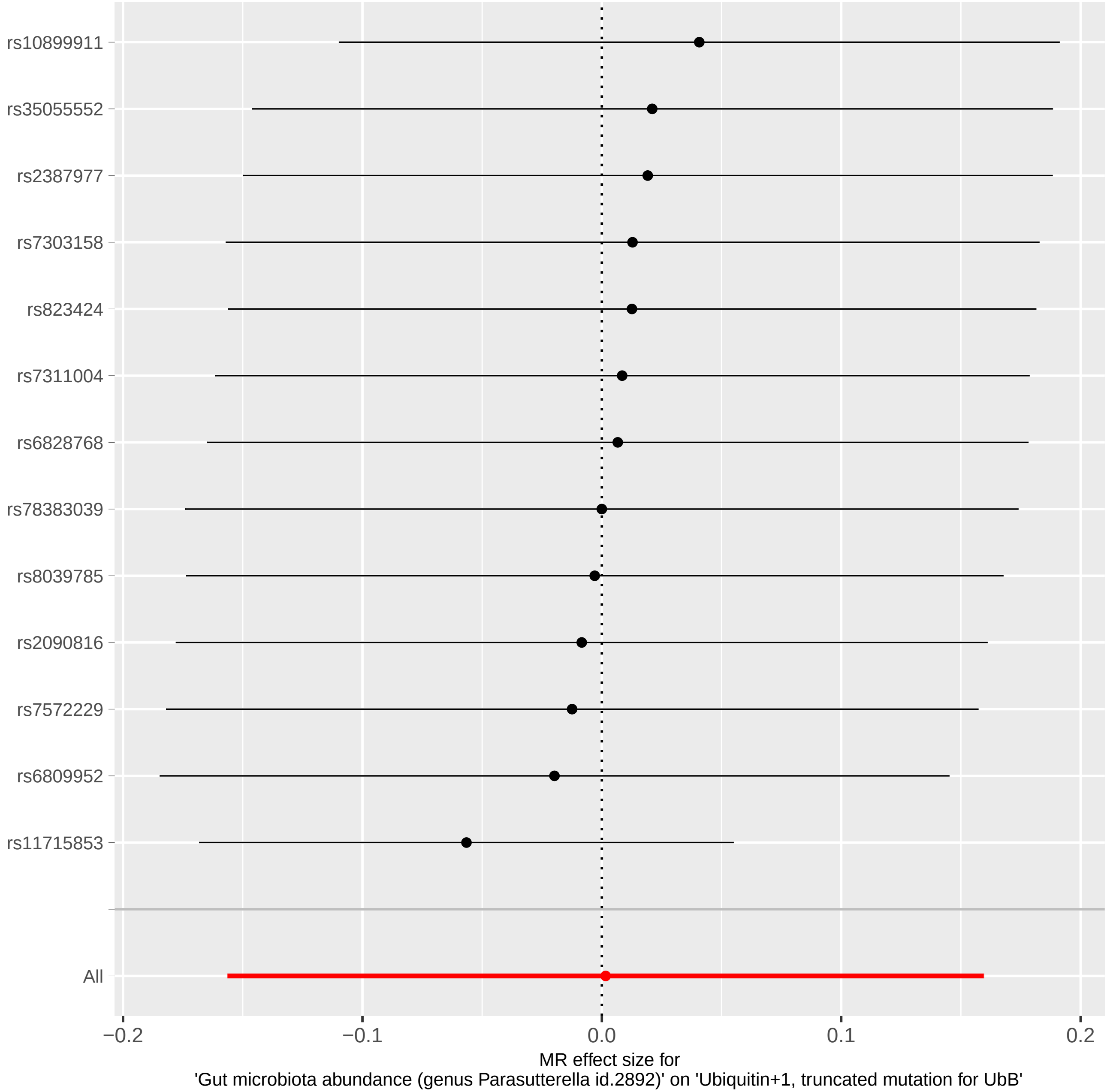

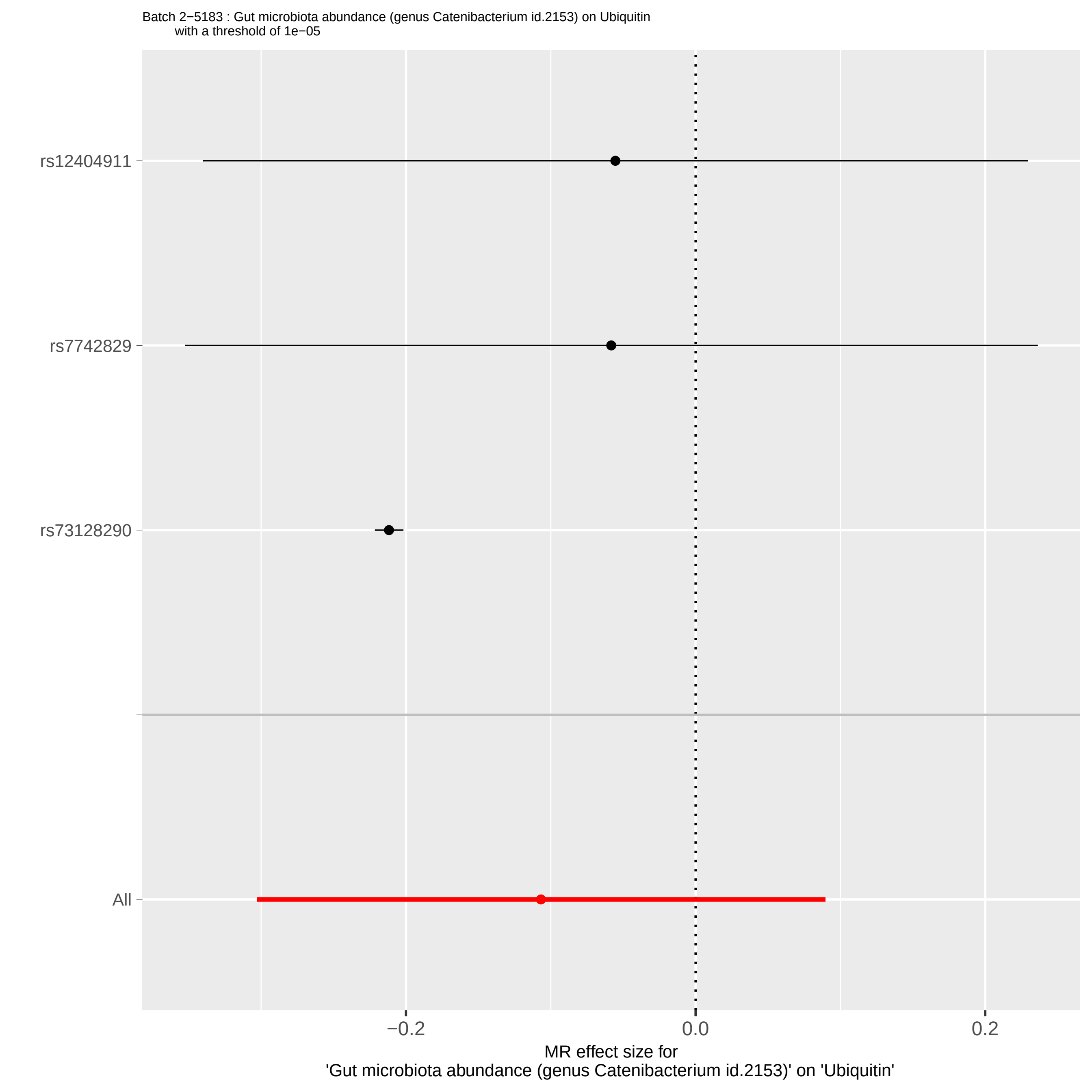
Batch 2−5183 : Gut microbiota abundance (genus Catenibacterium id.2153) on Ubiquitin
with a threshold of 1e−05
rs12404911
rs7742829
rs73128290
All
−0.2
0.0
0.2
MR effect size for
'Gut microbiota abundance (genus Catenibacterium id.2153)' on 'Ubiquitin'

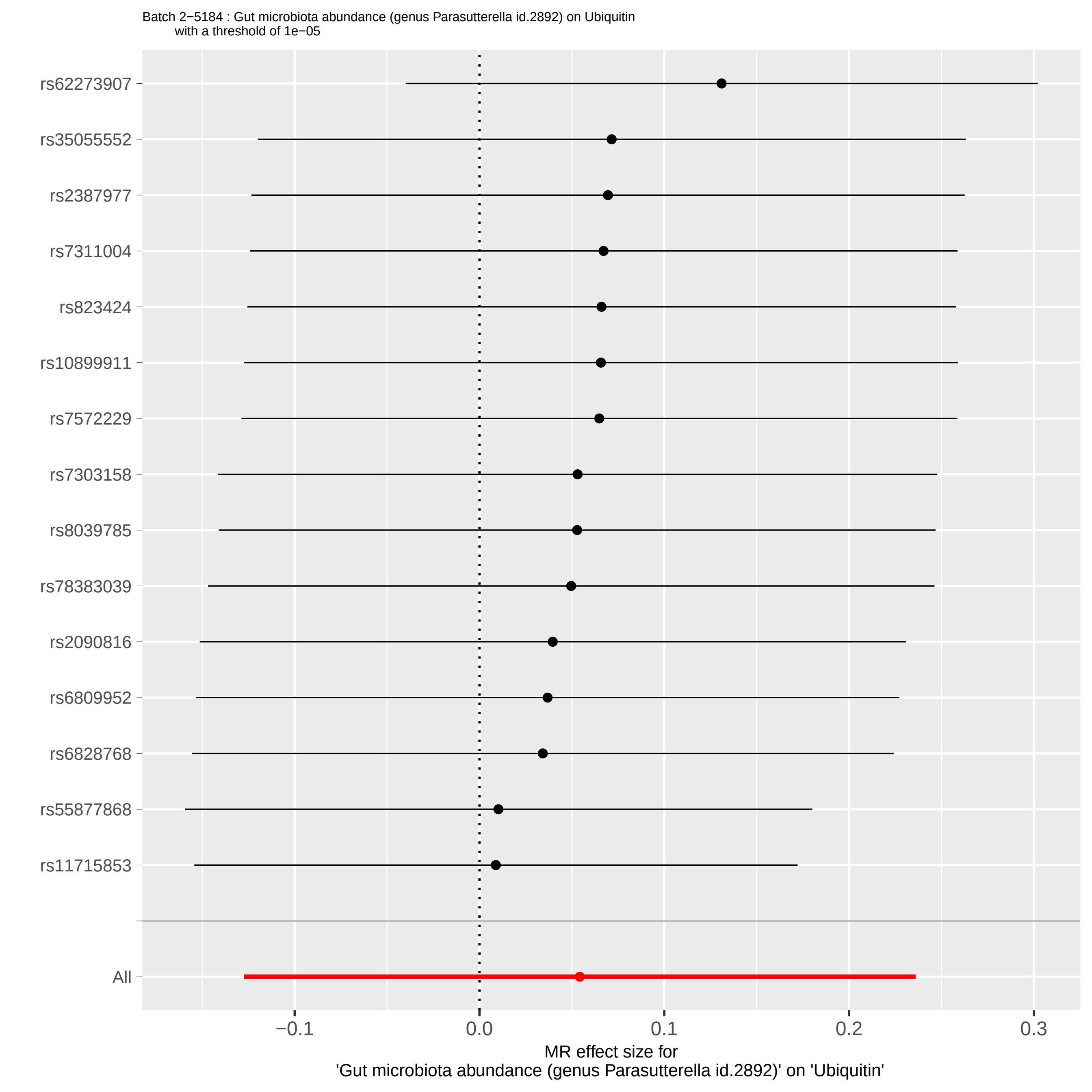
Batch 2−5184 : Gut microbiota abundance (genus Parasutterella id.2892) on Ubiquitin
with a threshold of 1e−05
rs62273907
rs35055552
rs2387977
rs7311004
rs823424
rs10899911
rs7572229
rs7303158
rs8039785
rs78383039
rs2090816
rs6809952
rs6828768
rs55877868
rs11715853
All
−0.1
0.0
0.1
0.2
0.3
MR effect size for
'Gut microbiota abundance (genus Parasutterella id.2892)' on 'Ubiquitin'

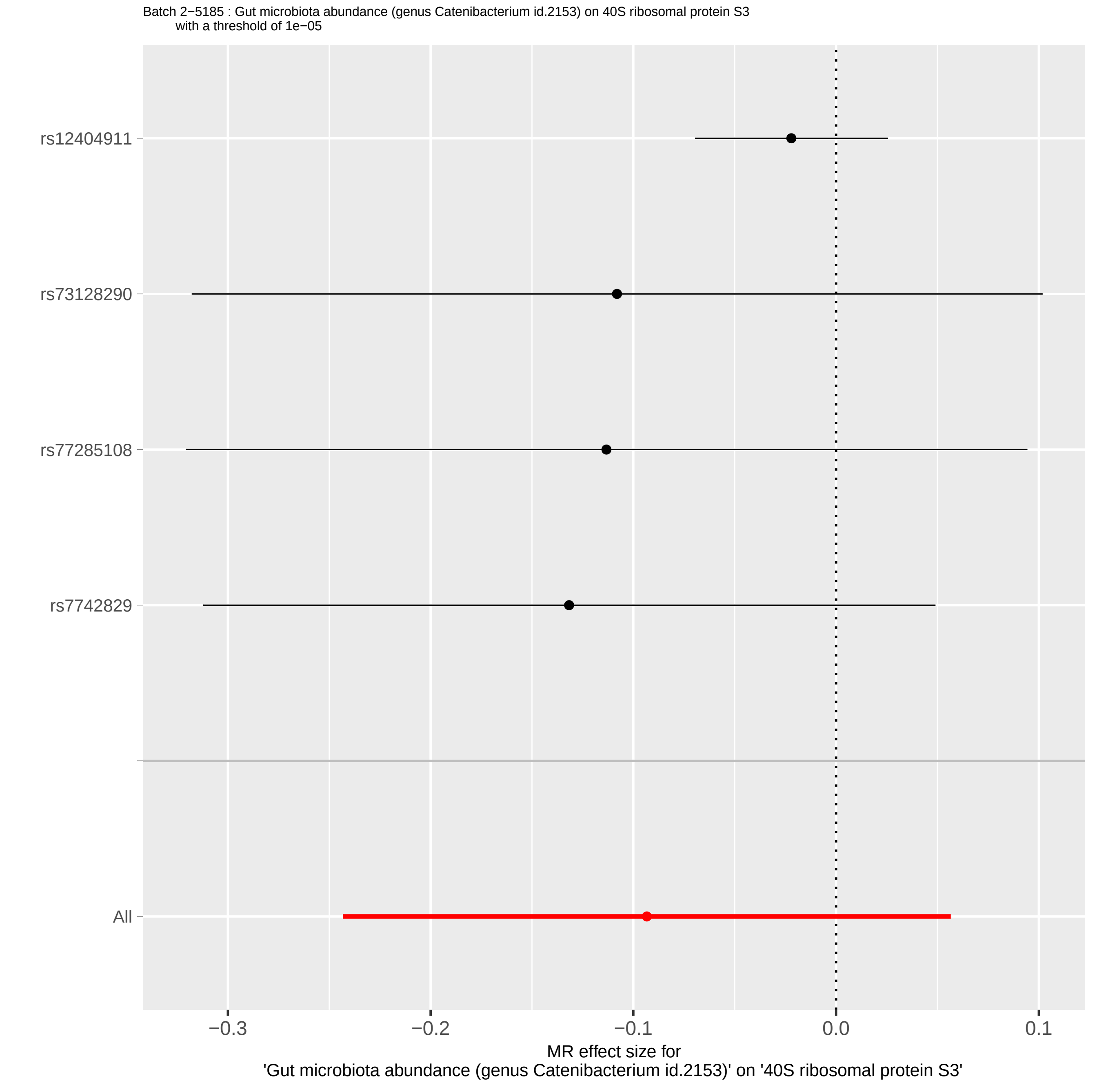
Batch 2−5185 : Gut microbiota abundance (genus Catenibacterium id.2153) on 40S ribosomal protein S3
with a threshold of 1e−05
rs12404911
rs73128290
rs77285108
rs7742829
All
−0.3
−0.2
−0.1
0.0
0.1
MR effect size for
'Gut microbiota abundance (genus Catenibacterium id.2153)' on '40S ribosomal protein S3'

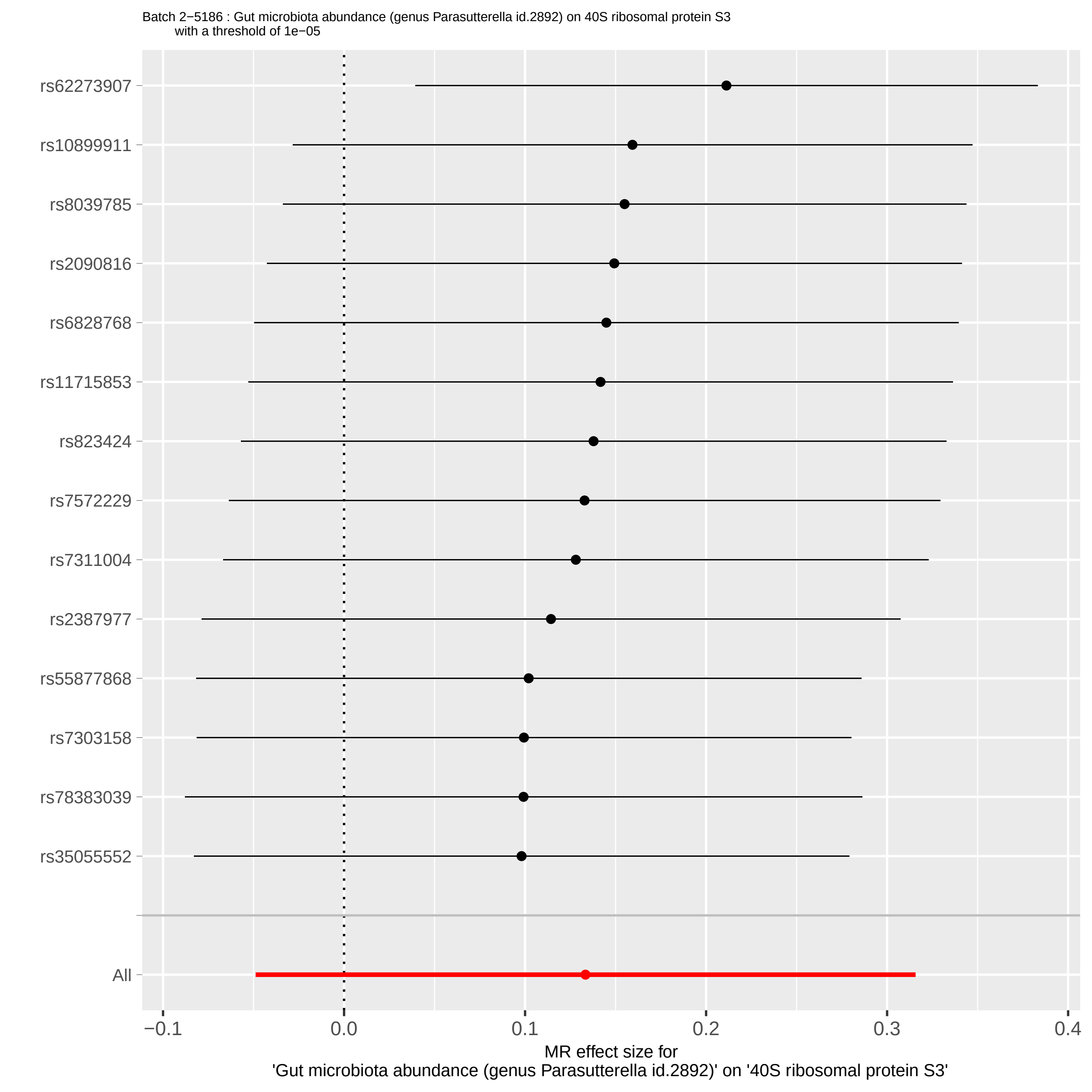

Batch 2−5186 : Gut microbiota abundance (genus Parasutterella id.2892) on 40S ribosomal protein S3
with a threshold of 1e−05
rs62273907
rs10899911
rs8039785
rs2090816
rs6828768
rs11715853
rs823424
rs7572229
rs7311004
rs2387977
rs55877868
rs7303158
rs78383039
rs35055552
All
−0.1
0.0
0.1
0.2
0.3
0.4
MR effect size for
'Gut microbiota abundance (genus Parasutterella id.2892)' on '40S ribosomal protein S3'

Batch 2−5187 : Gut microbiota abundance (genus Catenibacterium id.2153) on 40S ribosomal protein S3a
with a threshold of 1e−05

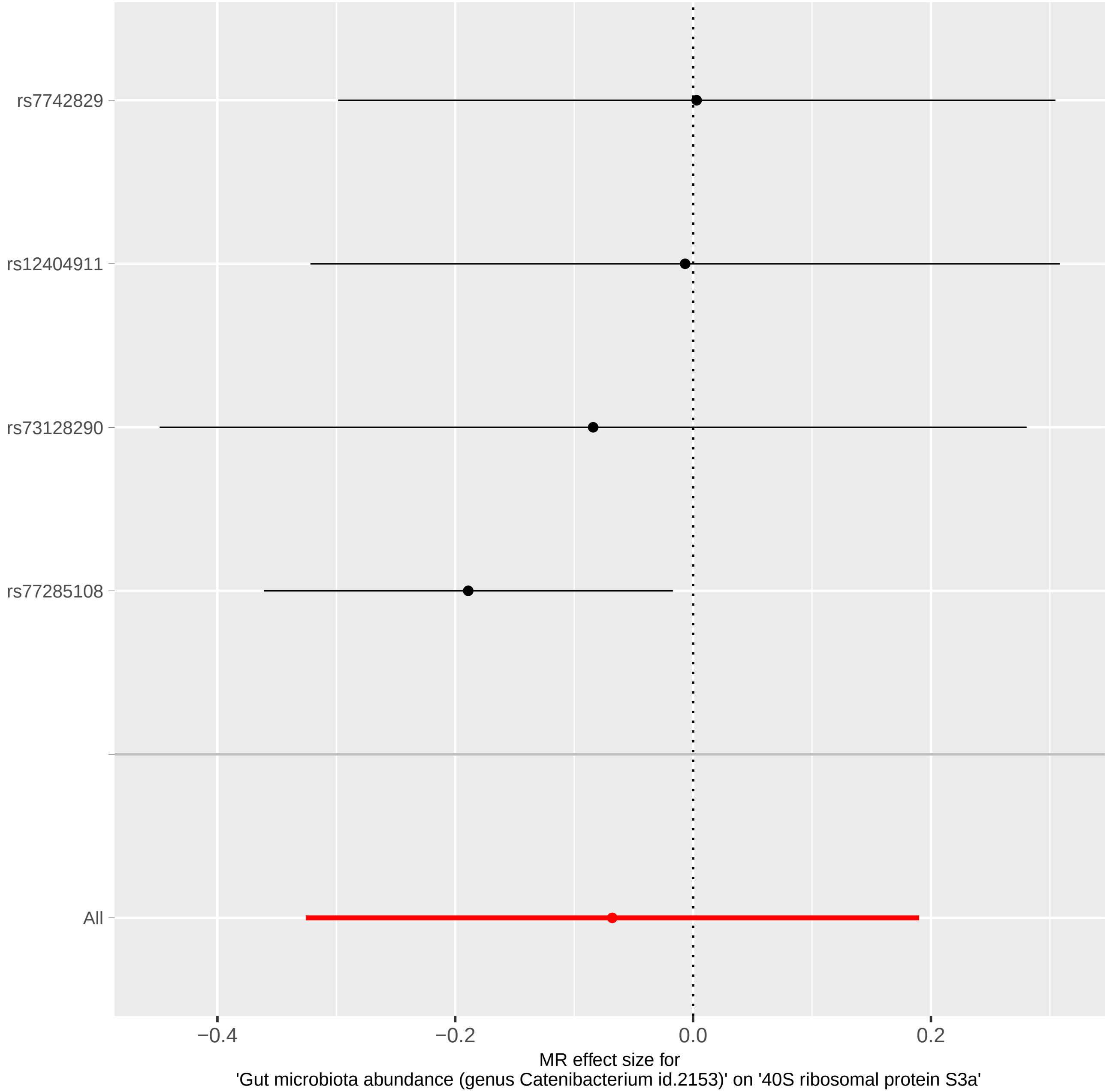

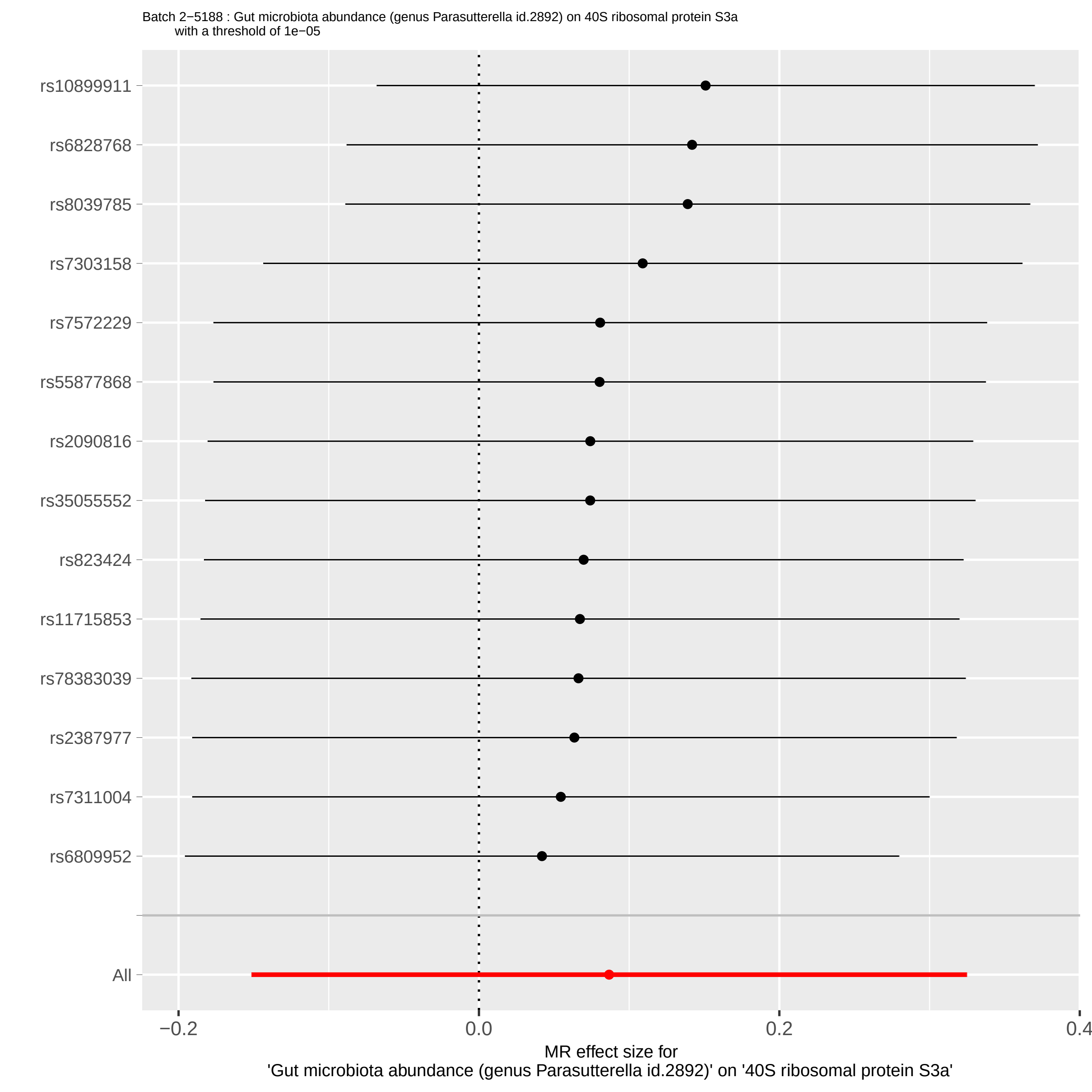

Batch 2−5188 : Gut microbiota abundance (genus Parasutterella id.2892) on 40S ribosomal protein S3a
with a threshold of 1e−05
rs10899911
rs6828768
rs8039785
rs7303158
rs7572229
rs55877868
rs2090816
rs35055552
rs823424
rs11715853
rs78383039
rs2387977
rs7311004
rs6809952
All
−0.2
0.0
0.2
0.4
MR effect size for
'Gut microbiota abundance (genus Parasutterella id.2892)' on '40S ribosomal protein S3a'

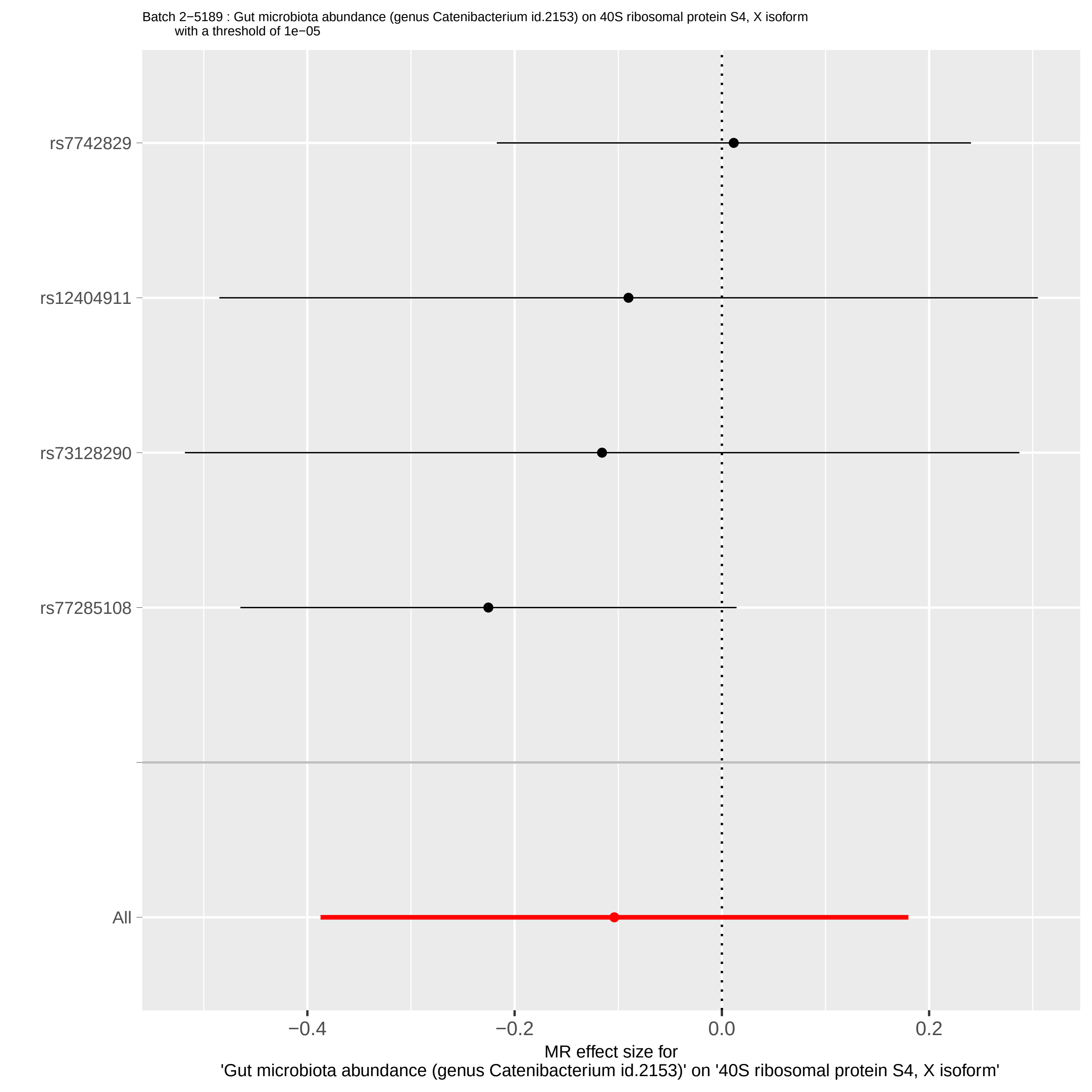
Batch 2−5189 : Gut microbiota abundance (genus Catenibacterium id.2153) on 40S ribosomal protein S4, X isoform
with a threshold of 1e−05
rs7742829
rs12404911
rs73128290
rs77285108
All
−0.4
−0.2
0.0
0.2
MR effect size for
'Gut microbiota abundance (genus Catenibacterium id.2153)' on '40S ribosomal protein S4, X isoform'

Batch 2−5190 : Gut microbiota abundance (genus Parasutterella id.2892) on 40S ribosomal protein S4, X isoform
with a threshold of 1e−05

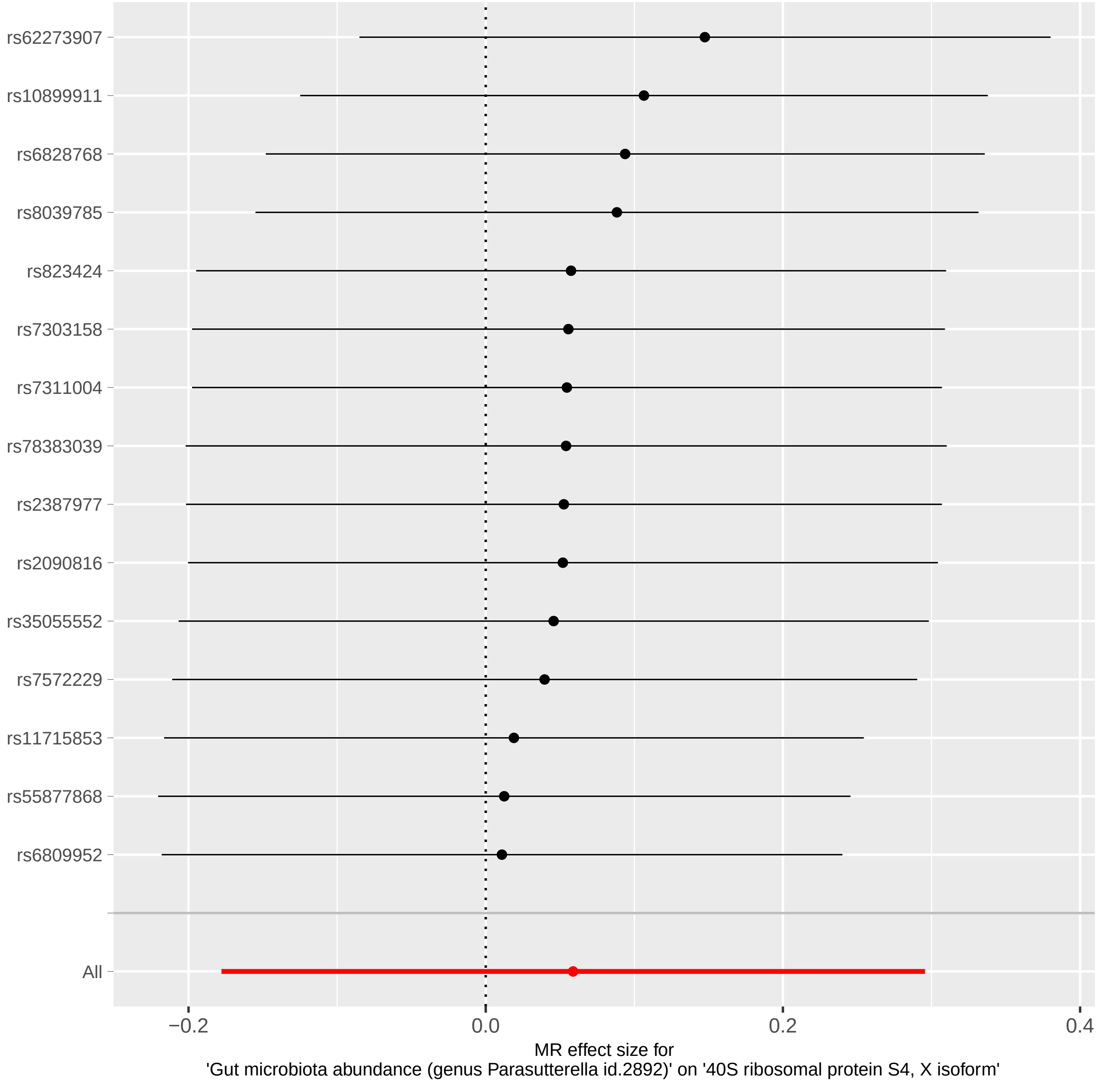

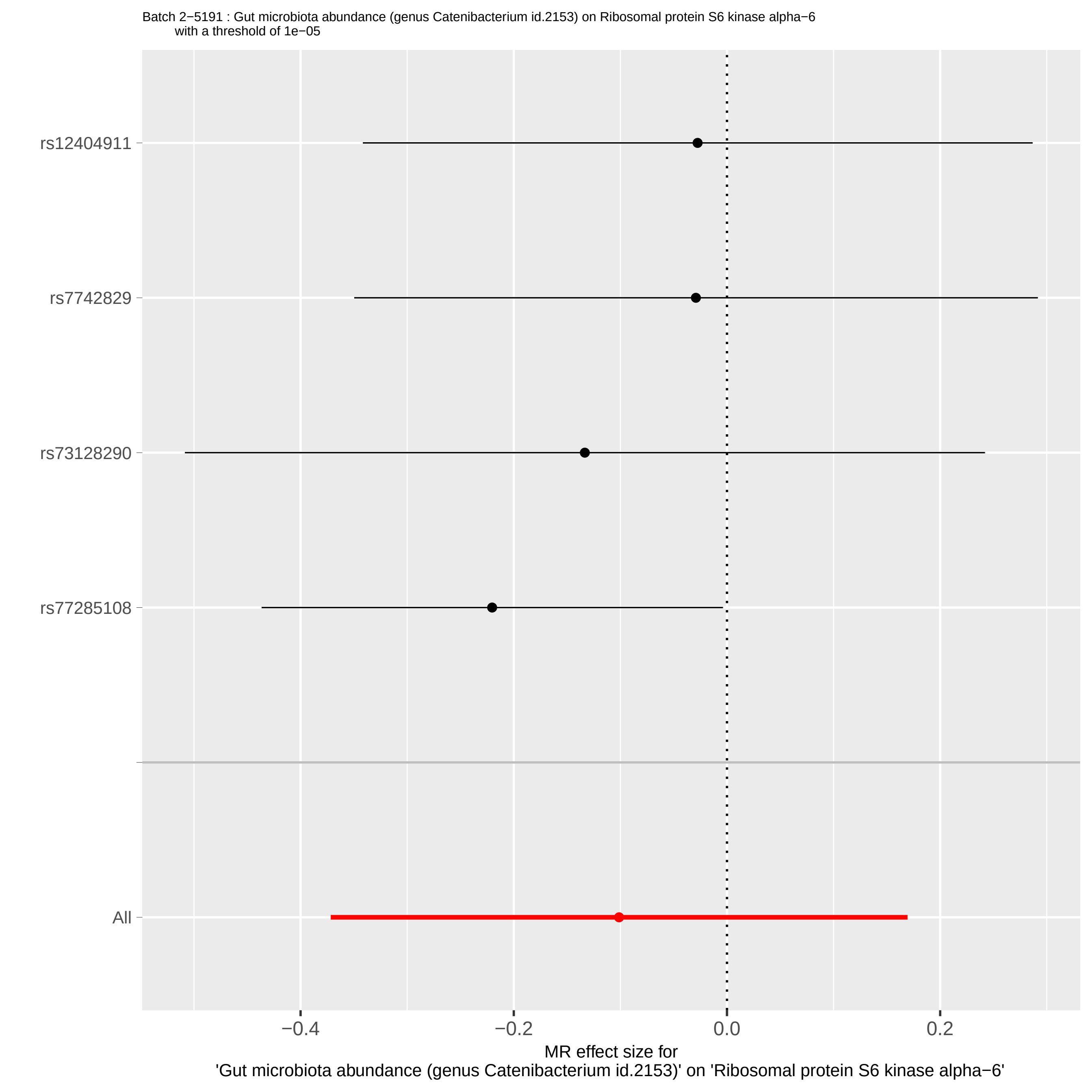

Batch 2−5191 : Gut microbiota abundance (genus Catenibacterium id.2153) on Ribosomal protein S6 kinase alpha−6
with a threshold of 1e−05
rs12404911
rs7742829
rs73128290
rs77285108
All
−0.4
−0.2
0.0
0.2
MR effect size for
'Gut microbiota abundance (genus Catenibacterium id.2153)' on 'Ribosomal protein S6 kinase alpha−6'

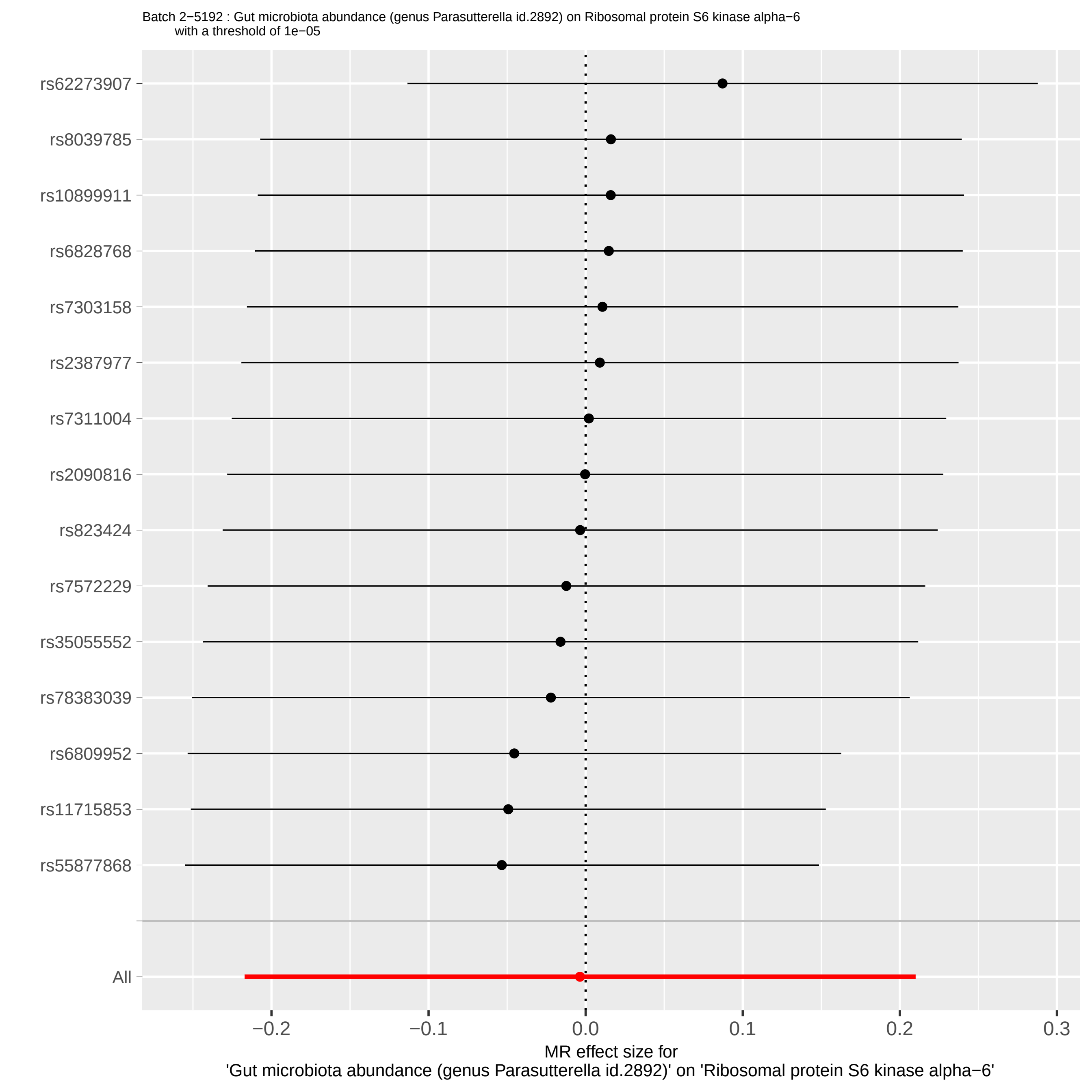

Batch 2−5192 : Gut microbiota abundance (genus Parasutterella id.2892) on Ribosomal protein S6 kinase alpha−6
with a threshold of 1e−05
rs62273907
rs8039785
rs10899911
rs6828768
rs7303158
rs2387977
rs7311004
rs2090816
rs823424
rs7572229
rs35055552
rs78383039
rs6809952
rs11715853
rs55877868
All
−0.2
−0.1
0.0
0.1
0.2
0.3
MR effect size for
'Gut microbiota abundance (genus Parasutterella id.2892)' on 'Ribosomal protein S6 kinase alpha−6'

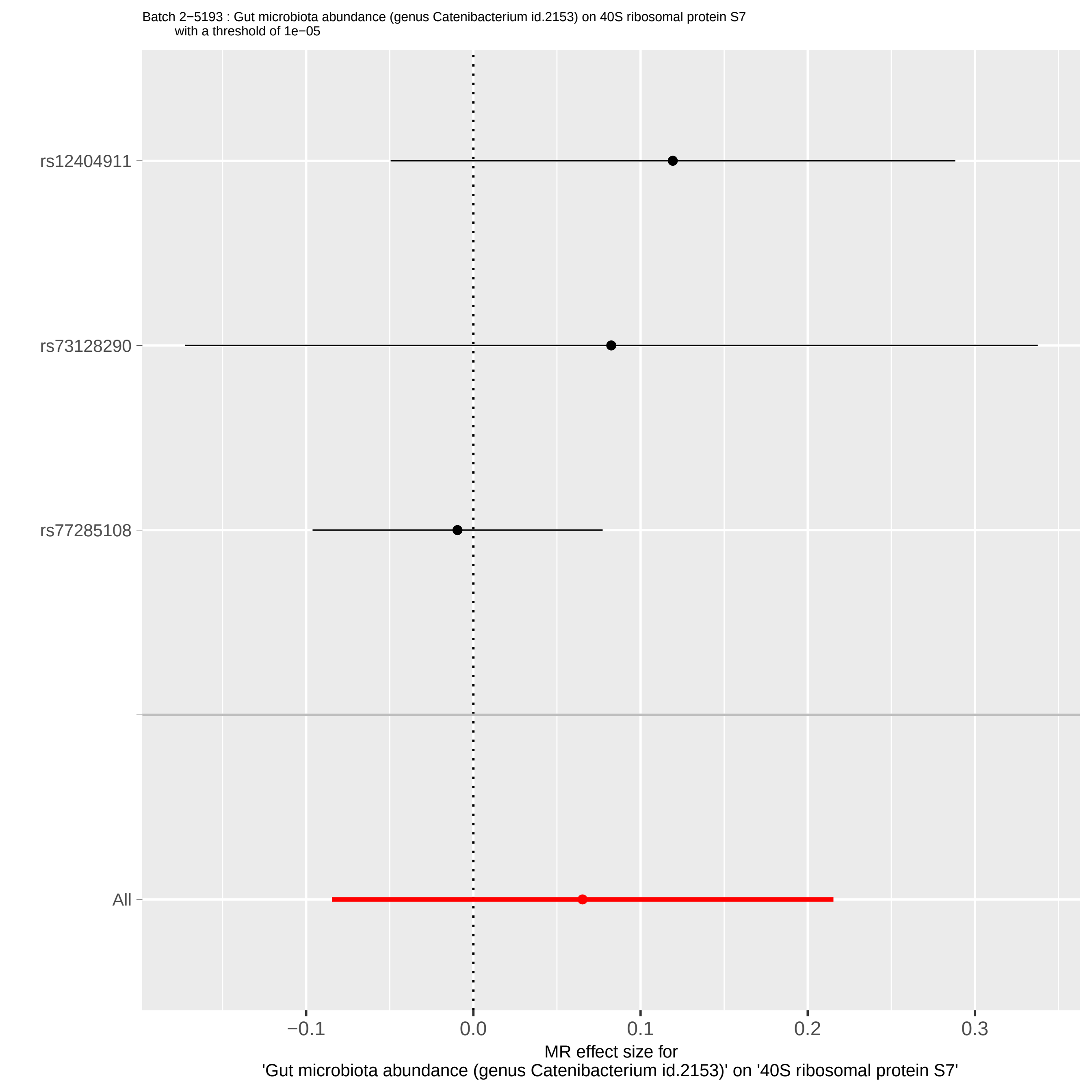
Batch 2−5193 : Gut microbiota abundance (genus Catenibacterium id.2153) on 40S ribosomal protein S7
with a threshold of 1e−05
rs12404911
rs73128290
rs77285108
All
−0.1
0.0
0.1
0.2
0.3
MR effect size for
'Gut microbiota abundance (genus Catenibacterium id.2153)' on '40S ribosomal protein S7'

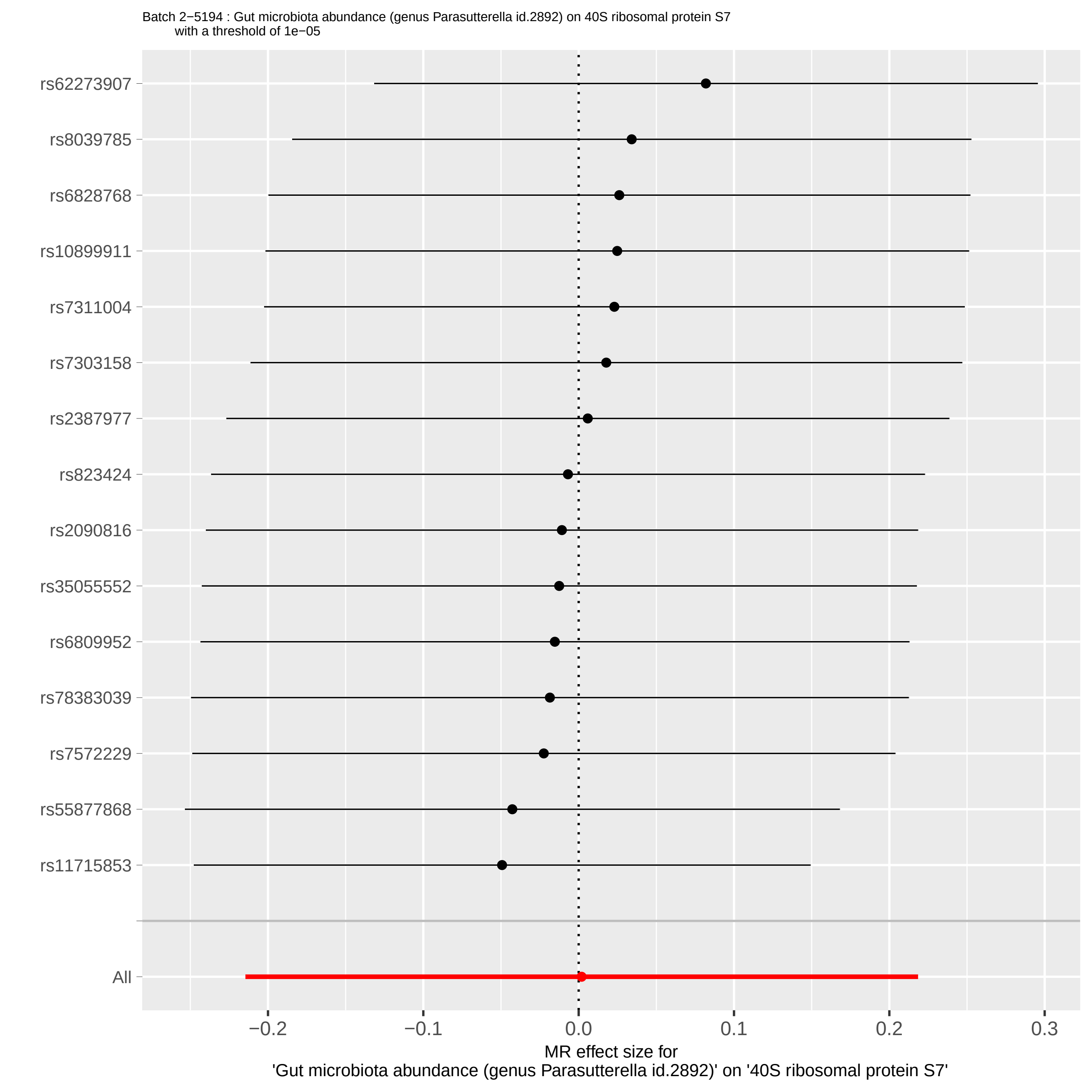

Batch 2−5194 : Gut microbiota abundance (genus Parasutterella id.2892) on 40S ribosomal protein S7
with a threshold of 1e−05
rs62273907
rs8039785
rs6828768
rs10899911
rs7311004
rs7303158
rs2387977
rs823424
rs2090816
rs35055552
rs6809952
rs78383039
rs7572229
rs55877868
rs11715853
All
−0.2
−0.1
0.0
0.1
0.2
0.3
MR effect size for
'Gut microbiota abundance (genus Parasutterella id.2892)' on '40S ribosomal protein S7'

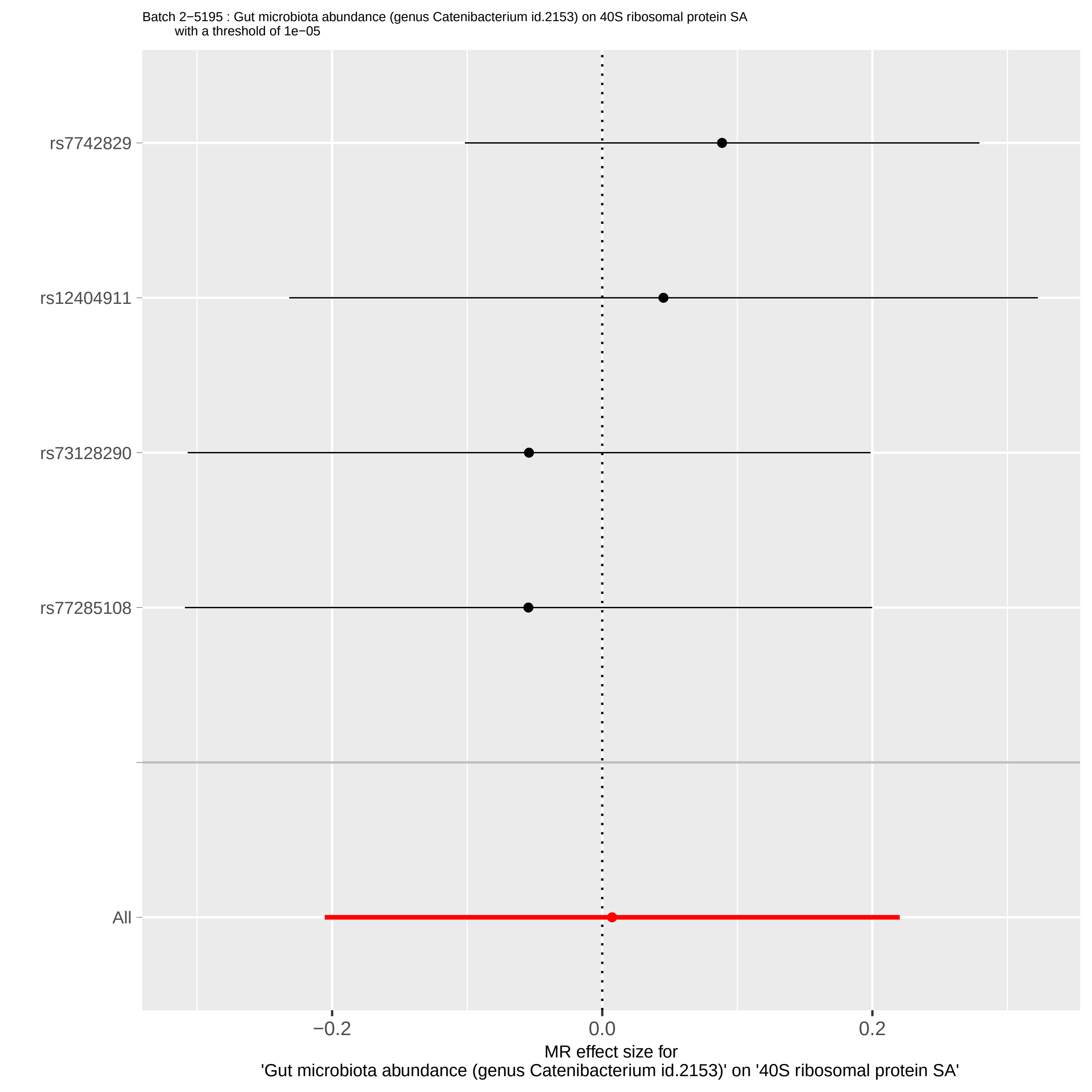
Batch 2−5195 : Gut microbiota abundance (genus Catenibacterium id.2153) on 40S ribosomal protein SA
with a threshold of 1e−05
rs7742829
rs12404911
rs73128290
rs77285108
All
−0.2
0.0
0.2
MR effect size for
'Gut microbiota abundance (genus Catenibacterium id.2153)' on '40S ribosomal protein SA'

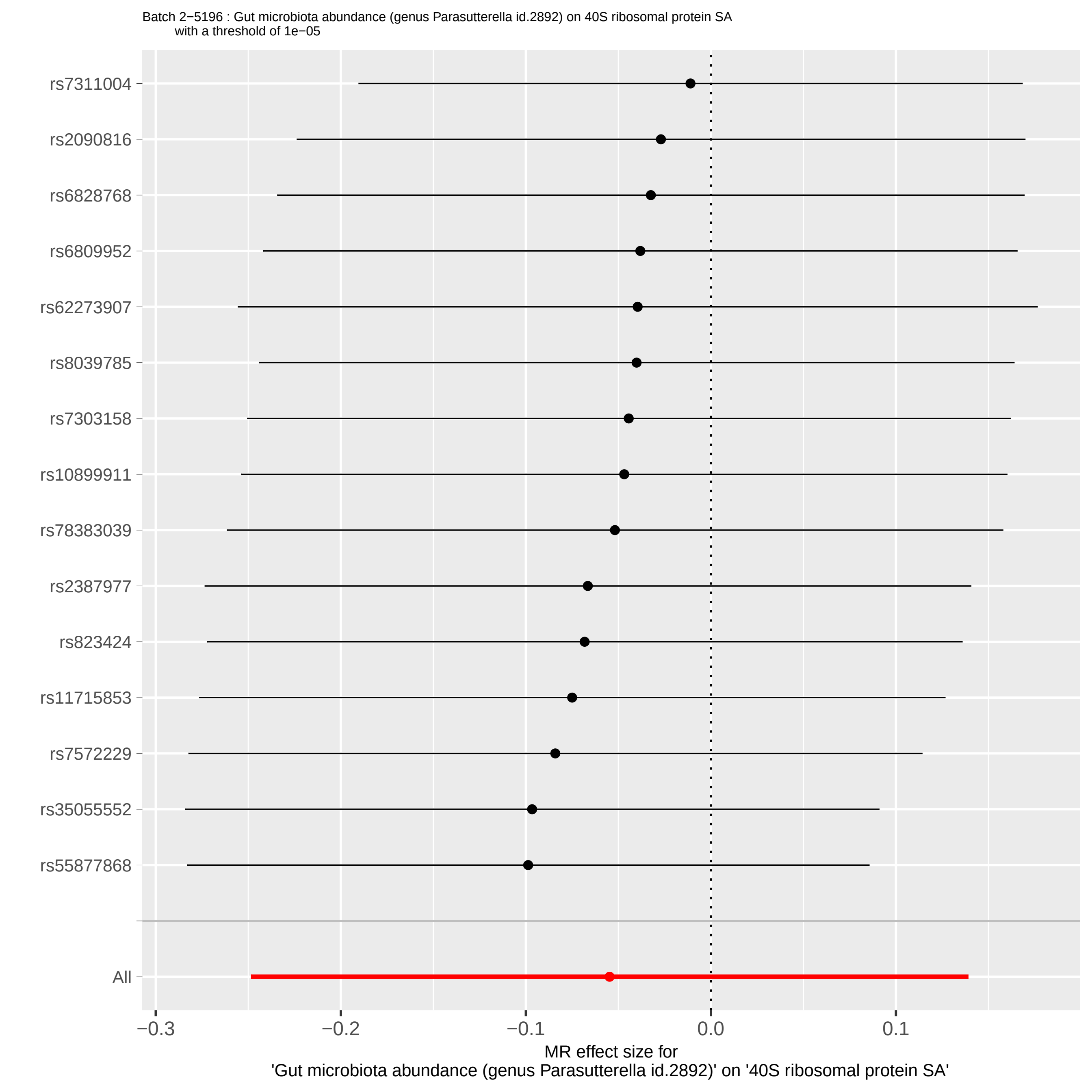

Batch 2−5196 : Gut microbiota abundance (genus Parasutterella id.2892) on 40S ribosomal protein SA
with a threshold of 1e−05
rs7311004
rs2090816
rs6828768
rs6809952
rs62273907
rs8039785
rs7303158
rs10899911
rs78383039
rs2387977
rs823424
rs11715853
rs7572229
rs35055552
rs55877868
All
−0.3
−0.2
−0.1
0.0
0.1
MR effect size for
'Gut microbiota abundance (genus Parasutterella id.2892)' on '40S ribosomal protein SA'

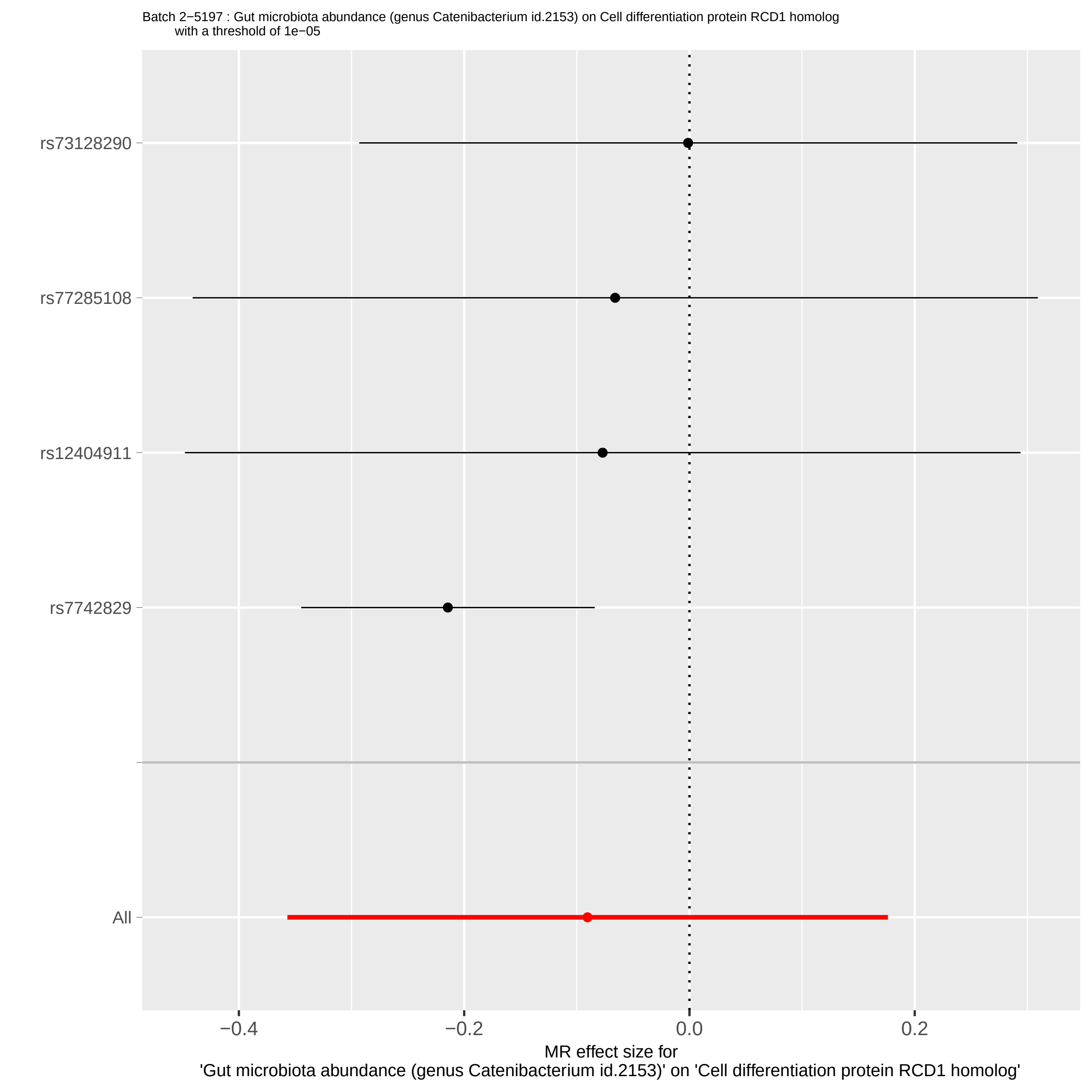
Batch 2−5197 : Gut microbiota abundance (genus Catenibacterium id.2153) on Cell differentiation protein RCD1 homolog
with a threshold of 1e−05
rs73128290
rs77285108
rs12404911
rs7742829
All
−0.4
−0.2
0.0
0.2
MR effect size for
'Gut microbiota abundance (genus Catenibacterium id.2153)' on 'Cell differentiation protein RCD1 homolog'

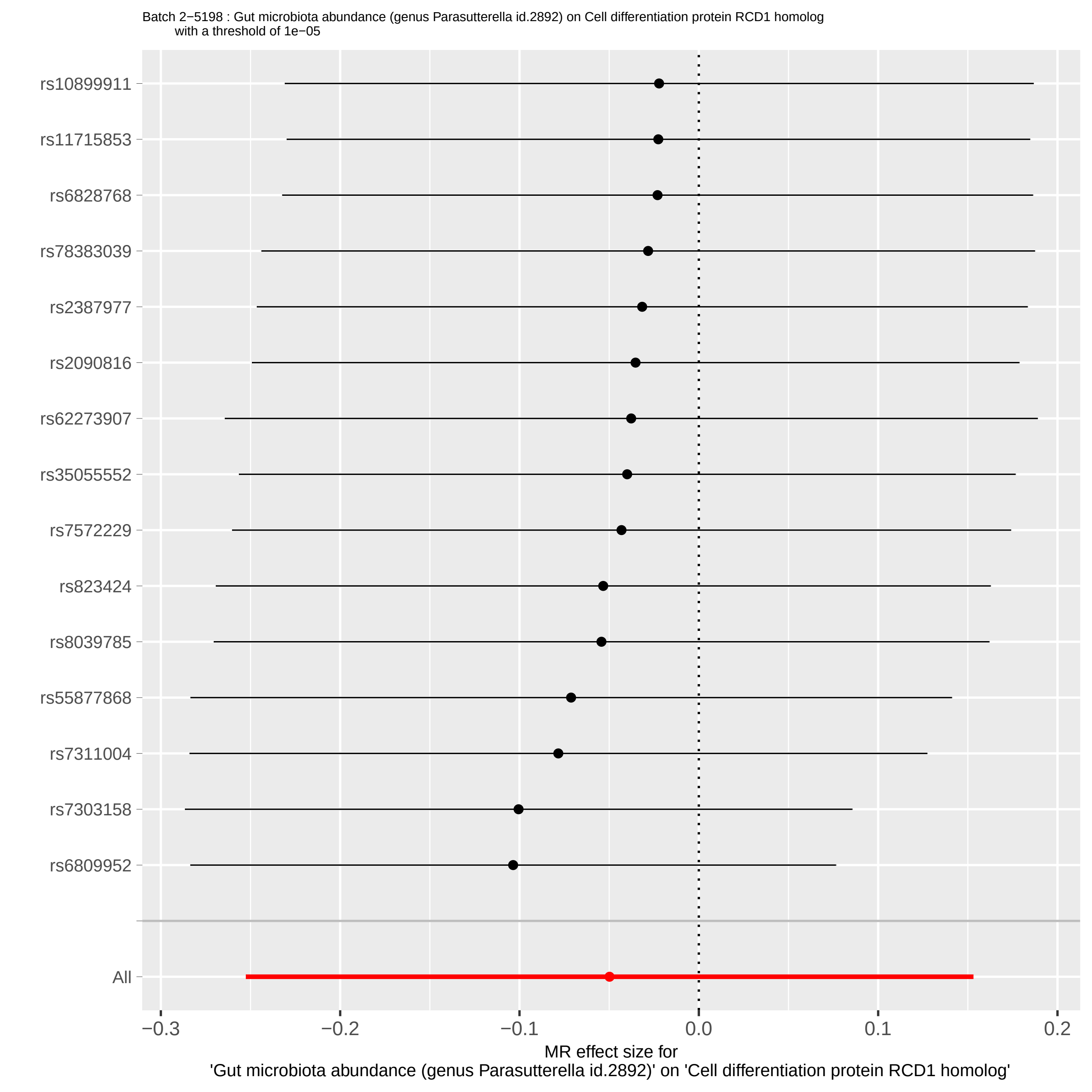

Batch 2−5198 : Gut microbiota abundance (genus Parasutterella id.2892) on Cell differentiation protein RCD1 homolog
with a threshold of 1e−05
rs10899911
rs11715853
rs6828768
rs78383039
rs2387977
rs2090816
rs62273907
rs35055552
rs7572229
rs823424
rs8039785
rs55877868
rs7311004
rs7303158
rs6809952
All
−0.3
−0.2
−0.1
0.0
0.1
0.2
MR effect size for
'Gut microbiota abundance (genus Parasutterella id.2892)' on 'Cell differentiation protein RCD1 homolog'

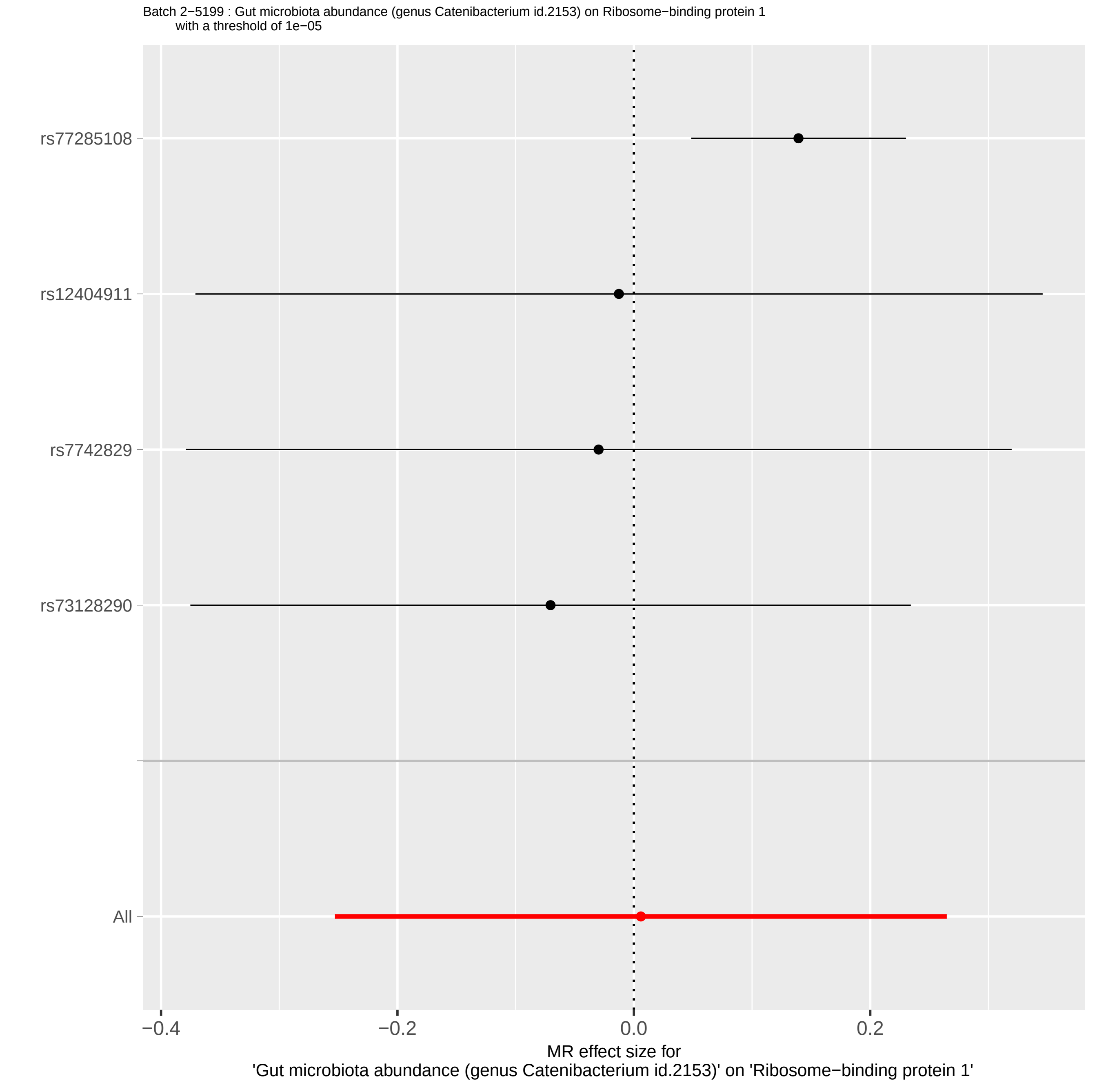

Batch 2−5199 : Gut microbiota abundance (genus Catenibacterium id.2153) on Ribosome−binding protein 1
with a threshold of 1e−05
rs77285108
rs12404911
rs7742829
rs73128290
All
−0.4
−0.2
0.0
0.2
MR effect size for
'Gut microbiota abundance (genus Catenibacterium id.2153)' on 'Ribosome−binding protein 1'

Batch 2−5200 : Gut microbiota abundance (genus Parasutterella id.2892) on Ribosome−binding protein 1
with a threshold of 1e−05

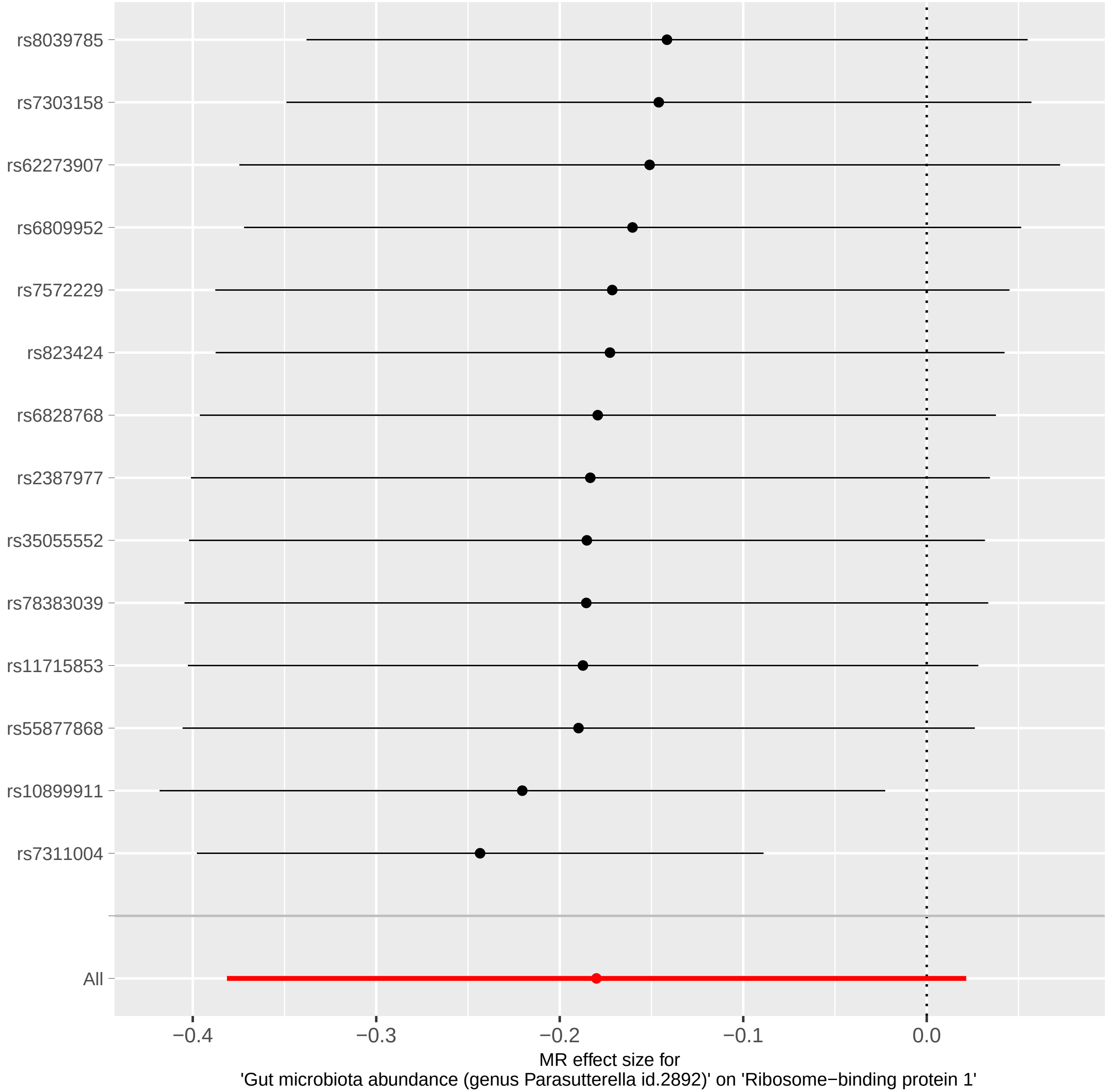

Batch 2−5201 : Gut microbiota abundance (genus Catenibacterium id.2153) on Ribonucleoside−diphosphate reductase large subunit with a threshold of 1e−05

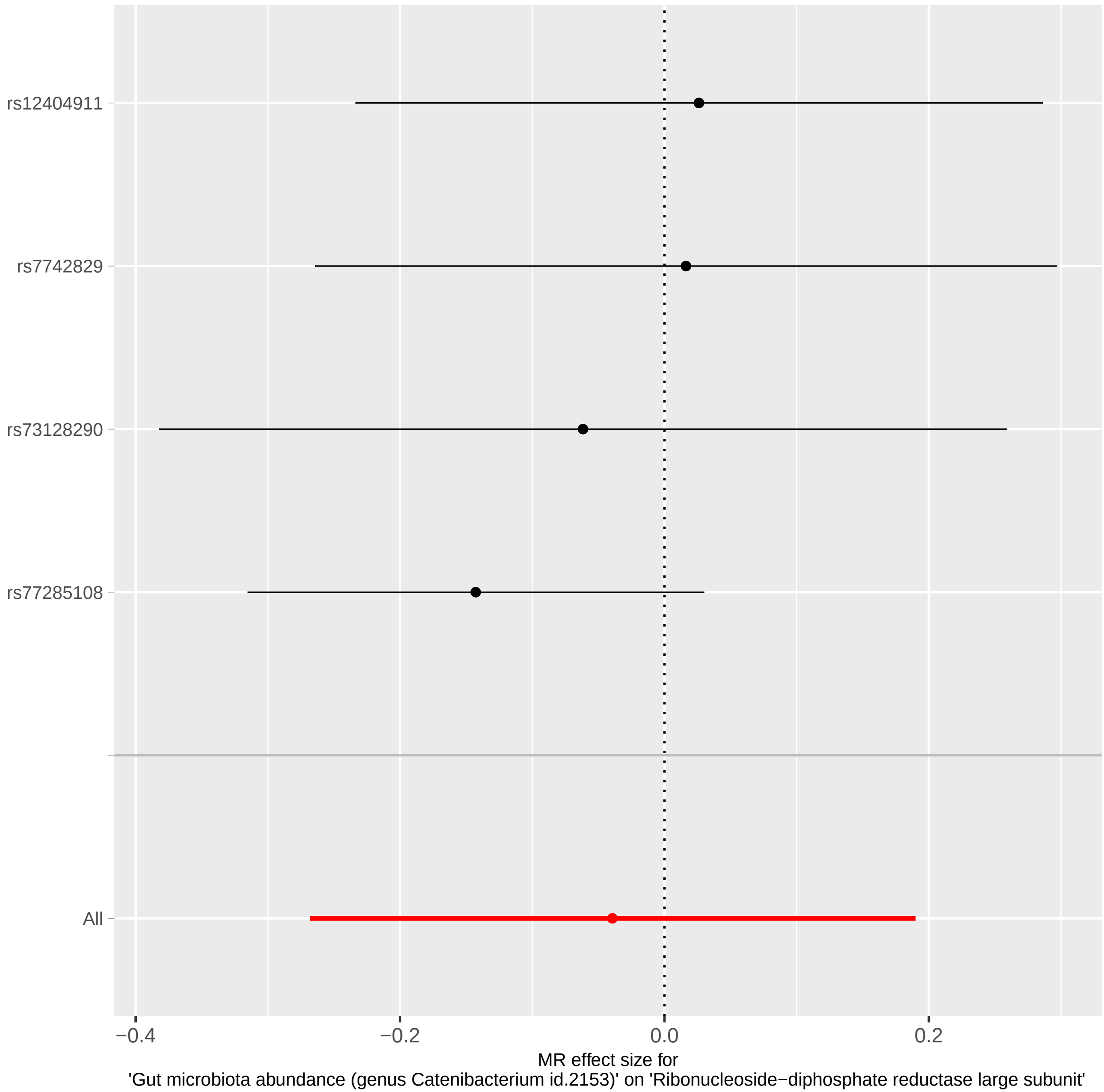

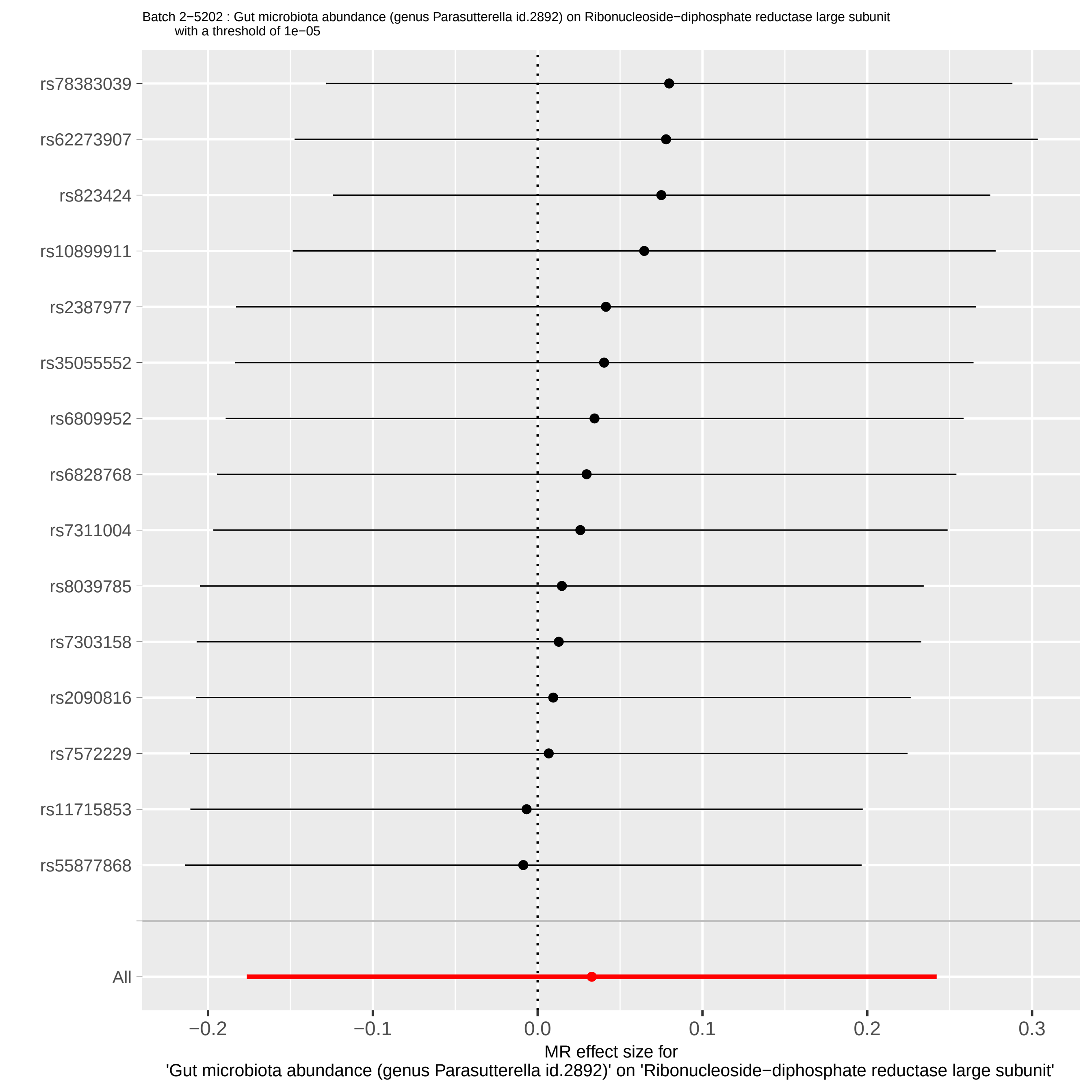

Batch 2−5202 : Gut microbiota abundance (genus Parasutterella id.2892) on Ribonucleoside−diphosphate reductase large subunit
with a threshold of 1e−05
rs78383039
rs62273907
rs823424
rs10899911
rs2387977
rs35055552
rs6809952
rs6828768
rs7311004
rs8039785
rs7303158
rs2090816
rs7572229
rs11715853
rs55877868
All
−0.2
−0.1
0.0
0.1
0.2
0.3
MR effect size for
'Gut microbiota abundance (genus Parasutterella id.2892)' on 'Ribonucleoside−diphosphate reductase large subunit'

Batch 2−5203 : Gut microbiota abundance (genus Catenibacterium id.2153) on Ribonucleoside−diphosphate reductase subunit M2 B
with a threshold of 1e−05

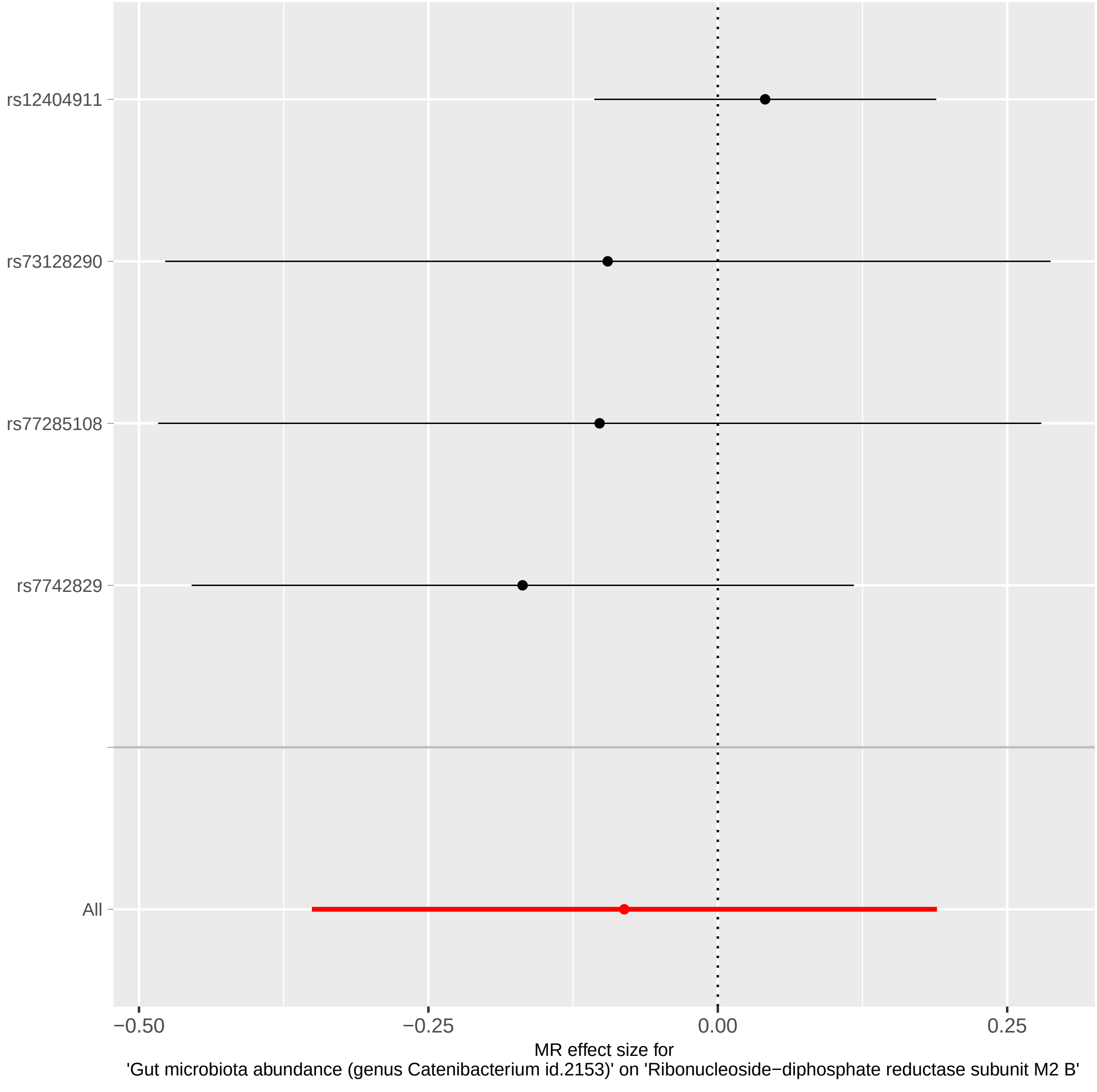

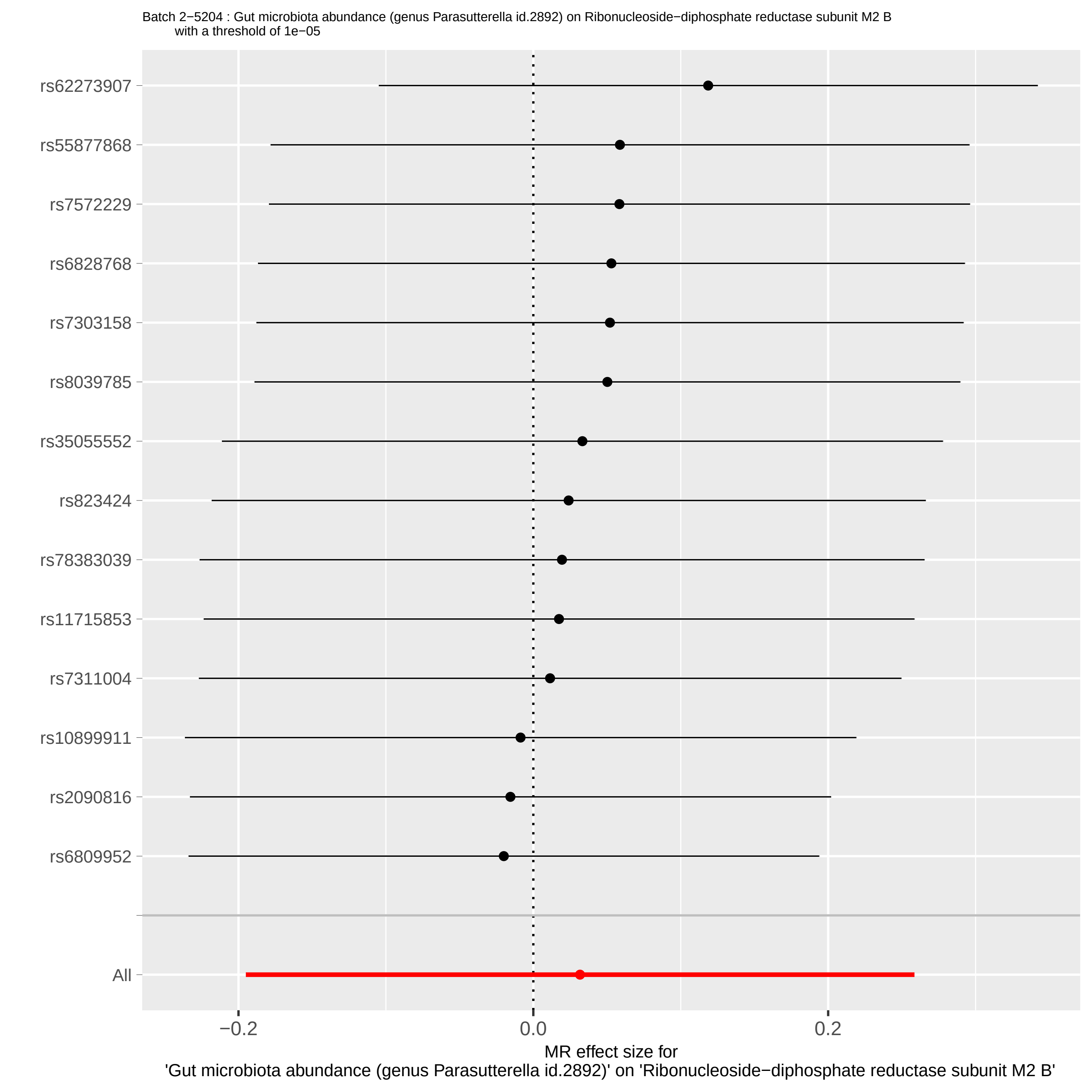
Batch 2−5204 : Gut microbiota abundance (genus Parasutterella id.2892) on Ribonucleoside−diphosphate reductase subunit M2 B
with a threshold of 1e−05
rs62273907
rs55877868
rs7572229
rs6828768
rs7303158
rs8039785
rs35055552
rs823424
rs78383039
rs11715853
rs7311004
rs10899911
rs2090816
rs6809952
All
−0.2
0.0
0.2
MR effect size for
'Gut microbiota abundance (genus Parasutterella id.2892)' on 'Ribonucleoside−diphosphate reductase subunit M2 B'

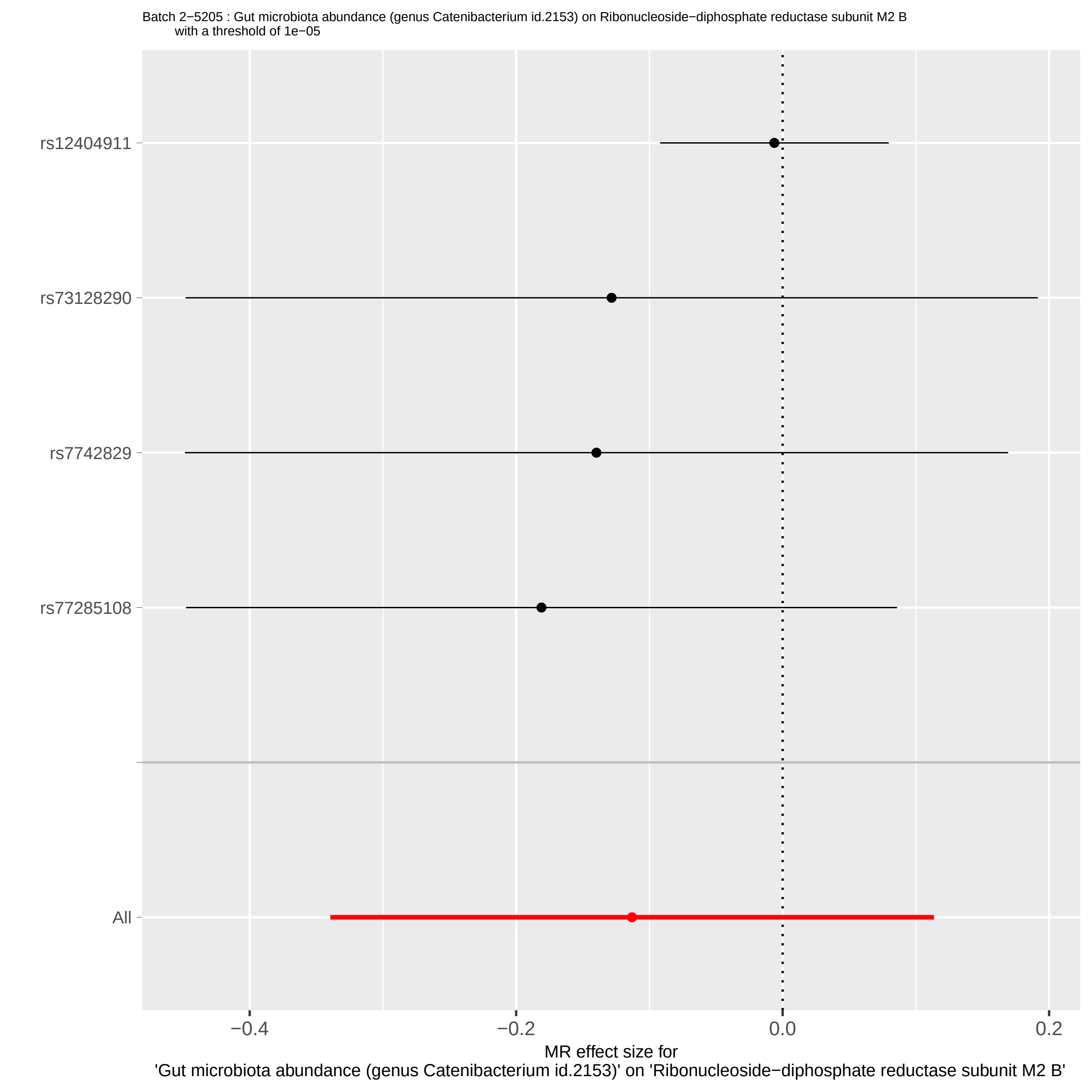

Batch 2−5205 : Gut microbiota abundance (genus Catenibacterium id.2153) on Ribonucleoside−diphosphate reductase subunit M2 B
with a threshold of 1e−05
rs12404911
rs73128290
rs7742829
rs77285108
All
−0.4
−0.2
0.0
0.2
MR effect size for
'Gut microbiota abundance (genus Catenibacterium id.2153)' on 'Ribonucleoside−diphosphate reductase subunit M2 B'

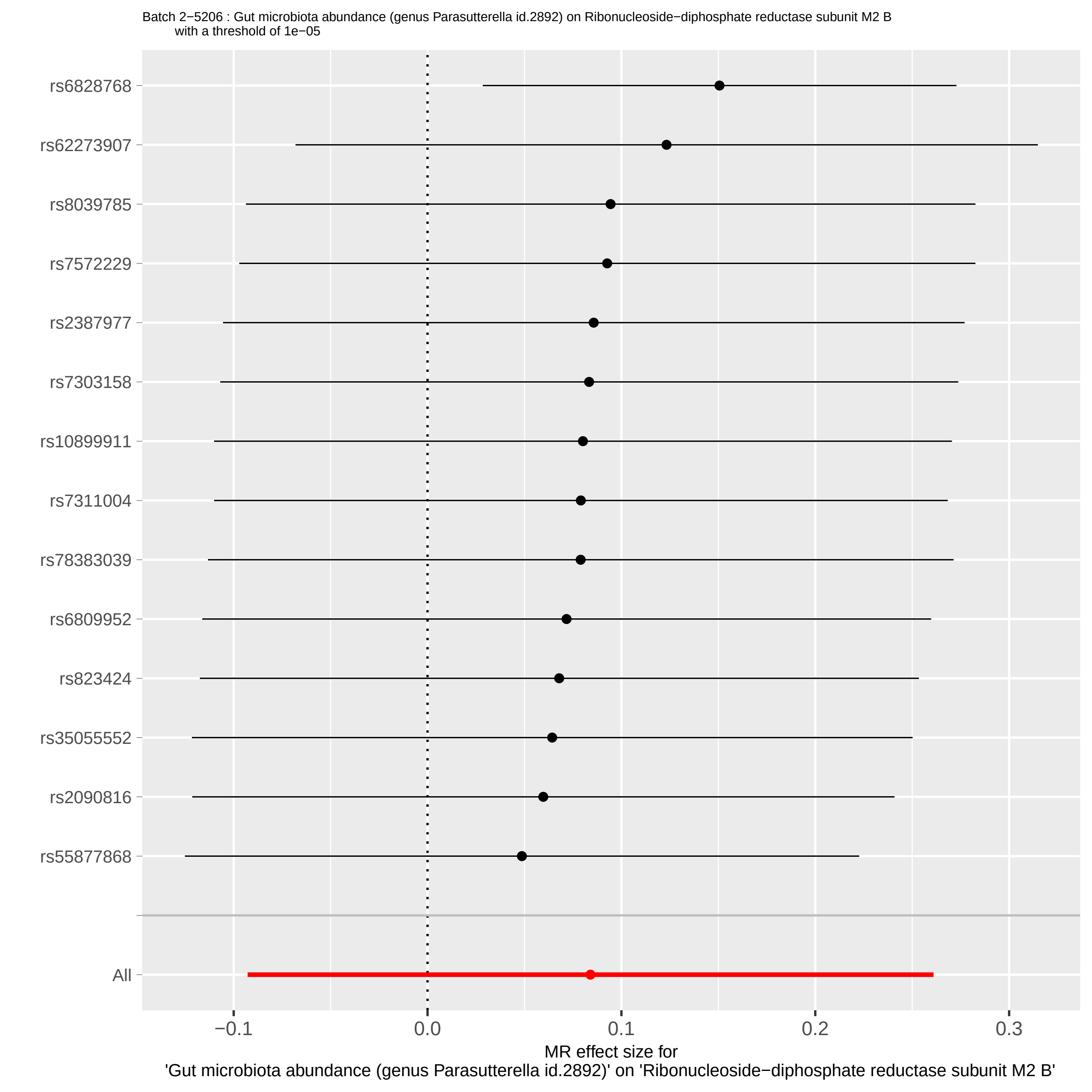

Batch 2−5206 : Gut microbiota abundance (genus Parasutterella id.2892) on Ribonucleoside−diphosphate reductase subunit M2 B
with a threshold of 1e−05
rs6828768
rs62273907
rs8039785
rs7572229
rs2387977
rs7303158
rs10899911
rs7311004
rs78383039
rs6809952
rs823424
rs35055552
rs2090816
rs55877868
All
−0.1
0.0
0.1
0.2
0.3
MR effect size for
'Gut microbiota abundance (genus Parasutterella id.2892)' on 'Ribonucleoside−diphosphate reductase subunit M2 B'

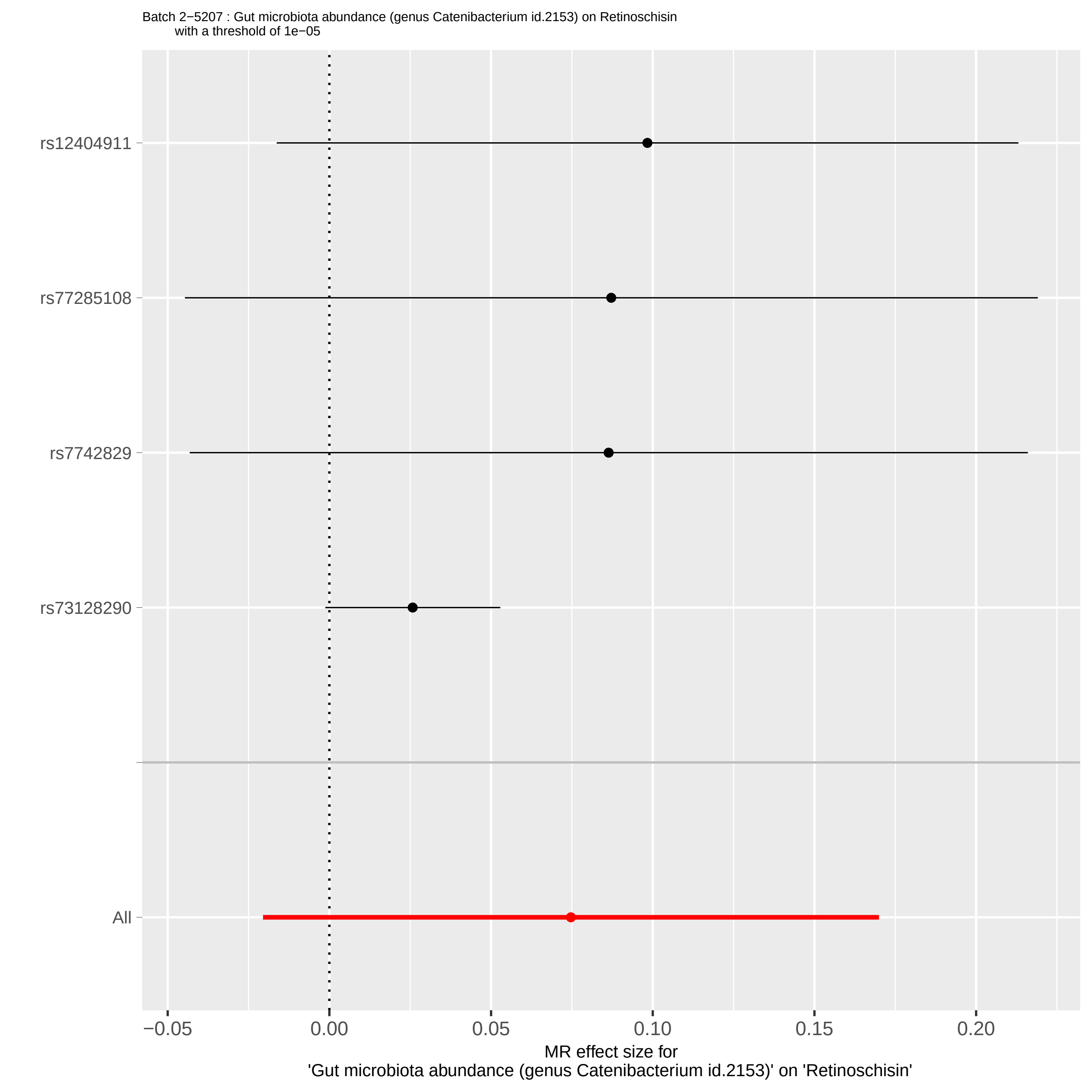

Batch 2−5207 : Gut microbiota abundance (genus Catenibacterium id.2153) on Retinoschisin
with a threshold of 1e−05
rs12404911
rs77285108
rs7742829
rs73128290
All
−0.05
0.00
0.05
0.10
0.15
0.20
MR effect size for
'Gut microbiota abundance (genus Catenibacterium id.2153)' on 'Retinoschisin'

Batch 2−5208 : Gut microbiota abundance (genus Parasutterella id.2892) on Retinoschisin
with a threshold of 1e−05

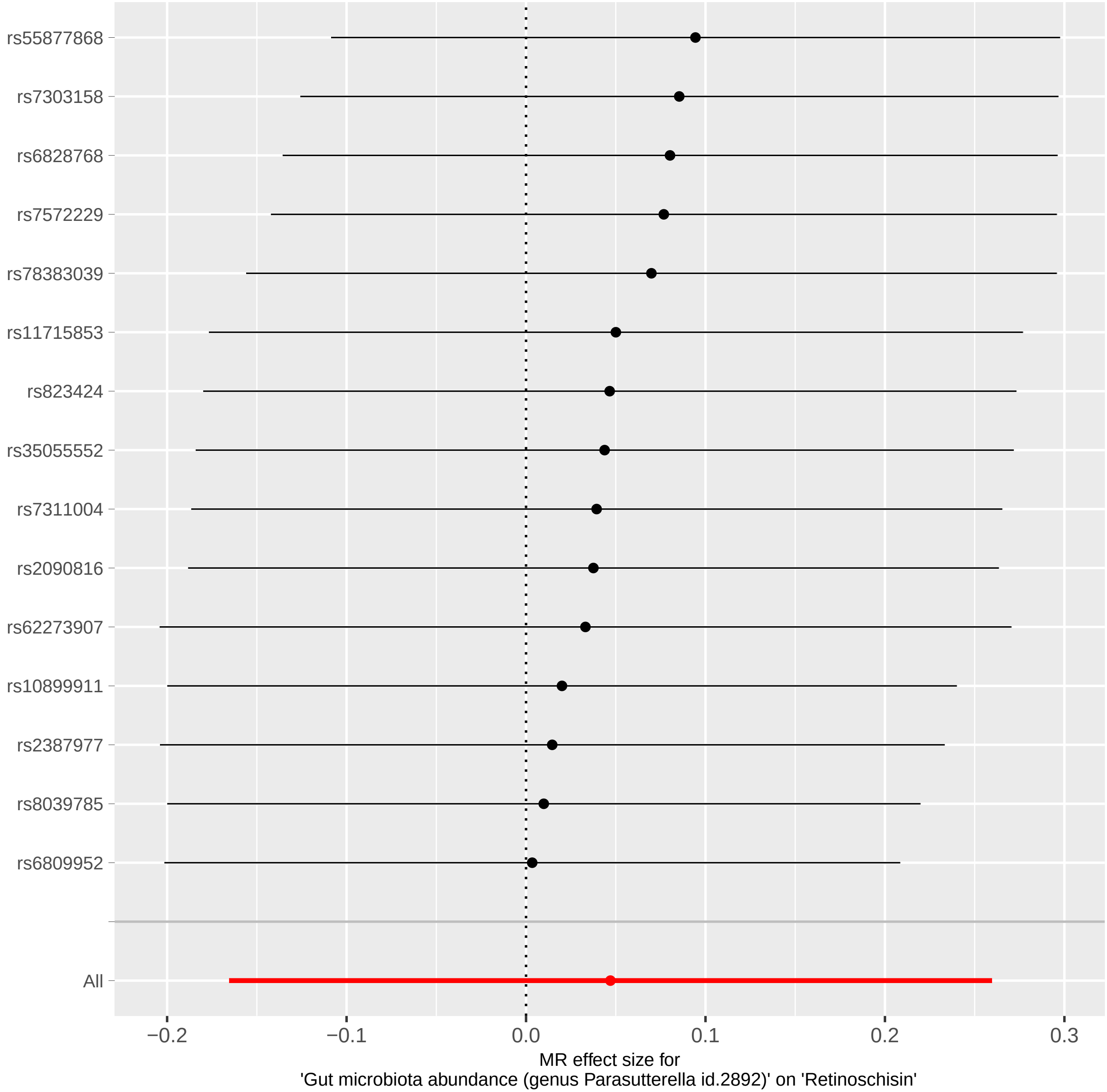

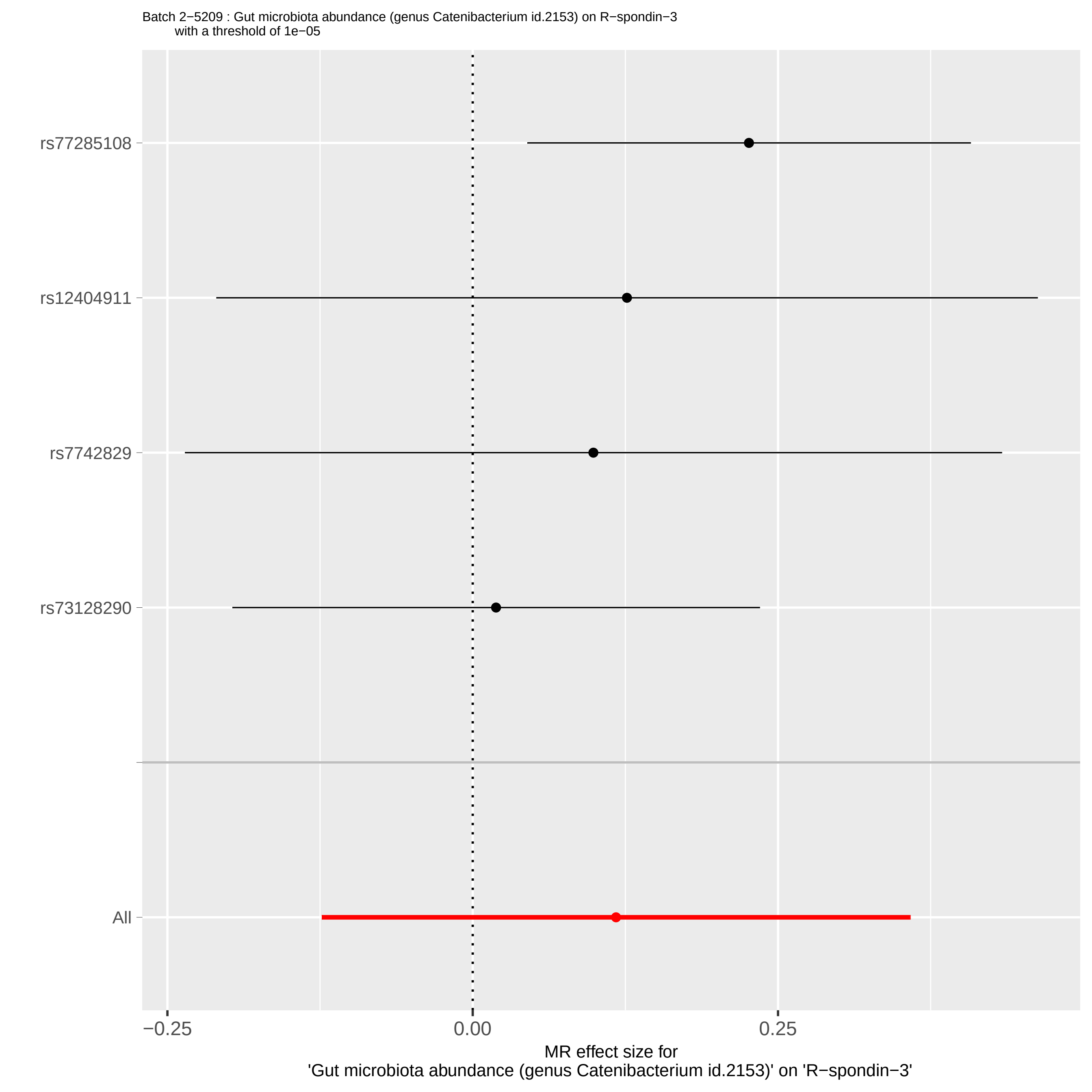

Batch 2−5209 : Gut microbiota abundance (genus Catenibacterium id.2153) on R−spondin−3
with a threshold of 1e−05
rs77285108
rs12404911
rs7742829
rs73128290
All
−0.25
0.00
0.25
MR effect size for
'Gut microbiota abundance (genus Catenibacterium id.2153)' on 'R−spondin−3'

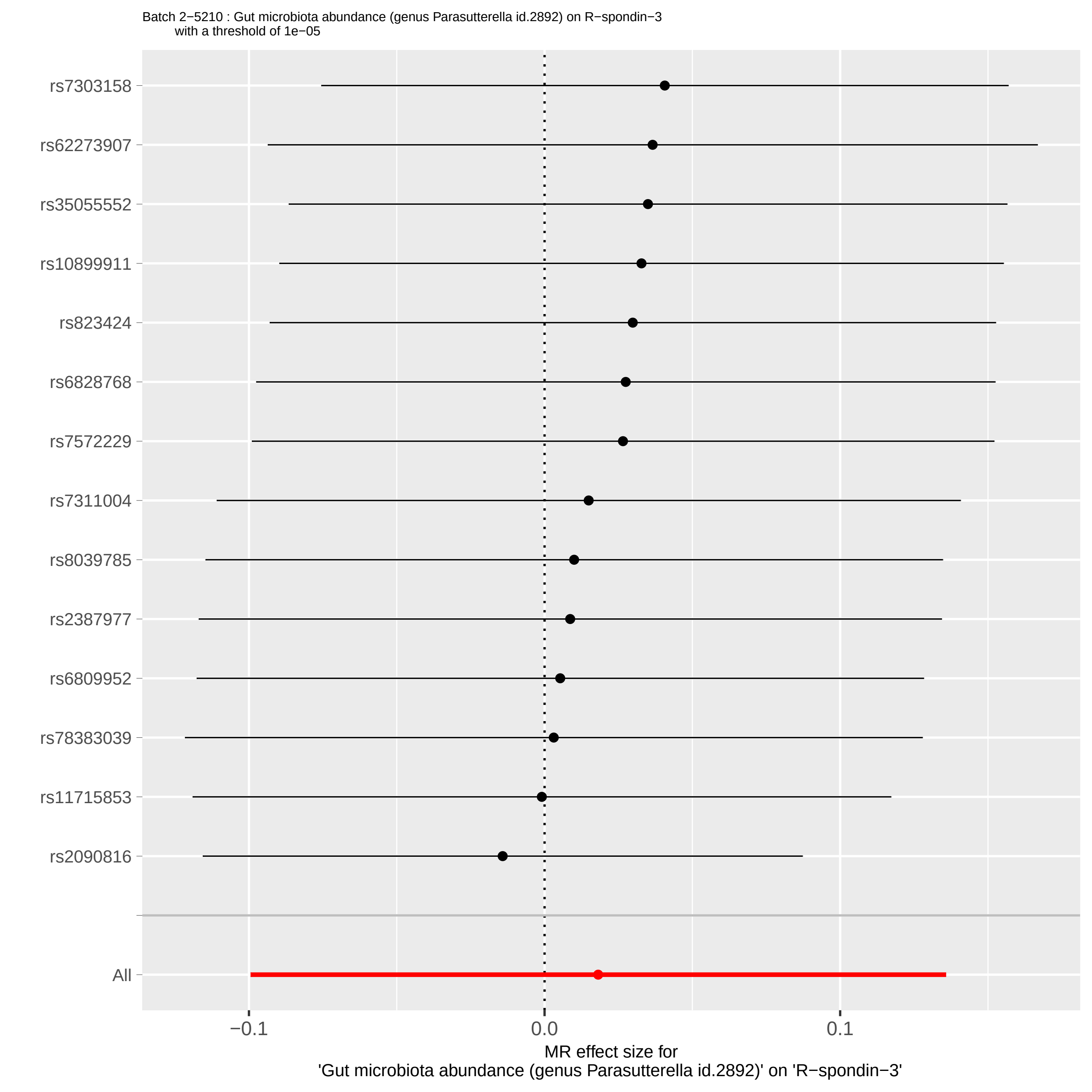

Batch 2−5210 : Gut microbiota abundance (genus Parasutterella id.2892) on R−spondin−3
with a threshold of 1e−05
rs7303158
rs62273907
rs35055552
rs10899911
rs823424
rs6828768
rs7572229
rs7311004
rs8039785
rs2387977
rs6809952
rs78383039
rs11715853
rs2090816
All
−0.1
0.0
0.1
MR effect size for
'Gut microbiota abundance (genus Parasutterella id.2892)' on 'R−spondin−3'

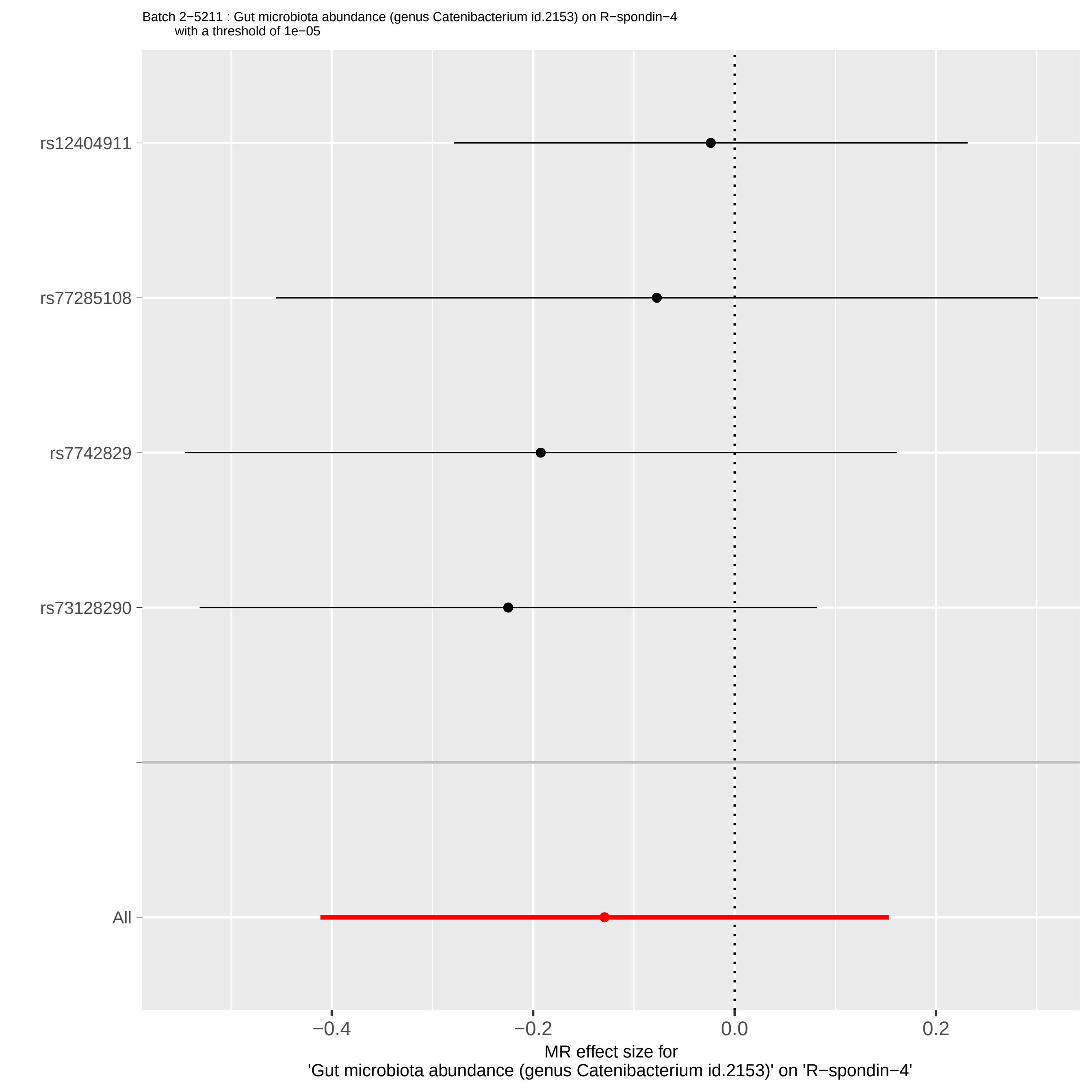

Batch 2−5211 : Gut microbiota abundance (genus Catenibacterium id.2153) on R−spondin−4
with a threshold of 1e−05
rs12404911
rs77285108
rs7742829
rs73128290
All
−0.4
−0.2
0.0
0.2
MR effect size for
'Gut microbiota abundance (genus Catenibacterium id.2153)' on 'R−spondin−4'

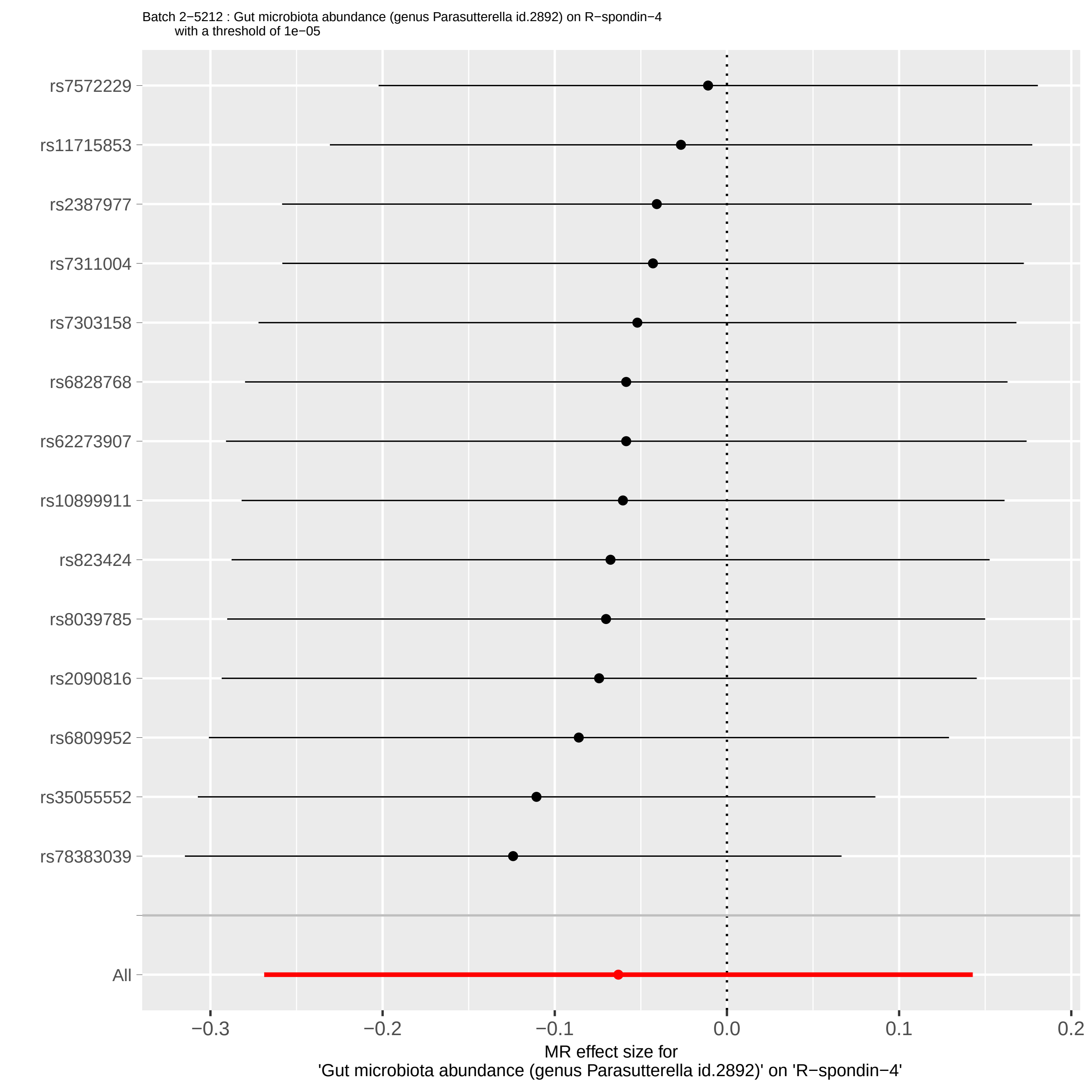
Batch 2−5212 : Gut microbiota abundance (genus Parasutterella id.2892) on R−spondin−4
with a threshold of 1e−05
rs7572229
rs11715853
rs2387977
rs7311004
rs7303158
rs6828768
rs62273907
rs10899911
rs823424
rs8039785
rs2090816
rs6809952
rs35055552
rs78383039
All
−0.3
−0.2
−0.1
0.0
0.1
0.2
MR effect size for
'Gut microbiota abundance (genus Parasutterella id.2892)' on 'R−spondin−4'

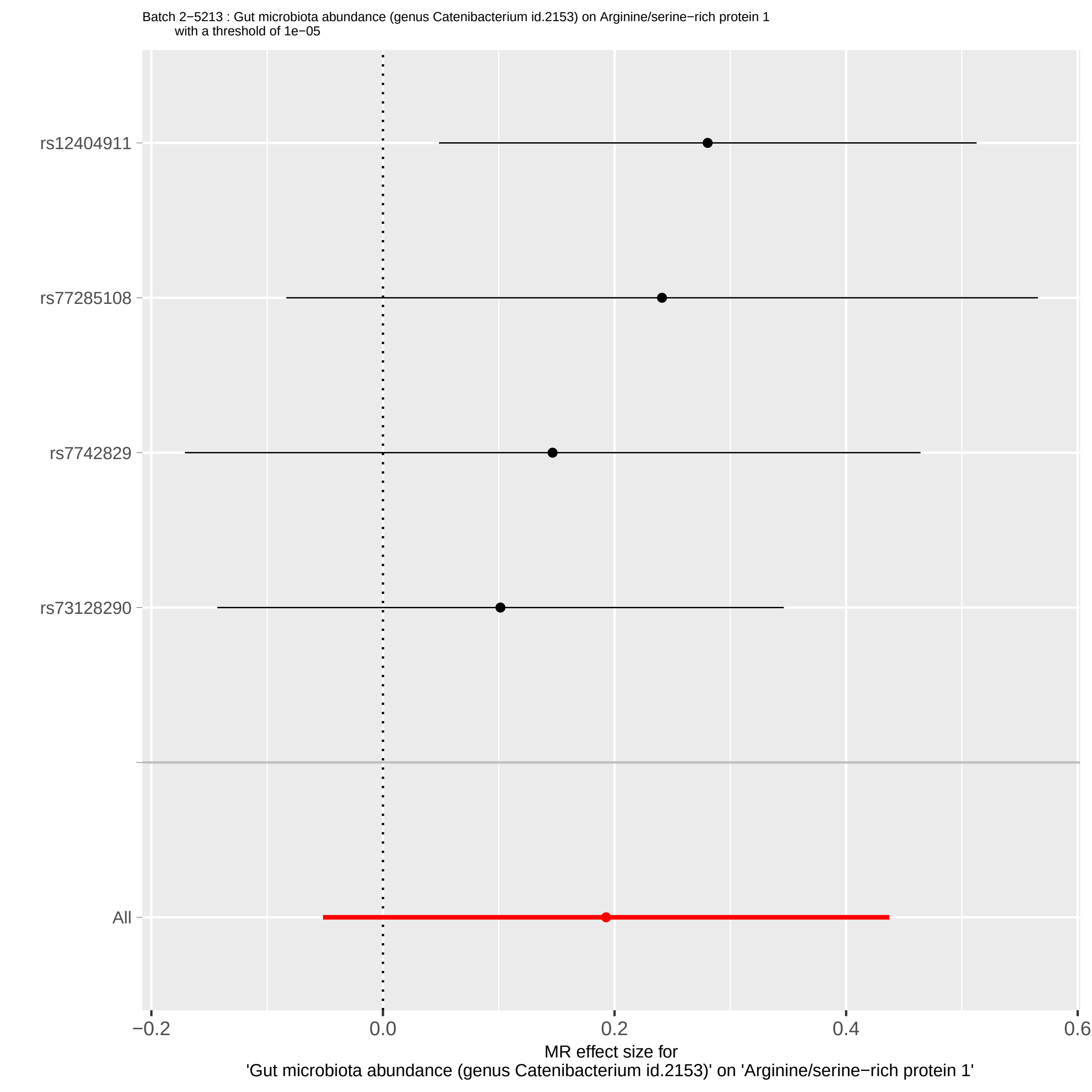

Batch 2−5213 : Gut microbiota abundance (genus Catenibacterium id.2153) on Arginine/serine−rich protein 1
with a threshold of 1e−05
rs12404911
rs77285108
rs7742829
rs73128290
All
−0.2
0.0
0.2
0.4
0.6
MR effect size for
'Gut microbiota abundance (genus Catenibacterium id.2153)' on 'Arginine/serine−rich protein 1'

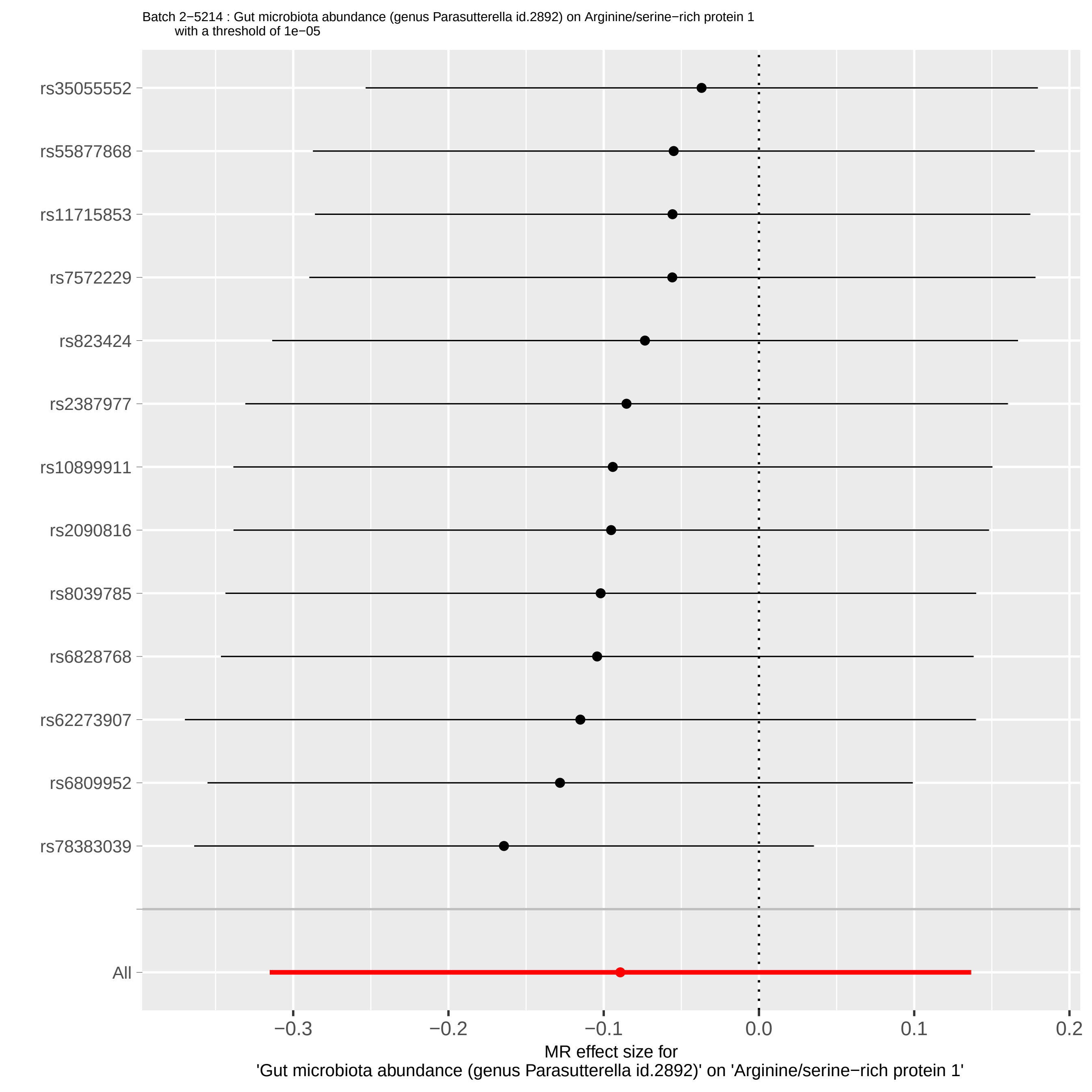

Batch 2−5214 : Gut microbiota abundance (genus Parasutterella id.2892) on Arginine/serine−rich protein 1
with a threshold of 1e−05
rs35055552
rs55877868
rs11715853
rs7572229
rs823424
rs2387977
rs10899911
rs2090816
rs8039785
rs6828768
rs62273907
rs6809952
rs78383039
All
−0.3
−0.2
−0.1
0.0
0.1
0.2
MR effect size for
'Gut microbiota abundance (genus Parasutterella id.2892)' on 'Arginine/serine−rich protein 1'

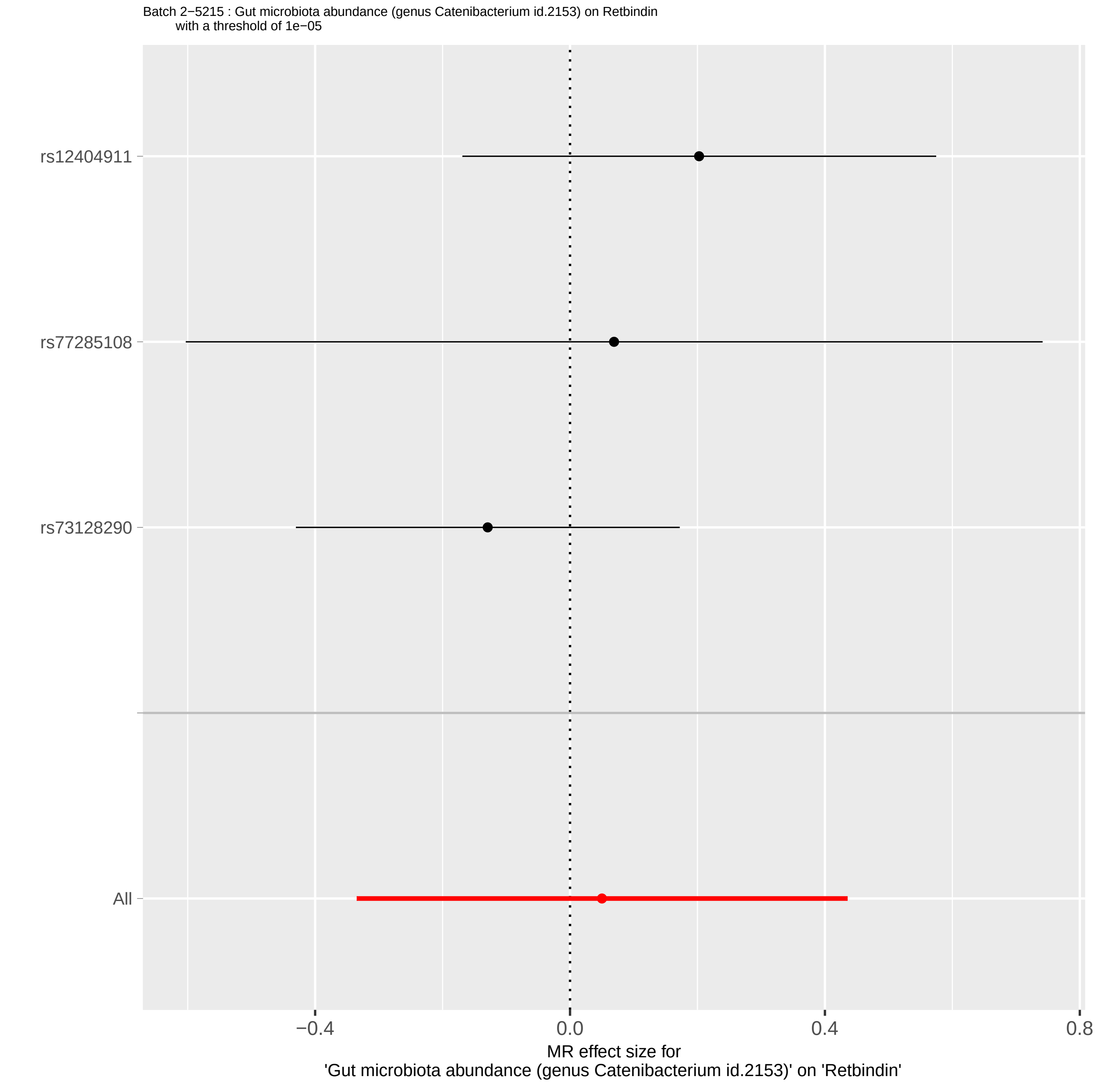

Batch 2−5215 : Gut microbiota abundance (genus Catenibacterium id.2153) on Retbindin
with a threshold of 1e−05
rs12404911
rs77285108
rs73128290
All
−0.4
0.0
0.4
0.8
MR effect size for
'Gut microbiota abundance (genus Catenibacterium id.2153)' on 'Retbindin'

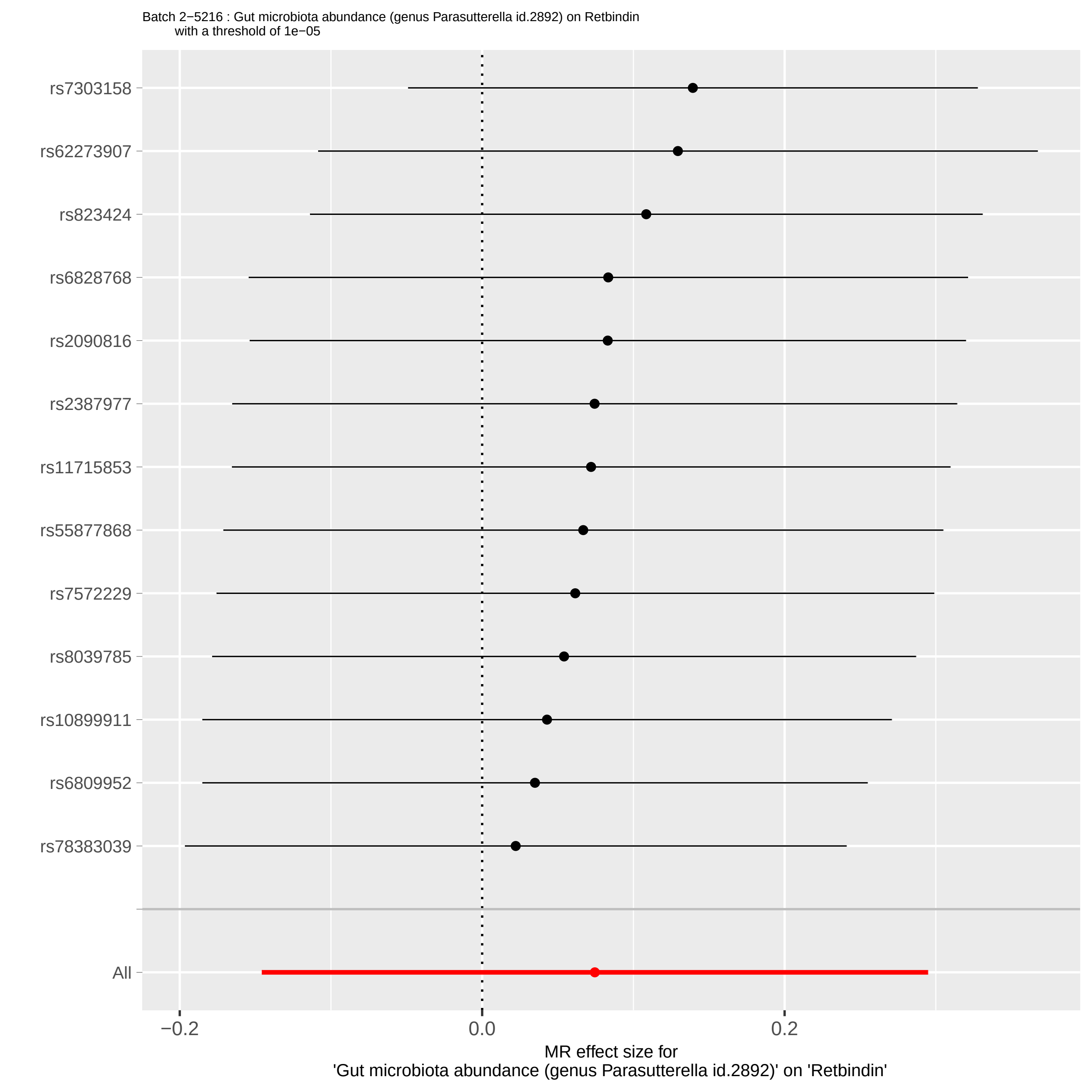
Batch 2−5216 : Gut microbiota abundance (genus Parasutterella id.2892) on Retbindin
with a threshold of 1e−05
rs7303158
rs62273907
rs823424
rs6828768
rs2090816
rs2387977
rs11715853
rs55877868
rs7572229
rs8039785
rs10899911
rs6809952
rs78383039
All
−0.2
0.0
0.2
MR effect size for
'Gut microbiota abundance (genus Parasutterella id.2892)' on 'Retbindin'

Batch 2−5217 : Gut microbiota abundance (genus Catenibacterium id.2153) on Reticulon−4 receptor
with a threshold of 1e−05

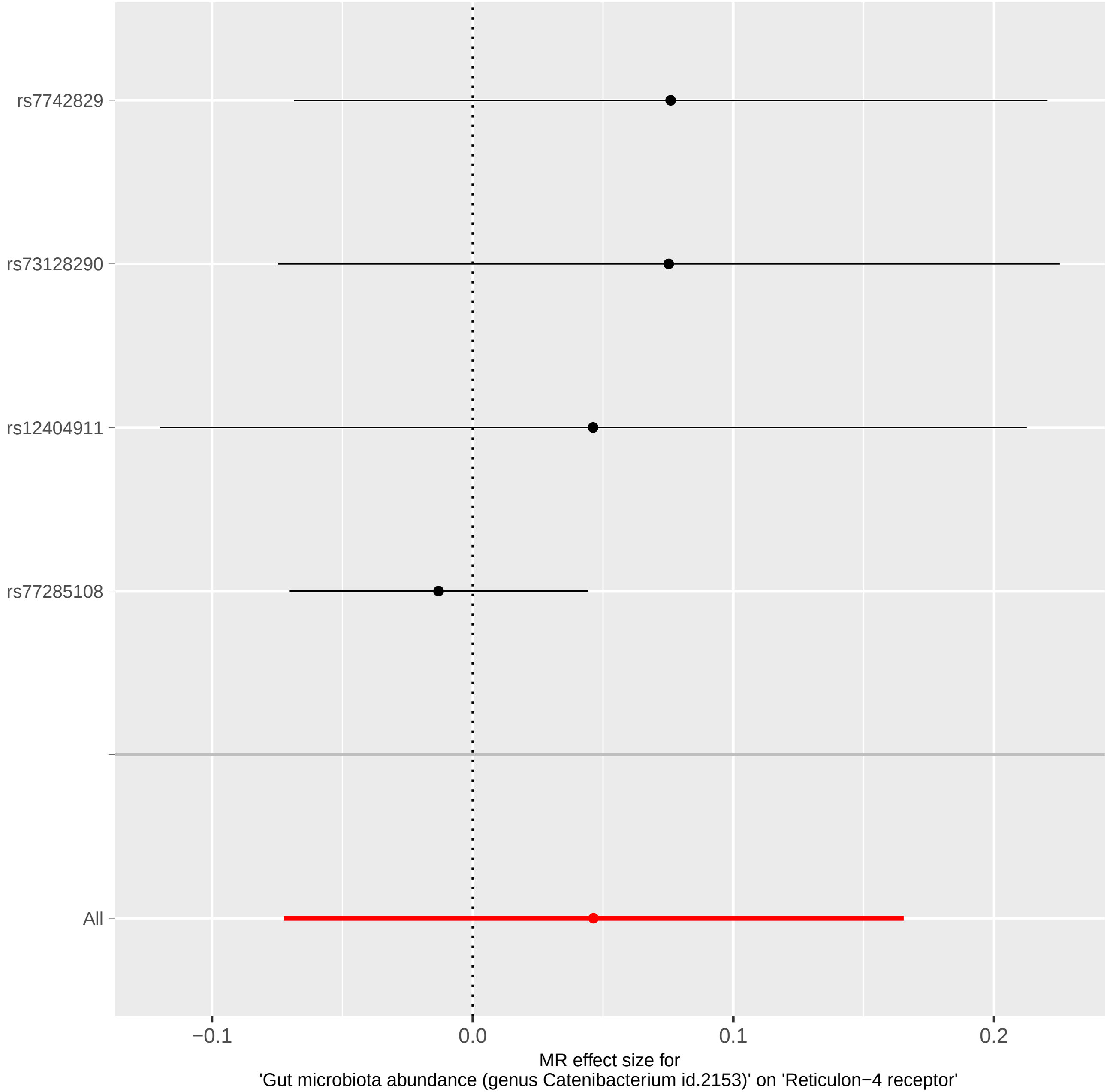

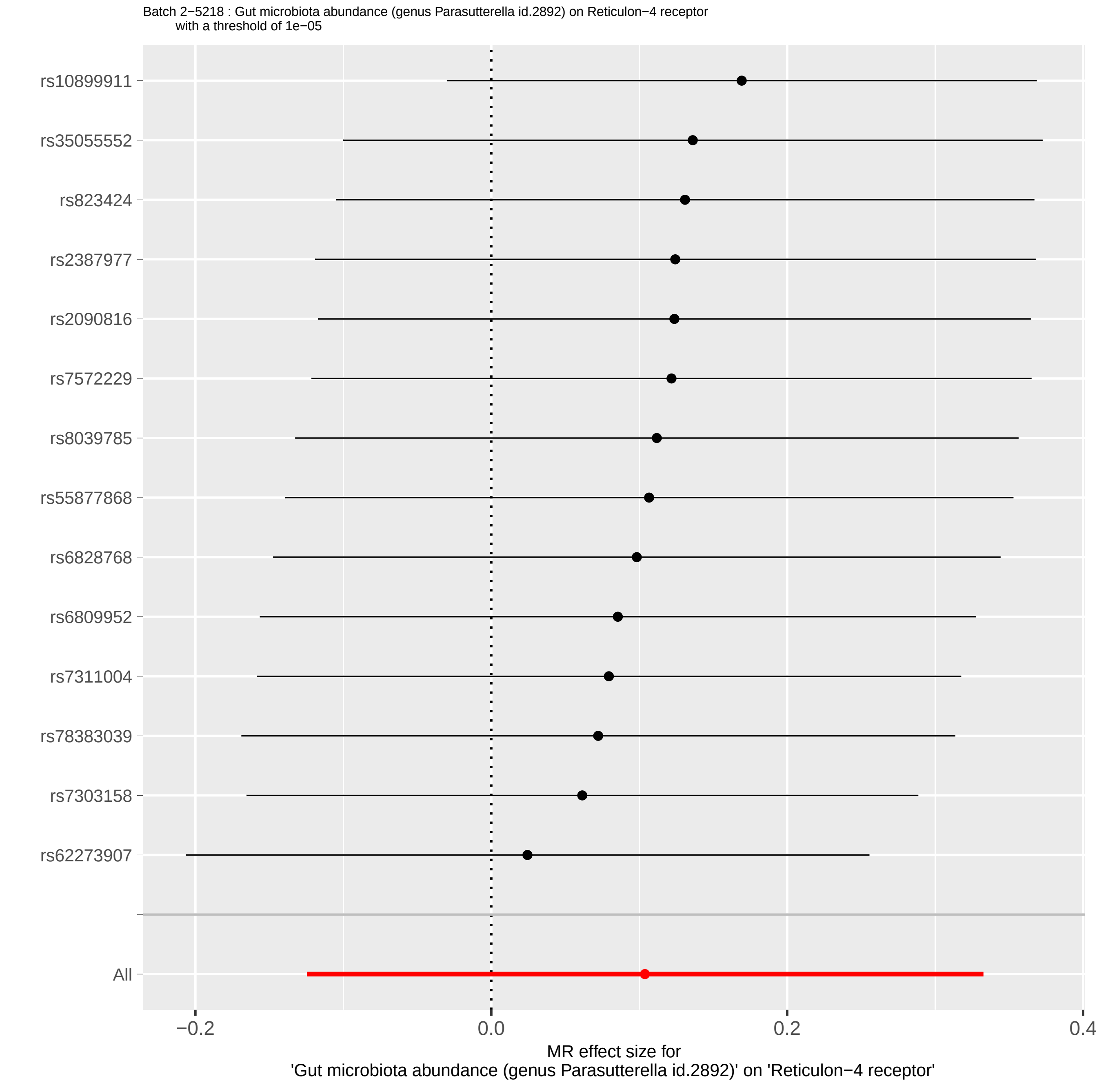

Batch 2−5218 : Gut microbiota abundance (genus Parasutterella id.2892) on Reticulon−4 receptor
with a threshold of 1e−05
rs10899911
rs35055552
rs823424
rs2387977
rs2090816
rs7572229
rs8039785
rs55877868
rs6828768
rs6809952
rs7311004
rs78383039
rs7303158
rs62273907
All
−0.2
0.0
0.2
0.4
MR effect size for
'Gut microbiota abundance (genus Parasutterella id.2892)' on 'Reticulon−4 receptor'

Batch 2−5219 : Gut microbiota abundance (genus Catenibacterium id.2153) on RUN and FYVE domain−containing protein 1
with a threshold of 1e−05

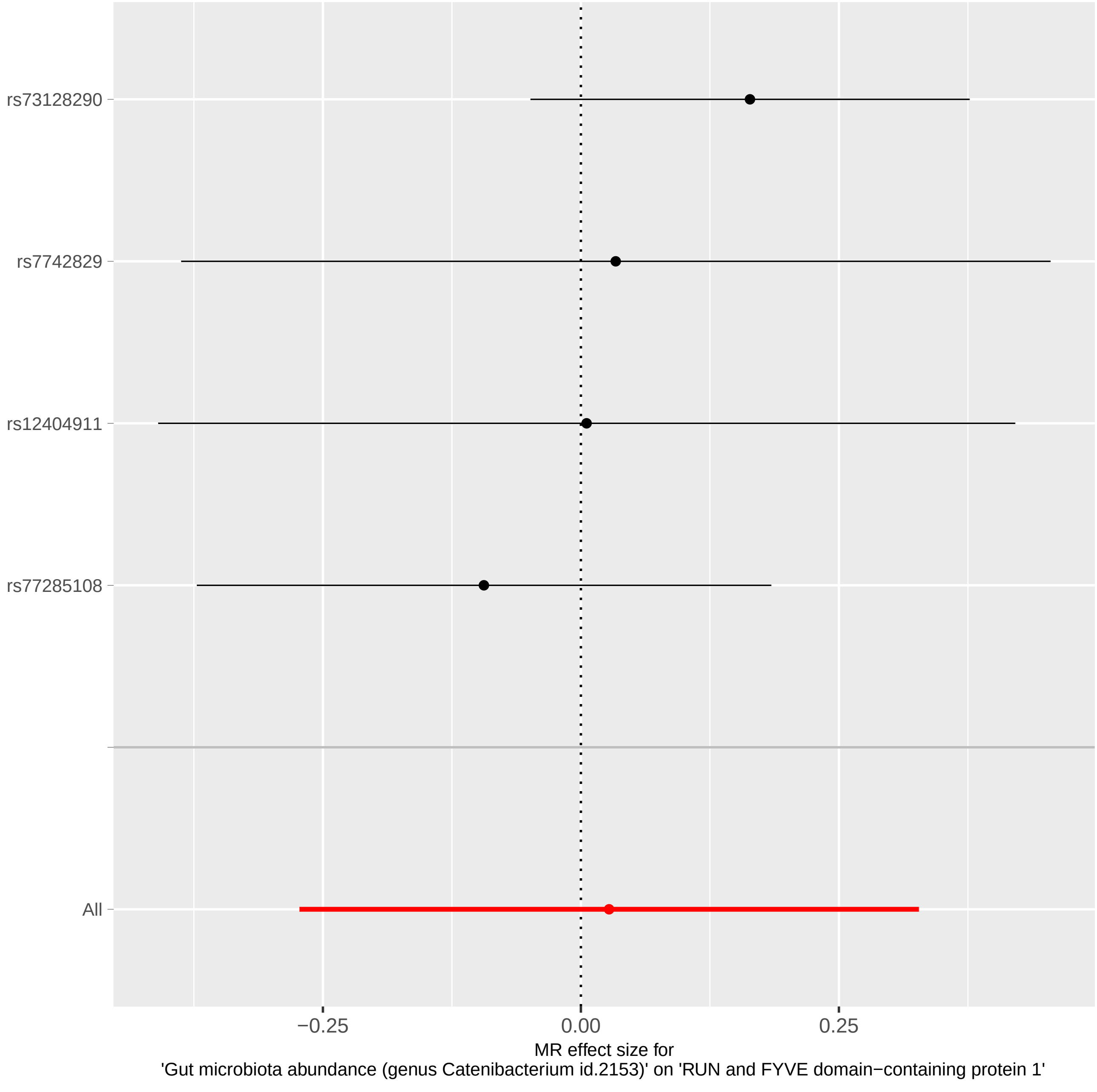

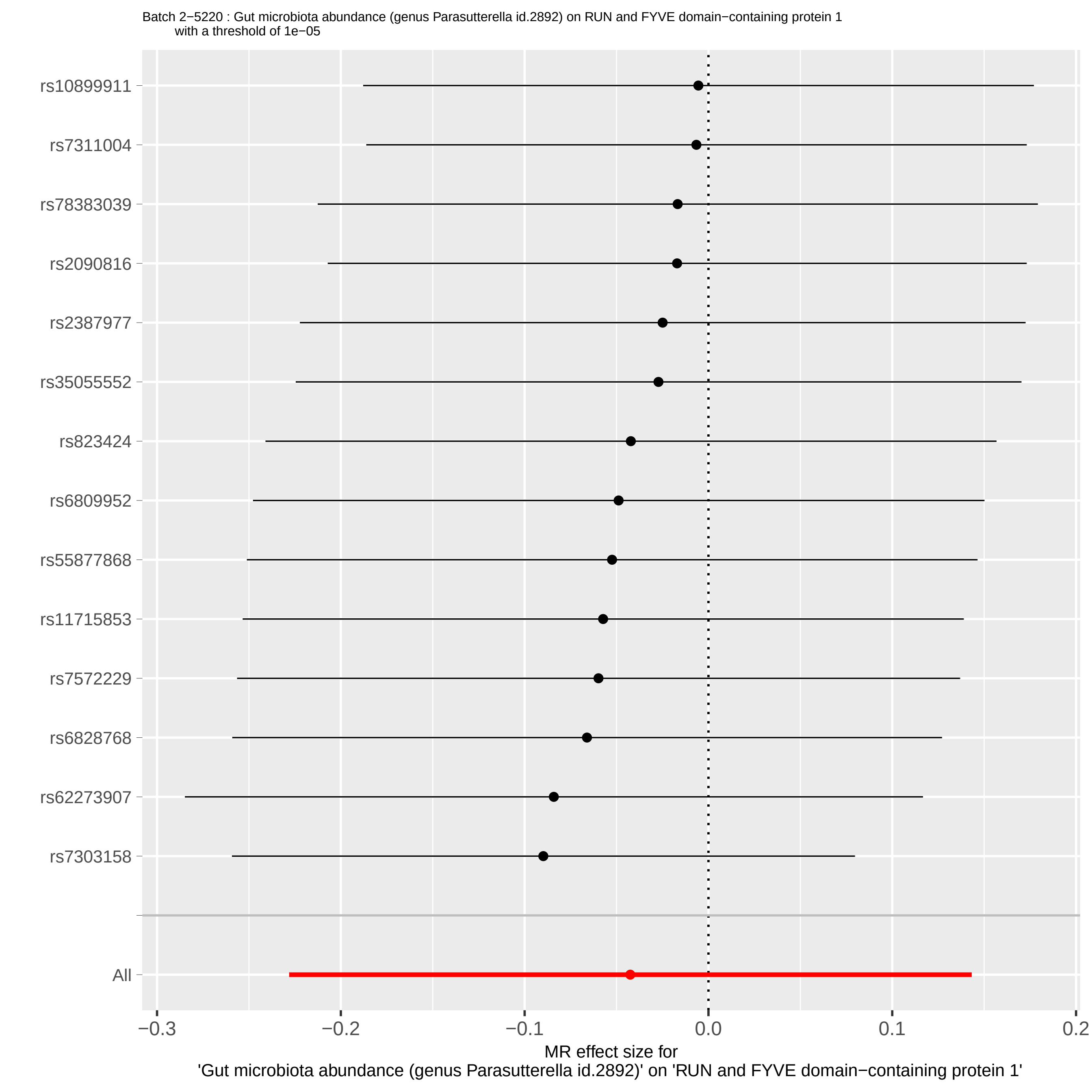
Batch 2−5220 : Gut microbiota abundance (genus Parasutterella id.2892) on RUN and FYVE domain−containing protein 1
with a threshold of 1e−05
rs10899911
rs7311004
rs78383039
rs2090816
rs2387977
rs35055552
rs823424
rs6809952
rs55877868
rs11715853
rs7572229
rs6828768
rs62273907
rs7303158
All
−0.3
−0.2
−0.1
0.0
0.1
0.2
MR effect size for
'Gut microbiota abundance (genus Parasutterella id.2892)' on 'RUN and FYVE domain−containing protein 1'

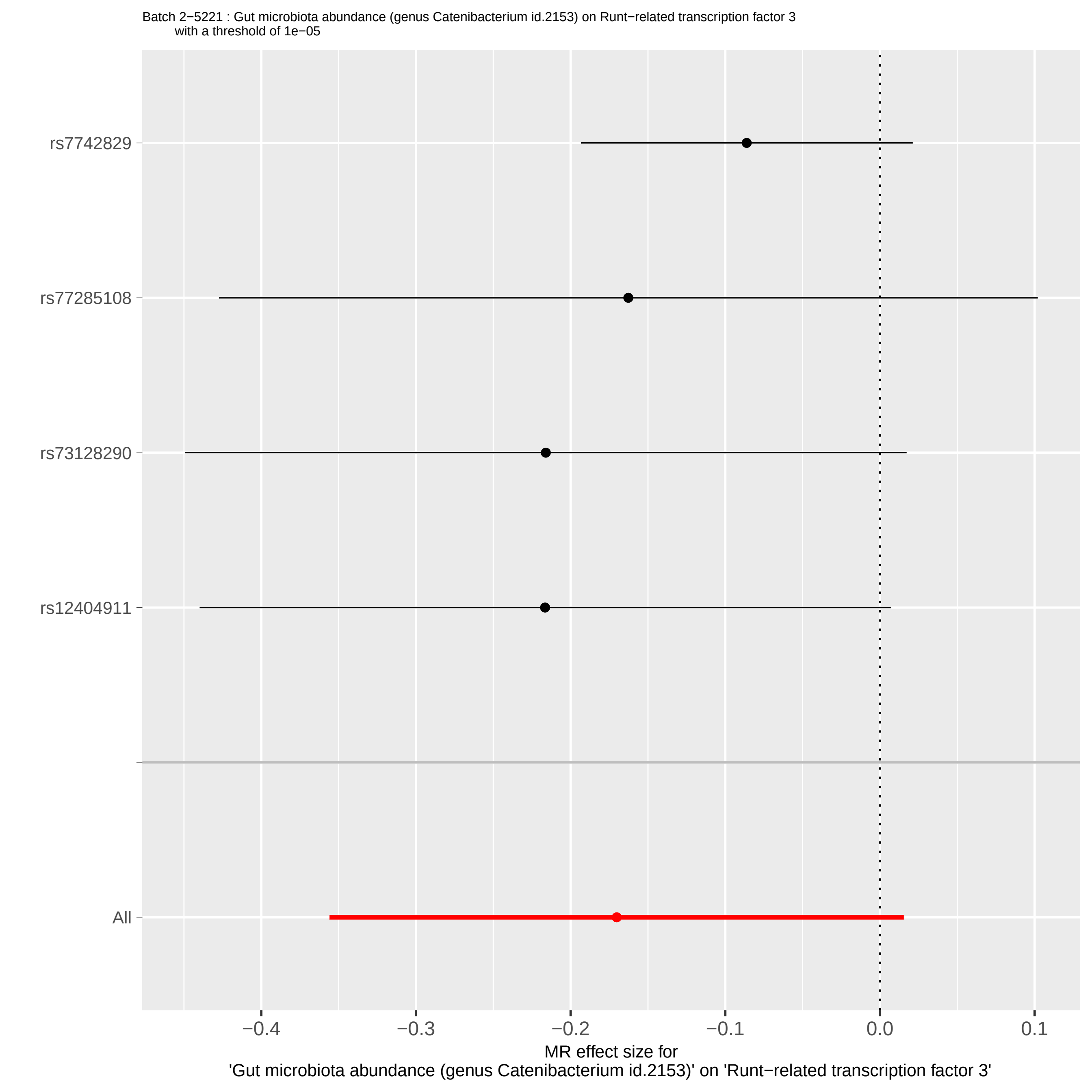
Batch 2−5221 : Gut microbiota abundance (genus Catenibacterium id.2153) on Runt−related transcription factor 3
with a threshold of 1e−05
rs7742829
rs77285108
rs73128290
rs12404911
All
−0.4
−0.3
−0.2
−0.1
0.0
0.1
MR effect size for
'Gut microbiota abundance (genus Catenibacterium id.2153)' on 'Runt−related transcription factor 3'

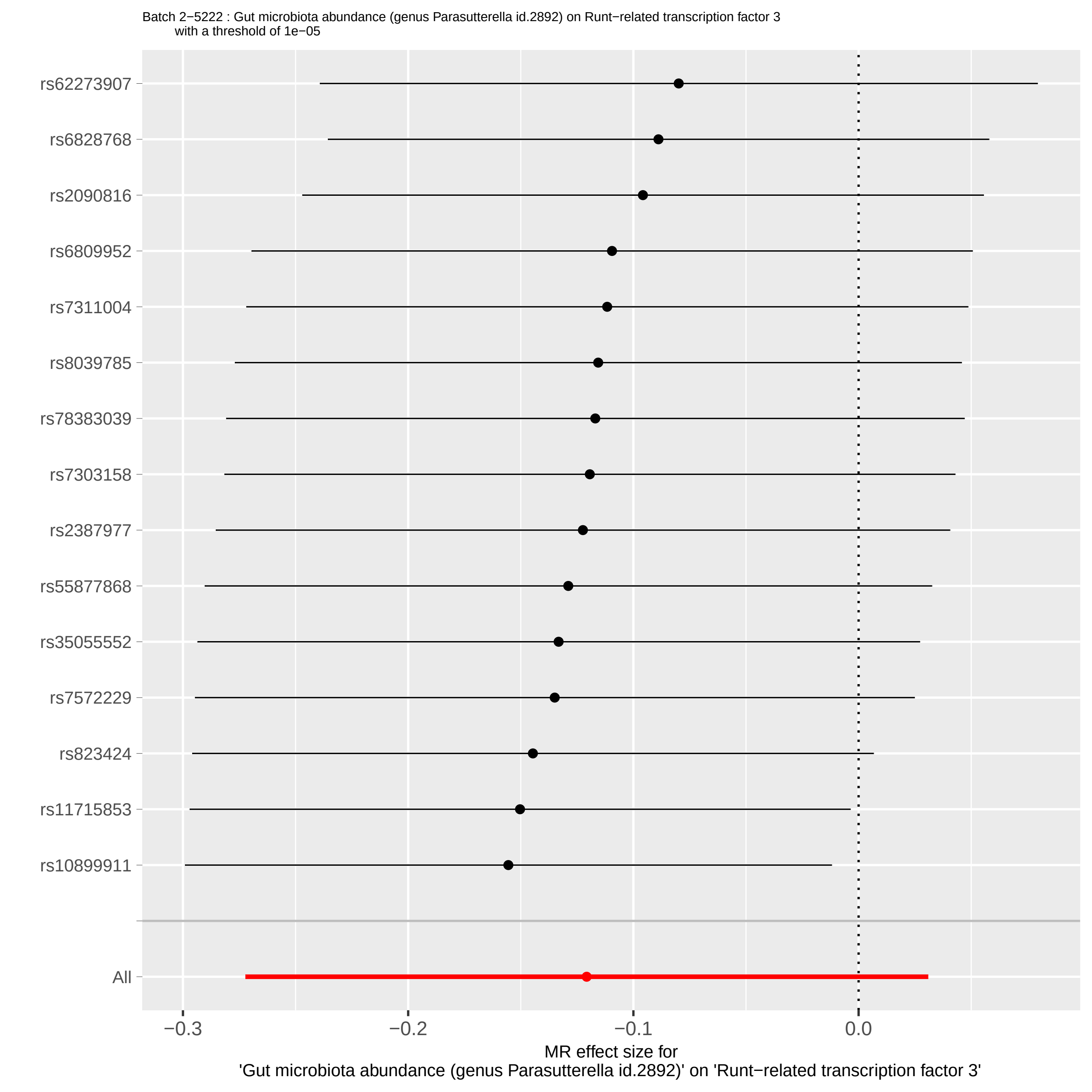
Batch 2−5222 : Gut microbiota abundance (genus Parasutterella id.2892) on Runt−related transcription factor 3
with a threshold of 1e−05
rs62273907
rs6828768
rs2090816
rs6809952
rs7311004
rs8039785
rs78383039
rs7303158
rs2387977
rs55877868
rs35055552
rs7572229
rs823424
rs11715853
rs10899911
All
−0.3
−0.2
−0.1
0.0
MR effect size for
'Gut microbiota abundance (genus Parasutterella id.2892)' on 'Runt−related transcription factor 3'

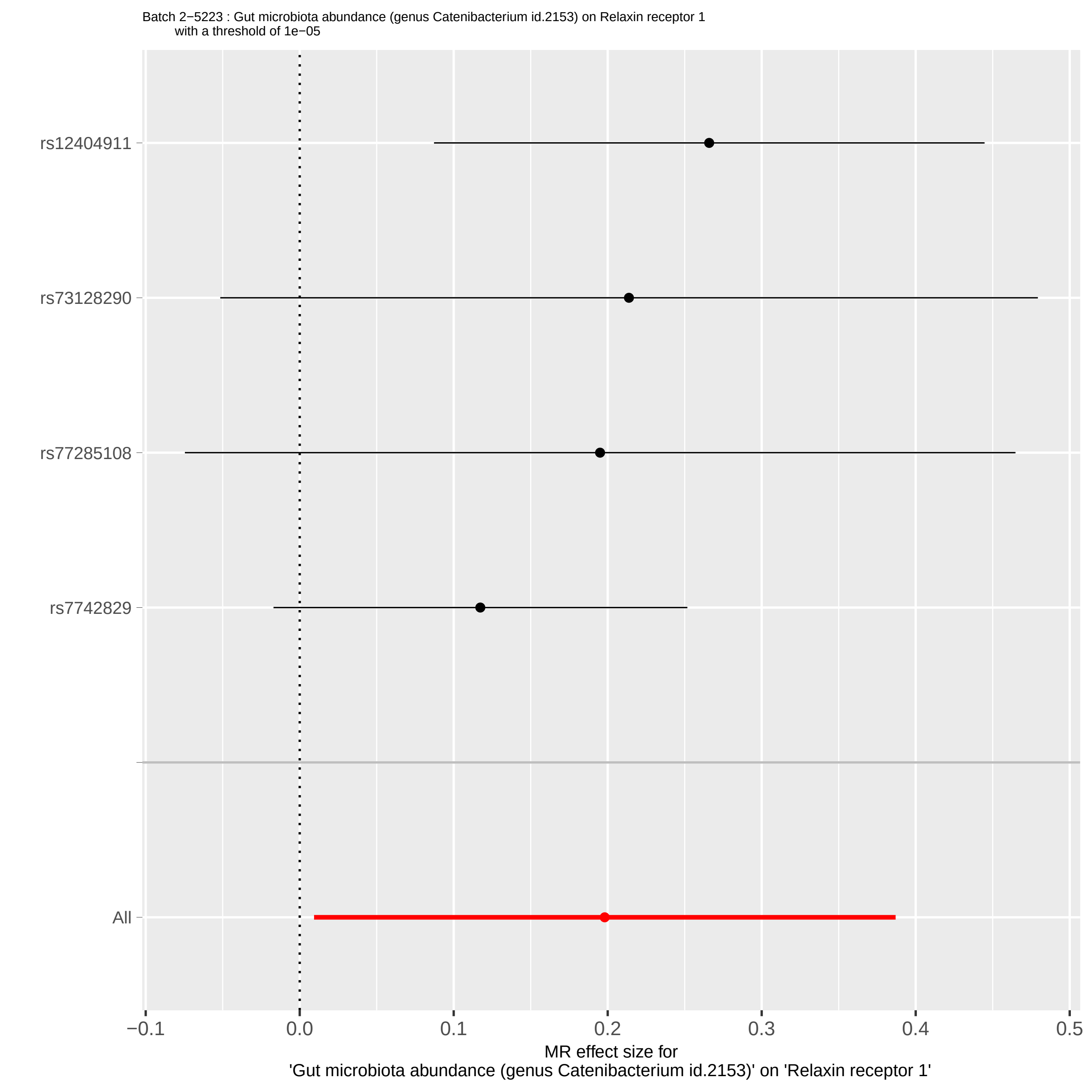
Batch 2−5223 : Gut microbiota abundance (genus Catenibacterium id.2153) on Relaxin receptor 1
with a threshold of 1e−05
rs12404911
rs73128290
rs77285108
rs7742829
All
−0.1
0.0
0.1
0.2
0.3
0.4
0.5
MR effect size for
'Gut microbiota abundance (genus Catenibacterium id.2153)' on 'Relaxin receptor 1'

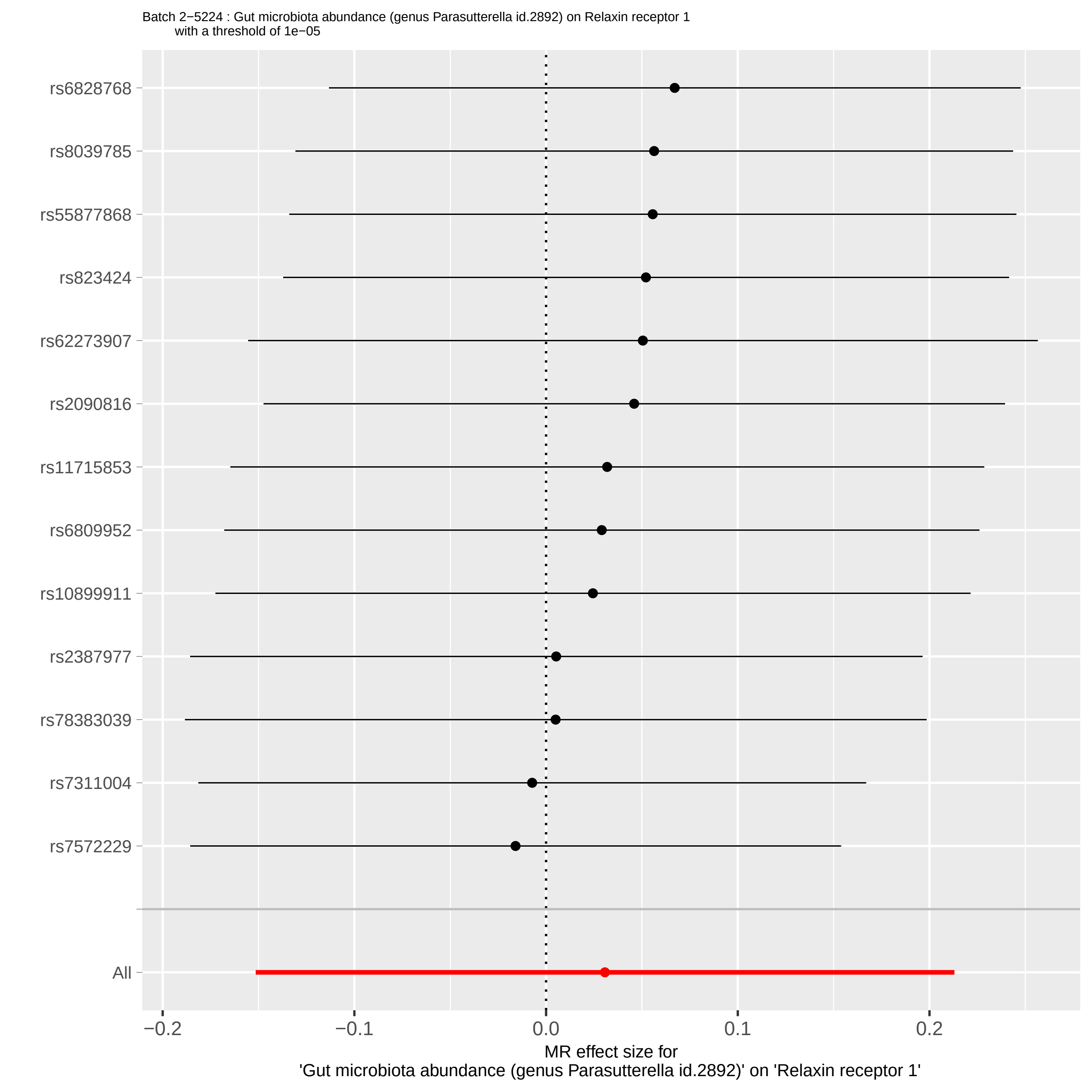
Batch 2−5224 : Gut microbiota abundance (genus Parasutterella id.2892) on Relaxin receptor 1
with a threshold of 1e−05
rs6828768
rs8039785
rs55877868
rs823424
rs62273907
rs2090816
rs11715853
rs6809952
rs10899911
rs2387977
rs78383039
rs7311004
rs7572229
All
−0.2
−0.1
0.0
0.1
0.2
MR effect size for
'Gut microbiota abundance (genus Parasutterella id.2892)' on 'Relaxin receptor 1'

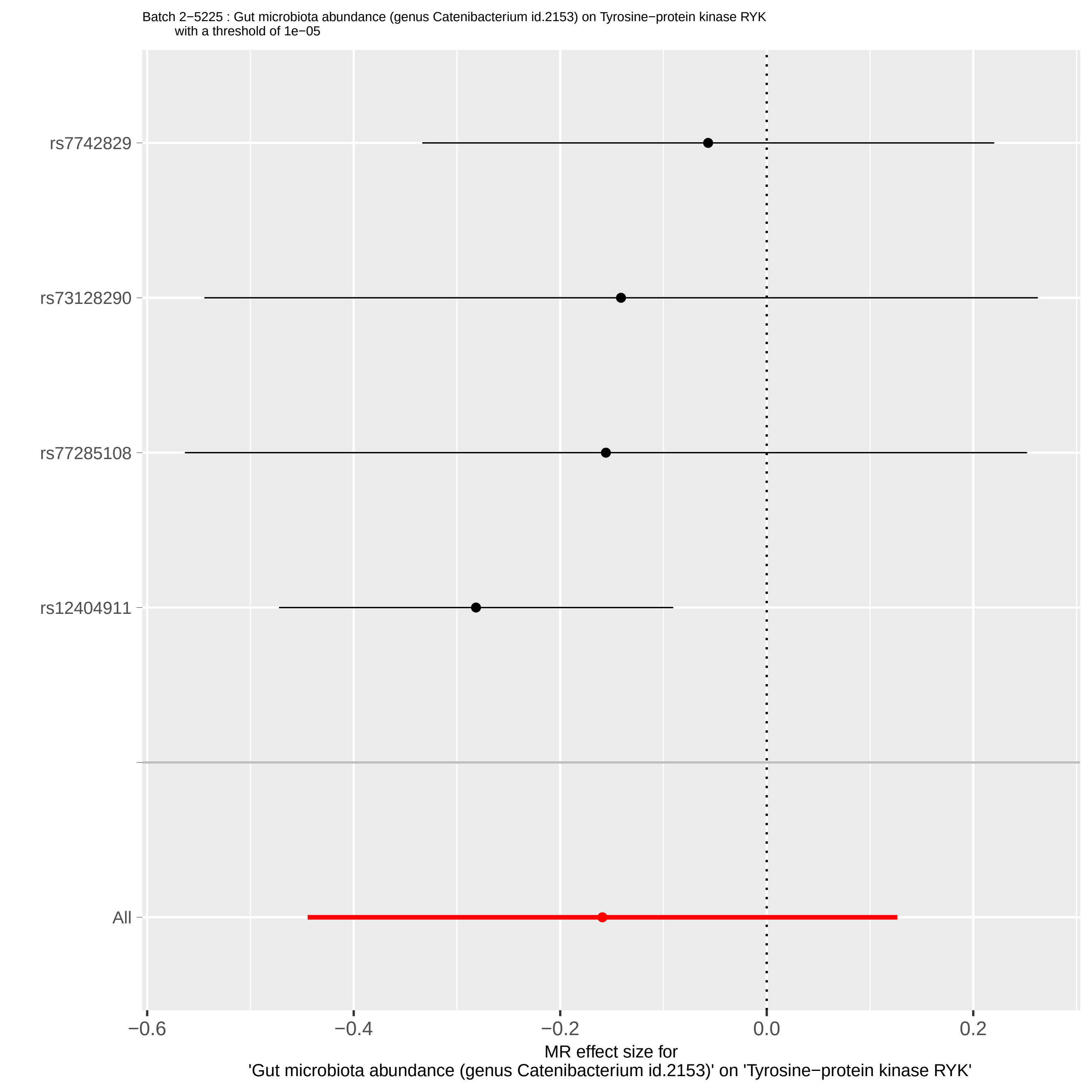

Batch 2−5225 : Gut microbiota abundance (genus Catenibacterium id.2153) on Tyrosine−protein kinase RYK
with a threshold of 1e−05
rs7742829
rs73128290
rs77285108
rs12404911
All
−0.6
−0.4
−0.2
0.0
0.2
MR effect size for
'Gut microbiota abundance (genus Catenibacterium id.2153)' on 'Tyrosine−protein kinase RYK'

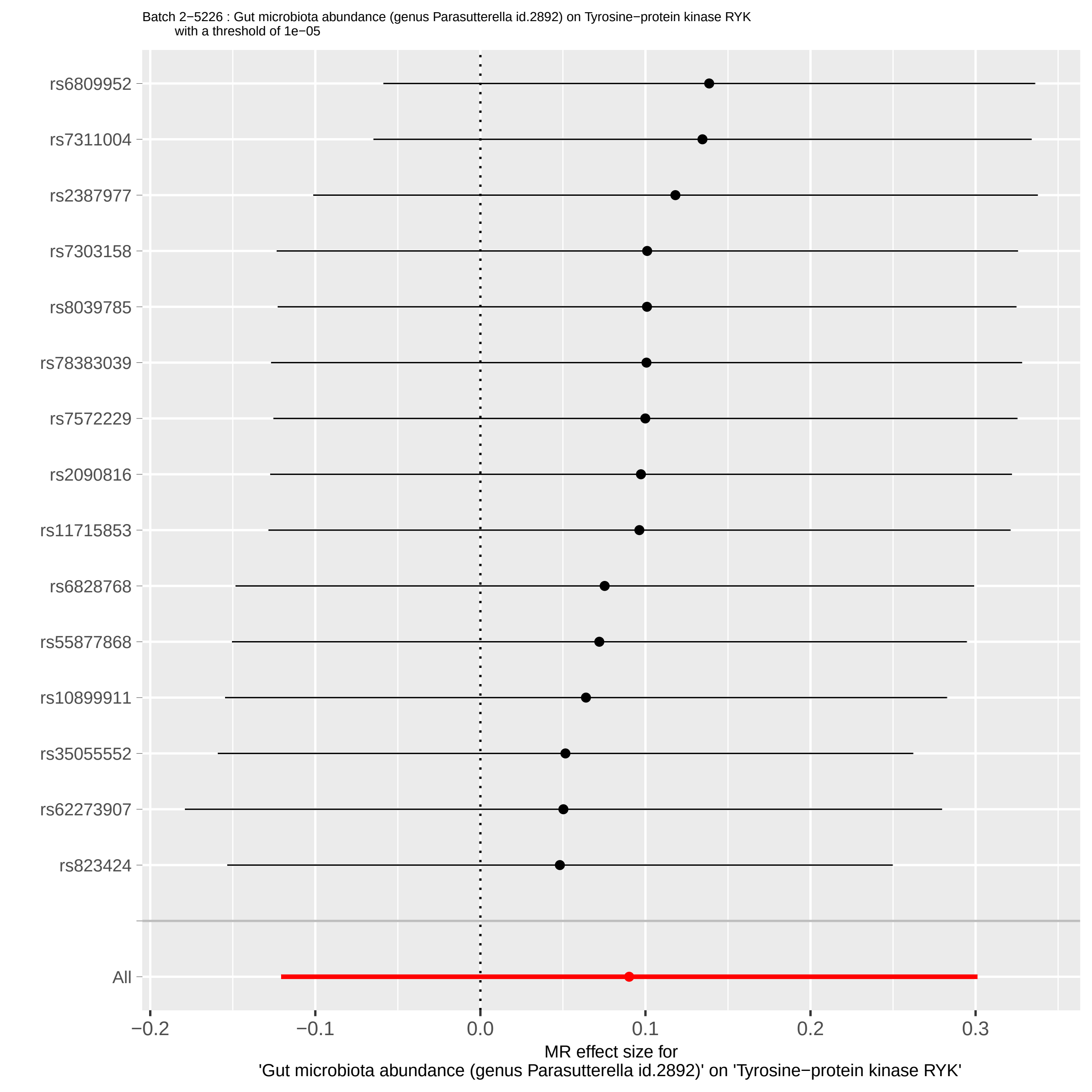

Batch 2−5226 : Gut microbiota abundance (genus Parasutterella id.2892) on Tyrosine−protein kinase RYK
with a threshold of 1e−05
rs6809952
rs7311004
rs2387977
rs7303158
rs8039785
rs78383039
rs7572229
rs2090816
rs11715853
rs6828768
rs55877868
rs10899911
rs35055552
rs62273907
rs823424
All
−0.2
−0.1
0.0
0.1
0.2
0.3
MR effect size for
'Gut microbiota abundance (genus Parasutterella id.2892)' on 'Tyrosine−protein kinase RYK'

Batch 2−5227 : Gut microbiota abundance (genus Catenibacterium id.2153) on Protein S100−A11
with a threshold of 1e−05

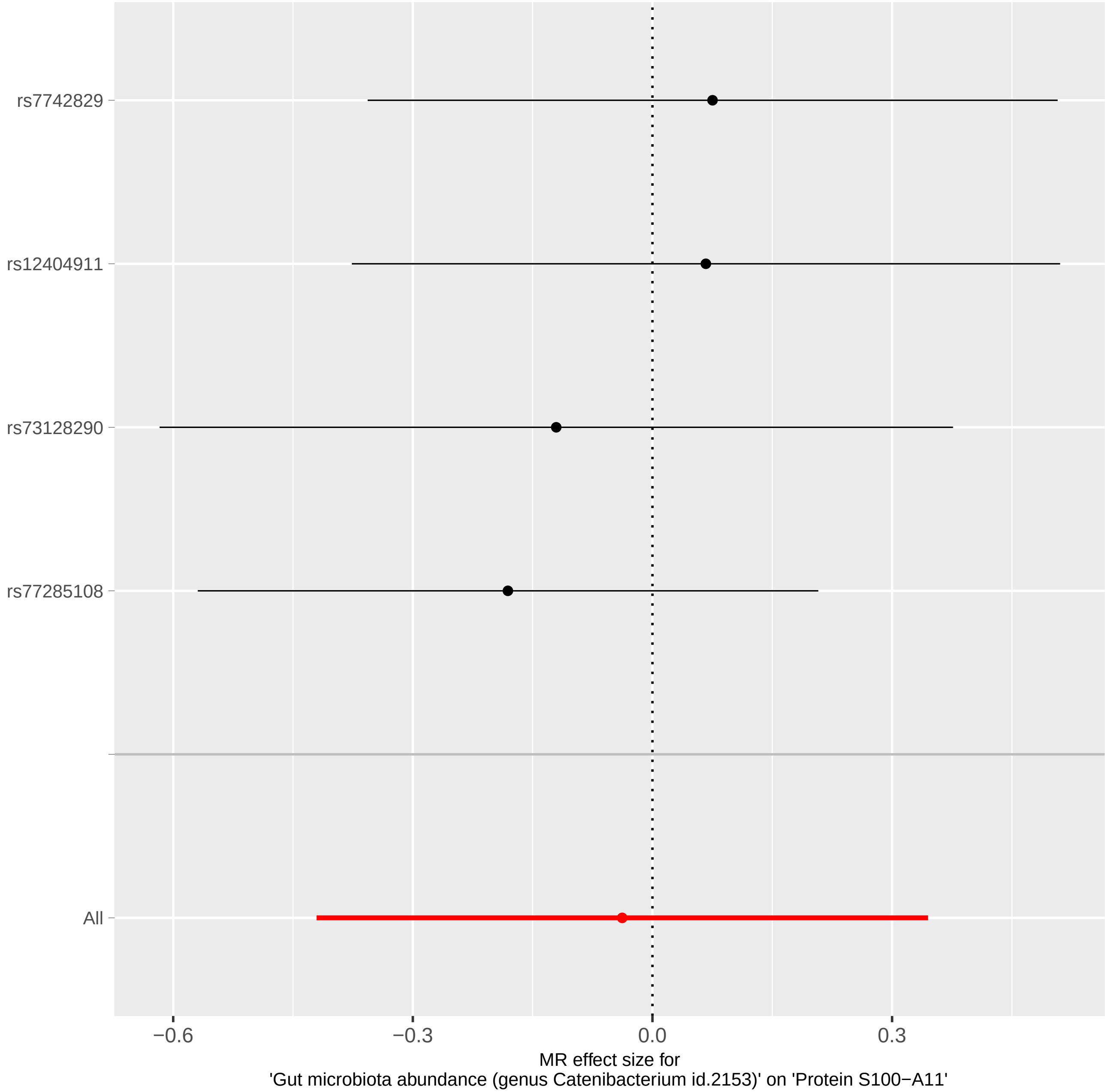

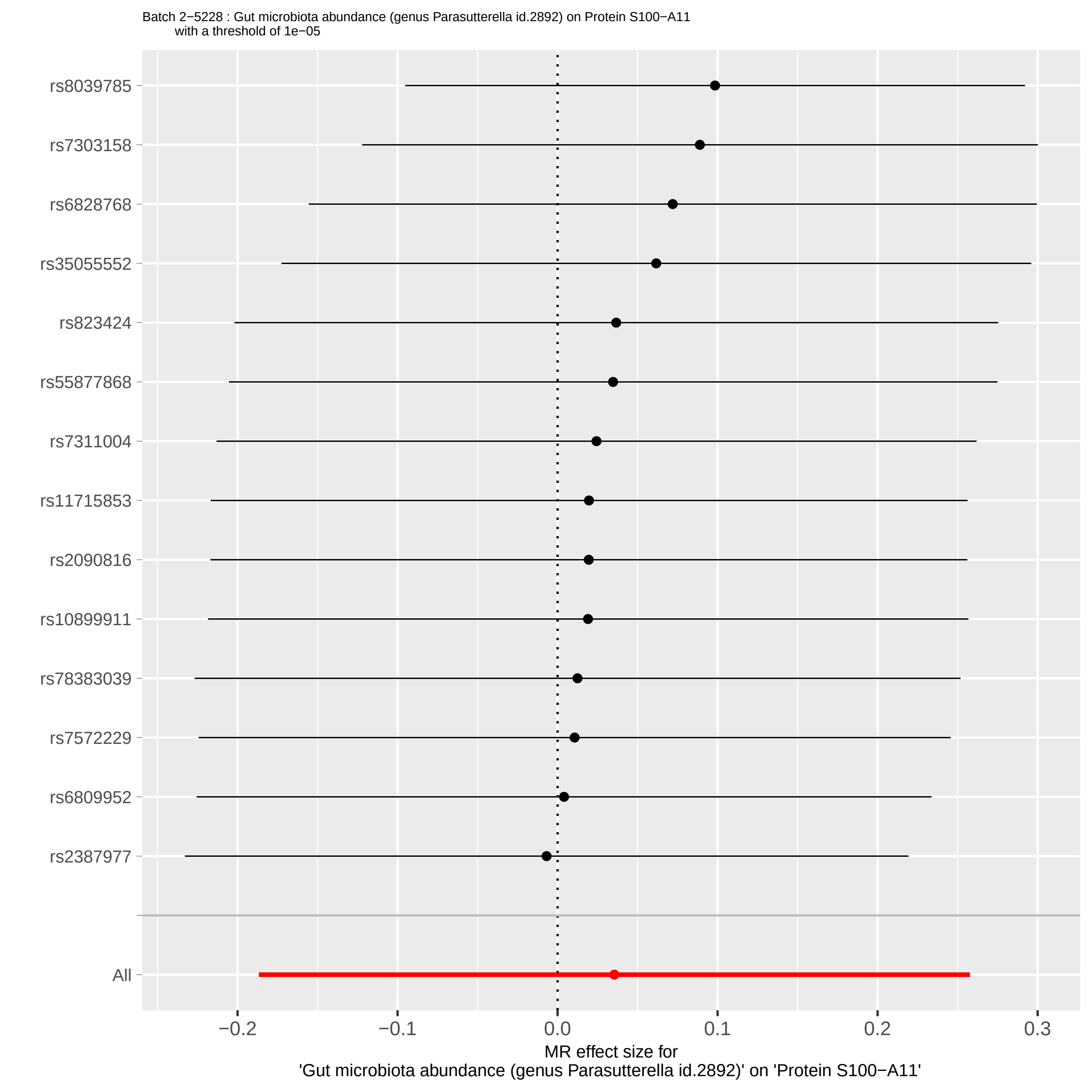

Batch 2−5228 : Gut microbiota abundance (genus Parasutterella id.2892) on Protein S100−A11
with a threshold of 1e−05
rs8039785
rs7303158
rs6828768
rs35055552
rs823424
rs55877868
rs7311004
rs11715853
rs2090816
rs10899911
rs78383039
rs7572229
rs6809952
rs2387977
All
−0.2
−0.1
0.0
0.1
0.2
0.3
MR effect size for
'Gut microbiota abundance (genus Parasutterella id.2892)' on 'Protein S100−A11'

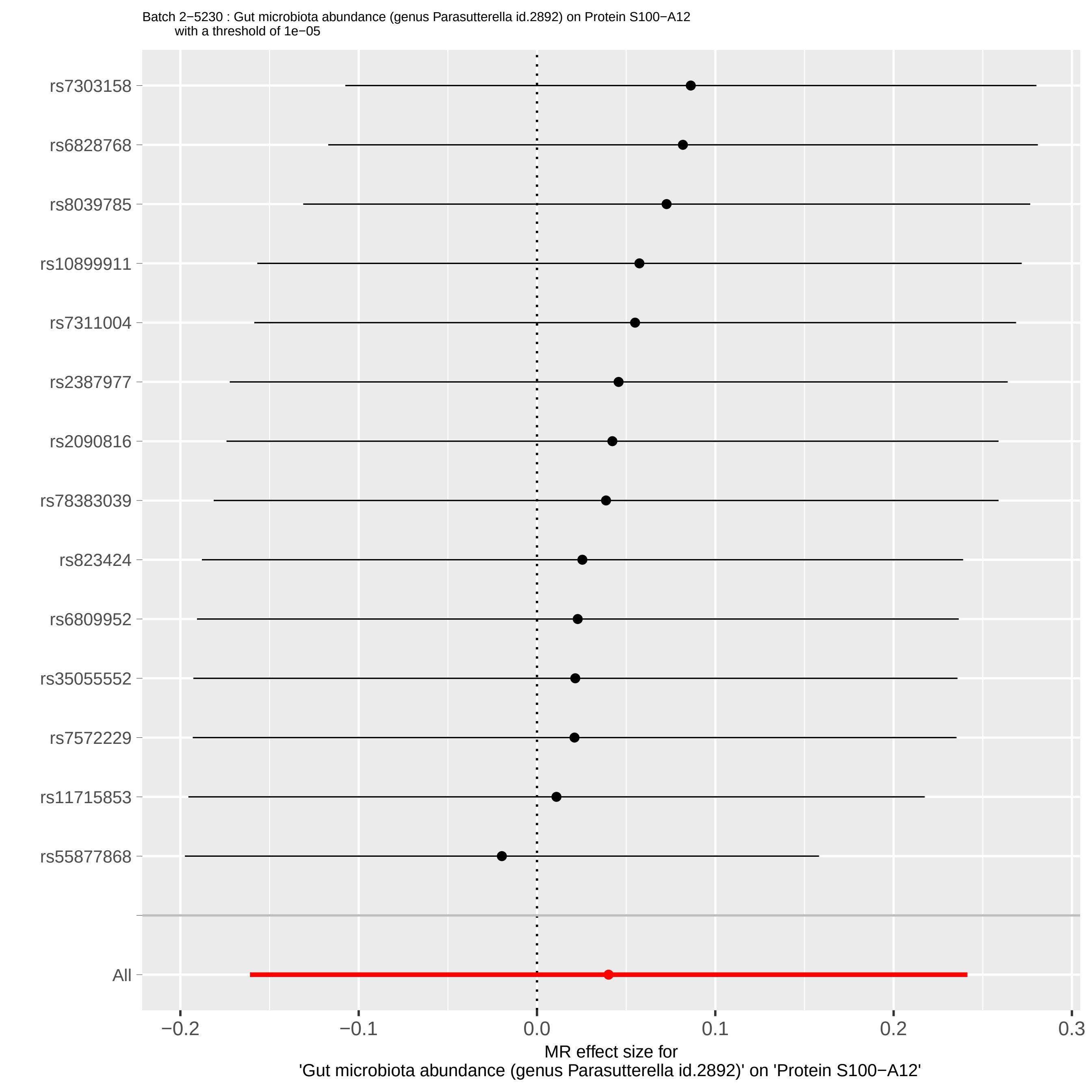
Batch 2−5230 : Gut microbiota abundance (genus Parasutterella id.2892) on Protein S100−A12
with a threshold of 1e−05
rs7303158
rs6828768
rs8039785
rs10899911
rs7311004
rs2387977
rs2090816
rs78383039
rs823424
rs6809952
rs35055552
rs7572229
rs11715853
rs55877868
All
−0.2
−0.1
0.0
0.1
0.2
0.3
MR effect size for
'Gut microbiota abundance (genus Parasutterella id.2892)' on 'Protein S100−A12'

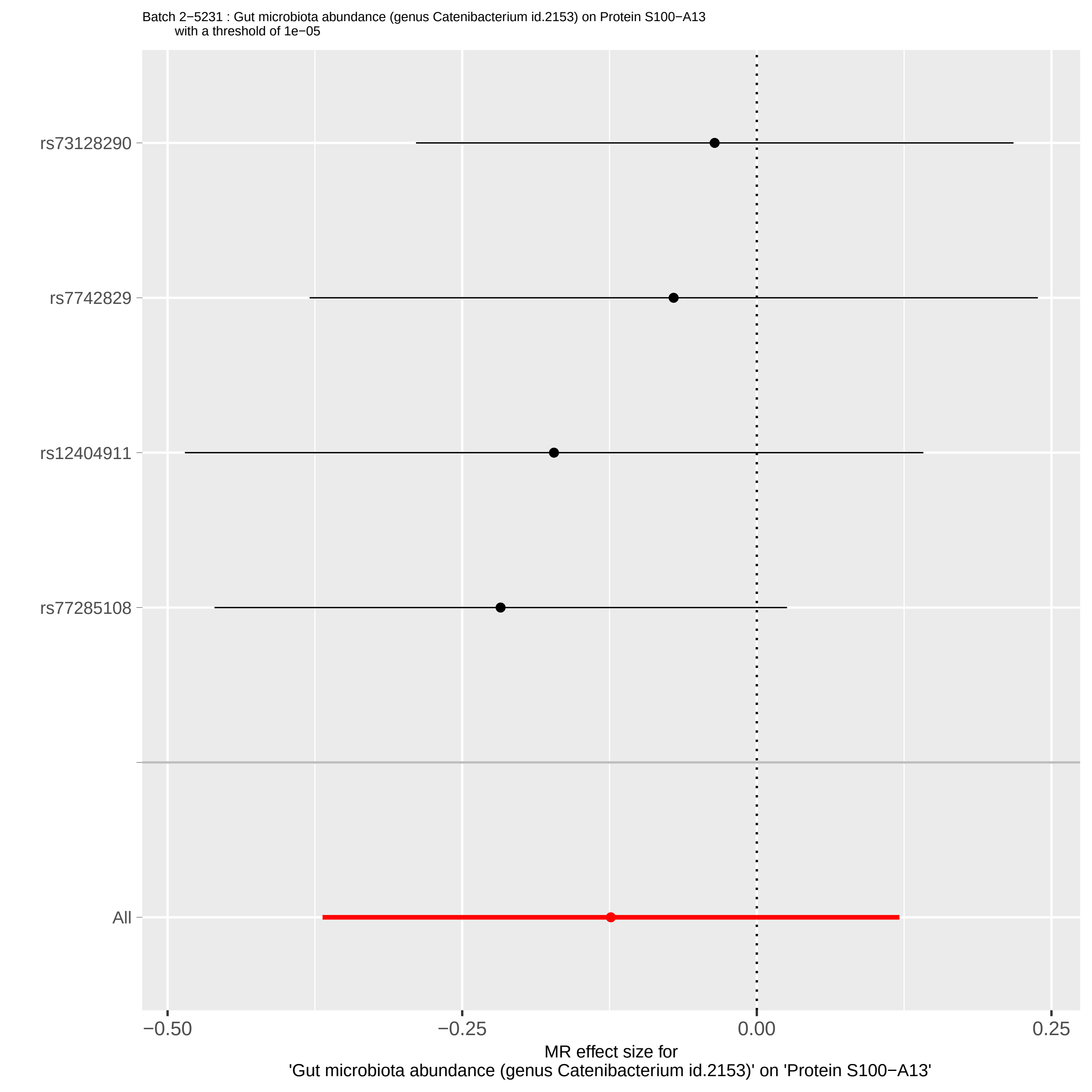

Batch 2−5231 : Gut microbiota abundance (genus Catenibacterium id.2153) on Protein S100−A13
with a threshold of 1e−05
rs73128290
rs7742829
rs12404911
rs77285108
All
−0.50
−0.25
0.00
0.25
MR effect size for
'Gut microbiota abundance (genus Catenibacterium id.2153)' on 'Protein S100−A13'

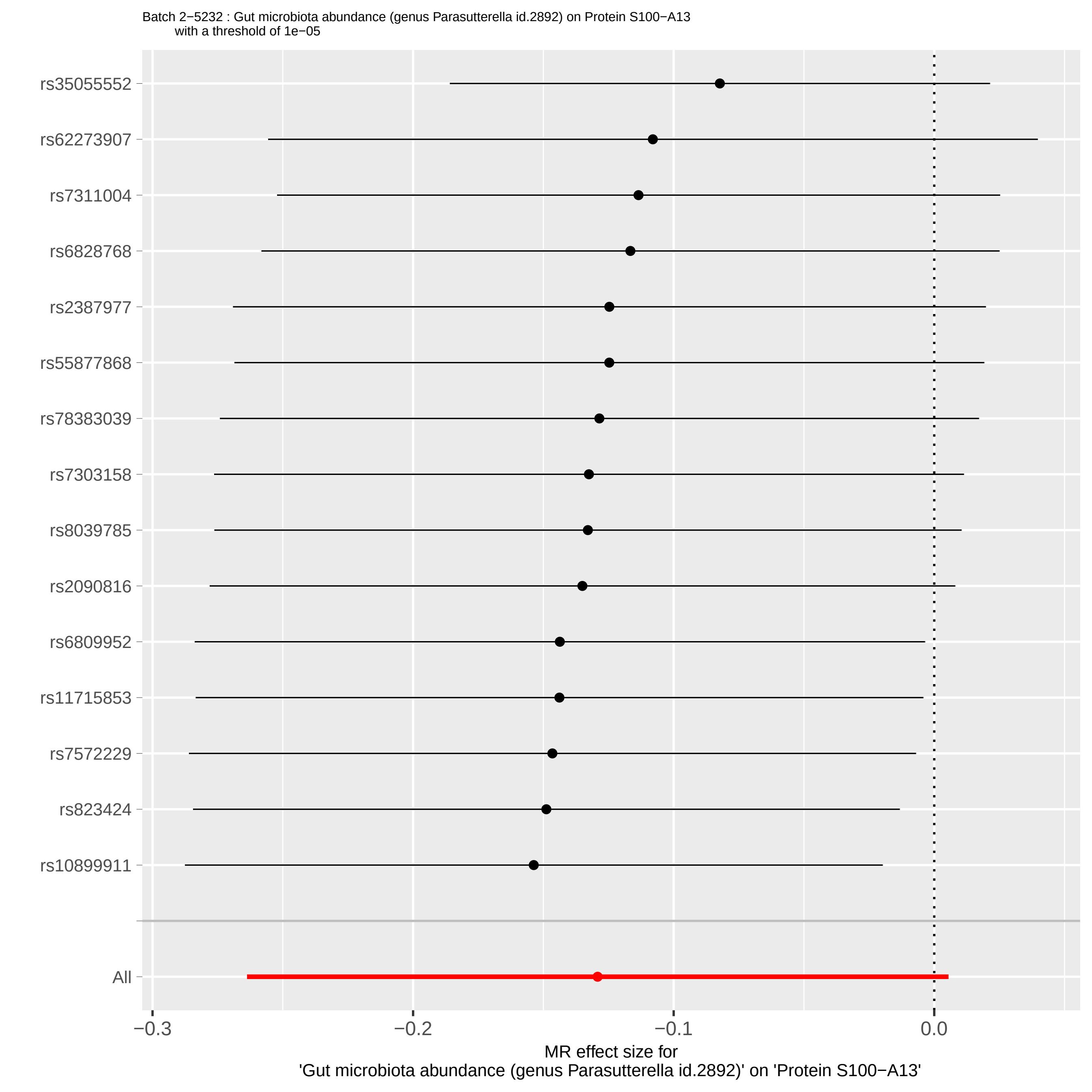

Batch 2−5232 : Gut microbiota abundance (genus Parasutterella id.2892) on Protein S100−A13
with a threshold of 1e−05
rs35055552
rs62273907
rs7311004
rs6828768
rs2387977
rs55877868
rs78383039
rs7303158
rs8039785
rs2090816
rs6809952
rs11715853
rs7572229
rs823424
rs10899911
All
−0.3
−0.2
−0.1
0.0
MR effect size for
'Gut microbiota abundance (genus Parasutterella id.2892)' on 'Protein S100−A13'

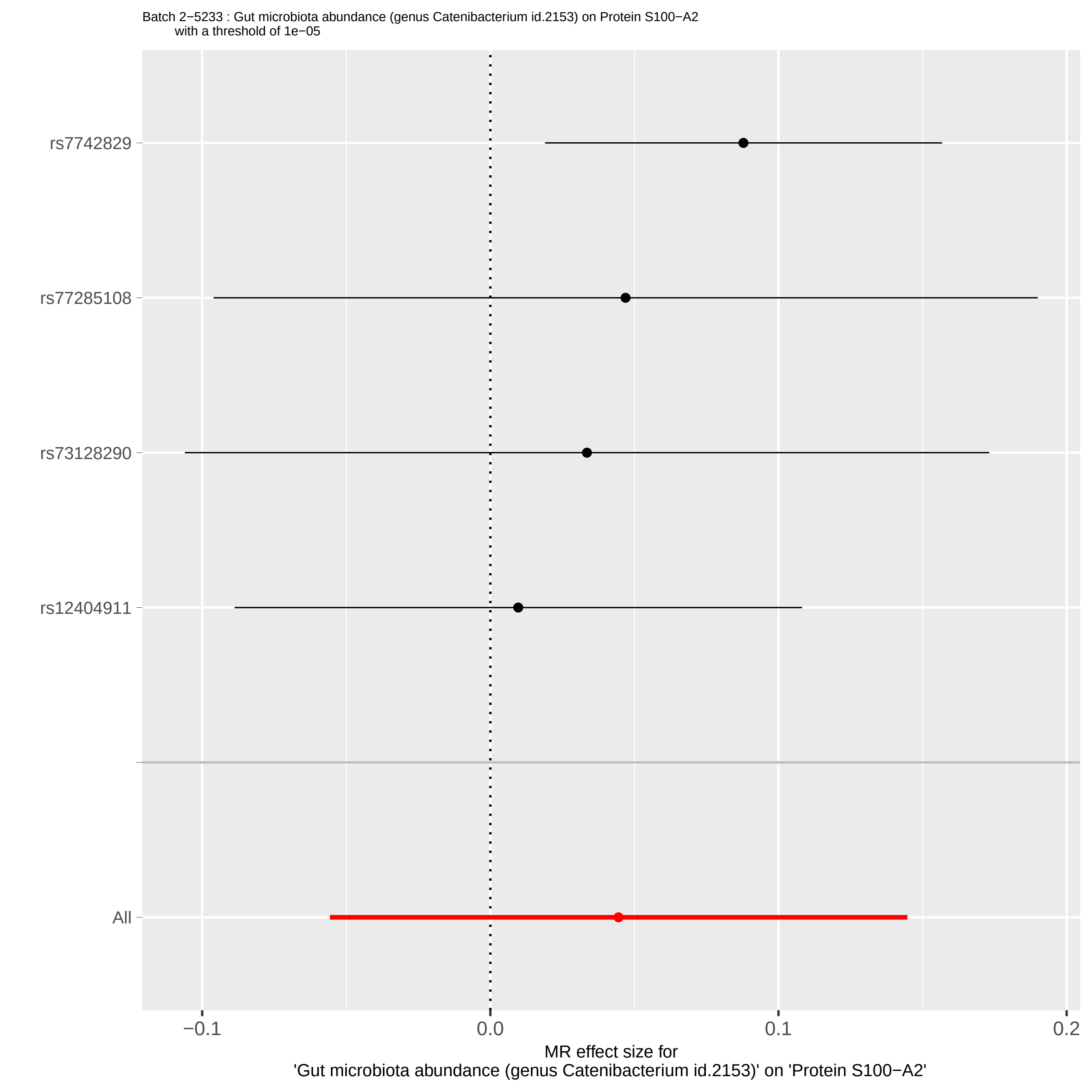

Batch 2−5233 : Gut microbiota abundance (genus Catenibacterium id.2153) on Protein S100−A2
with a threshold of 1e−05
rs7742829
rs77285108
rs73128290
rs12404911
All
−0.1
0.0
0.1
0.2
MR effect size for
'Gut microbiota abundance (genus Catenibacterium id.2153)' on 'Protein S100−A2'

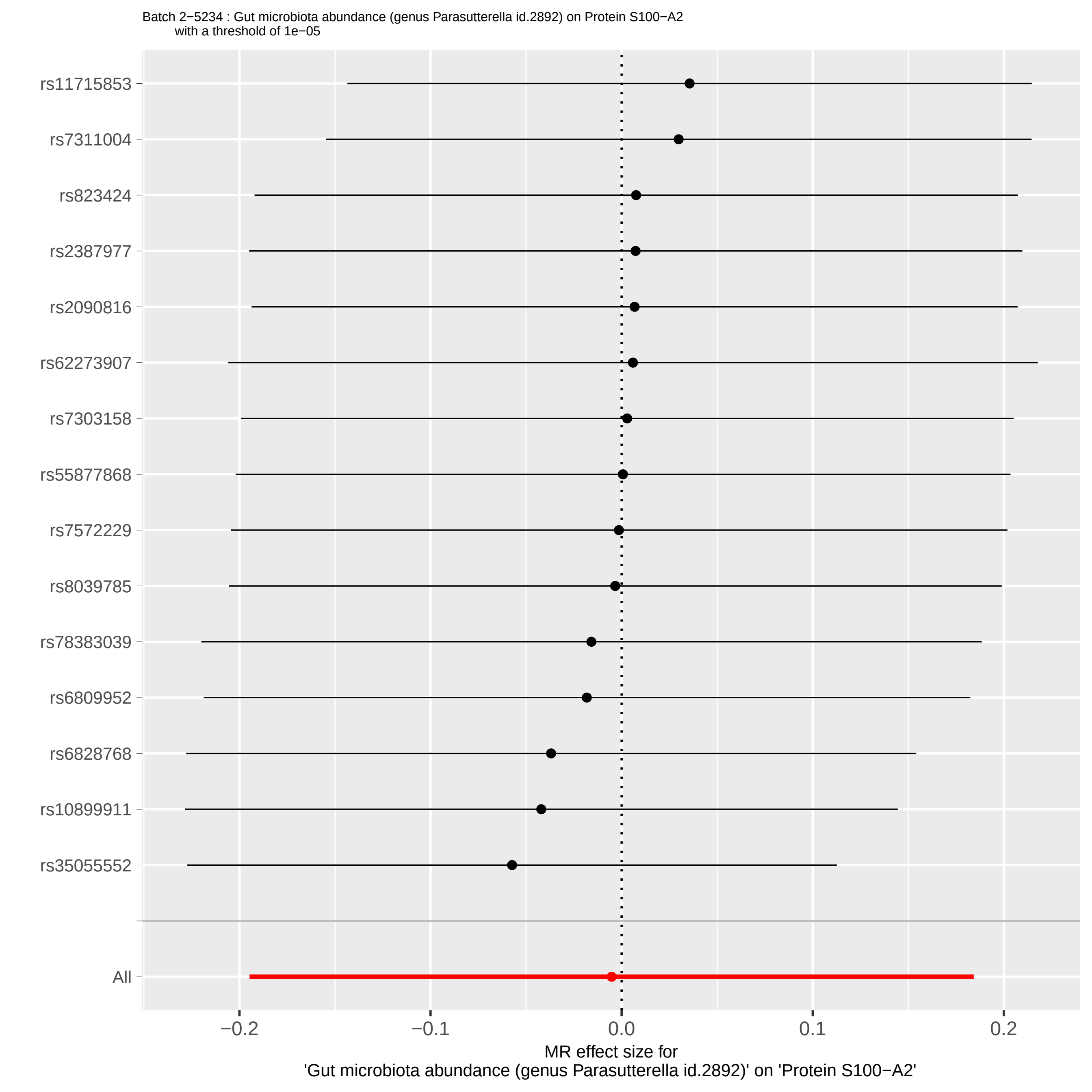
Batch 2−5234 : Gut microbiota abundance (genus Parasutterella id.2892) on Protein S100−A2
with a threshold of 1e−05
rs11715853
rs7311004
rs823424
rs2387977
rs2090816
rs62273907
rs7303158
rs55877868
rs7572229
rs8039785
rs78383039
rs6809952
rs6828768
rs10899911
rs35055552
All
−0.2
−0.1
0.0
0.1
0.2
MR effect size for
'Gut microbiota abundance (genus Parasutterella id.2892)' on 'Protein S100−A2'

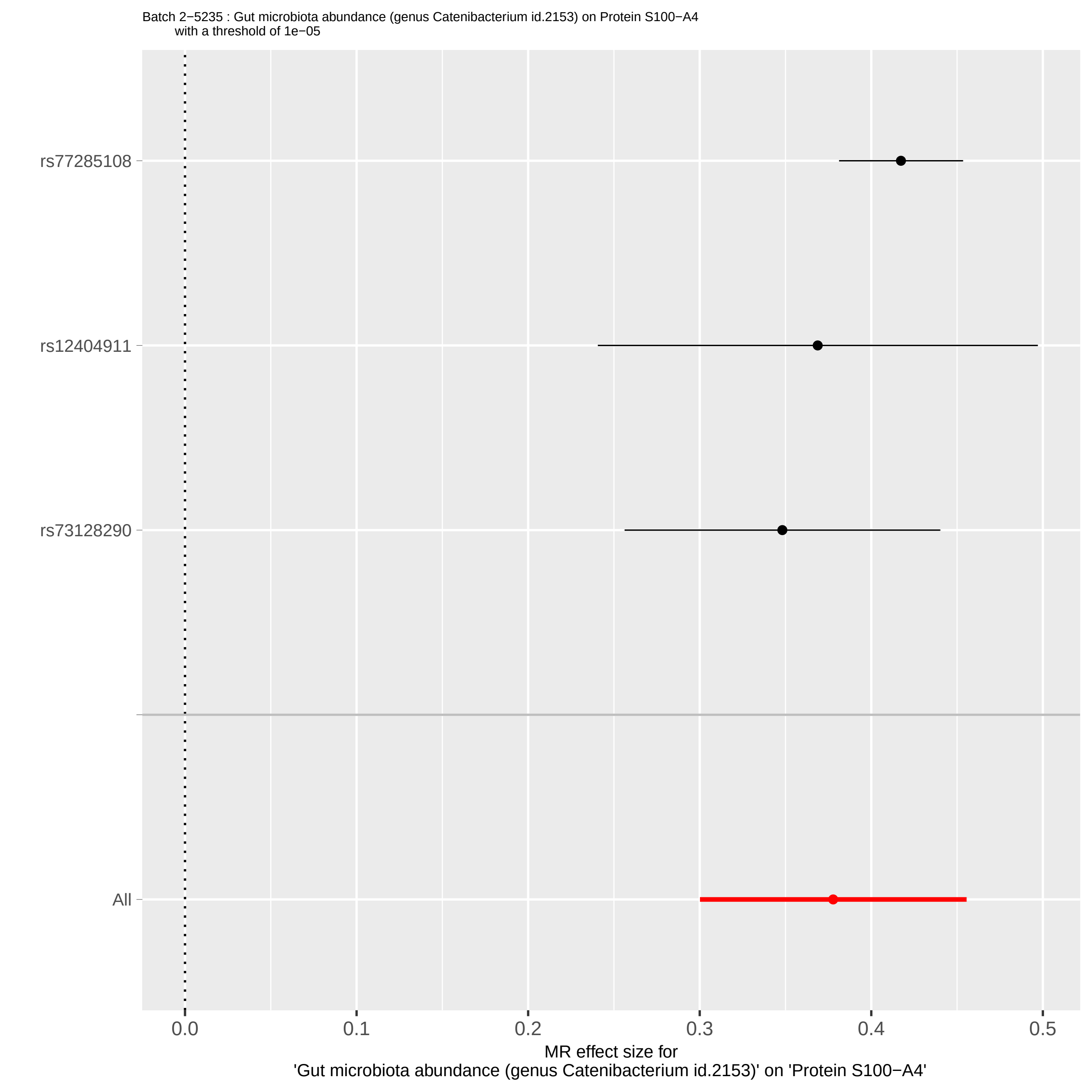
Batch 2−5235 : Gut microbiota abundance (genus Catenibacterium id.2153) on Protein S100−A4
with a threshold of 1e−05
rs77285108
rs12404911
rs73128290
All
0.0
0.1
0.2
0.3
0.4
0.5
MR effect size for
'Gut microbiota abundance (genus Catenibacterium id.2153)' on 'Protein S100−A4'

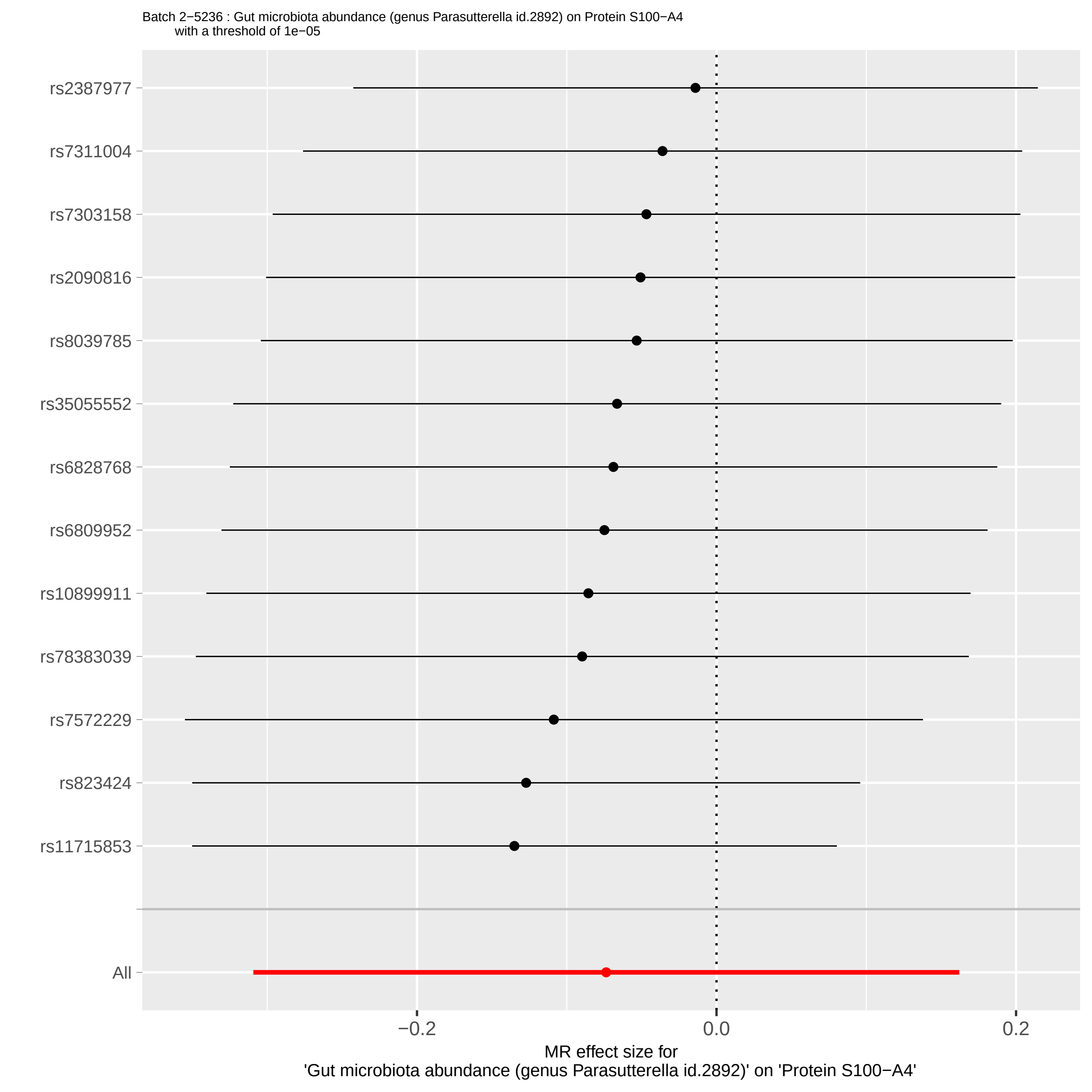

Batch 2−5236 : Gut microbiota abundance (genus Parasutterella id.2892) on Protein S100−A4
with a threshold of 1e−05
rs2387977
rs7311004
rs7303158
rs2090816
rs8039785
rs35055552
rs6828768
rs6809952
rs10899911
rs78383039
rs7572229
rs823424
rs11715853
All
−0.2
0.0
0.2
MR effect size for
'Gut microbiota abundance (genus Parasutterella id.2892)' on 'Protein S100−A4'

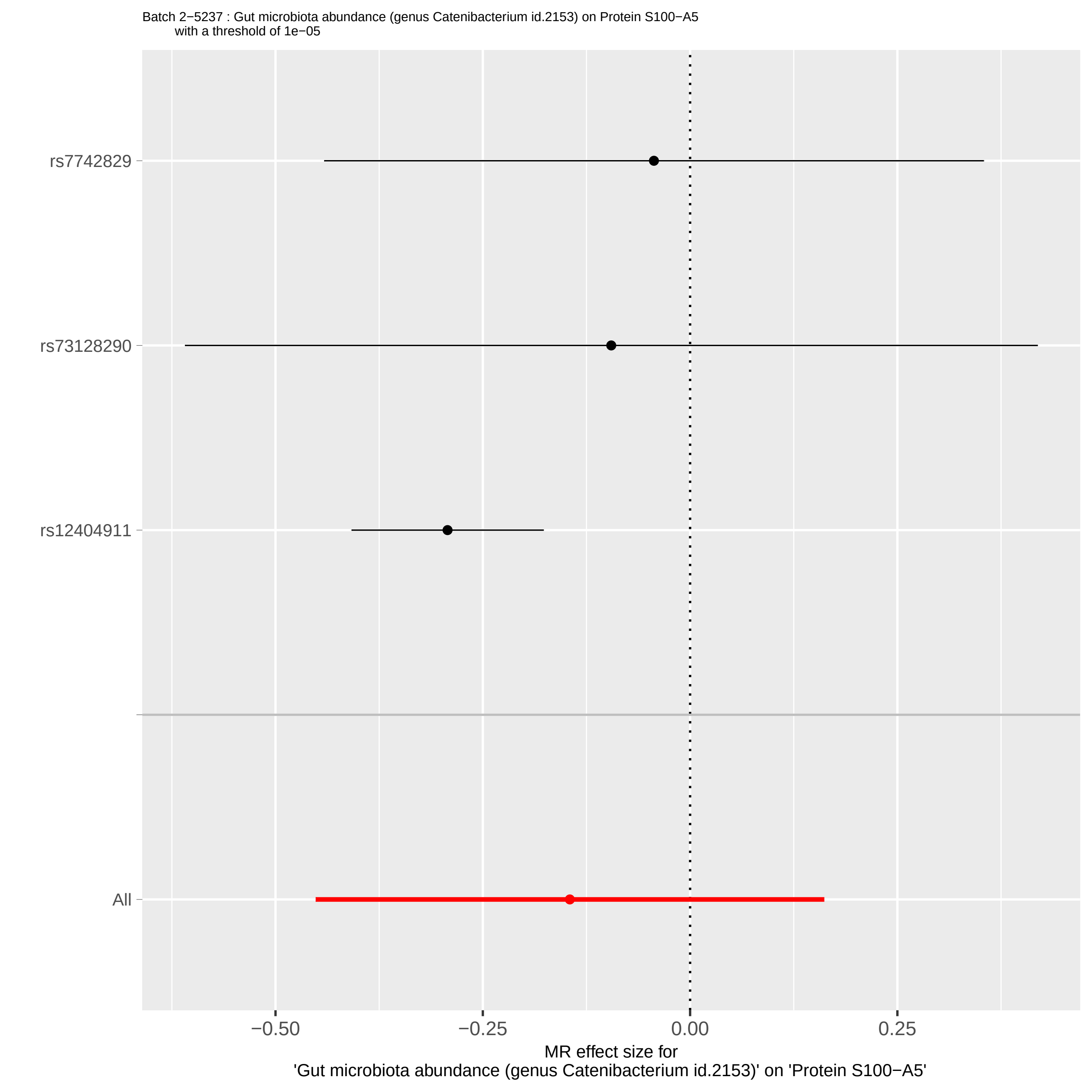
Batch 2−5237 : Gut microbiota abundance (genus Catenibacterium id.2153) on Protein S100−A5
with a threshold of 1e−05
rs7742829
rs73128290
rs12404911
All
−0.50
−0.25
0.00
0.25
MR effect size for
'Gut microbiota abundance (genus Catenibacterium id.2153)' on 'Protein S100−A5'

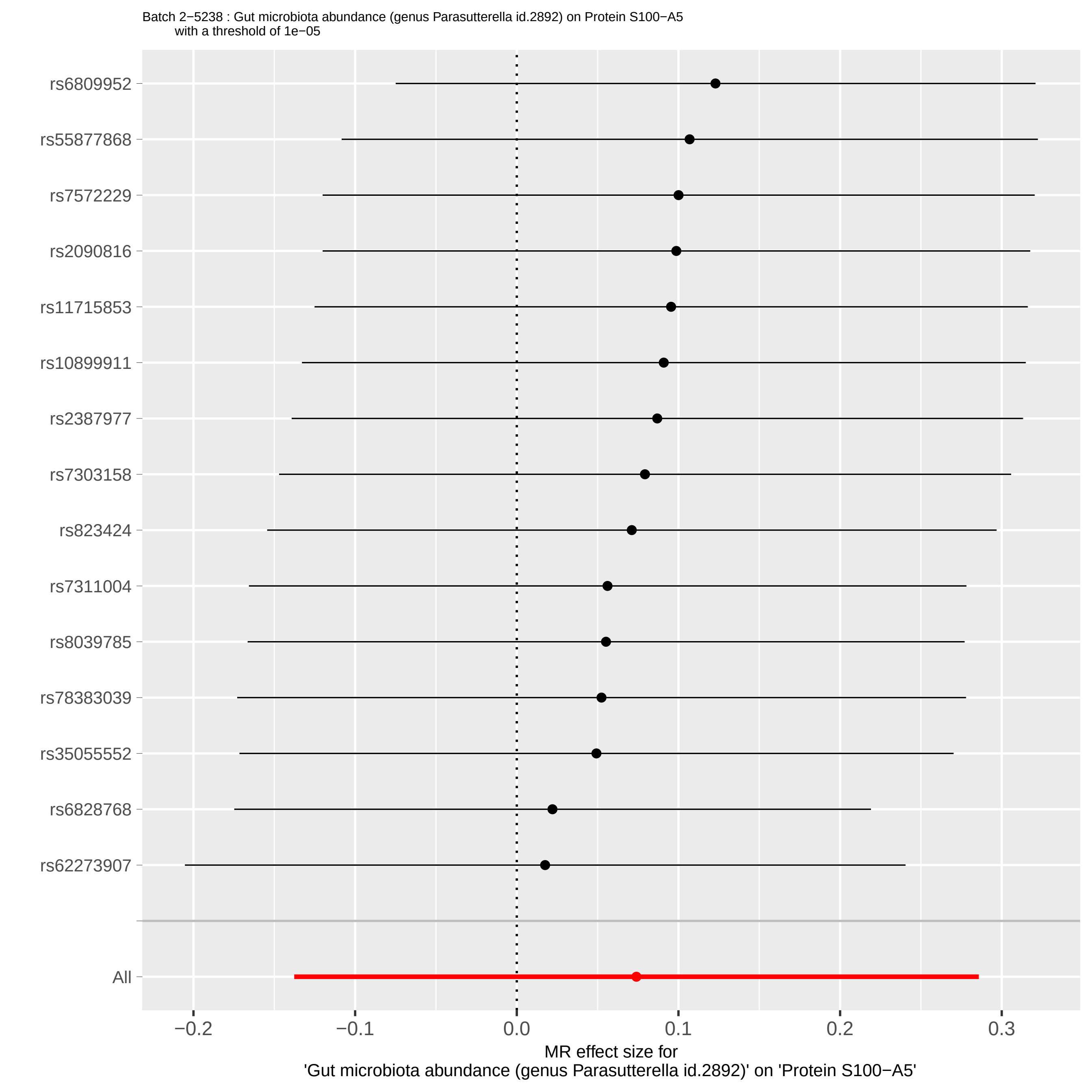

Batch 2−5238 : Gut microbiota abundance (genus Parasutterella id.2892) on Protein S100−A5
with a threshold of 1e−05
rs6809952
rs55877868
rs7572229
rs2090816
rs11715853
rs10899911
rs2387977
rs7303158
rs823424
rs7311004
rs8039785
rs78383039
rs35055552
rs6828768
rs62273907
All
−0.2
−0.1
0.0
0.1
0.2
0.3
MR effect size for
'Gut microbiota abundance (genus Parasutterella id.2892)' on 'Protein S100−A5'

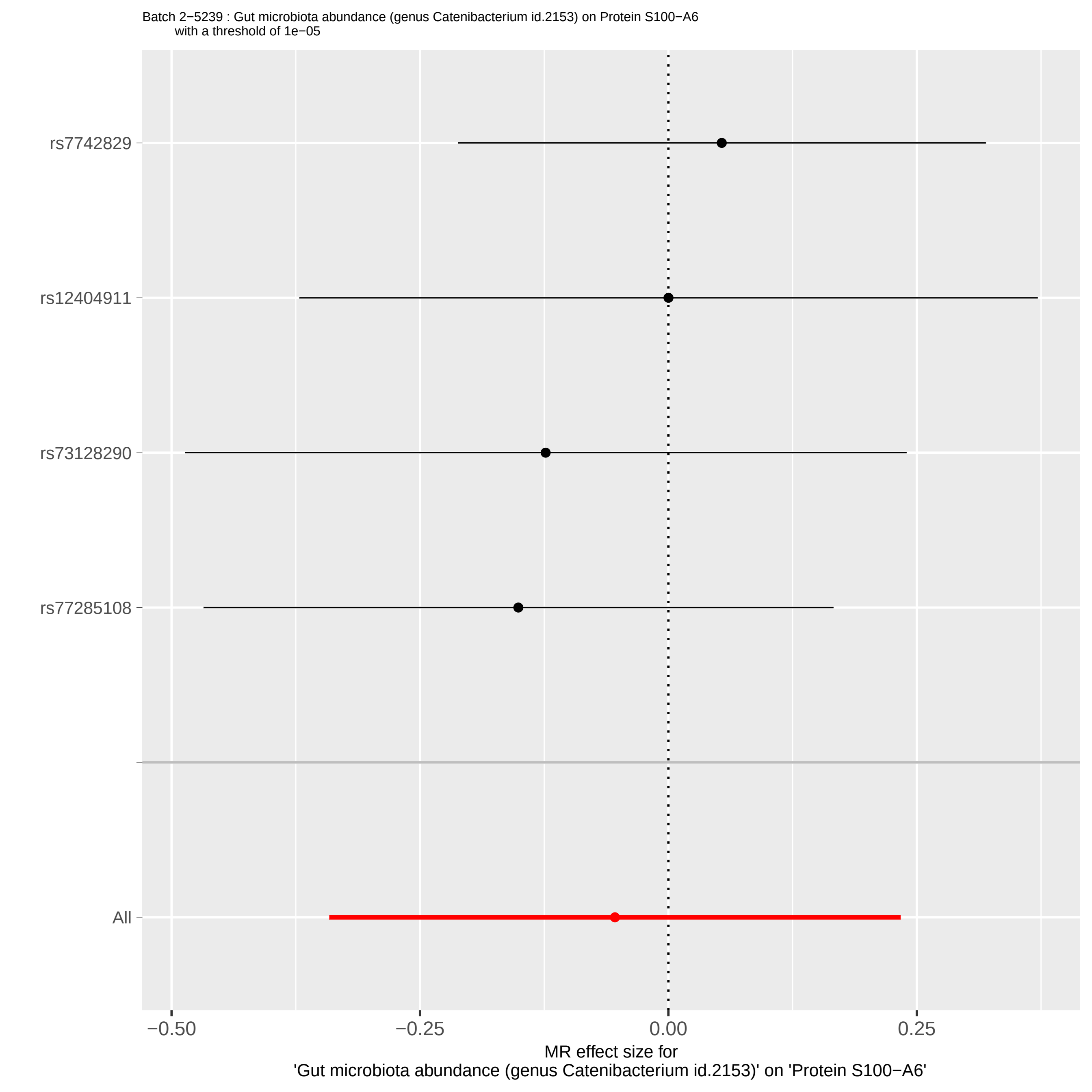

Batch 2−5239 : Gut microbiota abundance (genus Catenibacterium id.2153) on Protein S100−A6
with a threshold of 1e−05
rs7742829
rs12404911
rs73128290
rs77285108
All
−0.50
−0.25
0.00
0.25
MR effect size for
'Gut microbiota abundance (genus Catenibacterium id.2153)' on 'Protein S100−A6'

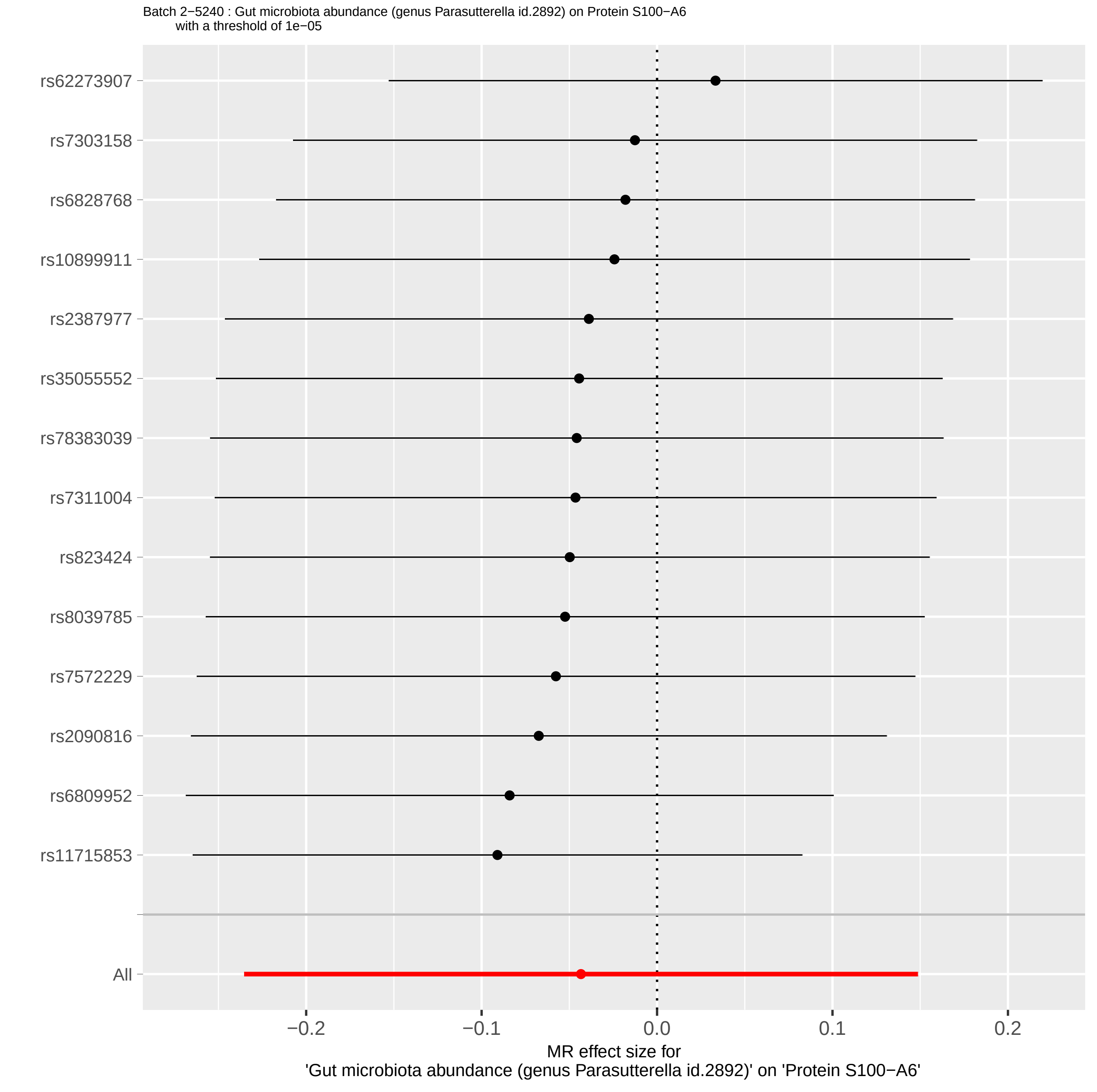

Batch 2−5240 : Gut microbiota abundance (genus Parasutterella id.2892) on Protein S100−A6
with a threshold of 1e−05
rs62273907
rs7303158
rs6828768
rs10899911
rs2387977
rs35055552
rs78383039
rs7311004
rs823424
rs8039785
rs7572229
rs2090816
rs6809952
rs11715853
All
−0.2
−0.1
0.0
0.1
0.2
MR effect size for
'Gut microbiota abundance (genus Parasutterella id.2892)' on 'Protein S100−A6'

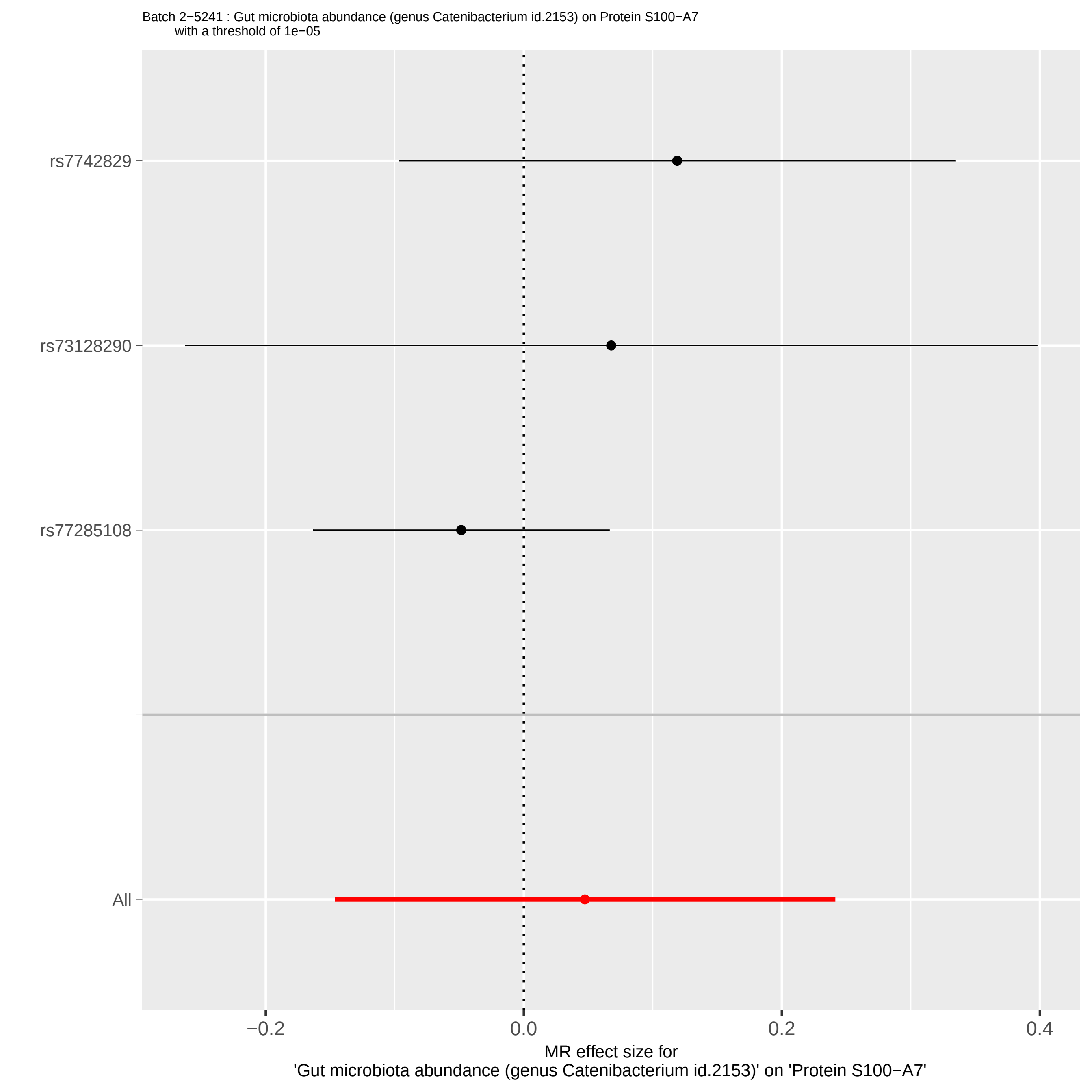

Batch 2−5241 : Gut microbiota abundance (genus Catenibacterium id.2153) on Protein S100−A7
with a threshold of 1e−05
rs7742829
rs73128290
rs77285108
All
−0.2
0.0
0.2
0.4
MR effect size for
'Gut microbiota abundance (genus Catenibacterium id.2153)' on 'Protein S100−A7'

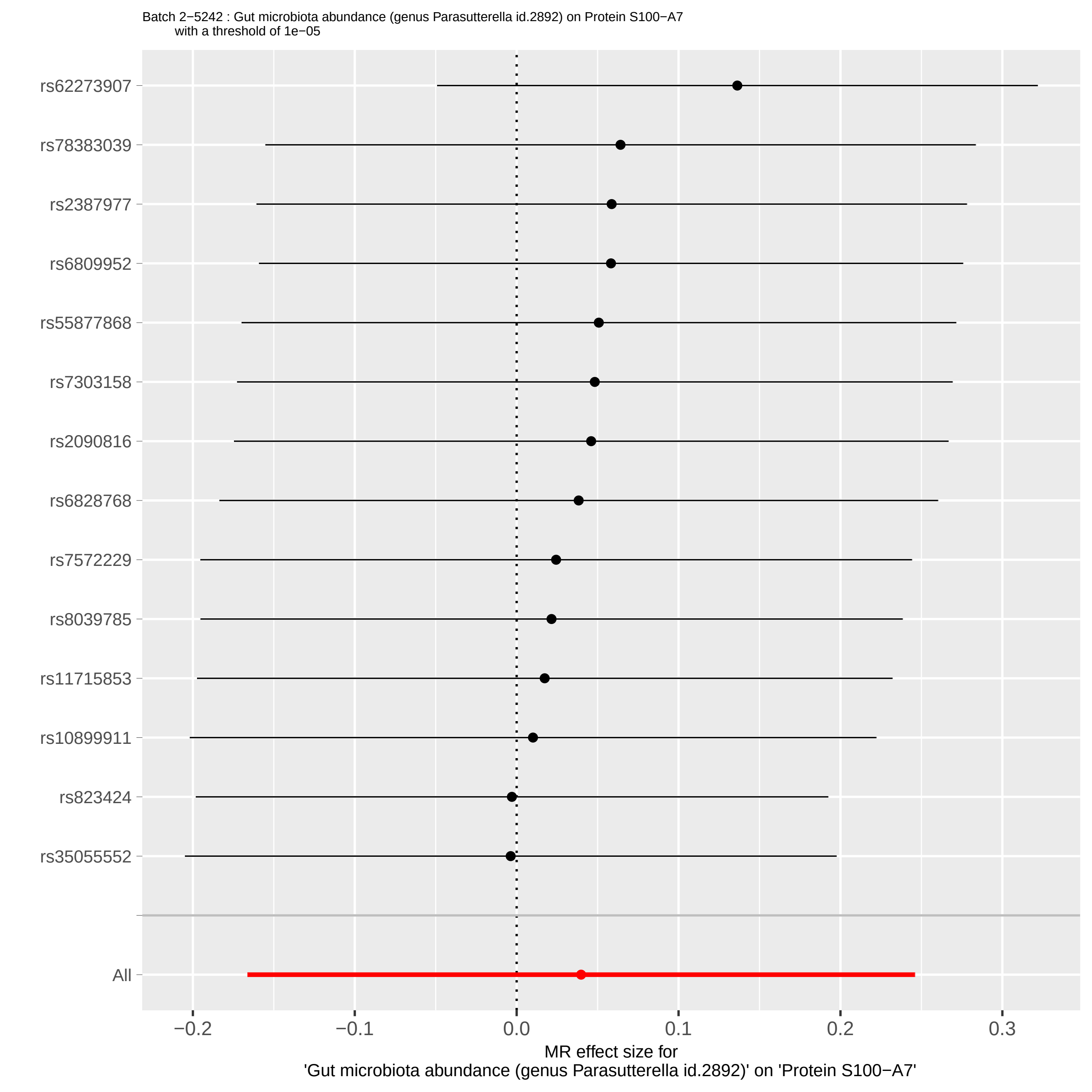

Batch 2−5242 : Gut microbiota abundance (genus Parasutterella id.2892) on Protein S100−A7
with a threshold of 1e−05
rs62273907
rs78383039
rs2387977
rs6809952
rs55877868
rs7303158
rs2090816
rs6828768
rs7572229
rs8039785
rs11715853
rs10899911
rs823424
rs35055552
All
−0.2
−0.1
0.0
0.1
0.2
0.3
MR effect size for
'Gut microbiota abundance (genus Parasutterella id.2892)' on 'Protein S100−A7'

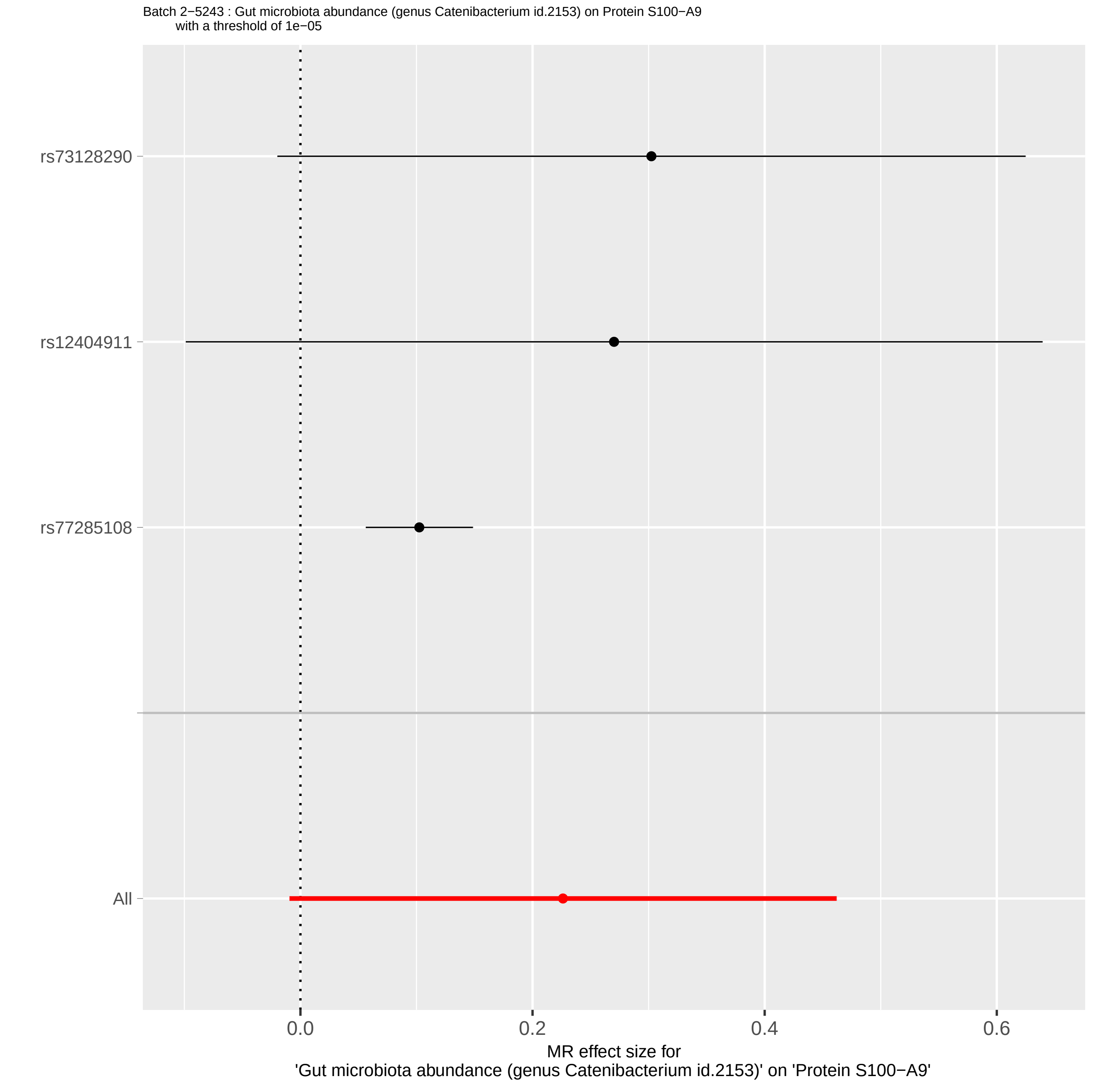
Batch 2−5243 : Gut microbiota abundance (genus Catenibacterium id.2153) on Protein S100−A9
with a threshold of 1e−05
rs73128290
rs12404911
rs77285108
All
0.0
0.2
0.4
0.6
MR effect size for
'Gut microbiota abundance (genus Catenibacterium id.2153)' on 'Protein S100−A9'

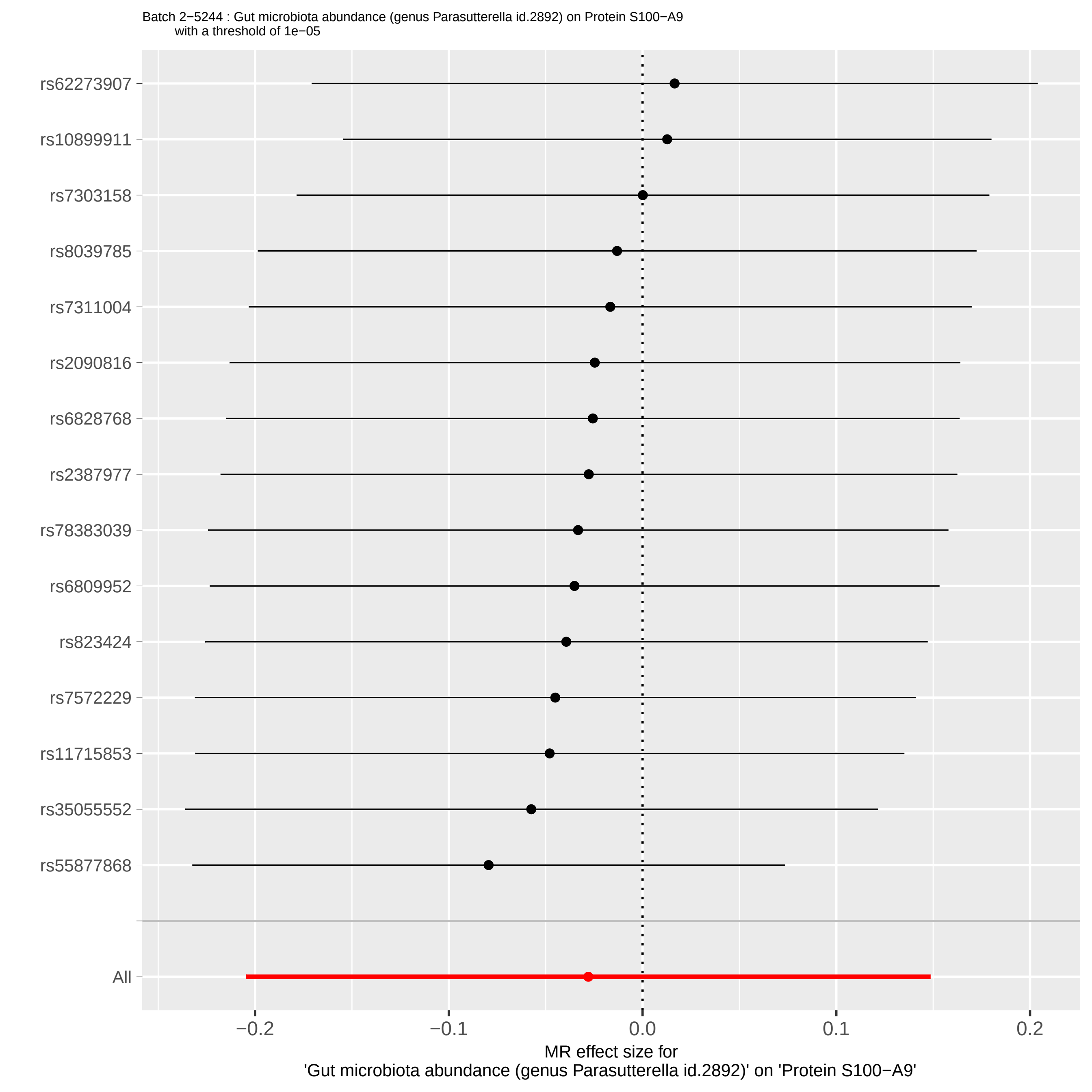

Batch 2−5244 : Gut microbiota abundance (genus Parasutterella id.2892) on Protein S100−A9
with a threshold of 1e−05
rs62273907
rs10899911
rs7303158
rs8039785
rs7311004
rs2090816
rs6828768
rs2387977
rs78383039
rs6809952
rs823424
rs7572229
rs11715853
rs35055552
rs55877868
All
−0.2
−0.1
0.0
0.1
0.2
MR effect size for
'Gut microbiota abundance (genus Parasutterella id.2892)' on 'Protein S100−A9'

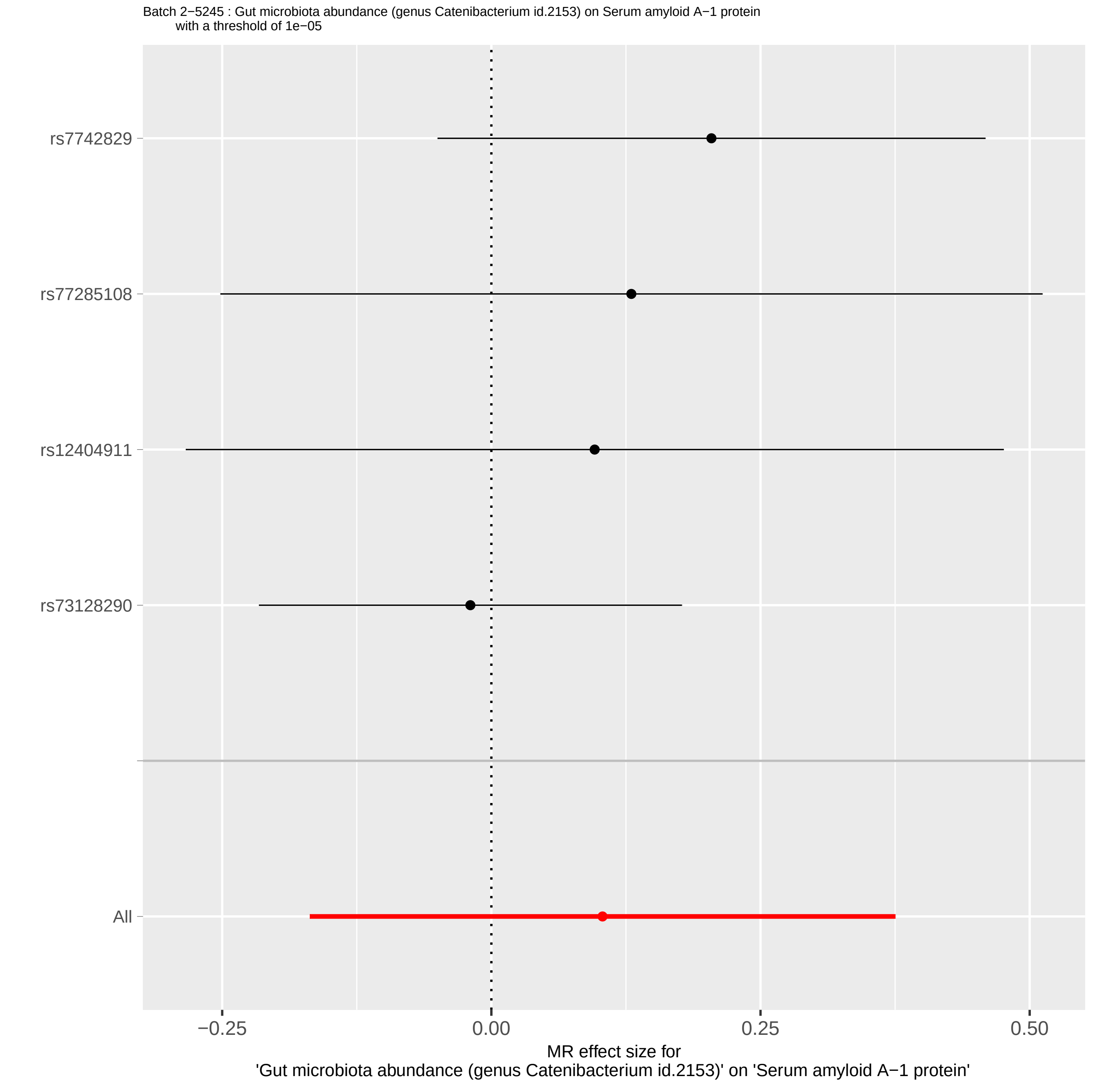

Batch 2−5245 : Gut microbiota abundance (genus Catenibacterium id.2153) on Serum amyloid A−1 protein
with a threshold of 1e−05
rs7742829
rs77285108
rs12404911
rs73128290
All
−0.25
0.00
0.25
0.50
MR effect size for
'Gut microbiota abundance (genus Catenibacterium id.2153)' on 'Serum amyloid A−1 protein'

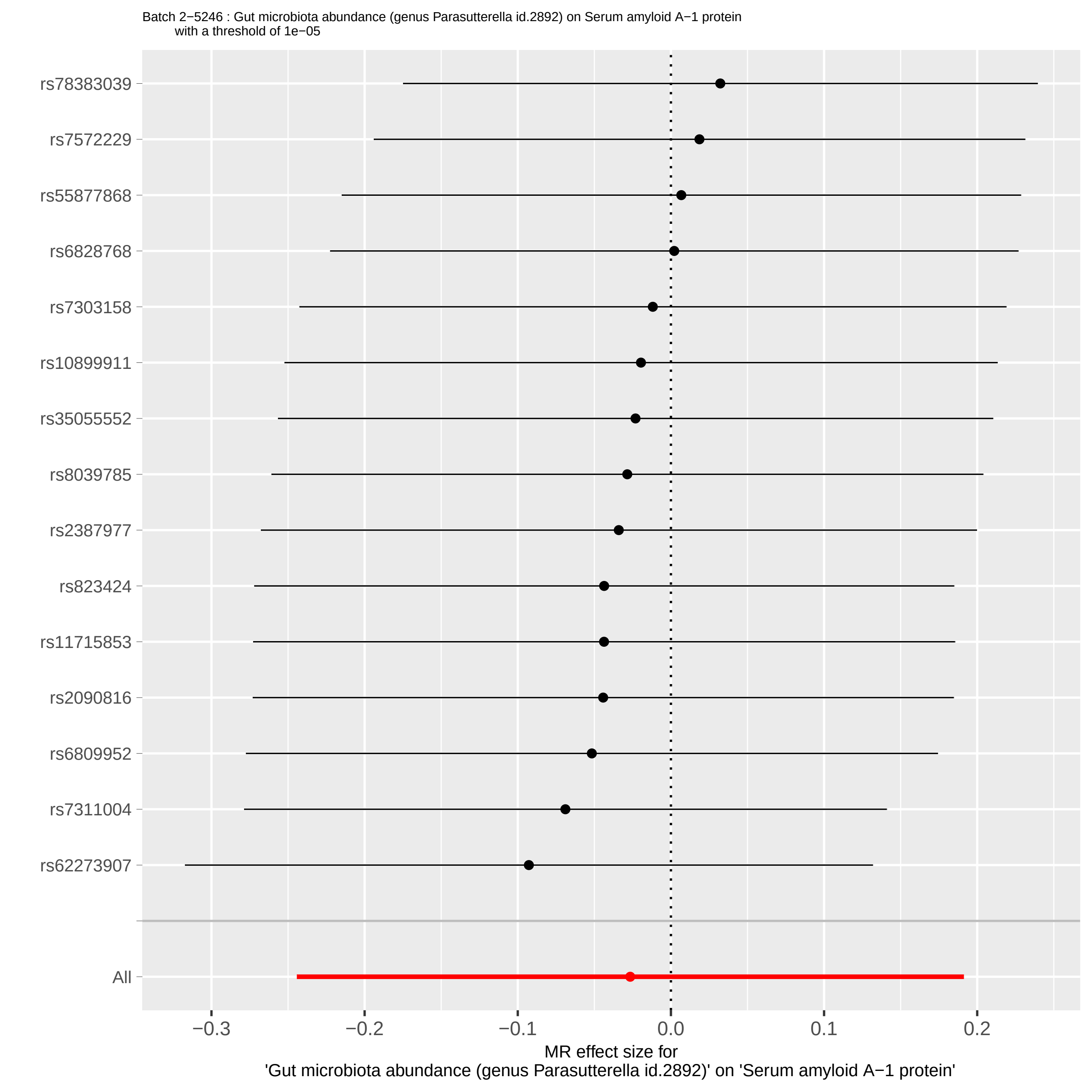

Batch 2−5246 : Gut microbiota abundance (genus Parasutterella id.2892) on Serum amyloid A−1 protein
with a threshold of 1e−05
rs78383039
rs7572229
rs55877868
rs6828768
rs7303158
rs10899911
rs35055552
rs8039785
rs2387977
rs823424
rs11715853
rs2090816
rs6809952
rs7311004
rs62273907
All
−0.3
−0.2
−0.1
0.0
0.1
0.2
MR effect size for
'Gut microbiota abundance (genus Parasutterella id.2892)' on 'Serum amyloid A−1 protein'

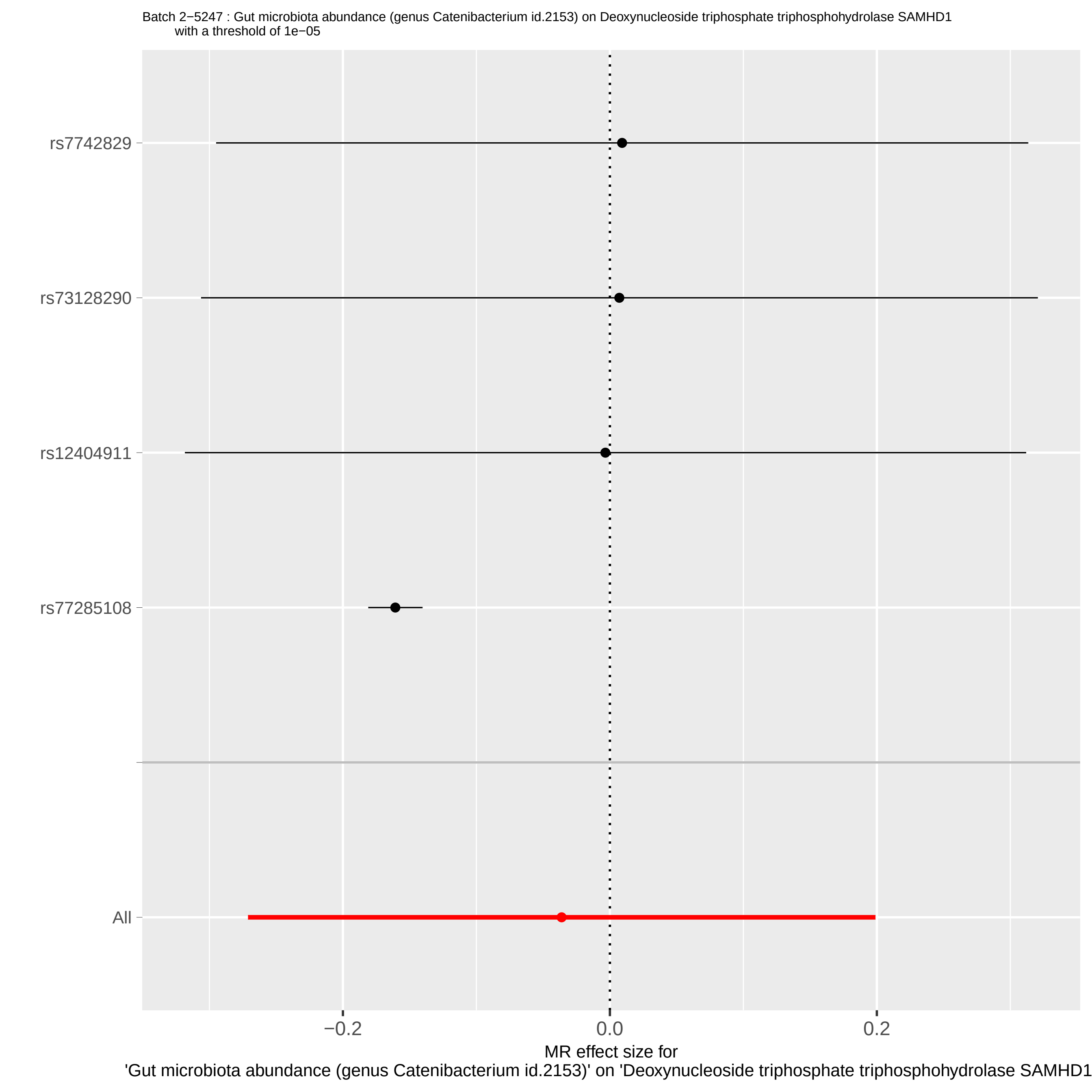
Batch 2−5247 : Gut microbiota abundance (genus Catenibacterium id.2153) on Deoxynucleoside triphosphate triphosphohydrolase SAMHD1
with a threshold of 1e−05
rs7742829
rs73128290
rs12404911
rs77285108
All
−0.2
0.0
0.2
MR effect size for
'Gut microbiota abundance (genus Catenibacterium id.2153)' on 'Deoxynucleoside triphosphate triphosphohydrolase SAMHD1'

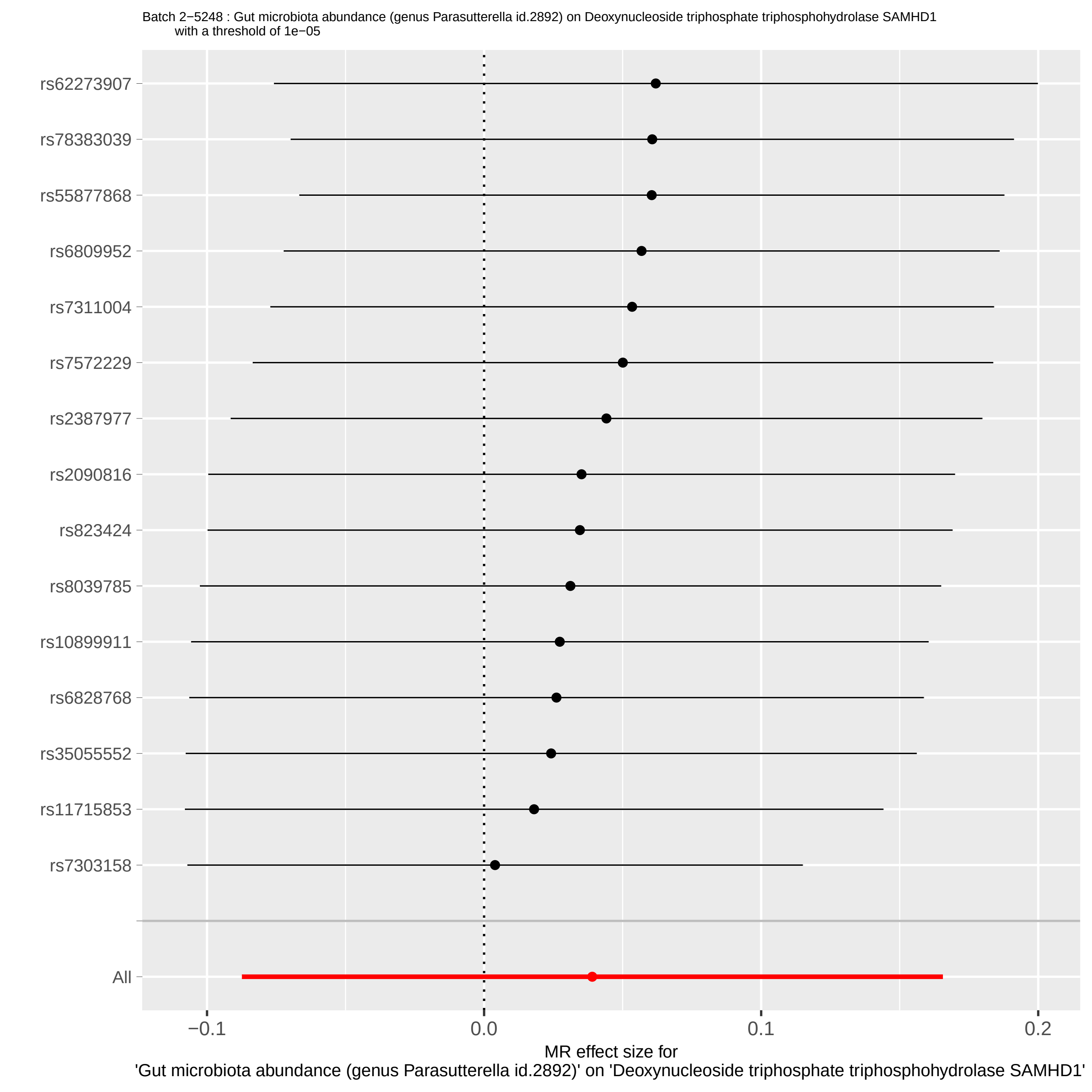

Batch 2−5248 : Gut microbiota abundance (genus Parasutterella id.2892) on Deoxynucleoside triphosphate triphosphohydrolase SAMHD1 with a threshold of 1e−05
rs62273907
rs78383039
rs55877868
rs6809952
rs7311004
rs7572229
rs2387977
rs2090816
rs823424
rs8039785
rs10899911
rs6828768
rs35055552
rs11715853
rs7303158
All
−0.1
0.0
0.1
0.2
MR effect size for
'Gut microbiota abundance (genus Parasutterella id.2892)' on 'Deoxynucleoside triphosphate triphosphohydrolase SAMHD1'

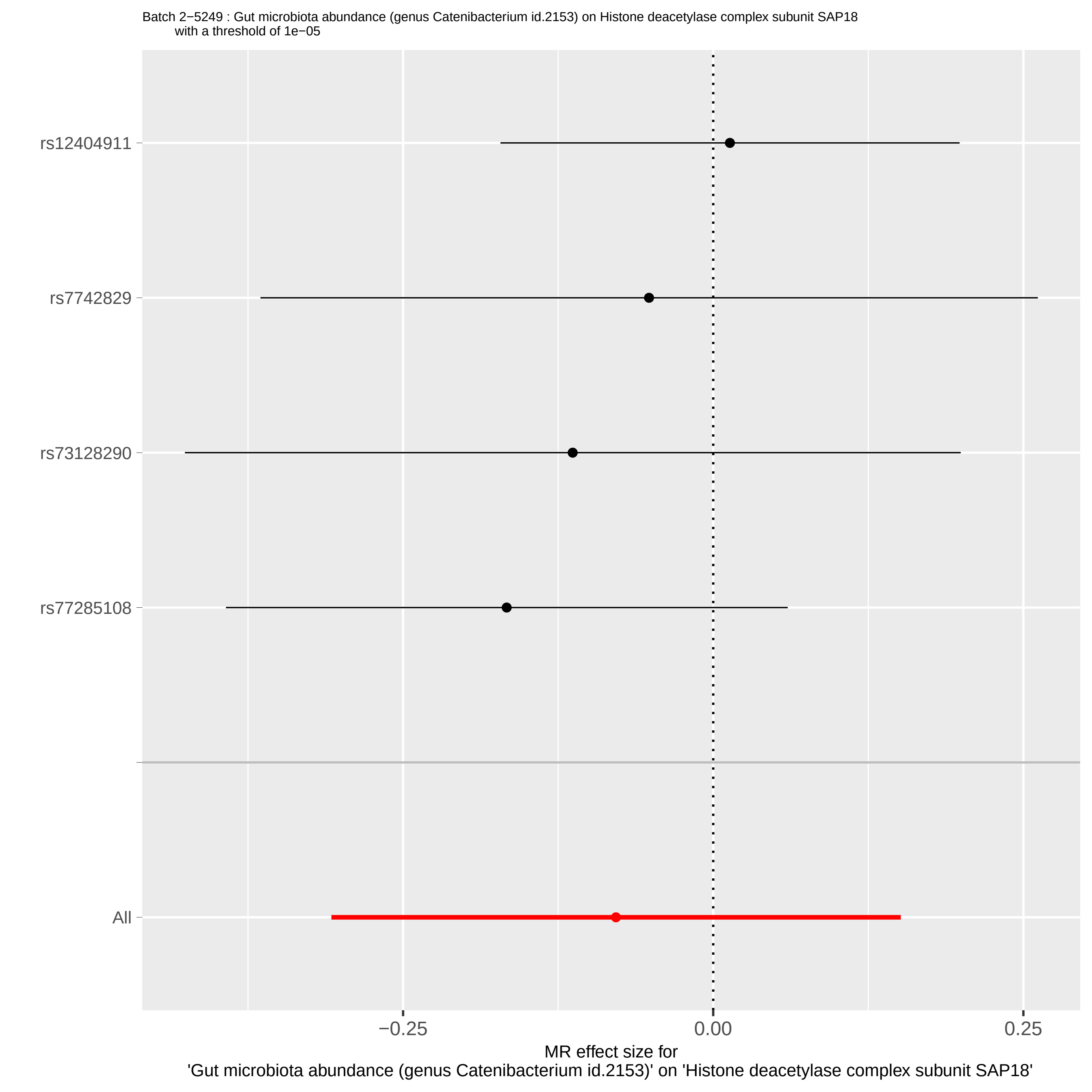
Batch 2−5249 : Gut microbiota abundance (genus Catenibacterium id.2153) on Histone deacetylase complex subunit SAP18
with a threshold of 1e−05
rs12404911
rs7742829
rs73128290
rs77285108
All
−0.25
0.00
0.25
MR effect size for
'Gut microbiota abundance (genus Catenibacterium id.2153)' on 'Histone deacetylase complex subunit SAP18'

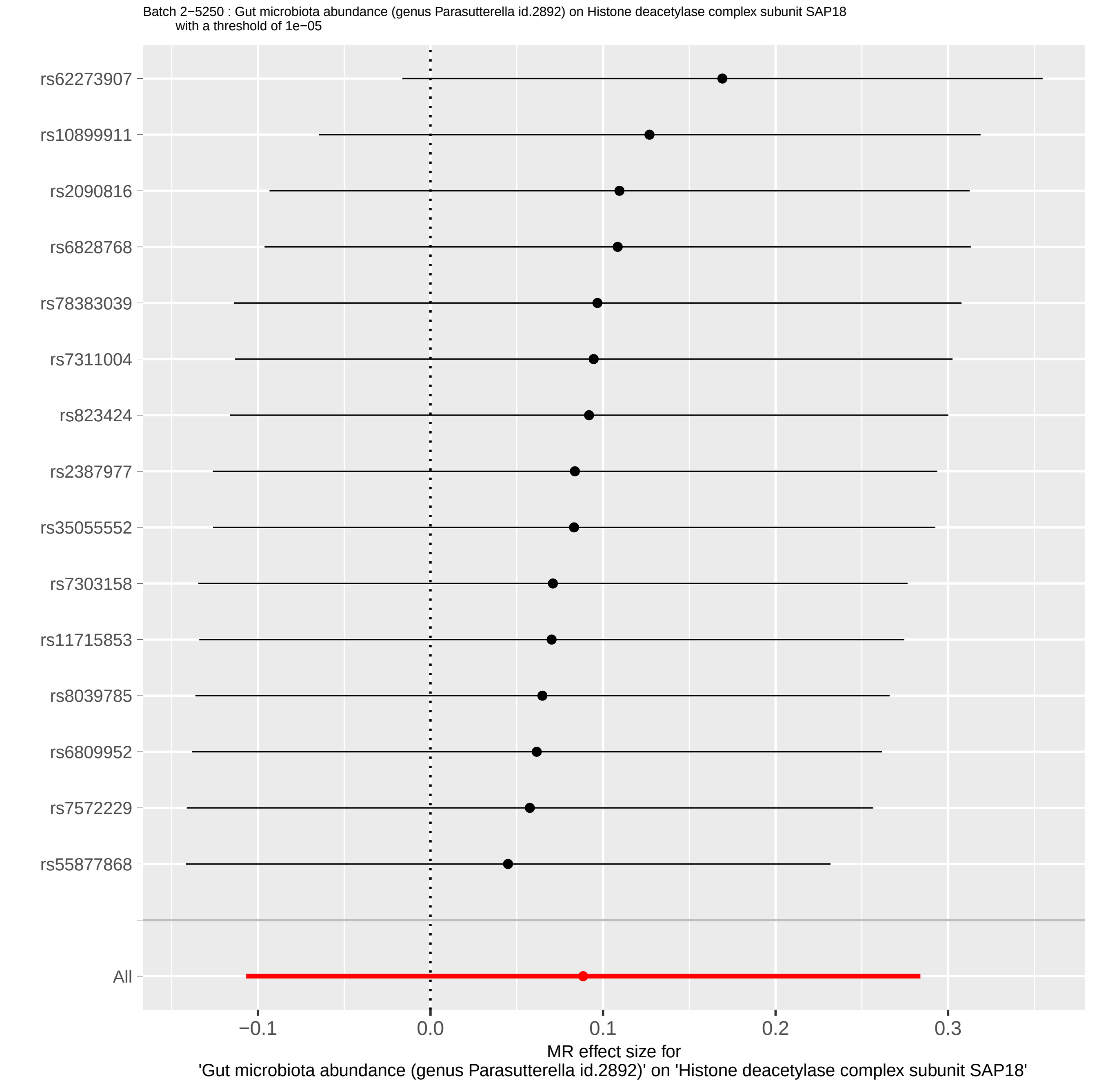

Batch 2−5250 : Gut microbiota abundance (genus Parasutterella id.2892) on Histone deacetylase complex subunit SAP18
with a threshold of 1e−05
rs62273907
rs10899911
rs2090816
rs6828768
rs78383039
rs7311004
rs823424
rs2387977
rs35055552
rs7303158
rs11715853
rs8039785
rs6809952
rs7572229
rs55877868
All
−0.1
0.0
0.1
0.2
0.3
MR effect size for
'Gut microbiota abundance (genus Parasutterella id.2892)' on 'Histone deacetylase complex subunit SAP18'

Batch 2−5251 : Gut microbiota abundance (genus Catenibacterium id.2153) on Histone deacetylase complex subunit SAP30
with a threshold of 1e−05

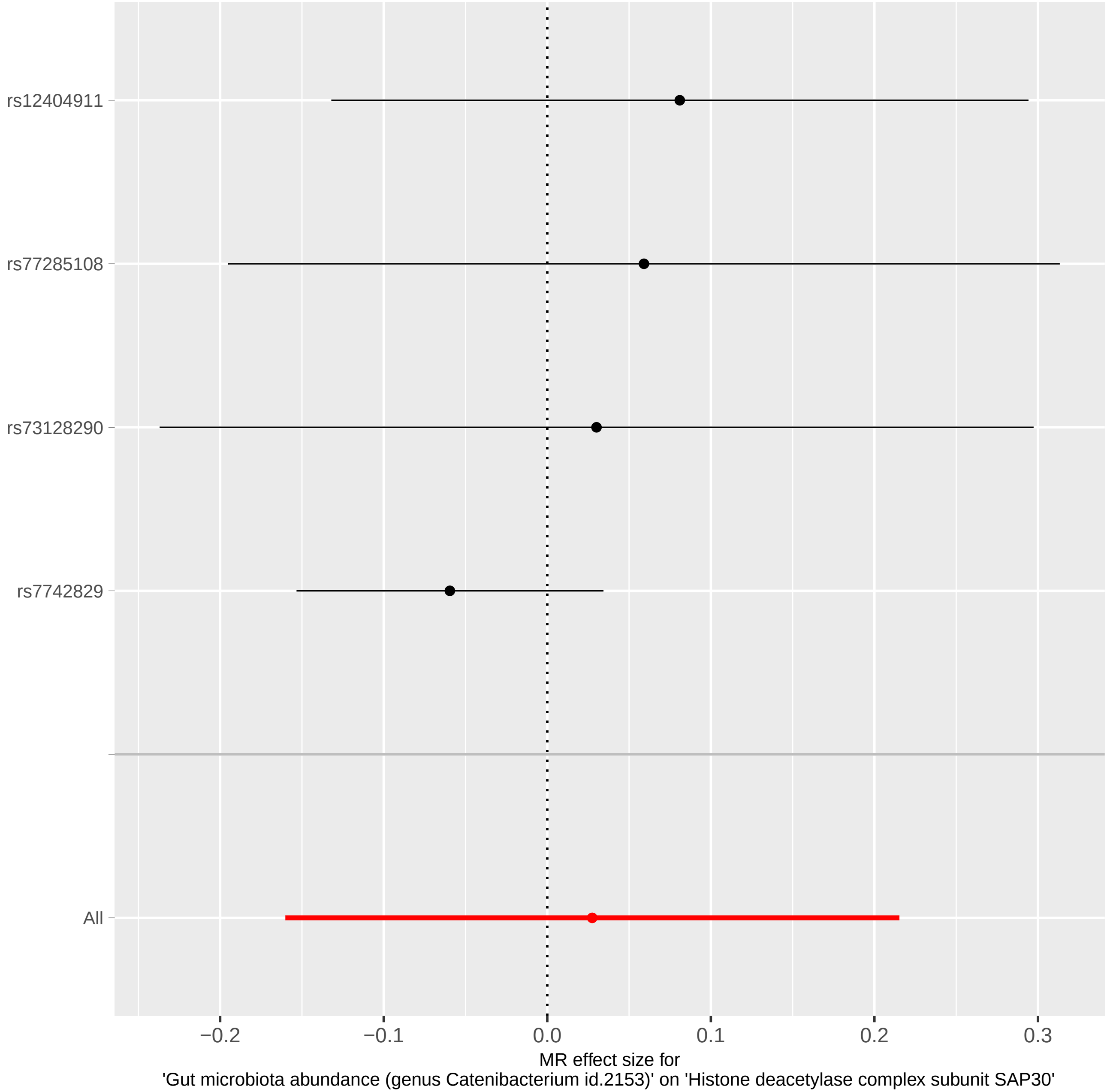

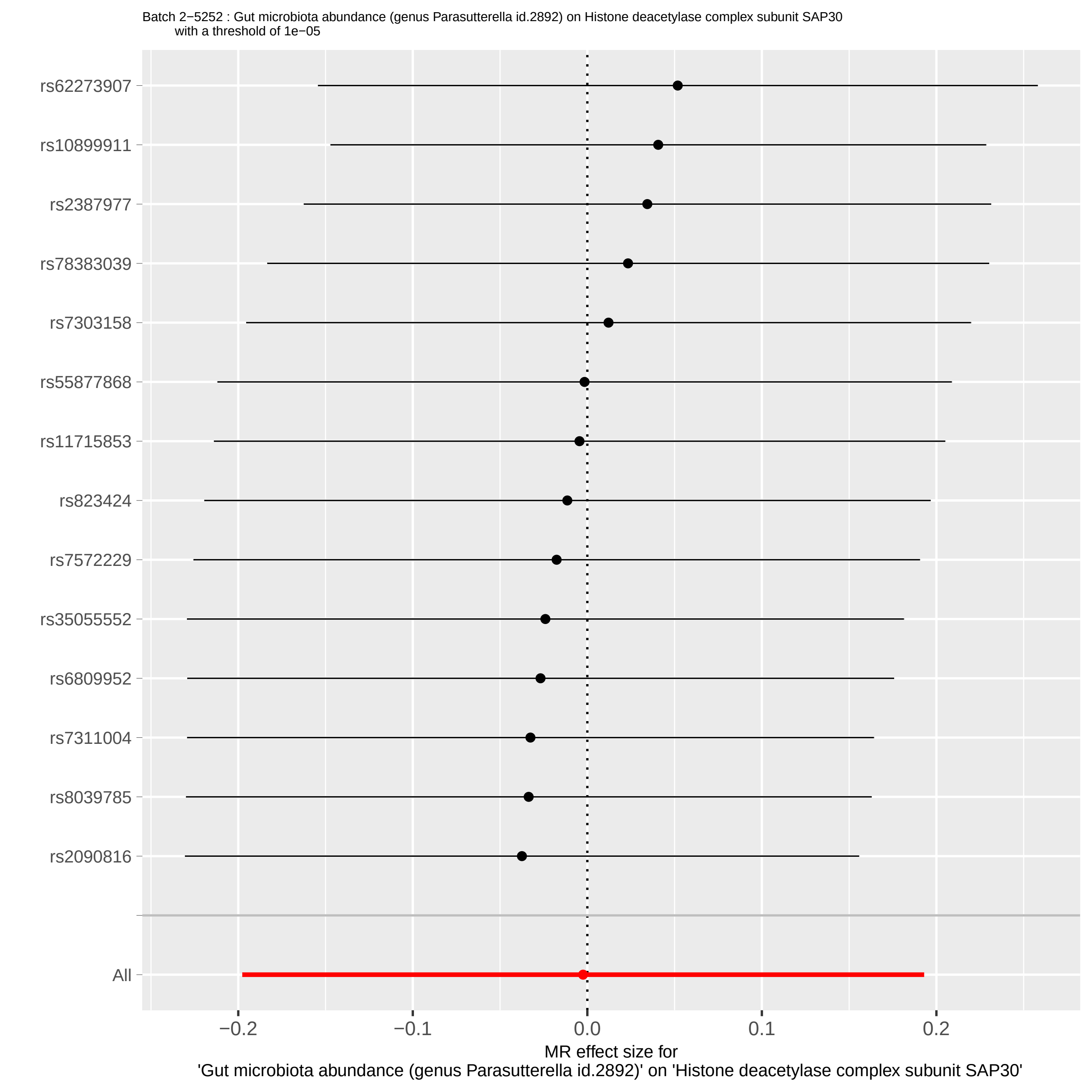

Batch 2−5252 : Gut microbiota abundance (genus Parasutterella id.2892) on Histone deacetylase complex subunit SAP30
with a threshold of 1e−05
rs62273907
rs10899911
rs2387977
rs78383039
rs7303158
rs55877868
rs11715853
rs823424
rs7572229
rs35055552
rs6809952
rs7311004
rs8039785
rs2090816
All
−0.2
−0.1
0.0
0.1
0.2
MR effect size for
'Gut microbiota abundance (genus Parasutterella id.2892)' on 'Histone deacetylase complex subunit SAP30'

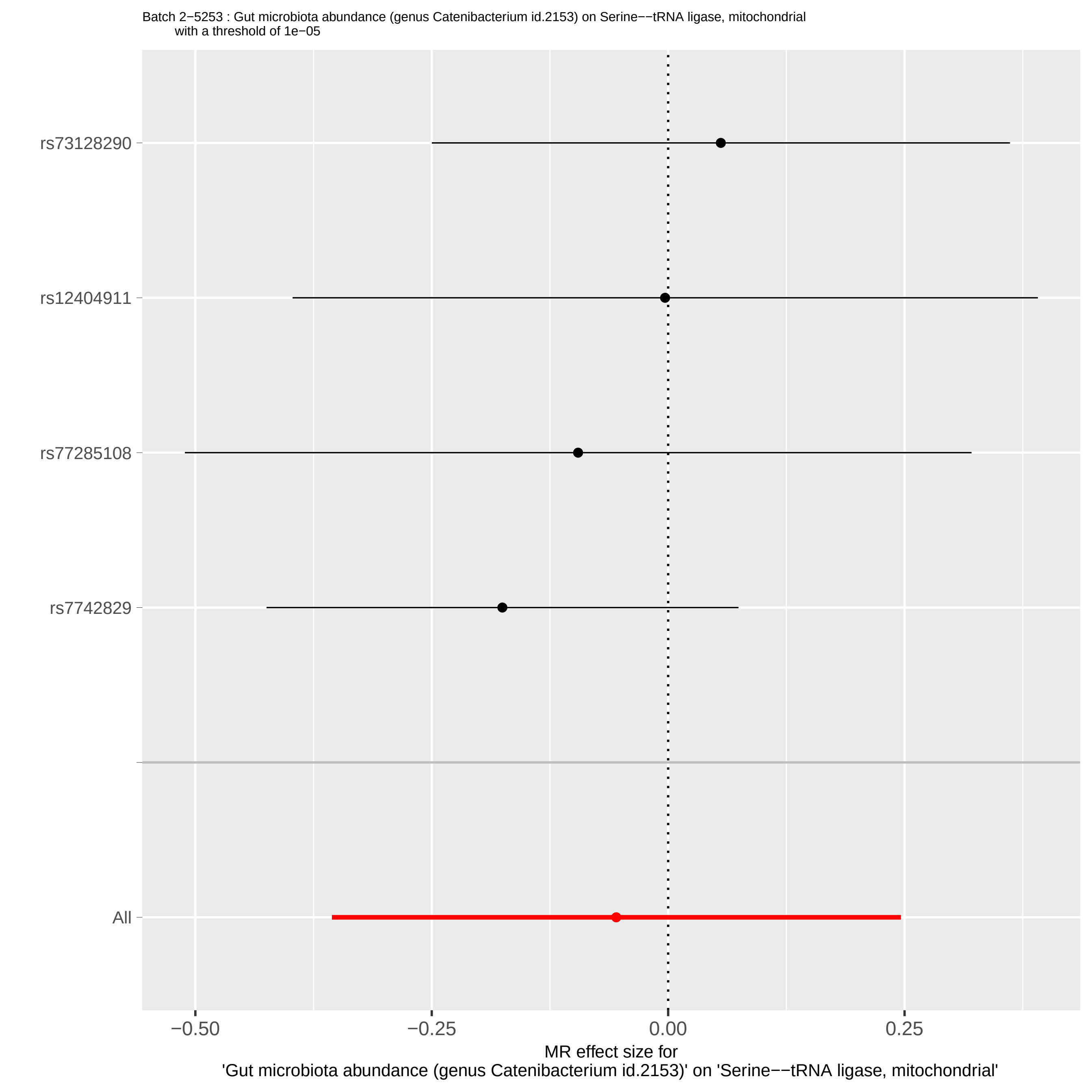
Batch 2−5253 : Gut microbiota abundance (genus Catenibacterium id.2153) on Serine−−tRNA ligase, mitochondrial
with a threshold of 1e−05
rs73128290
rs12404911
rs77285108
rs7742829
All
−0.50
−0.25
0.00
0.25
MR effect size for
'Gut microbiota abundance (genus Catenibacterium id.2153)' on 'Serine−−tRNA ligase, mitochondrial'

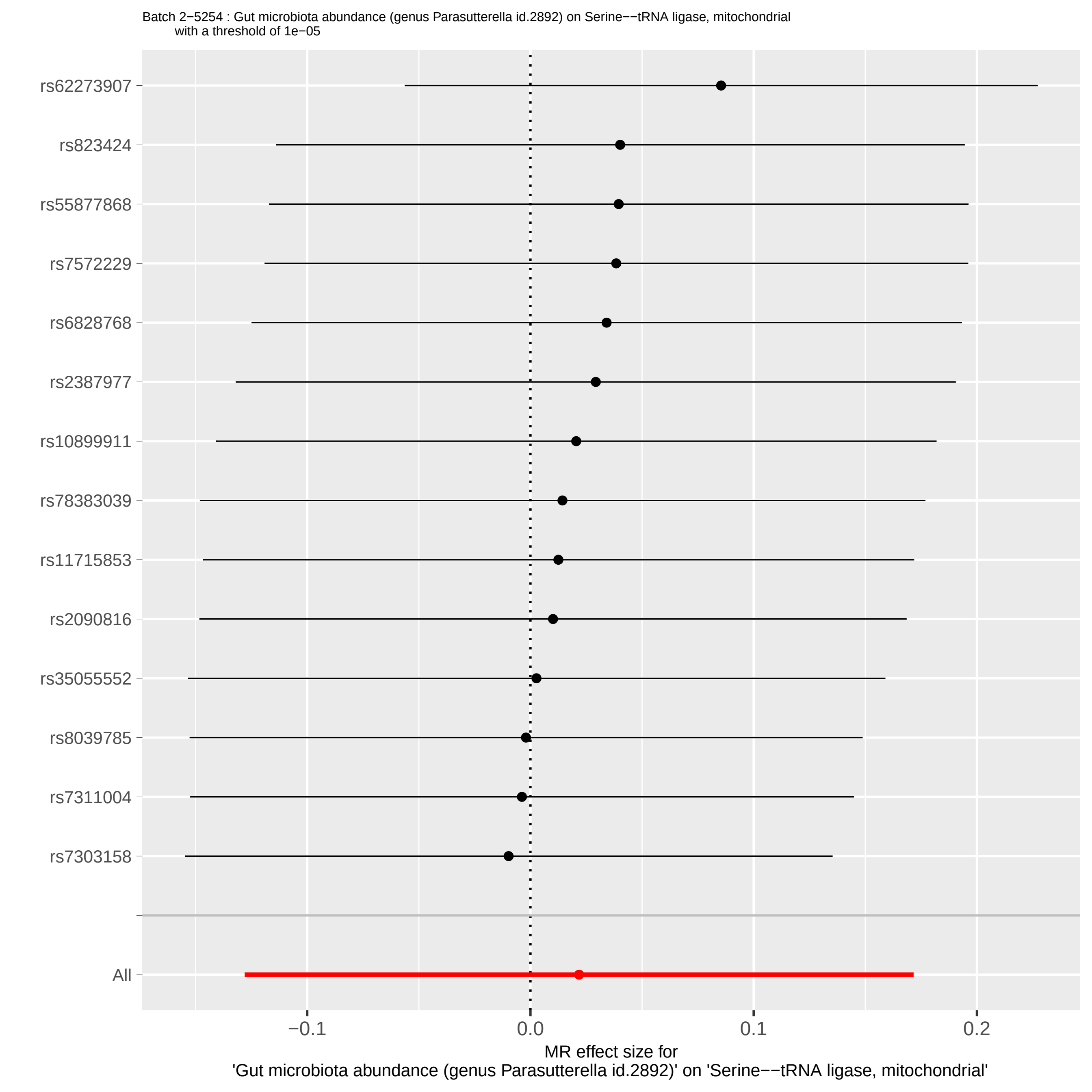

Batch 2−5254 : Gut microbiota abundance (genus Parasutterella id.2892) on Serine−−tRNA ligase, mitochondrial
with a threshold of 1e−05
rs62273907
rs823424
rs55877868
rs7572229
rs6828768
rs2387977
rs10899911
rs78383039
rs11715853
rs2090816
rs35055552
rs8039785
rs7311004
rs7303158
All
−0.1
0.0
0.1
0.2
MR effect size for
'Gut microbiota abundance (genus Parasutterella id.2892)' on 'Serine−−tRNA ligase, mitochondrial'

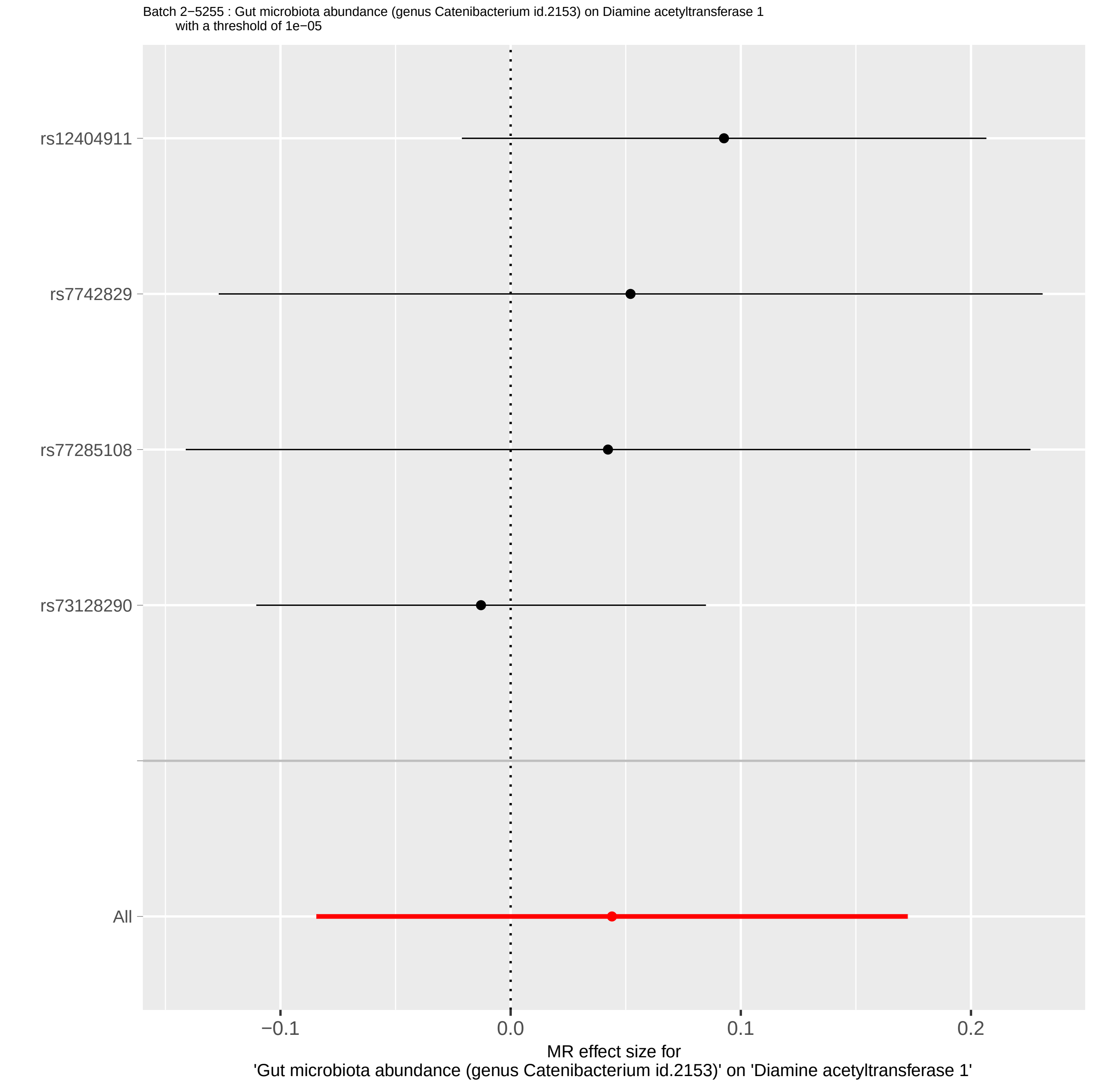
Batch 2−5255 : Gut microbiota abundance (genus Catenibacterium id.2153) on Diamine acetyltransferase 1
with a threshold of 1e−05
rs12404911
rs7742829
rs77285108
rs73128290
All
−0.1
0.0
0.1
0.2
MR effect size for
'Gut microbiota abundance (genus Catenibacterium id.2153)' on 'Diamine acetyltransferase 1'

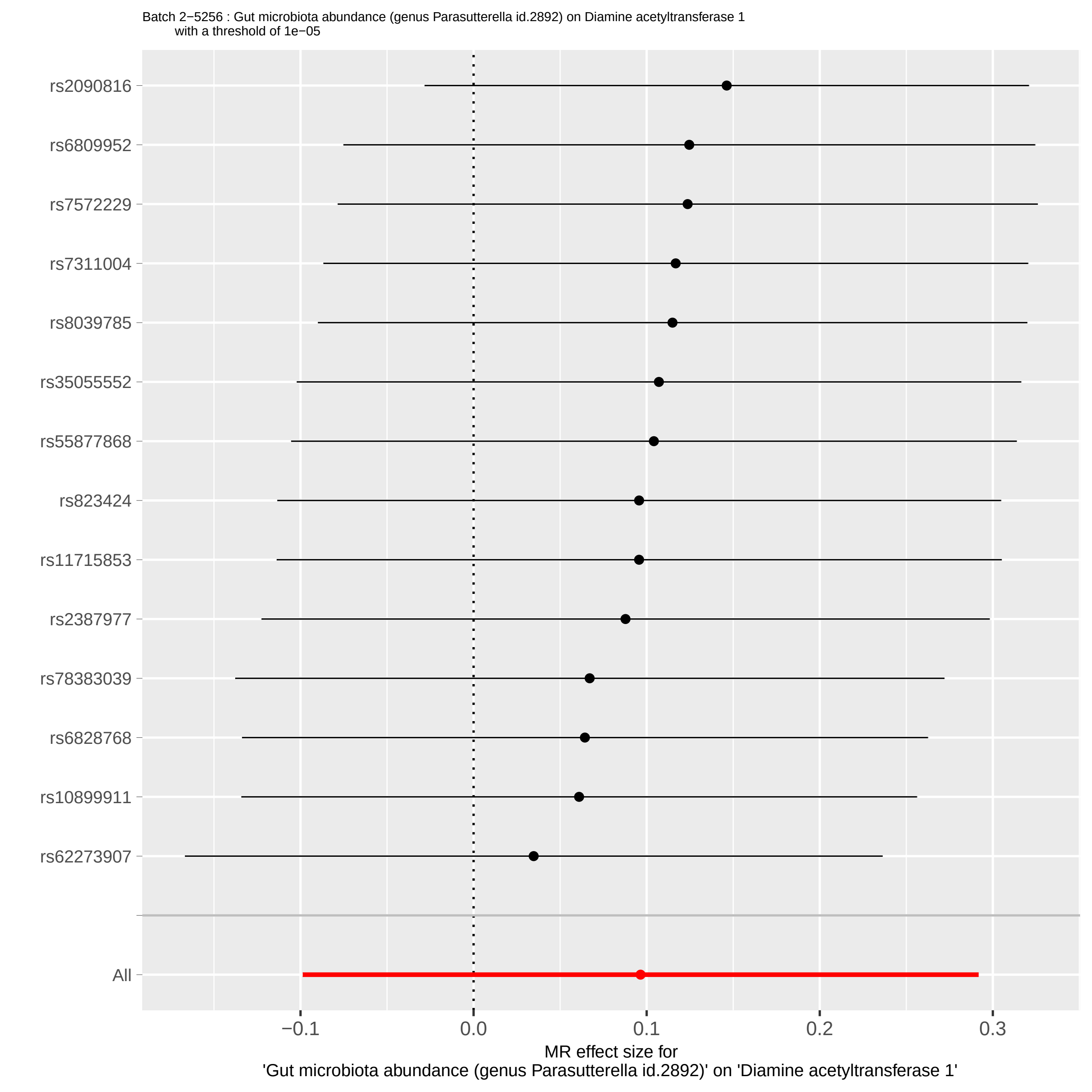
Batch 2−5256 : Gut microbiota abundance (genus Parasutterella id.2892) on Diamine acetyltransferase 1
with a threshold of 1e−05
rs2090816
rs6809952
rs7572229
rs7311004
rs8039785
rs35055552
rs55877868
rs823424
rs11715853
rs2387977
rs78383039
rs6828768
rs10899911
rs62273907
All
−0.1
0.0
0.1
0.2
0.3
MR effect size for
'Gut microbiota abundance (genus Parasutterella id.2892)' on 'Diamine acetyltransferase 1'

Batch 2−5257 : Gut microbiota abundance (genus Catenibacterium id.2153) on Diamine acetyltransferase 2
with a threshold of 1e−05

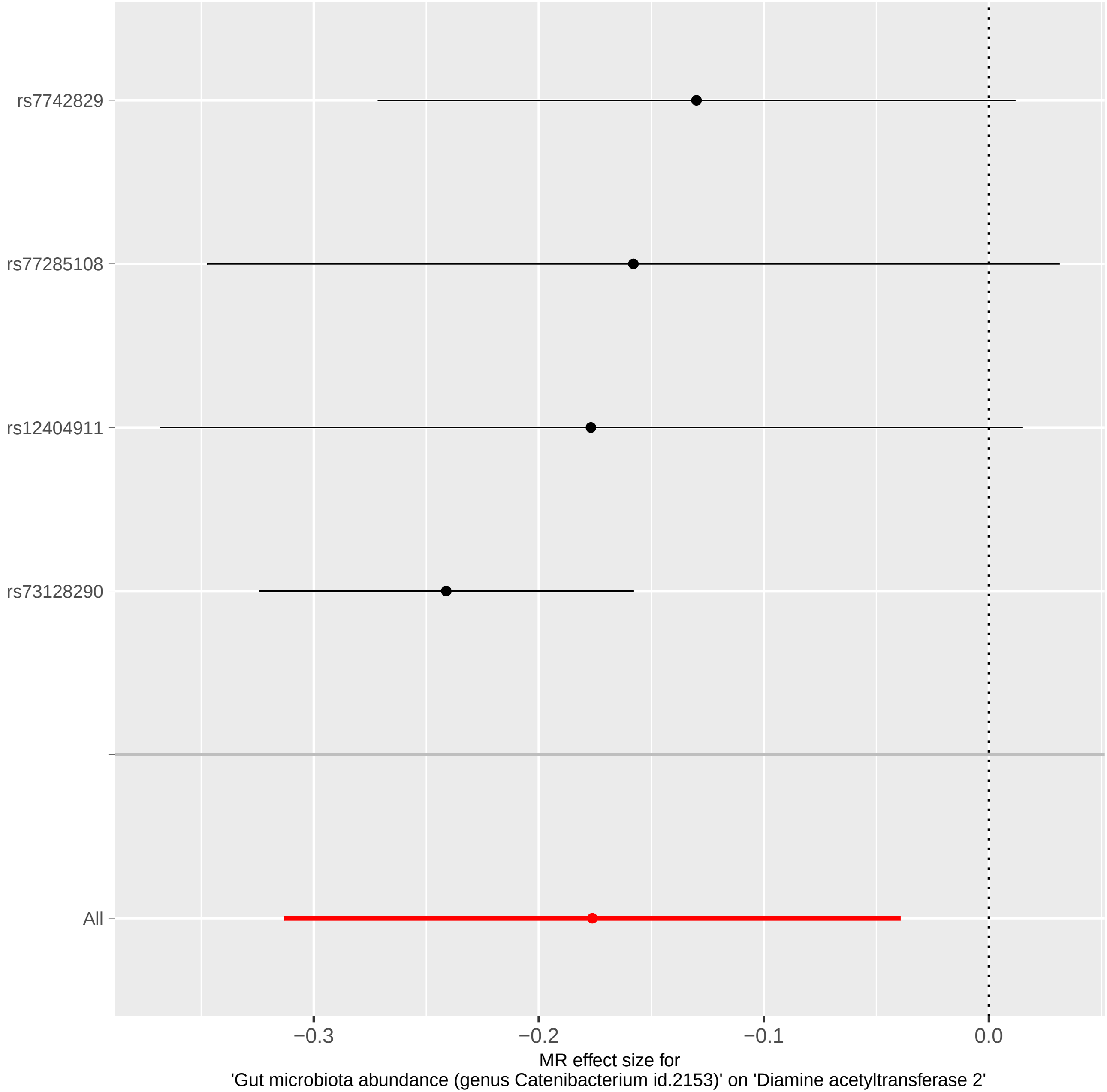

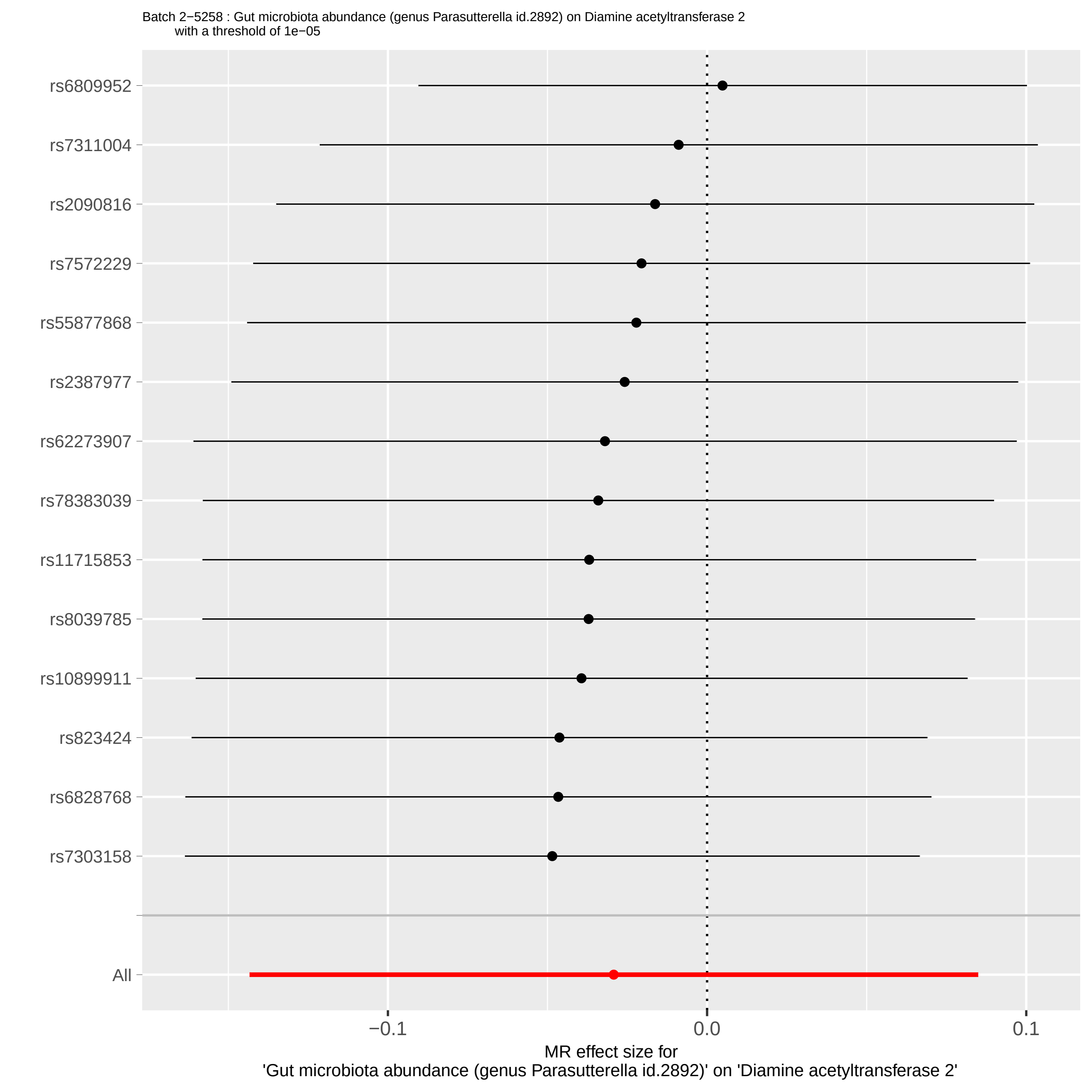
Batch 2−5258 : Gut microbiota abundance (genus Parasutterella id.2892) on Diamine acetyltransferase 2
with a threshold of 1e−05
rs6809952
rs7311004
rs2090816
rs7572229
rs55877868
rs2387977
rs62273907
rs78383039
rs11715853
rs8039785
rs10899911
rs823424
rs6828768
rs7303158
All
−0.1
0.0
0.1
MR effect size for
'Gut microbiota abundance (genus Parasutterella id.2892)' on 'Diamine acetyltransferase 2'

Batch 2−5259 : Gut microbiota abundance (genus Catenibacterium id.2153) on DNA−binding protein SATB1
with a threshold of 1e−05

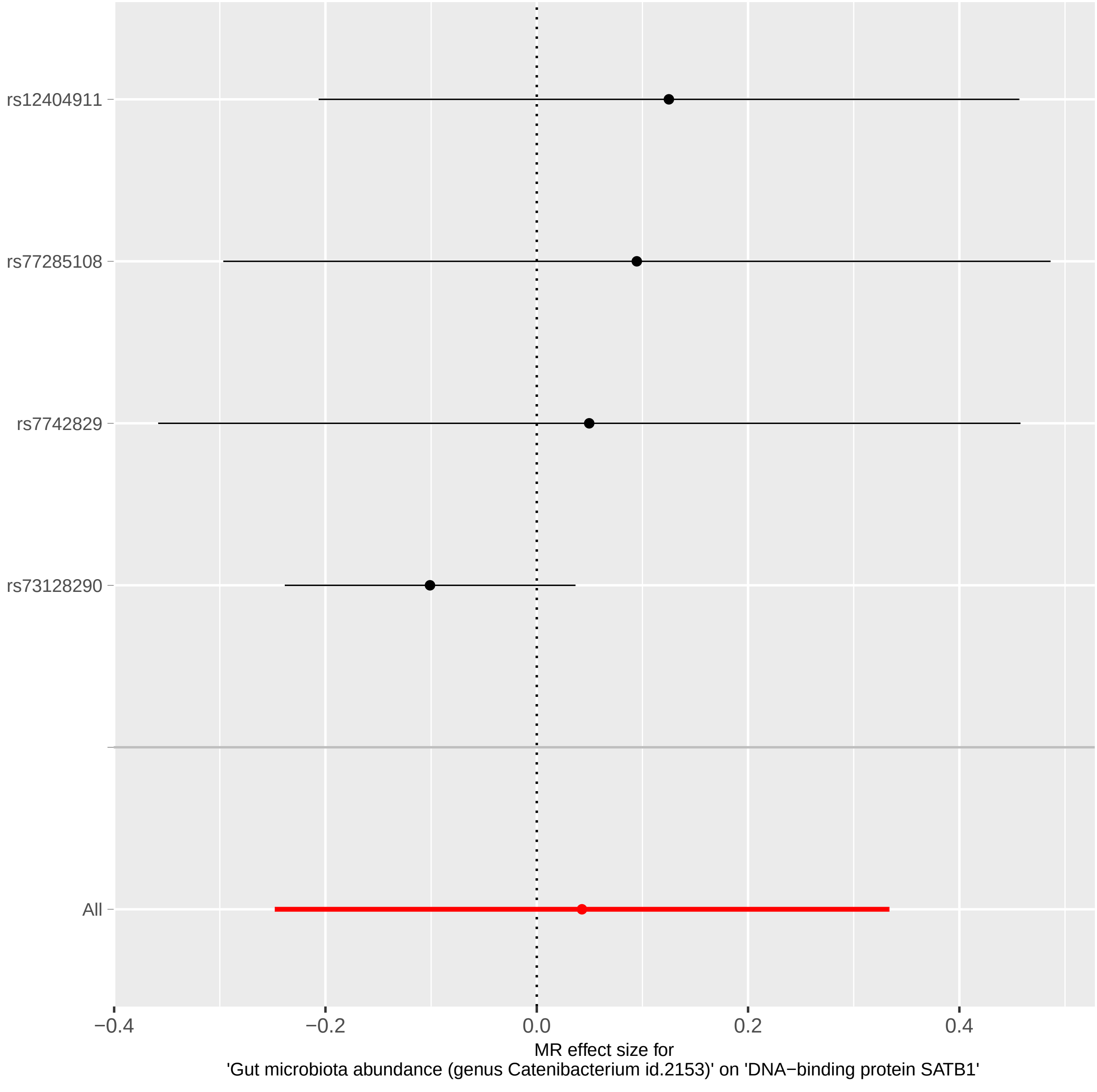

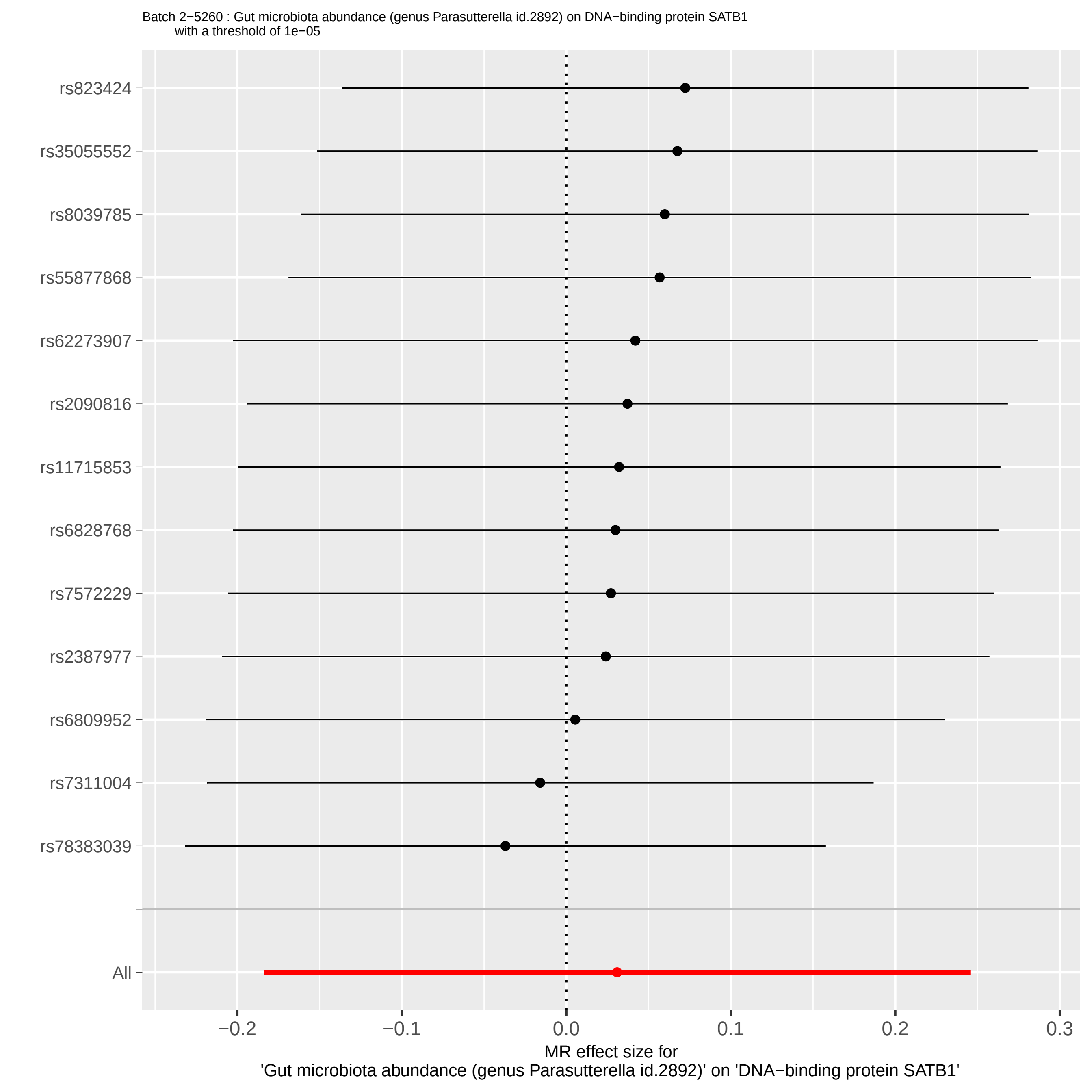
Batch 2−5260 : Gut microbiota abundance (genus Parasutterella id.2892) on DNA−binding protein SATB1
with a threshold of 1e−05
rs823424
rs35055552
rs8039785
rs55877868
rs62273907
rs2090816
rs11715853
rs6828768
rs7572229
rs2387977
rs6809952
rs7311004
rs78383039
All
−0.2
−0.1
0.0
0.1
0.2
0.3
MR effect size for
'Gut microbiota abundance (genus Parasutterella id.2892)' on 'DNA−binding protein SATB1'

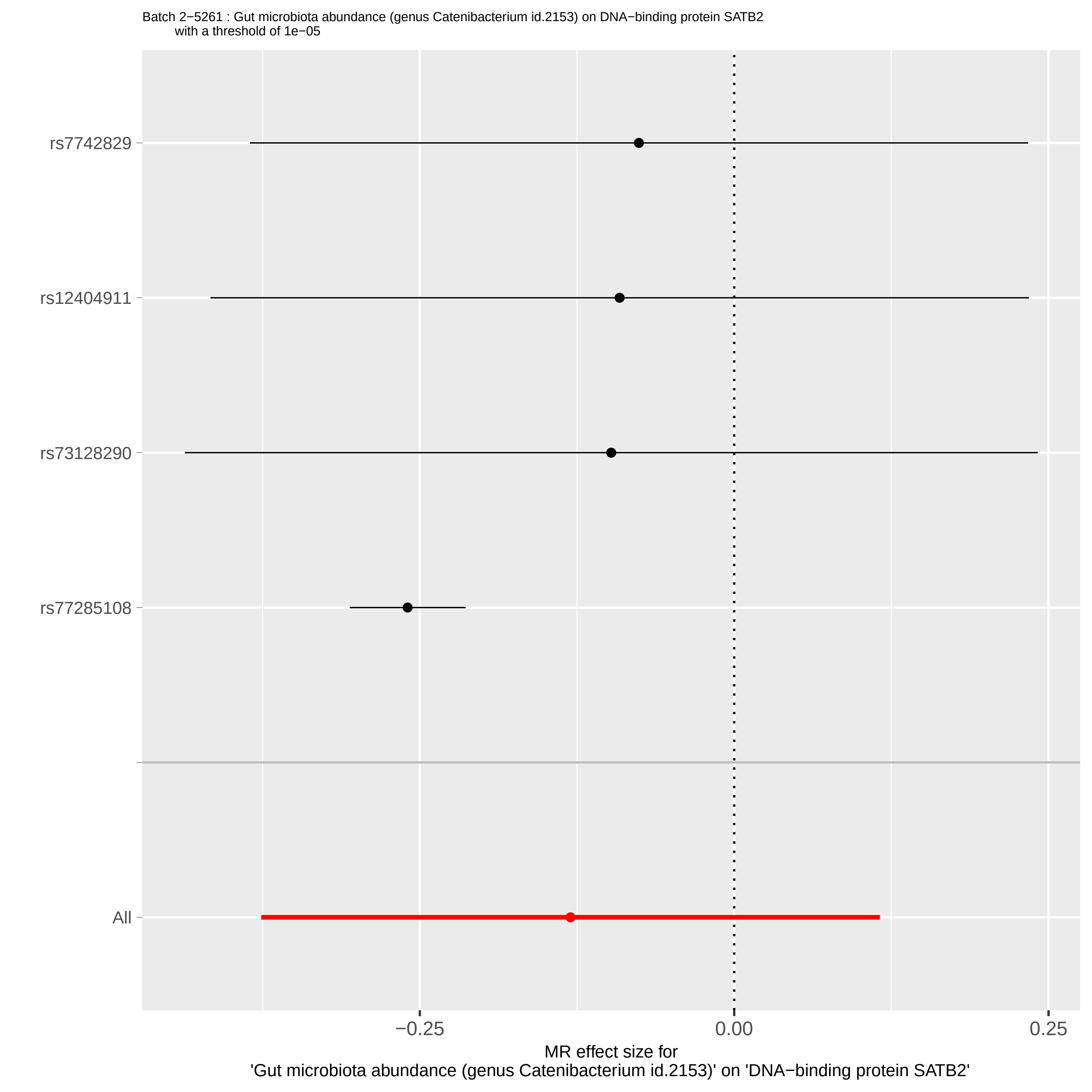
Batch 2−5261 : Gut microbiota abundance (genus Catenibacterium id.2153) on DNA−binding protein SATB2
with a threshold of 1e−05
rs7742829
rs12404911
rs73128290
rs77285108
All
−0.25
0.00
0.25
MR effect size for
'Gut microbiota abundance (genus Catenibacterium id.2153)' on 'DNA−binding protein SATB2'

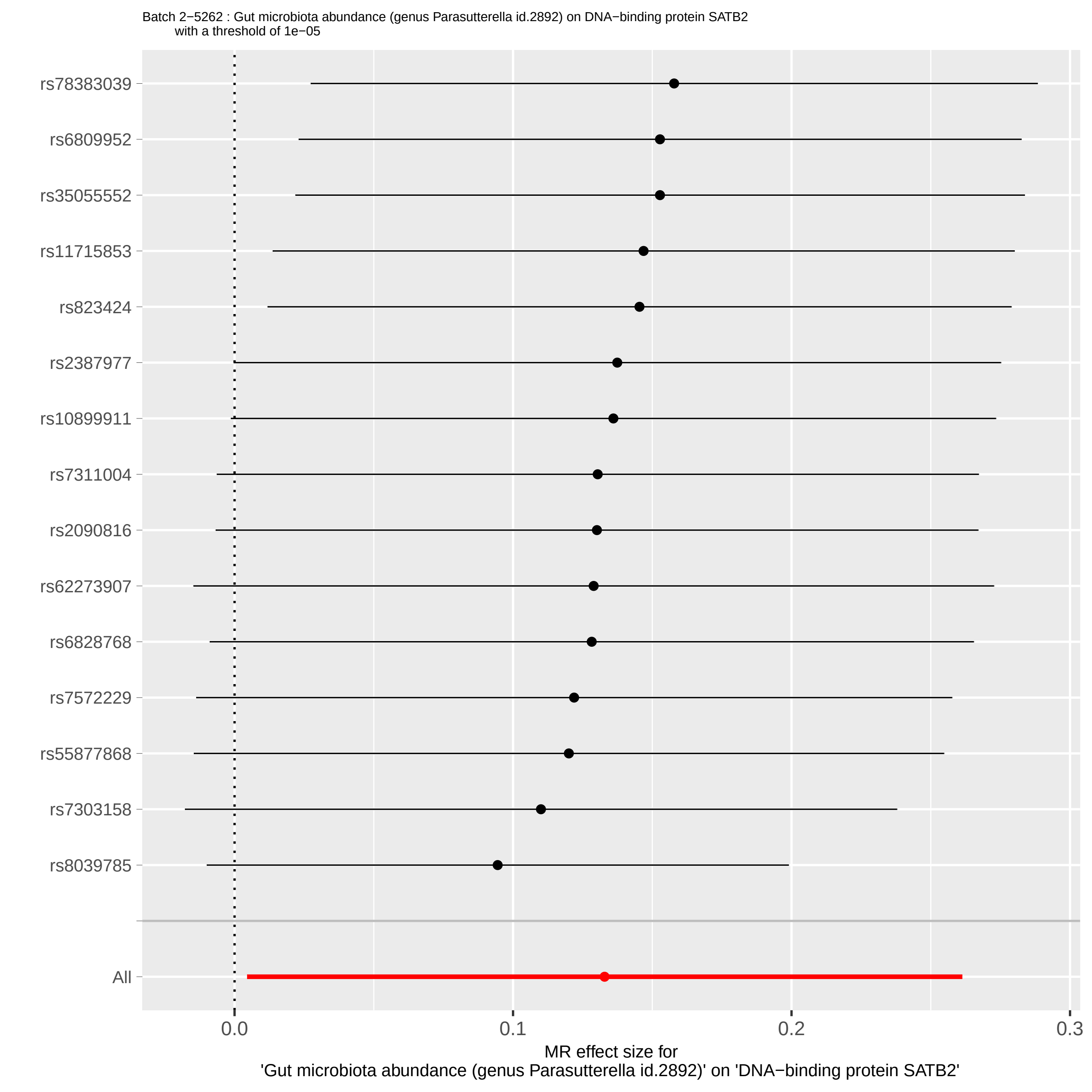

Batch 2−5262 : Gut microbiota abundance (genus Parasutterella id.2892) on DNA−binding protein SATB2
with a threshold of 1e−05
rs78383039
rs6809952
rs35055552
rs11715853
rs823424
rs2387977
rs10899911
rs7311004
rs2090816
rs62273907
rs6828768
rs7572229
rs55877868
rs7303158
rs8039785
All
0.0
0.1
0.2
0.3
MR effect size for
'Gut microbiota abundance (genus Parasutterella id.2892)' on 'DNA−binding protein SATB2'

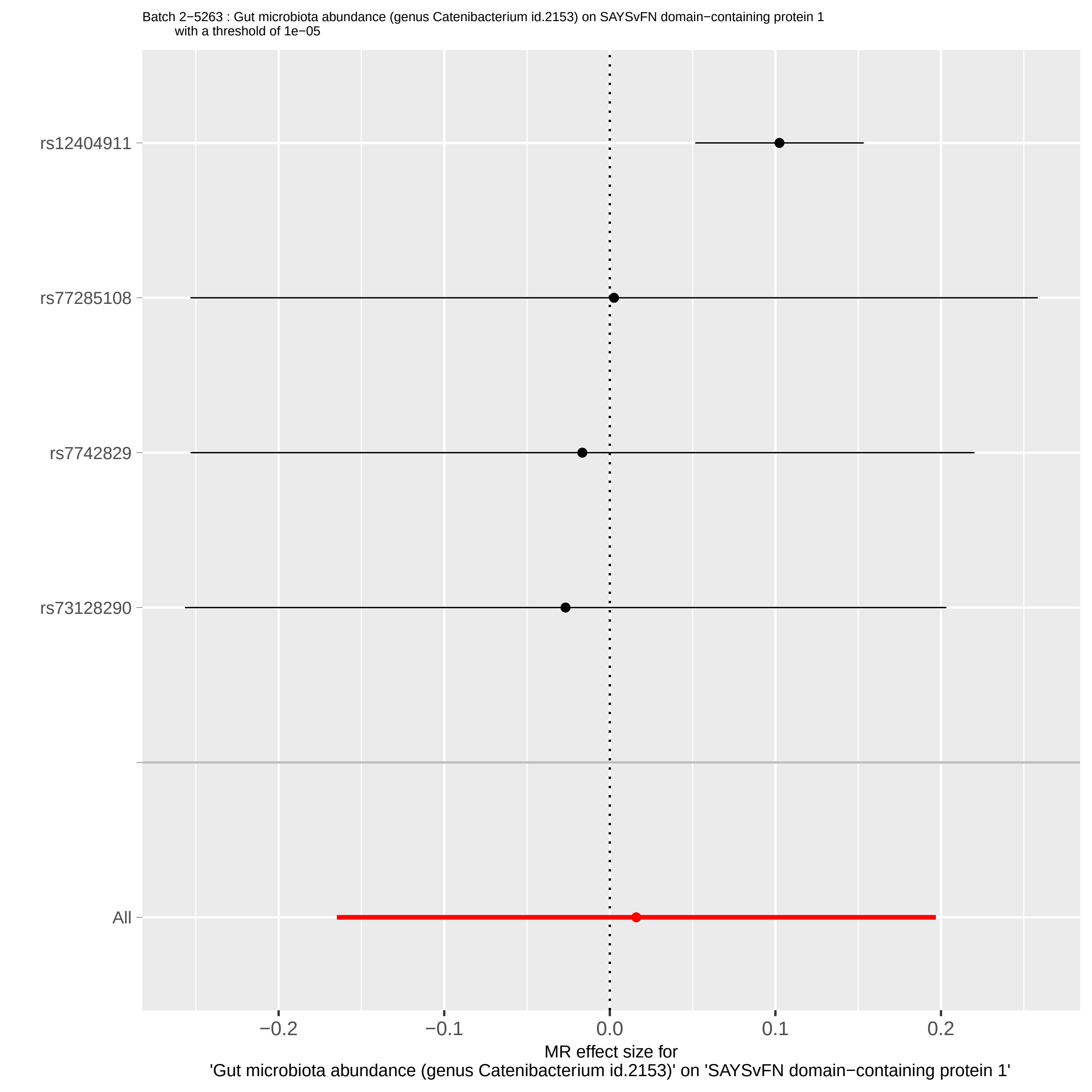
Batch 2−5263 : Gut microbiota abundance (genus Catenibacterium id.2153) on SAYSvFN domain−containing protein 1
with a threshold of 1e−05
rs12404911
rs77285108
rs7742829
rs73128290
All
−0.2
−0.1
0.0
0.1
0.2
MR effect size for
'Gut microbiota abundance (genus Catenibacterium id.2153)' on 'SAYSvFN domain−containing protein 1'

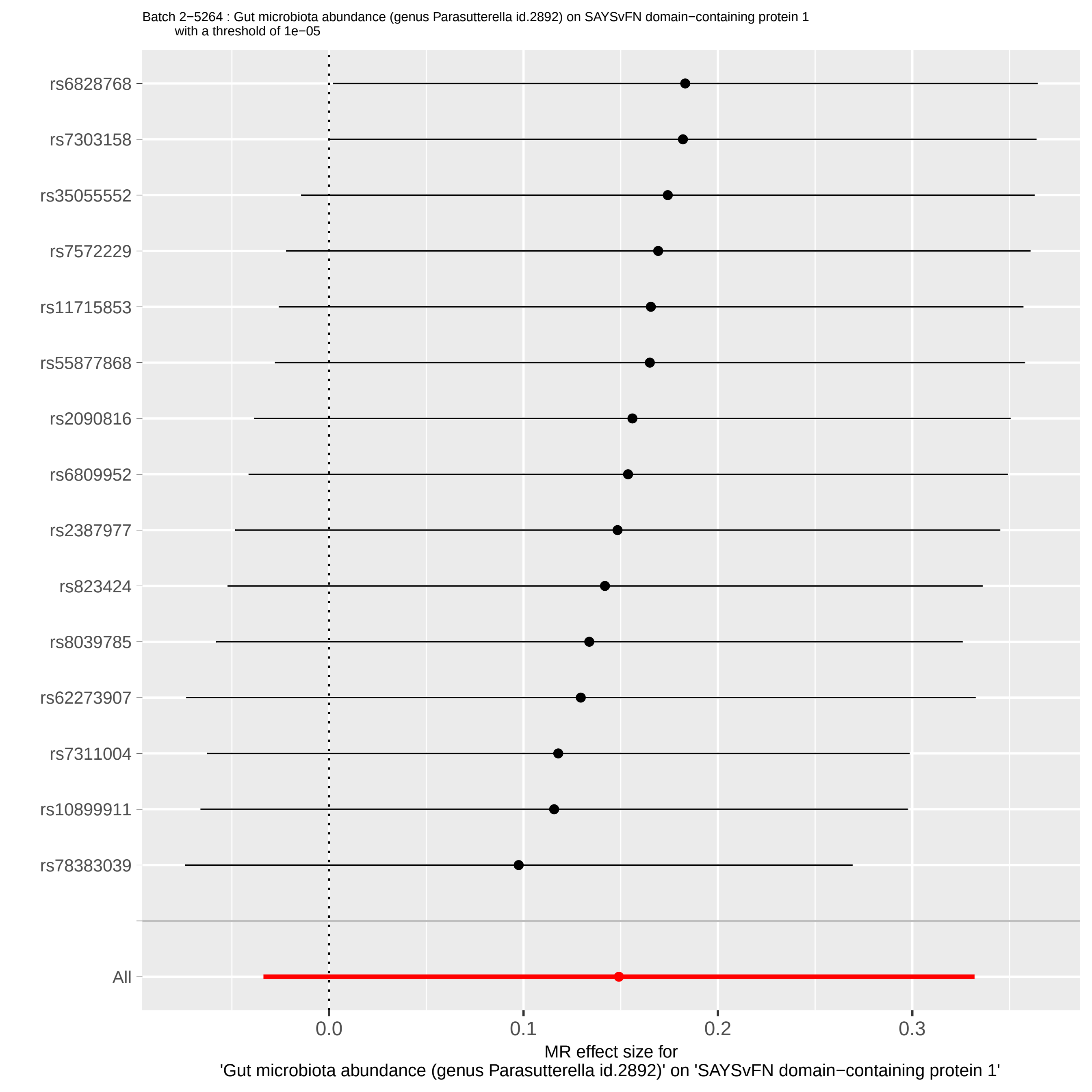

Batch 2−5264 : Gut microbiota abundance (genus Parasutterella id.2892) on SAYSvFN domain−containing protein 1
with a threshold of 1e−05
rs6828768
rs7303158
rs35055552
rs7572229
rs11715853
rs55877868
rs2090816
rs6809952
rs2387977
rs823424
rs8039785
rs62273907
rs7311004
rs10899911
rs78383039
All
0.0
0.1
0.2
0.3
MR effect size for
'Gut microbiota abundance (genus Parasutterella id.2892)' on 'SAYSvFN domain−containing protein 1'

Batch 2−5265 : Gut microbiota abundance (genus Catenibacterium id.2153) on Suprabasin
with a threshold of 1e−05

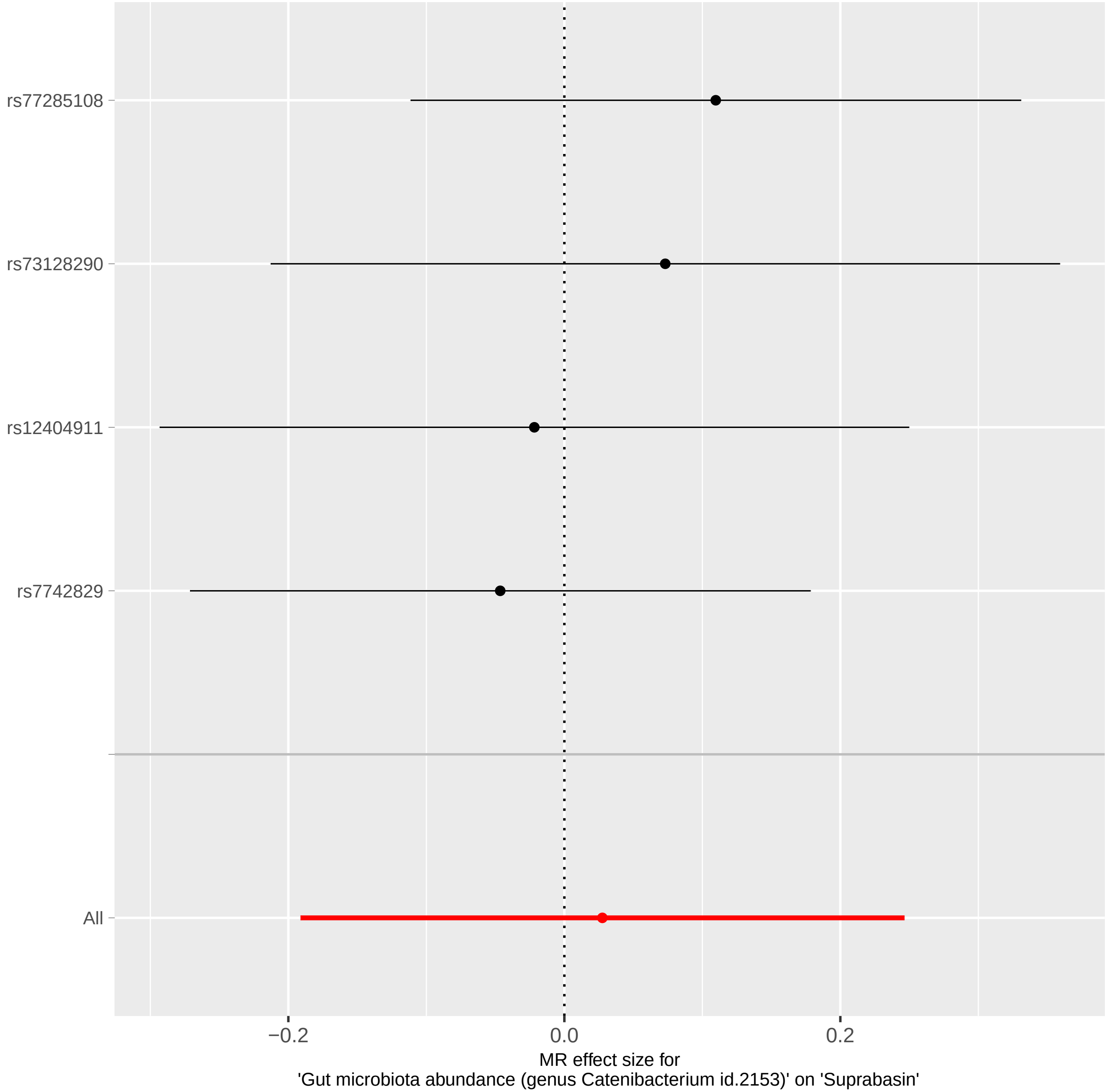

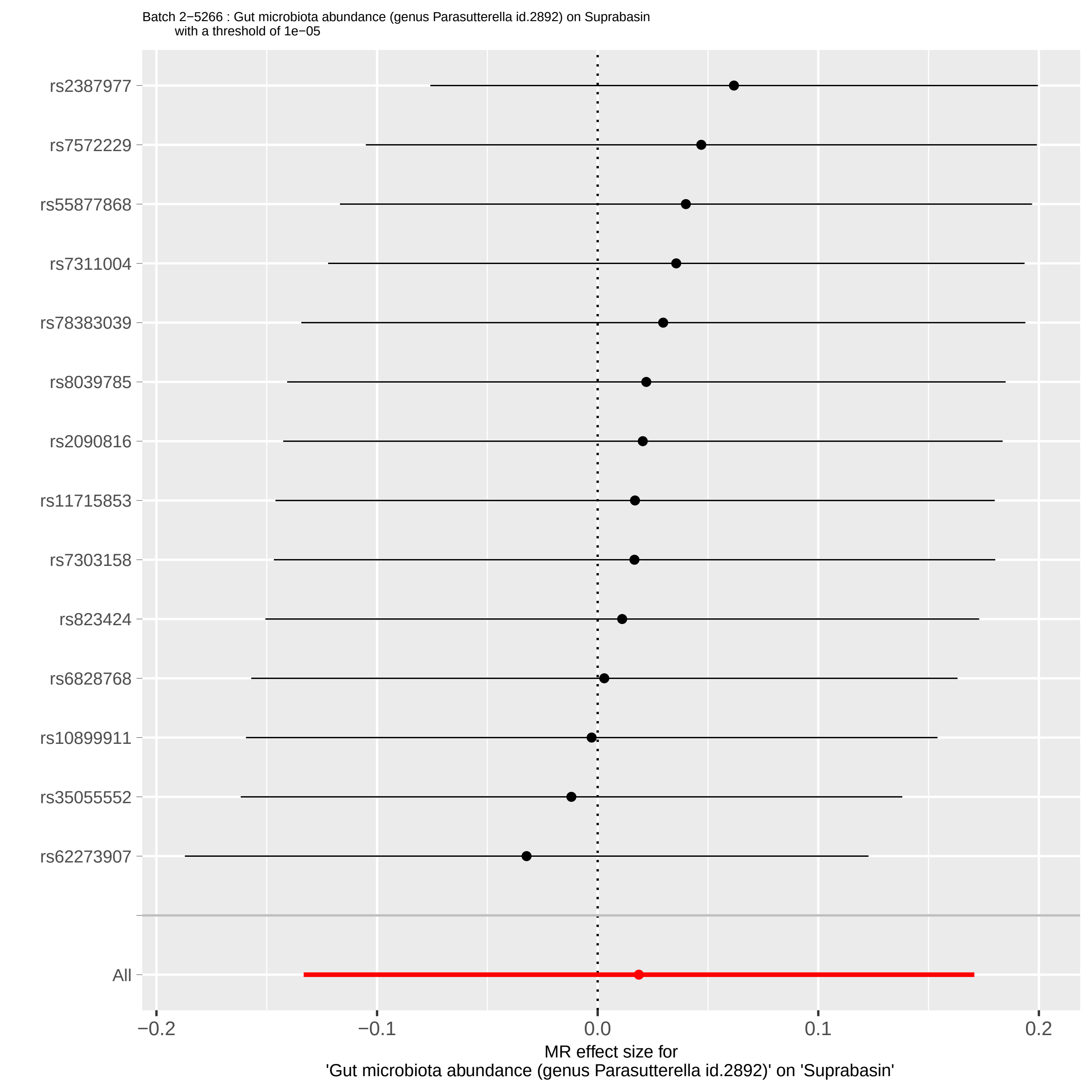
Batch 2−5266 : Gut microbiota abundance (genus Parasutterella id.2892) on Suprabasin
with a threshold of 1e−05
rs2387977
rs7572229
rs55877868
rs7311004
rs78383039
rs8039785
rs2090816
rs11715853
rs7303158
rs823424
rs6828768
rs10899911
rs35055552
rs62273907
All
−0.2
−0.1
0.0
0.1
0.2
MR effect size for
'Gut microbiota abundance (genus Parasutterella id.2892)' on 'Suprabasin'

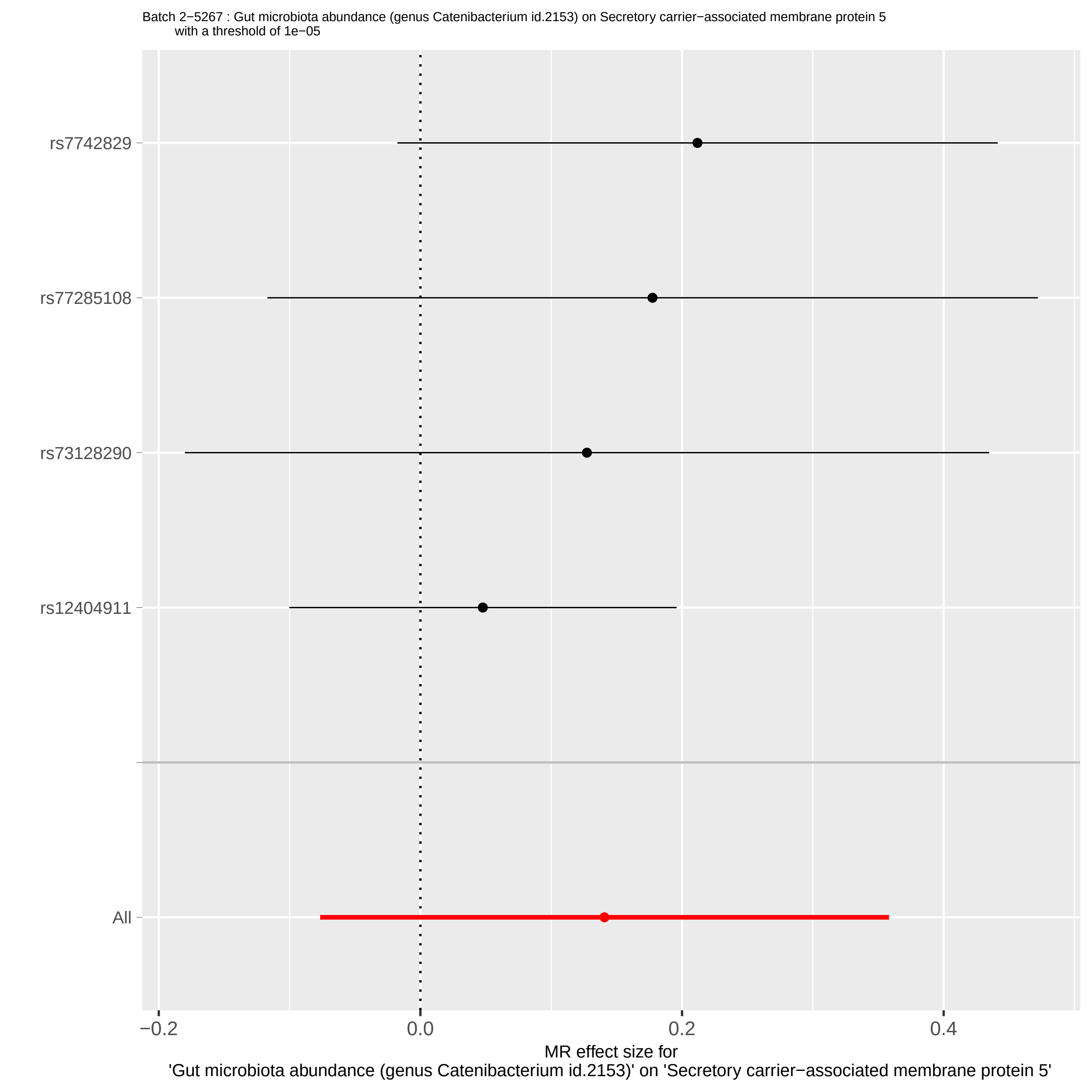

Batch 2−5267 : Gut microbiota abundance (genus Catenibacterium id.2153) on Secretory carrier−associated membrane protein 5
with a threshold of 1e−05
rs7742829
rs77285108
rs73128290
rs12404911
All
−0.2
0.0
0.2
0.4
MR effect size for
'Gut microbiota abundance (genus Catenibacterium id.2153)' on 'Secretory carrier−associated membrane protein 5'

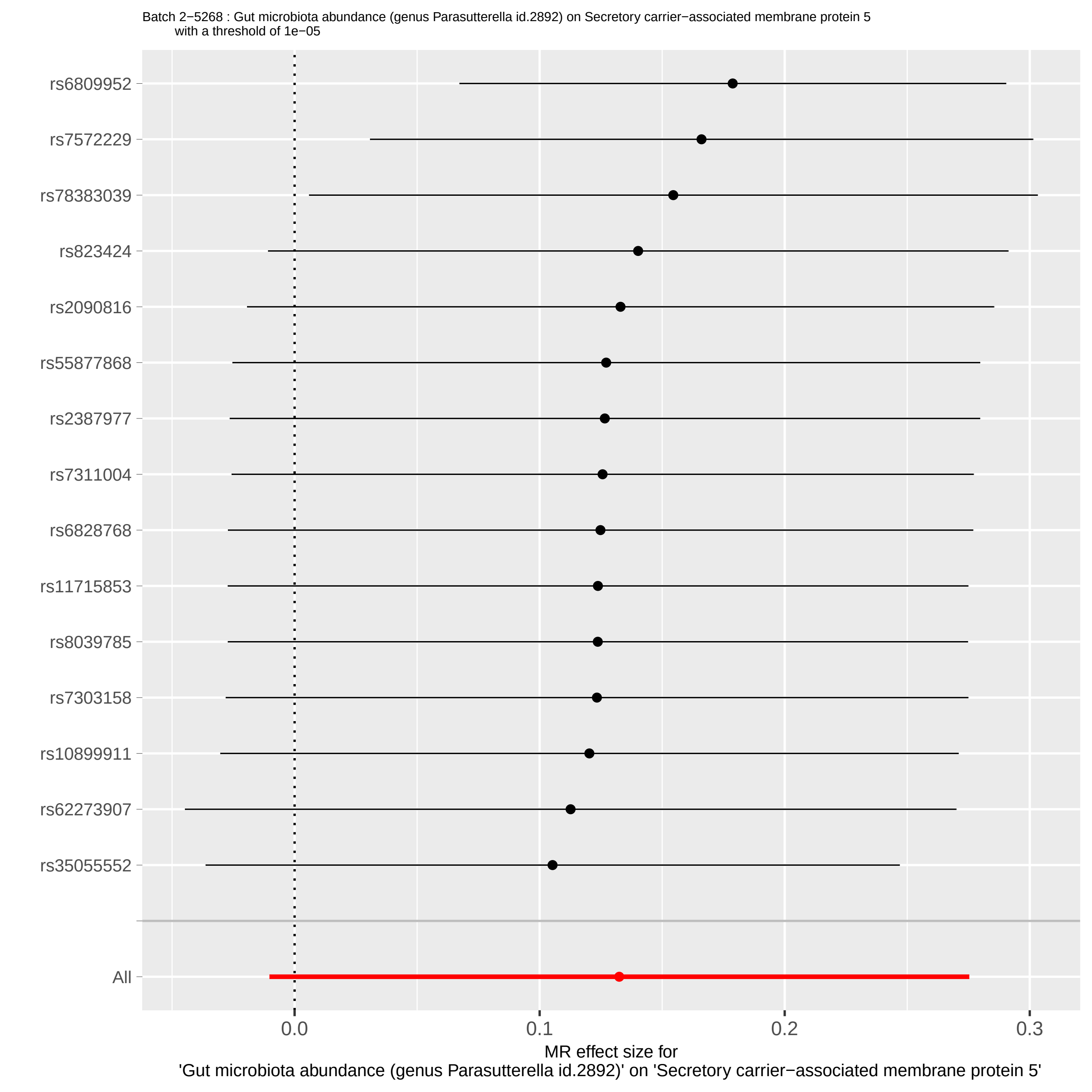

Batch 2−5268 : Gut microbiota abundance (genus Parasutterella id.2892) on Secretory carrier−associated membrane protein 5
with a threshold of 1e−05
rs6809952
rs7572229
rs78383039
rs823424
rs2090816
rs55877868
rs2387977
rs7311004
rs6828768
rs11715853
rs8039785
rs7303158
rs10899911
rs62273907
rs35055552
All
0.0
0.1
0.2
0.3
MR effect size for
'Gut microbiota abundance (genus Parasutterella id.2892)' on 'Secretory carrier−associated membrane protein 5'

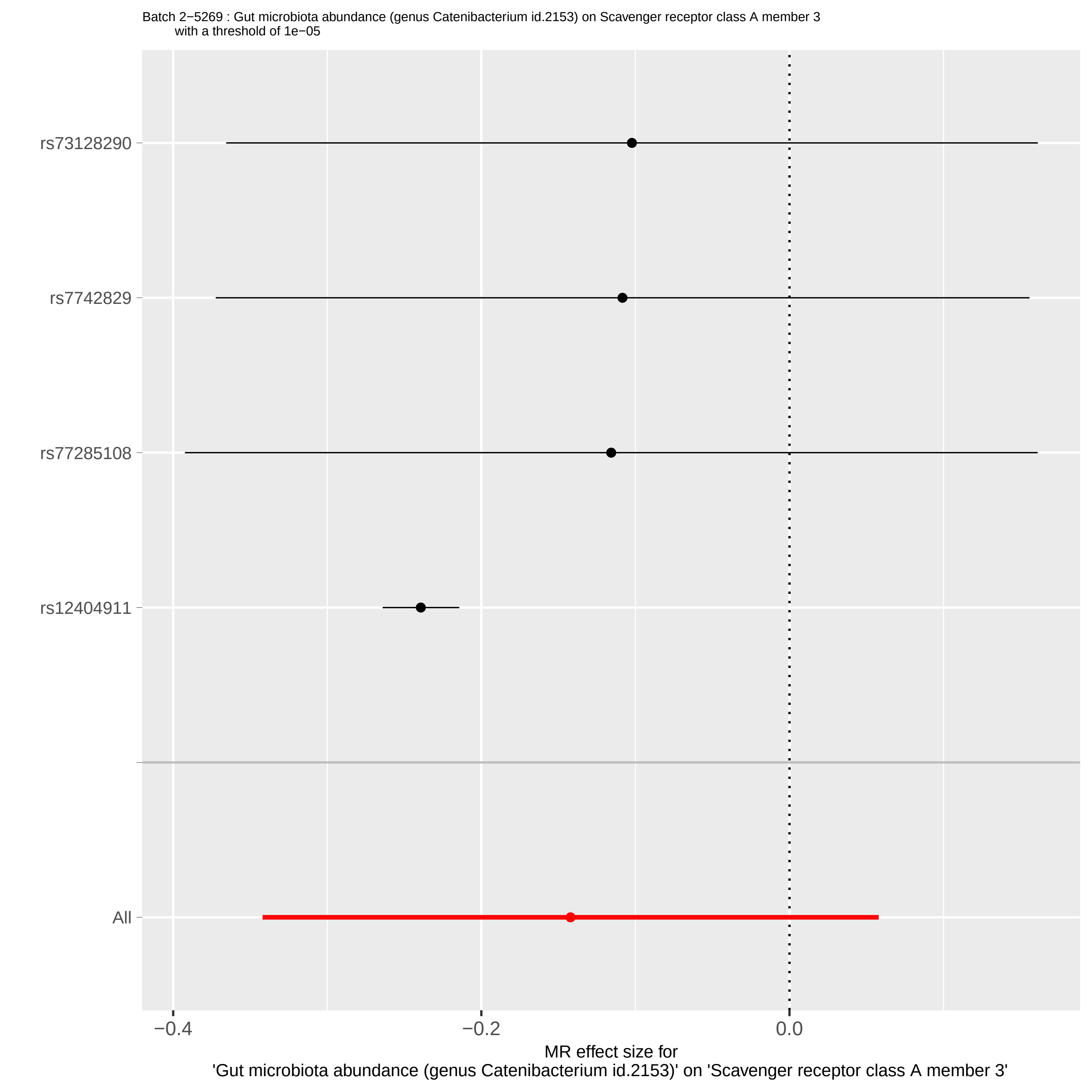

Batch 2−5269 : Gut microbiota abundance (genus Catenibacterium id.2153) on Scavenger receptor class A member 3
with a threshold of 1e−05
rs73128290
rs7742829
rs77285108
rs12404911
All
−0.4
−0.2
0.0
MR effect size for
'Gut microbiota abundance (genus Catenibacterium id.2153)' on 'Scavenger receptor class A member 3'

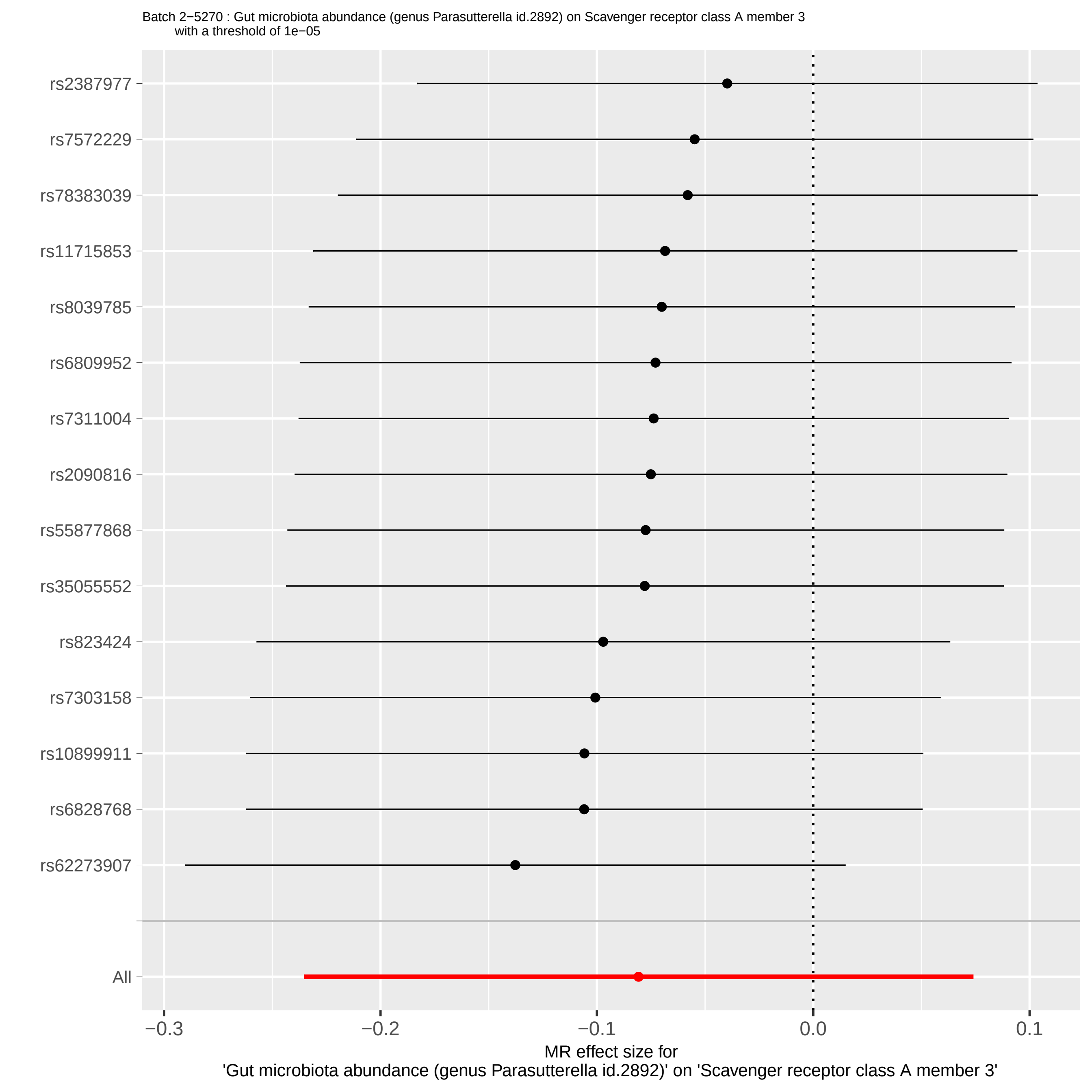

Batch 2−5270 : Gut microbiota abundance (genus Parasutterella id.2892) on Scavenger receptor class A member 3
with a threshold of 1e−05
rs2387977
rs7572229
rs78383039
rs11715853
rs8039785
rs6809952
rs7311004
rs2090816
rs55877868
rs35055552
rs823424
rs7303158
rs10899911
rs6828768
rs62273907
All
−0.3
−0.2
−0.1
0.0
0.1
MR effect size for
'Gut microbiota abundance (genus Parasutterella id.2892)' on 'Scavenger receptor class A member 3'

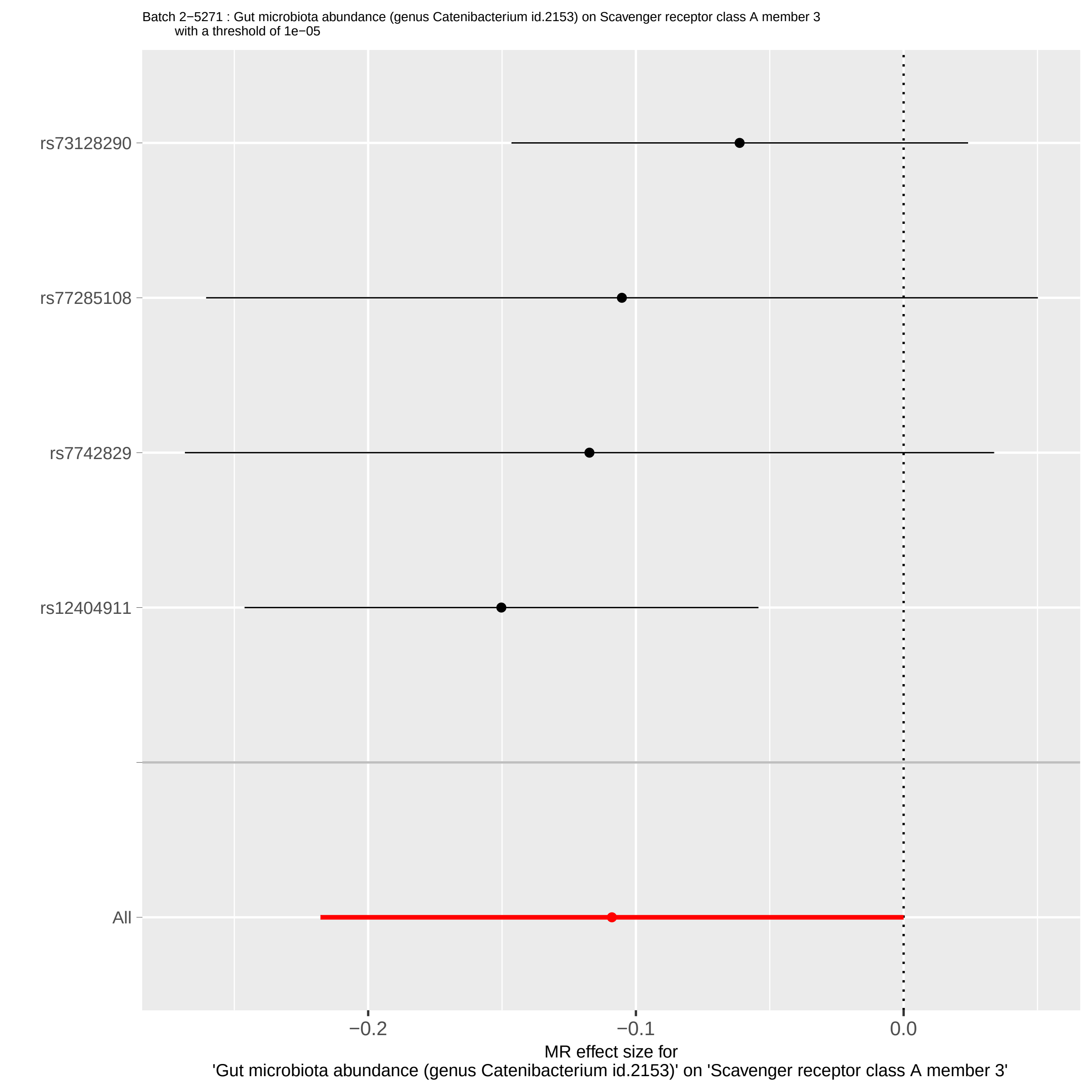
Batch 2−5271 : Gut microbiota abundance (genus Catenibacterium id.2153) on Scavenger receptor class A member 3
with a threshold of 1e−05
rs73128290
rs77285108
rs7742829
rs12404911
All
−0.2
−0.1
0.0
MR effect size for
'Gut microbiota abundance (genus Catenibacterium id.2153)' on 'Scavenger receptor class A member 3'

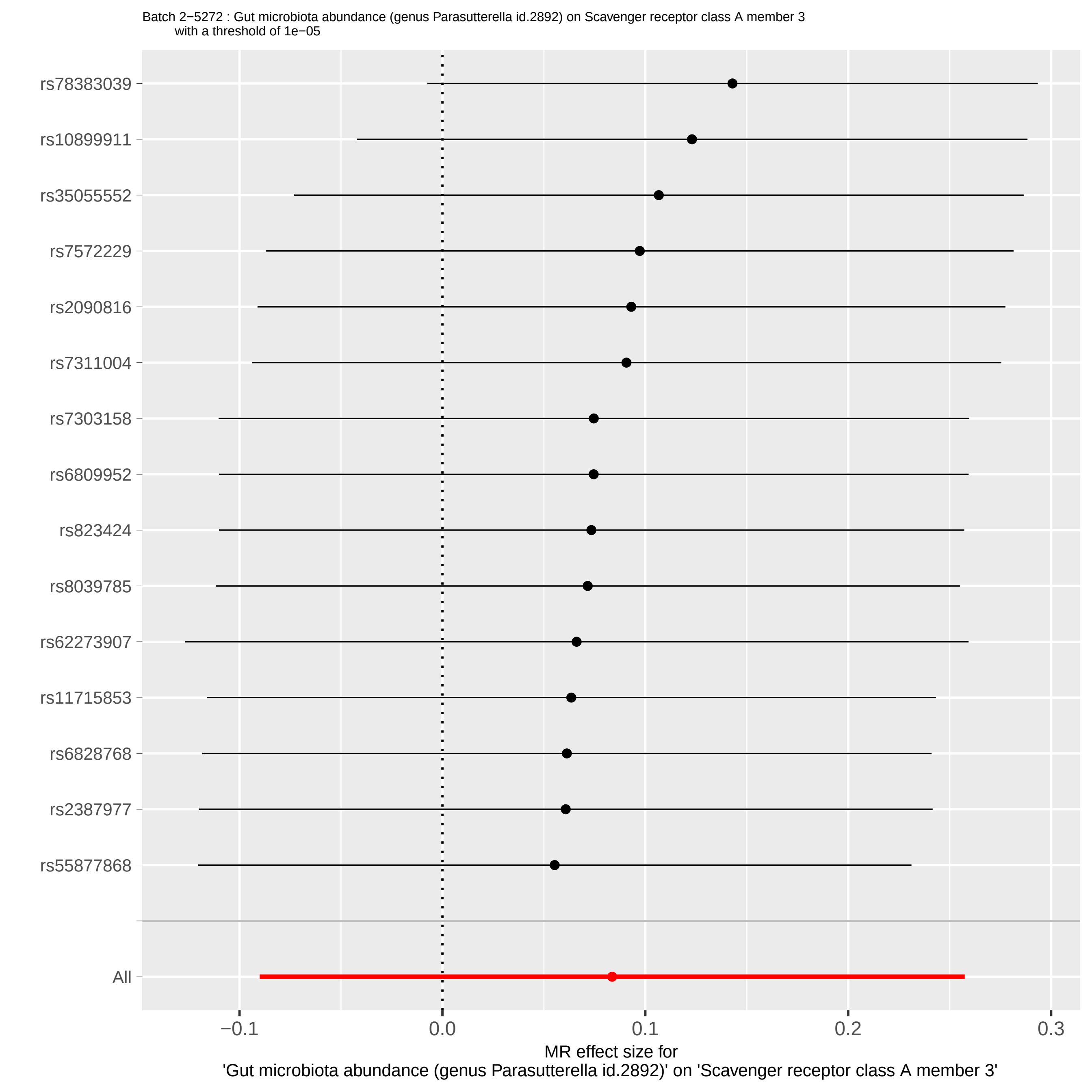

Batch 2−5272 : Gut microbiota abundance (genus Parasutterella id.2892) on Scavenger receptor class A member 3
with a threshold of 1e−05
rs78383039
rs10899911
rs35055552
rs7572229
rs2090816
rs7311004
rs7303158
rs6809952
rs823424
rs8039785
rs62273907
rs11715853
rs6828768
rs2387977
rs55877868
All
−0.1
0.0
0.1
0.2
0.3
MR effect size for
'Gut microbiota abundance (genus Parasutterella id.2892)' on 'Scavenger receptor class A member 3'

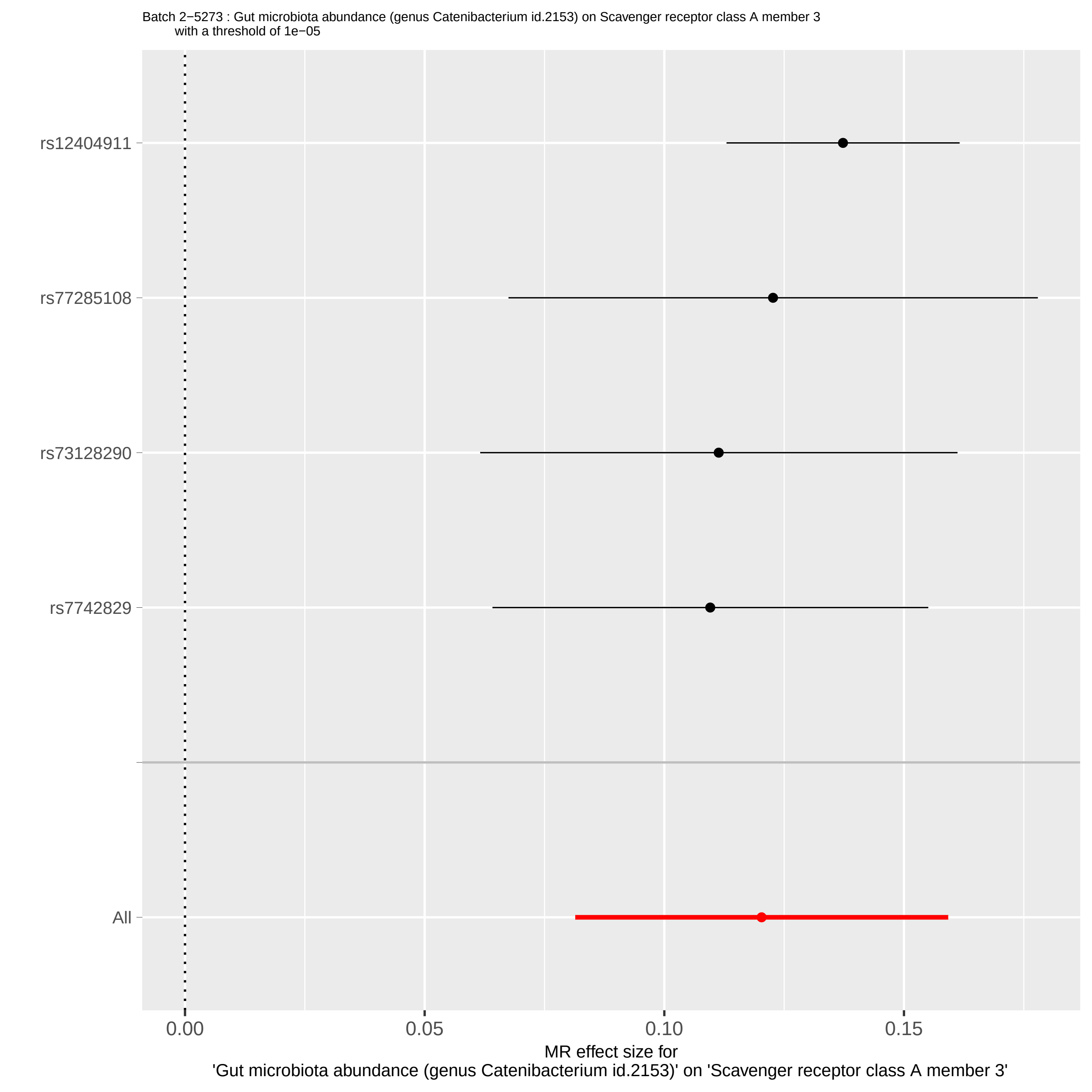

Batch 2−5273 : Gut microbiota abundance (genus Catenibacterium id.2153) on Scavenger receptor class A member 3
with a threshold of 1e−05
rs12404911
rs77285108
rs73128290
rs7742829
All
0.00
0.05
0.10
0.15
MR effect size for
'Gut microbiota abundance (genus Catenibacterium id.2153)' on 'Scavenger receptor class A member 3'

Batch 2−5274 : Gut microbiota abundance (genus Parasutterella id.2892) on Scavenger receptor class A member 3
with a threshold of 1e−05

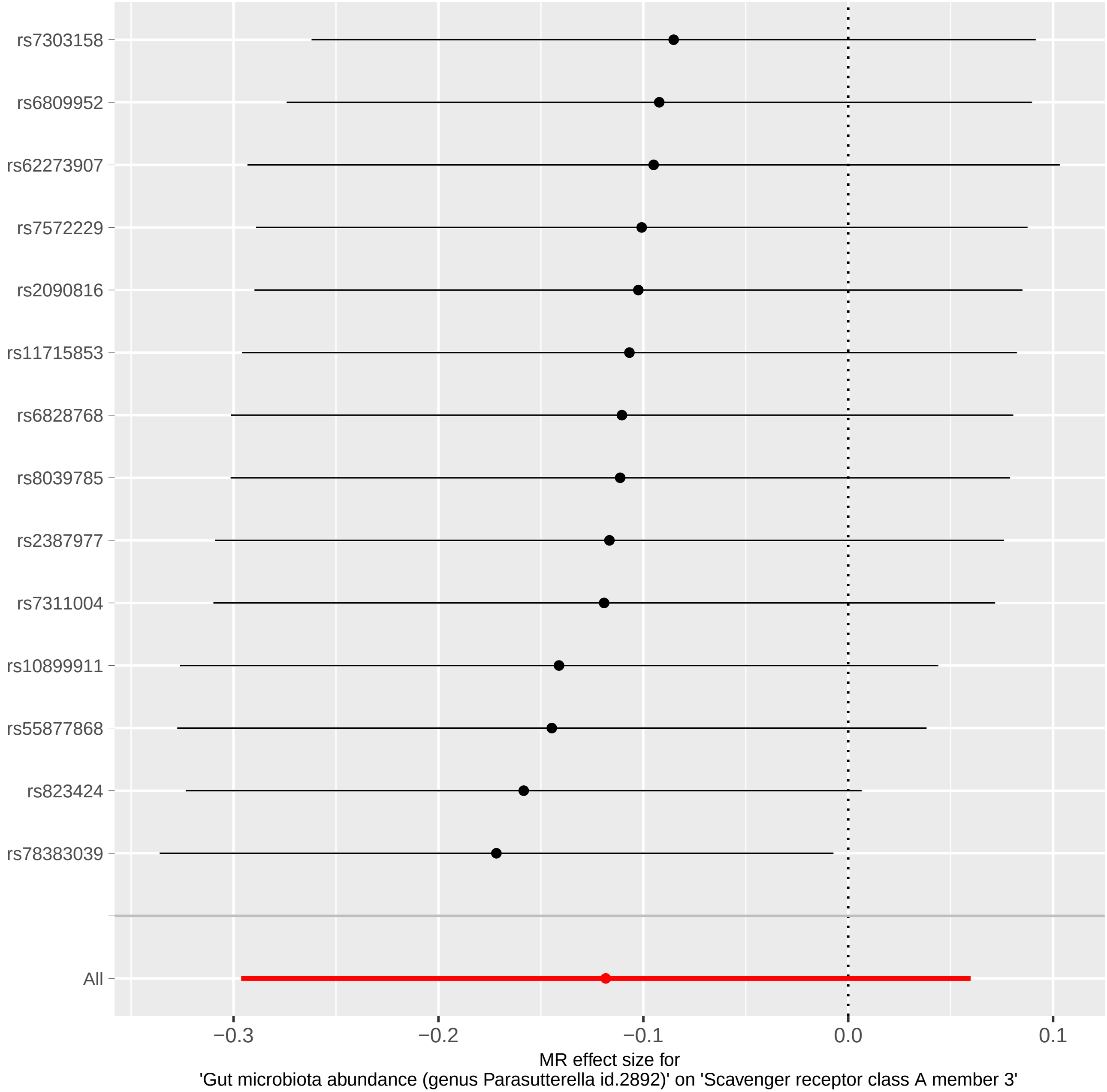

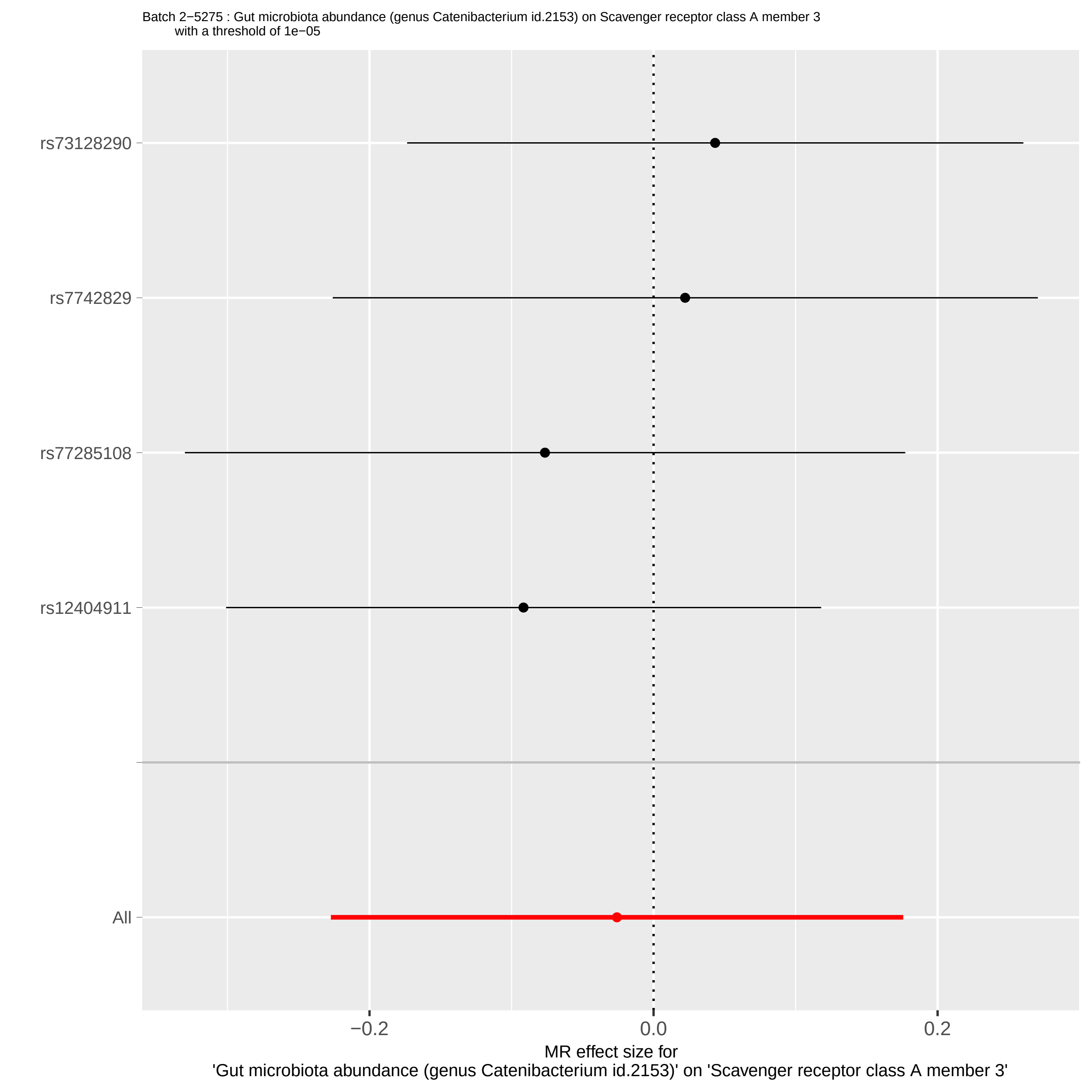

Batch 2−5275 : Gut microbiota abundance (genus Catenibacterium id.2153) on Scavenger receptor class A member 3
with a threshold of 1e−05
rs73128290
rs7742829
rs77285108
rs12404911
All
−0.2
0.0
0.2
MR effect size for
'Gut microbiota abundance (genus Catenibacterium id.2153)' on 'Scavenger receptor class A member 3'

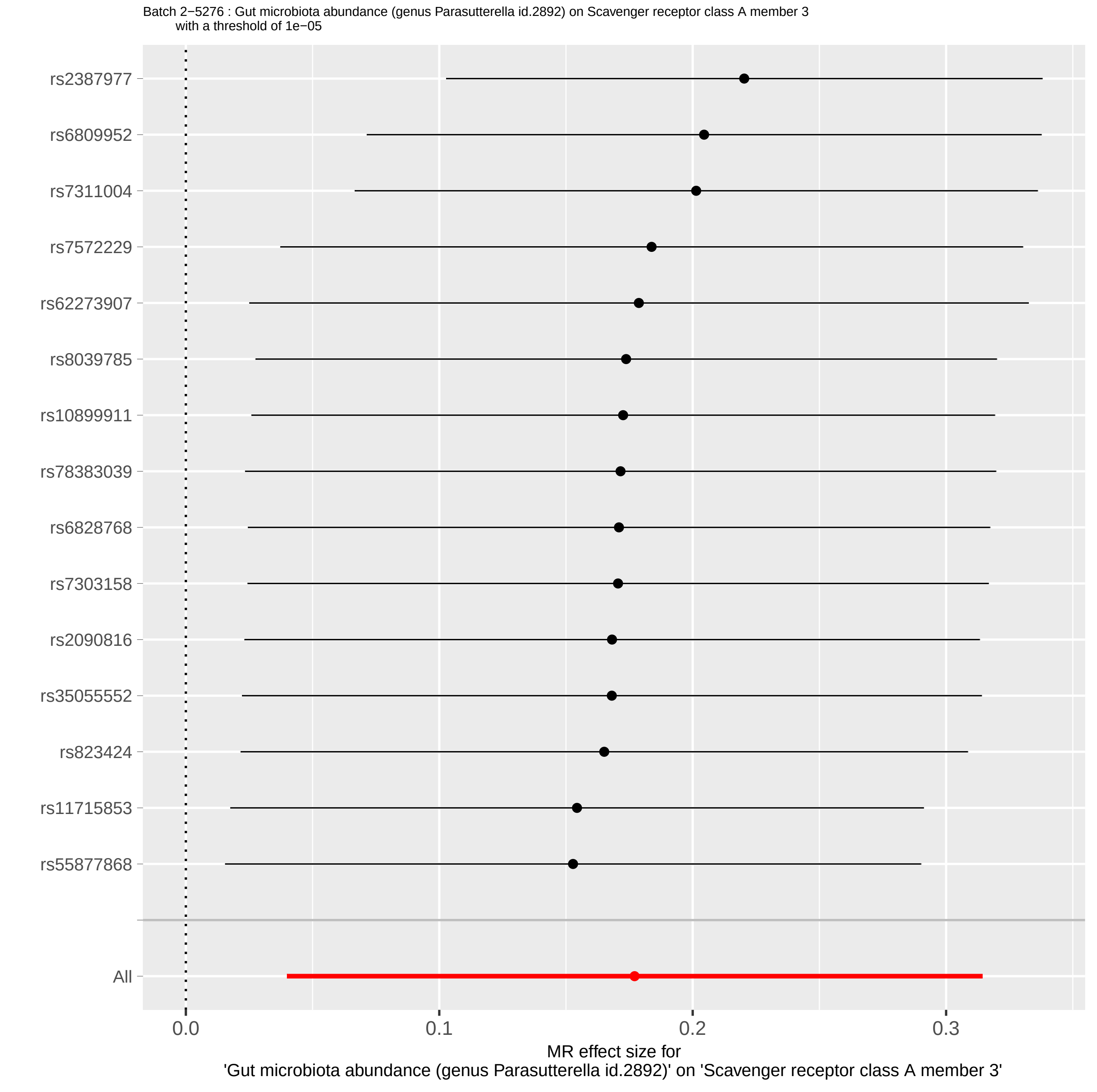
Batch 2−5276 : Gut microbiota abundance (genus Parasutterella id.2892) on Scavenger receptor class A member 3
with a threshold of 1e−05
rs2387977
rs6809952
rs7311004
rs7572229
rs62273907
rs8039785
rs10899911
rs78383039
rs6828768
rs7303158
rs2090816
rs35055552
rs823424
rs11715853
rs55877868
All
0.0
0.1
0.2
0.3
MR effect size for
'Gut microbiota abundance (genus Parasutterella id.2892)' on 'Scavenger receptor class A member 3'

Batch 2−5277 : Gut microbiota abundance (genus Catenibacterium id.2153) on Scavenger receptor class A member 5
with a threshold of 1e−05

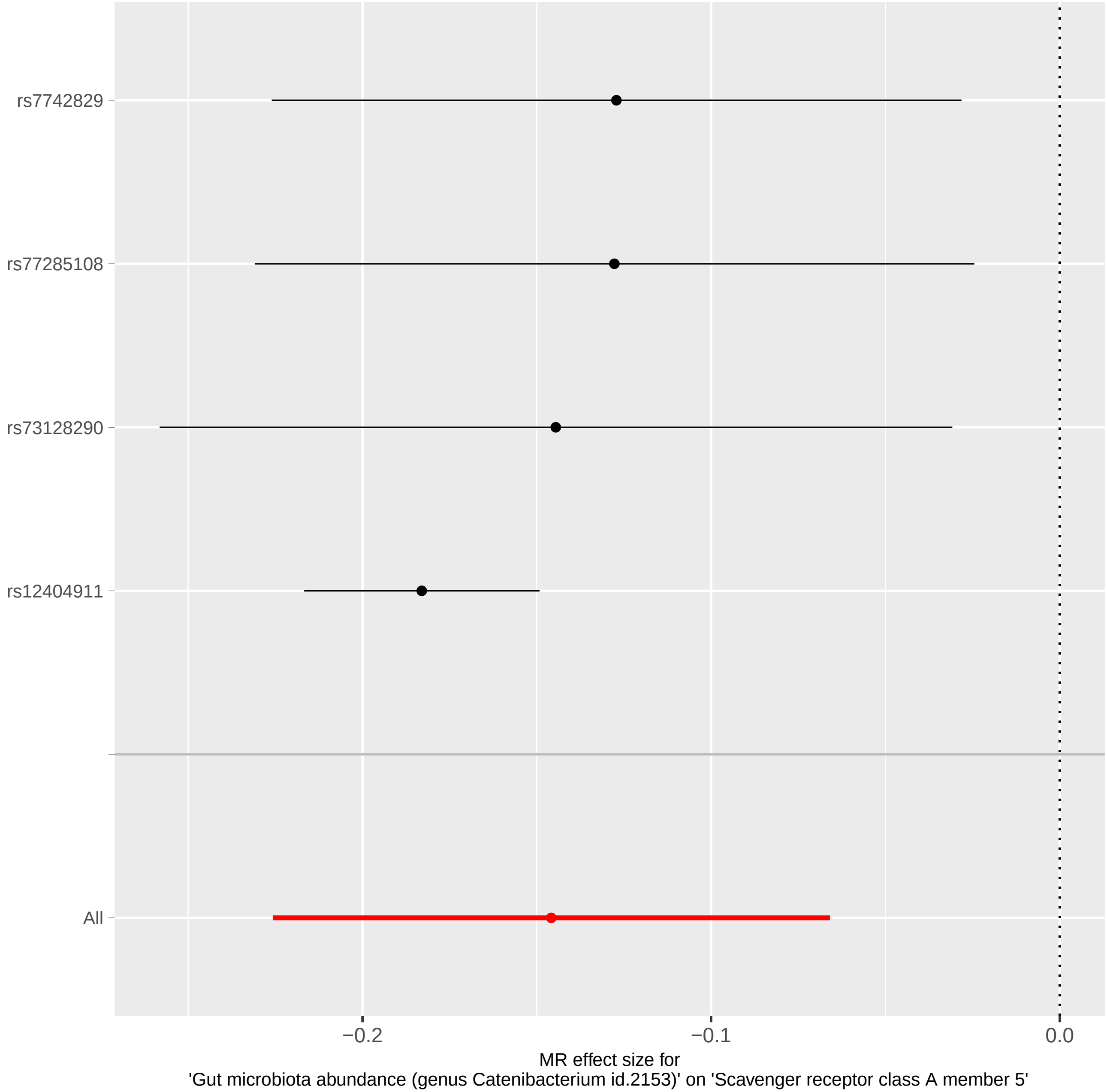

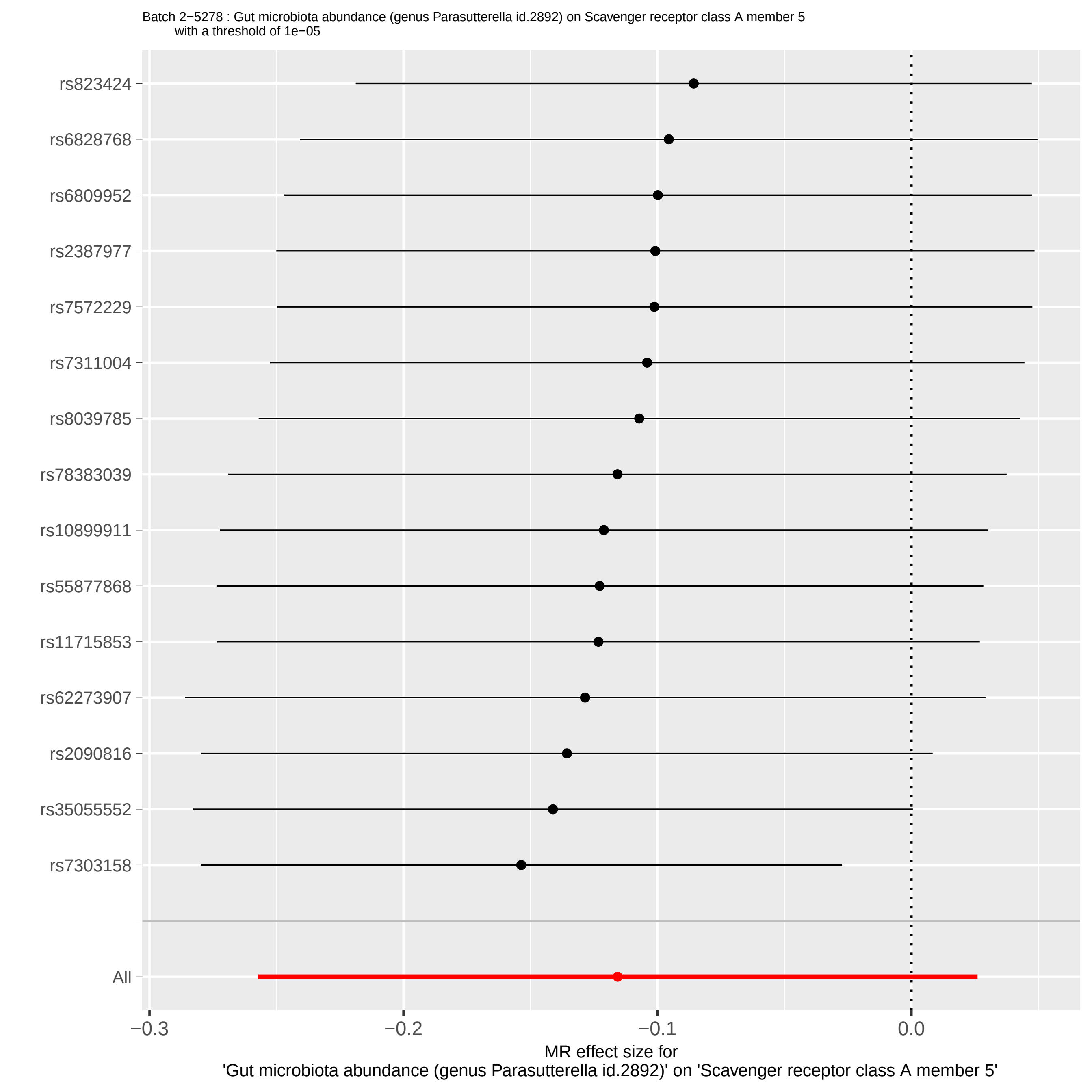
Batch 2−5278 : Gut microbiota abundance (genus Parasutterella id.2892) on Scavenger receptor class A member 5
with a threshold of 1e−05
rs823424
rs6828768
rs6809952
rs2387977
rs7572229
rs7311004
rs8039785
rs78383039
rs10899911
rs55877868
rs11715853
rs62273907
rs2090816
rs35055552
rs7303158
All
−0.3
−0.2
−0.1
0.0
MR effect size for
'Gut microbiota abundance (genus Parasutterella id.2892)' on 'Scavenger receptor class A member 5'

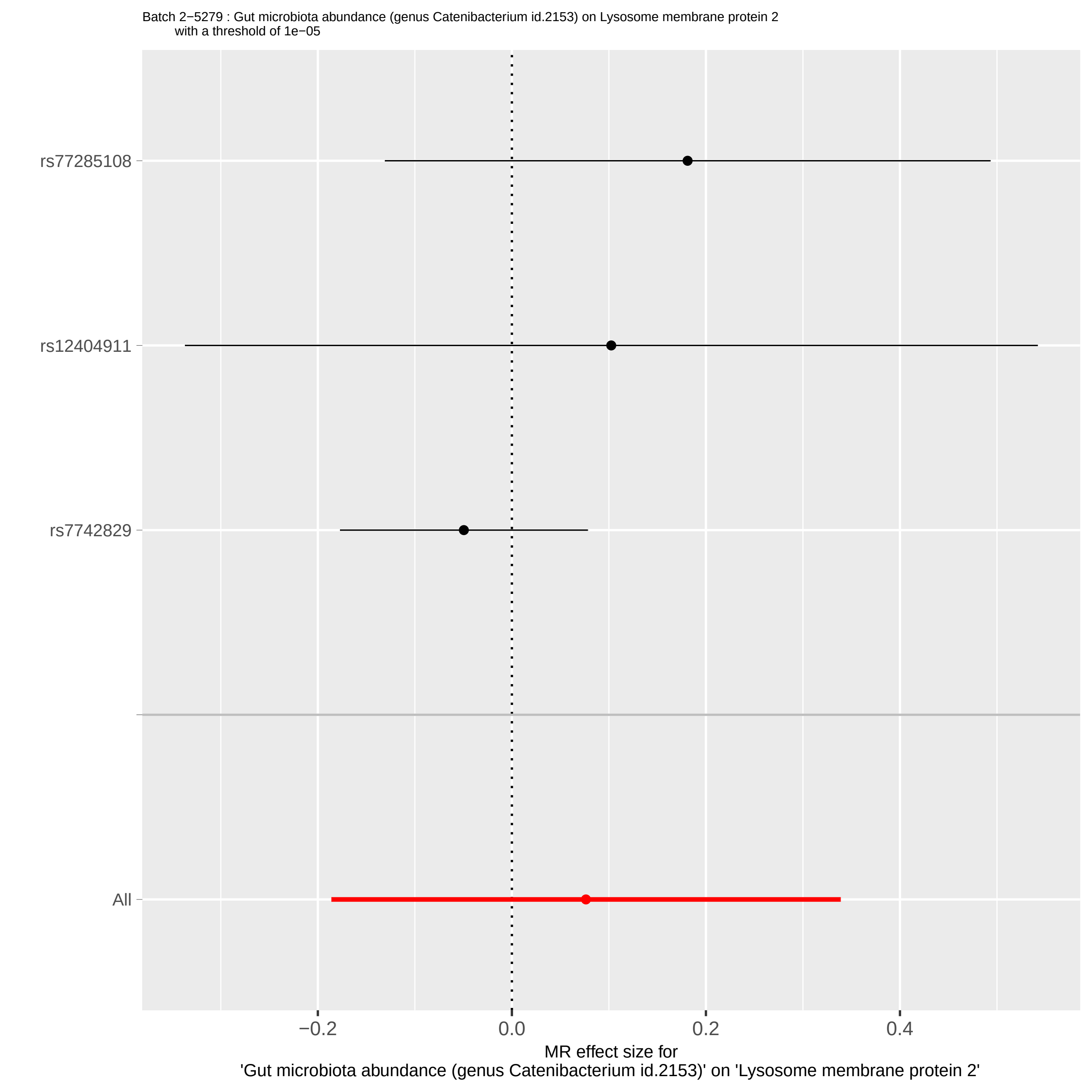

Batch 2−5279 : Gut microbiota abundance (genus Catenibacterium id.2153) on Lysosome membrane protein 2
with a threshold of 1e−05
rs77285108
rs12404911
rs7742829
All
−0.2
0.0
0.2
0.4
MR effect size for
'Gut microbiota abundance (genus Catenibacterium id.2153)' on 'Lysosome membrane protein 2'

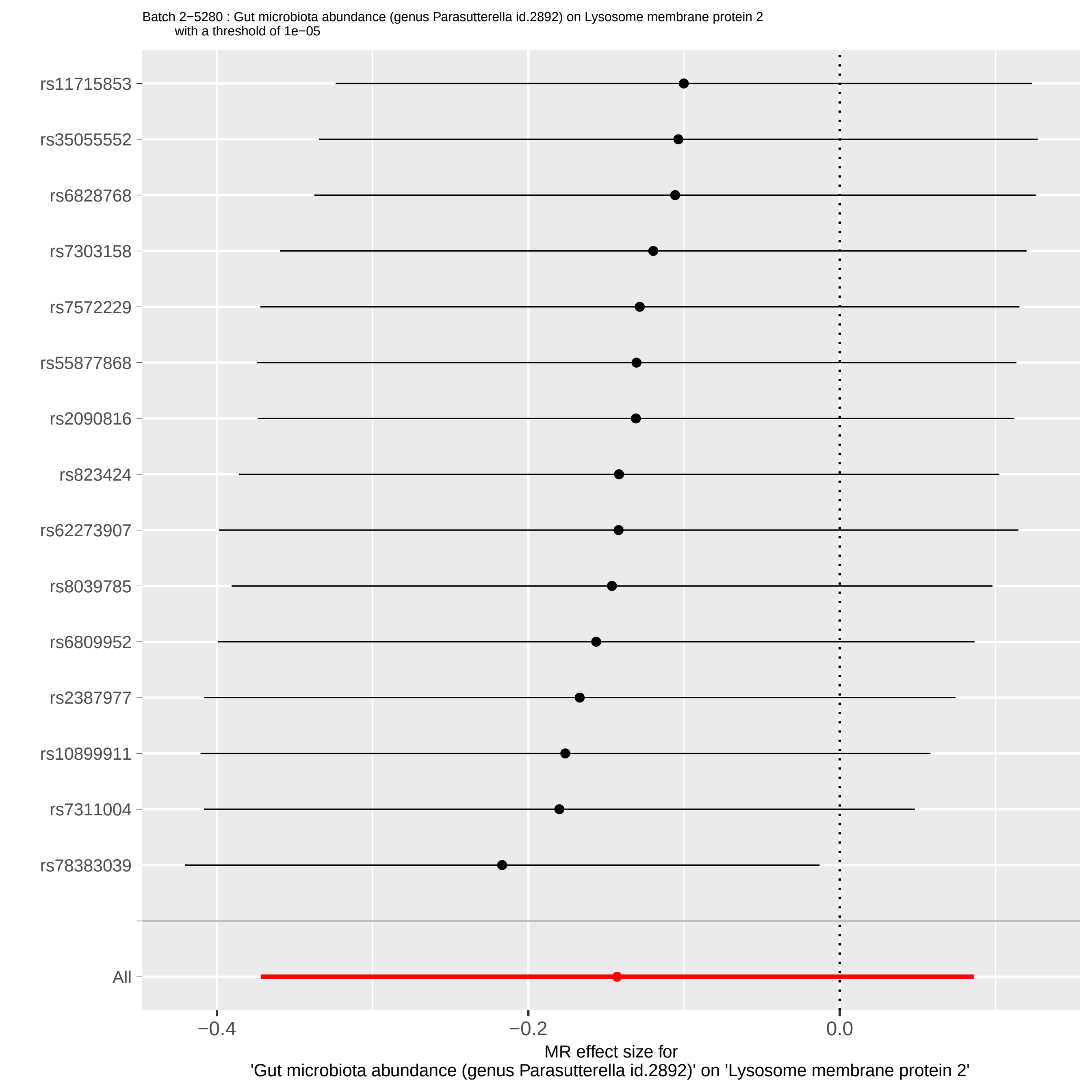

Batch 2−5280 : Gut microbiota abundance (genus Parasutterella id.2892) on Lysosome membrane protein 2
with a threshold of 1e−05
rs11715853
rs35055552
rs6828768
rs7303158
rs7572229
rs55877868
rs2090816
rs823424
rs62273907
rs8039785
rs6809952
rs2387977
rs10899911
rs7311004
rs78383039
All
−0.4
−0.2
0.0
MR effect size for
'Gut microbiota abundance (genus Parasutterella id.2892)' on 'Lysosome membrane protein 2'

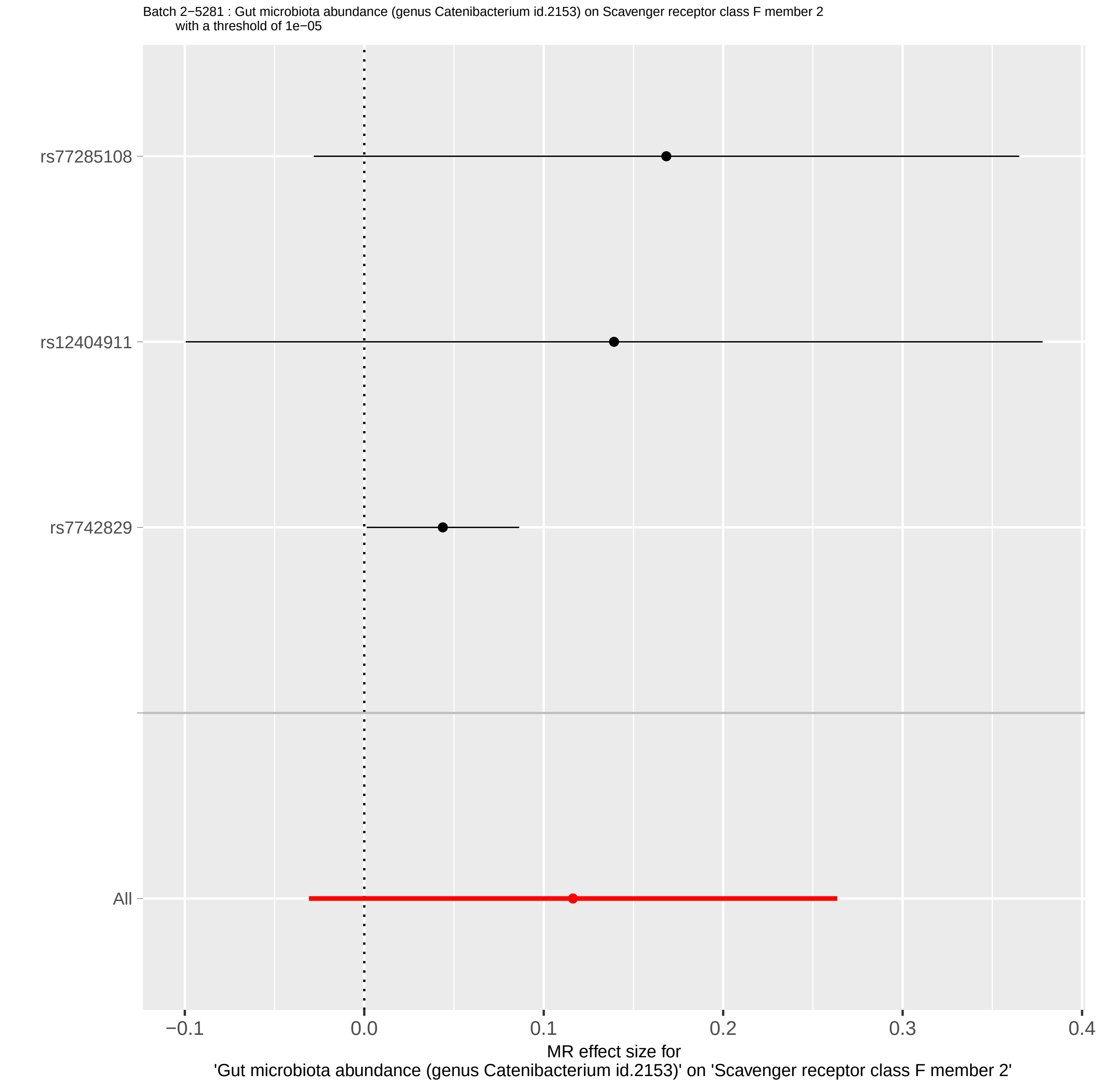

Batch 2−5281 : Gut microbiota abundance (genus Catenibacterium id.2153) on Scavenger receptor class F member 2
with a threshold of 1e−05
rs77285108
rs12404911
rs7742829
All
−0.1
0.0
0.1
0.2
0.3
0.4
MR effect size for
'Gut microbiota abundance (genus Catenibacterium id.2153)' on 'Scavenger receptor class F member 2'

Batch 2−5282 : Gut microbiota abundance (genus Parasutterella id.2892) on Scavenger receptor class F member 2
with a threshold of 1e−05

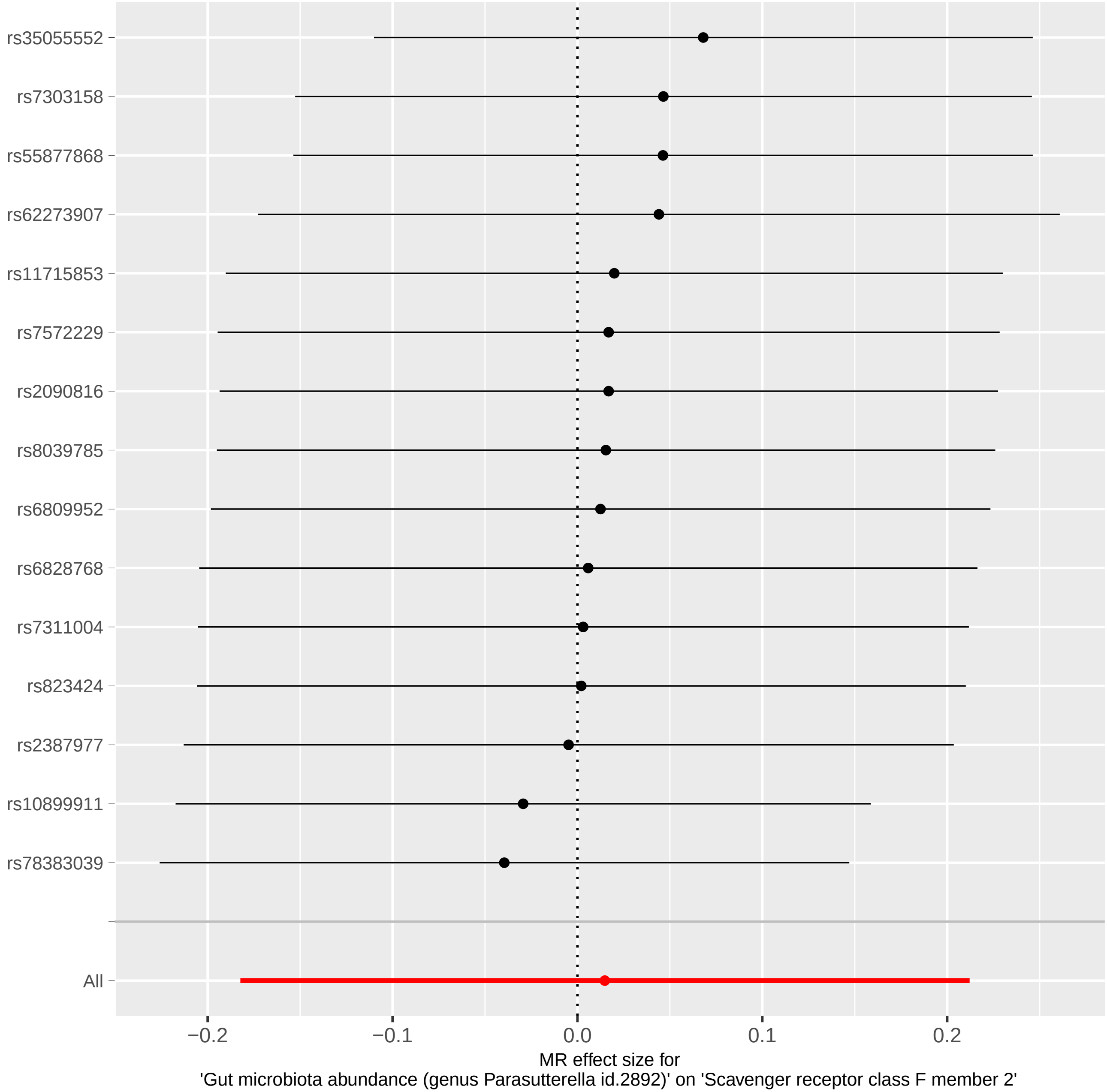

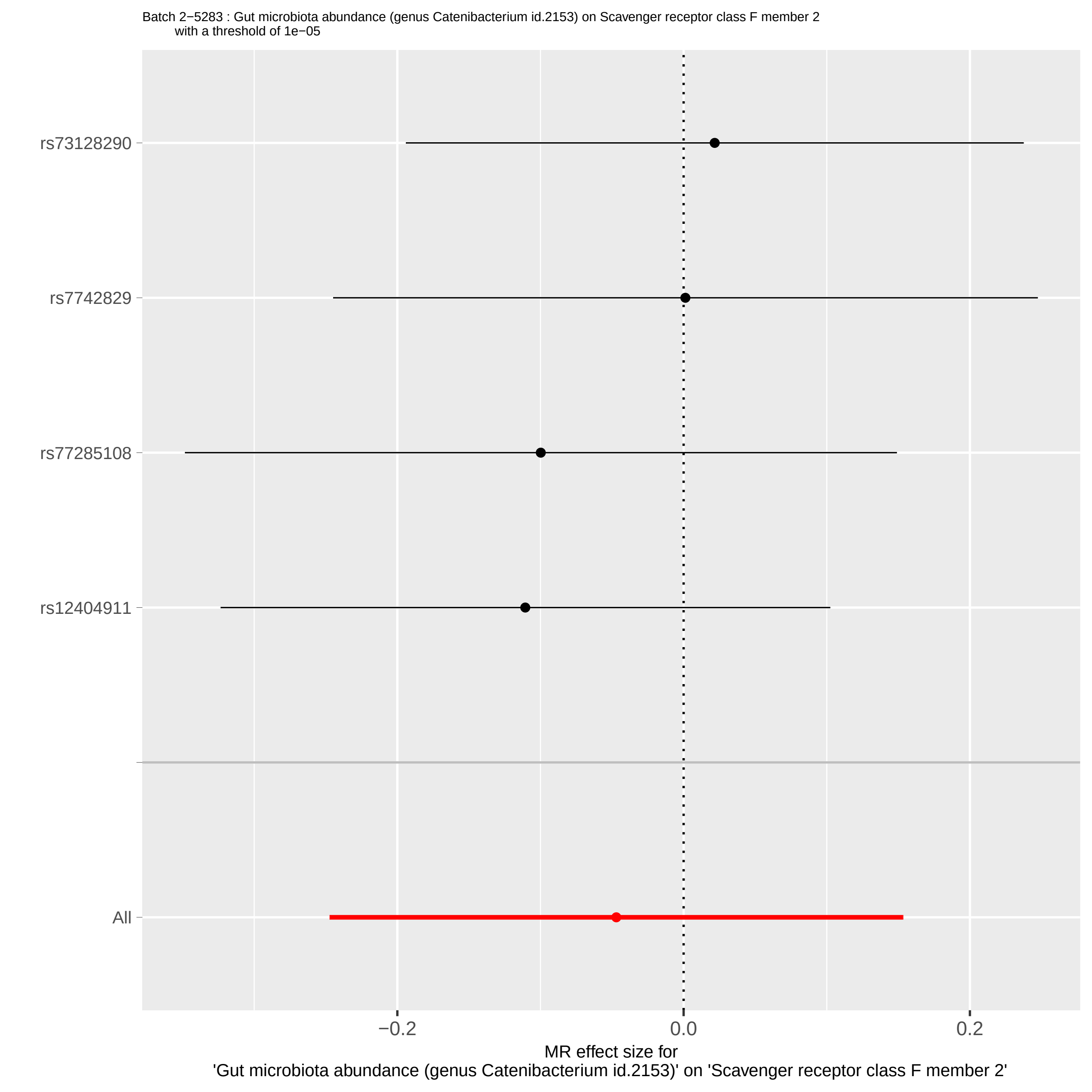
Batch 2−5283 : Gut microbiota abundance (genus Catenibacterium id.2153) on Scavenger receptor class F member 2
with a threshold of 1e−05
rs73128290
rs7742829
rs77285108
rs12404911
All
−0.2
0.0
0.2
MR effect size for
'Gut microbiota abundance (genus Catenibacterium id.2153)' on 'Scavenger receptor class F member 2'

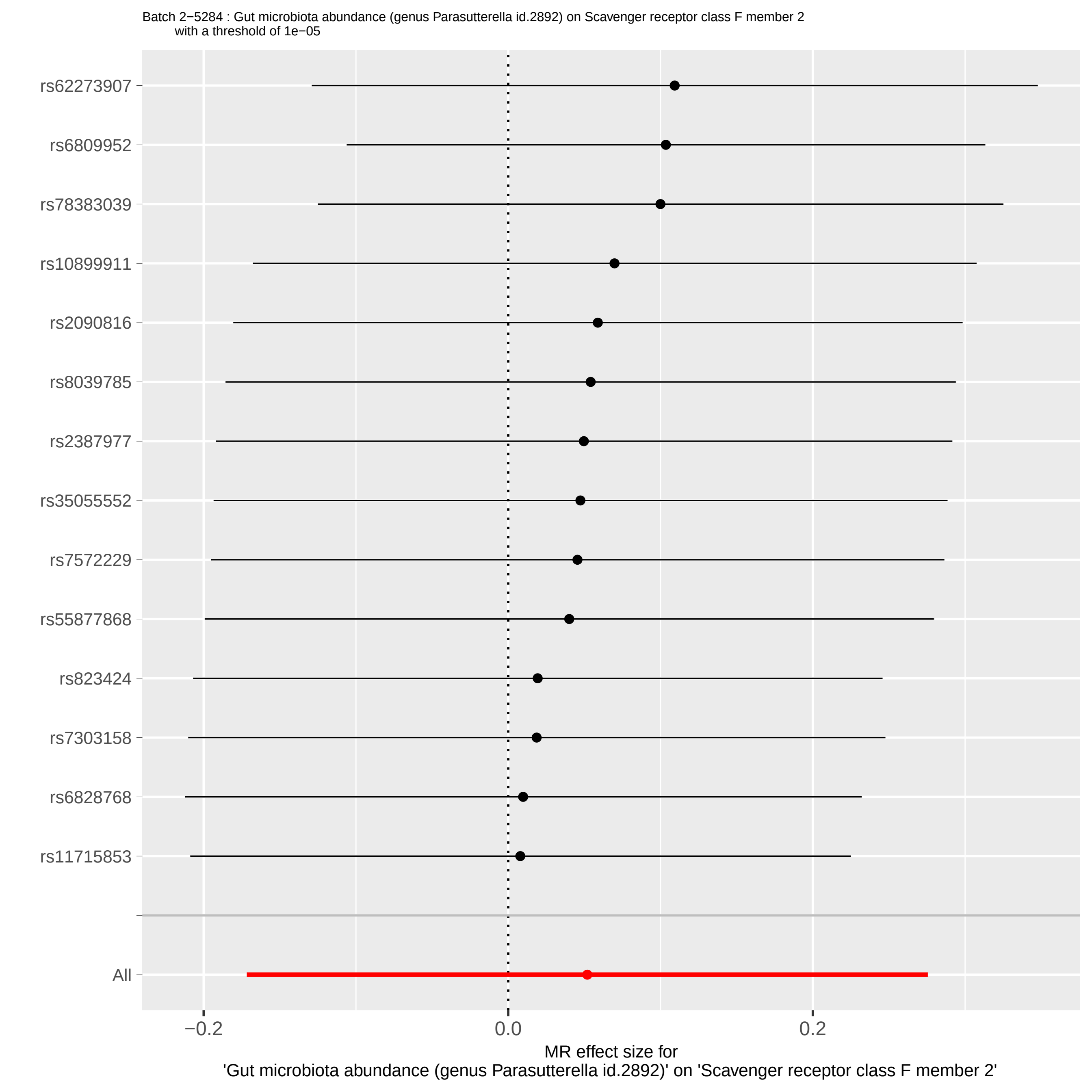

Batch 2−5284 : Gut microbiota abundance (genus Parasutterella id.2892) on Scavenger receptor class F member 2
with a threshold of 1e−05
rs62273907
rs6809952
rs78383039
rs10899911
rs2090816
rs8039785
rs2387977
rs35055552
rs7572229
rs55877868
rs823424
rs7303158
rs6828768
rs11715853
All
−0.2
0.0
0.2
MR effect size for
'Gut microbiota abundance (genus Parasutterella id.2892)' on 'Scavenger receptor class F member 2'

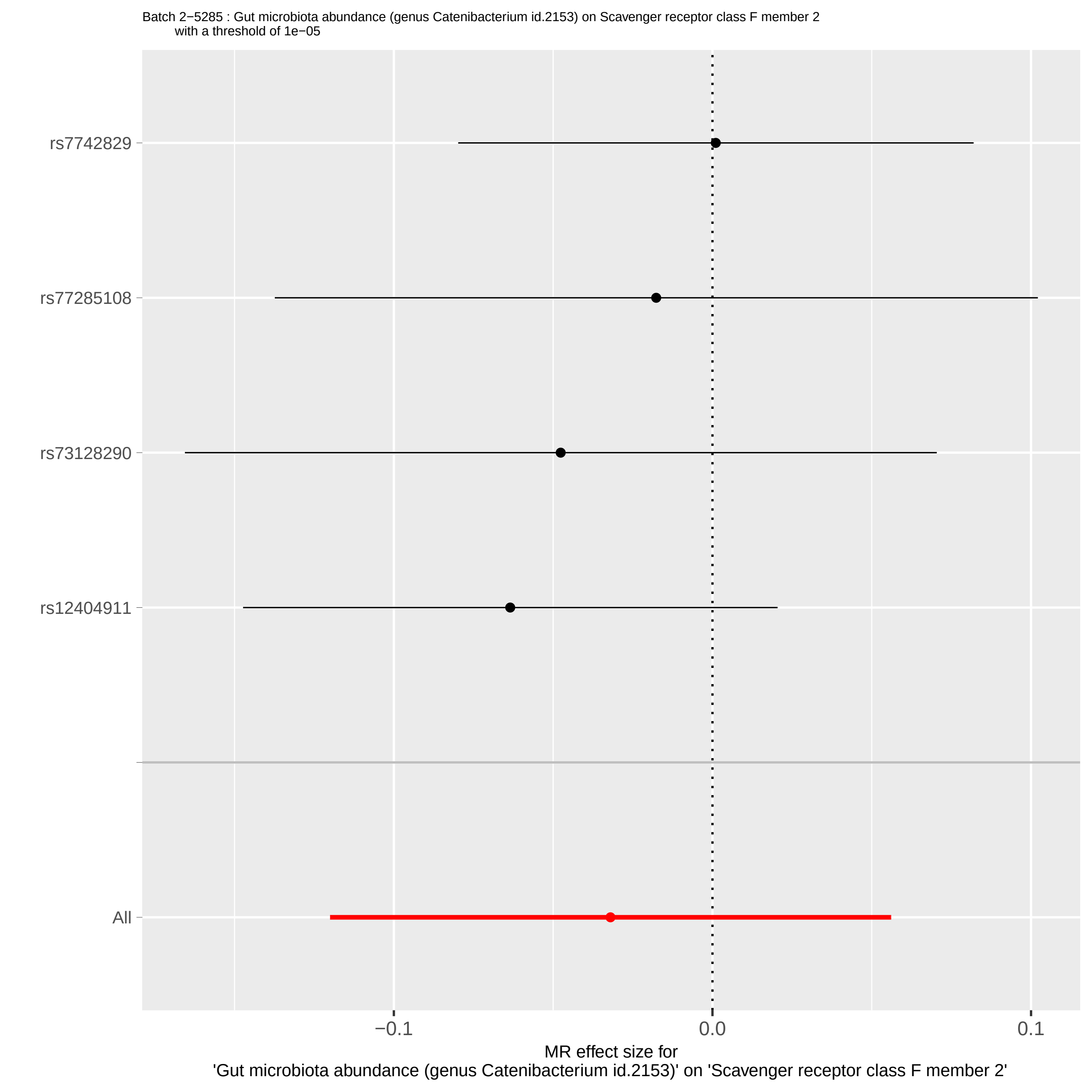

Batch 2−5285 : Gut microbiota abundance (genus Catenibacterium id.2153) on Scavenger receptor class F member 2
with a threshold of 1e−05
rs7742829
rs77285108
rs73128290
rs12404911
All
−0.1
0.0
0.1
MR effect size for
'Gut microbiota abundance (genus Catenibacterium id.2153)' on 'Scavenger receptor class F member 2'

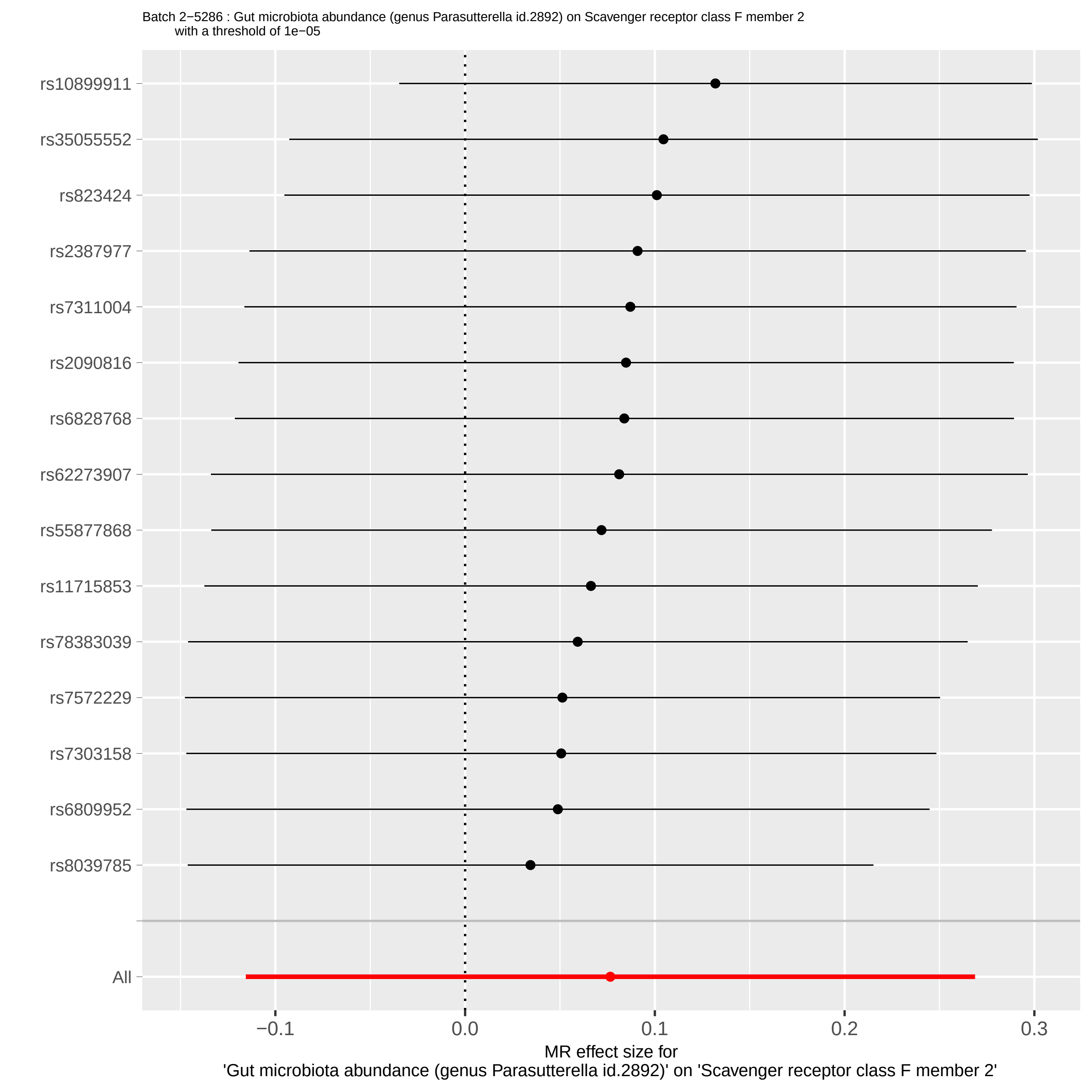

Batch 2−5286 : Gut microbiota abundance (genus Parasutterella id.2892) on Scavenger receptor class F member 2
with a threshold of 1e−05
rs10899911
rs35055552
rs823424
rs2387977
rs7311004
rs2090816
rs6828768
rs62273907
rs55877868
rs11715853
rs78383039
rs7572229
rs7303158
rs6809952
rs8039785
All
−0.1
0.0
0.1
0.2
0.3
MR effect size for
'Gut microbiota abundance (genus Parasutterella id.2892)' on 'Scavenger receptor class F member 2'

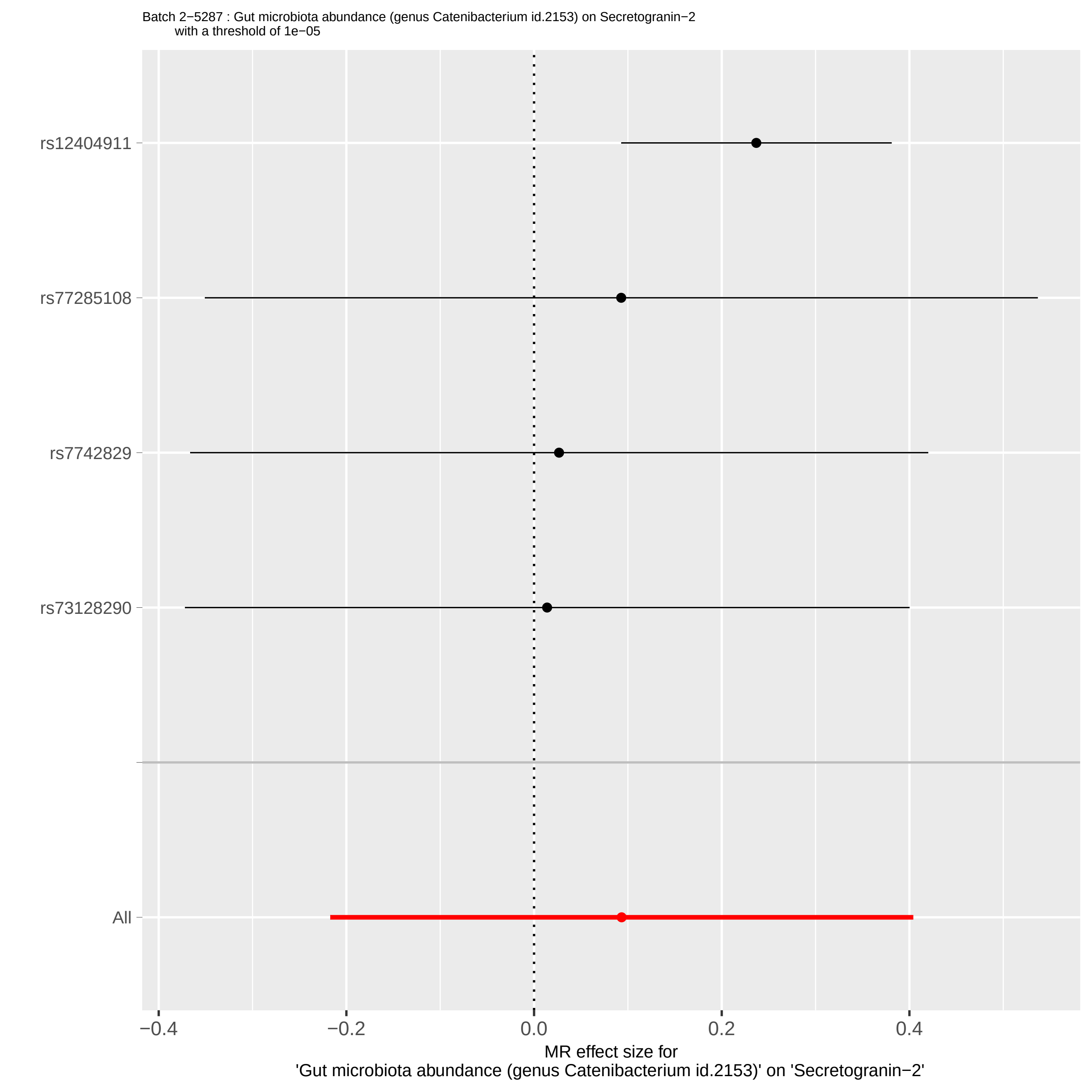
Batch 2−5287 : Gut microbiota abundance (genus Catenibacterium id.2153) on Secretogranin−2
with a threshold of 1e−05
rs12404911
rs77285108
rs7742829
rs73128290
All
−0.4
−0.2
0.0
0.2
0.4
MR effect size for
'Gut microbiota abundance (genus Catenibacterium id.2153)' on 'Secretogranin−2'

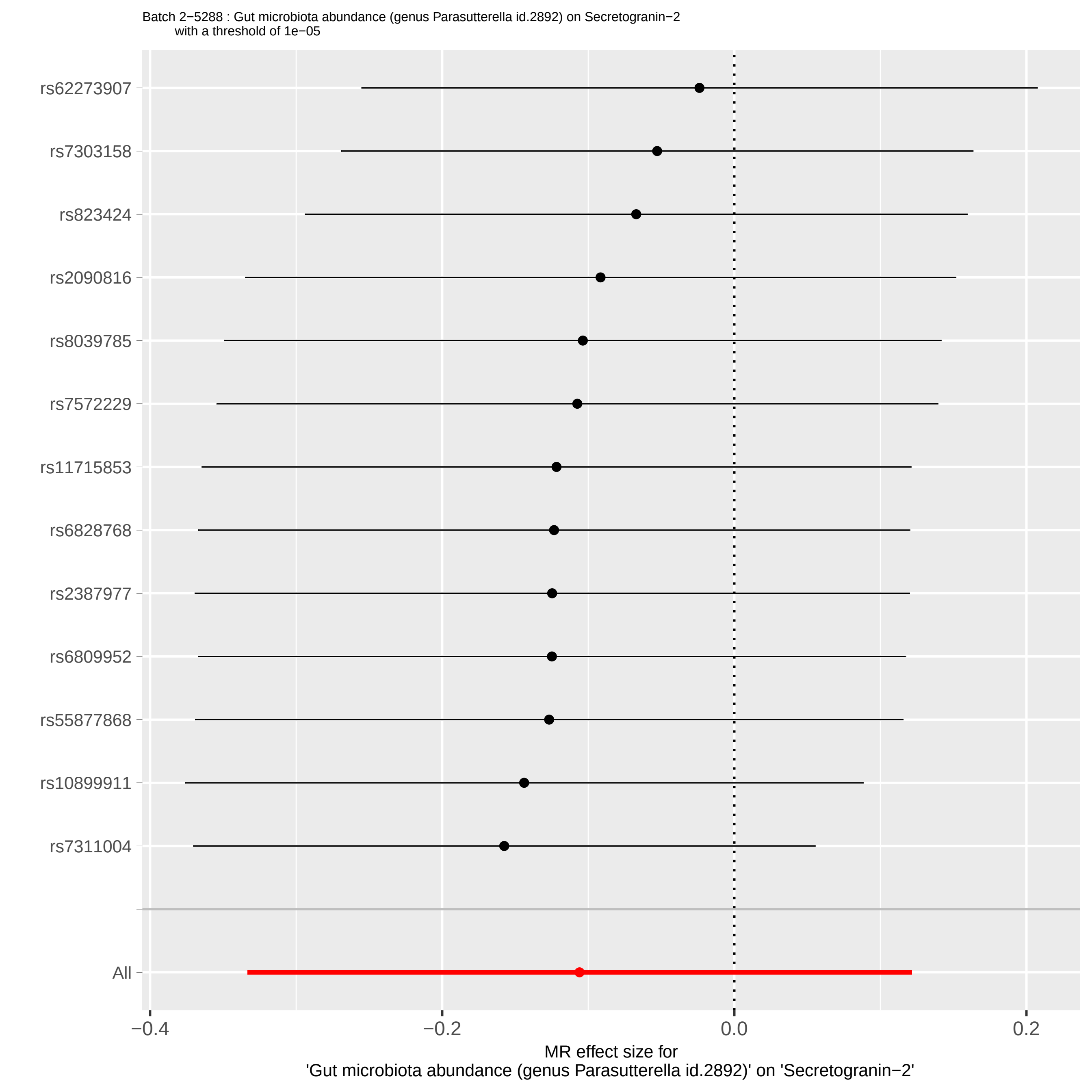
Batch 2−5288 : Gut microbiota abundance (genus Parasutterella id.2892) on Secretogranin−2
with a threshold of 1e−05
rs62273907
rs7303158
rs823424
rs2090816
rs8039785
rs7572229
rs11715853
rs6828768
rs2387977
rs6809952
rs55877868
rs10899911
rs7311004
All
−0.4
−0.2
0.0
0.2
MR effect size for
'Gut microbiota abundance (genus Parasutterella id.2892)' on 'Secretogranin−2'

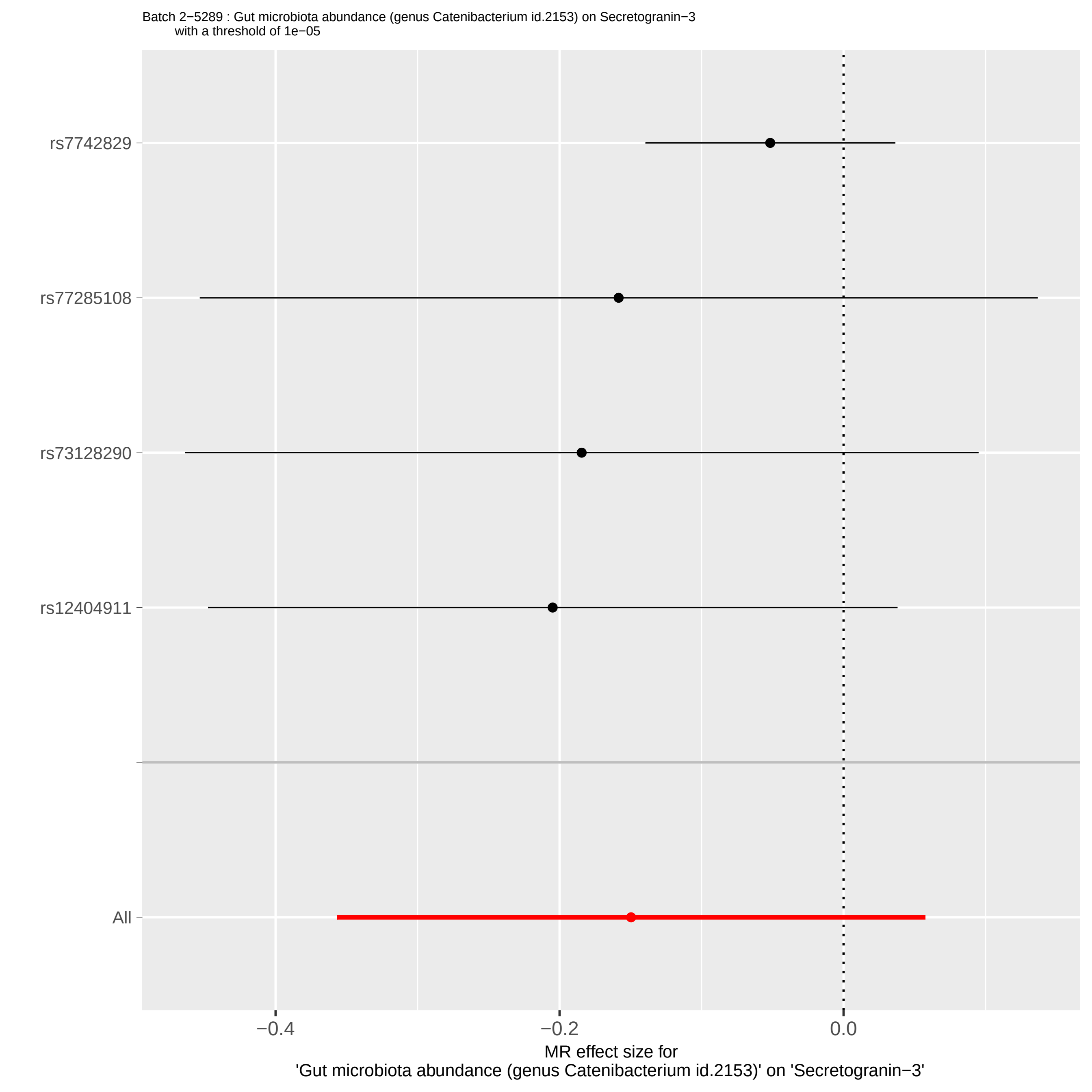
Batch 2−5289 : Gut microbiota abundance (genus Catenibacterium id.2153) on Secretogranin−3
with a threshold of 1e−05
rs7742829
rs77285108
rs73128290
rs12404911
All
−0.4
−0.2
0.0
MR effect size for
'Gut microbiota abundance (genus Catenibacterium id.2153)' on 'Secretogranin−3'

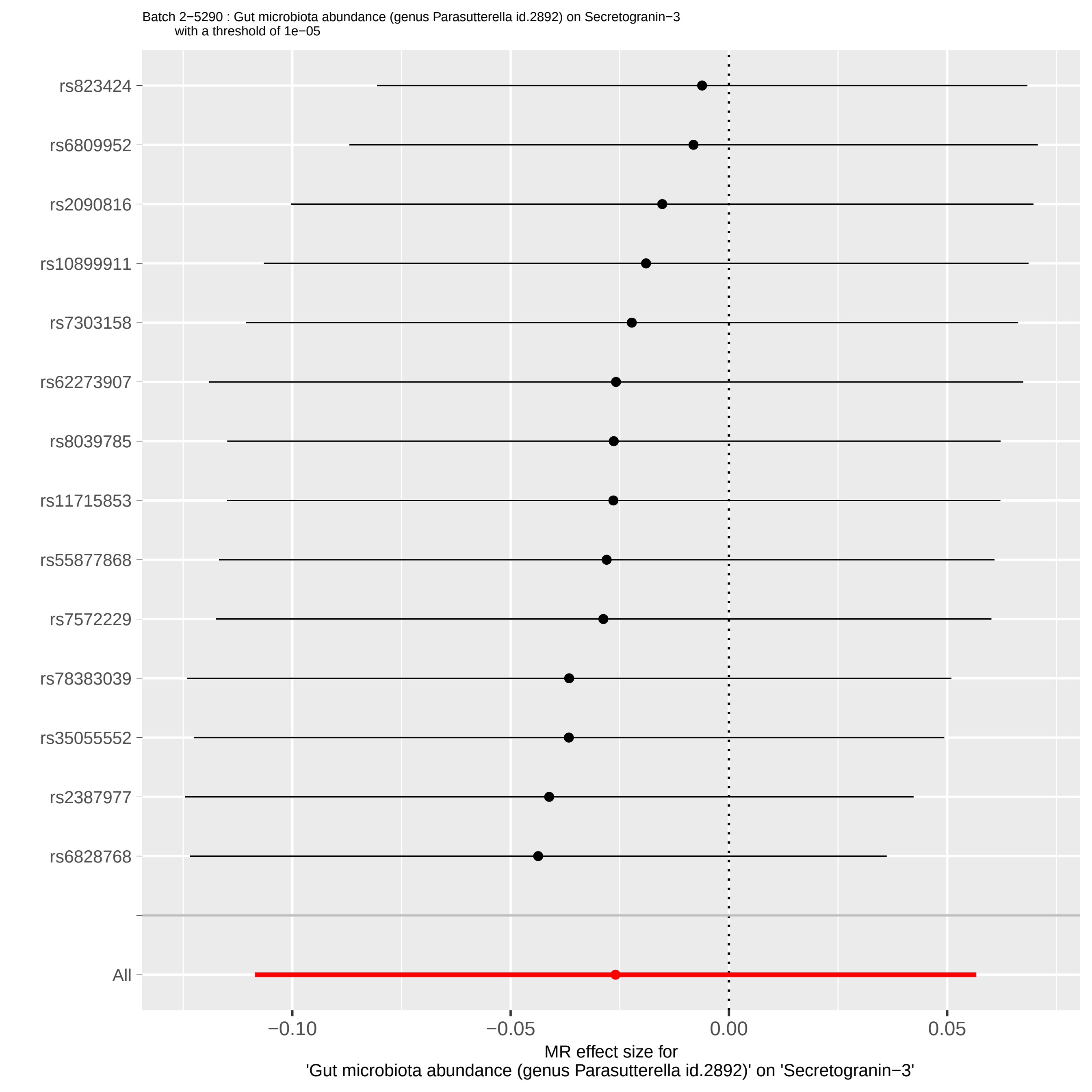

Batch 2−5290 : Gut microbiota abundance (genus Parasutterella id.2892) on Secretogranin−3
with a threshold of 1e−05
rs823424
rs6809952
rs2090816
rs10899911
rs7303158
rs62273907
rs8039785
rs11715853
rs55877868
rs7572229
rs78383039
rs35055552
rs2387977
rs6828768
All
−0.10
−0.05
0.00
0.05
MR effect size for
'Gut microbiota abundance (genus Parasutterella id.2892)' on 'Secretogranin−3'

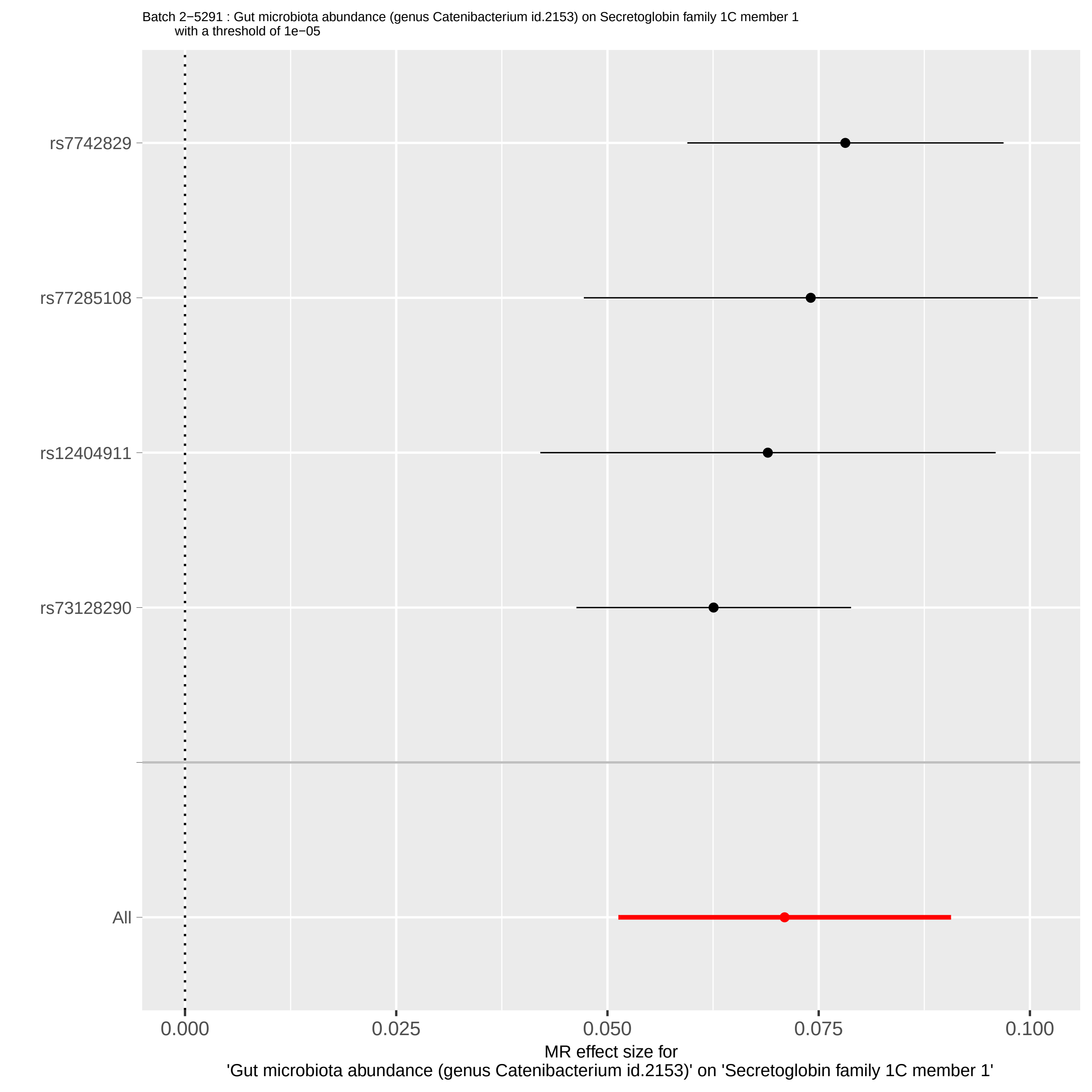
Batch 2−5291 : Gut microbiota abundance (genus Catenibacterium id.2153) on Secretoglobin family 1C member 1
with a threshold of 1e−05
rs7742829
rs77285108
rs12404911
rs73128290
All
0.000
0.025
0.050
0.075
0.100
MR effect size for
'Gut microbiota abundance (genus Catenibacterium id.2153)' on 'Secretoglobin family 1C member 1'

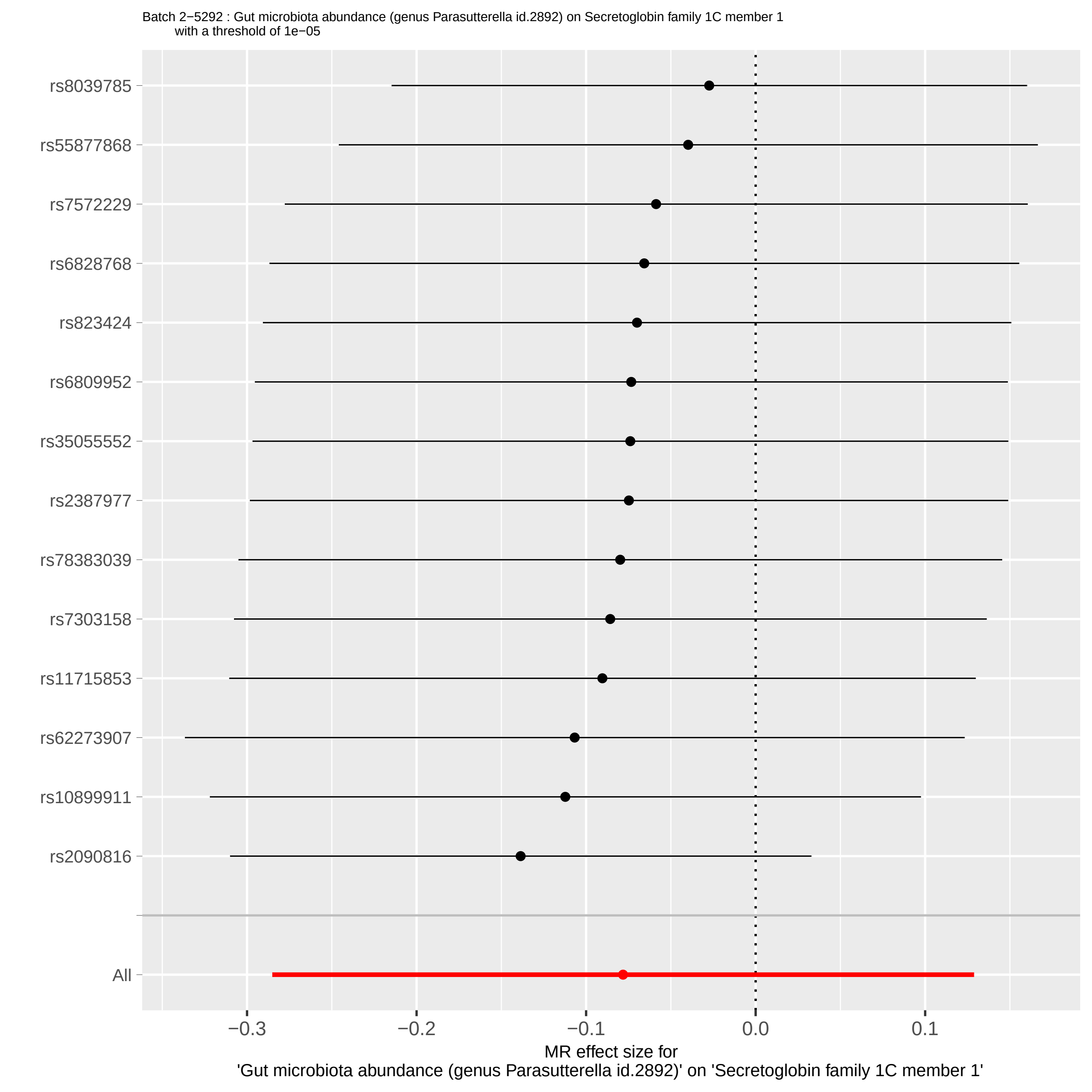

Batch 2−5292 : Gut microbiota abundance (genus Parasutterella id.2892) on Secretoglobin family 1C member 1
with a threshold of 1e−05
rs8039785
rs55877868
rs7572229
rs6828768
rs823424
rs6809952
rs35055552
rs2387977
rs78383039
rs7303158
rs11715853
rs62273907
rs10899911
rs2090816
All
−0.3
−0.2
−0.1
0.0
0.1
MR effect size for
'Gut microbiota abundance (genus Parasutterella id.2892)' on 'Secretoglobin family 1C member 1'

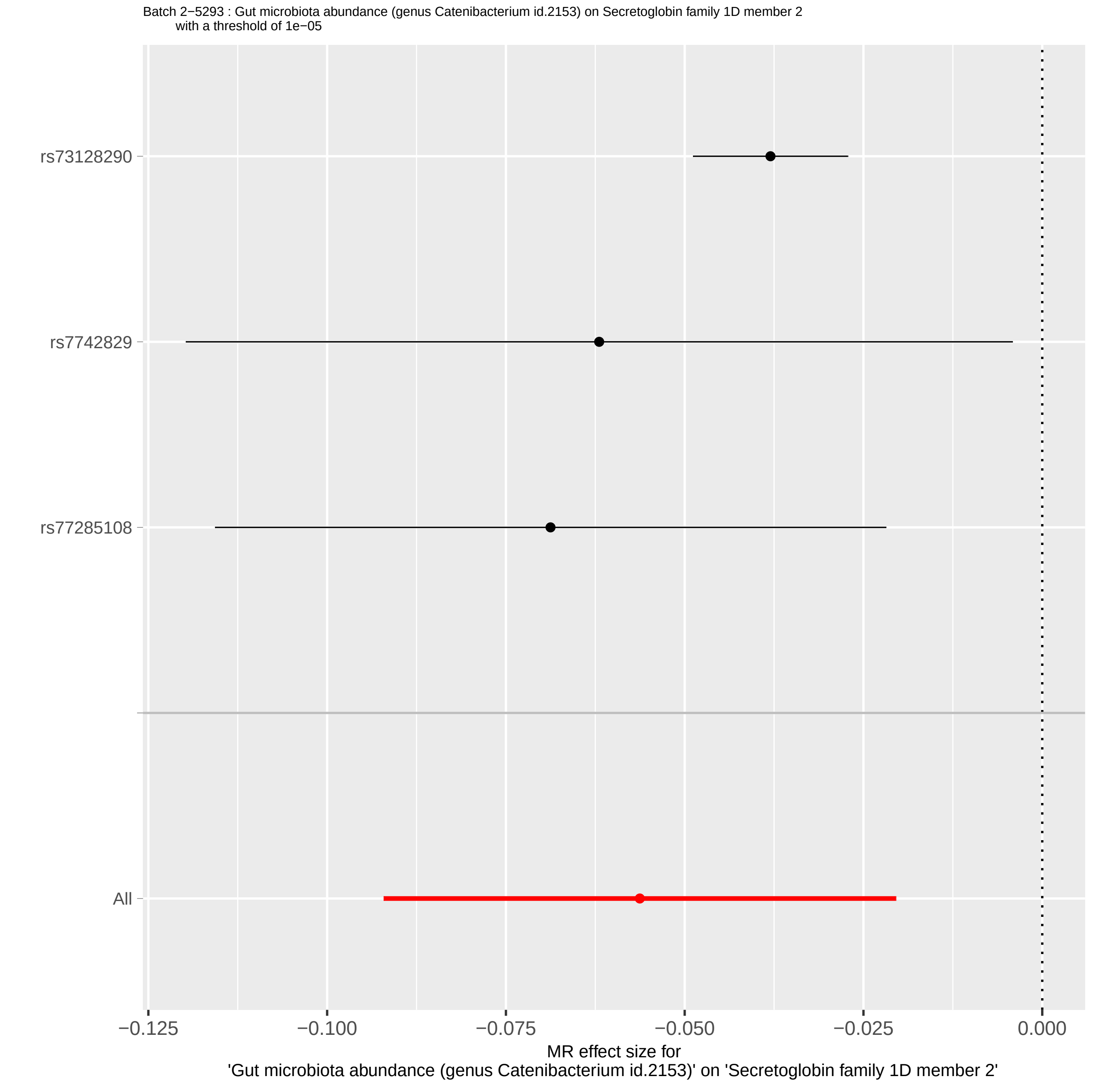

Batch 2−5293 : Gut microbiota abundance (genus Catenibacterium id.2153) on Secretoglobin family 1D member 2
with a threshold of 1e−05
rs73128290
rs7742829
rs77285108
All
−0.125
−0.100
−0.075
−0.050
−0.025
0.000
MR effect size for
'Gut microbiota abundance (genus Catenibacterium id.2153)' on 'Secretoglobin family 1D member 2'

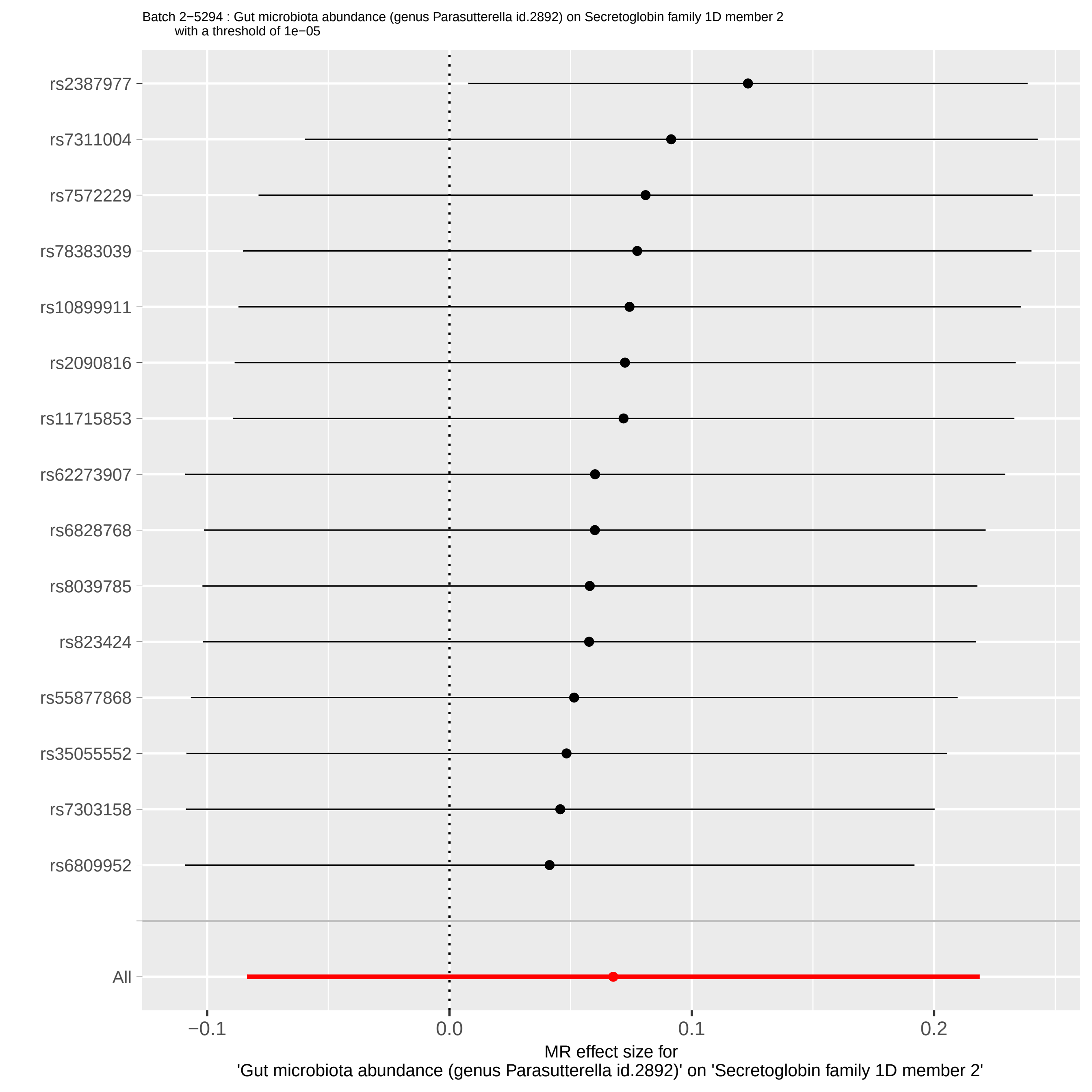
Batch 2−5294 : Gut microbiota abundance (genus Parasutterella id.2892) on Secretoglobin family 1D member 2
with a threshold of 1e−05
rs2387977
rs7311004
rs7572229
rs78383039
rs10899911
rs2090816
rs11715853
rs62273907
rs6828768
rs8039785
rs823424
rs55877868
rs35055552
rs7303158
rs6809952
All
−0.1
0.0
0.1
0.2
MR effect size for
'Gut microbiota abundance (genus Parasutterella id.2892)' on 'Secretoglobin family 1D member 2'

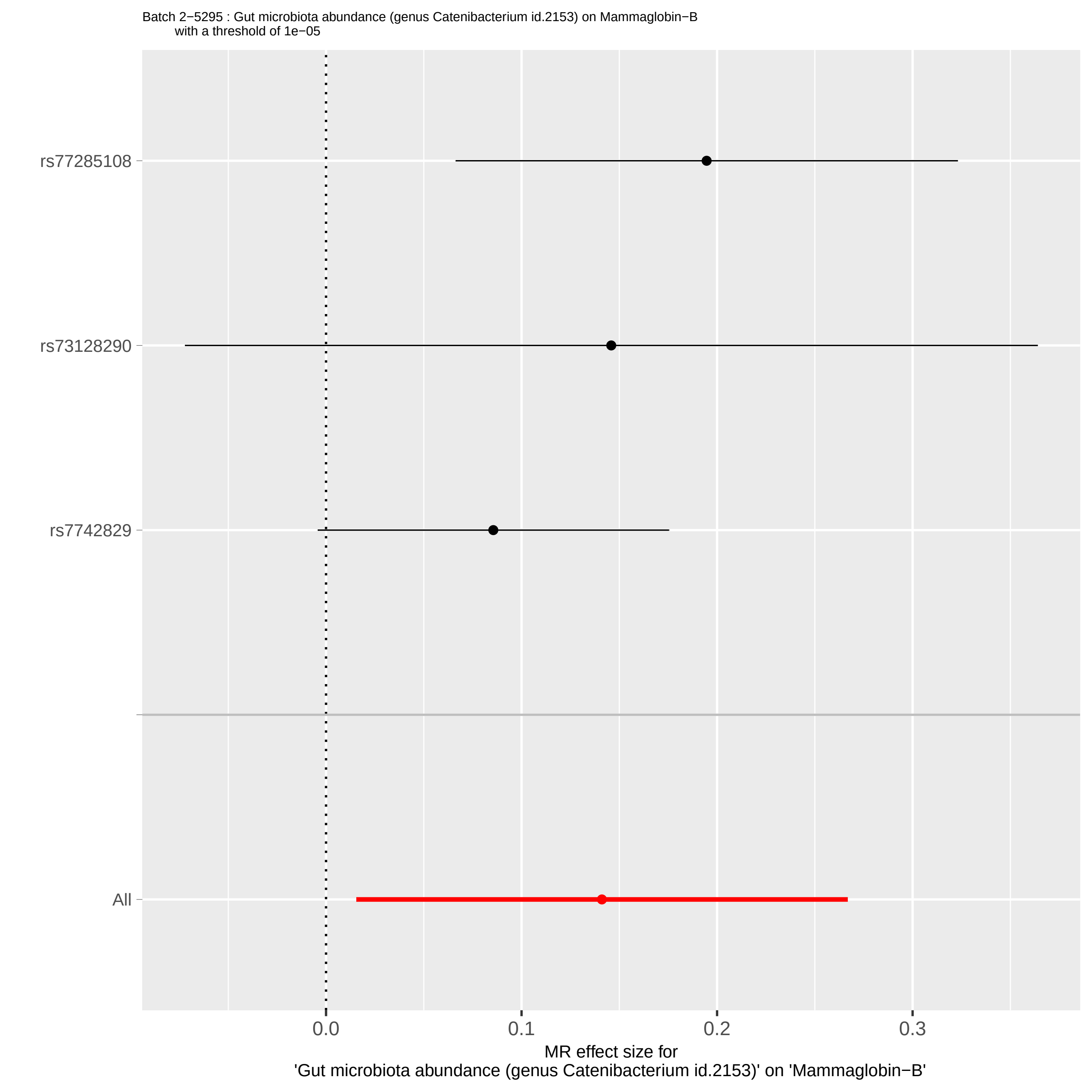
Batch 2−5295 : Gut microbiota abundance (genus Catenibacterium id.2153) on Mammaglobin−B
with a threshold of 1e−05
rs77285108
rs73128290
rs7742829
All
0.0
0.1
0.2
0.3
MR effect size for
'Gut microbiota abundance (genus Catenibacterium id.2153)' on 'Mammaglobin−B'

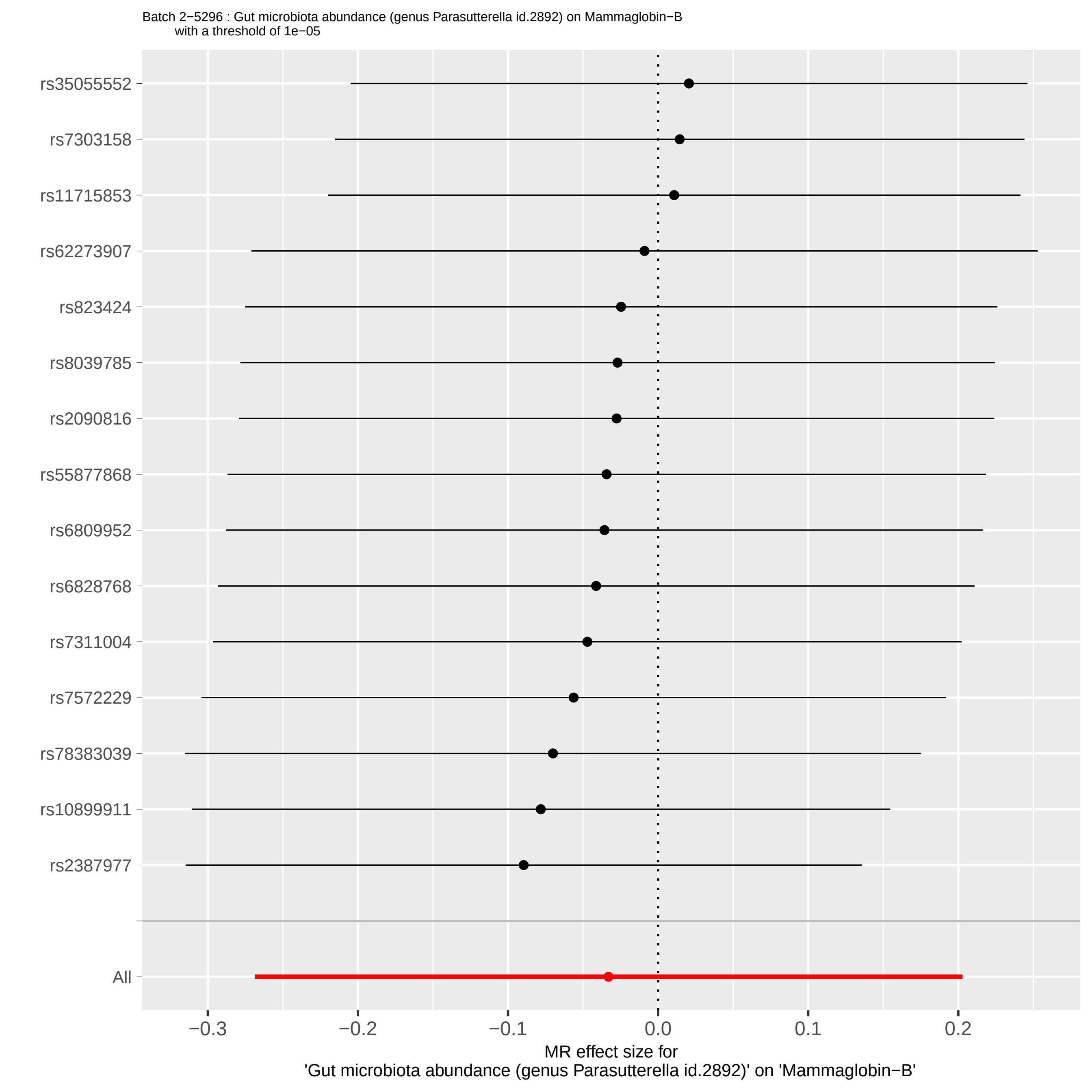

Batch 2−5296 : Gut microbiota abundance (genus Parasutterella id.2892) on Mammaglobin−B
with a threshold of 1e−05
rs35055552
rs7303158
rs11715853
rs62273907
rs823424
rs8039785
rs2090816
rs55877868
rs6809952
rs6828768
rs7311004
rs7572229
rs78383039
rs10899911
rs2387977
All
−0.3
−0.2
−0.1
0.0
0.1
0.2
MR effect size for
'Gut microbiota abundance (genus Parasutterella id.2892)' on 'Mammaglobin−B'

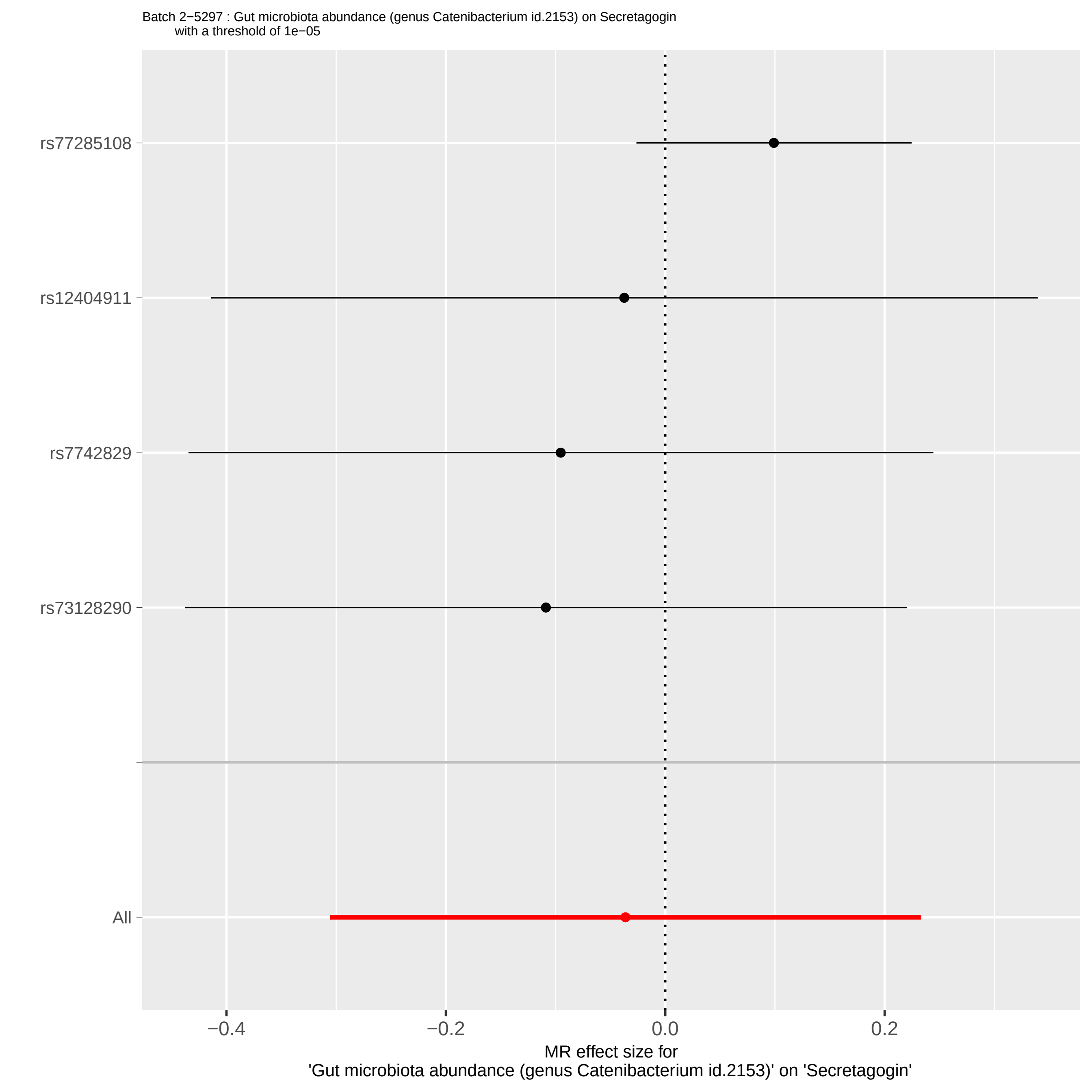
Batch 2−5297 : Gut microbiota abundance (genus Catenibacterium id.2153) on Secretagogin
with a threshold of 1e−05
rs77285108
rs12404911
rs7742829
rs73128290
All
−0.4
−0.2
0.0
0.2
MR effect size for
'Gut microbiota abundance (genus Catenibacterium id.2153)' on 'Secretagogin'

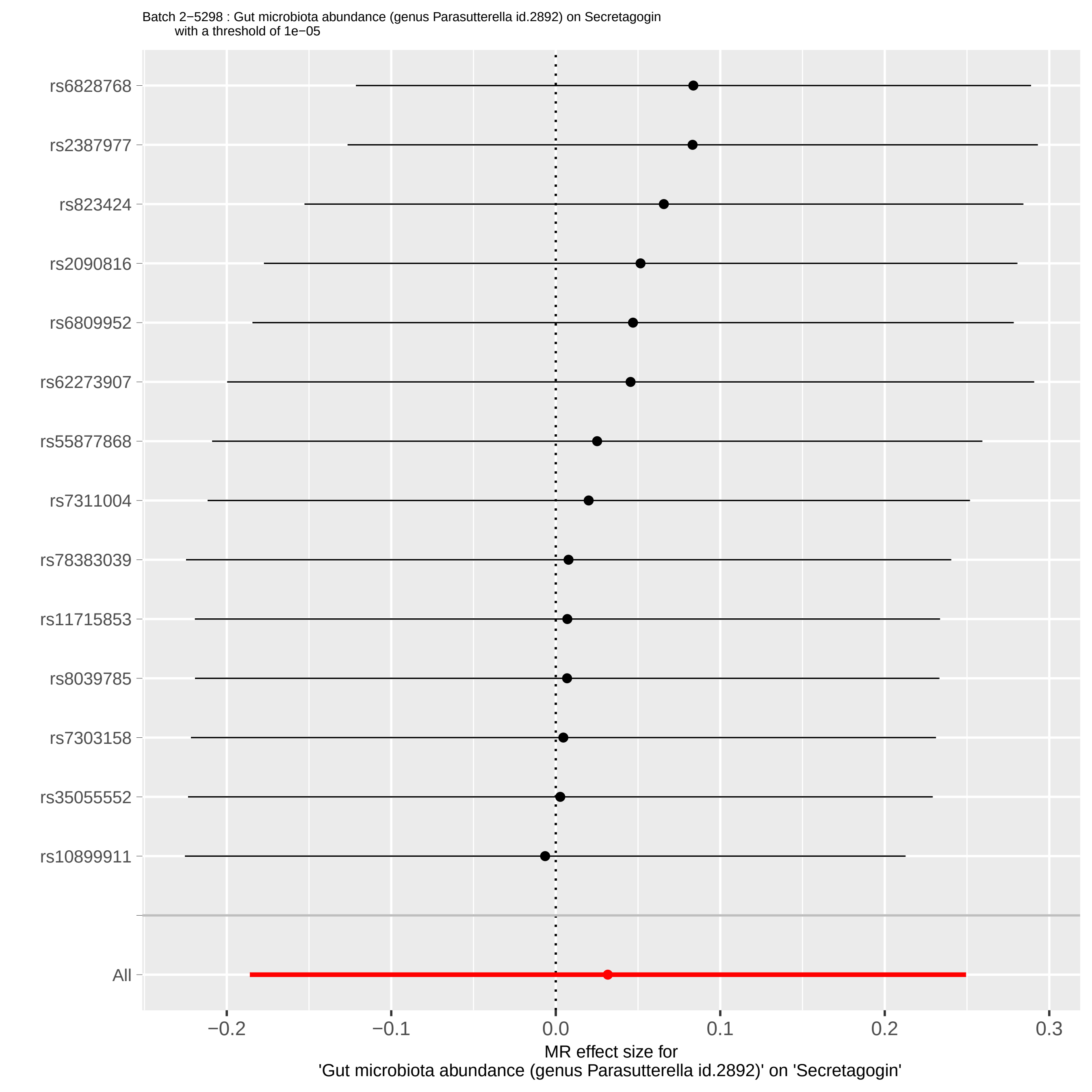
Batch 2−5298 : Gut microbiota abundance (genus Parasutterella id.2892) on Secretagogin
with a threshold of 1e−05
rs6828768
rs2387977
rs823424
rs2090816
rs6809952
rs62273907
rs55877868
rs7311004
rs78383039
rs11715853
rs8039785
rs7303158
rs35055552
rs10899911
All
−0.2
−0.1
0.0
0.1
0.2
0.3
MR effect size for
'Gut microbiota abundance (genus Parasutterella id.2892)' on 'Secretagogin'

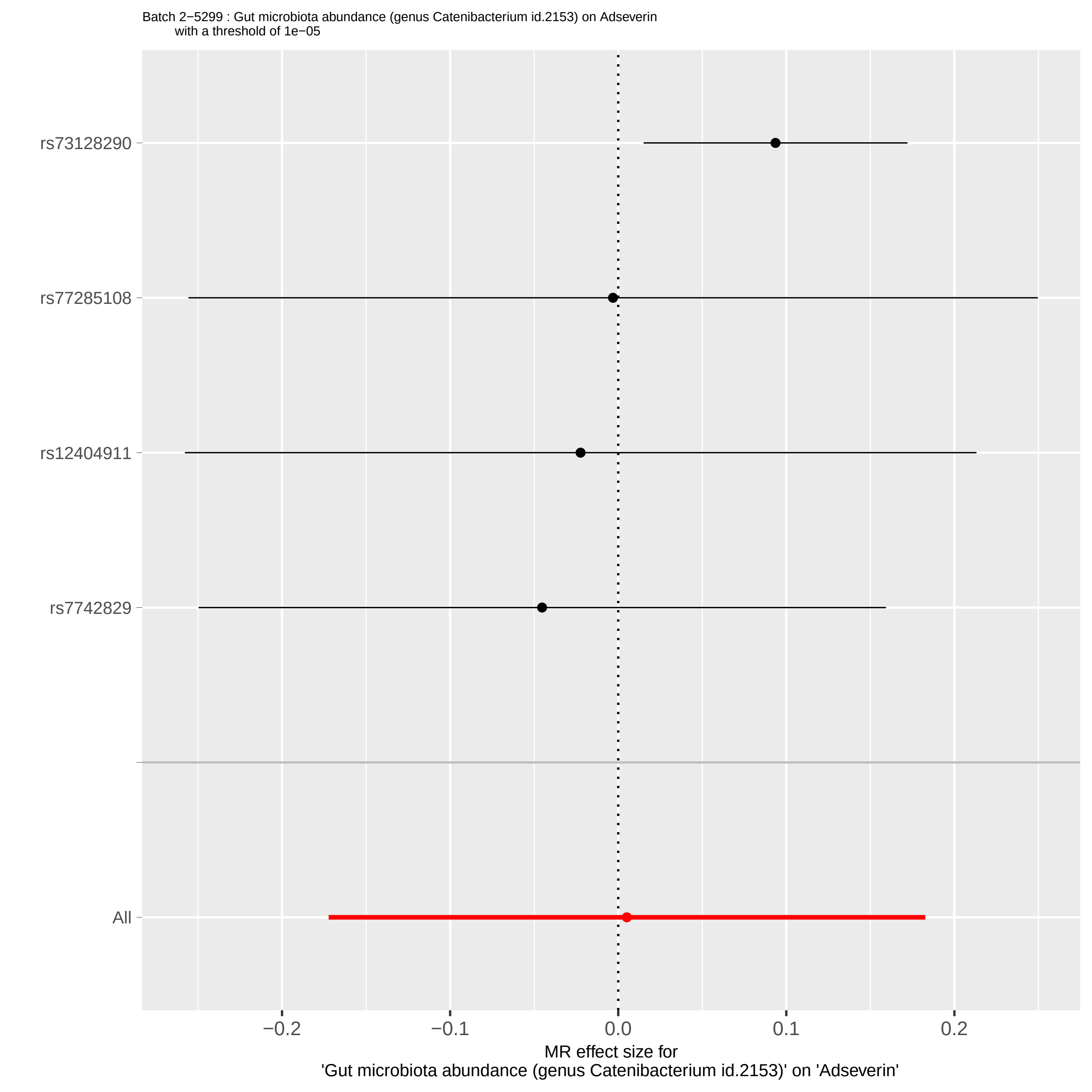
Batch 2−5299 : Gut microbiota abundance (genus Catenibacterium id.2153) on Adseverin
with a threshold of 1e−05
rs73128290
rs77285108
rs12404911
rs7742829
All
−0.2
−0.1
0.0
0.1
0.2
MR effect size for
'Gut microbiota abundance (genus Catenibacterium id.2153)' on 'Adseverin'

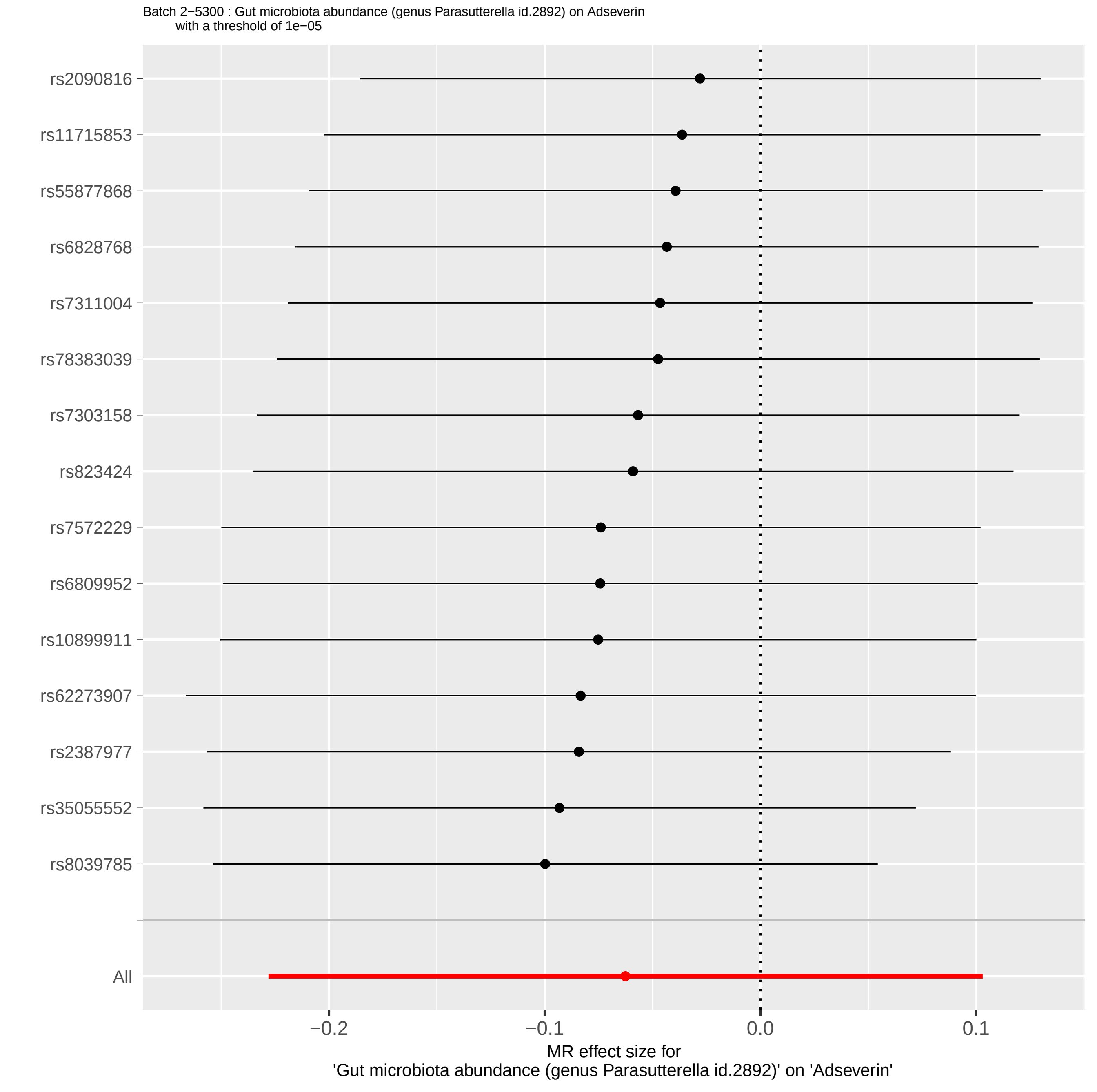

Batch 2−5300 : Gut microbiota abundance (genus Parasutterella id.2892) on Adseverin
with a threshold of 1e−05
rs2090816
rs11715853
rs55877868
rs6828768
rs7311004
rs78383039
rs7303158
rs823424
rs7572229
rs6809952
rs10899911
rs62273907
rs2387977
rs35055552
rs8039785
All
−0.2
−0.1
0.0
0.1
MR effect size for
'Gut microbiota abundance (genus Parasutterella id.2892)' on 'Adseverin'

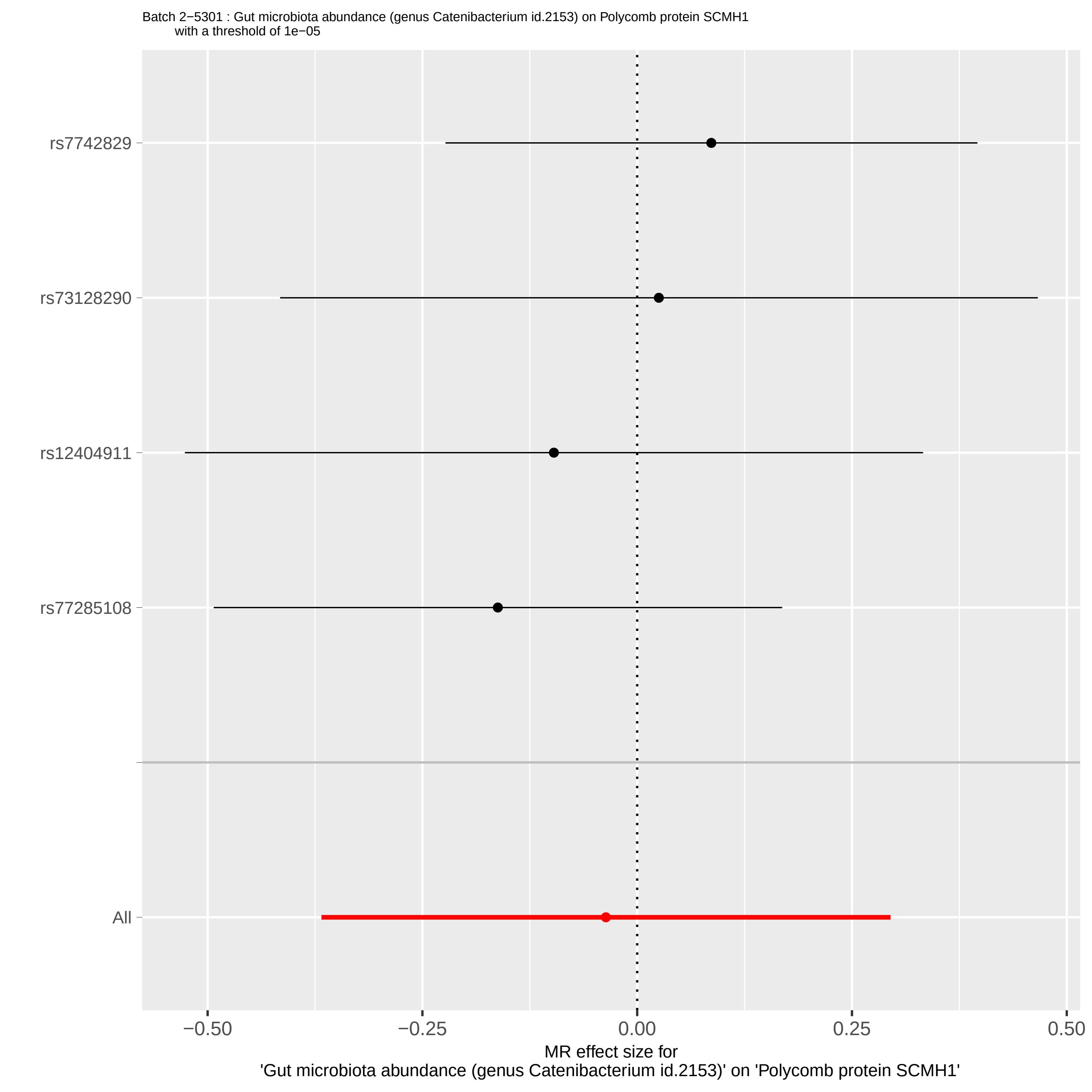

Batch 2−5301 : Gut microbiota abundance (genus Catenibacterium id.2153) on Polycomb protein SCMH1
with a threshold of 1e−05
rs7742829
rs73128290
rs12404911
rs77285108
All
−0.50
−0.25
0.00
0.25
0.50
MR effect size for
'Gut microbiota abundance (genus Catenibacterium id.2153)' on 'Polycomb protein SCMH1'

Batch 2−5302 : Gut microbiota abundance (genus Parasutterella id.2892) on Polycomb protein SCMH1
with a threshold of 1e−05

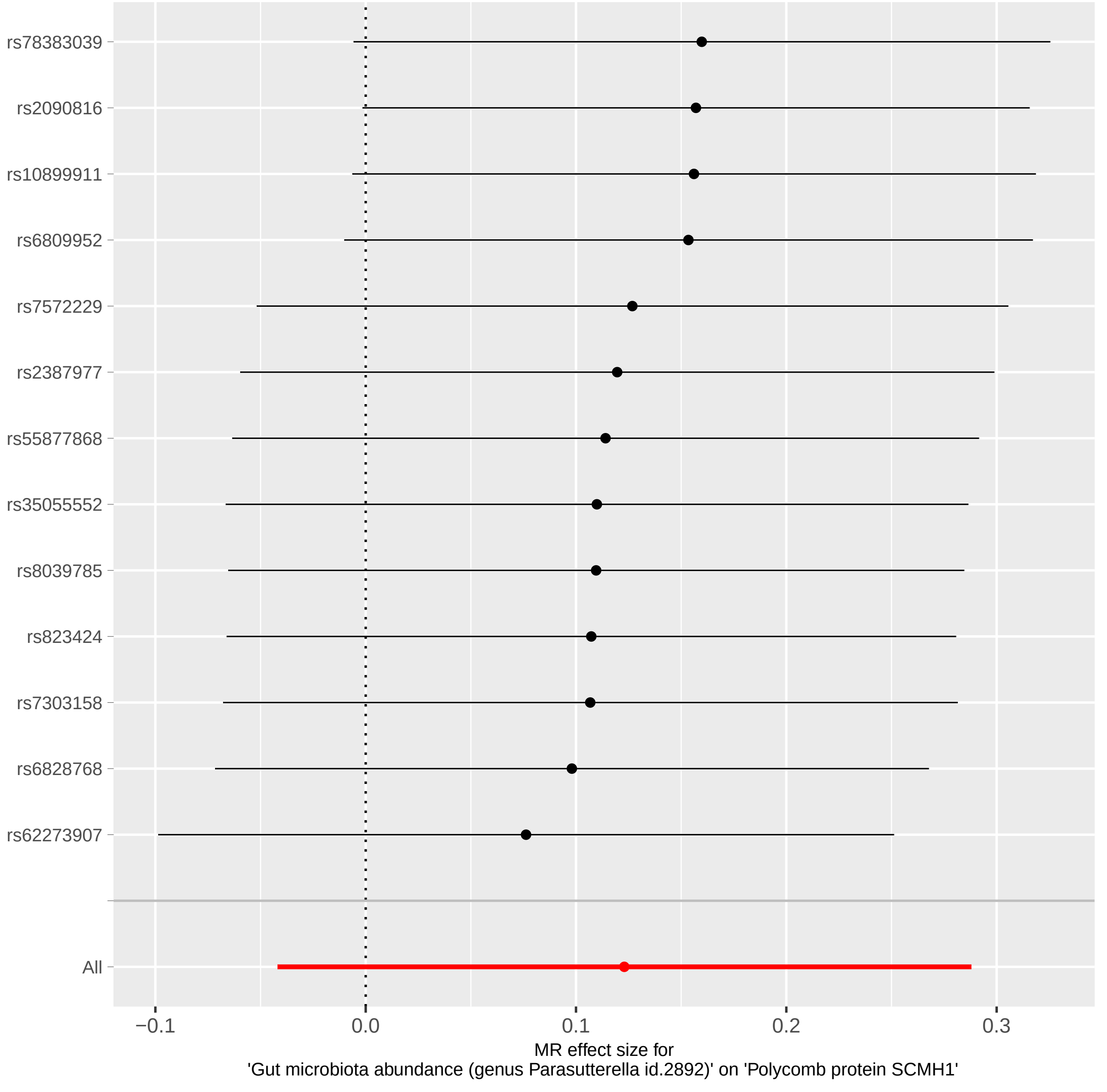

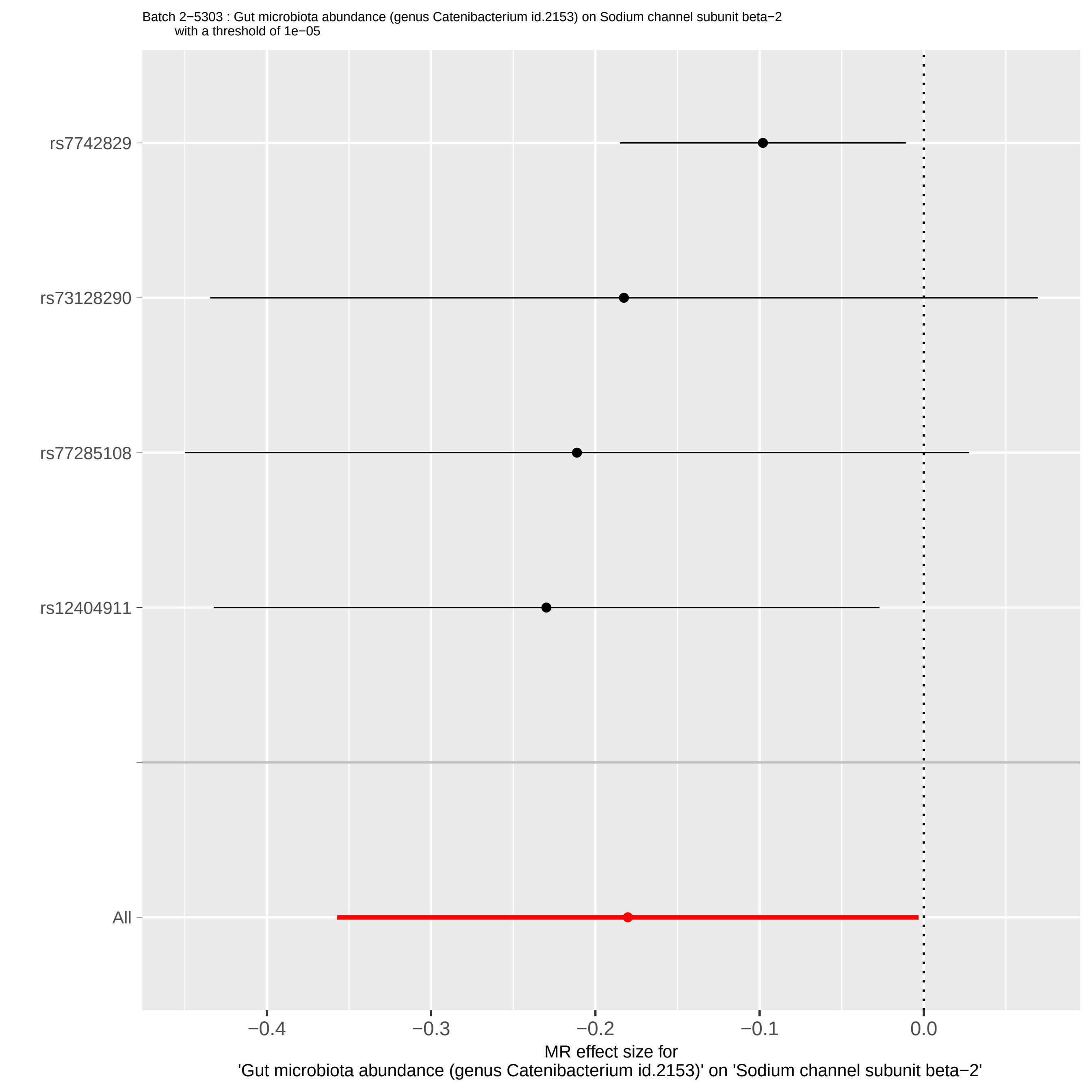

Batch 2−5303 : Gut microbiota abundance (genus Catenibacterium id.2153) on Sodium channel subunit beta−2
with a threshold of 1e−05
rs7742829
rs73128290
rs77285108
rs12404911
All
−0.4
−0.3
−0.2
−0.1
0.0
MR effect size for
'Gut microbiota abundance (genus Catenibacterium id.2153)' on 'Sodium channel subunit beta−2'

Batch 2−5304 : Gut microbiota abundance (genus Parasutterella id.2892) on Sodium channel subunit beta−2
with a threshold of 1e−05

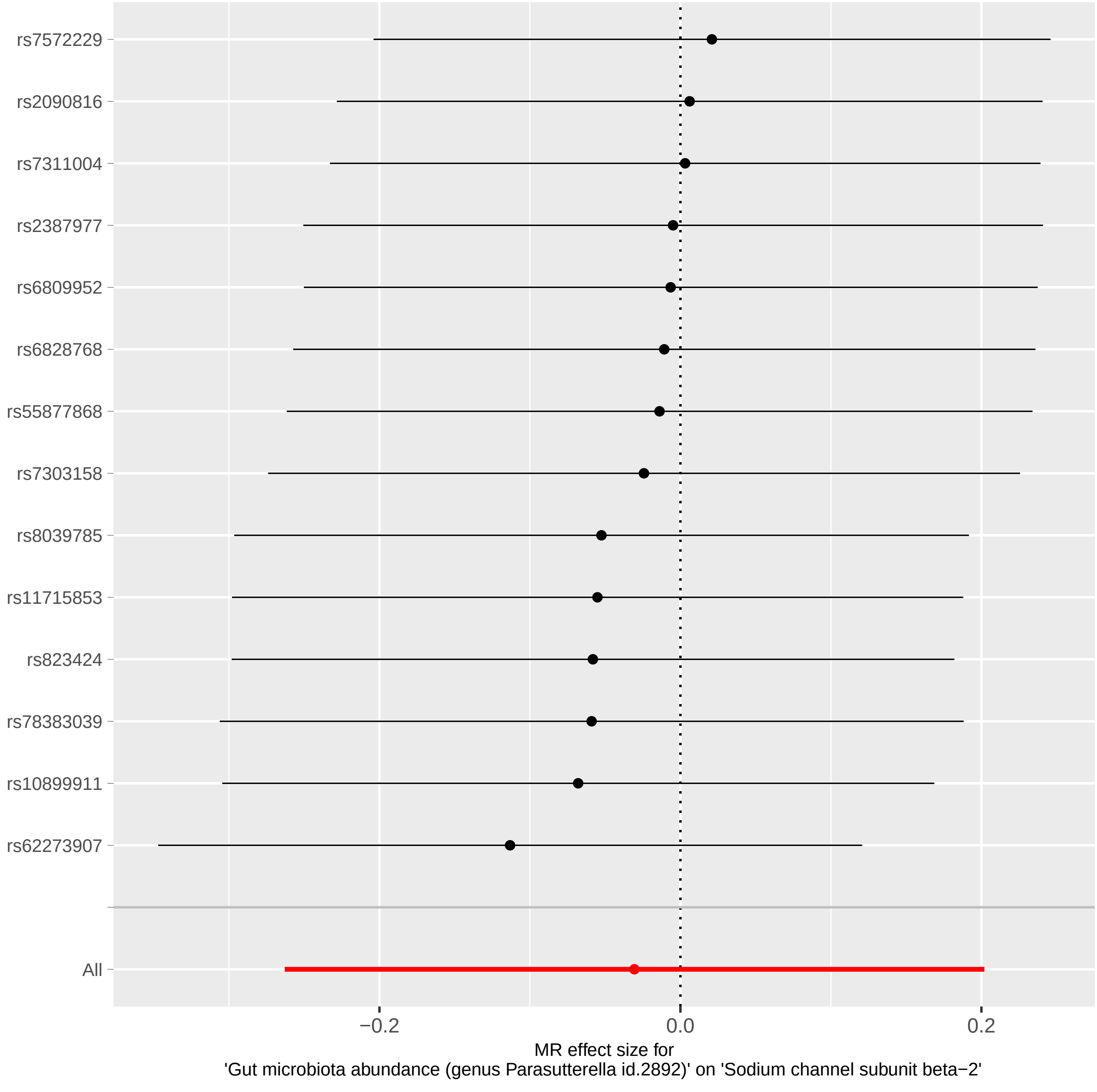

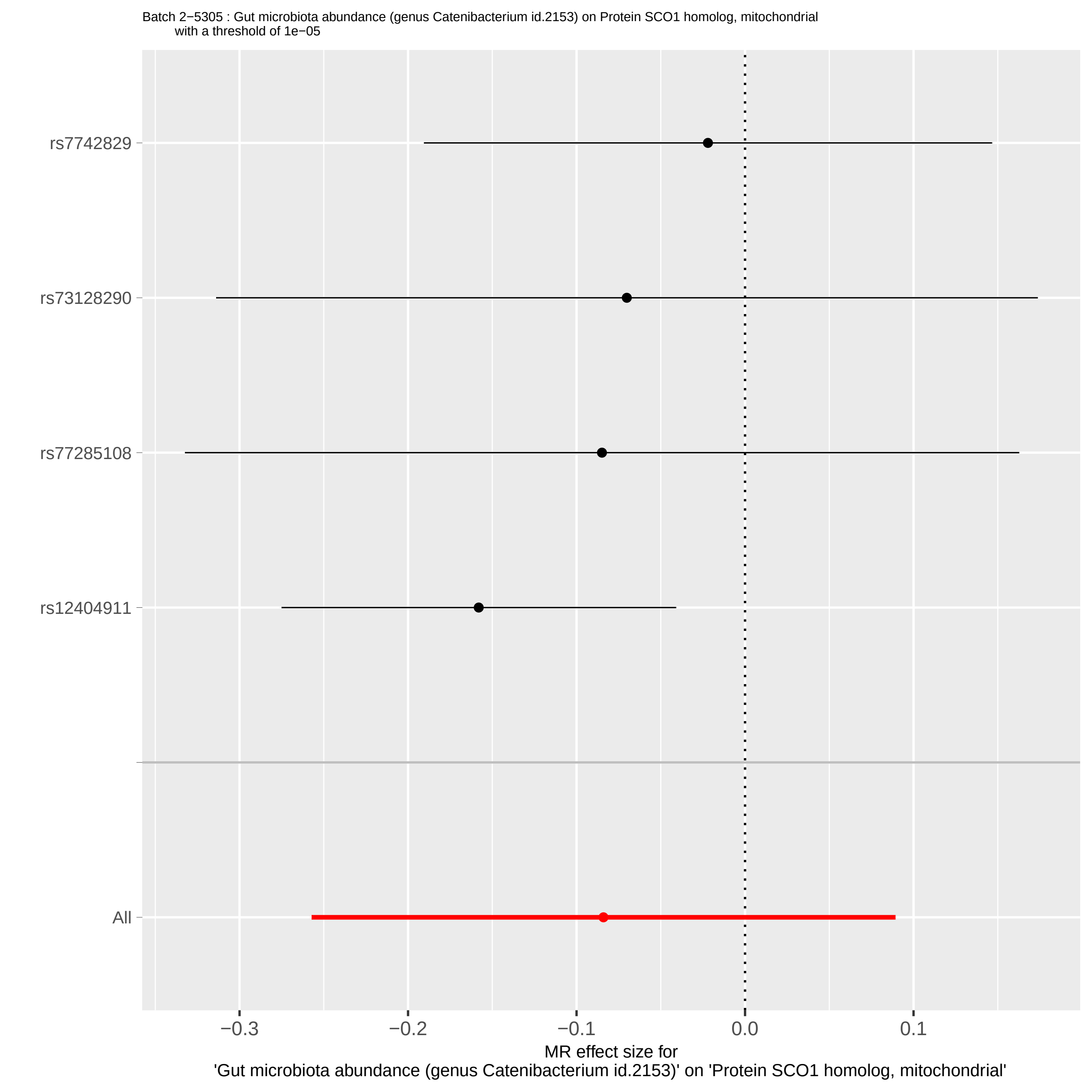
Batch 2−5305 : Gut microbiota abundance (genus Catenibacterium id.2153) on Protein SCO1 homolog, mitochondrial
with a threshold of 1e−05
rs7742829
rs73128290
rs77285108
rs12404911
All
−0.3
−0.2
−0.1
0.0
0.1
MR effect size for
'Gut microbiota abundance (genus Catenibacterium id.2153)' on 'Protein SCO1 homolog, mitochondrial'

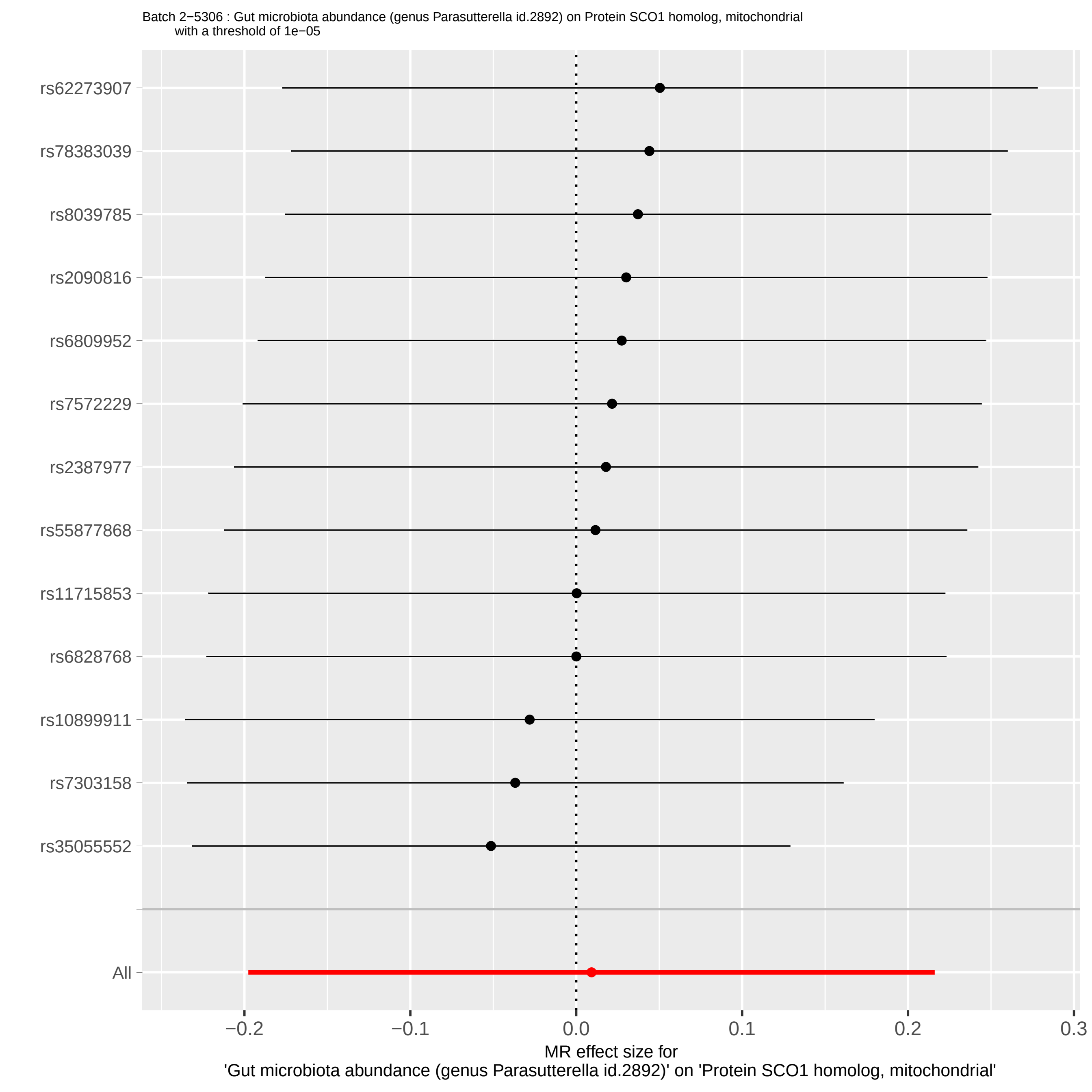
Batch 2−5306 : Gut microbiota abundance (genus Parasutterella id.2892) on Protein SCO1 homolog, mitochondrial
with a threshold of 1e−05
rs62273907
rs78383039
rs8039785
rs2090816
rs6809952
rs7572229
rs2387977
rs55877868
rs11715853
rs6828768
rs10899911
rs7303158
rs35055552
All
−0.2
−0.1
0.0
0.1
0.2
0.3
MR effect size for
'Gut microbiota abundance (genus Parasutterella id.2892)' on 'Protein SCO1 homolog, mitochondrial'

Batch 2−5307 : Gut microbiota abundance (genus Catenibacterium id.2153) on SCP2 sterol−binding domain−containing protein 1
with a threshold of 1e−05

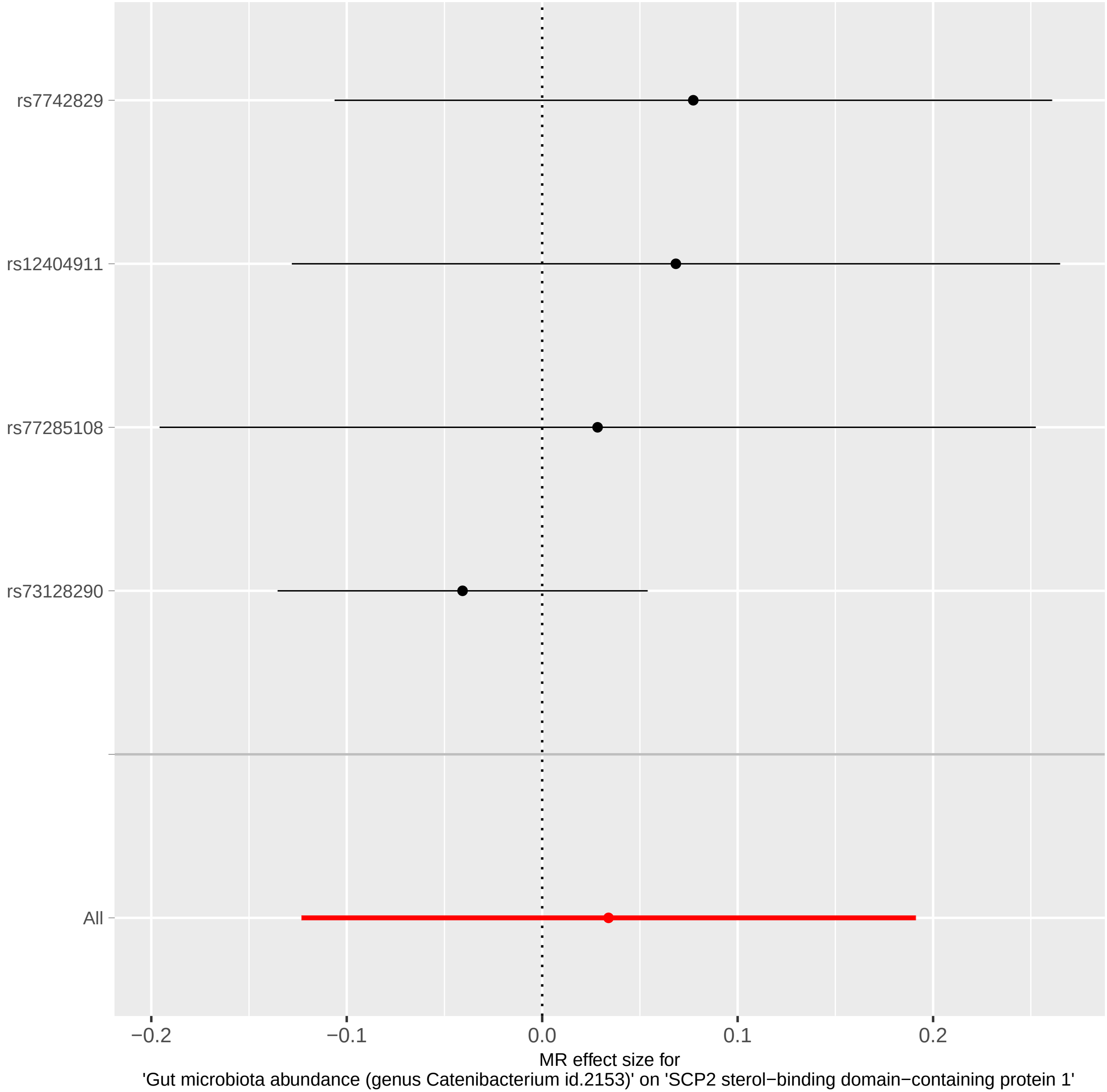

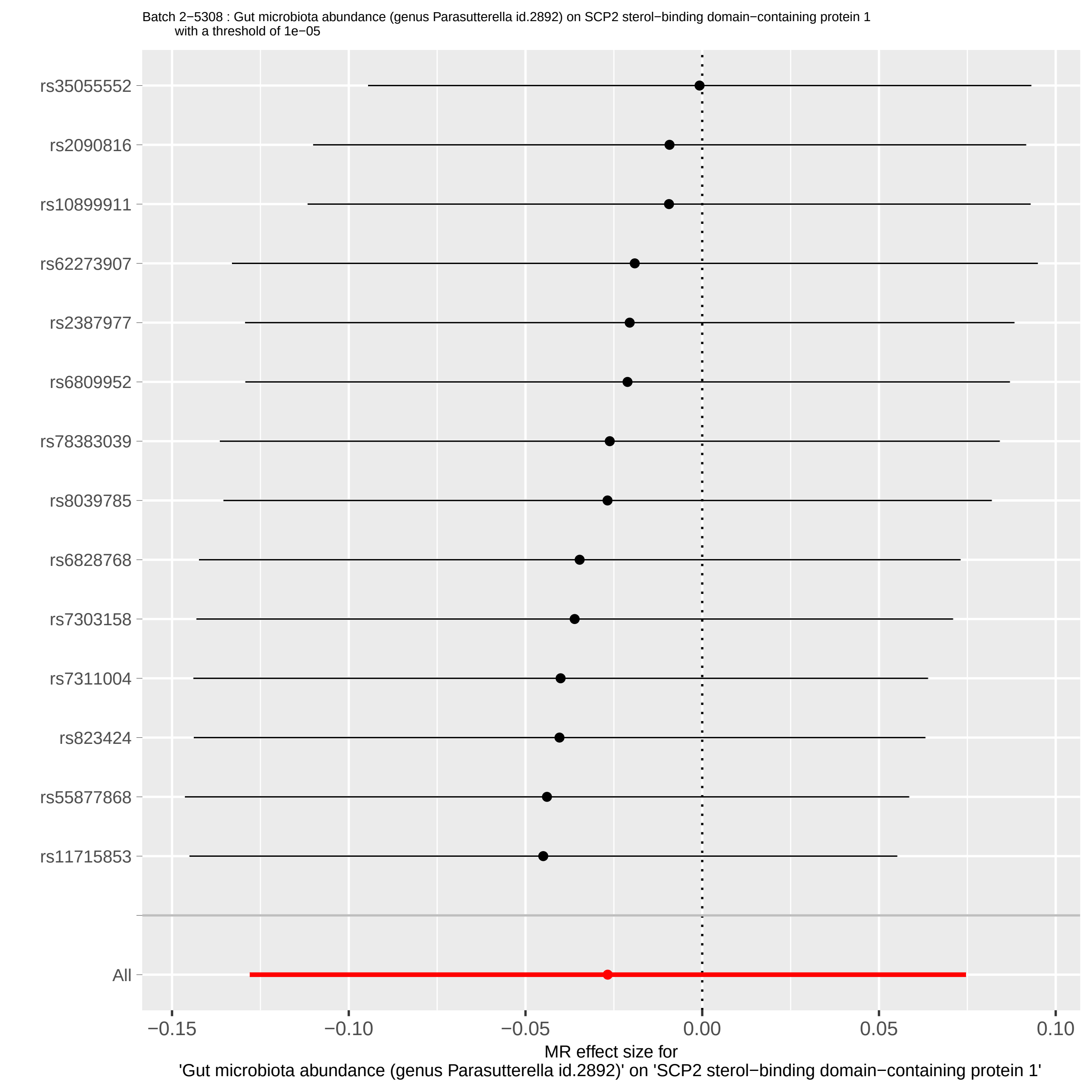

Batch 2−5308 : Gut microbiota abundance (genus Parasutterella id.2892) on SCP2 sterol−binding domain−containing protein 1
with a threshold of 1e−05
rs35055552
rs2090816
rs10899911
rs62273907
rs2387977
rs6809952
rs78383039
rs8039785
rs6828768
rs7303158
rs7311004
rs823424
rs55877868
rs11715853
All
−0.15
−0.10
−0.05
0.00
0.05
0.10
MR effect size for
'Gut microbiota abundance (genus Parasutterella id.2892)' on 'SCP2 sterol−binding domain−containing protein 1'

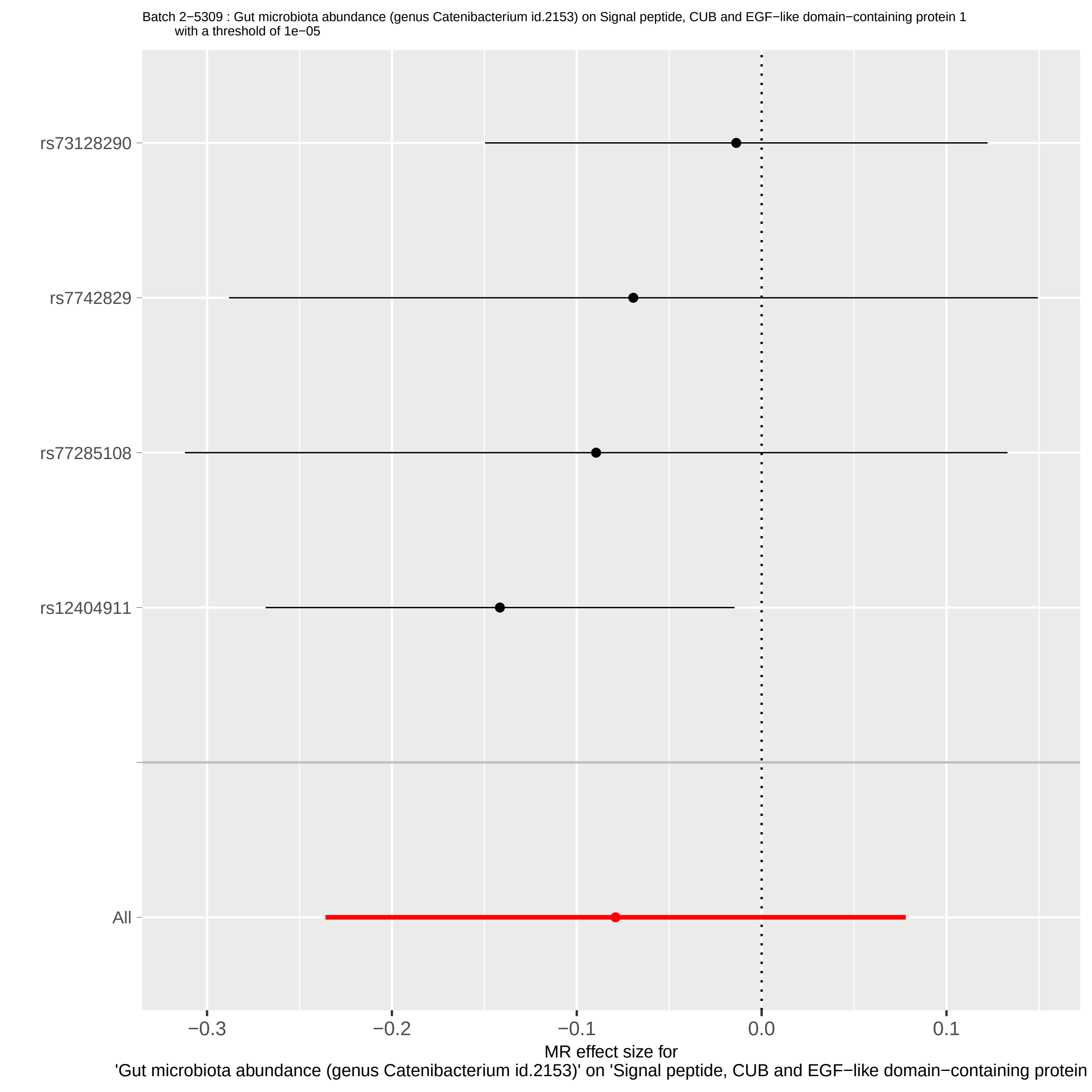
Batch 2−5309 : Gut microbiota abundance (genus Catenibacterium id.2153) on Signal peptide, CUB and EGF−like domain−containing protein 1
with a threshold of 1e−05
rs73128290
rs7742829
rs77285108
rs12404911
All
−0.3
−0.2
−0.1
0.0
0.1
MR effect size for
'Gut microbiota abundance (genus Catenibacterium id.2153)' on 'Signal peptide, CUB and EGF−like domain−containing protein

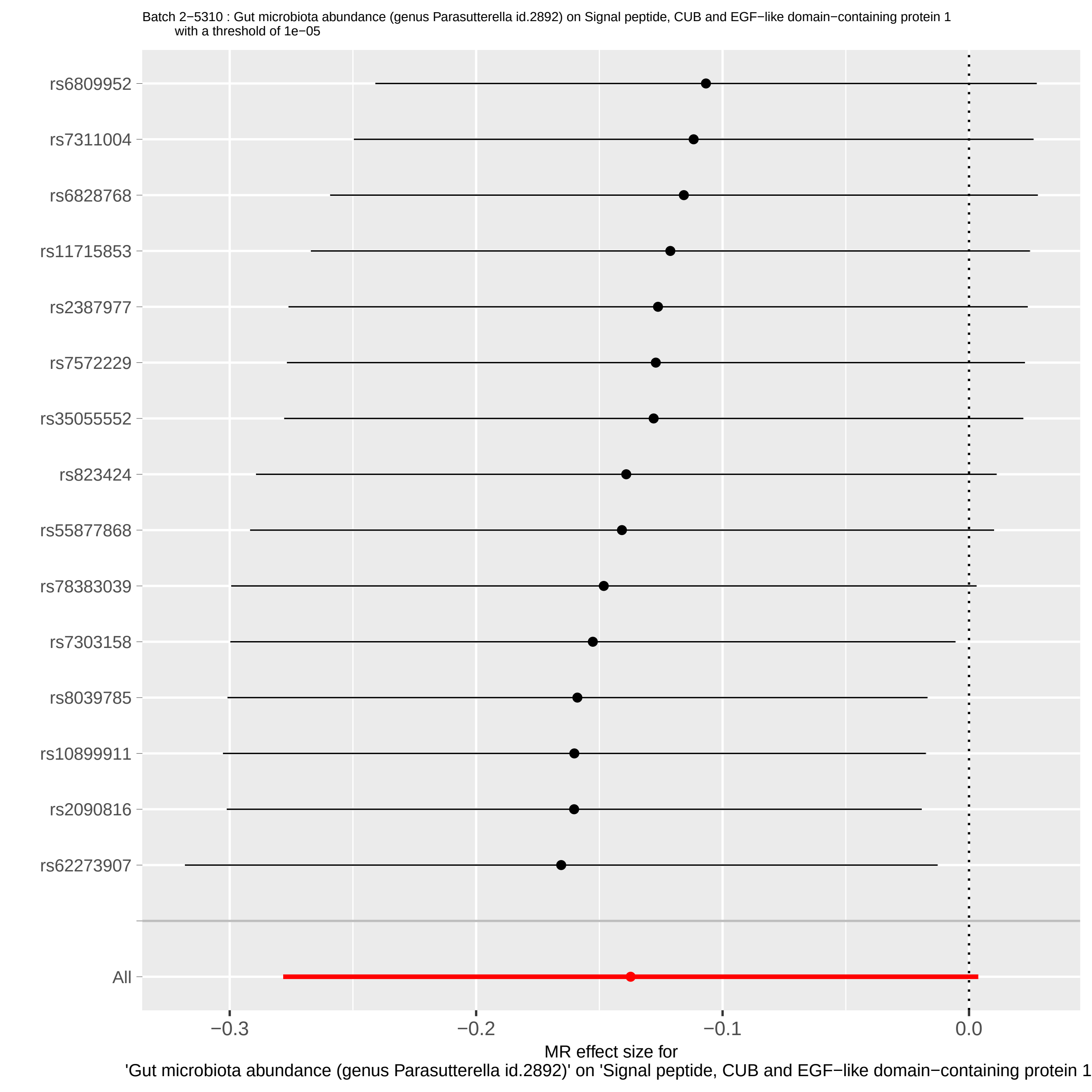
Batch 2−5310 : Gut microbiota abundance (genus Parasutterella id.2892) on Signal peptide, CUB and EGF−like domain−containing protein 1
with a threshold of 1e−05
rs6809952
rs7311004
rs6828768
rs11715853
rs2387977
rs7572229
rs35055552
rs823424
rs55877868
rs78383039
rs7303158
rs8039785
rs10899911
rs2090816
rs62273907
All
−0.3
−0.2
−0.1
0.0
MR effect size for
'Gut microbiota abundance (genus Parasutterella id.2892)' on 'Signal peptide, CUB and EGF−like domain−containing protein 1

Batch 2−5311 : Gut microbiota abundance (genus Catenibacterium id.2153) on Stromal cell−derived factor 2−like protein 1
with a threshold of 1e−05

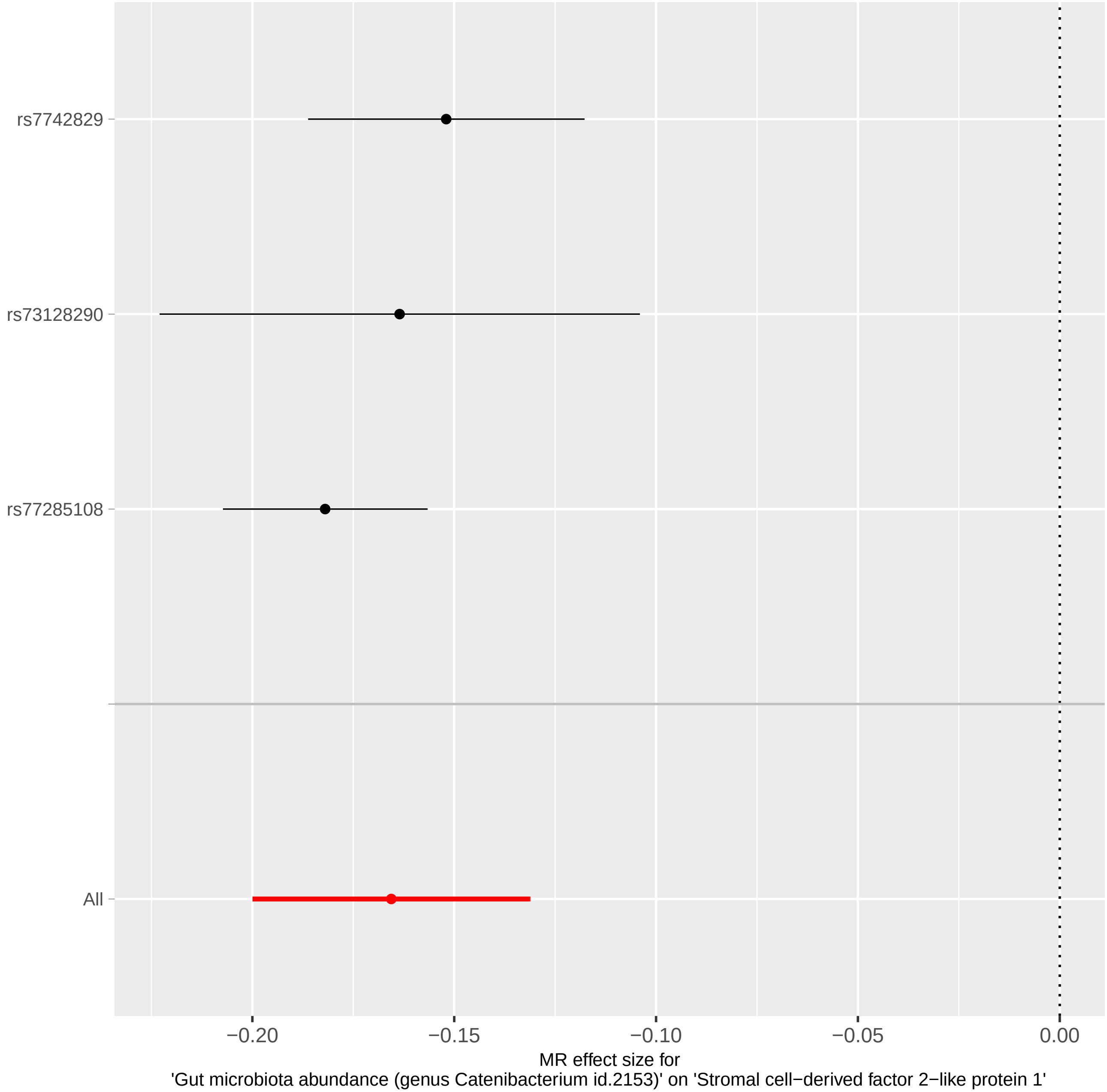

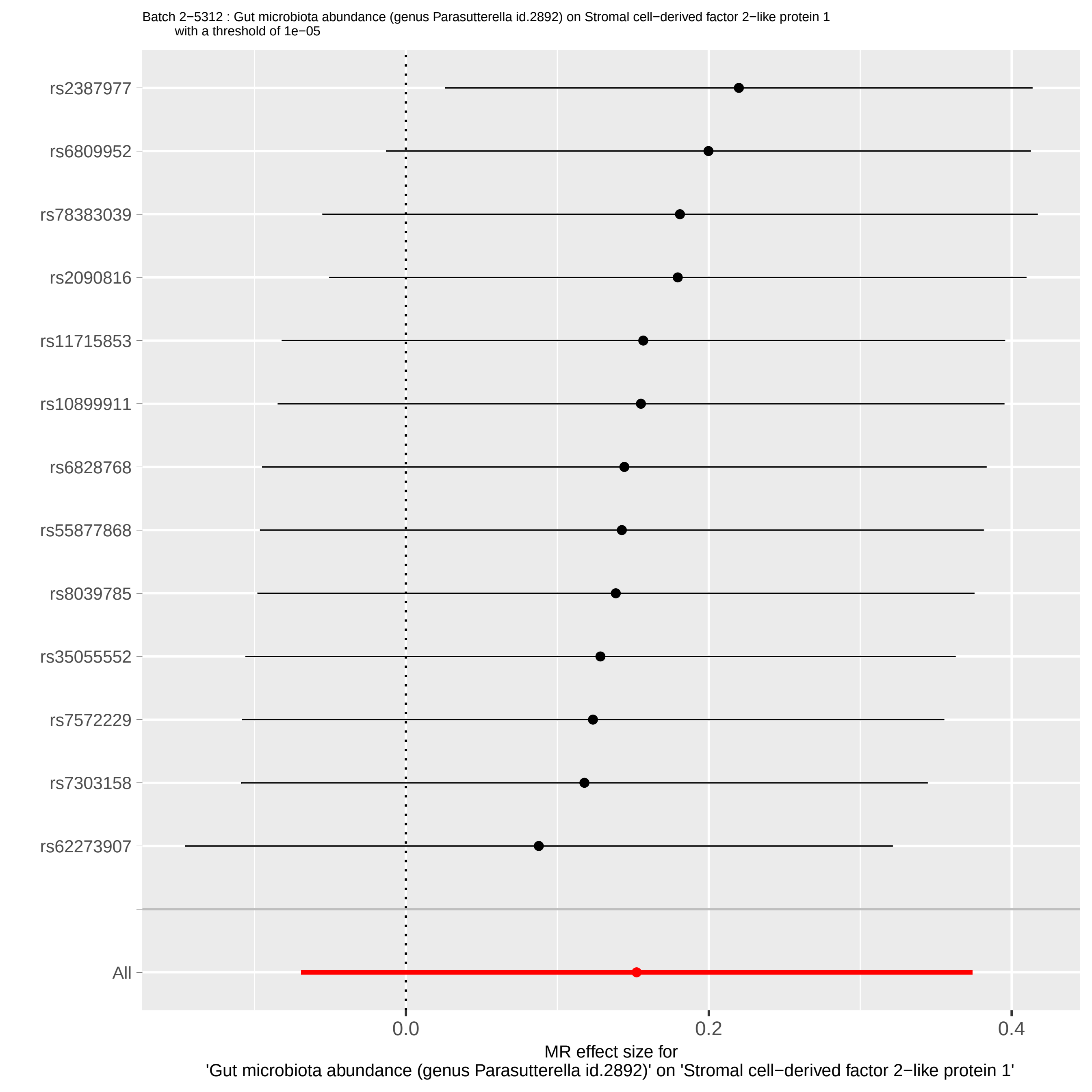
Batch 2−5312 : Gut microbiota abundance (genus Parasutterella id.2892) on Stromal cell−derived factor 2−like protein 1
with a threshold of 1e−05
rs2387977
rs6809952
rs78383039
rs2090816
rs11715853
rs10899911
rs6828768
rs55877868
rs8039785
rs35055552
rs7572229
rs7303158
rs62273907
All
0.0
0.2
0.4
MR effect size for
'Gut microbiota abundance (genus Parasutterella id.2892)' on 'Stromal cell−derived factor 2−like protein 1'

Batch 2−5313 : Gut microbiota abundance (genus Catenibacterium id.2153) on Succinate dehydrogenase assembly factor 2, mitochondrial
with a threshold of 1e−05

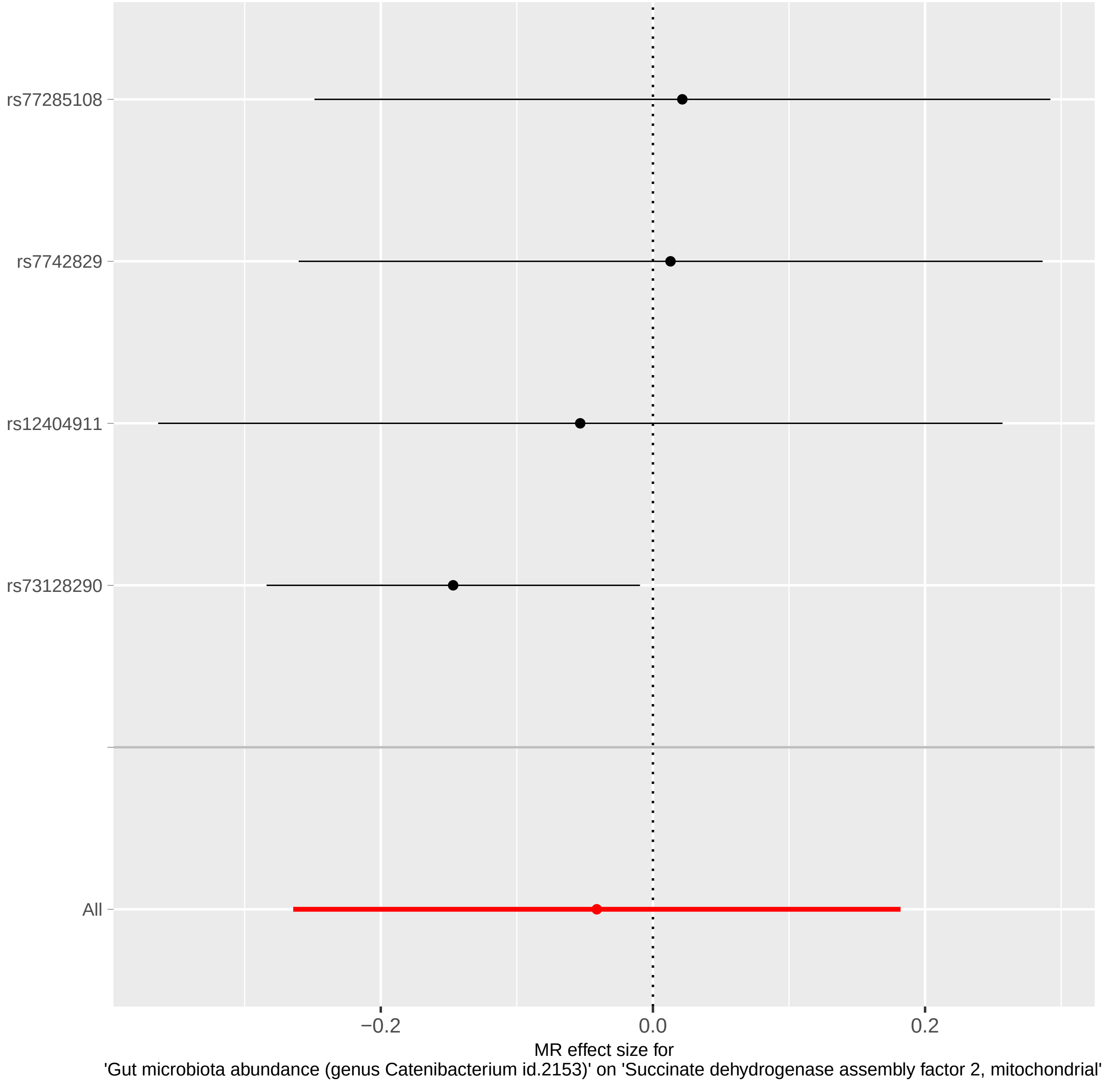

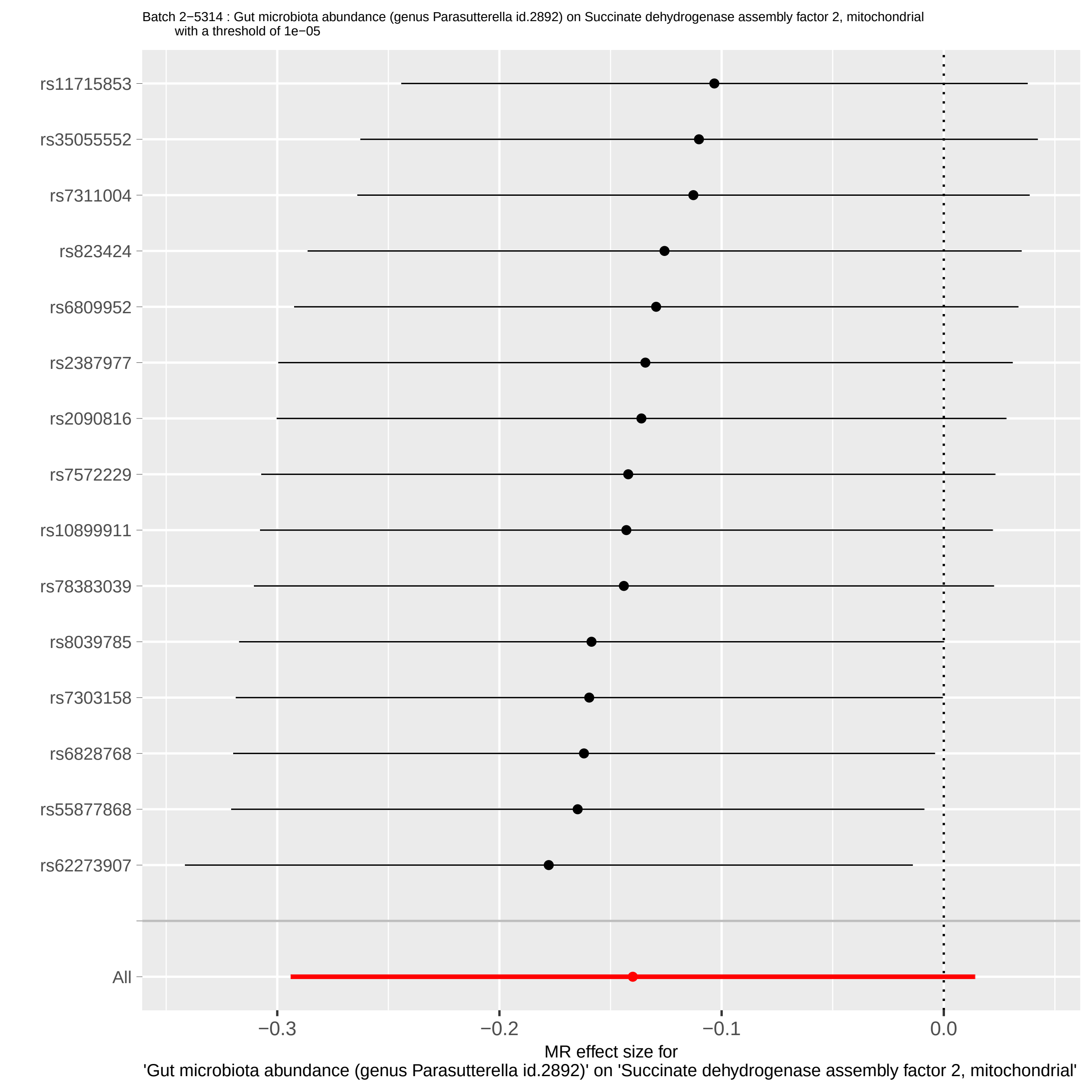

Batch 2−5314 : Gut microbiota abundance (genus Parasutterella id.2892) on Succinate dehydrogenase assembly factor 2, mitochondrial
with a threshold of 1e−05
rs11715853
rs35055552
rs7311004
rs823424
rs6809952
rs2387977
rs2090816
rs7572229
rs10899911
rs78383039
rs8039785
rs7303158
rs6828768
rs55877868
rs62273907
All
−0.3
−0.2
−0.1
0.0
MR effect size for
'Gut microbiota abundance (genus Parasutterella id.2892)' on 'Succinate dehydrogenase assembly factor 2, mitochondrial'

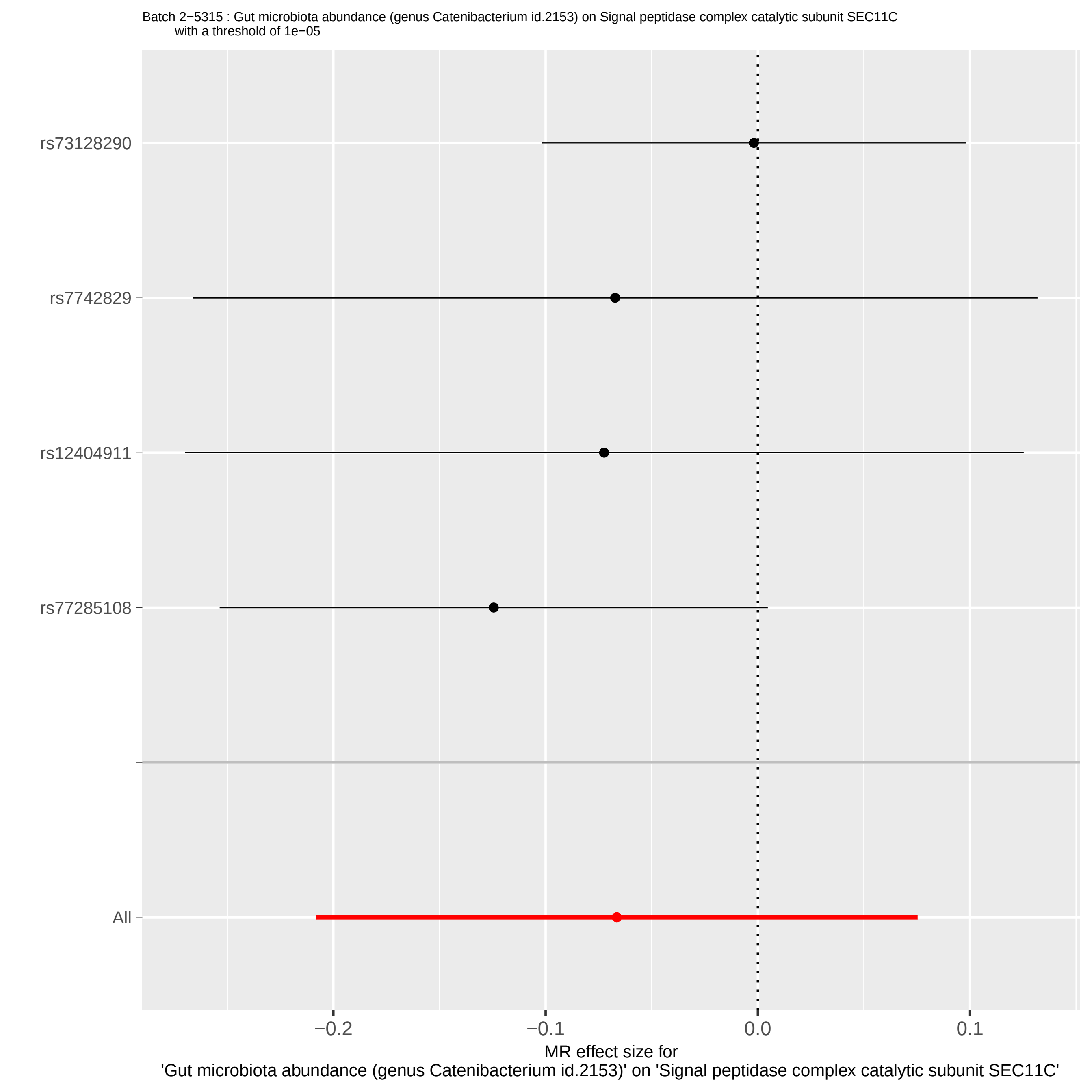
Batch 2−5315 : Gut microbiota abundance (genus Catenibacterium id.2153) on Signal peptidase complex catalytic subunit SEC11C
with a threshold of 1e−05
rs73128290
rs7742829
rs12404911
rs77285108
All
−0.2
−0.1
0.0
0.1
MR effect size for
'Gut microbiota abundance (genus Catenibacterium id.2153)' on 'Signal peptidase complex catalytic subunit SEC11C'

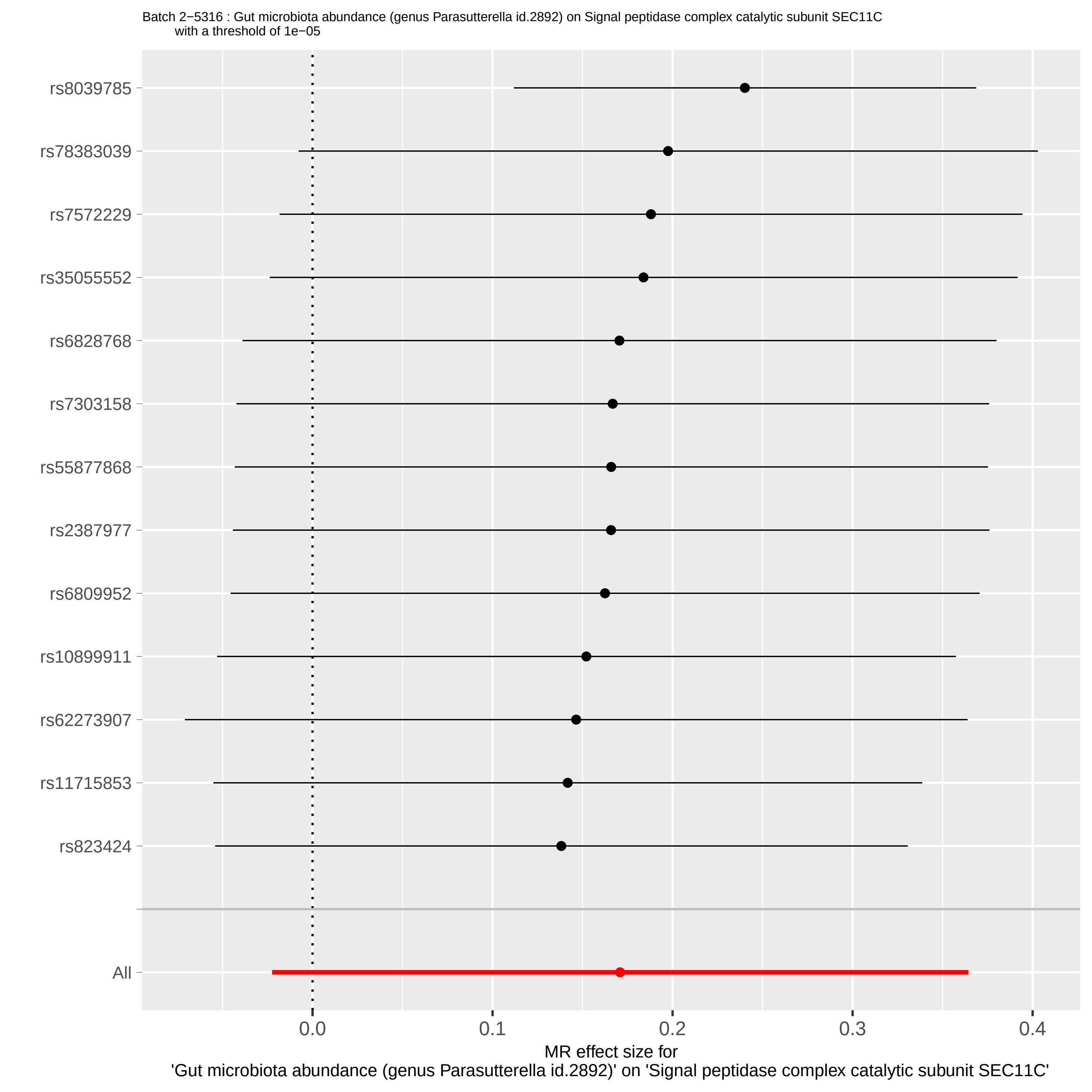
Batch 2−5316 : Gut microbiota abundance (genus Parasutterella id.2892) on Signal peptidase complex catalytic subunit SEC11C
with a threshold of 1e−05
rs8039785
rs78383039
rs7572229
rs35055552
rs6828768
rs7303158
rs55877868
rs2387977
rs6809952
rs10899911
rs62273907
rs11715853
rs823424
All
0.0
0.1
0.2
0.3
0.4
MR effect size for
'Gut microbiota abundance (genus Parasutterella id.2892)' on 'Signal peptidase complex catalytic subunit SEC11C'

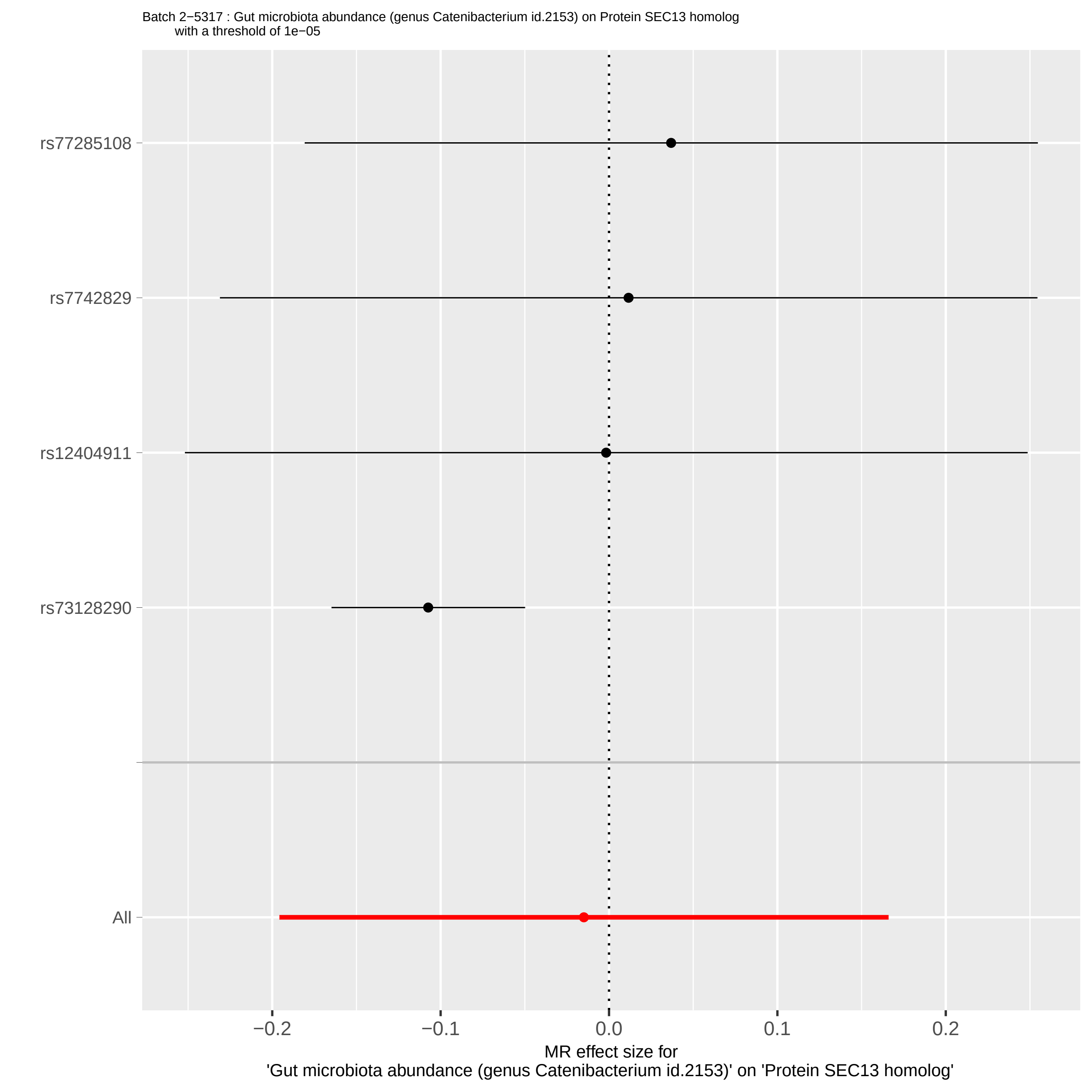
Batch 2−5317 : Gut microbiota abundance (genus Catenibacterium id.2153) on Protein SEC13 homolog
with a threshold of 1e−05
rs77285108
rs7742829
rs12404911
rs73128290
All
−0.2
−0.1
0.0
0.1
0.2
MR effect size for
'Gut microbiota abundance (genus Catenibacterium id.2153)' on 'Protein SEC13 homolog'

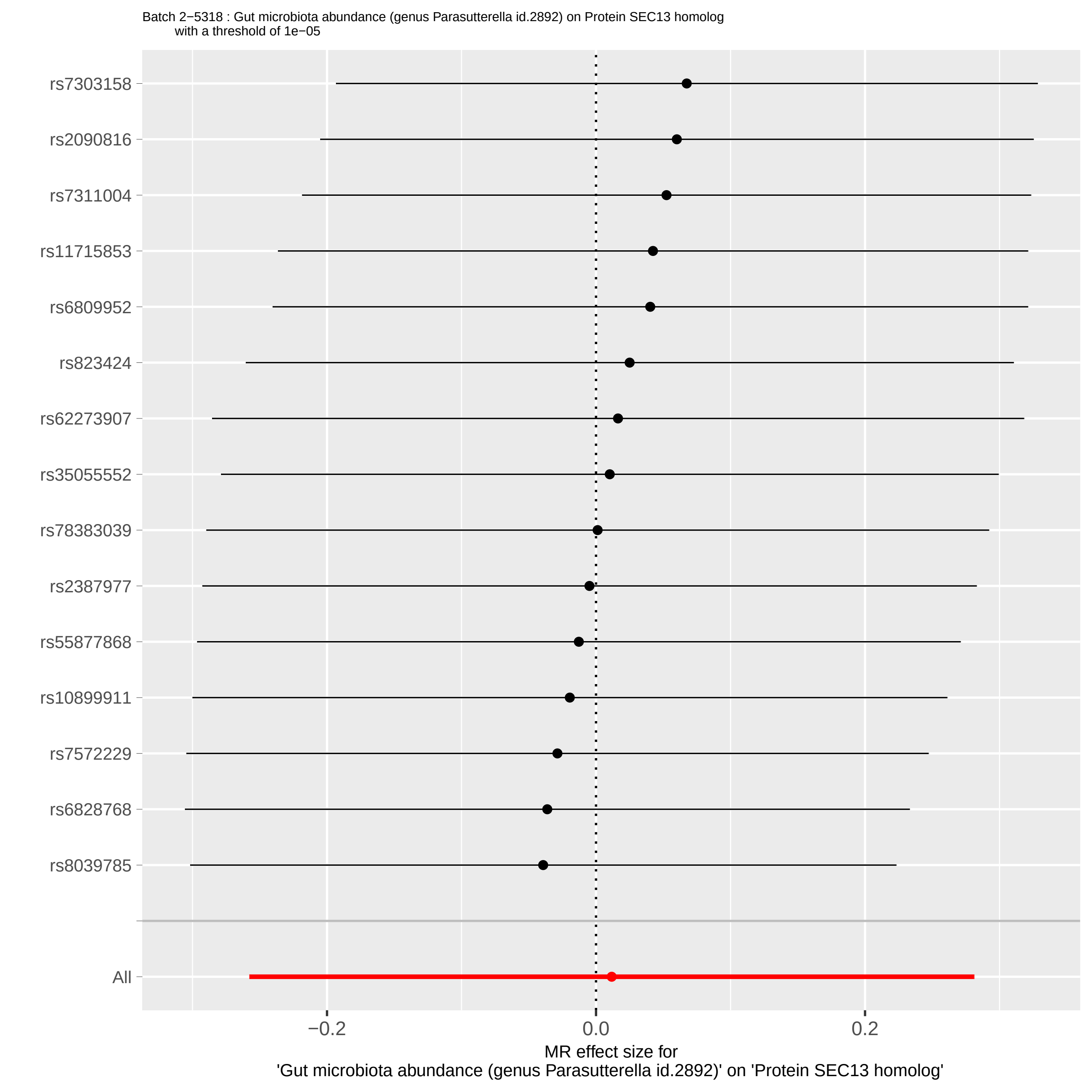

Batch 2−5318 : Gut microbiota abundance (genus Parasutterella id.2892) on Protein SEC13 homolog
with a threshold of 1e−05
rs7303158
rs2090816
rs7311004
rs11715853
rs6809952
rs823424
rs62273907
rs35055552
rs78383039
rs2387977
rs55877868
rs10899911
rs7572229
rs6828768
rs8039785
All
−0.2
0.0
0.2
MR effect size for
'Gut microbiota abundance (genus Parasutterella id.2892)' on 'Protein SEC13 homolog'

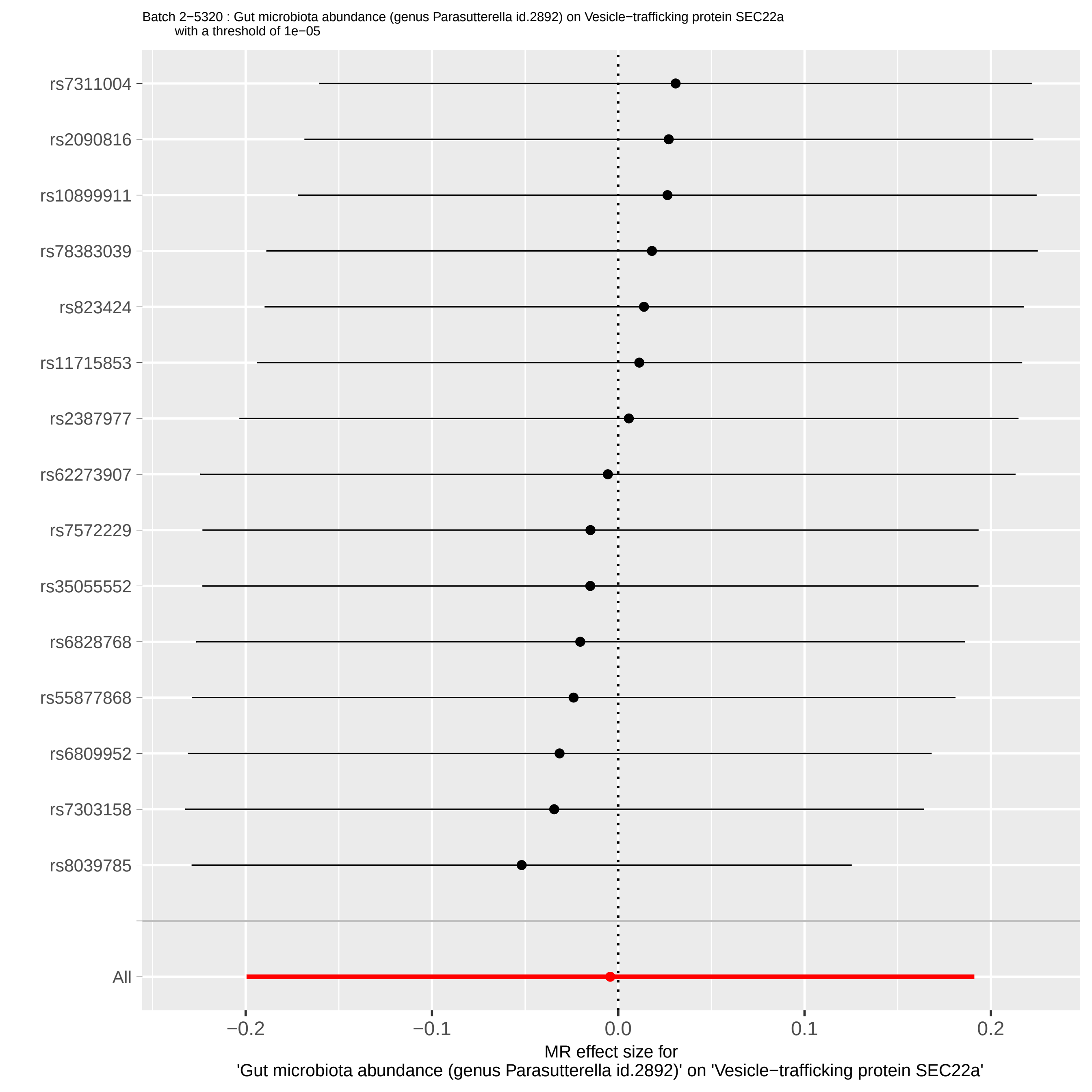

Batch 2−5320 : Gut microbiota abundance (genus Parasutterella id.2892) on Vesicle−trafficking protein SEC22a
with a threshold of 1e−05
rs7311004
rs2090816
rs10899911
rs78383039
rs823424
rs11715853
rs2387977
rs62273907
rs7572229
rs35055552
rs6828768
rs55877868
rs6809952
rs7303158
rs8039785
All
−0.2
−0.1
0.0
0.1
0.2
MR effect size for
'Gut microbiota abundance (genus Parasutterella id.2892)' on 'Vesicle−trafficking protein SEC22a'

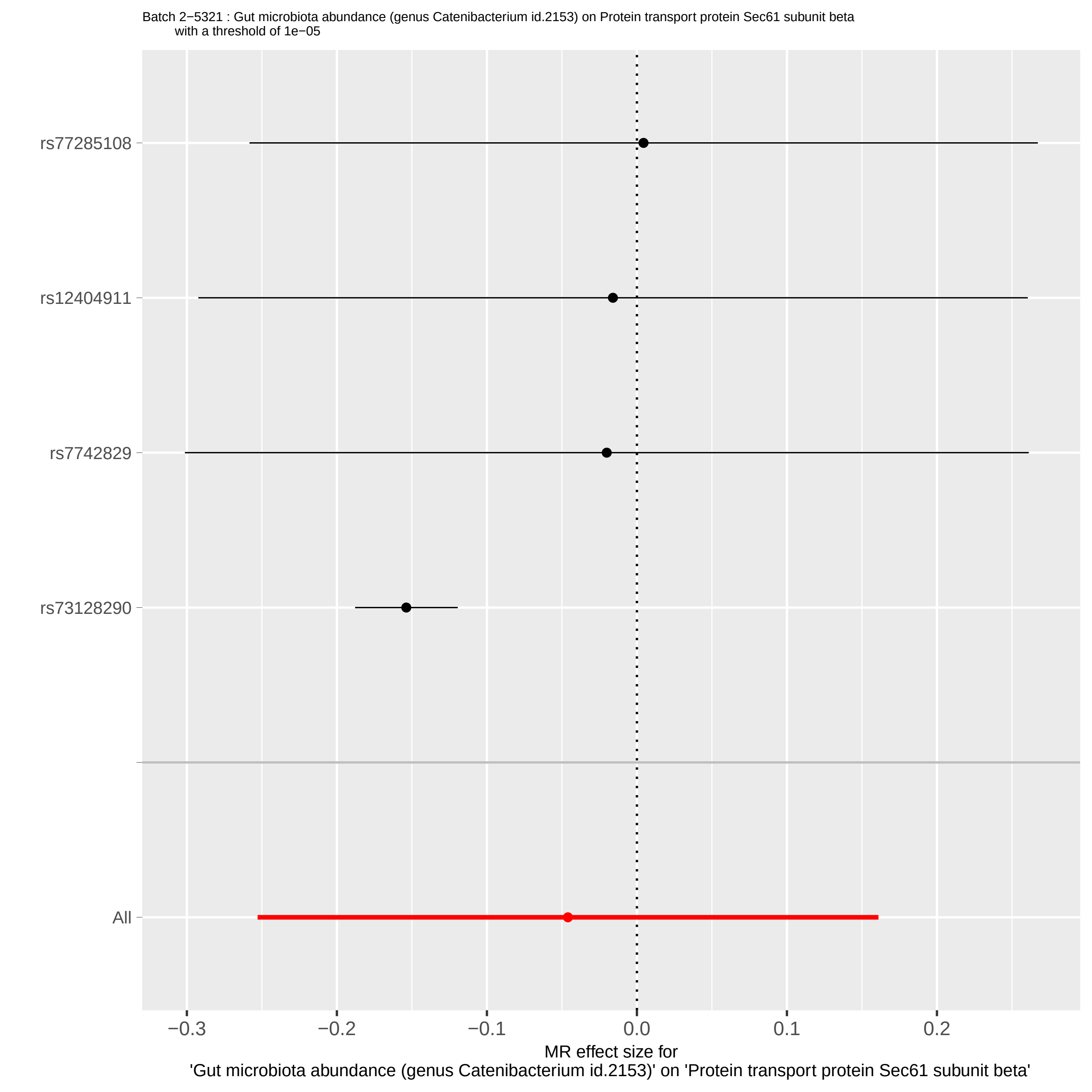
Batch 2−5321 : Gut microbiota abundance (genus Catenibacterium id.2153) on Protein transport protein Sec61 subunit beta
with a threshold of 1e−05
rs77285108
rs12404911
rs7742829
rs73128290
All
−0.3
−0.2
−0.1
0.0
0.1
0.2
MR effect size for
'Gut microbiota abundance (genus Catenibacterium id.2153)' on 'Protein transport protein Sec61 subunit beta'

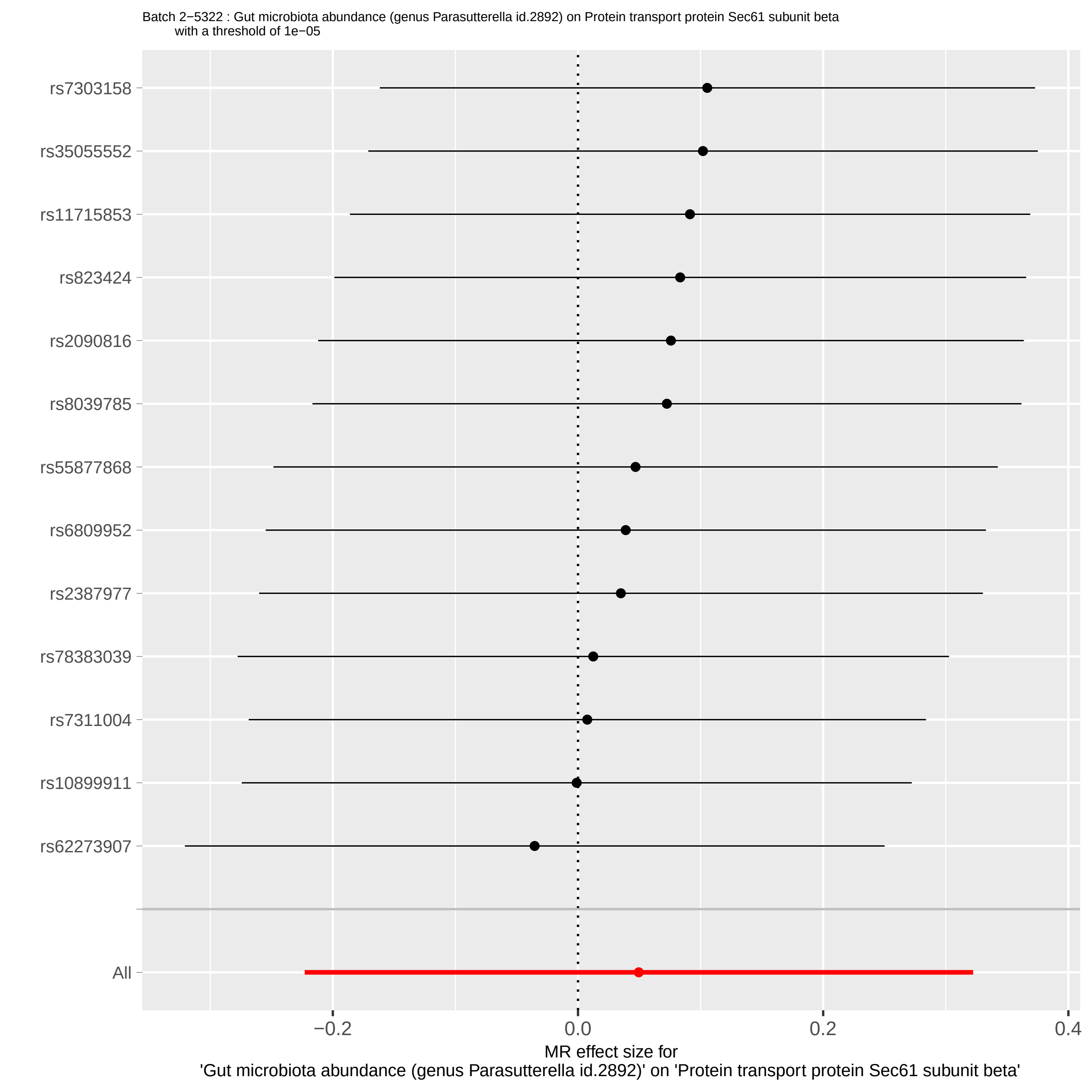
Batch 2−5322 : Gut microbiota abundance (genus Parasutterella id.2892) on Protein transport protein Sec61 subunit beta
with a threshold of 1e−05
rs7303158
rs35055552
rs11715853
rs823424
rs2090816
rs8039785
rs55877868
rs6809952
rs2387977
rs78383039
rs7311004
rs10899911
rs62273907
All
−0.2
0.0
0.2
0.4
MR effect size for
'Gut microbiota abundance (genus Parasutterella id.2892)' on 'Protein transport protein Sec61 subunit beta'

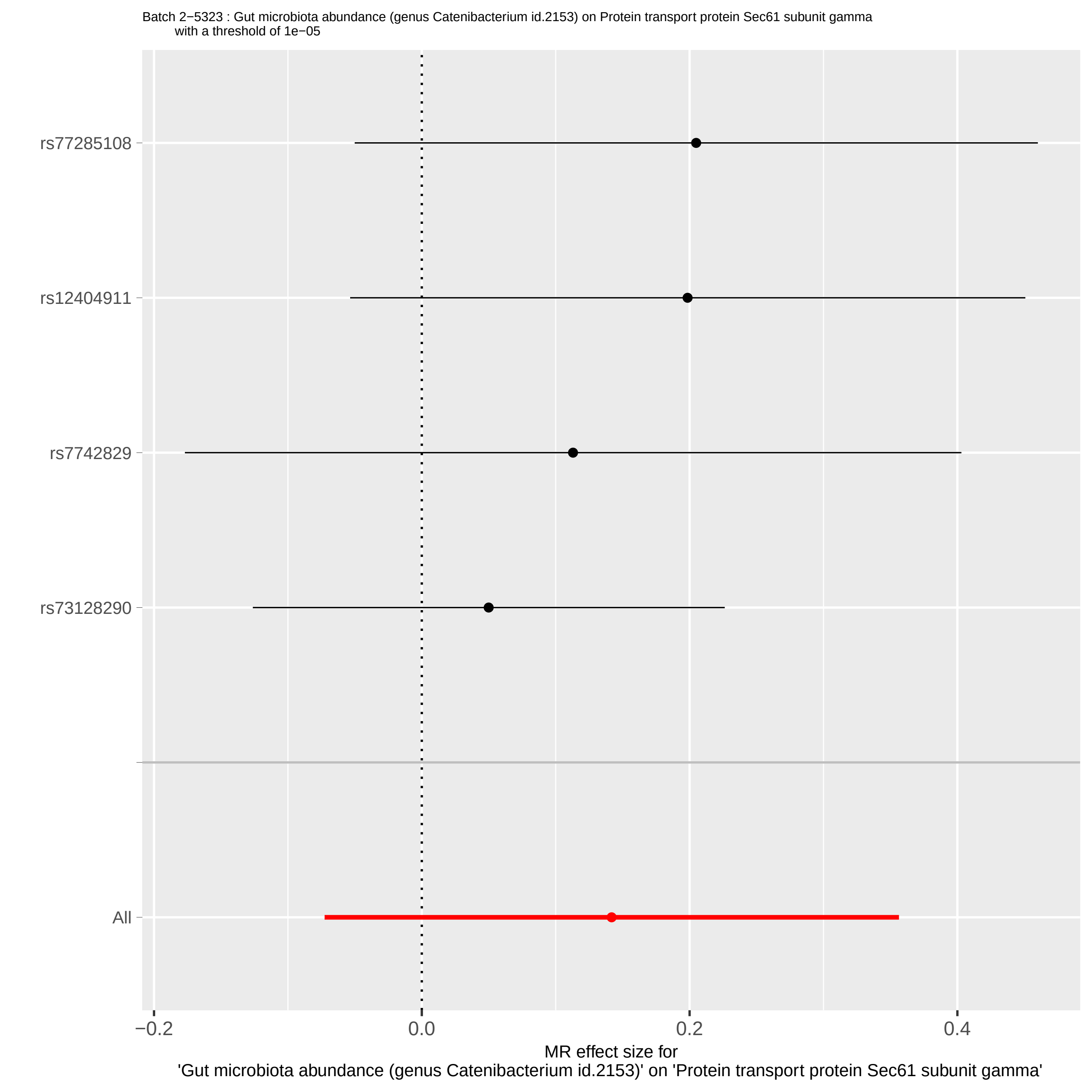

Batch 2−5323 : Gut microbiota abundance (genus Catenibacterium id.2153) on Protein transport protein Sec61 subunit gamma
with a threshold of 1e−05
rs77285108
rs12404911
rs7742829
rs73128290
All
−0.2
0.0
0.2
0.4
MR effect size for
'Gut microbiota abundance (genus Catenibacterium id.2153)' on 'Protein transport protein Sec61 subunit gamma'

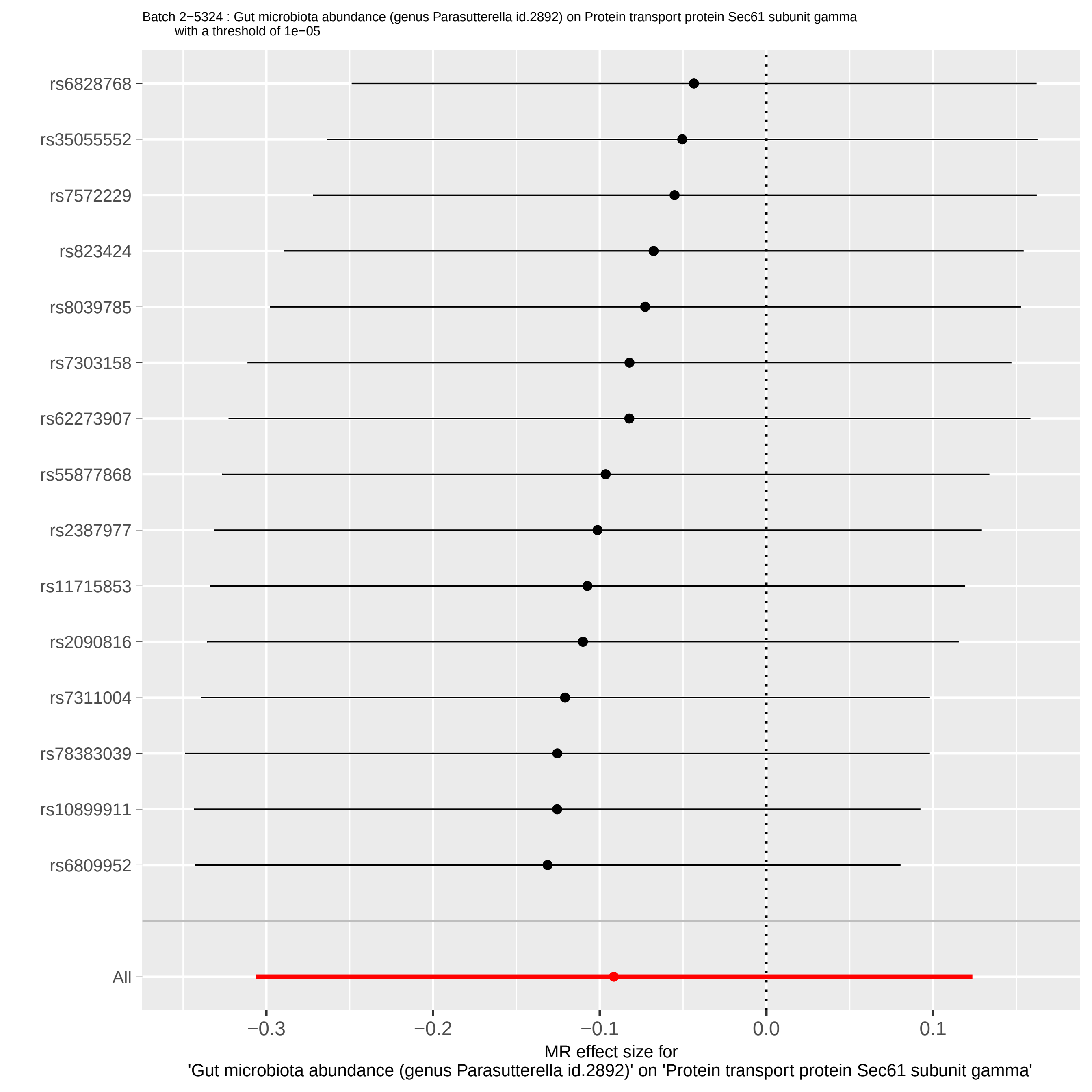
Batch 2−5324 : Gut microbiota abundance (genus Parasutterella id.2892) on Protein transport protein Sec61 subunit gamma
with a threshold of 1e−05
rs6828768
rs35055552
rs7572229
rs823424
rs8039785
rs7303158
rs62273907
rs55877868
rs2387977
rs11715853
rs2090816
rs7311004
rs78383039
rs10899911
rs6809952
All
−0.3
−0.2
−0.1
0.0
0.1
MR effect size for
'Gut microbiota abundance (genus Parasutterella id.2892)' on 'Protein transport protein Sec61 subunit gamma'

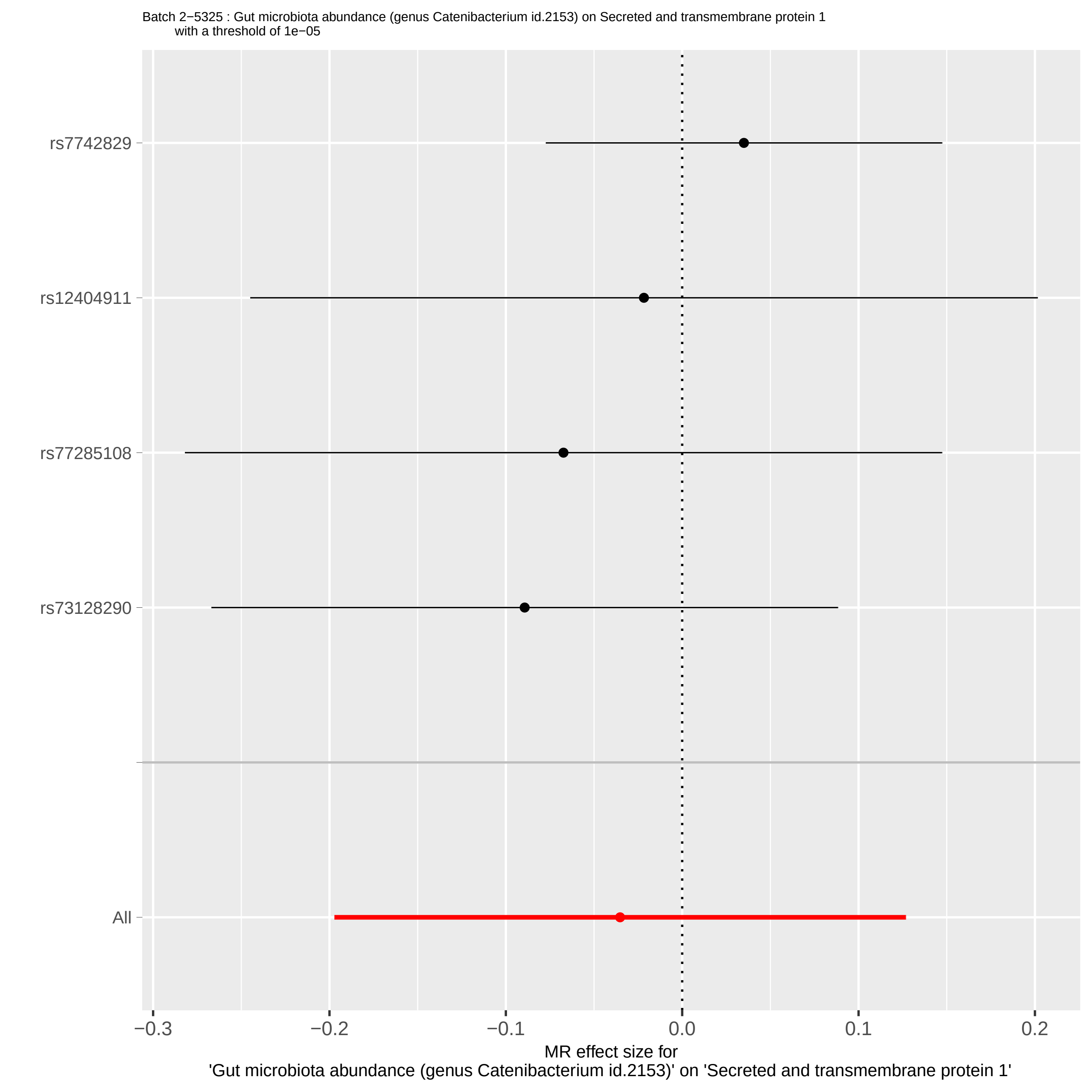

Batch 2−5325 : Gut microbiota abundance (genus Catenibacterium id.2153) on Secreted and transmembrane protein 1
with a threshold of 1e−05
rs7742829
rs12404911
rs77285108
rs73128290
All
−0.3
−0.2
−0.1
0.0
0.1
0.2
MR effect size for
'Gut microbiota abundance (genus Catenibacterium id.2153)' on 'Secreted and transmembrane protein 1'

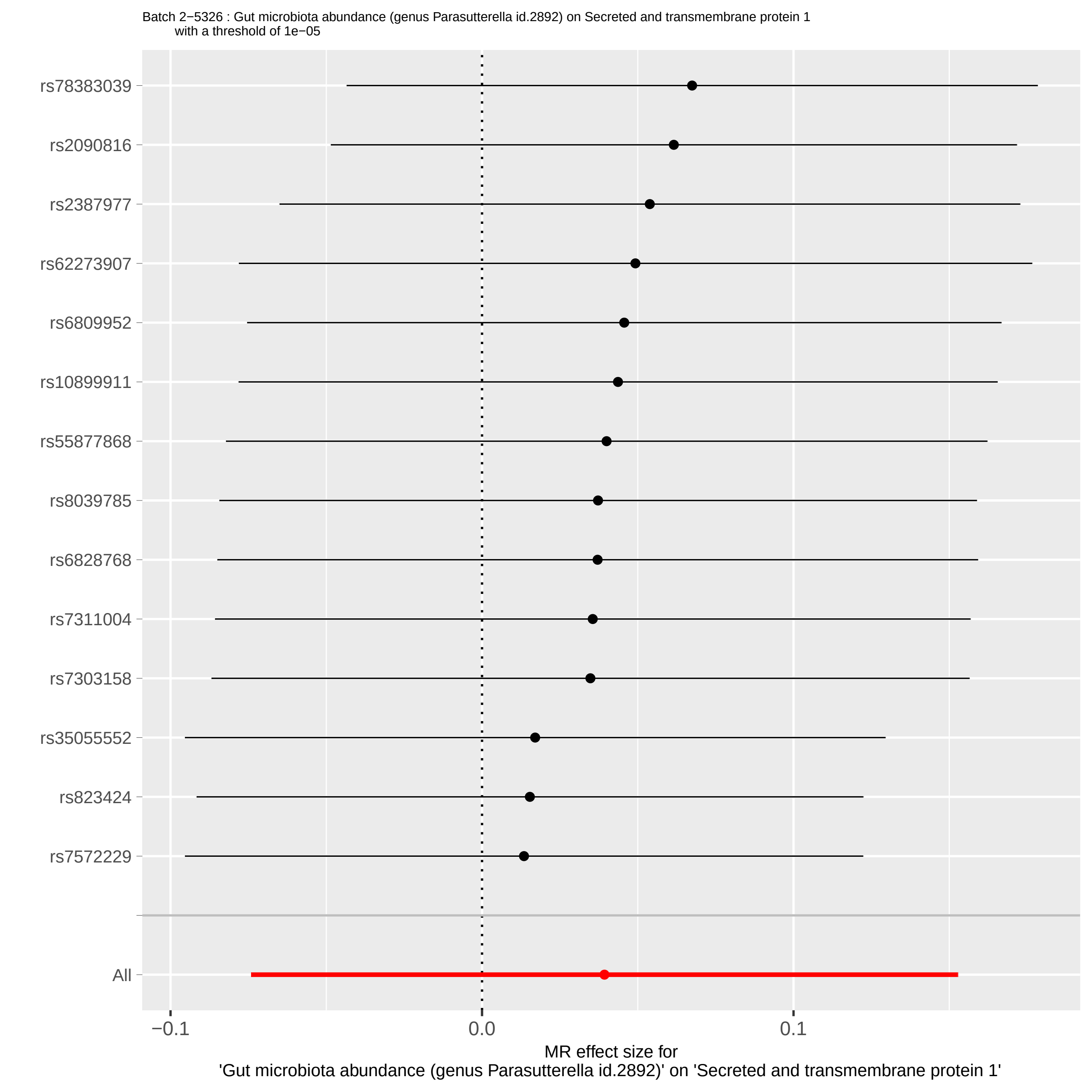
Batch 2−5326 : Gut microbiota abundance (genus Parasutterella id.2892) on Secreted and transmembrane protein 1
with a threshold of 1e−05
rs78383039
rs2090816
rs2387977
rs62273907
rs6809952
rs10899911
rs55877868
rs8039785
rs6828768
rs7311004
rs7303158
rs35055552
rs823424
rs7572229
All
−0.1
0.0
0.1
MR effect size for
'Gut microbiota abundance (genus Parasutterella id.2892)' on 'Secreted and transmembrane protein 1'

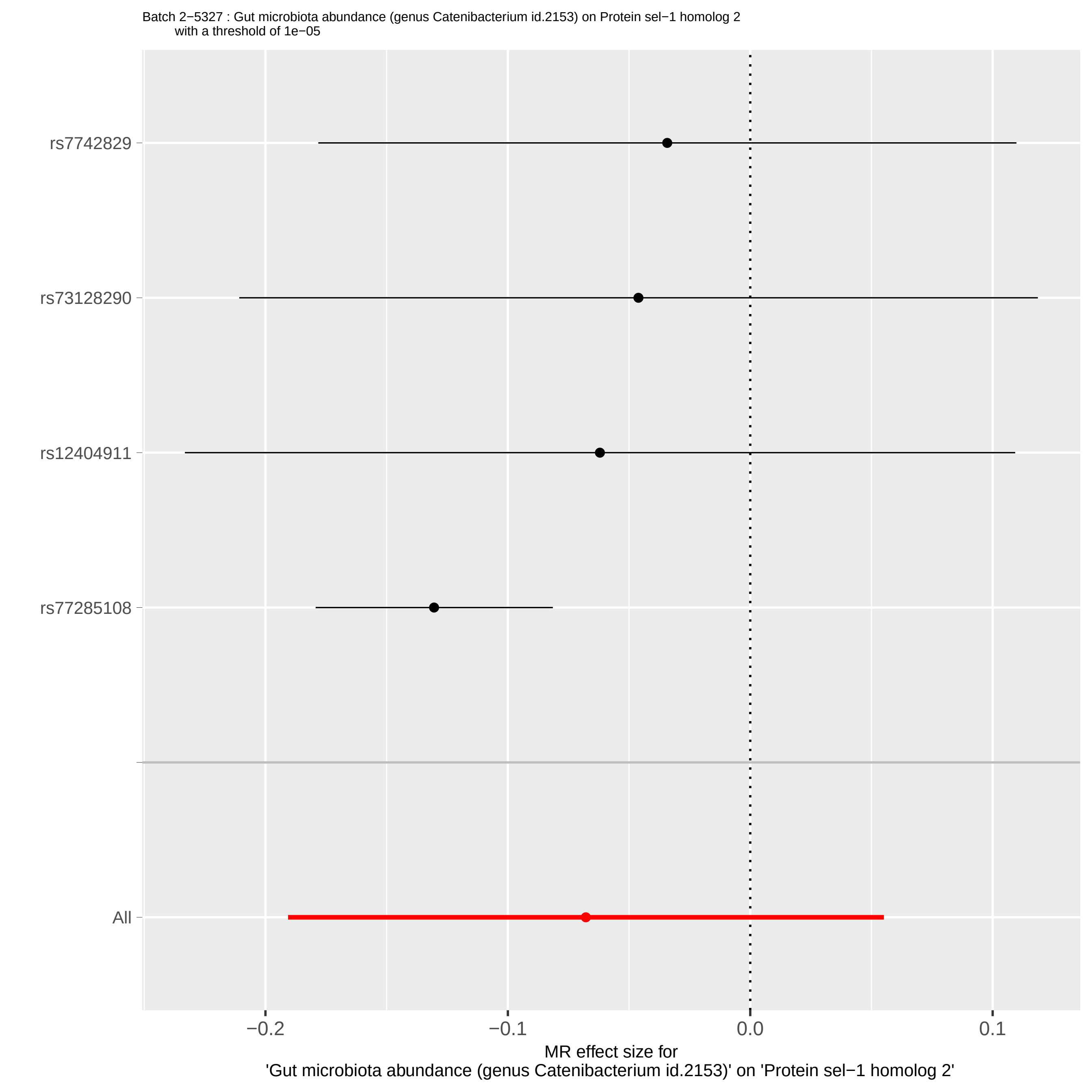

Batch 2−5327 : Gut microbiota abundance (genus Catenibacterium id.2153) on Protein sel−1 homolog 2
with a threshold of 1e−05
rs7742829
rs73128290
rs12404911
rs77285108
All
−0.2
−0.1
0.0
0.1
MR effect size for
'Gut microbiota abundance (genus Catenibacterium id.2153)' on 'Protein sel−1 homolog 2'

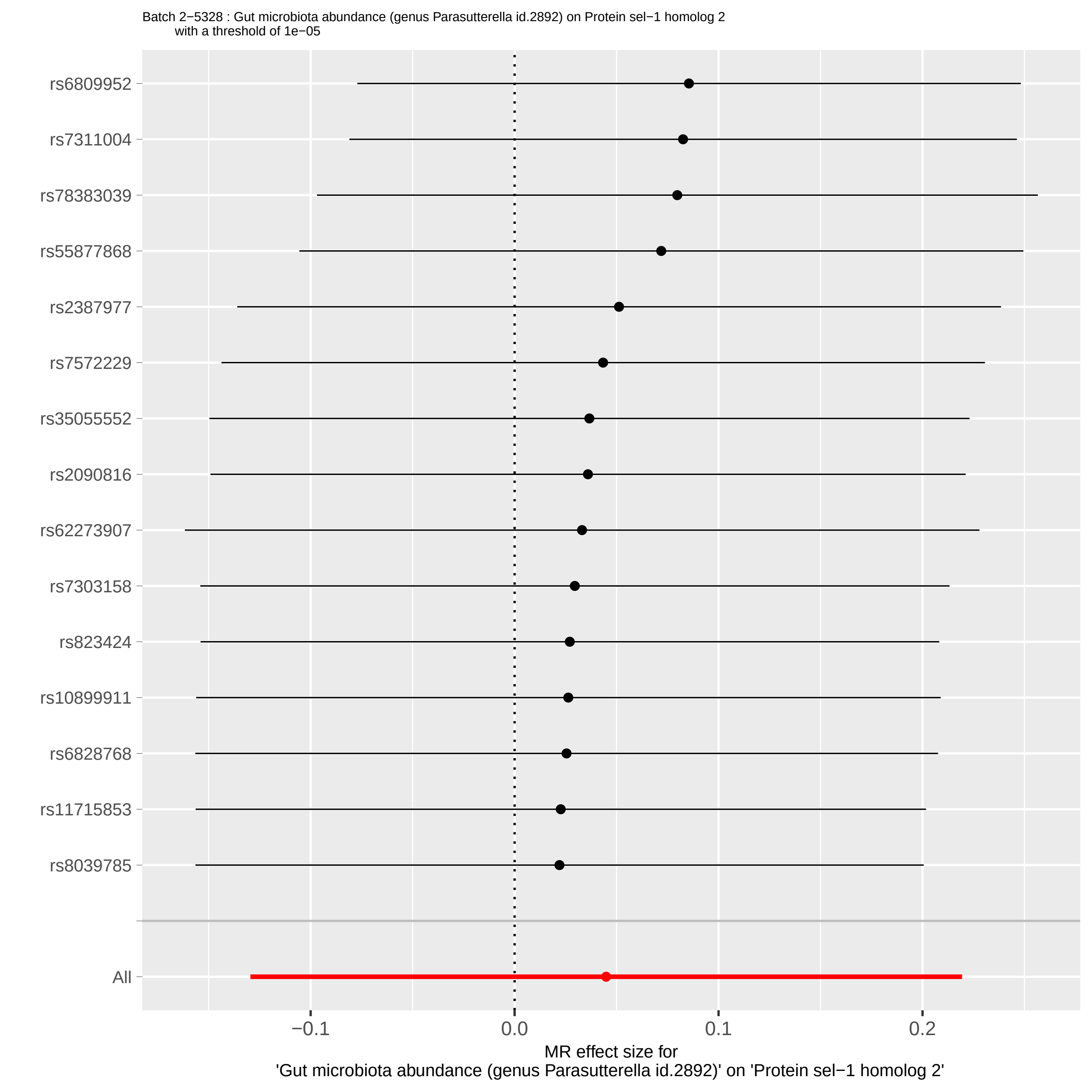
Batch 2−5328 : Gut microbiota abundance (genus Parasutterella id.2892) on Protein sel−1 homolog 2
with a threshold of 1e−05
rs6809952
rs7311004
rs78383039
rs55877868
rs2387977
rs7572229
rs35055552
rs2090816
rs62273907
rs7303158
rs823424
rs10899911
rs6828768
rs11715853
rs8039785
All
−0.1
0.0
0.1
0.2
MR effect size for
'Gut microbiota abundance (genus Parasutterella id.2892)' on 'Protein sel−1 homolog 2'

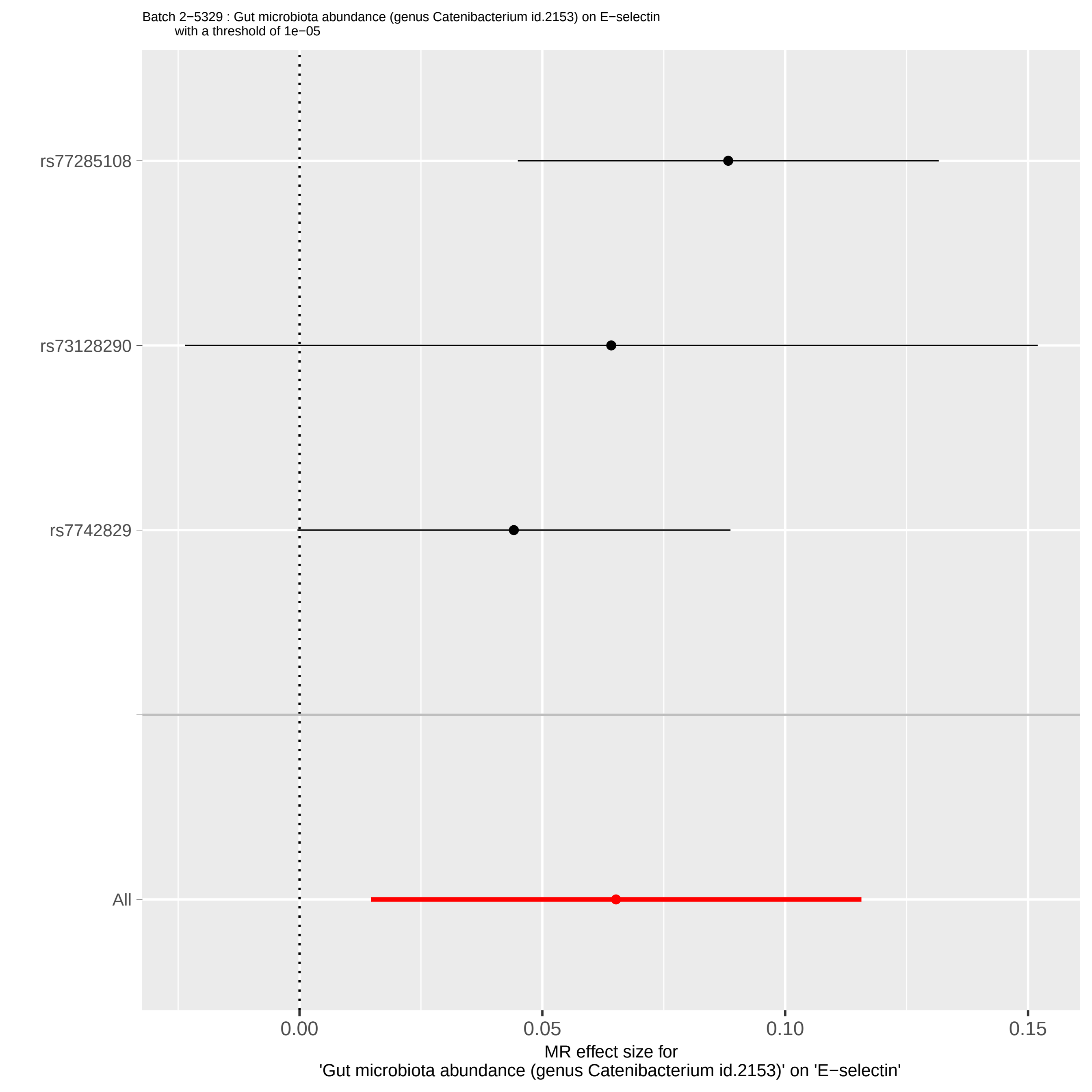
Batch 2−5329 : Gut microbiota abundance (genus Catenibacterium id.2153) on E−selectin
with a threshold of 1e−05
rs77285108
rs73128290
rs7742829
All
0.00
0.05
0.10
0.15
MR effect size for
'Gut microbiota abundance (genus Catenibacterium id.2153)' on 'E−selectin'

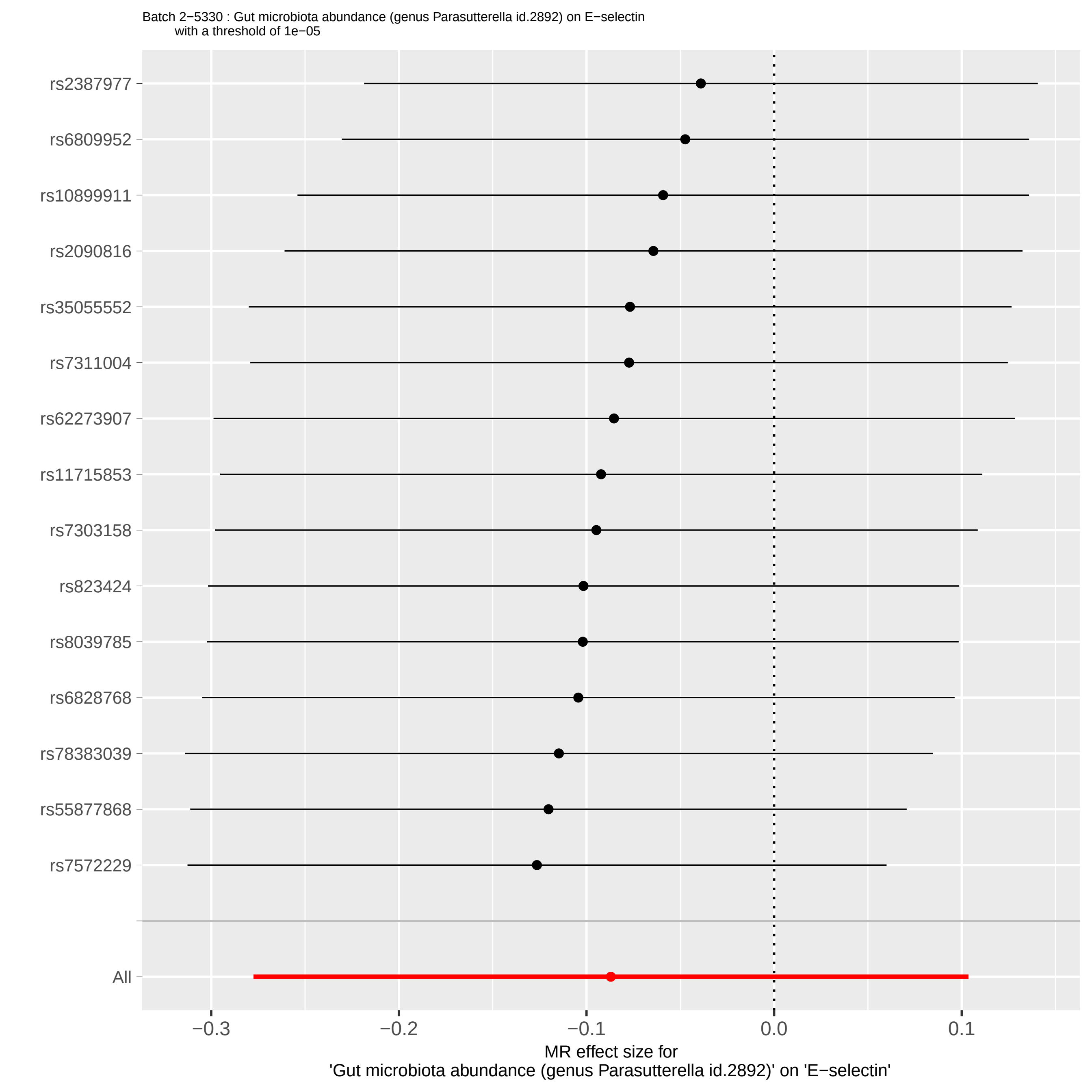
Batch 2−5330 : Gut microbiota abundance (genus Parasutterella id.2892) on E−selectin
with a threshold of 1e−05
rs2387977
rs6809952
rs10899911
rs2090816
rs35055552
rs7311004
rs62273907
rs11715853
rs7303158
rs823424
rs8039785
rs6828768
rs78383039
rs55877868
rs7572229
All
−0.3
−0.2
−0.1
0.0
0.1
MR effect size for
'Gut microbiota abundance (genus Parasutterella id.2892)' on 'E−selectin'

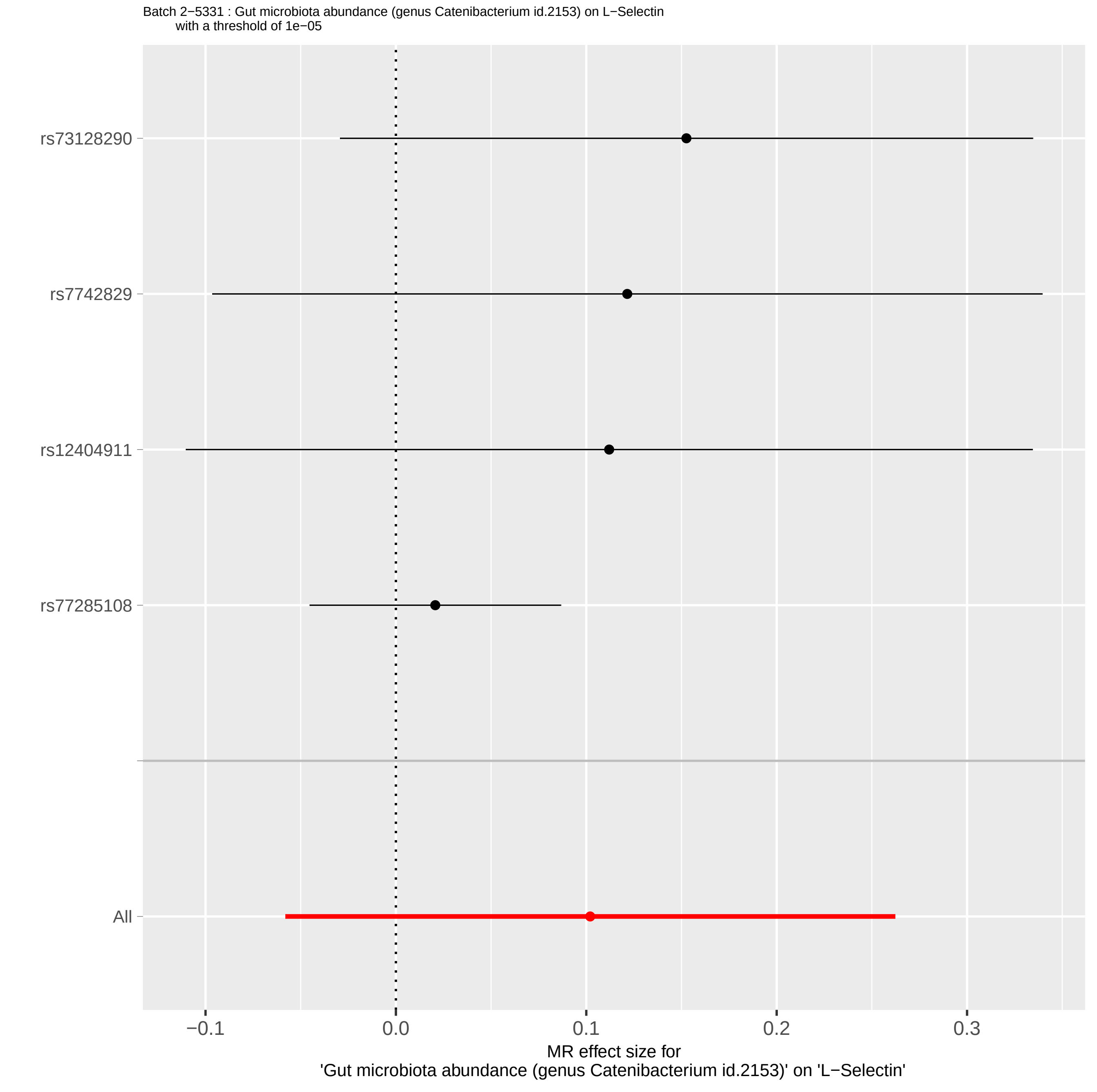

Batch 2−5331 : Gut microbiota abundance (genus Catenibacterium id.2153) on L−Selectin
with a threshold of 1e−05
rs73128290
rs7742829
rs12404911
rs77285108
All
−0.1
0.0
0.1
0.2
0.3
MR effect size for
'Gut microbiota abundance (genus Catenibacterium id.2153)' on 'L−Selectin'

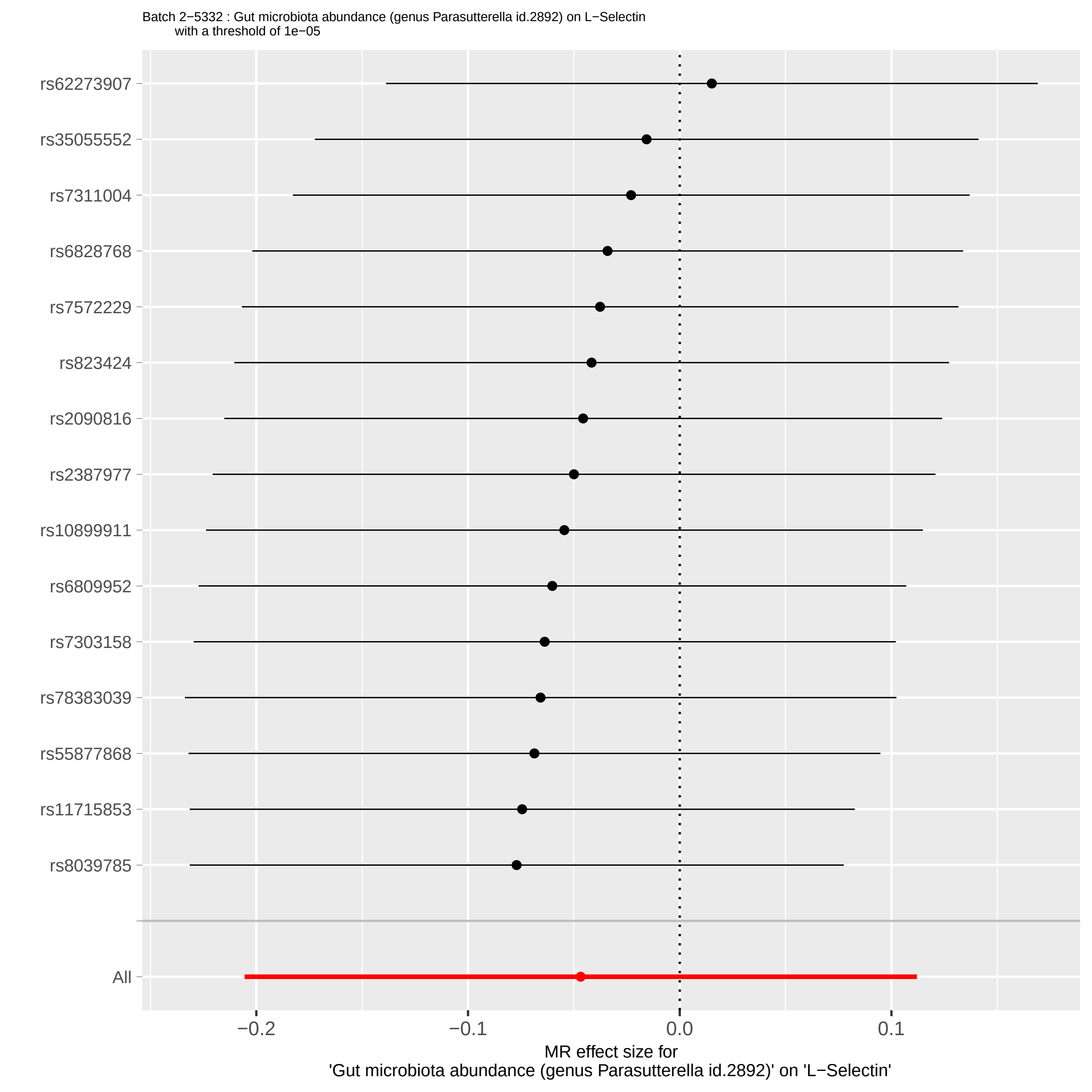

Batch 2−5332 : Gut microbiota abundance (genus Parasutterella id.2892) on L−Selectin
with a threshold of 1e−05
rs62273907
rs35055552
rs7311004
rs6828768
rs7572229
rs823424
rs2090816
rs2387977
rs10899911
rs6809952
rs7303158
rs78383039
rs55877868
rs11715853
rs8039785
All
−0.2
−0.1
0.0
0.1
MR effect size for
'Gut microbiota abundance (genus Parasutterella id.2892)' on 'L−Selectin'

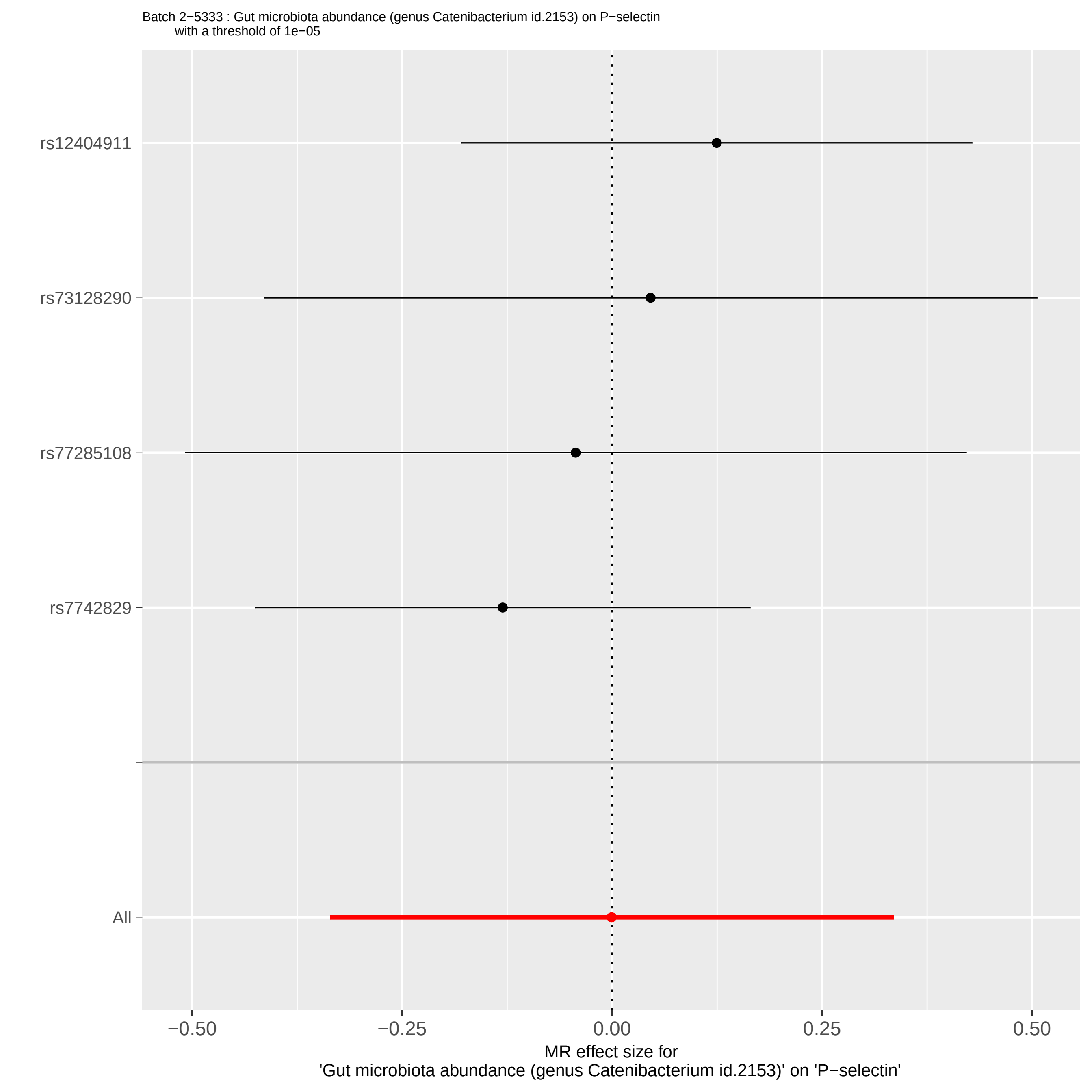

Batch 2−5333 : Gut microbiota abundance (genus Catenibacterium id.2153) on P−selectin
with a threshold of 1e−05
rs12404911
rs73128290
rs77285108
rs7742829
All
−0.50
−0.25
0.00
0.25
0.50
MR effect size for
'Gut microbiota abundance (genus Catenibacterium id.2153)' on 'P−selectin'

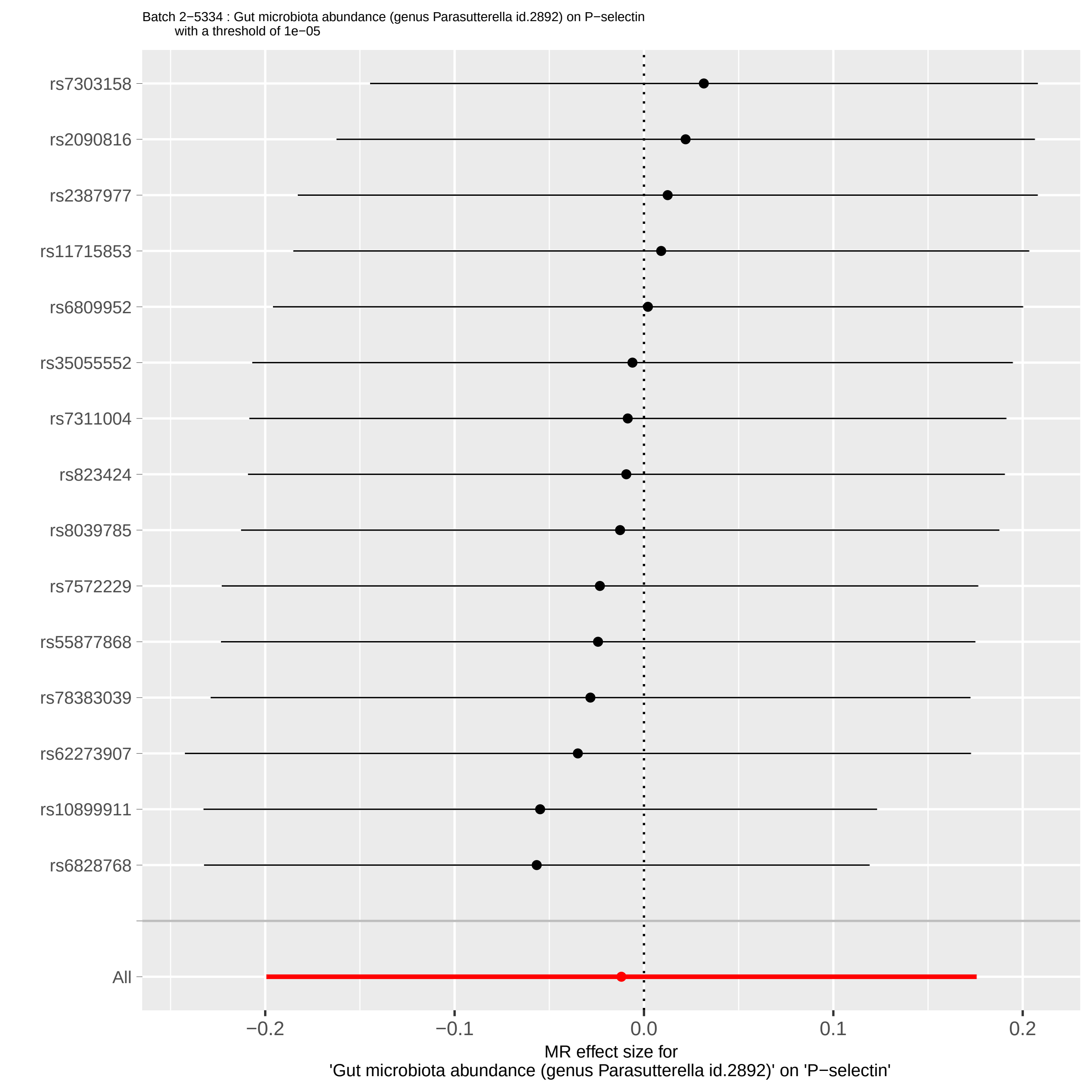
Batch 2−5334 : Gut microbiota abundance (genus Parasutterella id.2892) on P−selectin
with a threshold of 1e−05
rs7303158
rs2090816
rs2387977
rs11715853
rs6809952
rs35055552
rs7311004
rs823424
rs8039785
rs7572229
rs55877868
rs78383039
rs62273907
rs10899911
rs6828768
All
−0.2
−0.1
0.0
0.1
0.2
MR effect size for
'Gut microbiota abundance (genus Parasutterella id.2892)' on 'P−selectin'

Batch 2−5335 : Gut microbiota abundance (genus Catenibacterium id.2153) on P−selectin glycoprotein ligand 1
with a threshold of 1e−05

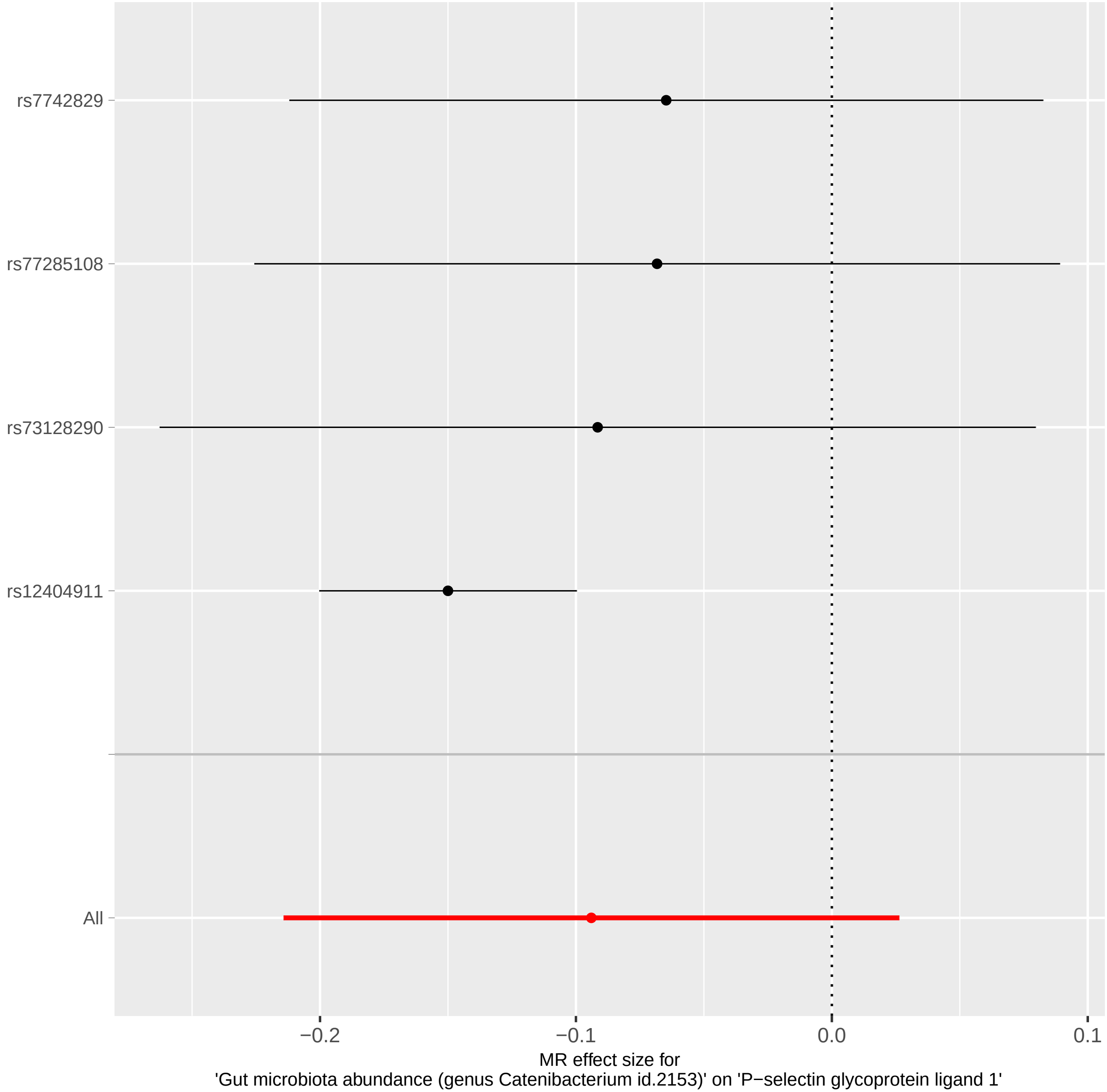

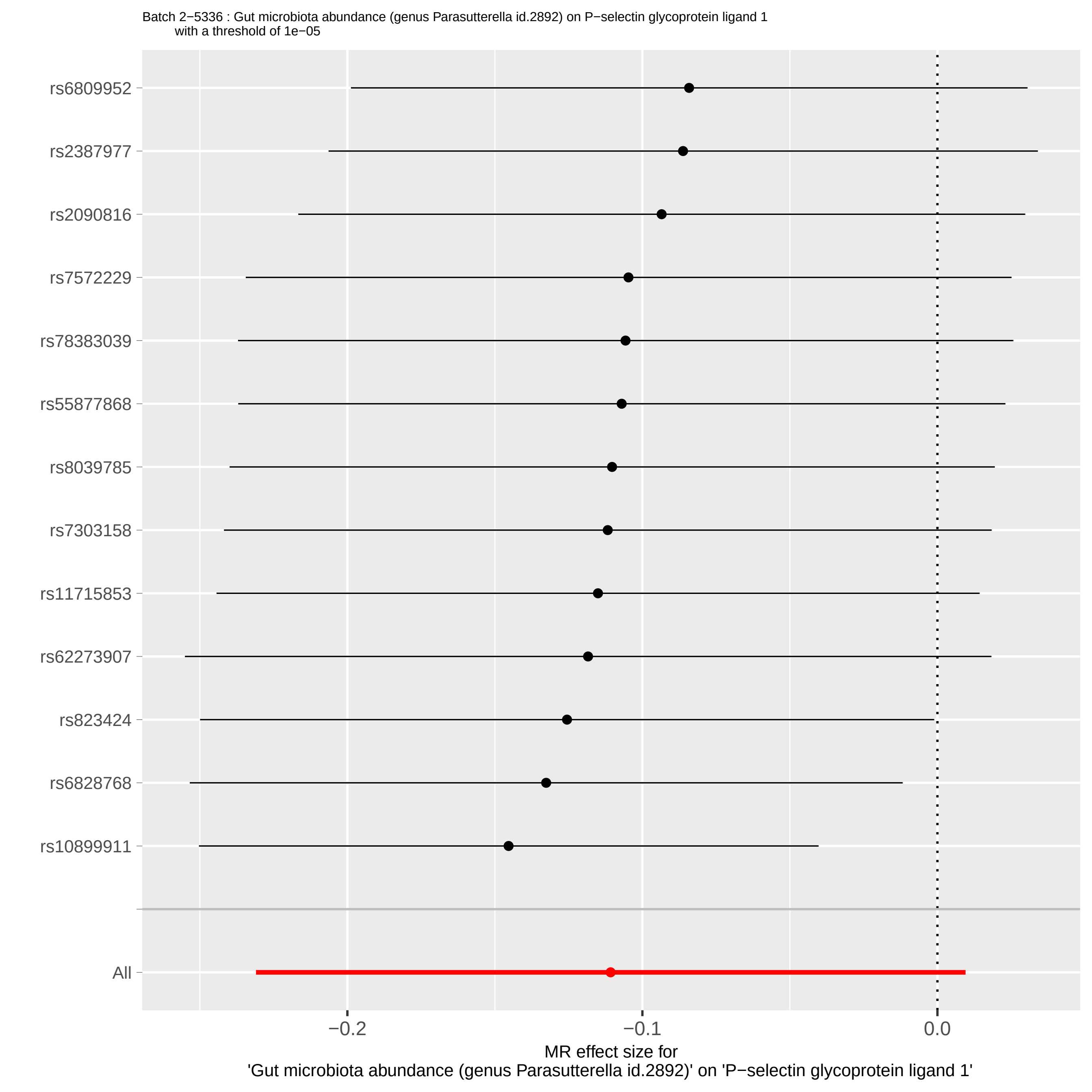
Batch 2−5336 : Gut microbiota abundance (genus Parasutterella id.2892) on P−selectin glycoprotein ligand 1
with a threshold of 1e−05
rs6809952
rs2387977
rs2090816
rs7572229
rs78383039
rs55877868
rs8039785
rs7303158
rs11715853
rs62273907
rs823424
rs6828768
rs10899911
All
−0.2
−0.1
0.0
MR effect size for
'Gut microbiota abundance (genus Parasutterella id.2892)' on 'P−selectin glycoprotein ligand 1'

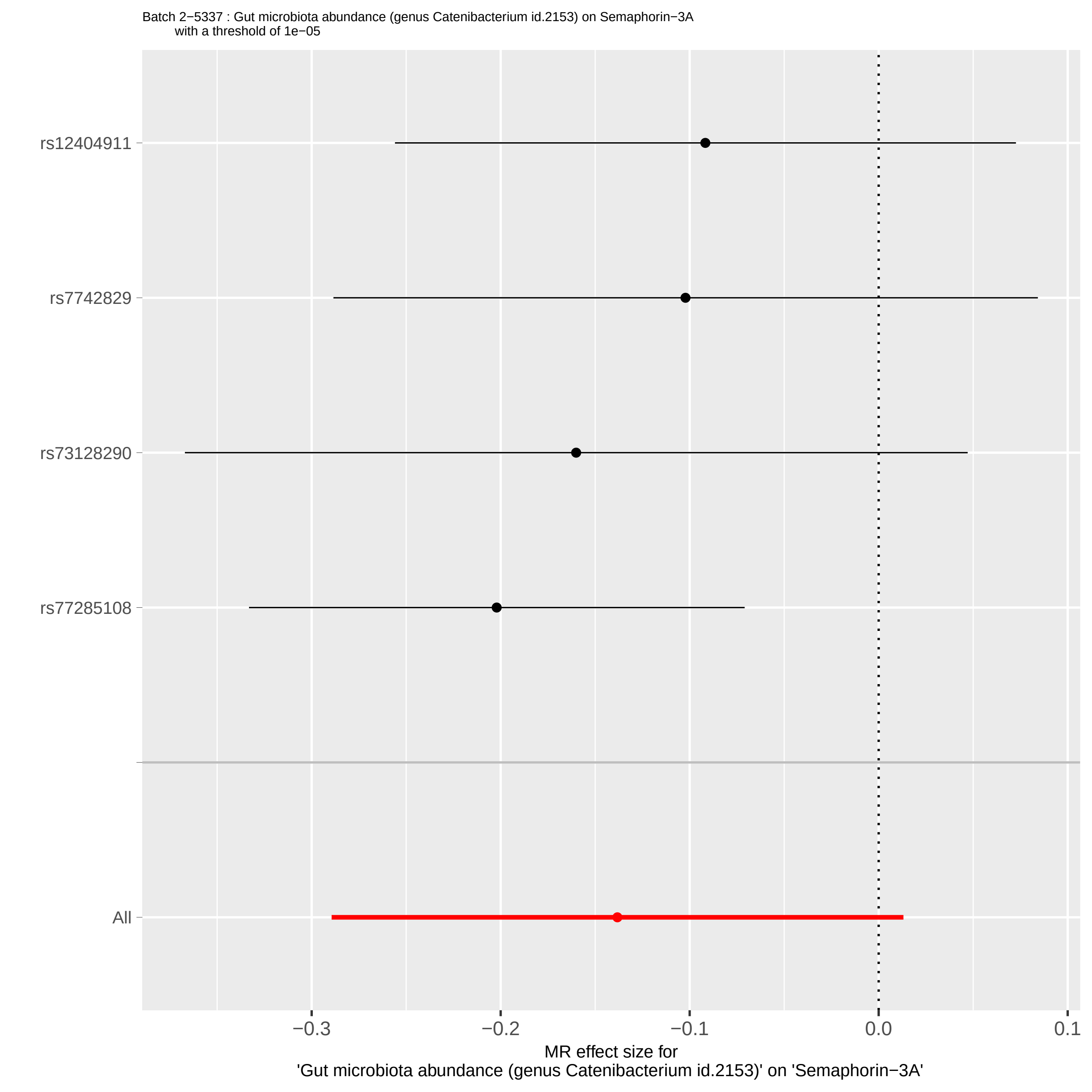

Batch 2−5337 : Gut microbiota abundance (genus Catenibacterium id.2153) on Semaphorin−3A
with a threshold of 1e−05
rs12404911
rs7742829
rs73128290
rs77285108
All
−0.3
−0.2
−0.1
0.0
0.1
MR effect size for
'Gut microbiota abundance (genus Catenibacterium id.2153)' on 'Semaphorin−3A'

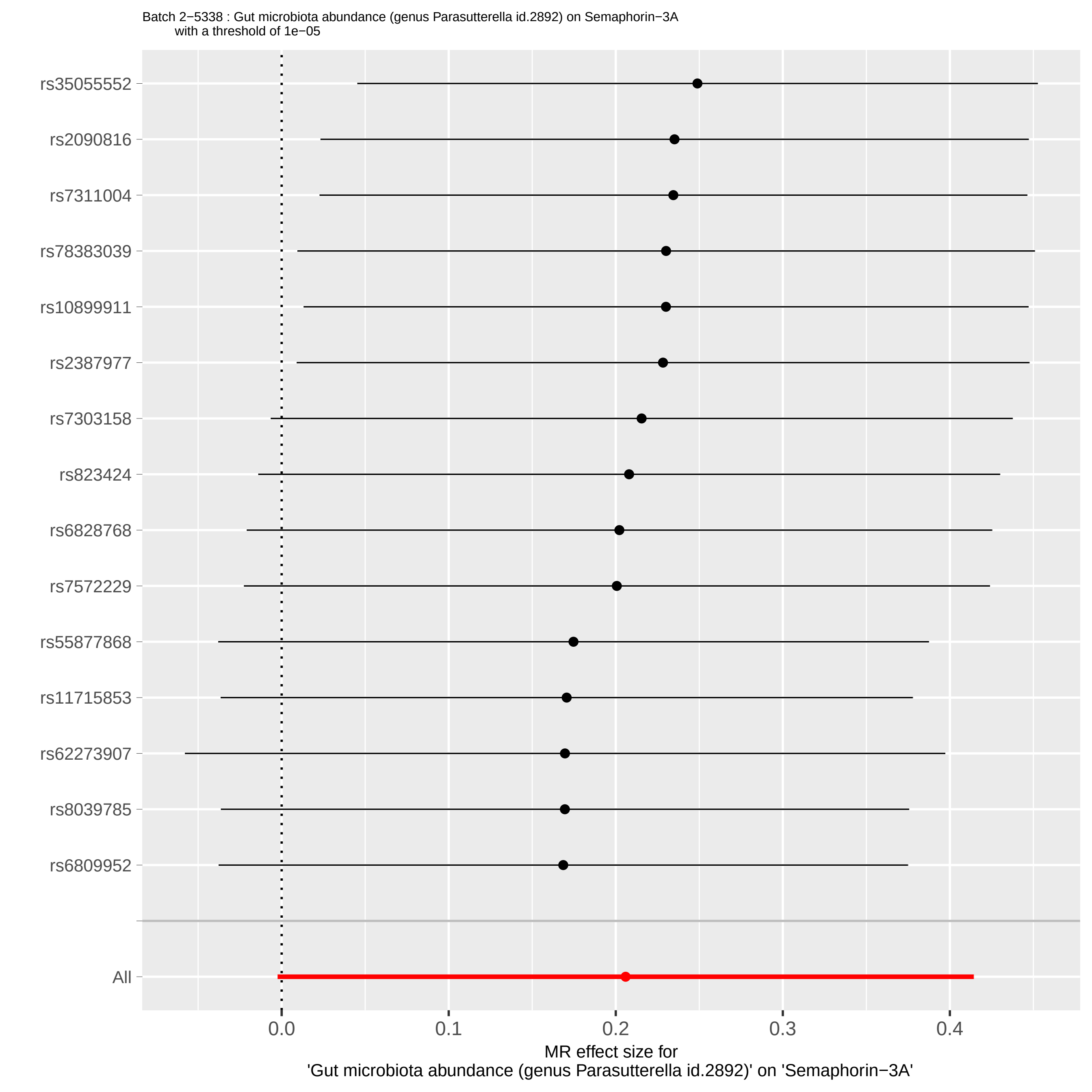

Batch 2−5338 : Gut microbiota abundance (genus Parasutterella id.2892) on Semaphorin−3A
with a threshold of 1e−05
rs35055552
rs2090816
rs7311004
rs78383039
rs10899911
rs2387977
rs7303158
rs823424
rs6828768
rs7572229
rs55877868
rs11715853
rs62273907
rs8039785
rs6809952
All
0.0
0.1
0.2
0.3
0.4
MR effect size for
'Gut microbiota abundance (genus Parasutterella id.2892)' on 'Semaphorin−3A'

Batch 2−5339 : Gut microbiota abundance (genus Catenibacterium id.2153) on Semaphorin−3B
with a threshold of 1e−05

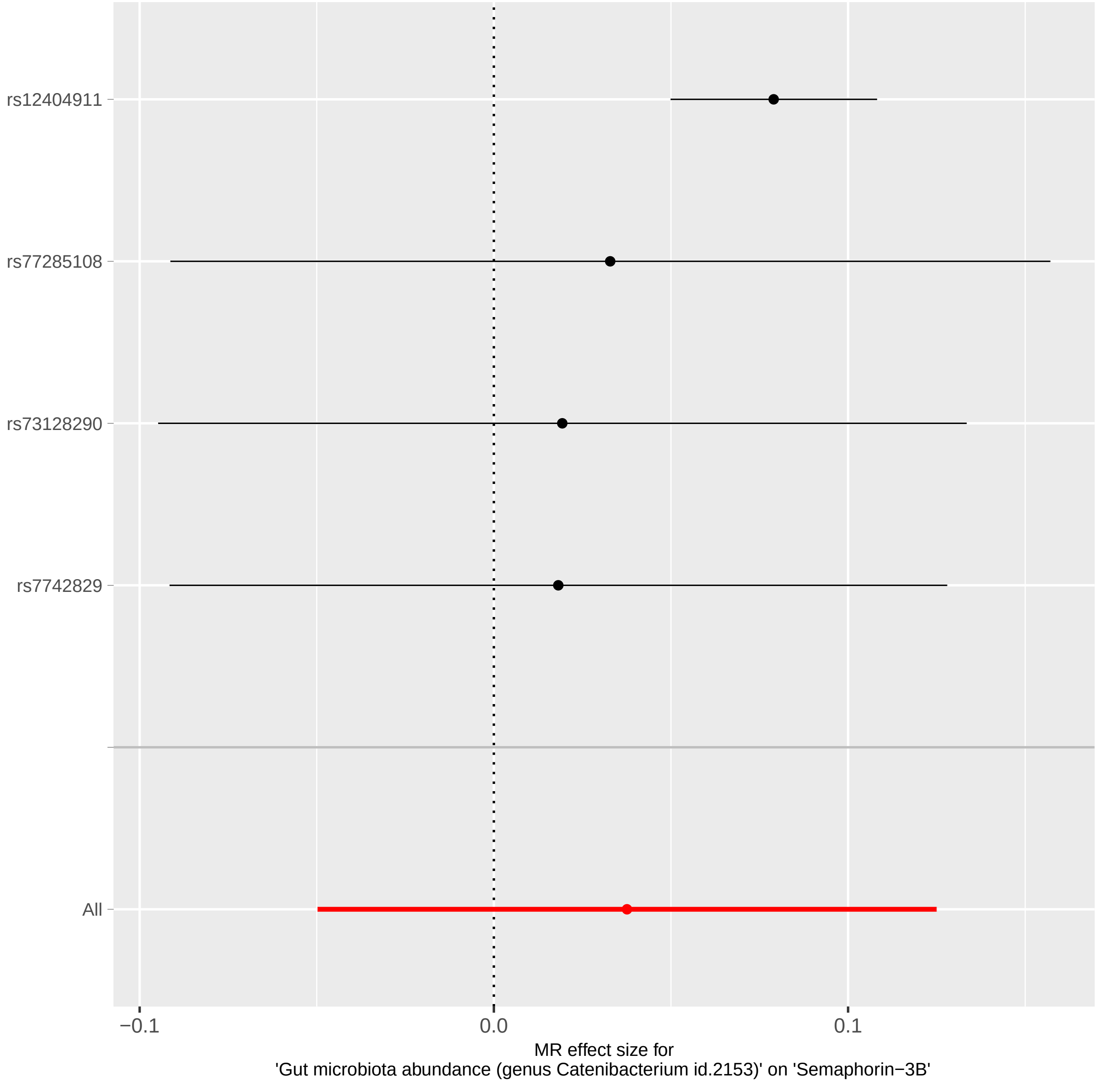

Batch 2−5340 : Gut microbiota abundance (genus Parasutterella id.2892) on Semaphorin−3B
with a threshold of 1e−05

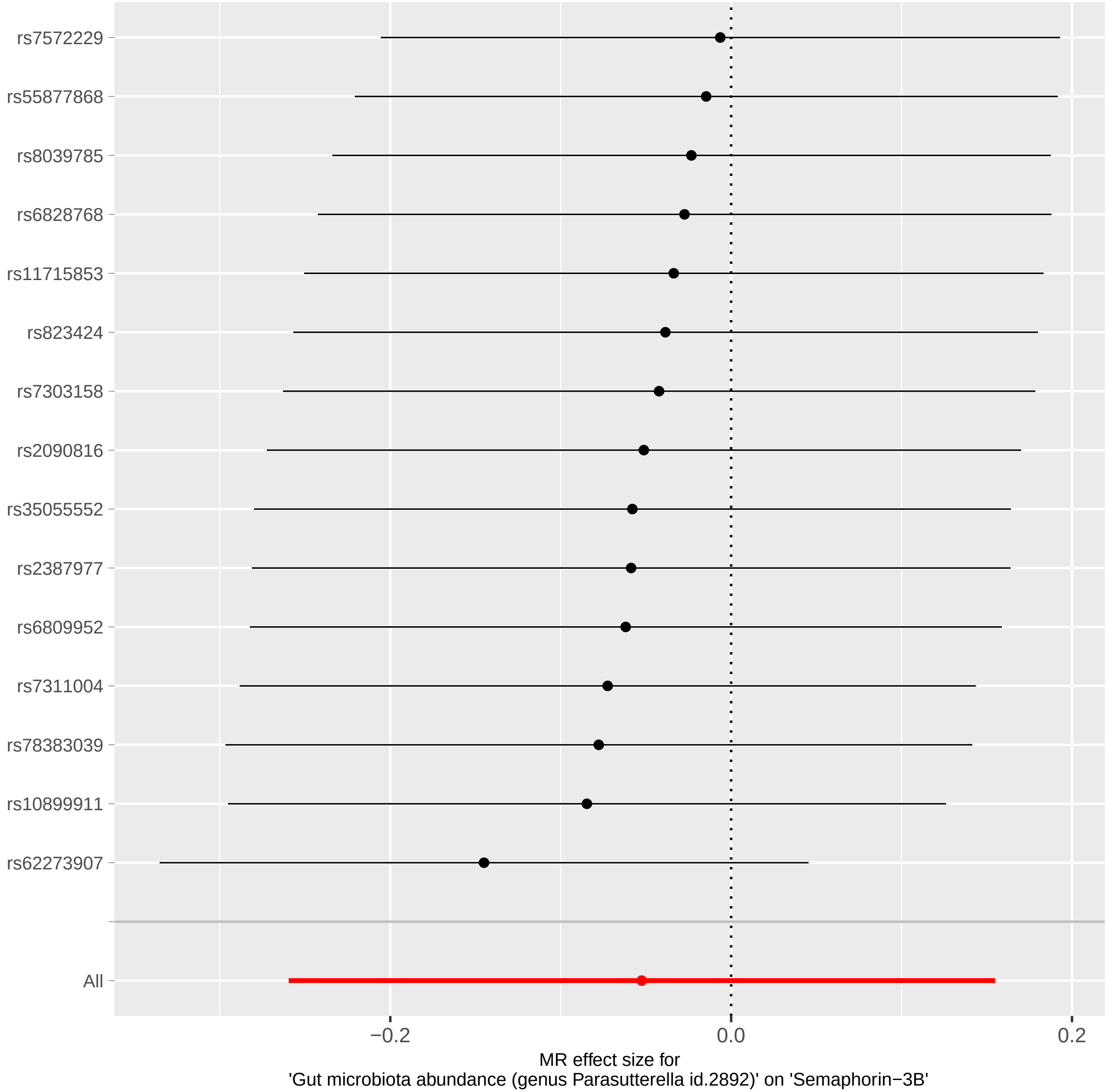

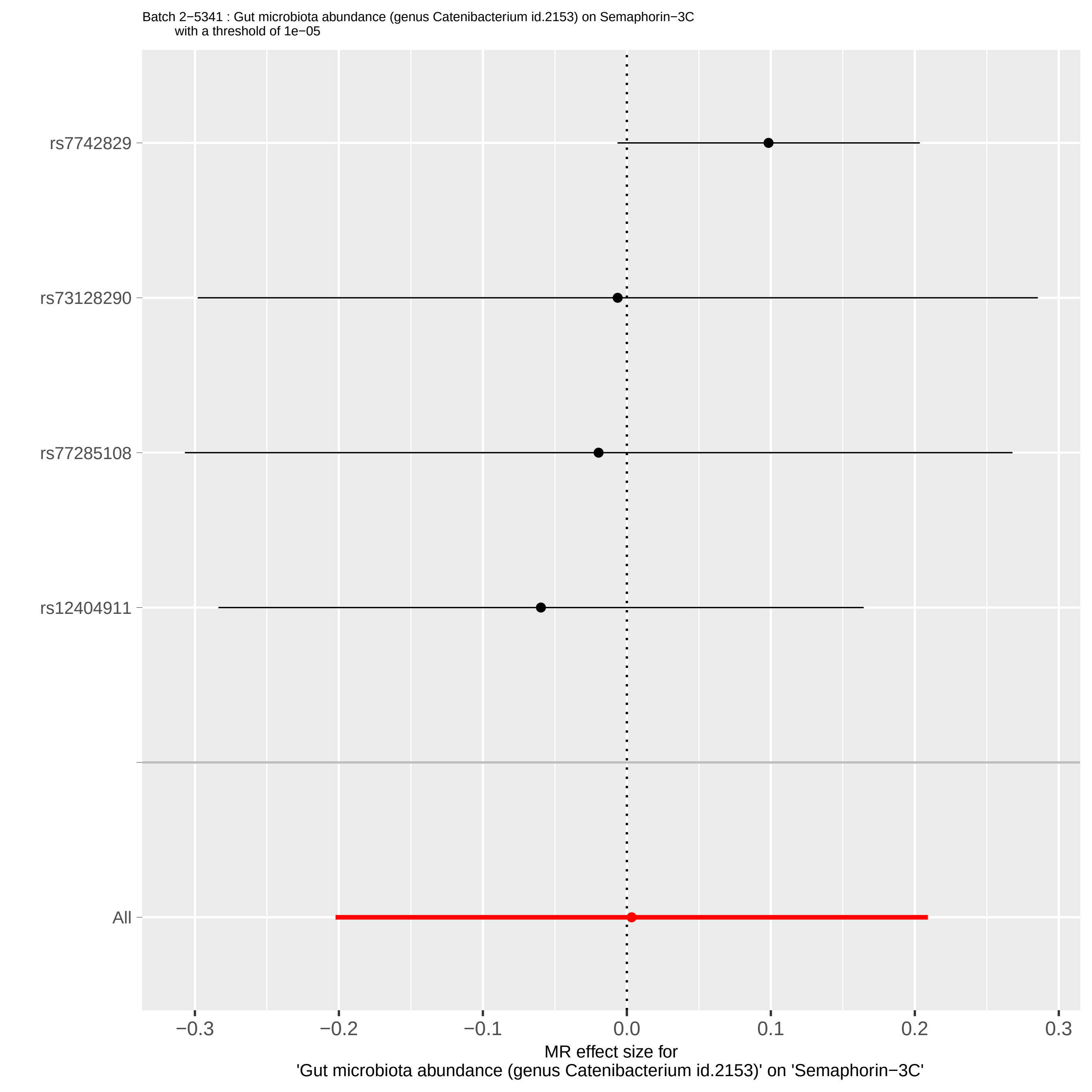
Batch 2−5341 : Gut microbiota abundance (genus Catenibacterium id.2153) on Semaphorin−3C
with a threshold of 1e−05
rs7742829
rs73128290
rs77285108
rs12404911
All
−0.3
−0.2
−0.1
0.0
0.1
0.2
0.3
MR effect size for
'Gut microbiota abundance (genus Catenibacterium id.2153)' on 'Semaphorin−3C'

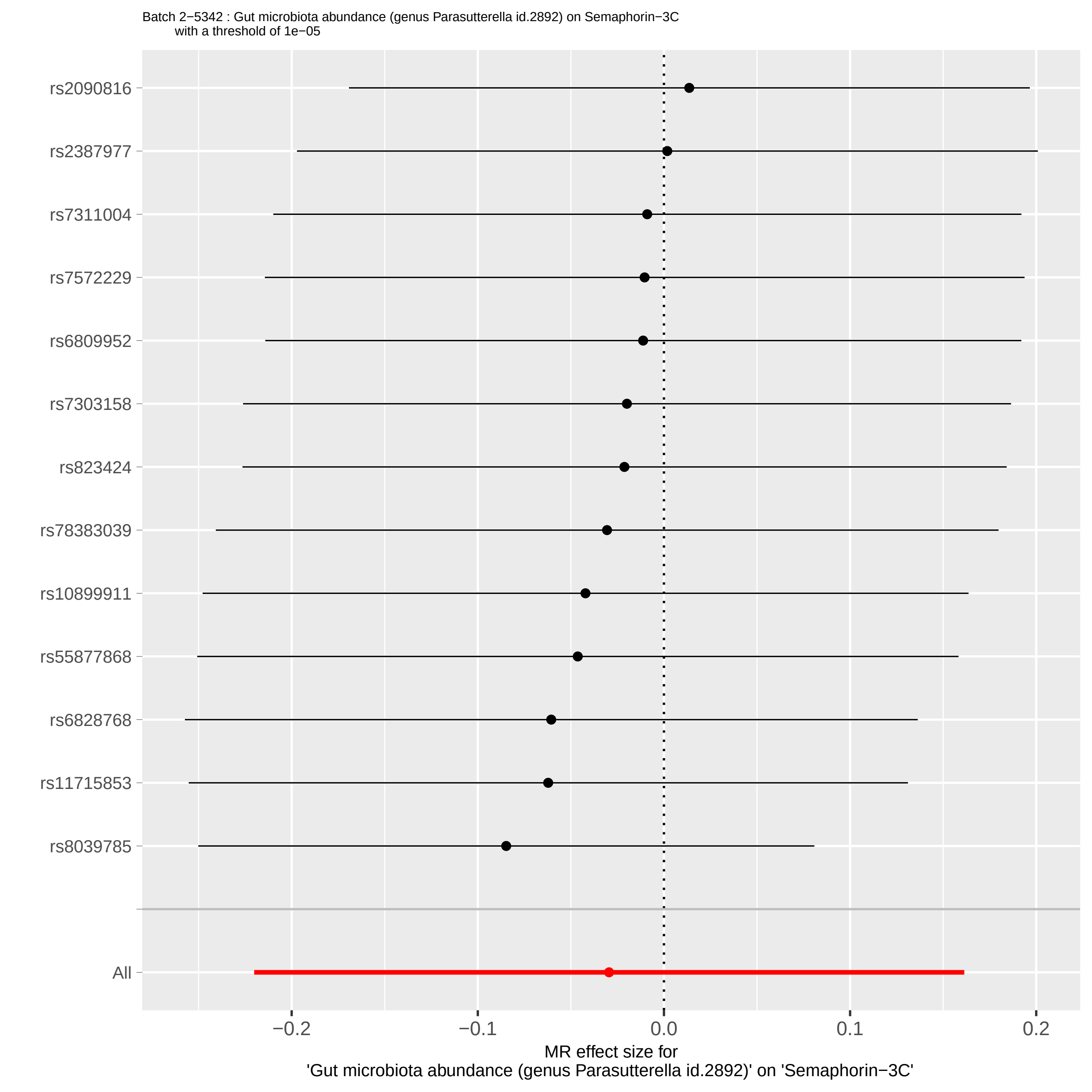
Batch 2−5342 : Gut microbiota abundance (genus Parasutterella id.2892) on Semaphorin−3C
with a threshold of 1e−05
rs2090816
rs2387977
rs7311004
rs7572229
rs6809952
rs7303158
rs823424
rs78383039
rs10899911
rs55877868
rs6828768
rs11715853
rs8039785
All
−0.2
−0.1
0.0
0.1
0.2
MR effect size for
'Gut microbiota abundance (genus Parasutterella id.2892)' on 'Semaphorin−3C'

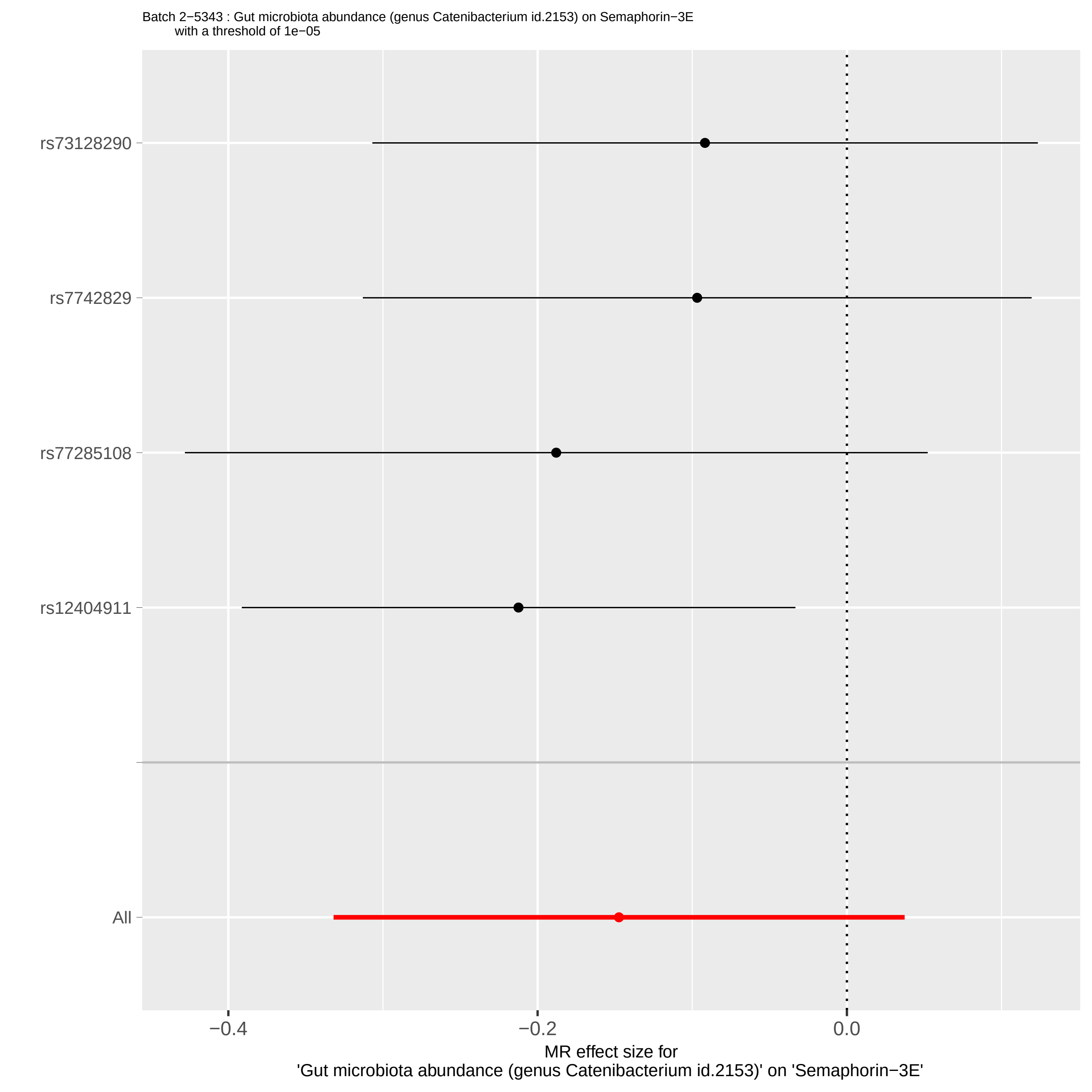
Batch 2−5343 : Gut microbiota abundance (genus Catenibacterium id.2153) on Semaphorin−3E
with a threshold of 1e−05
rs73128290
rs7742829
rs77285108
rs12404911
All
−0.4
−0.2
0.0
MR effect size for
'Gut microbiota abundance (genus Catenibacterium id.2153)' on 'Semaphorin−3E'

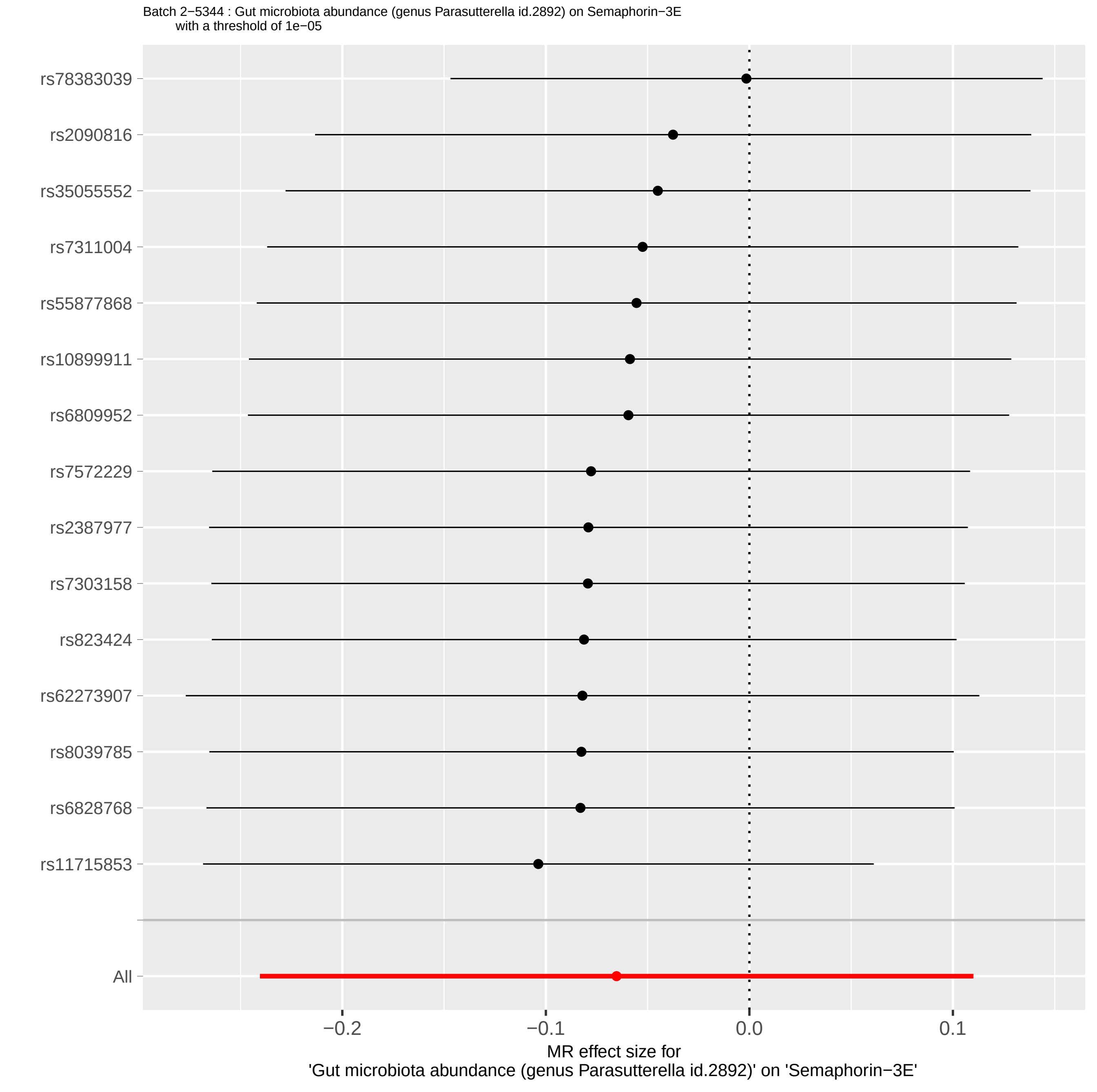

Batch 2−5344 : Gut microbiota abundance (genus Parasutterella id.2892) on Semaphorin−3E
with a threshold of 1e−05
rs78383039
rs2090816
rs35055552
rs7311004
rs55877868
rs10899911
rs6809952
rs7572229
rs2387977
rs7303158
rs823424
rs62273907
rs8039785
rs6828768
rs11715853
All
−0.2
−0.1
0.0
0.1
MR effect size for
'Gut microbiota abundance (genus Parasutterella id.2892)' on 'Semaphorin−3E'

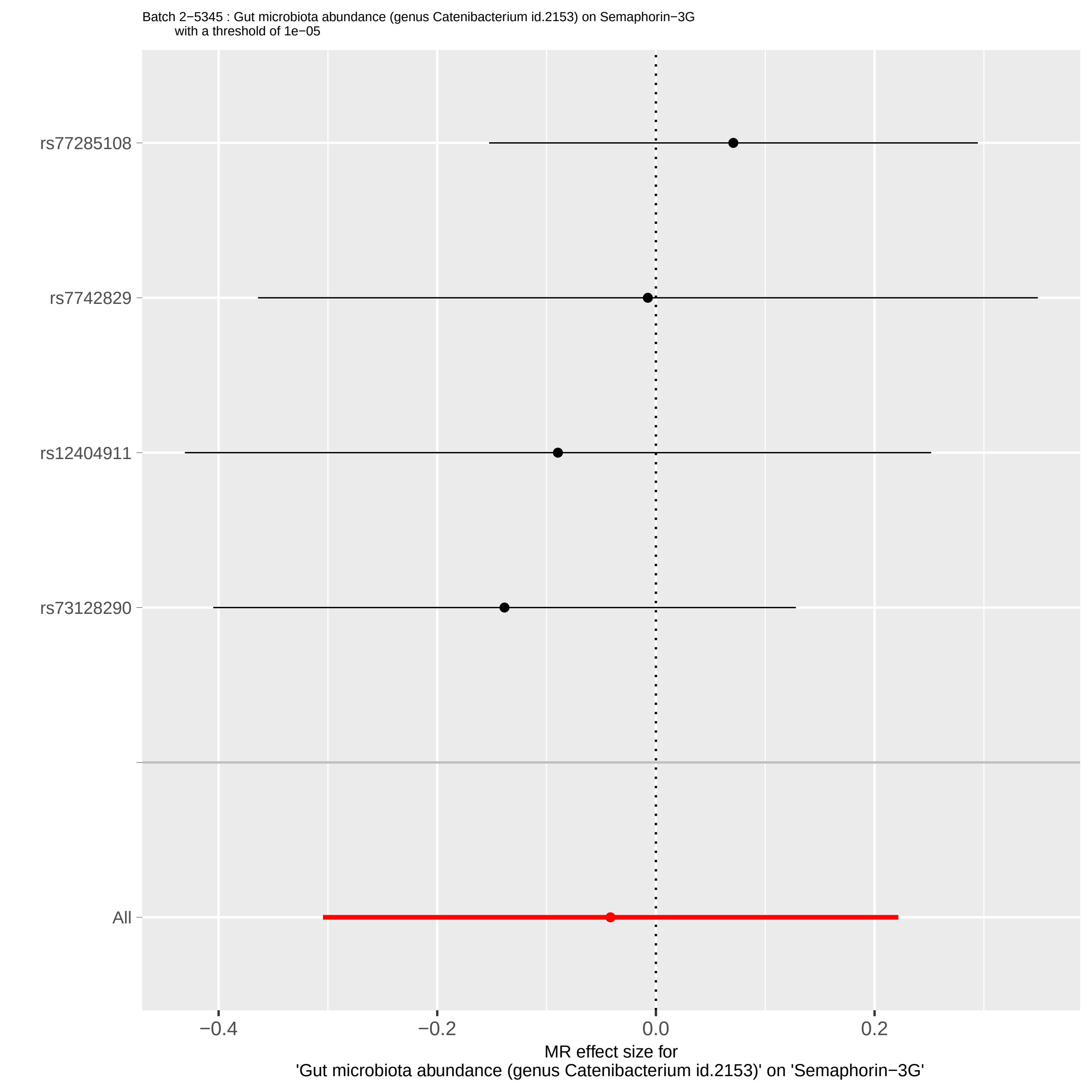
Batch 2−5345 : Gut microbiota abundance (genus Catenibacterium id.2153) on Semaphorin−3G
with a threshold of 1e−05
rs77285108
rs7742829
rs12404911
rs73128290
All
−0.4
−0.2
0.0
0.2
MR effect size for
'Gut microbiota abundance (genus Catenibacterium id.2153)' on 'Semaphorin−3G'

Batch 2−5346 : Gut microbiota abundance (genus Parasutterella id.2892) on Semaphorin−3G
with a threshold of 1e−05

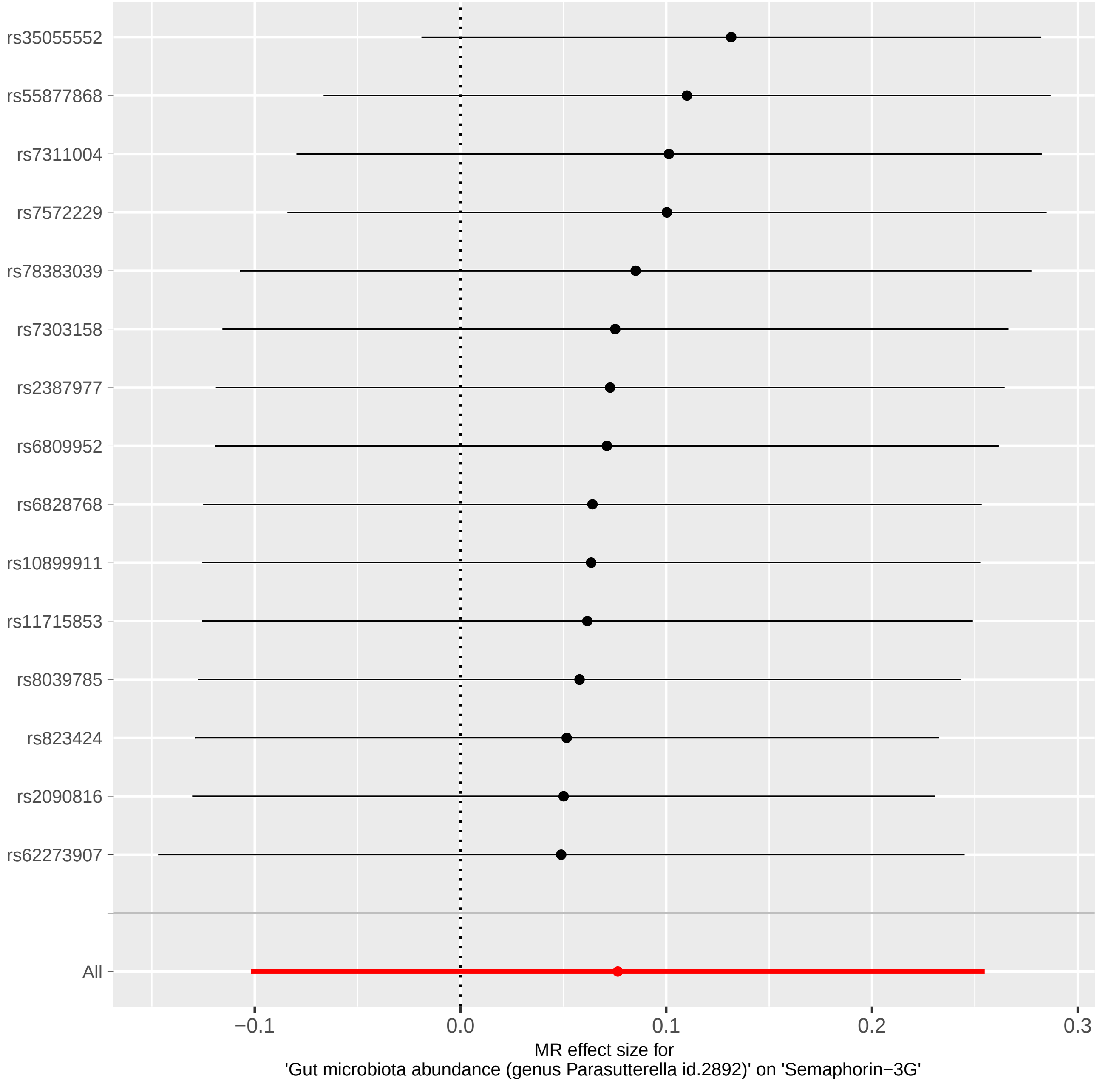

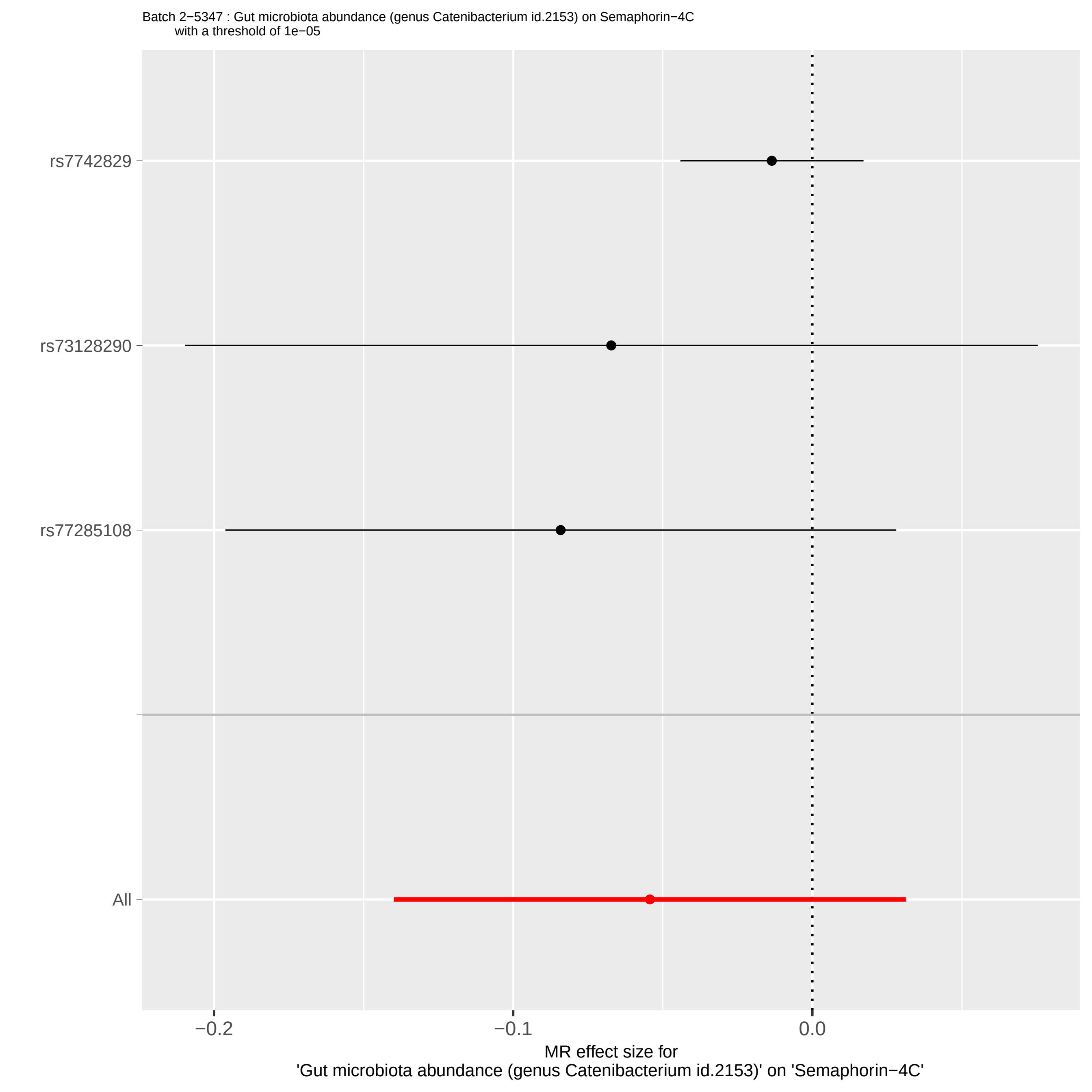

Batch 2−5347 : Gut microbiota abundance (genus Catenibacterium id.2153) on Semaphorin−4C
with a threshold of 1e−05
rs7742829
rs73128290
rs77285108
All
−0.2
−0.1
0.0
MR effect size for
'Gut microbiota abundance (genus Catenibacterium id.2153)' on 'Semaphorin−4C'

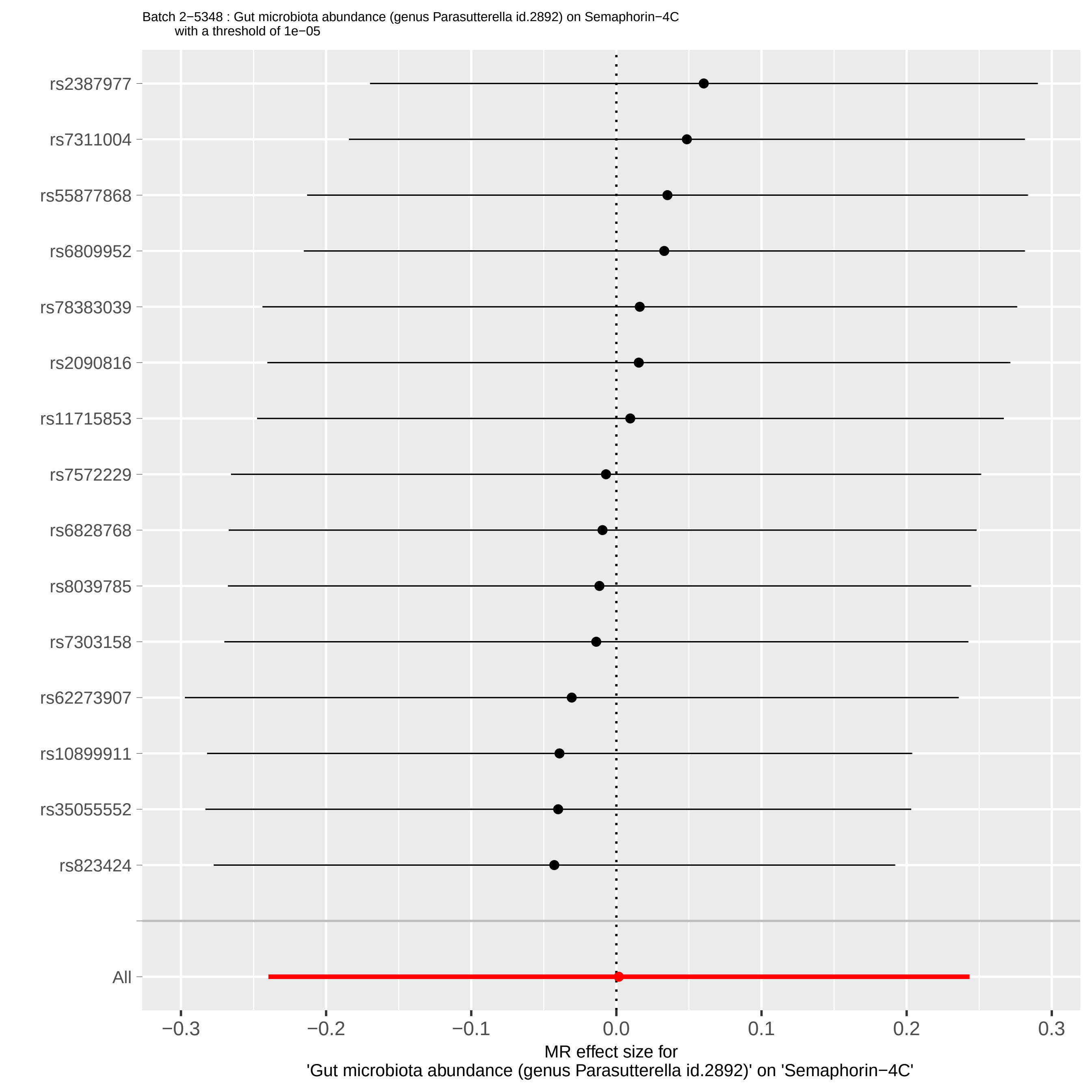

Batch 2−5348 : Gut microbiota abundance (genus Parasutterella id.2892) on Semaphorin−4C
with a threshold of 1e−05
rs2387977
rs7311004
rs55877868
rs6809952
rs78383039
rs2090816
rs11715853
rs7572229
rs6828768
rs8039785
rs7303158
rs62273907
rs10899911
rs35055552
rs823424
All
−0.3
−0.2
−0.1
0.0
0.1
0.2
0.3
MR effect size for
'Gut microbiota abundance (genus Parasutterella id.2892)' on 'Semaphorin−4C'

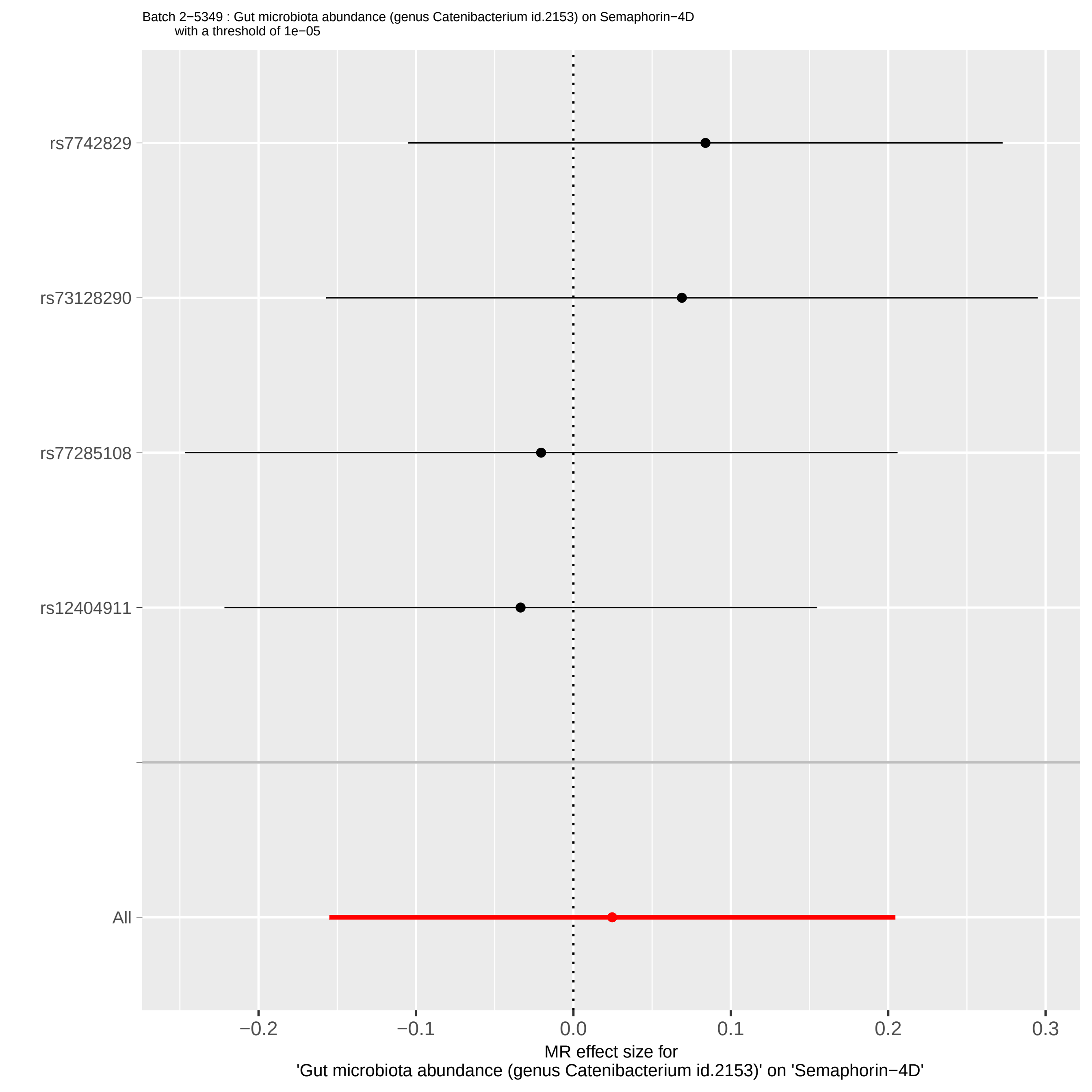

Batch 2−5349 : Gut microbiota abundance (genus Catenibacterium id.2153) on Semaphorin−4D
with a threshold of 1e−05
rs7742829
rs73128290
rs77285108
rs12404911
All
−0.2
−0.1
0.0
0.1
0.2
0.3
MR effect size for
'Gut microbiota abundance (genus Catenibacterium id.2153)' on 'Semaphorin−4D'

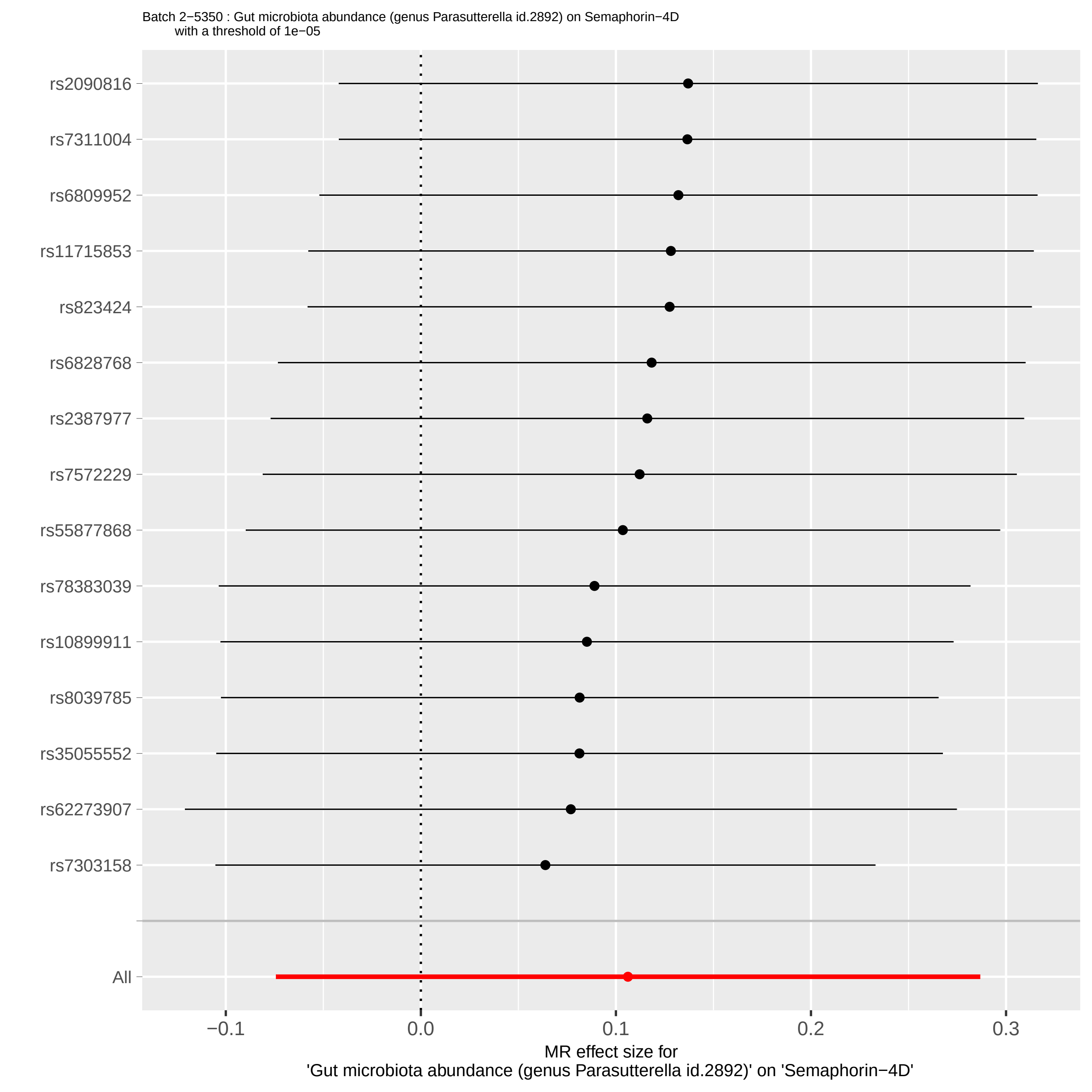

Batch 2−5350 : Gut microbiota abundance (genus Parasutterella id.2892) on Semaphorin−4D
with a threshold of 1e−05
rs2090816
rs7311004
rs6809952
rs11715853
rs823424
rs6828768
rs2387977
rs7572229
rs55877868
rs78383039
rs10899911
rs8039785
rs35055552
rs62273907
rs7303158
All
−0.1
0.0
0.1
0.2
0.3
MR effect size for
'Gut microbiota abundance (genus Parasutterella id.2892)' on 'Semaphorin−4D'

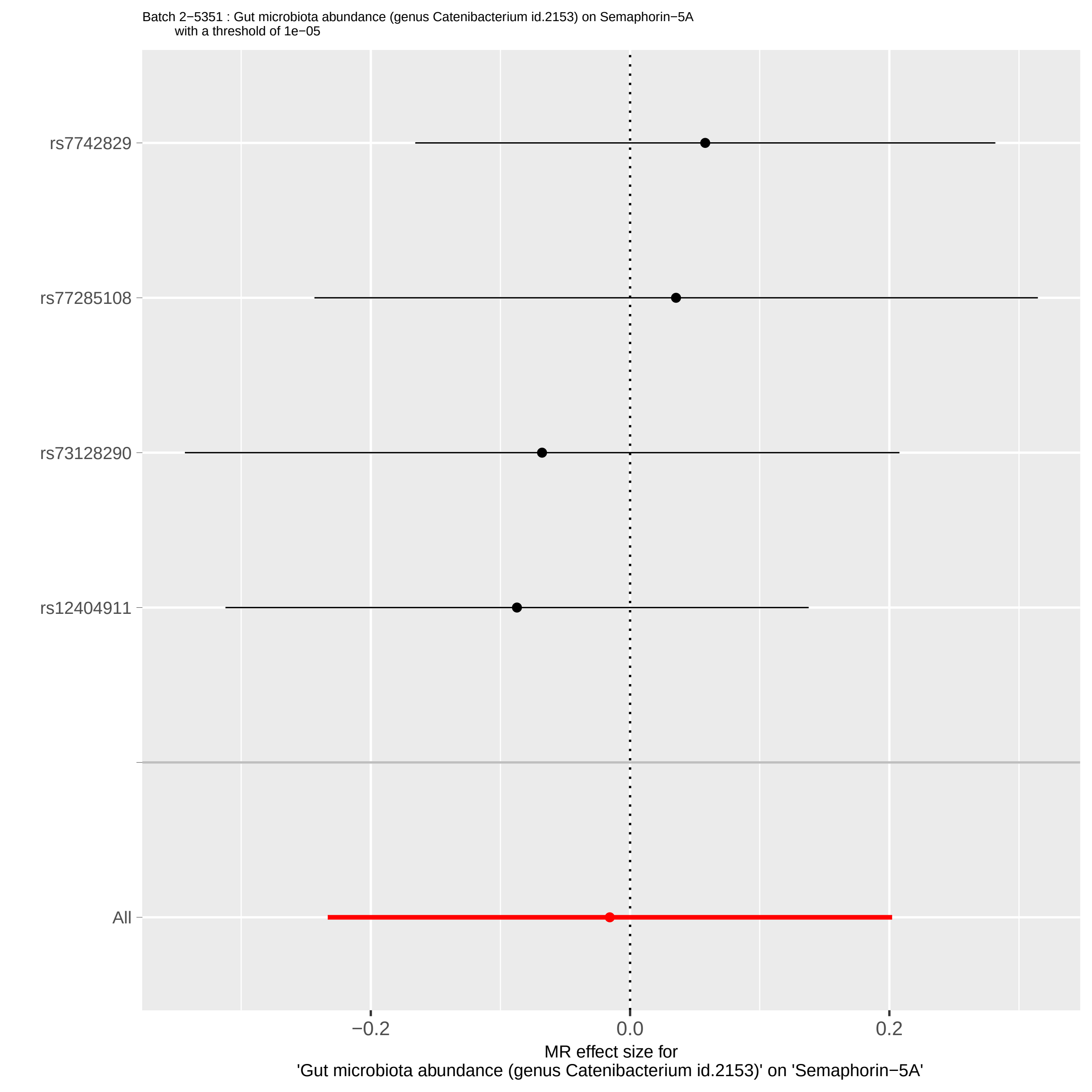

Batch 2−5351 : Gut microbiota abundance (genus Catenibacterium id.2153) on Semaphorin−5A
with a threshold of 1e−05
rs7742829
rs77285108
rs73128290
rs12404911
All
−0.2
0.0
0.2
MR effect size for
'Gut microbiota abundance (genus Catenibacterium id.2153)' on 'Semaphorin−5A'

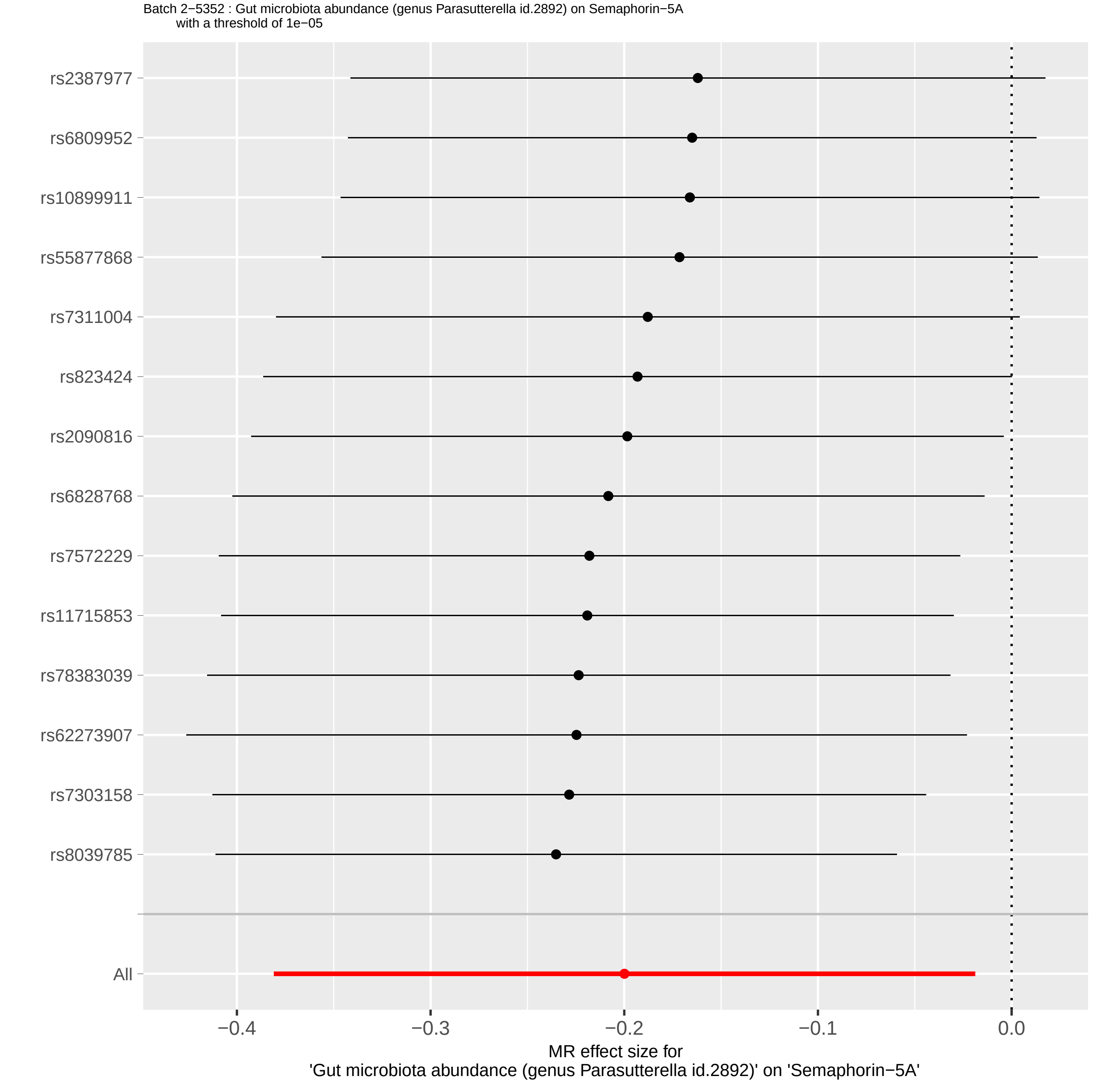
Batch 2−5352 : Gut microbiota abundance (genus Parasutterella id.2892) on Semaphorin−5A
with a threshold of 1e−05
rs2387977
rs6809952
rs10899911
rs55877868
rs7311004
rs823424
rs2090816
rs6828768
rs7572229
rs11715853
rs78383039
rs62273907
rs7303158
rs8039785
All
−0.4
−0.3
−0.2
−0.1
0.0
MR effect size for
'Gut microbiota abundance (genus Parasutterella id.2892)' on 'Semaphorin−5A'

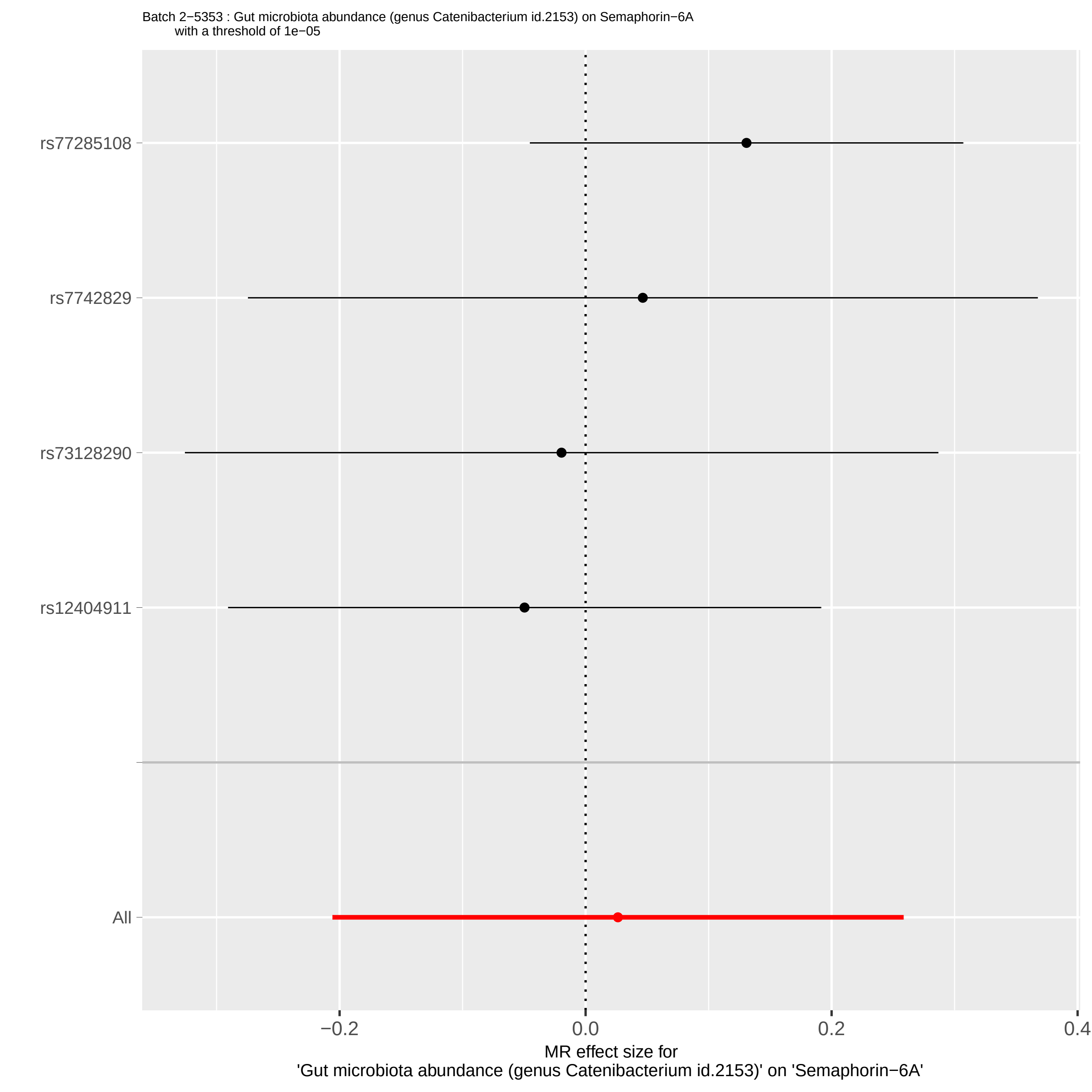
Batch 2−5353 : Gut microbiota abundance (genus Catenibacterium id.2153) on Semaphorin−6A
with a threshold of 1e−05
rs77285108
rs7742829
rs73128290
rs12404911
All
−0.2
0.0
0.2
0.4
MR effect size for
'Gut microbiota abundance (genus Catenibacterium id.2153)' on 'Semaphorin−6A'

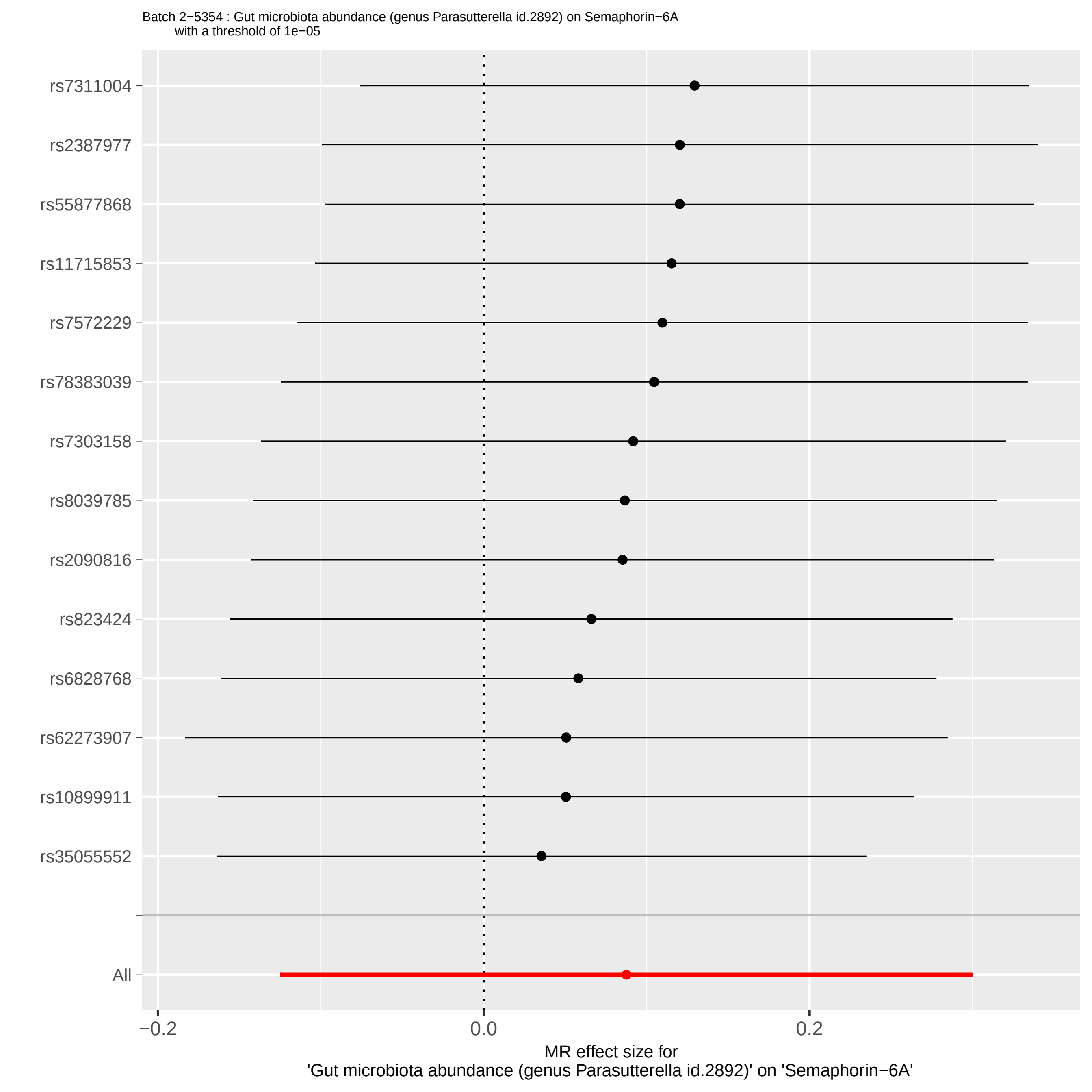
Batch 2−5354 : Gut microbiota abundance (genus Parasutterella id.2892) on Semaphorin−6A
with a threshold of 1e−05
rs7311004
rs2387977
rs55877868
rs11715853
rs7572229
rs78383039
rs7303158
rs8039785
rs2090816
rs823424
rs6828768
rs62273907
rs10899911
rs35055552
All
−0.2
0.0
0.2
MR effect size for
'Gut microbiota abundance (genus Parasutterella id.2892)' on 'Semaphorin−6A'

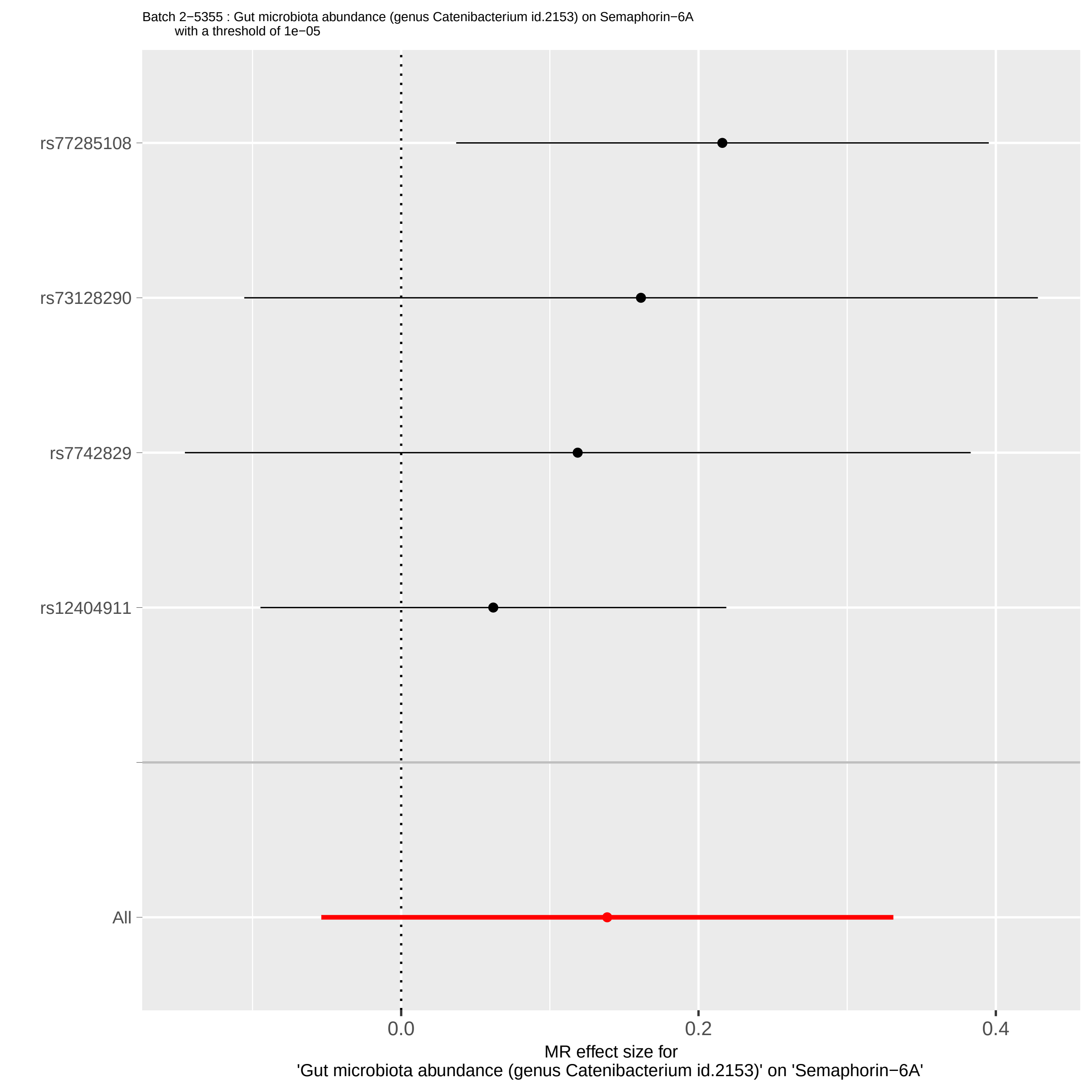
Batch 2−5355 : Gut microbiota abundance (genus Catenibacterium id.2153) on Semaphorin−6A
with a threshold of 1e−05
rs77285108
rs73128290
rs7742829
rs12404911
All
0.0
0.2
0.4
MR effect size for
'Gut microbiota abundance (genus Catenibacterium id.2153)' on 'Semaphorin−6A'

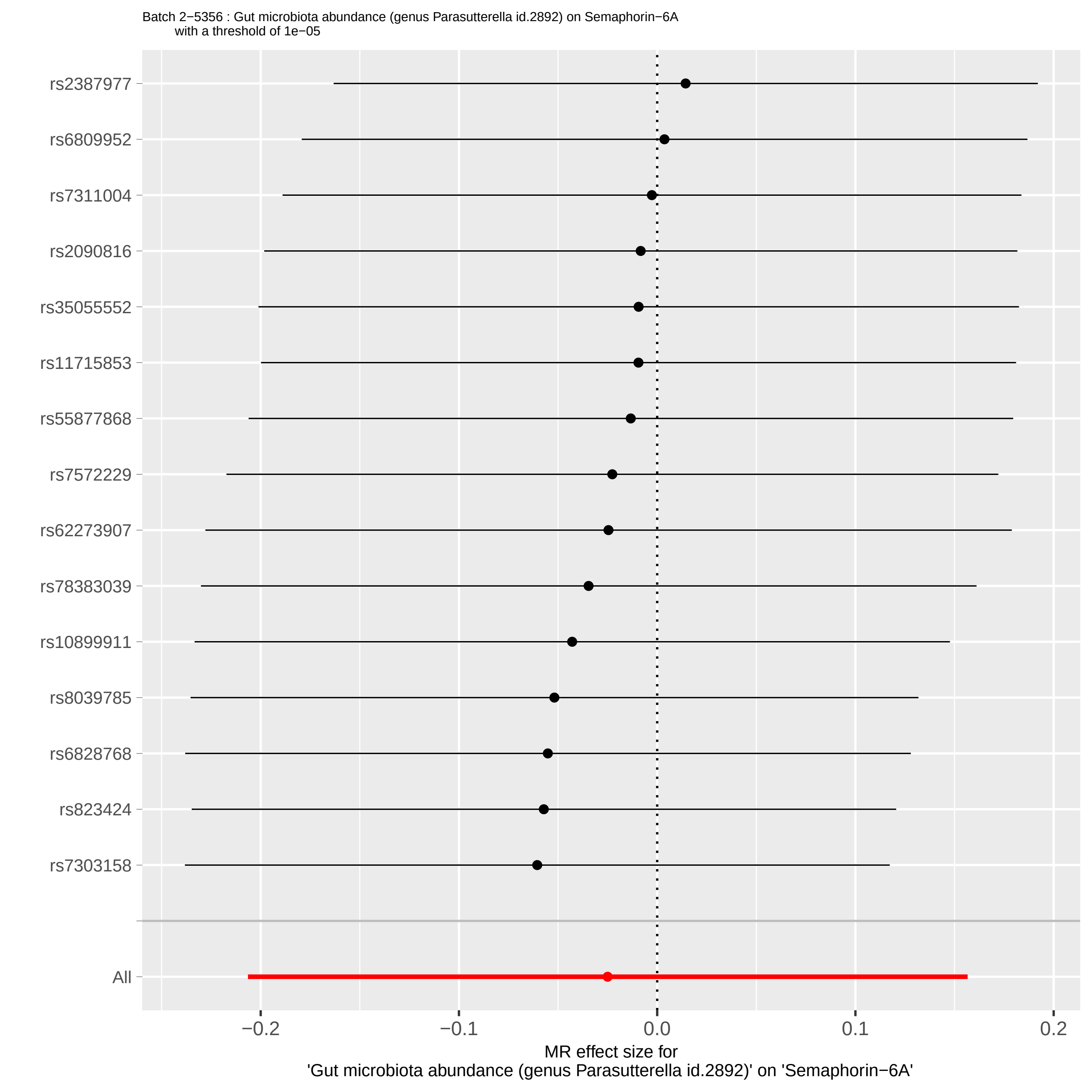

Batch 2−5356 : Gut microbiota abundance (genus Parasutterella id.2892) on Semaphorin−6A
with a threshold of 1e−05
rs2387977
rs6809952
rs7311004
rs2090816
rs35055552
rs11715853
rs55877868
rs7572229
rs62273907
rs78383039
rs10899911
rs8039785
rs6828768
rs823424
rs7303158
All
−0.2
−0.1
0.0
0.1
0.2
MR effect size for
'Gut microbiota abundance (genus Parasutterella id.2892)' on 'Semaphorin−6A'

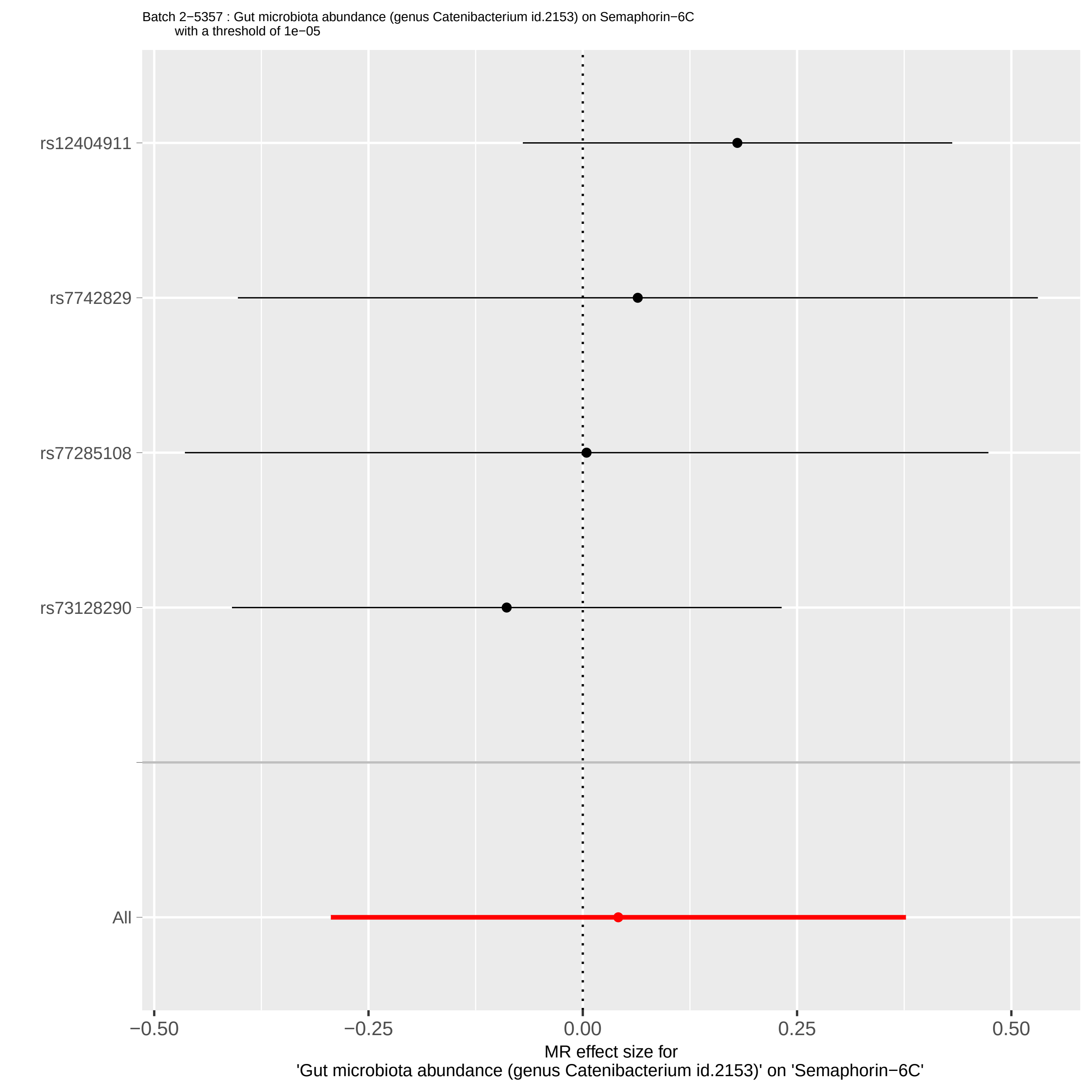
Batch 2−5357 : Gut microbiota abundance (genus Catenibacterium id.2153) on Semaphorin−6C
with a threshold of 1e−05
rs12404911
rs7742829
rs77285108
rs73128290
All
−0.50
−0.25
0.00
0.25
0.50
MR effect size for
'Gut microbiota abundance (genus Catenibacterium id.2153)' on 'Semaphorin−6C'

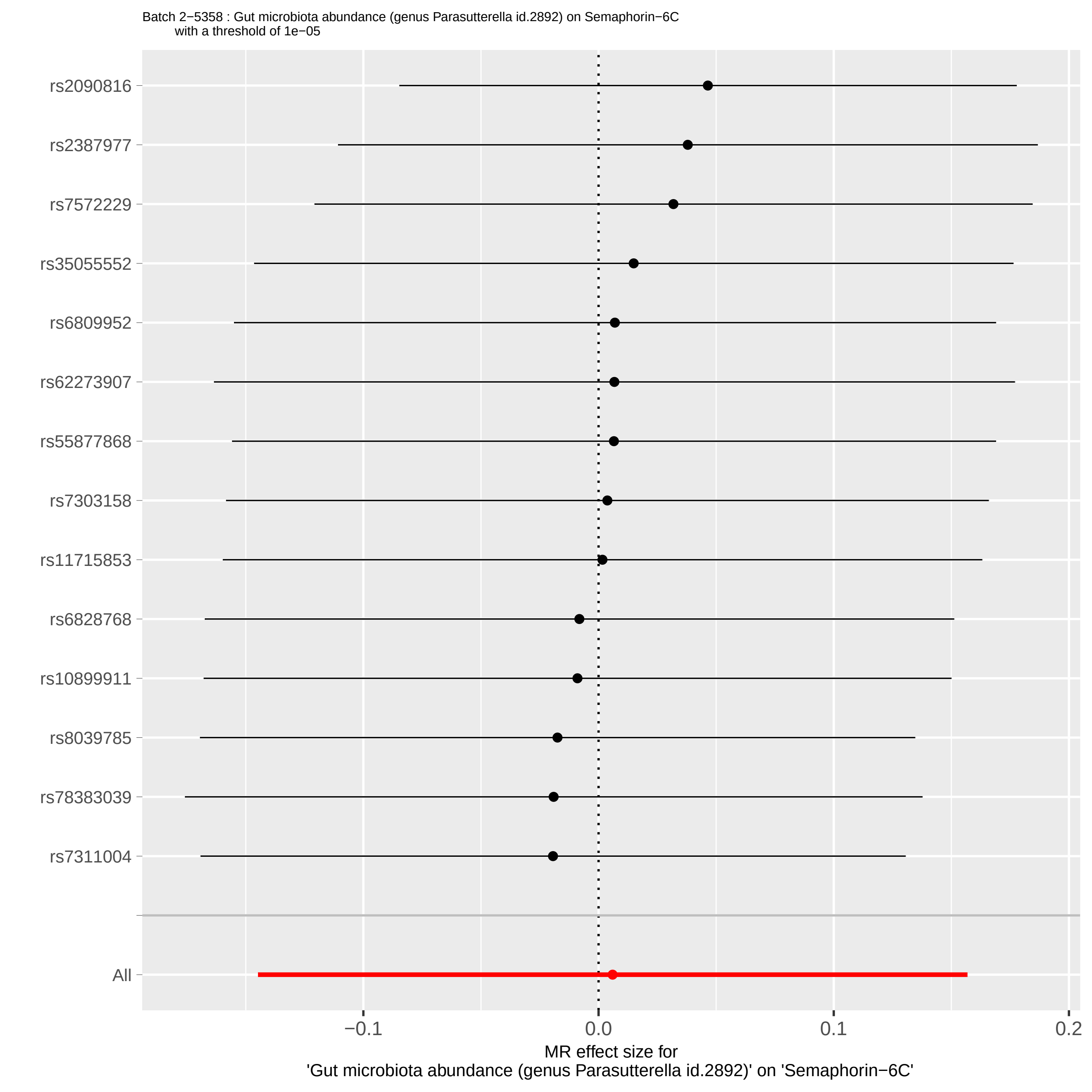
Batch 2−5358 : Gut microbiota abundance (genus Parasutterella id.2892) on Semaphorin−6C
with a threshold of 1e−05
rs2090816
rs2387977
rs7572229
rs35055552
rs6809952
rs62273907
rs55877868
rs7303158
rs11715853
rs6828768
rs10899911
rs8039785
rs78383039
rs7311004
All
−0.1
0.0
0.1
0.2
MR effect size for
'Gut microbiota abundance (genus Parasutterella id.2892)' on 'Semaphorin−6C'

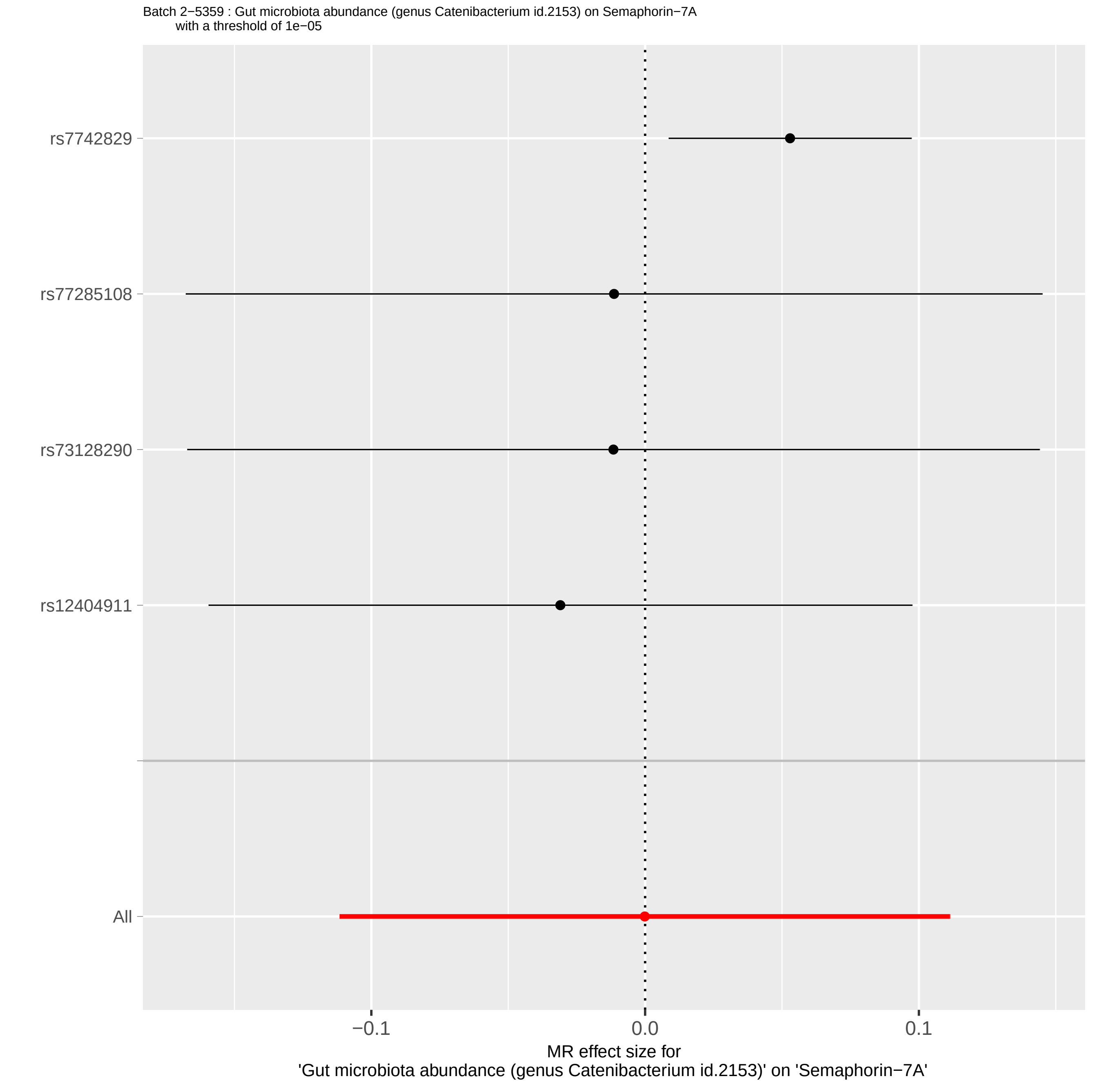

Batch 2−5359 : Gut microbiota abundance (genus Catenibacterium id.2153) on Semaphorin−7A
with a threshold of 1e−05
rs7742829
rs77285108
rs73128290
rs12404911
All
−0.1
0.0
0.1
MR effect size for
'Gut microbiota abundance (genus Catenibacterium id.2153)' on 'Semaphorin−7A'

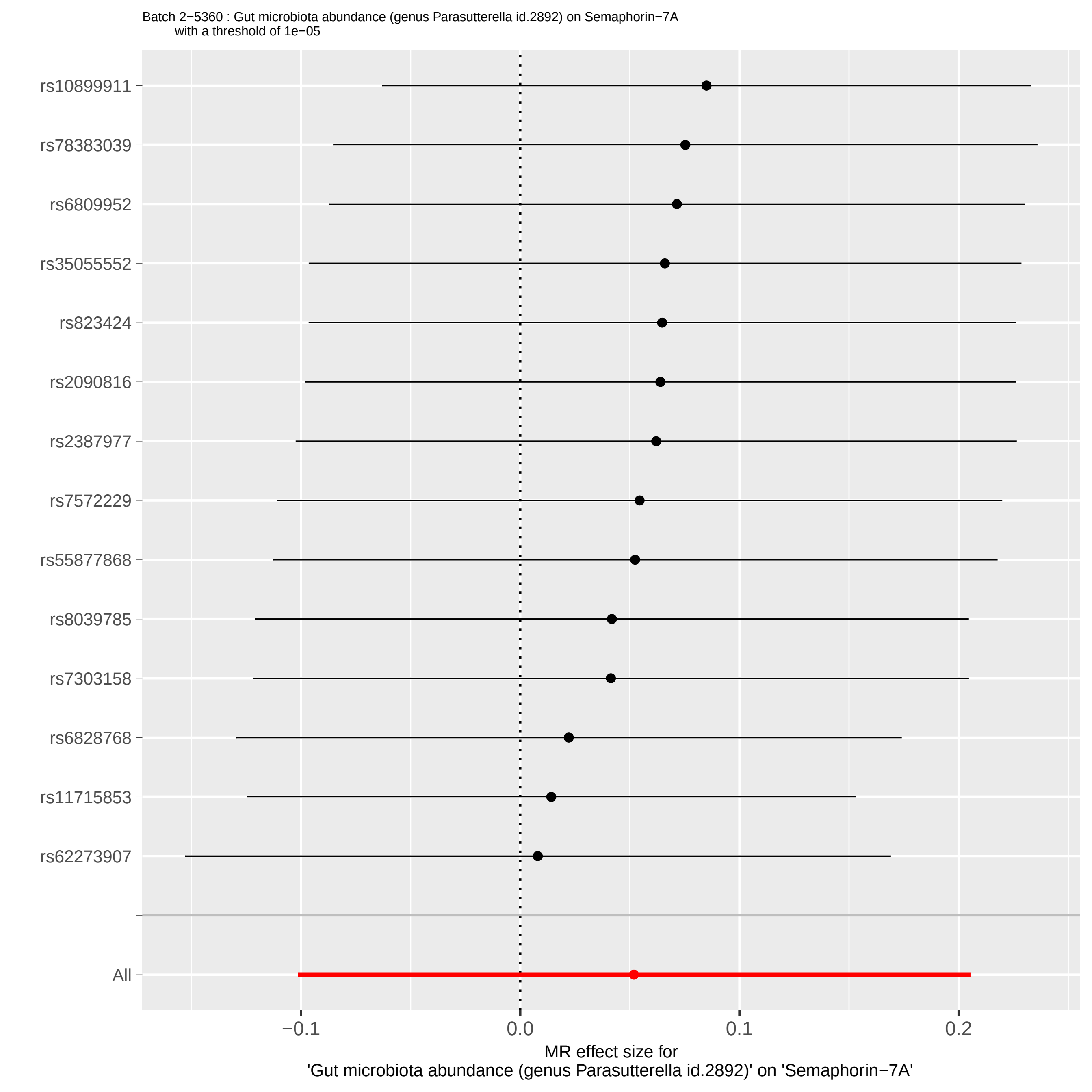

Batch 2−5360 : Gut microbiota abundance (genus Parasutterella id.2892) on Semaphorin−7A
with a threshold of 1e−05
rs10899911
rs78383039
rs6809952
rs35055552
rs823424
rs2090816
rs2387977
rs7572229
rs55877868
rs8039785
rs7303158
rs6828768
rs11715853
rs62273907
All
−0.1
0.0
0.1
0.2
MR effect size for
'Gut microbiota abundance (genus Parasutterella id.2892)' on 'Semaphorin−7A'

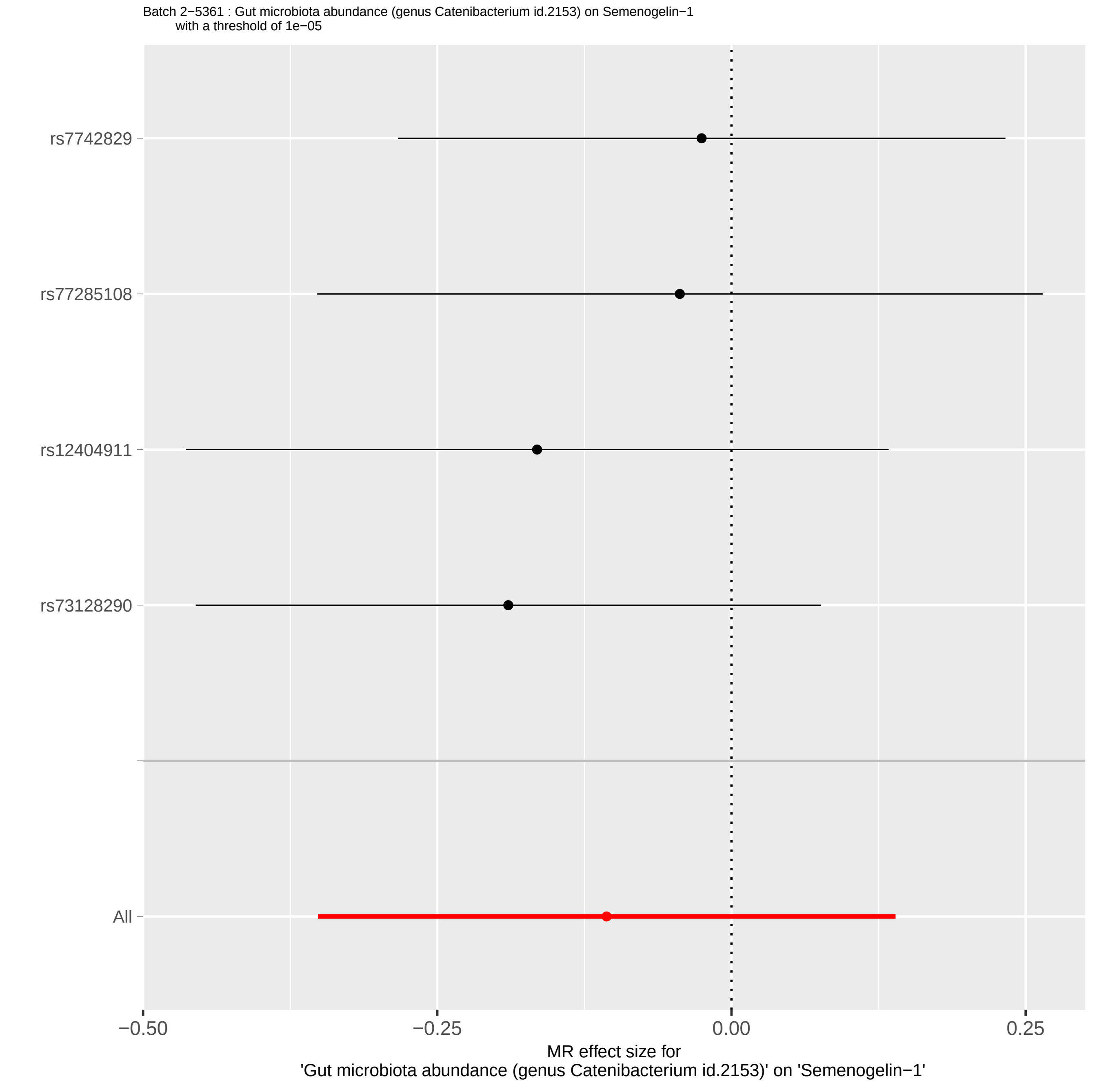
Batch 2−5361 : Gut microbiota abundance (genus Catenibacterium id.2153) on Semenogelin−1
with a threshold of 1e−05
rs7742829
rs77285108
rs12404911
rs73128290
All
−0.50
−0.25
0.00
0.25
MR effect size for
'Gut microbiota abundance (genus Catenibacterium id.2153)' on 'Semenogelin−1'

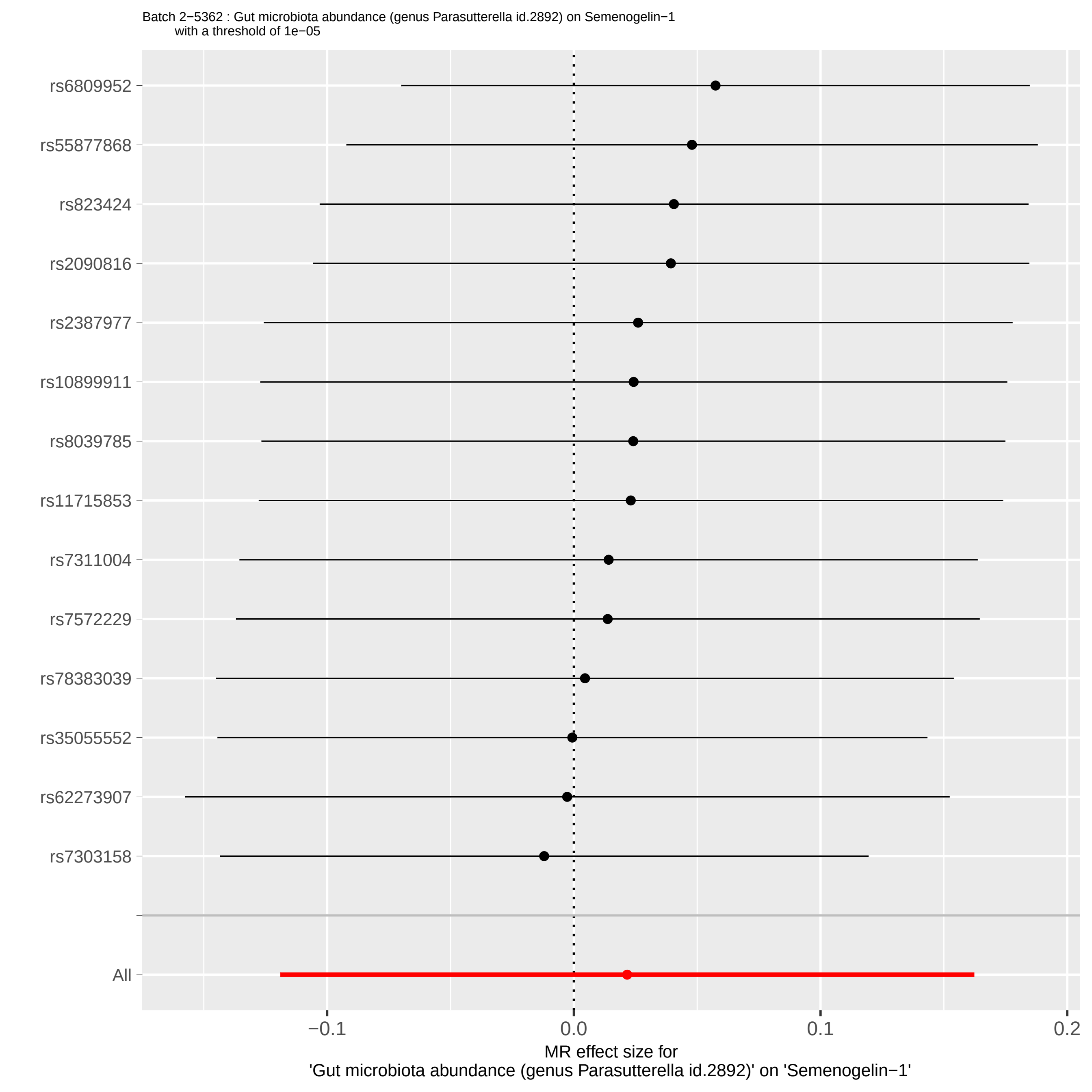

Batch 2−5362 : Gut microbiota abundance (genus Parasutterella id.2892) on Semenogelin−1
with a threshold of 1e−05
rs6809952
rs55877868
rs823424
rs2090816
rs2387977
rs10899911
rs8039785
rs11715853
rs7311004
rs7572229
rs78383039
rs35055552
rs62273907
rs7303158
All
−0.1
0.0
0.1
0.2
MR effect size for
'Gut microbiota abundance (genus Parasutterella id.2892)' on 'Semenogelin−1'

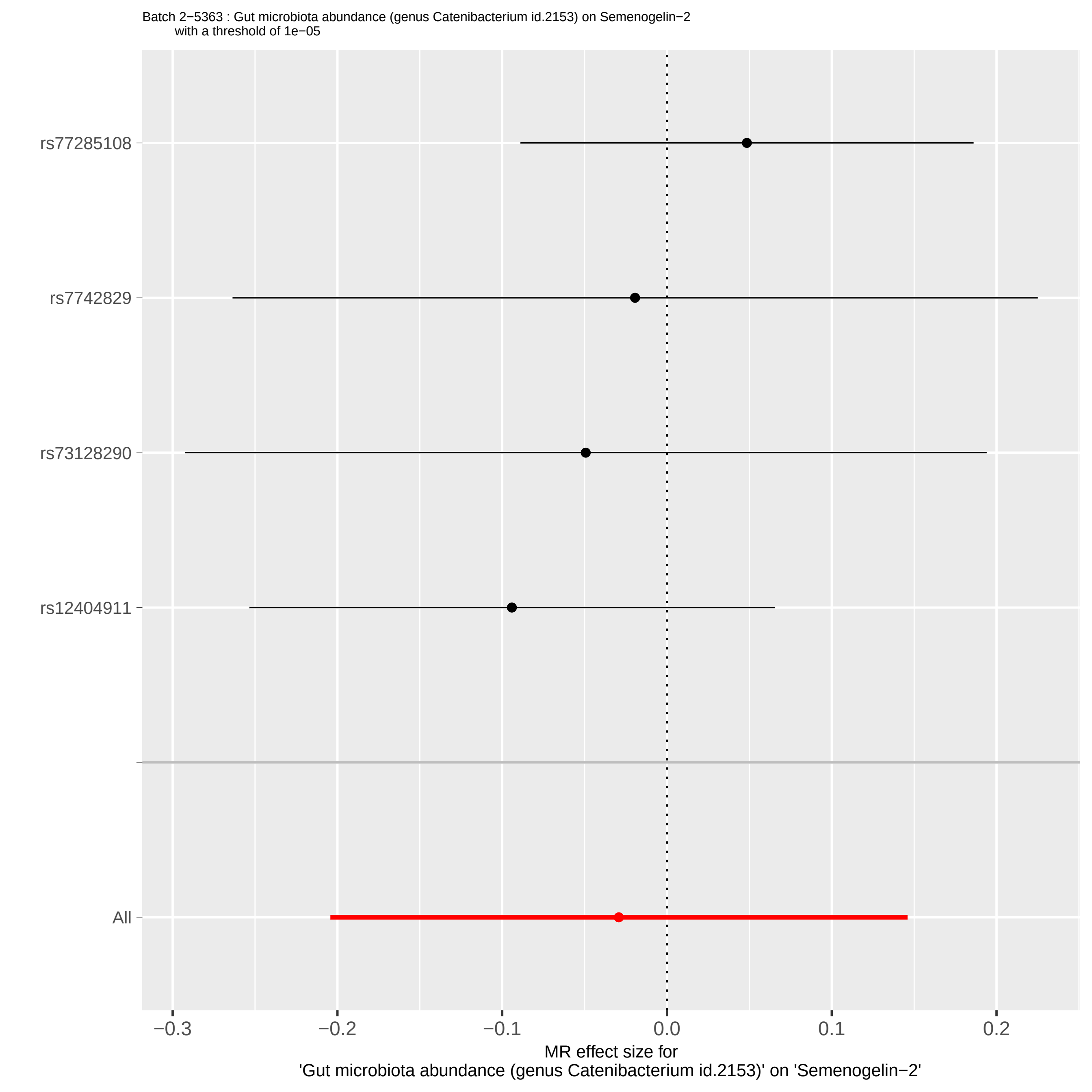
Batch 2−5363 : Gut microbiota abundance (genus Catenibacterium id.2153) on Semenogelin−2
with a threshold of 1e−05
rs77285108
rs7742829
rs73128290
rs12404911
All
−0.3
−0.2
−0.1
0.0
0.1
0.2
MR effect size for
'Gut microbiota abundance (genus Catenibacterium id.2153)' on 'Semenogelin−2'

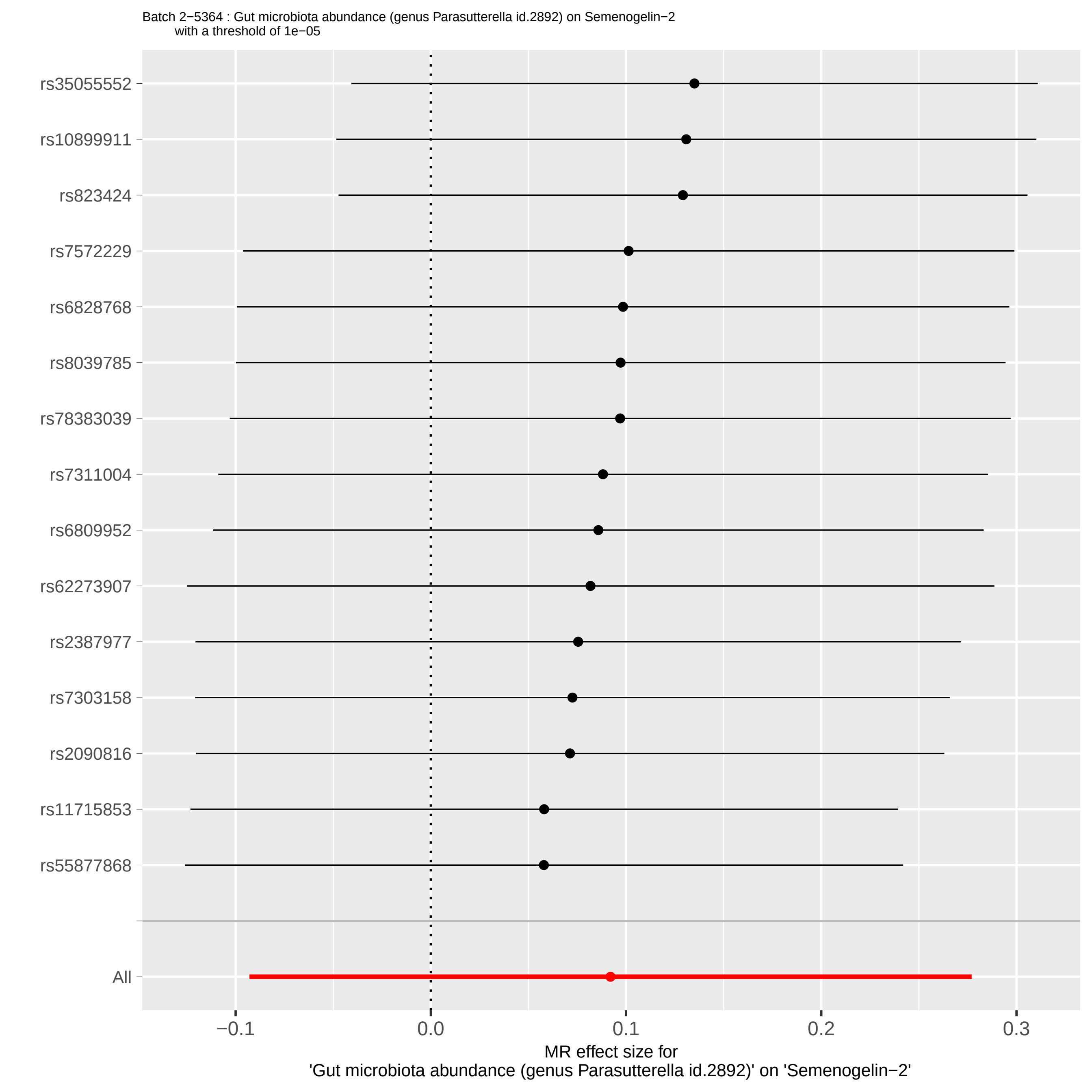
Batch 2−5364 : Gut microbiota abundance (genus Parasutterella id.2892) on Semenogelin−2
with a threshold of 1e−05
rs35055552
rs10899911
rs823424
rs7572229
rs6828768
rs8039785
rs78383039
rs7311004
rs6809952
rs62273907
rs2387977
rs7303158
rs2090816
rs11715853
rs55877868
All
−0.1
0.0
0.1
0.2
0.3
MR effect size for
'Gut microbiota abundance (genus Parasutterella id.2892)' on 'Semenogelin−2'

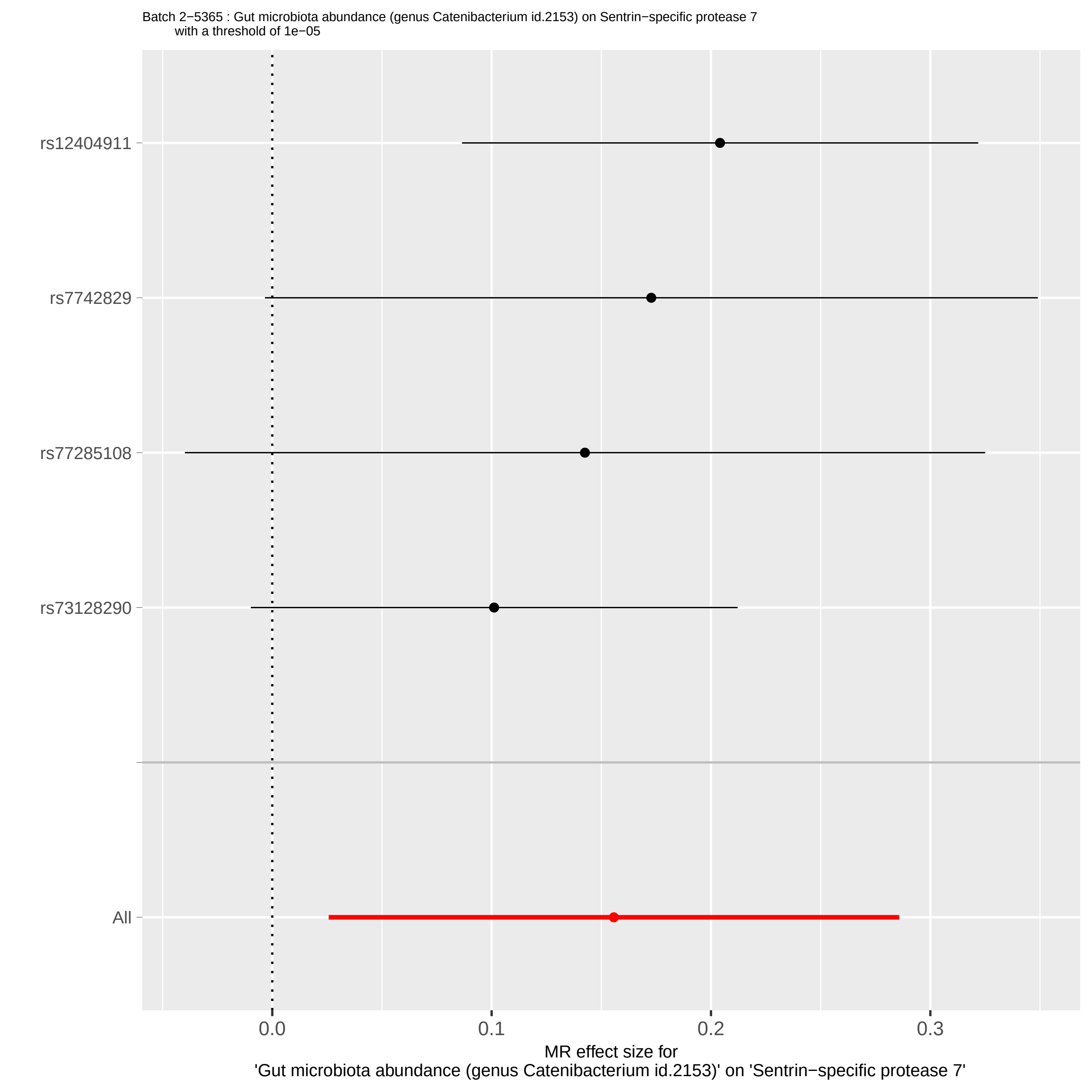
Batch 2−5365 : Gut microbiota abundance (genus Catenibacterium id.2153) on Sentrin−specific protease 7
with a threshold of 1e−05
rs12404911
rs7742829
rs77285108
rs73128290
All
0.0
0.1
0.2
0.3
MR effect size for
'Gut microbiota abundance (genus Catenibacterium id.2153)' on 'Sentrin−specific protease 7'

Batch 2−5366 : Gut microbiota abundance (genus Parasutterella id.2892) on Sentrin−specific protease 7
with a threshold of 1e−05

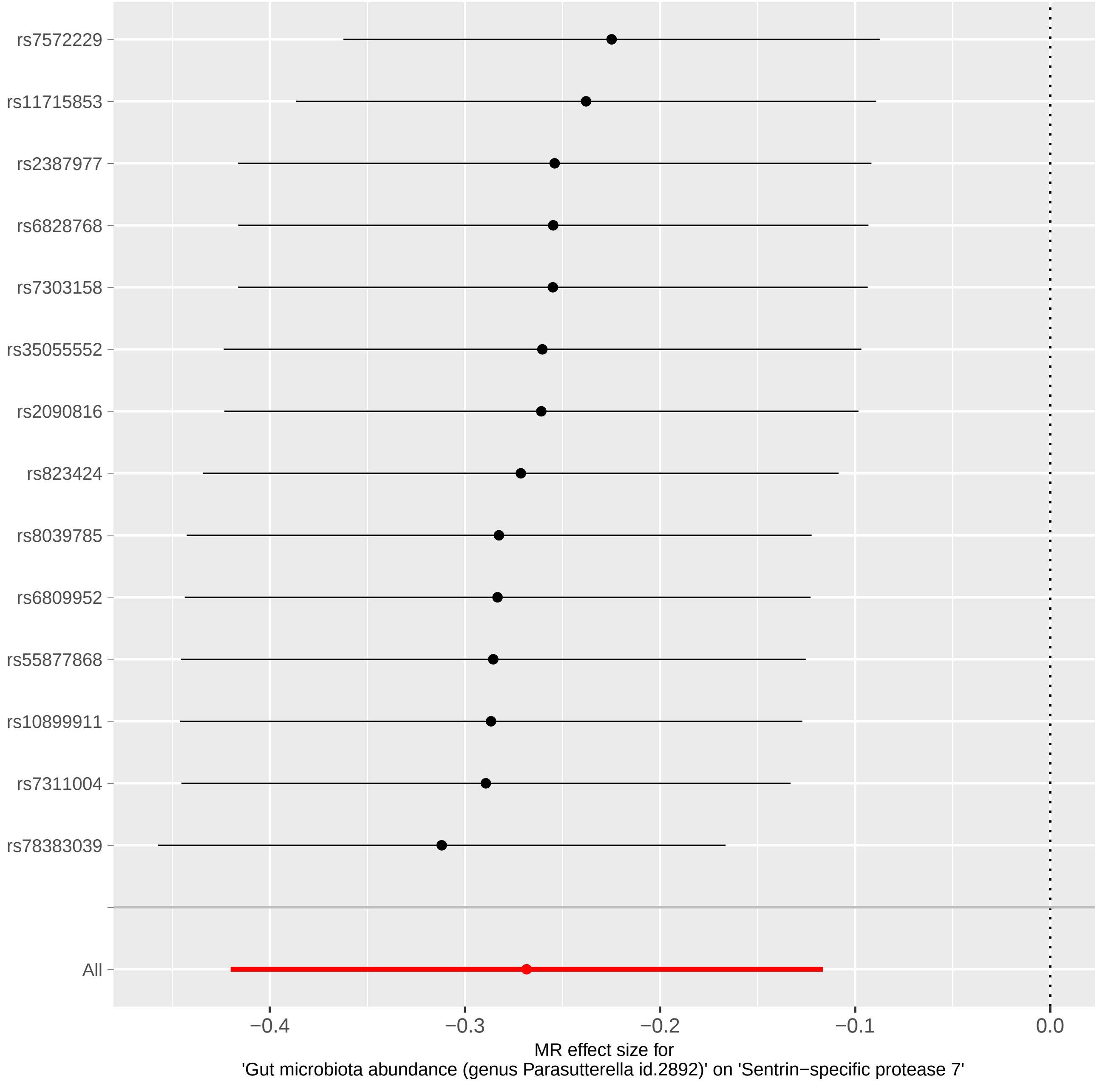

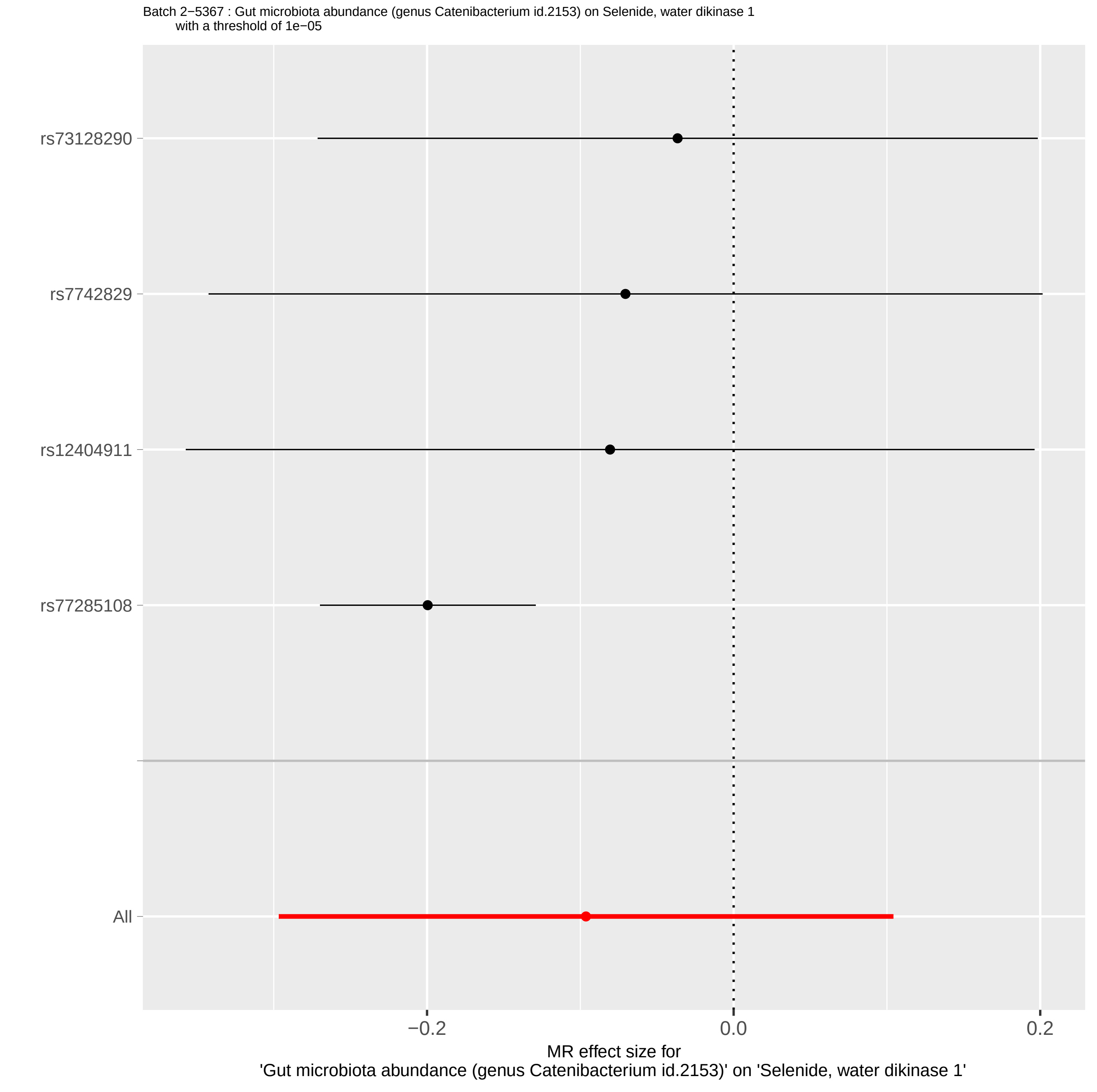
Batch 2−5367 : Gut microbiota abundance (genus Catenibacterium id.2153) on Selenide, water dikinase 1
with a threshold of 1e−05
rs73128290
rs7742829
rs12404911
rs77285108
All
−0.2
0.0
0.2
MR effect size for
'Gut microbiota abundance (genus Catenibacterium id.2153)' on 'Selenide, water dikinase 1'

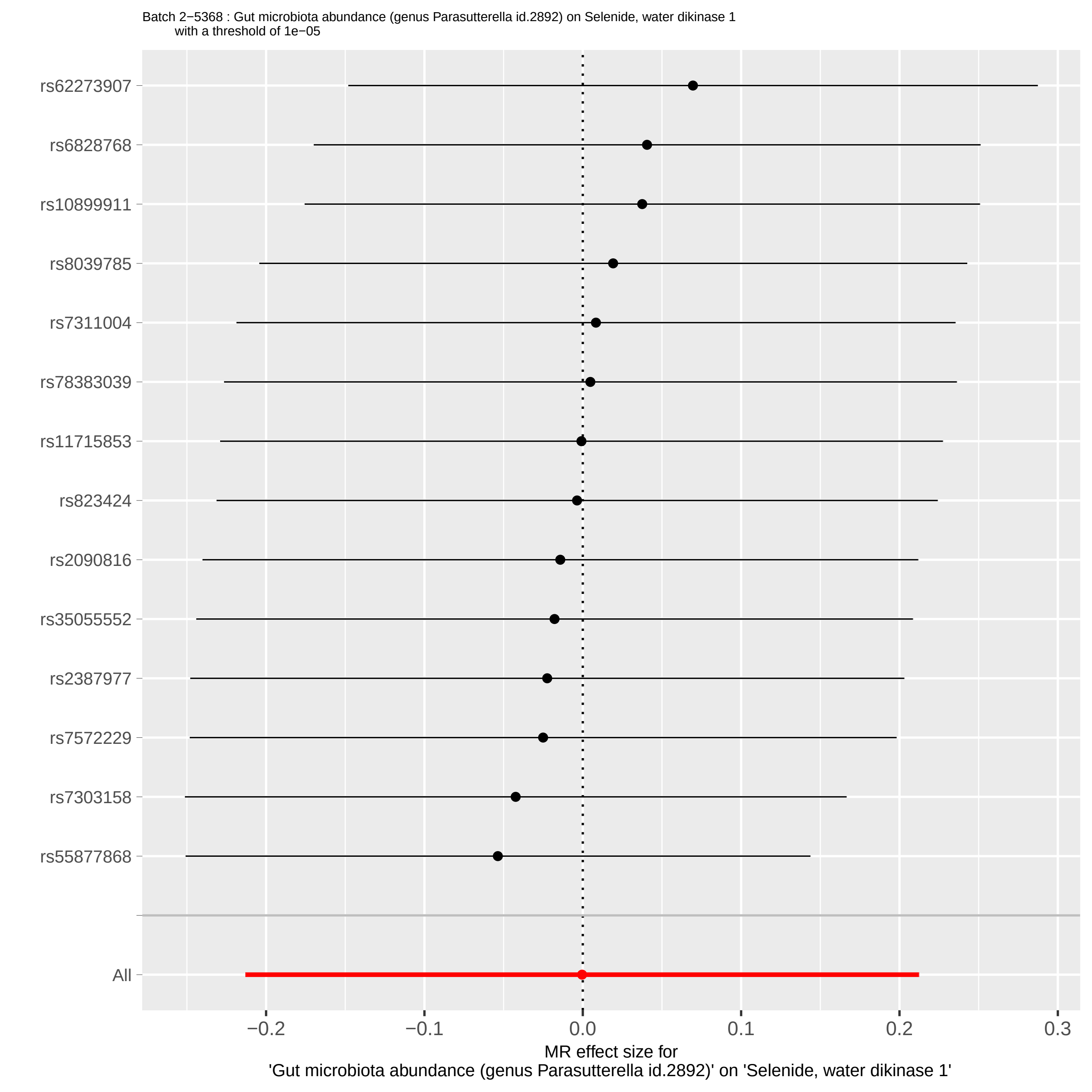

Batch 2−5368 : Gut microbiota abundance (genus Parasutterella id.2892) on Selenide, water dikinase 1
with a threshold of 1e−05
rs62273907
rs6828768
rs10899911
rs8039785
rs7311004
rs78383039
rs11715853
rs823424
rs2090816
rs35055552
rs2387977
rs7572229
rs7303158
rs55877868
All
−0.2
−0.1
0.0
0.1
0.2
0.3
MR effect size for
'Gut microbiota abundance (genus Parasutterella id.2892)' on 'Selenide, water dikinase 1'

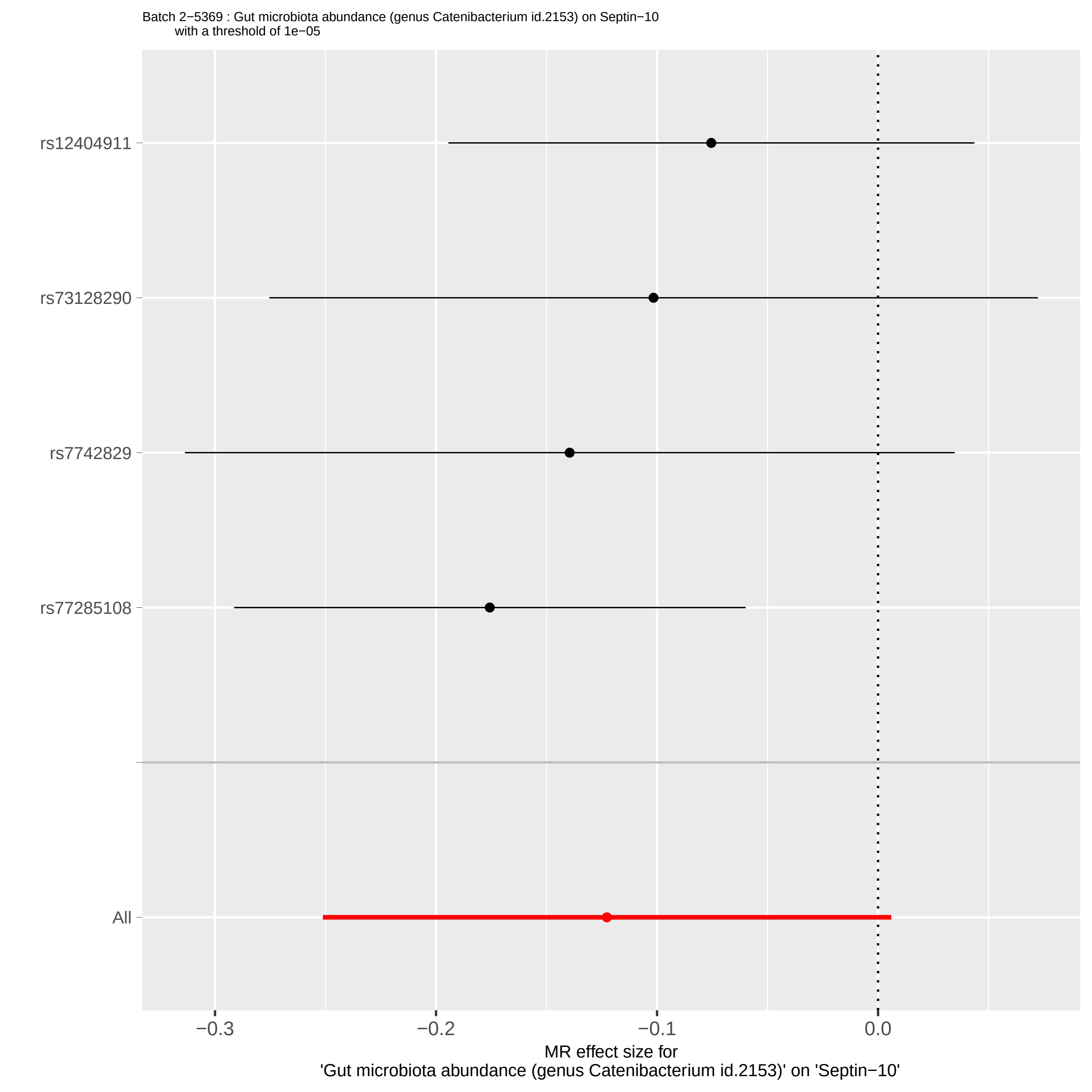
Batch 2−5369 : Gut microbiota abundance (genus Catenibacterium id.2153) on Septin−10
with a threshold of 1e−05
rs12404911
rs73128290
rs7742829
rs77285108
All
−0.3
−0.2
−0.1
0.0
MR effect size for
'Gut microbiota abundance (genus Catenibacterium id.2153)' on 'Septin−10'

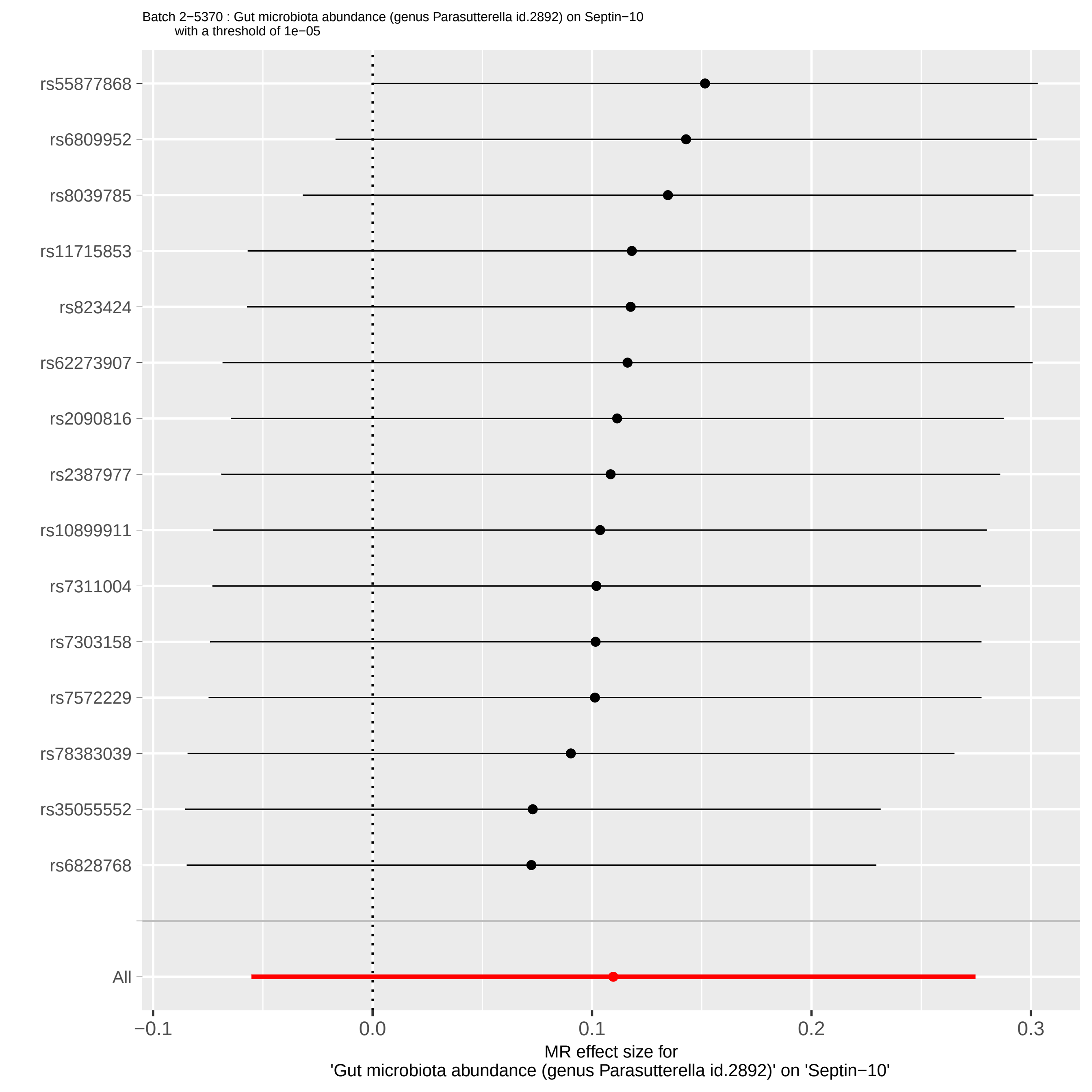
Batch 2−5370 : Gut microbiota abundance (genus Parasutterella id.2892) on Septin−10
with a threshold of 1e−05
rs55877868
rs6809952
rs8039785
rs11715853
rs823424
rs62273907
rs2090816
rs2387977
rs10899911
rs7311004
rs7303158
rs7572229
rs78383039
rs35055552
rs6828768
All
−0.1
0.0
0.1
0.2
0.3
MR effect size for
'Gut microbiota abundance (genus Parasutterella id.2892)' on 'Septin−10'

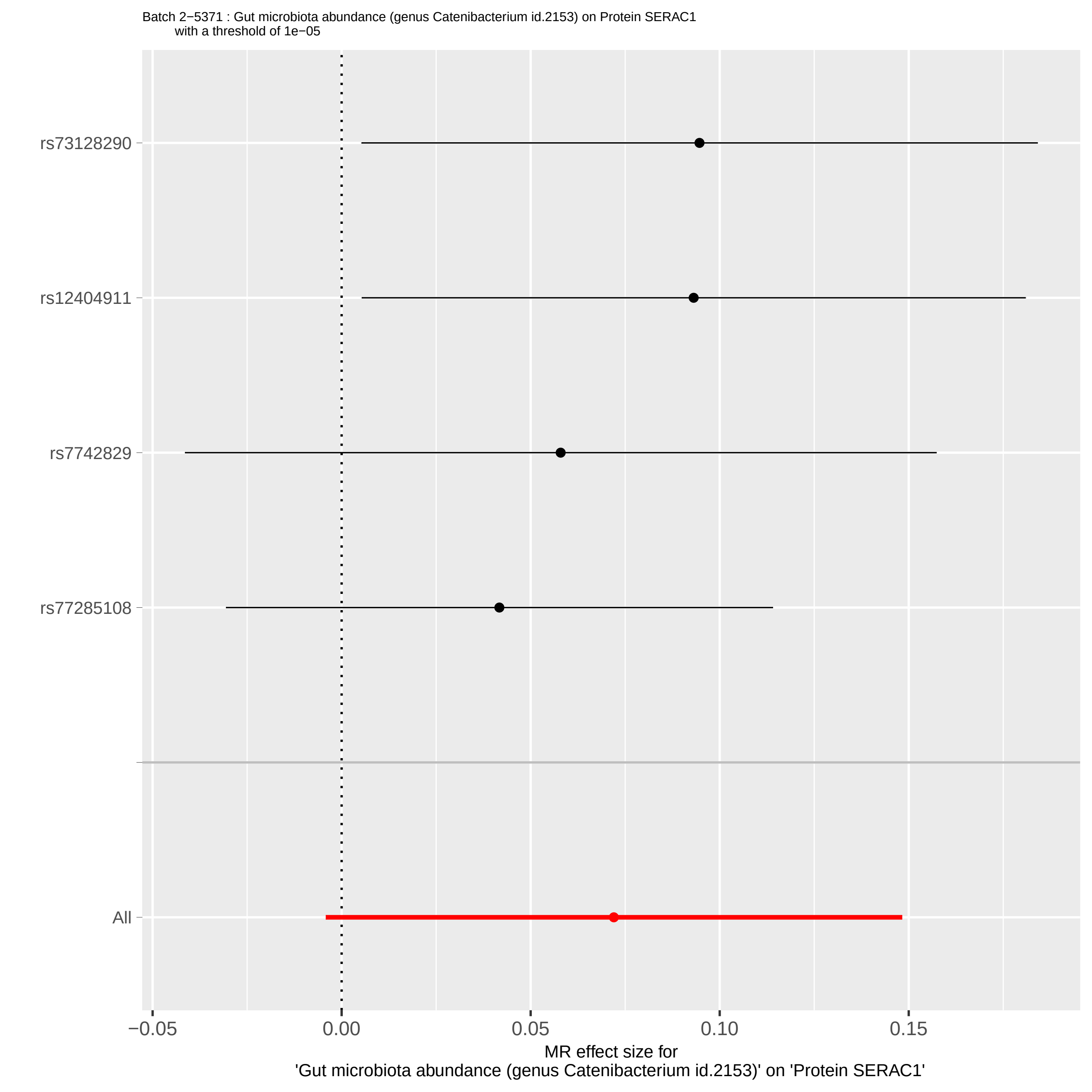

Batch 2−5371 : Gut microbiota abundance (genus Catenibacterium id.2153) on Protein SERAC1
with a threshold of 1e−05
rs73128290
rs12404911
rs7742829
rs77285108
All
−0.05
0.00
0.05
0.10
0.15
MR effect size for
'Gut microbiota abundance (genus Catenibacterium id.2153)' on 'Protein SERAC1'

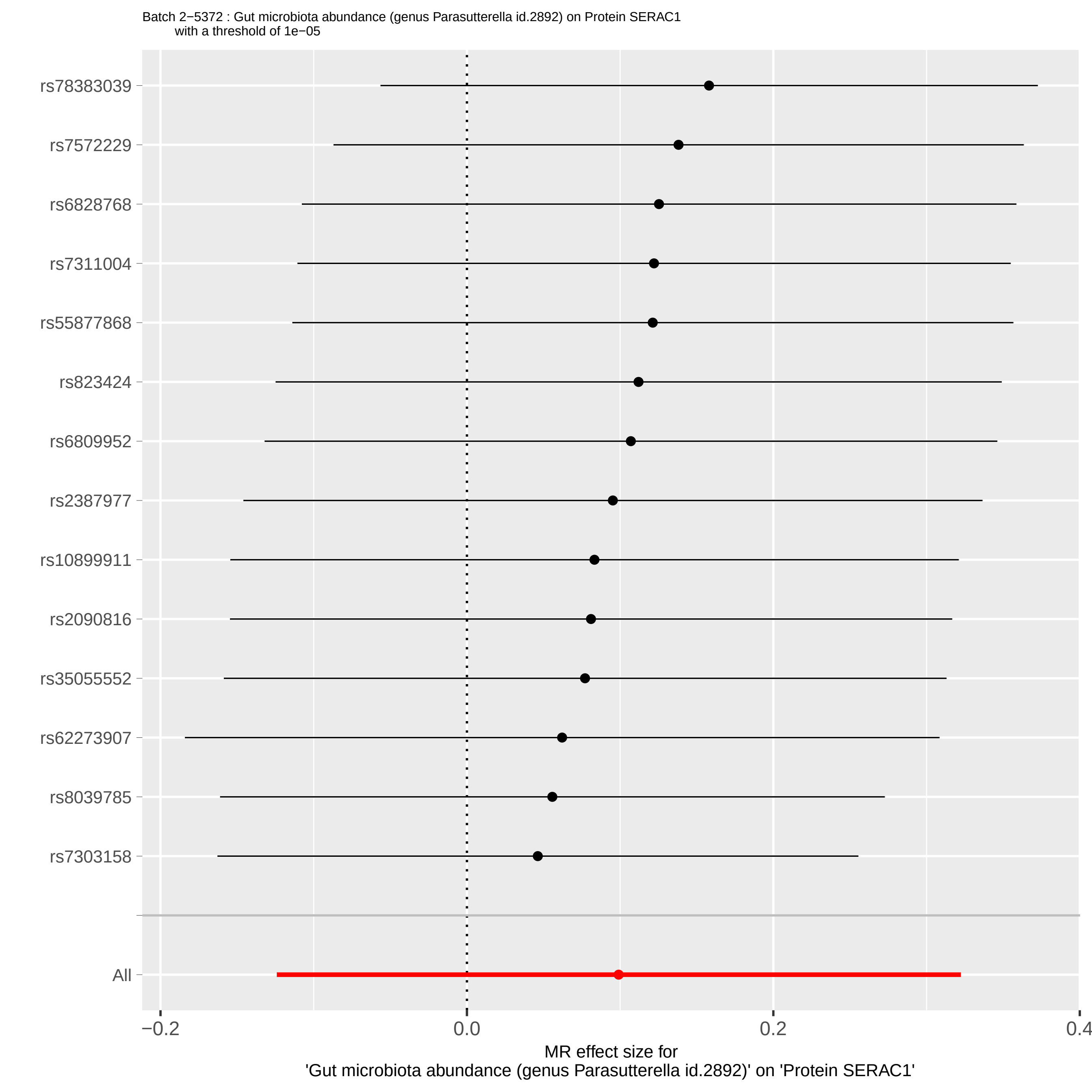
Batch 2−5372 : Gut microbiota abundance (genus Parasutterella id.2892) on Protein SERAC1
with a threshold of 1e−05
rs78383039
rs7572229
rs6828768
rs7311004
rs55877868
rs823424
rs6809952
rs2387977
rs10899911
rs2090816
rs35055552
rs62273907
rs8039785
rs7303158
All
−0.2
0.0
0.2
0.4
MR effect size for
'Gut microbiota abundance (genus Parasutterella id.2892)' on 'Protein SERAC1'

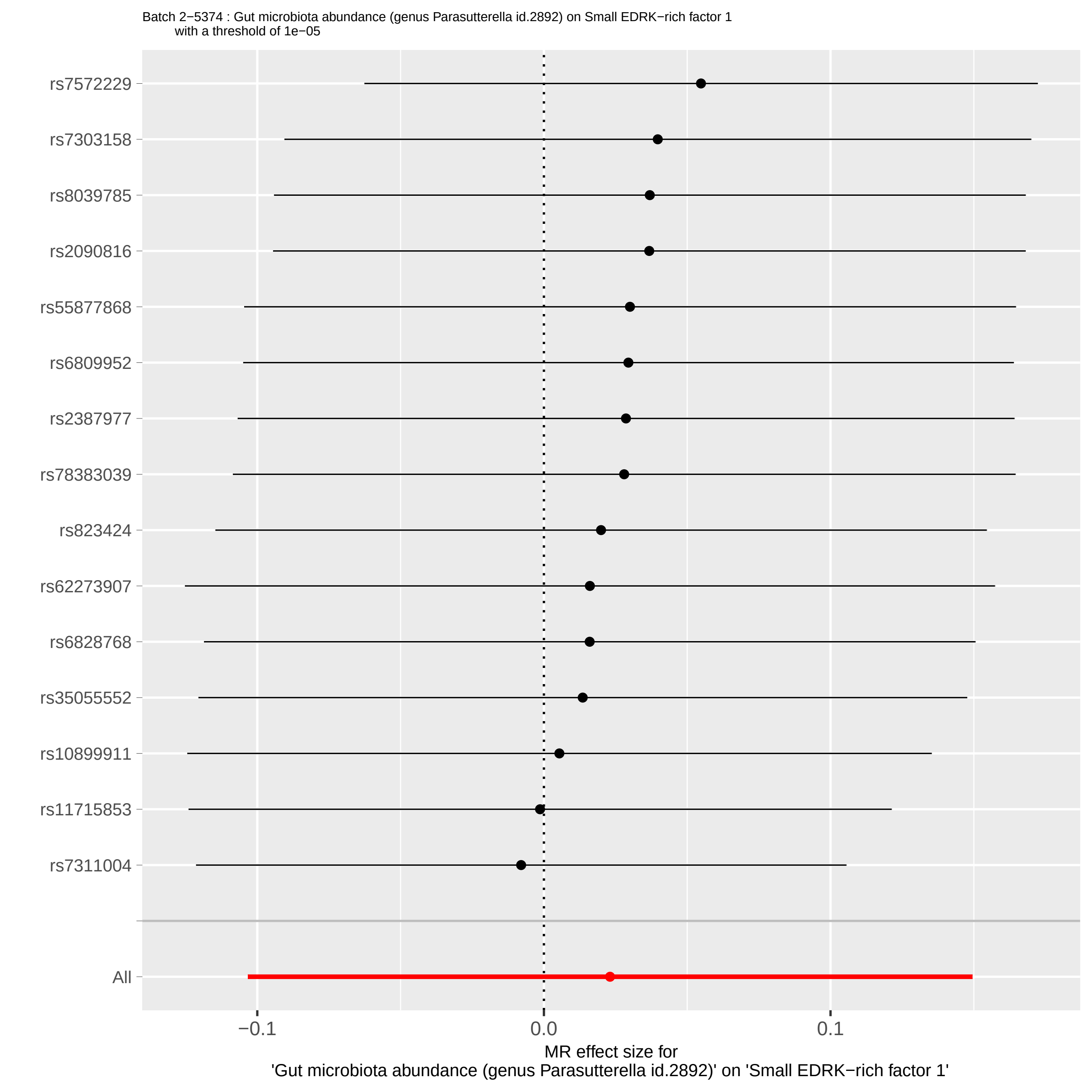

Batch 2−5374 : Gut microbiota abundance (genus Parasutterella id.2892) on Small EDRK−rich factor 1
with a threshold of 1e−05
rs7572229
rs7303158
rs8039785
rs2090816
rs55877868
rs6809952
rs2387977
rs78383039
rs823424
rs62273907
rs6828768
rs35055552
rs10899911
rs11715853
rs7311004
All
−0.1
0.0
0.1
MR effect size for
'Gut microbiota abundance (genus Parasutterella id.2892)' on 'Small EDRK−rich factor 1'

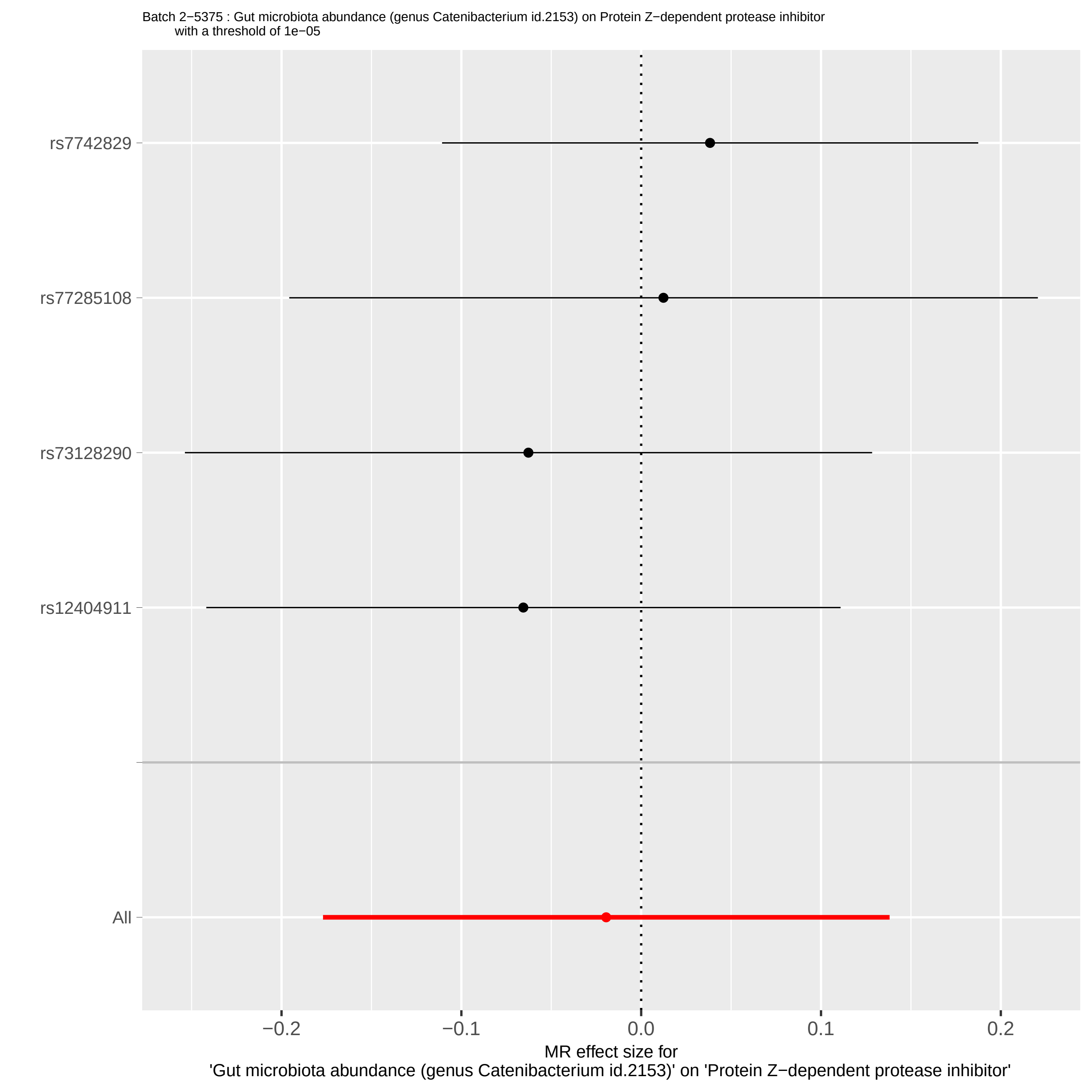
Batch 2−5375 : Gut microbiota abundance (genus Catenibacterium id.2153) on Protein Z−dependent protease inhibitor
with a threshold of 1e−05
rs7742829
rs77285108
rs73128290
rs12404911
All
−0.2
−0.1
0.0
0.1
0.2
MR effect size for
'Gut microbiota abundance (genus Catenibacterium id.2153)' on 'Protein Z−dependent protease inhibitor'

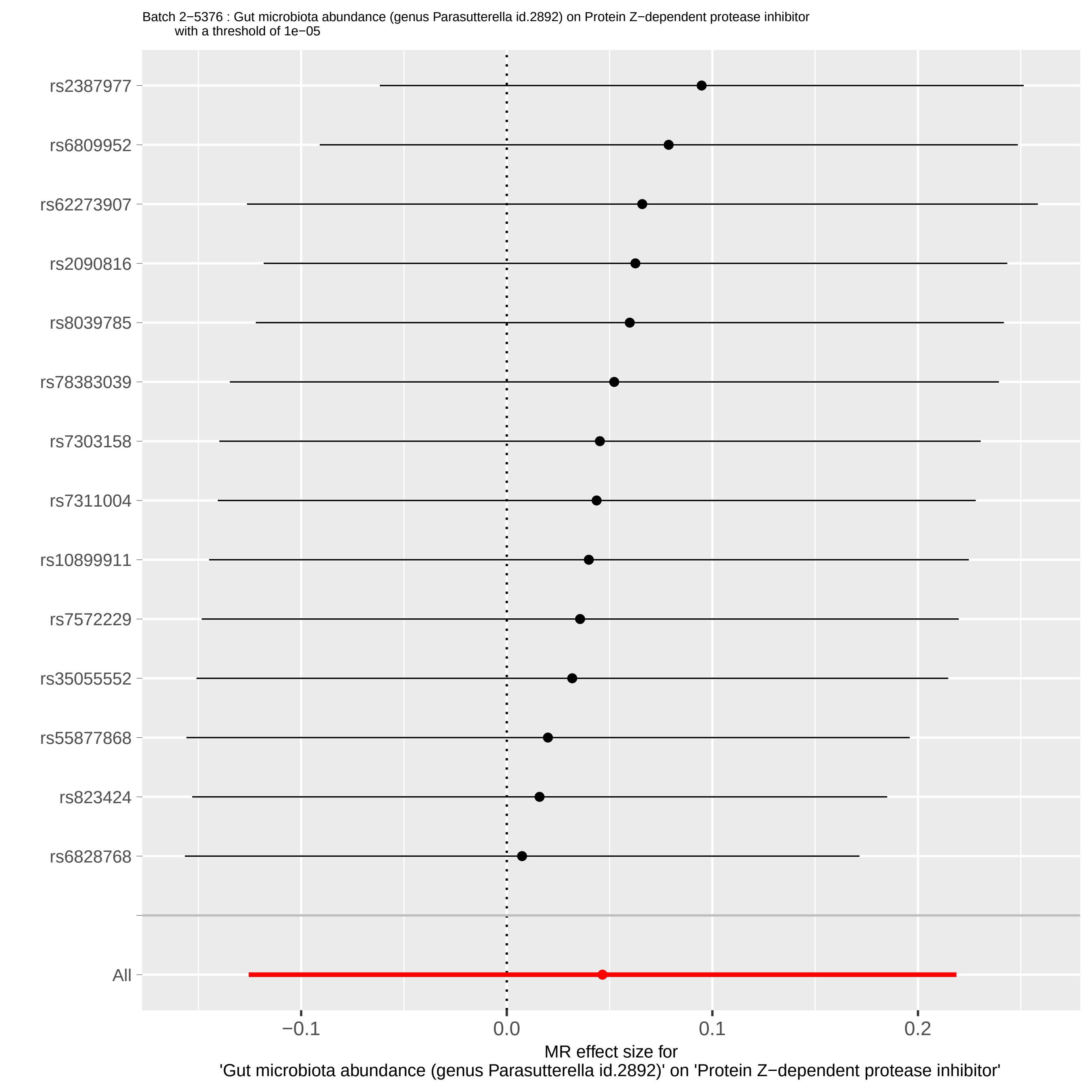
Batch 2−5376 : Gut microbiota abundance (genus Parasutterella id.2892) on Protein Z−dependent protease inhibitor
with a threshold of 1e−05
rs2387977
rs6809952
rs62273907
rs2090816
rs8039785
rs78383039
rs7303158
rs7311004
rs10899911
rs7572229
rs35055552
rs55877868
rs823424
rs6828768
All
−0.1
0.0
0.1
0.2
MR effect size for
'Gut microbiota abundance (genus Parasutterella id.2892)' on 'Protein Z−dependent protease inhibitor'

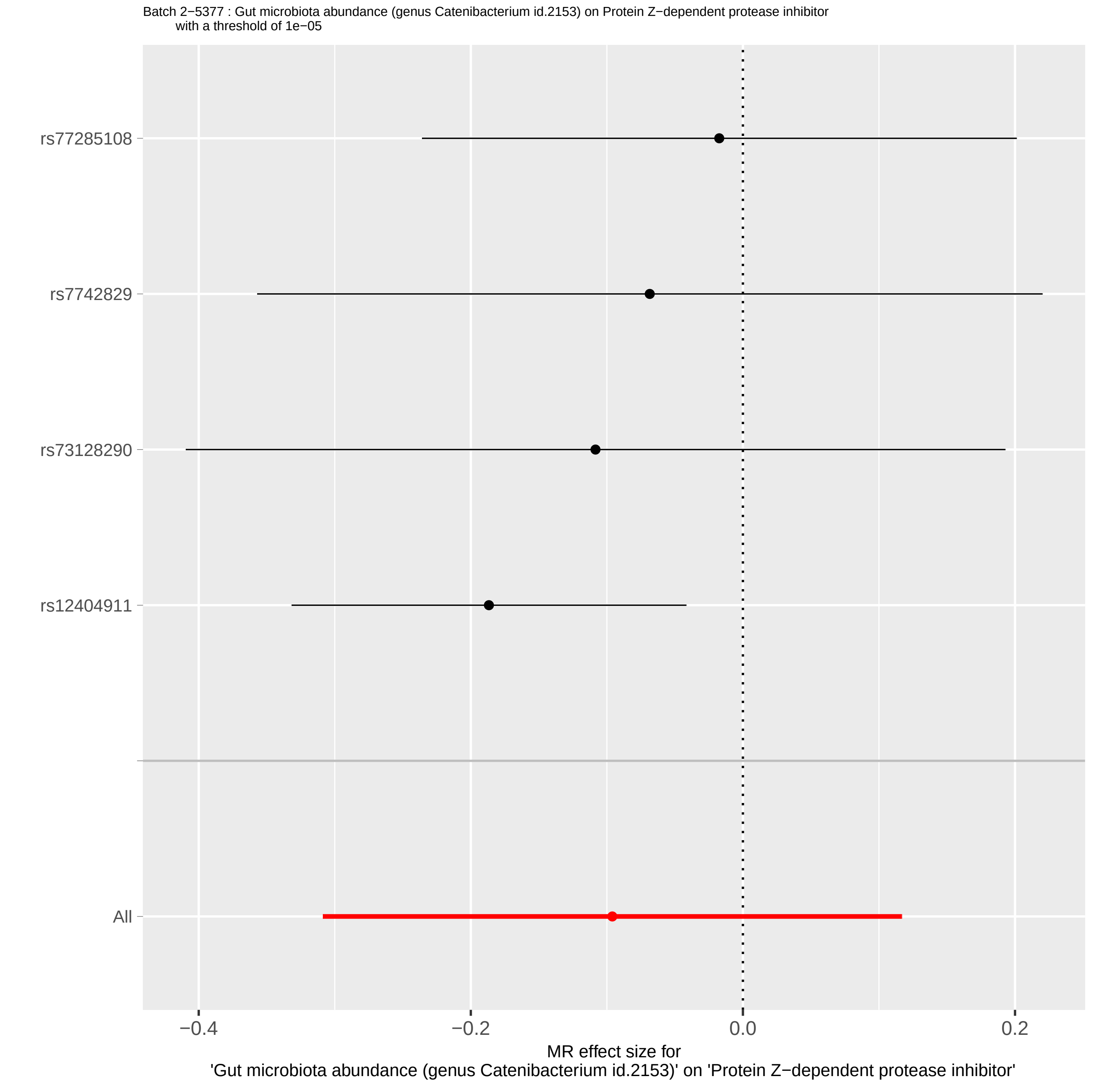
Batch 2−5377 : Gut microbiota abundance (genus Catenibacterium id.2153) on Protein Z−dependent protease inhibitor
with a threshold of 1e−05
rs77285108
rs7742829
rs73128290
rs12404911
All
−0.4
−0.2
0.0
0.2
MR effect size for
'Gut microbiota abundance (genus Catenibacterium id.2153)' on 'Protein Z−dependent protease inhibitor'

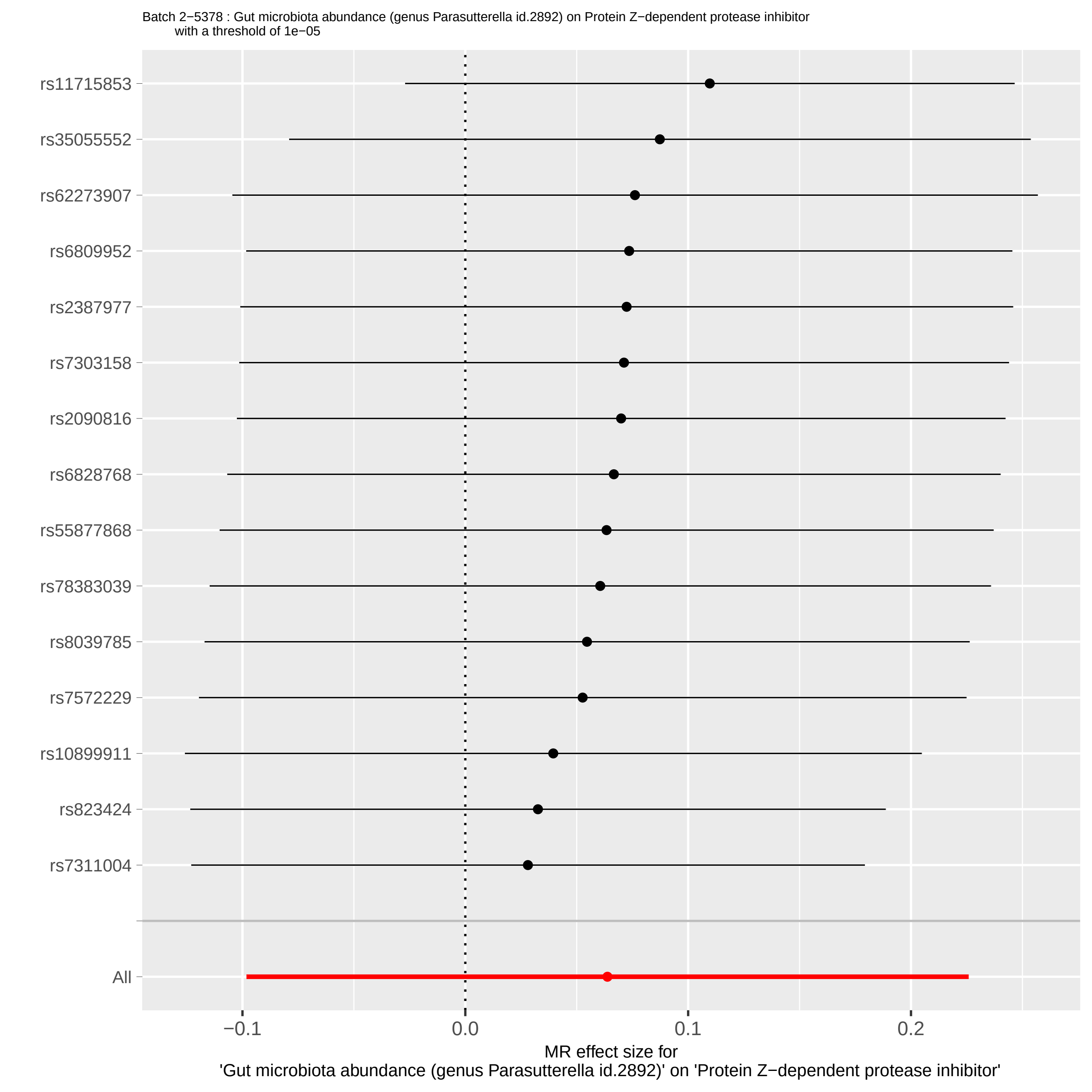
Batch 2−5378 : Gut microbiota abundance (genus Parasutterella id.2892) on Protein Z−dependent protease inhibitor
with a threshold of 1e−05
rs11715853
rs35055552
rs62273907
rs6809952
rs2387977
rs7303158
rs2090816
rs6828768
rs55877868
rs78383039
rs8039785
rs7572229
rs10899911
rs823424
rs7311004
All
−0.1
0.0
0.1
0.2
MR effect size for
'Gut microbiota abundance (genus Parasutterella id.2892)' on 'Protein Z−dependent protease inhibitor'

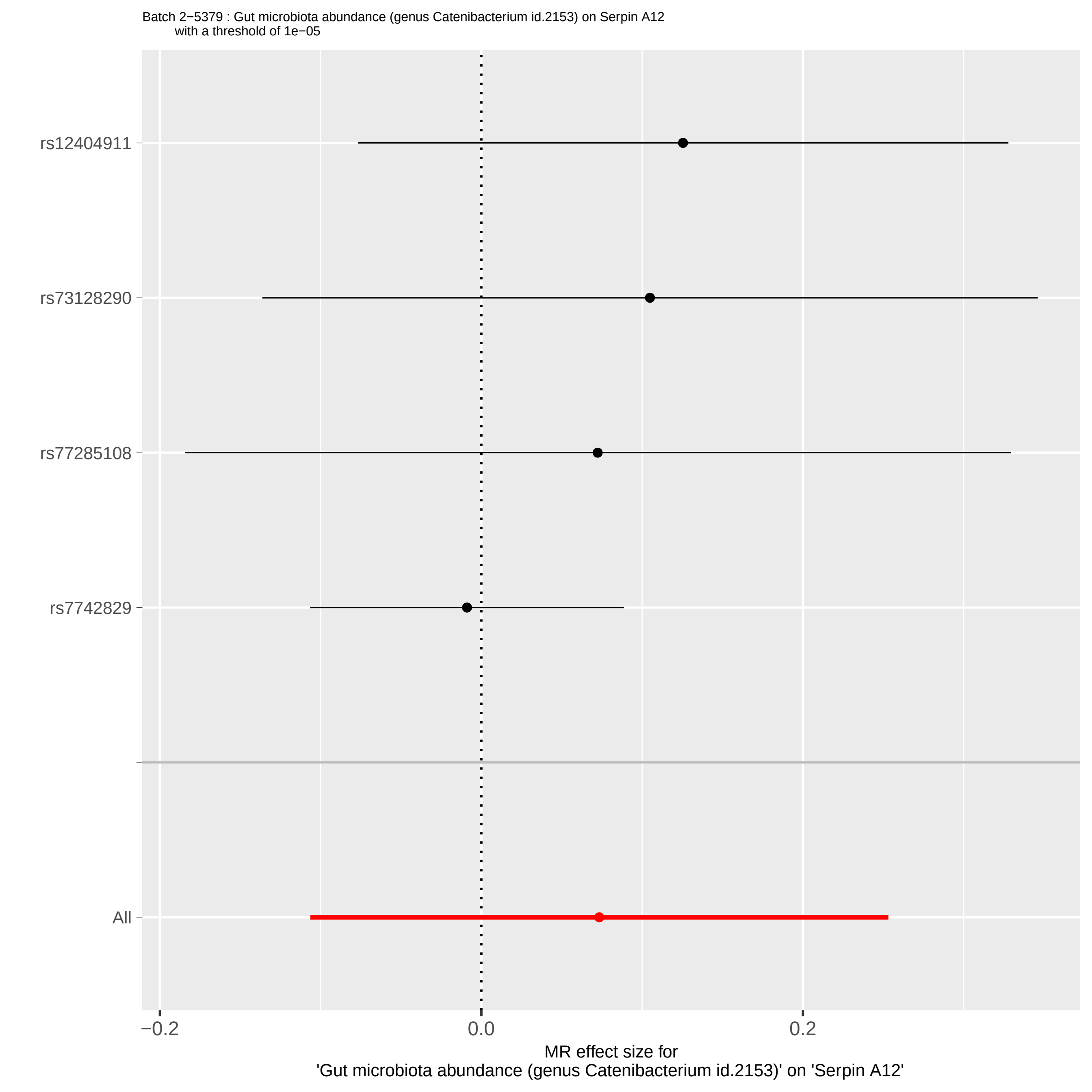

Batch 2−5379 : Gut microbiota abundance (genus Catenibacterium id.2153) on Serpin A12
with a threshold of 1e−05
rs12404911
rs73128290
rs77285108
rs7742829
All
−0.2
0.0
0.2
MR effect size for
'Gut microbiota abundance (genus Catenibacterium id.2153)' on 'Serpin A12'

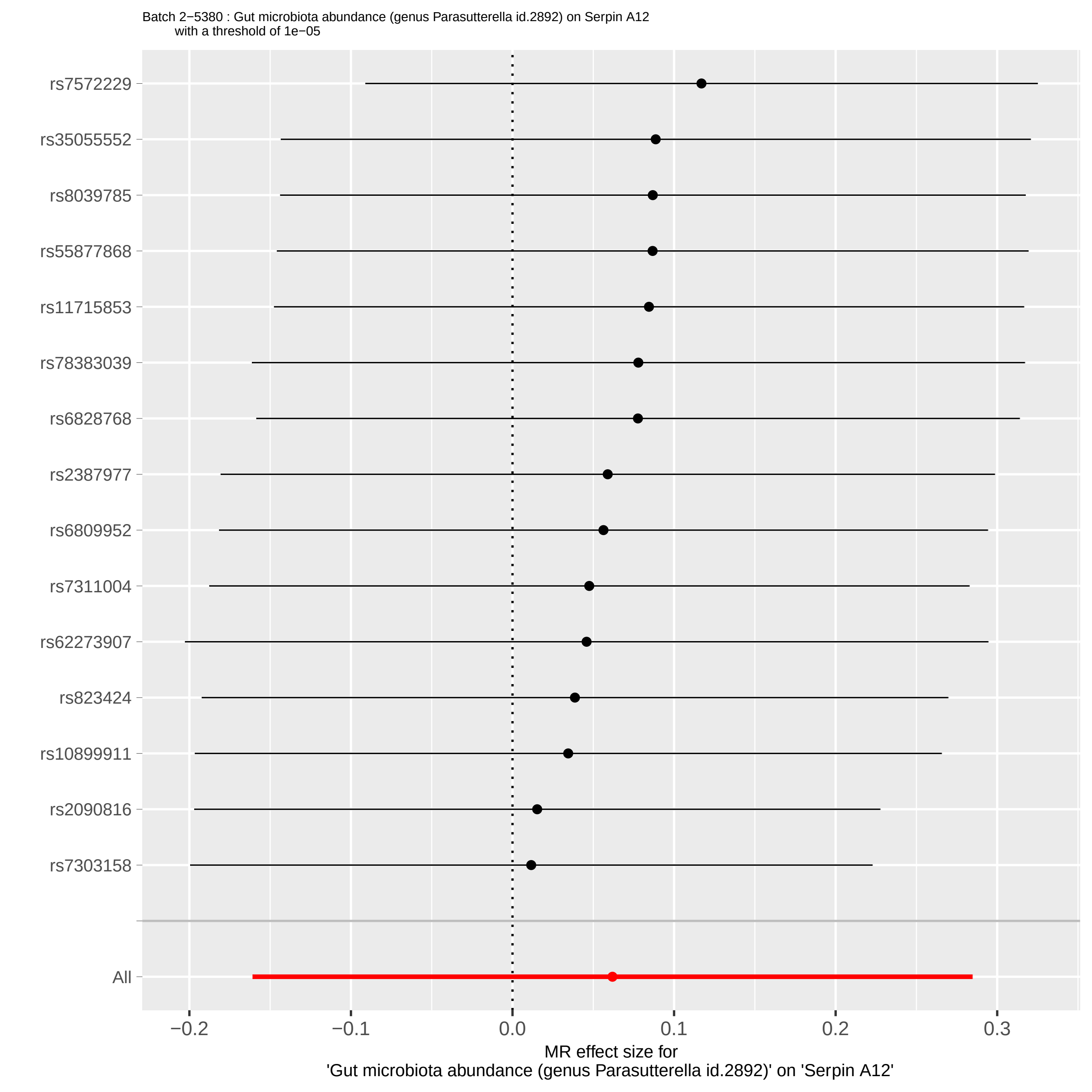

Batch 2−5380 : Gut microbiota abundance (genus Parasutterella id.2892) on Serpin A12
with a threshold of 1e−05
rs7572229
rs35055552
rs8039785
rs55877868
rs11715853
rs78383039
rs6828768
rs2387977
rs6809952
rs7311004
rs62273907
rs823424
rs10899911
rs2090816
rs7303158
All
−0.2
−0.1
0.0
0.1
0.2
0.3
MR effect size for
'Gut microbiota abundance (genus Parasutterella id.2892)' on 'Serpin A12'

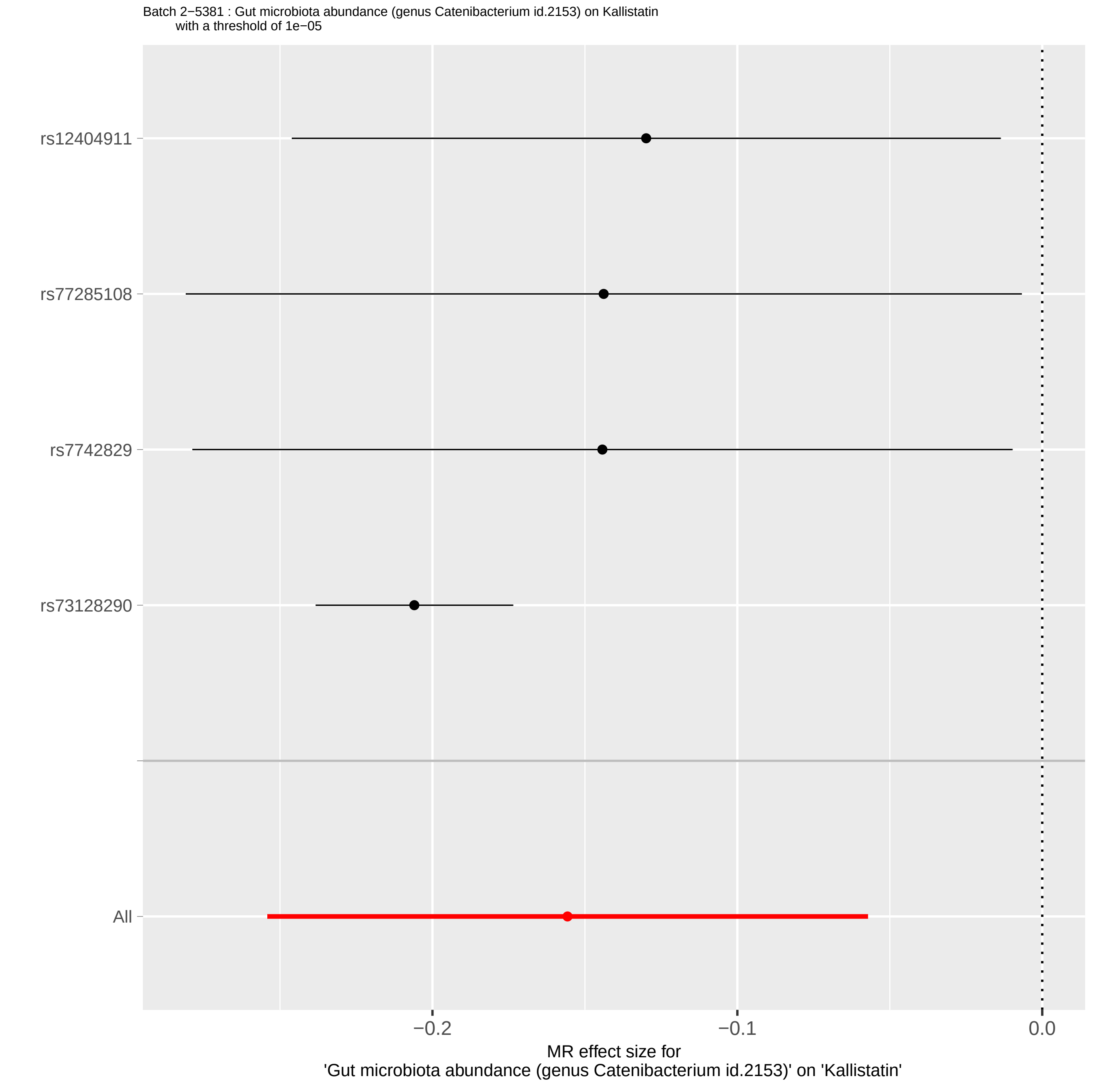

Batch 2−5381 : Gut microbiota abundance (genus Catenibacterium id.2153) on Kallistatin
with a threshold of 1e−05
rs12404911
rs77285108
rs7742829
rs73128290
All
−0.2
−0.1
0.0
MR effect size for
'Gut microbiota abundance (genus Catenibacterium id.2153)' on 'Kallistatin'

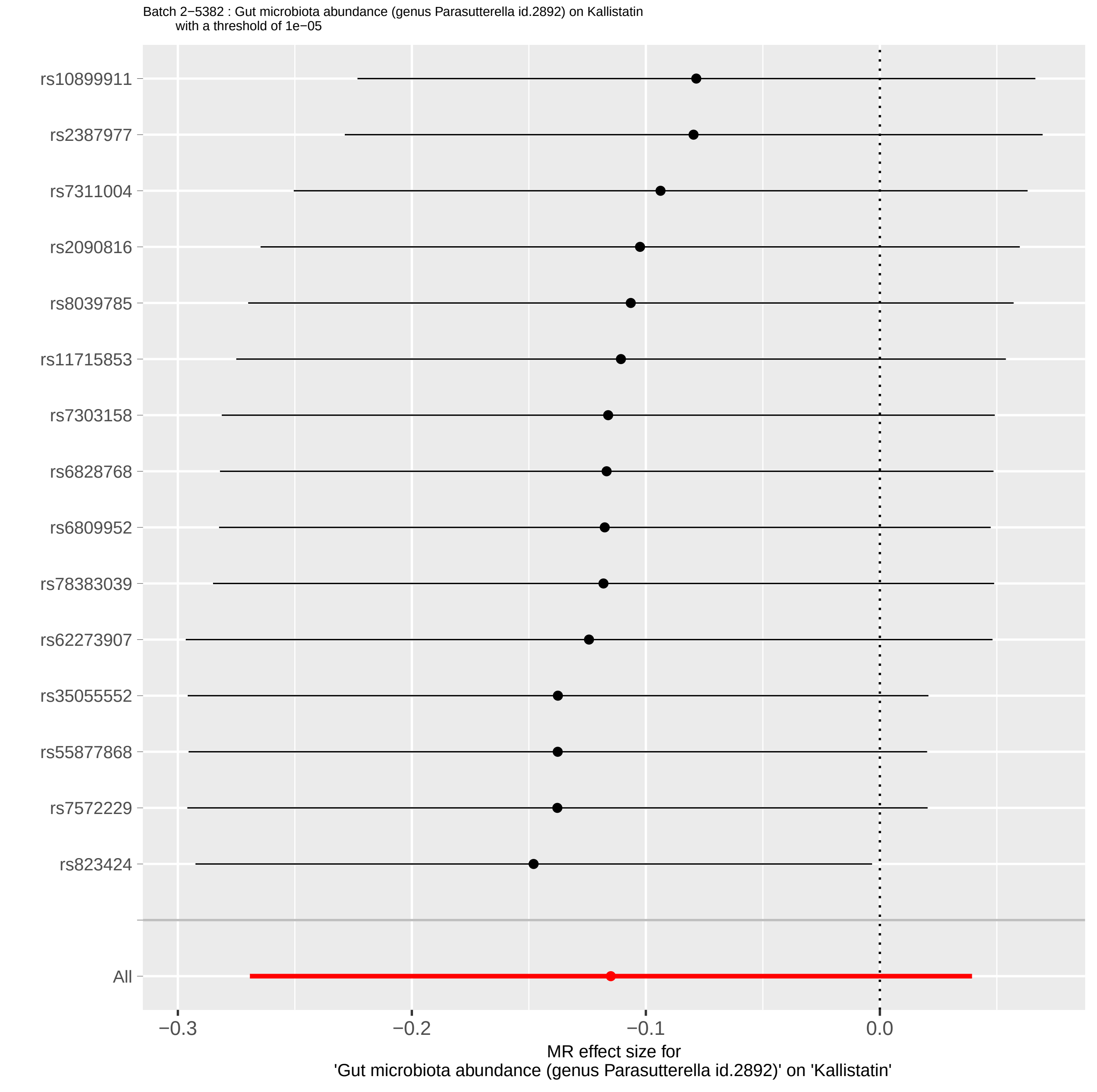

Batch 2−5382 : Gut microbiota abundance (genus Parasutterella id.2892) on Kallistatin
with a threshold of 1e−05
rs10899911
rs2387977
rs7311004
rs2090816
rs8039785
rs11715853
rs7303158
rs6828768
rs6809952
rs78383039
rs62273907
rs35055552
rs55877868
rs7572229
rs823424
All
−0.3
−0.2
−0.1
0.0
MR effect size for
'Gut microbiota abundance (genus Parasutterella id.2892)' on 'Kallistatin'

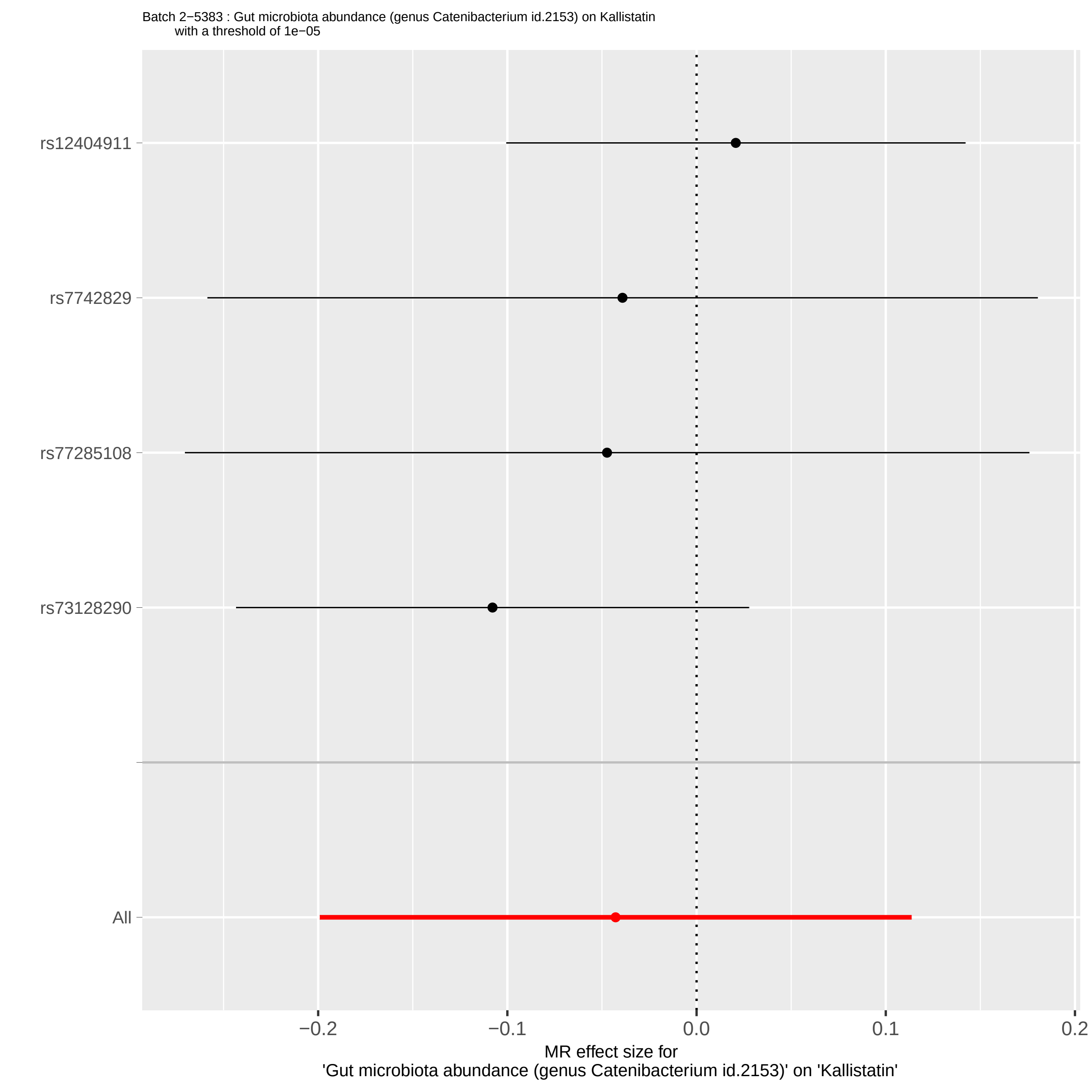
Batch 2−5383 : Gut microbiota abundance (genus Catenibacterium id.2153) on Kallistatin
with a threshold of 1e−05
rs12404911
rs7742829
rs77285108
rs73128290
All
−0.2
−0.1
0.0
0.1
0.2
MR effect size for
'Gut microbiota abundance (genus Catenibacterium id.2153)' on 'Kallistatin'

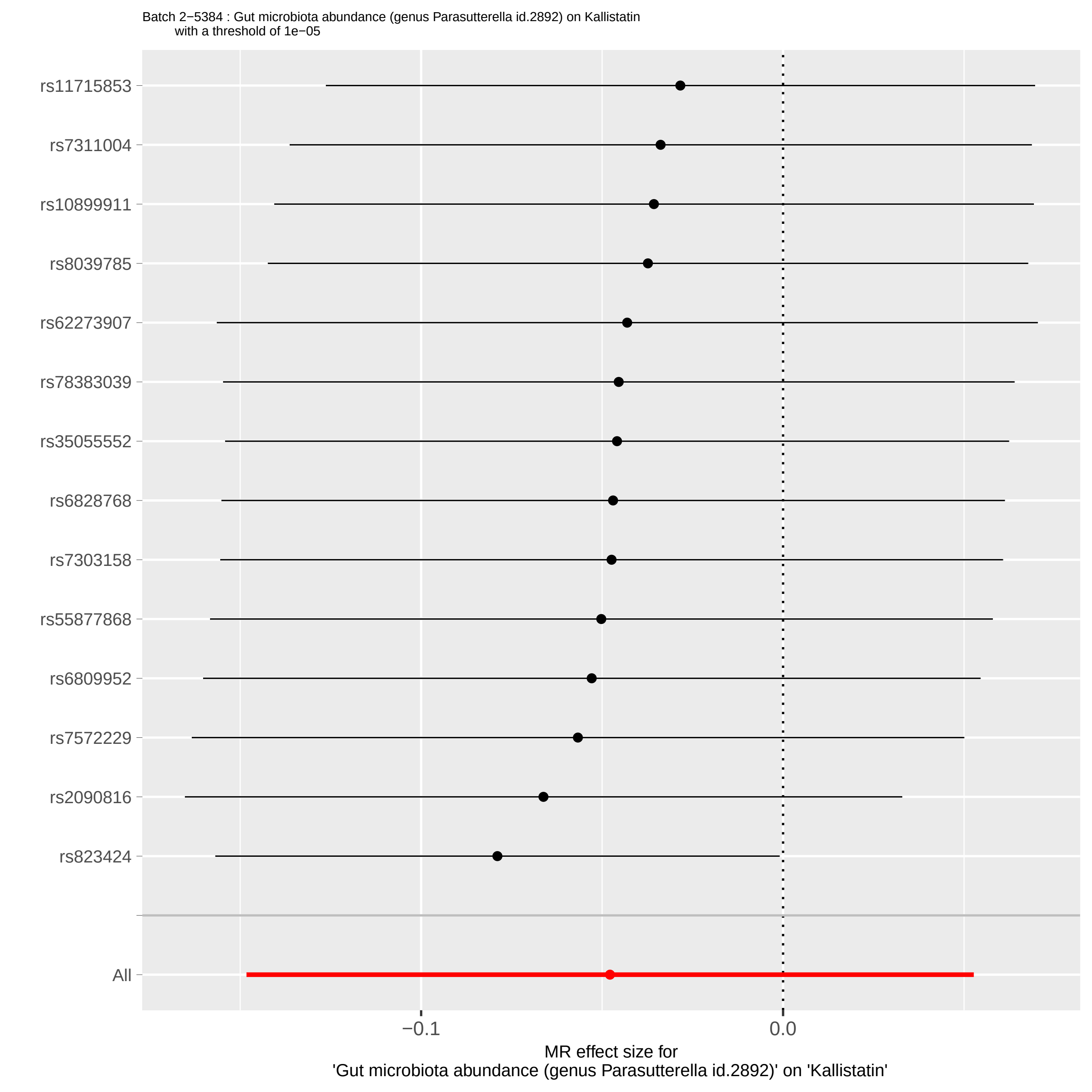

Batch 2−5384 : Gut microbiota abundance (genus Parasutterella id.2892) on Kallistatin
with a threshold of 1e−05
rs11715853
rs7311004
rs10899911
rs8039785
rs62273907
rs78383039
rs35055552
rs6828768
rs7303158
rs55877868
rs6809952
rs7572229
rs2090816
rs823424
All
−0.1
0.0
MR effect size for
'Gut microbiota abundance (genus Parasutterella id.2892)' on 'Kallistatin'

Batch 2−5385 : Gut microbiota abundance (genus Catenibacterium id.2153) on Corticosteroid−binding globulin
with a threshold of 1e−05

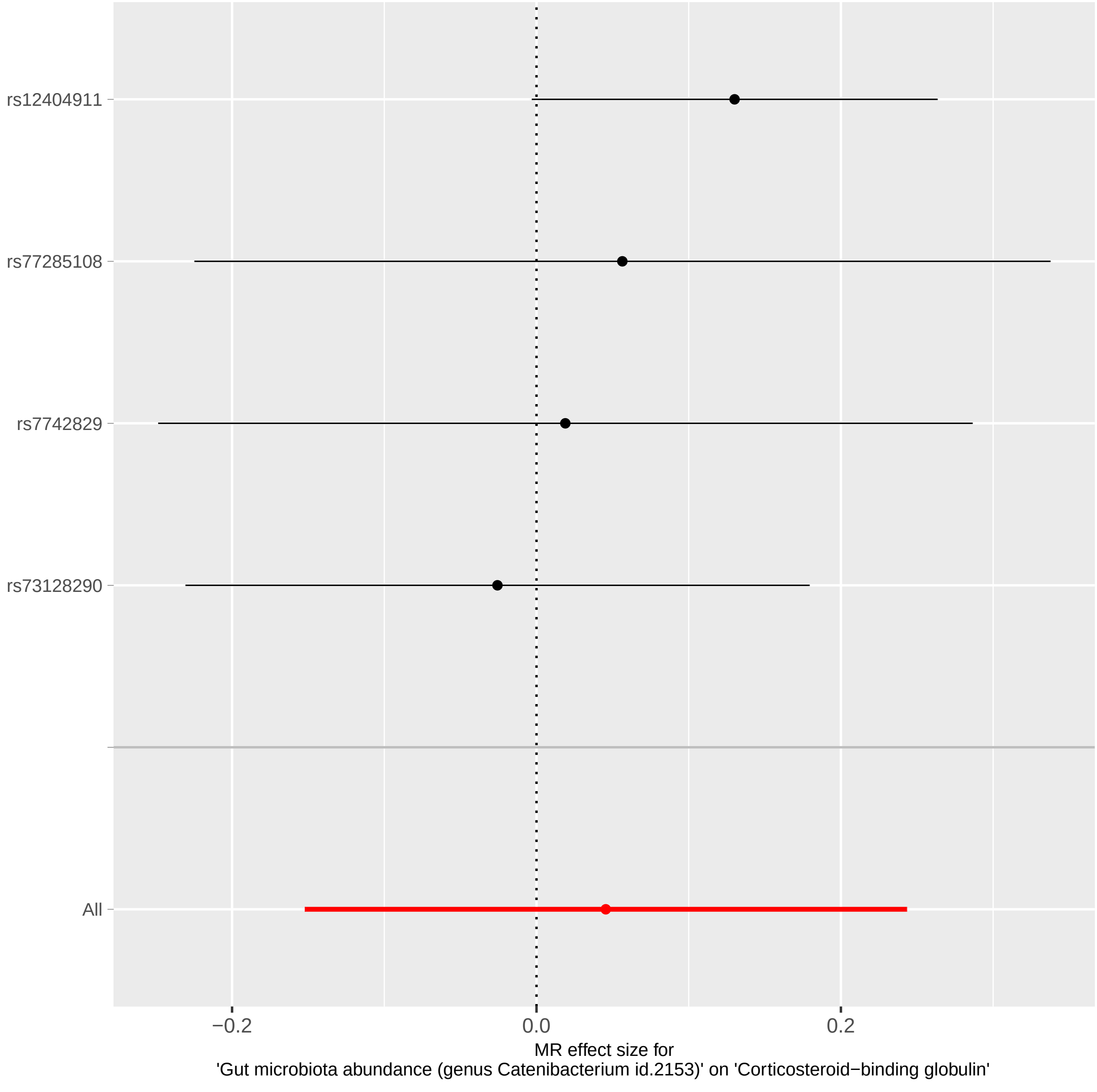

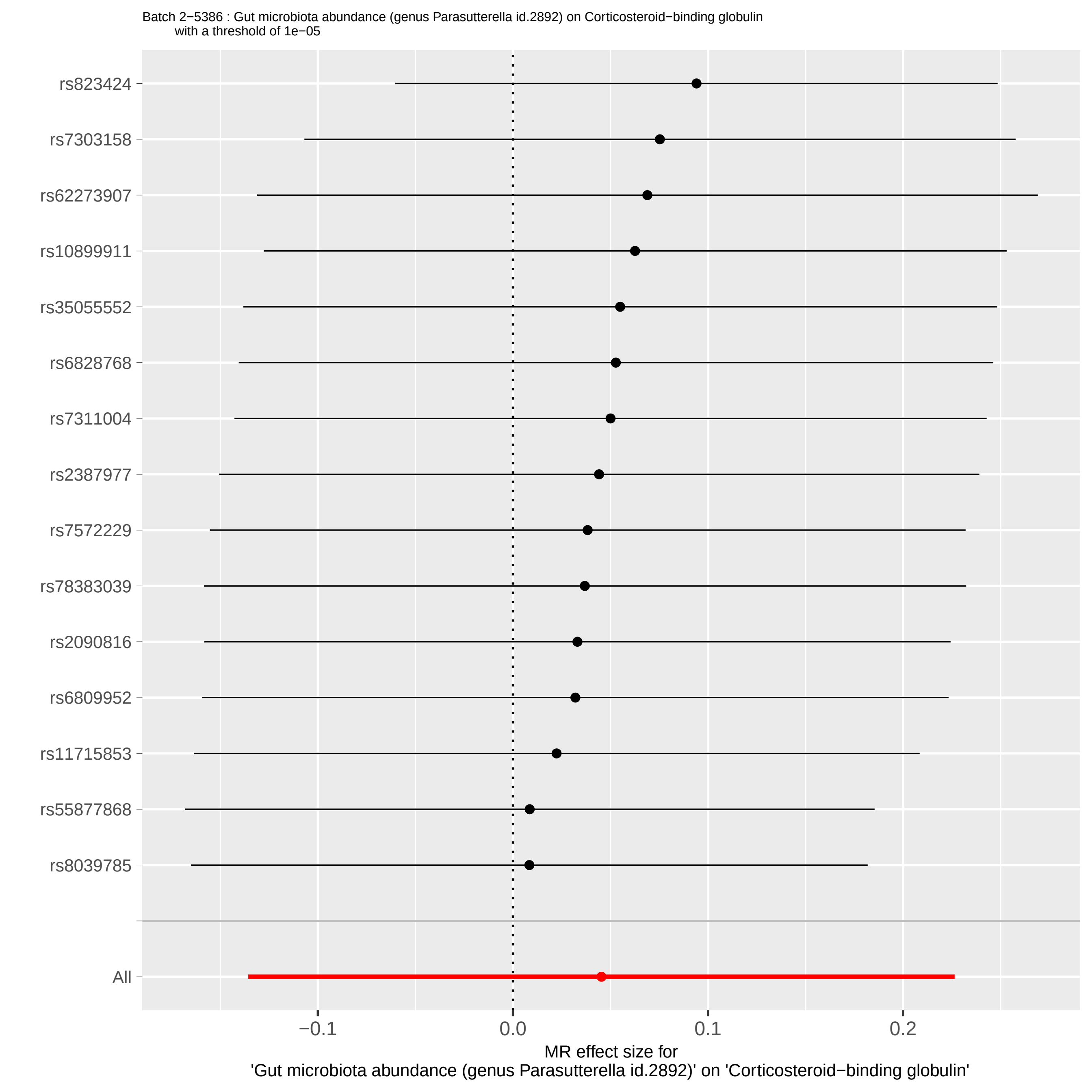

Batch 2−5386 : Gut microbiota abundance (genus Parasutterella id.2892) on Corticosteroid−binding globulin
with a threshold of 1e−05
rs823424
rs7303158
rs62273907
rs10899911
rs35055552
rs6828768
rs7311004
rs2387977
rs7572229
rs78383039
rs2090816
rs6809952
rs11715853
rs55877868
rs8039785
All
−0.1
0.0
0.1
0.2
MR effect size for
'Gut microbiota abundance (genus Parasutterella id.2892)' on 'Corticosteroid−binding globulin'

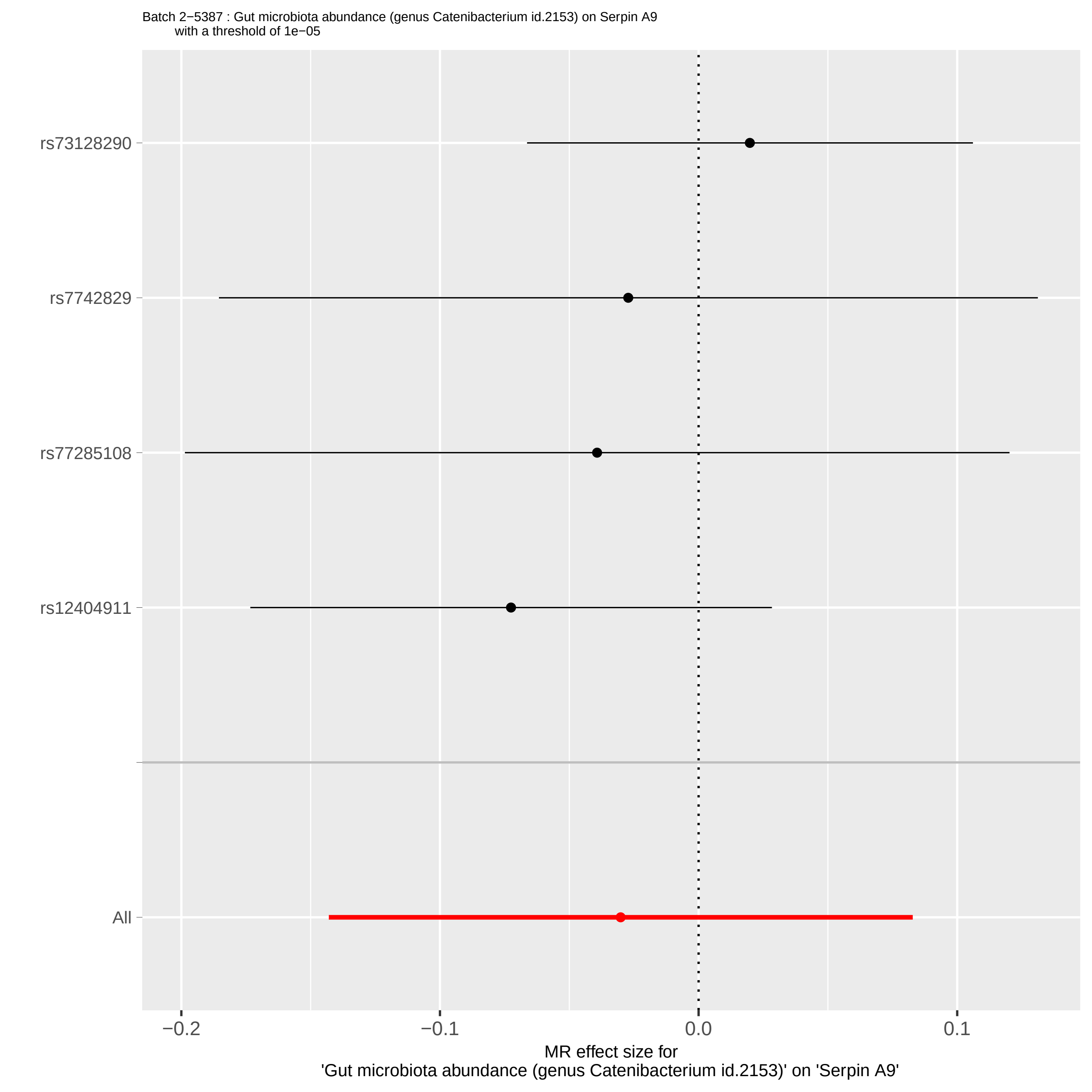
Batch 2−5387 : Gut microbiota abundance (genus Catenibacterium id.2153) on Serpin A9
with a threshold of 1e−05
rs73128290
rs7742829
rs77285108
rs12404911
All
−0.2
−0.1
0.0
0.1
MR effect size for
'Gut microbiota abundance (genus Catenibacterium id.2153)' on 'Serpin A9'

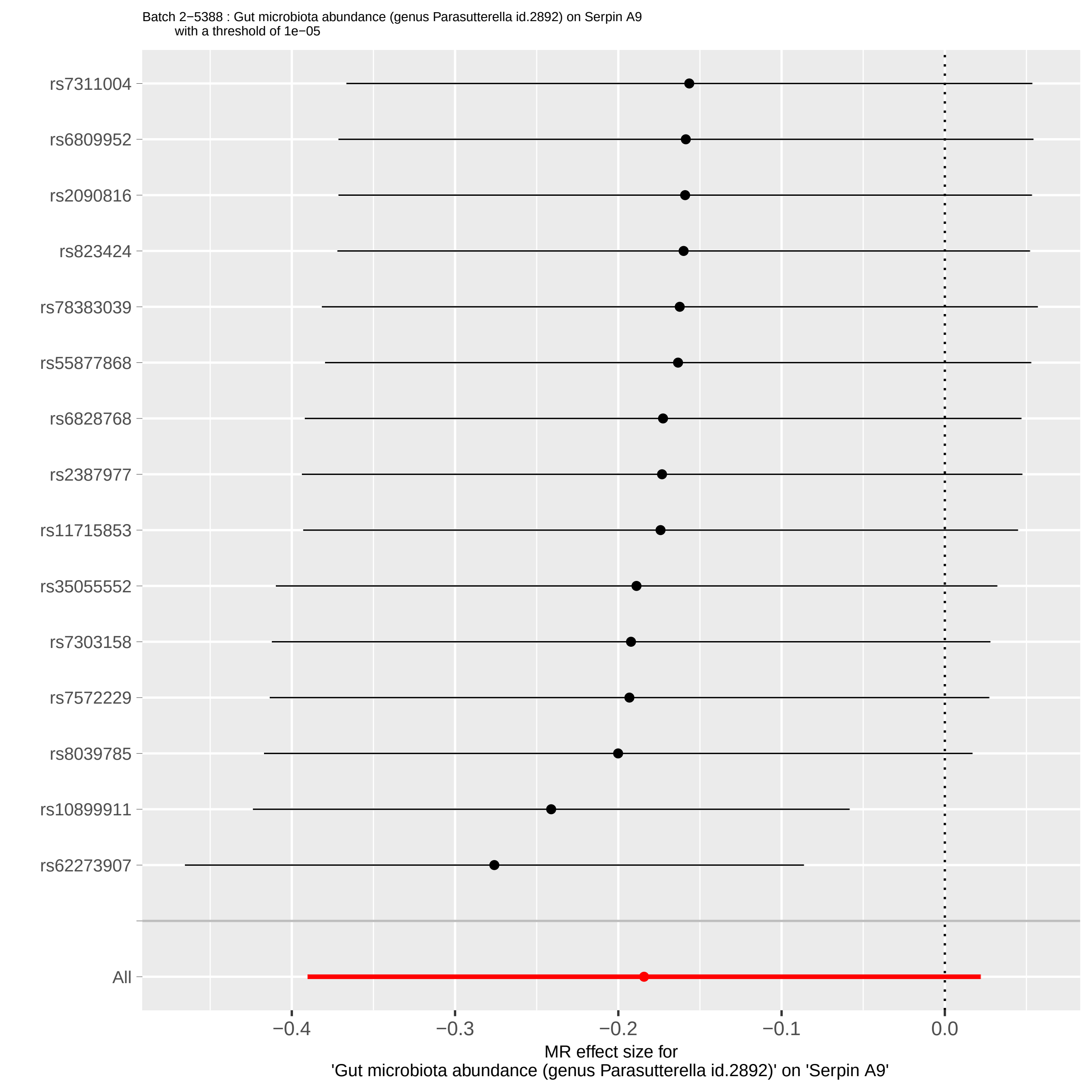

Batch 2−5388 : Gut microbiota abundance (genus Parasutterella id.2892) on Serpin A9
with a threshold of 1e−05
rs7311004
rs6809952
rs2090816
rs823424
rs78383039
rs55877868
rs6828768
rs2387977
rs11715853
rs35055552
rs7303158
rs7572229
rs8039785
rs10899911
rs62273907
All
−0.4
−0.3
−0.2
−0.1
0.0
MR effect size for
'Gut microbiota abundance (genus Parasutterella id.2892)' on 'Serpin A9'

Batch 2−5389 : Gut microbiota abundance (genus Catenibacterium id.2153) on Antithrombin−III
with a threshold of 1e−05

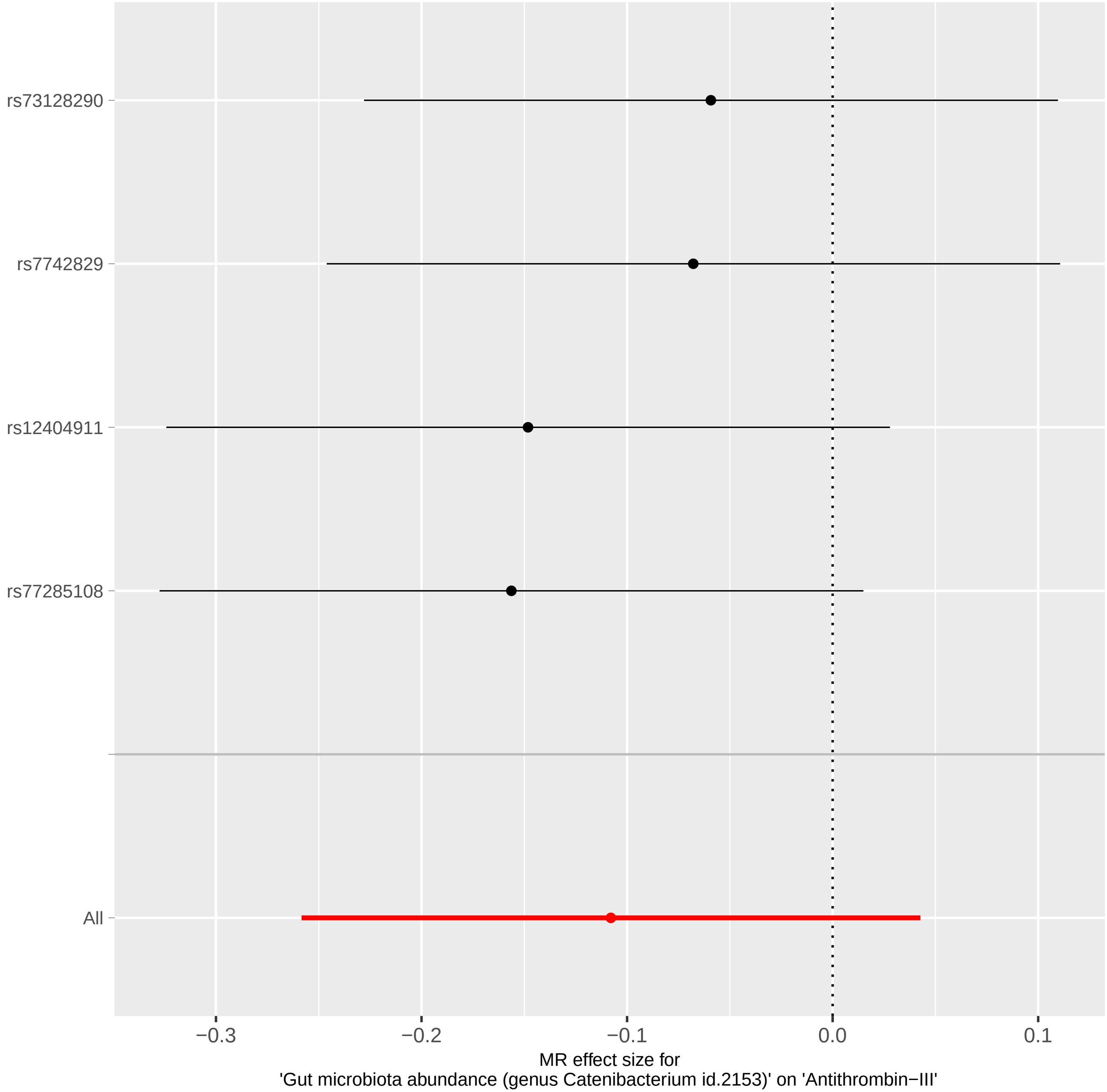

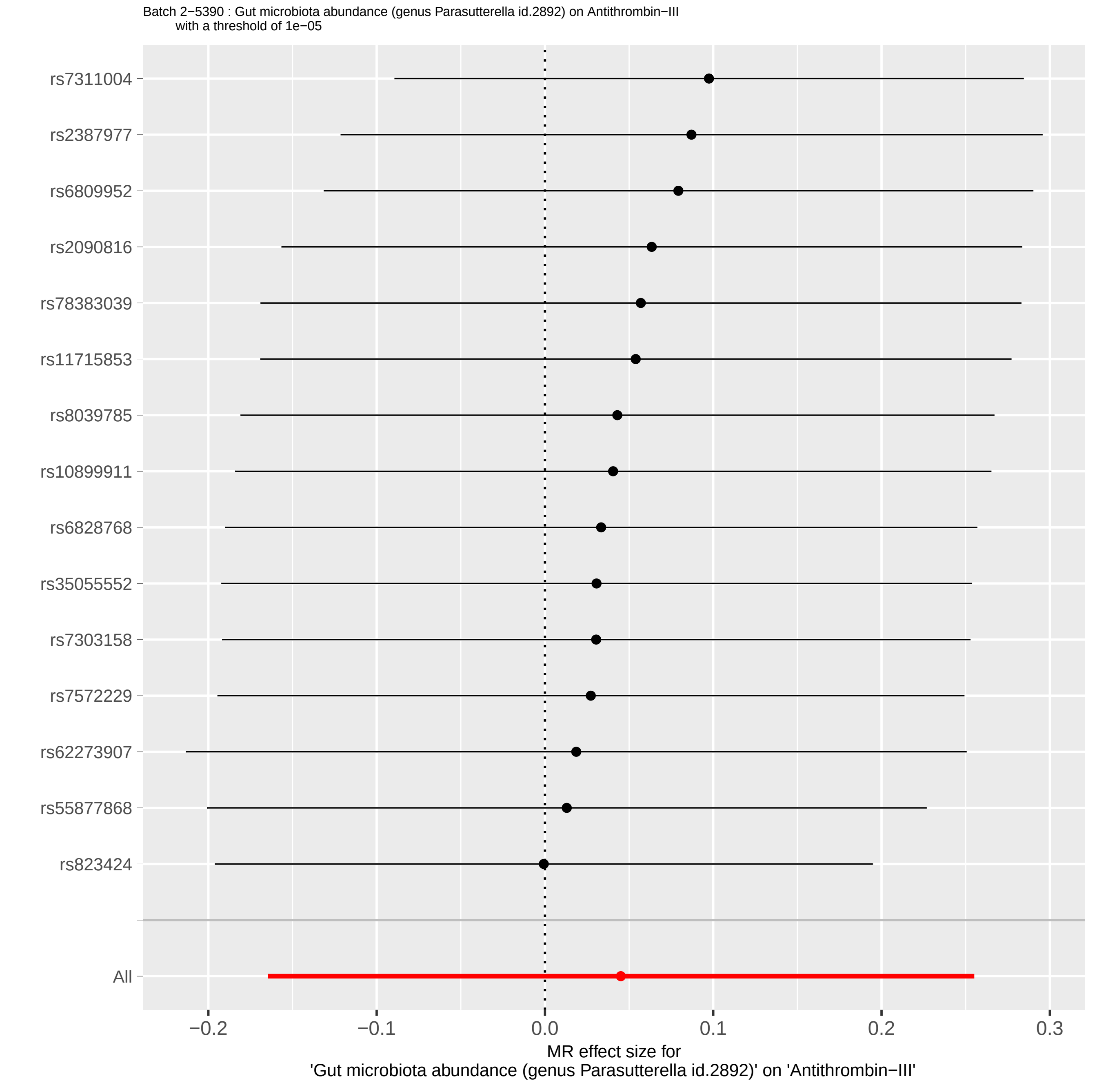
Batch 2−5390 : Gut microbiota abundance (genus Parasutterella id.2892) on Antithrombin−III
with a threshold of 1e−05
rs7311004
rs2387977
rs6809952
rs2090816
rs78383039
rs11715853
rs8039785
rs10899911
rs6828768
rs35055552
rs7303158
rs7572229
rs62273907
rs55877868
rs823424
All
−0.2
−0.1
0.0
0.1
0.2
0.3
MR effect size for
'Gut microbiota abundance (genus Parasutterella id.2892)' on 'Antithrombin−III'

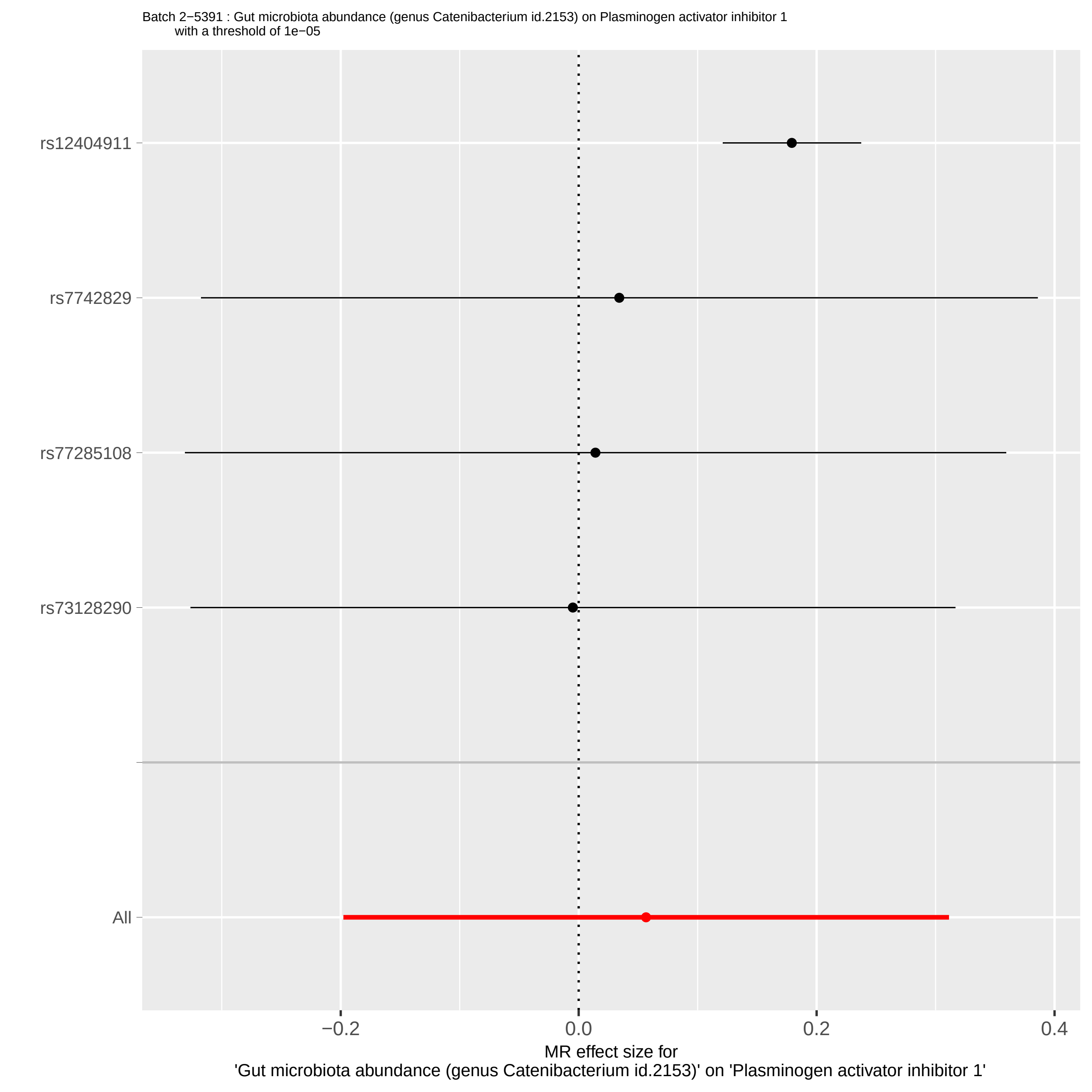
Batch 2−5391 : Gut microbiota abundance (genus Catenibacterium id.2153) on Plasminogen activator inhibitor 1
with a threshold of 1e−05
rs12404911
rs7742829
rs77285108
rs73128290
All
−0.2
0.0
0.2
0.4
MR effect size for
'Gut microbiota abundance (genus Catenibacterium id.2153)' on 'Plasminogen activator inhibitor 1'

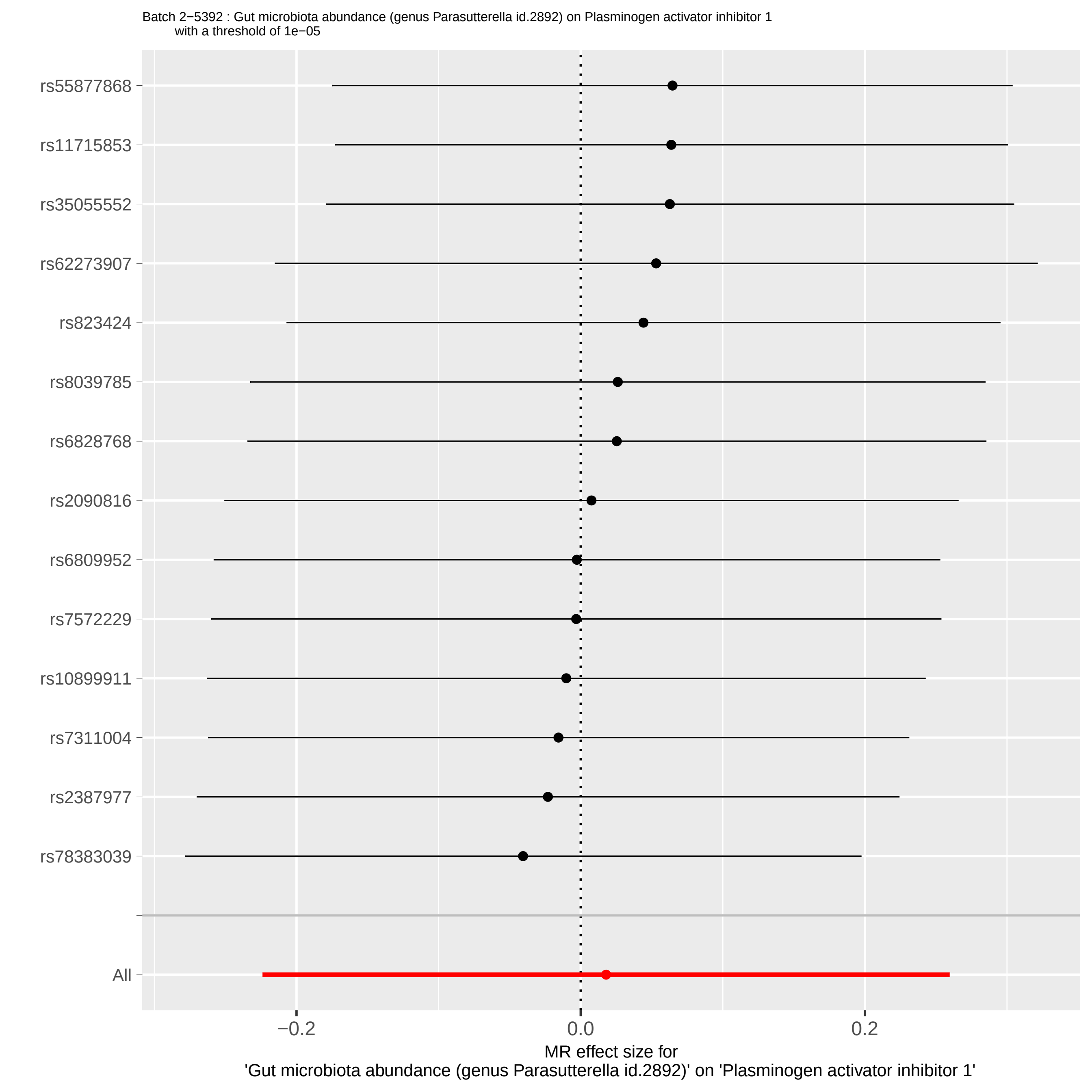

Batch 2−5392 : Gut microbiota abundance (genus Parasutterella id.2892) on Plasminogen activator inhibitor 1
with a threshold of 1e−05
rs55877868
rs11715853
rs35055552
rs62273907
rs823424
rs8039785
rs6828768
rs2090816
rs6809952
rs7572229
rs10899911
rs7311004
rs2387977
rs78383039
All
−0.2
0.0
0.2
MR effect size for
'Gut microbiota abundance (genus Parasutterella id.2892)' on 'Plasminogen activator inhibitor 1'

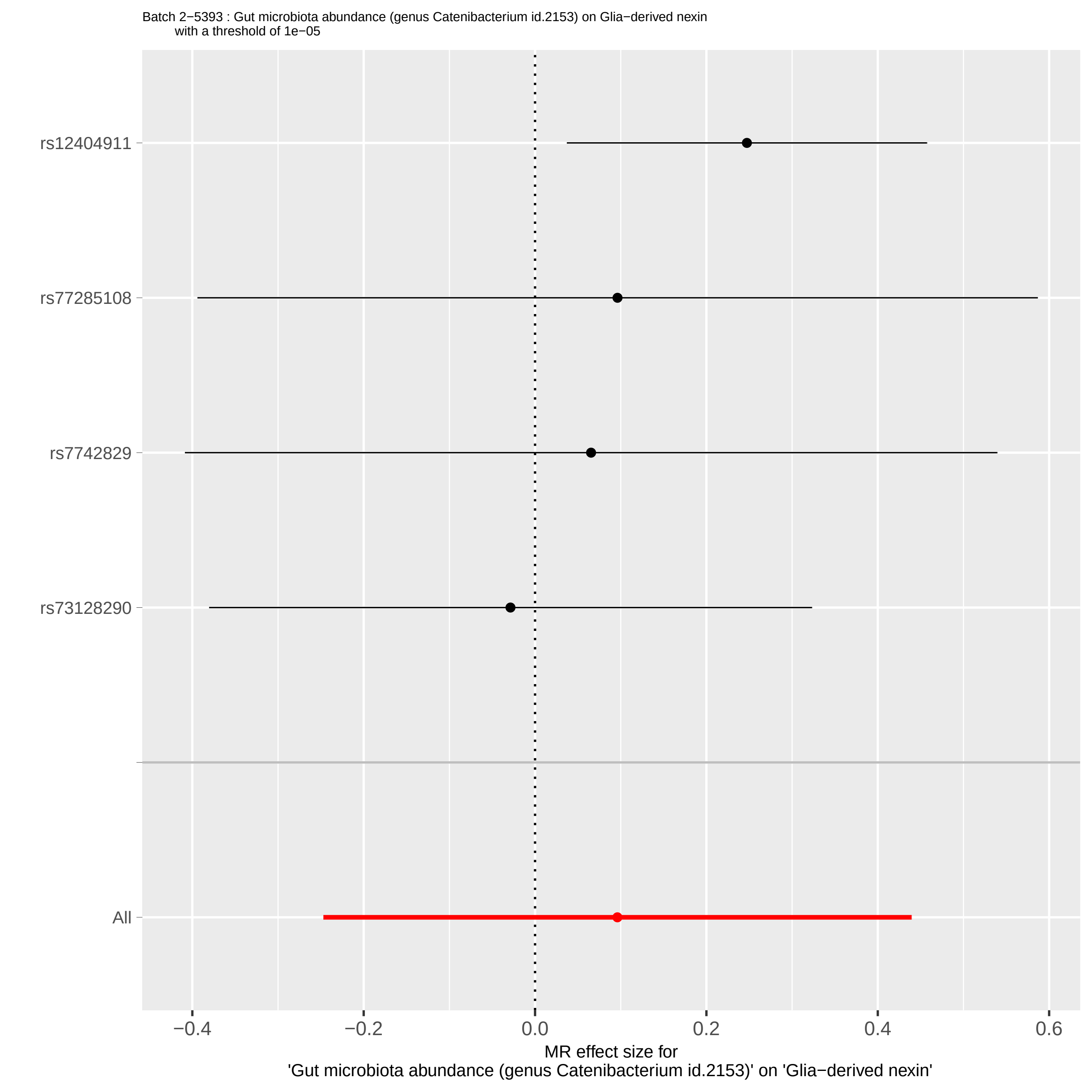
Batch 2−5393 : Gut microbiota abundance (genus Catenibacterium id.2153) on Glia−derived nexin
with a threshold of 1e−05
rs12404911
rs77285108
rs7742829
rs73128290
All
−0.4
−0.2
0.0
0.2
0.4
0.6
MR effect size for
'Gut microbiota abundance (genus Catenibacterium id.2153)' on 'Glia−derived nexin'

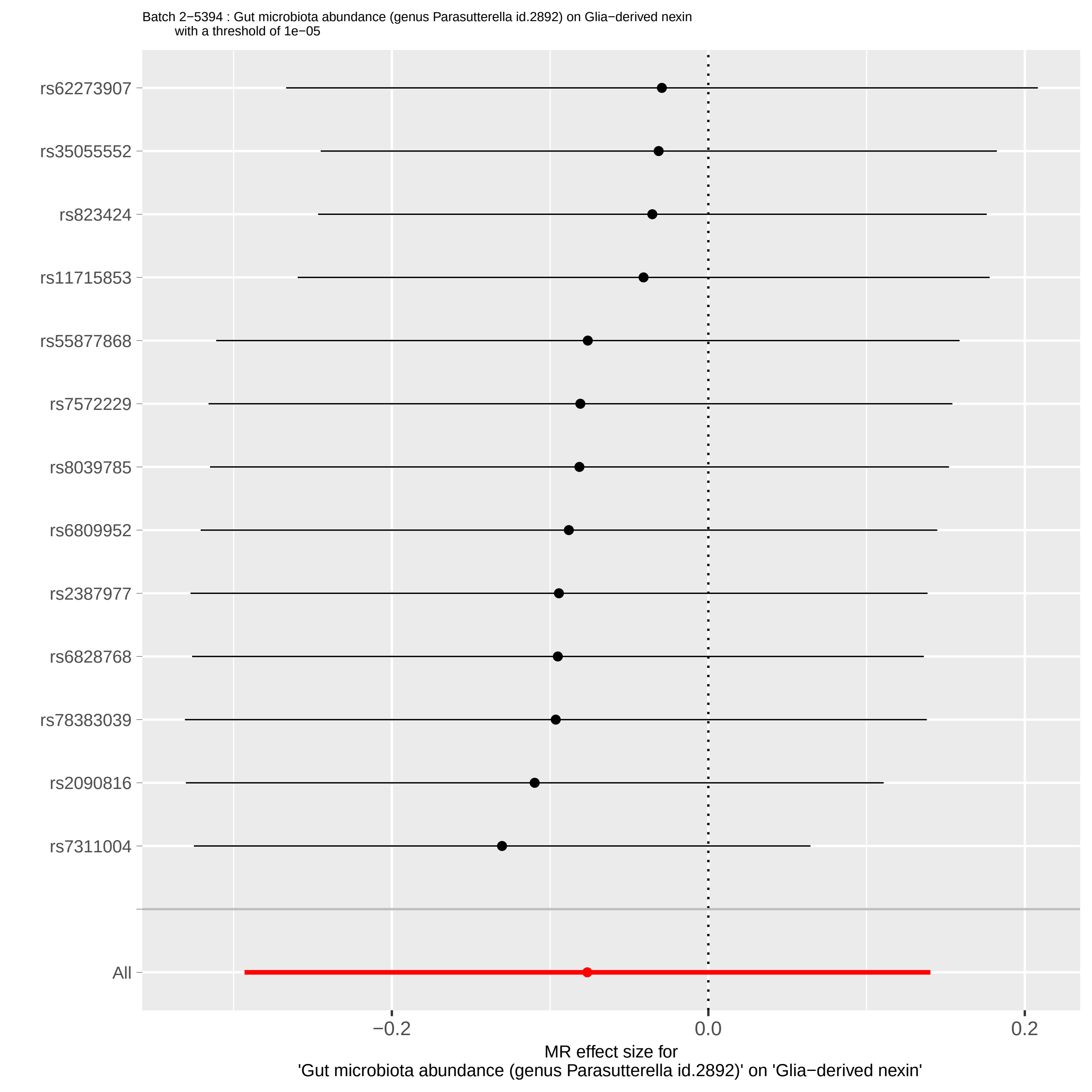
Batch 2−5394 : Gut microbiota abundance (genus Parasutterella id.2892) on Glia−derived nexin
with a threshold of 1e−05
rs62273907
rs35055552
rs823424
rs11715853
rs55877868
rs7572229
rs8039785
rs6809952
rs2387977
rs6828768
rs78383039
rs2090816
rs7311004
All
−0.2
0.0
0.2
MR effect size for
'Gut microbiota abundance (genus Parasutterella id.2892)' on 'Glia−derived nexin'

Batch 2−5395 : Gut microbiota abundance (genus Catenibacterium id.2153) on Pigment epithelium−derived factor
with a threshold of 1e−05

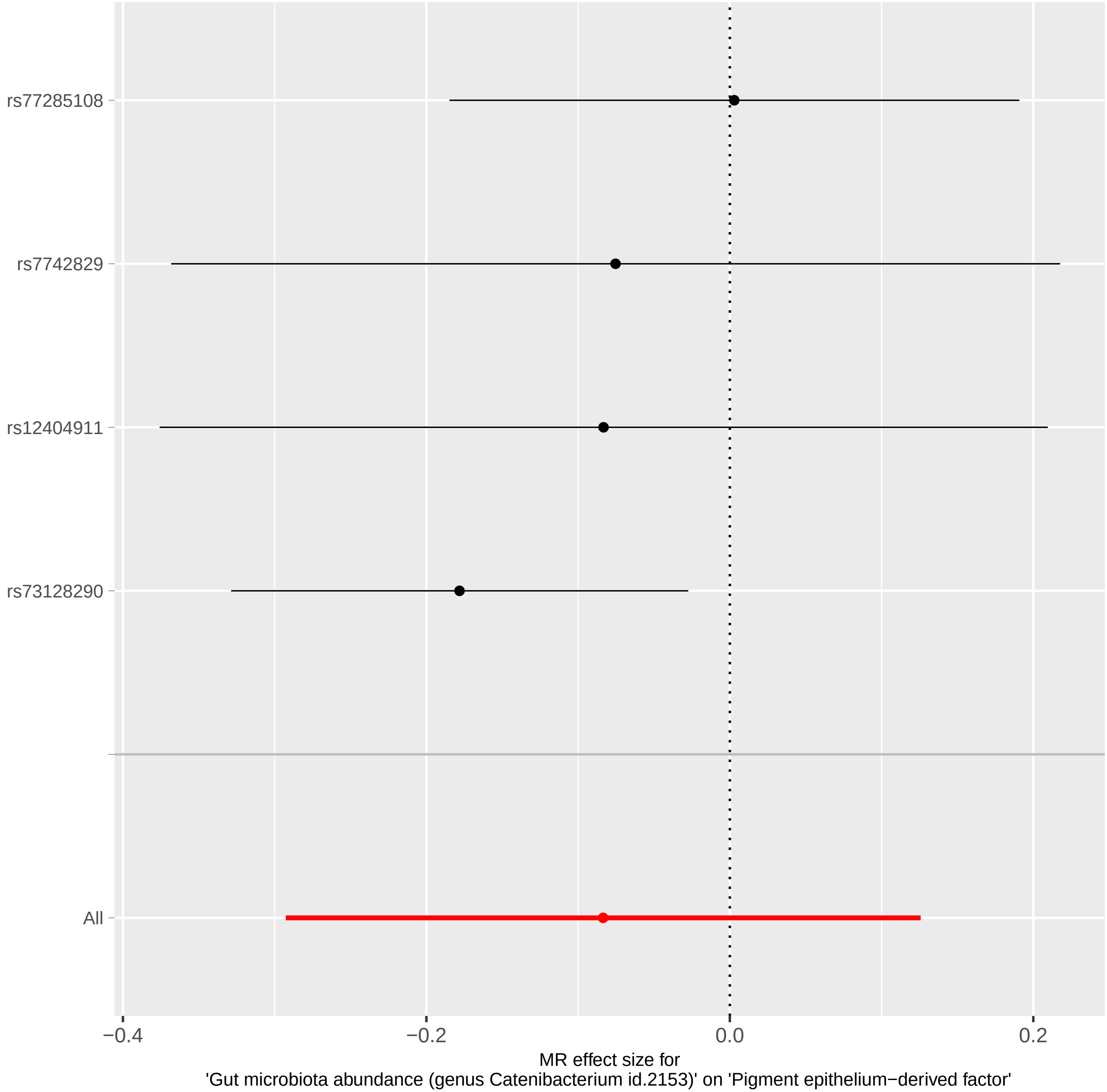

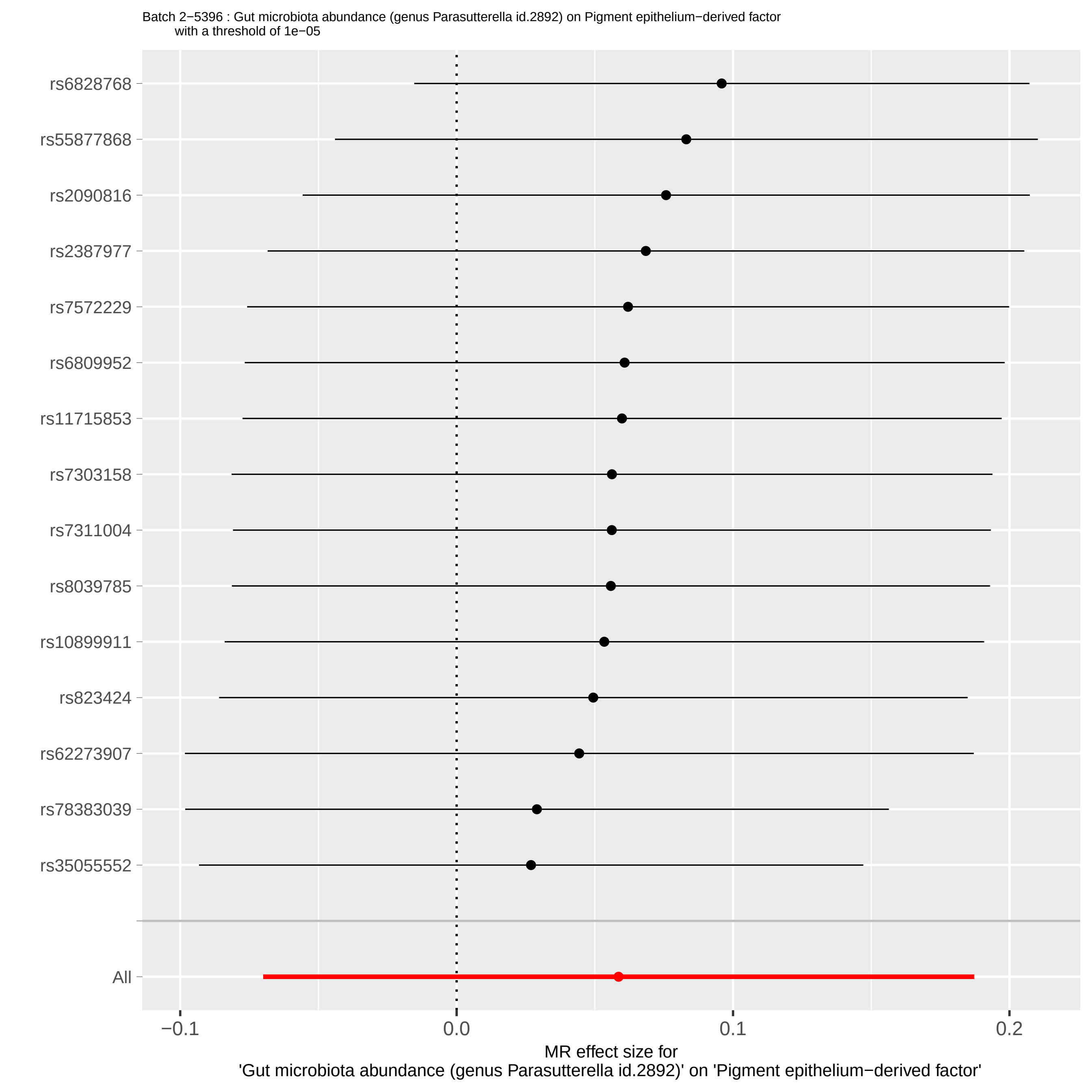
Batch 2−5396 : Gut microbiota abundance (genus Parasutterella id.2892) on Pigment epithelium−derived factor
with a threshold of 1e−05
rs6828768
rs55877868
rs2090816
rs2387977
rs7572229
rs6809952
rs11715853
rs7303158
rs7311004
rs8039785
rs10899911
rs823424
rs62273907
rs78383039
rs35055552
All
−0.1
0.0
0.1
0.2
MR effect size for
'Gut microbiota abundance (genus Parasutterella id.2892)' on 'Pigment epithelium−derived factor'

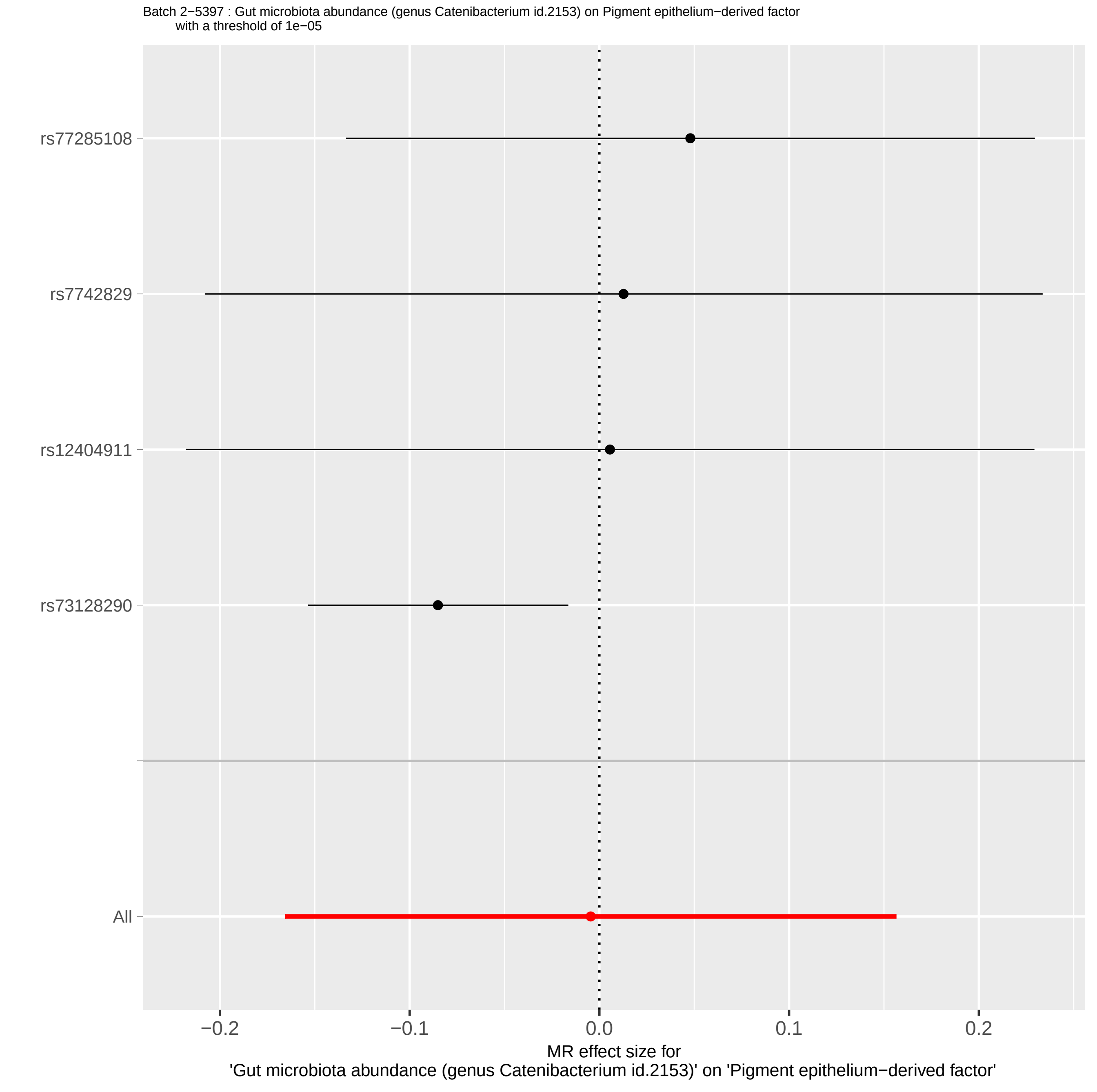
Batch 2−5397 : Gut microbiota abundance (genus Catenibacterium id.2153) on Pigment epithelium−derived factor
with a threshold of 1e−05
rs77285108
rs7742829
rs12404911
rs73128290
All
−0.2
−0.1
0.0
0.1
0.2
MR effect size for
'Gut microbiota abundance (genus Catenibacterium id.2153)' on 'Pigment epithelium−derived factor'

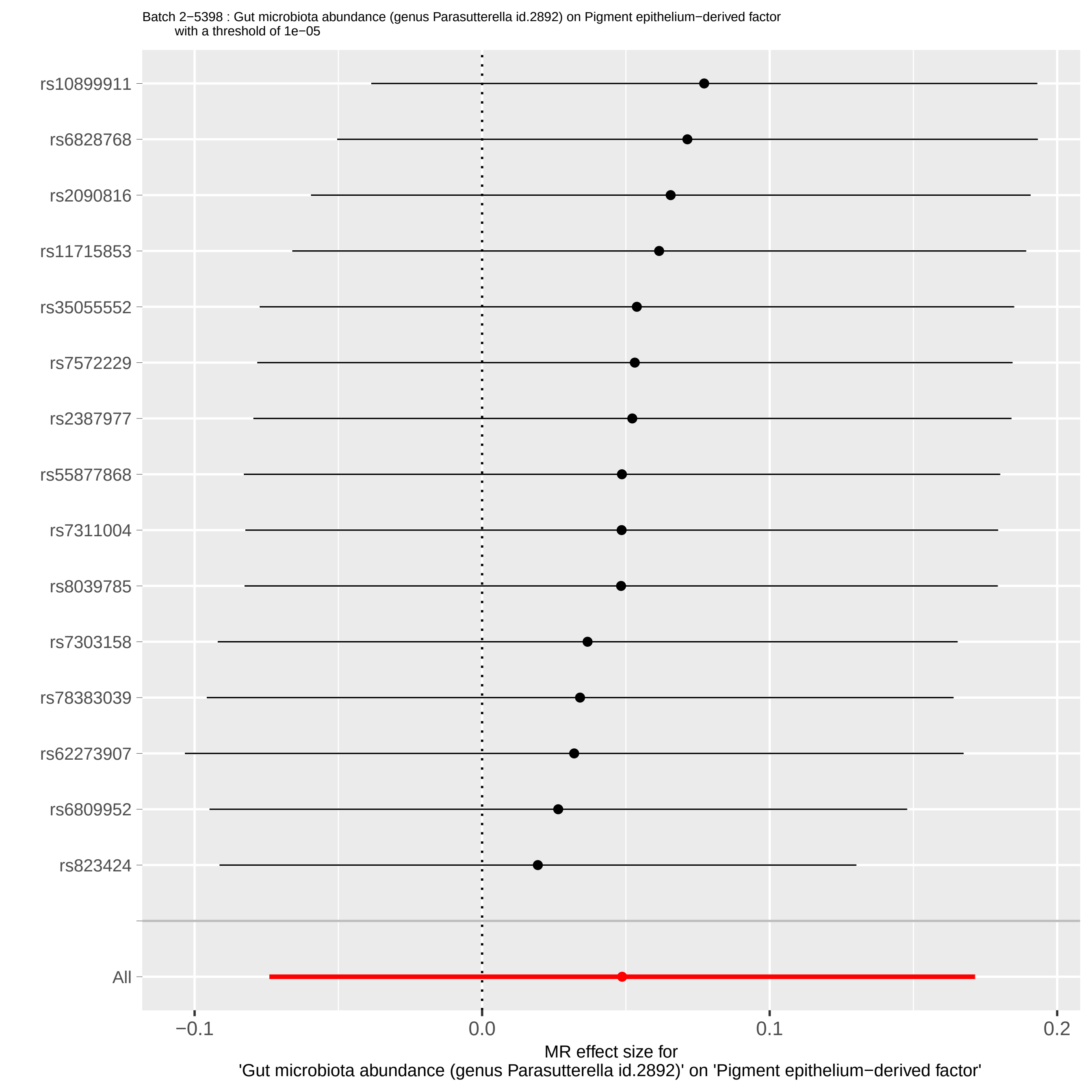
Batch 2−5398 : Gut microbiota abundance (genus Parasutterella id.2892) on Pigment epithelium−derived factor
with a threshold of 1e−05
rs10899911
rs6828768
rs2090816
rs11715853
rs35055552
rs7572229
rs2387977
rs55877868
rs7311004
rs8039785
rs7303158
rs78383039
rs62273907
rs6809952
rs823424
All
−0.1
0.0
0.1
0.2
MR effect size for
'Gut microbiota abundance (genus Parasutterella id.2892)' on 'Pigment epithelium−derived factor'

Batch 2−5399 : Gut microbiota abundance (genus Catenibacterium id.2153) on Alpha−2−antiplasmin
with a threshold of 1e−05

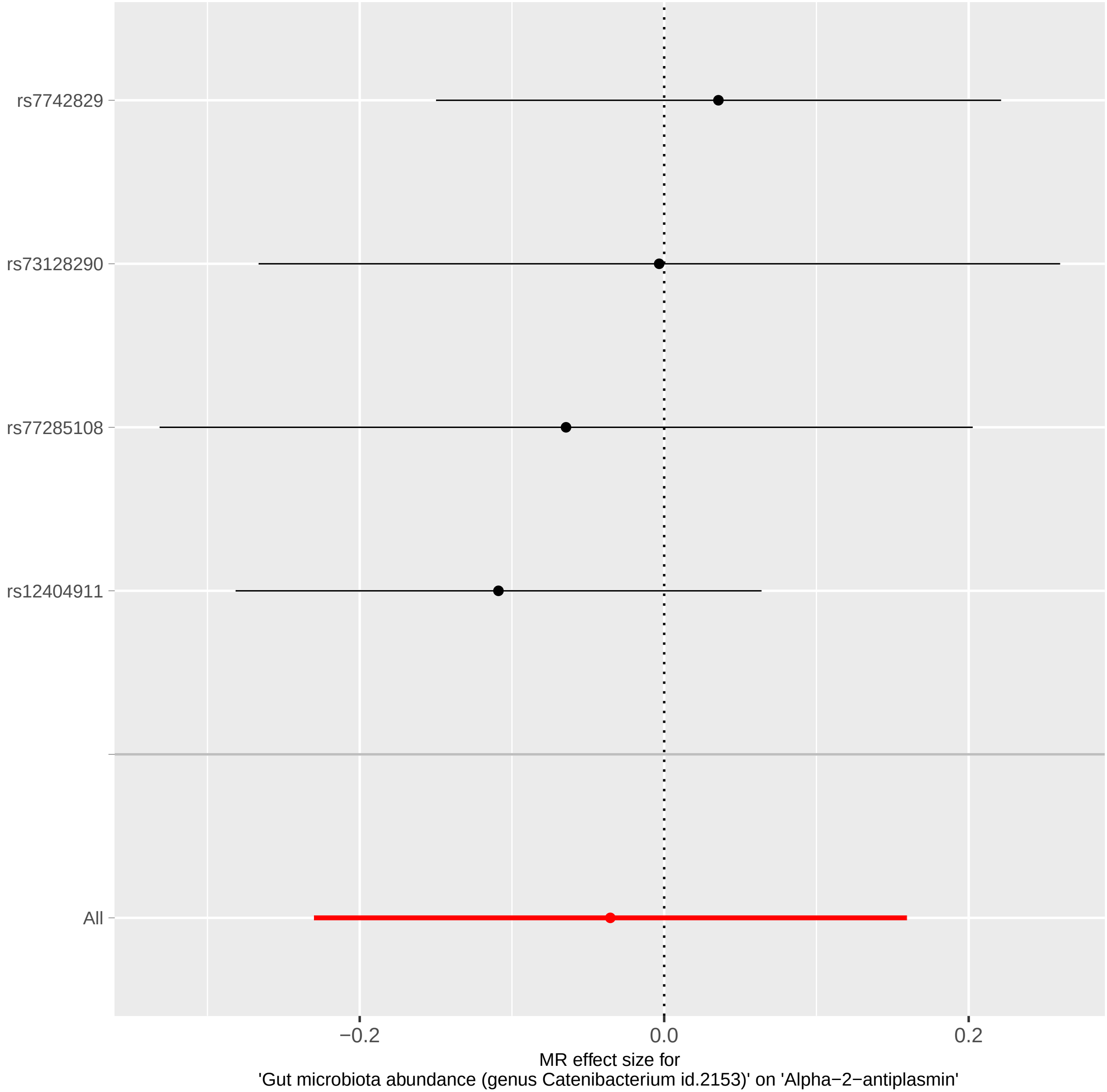

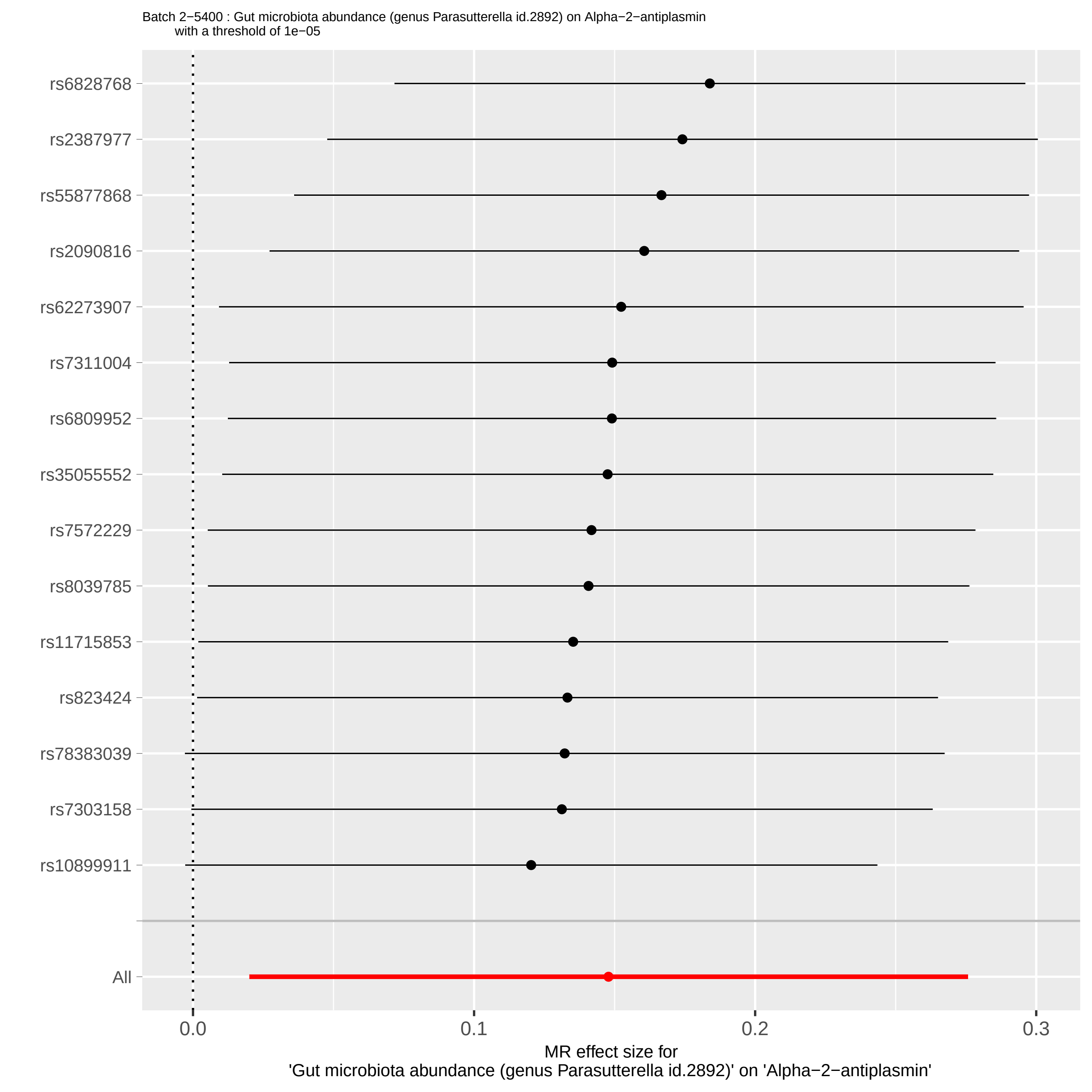

Batch 2−5400 : Gut microbiota abundance (genus Parasutterella id.2892) on Alpha−2−antiplasmin
with a threshold of 1e−05
rs6828768
rs2387977
rs55877868
rs2090816
rs62273907
rs7311004
rs6809952
rs35055552
rs7572229
rs8039785
rs11715853
rs823424
rs78383039
rs7303158
rs10899911
All
0.0
0.1
0.2
0.3
MR effect size for
'Gut microbiota abundance (genus Parasutterella id.2892)' on 'Alpha−2−antiplasmin'

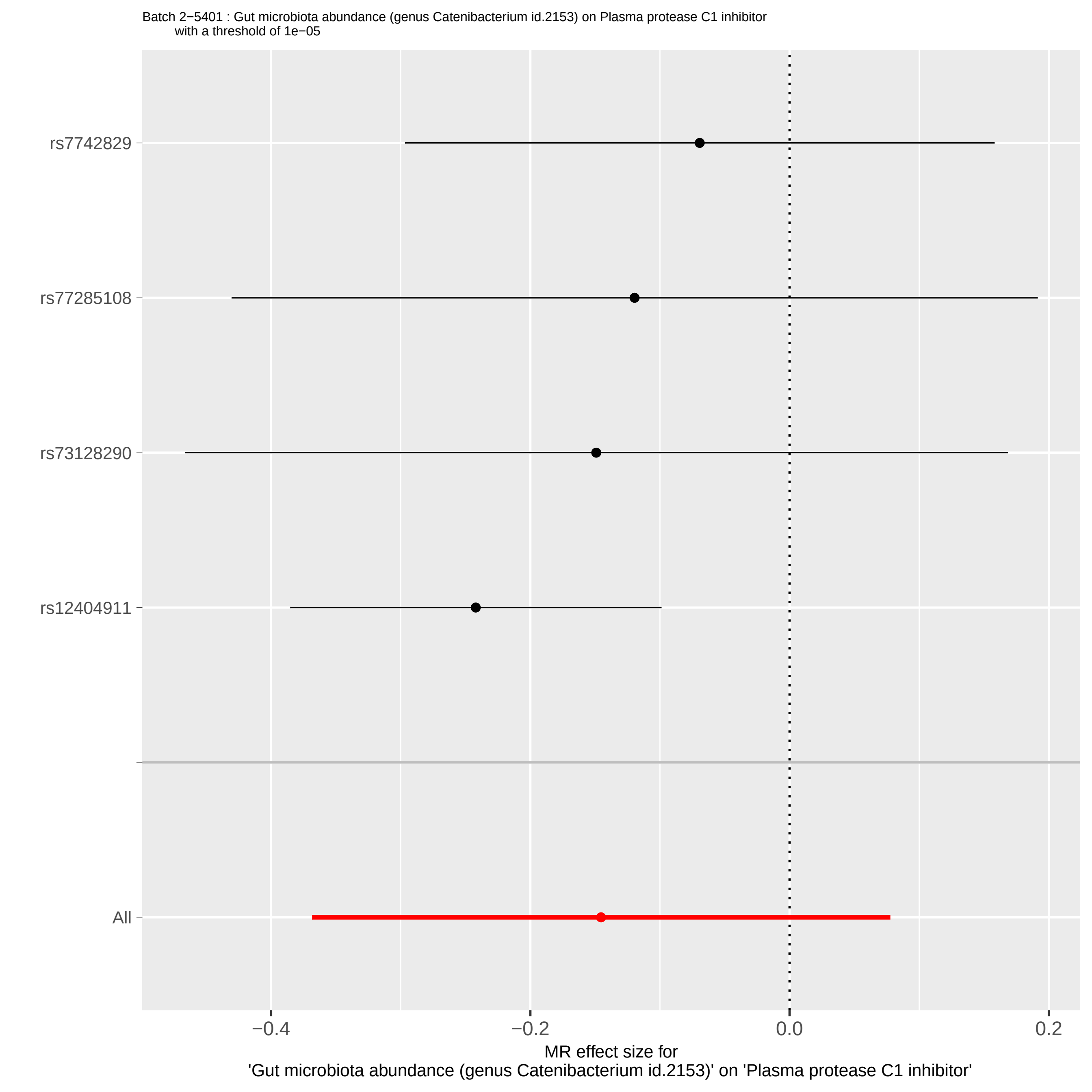
Batch 2−5401 : Gut microbiota abundance (genus Catenibacterium id.2153) on Plasma protease C1 inhibitor
with a threshold of 1e−05
rs7742829
rs77285108
rs73128290
rs12404911
All
−0.4
−0.2
0.0
0.2
MR effect size for
'Gut microbiota abundance (genus Catenibacterium id.2153)' on 'Plasma protease C1 inhibitor'

Batch 2−5402 : Gut microbiota abundance (genus Parasutterella id.2892) on Plasma protease C1 inhibitor
with a threshold of 1e−05

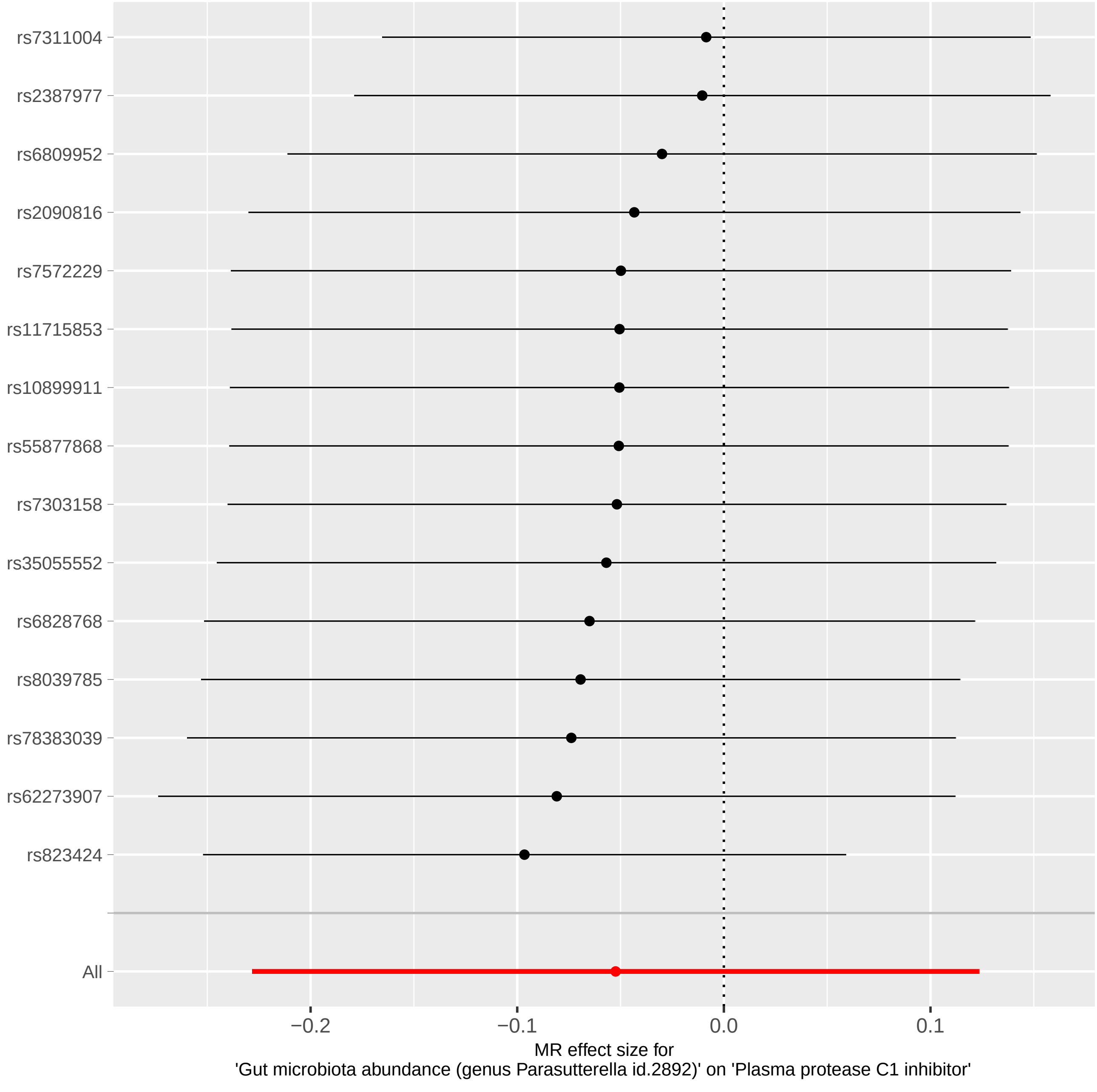

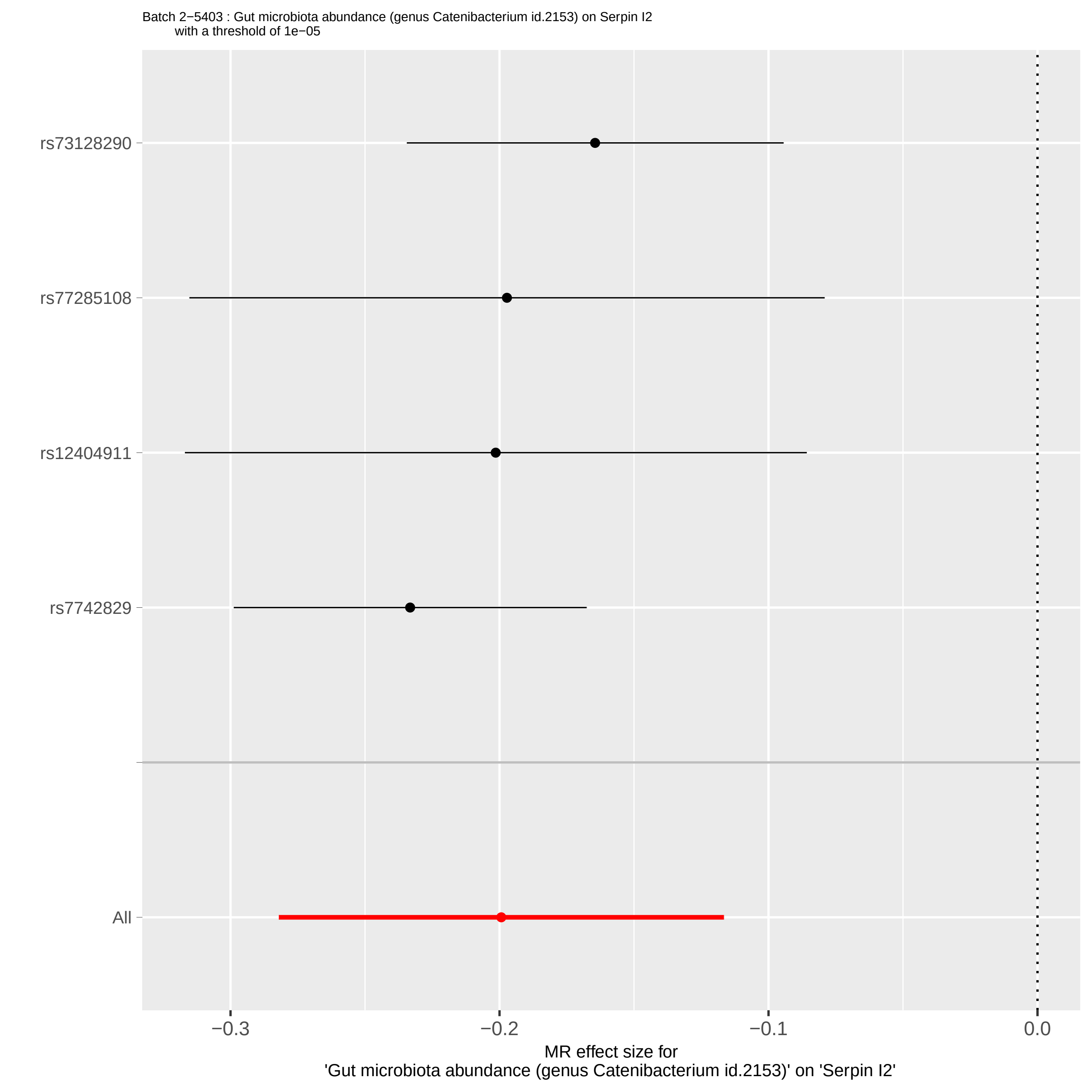
Batch 2−5403 : Gut microbiota abundance (genus Catenibacterium id.2153) on Serpin I2
with a threshold of 1e−05
rs73128290
rs77285108
rs12404911
rs7742829
All
−0.3
−0.2
−0.1
0.0
MR effect size for
'Gut microbiota abundance (genus Catenibacterium id.2153)' on 'Serpin I2'

Batch 2−5404 : Gut microbiota abundance (genus Parasutterella id.2892) on Serpin I2
with a threshold of 1e−05

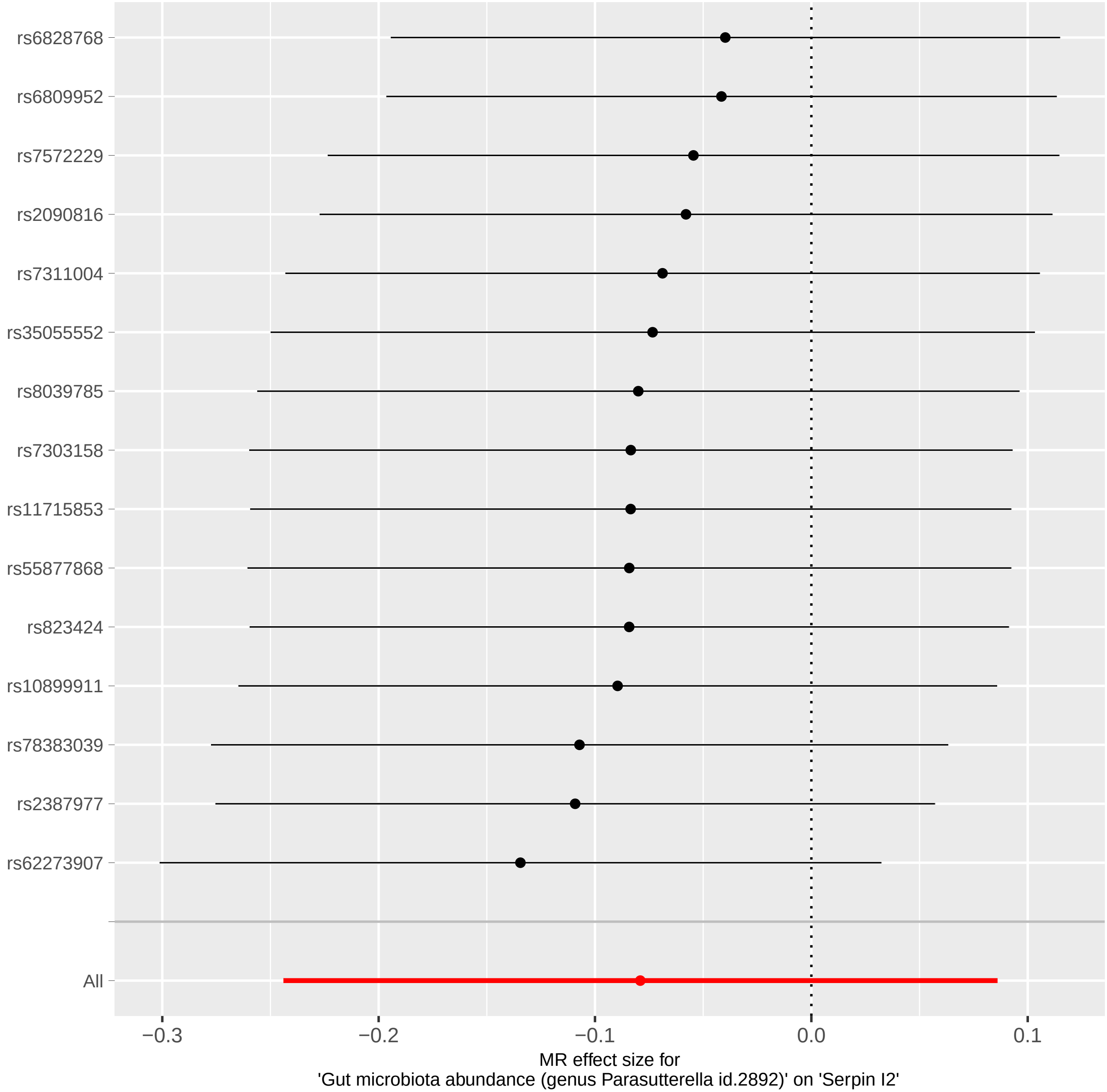

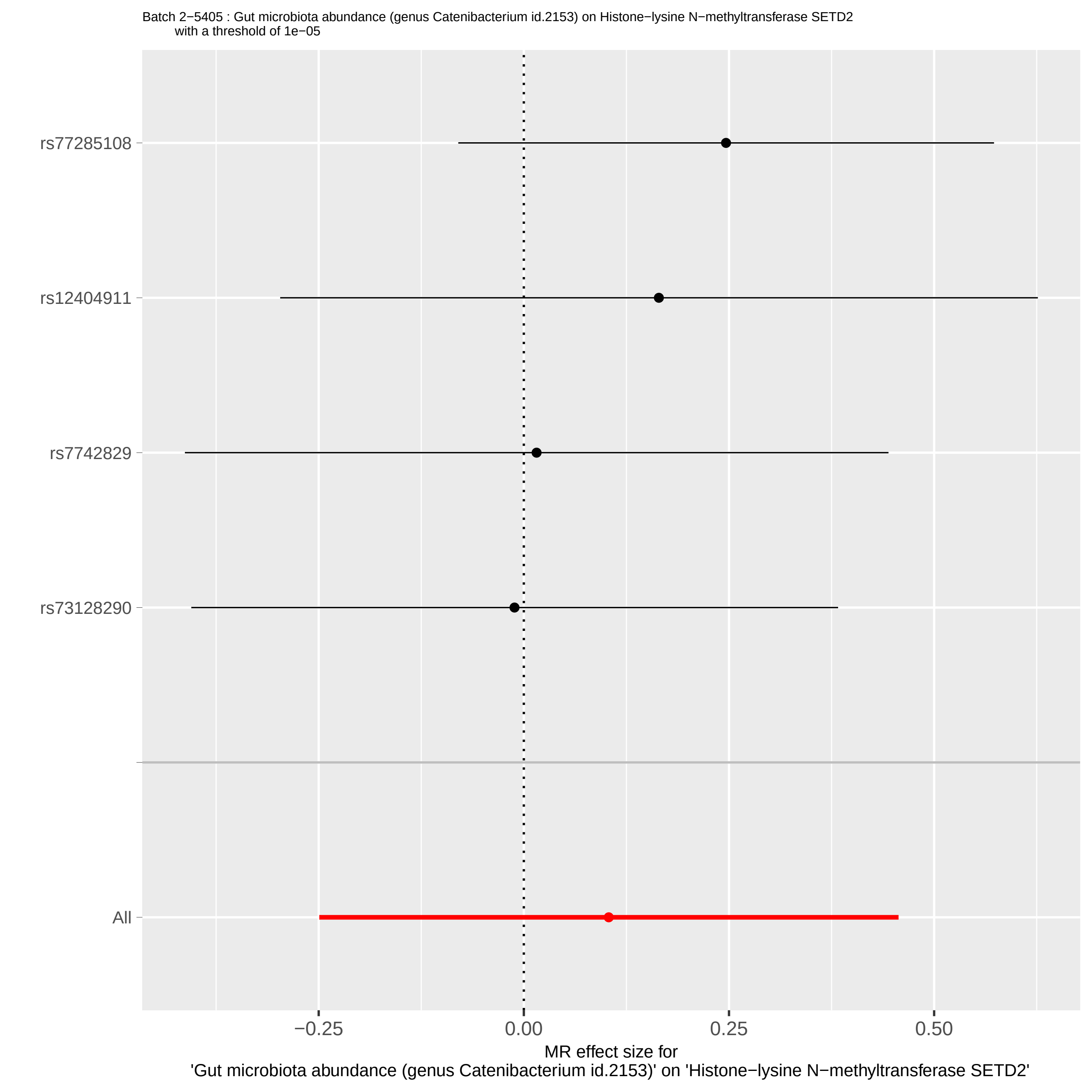
Batch 2−5405 : Gut microbiota abundance (genus Catenibacterium id.2153) on Histone−lysine N−methyltransferase SETD2
with a threshold of 1e−05
rs77285108
rs12404911
rs7742829
rs73128290
All
−0.25
0.00
0.25
0.50
MR effect size for
'Gut microbiota abundance (genus Catenibacterium id.2153)' on 'Histone−lysine N−methyltransferase SETD2'

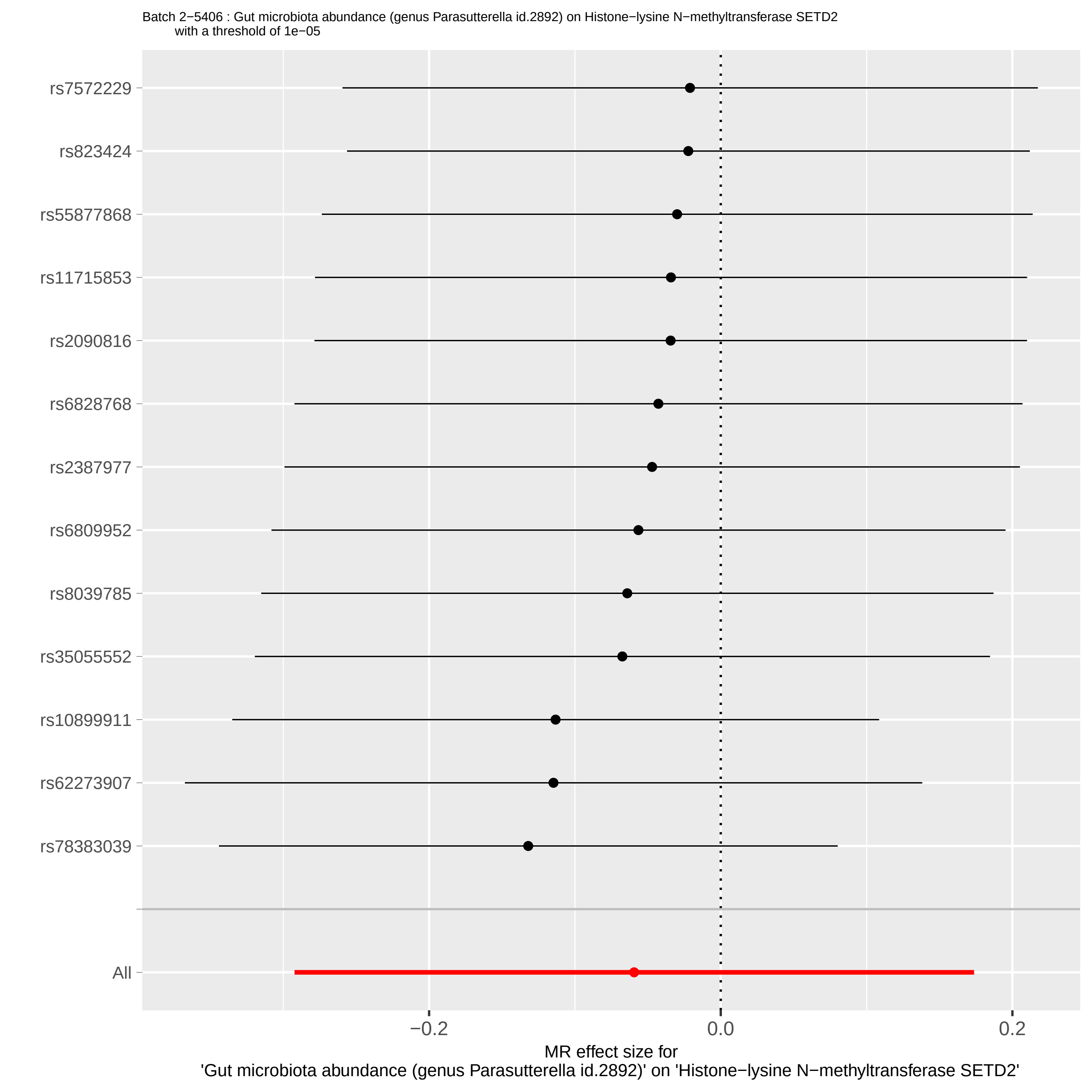
Batch 2−5406 : Gut microbiota abundance (genus Parasutterella id.2892) on Histone−lysine N−methyltransferase SETD2
with a threshold of 1e−05
rs7572229
rs823424
rs55877868
rs11715853
rs2090816
rs6828768
rs2387977
rs6809952
rs8039785
rs35055552
rs10899911
rs62273907
rs78383039
All
−0.2
0.0
0.2
MR effect size for
'Gut microbiota abundance (genus Parasutterella id.2892)' on 'Histone−lysine N−methyltransferase SETD2'

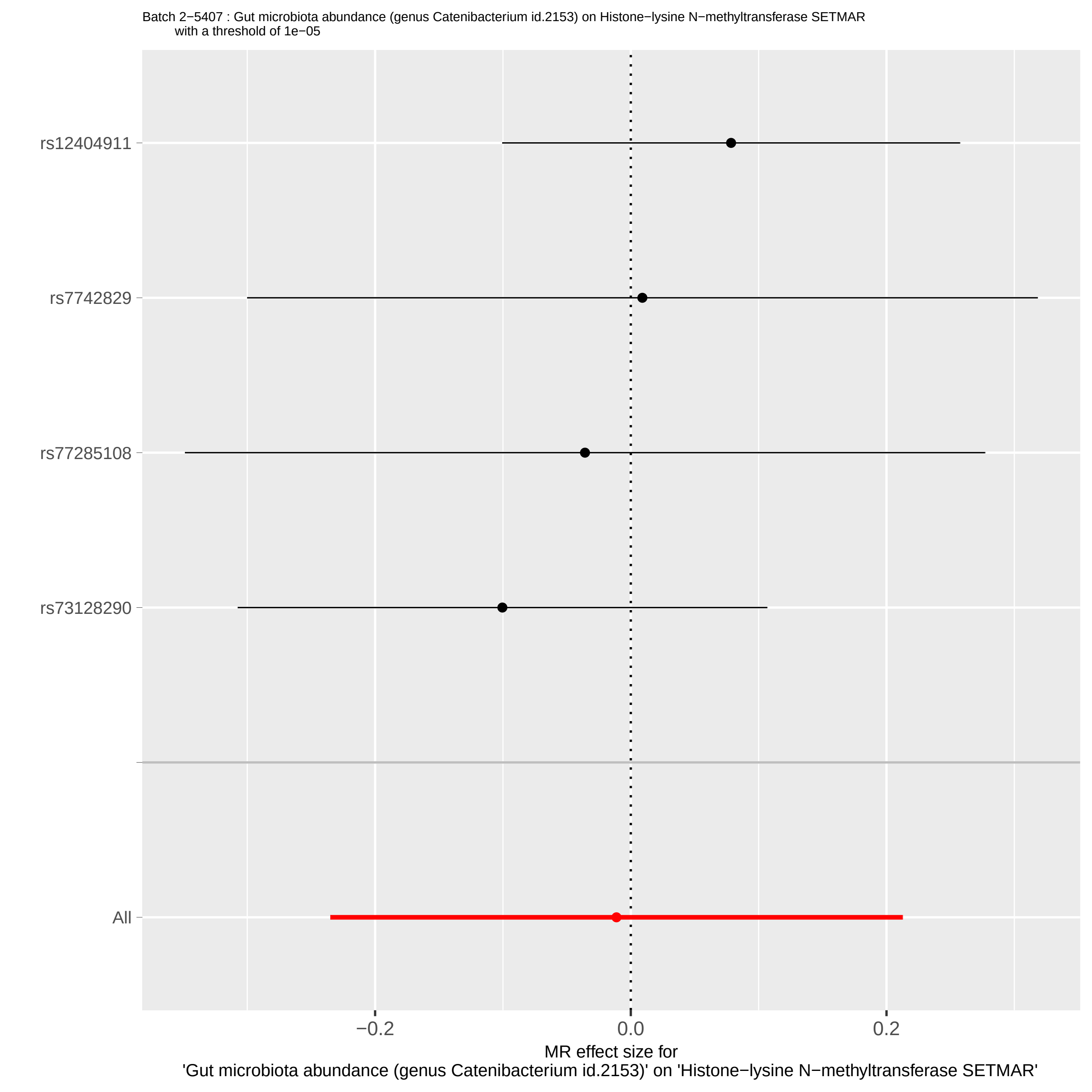

Batch 2−5407 : Gut microbiota abundance (genus Catenibacterium id.2153) on Histone−lysine N−methyltransferase SETMAR
with a threshold of 1e−05
rs12404911
rs7742829
rs77285108
rs73128290
All
−0.2
0.0
0.2
MR effect size for
'Gut microbiota abundance (genus Catenibacterium id.2153)' on 'Histone−lysine N−methyltransferase SETMAR'

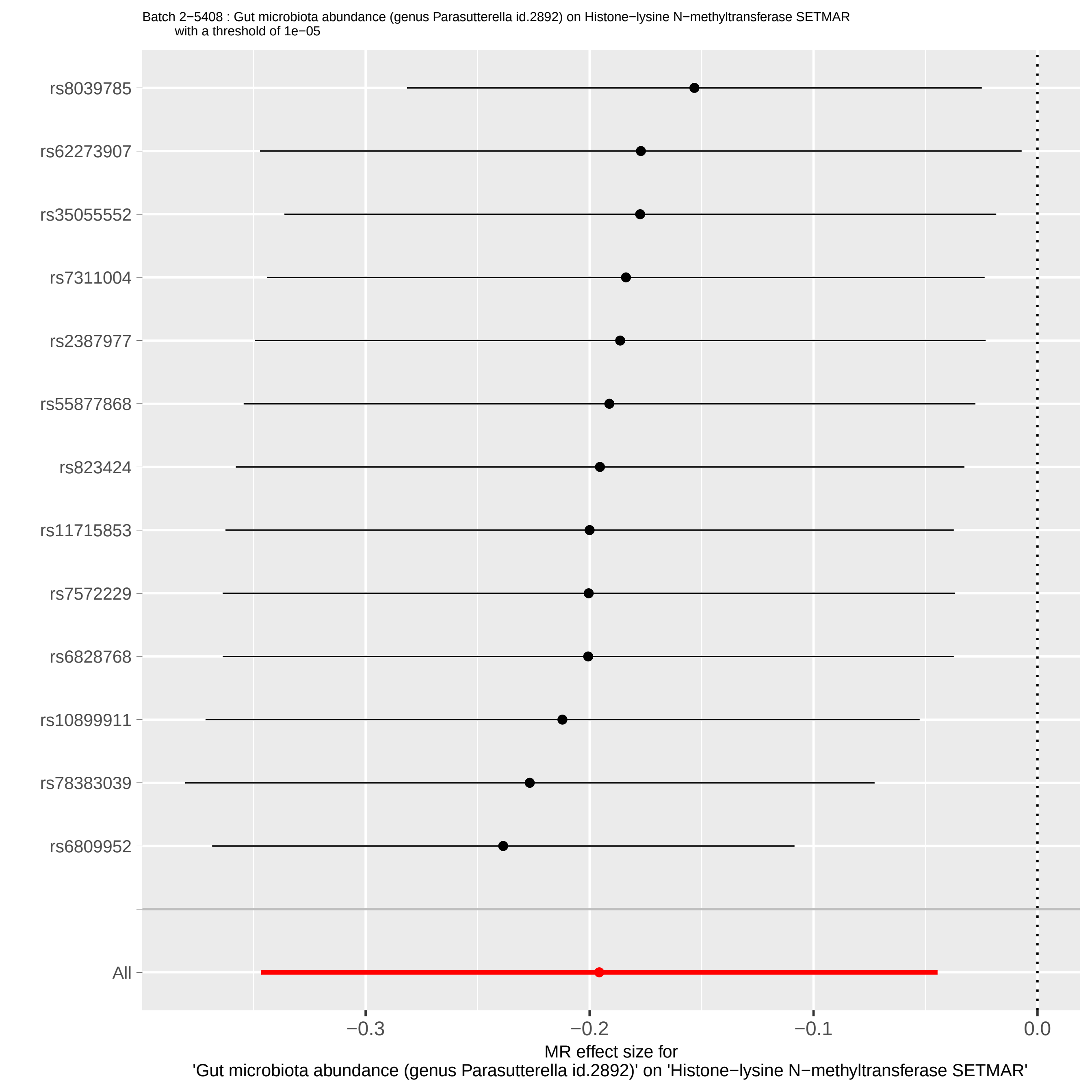

Batch 2−5408 : Gut microbiota abundance (genus Parasutterella id.2892) on Histone−lysine N−methyltransferase SETMAR
with a threshold of 1e−05
rs8039785
rs62273907
rs35055552
rs7311004
rs2387977
rs55877868
rs823424
rs11715853
rs7572229
rs6828768
rs10899911
rs78383039
rs6809952
All
−0.3
−0.2
−0.1
0.0
MR effect size for
'Gut microbiota abundance (genus Parasutterella id.2892)' on 'Histone−lysine N−methyltransferase SETMAR'

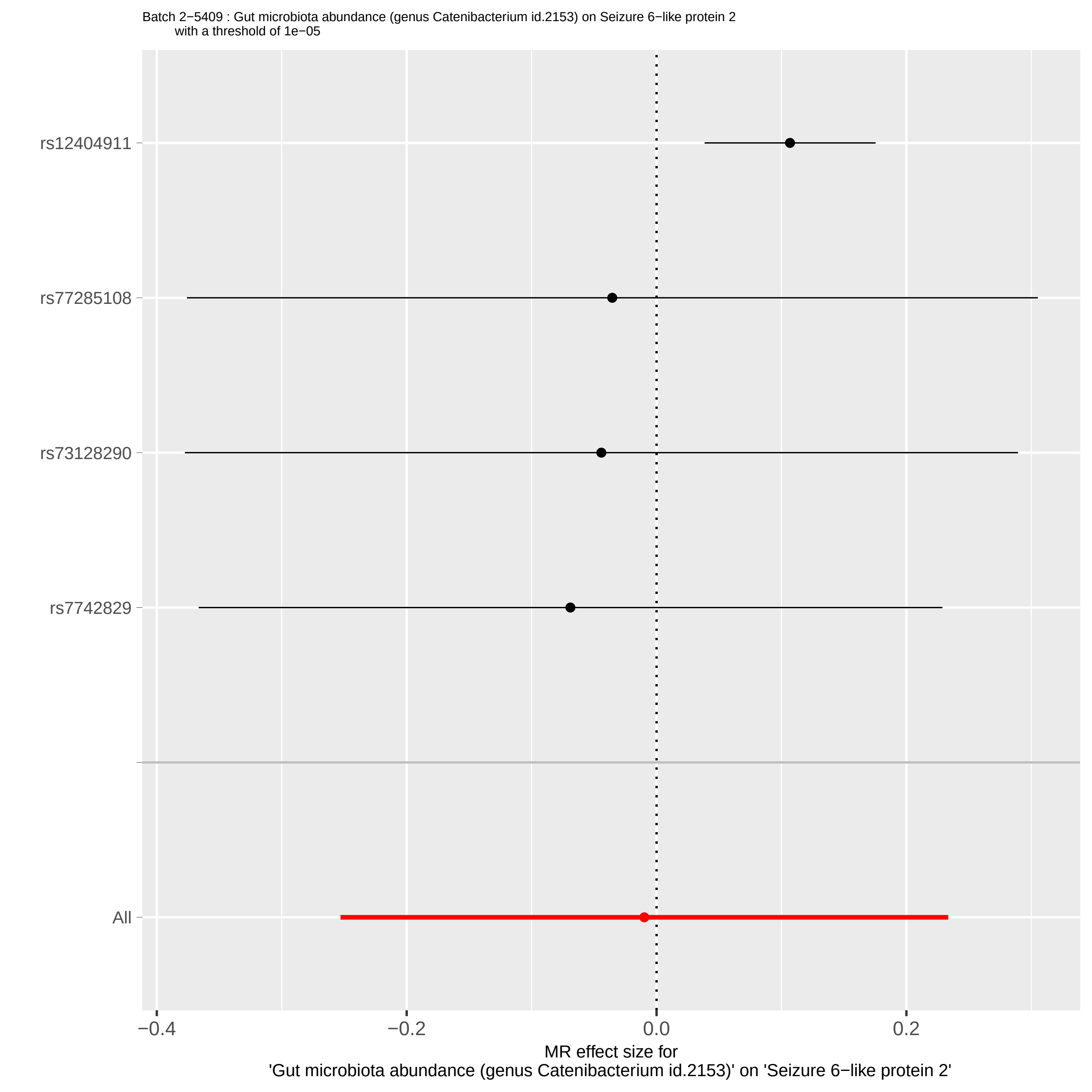
Batch 2−5409 : Gut microbiota abundance (genus Catenibacterium id.2153) on Seizure 6−like protein 2
with a threshold of 1e−05
rs12404911
rs77285108
rs73128290
rs7742829
All
−0.4
−0.2
0.0
0.2
MR effect size for
'Gut microbiota abundance (genus Catenibacterium id.2153)' on 'Seizure 6−like protein 2'

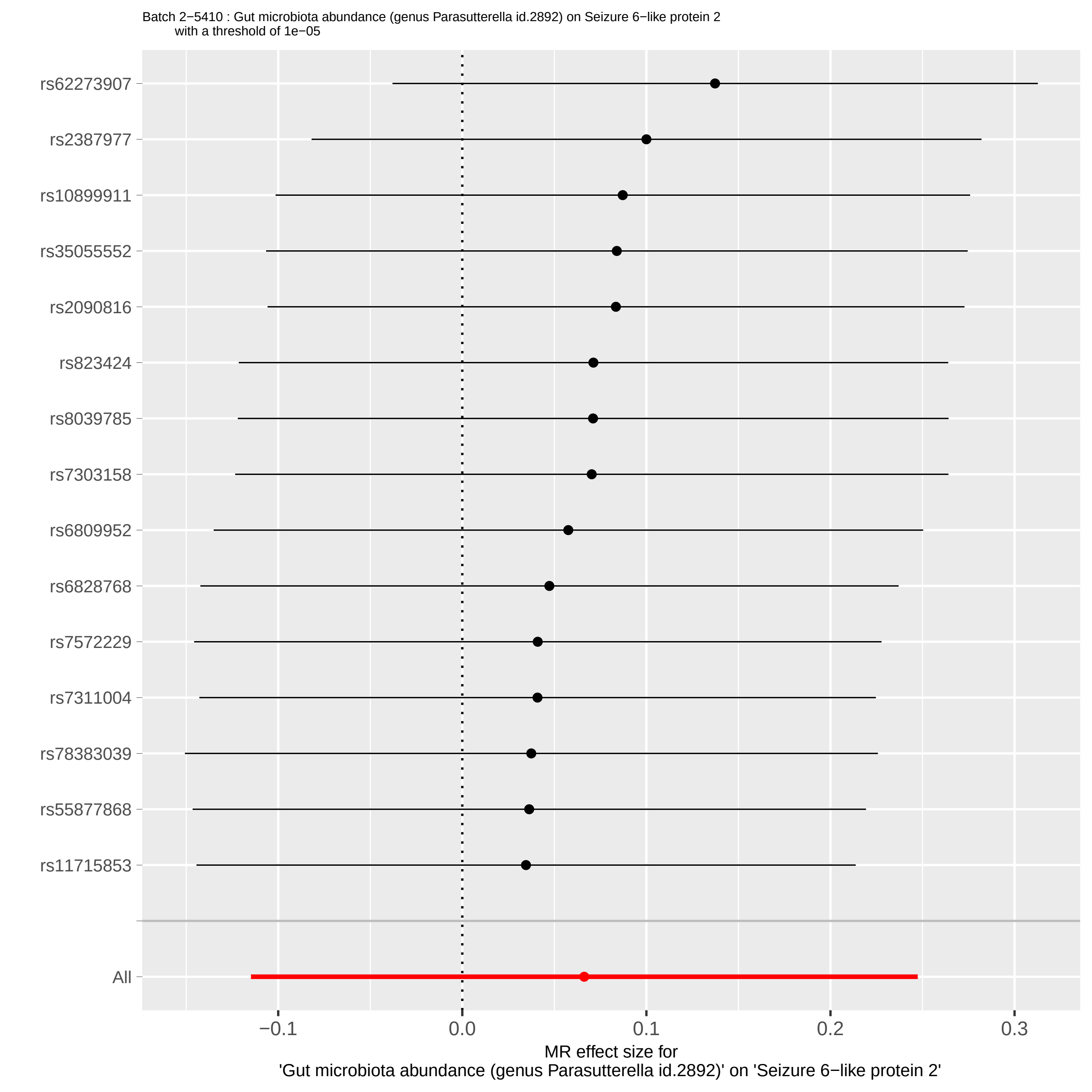

Batch 2−5410 : Gut microbiota abundance (genus Parasutterella id.2892) on Seizure 6−like protein 2
with a threshold of 1e−05
rs62273907
rs2387977
rs10899911
rs35055552
rs2090816
rs823424
rs8039785
rs7303158
rs6809952
rs6828768
rs7572229
rs7311004
rs78383039
rs55877868
rs11715853
All
−0.1
0.0
0.1
0.2
0.3
MR effect size for
'Gut microbiota abundance (genus Parasutterella id.2892)' on 'Seizure 6−like protein 2'

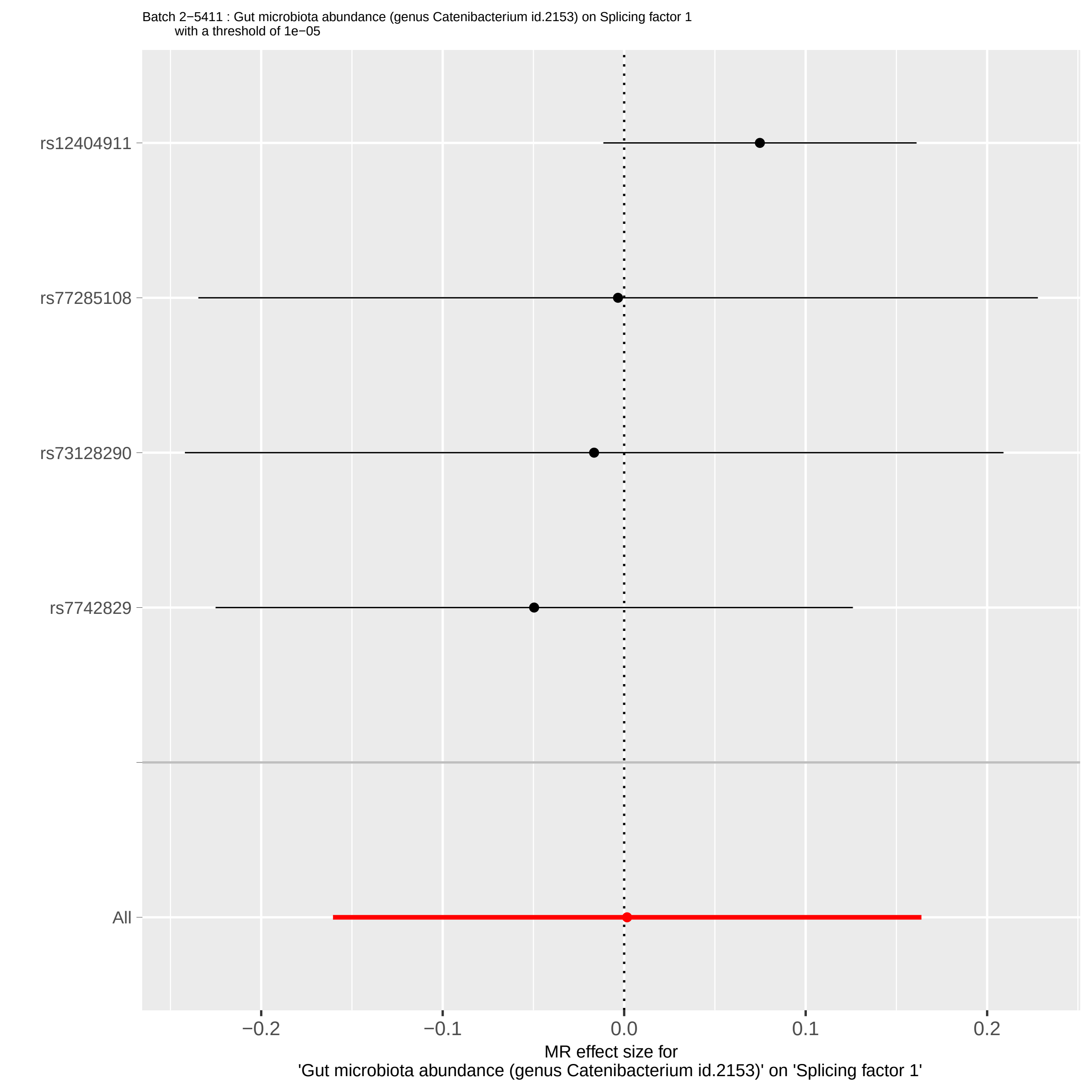
Batch 2−5411 : Gut microbiota abundance (genus Catenibacterium id.2153) on Splicing factor 1
with a threshold of 1e−05
rs12404911
rs77285108
rs73128290
rs7742829
All
−0.2
−0.1
0.0
0.1
0.2
MR effect size for
'Gut microbiota abundance (genus Catenibacterium id.2153)' on 'Splicing factor 1'

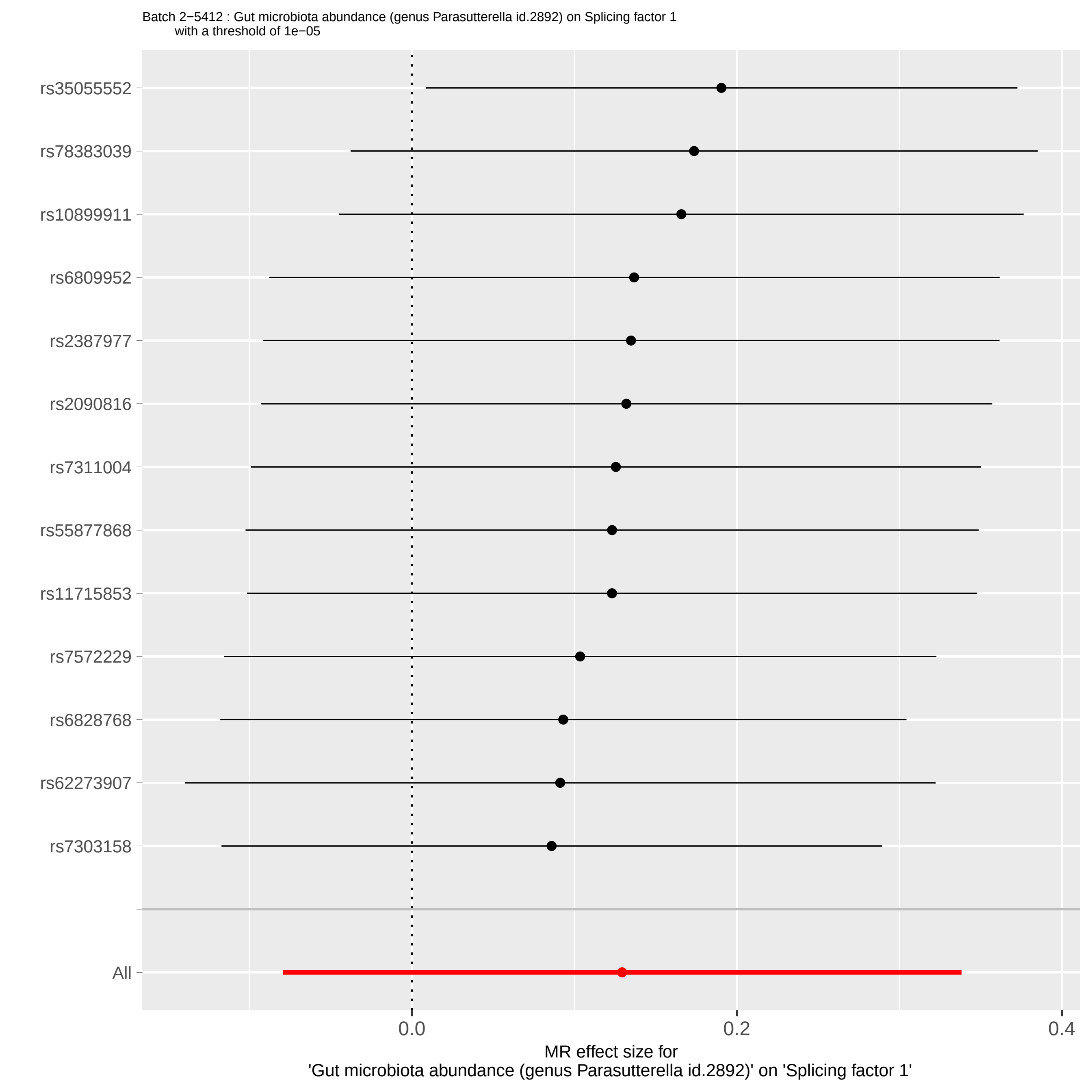
Batch 2−5412 : Gut microbiota abundance (genus Parasutterella id.2892) on Splicing factor 1
with a threshold of 1e−05
rs35055552
rs78383039
rs10899911
rs6809952
rs2387977
rs2090816
rs7311004
rs55877868
rs11715853
rs7572229
rs6828768
rs62273907
rs7303158
All
0.0
0.2
0.4
MR effect size for
'Gut microbiota abundance (genus Parasutterella id.2892)' on 'Splicing factor 1'

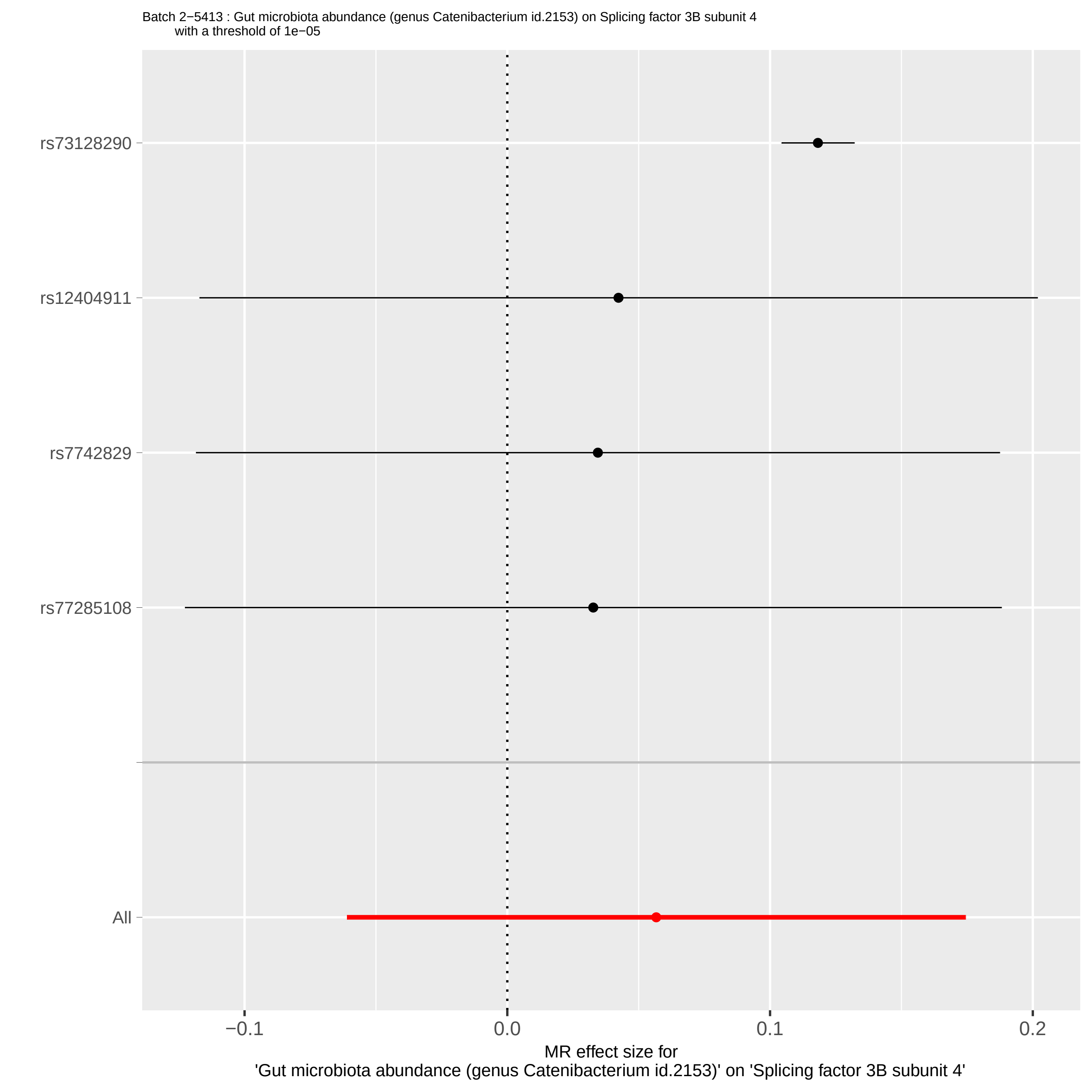
Batch 2−5413 : Gut microbiota abundance (genus Catenibacterium id.2153) on Splicing factor 3B subunit 4
with a threshold of 1e−05
rs73128290
rs12404911
rs7742829
rs77285108
All
−0.1
0.0
0.1
0.2
MR effect size for
'Gut microbiota abundance (genus Catenibacterium id.2153)' on 'Splicing factor 3B subunit 4'

Batch 2−5414 : Gut microbiota abundance (genus Parasutterella id.2892) on Splicing factor 3B subunit 4
with a threshold of 1e−05

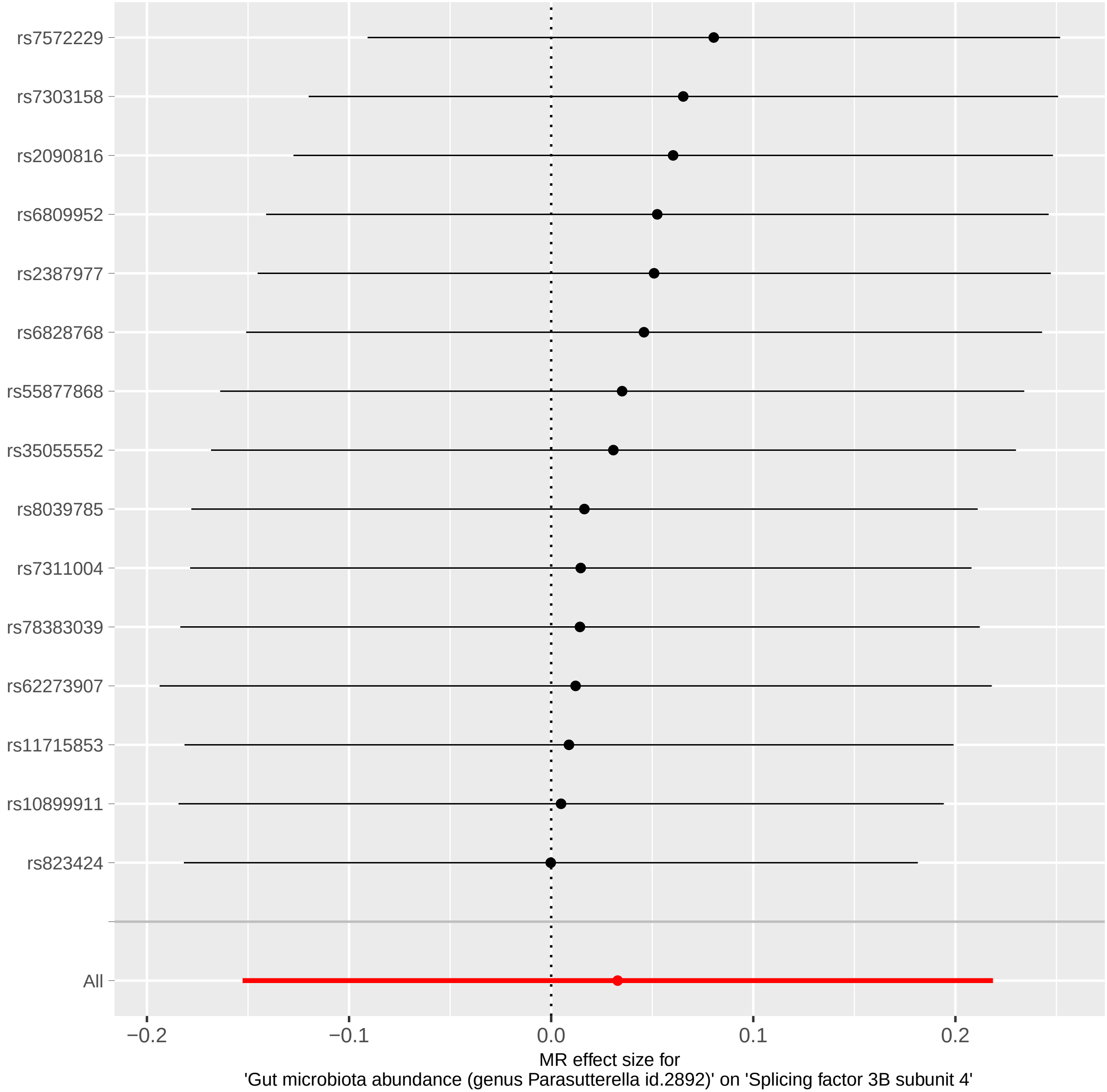

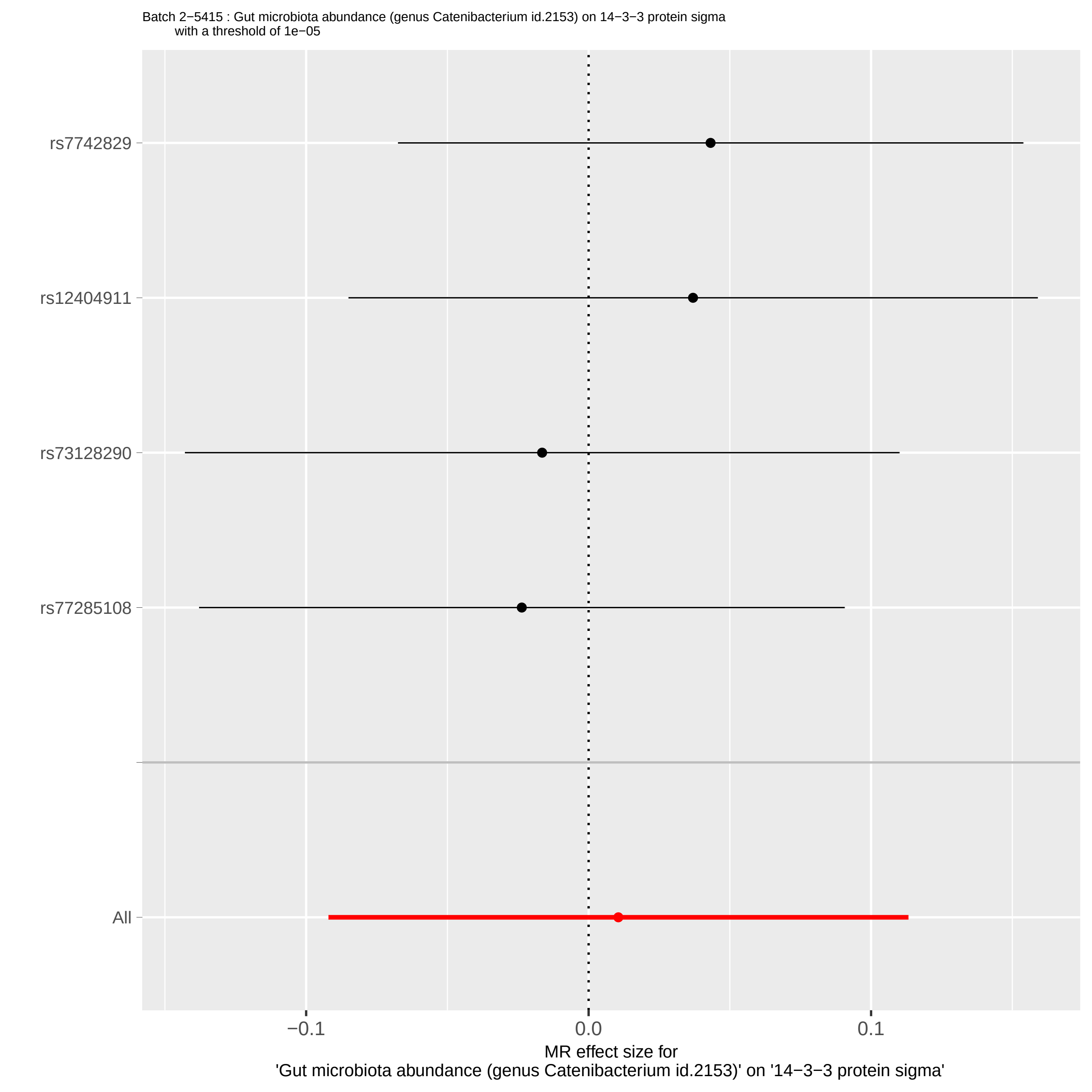
Batch 2−5415 : Gut microbiota abundance (genus Catenibacterium id.2153) on 14−3−3 protein sigma
with a threshold of 1e−05
rs7742829
rs12404911
rs73128290
rs77285108
All
−0.1
0.0
0.1
MR effect size for
'Gut microbiota abundance (genus Catenibacterium id.2153)' on '14−3−3 protein sigma'

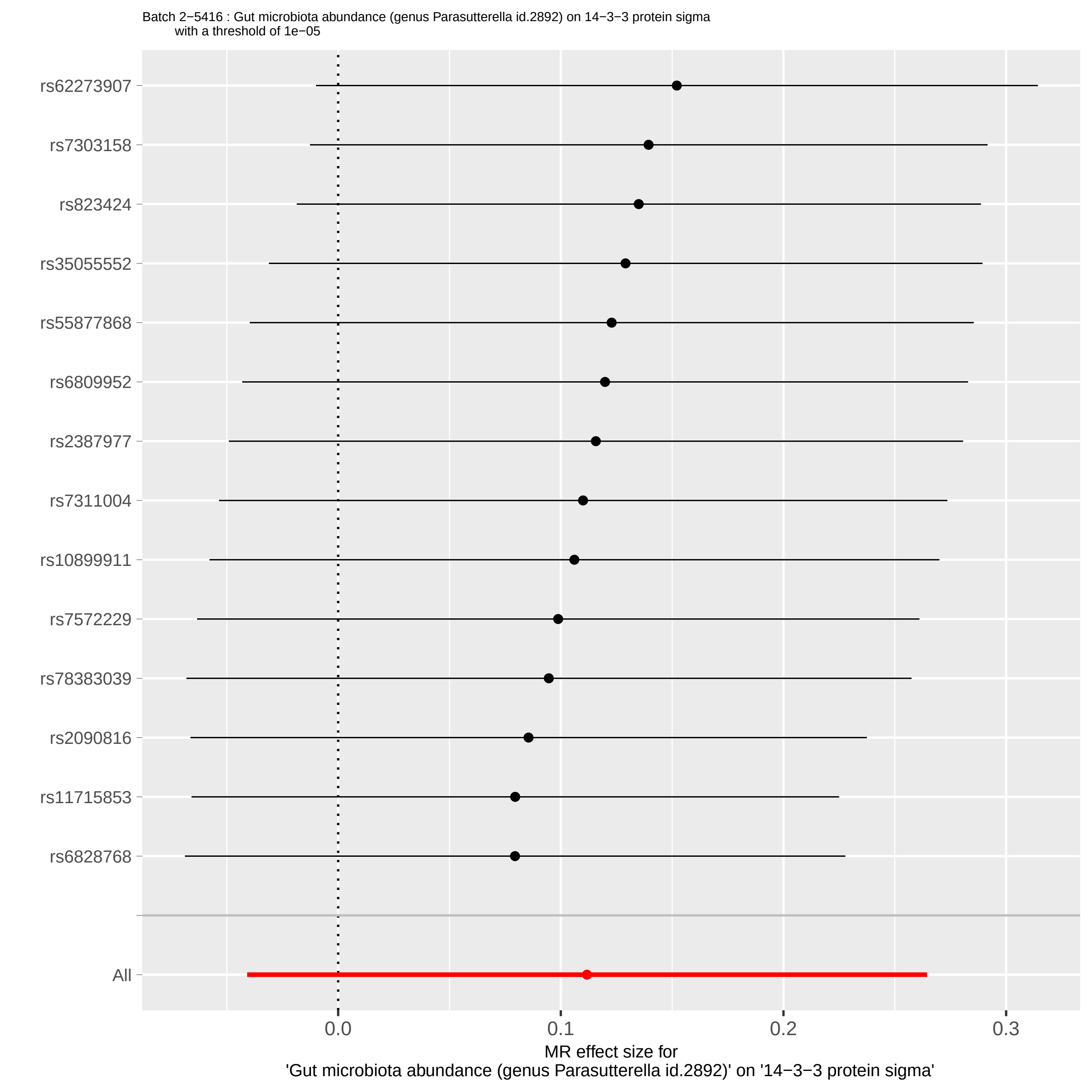

Batch 2−5416 : Gut microbiota abundance (genus Parasutterella id.2892) on 14−3−3 protein sigma
with a threshold of 1e−05
rs62273907
rs7303158
rs823424
rs35055552
rs55877868
rs6809952
rs2387977
rs7311004
rs10899911
rs7572229
rs78383039
rs2090816
rs11715853
rs6828768
All
0.0
0.1
0.2
0.3
MR effect size for
'Gut microbiota abundance (genus Parasutterella id.2892)' on '14−3−3 protein sigma'

Batch 2−5417 : Gut microbiota abundance (genus Catenibacterium id.2153) on Secreted frizzled−related protein 1
with a threshold of 1e−05

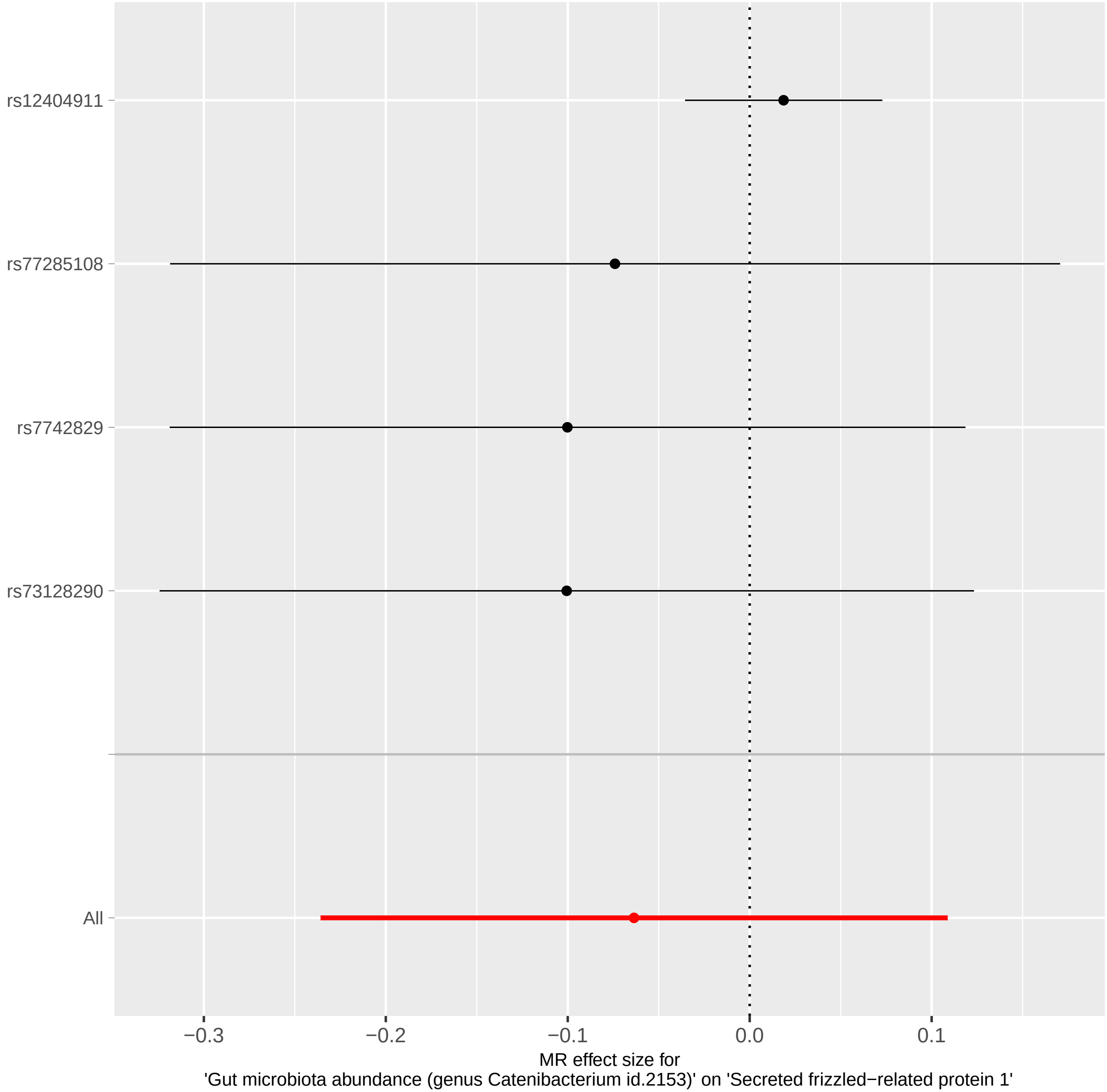

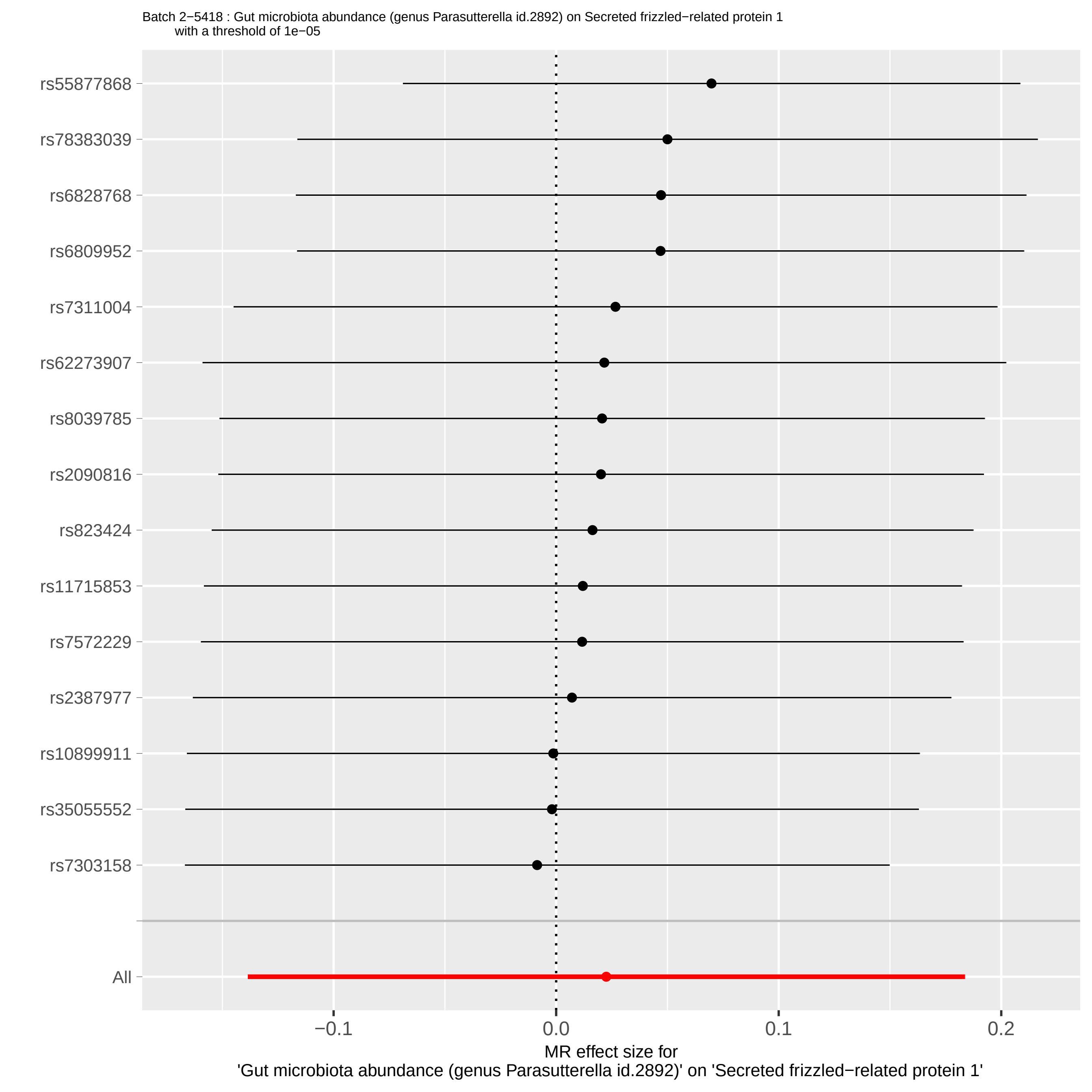
Batch 2−5418 : Gut microbiota abundance (genus Parasutterella id.2892) on Secreted frizzled−related protein 1
with a threshold of 1e−05
rs55877868
rs78383039
rs6828768
rs6809952
rs7311004
rs62273907
rs8039785
rs2090816
rs823424
rs11715853
rs7572229
rs2387977
rs10899911
rs35055552
rs7303158
All
−0.1
0.0
0.1
0.2
MR effect size for
'Gut microbiota abundance (genus Parasutterella id.2892)' on 'Secreted frizzled−related protein 1'

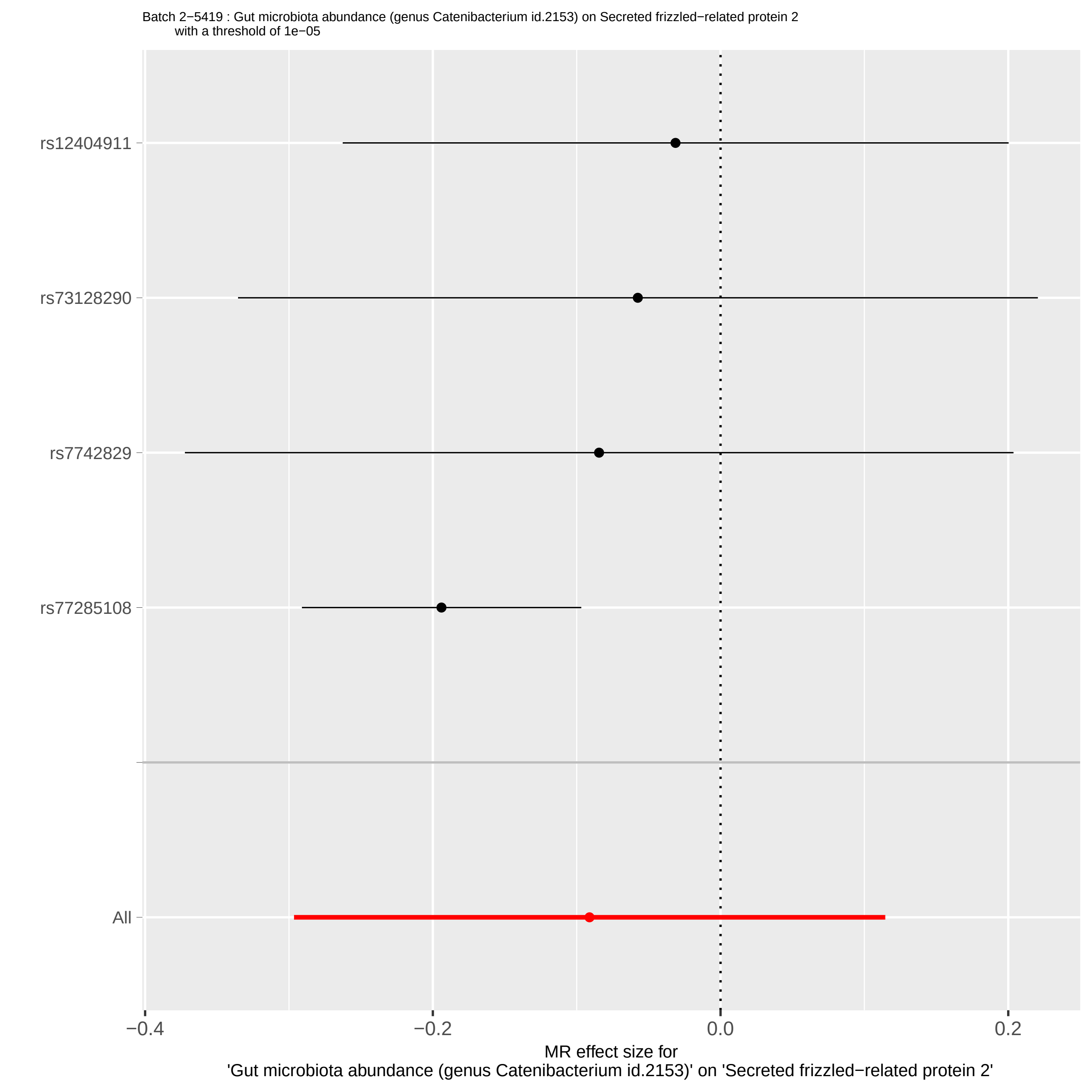
Batch 2−5419 : Gut microbiota abundance (genus Catenibacterium id.2153) on Secreted frizzled−related protein 2
with a threshold of 1e−05
rs12404911
rs73128290
rs7742829
rs77285108
All
−0.4
−0.2
0.0
0.2
MR effect size for
'Gut microbiota abundance (genus Catenibacterium id.2153)' on 'Secreted frizzled−related protein 2'

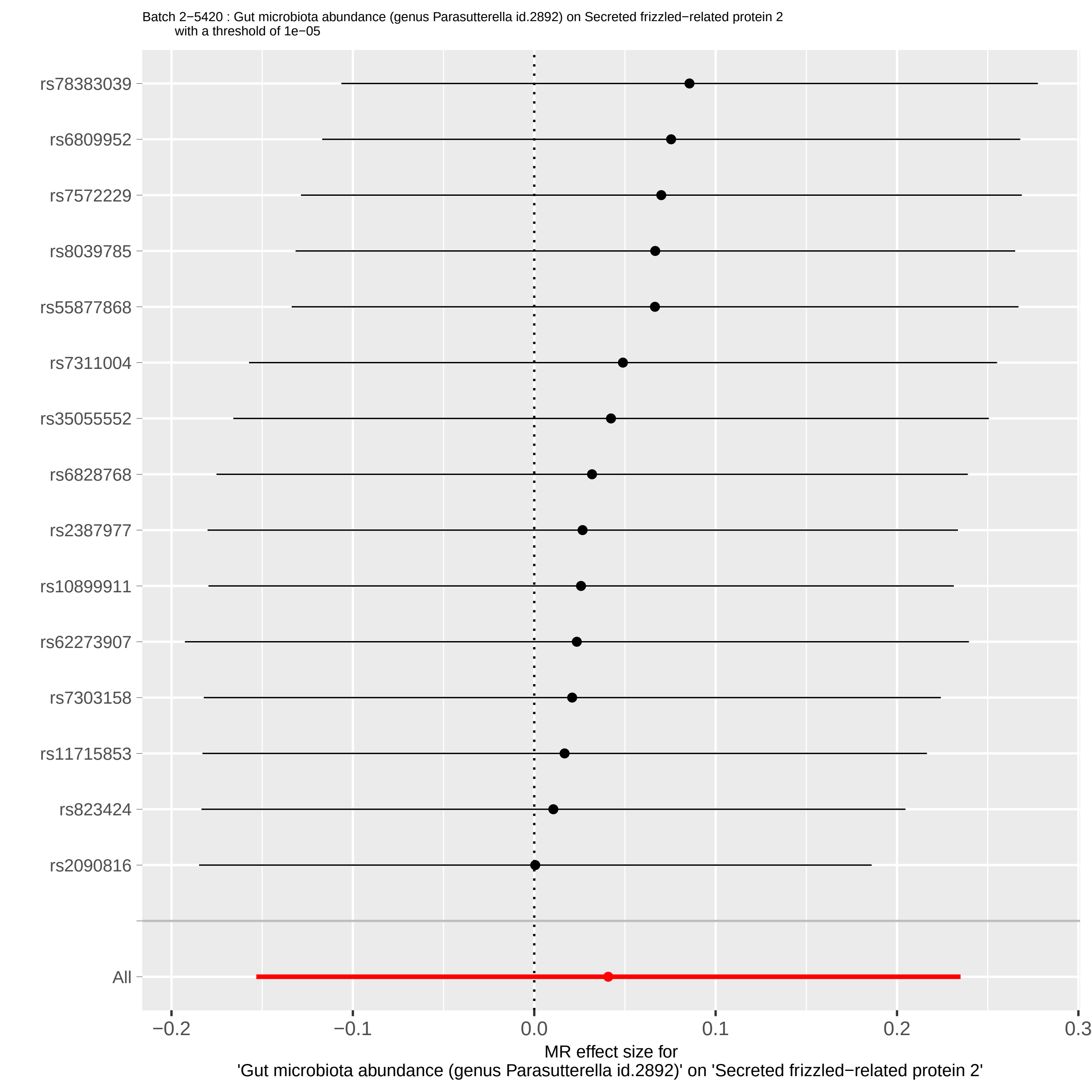

Batch 2−5420 : Gut microbiota abundance (genus Parasutterella id.2892) on Secreted frizzled−related protein 2
with a threshold of 1e−05
rs78383039
rs6809952
rs7572229
rs8039785
rs55877868
rs7311004
rs35055552
rs6828768
rs2387977
rs10899911
rs62273907
rs7303158
rs11715853
rs823424
rs2090816
All
−0.2
−0.1
0.0
0.1
0.2
0.3
MR effect size for
'Gut microbiota abundance (genus Parasutterella id.2892)' on 'Secreted frizzled−related protein 2'

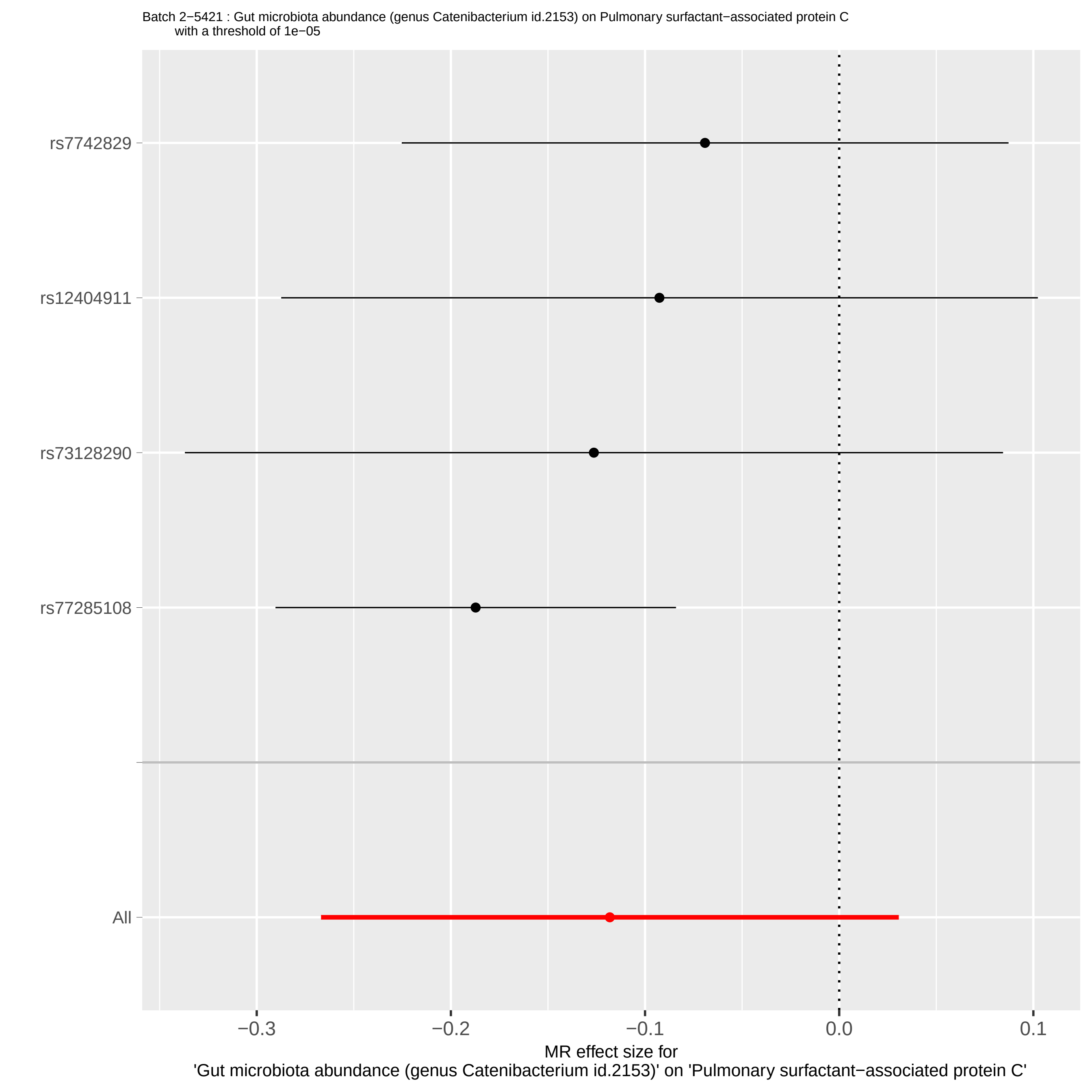
Batch 2−5421 : Gut microbiota abundance (genus Catenibacterium id.2153) on Pulmonary surfactant−associated protein C
with a threshold of 1e−05
rs7742829
rs12404911
rs73128290
rs77285108
All
−0.3
−0.2
−0.1
0.0
0.1
MR effect size for
'Gut microbiota abundance (genus Catenibacterium id.2153)' on 'Pulmonary surfactant−associated protein C'

Batch 2−5422 : Gut microbiota abundance (genus Parasutterella id.2892) on Pulmonary surfactant−associated protein C
with a threshold of 1e−05

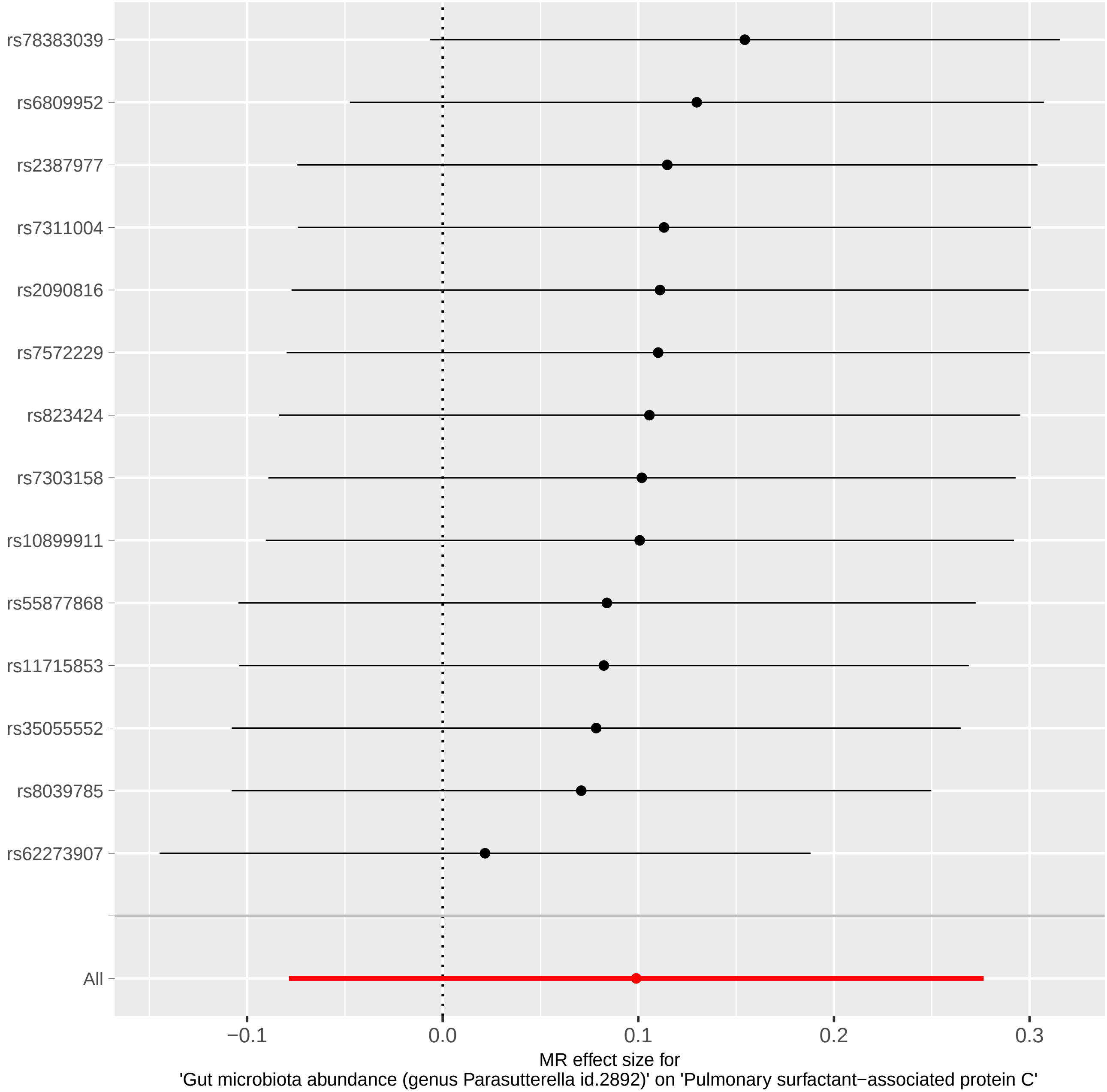

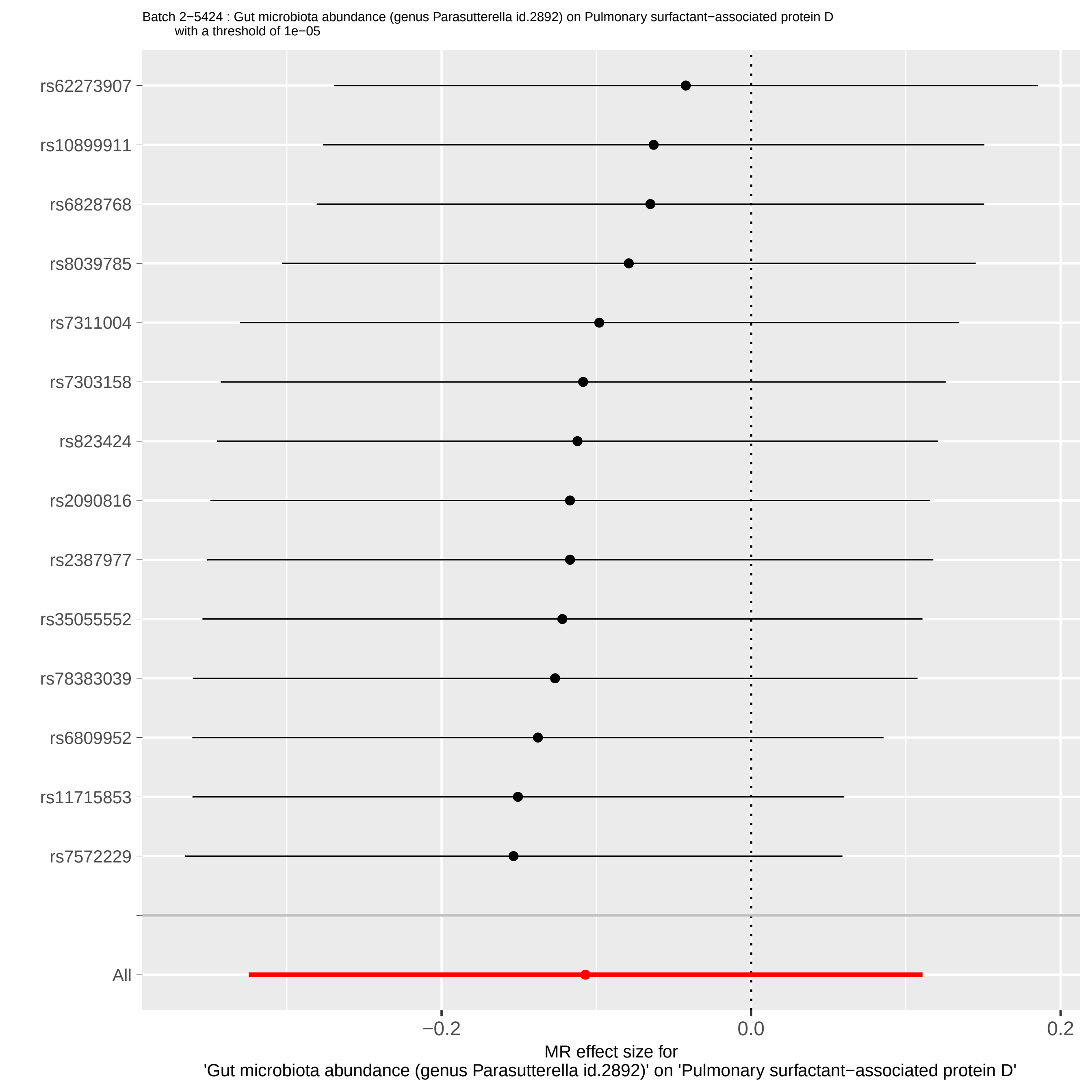
Batch 2−5424 : Gut microbiota abundance (genus Parasutterella id.2892) on Pulmonary surfactant−associated protein D
with a threshold of 1e−05
rs62273907
rs10899911
rs6828768
rs8039785
rs7311004
rs7303158
rs823424
rs2090816
rs2387977
rs35055552
rs78383039
rs6809952
rs11715853
rs7572229
All
−0.2
0.0
0.2
MR effect size for
'Gut microbiota abundance (genus Parasutterella id.2892)' on 'Pulmonary surfactant−associated protein D'

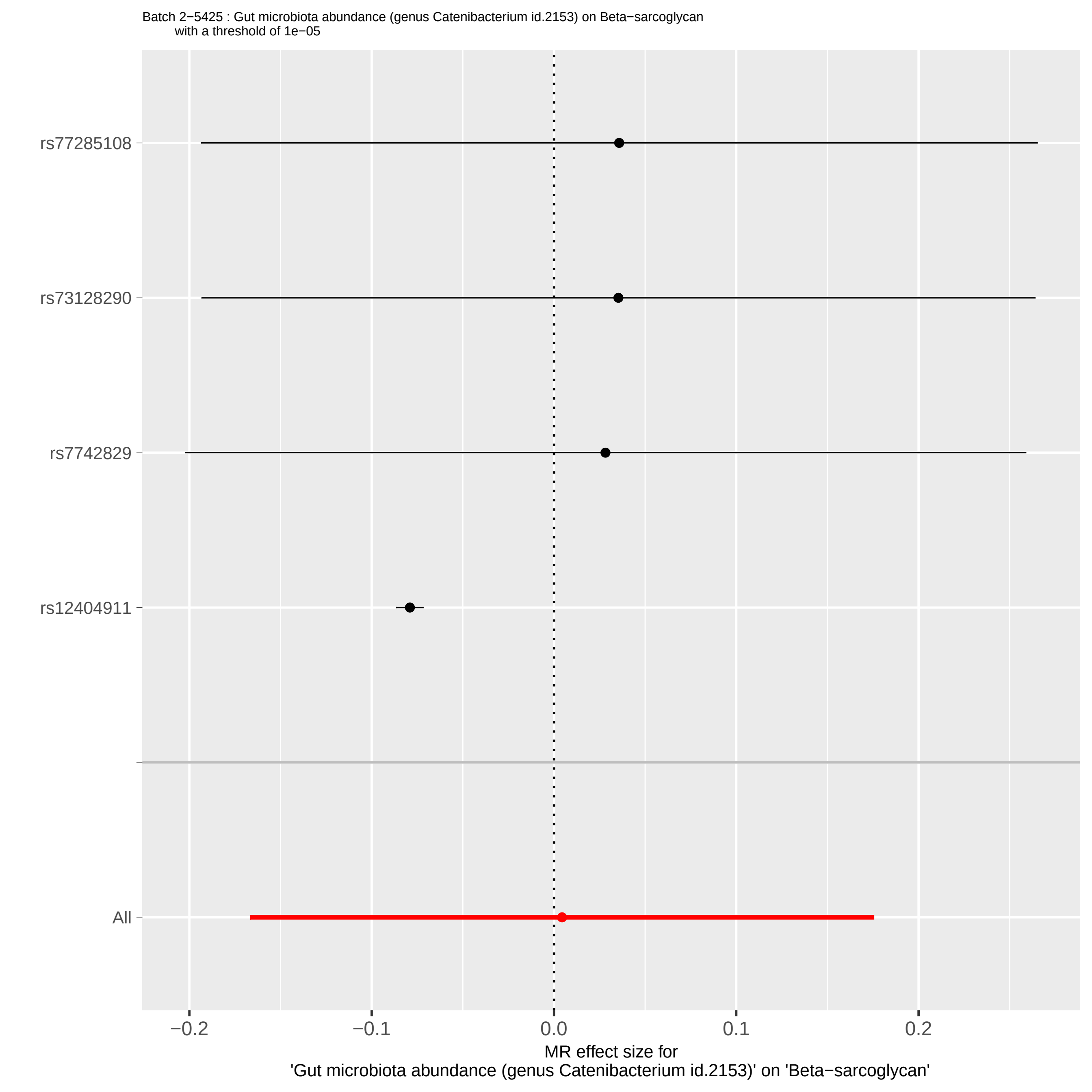

Batch 2−5425 : Gut microbiota abundance (genus Catenibacterium id.2153) on Beta−sarcoglycan
with a threshold of 1e−05
rs77285108
rs73128290
rs7742829
rs12404911
All
−0.2
−0.1
0.0
0.1
0.2
MR effect size for
'Gut microbiota abundance (genus Catenibacterium id.2153)' on 'Beta−sarcoglycan'

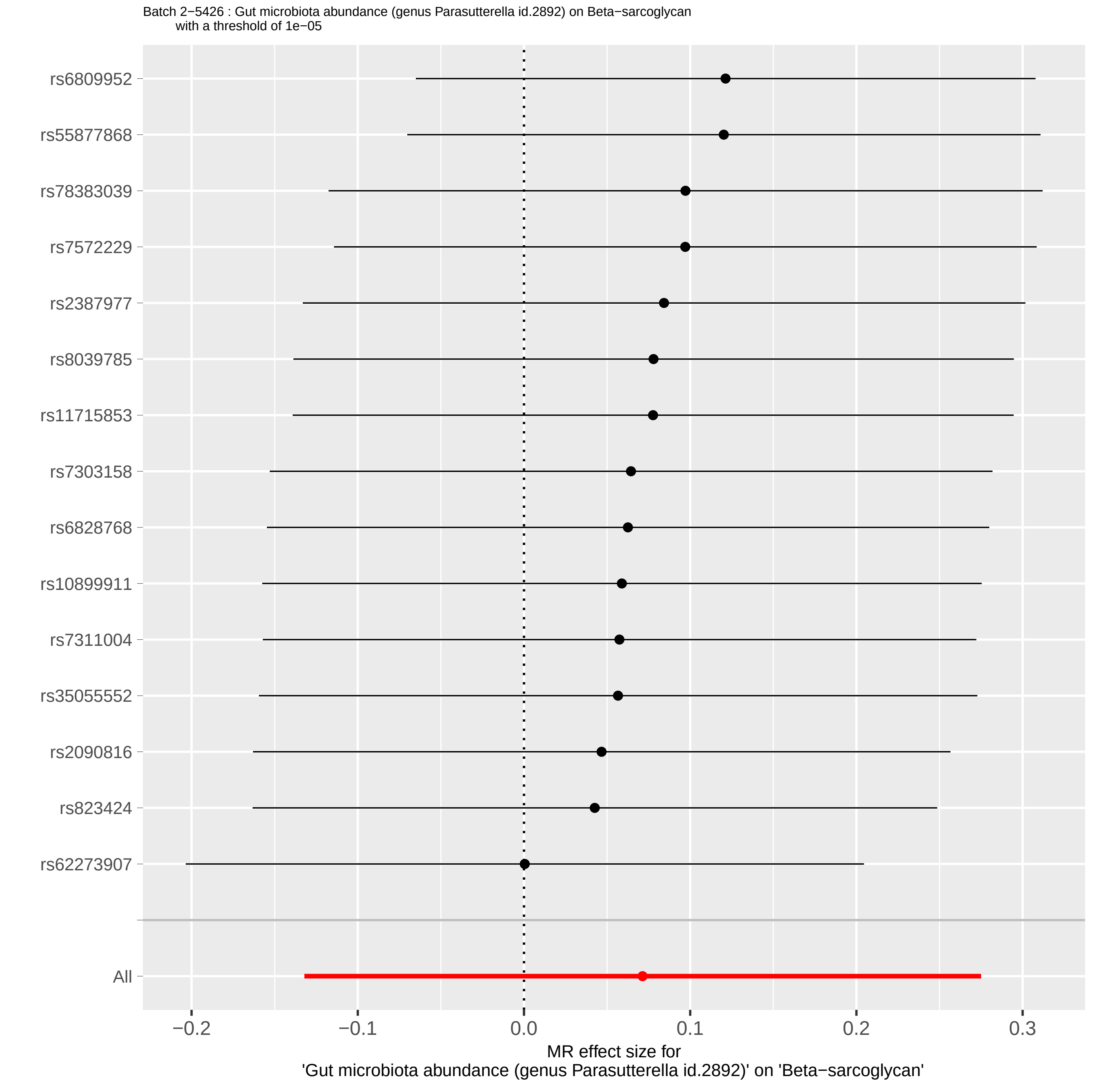
Batch 2−5426 : Gut microbiota abundance (genus Parasutterella id.2892) on Beta−sarcoglycan
with a threshold of 1e−05
rs6809952
rs55877868
rs78383039
rs7572229
rs2387977
rs8039785
rs11715853
rs7303158
rs6828768
rs10899911
rs7311004
rs35055552
rs2090816
rs823424
rs62273907
All
−0.2
−0.1
0.0
0.1
0.2
0.3
MR effect size for
'Gut microbiota abundance (genus Parasutterella id.2892)' on 'Beta−sarcoglycan'

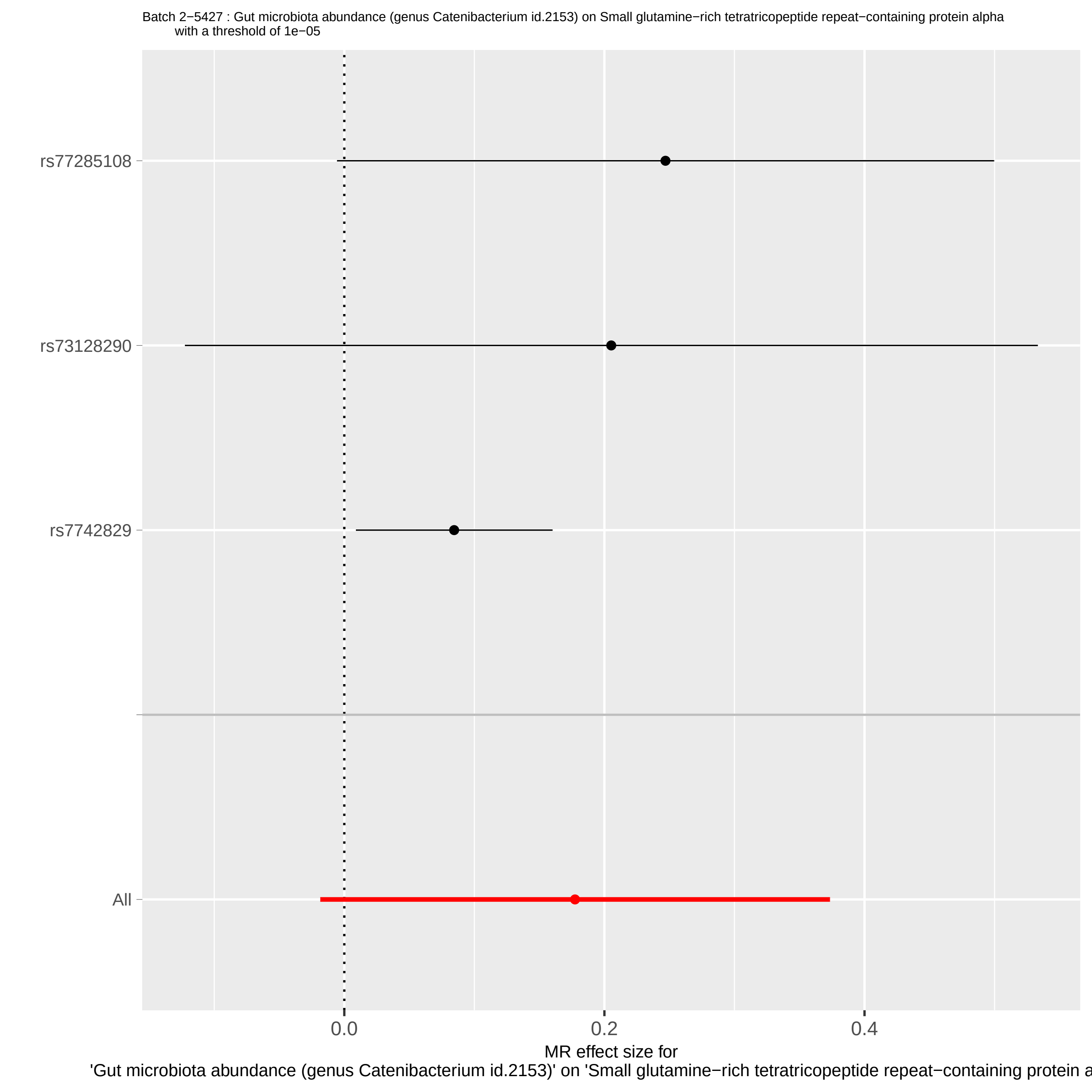

Batch 2−5427 : Gut microbiota abundance (genus Catenibacterium id.2153) on Small glutamine−rich tetratricopeptide repeat−containing protein alpha
with a threshold of 1e−05
rs77285108
rs73128290
rs7742829
All
0.0
0.2
0.4
MR effect size for
'Gut microbiota abundance (genus Catenibacterium id.2153)' on 'Small glutamine−rich tetratricopeptide repeat−containing protein a

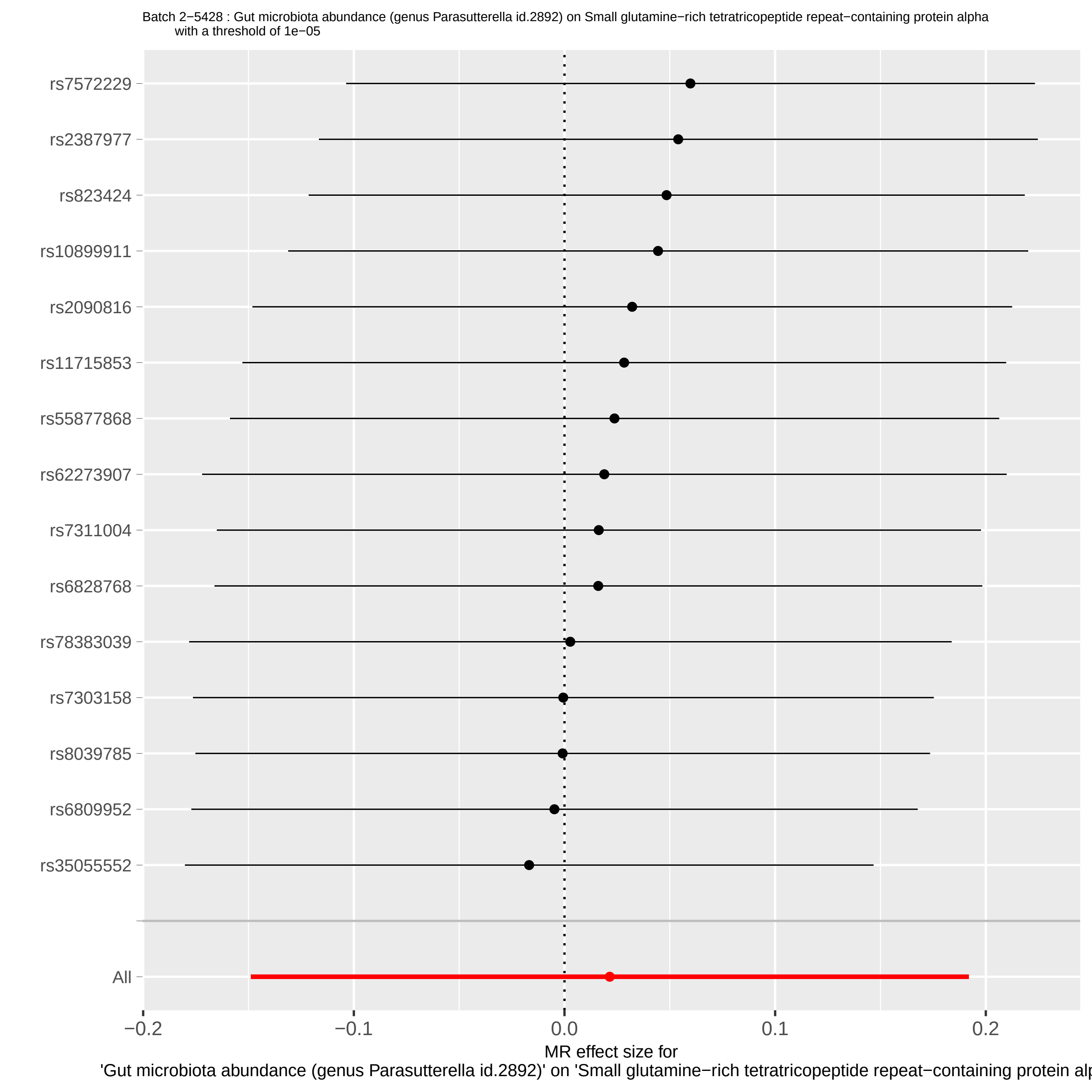
Batch 2−5428 : Gut microbiota abundance (genus Parasutterella id.2892) on Small glutamine−rich tetratricopeptide repeat−containing protein alpha
with a threshold of 1e−05
rs7572229
rs2387977
rs823424
rs10899911
rs2090816
rs11715853
rs55877868
rs62273907
rs7311004
rs6828768
rs78383039
rs7303158
rs8039785
rs6809952
rs35055552
All
−0.2
−0.1
0.0
0.1
0.2
MR effect size for
'Gut microbiota abundance (genus Parasutterella id.2892)' on 'Small glutamine−rich tetratricopeptide repeat−containing protein al

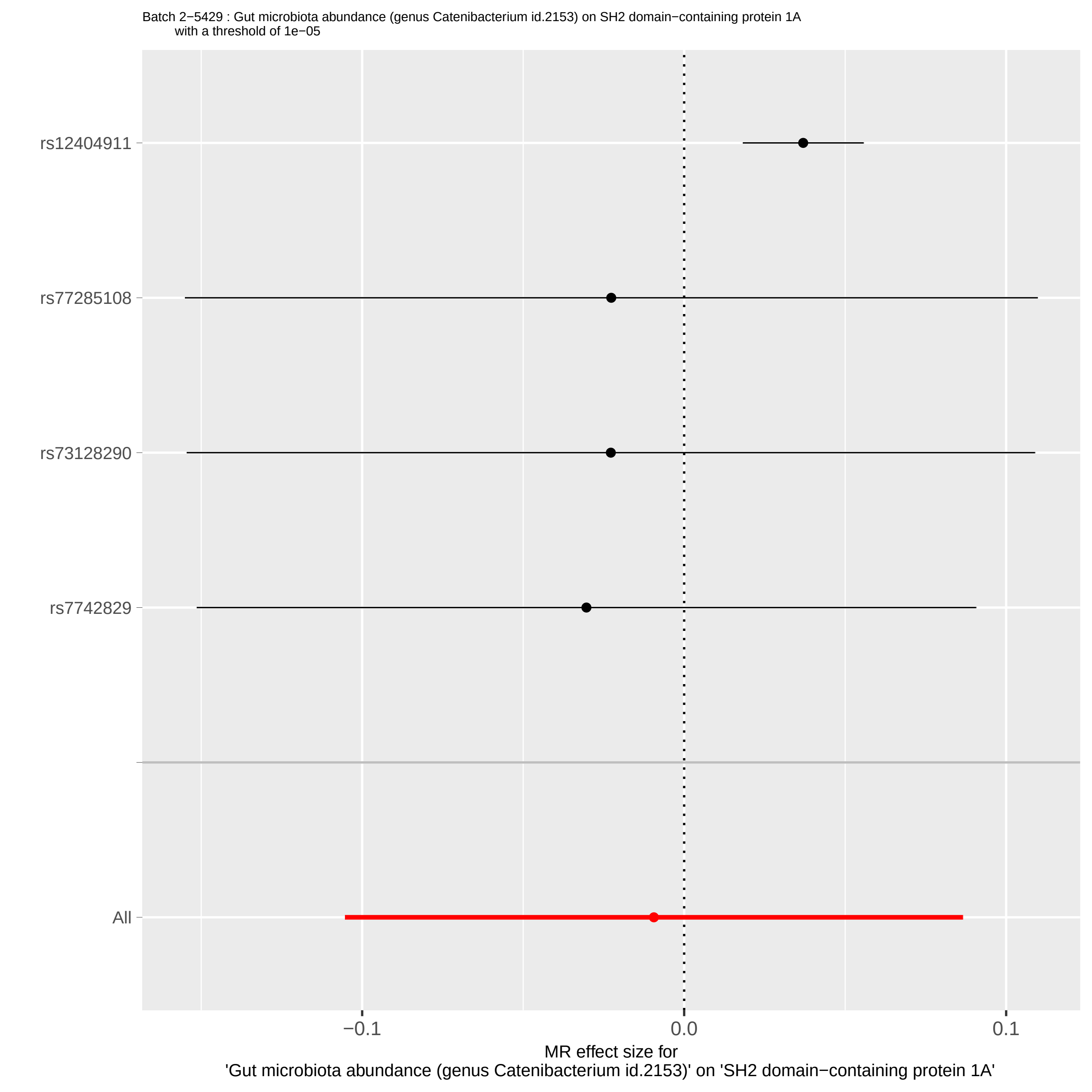
Batch 2−5429 : Gut microbiota abundance (genus Catenibacterium id.2153) on SH2 domain−containing protein 1A
with a threshold of 1e−05
rs12404911
rs77285108
rs73128290
rs7742829
All
−0.1
0.0
0.1
MR effect size for
'Gut microbiota abundance (genus Catenibacterium id.2153)' on 'SH2 domain−containing protein 1A'

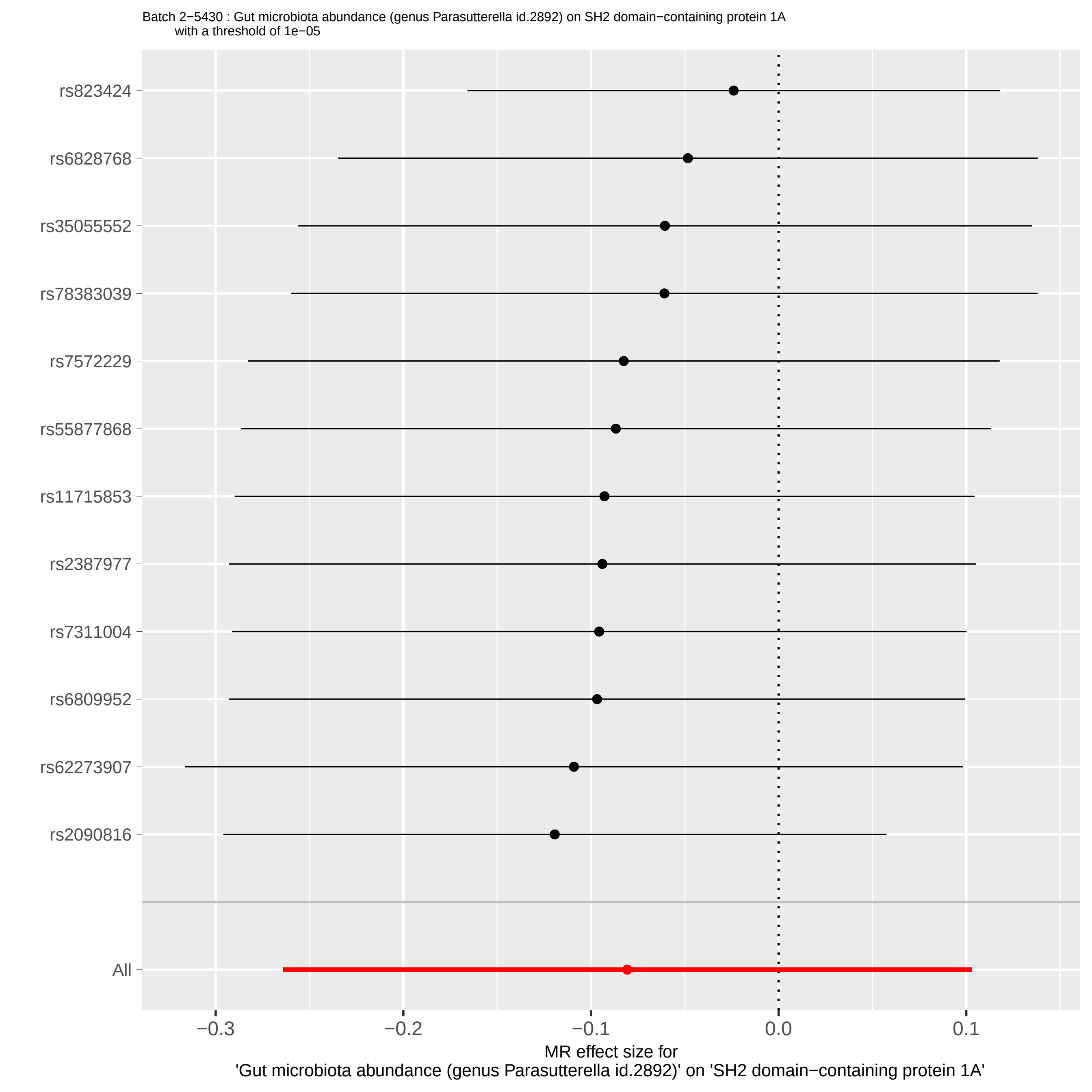
Batch 2−5430 : Gut microbiota abundance (genus Parasutterella id.2892) on SH2 domain−containing protein 1A
with a threshold of 1e−05
rs823424
rs6828768
rs35055552
rs78383039
rs7572229
rs55877868
rs11715853
rs2387977
rs7311004
rs6809952
rs62273907
rs2090816
All
−0.3
−0.2
−0.1
0.0
0.1
MR effect size for
'Gut microbiota abundance (genus Parasutterella id.2892)' on 'SH2 domain−containing protein 1A'

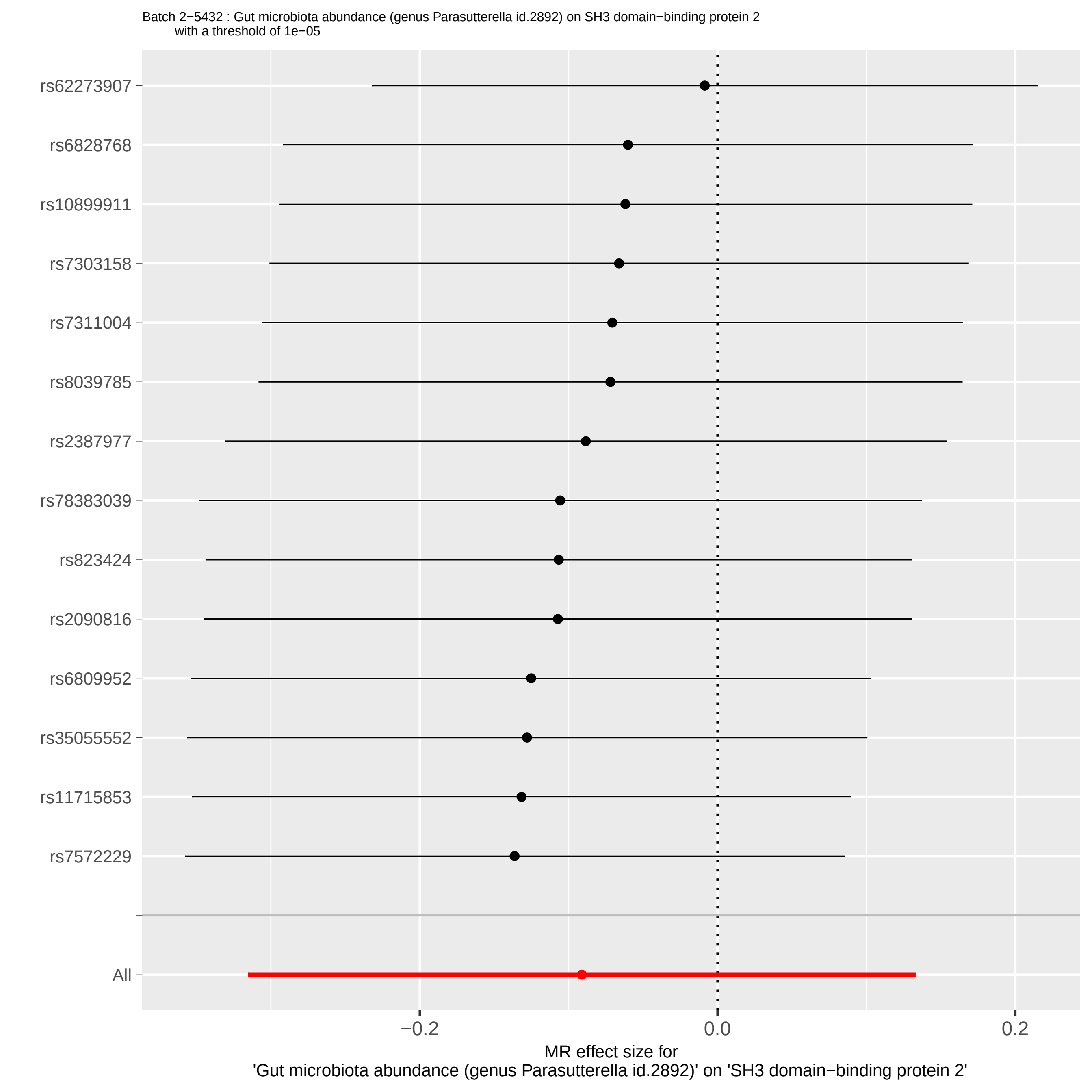

Batch 2−5432 : Gut microbiota abundance (genus Parasutterella id.2892) on SH3 domain−binding protein 2
with a threshold of 1e−05
rs62273907
rs6828768
rs10899911
rs7303158
rs7311004
rs8039785
rs2387977
rs78383039
rs823424
rs2090816
rs6809952
rs35055552
rs11715853
rs7572229
All
−0.2
0.0
0.2
MR effect size for
'Gut microbiota abundance (genus Parasutterella id.2892)' on 'SH3 domain−binding protein 2'

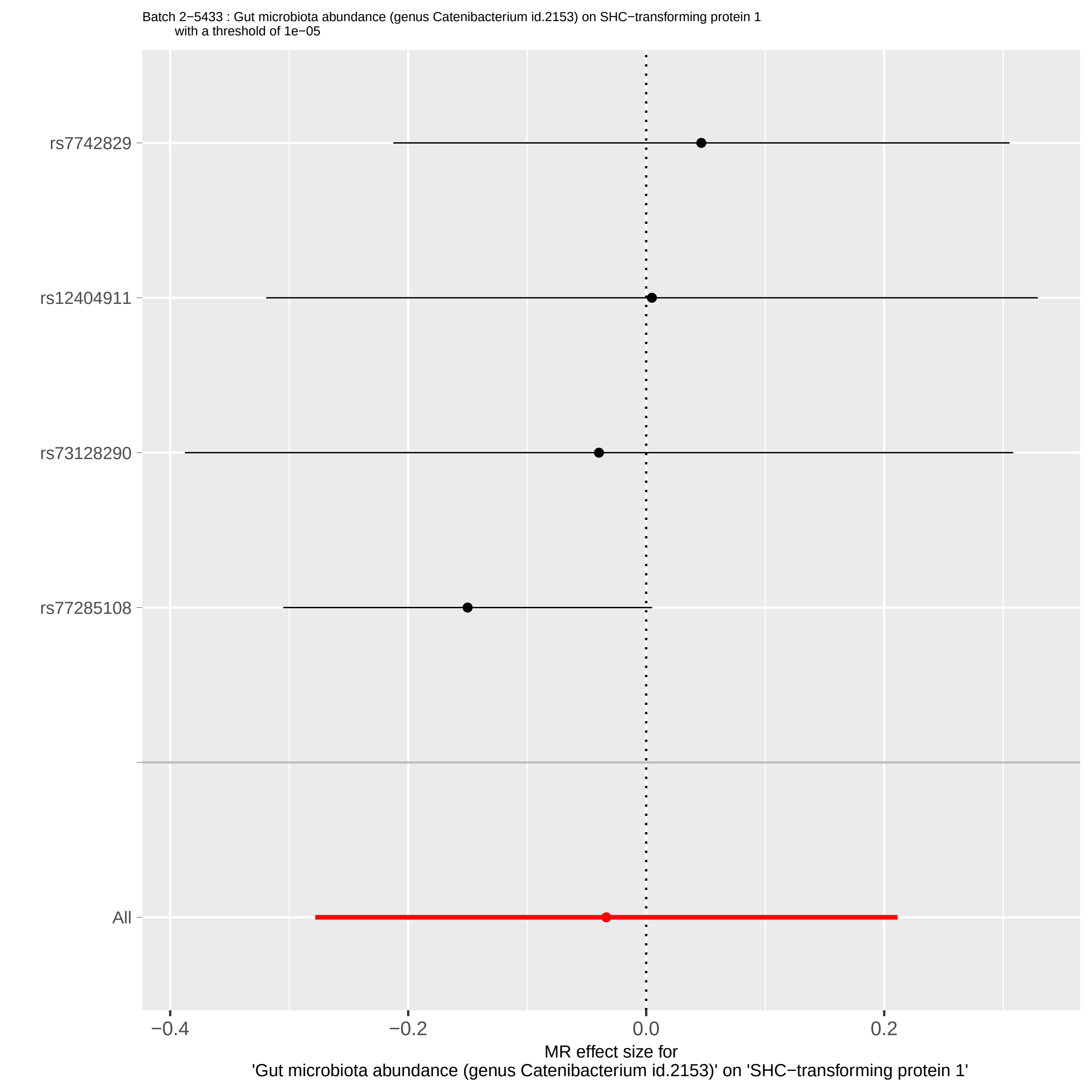
Batch 2−5433 : Gut microbiota abundance (genus Catenibacterium id.2153) on SHC−transforming protein 1
with a threshold of 1e−05
rs7742829
rs12404911
rs73128290
rs77285108
All
−0.4
−0.2
0.0
0.2
MR effect size for
'Gut microbiota abundance (genus Catenibacterium id.2153)' on 'SHC−transforming protein 1'

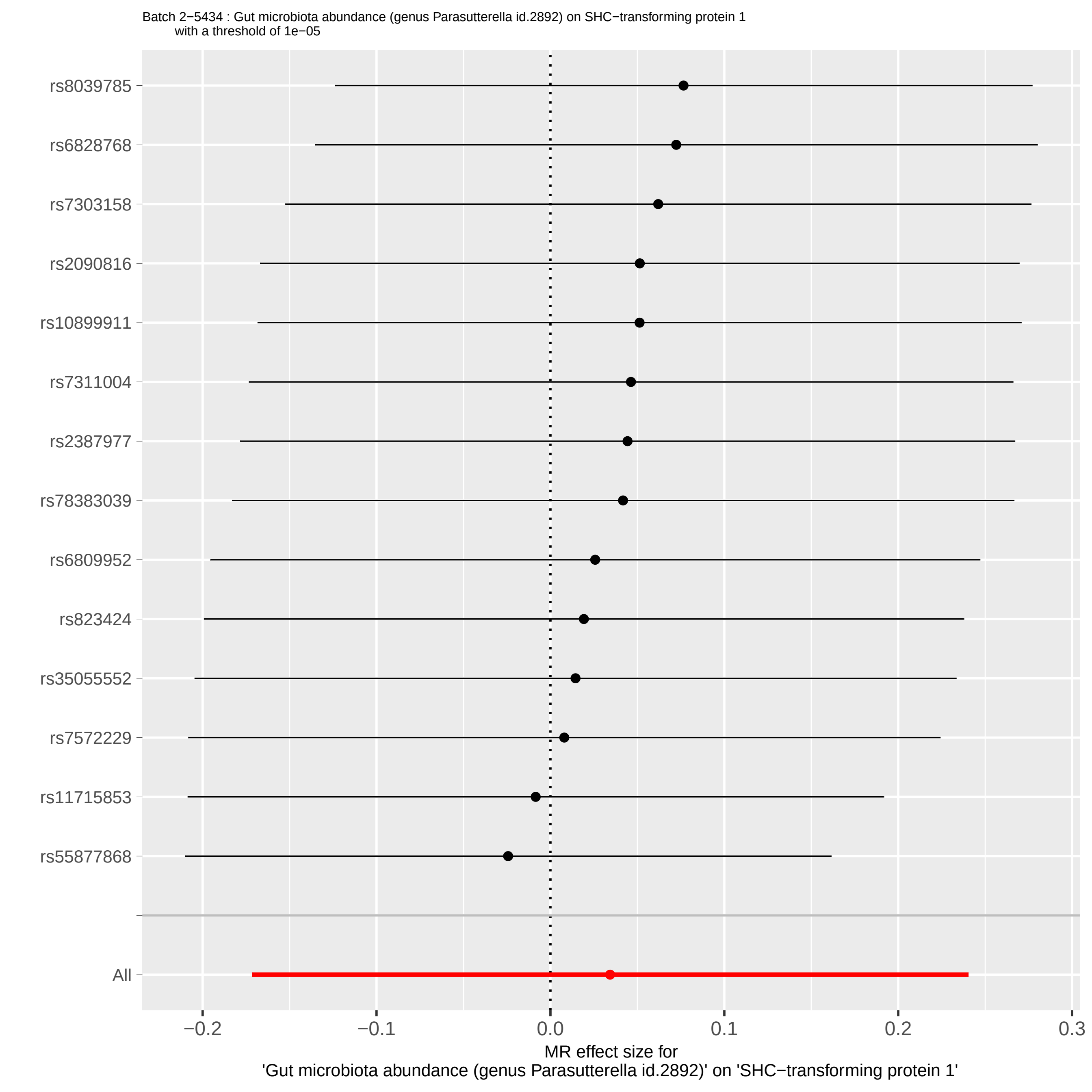

Batch 2−5434 : Gut microbiota abundance (genus Parasutterella id.2892) on SHC−transforming protein 1
with a threshold of 1e−05
rs8039785
rs6828768
rs7303158
rs2090816
rs10899911
rs7311004
rs2387977
rs78383039
rs6809952
rs823424
rs35055552
rs7572229
rs11715853
rs55877868
All
−0.2
−0.1
0.0
0.1
0.2
0.3
MR effect size for
'Gut microbiota abundance (genus Parasutterella id.2892)' on 'SHC−transforming protein 1'

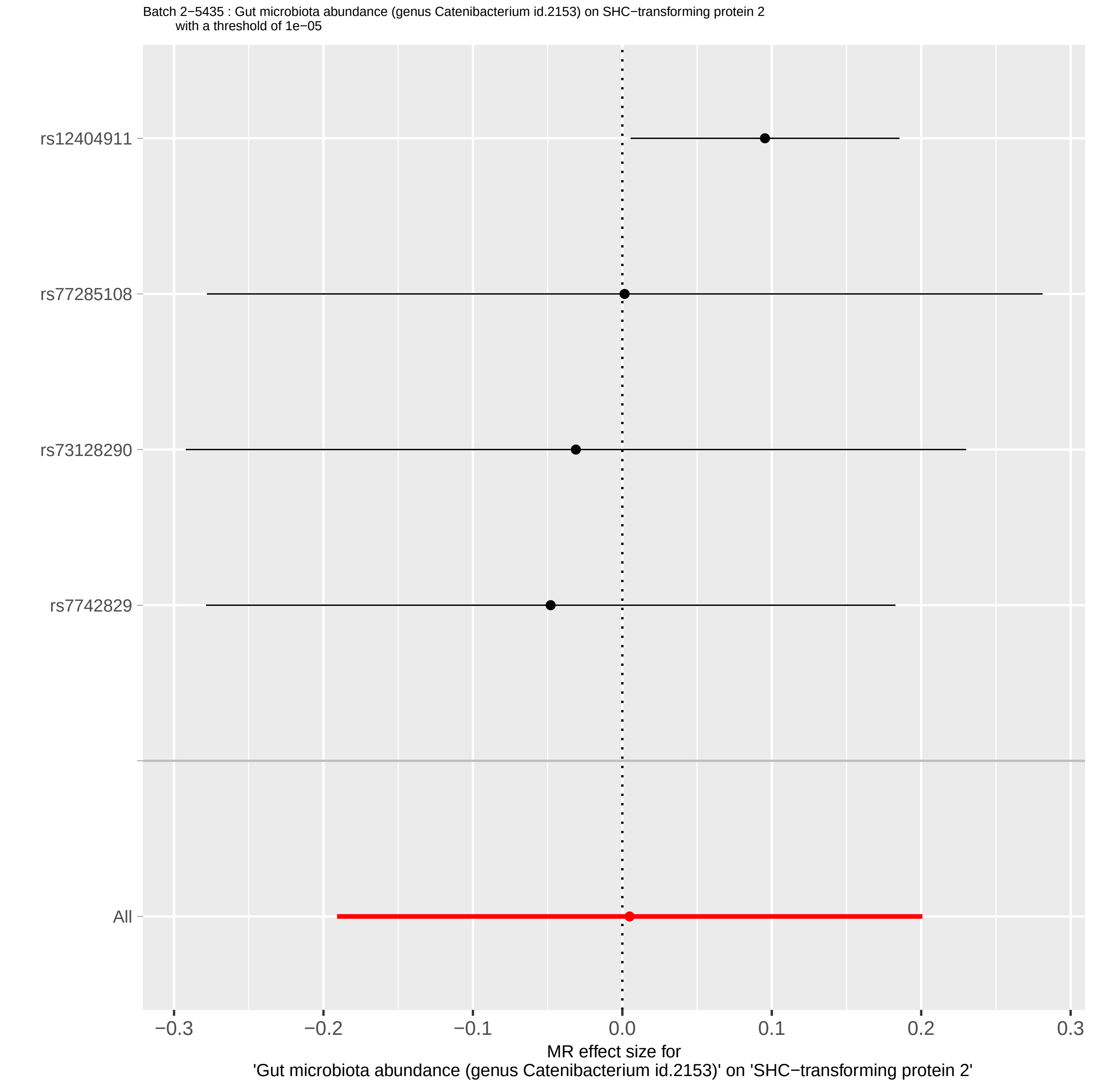

Batch 2−5435 : Gut microbiota abundance (genus Catenibacterium id.2153) on SHC−transforming protein 2
with a threshold of 1e−05
rs12404911
rs77285108
rs73128290
rs7742829
All
−0.3
−0.2
−0.1
0.0
0.1
0.2
0.3
MR effect size for
'Gut microbiota abundance (genus Catenibacterium id.2153)' on 'SHC−transforming protein 2'

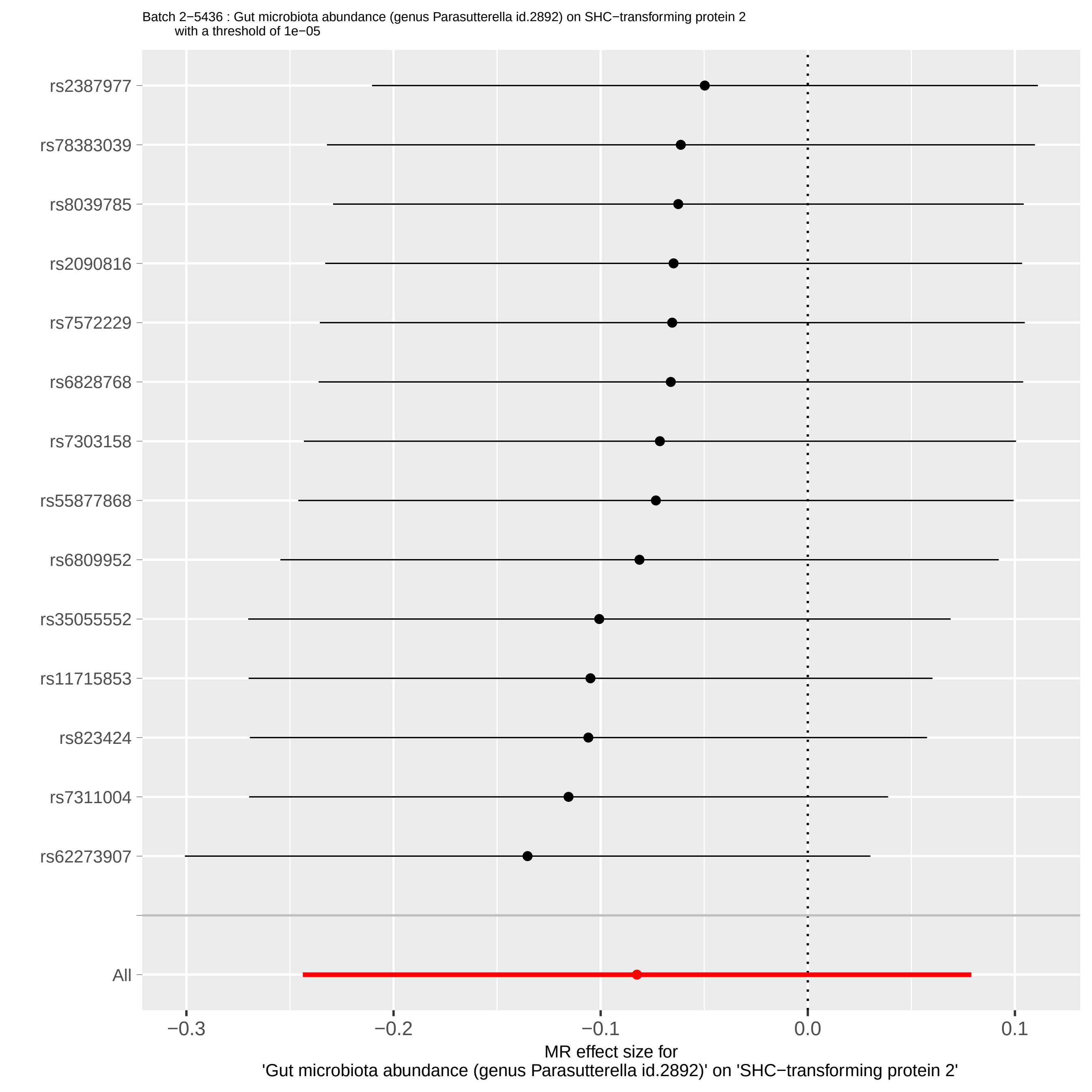

Batch 2−5436 : Gut microbiota abundance (genus Parasutterella id.2892) on SHC−transforming protein 2
with a threshold of 1e−05
rs2387977
rs78383039
rs8039785
rs2090816
rs7572229
rs6828768
rs7303158
rs55877868
rs6809952
rs35055552
rs11715853
rs823424
rs7311004
rs62273907
All
−0.3
−0.2
−0.1
0.0
0.1
MR effect size for
'Gut microbiota abundance (genus Parasutterella id.2892)' on 'SHC−transforming protein 2'

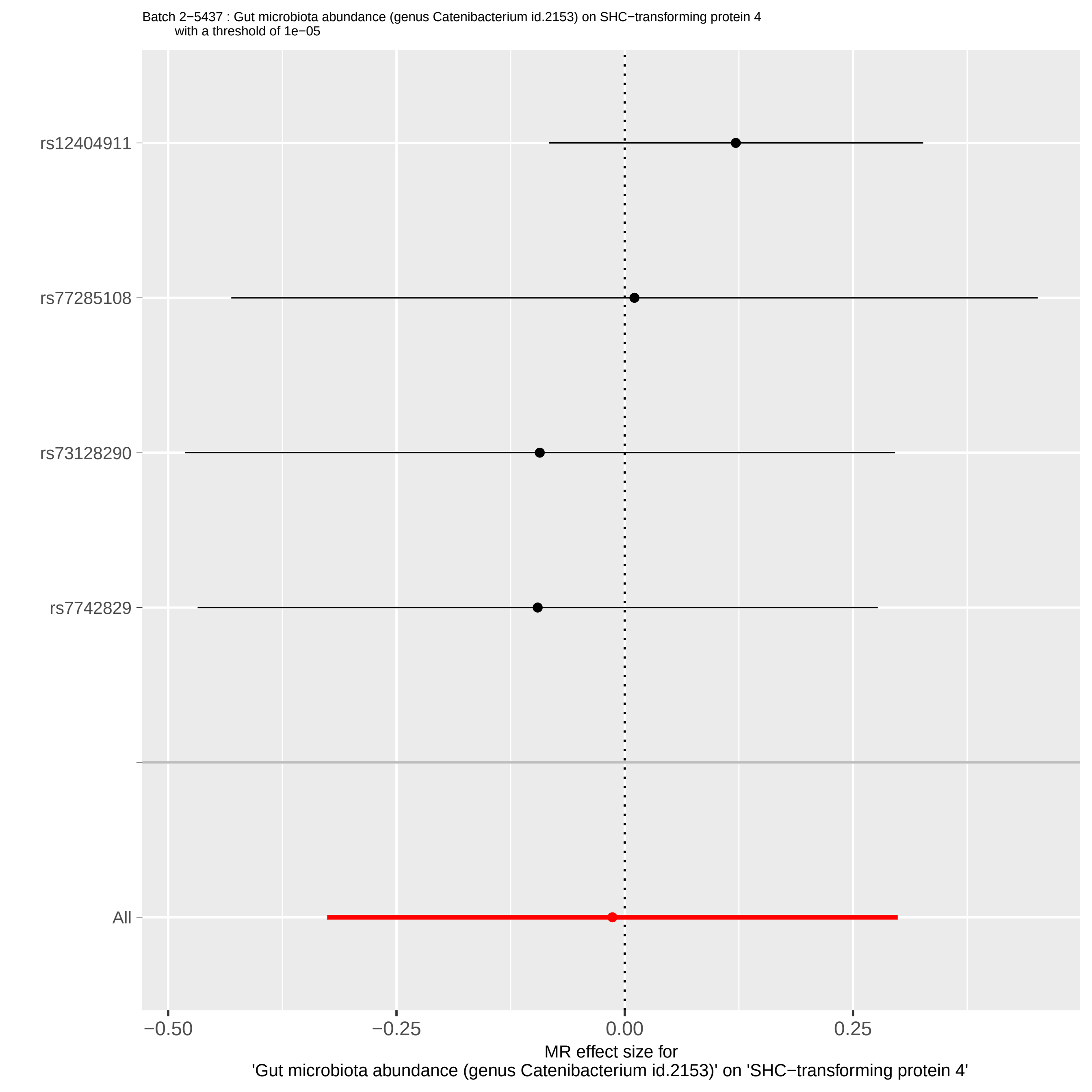
Batch 2−5437 : Gut microbiota abundance (genus Catenibacterium id.2153) on SHC−transforming protein 4
with a threshold of 1e−05
rs12404911
rs77285108
rs73128290
rs7742829
All
−0.50
−0.25
0.00
0.25
MR effect size for
'Gut microbiota abundance (genus Catenibacterium id.2153)' on 'SHC−transforming protein 4'

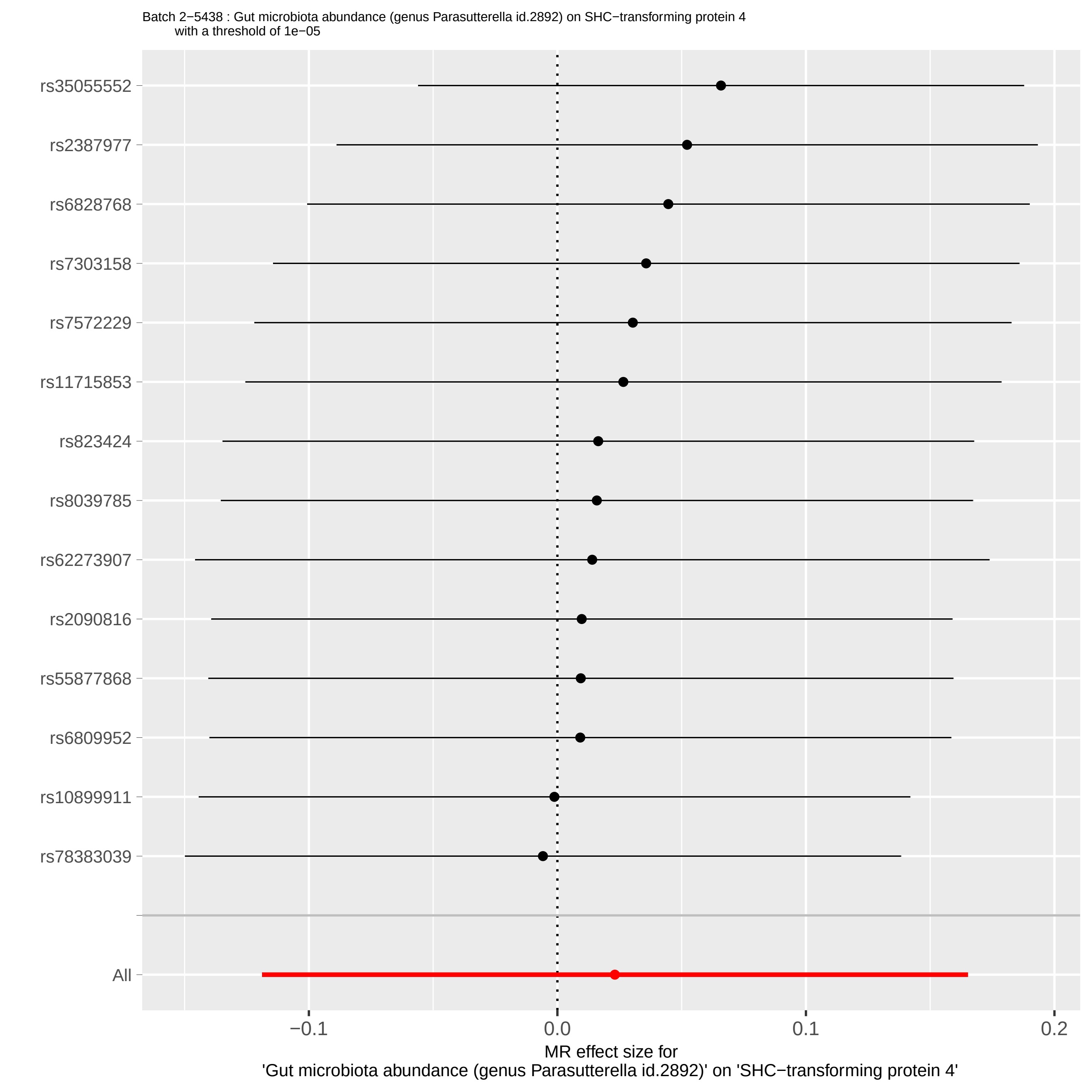

Batch 2−5438 : Gut microbiota abundance (genus Parasutterella id.2892) on SHC−transforming protein 4
with a threshold of 1e−05
rs35055552
rs2387977
rs6828768
rs7303158
rs7572229
rs11715853
rs823424
rs8039785
rs62273907
rs2090816
rs55877868
rs6809952
rs10899911
rs78383039
All
−0.1
0.0
0.1
0.2
MR effect size for
'Gut microbiota abundance (genus Parasutterella id.2892)' on 'SHC−transforming protein 4'

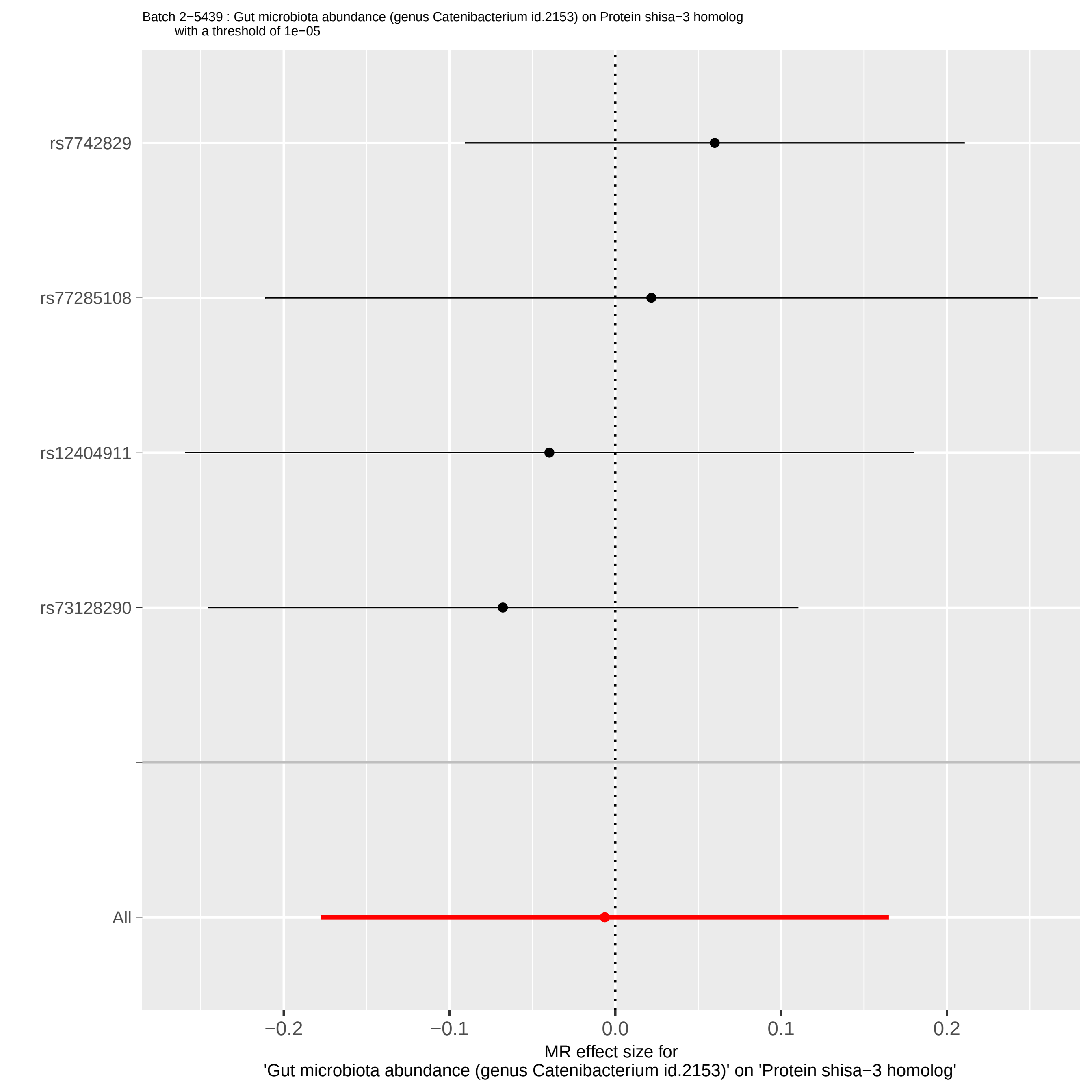

Batch 2−5439 : Gut microbiota abundance (genus Catenibacterium id.2153) on Protein shisa−3 homolog
with a threshold of 1e−05
rs7742829
rs77285108
rs12404911
rs73128290
All
−0.2
−0.1
0.0
0.1
0.2
MR effect size for
'Gut microbiota abundance (genus Catenibacterium id.2153)' on 'Protein shisa−3 homolog'

Batch 2−5440 : Gut microbiota abundance (genus Parasutterella id.2892) on Protein shisa−3 homolog
with a threshold of 1e−05

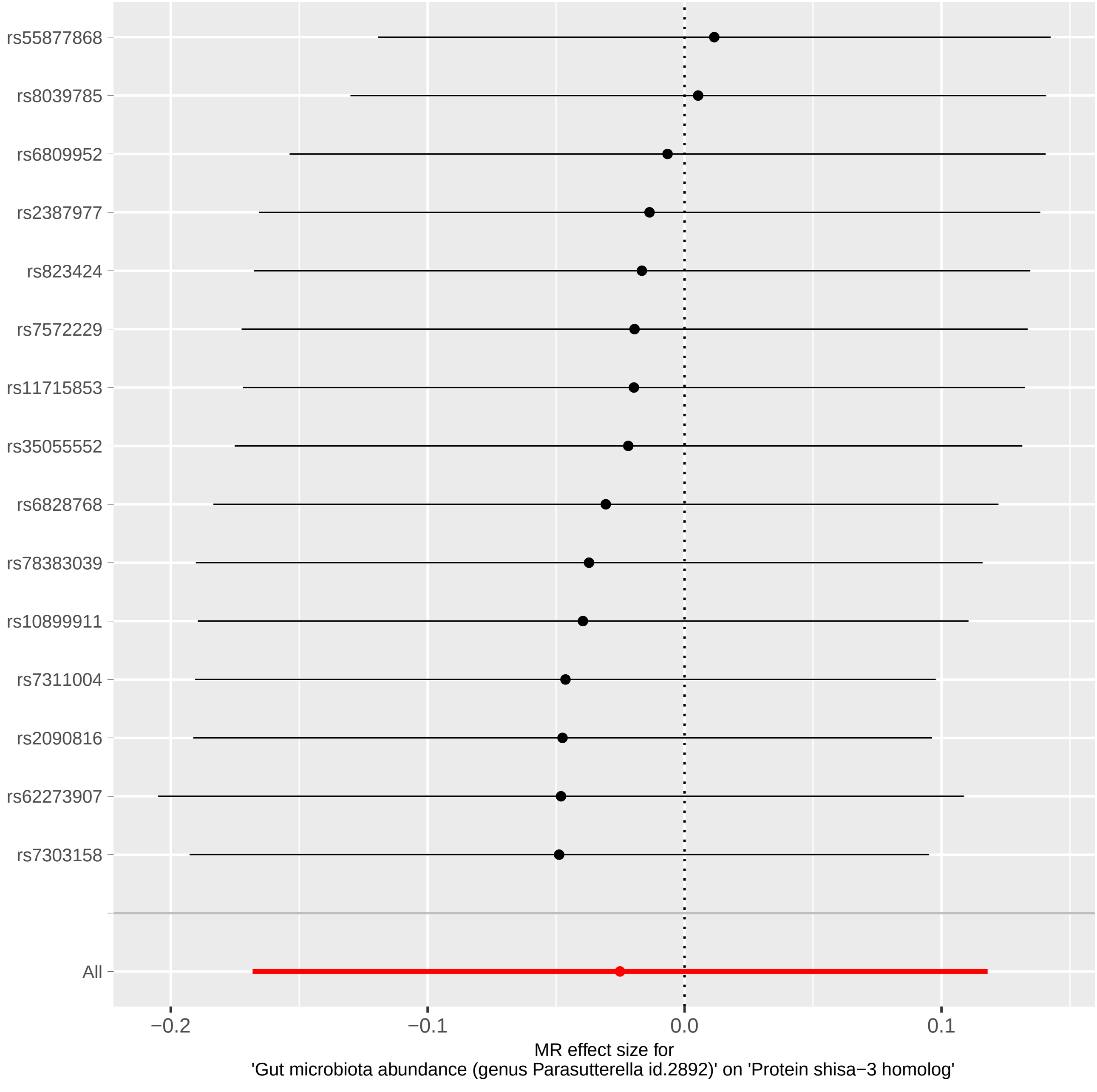

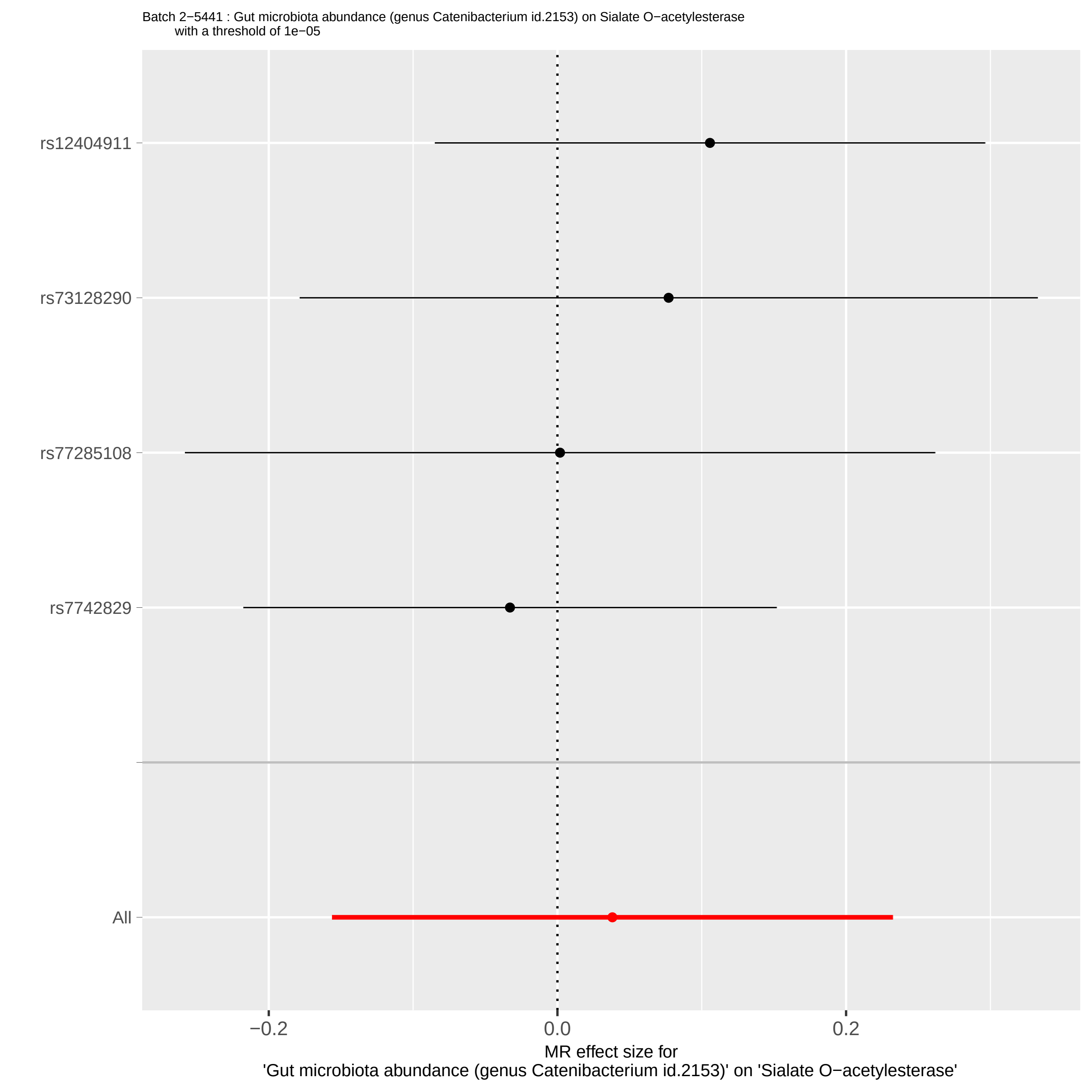
Batch 2−5441 : Gut microbiota abundance (genus Catenibacterium id.2153) on Sialate O−acetylesterase
with a threshold of 1e−05
rs12404911
rs73128290
rs77285108
rs7742829
All
−0.2
0.0
0.2
MR effect size for
'Gut microbiota abundance (genus Catenibacterium id.2153)' on 'Sialate O−acetylesterase'

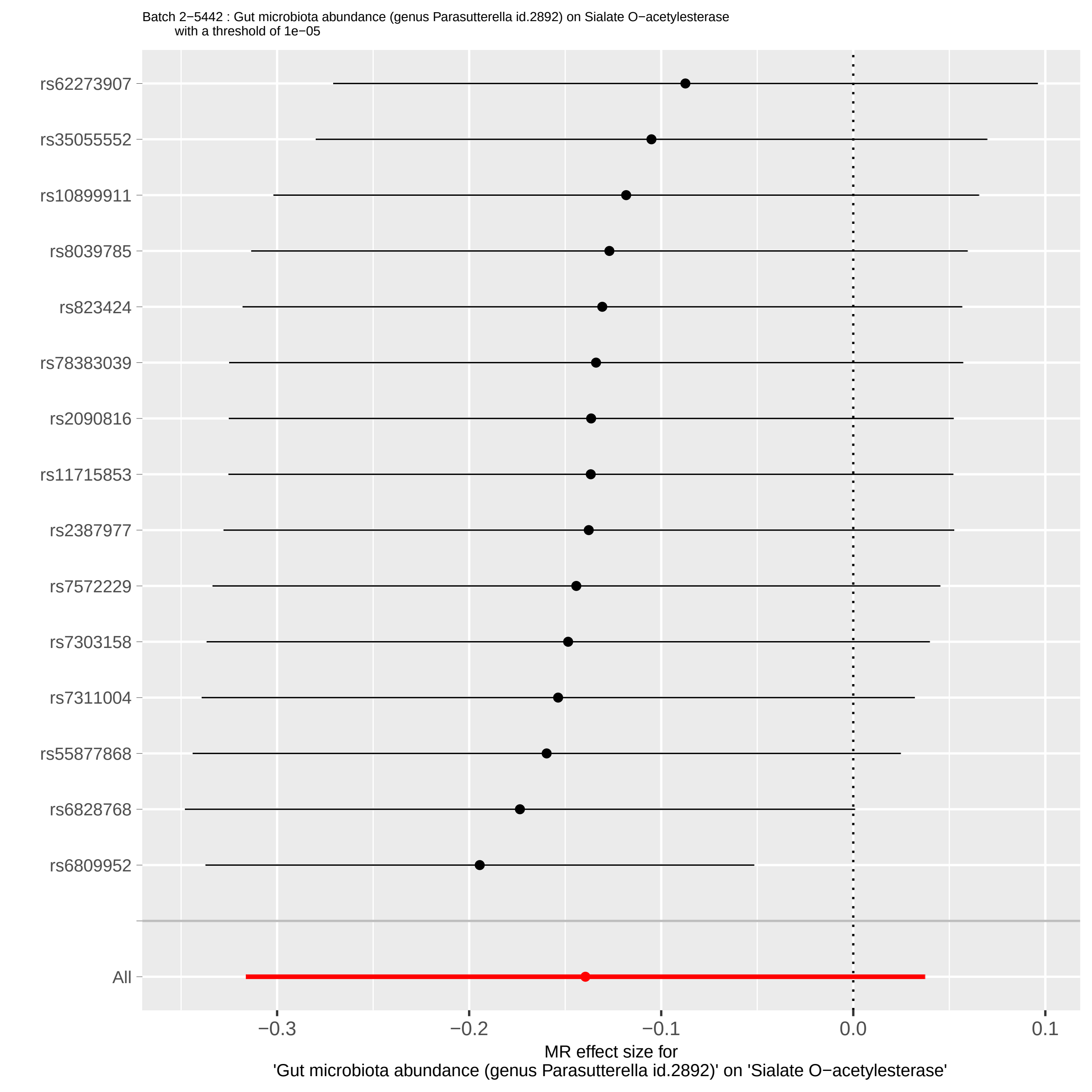
Batch 2−5442 : Gut microbiota abundance (genus Parasutterella id.2892) on Sialate O−acetylesterase
with a threshold of 1e−05
rs62273907
rs35055552
rs10899911
rs8039785
rs823424
rs78383039
rs2090816
rs11715853
rs2387977
rs7572229
rs7303158
rs7311004
rs55877868
rs6828768
rs6809952
All
−0.3
−0.2
−0.1
0.0
0.1
MR effect size for
'Gut microbiota abundance (genus Parasutterella id.2892)' on 'Sialate O−acetylesterase'

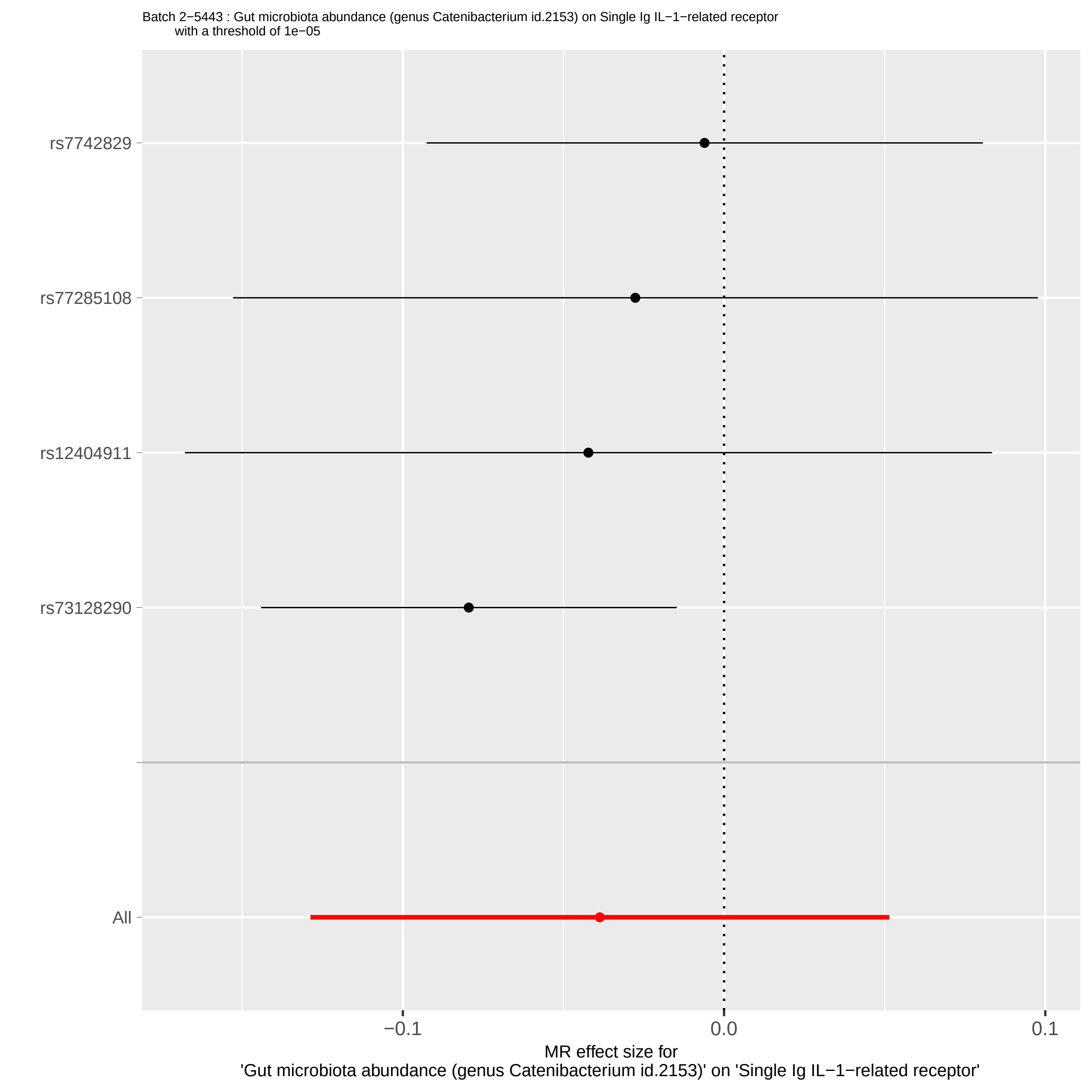
Batch 2−5443 : Gut microbiota abundance (genus Catenibacterium id.2153) on Single Ig IL−1−related receptor
with a threshold of 1e−05
rs7742829
rs77285108
rs12404911
rs73128290
All
−0.1
0.0
0.1
MR effect size for
'Gut microbiota abundance (genus Catenibacterium id.2153)' on 'Single Ig IL−1−related receptor'

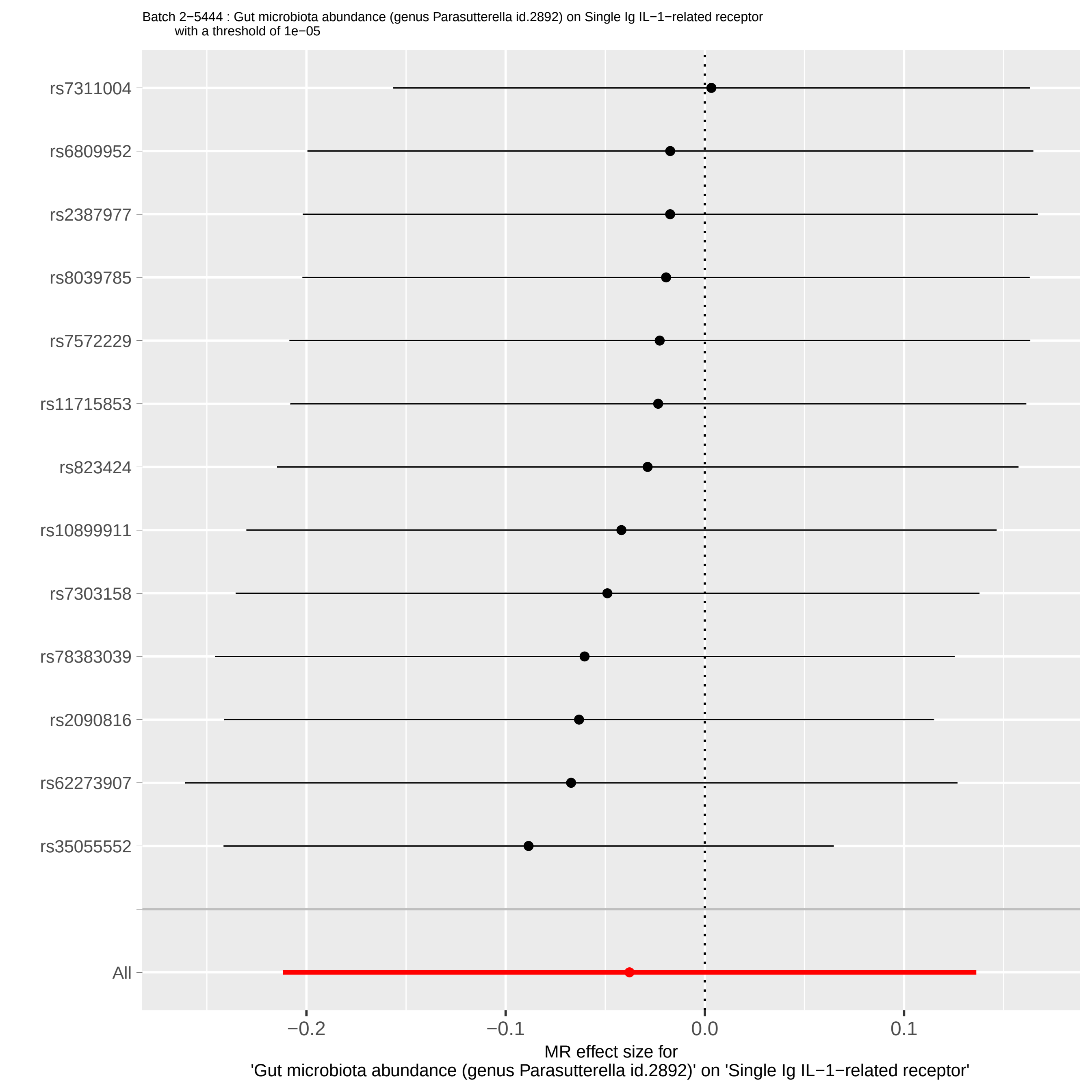

Batch 2−5444 : Gut microbiota abundance (genus Parasutterella id.2892) on Single Ig IL−1−related receptor
with a threshold of 1e−05
rs7311004
rs6809952
rs2387977
rs8039785
rs7572229
rs11715853
rs823424
rs10899911
rs7303158
rs78383039
rs2090816
rs62273907
rs35055552
All
−0.2
−0.1
0.0
0.1
MR effect size for
'Gut microbiota abundance (genus Parasutterella id.2892)' on 'Single Ig IL−1−related receptor'

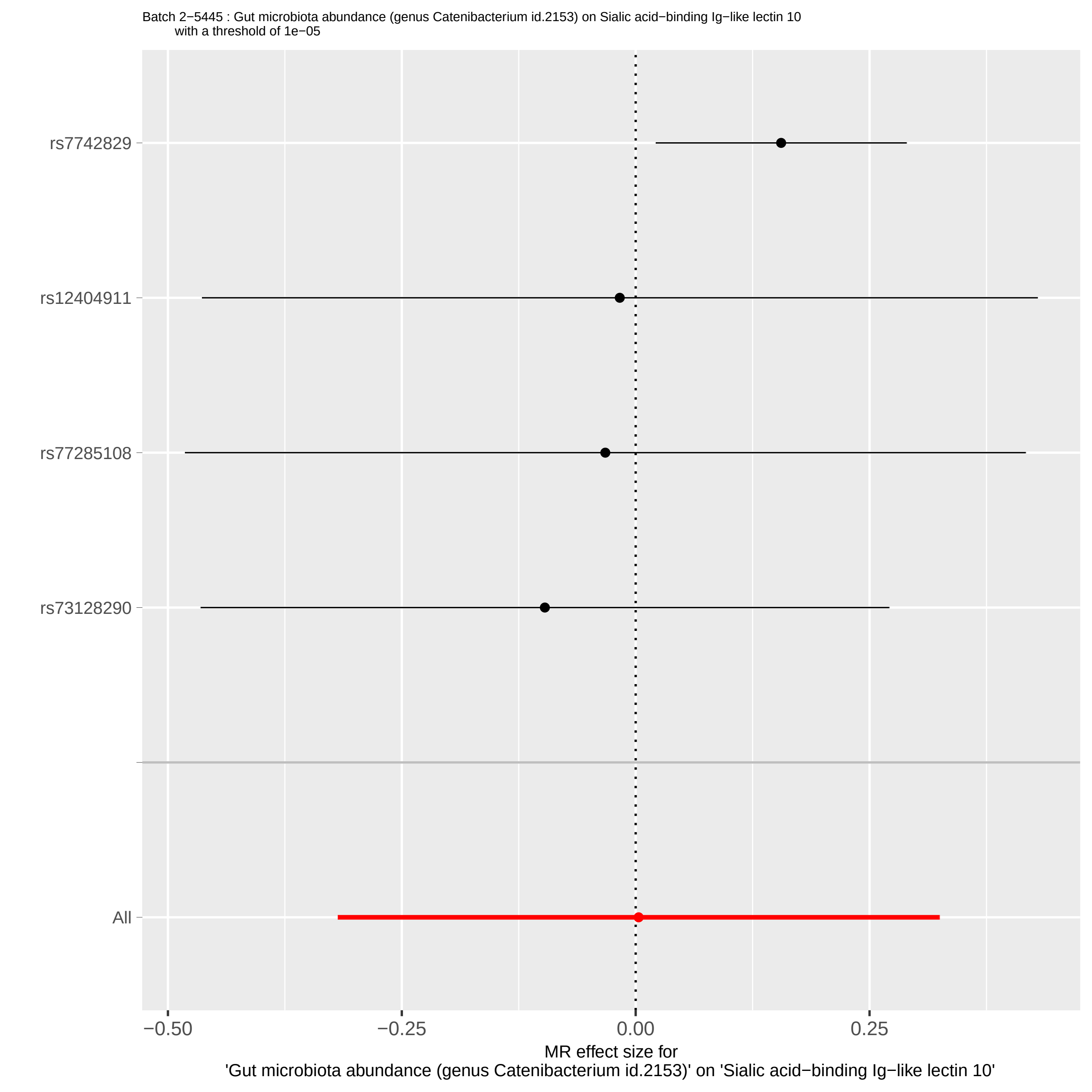
Batch 2−5445 : Gut microbiota abundance (genus Catenibacterium id.2153) on Sialic acid−binding Ig−like lectin 10
with a threshold of 1e−05
rs7742829
rs12404911
rs77285108
rs73128290
All
−0.50
−0.25
0.00
0.25
MR effect size for
'Gut microbiota abundance (genus Catenibacterium id.2153)' on 'Sialic acid−binding Ig−like lectin 10'

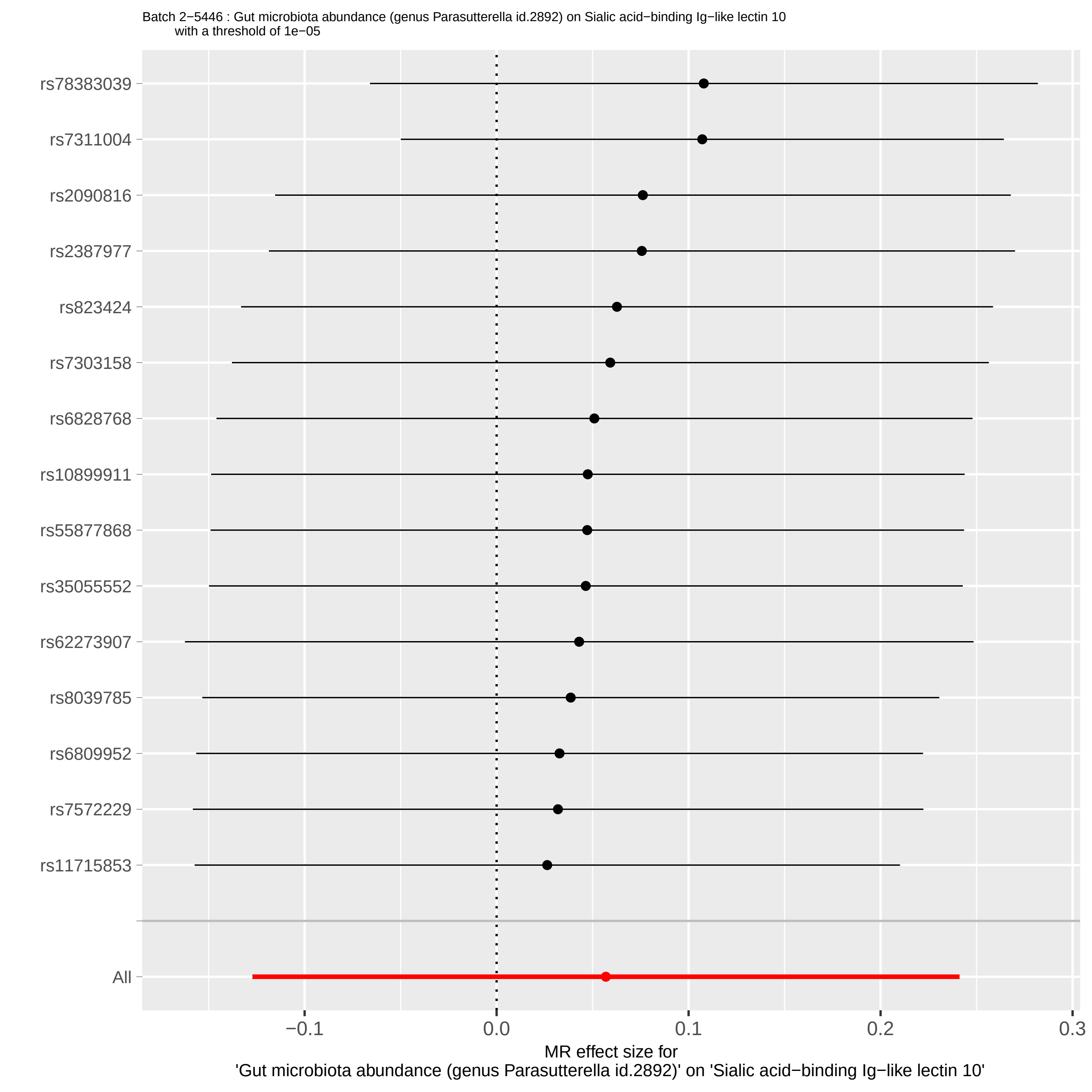

Batch 2−5446 : Gut microbiota abundance (genus Parasutterella id.2892) on Sialic acid−binding Ig−like lectin 10
with a threshold of 1e−05
rs78383039
rs7311004
rs2090816
rs2387977
rs823424
rs7303158
rs6828768
rs10899911
rs55877868
rs35055552
rs62273907
rs8039785
rs6809952
rs7572229
rs11715853
All
−0.1
0.0
0.1
0.2
0.3
MR effect size for
'Gut microbiota abundance (genus Parasutterella id.2892)' on 'Sialic acid−binding Ig−like lectin 10'

Batch 2−5447 : Gut microbiota abundance (genus Catenibacterium id.2153) on Sialic acid−binding Ig−like lectin 11
with a threshold of 1e−05

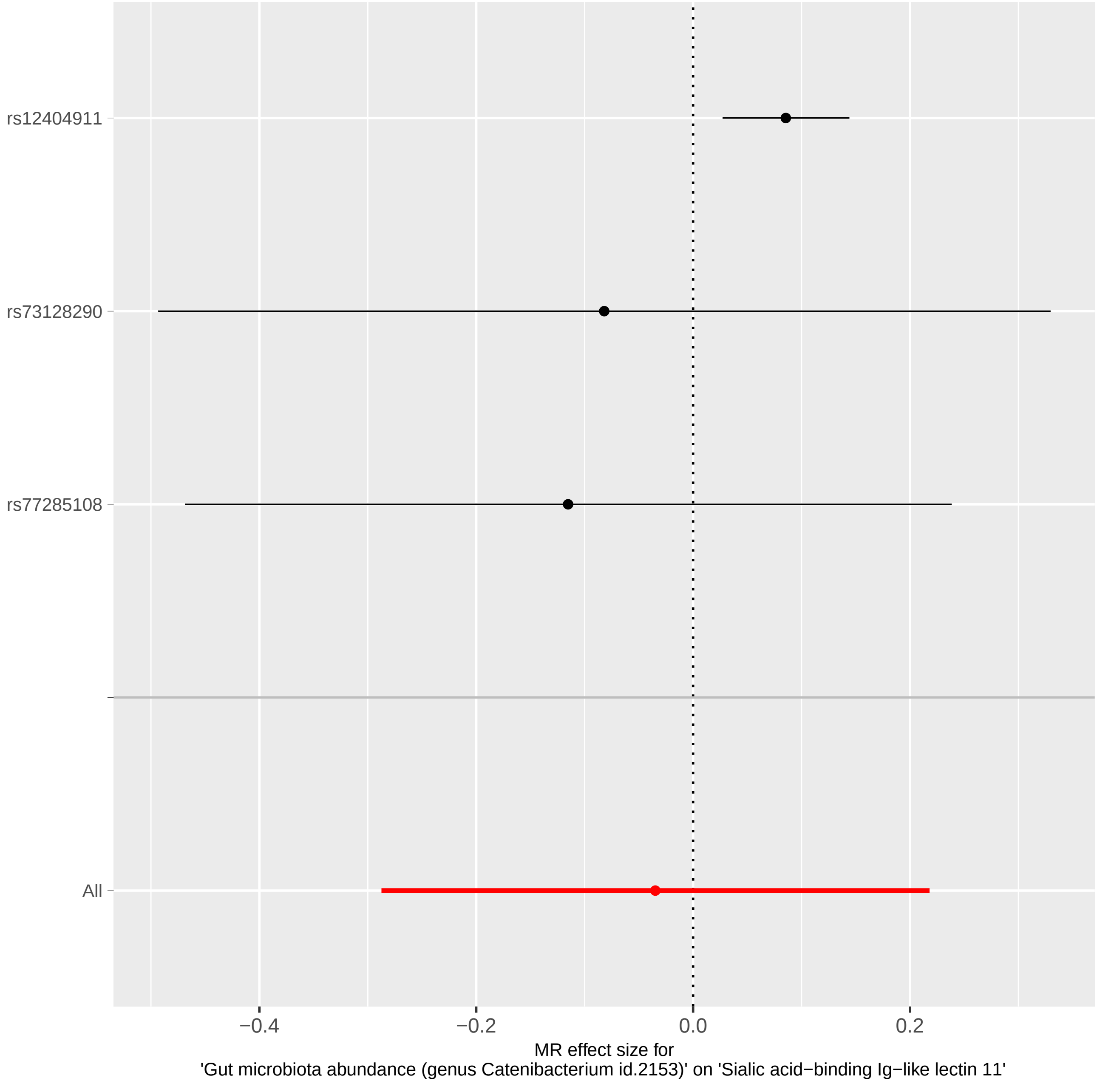

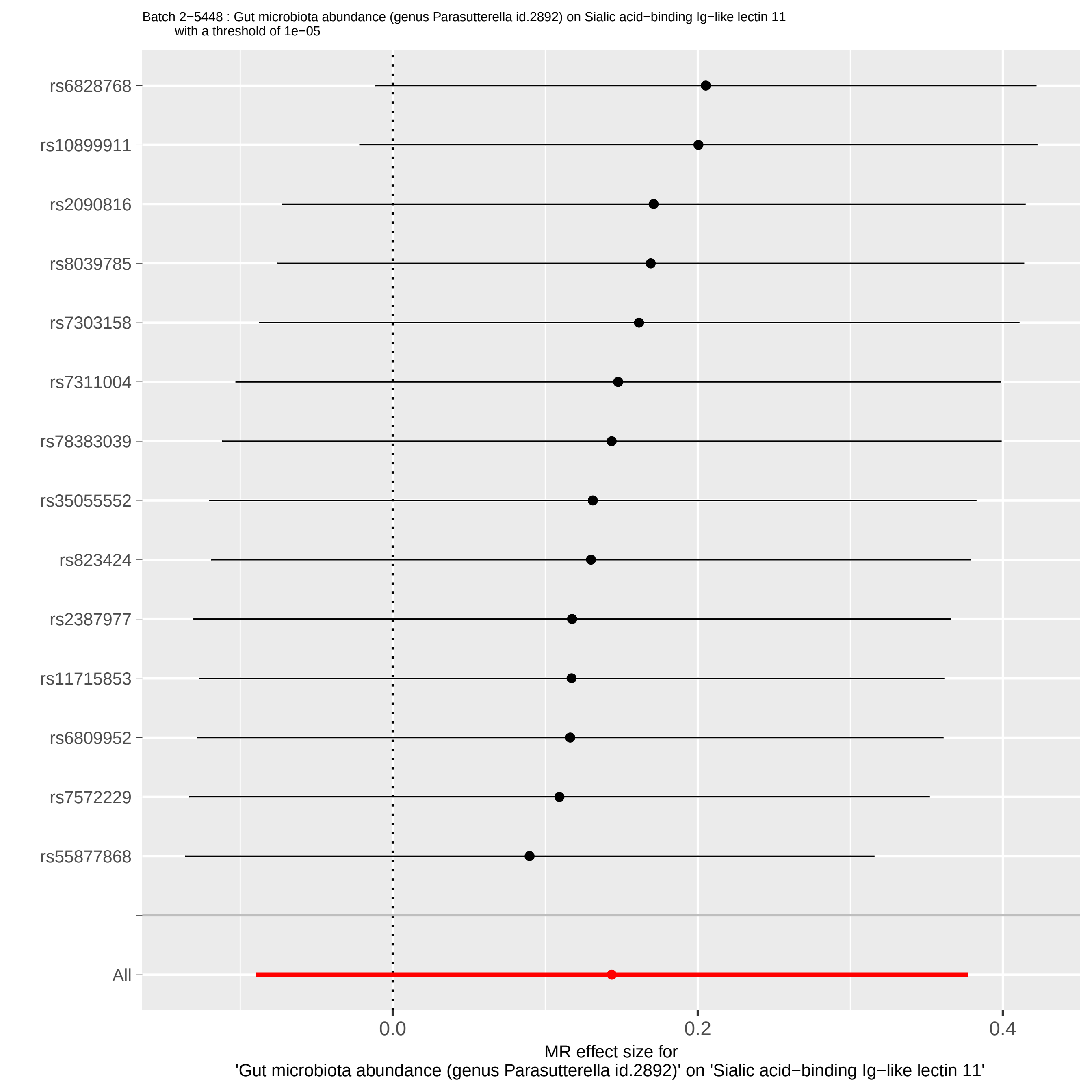

Batch 2−5448 : Gut microbiota abundance (genus Parasutterella id.2892) on Sialic acid−binding Ig−like lectin 11
with a threshold of 1e−05
rs6828768
rs10899911
rs2090816
rs8039785
rs7303158
rs7311004
rs78383039
rs35055552
rs823424
rs2387977
rs11715853
rs6809952
rs7572229
rs55877868
All
0.0
0.2
0.4
MR effect size for
'Gut microbiota abundance (genus Parasutterella id.2892)' on 'Sialic acid−binding Ig−like lectin 11'

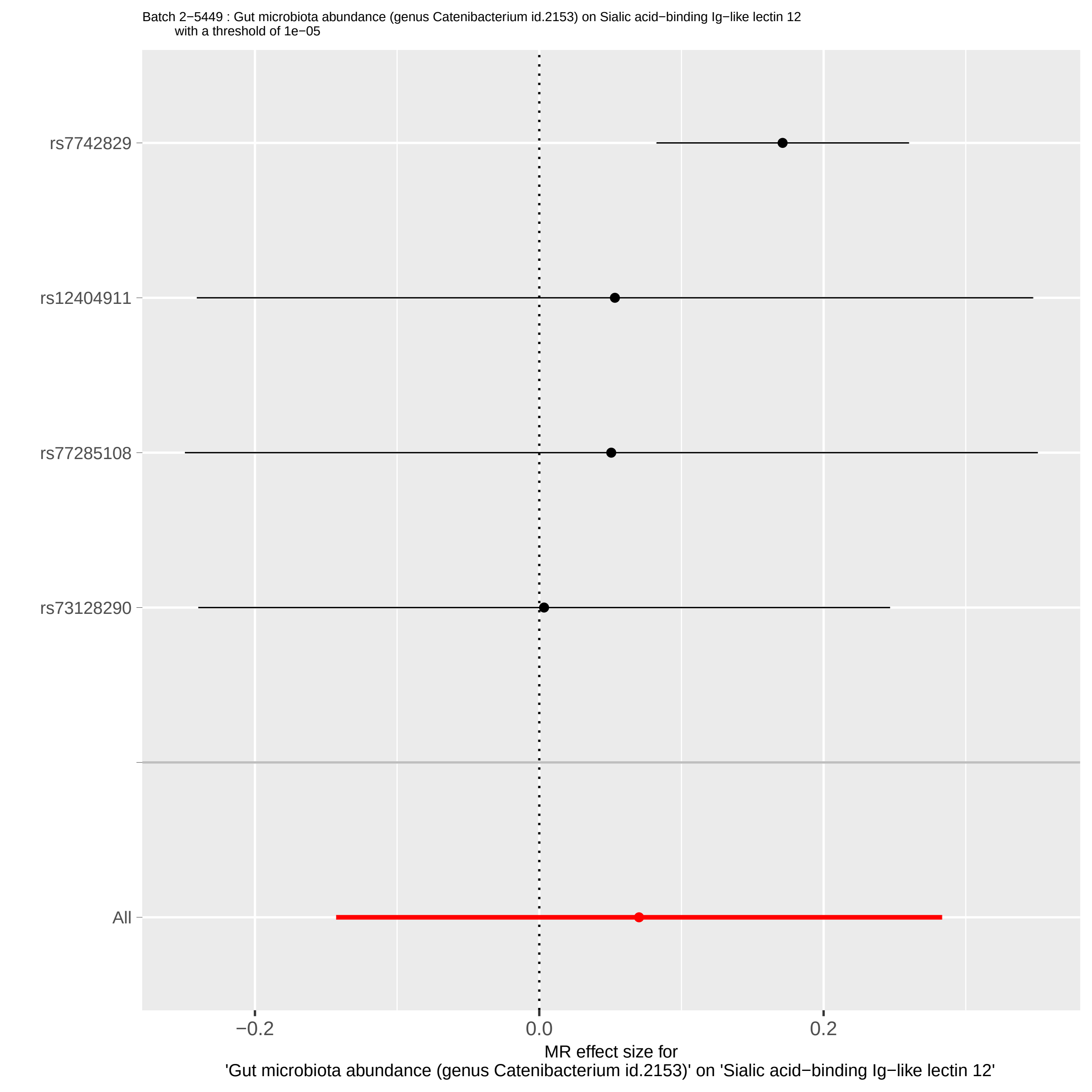
Batch 2−5449 : Gut microbiota abundance (genus Catenibacterium id.2153) on Sialic acid−binding Ig−like lectin 12
with a threshold of 1e−05
rs7742829
rs12404911
rs77285108
rs73128290
All
−0.2
0.0
0.2
MR effect size for
'Gut microbiota abundance (genus Catenibacterium id.2153)' on 'Sialic acid−binding Ig−like lectin 12'

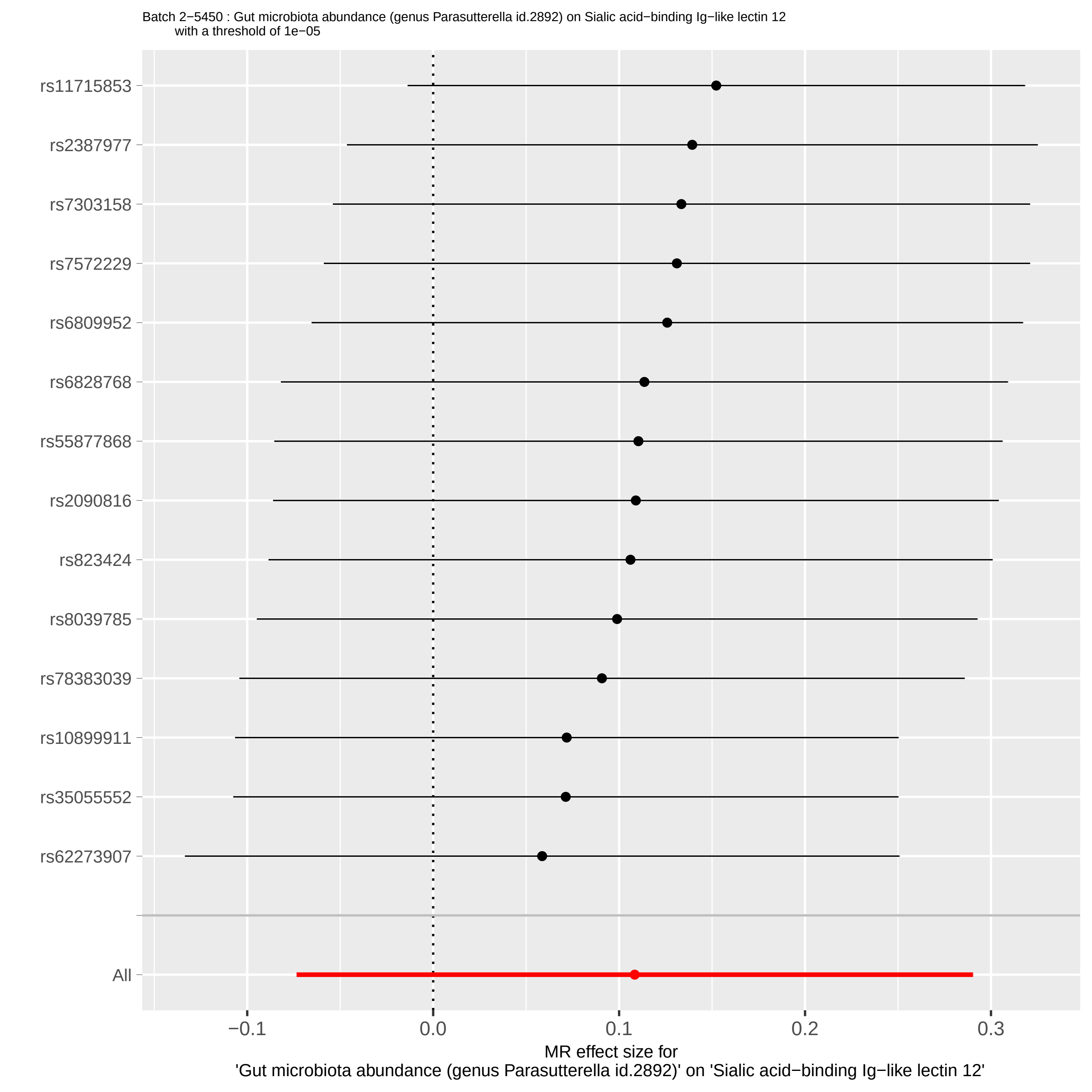

Batch 2−5450 : Gut microbiota abundance (genus Parasutterella id.2892) on Sialic acid−binding Ig−like lectin 12
with a threshold of 1e−05
rs11715853
rs2387977
rs7303158
rs7572229
rs6809952
rs6828768
rs55877868
rs2090816
rs823424
rs8039785
rs78383039
rs10899911
rs35055552
rs62273907
All
−0.1
0.0
0.1
0.2
0.3
MR effect size for
'Gut microbiota abundance (genus Parasutterella id.2892)' on 'Sialic acid−binding Ig−like lectin 12'

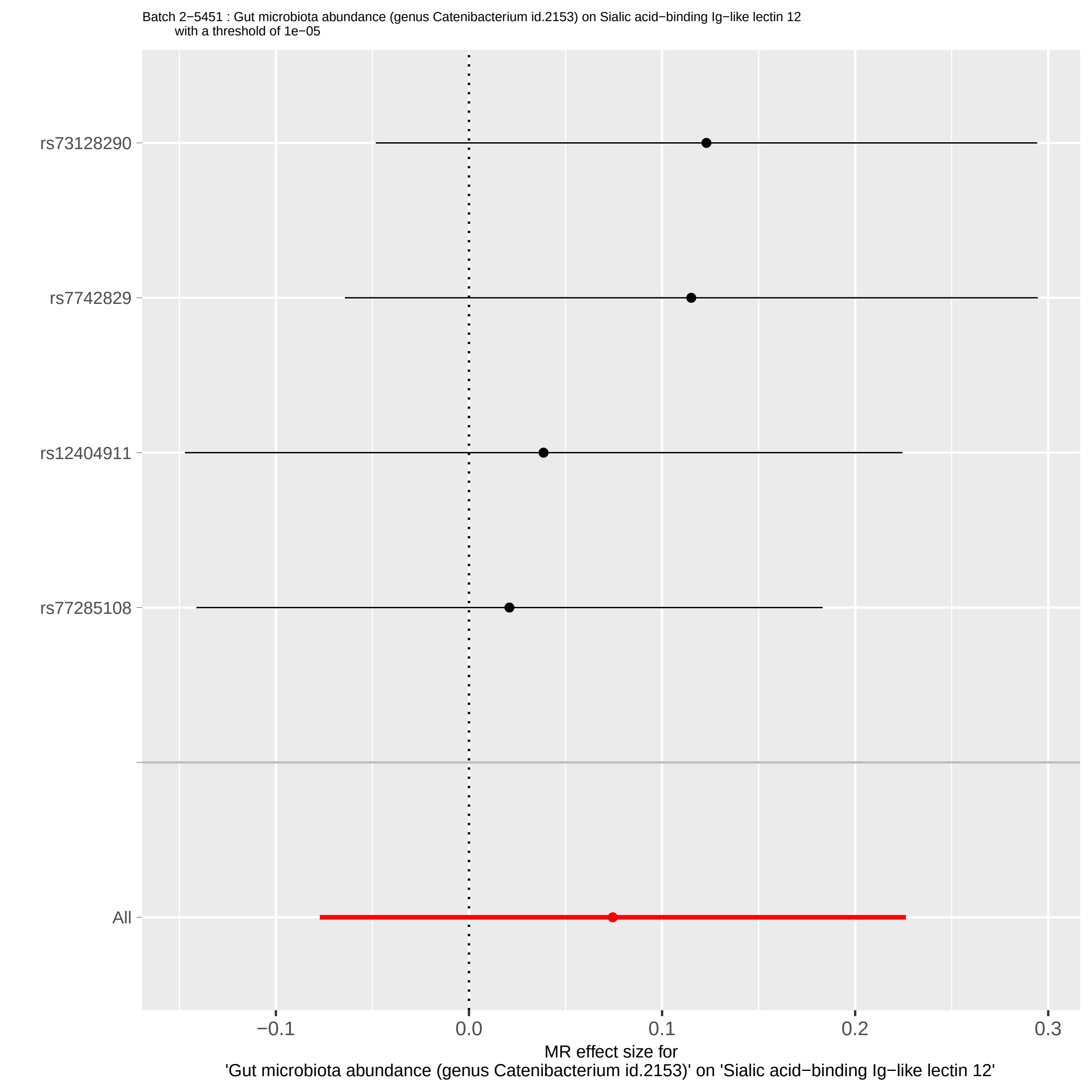

Batch 2−5451 : Gut microbiota abundance (genus Catenibacterium id.2153) on Sialic acid−binding Ig−like lectin 12
with a threshold of 1e−05
rs73128290
rs7742829
rs12404911
rs77285108
All
−0.1
0.0
0.1
0.2
0.3
MR effect size for
'Gut microbiota abundance (genus Catenibacterium id.2153)' on 'Sialic acid−binding Ig−like lectin 12'

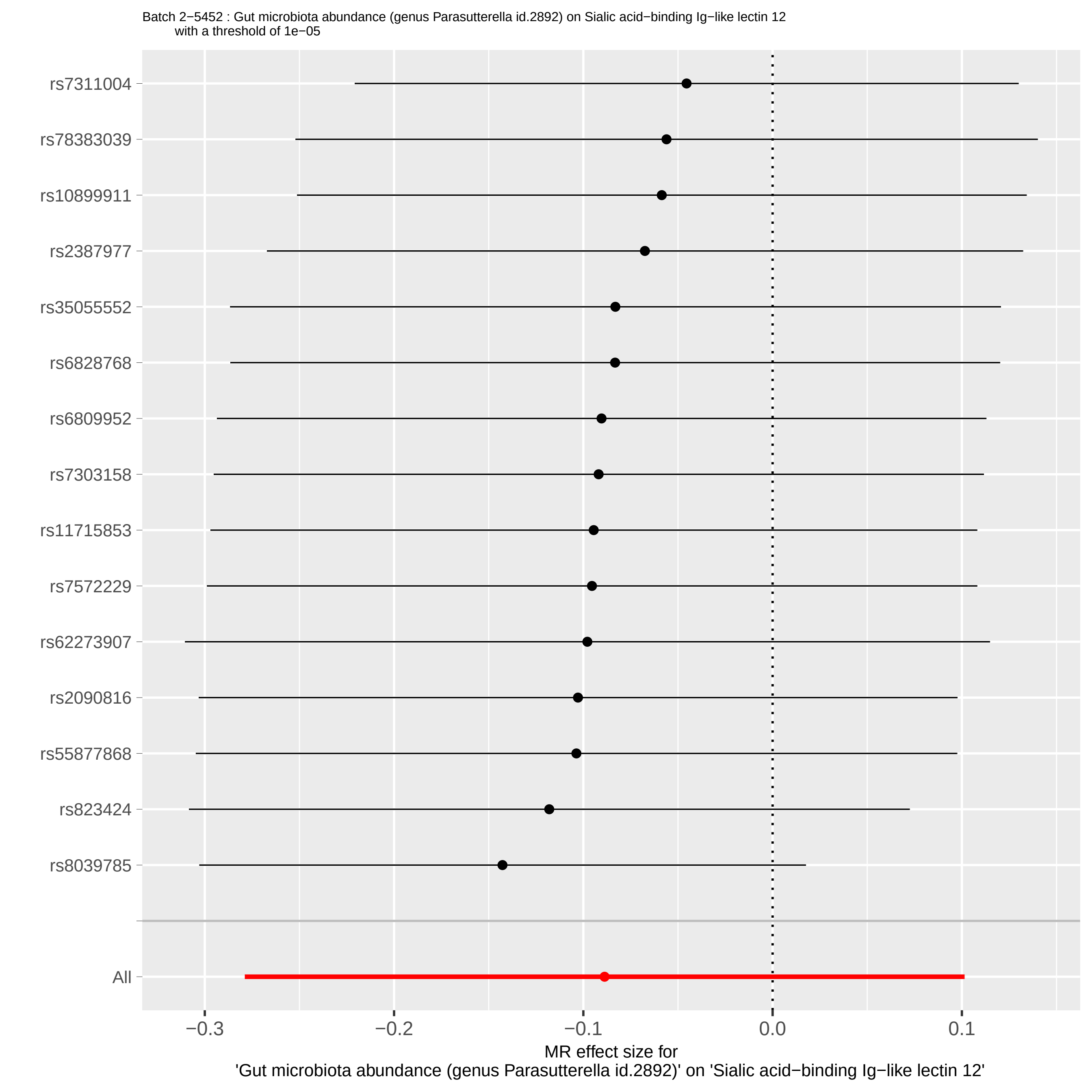
Batch 2−5452 : Gut microbiota abundance (genus Parasutterella id.2892) on Sialic acid−binding Ig−like lectin 12
with a threshold of 1e−05
rs7311004
rs78383039
rs10899911
rs2387977
rs35055552
rs6828768
rs6809952
rs7303158
rs11715853
rs7572229
rs62273907
rs2090816
rs55877868
rs823424
rs8039785
All
−0.3
−0.2
−0.1
0.0
0.1
MR effect size for
'Gut microbiota abundance (genus Parasutterella id.2892)' on 'Sialic acid−binding Ig−like lectin 12'

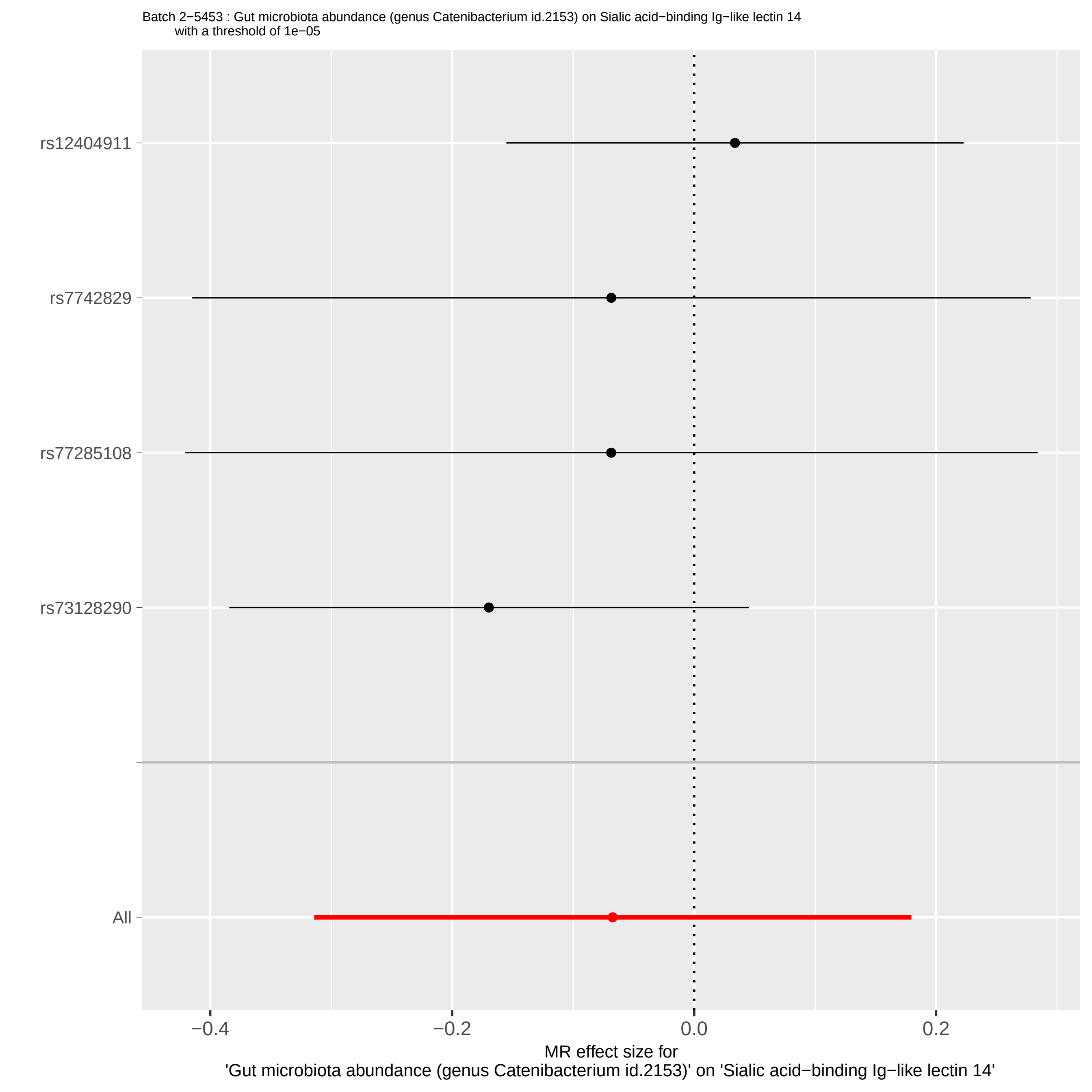
Batch 2−5453 : Gut microbiota abundance (genus Catenibacterium id.2153) on Sialic acid−binding Ig−like lectin 14
with a threshold of 1e−05
rs12404911
rs7742829
rs77285108
rs73128290
All
−0.4
−0.2
0.0
0.2
MR effect size for
'Gut microbiota abundance (genus Catenibacterium id.2153)' on 'Sialic acid−binding Ig−like lectin 14'

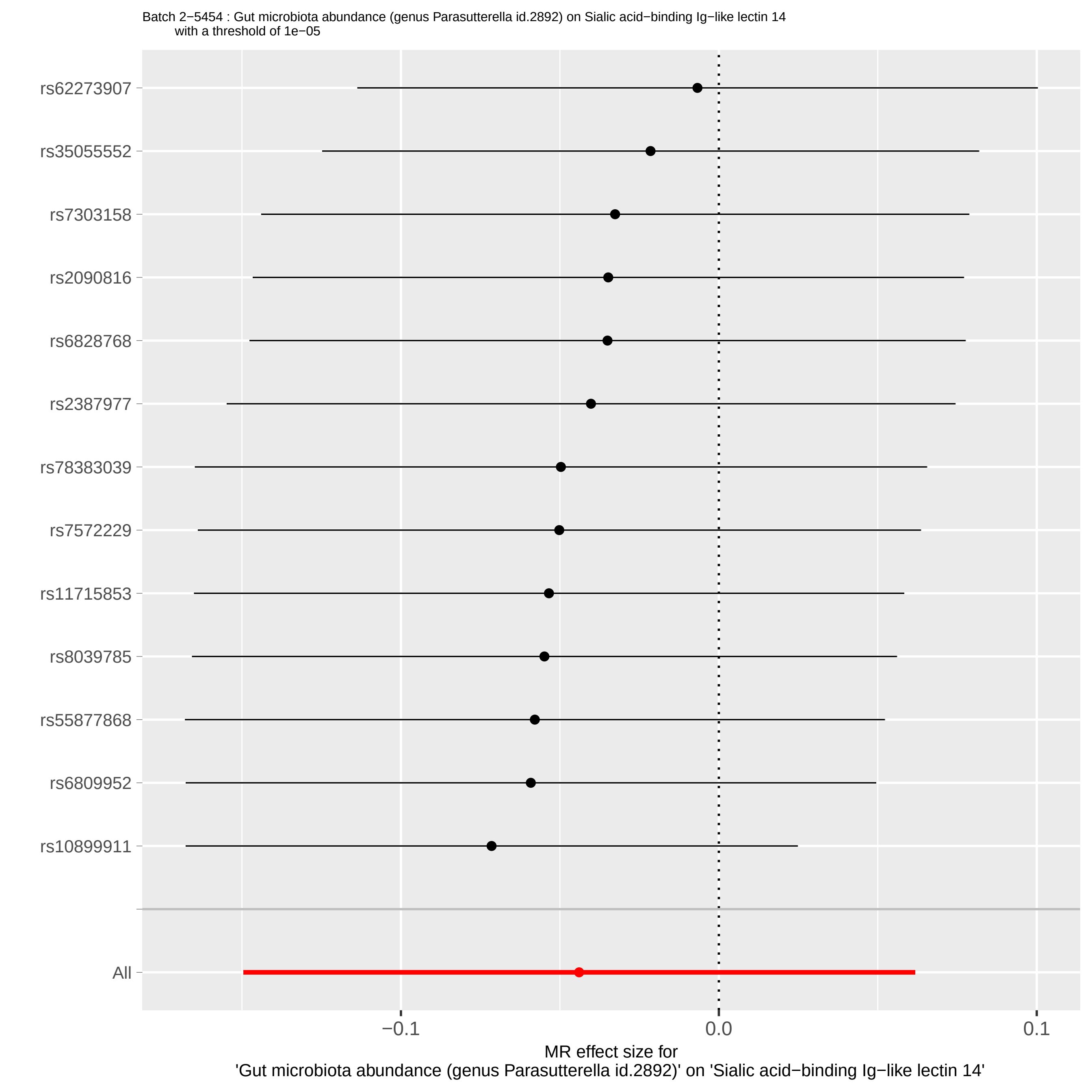

Batch 2−5454 : Gut microbiota abundance (genus Parasutterella id.2892) on Sialic acid−binding Ig−like lectin 14
with a threshold of 1e−05
rs62273907
rs35055552
rs7303158
rs2090816
rs6828768
rs2387977
rs78383039
rs7572229
rs11715853
rs8039785
rs55877868
rs6809952
rs10899911
All
−0.1
0.0
0.1
MR effect size for
'Gut microbiota abundance (genus Parasutterella id.2892)' on 'Sialic acid−binding Ig−like lectin 14'

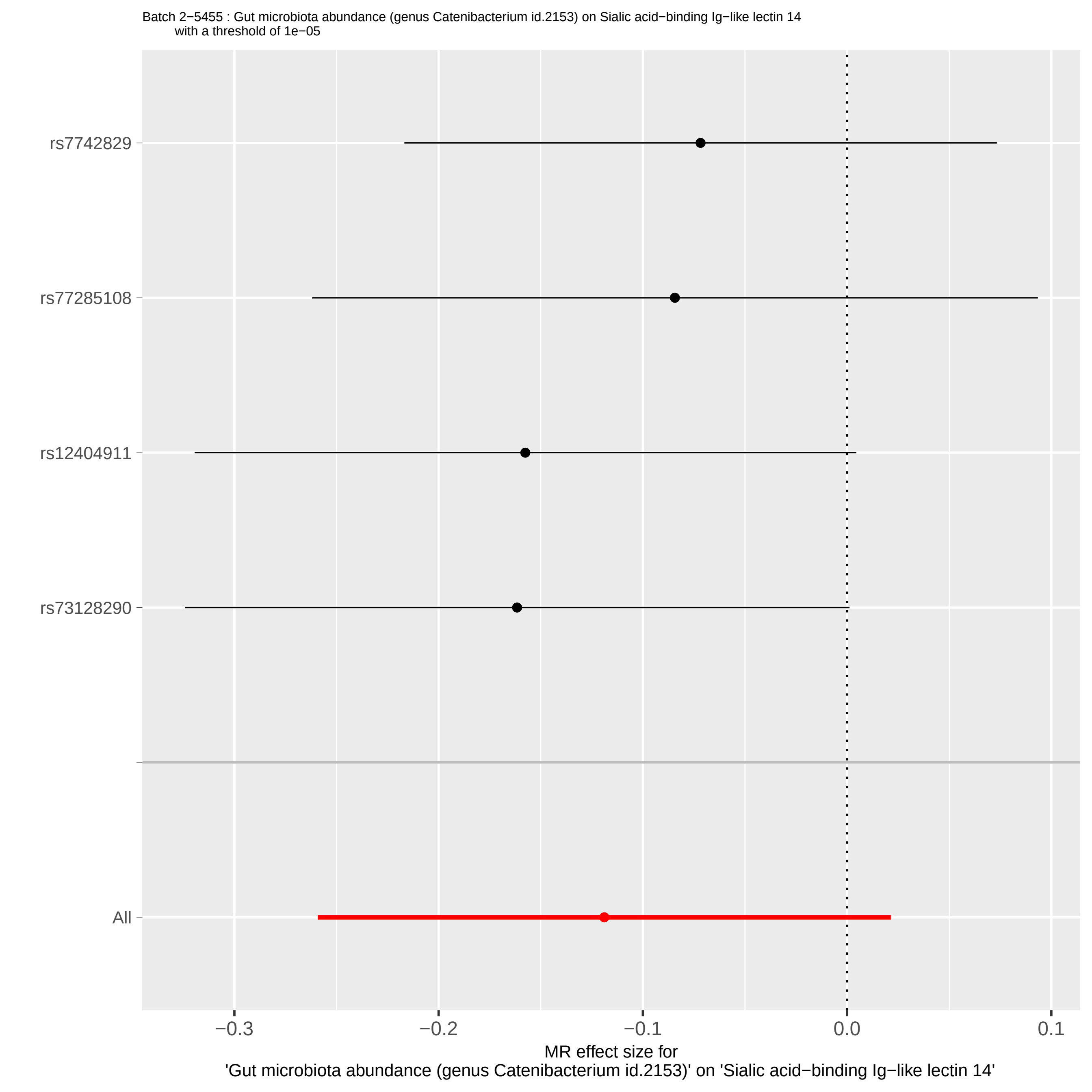
Batch 2−5455 : Gut microbiota abundance (genus Catenibacterium id.2153) on Sialic acid−binding Ig−like lectin 14
with a threshold of 1e−05
rs7742829
rs77285108
rs12404911
rs73128290
All
−0.3
−0.2
−0.1
0.0
0.1
MR effect size for
'Gut microbiota abundance (genus Catenibacterium id.2153)' on 'Sialic acid−binding Ig−like lectin 14'

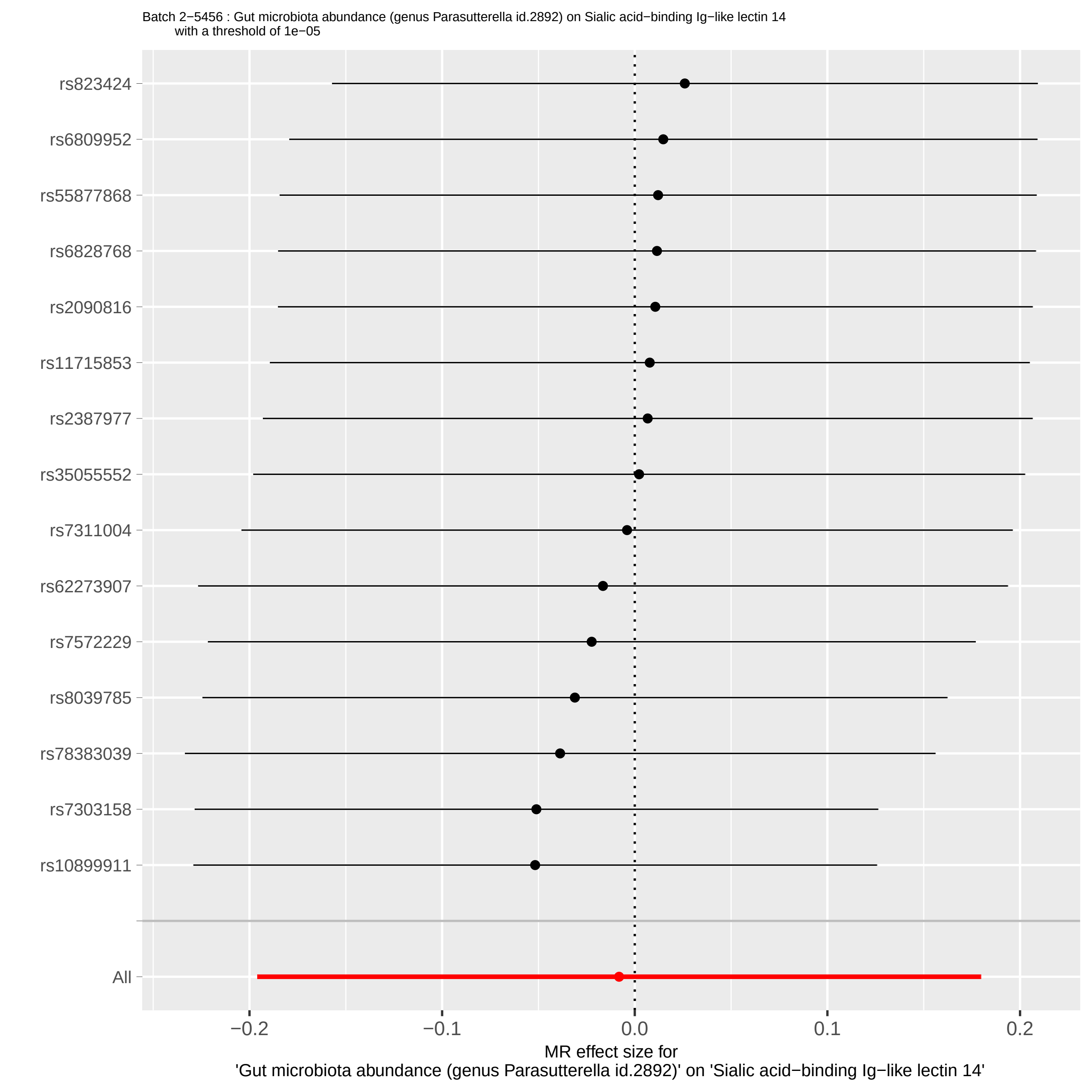
Batch 2−5456 : Gut microbiota abundance (genus Parasutterella id.2892) on Sialic acid−binding Ig−like lectin 14
with a threshold of 1e−05
rs823424
rs6809952
rs55877868
rs6828768
rs2090816
rs11715853
rs2387977
rs35055552
rs7311004
rs62273907
rs7572229
rs8039785
rs78383039
rs7303158
rs10899911
All
−0.2
−0.1
0.0
0.1
0.2
MR effect size for
'Gut microbiota abundance (genus Parasutterella id.2892)' on 'Sialic acid−binding Ig−like lectin 14'

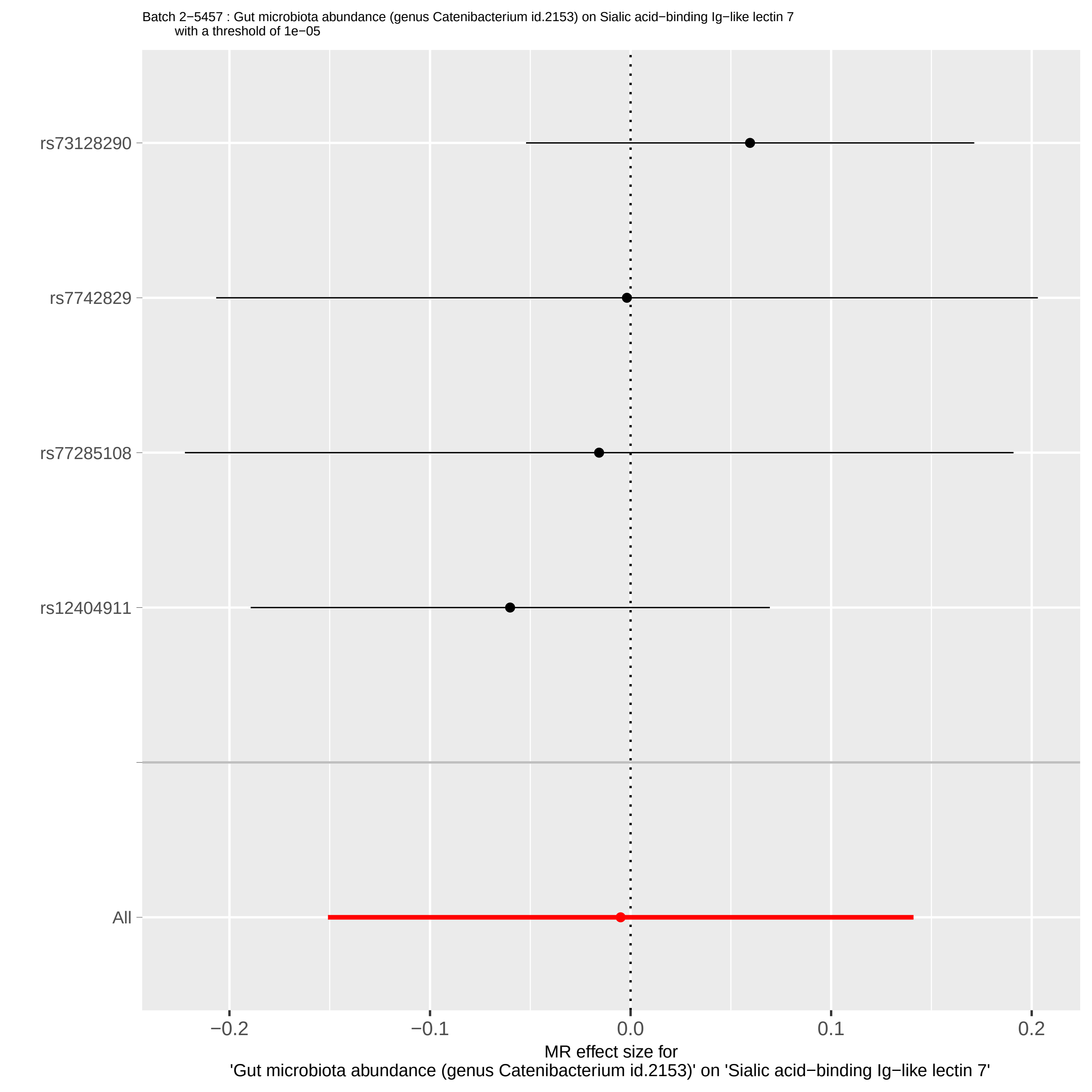
Batch 2−5457 : Gut microbiota abundance (genus Catenibacterium id.2153) on Sialic acid−binding Ig−like lectin 7
with a threshold of 1e−05
rs73128290
rs7742829
rs77285108
rs12404911
All
−0.2
−0.1
0.0
0.1
0.2
MR effect size for
'Gut microbiota abundance (genus Catenibacterium id.2153)' on 'Sialic acid−binding Ig−like lectin 7'

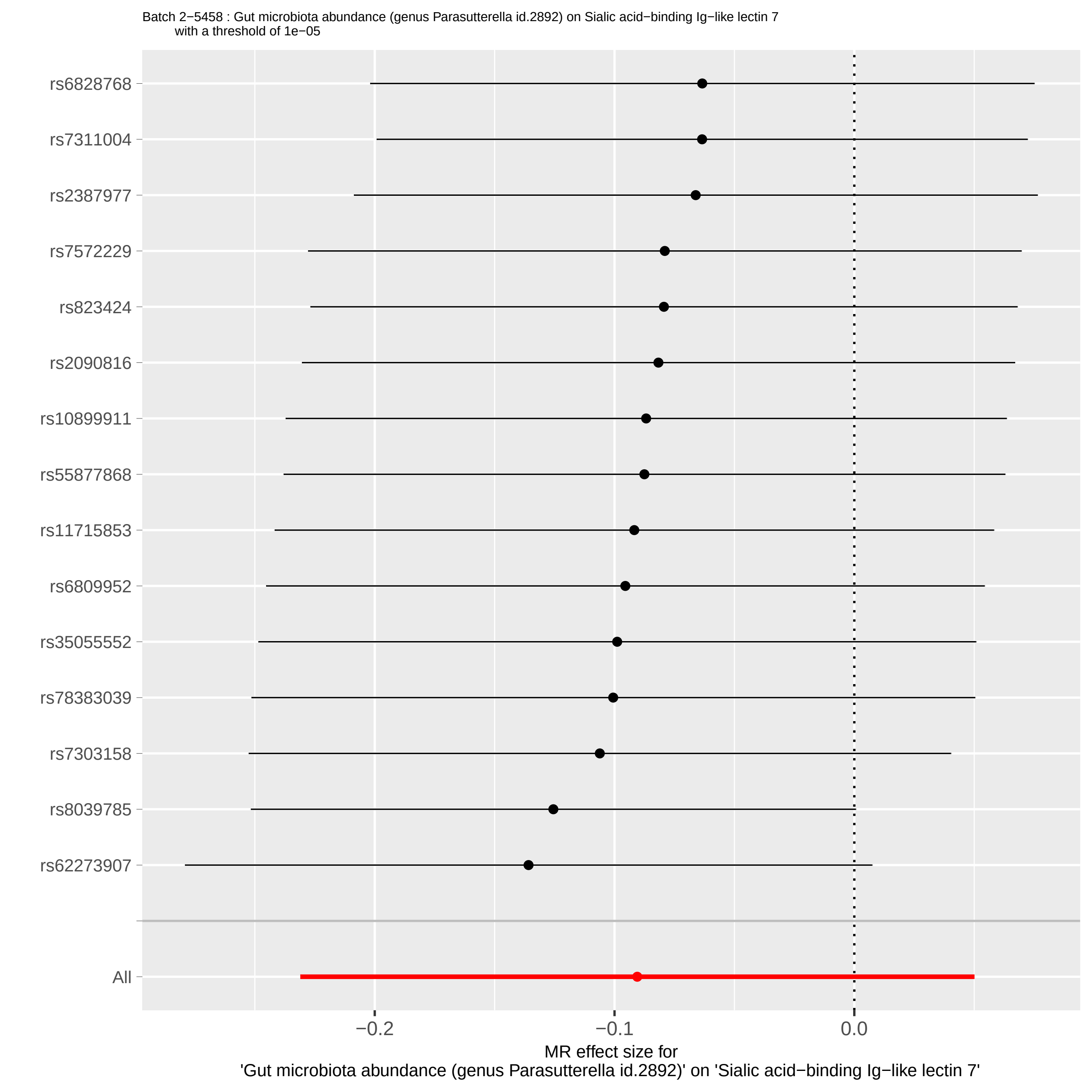
Batch 2−5458 : Gut microbiota abundance (genus Parasutterella id.2892) on Sialic acid−binding Ig−like lectin 7
with a threshold of 1e−05
rs6828768
rs7311004
rs2387977
rs7572229
rs823424
rs2090816
rs10899911
rs55877868
rs11715853
rs6809952
rs35055552
rs78383039
rs7303158
rs8039785
rs62273907
All
−0.2
−0.1
0.0
MR effect size for
'Gut microbiota abundance (genus Parasutterella id.2892)' on 'Sialic acid−binding Ig−like lectin 7'

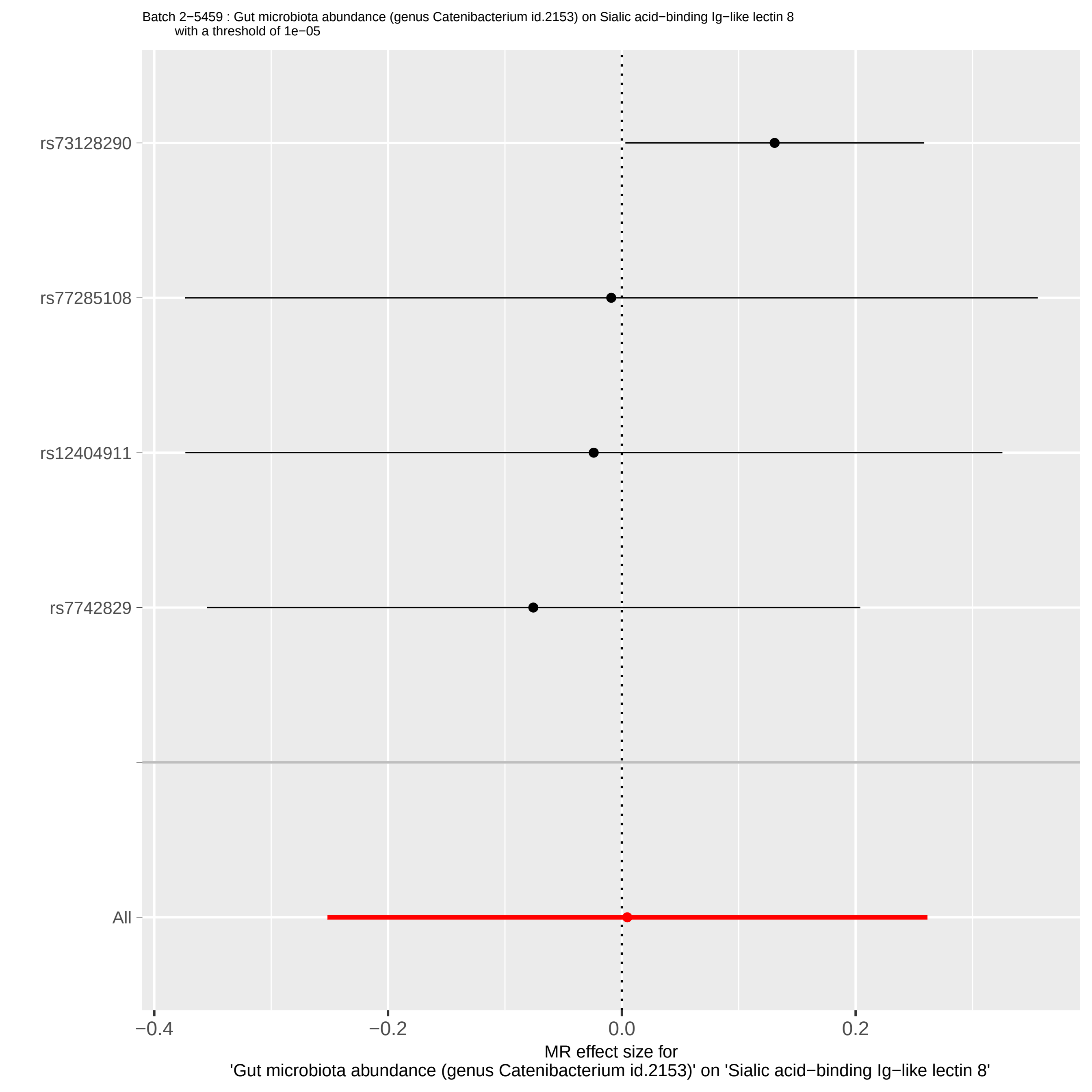
Batch 2−5459 : Gut microbiota abundance (genus Catenibacterium id.2153) on Sialic acid−binding Ig−like lectin 8
with a threshold of 1e−05
rs73128290
rs77285108
rs12404911
rs7742829
All
−0.4
−0.2
0.0
0.2
MR effect size for
'Gut microbiota abundance (genus Catenibacterium id.2153)' on 'Sialic acid−binding Ig−like lectin 8'

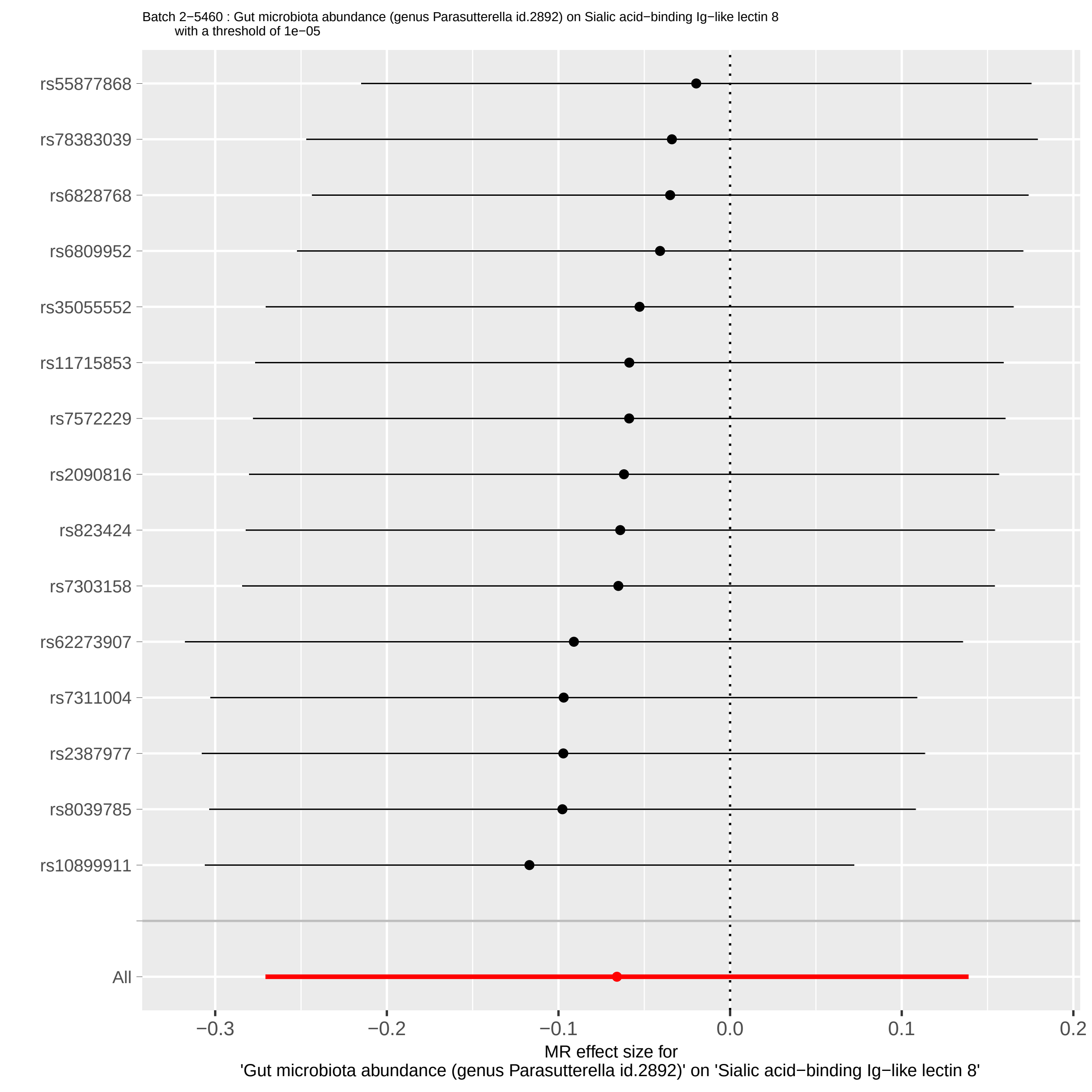
Batch 2−5460 : Gut microbiota abundance (genus Parasutterella id.2892) on Sialic acid−binding Ig−like lectin 8
with a threshold of 1e−05
rs55877868
rs78383039
rs6828768
rs6809952
rs35055552
rs11715853
rs7572229
rs2090816
rs823424
rs7303158
rs62273907
rs7311004
rs2387977
rs8039785
rs10899911
All
−0.3
−0.2
−0.1
0.0
0.1
0.2
MR effect size for
'Gut microbiota abundance (genus Parasutterella id.2892)' on 'Sialic acid−binding Ig−like lectin 8'

Batch 2−5461 : Gut microbiota abundance (genus Catenibacterium id.2153) on Sialic acid−binding Ig−like lectin 9
with a threshold of 1e−05

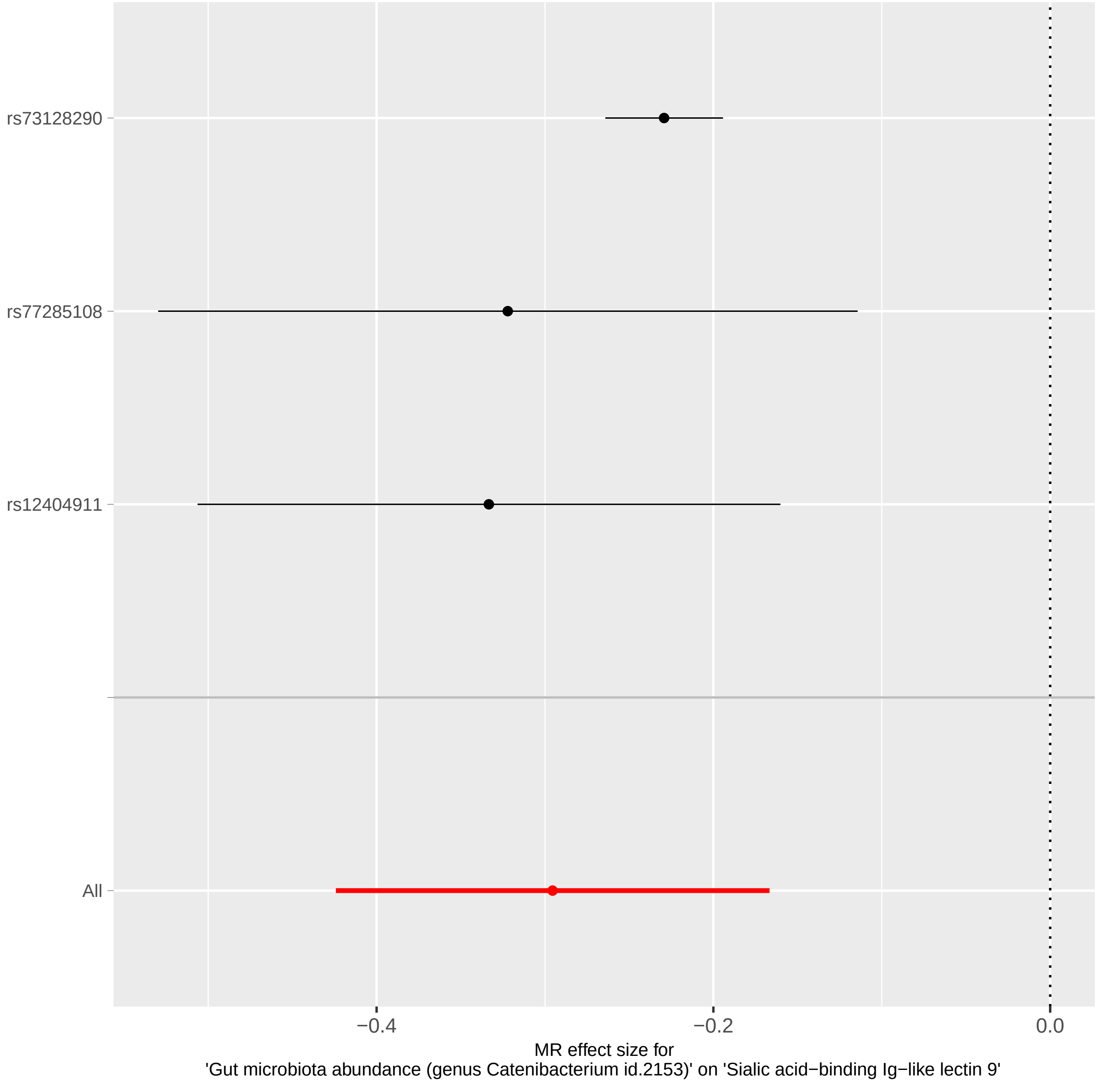

Batch 2−5462 : Gut microbiota abundance (genus Parasutterella id.2892) on Sialic acid−binding Ig−like lectin 9
with a threshold of 1e−05

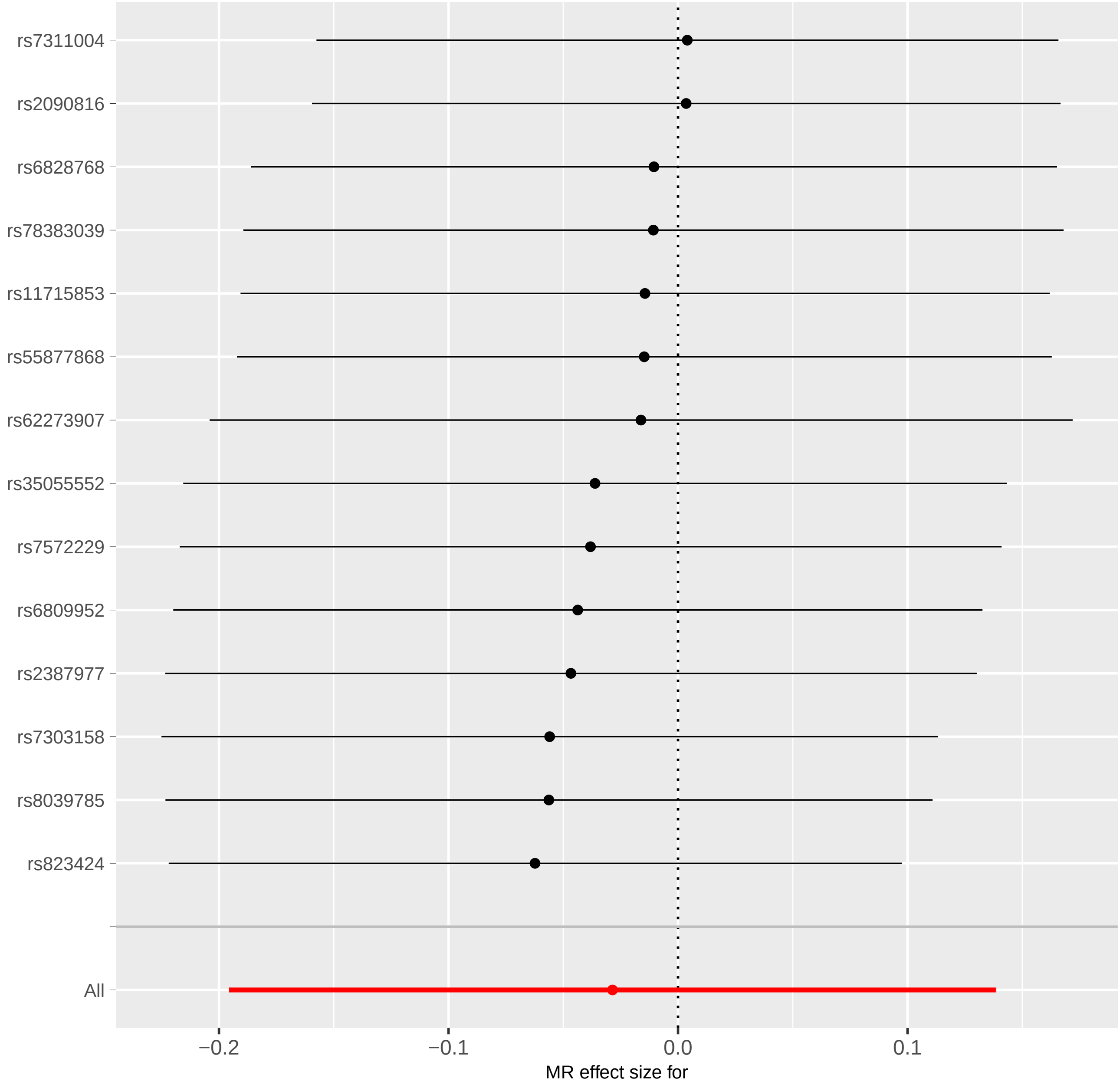

MR effect size for
'Gut microbiota abundance (genus Parasutterella id.2892)' on 'Sialic acid−binding Ig−like lectin 9'

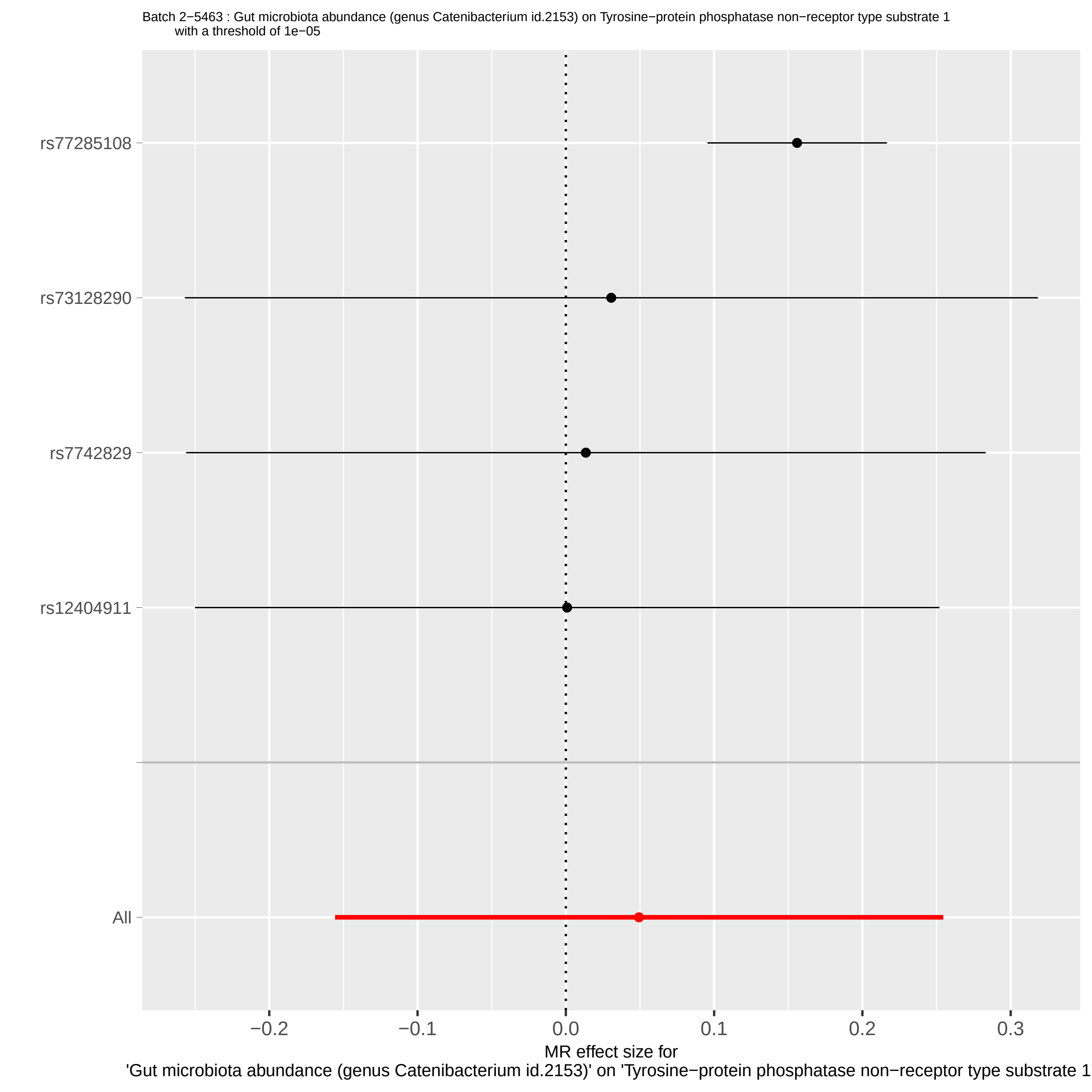
Batch 2−5463 : Gut microbiota abundance (genus Catenibacterium id.2153) on Tyrosine−protein phosphatase non−receptor type substrate 1
with a threshold of 1e−05
rs77285108
rs73128290
rs7742829
rs12404911
All
−0.2
−0.1
0.0
0.1
0.2
0.3
MR effect size for
'Gut microbiota abundance (genus Catenibacterium id.2153)' on 'Tyrosine−protein phosphatase non−receptor type substrate 1

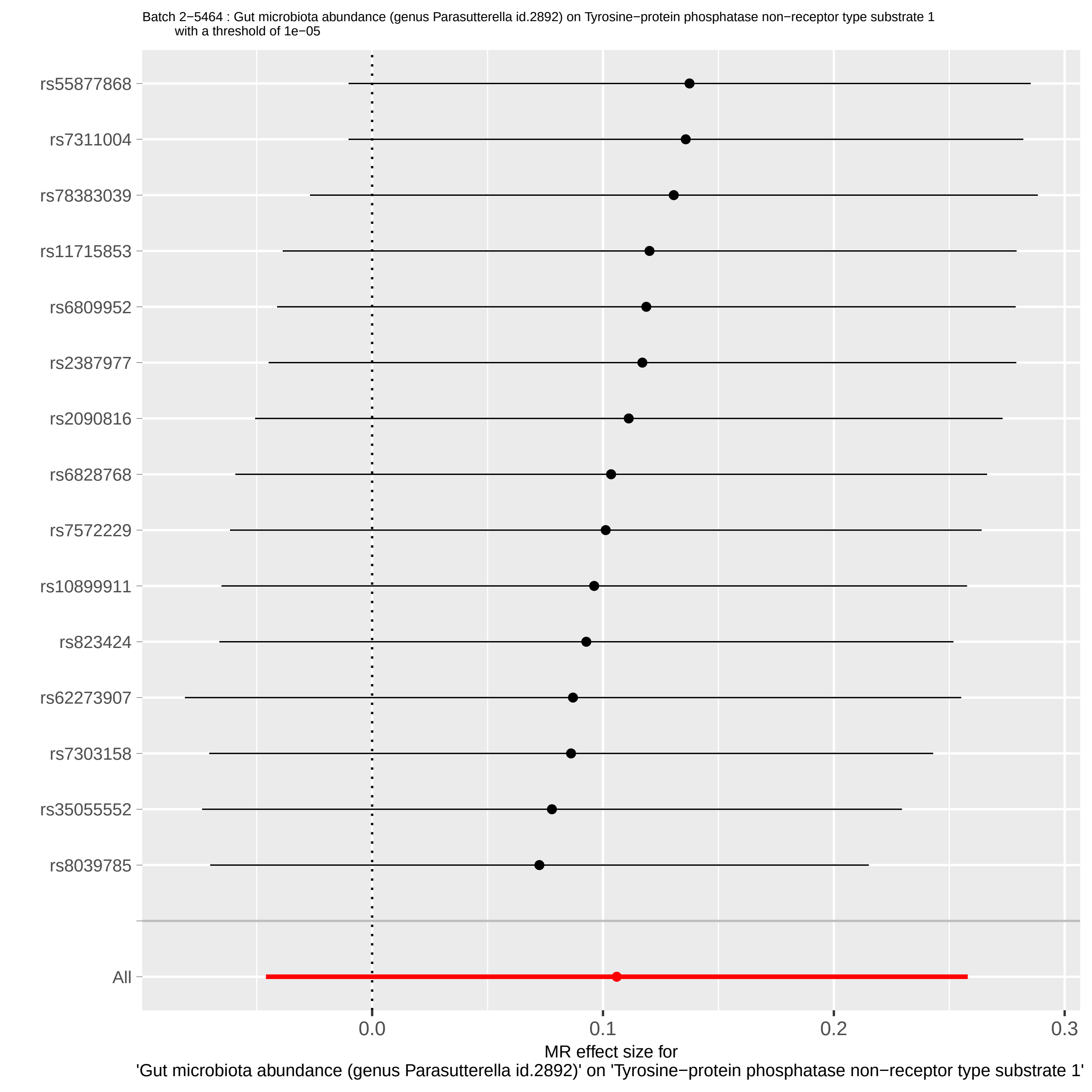

Batch 2−5464 : Gut microbiota abundance (genus Parasutterella id.2892) on Tyrosine−protein phosphatase non−receptor type substrate 1
with a threshold of 1e−05
rs55877868
rs7311004
rs78383039
rs11715853
rs6809952
rs2387977
rs2090816
rs6828768
rs7572229
rs10899911
rs823424
rs62273907
rs7303158
rs35055552
rs8039785
All
0.0
0.1
0.2
0.3
MR effect size for
'Gut microbiota abundance (genus Parasutterella id.2892)' on 'Tyrosine−protein phosphatase non−receptor type substrate 1'

Batch 2−5465 : Gut microbiota abundance (genus Catenibacterium id.2153) on Signal−regulatory protein beta−1
with a threshold of 1e−05

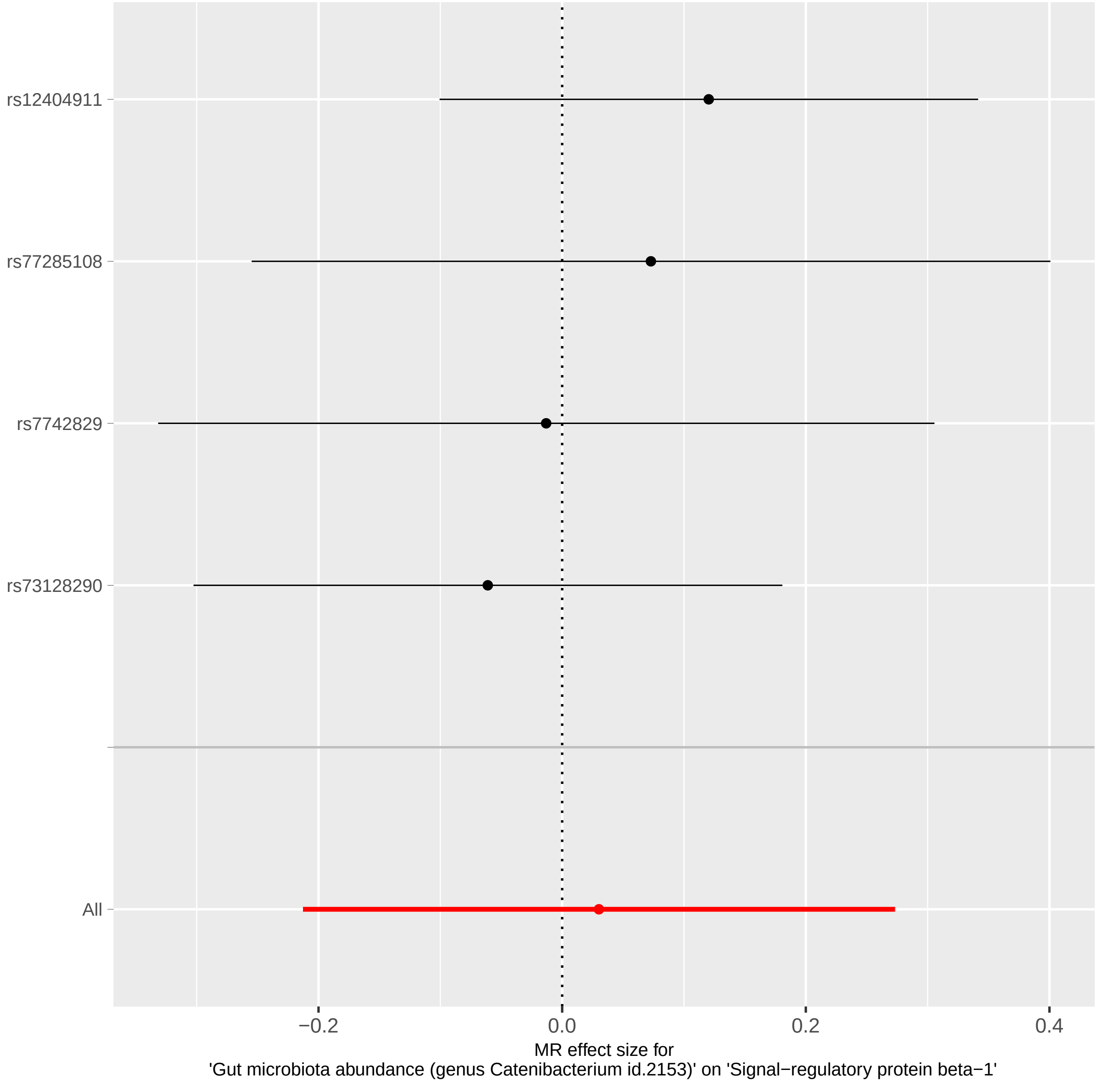

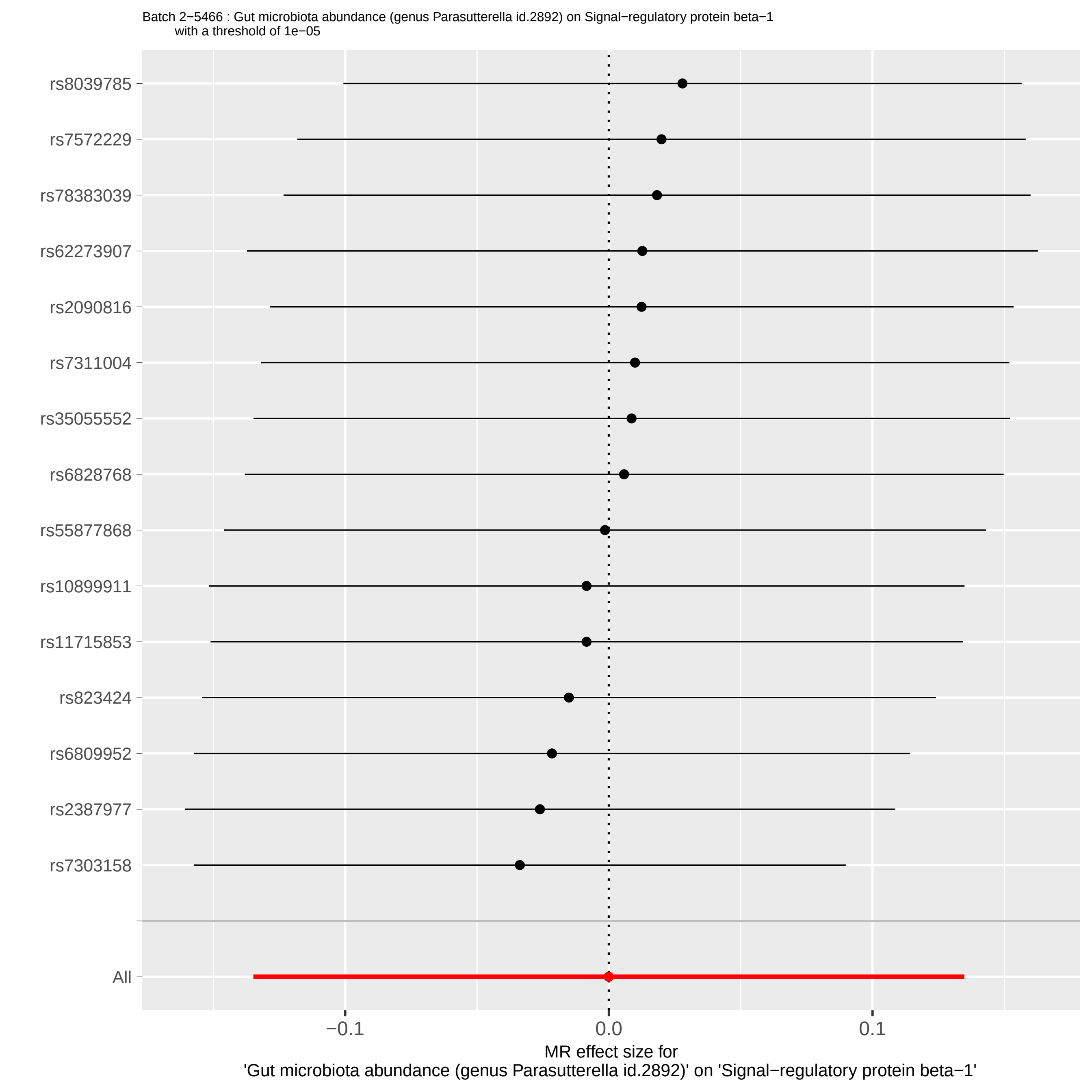

Batch 2−5466 : Gut microbiota abundance (genus Parasutterella id.2892) on Signal−regulatory protein beta−1
with a threshold of 1e−05
rs8039785
rs7572229
rs78383039
rs62273907
rs2090816
rs7311004
rs35055552
rs6828768
rs55877868
rs10899911
rs11715853
rs823424
rs6809952
rs2387977
rs7303158
All
−0.1
0.0
0.1
MR effect size for
'Gut microbiota abundance (genus Parasutterella id.2892)' on 'Signal−regulatory protein beta−1'

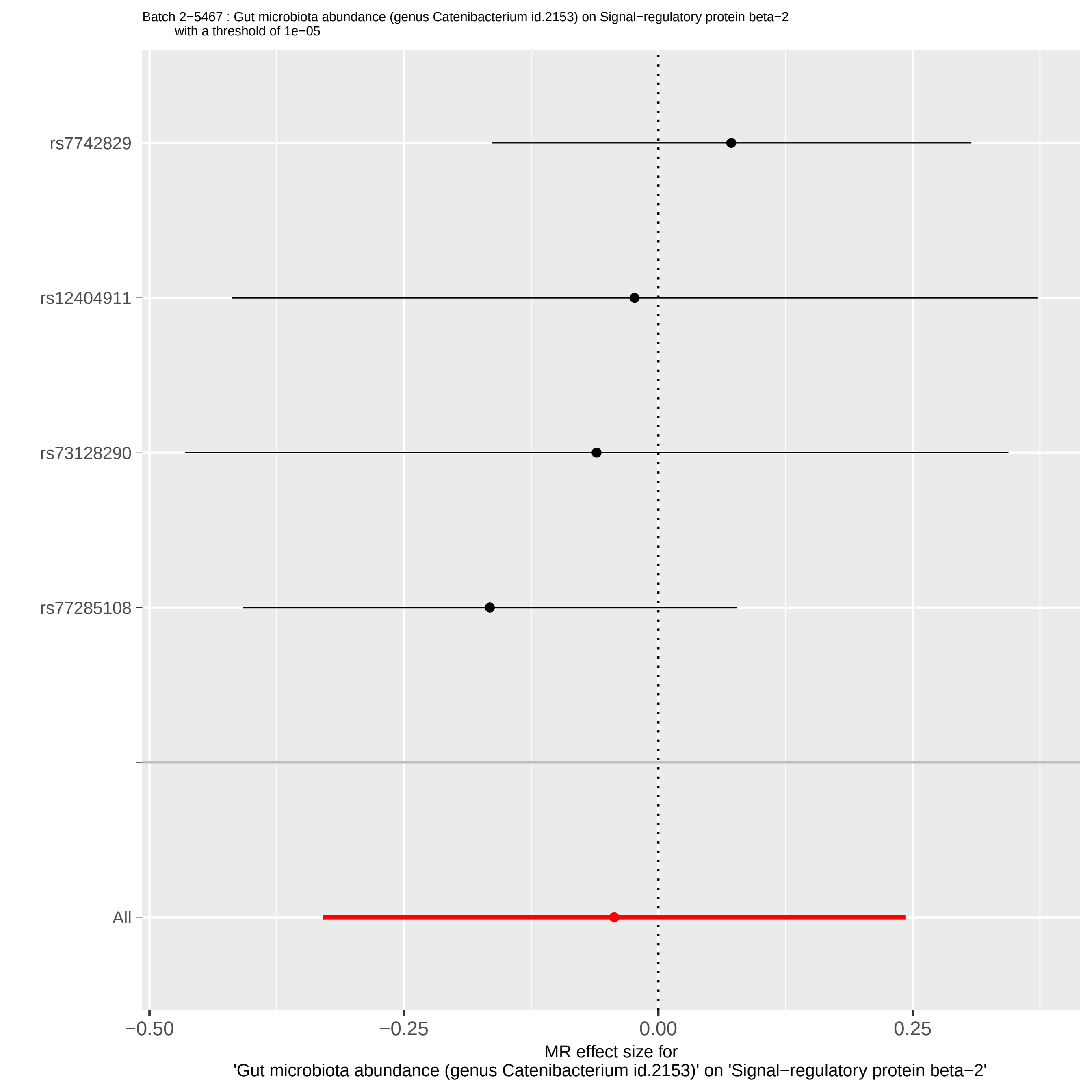

Batch 2−5467 : Gut microbiota abundance (genus Catenibacterium id.2153) on Signal−regulatory protein beta−2
with a threshold of 1e−05
rs7742829
rs12404911
rs73128290
rs77285108
All
−0.50
−0.25
0.00
0.25
MR effect size for
'Gut microbiota abundance (genus Catenibacterium id.2153)' on 'Signal−regulatory protein beta−2'

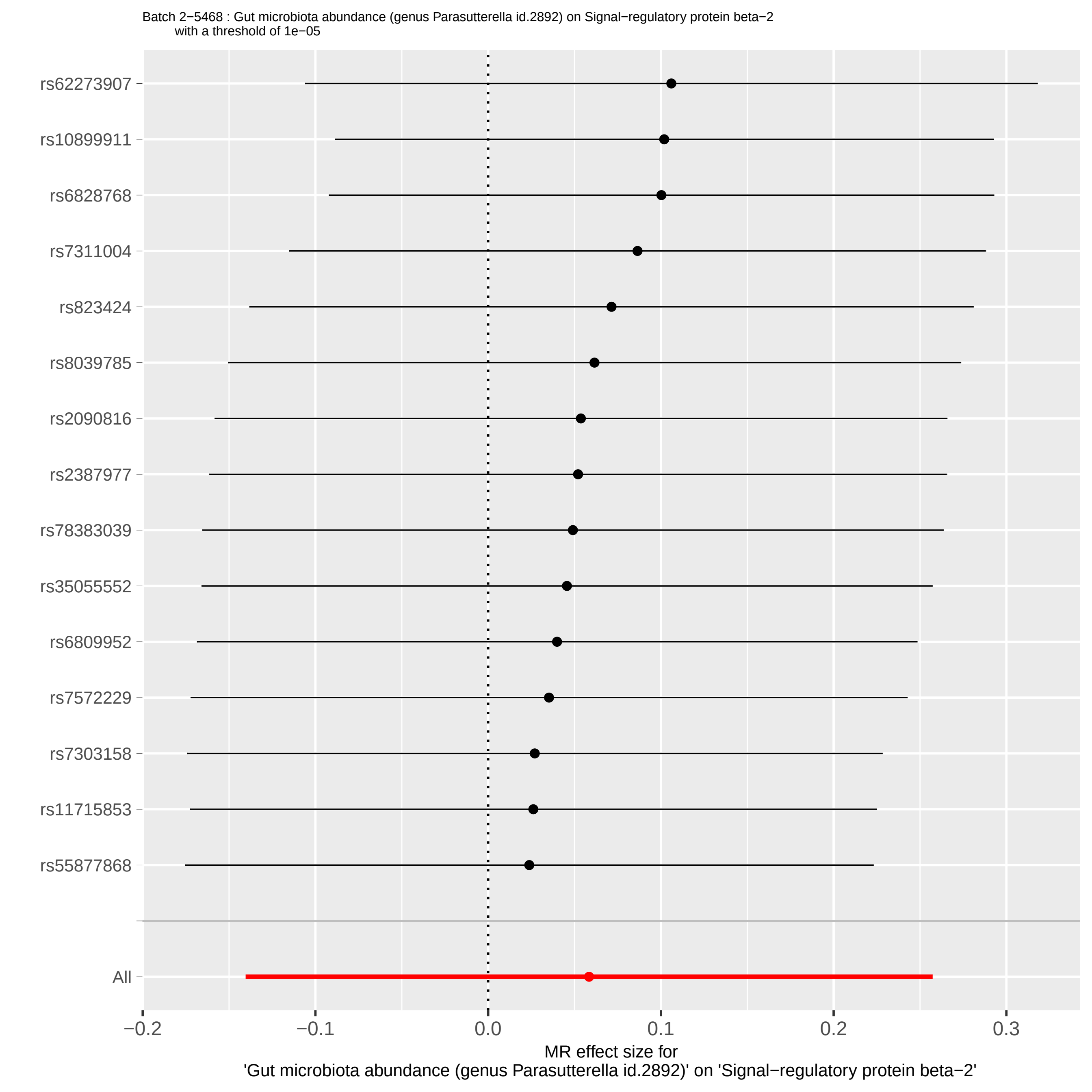
Batch 2−5468 : Gut microbiota abundance (genus Parasutterella id.2892) on Signal−regulatory protein beta−2
with a threshold of 1e−05
rs62273907
rs10899911
rs6828768
rs7311004
rs823424
rs8039785
rs2090816
rs2387977
rs78383039
rs35055552
rs6809952
rs7572229
rs7303158
rs11715853
rs55877868
All
−0.2
−0.1
0.0
0.1
0.2
0.3
MR effect size for
'Gut microbiota abundance (genus Parasutterella id.2892)' on 'Signal−regulatory protein beta−2'

Batch 2−5469 : Gut microbiota abundance (genus Catenibacterium id.2153) on Signal−regulatory protein gamma
with a threshold of 1e−05

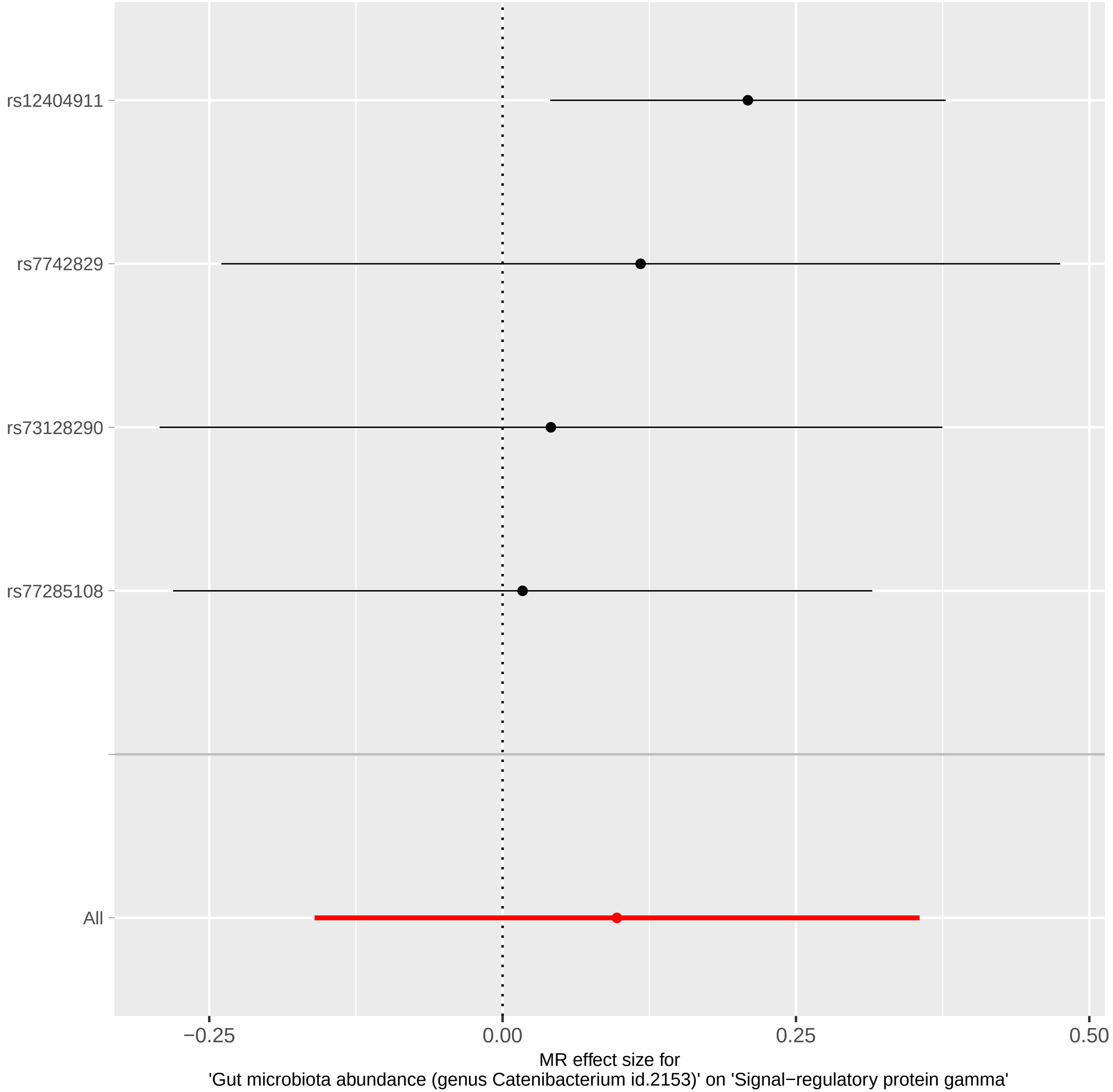

Batch 2−5470 : Gut microbiota abundance (genus Parasutterella id.2892) on Signal−regulatory protein gamma
with a threshold of 1e−05

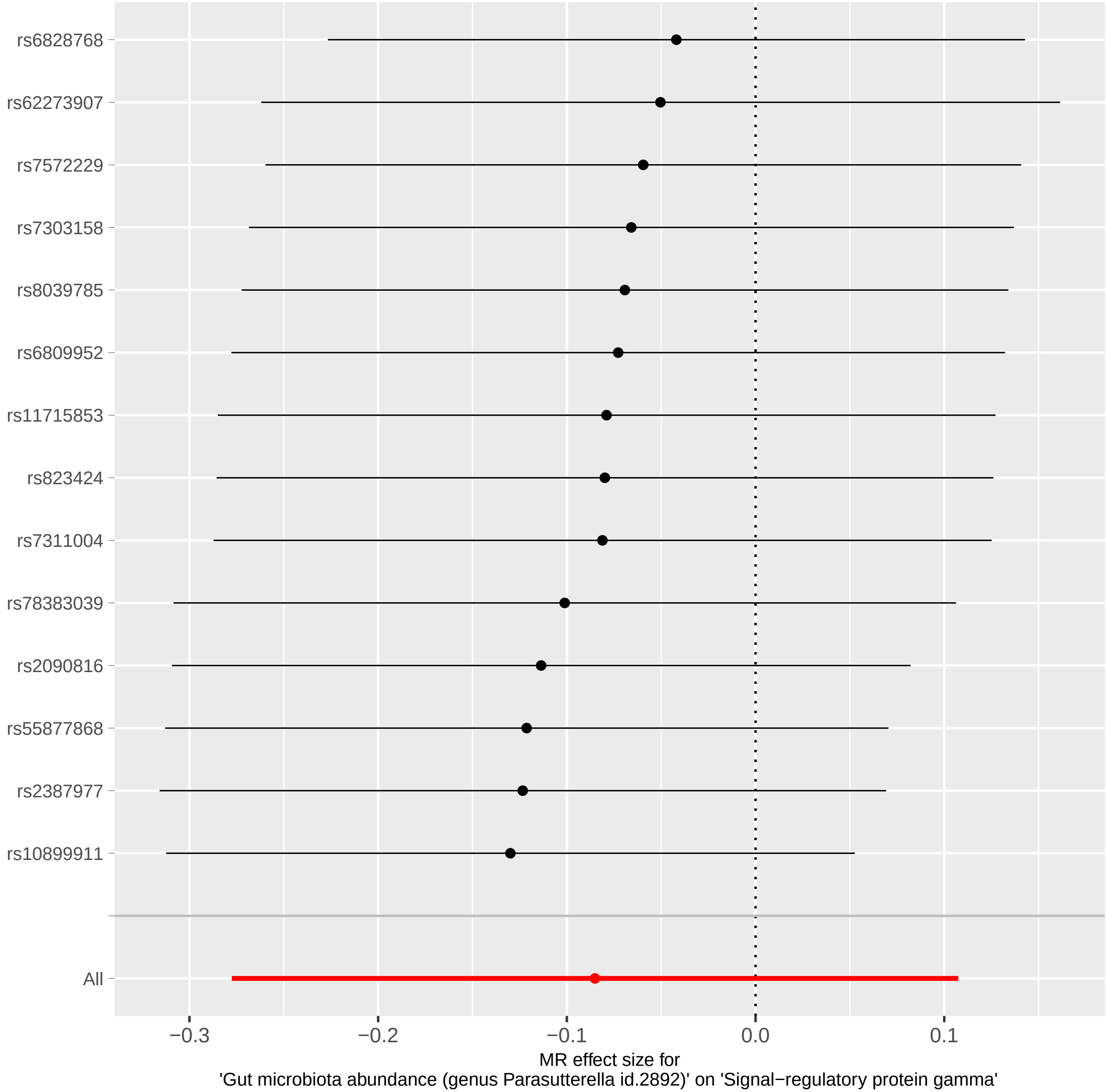

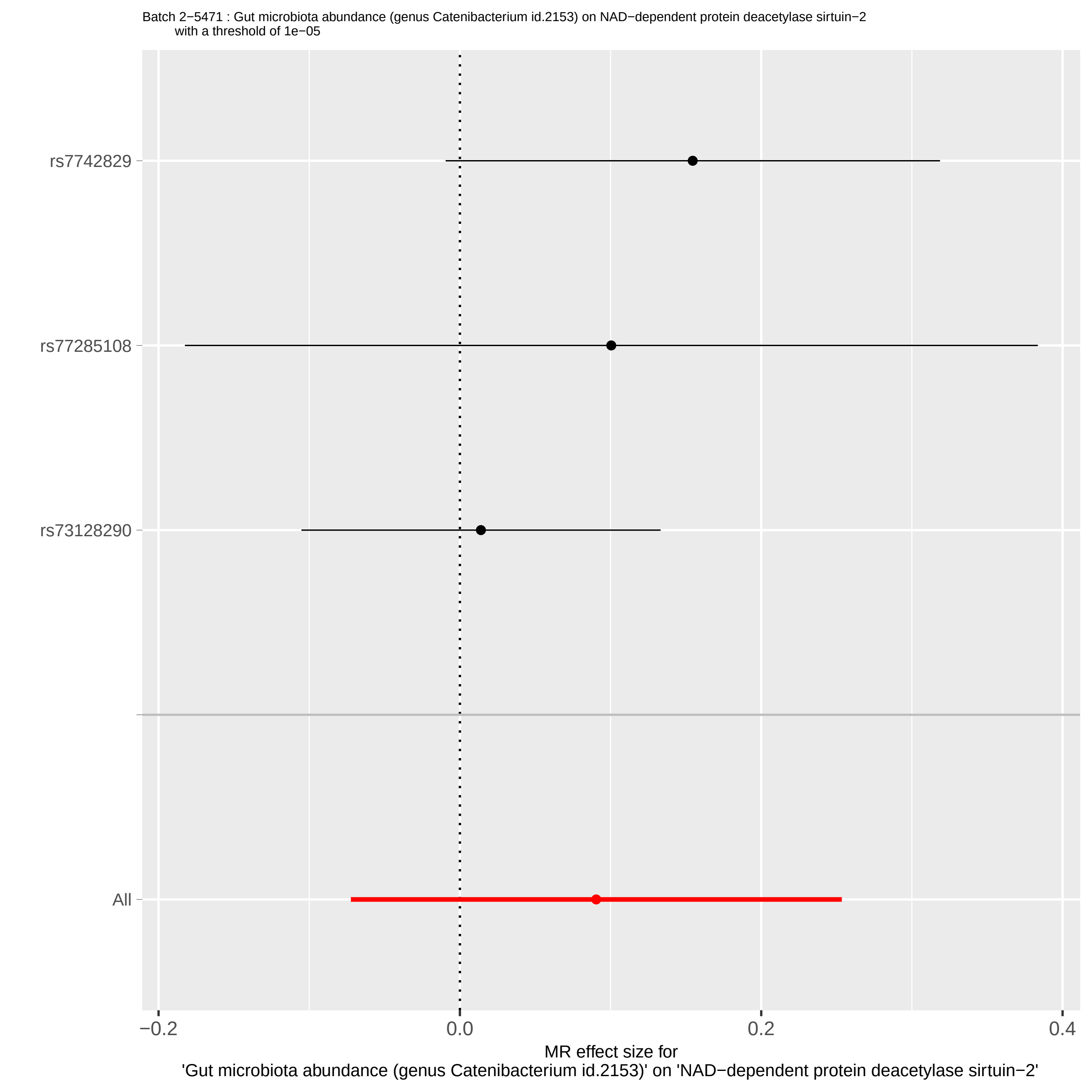

Batch 2−5471 : Gut microbiota abundance (genus Catenibacterium id.2153) on NAD−dependent protein deacetylase sirtuin−2
with a threshold of 1e−05
rs7742829
rs77285108
rs73128290
All
−0.2
0.0
0.2
0.4
MR effect size for
'Gut microbiota abundance (genus Catenibacterium id.2153)' on 'NAD−dependent protein deacetylase sirtuin−2'

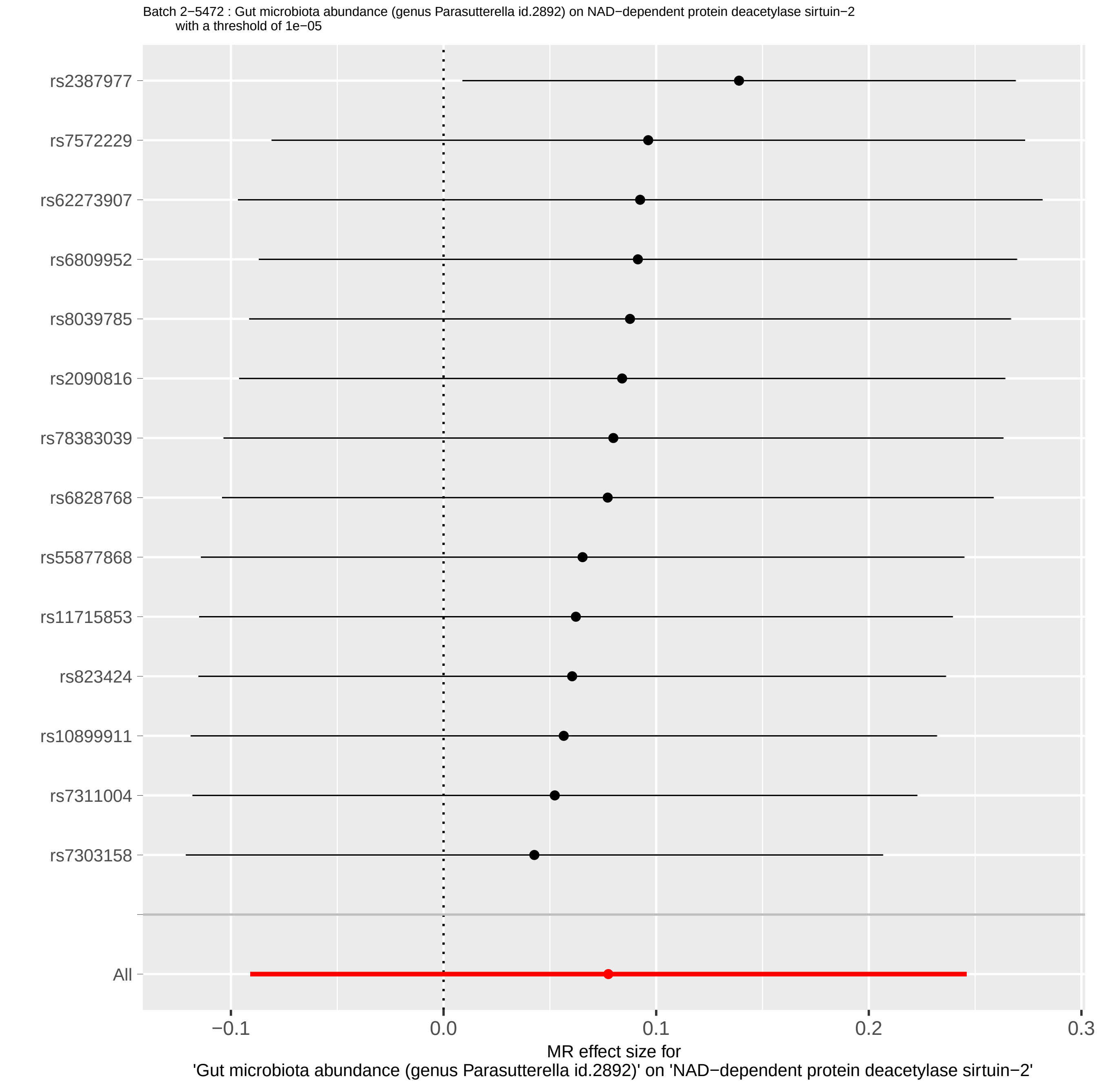

Batch 2−5472 : Gut microbiota abundance (genus Parasutterella id.2892) on NAD−dependent protein deacetylase sirtuin−2
with a threshold of 1e−05
rs2387977
rs7572229
rs62273907
rs6809952
rs8039785
rs2090816
rs78383039
rs6828768
rs55877868
rs11715853
rs823424
rs10899911
rs7311004
rs7303158
All
−0.1
0.0
0.1
0.2
0.3
MR effect size for
'Gut microbiota abundance (genus Parasutterella id.2892)' on 'NAD−dependent protein deacetylase sirtuin−2'

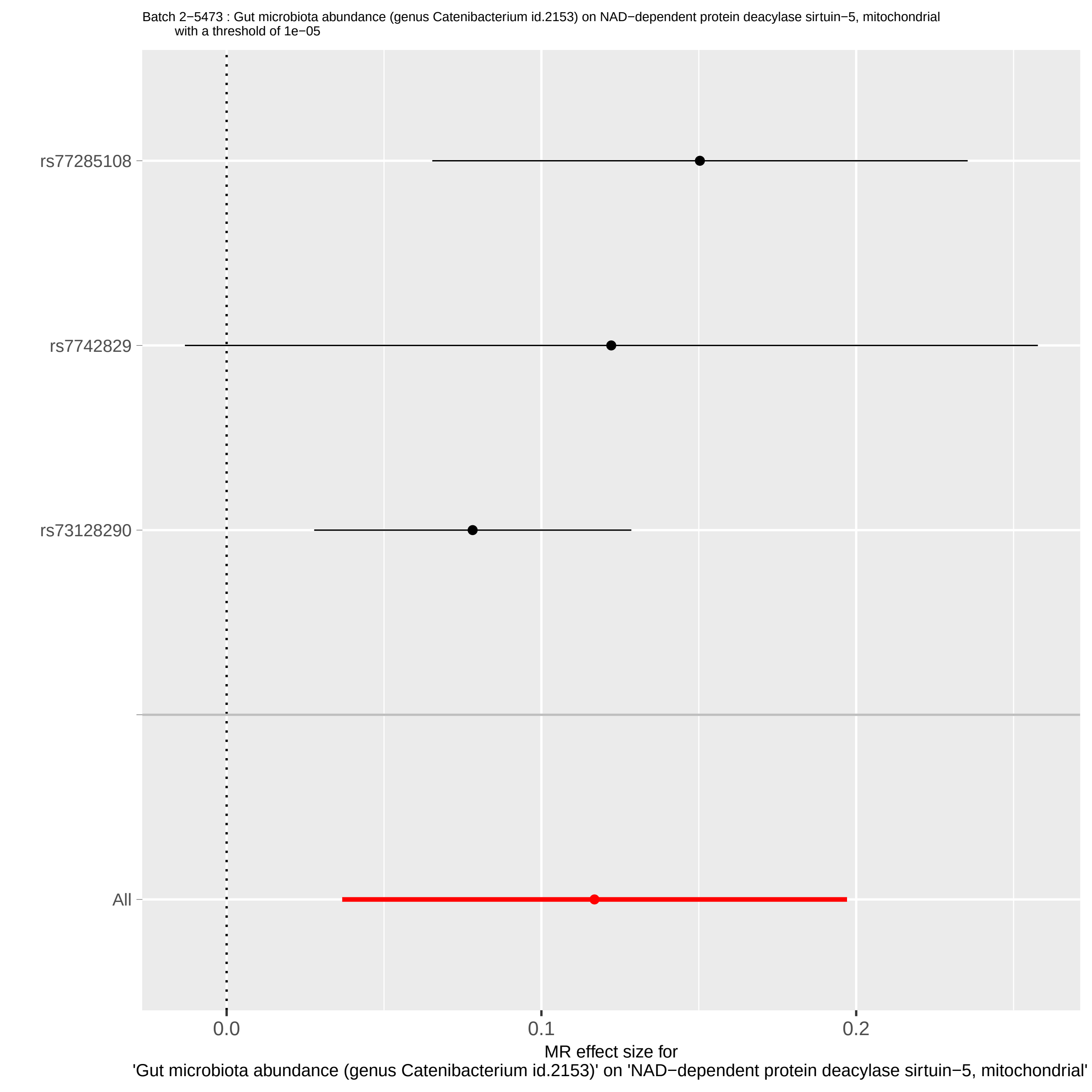
Batch 2−5473 : Gut microbiota abundance (genus Catenibacterium id.2153) on NAD−dependent protein deacylase sirtuin−5, mitochondrial
with a threshold of 1e−05
rs77285108
rs7742829
rs73128290
All
0.0
0.1
0.2
MR effect size for
'Gut microbiota abundance (genus Catenibacterium id.2153)' on 'NAD−dependent protein deacylase sirtuin−5, mitochondrial'

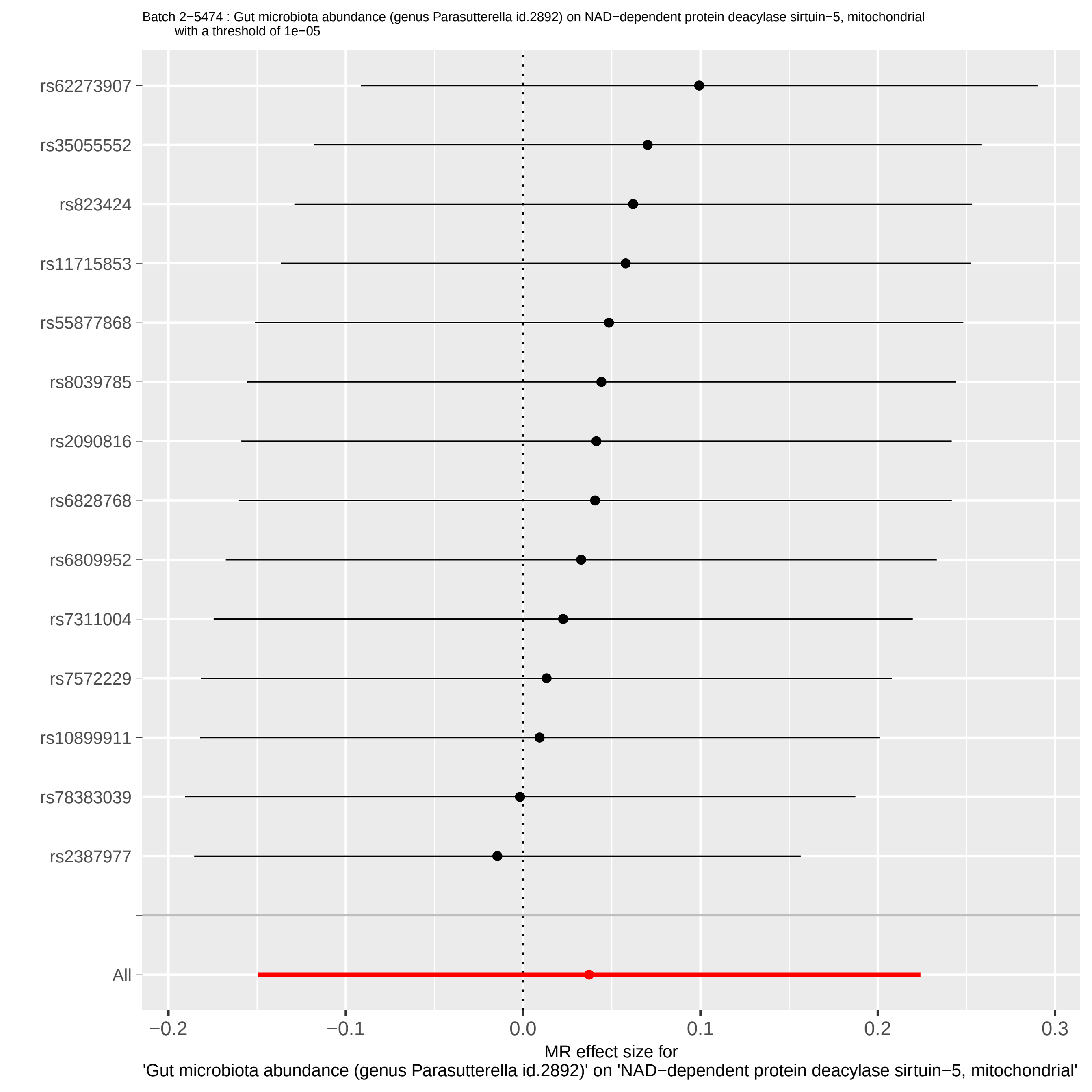

Batch 2−5474 : Gut microbiota abundance (genus Parasutterella id.2892) on NAD−dependent protein deacylase sirtuin−5, mitochondrial
with a threshold of 1e−05
rs62273907
rs35055552
rs823424
rs11715853
rs55877868
rs8039785
rs2090816
rs6828768
rs6809952
rs7311004
rs7572229
rs10899911
rs78383039
rs2387977
All
−0.2
−0.1
0.0
0.1
0.2
0.3
MR effect size for
'Gut microbiota abundance (genus Parasutterella id.2892)' on 'NAD−dependent protein deacylase sirtuin−5, mitochondrial'

Batch 2−5475 : Gut microbiota abundance (genus Catenibacterium id.2153) on Signaling threshold−regulating transmembrane adapter 1 with a threshold of 1e−05

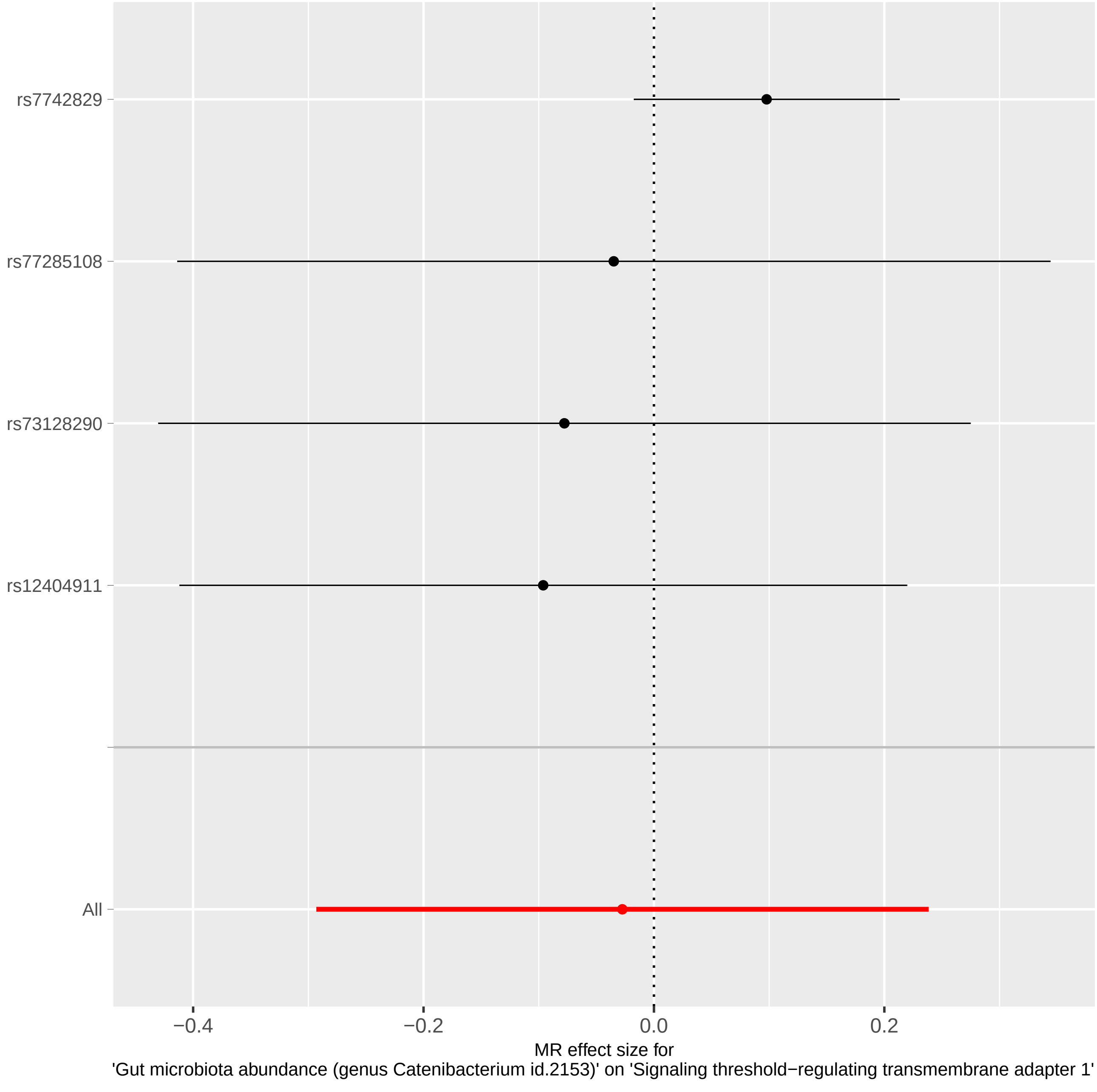

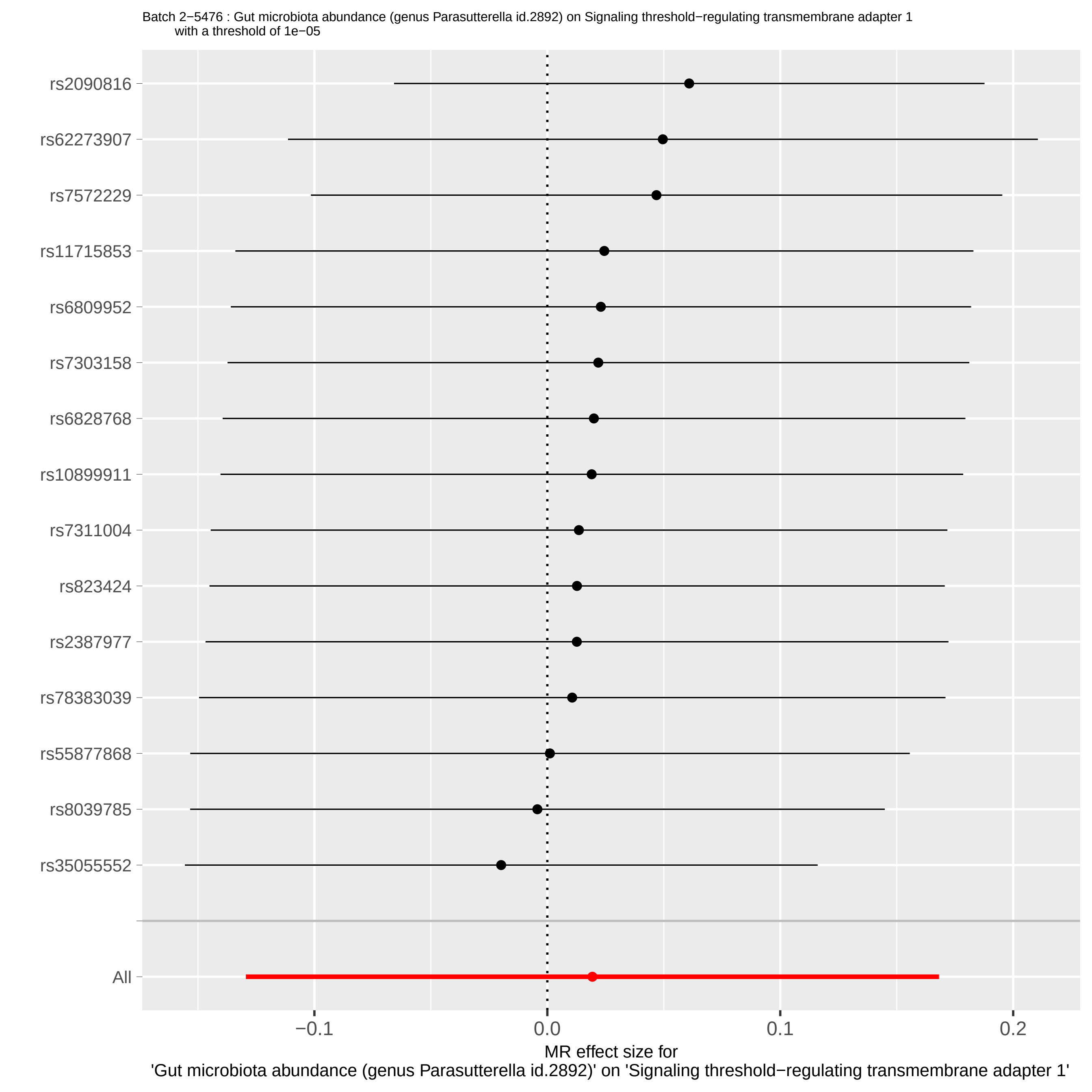

Batch 2−5476 : Gut microbiota abundance (genus Parasutterella id.2892) on Signaling threshold−regulating transmembrane adapter 1
with a threshold of 1e−05
rs2090816
rs62273907
rs7572229
rs11715853
rs6809952
rs7303158
rs6828768
rs10899911
rs7311004
rs823424
rs2387977
rs78383039
rs55877868
rs8039785
rs35055552
All
−0.1
0.0
0.1
0.2
MR effect size for
'Gut microbiota abundance (genus Parasutterella id.2892)' on 'Signaling threshold−regulating transmembrane adapter 1'

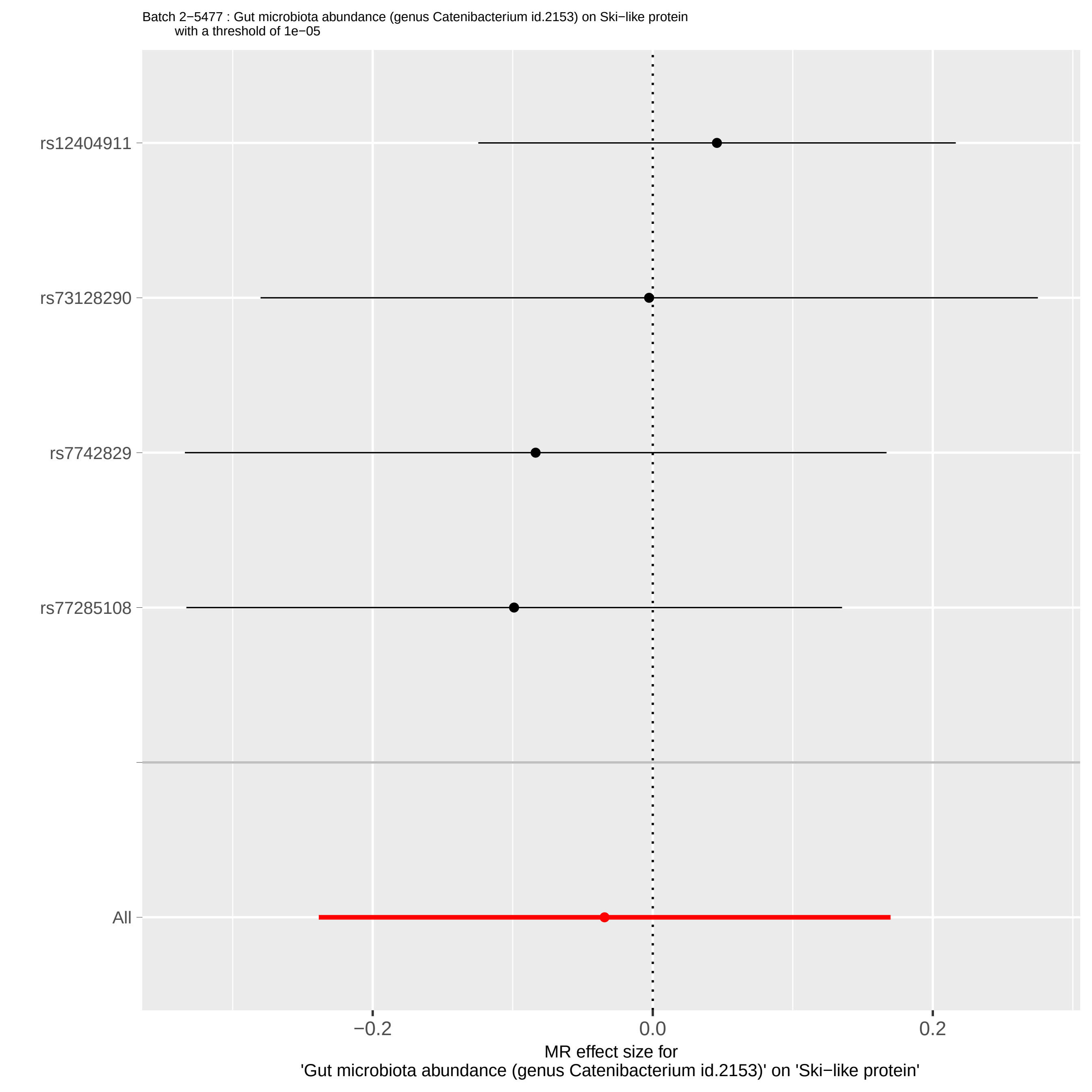
Batch 2−5477 : Gut microbiota abundance (genus Catenibacterium id.2153) on Ski−like protein
with a threshold of 1e−05
rs12404911
rs73128290
rs7742829
rs77285108
All
−0.2
0.0
0.2
MR effect size for
'Gut microbiota abundance (genus Catenibacterium id.2153)' on 'Ski−like protein'

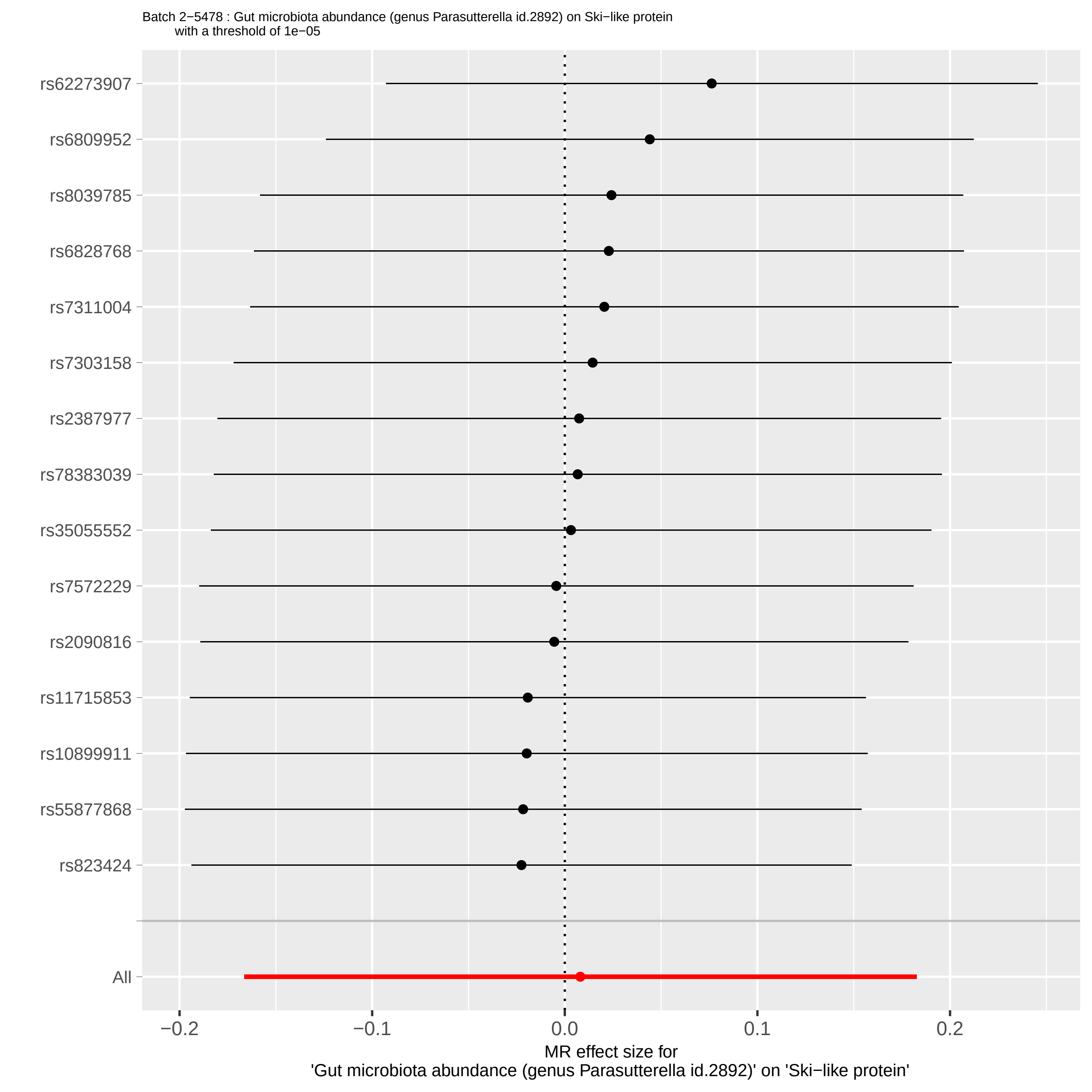

Batch 2−5478 : Gut microbiota abundance (genus Parasutterella id.2892) on Ski−like protein
with a threshold of 1e−05
rs62273907
rs6809952
rs8039785
rs6828768
rs7311004
rs7303158
rs2387977
rs78383039
rs35055552
rs7572229
rs2090816
rs11715853
rs10899911
rs55877868
rs823424
All
−0.2
−0.1
0.0
0.1
0.2
MR effect size for
'Gut microbiota abundance (genus Parasutterella id.2892)' on 'Ski−like protein'

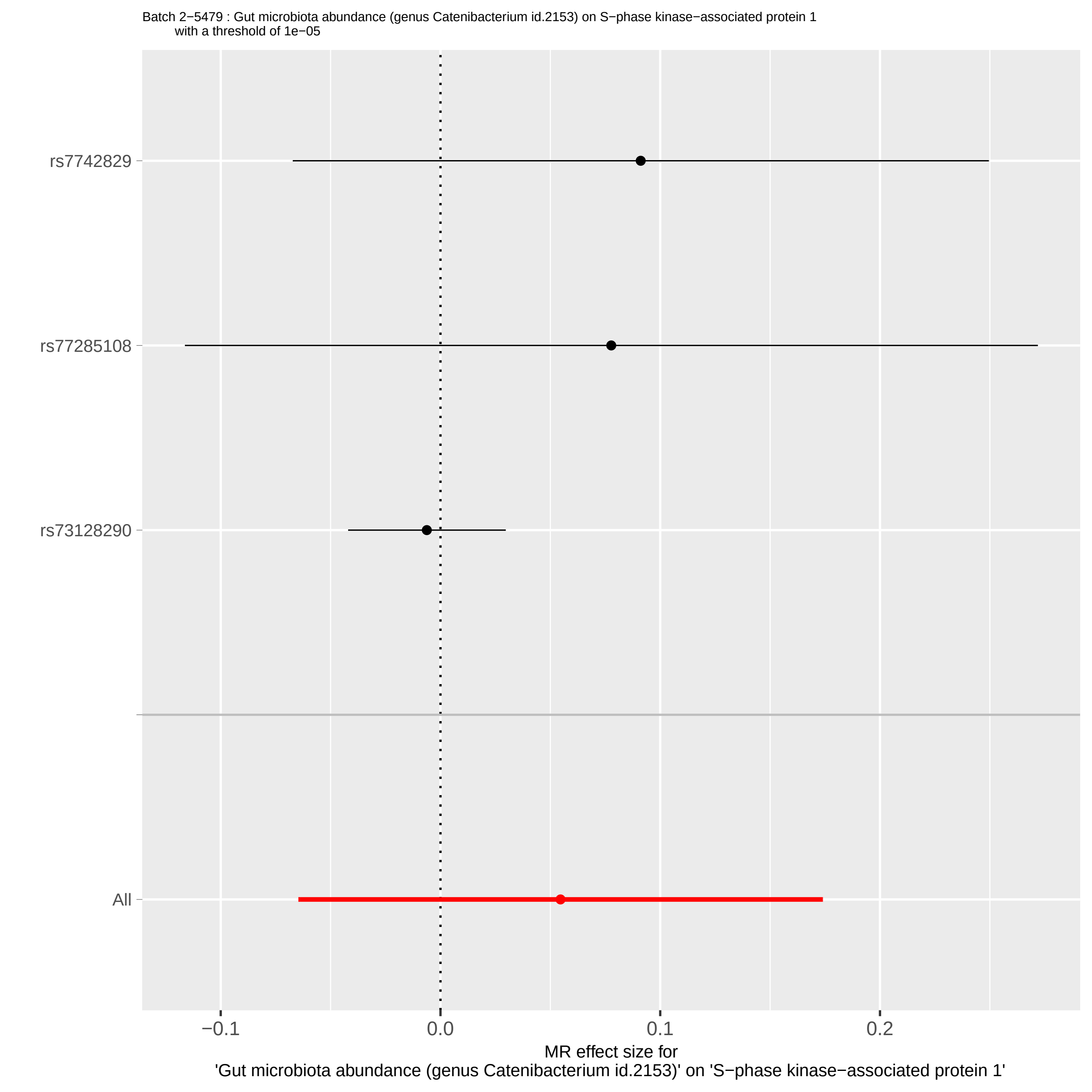
Batch 2−5479 : Gut microbiota abundance (genus Catenibacterium id.2153) on S−phase kinase−associated protein 1
with a threshold of 1e−05
rs7742829
rs77285108
rs73128290
All
−0.1
0.0
0.1
0.2
MR effect size for
'Gut microbiota abundance (genus Catenibacterium id.2153)' on 'S−phase kinase−associated protein 1'

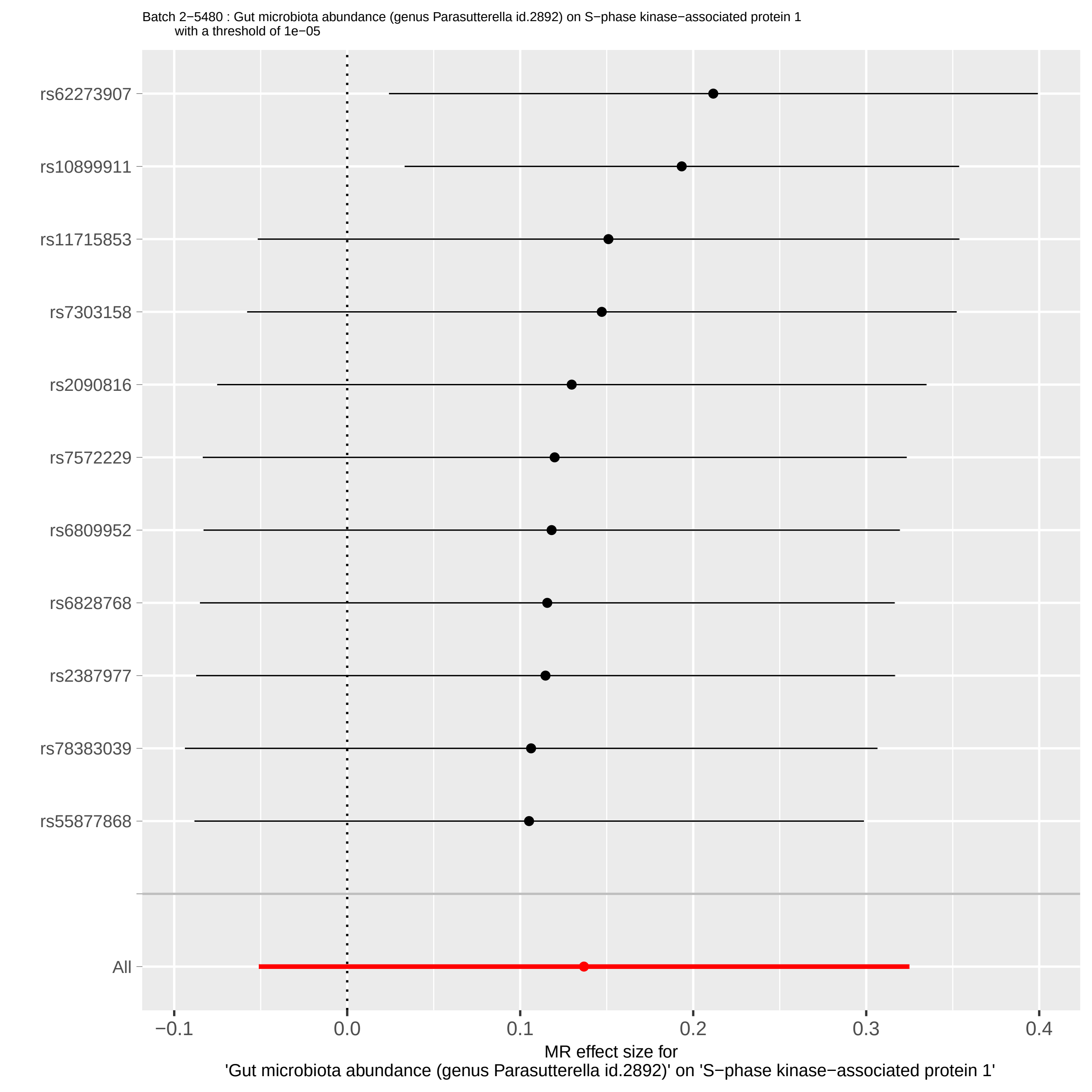

Batch 2−5480 : Gut microbiota abundance (genus Parasutterella id.2892) on S−phase kinase−associated protein 1
with a threshold of 1e−05
rs62273907
rs10899911
rs11715853
rs7303158
rs2090816
rs7572229
rs6809952
rs6828768
rs2387977
rs78383039
rs55877868
All
−0.1
0.0
0.1
0.2
0.3
0.4
MR effect size for
'Gut microbiota abundance (genus Parasutterella id.2892)' on 'S−phase kinase−associated protein 1'

Batch 2−5481 : Gut microbiota abundance (genus Catenibacterium id.2153) on Signaling lymphocytic activation molecule
with a threshold of 1e−05

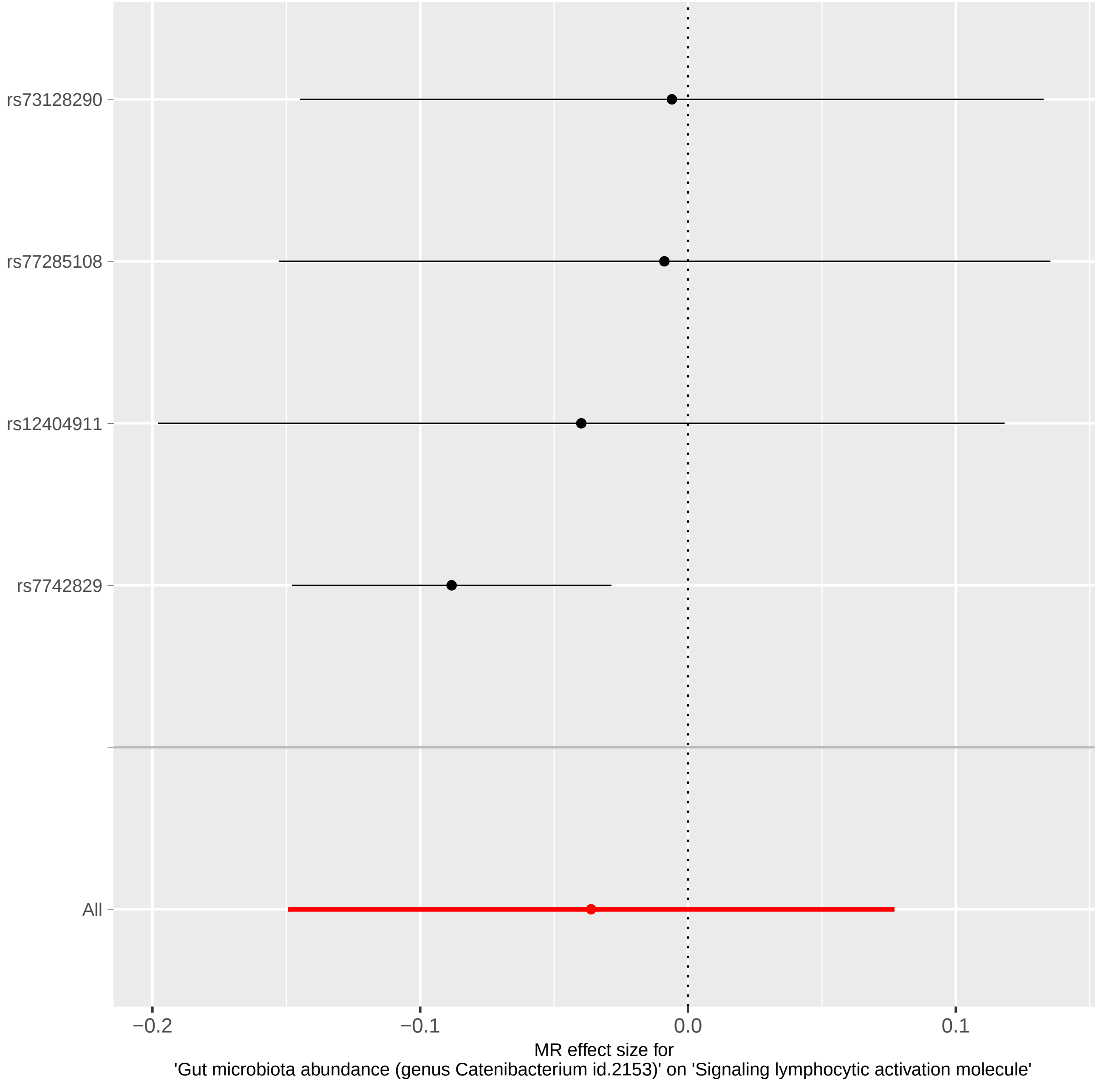

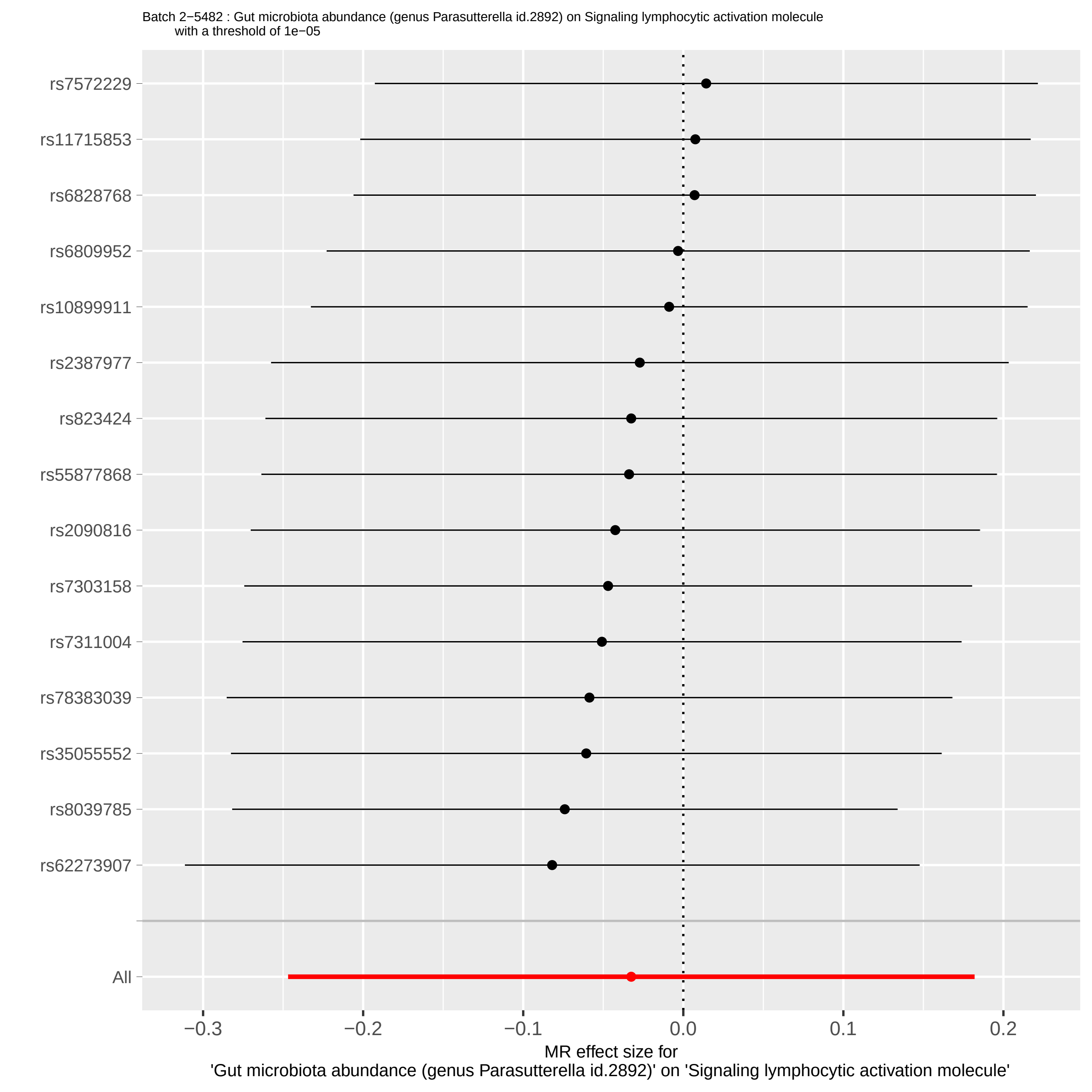

Batch 2−5482 : Gut microbiota abundance (genus Parasutterella id.2892) on Signaling lymphocytic activation molecule
with a threshold of 1e−05
rs7572229
rs11715853
rs6828768
rs6809952
rs10899911
rs2387977
rs823424
rs55877868
rs2090816
rs7303158
rs7311004
rs78383039
rs35055552
rs8039785
rs62273907
All
−0.3
−0.2
−0.1
0.0
0.1
0.2
MR effect size for
'Gut microbiota abundance (genus Parasutterella id.2892)' on 'Signaling lymphocytic activation molecule'

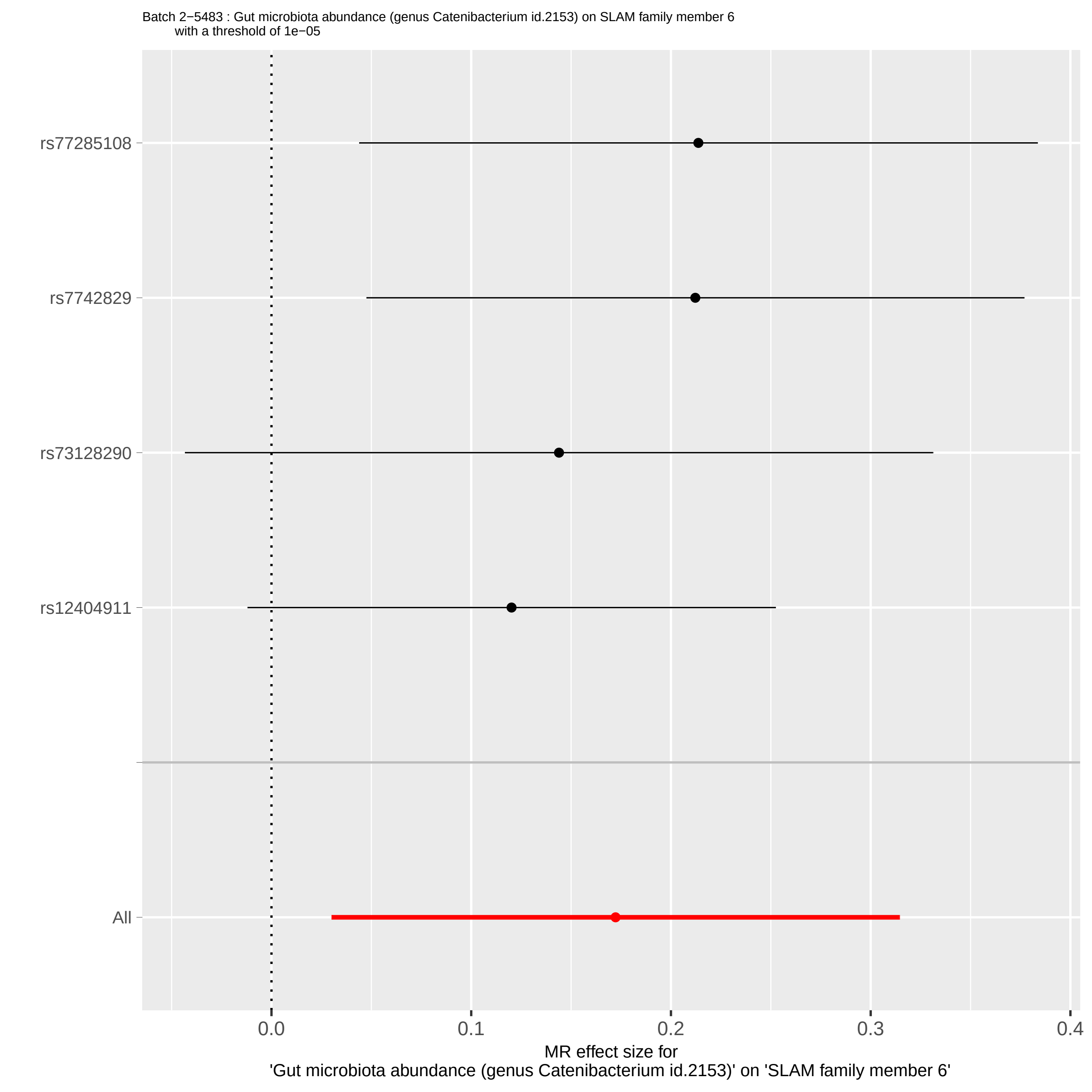

Batch 2−5483 : Gut microbiota abundance (genus Catenibacterium id.2153) on SLAM family member 6
with a threshold of 1e−05
rs77285108
rs7742829
rs73128290
rs12404911
All
0.0
0.1
0.2
0.3
0.4
MR effect size for
'Gut microbiota abundance (genus Catenibacterium id.2153)' on 'SLAM family member 6'

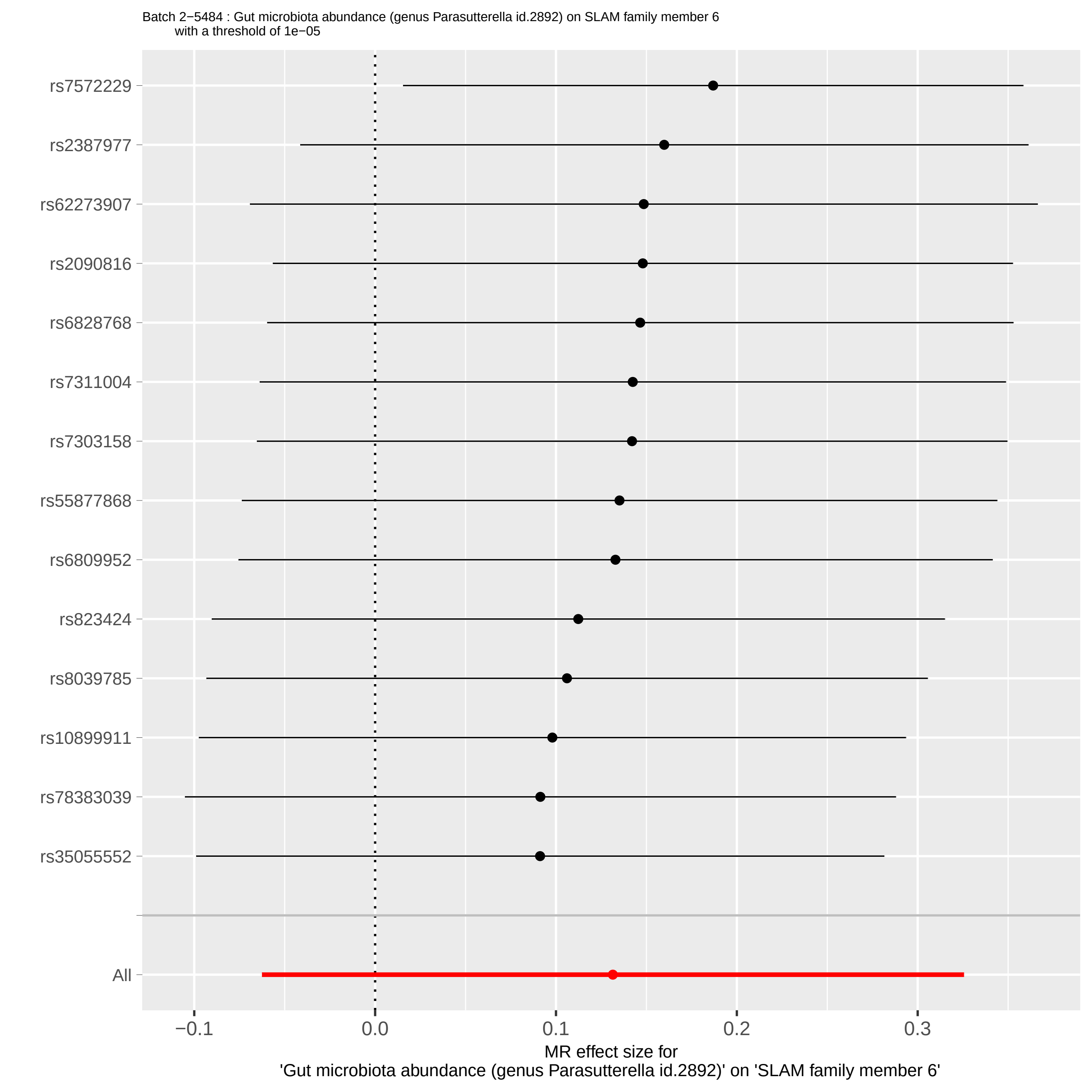

Batch 2−5484 : Gut microbiota abundance (genus Parasutterella id.2892) on SLAM family member 6
with a threshold of 1e−05
rs7572229
rs2387977
rs62273907
rs2090816
rs6828768
rs7311004
rs7303158
rs55877868
rs6809952
rs823424
rs8039785
rs10899911
rs78383039
rs35055552
All
−0.1
0.0
0.1
0.2
0.3
MR effect size for
'Gut microbiota abundance (genus Parasutterella id.2892)' on 'SLAM family member 6'

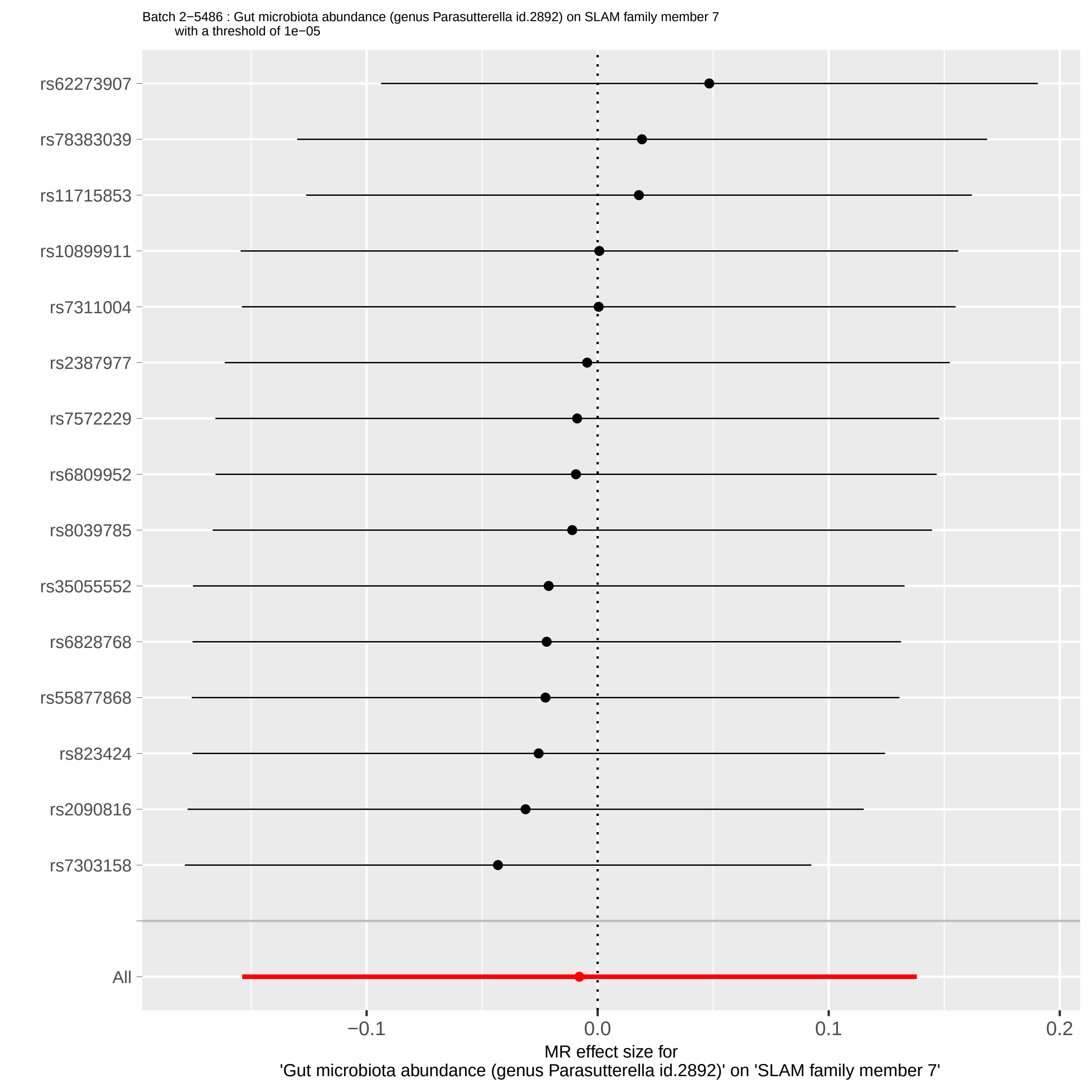
Batch 2−5486 : Gut microbiota abundance (genus Parasutterella id.2892) on SLAM family member 7
with a threshold of 1e−05
rs62273907
rs78383039
rs11715853
rs10899911
rs7311004
rs2387977
rs7572229
rs6809952
rs8039785
rs35055552
rs6828768
rs55877868
rs823424
rs2090816
rs7303158
All
−0.1
0.0
0.1
0.2
MR effect size for
'Gut microbiota abundance (genus Parasutterella id.2892)' on 'SLAM family member 7'

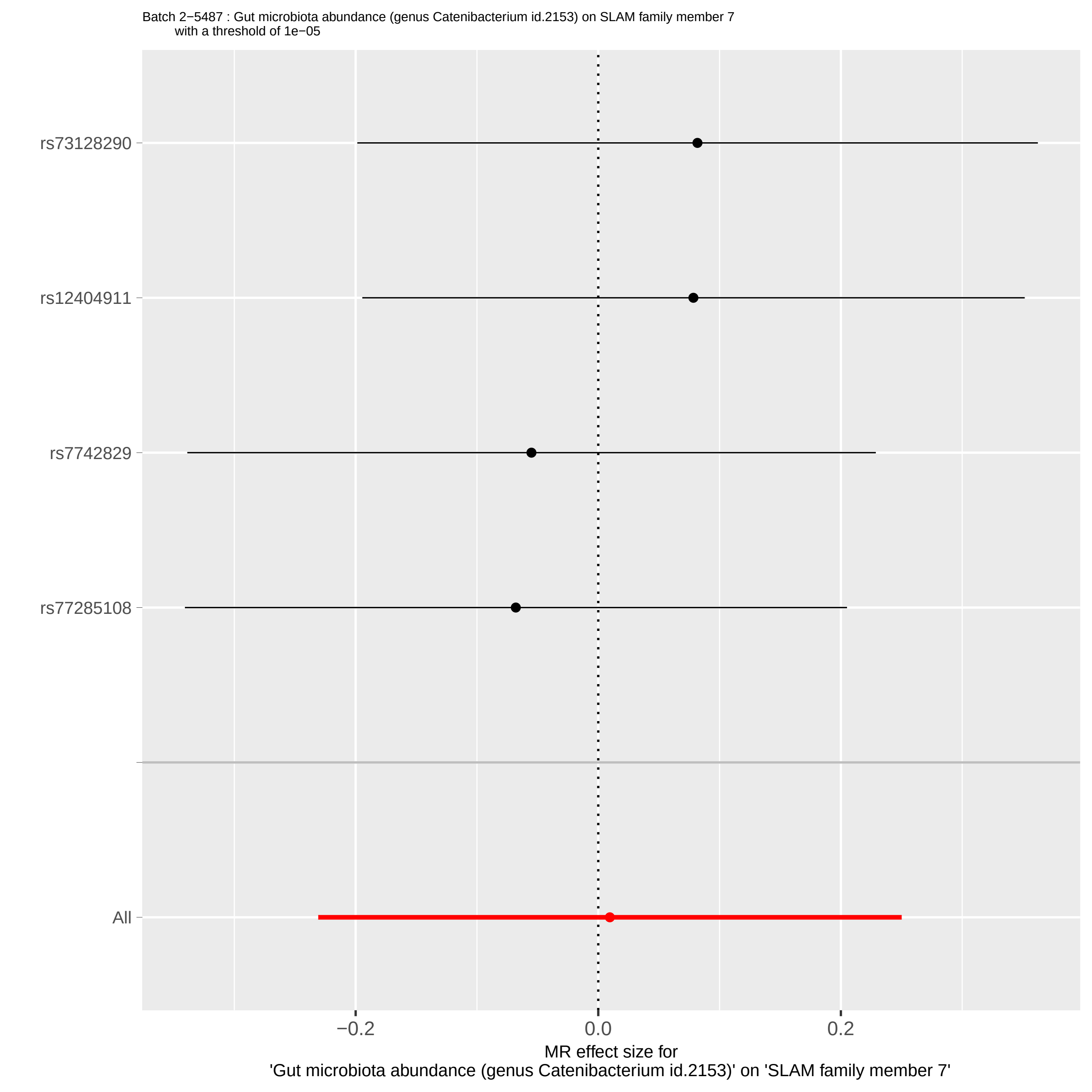

Batch 2−5487 : Gut microbiota abundance (genus Catenibacterium id.2153) on SLAM family member 7
with a threshold of 1e−05
rs73128290
rs12404911
rs7742829
rs77285108
All
−0.2
0.0
0.2
MR effect size for
'Gut microbiota abundance (genus Catenibacterium id.2153)' on 'SLAM family member 7'

Batch 2−5488 : Gut microbiota abundance (genus Parasutterella id.2892) on SLAM family member 7
with a threshold of 1e−05

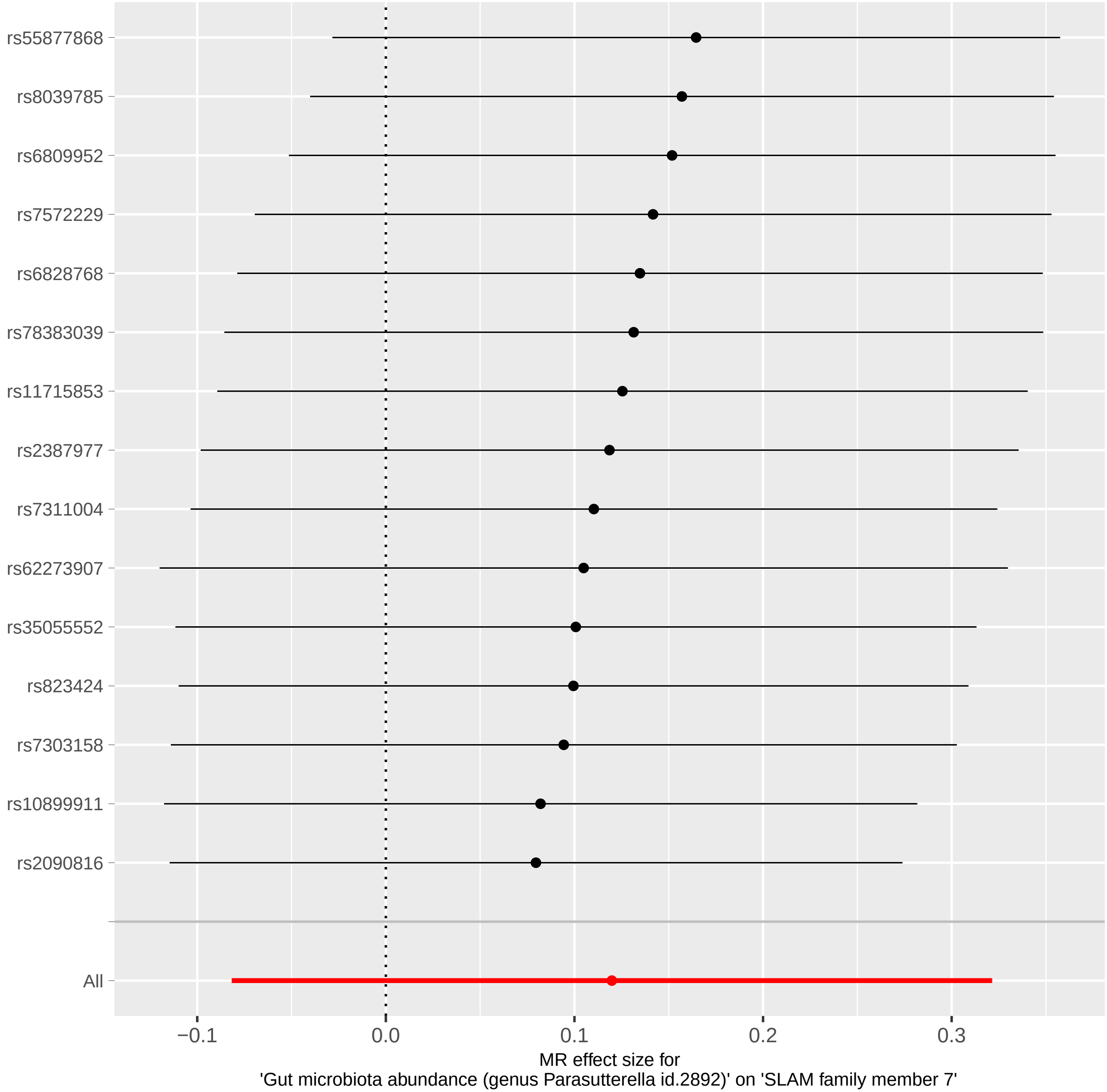

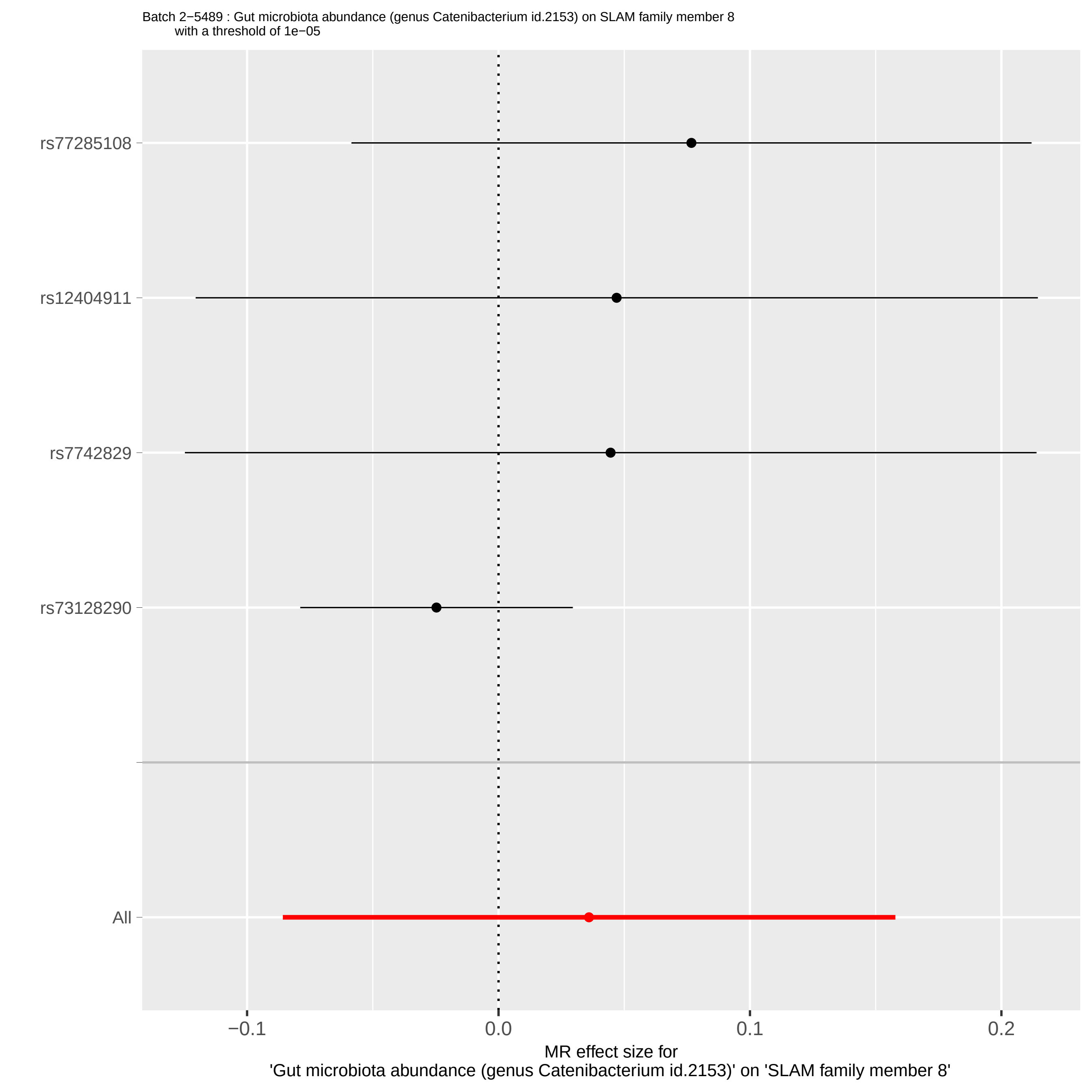

Batch 2−5489 : Gut microbiota abundance (genus Catenibacterium id.2153) on SLAM family member 8
with a threshold of 1e−05
rs77285108
rs12404911
rs7742829
rs73128290
All
−0.1
0.0
0.1
0.2
MR effect size for
'Gut microbiota abundance (genus Catenibacterium id.2153)' on 'SLAM family member 8'

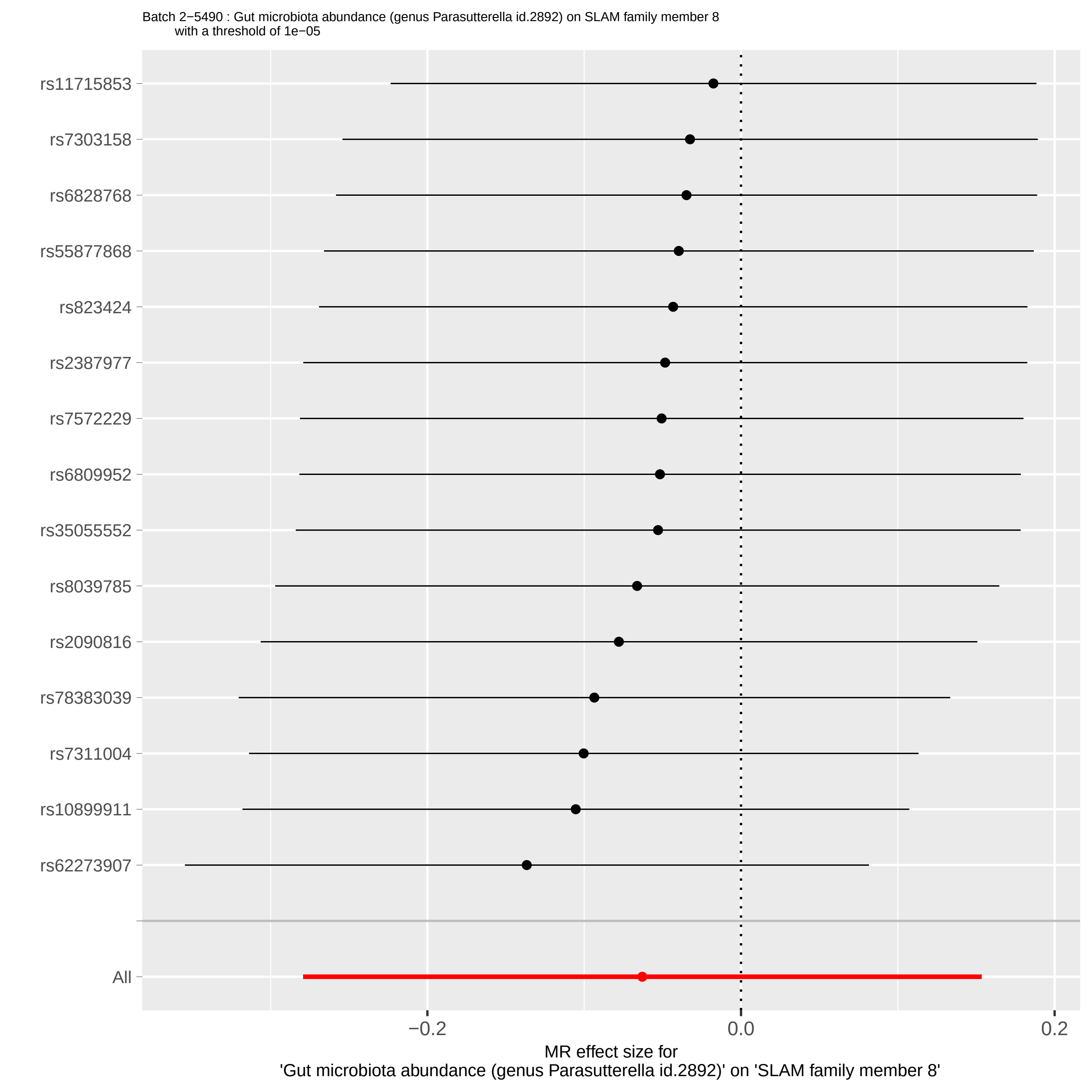

Batch 2−5490 : Gut microbiota abundance (genus Parasutterella id.2892) on SLAM family member 8
with a threshold of 1e−05
rs11715853
rs7303158
rs6828768
rs55877868
rs823424
rs2387977
rs7572229
rs6809952
rs35055552
rs8039785
rs2090816
rs78383039
rs7311004
rs10899911
rs62273907
All
−0.2
0.0
0.2
MR effect size for
'Gut microbiota abundance (genus Parasutterella id.2892)' on 'SLAM family member 8'

Batch 2−5491 : Gut microbiota abundance (genus Catenibacterium id.2153) on Urea transporter 1
with a threshold of 1e−05

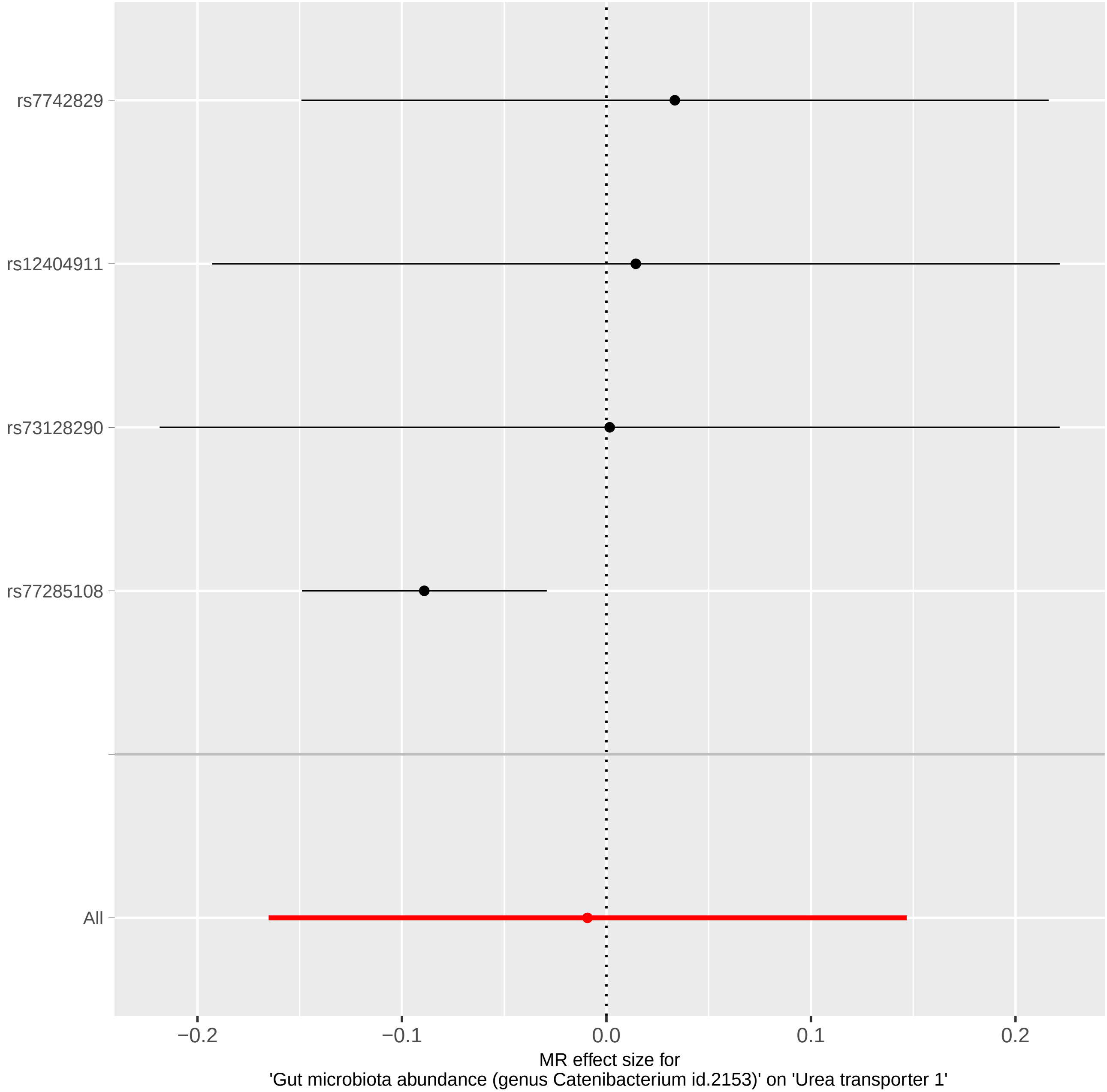

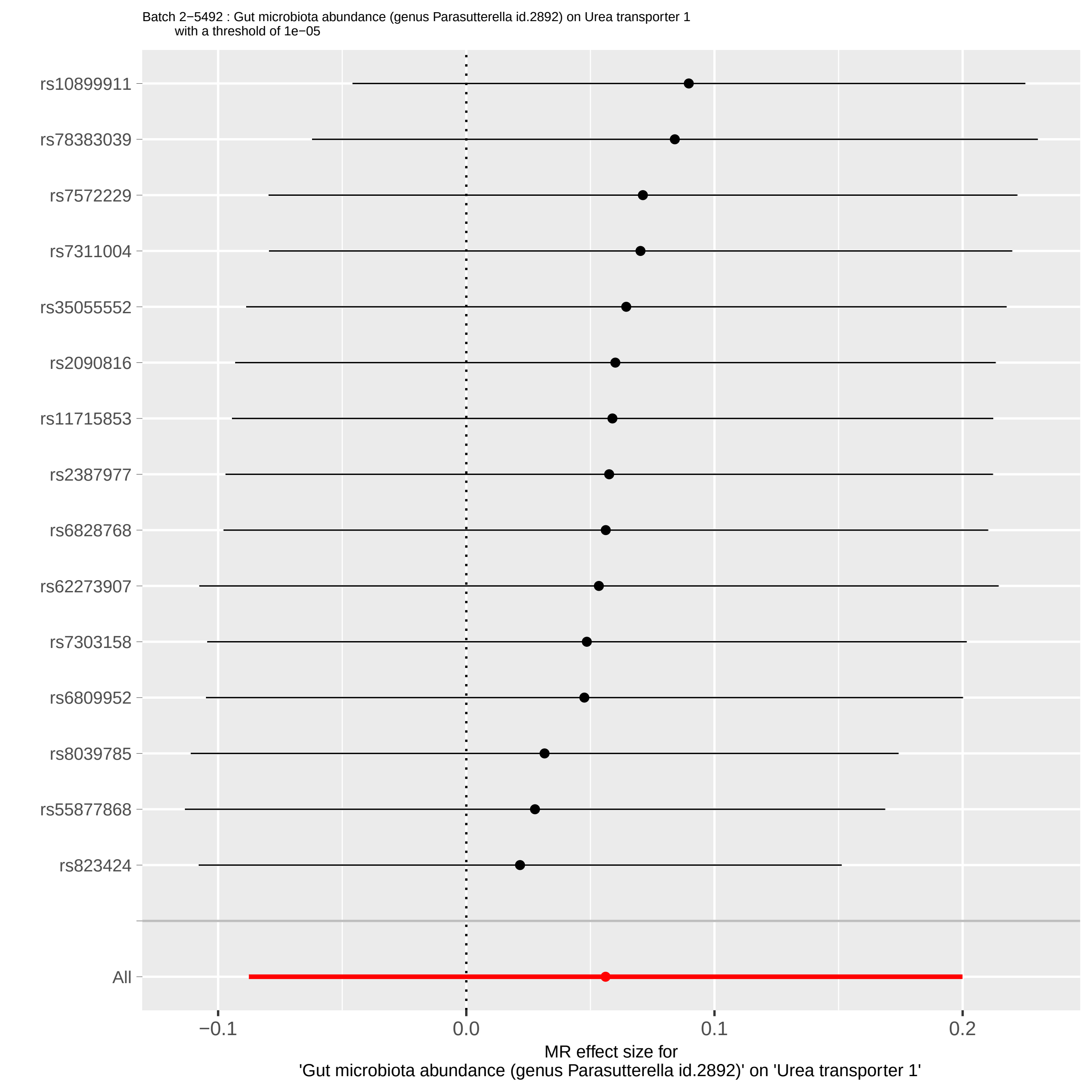
Batch 2−5492 : Gut microbiota abundance (genus Parasutterella id.2892) on Urea transporter 1
with a threshold of 1e−05
rs10899911
rs78383039
rs7572229
rs7311004
rs35055552
rs2090816
rs11715853
rs2387977
rs6828768
rs62273907
rs7303158
rs6809952
rs8039785
rs55877868
rs823424
All
−0.1
0.0
0.1
0.2
MR effect size for
'Gut microbiota abundance (genus Parasutterella id.2892)' on 'Urea transporter 1'

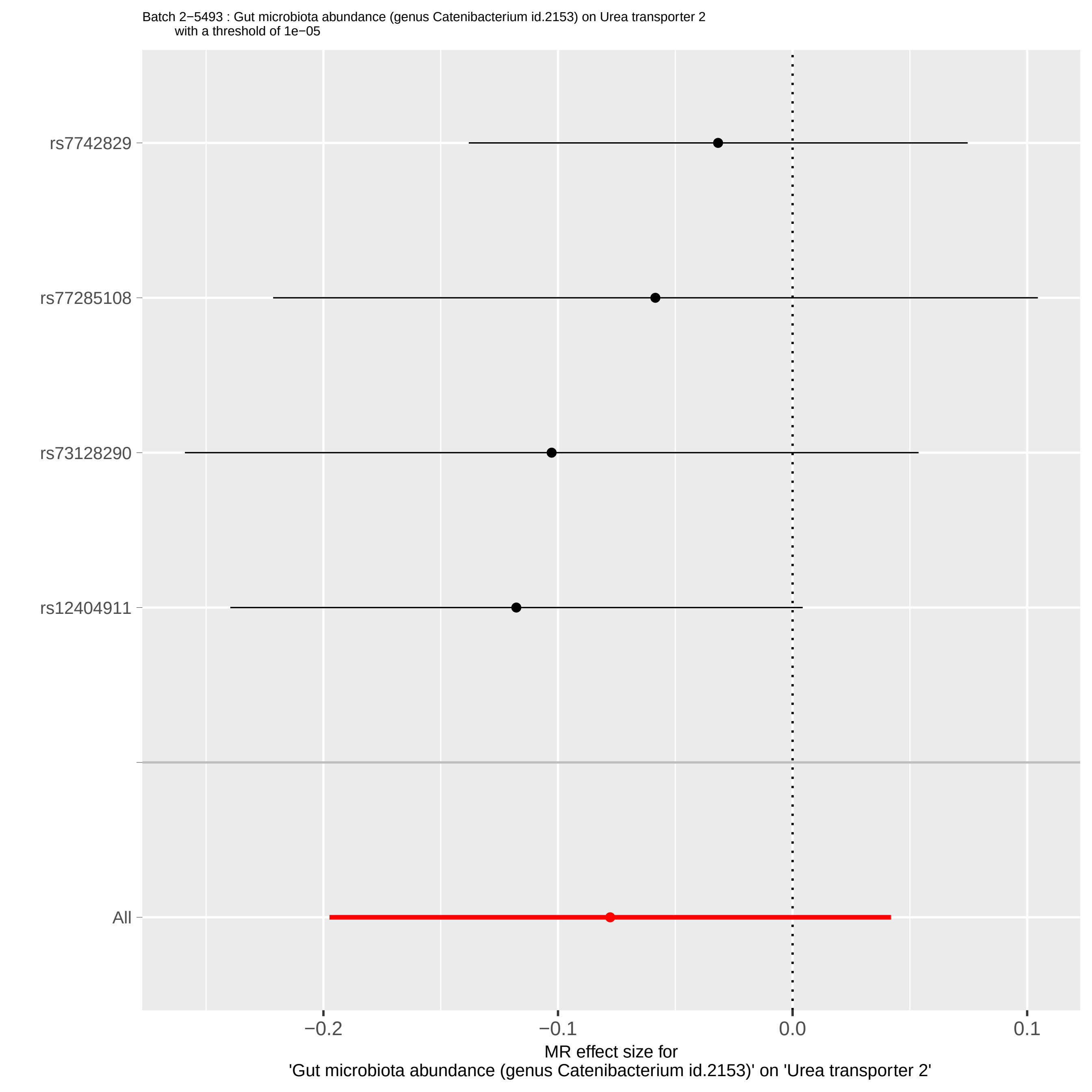
Batch 2−5493 : Gut microbiota abundance (genus Catenibacterium id.2153) on Urea transporter 2
with a threshold of 1e−05
rs7742829
rs77285108
rs73128290
rs12404911
All
−0.2
−0.1
0.0
0.1
MR effect size for
'Gut microbiota abundance (genus Catenibacterium id.2153)' on 'Urea transporter 2'

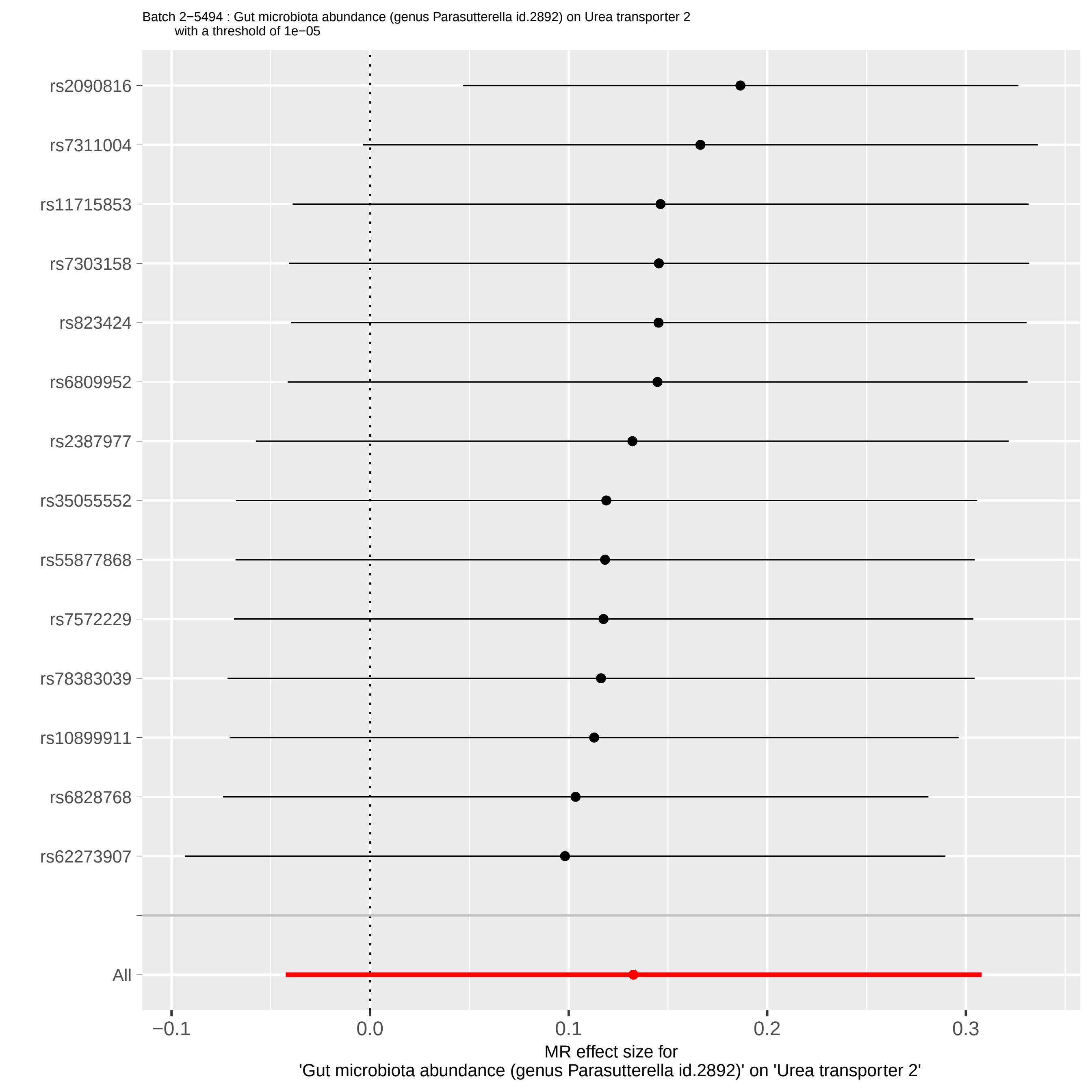
Batch 2−5494 : Gut microbiota abundance (genus Parasutterella id.2892) on Urea transporter 2
with a threshold of 1e−05
rs2090816
rs7311004
rs11715853
rs7303158
rs823424
rs6809952
rs2387977
rs35055552
rs55877868
rs7572229
rs78383039
rs10899911
rs6828768
rs62273907
All
−0.1
0.0
0.1
0.2
0.3
MR effect size for
'Gut microbiota abundance (genus Parasutterella id.2892)' on 'Urea transporter 2'

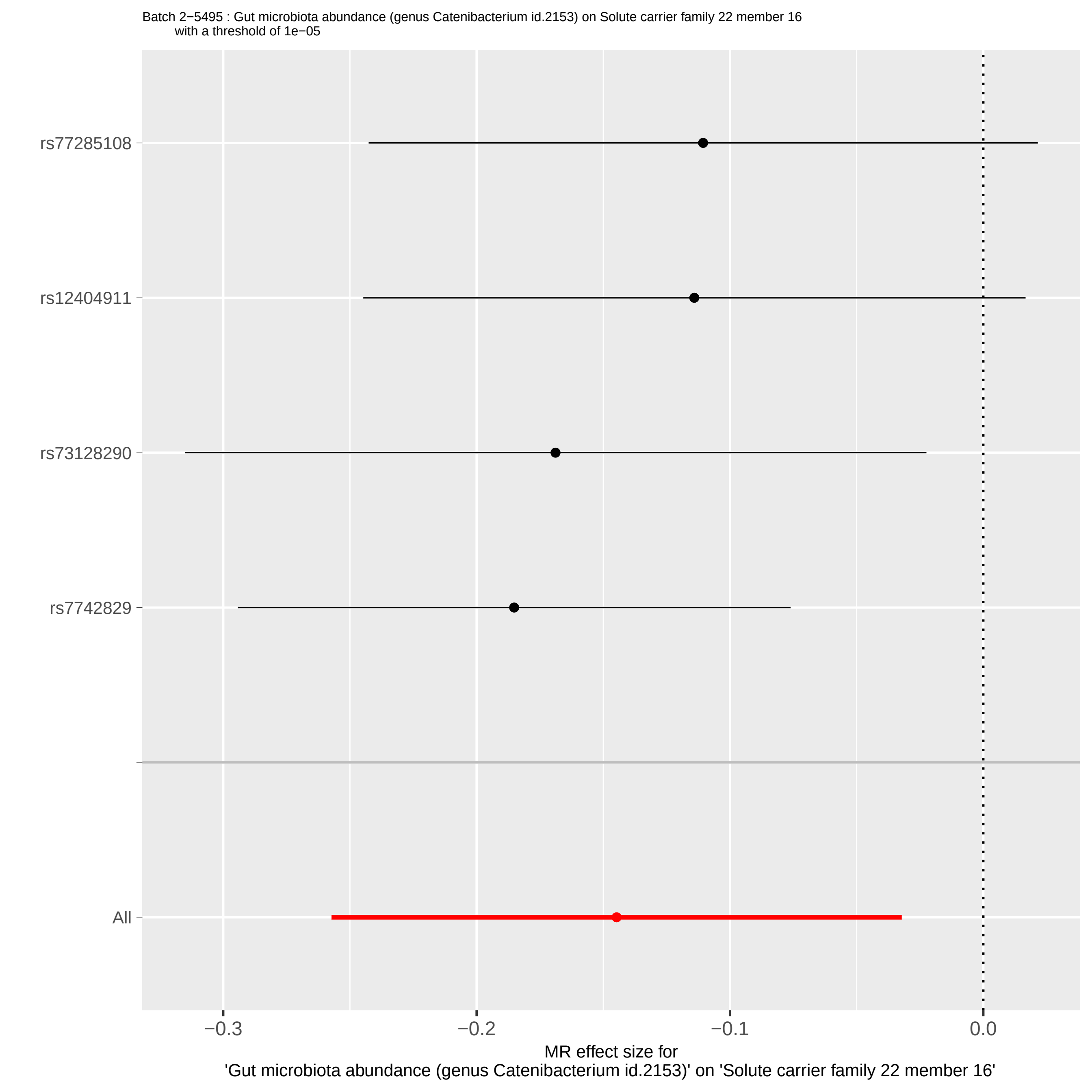
Batch 2−5495 : Gut microbiota abundance (genus Catenibacterium id.2153) on Solute carrier family 22 member 16
with a threshold of 1e−05
rs77285108
rs12404911
rs73128290
rs7742829
All
−0.3
−0.2
−0.1
0.0
MR effect size for
'Gut microbiota abundance (genus Catenibacterium id.2153)' on 'Solute carrier family 22 member 16'

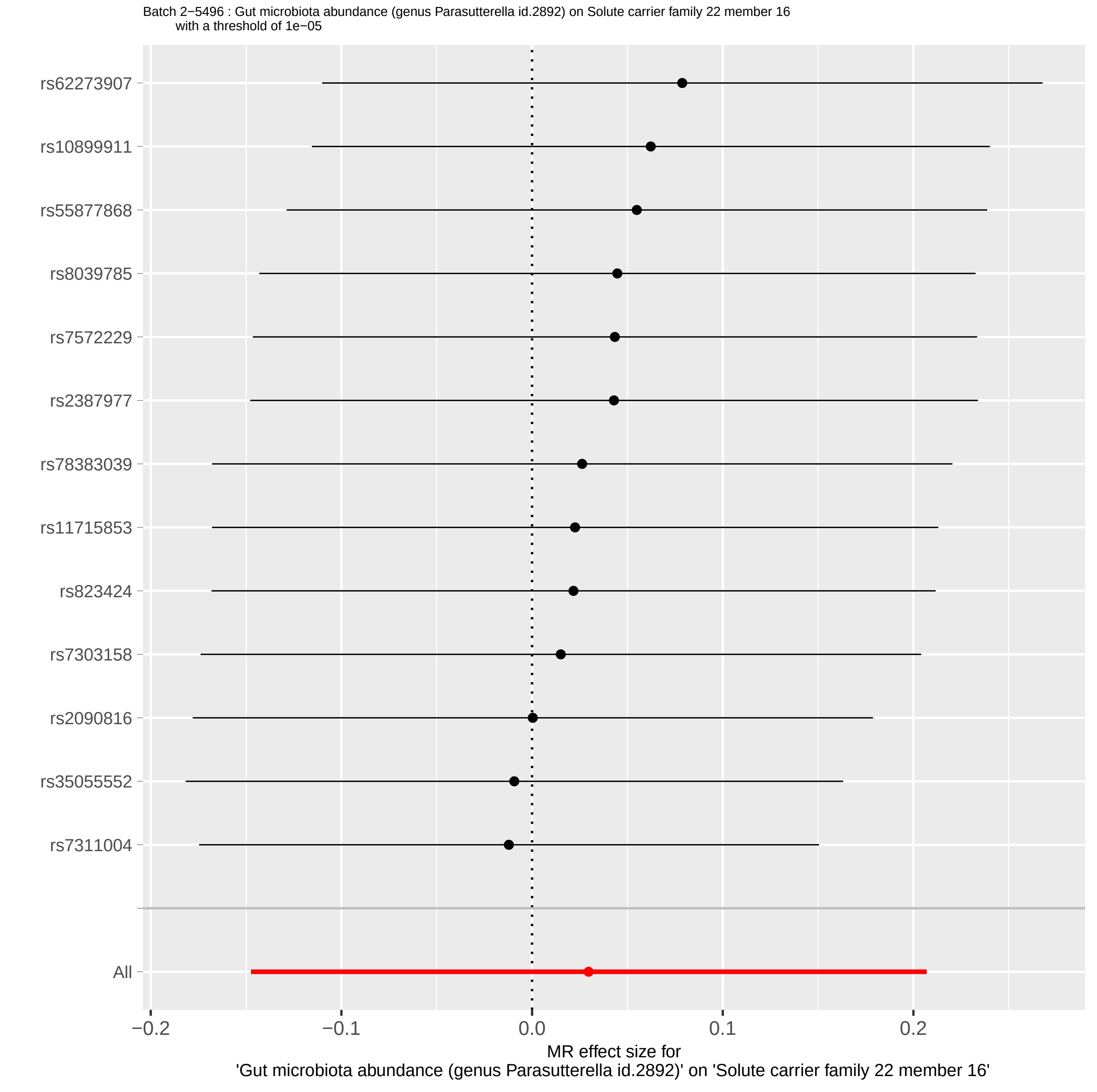
Batch 2−5496 : Gut microbiota abundance (genus Parasutterella id.2892) on Solute carrier family 22 member 16
with a threshold of 1e−05
rs62273907
rs10899911
rs55877868
rs8039785
rs7572229
rs2387977
rs78383039
rs11715853
rs823424
rs7303158
rs2090816
rs35055552
rs7311004
All
−0.2
−0.1
0.0
0.1
0.2
MR effect size for
'Gut microbiota abundance (genus Parasutterella id.2892)' on 'Solute carrier family 22 member 16'

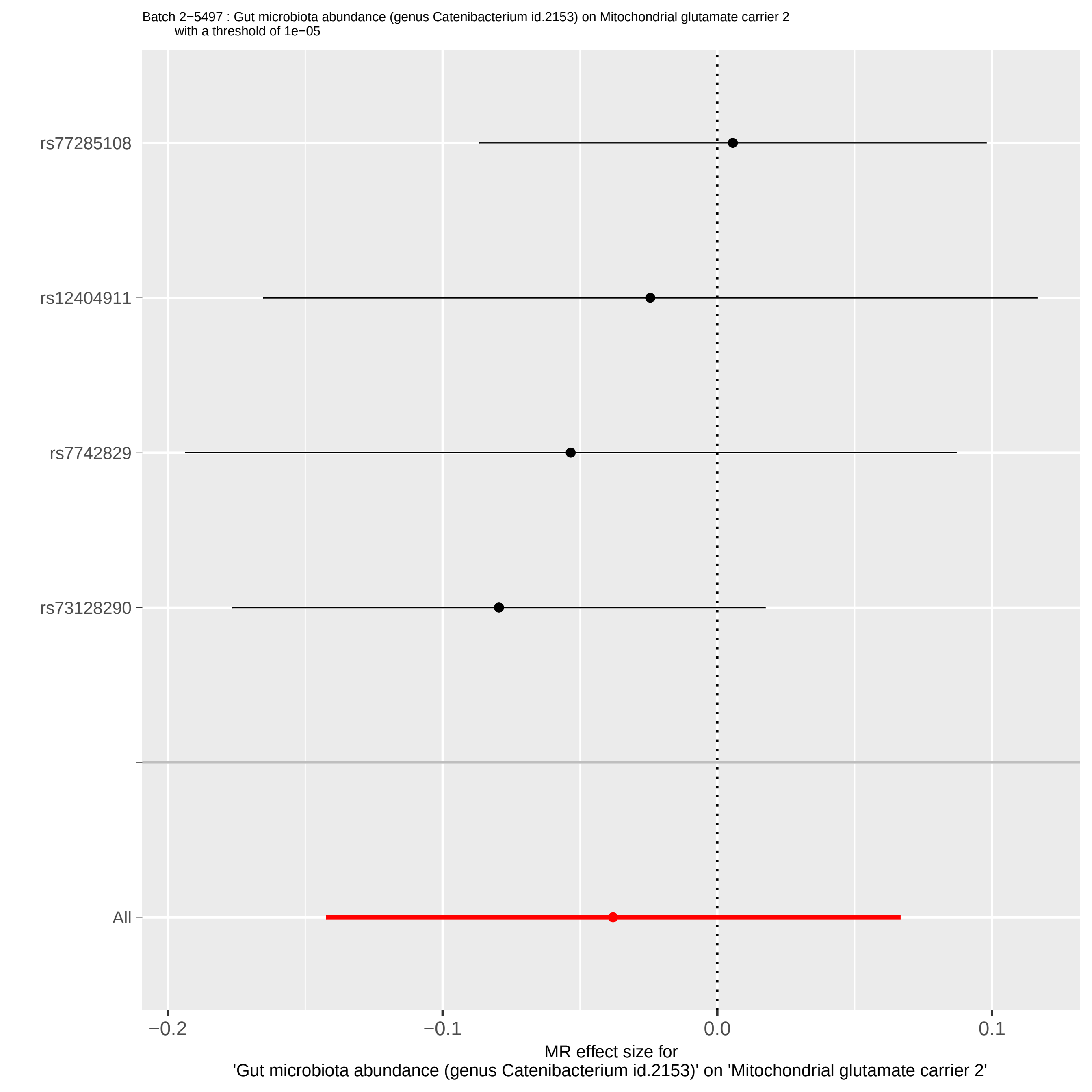
Batch 2−5497 : Gut microbiota abundance (genus Catenibacterium id.2153) on Mitochondrial glutamate carrier 2
with a threshold of 1e−05
rs77285108
rs12404911
rs7742829
rs73128290
All
−0.2
−0.1
0.0
0.1
MR effect size for
'Gut microbiota abundance (genus Catenibacterium id.2153)' on 'Mitochondrial glutamate carrier 2'

Batch 2−5498 : Gut microbiota abundance (genus Parasutterella id.2892) on Mitochondrial glutamate carrier 2
with a threshold of 1e−05

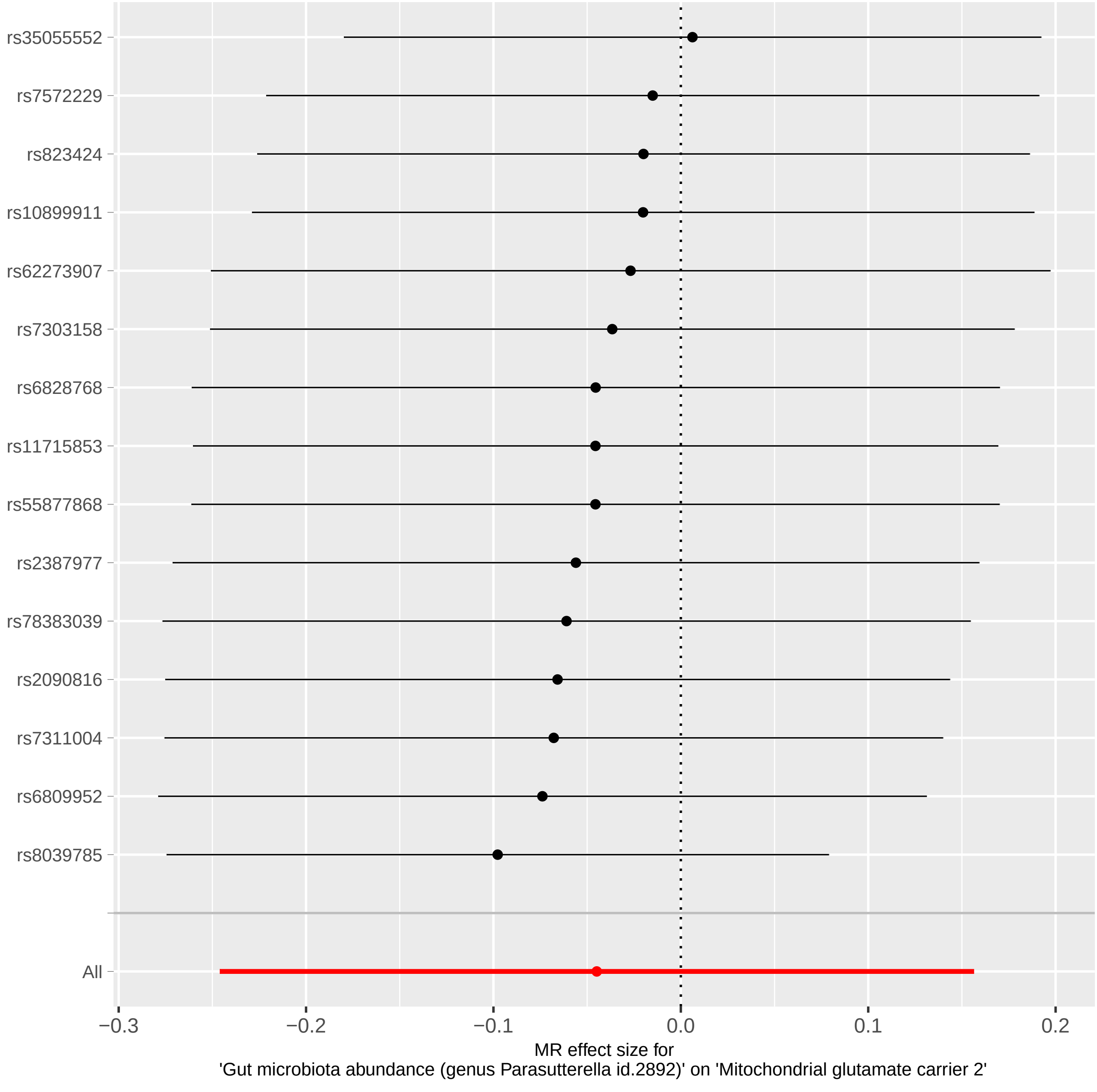

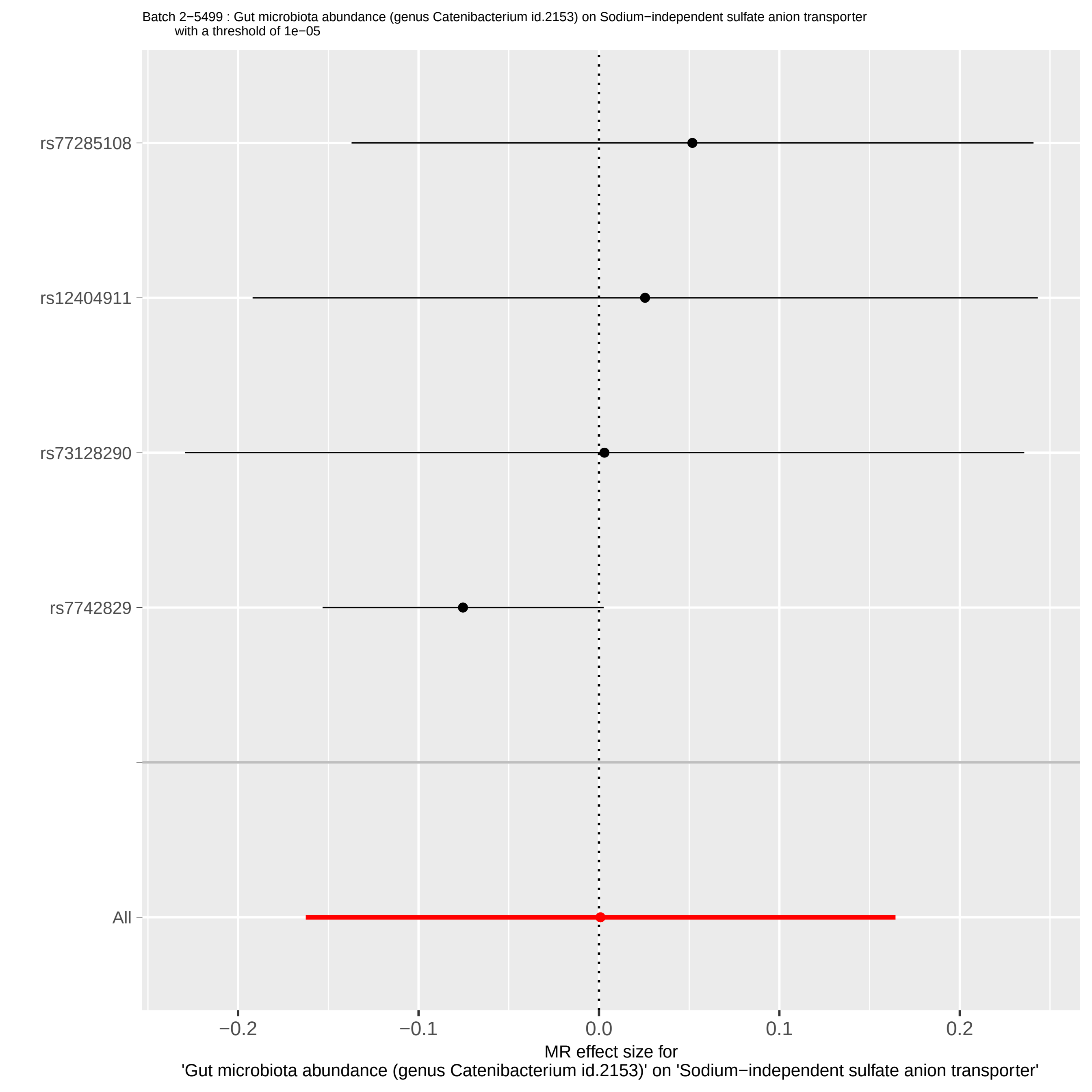
Batch 2−5499 : Gut microbiota abundance (genus Catenibacterium id.2153) on Sodium−independent sulfate anion transporter
with a threshold of 1e−05
rs77285108
rs12404911
rs73128290
rs7742829
All
−0.2
−0.1
0.0
0.1
0.2
MR effect size for
'Gut microbiota abundance (genus Catenibacterium id.2153)' on 'Sodium−independent sulfate anion transporter'

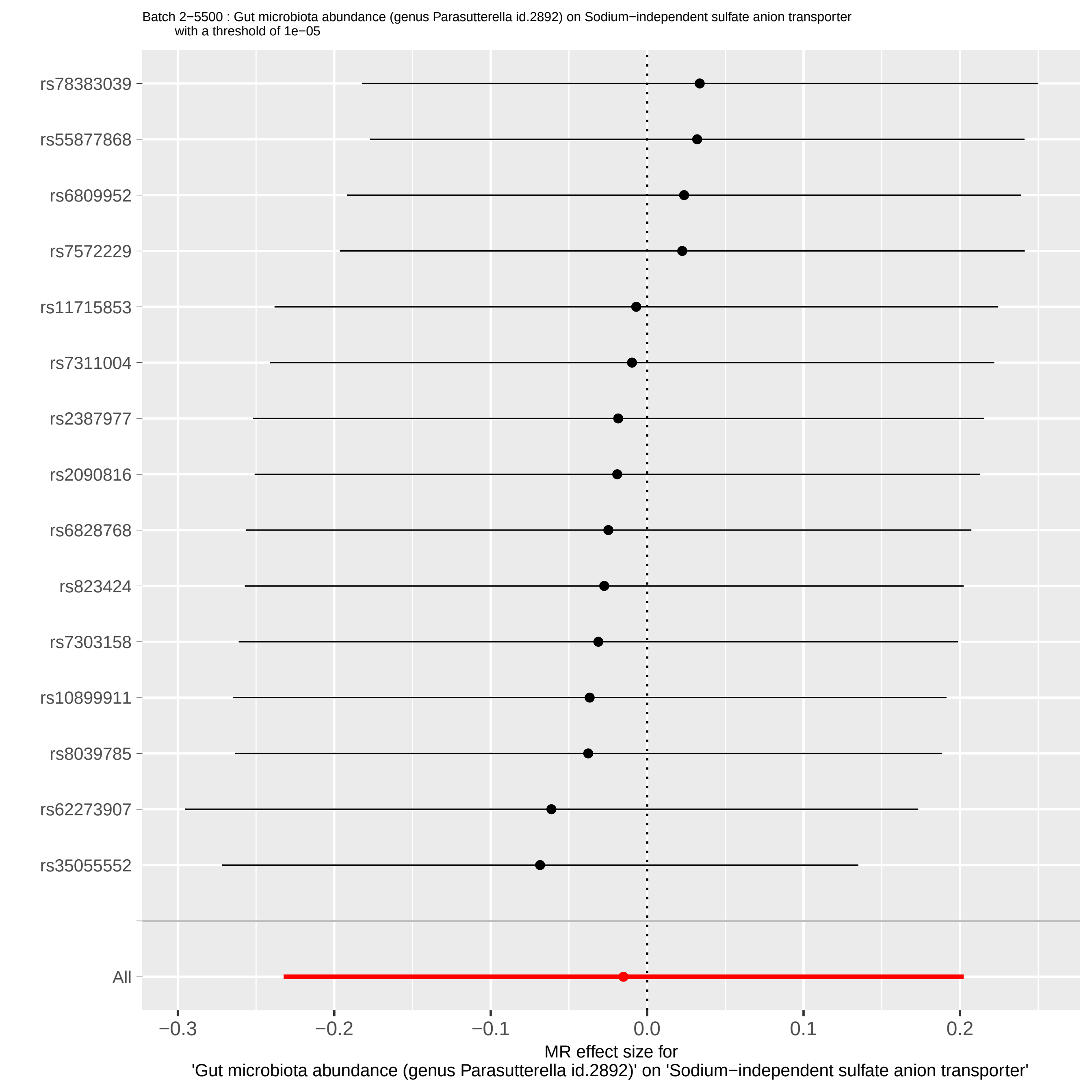
Batch 2−5500 : Gut microbiota abundance (genus Parasutterella id.2892) on Sodium−independent sulfate anion transporter
with a threshold of 1e−05
rs78383039
rs55877868
rs6809952
rs7572229
rs11715853
rs7311004
rs2387977
rs2090816
rs6828768
rs823424
rs7303158
rs10899911
rs8039785
rs62273907
rs35055552
All
−0.3
−0.2
−0.1
0.0
0.1
0.2
MR effect size for
'Gut microbiota abundance (genus Parasutterella id.2892)' on 'Sodium−independent sulfate anion transporter'

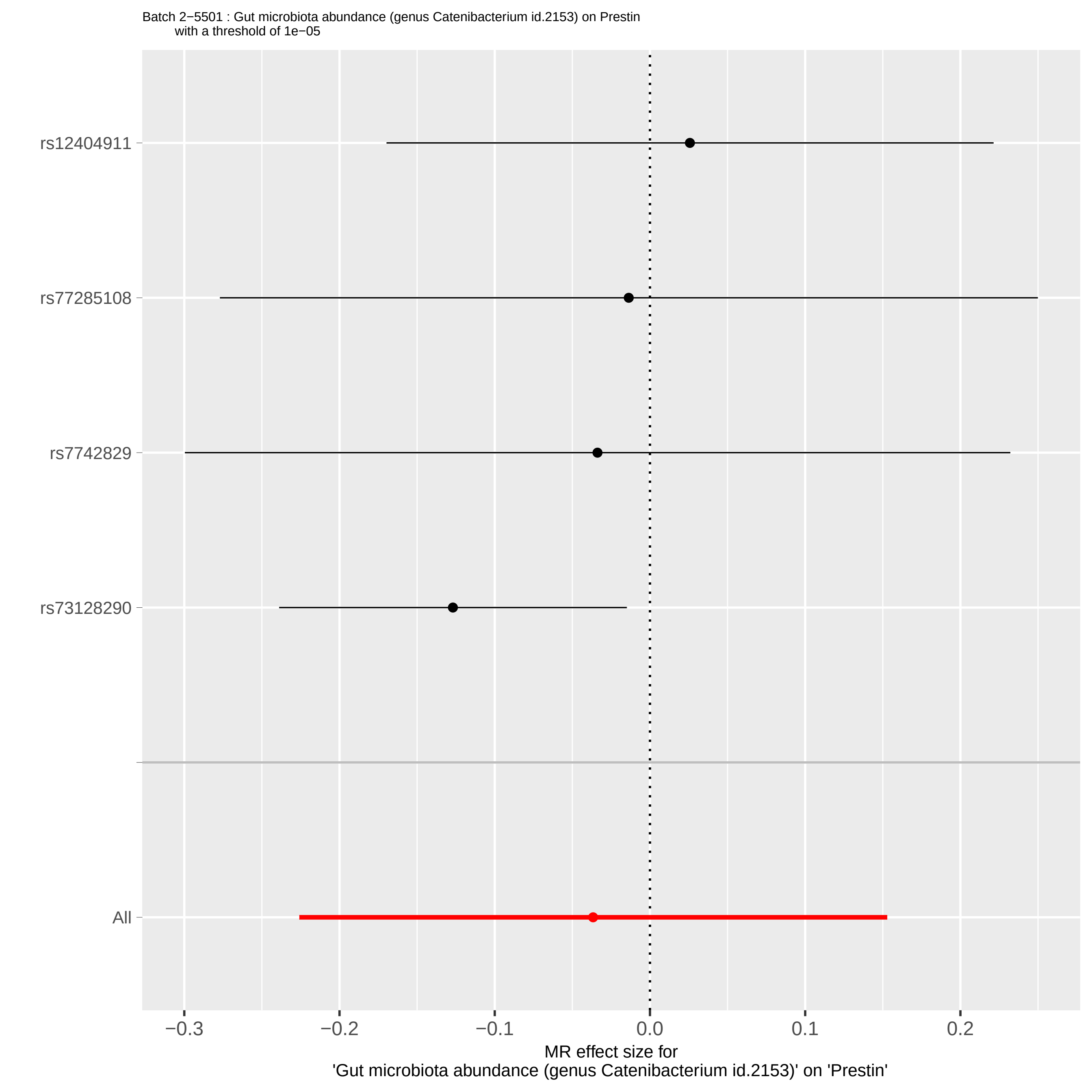

Batch 2−5501 : Gut microbiota abundance (genus Catenibacterium id.2153) on Prestin
with a threshold of 1e−05
rs12404911
rs77285108
rs7742829
rs73128290
All
−0.3
−0.2
−0.1
0.0
0.1
0.2
MR effect size for
'Gut microbiota abundance (genus Catenibacterium id.2153)' on 'Prestin'

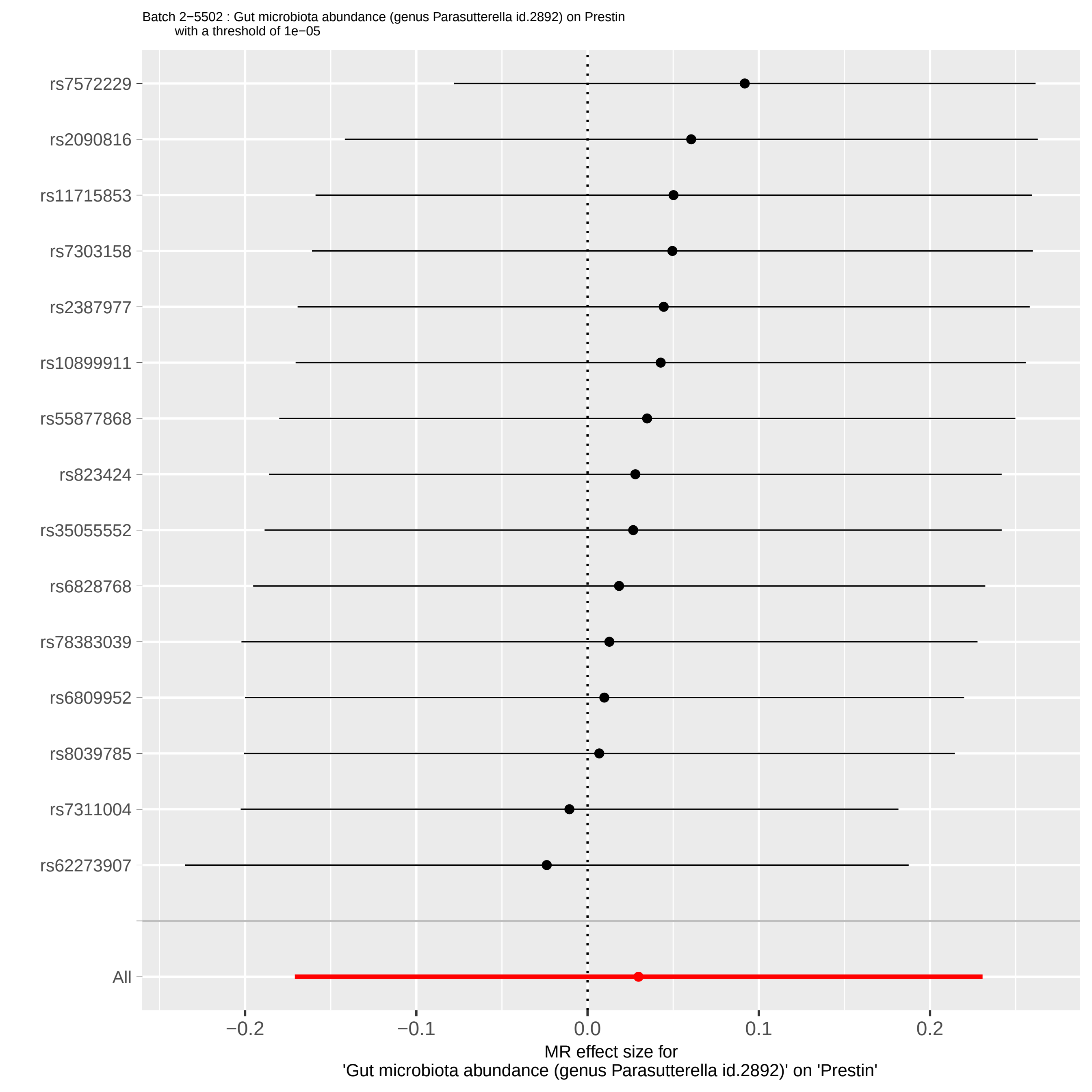

Batch 2−5502 : Gut microbiota abundance (genus Parasutterella id.2892) on Prestin
with a threshold of 1e−05
rs7572229
rs2090816
rs11715853
rs7303158
rs2387977
rs10899911
rs55877868
rs823424
rs35055552
rs6828768
rs78383039
rs6809952
rs8039785
rs7311004
rs62273907
All
−0.2
−0.1
0.0
0.1
0.2
MR effect size for
'Gut microbiota abundance (genus Parasutterella id.2892)' on 'Prestin'

Batch 2−5503 : Gut microbiota abundance (genus Catenibacterium id.2153) on Very long−chain acyl−CoA synthetase
with a threshold of 1e−05

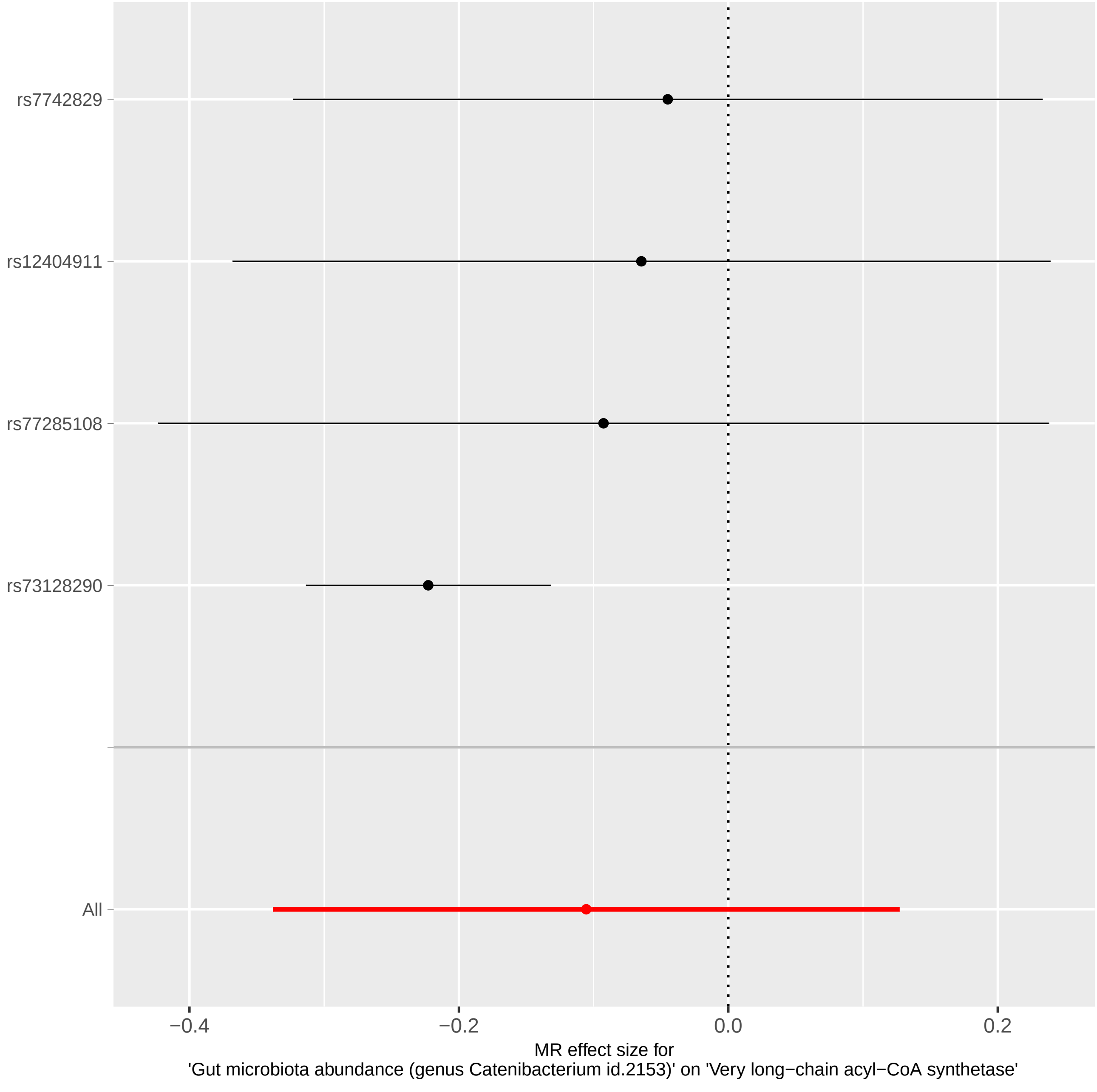

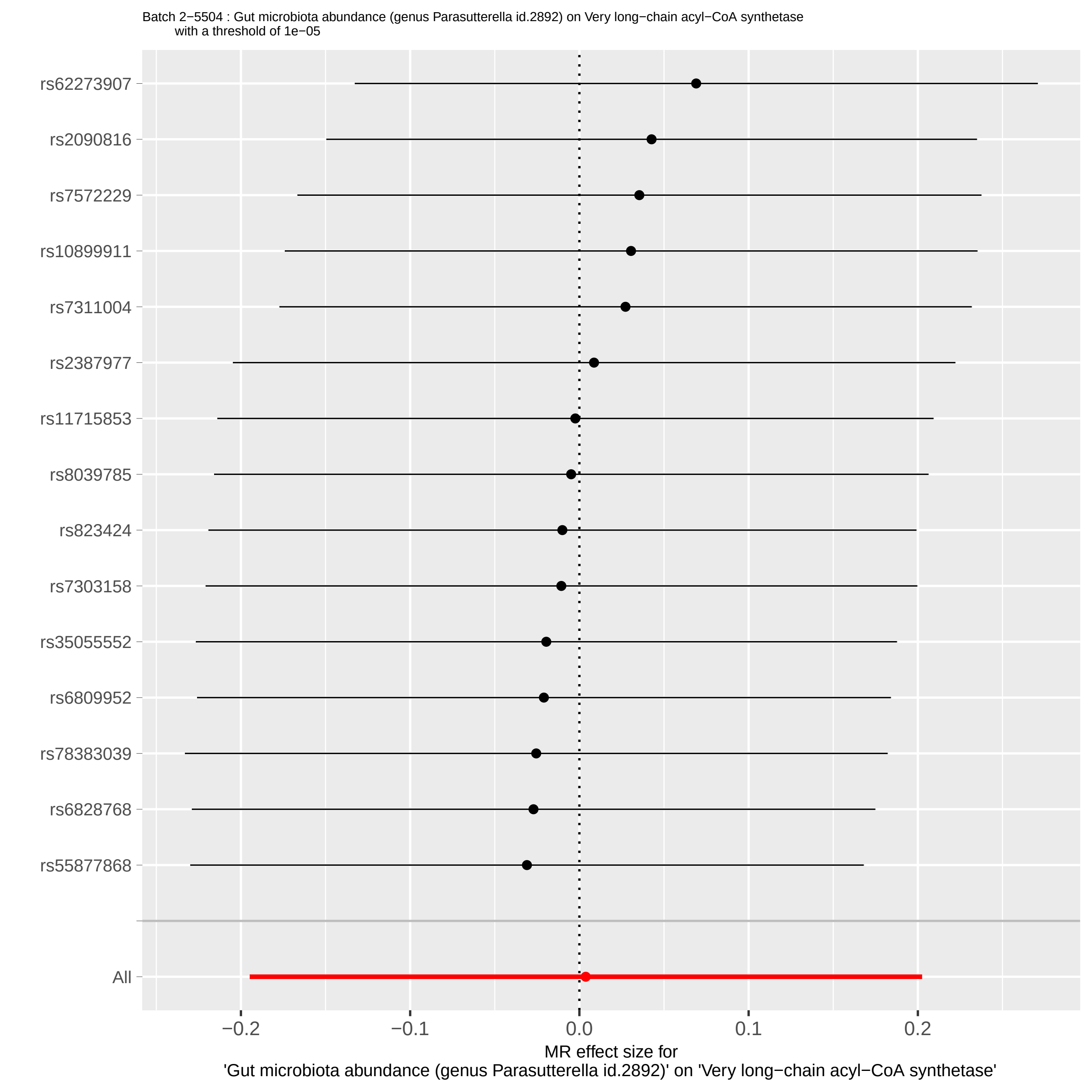

Batch 2−5504 : Gut microbiota abundance (genus Parasutterella id.2892) on Very long−chain acyl−CoA synthetase
with a threshold of 1e−05
rs62273907
rs2090816
rs7572229
rs10899911
rs7311004
rs2387977
rs11715853
rs8039785
rs823424
rs7303158
rs35055552
rs6809952
rs78383039
rs6828768
rs55877868
All
−0.2
−0.1
0.0
0.1
0.2
MR effect size for
'Gut microbiota abundance (genus Parasutterella id.2892)' on 'Very long−chain acyl−CoA synthetase'

Batch 2−5505 : Gut microbiota abundance (genus Catenibacterium id.2153) on Zinc transporter 3
with a threshold of 1e−05

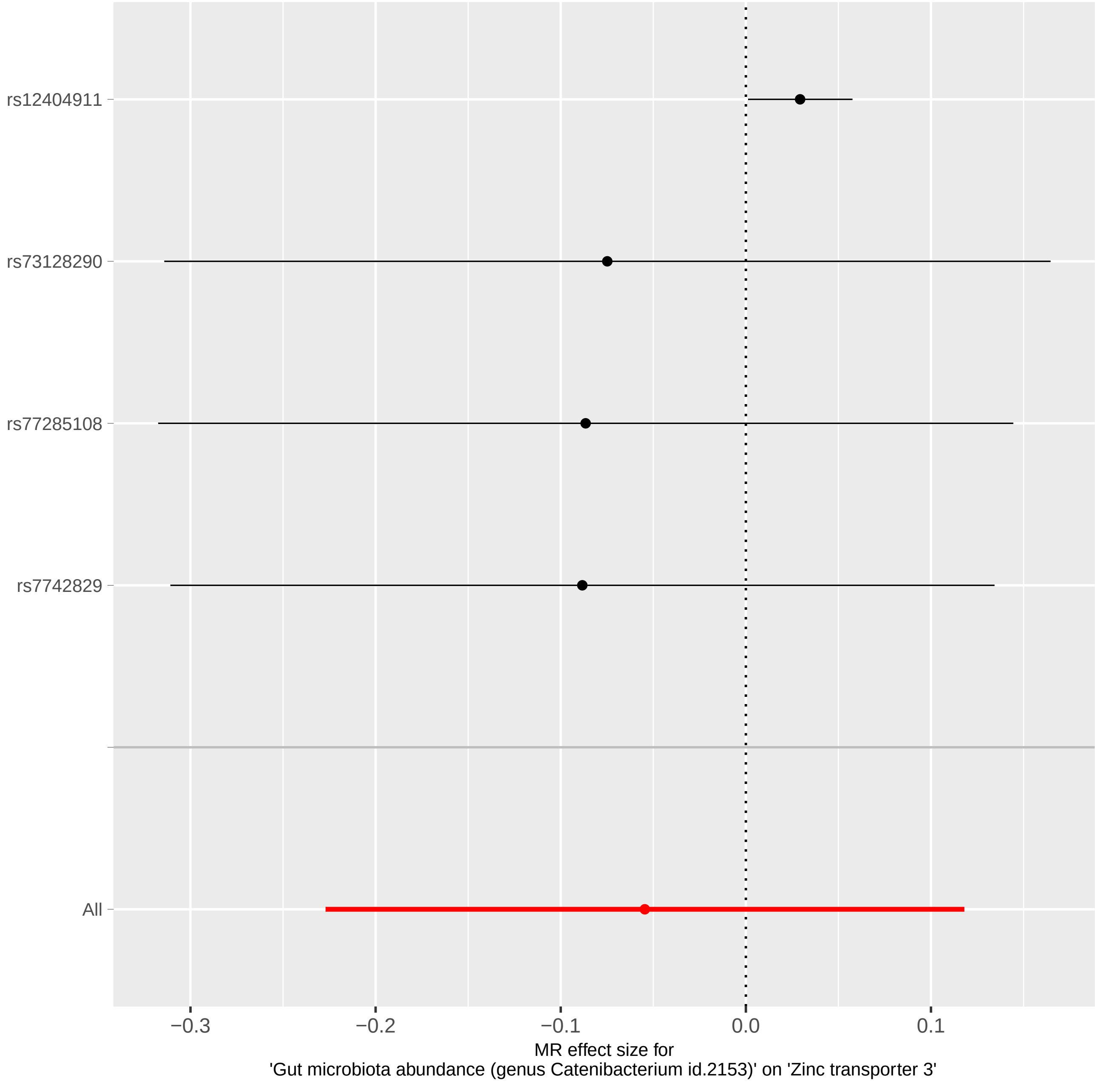

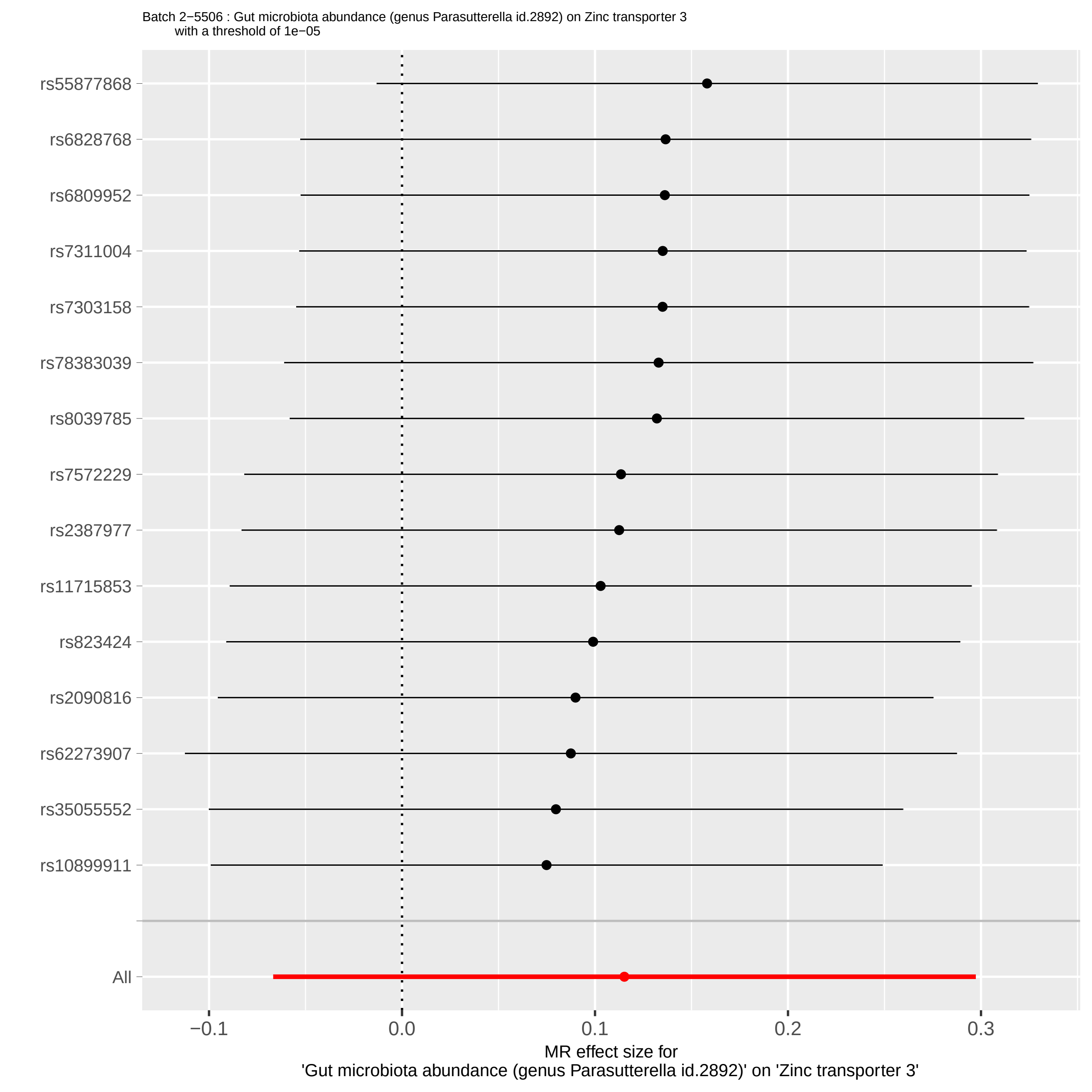

Batch 2−5506 : Gut microbiota abundance (genus Parasutterella id.2892) on Zinc transporter 3
with a threshold of 1e−05
rs55877868
rs6828768
rs6809952
rs7311004
rs7303158
rs78383039
rs8039785
rs7572229
rs2387977
rs11715853
rs823424
rs2090816
rs62273907
rs35055552
rs10899911
All
−0.1
0.0
0.1
0.2
0.3
MR effect size for
'Gut microbiota abundance (genus Parasutterella id.2892)' on 'Zinc transporter 3'

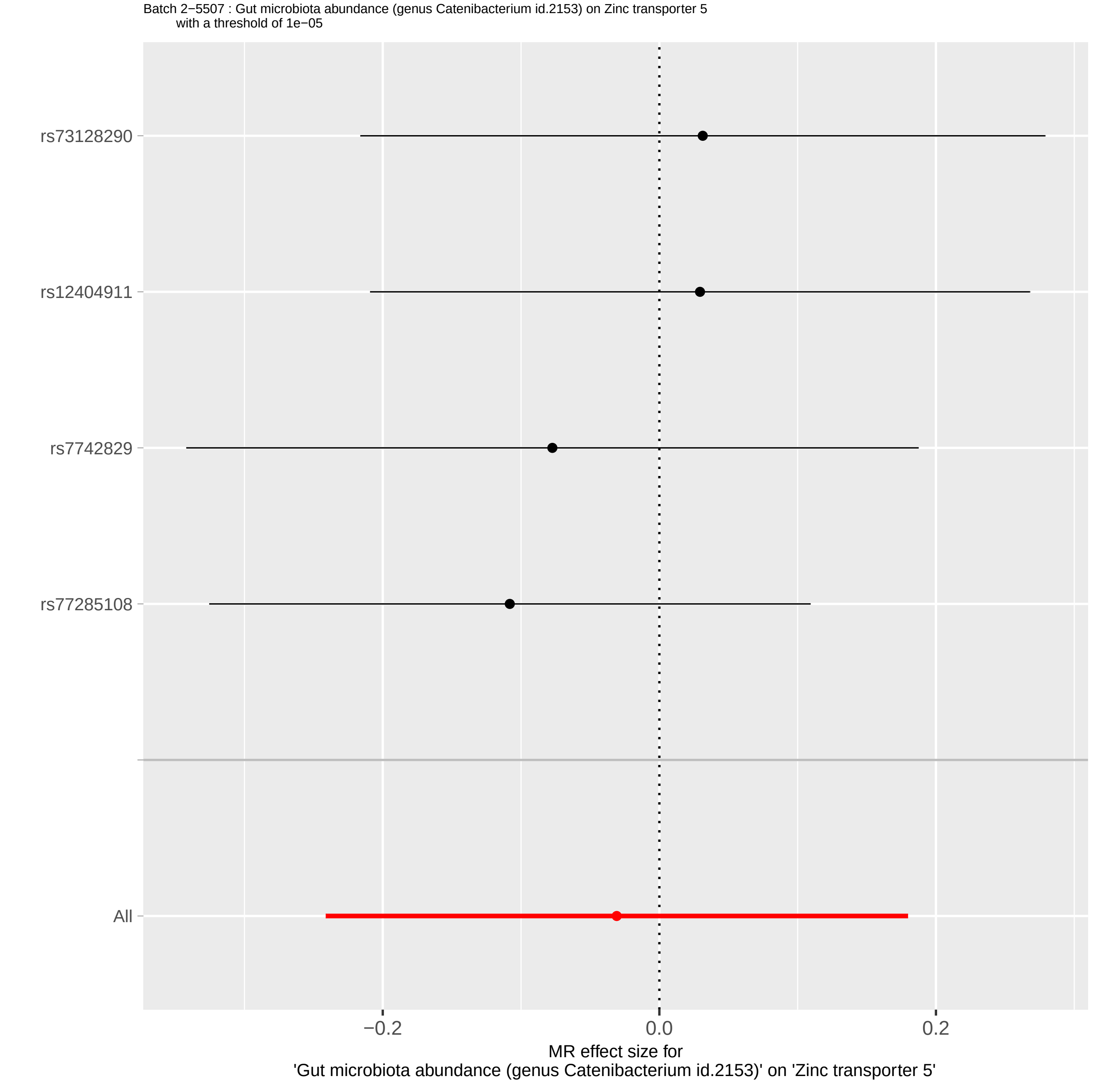

Batch 2−5507 : Gut microbiota abundance (genus Catenibacterium id.2153) on Zinc transporter 5
with a threshold of 1e−05
rs73128290
rs12404911
rs7742829
rs77285108
All
−0.2
0.0
0.2
MR effect size for
'Gut microbiota abundance (genus Catenibacterium id.2153)' on 'Zinc transporter 5'

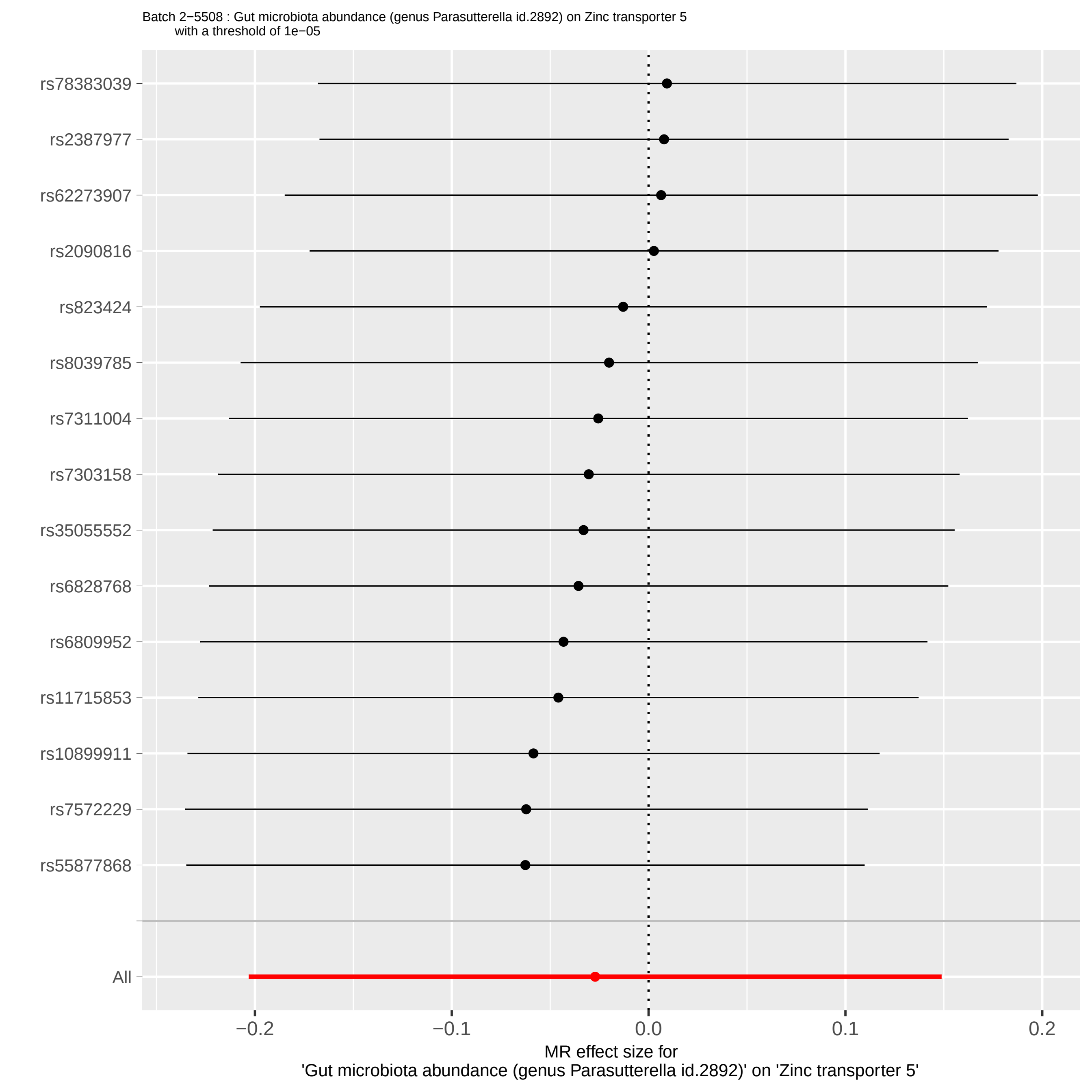

Batch 2−5508 : Gut microbiota abundance (genus Parasutterella id.2892) on Zinc transporter 5
with a threshold of 1e−05
rs78383039
rs2387977
rs62273907
rs2090816
rs823424
rs8039785
rs7311004
rs7303158
rs35055552
rs6828768
rs6809952
rs11715853
rs10899911
rs7572229
rs55877868
All
−0.2
−0.1
0.0
0.1
0.2
MR effect size for
'Gut microbiota abundance (genus Parasutterella id.2892)' on 'Zinc transporter 5'

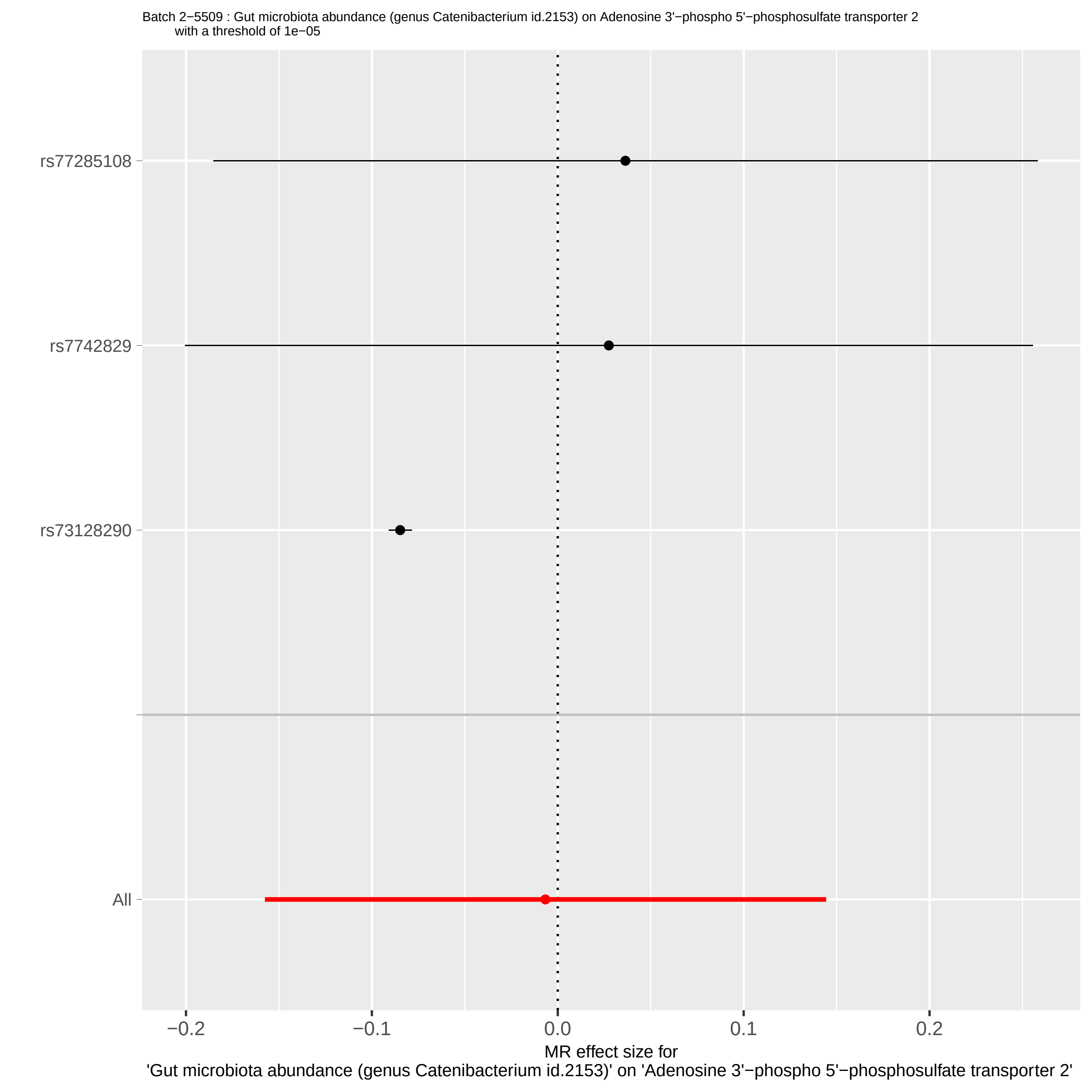
Batch 2−5509 : Gut microbiota abundance (genus Catenibacterium id.2153) on Adenosine 3'−phospho 5'−phosphosulfate transporter 2
with a threshold of 1e−05
rs77285108
rs7742829
rs73128290
All
−0.2
−0.1
0.0
0.1
0.2
MR effect size for
'Gut microbiota abundance (genus Catenibacterium id.2153)' on 'Adenosine 3'−phospho 5'−phosphosulfate transporter 2'

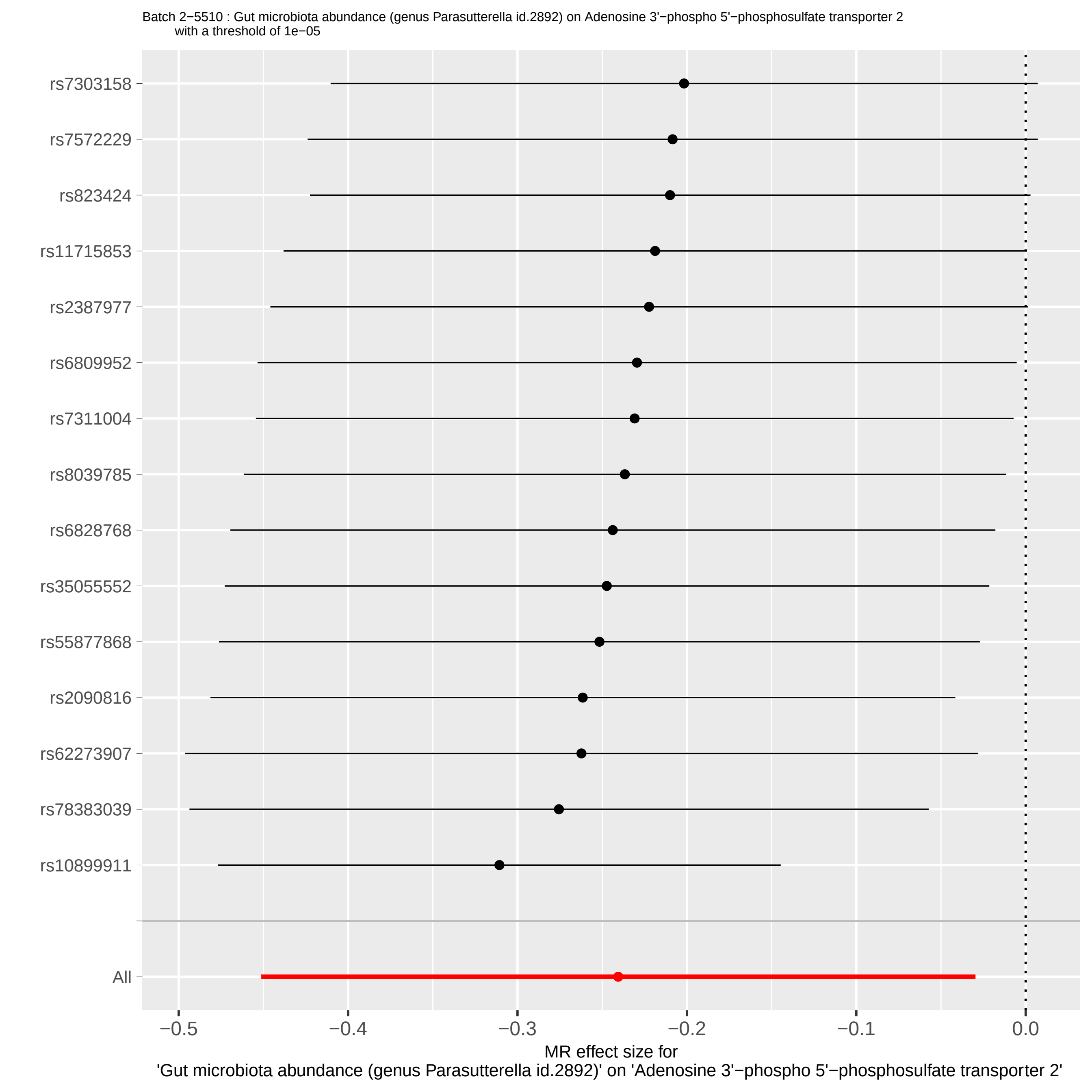
Batch 2−5510 : Gut microbiota abundance (genus Parasutterella id.2892) on Adenosine 3'−phospho 5'−phosphosulfate transporter 2
with a threshold of 1e−05
rs7303158
rs7572229
rs823424
rs11715853
rs2387977
rs6809952
rs7311004
rs8039785
rs6828768
rs35055552
rs55877868
rs2090816
rs62273907
rs78383039
rs10899911
All
−0.5
−0.4
−0.3
−0.2
−0.1
0.0
MR effect size for
'Gut microbiota abundance (genus Parasutterella id.2892)' on 'Adenosine 3'−phospho 5'−phosphosulfate transporter 2'

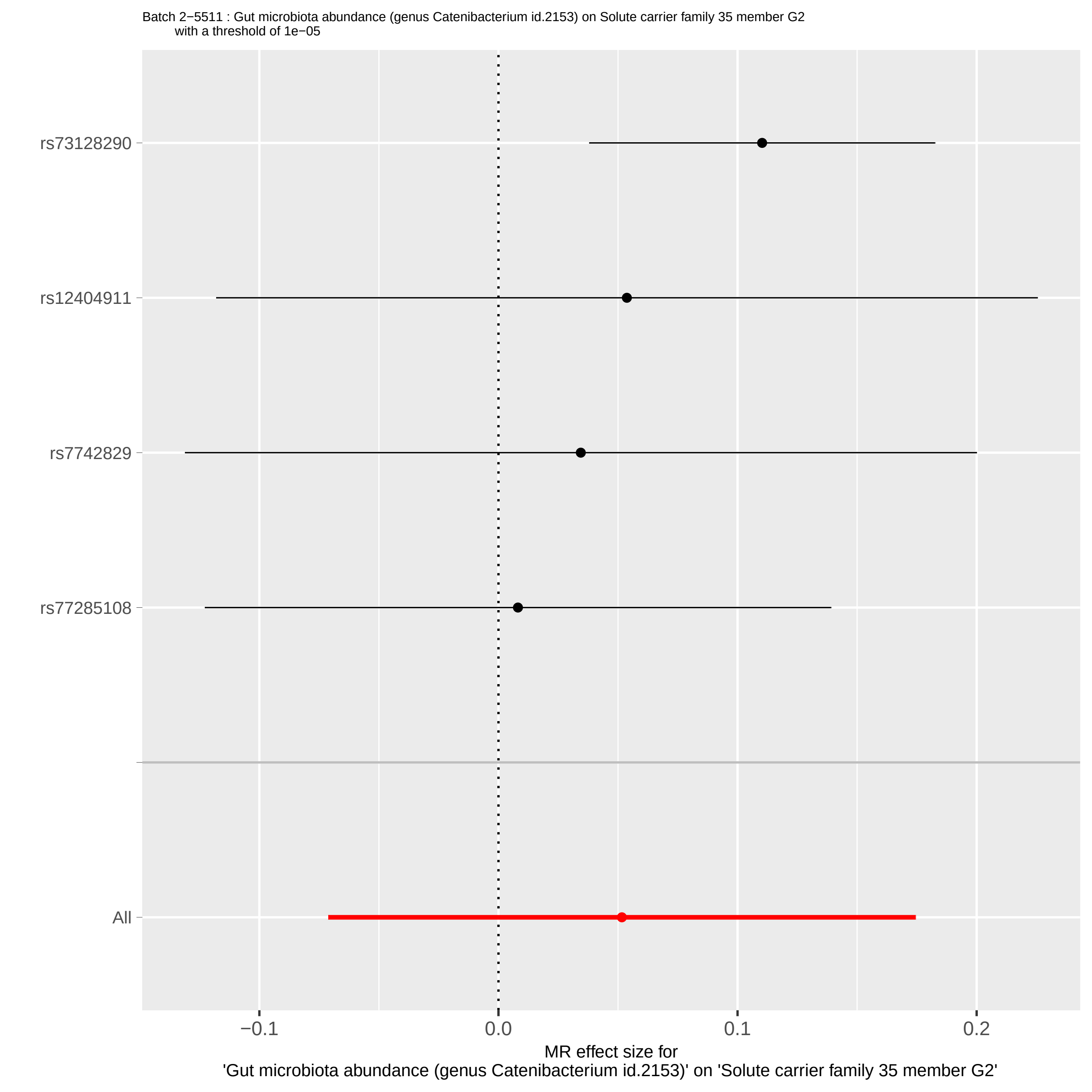
Batch 2−5511 : Gut microbiota abundance (genus Catenibacterium id.2153) on Solute carrier family 35 member G2
with a threshold of 1e−05
rs73128290
rs12404911
rs7742829
rs77285108
All
−0.1
0.0
0.1
0.2
MR effect size for
'Gut microbiota abundance (genus Catenibacterium id.2153)' on 'Solute carrier family 35 member G2'

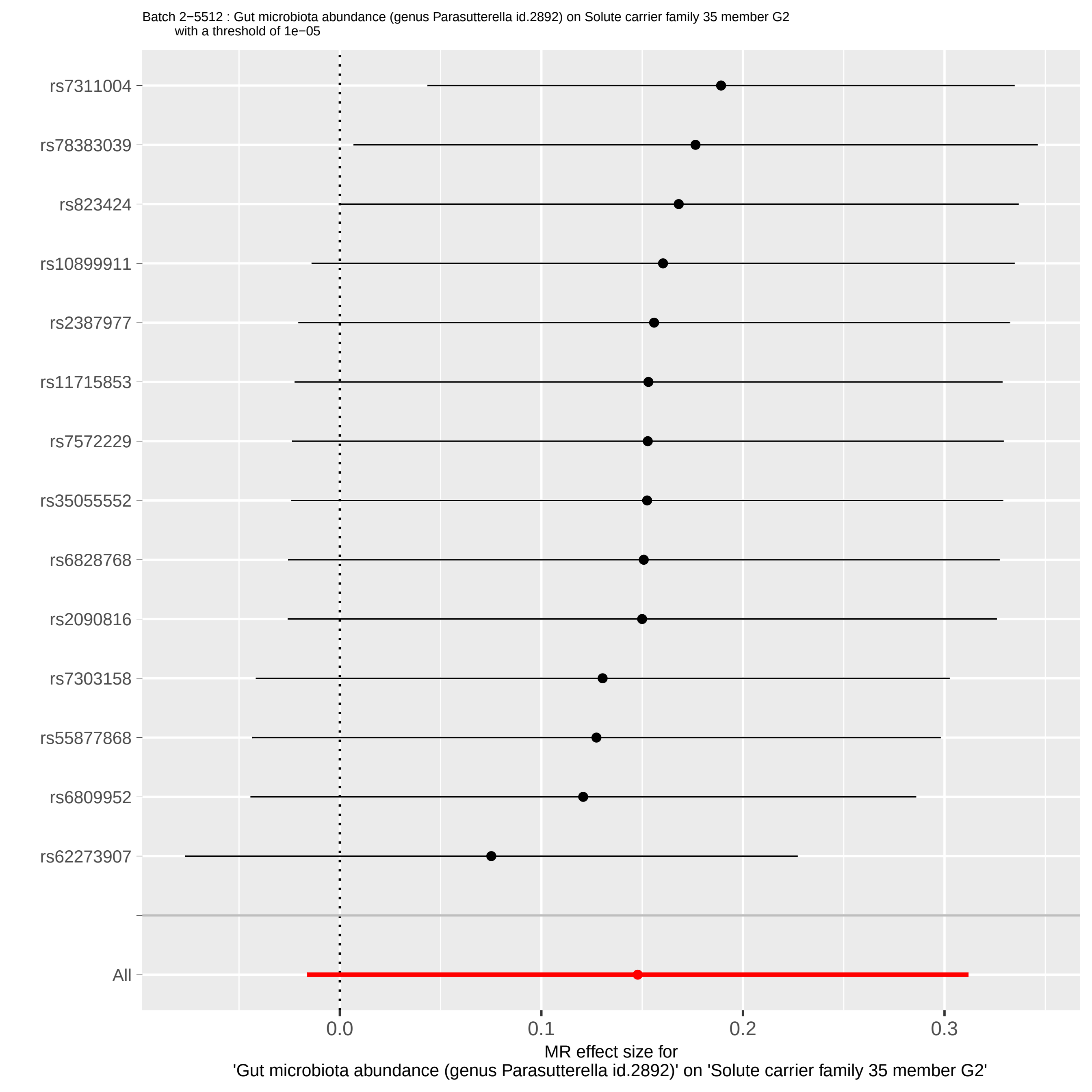
Batch 2−5512 : Gut microbiota abundance (genus Parasutterella id.2892) on Solute carrier family 35 member G2
with a threshold of 1e−05
rs7311004
rs78383039
rs823424
rs10899911
rs2387977
rs11715853
rs7572229
rs35055552
rs6828768
rs2090816
rs7303158
rs55877868
rs6809952
rs62273907
All
0.0
0.1
0.2
0.3
MR effect size for
'Gut microbiota abundance (genus Parasutterella id.2892)' on 'Solute carrier family 35 member G2'

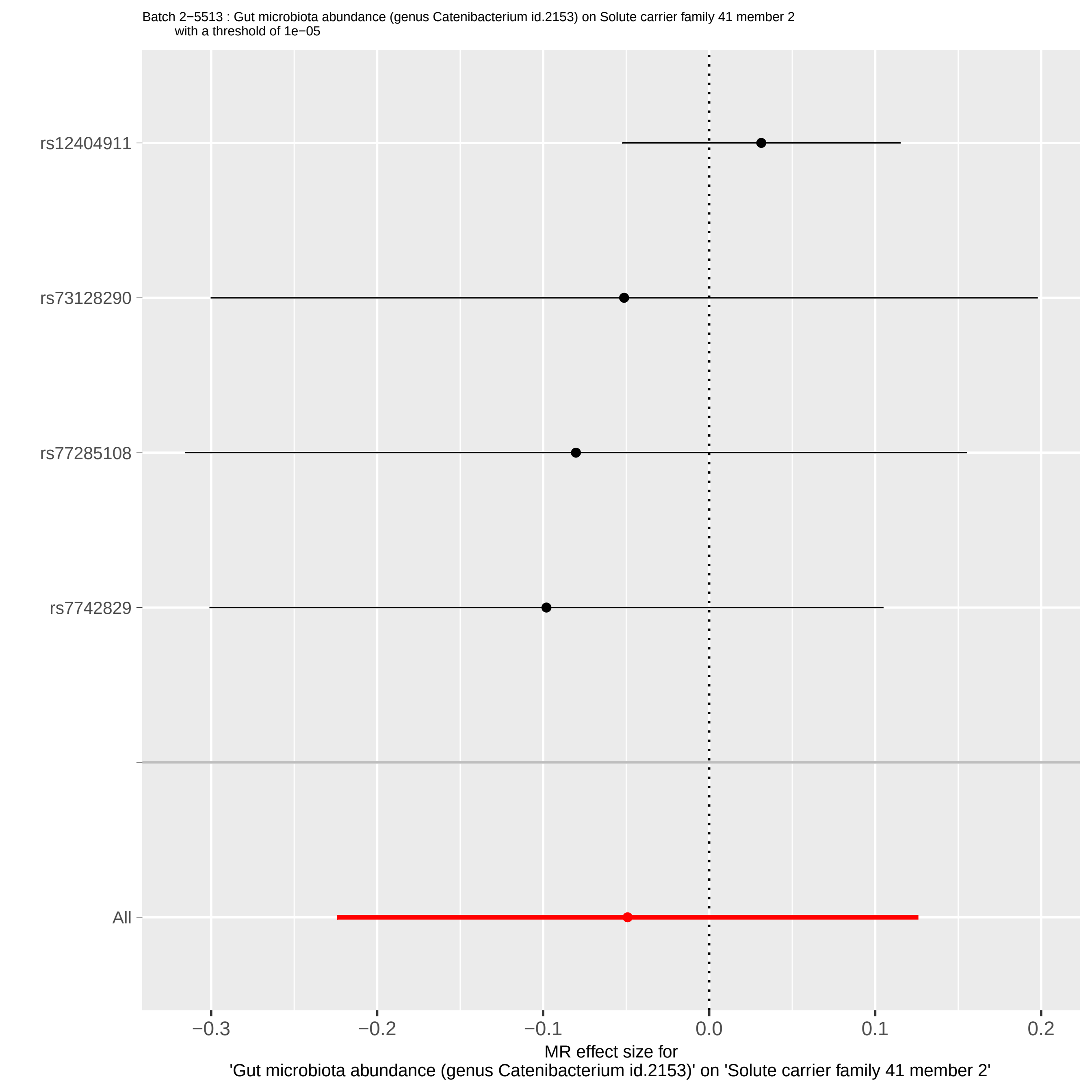
Batch 2−5513 : Gut microbiota abundance (genus Catenibacterium id.2153) on Solute carrier family 41 member 2
with a threshold of 1e−05
rs12404911
rs73128290
rs77285108
rs7742829
All
−0.3
−0.2
−0.1
0.0
0.1
0.2
MR effect size for
'Gut microbiota abundance (genus Catenibacterium id.2153)' on 'Solute carrier family 41 member 2'

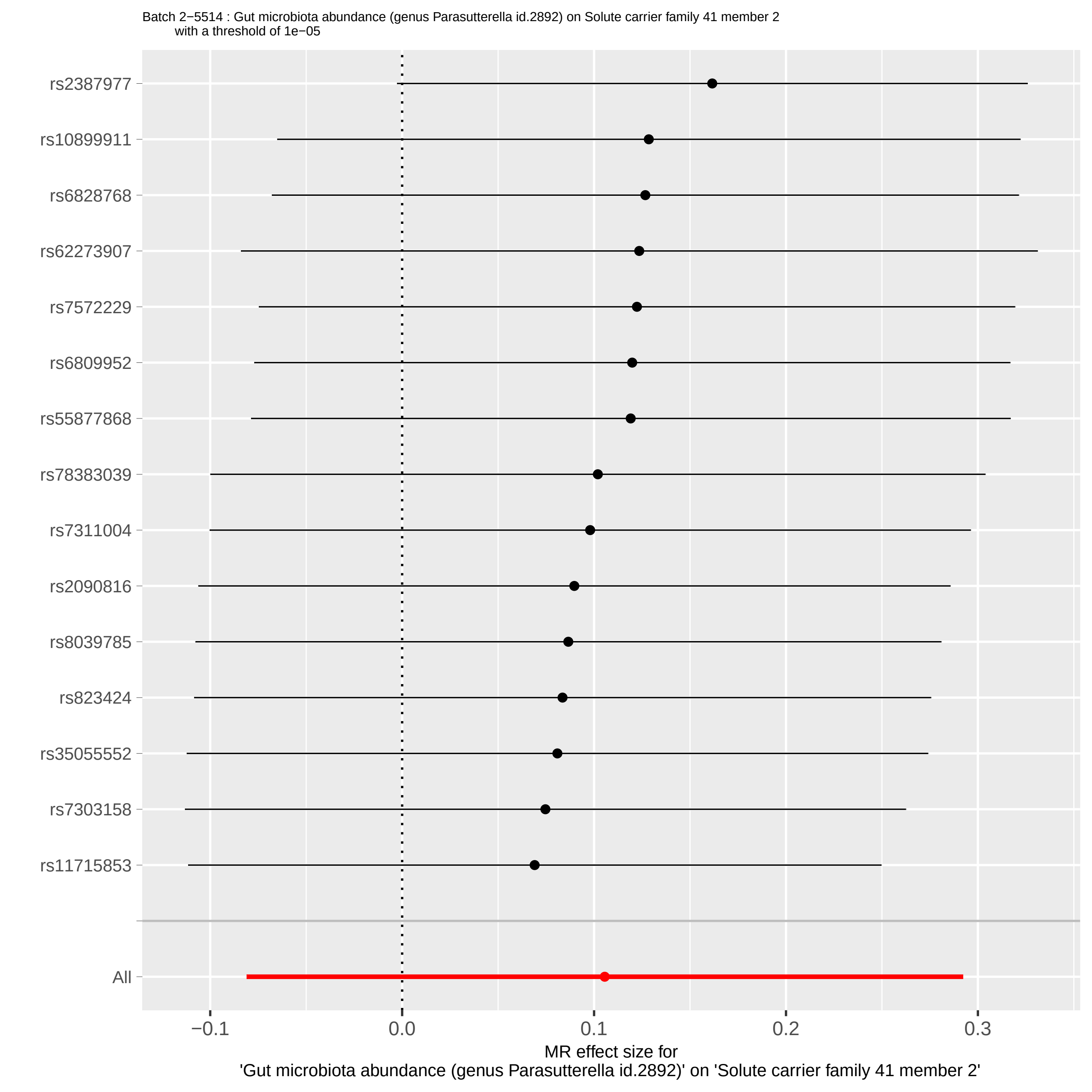

Batch 2−5514 : Gut microbiota abundance (genus Parasutterella id.2892) on Solute carrier family 41 member 2
with a threshold of 1e−05
rs2387977
rs10899911
rs6828768
rs62273907
rs7572229
rs6809952
rs55877868
rs78383039
rs7311004
rs2090816
rs8039785
rs823424
rs35055552
rs7303158
rs11715853
All
−0.1
0.0
0.1
0.2
0.3
MR effect size for
'Gut microbiota abundance (genus Parasutterella id.2892)' on 'Solute carrier family 41 member 2'

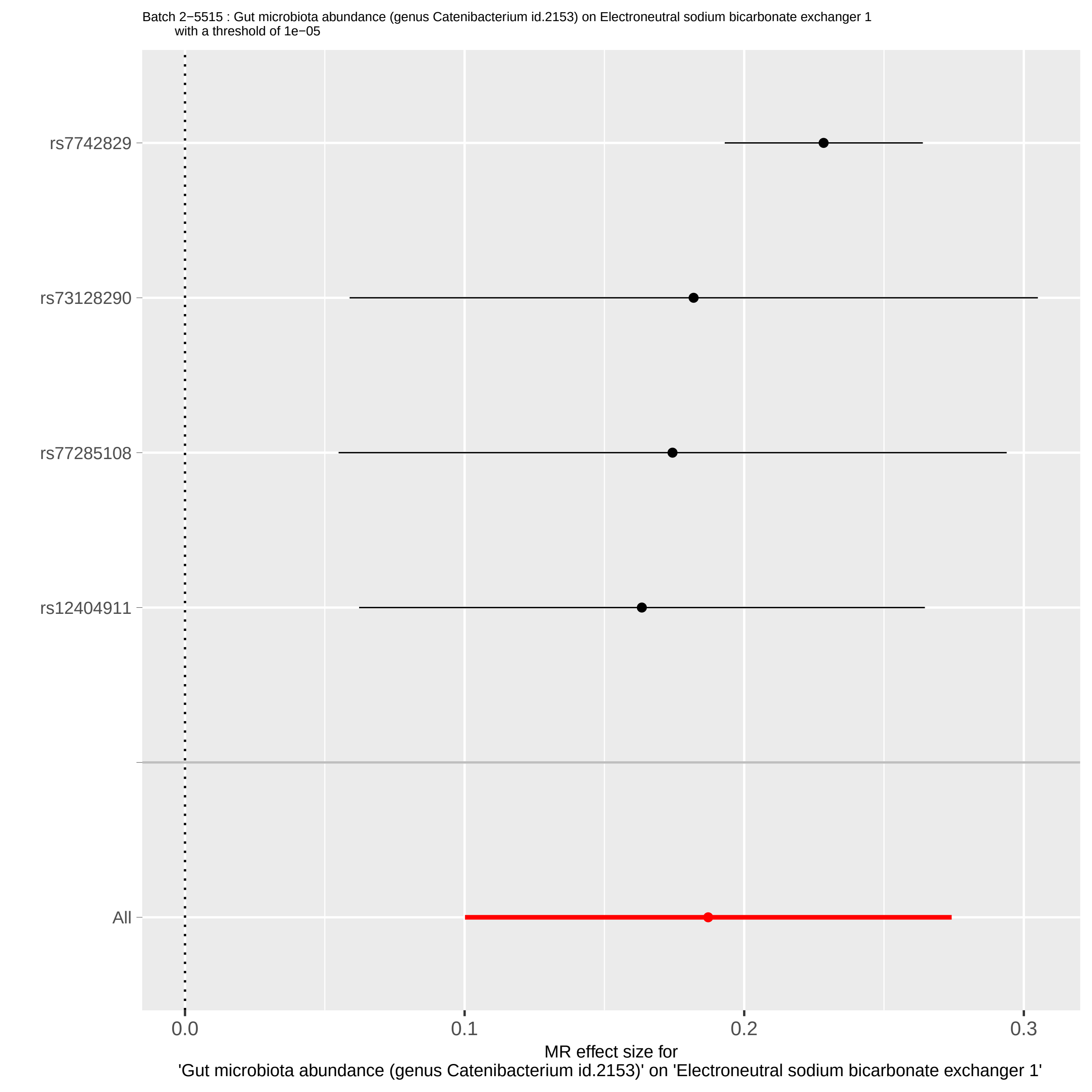
Batch 2−5515 : Gut microbiota abundance (genus Catenibacterium id.2153) on Electroneutral sodium bicarbonate exchanger 1
with a threshold of 1e−05
rs7742829
rs73128290
rs77285108
rs12404911
All
0.0
0.1
0.2
0.3
MR effect size for
'Gut microbiota abundance (genus Catenibacterium id.2153)' on 'Electroneutral sodium bicarbonate exchanger 1'

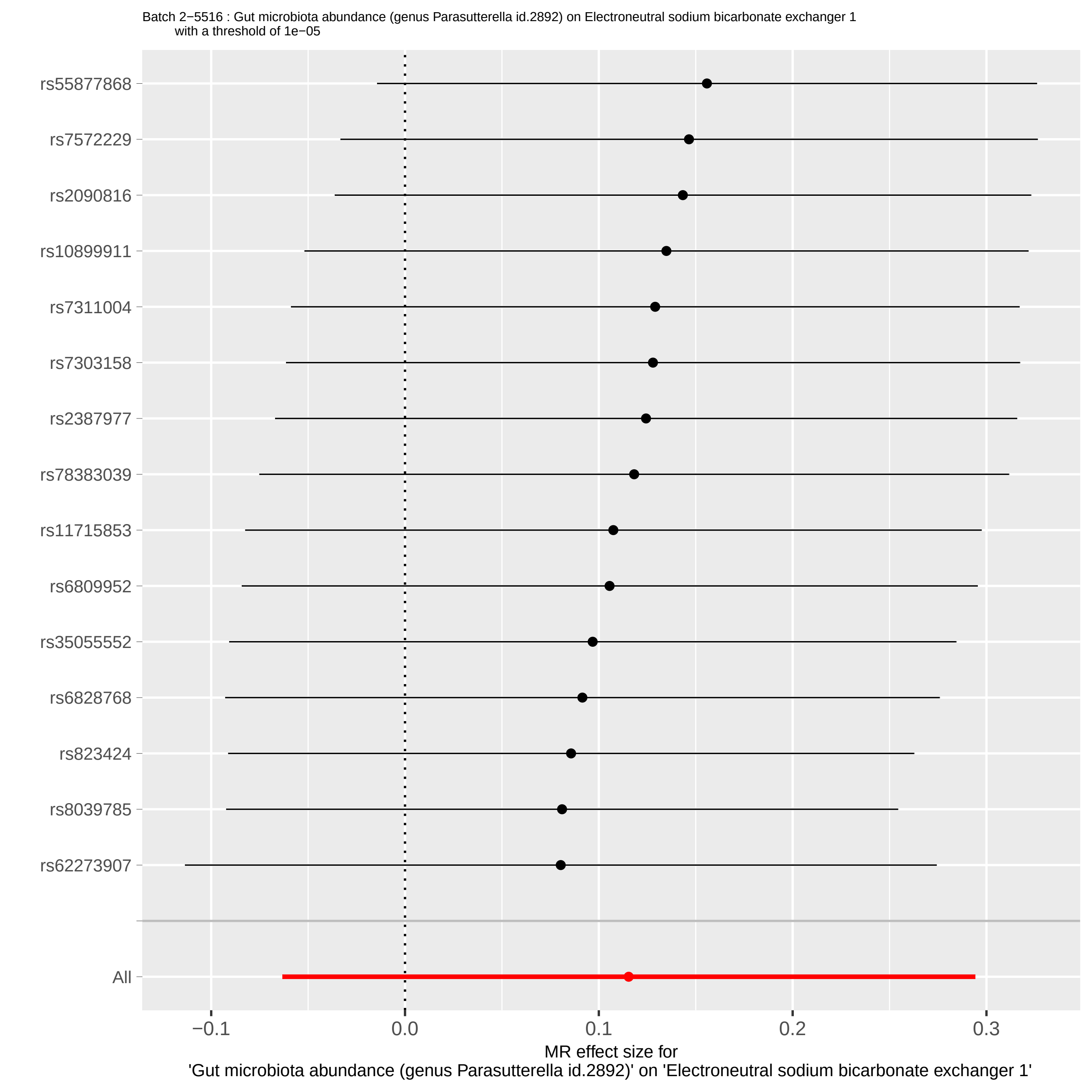

Batch 2−5516 : Gut microbiota abundance (genus Parasutterella id.2892) on Electroneutral sodium bicarbonate exchanger 1
with a threshold of 1e−05
rs55877868
rs7572229
rs2090816
rs10899911
rs7311004
rs7303158
rs2387977
rs78383039
rs11715853
rs6809952
rs35055552
rs6828768
rs823424
rs8039785
rs62273907
All
−0.1
0.0
0.1
0.2
0.3
MR effect size for
'Gut microbiota abundance (genus Parasutterella id.2892)' on 'Electroneutral sodium bicarbonate exchanger 1'

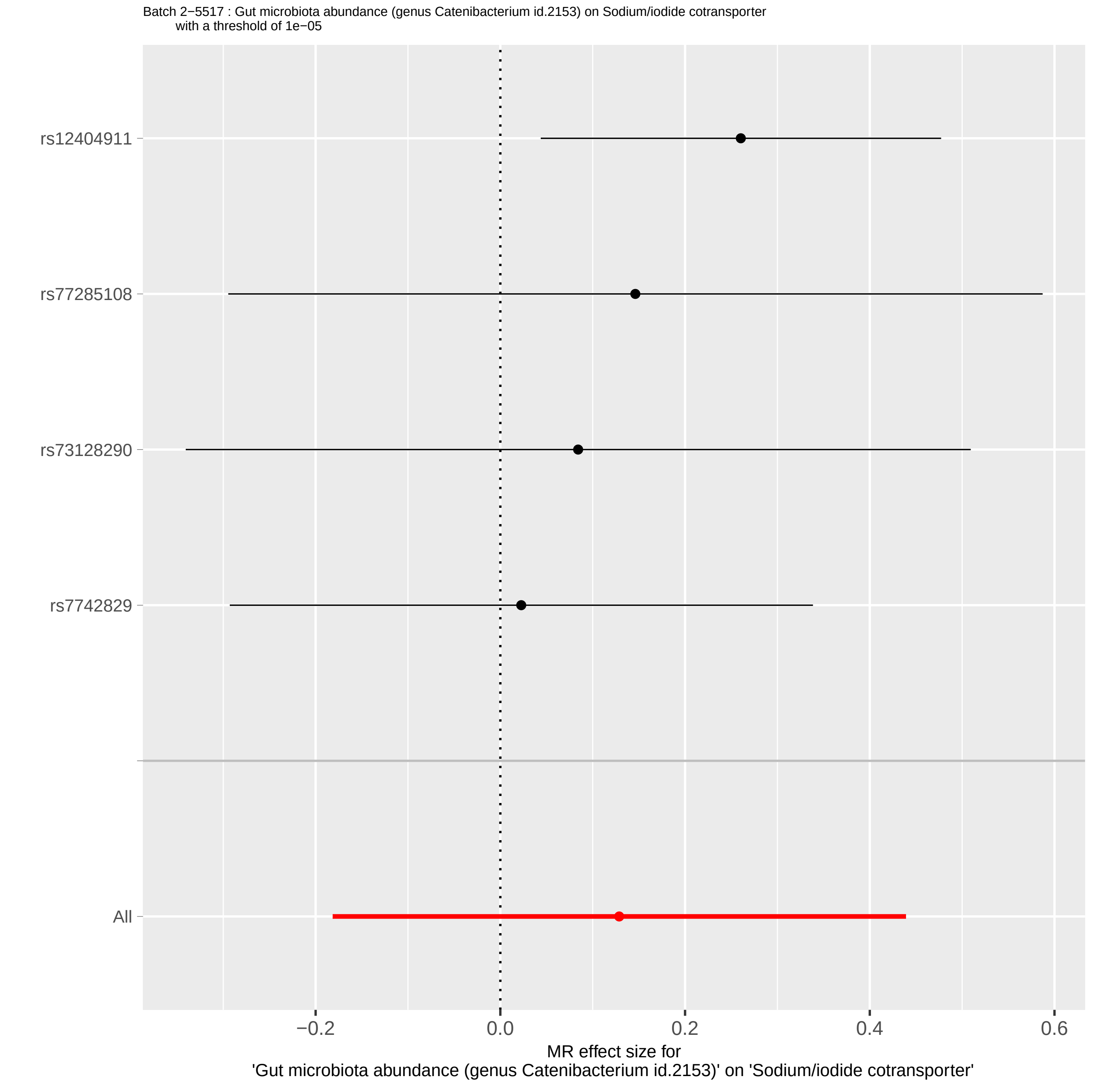

Batch 2−5517 : Gut microbiota abundance (genus Catenibacterium id.2153) on Sodium/iodide cotransporter
with a threshold of 1e−05
rs12404911
rs77285108
rs73128290
rs7742829
All
−0.2
0.0
0.2
0.4
0.6
MR effect size for
'Gut microbiota abundance (genus Catenibacterium id.2153)' on 'Sodium/iodide cotransporter'

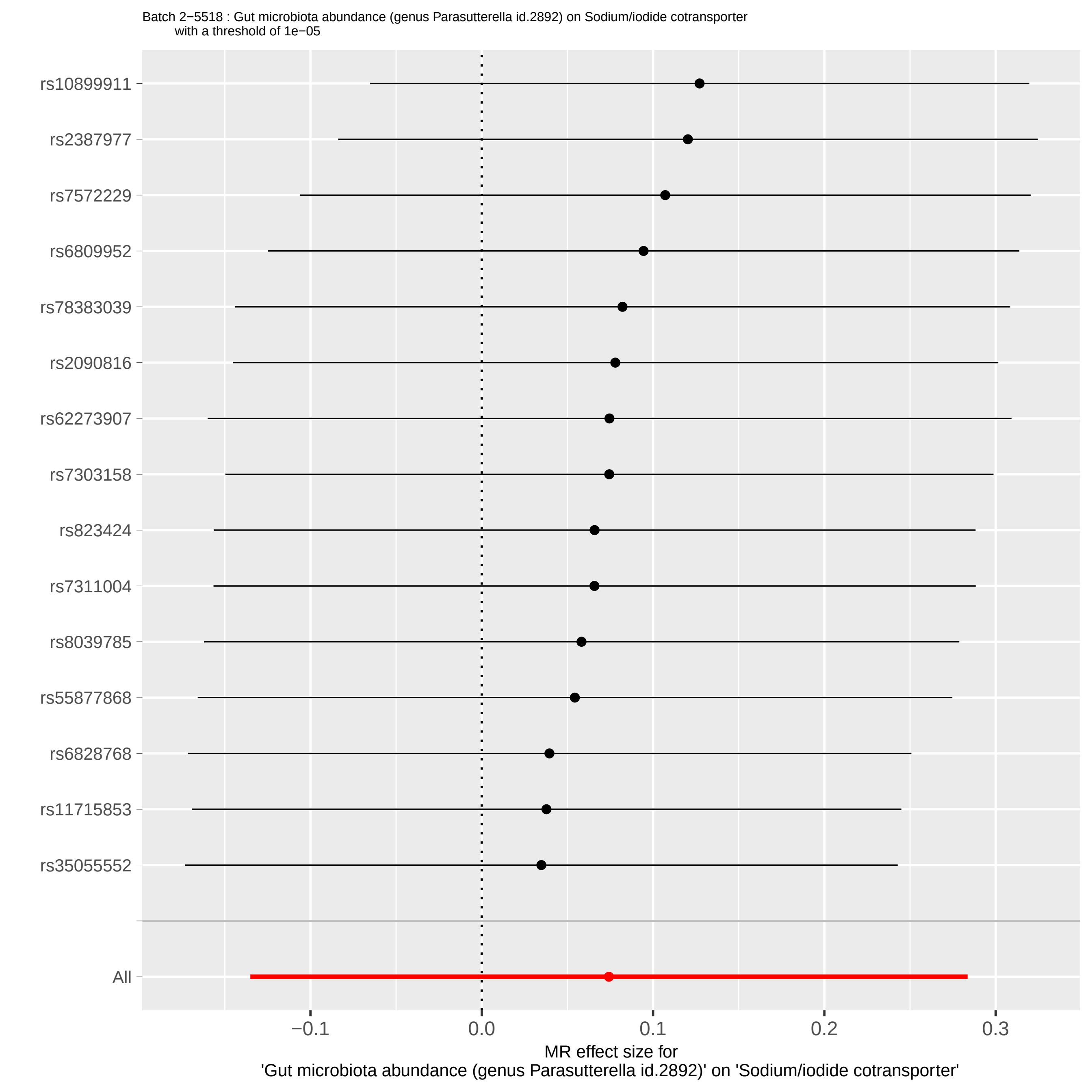
Batch 2−5518 : Gut microbiota abundance (genus Parasutterella id.2892) on Sodium/iodide cotransporter
with a threshold of 1e−05
rs10899911
rs2387977
rs7572229
rs6809952
rs78383039
rs2090816
rs62273907
rs7303158
rs823424
rs7311004
rs8039785
rs55877868
rs6828768
rs11715853
rs35055552
All
−0.1
0.0
0.1
0.2
0.3
MR effect size for
'Gut microbiota abundance (genus Parasutterella id.2892)' on 'Sodium/iodide cotransporter'

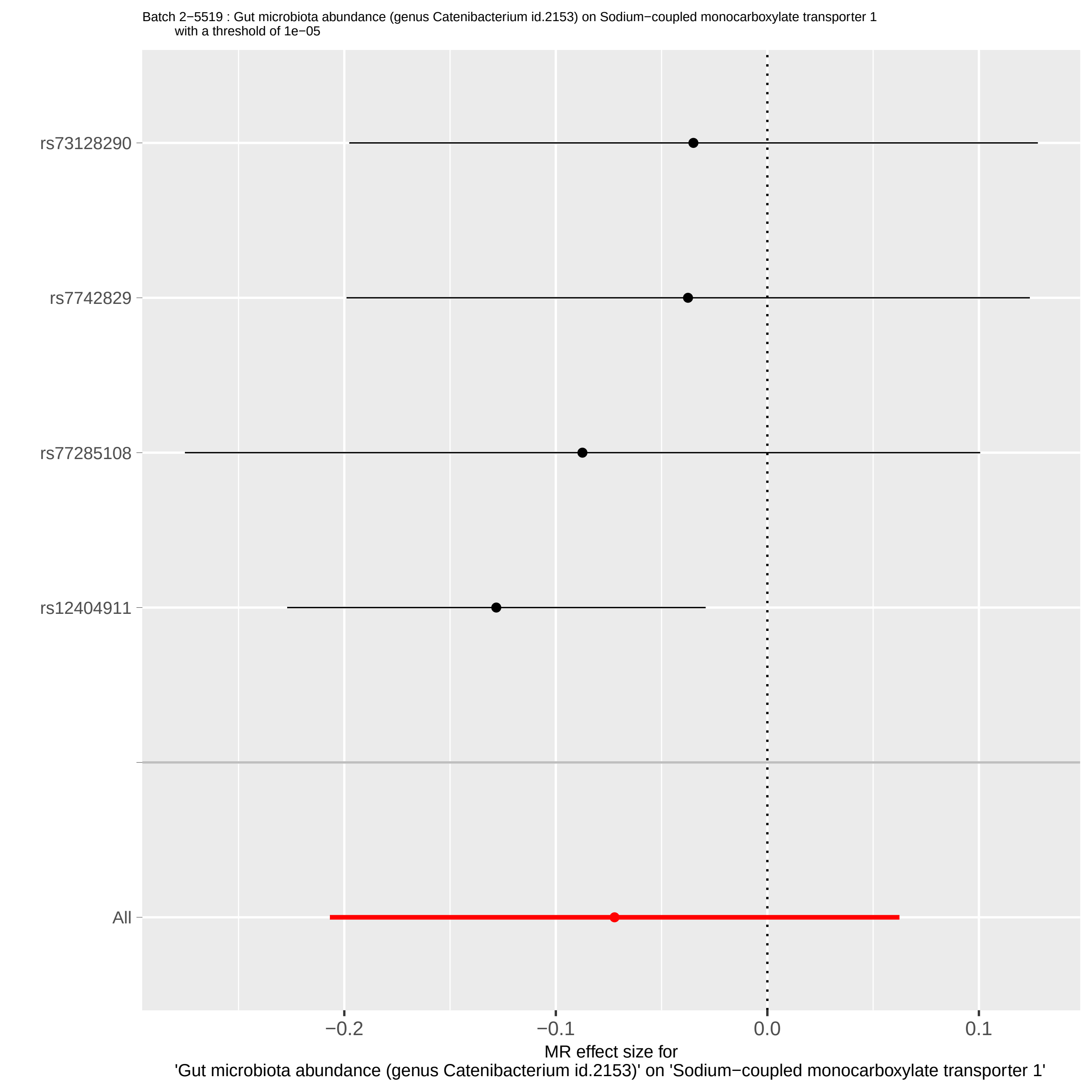

Batch 2−5519 : Gut microbiota abundance (genus Catenibacterium id.2153) on Sodium−coupled monocarboxylate transporter 1
with a threshold of 1e−05
rs73128290
rs7742829
rs77285108
rs12404911
All
−0.2
−0.1
0.0
0.1
MR effect size for
'Gut microbiota abundance (genus Catenibacterium id.2153)' on 'Sodium−coupled monocarboxylate transporter 1'

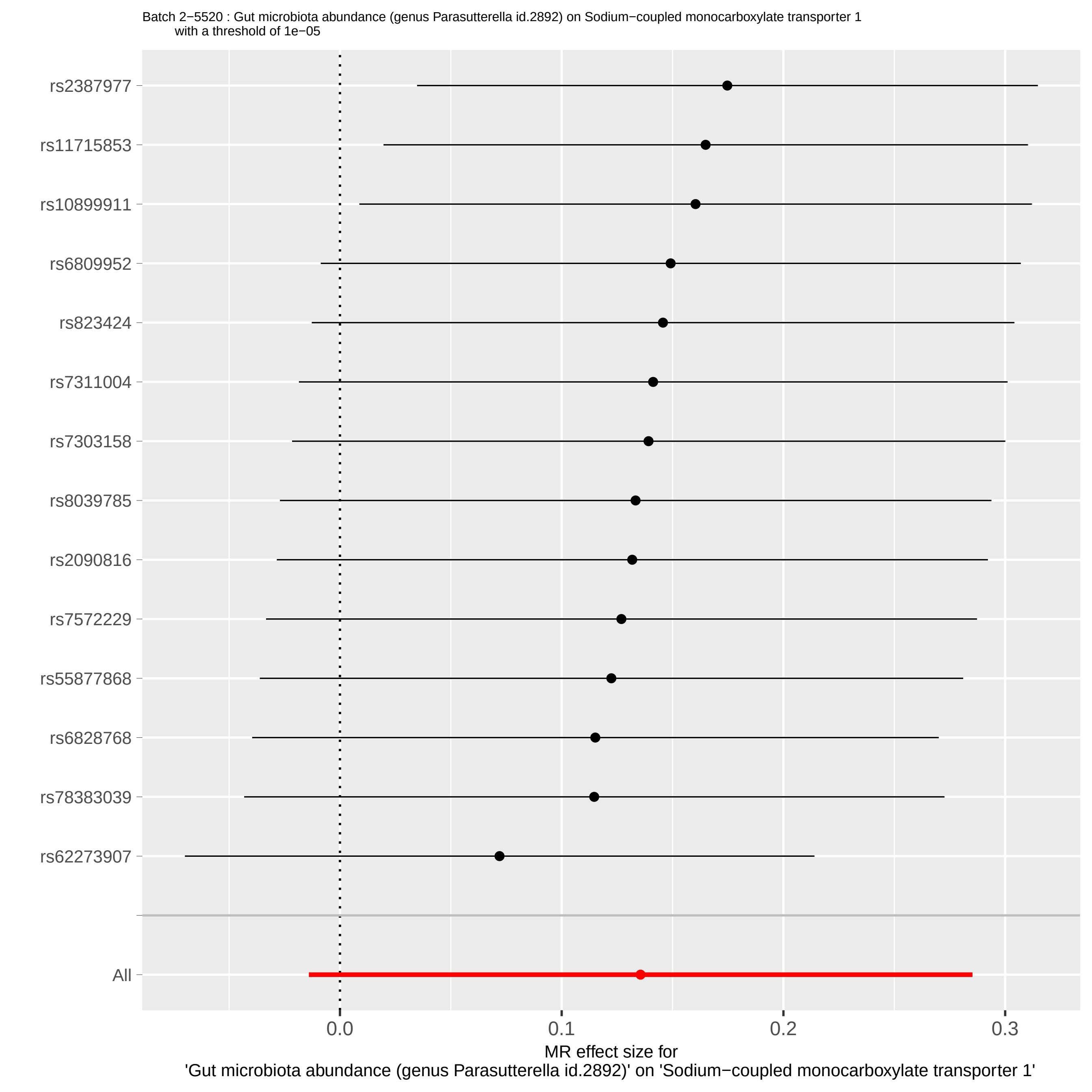
Batch 2−5520 : Gut microbiota abundance (genus Parasutterella id.2892) on Sodium−coupled monocarboxylate transporter 1
with a threshold of 1e−05
rs2387977
rs11715853
rs10899911
rs6809952
rs823424
rs7311004
rs7303158
rs8039785
rs2090816
rs7572229
rs55877868
rs6828768
rs78383039
rs62273907
All
0.0
0.1
0.2
0.3
MR effect size for
'Gut microbiota abundance (genus Parasutterella id.2892)' on 'Sodium−coupled monocarboxylate transporter 1'

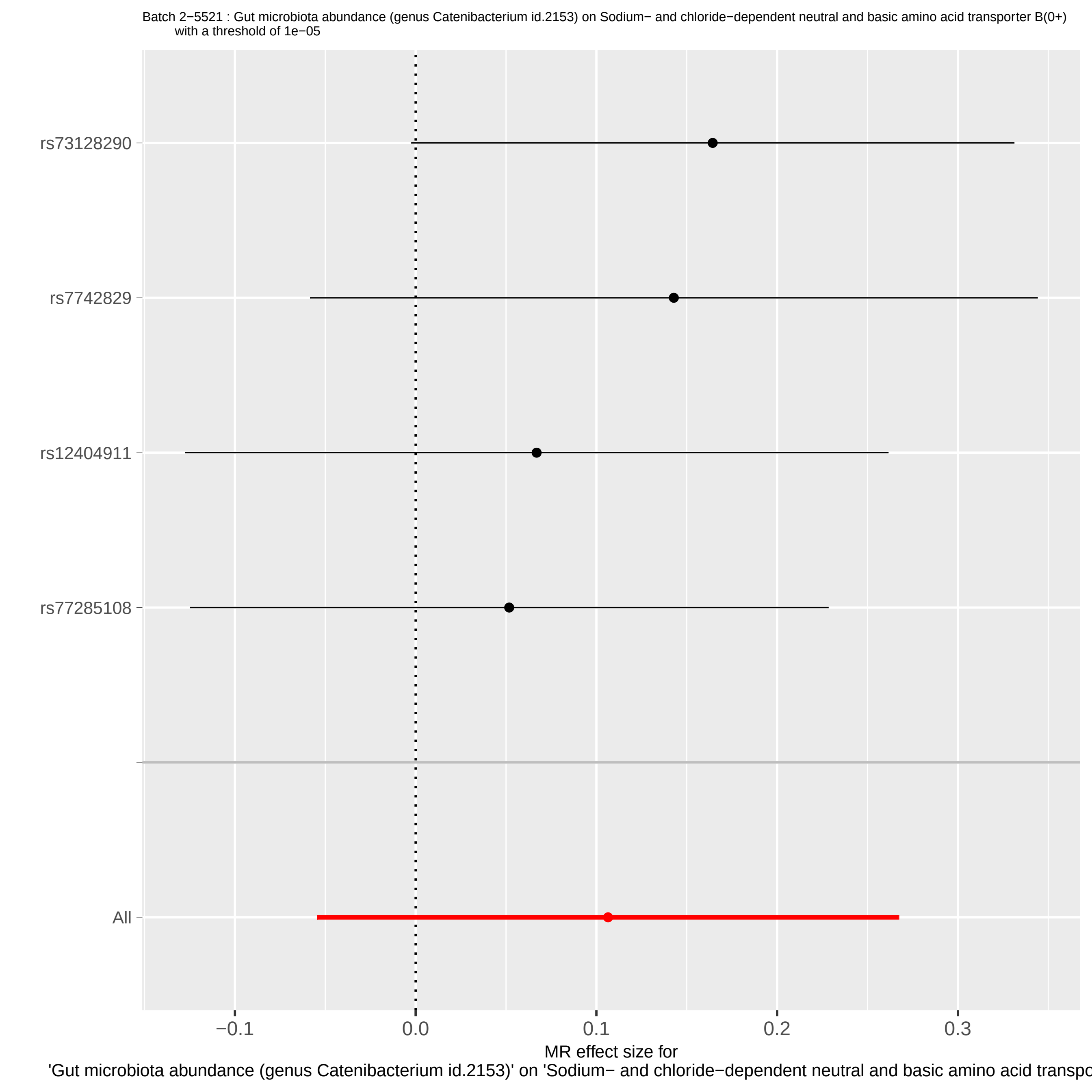
Batch 2−5521 : Gut microbiota abundance (genus Catenibacterium id.2153) on Sodium− and chloride−dependent neutral and basic amino acid transporter B(0+)
with a threshold of 1e−05
rs73128290
rs7742829
rs12404911
rs77285108
All
−0.1
0.0
0.1
0.2
0.3
MR effect size for
'Gut microbiota abundance (genus Catenibacterium id.2153)' on 'Sodium− and chloride−dependent neutral and basic amino acid transpo

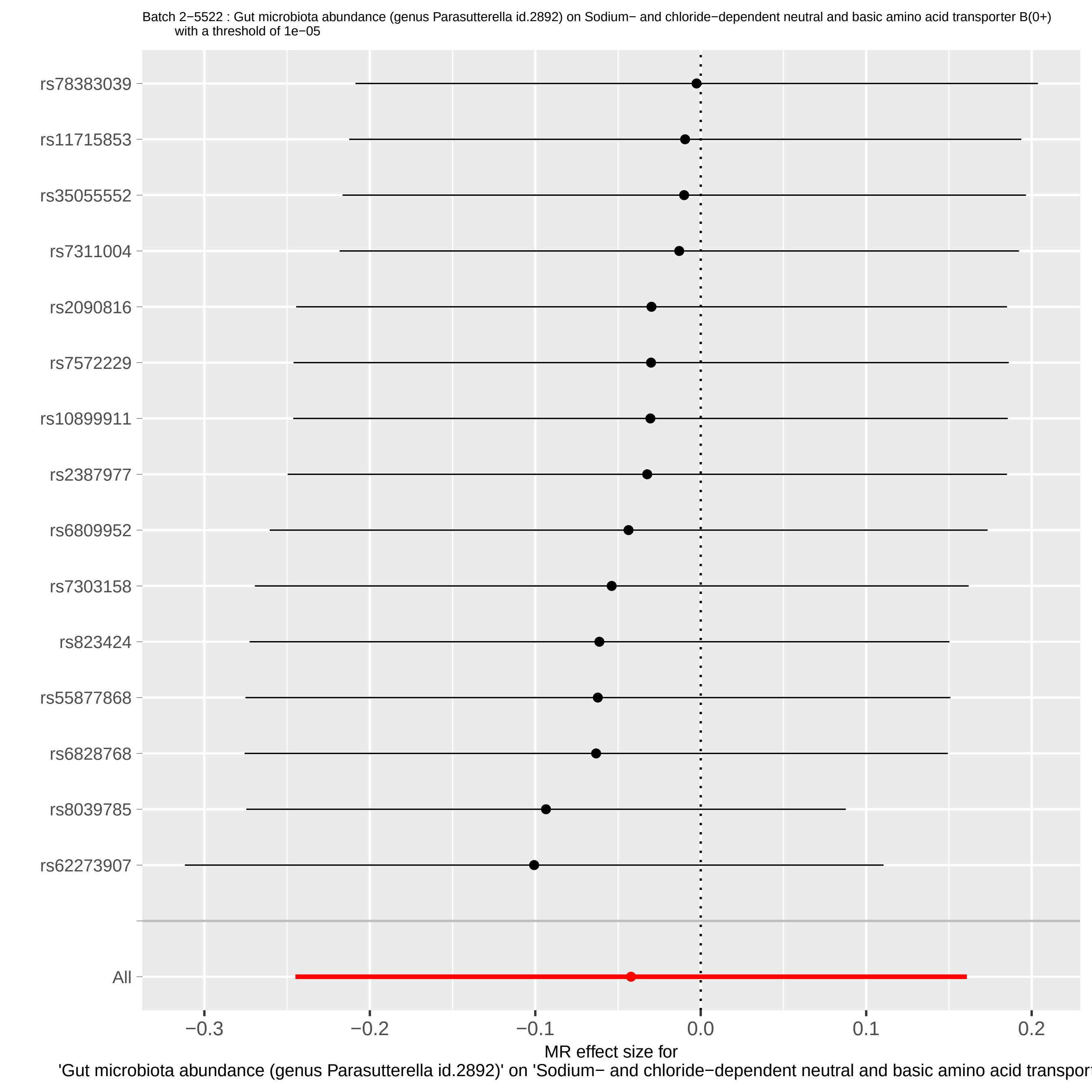

Batch 2−5522 : Gut microbiota abundance (genus Parasutterella id.2892) on Sodium− and chloride−dependent neutral and basic amino acid transporter B(0+)
with a threshold of 1e−05
rs78383039
rs11715853
rs35055552
rs7311004
rs2090816
rs7572229
rs10899911
rs2387977
rs6809952
rs7303158
rs823424
rs55877868
rs6828768
rs8039785
rs62273907
All
−0.3
−0.2
−0.1
0.0
0.1
0.2
MR effect size for
'Gut microbiota abundance (genus Parasutterella id.2892)' on 'Sodium− and chloride−dependent neutral and basic amino acid transpor

Batch 2−5523 : Gut microbiota abundance (genus Catenibacterium id.2153) on Orphan sodium− and chloride−dependent neurotransmitter transporter NTT5
with a threshold of 1e−05

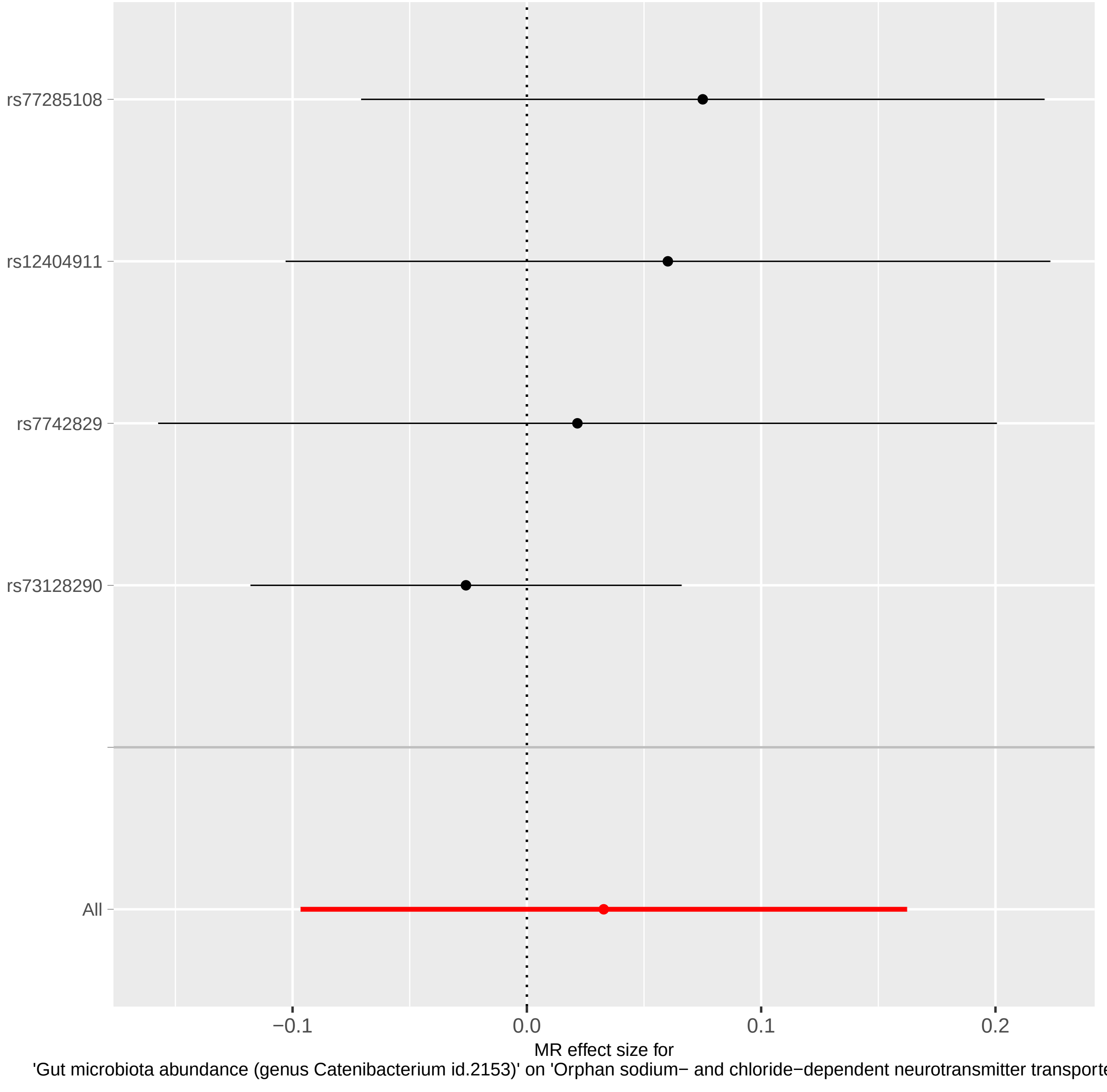

Batch 2−5524 : Gut microbiota abundance (genus Parasutterella id.2892) on Orphan sodium− and chloride−dependent neurotransmitter transporter NTT5 with a threshold of 1e−05

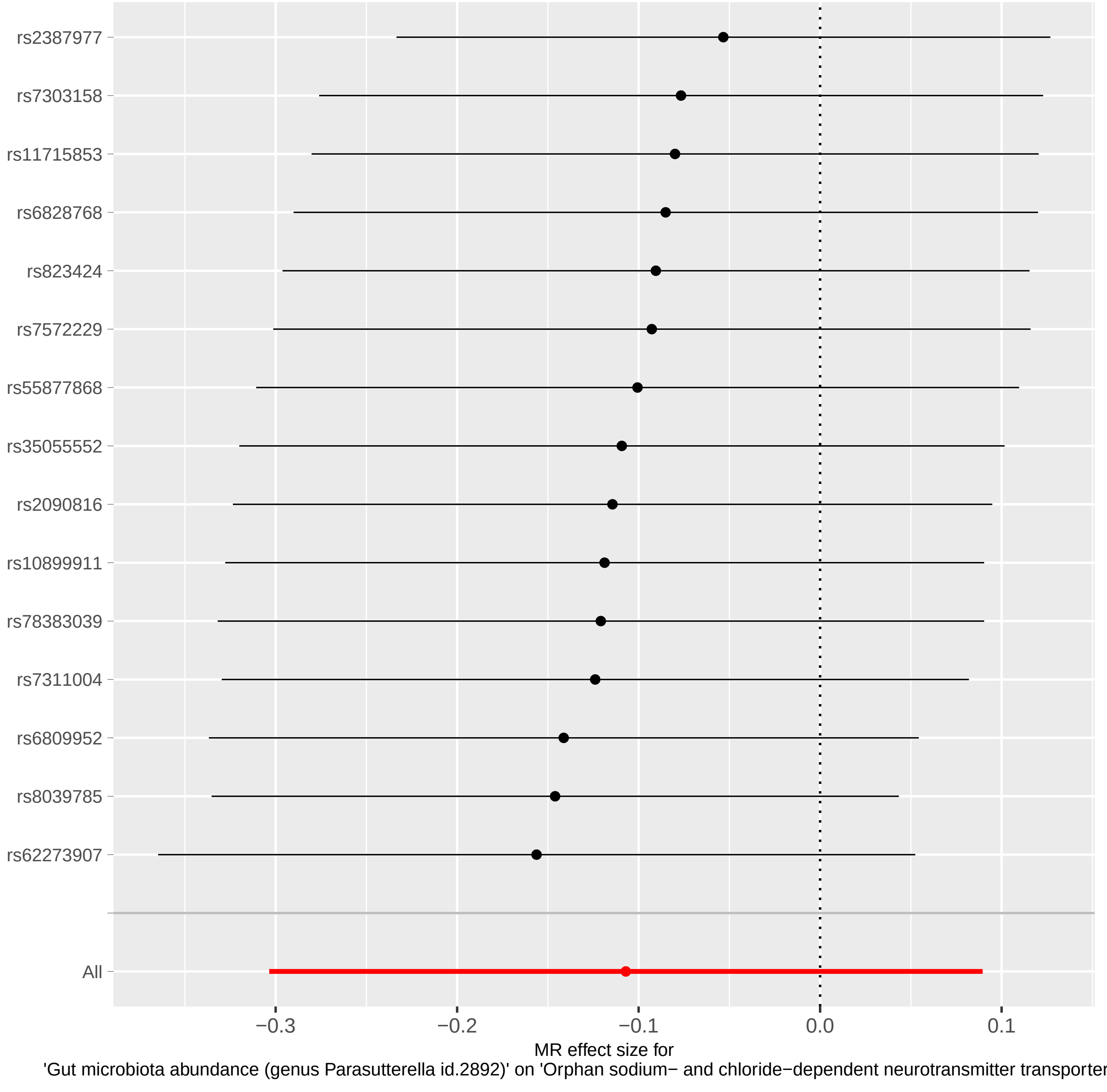

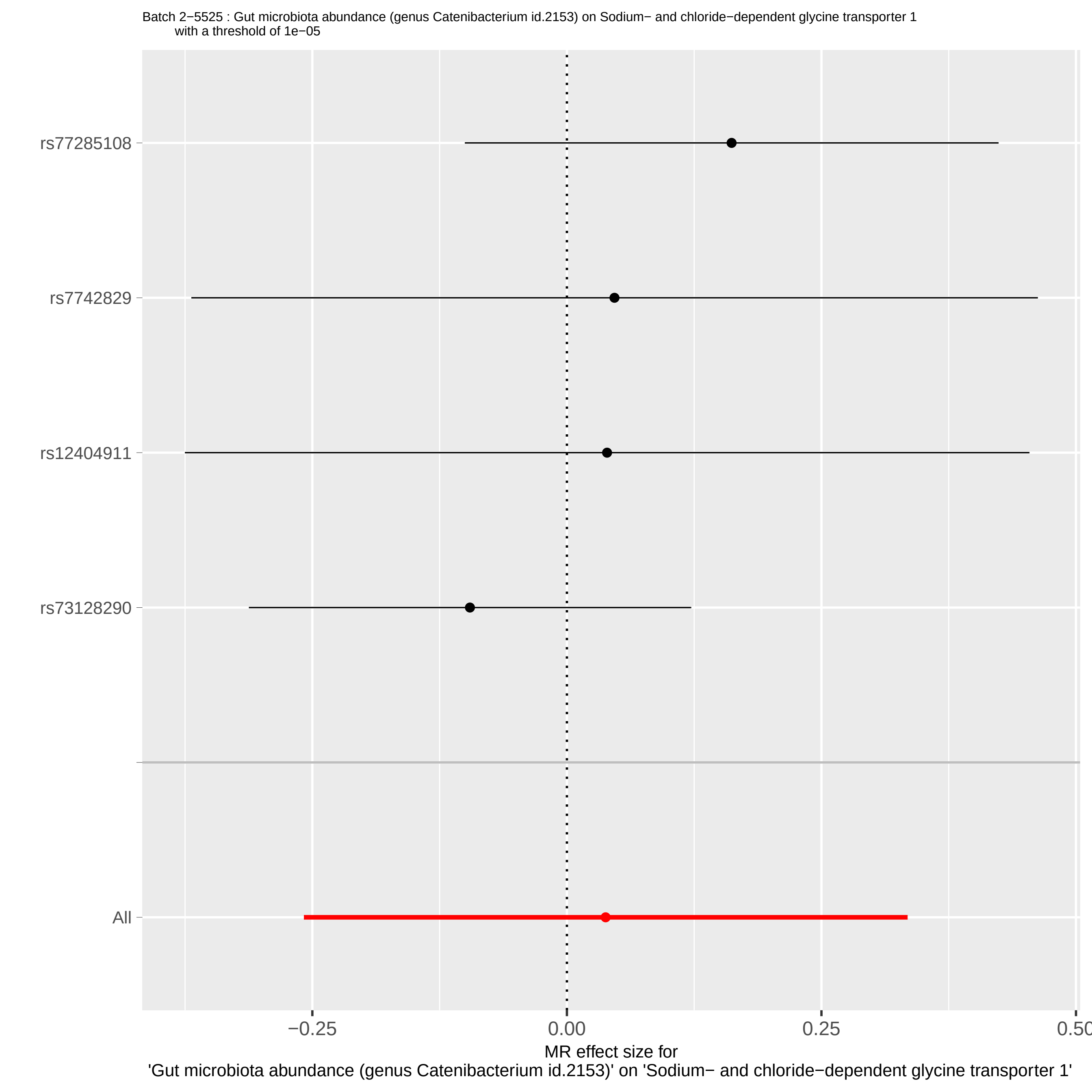

Batch 2−5525 : Gut microbiota abundance (genus Catenibacterium id.2153) on Sodium− and chloride−dependent glycine transporter 1
with a threshold of 1e−05
rs77285108
rs7742829
rs12404911
rs73128290
All
−0.25
0.00
0.25
0.50
MR effect size for
'Gut microbiota abundance (genus Catenibacterium id.2153)' on 'Sodium− and chloride−dependent glycine transporter 1'

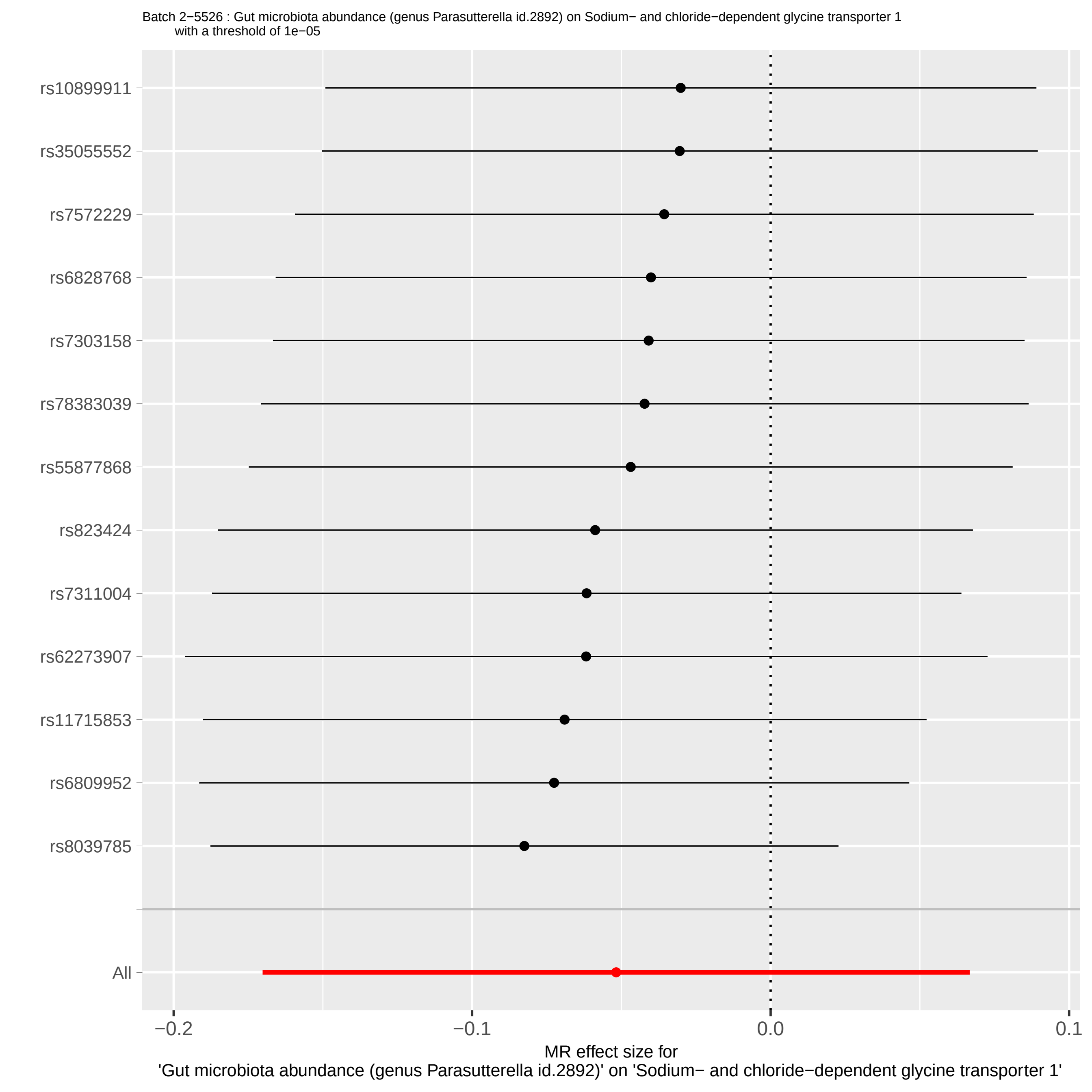

Batch 2−5526 : Gut microbiota abundance (genus Parasutterella id.2892) on Sodium− and chloride−dependent glycine transporter 1 with a threshold of 1e−05
rs10899911
rs35055552
rs7572229
rs6828768
rs7303158
rs78383039
rs55877868
rs823424
rs7311004
rs62273907
rs11715853
rs6809952
rs8039785
All
−0.2
−0.1
0.0
0.1
MR effect size for
'Gut microbiota abundance (genus Parasutterella id.2892)' on 'Sodium− and chloride−dependent glycine transporter 1'

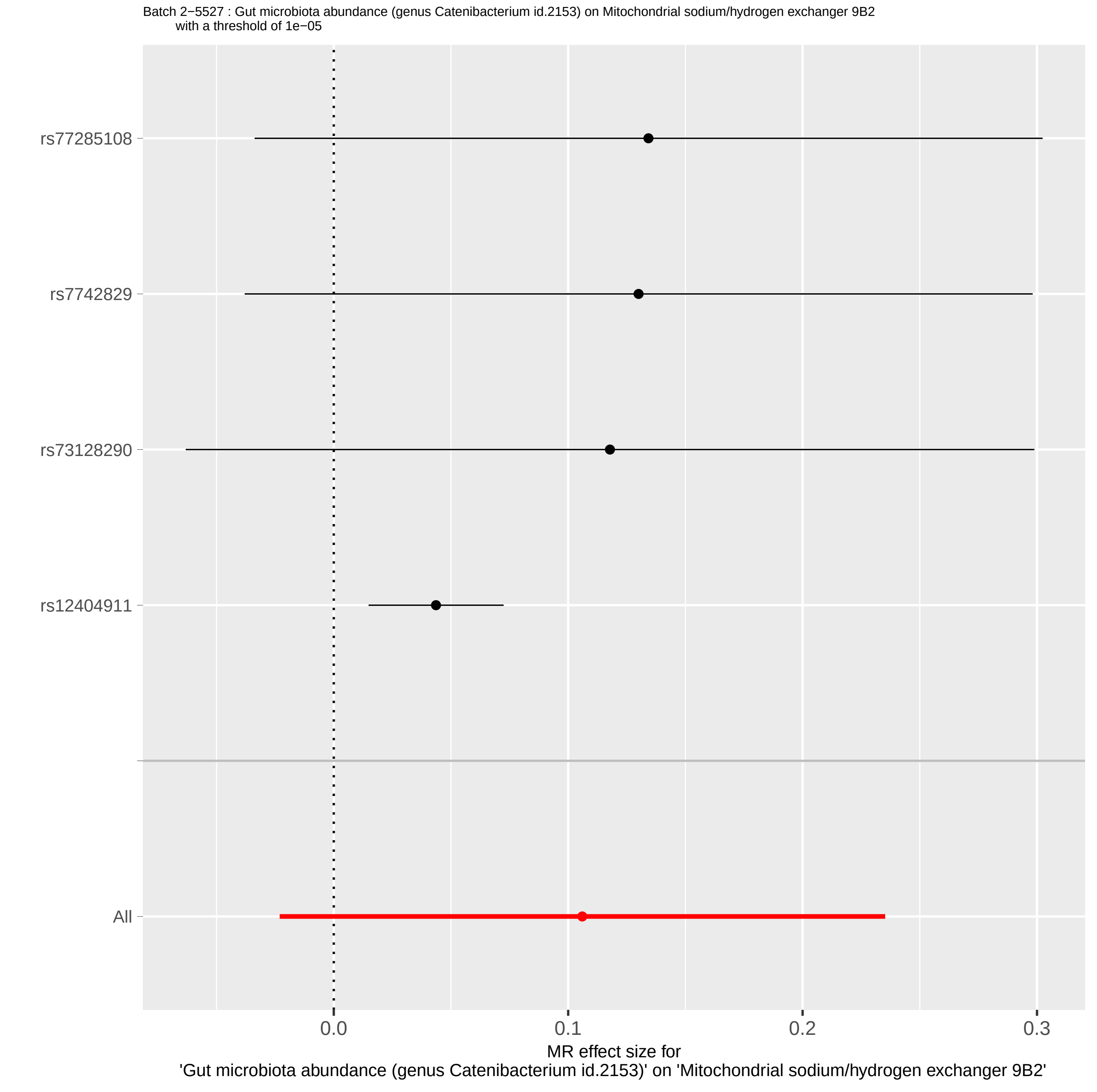

Batch 2−5527 : Gut microbiota abundance (genus Catenibacterium id.2153) on Mitochondrial sodium/hydrogen exchanger 9B2
with a threshold of 1e−05
rs77285108
rs7742829
rs73128290
rs12404911
All
0.0
0.1
0.2
0.3
MR effect size for
'Gut microbiota abundance (genus Catenibacterium id.2153)' on 'Mitochondrial sodium/hydrogen exchanger 9B2'

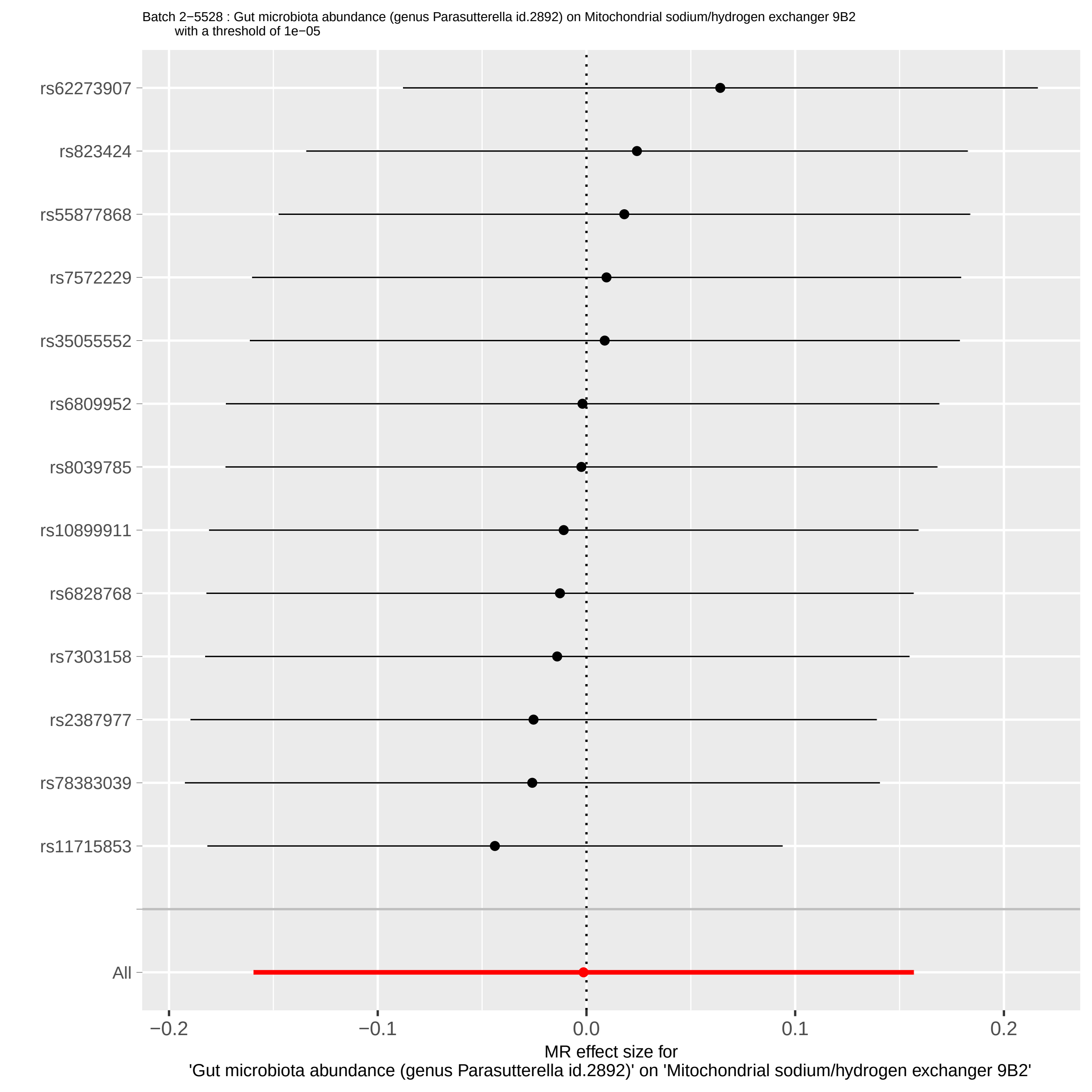
Batch 2−5528 : Gut microbiota abundance (genus Parasutterella id.2892) on Mitochondrial sodium/hydrogen exchanger 9B2
with a threshold of 1e−05
rs62273907
rs823424
rs55877868
rs7572229
rs35055552
rs6809952
rs8039785
rs10899911
rs6828768
rs7303158
rs2387977
rs78383039
rs11715853
All
−0.2
−0.1
0.0
0.1
0.2
MR effect size for
'Gut microbiota abundance (genus Parasutterella id.2892)' on 'Mitochondrial sodium/hydrogen exchanger 9B2'

Batch 2−5529 : Gut microbiota abundance (genus Catenibacterium id.2153) on Solute carrier organic anion transporter family member 5A1 with a threshold of 1e−05

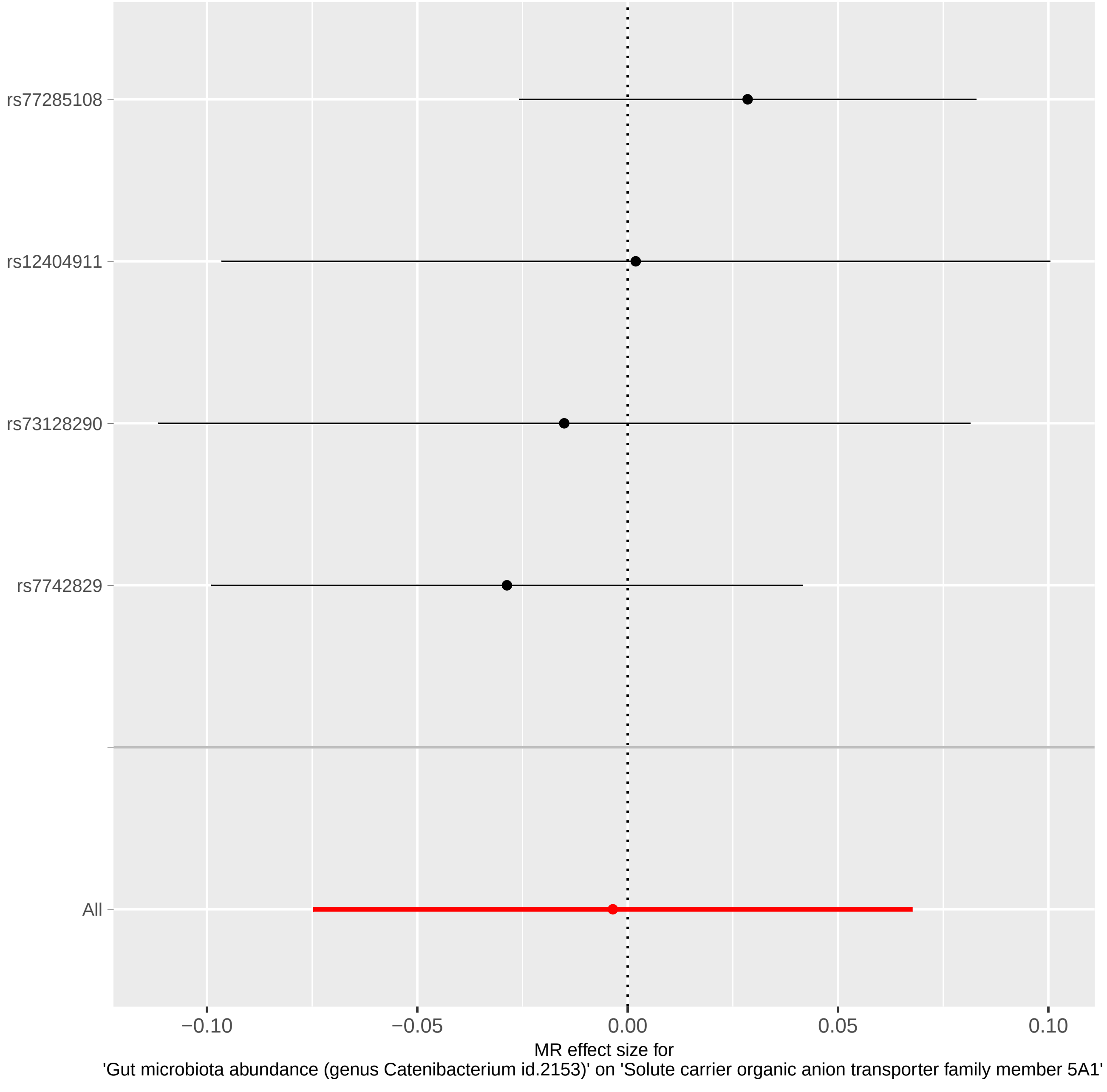

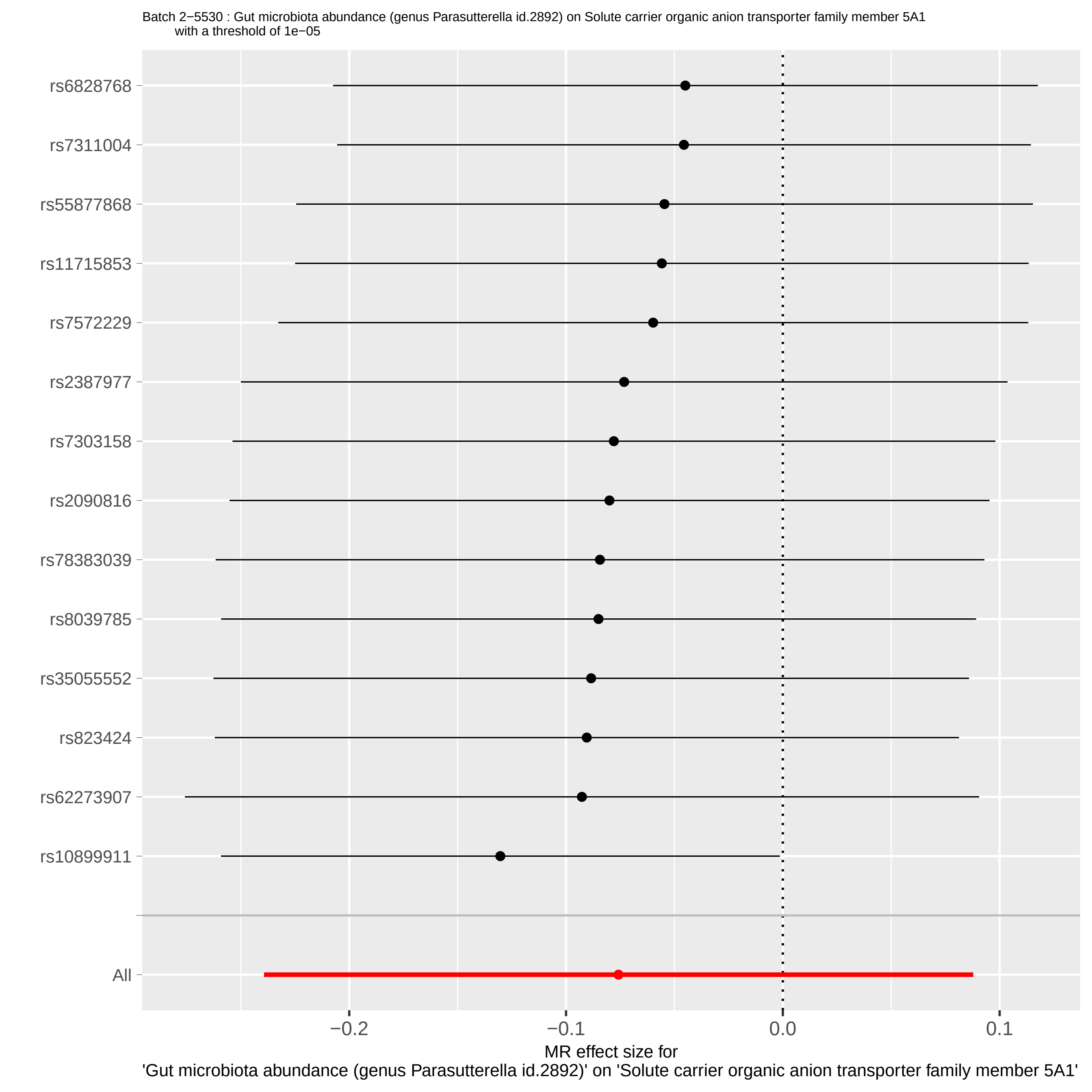
Batch 2−5530 : Gut microbiota abundance (genus Parasutterella id.2892) on Solute carrier organic anion transporter family member 5A1
with a threshold of 1e−05
rs6828768
rs7311004
rs55877868
rs11715853
rs7572229
rs2387977
rs7303158
rs2090816
rs78383039
rs8039785
rs35055552
rs823424
rs62273907
rs10899911
All
−0.2
−0.1
0.0
0.1
MR effect size for
'Gut microbiota abundance (genus Parasutterella id.2892)' on 'Solute carrier organic anion transporter family member 5A1'

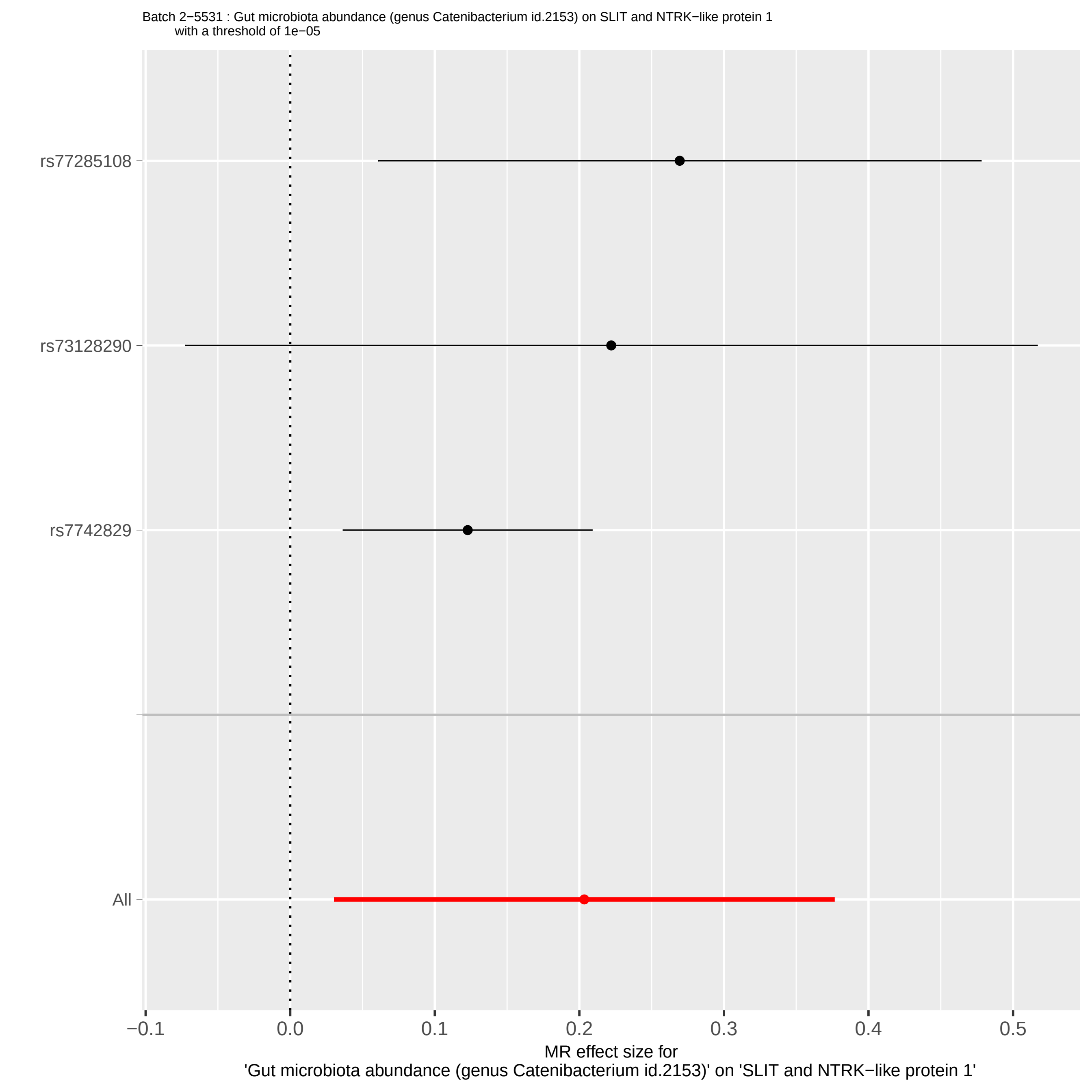

Batch 2−5531 : Gut microbiota abundance (genus Catenibacterium id.2153) on SLIT and NTRK−like protein 1
with a threshold of 1e−05
rs77285108
rs73128290
rs7742829
All
−0.1
0.0
0.1
0.2
0.3
0.4
0.5
MR effect size for
'Gut microbiota abundance (genus Catenibacterium id.2153)' on 'SLIT and NTRK−like protein 1'

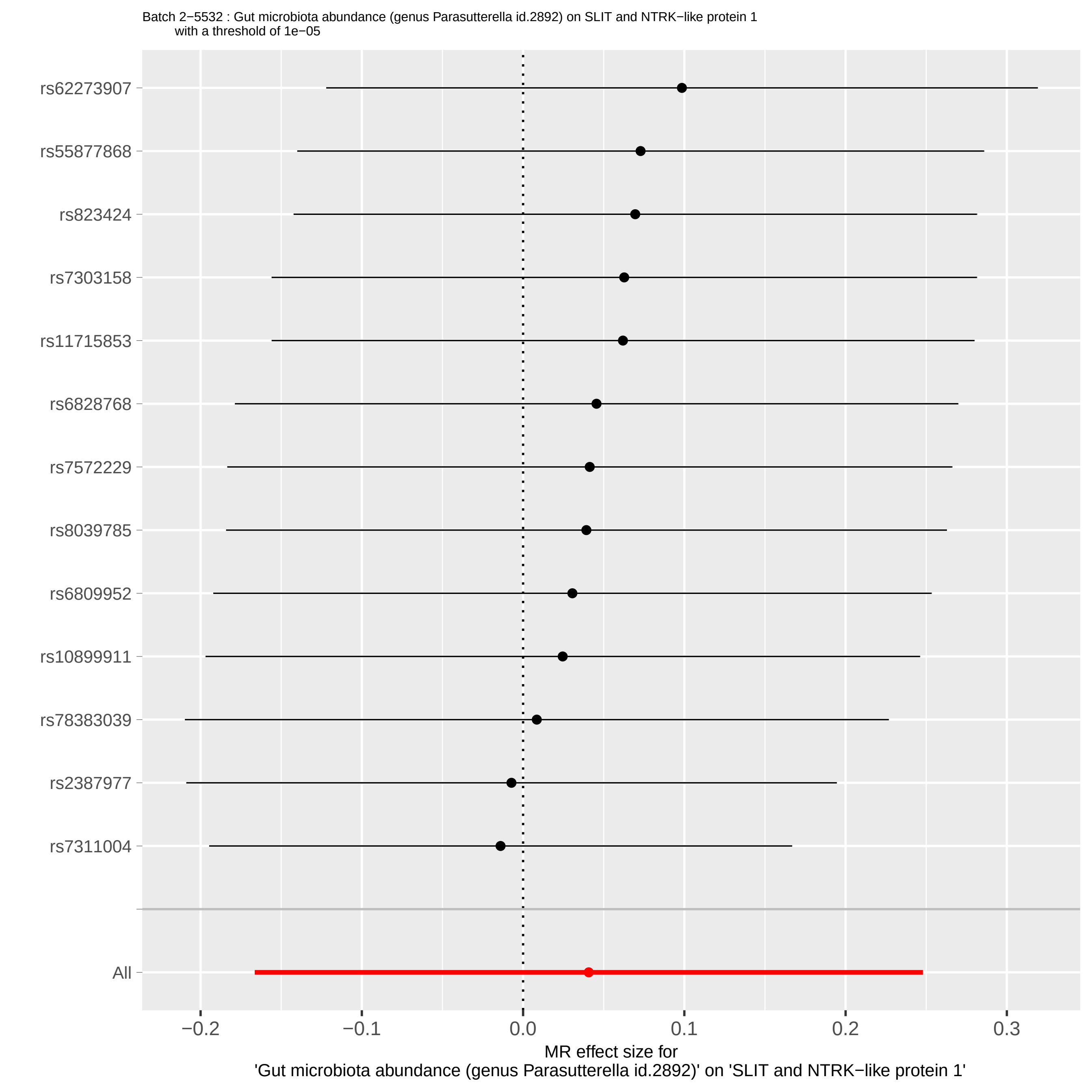

Batch 2−5532 : Gut microbiota abundance (genus Parasutterella id.2892) on SLIT and NTRK−like protein 1
with a threshold of 1e−05
rs62273907
rs55877868
rs823424
rs7303158
rs11715853
rs6828768
rs7572229
rs8039785
rs6809952
rs10899911
rs78383039
rs2387977
rs7311004
All
−0.2
−0.1
0.0
0.1
0.2
0.3
MR effect size for
'Gut microbiota abundance (genus Parasutterella id.2892)' on 'SLIT and NTRK−like protein 1'

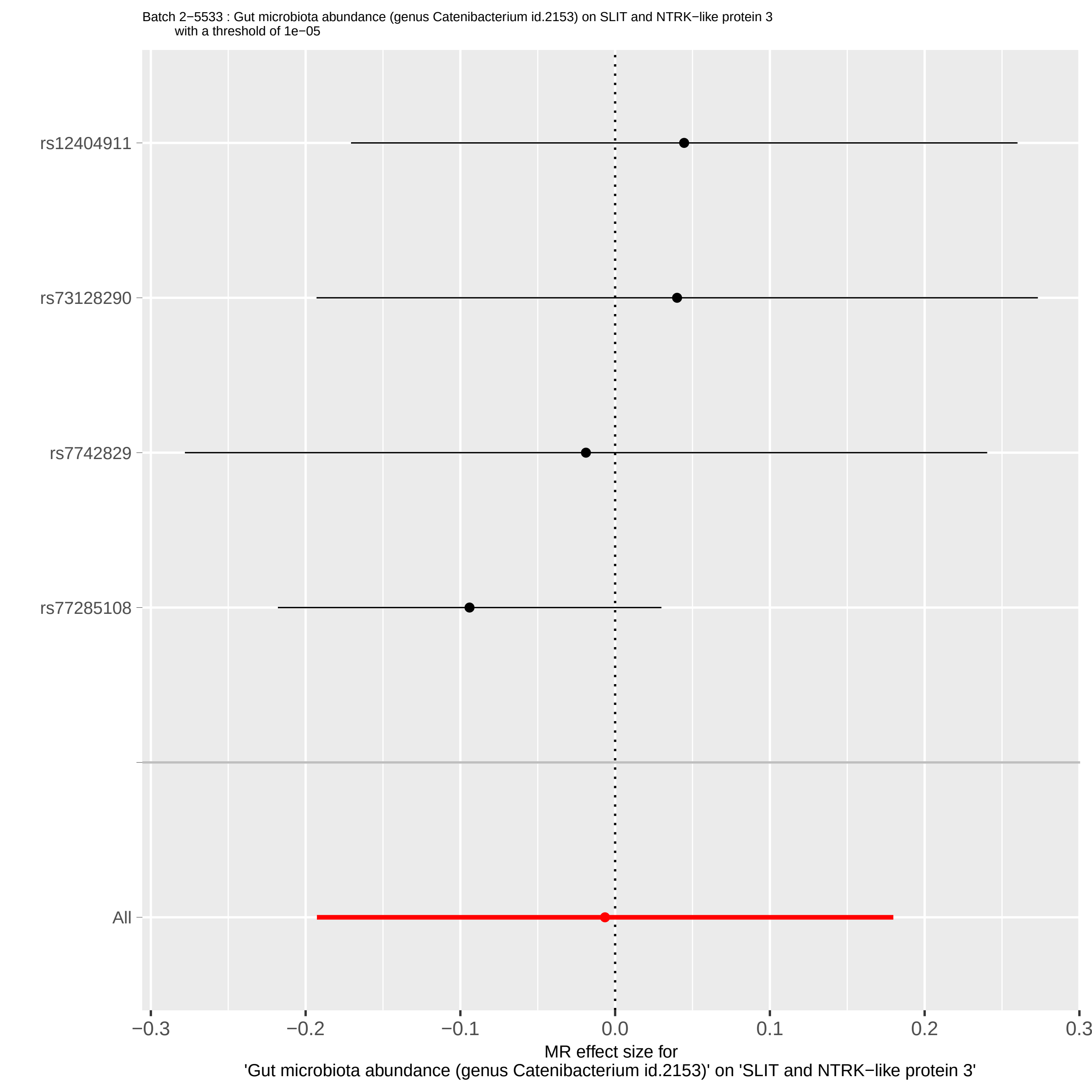
Batch 2−5533 : Gut microbiota abundance (genus Catenibacterium id.2153) on SLIT and NTRK−like protein 3
with a threshold of 1e−05
rs12404911
rs73128290
rs7742829
rs77285108
All
−0.3
−0.2
−0.1
0.0
0.1
0.2
0.3
MR effect size for
'Gut microbiota abundance (genus Catenibacterium id.2153)' on 'SLIT and NTRK−like protein 3'

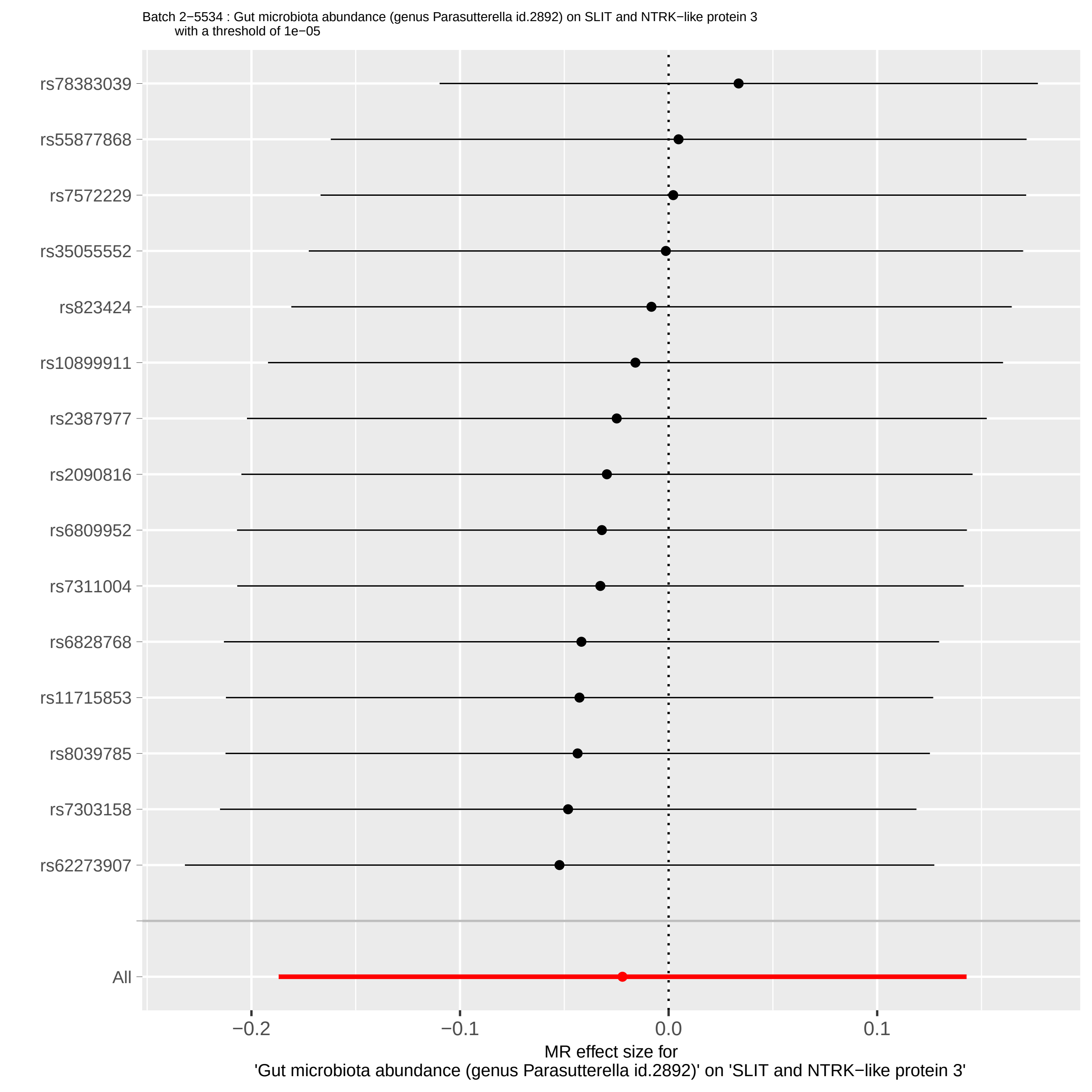
Batch 2−5534 : Gut microbiota abundance (genus Parasutterella id.2892) on SLIT and NTRK−like protein 3
with a threshold of 1e−05
rs78383039
rs55877868
rs7572229
rs35055552
rs823424
rs10899911
rs2387977
rs2090816
rs6809952
rs7311004
rs6828768
rs11715853
rs8039785
rs7303158
rs62273907
All
−0.2
−0.1
0.0
0.1
MR effect size for
'Gut microbiota abundance (genus Parasutterella id.2892)' on 'SLIT and NTRK−like protein 3'

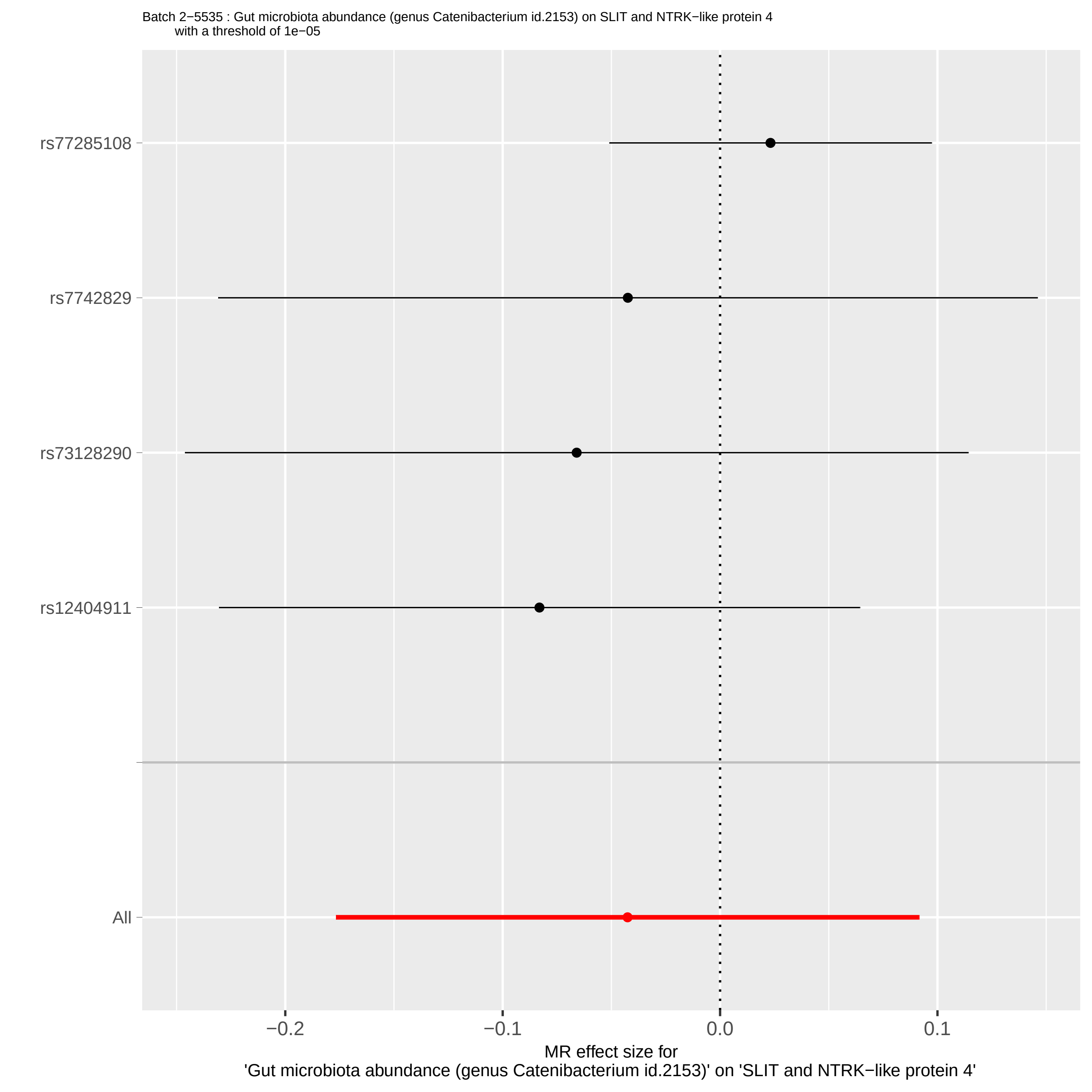
Batch 2−5535 : Gut microbiota abundance (genus Catenibacterium id.2153) on SLIT and NTRK−like protein 4
with a threshold of 1e−05
rs77285108
rs7742829
rs73128290
rs12404911
All
−0.2
−0.1
0.0
0.1
MR effect size for
'Gut microbiota abundance (genus Catenibacterium id.2153)' on 'SLIT and NTRK−like protein 4'

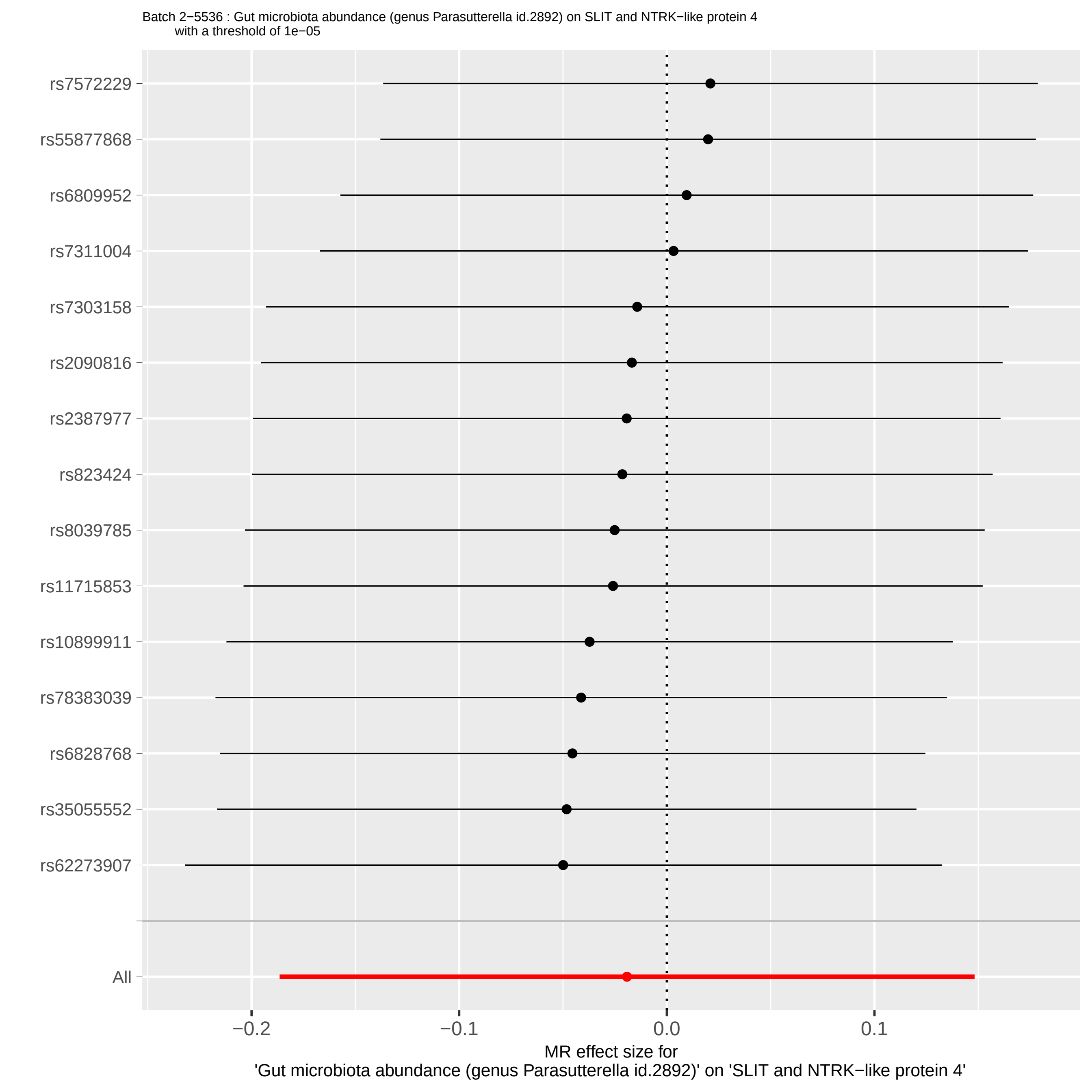
Batch 2−5536 : Gut microbiota abundance (genus Parasutterella id.2892) on SLIT and NTRK−like protein 4
with a threshold of 1e−05
rs7572229
rs55877868
rs6809952
rs7311004
rs7303158
rs2090816
rs2387977
rs823424
rs8039785
rs11715853
rs10899911
rs78383039
rs6828768
rs35055552
rs62273907
All
−0.2
−0.1
0.0
0.1
MR effect size for
'Gut microbiota abundance (genus Parasutterella id.2892)' on 'SLIT and NTRK−like protein 4'

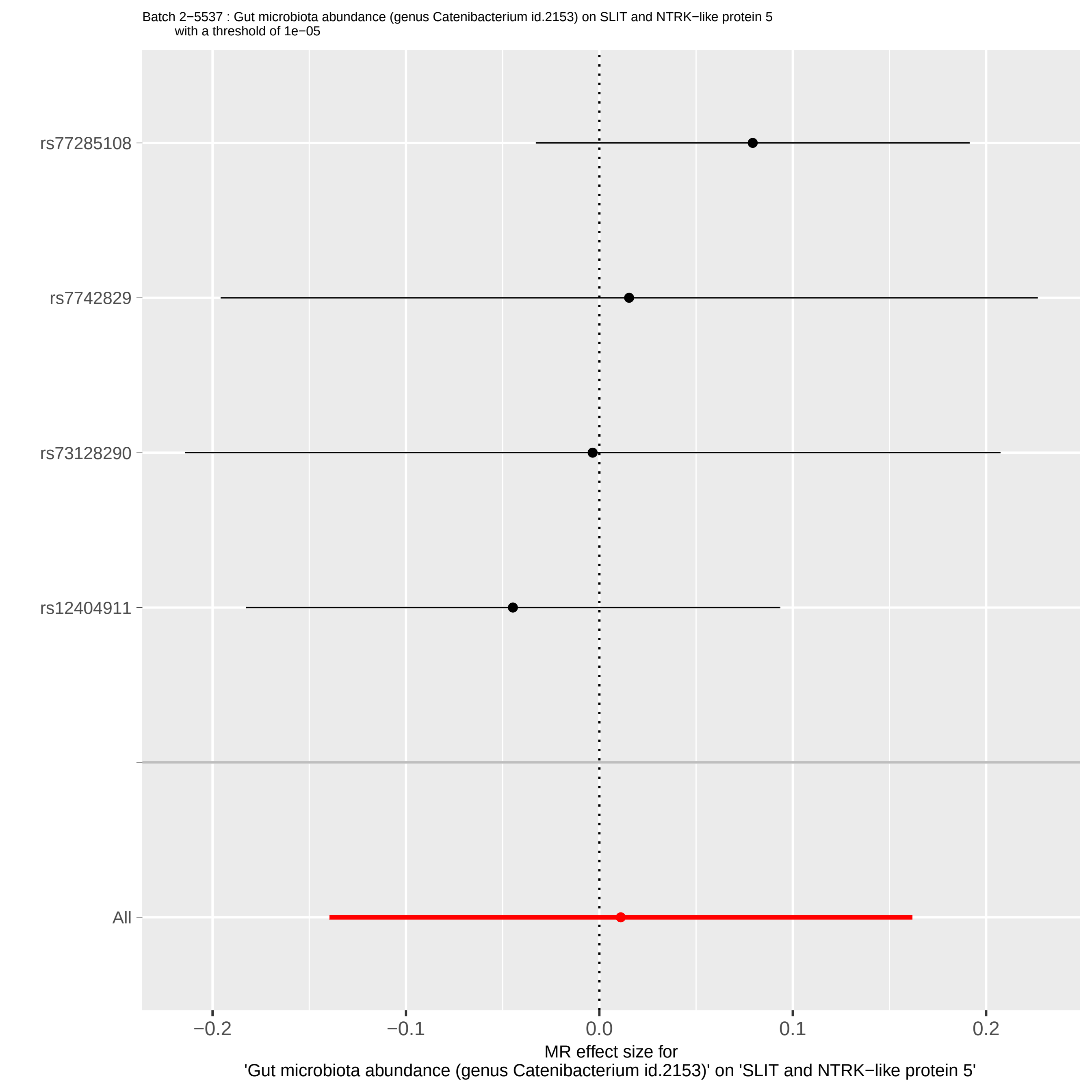
Batch 2−5537 : Gut microbiota abundance (genus Catenibacterium id.2153) on SLIT and NTRK−like protein 5
with a threshold of 1e−05
rs77285108
rs7742829
rs73128290
rs12404911
All
−0.2
−0.1
0.0
0.1
0.2
MR effect size for
'Gut microbiota abundance (genus Catenibacterium id.2153)' on 'SLIT and NTRK−like protein 5'

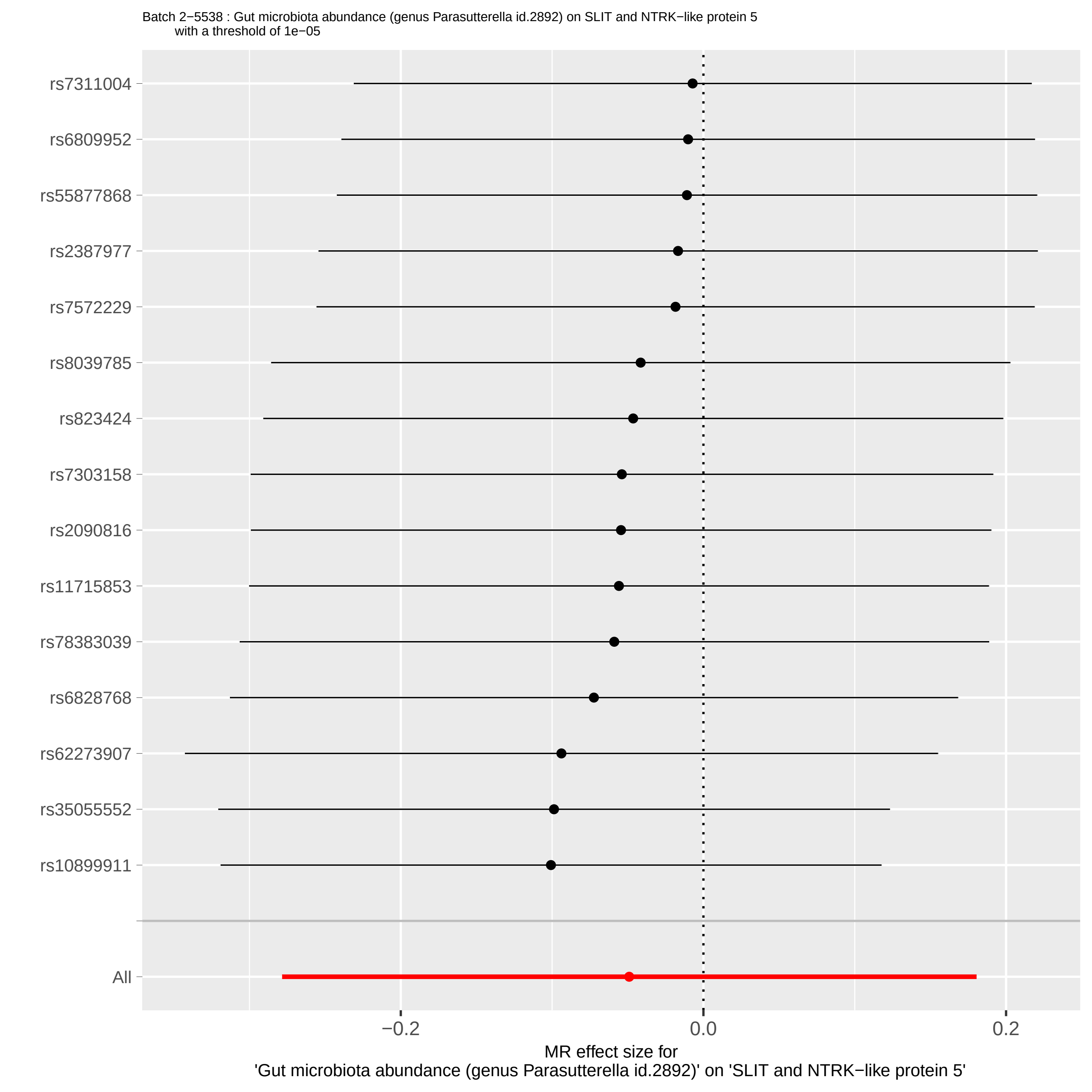
Batch 2−5538 : Gut microbiota abundance (genus Parasutterella id.2892) on SLIT and NTRK−like protein 5
with a threshold of 1e−05
rs7311004
rs6809952
rs55877868
rs2387977
rs7572229
rs8039785
rs823424
rs7303158
rs2090816
rs11715853
rs78383039
rs6828768
rs62273907
rs35055552
rs10899911
All
−0.2
0.0
0.2
MR effect size for
'Gut microbiota abundance (genus Parasutterella id.2892)' on 'SLIT and NTRK−like protein 5'

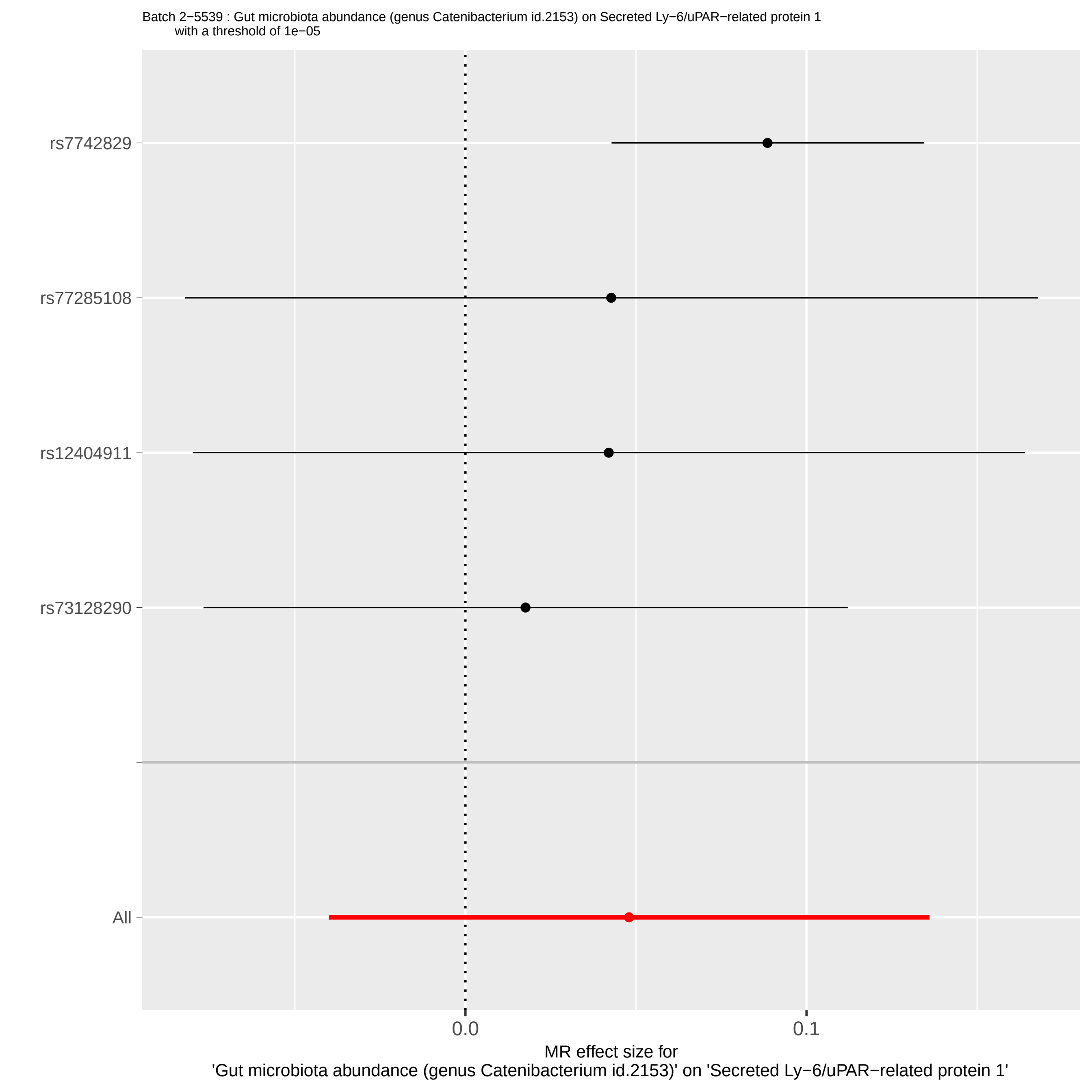
Batch 2−5539 : Gut microbiota abundance (genus Catenibacterium id.2153) on Secreted Ly−6/uPAR−related protein 1
with a threshold of 1e−05
rs7742829
rs77285108
rs12404911
rs73128290
All
0.0
0.1
MR effect size for
'Gut microbiota abundance (genus Catenibacterium id.2153)' on 'Secreted Ly−6/uPAR−related protein 1'

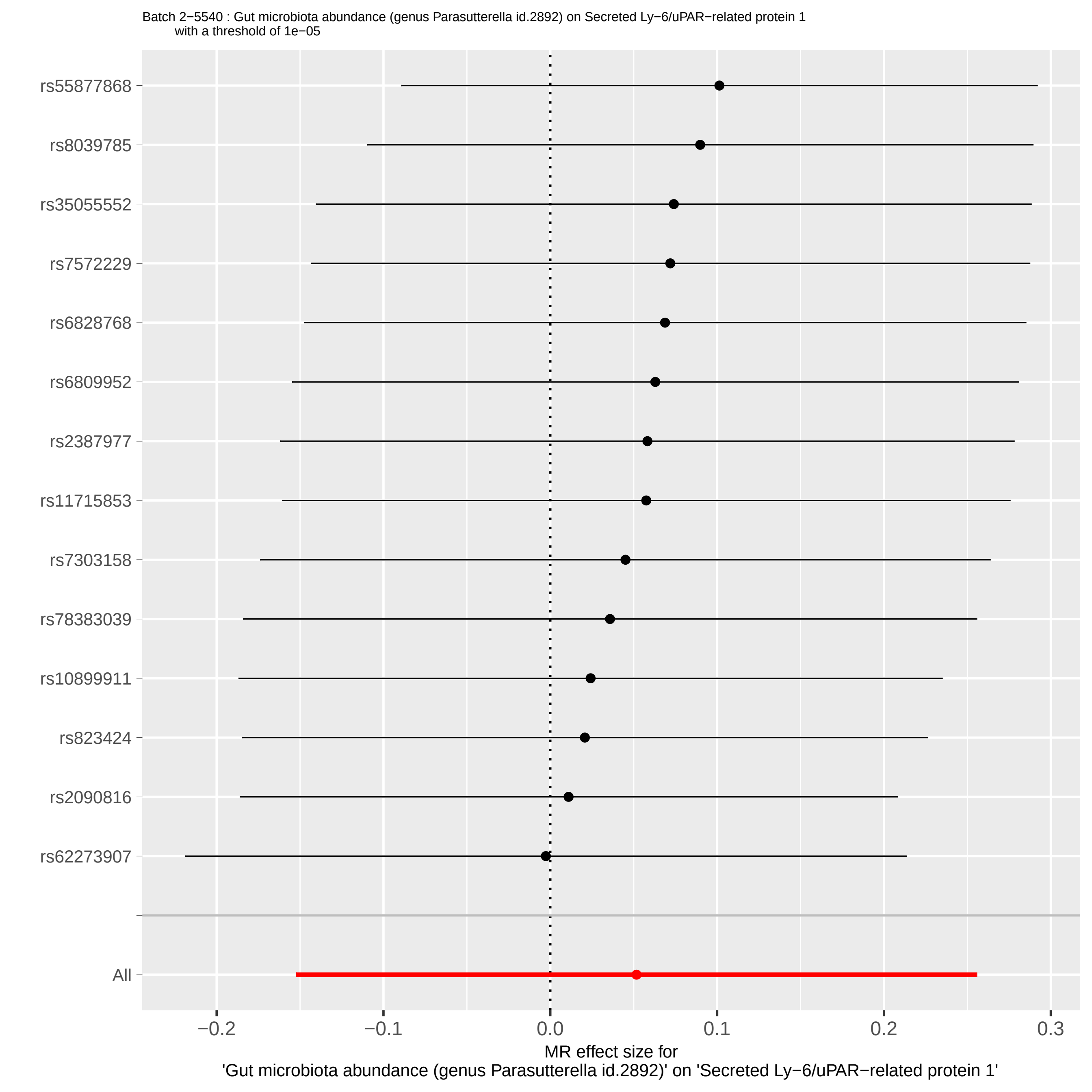

Batch 2−5540 : Gut microbiota abundance (genus Parasutterella id.2892) on Secreted Ly−6/uPAR−related protein 1
with a threshold of 1e−05
rs55877868
rs8039785
rs35055552
rs7572229
rs6828768
rs6809952
rs2387977
rs11715853
rs7303158
rs78383039
rs10899911
rs823424
rs2090816
rs62273907
All
−0.2
−0.1
0.0
0.1
0.2
0.3
MR effect size for
'Gut microbiota abundance (genus Parasutterella id.2892)' on 'Secreted Ly−6/uPAR−related protein 1'

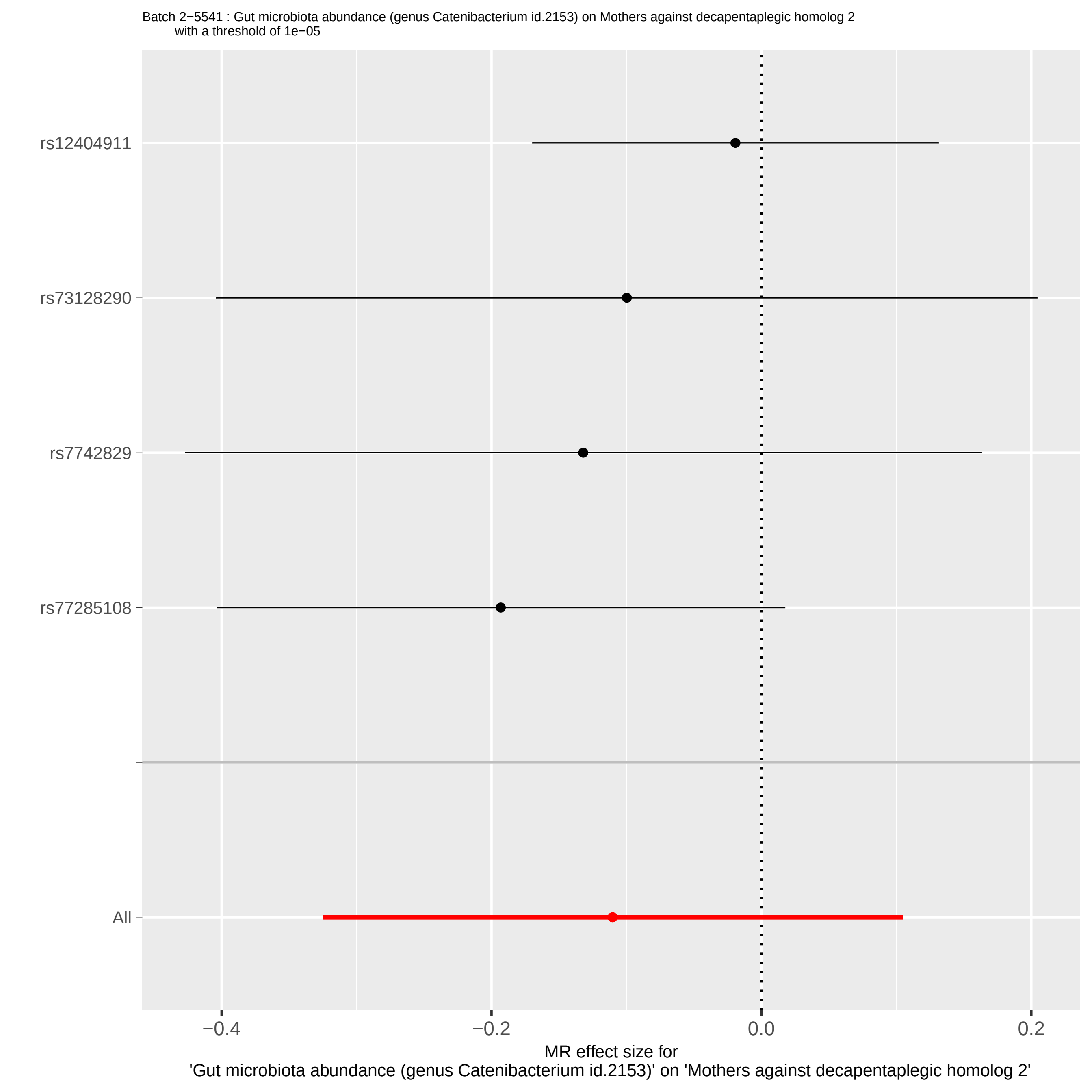
Batch 2−5541 : Gut microbiota abundance (genus Catenibacterium id.2153) on Mothers against decapentaplegic homolog 2
with a threshold of 1e−05
rs12404911
rs73128290
rs7742829
rs77285108
All
−0.4
−0.2
0.0
0.2
MR effect size for
'Gut microbiota abundance (genus Catenibacterium id.2153)' on 'Mothers against decapentaplegic homolog 2'

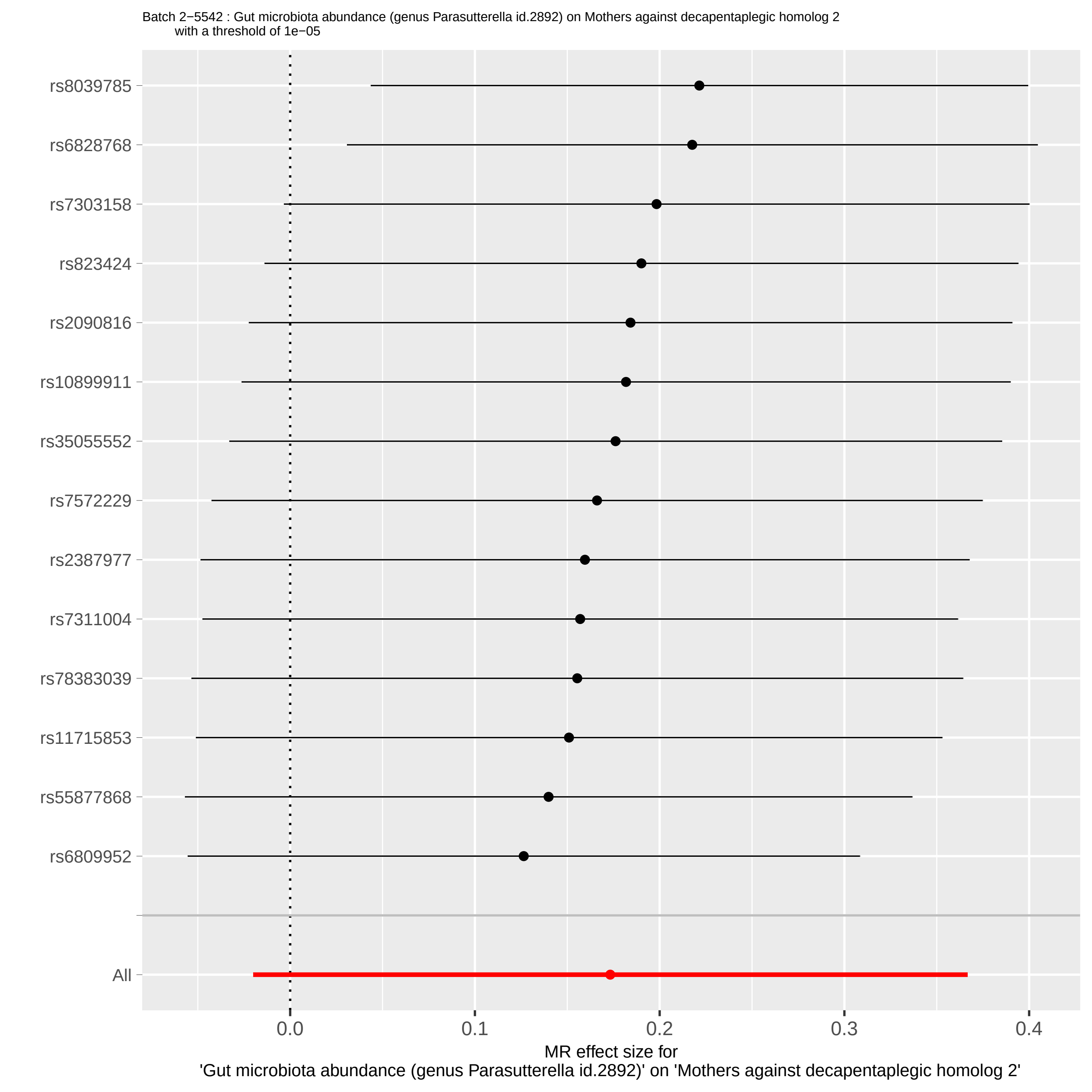
Batch 2−5542 : Gut microbiota abundance (genus Parasutterella id.2892) on Mothers against decapentaplegic homolog 2
with a threshold of 1e−05
rs8039785
rs6828768
rs7303158
rs823424
rs2090816
rs10899911
rs35055552
rs7572229
rs2387977
rs7311004
rs78383039
rs11715853
rs55877868
rs6809952
All
0.0
0.1
0.2
0.3
0.4
MR effect size for
'Gut microbiota abundance (genus Parasutterella id.2892)' on 'Mothers against decapentaplegic homolog 2'

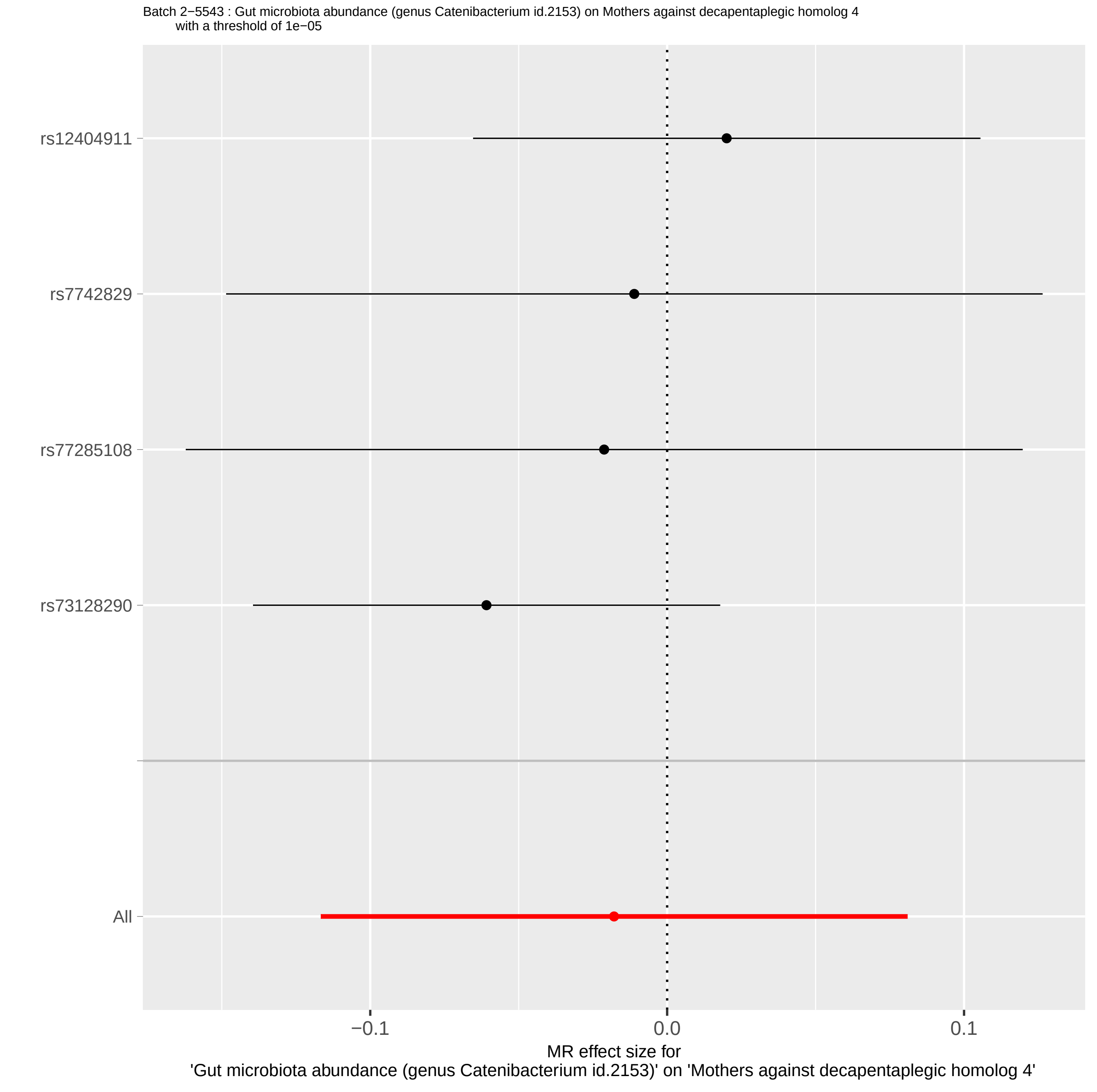
Batch 2−5543 : Gut microbiota abundance (genus Catenibacterium id.2153) on Mothers against decapentaplegic homolog 4
with a threshold of 1e−05
rs12404911
rs7742829
rs77285108
rs73128290
All
−0.1
0.0
0.1
MR effect size for
'Gut microbiota abundance (genus Catenibacterium id.2153)' on 'Mothers against decapentaplegic homolog 4'

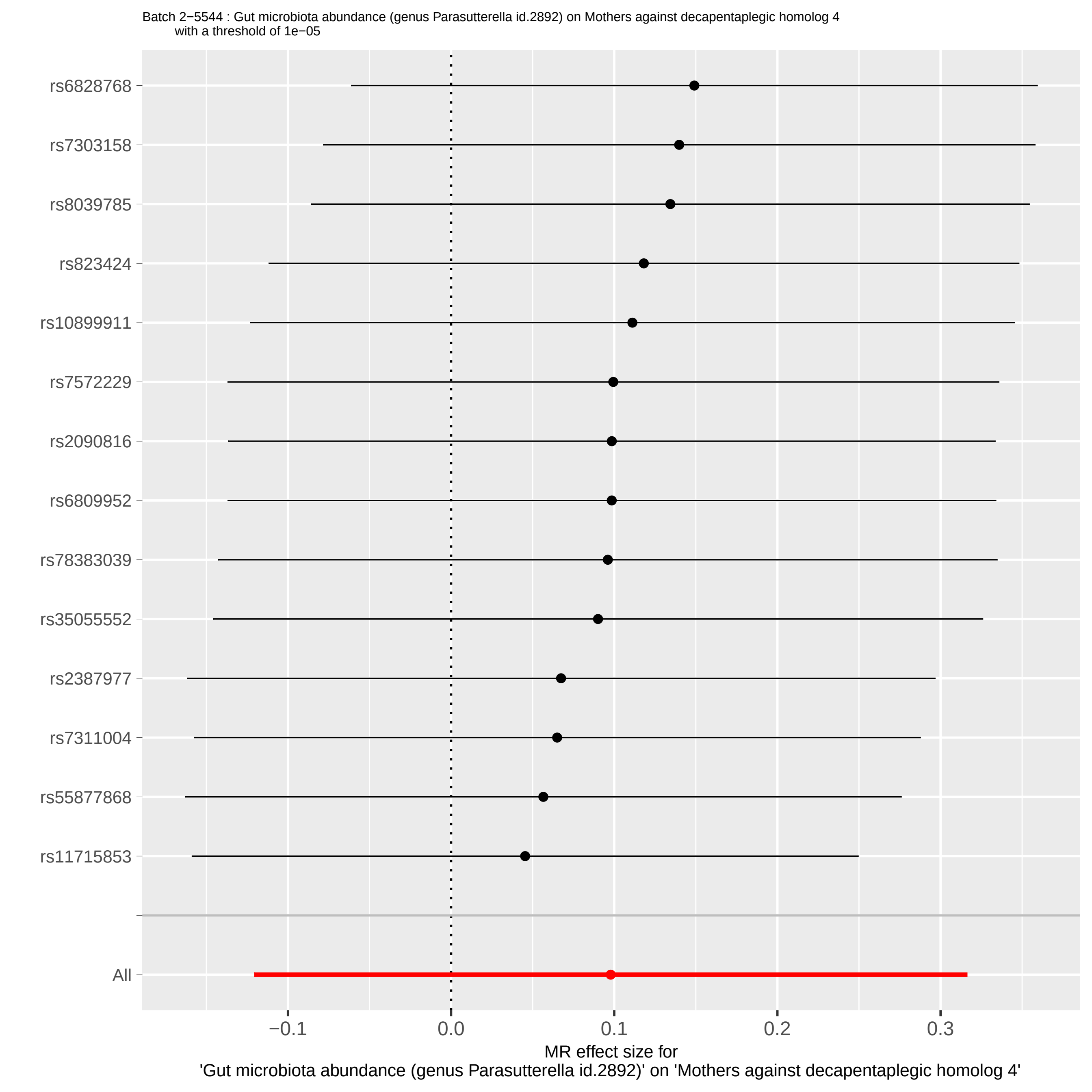
Batch 2−5544 : Gut microbiota abundance (genus Parasutterella id.2892) on Mothers against decapentaplegic homolog 4
with a threshold of 1e−05
rs6828768
rs7303158
rs8039785
rs823424
rs10899911
rs7572229
rs2090816
rs6809952
rs78383039
rs35055552
rs2387977
rs7311004
rs55877868
rs11715853
All
−0.1
0.0
0.1
0.2
0.3
MR effect size for
'Gut microbiota abundance (genus Parasutterella id.2892)' on 'Mothers against decapentaplegic homolog 4'

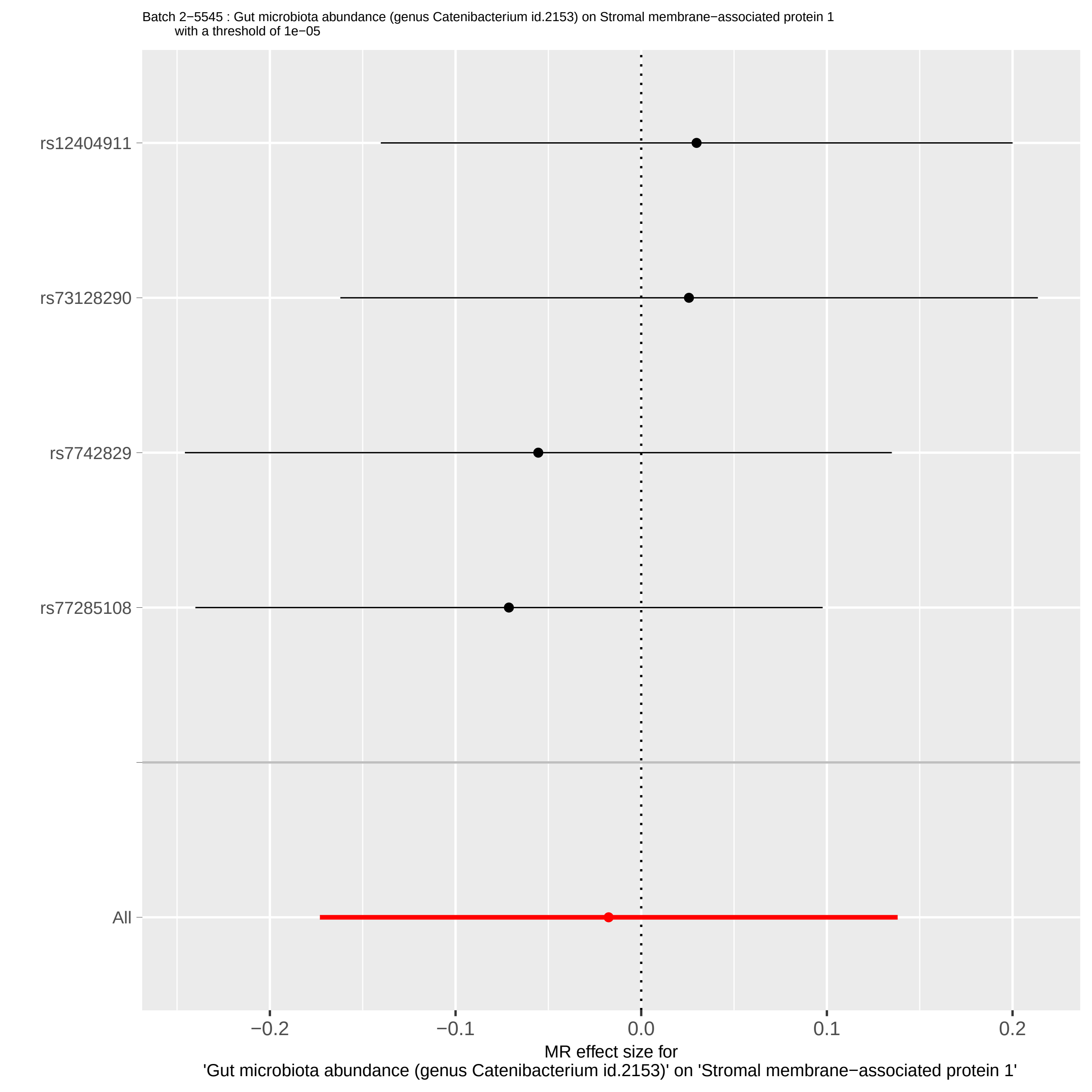
Batch 2−5545 : Gut microbiota abundance (genus Catenibacterium id.2153) on Stromal membrane−associated protein 1
with a threshold of 1e−05
rs12404911
rs73128290
rs7742829
rs77285108
All
−0.2
−0.1
0.0
0.1
0.2
MR effect size for
'Gut microbiota abundance (genus Catenibacterium id.2153)' on 'Stromal membrane−associated protein 1'

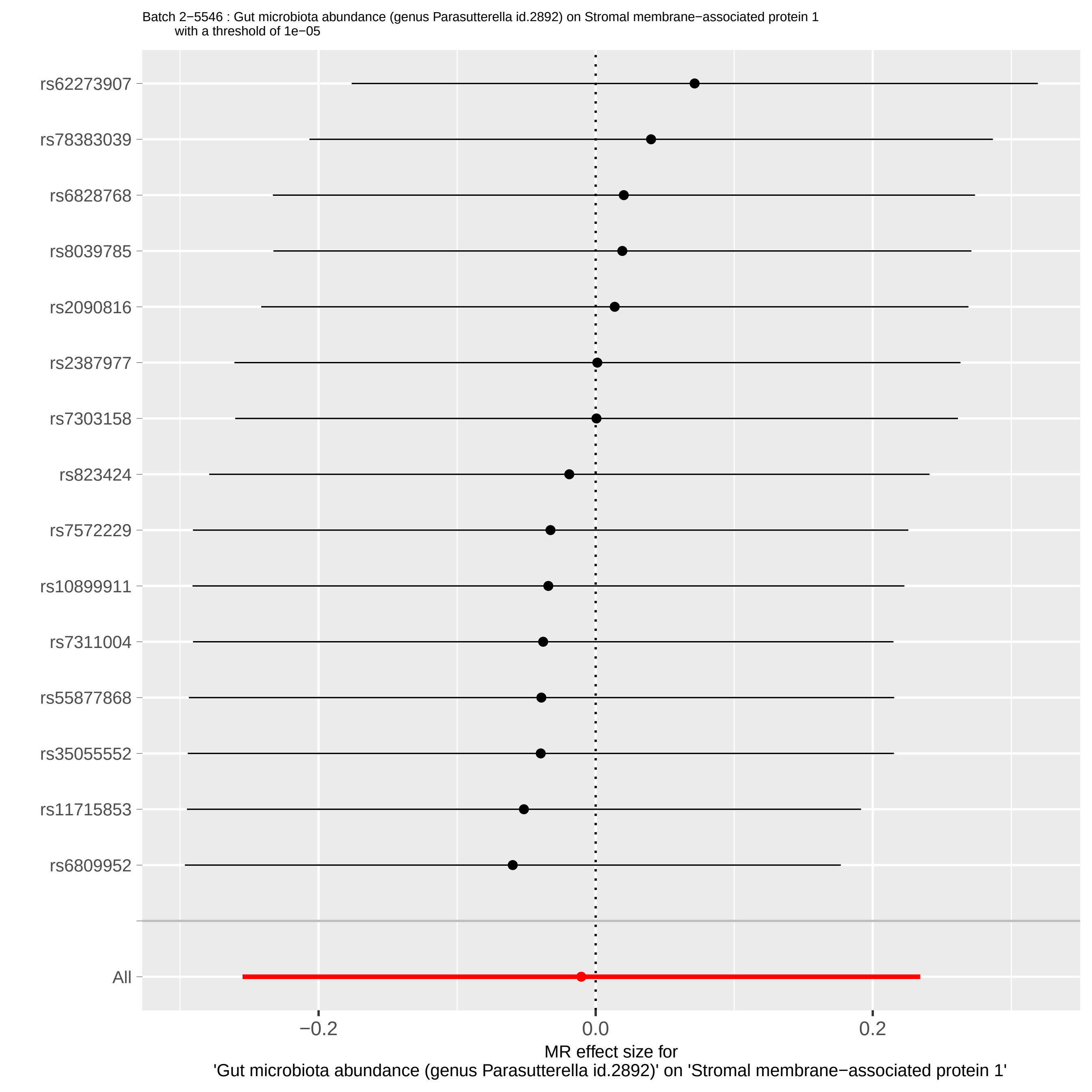
Batch 2−5546 : Gut microbiota abundance (genus Parasutterella id.2892) on Stromal membrane−associated protein 1
with a threshold of 1e−05
rs62273907
rs78383039
rs6828768
rs8039785
rs2090816
rs2387977
rs7303158
rs823424
rs7572229
rs10899911
rs7311004
rs55877868
rs35055552
rs11715853
rs6809952
All
−0.2
0.0
0.2
MR effect size for
'Gut microbiota abundance (genus Parasutterella id.2892)' on 'Stromal membrane−associated protein 1'

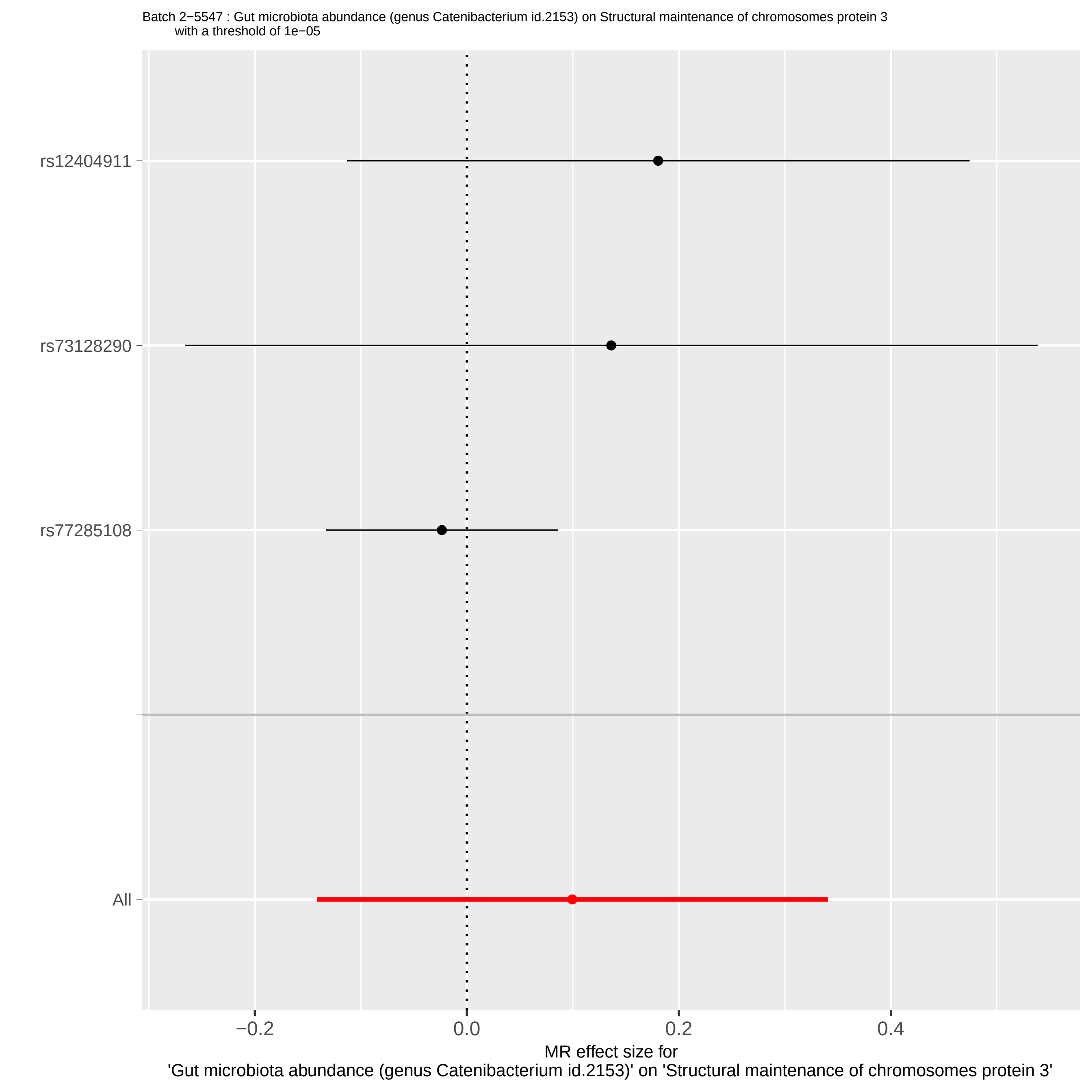
Batch 2−5547 : Gut microbiota abundance (genus Catenibacterium id.2153) on Structural maintenance of chromosomes protein 3
with a threshold of 1e−05
rs12404911
rs73128290
rs77285108
All
−0.2
0.0
0.2
0.4
MR effect size for
'Gut microbiota abundance (genus Catenibacterium id.2153)' on 'Structural maintenance of chromosomes protein 3'

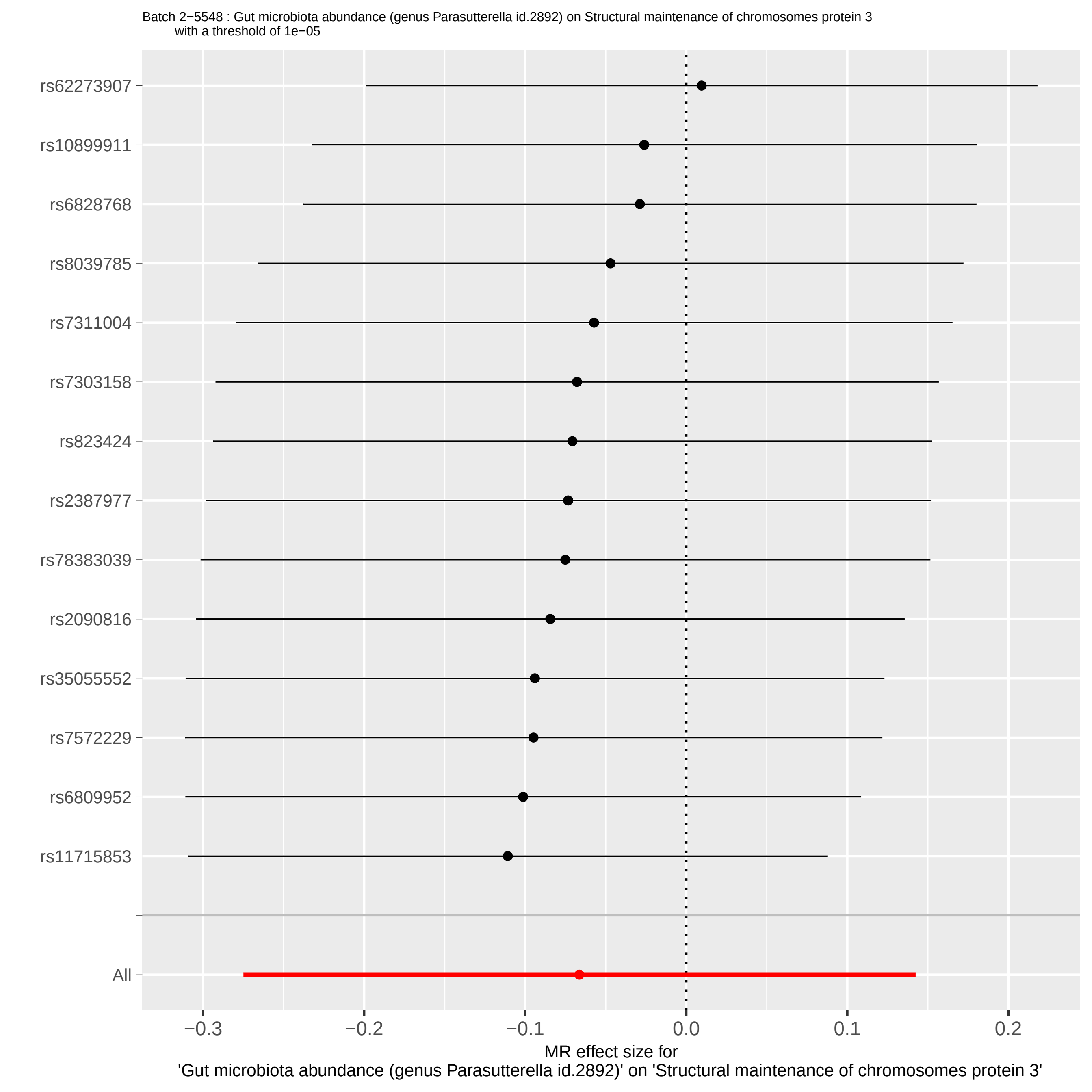

Batch 2−5548 : Gut microbiota abundance (genus Parasutterella id.2892) on Structural maintenance of chromosomes protein 3
with a threshold of 1e−05
rs62273907
rs10899911
rs6828768
rs8039785
rs7311004
rs7303158
rs823424
rs2387977
rs78383039
rs2090816
rs35055552
rs7572229
rs6809952
rs11715853
All
−0.3
−0.2
−0.1
0.0
0.1
0.2
MR effect size for
'Gut microbiota abundance (genus Parasutterella id.2892)' on 'Structural maintenance of chromosomes protein 3'

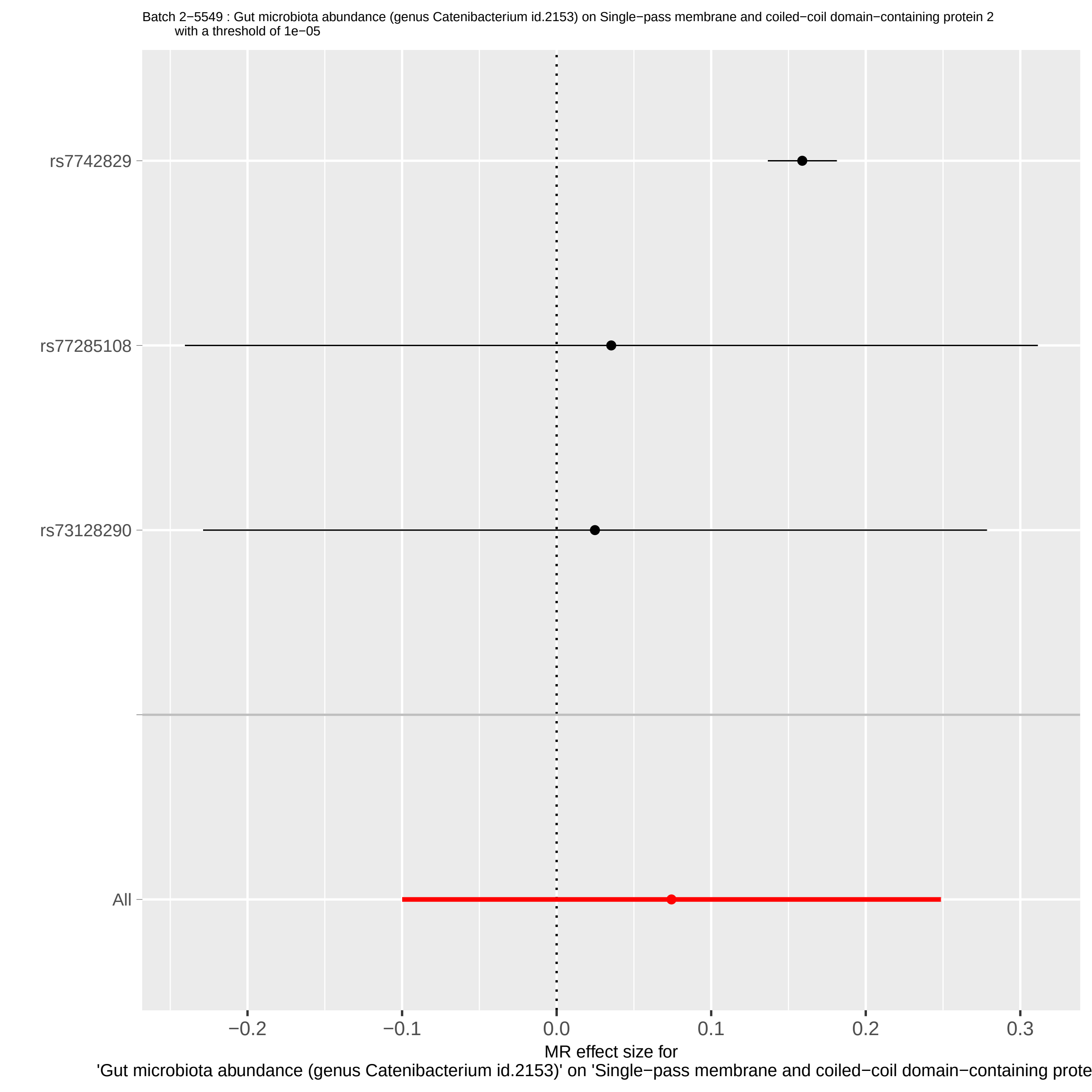

Batch 2−5549 : Gut microbiota abundance (genus Catenibacterium id.2153) on Single−pass membrane and coiled−coil domain−containing protein 2
with a threshold of 1e−05
rs7742829
rs77285108
rs73128290
All
−0.2
−0.1
0.0
0.1
0.2
0.3
MR effect size for
'Gut microbiota abundance (genus Catenibacterium id.2153)' on 'Single−pass membrane and coiled−coil domain−containing prote

Batch 2−5550 : Gut microbiota abundance (genus Parasutterella id.2892) on Single−pass membrane and coiled−coil domain−containing protein 2
with a threshold of 1e−05

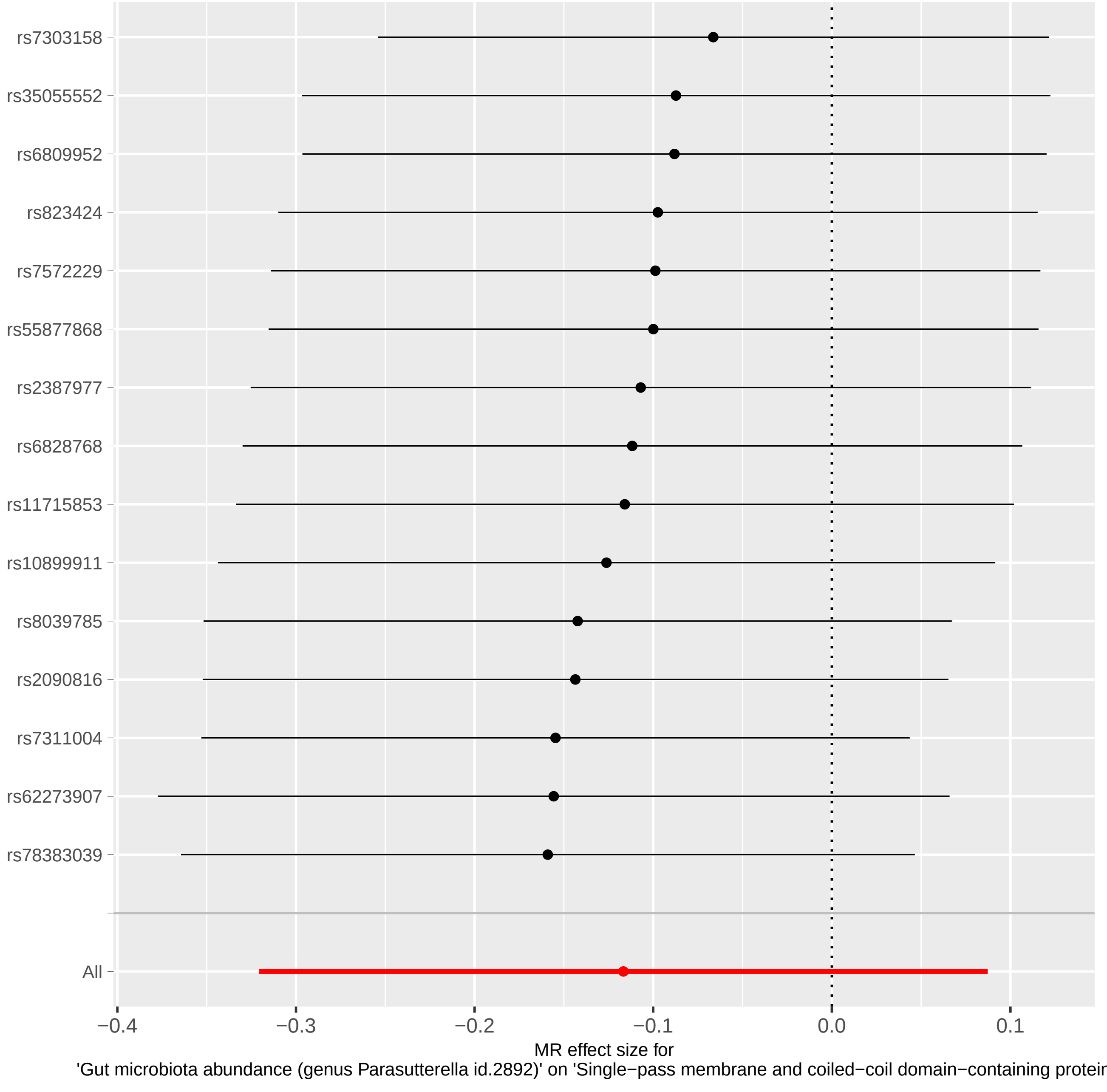

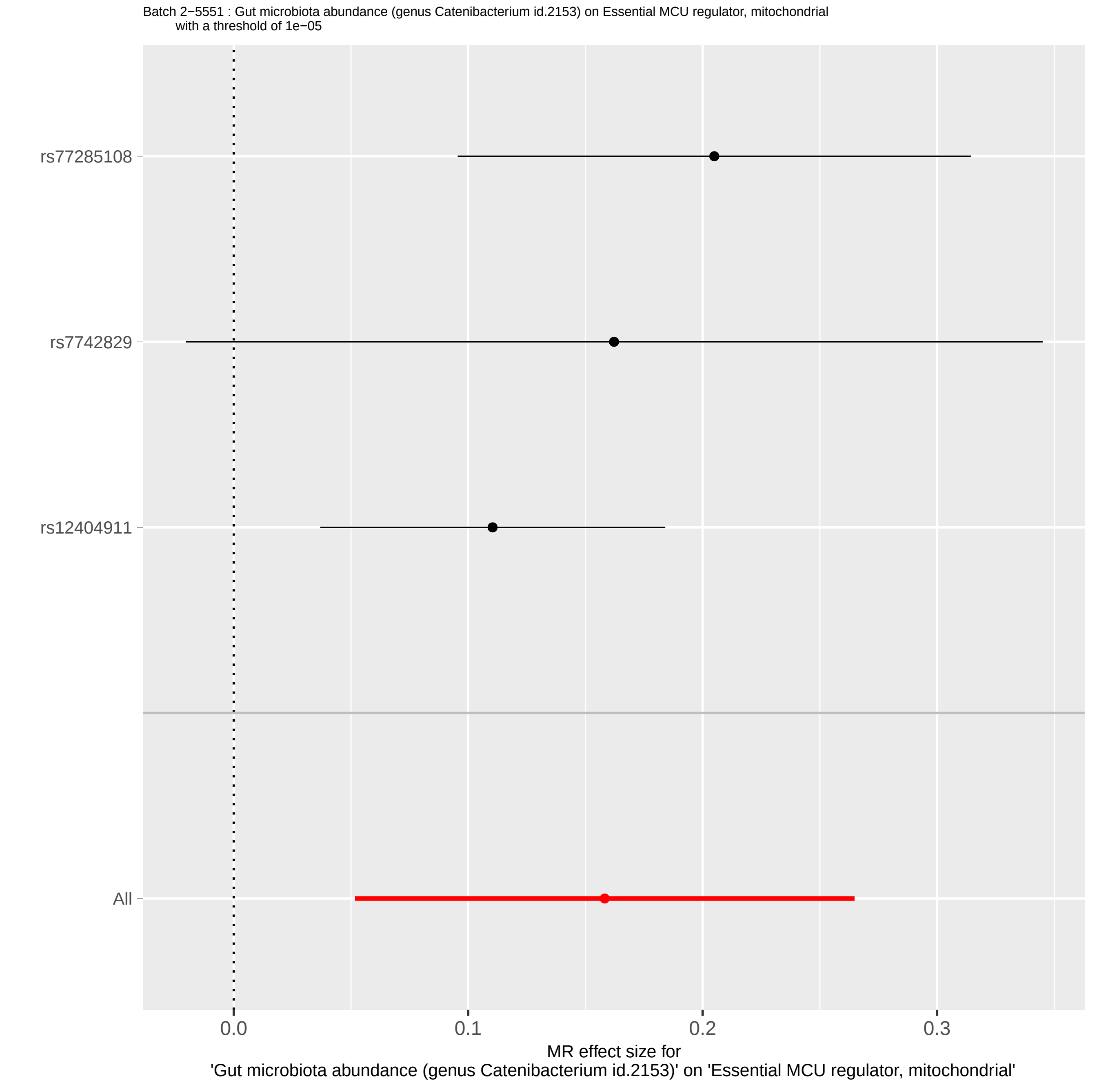
Batch 2−5551 : Gut microbiota abundance (genus Catenibacterium id.2153) on Essential MCU regulator, mitochondrial
with a threshold of 1e−05
rs77285108
rs7742829
rs12404911
All
0.0
0.1
0.2
0.3
MR effect size for
'Gut microbiota abundance (genus Catenibacterium id.2153)' on 'Essential MCU regulator, mitochondrial'

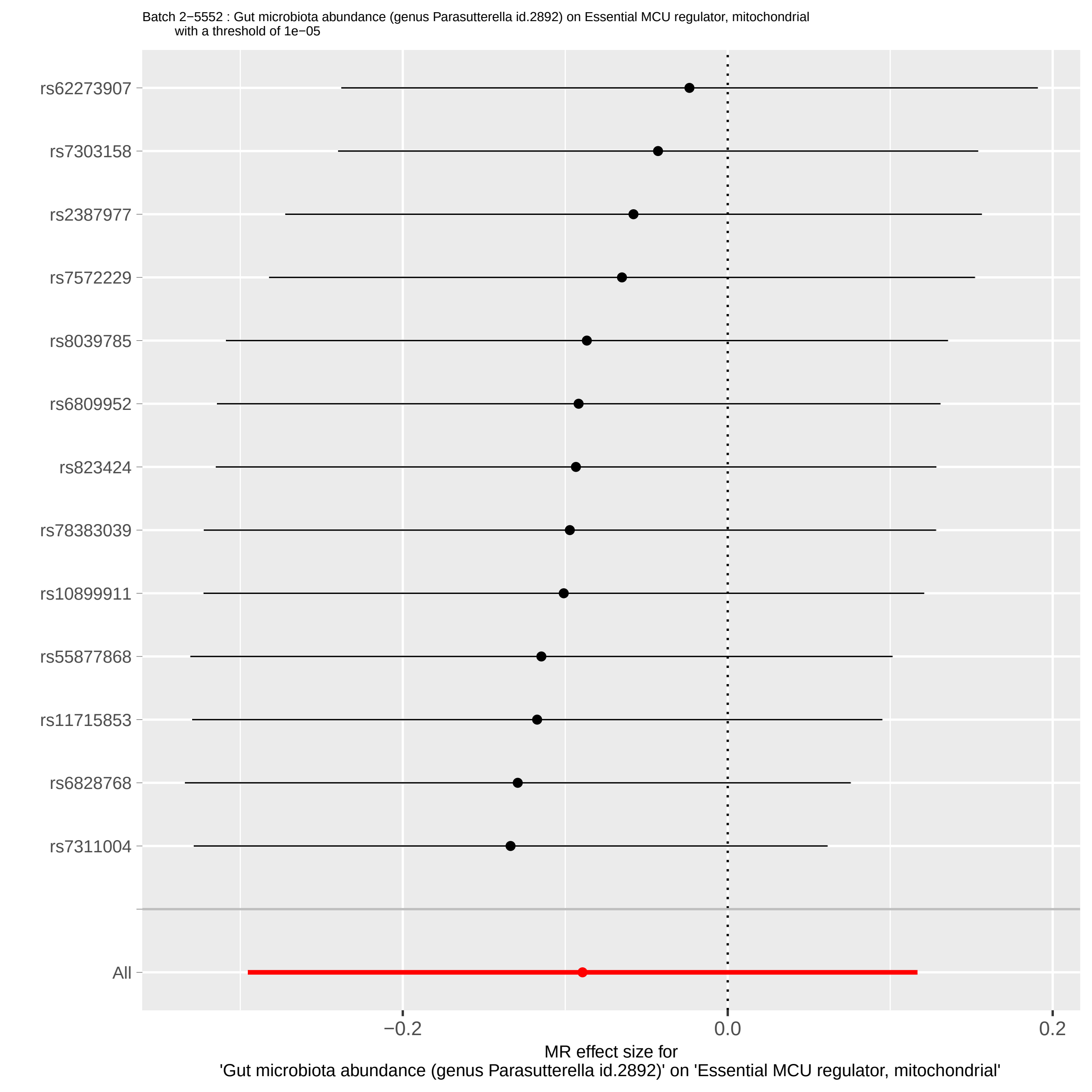

Batch 2−5552 : Gut microbiota abundance (genus Parasutterella id.2892) on Essential MCU regulator, mitochondrial
with a threshold of 1e−05
rs62273907
rs7303158
rs2387977
rs7572229
rs8039785
rs6809952
rs823424
rs78383039
rs10899911
rs55877868
rs11715853
rs6828768
rs7311004
All
−0.2
0.0
0.2
MR effect size for
'Gut microbiota abundance (genus Parasutterella id.2892)' on 'Essential MCU regulator, mitochondrial'

Batch 2−5553 : Gut microbiota abundance (genus Catenibacterium id.2153) on Small integral membrane protein 10
with a threshold of 1e−05

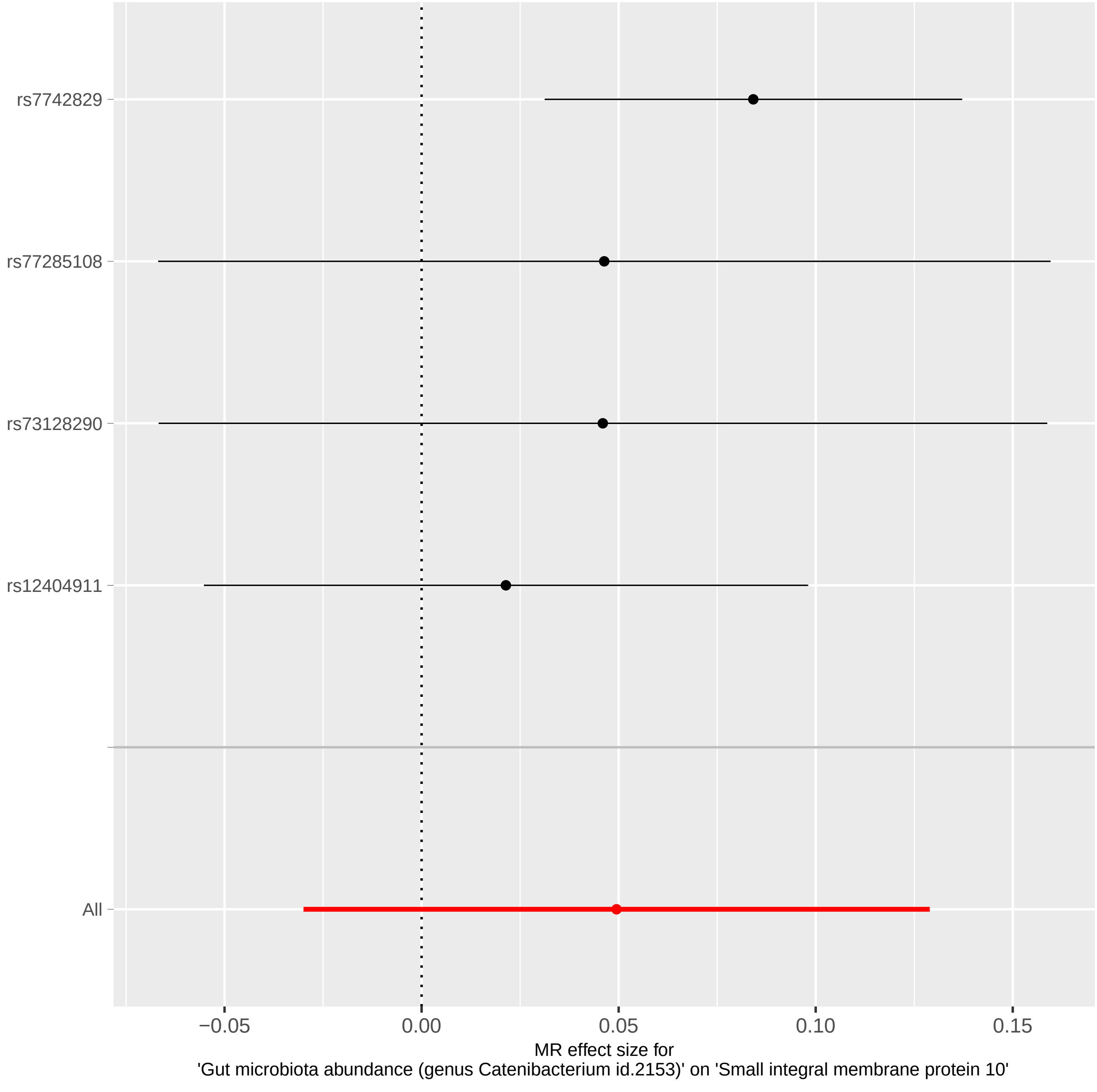

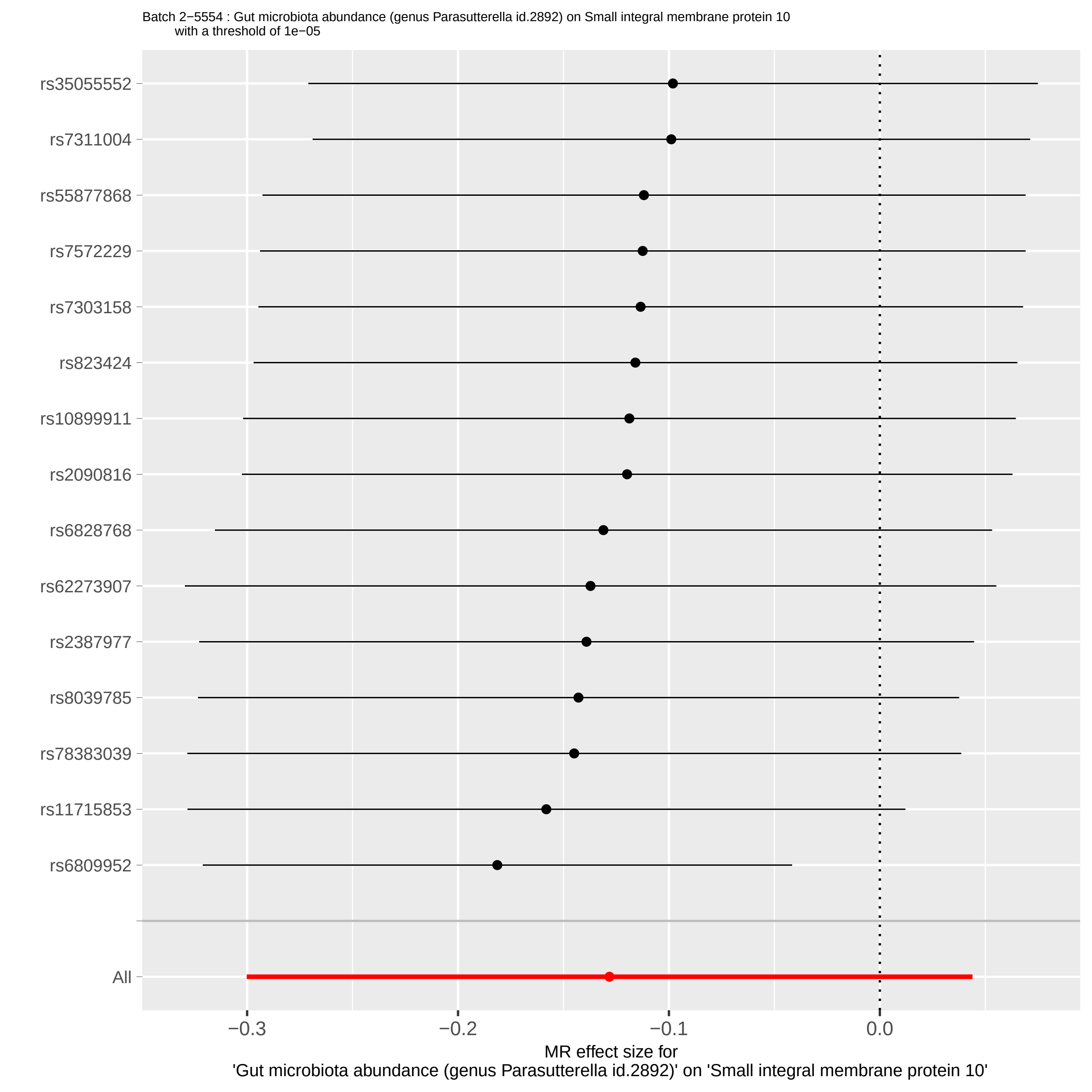
Batch 2−5554 : Gut microbiota abundance (genus Parasutterella id.2892) on Small integral membrane protein 10
with a threshold of 1e−05
rs35055552
rs7311004
rs55877868
rs7572229
rs7303158
rs823424
rs10899911
rs2090816
rs6828768
rs62273907
rs2387977
rs8039785
rs78383039
rs11715853
rs6809952
All
−0.3
−0.2
−0.1
0.0
MR effect size for
'Gut microbiota abundance (genus Parasutterella id.2892)' on 'Small integral membrane protein 10'

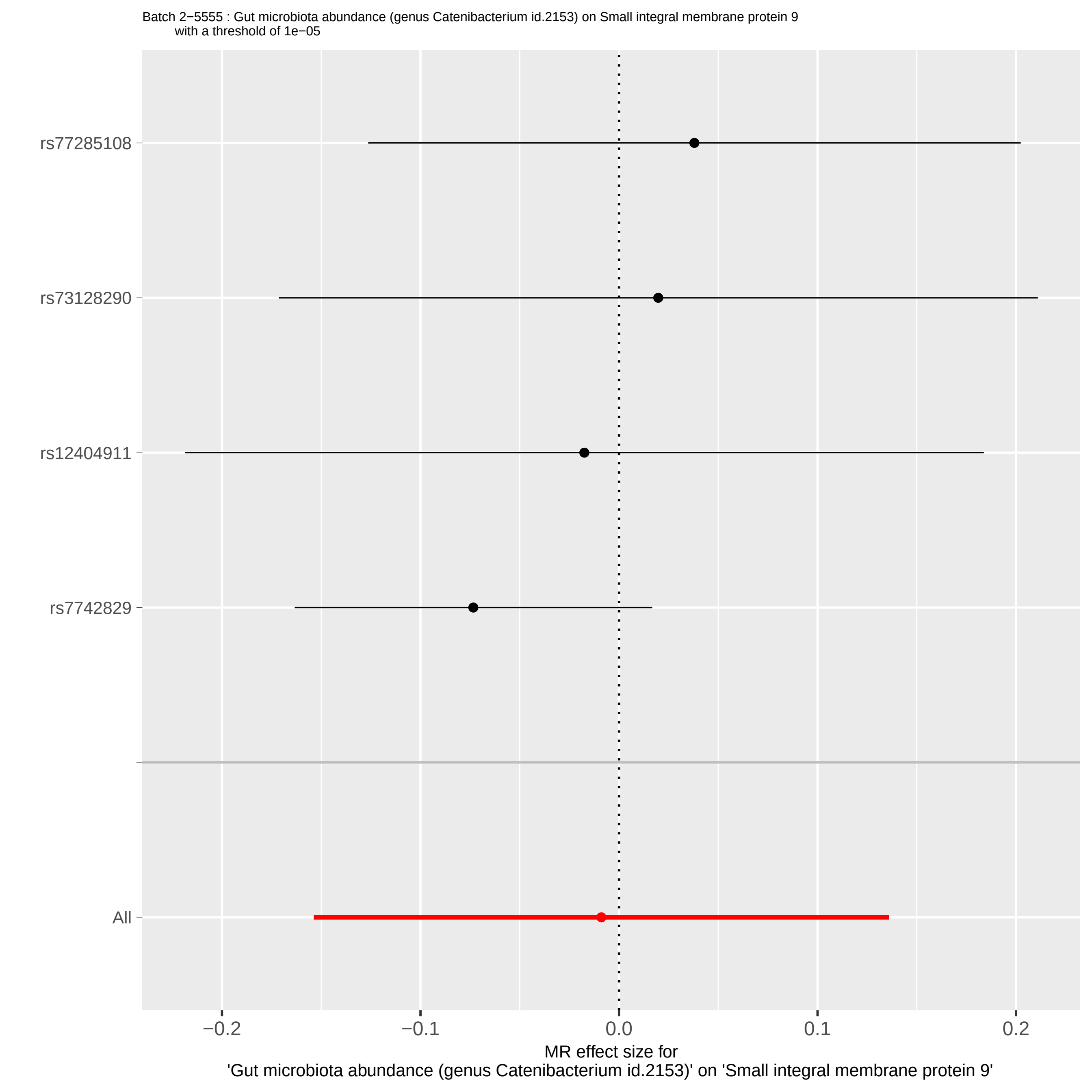

Batch 2−5555 : Gut microbiota abundance (genus Catenibacterium id.2153) on Small integral membrane protein 9
with a threshold of 1e−05
rs77285108
rs73128290
rs12404911
rs7742829
All
−0.2
−0.1
0.0
0.1
0.2
MR effect size for
'Gut microbiota abundance (genus Catenibacterium id.2153)' on 'Small integral membrane protein 9'

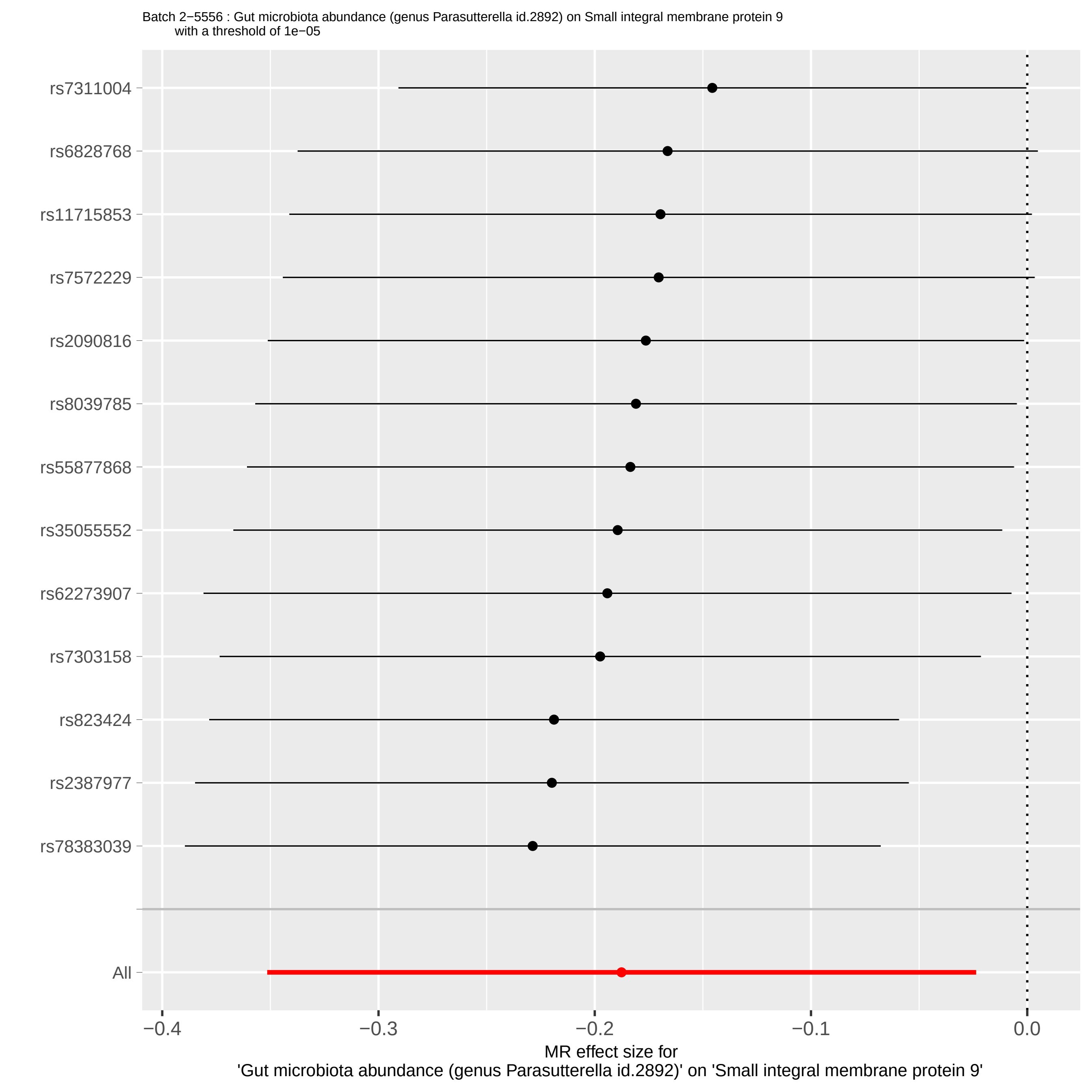

Batch 2−5556 : Gut microbiota abundance (genus Parasutterella id.2892) on Small integral membrane protein 9
with a threshold of 1e−05
rs7311004
rs6828768
rs11715853
rs7572229
rs2090816
rs8039785
rs55877868
rs35055552
rs62273907
rs7303158
rs823424
rs2387977
rs78383039
All
−0.4
−0.3
−0.2
−0.1
0.0
MR effect size for
'Gut microbiota abundance (genus Parasutterella id.2892)' on 'Small integral membrane protein 9'

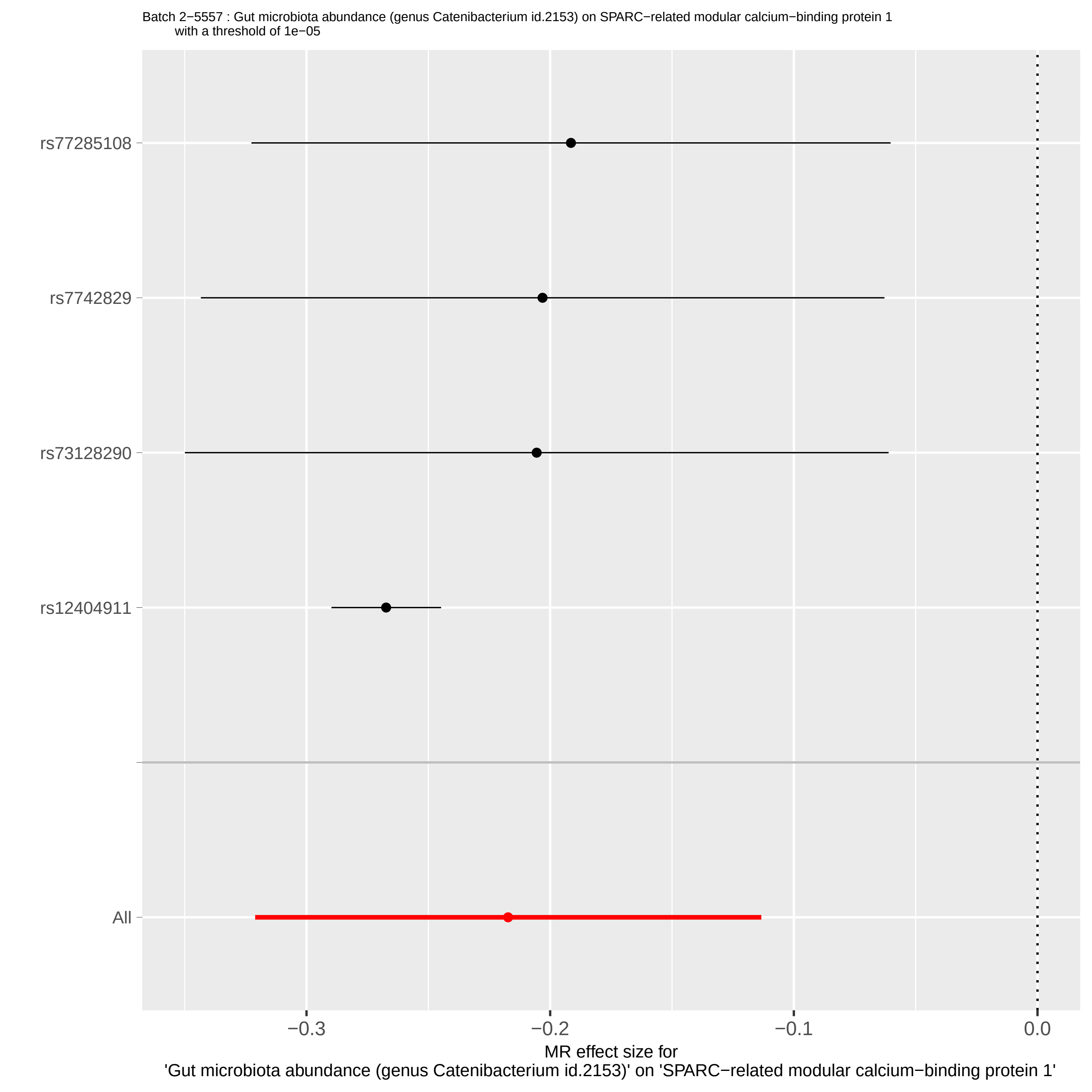

Batch 2−5557 : Gut microbiota abundance (genus Catenibacterium id.2153) on SPARC−related modular calcium−binding protein 1
with a threshold of 1e−05
rs77285108
rs7742829
rs73128290
rs12404911
All
−0.3
−0.2
−0.1
0.0
MR effect size for
'Gut microbiota abundance (genus Catenibacterium id.2153)' on 'SPARC−related modular calcium−binding protein 1'

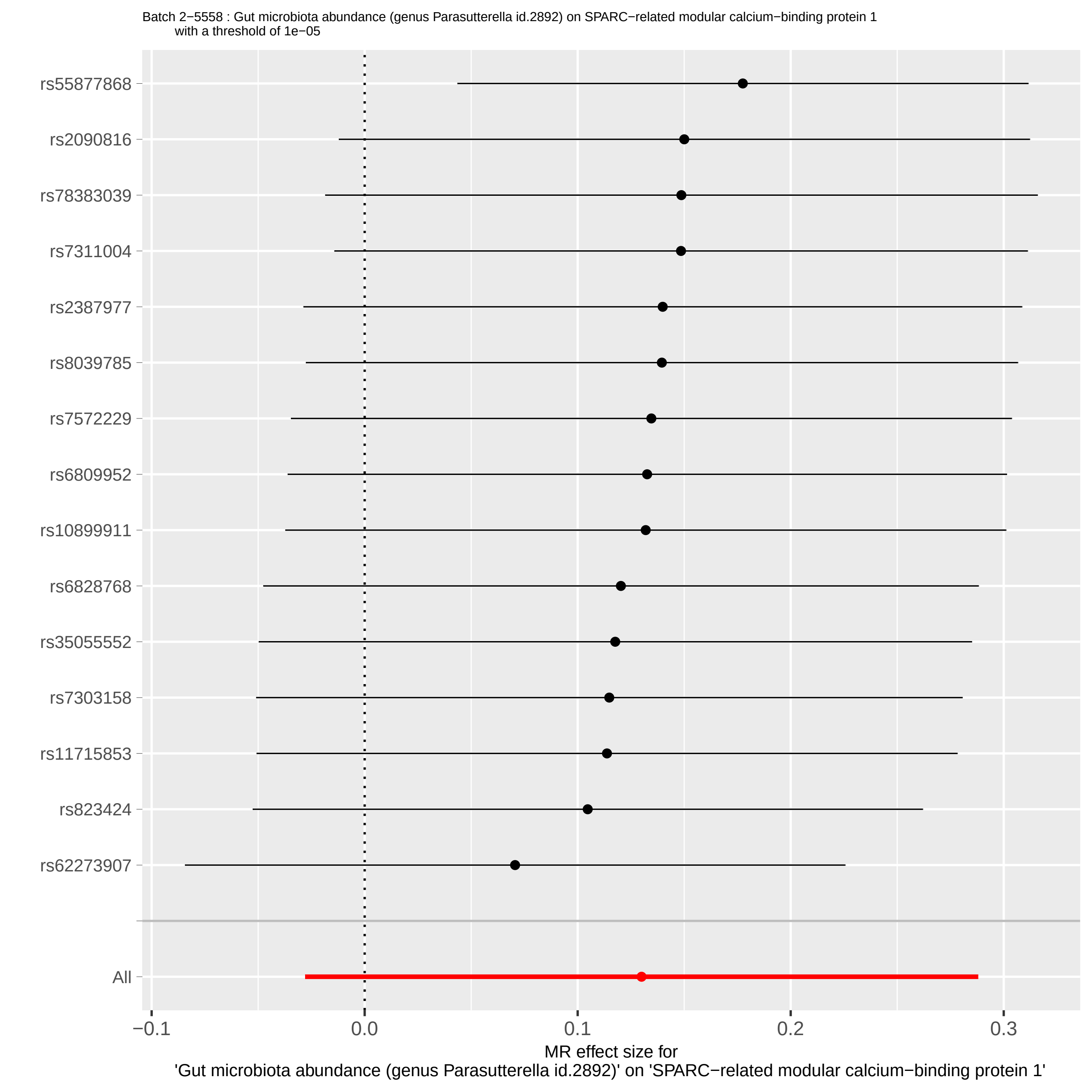

Batch 2−5558 : Gut microbiota abundance (genus Parasutterella id.2892) on SPARC−related modular calcium−binding protein 1
with a threshold of 1e−05
rs55877868
rs2090816
rs78383039
rs7311004
rs2387977
rs8039785
rs7572229
rs6809952
rs10899911
rs6828768
rs35055552
rs7303158
rs11715853
rs823424
rs62273907
All
−0.1
0.0
0.1
0.2
0.3
MR effect size for
'Gut microbiota abundance (genus Parasutterella id.2892)' on 'SPARC−related modular calcium−binding protein 1'

Batch 2−5559 : Gut microbiota abundance (genus Catenibacterium id.2153) on Acid sphingomyelinase−like phosphodiesterase 3a
with a threshold of 1e−05

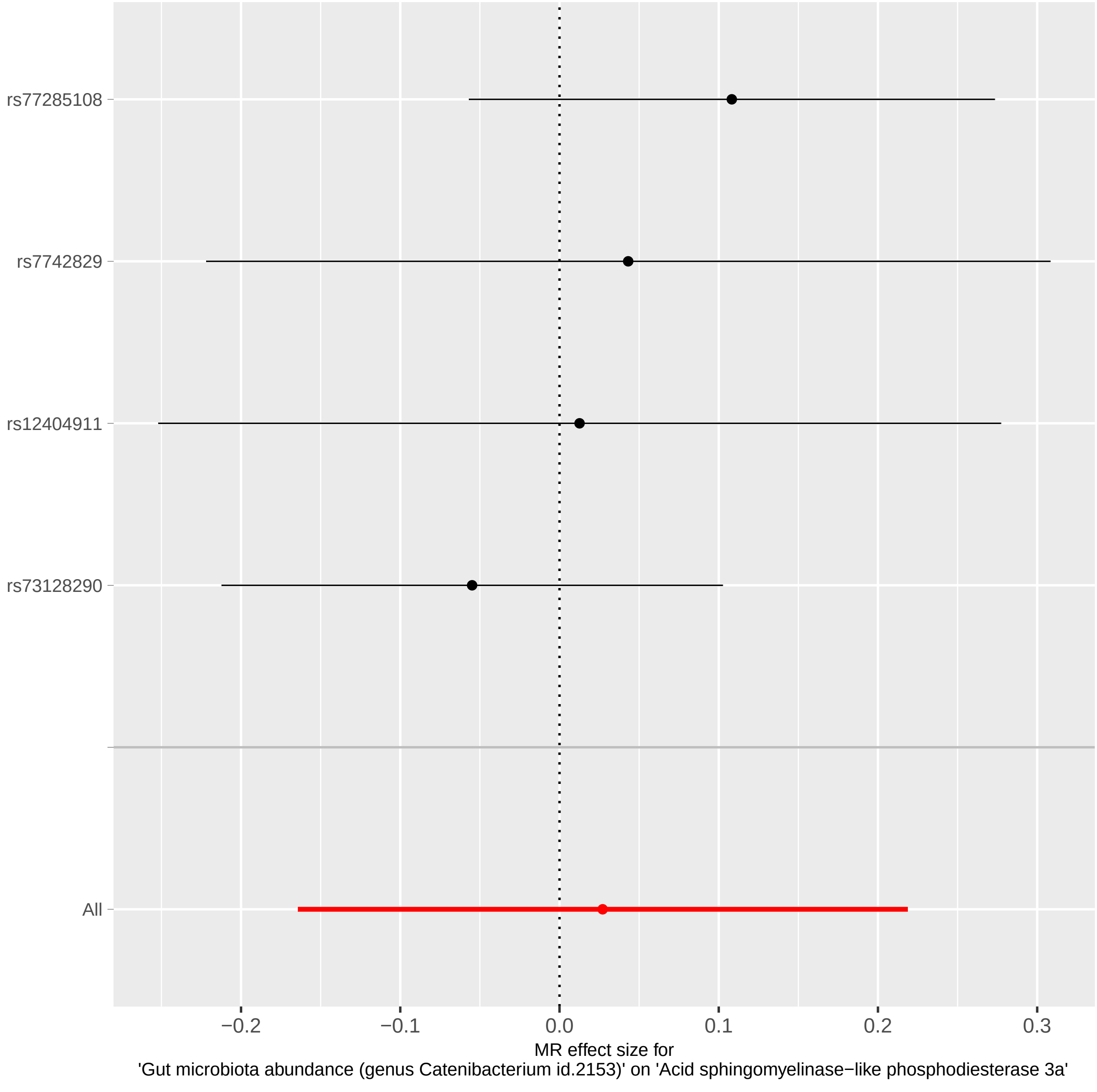

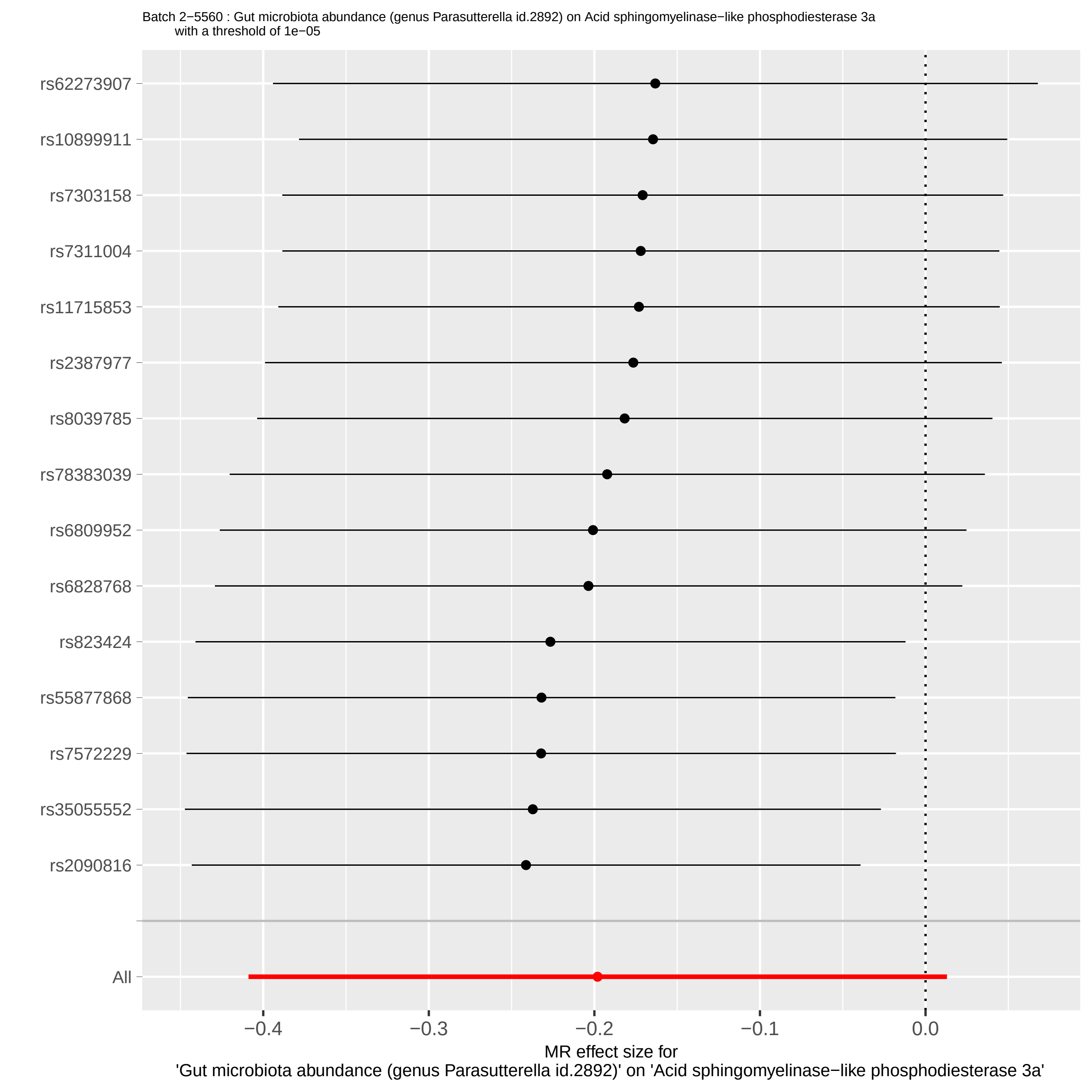
Batch 2−5560 : Gut microbiota abundance (genus Parasutterella id.2892) on Acid sphingomyelinase−like phosphodiesterase 3a
with a threshold of 1e−05
rs62273907
rs10899911
rs7303158
rs7311004
rs11715853
rs2387977
rs8039785
rs78383039
rs6809952
rs6828768
rs823424
rs55877868
rs7572229
rs35055552
rs2090816
All
−0.4
−0.3
−0.2
−0.1
0.0
MR effect size for
'Gut microbiota abundance (genus Parasutterella id.2892)' on 'Acid sphingomyelinase−like phosphodiesterase 3a'

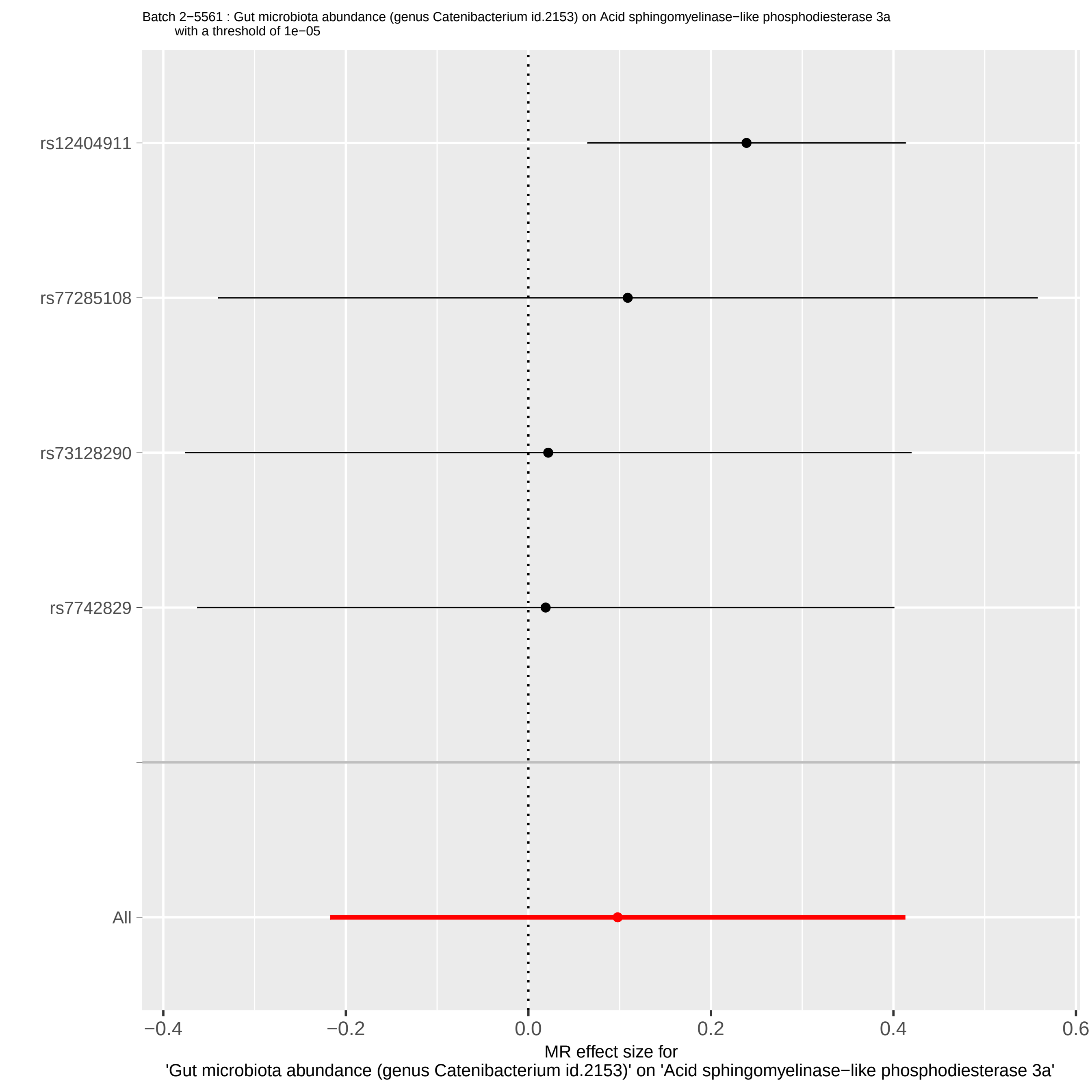
Batch 2−5561 : Gut microbiota abundance (genus Catenibacterium id.2153) on Acid sphingomyelinase−like phosphodiesterase 3a
with a threshold of 1e−05
rs12404911
rs77285108
rs73128290
rs7742829
All
−0.4
−0.2
0.0
0.2
0.4
0.6
MR effect size for
'Gut microbiota abundance (genus Catenibacterium id.2153)' on 'Acid sphingomyelinase−like phosphodiesterase 3a'

Batch 2−5562 : Gut microbiota abundance (genus Parasutterella id.2892) on Acid sphingomyelinase−like phosphodiesterase 3a
with a threshold of 1e−05

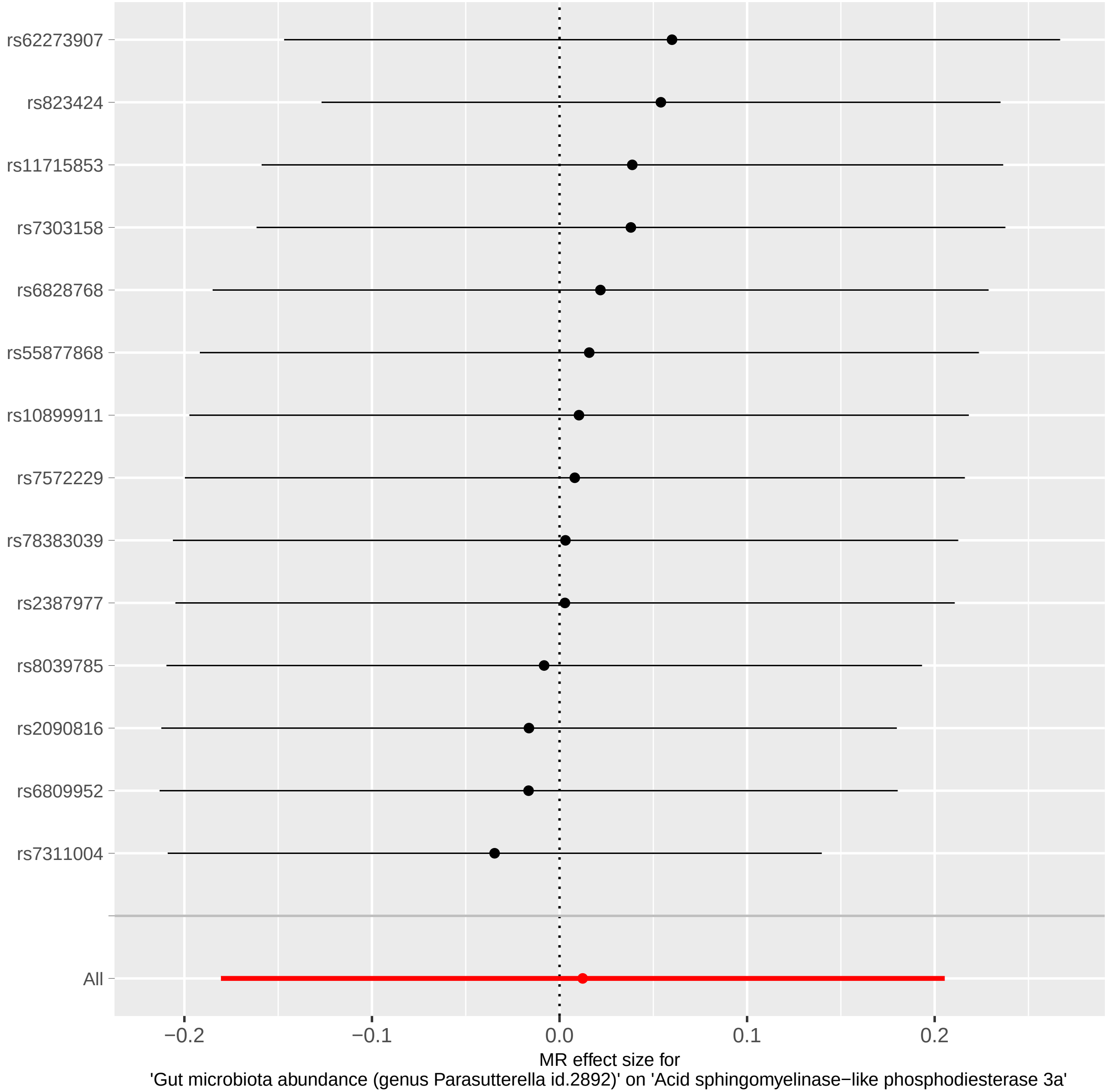

Batch 2−5563 : Gut microbiota abundance (genus Catenibacterium id.2153) on Submaxillary gland androgen−regulated protein 3A
with a threshold of 1e−05

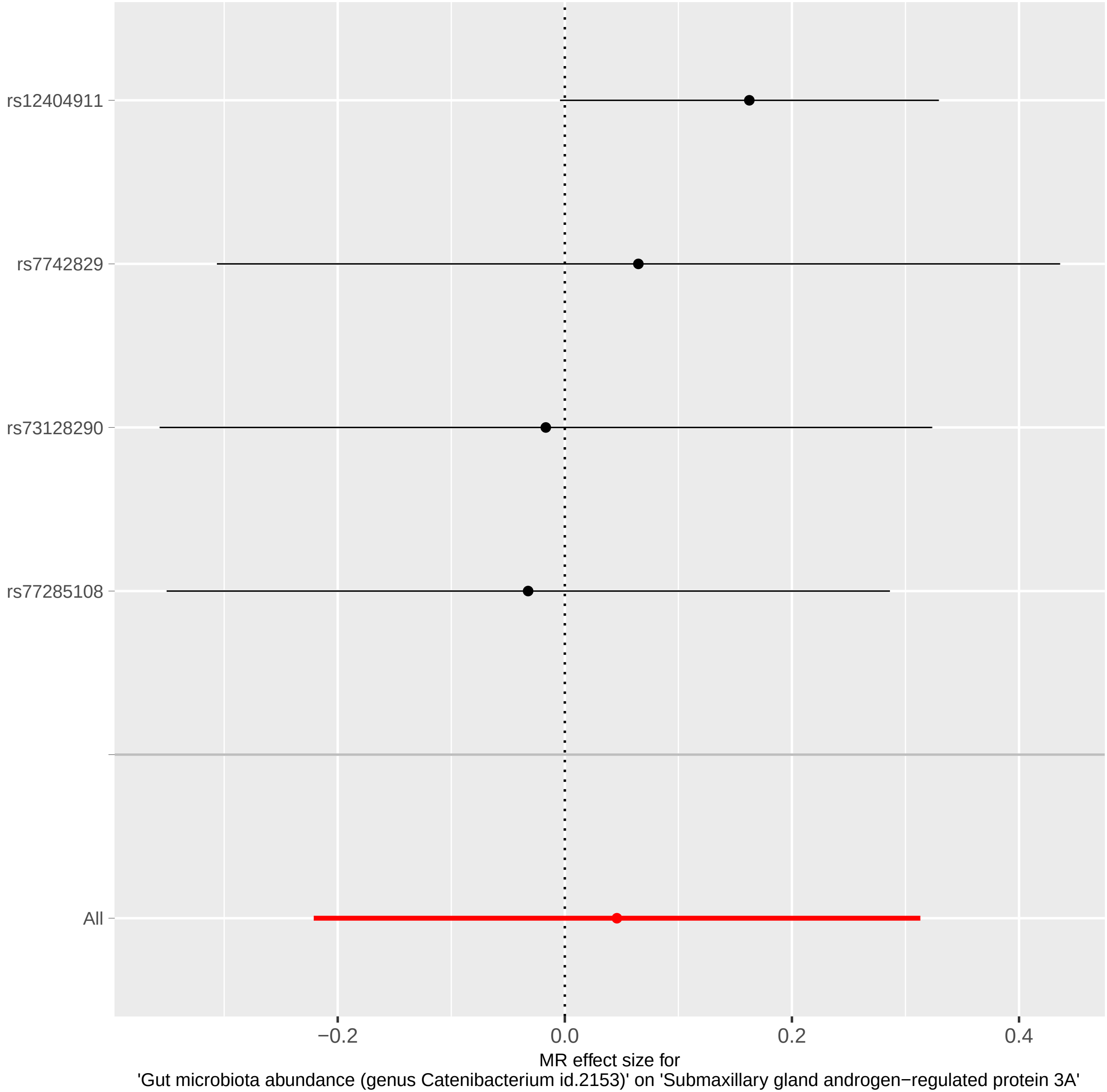

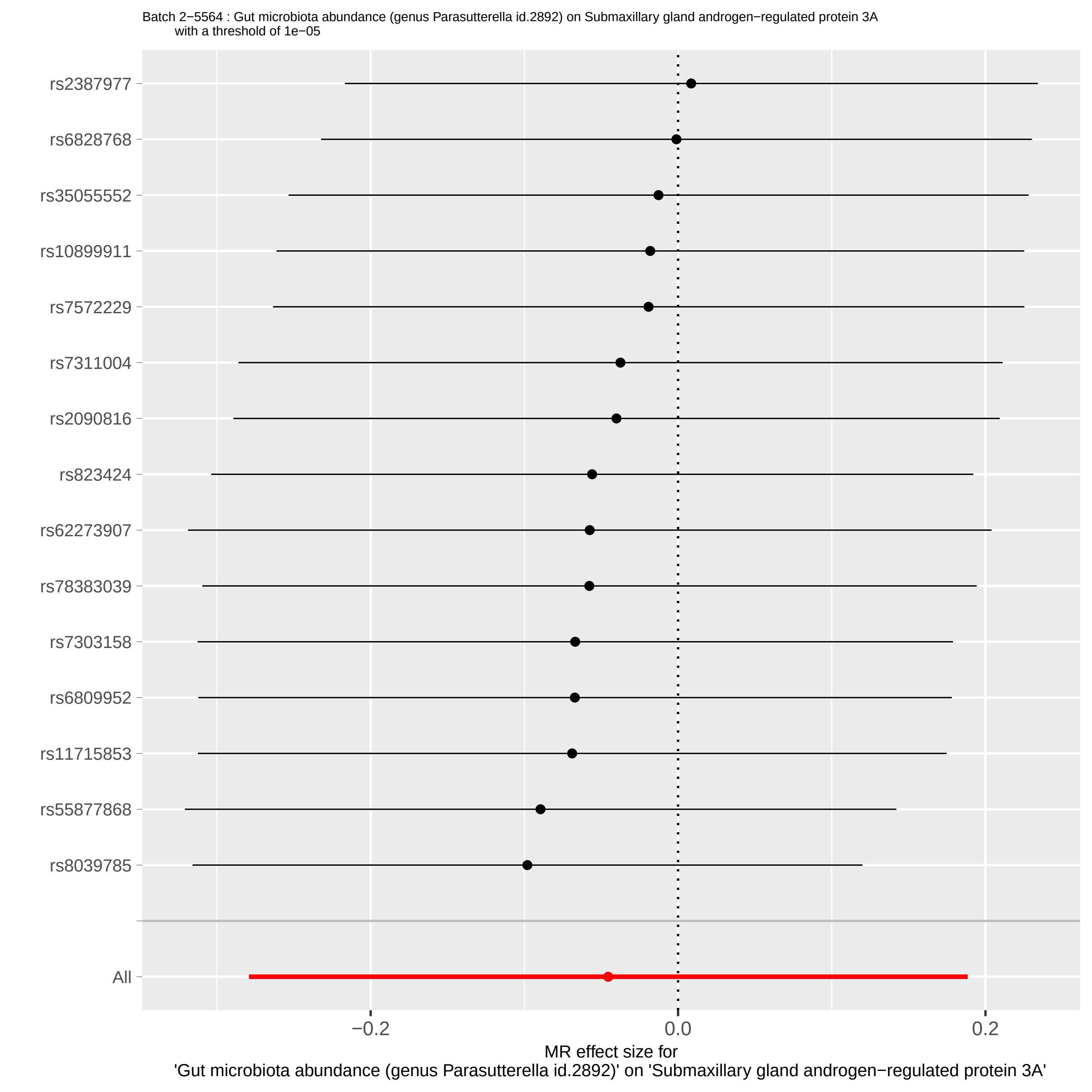

Batch 2−5564 : Gut microbiota abundance (genus Parasutterella id.2892) on Submaxillary gland androgen−regulated protein 3A
with a threshold of 1e−05
rs2387977
rs6828768
rs35055552
rs10899911
rs7572229
rs7311004
rs2090816
rs823424
rs62273907
rs78383039
rs7303158
rs6809952
rs11715853
rs55877868
rs8039785
All
−0.2
0.0
0.2
MR effect size for
'Gut microbiota abundance (genus Parasutterella id.2892)' on 'Submaxillary gland androgen−regulated protein 3A'

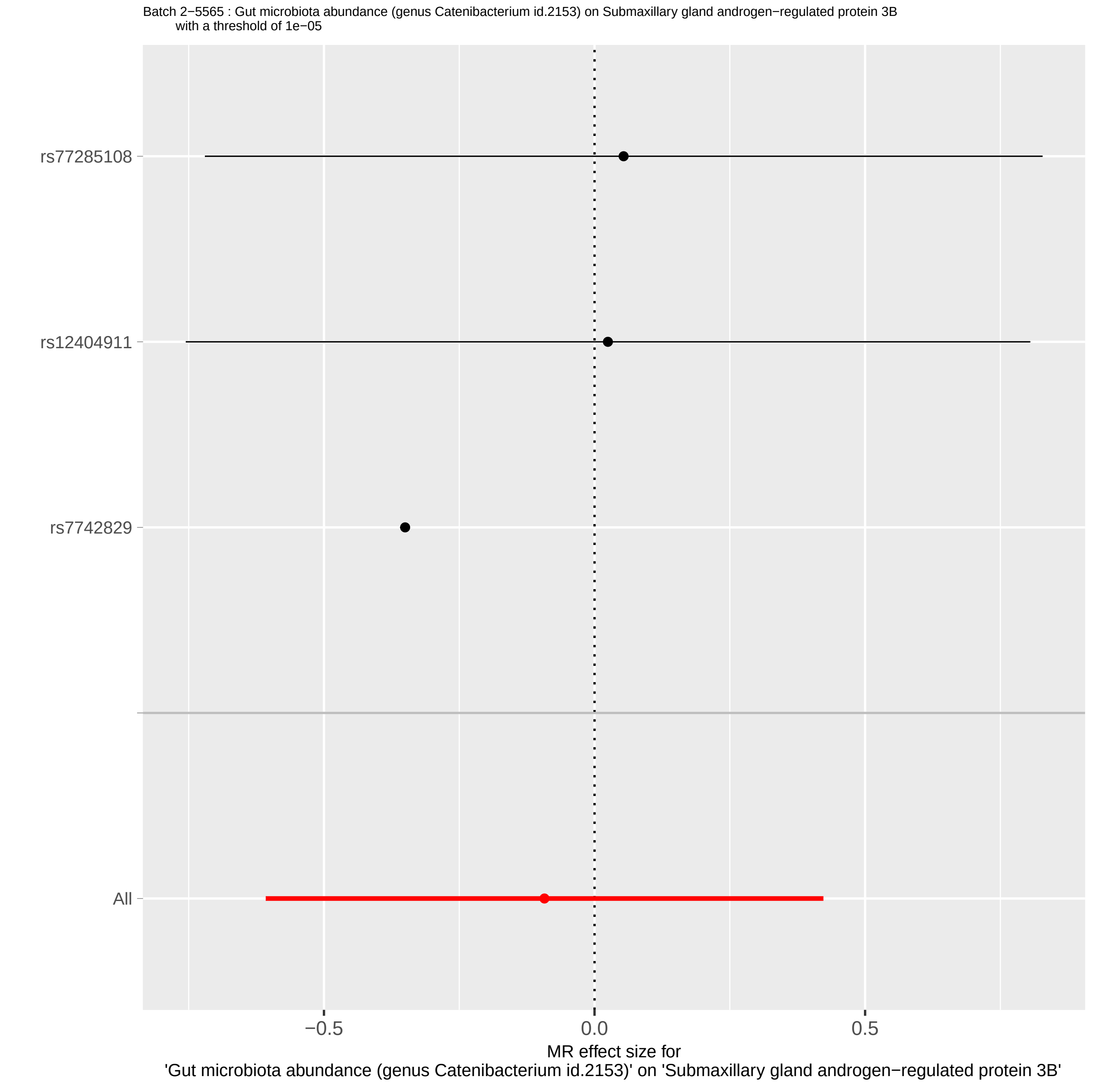

Batch 2−5565 : Gut microbiota abundance (genus Catenibacterium id.2153) on Submaxillary gland androgen−regulated protein 3B
with a threshold of 1e−05
rs77285108
rs12404911
rs7742829
All
−0.5
0.0
0.5
MR effect size for
'Gut microbiota abundance (genus Catenibacterium id.2153)' on 'Submaxillary gland androgen−regulated protein 3B'

Batch 2−5566 : Gut microbiota abundance (genus Parasutterella id.2892) on Submaxillary gland androgen−regulated protein 3B
with a threshold of 1e−05

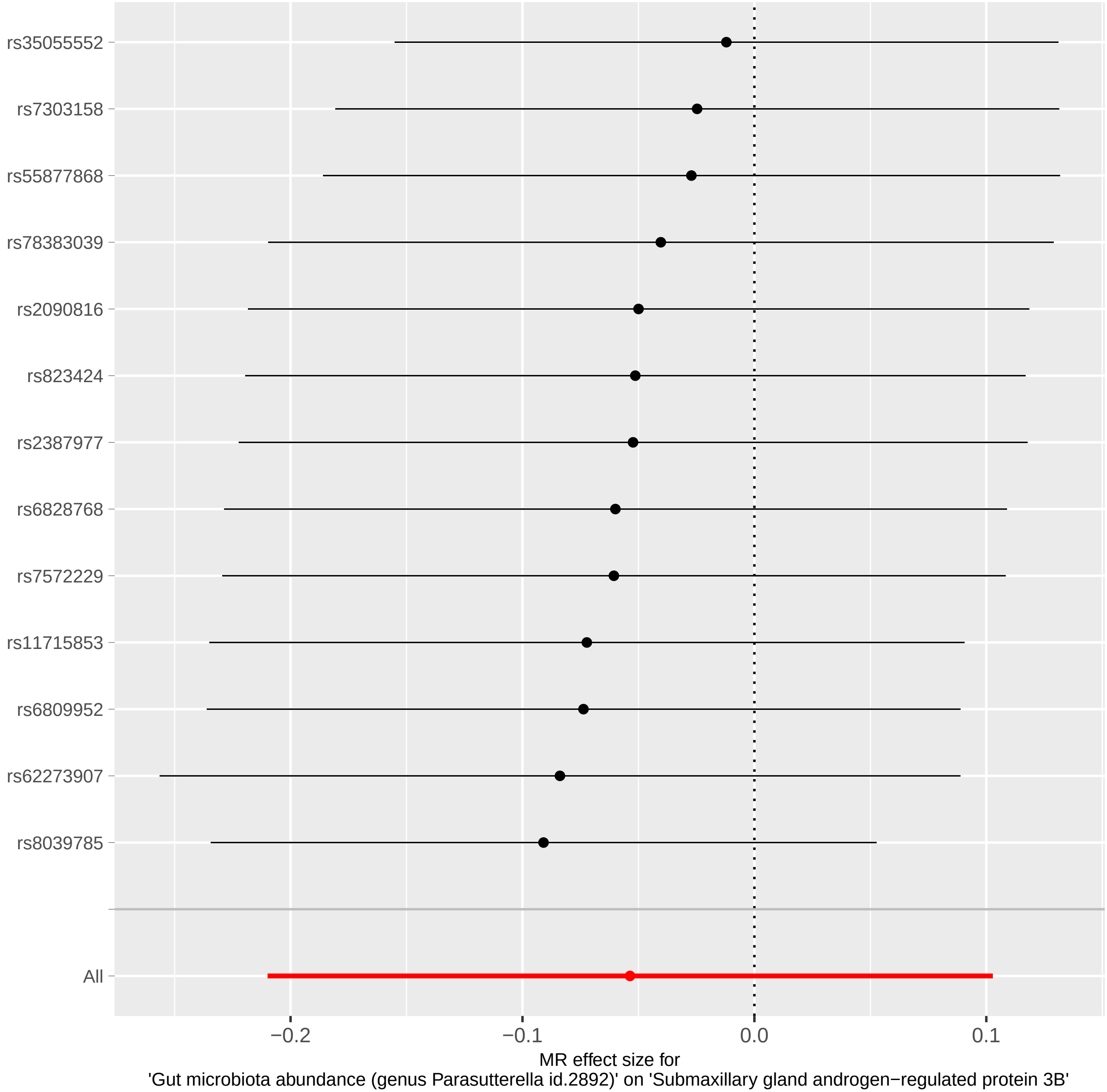

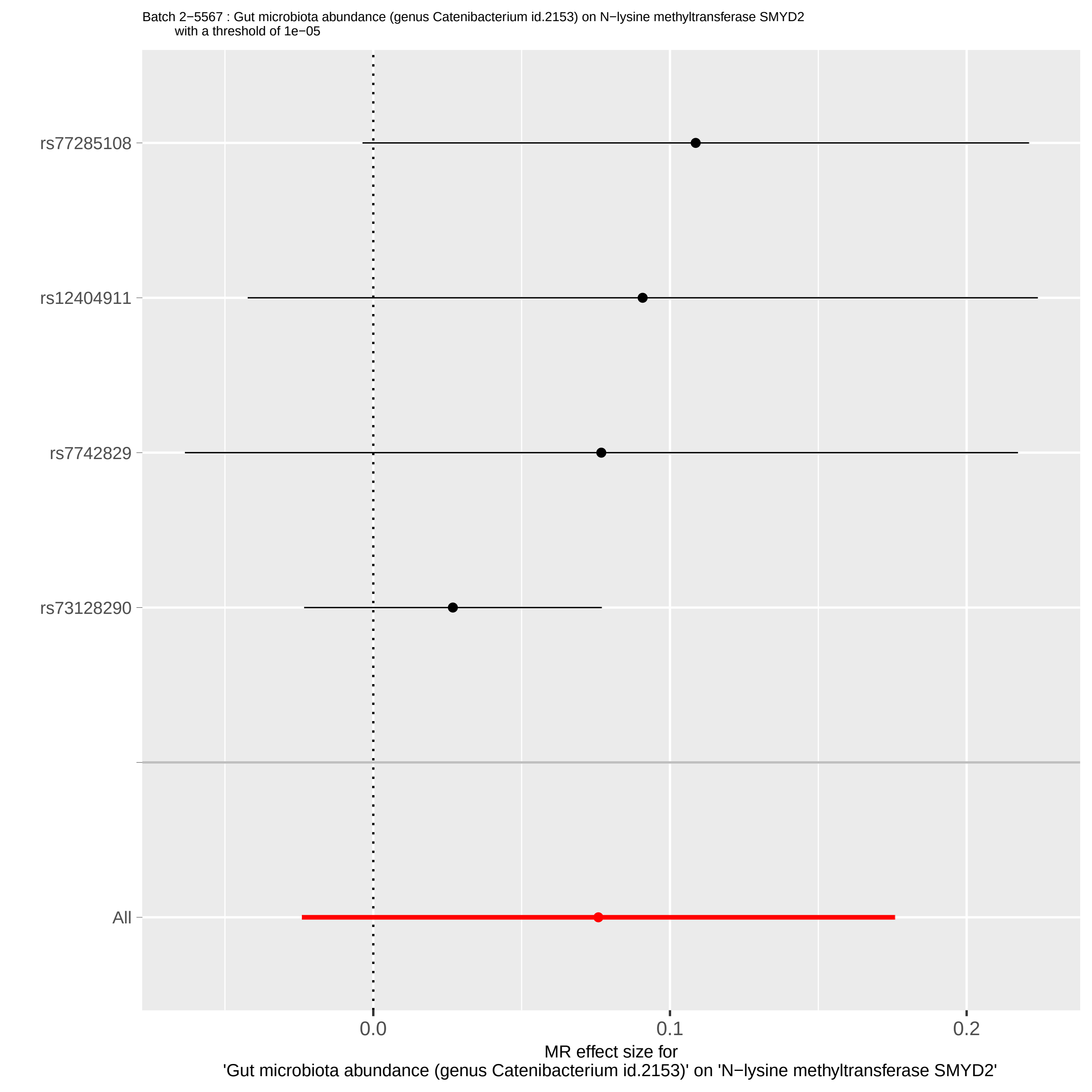

Batch 2−5567 : Gut microbiota abundance (genus Catenibacterium id.2153) on N−lysine methyltransferase SMYD2
with a threshold of 1e−05
rs77285108
rs12404911
rs7742829
rs73128290
All
0.0
0.1
0.2
MR effect size for
'Gut microbiota abundance (genus Catenibacterium id.2153)' on 'N−lysine methyltransferase SMYD2'

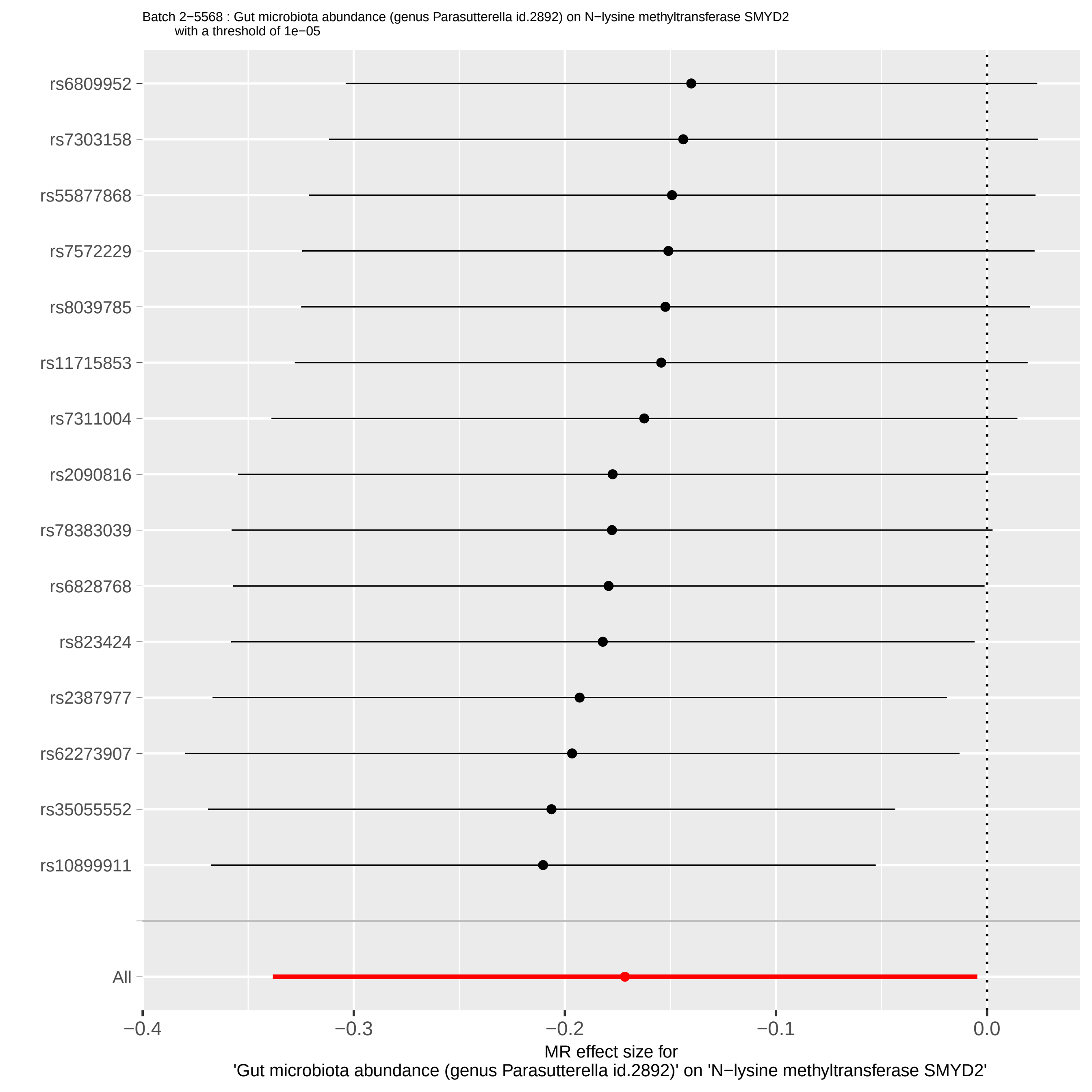

Batch 2−5568 : Gut microbiota abundance (genus Parasutterella id.2892) on N−lysine methyltransferase SMYD2
with a threshold of 1e−05
rs6809952
rs7303158
rs55877868
rs7572229
rs8039785
rs11715853
rs7311004
rs2090816
rs78383039
rs6828768
rs823424
rs2387977
rs62273907
rs35055552
rs10899911
All
−0.4
−0.3
−0.2
−0.1
0.0
MR effect size for
'Gut microbiota abundance (genus Parasutterella id.2892)' on 'N−lysine methyltransferase SMYD2'

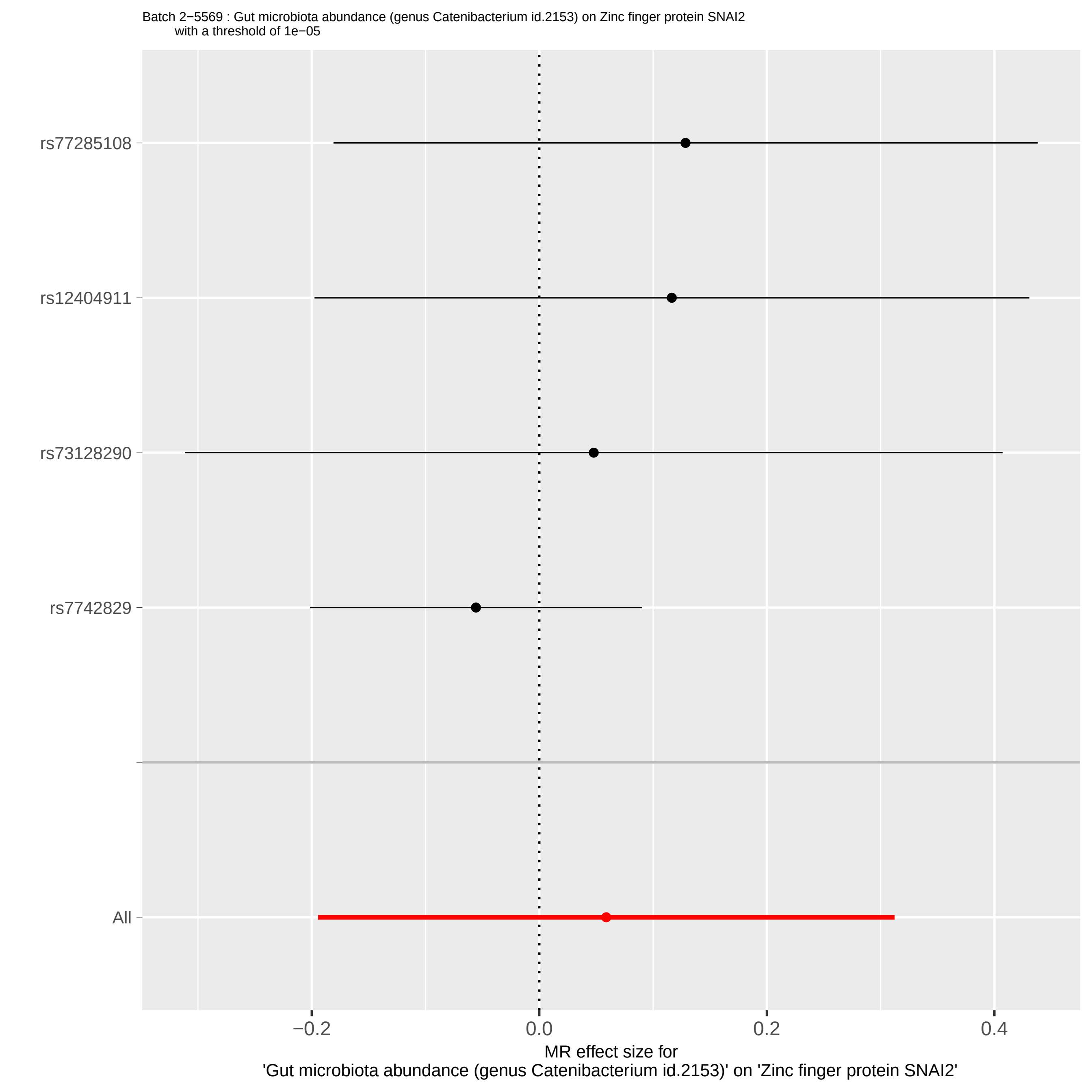

Batch 2−5569 : Gut microbiota abundance (genus Catenibacterium id.2153) on Zinc finger protein SNAI2
with a threshold of 1e−05
rs77285108
rs12404911
rs73128290
rs7742829
All
−0.2
0.0
0.2
0.4
MR effect size for
'Gut microbiota abundance (genus Catenibacterium id.2153)' on 'Zinc finger protein SNAI2'

Batch 2−5570 : Gut microbiota abundance (genus Parasutterella id.2892) on Zinc finger protein SNAI2
with a threshold of 1e−05

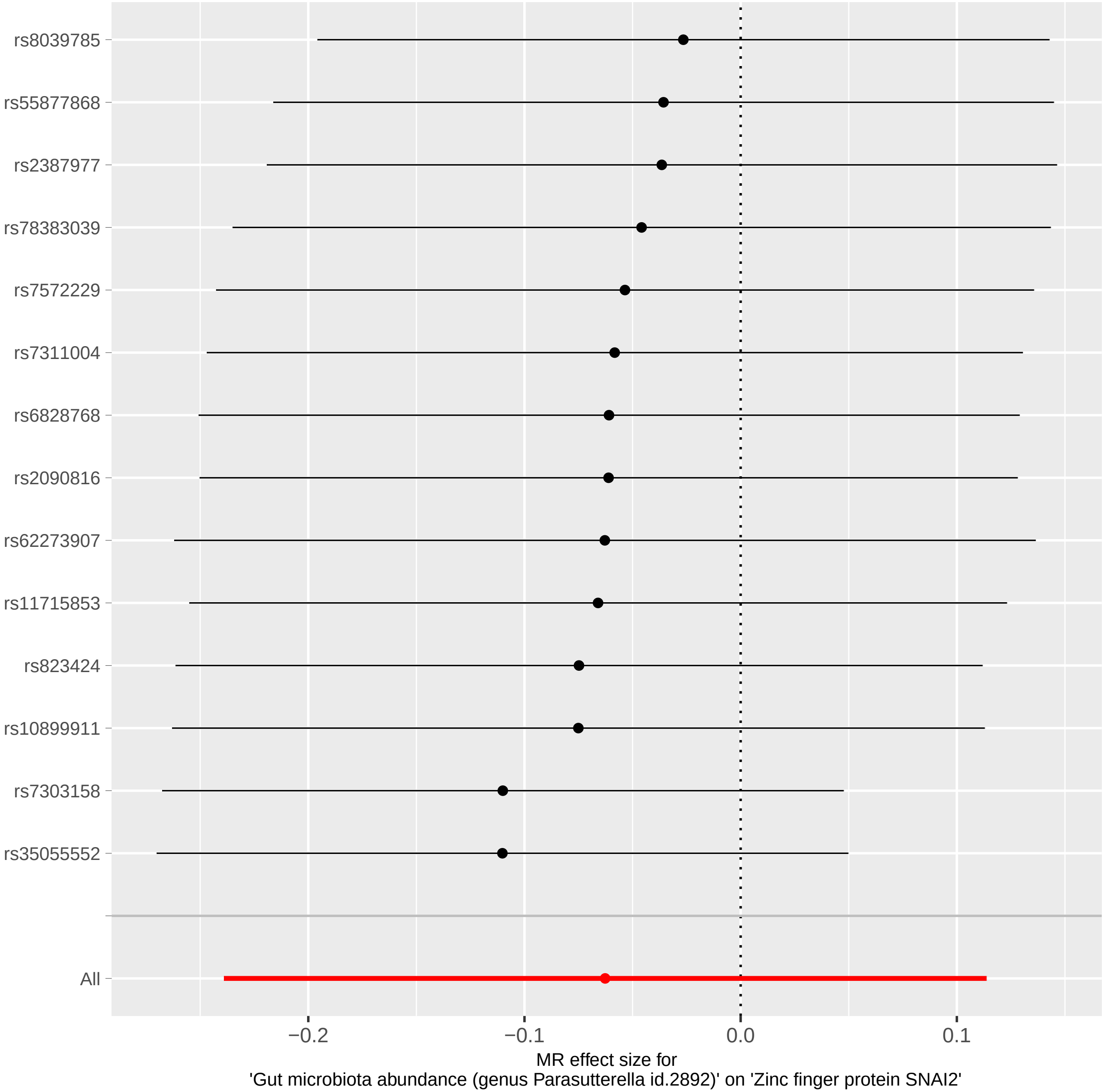

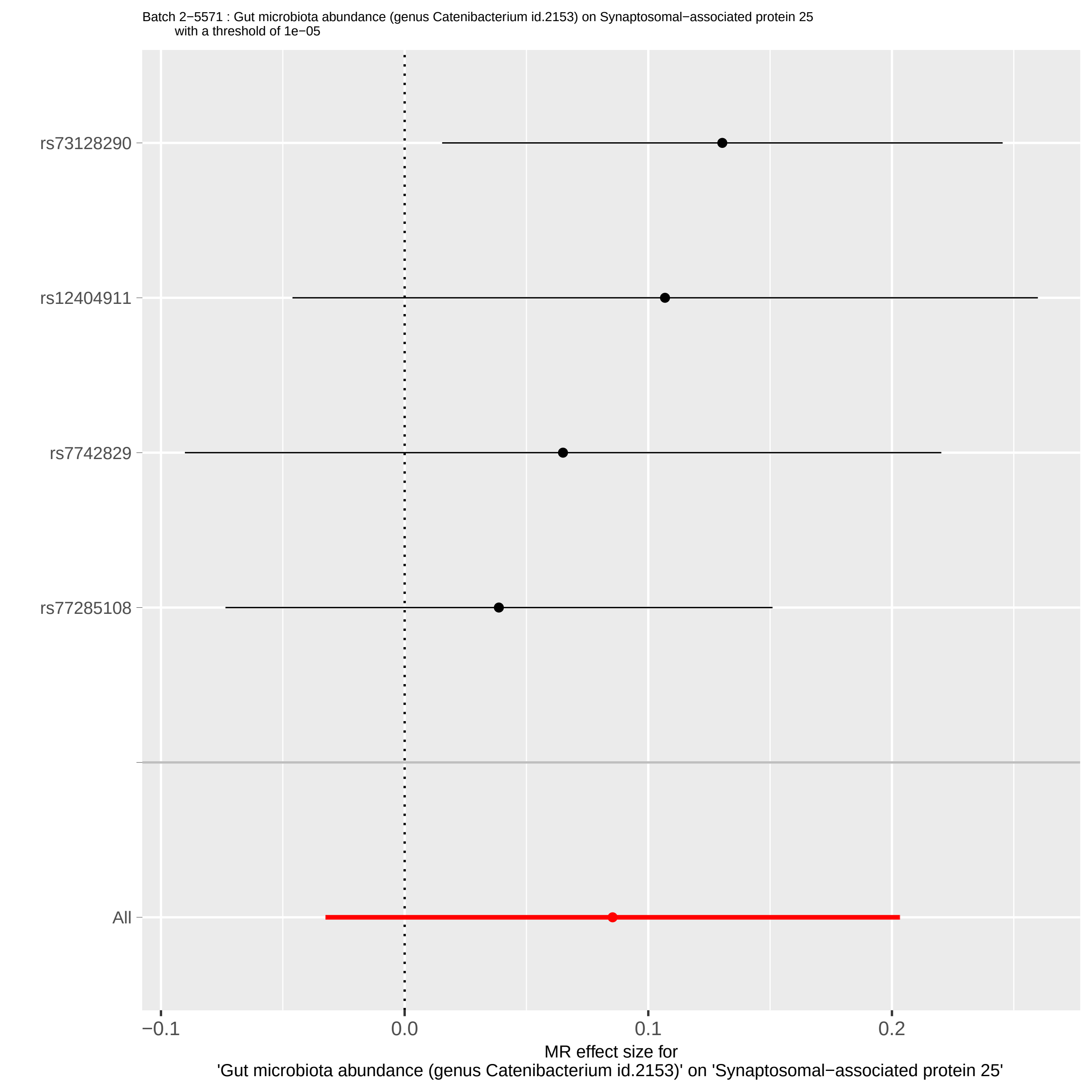
Batch 2−5571 : Gut microbiota abundance (genus Catenibacterium id.2153) on Synaptosomal−associated protein 25
with a threshold of 1e−05
rs73128290
rs12404911
rs7742829
rs77285108
All
−0.1
0.0
0.1
0.2
MR effect size for
'Gut microbiota abundance (genus Catenibacterium id.2153)' on 'Synaptosomal−associated protein 25'

Batch 2−5572 : Gut microbiota abundance (genus Parasutterella id.2892) on Synaptosomal−associated protein 25
with a threshold of 1e−05

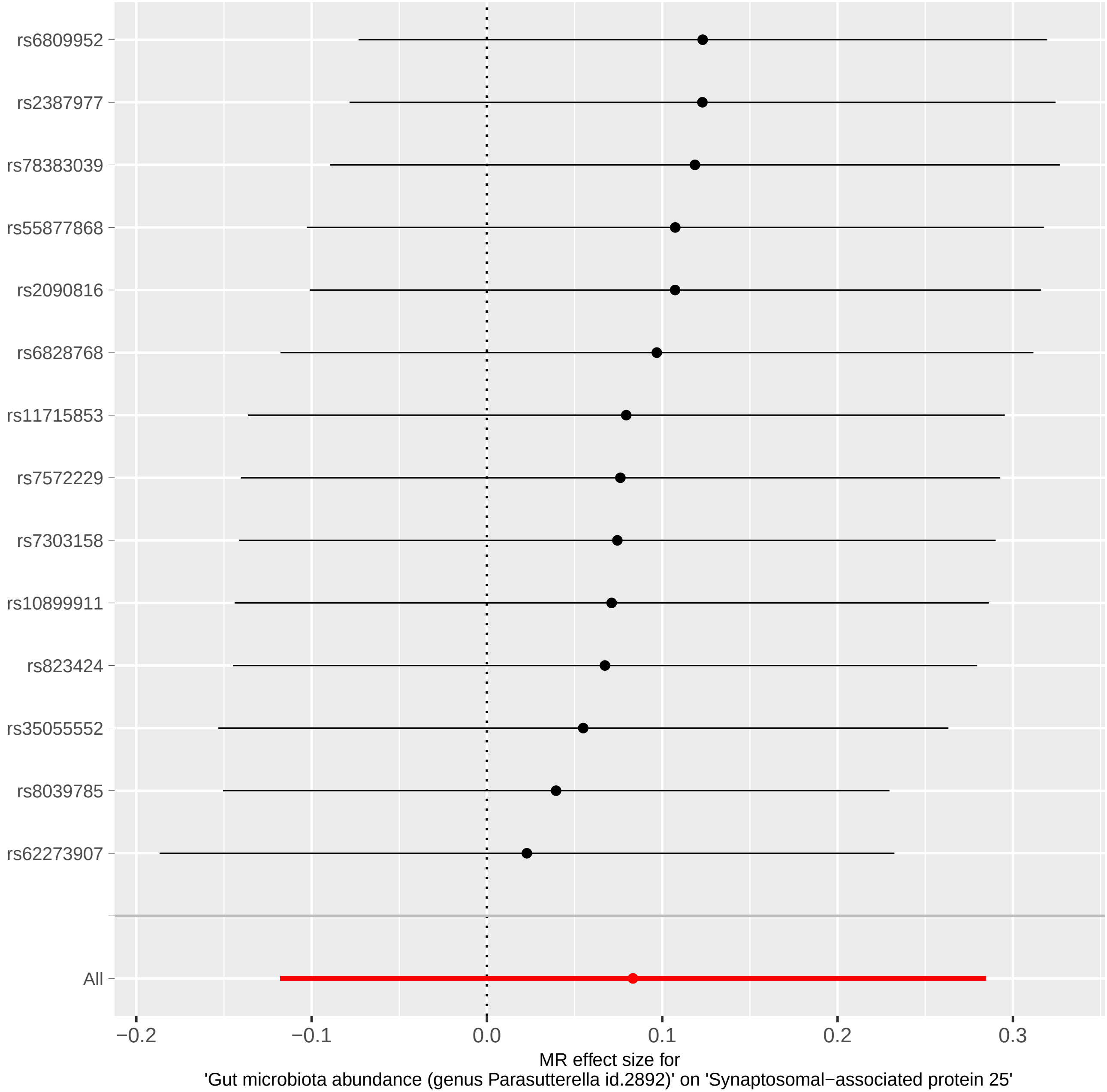

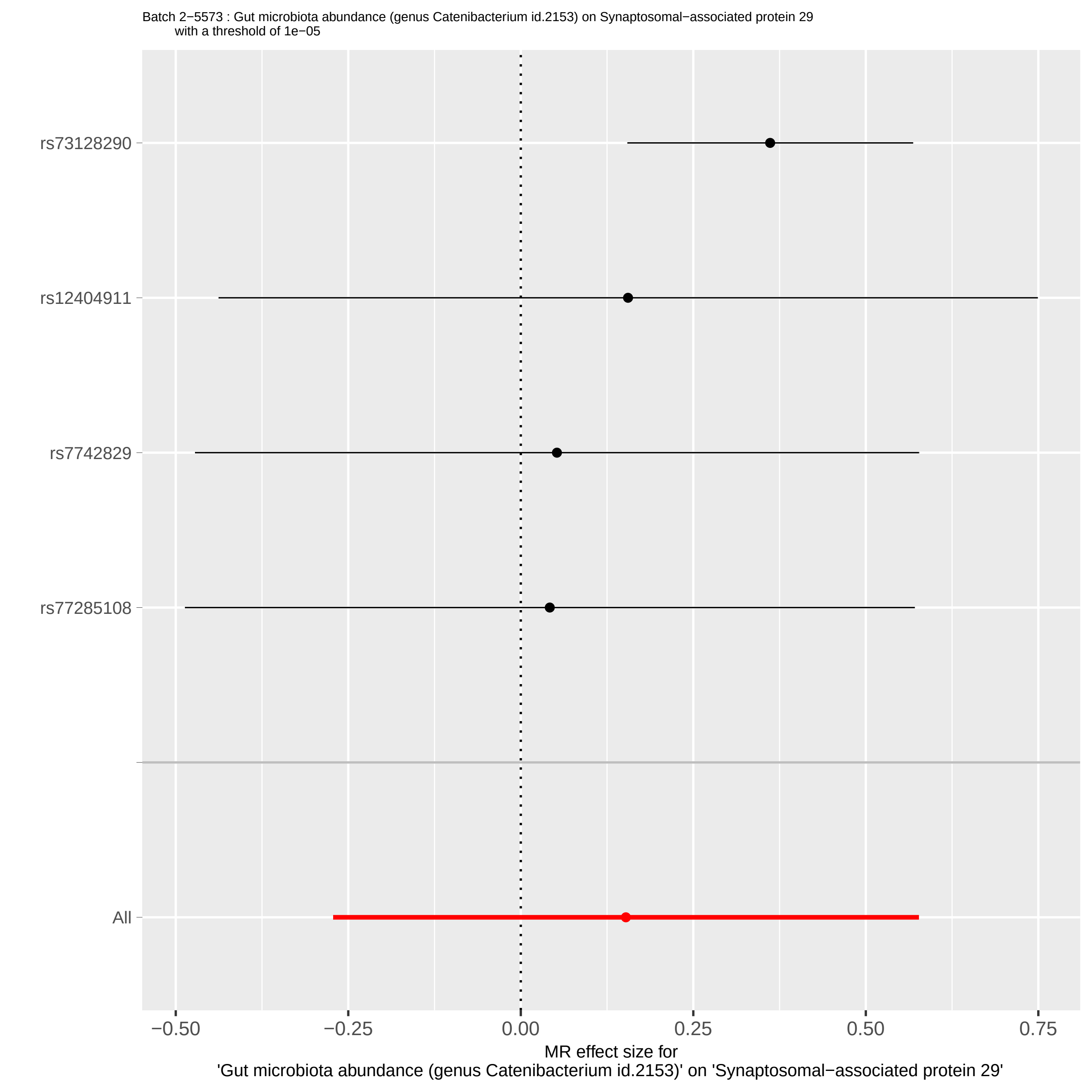
Batch 2−5573 : Gut microbiota abundance (genus Catenibacterium id.2153) on Synaptosomal−associated protein 29
with a threshold of 1e−05
rs73128290
rs12404911
rs7742829
rs77285108
All
−0.50
−0.25
0.00
0.25
0.50
0.75
MR effect size for
'Gut microbiota abundance (genus Catenibacterium id.2153)' on 'Synaptosomal−associated protein 29'

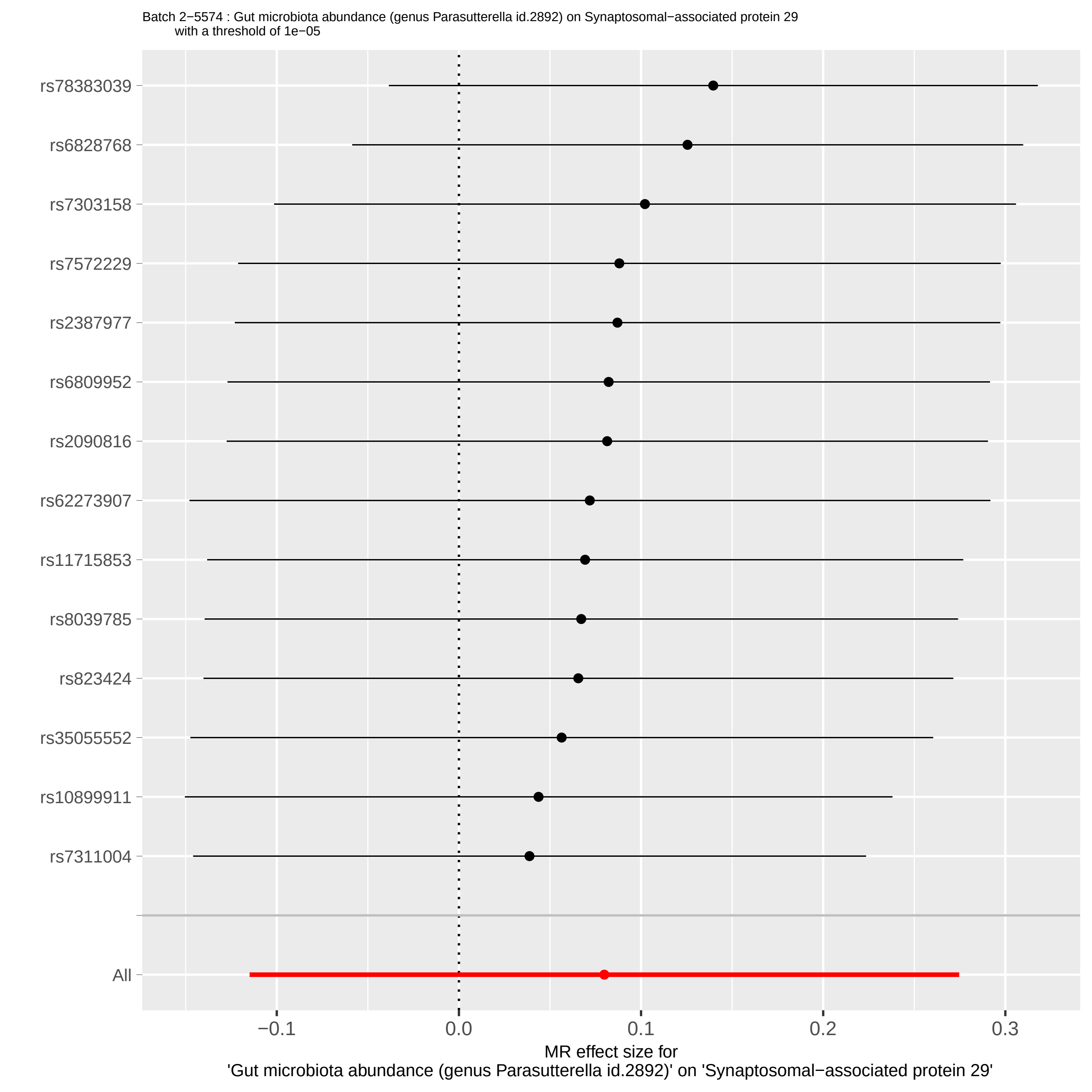

Batch 2−5574 : Gut microbiota abundance (genus Parasutterella id.2892) on Synaptosomal−associated protein 29
with a threshold of 1e−05
rs78383039
rs6828768
rs7303158
rs7572229
rs2387977
rs6809952
rs2090816
rs62273907
rs11715853
rs8039785
rs823424
rs35055552
rs10899911
rs7311004
All
−0.1
0.0
0.1
0.2
0.3
MR effect size for
'Gut microbiota abundance (genus Parasutterella id.2892)' on 'Synaptosomal−associated protein 29'

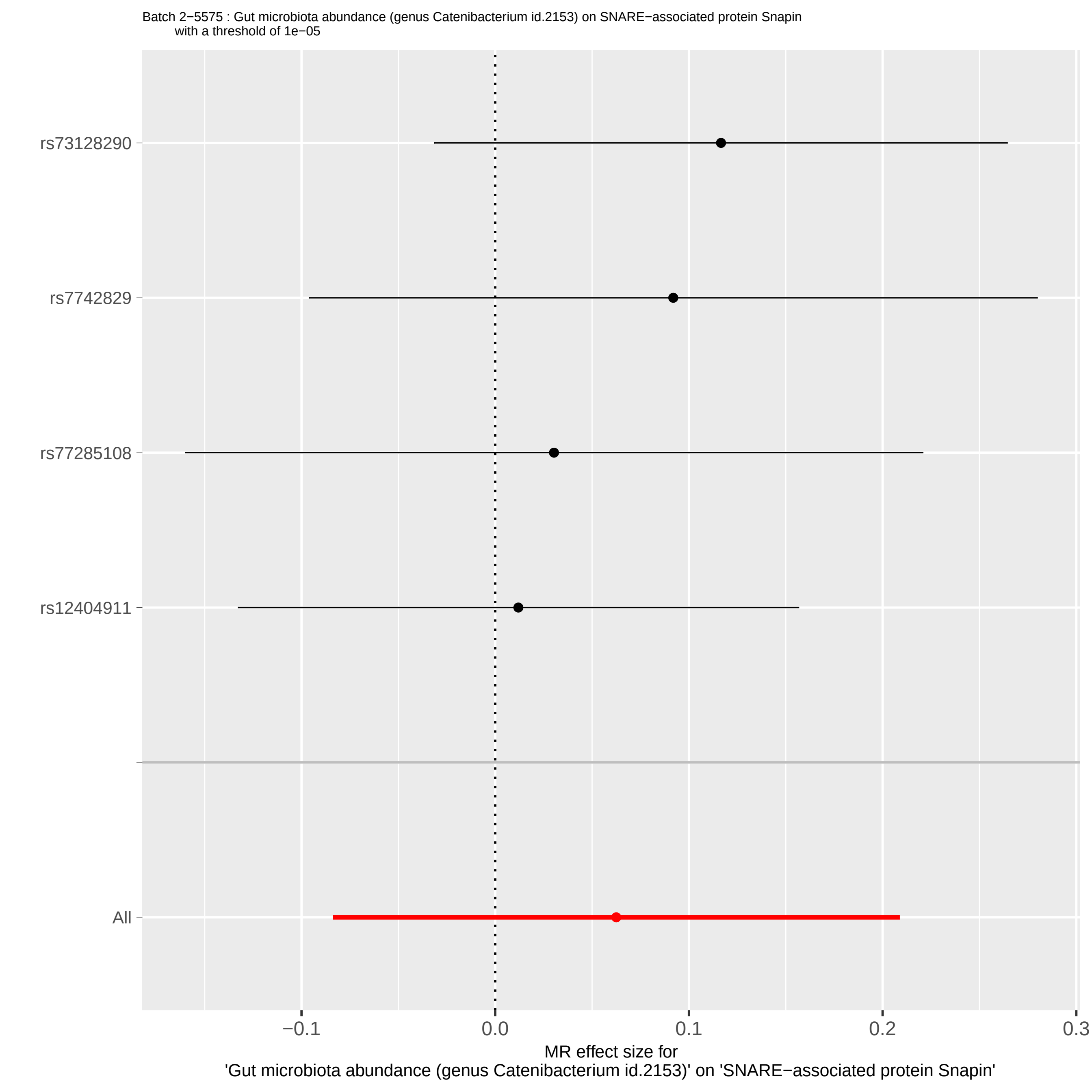
Batch 2−5575 : Gut microbiota abundance (genus Catenibacterium id.2153) on SNARE−associated protein Snapin
with a threshold of 1e−05
rs73128290
rs7742829
rs77285108
rs12404911
All
−0.1
0.0
0.1
0.2
0.3
MR effect size for
'Gut microbiota abundance (genus Catenibacterium id.2153)' on 'SNARE−associated protein Snapin'

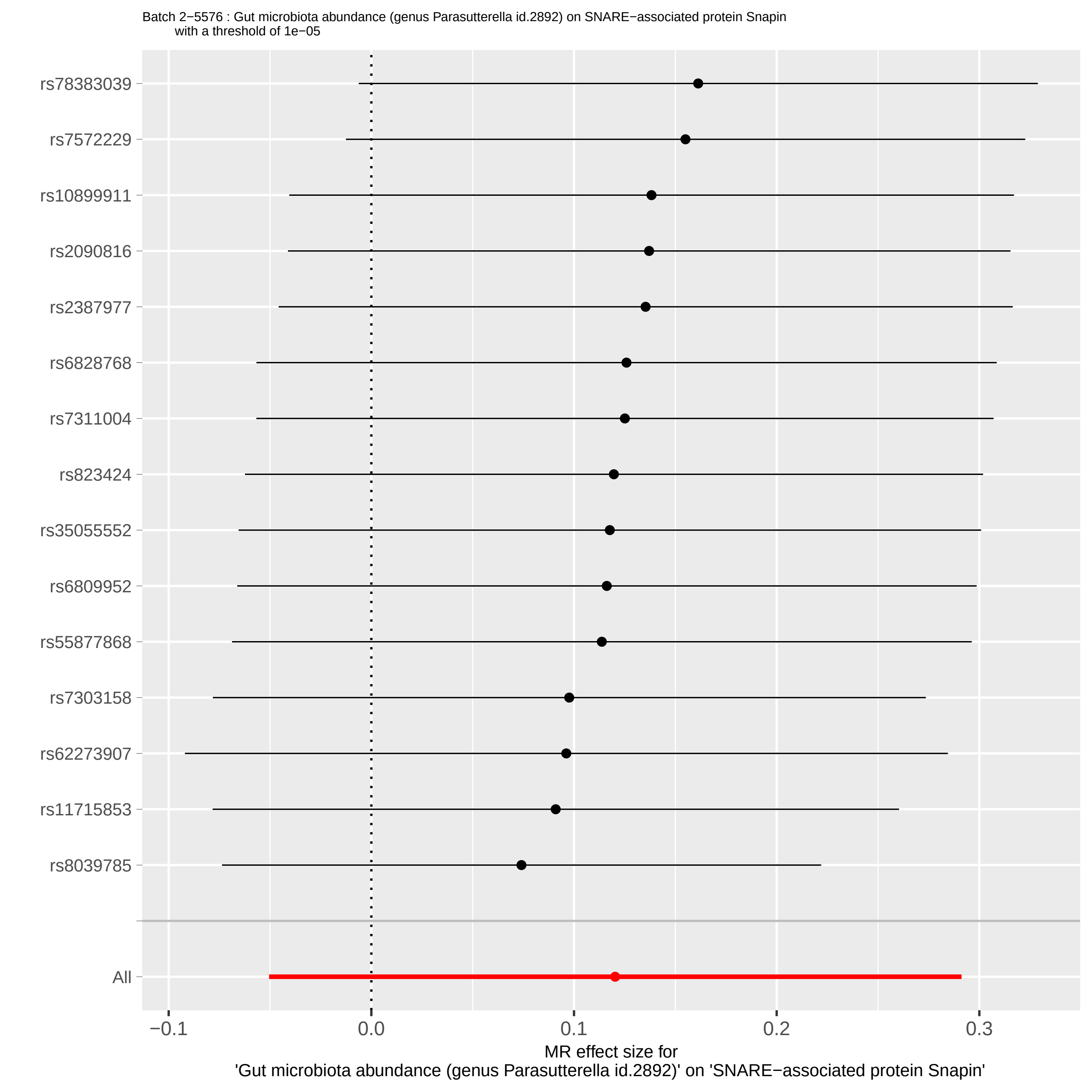
Batch 2−5576 : Gut microbiota abundance (genus Parasutterella id.2892) on SNARE−associated protein Snapin
with a threshold of 1e−05
rs78383039
rs7572229
rs10899911
rs2090816
rs2387977
rs6828768
rs7311004
rs823424
rs35055552
rs6809952
rs55877868
rs7303158
rs62273907
rs11715853
rs8039785
All
−0.1
0.0
0.1
0.2
0.3
MR effect size for
'Gut microbiota abundance (genus Parasutterella id.2892)' on 'SNARE−associated protein Snapin'

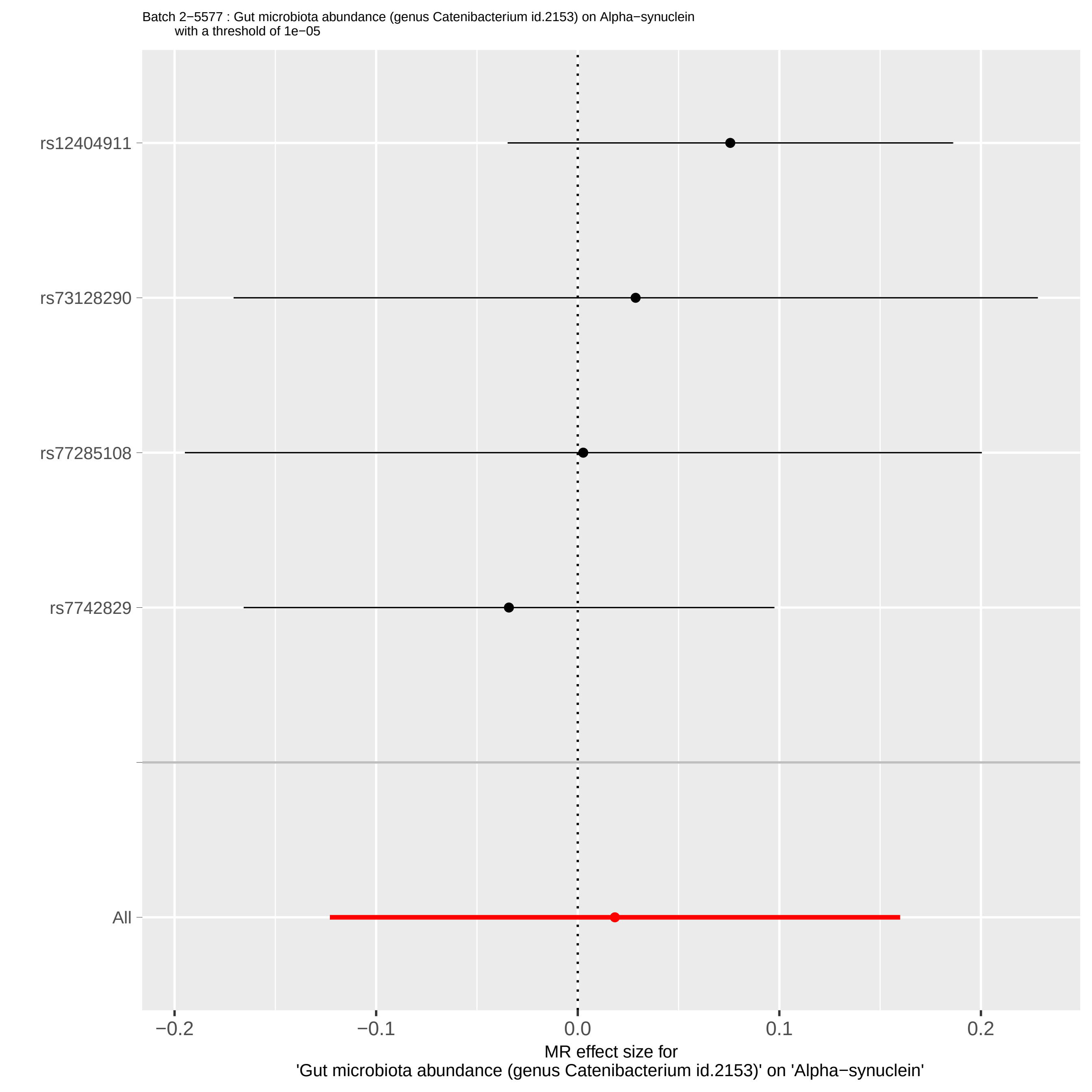
Batch 2−5577 : Gut microbiota abundance (genus Catenibacterium id.2153) on Alpha−synuclein
with a threshold of 1e−05
rs12404911
rs73128290
rs77285108
rs7742829
All
−0.2
−0.1
0.0
0.1
0.2
MR effect size for
'Gut microbiota abundance (genus Catenibacterium id.2153)' on 'Alpha−synuclein'

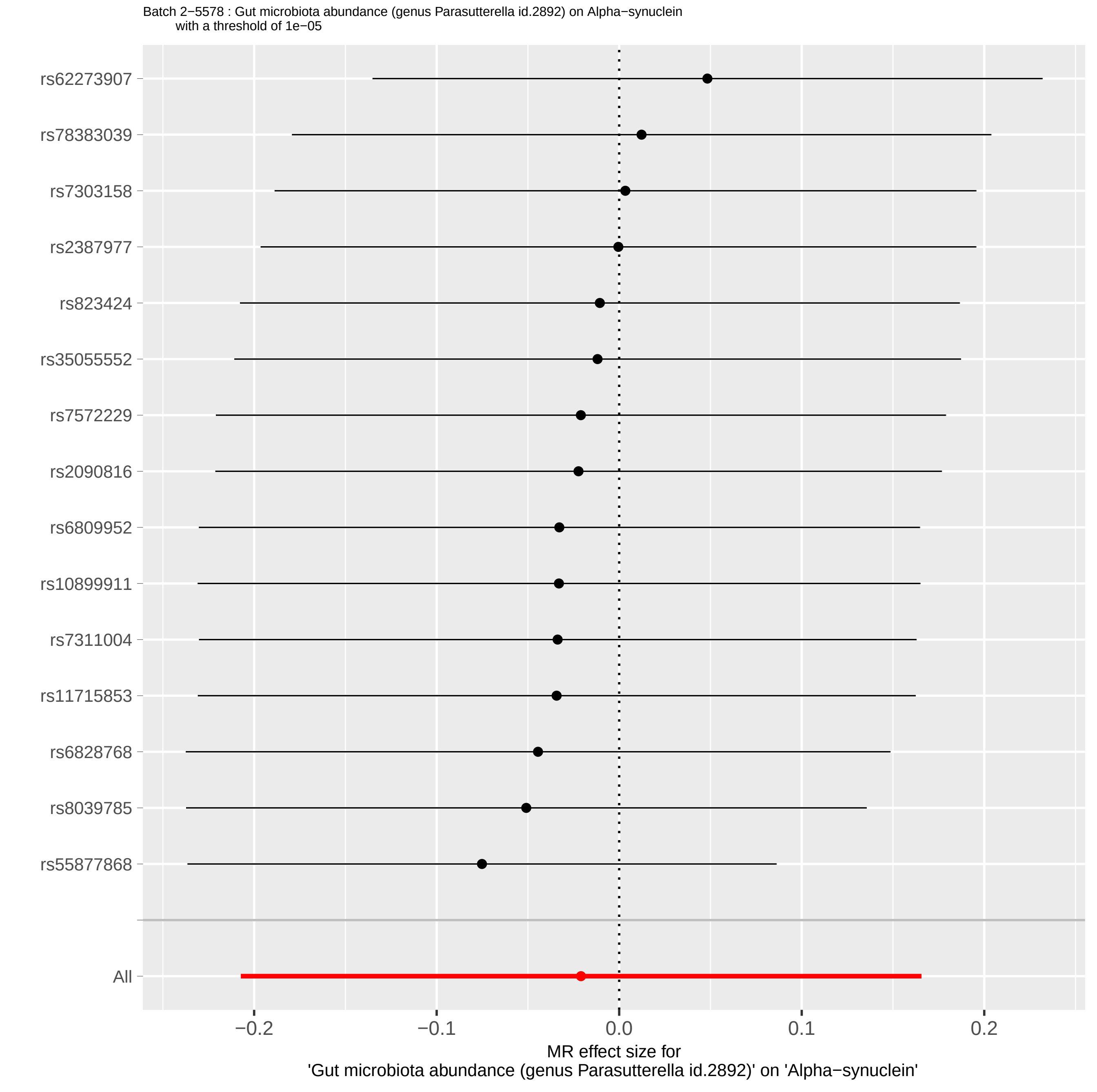
Batch 2−5578 : Gut microbiota abundance (genus Parasutterella id.2892) on Alpha−synuclein
with a threshold of 1e−05
rs62273907
rs78383039
rs7303158
rs2387977
rs823424
rs35055552
rs7572229
rs2090816
rs6809952
rs10899911
rs7311004
rs11715853
rs6828768
rs8039785
rs55877868
All
−0.2
−0.1
0.0
0.1
0.2
MR effect size for
'Gut microbiota abundance (genus Parasutterella id.2892)' on 'Alpha−synuclein'

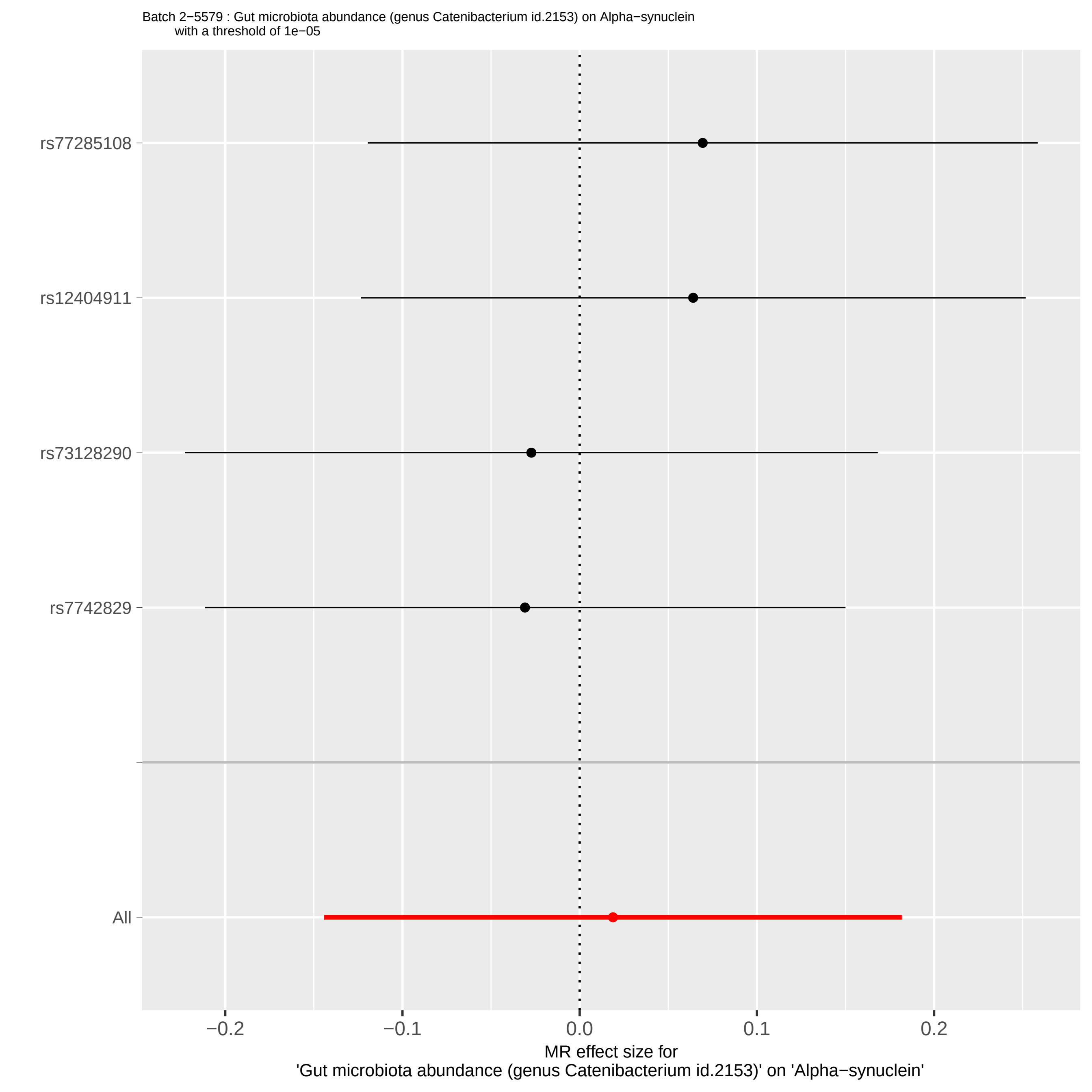
Batch 2−5579 : Gut microbiota abundance (genus Catenibacterium id.2153) on Alpha−synuclein
with a threshold of 1e−05
rs77285108
rs12404911
rs73128290
rs7742829
All
−0.2
−0.1
0.0
0.1
0.2
MR effect size for
'Gut microbiota abundance (genus Catenibacterium id.2153)' on 'Alpha−synuclein'

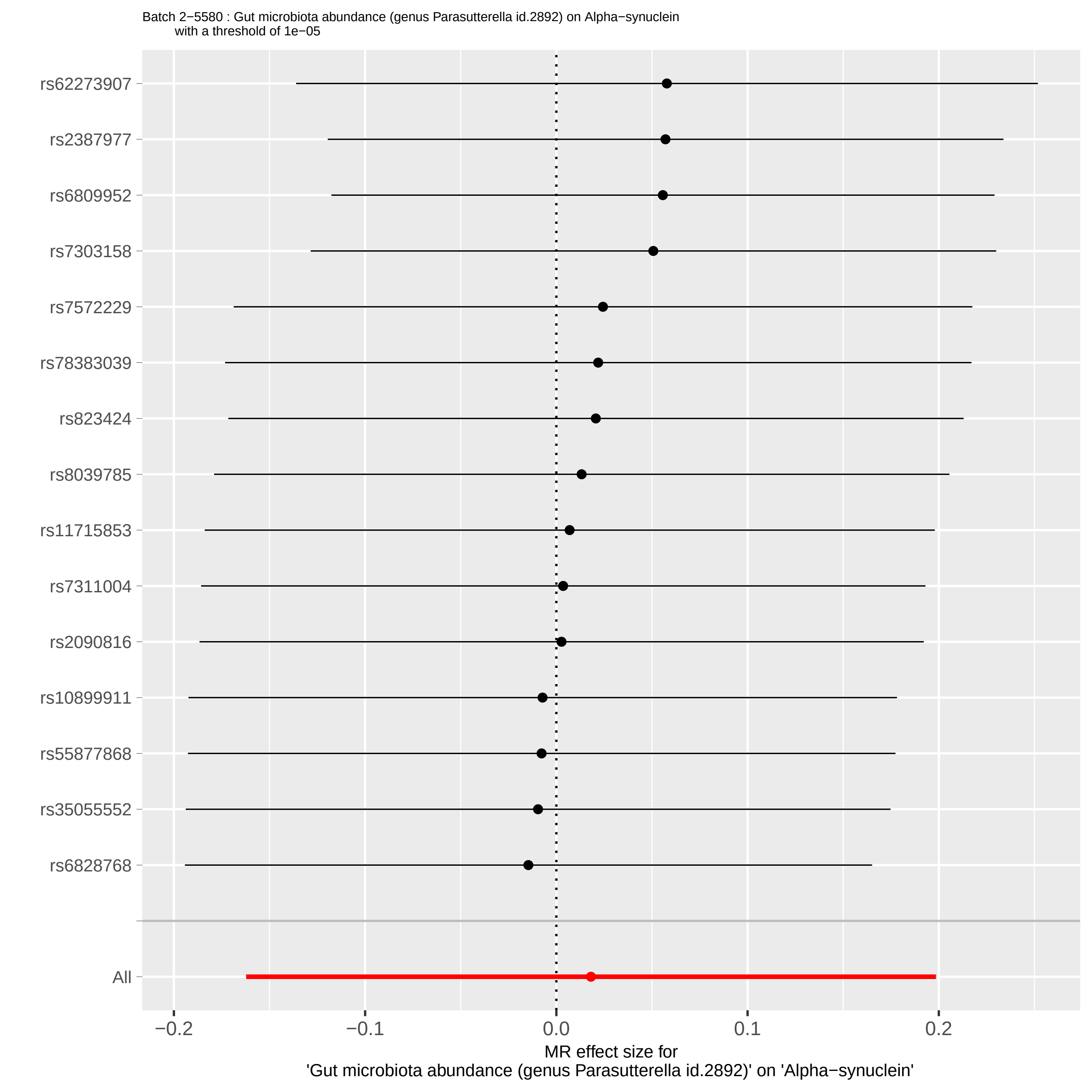
Batch 2−5580 : Gut microbiota abundance (genus Parasutterella id.2892) on Alpha−synuclein
with a threshold of 1e−05
rs62273907
rs2387977
rs6809952
rs7303158
rs7572229
rs78383039
rs823424
rs8039785
rs11715853
rs7311004
rs2090816
rs10899911
rs55877868
rs35055552
rs6828768
All
−0.2
−0.1
0.0
0.1
0.2
MR effect size for
'Gut microbiota abundance (genus Parasutterella id.2892)' on 'Alpha−synuclein'

Batch 2−5581 : Gut microbiota abundance (genus Catenibacterium id.2153) on U1 small nuclear ribonucleoprotein A
with a threshold of 1e−05

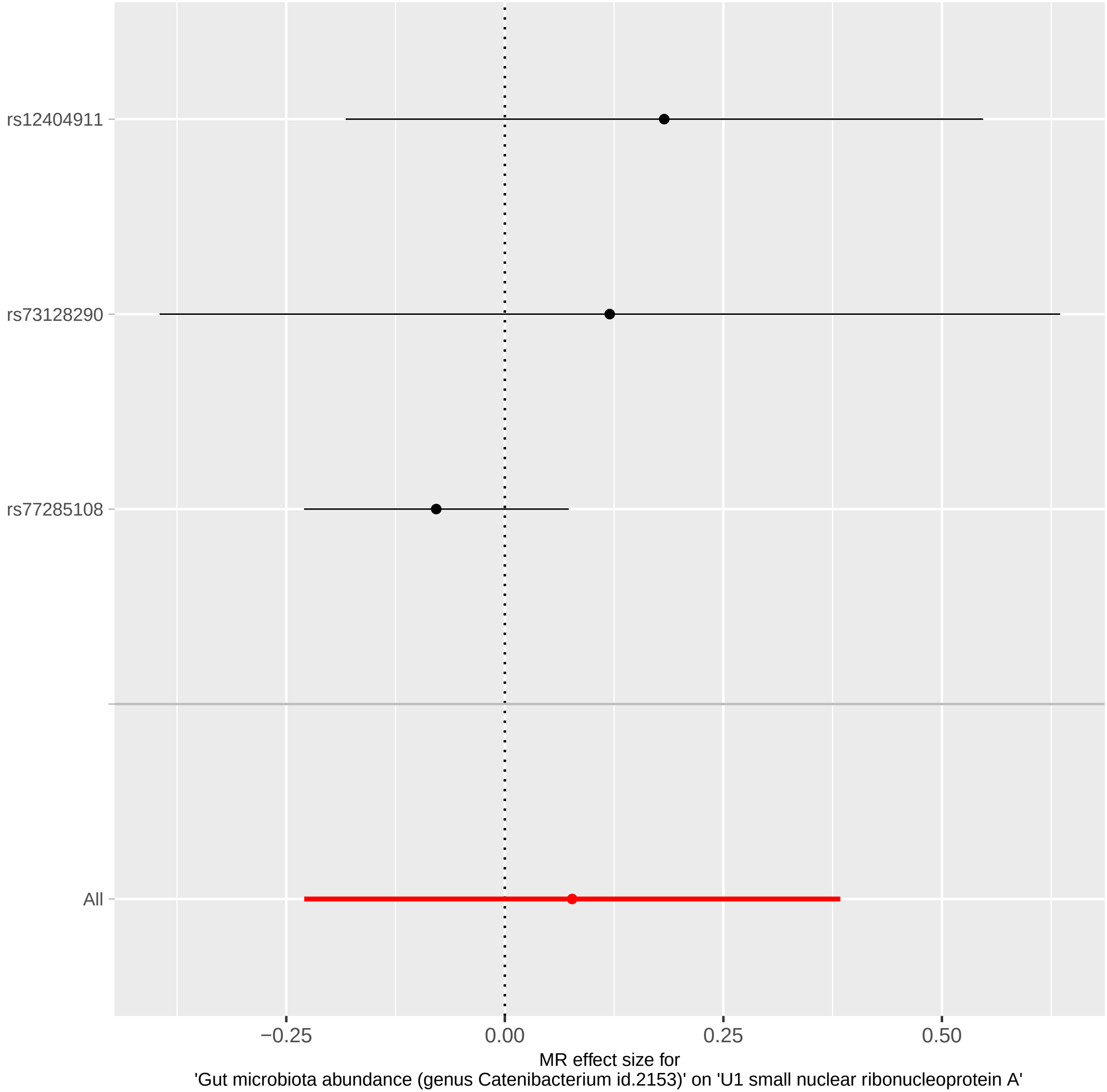

Batch 2−5582 : Gut microbiota abundance (genus Parasutterella id.2892) on U1 small nuclear ribonucleoprotein A
with a threshold of 1e−05

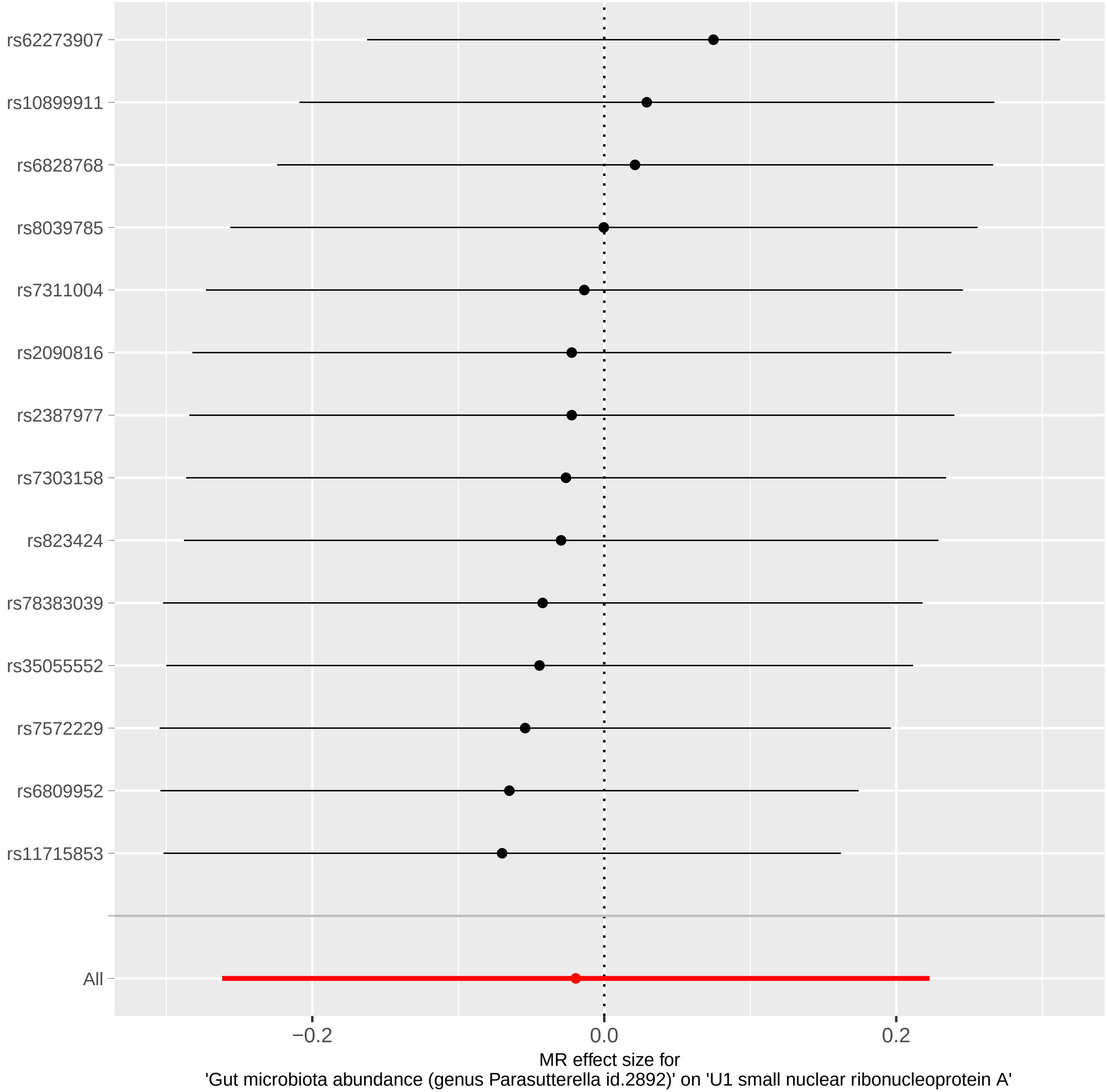

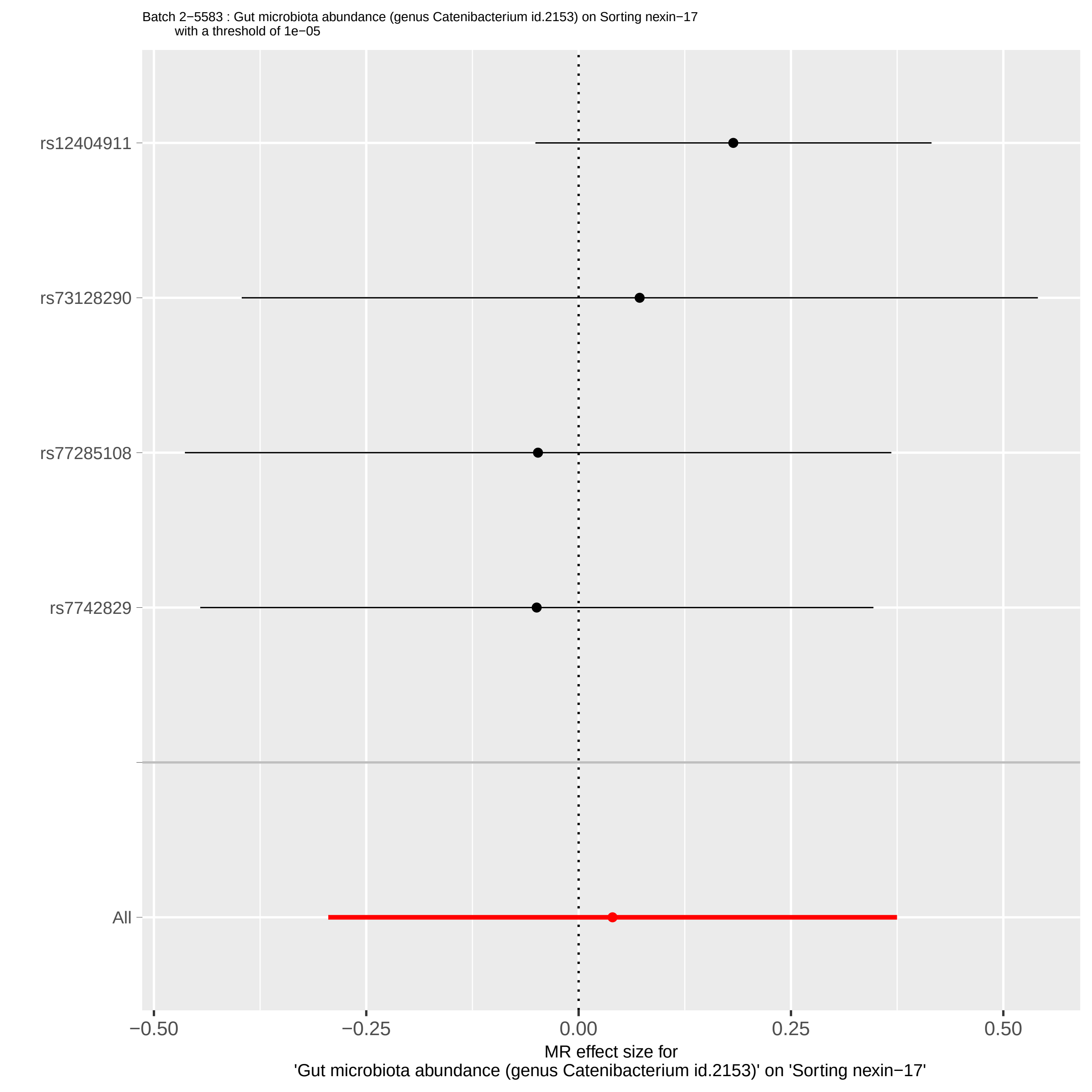

Batch 2−5583 : Gut microbiota abundance (genus Catenibacterium id.2153) on Sorting nexin−17
with a threshold of 1e−05
rs12404911
rs73128290
rs77285108
rs7742829
All
−0.50
−0.25
0.00
0.25
0.50
MR effect size for
'Gut microbiota abundance (genus Catenibacterium id.2153)' on 'Sorting nexin−17'

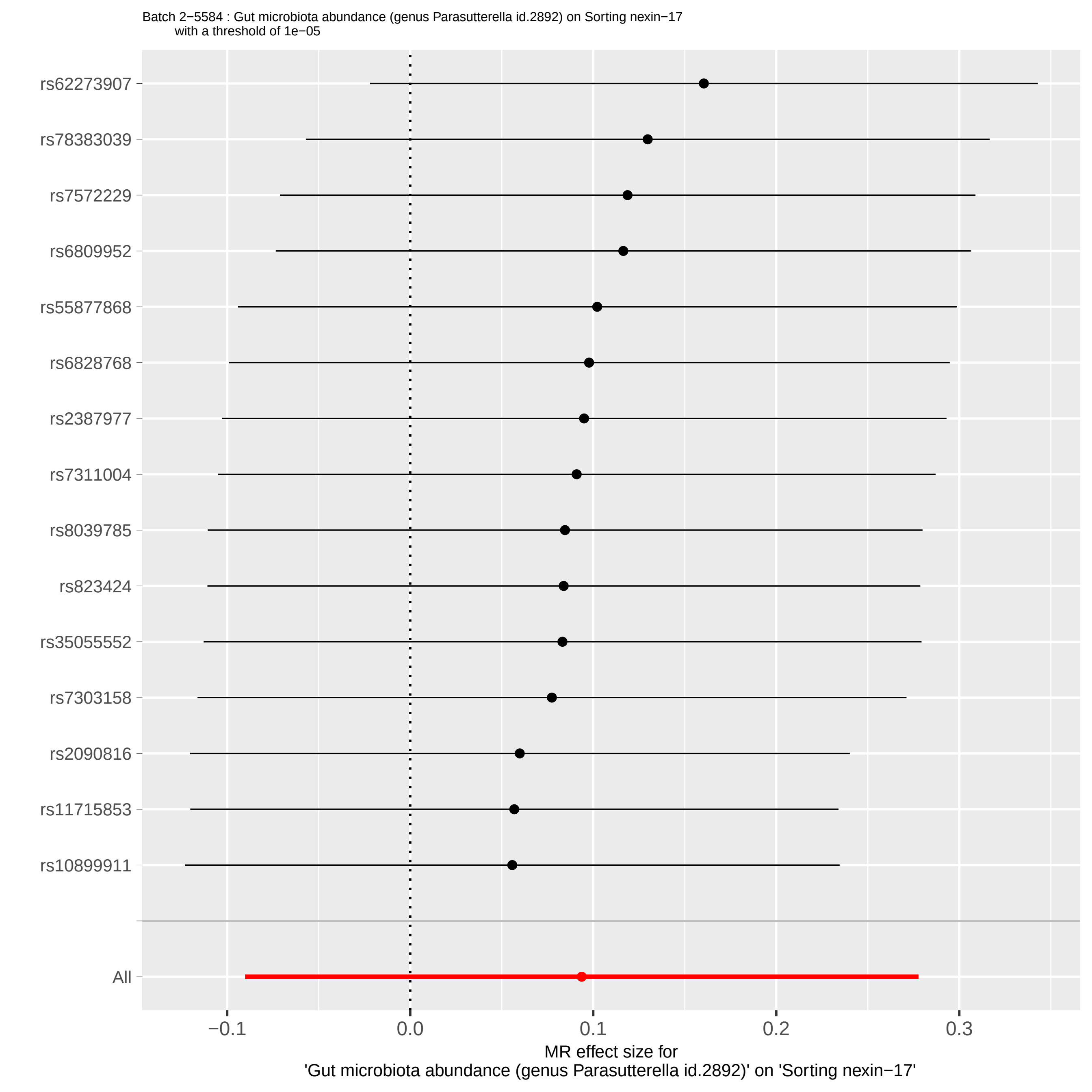
Batch 2−5584 : Gut microbiota abundance (genus Parasutterella id.2892) on Sorting nexin−17
with a threshold of 1e−05
rs62273907
rs78383039
rs7572229
rs6809952
rs55877868
rs6828768
rs2387977
rs7311004
rs8039785
rs823424
rs35055552
rs7303158
rs2090816
rs11715853
rs10899911
All
−0.1
0.0
0.1
0.2
0.3
MR effect size for
'Gut microbiota abundance (genus Parasutterella id.2892)' on 'Sorting nexin−17'

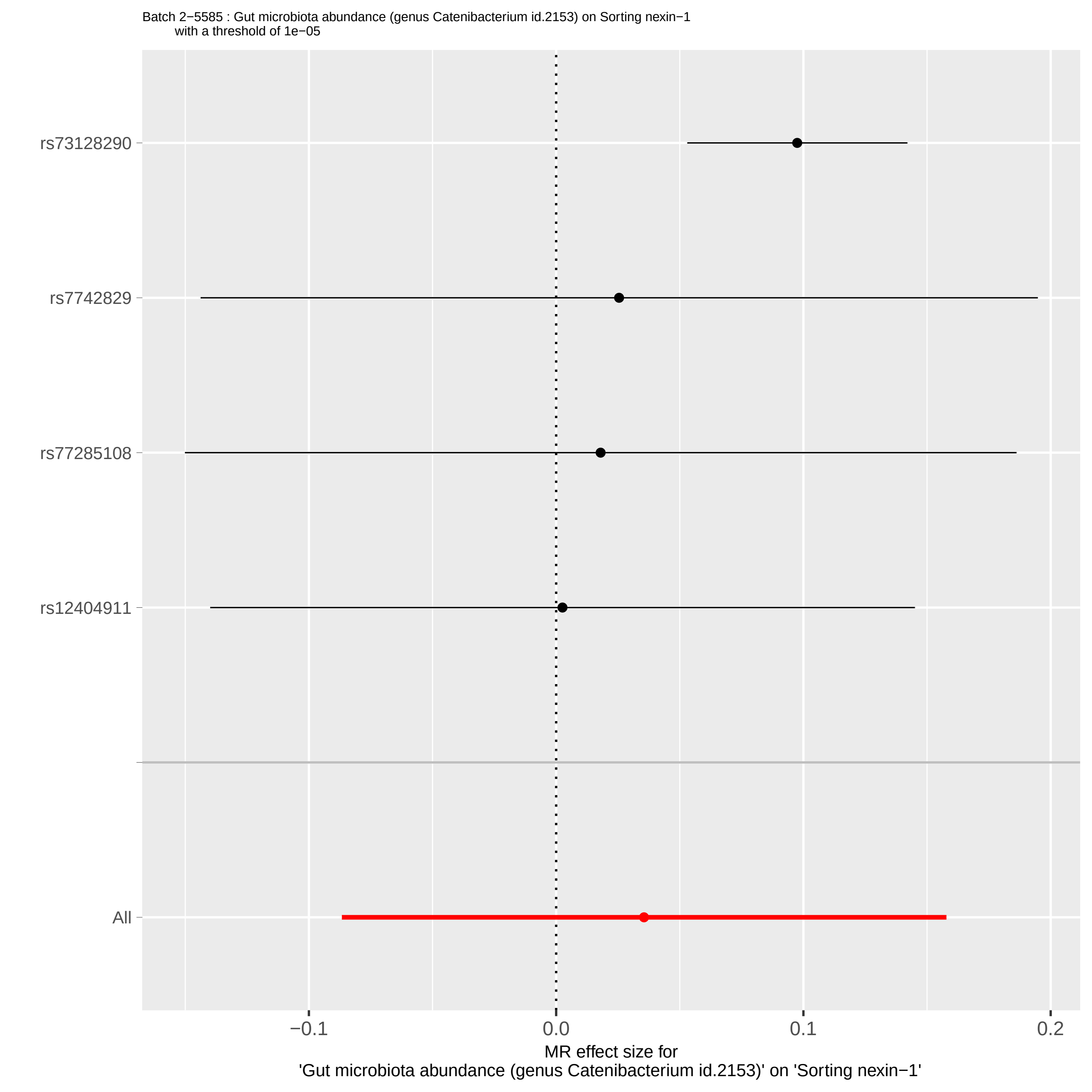
Batch 2−5585 : Gut microbiota abundance (genus Catenibacterium id.2153) on Sorting nexin−1
with a threshold of 1e−05
rs73128290
rs7742829
rs77285108
rs12404911
All
−0.1
0.0
0.1
0.2
MR effect size for
'Gut microbiota abundance (genus Catenibacterium id.2153)' on 'Sorting nexin−1'

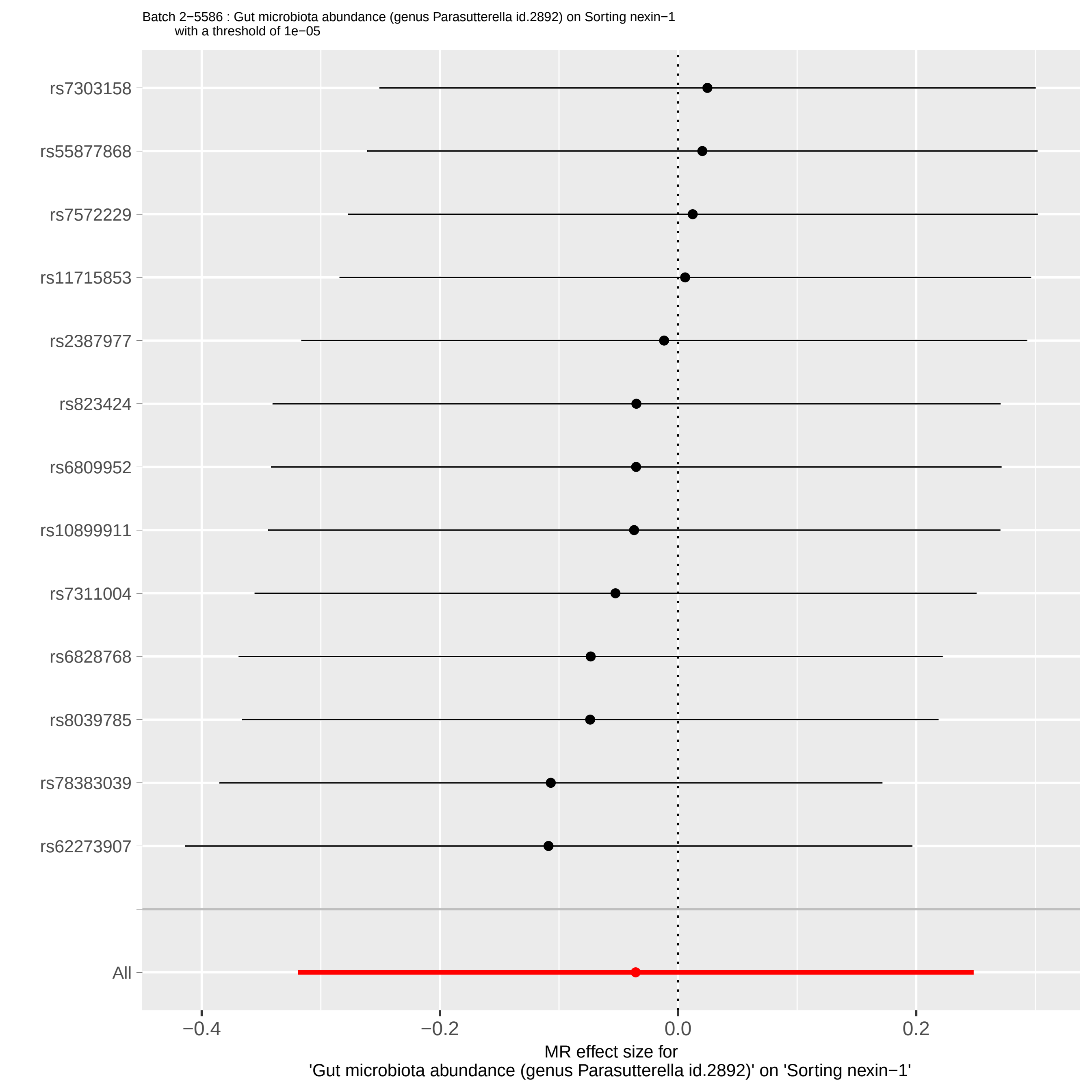

Batch 2−5586 : Gut microbiota abundance (genus Parasutterella id.2892) on Sorting nexin−1
with a threshold of 1e−05
rs7303158
rs55877868
rs7572229
rs11715853
rs2387977
rs823424
rs6809952
rs10899911
rs7311004
rs6828768
rs8039785
rs78383039
rs62273907
All
−0.4
−0.2
0.0
0.2
MR effect size for
'Gut microbiota abundance (genus Parasutterella id.2892)' on 'Sorting nexin−1'

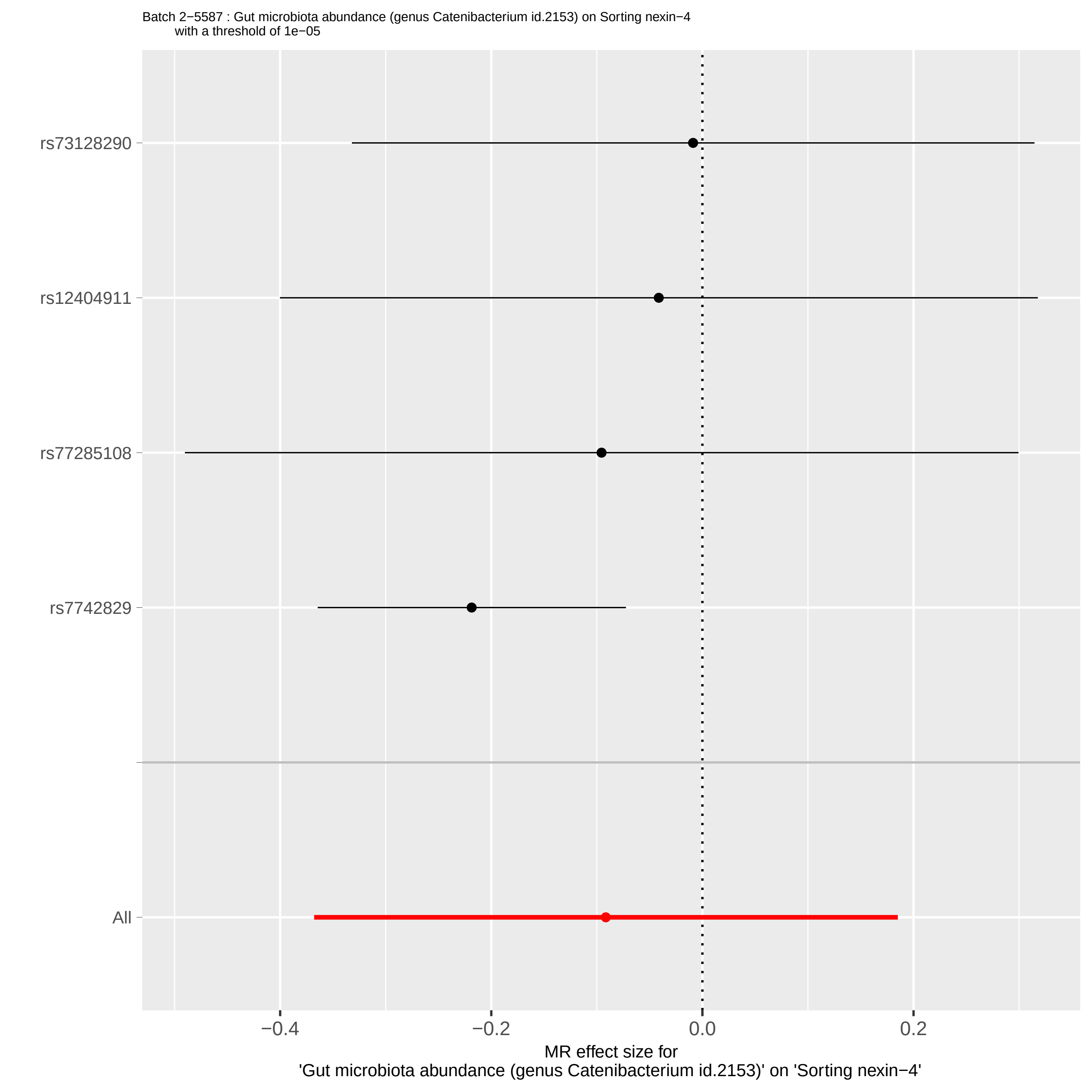

Batch 2−5587 : Gut microbiota abundance (genus Catenibacterium id.2153) on Sorting nexin−4
with a threshold of 1e−05
rs73128290
rs12404911
rs77285108
rs7742829
All
−0.4
−0.2
0.0
0.2
MR effect size for
'Gut microbiota abundance (genus Catenibacterium id.2153)' on 'Sorting nexin−4'

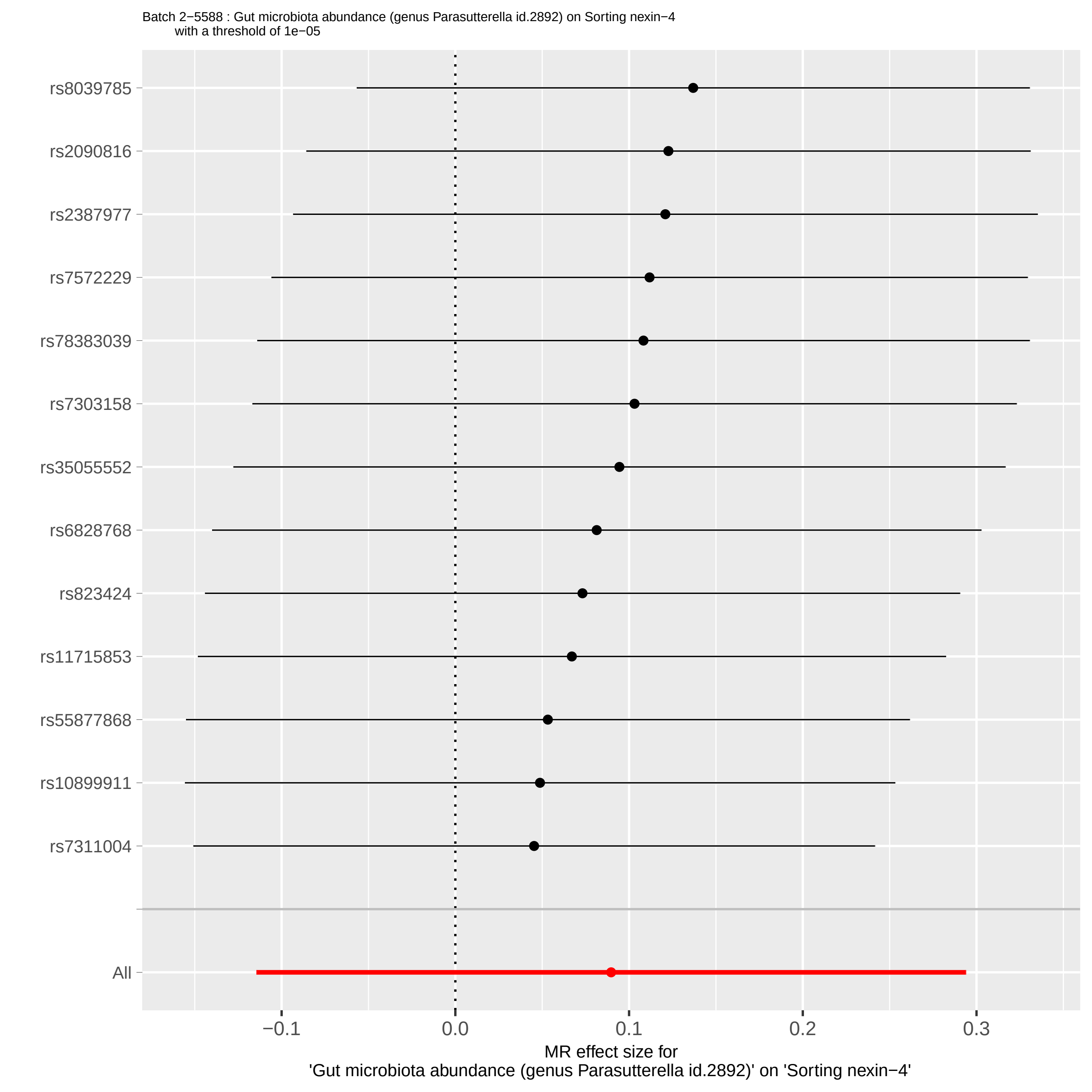

Batch 2−5588 : Gut microbiota abundance (genus Parasutterella id.2892) on Sorting nexin−4
with a threshold of 1e−05
rs8039785
rs2090816
rs2387977
rs7572229
rs78383039
rs7303158
rs35055552
rs6828768
rs823424
rs11715853
rs55877868
rs10899911
rs7311004
All
−0.1
0.0
0.1
0.2
0.3
MR effect size for
'Gut microbiota abundance (genus Parasutterella id.2892)' on 'Sorting nexin−4'

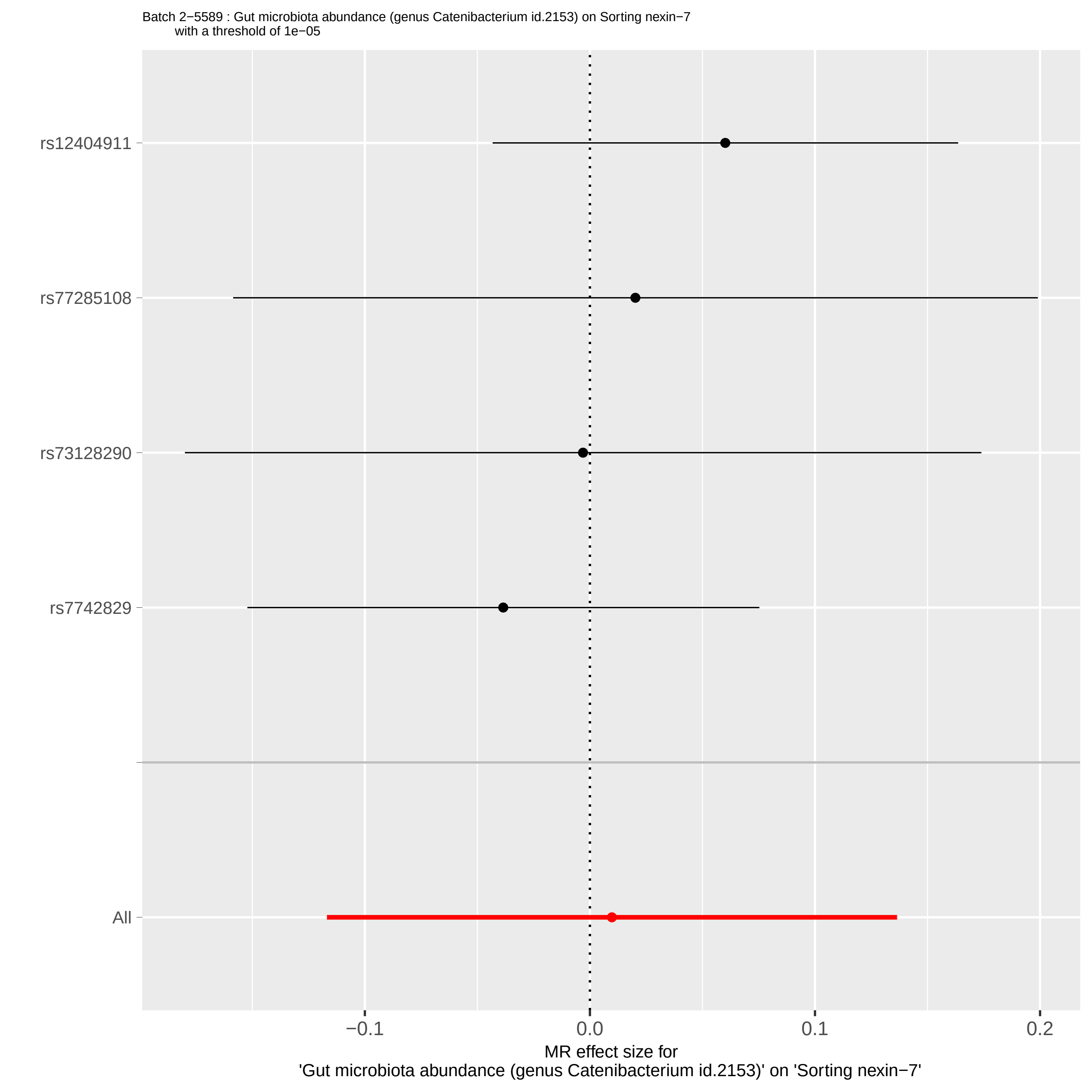

Batch 2−5589 : Gut microbiota abundance (genus Catenibacterium id.2153) on Sorting nexin−7
with a threshold of 1e−05
rs12404911
rs77285108
rs73128290
rs7742829
All
−0.1
0.0
0.1
0.2
MR effect size for
'Gut microbiota abundance (genus Catenibacterium id.2153)' on 'Sorting nexin−7'

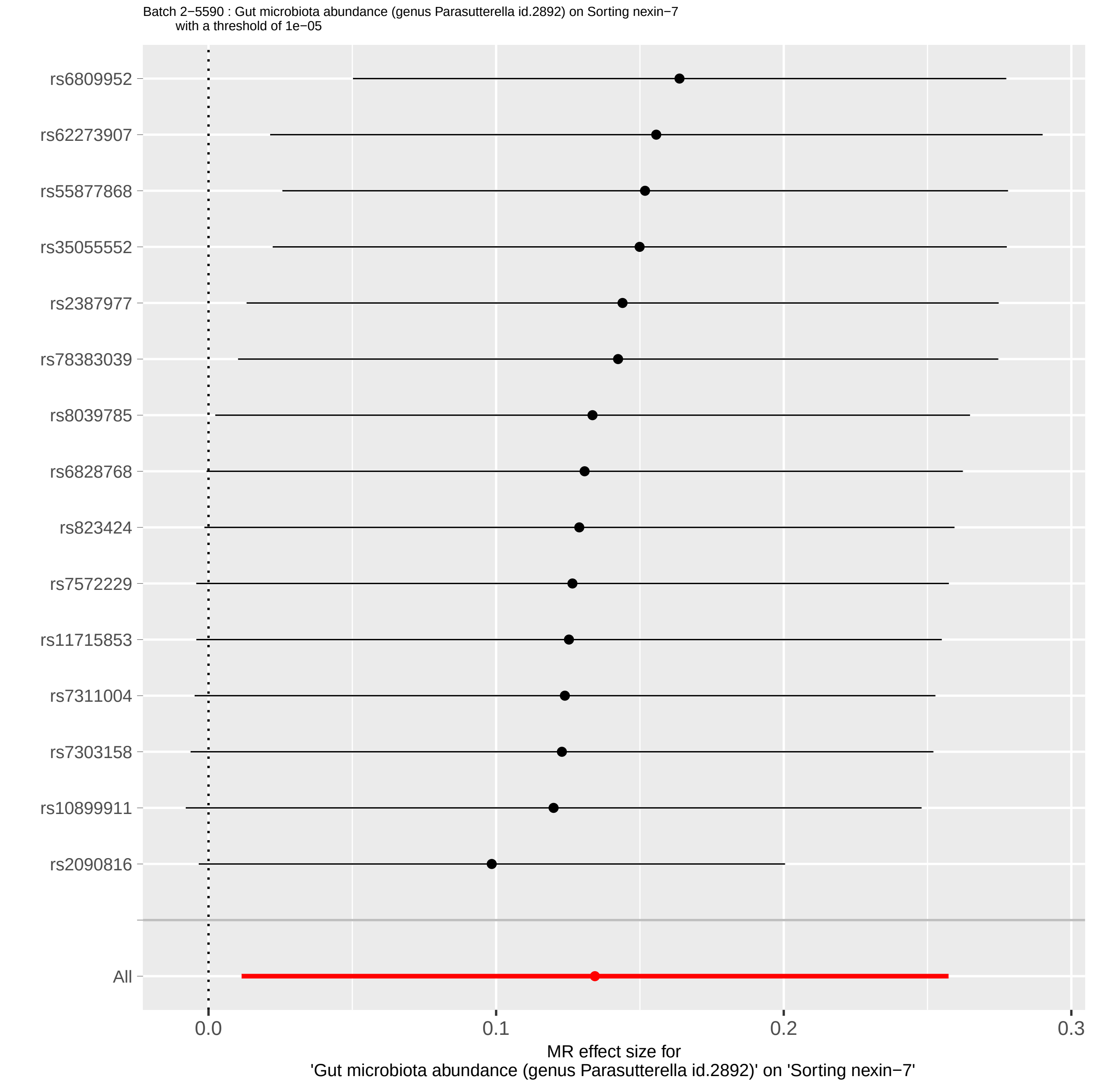

Batch 2−5590 : Gut microbiota abundance (genus Parasutterella id.2892) on Sorting nexin−7
with a threshold of 1e−05
rs6809952
rs62273907
rs55877868
rs35055552
rs2387977
rs78383039
rs8039785
rs6828768
rs823424
rs7572229
rs11715853
rs7311004
rs7303158
rs10899911
rs2090816
All
0.0
0.1
0.2
0.3
MR effect size for
'Gut microbiota abundance (genus Parasutterella id.2892)' on 'Sorting nexin−7'

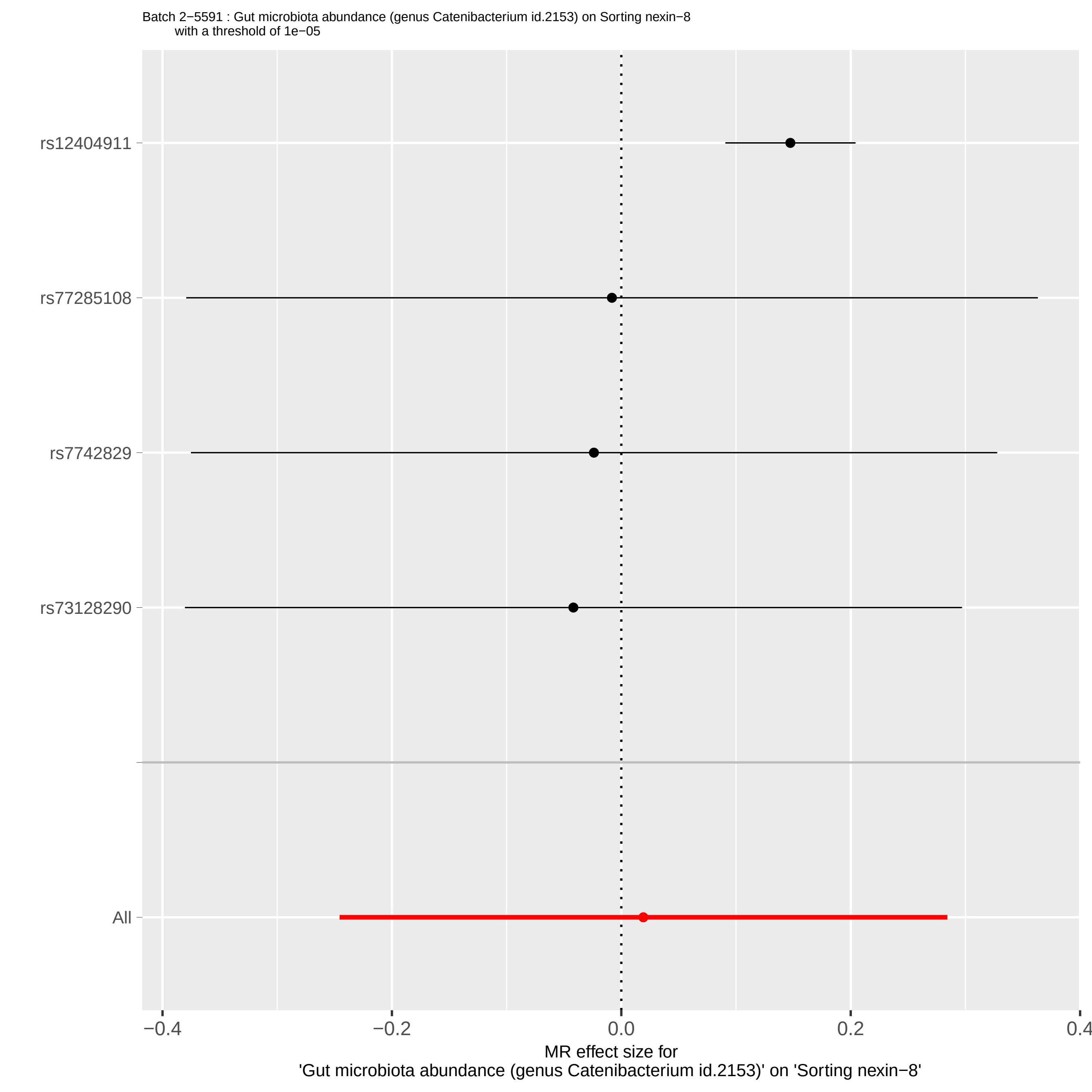

Batch 2−5591 : Gut microbiota abundance (genus Catenibacterium id.2153) on Sorting nexin−8
with a threshold of 1e−05
rs12404911
rs77285108
rs7742829
rs73128290
All
−0.4
−0.2
0.0
0.2
0.4
MR effect size for
'Gut microbiota abundance (genus Catenibacterium id.2153)' on 'Sorting nexin−8'

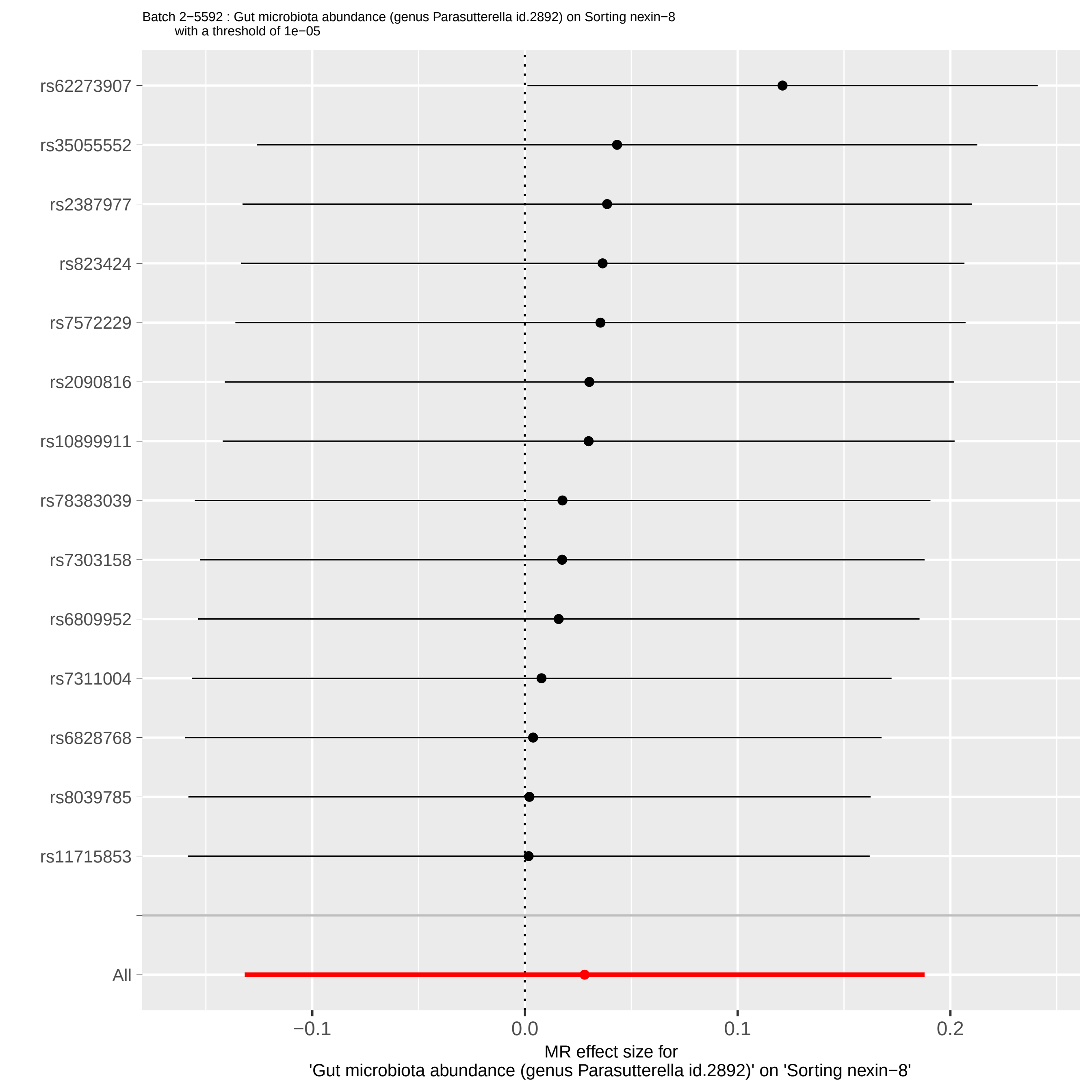
Batch 2−5592 : Gut microbiota abundance (genus Parasutterella id.2892) on Sorting nexin−8
with a threshold of 1e−05
rs62273907
rs35055552
rs2387977
rs823424
rs7572229
rs2090816
rs10899911
rs78383039
rs7303158
rs6809952
rs7311004
rs6828768
rs8039785
rs11715853
All
−0.1
0.0
0.1
0.2
MR effect size for
'Gut microbiota abundance (genus Parasutterella id.2892)' on 'Sorting nexin−8'

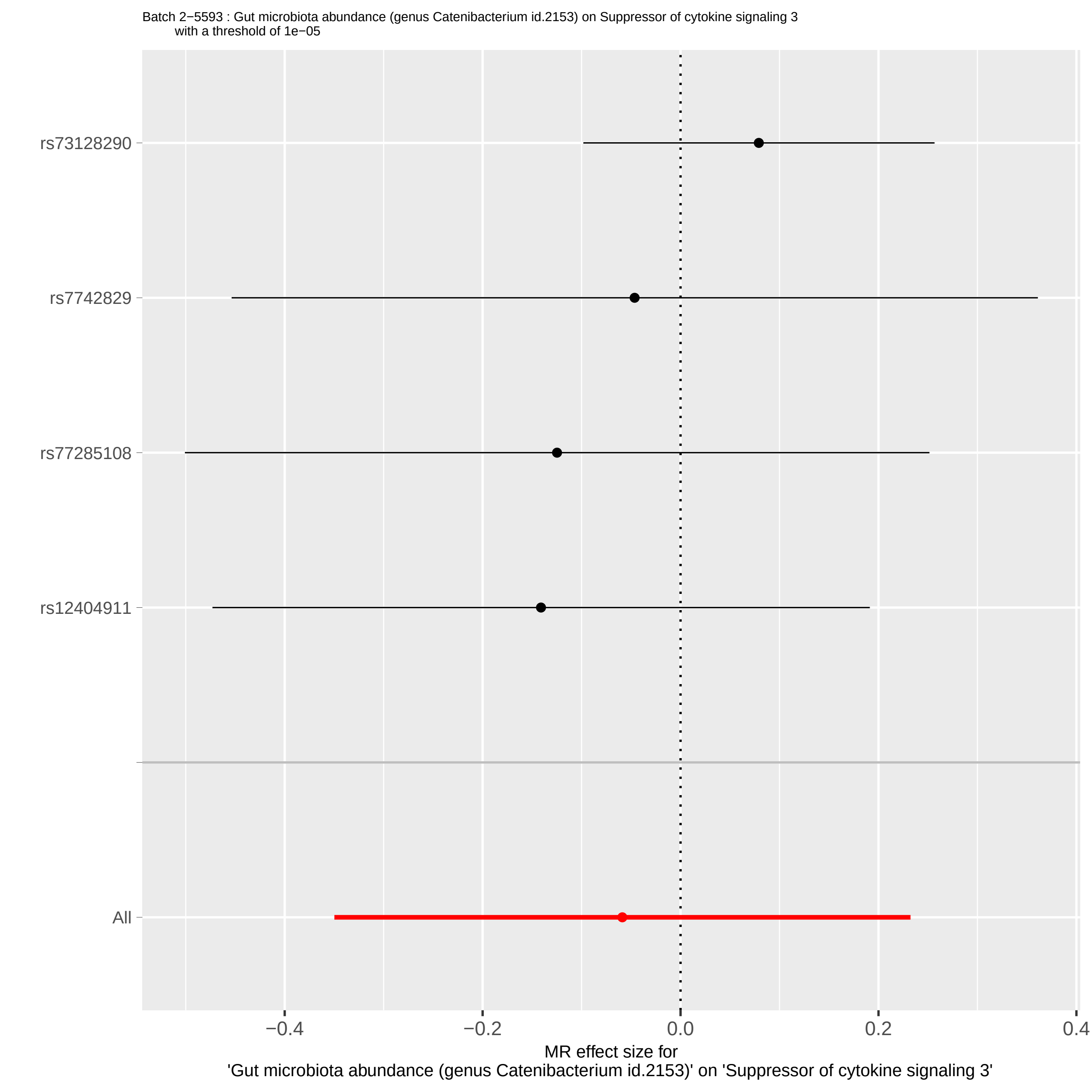
Batch 2−5593 : Gut microbiota abundance (genus Catenibacterium id.2153) on Suppressor of cytokine signaling 3
with a threshold of 1e−05
rs73128290
rs7742829
rs77285108
rs12404911
All
−0.4
−0.2
0.0
0.2
0.4
MR effect size for
'Gut microbiota abundance (genus Catenibacterium id.2153)' on 'Suppressor of cytokine signaling 3'

Batch 2−5594 : Gut microbiota abundance (genus Parasutterella id.2892) on Suppressor of cytokine signaling 3
with a threshold of 1e−05

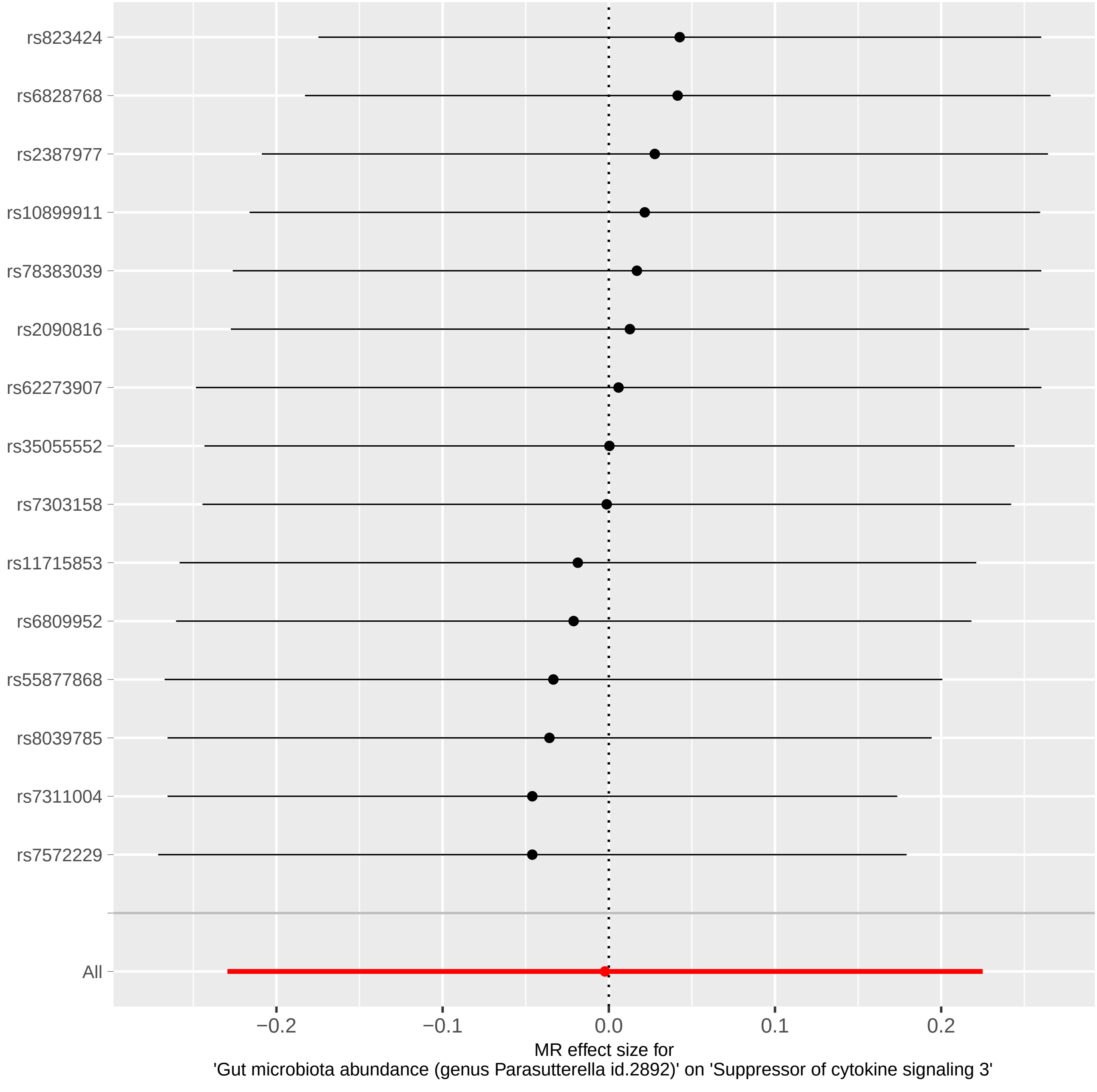

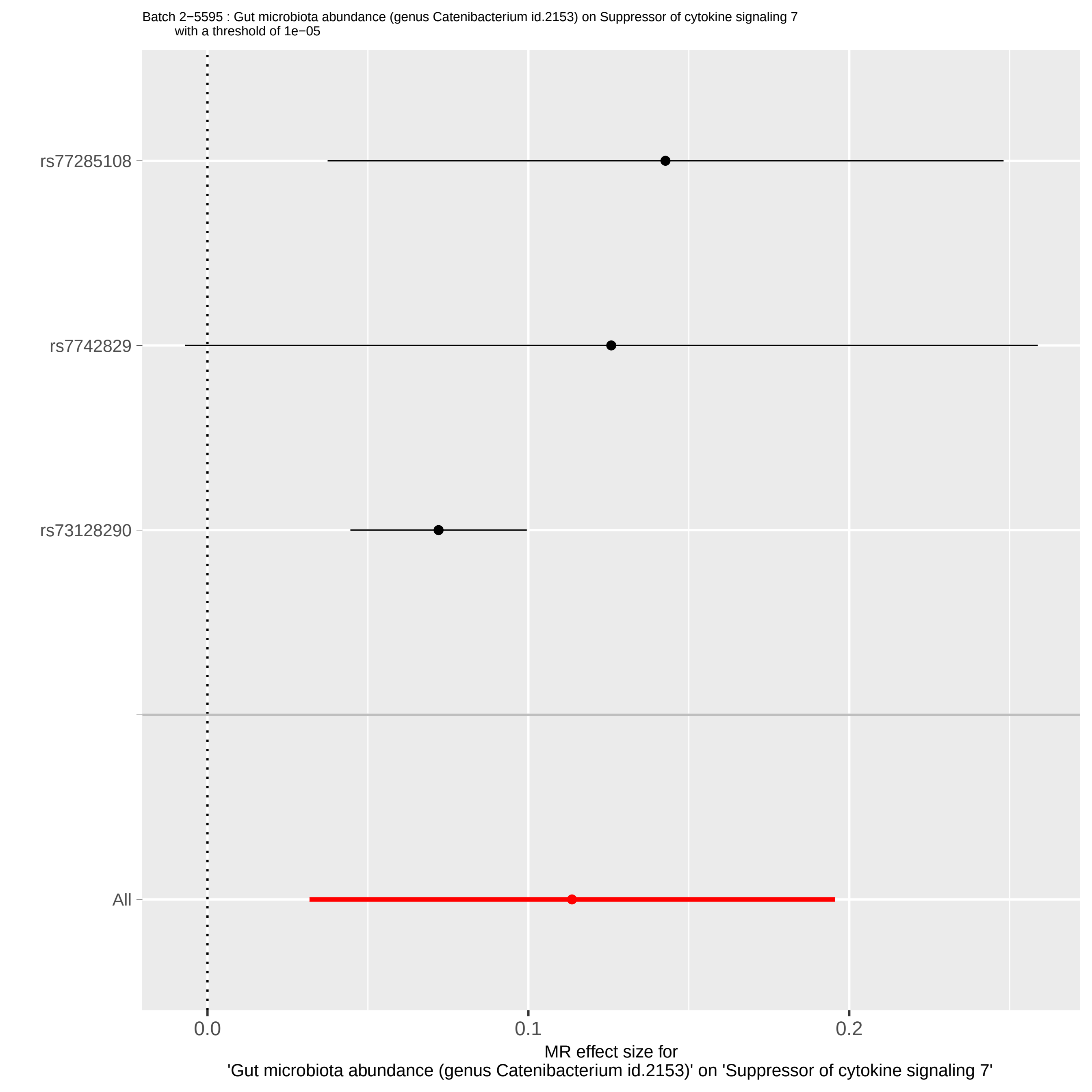
Batch 2−5595 : Gut microbiota abundance (genus Catenibacterium id.2153) on Suppressor of cytokine signaling 7
with a threshold of 1e−05
rs77285108
rs7742829
rs73128290
All
0.0
0.1
0.2
MR effect size for
'Gut microbiota abundance (genus Catenibacterium id.2153)' on 'Suppressor of cytokine signaling 7'

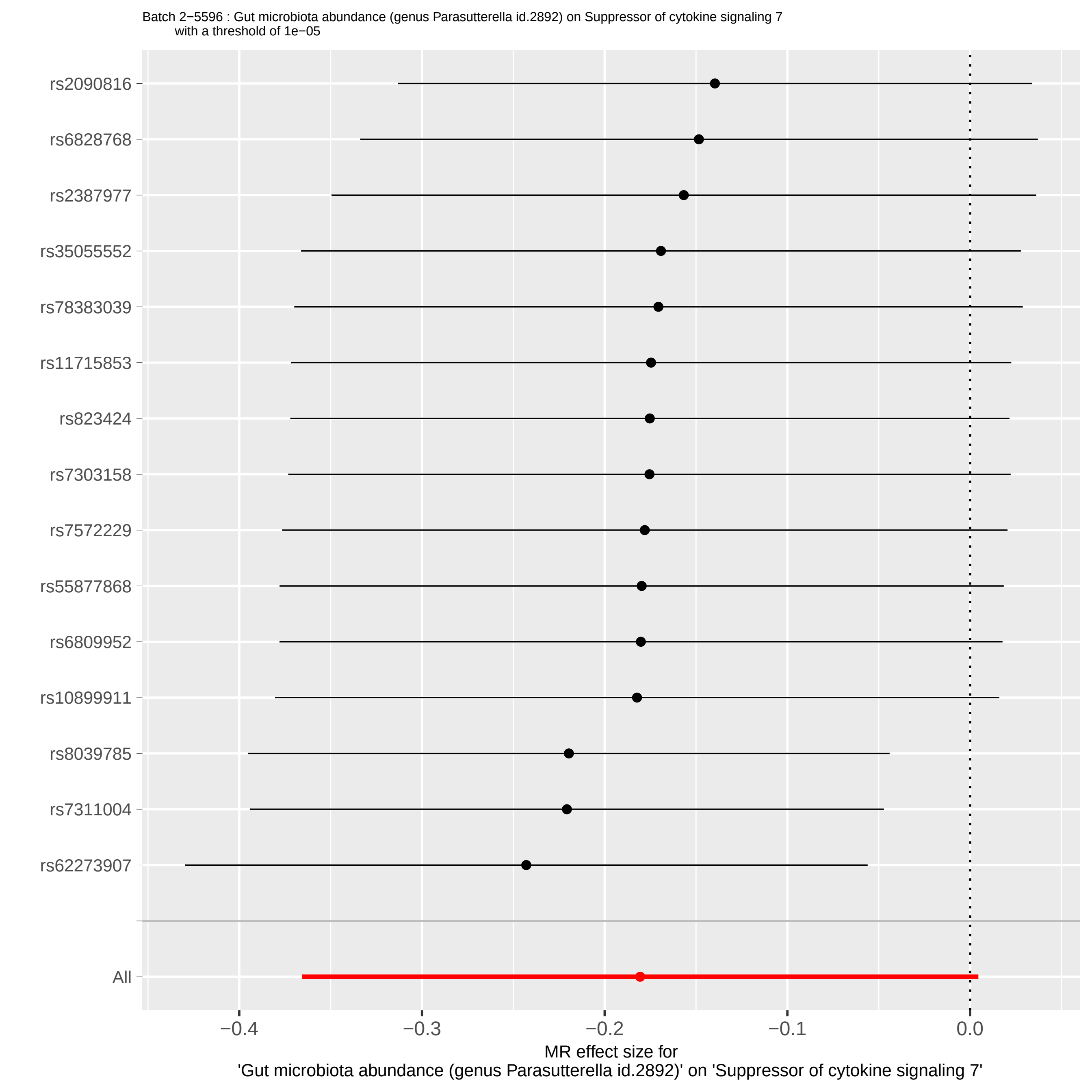

Batch 2−5596 : Gut microbiota abundance (genus Parasutterella id.2892) on Suppressor of cytokine signaling 7
with a threshold of 1e−05
rs2090816
rs6828768
rs2387977
rs35055552
rs78383039
rs11715853
rs823424
rs7303158
rs7572229
rs55877868
rs6809952
rs10899911
rs8039785
rs7311004
rs62273907
All
−0.4
−0.3
−0.2
−0.1
0.0
MR effect size for
'Gut microbiota abundance (genus Parasutterella id.2892)' on 'Suppressor of cytokine signaling 7'

Batch 2−5597 : Gut microbiota abundance (genus Catenibacterium id.2153) on Superoxide dismutase [Mn], mitochondrial
with a threshold of 1e−05

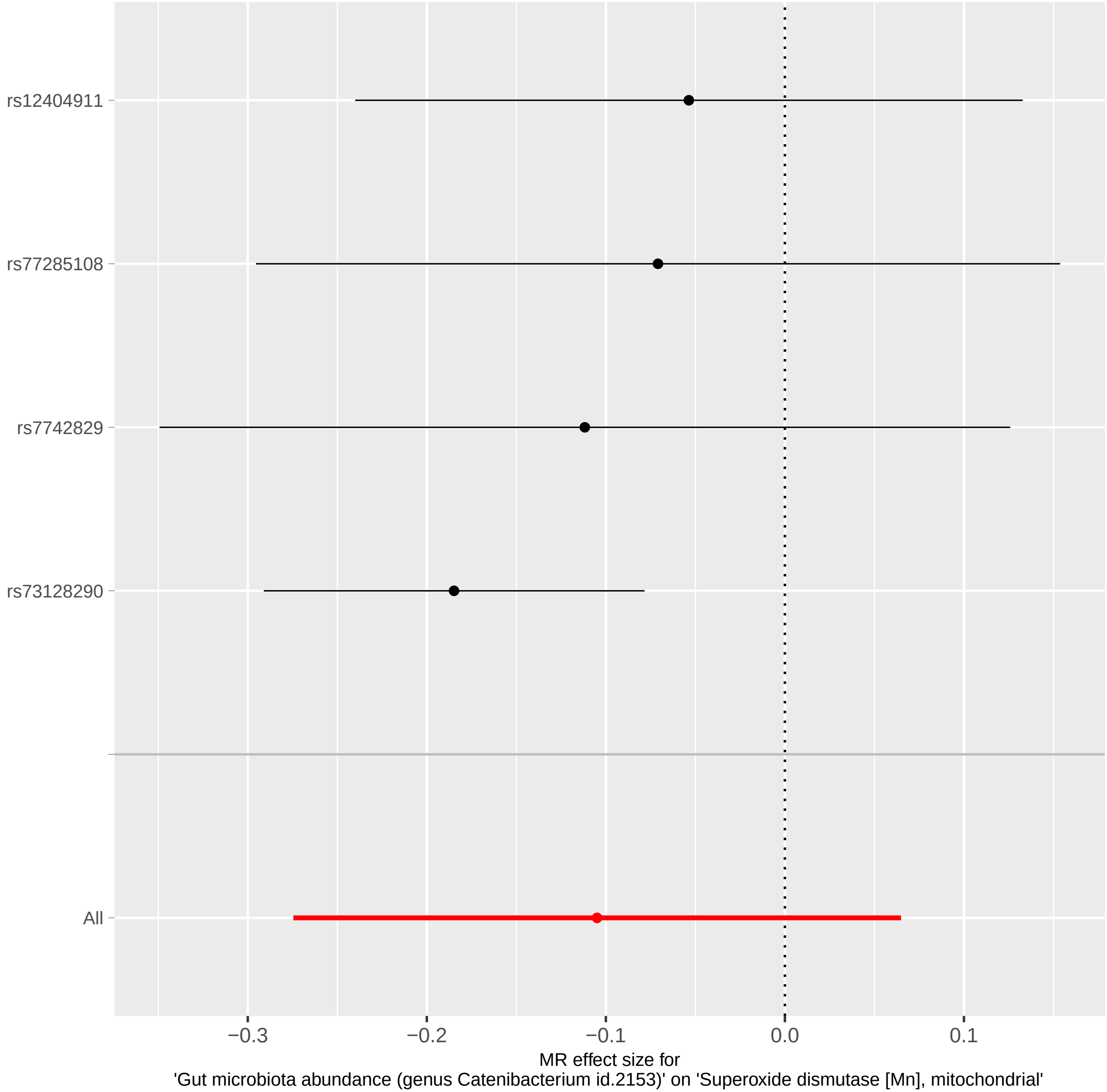

Batch 2−5598 : Gut microbiota abundance (genus Parasutterella id.2892) on Superoxide dismutase [Mn], mitochondrial with a threshold of 1e−05

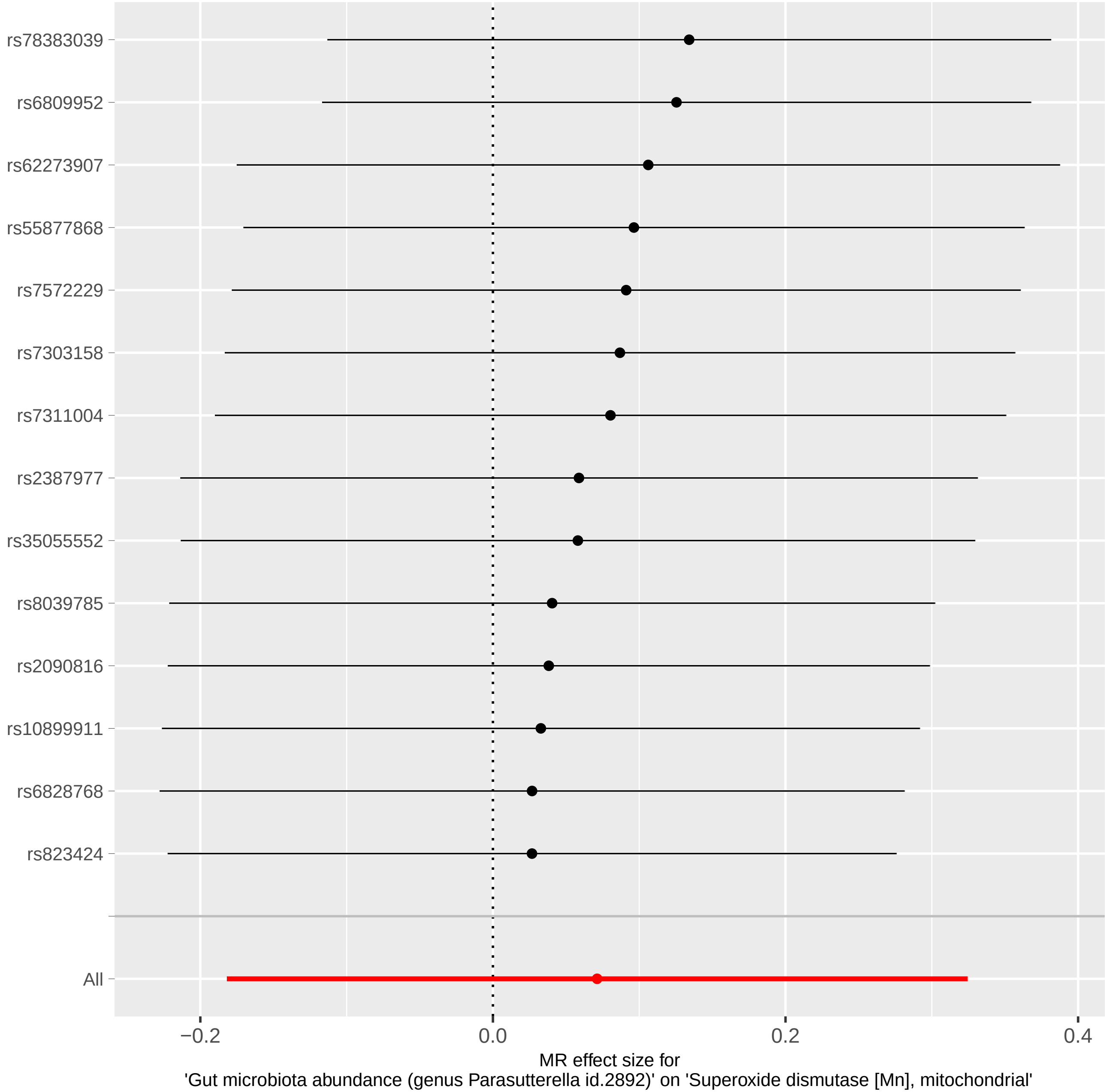

Batch 2−5599 : Gut microbiota abundance (genus Catenibacterium id.2153) on Extracellular superoxide dismutase [Cu−Zn] with a threshold of 1e−05

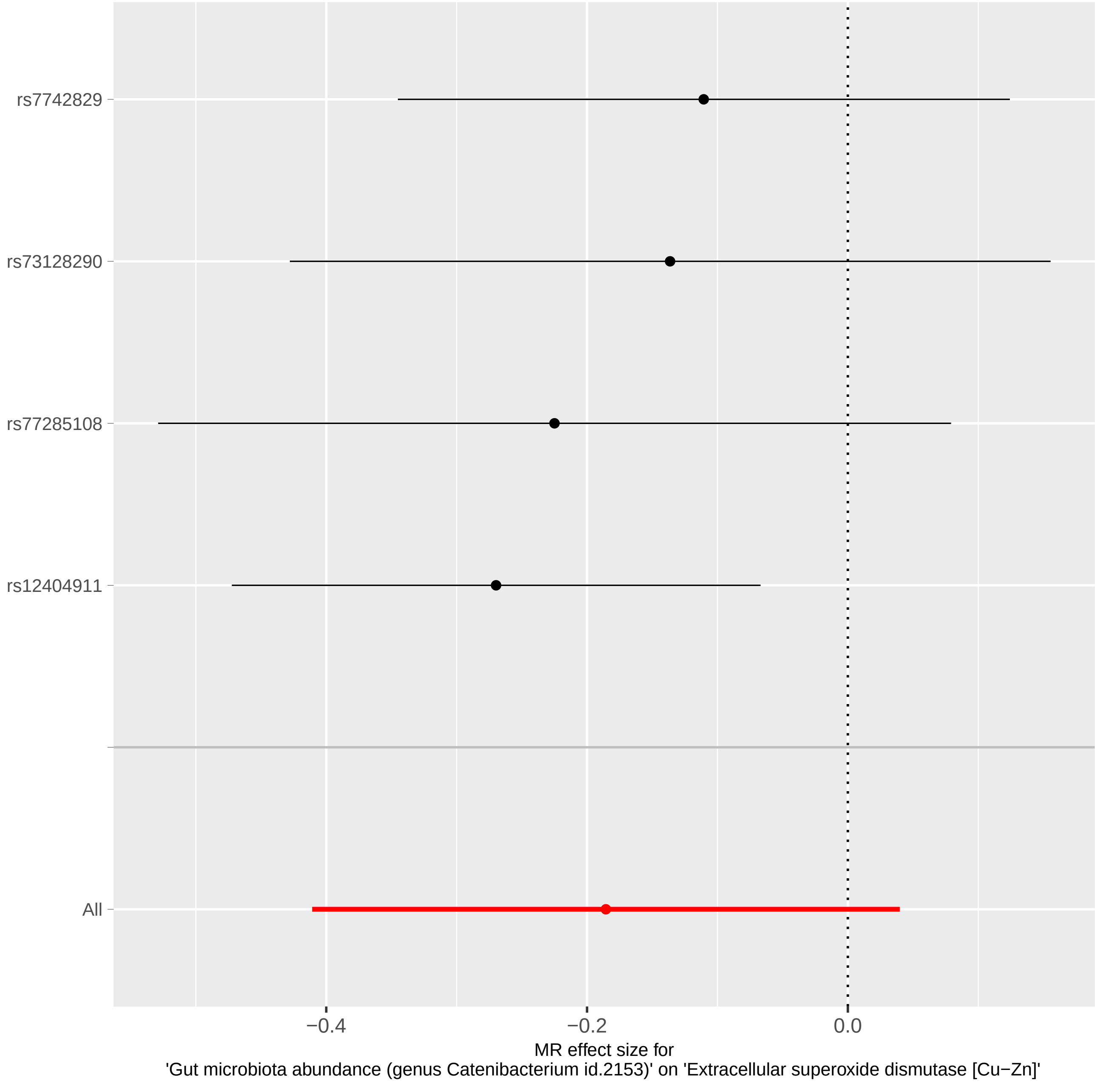

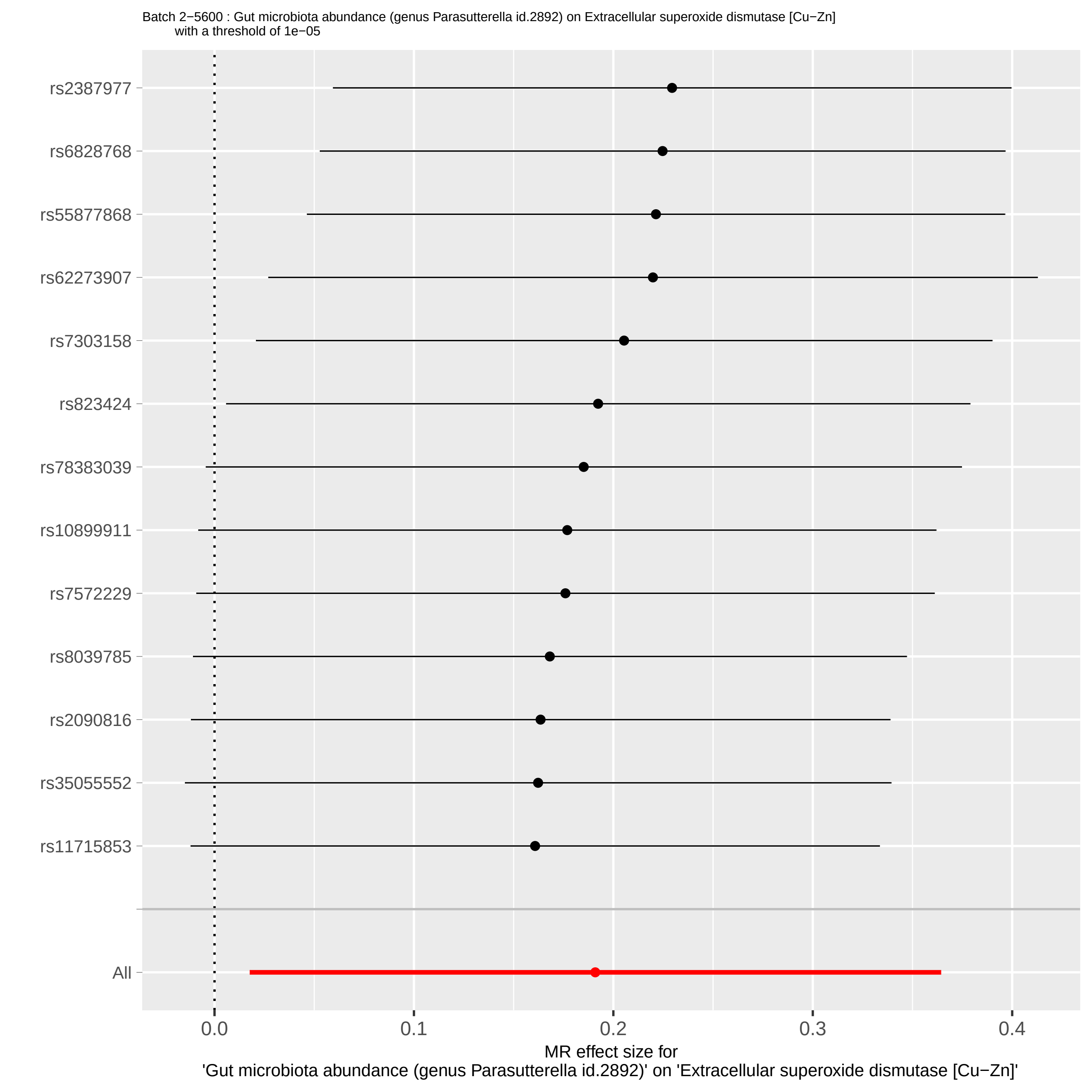

Batch 2−5600 : Gut microbiota abundance (genus Parasutterella id.2892) on Extracellular superoxide dismutase [Cu−Zn]
with a threshold of 1e−05
rs2387977
rs6828768
rs55877868
rs62273907
rs7303158
rs823424
rs78383039
rs10899911
rs7572229
rs8039785
rs2090816
rs35055552
rs11715853
All
0.0
0.1
0.2
0.3
0.4
MR effect size for
'Gut microbiota abundance (genus Parasutterella id.2892)' on 'Extracellular superoxide dismutase [Cu−Zn]'

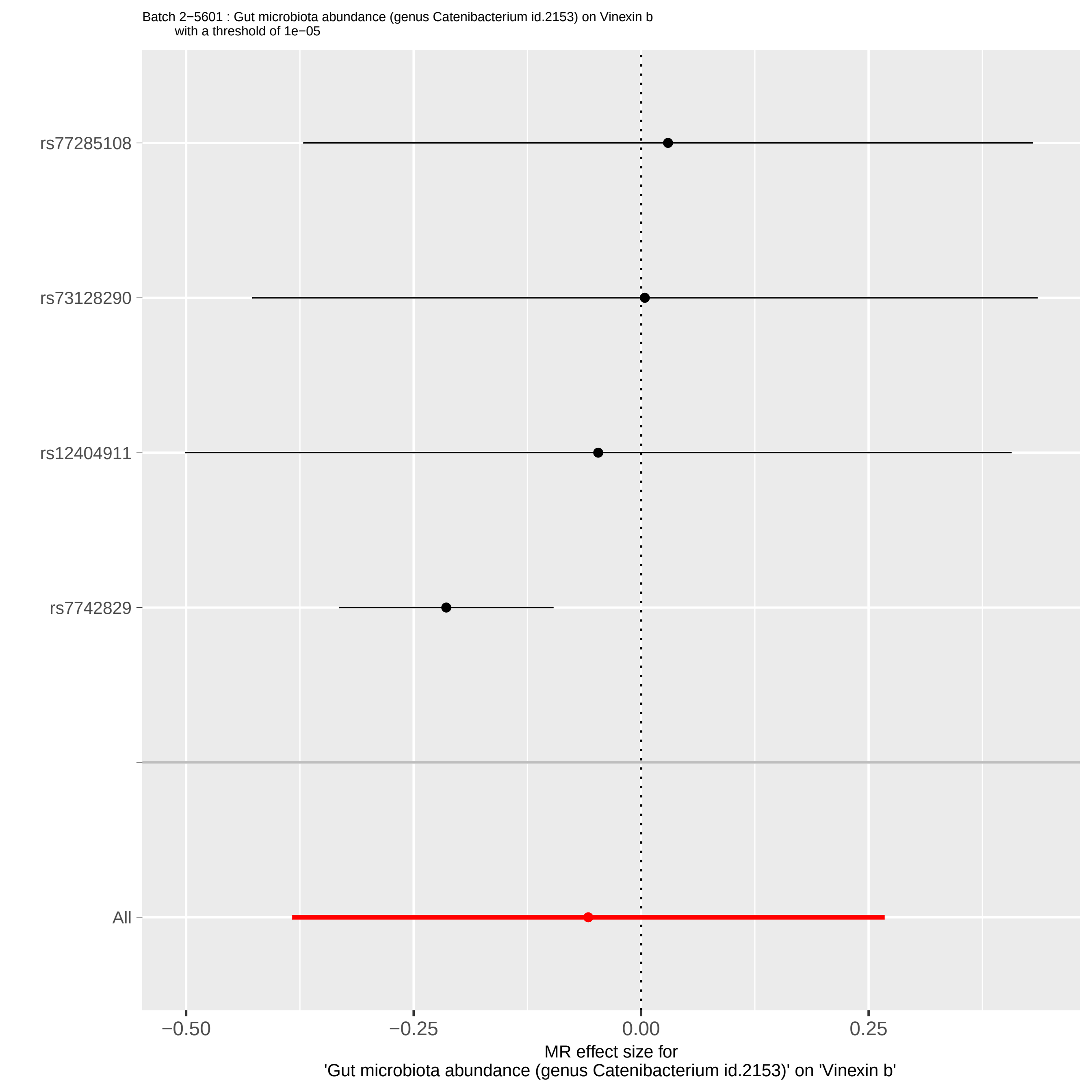

Batch 2−5601 : Gut microbiota abundance (genus Catenibacterium id.2153) on Vinexin b
with a threshold of 1e−05
rs77285108
rs73128290
rs12404911
rs7742829
All
−0.50
−0.25
0.00
0.25
MR effect size for
'Gut microbiota abundance (genus Catenibacterium id.2153)' on 'Vinexin b'

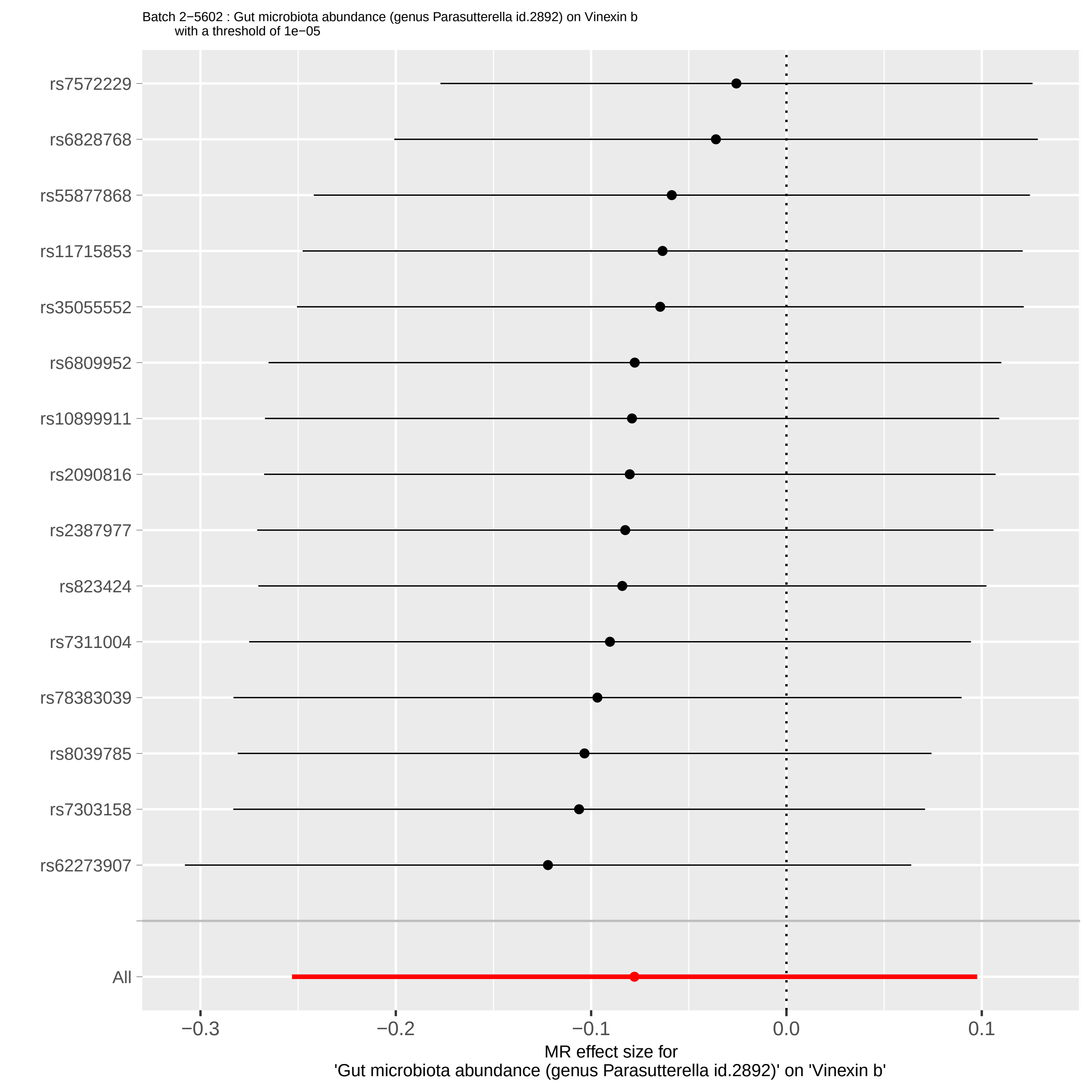

Batch 2−5602 : Gut microbiota abundance (genus Parasutterella id.2892) on Vinexin b
with a threshold of 1e−05
rs7572229
rs6828768
rs55877868
rs11715853
rs35055552
rs6809952
rs10899911
rs2090816
rs2387977
rs823424
rs7311004
rs78383039
rs8039785
rs7303158
rs62273907
All
−0.3
−0.2
−0.1
0.0
0.1
MR effect size for
'Gut microbiota abundance (genus Parasutterella id.2892)' on 'Vinexin b'

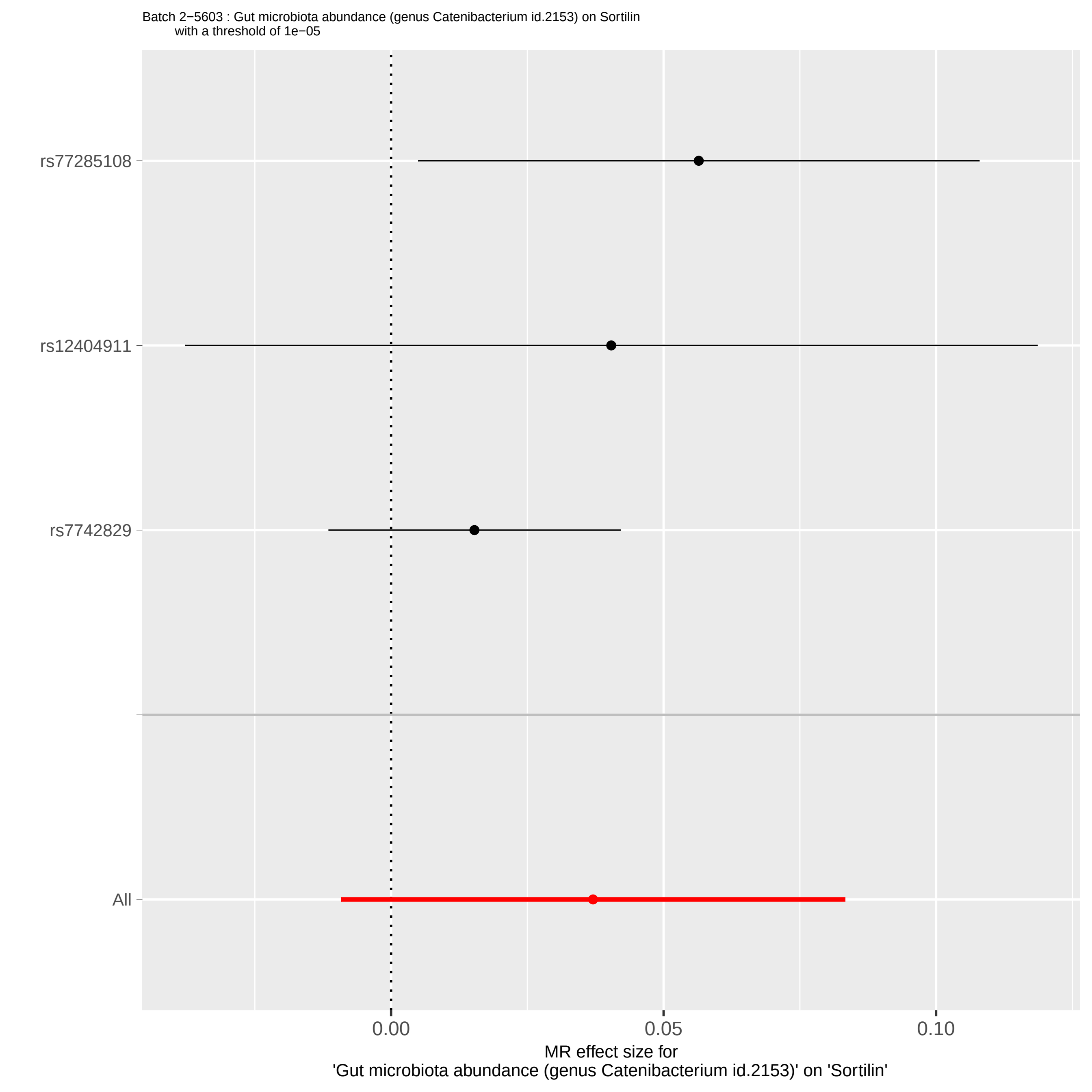
Batch 2−5603 : Gut microbiota abundance (genus Catenibacterium id.2153) on Sortilin
with a threshold of 1e−05
rs77285108
rs12404911
rs7742829
All
0.00
0.05
0.10
MR effect size for
'Gut microbiota abundance (genus Catenibacterium id.2153)' on 'Sortilin'

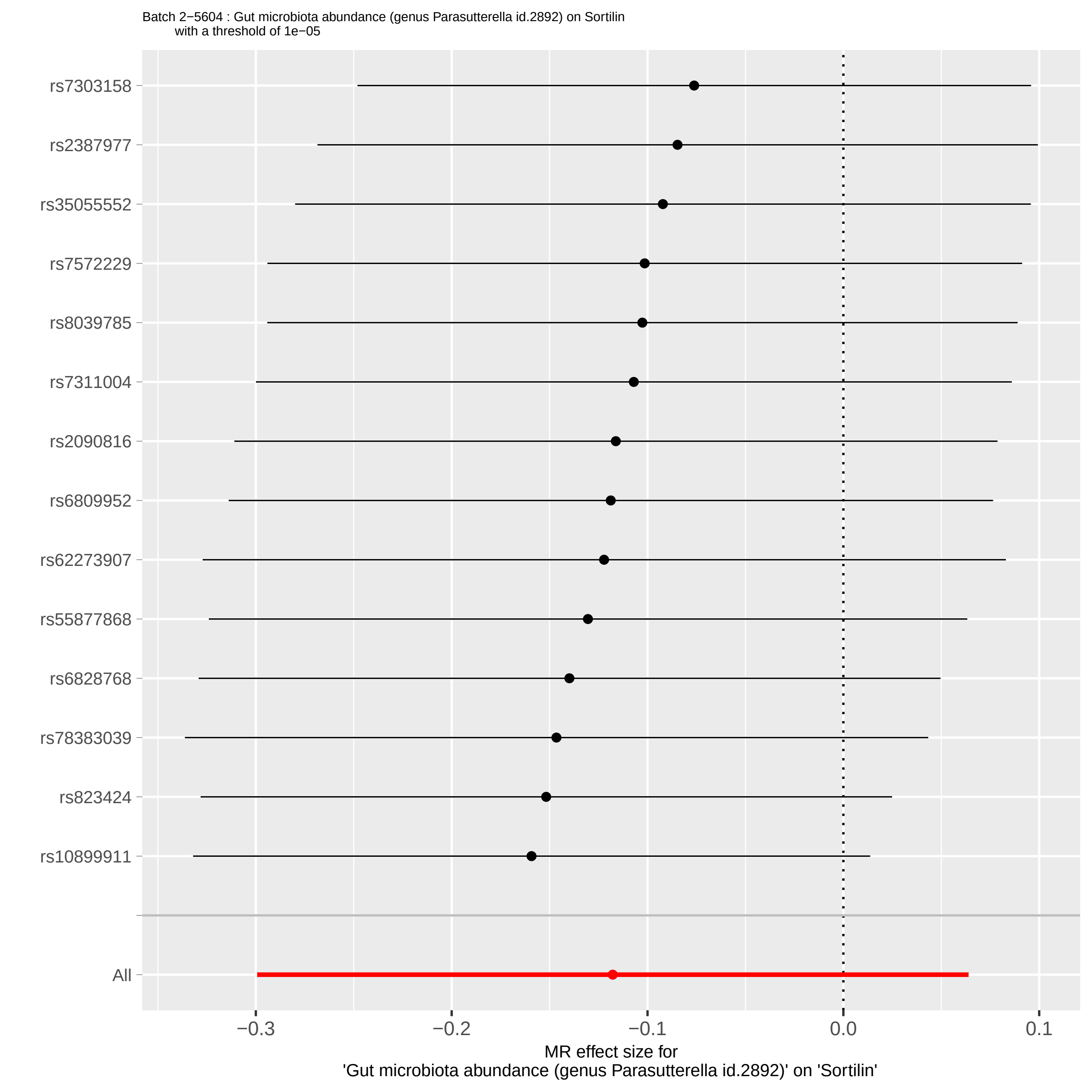
Batch 2−5604 : Gut microbiota abundance (genus Parasutterella id.2892) on Sortilin
with a threshold of 1e−05
rs7303158
rs2387977
rs35055552
rs7572229
rs8039785
rs7311004
rs2090816
rs6809952
rs62273907
rs55877868
rs6828768
rs78383039
rs823424
rs10899911
All
−0.3
−0.2
−0.1
0.0
0.1
MR effect size for
'Gut microbiota abundance (genus Parasutterella id.2892)' on 'Sortilin'

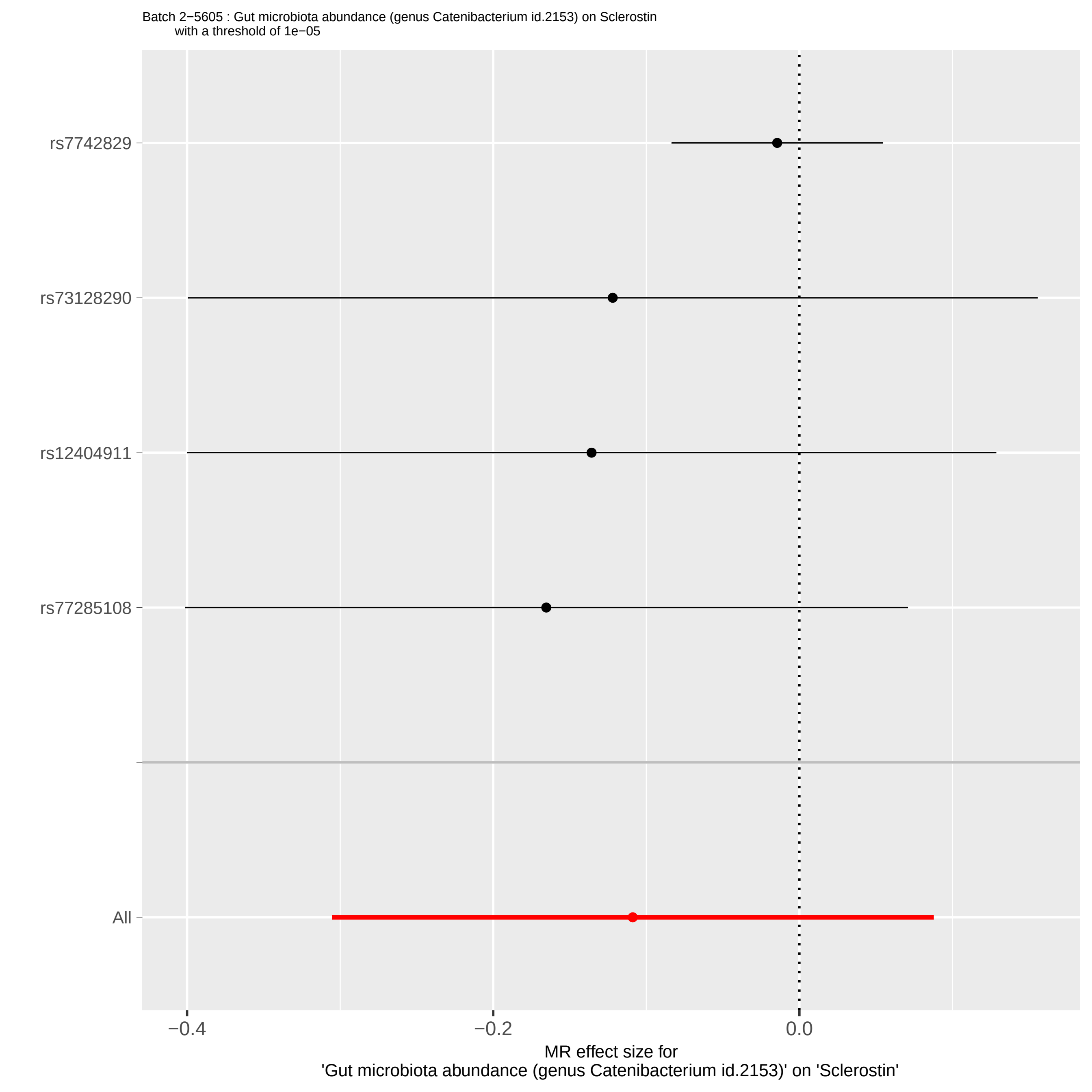
Batch 2−5605 : Gut microbiota abundance (genus Catenibacterium id.2153) on Sclerostin
with a threshold of 1e−05
rs7742829
rs73128290
rs12404911
rs77285108
All
−0.4
−0.2
0.0
MR effect size for
'Gut microbiota abundance (genus Catenibacterium id.2153)' on 'Sclerostin'

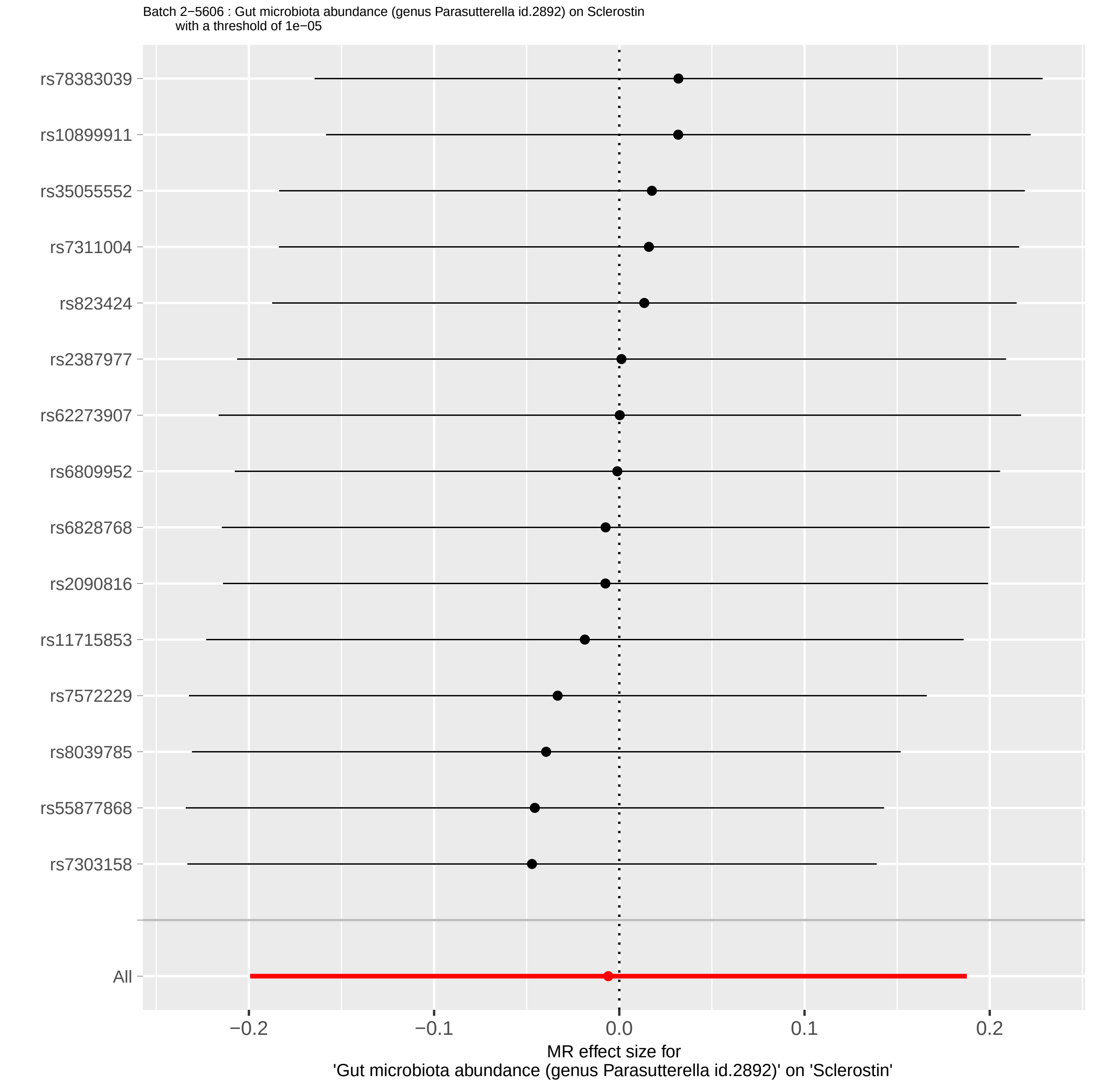
Batch 2−5606 : Gut microbiota abundance (genus Parasutterella id.2892) on Sclerostin
with a threshold of 1e−05
rs78383039
rs10899911
rs35055552
rs7311004
rs823424
rs2387977
rs62273907
rs6809952
rs6828768
rs2090816
rs11715853
rs7572229
rs8039785
rs55877868
rs7303158
All
−0.2
−0.1
0.0
0.1
0.2
MR effect size for
'Gut microbiota abundance (genus Parasutterella id.2892)' on 'Sclerostin'

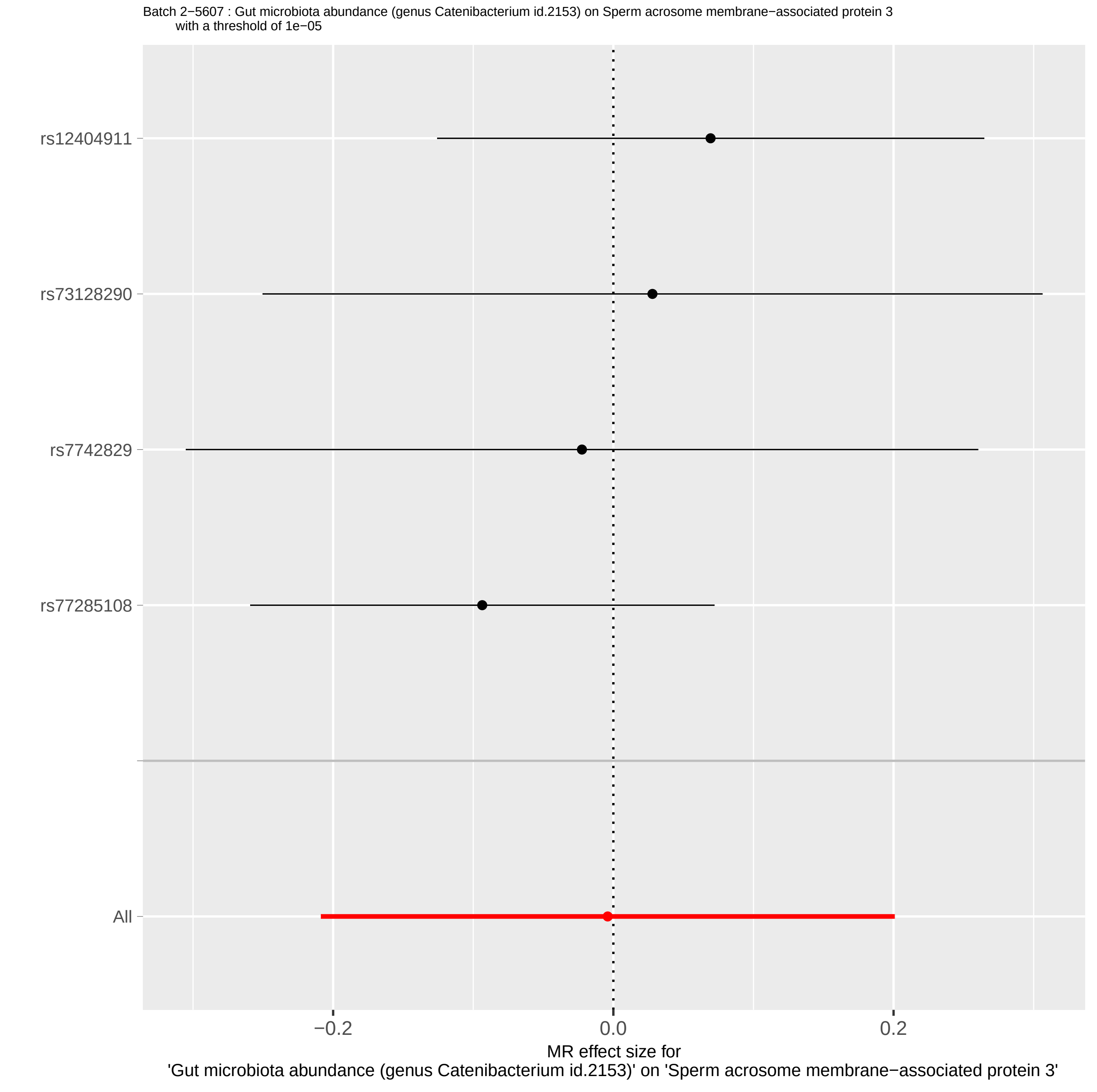
Batch 2−5607 : Gut microbiota abundance (genus Catenibacterium id.2153) on Sperm acrosome membrane−associated protein 3
with a threshold of 1e−05
rs12404911
rs73128290
rs7742829
rs77285108
All
−0.2
0.0
0.2
MR effect size for
'Gut microbiota abundance (genus Catenibacterium id.2153)' on 'Sperm acrosome membrane−associated protein 3'

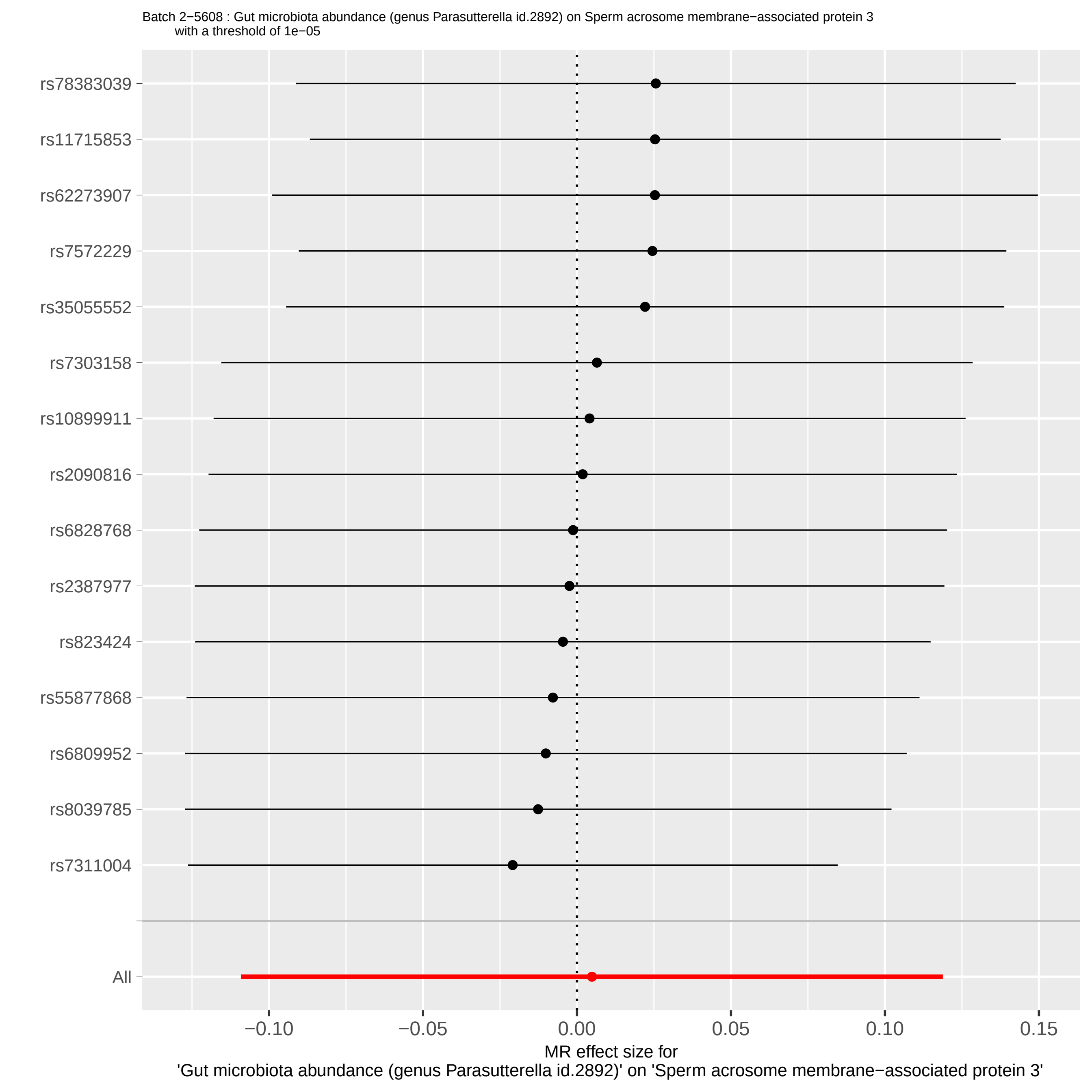

Batch 2−5608 : Gut microbiota abundance (genus Parasutterella id.2892) on Sperm acrosome membrane−associated protein 3
with a threshold of 1e−05
rs78383039
rs11715853
rs62273907
rs7572229
rs35055552
rs7303158
rs10899911
rs2090816
rs6828768
rs2387977
rs823424
rs55877868
rs6809952
rs8039785
rs7311004
All
−0.10
−0.05
0.00
0.05
0.10
0.15
MR effect size for
'Gut microbiota abundance (genus Parasutterella id.2892)' on 'Sperm acrosome membrane−associated protein 3'

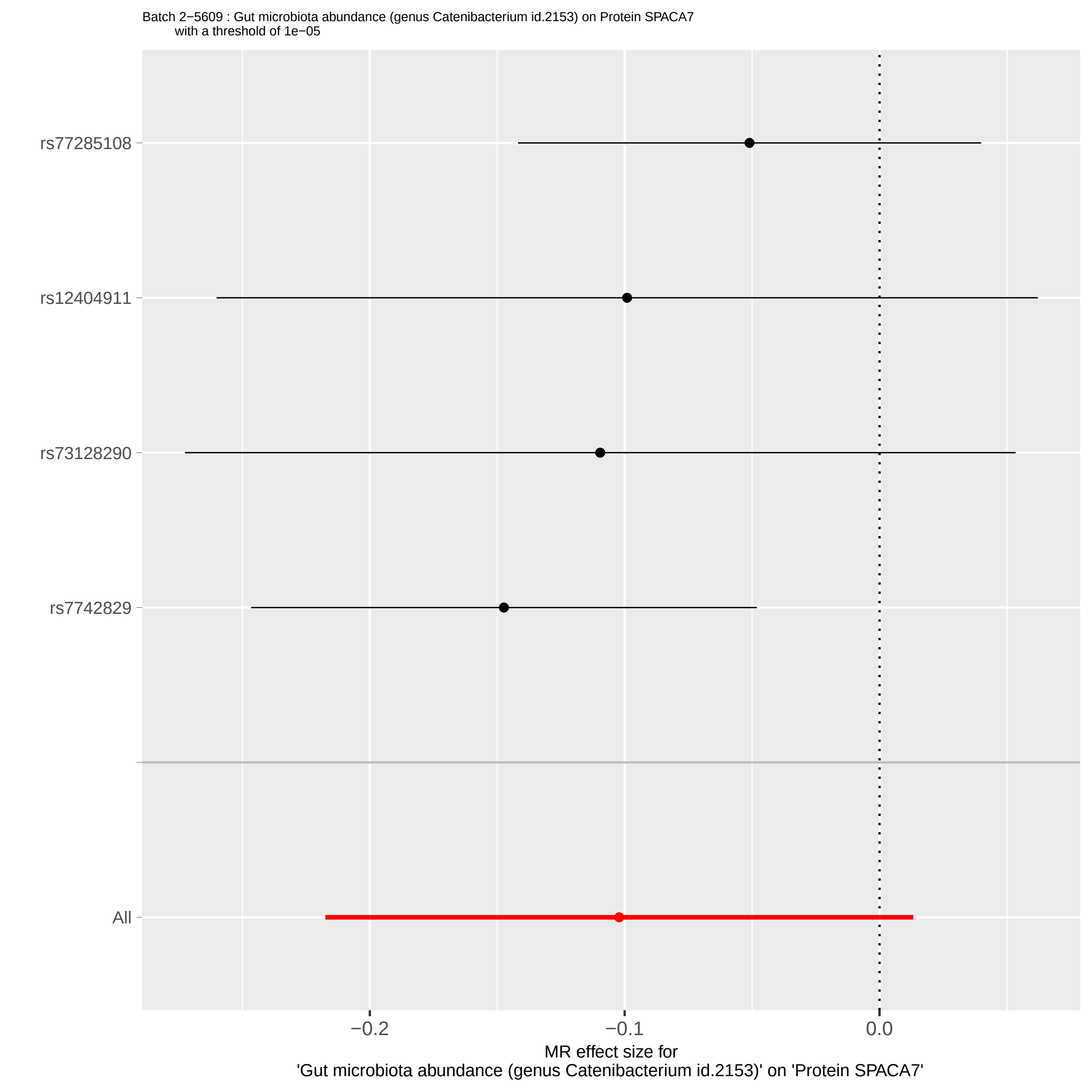
Batch 2−5609 : Gut microbiota abundance (genus Catenibacterium id.2153) on Protein SPACA7
with a threshold of 1e−05
rs77285108
rs12404911
rs73128290
rs7742829
All
−0.2
−0.1
0.0
MR effect size for
'Gut microbiota abundance (genus Catenibacterium id.2153)' on 'Protein SPACA7'

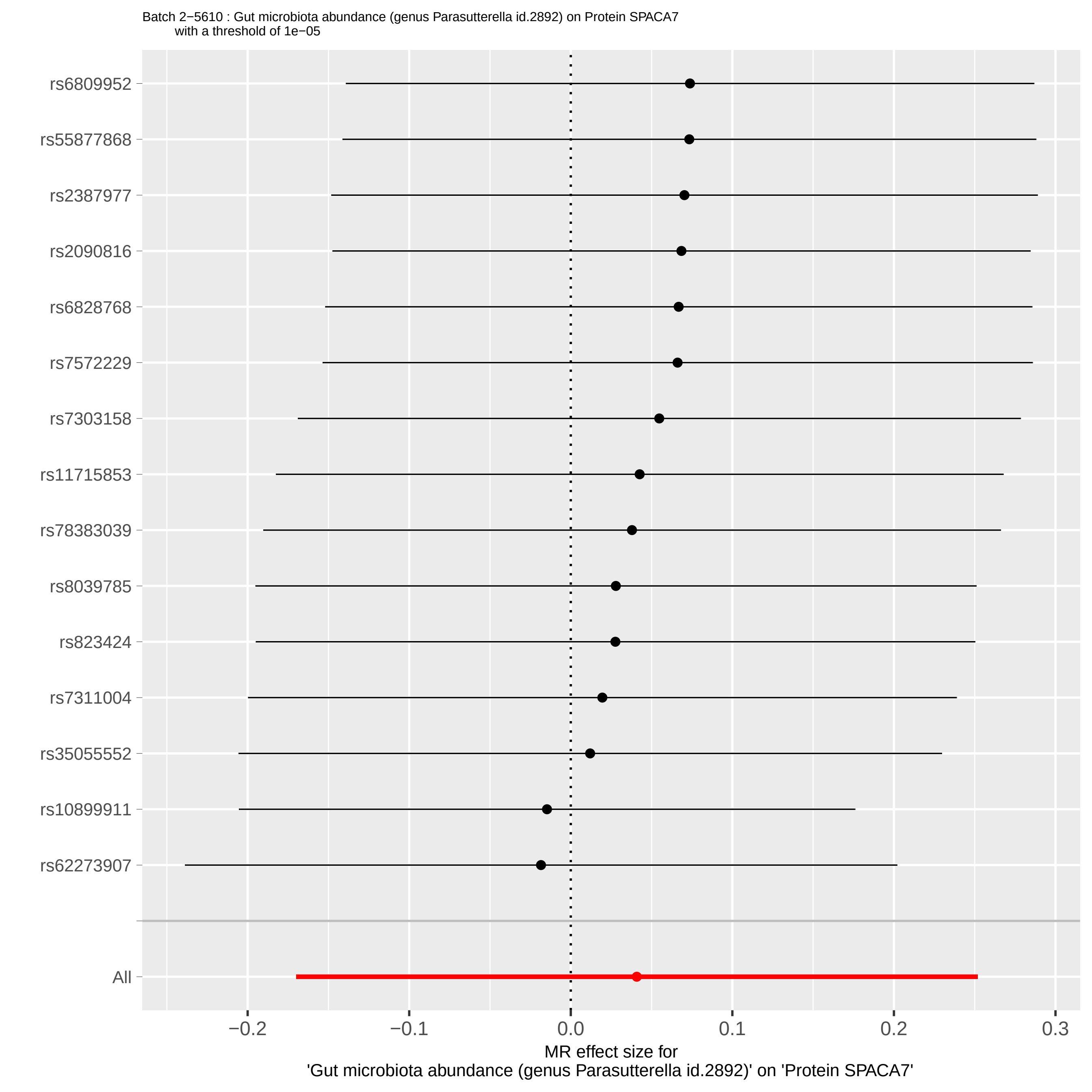
Batch 2−5610 : Gut microbiota abundance (genus Parasutterella id.2892) on Protein SPACA7
with a threshold of 1e−05
rs6809952
rs55877868
rs2387977
rs2090816
rs6828768
rs7572229
rs7303158
rs11715853
rs78383039
rs8039785
rs823424
rs7311004
rs35055552
rs10899911
rs62273907
All
−0.2
−0.1
0.0
0.1
0.2
0.3
MR effect size for
'Gut microbiota abundance (genus Parasutterella id.2892)' on 'Protein SPACA7'

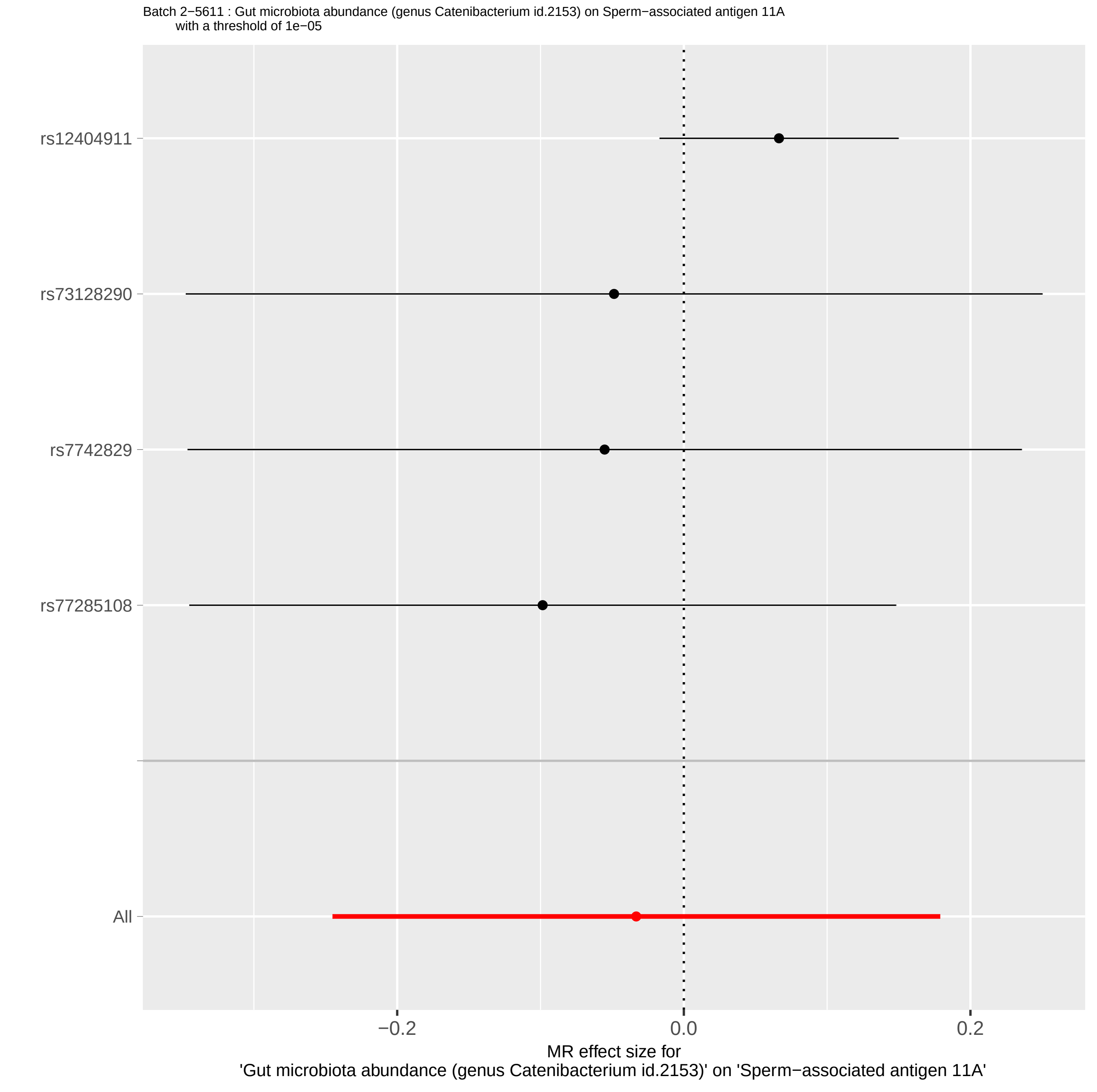
Batch 2−5611 : Gut microbiota abundance (genus Catenibacterium id.2153) on Sperm−associated antigen 11A
with a threshold of 1e−05
rs12404911
rs73128290
rs7742829
rs77285108
All
−0.2
0.0
0.2
MR effect size for
'Gut microbiota abundance (genus Catenibacterium id.2153)' on 'Sperm−associated antigen 11A'

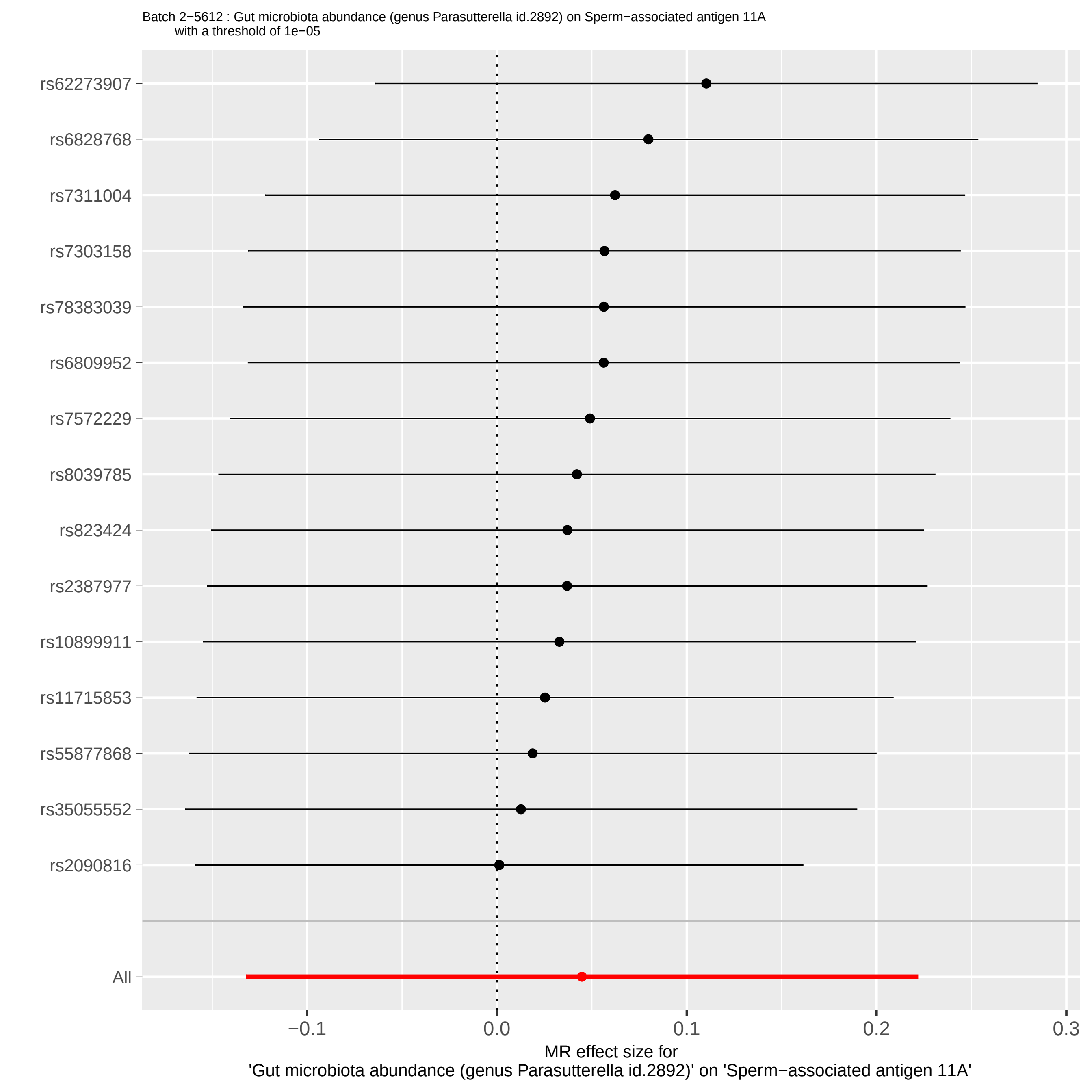
Batch 2−5612 : Gut microbiota abundance (genus Parasutterella id.2892) on Sperm−associated antigen 11A
with a threshold of 1e−05
rs62273907
rs6828768
rs7311004
rs7303158
rs78383039
rs6809952
rs7572229
rs8039785
rs823424
rs2387977
rs10899911
rs11715853
rs55877868
rs35055552
rs2090816
All
−0.1
0.0
0.1
0.2
0.3
MR effect size for
'Gut microbiota abundance (genus Parasutterella id.2892)' on 'Sperm−associated antigen 11A'

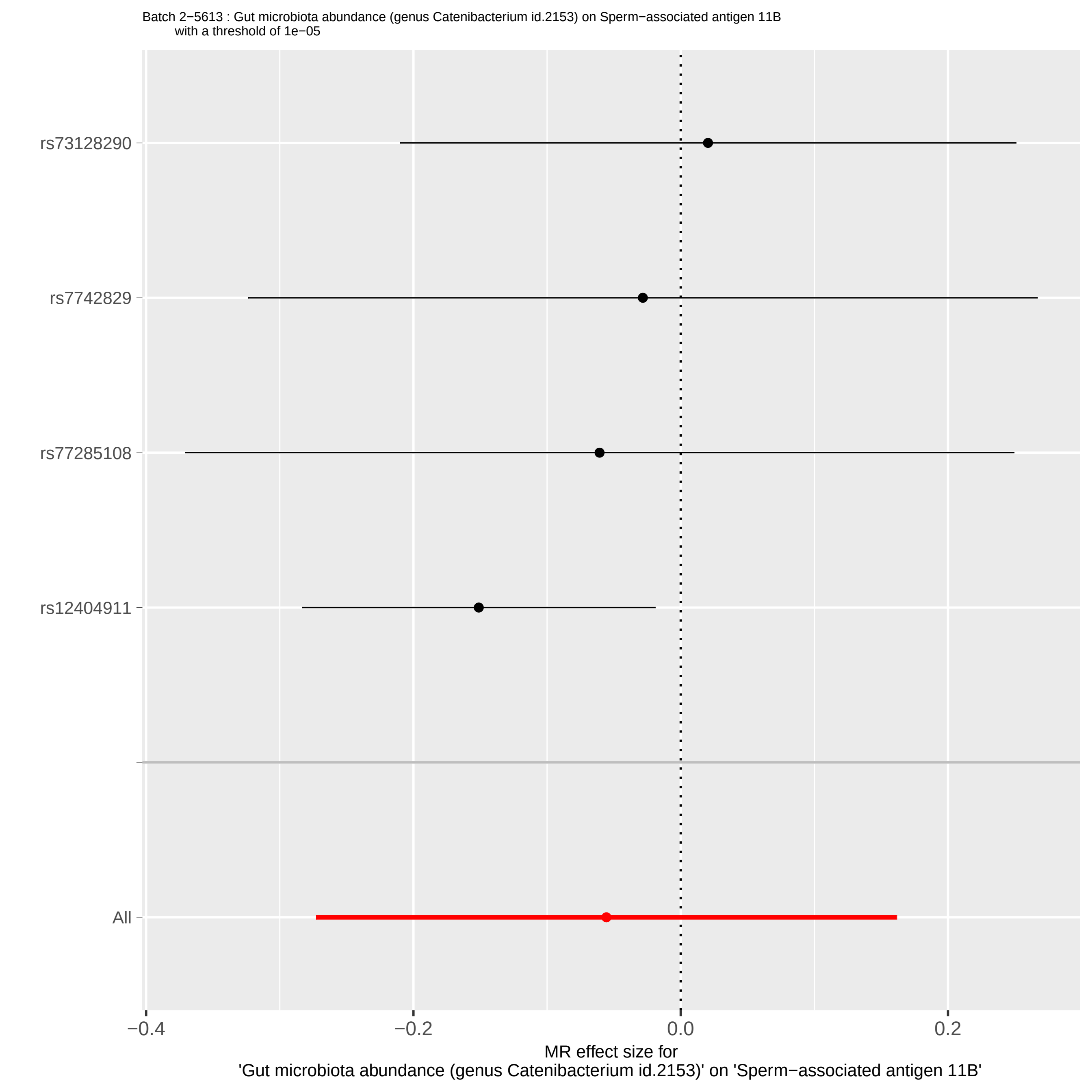
Batch 2−5613 : Gut microbiota abundance (genus Catenibacterium id.2153) on Sperm−associated antigen 11B
with a threshold of 1e−05
rs73128290
rs7742829
rs77285108
rs12404911
All
−0.4
−0.2
0.0
0.2
MR effect size for
'Gut microbiota abundance (genus Catenibacterium id.2153)' on 'Sperm−associated antigen 11B'

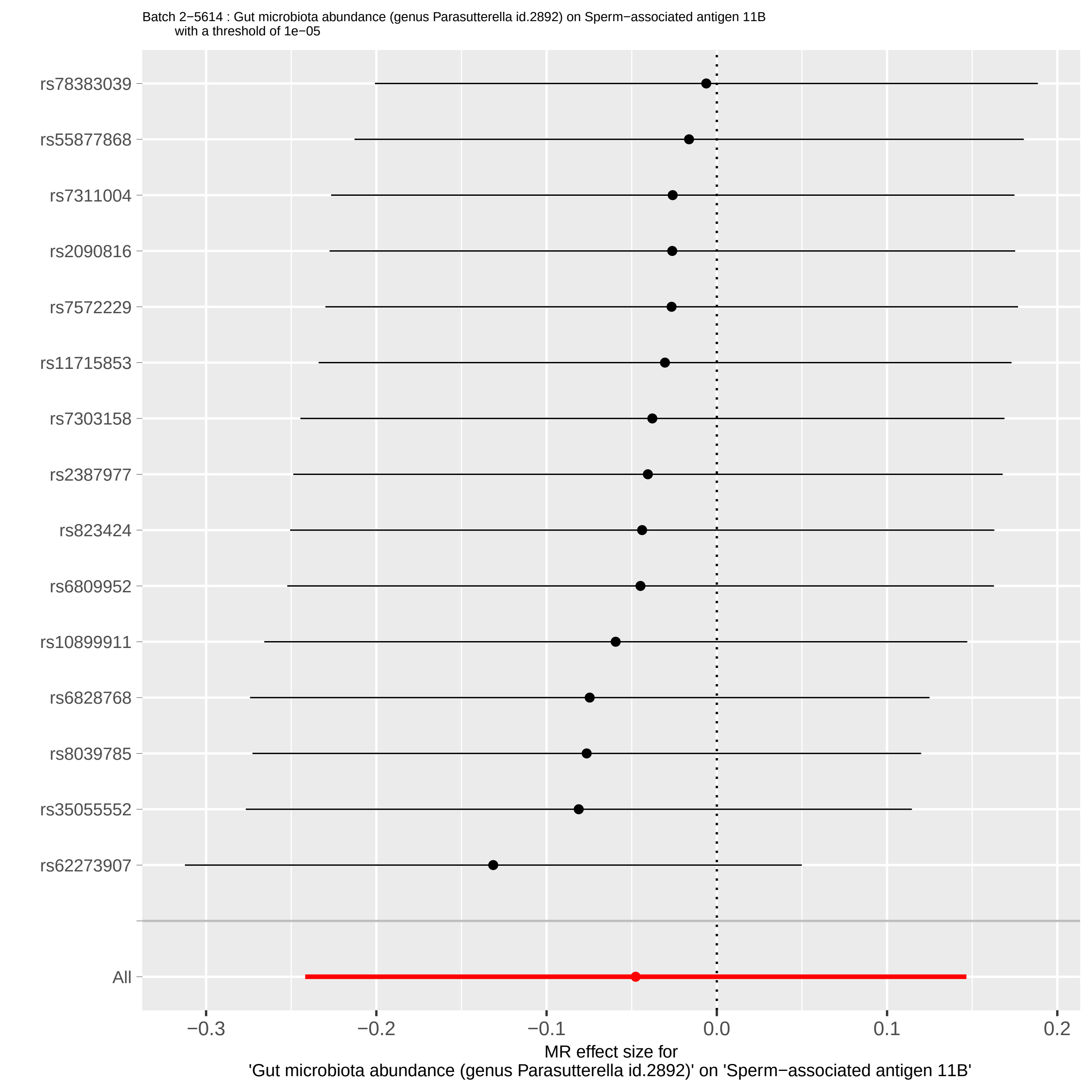
Batch 2−5614 : Gut microbiota abundance (genus Parasutterella id.2892) on Sperm−associated antigen 11B
with a threshold of 1e−05
rs78383039
rs55877868
rs7311004
rs2090816
rs7572229
rs11715853
rs7303158
rs2387977
rs823424
rs6809952
rs10899911
rs6828768
rs8039785
rs35055552
rs62273907
All
−0.3
−0.2
−0.1
0.0
0.1
0.2
MR effect size for
'Gut microbiota abundance (genus Parasutterella id.2892)' on 'Sperm−associated antigen 11B'

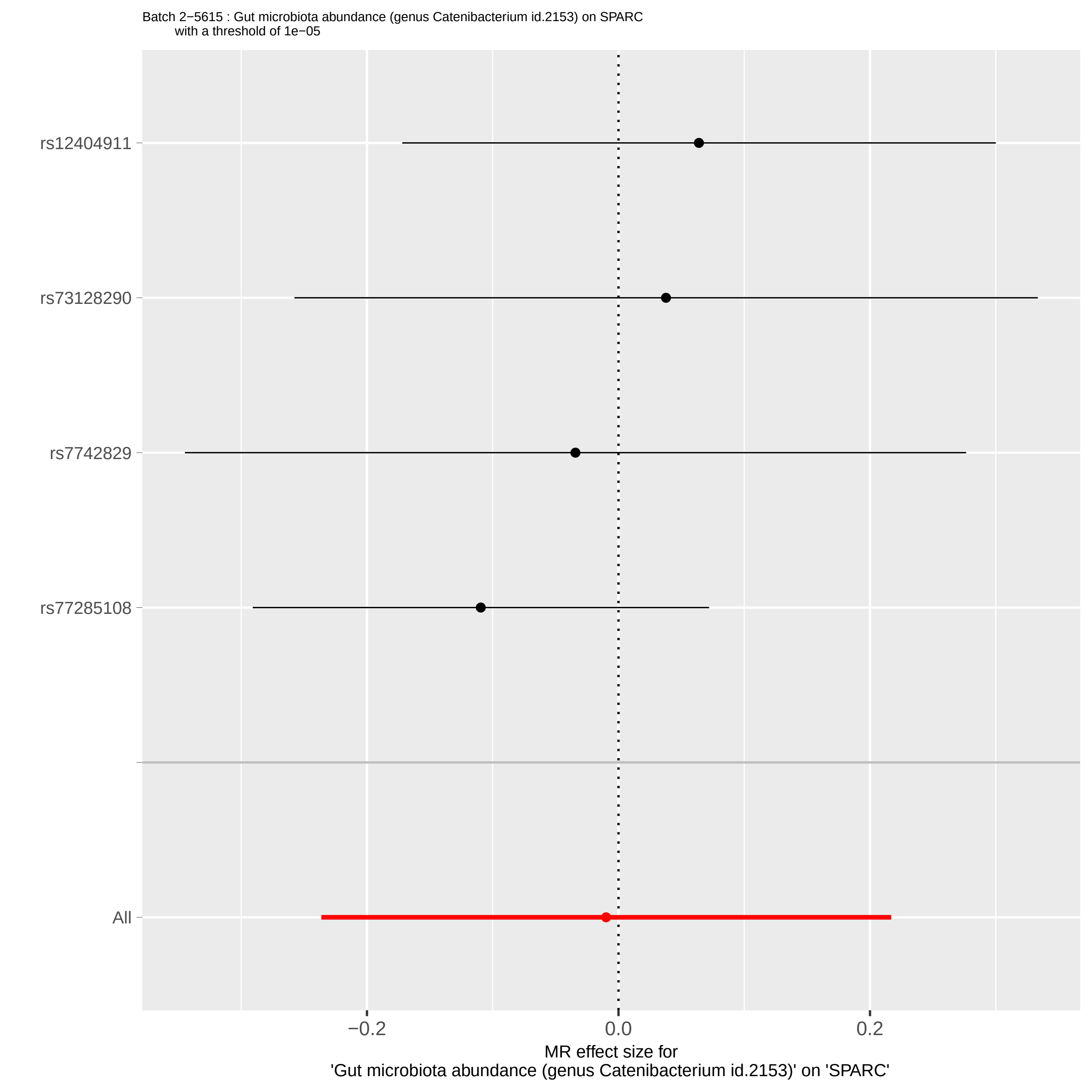

Batch 2−5615 : Gut microbiota abundance (genus Catenibacterium id.2153) on SPARC
with a threshold of 1e−05
rs12404911
rs73128290
rs7742829
rs77285108
All
−0.2
0.0
0.2
MR effect size for
'Gut microbiota abundance (genus Catenibacterium id.2153)' on 'SPARC'

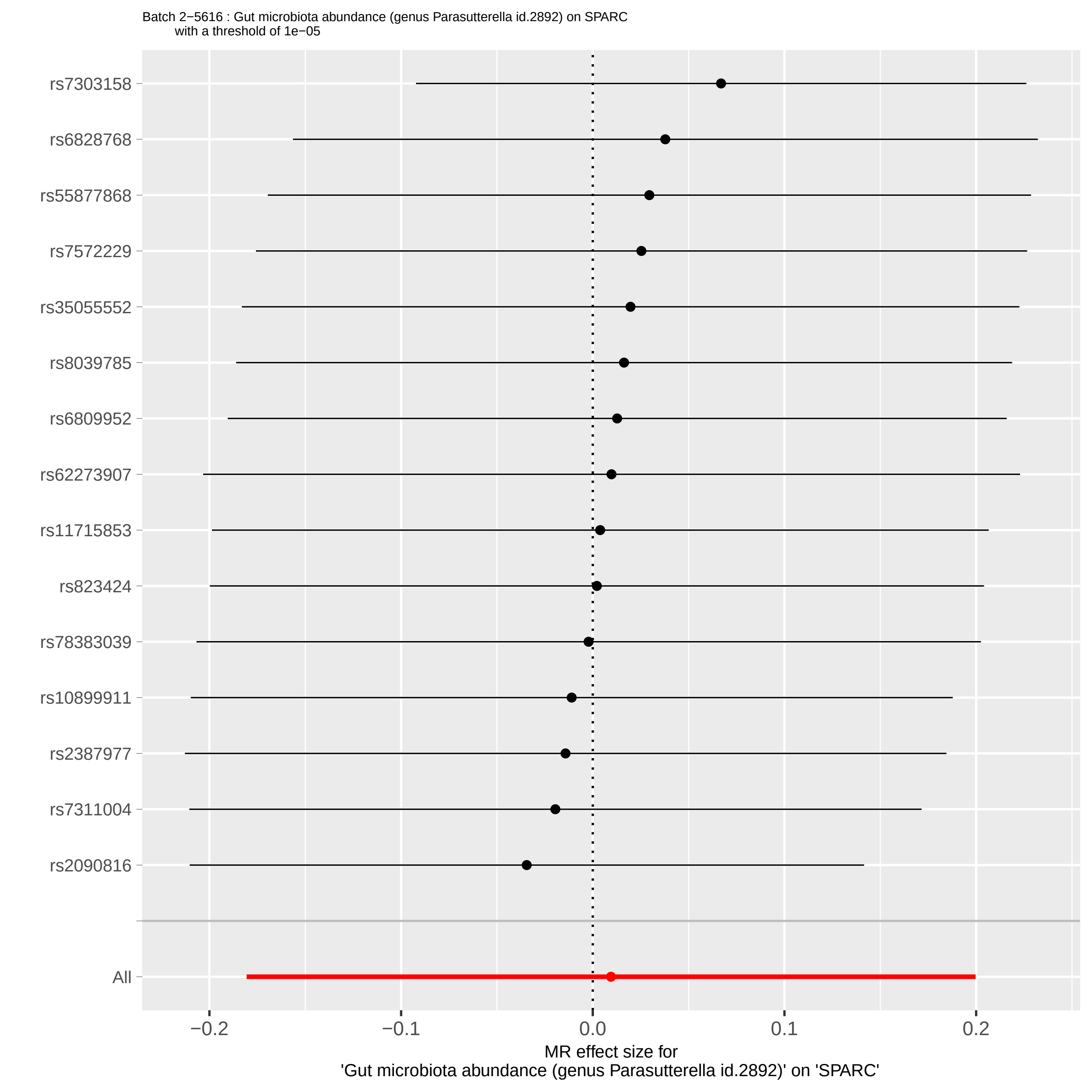
Batch 2−5616 : Gut microbiota abundance (genus Parasutterella id.2892) on SPARC
with a threshold of 1e−05
rs7303158
rs6828768
rs55877868
rs7572229
rs35055552
rs8039785
rs6809952
rs62273907
rs11715853
rs823424
rs78383039
rs10899911
rs2387977
rs7311004
rs2090816
All
−0.2
−0.1
0.0
0.1
0.2
MR effect size for
'Gut microbiota abundance (genus Parasutterella id.2892)' on 'SPARC'

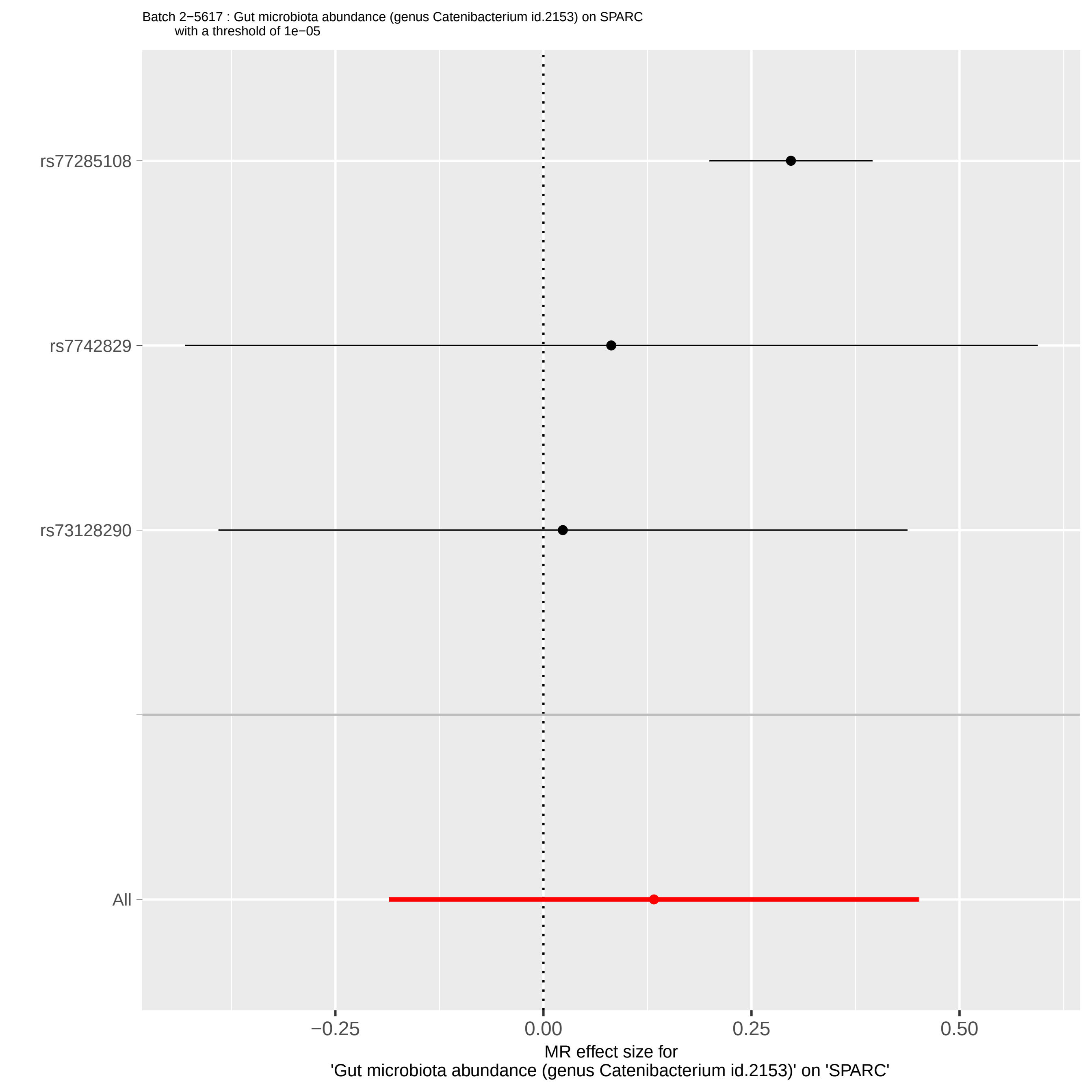

Batch 2−5617 : Gut microbiota abundance (genus Catenibacterium id.2153) on SPARC
with a threshold of 1e−05
rs77285108
rs7742829
rs73128290
All
−0.25
0.00
0.25
0.50
MR effect size for
'Gut microbiota abundance (genus Catenibacterium id.2153)' on 'SPARC'

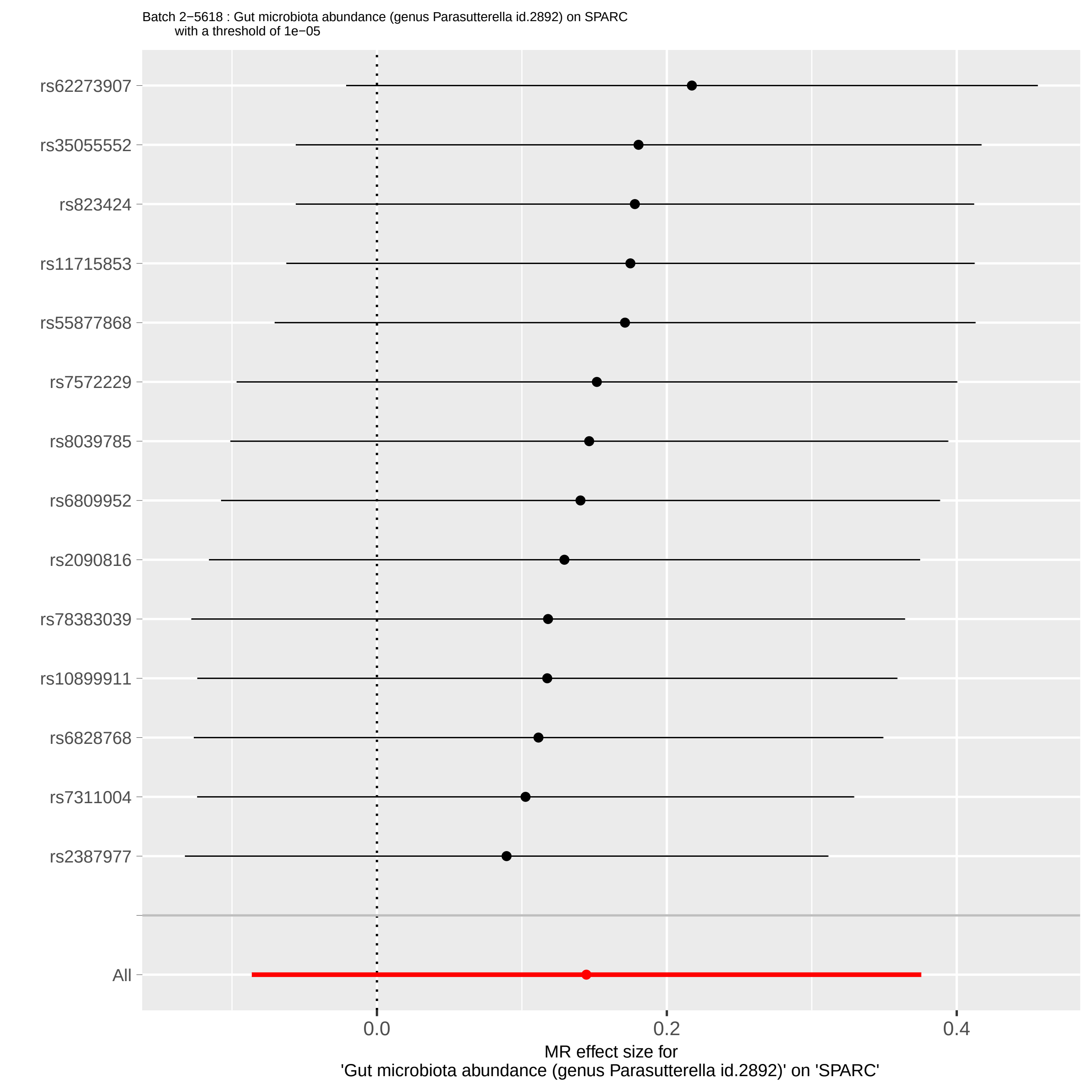
Batch 2−5618 : Gut microbiota abundance (genus Parasutterella id.2892) on SPARC
with a threshold of 1e−05
rs62273907
rs35055552
rs823424
rs11715853
rs55877868
rs7572229
rs8039785
rs6809952
rs2090816
rs78383039
rs10899911
rs6828768
rs7311004
rs2387977
All
0.0
0.2
0.4
MR effect size for
'Gut microbiota abundance (genus Parasutterella id.2892)' on 'SPARC'

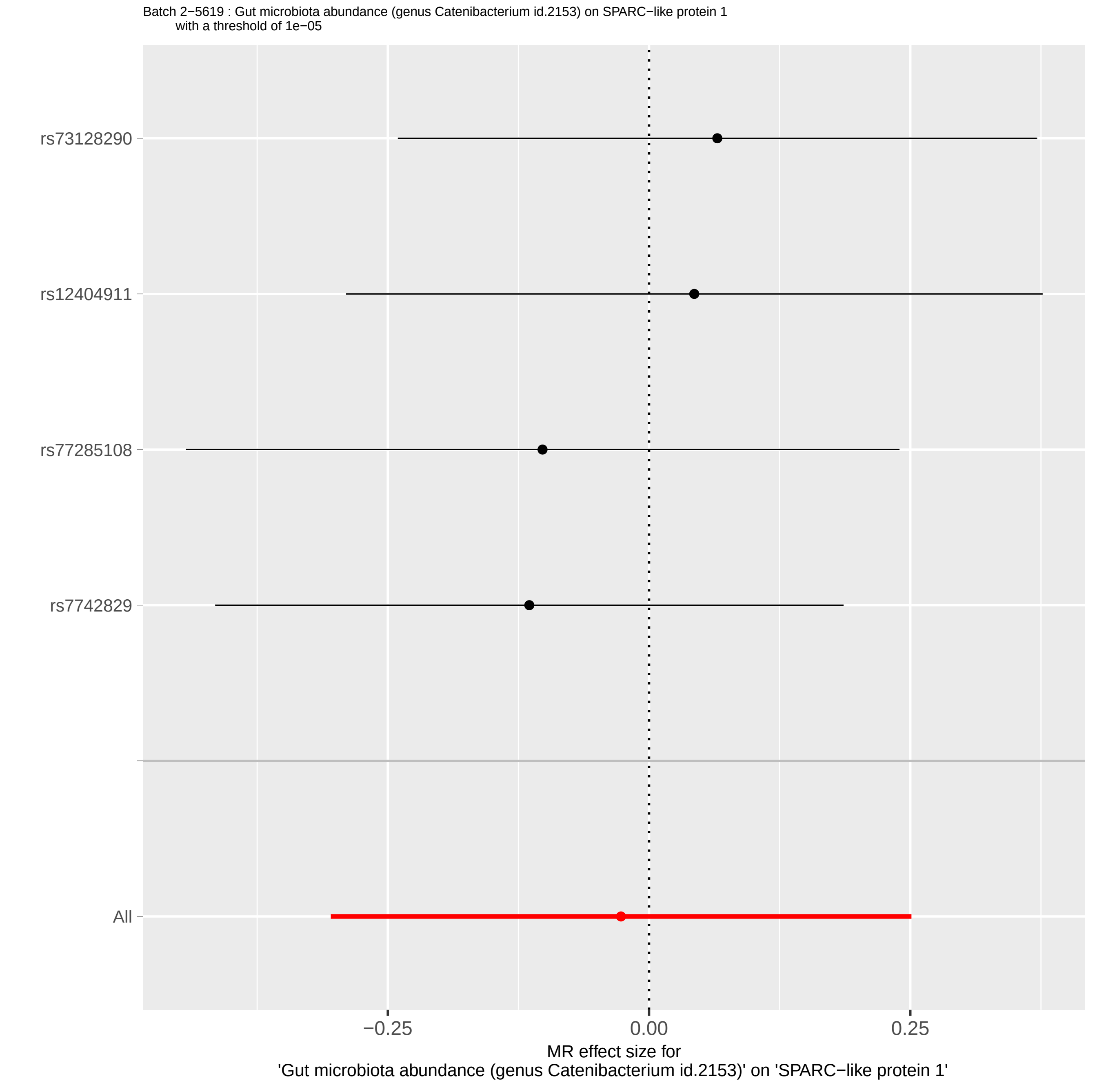
Batch 2−5619 : Gut microbiota abundance (genus Catenibacterium id.2153) on SPARC−like protein 1
with a threshold of 1e−05
rs73128290
rs12404911
rs77285108
rs7742829
All
−0.25
0.00
0.25
MR effect size for
'Gut microbiota abundance (genus Catenibacterium id.2153)' on 'SPARC−like protein 1'

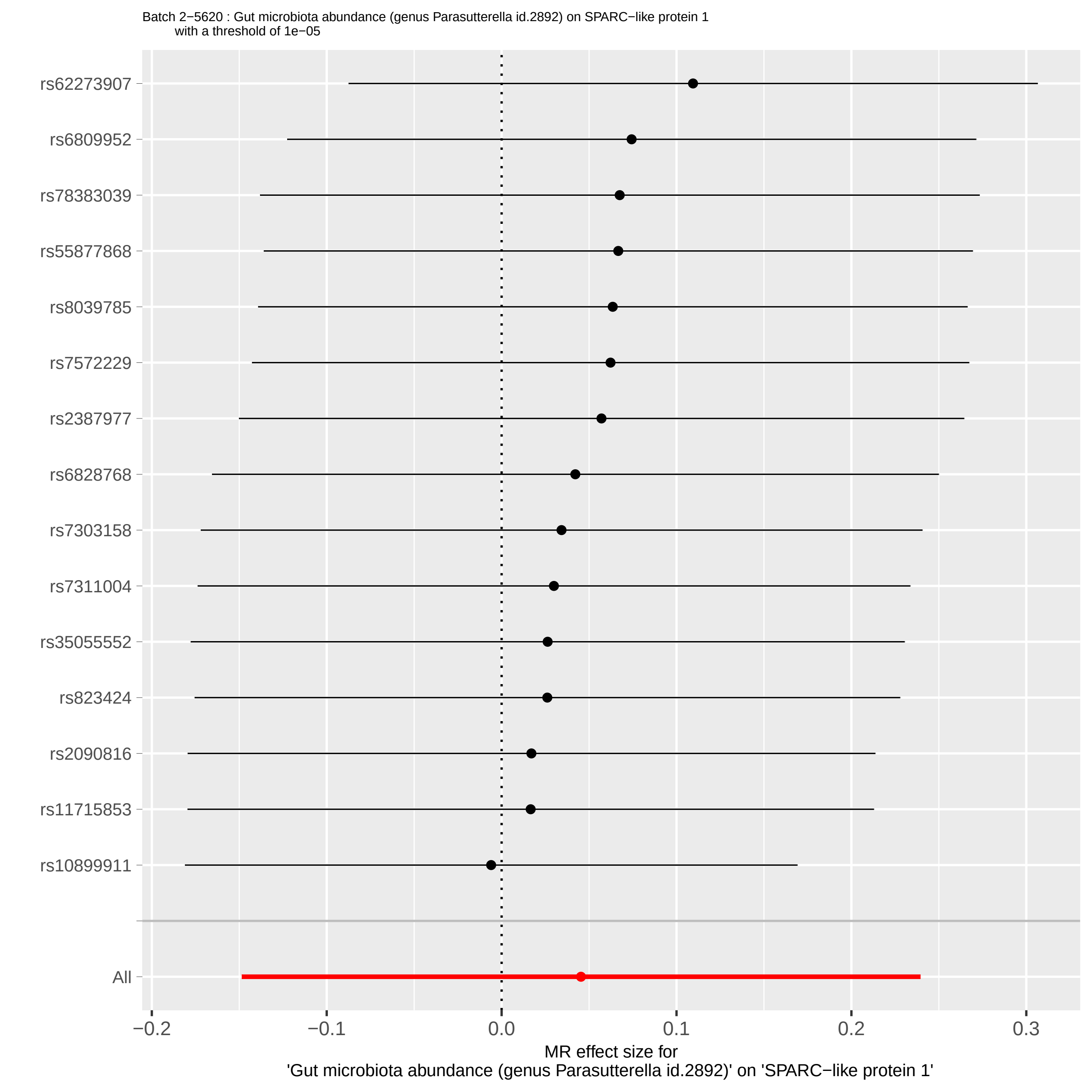
Batch 2−5620 : Gut microbiota abundance (genus Parasutterella id.2892) on SPARC−like protein 1
with a threshold of 1e−05
rs62273907
rs6809952
rs78383039
rs55877868
rs8039785
rs7572229
rs2387977
rs6828768
rs7303158
rs7311004
rs35055552
rs823424
rs2090816
rs11715853
rs10899911
All
−0.2
−0.1
0.0
0.1
0.2
0.3
MR effect size for
'Gut microbiota abundance (genus Parasutterella id.2892)' on 'SPARC−like protein 1'

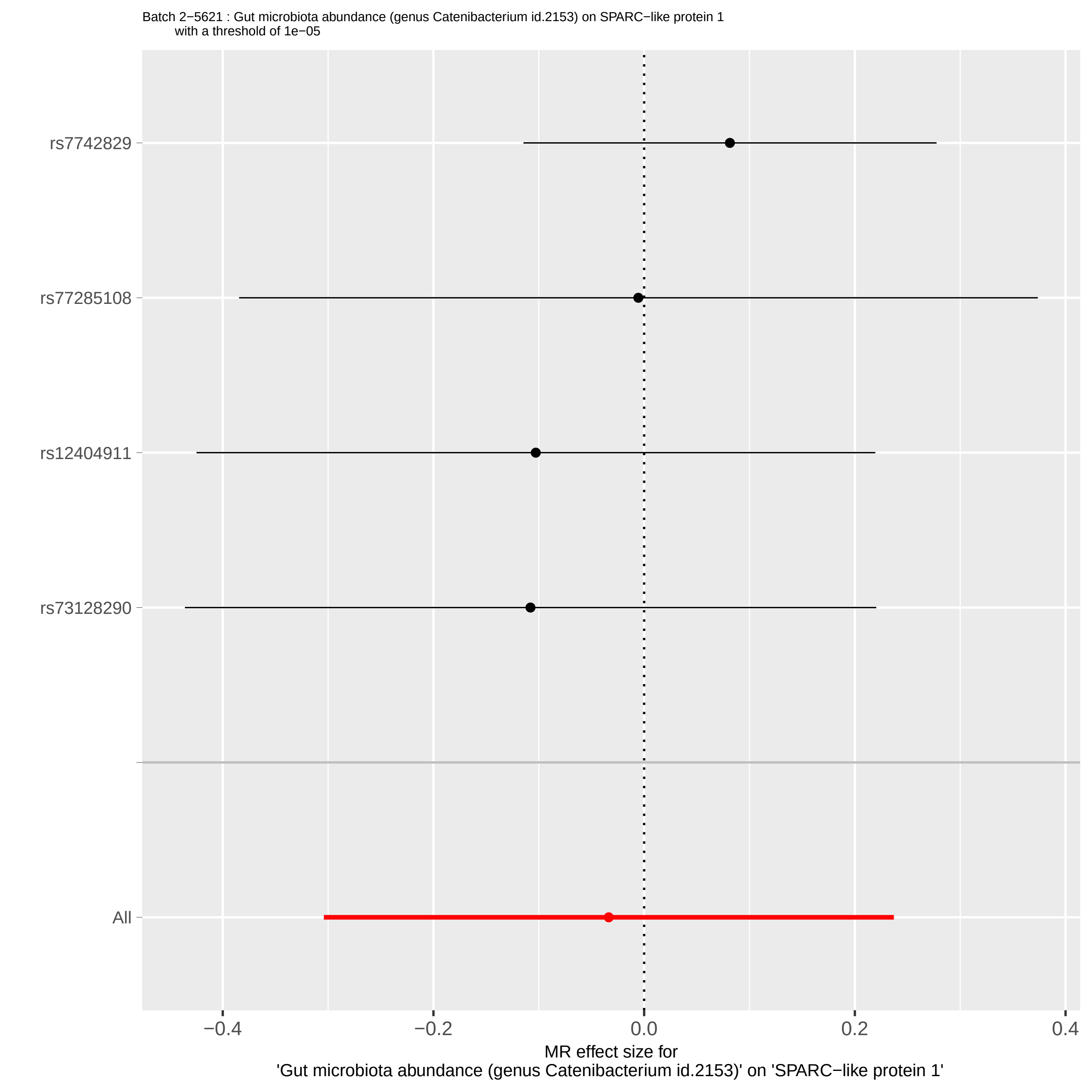
Batch 2−5621 : Gut microbiota abundance (genus Catenibacterium id.2153) on SPARC−like protein 1
with a threshold of 1e−05
rs7742829
rs77285108
rs12404911
rs73128290
All
−0.4
−0.2
0.0
0.2
0.4
MR effect size for
'Gut microbiota abundance (genus Catenibacterium id.2153)' on 'SPARC−like protein 1'

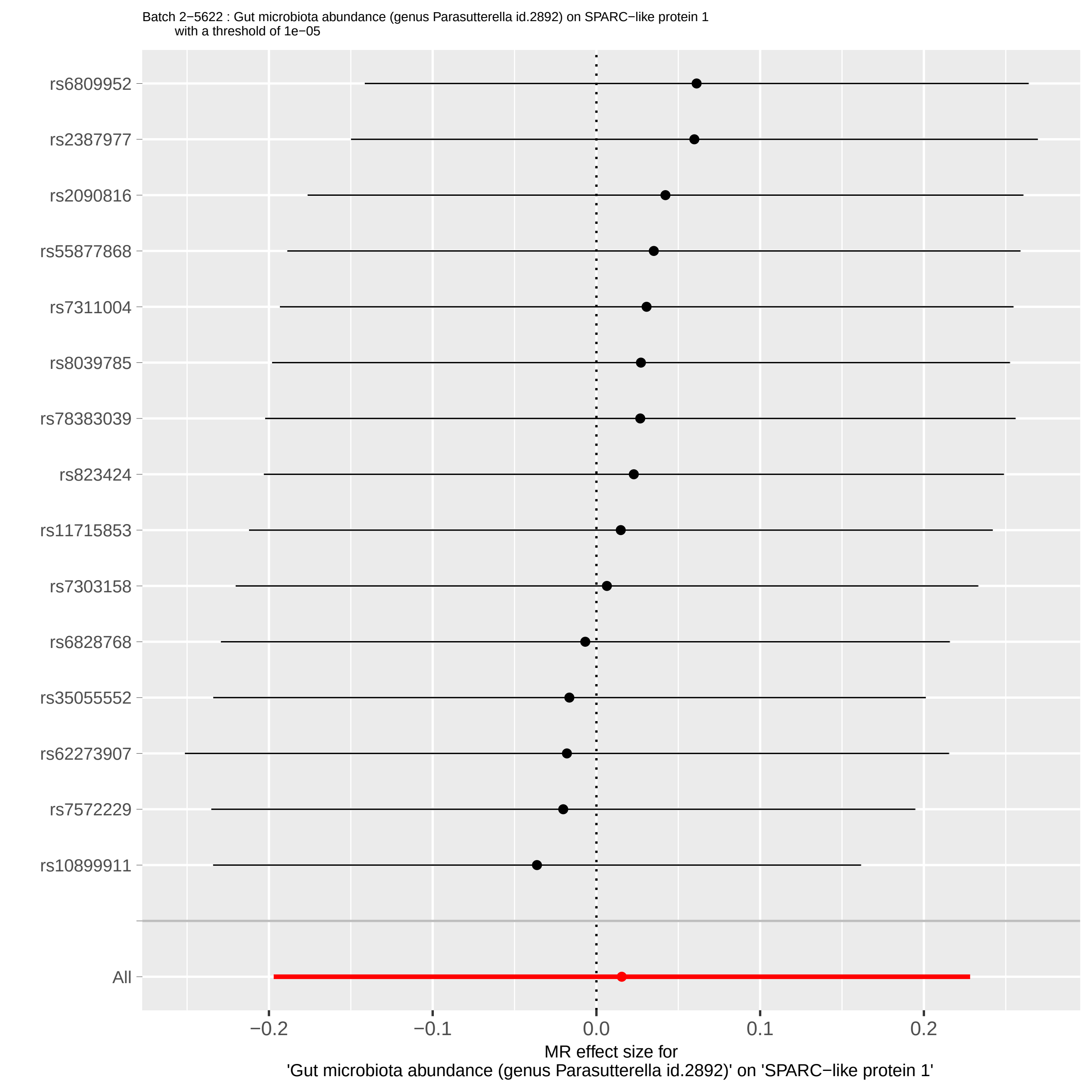

Batch 2−5622 : Gut microbiota abundance (genus Parasutterella id.2892) on SPARC−like protein 1
with a threshold of 1e−05
rs6809952
rs2387977
rs2090816
rs55877868
rs7311004
rs8039785
rs78383039
rs823424
rs11715853
rs7303158
rs6828768
rs35055552
rs62273907
rs7572229
rs10899911
All
−0.2
−0.1
0.0
0.1
0.2
MR effect size for
'Gut microbiota abundance (genus Parasutterella id.2892)' on 'SPARC−like protein 1'

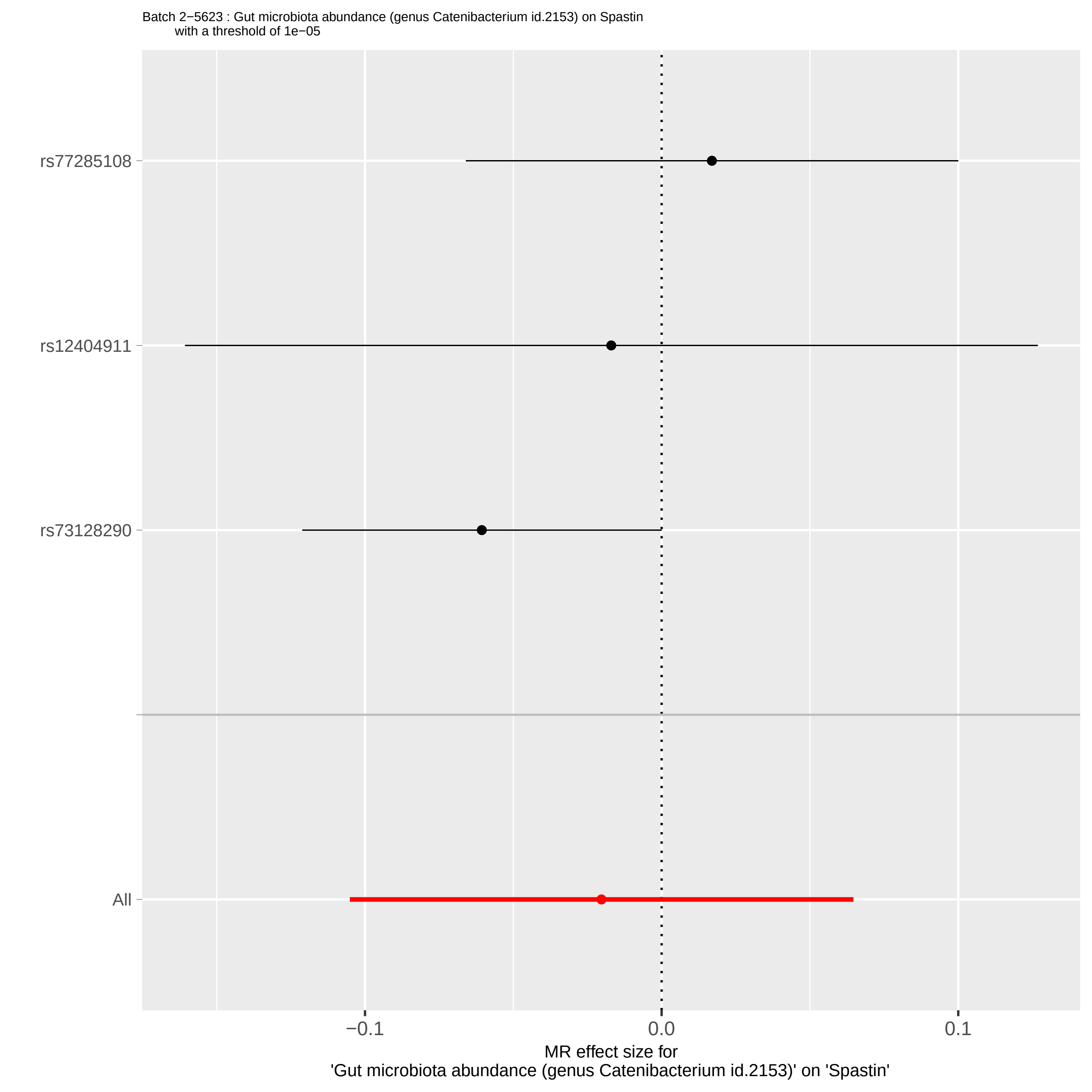

Batch 2−5623 : Gut microbiota abundance (genus Catenibacterium id.2153) on Spastin
with a threshold of 1e−05
rs77285108
rs12404911
rs73128290
All
−0.1
0.0
0.1
MR effect size for
'Gut microbiota abundance (genus Catenibacterium id.2153)' on 'Spastin'

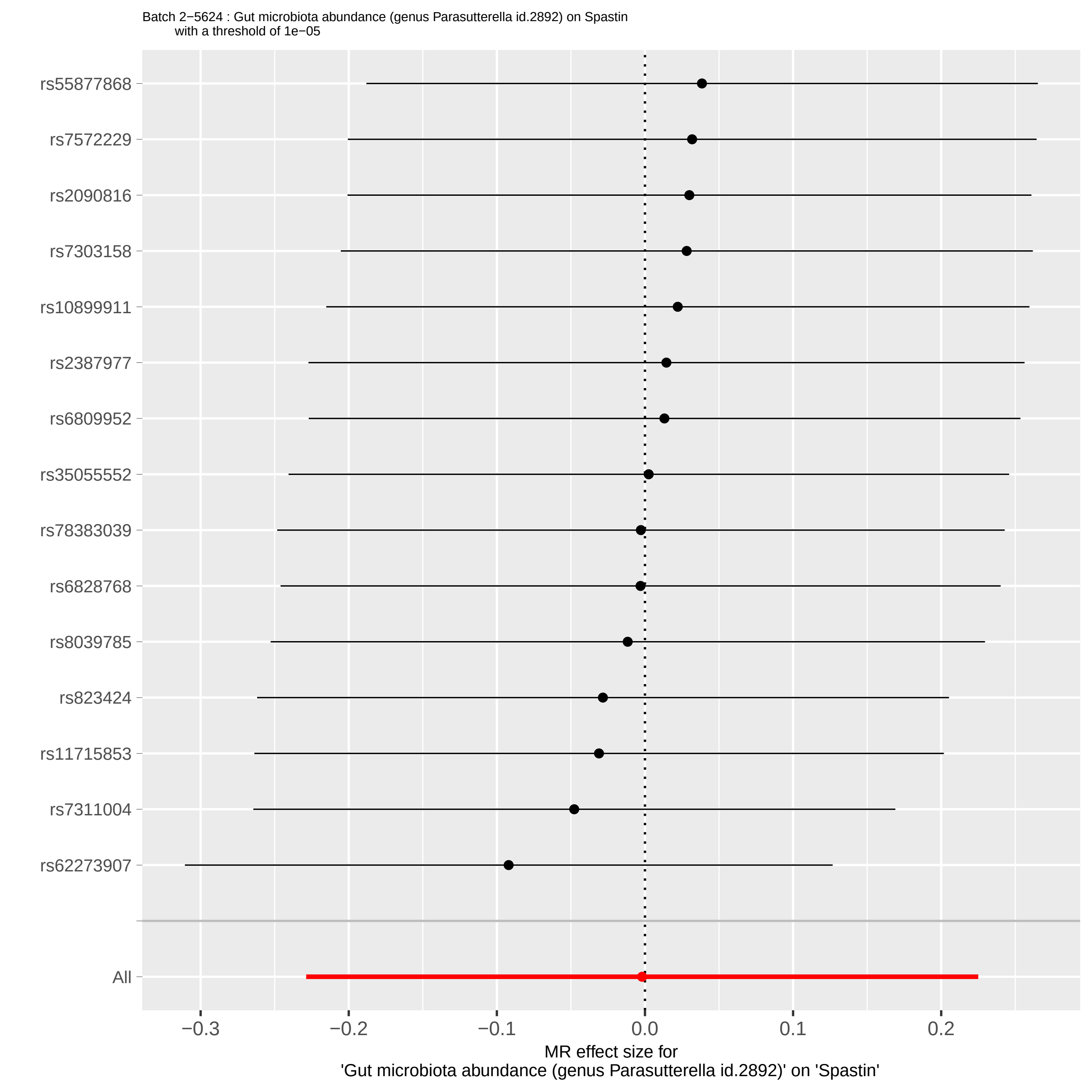

Batch 2−5624 : Gut microbiota abundance (genus Parasutterella id.2892) on Spastin
with a threshold of 1e−05
rs55877868
rs7572229
rs2090816
rs7303158
rs10899911
rs2387977
rs6809952
rs35055552
rs78383039
rs6828768
rs8039785
rs823424
rs11715853
rs7311004
rs62273907
All
−0.3
−0.2
−0.1
0.0
0.1
0.2
MR effect size for
'Gut microbiota abundance (genus Parasutterella id.2892)' on 'Spastin'

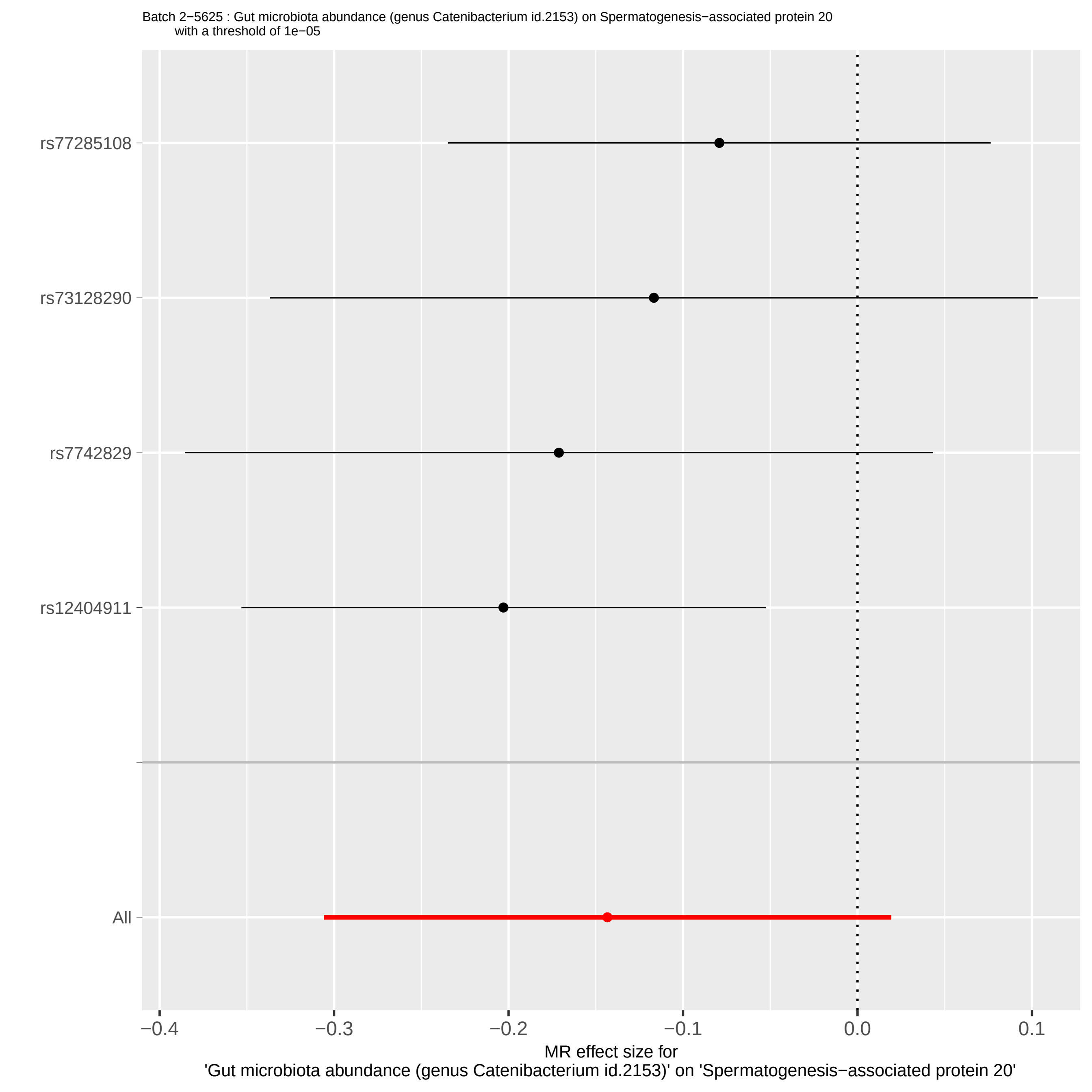
Batch 2−5625 : Gut microbiota abundance (genus Catenibacterium id.2153) on Spermatogenesis−associated protein 20
with a threshold of 1e−05
rs77285108
rs73128290
rs7742829
rs12404911
All
−0.4
−0.3
−0.2
−0.1
0.0
0.1
MR effect size for
'Gut microbiota abundance (genus Catenibacterium id.2153)' on 'Spermatogenesis−associated protein 20'

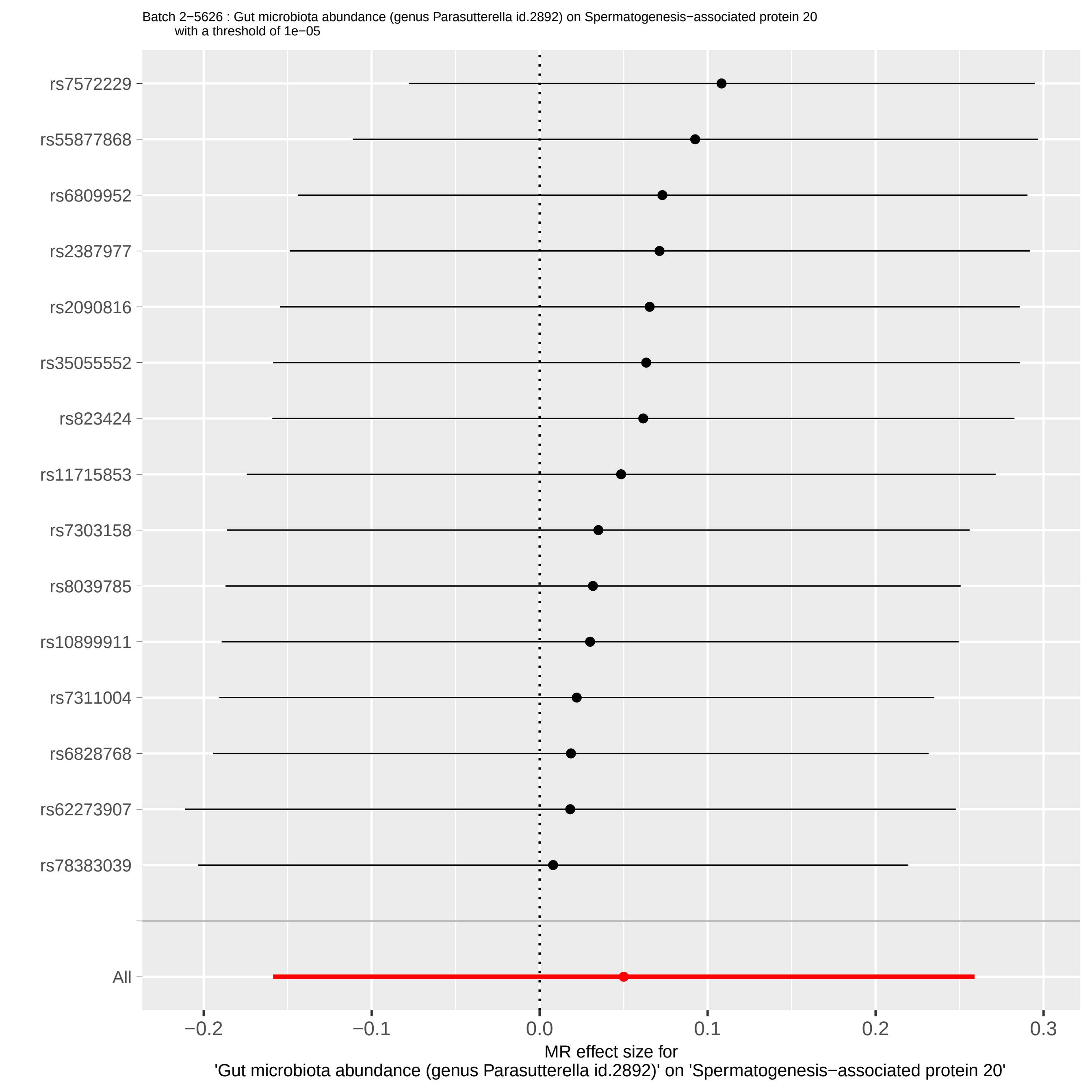
Batch 2−5626 : Gut microbiota abundance (genus Parasutterella id.2892) on Spermatogenesis−associated protein 20
with a threshold of 1e−05
rs7572229
rs55877868
rs6809952
rs2387977
rs2090816
rs35055552
rs823424
rs11715853
rs7303158
rs8039785
rs10899911
rs7311004
rs6828768
rs62273907
rs78383039
All
−0.2
−0.1
0.0
0.1
0.2
0.3
MR effect size for
'Gut microbiota abundance (genus Parasutterella id.2892)' on 'Spermatogenesis−associated protein 20'

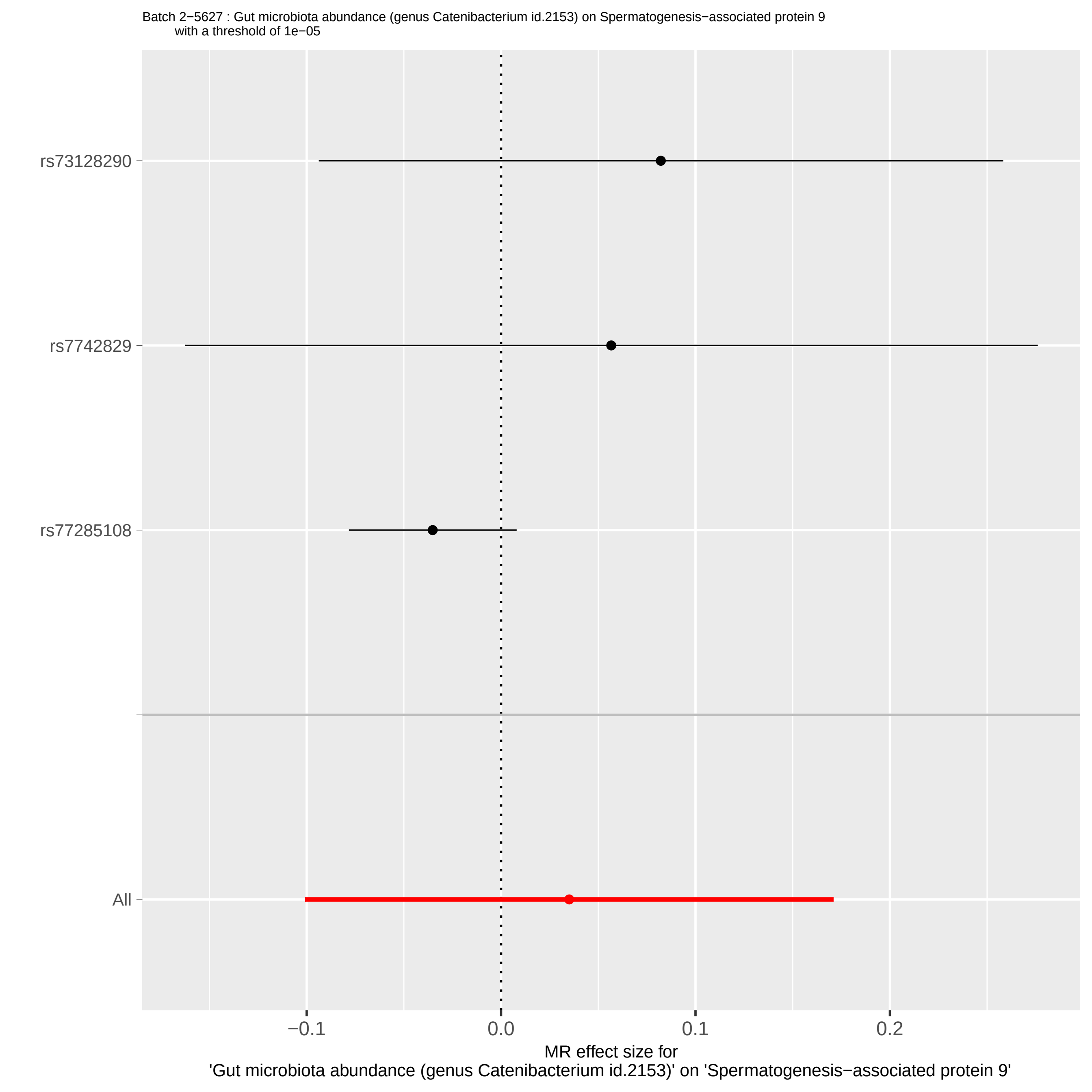
Batch 2−5627 : Gut microbiota abundance (genus Catenibacterium id.2153) on Spermatogenesis−associated protein 9
with a threshold of 1e−05
rs73128290
rs7742829
rs77285108
All
−0.1
0.0
0.1
0.2
MR effect size for
'Gut microbiota abundance (genus Catenibacterium id.2153)' on 'Spermatogenesis−associated protein 9'

Batch 2−5628 : Gut microbiota abundance (genus Parasutterella id.2892) on Spermatogenesis−associated protein 9
with a threshold of 1e−05

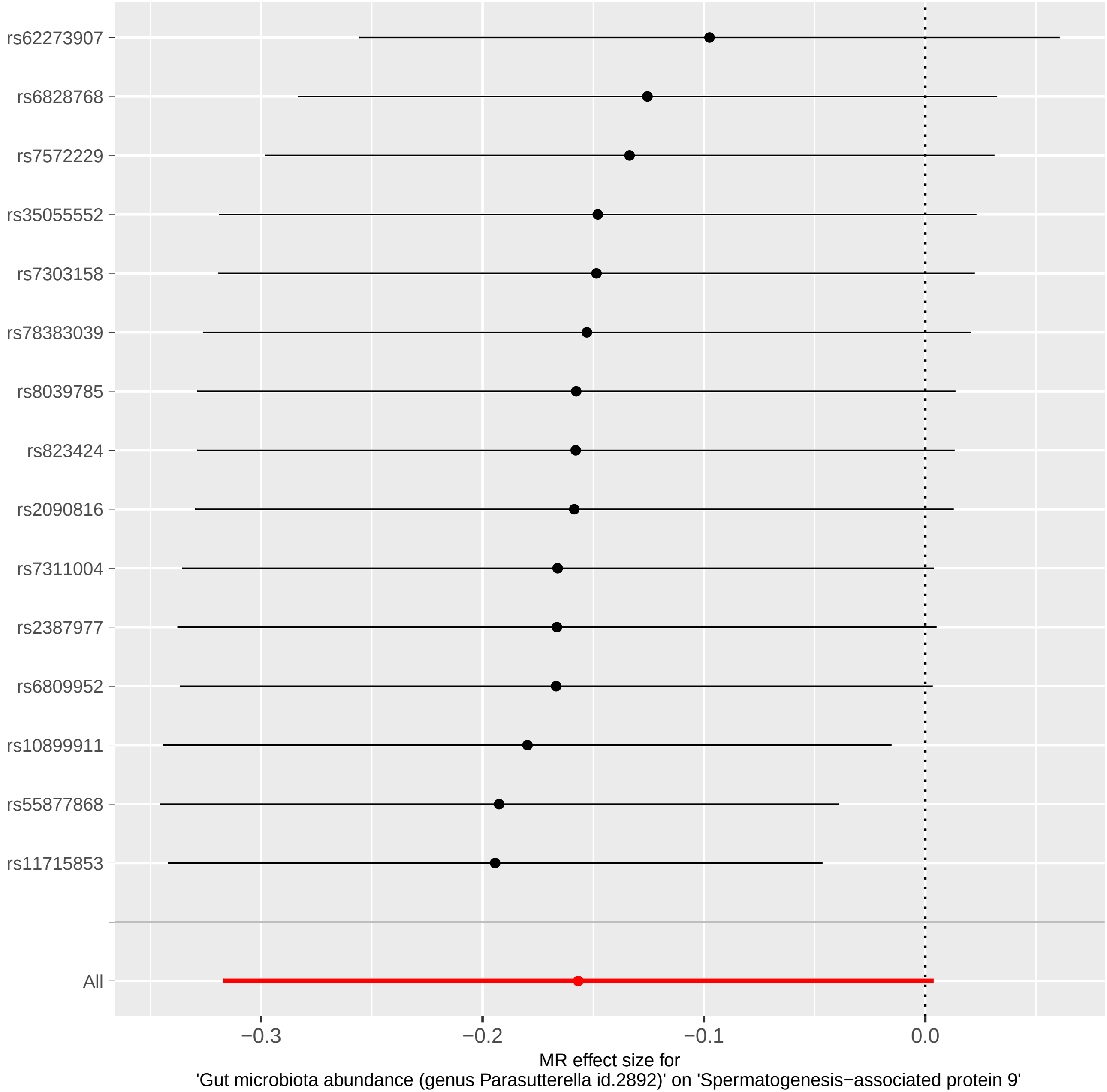

Batch 2−5629 : Gut microbiota abundance (genus Catenibacterium id.2153) on Maspardin
with a threshold of 1e−05

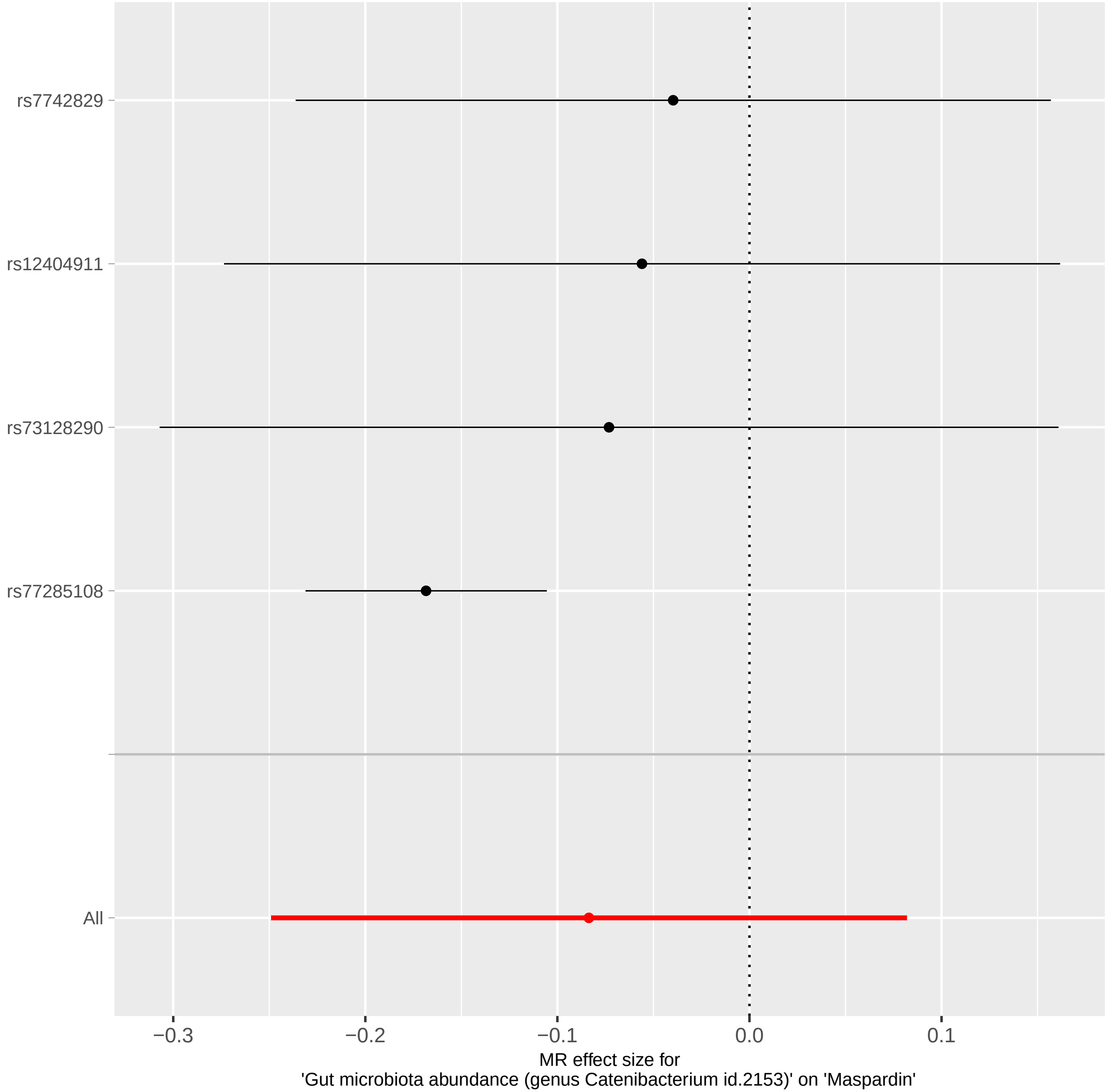

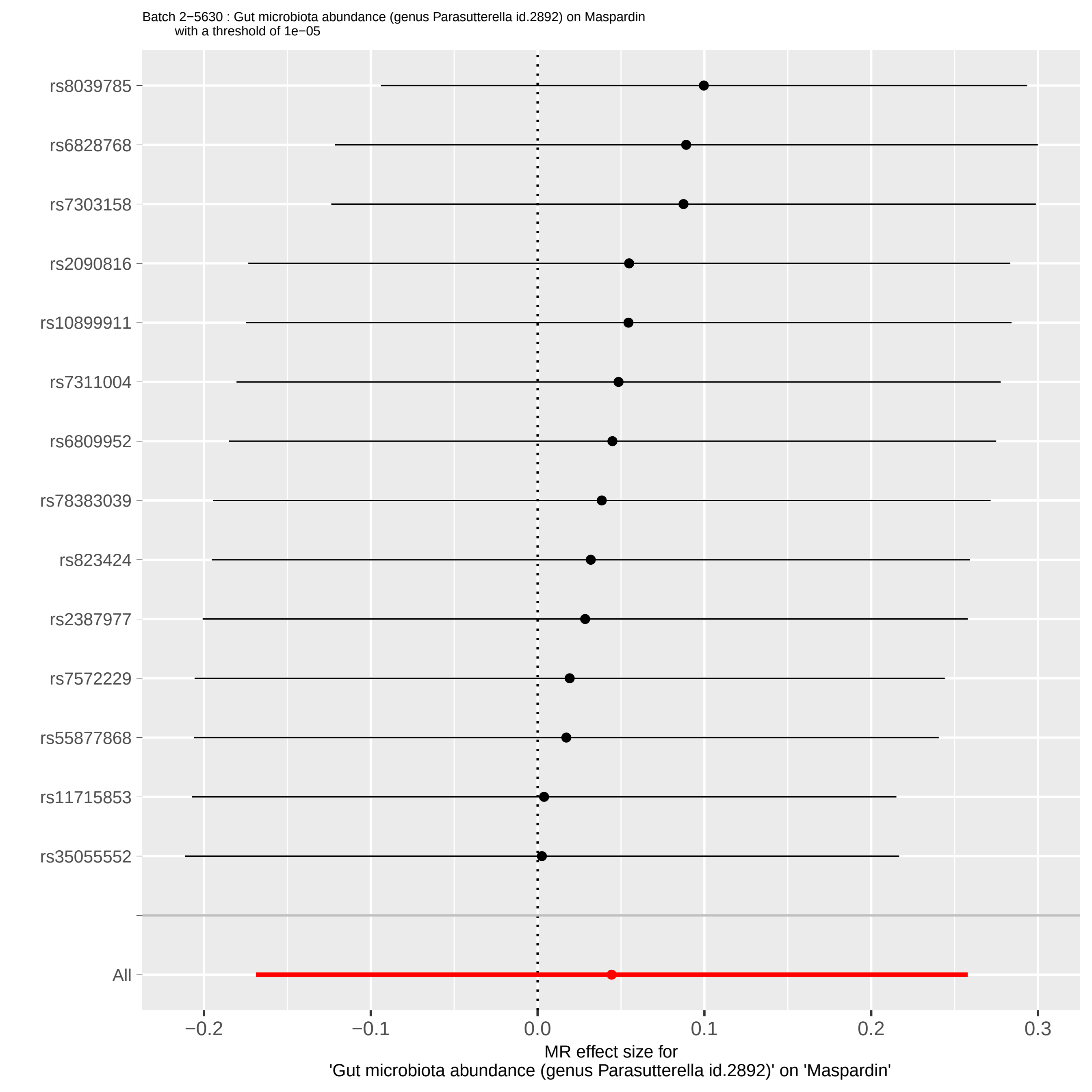
Batch 2−5630 : Gut microbiota abundance (genus Parasutterella id.2892) on Maspardin
with a threshold of 1e−05
rs8039785
rs6828768
rs7303158
rs2090816
rs10899911
rs7311004
rs6809952
rs78383039
rs823424
rs2387977
rs7572229
rs55877868
rs11715853
rs35055552
All
−0.2
−0.1
0.0
0.1
0.2
0.3
MR effect size for
'Gut microbiota abundance (genus Parasutterella id.2892)' on 'Maspardin'

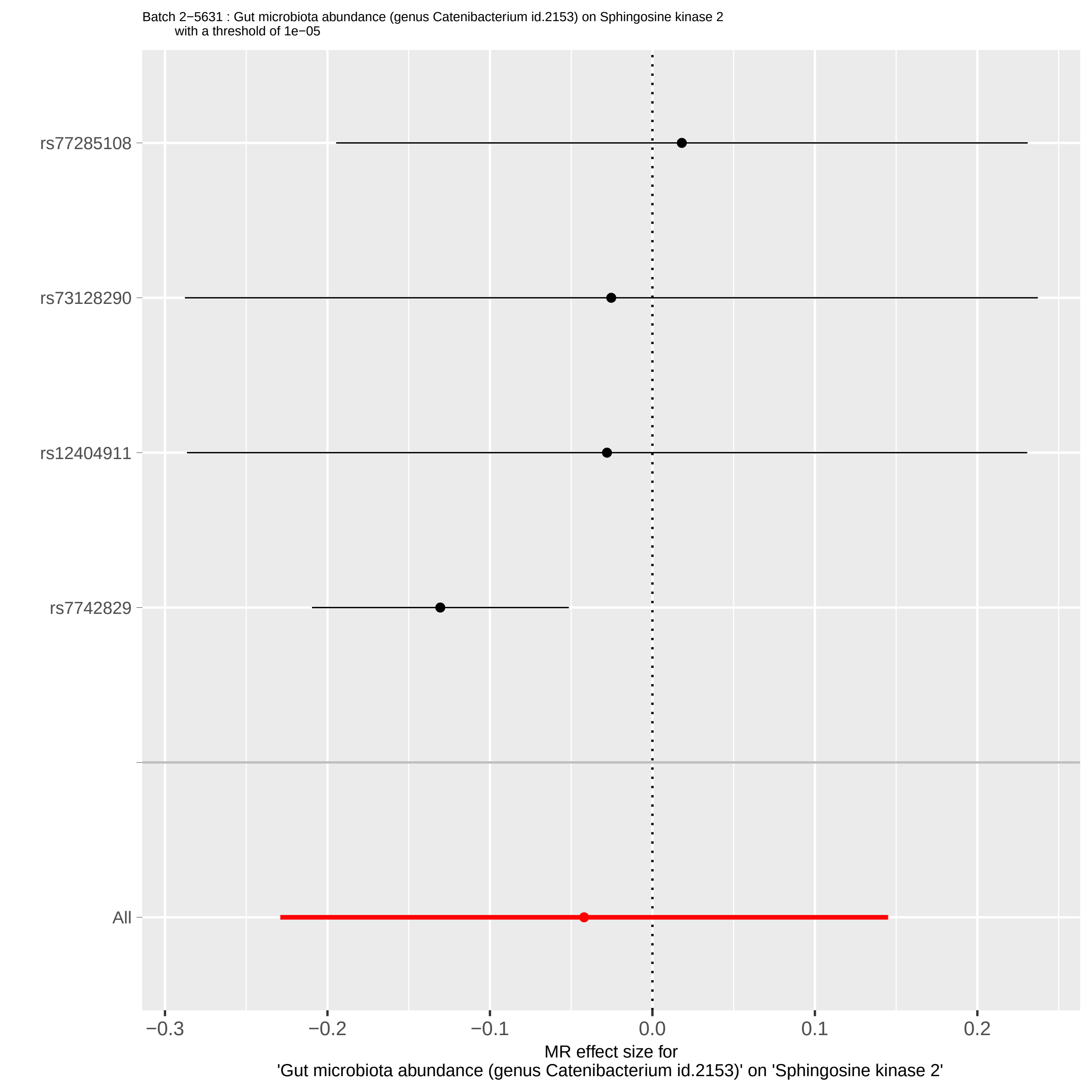
Batch 2−5631 : Gut microbiota abundance (genus Catenibacterium id.2153) on Sphingosine kinase 2
with a threshold of 1e−05
rs77285108
rs73128290
rs12404911
rs7742829
All
−0.3
−0.2
−0.1
0.0
0.1
0.2
MR effect size for
'Gut microbiota abundance (genus Catenibacterium id.2153)' on 'Sphingosine kinase 2'

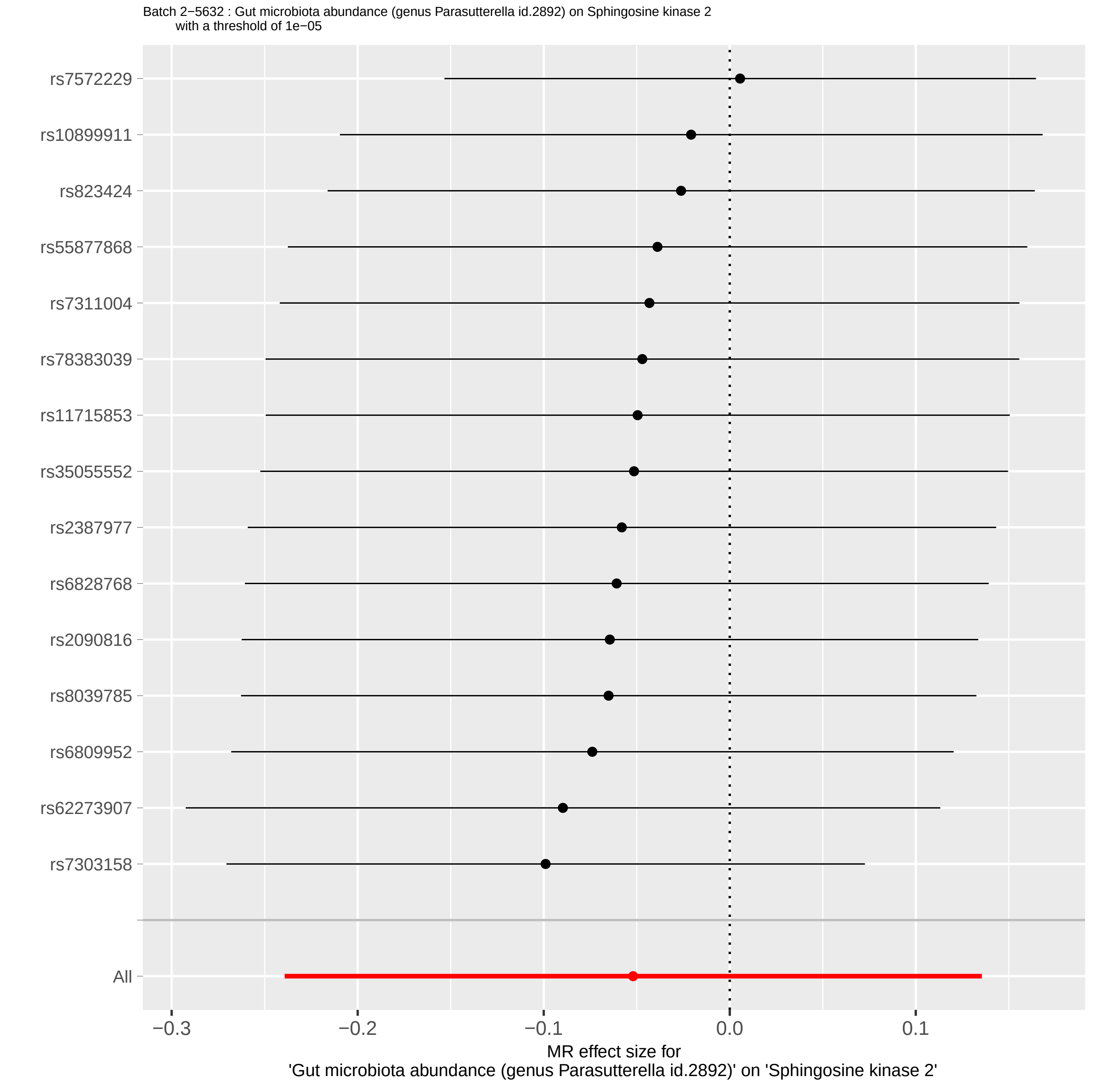

Batch 2−5632 : Gut microbiota abundance (genus Parasutterella id.2892) on Sphingosine kinase 2
with a threshold of 1e−05
rs7572229
rs10899911
rs823424
rs55877868
rs7311004
rs78383039
rs11715853
rs35055552
rs2387977
rs6828768
rs2090816
rs8039785
rs6809952
rs62273907
rs7303158
All
−0.3
−0.2
−0.1
0.0
0.1
MR effect size for
'Gut microbiota abundance (genus Parasutterella id.2892)' on 'Sphingosine kinase 2'

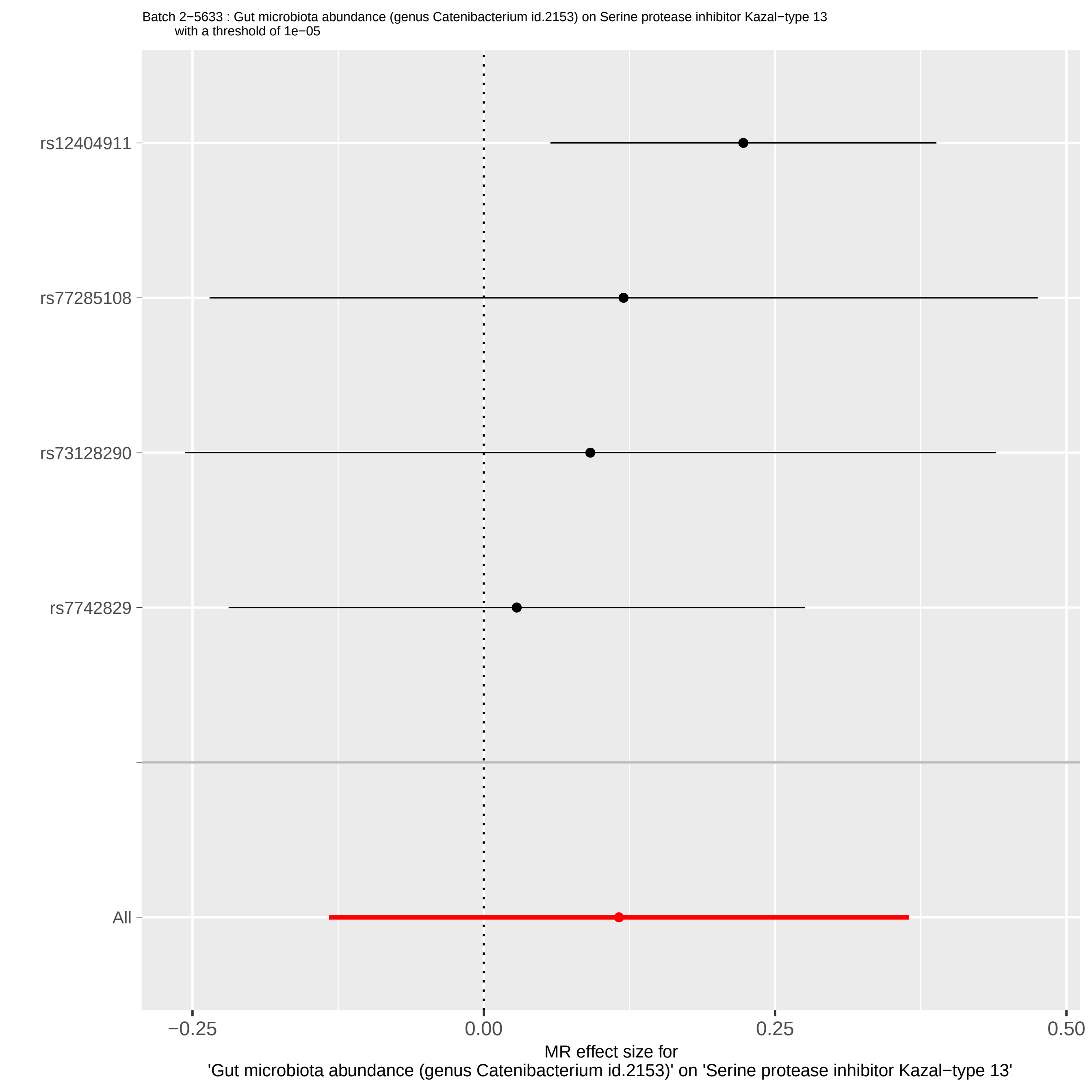
Batch 2−5633 : Gut microbiota abundance (genus Catenibacterium id.2153) on Serine protease inhibitor Kazal−type 13
with a threshold of 1e−05
rs12404911
rs77285108
rs73128290
rs7742829
All
−0.25
0.00
0.25
0.50
MR effect size for
'Gut microbiota abundance (genus Catenibacterium id.2153)' on 'Serine protease inhibitor Kazal−type 13'

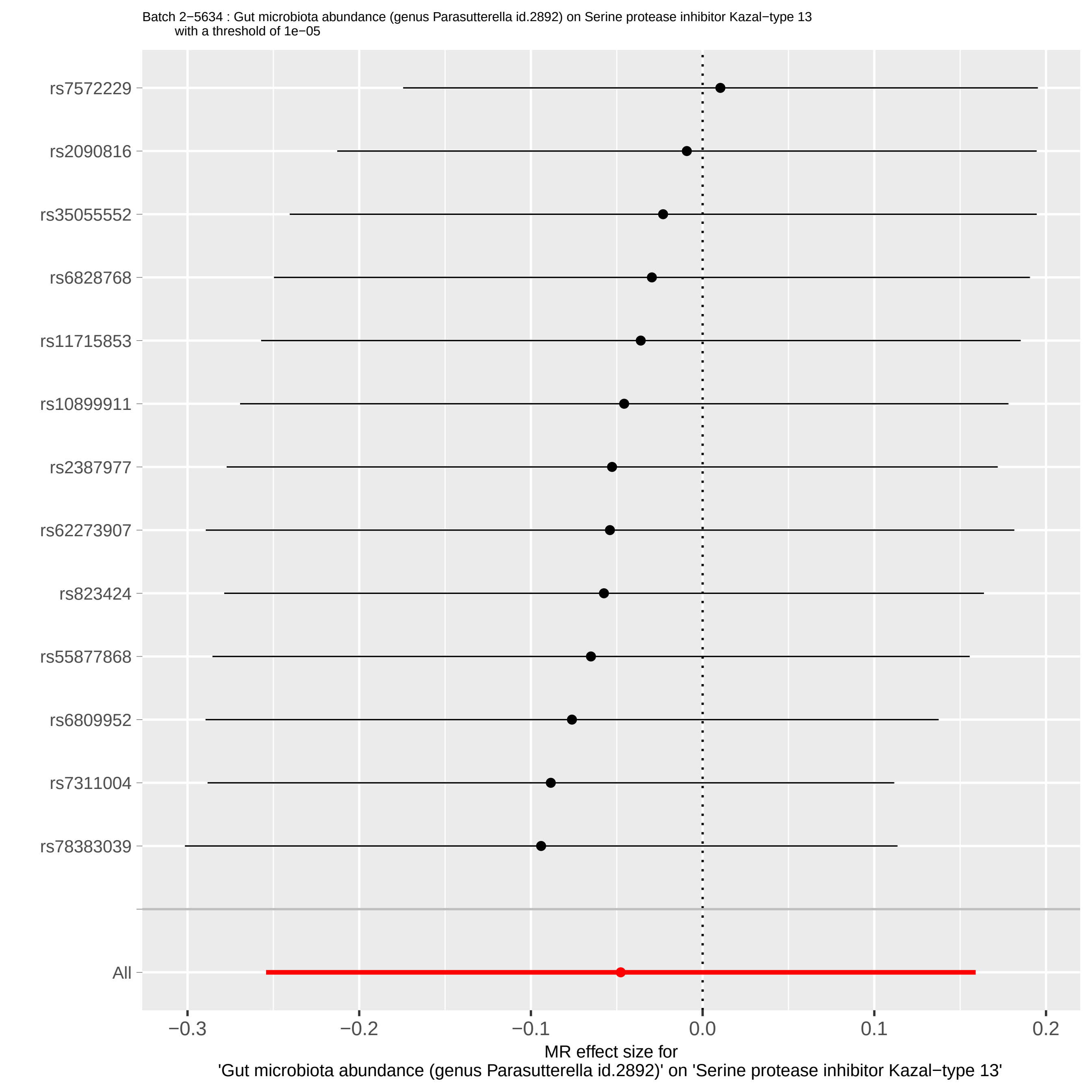

Batch 2−5634 : Gut microbiota abundance (genus Parasutterella id.2892) on Serine protease inhibitor Kazal−type 13
with a threshold of 1e−05
rs7572229
rs2090816
rs35055552
rs6828768
rs11715853
rs10899911
rs2387977
rs62273907
rs823424
rs55877868
rs6809952
rs7311004
rs78383039
All
−0.3
−0.2
−0.1
0.0
0.1
0.2
MR effect size for
'Gut microbiota abundance (genus Parasutterella id.2892)' on 'Serine protease inhibitor Kazal−type 13'

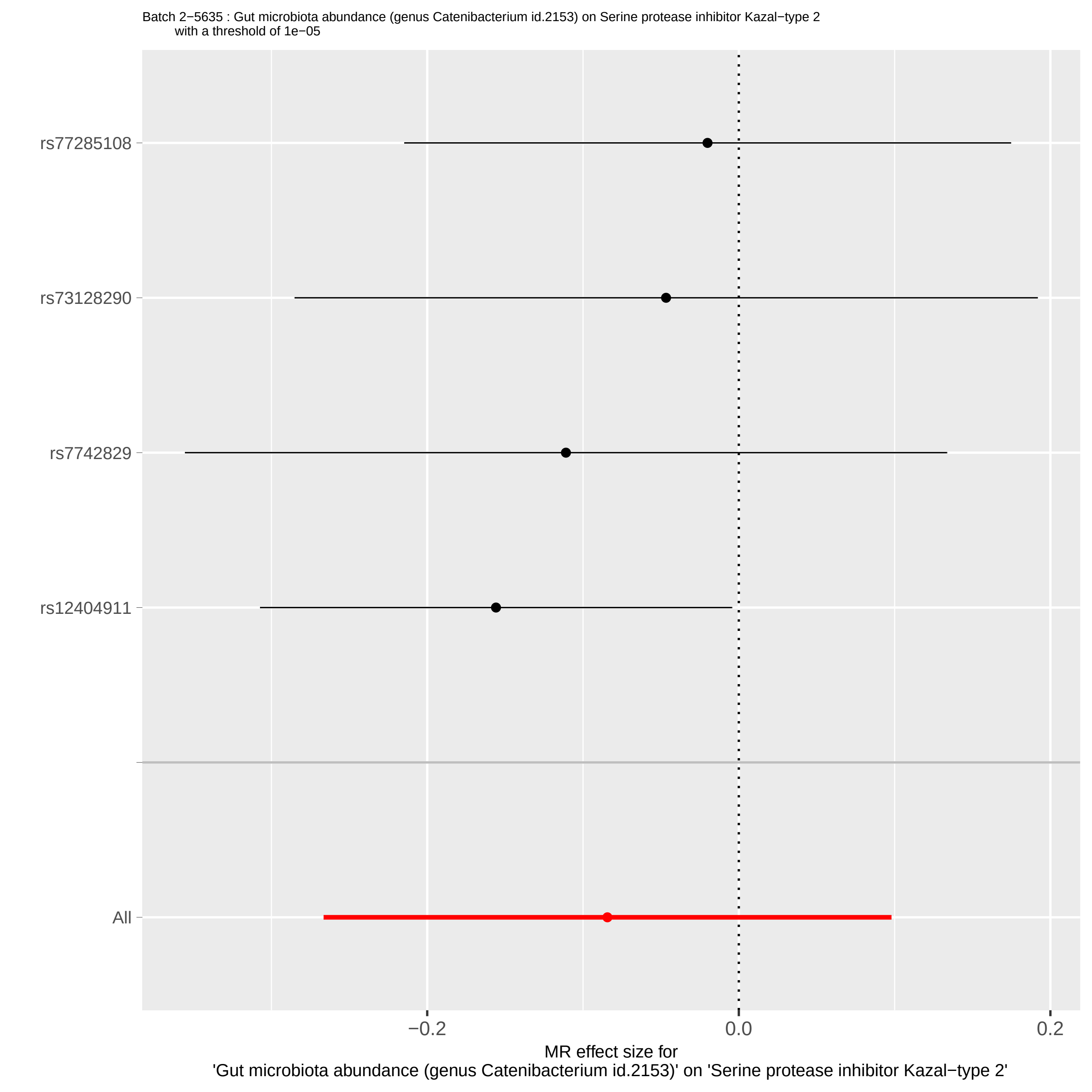
Batch 2−5635 : Gut microbiota abundance (genus Catenibacterium id.2153) on Serine protease inhibitor Kazal−type 2
with a threshold of 1e−05
rs77285108
rs73128290
rs7742829
rs12404911
All
−0.2
0.0
0.2
MR effect size for
'Gut microbiota abundance (genus Catenibacterium id.2153)' on 'Serine protease inhibitor Kazal−type 2'

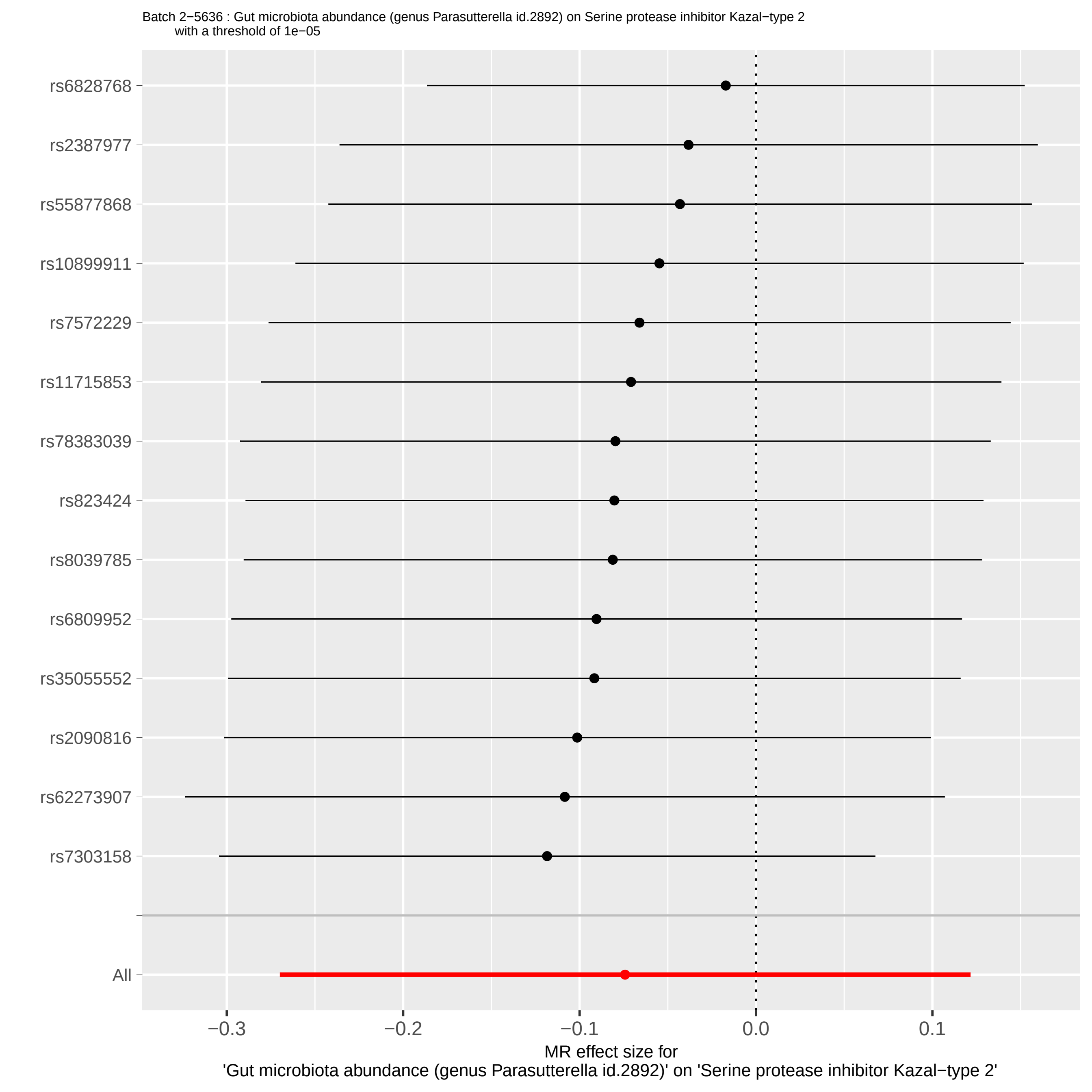
Batch 2−5636 : Gut microbiota abundance (genus Parasutterella id.2892) on Serine protease inhibitor Kazal−type 2
with a threshold of 1e−05
rs6828768
rs2387977
rs55877868
rs10899911
rs7572229
rs11715853
rs78383039
rs823424
rs8039785
rs6809952
rs35055552
rs2090816
rs62273907
rs7303158
All
−0.3
−0.2
−0.1
0.0
0.1
MR effect size for
'Gut microbiota abundance (genus Parasutterella id.2892)' on 'Serine protease inhibitor Kazal−type 2'

Batch 2−5637 : Gut microbiota abundance (genus Catenibacterium id.2153) on Serine protease inhibitor Kazal−type 5
with a threshold of 1e−05

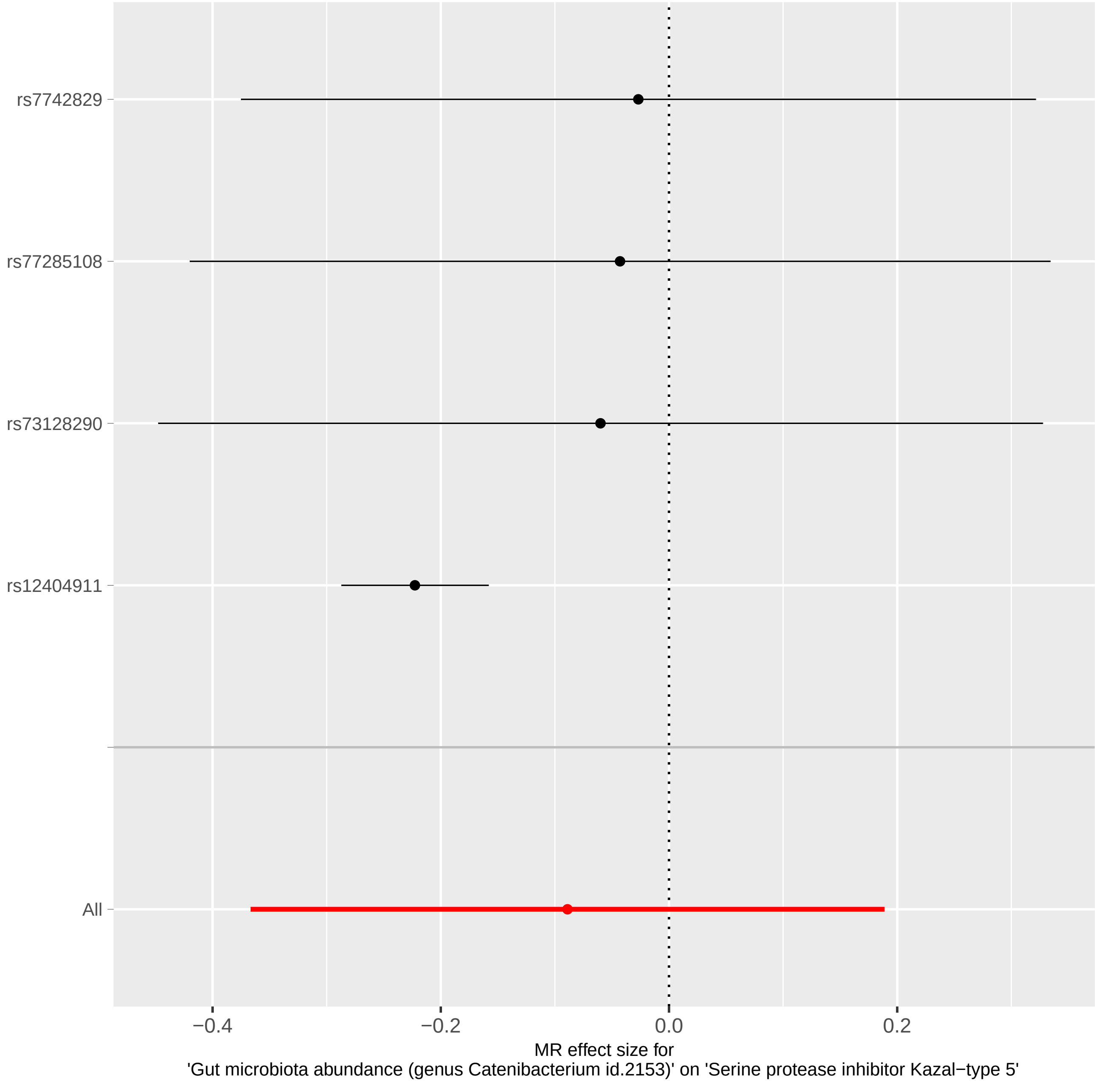

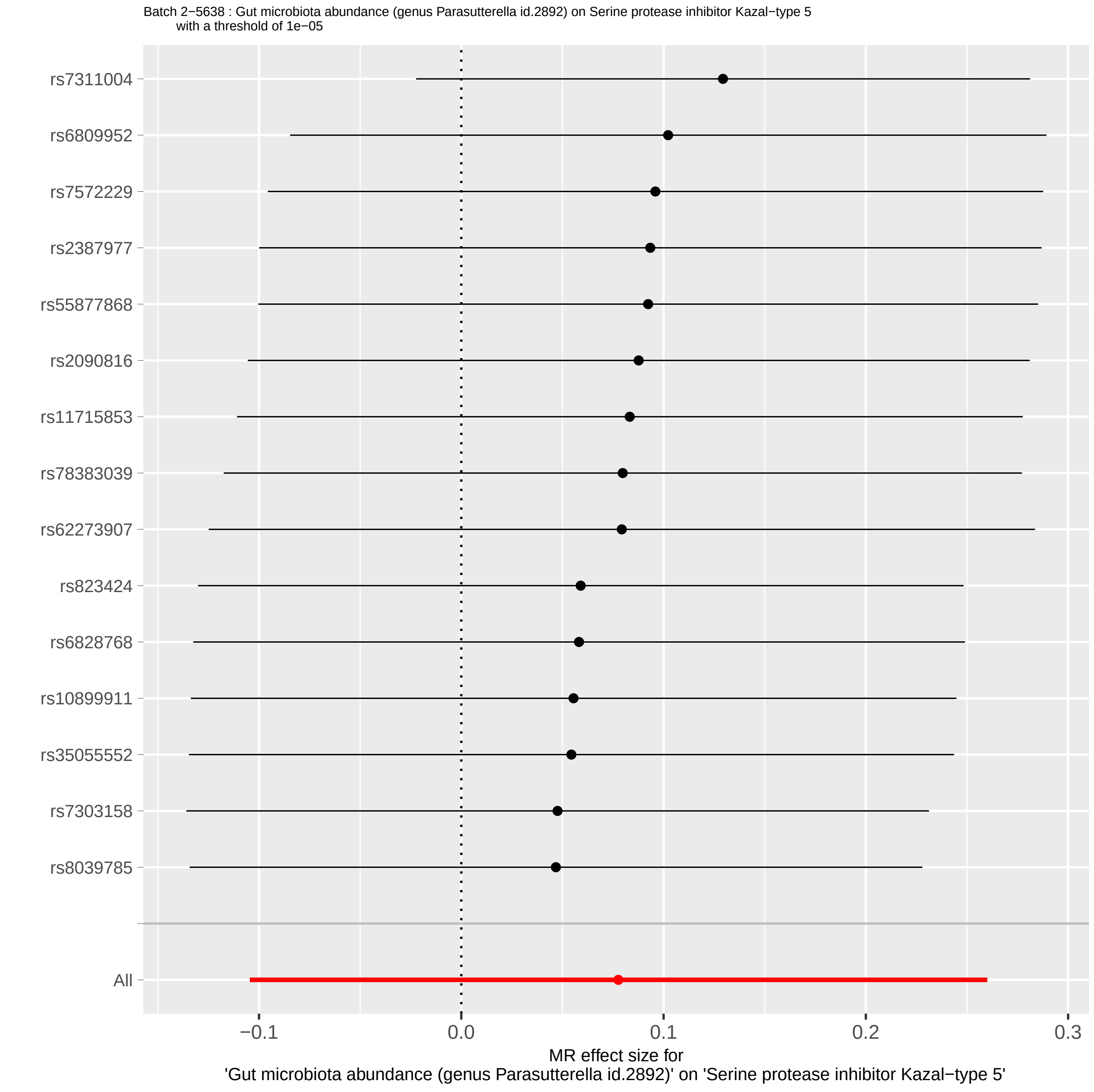
Batch 2−5638 : Gut microbiota abundance (genus Parasutterella id.2892) on Serine protease inhibitor Kazal−type 5
with a threshold of 1e−05
rs7311004
rs6809952
rs7572229
rs2387977
rs55877868
rs2090816
rs11715853
rs78383039
rs62273907
rs823424
rs6828768
rs10899911
rs35055552
rs7303158
rs8039785
All
−0.1
0.0
0.1
0.2
0.3
MR effect size for
'Gut microbiota abundance (genus Parasutterella id.2892)' on 'Serine protease inhibitor Kazal−type 5'

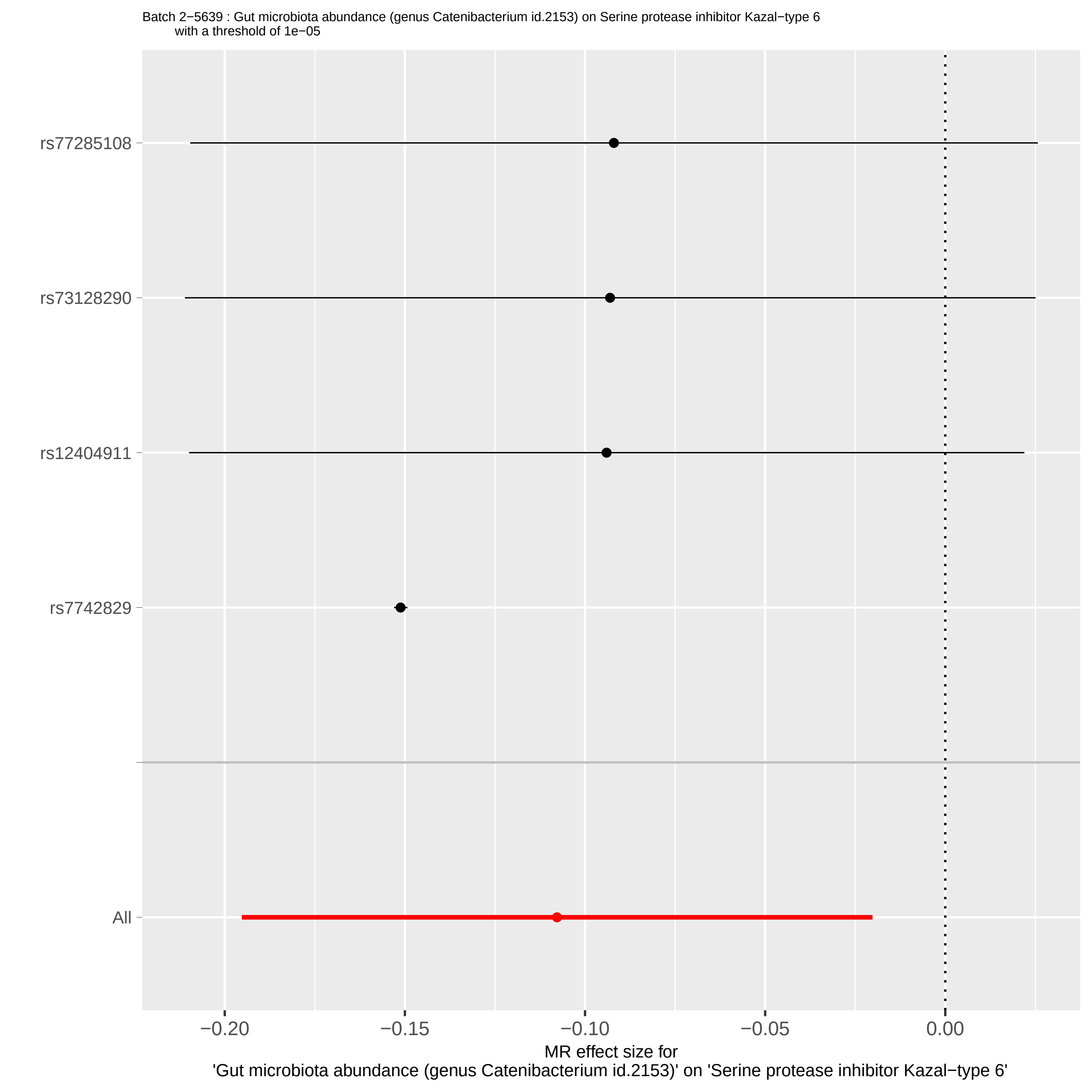

Batch 2−5639 : Gut microbiota abundance (genus Catenibacterium id.2153) on Serine protease inhibitor Kazal−type 6
with a threshold of 1e−05
rs77285108
rs73128290
rs12404911
rs7742829
All
−0.20
−0.15
−0.10
−0.05
0.00
MR effect size for
'Gut microbiota abundance (genus Catenibacterium id.2153)' on 'Serine protease inhibitor Kazal−type 6'

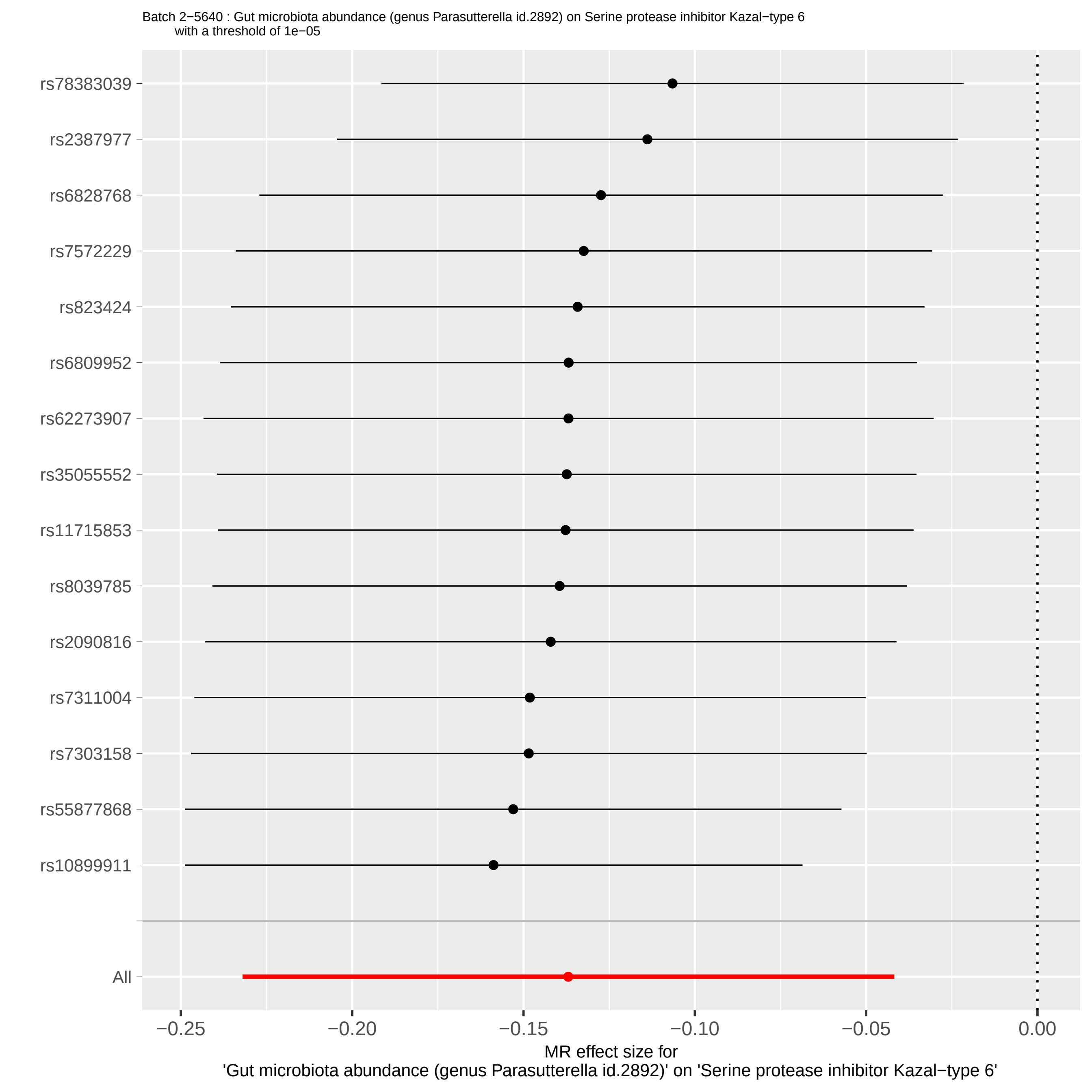
Batch 2−5640 : Gut microbiota abundance (genus Parasutterella id.2892) on Serine protease inhibitor Kazal−type 6
with a threshold of 1e−05
rs78383039
rs2387977
rs6828768
rs7572229
rs823424
rs6809952
rs62273907
rs35055552
rs11715853
rs8039785
rs2090816
rs7311004
rs7303158
rs55877868
rs10899911
All
−0.25
−0.20
−0.15
−0.10
−0.05
0.00
MR effect size for
'Gut microbiota abundance (genus Parasutterella id.2892)' on 'Serine protease inhibitor Kazal−type 6'

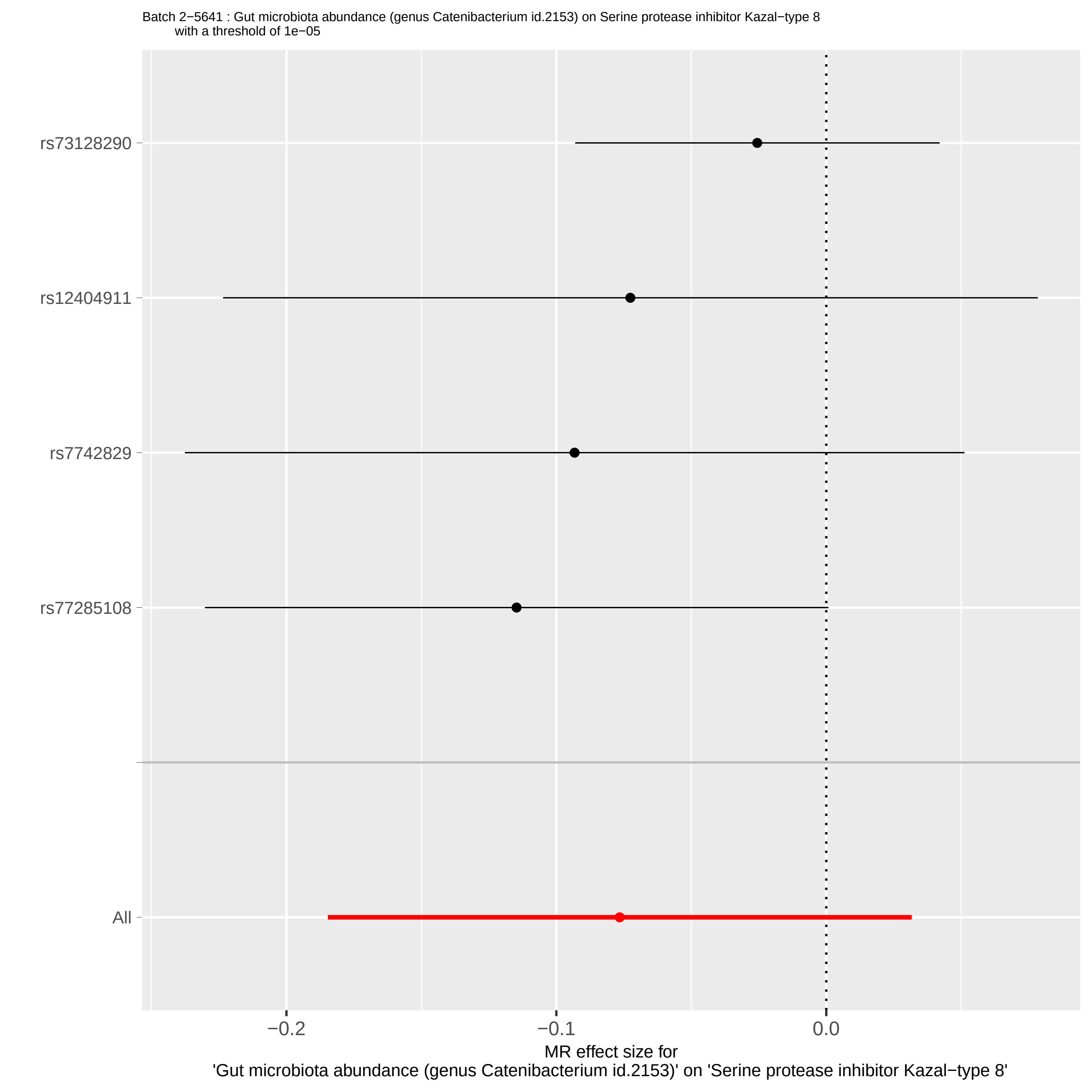
Batch 2−5641 : Gut microbiota abundance (genus Catenibacterium id.2153) on Serine protease inhibitor Kazal−type 8
with a threshold of 1e−05
rs73128290
rs12404911
rs7742829
rs77285108
All
−0.2
−0.1
0.0
MR effect size for
'Gut microbiota abundance (genus Catenibacterium id.2153)' on 'Serine protease inhibitor Kazal−type 8'

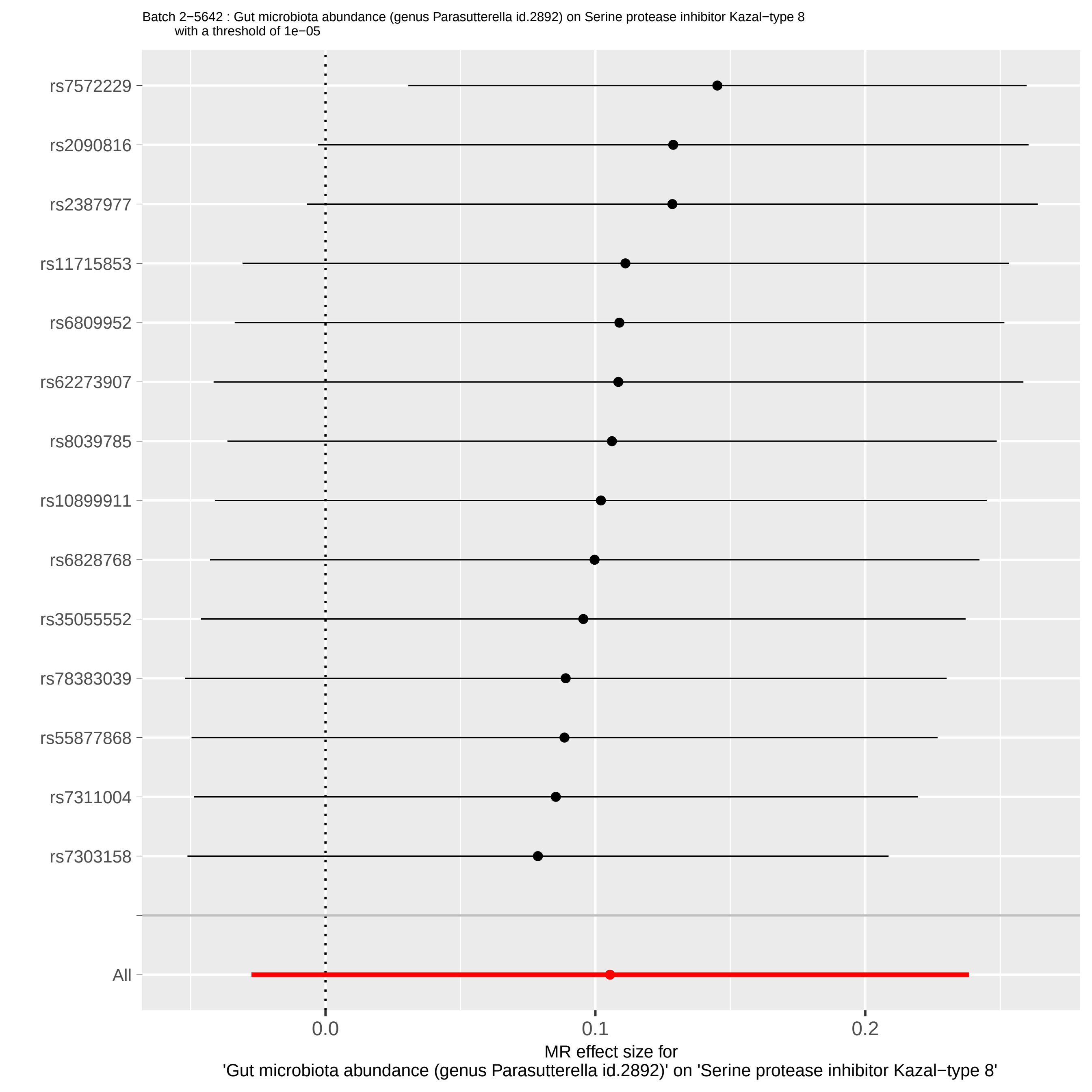

Batch 2−5642 : Gut microbiota abundance (genus Parasutterella id.2892) on Serine protease inhibitor Kazal−type 8
with a threshold of 1e−05
rs7572229
rs2090816
rs2387977
rs11715853
rs6809952
rs62273907
rs8039785
rs10899911
rs6828768
rs35055552
rs78383039
rs55877868
rs7311004
rs7303158
All
0.0
0.1
0.2
MR effect size for
'Gut microbiota abundance (genus Parasutterella id.2892)' on 'Serine protease inhibitor Kazal−type 8'

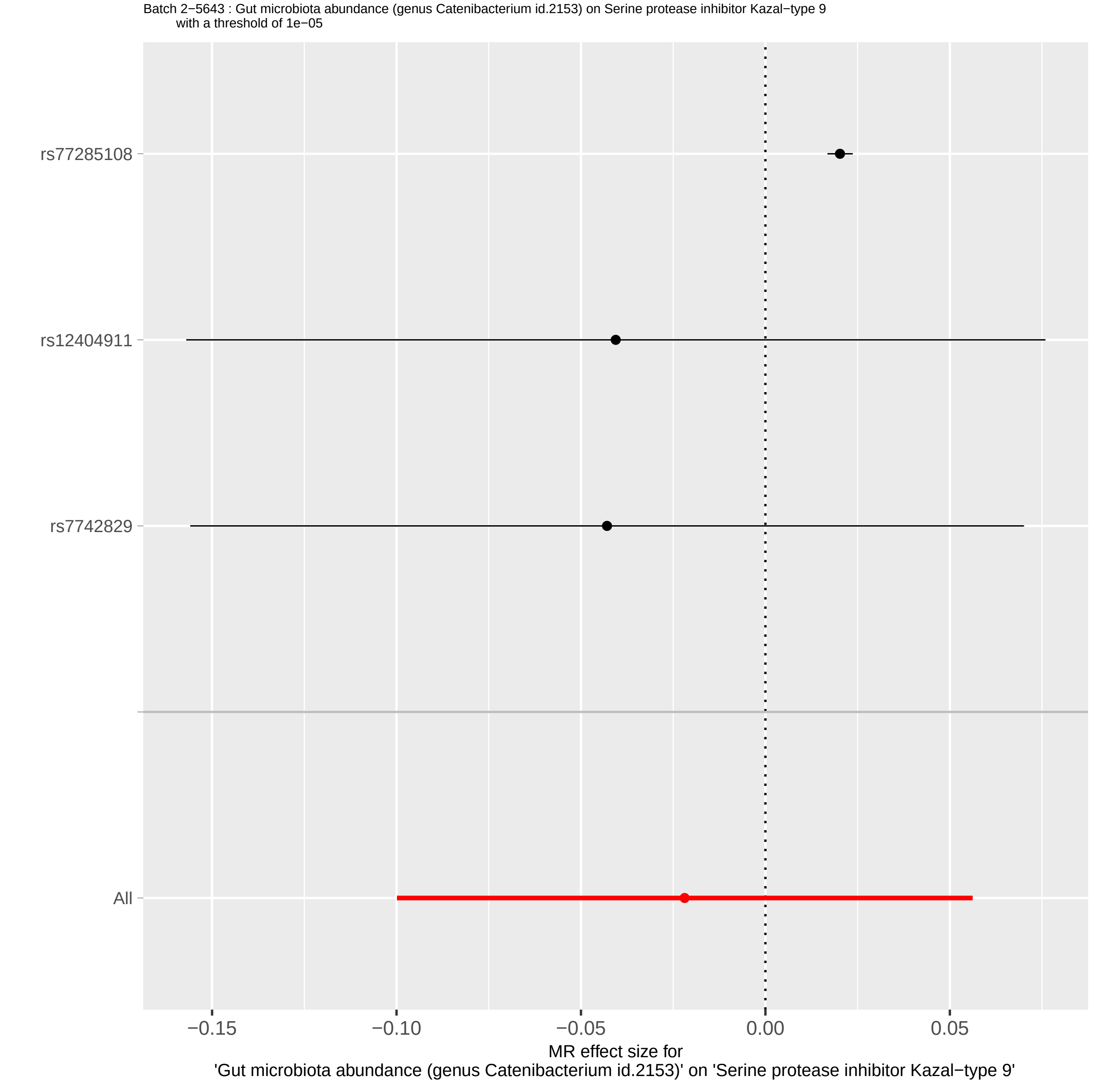

Batch 2−5643 : Gut microbiota abundance (genus Catenibacterium id.2153) on Serine protease inhibitor Kazal−type 9
with a threshold of 1e−05
rs77285108
rs12404911
rs7742829
All
−0.15
−0.10
−0.05
0.00
0.05
MR effect size for
'Gut microbiota abundance (genus Catenibacterium id.2153)' on 'Serine protease inhibitor Kazal−type 9'

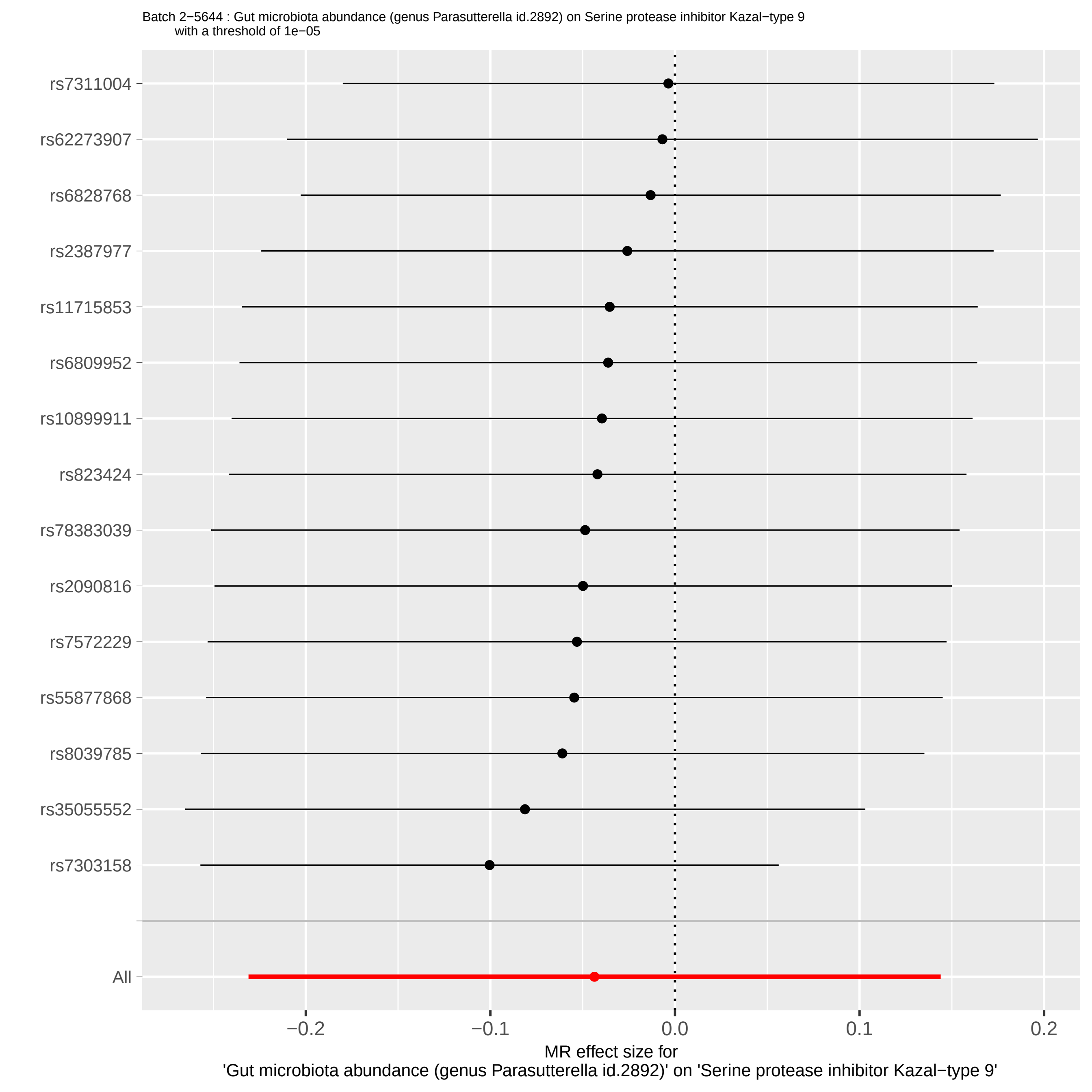

Batch 2−5644 : Gut microbiota abundance (genus Parasutterella id.2892) on Serine protease inhibitor Kazal−type 9
with a threshold of 1e−05
rs7311004
rs62273907
rs6828768
rs2387977
rs11715853
rs6809952
rs10899911
rs823424
rs78383039
rs2090816
rs7572229
rs55877868
rs8039785
rs35055552
rs7303158
All
−0.2
−0.1
0.0
0.1
0.2
MR effect size for
'Gut microbiota abundance (genus Parasutterella id.2892)' on 'Serine protease inhibitor Kazal−type 9'

Batch 2−5645 : Gut microbiota abundance (genus Catenibacterium id.2153) on Kunitz−type protease inhibitor 1
with a threshold of 1e−05

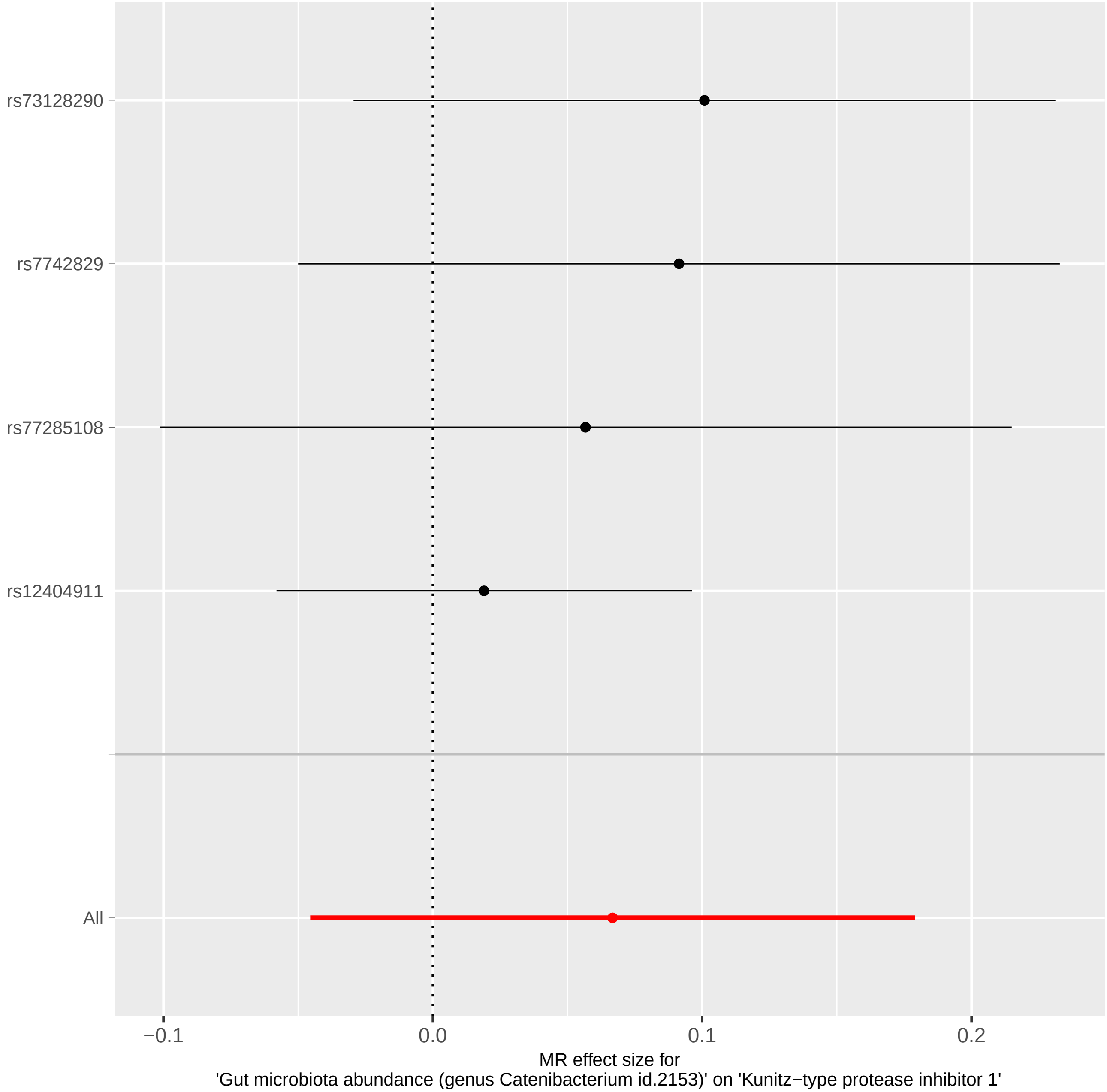

Batch 2−5646 : Gut microbiota abundance (genus Parasutterella id.2892) on Kunitz−type protease inhibitor 1
with a threshold of 1e−05

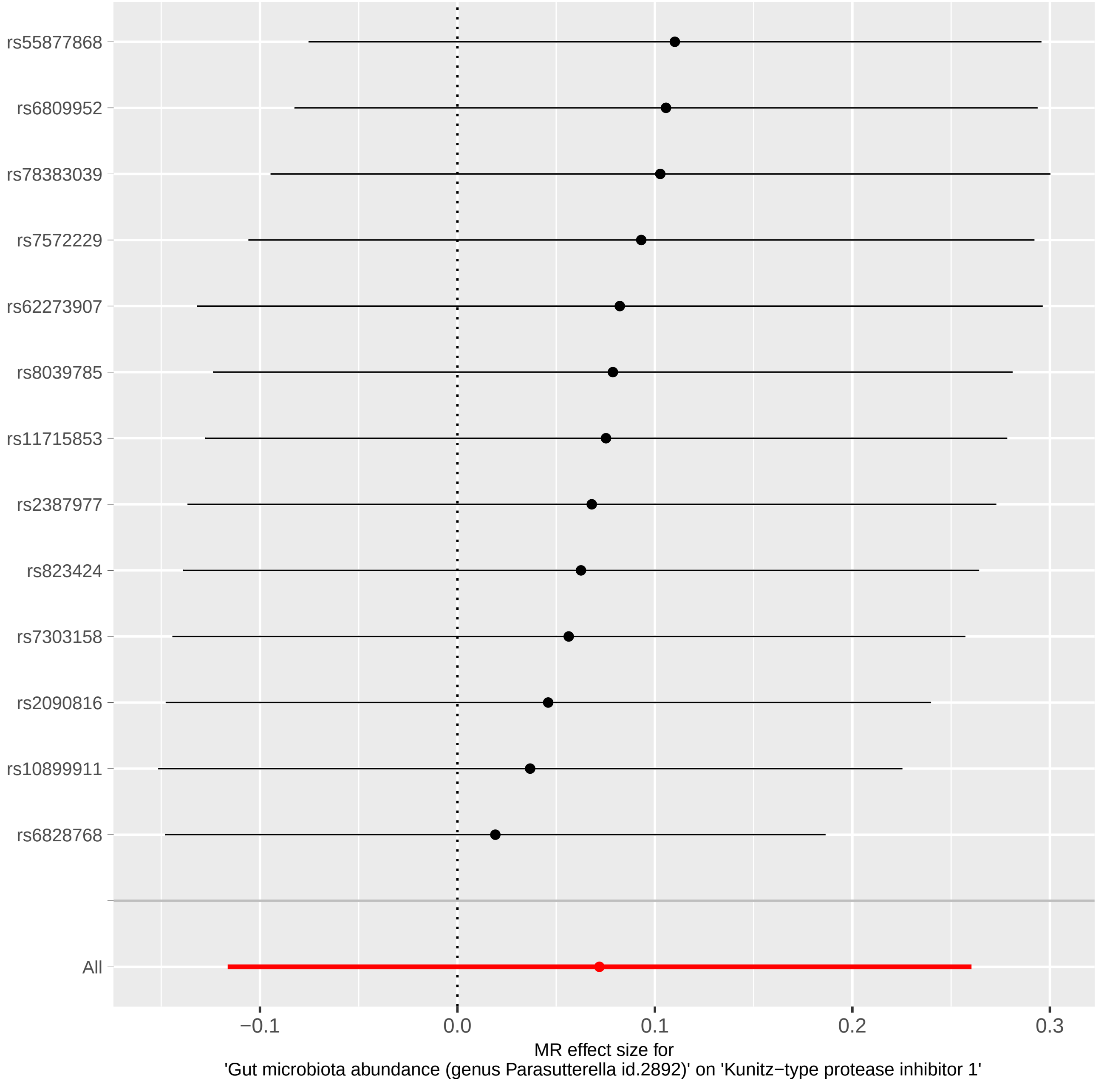

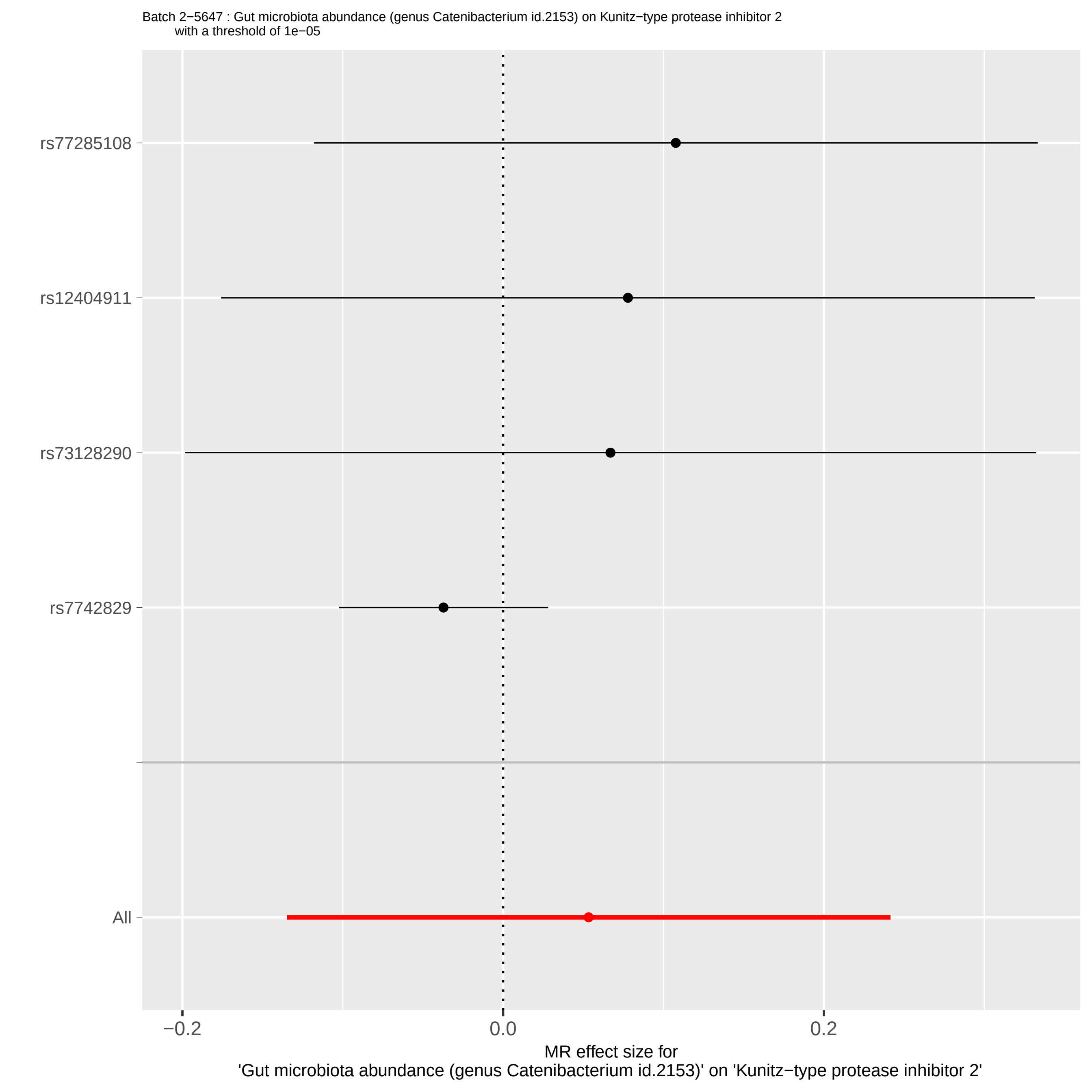

Batch 2−5647 : Gut microbiota abundance (genus Catenibacterium id.2153) on Kunitz−type protease inhibitor 2
with a threshold of 1e−05
rs77285108
rs12404911
rs73128290
rs7742829
All
−0.2
0.0
0.2
MR effect size for
'Gut microbiota abundance (genus Catenibacterium id.2153)' on 'Kunitz−type protease inhibitor 2'

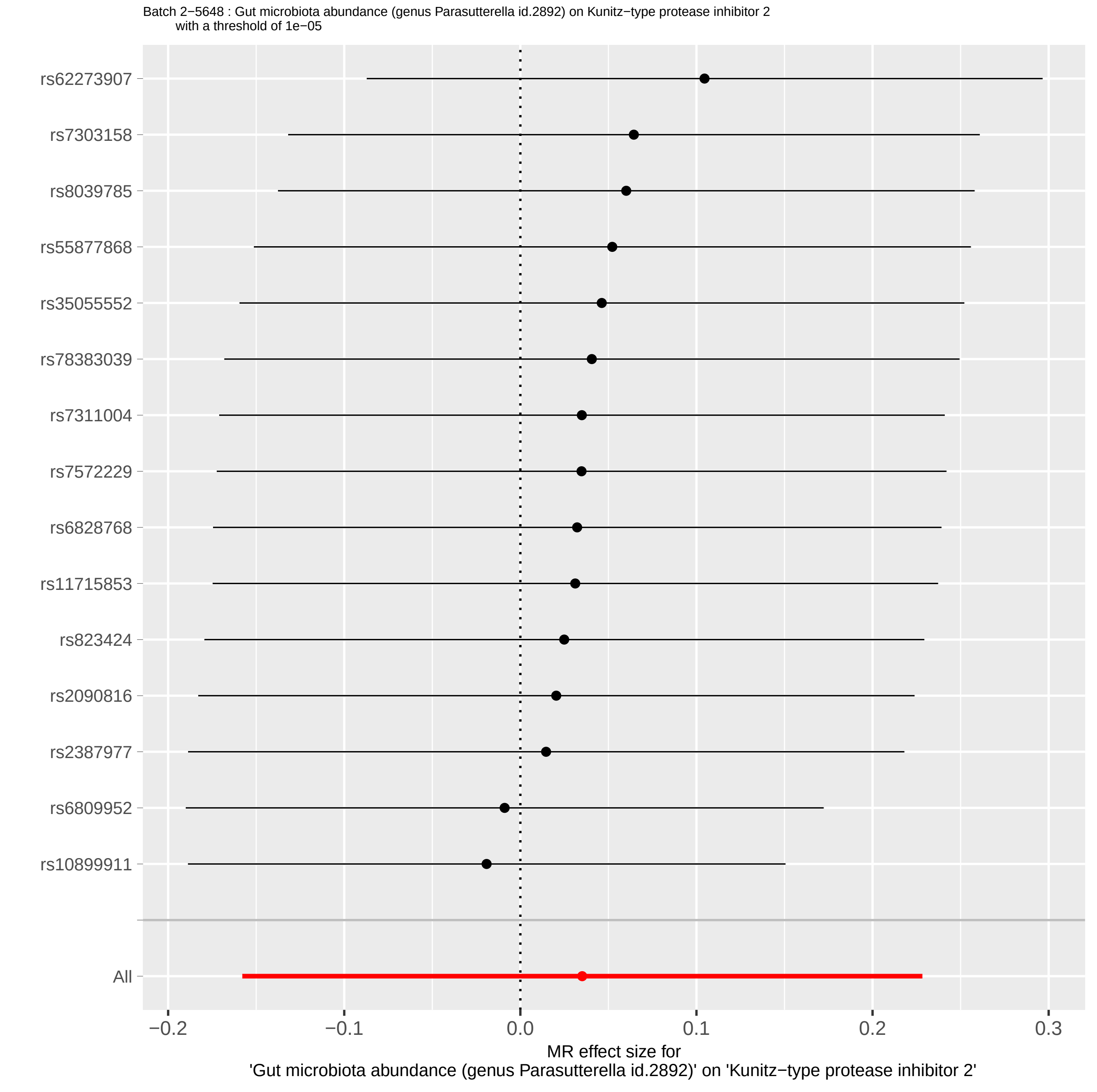
Batch 2−5648 : Gut microbiota abundance (genus Parasutterella id.2892) on Kunitz−type protease inhibitor 2
with a threshold of 1e−05
rs62273907
rs7303158
rs8039785
rs55877868
rs35055552
rs78383039
rs7311004
rs7572229
rs6828768
rs11715853
rs823424
rs2090816
rs2387977
rs6809952
rs10899911
All
−0.2
−0.1
0.0
0.1
0.2
0.3
MR effect size for
'Gut microbiota abundance (genus Parasutterella id.2892)' on 'Kunitz−type protease inhibitor 2'

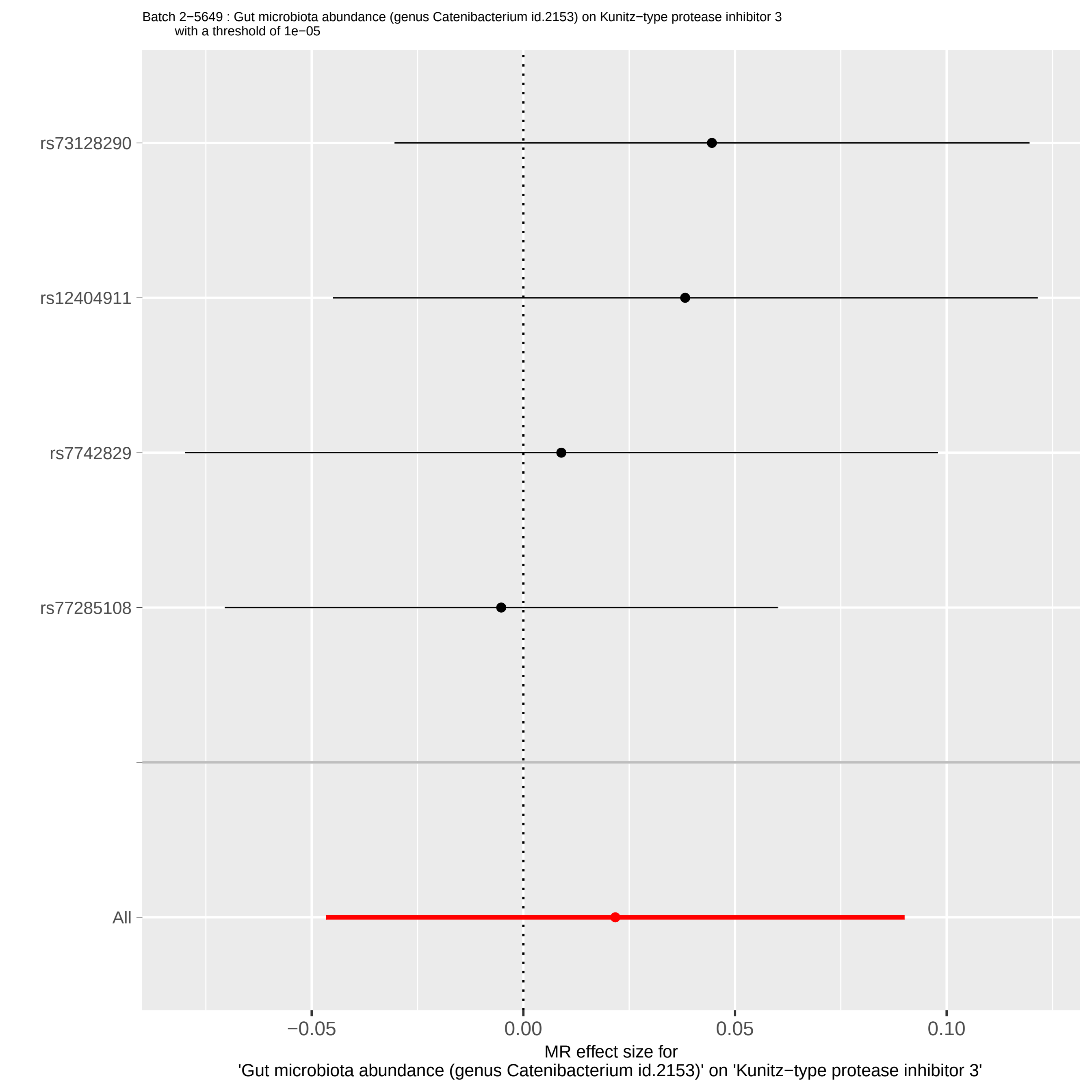
Batch 2−5649 : Gut microbiota abundance (genus Catenibacterium id.2153) on Kunitz−type protease inhibitor 3
with a threshold of 1e−05
rs73128290
rs12404911
rs7742829
rs77285108
All
−0.05
0.00
0.05
0.10
MR effect size for
'Gut microbiota abundance (genus Catenibacterium id.2153)' on 'Kunitz−type protease inhibitor 3'

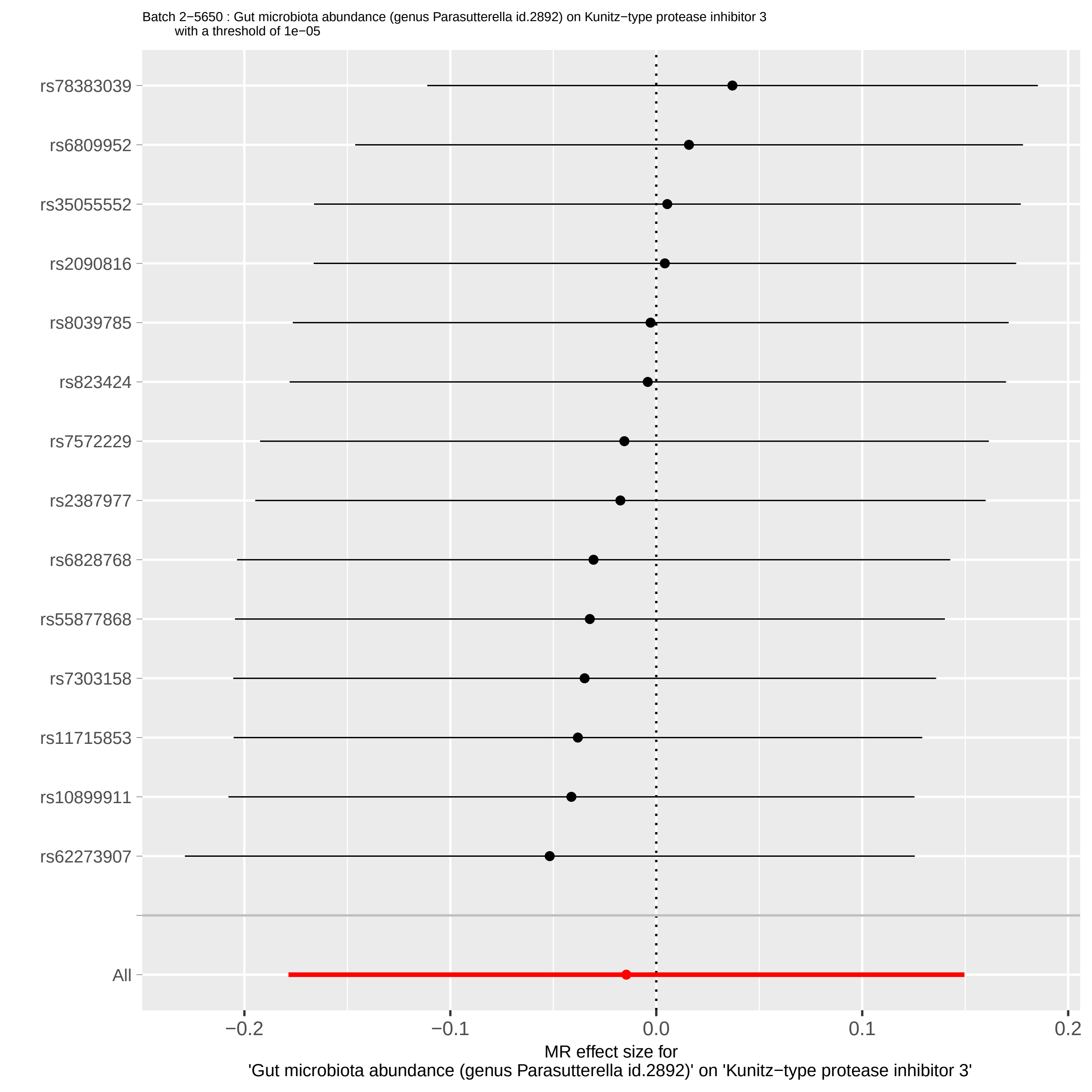
Batch 2−5650 : Gut microbiota abundance (genus Parasutterella id.2892) on Kunitz−type protease inhibitor 3
with a threshold of 1e−05
rs78383039
rs6809952
rs35055552
rs2090816
rs8039785
rs823424
rs7572229
rs2387977
rs6828768
rs55877868
rs7303158
rs11715853
rs10899911
rs62273907
All
−0.2
−0.1
0.0
0.1
0.2
MR effect size for
'Gut microbiota abundance (genus Parasutterella id.2892)' on 'Kunitz−type protease inhibitor 3'

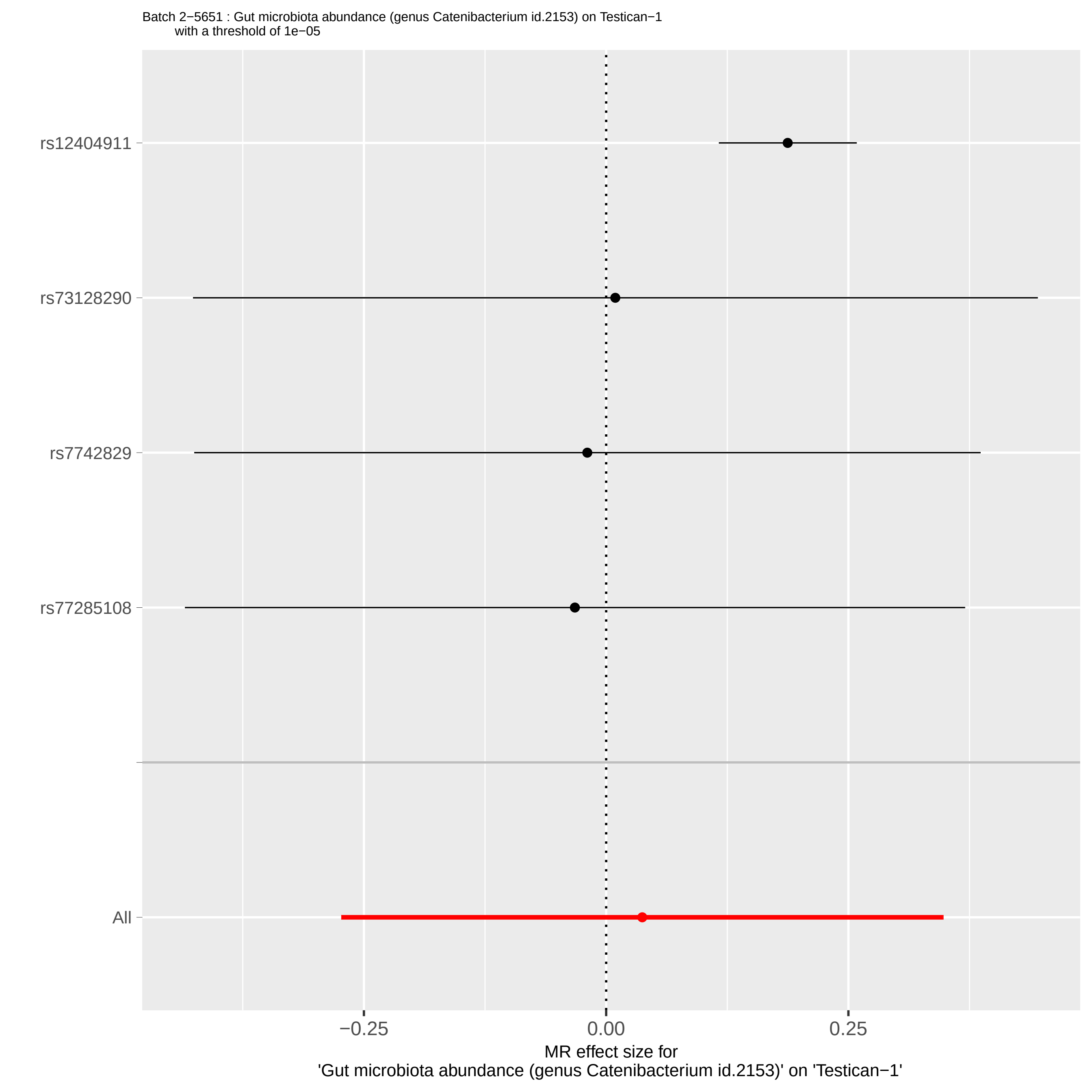
Batch 2−5651 : Gut microbiota abundance (genus Catenibacterium id.2153) on Testican−1
with a threshold of 1e−05
rs12404911
rs73128290
rs7742829
rs77285108
All
−0.25
0.00
0.25
MR effect size for
'Gut microbiota abundance (genus Catenibacterium id.2153)' on 'Testican−1'

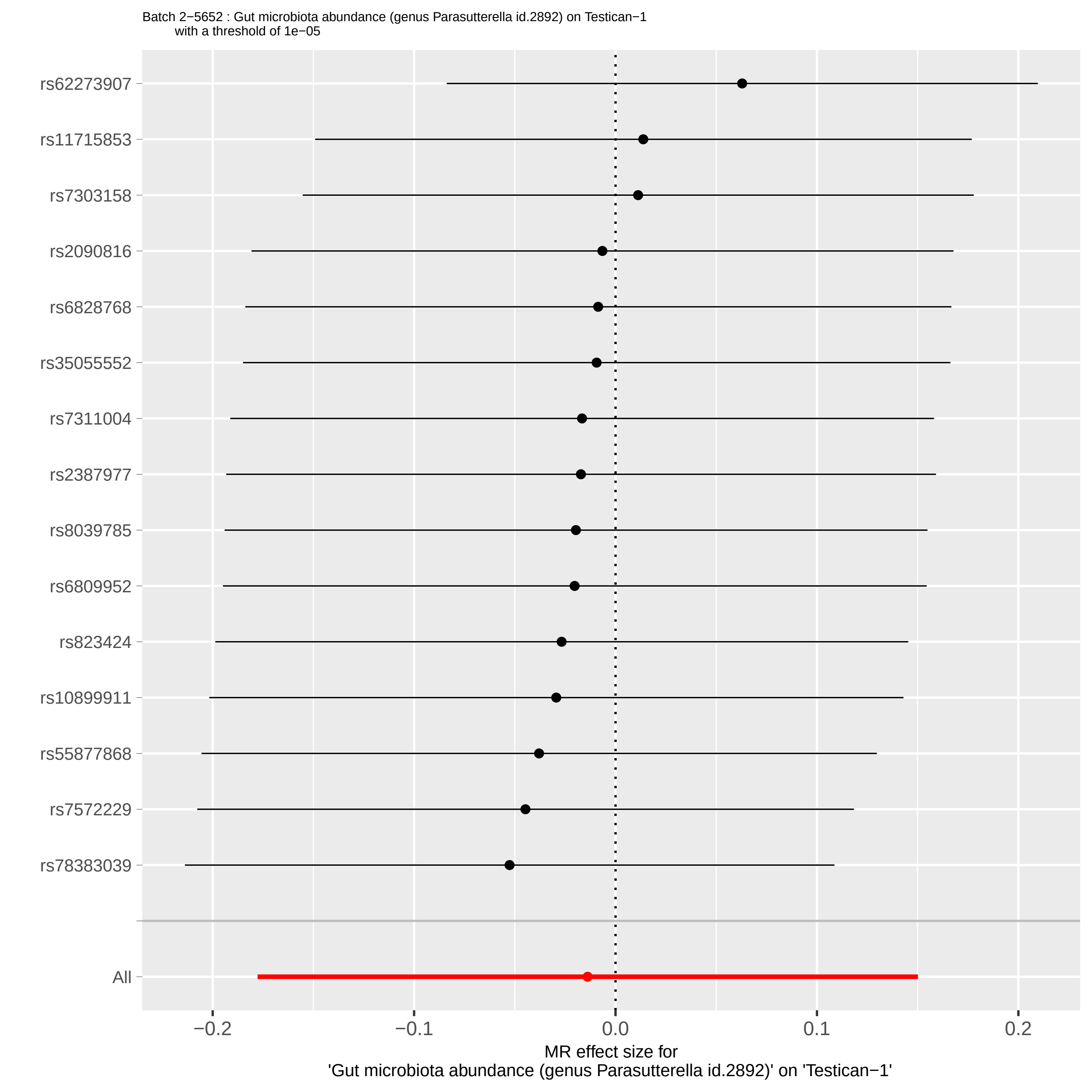

Batch 2−5652 : Gut microbiota abundance (genus Parasutterella id.2892) on Testican−1
with a threshold of 1e−05
rs62273907
rs11715853
rs7303158
rs2090816
rs6828768
rs35055552
rs7311004
rs2387977
rs8039785
rs6809952
rs823424
rs10899911
rs55877868
rs7572229
rs78383039
All
−0.2
−0.1
0.0
0.1
0.2
MR effect size for
'Gut microbiota abundance (genus Parasutterella id.2892)' on 'Testican−1'

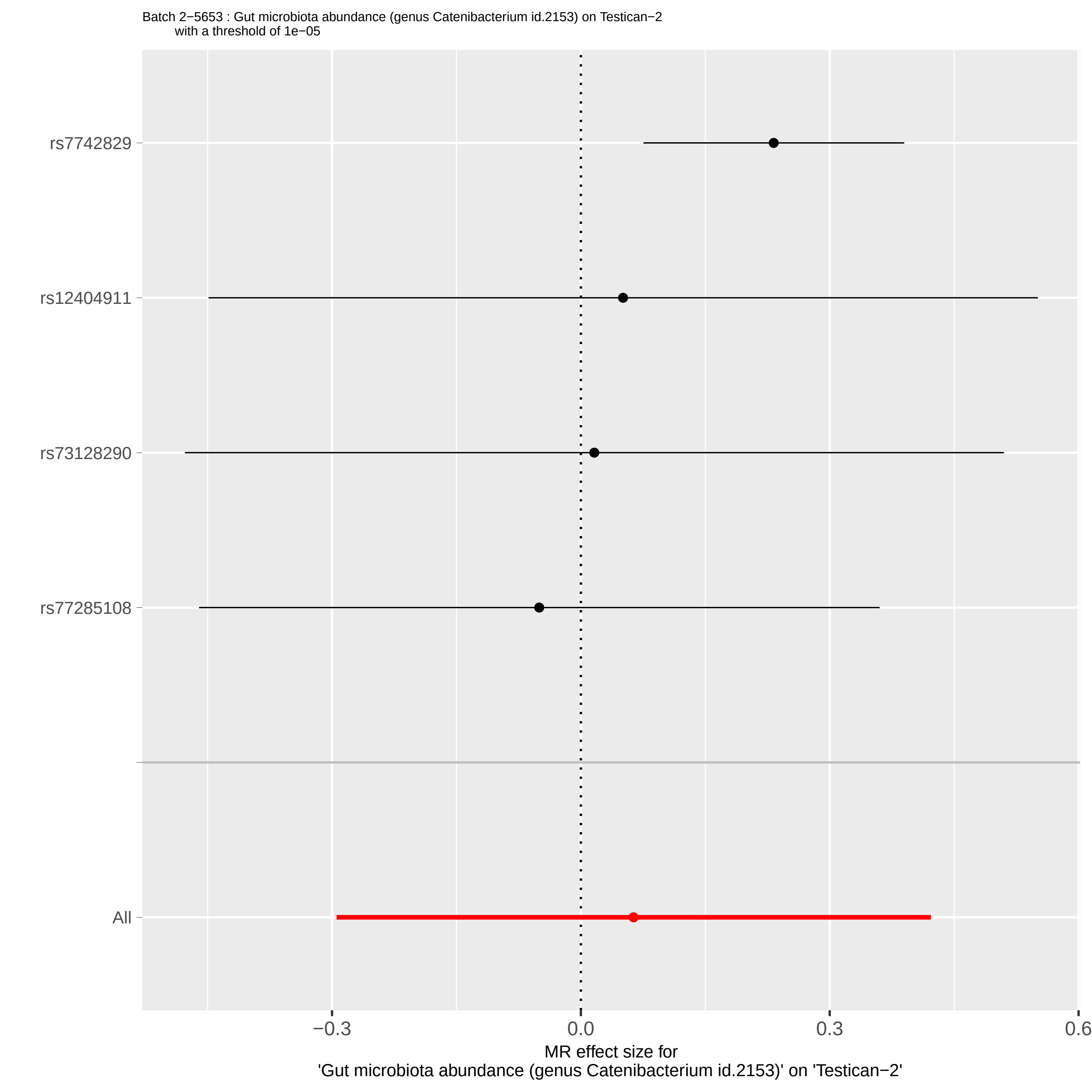

Batch 2−5653 : Gut microbiota abundance (genus Catenibacterium id.2153) on Testican−2
with a threshold of 1e−05
rs7742829
rs12404911
rs73128290
rs77285108
All
−0.3
0.0
0.3
0.6
MR effect size for
'Gut microbiota abundance (genus Catenibacterium id.2153)' on 'Testican−2'

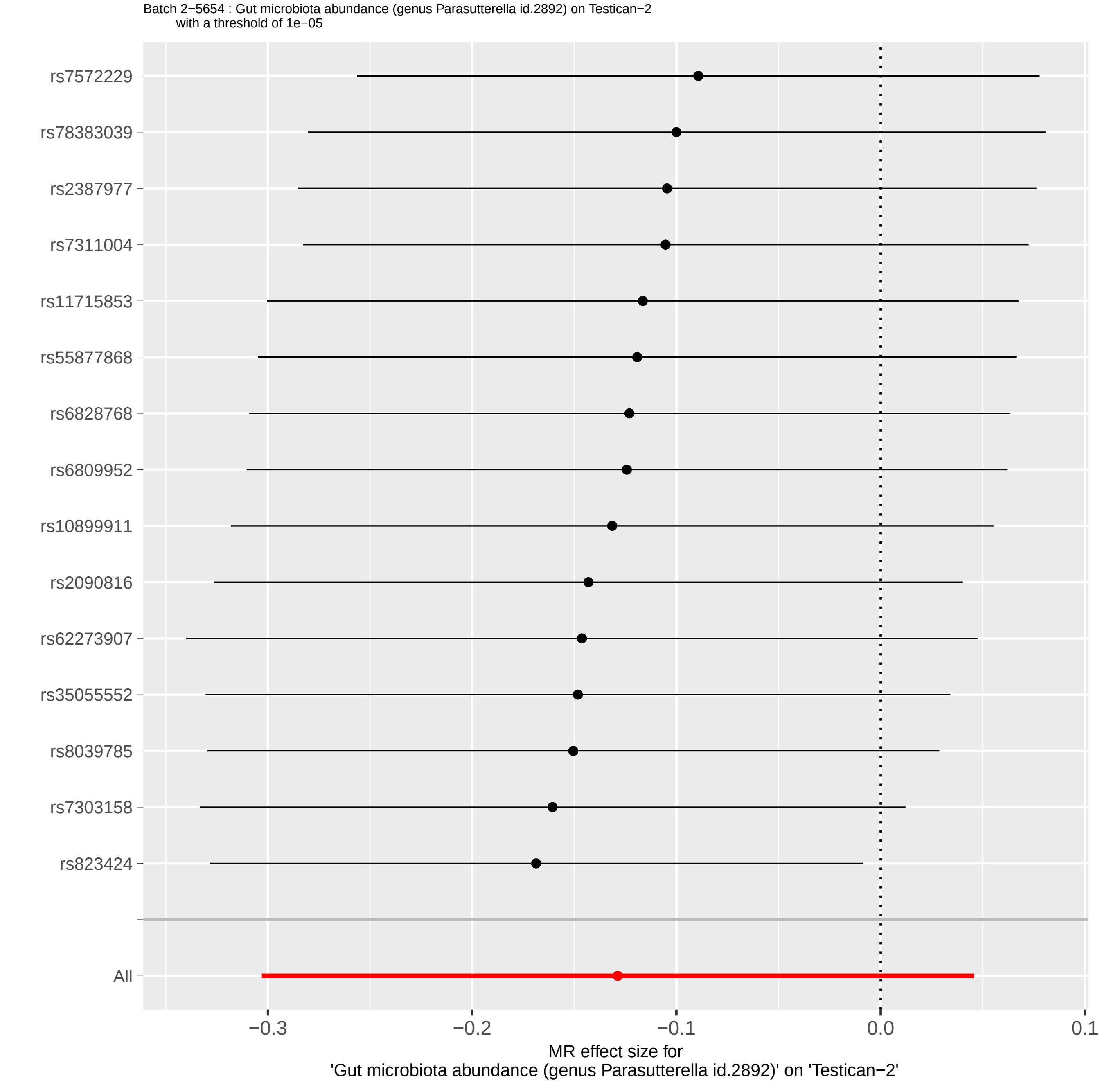

Batch 2−5654 : Gut microbiota abundance (genus Parasutterella id.2892) on Testican−2
with a threshold of 1e−05
rs7572229
rs78383039
rs2387977
rs7311004
rs11715853
rs55877868
rs6828768
rs6809952
rs10899911
rs2090816
rs62273907
rs35055552
rs8039785
rs7303158
rs823424
All
−0.3
−0.2
−0.1
0.0
0.1
MR effect size for
'Gut microbiota abundance (genus Parasutterella id.2892)' on 'Testican−2'

Batch 2−5655 : Gut microbiota abundance (genus Catenibacterium id.2153) on Testican−3
with a threshold of 1e−05

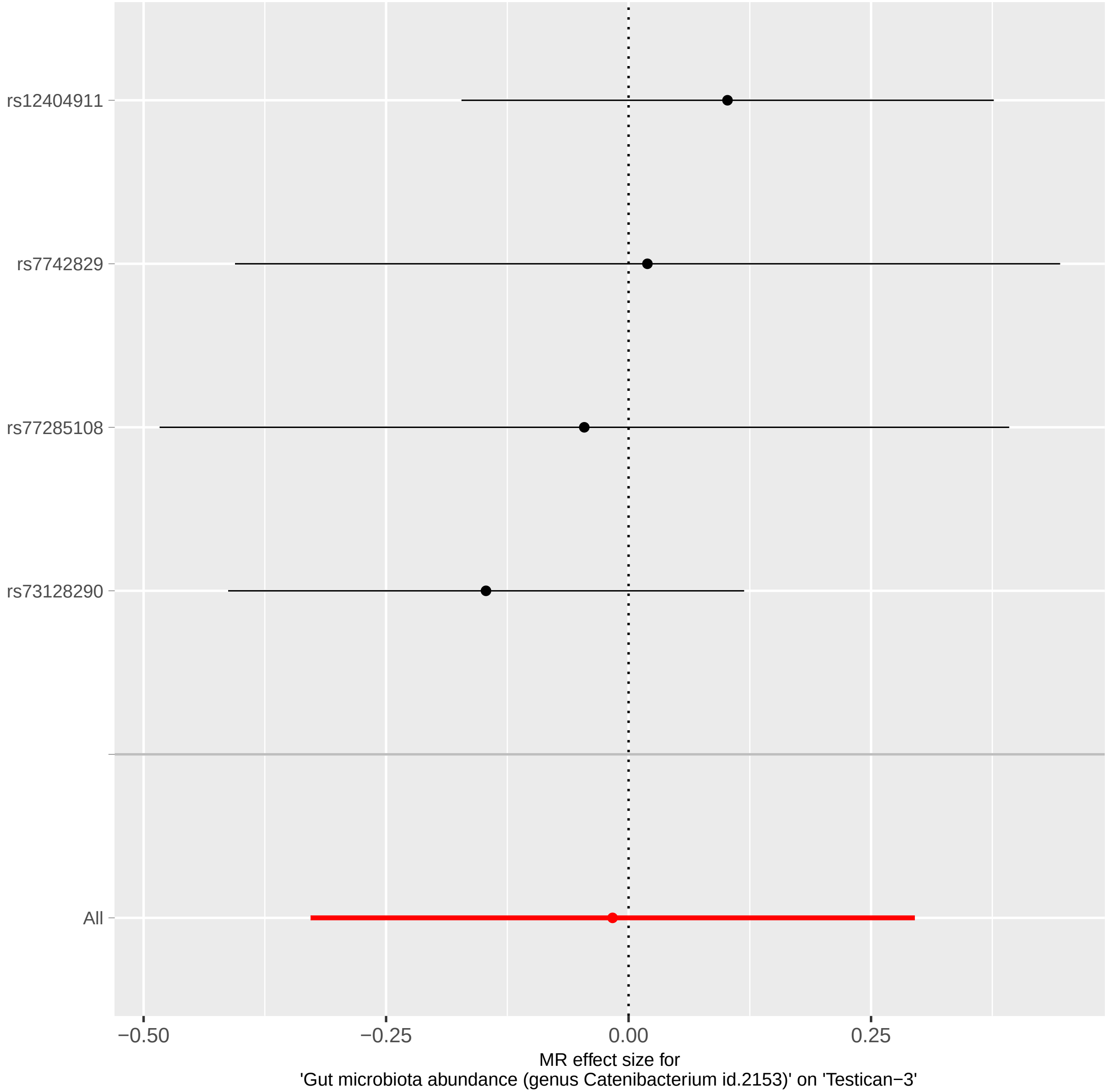

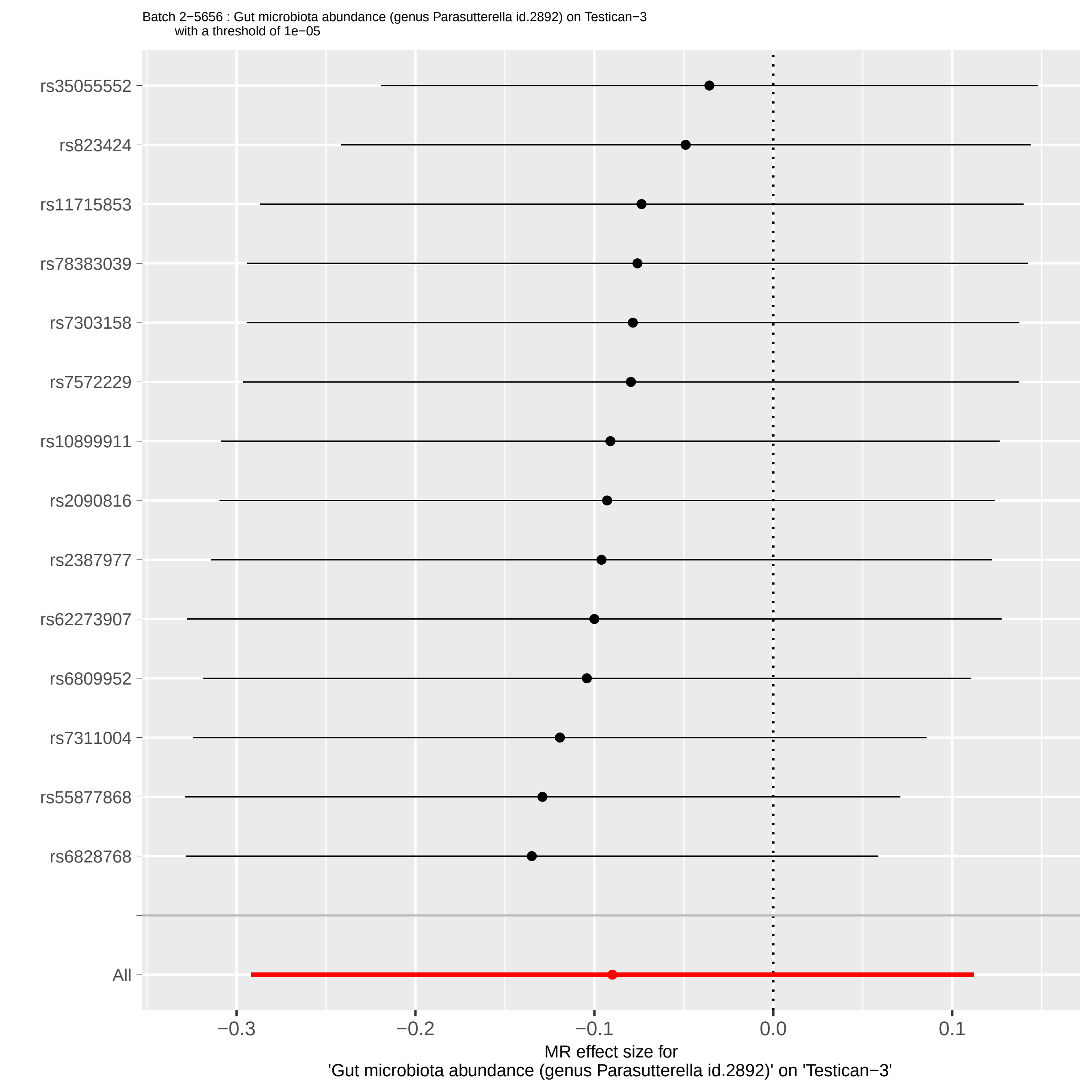
Batch 2−5656 : Gut microbiota abundance (genus Parasutterella id.2892) on Testican−3
with a threshold of 1e−05
rs35055552
rs823424
rs11715853
rs78383039
rs7303158
rs7572229
rs10899911
rs2090816
rs2387977
rs62273907
rs6809952
rs7311004
rs55877868
rs6828768
All
−0.3
−0.2
−0.1
0.0
0.1
MR effect size for
'Gut microbiota abundance (genus Parasutterella id.2892)' on 'Testican−3'

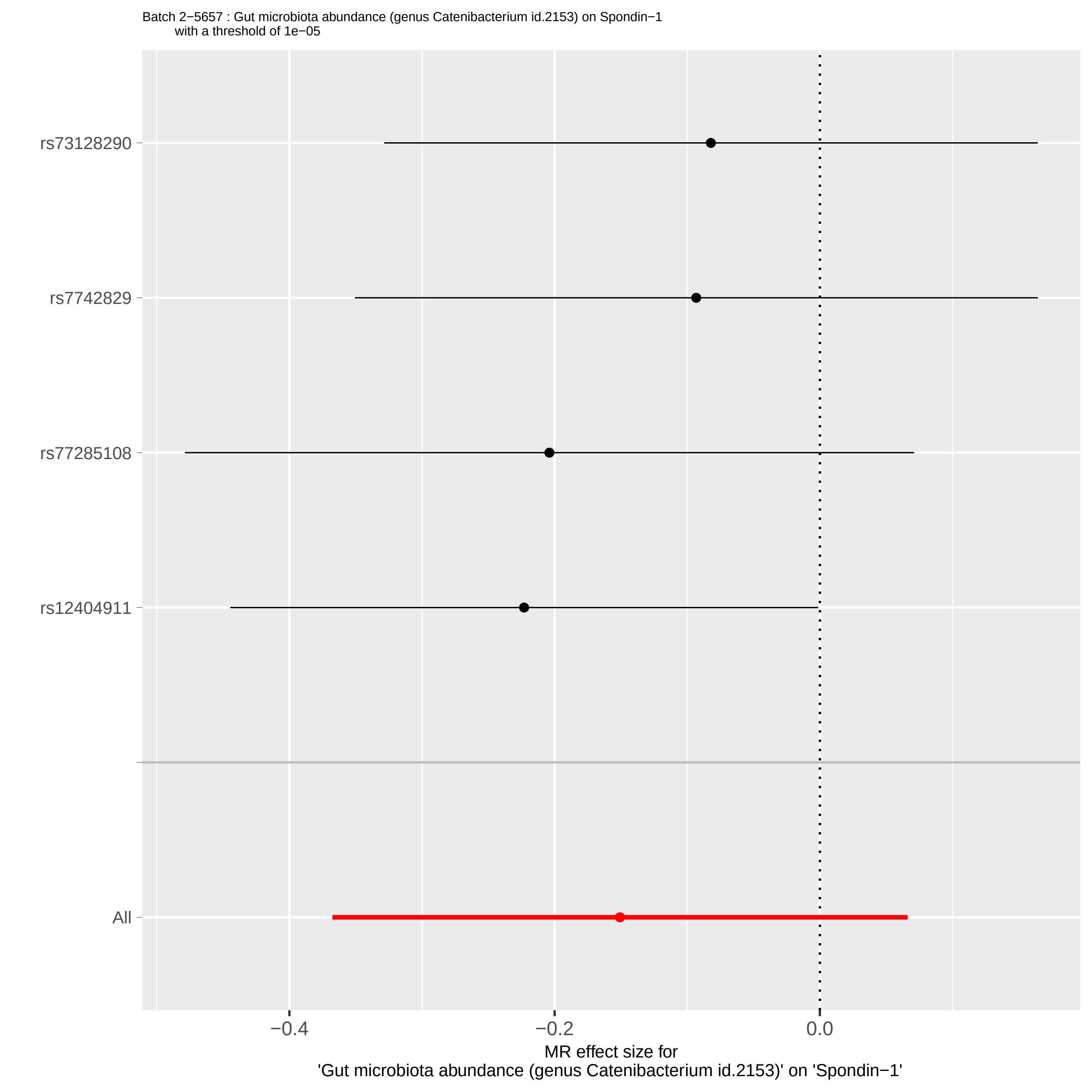
Batch 2−5657 : Gut microbiota abundance (genus Catenibacterium id.2153) on Spondin−1
with a threshold of 1e−05
rs73128290
rs7742829
rs77285108
rs12404911
All
−0.4
−0.2
0.0
MR effect size for
'Gut microbiota abundance (genus Catenibacterium id.2153)' on 'Spondin−1'

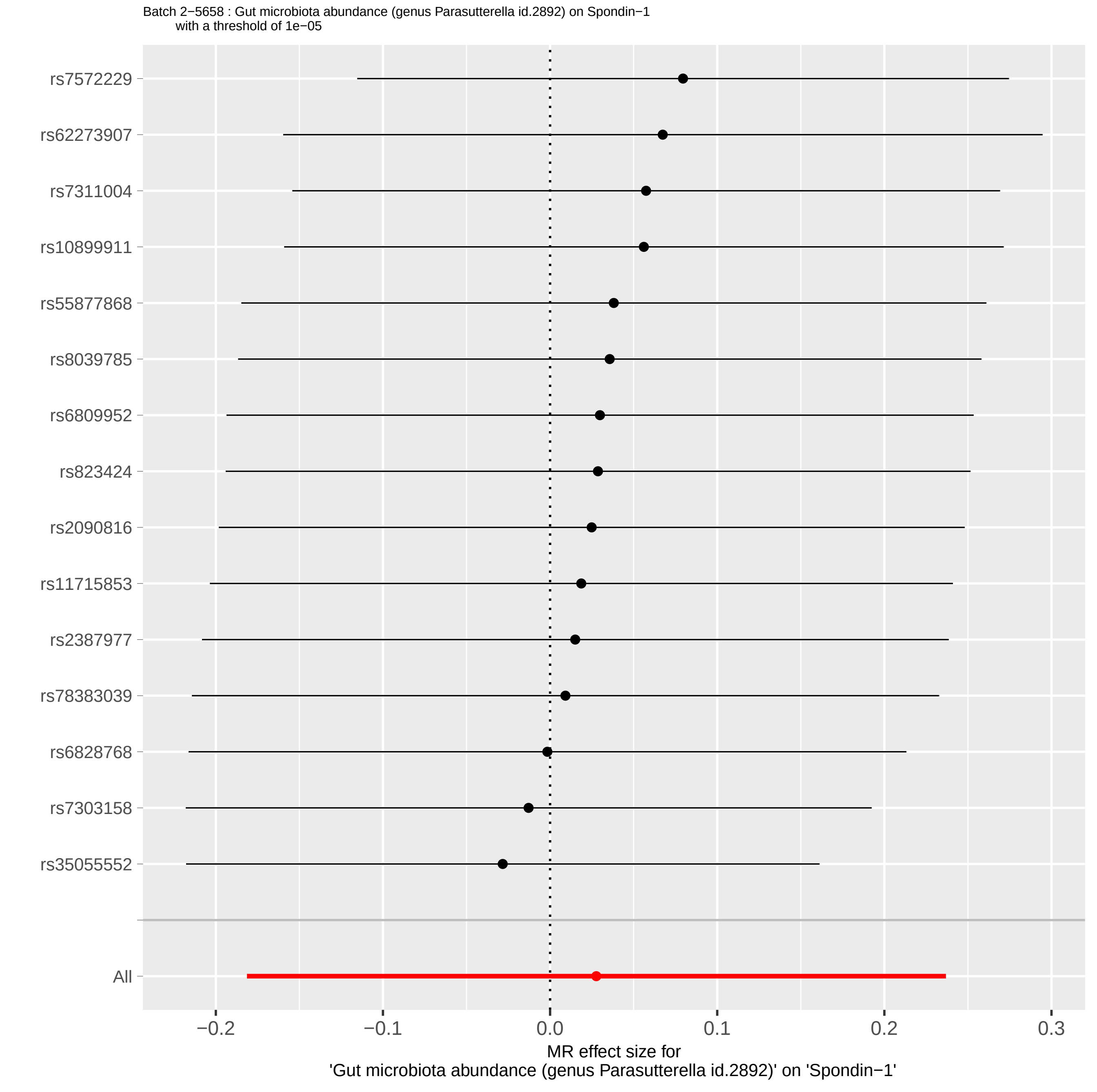

Batch 2−5658 : Gut microbiota abundance (genus Parasutterella id.2892) on Spondin−1
with a threshold of 1e−05
rs7572229
rs62273907
rs7311004
rs10899911
rs55877868
rs8039785
rs6809952
rs823424
rs2090816
rs11715853
rs2387977
rs78383039
rs6828768
rs7303158
rs35055552
All
−0.2
−0.1
0.0
0.1
0.2
0.3
MR effect size for
'Gut microbiota abundance (genus Parasutterella id.2892)' on 'Spondin−1'

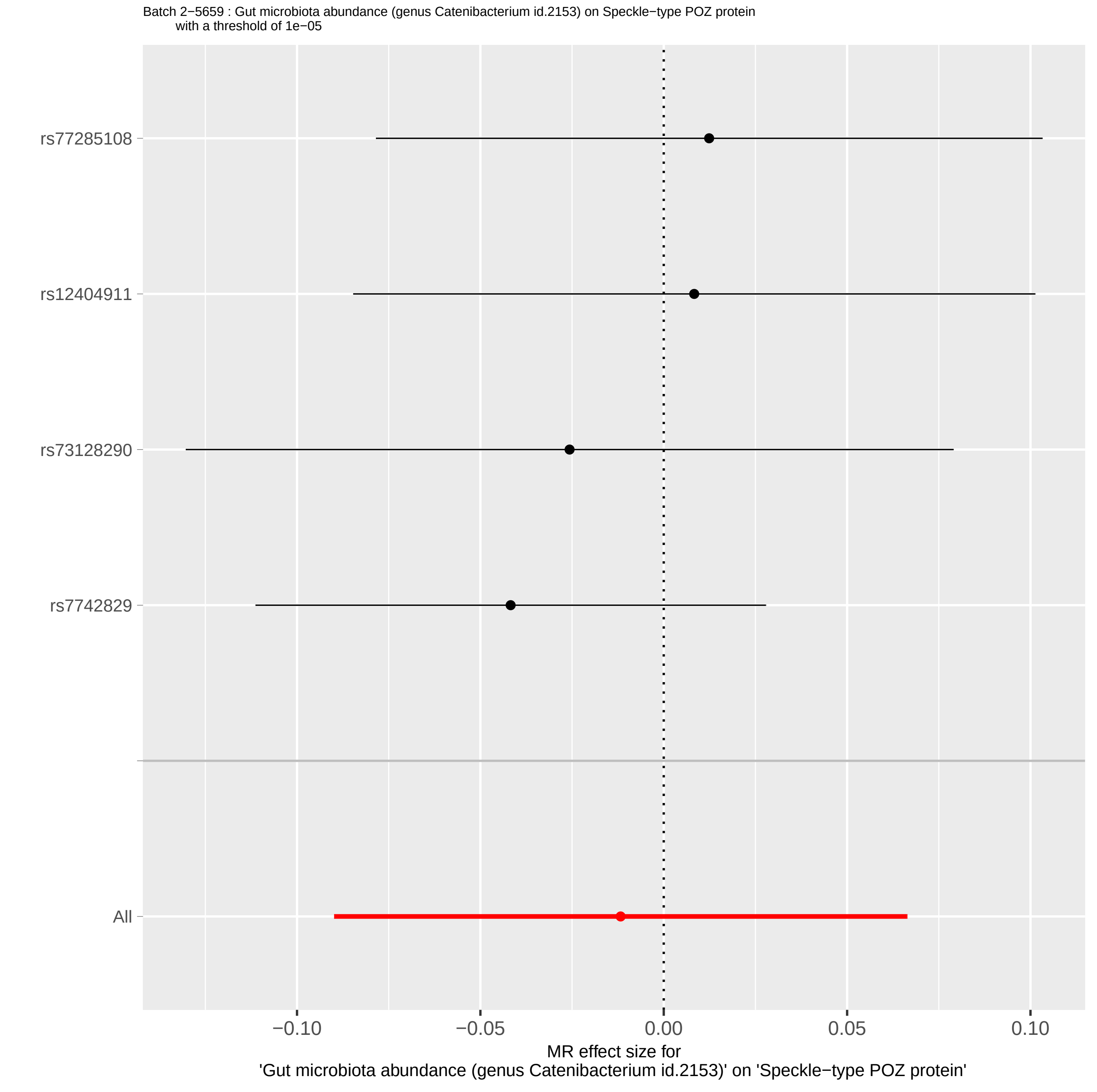
Batch 2−5659 : Gut microbiota abundance (genus Catenibacterium id.2153) on Speckle−type POZ protein
with a threshold of 1e−05
rs77285108
rs12404911
rs73128290
rs7742829
All
−0.10
−0.05
0.00
0.05
0.10
MR effect size for
'Gut microbiota abundance (genus Catenibacterium id.2153)' on 'Speckle−type POZ protein'

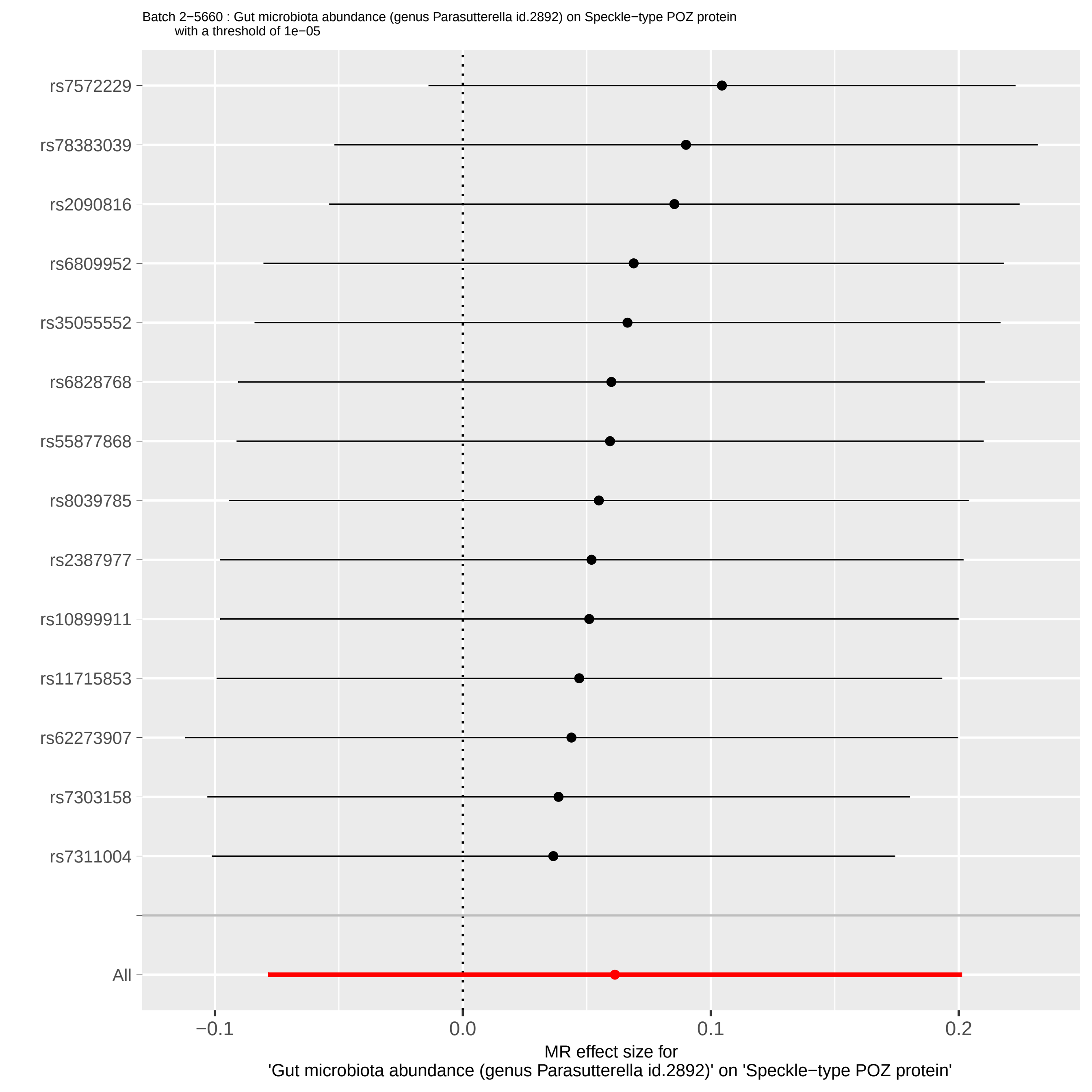

Batch 2−5660 : Gut microbiota abundance (genus Parasutterella id.2892) on Speckle−type POZ protein
with a threshold of 1e−05
rs7572229
rs78383039
rs2090816
rs6809952
rs35055552
rs6828768
rs55877868
rs8039785
rs2387977
rs10899911
rs11715853
rs62273907
rs7303158
rs7311004
All
−0.1
0.0
0.1
0.2
MR effect size for
'Gut microbiota abundance (genus Parasutterella id.2892)' on 'Speckle−type POZ protein'

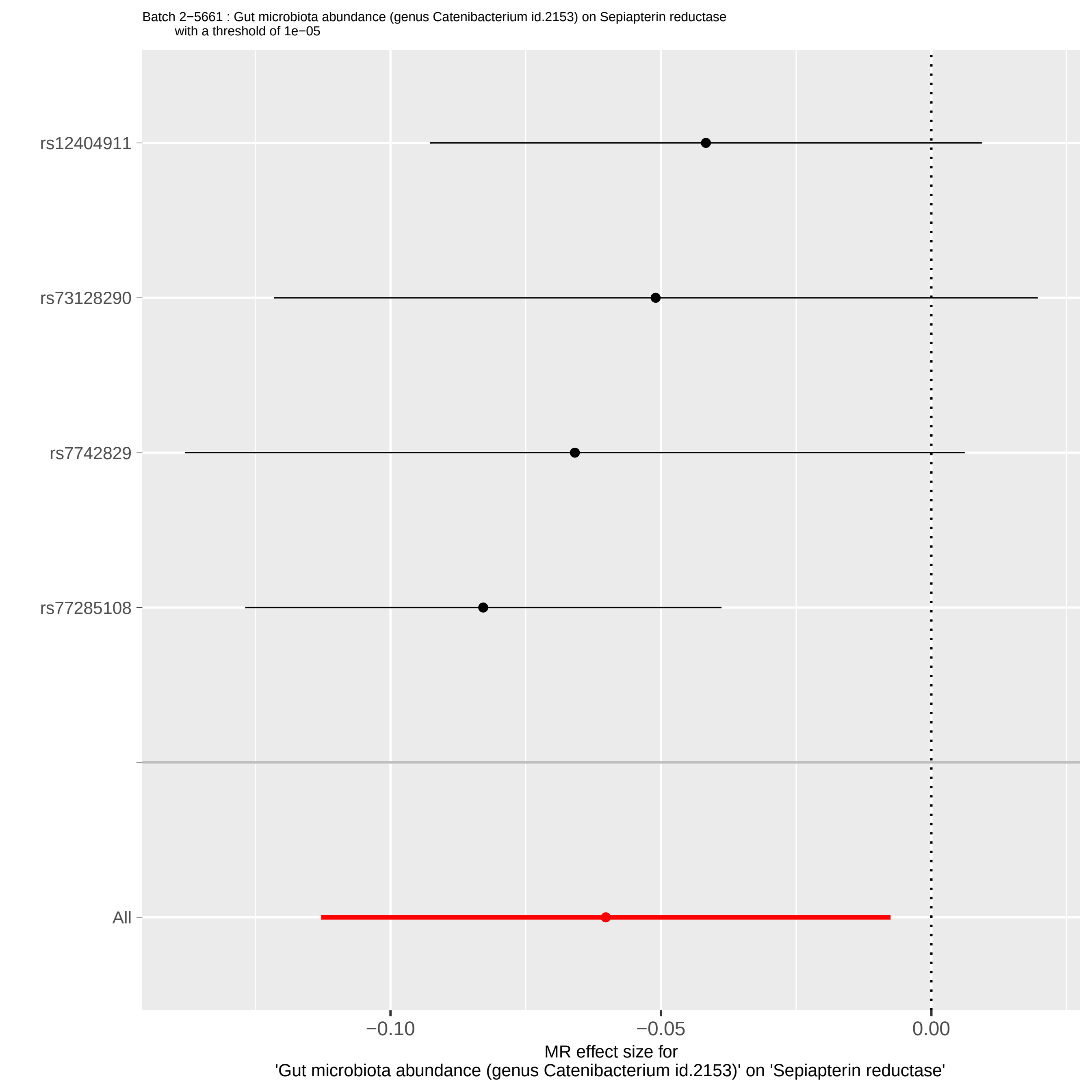
Batch 2−5661 : Gut microbiota abundance (genus Catenibacterium id.2153) on Sepiapterin reductase
with a threshold of 1e−05
rs12404911
rs73128290
rs7742829
rs77285108
All
−0.10
−0.05
0.00
MR effect size for
'Gut microbiota abundance (genus Catenibacterium id.2153)' on 'Sepiapterin reductase'

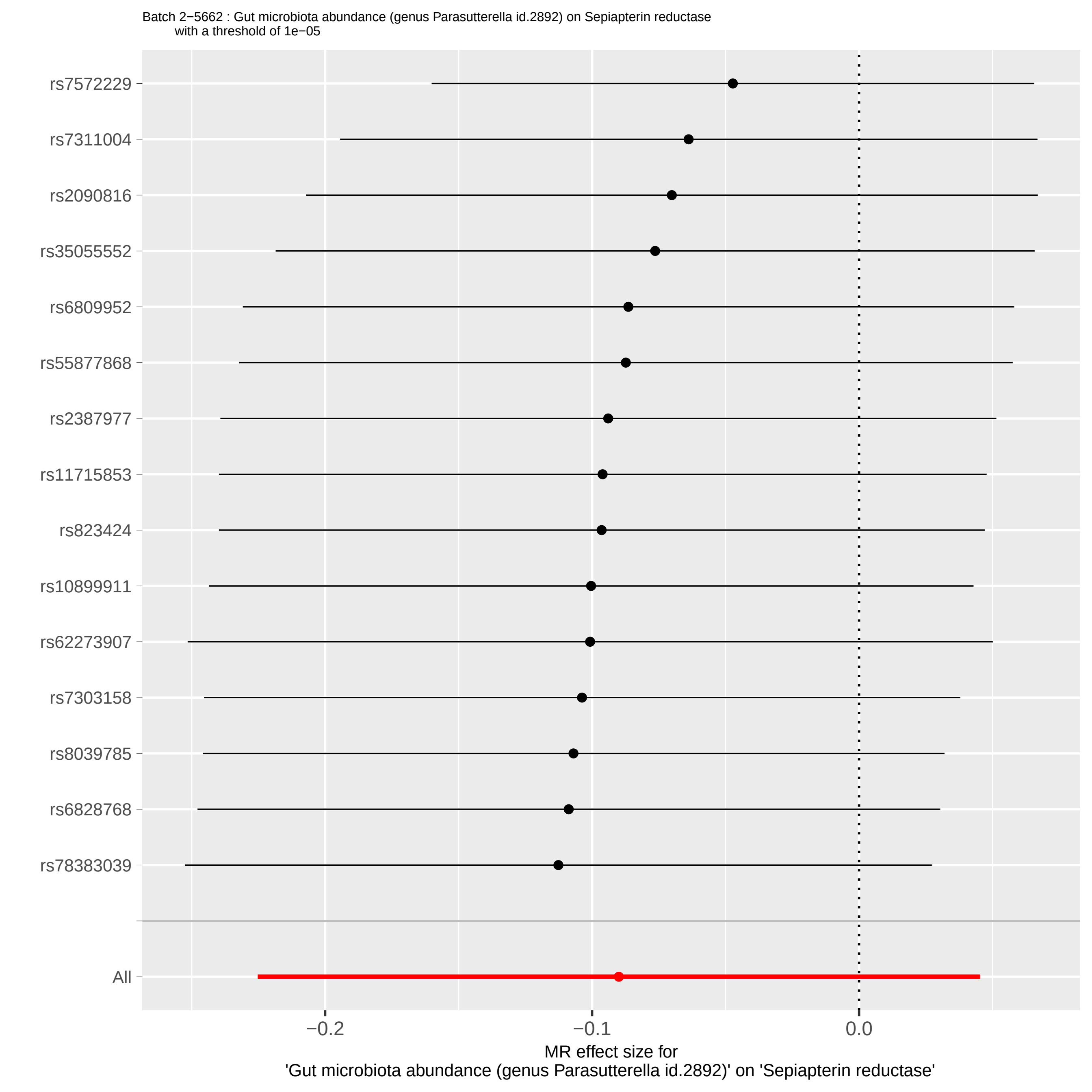

Batch 2−5662 : Gut microbiota abundance (genus Parasutterella id.2892) on Sepiapterin reductase
with a threshold of 1e−05
rs7572229
rs7311004
rs2090816
rs35055552
rs6809952
rs55877868
rs2387977
rs11715853
rs823424
rs10899911
rs62273907
rs7303158
rs8039785
rs6828768
rs78383039
All
−0.2
−0.1
0.0
MR effect size for
'Gut microbiota abundance (genus Parasutterella id.2892)' on 'Sepiapterin reductase'

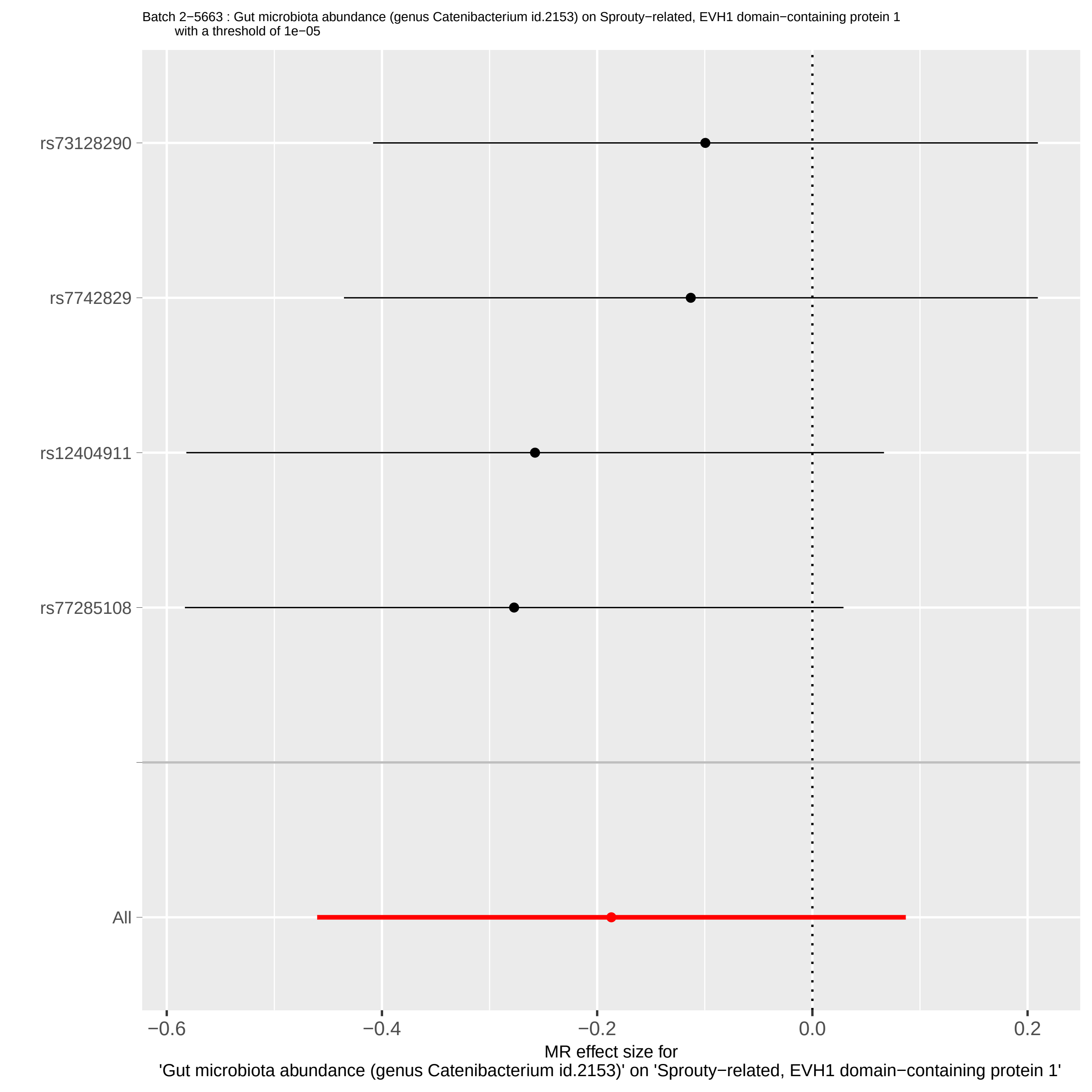

Batch 2−5663 : Gut microbiota abundance (genus Catenibacterium id.2153) on Sprouty−related, EVH1 domain−containing protein 1
with a threshold of 1e−05
rs73128290
rs7742829
rs12404911
rs77285108
All
−0.6
−0.4
−0.2
0.0
0.2
MR effect size for
'Gut microbiota abundance (genus Catenibacterium id.2153)' on 'Sprouty−related, EVH1 domain−containing protein 1'

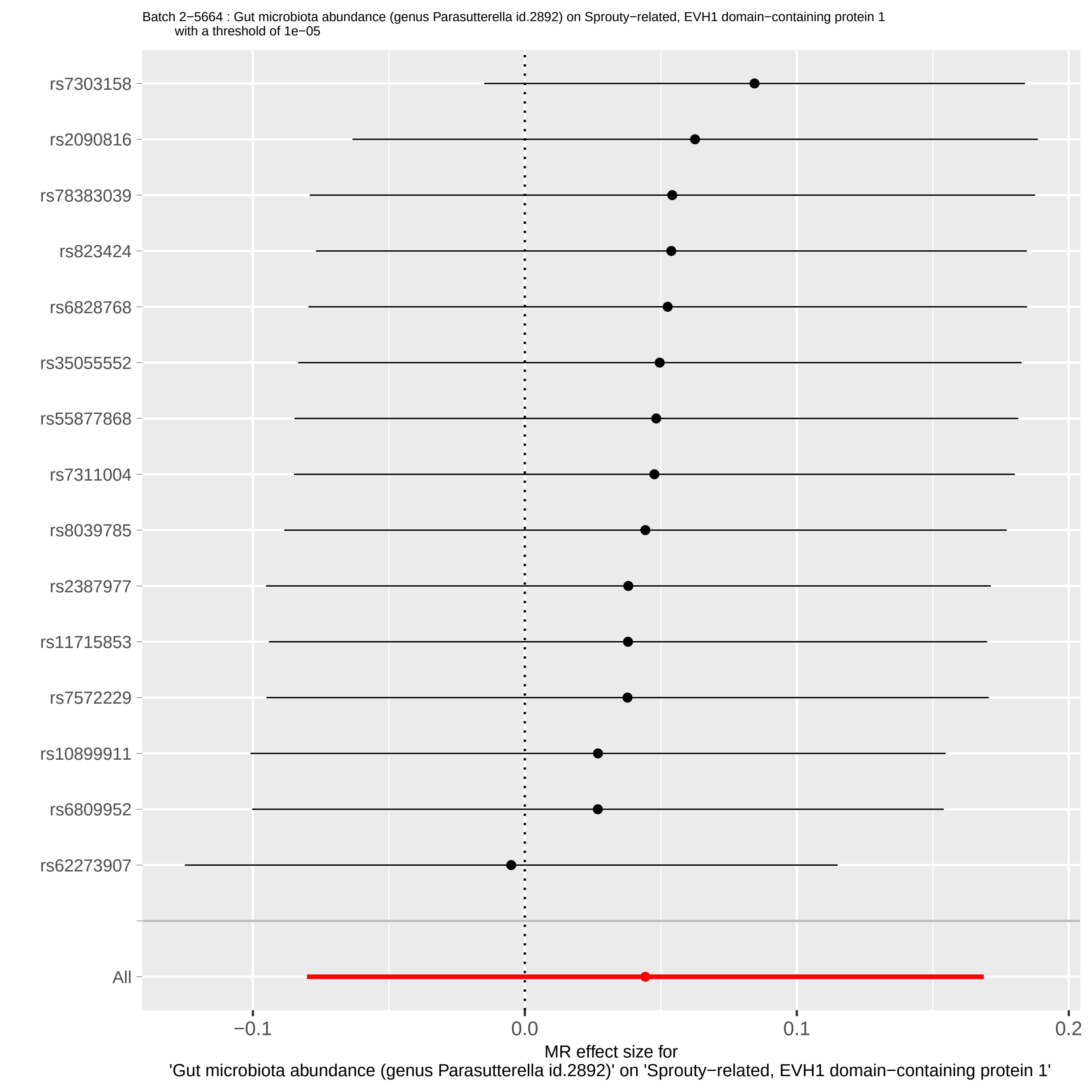

Batch 2−5664 : Gut microbiota abundance (genus Parasutterella id.2892) on Sprouty−related, EVH1 domain−containing protein 1
with a threshold of 1e−05
rs7303158
rs2090816
rs78383039
rs823424
rs6828768
rs35055552
rs55877868
rs7311004
rs8039785
rs2387977
rs11715853
rs7572229
rs10899911
rs6809952
rs62273907
All
−0.1
0.0
0.1
0.2
MR effect size for
'Gut microbiota abundance (genus Parasutterella id.2892)' on 'Sprouty−related, EVH1 domain−containing protein 1'

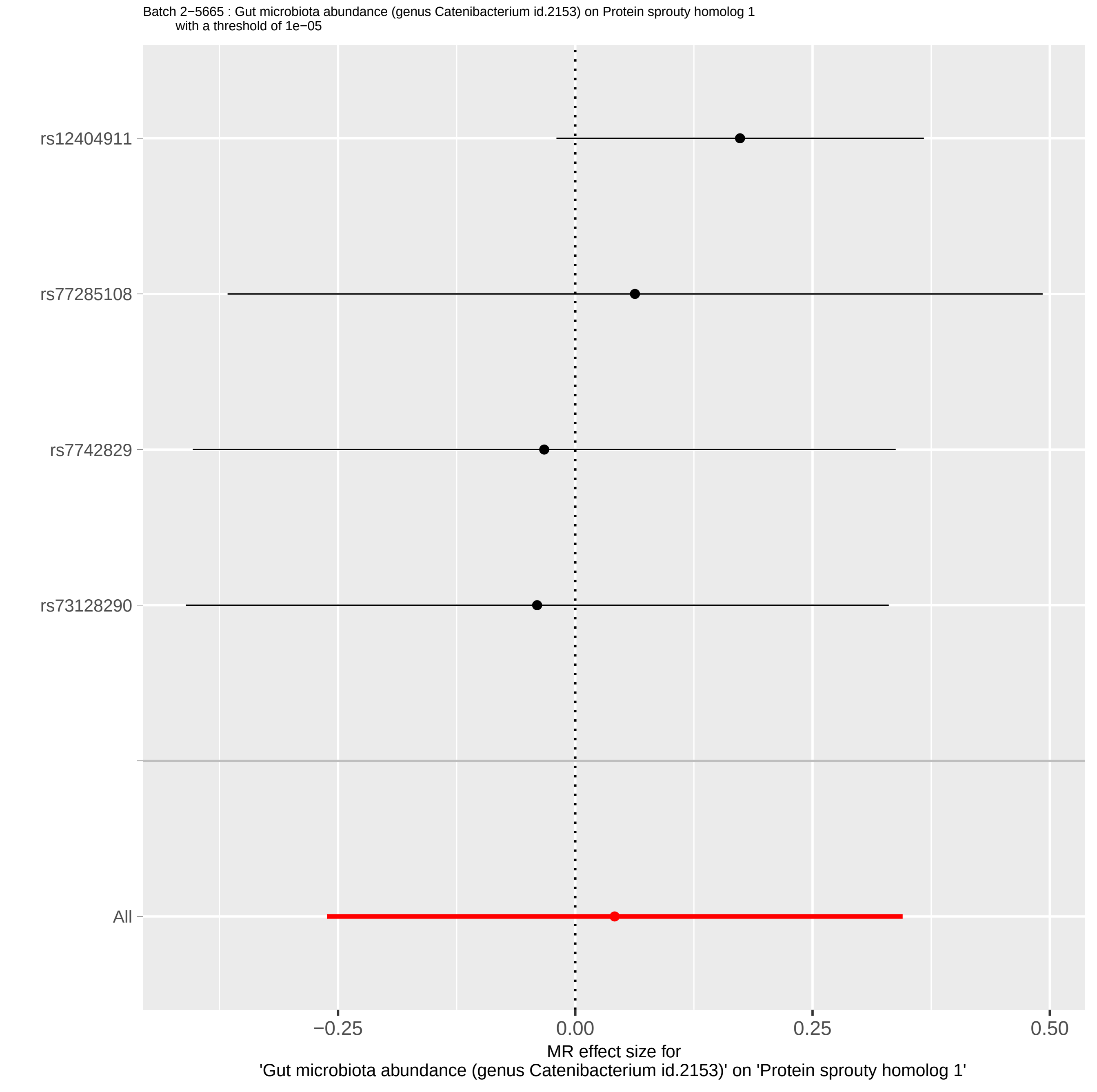

Batch 2−5665 : Gut microbiota abundance (genus Catenibacterium id.2153) on Protein sprouty homolog 1
with a threshold of 1e−05
rs12404911
rs77285108
rs7742829
rs73128290
All
−0.25
0.00
0.25
0.50
MR effect size for
'Gut microbiota abundance (genus Catenibacterium id.2153)' on 'Protein sprouty homolog 1'

Batch 2−5666 : Gut microbiota abundance (genus Parasutterella id.2892) on Protein sprouty homolog 1
with a threshold of 1e−05

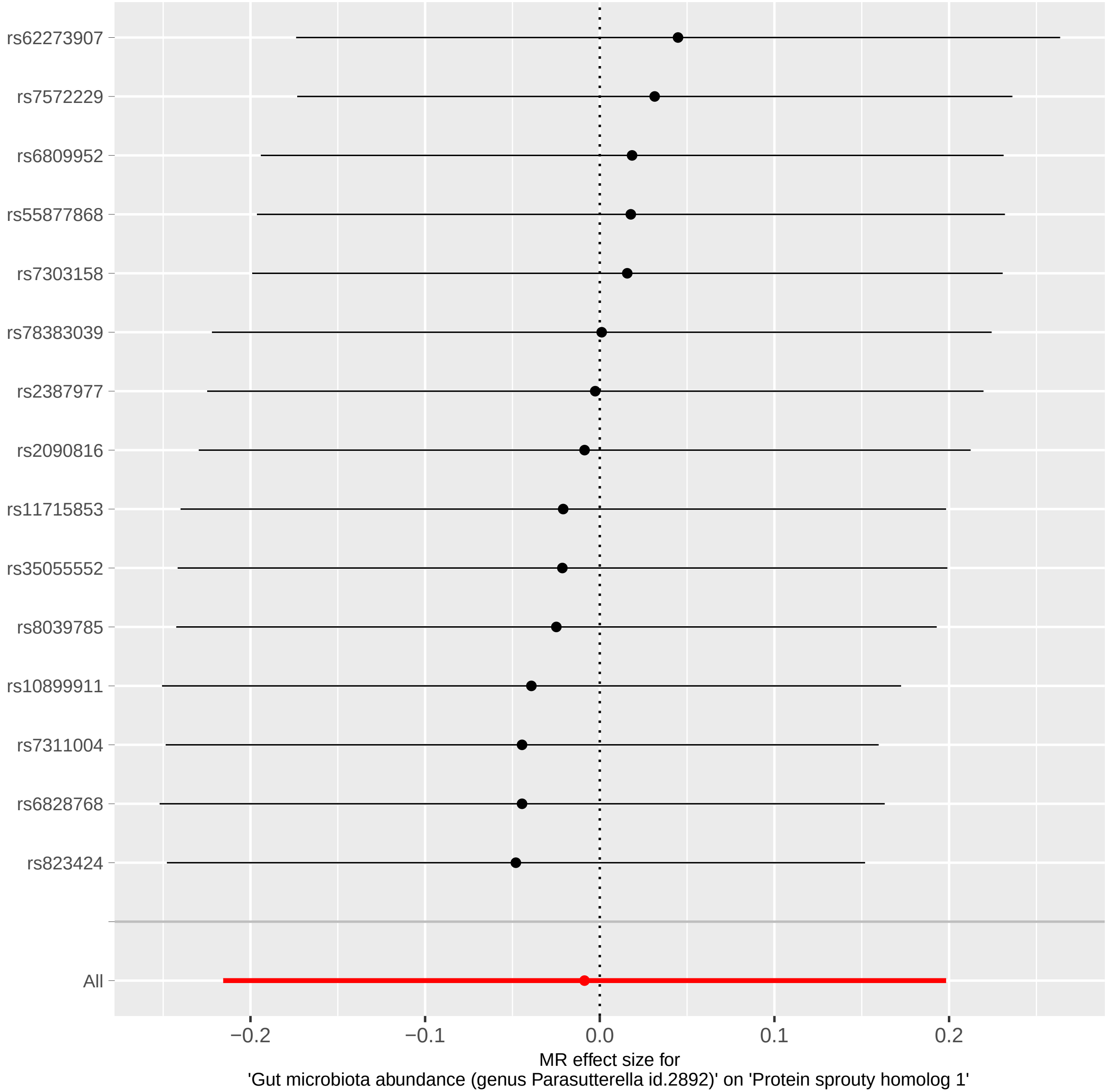

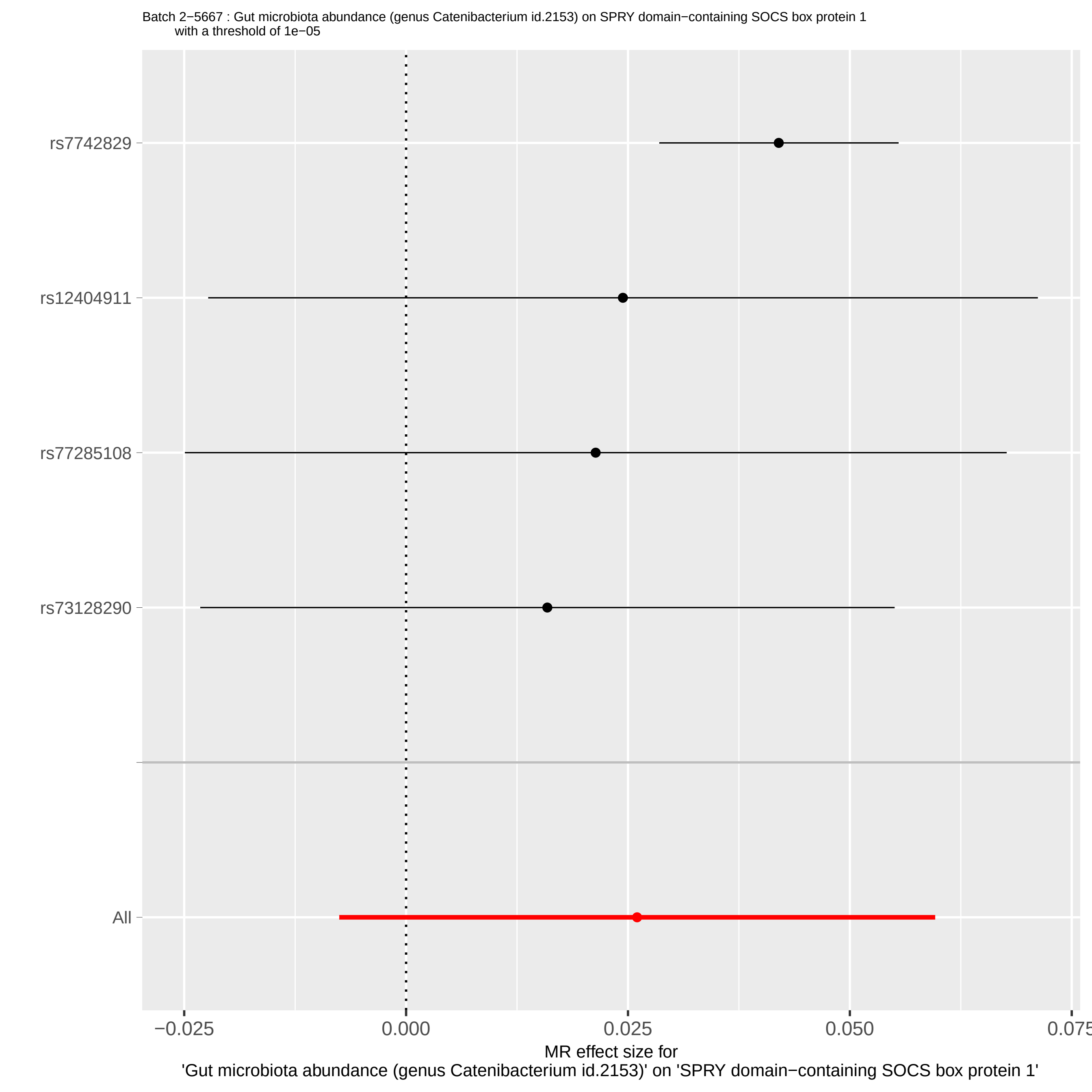
Batch 2−5667 : Gut microbiota abundance (genus Catenibacterium id.2153) on SPRY domain−containing SOCS box protein 1
with a threshold of 1e−05
rs7742829
rs12404911
rs77285108
rs73128290
All
−0.025
0.000
0.025
0.050
0.075
MR effect size for
'Gut microbiota abundance (genus Catenibacterium id.2153)' on 'SPRY domain−containing SOCS box protein 1'

Batch 2−5668 : Gut microbiota abundance (genus Parasutterella id.2892) on SPRY domain−containing SOCS box protein 1
with a threshold of 1e−05

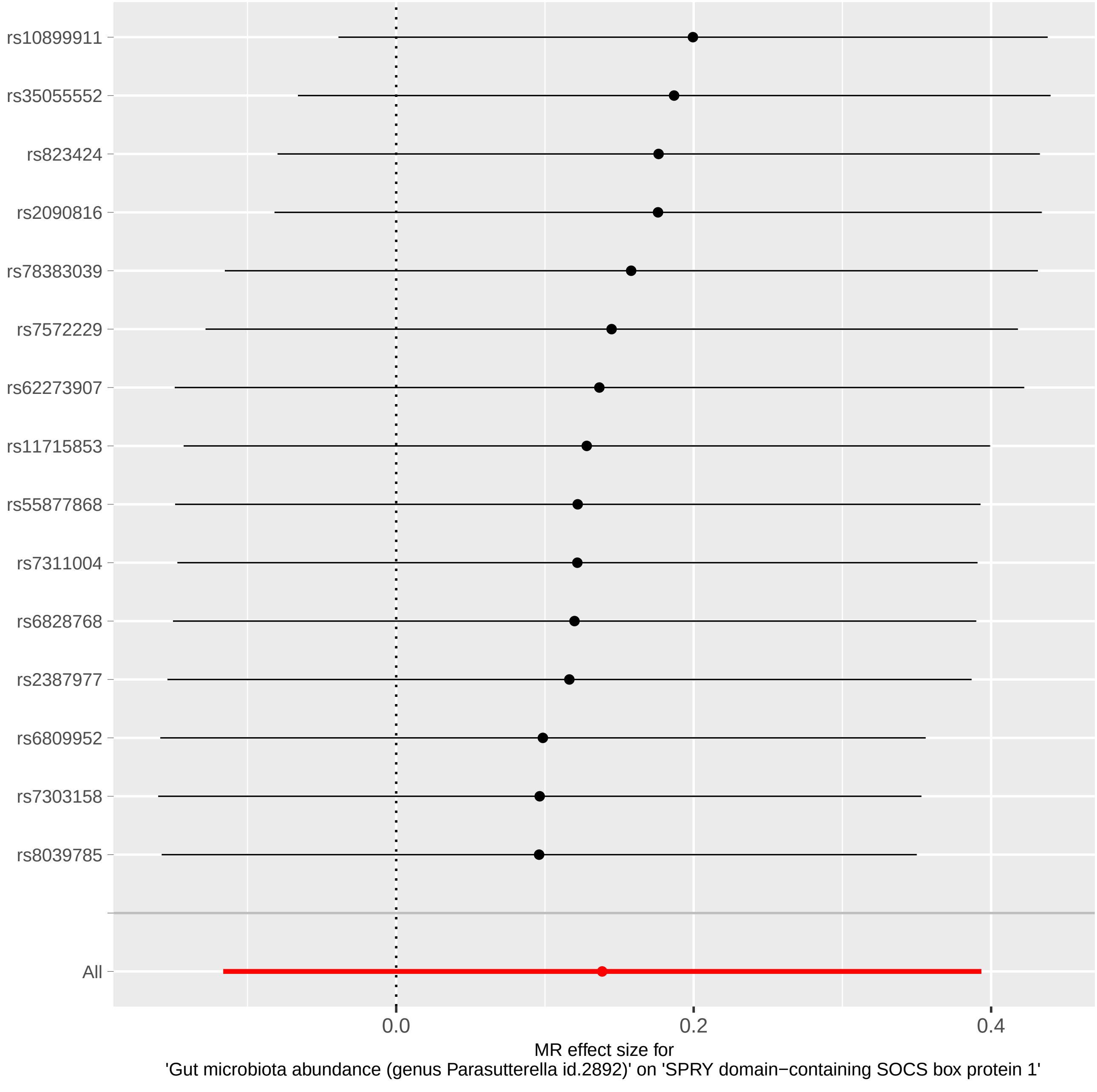

Batch 2−5669 : Gut microbiota abundance (genus Catenibacterium id.2153) on Spectrin alpha chain, non−erythrocytic 1
with a threshold of 1e−05

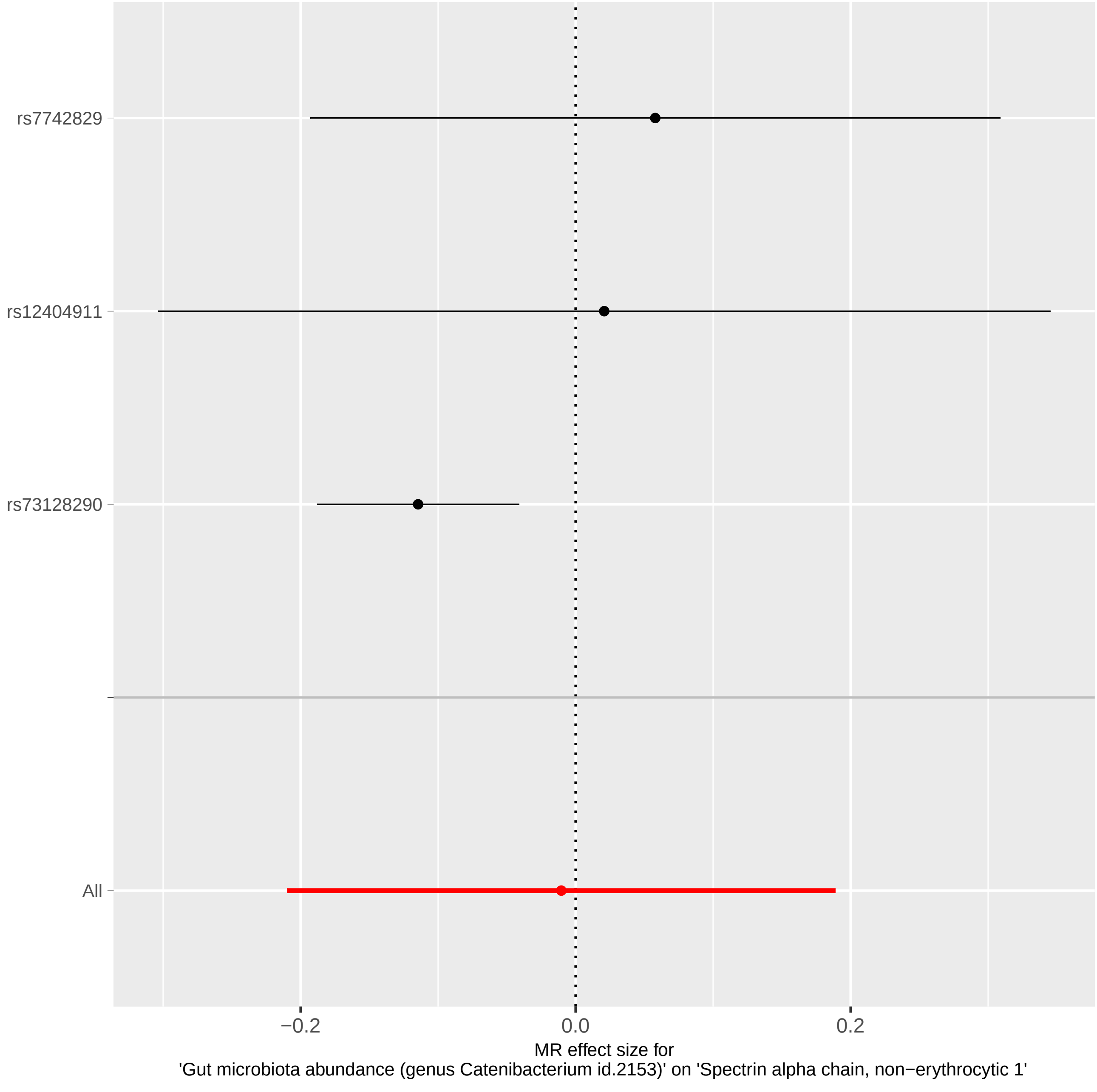

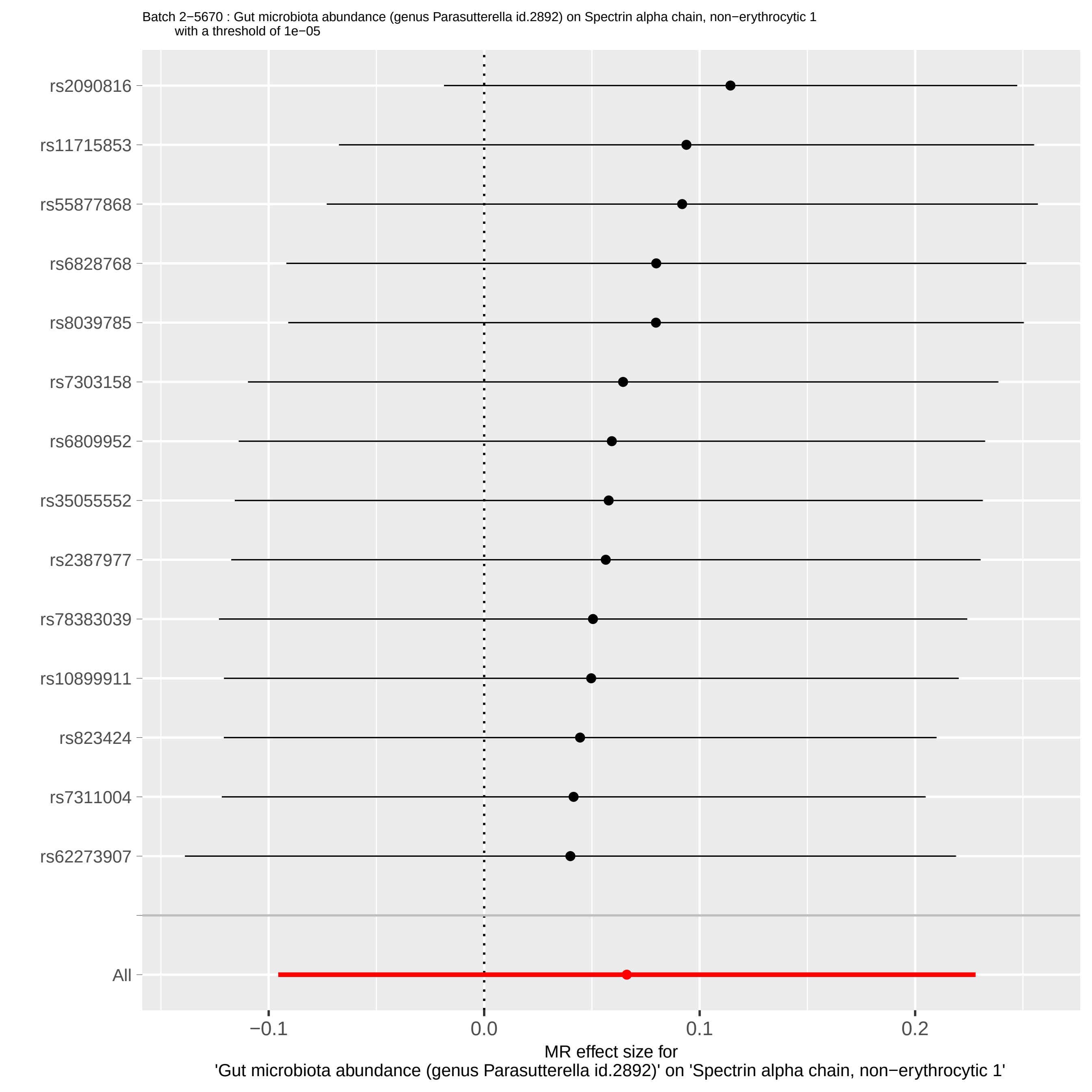
Batch 2−5670 : Gut microbiota abundance (genus Parasutterella id.2892) on Spectrin alpha chain, non−erythrocytic 1
with a threshold of 1e−05
rs2090816
rs11715853
rs55877868
rs6828768
rs8039785
rs7303158
rs6809952
rs35055552
rs2387977
rs78383039
rs10899911
rs823424
rs7311004
rs62273907
All
−0.1
0.0
0.1
0.2
MR effect size for
'Gut microbiota abundance (genus Parasutterella id.2892)' on 'Spectrin alpha chain, non−erythrocytic 1'

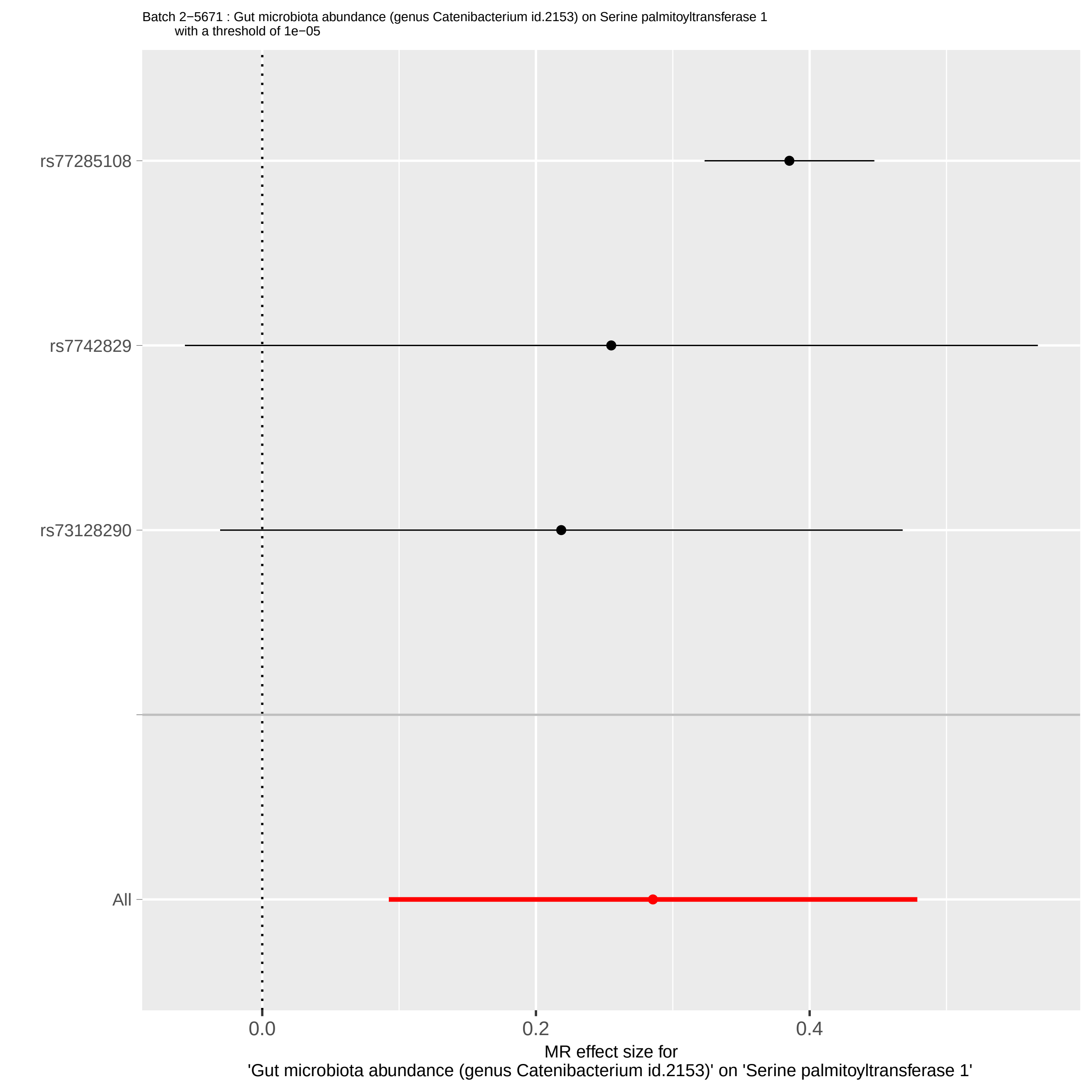

Batch 2−5671 : Gut microbiota abundance (genus Catenibacterium id.2153) on Serine palmitoyltransferase 1
with a threshold of 1e−05
rs77285108
rs7742829
rs73128290
All
0.0
0.2
0.4
MR effect size for
'Gut microbiota abundance (genus Catenibacterium id.2153)' on 'Serine palmitoyltransferase 1'

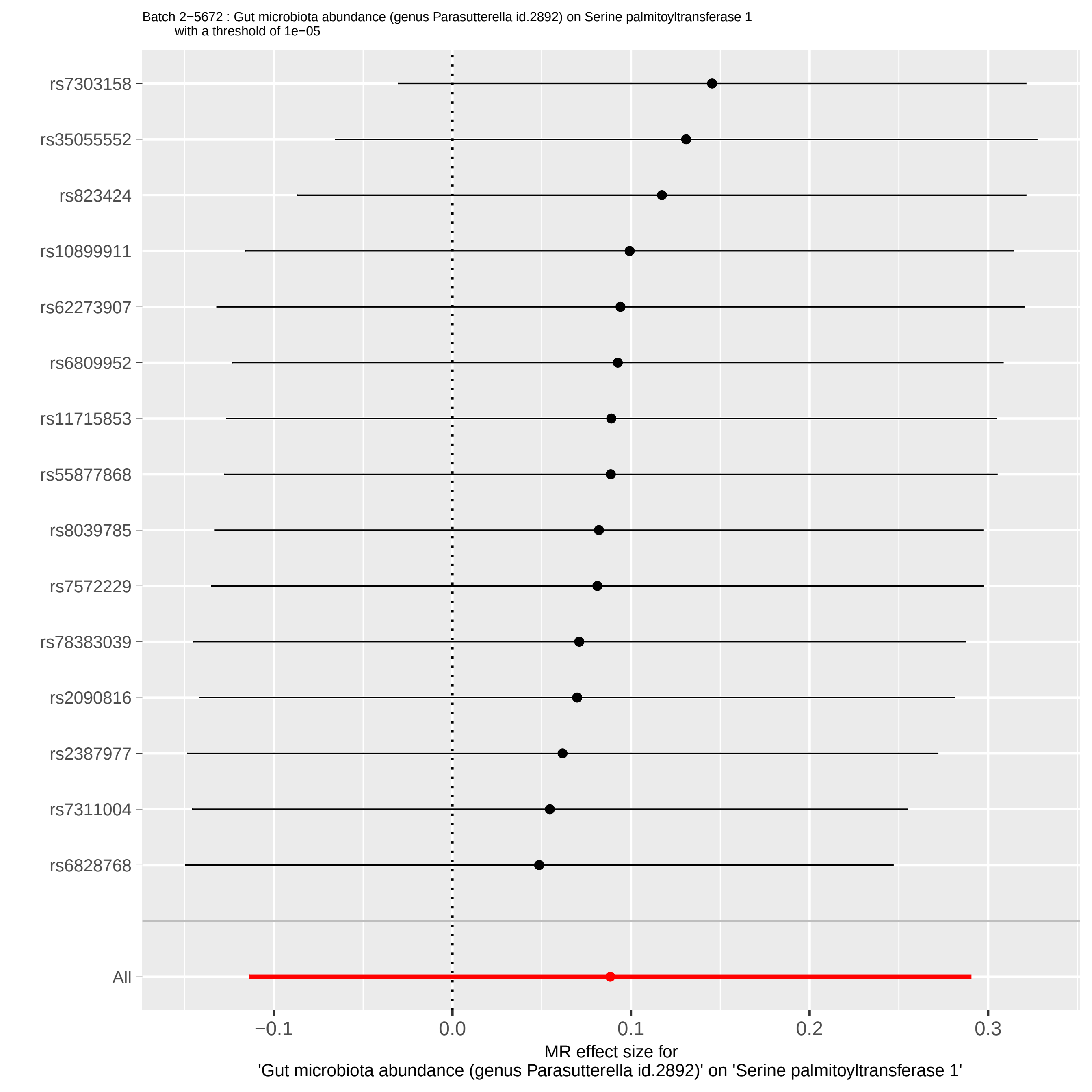
Batch 2−5672 : Gut microbiota abundance (genus Parasutterella id.2892) on Serine palmitoyltransferase 1
with a threshold of 1e−05
rs7303158
rs35055552
rs823424
rs10899911
rs62273907
rs6809952
rs11715853
rs55877868
rs8039785
rs7572229
rs78383039
rs2090816
rs2387977
rs7311004
rs6828768
All
−0.1
0.0
0.1
0.2
0.3
MR effect size for
'Gut microbiota abundance (genus Parasutterella id.2892)' on 'Serine palmitoyltransferase 1'

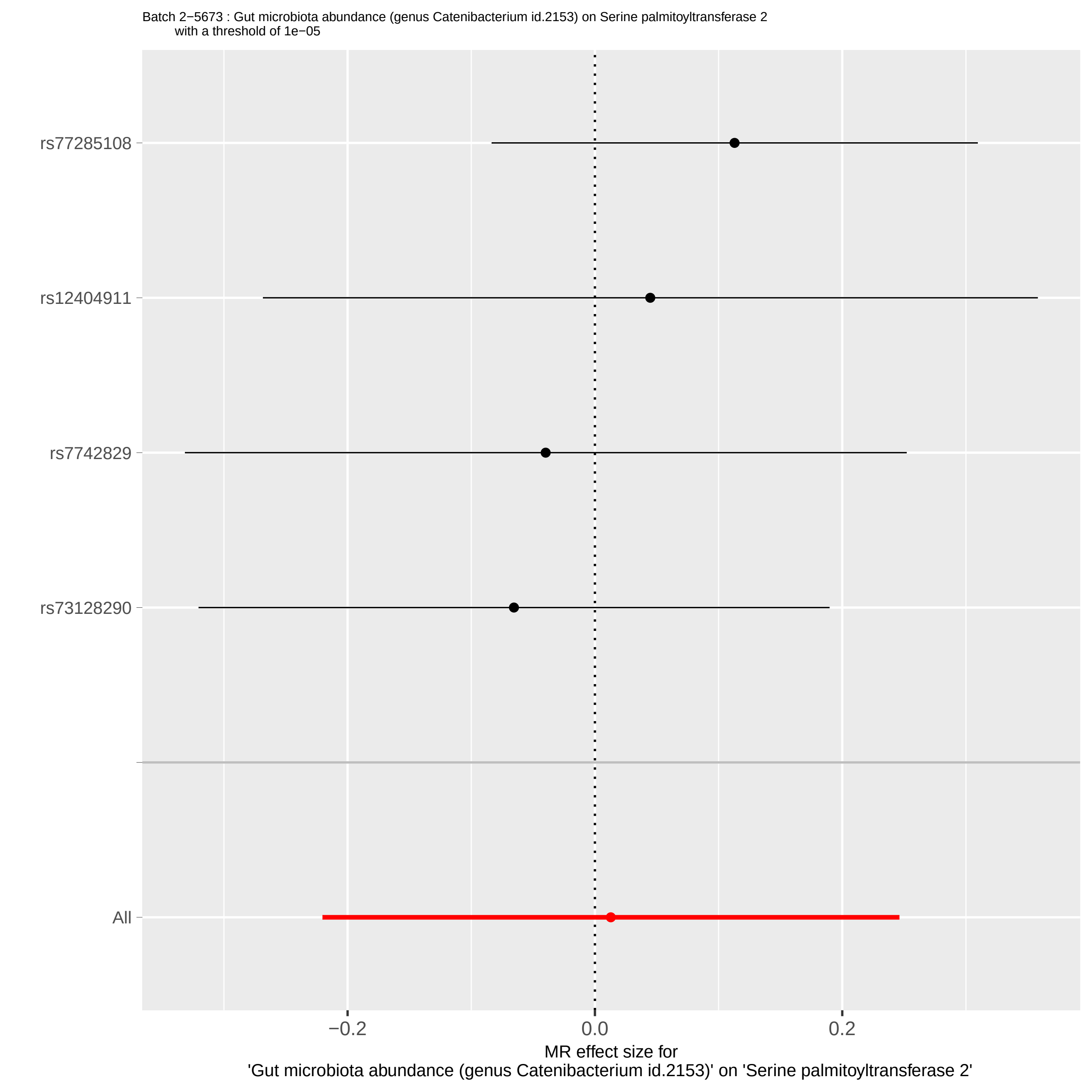
Batch 2−5673 : Gut microbiota abundance (genus Catenibacterium id.2153) on Serine palmitoyltransferase 2
with a threshold of 1e−05
rs77285108
rs12404911
rs7742829
rs73128290
All
−0.2
0.0
0.2
MR effect size for
'Gut microbiota abundance (genus Catenibacterium id.2153)' on 'Serine palmitoyltransferase 2'

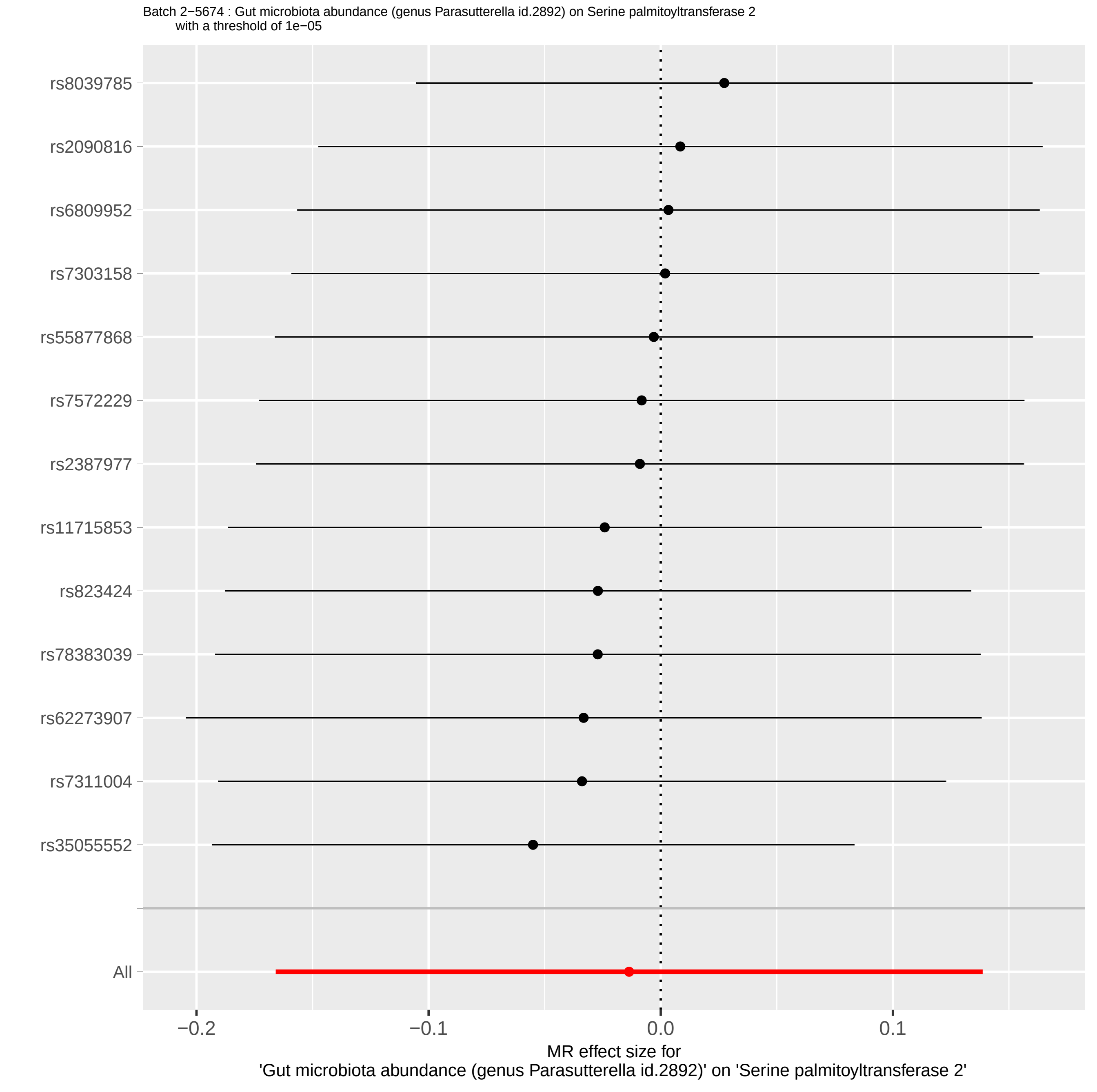
Batch 2−5674 : Gut microbiota abundance (genus Parasutterella id.2892) on Serine palmitoyltransferase 2
with a threshold of 1e−05
rs8039785
rs2090816
rs6809952
rs7303158
rs55877868
rs7572229
rs2387977
rs11715853
rs823424
rs78383039
rs62273907
rs7311004
rs35055552
All
−0.2
−0.1
0.0
0.1
MR effect size for
'Gut microbiota abundance (genus Parasutterella id.2892)' on 'Serine palmitoyltransferase 2'

Batch 2−5675 : Gut microbiota abundance (genus Catenibacterium id.2153) on Serine/arginine−rich splicing factor 6
with a threshold of 1e−05

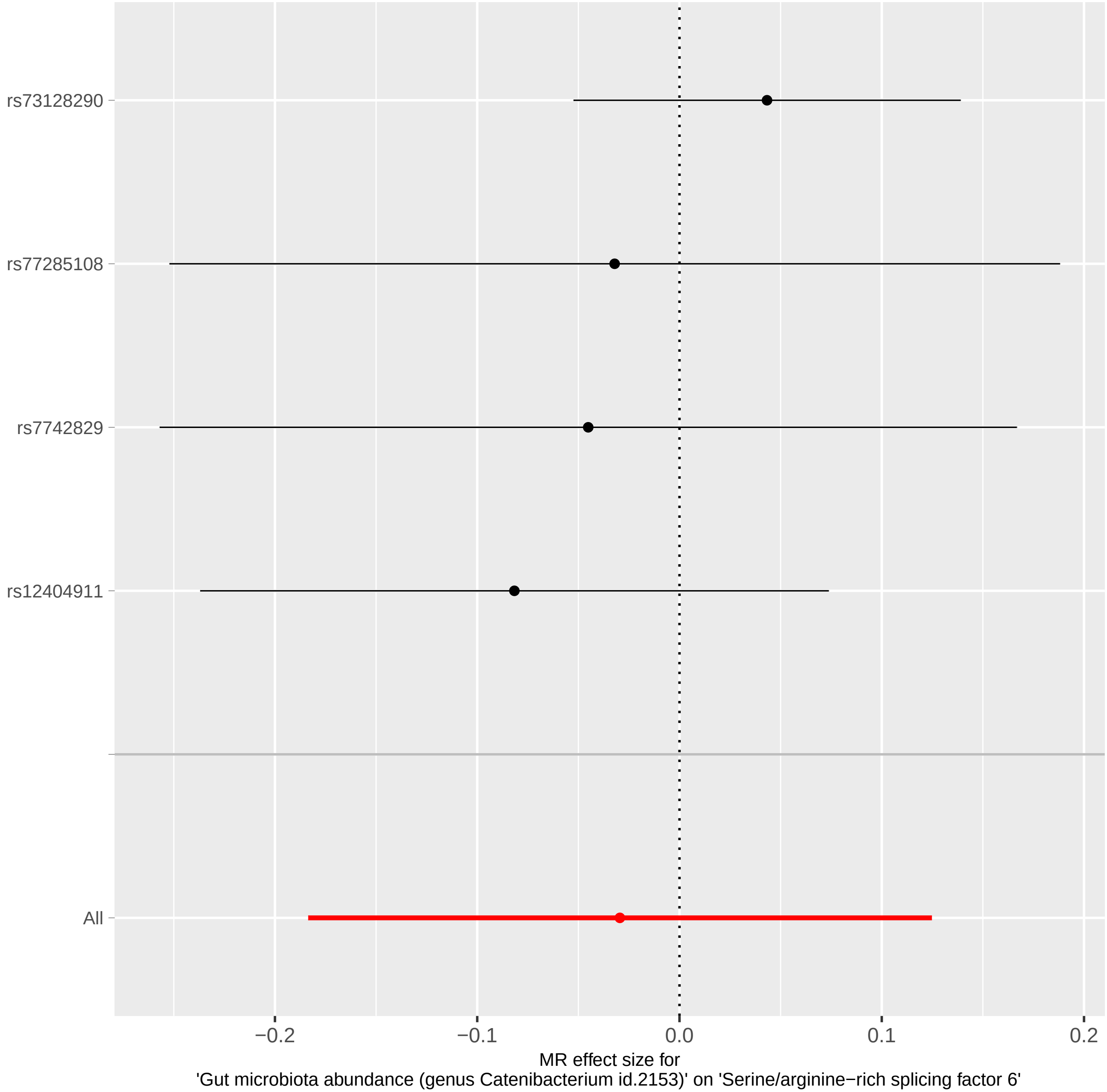

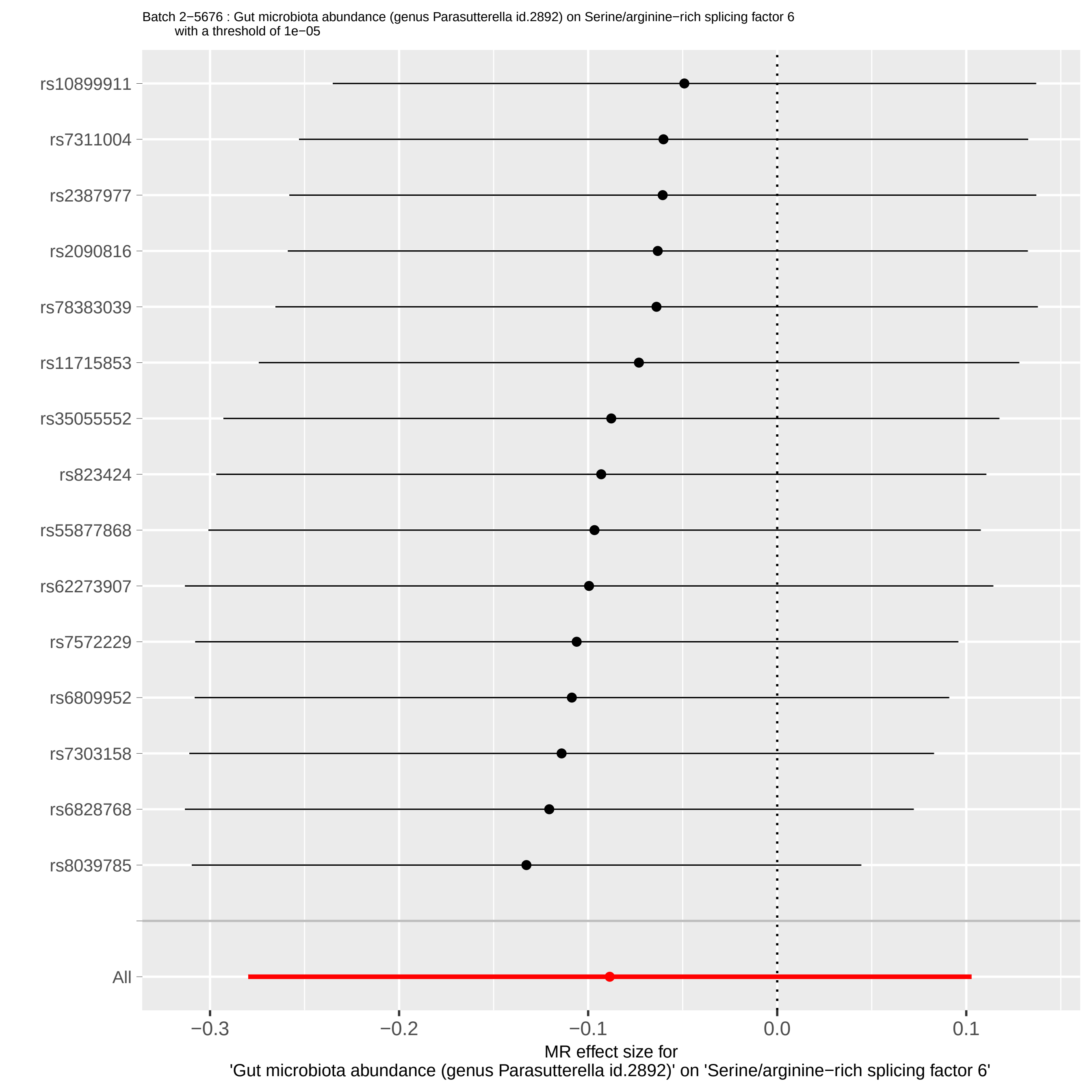

Batch 2−5676 : Gut microbiota abundance (genus Parasutterella id.2892) on Serine/arginine−rich splicing factor 6
with a threshold of 1e−05
rs10899911
rs7311004
rs2387977
rs2090816
rs78383039
rs11715853
rs35055552
rs823424
rs55877868
rs62273907
rs7572229
rs6809952
rs7303158
rs6828768
rs8039785
All
−0.3
−0.2
−0.1
0.0
0.1
MR effect size for
'Gut microbiota abundance (genus Parasutterella id.2892)' on 'Serine/arginine−rich splicing factor 6'

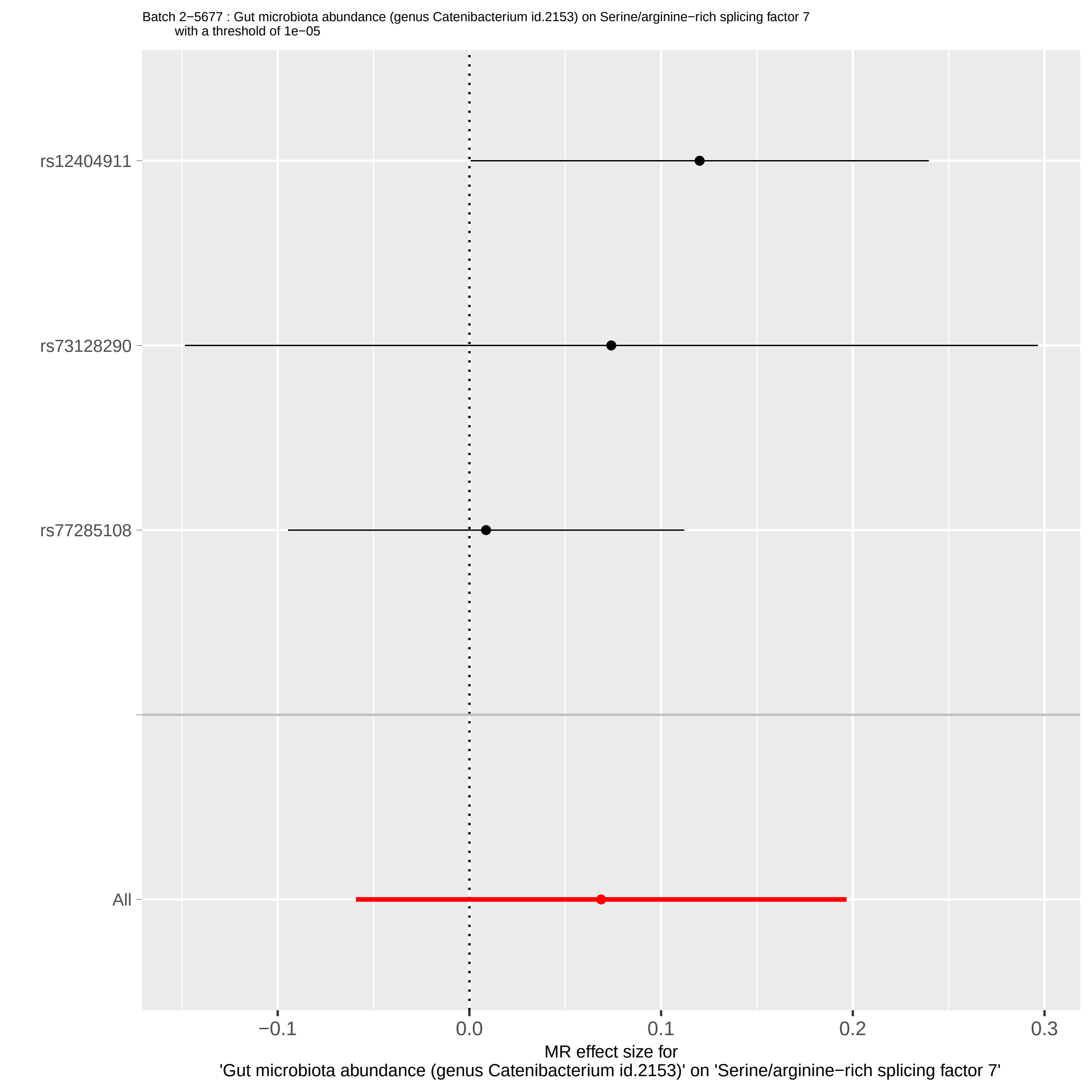
Batch 2−5677 : Gut microbiota abundance (genus Catenibacterium id.2153) on Serine/arginine−rich splicing factor 7
with a threshold of 1e−05
rs12404911
rs73128290
rs77285108
All
−0.1
0.0
0.1
0.2
0.3
MR effect size for
'Gut microbiota abundance (genus Catenibacterium id.2153)' on 'Serine/arginine−rich splicing factor 7'

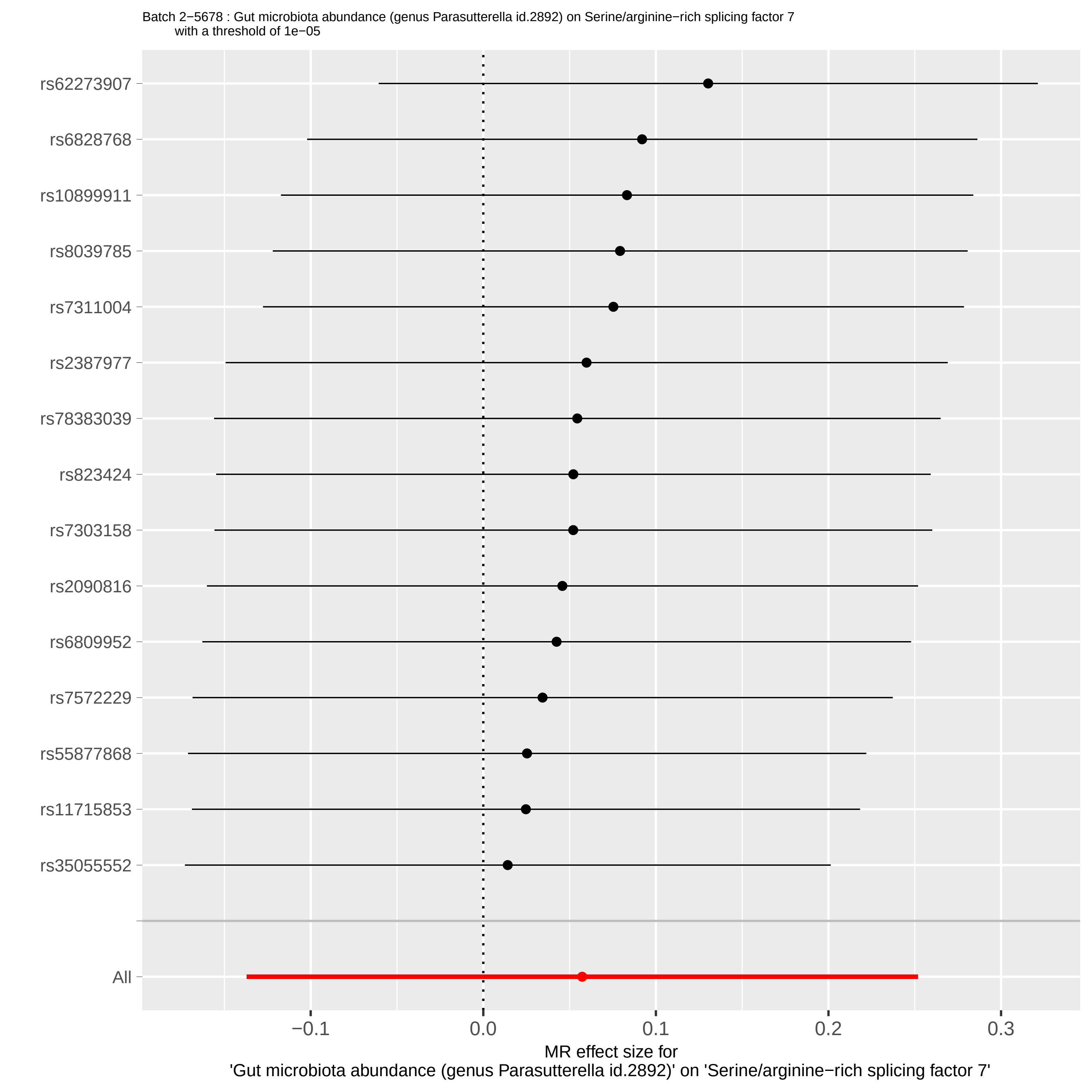

Batch 2−5678 : Gut microbiota abundance (genus Parasutterella id.2892) on Serine/arginine−rich splicing factor 7
with a threshold of 1e−05
rs62273907
rs6828768
rs10899911
rs8039785
rs7311004
rs2387977
rs78383039
rs823424
rs7303158
rs2090816
rs6809952
rs7572229
rs55877868
rs11715853
rs35055552
All
−0.1
0.0
0.1
0.2
0.3
MR effect size for
'Gut microbiota abundance (genus Parasutterella id.2892)' on 'Serine/arginine−rich splicing factor 7'

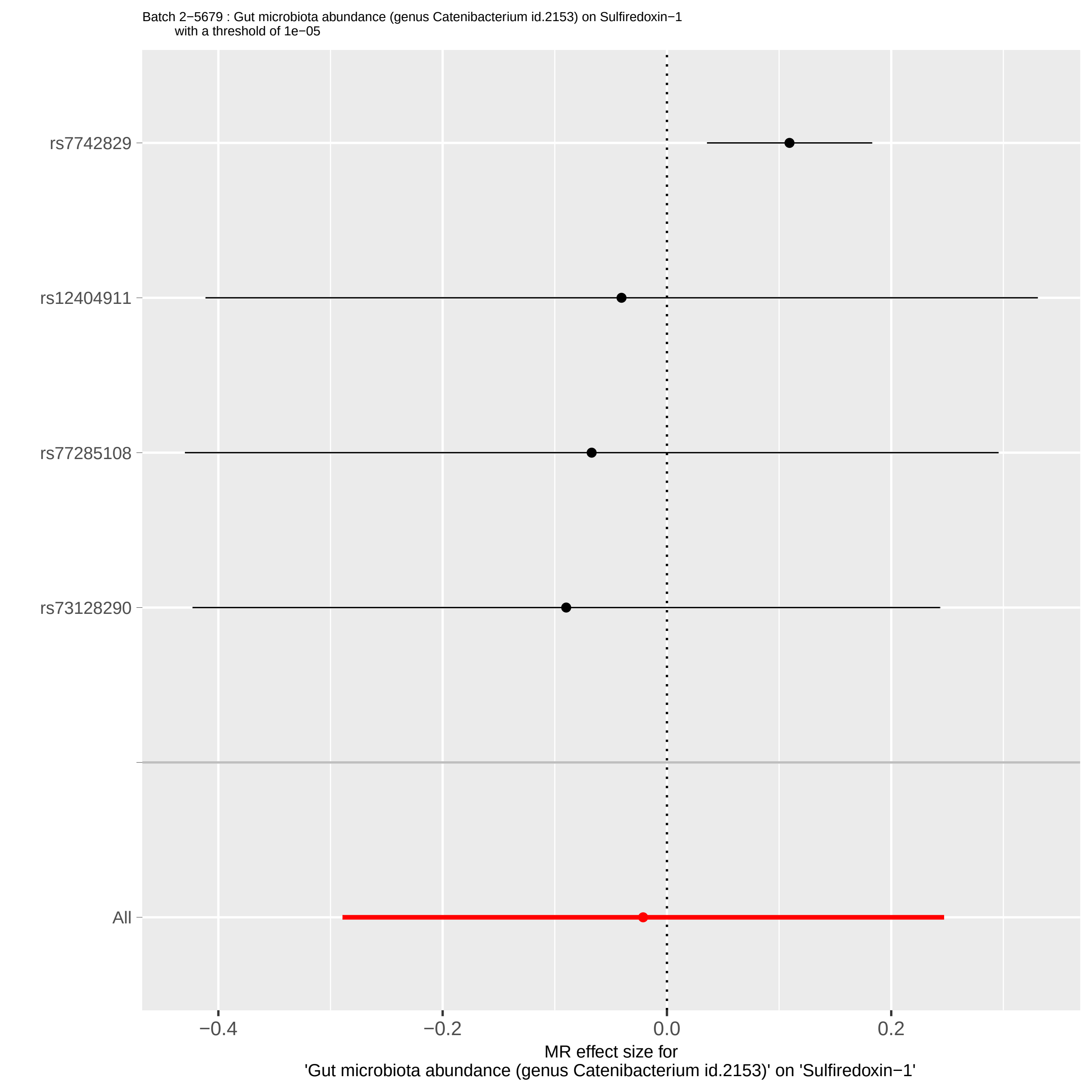
Batch 2−5679 : Gut microbiota abundance (genus Catenibacterium id.2153) on Sulfiredoxin−1
with a threshold of 1e−05
rs7742829
rs12404911
rs77285108
rs73128290
All
−0.4
−0.2
0.0
0.2
MR effect size for
'Gut microbiota abundance (genus Catenibacterium id.2153)' on 'Sulfiredoxin−1'

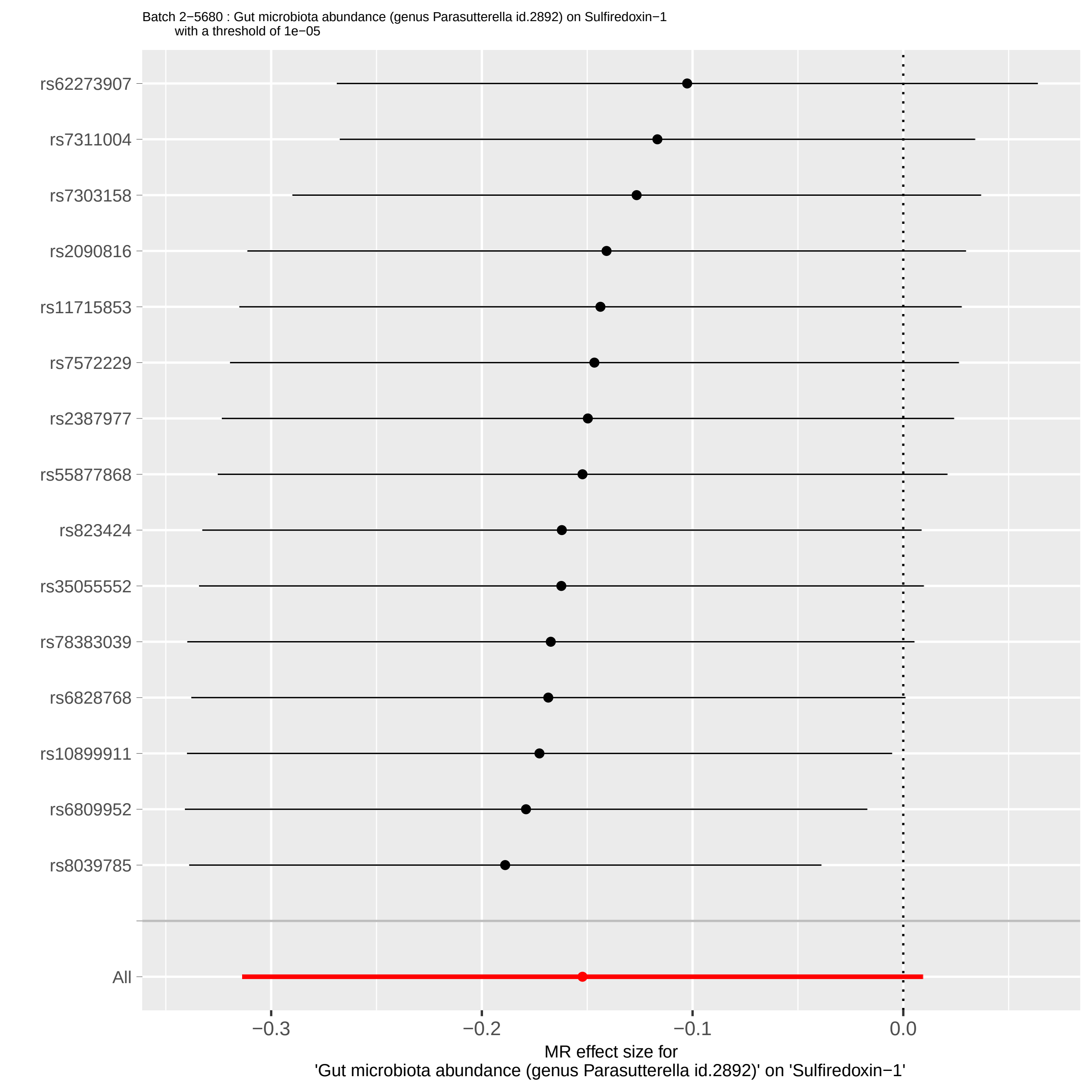

Batch 2−5680 : Gut microbiota abundance (genus Parasutterella id.2892) on Sulfiredoxin−1
with a threshold of 1e−05
rs62273907
rs7311004
rs7303158
rs2090816
rs11715853
rs7572229
rs2387977
rs55877868
rs823424
rs35055552
rs78383039
rs6828768
rs10899911
rs6809952
rs8039785
All
−0.3
−0.2
−0.1
0.0
MR effect size for
'Gut microbiota abundance (genus Parasutterella id.2892)' on 'Sulfiredoxin−1'

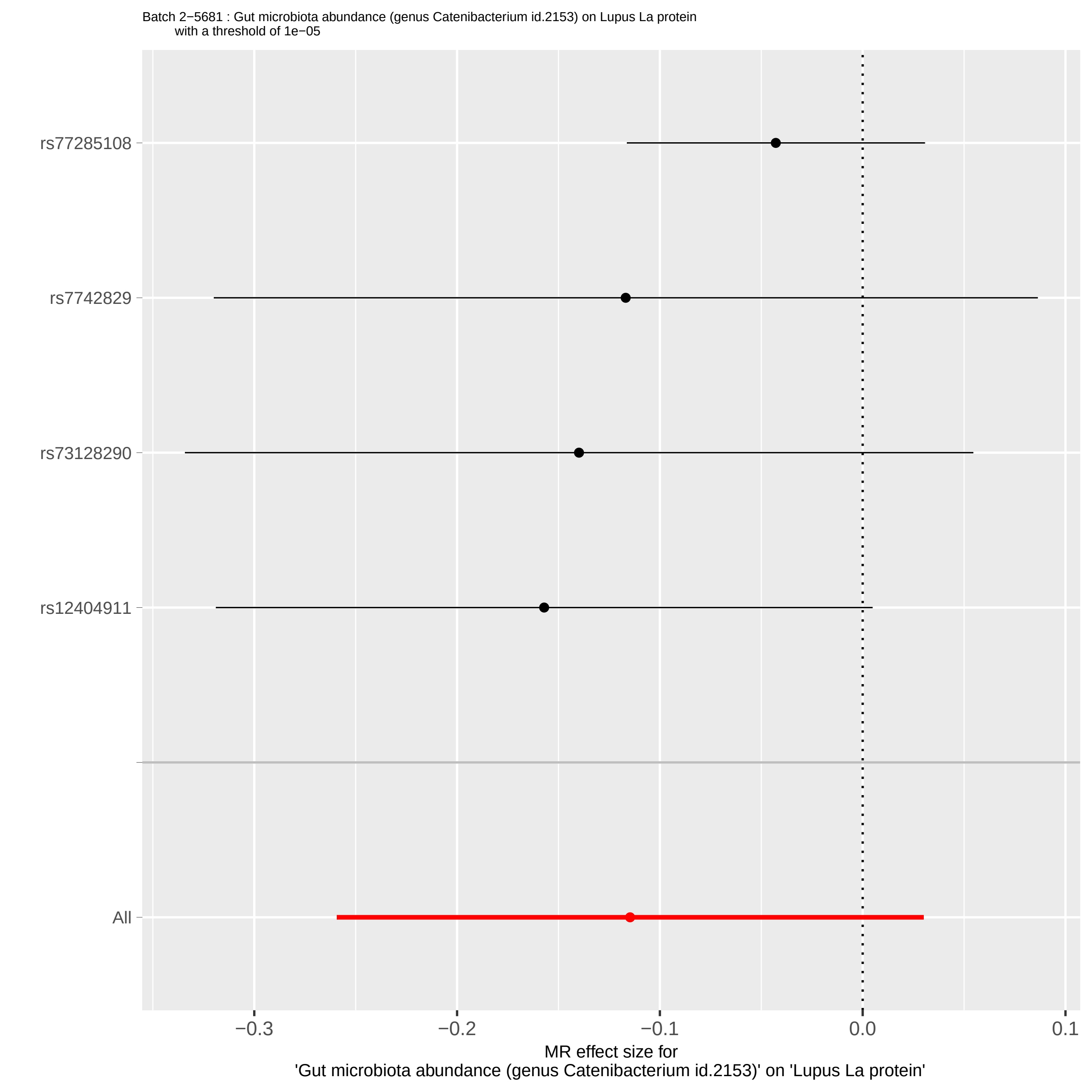

Batch 2−5681 : Gut microbiota abundance (genus Catenibacterium id.2153) on Lupus La protein
with a threshold of 1e−05
rs77285108
rs7742829
rs73128290
rs12404911
All
−0.3
−0.2
−0.1
0.0
0.1
MR effect size for
'Gut microbiota abundance (genus Catenibacterium id.2153)' on 'Lupus La protein'

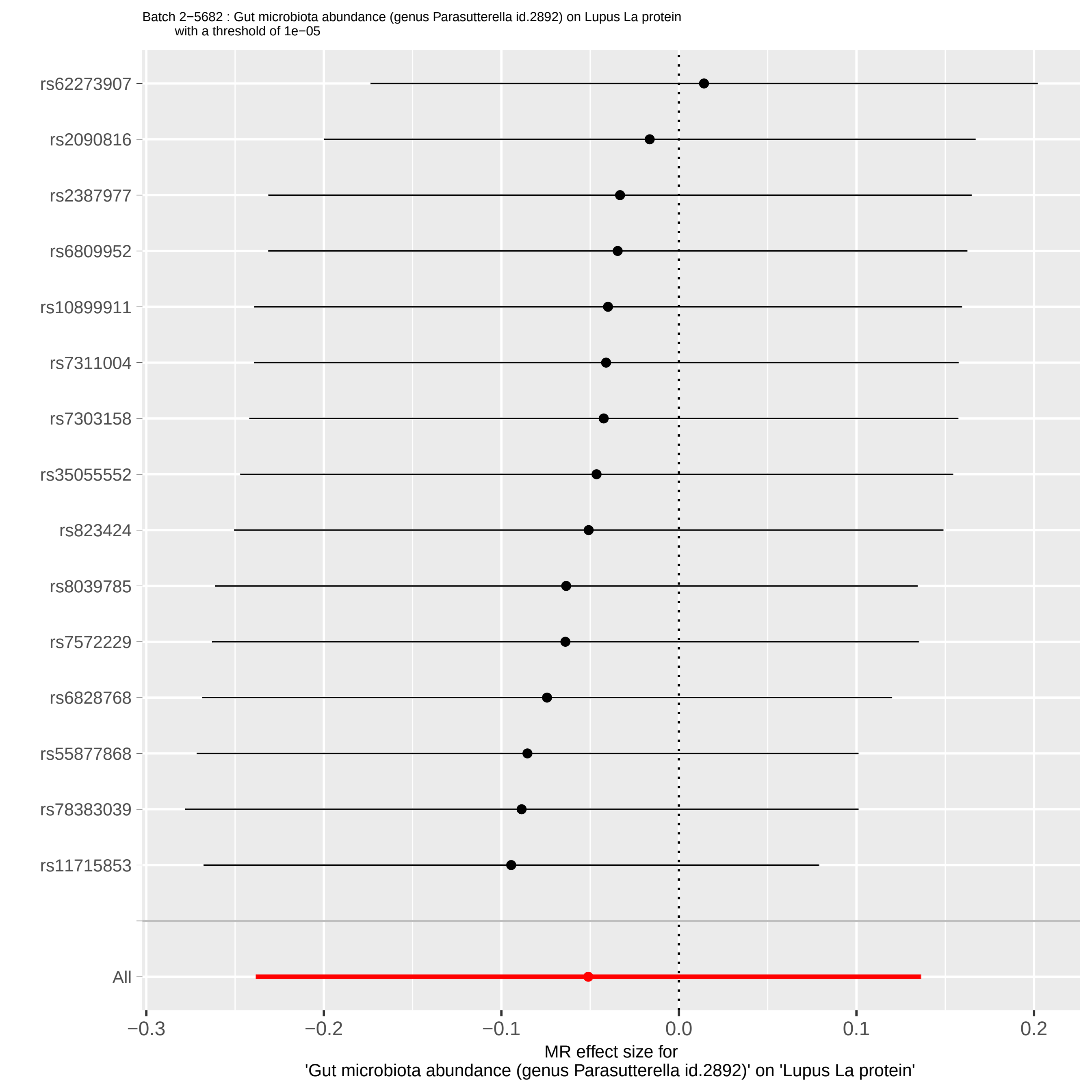
Batch 2−5682 : Gut microbiota abundance (genus Parasutterella id.2892) on Lupus La protein
with a threshold of 1e−05
rs62273907
rs2090816
rs2387977
rs6809952
rs10899911
rs7311004
rs7303158
rs35055552
rs823424
rs8039785
rs7572229
rs6828768
rs55877868
rs78383039
rs11715853
All
−0.3
−0.2
−0.1
0.0
0.1
0.2
MR effect size for
'Gut microbiota abundance (genus Parasutterella id.2892)' on 'Lupus La protein'

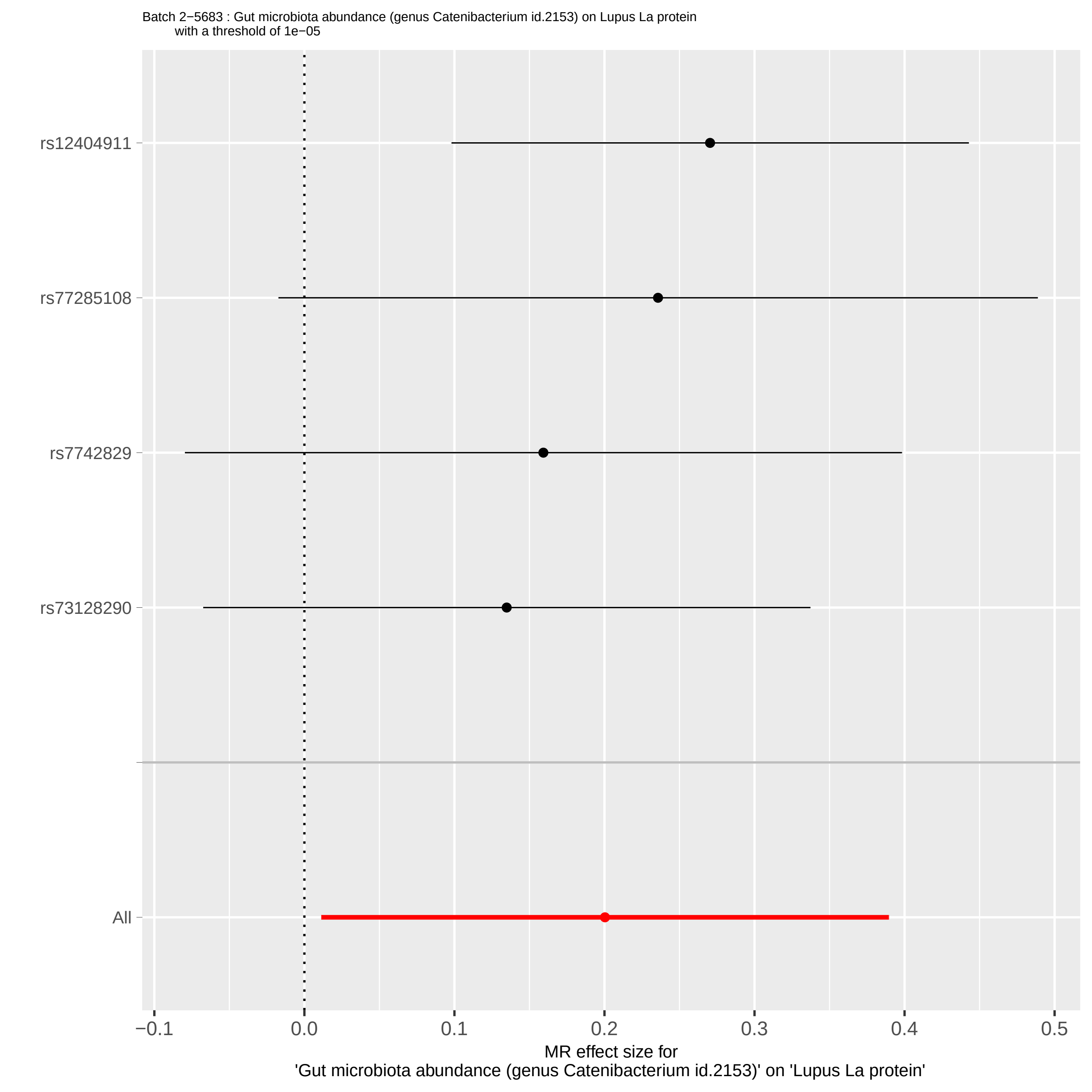
Batch 2−5683 : Gut microbiota abundance (genus Catenibacterium id.2153) on Lupus La protein
with a threshold of 1e−05
rs12404911
rs77285108
rs7742829
rs73128290
All
−0.1
0.0
0.1
0.2
0.3
0.4
0.5
MR effect size for
'Gut microbiota abundance (genus Catenibacterium id.2153)' on 'Lupus La protein'

Batch 2−5684 : Gut microbiota abundance (genus Parasutterella id.2892) on Lupus La protein
with a threshold of 1e−05

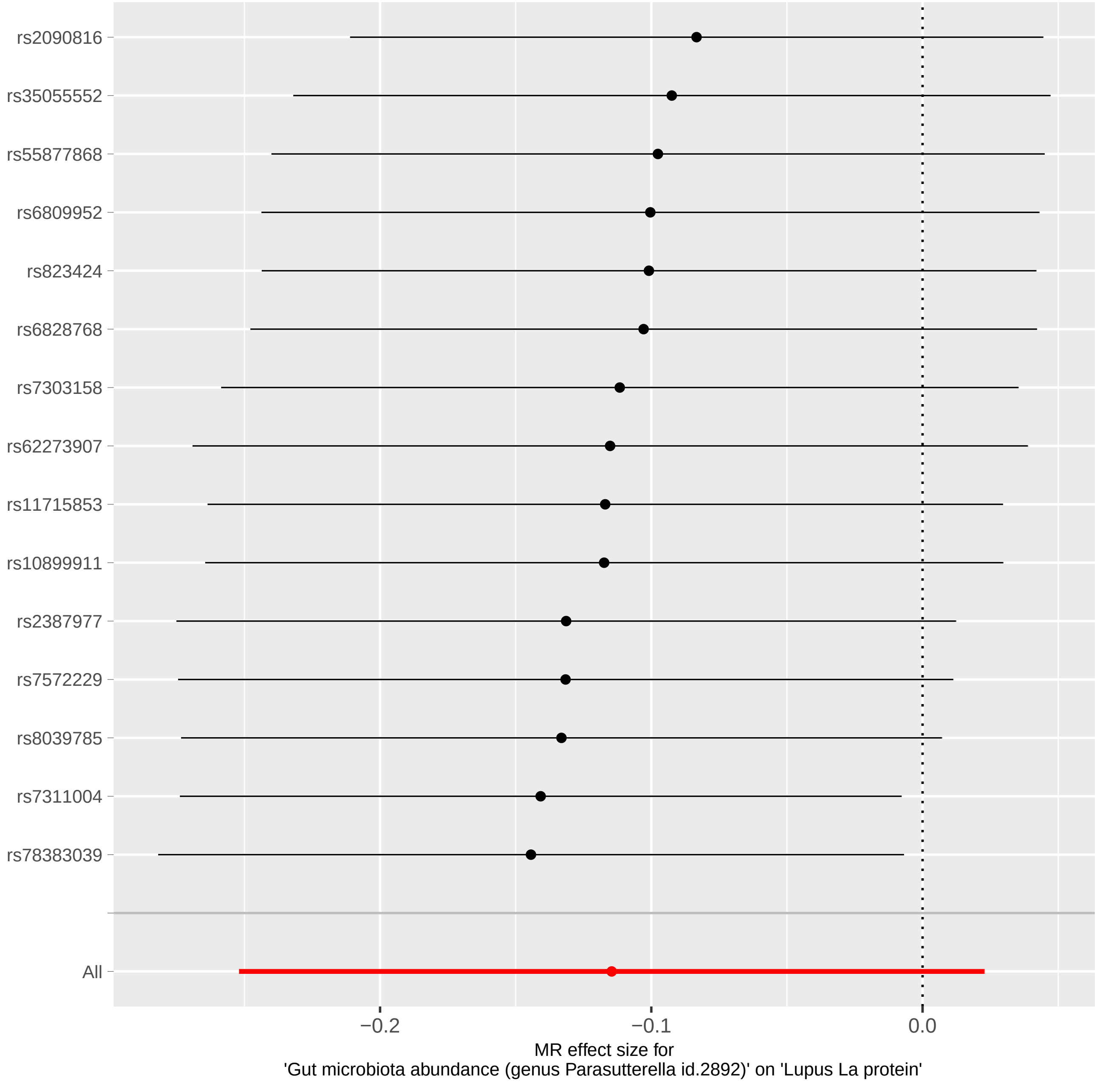

Batch 2−5685 : Gut microbiota abundance (genus Catenibacterium id.2153) on Serine−rich single−pass membrane protein 1
with a threshold of 1e−05

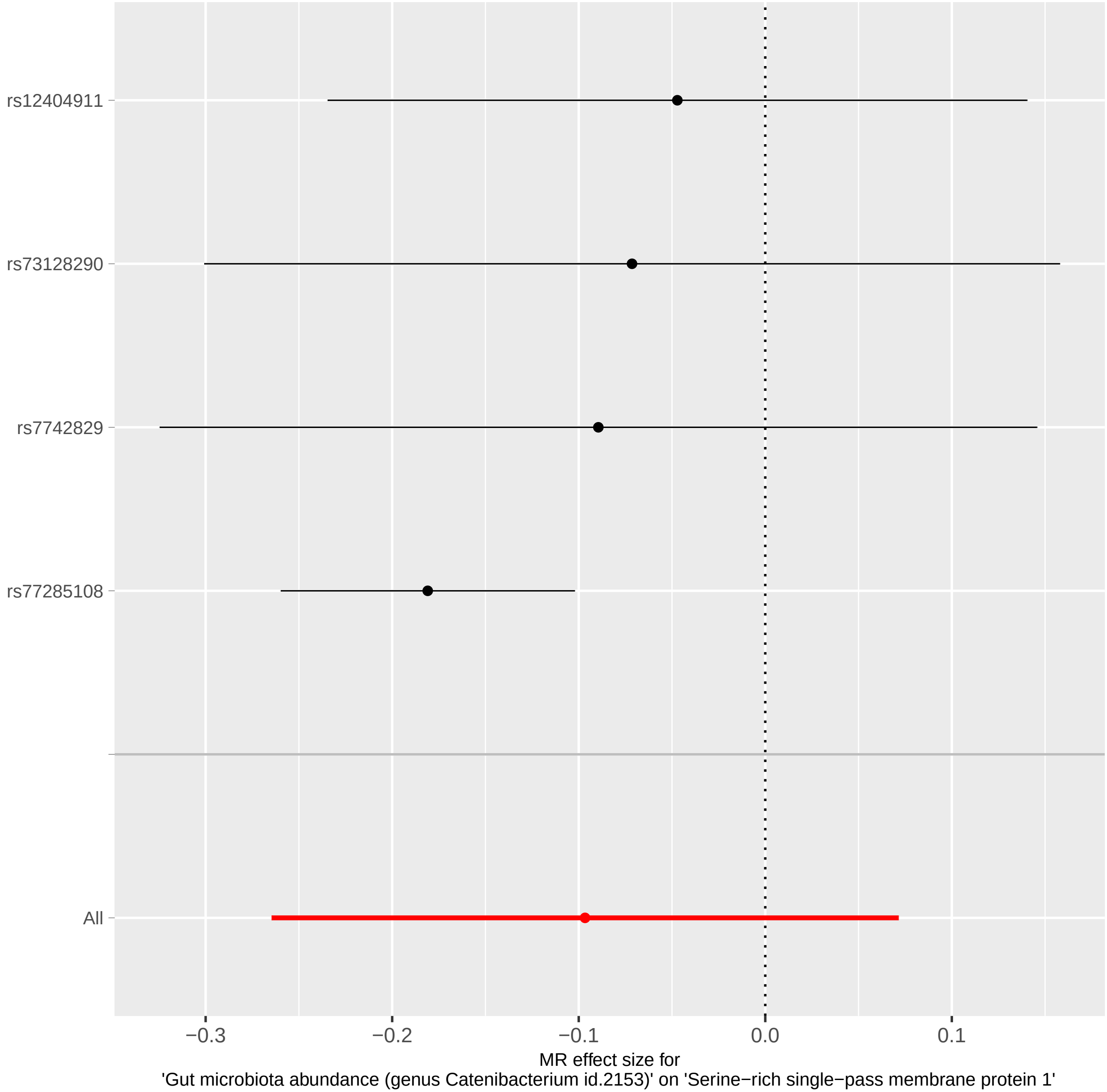

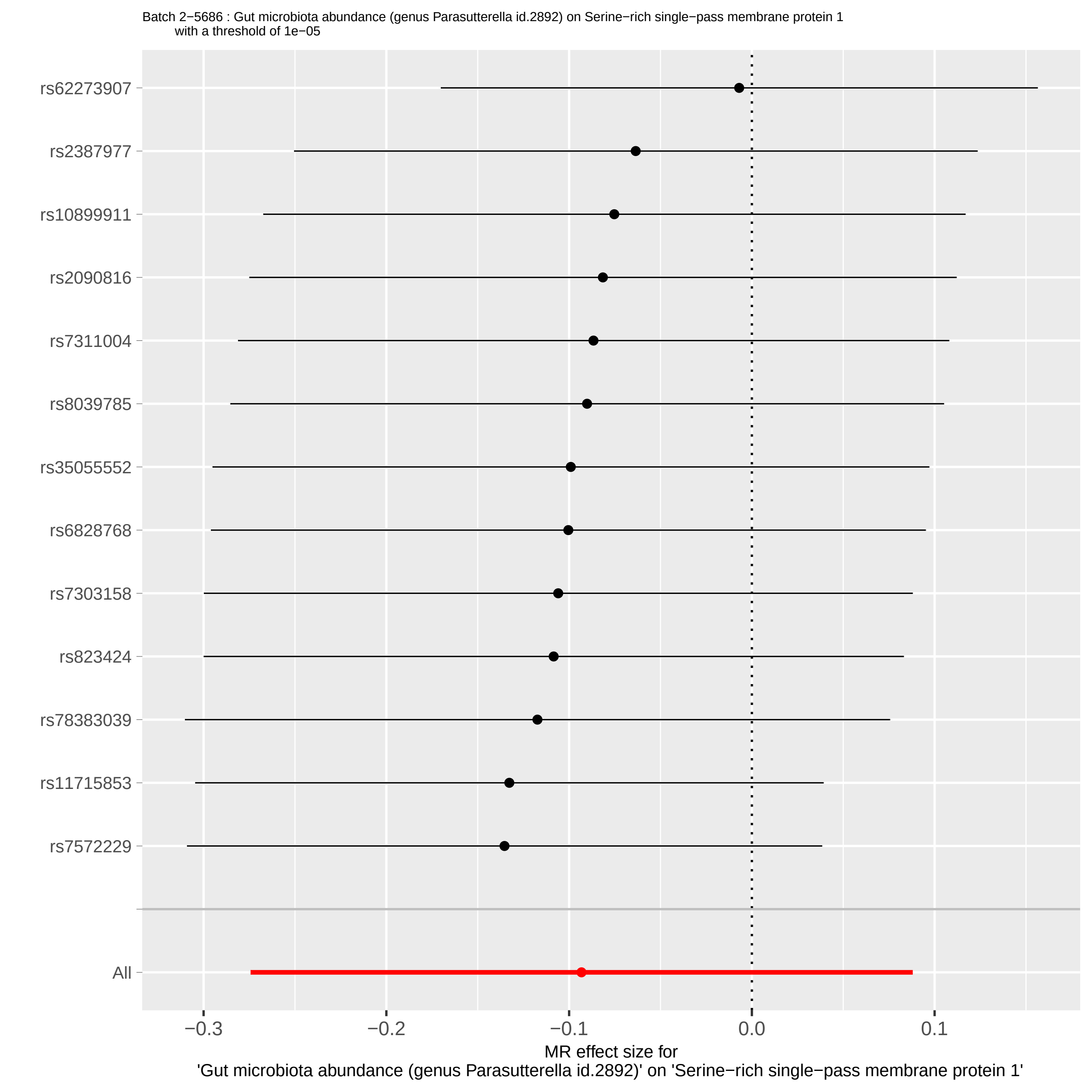

Batch 2−5686 : Gut microbiota abundance (genus Parasutterella id.2892) on Serine−rich single−pass membrane protein 1
with a threshold of 1e−05
rs62273907
rs2387977
rs10899911
rs2090816
rs7311004
rs8039785
rs35055552
rs6828768
rs7303158
rs823424
rs78383039
rs11715853
rs7572229
All
−0.3
−0.2
−0.1
0.0
0.1
MR effect size for
'Gut microbiota abundance (genus Parasutterella id.2892)' on 'Serine−rich single−pass membrane protein 1'

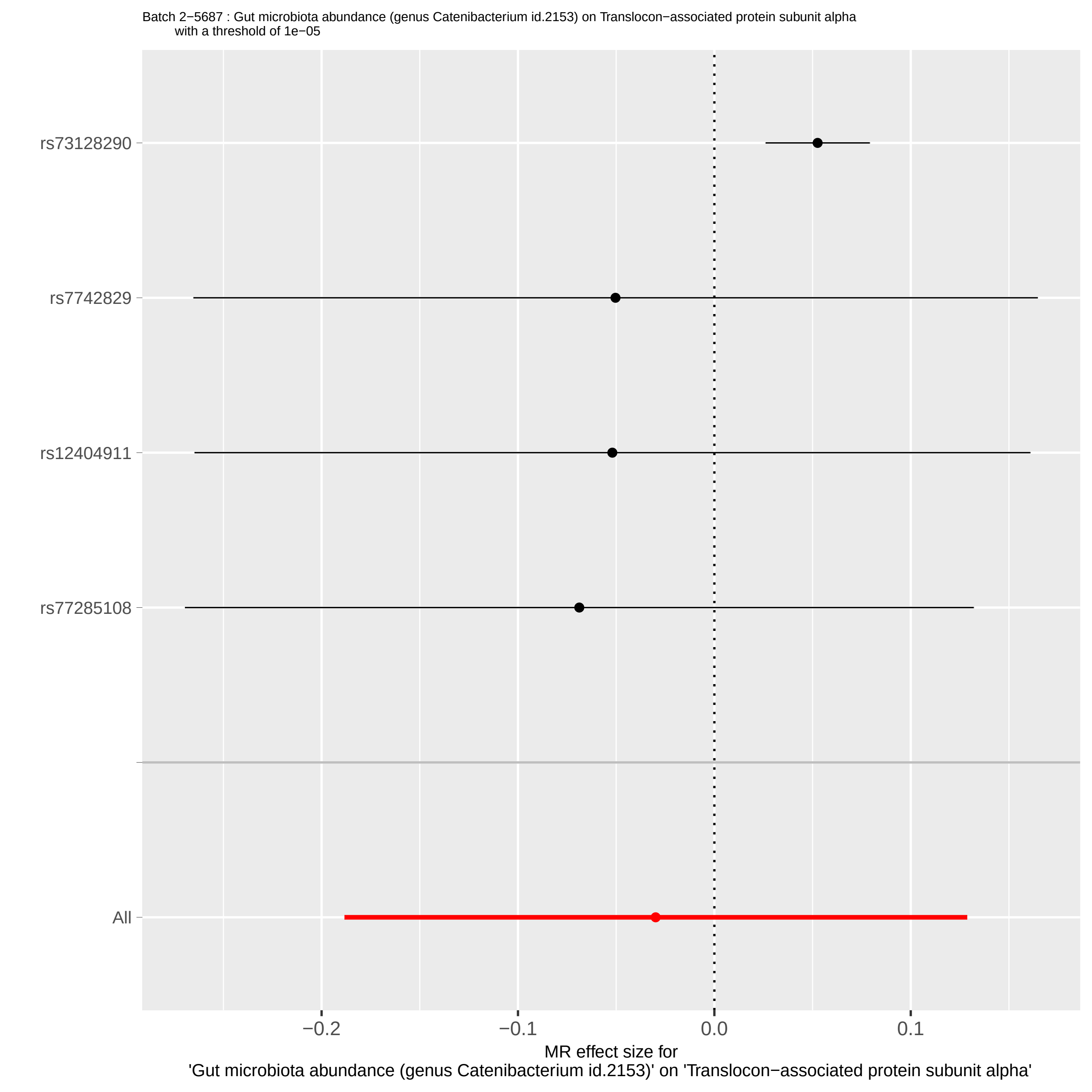
Batch 2−5687 : Gut microbiota abundance (genus Catenibacterium id.2153) on Translocon−associated protein subunit alpha
with a threshold of 1e−05
rs73128290
rs7742829
rs12404911
rs77285108
All
−0.2
−0.1
0.0
0.1
MR effect size for
'Gut microbiota abundance (genus Catenibacterium id.2153)' on 'Translocon−associated protein subunit alpha'

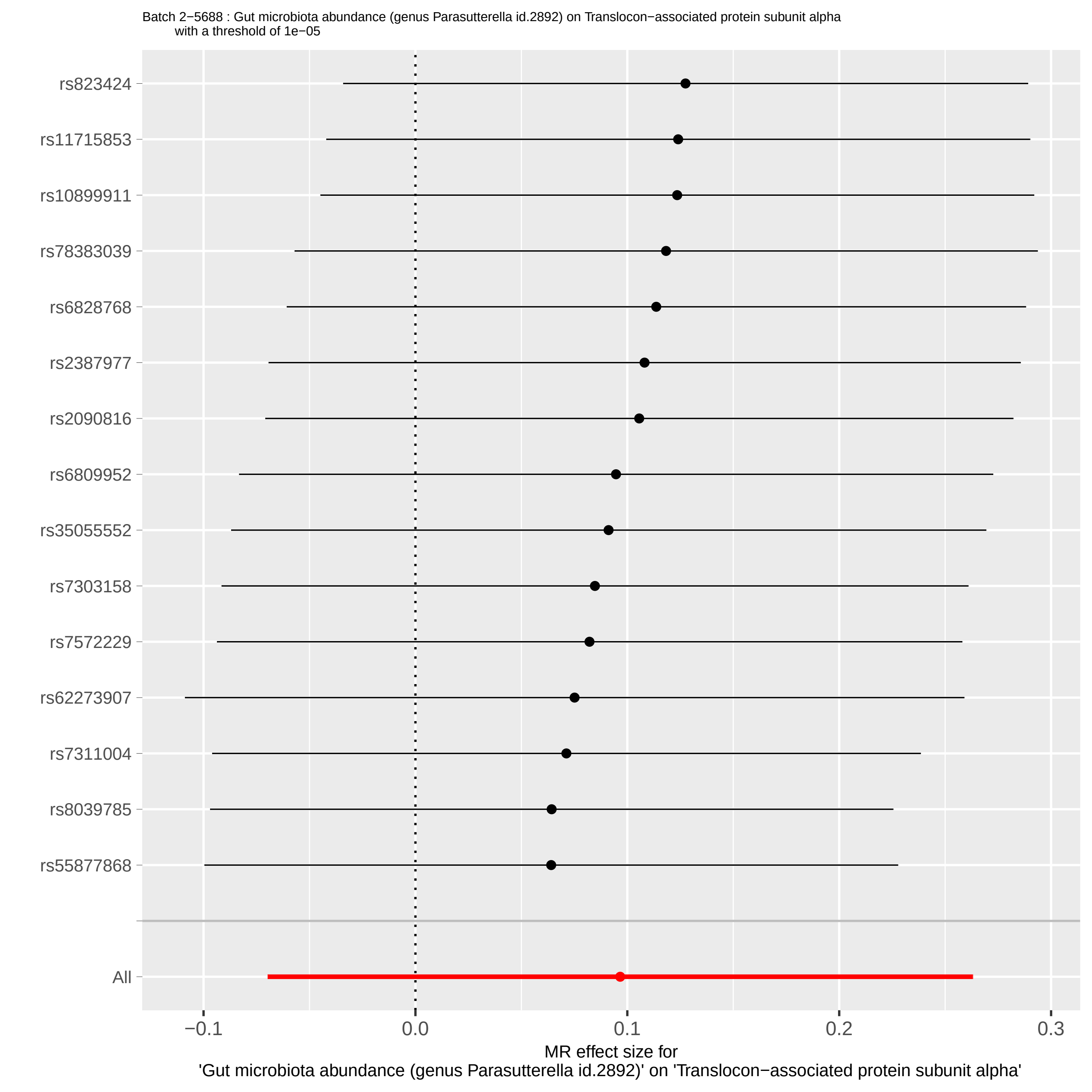
Batch 2−5688 : Gut microbiota abundance (genus Parasutterella id.2892) on Translocon−associated protein subunit alpha
with a threshold of 1e−05
rs823424
rs11715853
rs10899911
rs78383039
rs6828768
rs2387977
rs2090816
rs6809952
rs35055552
rs7303158
rs7572229
rs62273907
rs7311004
rs8039785
rs55877868
All
−0.1
0.0
0.1
0.2
0.3
MR effect size for
'Gut microbiota abundance (genus Parasutterella id.2892)' on 'Translocon−associated protein subunit alpha'

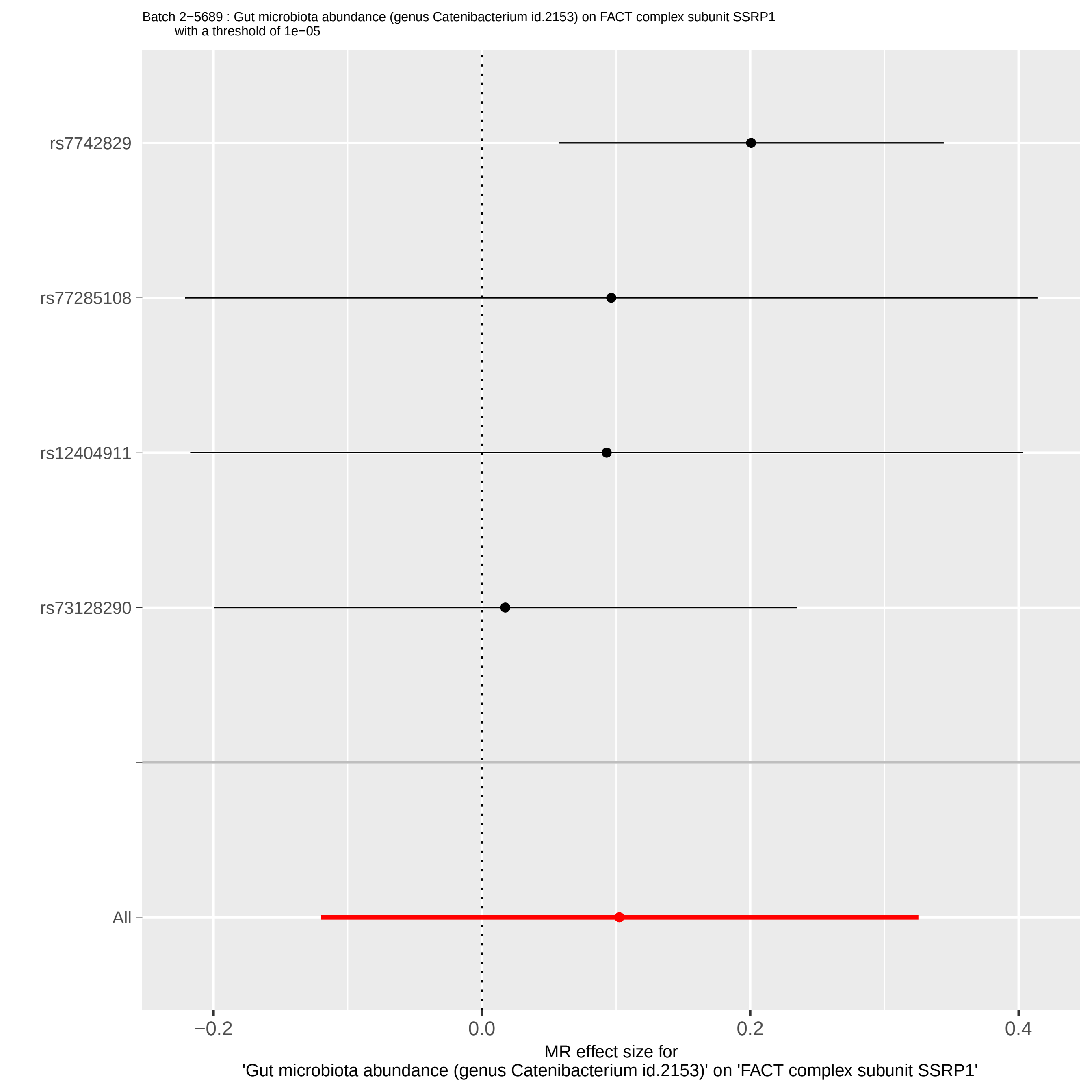

Batch 2−5689 : Gut microbiota abundance (genus Catenibacterium id.2153) on FACT complex subunit SSRP1
with a threshold of 1e−05
rs7742829
rs77285108
rs12404911
rs73128290
All
−0.2
0.0
0.2
0.4
MR effect size for
'Gut microbiota abundance (genus Catenibacterium id.2153)' on 'FACT complex subunit SSRP1'

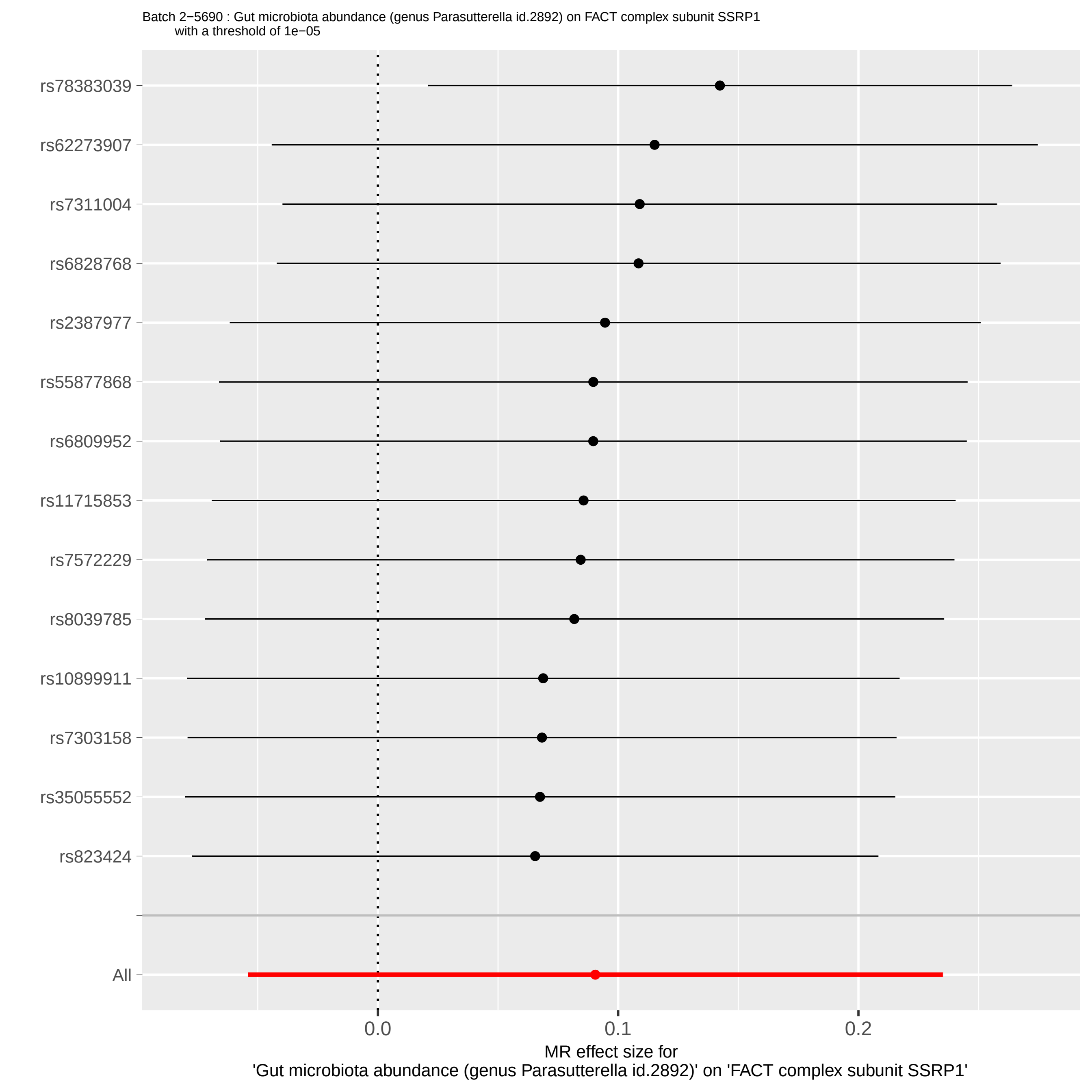
Batch 2−5690 : Gut microbiota abundance (genus Parasutterella id.2892) on FACT complex subunit SSRP1
with a threshold of 1e−05
rs78383039
rs62273907
rs7311004
rs6828768
rs2387977
rs55877868
rs6809952
rs11715853
rs7572229
rs8039785
rs10899911
rs7303158
rs35055552
rs823424
All
0.0
0.1
0.2
MR effect size for
'Gut microbiota abundance (genus Parasutterella id.2892)' on 'FACT complex subunit SSRP1'

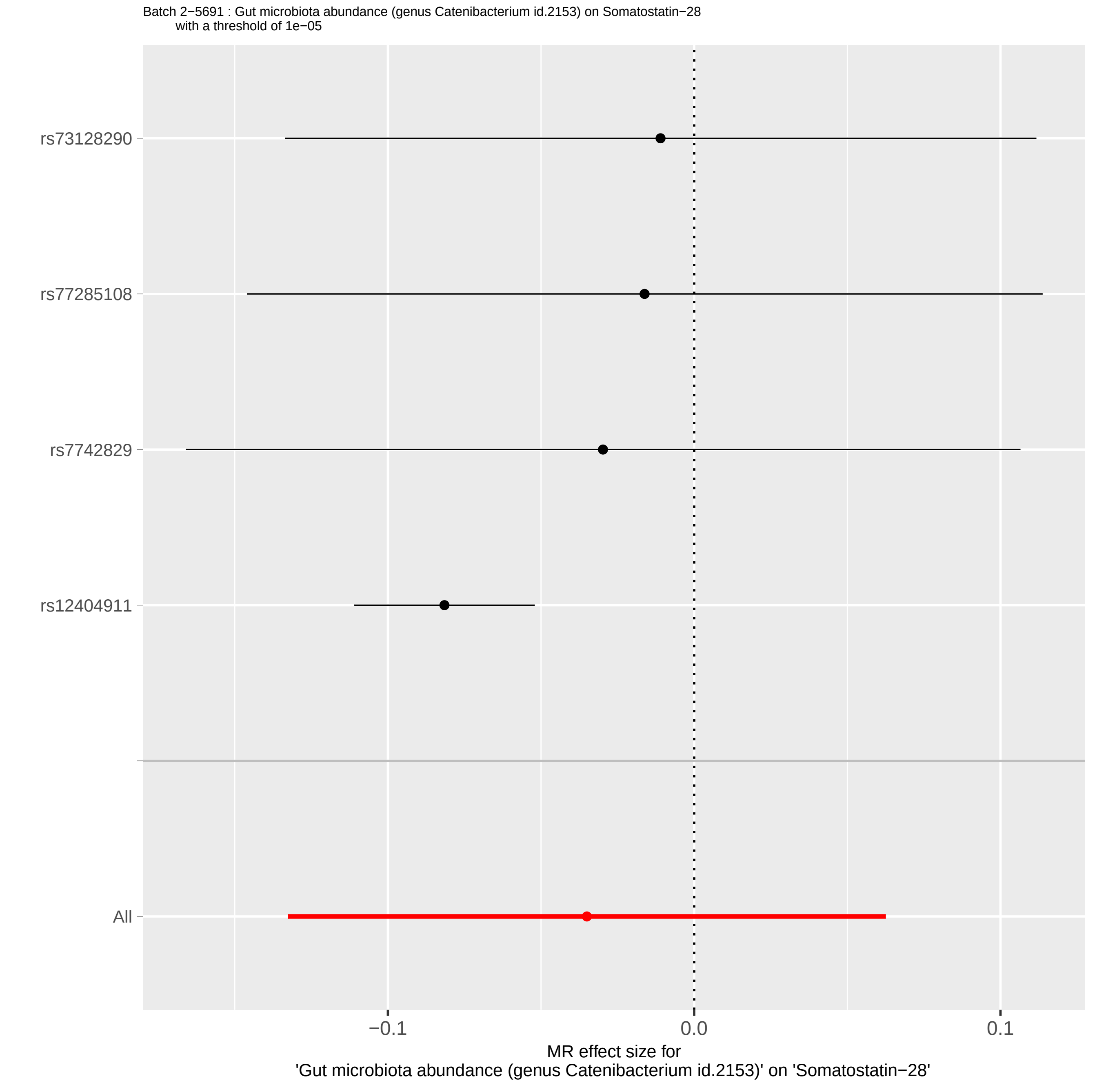
Batch 2−5691 : Gut microbiota abundance (genus Catenibacterium id.2153) on Somatostatin−28
with a threshold of 1e−05
rs73128290
rs77285108
rs7742829
rs12404911
All
−0.1
0.0
0.1
MR effect size for
'Gut microbiota abundance (genus Catenibacterium id.2153)' on 'Somatostatin−28'

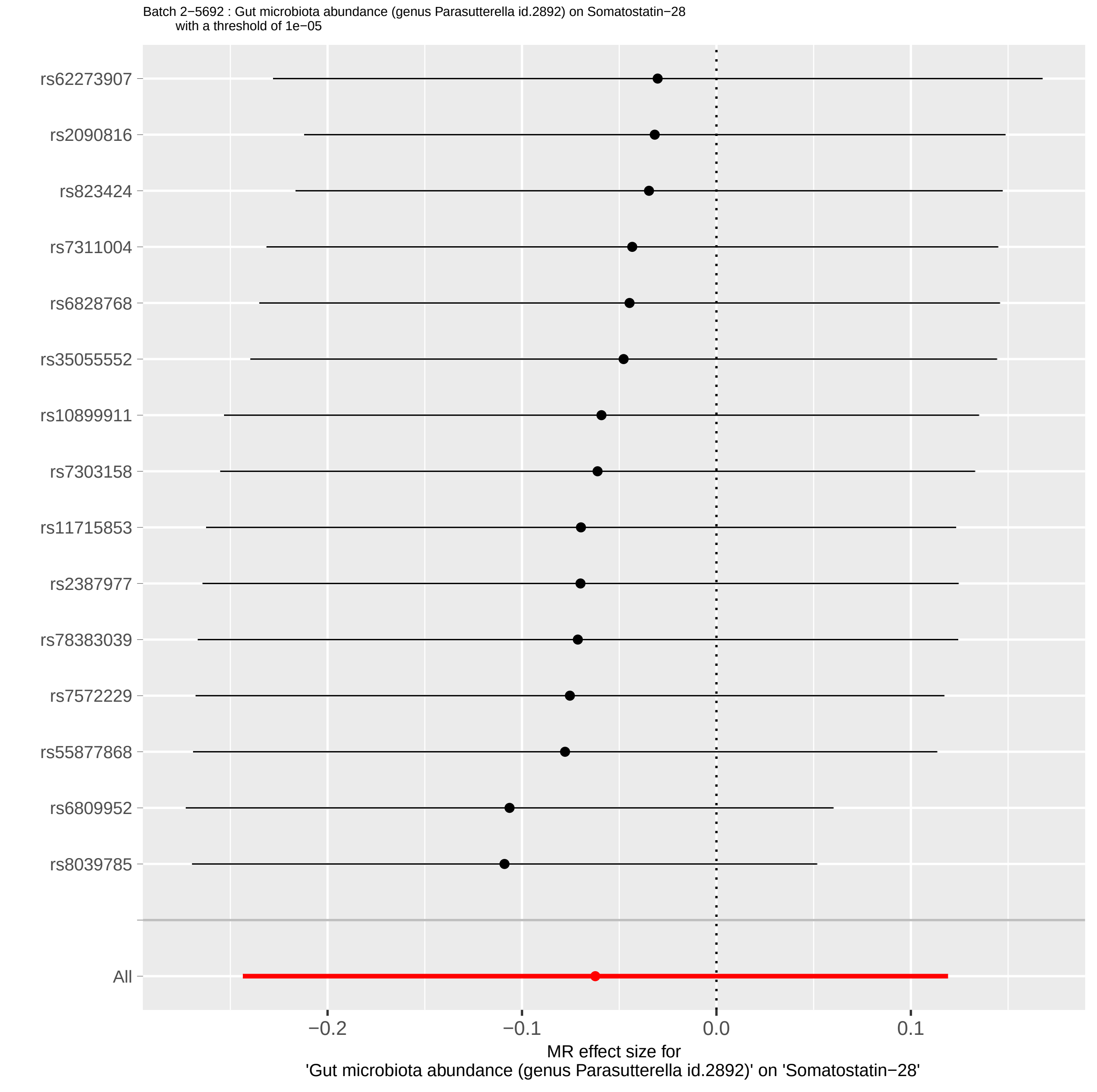
Batch 2−5692 : Gut microbiota abundance (genus Parasutterella id.2892) on Somatostatin−28
with a threshold of 1e−05
rs62273907
rs2090816
rs823424
rs7311004
rs6828768
rs35055552
rs10899911
rs7303158
rs11715853
rs2387977
rs78383039
rs7572229
rs55877868
rs6809952
rs8039785
All
−0.2
−0.1
0.0
0.1
MR effect size for
'Gut microbiota abundance (genus Parasutterella id.2892)' on 'Somatostatin−28'

Batch 2−5693 : Gut microbiota abundance (genus Catenibacterium id.2153) on RNA polymerase II subunit A C−terminal domain phosphatase SSU72
with a threshold of 1e−05

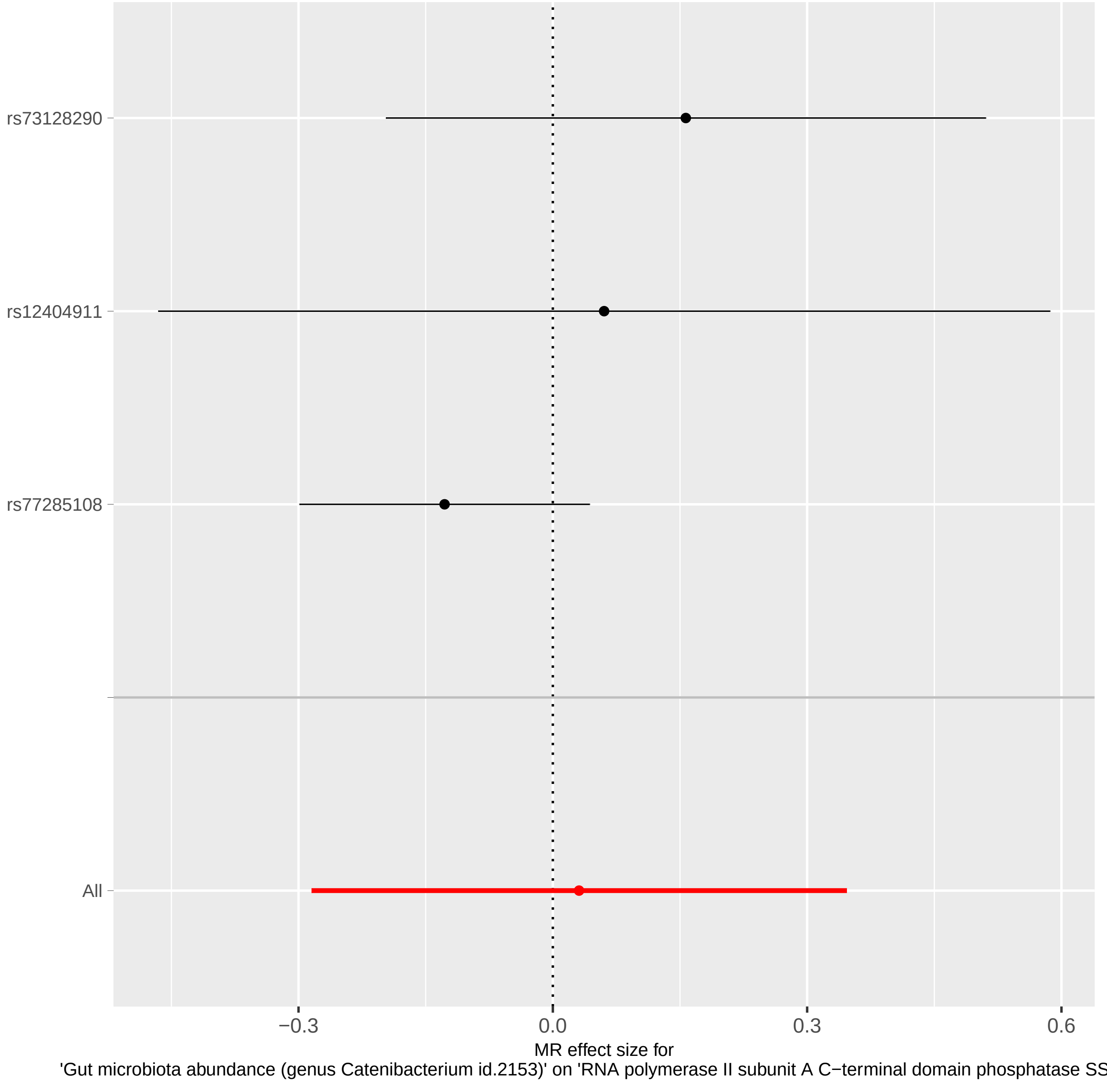

Batch 2−5694 : Gut microbiota abundance (genus Parasutterella id.2892) on RNA polymerase II subunit A C−terminal domain phosphatase SSU72
with a threshold of 1e−05

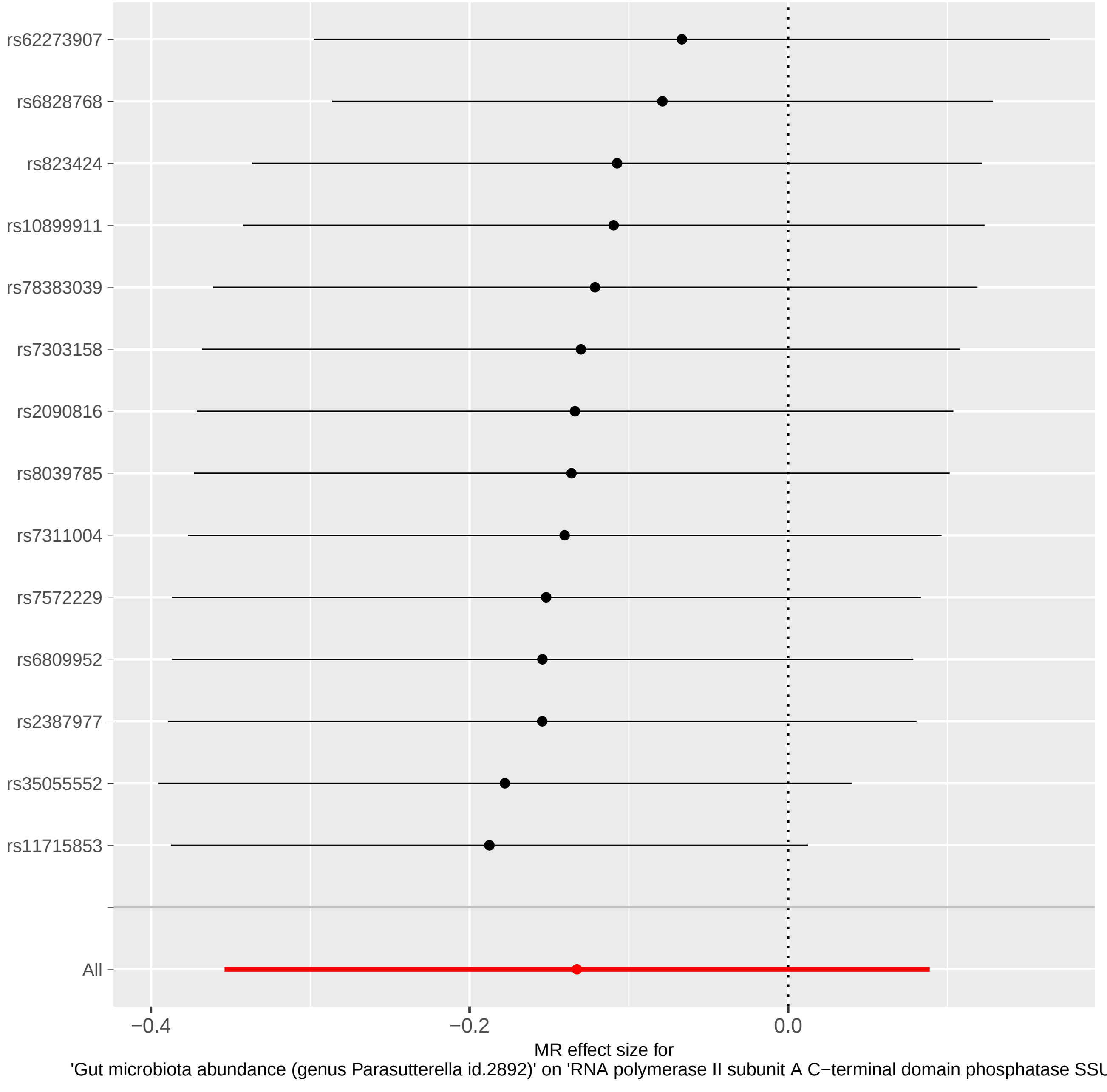

Batch 2−5695 : Gut microbiota abundance (genus Catenibacterium id.2153) on CMP−N−acetylneuraminate−beta−galactosamide−alpha−2,3−sialyltransferase 1 with a threshold of 1e−05

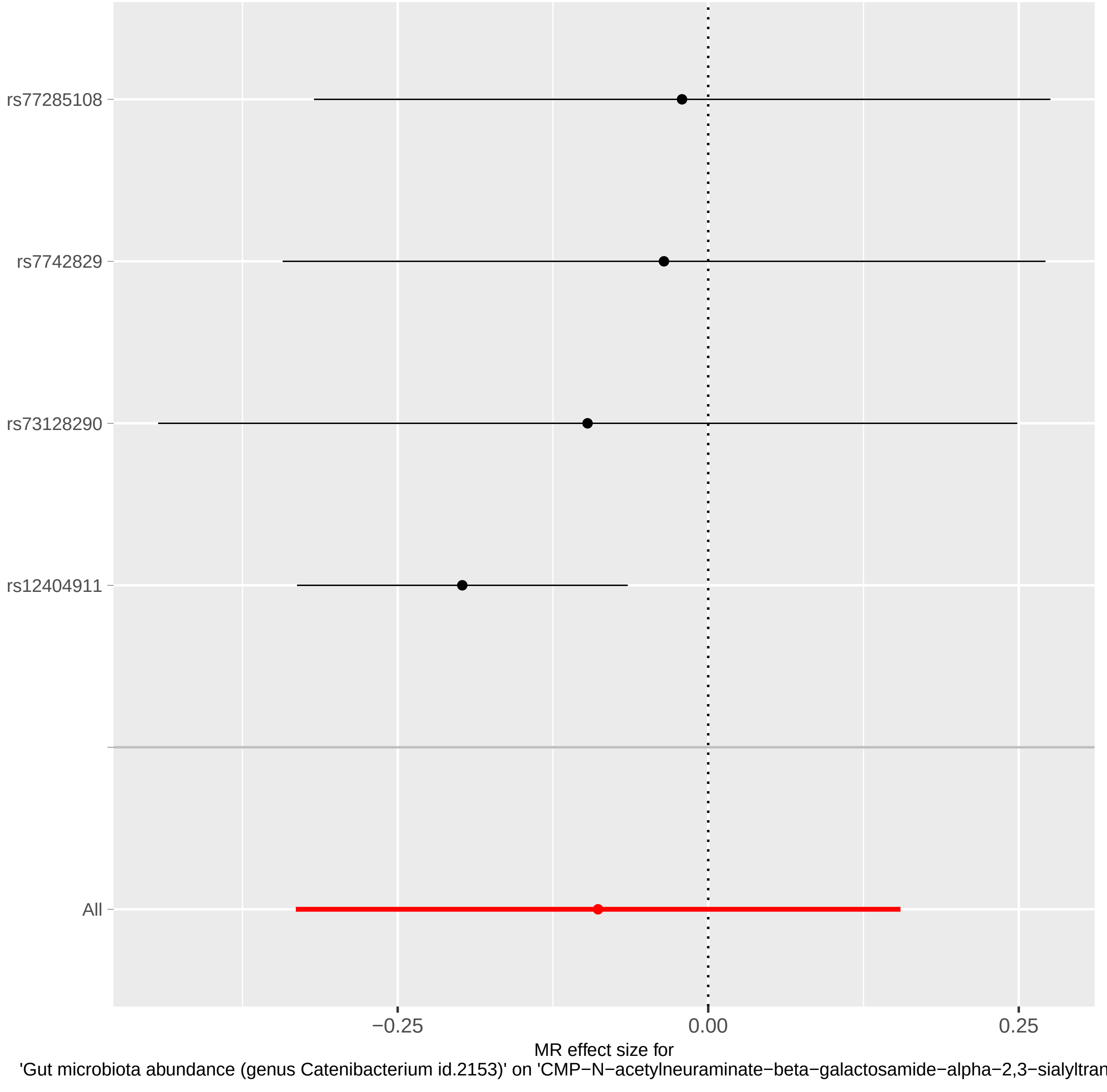

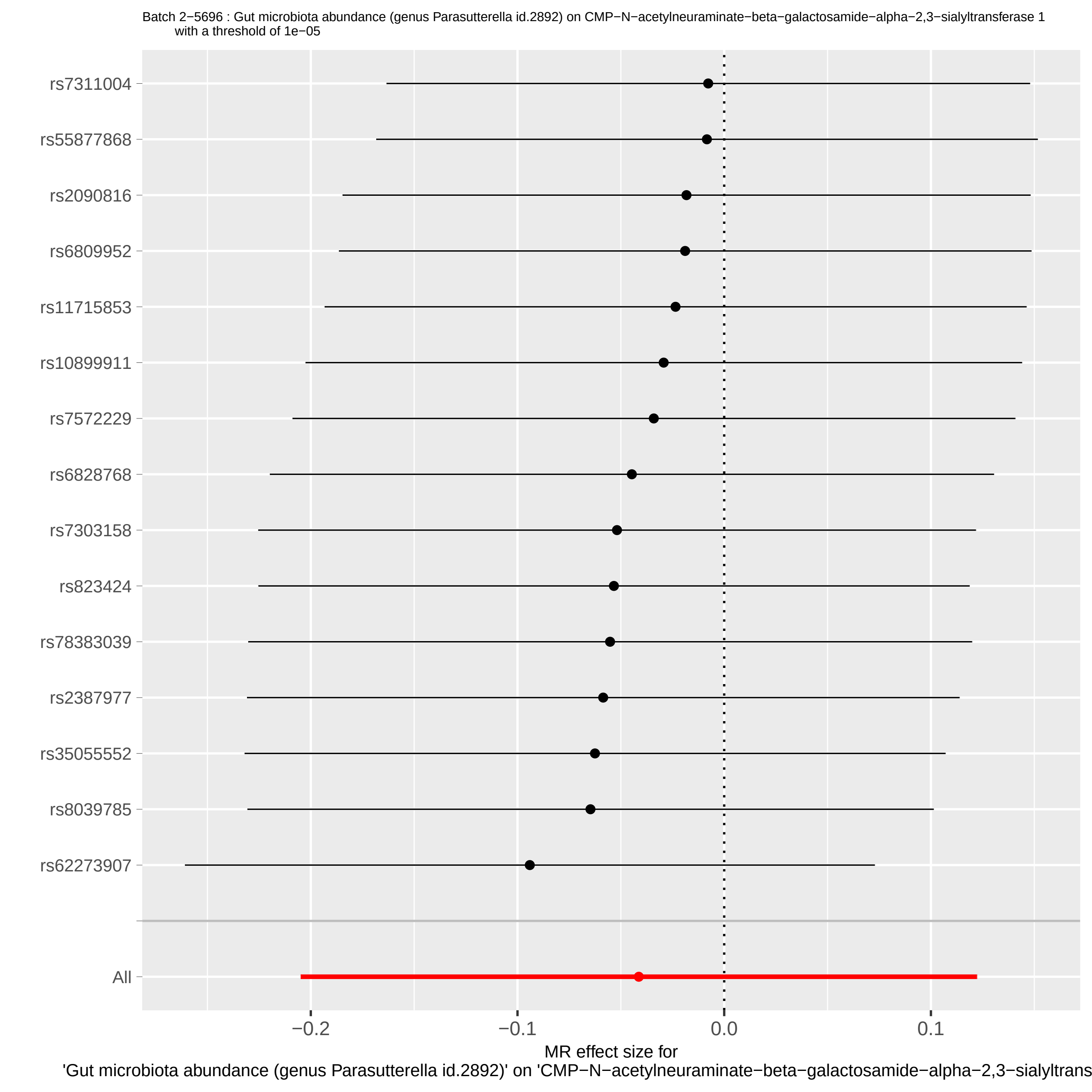

Batch 2−5696 : Gut microbiota abundance (genus Parasutterella id.2892) on CMP−N−acetylneuraminate−beta−galactosamide−alpha−2,3−sialyltransferase 1 with a threshold of 1e−05
rs7311004
rs55877868
rs2090816
rs6809952
rs11715853
rs10899911
rs7572229
rs6828768
rs7303158
rs823424
rs78383039
rs2387977
rs35055552
rs8039785
rs62273907
All
−0.2
−0.1
0.0
0.1
MR effect size for
'Gut microbiota abundance (genus Parasutterella id.2892)' on 'CMP−N−acetylneuraminate−beta−galactosamide−alpha−2,3−sialyltrans

Batch 2−5697 : Gut microbiota abundance (genus Catenibacterium id.2153) on CMP−N−acetylneuraminate−beta−galactosamide−alpha−2,3−sialyltransferase 2 with a threshold of 1e−05

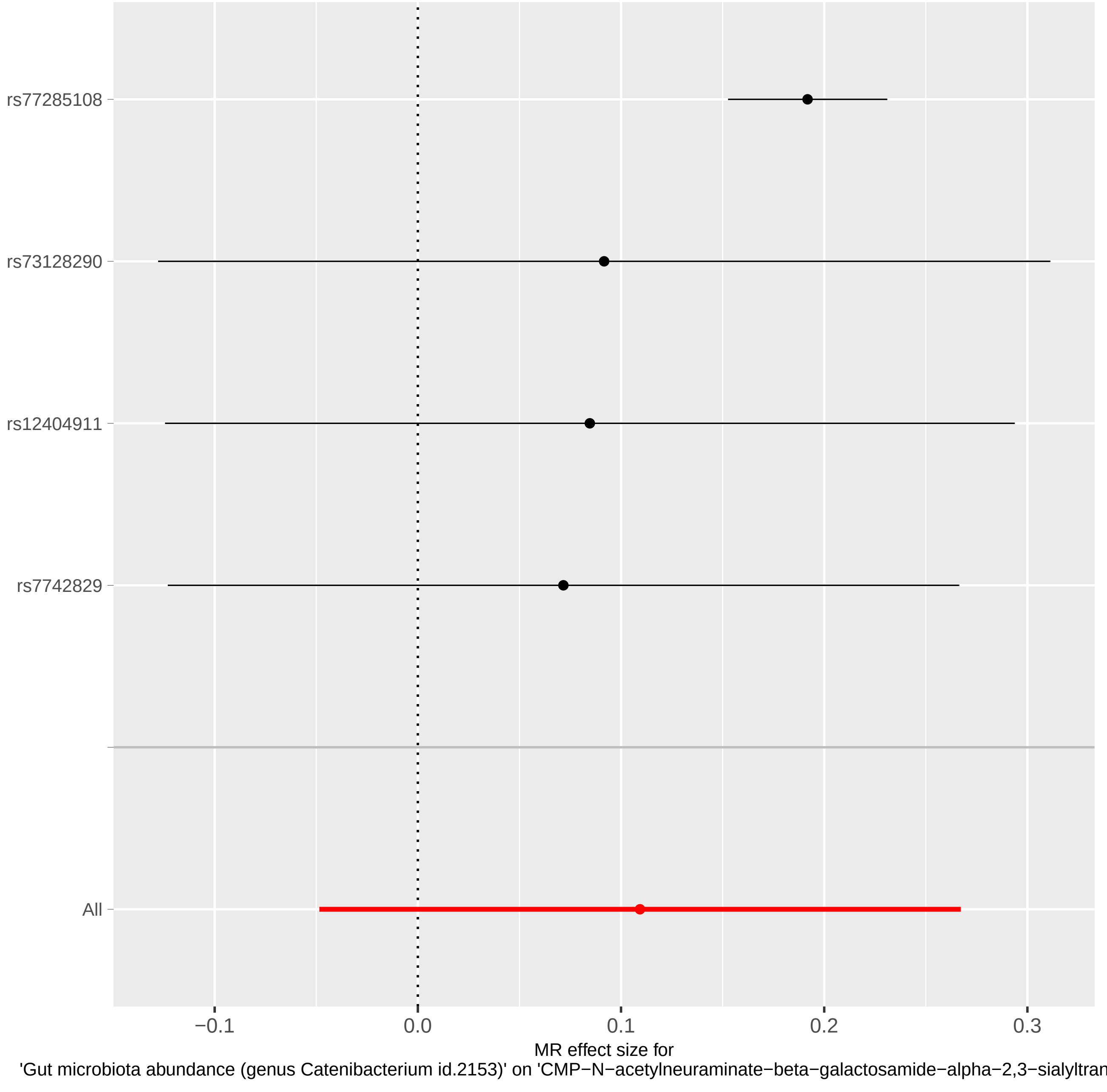

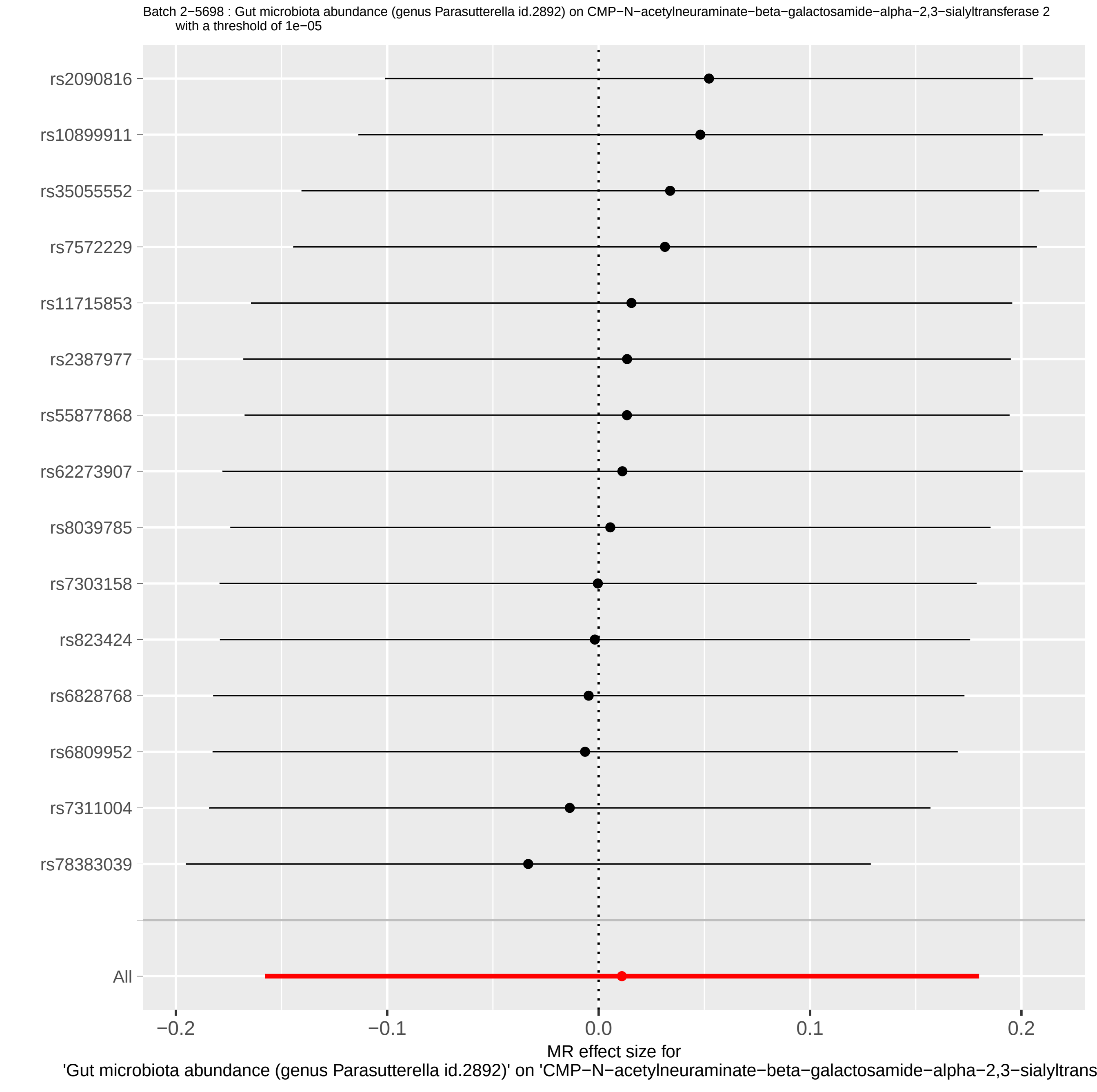

Batch 2−5698 : Gut microbiota abundance (genus Parasutterella id.2892) on CMP−N−acetylneuraminate−beta−galactosamide−alpha−2,3−sialyltransferase 2 with a threshold of 1e−05
rs2090816
rs10899911
rs35055552
rs7572229
rs11715853
rs2387977
rs55877868
rs62273907
rs8039785
rs7303158
rs823424
rs6828768
rs6809952
rs7311004
rs78383039
All
−0.2
−0.1
0.0
0.1
0.2
MR effect size for
'Gut microbiota abundance (genus Parasutterella id.2892)' on 'CMP−N−acetylneuraminate−beta−galactosamide−alpha−2,3−sialyltrans

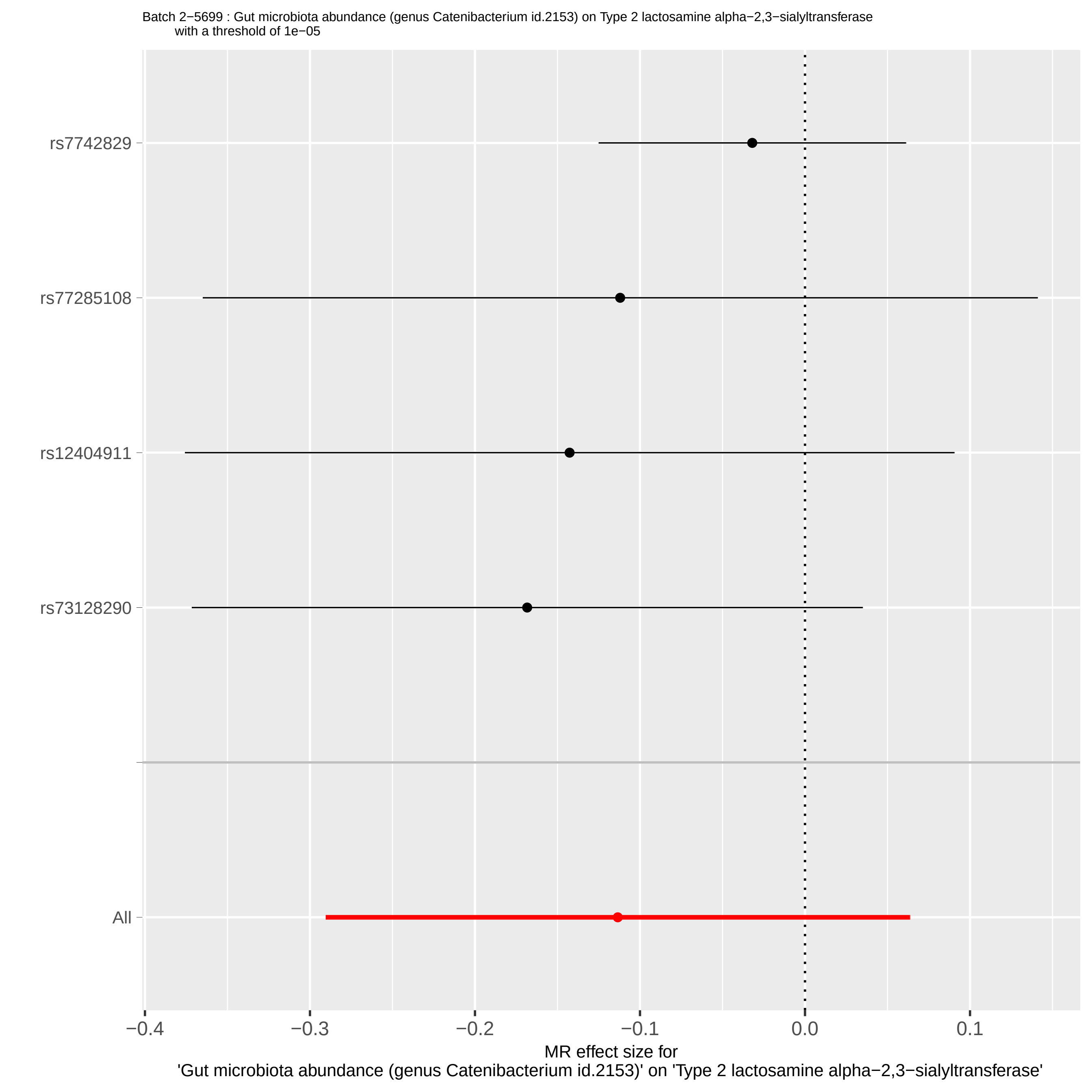
Batch 2−5699 : Gut microbiota abundance (genus Catenibacterium id.2153) on Type 2 lactosamine alpha−2,3−sialyltransferase
with a threshold of 1e−05
rs7742829
rs77285108
rs12404911
rs73128290
All
−0.4
−0.3
−0.2
−0.1
0.0
0.1
MR effect size for
'Gut microbiota abundance (genus Catenibacterium id.2153)' on 'Type 2 lactosamine alpha−2,3−sialyltransferase'

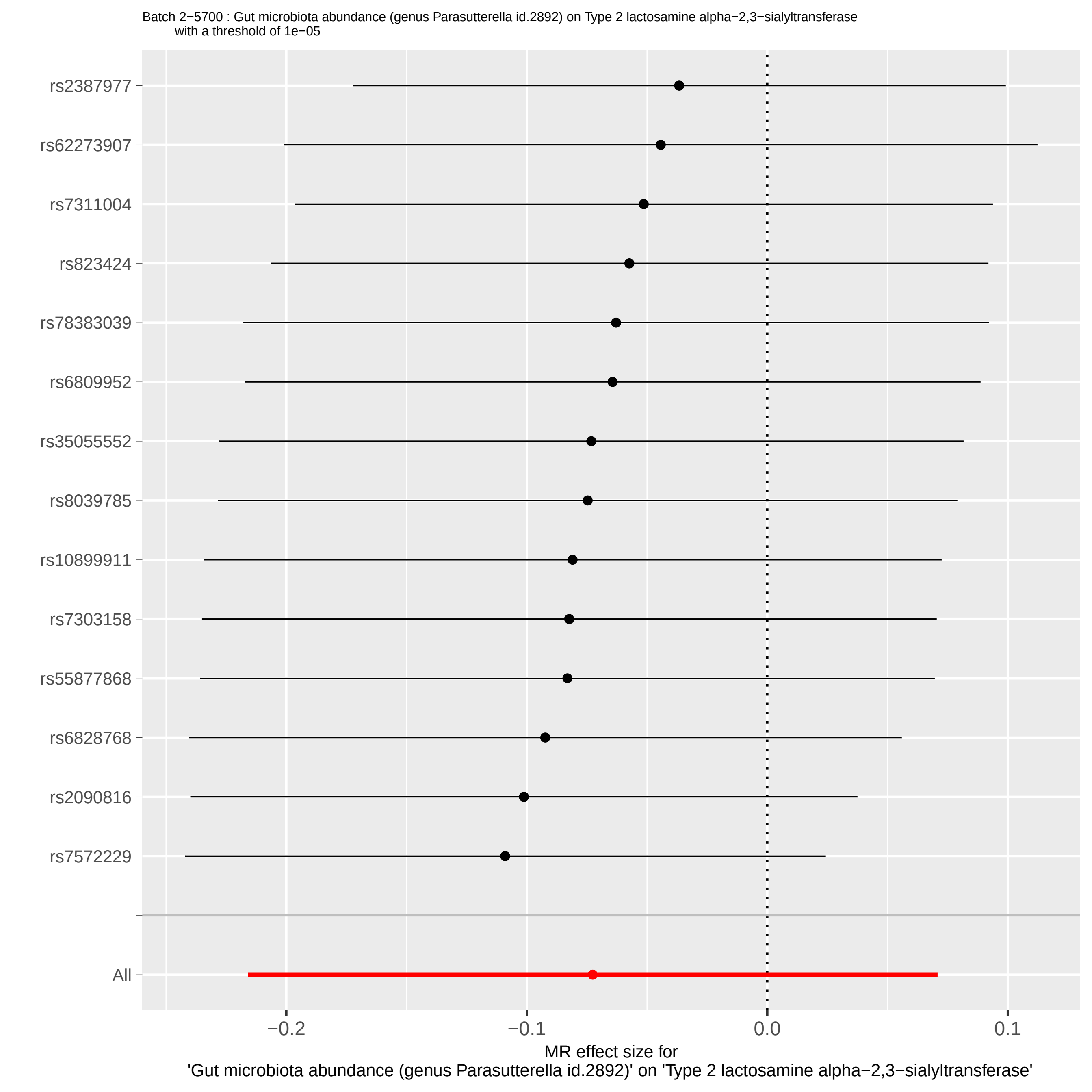

Batch 2−5700 : Gut microbiota abundance (genus Parasutterella id.2892) on Type 2 lactosamine alpha−2,3−sialyltransferase
with a threshold of 1e−05
rs2387977
rs62273907
rs7311004
rs823424
rs78383039
rs6809952
rs35055552
rs8039785
rs10899911
rs7303158
rs55877868
rs6828768
rs2090816
rs7572229
All
−0.2
−0.1
0.0
0.1
MR effect size for
'Gut microbiota abundance (genus Parasutterella id.2892)' on 'Type 2 lactosamine alpha−2,3−sialyltransferase'

Batch 2−5701 : Gut microbiota abundance (genus Catenibacterium id.2153) on Beta−galactoside alpha−2,6−sialyltransferase 1
with a threshold of 1e−05

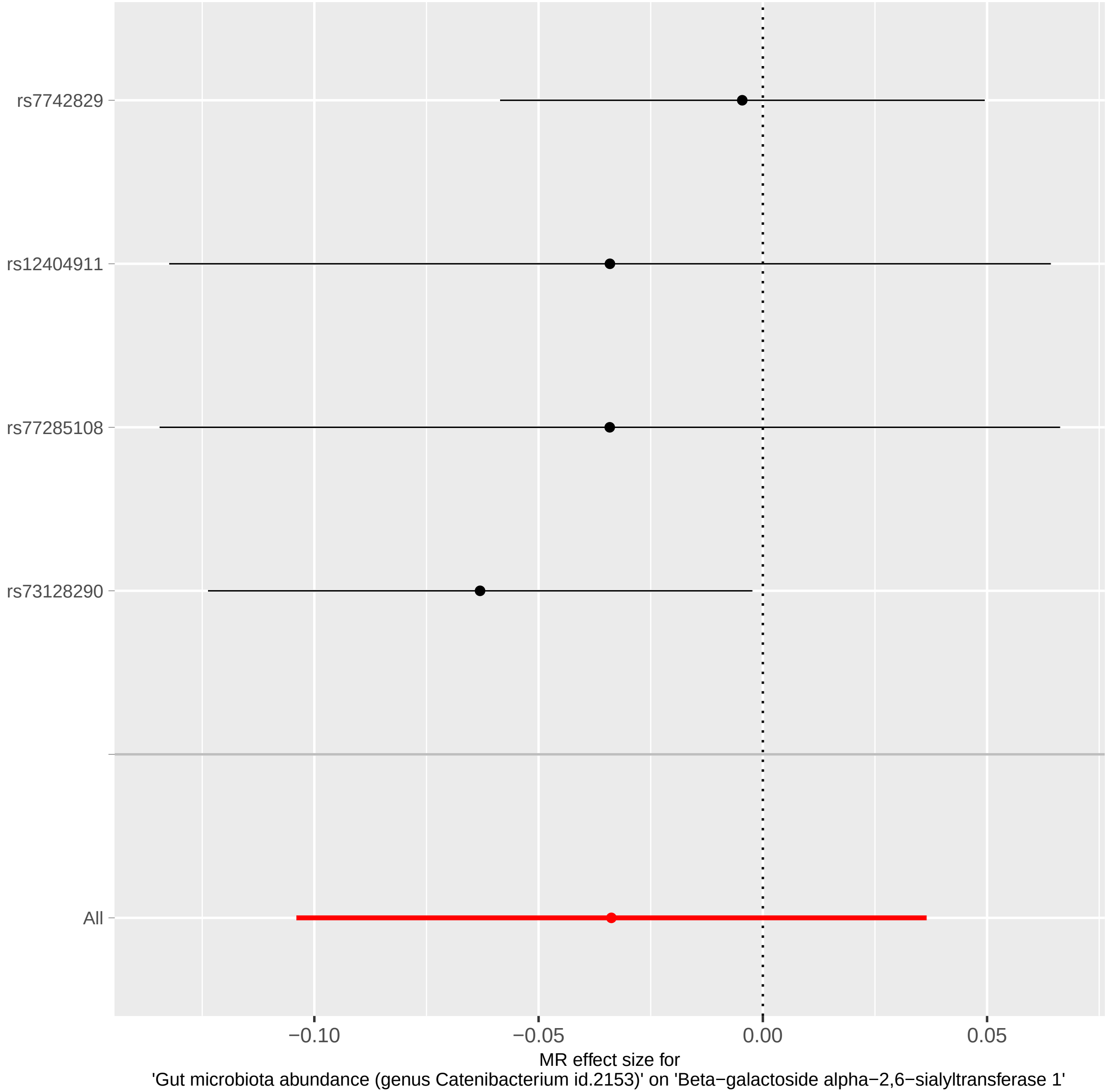

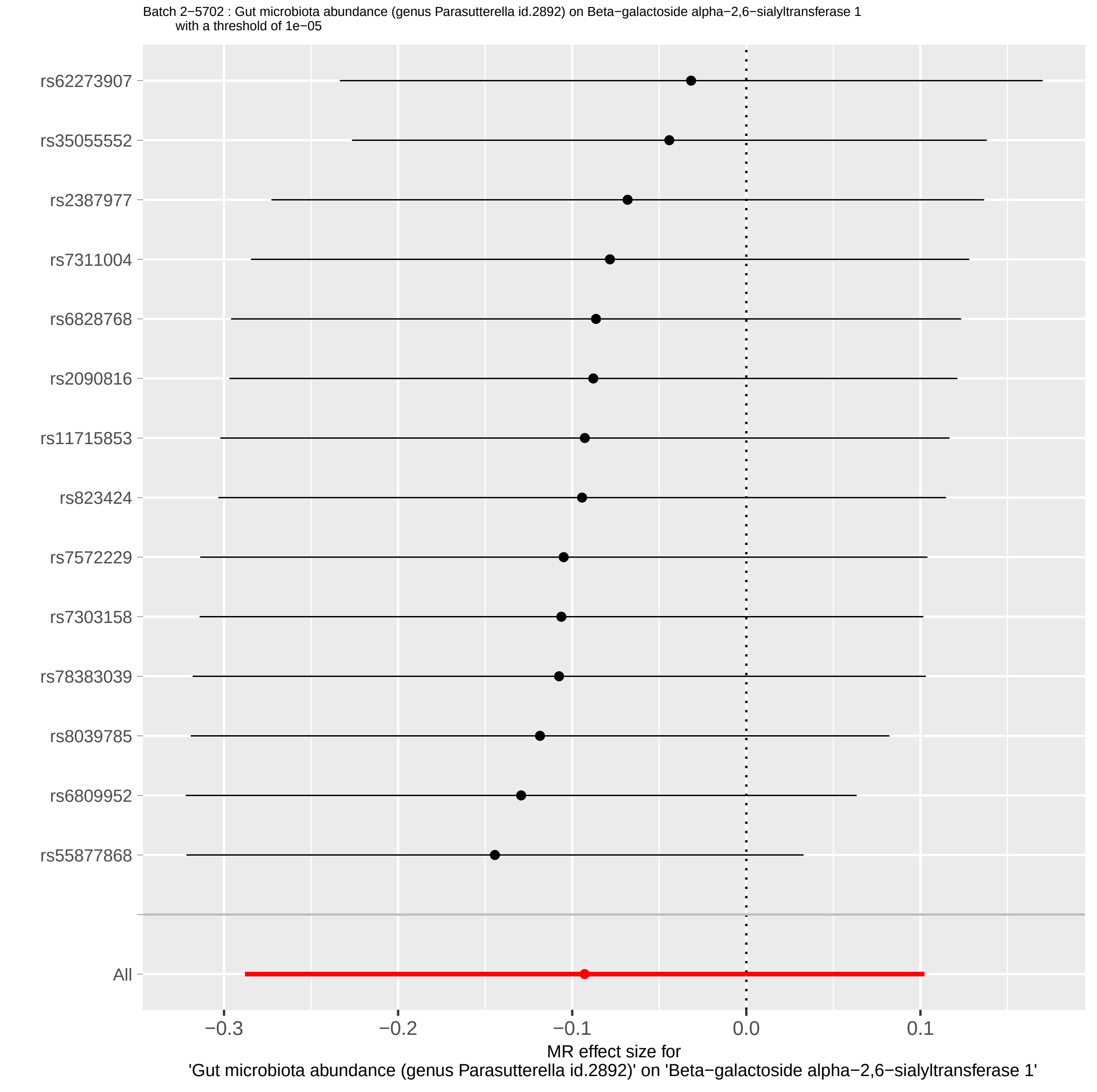

Batch 2−5702 : Gut microbiota abundance (genus Parasutterella id.2892) on Beta−galactoside alpha−2,6−sialyltransferase 1
with a threshold of 1e−05
rs62273907
rs35055552
rs2387977
rs7311004
rs6828768
rs2090816
rs11715853
rs823424
rs7572229
rs7303158
rs78383039
rs8039785
rs6809952
rs55877868
All
−0.3
−0.2
−0.1
0.0
0.1
MR effect size for
'Gut microbiota abundance (genus Parasutterella id.2892)' on 'Beta−galactoside alpha−2,6−sialyltransferase 1'

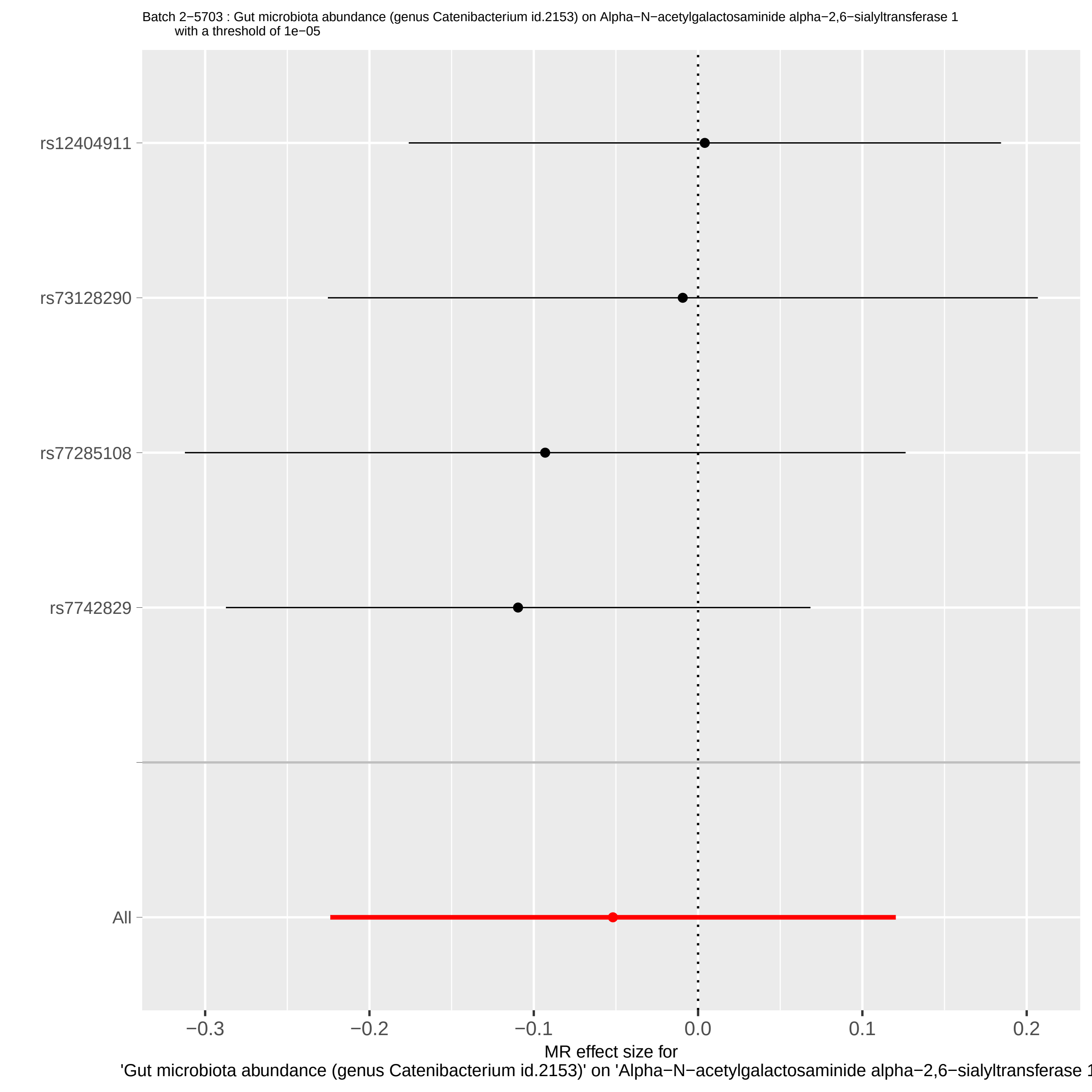
Batch 2−5703 : Gut microbiota abundance (genus Catenibacterium id.2153) on Alpha−N−acetylgalactosaminide alpha−2,6−sialyltransferase 1 with a threshold of 1e−05
rs12404911
rs73128290
rs77285108
rs7742829
All
−0.3
−0.2
−0.1
0.0
0.1
0.2
MR effect size for
'Gut microbiota abundance (genus Catenibacterium id.2153)' on 'Alpha−N−acetylgalactosaminide alpha−2,6−sialyltransferase 1

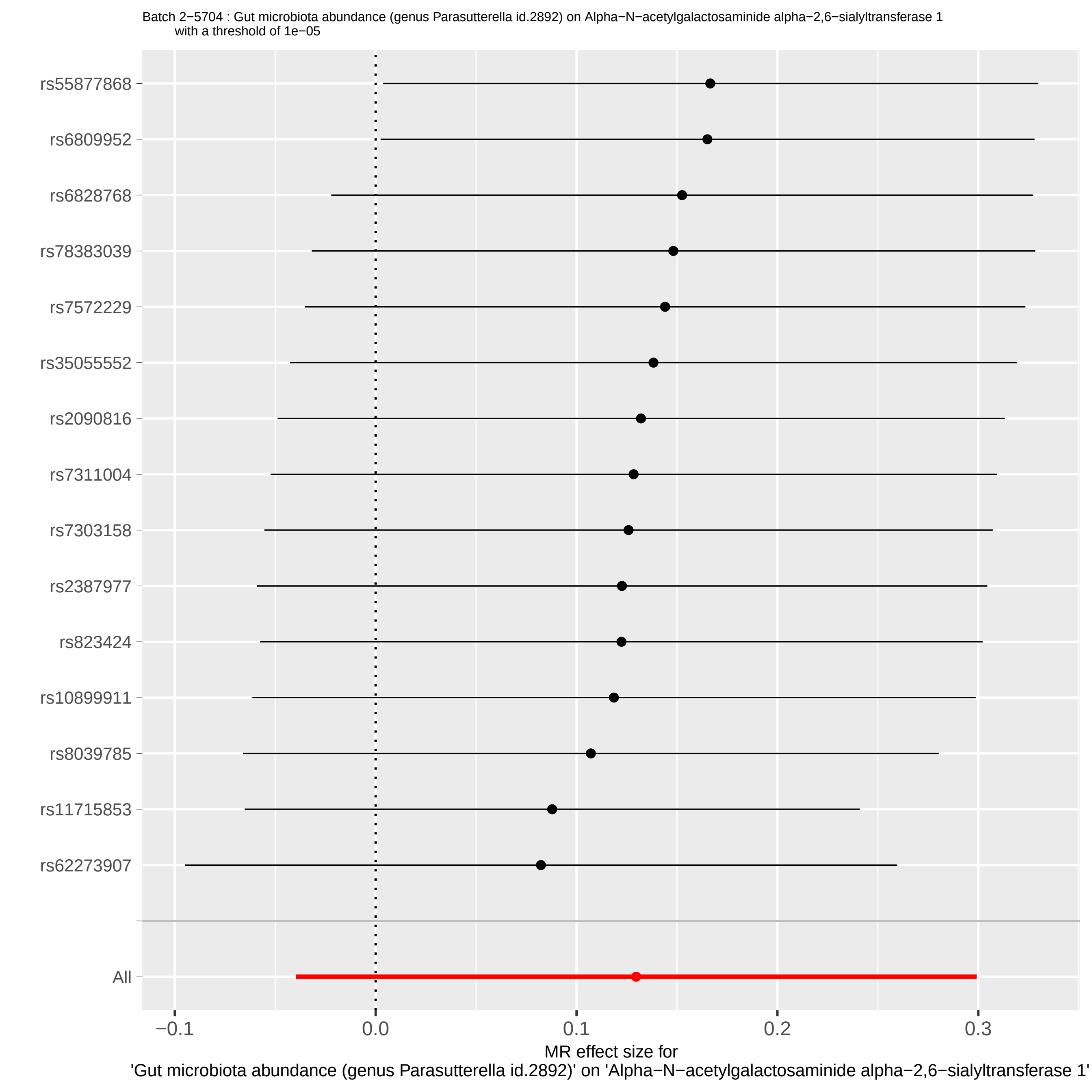
Batch 2−5704 : Gut microbiota abundance (genus Parasutterella id.2892) on Alpha−N−acetylgalactosaminide alpha−2,6−sialyltransferase 1
with a threshold of 1e−05
rs55877868
rs6809952
rs6828768
rs78383039
rs7572229
rs35055552
rs2090816
rs7311004
rs7303158
rs2387977
rs823424
rs10899911
rs8039785
rs11715853
rs62273907
All
−0.1
0.0
0.1
0.2
0.3
MR effect size for
'Gut microbiota abundance (genus Parasutterella id.2892)' on 'Alpha−N−acetylgalactosaminide alpha−2,6−sialyltransferase 1'

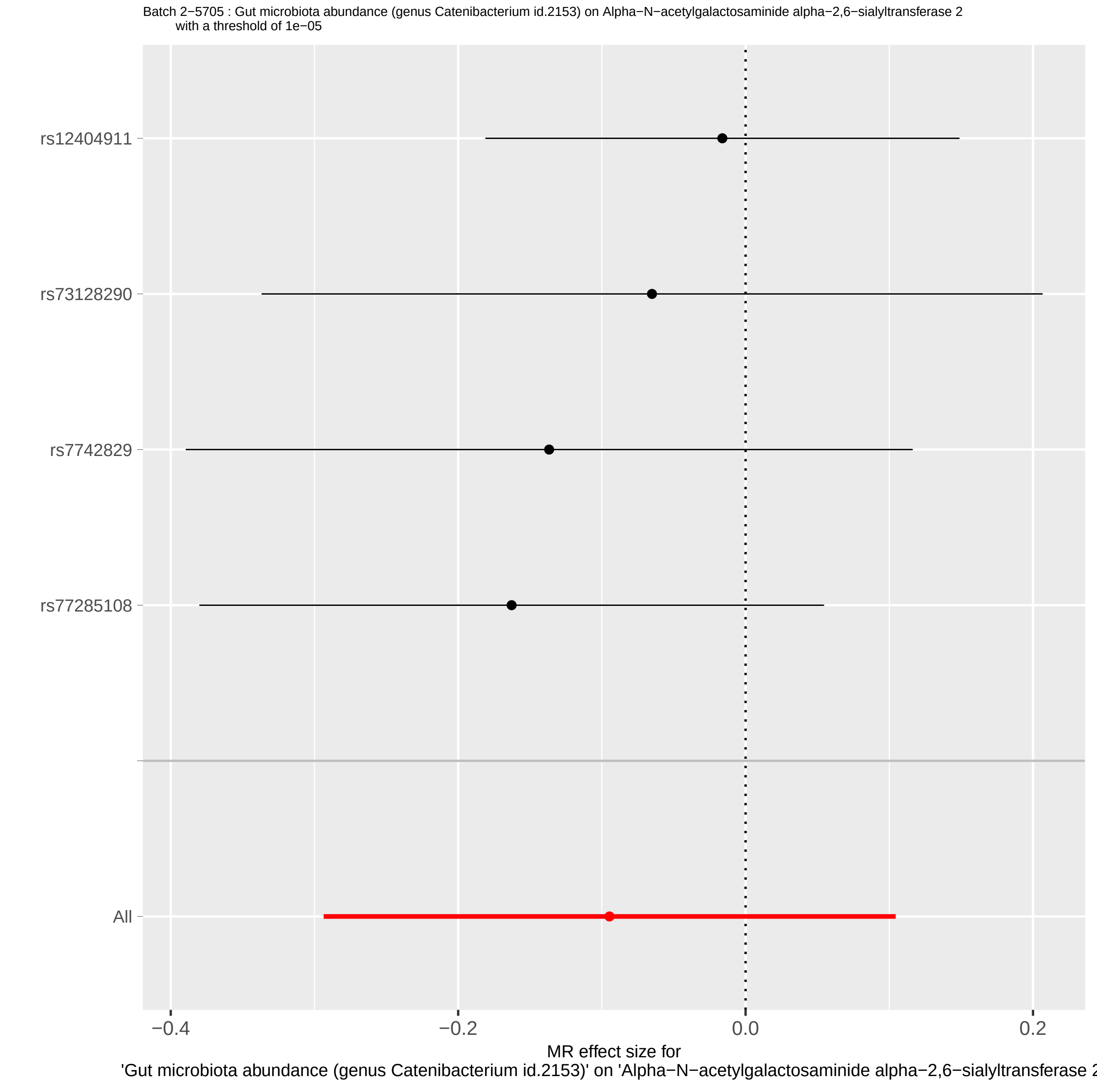

Batch 2−5705 : Gut microbiota abundance (genus Catenibacterium id.2153) on Alpha−N−acetylgalactosaminide alpha−2,6−sialyltransferase 2
with a threshold of 1e−05
rs12404911
rs73128290
rs7742829
rs77285108
All
−0.4
−0.2
0.0
0.2
MR effect size for
'Gut microbiota abundance (genus Catenibacterium id.2153)' on 'Alpha−N−acetylgalactosaminide alpha−2,6−sialyltransferase 2

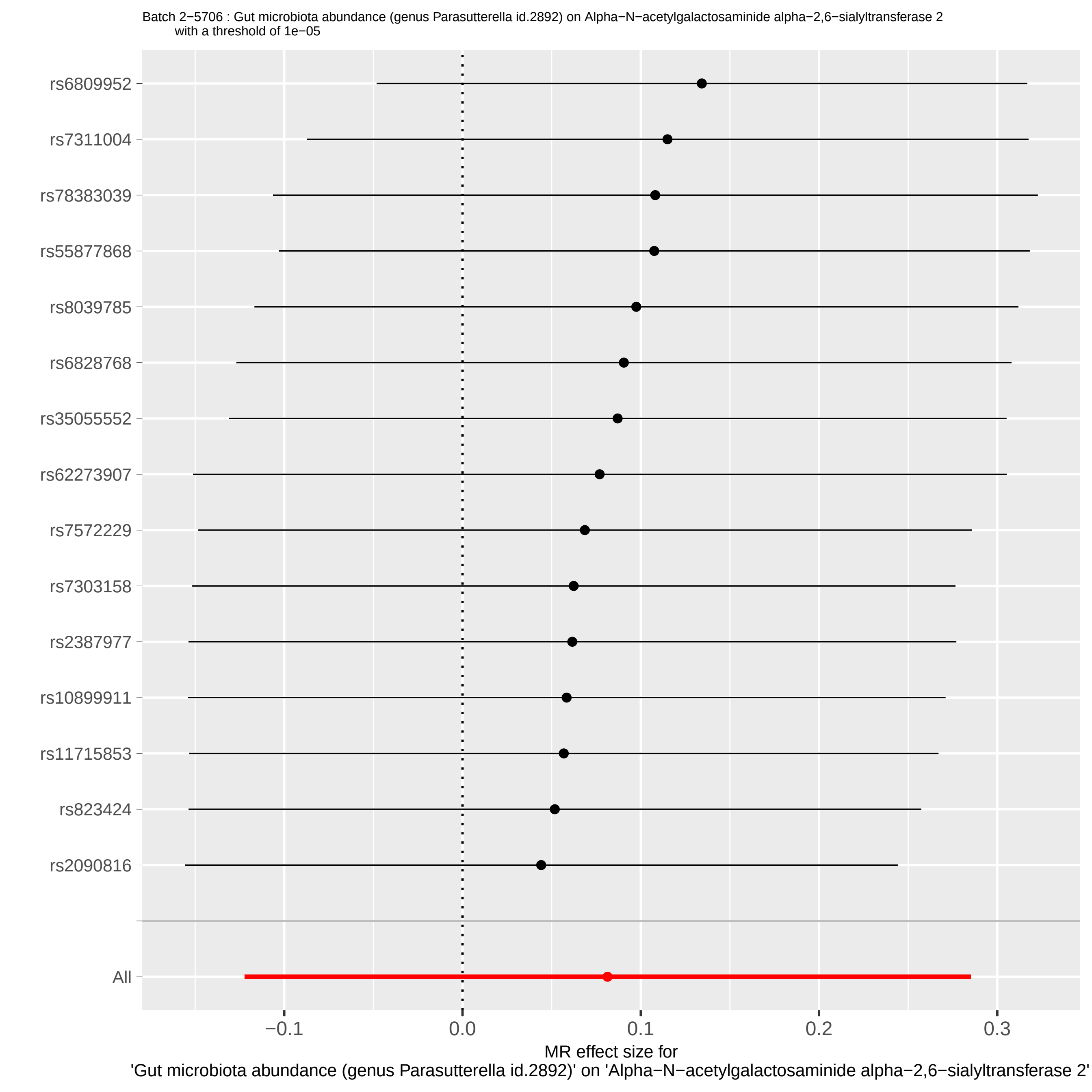

Batch 2−5706 : Gut microbiota abundance (genus Parasutterella id.2892) on Alpha−N−acetylgalactosaminide alpha−2,6−sialyltransferase 2
with a threshold of 1e−05
rs6809952
rs7311004
rs78383039
rs55877868
rs8039785
rs6828768
rs35055552
rs62273907
rs7572229
rs7303158
rs2387977
rs10899911
rs11715853
rs823424
rs2090816
All
−0.1
0.0
0.1
0.2
0.3
MR effect size for
'Gut microbiota abundance (genus Parasutterella id.2892)' on 'Alpha−N−acetylgalactosaminide alpha−2,6−sialyltransferase 2'

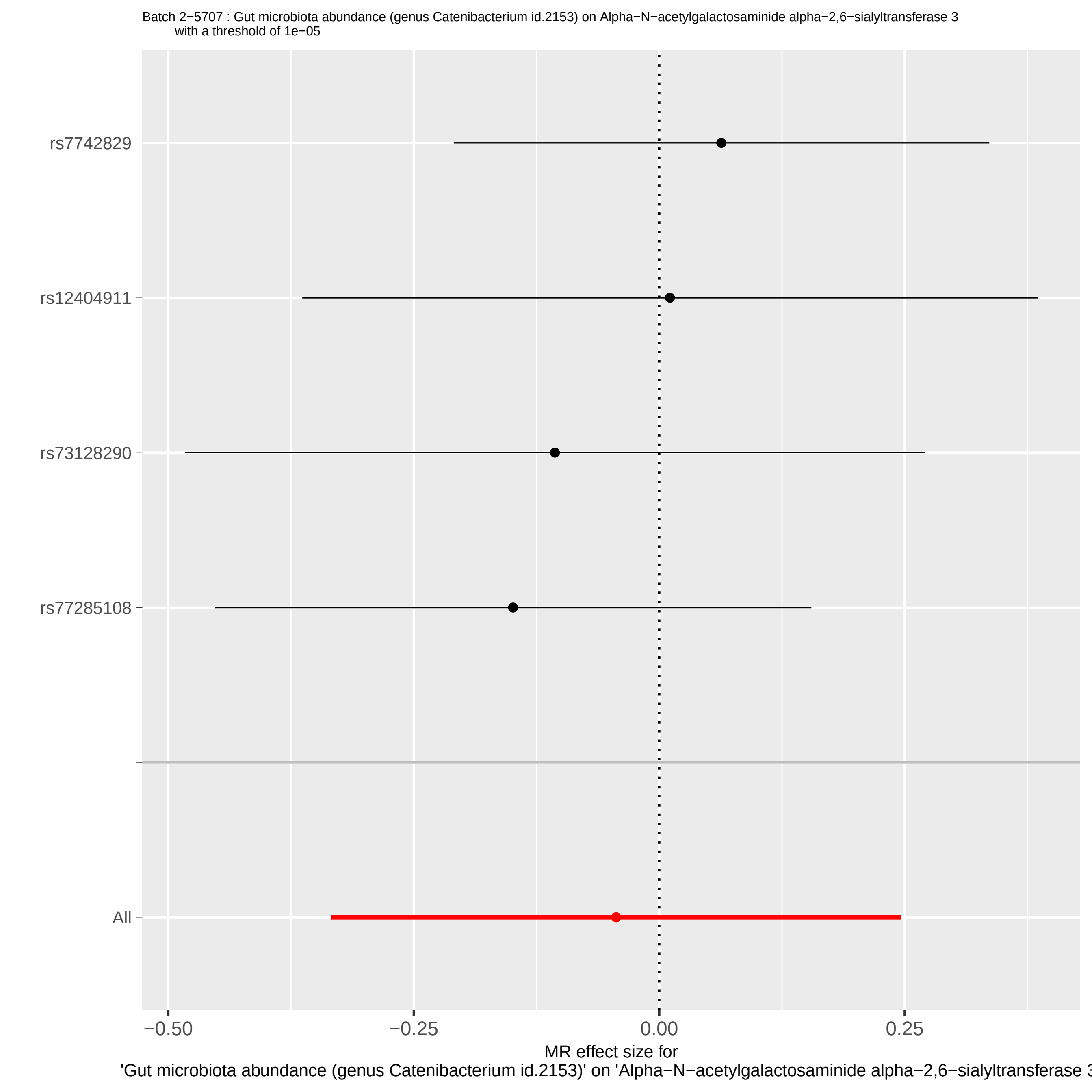
Batch 2−5707 : Gut microbiota abundance (genus Catenibacterium id.2153) on Alpha−N−acetylgalactosaminide alpha−2,6−sialyltransferase 3
with a threshold of 1e−05
rs7742829
rs12404911
rs73128290
rs77285108
All
−0.50
−0.25
0.00
0.25
MR effect size for
'Gut microbiota abundance (genus Catenibacterium id.2153)' on 'Alpha−N−acetylgalactosaminide alpha−2,6−sialyltransferase 3

Batch 2−5708 : Gut microbiota abundance (genus Parasutterella id.2892) on Alpha−N−acetylgalactosaminide alpha−2,6−sialyltransferase 3 with a threshold of 1e−05

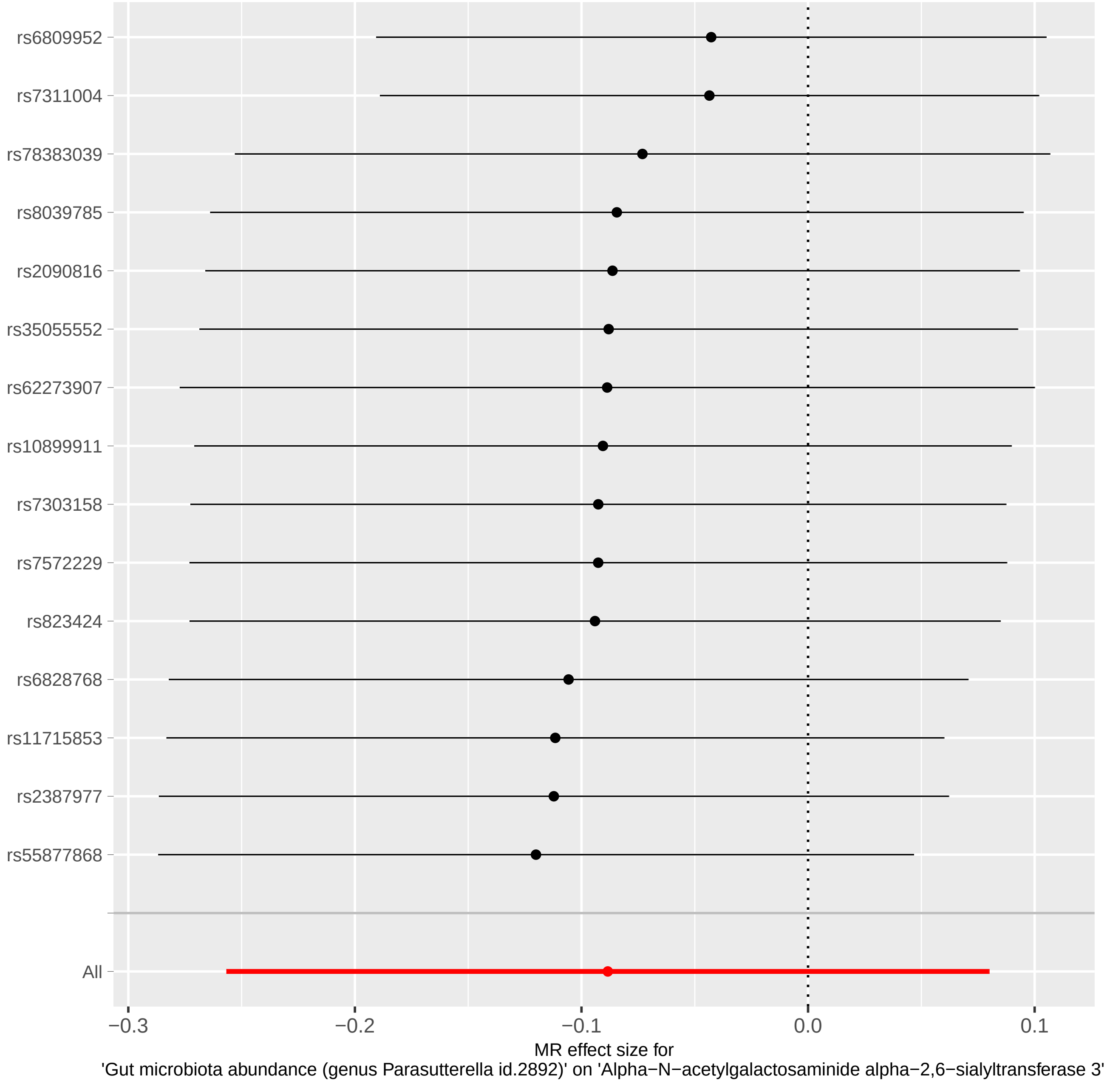

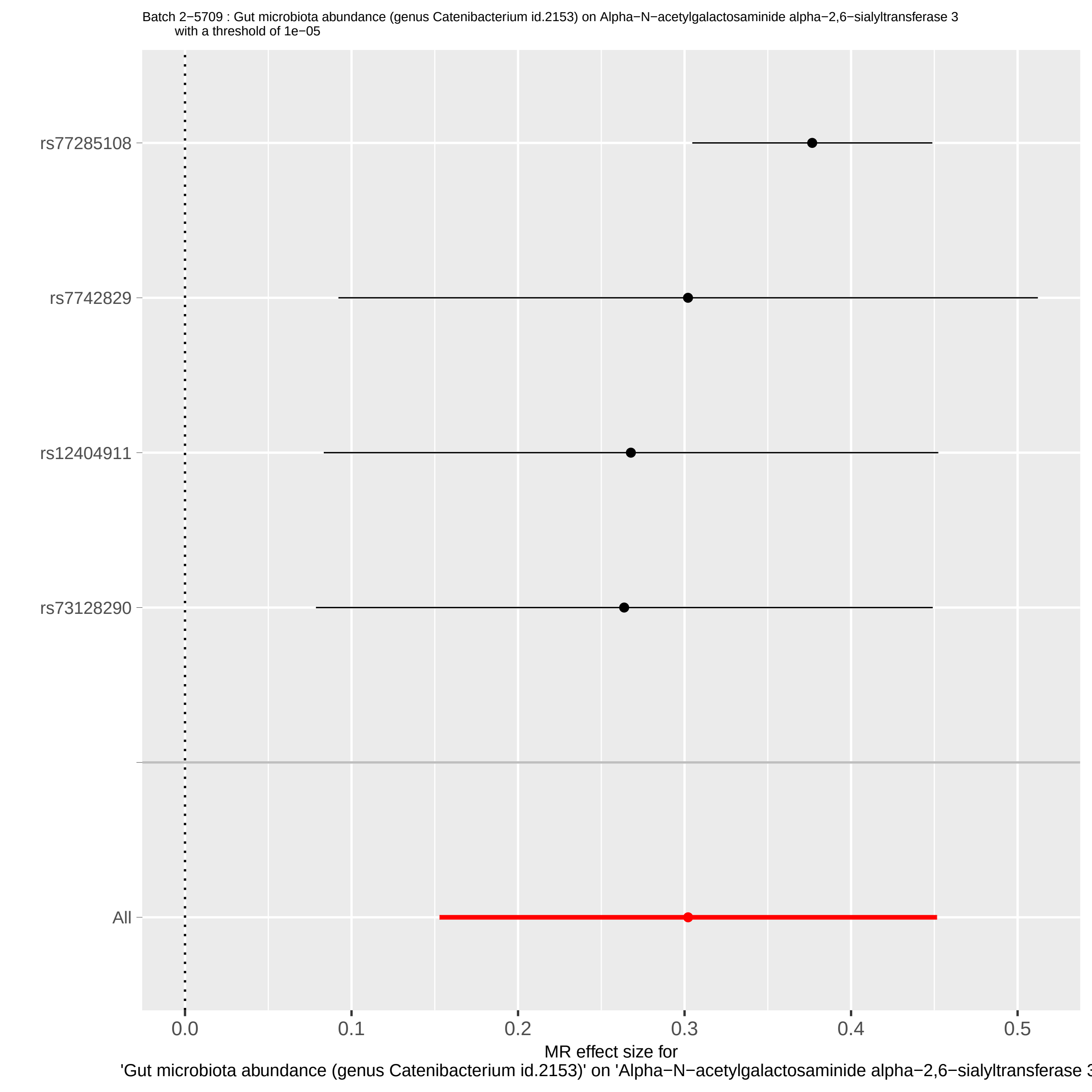
Batch 2−5709 : Gut microbiota abundance (genus Catenibacterium id.2153) on Alpha−N−acetylgalactosaminide alpha−2,6−sialyltransferase 3
with a threshold of 1e−05
rs77285108
rs7742829
rs12404911
rs73128290
All
0.0
0.1
0.2
0.3
0.4
0.5
MR effect size for
'Gut microbiota abundance (genus Catenibacterium id.2153)' on 'Alpha−N−acetylgalactosaminide alpha−2,6−sialyltransferase 3

Batch 2−5710 : Gut microbiota abundance (genus Parasutterella id.2892) on Alpha−N−acetylgalactosaminide alpha−2,6−sialyltransferase 3 with a threshold of 1e−05

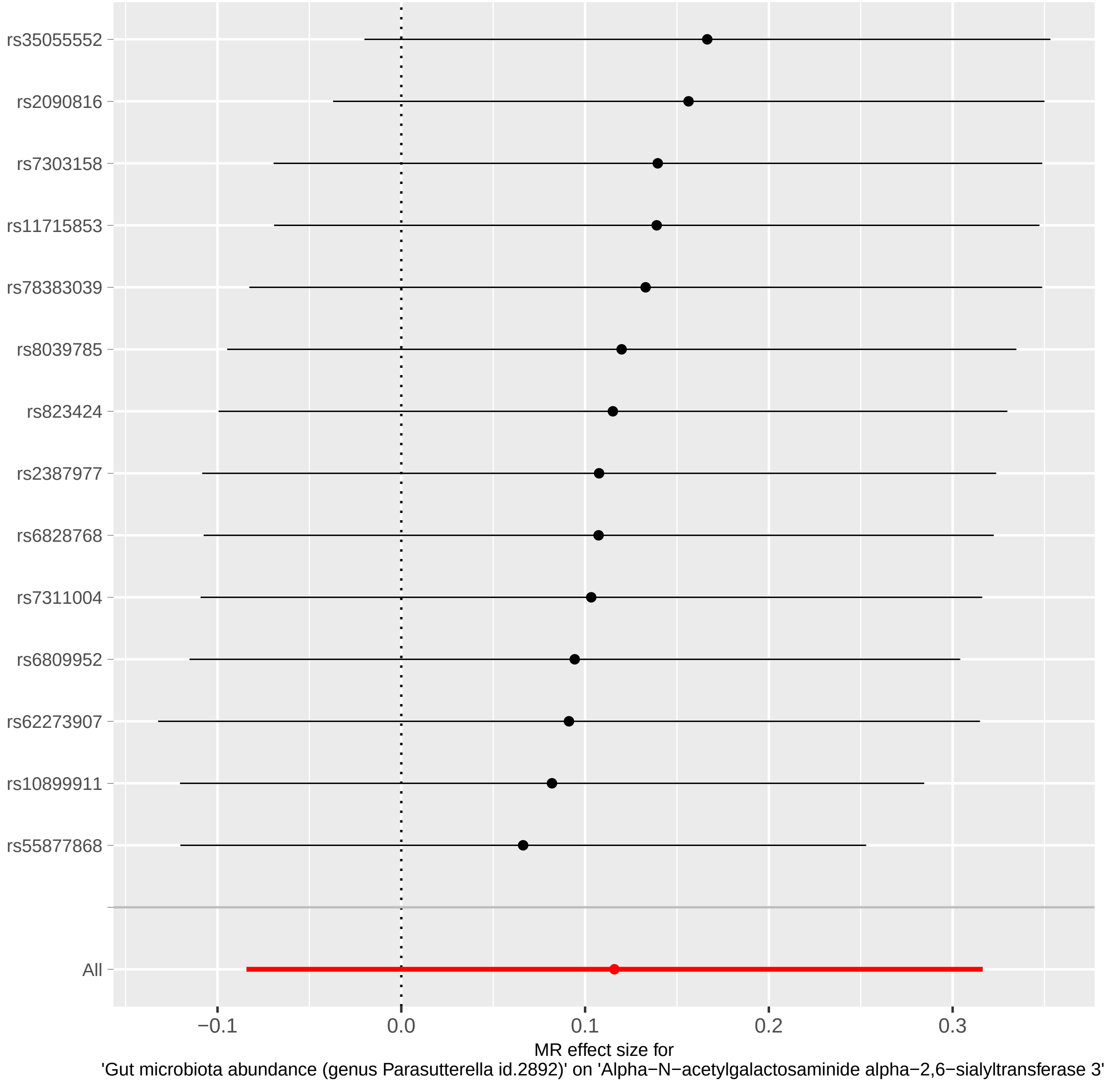

Batch 2−5711 : Gut microbiota abundance (genus Catenibacterium id.2153) on Alpha−N−acetylgalactosaminide alpha−2,6−sialyltransferase 5 with a threshold of 1e−05

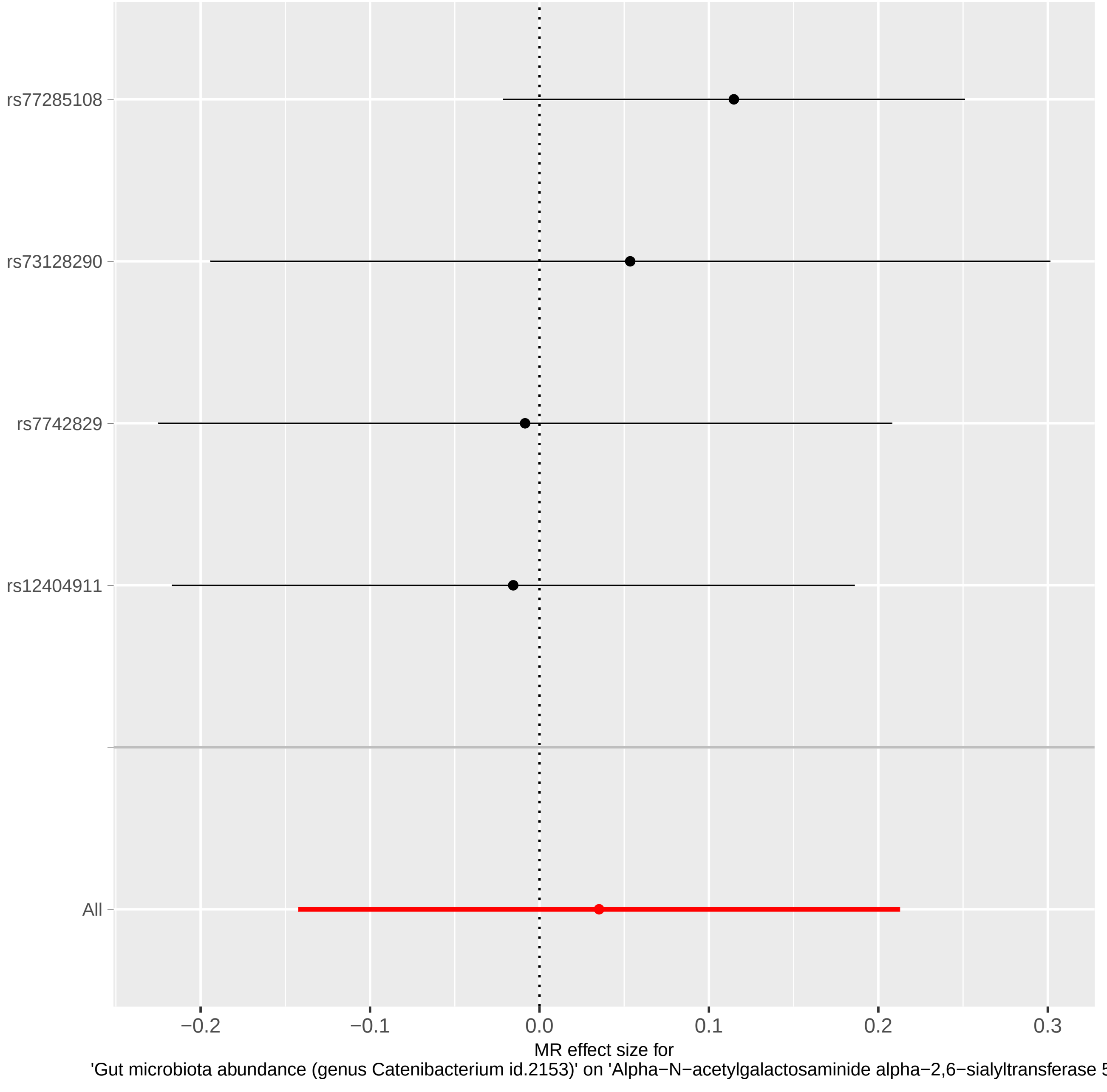

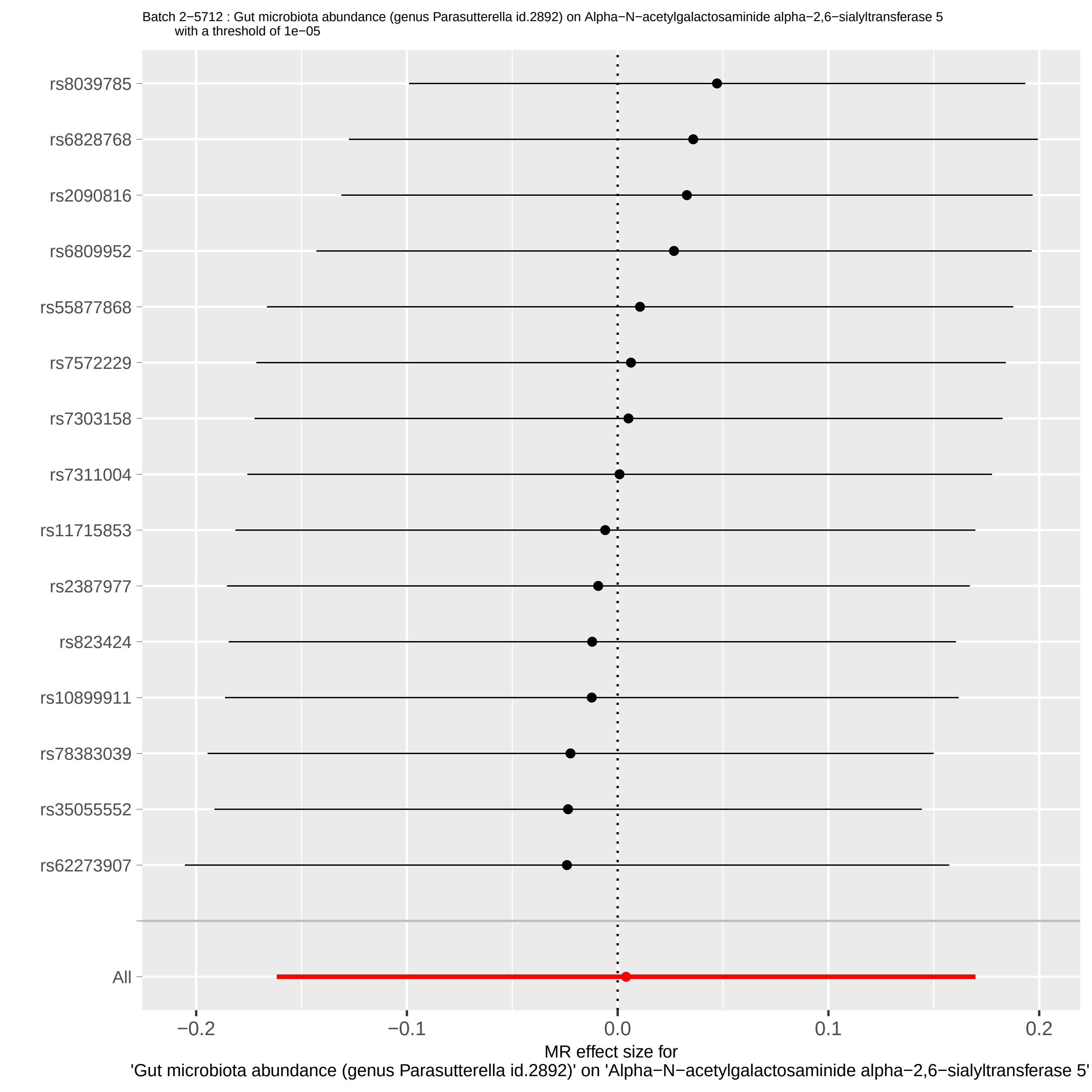
Batch 2−5712 : Gut microbiota abundance (genus Parasutterella id.2892) on Alpha−N−acetylgalactosaminide alpha−2,6−sialyltransferase 5
with a threshold of 1e−05
rs8039785
rs6828768
rs2090816
rs6809952
rs55877868
rs7572229
rs7303158
rs7311004
rs11715853
rs2387977
rs823424
rs10899911
rs78383039
rs35055552
rs62273907
All
−0.2
−0.1
0.0
0.1
0.2
MR effect size for
'Gut microbiota abundance (genus Parasutterella id.2892)' on 'Alpha−N−acetylgalactosaminide alpha−2,6−sialyltransferase 5'

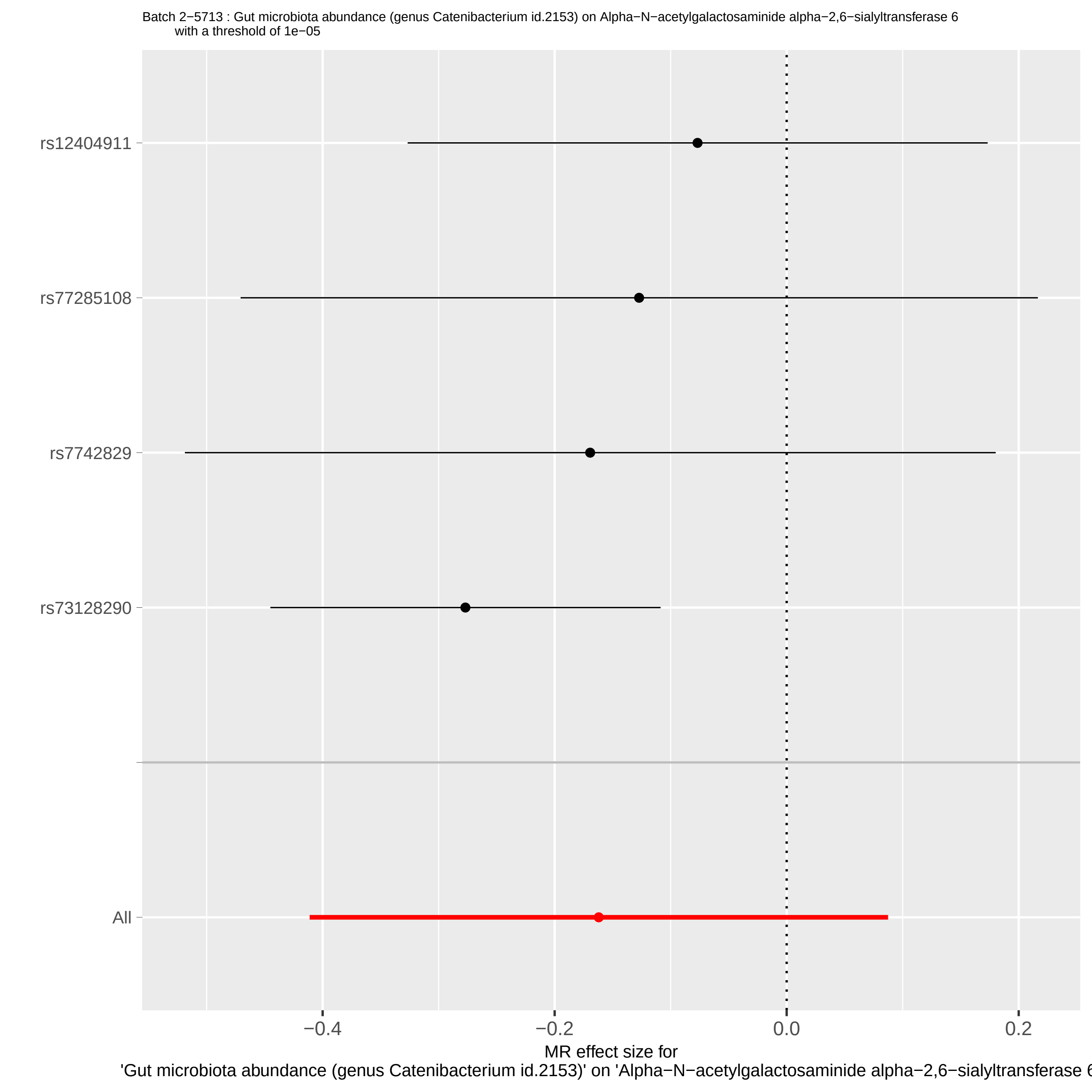

Batch 2−5713 : Gut microbiota abundance (genus Catenibacterium id.2153) on Alpha−N−acetylgalactosaminide alpha−2,6−sialyltransferase 6
with a threshold of 1e−05
rs12404911
rs77285108
rs7742829
rs73128290
All
−0.4
−0.2
0.0
0.2
MR effect size for
'Gut microbiota abundance (genus Catenibacterium id.2153)' on 'Alpha−N−acetylgalactosaminide alpha−2,6−sialyltransferase 6

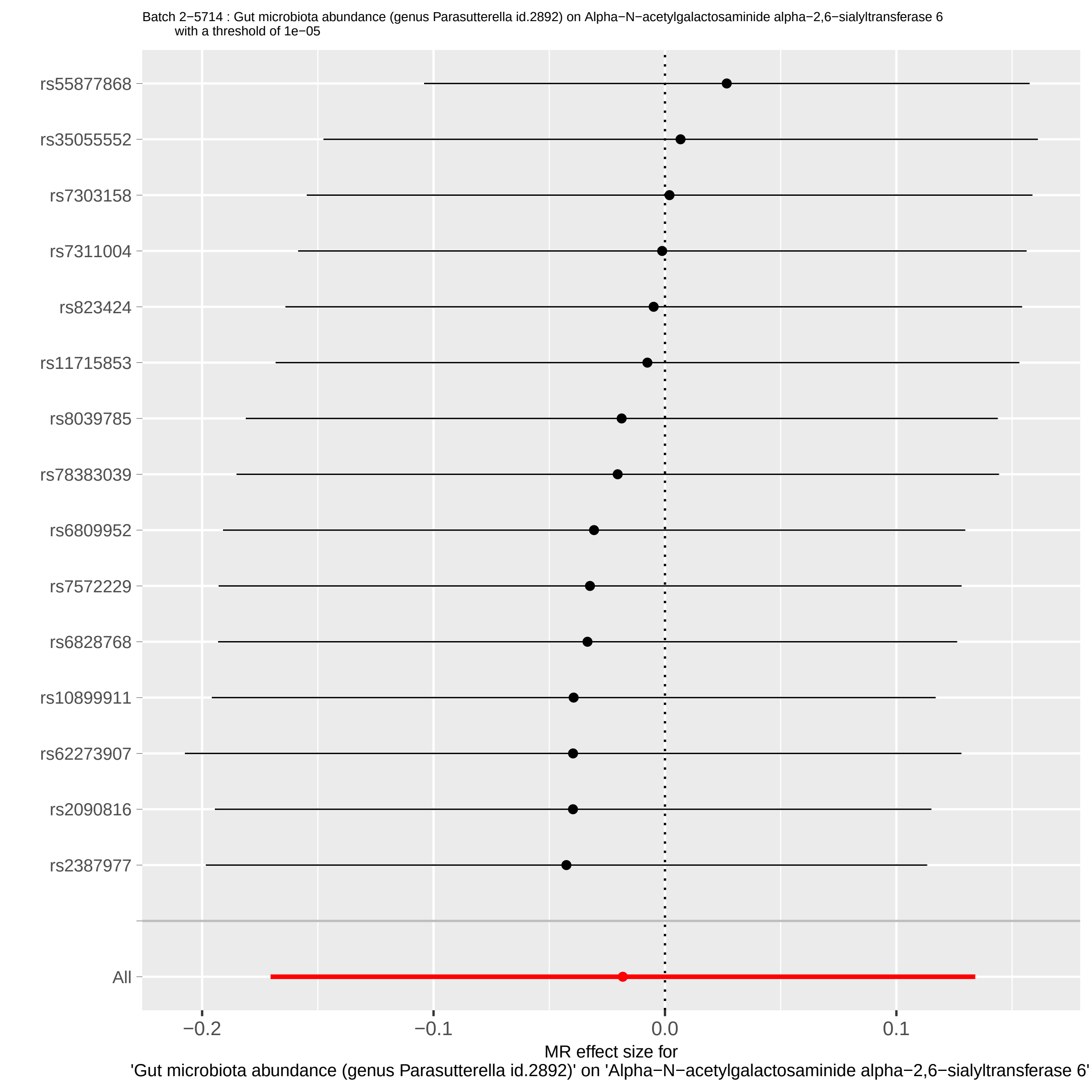

Batch 2−5714 : Gut microbiota abundance (genus Parasutterella id.2892) on Alpha−N−acetylgalactosaminide alpha−2,6−sialyltransferase 6
with a threshold of 1e−05
rs55877868
rs35055552
rs7303158
rs7311004
rs823424
rs11715853
rs8039785
rs78383039
rs6809952
rs7572229
rs6828768
rs10899911
rs62273907
rs2090816
rs2387977
All
−0.2
−0.1
0.0
0.1
MR effect size for
'Gut microbiota abundance (genus Parasutterella id.2892)' on 'Alpha−N−acetylgalactosaminide alpha−2,6−sialyltransferase 6'

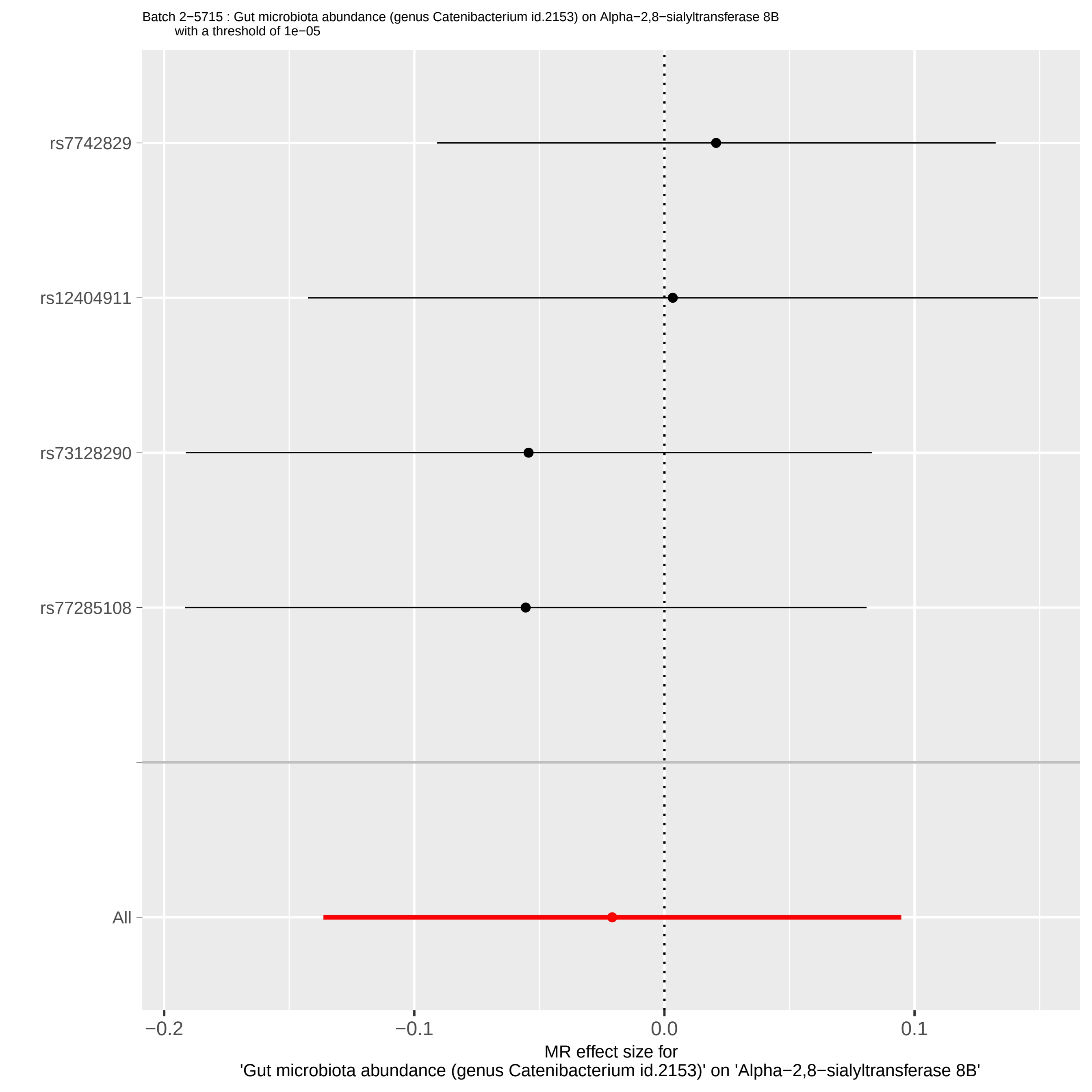

Batch 2−5715 : Gut microbiota abundance (genus Catenibacterium id.2153) on Alpha−2,8−sialyltransferase 8B
with a threshold of 1e−05
rs7742829
rs12404911
rs73128290
rs77285108
All
−0.2
−0.1
0.0
0.1
MR effect size for
'Gut microbiota abundance (genus Catenibacterium id.2153)' on 'Alpha−2,8−sialyltransferase 8B'

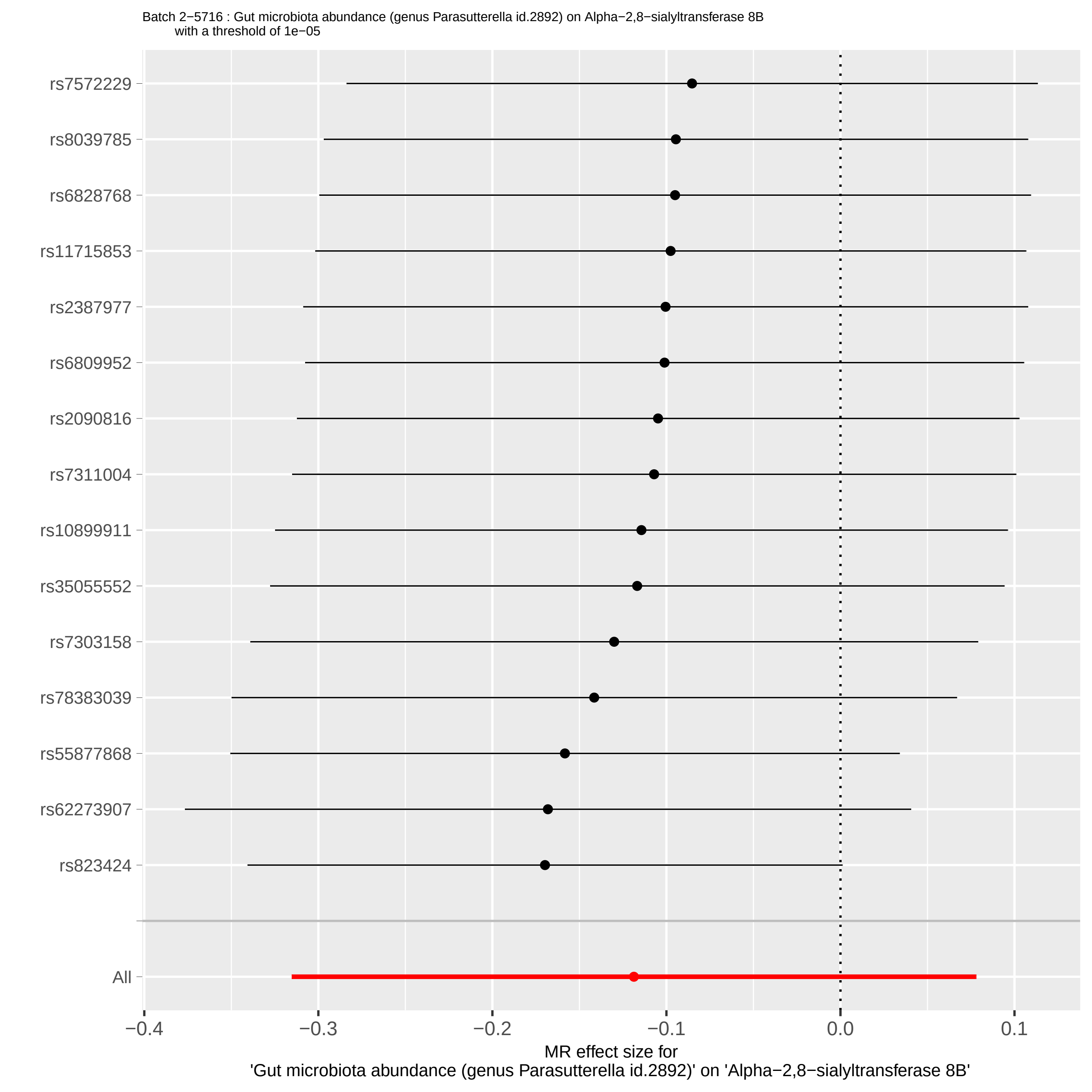
Batch 2−5716 : Gut microbiota abundance (genus Parasutterella id.2892) on Alpha−2,8−sialyltransferase 8B
with a threshold of 1e−05
rs7572229
rs8039785
rs6828768
rs11715853
rs2387977
rs6809952
rs2090816
rs7311004
rs10899911
rs35055552
rs7303158
rs78383039
rs55877868
rs62273907
rs823424
All
−0.4
−0.3
−0.2
−0.1
0.0
0.1
MR effect size for
'Gut microbiota abundance (genus Parasutterella id.2892)' on 'Alpha−2,8−sialyltransferase 8B'

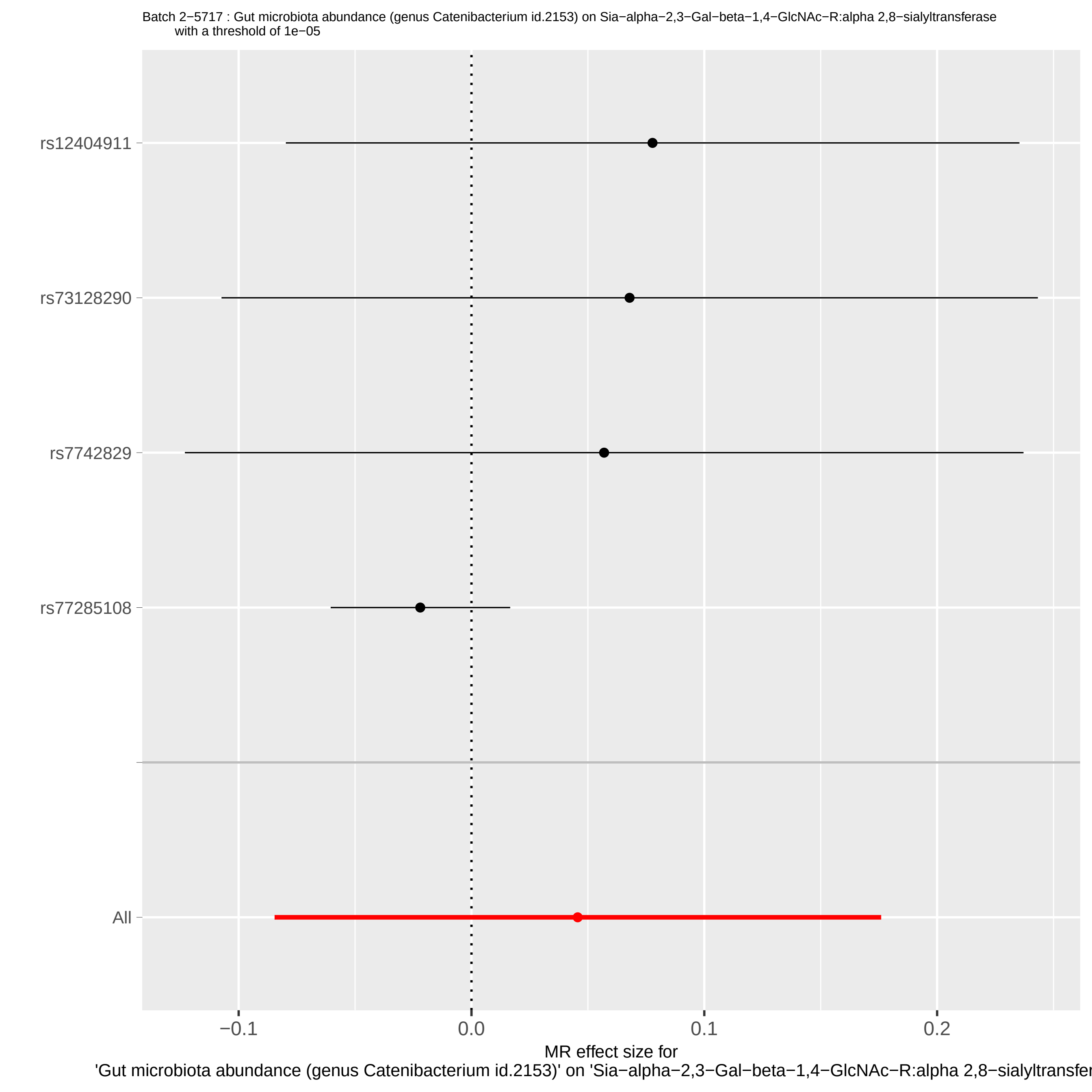

Batch 2−5717 : Gut microbiota abundance (genus Catenibacterium id.2153) on Sia−alpha−2,3−Gal−beta−1,4−GlcNAc−R:alpha 2,8−sialyltransferase with a threshold of 1e−05
rs12404911
rs73128290
rs7742829
rs77285108
All
−0.1
0.0
0.1
0.2
MR effect size for
'Gut microbiota abundance (genus Catenibacterium id.2153)' on 'Sia−alpha−2,3−Gal−beta−1,4−GlcNAc−R:alpha 2,8−sialyltransfer

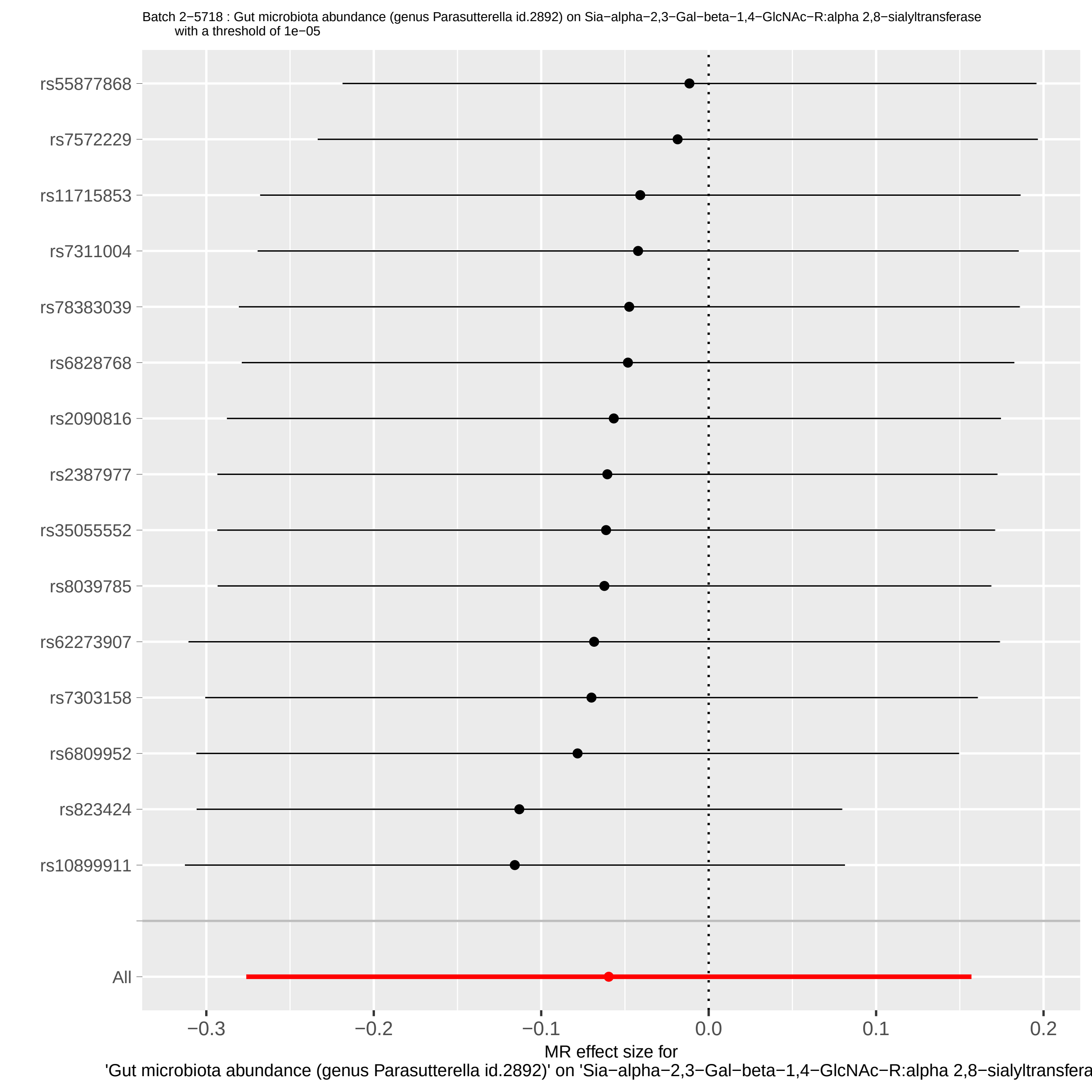

Batch 2−5718 : Gut microbiota abundance (genus Parasutterella id.2892) on Sia−alpha−2,3−Gal−beta−1,4−GlcNAc−R:alpha 2,8−sialyltransferase
with a threshold of 1e−05
rs55877868
rs7572229
rs11715853
rs7311004
rs78383039
rs6828768
rs2090816
rs2387977
rs35055552
rs8039785
rs62273907
rs7303158
rs6809952
rs823424
rs10899911
All
−0.3
−0.2
−0.1
0.0
0.1
0.2
MR effect size for
'Gut microbiota abundance (genus Parasutterella id.2892)' on 'Sia−alpha−2,3−Gal−beta−1,4−GlcNAc−R:alpha 2,8−sialyltransfera

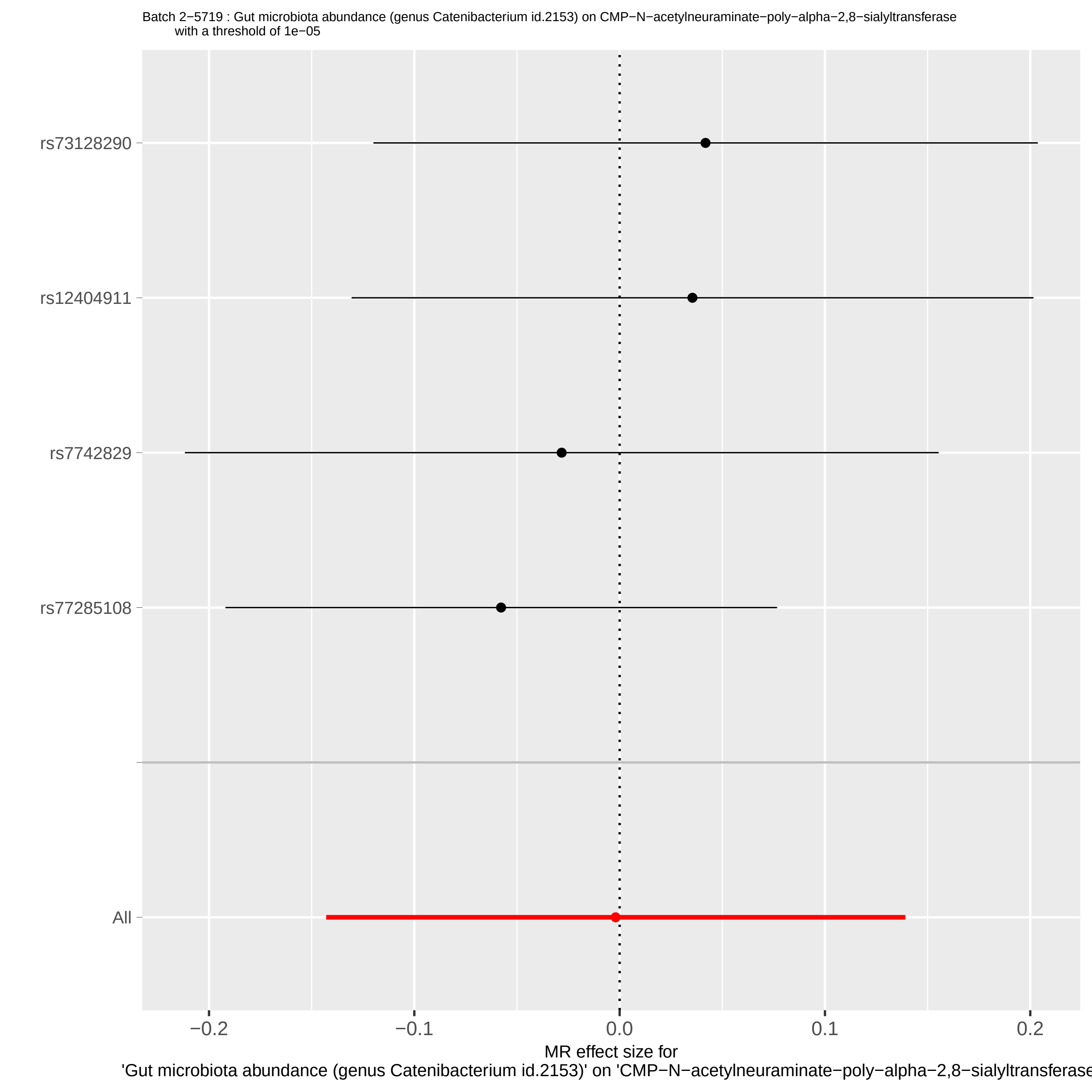

Batch 2−5719 : Gut microbiota abundance (genus Catenibacterium id.2153) on CMP−N−acetylneuraminate−poly−alpha−2,8−sialyltransferase
with a threshold of 1e−05
rs73128290
rs12404911
rs7742829
rs77285108
All
−0.2
−0.1
0.0
0.1
0.2
MR effect size for
'Gut microbiota abundance (genus Catenibacterium id.2153)' on 'CMP−N−acetylneuraminate−poly−alpha−2,8−sialyltransferase

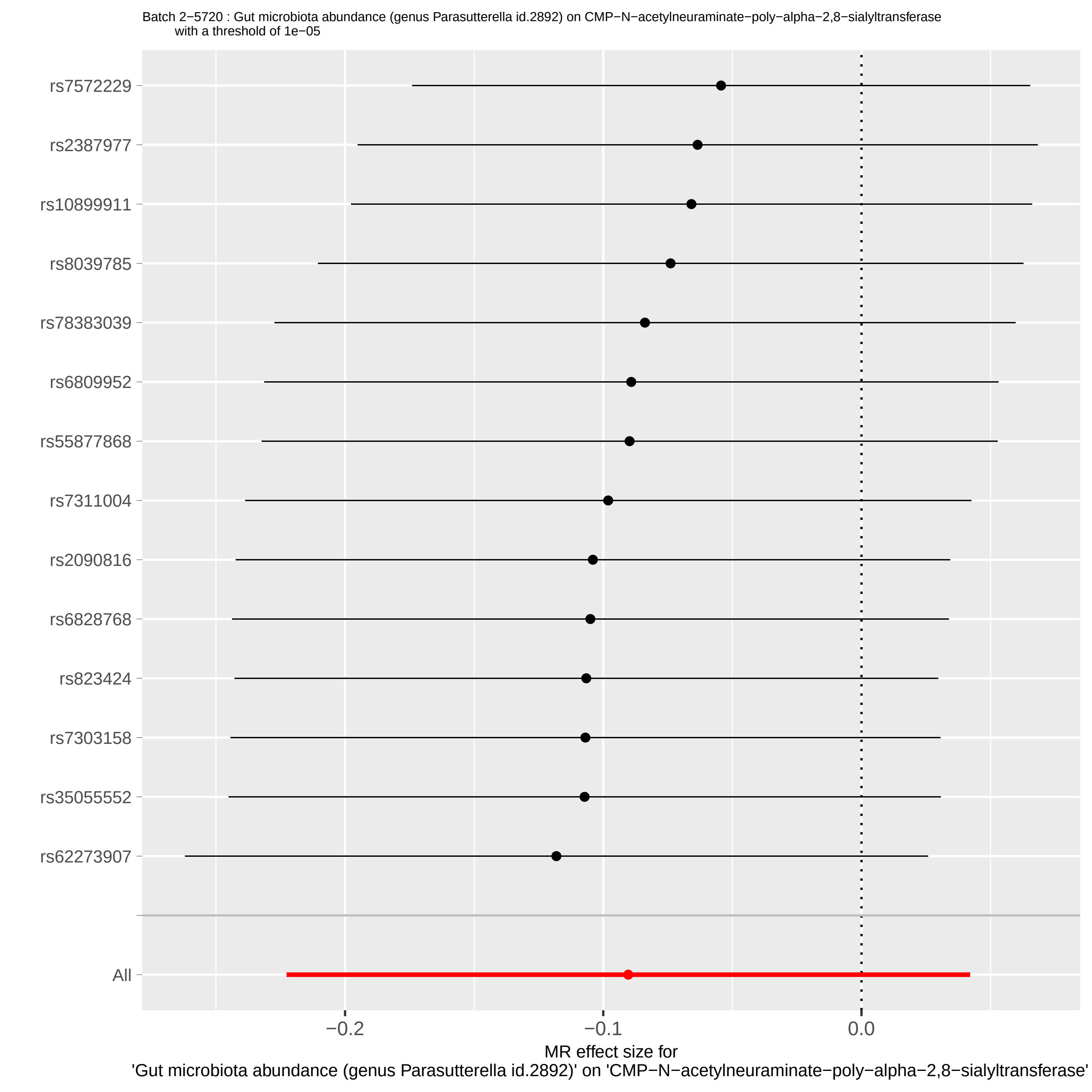

Batch 2−5720 : Gut microbiota abundance (genus Parasutterella id.2892) on CMP−N−acetylneuraminate−poly−alpha−2,8−sialyltransferase
with a threshold of 1e−05
rs7572229
rs2387977
rs10899911
rs8039785
rs78383039
rs6809952
rs55877868
rs7311004
rs2090816
rs6828768
rs823424
rs7303158
rs35055552
rs62273907
All
−0.2
−0.1
0.0
MR effect size for
'Gut microbiota abundance (genus Parasutterella id.2892)' on 'CMP−N−acetylneuraminate−poly−alpha−2,8−sialyltransferase'

Batch 2−5721 : Gut microbiota abundance (genus Catenibacterium id.2153) on Alpha−2,8−sialyltransferase 8F
with a threshold of 1e−05

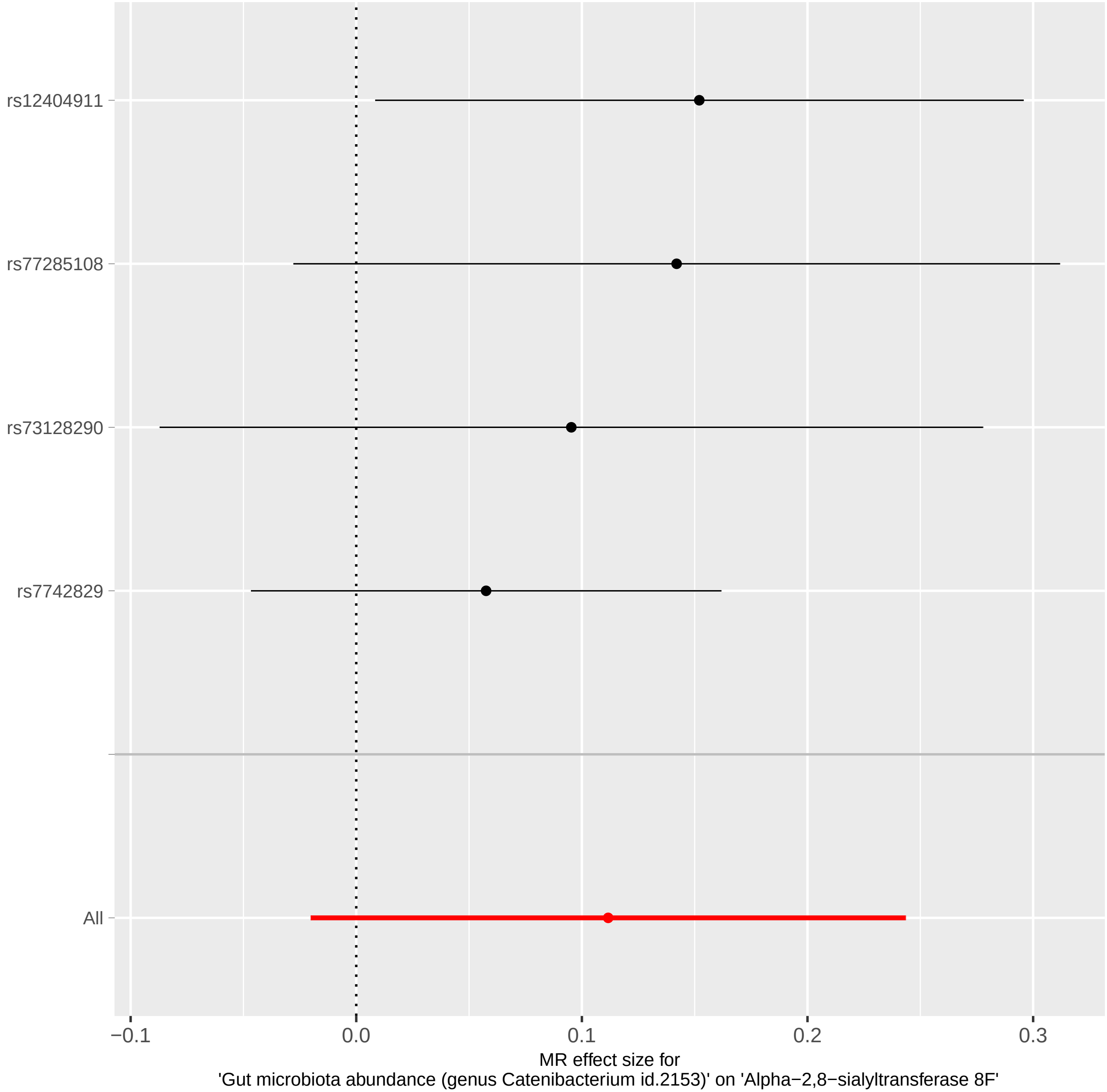

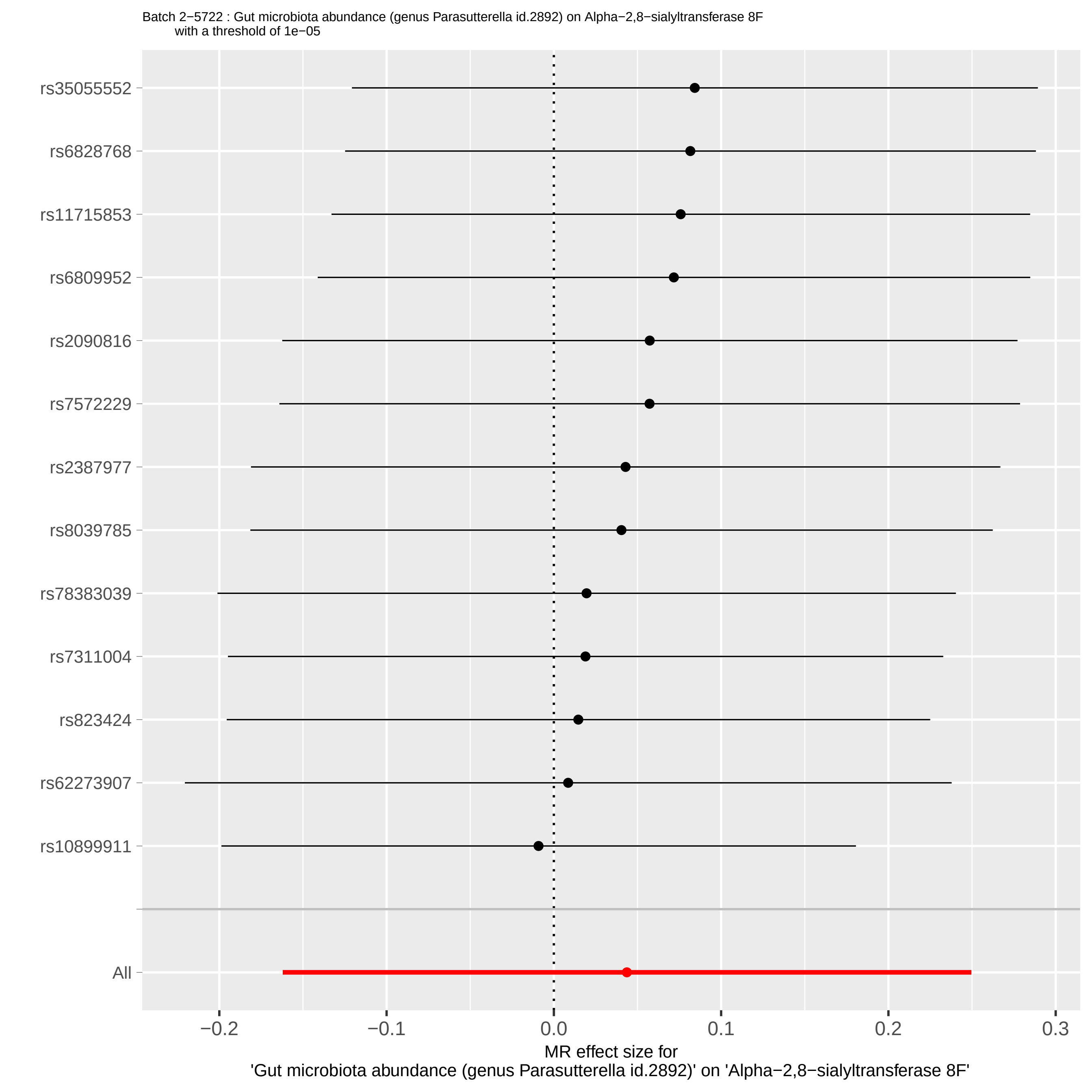

Batch 2−5722 : Gut microbiota abundance (genus Parasutterella id.2892) on Alpha−2,8−sialyltransferase 8F
with a threshold of 1e−05
rs35055552
rs6828768
rs11715853
rs6809952
rs2090816
rs7572229
rs2387977
rs8039785
rs78383039
rs7311004
rs823424
rs62273907
rs10899911
All
−0.2
−0.1
0.0
0.1
0.2
0.3
MR effect size for
'Gut microbiota abundance (genus Parasutterella id.2892)' on 'Alpha−2,8−sialyltransferase 8F'

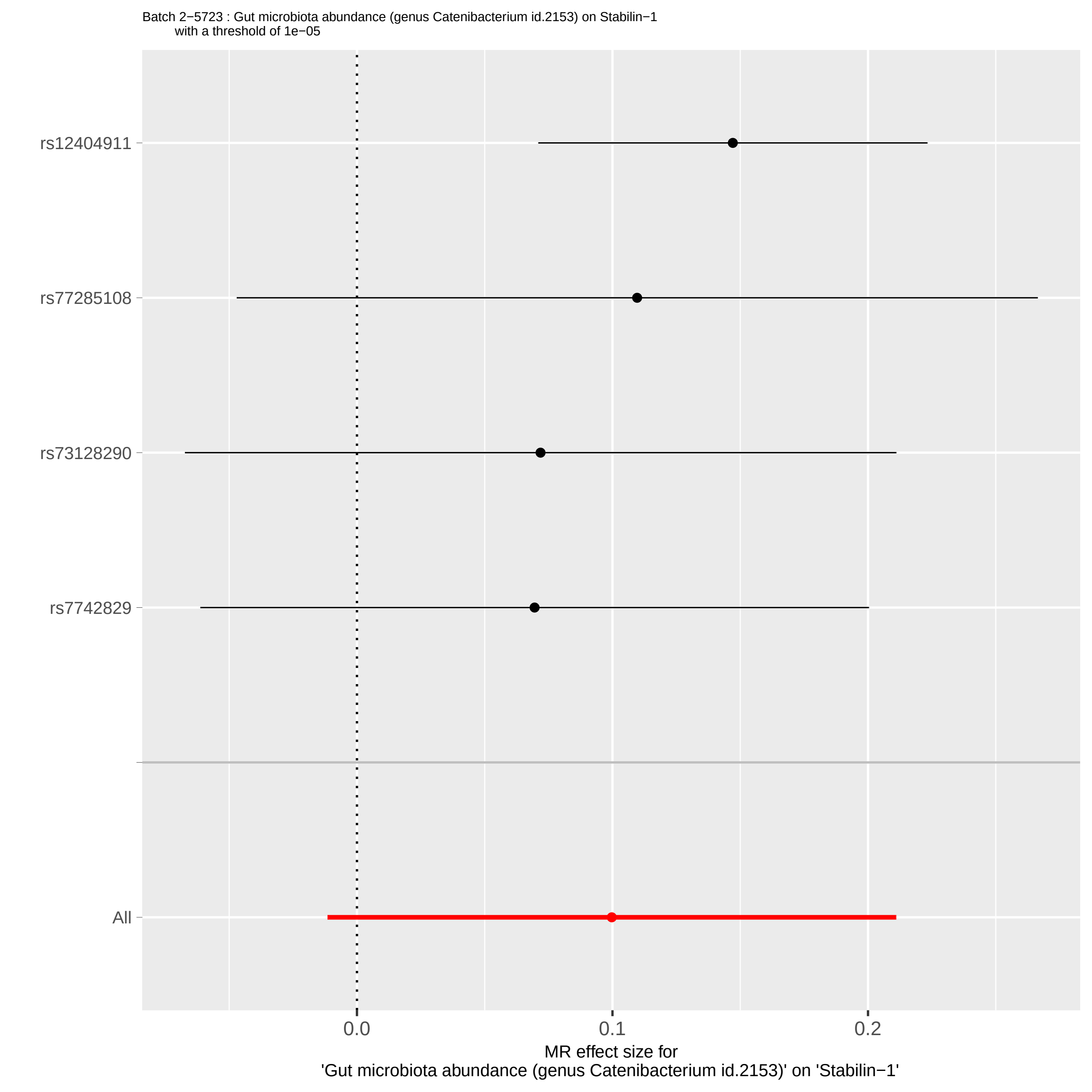
Batch 2−5723 : Gut microbiota abundance (genus Catenibacterium id.2153) on Stabilin−1
with a threshold of 1e−05
rs12404911
rs77285108
rs73128290
rs7742829
All
0.0
0.1
0.2
MR effect size for
'Gut microbiota abundance (genus Catenibacterium id.2153)' on 'Stabilin−1'

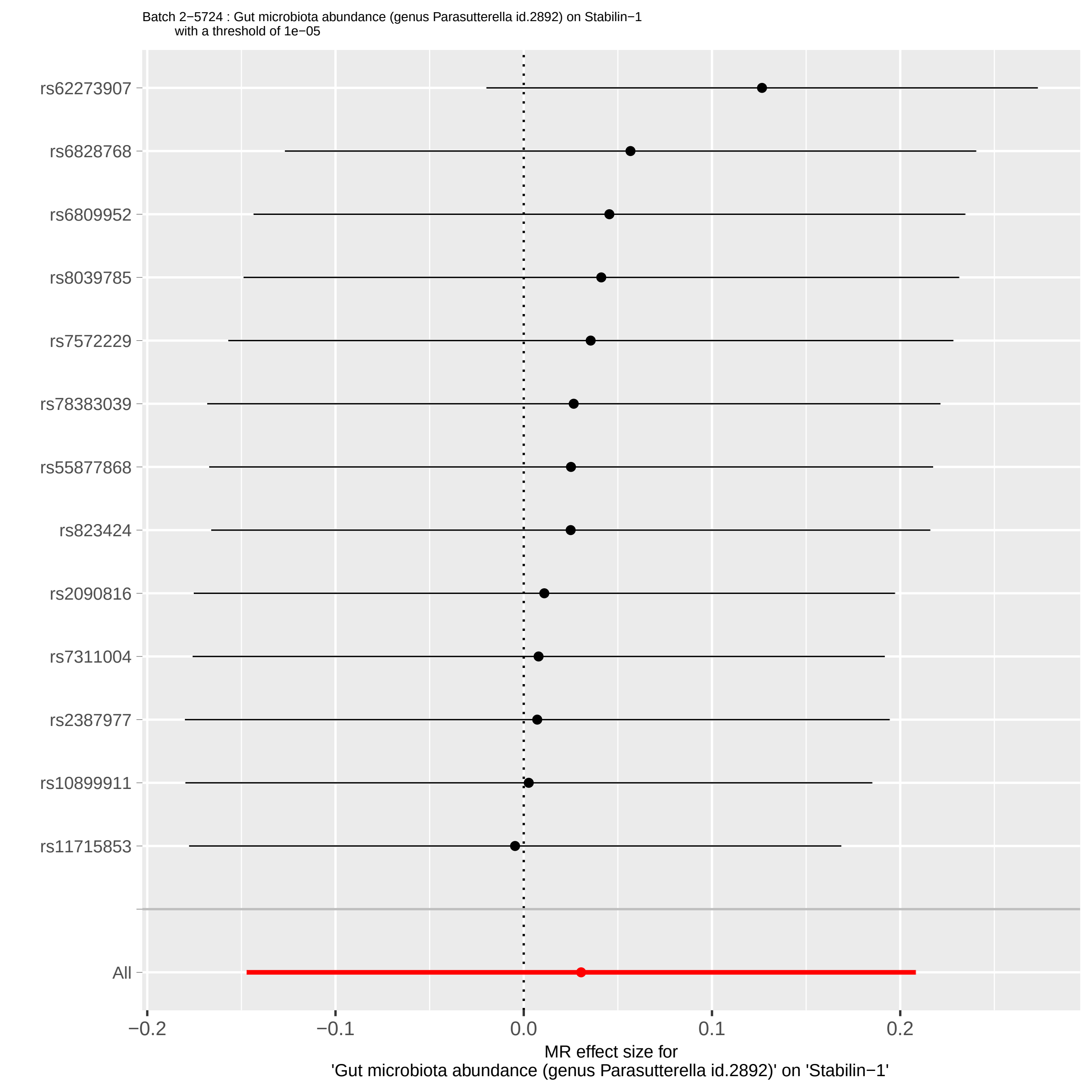

Batch 2−5724 : Gut microbiota abundance (genus Parasutterella id.2892) on Stabilin−1
with a threshold of 1e−05
rs62273907
rs6828768
rs6809952
rs8039785
rs7572229
rs78383039
rs55877868
rs823424
rs2090816
rs7311004
rs2387977
rs10899911
rs11715853
All
−0.2
−0.1
0.0
0.1
0.2
MR effect size for
'Gut microbiota abundance (genus Parasutterella id.2892)' on 'Stabilin−1'

Batch 2−5725 : Gut microbiota abundance (genus Catenibacterium id.2153) on Stabilin−2
with a threshold of 1e−05

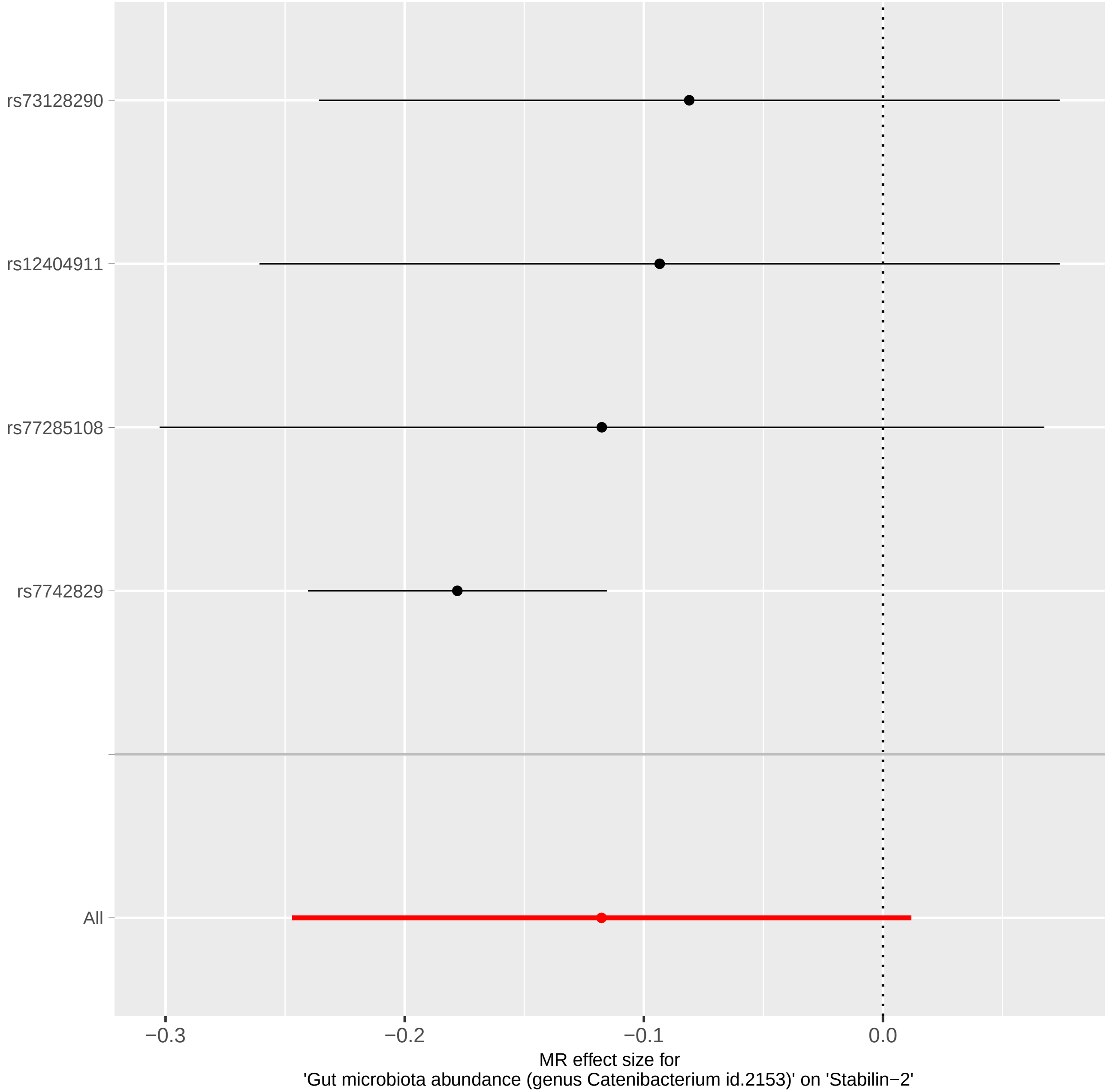

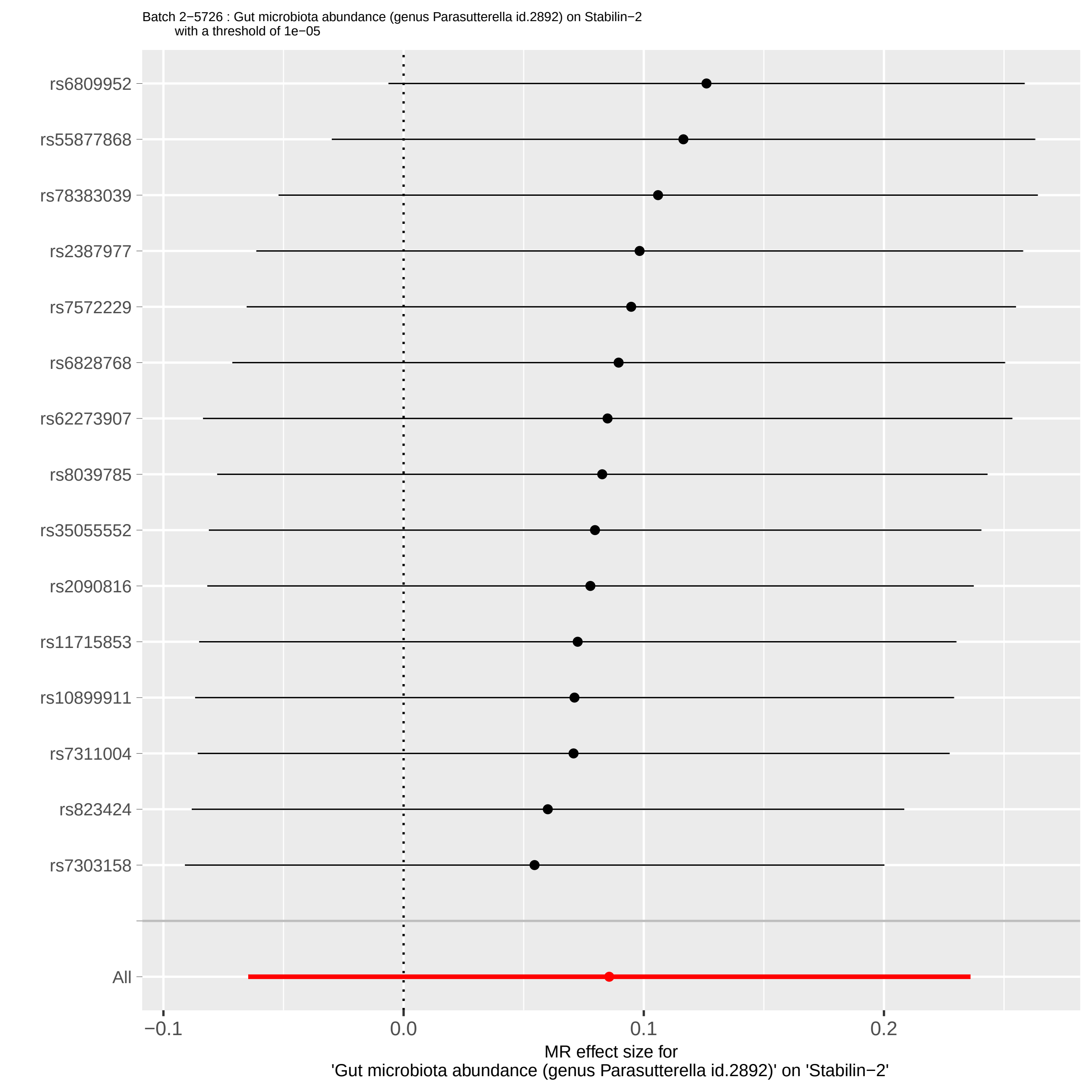

Batch 2−5726 : Gut microbiota abundance (genus Parasutterella id.2892) on Stabilin−2
with a threshold of 1e−05
rs6809952
rs55877868
rs78383039
rs2387977
rs7572229
rs6828768
rs62273907
rs8039785
rs35055552
rs2090816
rs11715853
rs10899911
rs7311004
rs823424
rs7303158
All
−0.1
0.0
0.1
0.2
MR effect size for
'Gut microbiota abundance (genus Parasutterella id.2892)' on 'Stabilin−2'

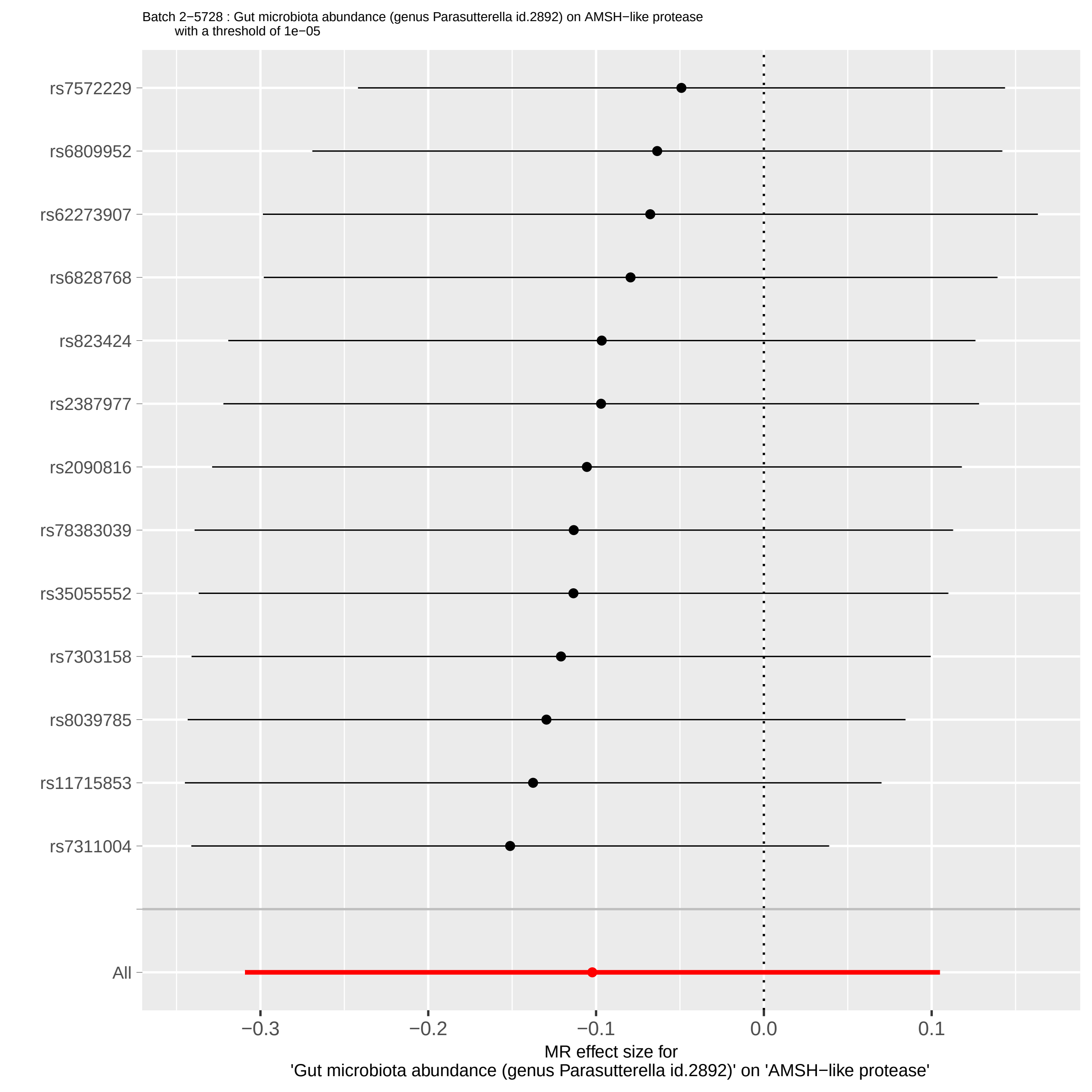
Batch 2−5728 : Gut microbiota abundance (genus Parasutterella id.2892) on AMSH−like protease
with a threshold of 1e−05
rs7572229
rs6809952
rs62273907
rs6828768
rs823424
rs2387977
rs2090816
rs78383039
rs35055552
rs7303158
rs8039785
rs11715853
rs7311004
All
−0.3
−0.2
−0.1
0.0
0.1
MR effect size for
'Gut microbiota abundance (genus Parasutterella id.2892)' on 'AMSH−like protease'

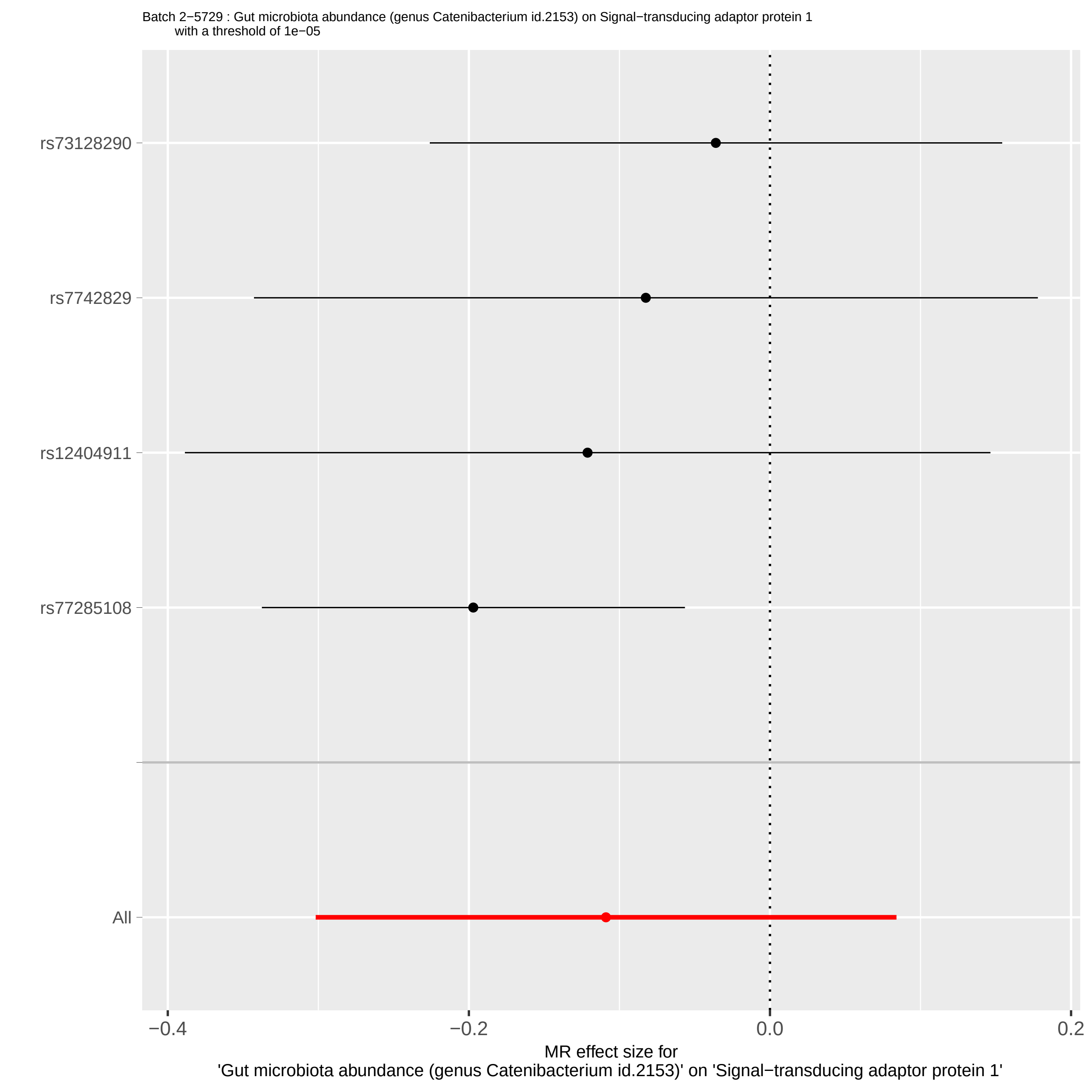

Batch 2−5729 : Gut microbiota abundance (genus Catenibacterium id.2153) on Signal−transducing adaptor protein 1
with a threshold of 1e−05
rs73128290
rs7742829
rs12404911
rs77285108
All
−0.4
−0.2
0.0
0.2
MR effect size for
'Gut microbiota abundance (genus Catenibacterium id.2153)' on 'Signal−transducing adaptor protein 1'

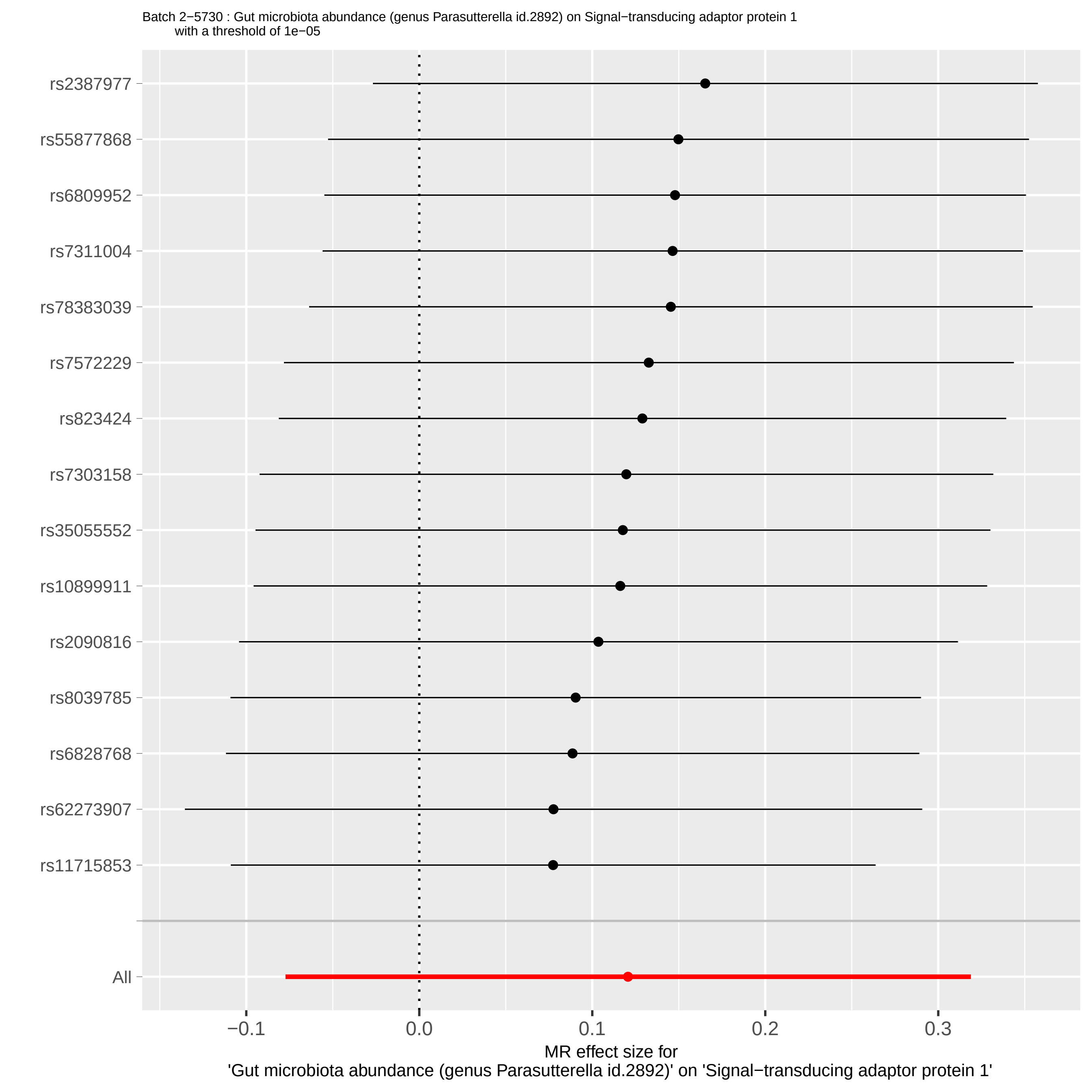
Batch 2−5730 : Gut microbiota abundance (genus Parasutterella id.2892) on Signal−transducing adaptor protein 1
with a threshold of 1e−05
rs2387977
rs55877868
rs6809952
rs7311004
rs78383039
rs7572229
rs823424
rs7303158
rs35055552
rs10899911
rs2090816
rs8039785
rs6828768
rs62273907
rs11715853
All
−0.1
0.0
0.1
0.2
0.3
MR effect size for
'Gut microbiota abundance (genus Parasutterella id.2892)' on 'Signal−transducing adaptor protein 1'

Batch 2−5731 : Gut microbiota abundance (genus Catenibacterium id.2153) on Steroidogenic acute regulatory protein, mitochondrial
with a threshold of 1e−05

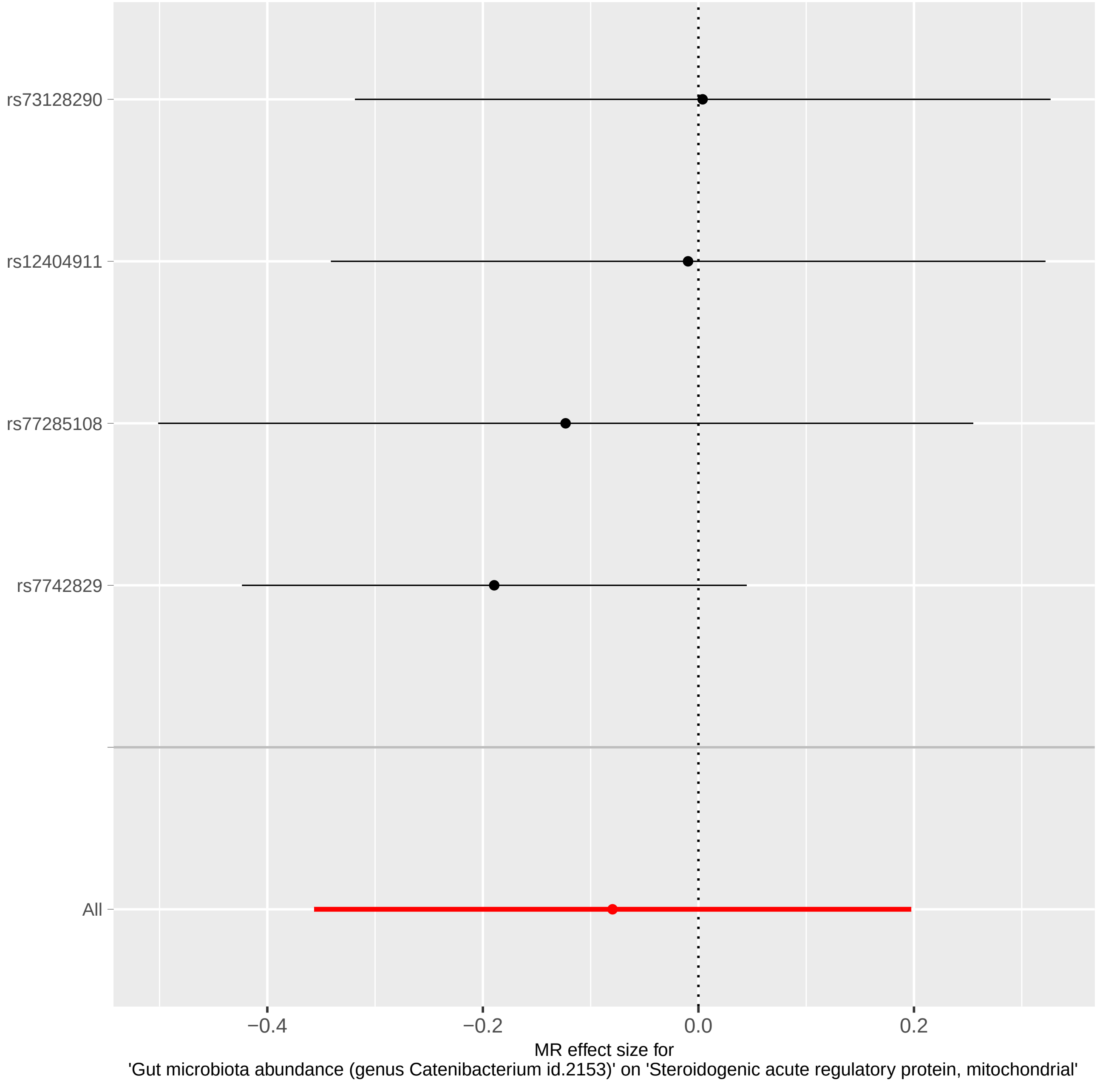

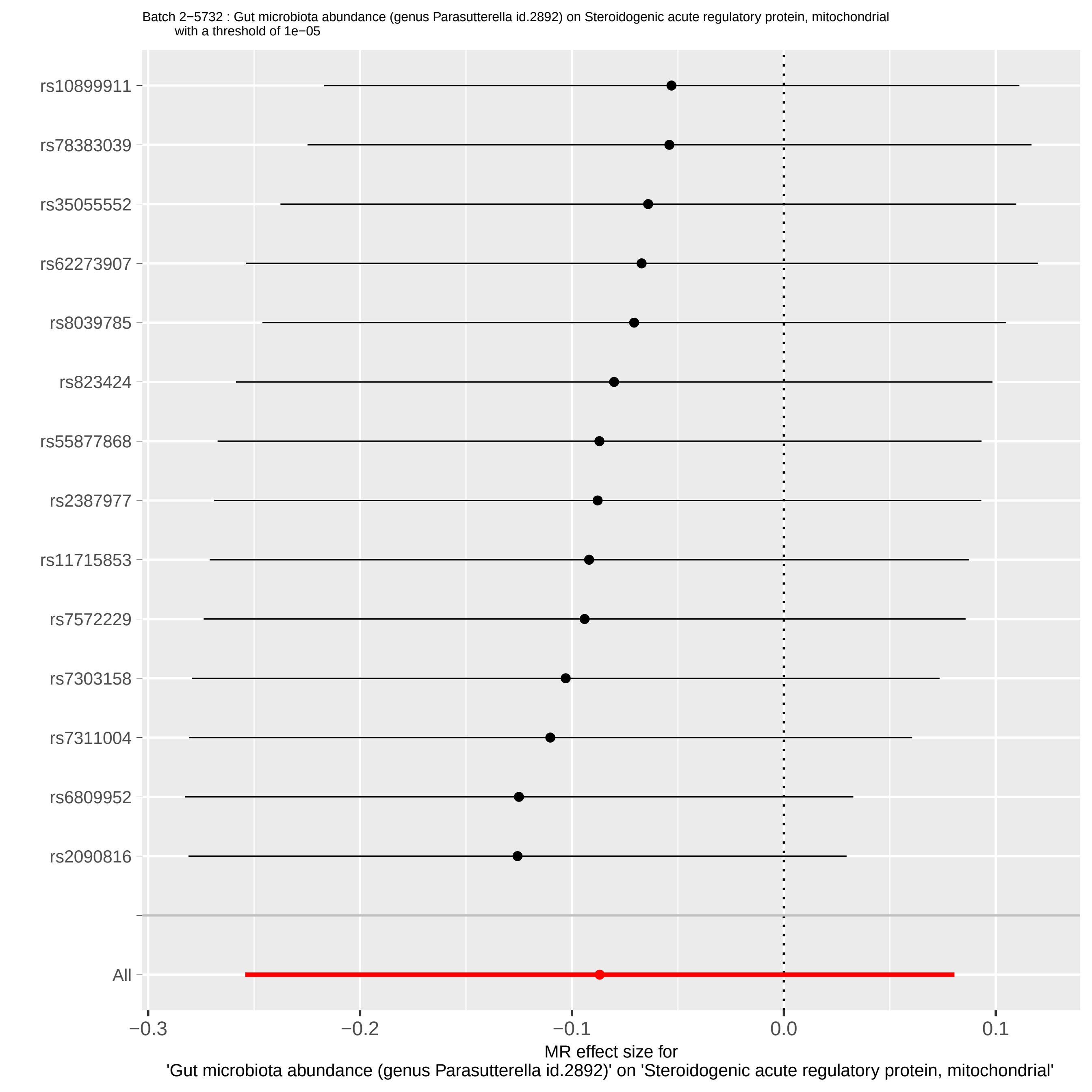
Batch 2−5732 : Gut microbiota abundance (genus Parasutterella id.2892) on Steroidogenic acute regulatory protein, mitochondrial
with a threshold of 1e−05
rs10899911
rs78383039
rs35055552
rs62273907
rs8039785
rs823424
rs55877868
rs2387977
rs11715853
rs7572229
rs7303158
rs7311004
rs6809952
rs2090816
All
−0.3
−0.2
−0.1
0.0
0.1
MR effect size for
'Gut microbiota abundance (genus Parasutterella id.2892)' on 'Steroidogenic acute regulatory protein, mitochondrial'

Batch 2−5733 : Gut microbiota abundance (genus Catenibacterium id.2153) on Signal transducer and activator of transcription 1−alpha/beta with a threshold of 1e−05

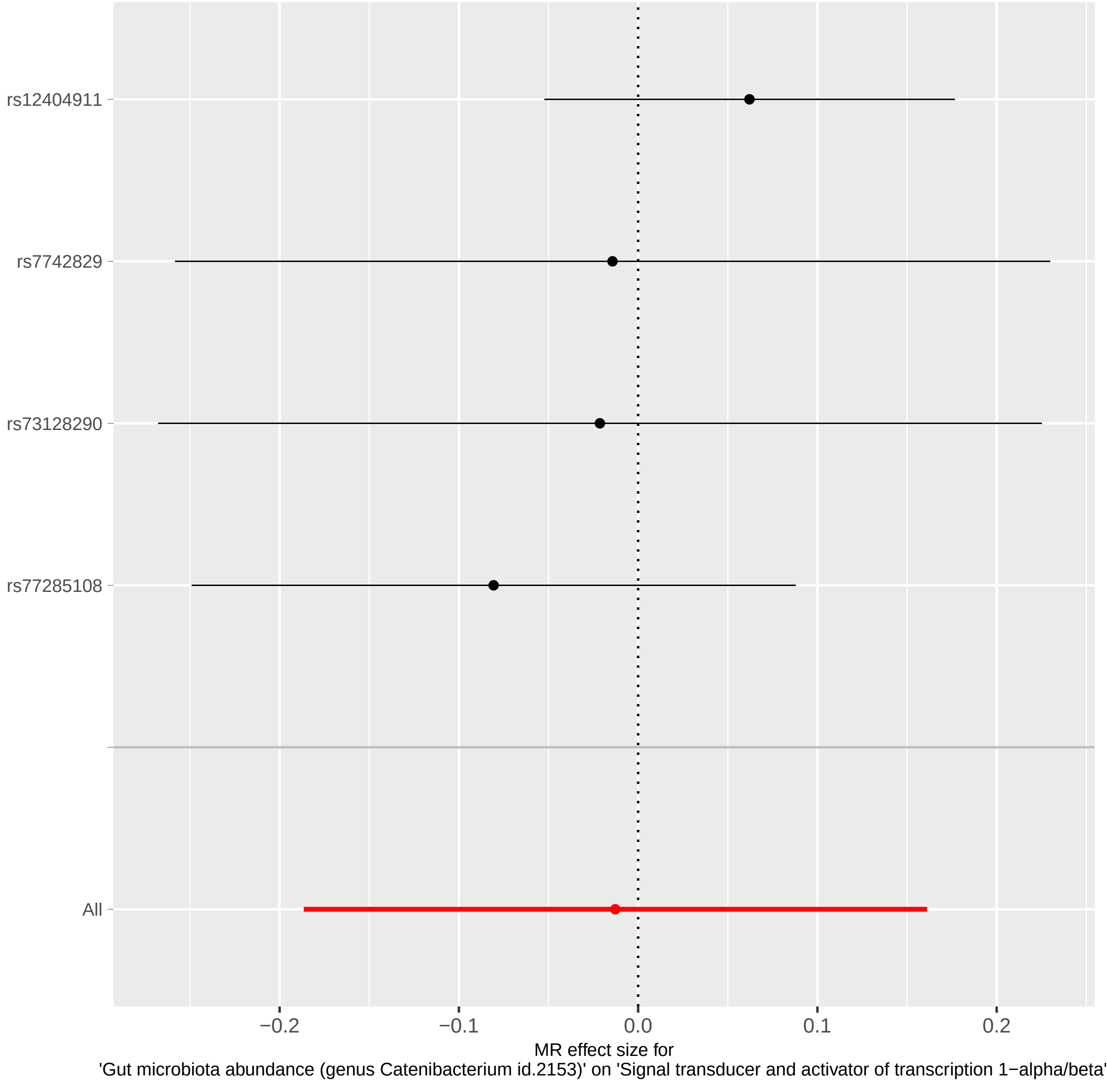

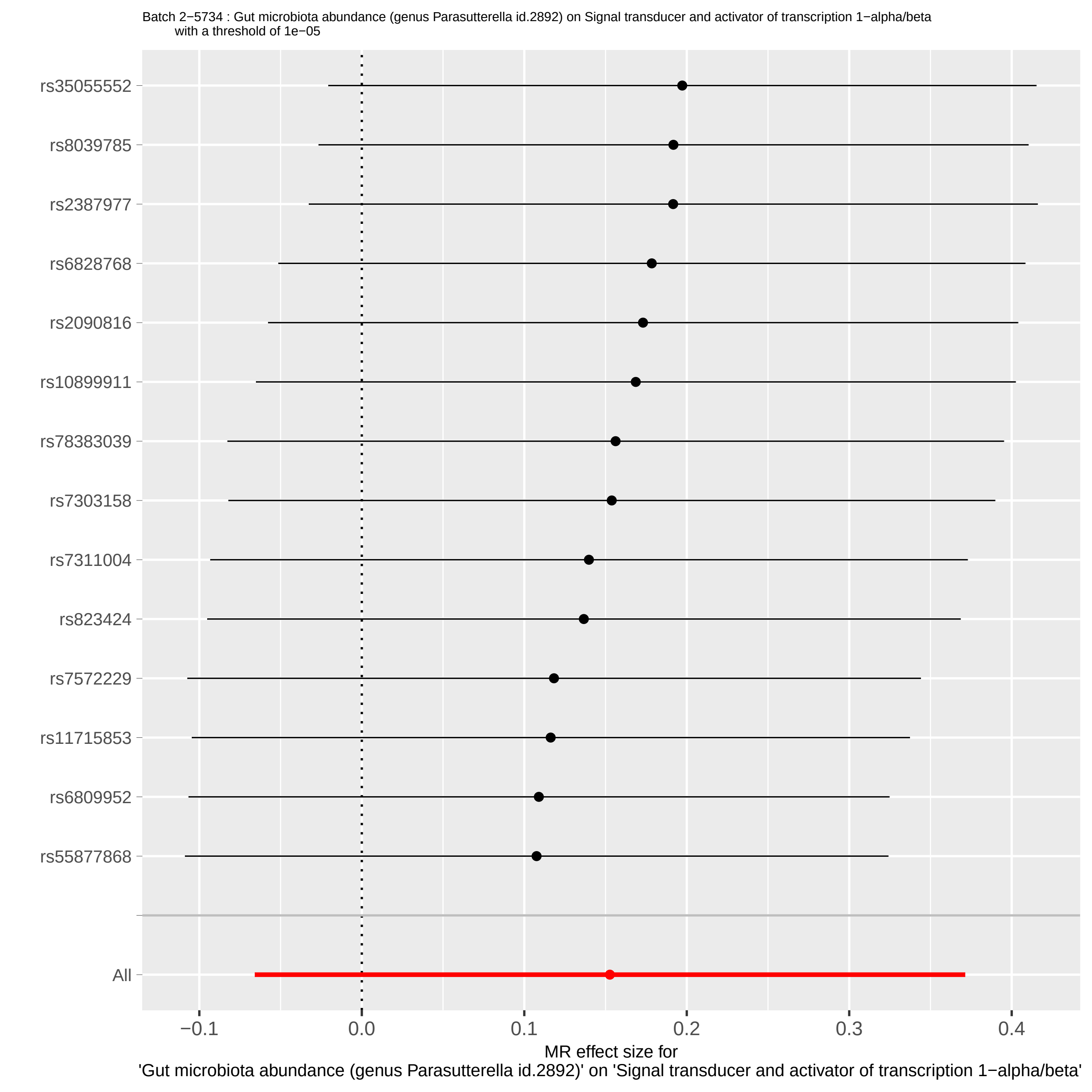

Batch 2−5734 : Gut microbiota abundance (genus Parasutterella id.2892) on Signal transducer and activator of transcription 1−alpha/beta
with a threshold of 1e−05
rs35055552
rs8039785
rs2387977
rs6828768
rs2090816
rs10899911
rs78383039
rs7303158
rs7311004
rs823424
rs7572229
rs11715853
rs6809952
rs55877868
All
−0.1
0.0
0.1
0.2
0.3
0.4
MR effect size for
'Gut microbiota abundance (genus Parasutterella id.2892)' on 'Signal transducer and activator of transcription 1−alpha/beta'

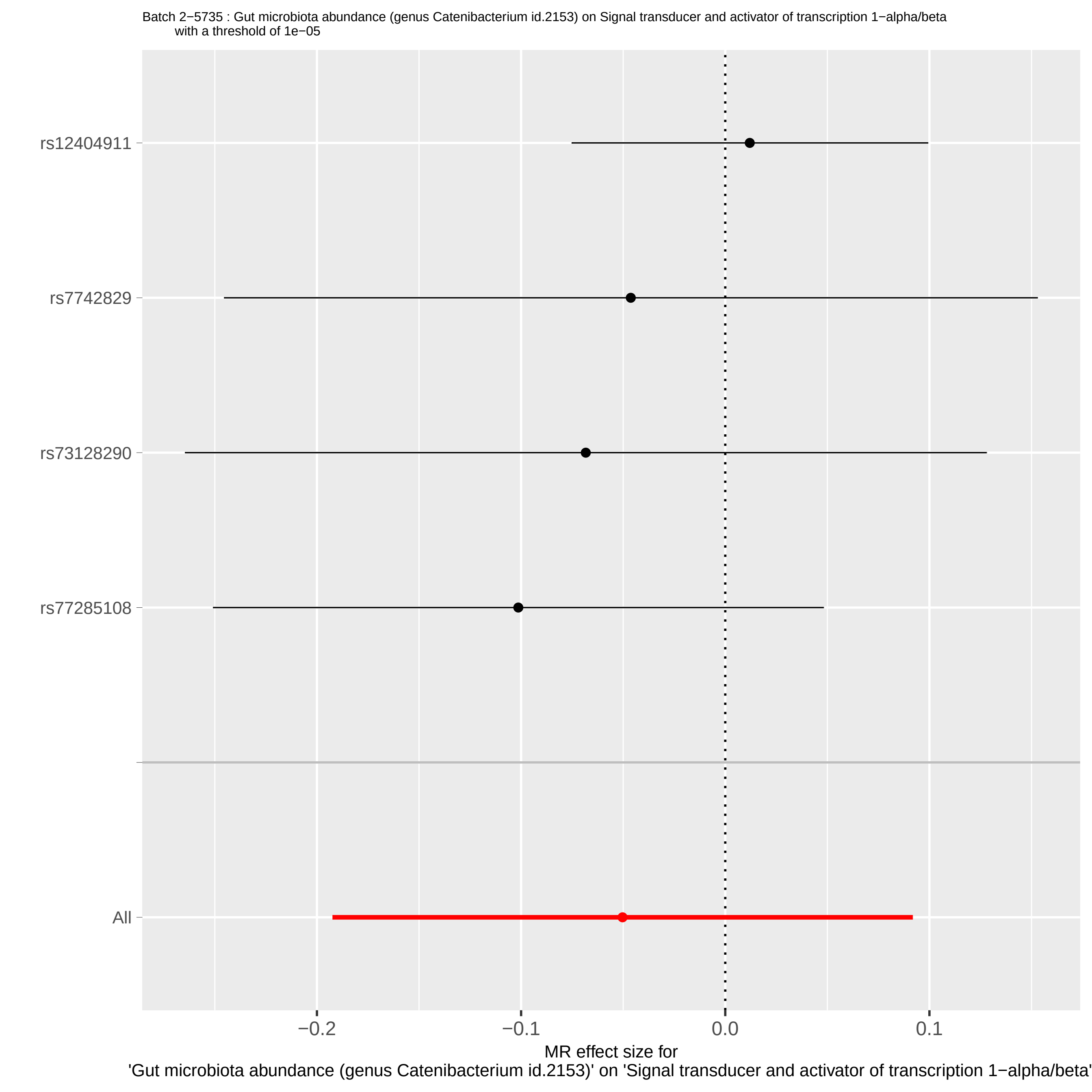

Batch 2−5735 : Gut microbiota abundance (genus Catenibacterium id.2153) on Signal transducer and activator of transcription 1−alpha/beta
with a threshold of 1e−05
rs12404911
rs7742829
rs73128290
rs77285108
All
−0.2
−0.1
0.0
0.1
MR effect size for
'Gut microbiota abundance (genus Catenibacterium id.2153)' on 'Signal transducer and activator of transcription 1−alpha/beta'

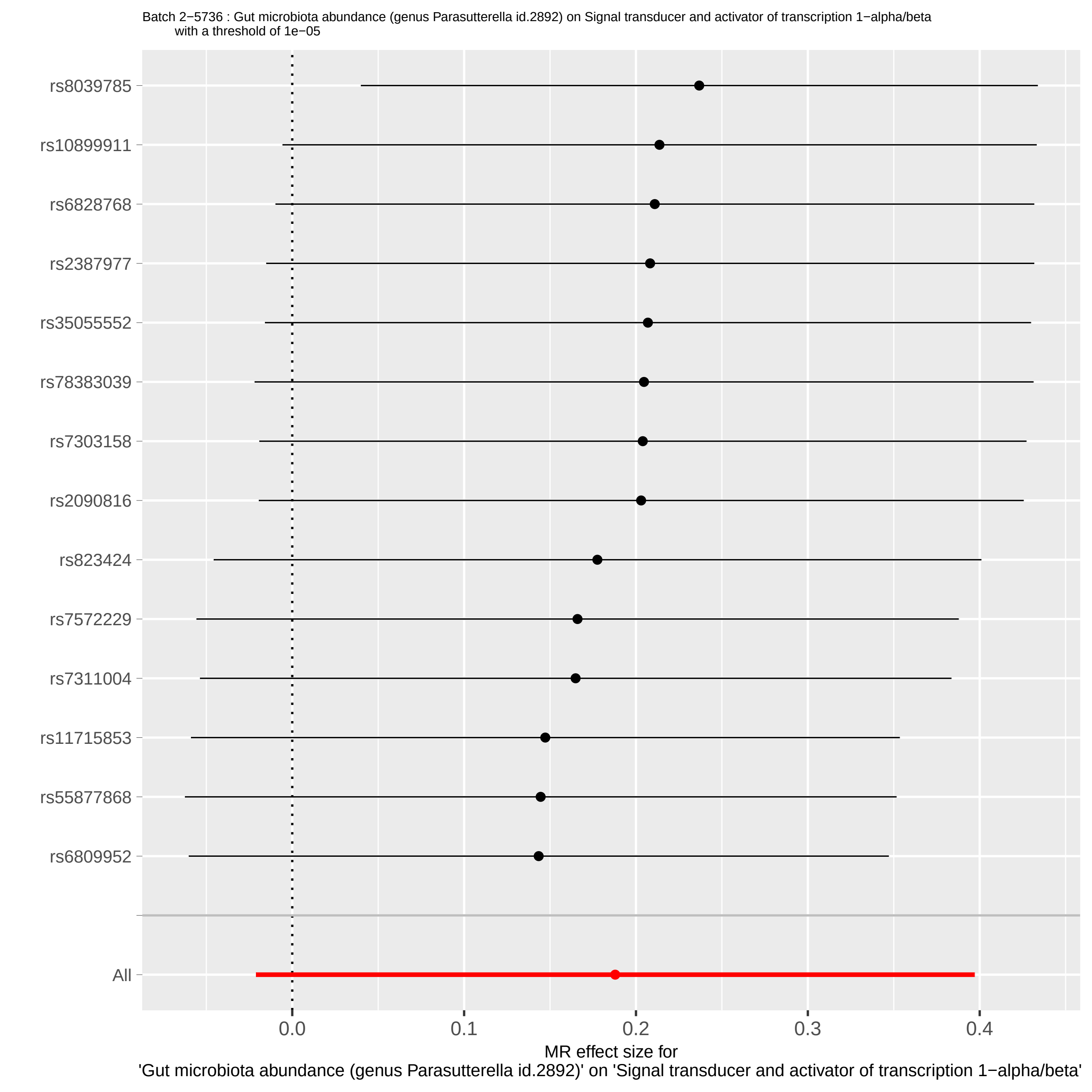

Batch 2−5736 : Gut microbiota abundance (genus Parasutterella id.2892) on Signal transducer and activator of transcription 1−alpha/beta with a threshold of 1e−05
rs8039785
rs10899911
rs6828768
rs2387977
rs35055552
rs78383039
rs7303158
rs2090816
rs823424
rs7572229
rs7311004
rs11715853
rs55877868
rs6809952
All
0.0
0.1
0.2
0.3
0.4
MR effect size for
'Gut microbiota abundance (genus Parasutterella id.2892)' on 'Signal transducer and activator of transcription 1−alpha/beta'

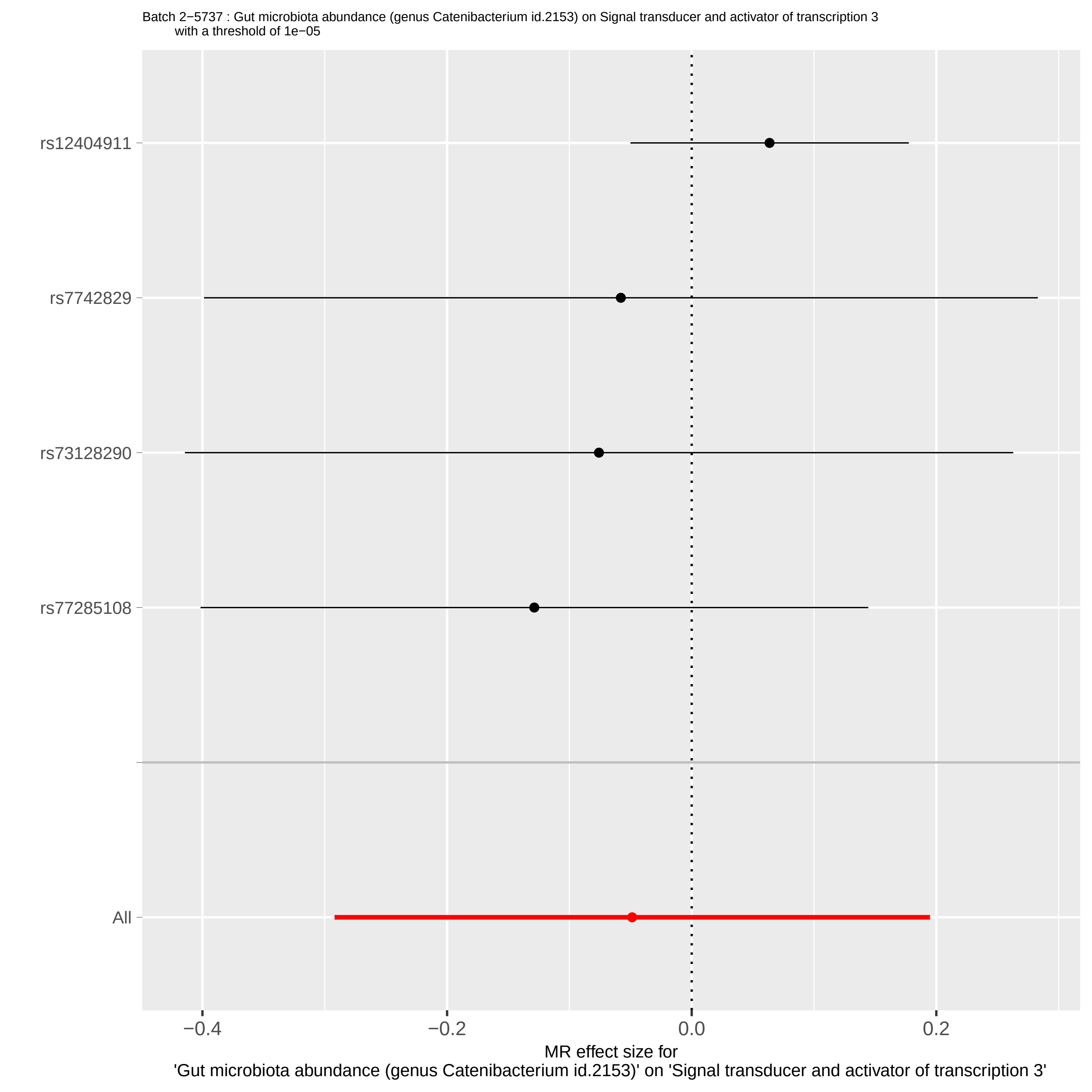
Batch 2−5737 : Gut microbiota abundance (genus Catenibacterium id.2153) on Signal transducer and activator of transcription 3
with a threshold of 1e−05
rs12404911
rs7742829
rs73128290
rs77285108
All
−0.4
−0.2
0.0
0.2
MR effect size for
'Gut microbiota abundance (genus Catenibacterium id.2153)' on 'Signal transducer and activator of transcription 3'

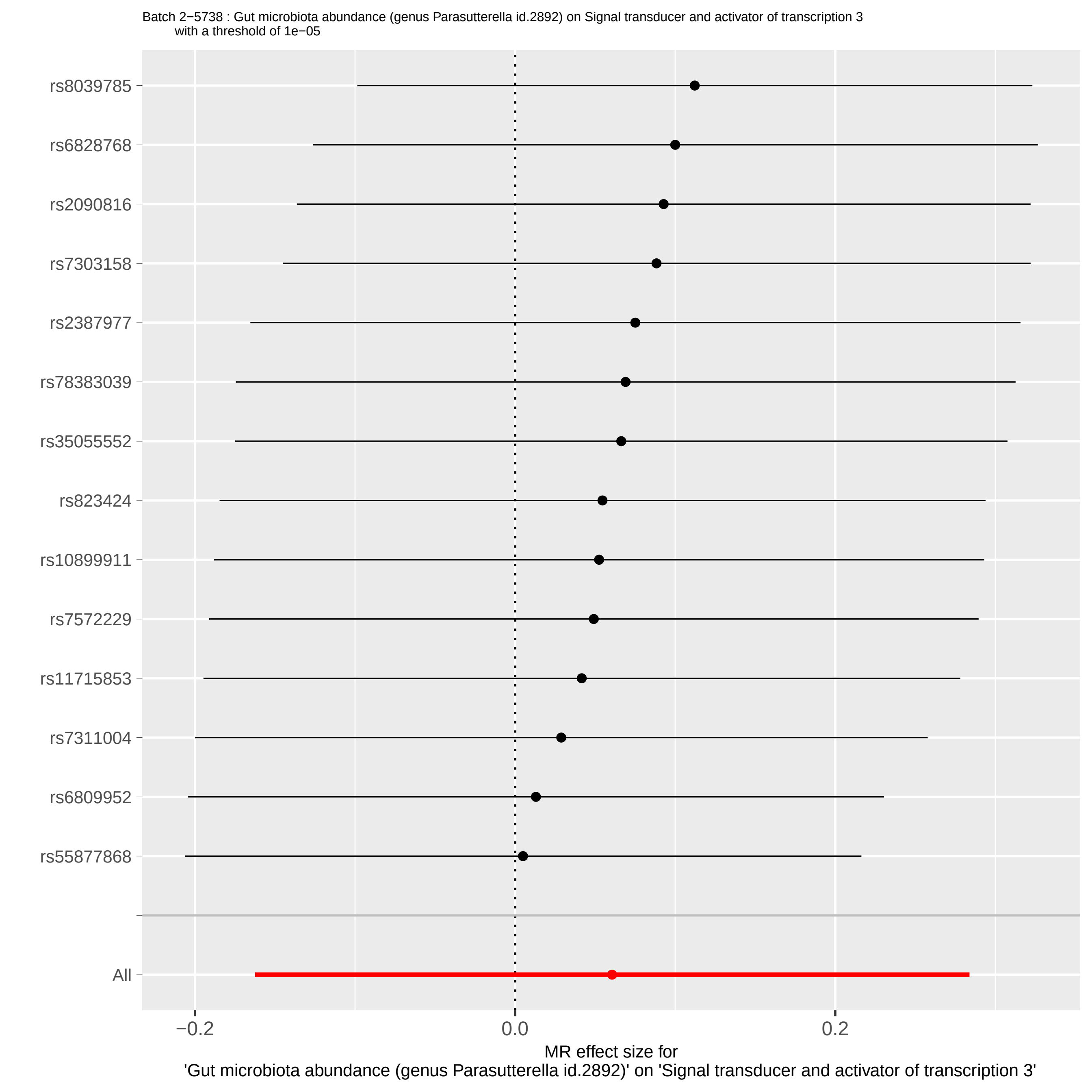
Batch 2−5738 : Gut microbiota abundance (genus Parasutterella id.2892) on Signal transducer and activator of transcription 3
with a threshold of 1e−05
rs8039785
rs6828768
rs2090816
rs7303158
rs2387977
rs78383039
rs35055552
rs823424
rs10899911
rs7572229
rs11715853
rs7311004
rs6809952
rs55877868
All
−0.2
0.0
0.2
MR effect size for
'Gut microbiota abundance (genus Parasutterella id.2892)' on 'Signal transducer and activator of transcription 3'

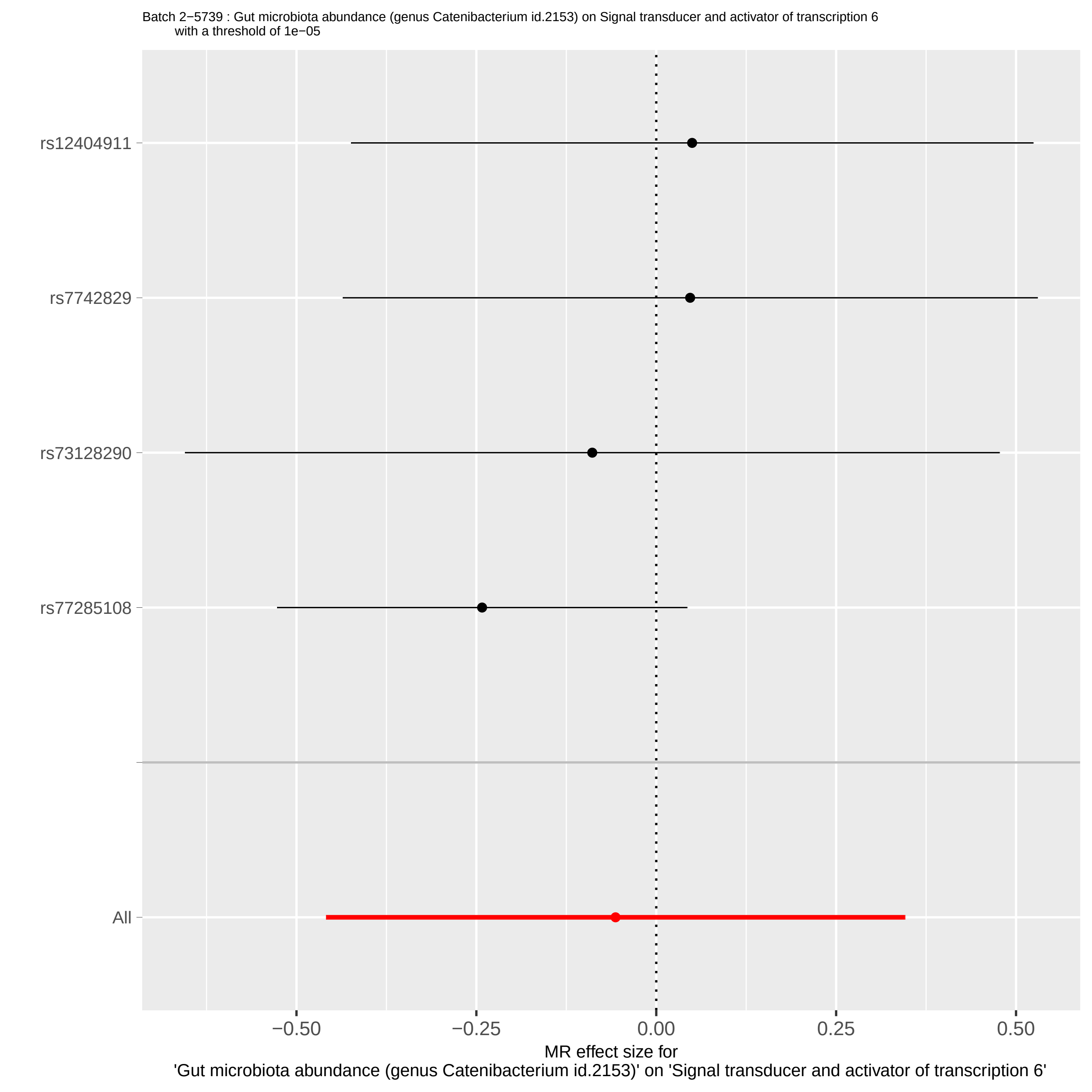
Batch 2−5739 : Gut microbiota abundance (genus Catenibacterium id.2153) on Signal transducer and activator of transcription 6
with a threshold of 1e−05
rs12404911
rs7742829
rs73128290
rs77285108
All
−0.50
−0.25
0.00
0.25
0.50
MR effect size for
'Gut microbiota abundance (genus Catenibacterium id.2153)' on 'Signal transducer and activator of transcription 6'

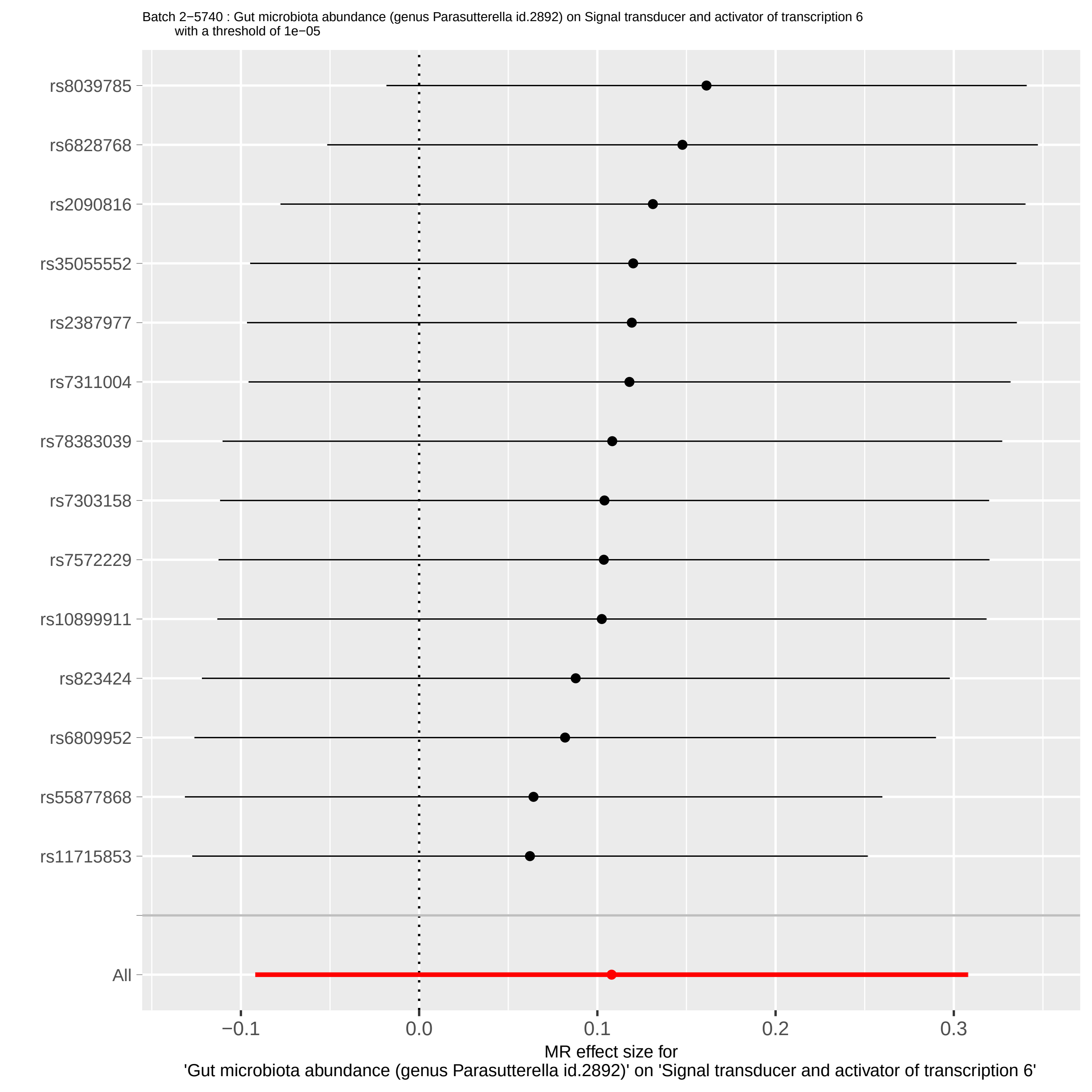

Batch 2−5740 : Gut microbiota abundance (genus Parasutterella id.2892) on Signal transducer and activator of transcription 6
with a threshold of 1e−05
rs8039785
rs6828768
rs2090816
rs35055552
rs2387977
rs7311004
rs78383039
rs7303158
rs7572229
rs10899911
rs823424
rs6809952
rs55877868
rs11715853
All
−0.1
0.0
0.1
0.2
0.3
MR effect size for
'Gut microbiota abundance (genus Parasutterella id.2892)' on 'Signal transducer and activator of transcription 6'

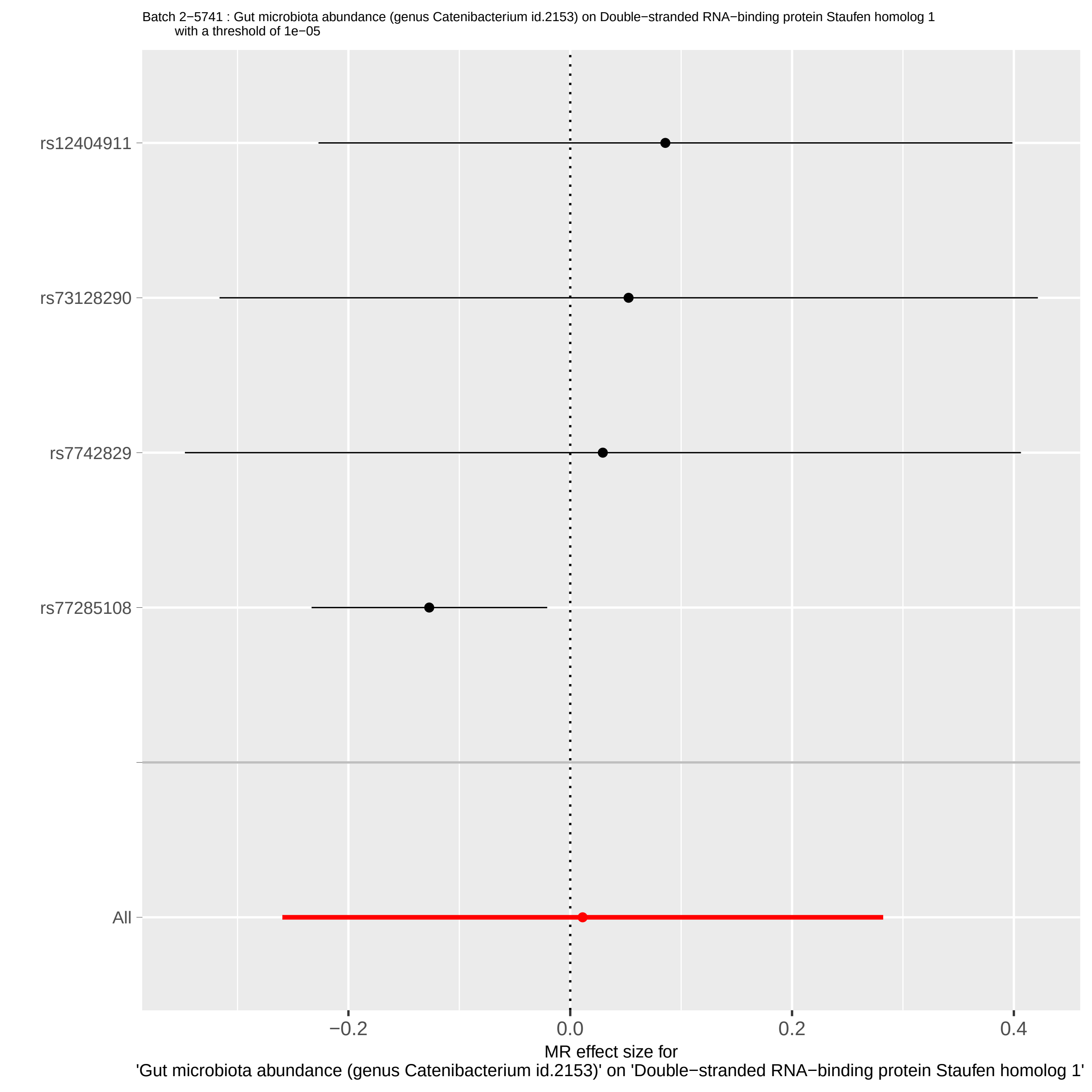

Batch 2−5741 : Gut microbiota abundance (genus Catenibacterium id.2153) on Double−stranded RNA−binding protein Staufen homolog 1
with a threshold of 1e−05
rs12404911
rs73128290
rs7742829
rs77285108
All
−0.2
0.0
0.2
0.4
MR effect size for
'Gut microbiota abundance (genus Catenibacterium id.2153)' on 'Double−stranded RNA−binding protein Staufen homolog 1'

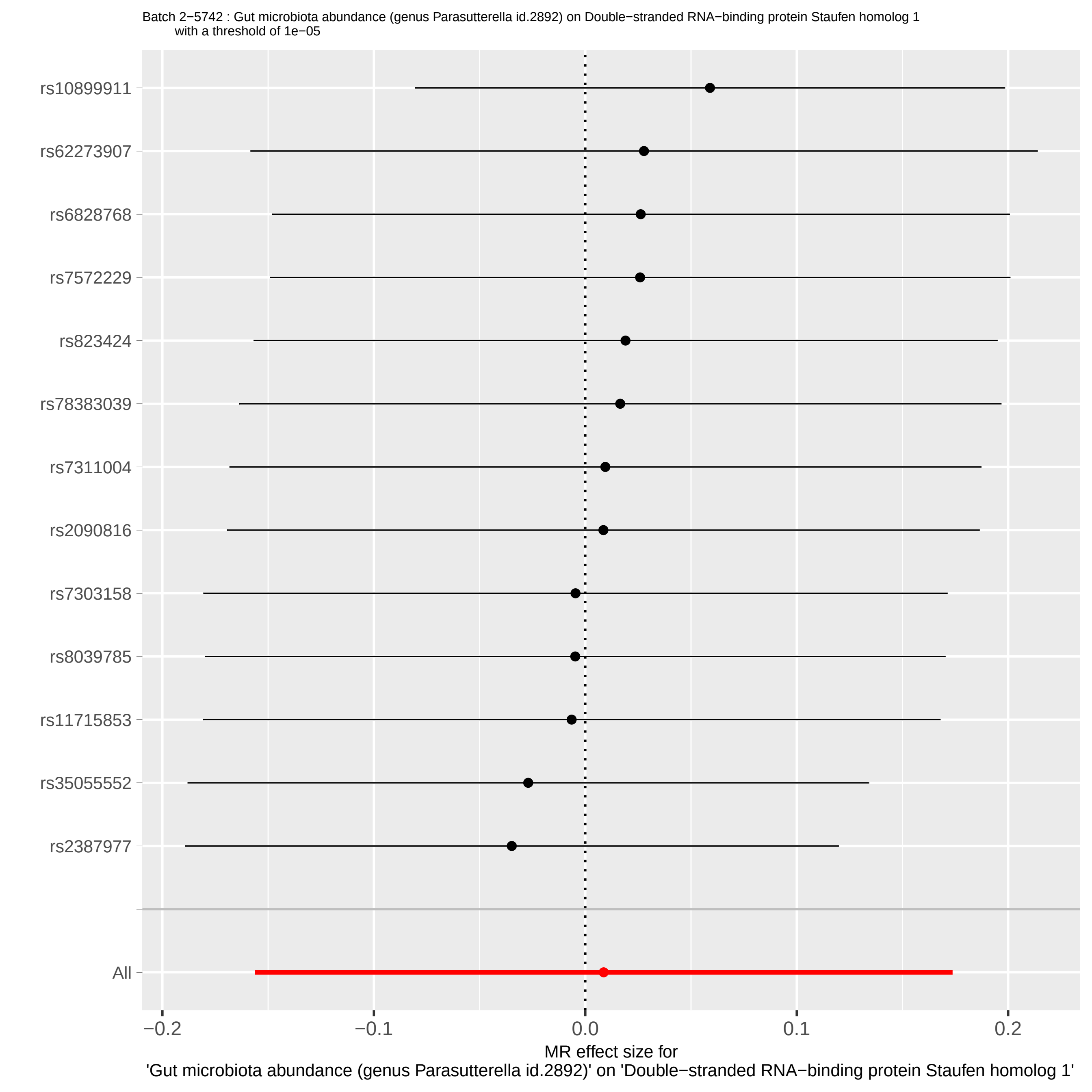
Batch 2−5742 : Gut microbiota abundance (genus Parasutterella id.2892) on Double−stranded RNA−binding protein Staufen homolog 1
with a threshold of 1e−05
rs10899911
rs62273907
rs6828768
rs7572229
rs823424
rs78383039
rs7311004
rs2090816
rs7303158
rs8039785
rs11715853
rs35055552
rs2387977
All
−0.2
−0.1
0.0
0.1
0.2
MR effect size for
'Gut microbiota abundance (genus Parasutterella id.2892)' on 'Double−stranded RNA−binding protein Staufen homolog 1'

Batch 2−5743 : Gut microbiota abundance (genus Catenibacterium id.2153) on Double−stranded RNA−binding protein Staufen homolog 2
with a threshold of 1e−05

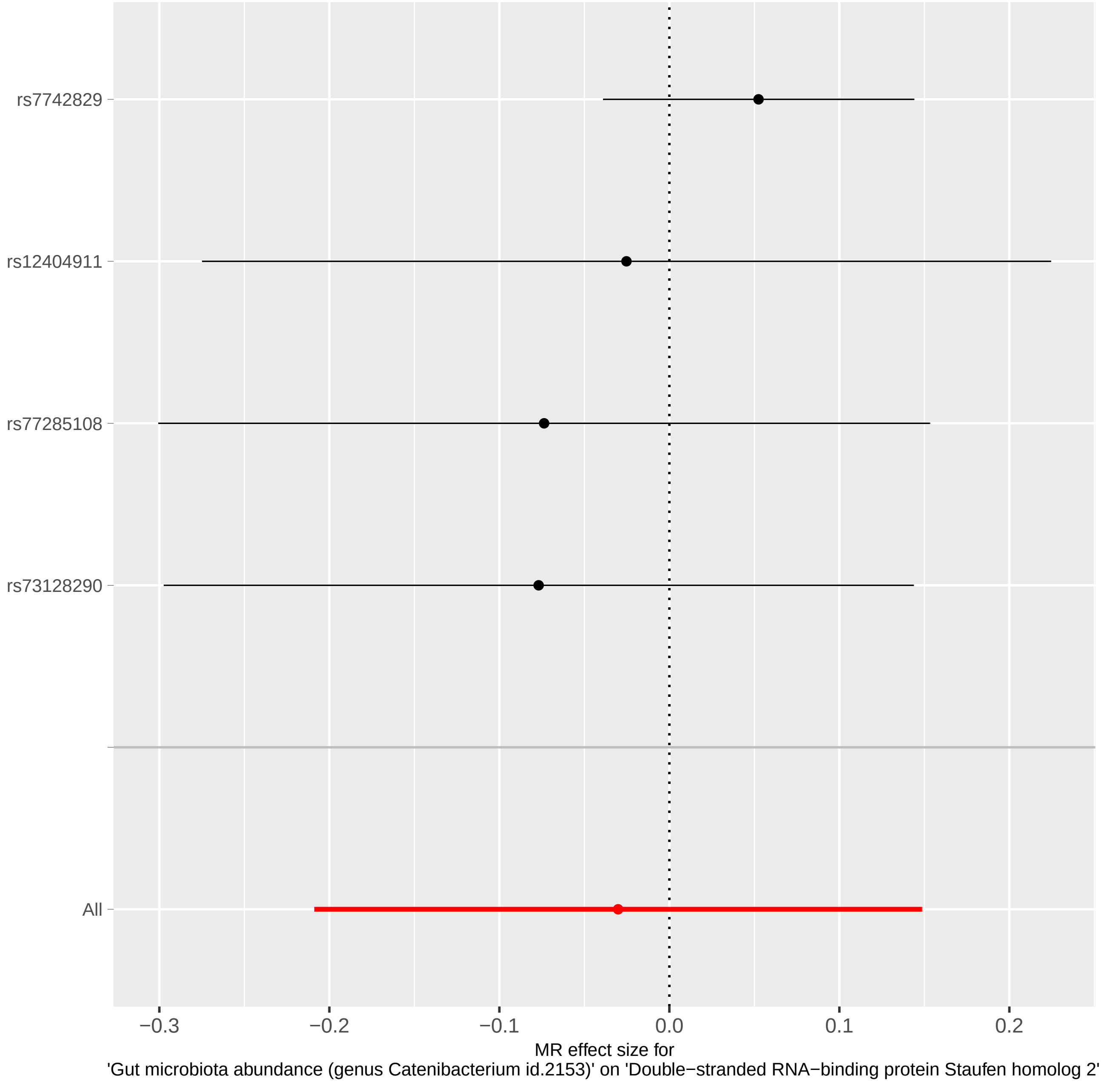

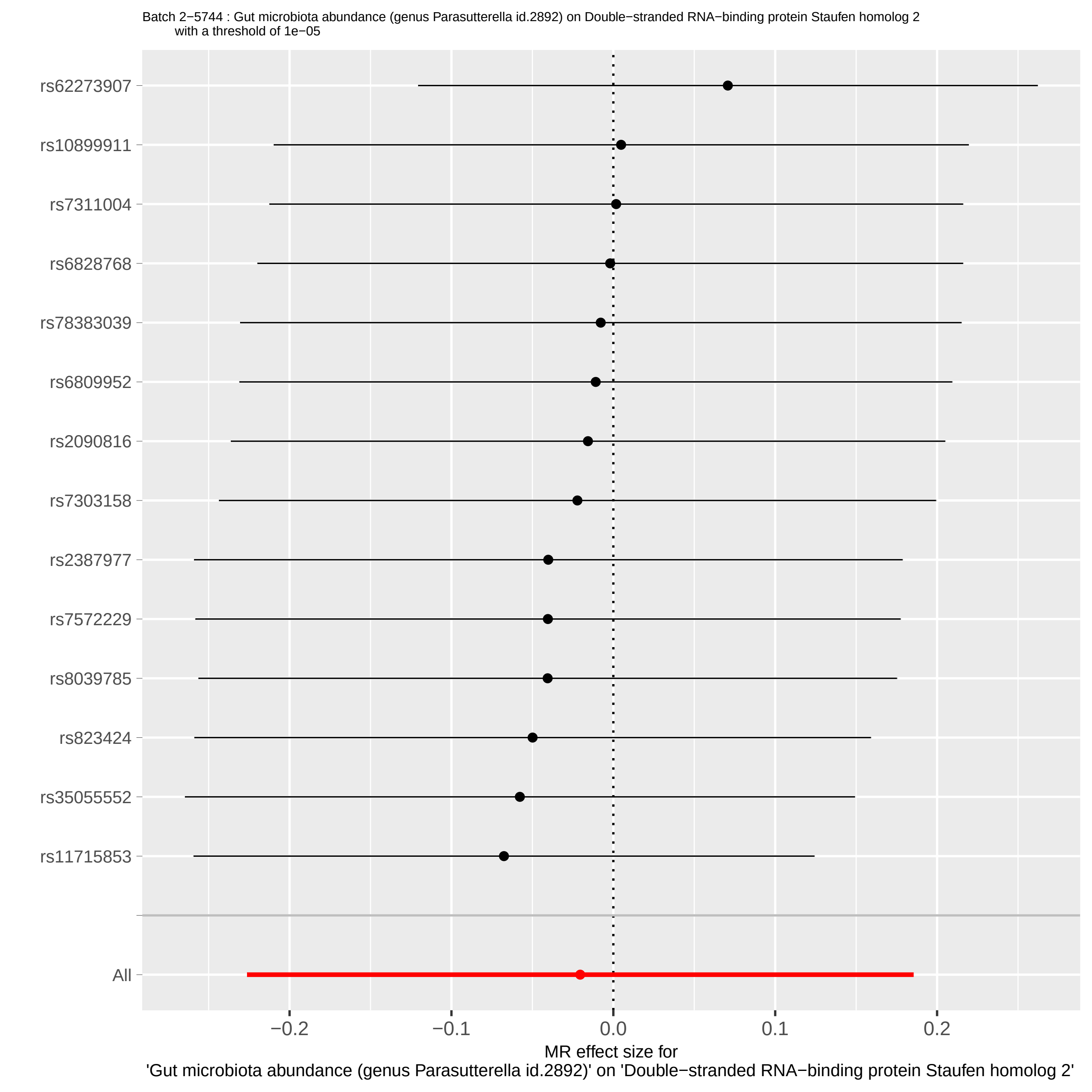

Batch 2−5744 : Gut microbiota abundance (genus Parasutterella id.2892) on Double−stranded RNA−binding protein Staufen homolog 2
with a threshold of 1e−05
rs62273907
rs10899911
rs7311004
rs6828768
rs78383039
rs6809952
rs2090816
rs7303158
rs2387977
rs7572229
rs8039785
rs823424
rs35055552
rs11715853
All
−0.2
−0.1
0.0
0.1
0.2
MR effect size for
'Gut microbiota abundance (genus Parasutterella id.2892)' on 'Double−stranded RNA−binding protein Staufen homolog 2'

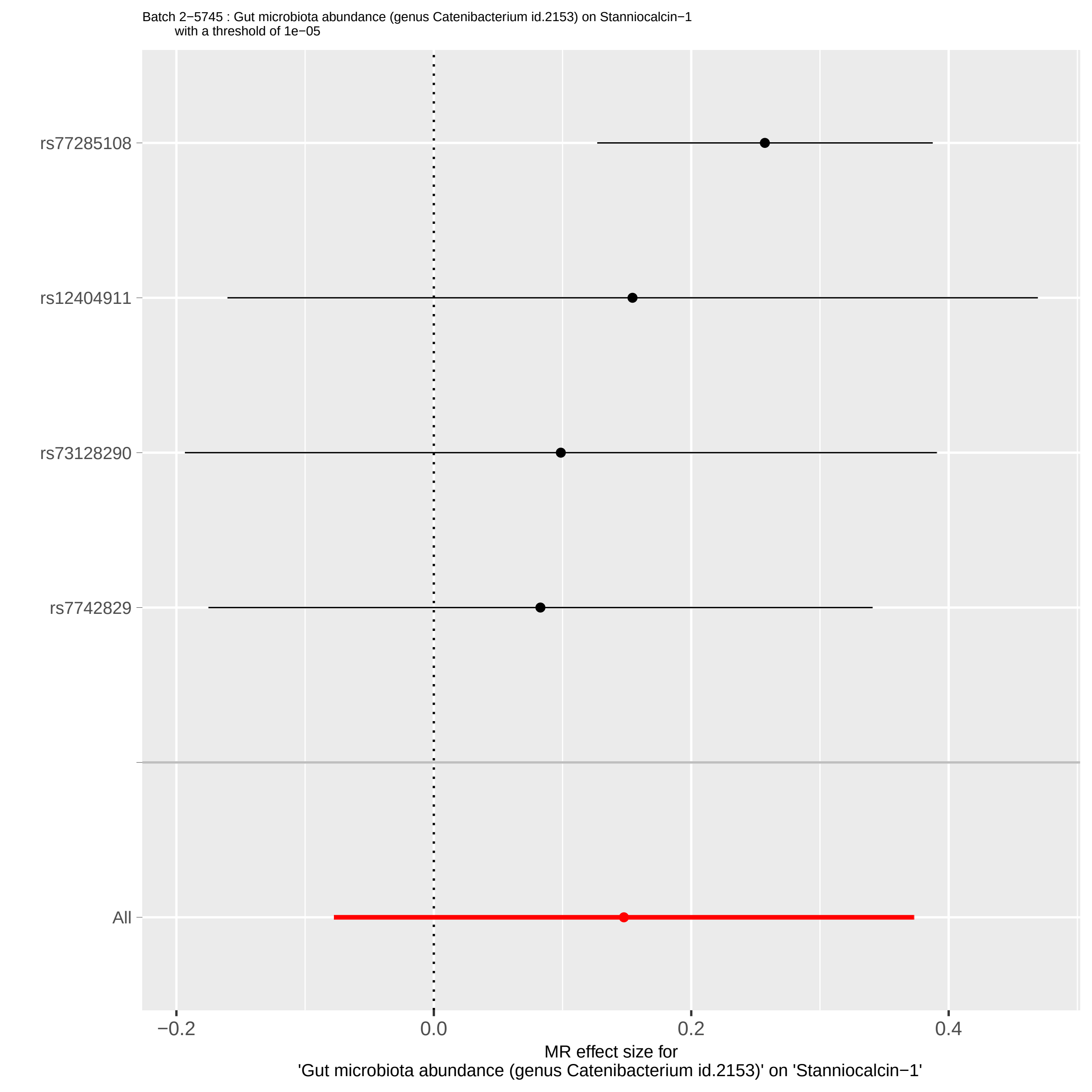
Batch 2−5745 : Gut microbiota abundance (genus Catenibacterium id.2153) on Stanniocalcin−1
with a threshold of 1e−05
rs77285108
rs12404911
rs73128290
rs7742829
All
−0.2
0.0
0.2
0.4
MR effect size for
'Gut microbiota abundance (genus Catenibacterium id.2153)' on 'Stanniocalcin−1'

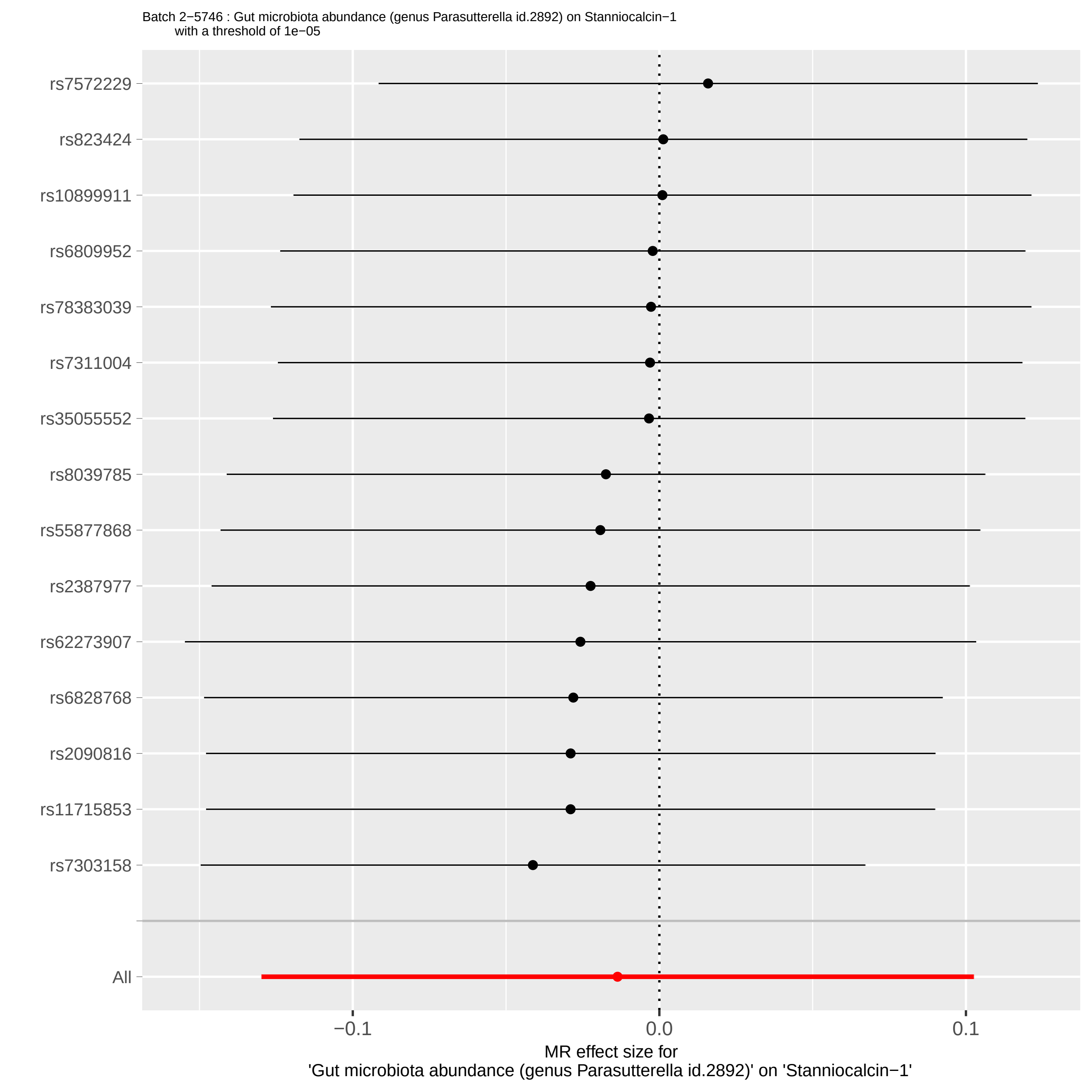
Batch 2−5746 : Gut microbiota abundance (genus Parasutterella id.2892) on Stanniocalcin−1
with a threshold of 1e−05
rs7572229
rs823424
rs10899911
rs6809952
rs78383039
rs7311004
rs35055552
rs8039785
rs55877868
rs2387977
rs62273907
rs6828768
rs2090816
rs11715853
rs7303158
All
−0.1
0.0
0.1
MR effect size for
'Gut microbiota abundance (genus Parasutterella id.2892)' on 'Stanniocalcin−1'

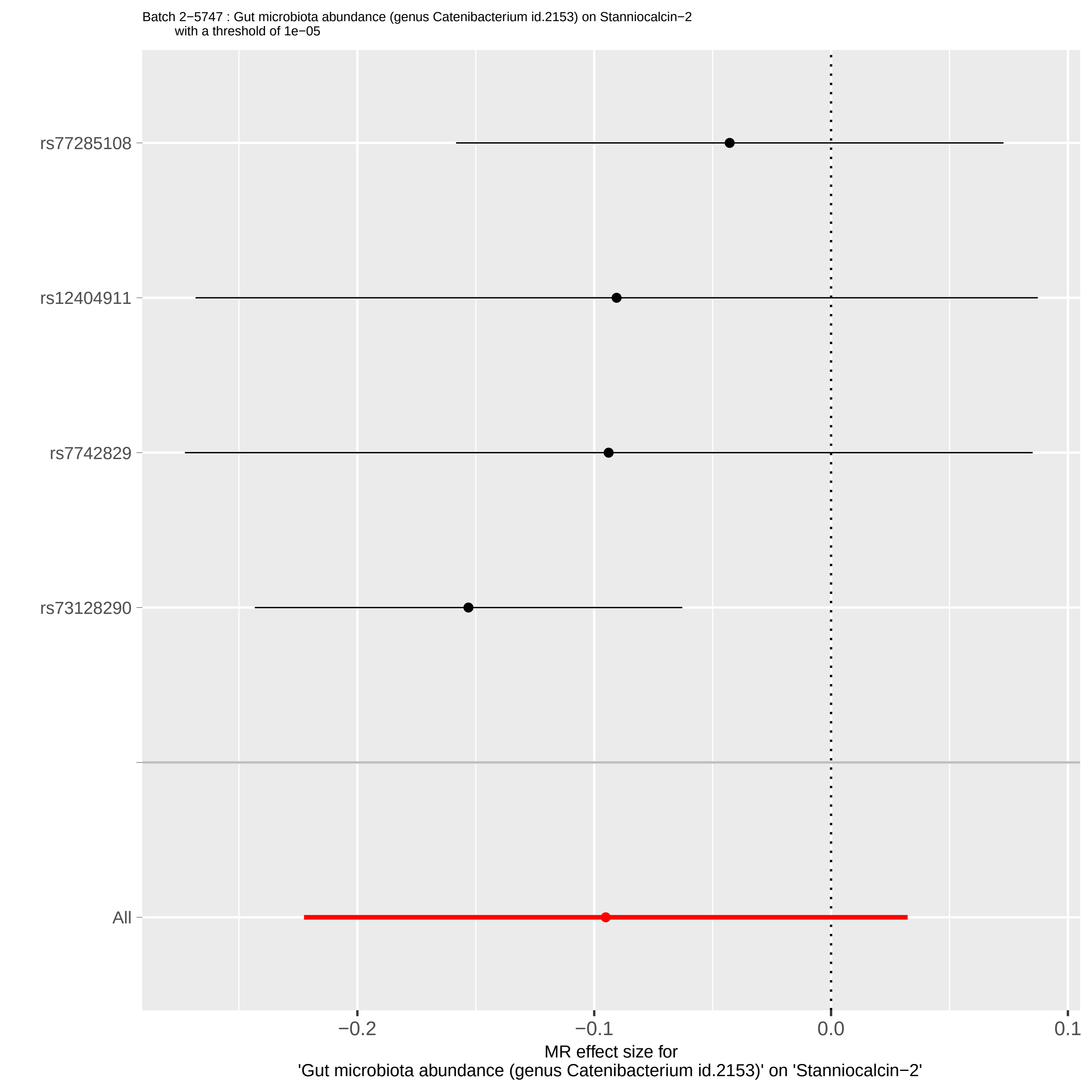

Batch 2−5747 : Gut microbiota abundance (genus Catenibacterium id.2153) on Stanniocalcin−2
with a threshold of 1e−05
rs77285108
rs12404911
rs7742829
rs73128290
All
−0.2
−0.1
0.0
0.1
MR effect size for
'Gut microbiota abundance (genus Catenibacterium id.2153)' on 'Stanniocalcin−2'

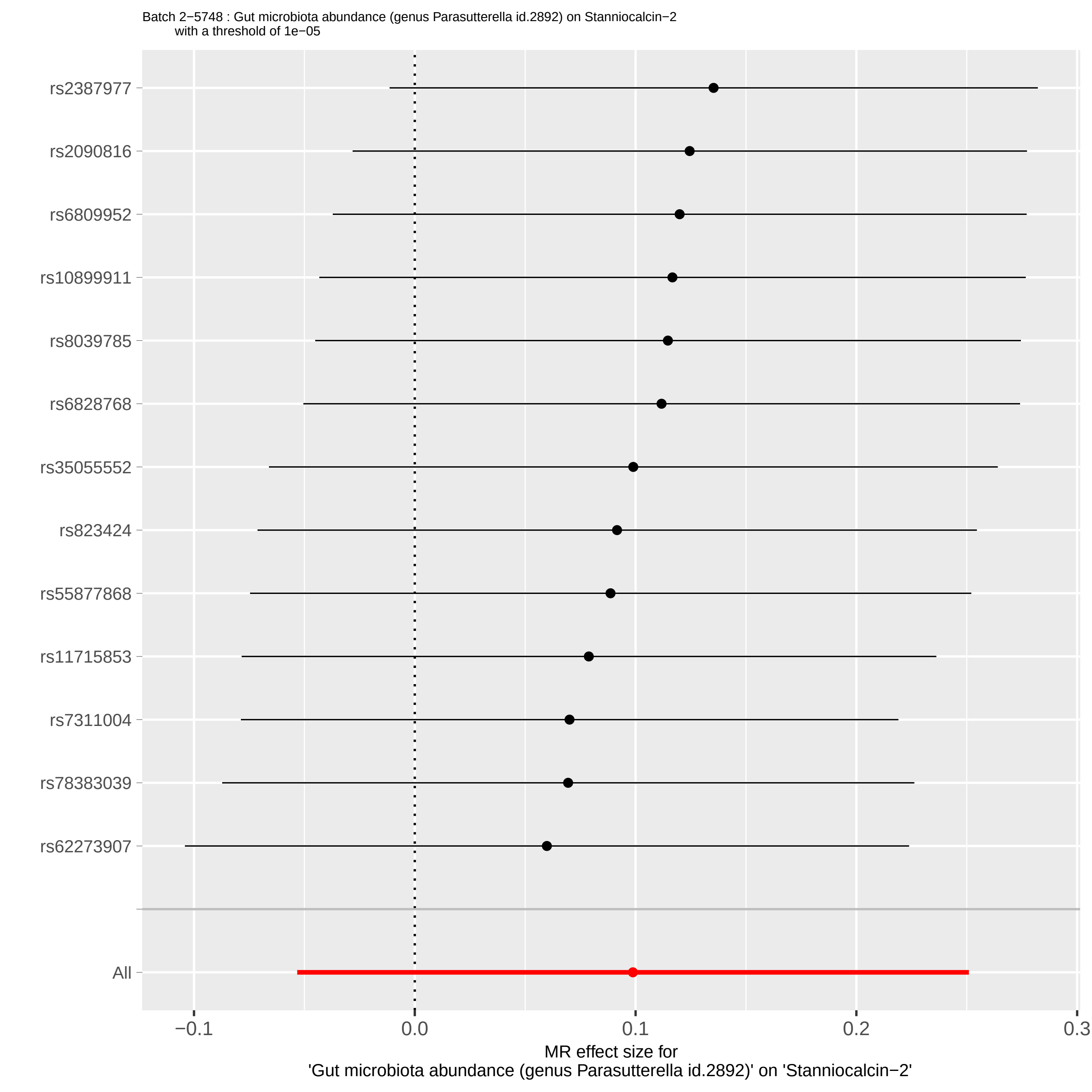
Batch 2−5748 : Gut microbiota abundance (genus Parasutterella id.2892) on Stanniocalcin−2
with a threshold of 1e−05
rs2387977
rs2090816
rs6809952
rs10899911
rs8039785
rs6828768
rs35055552
rs823424
rs55877868
rs11715853
rs7311004
rs78383039
rs62273907
All
−0.1
0.0
0.1
0.2
0.3
MR effect size for
'Gut microbiota abundance (genus Parasutterella id.2892)' on 'Stanniocalcin−2'

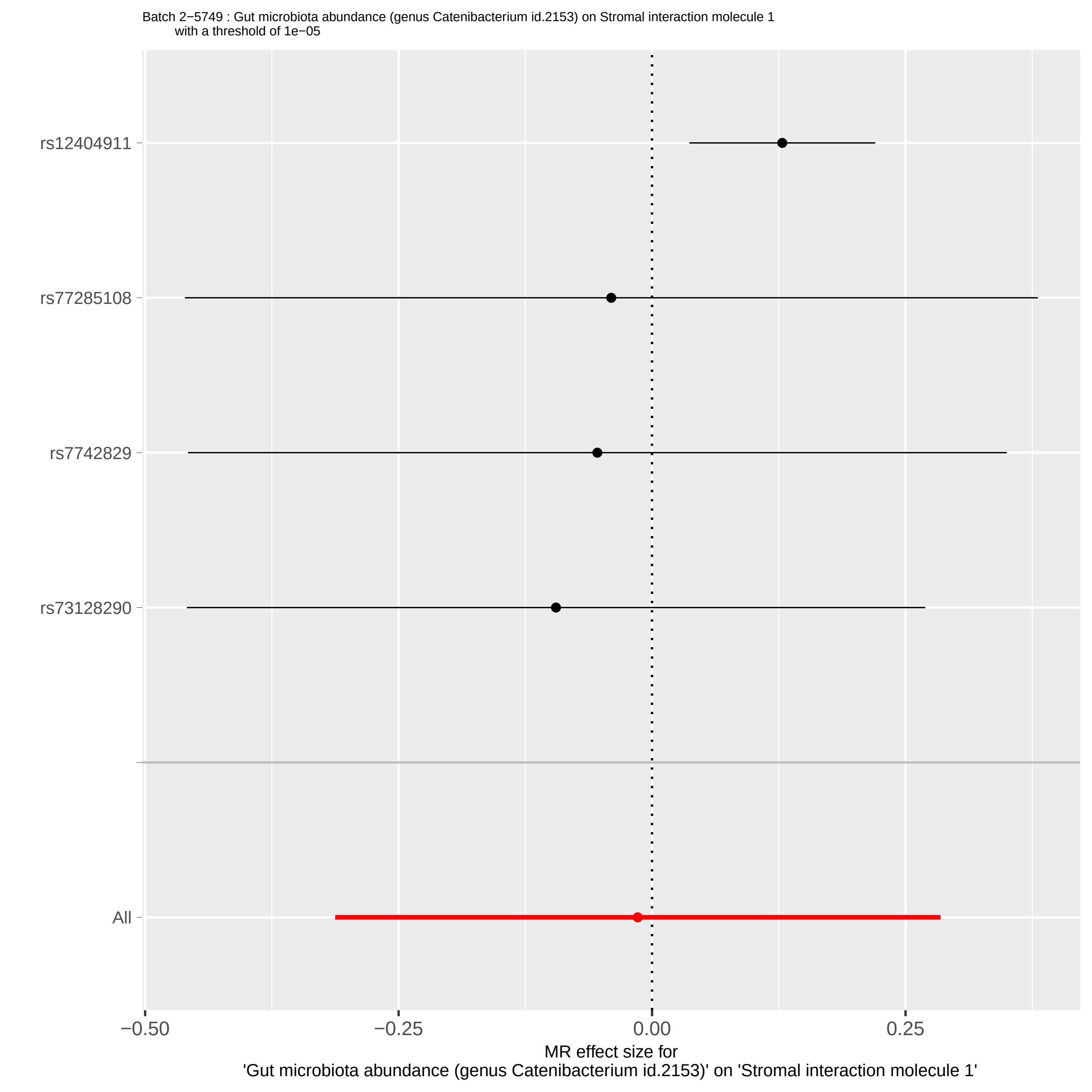
Batch 2−5749 : Gut microbiota abundance (genus Catenibacterium id.2153) on Stromal interaction molecule 1
with a threshold of 1e−05
rs12404911
rs77285108
rs7742829
rs73128290
All
−0.50
−0.25
0.00
0.25
MR effect size for
'Gut microbiota abundance (genus Catenibacterium id.2153)' on 'Stromal interaction molecule 1'

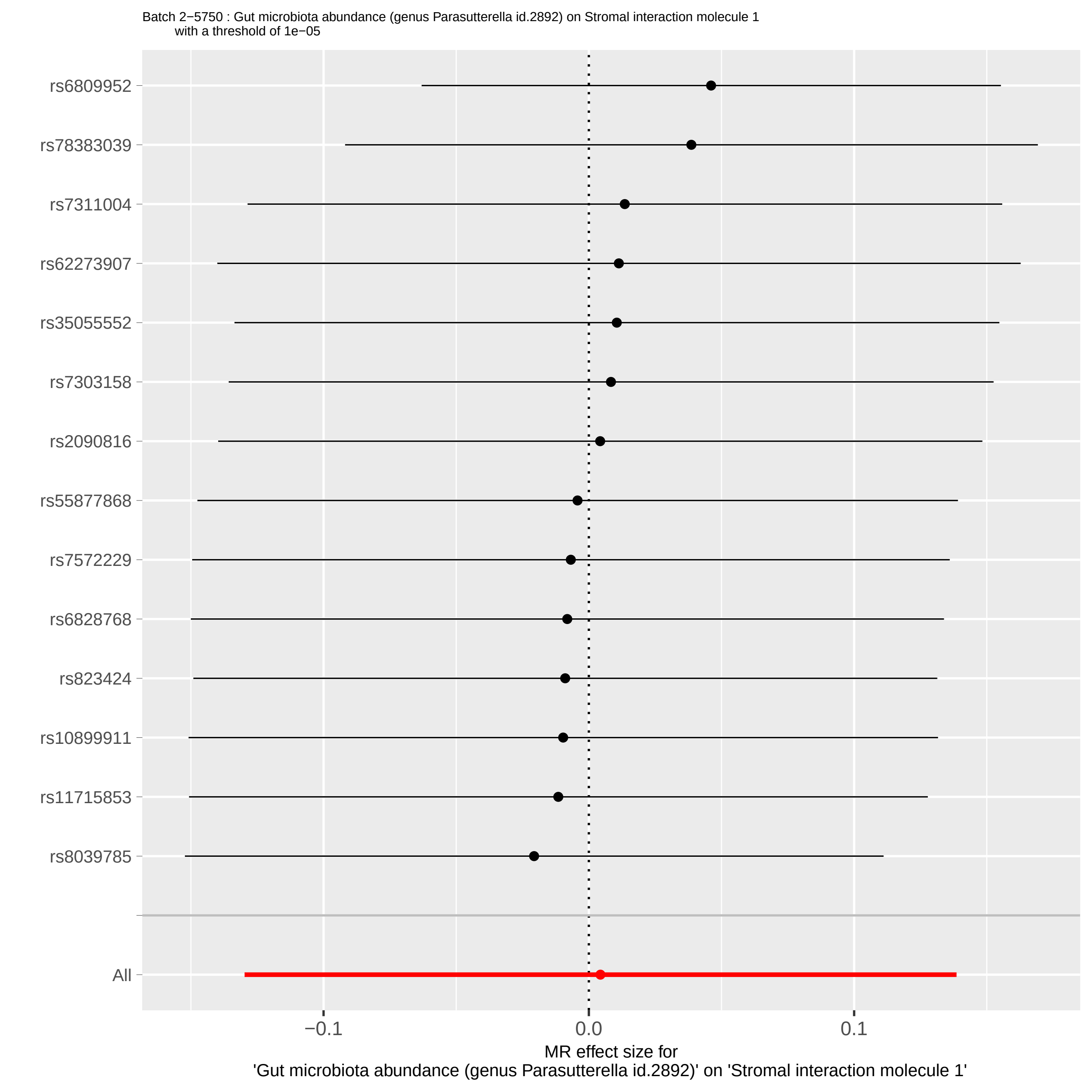

Batch 2−5750 : Gut microbiota abundance (genus Parasutterella id.2892) on Stromal interaction molecule 1
with a threshold of 1e−05
rs6809952
rs78383039
rs7311004
rs62273907
rs35055552
rs7303158
rs2090816
rs55877868
rs7572229
rs6828768
rs823424
rs10899911
rs11715853
rs8039785
All
−0.1
0.0
0.1
MR effect size for
'Gut microbiota abundance (genus Parasutterella id.2892)' on 'Stromal interaction molecule 1'

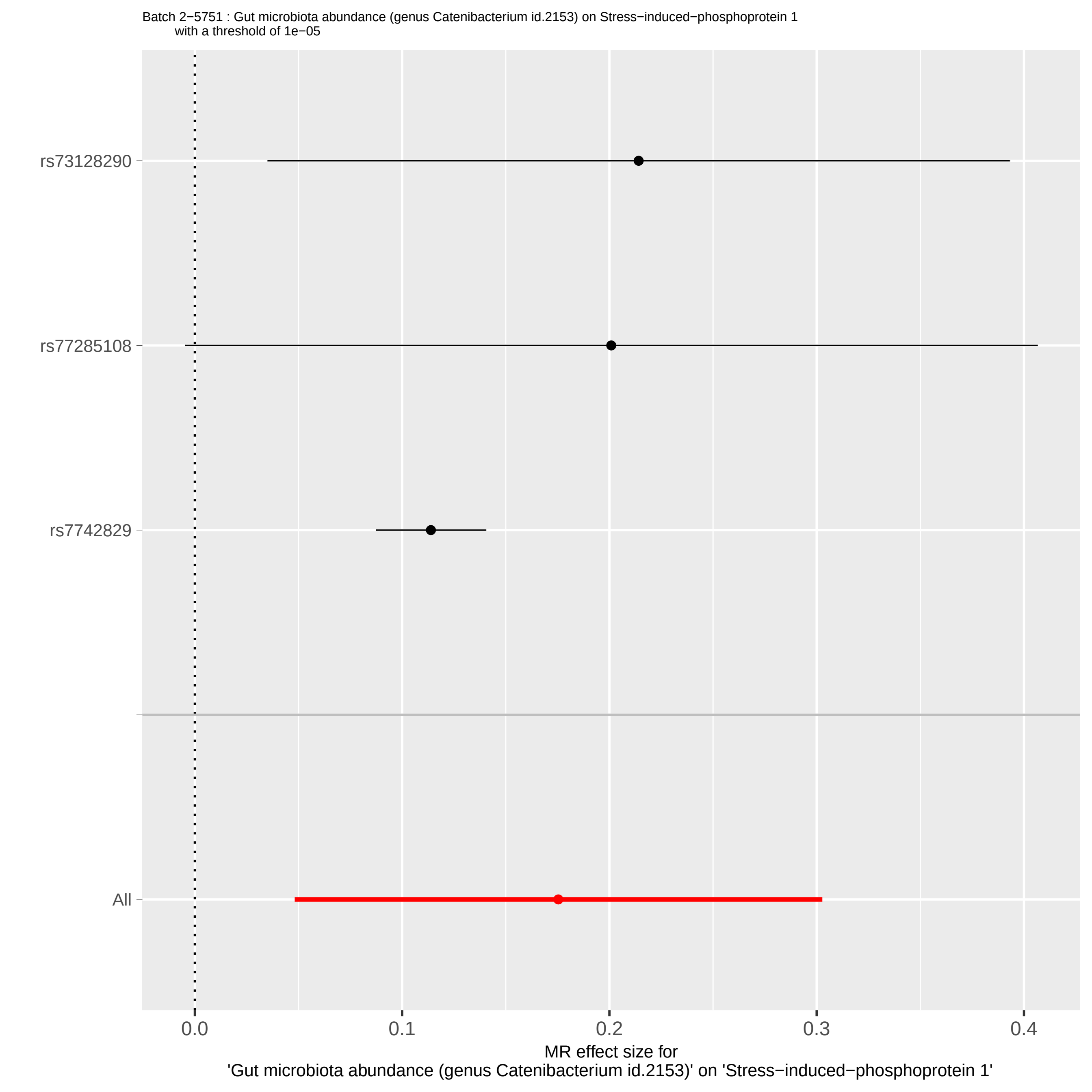
Batch 2−5751 : Gut microbiota abundance (genus Catenibacterium id.2153) on Stress−induced−phosphoprotein 1
with a threshold of 1e−05
rs73128290
rs77285108
rs7742829
All
0.0
0.1
0.2
0.3
0.4
MR effect size for
'Gut microbiota abundance (genus Catenibacterium id.2153)' on 'Stress−induced−phosphoprotein 1'

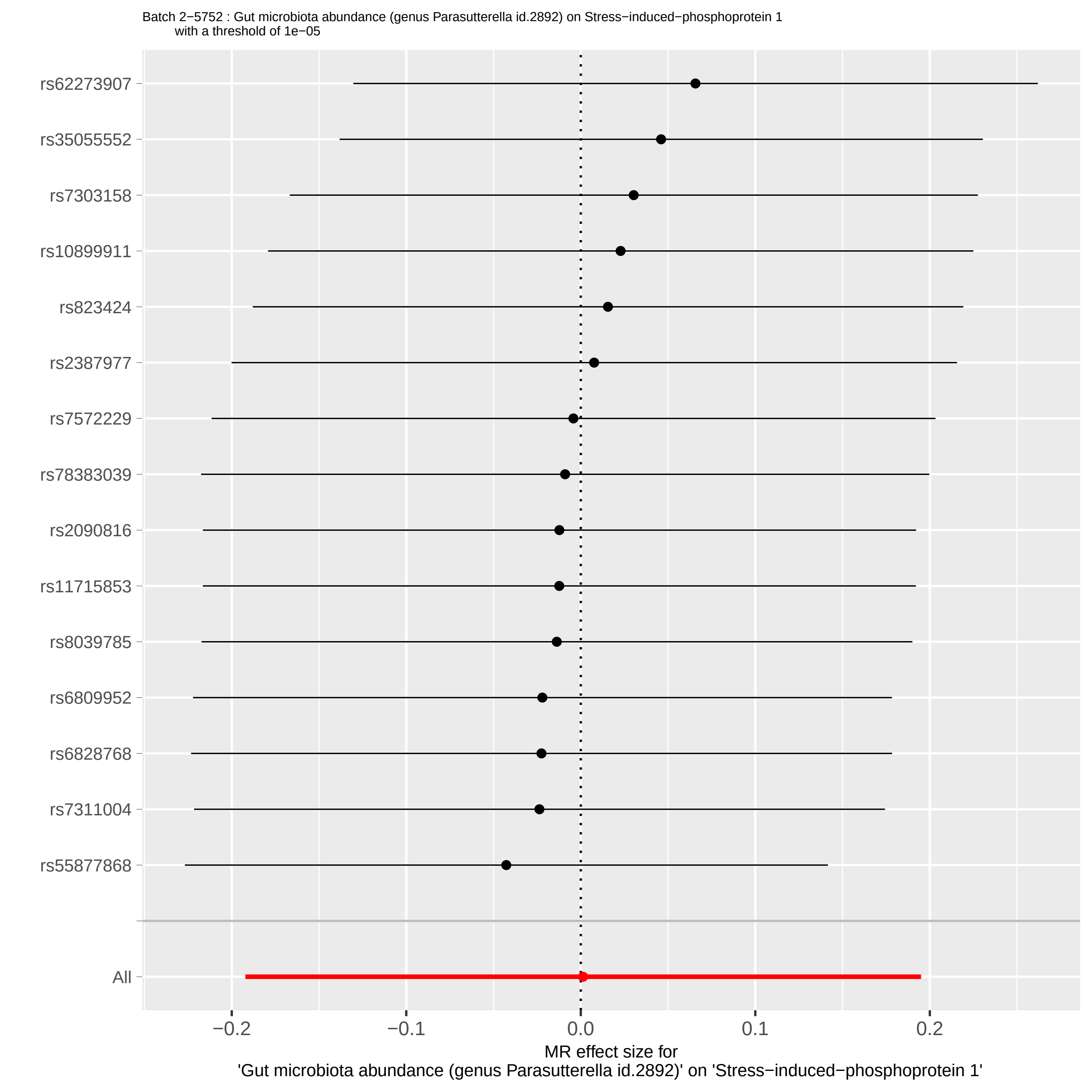
Batch 2−5752 : Gut microbiota abundance (genus Parasutterella id.2892) on Stress−induced−phosphoprotein 1
with a threshold of 1e−05
rs62273907
rs35055552
rs7303158
rs10899911
rs823424
rs2387977
rs7572229
rs78383039
rs2090816
rs11715853
rs8039785
rs6809952
rs6828768
rs7311004
rs55877868
All
−0.2
−0.1
0.0
0.1
0.2
MR effect size for
'Gut microbiota abundance (genus Parasutterella id.2892)' on 'Stress−induced−phosphoprotein 1'

Batch 2−5753 : Gut microbiota abundance (genus Catenibacterium id.2153) on Serine/threonine−protein kinase 16
with a threshold of 1e−05

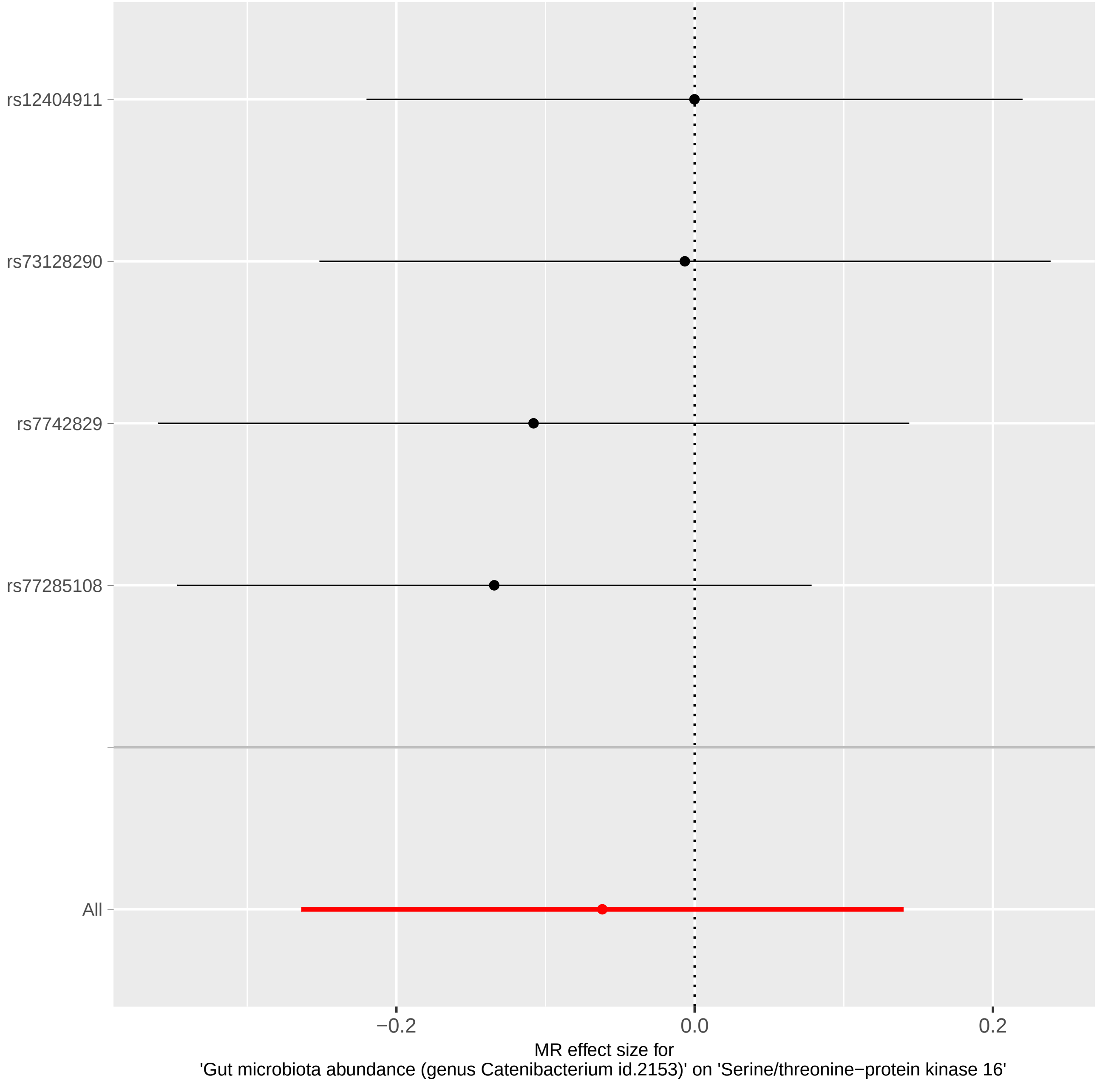

Batch 2−5754 : Gut microbiota abundance (genus Parasutterella id.2892) on Serine/threonine−protein kinase 16
with a threshold of 1e−05

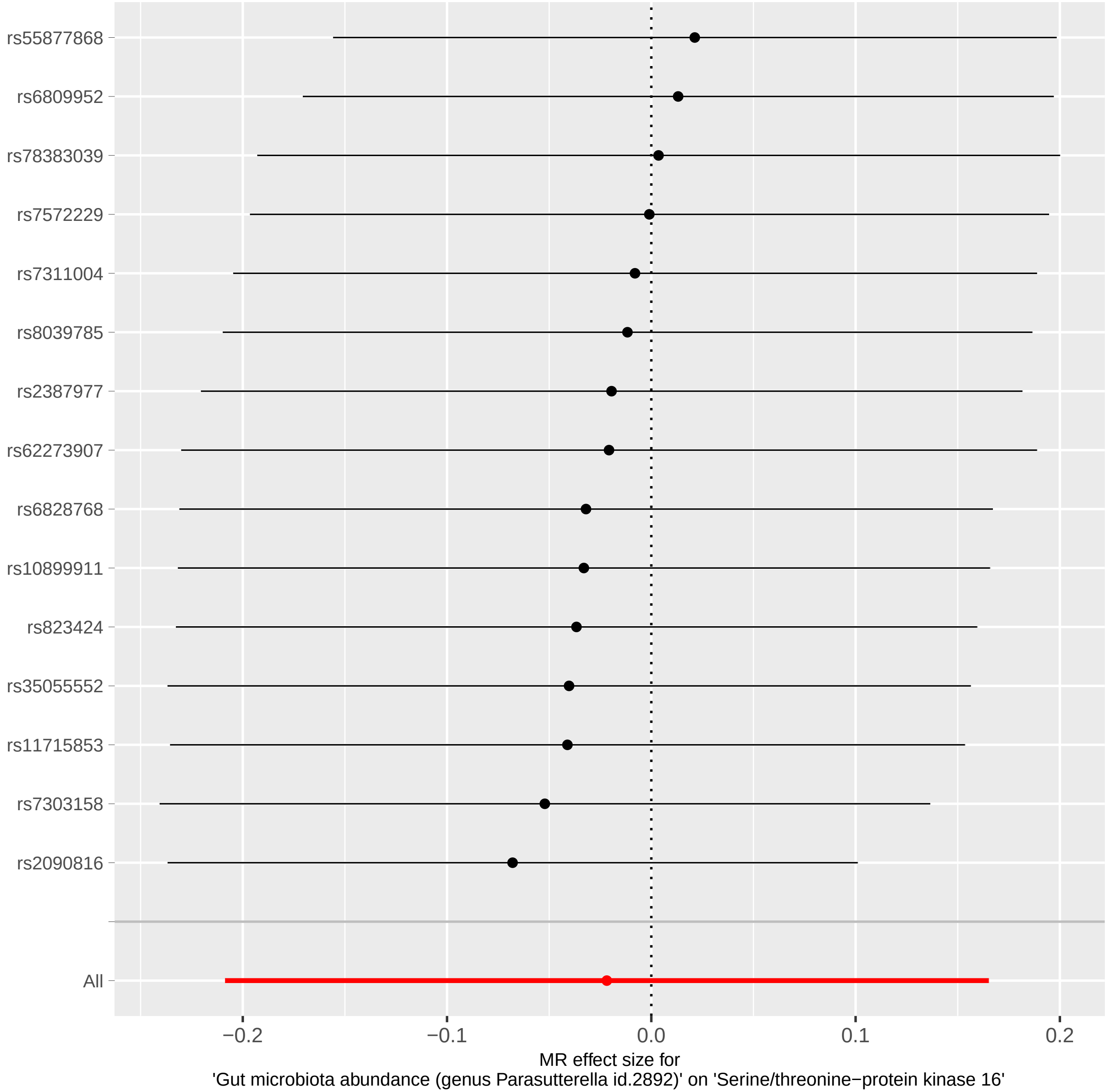

Batch 2−5755 : Gut microbiota abundance (genus Catenibacterium id.2153) on Serine/threonine−protein kinase 17B
with a threshold of 1e−05

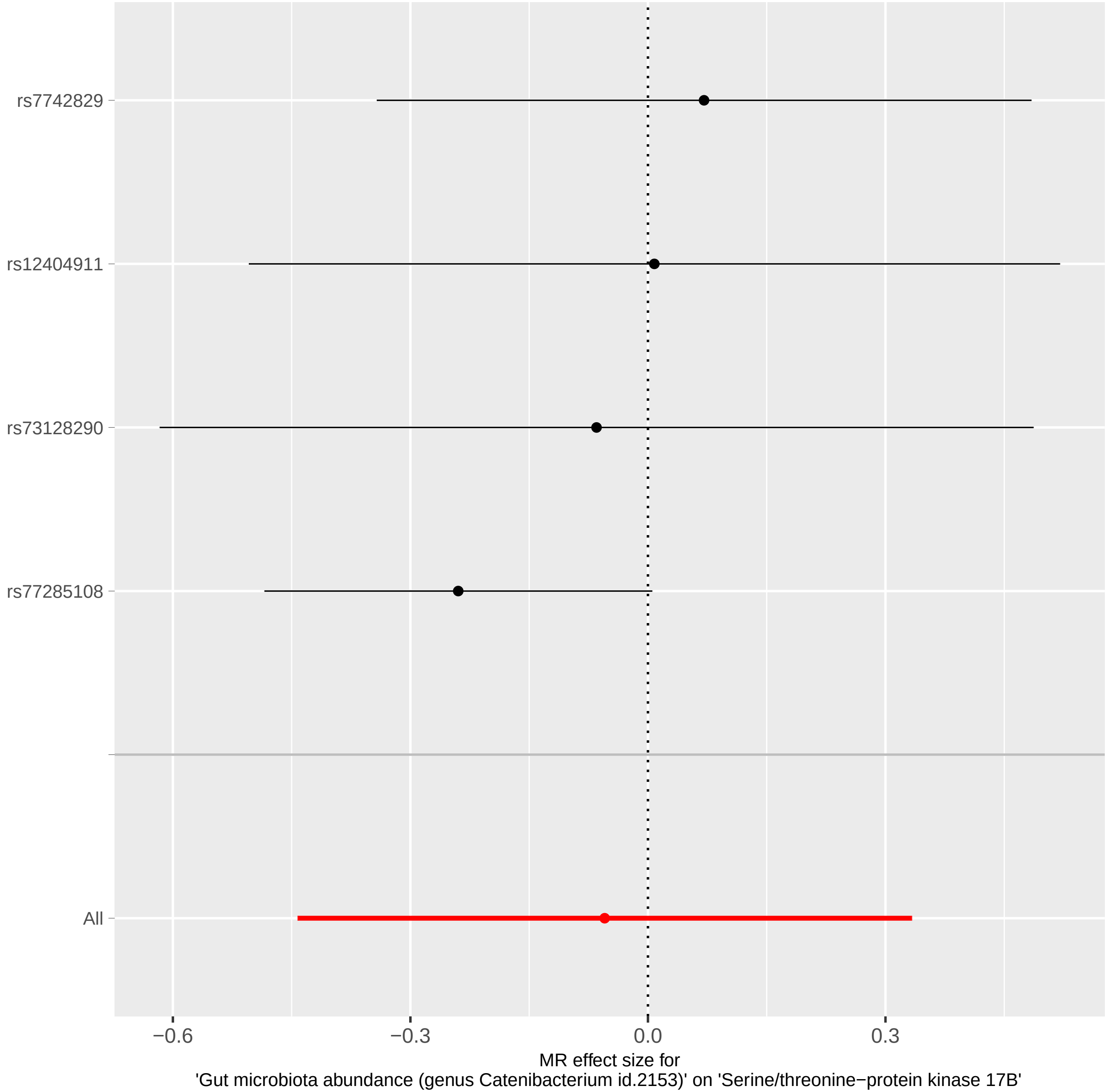

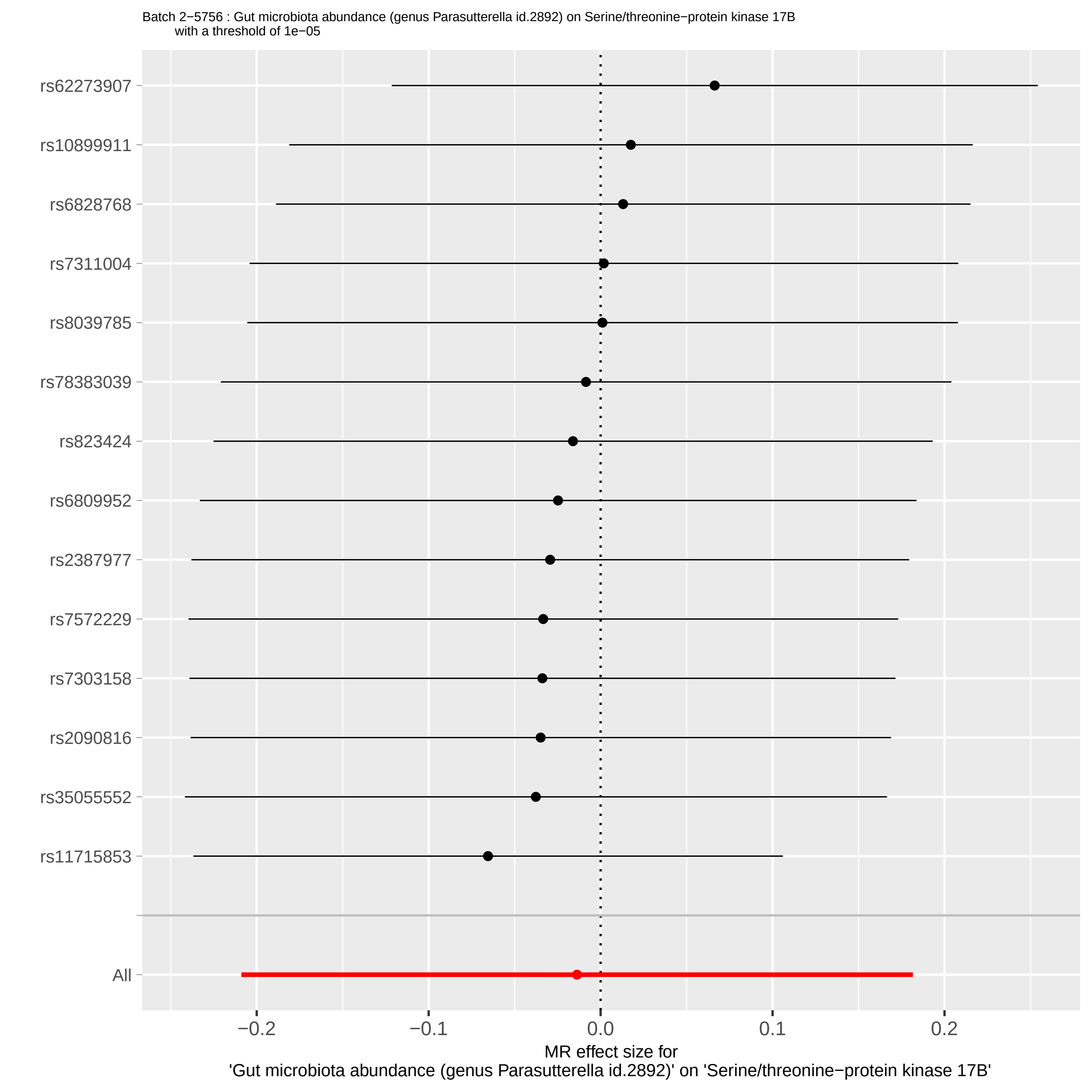

Batch 2−5756 : Gut microbiota abundance (genus Parasutterella id.2892) on Serine/threonine−protein kinase 17B
with a threshold of 1e−05
rs62273907
rs10899911
rs6828768
rs7311004
rs8039785
rs78383039
rs823424
rs6809952
rs2387977
rs7572229
rs7303158
rs2090816
rs35055552
rs11715853
All
−0.2
−0.1
0.0
0.1
0.2
MR effect size for
'Gut microbiota abundance (genus Parasutterella id.2892)' on 'Serine/threonine−protein kinase 17B'

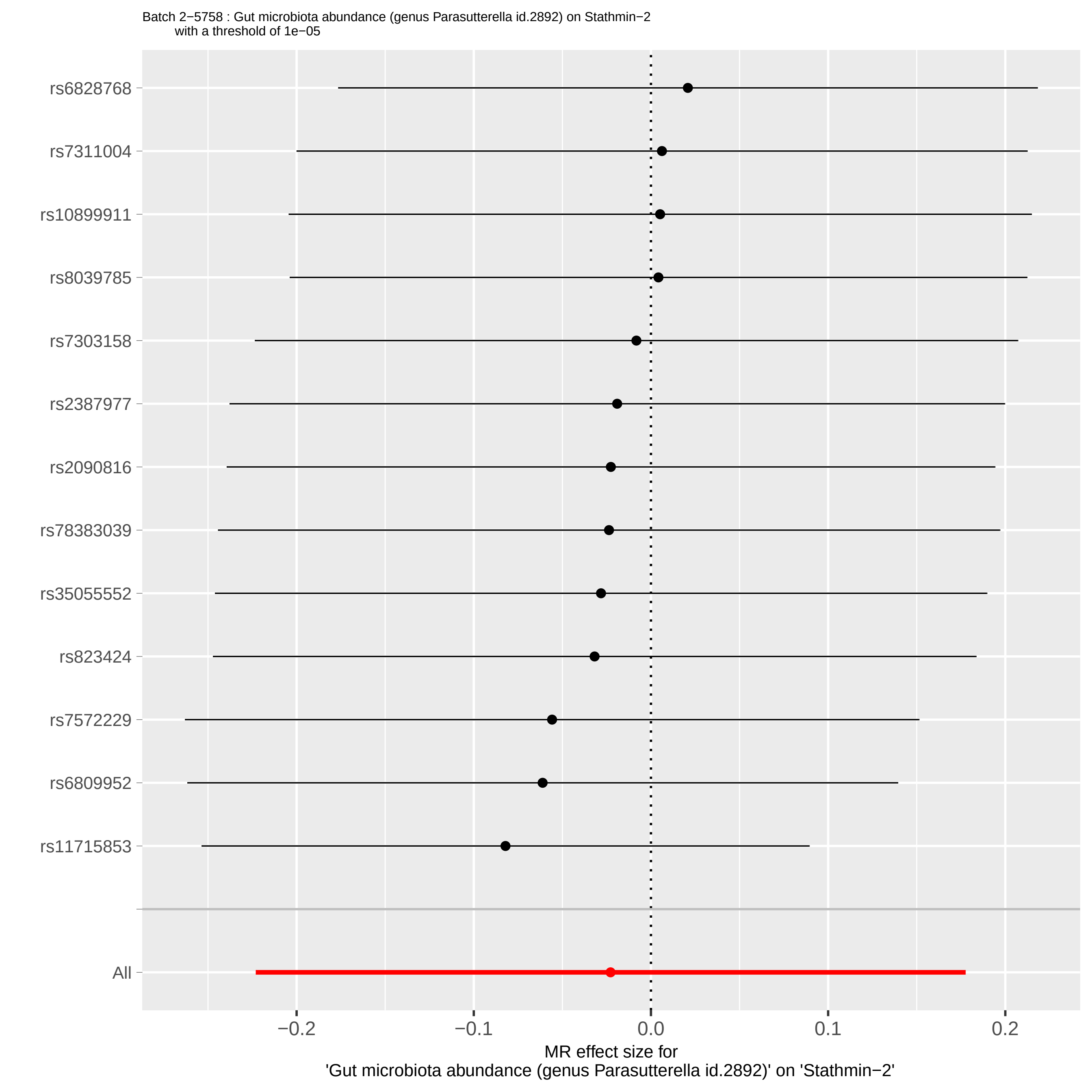

Batch 2−5758 : Gut microbiota abundance (genus Parasutterella id.2892) on Stathmin−2
with a threshold of 1e−05
rs6828768
rs7311004
rs10899911
rs8039785
rs7303158
rs2387977
rs2090816
rs78383039
rs35055552
rs823424
rs7572229
rs6809952
rs11715853
All
−0.2
−0.1
0.0
0.1
0.2
MR effect size for
'Gut microbiota abundance (genus Parasutterella id.2892)' on 'Stathmin−2'

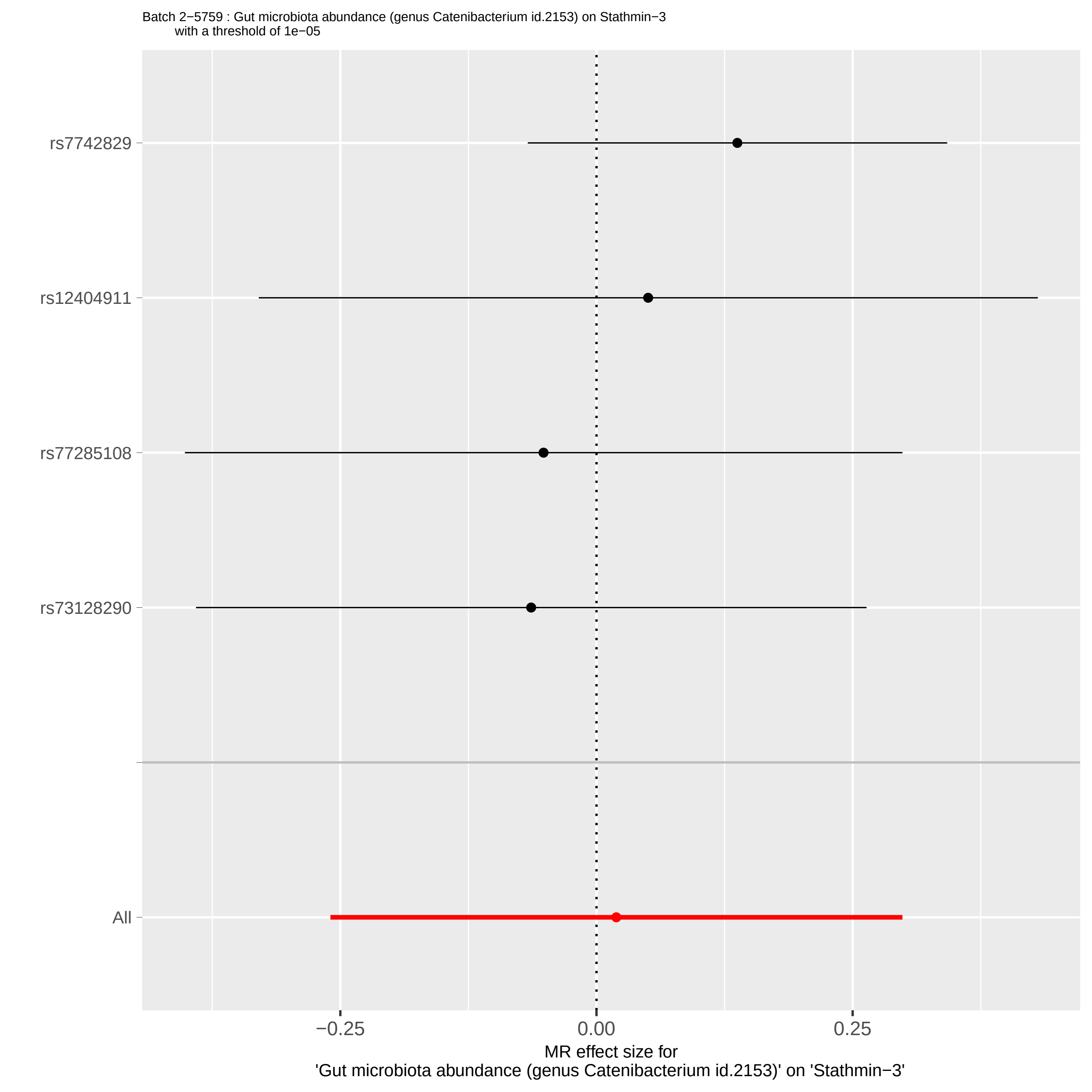
Batch 2−5759 : Gut microbiota abundance (genus Catenibacterium id.2153) on Stathmin−3
with a threshold of 1e−05
rs7742829
rs12404911
rs77285108
rs73128290
All
−0.25
0.00
0.25
MR effect size for
'Gut microbiota abundance (genus Catenibacterium id.2153)' on 'Stathmin−3'

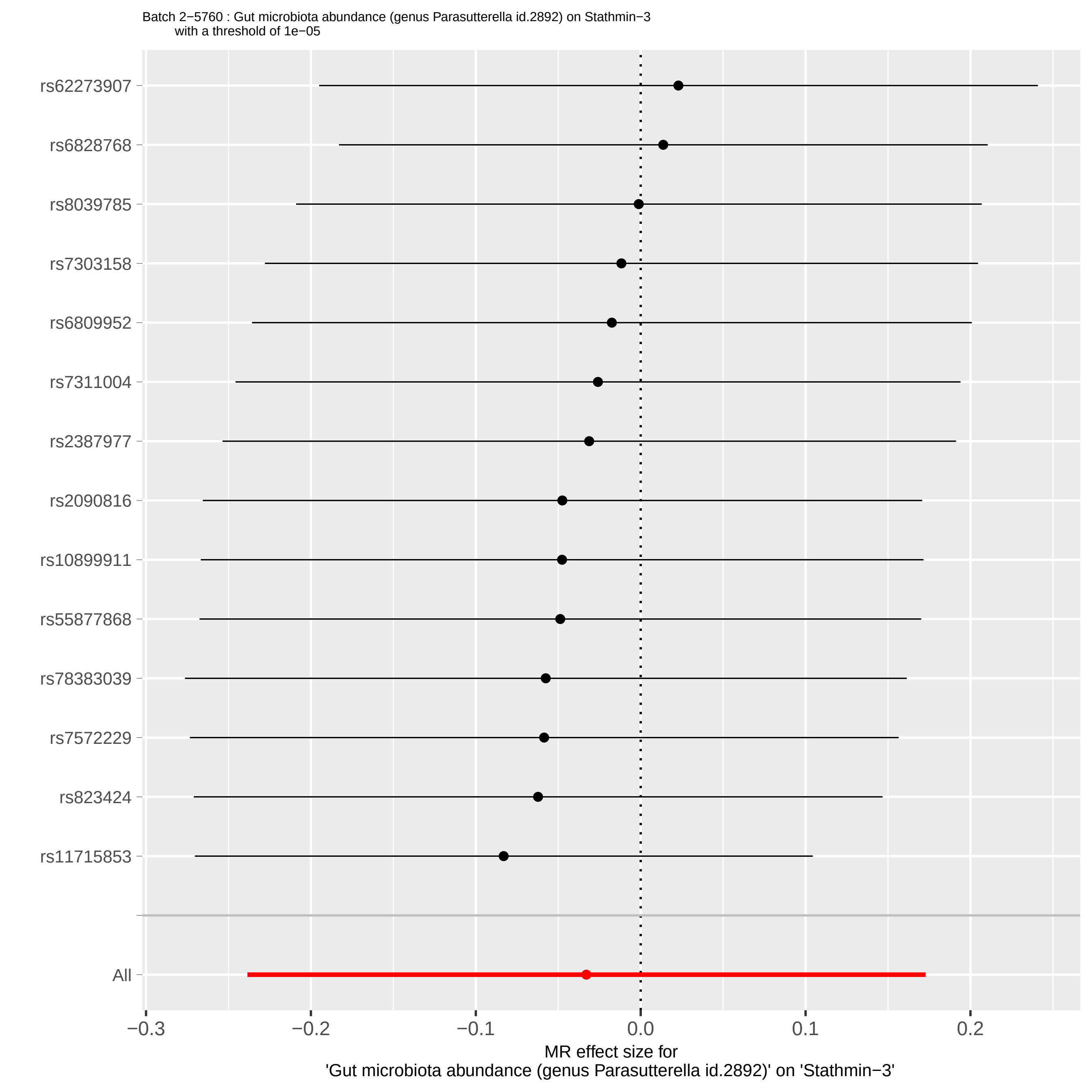

Batch 2−5760 : Gut microbiota abundance (genus Parasutterella id.2892) on Stathmin−3
with a threshold of 1e−05
rs62273907
rs6828768
rs8039785
rs7303158
rs6809952
rs7311004
rs2387977
rs2090816
rs10899911
rs55877868
rs78383039
rs7572229
rs823424
rs11715853
All
−0.3
−0.2
−0.1
0.0
0.1
0.2
MR effect size for
'Gut microbiota abundance (genus Parasutterella id.2892)' on 'Stathmin−3'

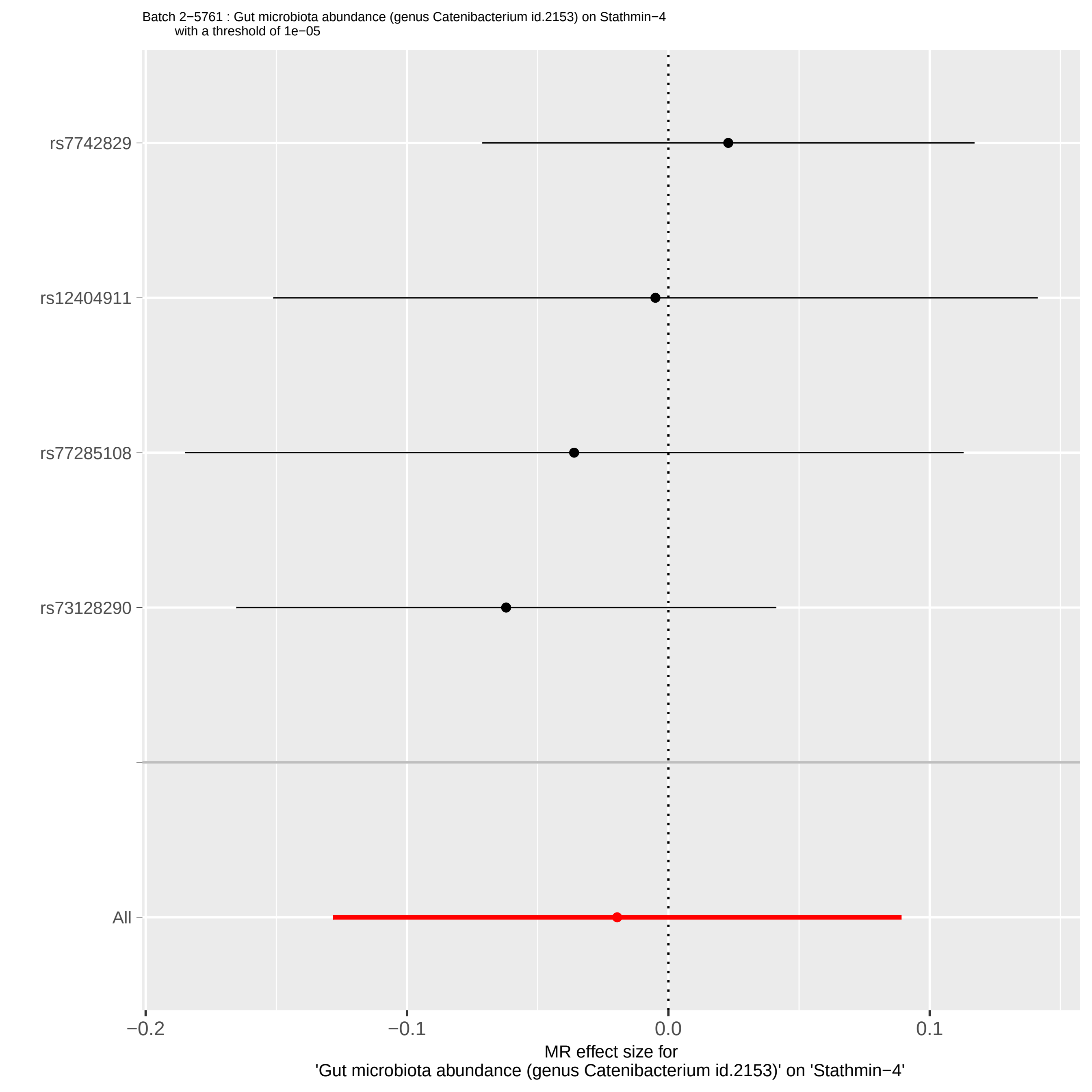
Batch 2−5761 : Gut microbiota abundance (genus Catenibacterium id.2153) on Stathmin−4
with a threshold of 1e−05
rs7742829
rs12404911
rs77285108
rs73128290
All
−0.2
−0.1
0.0
0.1
MR effect size for
'Gut microbiota abundance (genus Catenibacterium id.2153)' on 'Stathmin−4'

Batch 2−5762 : Gut microbiota abundance (genus Parasutterella id.2892) on Stathmin−4
with a threshold of 1e−05

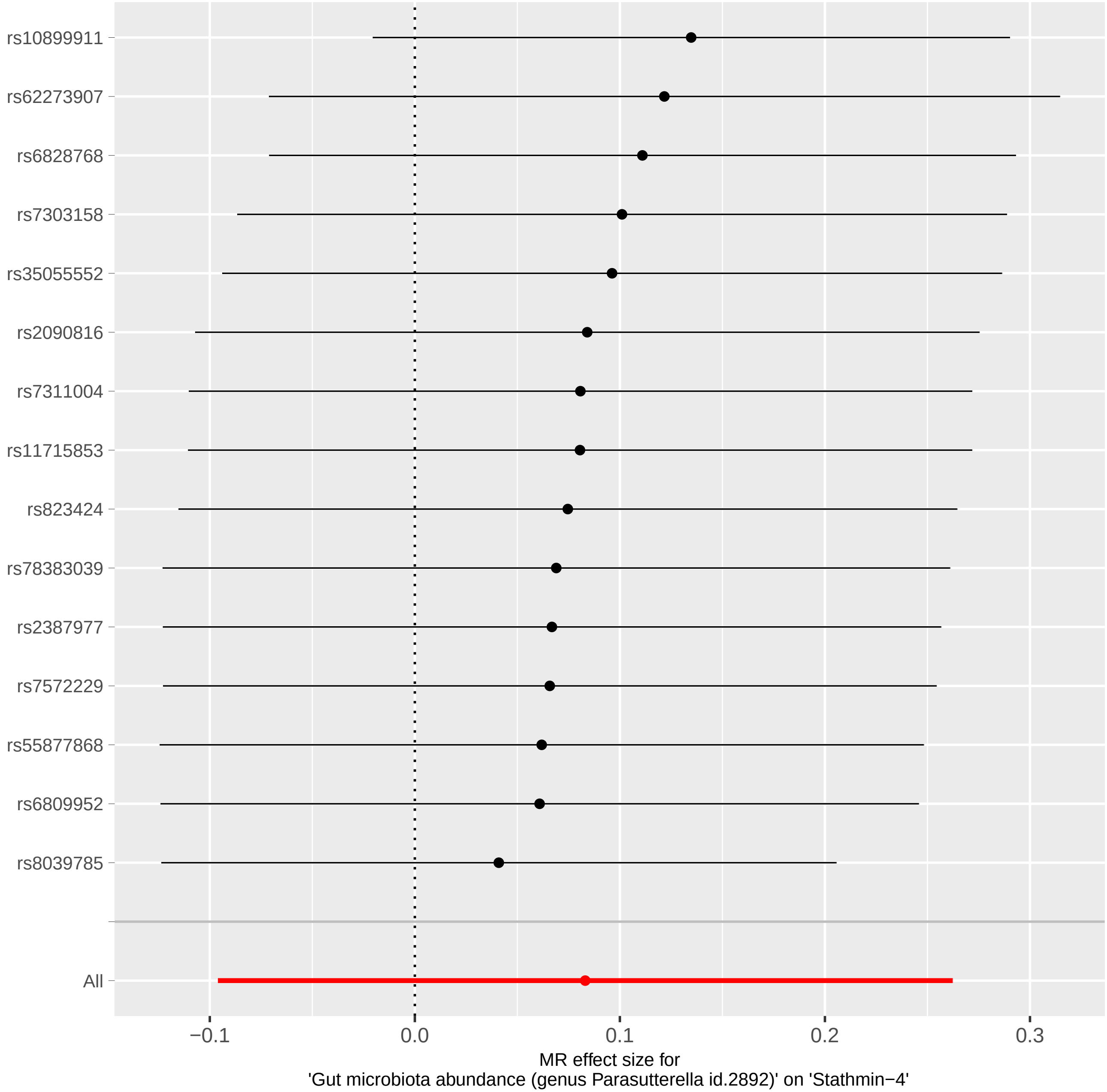

Batch 2−5763 : Gut microbiota abundance (genus Catenibacterium id.2153) on Erythrocyte band 7 integral membrane protein
with a threshold of 1e−05

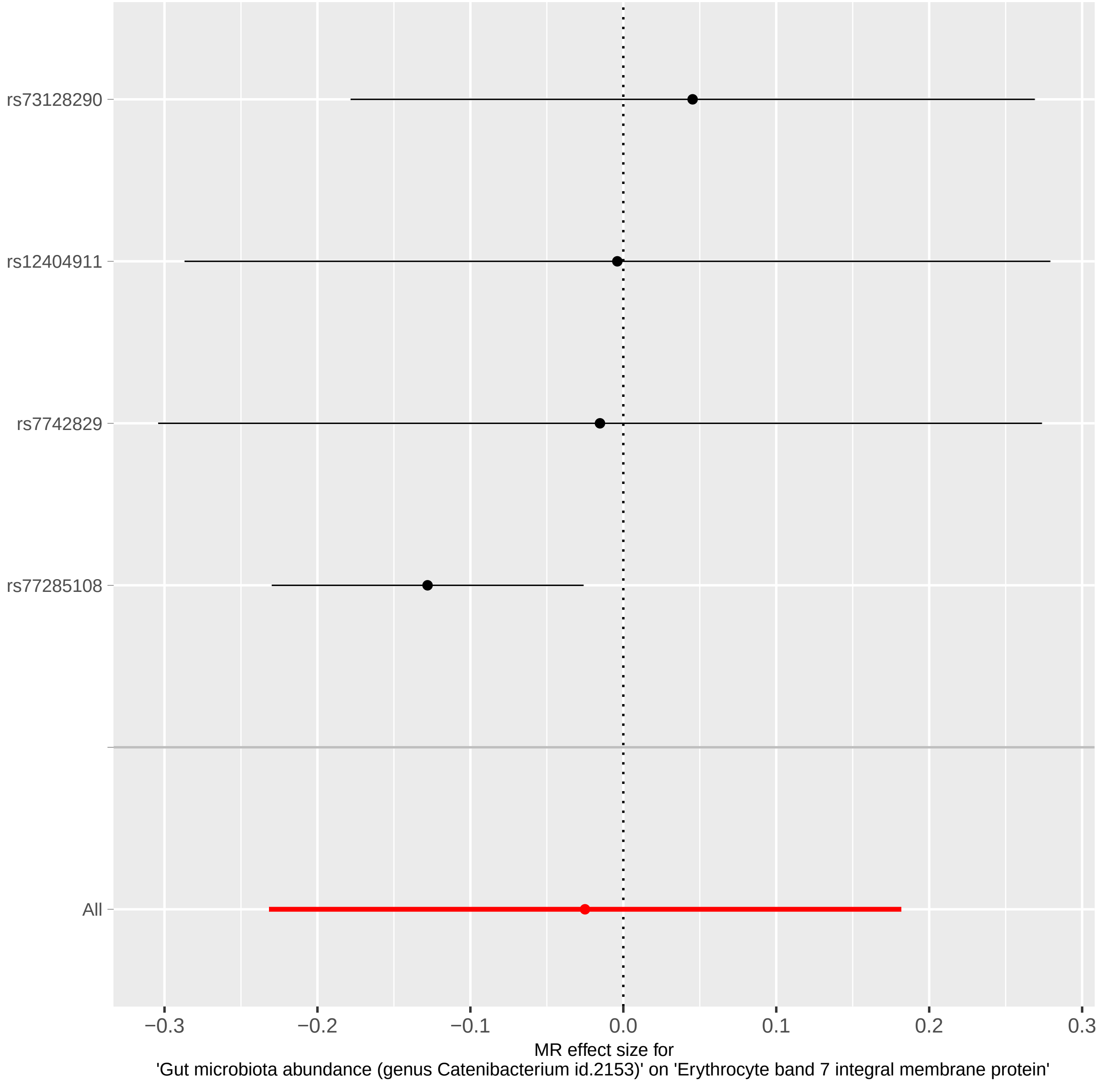

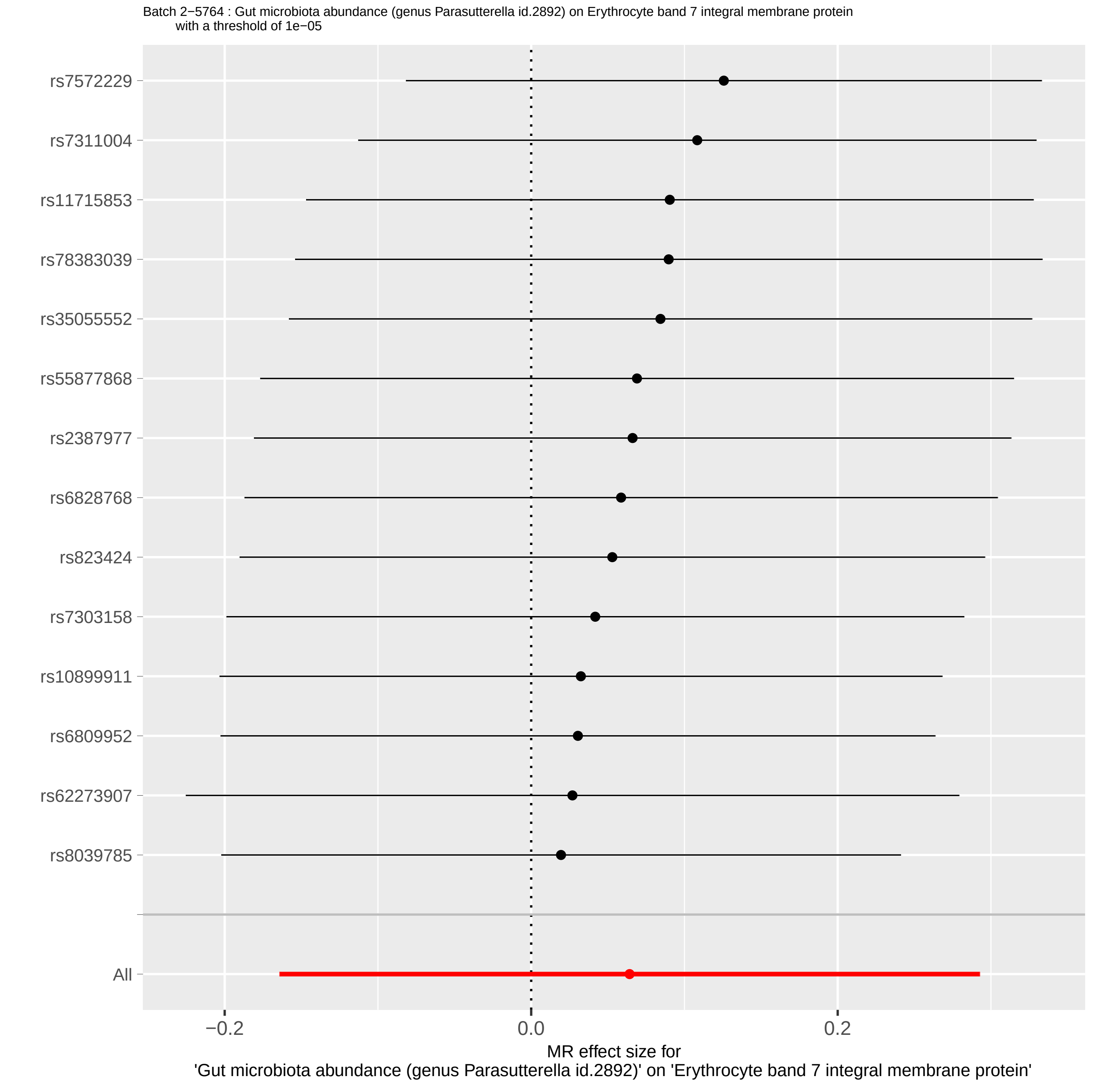
Batch 2−5764 : Gut microbiota abundance (genus Parasutterella id.2892) on Erythrocyte band 7 integral membrane protein
with a threshold of 1e−05
rs7572229
rs7311004
rs11715853
rs78383039
rs35055552
rs55877868
rs2387977
rs6828768
rs823424
rs7303158
rs10899911
rs6809952
rs62273907
rs8039785
All
−0.2
0.0
0.2
MR effect size for
'Gut microbiota abundance (genus Parasutterella id.2892)' on 'Erythrocyte band 7 integral membrane protein'

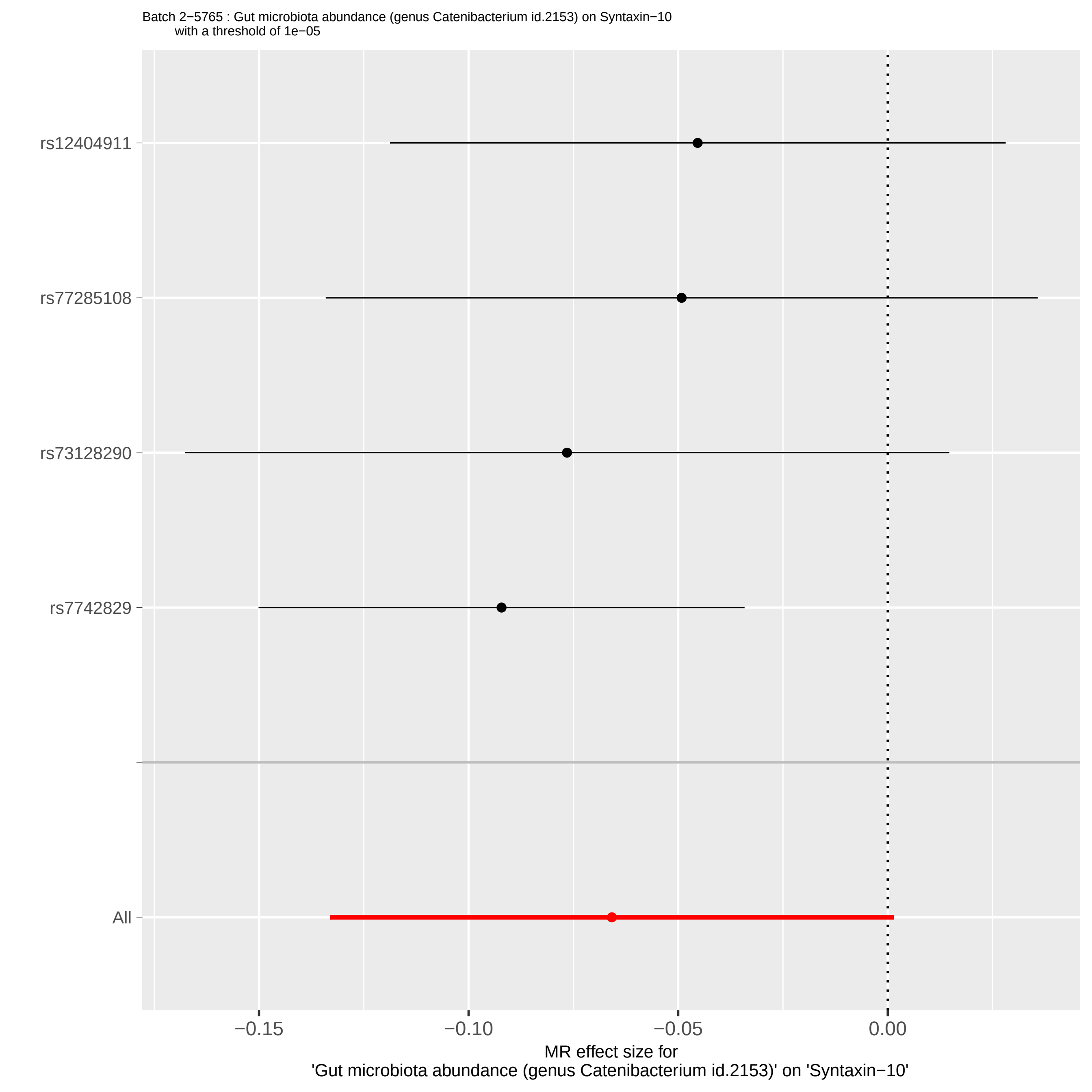
Batch 2−5765 : Gut microbiota abundance (genus Catenibacterium id.2153) on Syntaxin−10
with a threshold of 1e−05
rs12404911
rs77285108
rs73128290
rs7742829
All
−0.15
−0.10
−0.05
0.00
MR effect size for
'Gut microbiota abundance (genus Catenibacterium id.2153)' on 'Syntaxin−10'

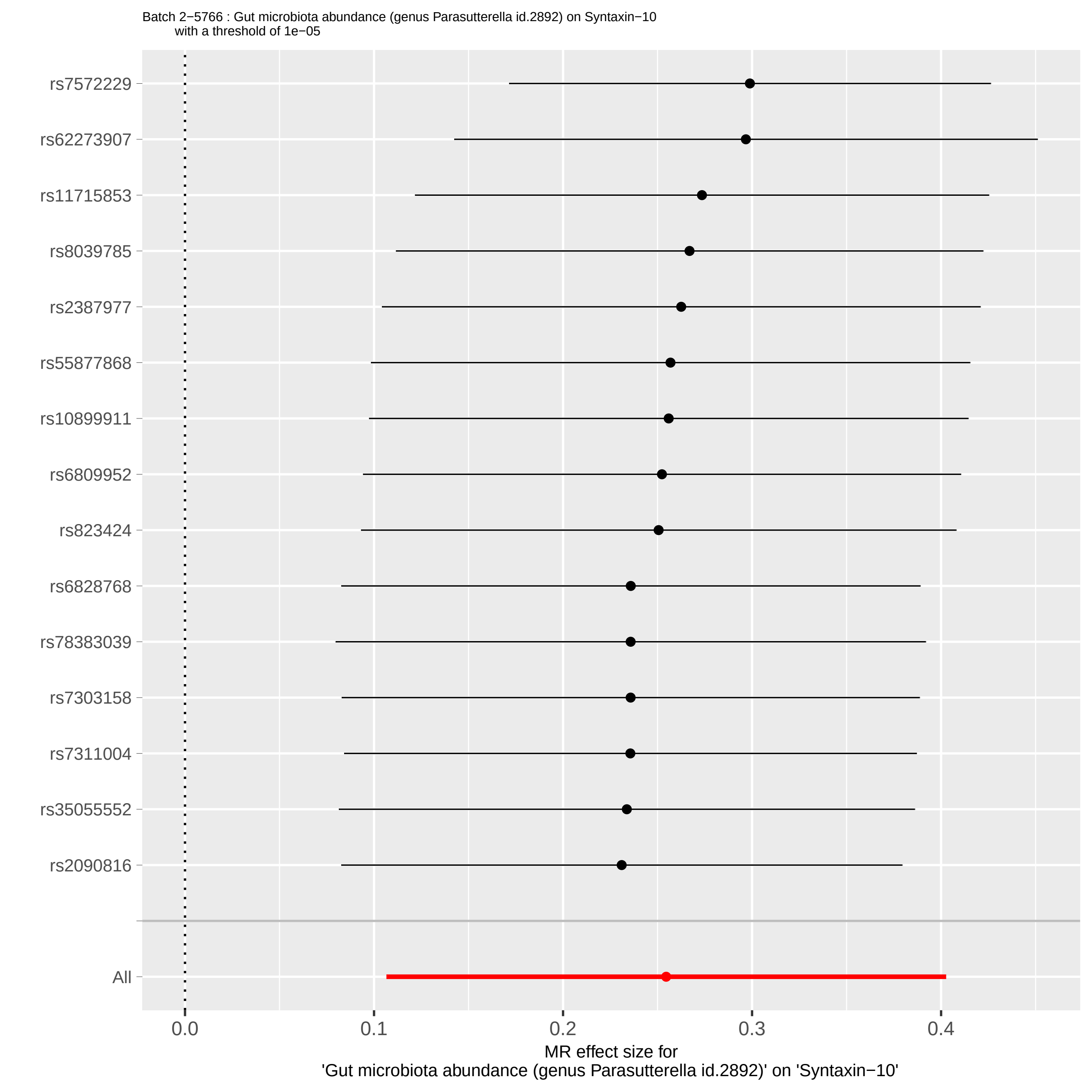
Batch 2−5766 : Gut microbiota abundance (genus Parasutterella id.2892) on Syntaxin−10
with a threshold of 1e−05
rs7572229
rs62273907
rs11715853
rs8039785
rs2387977
rs55877868
rs10899911
rs6809952
rs823424
rs6828768
rs78383039
rs7303158
rs7311004
rs35055552
rs2090816
All
0.0
0.1
0.2
0.3
0.4
MR effect size for
'Gut microbiota abundance (genus Parasutterella id.2892)' on 'Syntaxin−10'

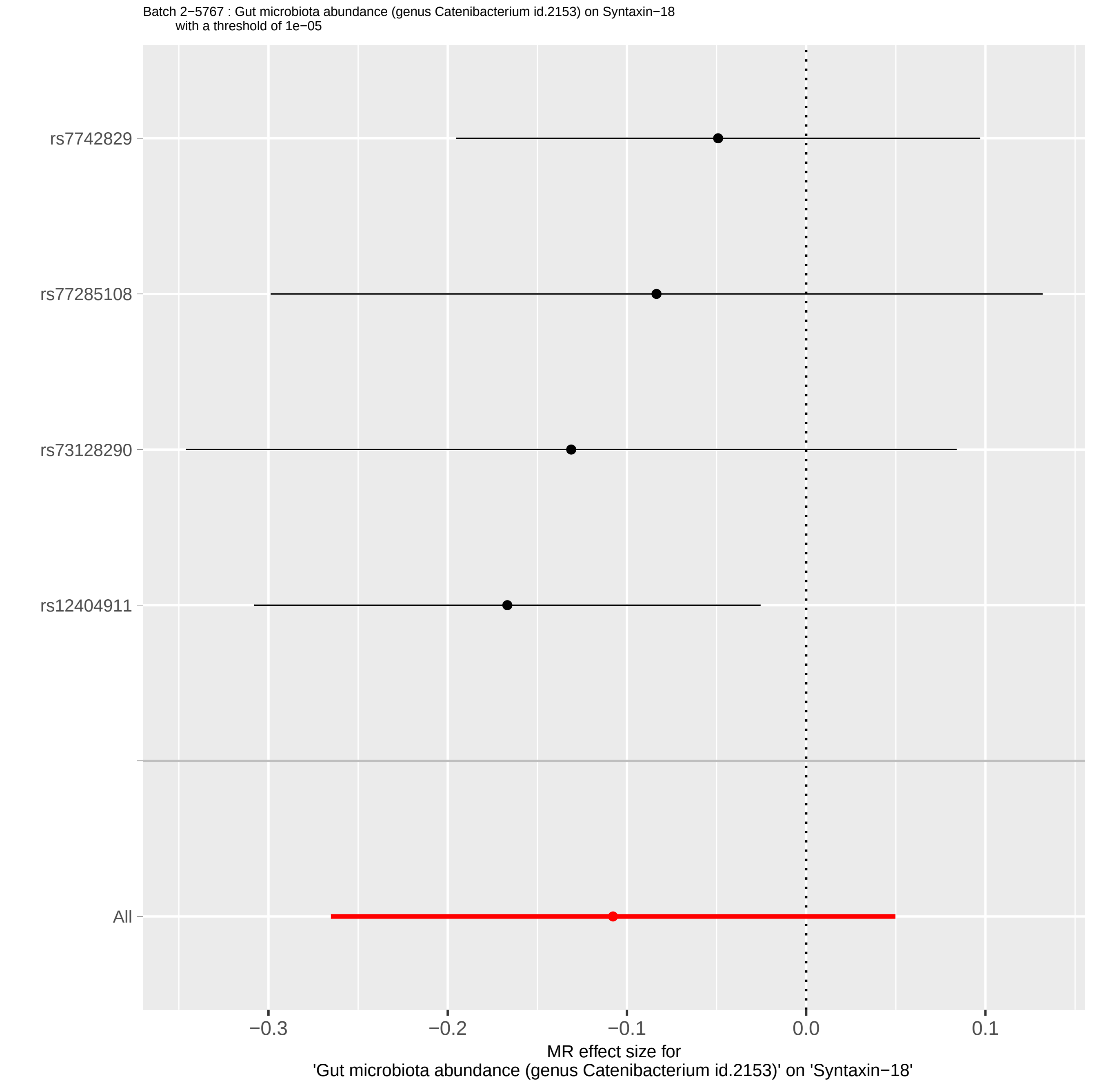

Batch 2−5767 : Gut microbiota abundance (genus Catenibacterium id.2153) on Syntaxin−18
with a threshold of 1e−05
rs7742829
rs77285108
rs73128290
rs12404911
All
−0.3
−0.2
−0.1
0.0
0.1
MR effect size for
'Gut microbiota abundance (genus Catenibacterium id.2153)' on 'Syntaxin−18'

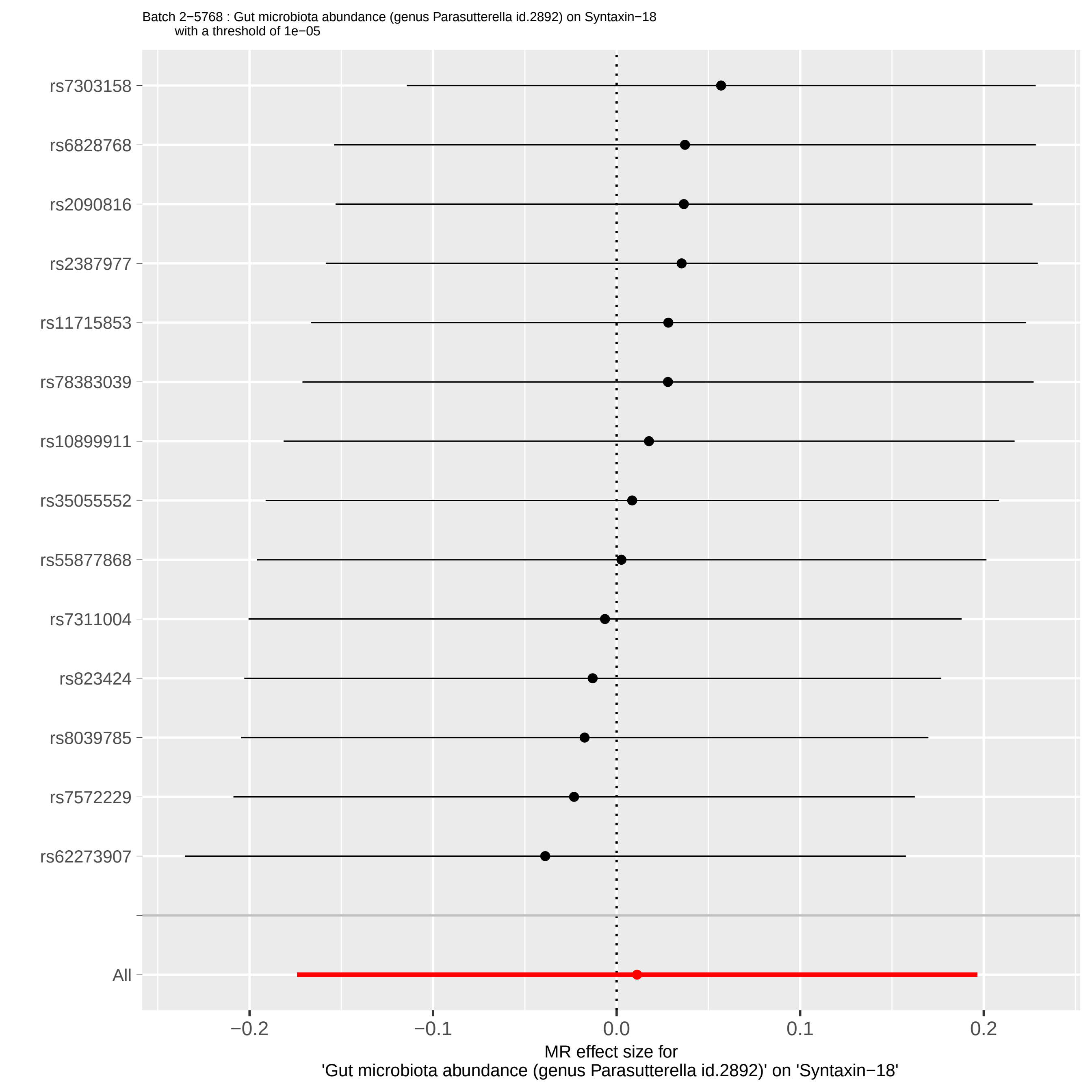

Batch 2−5768 : Gut microbiota abundance (genus Parasutterella id.2892) on Syntaxin−18
with a threshold of 1e−05
rs7303158
rs6828768
rs2090816
rs2387977
rs11715853
rs78383039
rs10899911
rs35055552
rs55877868
rs7311004
rs823424
rs8039785
rs7572229
rs62273907
All
−0.2
−0.1
0.0
0.1
0.2
MR effect size for
'Gut microbiota abundance (genus Parasutterella id.2892)' on 'Syntaxin−18'

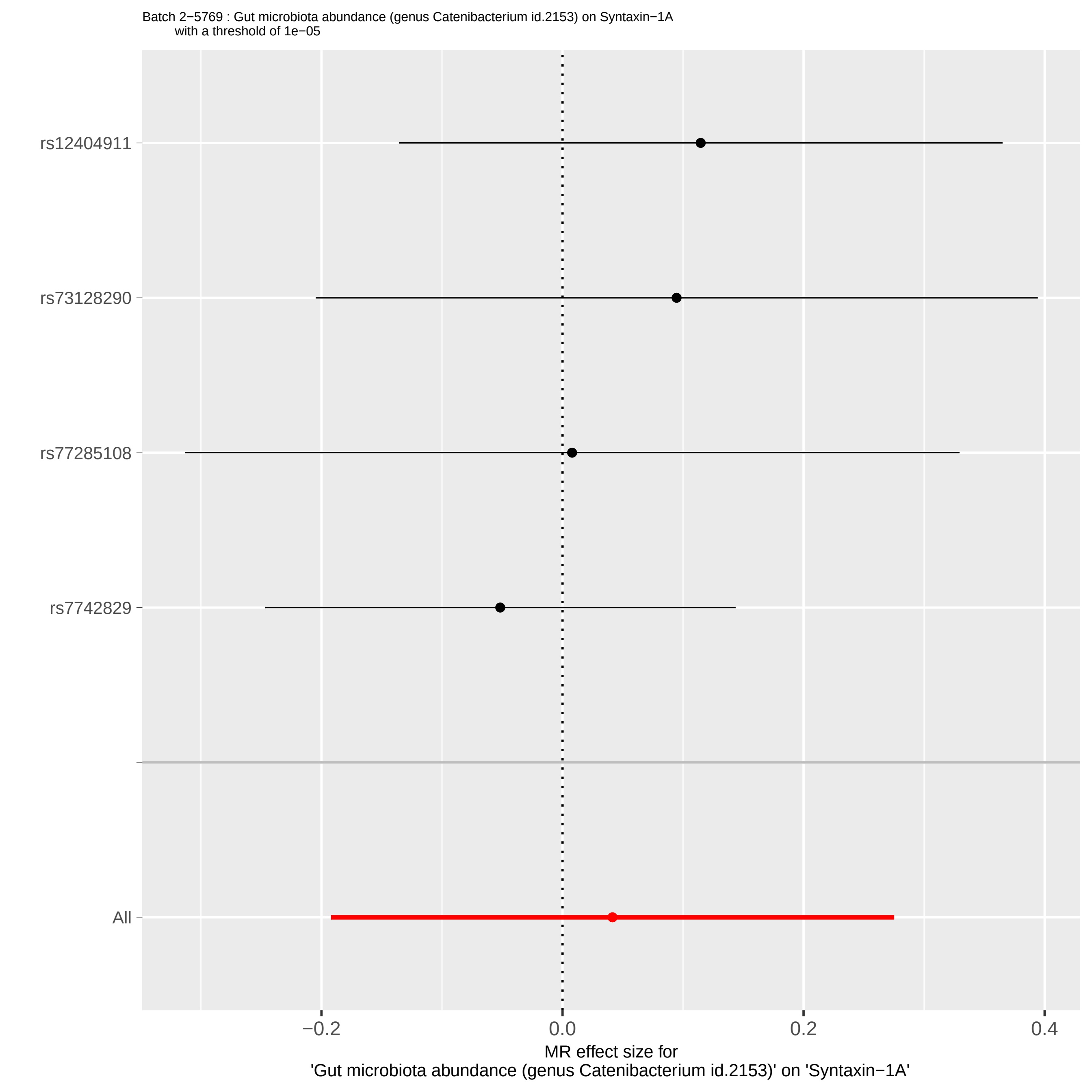

Batch 2−5769 : Gut microbiota abundance (genus Catenibacterium id.2153) on Syntaxin−1A
with a threshold of 1e−05
rs12404911
rs73128290
rs77285108
rs7742829
All
−0.2
0.0
0.2
0.4
MR effect size for
'Gut microbiota abundance (genus Catenibacterium id.2153)' on 'Syntaxin−1A'

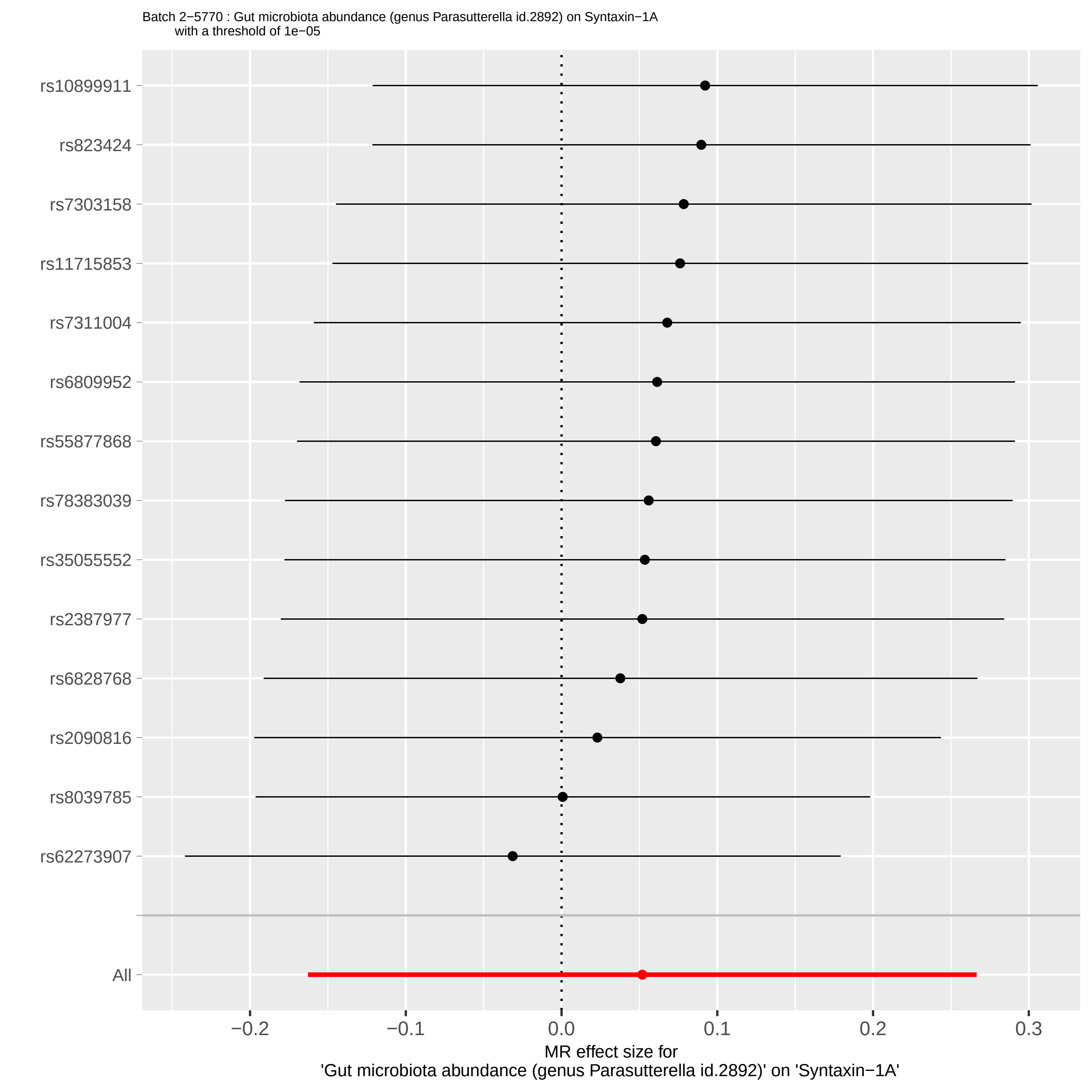
Batch 2−5770 : Gut microbiota abundance (genus Parasutterella id.2892) on Syntaxin−1A
with a threshold of 1e−05
rs10899911
rs823424
rs7303158
rs11715853
rs7311004
rs6809952
rs55877868
rs78383039
rs35055552
rs2387977
rs6828768
rs2090816
rs8039785
rs62273907
All
−0.2
−0.1
0.0
0.1
0.2
0.3
MR effect size for
'Gut microbiota abundance (genus Parasutterella id.2892)' on 'Syntaxin−1A'

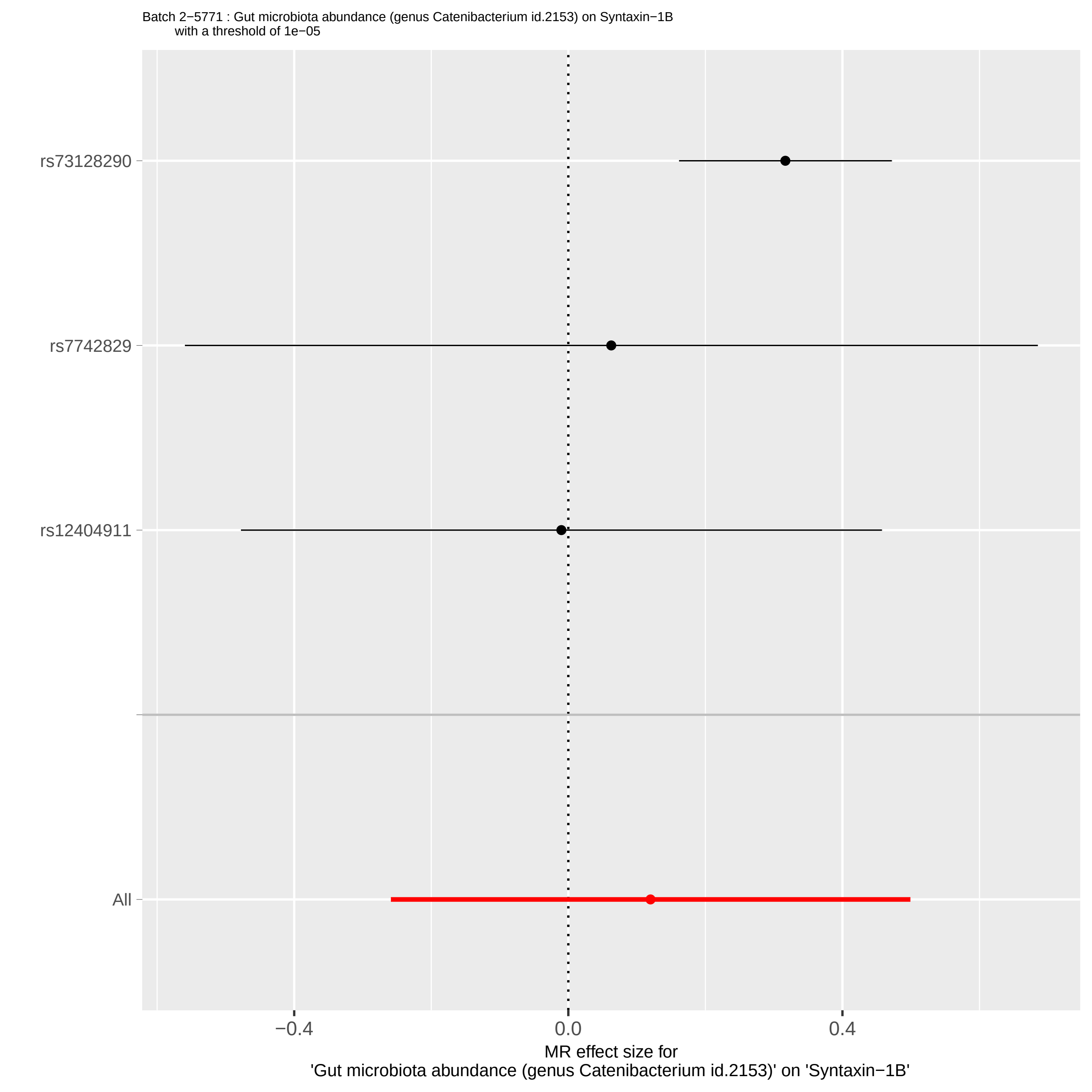

Batch 2−5771 : Gut microbiota abundance (genus Catenibacterium id.2153) on Syntaxin−1B
with a threshold of 1e−05
rs73128290
rs7742829
rs12404911
All
−0.4
0.0
0.4
MR effect size for
'Gut microbiota abundance (genus Catenibacterium id.2153)' on 'Syntaxin−1B'

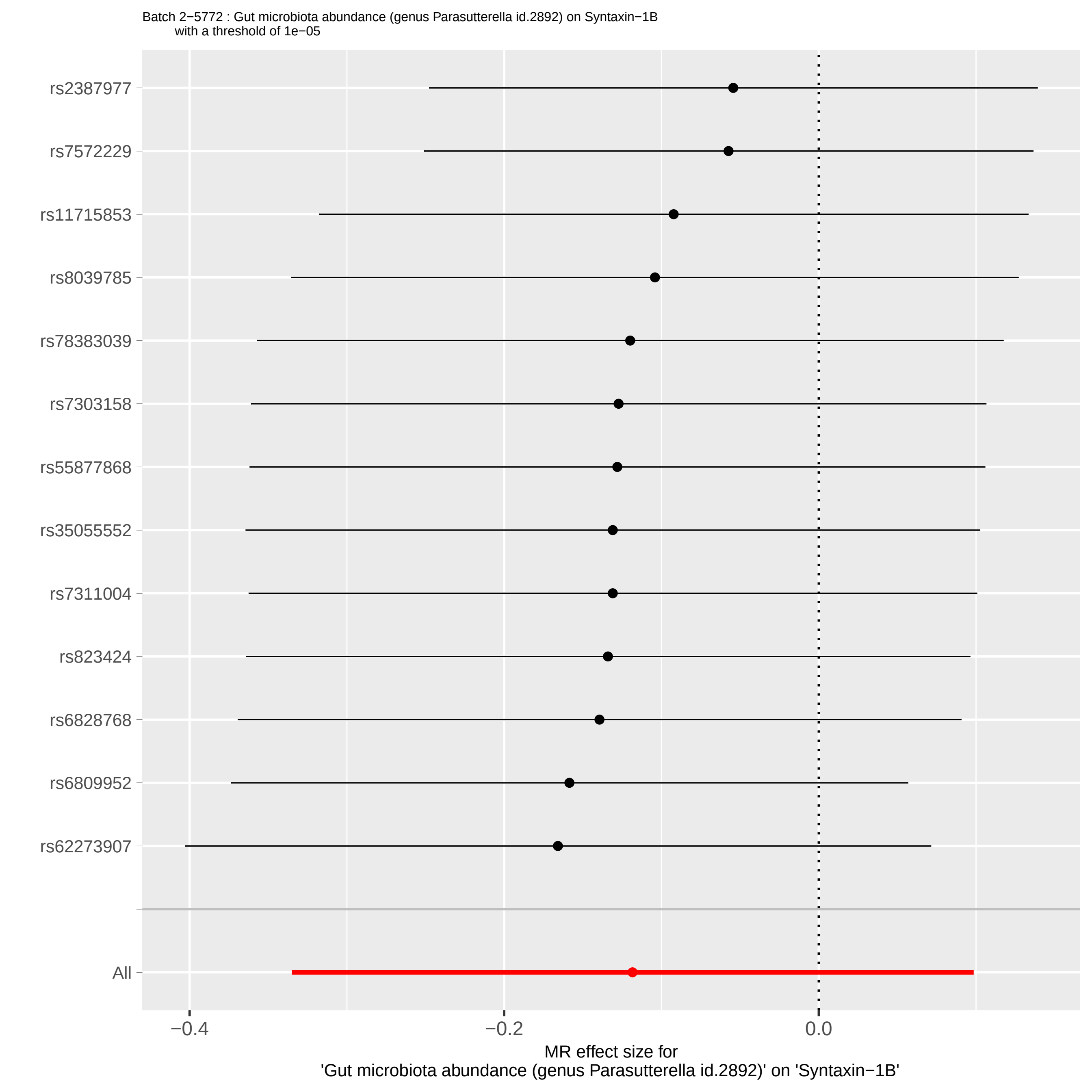
Batch 2−5772 : Gut microbiota abundance (genus Parasutterella id.2892) on Syntaxin−1B
with a threshold of 1e−05
rs2387977
rs7572229
rs11715853
rs8039785
rs78383039
rs7303158
rs55877868
rs35055552
rs7311004
rs823424
rs6828768
rs6809952
rs62273907
All
−0.4
−0.2
0.0
MR effect size for
'Gut microbiota abundance (genus Parasutterella id.2892)' on 'Syntaxin−1B'

Batch 2−5773 : Gut microbiota abundance (genus Catenibacterium id.2153) on Syntaxin−2
with a threshold of 1e−05

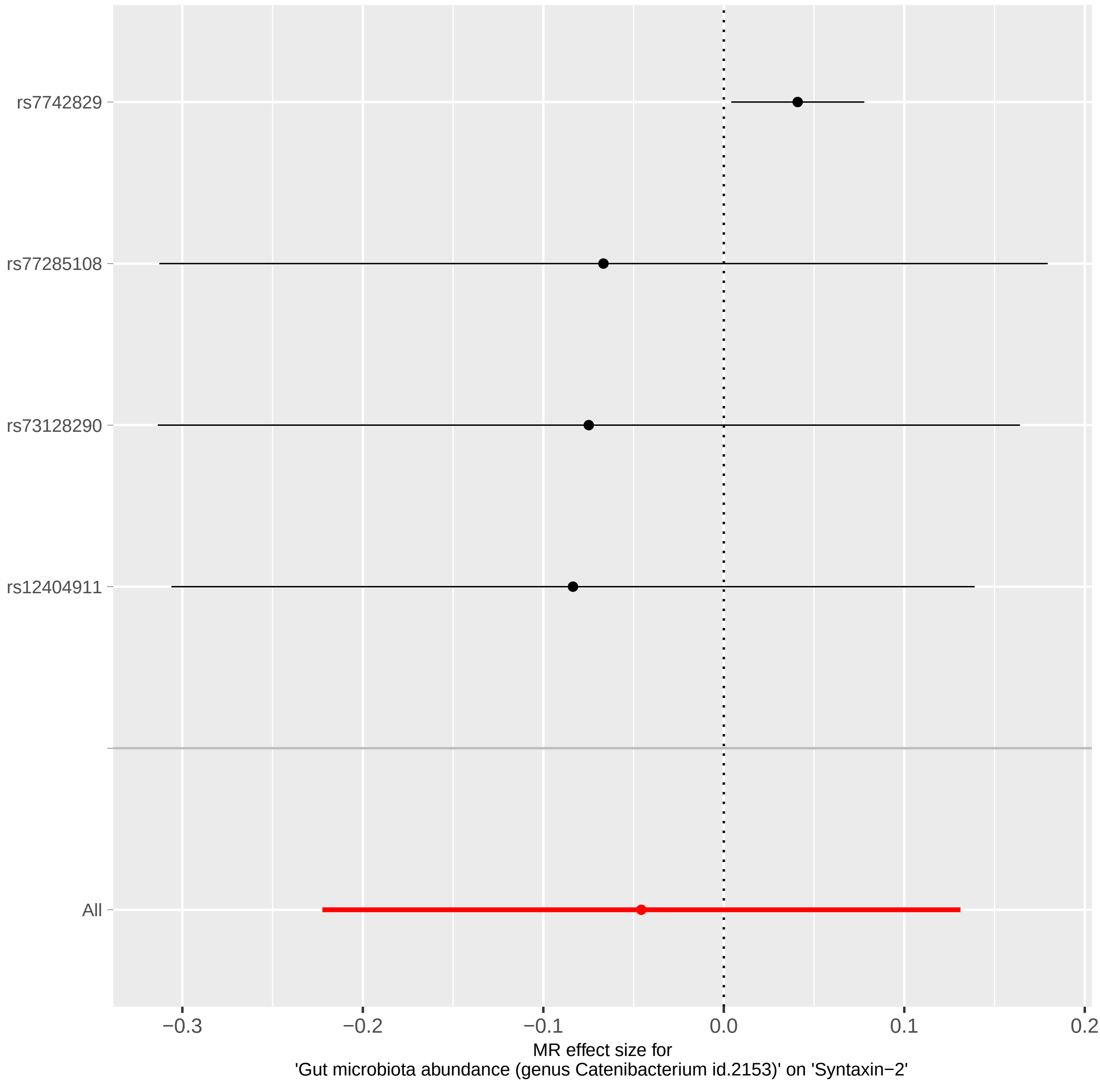

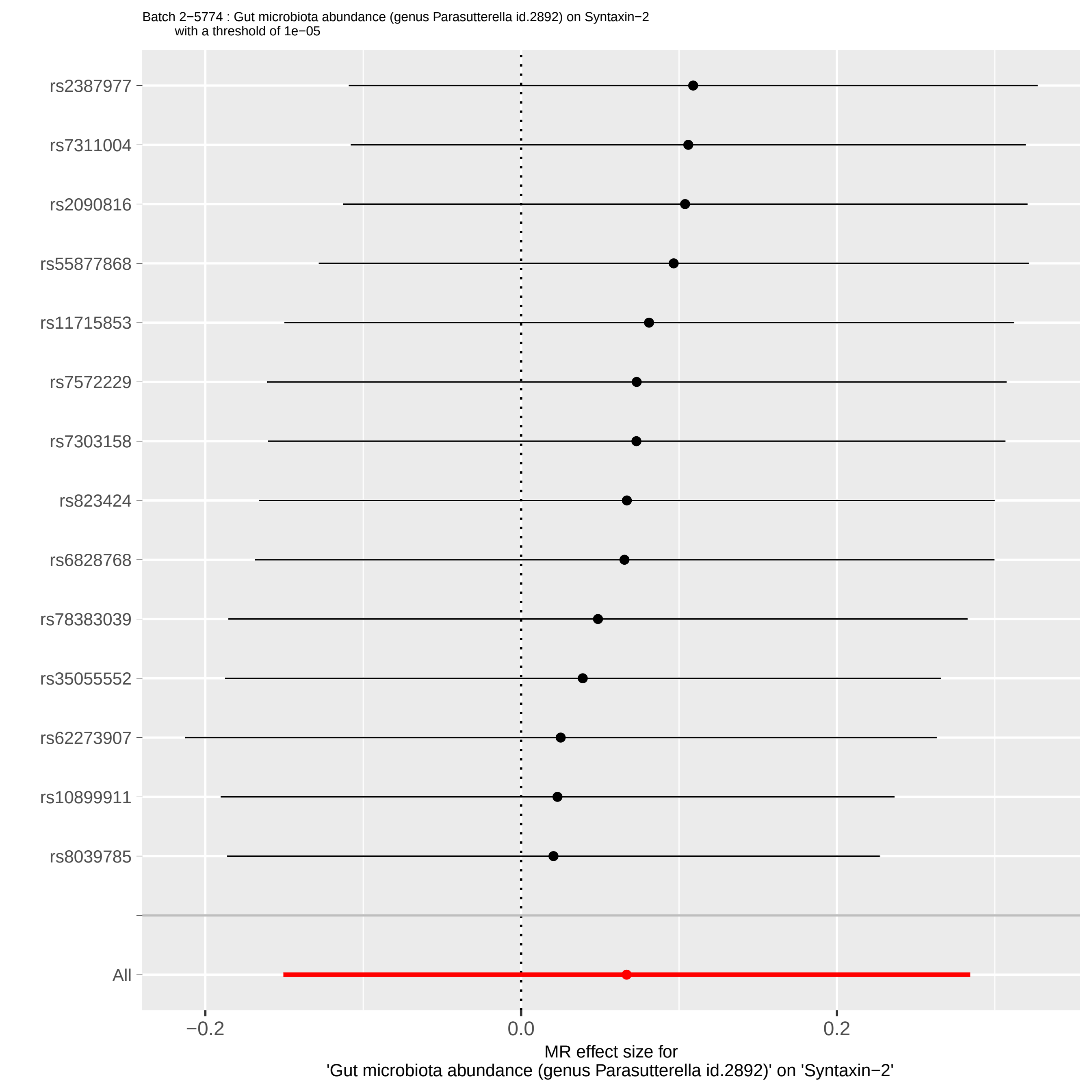
Batch 2−5774 : Gut microbiota abundance (genus Parasutterella id.2892) on Syntaxin−2
with a threshold of 1e−05
rs2387977
rs7311004
rs2090816
rs55877868
rs11715853
rs7572229
rs7303158
rs823424
rs6828768
rs78383039
rs35055552
rs62273907
rs10899911
rs8039785
All
−0.2
0.0
0.2
MR effect size for
'Gut microbiota abundance (genus Parasutterella id.2892)' on 'Syntaxin−2'

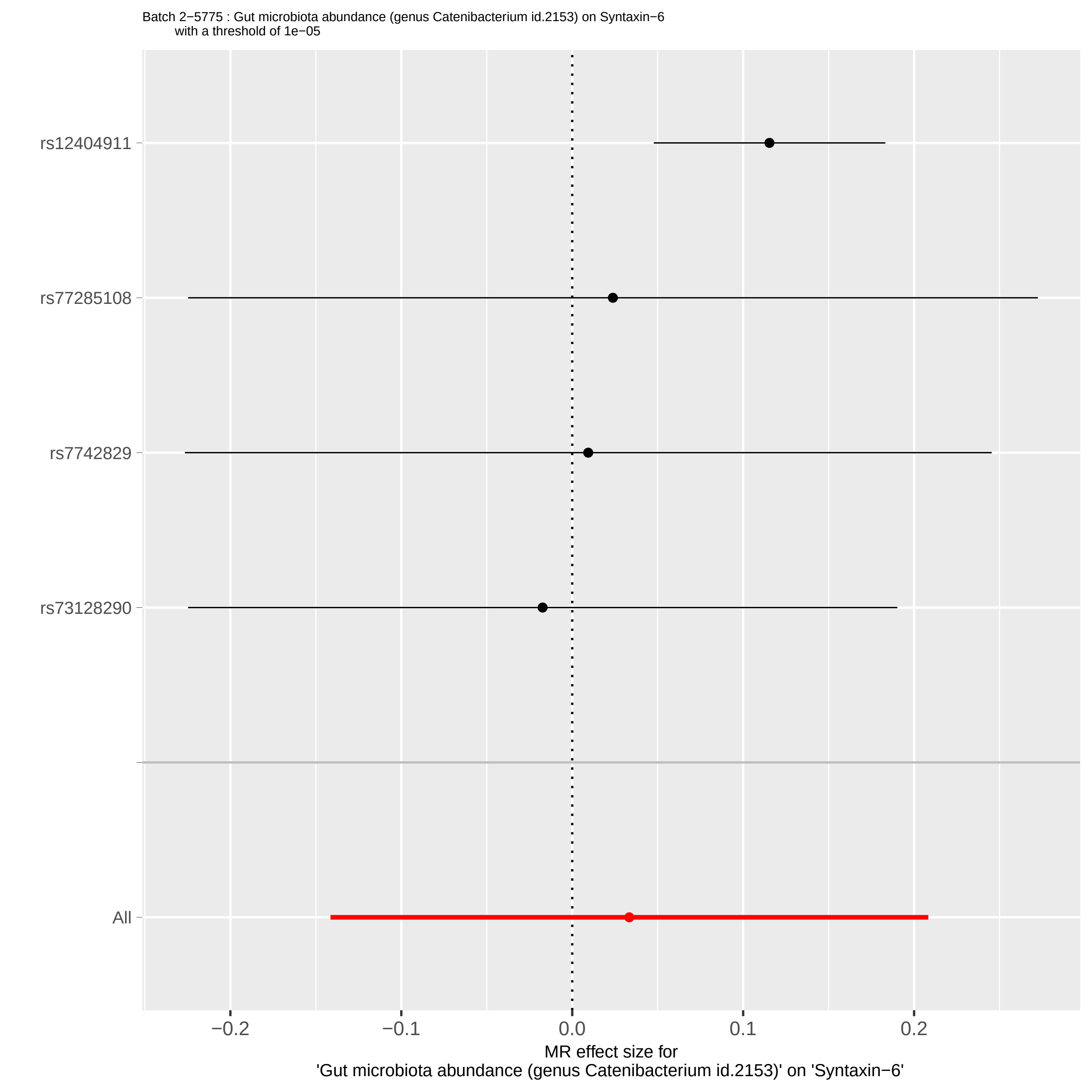
Batch 2−5775 : Gut microbiota abundance (genus Catenibacterium id.2153) on Syntaxin−6
with a threshold of 1e−05
rs12404911
rs77285108
rs7742829
rs73128290
All
−0.2
−0.1
0.0
0.1
0.2
MR effect size for
'Gut microbiota abundance (genus Catenibacterium id.2153)' on 'Syntaxin−6'

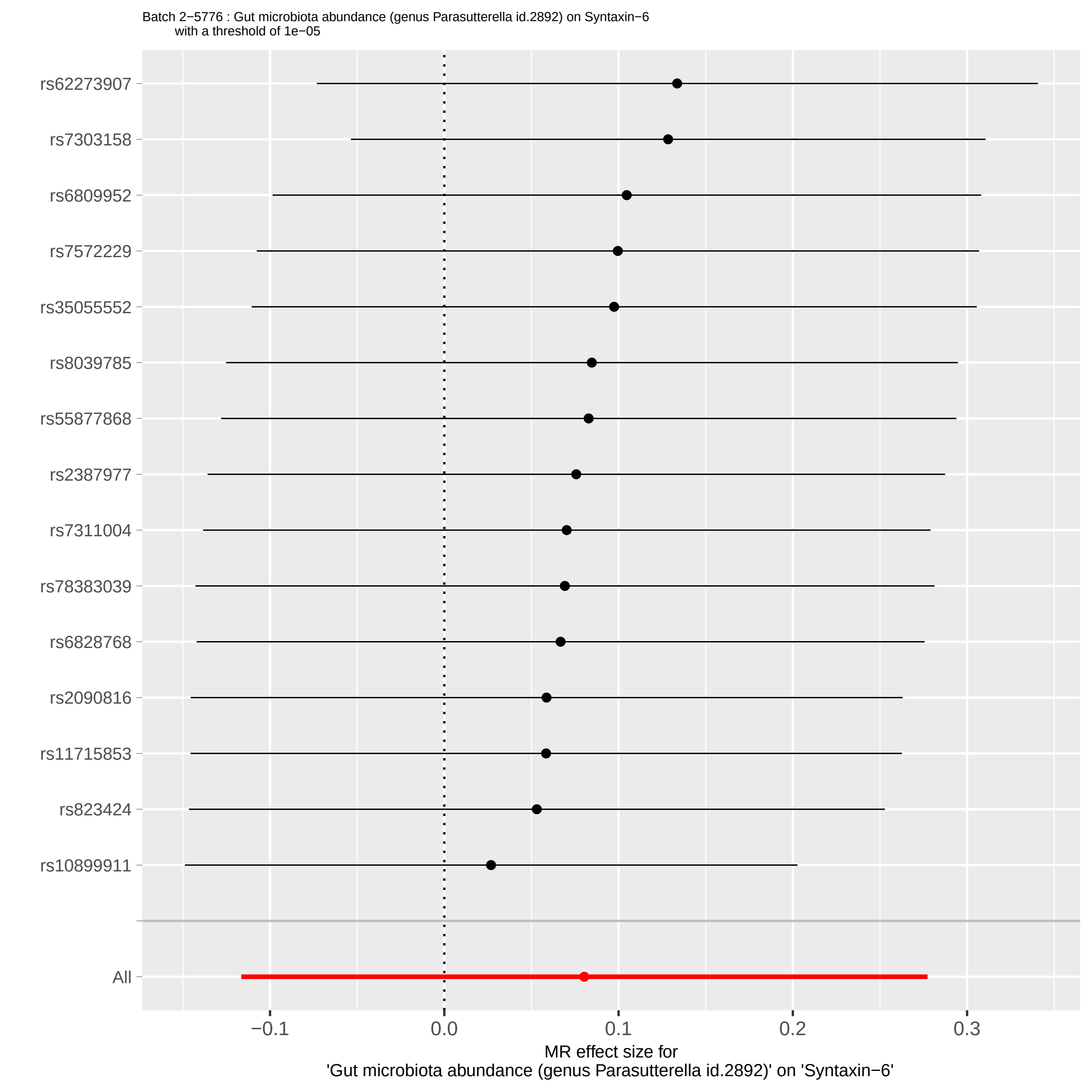

Batch 2−5776 : Gut microbiota abundance (genus Parasutterella id.2892) on Syntaxin−6
with a threshold of 1e−05
rs62273907
rs7303158
rs6809952
rs7572229
rs35055552
rs8039785
rs55877868
rs2387977
rs7311004
rs78383039
rs6828768
rs2090816
rs11715853
rs823424
rs10899911
All
−0.1
0.0
0.1
0.2
0.3
MR effect size for
'Gut microbiota abundance (genus Parasutterella id.2892)' on 'Syntaxin−6'

Batch 2−5777 : Gut microbiota abundance (genus Catenibacterium id.2153) on Syntaxin−7
with a threshold of 1e−05

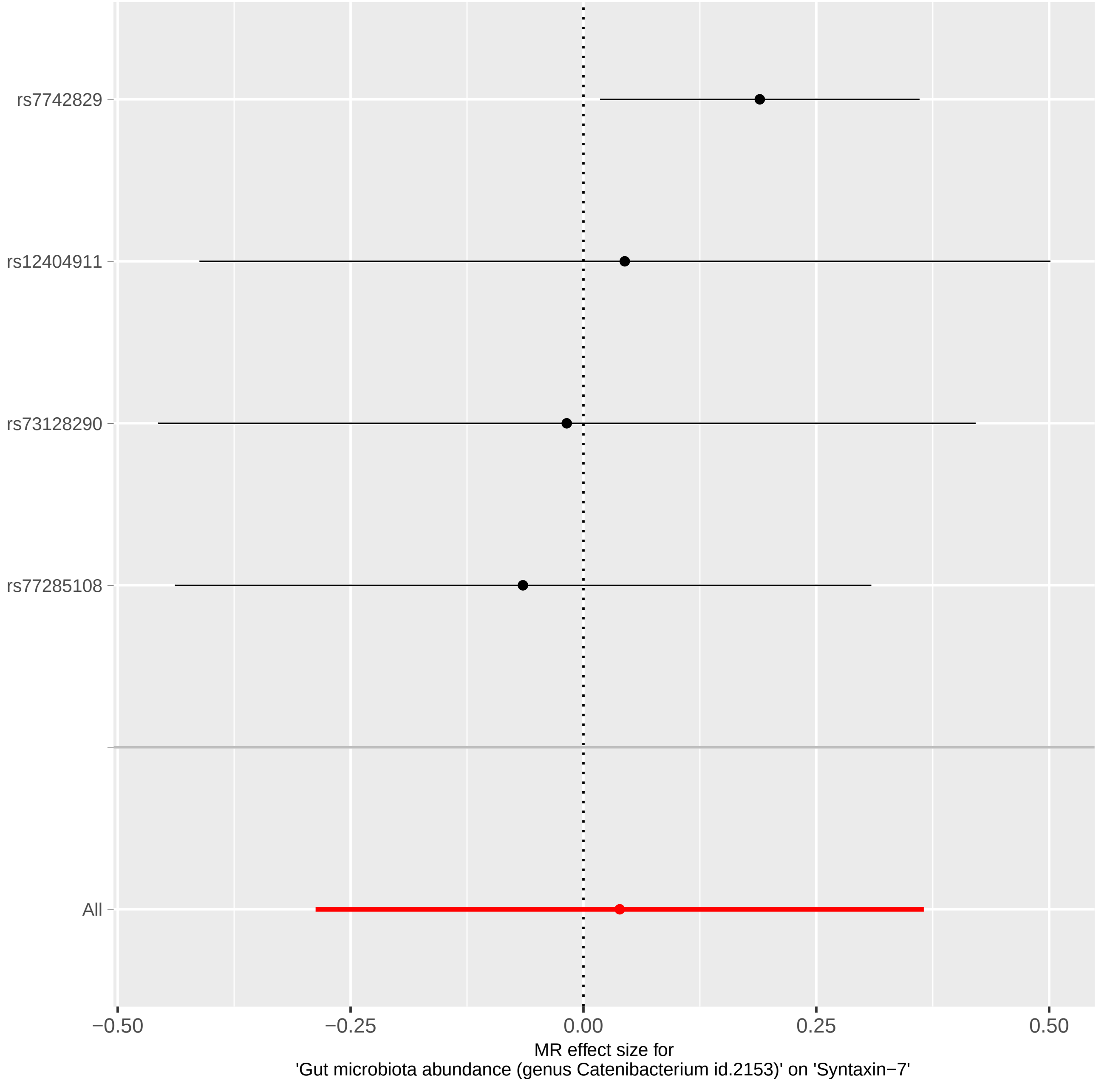

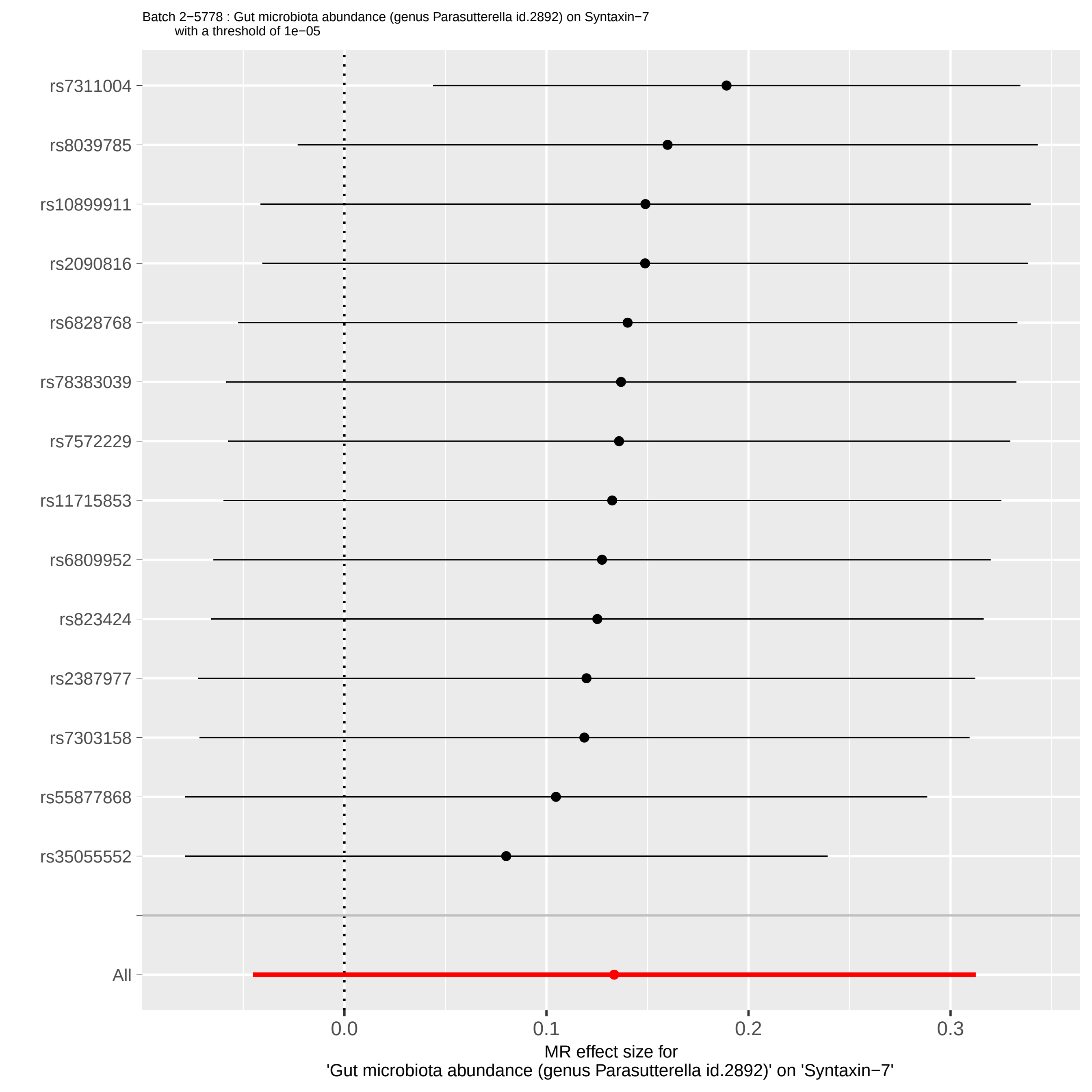
Batch 2−5778 : Gut microbiota abundance (genus Parasutterella id.2892) on Syntaxin−7
with a threshold of 1e−05
rs7311004
rs8039785
rs10899911
rs2090816
rs6828768
rs78383039
rs7572229
rs11715853
rs6809952
rs823424
rs2387977
rs7303158
rs55877868
rs35055552
All
0.0
0.1
0.2
0.3
MR effect size for
'Gut microbiota abundance (genus Parasutterella id.2892)' on 'Syntaxin−7'

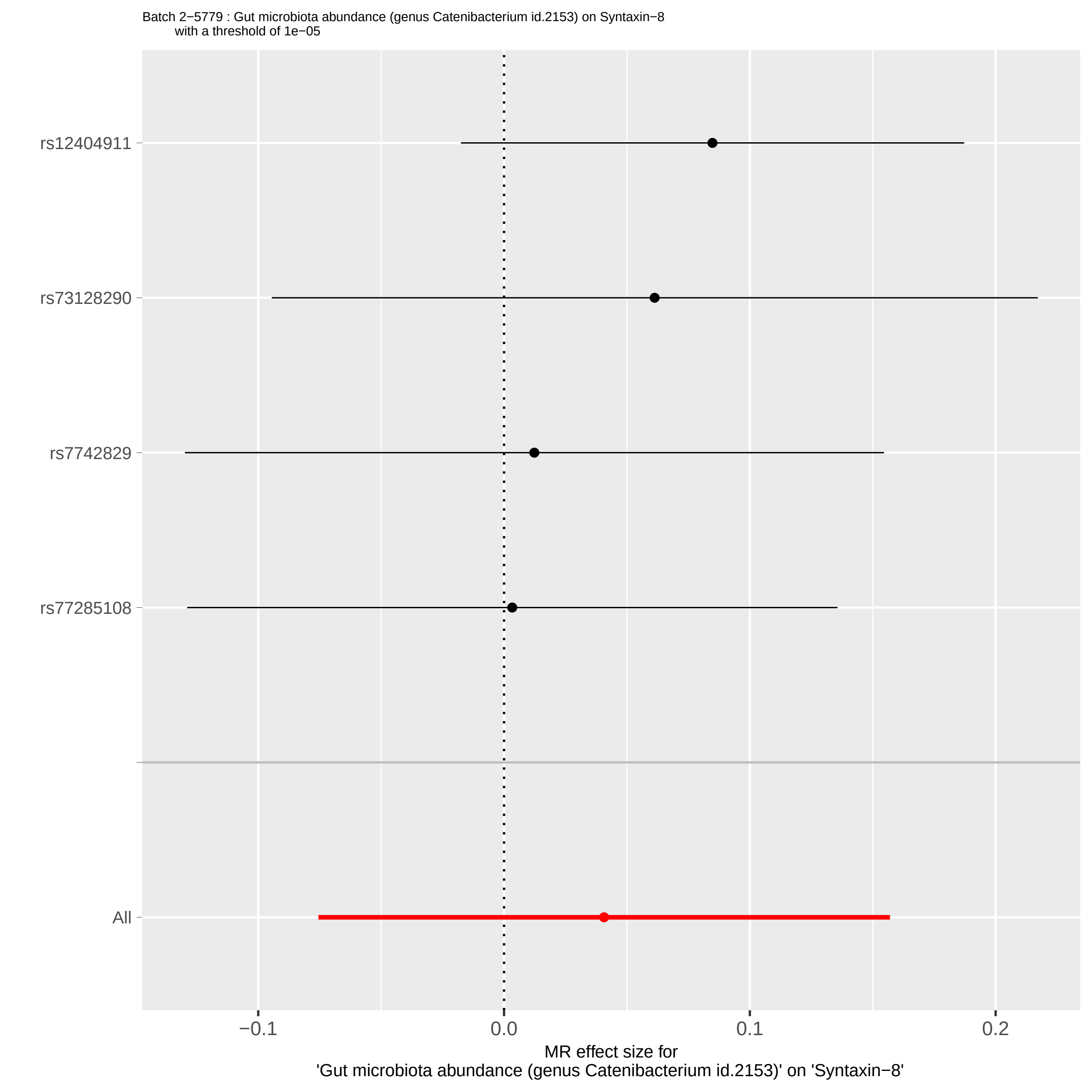
Batch 2−5779 : Gut microbiota abundance (genus Catenibacterium id.2153) on Syntaxin−8
with a threshold of 1e−05
rs12404911
rs73128290
rs7742829
rs77285108
All
−0.1
0.0
0.1
0.2
MR effect size for
'Gut microbiota abundance (genus Catenibacterium id.2153)' on 'Syntaxin−8'

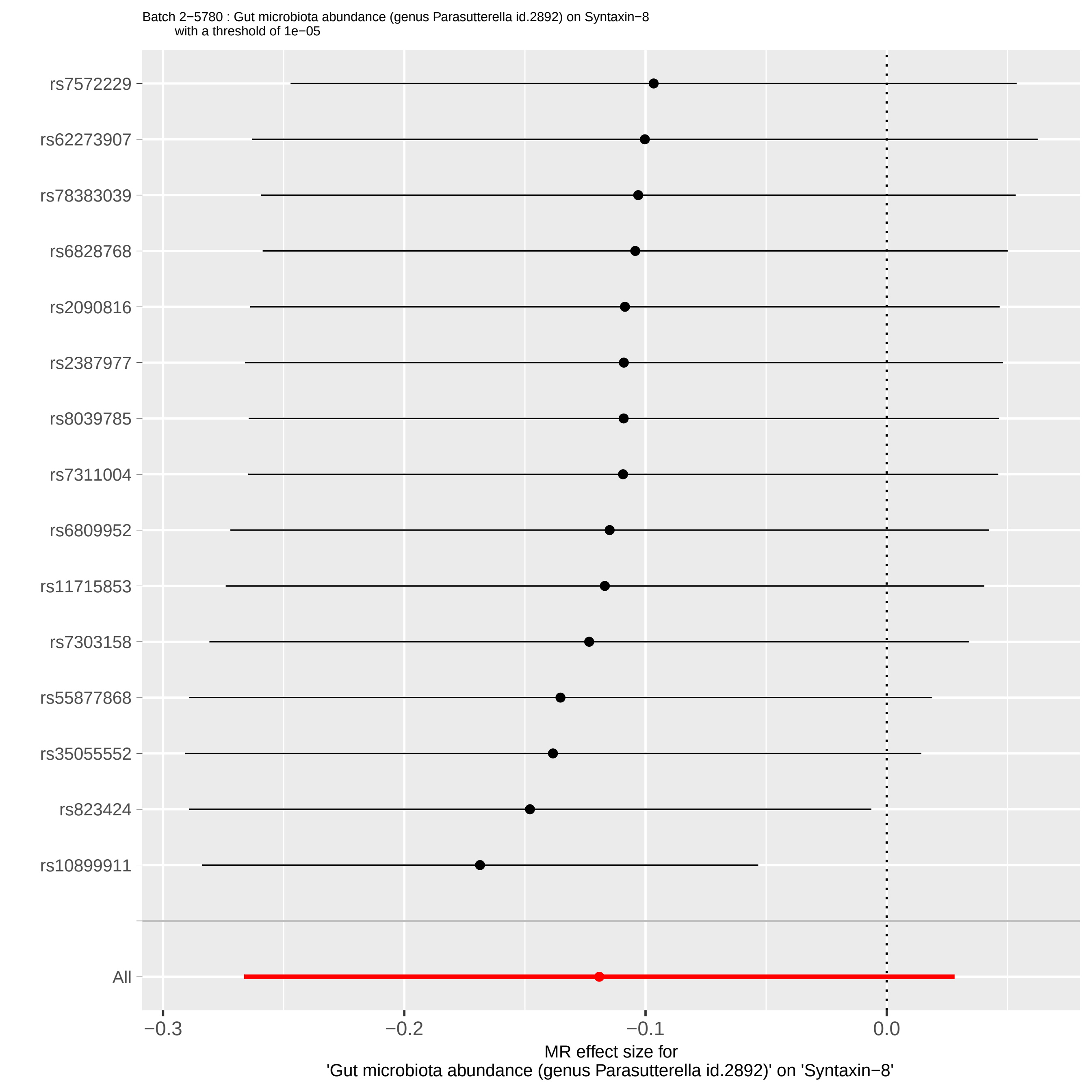

Batch 2−5780 : Gut microbiota abundance (genus Parasutterella id.2892) on Syntaxin−8
with a threshold of 1e−05
rs7572229
rs62273907
rs78383039
rs6828768
rs2090816
rs2387977
rs8039785
rs7311004
rs6809952
rs11715853
rs7303158
rs55877868
rs35055552
rs823424
rs10899911
All
−0.3
−0.2
−0.1
0.0
MR effect size for
'Gut microbiota abundance (genus Parasutterella id.2892)' on 'Syntaxin−8'

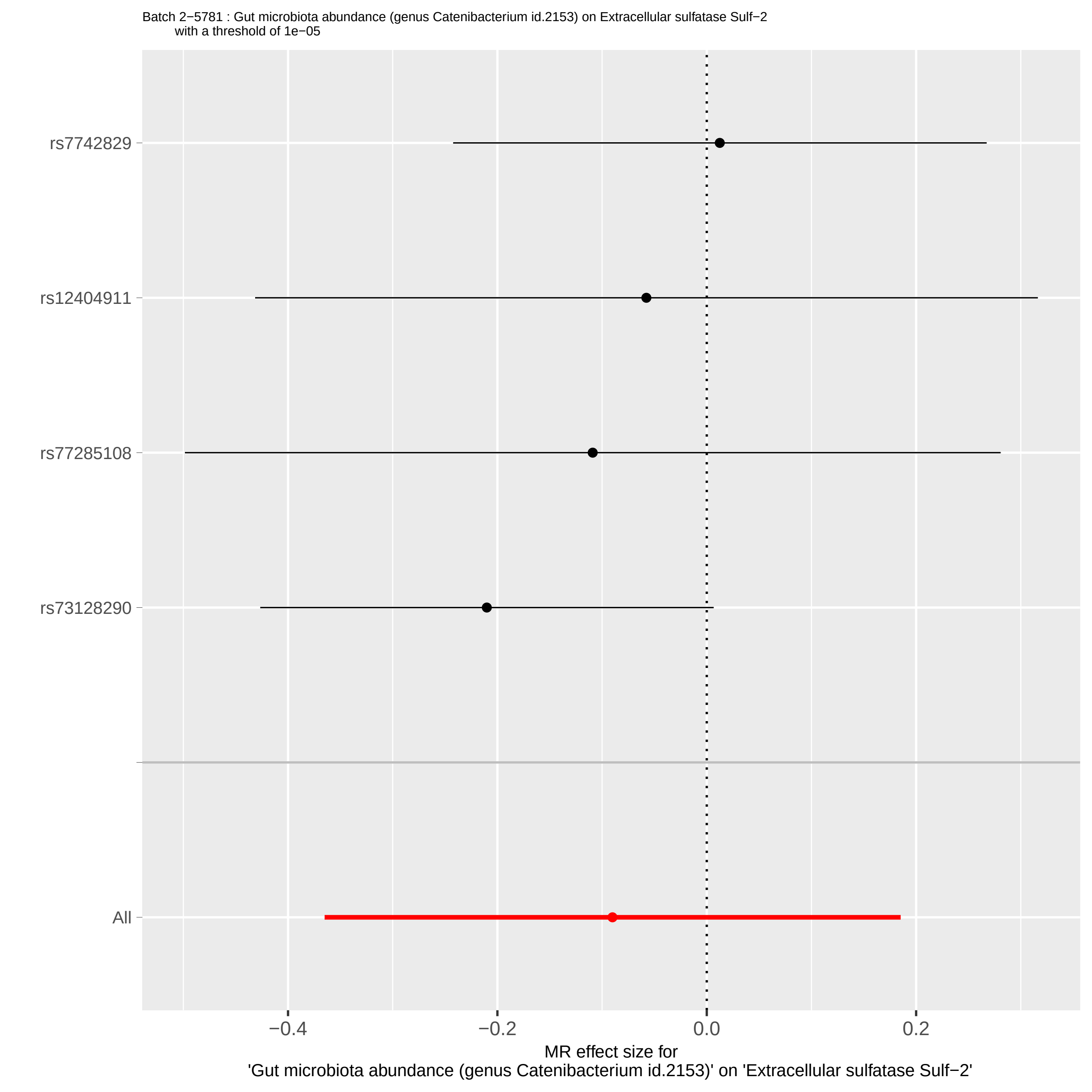

Batch 2−5781 : Gut microbiota abundance (genus Catenibacterium id.2153) on Extracellular sulfatase Sulf−2
with a threshold of 1e−05
rs7742829
rs12404911
rs77285108
rs73128290
All
−0.4
−0.2
0.0
0.2
MR effect size for
'Gut microbiota abundance (genus Catenibacterium id.2153)' on 'Extracellular sulfatase Sulf−2'

Batch 2−5782 : Gut microbiota abundance (genus Parasutterella id.2892) on Extracellular sulfatase Sulf−2
with a threshold of 1e−05

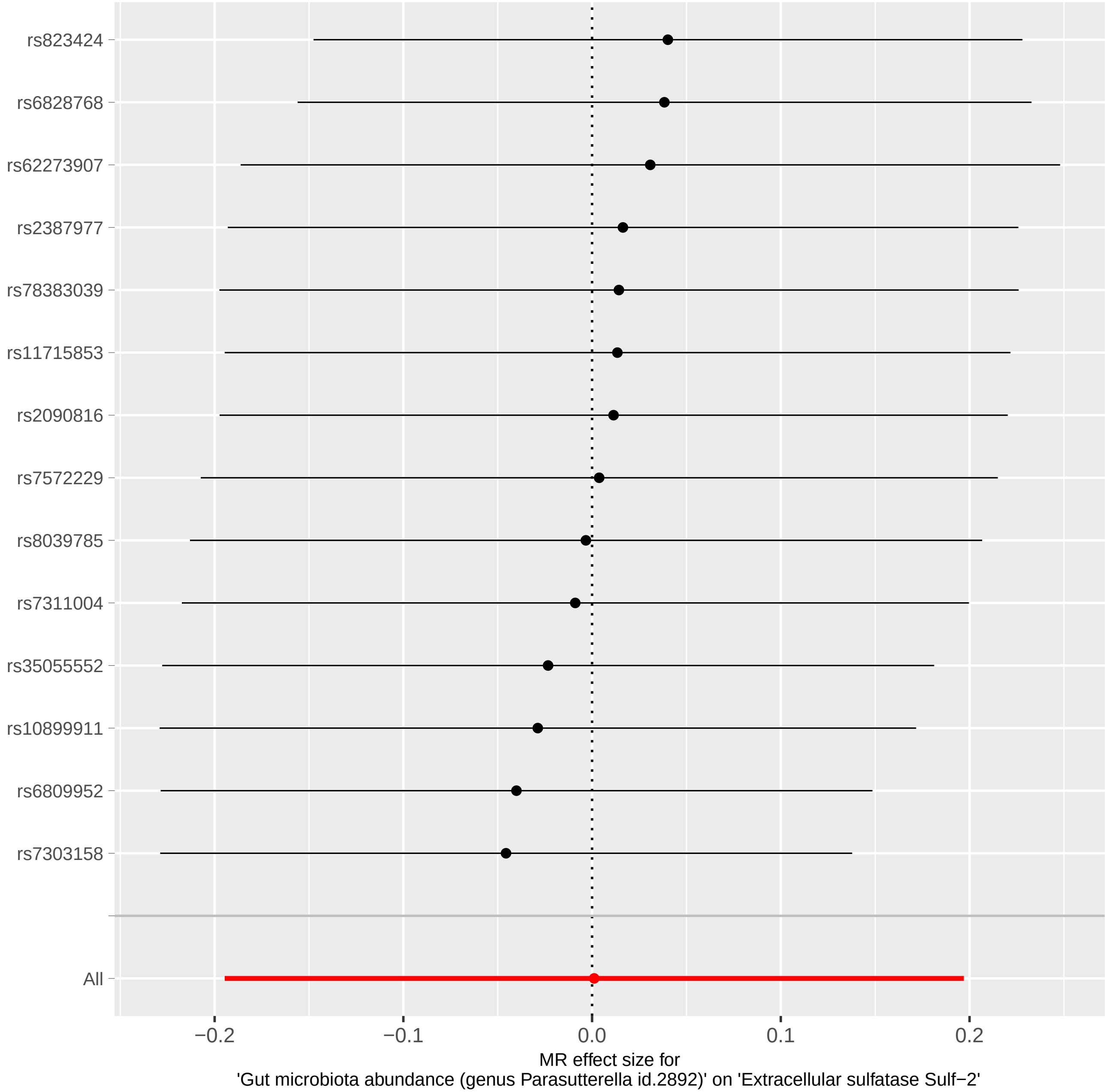

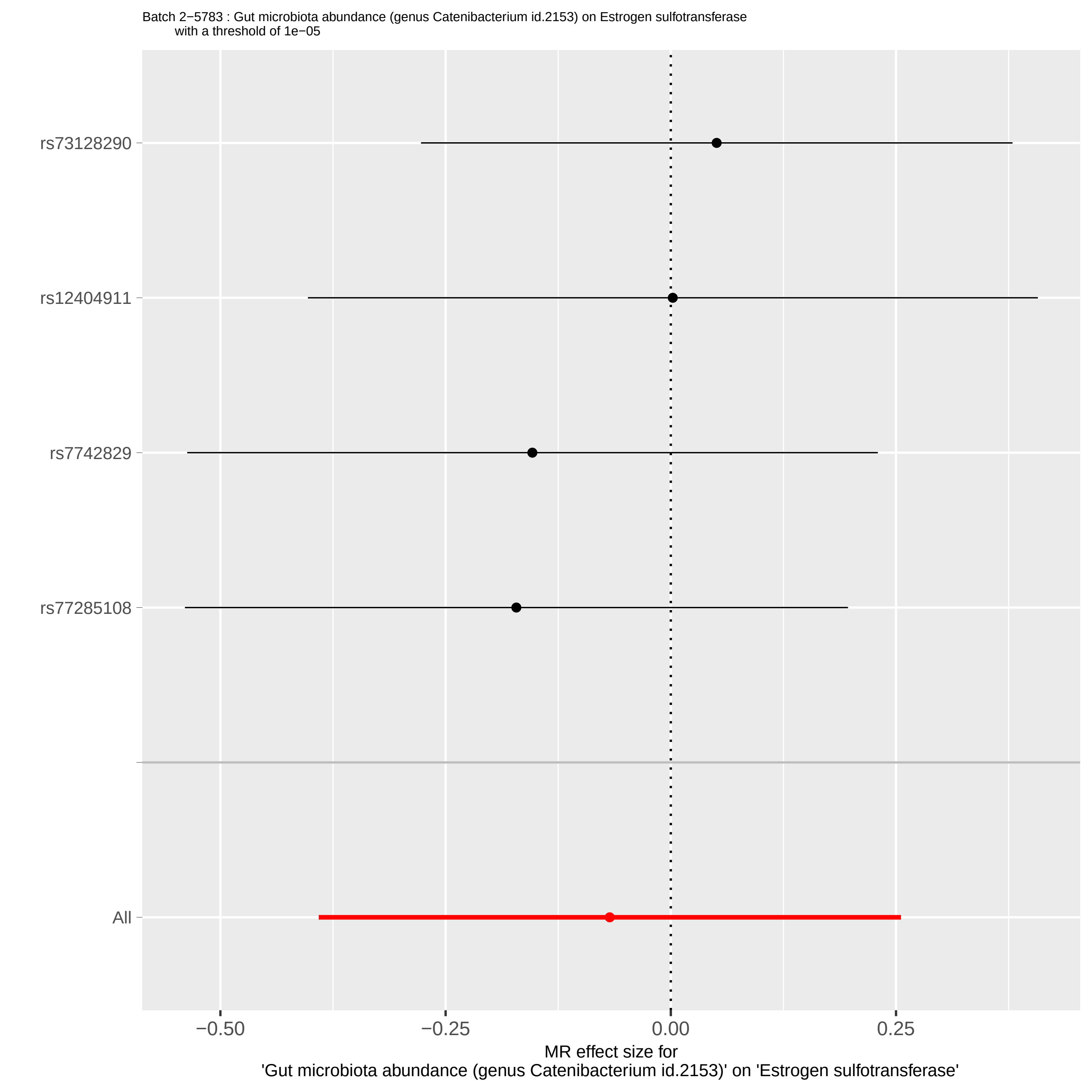
Batch 2−5783 : Gut microbiota abundance (genus Catenibacterium id.2153) on Estrogen sulfotransferase
with a threshold of 1e−05
rs73128290
rs12404911
rs7742829
rs77285108
All
−0.50
−0.25
0.00
0.25
MR effect size for
'Gut microbiota abundance (genus Catenibacterium id.2153)' on 'Estrogen sulfotransferase'

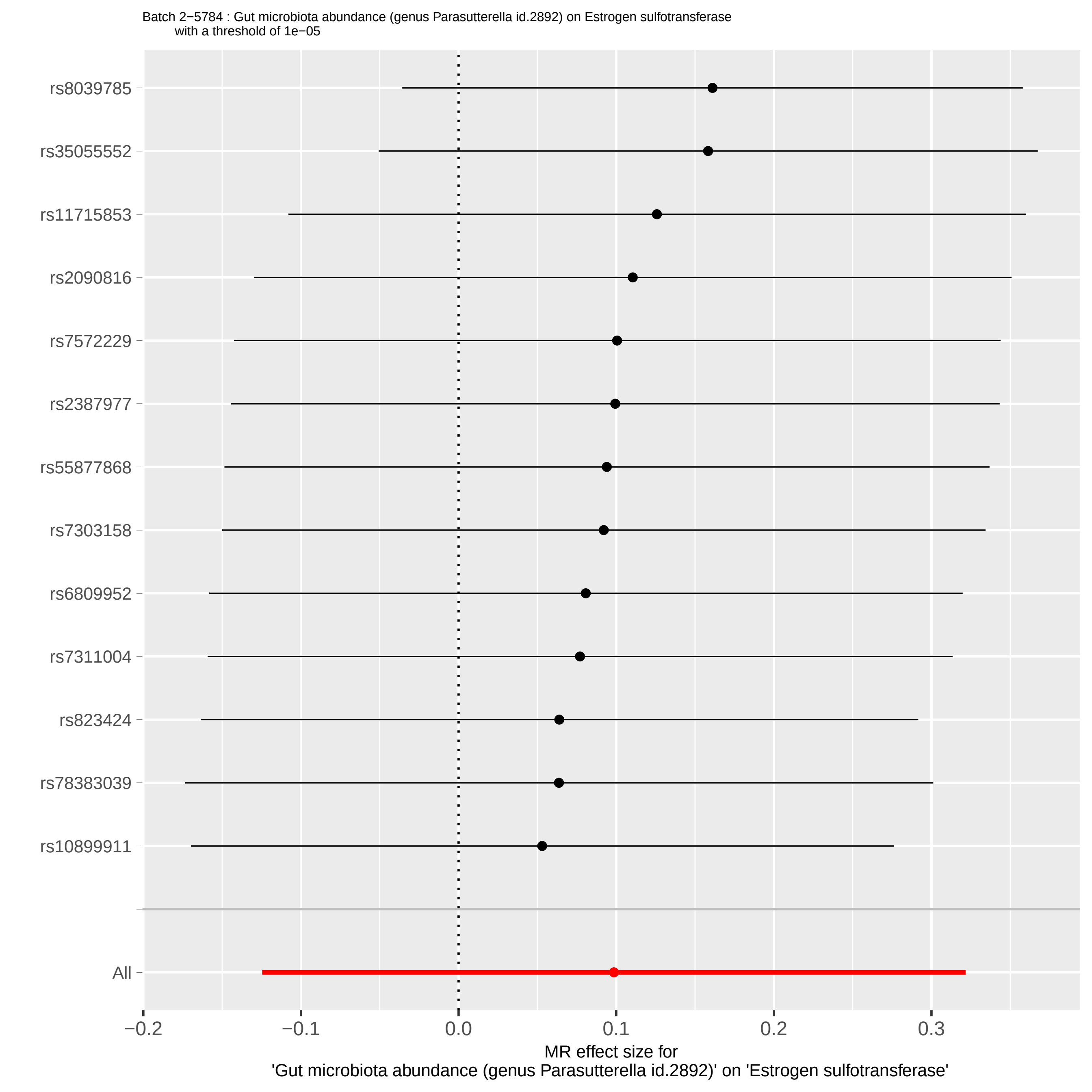

Batch 2−5784 : Gut microbiota abundance (genus Parasutterella id.2892) on Estrogen sulfotransferase
with a threshold of 1e−05
rs8039785
rs35055552
rs11715853
rs2090816
rs7572229
rs2387977
rs55877868
rs7303158
rs6809952
rs7311004
rs823424
rs78383039
rs10899911
All
−0.2
−0.1
0.0
0.1
0.2
0.3
MR effect size for
'Gut microbiota abundance (genus Parasutterella id.2892)' on 'Estrogen sulfotransferase'

Batch 2−5785 : Gut microbiota abundance (genus Catenibacterium id.2153) on Bile salt sulfotransferase
with a threshold of 1e−05

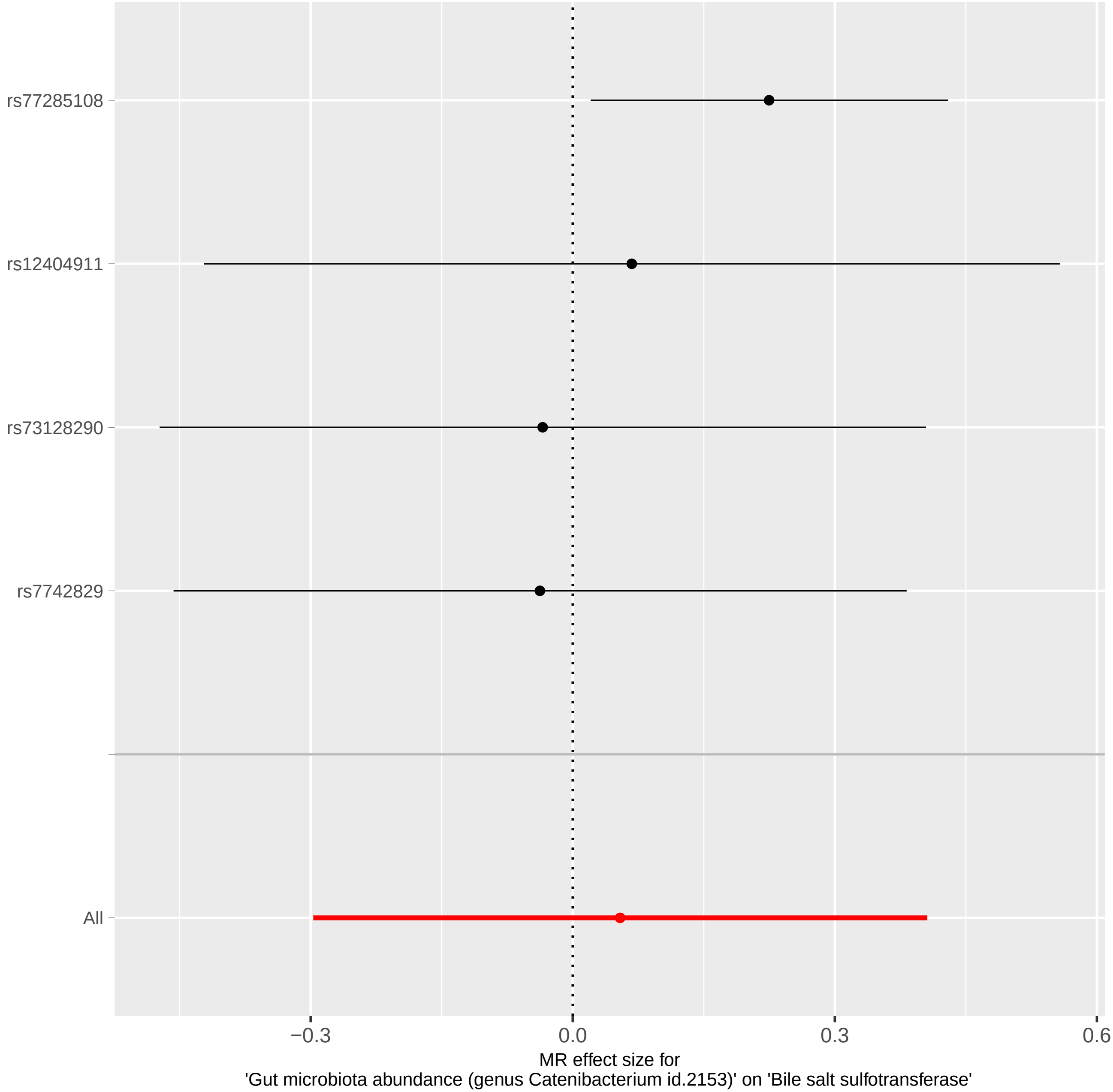

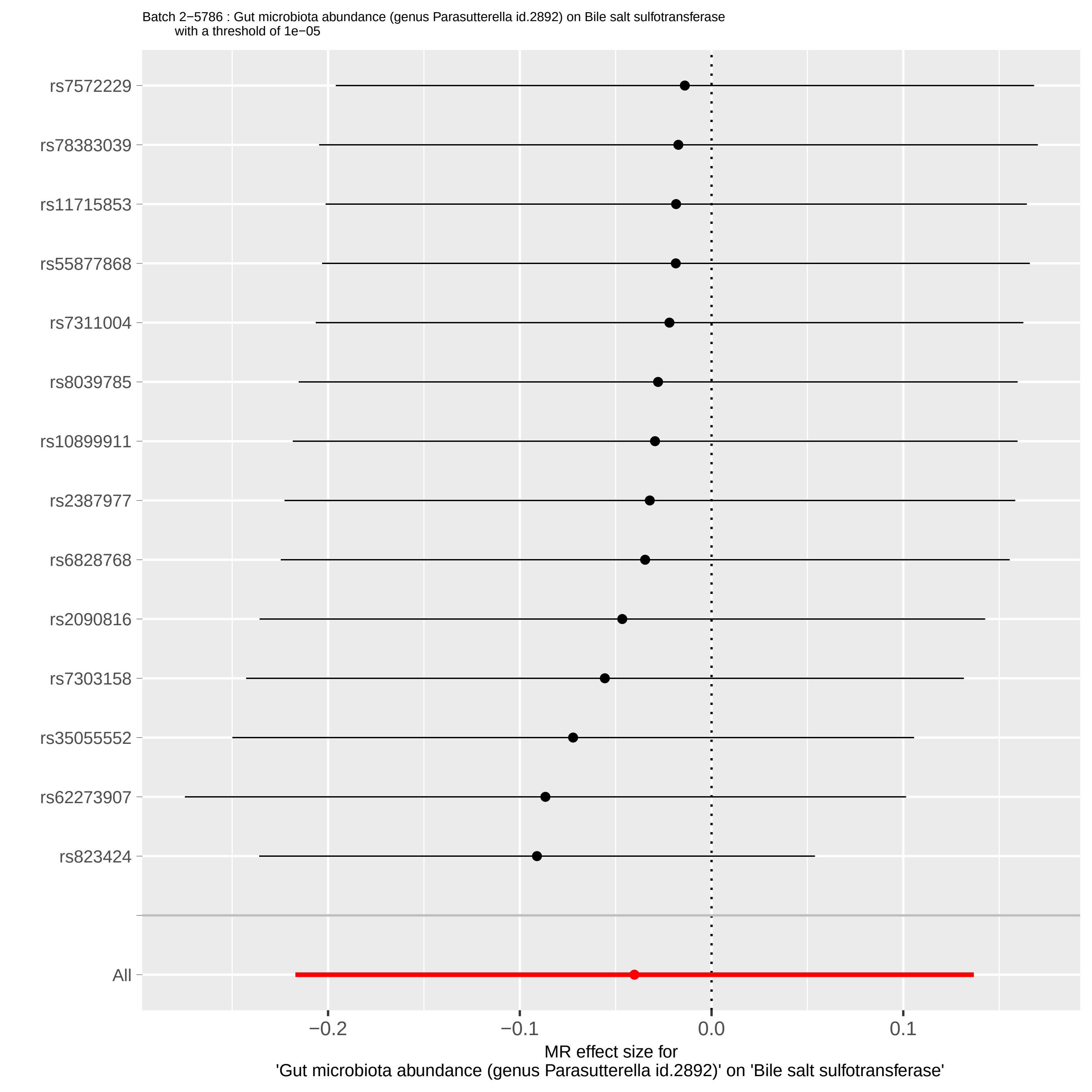
Batch 2−5786 : Gut microbiota abundance (genus Parasutterella id.2892) on Bile salt sulfotransferase
with a threshold of 1e−05
rs7572229
rs78383039
rs11715853
rs55877868
rs7311004
rs8039785
rs10899911
rs2387977
rs6828768
rs2090816
rs7303158
rs35055552
rs62273907
rs823424
All
−0.2
−0.1
0.0
0.1
MR effect size for
'Gut microbiota abundance (genus Parasutterella id.2892)' on 'Bile salt sulfotransferase'

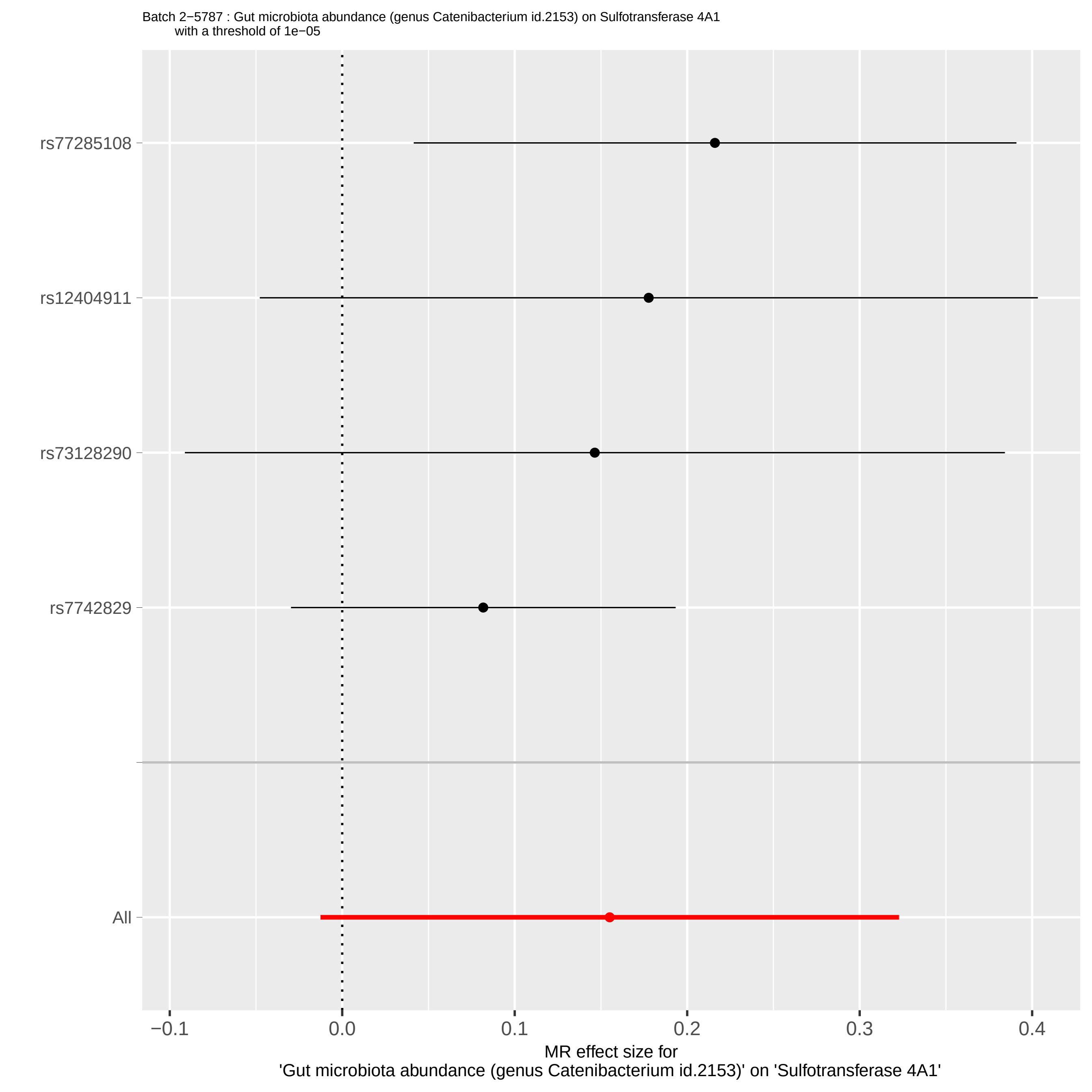
Batch 2−5787 : Gut microbiota abundance (genus Catenibacterium id.2153) on Sulfotransferase 4A1
with a threshold of 1e−05
rs77285108
rs12404911
rs73128290
rs7742829
All
−0.1
0.0
0.1
0.2
0.3
0.4
MR effect size for
'Gut microbiota abundance (genus Catenibacterium id.2153)' on 'Sulfotransferase 4A1'

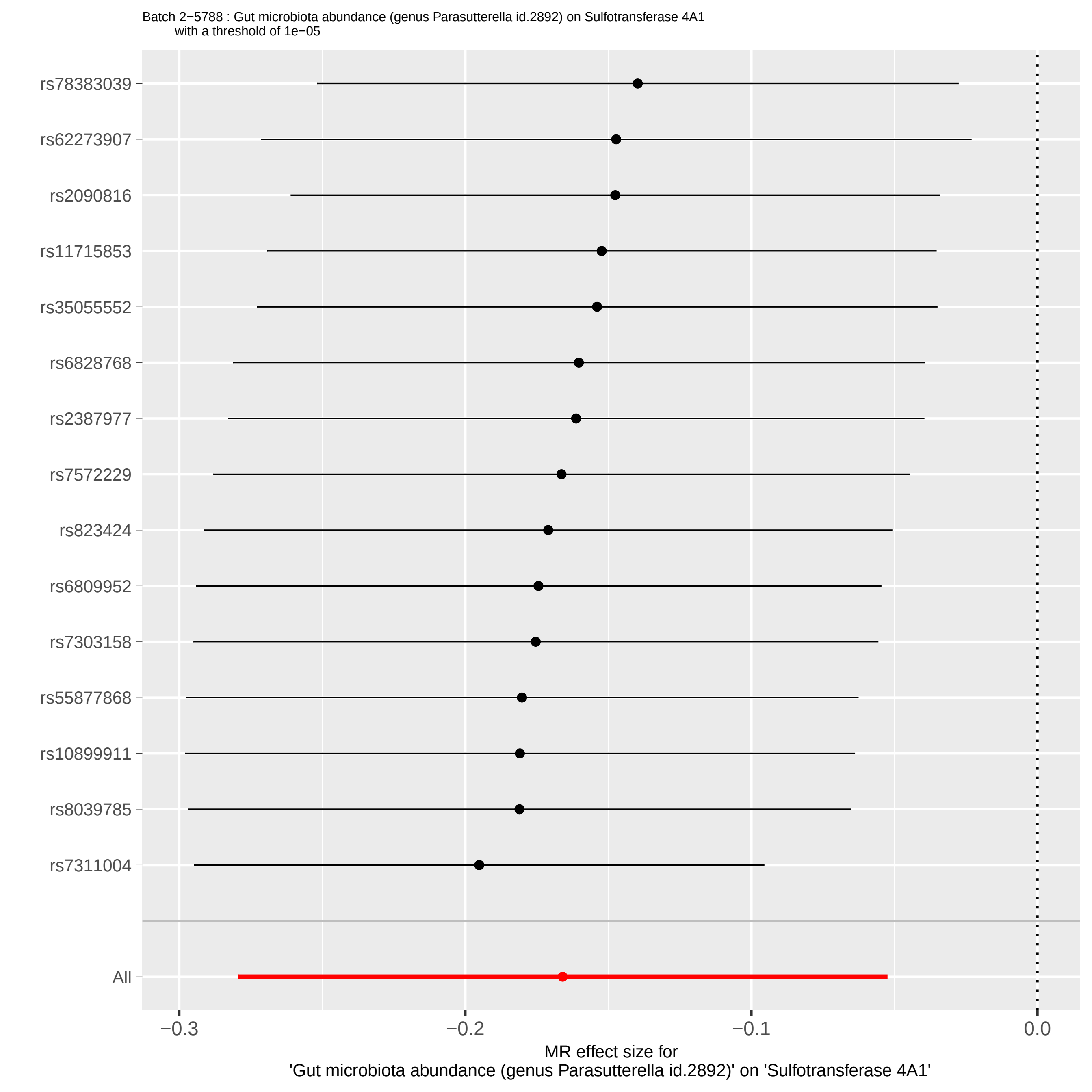
Batch 2−5788 : Gut microbiota abundance (genus Parasutterella id.2892) on Sulfotransferase 4A1
with a threshold of 1e−05
rs78383039
rs62273907
rs2090816
rs11715853
rs35055552
rs6828768
rs2387977
rs7572229
rs823424
rs6809952
rs7303158
rs55877868
rs10899911
rs8039785
rs7311004
All
−0.3
−0.2
−0.1
0.0
MR effect size for
'Gut microbiota abundance (genus Parasutterella id.2892)' on 'Sulfotransferase 4A1'

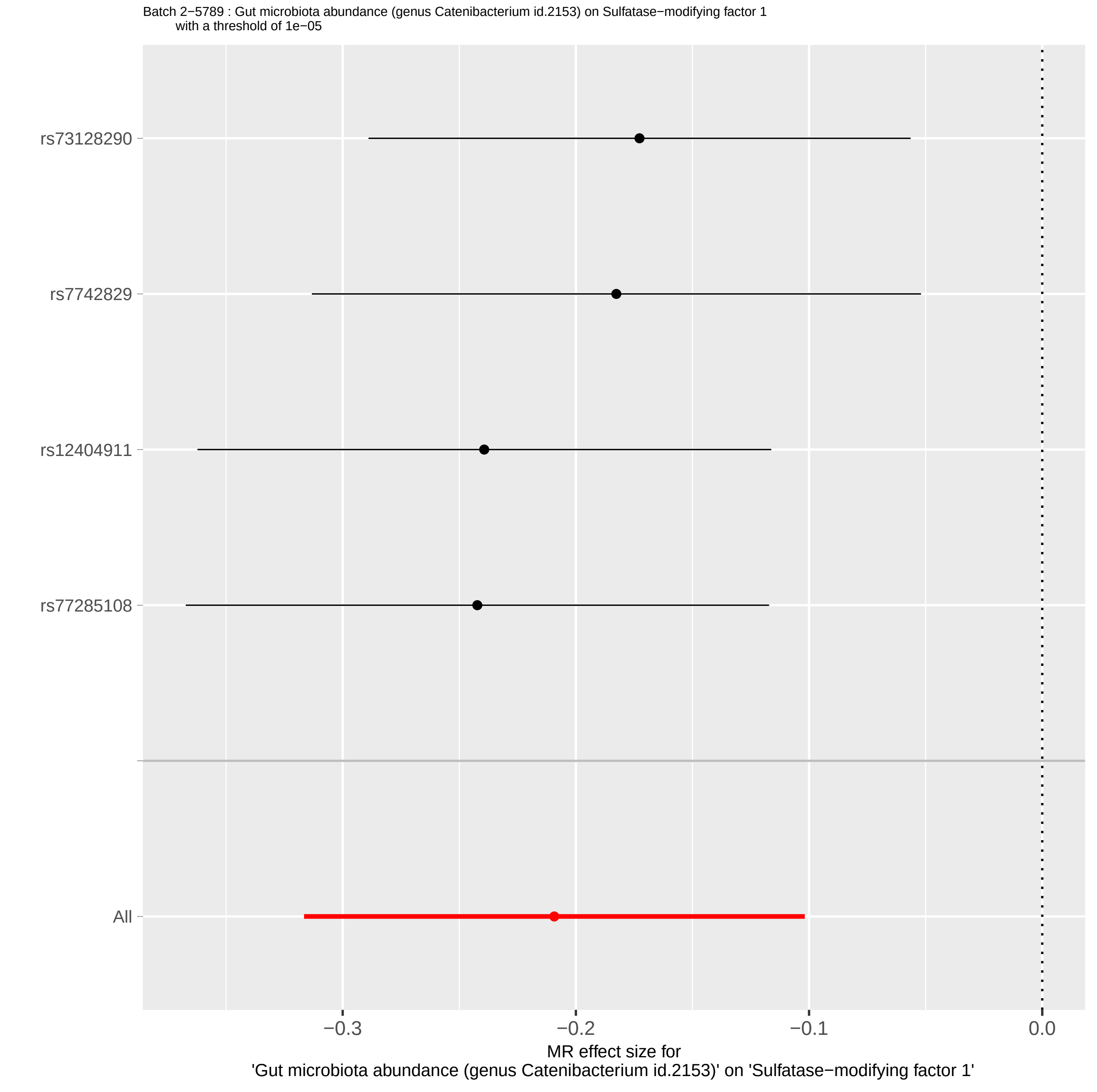

Batch 2−5789 : Gut microbiota abundance (genus Catenibacterium id.2153) on Sulfatase−modifying factor 1
with a threshold of 1e−05
rs73128290
rs7742829
rs12404911
rs77285108
All
−0.3
−0.2
−0.1
0.0
MR effect size for
'Gut microbiota abundance (genus Catenibacterium id.2153)' on 'Sulfatase−modifying factor 1'

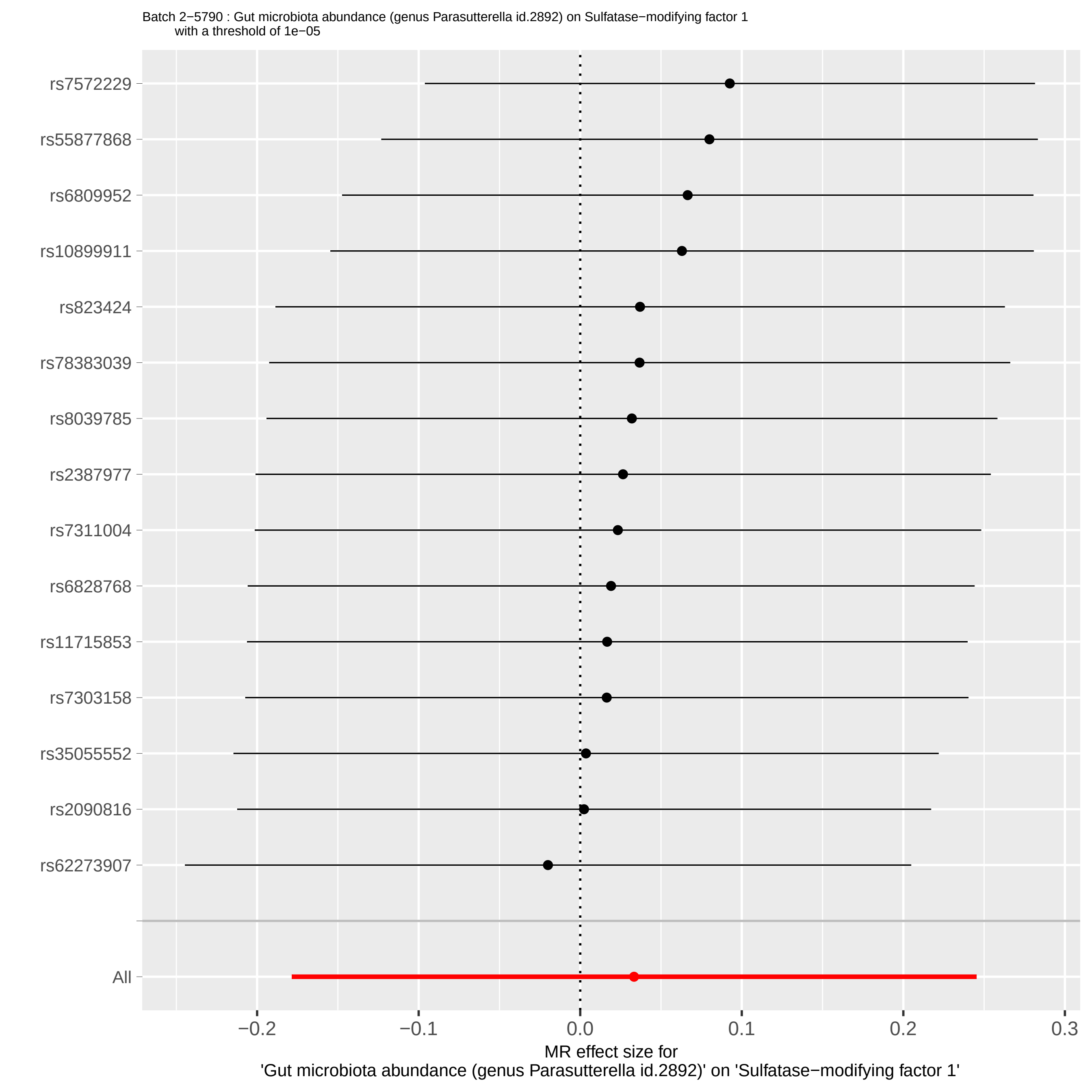

Batch 2−5790 : Gut microbiota abundance (genus Parasutterella id.2892) on Sulfatase−modifying factor 1
with a threshold of 1e−05
rs7572229
rs55877868
rs6809952
rs10899911
rs823424
rs78383039
rs8039785
rs2387977
rs7311004
rs6828768
rs11715853
rs7303158
rs35055552
rs2090816
rs62273907
All
−0.2
−0.1
0.0
0.1
0.2
0.3
MR effect size for
'Gut microbiota abundance (genus Parasutterella id.2892)' on 'Sulfatase−modifying factor 1'

Batch 2−5791 : Gut microbiota abundance (genus Catenibacterium id.2153) on Small ubiquitin−related modifier 3
with a threshold of 1e−05

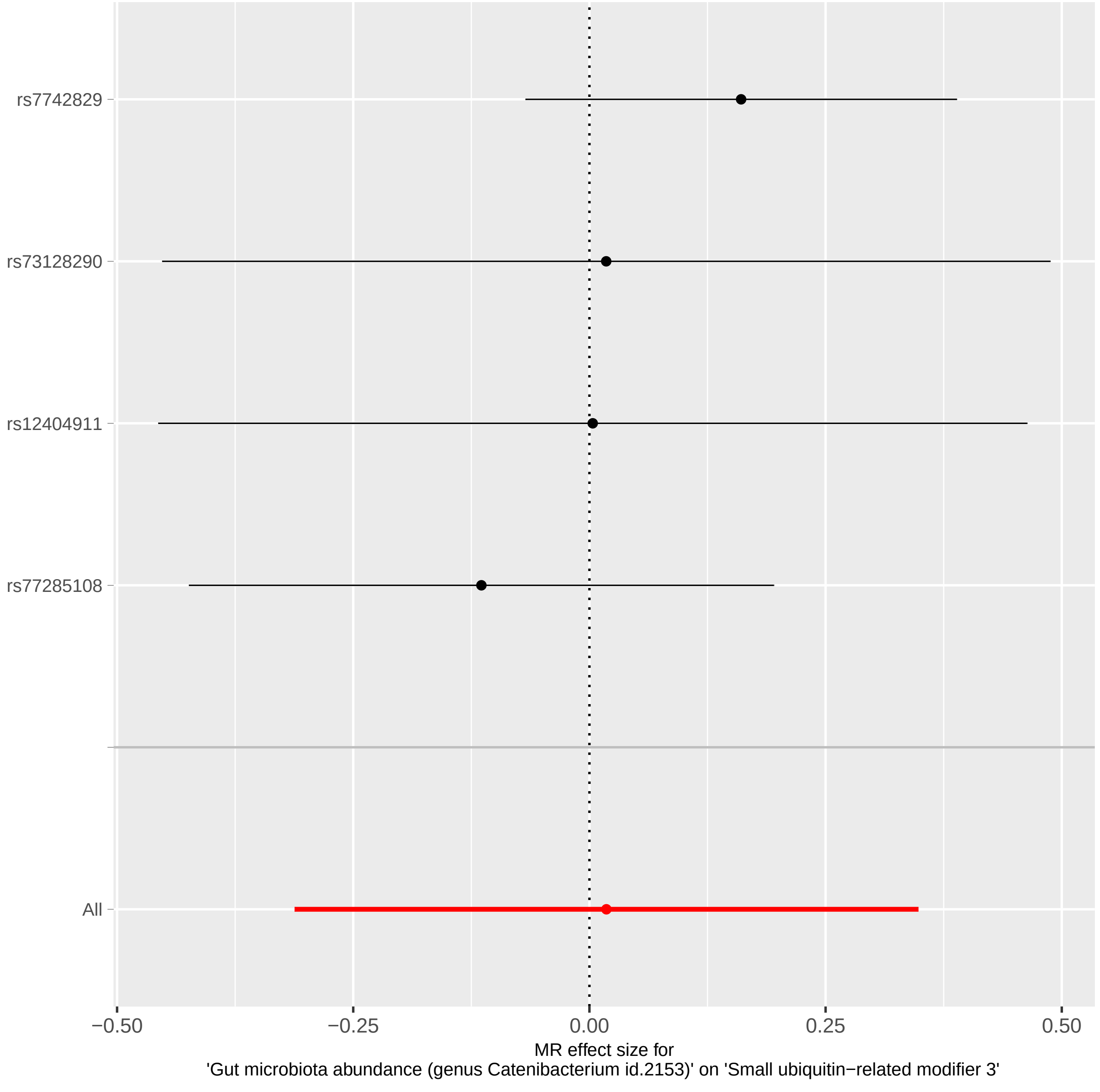

Batch 2−5792 : Gut microbiota abundance (genus Parasutterella id.2892) on Small ubiquitin−related modifier 3
with a threshold of 1e−05

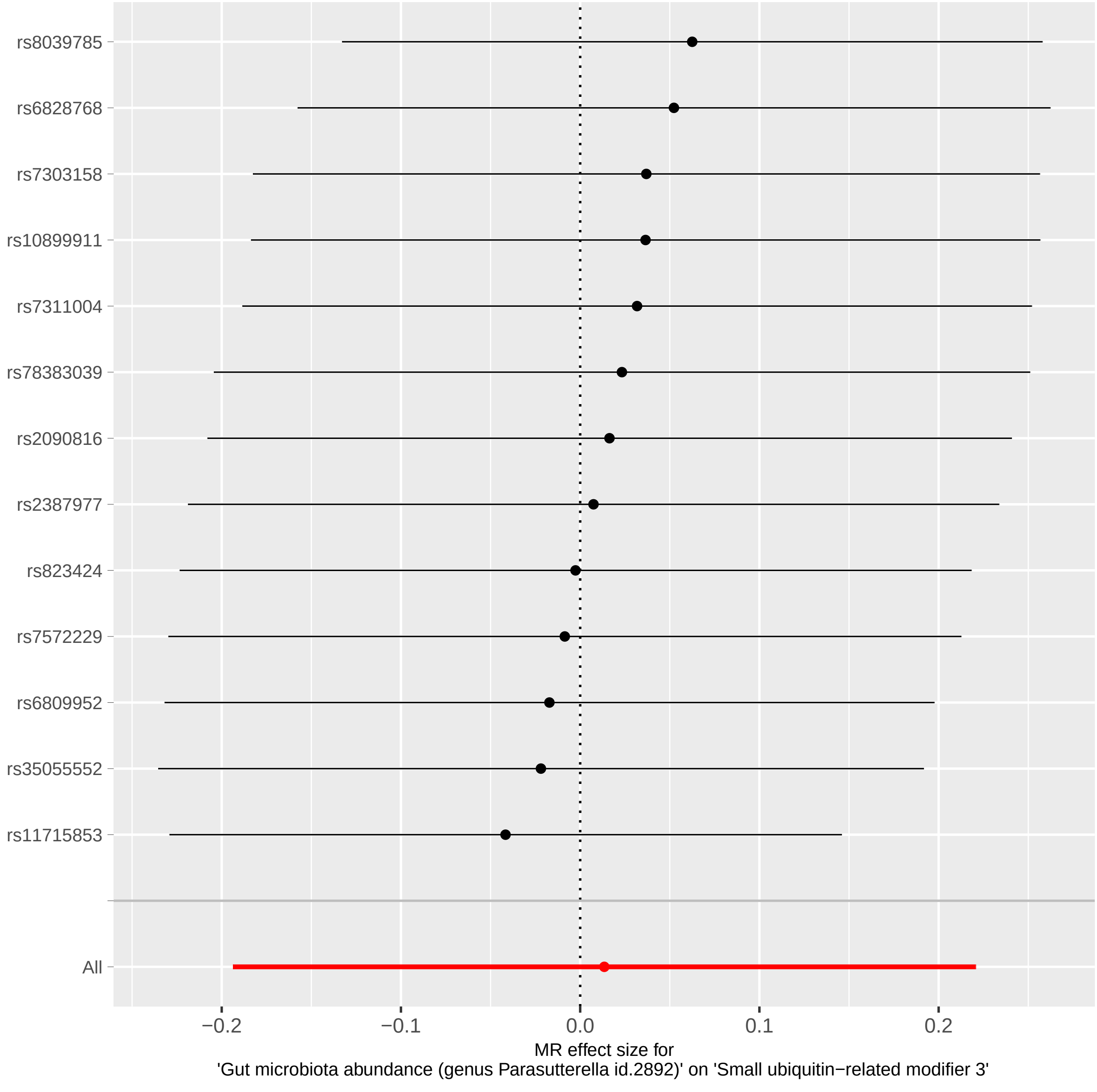

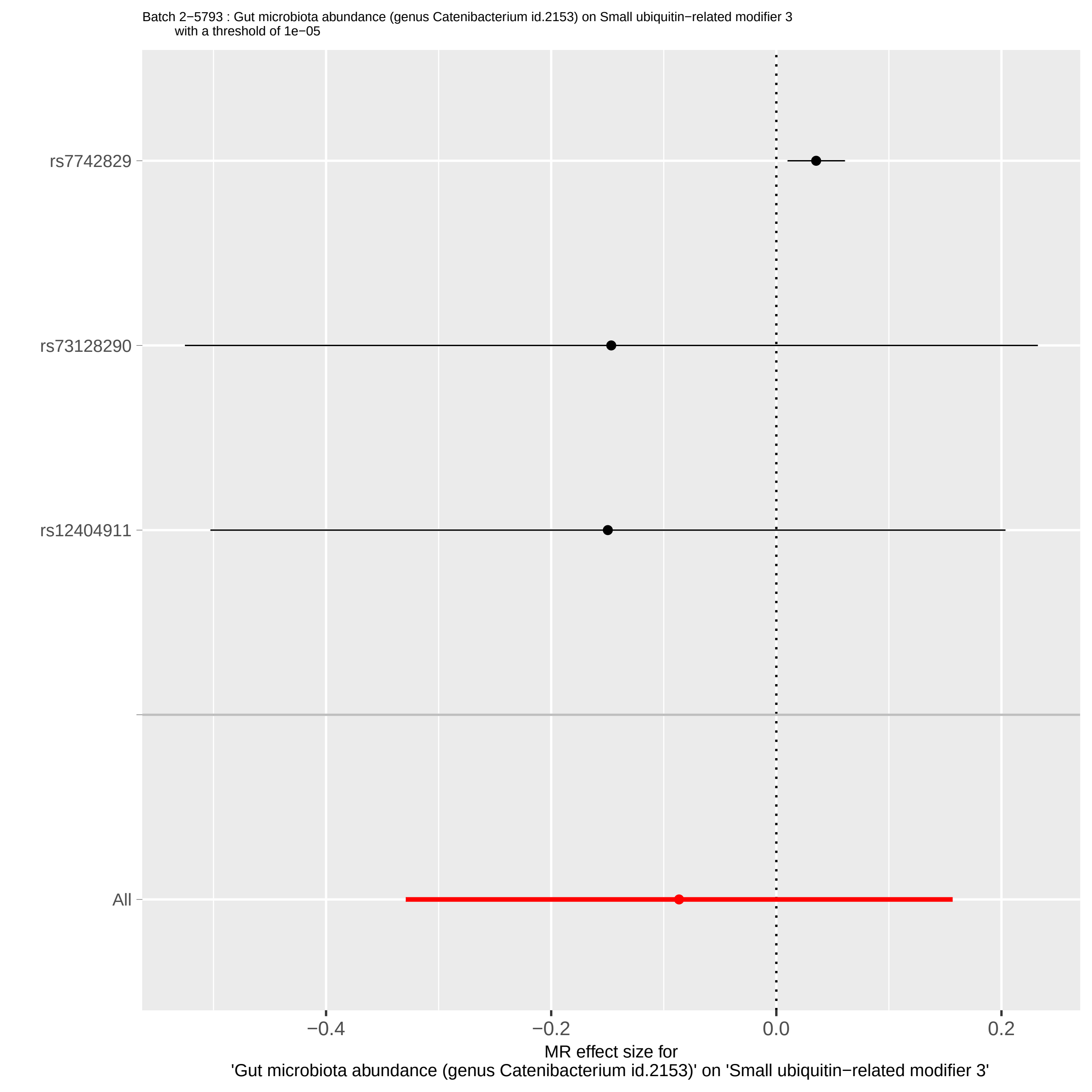

Batch 2−5793 : Gut microbiota abundance (genus Catenibacterium id.2153) on Small ubiquitin−related modifier 3
with a threshold of 1e−05
rs7742829
rs73128290
rs12404911
All
−0.4
−0.2
0.0
0.2
MR effect size for
'Gut microbiota abundance (genus Catenibacterium id.2153)' on 'Small ubiquitin−related modifier 3'

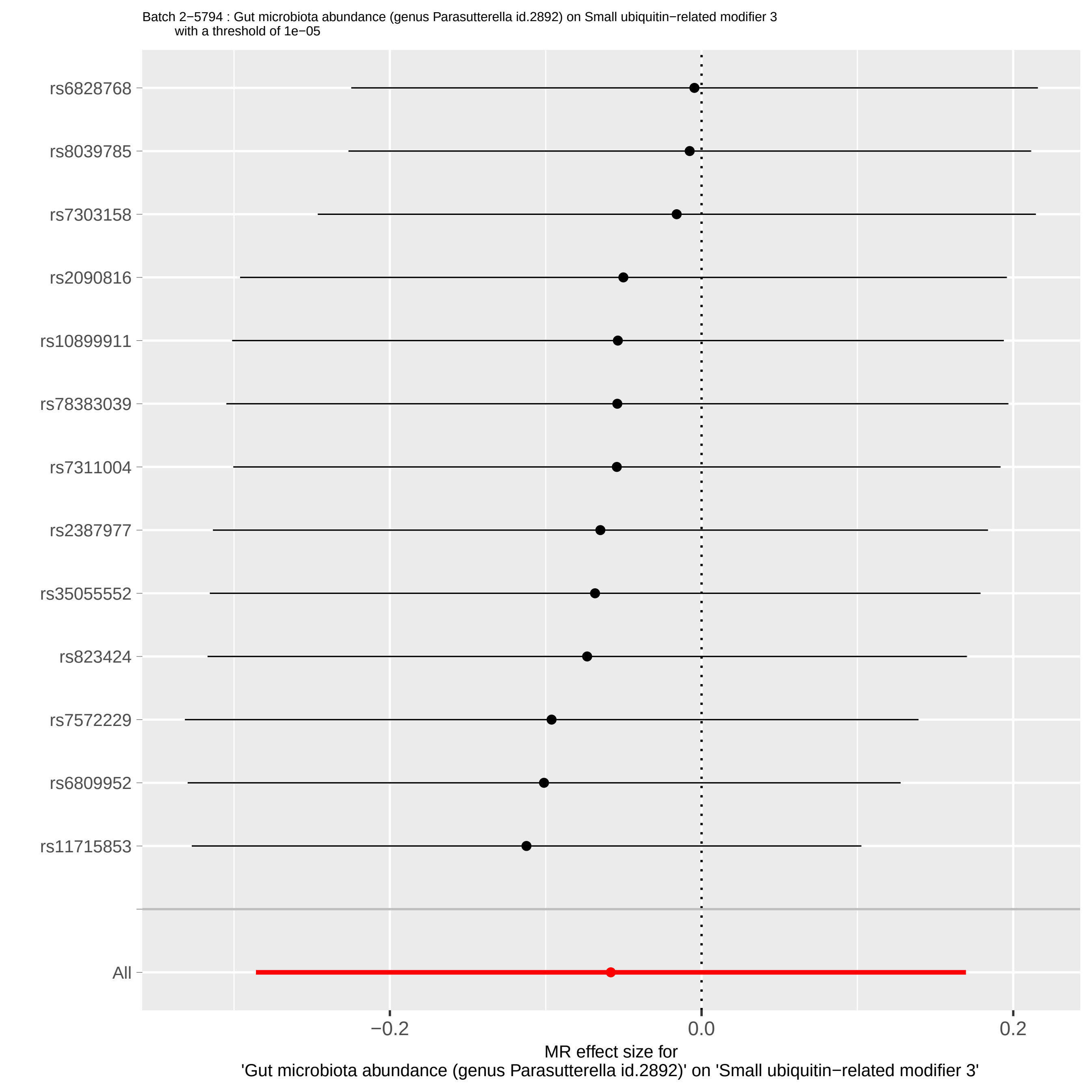

Batch 2−5794 : Gut microbiota abundance (genus Parasutterella id.2892) on Small ubiquitin−related modifier 3
with a threshold of 1e−05
rs6828768
rs8039785
rs7303158
rs2090816
rs10899911
rs78383039
rs7311004
rs2387977
rs35055552
rs823424
rs7572229
rs6809952
rs11715853
All
−0.2
0.0
0.2
MR effect size for
'Gut microbiota abundance (genus Parasutterella id.2892)' on 'Small ubiquitin−related modifier 3'

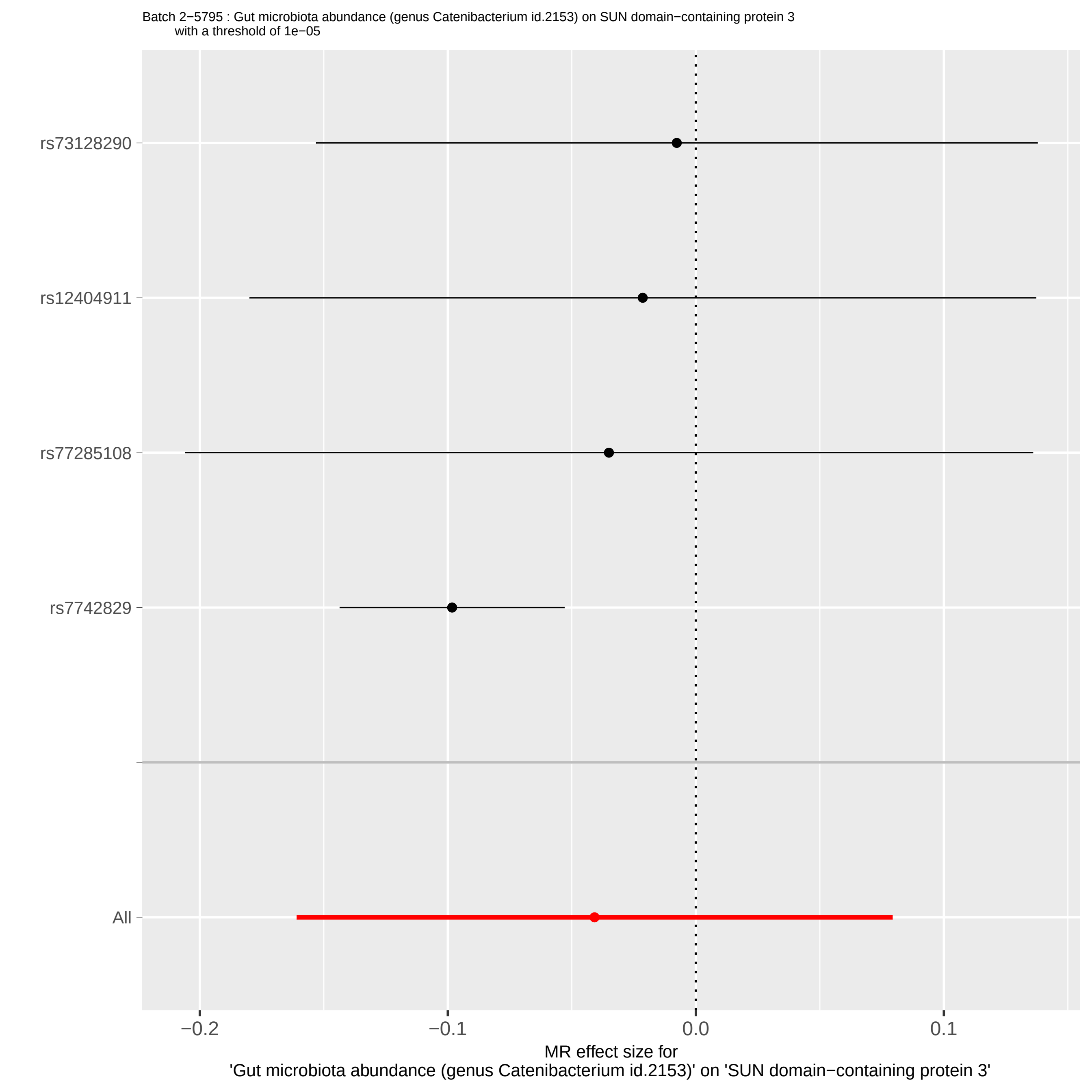

Batch 2−5795 : Gut microbiota abundance (genus Catenibacterium id.2153) on SUN domain−containing protein 3
with a threshold of 1e−05
rs73128290
rs12404911
rs77285108
rs7742829
All
−0.2
−0.1
0.0
0.1
MR effect size for
'Gut microbiota abundance (genus Catenibacterium id.2153)' on 'SUN domain−containing protein 3'

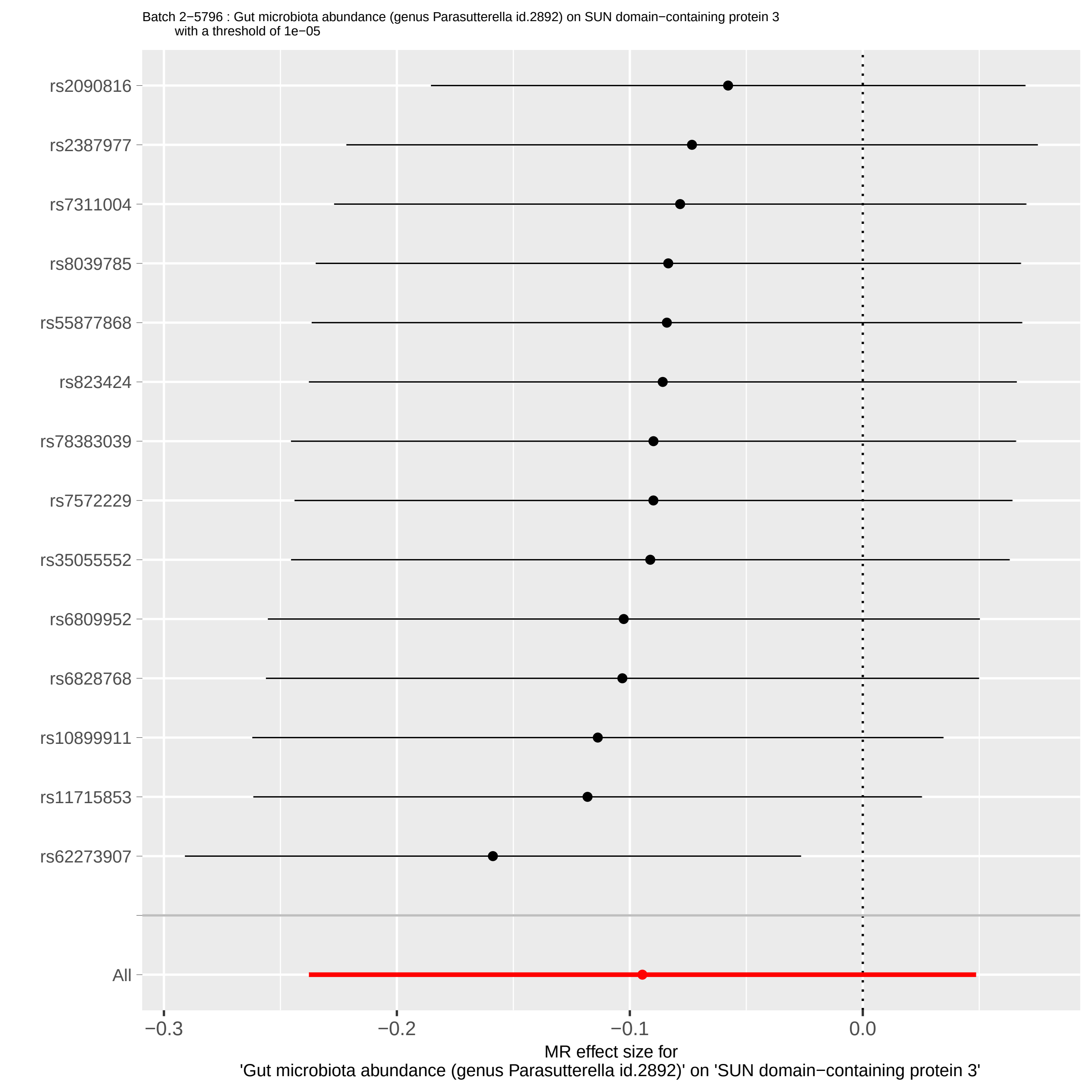
Batch 2−5796 : Gut microbiota abundance (genus Parasutterella id.2892) on SUN domain−containing protein 3
with a threshold of 1e−05
rs2090816
rs2387977
rs7311004
rs8039785
rs55877868
rs823424
rs78383039
rs7572229
rs35055552
rs6809952
rs6828768
rs10899911
rs11715853
rs62273907
All
−0.3
−0.2
−0.1
0.0
MR effect size for
'Gut microbiota abundance (genus Parasutterella id.2892)' on 'SUN domain−containing protein 3'

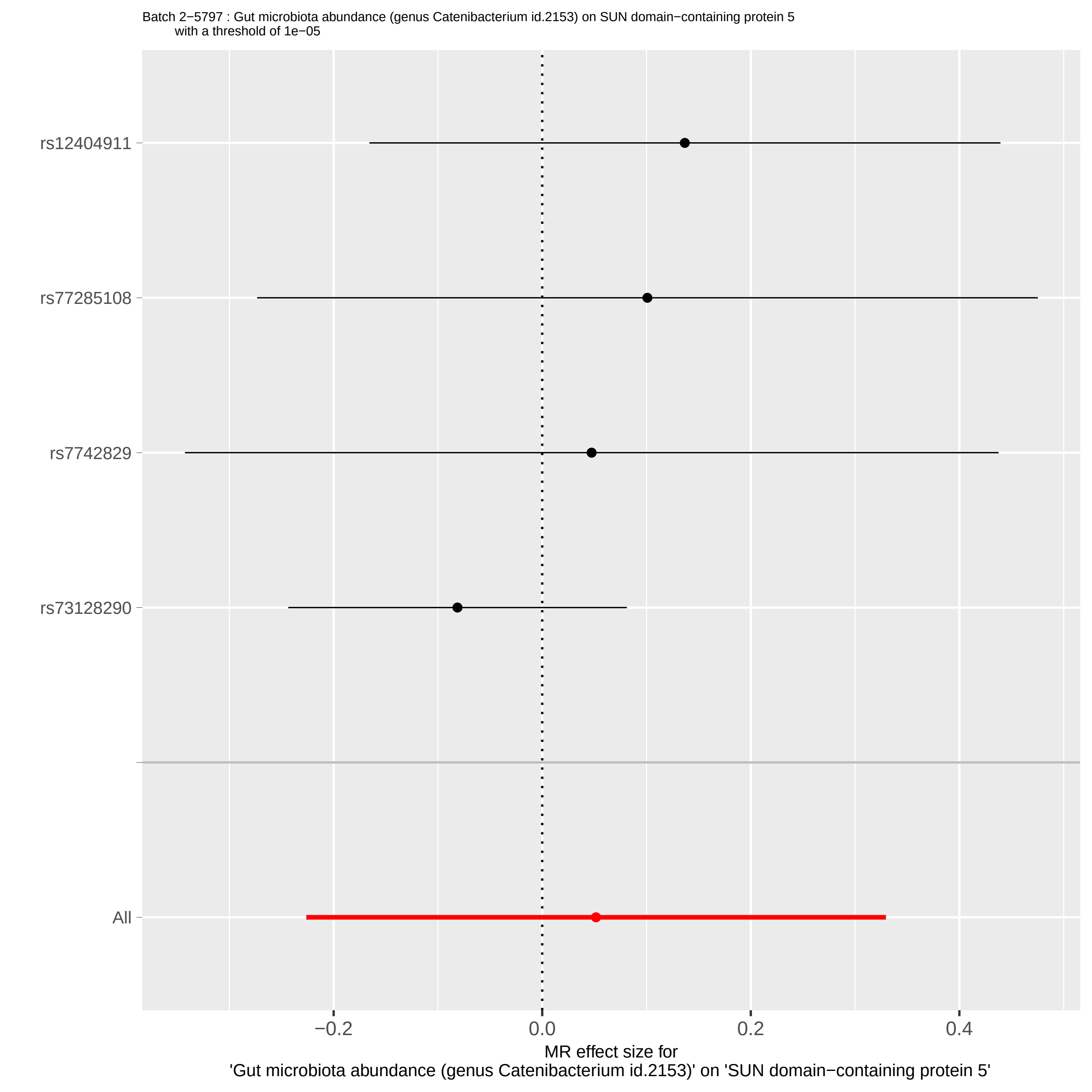

Batch 2−5797 : Gut microbiota abundance (genus Catenibacterium id.2153) on SUN domain−containing protein 5
with a threshold of 1e−05
rs12404911
rs77285108
rs7742829
rs73128290
All
−0.2
0.0
0.2
0.4
MR effect size for
'Gut microbiota abundance (genus Catenibacterium id.2153)' on 'SUN domain−containing protein 5'

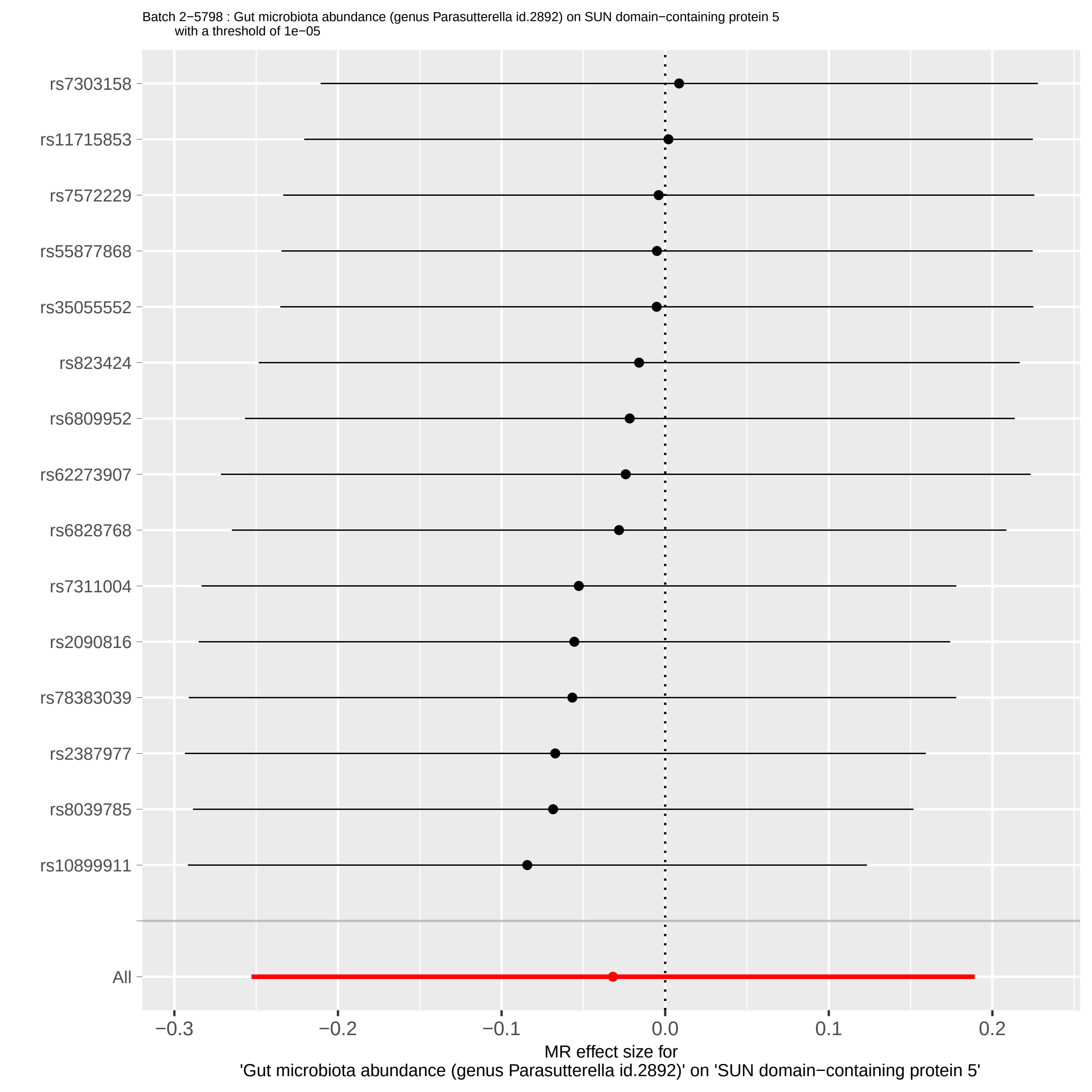

Batch 2−5798 : Gut microbiota abundance (genus Parasutterella id.2892) on SUN domain−containing protein 5
with a threshold of 1e−05
rs7303158
rs11715853
rs7572229
rs55877868
rs35055552
rs823424
rs6809952
rs62273907
rs6828768
rs7311004
rs2090816
rs78383039
rs2387977
rs8039785
rs10899911
All
−0.3
−0.2
−0.1
0.0
0.1
0.2
MR effect size for
'Gut microbiota abundance (genus Parasutterella id.2892)' on 'SUN domain−containing protein 5'

Batch 2−5799 : Gut microbiota abundance (genus Catenibacterium id.2153) on Surfeit locus protein 1
with a threshold of 1e−05

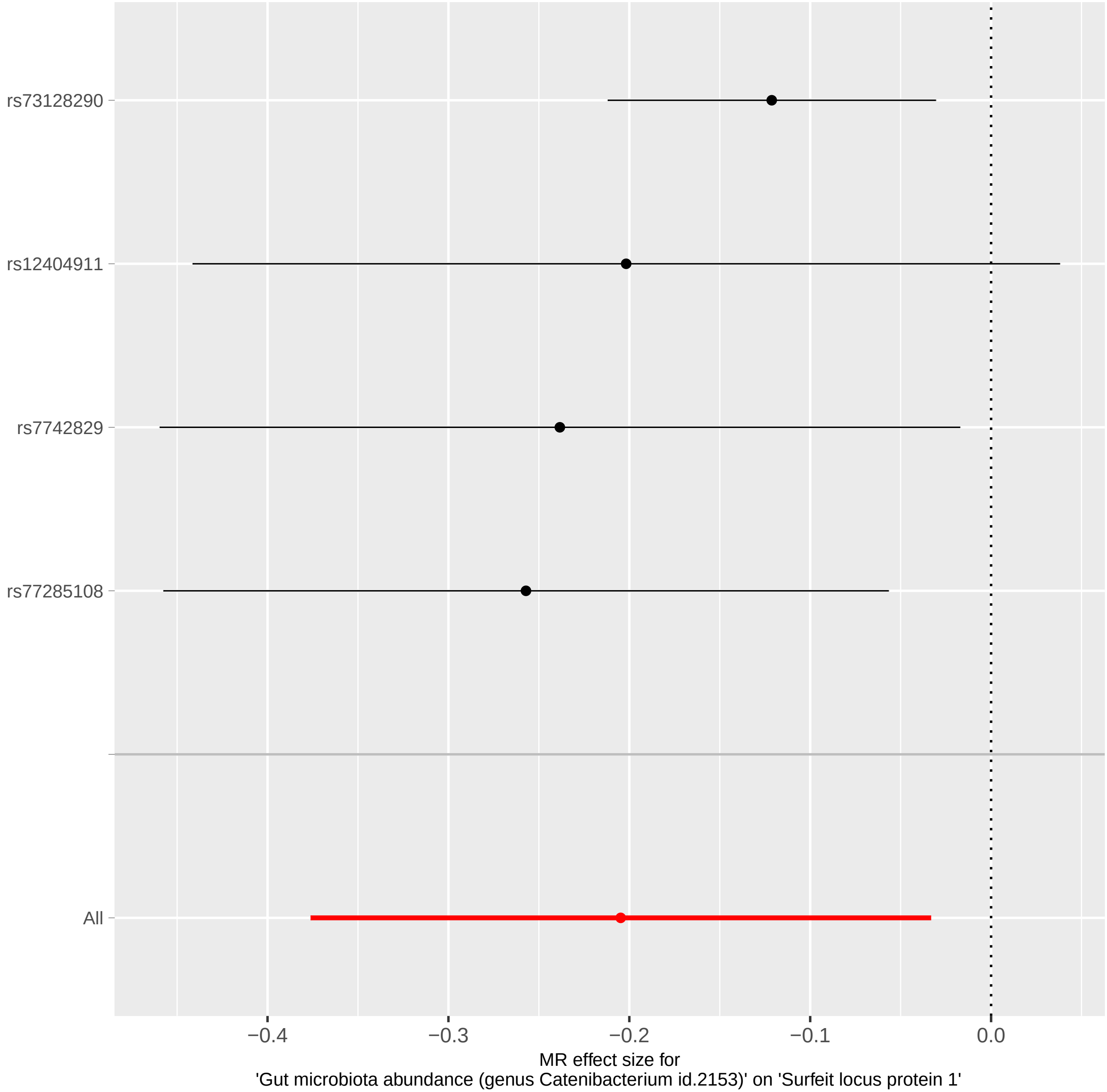

Batch 2−5800 : Gut microbiota abundance (genus Parasutterella id.2892) on Surfeit locus protein 1
with a threshold of 1e−05

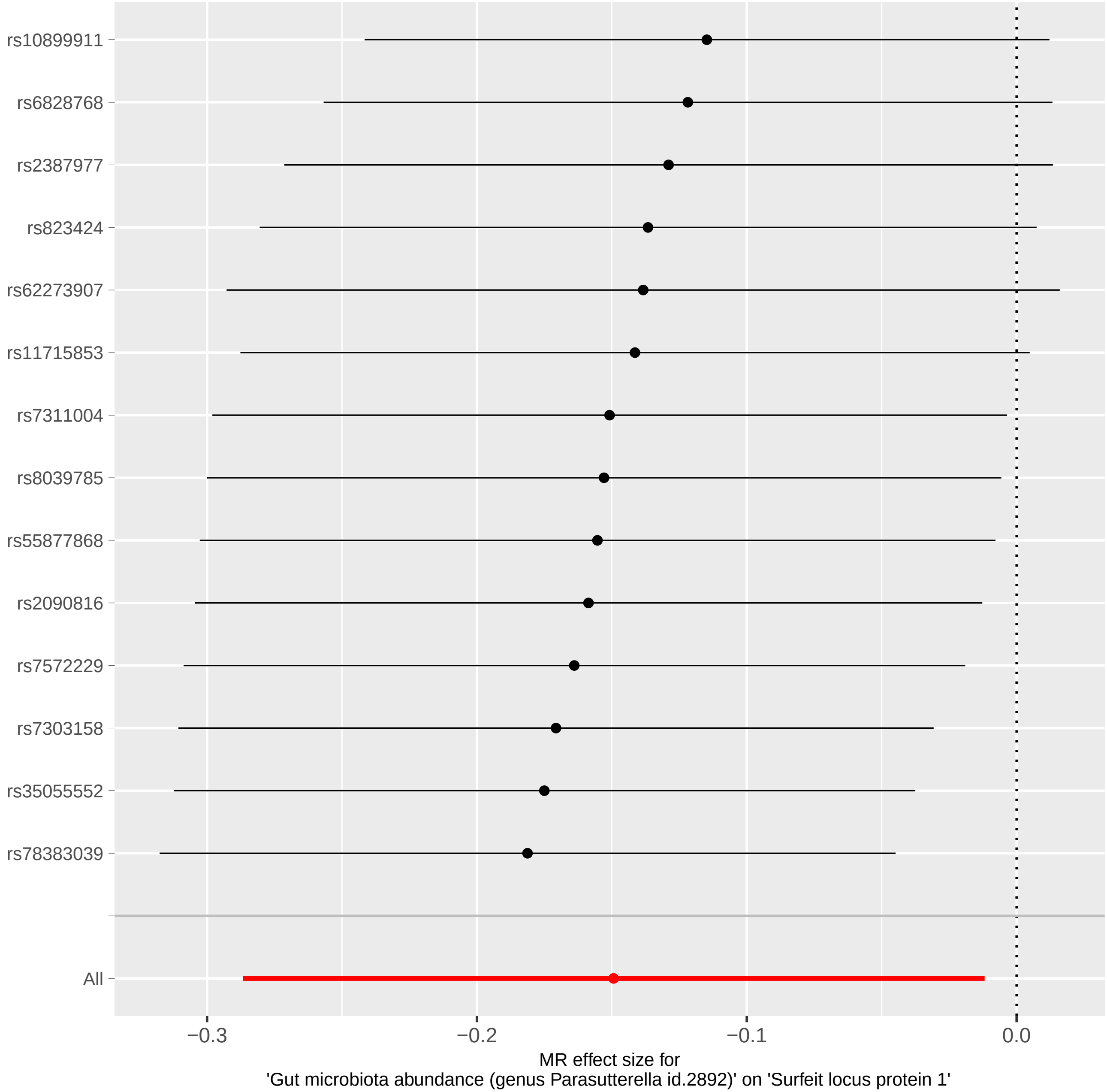

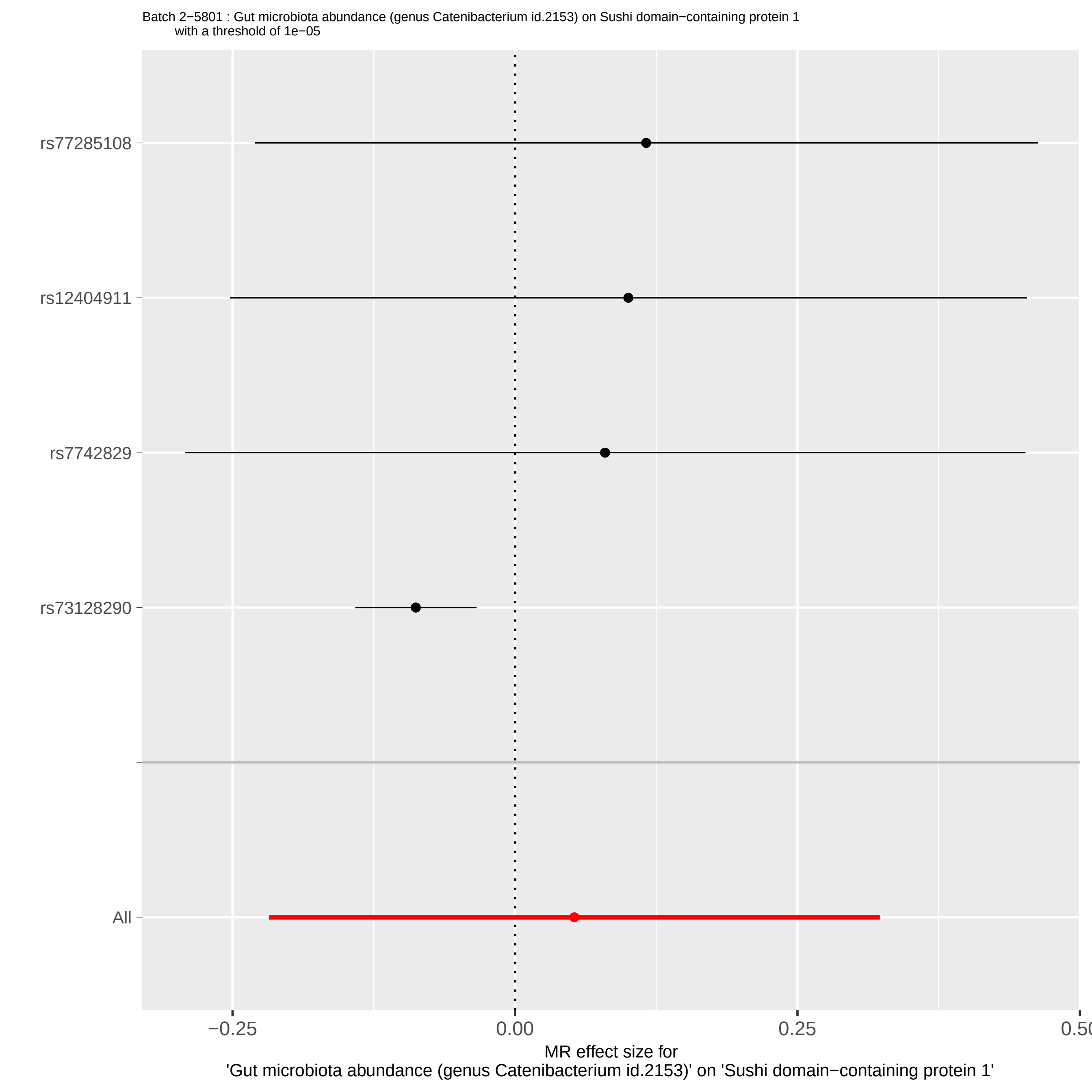

Batch 2−5801 : Gut microbiota abundance (genus Catenibacterium id.2153) on Sushi domain−containing protein 1
with a threshold of 1e−05
rs77285108
rs12404911
rs7742829
rs73128290
All
−0.25
0.00
0.25
0.50
MR effect size for
'Gut microbiota abundance (genus Catenibacterium id.2153)' on 'Sushi domain−containing protein 1'

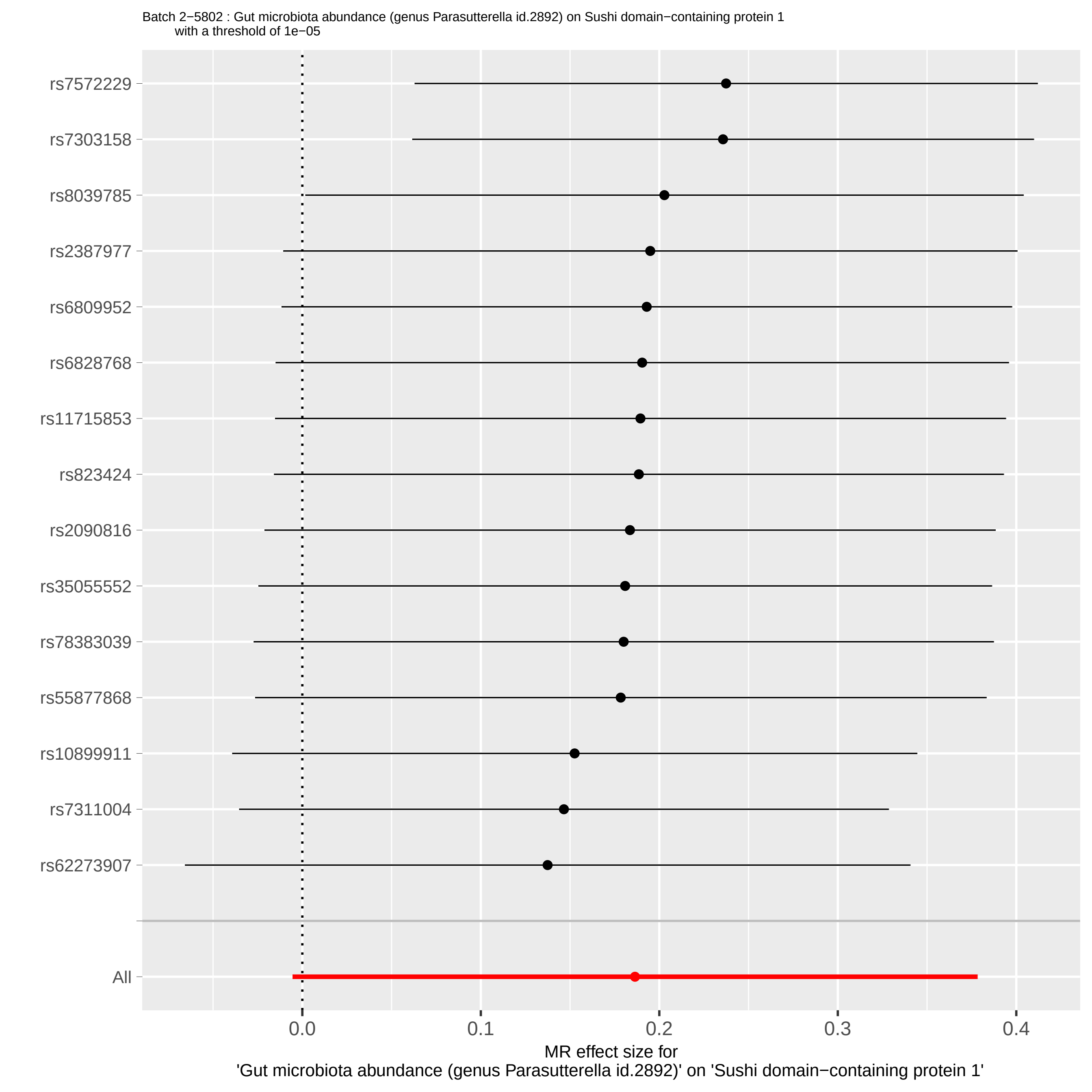

Batch 2−5802 : Gut microbiota abundance (genus Parasutterella id.2892) on Sushi domain−containing protein 1
with a threshold of 1e−05
rs7572229
rs7303158
rs8039785
rs2387977
rs6809952
rs6828768
rs11715853
rs823424
rs2090816
rs35055552
rs78383039
rs55877868
rs10899911
rs7311004
rs62273907
All
0.0
0.1
0.2
0.3
0.4
MR effect size for
'Gut microbiota abundance (genus Parasutterella id.2892)' on 'Sushi domain−containing protein 1'

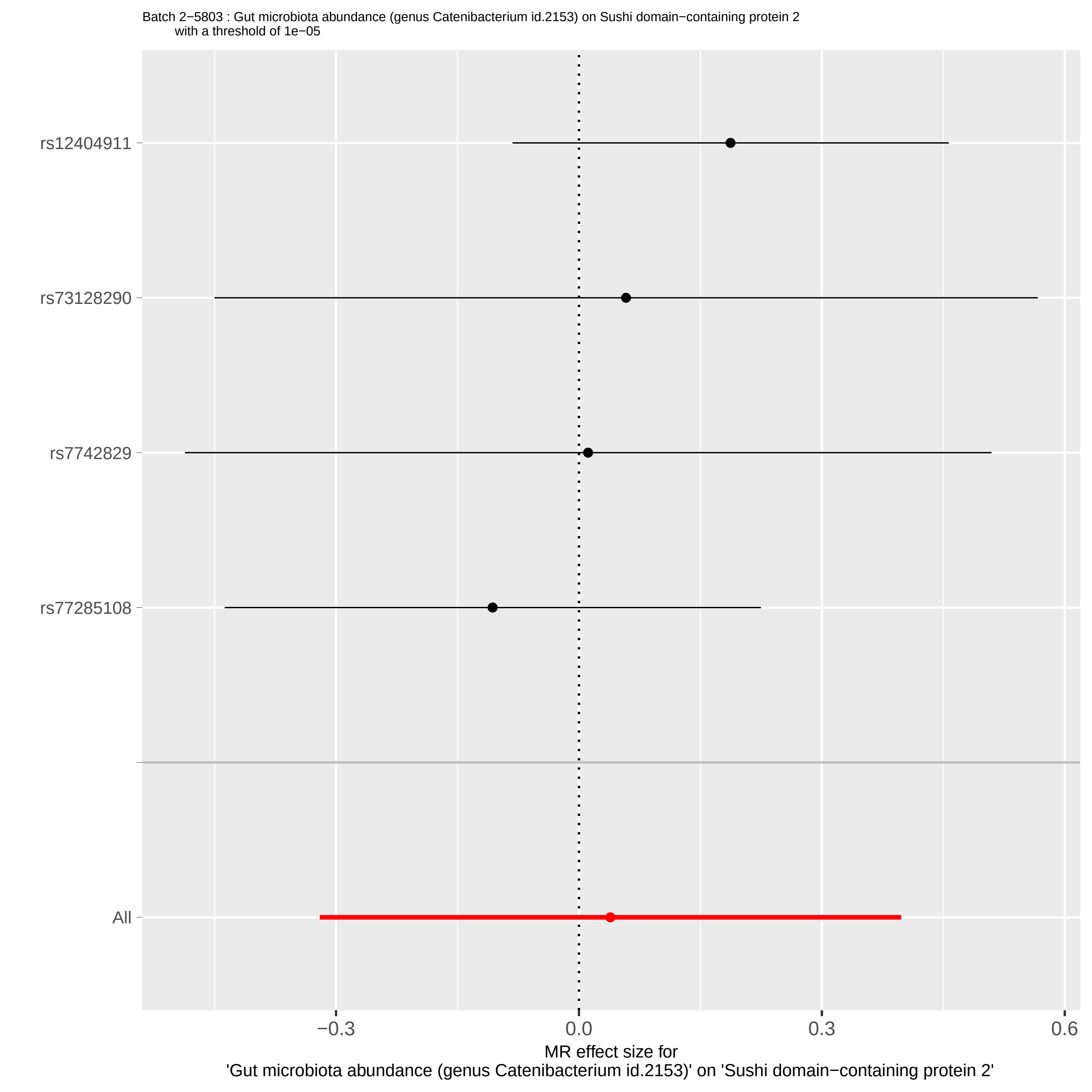

Batch 2−5803 : Gut microbiota abundance (genus Catenibacterium id.2153) on Sushi domain−containing protein 2
with a threshold of 1e−05
rs12404911
rs73128290
rs7742829
rs77285108
All
−0.3
0.0
0.3
0.6
MR effect size for
'Gut microbiota abundance (genus Catenibacterium id.2153)' on 'Sushi domain−containing protein 2'

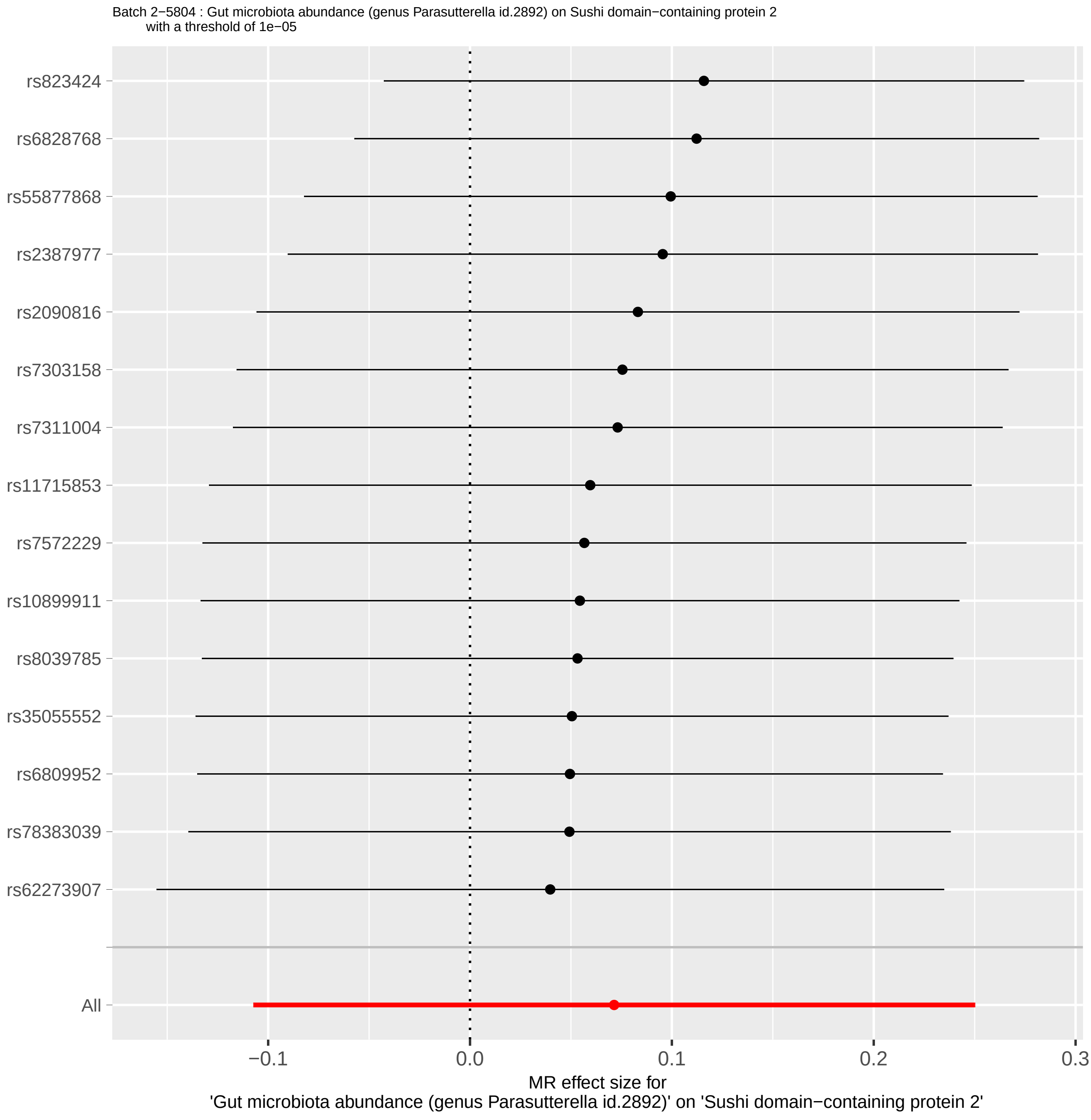
Batch 2−5804 : Gut microbiota abundance (genus Parasutterella id.2892) on Sushi domain−containing protein 2
with a threshold of 1e−05
rs823424
rs6828768
rs55877868
rs2387977
rs2090816
rs7303158
rs7311004
rs11715853
rs7572229
rs10899911
rs8039785
rs35055552
rs6809952
rs78383039
rs62273907
All
−0.1
0.0
0.1
0.2
0.3
MR effect size for
'Gut microbiota abundance (genus Parasutterella id.2892)' on 'Sushi domain−containing protein 2'

Batch 2−5805 : Gut microbiota abundance (genus Catenibacterium id.2153) on Sushi domain−containing protein 3
with a threshold of 1e−05

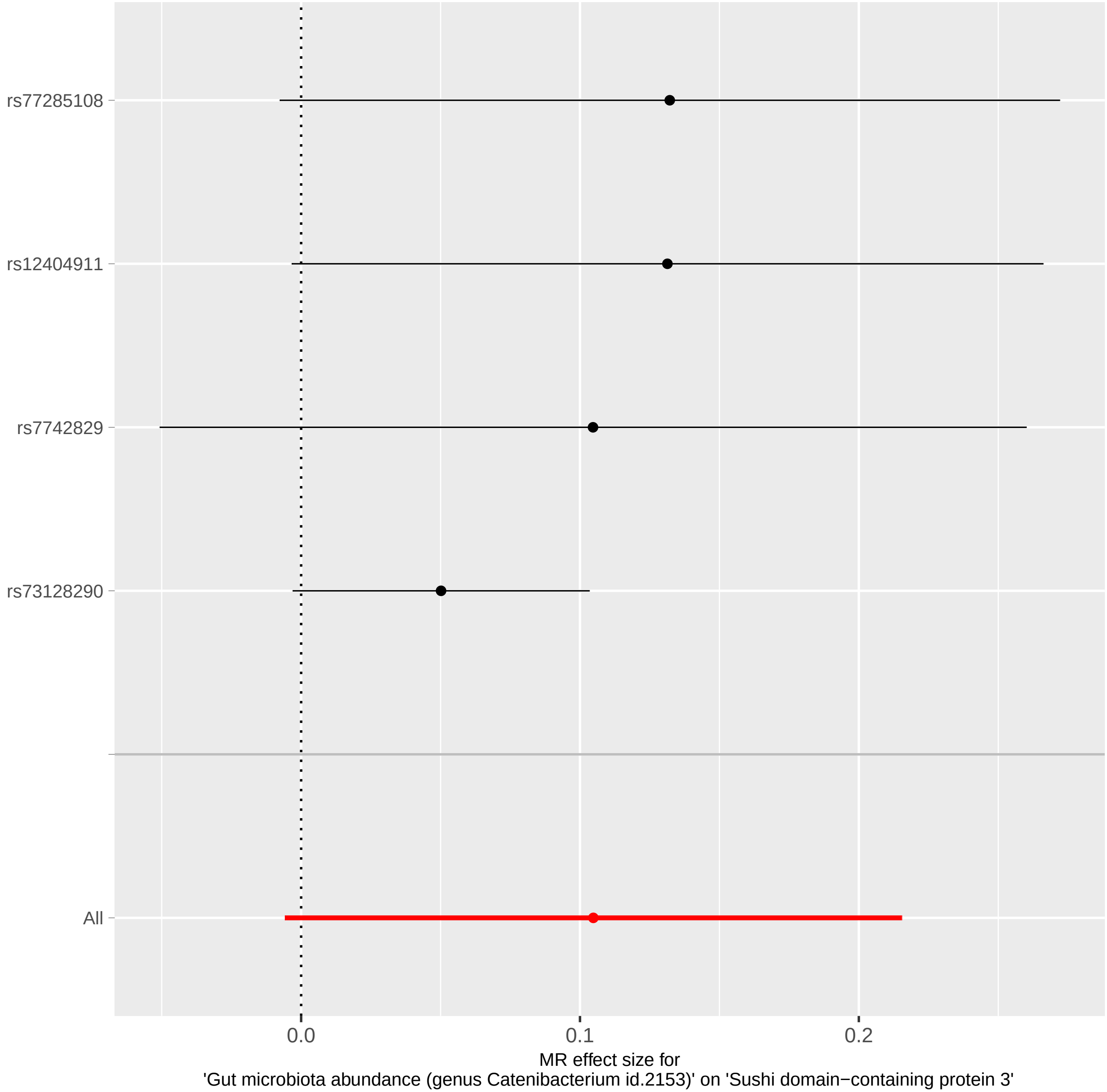

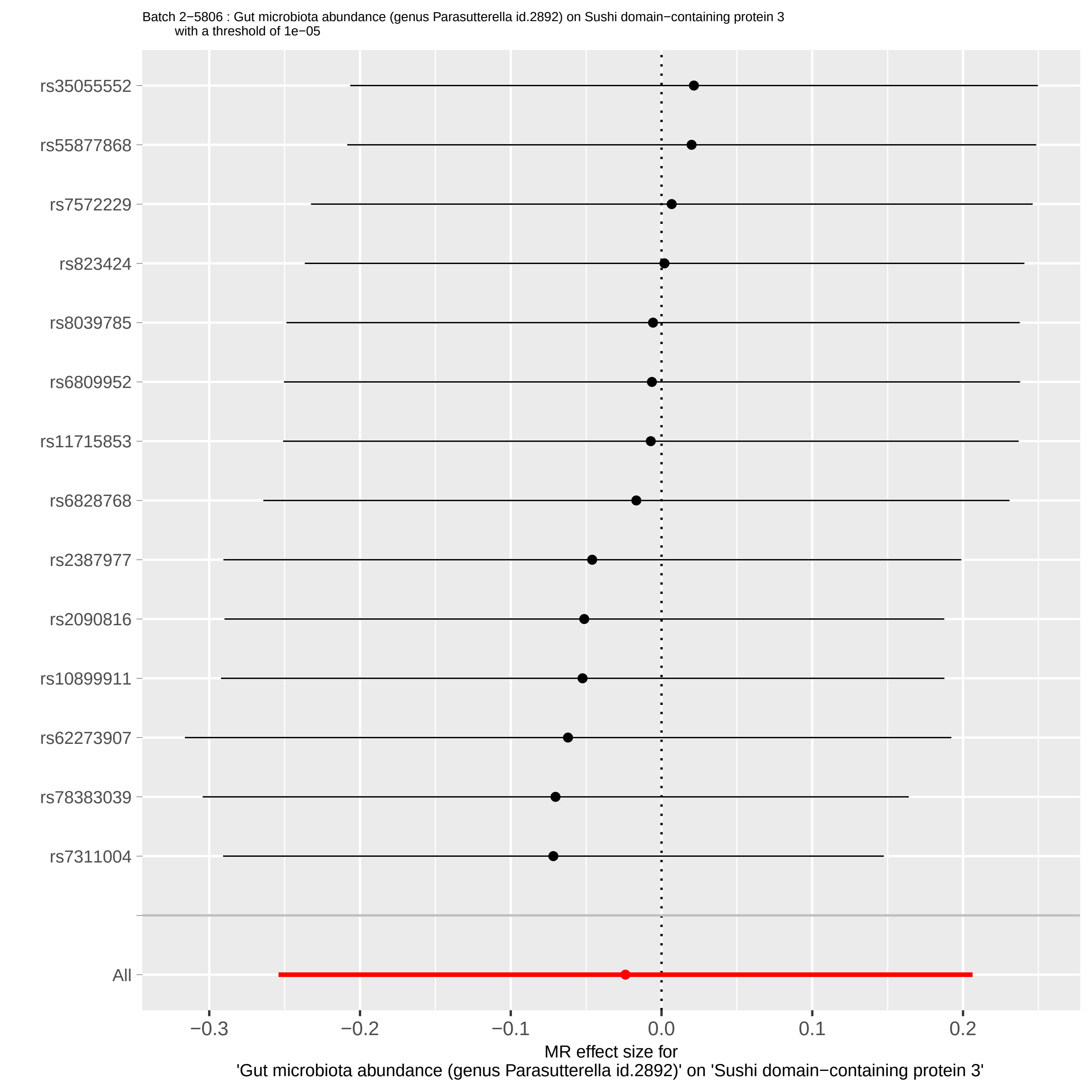

Batch 2−5806 : Gut microbiota abundance (genus Parasutterella id.2892) on Sushi domain−containing protein 3
with a threshold of 1e−05
rs35055552
rs55877868
rs7572229
rs823424
rs8039785
rs6809952
rs11715853
rs6828768
rs2387977
rs2090816
rs10899911
rs62273907
rs78383039
rs7311004
All
−0.3
−0.2
−0.1
0.0
0.1
0.2
MR effect size for
'Gut microbiota abundance (genus Parasutterella id.2892)' on 'Sushi domain−containing protein 3'

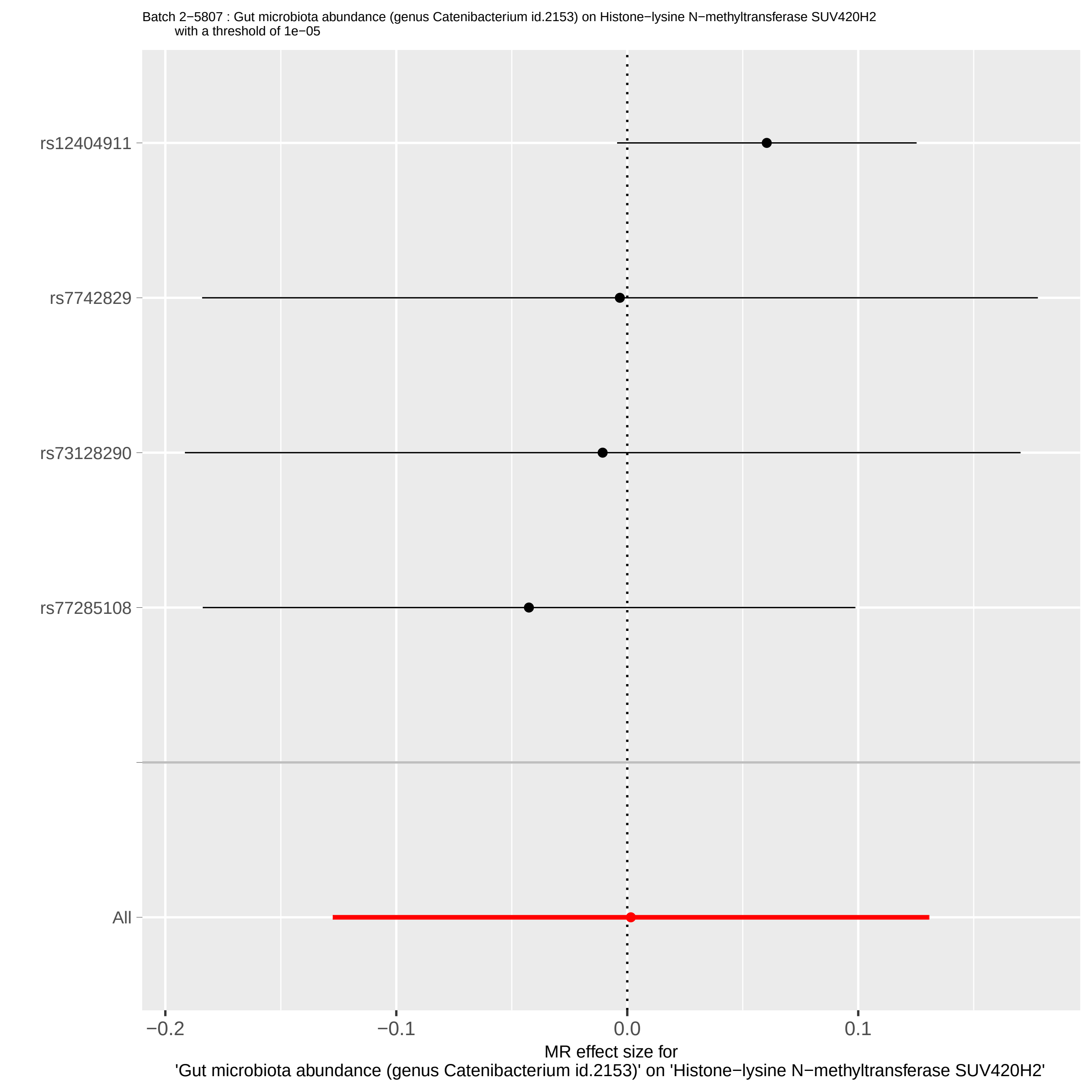

Batch 2−5807 : Gut microbiota abundance (genus Catenibacterium id.2153) on Histone−lysine N−methyltransferase SUV420H2
with a threshold of 1e−05
rs12404911
rs7742829
rs73128290
rs77285108
All
−0.2
−0.1
0.0
0.1
MR effect size for
'Gut microbiota abundance (genus Catenibacterium id.2153)' on 'Histone−lysine N−methyltransferase SUV420H2'

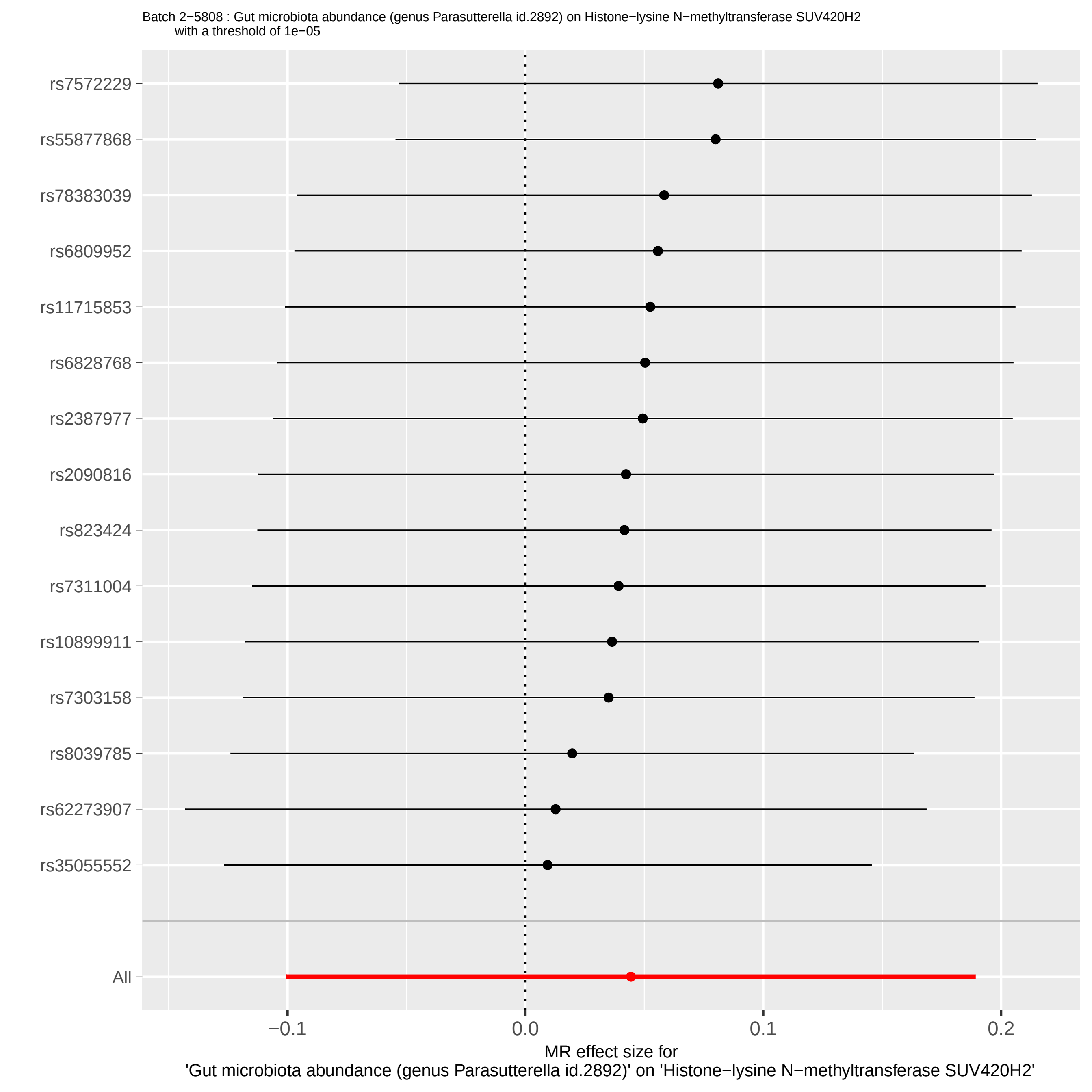

Batch 2−5808 : Gut microbiota abundance (genus Parasutterella id.2892) on Histone−lysine N−methyltransferase SUV420H2
with a threshold of 1e−05
rs7572229
rs55877868
rs78383039
rs6809952
rs11715853
rs6828768
rs2387977
rs2090816
rs823424
rs7311004
rs10899911
rs7303158
rs8039785
rs62273907
rs35055552
All
−0.1
0.0
0.1
0.2
MR effect size for
'Gut microbiota abundance (genus Parasutterella id.2892)' on 'Histone−lysine N−methyltransferase SUV420H2'

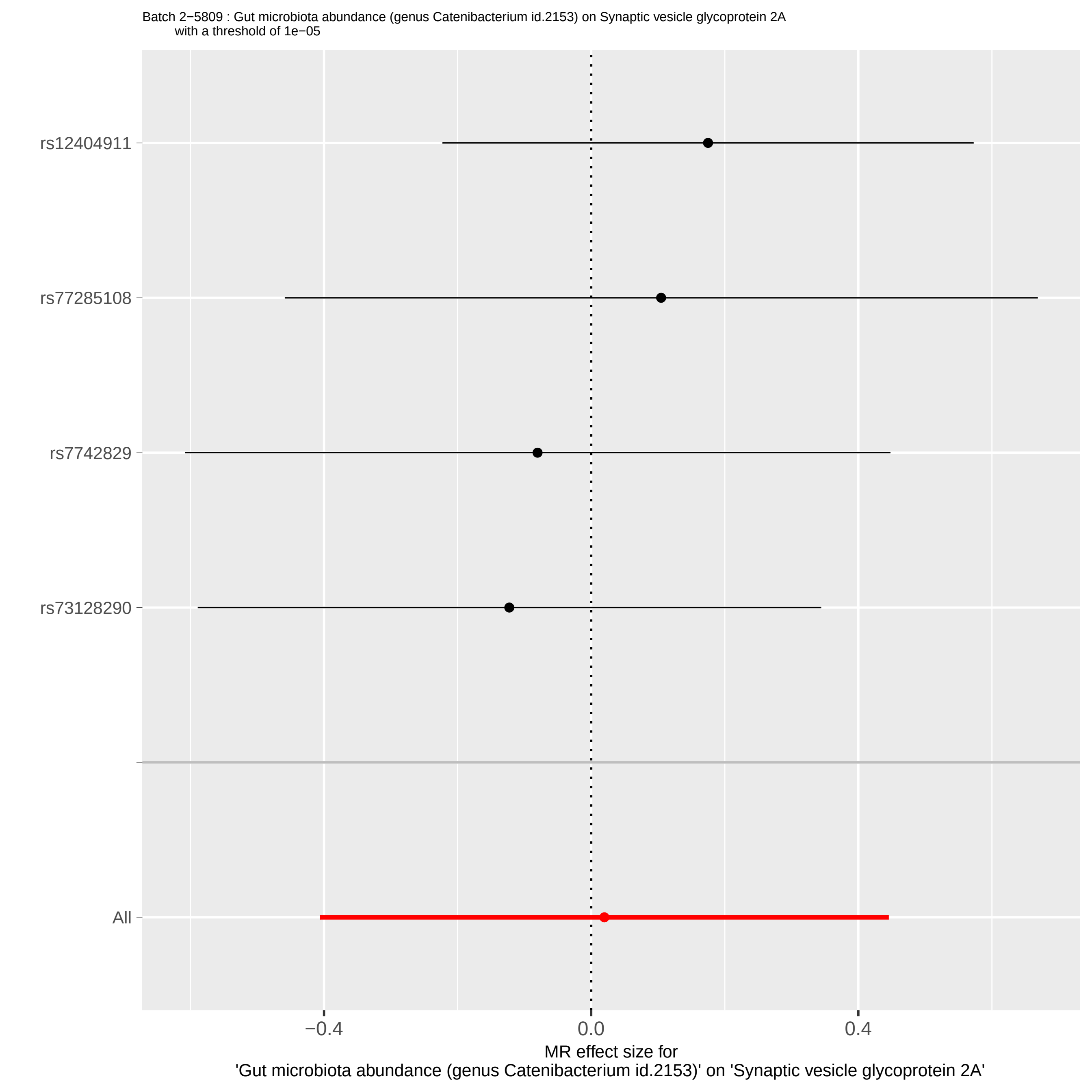
Batch 2−5809 : Gut microbiota abundance (genus Catenibacterium id.2153) on Synaptic vesicle glycoprotein 2A
with a threshold of 1e−05
rs12404911
rs77285108
rs7742829
rs73128290
All
−0.4
0.0
0.4
MR effect size for
'Gut microbiota abundance (genus Catenibacterium id.2153)' on 'Synaptic vesicle glycoprotein 2A'

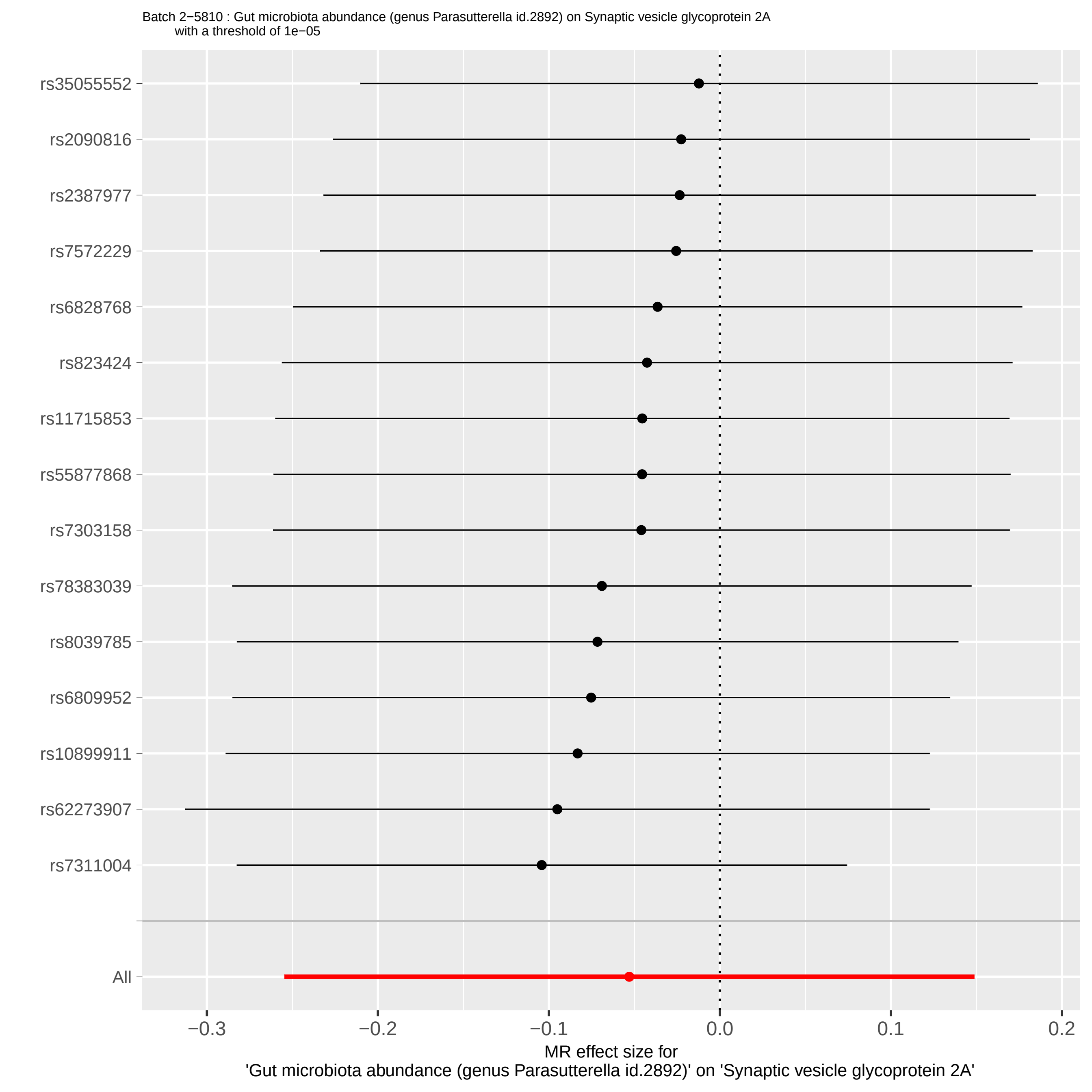

Batch 2−5810 : Gut microbiota abundance (genus Parasutterella id.2892) on Synaptic vesicle glycoprotein 2A
with a threshold of 1e−05
rs35055552
rs2090816
rs2387977
rs7572229
rs6828768
rs823424
rs11715853
rs55877868
rs7303158
rs78383039
rs8039785
rs6809952
rs10899911
rs62273907
rs7311004
All
−0.3
−0.2
−0.1
0.0
0.1
0.2
MR effect size for
'Gut microbiota abundance (genus Parasutterella id.2892)' on 'Synaptic vesicle glycoprotein 2A'

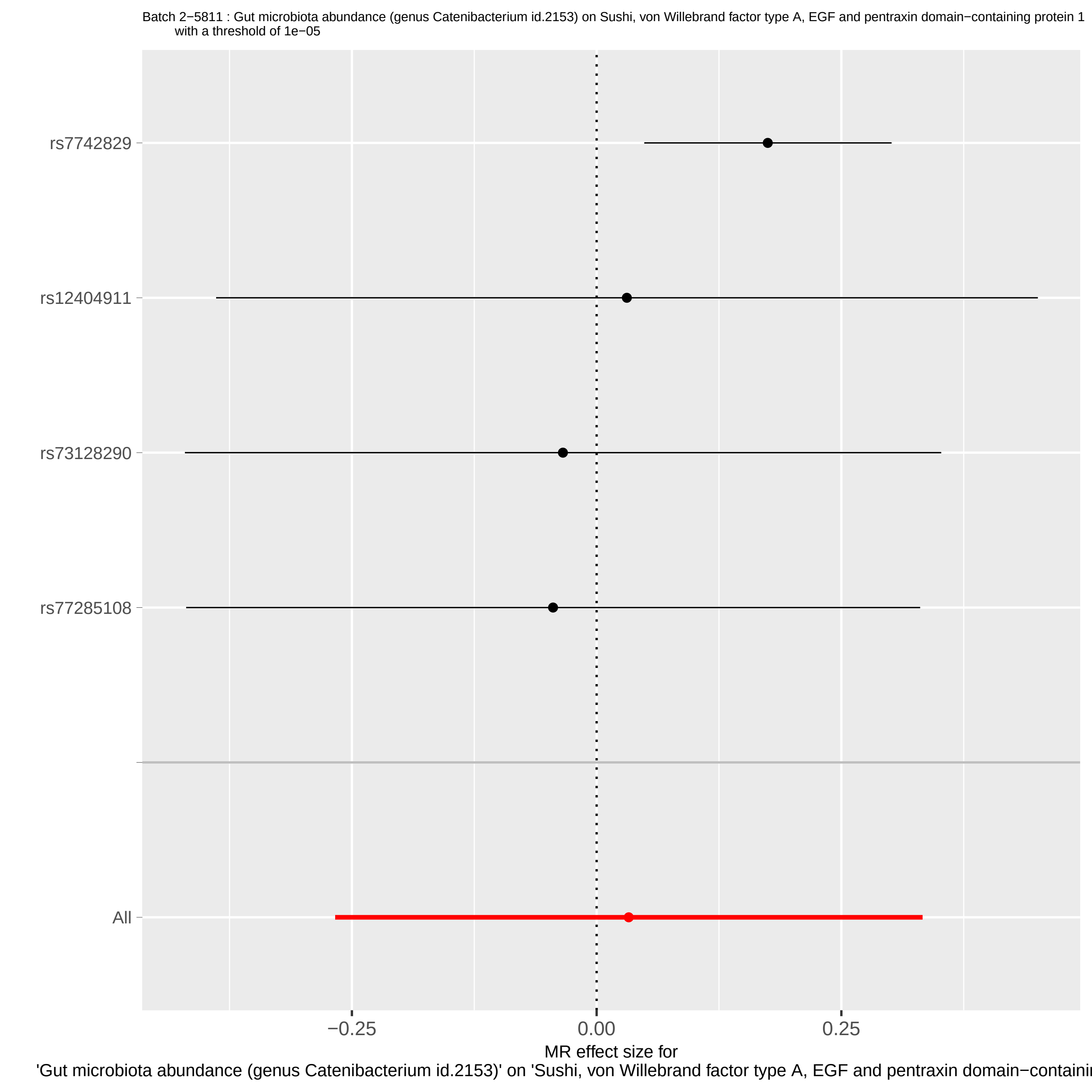

Batch 2−5811 : Gut microbiota abundance (genus Catenibacterium id.2153) on Sushi, von Willebrand factor type A, EGF and pentraxin domain−containing protein 1
with a threshold of 1e−05
rs7742829
rs12404911
rs73128290
rs77285108
All
−0.25
0.00
0.25
MR effect size for
'Gut microbiota abundance (genus Catenibacterium id.2153)' on 'Sushi, von Willebrand factor type A, EGF and pentraxin domain−containi

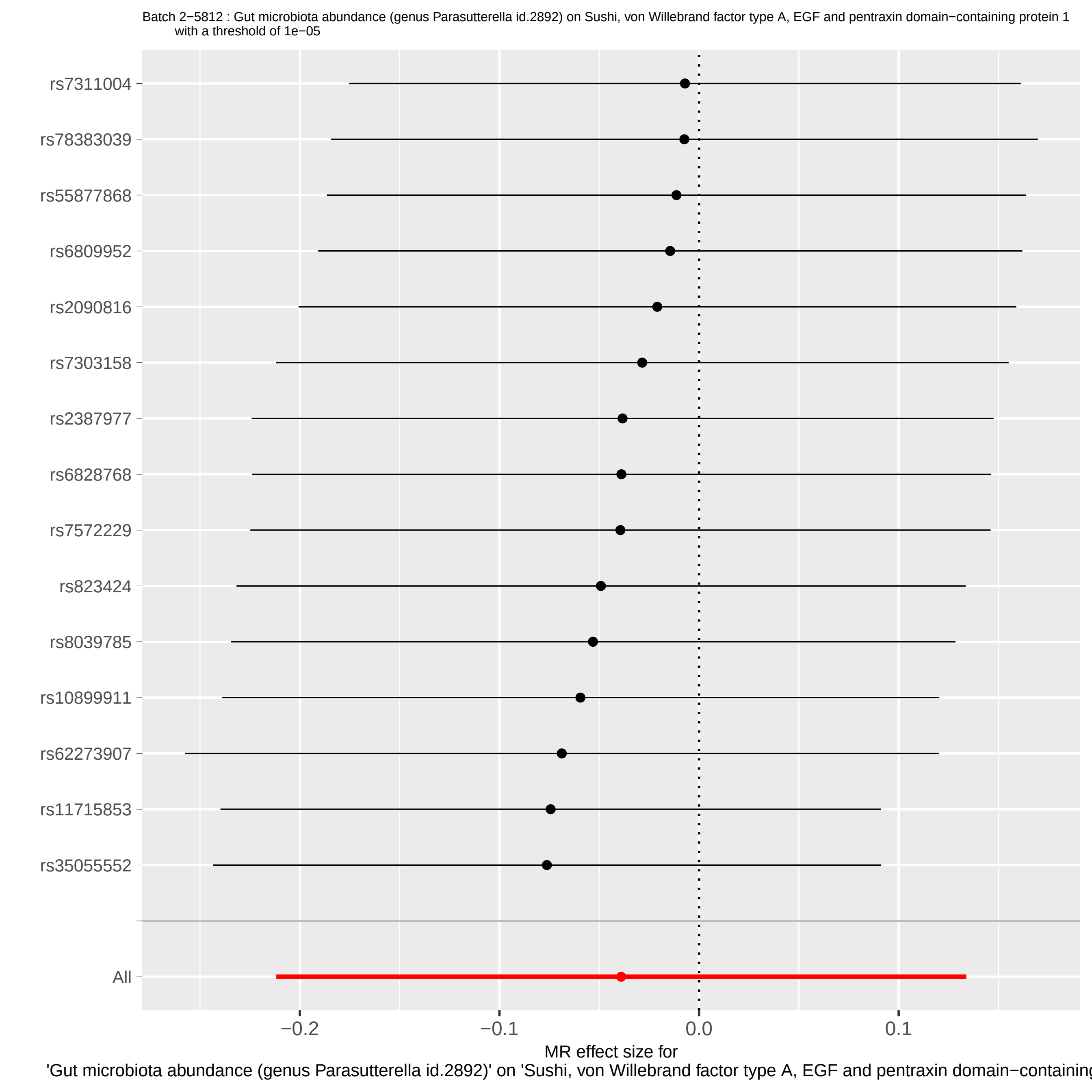
Batch 2−5812 : Gut microbiota abundance (genus Parasutterella id.2892) on Sushi, von Willebrand factor type A, EGF and pentraxin domain−containing protein 1 with a threshold of 1e−05
rs7311004
rs78383039
rs55877868
rs6809952
rs2090816
rs7303158
rs2387977
rs6828768
rs7572229
rs823424
rs8039785
rs10899911
rs62273907
rs11715853
rs35055552
All
−0.2
−0.1
0.0
0.1
MR effect size for
'Gut microbiota abundance (genus Parasutterella id.2892)' on 'Sushi, von Willebrand factor type A, EGF and pentraxin domain−containing

Batch 2−5813 : Gut microbiota abundance (genus Catenibacterium id.2153) on Sushi, von Willebrand factor type A, EGF and pentraxin domain−containing protein 1
with a threshold of 1e−05

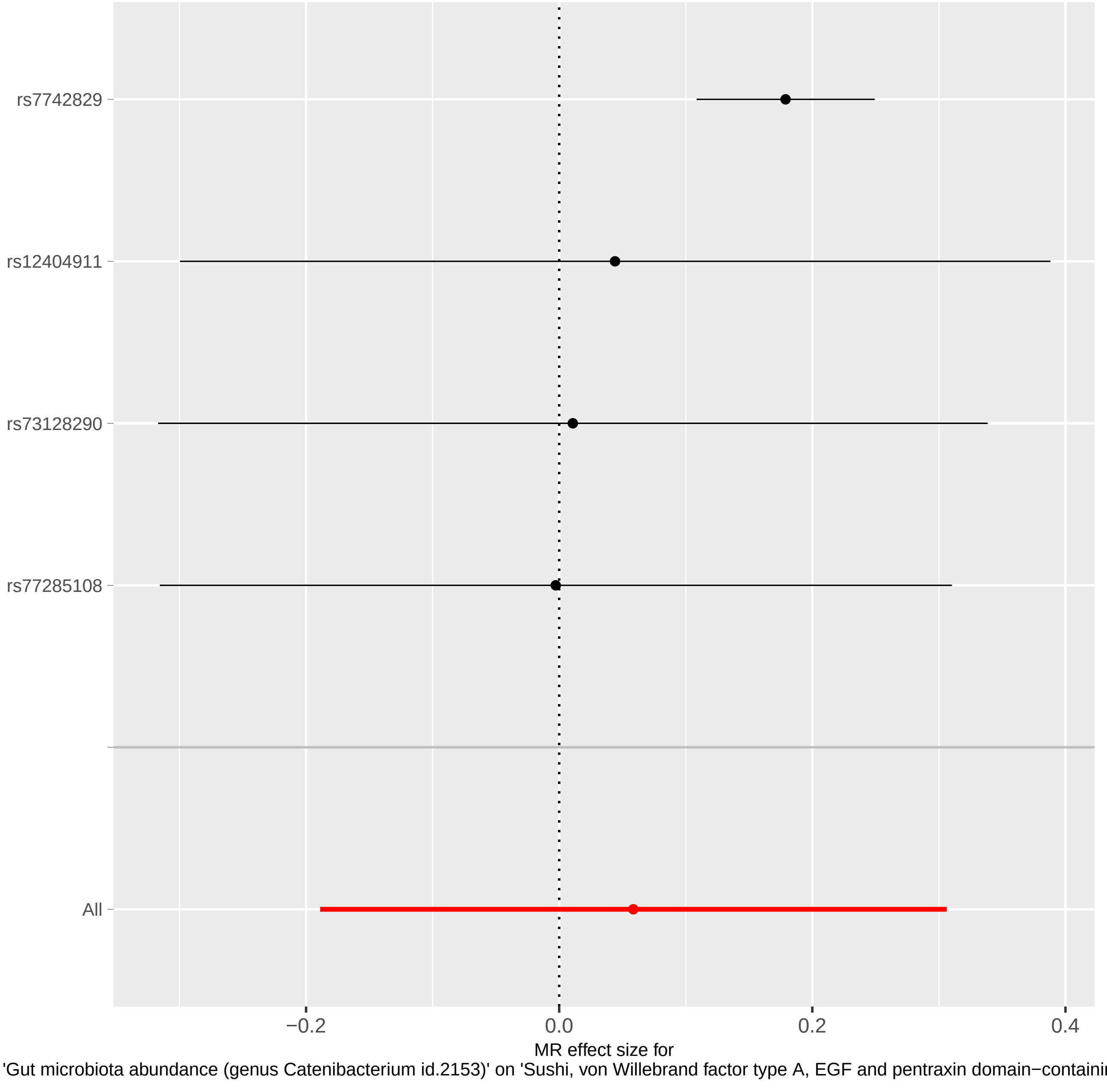

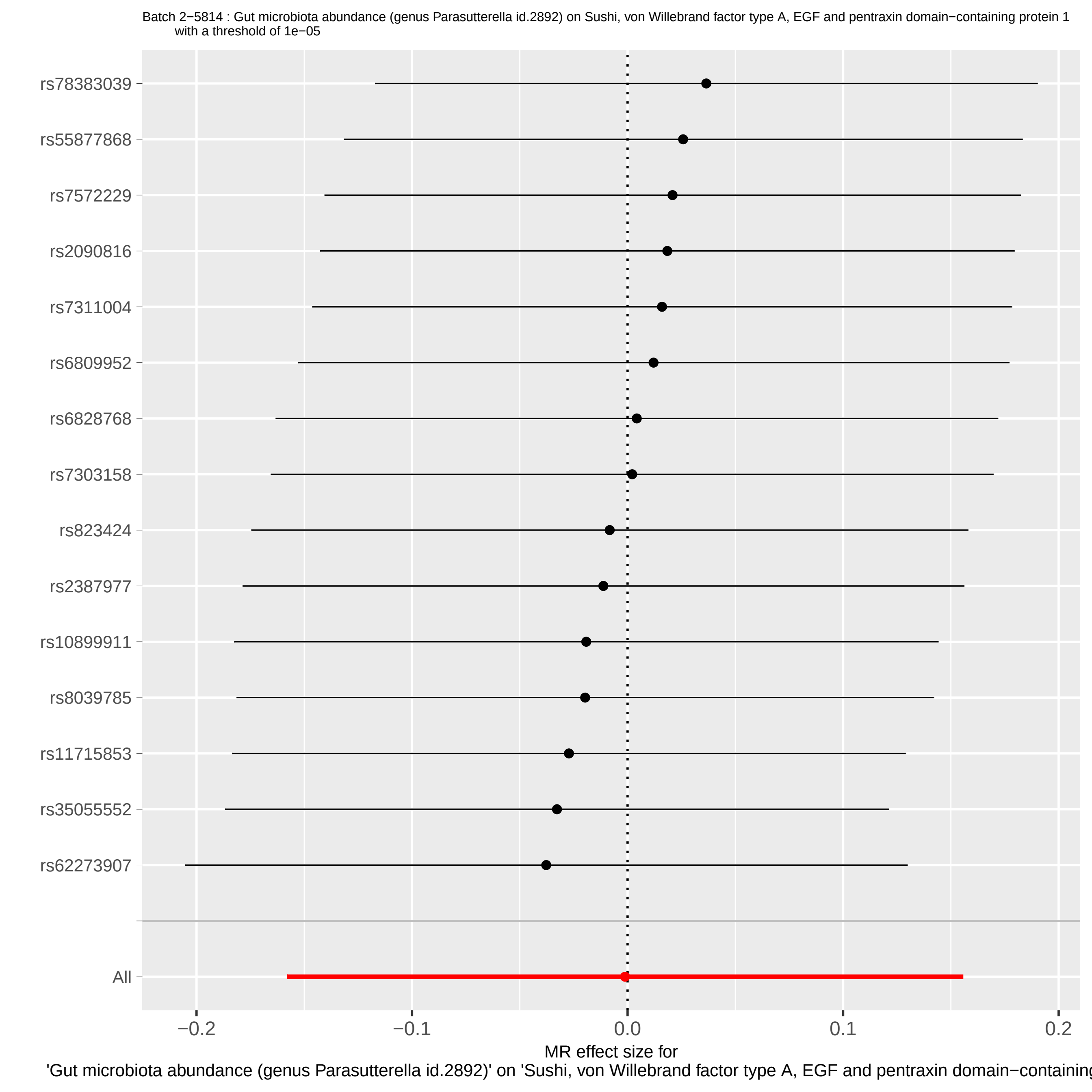
Batch 2−5814 : Gut microbiota abundance (genus Parasutterella id.2892) on Sushi, von Willebrand factor type A, EGF and pentraxin domain−containing protein 1 with a threshold of 1e−05
rs78383039
rs55877868
rs7572229
rs2090816
rs7311004
rs6809952
rs6828768
rs7303158
rs823424
rs2387977
rs10899911
rs8039785
rs11715853
rs35055552
rs62273907
All
−0.2
−0.1
0.0
0.1
0.2
MR effect size for
'Gut microbiota abundance (genus Parasutterella id.2892)' on 'Sushi, von Willebrand factor type A, EGF and pentraxin domain−containing

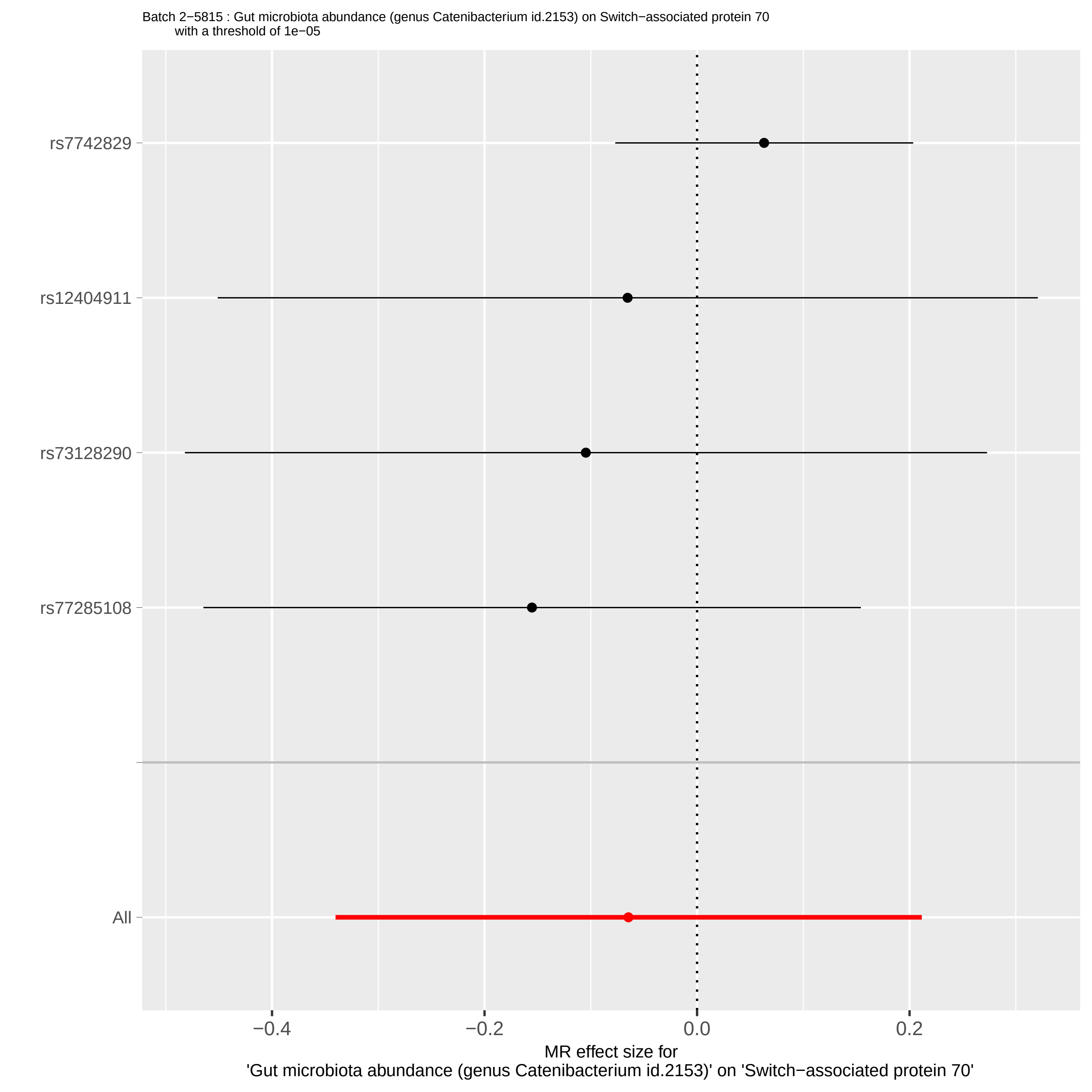
Batch 2−5815 : Gut microbiota abundance (genus Catenibacterium id.2153) on Switch−associated protein 70
with a threshold of 1e−05
rs7742829
rs12404911
rs73128290
rs77285108
All
−0.4
−0.2
0.0
0.2
MR effect size for
'Gut microbiota abundance (genus Catenibacterium id.2153)' on 'Switch−associated protein 70'

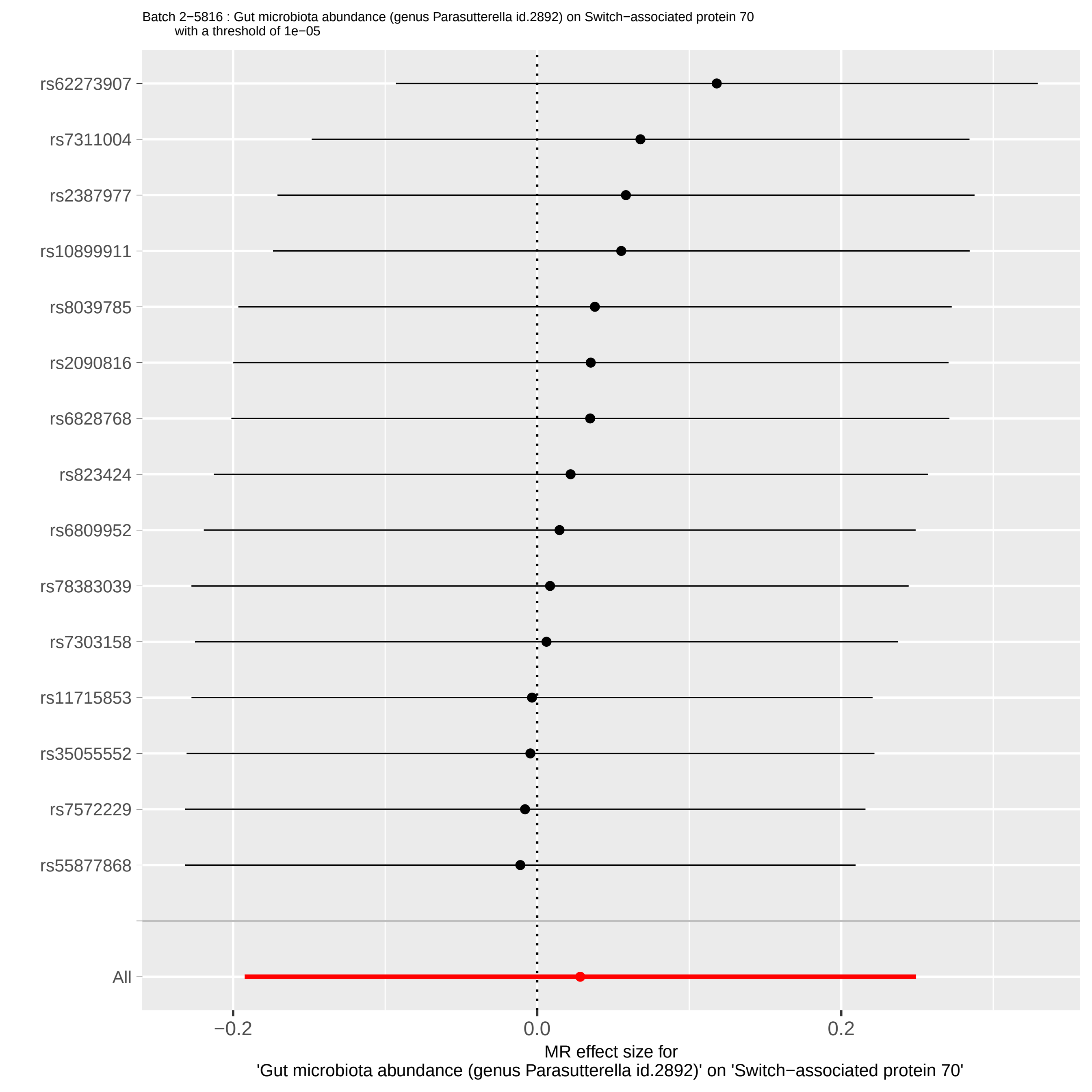

Batch 2−5816 : Gut microbiota abundance (genus Parasutterella id.2892) on Switch−associated protein 70
with a threshold of 1e−05
rs62273907
rs7311004
rs2387977
rs10899911
rs8039785
rs2090816
rs6828768
rs823424
rs6809952
rs78383039
rs7303158
rs11715853
rs35055552
rs7572229
rs55877868
All
−0.2
0.0
0.2
MR effect size for
'Gut microbiota abundance (genus Parasutterella id.2892)' on 'Switch−associated protein 70'

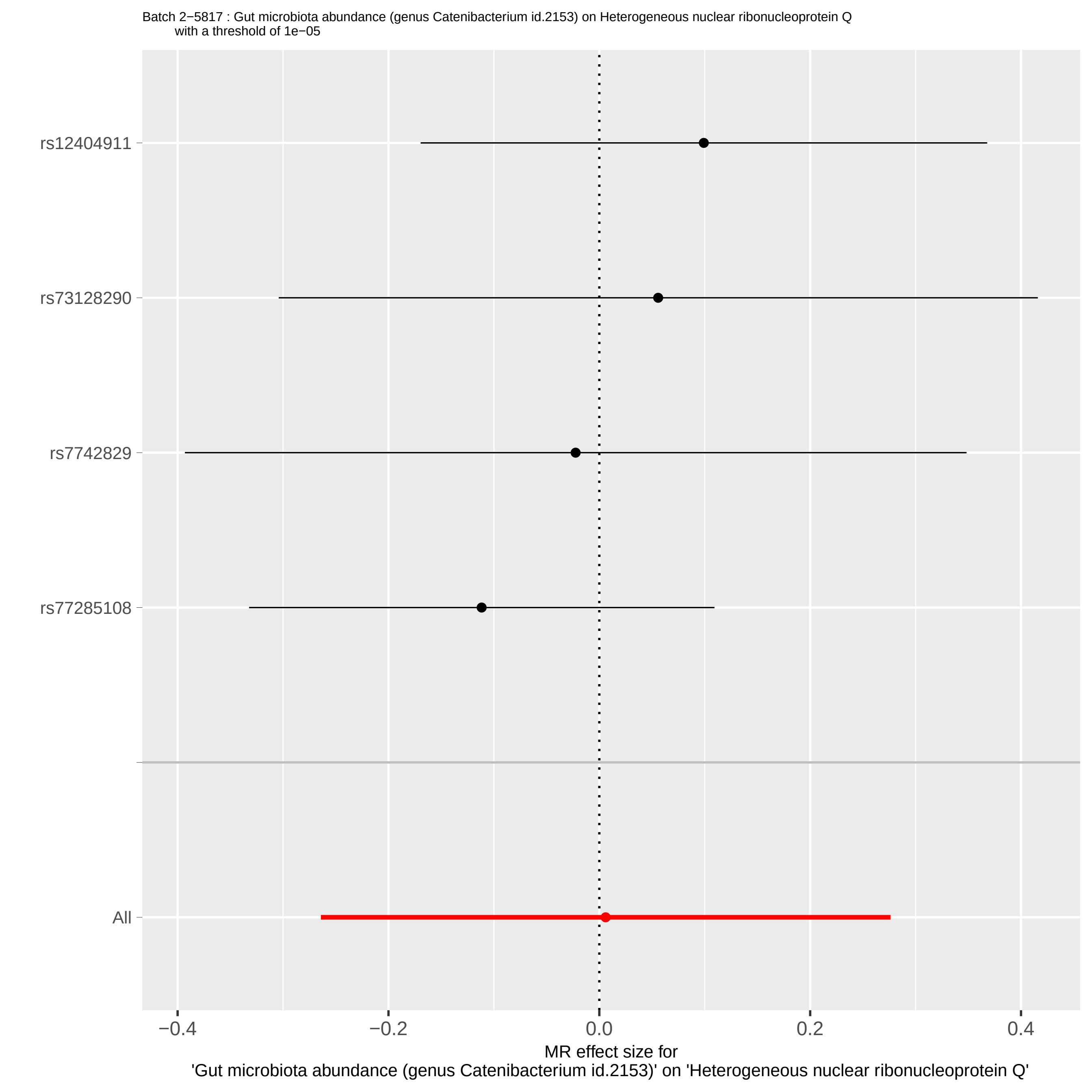
Batch 2−5817 : Gut microbiota abundance (genus Catenibacterium id.2153) on Heterogeneous nuclear ribonucleoprotein Q
with a threshold of 1e−05
rs12404911
rs73128290
rs7742829
rs77285108
All
−0.4
−0.2
0.0
0.2
0.4
MR effect size for
'Gut microbiota abundance (genus Catenibacterium id.2153)' on 'Heterogeneous nuclear ribonucleoprotein Q'

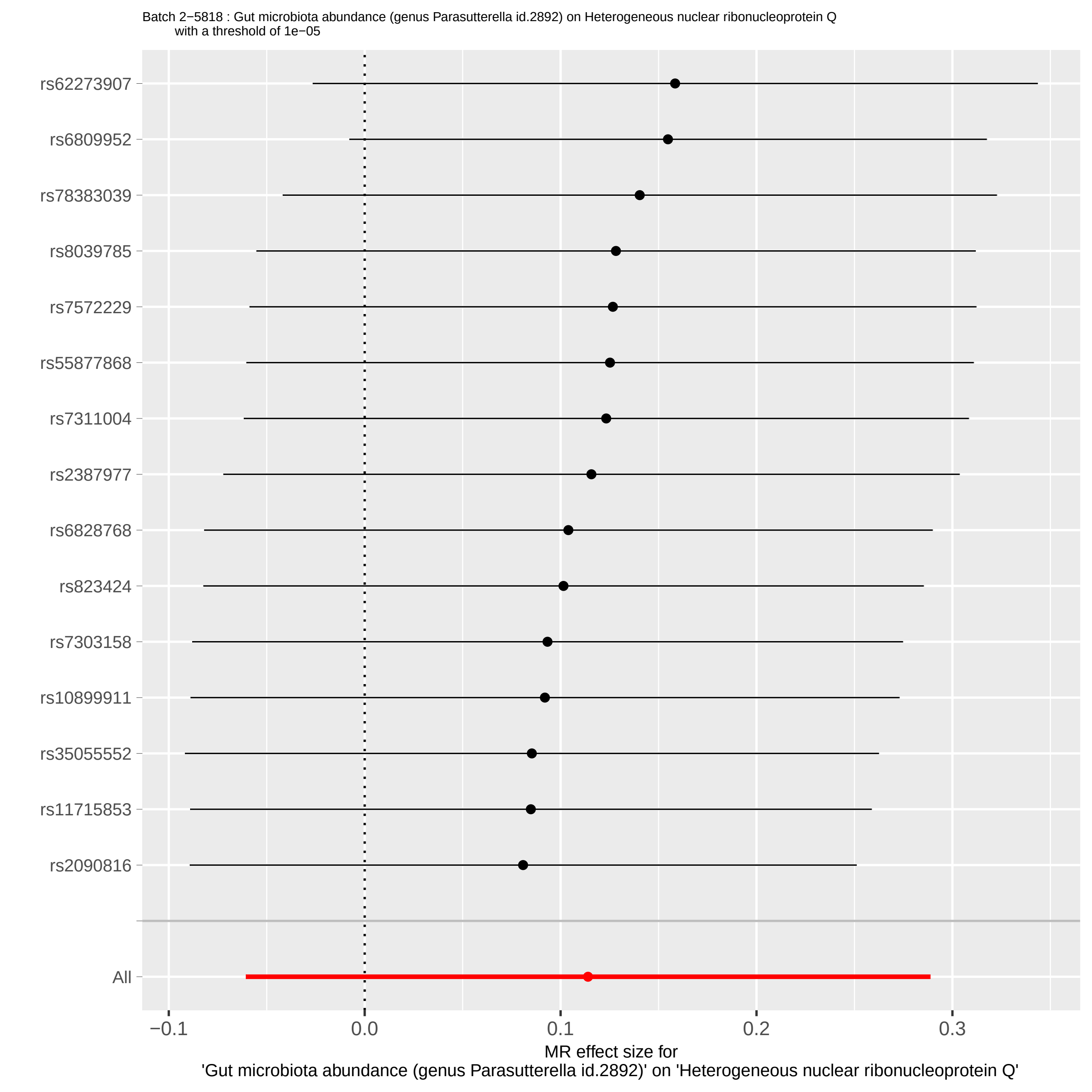

Batch 2−5818 : Gut microbiota abundance (genus Parasutterella id.2892) on Heterogeneous nuclear ribonucleoprotein Q
with a threshold of 1e−05
rs62273907
rs6809952
rs78383039
rs8039785
rs7572229
rs55877868
rs7311004
rs2387977
rs6828768
rs823424
rs7303158
rs10899911
rs35055552
rs11715853
rs2090816
All
−0.1
0.0
0.1
0.2
0.3
MR effect size for
'Gut microbiota abundance (genus Parasutterella id.2892)' on 'Heterogeneous nuclear ribonucleoprotein Q'

Batch 2−5819 : Gut microbiota abundance (genus Catenibacterium id.2153) on Nesprin−2
with a threshold of 1e−05

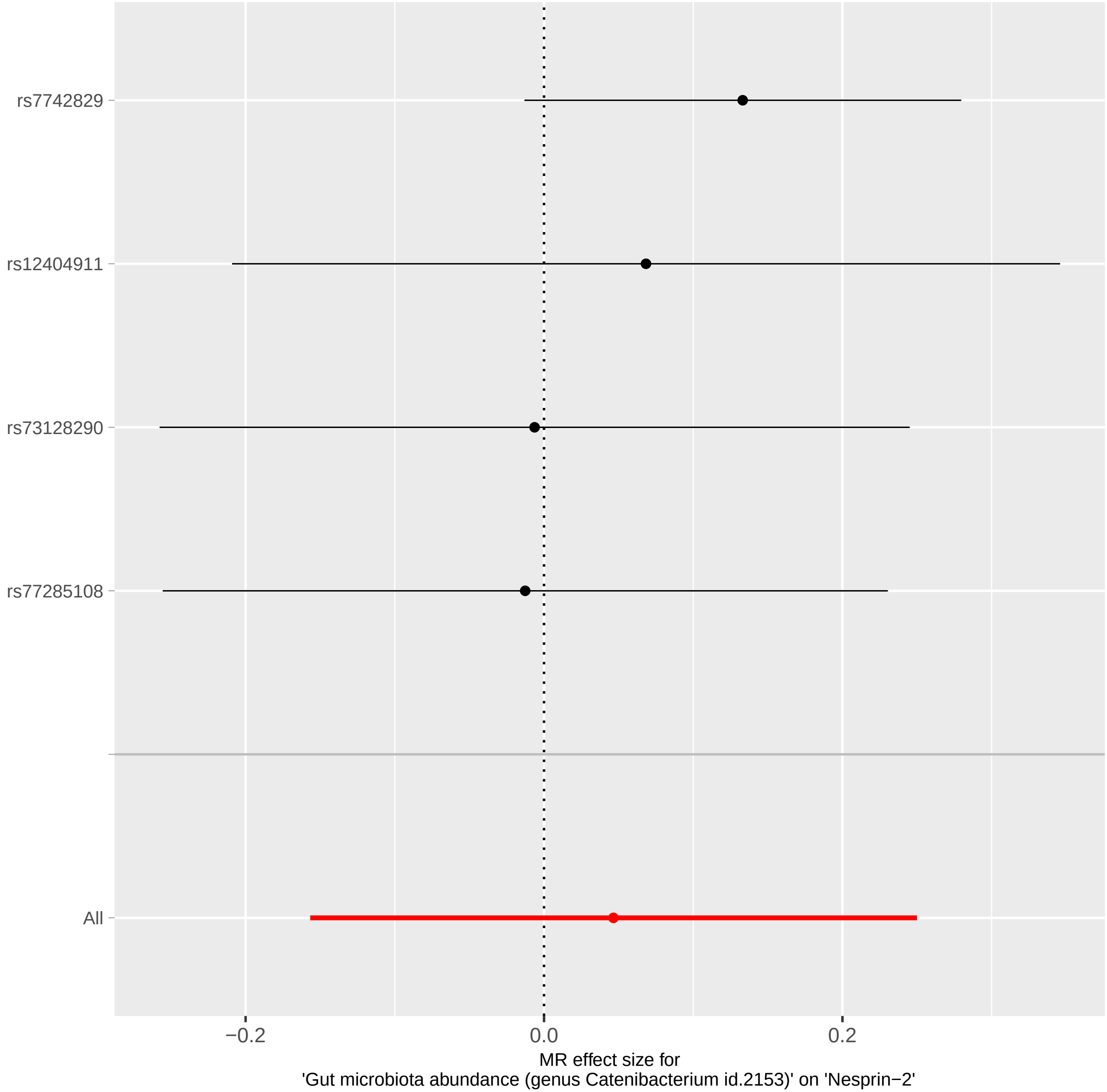

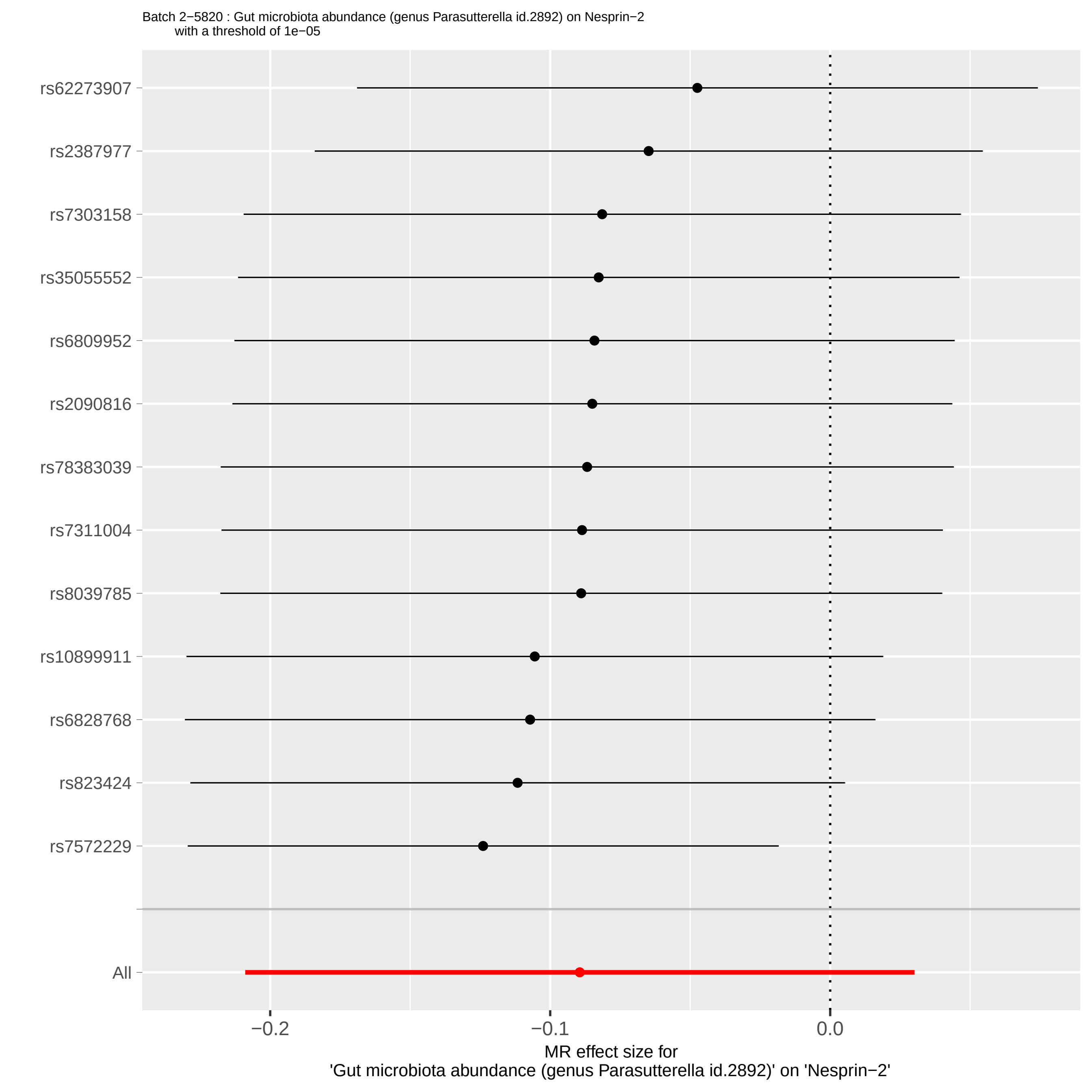

Batch 2−5820 : Gut microbiota abundance (genus Parasutterella id.2892) on Nesprin−2
with a threshold of 1e−05
rs62273907
rs2387977
rs7303158
rs35055552
rs6809952
rs2090816
rs78383039
rs7311004
rs8039785
rs10899911
rs6828768
rs823424
rs7572229
All
−0.2
−0.1
0.0
MR effect size for
'Gut microbiota abundance (genus Parasutterella id.2892)' on 'Nesprin−2'

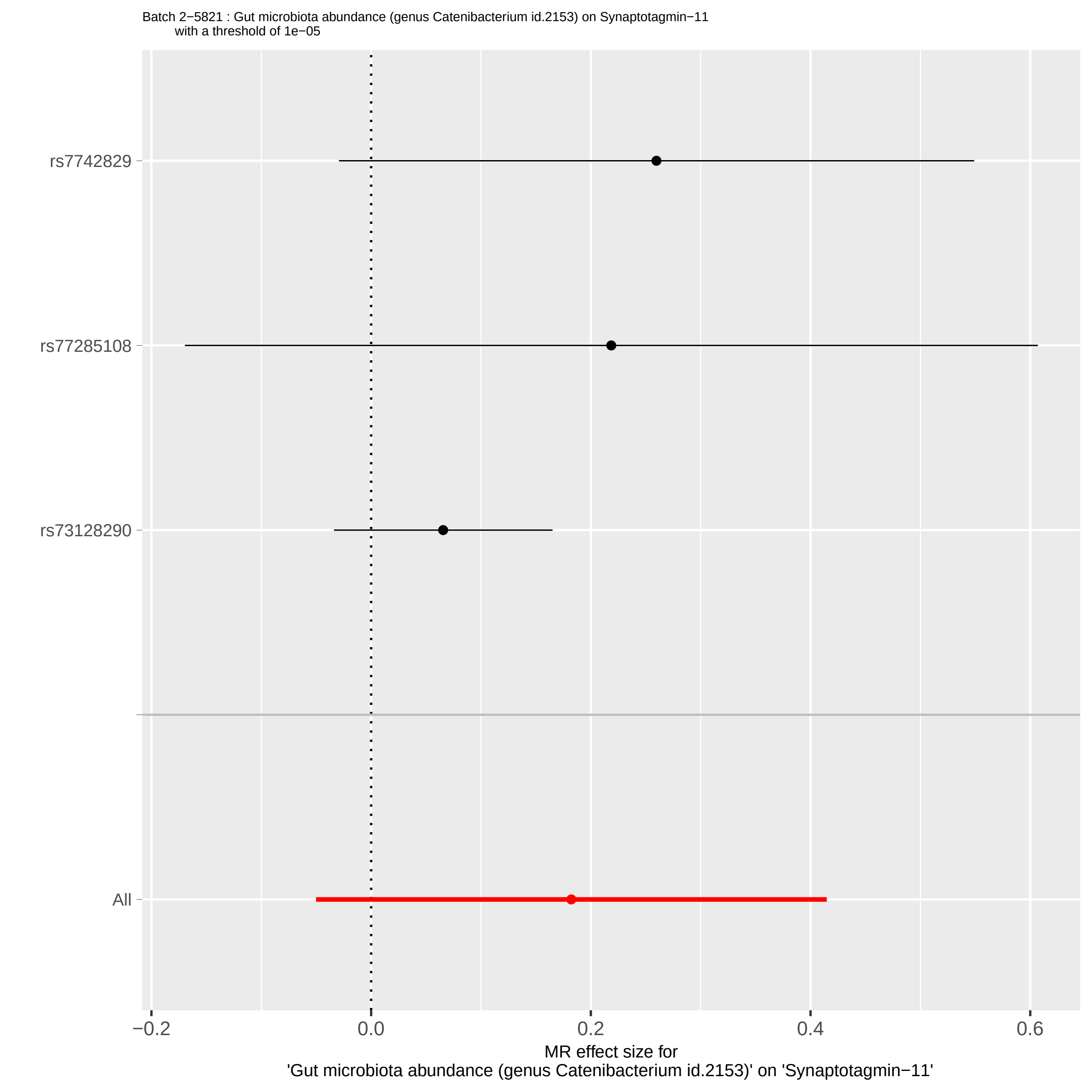
Batch 2−5821 : Gut microbiota abundance (genus Catenibacterium id.2153) on Synaptotagmin−11
with a threshold of 1e−05
rs7742829
rs77285108
rs73128290
All
−0.2
0.0
0.2
0.4
0.6
MR effect size for
'Gut microbiota abundance (genus Catenibacterium id.2153)' on 'Synaptotagmin−11'

Batch 2−5822 : Gut microbiota abundance (genus Parasutterella id.2892) on Synaptotagmin−11
with a threshold of 1e−05

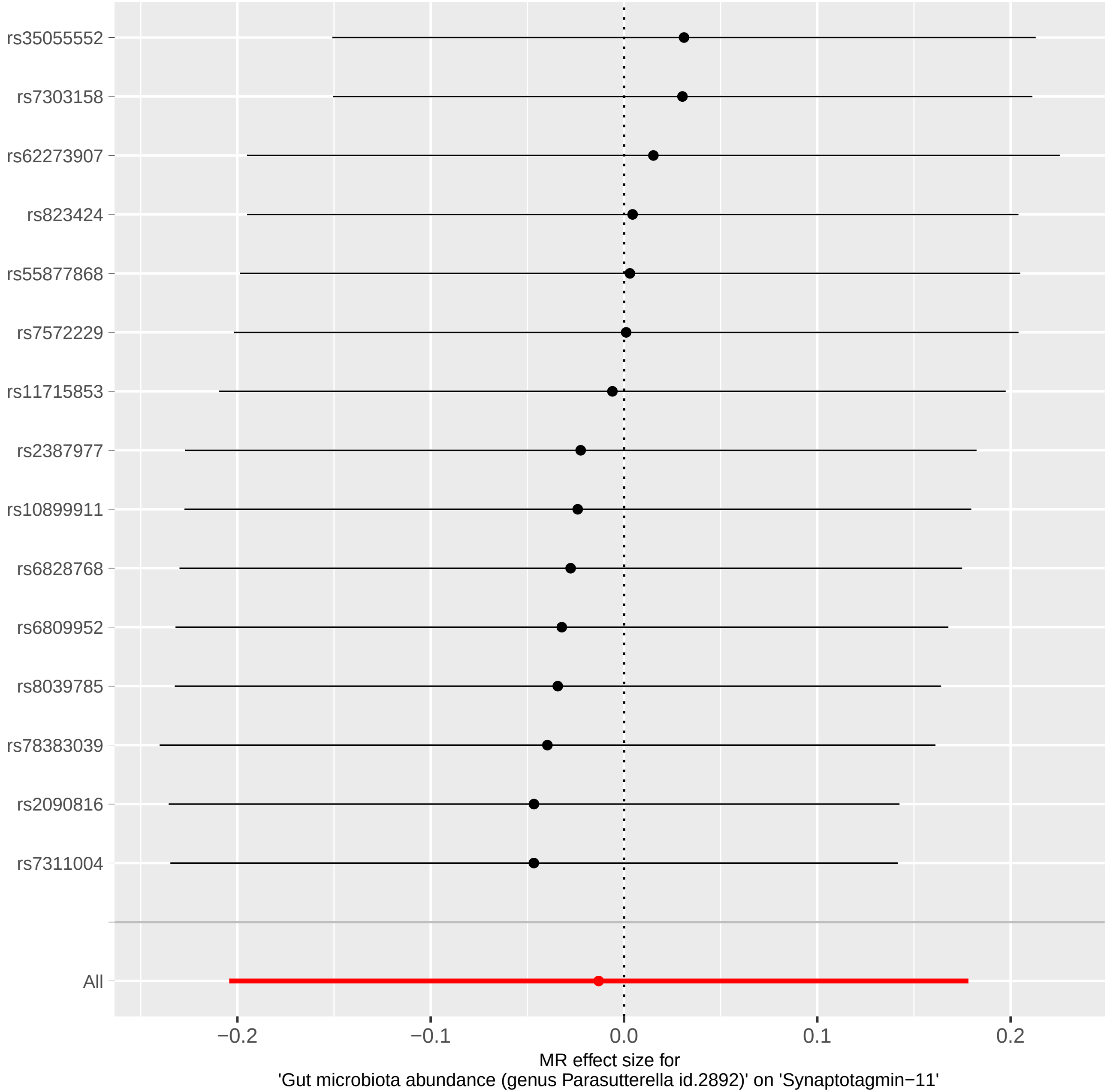

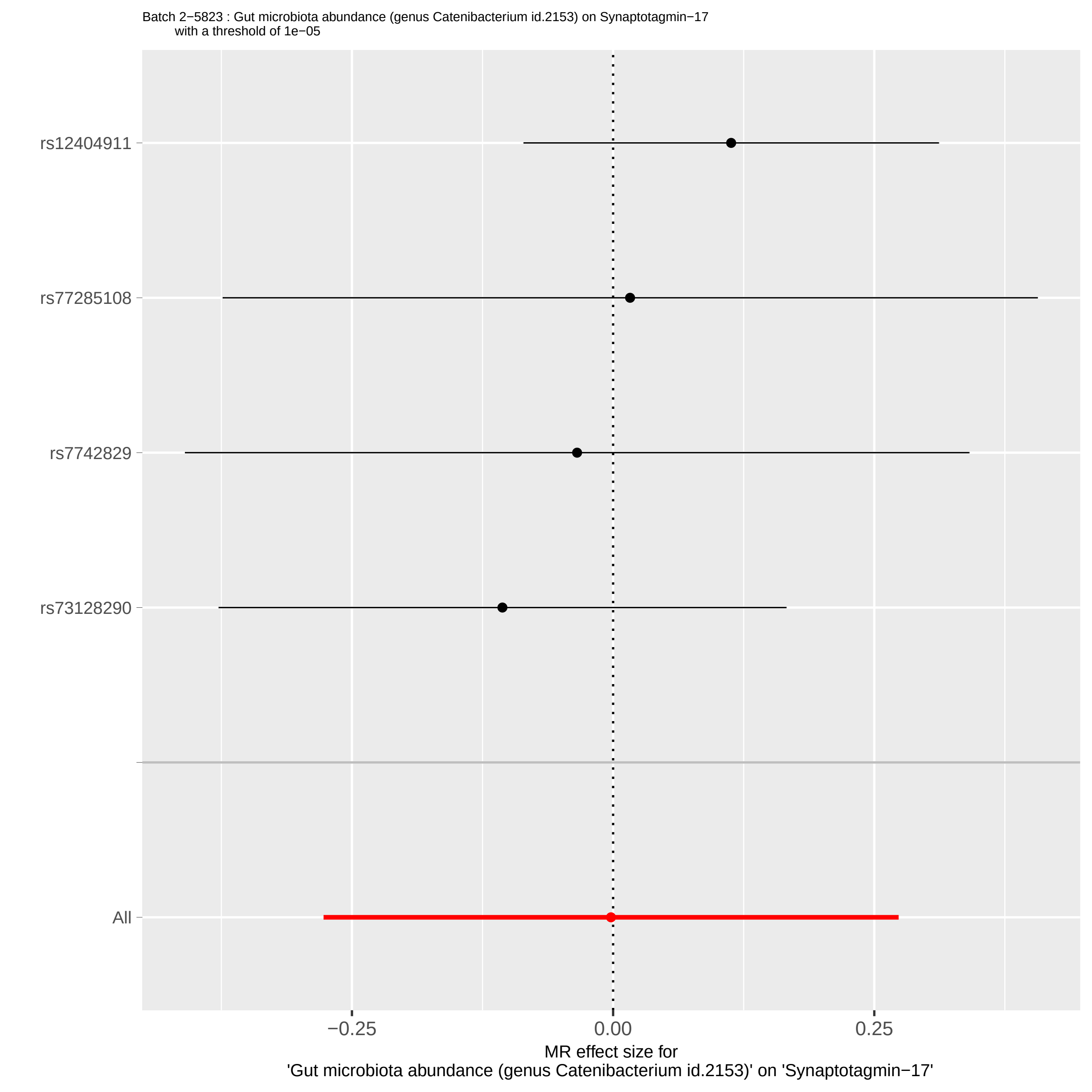
Batch 2−5823 : Gut microbiota abundance (genus Catenibacterium id.2153) on Synaptotagmin−17
with a threshold of 1e−05
rs12404911
rs77285108
rs7742829
rs73128290
All
−0.25
0.00
0.25
MR effect size for
'Gut microbiota abundance (genus Catenibacterium id.2153)' on 'Synaptotagmin−17'

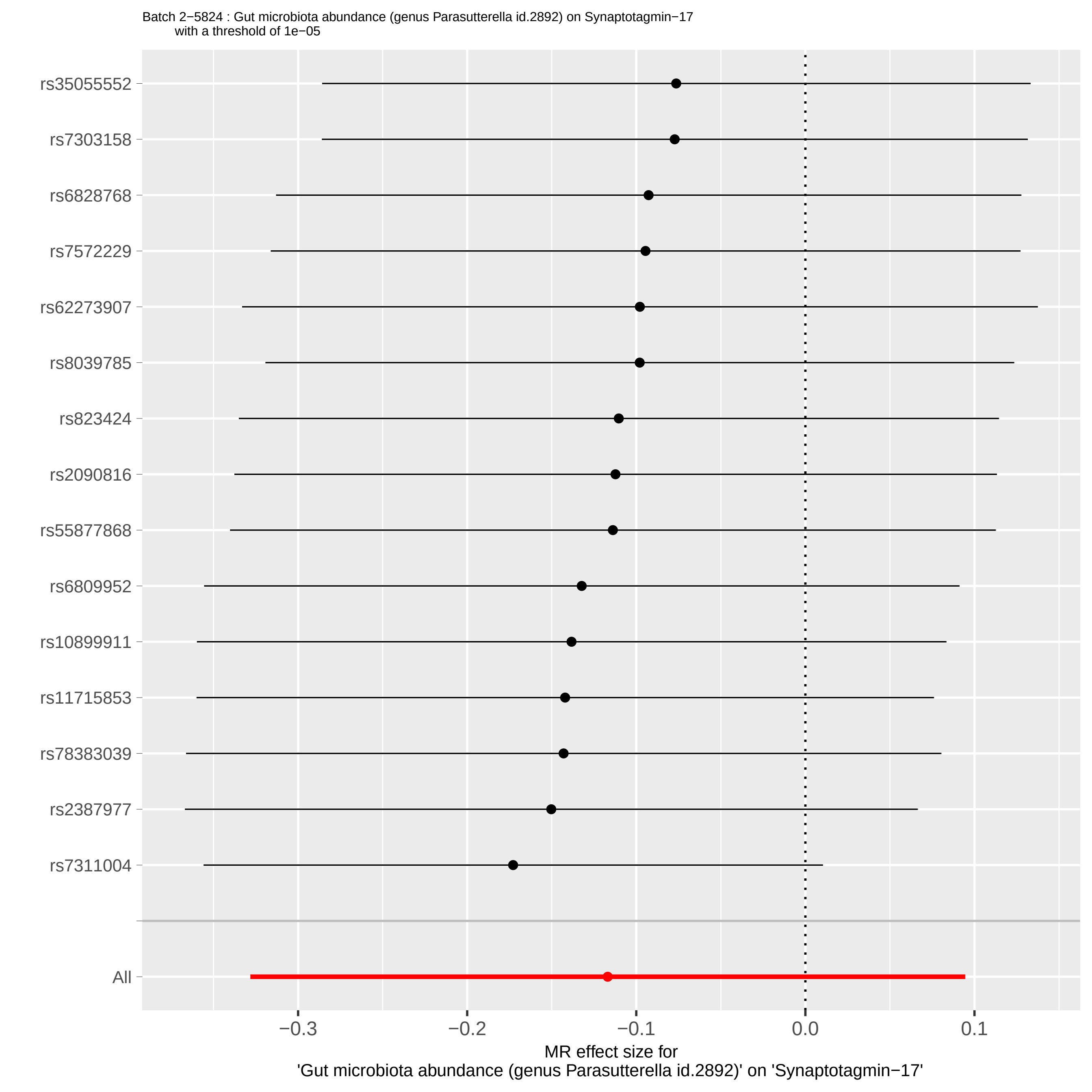

Batch 2−5824 : Gut microbiota abundance (genus Parasutterella id.2892) on Synaptotagmin−17
with a threshold of 1e−05
rs35055552
rs7303158
rs6828768
rs7572229
rs62273907
rs8039785
rs823424
rs2090816
rs55877868
rs6809952
rs10899911
rs11715853
rs78383039
rs2387977
rs7311004
All
−0.3
−0.2
−0.1
0.0
0.1
MR effect size for
'Gut microbiota abundance (genus Parasutterella id.2892)' on 'Synaptotagmin−17'

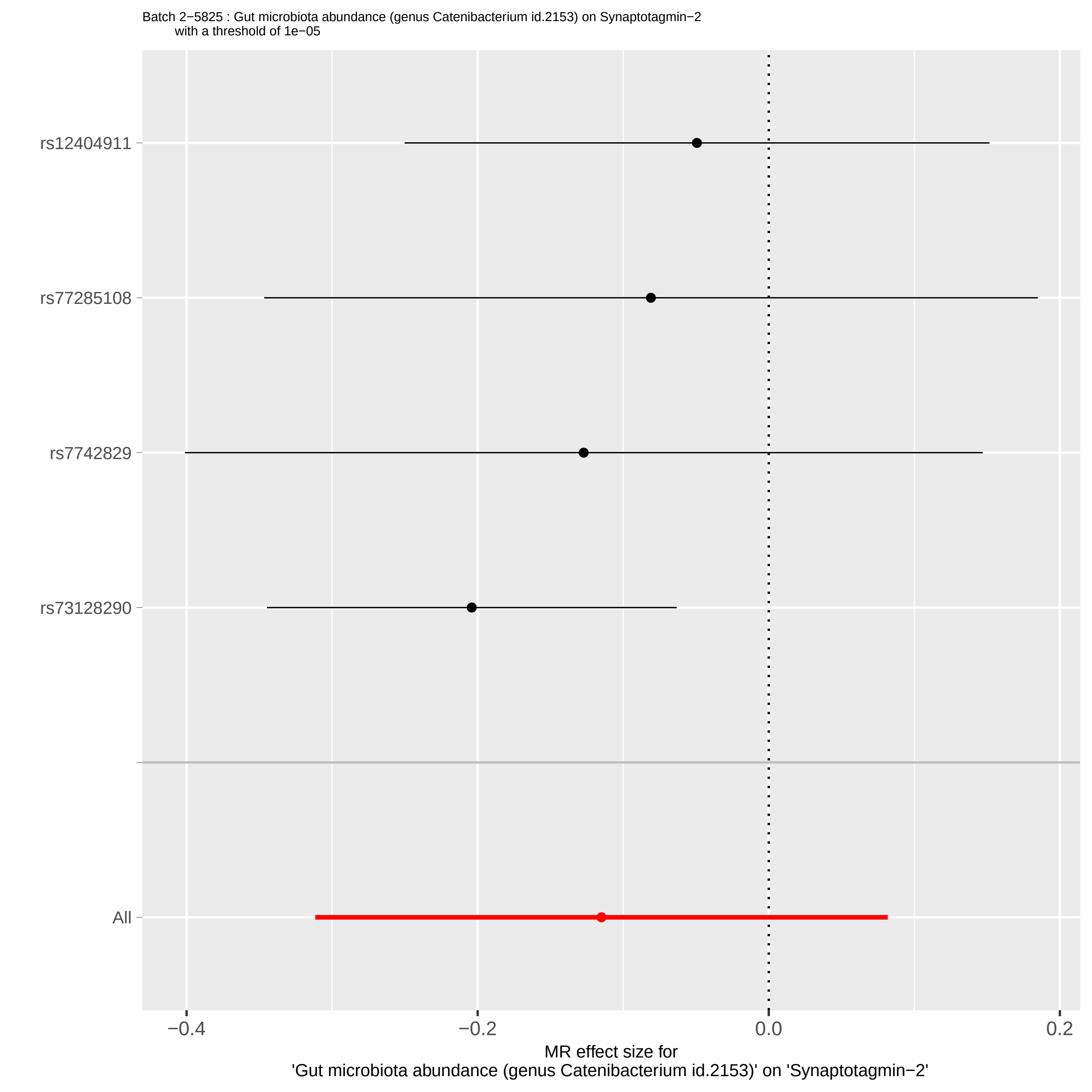

Batch 2−5825 : Gut microbiota abundance (genus Catenibacterium id.2153) on Synaptotagmin−2
with a threshold of 1e−05
rs12404911
rs77285108
rs7742829
rs73128290
All
−0.4
−0.2
0.0
0.2
MR effect size for
'Gut microbiota abundance (genus Catenibacterium id.2153)' on 'Synaptotagmin−2'

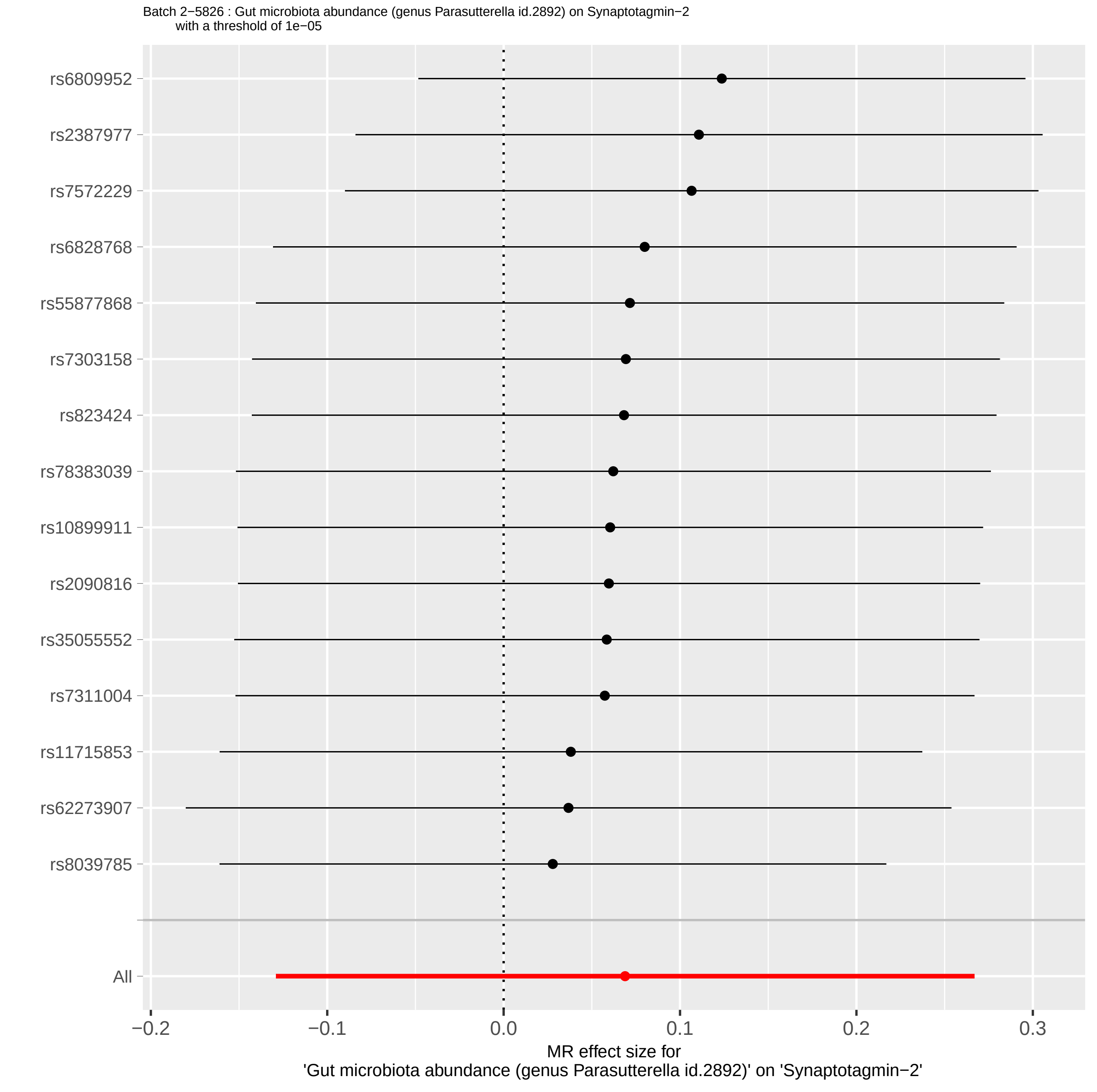
Batch 2−5826 : Gut microbiota abundance (genus Parasutterella id.2892) on Synaptotagmin−2
with a threshold of 1e−05
rs6809952
rs2387977
rs7572229
rs6828768
rs55877868
rs7303158
rs823424
rs78383039
rs10899911
rs2090816
rs35055552
rs7311004
rs11715853
rs62273907
rs8039785
All
−0.2
−0.1
0.0
0.1
0.2
0.3
MR effect size for
'Gut microbiota abundance (genus Parasutterella id.2892)' on 'Synaptotagmin−2'

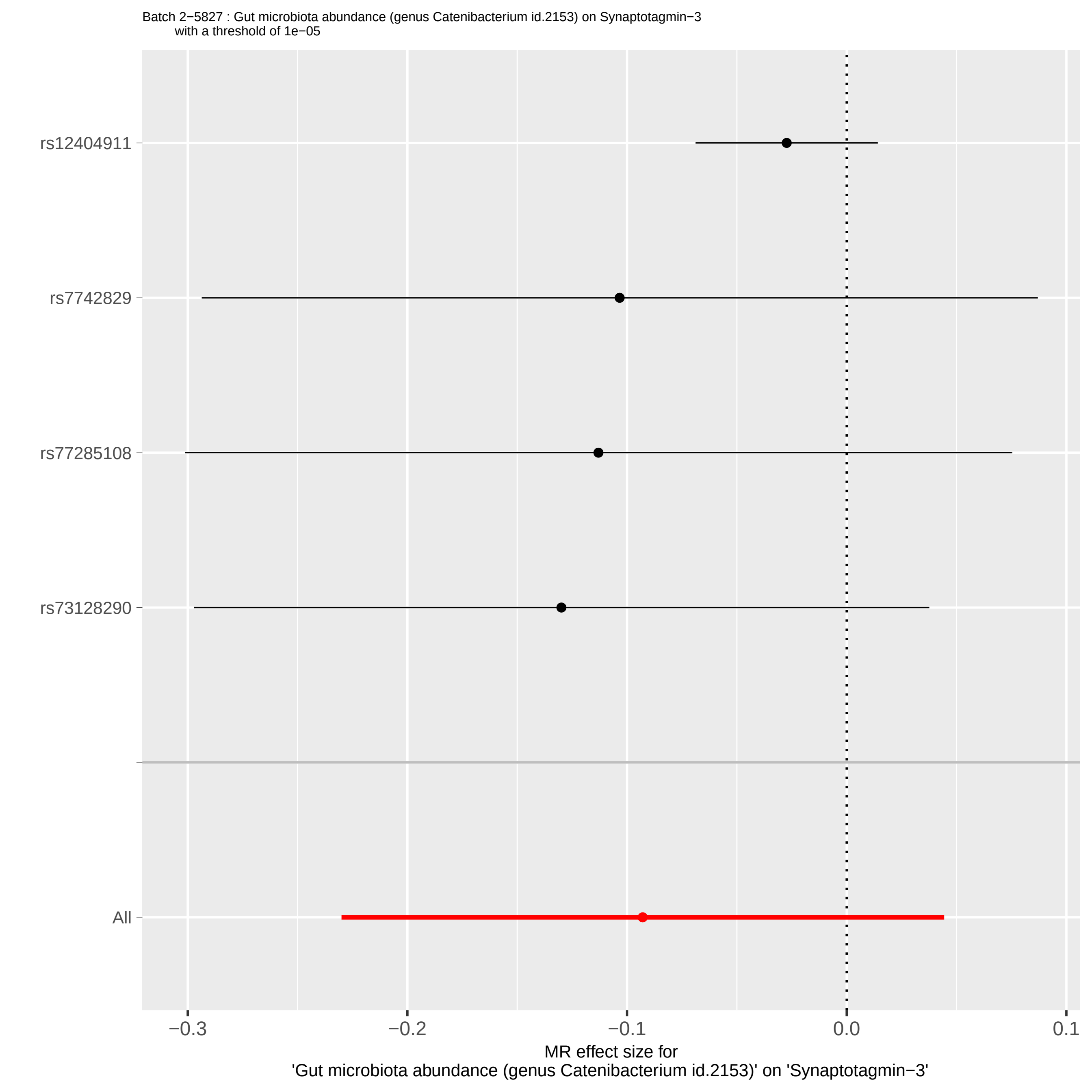
Batch 2−5827 : Gut microbiota abundance (genus Catenibacterium id.2153) on Synaptotagmin−3
with a threshold of 1e−05
rs12404911
rs7742829
rs77285108
rs73128290
All
−0.3
−0.2
−0.1
0.0
0.1
MR effect size for
'Gut microbiota abundance (genus Catenibacterium id.2153)' on 'Synaptotagmin−3'

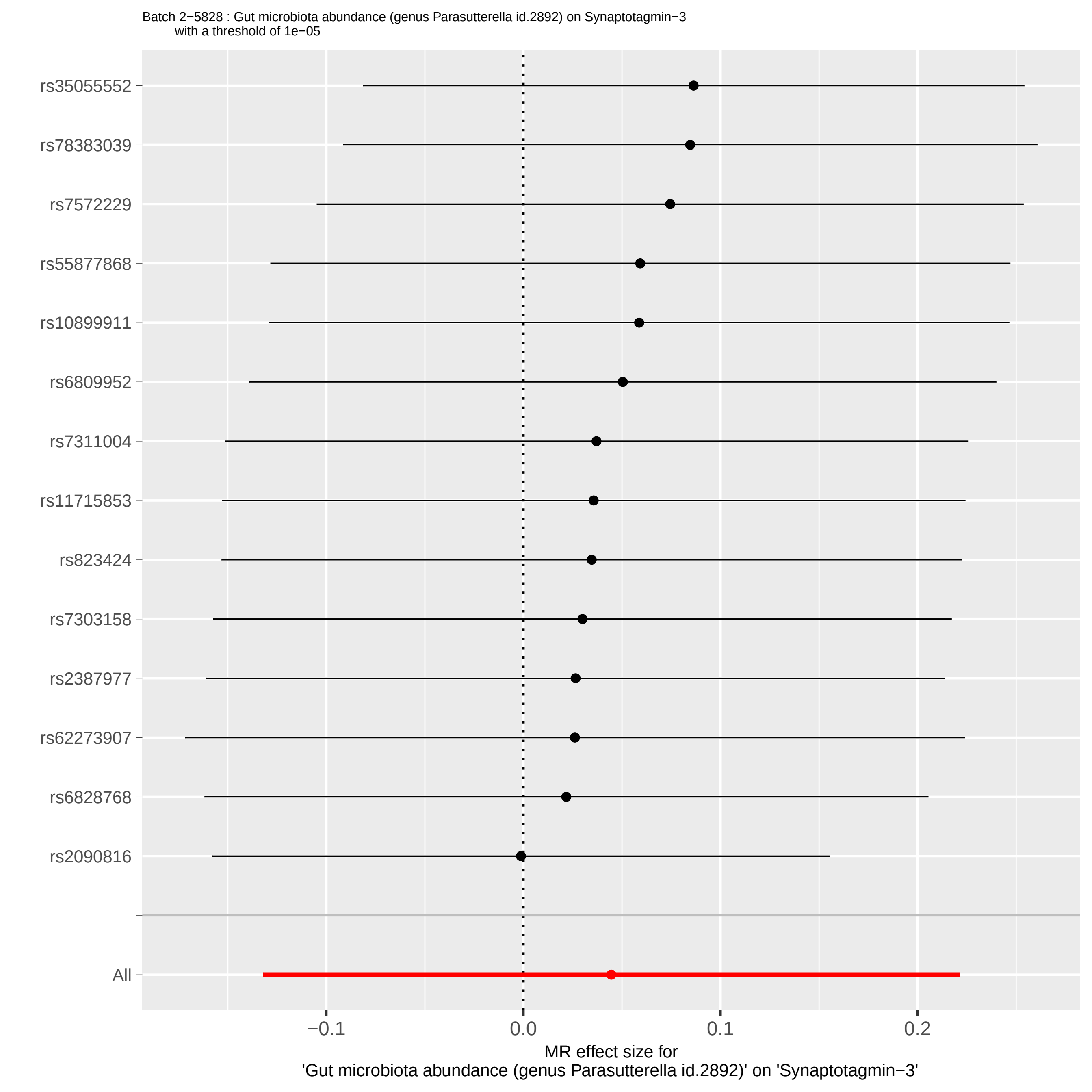

Batch 2−5828 : Gut microbiota abundance (genus Parasutterella id.2892) on Synaptotagmin−3
with a threshold of 1e−05
rs35055552
rs78383039
rs7572229
rs55877868
rs10899911
rs6809952
rs7311004
rs11715853
rs823424
rs7303158
rs2387977
rs62273907
rs6828768
rs2090816
All
−0.1
0.0
0.1
0.2
MR effect size for
'Gut microbiota abundance (genus Parasutterella id.2892)' on 'Synaptotagmin−3'

Batch 2−5829 : Gut microbiota abundance (genus Catenibacterium id.2153) on Synaptotagmin−5
with a threshold of 1e−05

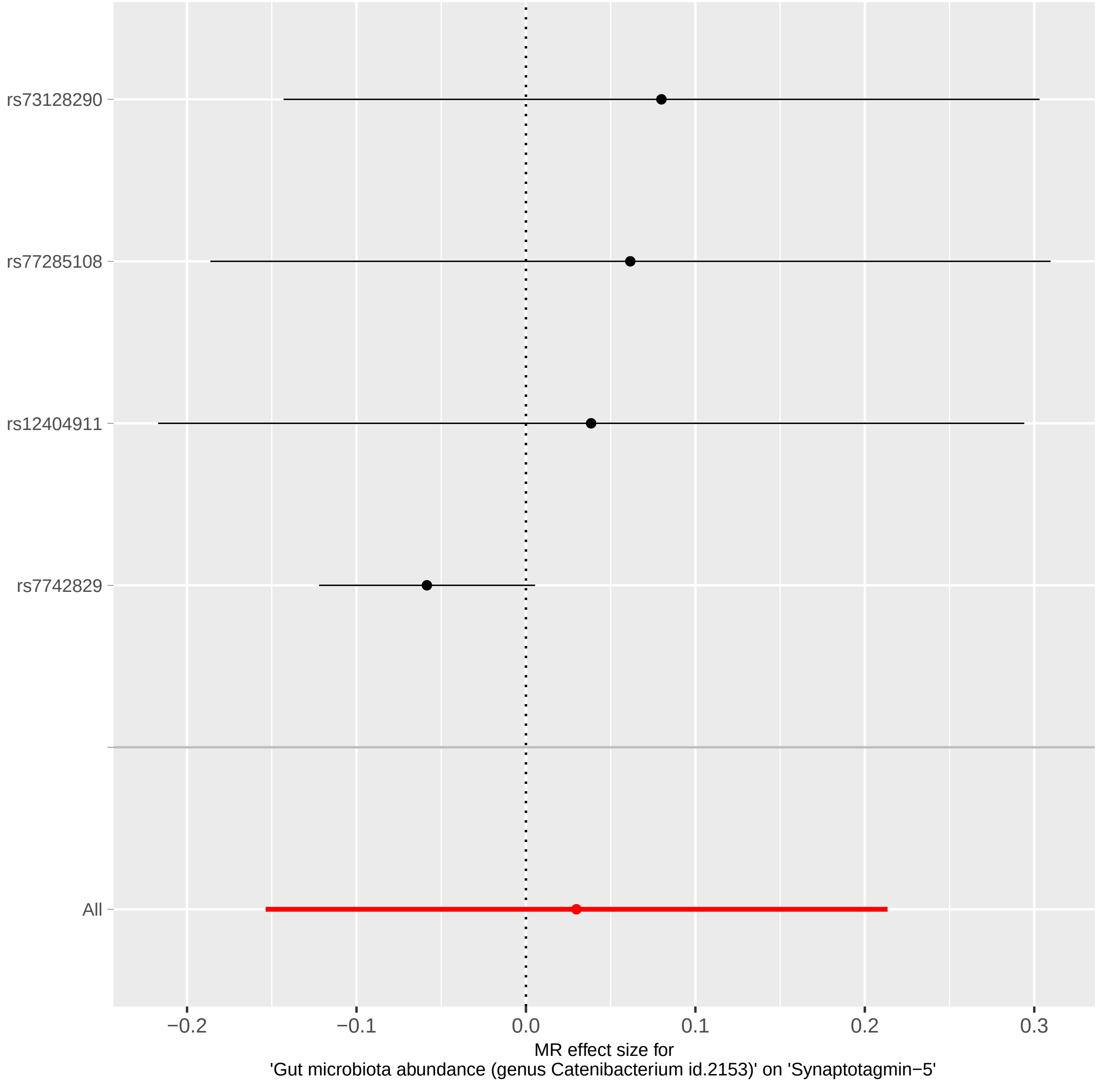

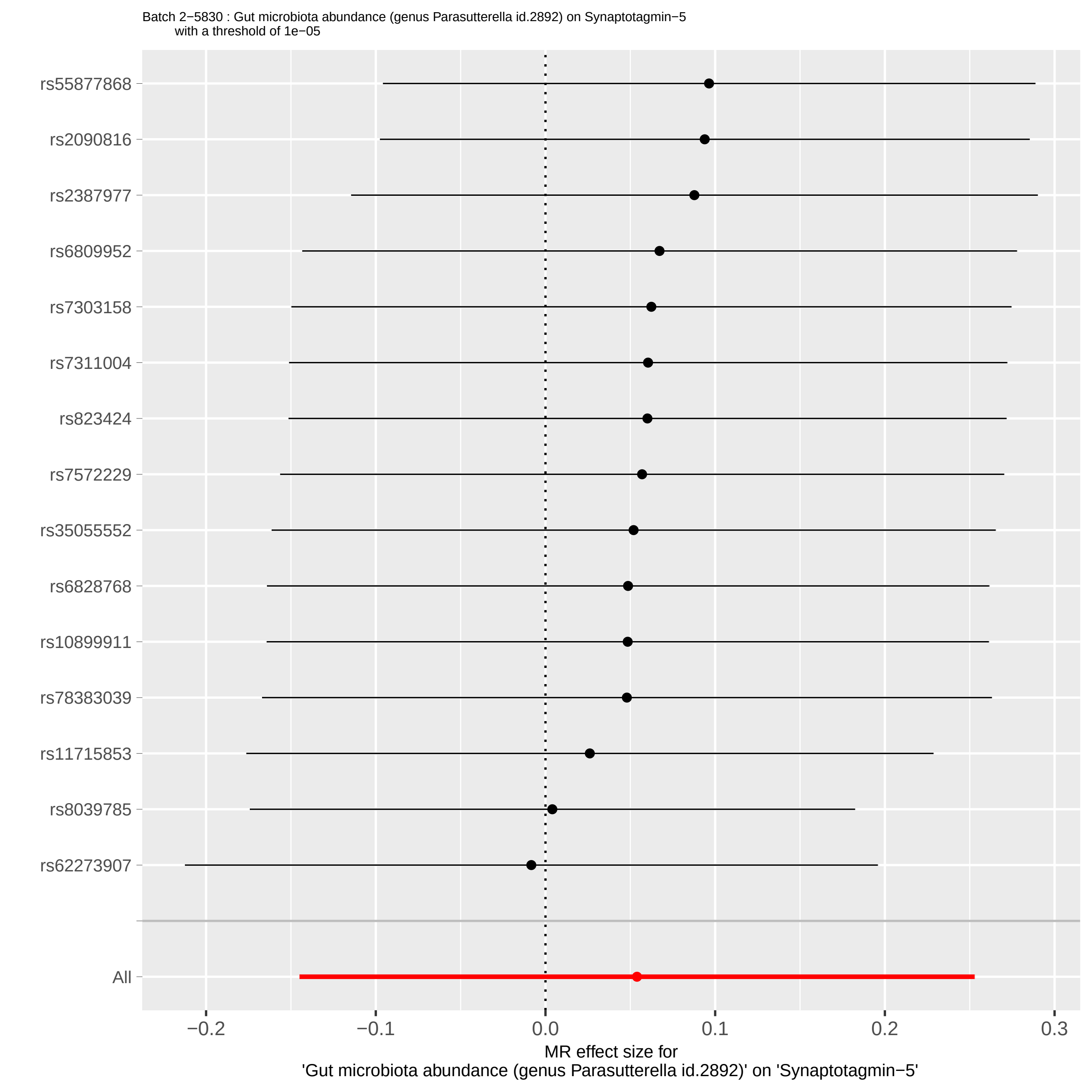

Batch 2−5830 : Gut microbiota abundance (genus Parasutterella id.2892) on Synaptotagmin−5
with a threshold of 1e−05
rs55877868
rs2090816
rs2387977
rs6809952
rs7303158
rs7311004
rs823424
rs7572229
rs35055552
rs6828768
rs10899911
rs78383039
rs11715853
rs8039785
rs62273907
All
−0.2
−0.1
0.0
0.1
0.2
0.3
MR effect size for
'Gut microbiota abundance (genus Parasutterella id.2892)' on 'Synaptotagmin−5'

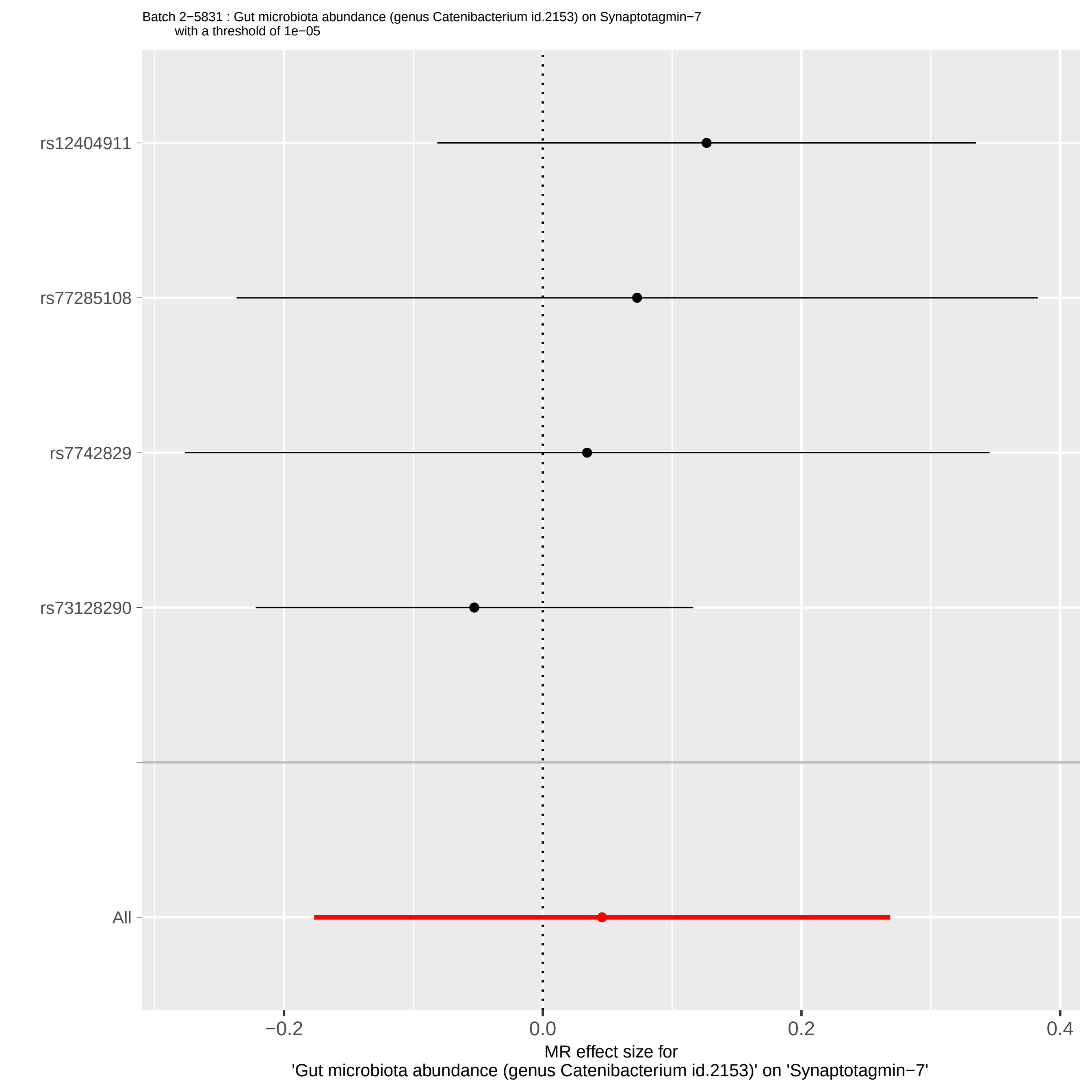
Batch 2−5831 : Gut microbiota abundance (genus Catenibacterium id.2153) on Synaptotagmin−7
with a threshold of 1e−05
rs12404911
rs77285108
rs7742829
rs73128290
All
−0.2
0.0
0.2
0.4
MR effect size for
'Gut microbiota abundance (genus Catenibacterium id.2153)' on 'Synaptotagmin−7'

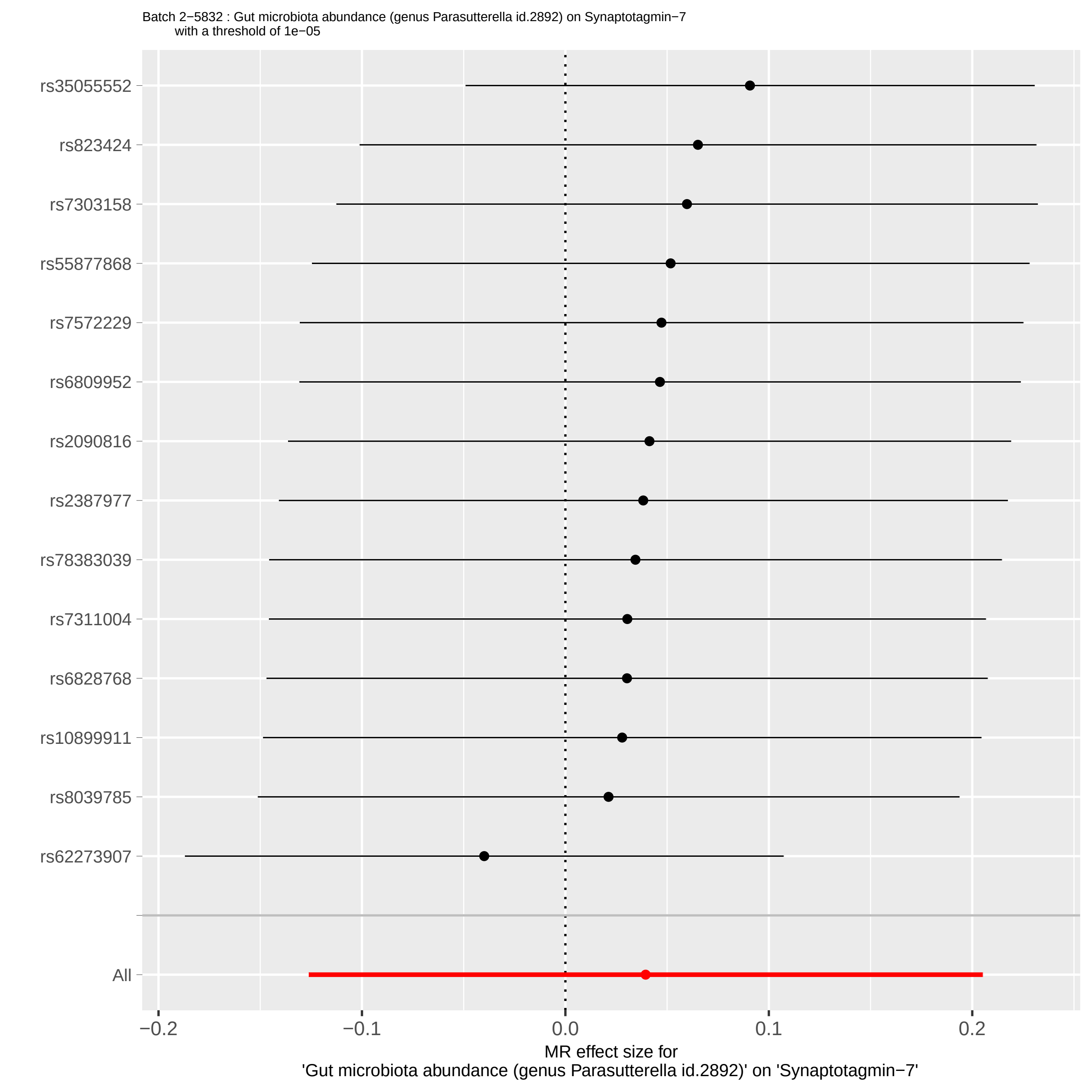
Batch 2−5832 : Gut microbiota abundance (genus Parasutterella id.2892) on Synaptotagmin−7
with a threshold of 1e−05
rs35055552
rs823424
rs7303158
rs55877868
rs7572229
rs6809952
rs2090816
rs2387977
rs78383039
rs7311004
rs6828768
rs10899911
rs8039785
rs62273907
All
−0.2
−0.1
0.0
0.1
0.2
MR effect size for
'Gut microbiota abundance (genus Parasutterella id.2892)' on 'Synaptotagmin−7'

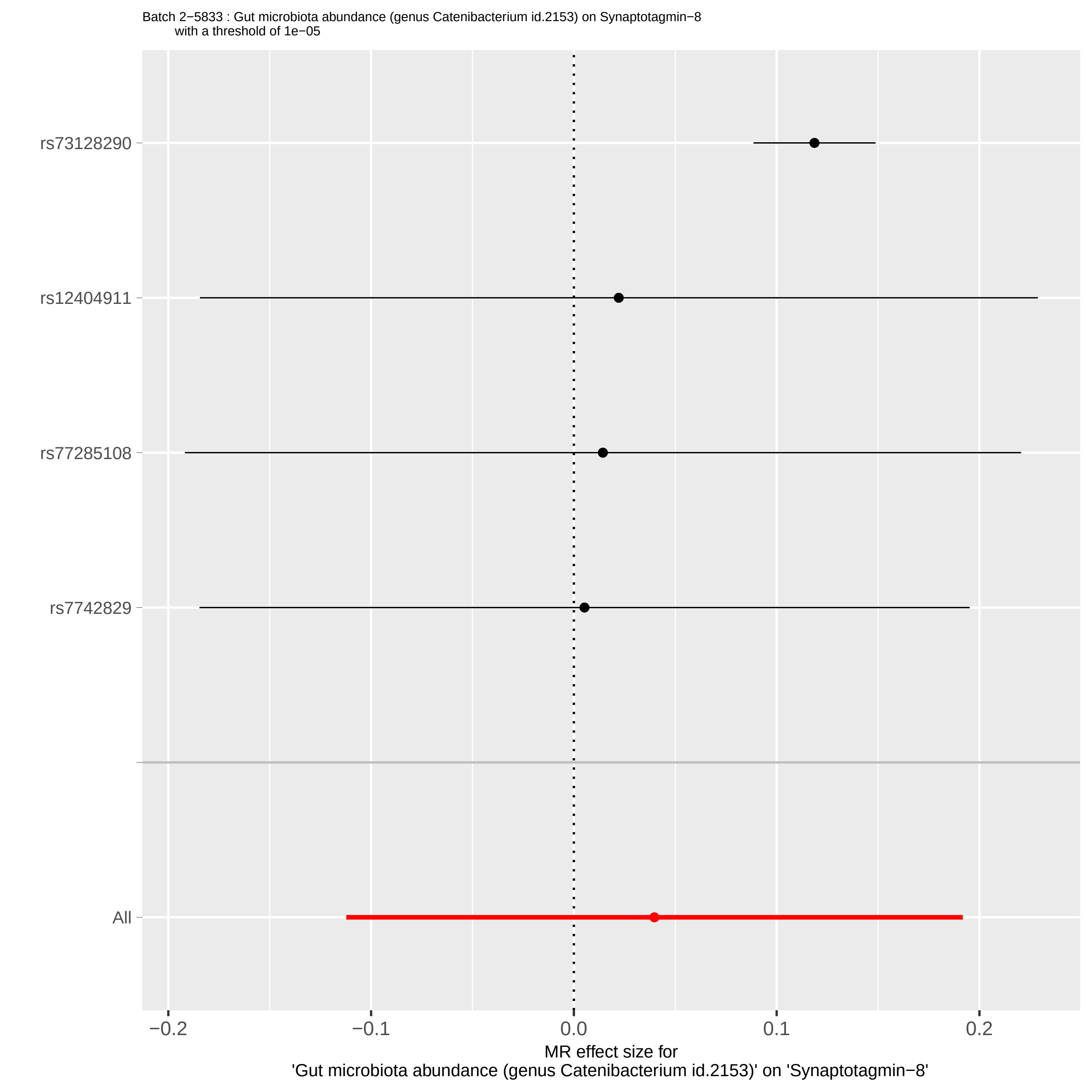
Batch 2−5833 : Gut microbiota abundance (genus Catenibacterium id.2153) on Synaptotagmin−8
with a threshold of 1e−05
rs73128290
rs12404911
rs77285108
rs7742829
All
−0.2
−0.1
0.0
0.1
0.2
MR effect size for
'Gut microbiota abundance (genus Catenibacterium id.2153)' on 'Synaptotagmin−8'

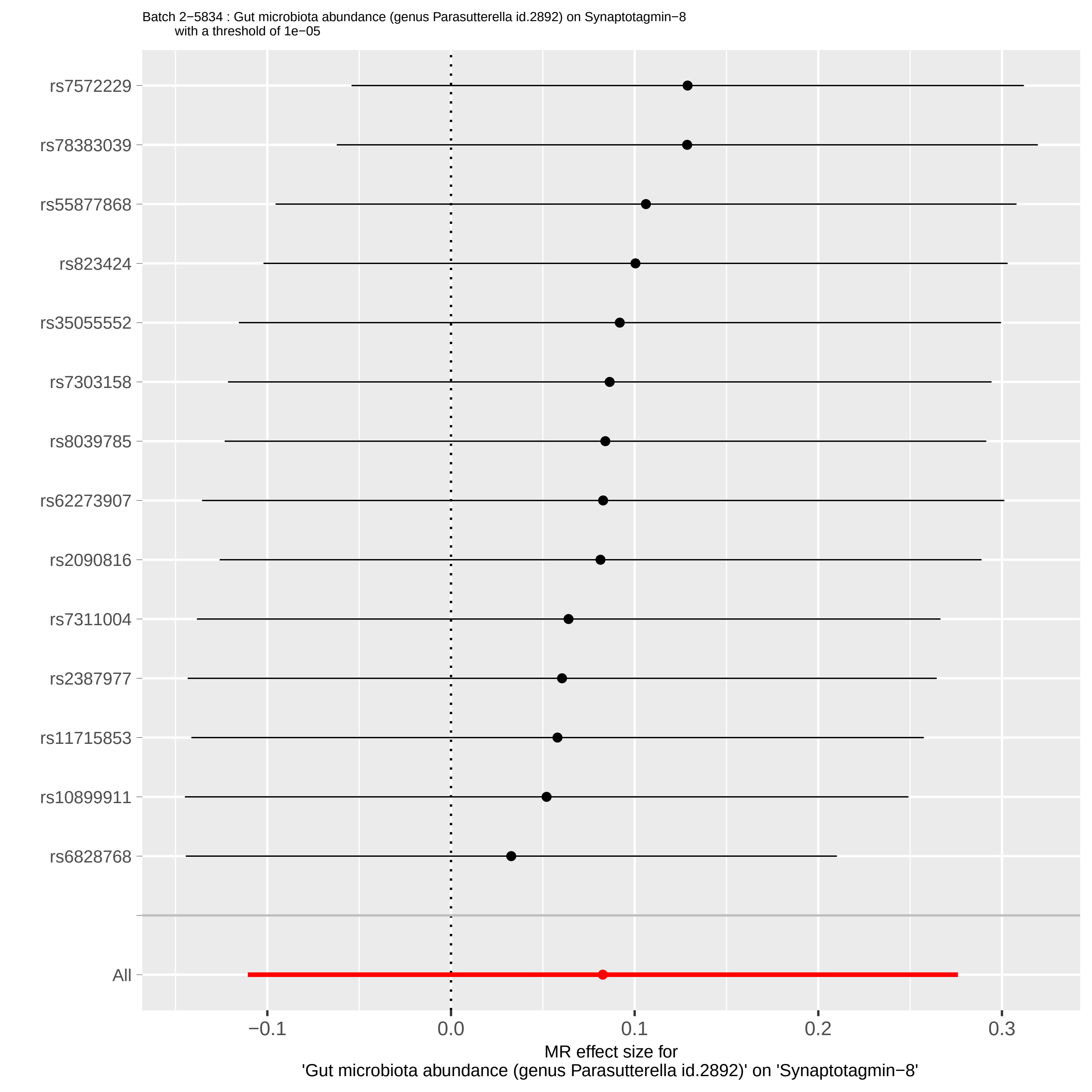
Batch 2−5834 : Gut microbiota abundance (genus Parasutterella id.2892) on Synaptotagmin−8
with a threshold of 1e−05
rs7572229
rs78383039
rs55877868
rs823424
rs35055552
rs7303158
rs8039785
rs62273907
rs2090816
rs7311004
rs2387977
rs11715853
rs10899911
rs6828768
All
−0.1
0.0
0.1
0.2
0.3
MR effect size for
'Gut microbiota abundance (genus Parasutterella id.2892)' on 'Synaptotagmin−8'

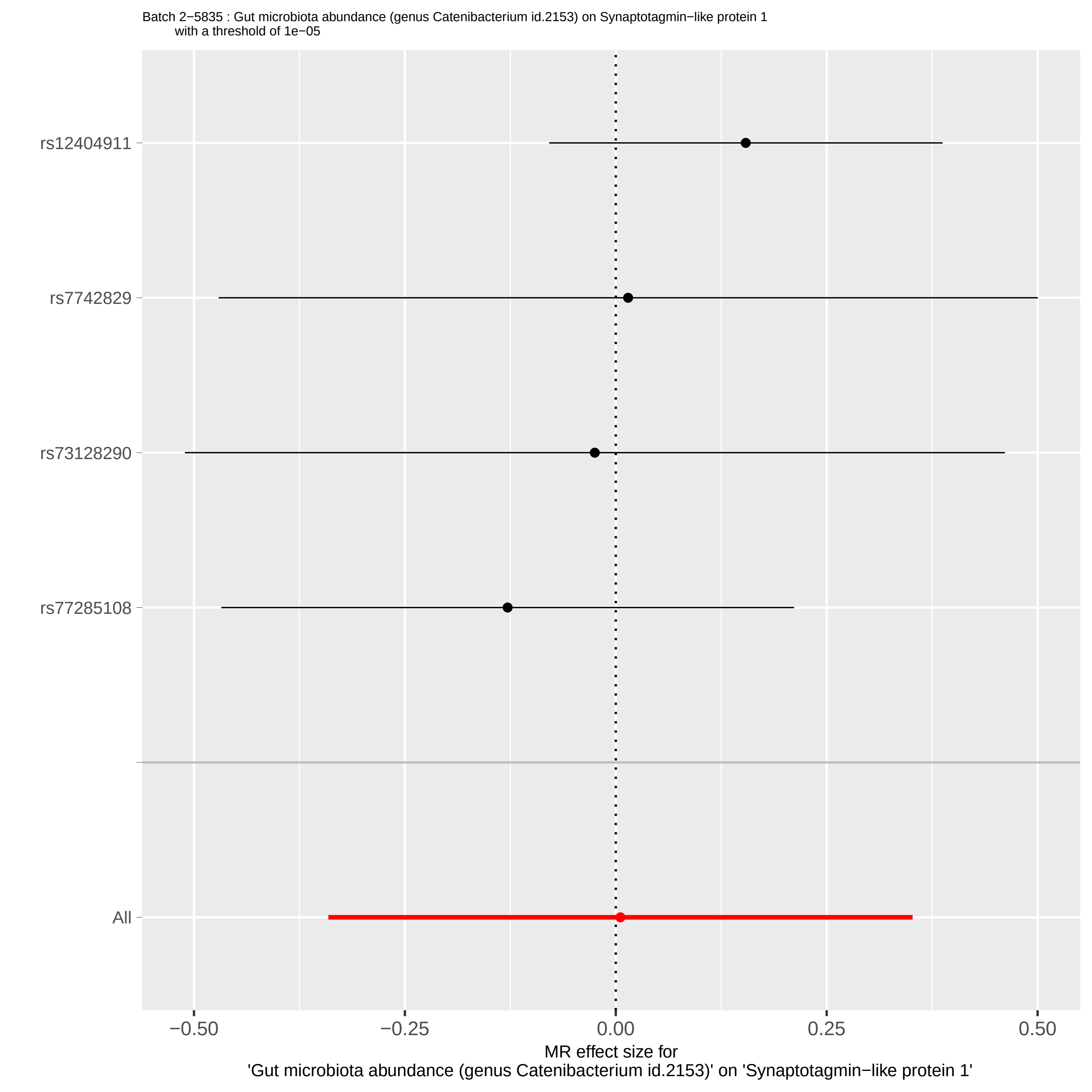

Batch 2−5835 : Gut microbiota abundance (genus Catenibacterium id.2153) on Synaptotagmin−like protein 1
with a threshold of 1e−05
rs12404911
rs7742829
rs73128290
rs77285108
All
−0.50
−0.25
0.00
0.25
0.50
MR effect size for
'Gut microbiota abundance (genus Catenibacterium id.2153)' on 'Synaptotagmin−like protein 1'

Batch 2−5836 : Gut microbiota abundance (genus Parasutterella id.2892) on Synaptotagmin−like protein 1
with a threshold of 1e−05

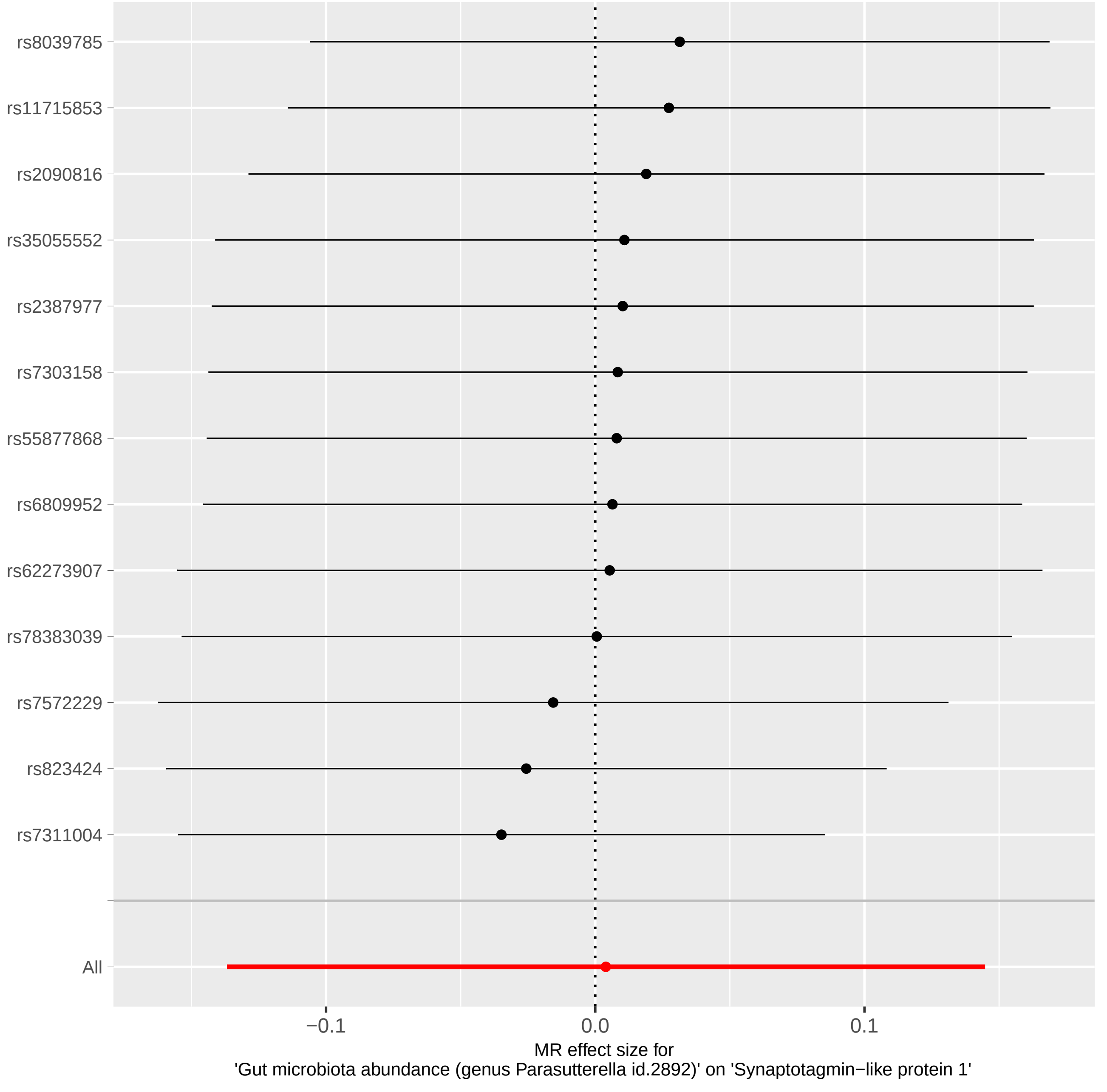

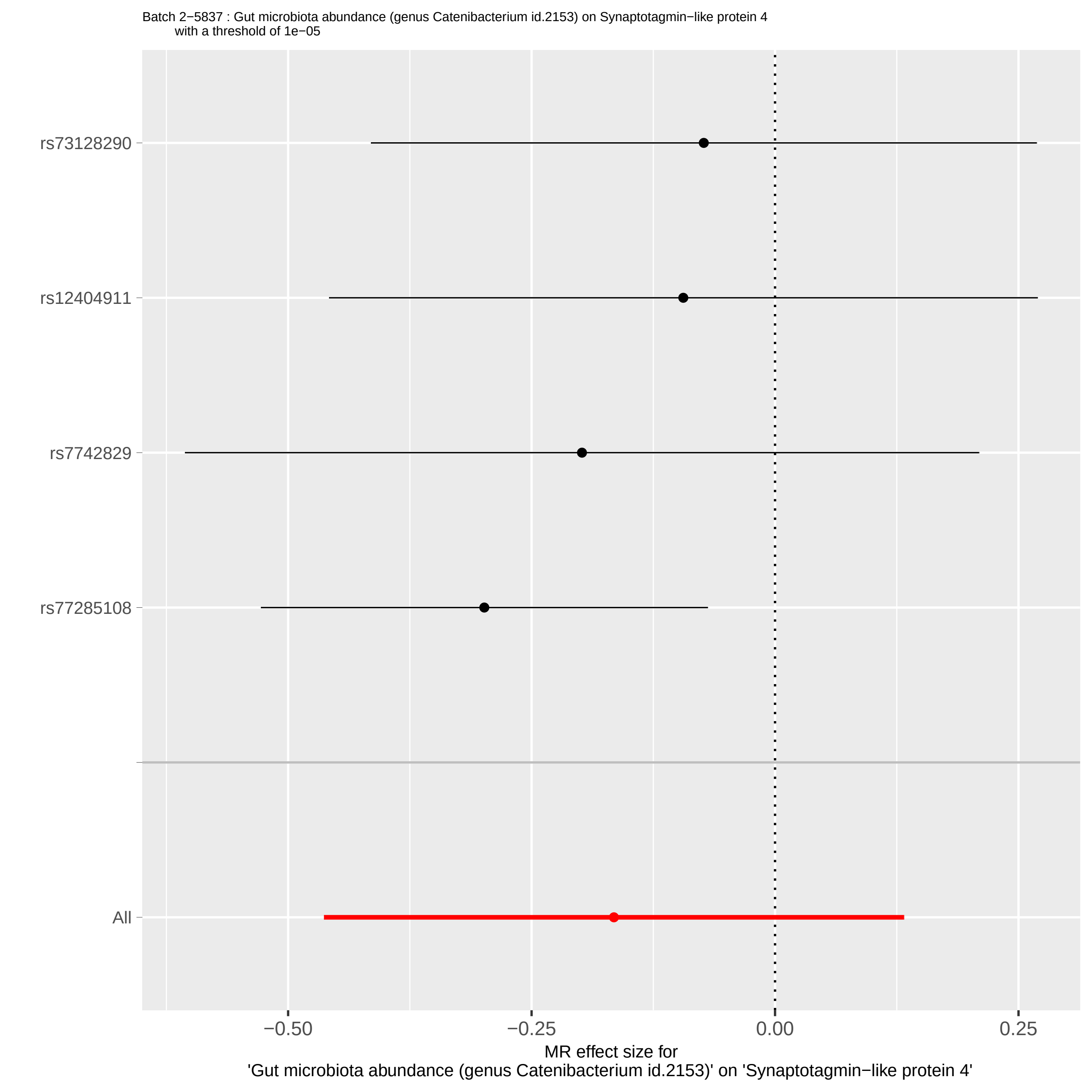

Batch 2−5837 : Gut microbiota abundance (genus Catenibacterium id.2153) on Synaptotagmin−like protein 4
with a threshold of 1e−05
rs73128290
rs12404911
rs7742829
rs77285108
All
−0.50
−0.25
0.00
0.25
MR effect size for
'Gut microbiota abundance (genus Catenibacterium id.2153)' on 'Synaptotagmin−like protein 4'

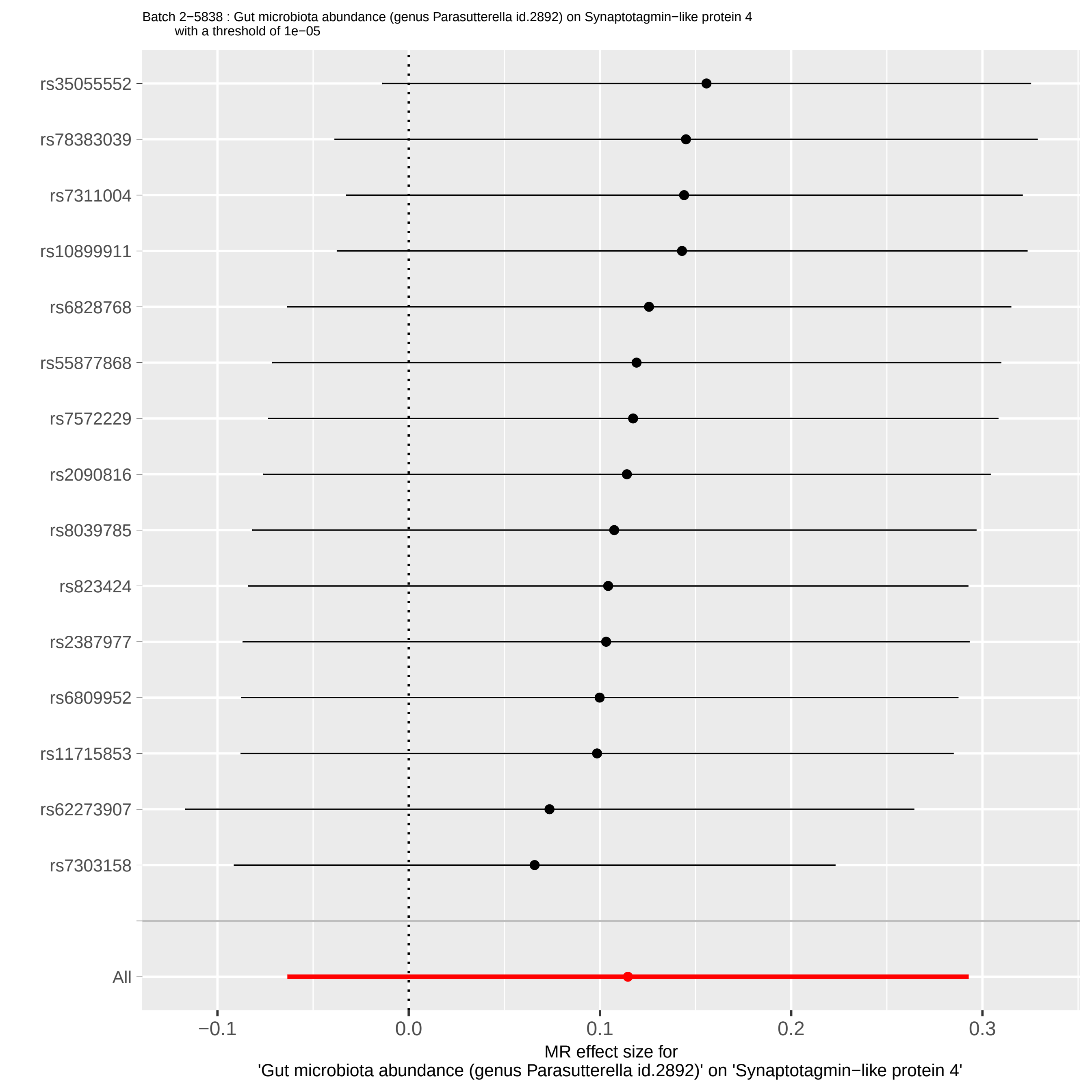

Batch 2−5838 : Gut microbiota abundance (genus Parasutterella id.2892) on Synaptotagmin−like protein 4
with a threshold of 1e−05
rs35055552
rs78383039
rs7311004
rs10899911
rs6828768
rs55877868
rs7572229
rs2090816
rs8039785
rs823424
rs2387977
rs6809952
rs11715853
rs62273907
rs7303158
All
−0.1
0.0
0.1
0.2
0.3
MR effect size for
'Gut microbiota abundance (genus Parasutterella id.2892)' on 'Synaptotagmin−like protein 4'

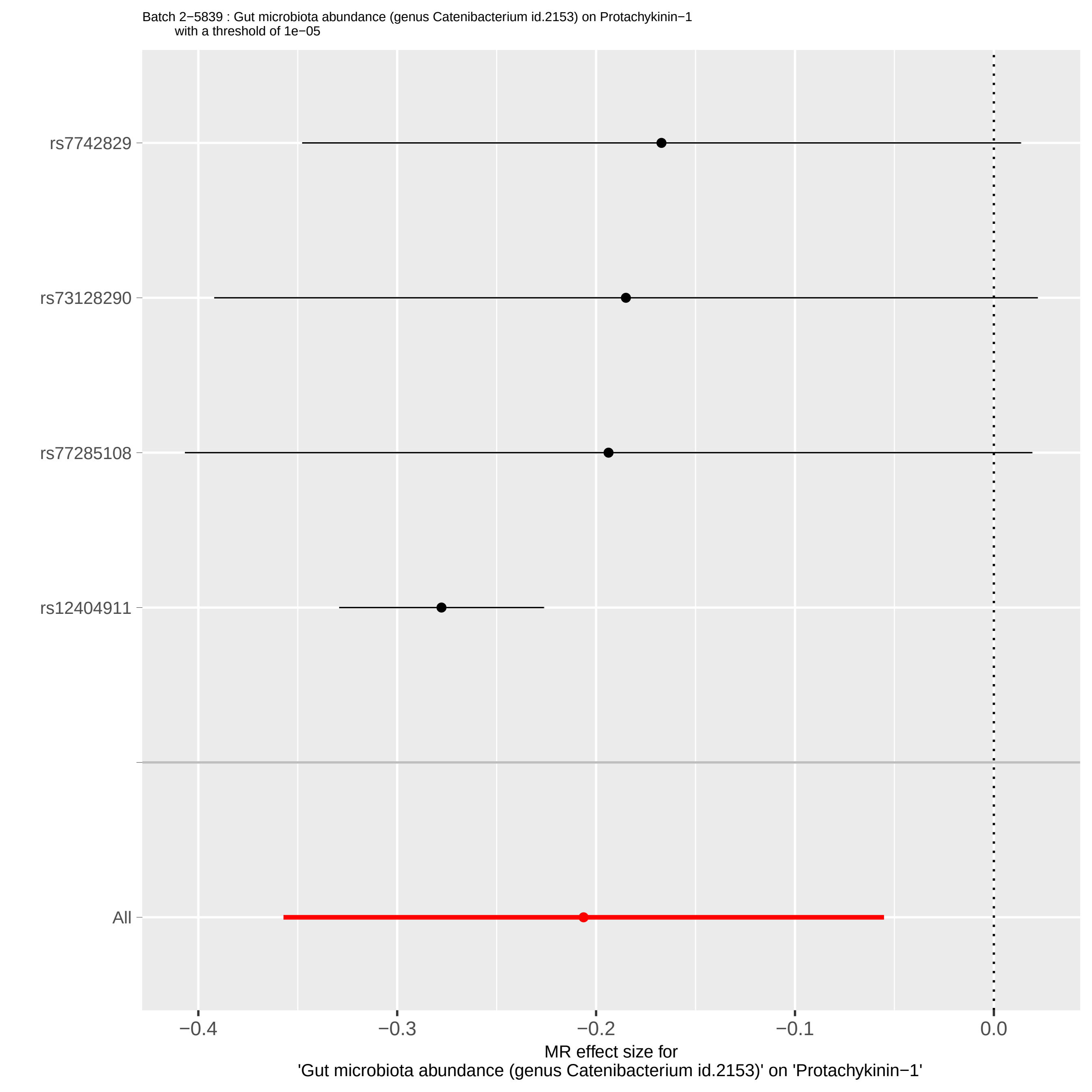
Batch 2−5839 : Gut microbiota abundance (genus Catenibacterium id.2153) on Protachykinin−1
with a threshold of 1e−05
rs7742829
rs73128290
rs77285108
rs12404911
All
−0.4
−0.3
−0.2
−0.1
0.0
MR effect size for
'Gut microbiota abundance (genus Catenibacterium id.2153)' on 'Protachykinin−1'

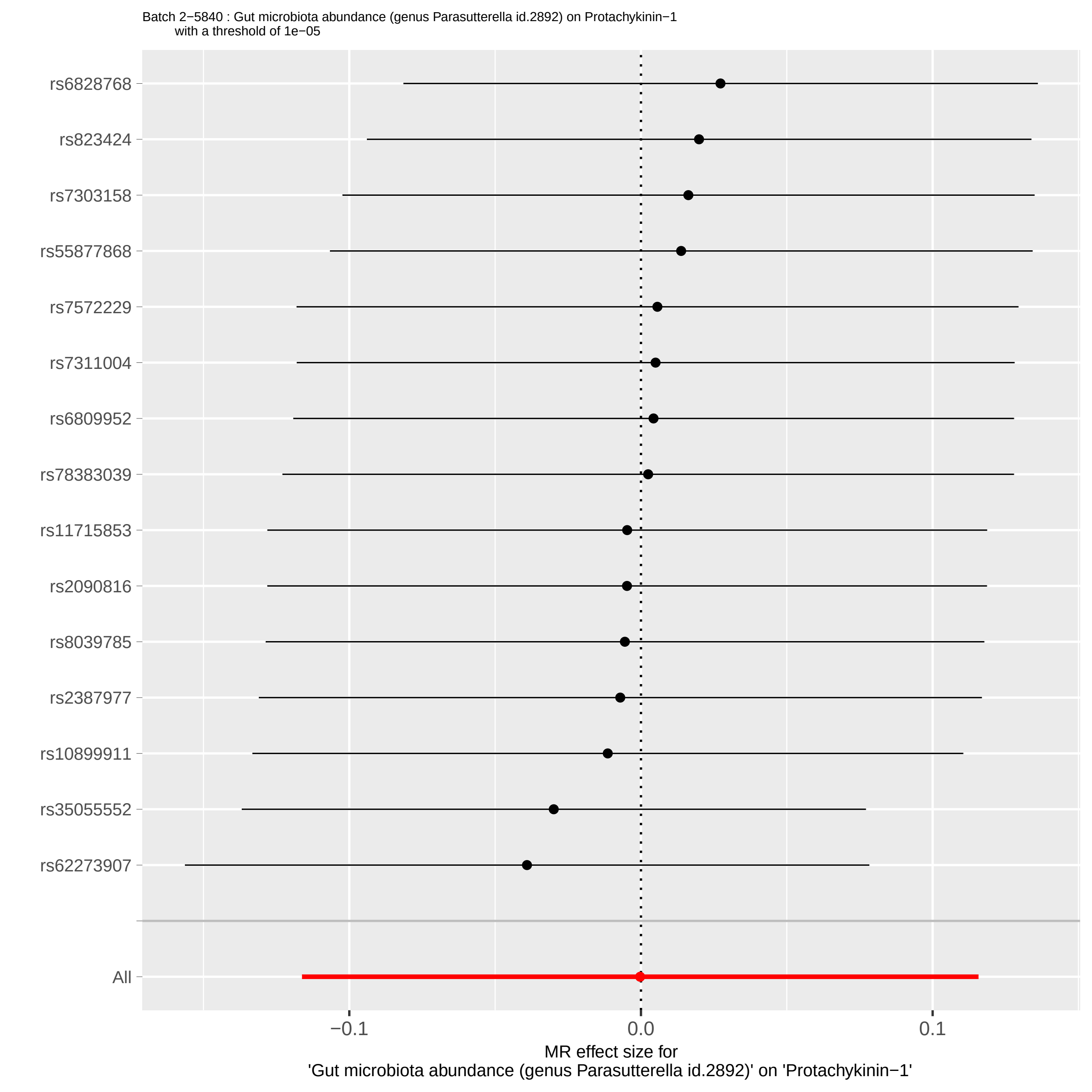

Batch 2−5840 : Gut microbiota abundance (genus Parasutterella id.2892) on Protachykinin−1
with a threshold of 1e−05
rs6828768
rs823424
rs7303158
rs55877868
rs7572229
rs7311004
rs6809952
rs78383039
rs11715853
rs2090816
rs8039785
rs2387977
rs10899911
rs35055552
rs62273907
All
−0.1
0.0
0.1
MR effect size for
'Gut microbiota abundance (genus Parasutterella id.2892)' on 'Protachykinin−1'

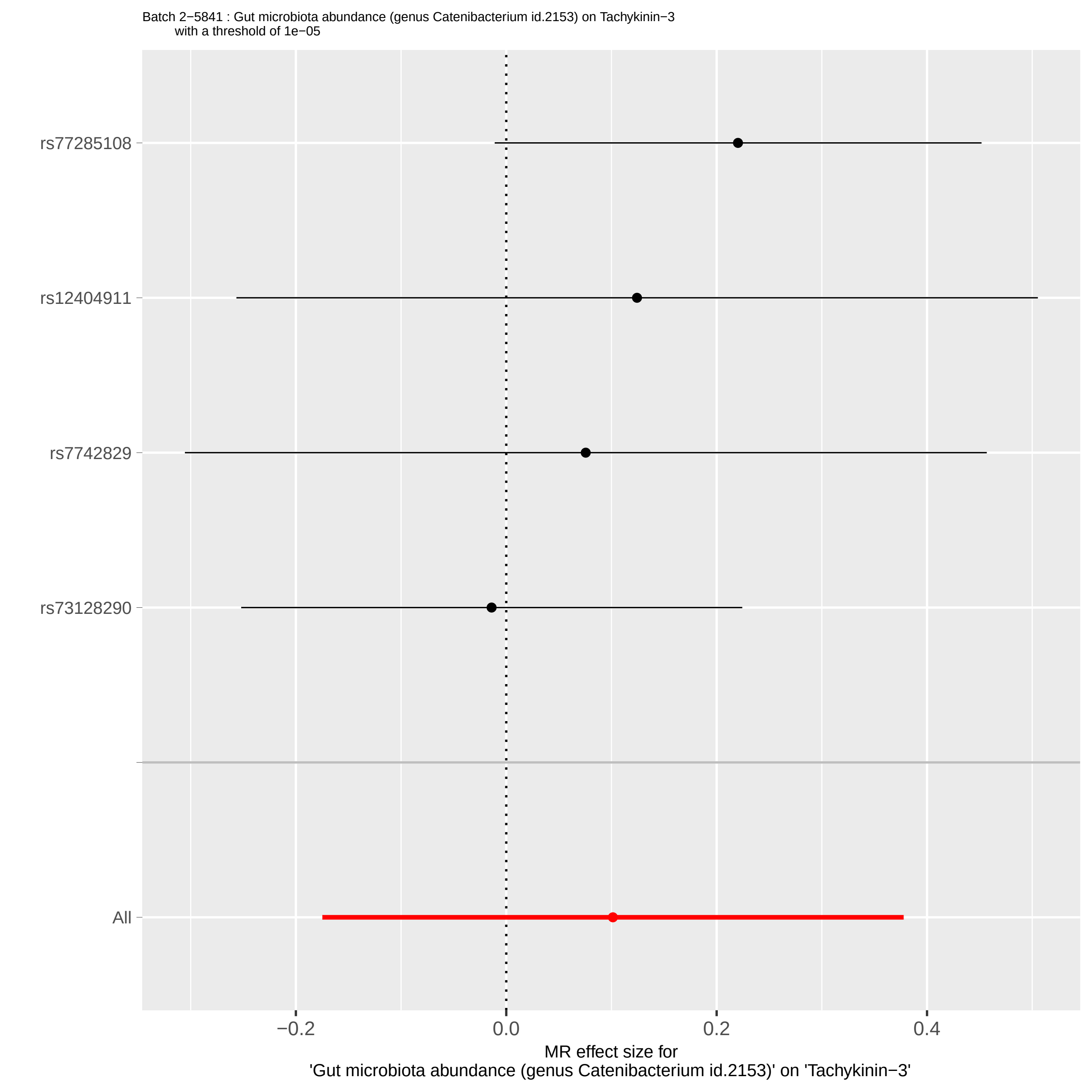

Batch 2−5841 : Gut microbiota abundance (genus Catenibacterium id.2153) on Tachykinin−3
with a threshold of 1e−05
rs77285108
rs12404911
rs7742829
rs73128290
All
−0.2
0.0
0.2
0.4
MR effect size for
'Gut microbiota abundance (genus Catenibacterium id.2153)' on 'Tachykinin−3'

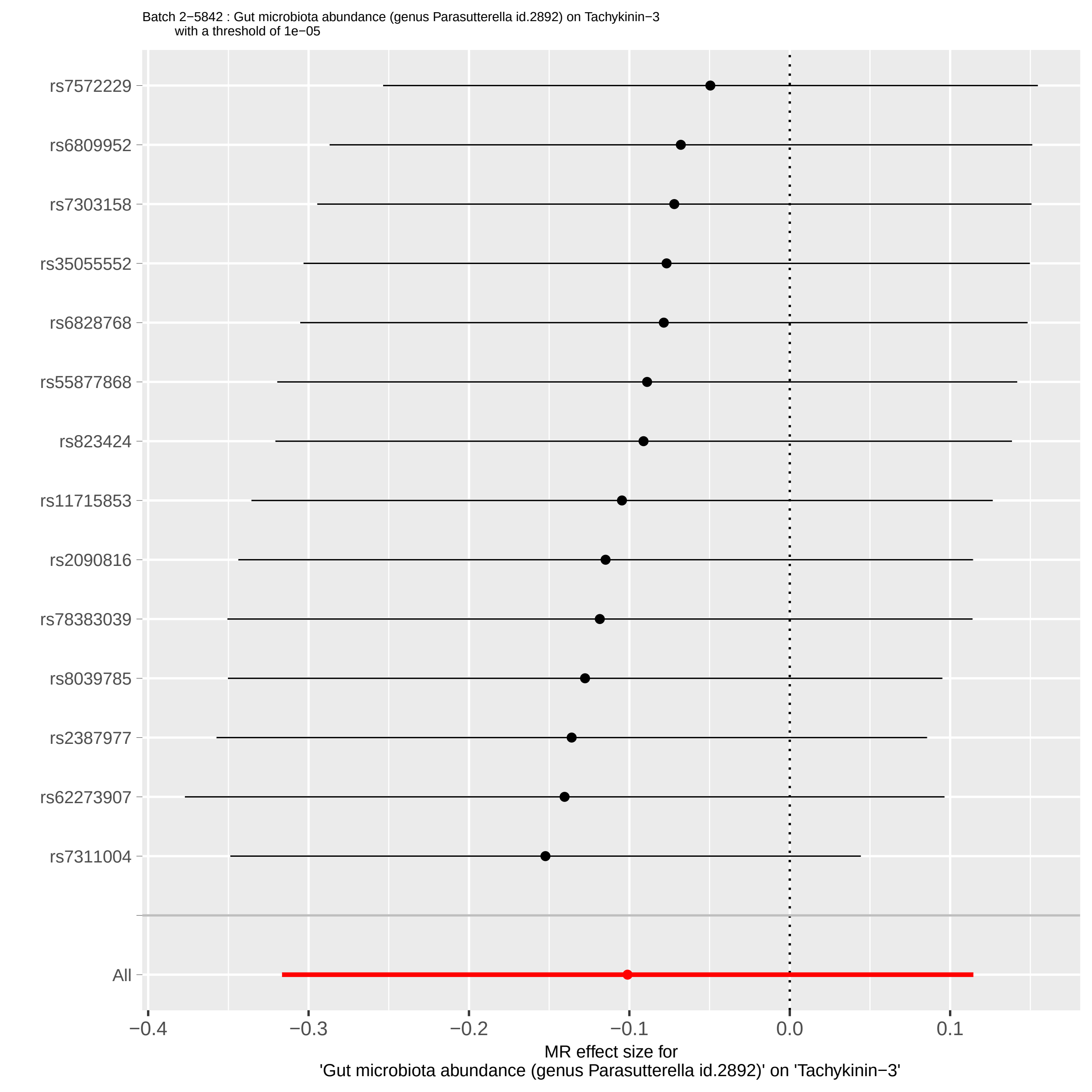

Batch 2−5842 : Gut microbiota abundance (genus Parasutterella id.2892) on Tachykinin−3
with a threshold of 1e−05
rs7572229
rs6809952
rs7303158
rs35055552
rs6828768
rs55877868
rs823424
rs11715853
rs2090816
rs78383039
rs8039785
rs2387977
rs62273907
rs7311004
All
−0.4
−0.3
−0.2
−0.1
0.0
0.1
MR effect size for
'Gut microbiota abundance (genus Parasutterella id.2892)' on 'Tachykinin−3'

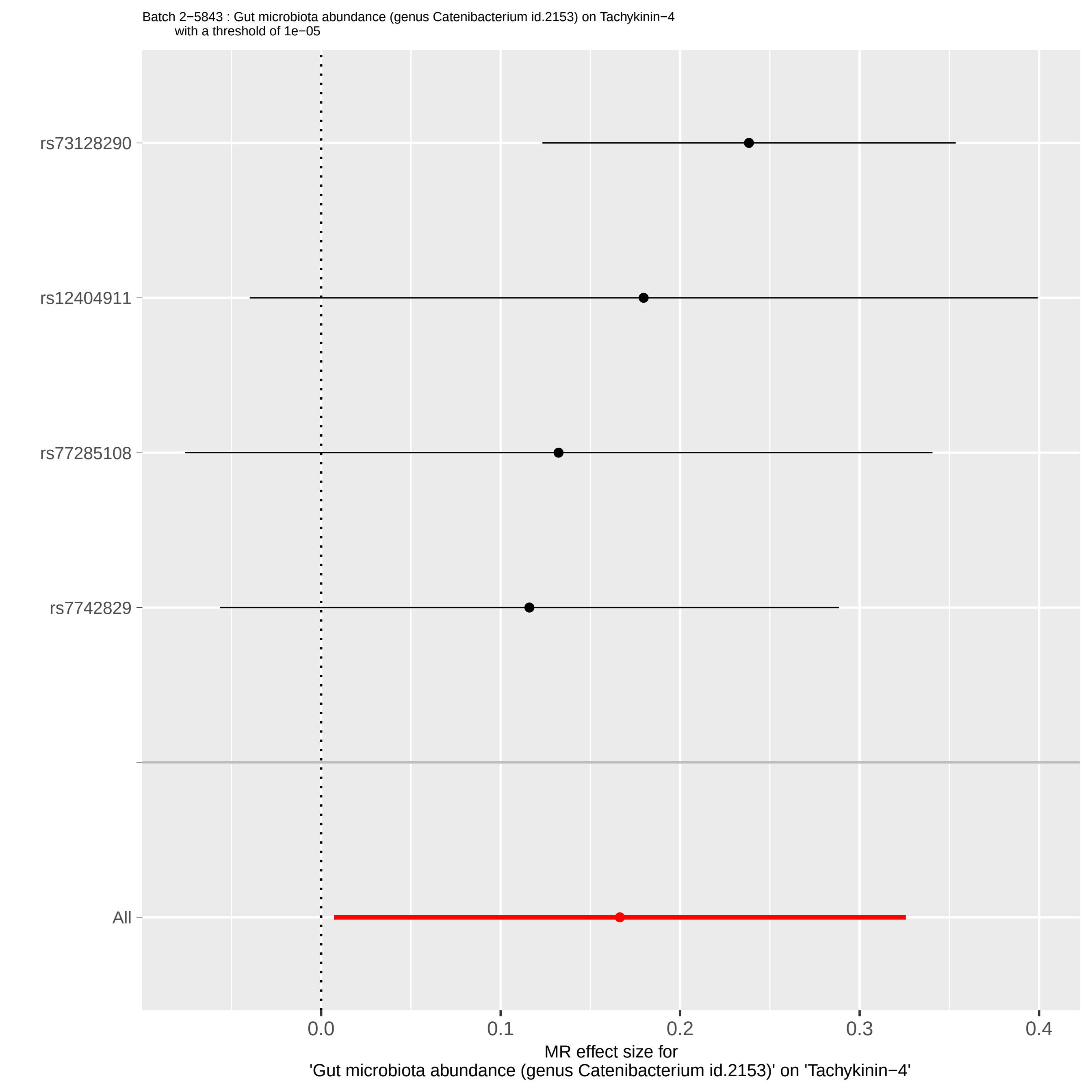
Batch 2−5843 : Gut microbiota abundance (genus Catenibacterium id.2153) on Tachykinin−4
with a threshold of 1e−05
rs73128290
rs12404911
rs77285108
rs7742829
All
0.0
0.1
0.2
0.3
0.4
MR effect size for
'Gut microbiota abundance (genus Catenibacterium id.2153)' on 'Tachykinin−4'

Batch 2−5844 : Gut microbiota abundance (genus Parasutterella id.2892) on Tachykinin−4
with a threshold of 1e−05

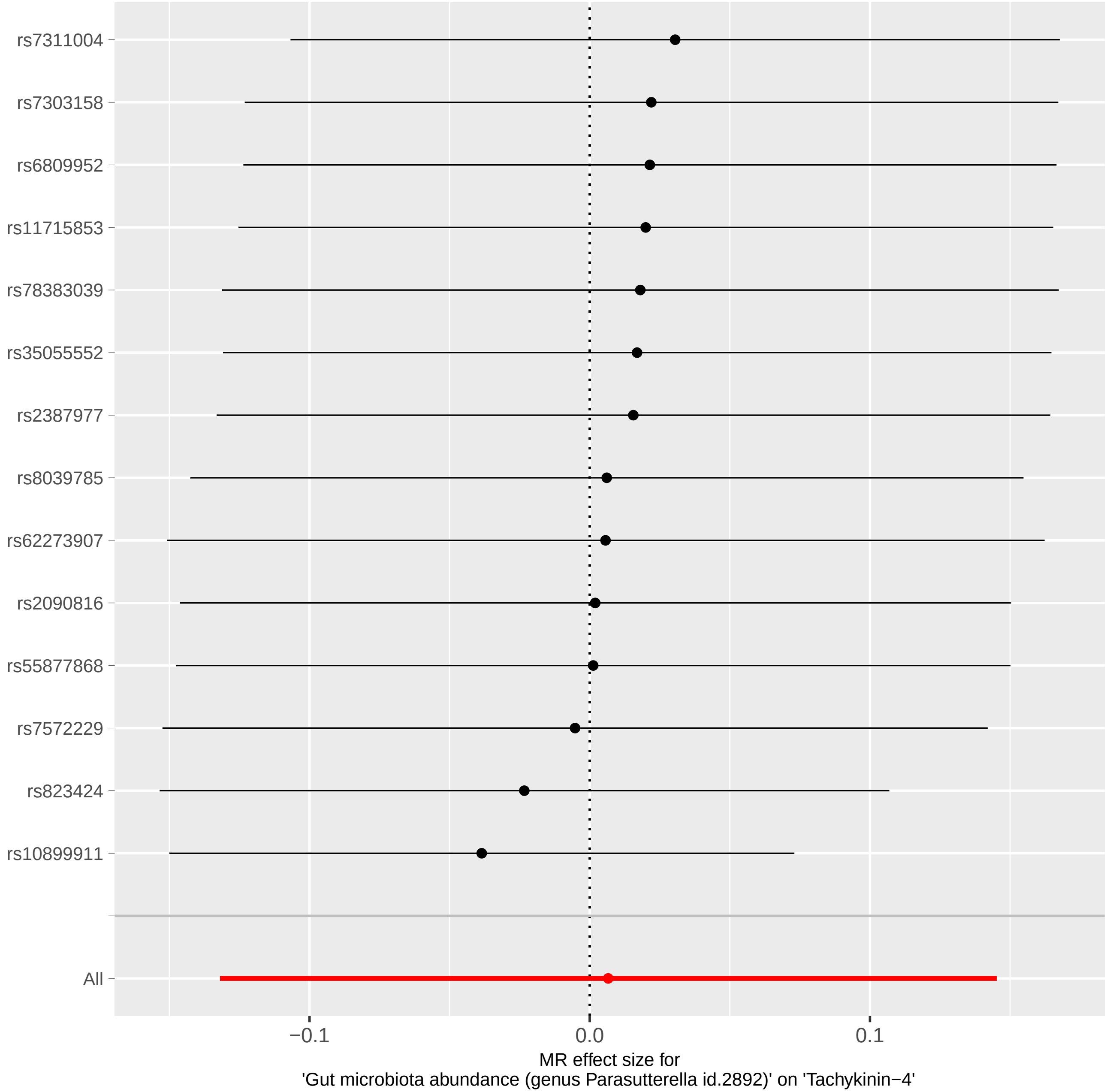

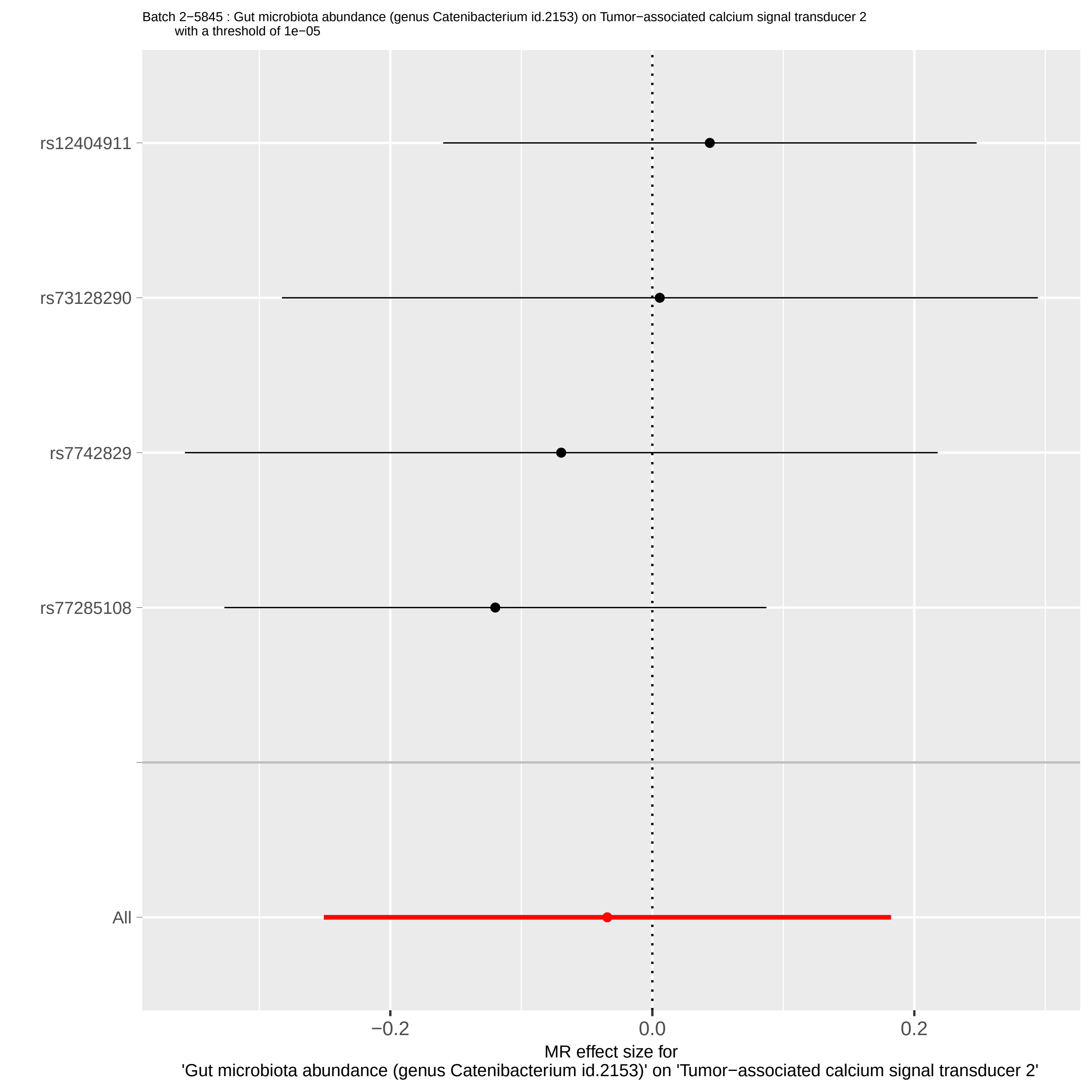
Batch 2−5845 : Gut microbiota abundance (genus Catenibacterium id.2153) on Tumor−associated calcium signal transducer 2
with a threshold of 1e−05
rs12404911
rs73128290
rs7742829
rs77285108
All
−0.2
0.0
0.2
MR effect size for
'Gut microbiota abundance (genus Catenibacterium id.2153)' on 'Tumor−associated calcium signal transducer 2'

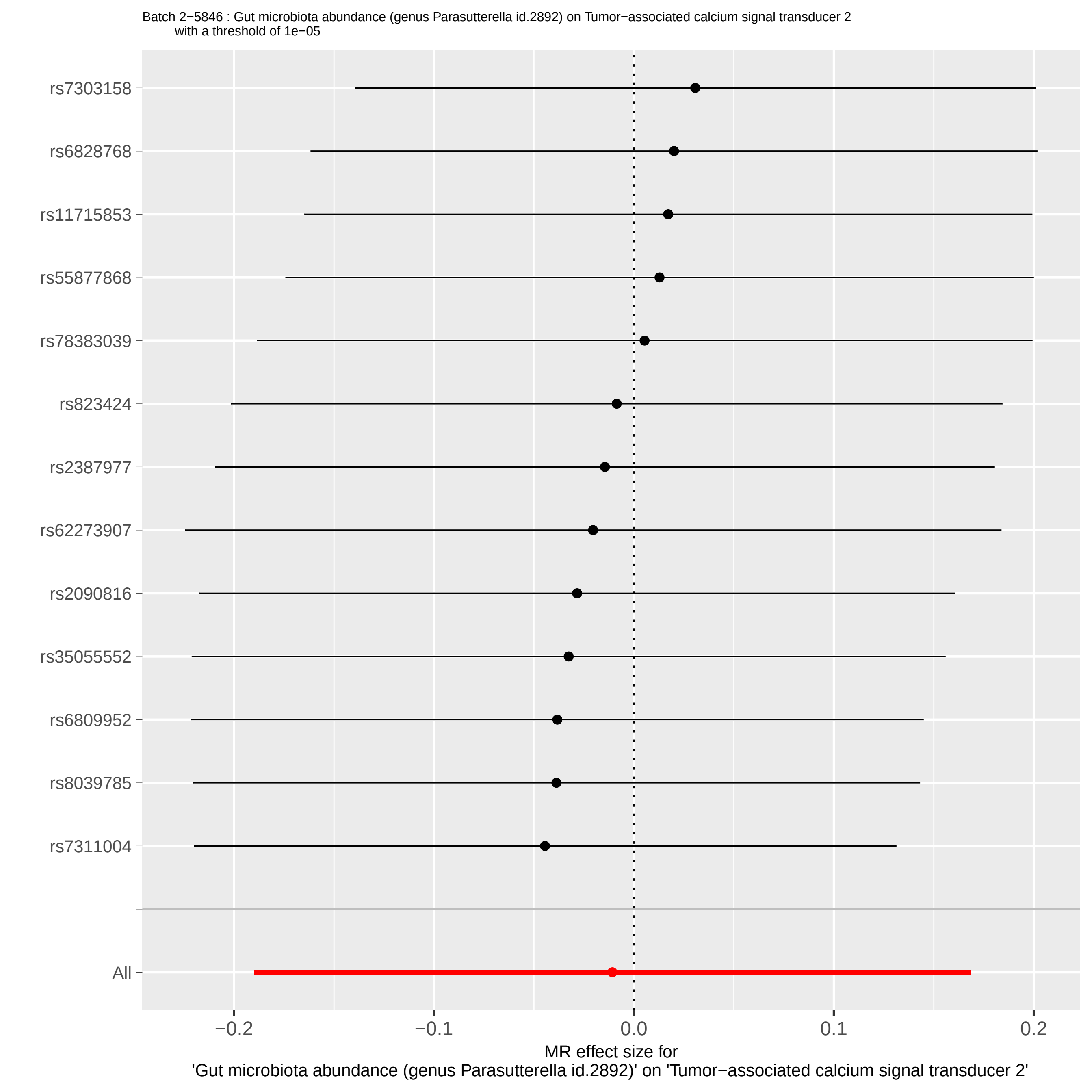
Batch 2−5846 : Gut microbiota abundance (genus Parasutterella id.2892) on Tumor−associated calcium signal transducer 2
with a threshold of 1e−05
rs7303158
rs6828768
rs11715853
rs55877868
rs78383039
rs823424
rs2387977
rs62273907
rs2090816
rs35055552
rs6809952
rs8039785
rs7311004
All
−0.2
−0.1
0.0
0.1
0.2
MR effect size for
'Gut microbiota abundance (genus Parasutterella id.2892)' on 'Tumor−associated calcium signal transducer 2'

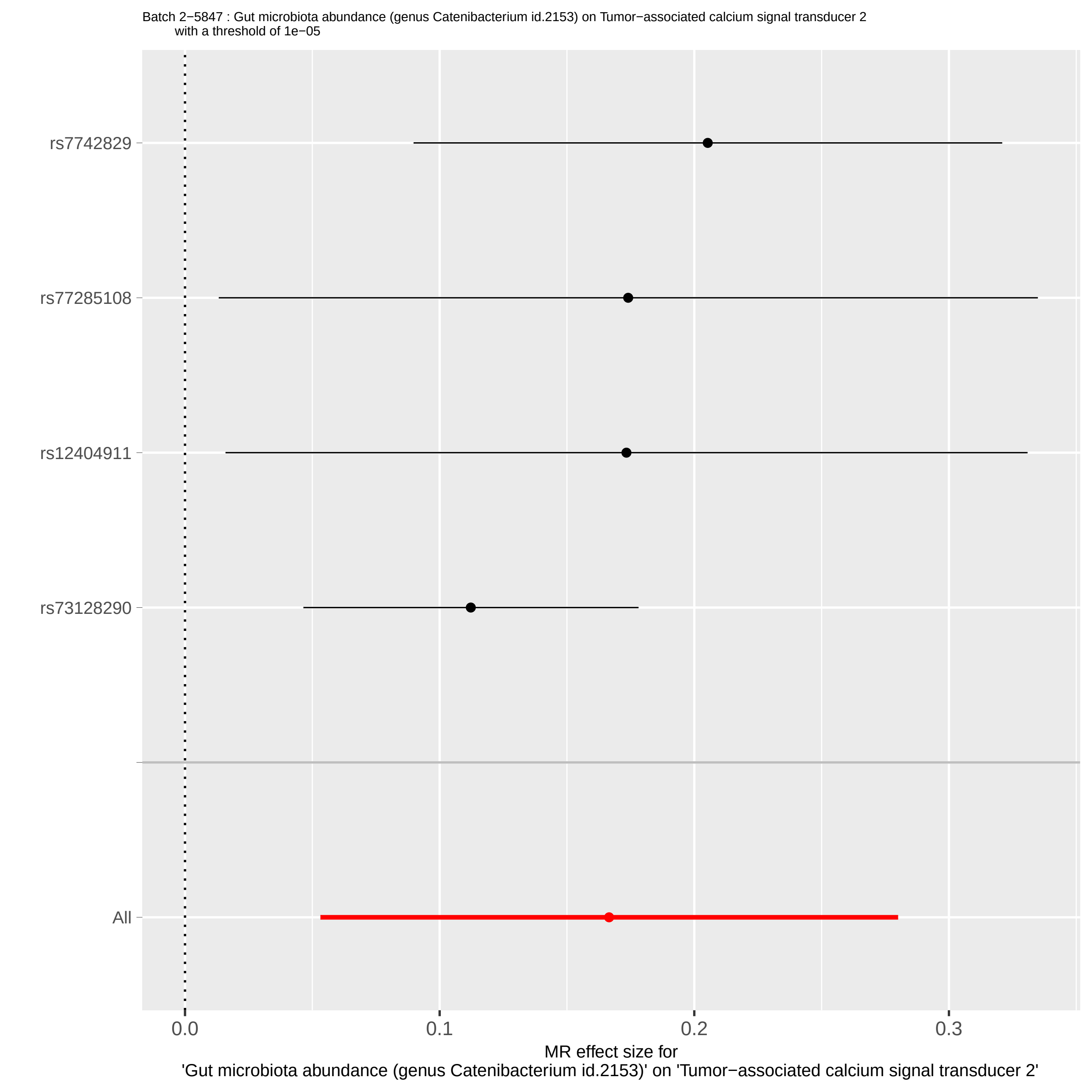

Batch 2−5847 : Gut microbiota abundance (genus Catenibacterium id.2153) on Tumor−associated calcium signal transducer 2
with a threshold of 1e−05
rs7742829
rs77285108
rs12404911
rs73128290
All
0.0
0.1
0.2
0.3
MR effect size for
'Gut microbiota abundance (genus Catenibacterium id.2153)' on 'Tumor−associated calcium signal transducer 2'

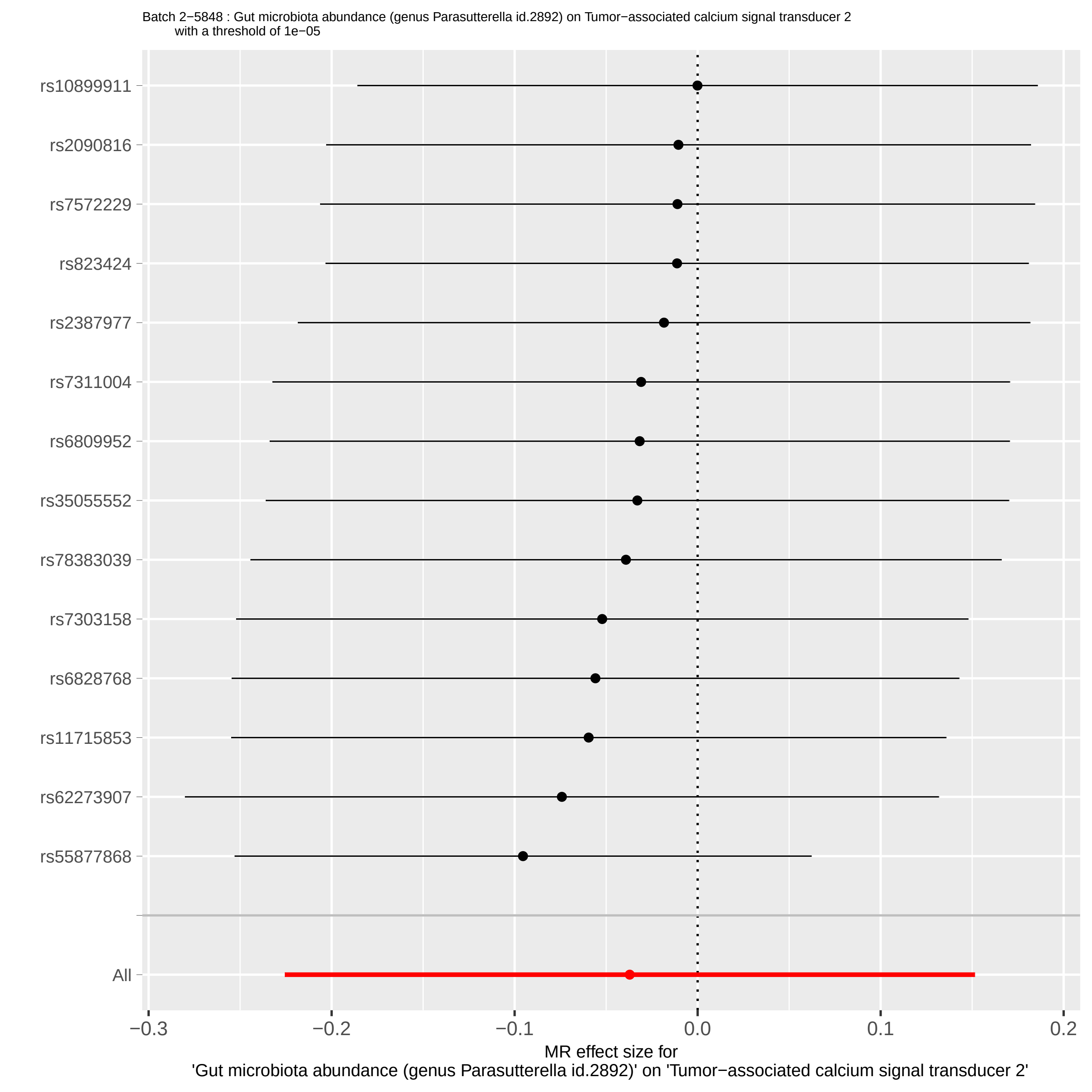

Batch 2−5848 : Gut microbiota abundance (genus Parasutterella id.2892) on Tumor−associated calcium signal transducer 2
with a threshold of 1e−05
rs10899911
rs2090816
rs7572229
rs823424
rs2387977
rs7311004
rs6809952
rs35055552
rs78383039
rs7303158
rs6828768
rs11715853
rs62273907
rs55877868
All
−0.3
−0.2
−0.1
0.0
0.1
0.2
MR effect size for
'Gut microbiota abundance (genus Parasutterella id.2892)' on 'Tumor−associated calcium signal transducer 2'

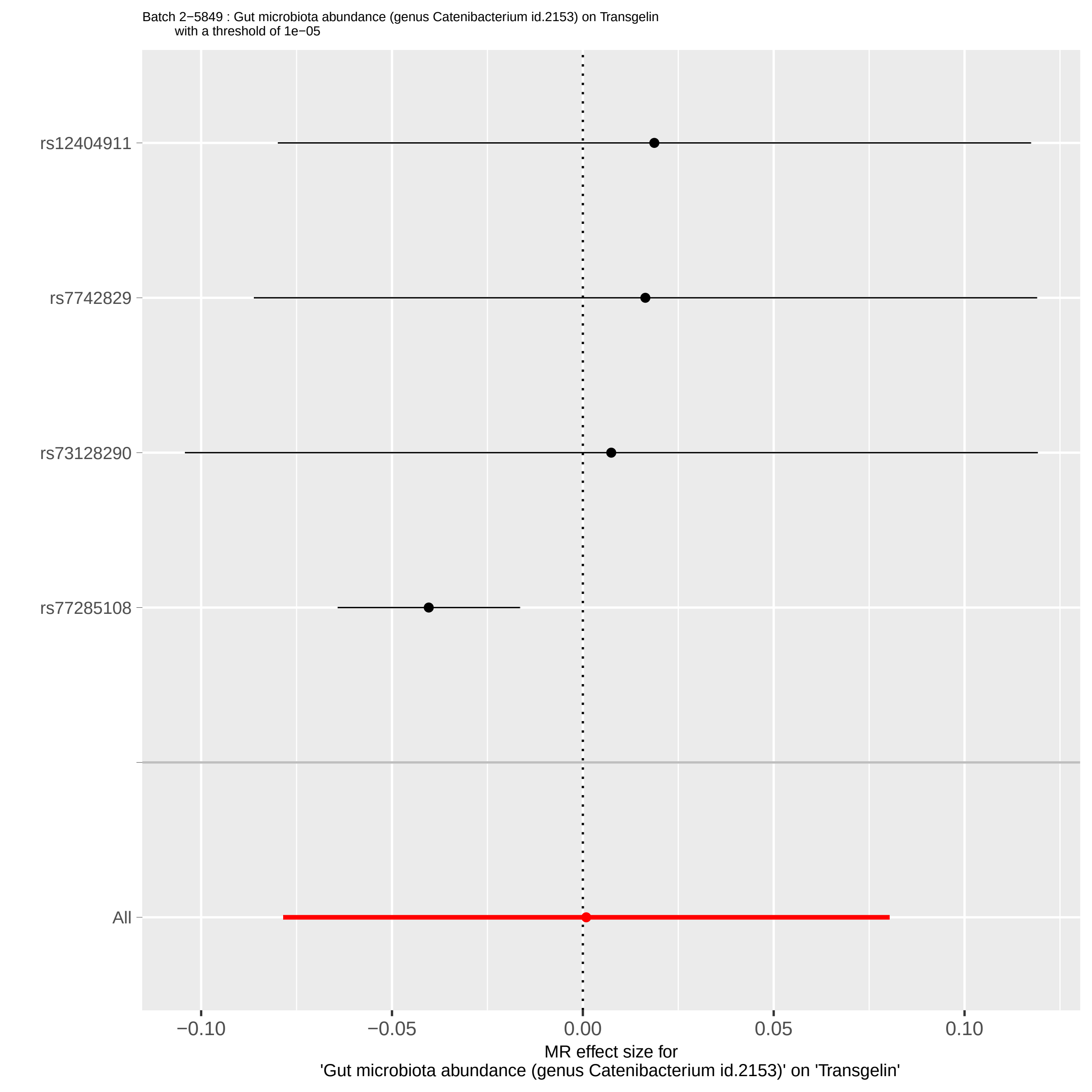
Batch 2−5849 : Gut microbiota abundance (genus Catenibacterium id.2153) on Transgelin
with a threshold of 1e−05
rs12404911
rs7742829
rs73128290
rs77285108
All
−0.10
−0.05
0.00
0.05
0.10
MR effect size for
'Gut microbiota abundance (genus Catenibacterium id.2153)' on 'Transgelin'

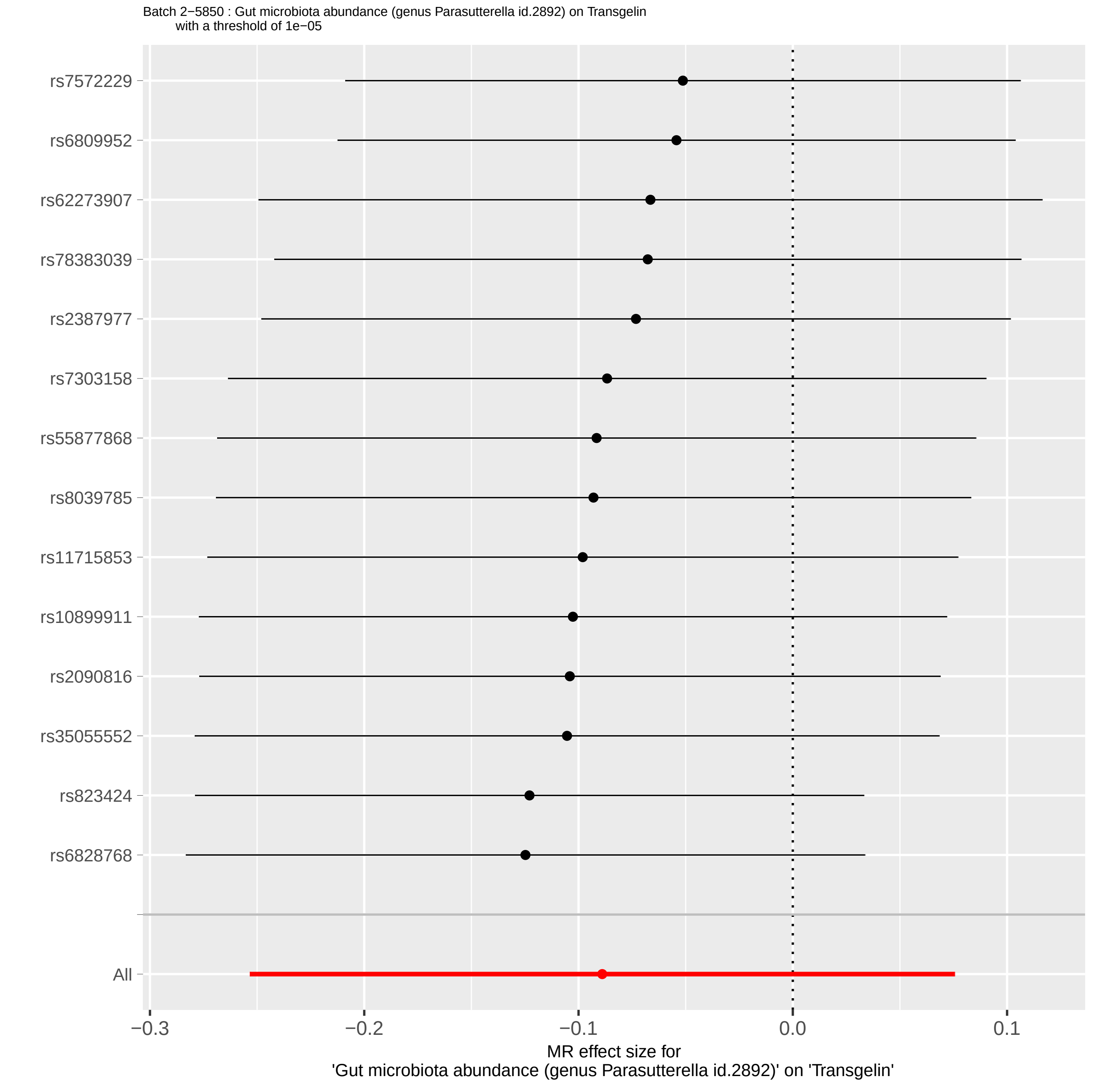
Batch 2−5850 : Gut microbiota abundance (genus Parasutterella id.2892) on Transgelin
with a threshold of 1e−05
rs7572229
rs6809952
rs62273907
rs78383039
rs2387977
rs7303158
rs55877868
rs8039785
rs11715853
rs10899911
rs2090816
rs35055552
rs823424
rs6828768
All
−0.3
−0.2
−0.1
0.0
0.1
MR effect size for
'Gut microbiota abundance (genus Parasutterella id.2892)' on 'Transgelin'

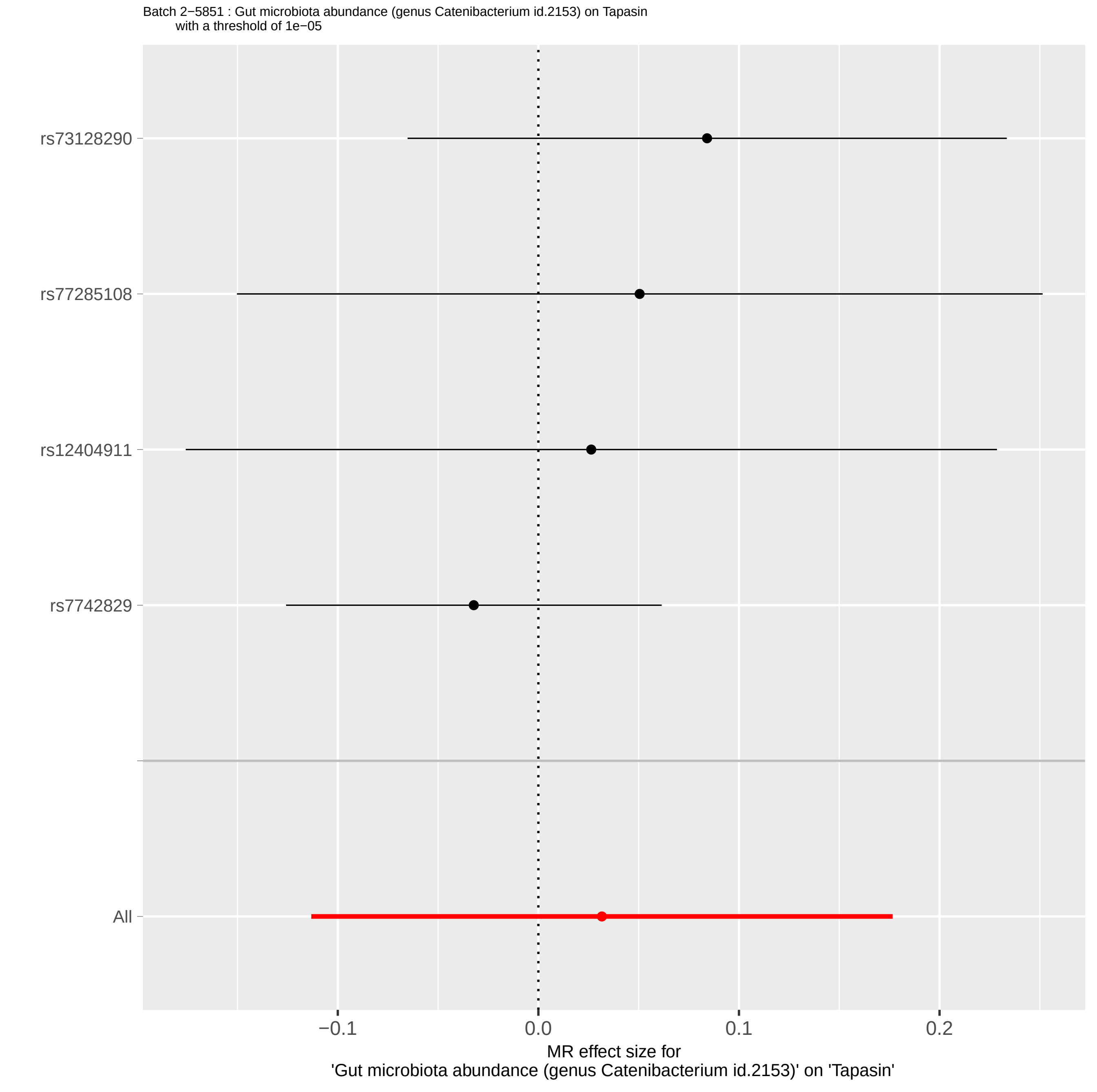
Batch 2−5851 : Gut microbiota abundance (genus Catenibacterium id.2153) on Tapasin
with a threshold of 1e−05
rs73128290
rs77285108
rs12404911
rs7742829
All
−0.1
0.0
0.1
0.2
MR effect size for
'Gut microbiota abundance (genus Catenibacterium id.2153)' on 'Tapasin'

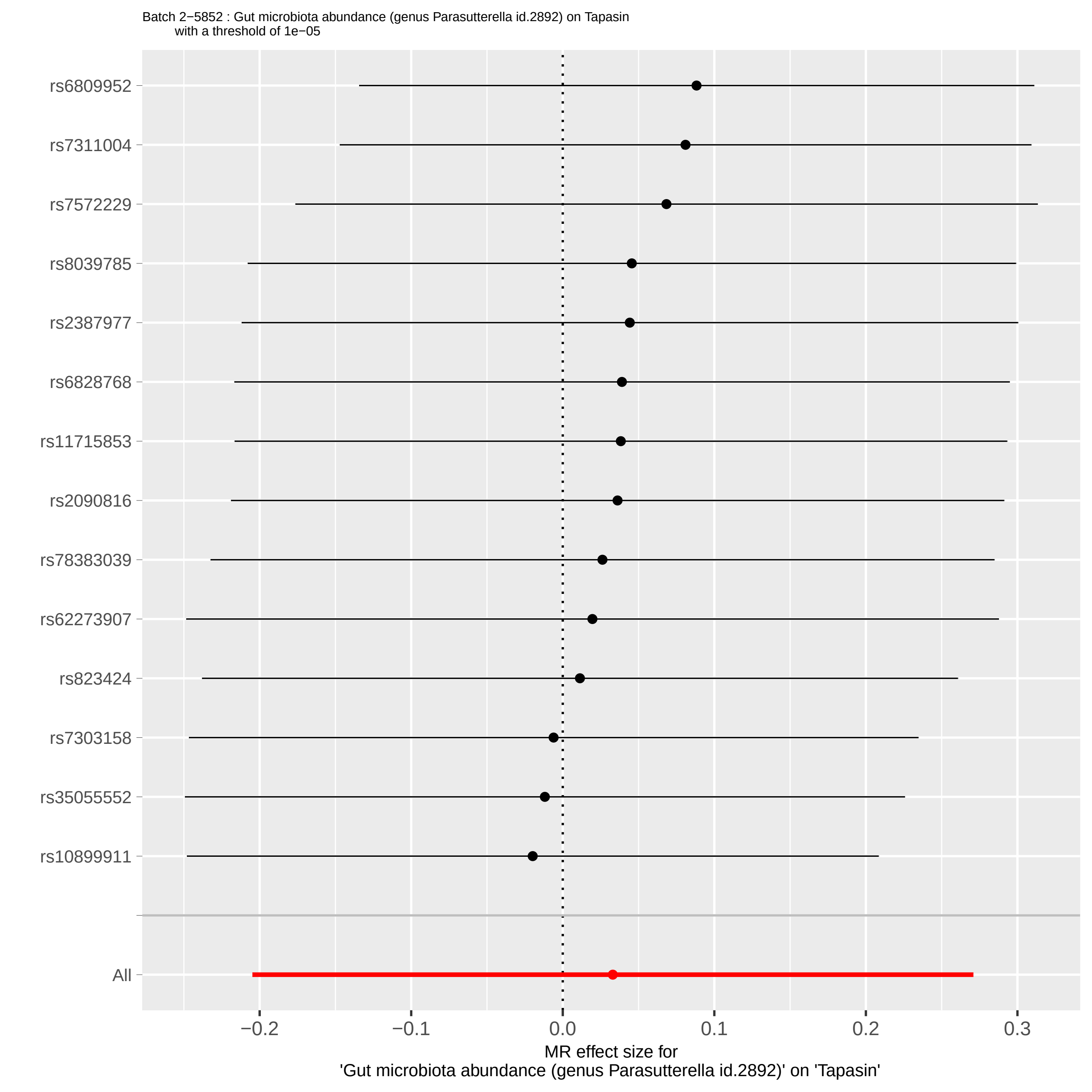
Batch 2−5852 : Gut microbiota abundance (genus Parasutterella id.2892) on Tapasin
with a threshold of 1e−05
rs6809952
rs7311004
rs7572229
rs8039785
rs2387977
rs6828768
rs11715853
rs2090816
rs78383039
rs62273907
rs823424
rs7303158
rs35055552
rs10899911
All
−0.2
−0.1
0.0
0.1
0.2
0.3
MR effect size for
[truncated: 488,263 more chars]
